# Supplementary material for: Genome-wide analysis of the WRKY gene family in drumstick (Moringa oleifera Lam.)
Source: PeerJ. 2019 Jun 10;7:e7063. doi: 10.7717/peerj.7063 (PMC6563795; doi:10.7717/peerj.7063)
Supplement: Supplemental Information 1 [file peerj-07-7063-s003.gz › MoWRKY10_plantcare.html]

Content-Type: text/html; charset=ISO-8859-1


CallMat\_Firefox


Webmaster Firefox specific output  
To save the result:
click on the frame with the right mouse button and save the source code as a text file with extension .html  
REFERENCE:PlantCARE: a database of plant cis-acting regulatory elements and a portal to tools for in silico analysis of promoter sequences.  
Lescot, M., Déhais, P., Moreau, Y., De Moor, B., Rouzé ,P.,and Rombauts, S.  
Nucleic Acids Res., Database issue(2002), 30(1):325-327.   


---

> 2018/04/13 10:10:12  
+ TTAAAAAACT GACTGTTTAT ATCAGTTGTA TTTTCATAAA ATTTGTCATT CGATTTTATG GGTTTTTGTT   
  
  
+ TTAAGTTAGA ATTTATATCT ATATTTTTAC ATTATAACAT AAAAATTCTG ATTTTTTTAA AAATTAAATG   
  
  
+ TAATAATAGA ATATATCTAT GGCATTAAAA CTATCATTCT GGTTGATAAA ACACCCACAG CCACAAGGGA   
  
  
+ AGAGTAAGTA TTTTATATTT CCCCTTCCAC ATAATCCATA TCCAGGTGTG TCTCCTGATG ATTAATGGAC   
  
  
+ ATCACCATCC TCTGGGTCCC ATGAGAGTGA ATGCCCCAAT CAGAACGGCT TGCTCTGGGC TGTAATAAAA   
  
  
+ TCCAGCTCGT GAATTGTGAG GGGCGTACAC GGCAACTTGA CCGCCTTTGC TTTTTTCTCG GTGGGTCCCT   
  
  
+ GTTGGGGCAA CATCGCTGTC ATCGTCTGTG ATTTTGATGT GGTAGGAAGC ATCCGATTTG TAACTGTTAA   
  
  
+ GAGTTTCTTA TGCAACGTCG TTATGGAGAG GTCCTGTACG CCCACTGGAG CATTTGATGC TGCAACAGCA   
  
  
+ AACCAAAACA AAAGGAGTCA GATTTTTGGA ATTTCTACTC TGTCGGCAGT TTAGCCCCCC AGTCTGTTAA   
  
  
+ TCTTGCAAGT TCCAGGATTC TTTTGGGTTG CCCAGAAGTC AATCCCCTCT TCATGCCTAT CGGTTCATCT   
  
  
+ CACCTGTTGA CATGACAACT ATCTGACCAA AAATAAAATT TTTATGCTTT ATCTTGCAGA CATTTTAAAA   
  
  
+ GGGTTTTCGT TCATTTAATA GAATTTTTCT TTTTTTTTAA ATGAATTTGA GGTTCCAAAT ATTTTAGGGA   
  
  
+ TCTTTAAGGG GGAGAAGAGG AGTAATGCAC ATGGTAAAGA GAGTGAAAGT TCTGACACTG TTAAGTGTAG   
  
  
+ GACTGTAGGT GGGTTCCGGT CTTGGAGGGC AACAAGTCAA TTGAAATGAG AAAGTTTCGT GCTAGAAATC   
  
  
+ ATTTGACATT TGCGTTGGGG CAGGGATTCC TGTTTTTCCT TATCTCATTG CTAGTTCATT TGGGTAGGGA   
  
  
+ TTAAATTGGC AGGAACTTAA GTTTGTCTTC CTTTGATTTC ATCGAAAGCT CCAAATCAGT CTTATCAGCA   
  
  
+ ATGGCAGCTT AGCCATTGTG TGATTAGTTT AGTTGCGAGA ATGGAATCTT TAGTGGGTGG GGGGGCACTT   
  
  
+ AGAAGCATGC CAGGTGGAGG GAAACTTCAA GGCTTACGGC CGTGAAACTG TATTTCGTCT TTAAAAAAAA   
  
  
+ AAAAAATATT AGCTCACAAG AATTCTTGAA AAAAAAAAAA AAAAAAGGGA AAGAGGATGT CGTTTTCCAT   
  
  
+ TTCTCTGCTC TGTGGACTTT TTGGGTTCCA CTGGTTTTCA GGGTAGCACG AAACTGTCAA AAAGCCGACT   
  
  
+ GATTTAGAGA GAGGAAAACA ACAATTAGGG AAGGCTGTAC AGAGACAAGC TTTATAATAT CTGTATTTGG   
  
  
+ AGGGAGAAAC AAAGAAGTTG ATAGCTCAA  

- AATTTTTTGA CTGACAAATA TAGTCAACAT AAAAGTATTT TAAACAGTAA GCTAAAATAC CCAAAAACAA   
  
  
- AATTCAATCT TAAATATAGA TATAAAAATG TAATATTGTA TTTTTAAGAC TAAAAAAATT TTTAATTTAC   
  
  
- ATTATTATCT TATATAGATA CCGTAATTTT GATAGTAAGA CCAACTATTT TGTGGGTGTC GGTGTTCCCT   
  
  
- TCTCATTCAT AAAATATAAA GGGGAAGGTG TATTAGGTAT AGGTCCACAC AGAGGACTAC TAATTACCTG   
  
  
- TAGTGGTAGG AGACCCAGGG TACTCTCACT TACGGGGTTA GTCTTGCCGA ACGAGACCCG ACATTATTTT   
  
  
- AGGTCGAGCA CTTAACACTC CCCGCATGTG CCGTTGAACT GGCGGAAACG AAAAAAGAGC CACCCAGGGA   
  
  
- CAACCCCGTT GTAGCGACAG TAGCAGACAC TAAAACTACA CCATCCTTCG TAGGCTAAAC ATTGACAATT   
  
  
- CTCAAAGAAT ACGTTGCAGC AATACCTCTC CAGGACATGC GGGTGACCTC GTAAACTACG ACGTTGTCGT   
  
  
- TTGGTTTTGT TTTCCTCAGT CTAAAAACCT TAAAGATGAG ACAGCCGTCA AATCGGGGGG TCAGACAATT   
  
  
- AGAACGTTCA AGGTCCTAAG AAAACCCAAC GGGTCTTCAG TTAGGGGAGA AGTACGGATA GCCAAGTAGA   
  
  
- GTGGACAACT GTACTGTTGA TAGACTGGTT TTTATTTTAA AAATACGAAA TAGAACGTCT GTAAAATTTT   
  
  
- CCCAAAAGCA AGTAAATTAT CTTAAAAAGA AAAAAAAATT TACTTAAACT CCAAGGTTTA TAAAATCCCT   
  
  
- AGAAATTCCC CCTCTTCTCC TCATTACGTG TACCATTTCT CTCACTTTCA AGACTGTGAC AATTCACATC   
  
  
- CTGACATCCA CCCAAGGCCA GAACCTCCCG TTGTTCAGTT AACTTTACTC TTTCAAAGCA CGATCTTTAG   
  
  
- TAAACTGTAA ACGCAACCCC GTCCCTAAGG ACAAAAAGGA ATAGAGTAAC GATCAAGTAA ACCCATCCCT   
  
  
- AATTTAACCG TCCTTGAATT CAAACAGAAG GAAACTAAAG TAGCTTTCGA GGTTTAGTCA GAATAGTCGT   
  
  
- TACCGTCGAA TCGGTAACAC ACTAATCAAA TCAACGCTCT TACCTTAGAA ATCACCCACC CCCCCGTGAA   
  
  
- TCTTCGTACG GTCCACCTCC CTTTGAAGTT CCGAATGCCG GCACTTTGAC ATAAAGCAGA AATTTTTTTT   
  
  
- TTTTTTATAA TCGAGTGTTC TTAAGAACTT TTTTTTTTTT TTTTTTCCCT TTCTCCTACA GCAAAAGGTA   
  
  
- AAGAGACGAG ACACCTGAAA AACCCAAGGT GACCAAAAGT CCCATCGTGC TTTGACAGTT TTTCGGCTGA   
  
  
- CTAAATCTCT CTCCTTTTGT TGTTAATCCC TTCCGACATG TCTCTGTTCG AAATATTATA GACATAAACC   
  
  
- TCCCTCTTTG TTTCTTCAAC TATCGAGTT

  
  
Motifs Found  

+     5UTR Py-rich stretch

| Site Name | Organism | Position | Strand | Matrix score. | sequence | function |
| --- | --- | --- | --- | --- | --- | --- |
| 5UTR Py-rich stretch | Lycopersicon esculentum | 592 | + | 9 | TTTCTTCTCT | cis-acting element conferring high transcription levels |
| 5UTR Py-rich stretch | Lycopersicon esculentum | 1408 | - | 9 | TTTCTTCTCT | cis-acting element conferring high transcription levels |

> 2018/04/13 10:10:12  
+ TTAAAAAACT GACTGTTTAT ATCAGTTGTA TTTTCATAAA ATTTGTCATT CGATTTTATG GGTTTTTGTT   
  
  
+ TTAAGTTAGA ATTTATATCT ATATTTTTAC ATTATAACAT AAAAATTCTG ATTTTTTTAA AAATTAAATG   
  
  
+ TAATAATAGA ATATATCTAT GGCATTAAAA CTATCATTCT GGTTGATAAA ACACCCACAG CCACAAGGGA   
  
  
+ AGAGTAAGTA TTTTATATTT CCCCTTCCAC ATAATCCATA TCCAGGTGTG TCTCCTGATG ATTAATGGAC   
  
  
+ ATCACCATCC TCTGGGTCCC ATGAGAGTGA ATGCCCCAAT CAGAACGGCT TGCTCTGGGC TGTAATAAAA   
  
  
+ TCCAGCTCGT GAATTGTGAG GGGCGTACAC GGCAACTTGA CCGCCTTTGC TTTTTTCTCG GTGGGTCCCT   
  
  
+ GTTGGGGCAA CATCGCTGTC ATCGTCTGTG ATTTTGATGT GGTAGGAAGC ATCCGATTTG TAACTGTTAA   
  
  
+ GAGTTTCTTA TGCAACGTCG TTATGGAGAG GTCCTGTACG CCCACTGGAG CATTTGATGC TGCAACAGCA   
  
  
+ AACCAAAACA AAAGGAGTCA GATTTTTGGA ATTTCTACTC TGTCGGCAGT TTAGCCCCCC AGTCTGTTAA   
  
  
+ TCTTGCAAGT TCCAGGATTC TTTTGGGTTG CCCAGAAGTC AATCCCCTCT TCATGCCTAT CGGTTCATCT   
  
  
+ CACCTGTTGA CATGACAACT ATCTGACCAA AAATAAAATT TTTATGCTTT ATCTTGCAGA CATTTTAAAA   
  
  
+ GGGTTTTCGT TCATTTAATA GAATTTTTCT TTTTTTTTAA ATGAATTTGA GGTTCCAAAT ATTTTAGGGA   
  
  
+ TCTTTAAGGG GGAGAAGAGG AGTAATGCAC ATGGTAAAGA GAGTGAAAGT TCTGACACTG TTAAGTGTAG   
  
  
+ GACTGTAGGT GGGTTCCGGT CTTGGAGGGC AACAAGTCAA TTGAAATGAG AAAGTTTCGT GCTAGAAATC   
  
  
+ ATTTGACATT TGCGTTGGGG CAGGGATTCC TGTTTTTCCT TATCTCATTG CTAGTTCATT TGGGTAGGGA   
  
  
+ TTAAATTGGC AGGAACTTAA GTTTGTCTTC CTTTGATTTC ATCGAAAGCT CCAAATCAGT CTTATCAGCA   
  
  
+ ATGGCAGCTT AGCCATTGTG TGATTAGTTT AGTTGCGAGA ATGGAATCTT TAGTGGGTGG GGGGGCACTT   
  
  
+ AGAAGCATGC CAGGTGGAGG GAAACTTCAA GGCTTACGGC CGTGAAACTG TATTTCGTCT TTAAAAAAAA   
  
  
+ AAAAAATATT AGCTCACAAG AATTCTTGAA AAAAAAAAAA AAAAAAGGGA AAGAGGATGT CGTTTTCCAT   
  
  
+ TTCTCTGCTC TGTGGACTTT TTGGGTTCCA CTGGTTTTCA GGGTAGCACG AAACTGTCAA AAAGCCGACT   
  
  
+ GATTTAGAGA GAGGAAAACA ACAATTAGGG AAGGCTGTAC AGAGACAAGC TTTATAATAT CTGTATTTGG   
  
  
+ AGGGAGAAAC AAAGAAGTTG ATAGCTCAA  

- AATTTTTTGA CTGACAAATA TAGTCAACAT AAAAGTATTT TAAACAGTAA GCTAAAATAC CCAAAAACAA   
  
  
- AATTCAATCT TAAATATAGA TATAAAAATG TAATATTGTA TTTTTAAGAC TAAAAAAATT TTTAATTTAC   
  
  
- ATTATTATCT TATATAGATA CCGTAATTTT GATAGTAAGA CCAACTATTT TGTGGGTGTC GGTGTTCCCT   
  
  
- TCTCATTCAT AAAATATAAA GGGGAAGGTG TATTAGGTAT AGGTCCACAC AGAGGACTAC TAATTACCTG   
  
  
- TAGTGGTAGG AGACCCAGGG TACTCTCACT TACGGGGTTA GTCTTGCCGA ACGAGACCCG ACATTATTTT   
  
  
- AGGTCGAGCA CTTAACACTC CCCGCATGTG CCGTTGAACT GGCGGAAACG AAAAAAGAGC CACCCAGGGA   
  
  
- CAACCCCGTT GTAGCGACAG TAGCAGACAC TAAAACTACA CCATCCTTCG TAGGCTAAAC ATTGACAATT   
  
  
- CTCAAAGAAT ACGTTGCAGC AATACCTCTC CAGGACATGC GGGTGACCTC GTAAACTACG ACGTTGTCGT   
  
  
- TTGGTTTTGT TTTCCTCAGT CTAAAAACCT TAAAGATGAG ACAGCCGTCA AATCGGGGGG TCAGACAATT   
  
  
- AGAACGTTCA AGGTCCTAAG AAAACCCAAC GGGTCTTCAG TTAGGGGAGA AGTACGGATA GCCAAGTAGA   
  
  
- GTGGACAACT GTACTGTTGA TAGACTGGTT TTTATTTTAA AAATACGAAA TAGAACGTCT GTAAAATTTT   
  
  
- CCCAAAAGCA AGTAAATTAT CTTAAAAAGA AAAAAAAATT TACTTAAACT CCAAGGTTTA TAAAATCCCT   
  
  
- AGAAATTCCC CCTCTTCTCC TCATTACGTG TACCATTTCT CTCACTTTCA AGACTGTGAC AATTCACATC   
  
  
- CTGACATCCA CCCAAGGCCA GAACCTCCCG TTGTTCAGTT AACTTTACTC TTTCAAAGCA CGATCTTTAG   
  
  
- TAAACTGTAA ACGCAACCCC GTCCCTAAGG ACAAAAAGGA ATAGAGTAAC GATCAAGTAA ACCCATCCCT   
  
  
- AATTTAACCG TCCTTGAATT CAAACAGAAG GAAACTAAAG TAGCTTTCGA GGTTTAGTCA GAATAGTCGT   
  
  
- TACCGTCGAA TCGGTAACAC ACTAATCAAA TCAACGCTCT TACCTTAGAA ATCACCCACC CCCCCGTGAA   
  
  
- TCTTCGTACG GTCCACCTCC CTTTGAAGTT CCGAATGCCG GCACTTTGAC ATAAAGCAGA AATTTTTTTT   
  
  
- TTTTTTATAA TCGAGTGTTC TTAAGAACTT TTTTTTTTTT TTTTTTCCCT TTCTCCTACA GCAAAAGGTA   
  
  
- AAGAGACGAG ACACCTGAAA AACCCAAGGT GACCAAAAGT CCCATCGTGC TTTGACAGTT TTTCGGCTGA   
  
  
- CTAAATCTCT CTCCTTTTGT TGTTAATCCC TTCCGACATG TCTCTGTTCG AAATATTATA GACATAAACC   
  
  
- TCCCTCTTTG TTTCTTCAAC TATCGAGTT

+     AAGAA-motif

| Site Name | Organism | Position | Strand | Matrix score. | sequence | function |
| --- | --- | --- | --- | --- | --- | --- |
| AAGAA-motif | Avena sativa | 873 | + | 9 | gGTAAAGAAA |  |

> 2018/04/13 10:10:12  
+ TTAAAAAACT GACTGTTTAT ATCAGTTGTA TTTTCATAAA ATTTGTCATT CGATTTTATG GGTTTTTGTT   
  
  
+ TTAAGTTAGA ATTTATATCT ATATTTTTAC ATTATAACAT AAAAATTCTG ATTTTTTTAA AAATTAAATG   
  
  
+ TAATAATAGA ATATATCTAT GGCATTAAAA CTATCATTCT GGTTGATAAA ACACCCACAG CCACAAGGGA   
  
  
+ AGAGTAAGTA TTTTATATTT CCCCTTCCAC ATAATCCATA TCCAGGTGTG TCTCCTGATG ATTAATGGAC   
  
  
+ ATCACCATCC TCTGGGTCCC ATGAGAGTGA ATGCCCCAAT CAGAACGGCT TGCTCTGGGC TGTAATAAAA   
  
  
+ TCCAGCTCGT GAATTGTGAG GGGCGTACAC GGCAACTTGA CCGCCTTTGC TTTTTTCTCG GTGGGTCCCT   
  
  
+ GTTGGGGCAA CATCGCTGTC ATCGTCTGTG ATTTTGATGT GGTAGGAAGC ATCCGATTTG TAACTGTTAA   
  
  
+ GAGTTTCTTA TGCAACGTCG TTATGGAGAG GTCCTGTACG CCCACTGGAG CATTTGATGC TGCAACAGCA   
  
  
+ AACCAAAACA AAAGGAGTCA GATTTTTGGA ATTTCTACTC TGTCGGCAGT TTAGCCCCCC AGTCTGTTAA   
  
  
+ TCTTGCAAGT TCCAGGATTC TTTTGGGTTG CCCAGAAGTC AATCCCCTCT TCATGCCTAT CGGTTCATCT   
  
  
+ CACCTGTTGA CATGACAACT ATCTGACCAA AAATAAAATT TTTATGCTTT ATCTTGCAGA CATTTTAAAA   
  
  
+ GGGTTTTCGT TCATTTAATA GAATTTTTCT TTTTTTTTAA ATGAATTTGA GGTTCCAAAT ATTTTAGGGA   
  
  
+ TCTTTAAGGG GGAGAAGAGG AGTAATGCAC ATGGTAAAGA GAGTGAAAGT TCTGACACTG TTAAGTGTAG   
  
  
+ GACTGTAGGT GGGTTCCGGT CTTGGAGGGC AACAAGTCAA TTGAAATGAG AAAGTTTCGT GCTAGAAATC   
  
  
+ ATTTGACATT TGCGTTGGGG CAGGGATTCC TGTTTTTCCT TATCTCATTG CTAGTTCATT TGGGTAGGGA   
  
  
+ TTAAATTGGC AGGAACTTAA GTTTGTCTTC CTTTGATTTC ATCGAAAGCT CCAAATCAGT CTTATCAGCA   
  
  
+ ATGGCAGCTT AGCCATTGTG TGATTAGTTT AGTTGCGAGA ATGGAATCTT TAGTGGGTGG GGGGGCACTT   
  
  
+ AGAAGCATGC CAGGTGGAGG GAAACTTCAA GGCTTACGGC CGTGAAACTG TATTTCGTCT TTAAAAAAAA   
  
  
+ AAAAAATATT AGCTCACAAG AATTCTTGAA AAAAAAAAAA AAAAAAGGGA AAGAGGATGT CGTTTTCCAT   
  
  
+ TTCTCTGCTC TGTGGACTTT TTGGGTTCCA CTGGTTTTCA GGGTAGCACG AAACTGTCAA AAAGCCGACT   
  
  
+ GATTTAGAGA GAGGAAAACA ACAATTAGGG AAGGCTGTAC AGAGACAAGC TTTATAATAT CTGTATTTGG   
  
  
+ AGGGAGAAAC AAAGAAGTTG ATAGCTCAA  

- AATTTTTTGA CTGACAAATA TAGTCAACAT AAAAGTATTT TAAACAGTAA GCTAAAATAC CCAAAAACAA   
  
  
- AATTCAATCT TAAATATAGA TATAAAAATG TAATATTGTA TTTTTAAGAC TAAAAAAATT TTTAATTTAC   
  
  
- ATTATTATCT TATATAGATA CCGTAATTTT GATAGTAAGA CCAACTATTT TGTGGGTGTC GGTGTTCCCT   
  
  
- TCTCATTCAT AAAATATAAA GGGGAAGGTG TATTAGGTAT AGGTCCACAC AGAGGACTAC TAATTACCTG   
  
  
- TAGTGGTAGG AGACCCAGGG TACTCTCACT TACGGGGTTA GTCTTGCCGA ACGAGACCCG ACATTATTTT   
  
  
- AGGTCGAGCA CTTAACACTC CCCGCATGTG CCGTTGAACT GGCGGAAACG AAAAAAGAGC CACCCAGGGA   
  
  
- CAACCCCGTT GTAGCGACAG TAGCAGACAC TAAAACTACA CCATCCTTCG TAGGCTAAAC ATTGACAATT   
  
  
- CTCAAAGAAT ACGTTGCAGC AATACCTCTC CAGGACATGC GGGTGACCTC GTAAACTACG ACGTTGTCGT   
  
  
- TTGGTTTTGT TTTCCTCAGT CTAAAAACCT TAAAGATGAG ACAGCCGTCA AATCGGGGGG TCAGACAATT   
  
  
- AGAACGTTCA AGGTCCTAAG AAAACCCAAC GGGTCTTCAG TTAGGGGAGA AGTACGGATA GCCAAGTAGA   
  
  
- GTGGACAACT GTACTGTTGA TAGACTGGTT TTTATTTTAA AAATACGAAA TAGAACGTCT GTAAAATTTT   
  
  
- CCCAAAAGCA AGTAAATTAT CTTAAAAAGA AAAAAAAATT TACTTAAACT CCAAGGTTTA TAAAATCCCT   
  
  
- AGAAATTCCC CCTCTTCTCC TCATTACGTG TACCATTTCT CTCACTTTCA AGACTGTGAC AATTCACATC   
  
  
- CTGACATCCA CCCAAGGCCA GAACCTCCCG TTGTTCAGTT AACTTTACTC TTTCAAAGCA CGATCTTTAG   
  
  
- TAAACTGTAA ACGCAACCCC GTCCCTAAGG ACAAAAAGGA ATAGAGTAAC GATCAAGTAA ACCCATCCCT   
  
  
- AATTTAACCG TCCTTGAATT CAAACAGAAG GAAACTAAAG TAGCTTTCGA GGTTTAGTCA GAATAGTCGT   
  
  
- TACCGTCGAA TCGGTAACAC ACTAATCAAA TCAACGCTCT TACCTTAGAA ATCACCCACC CCCCCGTGAA   
  
  
- TCTTCGTACG GTCCACCTCC CTTTGAAGTT CCGAATGCCG GCACTTTGAC ATAAAGCAGA AATTTTTTTT   
  
  
- TTTTTTATAA TCGAGTGTTC TTAAGAACTT TTTTTTTTTT TTTTTTCCCT TTCTCCTACA GCAAAAGGTA   
  
  
- AAGAGACGAG ACACCTGAAA AACCCAAGGT GACCAAAAGT CCCATCGTGC TTTGACAGTT TTTCGGCTGA   
  
  
- CTAAATCTCT CTCCTTTTGT TGTTAATCCC TTCCGACATG TCTCTGTTCG AAATATTATA GACATAAACC   
  
  
- TCCCTCTTTG TTTCTTCAAC TATCGAGTT

+     AC-I

| Site Name | Organism | Position | Strand | Matrix score. | sequence | function |
| --- | --- | --- | --- | --- | --- | --- |
| AC-I | Phaseolus vulgaris | 914 | - | 9 | CCCACCTACC |  |

> 2018/04/13 10:10:12  
+ TTAAAAAACT GACTGTTTAT ATCAGTTGTA TTTTCATAAA ATTTGTCATT CGATTTTATG GGTTTTTGTT   
  
  
+ TTAAGTTAGA ATTTATATCT ATATTTTTAC ATTATAACAT AAAAATTCTG ATTTTTTTAA AAATTAAATG   
  
  
+ TAATAATAGA ATATATCTAT GGCATTAAAA CTATCATTCT GGTTGATAAA ACACCCACAG CCACAAGGGA   
  
  
+ AGAGTAAGTA TTTTATATTT CCCCTTCCAC ATAATCCATA TCCAGGTGTG TCTCCTGATG ATTAATGGAC   
  
  
+ ATCACCATCC TCTGGGTCCC ATGAGAGTGA ATGCCCCAAT CAGAACGGCT TGCTCTGGGC TGTAATAAAA   
  
  
+ TCCAGCTCGT GAATTGTGAG GGGCGTACAC GGCAACTTGA CCGCCTTTGC TTTTTTCTCG GTGGGTCCCT   
  
  
+ GTTGGGGCAA CATCGCTGTC ATCGTCTGTG ATTTTGATGT GGTAGGAAGC ATCCGATTTG TAACTGTTAA   
  
  
+ GAGTTTCTTA TGCAACGTCG TTATGGAGAG GTCCTGTACG CCCACTGGAG CATTTGATGC TGCAACAGCA   
  
  
+ AACCAAAACA AAAGGAGTCA GATTTTTGGA ATTTCTACTC TGTCGGCAGT TTAGCCCCCC AGTCTGTTAA   
  
  
+ TCTTGCAAGT TCCAGGATTC TTTTGGGTTG CCCAGAAGTC AATCCCCTCT TCATGCCTAT CGGTTCATCT   
  
  
+ CACCTGTTGA CATGACAACT ATCTGACCAA AAATAAAATT TTTATGCTTT ATCTTGCAGA CATTTTAAAA   
  
  
+ GGGTTTTCGT TCATTTAATA GAATTTTTCT TTTTTTTTAA ATGAATTTGA GGTTCCAAAT ATTTTAGGGA   
  
  
+ TCTTTAAGGG GGAGAAGAGG AGTAATGCAC ATGGTAAAGA GAGTGAAAGT TCTGACACTG TTAAGTGTAG   
  
  
+ GACTGTAGGT GGGTTCCGGT CTTGGAGGGC AACAAGTCAA TTGAAATGAG AAAGTTTCGT GCTAGAAATC   
  
  
+ ATTTGACATT TGCGTTGGGG CAGGGATTCC TGTTTTTCCT TATCTCATTG CTAGTTCATT TGGGTAGGGA   
  
  
+ TTAAATTGGC AGGAACTTAA GTTTGTCTTC CTTTGATTTC ATCGAAAGCT CCAAATCAGT CTTATCAGCA   
  
  
+ ATGGCAGCTT AGCCATTGTG TGATTAGTTT AGTTGCGAGA ATGGAATCTT TAGTGGGTGG GGGGGCACTT   
  
  
+ AGAAGCATGC CAGGTGGAGG GAAACTTCAA GGCTTACGGC CGTGAAACTG TATTTCGTCT TTAAAAAAAA   
  
  
+ AAAAAATATT AGCTCACAAG AATTCTTGAA AAAAAAAAAA AAAAAAGGGA AAGAGGATGT CGTTTTCCAT   
  
  
+ TTCTCTGCTC TGTGGACTTT TTGGGTTCCA CTGGTTTTCA GGGTAGCACG AAACTGTCAA AAAGCCGACT   
  
  
+ GATTTAGAGA GAGGAAAACA ACAATTAGGG AAGGCTGTAC AGAGACAAGC TTTATAATAT CTGTATTTGG   
  
  
+ AGGGAGAAAC AAAGAAGTTG ATAGCTCAA  

- AATTTTTTGA CTGACAAATA TAGTCAACAT AAAAGTATTT TAAACAGTAA GCTAAAATAC CCAAAAACAA   
  
  
- AATTCAATCT TAAATATAGA TATAAAAATG TAATATTGTA TTTTTAAGAC TAAAAAAATT TTTAATTTAC   
  
  
- ATTATTATCT TATATAGATA CCGTAATTTT GATAGTAAGA CCAACTATTT TGTGGGTGTC GGTGTTCCCT   
  
  
- TCTCATTCAT AAAATATAAA GGGGAAGGTG TATTAGGTAT AGGTCCACAC AGAGGACTAC TAATTACCTG   
  
  
- TAGTGGTAGG AGACCCAGGG TACTCTCACT TACGGGGTTA GTCTTGCCGA ACGAGACCCG ACATTATTTT   
  
  
- AGGTCGAGCA CTTAACACTC CCCGCATGTG CCGTTGAACT GGCGGAAACG AAAAAAGAGC CACCCAGGGA   
  
  
- CAACCCCGTT GTAGCGACAG TAGCAGACAC TAAAACTACA CCATCCTTCG TAGGCTAAAC ATTGACAATT   
  
  
- CTCAAAGAAT ACGTTGCAGC AATACCTCTC CAGGACATGC GGGTGACCTC GTAAACTACG ACGTTGTCGT   
  
  
- TTGGTTTTGT TTTCCTCAGT CTAAAAACCT TAAAGATGAG ACAGCCGTCA AATCGGGGGG TCAGACAATT   
  
  
- AGAACGTTCA AGGTCCTAAG AAAACCCAAC GGGTCTTCAG TTAGGGGAGA AGTACGGATA GCCAAGTAGA   
  
  
- GTGGACAACT GTACTGTTGA TAGACTGGTT TTTATTTTAA AAATACGAAA TAGAACGTCT GTAAAATTTT   
  
  
- CCCAAAAGCA AGTAAATTAT CTTAAAAAGA AAAAAAAATT TACTTAAACT CCAAGGTTTA TAAAATCCCT   
  
  
- AGAAATTCCC CCTCTTCTCC TCATTACGTG TACCATTTCT CTCACTTTCA AGACTGTGAC AATTCACATC   
  
  
- CTGACATCCA CCCAAGGCCA GAACCTCCCG TTGTTCAGTT AACTTTACTC TTTCAAAGCA CGATCTTTAG   
  
  
- TAAACTGTAA ACGCAACCCC GTCCCTAAGG ACAAAAAGGA ATAGAGTAAC GATCAAGTAA ACCCATCCCT   
  
  
- AATTTAACCG TCCTTGAATT CAAACAGAAG GAAACTAAAG TAGCTTTCGA GGTTTAGTCA GAATAGTCGT   
  
  
- TACCGTCGAA TCGGTAACAC ACTAATCAAA TCAACGCTCT TACCTTAGAA ATCACCCACC CCCCCGTGAA   
  
  
- TCTTCGTACG GTCCACCTCC CTTTGAAGTT CCGAATGCCG GCACTTTGAC ATAAAGCAGA AATTTTTTTT   
  
  
- TTTTTTATAA TCGAGTGTTC TTAAGAACTT TTTTTTTTTT TTTTTTCCCT TTCTCCTACA GCAAAAGGTA   
  
  
- AAGAGACGAG ACACCTGAAA AACCCAAGGT GACCAAAAGT CCCATCGTGC TTTGACAGTT TTTCGGCTGA   
  
  
- CTAAATCTCT CTCCTTTTGT TGTTAATCCC TTCCGACATG TCTCTGTTCG AAATATTATA GACATAAACC   
  
  
- TCCCTCTTTG TTTCTTCAAC TATCGAGTT

+     AE-box

| Site Name | Organism | Position | Strand | Matrix score. | sequence | function |
| --- | --- | --- | --- | --- | --- | --- |
| AE-box | Arabidopsis thaliana | 1475 | + | 8 | AGAAACAA | part of a module for light response |

> 2018/04/13 10:10:12  
+ TTAAAAAACT GACTGTTTAT ATCAGTTGTA TTTTCATAAA ATTTGTCATT CGATTTTATG GGTTTTTGTT   
  
  
+ TTAAGTTAGA ATTTATATCT ATATTTTTAC ATTATAACAT AAAAATTCTG ATTTTTTTAA AAATTAAATG   
  
  
+ TAATAATAGA ATATATCTAT GGCATTAAAA CTATCATTCT GGTTGATAAA ACACCCACAG CCACAAGGGA   
  
  
+ AGAGTAAGTA TTTTATATTT CCCCTTCCAC ATAATCCATA TCCAGGTGTG TCTCCTGATG ATTAATGGAC   
  
  
+ ATCACCATCC TCTGGGTCCC ATGAGAGTGA ATGCCCCAAT CAGAACGGCT TGCTCTGGGC TGTAATAAAA   
  
  
+ TCCAGCTCGT GAATTGTGAG GGGCGTACAC GGCAACTTGA CCGCCTTTGC TTTTTTCTCG GTGGGTCCCT   
  
  
+ GTTGGGGCAA CATCGCTGTC ATCGTCTGTG ATTTTGATGT GGTAGGAAGC ATCCGATTTG TAACTGTTAA   
  
  
+ GAGTTTCTTA TGCAACGTCG TTATGGAGAG GTCCTGTACG CCCACTGGAG CATTTGATGC TGCAACAGCA   
  
  
+ AACCAAAACA AAAGGAGTCA GATTTTTGGA ATTTCTACTC TGTCGGCAGT TTAGCCCCCC AGTCTGTTAA   
  
  
+ TCTTGCAAGT TCCAGGATTC TTTTGGGTTG CCCAGAAGTC AATCCCCTCT TCATGCCTAT CGGTTCATCT   
  
  
+ CACCTGTTGA CATGACAACT ATCTGACCAA AAATAAAATT TTTATGCTTT ATCTTGCAGA CATTTTAAAA   
  
  
+ GGGTTTTCGT TCATTTAATA GAATTTTTCT TTTTTTTTAA ATGAATTTGA GGTTCCAAAT ATTTTAGGGA   
  
  
+ TCTTTAAGGG GGAGAAGAGG AGTAATGCAC ATGGTAAAGA GAGTGAAAGT TCTGACACTG TTAAGTGTAG   
  
  
+ GACTGTAGGT GGGTTCCGGT CTTGGAGGGC AACAAGTCAA TTGAAATGAG AAAGTTTCGT GCTAGAAATC   
  
  
+ ATTTGACATT TGCGTTGGGG CAGGGATTCC TGTTTTTCCT TATCTCATTG CTAGTTCATT TGGGTAGGGA   
  
  
+ TTAAATTGGC AGGAACTTAA GTTTGTCTTC CTTTGATTTC ATCGAAAGCT CCAAATCAGT CTTATCAGCA   
  
  
+ ATGGCAGCTT AGCCATTGTG TGATTAGTTT AGTTGCGAGA ATGGAATCTT TAGTGGGTGG GGGGGCACTT   
  
  
+ AGAAGCATGC CAGGTGGAGG GAAACTTCAA GGCTTACGGC CGTGAAACTG TATTTCGTCT TTAAAAAAAA   
  
  
+ AAAAAATATT AGCTCACAAG AATTCTTGAA AAAAAAAAAA AAAAAAGGGA AAGAGGATGT CGTTTTCCAT   
  
  
+ TTCTCTGCTC TGTGGACTTT TTGGGTTCCA CTGGTTTTCA GGGTAGCACG AAACTGTCAA AAAGCCGACT   
  
  
+ GATTTAGAGA GAGGAAAACA ACAATTAGGG AAGGCTGTAC AGAGACAAGC TTTATAATAT CTGTATTTGG   
  
  
+ AGGGAGAAAC AAAGAAGTTG ATAGCTCAA  

- AATTTTTTGA CTGACAAATA TAGTCAACAT AAAAGTATTT TAAACAGTAA GCTAAAATAC CCAAAAACAA   
  
  
- AATTCAATCT TAAATATAGA TATAAAAATG TAATATTGTA TTTTTAAGAC TAAAAAAATT TTTAATTTAC   
  
  
- ATTATTATCT TATATAGATA CCGTAATTTT GATAGTAAGA CCAACTATTT TGTGGGTGTC GGTGTTCCCT   
  
  
- TCTCATTCAT AAAATATAAA GGGGAAGGTG TATTAGGTAT AGGTCCACAC AGAGGACTAC TAATTACCTG   
  
  
- TAGTGGTAGG AGACCCAGGG TACTCTCACT TACGGGGTTA GTCTTGCCGA ACGAGACCCG ACATTATTTT   
  
  
- AGGTCGAGCA CTTAACACTC CCCGCATGTG CCGTTGAACT GGCGGAAACG AAAAAAGAGC CACCCAGGGA   
  
  
- CAACCCCGTT GTAGCGACAG TAGCAGACAC TAAAACTACA CCATCCTTCG TAGGCTAAAC ATTGACAATT   
  
  
- CTCAAAGAAT ACGTTGCAGC AATACCTCTC CAGGACATGC GGGTGACCTC GTAAACTACG ACGTTGTCGT   
  
  
- TTGGTTTTGT TTTCCTCAGT CTAAAAACCT TAAAGATGAG ACAGCCGTCA AATCGGGGGG TCAGACAATT   
  
  
- AGAACGTTCA AGGTCCTAAG AAAACCCAAC GGGTCTTCAG TTAGGGGAGA AGTACGGATA GCCAAGTAGA   
  
  
- GTGGACAACT GTACTGTTGA TAGACTGGTT TTTATTTTAA AAATACGAAA TAGAACGTCT GTAAAATTTT   
  
  
- CCCAAAAGCA AGTAAATTAT CTTAAAAAGA AAAAAAAATT TACTTAAACT CCAAGGTTTA TAAAATCCCT   
  
  
- AGAAATTCCC CCTCTTCTCC TCATTACGTG TACCATTTCT CTCACTTTCA AGACTGTGAC AATTCACATC   
  
  
- CTGACATCCA CCCAAGGCCA GAACCTCCCG TTGTTCAGTT AACTTTACTC TTTCAAAGCA CGATCTTTAG   
  
  
- TAAACTGTAA ACGCAACCCC GTCCCTAAGG ACAAAAAGGA ATAGAGTAAC GATCAAGTAA ACCCATCCCT   
  
  
- AATTTAACCG TCCTTGAATT CAAACAGAAG GAAACTAAAG TAGCTTTCGA GGTTTAGTCA GAATAGTCGT   
  
  
- TACCGTCGAA TCGGTAACAC ACTAATCAAA TCAACGCTCT TACCTTAGAA ATCACCCACC CCCCCGTGAA   
  
  
- TCTTCGTACG GTCCACCTCC CTTTGAAGTT CCGAATGCCG GCACTTTGAC ATAAAGCAGA AATTTTTTTT   
  
  
- TTTTTTATAA TCGAGTGTTC TTAAGAACTT TTTTTTTTTT TTTTTTCCCT TTCTCCTACA GCAAAAGGTA   
  
  
- AAGAGACGAG ACACCTGAAA AACCCAAGGT GACCAAAAGT CCCATCGTGC TTTGACAGTT TTTCGGCTGA   
  
  
- CTAAATCTCT CTCCTTTTGT TGTTAATCCC TTCCGACATG TCTCTGTTCG AAATATTATA GACATAAACC   
  
  
- TCCCTCTTTG TTTCTTCAAC TATCGAGTT

+     ARE

| Site Name | Organism | Position | Strand | Matrix score. | sequence | function |
| --- | --- | --- | --- | --- | --- | --- |
| ARE | Zea mays | 1362 | + | 6 | TGGTTT | cis-acting regulatory element essential for the anaerobic induction |
| ARE | Zea mays | 560 | - | 6 | TGGTTT | cis-acting regulatory element essential for the anaerobic induction |

> 2018/04/13 10:10:12  
+ TTAAAAAACT GACTGTTTAT ATCAGTTGTA TTTTCATAAA ATTTGTCATT CGATTTTATG GGTTTTTGTT   
  
  
+ TTAAGTTAGA ATTTATATCT ATATTTTTAC ATTATAACAT AAAAATTCTG ATTTTTTTAA AAATTAAATG   
  
  
+ TAATAATAGA ATATATCTAT GGCATTAAAA CTATCATTCT GGTTGATAAA ACACCCACAG CCACAAGGGA   
  
  
+ AGAGTAAGTA TTTTATATTT CCCCTTCCAC ATAATCCATA TCCAGGTGTG TCTCCTGATG ATTAATGGAC   
  
  
+ ATCACCATCC TCTGGGTCCC ATGAGAGTGA ATGCCCCAAT CAGAACGGCT TGCTCTGGGC TGTAATAAAA   
  
  
+ TCCAGCTCGT GAATTGTGAG GGGCGTACAC GGCAACTTGA CCGCCTTTGC TTTTTTCTCG GTGGGTCCCT   
  
  
+ GTTGGGGCAA CATCGCTGTC ATCGTCTGTG ATTTTGATGT GGTAGGAAGC ATCCGATTTG TAACTGTTAA   
  
  
+ GAGTTTCTTA TGCAACGTCG TTATGGAGAG GTCCTGTACG CCCACTGGAG CATTTGATGC TGCAACAGCA   
  
  
+ AACCAAAACA AAAGGAGTCA GATTTTTGGA ATTTCTACTC TGTCGGCAGT TTAGCCCCCC AGTCTGTTAA   
  
  
+ TCTTGCAAGT TCCAGGATTC TTTTGGGTTG CCCAGAAGTC AATCCCCTCT TCATGCCTAT CGGTTCATCT   
  
  
+ CACCTGTTGA CATGACAACT ATCTGACCAA AAATAAAATT TTTATGCTTT ATCTTGCAGA CATTTTAAAA   
  
  
+ GGGTTTTCGT TCATTTAATA GAATTTTTCT TTTTTTTTAA ATGAATTTGA GGTTCCAAAT ATTTTAGGGA   
  
  
+ TCTTTAAGGG GGAGAAGAGG AGTAATGCAC ATGGTAAAGA GAGTGAAAGT TCTGACACTG TTAAGTGTAG   
  
  
+ GACTGTAGGT GGGTTCCGGT CTTGGAGGGC AACAAGTCAA TTGAAATGAG AAAGTTTCGT GCTAGAAATC   
  
  
+ ATTTGACATT TGCGTTGGGG CAGGGATTCC TGTTTTTCCT TATCTCATTG CTAGTTCATT TGGGTAGGGA   
  
  
+ TTAAATTGGC AGGAACTTAA GTTTGTCTTC CTTTGATTTC ATCGAAAGCT CCAAATCAGT CTTATCAGCA   
  
  
+ ATGGCAGCTT AGCCATTGTG TGATTAGTTT AGTTGCGAGA ATGGAATCTT TAGTGGGTGG GGGGGCACTT   
  
  
+ AGAAGCATGC CAGGTGGAGG GAAACTTCAA GGCTTACGGC CGTGAAACTG TATTTCGTCT TTAAAAAAAA   
  
  
+ AAAAAATATT AGCTCACAAG AATTCTTGAA AAAAAAAAAA AAAAAAGGGA AAGAGGATGT CGTTTTCCAT   
  
  
+ TTCTCTGCTC TGTGGACTTT TTGGGTTCCA CTGGTTTTCA GGGTAGCACG AAACTGTCAA AAAGCCGACT   
  
  
+ GATTTAGAGA GAGGAAAACA ACAATTAGGG AAGGCTGTAC AGAGACAAGC TTTATAATAT CTGTATTTGG   
  
  
+ AGGGAGAAAC AAAGAAGTTG ATAGCTCAA  

- AATTTTTTGA CTGACAAATA TAGTCAACAT AAAAGTATTT TAAACAGTAA GCTAAAATAC CCAAAAACAA   
  
  
- AATTCAATCT TAAATATAGA TATAAAAATG TAATATTGTA TTTTTAAGAC TAAAAAAATT TTTAATTTAC   
  
  
- ATTATTATCT TATATAGATA CCGTAATTTT GATAGTAAGA CCAACTATTT TGTGGGTGTC GGTGTTCCCT   
  
  
- TCTCATTCAT AAAATATAAA GGGGAAGGTG TATTAGGTAT AGGTCCACAC AGAGGACTAC TAATTACCTG   
  
  
- TAGTGGTAGG AGACCCAGGG TACTCTCACT TACGGGGTTA GTCTTGCCGA ACGAGACCCG ACATTATTTT   
  
  
- AGGTCGAGCA CTTAACACTC CCCGCATGTG CCGTTGAACT GGCGGAAACG AAAAAAGAGC CACCCAGGGA   
  
  
- CAACCCCGTT GTAGCGACAG TAGCAGACAC TAAAACTACA CCATCCTTCG TAGGCTAAAC ATTGACAATT   
  
  
- CTCAAAGAAT ACGTTGCAGC AATACCTCTC CAGGACATGC GGGTGACCTC GTAAACTACG ACGTTGTCGT   
  
  
- TTGGTTTTGT TTTCCTCAGT CTAAAAACCT TAAAGATGAG ACAGCCGTCA AATCGGGGGG TCAGACAATT   
  
  
- AGAACGTTCA AGGTCCTAAG AAAACCCAAC GGGTCTTCAG TTAGGGGAGA AGTACGGATA GCCAAGTAGA   
  
  
- GTGGACAACT GTACTGTTGA TAGACTGGTT TTTATTTTAA AAATACGAAA TAGAACGTCT GTAAAATTTT   
  
  
- CCCAAAAGCA AGTAAATTAT CTTAAAAAGA AAAAAAAATT TACTTAAACT CCAAGGTTTA TAAAATCCCT   
  
  
- AGAAATTCCC CCTCTTCTCC TCATTACGTG TACCATTTCT CTCACTTTCA AGACTGTGAC AATTCACATC   
  
  
- CTGACATCCA CCCAAGGCCA GAACCTCCCG TTGTTCAGTT AACTTTACTC TTTCAAAGCA CGATCTTTAG   
  
  
- TAAACTGTAA ACGCAACCCC GTCCCTAAGG ACAAAAAGGA ATAGAGTAAC GATCAAGTAA ACCCATCCCT   
  
  
- AATTTAACCG TCCTTGAATT CAAACAGAAG GAAACTAAAG TAGCTTTCGA GGTTTAGTCA GAATAGTCGT   
  
  
- TACCGTCGAA TCGGTAACAC ACTAATCAAA TCAACGCTCT TACCTTAGAA ATCACCCACC CCCCCGTGAA   
  
  
- TCTTCGTACG GTCCACCTCC CTTTGAAGTT CCGAATGCCG GCACTTTGAC ATAAAGCAGA AATTTTTTTT   
  
  
- TTTTTTATAA TCGAGTGTTC TTAAGAACTT TTTTTTTTTT TTTTTTCCCT TTCTCCTACA GCAAAAGGTA   
  
  
- AAGAGACGAG ACACCTGAAA AACCCAAGGT GACCAAAAGT CCCATCGTGC TTTGACAGTT TTTCGGCTGA   
  
  
- CTAAATCTCT CTCCTTTTGT TGTTAATCCC TTCCGACATG TCTCTGTTCG AAATATTATA GACATAAACC   
  
  
- TCCCTCTTTG TTTCTTCAAC TATCGAGTT

+     AT-rich sequence

| Site Name | Organism | Position | Strand | Matrix score. | sequence | function |
| --- | --- | --- | --- | --- | --- | --- |
| AT-rich sequence | Pisum sativum | 217 | - | 9 | TAAAATACT | element for maximal elicitor-mediated activation (2copies) |

> 2018/04/13 10:10:12  
+ TTAAAAAACT GACTGTTTAT ATCAGTTGTA TTTTCATAAA ATTTGTCATT CGATTTTATG GGTTTTTGTT   
  
  
+ TTAAGTTAGA ATTTATATCT ATATTTTTAC ATTATAACAT AAAAATTCTG ATTTTTTTAA AAATTAAATG   
  
  
+ TAATAATAGA ATATATCTAT GGCATTAAAA CTATCATTCT GGTTGATAAA ACACCCACAG CCACAAGGGA   
  
  
+ AGAGTAAGTA TTTTATATTT CCCCTTCCAC ATAATCCATA TCCAGGTGTG TCTCCTGATG ATTAATGGAC   
  
  
+ ATCACCATCC TCTGGGTCCC ATGAGAGTGA ATGCCCCAAT CAGAACGGCT TGCTCTGGGC TGTAATAAAA   
  
  
+ TCCAGCTCGT GAATTGTGAG GGGCGTACAC GGCAACTTGA CCGCCTTTGC TTTTTTCTCG GTGGGTCCCT   
  
  
+ GTTGGGGCAA CATCGCTGTC ATCGTCTGTG ATTTTGATGT GGTAGGAAGC ATCCGATTTG TAACTGTTAA   
  
  
+ GAGTTTCTTA TGCAACGTCG TTATGGAGAG GTCCTGTACG CCCACTGGAG CATTTGATGC TGCAACAGCA   
  
  
+ AACCAAAACA AAAGGAGTCA GATTTTTGGA ATTTCTACTC TGTCGGCAGT TTAGCCCCCC AGTCTGTTAA   
  
  
+ TCTTGCAAGT TCCAGGATTC TTTTGGGTTG CCCAGAAGTC AATCCCCTCT TCATGCCTAT CGGTTCATCT   
  
  
+ CACCTGTTGA CATGACAACT ATCTGACCAA AAATAAAATT TTTATGCTTT ATCTTGCAGA CATTTTAAAA   
  
  
+ GGGTTTTCGT TCATTTAATA GAATTTTTCT TTTTTTTTAA ATGAATTTGA GGTTCCAAAT ATTTTAGGGA   
  
  
+ TCTTTAAGGG GGAGAAGAGG AGTAATGCAC ATGGTAAAGA GAGTGAAAGT TCTGACACTG TTAAGTGTAG   
  
  
+ GACTGTAGGT GGGTTCCGGT CTTGGAGGGC AACAAGTCAA TTGAAATGAG AAAGTTTCGT GCTAGAAATC   
  
  
+ ATTTGACATT TGCGTTGGGG CAGGGATTCC TGTTTTTCCT TATCTCATTG CTAGTTCATT TGGGTAGGGA   
  
  
+ TTAAATTGGC AGGAACTTAA GTTTGTCTTC CTTTGATTTC ATCGAAAGCT CCAAATCAGT CTTATCAGCA   
  
  
+ ATGGCAGCTT AGCCATTGTG TGATTAGTTT AGTTGCGAGA ATGGAATCTT TAGTGGGTGG GGGGGCACTT   
  
  
+ AGAAGCATGC CAGGTGGAGG GAAACTTCAA GGCTTACGGC CGTGAAACTG TATTTCGTCT TTAAAAAAAA   
  
  
+ AAAAAATATT AGCTCACAAG AATTCTTGAA AAAAAAAAAA AAAAAAGGGA AAGAGGATGT CGTTTTCCAT   
  
  
+ TTCTCTGCTC TGTGGACTTT TTGGGTTCCA CTGGTTTTCA GGGTAGCACG AAACTGTCAA AAAGCCGACT   
  
  
+ GATTTAGAGA GAGGAAAACA ACAATTAGGG AAGGCTGTAC AGAGACAAGC TTTATAATAT CTGTATTTGG   
  
  
+ AGGGAGAAAC AAAGAAGTTG ATAGCTCAA  

- AATTTTTTGA CTGACAAATA TAGTCAACAT AAAAGTATTT TAAACAGTAA GCTAAAATAC CCAAAAACAA   
  
  
- AATTCAATCT TAAATATAGA TATAAAAATG TAATATTGTA TTTTTAAGAC TAAAAAAATT TTTAATTTAC   
  
  
- ATTATTATCT TATATAGATA CCGTAATTTT GATAGTAAGA CCAACTATTT TGTGGGTGTC GGTGTTCCCT   
  
  
- TCTCATTCAT AAAATATAAA GGGGAAGGTG TATTAGGTAT AGGTCCACAC AGAGGACTAC TAATTACCTG   
  
  
- TAGTGGTAGG AGACCCAGGG TACTCTCACT TACGGGGTTA GTCTTGCCGA ACGAGACCCG ACATTATTTT   
  
  
- AGGTCGAGCA CTTAACACTC CCCGCATGTG CCGTTGAACT GGCGGAAACG AAAAAAGAGC CACCCAGGGA   
  
  
- CAACCCCGTT GTAGCGACAG TAGCAGACAC TAAAACTACA CCATCCTTCG TAGGCTAAAC ATTGACAATT   
  
  
- CTCAAAGAAT ACGTTGCAGC AATACCTCTC CAGGACATGC GGGTGACCTC GTAAACTACG ACGTTGTCGT   
  
  
- TTGGTTTTGT TTTCCTCAGT CTAAAAACCT TAAAGATGAG ACAGCCGTCA AATCGGGGGG TCAGACAATT   
  
  
- AGAACGTTCA AGGTCCTAAG AAAACCCAAC GGGTCTTCAG TTAGGGGAGA AGTACGGATA GCCAAGTAGA   
  
  
- GTGGACAACT GTACTGTTGA TAGACTGGTT TTTATTTTAA AAATACGAAA TAGAACGTCT GTAAAATTTT   
  
  
- CCCAAAAGCA AGTAAATTAT CTTAAAAAGA AAAAAAAATT TACTTAAACT CCAAGGTTTA TAAAATCCCT   
  
  
- AGAAATTCCC CCTCTTCTCC TCATTACGTG TACCATTTCT CTCACTTTCA AGACTGTGAC AATTCACATC   
  
  
- CTGACATCCA CCCAAGGCCA GAACCTCCCG TTGTTCAGTT AACTTTACTC TTTCAAAGCA CGATCTTTAG   
  
  
- TAAACTGTAA ACGCAACCCC GTCCCTAAGG ACAAAAAGGA ATAGAGTAAC GATCAAGTAA ACCCATCCCT   
  
  
- AATTTAACCG TCCTTGAATT CAAACAGAAG GAAACTAAAG TAGCTTTCGA GGTTTAGTCA GAATAGTCGT   
  
  
- TACCGTCGAA TCGGTAACAC ACTAATCAAA TCAACGCTCT TACCTTAGAA ATCACCCACC CCCCCGTGAA   
  
  
- TCTTCGTACG GTCCACCTCC CTTTGAAGTT CCGAATGCCG GCACTTTGAC ATAAAGCAGA AATTTTTTTT   
  
  
- TTTTTTATAA TCGAGTGTTC TTAAGAACTT TTTTTTTTTT TTTTTTCCCT TTCTCCTACA GCAAAAGGTA   
  
  
- AAGAGACGAG ACACCTGAAA AACCCAAGGT GACCAAAAGT CCCATCGTGC TTTGACAGTT TTTCGGCTGA   
  
  
- CTAAATCTCT CTCCTTTTGT TGTTAATCCC TTCCGACATG TCTCTGTTCG AAATATTATA GACATAAACC   
  
  
- TCCCTCTTTG TTTCTTCAAC TATCGAGTT

+     ATCT-motif

| Site Name | Organism | Position | Strand | Matrix score. | sequence | function |
| --- | --- | --- | --- | --- | --- | --- |
| ATCT-motif | Pisum sativum | 1048 | - | 9 | AATCTAATCC | part of a conserved DNA module involved in light responsiveness |

> 2018/04/13 10:10:12  
+ TTAAAAAACT GACTGTTTAT ATCAGTTGTA TTTTCATAAA ATTTGTCATT CGATTTTATG GGTTTTTGTT   
  
  
+ TTAAGTTAGA ATTTATATCT ATATTTTTAC ATTATAACAT AAAAATTCTG ATTTTTTTAA AAATTAAATG   
  
  
+ TAATAATAGA ATATATCTAT GGCATTAAAA CTATCATTCT GGTTGATAAA ACACCCACAG CCACAAGGGA   
  
  
+ AGAGTAAGTA TTTTATATTT CCCCTTCCAC ATAATCCATA TCCAGGTGTG TCTCCTGATG ATTAATGGAC   
  
  
+ ATCACCATCC TCTGGGTCCC ATGAGAGTGA ATGCCCCAAT CAGAACGGCT TGCTCTGGGC TGTAATAAAA   
  
  
+ TCCAGCTCGT GAATTGTGAG GGGCGTACAC GGCAACTTGA CCGCCTTTGC TTTTTTCTCG GTGGGTCCCT   
  
  
+ GTTGGGGCAA CATCGCTGTC ATCGTCTGTG ATTTTGATGT GGTAGGAAGC ATCCGATTTG TAACTGTTAA   
  
  
+ GAGTTTCTTA TGCAACGTCG TTATGGAGAG GTCCTGTACG CCCACTGGAG CATTTGATGC TGCAACAGCA   
  
  
+ AACCAAAACA AAAGGAGTCA GATTTTTGGA ATTTCTACTC TGTCGGCAGT TTAGCCCCCC AGTCTGTTAA   
  
  
+ TCTTGCAAGT TCCAGGATTC TTTTGGGTTG CCCAGAAGTC AATCCCCTCT TCATGCCTAT CGGTTCATCT   
  
  
+ CACCTGTTGA CATGACAACT ATCTGACCAA AAATAAAATT TTTATGCTTT ATCTTGCAGA CATTTTAAAA   
  
  
+ GGGTTTTCGT TCATTTAATA GAATTTTTCT TTTTTTTTAA ATGAATTTGA GGTTCCAAAT ATTTTAGGGA   
  
  
+ TCTTTAAGGG GGAGAAGAGG AGTAATGCAC ATGGTAAAGA GAGTGAAAGT TCTGACACTG TTAAGTGTAG   
  
  
+ GACTGTAGGT GGGTTCCGGT CTTGGAGGGC AACAAGTCAA TTGAAATGAG AAAGTTTCGT GCTAGAAATC   
  
  
+ ATTTGACATT TGCGTTGGGG CAGGGATTCC TGTTTTTCCT TATCTCATTG CTAGTTCATT TGGGTAGGGA   
  
  
+ TTAAATTGGC AGGAACTTAA GTTTGTCTTC CTTTGATTTC ATCGAAAGCT CCAAATCAGT CTTATCAGCA   
  
  
+ ATGGCAGCTT AGCCATTGTG TGATTAGTTT AGTTGCGAGA ATGGAATCTT TAGTGGGTGG GGGGGCACTT   
  
  
+ AGAAGCATGC CAGGTGGAGG GAAACTTCAA GGCTTACGGC CGTGAAACTG TATTTCGTCT TTAAAAAAAA   
  
  
+ AAAAAATATT AGCTCACAAG AATTCTTGAA AAAAAAAAAA AAAAAAGGGA AAGAGGATGT CGTTTTCCAT   
  
  
+ TTCTCTGCTC TGTGGACTTT TTGGGTTCCA CTGGTTTTCA GGGTAGCACG AAACTGTCAA AAAGCCGACT   
  
  
+ GATTTAGAGA GAGGAAAACA ACAATTAGGG AAGGCTGTAC AGAGACAAGC TTTATAATAT CTGTATTTGG   
  
  
+ AGGGAGAAAC AAAGAAGTTG ATAGCTCAA  

- AATTTTTTGA CTGACAAATA TAGTCAACAT AAAAGTATTT TAAACAGTAA GCTAAAATAC CCAAAAACAA   
  
  
- AATTCAATCT TAAATATAGA TATAAAAATG TAATATTGTA TTTTTAAGAC TAAAAAAATT TTTAATTTAC   
  
  
- ATTATTATCT TATATAGATA CCGTAATTTT GATAGTAAGA CCAACTATTT TGTGGGTGTC GGTGTTCCCT   
  
  
- TCTCATTCAT AAAATATAAA GGGGAAGGTG TATTAGGTAT AGGTCCACAC AGAGGACTAC TAATTACCTG   
  
  
- TAGTGGTAGG AGACCCAGGG TACTCTCACT TACGGGGTTA GTCTTGCCGA ACGAGACCCG ACATTATTTT   
  
  
- AGGTCGAGCA CTTAACACTC CCCGCATGTG CCGTTGAACT GGCGGAAACG AAAAAAGAGC CACCCAGGGA   
  
  
- CAACCCCGTT GTAGCGACAG TAGCAGACAC TAAAACTACA CCATCCTTCG TAGGCTAAAC ATTGACAATT   
  
  
- CTCAAAGAAT ACGTTGCAGC AATACCTCTC CAGGACATGC GGGTGACCTC GTAAACTACG ACGTTGTCGT   
  
  
- TTGGTTTTGT TTTCCTCAGT CTAAAAACCT TAAAGATGAG ACAGCCGTCA AATCGGGGGG TCAGACAATT   
  
  
- AGAACGTTCA AGGTCCTAAG AAAACCCAAC GGGTCTTCAG TTAGGGGAGA AGTACGGATA GCCAAGTAGA   
  
  
- GTGGACAACT GTACTGTTGA TAGACTGGTT TTTATTTTAA AAATACGAAA TAGAACGTCT GTAAAATTTT   
  
  
- CCCAAAAGCA AGTAAATTAT CTTAAAAAGA AAAAAAAATT TACTTAAACT CCAAGGTTTA TAAAATCCCT   
  
  
- AGAAATTCCC CCTCTTCTCC TCATTACGTG TACCATTTCT CTCACTTTCA AGACTGTGAC AATTCACATC   
  
  
- CTGACATCCA CCCAAGGCCA GAACCTCCCG TTGTTCAGTT AACTTTACTC TTTCAAAGCA CGATCTTTAG   
  
  
- TAAACTGTAA ACGCAACCCC GTCCCTAAGG ACAAAAAGGA ATAGAGTAAC GATCAAGTAA ACCCATCCCT   
  
  
- AATTTAACCG TCCTTGAATT CAAACAGAAG GAAACTAAAG TAGCTTTCGA GGTTTAGTCA GAATAGTCGT   
  
  
- TACCGTCGAA TCGGTAACAC ACTAATCAAA TCAACGCTCT TACCTTAGAA ATCACCCACC CCCCCGTGAA   
  
  
- TCTTCGTACG GTCCACCTCC CTTTGAAGTT CCGAATGCCG GCACTTTGAC ATAAAGCAGA AATTTTTTTT   
  
  
- TTTTTTATAA TCGAGTGTTC TTAAGAACTT TTTTTTTTTT TTTTTTCCCT TTCTCCTACA GCAAAAGGTA   
  
  
- AAGAGACGAG ACACCTGAAA AACCCAAGGT GACCAAAAGT CCCATCGTGC TTTGACAGTT TTTCGGCTGA   
  
  
- CTAAATCTCT CTCCTTTTGT TGTTAATCCC TTCCGACATG TCTCTGTTCG AAATATTATA GACATAAACC   
  
  
- TCCCTCTTTG TTTCTTCAAC TATCGAGTT

+     Box 4

| Site Name | Organism | Position | Strand | Matrix score. | sequence | function |
| --- | --- | --- | --- | --- | --- | --- |
| Box 4 | Petroselinum crispum | 271 | + | 6 | ATTAAT | part of a conserved DNA module involved in light responsiveness |

> 2018/04/13 10:10:12  
+ TTAAAAAACT GACTGTTTAT ATCAGTTGTA TTTTCATAAA ATTTGTCATT CGATTTTATG GGTTTTTGTT   
  
  
+ TTAAGTTAGA ATTTATATCT ATATTTTTAC ATTATAACAT AAAAATTCTG ATTTTTTTAA AAATTAAATG   
  
  
+ TAATAATAGA ATATATCTAT GGCATTAAAA CTATCATTCT GGTTGATAAA ACACCCACAG CCACAAGGGA   
  
  
+ AGAGTAAGTA TTTTATATTT CCCCTTCCAC ATAATCCATA TCCAGGTGTG TCTCCTGATG ATTAATGGAC   
  
  
+ ATCACCATCC TCTGGGTCCC ATGAGAGTGA ATGCCCCAAT CAGAACGGCT TGCTCTGGGC TGTAATAAAA   
  
  
+ TCCAGCTCGT GAATTGTGAG GGGCGTACAC GGCAACTTGA CCGCCTTTGC TTTTTTCTCG GTGGGTCCCT   
  
  
+ GTTGGGGCAA CATCGCTGTC ATCGTCTGTG ATTTTGATGT GGTAGGAAGC ATCCGATTTG TAACTGTTAA   
  
  
+ GAGTTTCTTA TGCAACGTCG TTATGGAGAG GTCCTGTACG CCCACTGGAG CATTTGATGC TGCAACAGCA   
  
  
+ AACCAAAACA AAAGGAGTCA GATTTTTGGA ATTTCTACTC TGTCGGCAGT TTAGCCCCCC AGTCTGTTAA   
  
  
+ TCTTGCAAGT TCCAGGATTC TTTTGGGTTG CCCAGAAGTC AATCCCCTCT TCATGCCTAT CGGTTCATCT   
  
  
+ CACCTGTTGA CATGACAACT ATCTGACCAA AAATAAAATT TTTATGCTTT ATCTTGCAGA CATTTTAAAA   
  
  
+ GGGTTTTCGT TCATTTAATA GAATTTTTCT TTTTTTTTAA ATGAATTTGA GGTTCCAAAT ATTTTAGGGA   
  
  
+ TCTTTAAGGG GGAGAAGAGG AGTAATGCAC ATGGTAAAGA GAGTGAAAGT TCTGACACTG TTAAGTGTAG   
  
  
+ GACTGTAGGT GGGTTCCGGT CTTGGAGGGC AACAAGTCAA TTGAAATGAG AAAGTTTCGT GCTAGAAATC   
  
  
+ ATTTGACATT TGCGTTGGGG CAGGGATTCC TGTTTTTCCT TATCTCATTG CTAGTTCATT TGGGTAGGGA   
  
  
+ TTAAATTGGC AGGAACTTAA GTTTGTCTTC CTTTGATTTC ATCGAAAGCT CCAAATCAGT CTTATCAGCA   
  
  
+ ATGGCAGCTT AGCCATTGTG TGATTAGTTT AGTTGCGAGA ATGGAATCTT TAGTGGGTGG GGGGGCACTT   
  
  
+ AGAAGCATGC CAGGTGGAGG GAAACTTCAA GGCTTACGGC CGTGAAACTG TATTTCGTCT TTAAAAAAAA   
  
  
+ AAAAAATATT AGCTCACAAG AATTCTTGAA AAAAAAAAAA AAAAAAGGGA AAGAGGATGT CGTTTTCCAT   
  
  
+ TTCTCTGCTC TGTGGACTTT TTGGGTTCCA CTGGTTTTCA GGGTAGCACG AAACTGTCAA AAAGCCGACT   
  
  
+ GATTTAGAGA GAGGAAAACA ACAATTAGGG AAGGCTGTAC AGAGACAAGC TTTATAATAT CTGTATTTGG   
  
  
+ AGGGAGAAAC AAAGAAGTTG ATAGCTCAA  

- AATTTTTTGA CTGACAAATA TAGTCAACAT AAAAGTATTT TAAACAGTAA GCTAAAATAC CCAAAAACAA   
  
  
- AATTCAATCT TAAATATAGA TATAAAAATG TAATATTGTA TTTTTAAGAC TAAAAAAATT TTTAATTTAC   
  
  
- ATTATTATCT TATATAGATA CCGTAATTTT GATAGTAAGA CCAACTATTT TGTGGGTGTC GGTGTTCCCT   
  
  
- TCTCATTCAT AAAATATAAA GGGGAAGGTG TATTAGGTAT AGGTCCACAC AGAGGACTAC TAATTACCTG   
  
  
- TAGTGGTAGG AGACCCAGGG TACTCTCACT TACGGGGTTA GTCTTGCCGA ACGAGACCCG ACATTATTTT   
  
  
- AGGTCGAGCA CTTAACACTC CCCGCATGTG CCGTTGAACT GGCGGAAACG AAAAAAGAGC CACCCAGGGA   
  
  
- CAACCCCGTT GTAGCGACAG TAGCAGACAC TAAAACTACA CCATCCTTCG TAGGCTAAAC ATTGACAATT   
  
  
- CTCAAAGAAT ACGTTGCAGC AATACCTCTC CAGGACATGC GGGTGACCTC GTAAACTACG ACGTTGTCGT   
  
  
- TTGGTTTTGT TTTCCTCAGT CTAAAAACCT TAAAGATGAG ACAGCCGTCA AATCGGGGGG TCAGACAATT   
  
  
- AGAACGTTCA AGGTCCTAAG AAAACCCAAC GGGTCTTCAG TTAGGGGAGA AGTACGGATA GCCAAGTAGA   
  
  
- GTGGACAACT GTACTGTTGA TAGACTGGTT TTTATTTTAA AAATACGAAA TAGAACGTCT GTAAAATTTT   
  
  
- CCCAAAAGCA AGTAAATTAT CTTAAAAAGA AAAAAAAATT TACTTAAACT CCAAGGTTTA TAAAATCCCT   
  
  
- AGAAATTCCC CCTCTTCTCC TCATTACGTG TACCATTTCT CTCACTTTCA AGACTGTGAC AATTCACATC   
  
  
- CTGACATCCA CCCAAGGCCA GAACCTCCCG TTGTTCAGTT AACTTTACTC TTTCAAAGCA CGATCTTTAG   
  
  
- TAAACTGTAA ACGCAACCCC GTCCCTAAGG ACAAAAAGGA ATAGAGTAAC GATCAAGTAA ACCCATCCCT   
  
  
- AATTTAACCG TCCTTGAATT CAAACAGAAG GAAACTAAAG TAGCTTTCGA GGTTTAGTCA GAATAGTCGT   
  
  
- TACCGTCGAA TCGGTAACAC ACTAATCAAA TCAACGCTCT TACCTTAGAA ATCACCCACC CCCCCGTGAA   
  
  
- TCTTCGTACG GTCCACCTCC CTTTGAAGTT CCGAATGCCG GCACTTTGAC ATAAAGCAGA AATTTTTTTT   
  
  
- TTTTTTATAA TCGAGTGTTC TTAAGAACTT TTTTTTTTTT TTTTTTCCCT TTCTCCTACA GCAAAAGGTA   
  
  
- AAGAGACGAG ACACCTGAAA AACCCAAGGT GACCAAAAGT CCCATCGTGC TTTGACAGTT TTTCGGCTGA   
  
  
- CTAAATCTCT CTCCTTTTGT TGTTAATCCC TTCCGACATG TCTCTGTTCG AAATATTATA GACATAAACC   
  
  
- TCCCTCTTTG TTTCTTCAAC TATCGAGTT

+     Box II

| Site Name | Organism | Position | Strand | Matrix score. | sequence | function |
| --- | --- | --- | --- | --- | --- | --- |
| Box II | Pisum sativum | 1266 | - | 11 | GTGAGGTAATAT | part of a light responsive element |

> 2018/04/13 10:10:12  
+ TTAAAAAACT GACTGTTTAT ATCAGTTGTA TTTTCATAAA ATTTGTCATT CGATTTTATG GGTTTTTGTT   
  
  
+ TTAAGTTAGA ATTTATATCT ATATTTTTAC ATTATAACAT AAAAATTCTG ATTTTTTTAA AAATTAAATG   
  
  
+ TAATAATAGA ATATATCTAT GGCATTAAAA CTATCATTCT GGTTGATAAA ACACCCACAG CCACAAGGGA   
  
  
+ AGAGTAAGTA TTTTATATTT CCCCTTCCAC ATAATCCATA TCCAGGTGTG TCTCCTGATG ATTAATGGAC   
  
  
+ ATCACCATCC TCTGGGTCCC ATGAGAGTGA ATGCCCCAAT CAGAACGGCT TGCTCTGGGC TGTAATAAAA   
  
  
+ TCCAGCTCGT GAATTGTGAG GGGCGTACAC GGCAACTTGA CCGCCTTTGC TTTTTTCTCG GTGGGTCCCT   
  
  
+ GTTGGGGCAA CATCGCTGTC ATCGTCTGTG ATTTTGATGT GGTAGGAAGC ATCCGATTTG TAACTGTTAA   
  
  
+ GAGTTTCTTA TGCAACGTCG TTATGGAGAG GTCCTGTACG CCCACTGGAG CATTTGATGC TGCAACAGCA   
  
  
+ AACCAAAACA AAAGGAGTCA GATTTTTGGA ATTTCTACTC TGTCGGCAGT TTAGCCCCCC AGTCTGTTAA   
  
  
+ TCTTGCAAGT TCCAGGATTC TTTTGGGTTG CCCAGAAGTC AATCCCCTCT TCATGCCTAT CGGTTCATCT   
  
  
+ CACCTGTTGA CATGACAACT ATCTGACCAA AAATAAAATT TTTATGCTTT ATCTTGCAGA CATTTTAAAA   
  
  
+ GGGTTTTCGT TCATTTAATA GAATTTTTCT TTTTTTTTAA ATGAATTTGA GGTTCCAAAT ATTTTAGGGA   
  
  
+ TCTTTAAGGG GGAGAAGAGG AGTAATGCAC ATGGTAAAGA GAGTGAAAGT TCTGACACTG TTAAGTGTAG   
  
  
+ GACTGTAGGT GGGTTCCGGT CTTGGAGGGC AACAAGTCAA TTGAAATGAG AAAGTTTCGT GCTAGAAATC   
  
  
+ ATTTGACATT TGCGTTGGGG CAGGGATTCC TGTTTTTCCT TATCTCATTG CTAGTTCATT TGGGTAGGGA   
  
  
+ TTAAATTGGC AGGAACTTAA GTTTGTCTTC CTTTGATTTC ATCGAAAGCT CCAAATCAGT CTTATCAGCA   
  
  
+ ATGGCAGCTT AGCCATTGTG TGATTAGTTT AGTTGCGAGA ATGGAATCTT TAGTGGGTGG GGGGGCACTT   
  
  
+ AGAAGCATGC CAGGTGGAGG GAAACTTCAA GGCTTACGGC CGTGAAACTG TATTTCGTCT TTAAAAAAAA   
  
  
+ AAAAAATATT AGCTCACAAG AATTCTTGAA AAAAAAAAAA AAAAAAGGGA AAGAGGATGT CGTTTTCCAT   
  
  
+ TTCTCTGCTC TGTGGACTTT TTGGGTTCCA CTGGTTTTCA GGGTAGCACG AAACTGTCAA AAAGCCGACT   
  
  
+ GATTTAGAGA GAGGAAAACA ACAATTAGGG AAGGCTGTAC AGAGACAAGC TTTATAATAT CTGTATTTGG   
  
  
+ AGGGAGAAAC AAAGAAGTTG ATAGCTCAA  

- AATTTTTTGA CTGACAAATA TAGTCAACAT AAAAGTATTT TAAACAGTAA GCTAAAATAC CCAAAAACAA   
  
  
- AATTCAATCT TAAATATAGA TATAAAAATG TAATATTGTA TTTTTAAGAC TAAAAAAATT TTTAATTTAC   
  
  
- ATTATTATCT TATATAGATA CCGTAATTTT GATAGTAAGA CCAACTATTT TGTGGGTGTC GGTGTTCCCT   
  
  
- TCTCATTCAT AAAATATAAA GGGGAAGGTG TATTAGGTAT AGGTCCACAC AGAGGACTAC TAATTACCTG   
  
  
- TAGTGGTAGG AGACCCAGGG TACTCTCACT TACGGGGTTA GTCTTGCCGA ACGAGACCCG ACATTATTTT   
  
  
- AGGTCGAGCA CTTAACACTC CCCGCATGTG CCGTTGAACT GGCGGAAACG AAAAAAGAGC CACCCAGGGA   
  
  
- CAACCCCGTT GTAGCGACAG TAGCAGACAC TAAAACTACA CCATCCTTCG TAGGCTAAAC ATTGACAATT   
  
  
- CTCAAAGAAT ACGTTGCAGC AATACCTCTC CAGGACATGC GGGTGACCTC GTAAACTACG ACGTTGTCGT   
  
  
- TTGGTTTTGT TTTCCTCAGT CTAAAAACCT TAAAGATGAG ACAGCCGTCA AATCGGGGGG TCAGACAATT   
  
  
- AGAACGTTCA AGGTCCTAAG AAAACCCAAC GGGTCTTCAG TTAGGGGAGA AGTACGGATA GCCAAGTAGA   
  
  
- GTGGACAACT GTACTGTTGA TAGACTGGTT TTTATTTTAA AAATACGAAA TAGAACGTCT GTAAAATTTT   
  
  
- CCCAAAAGCA AGTAAATTAT CTTAAAAAGA AAAAAAAATT TACTTAAACT CCAAGGTTTA TAAAATCCCT   
  
  
- AGAAATTCCC CCTCTTCTCC TCATTACGTG TACCATTTCT CTCACTTTCA AGACTGTGAC AATTCACATC   
  
  
- CTGACATCCA CCCAAGGCCA GAACCTCCCG TTGTTCAGTT AACTTTACTC TTTCAAAGCA CGATCTTTAG   
  
  
- TAAACTGTAA ACGCAACCCC GTCCCTAAGG ACAAAAAGGA ATAGAGTAAC GATCAAGTAA ACCCATCCCT   
  
  
- AATTTAACCG TCCTTGAATT CAAACAGAAG GAAACTAAAG TAGCTTTCGA GGTTTAGTCA GAATAGTCGT   
  
  
- TACCGTCGAA TCGGTAACAC ACTAATCAAA TCAACGCTCT TACCTTAGAA ATCACCCACC CCCCCGTGAA   
  
  
- TCTTCGTACG GTCCACCTCC CTTTGAAGTT CCGAATGCCG GCACTTTGAC ATAAAGCAGA AATTTTTTTT   
  
  
- TTTTTTATAA TCGAGTGTTC TTAAGAACTT TTTTTTTTTT TTTTTTCCCT TTCTCCTACA GCAAAAGGTA   
  
  
- AAGAGACGAG ACACCTGAAA AACCCAAGGT GACCAAAAGT CCCATCGTGC TTTGACAGTT TTTCGGCTGA   
  
  
- CTAAATCTCT CTCCTTTTGT TGTTAATCCC TTCCGACATG TCTCTGTTCG AAATATTATA GACATAAACC   
  
  
- TCCCTCTTTG TTTCTTCAAC TATCGAGTT

+     Box-W1

| Site Name | Organism | Position | Strand | Matrix score. | sequence | function |
| --- | --- | --- | --- | --- | --- | --- |
| Box-W1 | Petroselinum crispum | 387 | + | 6 | TTGACC | fungal elicitor responsive element |

> 2018/04/13 10:10:12  
+ TTAAAAAACT GACTGTTTAT ATCAGTTGTA TTTTCATAAA ATTTGTCATT CGATTTTATG GGTTTTTGTT   
  
  
+ TTAAGTTAGA ATTTATATCT ATATTTTTAC ATTATAACAT AAAAATTCTG ATTTTTTTAA AAATTAAATG   
  
  
+ TAATAATAGA ATATATCTAT GGCATTAAAA CTATCATTCT GGTTGATAAA ACACCCACAG CCACAAGGGA   
  
  
+ AGAGTAAGTA TTTTATATTT CCCCTTCCAC ATAATCCATA TCCAGGTGTG TCTCCTGATG ATTAATGGAC   
  
  
+ ATCACCATCC TCTGGGTCCC ATGAGAGTGA ATGCCCCAAT CAGAACGGCT TGCTCTGGGC TGTAATAAAA   
  
  
+ TCCAGCTCGT GAATTGTGAG GGGCGTACAC GGCAACTTGA CCGCCTTTGC TTTTTTCTCG GTGGGTCCCT   
  
  
+ GTTGGGGCAA CATCGCTGTC ATCGTCTGTG ATTTTGATGT GGTAGGAAGC ATCCGATTTG TAACTGTTAA   
  
  
+ GAGTTTCTTA TGCAACGTCG TTATGGAGAG GTCCTGTACG CCCACTGGAG CATTTGATGC TGCAACAGCA   
  
  
+ AACCAAAACA AAAGGAGTCA GATTTTTGGA ATTTCTACTC TGTCGGCAGT TTAGCCCCCC AGTCTGTTAA   
  
  
+ TCTTGCAAGT TCCAGGATTC TTTTGGGTTG CCCAGAAGTC AATCCCCTCT TCATGCCTAT CGGTTCATCT   
  
  
+ CACCTGTTGA CATGACAACT ATCTGACCAA AAATAAAATT TTTATGCTTT ATCTTGCAGA CATTTTAAAA   
  
  
+ GGGTTTTCGT TCATTTAATA GAATTTTTCT TTTTTTTTAA ATGAATTTGA GGTTCCAAAT ATTTTAGGGA   
  
  
+ TCTTTAAGGG GGAGAAGAGG AGTAATGCAC ATGGTAAAGA GAGTGAAAGT TCTGACACTG TTAAGTGTAG   
  
  
+ GACTGTAGGT GGGTTCCGGT CTTGGAGGGC AACAAGTCAA TTGAAATGAG AAAGTTTCGT GCTAGAAATC   
  
  
+ ATTTGACATT TGCGTTGGGG CAGGGATTCC TGTTTTTCCT TATCTCATTG CTAGTTCATT TGGGTAGGGA   
  
  
+ TTAAATTGGC AGGAACTTAA GTTTGTCTTC CTTTGATTTC ATCGAAAGCT CCAAATCAGT CTTATCAGCA   
  
  
+ ATGGCAGCTT AGCCATTGTG TGATTAGTTT AGTTGCGAGA ATGGAATCTT TAGTGGGTGG GGGGGCACTT   
  
  
+ AGAAGCATGC CAGGTGGAGG GAAACTTCAA GGCTTACGGC CGTGAAACTG TATTTCGTCT TTAAAAAAAA   
  
  
+ AAAAAATATT AGCTCACAAG AATTCTTGAA AAAAAAAAAA AAAAAAGGGA AAGAGGATGT CGTTTTCCAT   
  
  
+ TTCTCTGCTC TGTGGACTTT TTGGGTTCCA CTGGTTTTCA GGGTAGCACG AAACTGTCAA AAAGCCGACT   
  
  
+ GATTTAGAGA GAGGAAAACA ACAATTAGGG AAGGCTGTAC AGAGACAAGC TTTATAATAT CTGTATTTGG   
  
  
+ AGGGAGAAAC AAAGAAGTTG ATAGCTCAA  

- AATTTTTTGA CTGACAAATA TAGTCAACAT AAAAGTATTT TAAACAGTAA GCTAAAATAC CCAAAAACAA   
  
  
- AATTCAATCT TAAATATAGA TATAAAAATG TAATATTGTA TTTTTAAGAC TAAAAAAATT TTTAATTTAC   
  
  
- ATTATTATCT TATATAGATA CCGTAATTTT GATAGTAAGA CCAACTATTT TGTGGGTGTC GGTGTTCCCT   
  
  
- TCTCATTCAT AAAATATAAA GGGGAAGGTG TATTAGGTAT AGGTCCACAC AGAGGACTAC TAATTACCTG   
  
  
- TAGTGGTAGG AGACCCAGGG TACTCTCACT TACGGGGTTA GTCTTGCCGA ACGAGACCCG ACATTATTTT   
  
  
- AGGTCGAGCA CTTAACACTC CCCGCATGTG CCGTTGAACT GGCGGAAACG AAAAAAGAGC CACCCAGGGA   
  
  
- CAACCCCGTT GTAGCGACAG TAGCAGACAC TAAAACTACA CCATCCTTCG TAGGCTAAAC ATTGACAATT   
  
  
- CTCAAAGAAT ACGTTGCAGC AATACCTCTC CAGGACATGC GGGTGACCTC GTAAACTACG ACGTTGTCGT   
  
  
- TTGGTTTTGT TTTCCTCAGT CTAAAAACCT TAAAGATGAG ACAGCCGTCA AATCGGGGGG TCAGACAATT   
  
  
- AGAACGTTCA AGGTCCTAAG AAAACCCAAC GGGTCTTCAG TTAGGGGAGA AGTACGGATA GCCAAGTAGA   
  
  
- GTGGACAACT GTACTGTTGA TAGACTGGTT TTTATTTTAA AAATACGAAA TAGAACGTCT GTAAAATTTT   
  
  
- CCCAAAAGCA AGTAAATTAT CTTAAAAAGA AAAAAAAATT TACTTAAACT CCAAGGTTTA TAAAATCCCT   
  
  
- AGAAATTCCC CCTCTTCTCC TCATTACGTG TACCATTTCT CTCACTTTCA AGACTGTGAC AATTCACATC   
  
  
- CTGACATCCA CCCAAGGCCA GAACCTCCCG TTGTTCAGTT AACTTTACTC TTTCAAAGCA CGATCTTTAG   
  
  
- TAAACTGTAA ACGCAACCCC GTCCCTAAGG ACAAAAAGGA ATAGAGTAAC GATCAAGTAA ACCCATCCCT   
  
  
- AATTTAACCG TCCTTGAATT CAAACAGAAG GAAACTAAAG TAGCTTTCGA GGTTTAGTCA GAATAGTCGT   
  
  
- TACCGTCGAA TCGGTAACAC ACTAATCAAA TCAACGCTCT TACCTTAGAA ATCACCCACC CCCCCGTGAA   
  
  
- TCTTCGTACG GTCCACCTCC CTTTGAAGTT CCGAATGCCG GCACTTTGAC ATAAAGCAGA AATTTTTTTT   
  
  
- TTTTTTATAA TCGAGTGTTC TTAAGAACTT TTTTTTTTTT TTTTTTCCCT TTCTCCTACA GCAAAAGGTA   
  
  
- AAGAGACGAG ACACCTGAAA AACCCAAGGT GACCAAAAGT CCCATCGTGC TTTGACAGTT TTTCGGCTGA   
  
  
- CTAAATCTCT CTCCTTTTGT TGTTAATCCC TTCCGACATG TCTCTGTTCG AAATATTATA GACATAAACC   
  
  
- TCCCTCTTTG TTTCTTCAAC TATCGAGTT

+     CAAT-box

| Site Name | Organism | Position | Strand | Matrix score. | sequence | function |
| --- | --- | --- | --- | --- | --- | --- |
| CAAT-box | Hordeum vulgare | 1119 | + | 4 | CAAT | common cis-acting element in promoter and enhancer regions |
| CAAT-box | Arabidopsis thaliana | 1055 | - | 5 | CCAAT | common cis-acting element in promoter and enhancer regions |
| CAAT-box | Brassica rapa | 981 | - | 5 | CAAAT | common cis-acting element in promoter and enhancer regions |
| CAAT-box | Hordeum vulgare | 670 | + | 4 | CAAT | common cis-acting element in promoter and enhancer regions |
| CAAT-box | Brassica rapa | 542 | - | 5 | CAAAT | common cis-acting element in promoter and enhancer regions |
| CAAT-box | Brassica rapa | 826 | + | 5 | CAAAT | common cis-acting element in promoter and enhancer regions |
| CAAT-box | Brassica rapa | 476 | - | 5 | CAAAT | common cis-acting element in promoter and enhancer regions |
| CAAT-box | Hordeum vulgare | 363 | - | 4 | CAAT | common cis-acting element in promoter and enhancer regions |
| CAAT-box | Hordeum vulgare | 317 | + | 4 | CAAT | common cis-acting element in promoter and enhancer regions |
| CAAT-box | Brassica rapa | 41 | - | 5 | CAAAT | common cis-acting element in promoter and enhancer regions |
| CAAT-box | Glycine max | 1422 | + | 5 | CAATT | common cis-acting element in promoter and enhancer regions |
| CAAT-box | Glycine max | 948 | + | 5 | CAATT | common cis-acting element in promoter and enhancer regions |
| CAAT-box | Brassica rapa | 1038 | - | 5 | CAAAT | common cis-acting element in promoter and enhancer regions |
| CAAT-box | Hordeum vulgare | 1027 | - | 4 | CAAT | common cis-acting element in promoter and enhancer regions |
| CAAT-box | Brassica rapa | 1102 | + | 5 | CAAAT | common cis-acting element in promoter and enhancer regions |
| CAAT-box | Arabidopsis thaliana | 316 | + | 5 | CCAAT | common cis-acting element in promoter and enhancer regions |
| CAAT-box | Glycine max | 949 | - | 5 | CAATT | common cis-acting element in promoter and enhancer regions |
| CAAT-box | Glycine max | 1054 | - | 5 | CAATT | common cis-acting element in promoter and enhancer regions |
| CAAT-box | Brassica rapa | 815 | - | 5 | CAAAT | common cis-acting element in promoter and enhancer regions |
| CAAT-box | Hordeum vulgare | 1135 | - | 4 | CAAT | common cis-acting element in promoter and enhancer regions |
| CAAT-box | Glycine max | 362 | - | 5 | CAATT | common cis-acting element in promoter and enhancer regions |
| CAAT-box | Hordeum vulgare | 950 | - | 4 | CAAT | common cis-acting element in promoter and enhancer regions |
| CAAT-box | Brassica rapa | 988 | - | 5 | CAAAT | common cis-acting element in promoter and enhancer regions |
| CAAT-box | Brassica rapa | 1465 | - | 5 | CAAAT | common cis-acting element in promoter and enhancer regions |

> 2018/04/13 10:10:12  
+ TTAAAAAACT GACTGTTTAT ATCAGTTGTA TTTTCATAAA ATTTGTCATT CGATTTTATG GGTTTTTGTT   
  
  
+ TTAAGTTAGA ATTTATATCT ATATTTTTAC ATTATAACAT AAAAATTCTG ATTTTTTTAA AAATTAAATG   
  
  
+ TAATAATAGA ATATATCTAT GGCATTAAAA CTATCATTCT GGTTGATAAA ACACCCACAG CCACAAGGGA   
  
  
+ AGAGTAAGTA TTTTATATTT CCCCTTCCAC ATAATCCATA TCCAGGTGTG TCTCCTGATG ATTAATGGAC   
  
  
+ ATCACCATCC TCTGGGTCCC ATGAGAGTGA ATGCCCCAAT CAGAACGGCT TGCTCTGGGC TGTAATAAAA   
  
  
+ TCCAGCTCGT GAATTGTGAG GGGCGTACAC GGCAACTTGA CCGCCTTTGC TTTTTTCTCG GTGGGTCCCT   
  
  
+ GTTGGGGCAA CATCGCTGTC ATCGTCTGTG ATTTTGATGT GGTAGGAAGC ATCCGATTTG TAACTGTTAA   
  
  
+ GAGTTTCTTA TGCAACGTCG TTATGGAGAG GTCCTGTACG CCCACTGGAG CATTTGATGC TGCAACAGCA   
  
  
+ AACCAAAACA AAAGGAGTCA GATTTTTGGA ATTTCTACTC TGTCGGCAGT TTAGCCCCCC AGTCTGTTAA   
  
  
+ TCTTGCAAGT TCCAGGATTC TTTTGGGTTG CCCAGAAGTC AATCCCCTCT TCATGCCTAT CGGTTCATCT   
  
  
+ CACCTGTTGA CATGACAACT ATCTGACCAA AAATAAAATT TTTATGCTTT ATCTTGCAGA CATTTTAAAA   
  
  
+ GGGTTTTCGT TCATTTAATA GAATTTTTCT TTTTTTTTAA ATGAATTTGA GGTTCCAAAT ATTTTAGGGA   
  
  
+ TCTTTAAGGG GGAGAAGAGG AGTAATGCAC ATGGTAAAGA GAGTGAAAGT TCTGACACTG TTAAGTGTAG   
  
  
+ GACTGTAGGT GGGTTCCGGT CTTGGAGGGC AACAAGTCAA TTGAAATGAG AAAGTTTCGT GCTAGAAATC   
  
  
+ ATTTGACATT TGCGTTGGGG CAGGGATTCC TGTTTTTCCT TATCTCATTG CTAGTTCATT TGGGTAGGGA   
  
  
+ TTAAATTGGC AGGAACTTAA GTTTGTCTTC CTTTGATTTC ATCGAAAGCT CCAAATCAGT CTTATCAGCA   
  
  
+ ATGGCAGCTT AGCCATTGTG TGATTAGTTT AGTTGCGAGA ATGGAATCTT TAGTGGGTGG GGGGGCACTT   
  
  
+ AGAAGCATGC CAGGTGGAGG GAAACTTCAA GGCTTACGGC CGTGAAACTG TATTTCGTCT TTAAAAAAAA   
  
  
+ AAAAAATATT AGCTCACAAG AATTCTTGAA AAAAAAAAAA AAAAAAGGGA AAGAGGATGT CGTTTTCCAT   
  
  
+ TTCTCTGCTC TGTGGACTTT TTGGGTTCCA CTGGTTTTCA GGGTAGCACG AAACTGTCAA AAAGCCGACT   
  
  
+ GATTTAGAGA GAGGAAAACA ACAATTAGGG AAGGCTGTAC AGAGACAAGC TTTATAATAT CTGTATTTGG   
  
  
+ AGGGAGAAAC AAAGAAGTTG ATAGCTCAA  

- AATTTTTTGA CTGACAAATA TAGTCAACAT AAAAGTATTT TAAACAGTAA GCTAAAATAC CCAAAAACAA   
  
  
- AATTCAATCT TAAATATAGA TATAAAAATG TAATATTGTA TTTTTAAGAC TAAAAAAATT TTTAATTTAC   
  
  
- ATTATTATCT TATATAGATA CCGTAATTTT GATAGTAAGA CCAACTATTT TGTGGGTGTC GGTGTTCCCT   
  
  
- TCTCATTCAT AAAATATAAA GGGGAAGGTG TATTAGGTAT AGGTCCACAC AGAGGACTAC TAATTACCTG   
  
  
- TAGTGGTAGG AGACCCAGGG TACTCTCACT TACGGGGTTA GTCTTGCCGA ACGAGACCCG ACATTATTTT   
  
  
- AGGTCGAGCA CTTAACACTC CCCGCATGTG CCGTTGAACT GGCGGAAACG AAAAAAGAGC CACCCAGGGA   
  
  
- CAACCCCGTT GTAGCGACAG TAGCAGACAC TAAAACTACA CCATCCTTCG TAGGCTAAAC ATTGACAATT   
  
  
- CTCAAAGAAT ACGTTGCAGC AATACCTCTC CAGGACATGC GGGTGACCTC GTAAACTACG ACGTTGTCGT   
  
  
- TTGGTTTTGT TTTCCTCAGT CTAAAAACCT TAAAGATGAG ACAGCCGTCA AATCGGGGGG TCAGACAATT   
  
  
- AGAACGTTCA AGGTCCTAAG AAAACCCAAC GGGTCTTCAG TTAGGGGAGA AGTACGGATA GCCAAGTAGA   
  
  
- GTGGACAACT GTACTGTTGA TAGACTGGTT TTTATTTTAA AAATACGAAA TAGAACGTCT GTAAAATTTT   
  
  
- CCCAAAAGCA AGTAAATTAT CTTAAAAAGA AAAAAAAATT TACTTAAACT CCAAGGTTTA TAAAATCCCT   
  
  
- AGAAATTCCC CCTCTTCTCC TCATTACGTG TACCATTTCT CTCACTTTCA AGACTGTGAC AATTCACATC   
  
  
- CTGACATCCA CCCAAGGCCA GAACCTCCCG TTGTTCAGTT AACTTTACTC TTTCAAAGCA CGATCTTTAG   
  
  
- TAAACTGTAA ACGCAACCCC GTCCCTAAGG ACAAAAAGGA ATAGAGTAAC GATCAAGTAA ACCCATCCCT   
  
  
- AATTTAACCG TCCTTGAATT CAAACAGAAG GAAACTAAAG TAGCTTTCGA GGTTTAGTCA GAATAGTCGT   
  
  
- TACCGTCGAA TCGGTAACAC ACTAATCAAA TCAACGCTCT TACCTTAGAA ATCACCCACC CCCCCGTGAA   
  
  
- TCTTCGTACG GTCCACCTCC CTTTGAAGTT CCGAATGCCG GCACTTTGAC ATAAAGCAGA AATTTTTTTT   
  
  
- TTTTTTATAA TCGAGTGTTC TTAAGAACTT TTTTTTTTTT TTTTTTCCCT TTCTCCTACA GCAAAAGGTA   
  
  
- AAGAGACGAG ACACCTGAAA AACCCAAGGT GACCAAAAGT CCCATCGTGC TTTGACAGTT TTTCGGCTGA   
  
  
- CTAAATCTCT CTCCTTTTGT TGTTAATCCC TTCCGACATG TCTCTGTTCG AAATATTATA GACATAAACC   
  
  
- TCCCTCTTTG TTTCTTCAAC TATCGAGTT

+     CATT-motif

| Site Name | Organism | Position | Strand | Matrix score. | sequence | function |
| --- | --- | --- | --- | --- | --- | --- |
| CATT-motif | Zea mays | 309 | - | 6 | GCATTC | part of a light responsive element |

> 2018/04/13 10:10:12  
+ TTAAAAAACT GACTGTTTAT ATCAGTTGTA TTTTCATAAA ATTTGTCATT CGATTTTATG GGTTTTTGTT   
  
  
+ TTAAGTTAGA ATTTATATCT ATATTTTTAC ATTATAACAT AAAAATTCTG ATTTTTTTAA AAATTAAATG   
  
  
+ TAATAATAGA ATATATCTAT GGCATTAAAA CTATCATTCT GGTTGATAAA ACACCCACAG CCACAAGGGA   
  
  
+ AGAGTAAGTA TTTTATATTT CCCCTTCCAC ATAATCCATA TCCAGGTGTG TCTCCTGATG ATTAATGGAC   
  
  
+ ATCACCATCC TCTGGGTCCC ATGAGAGTGA ATGCCCCAAT CAGAACGGCT TGCTCTGGGC TGTAATAAAA   
  
  
+ TCCAGCTCGT GAATTGTGAG GGGCGTACAC GGCAACTTGA CCGCCTTTGC TTTTTTCTCG GTGGGTCCCT   
  
  
+ GTTGGGGCAA CATCGCTGTC ATCGTCTGTG ATTTTGATGT GGTAGGAAGC ATCCGATTTG TAACTGTTAA   
  
  
+ GAGTTTCTTA TGCAACGTCG TTATGGAGAG GTCCTGTACG CCCACTGGAG CATTTGATGC TGCAACAGCA   
  
  
+ AACCAAAACA AAAGGAGTCA GATTTTTGGA ATTTCTACTC TGTCGGCAGT TTAGCCCCCC AGTCTGTTAA   
  
  
+ TCTTGCAAGT TCCAGGATTC TTTTGGGTTG CCCAGAAGTC AATCCCCTCT TCATGCCTAT CGGTTCATCT   
  
  
+ CACCTGTTGA CATGACAACT ATCTGACCAA AAATAAAATT TTTATGCTTT ATCTTGCAGA CATTTTAAAA   
  
  
+ GGGTTTTCGT TCATTTAATA GAATTTTTCT TTTTTTTTAA ATGAATTTGA GGTTCCAAAT ATTTTAGGGA   
  
  
+ TCTTTAAGGG GGAGAAGAGG AGTAATGCAC ATGGTAAAGA GAGTGAAAGT TCTGACACTG TTAAGTGTAG   
  
  
+ GACTGTAGGT GGGTTCCGGT CTTGGAGGGC AACAAGTCAA TTGAAATGAG AAAGTTTCGT GCTAGAAATC   
  
  
+ ATTTGACATT TGCGTTGGGG CAGGGATTCC TGTTTTTCCT TATCTCATTG CTAGTTCATT TGGGTAGGGA   
  
  
+ TTAAATTGGC AGGAACTTAA GTTTGTCTTC CTTTGATTTC ATCGAAAGCT CCAAATCAGT CTTATCAGCA   
  
  
+ ATGGCAGCTT AGCCATTGTG TGATTAGTTT AGTTGCGAGA ATGGAATCTT TAGTGGGTGG GGGGGCACTT   
  
  
+ AGAAGCATGC CAGGTGGAGG GAAACTTCAA GGCTTACGGC CGTGAAACTG TATTTCGTCT TTAAAAAAAA   
  
  
+ AAAAAATATT AGCTCACAAG AATTCTTGAA AAAAAAAAAA AAAAAAGGGA AAGAGGATGT CGTTTTCCAT   
  
  
+ TTCTCTGCTC TGTGGACTTT TTGGGTTCCA CTGGTTTTCA GGGTAGCACG AAACTGTCAA AAAGCCGACT   
  
  
+ GATTTAGAGA GAGGAAAACA ACAATTAGGG AAGGCTGTAC AGAGACAAGC TTTATAATAT CTGTATTTGG   
  
  
+ AGGGAGAAAC AAAGAAGTTG ATAGCTCAA  

- AATTTTTTGA CTGACAAATA TAGTCAACAT AAAAGTATTT TAAACAGTAA GCTAAAATAC CCAAAAACAA   
  
  
- AATTCAATCT TAAATATAGA TATAAAAATG TAATATTGTA TTTTTAAGAC TAAAAAAATT TTTAATTTAC   
  
  
- ATTATTATCT TATATAGATA CCGTAATTTT GATAGTAAGA CCAACTATTT TGTGGGTGTC GGTGTTCCCT   
  
  
- TCTCATTCAT AAAATATAAA GGGGAAGGTG TATTAGGTAT AGGTCCACAC AGAGGACTAC TAATTACCTG   
  
  
- TAGTGGTAGG AGACCCAGGG TACTCTCACT TACGGGGTTA GTCTTGCCGA ACGAGACCCG ACATTATTTT   
  
  
- AGGTCGAGCA CTTAACACTC CCCGCATGTG CCGTTGAACT GGCGGAAACG AAAAAAGAGC CACCCAGGGA   
  
  
- CAACCCCGTT GTAGCGACAG TAGCAGACAC TAAAACTACA CCATCCTTCG TAGGCTAAAC ATTGACAATT   
  
  
- CTCAAAGAAT ACGTTGCAGC AATACCTCTC CAGGACATGC GGGTGACCTC GTAAACTACG ACGTTGTCGT   
  
  
- TTGGTTTTGT TTTCCTCAGT CTAAAAACCT TAAAGATGAG ACAGCCGTCA AATCGGGGGG TCAGACAATT   
  
  
- AGAACGTTCA AGGTCCTAAG AAAACCCAAC GGGTCTTCAG TTAGGGGAGA AGTACGGATA GCCAAGTAGA   
  
  
- GTGGACAACT GTACTGTTGA TAGACTGGTT TTTATTTTAA AAATACGAAA TAGAACGTCT GTAAAATTTT   
  
  
- CCCAAAAGCA AGTAAATTAT CTTAAAAAGA AAAAAAAATT TACTTAAACT CCAAGGTTTA TAAAATCCCT   
  
  
- AGAAATTCCC CCTCTTCTCC TCATTACGTG TACCATTTCT CTCACTTTCA AGACTGTGAC AATTCACATC   
  
  
- CTGACATCCA CCCAAGGCCA GAACCTCCCG TTGTTCAGTT AACTTTACTC TTTCAAAGCA CGATCTTTAG   
  
  
- TAAACTGTAA ACGCAACCCC GTCCCTAAGG ACAAAAAGGA ATAGAGTAAC GATCAAGTAA ACCCATCCCT   
  
  
- AATTTAACCG TCCTTGAATT CAAACAGAAG GAAACTAAAG TAGCTTTCGA GGTTTAGTCA GAATAGTCGT   
  
  
- TACCGTCGAA TCGGTAACAC ACTAATCAAA TCAACGCTCT TACCTTAGAA ATCACCCACC CCCCCGTGAA   
  
  
- TCTTCGTACG GTCCACCTCC CTTTGAAGTT CCGAATGCCG GCACTTTGAC ATAAAGCAGA AATTTTTTTT   
  
  
- TTTTTTATAA TCGAGTGTTC TTAAGAACTT TTTTTTTTTT TTTTTTCCCT TTCTCCTACA GCAAAAGGTA   
  
  
- AAGAGACGAG ACACCTGAAA AACCCAAGGT GACCAAAAGT CCCATCGTGC TTTGACAGTT TTTCGGCTGA   
  
  
- CTAAATCTCT CTCCTTTTGT TGTTAATCCC TTCCGACATG TCTCTGTTCG AAATATTATA GACATAAACC   
  
  
- TCCCTCTTTG TTTCTTCAAC TATCGAGTT

+     G-Box

| Site Name | Organism | Position | Strand | Matrix score. | sequence | function |
| --- | --- | --- | --- | --- | --- | --- |
| G-Box | Pisum sativum | 1198 | - | 10 | GCCACCTGGCA | cis-acting regulatory element involved in light responsiveness |

> 2018/04/13 10:10:12  
+ TTAAAAAACT GACTGTTTAT ATCAGTTGTA TTTTCATAAA ATTTGTCATT CGATTTTATG GGTTTTTGTT   
  
  
+ TTAAGTTAGA ATTTATATCT ATATTTTTAC ATTATAACAT AAAAATTCTG ATTTTTTTAA AAATTAAATG   
  
  
+ TAATAATAGA ATATATCTAT GGCATTAAAA CTATCATTCT GGTTGATAAA ACACCCACAG CCACAAGGGA   
  
  
+ AGAGTAAGTA TTTTATATTT CCCCTTCCAC ATAATCCATA TCCAGGTGTG TCTCCTGATG ATTAATGGAC   
  
  
+ ATCACCATCC TCTGGGTCCC ATGAGAGTGA ATGCCCCAAT CAGAACGGCT TGCTCTGGGC TGTAATAAAA   
  
  
+ TCCAGCTCGT GAATTGTGAG GGGCGTACAC GGCAACTTGA CCGCCTTTGC TTTTTTCTCG GTGGGTCCCT   
  
  
+ GTTGGGGCAA CATCGCTGTC ATCGTCTGTG ATTTTGATGT GGTAGGAAGC ATCCGATTTG TAACTGTTAA   
  
  
+ GAGTTTCTTA TGCAACGTCG TTATGGAGAG GTCCTGTACG CCCACTGGAG CATTTGATGC TGCAACAGCA   
  
  
+ AACCAAAACA AAAGGAGTCA GATTTTTGGA ATTTCTACTC TGTCGGCAGT TTAGCCCCCC AGTCTGTTAA   
  
  
+ TCTTGCAAGT TCCAGGATTC TTTTGGGTTG CCCAGAAGTC AATCCCCTCT TCATGCCTAT CGGTTCATCT   
  
  
+ CACCTGTTGA CATGACAACT ATCTGACCAA AAATAAAATT TTTATGCTTT ATCTTGCAGA CATTTTAAAA   
  
  
+ GGGTTTTCGT TCATTTAATA GAATTTTTCT TTTTTTTTAA ATGAATTTGA GGTTCCAAAT ATTTTAGGGA   
  
  
+ TCTTTAAGGG GGAGAAGAGG AGTAATGCAC ATGGTAAAGA GAGTGAAAGT TCTGACACTG TTAAGTGTAG   
  
  
+ GACTGTAGGT GGGTTCCGGT CTTGGAGGGC AACAAGTCAA TTGAAATGAG AAAGTTTCGT GCTAGAAATC   
  
  
+ ATTTGACATT TGCGTTGGGG CAGGGATTCC TGTTTTTCCT TATCTCATTG CTAGTTCATT TGGGTAGGGA   
  
  
+ TTAAATTGGC AGGAACTTAA GTTTGTCTTC CTTTGATTTC ATCGAAAGCT CCAAATCAGT CTTATCAGCA   
  
  
+ ATGGCAGCTT AGCCATTGTG TGATTAGTTT AGTTGCGAGA ATGGAATCTT TAGTGGGTGG GGGGGCACTT   
  
  
+ AGAAGCATGC CAGGTGGAGG GAAACTTCAA GGCTTACGGC CGTGAAACTG TATTTCGTCT TTAAAAAAAA   
  
  
+ AAAAAATATT AGCTCACAAG AATTCTTGAA AAAAAAAAAA AAAAAAGGGA AAGAGGATGT CGTTTTCCAT   
  
  
+ TTCTCTGCTC TGTGGACTTT TTGGGTTCCA CTGGTTTTCA GGGTAGCACG AAACTGTCAA AAAGCCGACT   
  
  
+ GATTTAGAGA GAGGAAAACA ACAATTAGGG AAGGCTGTAC AGAGACAAGC TTTATAATAT CTGTATTTGG   
  
  
+ AGGGAGAAAC AAAGAAGTTG ATAGCTCAA  

- AATTTTTTGA CTGACAAATA TAGTCAACAT AAAAGTATTT TAAACAGTAA GCTAAAATAC CCAAAAACAA   
  
  
- AATTCAATCT TAAATATAGA TATAAAAATG TAATATTGTA TTTTTAAGAC TAAAAAAATT TTTAATTTAC   
  
  
- ATTATTATCT TATATAGATA CCGTAATTTT GATAGTAAGA CCAACTATTT TGTGGGTGTC GGTGTTCCCT   
  
  
- TCTCATTCAT AAAATATAAA GGGGAAGGTG TATTAGGTAT AGGTCCACAC AGAGGACTAC TAATTACCTG   
  
  
- TAGTGGTAGG AGACCCAGGG TACTCTCACT TACGGGGTTA GTCTTGCCGA ACGAGACCCG ACATTATTTT   
  
  
- AGGTCGAGCA CTTAACACTC CCCGCATGTG CCGTTGAACT GGCGGAAACG AAAAAAGAGC CACCCAGGGA   
  
  
- CAACCCCGTT GTAGCGACAG TAGCAGACAC TAAAACTACA CCATCCTTCG TAGGCTAAAC ATTGACAATT   
  
  
- CTCAAAGAAT ACGTTGCAGC AATACCTCTC CAGGACATGC GGGTGACCTC GTAAACTACG ACGTTGTCGT   
  
  
- TTGGTTTTGT TTTCCTCAGT CTAAAAACCT TAAAGATGAG ACAGCCGTCA AATCGGGGGG TCAGACAATT   
  
  
- AGAACGTTCA AGGTCCTAAG AAAACCCAAC GGGTCTTCAG TTAGGGGAGA AGTACGGATA GCCAAGTAGA   
  
  
- GTGGACAACT GTACTGTTGA TAGACTGGTT TTTATTTTAA AAATACGAAA TAGAACGTCT GTAAAATTTT   
  
  
- CCCAAAAGCA AGTAAATTAT CTTAAAAAGA AAAAAAAATT TACTTAAACT CCAAGGTTTA TAAAATCCCT   
  
  
- AGAAATTCCC CCTCTTCTCC TCATTACGTG TACCATTTCT CTCACTTTCA AGACTGTGAC AATTCACATC   
  
  
- CTGACATCCA CCCAAGGCCA GAACCTCCCG TTGTTCAGTT AACTTTACTC TTTCAAAGCA CGATCTTTAG   
  
  
- TAAACTGTAA ACGCAACCCC GTCCCTAAGG ACAAAAAGGA ATAGAGTAAC GATCAAGTAA ACCCATCCCT   
  
  
- AATTTAACCG TCCTTGAATT CAAACAGAAG GAAACTAAAG TAGCTTTCGA GGTTTAGTCA GAATAGTCGT   
  
  
- TACCGTCGAA TCGGTAACAC ACTAATCAAA TCAACGCTCT TACCTTAGAA ATCACCCACC CCCCCGTGAA   
  
  
- TCTTCGTACG GTCCACCTCC CTTTGAAGTT CCGAATGCCG GCACTTTGAC ATAAAGCAGA AATTTTTTTT   
  
  
- TTTTTTATAA TCGAGTGTTC TTAAGAACTT TTTTTTTTTT TTTTTTCCCT TTCTCCTACA GCAAAAGGTA   
  
  
- AAGAGACGAG ACACCTGAAA AACCCAAGGT GACCAAAAGT CCCATCGTGC TTTGACAGTT TTTCGGCTGA   
  
  
- CTAAATCTCT CTCCTTTTGT TGTTAATCCC TTCCGACATG TCTCTGTTCG AAATATTATA GACATAAACC   
  
  
- TCCCTCTTTG TTTCTTCAAC TATCGAGTT

+     G-box

| Site Name | Organism | Position | Strand | Matrix score. | sequence | function |
| --- | --- | --- | --- | --- | --- | --- |
| G-box | Arabidopsis thaliana | 1199 | + | 9 | GCCACGTGGA | cis-acting regulatory element involved in light responsiveness |
| G-box | Solanum tuberosum | 868 | + | 7 | CACATGG | cis-acting regulatory element involved in light responsiveness |

> 2018/04/13 10:10:12  
+ TTAAAAAACT GACTGTTTAT ATCAGTTGTA TTTTCATAAA ATTTGTCATT CGATTTTATG GGTTTTTGTT   
  
  
+ TTAAGTTAGA ATTTATATCT ATATTTTTAC ATTATAACAT AAAAATTCTG ATTTTTTTAA AAATTAAATG   
  
  
+ TAATAATAGA ATATATCTAT GGCATTAAAA CTATCATTCT GGTTGATAAA ACACCCACAG CCACAAGGGA   
  
  
+ AGAGTAAGTA TTTTATATTT CCCCTTCCAC ATAATCCATA TCCAGGTGTG TCTCCTGATG ATTAATGGAC   
  
  
+ ATCACCATCC TCTGGGTCCC ATGAGAGTGA ATGCCCCAAT CAGAACGGCT TGCTCTGGGC TGTAATAAAA   
  
  
+ TCCAGCTCGT GAATTGTGAG GGGCGTACAC GGCAACTTGA CCGCCTTTGC TTTTTTCTCG GTGGGTCCCT   
  
  
+ GTTGGGGCAA CATCGCTGTC ATCGTCTGTG ATTTTGATGT GGTAGGAAGC ATCCGATTTG TAACTGTTAA   
  
  
+ GAGTTTCTTA TGCAACGTCG TTATGGAGAG GTCCTGTACG CCCACTGGAG CATTTGATGC TGCAACAGCA   
  
  
+ AACCAAAACA AAAGGAGTCA GATTTTTGGA ATTTCTACTC TGTCGGCAGT TTAGCCCCCC AGTCTGTTAA   
  
  
+ TCTTGCAAGT TCCAGGATTC TTTTGGGTTG CCCAGAAGTC AATCCCCTCT TCATGCCTAT CGGTTCATCT   
  
  
+ CACCTGTTGA CATGACAACT ATCTGACCAA AAATAAAATT TTTATGCTTT ATCTTGCAGA CATTTTAAAA   
  
  
+ GGGTTTTCGT TCATTTAATA GAATTTTTCT TTTTTTTTAA ATGAATTTGA GGTTCCAAAT ATTTTAGGGA   
  
  
+ TCTTTAAGGG GGAGAAGAGG AGTAATGCAC ATGGTAAAGA GAGTGAAAGT TCTGACACTG TTAAGTGTAG   
  
  
+ GACTGTAGGT GGGTTCCGGT CTTGGAGGGC AACAAGTCAA TTGAAATGAG AAAGTTTCGT GCTAGAAATC   
  
  
+ ATTTGACATT TGCGTTGGGG CAGGGATTCC TGTTTTTCCT TATCTCATTG CTAGTTCATT TGGGTAGGGA   
  
  
+ TTAAATTGGC AGGAACTTAA GTTTGTCTTC CTTTGATTTC ATCGAAAGCT CCAAATCAGT CTTATCAGCA   
  
  
+ ATGGCAGCTT AGCCATTGTG TGATTAGTTT AGTTGCGAGA ATGGAATCTT TAGTGGGTGG GGGGGCACTT   
  
  
+ AGAAGCATGC CAGGTGGAGG GAAACTTCAA GGCTTACGGC CGTGAAACTG TATTTCGTCT TTAAAAAAAA   
  
  
+ AAAAAATATT AGCTCACAAG AATTCTTGAA AAAAAAAAAA AAAAAAGGGA AAGAGGATGT CGTTTTCCAT   
  
  
+ TTCTCTGCTC TGTGGACTTT TTGGGTTCCA CTGGTTTTCA GGGTAGCACG AAACTGTCAA AAAGCCGACT   
  
  
+ GATTTAGAGA GAGGAAAACA ACAATTAGGG AAGGCTGTAC AGAGACAAGC TTTATAATAT CTGTATTTGG   
  
  
+ AGGGAGAAAC AAAGAAGTTG ATAGCTCAA  

- AATTTTTTGA CTGACAAATA TAGTCAACAT AAAAGTATTT TAAACAGTAA GCTAAAATAC CCAAAAACAA   
  
  
- AATTCAATCT TAAATATAGA TATAAAAATG TAATATTGTA TTTTTAAGAC TAAAAAAATT TTTAATTTAC   
  
  
- ATTATTATCT TATATAGATA CCGTAATTTT GATAGTAAGA CCAACTATTT TGTGGGTGTC GGTGTTCCCT   
  
  
- TCTCATTCAT AAAATATAAA GGGGAAGGTG TATTAGGTAT AGGTCCACAC AGAGGACTAC TAATTACCTG   
  
  
- TAGTGGTAGG AGACCCAGGG TACTCTCACT TACGGGGTTA GTCTTGCCGA ACGAGACCCG ACATTATTTT   
  
  
- AGGTCGAGCA CTTAACACTC CCCGCATGTG CCGTTGAACT GGCGGAAACG AAAAAAGAGC CACCCAGGGA   
  
  
- CAACCCCGTT GTAGCGACAG TAGCAGACAC TAAAACTACA CCATCCTTCG TAGGCTAAAC ATTGACAATT   
  
  
- CTCAAAGAAT ACGTTGCAGC AATACCTCTC CAGGACATGC GGGTGACCTC GTAAACTACG ACGTTGTCGT   
  
  
- TTGGTTTTGT TTTCCTCAGT CTAAAAACCT TAAAGATGAG ACAGCCGTCA AATCGGGGGG TCAGACAATT   
  
  
- AGAACGTTCA AGGTCCTAAG AAAACCCAAC GGGTCTTCAG TTAGGGGAGA AGTACGGATA GCCAAGTAGA   
  
  
- GTGGACAACT GTACTGTTGA TAGACTGGTT TTTATTTTAA AAATACGAAA TAGAACGTCT GTAAAATTTT   
  
  
- CCCAAAAGCA AGTAAATTAT CTTAAAAAGA AAAAAAAATT TACTTAAACT CCAAGGTTTA TAAAATCCCT   
  
  
- AGAAATTCCC CCTCTTCTCC TCATTACGTG TACCATTTCT CTCACTTTCA AGACTGTGAC AATTCACATC   
  
  
- CTGACATCCA CCCAAGGCCA GAACCTCCCG TTGTTCAGTT AACTTTACTC TTTCAAAGCA CGATCTTTAG   
  
  
- TAAACTGTAA ACGCAACCCC GTCCCTAAGG ACAAAAAGGA ATAGAGTAAC GATCAAGTAA ACCCATCCCT   
  
  
- AATTTAACCG TCCTTGAATT CAAACAGAAG GAAACTAAAG TAGCTTTCGA GGTTTAGTCA GAATAGTCGT   
  
  
- TACCGTCGAA TCGGTAACAC ACTAATCAAA TCAACGCTCT TACCTTAGAA ATCACCCACC CCCCCGTGAA   
  
  
- TCTTCGTACG GTCCACCTCC CTTTGAAGTT CCGAATGCCG GCACTTTGAC ATAAAGCAGA AATTTTTTTT   
  
  
- TTTTTTATAA TCGAGTGTTC TTAAGAACTT TTTTTTTTTT TTTTTTCCCT TTCTCCTACA GCAAAAGGTA   
  
  
- AAGAGACGAG ACACCTGAAA AACCCAAGGT GACCAAAAGT CCCATCGTGC TTTGACAGTT TTTCGGCTGA   
  
  
- CTAAATCTCT CTCCTTTTGT TGTTAATCCC TTCCGACATG TCTCTGTTCG AAATATTATA GACATAAACC   
  
  
- TCCCTCTTTG TTTCTTCAAC TATCGAGTT

+     GA-motif

| Site Name | Organism | Position | Strand | Matrix score. | sequence | function |
| --- | --- | --- | --- | --- | --- | --- |
| GA-motif | Glycine max | 1076 | - | 8 | AAGGAAGA | part of a light responsive element |

> 2018/04/13 10:10:12  
+ TTAAAAAACT GACTGTTTAT ATCAGTTGTA TTTTCATAAA ATTTGTCATT CGATTTTATG GGTTTTTGTT   
  
  
+ TTAAGTTAGA ATTTATATCT ATATTTTTAC ATTATAACAT AAAAATTCTG ATTTTTTTAA AAATTAAATG   
  
  
+ TAATAATAGA ATATATCTAT GGCATTAAAA CTATCATTCT GGTTGATAAA ACACCCACAG CCACAAGGGA   
  
  
+ AGAGTAAGTA TTTTATATTT CCCCTTCCAC ATAATCCATA TCCAGGTGTG TCTCCTGATG ATTAATGGAC   
  
  
+ ATCACCATCC TCTGGGTCCC ATGAGAGTGA ATGCCCCAAT CAGAACGGCT TGCTCTGGGC TGTAATAAAA   
  
  
+ TCCAGCTCGT GAATTGTGAG GGGCGTACAC GGCAACTTGA CCGCCTTTGC TTTTTTCTCG GTGGGTCCCT   
  
  
+ GTTGGGGCAA CATCGCTGTC ATCGTCTGTG ATTTTGATGT GGTAGGAAGC ATCCGATTTG TAACTGTTAA   
  
  
+ GAGTTTCTTA TGCAACGTCG TTATGGAGAG GTCCTGTACG CCCACTGGAG CATTTGATGC TGCAACAGCA   
  
  
+ AACCAAAACA AAAGGAGTCA GATTTTTGGA ATTTCTACTC TGTCGGCAGT TTAGCCCCCC AGTCTGTTAA   
  
  
+ TCTTGCAAGT TCCAGGATTC TTTTGGGTTG CCCAGAAGTC AATCCCCTCT TCATGCCTAT CGGTTCATCT   
  
  
+ CACCTGTTGA CATGACAACT ATCTGACCAA AAATAAAATT TTTATGCTTT ATCTTGCAGA CATTTTAAAA   
  
  
+ GGGTTTTCGT TCATTTAATA GAATTTTTCT TTTTTTTTAA ATGAATTTGA GGTTCCAAAT ATTTTAGGGA   
  
  
+ TCTTTAAGGG GGAGAAGAGG AGTAATGCAC ATGGTAAAGA GAGTGAAAGT TCTGACACTG TTAAGTGTAG   
  
  
+ GACTGTAGGT GGGTTCCGGT CTTGGAGGGC AACAAGTCAA TTGAAATGAG AAAGTTTCGT GCTAGAAATC   
  
  
+ ATTTGACATT TGCGTTGGGG CAGGGATTCC TGTTTTTCCT TATCTCATTG CTAGTTCATT TGGGTAGGGA   
  
  
+ TTAAATTGGC AGGAACTTAA GTTTGTCTTC CTTTGATTTC ATCGAAAGCT CCAAATCAGT CTTATCAGCA   
  
  
+ ATGGCAGCTT AGCCATTGTG TGATTAGTTT AGTTGCGAGA ATGGAATCTT TAGTGGGTGG GGGGGCACTT   
  
  
+ AGAAGCATGC CAGGTGGAGG GAAACTTCAA GGCTTACGGC CGTGAAACTG TATTTCGTCT TTAAAAAAAA   
  
  
+ AAAAAATATT AGCTCACAAG AATTCTTGAA AAAAAAAAAA AAAAAAGGGA AAGAGGATGT CGTTTTCCAT   
  
  
+ TTCTCTGCTC TGTGGACTTT TTGGGTTCCA CTGGTTTTCA GGGTAGCACG AAACTGTCAA AAAGCCGACT   
  
  
+ GATTTAGAGA GAGGAAAACA ACAATTAGGG AAGGCTGTAC AGAGACAAGC TTTATAATAT CTGTATTTGG   
  
  
+ AGGGAGAAAC AAAGAAGTTG ATAGCTCAA  

- AATTTTTTGA CTGACAAATA TAGTCAACAT AAAAGTATTT TAAACAGTAA GCTAAAATAC CCAAAAACAA   
  
  
- AATTCAATCT TAAATATAGA TATAAAAATG TAATATTGTA TTTTTAAGAC TAAAAAAATT TTTAATTTAC   
  
  
- ATTATTATCT TATATAGATA CCGTAATTTT GATAGTAAGA CCAACTATTT TGTGGGTGTC GGTGTTCCCT   
  
  
- TCTCATTCAT AAAATATAAA GGGGAAGGTG TATTAGGTAT AGGTCCACAC AGAGGACTAC TAATTACCTG   
  
  
- TAGTGGTAGG AGACCCAGGG TACTCTCACT TACGGGGTTA GTCTTGCCGA ACGAGACCCG ACATTATTTT   
  
  
- AGGTCGAGCA CTTAACACTC CCCGCATGTG CCGTTGAACT GGCGGAAACG AAAAAAGAGC CACCCAGGGA   
  
  
- CAACCCCGTT GTAGCGACAG TAGCAGACAC TAAAACTACA CCATCCTTCG TAGGCTAAAC ATTGACAATT   
  
  
- CTCAAAGAAT ACGTTGCAGC AATACCTCTC CAGGACATGC GGGTGACCTC GTAAACTACG ACGTTGTCGT   
  
  
- TTGGTTTTGT TTTCCTCAGT CTAAAAACCT TAAAGATGAG ACAGCCGTCA AATCGGGGGG TCAGACAATT   
  
  
- AGAACGTTCA AGGTCCTAAG AAAACCCAAC GGGTCTTCAG TTAGGGGAGA AGTACGGATA GCCAAGTAGA   
  
  
- GTGGACAACT GTACTGTTGA TAGACTGGTT TTTATTTTAA AAATACGAAA TAGAACGTCT GTAAAATTTT   
  
  
- CCCAAAAGCA AGTAAATTAT CTTAAAAAGA AAAAAAAATT TACTTAAACT CCAAGGTTTA TAAAATCCCT   
  
  
- AGAAATTCCC CCTCTTCTCC TCATTACGTG TACCATTTCT CTCACTTTCA AGACTGTGAC AATTCACATC   
  
  
- CTGACATCCA CCCAAGGCCA GAACCTCCCG TTGTTCAGTT AACTTTACTC TTTCAAAGCA CGATCTTTAG   
  
  
- TAAACTGTAA ACGCAACCCC GTCCCTAAGG ACAAAAAGGA ATAGAGTAAC GATCAAGTAA ACCCATCCCT   
  
  
- AATTTAACCG TCCTTGAATT CAAACAGAAG GAAACTAAAG TAGCTTTCGA GGTTTAGTCA GAATAGTCGT   
  
  
- TACCGTCGAA TCGGTAACAC ACTAATCAAA TCAACGCTCT TACCTTAGAA ATCACCCACC CCCCCGTGAA   
  
  
- TCTTCGTACG GTCCACCTCC CTTTGAAGTT CCGAATGCCG GCACTTTGAC ATAAAGCAGA AATTTTTTTT   
  
  
- TTTTTTATAA TCGAGTGTTC TTAAGAACTT TTTTTTTTTT TTTTTTCCCT TTCTCCTACA GCAAAAGGTA   
  
  
- AAGAGACGAG ACACCTGAAA AACCCAAGGT GACCAAAAGT CCCATCGTGC TTTGACAGTT TTTCGGCTGA   
  
  
- CTAAATCTCT CTCCTTTTGT TGTTAATCCC TTCCGACATG TCTCTGTTCG AAATATTATA GACATAAACC   
  
  
- TCCCTCTTTG TTTCTTCAAC TATCGAGTT

+     GAG-motif

| Site Name | Organism | Position | Strand | Matrix score. | sequence | function |
| --- | --- | --- | --- | --- | --- | --- |
| GAG-motif | Arabidopsis thaliana | 878 | + | 7 | AGAGAGT | part of a light responsive element |

> 2018/04/13 10:10:12  
+ TTAAAAAACT GACTGTTTAT ATCAGTTGTA TTTTCATAAA ATTTGTCATT CGATTTTATG GGTTTTTGTT   
  
  
+ TTAAGTTAGA ATTTATATCT ATATTTTTAC ATTATAACAT AAAAATTCTG ATTTTTTTAA AAATTAAATG   
  
  
+ TAATAATAGA ATATATCTAT GGCATTAAAA CTATCATTCT GGTTGATAAA ACACCCACAG CCACAAGGGA   
  
  
+ AGAGTAAGTA TTTTATATTT CCCCTTCCAC ATAATCCATA TCCAGGTGTG TCTCCTGATG ATTAATGGAC   
  
  
+ ATCACCATCC TCTGGGTCCC ATGAGAGTGA ATGCCCCAAT CAGAACGGCT TGCTCTGGGC TGTAATAAAA   
  
  
+ TCCAGCTCGT GAATTGTGAG GGGCGTACAC GGCAACTTGA CCGCCTTTGC TTTTTTCTCG GTGGGTCCCT   
  
  
+ GTTGGGGCAA CATCGCTGTC ATCGTCTGTG ATTTTGATGT GGTAGGAAGC ATCCGATTTG TAACTGTTAA   
  
  
+ GAGTTTCTTA TGCAACGTCG TTATGGAGAG GTCCTGTACG CCCACTGGAG CATTTGATGC TGCAACAGCA   
  
  
+ AACCAAAACA AAAGGAGTCA GATTTTTGGA ATTTCTACTC TGTCGGCAGT TTAGCCCCCC AGTCTGTTAA   
  
  
+ TCTTGCAAGT TCCAGGATTC TTTTGGGTTG CCCAGAAGTC AATCCCCTCT TCATGCCTAT CGGTTCATCT   
  
  
+ CACCTGTTGA CATGACAACT ATCTGACCAA AAATAAAATT TTTATGCTTT ATCTTGCAGA CATTTTAAAA   
  
  
+ GGGTTTTCGT TCATTTAATA GAATTTTTCT TTTTTTTTAA ATGAATTTGA GGTTCCAAAT ATTTTAGGGA   
  
  
+ TCTTTAAGGG GGAGAAGAGG AGTAATGCAC ATGGTAAAGA GAGTGAAAGT TCTGACACTG TTAAGTGTAG   
  
  
+ GACTGTAGGT GGGTTCCGGT CTTGGAGGGC AACAAGTCAA TTGAAATGAG AAAGTTTCGT GCTAGAAATC   
  
  
+ ATTTGACATT TGCGTTGGGG CAGGGATTCC TGTTTTTCCT TATCTCATTG CTAGTTCATT TGGGTAGGGA   
  
  
+ TTAAATTGGC AGGAACTTAA GTTTGTCTTC CTTTGATTTC ATCGAAAGCT CCAAATCAGT CTTATCAGCA   
  
  
+ ATGGCAGCTT AGCCATTGTG TGATTAGTTT AGTTGCGAGA ATGGAATCTT TAGTGGGTGG GGGGGCACTT   
  
  
+ AGAAGCATGC CAGGTGGAGG GAAACTTCAA GGCTTACGGC CGTGAAACTG TATTTCGTCT TTAAAAAAAA   
  
  
+ AAAAAATATT AGCTCACAAG AATTCTTGAA AAAAAAAAAA AAAAAAGGGA AAGAGGATGT CGTTTTCCAT   
  
  
+ TTCTCTGCTC TGTGGACTTT TTGGGTTCCA CTGGTTTTCA GGGTAGCACG AAACTGTCAA AAAGCCGACT   
  
  
+ GATTTAGAGA GAGGAAAACA ACAATTAGGG AAGGCTGTAC AGAGACAAGC TTTATAATAT CTGTATTTGG   
  
  
+ AGGGAGAAAC AAAGAAGTTG ATAGCTCAA  

- AATTTTTTGA CTGACAAATA TAGTCAACAT AAAAGTATTT TAAACAGTAA GCTAAAATAC CCAAAAACAA   
  
  
- AATTCAATCT TAAATATAGA TATAAAAATG TAATATTGTA TTTTTAAGAC TAAAAAAATT TTTAATTTAC   
  
  
- ATTATTATCT TATATAGATA CCGTAATTTT GATAGTAAGA CCAACTATTT TGTGGGTGTC GGTGTTCCCT   
  
  
- TCTCATTCAT AAAATATAAA GGGGAAGGTG TATTAGGTAT AGGTCCACAC AGAGGACTAC TAATTACCTG   
  
  
- TAGTGGTAGG AGACCCAGGG TACTCTCACT TACGGGGTTA GTCTTGCCGA ACGAGACCCG ACATTATTTT   
  
  
- AGGTCGAGCA CTTAACACTC CCCGCATGTG CCGTTGAACT GGCGGAAACG AAAAAAGAGC CACCCAGGGA   
  
  
- CAACCCCGTT GTAGCGACAG TAGCAGACAC TAAAACTACA CCATCCTTCG TAGGCTAAAC ATTGACAATT   
  
  
- CTCAAAGAAT ACGTTGCAGC AATACCTCTC CAGGACATGC GGGTGACCTC GTAAACTACG ACGTTGTCGT   
  
  
- TTGGTTTTGT TTTCCTCAGT CTAAAAACCT TAAAGATGAG ACAGCCGTCA AATCGGGGGG TCAGACAATT   
  
  
- AGAACGTTCA AGGTCCTAAG AAAACCCAAC GGGTCTTCAG TTAGGGGAGA AGTACGGATA GCCAAGTAGA   
  
  
- GTGGACAACT GTACTGTTGA TAGACTGGTT TTTATTTTAA AAATACGAAA TAGAACGTCT GTAAAATTTT   
  
  
- CCCAAAAGCA AGTAAATTAT CTTAAAAAGA AAAAAAAATT TACTTAAACT CCAAGGTTTA TAAAATCCCT   
  
  
- AGAAATTCCC CCTCTTCTCC TCATTACGTG TACCATTTCT CTCACTTTCA AGACTGTGAC AATTCACATC   
  
  
- CTGACATCCA CCCAAGGCCA GAACCTCCCG TTGTTCAGTT AACTTTACTC TTTCAAAGCA CGATCTTTAG   
  
  
- TAAACTGTAA ACGCAACCCC GTCCCTAAGG ACAAAAAGGA ATAGAGTAAC GATCAAGTAA ACCCATCCCT   
  
  
- AATTTAACCG TCCTTGAATT CAAACAGAAG GAAACTAAAG TAGCTTTCGA GGTTTAGTCA GAATAGTCGT   
  
  
- TACCGTCGAA TCGGTAACAC ACTAATCAAA TCAACGCTCT TACCTTAGAA ATCACCCACC CCCCCGTGAA   
  
  
- TCTTCGTACG GTCCACCTCC CTTTGAAGTT CCGAATGCCG GCACTTTGAC ATAAAGCAGA AATTTTTTTT   
  
  
- TTTTTTATAA TCGAGTGTTC TTAAGAACTT TTTTTTTTTT TTTTTTCCCT TTCTCCTACA GCAAAAGGTA   
  
  
- AAGAGACGAG ACACCTGAAA AACCCAAGGT GACCAAAAGT CCCATCGTGC TTTGACAGTT TTTCGGCTGA   
  
  
- CTAAATCTCT CTCCTTTTGT TGTTAATCCC TTCCGACATG TCTCTGTTCG AAATATTATA GACATAAACC   
  
  
- TCCCTCTTTG TTTCTTCAAC TATCGAGTT

+     I-box

| Site Name | Organism | Position | Strand | Matrix score. | sequence | function |
| --- | --- | --- | --- | --- | --- | --- |
| I-box | Triticum aestivum | 1018 | - | 8 | AGATAAGG | part of a light responsive element |
| I-box | Flaveria trinervia | 246 | - | 7 | GATATGG | part of a light responsive element |

> 2018/04/13 10:10:12  
+ TTAAAAAACT GACTGTTTAT ATCAGTTGTA TTTTCATAAA ATTTGTCATT CGATTTTATG GGTTTTTGTT   
  
  
+ TTAAGTTAGA ATTTATATCT ATATTTTTAC ATTATAACAT AAAAATTCTG ATTTTTTTAA AAATTAAATG   
  
  
+ TAATAATAGA ATATATCTAT GGCATTAAAA CTATCATTCT GGTTGATAAA ACACCCACAG CCACAAGGGA   
  
  
+ AGAGTAAGTA TTTTATATTT CCCCTTCCAC ATAATCCATA TCCAGGTGTG TCTCCTGATG ATTAATGGAC   
  
  
+ ATCACCATCC TCTGGGTCCC ATGAGAGTGA ATGCCCCAAT CAGAACGGCT TGCTCTGGGC TGTAATAAAA   
  
  
+ TCCAGCTCGT GAATTGTGAG GGGCGTACAC GGCAACTTGA CCGCCTTTGC TTTTTTCTCG GTGGGTCCCT   
  
  
+ GTTGGGGCAA CATCGCTGTC ATCGTCTGTG ATTTTGATGT GGTAGGAAGC ATCCGATTTG TAACTGTTAA   
  
  
+ GAGTTTCTTA TGCAACGTCG TTATGGAGAG GTCCTGTACG CCCACTGGAG CATTTGATGC TGCAACAGCA   
  
  
+ AACCAAAACA AAAGGAGTCA GATTTTTGGA ATTTCTACTC TGTCGGCAGT TTAGCCCCCC AGTCTGTTAA   
  
  
+ TCTTGCAAGT TCCAGGATTC TTTTGGGTTG CCCAGAAGTC AATCCCCTCT TCATGCCTAT CGGTTCATCT   
  
  
+ CACCTGTTGA CATGACAACT ATCTGACCAA AAATAAAATT TTTATGCTTT ATCTTGCAGA CATTTTAAAA   
  
  
+ GGGTTTTCGT TCATTTAATA GAATTTTTCT TTTTTTTTAA ATGAATTTGA GGTTCCAAAT ATTTTAGGGA   
  
  
+ TCTTTAAGGG GGAGAAGAGG AGTAATGCAC ATGGTAAAGA GAGTGAAAGT TCTGACACTG TTAAGTGTAG   
  
  
+ GACTGTAGGT GGGTTCCGGT CTTGGAGGGC AACAAGTCAA TTGAAATGAG AAAGTTTCGT GCTAGAAATC   
  
  
+ ATTTGACATT TGCGTTGGGG CAGGGATTCC TGTTTTTCCT TATCTCATTG CTAGTTCATT TGGGTAGGGA   
  
  
+ TTAAATTGGC AGGAACTTAA GTTTGTCTTC CTTTGATTTC ATCGAAAGCT CCAAATCAGT CTTATCAGCA   
  
  
+ ATGGCAGCTT AGCCATTGTG TGATTAGTTT AGTTGCGAGA ATGGAATCTT TAGTGGGTGG GGGGGCACTT   
  
  
+ AGAAGCATGC CAGGTGGAGG GAAACTTCAA GGCTTACGGC CGTGAAACTG TATTTCGTCT TTAAAAAAAA   
  
  
+ AAAAAATATT AGCTCACAAG AATTCTTGAA AAAAAAAAAA AAAAAAGGGA AAGAGGATGT CGTTTTCCAT   
  
  
+ TTCTCTGCTC TGTGGACTTT TTGGGTTCCA CTGGTTTTCA GGGTAGCACG AAACTGTCAA AAAGCCGACT   
  
  
+ GATTTAGAGA GAGGAAAACA ACAATTAGGG AAGGCTGTAC AGAGACAAGC TTTATAATAT CTGTATTTGG   
  
  
+ AGGGAGAAAC AAAGAAGTTG ATAGCTCAA  

- AATTTTTTGA CTGACAAATA TAGTCAACAT AAAAGTATTT TAAACAGTAA GCTAAAATAC CCAAAAACAA   
  
  
- AATTCAATCT TAAATATAGA TATAAAAATG TAATATTGTA TTTTTAAGAC TAAAAAAATT TTTAATTTAC   
  
  
- ATTATTATCT TATATAGATA CCGTAATTTT GATAGTAAGA CCAACTATTT TGTGGGTGTC GGTGTTCCCT   
  
  
- TCTCATTCAT AAAATATAAA GGGGAAGGTG TATTAGGTAT AGGTCCACAC AGAGGACTAC TAATTACCTG   
  
  
- TAGTGGTAGG AGACCCAGGG TACTCTCACT TACGGGGTTA GTCTTGCCGA ACGAGACCCG ACATTATTTT   
  
  
- AGGTCGAGCA CTTAACACTC CCCGCATGTG CCGTTGAACT GGCGGAAACG AAAAAAGAGC CACCCAGGGA   
  
  
- CAACCCCGTT GTAGCGACAG TAGCAGACAC TAAAACTACA CCATCCTTCG TAGGCTAAAC ATTGACAATT   
  
  
- CTCAAAGAAT ACGTTGCAGC AATACCTCTC CAGGACATGC GGGTGACCTC GTAAACTACG ACGTTGTCGT   
  
  
- TTGGTTTTGT TTTCCTCAGT CTAAAAACCT TAAAGATGAG ACAGCCGTCA AATCGGGGGG TCAGACAATT   
  
  
- AGAACGTTCA AGGTCCTAAG AAAACCCAAC GGGTCTTCAG TTAGGGGAGA AGTACGGATA GCCAAGTAGA   
  
  
- GTGGACAACT GTACTGTTGA TAGACTGGTT TTTATTTTAA AAATACGAAA TAGAACGTCT GTAAAATTTT   
  
  
- CCCAAAAGCA AGTAAATTAT CTTAAAAAGA AAAAAAAATT TACTTAAACT CCAAGGTTTA TAAAATCCCT   
  
  
- AGAAATTCCC CCTCTTCTCC TCATTACGTG TACCATTTCT CTCACTTTCA AGACTGTGAC AATTCACATC   
  
  
- CTGACATCCA CCCAAGGCCA GAACCTCCCG TTGTTCAGTT AACTTTACTC TTTCAAAGCA CGATCTTTAG   
  
  
- TAAACTGTAA ACGCAACCCC GTCCCTAAGG ACAAAAAGGA ATAGAGTAAC GATCAAGTAA ACCCATCCCT   
  
  
- AATTTAACCG TCCTTGAATT CAAACAGAAG GAAACTAAAG TAGCTTTCGA GGTTTAGTCA GAATAGTCGT   
  
  
- TACCGTCGAA TCGGTAACAC ACTAATCAAA TCAACGCTCT TACCTTAGAA ATCACCCACC CCCCCGTGAA   
  
  
- TCTTCGTACG GTCCACCTCC CTTTGAAGTT CCGAATGCCG GCACTTTGAC ATAAAGCAGA AATTTTTTTT   
  
  
- TTTTTTATAA TCGAGTGTTC TTAAGAACTT TTTTTTTTTT TTTTTTCCCT TTCTCCTACA GCAAAAGGTA   
  
  
- AAGAGACGAG ACACCTGAAA AACCCAAGGT GACCAAAAGT CCCATCGTGC TTTGACAGTT TTTCGGCTGA   
  
  
- CTAAATCTCT CTCCTTTTGT TGTTAATCCC TTCCGACATG TCTCTGTTCG AAATATTATA GACATAAACC   
  
  
- TCCCTCTTTG TTTCTTCAAC TATCGAGTT

+     L-box

| Site Name | Organism | Position | Strand | Matrix score. | sequence | function |
| --- | --- | --- | --- | --- | --- | --- |
| L-box | Petroselinum crispum | 915 | - | 10 | ATCCCACCTAC | part of a light responsive element |

> 2018/04/13 10:10:12  
+ TTAAAAAACT GACTGTTTAT ATCAGTTGTA TTTTCATAAA ATTTGTCATT CGATTTTATG GGTTTTTGTT   
  
  
+ TTAAGTTAGA ATTTATATCT ATATTTTTAC ATTATAACAT AAAAATTCTG ATTTTTTTAA AAATTAAATG   
  
  
+ TAATAATAGA ATATATCTAT GGCATTAAAA CTATCATTCT GGTTGATAAA ACACCCACAG CCACAAGGGA   
  
  
+ AGAGTAAGTA TTTTATATTT CCCCTTCCAC ATAATCCATA TCCAGGTGTG TCTCCTGATG ATTAATGGAC   
  
  
+ ATCACCATCC TCTGGGTCCC ATGAGAGTGA ATGCCCCAAT CAGAACGGCT TGCTCTGGGC TGTAATAAAA   
  
  
+ TCCAGCTCGT GAATTGTGAG GGGCGTACAC GGCAACTTGA CCGCCTTTGC TTTTTTCTCG GTGGGTCCCT   
  
  
+ GTTGGGGCAA CATCGCTGTC ATCGTCTGTG ATTTTGATGT GGTAGGAAGC ATCCGATTTG TAACTGTTAA   
  
  
+ GAGTTTCTTA TGCAACGTCG TTATGGAGAG GTCCTGTACG CCCACTGGAG CATTTGATGC TGCAACAGCA   
  
  
+ AACCAAAACA AAAGGAGTCA GATTTTTGGA ATTTCTACTC TGTCGGCAGT TTAGCCCCCC AGTCTGTTAA   
  
  
+ TCTTGCAAGT TCCAGGATTC TTTTGGGTTG CCCAGAAGTC AATCCCCTCT TCATGCCTAT CGGTTCATCT   
  
  
+ CACCTGTTGA CATGACAACT ATCTGACCAA AAATAAAATT TTTATGCTTT ATCTTGCAGA CATTTTAAAA   
  
  
+ GGGTTTTCGT TCATTTAATA GAATTTTTCT TTTTTTTTAA ATGAATTTGA GGTTCCAAAT ATTTTAGGGA   
  
  
+ TCTTTAAGGG GGAGAAGAGG AGTAATGCAC ATGGTAAAGA GAGTGAAAGT TCTGACACTG TTAAGTGTAG   
  
  
+ GACTGTAGGT GGGTTCCGGT CTTGGAGGGC AACAAGTCAA TTGAAATGAG AAAGTTTCGT GCTAGAAATC   
  
  
+ ATTTGACATT TGCGTTGGGG CAGGGATTCC TGTTTTTCCT TATCTCATTG CTAGTTCATT TGGGTAGGGA   
  
  
+ TTAAATTGGC AGGAACTTAA GTTTGTCTTC CTTTGATTTC ATCGAAAGCT CCAAATCAGT CTTATCAGCA   
  
  
+ ATGGCAGCTT AGCCATTGTG TGATTAGTTT AGTTGCGAGA ATGGAATCTT TAGTGGGTGG GGGGGCACTT   
  
  
+ AGAAGCATGC CAGGTGGAGG GAAACTTCAA GGCTTACGGC CGTGAAACTG TATTTCGTCT TTAAAAAAAA   
  
  
+ AAAAAATATT AGCTCACAAG AATTCTTGAA AAAAAAAAAA AAAAAAGGGA AAGAGGATGT CGTTTTCCAT   
  
  
+ TTCTCTGCTC TGTGGACTTT TTGGGTTCCA CTGGTTTTCA GGGTAGCACG AAACTGTCAA AAAGCCGACT   
  
  
+ GATTTAGAGA GAGGAAAACA ACAATTAGGG AAGGCTGTAC AGAGACAAGC TTTATAATAT CTGTATTTGG   
  
  
+ AGGGAGAAAC AAAGAAGTTG ATAGCTCAA  

- AATTTTTTGA CTGACAAATA TAGTCAACAT AAAAGTATTT TAAACAGTAA GCTAAAATAC CCAAAAACAA   
  
  
- AATTCAATCT TAAATATAGA TATAAAAATG TAATATTGTA TTTTTAAGAC TAAAAAAATT TTTAATTTAC   
  
  
- ATTATTATCT TATATAGATA CCGTAATTTT GATAGTAAGA CCAACTATTT TGTGGGTGTC GGTGTTCCCT   
  
  
- TCTCATTCAT AAAATATAAA GGGGAAGGTG TATTAGGTAT AGGTCCACAC AGAGGACTAC TAATTACCTG   
  
  
- TAGTGGTAGG AGACCCAGGG TACTCTCACT TACGGGGTTA GTCTTGCCGA ACGAGACCCG ACATTATTTT   
  
  
- AGGTCGAGCA CTTAACACTC CCCGCATGTG CCGTTGAACT GGCGGAAACG AAAAAAGAGC CACCCAGGGA   
  
  
- CAACCCCGTT GTAGCGACAG TAGCAGACAC TAAAACTACA CCATCCTTCG TAGGCTAAAC ATTGACAATT   
  
  
- CTCAAAGAAT ACGTTGCAGC AATACCTCTC CAGGACATGC GGGTGACCTC GTAAACTACG ACGTTGTCGT   
  
  
- TTGGTTTTGT TTTCCTCAGT CTAAAAACCT TAAAGATGAG ACAGCCGTCA AATCGGGGGG TCAGACAATT   
  
  
- AGAACGTTCA AGGTCCTAAG AAAACCCAAC GGGTCTTCAG TTAGGGGAGA AGTACGGATA GCCAAGTAGA   
  
  
- GTGGACAACT GTACTGTTGA TAGACTGGTT TTTATTTTAA AAATACGAAA TAGAACGTCT GTAAAATTTT   
  
  
- CCCAAAAGCA AGTAAATTAT CTTAAAAAGA AAAAAAAATT TACTTAAACT CCAAGGTTTA TAAAATCCCT   
  
  
- AGAAATTCCC CCTCTTCTCC TCATTACGTG TACCATTTCT CTCACTTTCA AGACTGTGAC AATTCACATC   
  
  
- CTGACATCCA CCCAAGGCCA GAACCTCCCG TTGTTCAGTT AACTTTACTC TTTCAAAGCA CGATCTTTAG   
  
  
- TAAACTGTAA ACGCAACCCC GTCCCTAAGG ACAAAAAGGA ATAGAGTAAC GATCAAGTAA ACCCATCCCT   
  
  
- AATTTAACCG TCCTTGAATT CAAACAGAAG GAAACTAAAG TAGCTTTCGA GGTTTAGTCA GAATAGTCGT   
  
  
- TACCGTCGAA TCGGTAACAC ACTAATCAAA TCAACGCTCT TACCTTAGAA ATCACCCACC CCCCCGTGAA   
  
  
- TCTTCGTACG GTCCACCTCC CTTTGAAGTT CCGAATGCCG GCACTTTGAC ATAAAGCAGA AATTTTTTTT   
  
  
- TTTTTTATAA TCGAGTGTTC TTAAGAACTT TTTTTTTTTT TTTTTTCCCT TTCTCCTACA GCAAAAGGTA   
  
  
- AAGAGACGAG ACACCTGAAA AACCCAAGGT GACCAAAAGT CCCATCGTGC TTTGACAGTT TTTCGGCTGA   
  
  
- CTAAATCTCT CTCCTTTTGT TGTTAATCCC TTCCGACATG TCTCTGTTCG AAATATTATA GACATAAACC   
  
  
- TCCCTCTTTG TTTCTTCAAC TATCGAGTT

+     MBS

| Site Name | Organism | Position | Strand | Matrix score. | sequence | function |
| --- | --- | --- | --- | --- | --- | --- |
| MBS | Arabidopsis thaliana | 481 | + | 6 | TAACTG | MYB binding site involved in drought-inducibility |
| MBS | Zea mays | 388 | - | 6 | CGGTCA | MYB Binding Site |
| MBS | Arabidopsis thaliana | 23 | - | 6 | CAACTG | MYB binding site involved in drought-inducibility |

> 2018/04/13 10:10:12  
+ TTAAAAAACT GACTGTTTAT ATCAGTTGTA TTTTCATAAA ATTTGTCATT CGATTTTATG GGTTTTTGTT   
  
  
+ TTAAGTTAGA ATTTATATCT ATATTTTTAC ATTATAACAT AAAAATTCTG ATTTTTTTAA AAATTAAATG   
  
  
+ TAATAATAGA ATATATCTAT GGCATTAAAA CTATCATTCT GGTTGATAAA ACACCCACAG CCACAAGGGA   
  
  
+ AGAGTAAGTA TTTTATATTT CCCCTTCCAC ATAATCCATA TCCAGGTGTG TCTCCTGATG ATTAATGGAC   
  
  
+ ATCACCATCC TCTGGGTCCC ATGAGAGTGA ATGCCCCAAT CAGAACGGCT TGCTCTGGGC TGTAATAAAA   
  
  
+ TCCAGCTCGT GAATTGTGAG GGGCGTACAC GGCAACTTGA CCGCCTTTGC TTTTTTCTCG GTGGGTCCCT   
  
  
+ GTTGGGGCAA CATCGCTGTC ATCGTCTGTG ATTTTGATGT GGTAGGAAGC ATCCGATTTG TAACTGTTAA   
  
  
+ GAGTTTCTTA TGCAACGTCG TTATGGAGAG GTCCTGTACG CCCACTGGAG CATTTGATGC TGCAACAGCA   
  
  
+ AACCAAAACA AAAGGAGTCA GATTTTTGGA ATTTCTACTC TGTCGGCAGT TTAGCCCCCC AGTCTGTTAA   
  
  
+ TCTTGCAAGT TCCAGGATTC TTTTGGGTTG CCCAGAAGTC AATCCCCTCT TCATGCCTAT CGGTTCATCT   
  
  
+ CACCTGTTGA CATGACAACT ATCTGACCAA AAATAAAATT TTTATGCTTT ATCTTGCAGA CATTTTAAAA   
  
  
+ GGGTTTTCGT TCATTTAATA GAATTTTTCT TTTTTTTTAA ATGAATTTGA GGTTCCAAAT ATTTTAGGGA   
  
  
+ TCTTTAAGGG GGAGAAGAGG AGTAATGCAC ATGGTAAAGA GAGTGAAAGT TCTGACACTG TTAAGTGTAG   
  
  
+ GACTGTAGGT GGGTTCCGGT CTTGGAGGGC AACAAGTCAA TTGAAATGAG AAAGTTTCGT GCTAGAAATC   
  
  
+ ATTTGACATT TGCGTTGGGG CAGGGATTCC TGTTTTTCCT TATCTCATTG CTAGTTCATT TGGGTAGGGA   
  
  
+ TTAAATTGGC AGGAACTTAA GTTTGTCTTC CTTTGATTTC ATCGAAAGCT CCAAATCAGT CTTATCAGCA   
  
  
+ ATGGCAGCTT AGCCATTGTG TGATTAGTTT AGTTGCGAGA ATGGAATCTT TAGTGGGTGG GGGGGCACTT   
  
  
+ AGAAGCATGC CAGGTGGAGG GAAACTTCAA GGCTTACGGC CGTGAAACTG TATTTCGTCT TTAAAAAAAA   
  
  
+ AAAAAATATT AGCTCACAAG AATTCTTGAA AAAAAAAAAA AAAAAAGGGA AAGAGGATGT CGTTTTCCAT   
  
  
+ TTCTCTGCTC TGTGGACTTT TTGGGTTCCA CTGGTTTTCA GGGTAGCACG AAACTGTCAA AAAGCCGACT   
  
  
+ GATTTAGAGA GAGGAAAACA ACAATTAGGG AAGGCTGTAC AGAGACAAGC TTTATAATAT CTGTATTTGG   
  
  
+ AGGGAGAAAC AAAGAAGTTG ATAGCTCAA  

- AATTTTTTGA CTGACAAATA TAGTCAACAT AAAAGTATTT TAAACAGTAA GCTAAAATAC CCAAAAACAA   
  
  
- AATTCAATCT TAAATATAGA TATAAAAATG TAATATTGTA TTTTTAAGAC TAAAAAAATT TTTAATTTAC   
  
  
- ATTATTATCT TATATAGATA CCGTAATTTT GATAGTAAGA CCAACTATTT TGTGGGTGTC GGTGTTCCCT   
  
  
- TCTCATTCAT AAAATATAAA GGGGAAGGTG TATTAGGTAT AGGTCCACAC AGAGGACTAC TAATTACCTG   
  
  
- TAGTGGTAGG AGACCCAGGG TACTCTCACT TACGGGGTTA GTCTTGCCGA ACGAGACCCG ACATTATTTT   
  
  
- AGGTCGAGCA CTTAACACTC CCCGCATGTG CCGTTGAACT GGCGGAAACG AAAAAAGAGC CACCCAGGGA   
  
  
- CAACCCCGTT GTAGCGACAG TAGCAGACAC TAAAACTACA CCATCCTTCG TAGGCTAAAC ATTGACAATT   
  
  
- CTCAAAGAAT ACGTTGCAGC AATACCTCTC CAGGACATGC GGGTGACCTC GTAAACTACG ACGTTGTCGT   
  
  
- TTGGTTTTGT TTTCCTCAGT CTAAAAACCT TAAAGATGAG ACAGCCGTCA AATCGGGGGG TCAGACAATT   
  
  
- AGAACGTTCA AGGTCCTAAG AAAACCCAAC GGGTCTTCAG TTAGGGGAGA AGTACGGATA GCCAAGTAGA   
  
  
- GTGGACAACT GTACTGTTGA TAGACTGGTT TTTATTTTAA AAATACGAAA TAGAACGTCT GTAAAATTTT   
  
  
- CCCAAAAGCA AGTAAATTAT CTTAAAAAGA AAAAAAAATT TACTTAAACT CCAAGGTTTA TAAAATCCCT   
  
  
- AGAAATTCCC CCTCTTCTCC TCATTACGTG TACCATTTCT CTCACTTTCA AGACTGTGAC AATTCACATC   
  
  
- CTGACATCCA CCCAAGGCCA GAACCTCCCG TTGTTCAGTT AACTTTACTC TTTCAAAGCA CGATCTTTAG   
  
  
- TAAACTGTAA ACGCAACCCC GTCCCTAAGG ACAAAAAGGA ATAGAGTAAC GATCAAGTAA ACCCATCCCT   
  
  
- AATTTAACCG TCCTTGAATT CAAACAGAAG GAAACTAAAG TAGCTTTCGA GGTTTAGTCA GAATAGTCGT   
  
  
- TACCGTCGAA TCGGTAACAC ACTAATCAAA TCAACGCTCT TACCTTAGAA ATCACCCACC CCCCCGTGAA   
  
  
- TCTTCGTACG GTCCACCTCC CTTTGAAGTT CCGAATGCCG GCACTTTGAC ATAAAGCAGA AATTTTTTTT   
  
  
- TTTTTTATAA TCGAGTGTTC TTAAGAACTT TTTTTTTTTT TTTTTTCCCT TTCTCCTACA GCAAAAGGTA   
  
  
- AAGAGACGAG ACACCTGAAA AACCCAAGGT GACCAAAAGT CCCATCGTGC TTTGACAGTT TTTCGGCTGA   
  
  
- CTAAATCTCT CTCCTTTTGT TGTTAATCCC TTCCGACATG TCTCTGTTCG AAATATTATA GACATAAACC   
  
  
- TCCCTCTTTG TTTCTTCAAC TATCGAGTT

+     O2-site

| Site Name | Organism | Position | Strand | Matrix score. | sequence | function |
| --- | --- | --- | --- | --- | --- | --- |
| O2-site | Zea mays | 706 | + | 9 | GTTGACGTGA | cis-acting regulatory element involved in zein metabolism regulation |
| O2-site | Zea mays | 237 | - | 9 | GATGATGTGG | cis-acting regulatory element involved in zein metabolism regulation |

> 2018/04/13 10:10:12  
+ TTAAAAAACT GACTGTTTAT ATCAGTTGTA TTTTCATAAA ATTTGTCATT CGATTTTATG GGTTTTTGTT   
  
  
+ TTAAGTTAGA ATTTATATCT ATATTTTTAC ATTATAACAT AAAAATTCTG ATTTTTTTAA AAATTAAATG   
  
  
+ TAATAATAGA ATATATCTAT GGCATTAAAA CTATCATTCT GGTTGATAAA ACACCCACAG CCACAAGGGA   
  
  
+ AGAGTAAGTA TTTTATATTT CCCCTTCCAC ATAATCCATA TCCAGGTGTG TCTCCTGATG ATTAATGGAC   
  
  
+ ATCACCATCC TCTGGGTCCC ATGAGAGTGA ATGCCCCAAT CAGAACGGCT TGCTCTGGGC TGTAATAAAA   
  
  
+ TCCAGCTCGT GAATTGTGAG GGGCGTACAC GGCAACTTGA CCGCCTTTGC TTTTTTCTCG GTGGGTCCCT   
  
  
+ GTTGGGGCAA CATCGCTGTC ATCGTCTGTG ATTTTGATGT GGTAGGAAGC ATCCGATTTG TAACTGTTAA   
  
  
+ GAGTTTCTTA TGCAACGTCG TTATGGAGAG GTCCTGTACG CCCACTGGAG CATTTGATGC TGCAACAGCA   
  
  
+ AACCAAAACA AAAGGAGTCA GATTTTTGGA ATTTCTACTC TGTCGGCAGT TTAGCCCCCC AGTCTGTTAA   
  
  
+ TCTTGCAAGT TCCAGGATTC TTTTGGGTTG CCCAGAAGTC AATCCCCTCT TCATGCCTAT CGGTTCATCT   
  
  
+ CACCTGTTGA CATGACAACT ATCTGACCAA AAATAAAATT TTTATGCTTT ATCTTGCAGA CATTTTAAAA   
  
  
+ GGGTTTTCGT TCATTTAATA GAATTTTTCT TTTTTTTTAA ATGAATTTGA GGTTCCAAAT ATTTTAGGGA   
  
  
+ TCTTTAAGGG GGAGAAGAGG AGTAATGCAC ATGGTAAAGA GAGTGAAAGT TCTGACACTG TTAAGTGTAG   
  
  
+ GACTGTAGGT GGGTTCCGGT CTTGGAGGGC AACAAGTCAA TTGAAATGAG AAAGTTTCGT GCTAGAAATC   
  
  
+ ATTTGACATT TGCGTTGGGG CAGGGATTCC TGTTTTTCCT TATCTCATTG CTAGTTCATT TGGGTAGGGA   
  
  
+ TTAAATTGGC AGGAACTTAA GTTTGTCTTC CTTTGATTTC ATCGAAAGCT CCAAATCAGT CTTATCAGCA   
  
  
+ ATGGCAGCTT AGCCATTGTG TGATTAGTTT AGTTGCGAGA ATGGAATCTT TAGTGGGTGG GGGGGCACTT   
  
  
+ AGAAGCATGC CAGGTGGAGG GAAACTTCAA GGCTTACGGC CGTGAAACTG TATTTCGTCT TTAAAAAAAA   
  
  
+ AAAAAATATT AGCTCACAAG AATTCTTGAA AAAAAAAAAA AAAAAAGGGA AAGAGGATGT CGTTTTCCAT   
  
  
+ TTCTCTGCTC TGTGGACTTT TTGGGTTCCA CTGGTTTTCA GGGTAGCACG AAACTGTCAA AAAGCCGACT   
  
  
+ GATTTAGAGA GAGGAAAACA ACAATTAGGG AAGGCTGTAC AGAGACAAGC TTTATAATAT CTGTATTTGG   
  
  
+ AGGGAGAAAC AAAGAAGTTG ATAGCTCAA  

- AATTTTTTGA CTGACAAATA TAGTCAACAT AAAAGTATTT TAAACAGTAA GCTAAAATAC CCAAAAACAA   
  
  
- AATTCAATCT TAAATATAGA TATAAAAATG TAATATTGTA TTTTTAAGAC TAAAAAAATT TTTAATTTAC   
  
  
- ATTATTATCT TATATAGATA CCGTAATTTT GATAGTAAGA CCAACTATTT TGTGGGTGTC GGTGTTCCCT   
  
  
- TCTCATTCAT AAAATATAAA GGGGAAGGTG TATTAGGTAT AGGTCCACAC AGAGGACTAC TAATTACCTG   
  
  
- TAGTGGTAGG AGACCCAGGG TACTCTCACT TACGGGGTTA GTCTTGCCGA ACGAGACCCG ACATTATTTT   
  
  
- AGGTCGAGCA CTTAACACTC CCCGCATGTG CCGTTGAACT GGCGGAAACG AAAAAAGAGC CACCCAGGGA   
  
  
- CAACCCCGTT GTAGCGACAG TAGCAGACAC TAAAACTACA CCATCCTTCG TAGGCTAAAC ATTGACAATT   
  
  
- CTCAAAGAAT ACGTTGCAGC AATACCTCTC CAGGACATGC GGGTGACCTC GTAAACTACG ACGTTGTCGT   
  
  
- TTGGTTTTGT TTTCCTCAGT CTAAAAACCT TAAAGATGAG ACAGCCGTCA AATCGGGGGG TCAGACAATT   
  
  
- AGAACGTTCA AGGTCCTAAG AAAACCCAAC GGGTCTTCAG TTAGGGGAGA AGTACGGATA GCCAAGTAGA   
  
  
- GTGGACAACT GTACTGTTGA TAGACTGGTT TTTATTTTAA AAATACGAAA TAGAACGTCT GTAAAATTTT   
  
  
- CCCAAAAGCA AGTAAATTAT CTTAAAAAGA AAAAAAAATT TACTTAAACT CCAAGGTTTA TAAAATCCCT   
  
  
- AGAAATTCCC CCTCTTCTCC TCATTACGTG TACCATTTCT CTCACTTTCA AGACTGTGAC AATTCACATC   
  
  
- CTGACATCCA CCCAAGGCCA GAACCTCCCG TTGTTCAGTT AACTTTACTC TTTCAAAGCA CGATCTTTAG   
  
  
- TAAACTGTAA ACGCAACCCC GTCCCTAAGG ACAAAAAGGA ATAGAGTAAC GATCAAGTAA ACCCATCCCT   
  
  
- AATTTAACCG TCCTTGAATT CAAACAGAAG GAAACTAAAG TAGCTTTCGA GGTTTAGTCA GAATAGTCGT   
  
  
- TACCGTCGAA TCGGTAACAC ACTAATCAAA TCAACGCTCT TACCTTAGAA ATCACCCACC CCCCCGTGAA   
  
  
- TCTTCGTACG GTCCACCTCC CTTTGAAGTT CCGAATGCCG GCACTTTGAC ATAAAGCAGA AATTTTTTTT   
  
  
- TTTTTTATAA TCGAGTGTTC TTAAGAACTT TTTTTTTTTT TTTTTTCCCT TTCTCCTACA GCAAAAGGTA   
  
  
- AAGAGACGAG ACACCTGAAA AACCCAAGGT GACCAAAAGT CCCATCGTGC TTTGACAGTT TTTCGGCTGA   
  
  
- CTAAATCTCT CTCCTTTTGT TGTTAATCCC TTCCGACATG TCTCTGTTCG AAATATTATA GACATAAACC   
  
  
- TCCCTCTTTG TTTCTTCAAC TATCGAGTT

+     P-box

| Site Name | Organism | Position | Strand | Matrix score. | sequence | function |
| --- | --- | --- | --- | --- | --- | --- |
| P-box | Oryza sativa | 569 | - | 7 | CCTTTTG | gibberellin-responsive element |

> 2018/04/13 10:10:12  
+ TTAAAAAACT GACTGTTTAT ATCAGTTGTA TTTTCATAAA ATTTGTCATT CGATTTTATG GGTTTTTGTT   
  
  
+ TTAAGTTAGA ATTTATATCT ATATTTTTAC ATTATAACAT AAAAATTCTG ATTTTTTTAA AAATTAAATG   
  
  
+ TAATAATAGA ATATATCTAT GGCATTAAAA CTATCATTCT GGTTGATAAA ACACCCACAG CCACAAGGGA   
  
  
+ AGAGTAAGTA TTTTATATTT CCCCTTCCAC ATAATCCATA TCCAGGTGTG TCTCCTGATG ATTAATGGAC   
  
  
+ ATCACCATCC TCTGGGTCCC ATGAGAGTGA ATGCCCCAAT CAGAACGGCT TGCTCTGGGC TGTAATAAAA   
  
  
+ TCCAGCTCGT GAATTGTGAG GGGCGTACAC GGCAACTTGA CCGCCTTTGC TTTTTTCTCG GTGGGTCCCT   
  
  
+ GTTGGGGCAA CATCGCTGTC ATCGTCTGTG ATTTTGATGT GGTAGGAAGC ATCCGATTTG TAACTGTTAA   
  
  
+ GAGTTTCTTA TGCAACGTCG TTATGGAGAG GTCCTGTACG CCCACTGGAG CATTTGATGC TGCAACAGCA   
  
  
+ AACCAAAACA AAAGGAGTCA GATTTTTGGA ATTTCTACTC TGTCGGCAGT TTAGCCCCCC AGTCTGTTAA   
  
  
+ TCTTGCAAGT TCCAGGATTC TTTTGGGTTG CCCAGAAGTC AATCCCCTCT TCATGCCTAT CGGTTCATCT   
  
  
+ CACCTGTTGA CATGACAACT ATCTGACCAA AAATAAAATT TTTATGCTTT ATCTTGCAGA CATTTTAAAA   
  
  
+ GGGTTTTCGT TCATTTAATA GAATTTTTCT TTTTTTTTAA ATGAATTTGA GGTTCCAAAT ATTTTAGGGA   
  
  
+ TCTTTAAGGG GGAGAAGAGG AGTAATGCAC ATGGTAAAGA GAGTGAAAGT TCTGACACTG TTAAGTGTAG   
  
  
+ GACTGTAGGT GGGTTCCGGT CTTGGAGGGC AACAAGTCAA TTGAAATGAG AAAGTTTCGT GCTAGAAATC   
  
  
+ ATTTGACATT TGCGTTGGGG CAGGGATTCC TGTTTTTCCT TATCTCATTG CTAGTTCATT TGGGTAGGGA   
  
  
+ TTAAATTGGC AGGAACTTAA GTTTGTCTTC CTTTGATTTC ATCGAAAGCT CCAAATCAGT CTTATCAGCA   
  
  
+ ATGGCAGCTT AGCCATTGTG TGATTAGTTT AGTTGCGAGA ATGGAATCTT TAGTGGGTGG GGGGGCACTT   
  
  
+ AGAAGCATGC CAGGTGGAGG GAAACTTCAA GGCTTACGGC CGTGAAACTG TATTTCGTCT TTAAAAAAAA   
  
  
+ AAAAAATATT AGCTCACAAG AATTCTTGAA AAAAAAAAAA AAAAAAGGGA AAGAGGATGT CGTTTTCCAT   
  
  
+ TTCTCTGCTC TGTGGACTTT TTGGGTTCCA CTGGTTTTCA GGGTAGCACG AAACTGTCAA AAAGCCGACT   
  
  
+ GATTTAGAGA GAGGAAAACA ACAATTAGGG AAGGCTGTAC AGAGACAAGC TTTATAATAT CTGTATTTGG   
  
  
+ AGGGAGAAAC AAAGAAGTTG ATAGCTCAA  

- AATTTTTTGA CTGACAAATA TAGTCAACAT AAAAGTATTT TAAACAGTAA GCTAAAATAC CCAAAAACAA   
  
  
- AATTCAATCT TAAATATAGA TATAAAAATG TAATATTGTA TTTTTAAGAC TAAAAAAATT TTTAATTTAC   
  
  
- ATTATTATCT TATATAGATA CCGTAATTTT GATAGTAAGA CCAACTATTT TGTGGGTGTC GGTGTTCCCT   
  
  
- TCTCATTCAT AAAATATAAA GGGGAAGGTG TATTAGGTAT AGGTCCACAC AGAGGACTAC TAATTACCTG   
  
  
- TAGTGGTAGG AGACCCAGGG TACTCTCACT TACGGGGTTA GTCTTGCCGA ACGAGACCCG ACATTATTTT   
  
  
- AGGTCGAGCA CTTAACACTC CCCGCATGTG CCGTTGAACT GGCGGAAACG AAAAAAGAGC CACCCAGGGA   
  
  
- CAACCCCGTT GTAGCGACAG TAGCAGACAC TAAAACTACA CCATCCTTCG TAGGCTAAAC ATTGACAATT   
  
  
- CTCAAAGAAT ACGTTGCAGC AATACCTCTC CAGGACATGC GGGTGACCTC GTAAACTACG ACGTTGTCGT   
  
  
- TTGGTTTTGT TTTCCTCAGT CTAAAAACCT TAAAGATGAG ACAGCCGTCA AATCGGGGGG TCAGACAATT   
  
  
- AGAACGTTCA AGGTCCTAAG AAAACCCAAC GGGTCTTCAG TTAGGGGAGA AGTACGGATA GCCAAGTAGA   
  
  
- GTGGACAACT GTACTGTTGA TAGACTGGTT TTTATTTTAA AAATACGAAA TAGAACGTCT GTAAAATTTT   
  
  
- CCCAAAAGCA AGTAAATTAT CTTAAAAAGA AAAAAAAATT TACTTAAACT CCAAGGTTTA TAAAATCCCT   
  
  
- AGAAATTCCC CCTCTTCTCC TCATTACGTG TACCATTTCT CTCACTTTCA AGACTGTGAC AATTCACATC   
  
  
- CTGACATCCA CCCAAGGCCA GAACCTCCCG TTGTTCAGTT AACTTTACTC TTTCAAAGCA CGATCTTTAG   
  
  
- TAAACTGTAA ACGCAACCCC GTCCCTAAGG ACAAAAAGGA ATAGAGTAAC GATCAAGTAA ACCCATCCCT   
  
  
- AATTTAACCG TCCTTGAATT CAAACAGAAG GAAACTAAAG TAGCTTTCGA GGTTTAGTCA GAATAGTCGT   
  
  
- TACCGTCGAA TCGGTAACAC ACTAATCAAA TCAACGCTCT TACCTTAGAA ATCACCCACC CCCCCGTGAA   
  
  
- TCTTCGTACG GTCCACCTCC CTTTGAAGTT CCGAATGCCG GCACTTTGAC ATAAAGCAGA AATTTTTTTT   
  
  
- TTTTTTATAA TCGAGTGTTC TTAAGAACTT TTTTTTTTTT TTTTTTCCCT TTCTCCTACA GCAAAAGGTA   
  
  
- AAGAGACGAG ACACCTGAAA AACCCAAGGT GACCAAAAGT CCCATCGTGC TTTGACAGTT TTTCGGCTGA   
  
  
- CTAAATCTCT CTCCTTTTGT TGTTAATCCC TTCCGACATG TCTCTGTTCG AAATATTATA GACATAAACC   
  
  
- TCCCTCTTTG TTTCTTCAAC TATCGAGTT

+     Skn-1\_motif

| Site Name | Organism | Position | Strand | Matrix score. | sequence | function |
| --- | --- | --- | --- | --- | --- | --- |
| Skn-1\_motif | Oryza sativa | 438 | + | 5 | GTCAT | cis-acting regulatory element required for endosperm expression |
| Skn-1\_motif | Oryza sativa | 712 | - | 5 | GTCAT | cis-acting regulatory element required for endosperm expression |
| Skn-1\_motif | Oryza sativa | 45 | + | 5 | GTCAT | cis-acting regulatory element required for endosperm expression |

> 2018/04/13 10:10:12  
+ TTAAAAAACT GACTGTTTAT ATCAGTTGTA TTTTCATAAA ATTTGTCATT CGATTTTATG GGTTTTTGTT   
  
  
+ TTAAGTTAGA ATTTATATCT ATATTTTTAC ATTATAACAT AAAAATTCTG ATTTTTTTAA AAATTAAATG   
  
  
+ TAATAATAGA ATATATCTAT GGCATTAAAA CTATCATTCT GGTTGATAAA ACACCCACAG CCACAAGGGA   
  
  
+ AGAGTAAGTA TTTTATATTT CCCCTTCCAC ATAATCCATA TCCAGGTGTG TCTCCTGATG ATTAATGGAC   
  
  
+ ATCACCATCC TCTGGGTCCC ATGAGAGTGA ATGCCCCAAT CAGAACGGCT TGCTCTGGGC TGTAATAAAA   
  
  
+ TCCAGCTCGT GAATTGTGAG GGGCGTACAC GGCAACTTGA CCGCCTTTGC TTTTTTCTCG GTGGGTCCCT   
  
  
+ GTTGGGGCAA CATCGCTGTC ATCGTCTGTG ATTTTGATGT GGTAGGAAGC ATCCGATTTG TAACTGTTAA   
  
  
+ GAGTTTCTTA TGCAACGTCG TTATGGAGAG GTCCTGTACG CCCACTGGAG CATTTGATGC TGCAACAGCA   
  
  
+ AACCAAAACA AAAGGAGTCA GATTTTTGGA ATTTCTACTC TGTCGGCAGT TTAGCCCCCC AGTCTGTTAA   
  
  
+ TCTTGCAAGT TCCAGGATTC TTTTGGGTTG CCCAGAAGTC AATCCCCTCT TCATGCCTAT CGGTTCATCT   
  
  
+ CACCTGTTGA CATGACAACT ATCTGACCAA AAATAAAATT TTTATGCTTT ATCTTGCAGA CATTTTAAAA   
  
  
+ GGGTTTTCGT TCATTTAATA GAATTTTTCT TTTTTTTTAA ATGAATTTGA GGTTCCAAAT ATTTTAGGGA   
  
  
+ TCTTTAAGGG GGAGAAGAGG AGTAATGCAC ATGGTAAAGA GAGTGAAAGT TCTGACACTG TTAAGTGTAG   
  
  
+ GACTGTAGGT GGGTTCCGGT CTTGGAGGGC AACAAGTCAA TTGAAATGAG AAAGTTTCGT GCTAGAAATC   
  
  
+ ATTTGACATT TGCGTTGGGG CAGGGATTCC TGTTTTTCCT TATCTCATTG CTAGTTCATT TGGGTAGGGA   
  
  
+ TTAAATTGGC AGGAACTTAA GTTTGTCTTC CTTTGATTTC ATCGAAAGCT CCAAATCAGT CTTATCAGCA   
  
  
+ ATGGCAGCTT AGCCATTGTG TGATTAGTTT AGTTGCGAGA ATGGAATCTT TAGTGGGTGG GGGGGCACTT   
  
  
+ AGAAGCATGC CAGGTGGAGG GAAACTTCAA GGCTTACGGC CGTGAAACTG TATTTCGTCT TTAAAAAAAA   
  
  
+ AAAAAATATT AGCTCACAAG AATTCTTGAA AAAAAAAAAA AAAAAAGGGA AAGAGGATGT CGTTTTCCAT   
  
  
+ TTCTCTGCTC TGTGGACTTT TTGGGTTCCA CTGGTTTTCA GGGTAGCACG AAACTGTCAA AAAGCCGACT   
  
  
+ GATTTAGAGA GAGGAAAACA ACAATTAGGG AAGGCTGTAC AGAGACAAGC TTTATAATAT CTGTATTTGG   
  
  
+ AGGGAGAAAC AAAGAAGTTG ATAGCTCAA  

- AATTTTTTGA CTGACAAATA TAGTCAACAT AAAAGTATTT TAAACAGTAA GCTAAAATAC CCAAAAACAA   
  
  
- AATTCAATCT TAAATATAGA TATAAAAATG TAATATTGTA TTTTTAAGAC TAAAAAAATT TTTAATTTAC   
  
  
- ATTATTATCT TATATAGATA CCGTAATTTT GATAGTAAGA CCAACTATTT TGTGGGTGTC GGTGTTCCCT   
  
  
- TCTCATTCAT AAAATATAAA GGGGAAGGTG TATTAGGTAT AGGTCCACAC AGAGGACTAC TAATTACCTG   
  
  
- TAGTGGTAGG AGACCCAGGG TACTCTCACT TACGGGGTTA GTCTTGCCGA ACGAGACCCG ACATTATTTT   
  
  
- AGGTCGAGCA CTTAACACTC CCCGCATGTG CCGTTGAACT GGCGGAAACG AAAAAAGAGC CACCCAGGGA   
  
  
- CAACCCCGTT GTAGCGACAG TAGCAGACAC TAAAACTACA CCATCCTTCG TAGGCTAAAC ATTGACAATT   
  
  
- CTCAAAGAAT ACGTTGCAGC AATACCTCTC CAGGACATGC GGGTGACCTC GTAAACTACG ACGTTGTCGT   
  
  
- TTGGTTTTGT TTTCCTCAGT CTAAAAACCT TAAAGATGAG ACAGCCGTCA AATCGGGGGG TCAGACAATT   
  
  
- AGAACGTTCA AGGTCCTAAG AAAACCCAAC GGGTCTTCAG TTAGGGGAGA AGTACGGATA GCCAAGTAGA   
  
  
- GTGGACAACT GTACTGTTGA TAGACTGGTT TTTATTTTAA AAATACGAAA TAGAACGTCT GTAAAATTTT   
  
  
- CCCAAAAGCA AGTAAATTAT CTTAAAAAGA AAAAAAAATT TACTTAAACT CCAAGGTTTA TAAAATCCCT   
  
  
- AGAAATTCCC CCTCTTCTCC TCATTACGTG TACCATTTCT CTCACTTTCA AGACTGTGAC AATTCACATC   
  
  
- CTGACATCCA CCCAAGGCCA GAACCTCCCG TTGTTCAGTT AACTTTACTC TTTCAAAGCA CGATCTTTAG   
  
  
- TAAACTGTAA ACGCAACCCC GTCCCTAAGG ACAAAAAGGA ATAGAGTAAC GATCAAGTAA ACCCATCCCT   
  
  
- AATTTAACCG TCCTTGAATT CAAACAGAAG GAAACTAAAG TAGCTTTCGA GGTTTAGTCA GAATAGTCGT   
  
  
- TACCGTCGAA TCGGTAACAC ACTAATCAAA TCAACGCTCT TACCTTAGAA ATCACCCACC CCCCCGTGAA   
  
  
- TCTTCGTACG GTCCACCTCC CTTTGAAGTT CCGAATGCCG GCACTTTGAC ATAAAGCAGA AATTTTTTTT   
  
  
- TTTTTTATAA TCGAGTGTTC TTAAGAACTT TTTTTTTTTT TTTTTTCCCT TTCTCCTACA GCAAAAGGTA   
  
  
- AAGAGACGAG ACACCTGAAA AACCCAAGGT GACCAAAAGT CCCATCGTGC TTTGACAGTT TTTCGGCTGA   
  
  
- CTAAATCTCT CTCCTTTTGT TGTTAATCCC TTCCGACATG TCTCTGTTCG AAATATTATA GACATAAACC   
  
  
- TCCCTCTTTG TTTCTTCAAC TATCGAGTT

+     Sp1

| Site Name | Organism | Position | Strand | Matrix score. | sequence | function |
| --- | --- | --- | --- | --- | --- | --- |
| Sp1 | Zea mays | 1179 | - | 5 | CC(G/A)CCC | light responsive element |
| Sp1 | Zea mays | 1180 | - | 5 | CC(G/A)CCC | light responsive element |
| Sp1 | Zea mays | 1175 | - | 5.5 | CC(G/A)CCC | light responsive element |
| Sp1 | Zea mays | 615 | + | 5 | CC(G/A)CCC | light responsive element |

> 2018/04/13 10:10:12  
+ TTAAAAAACT GACTGTTTAT ATCAGTTGTA TTTTCATAAA ATTTGTCATT CGATTTTATG GGTTTTTGTT   
  
  
+ TTAAGTTAGA ATTTATATCT ATATTTTTAC ATTATAACAT AAAAATTCTG ATTTTTTTAA AAATTAAATG   
  
  
+ TAATAATAGA ATATATCTAT GGCATTAAAA CTATCATTCT GGTTGATAAA ACACCCACAG CCACAAGGGA   
  
  
+ AGAGTAAGTA TTTTATATTT CCCCTTCCAC ATAATCCATA TCCAGGTGTG TCTCCTGATG ATTAATGGAC   
  
  
+ ATCACCATCC TCTGGGTCCC ATGAGAGTGA ATGCCCCAAT CAGAACGGCT TGCTCTGGGC TGTAATAAAA   
  
  
+ TCCAGCTCGT GAATTGTGAG GGGCGTACAC GGCAACTTGA CCGCCTTTGC TTTTTTCTCG GTGGGTCCCT   
  
  
+ GTTGGGGCAA CATCGCTGTC ATCGTCTGTG ATTTTGATGT GGTAGGAAGC ATCCGATTTG TAACTGTTAA   
  
  
+ GAGTTTCTTA TGCAACGTCG TTATGGAGAG GTCCTGTACG CCCACTGGAG CATTTGATGC TGCAACAGCA   
  
  
+ AACCAAAACA AAAGGAGTCA GATTTTTGGA ATTTCTACTC TGTCGGCAGT TTAGCCCCCC AGTCTGTTAA   
  
  
+ TCTTGCAAGT TCCAGGATTC TTTTGGGTTG CCCAGAAGTC AATCCCCTCT TCATGCCTAT CGGTTCATCT   
  
  
+ CACCTGTTGA CATGACAACT ATCTGACCAA AAATAAAATT TTTATGCTTT ATCTTGCAGA CATTTTAAAA   
  
  
+ GGGTTTTCGT TCATTTAATA GAATTTTTCT TTTTTTTTAA ATGAATTTGA GGTTCCAAAT ATTTTAGGGA   
  
  
+ TCTTTAAGGG GGAGAAGAGG AGTAATGCAC ATGGTAAAGA GAGTGAAAGT TCTGACACTG TTAAGTGTAG   
  
  
+ GACTGTAGGT GGGTTCCGGT CTTGGAGGGC AACAAGTCAA TTGAAATGAG AAAGTTTCGT GCTAGAAATC   
  
  
+ ATTTGACATT TGCGTTGGGG CAGGGATTCC TGTTTTTCCT TATCTCATTG CTAGTTCATT TGGGTAGGGA   
  
  
+ TTAAATTGGC AGGAACTTAA GTTTGTCTTC CTTTGATTTC ATCGAAAGCT CCAAATCAGT CTTATCAGCA   
  
  
+ ATGGCAGCTT AGCCATTGTG TGATTAGTTT AGTTGCGAGA ATGGAATCTT TAGTGGGTGG GGGGGCACTT   
  
  
+ AGAAGCATGC CAGGTGGAGG GAAACTTCAA GGCTTACGGC CGTGAAACTG TATTTCGTCT TTAAAAAAAA   
  
  
+ AAAAAATATT AGCTCACAAG AATTCTTGAA AAAAAAAAAA AAAAAAGGGA AAGAGGATGT CGTTTTCCAT   
  
  
+ TTCTCTGCTC TGTGGACTTT TTGGGTTCCA CTGGTTTTCA GGGTAGCACG AAACTGTCAA AAAGCCGACT   
  
  
+ GATTTAGAGA GAGGAAAACA ACAATTAGGG AAGGCTGTAC AGAGACAAGC TTTATAATAT CTGTATTTGG   
  
  
+ AGGGAGAAAC AAAGAAGTTG ATAGCTCAA  

- AATTTTTTGA CTGACAAATA TAGTCAACAT AAAAGTATTT TAAACAGTAA GCTAAAATAC CCAAAAACAA   
  
  
- AATTCAATCT TAAATATAGA TATAAAAATG TAATATTGTA TTTTTAAGAC TAAAAAAATT TTTAATTTAC   
  
  
- ATTATTATCT TATATAGATA CCGTAATTTT GATAGTAAGA CCAACTATTT TGTGGGTGTC GGTGTTCCCT   
  
  
- TCTCATTCAT AAAATATAAA GGGGAAGGTG TATTAGGTAT AGGTCCACAC AGAGGACTAC TAATTACCTG   
  
  
- TAGTGGTAGG AGACCCAGGG TACTCTCACT TACGGGGTTA GTCTTGCCGA ACGAGACCCG ACATTATTTT   
  
  
- AGGTCGAGCA CTTAACACTC CCCGCATGTG CCGTTGAACT GGCGGAAACG AAAAAAGAGC CACCCAGGGA   
  
  
- CAACCCCGTT GTAGCGACAG TAGCAGACAC TAAAACTACA CCATCCTTCG TAGGCTAAAC ATTGACAATT   
  
  
- CTCAAAGAAT ACGTTGCAGC AATACCTCTC CAGGACATGC GGGTGACCTC GTAAACTACG ACGTTGTCGT   
  
  
- TTGGTTTTGT TTTCCTCAGT CTAAAAACCT TAAAGATGAG ACAGCCGTCA AATCGGGGGG TCAGACAATT   
  
  
- AGAACGTTCA AGGTCCTAAG AAAACCCAAC GGGTCTTCAG TTAGGGGAGA AGTACGGATA GCCAAGTAGA   
  
  
- GTGGACAACT GTACTGTTGA TAGACTGGTT TTTATTTTAA AAATACGAAA TAGAACGTCT GTAAAATTTT   
  
  
- CCCAAAAGCA AGTAAATTAT CTTAAAAAGA AAAAAAAATT TACTTAAACT CCAAGGTTTA TAAAATCCCT   
  
  
- AGAAATTCCC CCTCTTCTCC TCATTACGTG TACCATTTCT CTCACTTTCA AGACTGTGAC AATTCACATC   
  
  
- CTGACATCCA CCCAAGGCCA GAACCTCCCG TTGTTCAGTT AACTTTACTC TTTCAAAGCA CGATCTTTAG   
  
  
- TAAACTGTAA ACGCAACCCC GTCCCTAAGG ACAAAAAGGA ATAGAGTAAC GATCAAGTAA ACCCATCCCT   
  
  
- AATTTAACCG TCCTTGAATT CAAACAGAAG GAAACTAAAG TAGCTTTCGA GGTTTAGTCA GAATAGTCGT   
  
  
- TACCGTCGAA TCGGTAACAC ACTAATCAAA TCAACGCTCT TACCTTAGAA ATCACCCACC CCCCCGTGAA   
  
  
- TCTTCGTACG GTCCACCTCC CTTTGAAGTT CCGAATGCCG GCACTTTGAC ATAAAGCAGA AATTTTTTTT   
  
  
- TTTTTTATAA TCGAGTGTTC TTAAGAACTT TTTTTTTTTT TTTTTTCCCT TTCTCCTACA GCAAAAGGTA   
  
  
- AAGAGACGAG ACACCTGAAA AACCCAAGGT GACCAAAAGT CCCATCGTGC TTTGACAGTT TTTCGGCTGA   
  
  
- CTAAATCTCT CTCCTTTTGT TGTTAATCCC TTCCGACATG TCTCTGTTCG AAATATTATA GACATAAACC   
  
  
- TCCCTCTTTG TTTCTTCAAC TATCGAGTT

+     TATA-box

| Site Name | Organism | Position | Strand | Matrix score. | sequence | function |
| --- | --- | --- | --- | --- | --- | --- |
| TATA-box | Arabidopsis thaliana | 1452 | - | 5 | TATAA | core promoter element around -30 of transcription start |
| TATA-box | Glycine max | 144 | + | 5 | TAATA | core promoter element around -30 of transcription start |
| TATA-box | Zea mays | 124 | - | 8 | TTTAAAAA | core promoter element around -30 of transcription start |
| TATA-box | Lycopersicon esculentum | 110 | - | 5 | TTTTA | core promoter element around -30 of transcription start |
| TATA-box | Arabidopsis thaliana | 103 | + | 4 | TATA | core promoter element around -30 of transcription start |
| TATA-box | Arabidopsis thaliana | 1451 | - | 6 | TATAAA | core promoter element around -30 of transcription start |
| TATA-box | Glycine max | 1267 | - | 5 | TAATA | core promoter element around -30 of transcription start |
| TATA-box | Lycopersicon esculentum | 763 | + | 5 | TTTTA | core promoter element around -30 of transcription start |
| TATA-box | Lycopersicon esculentum | 1252 | - | 5 | TTTTA | core promoter element around -30 of transcription start |
| TATA-box | Zea mays | 1250 | + | 8 | TTTAAAAA | core promoter element around -30 of transcription start |
| TATA-box | Arabidopsis thaliana | 1165 | - | 8 | TAAAGATT | core promoter element around -30 of transcription start |
| TATA-box | Brassica napus | 101 | + | 6 | ATTATA | core promoter element around -30 of transcription start |
| TATA-box | Antirrhinum majus | 80 | - | 8 | TATAAATT | core promoter element around -30 of transcription start |
| TATA-box | Glycine max | 1455 | + | 5 | TAATA | core promoter element around -30 of transcription start |
| TATA-box | Arabidopsis thaliana | 102 | - | 5 | TATAA | core promoter element around -30 of transcription start |
| TATA-box | Lycopersicon esculentum | 95 | + | 5 | TTTTA | core promoter element around -30 of transcription start |
| TATA-box | Lycopersicon esculentum | 69 | + | 5 | TTTTA | core promoter element around -30 of transcription start |
| TATA-box | Arabidopsis thaliana | 88 | + | 9 | tcTATATAtt | core promoter element around -30 of transcription start |
| TATA-box | Lycopersicon esculentum | 37 | - | 5 | TTTTA | core promoter element around -30 of transcription start |
| TATA-box | Avena sativa | 84 | + | 12 | TATATTTATATTT | core promoter element around -30 of transcription start |
| TATA-box | Lycopersicon esculentum | 734 | - | 5 | TTTTA | core promoter element around -30 of transcription start |
| TATA-box | Lycopersicon esculentum | 125 | + | 5 | TTTTA | core promoter element around -30 of transcription start |
| TATA-box | Arabidopsis thaliana | 1453 | - | 4 | TATA | core promoter element around -30 of transcription start |
| TATA-box | Lycopersicon esculentum | 805 | + | 5 | TTTTA | core promoter element around -30 of transcription start |
| TATA-box | Zea mays | 804 | - | 8 | TTTAAAAA | core promoter element around -30 of transcription start |
| TATA-box | Pisum sativum | 220 | - | 8 | TATAAAAT | core promoter element around -30 of transcription start |
| TATA-box | Lycopersicon esculentum | 187 | - | 5 | TTTTA | core promoter element around -30 of transcription start |
| TATA-box | Glycine max | 786 | + | 5 | TAATA | core promoter element around -30 of transcription start |
| TATA-box | Lycopersicon esculentum | 128 | - | 5 | TTTTA | core promoter element around -30 of transcription start |
| TATA-box | Arabidopsis thaliana | 224 | + | 4 | TATA | core promoter element around -30 of transcription start |
| TATA-box | Arabidopsis thaliana | 222 | - | 6 | TATAAA | core promoter element around -30 of transcription start |
| TATA-box | Arabidopsis thaliana | 221 | - | 7 | TATAAAA | core promoter element around -30 of transcription start |
| TATA-box | Arabidopsis thaliana | 17 | - | 5 | TATAA | core promoter element around -30 of transcription start |
| TATA-box | Arabidopsis thaliana | 18 | + | 4 | TATA | core promoter element around -30 of transcription start |
| TATA-box | Lycopersicon esculentum | 832 | + | 5 | TTTTA | core promoter element around -30 of transcription start |
| TATA-box | Arabidopsis thaliana | 82 | - | 11 | TATAAATATAAA | core promoter element around -30 of transcription start |
| TATA-box | Lycopersicon esculentum | 346 | - | 5 | TTTTA | core promoter element around -30 of transcription start |
| TATA-box | Lycopersicon esculentum | 166 | - | 5 | TTTTA | core promoter element around -30 of transcription start |
| TATA-box | Brassica napus | 151 | + | 6 | ATATAT | core promoter element around -30 of transcription start |
| TATA-box | Lycopersicon esculentum | 54 | + | 5 | TTTTA | core promoter element around -30 of transcription start |
| TATA-box | Lycopersicon esculentum | 766 | - | 5 | TTTTA | core promoter element around -30 of transcription start |
| TATA-box | Zea mays | 126 | + | 8 | TTTAAAAA | core promoter element around -30 of transcription start |
| TATA-box | Glycine max | 141 | + | 5 | TAATA | core promoter element around -30 of transcription start |
| TATA-box | Arabidopsis thaliana | 152 | + | 4 | TATA | core promoter element around -30 of transcription start |
| TATA-box | Glycine max | 343 | + | 5 | TAATA | core promoter element around -30 of transcription start |
| TATA-box | Arabidopsis thaliana | 16 | - | 6 | TATAAA | core promoter element around -30 of transcription start |
| TATA-box | Lycopersicon esculentum | 2 | - | 5 | TTTTA | core promoter element around -30 of transcription start |
| TATA-box | Arabidopsis thaliana | 90 | + | 4 | TATA | core promoter element around -30 of transcription start |
| TATA-box | Arabidopsis thaliana | 223 | - | 5 | TATAA | core promoter element around -30 of transcription start |
| TATA-box | Ac | 81 | - | 7 | TATAAAT | core promoter element around -30 of transcription start |
| TATA-box | Lycopersicon esculentum | 740 | + | 5 | TTTTA | core promoter element around -30 of transcription start |
| TATA-box | Arabidopsis thaliana | 83 | - | 5 | TATAA | core promoter element around -30 of transcription start |

> 2018/04/13 10:10:12  
+ TTAAAAAACT GACTGTTTAT ATCAGTTGTA TTTTCATAAA ATTTGTCATT CGATTTTATG GGTTTTTGTT   
  
  
+ TTAAGTTAGA ATTTATATCT ATATTTTTAC ATTATAACAT AAAAATTCTG ATTTTTTTAA AAATTAAATG   
  
  
+ TAATAATAGA ATATATCTAT GGCATTAAAA CTATCATTCT GGTTGATAAA ACACCCACAG CCACAAGGGA   
  
  
+ AGAGTAAGTA TTTTATATTT CCCCTTCCAC ATAATCCATA TCCAGGTGTG TCTCCTGATG ATTAATGGAC   
  
  
+ ATCACCATCC TCTGGGTCCC ATGAGAGTGA ATGCCCCAAT CAGAACGGCT TGCTCTGGGC TGTAATAAAA   
  
  
+ TCCAGCTCGT GAATTGTGAG GGGCGTACAC GGCAACTTGA CCGCCTTTGC TTTTTTCTCG GTGGGTCCCT   
  
  
+ GTTGGGGCAA CATCGCTGTC ATCGTCTGTG ATTTTGATGT GGTAGGAAGC ATCCGATTTG TAACTGTTAA   
  
  
+ GAGTTTCTTA TGCAACGTCG TTATGGAGAG GTCCTGTACG CCCACTGGAG CATTTGATGC TGCAACAGCA   
  
  
+ AACCAAAACA AAAGGAGTCA GATTTTTGGA ATTTCTACTC TGTCGGCAGT TTAGCCCCCC AGTCTGTTAA   
  
  
+ TCTTGCAAGT TCCAGGATTC TTTTGGGTTG CCCAGAAGTC AATCCCCTCT TCATGCCTAT CGGTTCATCT   
  
  
+ CACCTGTTGA CATGACAACT ATCTGACCAA AAATAAAATT TTTATGCTTT ATCTTGCAGA CATTTTAAAA   
  
  
+ GGGTTTTCGT TCATTTAATA GAATTTTTCT TTTTTTTTAA ATGAATTTGA GGTTCCAAAT ATTTTAGGGA   
  
  
+ TCTTTAAGGG GGAGAAGAGG AGTAATGCAC ATGGTAAAGA GAGTGAAAGT TCTGACACTG TTAAGTGTAG   
  
  
+ GACTGTAGGT GGGTTCCGGT CTTGGAGGGC AACAAGTCAA TTGAAATGAG AAAGTTTCGT GCTAGAAATC   
  
  
+ ATTTGACATT TGCGTTGGGG CAGGGATTCC TGTTTTTCCT TATCTCATTG CTAGTTCATT TGGGTAGGGA   
  
  
+ TTAAATTGGC AGGAACTTAA GTTTGTCTTC CTTTGATTTC ATCGAAAGCT CCAAATCAGT CTTATCAGCA   
  
  
+ ATGGCAGCTT AGCCATTGTG TGATTAGTTT AGTTGCGAGA ATGGAATCTT TAGTGGGTGG GGGGGCACTT   
  
  
+ AGAAGCATGC CAGGTGGAGG GAAACTTCAA GGCTTACGGC CGTGAAACTG TATTTCGTCT TTAAAAAAAA   
  
  
+ AAAAAATATT AGCTCACAAG AATTCTTGAA AAAAAAAAAA AAAAAAGGGA AAGAGGATGT CGTTTTCCAT   
  
  
+ TTCTCTGCTC TGTGGACTTT TTGGGTTCCA CTGGTTTTCA GGGTAGCACG AAACTGTCAA AAAGCCGACT   
  
  
+ GATTTAGAGA GAGGAAAACA ACAATTAGGG AAGGCTGTAC AGAGACAAGC TTTATAATAT CTGTATTTGG   
  
  
+ AGGGAGAAAC AAAGAAGTTG ATAGCTCAA  

- AATTTTTTGA CTGACAAATA TAGTCAACAT AAAAGTATTT TAAACAGTAA GCTAAAATAC CCAAAAACAA   
  
  
- AATTCAATCT TAAATATAGA TATAAAAATG TAATATTGTA TTTTTAAGAC TAAAAAAATT TTTAATTTAC   
  
  
- ATTATTATCT TATATAGATA CCGTAATTTT GATAGTAAGA CCAACTATTT TGTGGGTGTC GGTGTTCCCT   
  
  
- TCTCATTCAT AAAATATAAA GGGGAAGGTG TATTAGGTAT AGGTCCACAC AGAGGACTAC TAATTACCTG   
  
  
- TAGTGGTAGG AGACCCAGGG TACTCTCACT TACGGGGTTA GTCTTGCCGA ACGAGACCCG ACATTATTTT   
  
  
- AGGTCGAGCA CTTAACACTC CCCGCATGTG CCGTTGAACT GGCGGAAACG AAAAAAGAGC CACCCAGGGA   
  
  
- CAACCCCGTT GTAGCGACAG TAGCAGACAC TAAAACTACA CCATCCTTCG TAGGCTAAAC ATTGACAATT   
  
  
- CTCAAAGAAT ACGTTGCAGC AATACCTCTC CAGGACATGC GGGTGACCTC GTAAACTACG ACGTTGTCGT   
  
  
- TTGGTTTTGT TTTCCTCAGT CTAAAAACCT TAAAGATGAG ACAGCCGTCA AATCGGGGGG TCAGACAATT   
  
  
- AGAACGTTCA AGGTCCTAAG AAAACCCAAC GGGTCTTCAG TTAGGGGAGA AGTACGGATA GCCAAGTAGA   
  
  
- GTGGACAACT GTACTGTTGA TAGACTGGTT TTTATTTTAA AAATACGAAA TAGAACGTCT GTAAAATTTT   
  
  
- CCCAAAAGCA AGTAAATTAT CTTAAAAAGA AAAAAAAATT TACTTAAACT CCAAGGTTTA TAAAATCCCT   
  
  
- AGAAATTCCC CCTCTTCTCC TCATTACGTG TACCATTTCT CTCACTTTCA AGACTGTGAC AATTCACATC   
  
  
- CTGACATCCA CCCAAGGCCA GAACCTCCCG TTGTTCAGTT AACTTTACTC TTTCAAAGCA CGATCTTTAG   
  
  
- TAAACTGTAA ACGCAACCCC GTCCCTAAGG ACAAAAAGGA ATAGAGTAAC GATCAAGTAA ACCCATCCCT   
  
  
- AATTTAACCG TCCTTGAATT CAAACAGAAG GAAACTAAAG TAGCTTTCGA GGTTTAGTCA GAATAGTCGT   
  
  
- TACCGTCGAA TCGGTAACAC ACTAATCAAA TCAACGCTCT TACCTTAGAA ATCACCCACC CCCCCGTGAA   
  
  
- TCTTCGTACG GTCCACCTCC CTTTGAAGTT CCGAATGCCG GCACTTTGAC ATAAAGCAGA AATTTTTTTT   
  
  
- TTTTTTATAA TCGAGTGTTC TTAAGAACTT TTTTTTTTTT TTTTTTCCCT TTCTCCTACA GCAAAAGGTA   
  
  
- AAGAGACGAG ACACCTGAAA AACCCAAGGT GACCAAAAGT CCCATCGTGC TTTGACAGTT TTTCGGCTGA   
  
  
- CTAAATCTCT CTCCTTTTGT TGTTAATCCC TTCCGACATG TCTCTGTTCG AAATATTATA GACATAAACC   
  
  
- TCCCTCTTTG TTTCTTCAAC TATCGAGTT

+     TCCACCT-motif

| Site Name | Organism | Position | Strand | Matrix score. | sequence | function |
| --- | --- | --- | --- | --- | --- | --- |
| TCCACCT-motif | Petroselinum hortense | 1202 | - | 7 | TCCACCT |  |

> 2018/04/13 10:10:12  
+ TTAAAAAACT GACTGTTTAT ATCAGTTGTA TTTTCATAAA ATTTGTCATT CGATTTTATG GGTTTTTGTT   
  
  
+ TTAAGTTAGA ATTTATATCT ATATTTTTAC ATTATAACAT AAAAATTCTG ATTTTTTTAA AAATTAAATG   
  
  
+ TAATAATAGA ATATATCTAT GGCATTAAAA CTATCATTCT GGTTGATAAA ACACCCACAG CCACAAGGGA   
  
  
+ AGAGTAAGTA TTTTATATTT CCCCTTCCAC ATAATCCATA TCCAGGTGTG TCTCCTGATG ATTAATGGAC   
  
  
+ ATCACCATCC TCTGGGTCCC ATGAGAGTGA ATGCCCCAAT CAGAACGGCT TGCTCTGGGC TGTAATAAAA   
  
  
+ TCCAGCTCGT GAATTGTGAG GGGCGTACAC GGCAACTTGA CCGCCTTTGC TTTTTTCTCG GTGGGTCCCT   
  
  
+ GTTGGGGCAA CATCGCTGTC ATCGTCTGTG ATTTTGATGT GGTAGGAAGC ATCCGATTTG TAACTGTTAA   
  
  
+ GAGTTTCTTA TGCAACGTCG TTATGGAGAG GTCCTGTACG CCCACTGGAG CATTTGATGC TGCAACAGCA   
  
  
+ AACCAAAACA AAAGGAGTCA GATTTTTGGA ATTTCTACTC TGTCGGCAGT TTAGCCCCCC AGTCTGTTAA   
  
  
+ TCTTGCAAGT TCCAGGATTC TTTTGGGTTG CCCAGAAGTC AATCCCCTCT TCATGCCTAT CGGTTCATCT   
  
  
+ CACCTGTTGA CATGACAACT ATCTGACCAA AAATAAAATT TTTATGCTTT ATCTTGCAGA CATTTTAAAA   
  
  
+ GGGTTTTCGT TCATTTAATA GAATTTTTCT TTTTTTTTAA ATGAATTTGA GGTTCCAAAT ATTTTAGGGA   
  
  
+ TCTTTAAGGG GGAGAAGAGG AGTAATGCAC ATGGTAAAGA GAGTGAAAGT TCTGACACTG TTAAGTGTAG   
  
  
+ GACTGTAGGT GGGTTCCGGT CTTGGAGGGC AACAAGTCAA TTGAAATGAG AAAGTTTCGT GCTAGAAATC   
  
  
+ ATTTGACATT TGCGTTGGGG CAGGGATTCC TGTTTTTCCT TATCTCATTG CTAGTTCATT TGGGTAGGGA   
  
  
+ TTAAATTGGC AGGAACTTAA GTTTGTCTTC CTTTGATTTC ATCGAAAGCT CCAAATCAGT CTTATCAGCA   
  
  
+ ATGGCAGCTT AGCCATTGTG TGATTAGTTT AGTTGCGAGA ATGGAATCTT TAGTGGGTGG GGGGGCACTT   
  
  
+ AGAAGCATGC CAGGTGGAGG GAAACTTCAA GGCTTACGGC CGTGAAACTG TATTTCGTCT TTAAAAAAAA   
  
  
+ AAAAAATATT AGCTCACAAG AATTCTTGAA AAAAAAAAAA AAAAAAGGGA AAGAGGATGT CGTTTTCCAT   
  
  
+ TTCTCTGCTC TGTGGACTTT TTGGGTTCCA CTGGTTTTCA GGGTAGCACG AAACTGTCAA AAAGCCGACT   
  
  
+ GATTTAGAGA GAGGAAAACA ACAATTAGGG AAGGCTGTAC AGAGACAAGC TTTATAATAT CTGTATTTGG   
  
  
+ AGGGAGAAAC AAAGAAGTTG ATAGCTCAA  

- AATTTTTTGA CTGACAAATA TAGTCAACAT AAAAGTATTT TAAACAGTAA GCTAAAATAC CCAAAAACAA   
  
  
- AATTCAATCT TAAATATAGA TATAAAAATG TAATATTGTA TTTTTAAGAC TAAAAAAATT TTTAATTTAC   
  
  
- ATTATTATCT TATATAGATA CCGTAATTTT GATAGTAAGA CCAACTATTT TGTGGGTGTC GGTGTTCCCT   
  
  
- TCTCATTCAT AAAATATAAA GGGGAAGGTG TATTAGGTAT AGGTCCACAC AGAGGACTAC TAATTACCTG   
  
  
- TAGTGGTAGG AGACCCAGGG TACTCTCACT TACGGGGTTA GTCTTGCCGA ACGAGACCCG ACATTATTTT   
  
  
- AGGTCGAGCA CTTAACACTC CCCGCATGTG CCGTTGAACT GGCGGAAACG AAAAAAGAGC CACCCAGGGA   
  
  
- CAACCCCGTT GTAGCGACAG TAGCAGACAC TAAAACTACA CCATCCTTCG TAGGCTAAAC ATTGACAATT   
  
  
- CTCAAAGAAT ACGTTGCAGC AATACCTCTC CAGGACATGC GGGTGACCTC GTAAACTACG ACGTTGTCGT   
  
  
- TTGGTTTTGT TTTCCTCAGT CTAAAAACCT TAAAGATGAG ACAGCCGTCA AATCGGGGGG TCAGACAATT   
  
  
- AGAACGTTCA AGGTCCTAAG AAAACCCAAC GGGTCTTCAG TTAGGGGAGA AGTACGGATA GCCAAGTAGA   
  
  
- GTGGACAACT GTACTGTTGA TAGACTGGTT TTTATTTTAA AAATACGAAA TAGAACGTCT GTAAAATTTT   
  
  
- CCCAAAAGCA AGTAAATTAT CTTAAAAAGA AAAAAAAATT TACTTAAACT CCAAGGTTTA TAAAATCCCT   
  
  
- AGAAATTCCC CCTCTTCTCC TCATTACGTG TACCATTTCT CTCACTTTCA AGACTGTGAC AATTCACATC   
  
  
- CTGACATCCA CCCAAGGCCA GAACCTCCCG TTGTTCAGTT AACTTTACTC TTTCAAAGCA CGATCTTTAG   
  
  
- TAAACTGTAA ACGCAACCCC GTCCCTAAGG ACAAAAAGGA ATAGAGTAAC GATCAAGTAA ACCCATCCCT   
  
  
- AATTTAACCG TCCTTGAATT CAAACAGAAG GAAACTAAAG TAGCTTTCGA GGTTTAGTCA GAATAGTCGT   
  
  
- TACCGTCGAA TCGGTAACAC ACTAATCAAA TCAACGCTCT TACCTTAGAA ATCACCCACC CCCCCGTGAA   
  
  
- TCTTCGTACG GTCCACCTCC CTTTGAAGTT CCGAATGCCG GCACTTTGAC ATAAAGCAGA AATTTTTTTT   
  
  
- TTTTTTATAA TCGAGTGTTC TTAAGAACTT TTTTTTTTTT TTTTTTCCCT TTCTCCTACA GCAAAAGGTA   
  
  
- AAGAGACGAG ACACCTGAAA AACCCAAGGT GACCAAAAGT CCCATCGTGC TTTGACAGTT TTTCGGCTGA   
  
  
- CTAAATCTCT CTCCTTTTGT TGTTAATCCC TTCCGACATG TCTCTGTTCG AAATATTATA GACATAAACC   
  
  
- TCCCTCTTTG TTTCTTCAAC TATCGAGTT

+     TCCC-motif

| Site Name | Organism | Position | Strand | Matrix score. | sequence | function |
| --- | --- | --- | --- | --- | --- | --- |
| TCCC-motif | Spinacia oleracea | 1471 | - | 7 | TCTCCCT | part of a light responsive element |

> 2018/04/13 10:10:12  
+ TTAAAAAACT GACTGTTTAT ATCAGTTGTA TTTTCATAAA ATTTGTCATT CGATTTTATG GGTTTTTGTT   
  
  
+ TTAAGTTAGA ATTTATATCT ATATTTTTAC ATTATAACAT AAAAATTCTG ATTTTTTTAA AAATTAAATG   
  
  
+ TAATAATAGA ATATATCTAT GGCATTAAAA CTATCATTCT GGTTGATAAA ACACCCACAG CCACAAGGGA   
  
  
+ AGAGTAAGTA TTTTATATTT CCCCTTCCAC ATAATCCATA TCCAGGTGTG TCTCCTGATG ATTAATGGAC   
  
  
+ ATCACCATCC TCTGGGTCCC ATGAGAGTGA ATGCCCCAAT CAGAACGGCT TGCTCTGGGC TGTAATAAAA   
  
  
+ TCCAGCTCGT GAATTGTGAG GGGCGTACAC GGCAACTTGA CCGCCTTTGC TTTTTTCTCG GTGGGTCCCT   
  
  
+ GTTGGGGCAA CATCGCTGTC ATCGTCTGTG ATTTTGATGT GGTAGGAAGC ATCCGATTTG TAACTGTTAA   
  
  
+ GAGTTTCTTA TGCAACGTCG TTATGGAGAG GTCCTGTACG CCCACTGGAG CATTTGATGC TGCAACAGCA   
  
  
+ AACCAAAACA AAAGGAGTCA GATTTTTGGA ATTTCTACTC TGTCGGCAGT TTAGCCCCCC AGTCTGTTAA   
  
  
+ TCTTGCAAGT TCCAGGATTC TTTTGGGTTG CCCAGAAGTC AATCCCCTCT TCATGCCTAT CGGTTCATCT   
  
  
+ CACCTGTTGA CATGACAACT ATCTGACCAA AAATAAAATT TTTATGCTTT ATCTTGCAGA CATTTTAAAA   
  
  
+ GGGTTTTCGT TCATTTAATA GAATTTTTCT TTTTTTTTAA ATGAATTTGA GGTTCCAAAT ATTTTAGGGA   
  
  
+ TCTTTAAGGG GGAGAAGAGG AGTAATGCAC ATGGTAAAGA GAGTGAAAGT TCTGACACTG TTAAGTGTAG   
  
  
+ GACTGTAGGT GGGTTCCGGT CTTGGAGGGC AACAAGTCAA TTGAAATGAG AAAGTTTCGT GCTAGAAATC   
  
  
+ ATTTGACATT TGCGTTGGGG CAGGGATTCC TGTTTTTCCT TATCTCATTG CTAGTTCATT TGGGTAGGGA   
  
  
+ TTAAATTGGC AGGAACTTAA GTTTGTCTTC CTTTGATTTC ATCGAAAGCT CCAAATCAGT CTTATCAGCA   
  
  
+ ATGGCAGCTT AGCCATTGTG TGATTAGTTT AGTTGCGAGA ATGGAATCTT TAGTGGGTGG GGGGGCACTT   
  
  
+ AGAAGCATGC CAGGTGGAGG GAAACTTCAA GGCTTACGGC CGTGAAACTG TATTTCGTCT TTAAAAAAAA   
  
  
+ AAAAAATATT AGCTCACAAG AATTCTTGAA AAAAAAAAAA AAAAAAGGGA AAGAGGATGT CGTTTTCCAT   
  
  
+ TTCTCTGCTC TGTGGACTTT TTGGGTTCCA CTGGTTTTCA GGGTAGCACG AAACTGTCAA AAAGCCGACT   
  
  
+ GATTTAGAGA GAGGAAAACA ACAATTAGGG AAGGCTGTAC AGAGACAAGC TTTATAATAT CTGTATTTGG   
  
  
+ AGGGAGAAAC AAAGAAGTTG ATAGCTCAA  

- AATTTTTTGA CTGACAAATA TAGTCAACAT AAAAGTATTT TAAACAGTAA GCTAAAATAC CCAAAAACAA   
  
  
- AATTCAATCT TAAATATAGA TATAAAAATG TAATATTGTA TTTTTAAGAC TAAAAAAATT TTTAATTTAC   
  
  
- ATTATTATCT TATATAGATA CCGTAATTTT GATAGTAAGA CCAACTATTT TGTGGGTGTC GGTGTTCCCT   
  
  
- TCTCATTCAT AAAATATAAA GGGGAAGGTG TATTAGGTAT AGGTCCACAC AGAGGACTAC TAATTACCTG   
  
  
- TAGTGGTAGG AGACCCAGGG TACTCTCACT TACGGGGTTA GTCTTGCCGA ACGAGACCCG ACATTATTTT   
  
  
- AGGTCGAGCA CTTAACACTC CCCGCATGTG CCGTTGAACT GGCGGAAACG AAAAAAGAGC CACCCAGGGA   
  
  
- CAACCCCGTT GTAGCGACAG TAGCAGACAC TAAAACTACA CCATCCTTCG TAGGCTAAAC ATTGACAATT   
  
  
- CTCAAAGAAT ACGTTGCAGC AATACCTCTC CAGGACATGC GGGTGACCTC GTAAACTACG ACGTTGTCGT   
  
  
- TTGGTTTTGT TTTCCTCAGT CTAAAAACCT TAAAGATGAG ACAGCCGTCA AATCGGGGGG TCAGACAATT   
  
  
- AGAACGTTCA AGGTCCTAAG AAAACCCAAC GGGTCTTCAG TTAGGGGAGA AGTACGGATA GCCAAGTAGA   
  
  
- GTGGACAACT GTACTGTTGA TAGACTGGTT TTTATTTTAA AAATACGAAA TAGAACGTCT GTAAAATTTT   
  
  
- CCCAAAAGCA AGTAAATTAT CTTAAAAAGA AAAAAAAATT TACTTAAACT CCAAGGTTTA TAAAATCCCT   
  
  
- AGAAATTCCC CCTCTTCTCC TCATTACGTG TACCATTTCT CTCACTTTCA AGACTGTGAC AATTCACATC   
  
  
- CTGACATCCA CCCAAGGCCA GAACCTCCCG TTGTTCAGTT AACTTTACTC TTTCAAAGCA CGATCTTTAG   
  
  
- TAAACTGTAA ACGCAACCCC GTCCCTAAGG ACAAAAAGGA ATAGAGTAAC GATCAAGTAA ACCCATCCCT   
  
  
- AATTTAACCG TCCTTGAATT CAAACAGAAG GAAACTAAAG TAGCTTTCGA GGTTTAGTCA GAATAGTCGT   
  
  
- TACCGTCGAA TCGGTAACAC ACTAATCAAA TCAACGCTCT TACCTTAGAA ATCACCCACC CCCCCGTGAA   
  
  
- TCTTCGTACG GTCCACCTCC CTTTGAAGTT CCGAATGCCG GCACTTTGAC ATAAAGCAGA AATTTTTTTT   
  
  
- TTTTTTATAA TCGAGTGTTC TTAAGAACTT TTTTTTTTTT TTTTTTCCCT TTCTCCTACA GCAAAAGGTA   
  
  
- AAGAGACGAG ACACCTGAAA AACCCAAGGT GACCAAAAGT CCCATCGTGC TTTGACAGTT TTTCGGCTGA   
  
  
- CTAAATCTCT CTCCTTTTGT TGTTAATCCC TTCCGACATG TCTCTGTTCG AAATATTATA GACATAAACC   
  
  
- TCCCTCTTTG TTTCTTCAAC TATCGAGTT

+     TGA-element

| Site Name | Organism | Position | Strand | Matrix score. | sequence | function |
| --- | --- | --- | --- | --- | --- | --- |
| TGA-element | Brassica oleracea | 507 | - | 6 | AACGAC | auxin-responsive element |
| TGA-element | Brassica oleracea | 1319 | - | 6 | AACGAC | auxin-responsive element |

> 2018/04/13 10:10:12  
+ TTAAAAAACT GACTGTTTAT ATCAGTTGTA TTTTCATAAA ATTTGTCATT CGATTTTATG GGTTTTTGTT   
  
  
+ TTAAGTTAGA ATTTATATCT ATATTTTTAC ATTATAACAT AAAAATTCTG ATTTTTTTAA AAATTAAATG   
  
  
+ TAATAATAGA ATATATCTAT GGCATTAAAA CTATCATTCT GGTTGATAAA ACACCCACAG CCACAAGGGA   
  
  
+ AGAGTAAGTA TTTTATATTT CCCCTTCCAC ATAATCCATA TCCAGGTGTG TCTCCTGATG ATTAATGGAC   
  
  
+ ATCACCATCC TCTGGGTCCC ATGAGAGTGA ATGCCCCAAT CAGAACGGCT TGCTCTGGGC TGTAATAAAA   
  
  
+ TCCAGCTCGT GAATTGTGAG GGGCGTACAC GGCAACTTGA CCGCCTTTGC TTTTTTCTCG GTGGGTCCCT   
  
  
+ GTTGGGGCAA CATCGCTGTC ATCGTCTGTG ATTTTGATGT GGTAGGAAGC ATCCGATTTG TAACTGTTAA   
  
  
+ GAGTTTCTTA TGCAACGTCG TTATGGAGAG GTCCTGTACG CCCACTGGAG CATTTGATGC TGCAACAGCA   
  
  
+ AACCAAAACA AAAGGAGTCA GATTTTTGGA ATTTCTACTC TGTCGGCAGT TTAGCCCCCC AGTCTGTTAA   
  
  
+ TCTTGCAAGT TCCAGGATTC TTTTGGGTTG CCCAGAAGTC AATCCCCTCT TCATGCCTAT CGGTTCATCT   
  
  
+ CACCTGTTGA CATGACAACT ATCTGACCAA AAATAAAATT TTTATGCTTT ATCTTGCAGA CATTTTAAAA   
  
  
+ GGGTTTTCGT TCATTTAATA GAATTTTTCT TTTTTTTTAA ATGAATTTGA GGTTCCAAAT ATTTTAGGGA   
  
  
+ TCTTTAAGGG GGAGAAGAGG AGTAATGCAC ATGGTAAAGA GAGTGAAAGT TCTGACACTG TTAAGTGTAG   
  
  
+ GACTGTAGGT GGGTTCCGGT CTTGGAGGGC AACAAGTCAA TTGAAATGAG AAAGTTTCGT GCTAGAAATC   
  
  
+ ATTTGACATT TGCGTTGGGG CAGGGATTCC TGTTTTTCCT TATCTCATTG CTAGTTCATT TGGGTAGGGA   
  
  
+ TTAAATTGGC AGGAACTTAA GTTTGTCTTC CTTTGATTTC ATCGAAAGCT CCAAATCAGT CTTATCAGCA   
  
  
+ ATGGCAGCTT AGCCATTGTG TGATTAGTTT AGTTGCGAGA ATGGAATCTT TAGTGGGTGG GGGGGCACTT   
  
  
+ AGAAGCATGC CAGGTGGAGG GAAACTTCAA GGCTTACGGC CGTGAAACTG TATTTCGTCT TTAAAAAAAA   
  
  
+ AAAAAATATT AGCTCACAAG AATTCTTGAA AAAAAAAAAA AAAAAAGGGA AAGAGGATGT CGTTTTCCAT   
  
  
+ TTCTCTGCTC TGTGGACTTT TTGGGTTCCA CTGGTTTTCA GGGTAGCACG AAACTGTCAA AAAGCCGACT   
  
  
+ GATTTAGAGA GAGGAAAACA ACAATTAGGG AAGGCTGTAC AGAGACAAGC TTTATAATAT CTGTATTTGG   
  
  
+ AGGGAGAAAC AAAGAAGTTG ATAGCTCAA  

- AATTTTTTGA CTGACAAATA TAGTCAACAT AAAAGTATTT TAAACAGTAA GCTAAAATAC CCAAAAACAA   
  
  
- AATTCAATCT TAAATATAGA TATAAAAATG TAATATTGTA TTTTTAAGAC TAAAAAAATT TTTAATTTAC   
  
  
- ATTATTATCT TATATAGATA CCGTAATTTT GATAGTAAGA CCAACTATTT TGTGGGTGTC GGTGTTCCCT   
  
  
- TCTCATTCAT AAAATATAAA GGGGAAGGTG TATTAGGTAT AGGTCCACAC AGAGGACTAC TAATTACCTG   
  
  
- TAGTGGTAGG AGACCCAGGG TACTCTCACT TACGGGGTTA GTCTTGCCGA ACGAGACCCG ACATTATTTT   
  
  
- AGGTCGAGCA CTTAACACTC CCCGCATGTG CCGTTGAACT GGCGGAAACG AAAAAAGAGC CACCCAGGGA   
  
  
- CAACCCCGTT GTAGCGACAG TAGCAGACAC TAAAACTACA CCATCCTTCG TAGGCTAAAC ATTGACAATT   
  
  
- CTCAAAGAAT ACGTTGCAGC AATACCTCTC CAGGACATGC GGGTGACCTC GTAAACTACG ACGTTGTCGT   
  
  
- TTGGTTTTGT TTTCCTCAGT CTAAAAACCT TAAAGATGAG ACAGCCGTCA AATCGGGGGG TCAGACAATT   
  
  
- AGAACGTTCA AGGTCCTAAG AAAACCCAAC GGGTCTTCAG TTAGGGGAGA AGTACGGATA GCCAAGTAGA   
  
  
- GTGGACAACT GTACTGTTGA TAGACTGGTT TTTATTTTAA AAATACGAAA TAGAACGTCT GTAAAATTTT   
  
  
- CCCAAAAGCA AGTAAATTAT CTTAAAAAGA AAAAAAAATT TACTTAAACT CCAAGGTTTA TAAAATCCCT   
  
  
- AGAAATTCCC CCTCTTCTCC TCATTACGTG TACCATTTCT CTCACTTTCA AGACTGTGAC AATTCACATC   
  
  
- CTGACATCCA CCCAAGGCCA GAACCTCCCG TTGTTCAGTT AACTTTACTC TTTCAAAGCA CGATCTTTAG   
  
  
- TAAACTGTAA ACGCAACCCC GTCCCTAAGG ACAAAAAGGA ATAGAGTAAC GATCAAGTAA ACCCATCCCT   
  
  
- AATTTAACCG TCCTTGAATT CAAACAGAAG GAAACTAAAG TAGCTTTCGA GGTTTAGTCA GAATAGTCGT   
  
  
- TACCGTCGAA TCGGTAACAC ACTAATCAAA TCAACGCTCT TACCTTAGAA ATCACCCACC CCCCCGTGAA   
  
  
- TCTTCGTACG GTCCACCTCC CTTTGAAGTT CCGAATGCCG GCACTTTGAC ATAAAGCAGA AATTTTTTTT   
  
  
- TTTTTTATAA TCGAGTGTTC TTAAGAACTT TTTTTTTTTT TTTTTTCCCT TTCTCCTACA GCAAAAGGTA   
  
  
- AAGAGACGAG ACACCTGAAA AACCCAAGGT GACCAAAAGT CCCATCGTGC TTTGACAGTT TTTCGGCTGA   
  
  
- CTAAATCTCT CTCCTTTTGT TGTTAATCCC TTCCGACATG TCTCTGTTCG AAATATTATA GACATAAACC   
  
  
- TCCCTCTTTG TTTCTTCAAC TATCGAGTT

+     Unnamed\_\_4

| Site Name | Organism | Position | Strand | Matrix score. | sequence | function |
| --- | --- | --- | --- | --- | --- | --- |
| Unnamed\_\_4 | Petroselinum hortense | 1206 | - | 4 | CTCC |  |
| Unnamed\_\_4 | Petroselinum hortense | 851 | - | 4 | CTCC |  |
| Unnamed\_\_4 | Petroselinum hortense | 515 | - | 4 | CTCC |  |
| Unnamed\_\_4 | Petroselinum hortense | 262 | + | 4 | CTCC |  |
| Unnamed\_\_4 | Petroselinum hortense | 859 | - | 4 | CTCC |  |
| Unnamed\_\_4 | Petroselinum hortense | 537 | - | 4 | CTCC |  |
| Unnamed\_\_4 | Petroselinum hortense | 1099 | + | 4 | CTCC |  |
| Unnamed\_\_4 | Petroselinum hortense | 934 | - | 4 | CTCC |  |
| Unnamed\_\_4 | Petroselinum hortense | 574 | - | 4 | CTCC |  |
| Unnamed\_\_4 | Petroselinum hortense | 1473 | - | 4 | CTCC |  |
| Unnamed\_\_4 | Petroselinum hortense | 1469 | - | 4 | CTCC |  |

> 2018/04/13 10:10:12  
+ TTAAAAAACT GACTGTTTAT ATCAGTTGTA TTTTCATAAA ATTTGTCATT CGATTTTATG GGTTTTTGTT   
  
  
+ TTAAGTTAGA ATTTATATCT ATATTTTTAC ATTATAACAT AAAAATTCTG ATTTTTTTAA AAATTAAATG   
  
  
+ TAATAATAGA ATATATCTAT GGCATTAAAA CTATCATTCT GGTTGATAAA ACACCCACAG CCACAAGGGA   
  
  
+ AGAGTAAGTA TTTTATATTT CCCCTTCCAC ATAATCCATA TCCAGGTGTG TCTCCTGATG ATTAATGGAC   
  
  
+ ATCACCATCC TCTGGGTCCC ATGAGAGTGA ATGCCCCAAT CAGAACGGCT TGCTCTGGGC TGTAATAAAA   
  
  
+ TCCAGCTCGT GAATTGTGAG GGGCGTACAC GGCAACTTGA CCGCCTTTGC TTTTTTCTCG GTGGGTCCCT   
  
  
+ GTTGGGGCAA CATCGCTGTC ATCGTCTGTG ATTTTGATGT GGTAGGAAGC ATCCGATTTG TAACTGTTAA   
  
  
+ GAGTTTCTTA TGCAACGTCG TTATGGAGAG GTCCTGTACG CCCACTGGAG CATTTGATGC TGCAACAGCA   
  
  
+ AACCAAAACA AAAGGAGTCA GATTTTTGGA ATTTCTACTC TGTCGGCAGT TTAGCCCCCC AGTCTGTTAA   
  
  
+ TCTTGCAAGT TCCAGGATTC TTTTGGGTTG CCCAGAAGTC AATCCCCTCT TCATGCCTAT CGGTTCATCT   
  
  
+ CACCTGTTGA CATGACAACT ATCTGACCAA AAATAAAATT TTTATGCTTT ATCTTGCAGA CATTTTAAAA   
  
  
+ GGGTTTTCGT TCATTTAATA GAATTTTTCT TTTTTTTTAA ATGAATTTGA GGTTCCAAAT ATTTTAGGGA   
  
  
+ TCTTTAAGGG GGAGAAGAGG AGTAATGCAC ATGGTAAAGA GAGTGAAAGT TCTGACACTG TTAAGTGTAG   
  
  
+ GACTGTAGGT GGGTTCCGGT CTTGGAGGGC AACAAGTCAA TTGAAATGAG AAAGTTTCGT GCTAGAAATC   
  
  
+ ATTTGACATT TGCGTTGGGG CAGGGATTCC TGTTTTTCCT TATCTCATTG CTAGTTCATT TGGGTAGGGA   
  
  
+ TTAAATTGGC AGGAACTTAA GTTTGTCTTC CTTTGATTTC ATCGAAAGCT CCAAATCAGT CTTATCAGCA   
  
  
+ ATGGCAGCTT AGCCATTGTG TGATTAGTTT AGTTGCGAGA ATGGAATCTT TAGTGGGTGG GGGGGCACTT   
  
  
+ AGAAGCATGC CAGGTGGAGG GAAACTTCAA GGCTTACGGC CGTGAAACTG TATTTCGTCT TTAAAAAAAA   
  
  
+ AAAAAATATT AGCTCACAAG AATTCTTGAA AAAAAAAAAA AAAAAAGGGA AAGAGGATGT CGTTTTCCAT   
  
  
+ TTCTCTGCTC TGTGGACTTT TTGGGTTCCA CTGGTTTTCA GGGTAGCACG AAACTGTCAA AAAGCCGACT   
  
  
+ GATTTAGAGA GAGGAAAACA ACAATTAGGG AAGGCTGTAC AGAGACAAGC TTTATAATAT CTGTATTTGG   
  
  
+ AGGGAGAAAC AAAGAAGTTG ATAGCTCAA  

- AATTTTTTGA CTGACAAATA TAGTCAACAT AAAAGTATTT TAAACAGTAA GCTAAAATAC CCAAAAACAA   
  
  
- AATTCAATCT TAAATATAGA TATAAAAATG TAATATTGTA TTTTTAAGAC TAAAAAAATT TTTAATTTAC   
  
  
- ATTATTATCT TATATAGATA CCGTAATTTT GATAGTAAGA CCAACTATTT TGTGGGTGTC GGTGTTCCCT   
  
  
- TCTCATTCAT AAAATATAAA GGGGAAGGTG TATTAGGTAT AGGTCCACAC AGAGGACTAC TAATTACCTG   
  
  
- TAGTGGTAGG AGACCCAGGG TACTCTCACT TACGGGGTTA GTCTTGCCGA ACGAGACCCG ACATTATTTT   
  
  
- AGGTCGAGCA CTTAACACTC CCCGCATGTG CCGTTGAACT GGCGGAAACG AAAAAAGAGC CACCCAGGGA   
  
  
- CAACCCCGTT GTAGCGACAG TAGCAGACAC TAAAACTACA CCATCCTTCG TAGGCTAAAC ATTGACAATT   
  
  
- CTCAAAGAAT ACGTTGCAGC AATACCTCTC CAGGACATGC GGGTGACCTC GTAAACTACG ACGTTGTCGT   
  
  
- TTGGTTTTGT TTTCCTCAGT CTAAAAACCT TAAAGATGAG ACAGCCGTCA AATCGGGGGG TCAGACAATT   
  
  
- AGAACGTTCA AGGTCCTAAG AAAACCCAAC GGGTCTTCAG TTAGGGGAGA AGTACGGATA GCCAAGTAGA   
  
  
- GTGGACAACT GTACTGTTGA TAGACTGGTT TTTATTTTAA AAATACGAAA TAGAACGTCT GTAAAATTTT   
  
  
- CCCAAAAGCA AGTAAATTAT CTTAAAAAGA AAAAAAAATT TACTTAAACT CCAAGGTTTA TAAAATCCCT   
  
  
- AGAAATTCCC CCTCTTCTCC TCATTACGTG TACCATTTCT CTCACTTTCA AGACTGTGAC AATTCACATC   
  
  
- CTGACATCCA CCCAAGGCCA GAACCTCCCG TTGTTCAGTT AACTTTACTC TTTCAAAGCA CGATCTTTAG   
  
  
- TAAACTGTAA ACGCAACCCC GTCCCTAAGG ACAAAAAGGA ATAGAGTAAC GATCAAGTAA ACCCATCCCT   
  
  
- AATTTAACCG TCCTTGAATT CAAACAGAAG GAAACTAAAG TAGCTTTCGA GGTTTAGTCA GAATAGTCGT   
  
  
- TACCGTCGAA TCGGTAACAC ACTAATCAAA TCAACGCTCT TACCTTAGAA ATCACCCACC CCCCCGTGAA   
  
  
- TCTTCGTACG GTCCACCTCC CTTTGAAGTT CCGAATGCCG GCACTTTGAC ATAAAGCAGA AATTTTTTTT   
  
  
- TTTTTTATAA TCGAGTGTTC TTAAGAACTT TTTTTTTTTT TTTTTTCCCT TTCTCCTACA GCAAAAGGTA   
  
  
- AAGAGACGAG ACACCTGAAA AACCCAAGGT GACCAAAAGT CCCATCGTGC TTTGACAGTT TTTCGGCTGA   
  
  
- CTAAATCTCT CTCCTTTTGT TGTTAATCCC TTCCGACATG TCTCTGTTCG AAATATTATA GACATAAACC   
  
  
- TCCCTCTTTG TTTCTTCAAC TATCGAGTT

+     W box

| Site Name | Organism | Position | Strand | Matrix score. | sequence | function |
| --- | --- | --- | --- | --- | --- | --- |
| W box | Arabidopsis thaliana | 387 | + | 6 | TTGACC |  |

> 2018/04/13 10:10:12  
+ TTAAAAAACT GACTGTTTAT ATCAGTTGTA TTTTCATAAA ATTTGTCATT CGATTTTATG GGTTTTTGTT   
  
  
+ TTAAGTTAGA ATTTATATCT ATATTTTTAC ATTATAACAT AAAAATTCTG ATTTTTTTAA AAATTAAATG   
  
  
+ TAATAATAGA ATATATCTAT GGCATTAAAA CTATCATTCT GGTTGATAAA ACACCCACAG CCACAAGGGA   
  
  
+ AGAGTAAGTA TTTTATATTT CCCCTTCCAC ATAATCCATA TCCAGGTGTG TCTCCTGATG ATTAATGGAC   
  
  
+ ATCACCATCC TCTGGGTCCC ATGAGAGTGA ATGCCCCAAT CAGAACGGCT TGCTCTGGGC TGTAATAAAA   
  
  
+ TCCAGCTCGT GAATTGTGAG GGGCGTACAC GGCAACTTGA CCGCCTTTGC TTTTTTCTCG GTGGGTCCCT   
  
  
+ GTTGGGGCAA CATCGCTGTC ATCGTCTGTG ATTTTGATGT GGTAGGAAGC ATCCGATTTG TAACTGTTAA   
  
  
+ GAGTTTCTTA TGCAACGTCG TTATGGAGAG GTCCTGTACG CCCACTGGAG CATTTGATGC TGCAACAGCA   
  
  
+ AACCAAAACA AAAGGAGTCA GATTTTTGGA ATTTCTACTC TGTCGGCAGT TTAGCCCCCC AGTCTGTTAA   
  
  
+ TCTTGCAAGT TCCAGGATTC TTTTGGGTTG CCCAGAAGTC AATCCCCTCT TCATGCCTAT CGGTTCATCT   
  
  
+ CACCTGTTGA CATGACAACT ATCTGACCAA AAATAAAATT TTTATGCTTT ATCTTGCAGA CATTTTAAAA   
  
  
+ GGGTTTTCGT TCATTTAATA GAATTTTTCT TTTTTTTTAA ATGAATTTGA GGTTCCAAAT ATTTTAGGGA   
  
  
+ TCTTTAAGGG GGAGAAGAGG AGTAATGCAC ATGGTAAAGA GAGTGAAAGT TCTGACACTG TTAAGTGTAG   
  
  
+ GACTGTAGGT GGGTTCCGGT CTTGGAGGGC AACAAGTCAA TTGAAATGAG AAAGTTTCGT GCTAGAAATC   
  
  
+ ATTTGACATT TGCGTTGGGG CAGGGATTCC TGTTTTTCCT TATCTCATTG CTAGTTCATT TGGGTAGGGA   
  
  
+ TTAAATTGGC AGGAACTTAA GTTTGTCTTC CTTTGATTTC ATCGAAAGCT CCAAATCAGT CTTATCAGCA   
  
  
+ ATGGCAGCTT AGCCATTGTG TGATTAGTTT AGTTGCGAGA ATGGAATCTT TAGTGGGTGG GGGGGCACTT   
  
  
+ AGAAGCATGC CAGGTGGAGG GAAACTTCAA GGCTTACGGC CGTGAAACTG TATTTCGTCT TTAAAAAAAA   
  
  
+ AAAAAATATT AGCTCACAAG AATTCTTGAA AAAAAAAAAA AAAAAAGGGA AAGAGGATGT CGTTTTCCAT   
  
  
+ TTCTCTGCTC TGTGGACTTT TTGGGTTCCA CTGGTTTTCA GGGTAGCACG AAACTGTCAA AAAGCCGACT   
  
  
+ GATTTAGAGA GAGGAAAACA ACAATTAGGG AAGGCTGTAC AGAGACAAGC TTTATAATAT CTGTATTTGG   
  
  
+ AGGGAGAAAC AAAGAAGTTG ATAGCTCAA  

- AATTTTTTGA CTGACAAATA TAGTCAACAT AAAAGTATTT TAAACAGTAA GCTAAAATAC CCAAAAACAA   
  
  
- AATTCAATCT TAAATATAGA TATAAAAATG TAATATTGTA TTTTTAAGAC TAAAAAAATT TTTAATTTAC   
  
  
- ATTATTATCT TATATAGATA CCGTAATTTT GATAGTAAGA CCAACTATTT TGTGGGTGTC GGTGTTCCCT   
  
  
- TCTCATTCAT AAAATATAAA GGGGAAGGTG TATTAGGTAT AGGTCCACAC AGAGGACTAC TAATTACCTG   
  
  
- TAGTGGTAGG AGACCCAGGG TACTCTCACT TACGGGGTTA GTCTTGCCGA ACGAGACCCG ACATTATTTT   
  
  
- AGGTCGAGCA CTTAACACTC CCCGCATGTG CCGTTGAACT GGCGGAAACG AAAAAAGAGC CACCCAGGGA   
  
  
- CAACCCCGTT GTAGCGACAG TAGCAGACAC TAAAACTACA CCATCCTTCG TAGGCTAAAC ATTGACAATT   
  
  
- CTCAAAGAAT ACGTTGCAGC AATACCTCTC CAGGACATGC GGGTGACCTC GTAAACTACG ACGTTGTCGT   
  
  
- TTGGTTTTGT TTTCCTCAGT CTAAAAACCT TAAAGATGAG ACAGCCGTCA AATCGGGGGG TCAGACAATT   
  
  
- AGAACGTTCA AGGTCCTAAG AAAACCCAAC GGGTCTTCAG TTAGGGGAGA AGTACGGATA GCCAAGTAGA   
  
  
- GTGGACAACT GTACTGTTGA TAGACTGGTT TTTATTTTAA AAATACGAAA TAGAACGTCT GTAAAATTTT   
  
  
- CCCAAAAGCA AGTAAATTAT CTTAAAAAGA AAAAAAAATT TACTTAAACT CCAAGGTTTA TAAAATCCCT   
  
  
- AGAAATTCCC CCTCTTCTCC TCATTACGTG TACCATTTCT CTCACTTTCA AGACTGTGAC AATTCACATC   
  
  
- CTGACATCCA CCCAAGGCCA GAACCTCCCG TTGTTCAGTT AACTTTACTC TTTCAAAGCA CGATCTTTAG   
  
  
- TAAACTGTAA ACGCAACCCC GTCCCTAAGG ACAAAAAGGA ATAGAGTAAC GATCAAGTAA ACCCATCCCT   
  
  
- AATTTAACCG TCCTTGAATT CAAACAGAAG GAAACTAAAG TAGCTTTCGA GGTTTAGTCA GAATAGTCGT   
  
  
- TACCGTCGAA TCGGTAACAC ACTAATCAAA TCAACGCTCT TACCTTAGAA ATCACCCACC CCCCCGTGAA   
  
  
- TCTTCGTACG GTCCACCTCC CTTTGAAGTT CCGAATGCCG GCACTTTGAC ATAAAGCAGA AATTTTTTTT   
  
  
- TTTTTTATAA TCGAGTGTTC TTAAGAACTT TTTTTTTTTT TTTTTTCCCT TTCTCCTACA GCAAAAGGTA   
  
  
- AAGAGACGAG ACACCTGAAA AACCCAAGGT GACCAAAAGT CCCATCGTGC TTTGACAGTT TTTCGGCTGA   
  
  
- CTAAATCTCT CTCCTTTTGT TGTTAATCCC TTCCGACATG TCTCTGTTCG AAATATTATA GACATAAACC   
  
  
- TCCCTCTTTG TTTCTTCAAC TATCGAGTT

+     box II

| Site Name | Organism | Position | Strand | Matrix score. | sequence | function |
| --- | --- | --- | --- | --- | --- | --- |
| box II | Petroselinum hortense | 1199 | - | 9 | TCCACGTGGC | part of a light responsive element |

> 2018/04/13 10:10:12  
+ TTAAAAAACT GACTGTTTAT ATCAGTTGTA TTTTCATAAA ATTTGTCATT CGATTTTATG GGTTTTTGTT   
  
  
+ TTAAGTTAGA ATTTATATCT ATATTTTTAC ATTATAACAT AAAAATTCTG ATTTTTTTAA AAATTAAATG   
  
  
+ TAATAATAGA ATATATCTAT GGCATTAAAA CTATCATTCT GGTTGATAAA ACACCCACAG CCACAAGGGA   
  
  
+ AGAGTAAGTA TTTTATATTT CCCCTTCCAC ATAATCCATA TCCAGGTGTG TCTCCTGATG ATTAATGGAC   
  
  
+ ATCACCATCC TCTGGGTCCC ATGAGAGTGA ATGCCCCAAT CAGAACGGCT TGCTCTGGGC TGTAATAAAA   
  
  
+ TCCAGCTCGT GAATTGTGAG GGGCGTACAC GGCAACTTGA CCGCCTTTGC TTTTTTCTCG GTGGGTCCCT   
  
  
+ GTTGGGGCAA CATCGCTGTC ATCGTCTGTG ATTTTGATGT GGTAGGAAGC ATCCGATTTG TAACTGTTAA   
  
  
+ GAGTTTCTTA TGCAACGTCG TTATGGAGAG GTCCTGTACG CCCACTGGAG CATTTGATGC TGCAACAGCA   
  
  
+ AACCAAAACA AAAGGAGTCA GATTTTTGGA ATTTCTACTC TGTCGGCAGT TTAGCCCCCC AGTCTGTTAA   
  
  
+ TCTTGCAAGT TCCAGGATTC TTTTGGGTTG CCCAGAAGTC AATCCCCTCT TCATGCCTAT CGGTTCATCT   
  
  
+ CACCTGTTGA CATGACAACT ATCTGACCAA AAATAAAATT TTTATGCTTT ATCTTGCAGA CATTTTAAAA   
  
  
+ GGGTTTTCGT TCATTTAATA GAATTTTTCT TTTTTTTTAA ATGAATTTGA GGTTCCAAAT ATTTTAGGGA   
  
  
+ TCTTTAAGGG GGAGAAGAGG AGTAATGCAC ATGGTAAAGA GAGTGAAAGT TCTGACACTG TTAAGTGTAG   
  
  
+ GACTGTAGGT GGGTTCCGGT CTTGGAGGGC AACAAGTCAA TTGAAATGAG AAAGTTTCGT GCTAGAAATC   
  
  
+ ATTTGACATT TGCGTTGGGG CAGGGATTCC TGTTTTTCCT TATCTCATTG CTAGTTCATT TGGGTAGGGA   
  
  
+ TTAAATTGGC AGGAACTTAA GTTTGTCTTC CTTTGATTTC ATCGAAAGCT CCAAATCAGT CTTATCAGCA   
  
  
+ ATGGCAGCTT AGCCATTGTG TGATTAGTTT AGTTGCGAGA ATGGAATCTT TAGTGGGTGG GGGGGCACTT   
  
  
+ AGAAGCATGC CAGGTGGAGG GAAACTTCAA GGCTTACGGC CGTGAAACTG TATTTCGTCT TTAAAAAAAA   
  
  
+ AAAAAATATT AGCTCACAAG AATTCTTGAA AAAAAAAAAA AAAAAAGGGA AAGAGGATGT CGTTTTCCAT   
  
  
+ TTCTCTGCTC TGTGGACTTT TTGGGTTCCA CTGGTTTTCA GGGTAGCACG AAACTGTCAA AAAGCCGACT   
  
  
+ GATTTAGAGA GAGGAAAACA ACAATTAGGG AAGGCTGTAC AGAGACAAGC TTTATAATAT CTGTATTTGG   
  
  
+ AGGGAGAAAC AAAGAAGTTG ATAGCTCAA  

- AATTTTTTGA CTGACAAATA TAGTCAACAT AAAAGTATTT TAAACAGTAA GCTAAAATAC CCAAAAACAA   
  
  
- AATTCAATCT TAAATATAGA TATAAAAATG TAATATTGTA TTTTTAAGAC TAAAAAAATT TTTAATTTAC   
  
  
- ATTATTATCT TATATAGATA CCGTAATTTT GATAGTAAGA CCAACTATTT TGTGGGTGTC GGTGTTCCCT   
  
  
- TCTCATTCAT AAAATATAAA GGGGAAGGTG TATTAGGTAT AGGTCCACAC AGAGGACTAC TAATTACCTG   
  
  
- TAGTGGTAGG AGACCCAGGG TACTCTCACT TACGGGGTTA GTCTTGCCGA ACGAGACCCG ACATTATTTT   
  
  
- AGGTCGAGCA CTTAACACTC CCCGCATGTG CCGTTGAACT GGCGGAAACG AAAAAAGAGC CACCCAGGGA   
  
  
- CAACCCCGTT GTAGCGACAG TAGCAGACAC TAAAACTACA CCATCCTTCG TAGGCTAAAC ATTGACAATT   
  
  
- CTCAAAGAAT ACGTTGCAGC AATACCTCTC CAGGACATGC GGGTGACCTC GTAAACTACG ACGTTGTCGT   
  
  
- TTGGTTTTGT TTTCCTCAGT CTAAAAACCT TAAAGATGAG ACAGCCGTCA AATCGGGGGG TCAGACAATT   
  
  
- AGAACGTTCA AGGTCCTAAG AAAACCCAAC GGGTCTTCAG TTAGGGGAGA AGTACGGATA GCCAAGTAGA   
  
  
- GTGGACAACT GTACTGTTGA TAGACTGGTT TTTATTTTAA AAATACGAAA TAGAACGTCT GTAAAATTTT   
  
  
- CCCAAAAGCA AGTAAATTAT CTTAAAAAGA AAAAAAAATT TACTTAAACT CCAAGGTTTA TAAAATCCCT   
  
  
- AGAAATTCCC CCTCTTCTCC TCATTACGTG TACCATTTCT CTCACTTTCA AGACTGTGAC AATTCACATC   
  
  
- CTGACATCCA CCCAAGGCCA GAACCTCCCG TTGTTCAGTT AACTTTACTC TTTCAAAGCA CGATCTTTAG   
  
  
- TAAACTGTAA ACGCAACCCC GTCCCTAAGG ACAAAAAGGA ATAGAGTAAC GATCAAGTAA ACCCATCCCT   
  
  
- AATTTAACCG TCCTTGAATT CAAACAGAAG GAAACTAAAG TAGCTTTCGA GGTTTAGTCA GAATAGTCGT   
  
  
- TACCGTCGAA TCGGTAACAC ACTAATCAAA TCAACGCTCT TACCTTAGAA ATCACCCACC CCCCCGTGAA   
  
  
- TCTTCGTACG GTCCACCTCC CTTTGAAGTT CCGAATGCCG GCACTTTGAC ATAAAGCAGA AATTTTTTTT   
  
  
- TTTTTTATAA TCGAGTGTTC TTAAGAACTT TTTTTTTTTT TTTTTTCCCT TTCTCCTACA GCAAAAGGTA   
  
  
- AAGAGACGAG ACACCTGAAA AACCCAAGGT GACCAAAAGT CCCATCGTGC TTTGACAGTT TTTCGGCTGA   
  
  
- CTAAATCTCT CTCCTTTTGT TGTTAATCCC TTCCGACATG TCTCTGTTCG AAATATTATA GACATAAACC   
  
  
- TCCCTCTTTG TTTCTTCAAC TATCGAGTT

+     circadian

| Site Name | Organism | Position | Strand | Matrix score. | sequence | function |
| --- | --- | --- | --- | --- | --- | --- |
| circadian | Lycopersicon esculentum | 646 | - | 6 | CAANNNNATC | cis-acting regulatory element involved in circadian control |
| circadian | Lycopersicon esculentum | 1049 | - | 6 | CAANNNNATC | cis-acting regulatory element involved in circadian control |

> 2018/04/13 10:10:12  
+ TTAAAAAACT GACTGTTTAT ATCAGTTGTA TTTTCATAAA ATTTGTCATT CGATTTTATG GGTTTTTGTT   
  
  
+ TTAAGTTAGA ATTTATATCT ATATTTTTAC ATTATAACAT AAAAATTCTG ATTTTTTTAA AAATTAAATG   
  
  
+ TAATAATAGA ATATATCTAT GGCATTAAAA CTATCATTCT GGTTGATAAA ACACCCACAG CCACAAGGGA   
  
  
+ AGAGTAAGTA TTTTATATTT CCCCTTCCAC ATAATCCATA TCCAGGTGTG TCTCCTGATG ATTAATGGAC   
  
  
+ ATCACCATCC TCTGGGTCCC ATGAGAGTGA ATGCCCCAAT CAGAACGGCT TGCTCTGGGC TGTAATAAAA   
  
  
+ TCCAGCTCGT GAATTGTGAG GGGCGTACAC GGCAACTTGA CCGCCTTTGC TTTTTTCTCG GTGGGTCCCT   
  
  
+ GTTGGGGCAA CATCGCTGTC ATCGTCTGTG ATTTTGATGT GGTAGGAAGC ATCCGATTTG TAACTGTTAA   
  
  
+ GAGTTTCTTA TGCAACGTCG TTATGGAGAG GTCCTGTACG CCCACTGGAG CATTTGATGC TGCAACAGCA   
  
  
+ AACCAAAACA AAAGGAGTCA GATTTTTGGA ATTTCTACTC TGTCGGCAGT TTAGCCCCCC AGTCTGTTAA   
  
  
+ TCTTGCAAGT TCCAGGATTC TTTTGGGTTG CCCAGAAGTC AATCCCCTCT TCATGCCTAT CGGTTCATCT   
  
  
+ CACCTGTTGA CATGACAACT ATCTGACCAA AAATAAAATT TTTATGCTTT ATCTTGCAGA CATTTTAAAA   
  
  
+ GGGTTTTCGT TCATTTAATA GAATTTTTCT TTTTTTTTAA ATGAATTTGA GGTTCCAAAT ATTTTAGGGA   
  
  
+ TCTTTAAGGG GGAGAAGAGG AGTAATGCAC ATGGTAAAGA GAGTGAAAGT TCTGACACTG TTAAGTGTAG   
  
  
+ GACTGTAGGT GGGTTCCGGT CTTGGAGGGC AACAAGTCAA TTGAAATGAG AAAGTTTCGT GCTAGAAATC   
  
  
+ ATTTGACATT TGCGTTGGGG CAGGGATTCC TGTTTTTCCT TATCTCATTG CTAGTTCATT TGGGTAGGGA   
  
  
+ TTAAATTGGC AGGAACTTAA GTTTGTCTTC CTTTGATTTC ATCGAAAGCT CCAAATCAGT CTTATCAGCA   
  
  
+ ATGGCAGCTT AGCCATTGTG TGATTAGTTT AGTTGCGAGA ATGGAATCTT TAGTGGGTGG GGGGGCACTT   
  
  
+ AGAAGCATGC CAGGTGGAGG GAAACTTCAA GGCTTACGGC CGTGAAACTG TATTTCGTCT TTAAAAAAAA   
  
  
+ AAAAAATATT AGCTCACAAG AATTCTTGAA AAAAAAAAAA AAAAAAGGGA AAGAGGATGT CGTTTTCCAT   
  
  
+ TTCTCTGCTC TGTGGACTTT TTGGGTTCCA CTGGTTTTCA GGGTAGCACG AAACTGTCAA AAAGCCGACT   
  
  
+ GATTTAGAGA GAGGAAAACA ACAATTAGGG AAGGCTGTAC AGAGACAAGC TTTATAATAT CTGTATTTGG   
  
  
+ AGGGAGAAAC AAAGAAGTTG ATAGCTCAA  

- AATTTTTTGA CTGACAAATA TAGTCAACAT AAAAGTATTT TAAACAGTAA GCTAAAATAC CCAAAAACAA   
  
  
- AATTCAATCT TAAATATAGA TATAAAAATG TAATATTGTA TTTTTAAGAC TAAAAAAATT TTTAATTTAC   
  
  
- ATTATTATCT TATATAGATA CCGTAATTTT GATAGTAAGA CCAACTATTT TGTGGGTGTC GGTGTTCCCT   
  
  
- TCTCATTCAT AAAATATAAA GGGGAAGGTG TATTAGGTAT AGGTCCACAC AGAGGACTAC TAATTACCTG   
  
  
- TAGTGGTAGG AGACCCAGGG TACTCTCACT TACGGGGTTA GTCTTGCCGA ACGAGACCCG ACATTATTTT   
  
  
- AGGTCGAGCA CTTAACACTC CCCGCATGTG CCGTTGAACT GGCGGAAACG AAAAAAGAGC CACCCAGGGA   
  
  
- CAACCCCGTT GTAGCGACAG TAGCAGACAC TAAAACTACA CCATCCTTCG TAGGCTAAAC ATTGACAATT   
  
  
- CTCAAAGAAT ACGTTGCAGC AATACCTCTC CAGGACATGC GGGTGACCTC GTAAACTACG ACGTTGTCGT   
  
  
- TTGGTTTTGT TTTCCTCAGT CTAAAAACCT TAAAGATGAG ACAGCCGTCA AATCGGGGGG TCAGACAATT   
  
  
- AGAACGTTCA AGGTCCTAAG AAAACCCAAC GGGTCTTCAG TTAGGGGAGA AGTACGGATA GCCAAGTAGA   
  
  
- GTGGACAACT GTACTGTTGA TAGACTGGTT TTTATTTTAA AAATACGAAA TAGAACGTCT GTAAAATTTT   
  
  
- CCCAAAAGCA AGTAAATTAT CTTAAAAAGA AAAAAAAATT TACTTAAACT CCAAGGTTTA TAAAATCCCT   
  
  
- AGAAATTCCC CCTCTTCTCC TCATTACGTG TACCATTTCT CTCACTTTCA AGACTGTGAC AATTCACATC   
  
  
- CTGACATCCA CCCAAGGCCA GAACCTCCCG TTGTTCAGTT AACTTTACTC TTTCAAAGCA CGATCTTTAG   
  
  
- TAAACTGTAA ACGCAACCCC GTCCCTAAGG ACAAAAAGGA ATAGAGTAAC GATCAAGTAA ACCCATCCCT   
  
  
- AATTTAACCG TCCTTGAATT CAAACAGAAG GAAACTAAAG TAGCTTTCGA GGTTTAGTCA GAATAGTCGT   
  
  
- TACCGTCGAA TCGGTAACAC ACTAATCAAA TCAACGCTCT TACCTTAGAA ATCACCCACC CCCCCGTGAA   
  
  
- TCTTCGTACG GTCCACCTCC CTTTGAAGTT CCGAATGCCG GCACTTTGAC ATAAAGCAGA AATTTTTTTT   
  
  
- TTTTTTATAA TCGAGTGTTC TTAAGAACTT TTTTTTTTTT TTTTTTCCCT TTCTCCTACA GCAAAAGGTA   
  
  
- AAGAGACGAG ACACCTGAAA AACCCAAGGT GACCAAAAGT CCCATCGTGC TTTGACAGTT TTTCGGCTGA   
  
  
- CTAAATCTCT CTCCTTTTGT TGTTAATCCC TTCCGACATG TCTCTGTTCG AAATATTATA GACATAAACC   
  
  
- TCCCTCTTTG TTTCTTCAAC TATCGAGTT
