# Supplementary material for: Genome-wide analysis of the WRKY gene family in drumstick (Moringa oleifera Lam.)
Source: PeerJ. 2019 Jun 10;7:e7063. doi: 10.7717/peerj.7063 (PMC6563795; doi:10.7717/peerj.7063)
Supplement: Supplemental Information 1 [file peerj-07-7063-s003.gz › MoWRKY11_plantcare.html]

Content-Type: text/html; charset=ISO-8859-1


CallMat\_Firefox


Webmaster Firefox specific output  
To save the result:
click on the frame with the right mouse button and save the source code as a text file with extension .html  
REFERENCE:PlantCARE: a database of plant cis-acting regulatory elements and a portal to tools for in silico analysis of promoter sequences.  
Lescot, M., Déhais, P., Moreau, Y., De Moor, B., Rouzé ,P.,and Rombauts, S.  
Nucleic Acids Res., Database issue(2002), 30(1):325-327.   


---

> 2018/04/13 10:10:12  
+ ATAACCTCGA TTGTTAGATT TGGCTCGAAA ATAAACAAAG GACATACTCC TTCTTGTTCT GTTTCAACTT   
  
  
+ TTCTCCTCTC CTCGTCCGAT CGAATAATAC GATTATCTTT ACCGTATTGT ACCCATCTTT TCCCACCCCC   
  
  
+ AATTACCTTT CCGCAGGTTG AATTGACTGA ACTGTCTTAA CTGGGTACAT TTCTCACTAA AGGTACCTAC   
  
  
+ GTAGGGGAGT GTTTCTAATT CTCATACACG GCATTTGCCG TGCGTTTAAC TGTTTGTCTT CCTACCAAAT   
  
  
+ CAAAGAACAG CTTTTCCAAC GGTTCTTATT TACTAAAATT CGGGTATTCA ATACTTTCAC CTGCTCGTAC   
  
  
+ CTTCAACTTG ACTACTTCTT CGGTACGGTT GGTCGAATTA GTATCTTAAT GAAGAACACA ATATAGACTT   
  
  
+ TATATCTACA TTGTAGTACC TGAAGGTACC TACTGATCGA GACCAACATC GAATCTCGAA CGAATTGCAT   
  
  
+ GAATTTGATG CTTCCTTTTC CTCATACGAA CGACAAATAA GAGTGTATCT TCGGTAGAAG TCGGTTAGTC   
  
  
+ GGTTATCGTA GTGAATTTGG CCATCCACTT AAAGTACGGA GCTCCAGTTT TTCGTCTATA AATTCAAAGT   
  
  
+ TGATTAGTTA ACTCACGTGT GAGGGGTGAA AGATGTACAT GTCAAGTAGC GTACTCGAAA TTGCGATTTA   
  
  
+ CTGAACCCGT ATGTATTCTC CTTAAAAGCG ACCCAAAAGG AGCCTCGAAG TTCCTTCTAG TTATAGAGTC   
  
  
+ TTTTTGTGAT GGACGACATC ACGTACCCTG TACAGCCGTA CCAAAATACC CCTATACTTC AGGATAACGA   
  
  
+ CAGACACCAA TTAATATTAC TAATTACTAC CATAATTCAT TTATGTAAGA GTTTAGTAAA CCTGAAGAAA   
  
  
+ GTTCTCCCCA GTGACCATGA GACAGAGTAG ATGGTTAAAA AGCCCGAAAG AAAAAGGCAT ACCCCCTCCT   
  
  
+ AATCTAACTA AACAGCGTCA CAGTGACCAA AACAAAGTTA GTTCGAGAGA TCCTTATTAA ATAATCCTTT   
  
  
+ TATGAACTTT TCTTCTCTTT CGTTCTTCTT TTACTTTTAG TTCCTAATGG TATCCTCTTA TCGTTCGTTT   
  
  
+ CCAGATACTT CTCTTCTACA TTGAGGTAAC CAGTGTGATT ATACATCGTA CCTCTTTCTT TCGACCTTCC   
  
  
+ ATTTCCGTTT AATATTATCG TATAGGTGTT CACTATATAG GTGAACACCA ACCTGAGACT TAGTTCGTCG   
  
  
+ ACGAAGACGA CCACACAACT TGAGGAGGGT CCGAAGTTAG GTTCACTGGT ACCGTAGACG TTCGAAGAGT   
  
  
+ TTCATTCCTA GTTATACTGG GTTAAAGAGA AACTGGACGG ACCTCTCCCG TTCCCTCGTG AAGTCCCGAC   
  
  
+ AACACGTCAG AAAACCGTCC TGTTGTCTAA CCTTGTTACC TTAGTCGACA AGGACGGTCA TATCTGGGAA   
  
  
+ CTTAAGGAAG AAGTTCAATC TTTTGCCCA  

- TATTGGAGCT AACAATCTAA ACCGAGCTTT TATTTGTTTC CTGTATGAGG AAGAACAAGA CAAAGTTGAA   
  
  
- AAGAGGAGAG GAGCAGGCTA GCTTATTATG CTAATAGAAA TGGCATAACA TGGGTAGAAA AGGGTGGGGG   
  
  
- TTAATGGAAA GGCGTCCAAC TTAACTGACT TGACAGAATT GACCCATGTA AAGAGTGATT TCCATGGATG   
  
  
- CATCCCCTCA CAAAGATTAA GAGTATGTGC CGTAAACGGC ACGCAAATTG ACAAACAGAA GGATGGTTTA   
  
  
- GTTTCTTGTC GAAAAGGTTG CCAAGAATAA ATGATTTTAA GCCCATAAGT TATGAAAGTG GACGAGCATG   
  
  
- GAAGTTGAAC TGATGAAGAA GCCATGCCAA CCAGCTTAAT CATAGAATTA CTTCTTGTGT TATATCTGAA   
  
  
- ATATAGATGT AACATCATGG ACTTCCATGG ATGACTAGCT CTGGTTGTAG CTTAGAGCTT GCTTAACGTA   
  
  
- CTTAAACTAC GAAGGAAAAG GAGTATGCTT GCTGTTTATT CTCACATAGA AGCCATCTTC AGCCAATCAG   
  
  
- CCAATAGCAT CACTTAAACC GGTAGGTGAA TTTCATGCCT CGAGGTCAAA AAGCAGATAT TTAAGTTTCA   
  
  
- ACTAATCAAT TGAGTGCACA CTCCCCACTT TCTACATGTA CAGTTCATCG CATGAGCTTT AACGCTAAAT   
  
  
- GACTTGGGCA TACATAAGAG GAATTTTCGC TGGGTTTTCC TCGGAGCTTC AAGGAAGATC AATATCTCAG   
  
  
- AAAAACACTA CCTGCTGTAG TGCATGGGAC ATGTCGGCAT GGTTTTATGG GGATATGAAG TCCTATTGCT   
  
  
- GTCTGTGGTT AATTATAATG ATTAATGATG GTATTAAGTA AATACATTCT CAAATCATTT GGACTTCTTT   
  
  
- CAAGAGGGGT CACTGGTACT CTGTCTCATC TACCAATTTT TCGGGCTTTC TTTTTCCGTA TGGGGGAGGA   
  
  
- TTAGATTGAT TTGTCGCAGT GTCACTGGTT TTGTTTCAAT CAAGCTCTCT AGGAATAATT TATTAGGAAA   
  
  
- ATACTTGAAA AGAAGAGAAA GCAAGAAGAA AATGAAAATC AAGGATTACC ATAGGAGAAT AGCAAGCAAA   
  
  
- GGTCTATGAA GAGAAGATGT AACTCCATTG GTCACACTAA TATGTAGCAT GGAGAAAGAA AGCTGGAAGG   
  
  
- TAAAGGCAAA TTATAATAGC ATATCCACAA GTGATATATC CACTTGTGGT TGGACTCTGA ATCAAGCAGC   
  
  
- TGCTTCTGCT GGTGTGTTGA ACTCCTCCCA GGCTTCAATC CAAGTGACCA TGGCATCTGC AAGCTTCTCA   
  
  
- AAGTAAGGAT CAATATGACC CAATTTCTCT TTGACCTGCC TGGAGAGGGC AAGGGAGCAC TTCAGGGCTG   
  
  
- TTGTGCAGTC TTTTGGCAGG ACAACAGATT GGAACAATGG AATCAGCTGT TCCTGCCAGT ATAGACCCTT   
  
  
- GAATTCCTTC TTCAAGTTAG AAAACGGGT

  
  
Motifs Found  

+     5UTR Py-rich stretch

| Site Name | Organism | Position | Strand | Matrix score. | sequence | function |
| --- | --- | --- | --- | --- | --- | --- |
| 5UTR Py-rich stretch | Lycopersicon esculentum | 1059 | + | 10 | TTTCTTCTCT | cis-acting element conferring high transcription levels |
| 5UTR Py-rich stretch | Lycopersicon esculentum | 70 | + | 9 | TTTCTTCTCT | cis-acting element conferring high transcription levels |

> 2018/04/13 10:10:12  
+ ATAACCTCGA TTGTTAGATT TGGCTCGAAA ATAAACAAAG GACATACTCC TTCTTGTTCT GTTTCAACTT   
  
  
+ TTCTCCTCTC CTCGTCCGAT CGAATAATAC GATTATCTTT ACCGTATTGT ACCCATCTTT TCCCACCCCC   
  
  
+ AATTACCTTT CCGCAGGTTG AATTGACTGA ACTGTCTTAA CTGGGTACAT TTCTCACTAA AGGTACCTAC   
  
  
+ GTAGGGGAGT GTTTCTAATT CTCATACACG GCATTTGCCG TGCGTTTAAC TGTTTGTCTT CCTACCAAAT   
  
  
+ CAAAGAACAG CTTTTCCAAC GGTTCTTATT TACTAAAATT CGGGTATTCA ATACTTTCAC CTGCTCGTAC   
  
  
+ CTTCAACTTG ACTACTTCTT CGGTACGGTT GGTCGAATTA GTATCTTAAT GAAGAACACA ATATAGACTT   
  
  
+ TATATCTACA TTGTAGTACC TGAAGGTACC TACTGATCGA GACCAACATC GAATCTCGAA CGAATTGCAT   
  
  
+ GAATTTGATG CTTCCTTTTC CTCATACGAA CGACAAATAA GAGTGTATCT TCGGTAGAAG TCGGTTAGTC   
  
  
+ GGTTATCGTA GTGAATTTGG CCATCCACTT AAAGTACGGA GCTCCAGTTT TTCGTCTATA AATTCAAAGT   
  
  
+ TGATTAGTTA ACTCACGTGT GAGGGGTGAA AGATGTACAT GTCAAGTAGC GTACTCGAAA TTGCGATTTA   
  
  
+ CTGAACCCGT ATGTATTCTC CTTAAAAGCG ACCCAAAAGG AGCCTCGAAG TTCCTTCTAG TTATAGAGTC   
  
  
+ TTTTTGTGAT GGACGACATC ACGTACCCTG TACAGCCGTA CCAAAATACC CCTATACTTC AGGATAACGA   
  
  
+ CAGACACCAA TTAATATTAC TAATTACTAC CATAATTCAT TTATGTAAGA GTTTAGTAAA CCTGAAGAAA   
  
  
+ GTTCTCCCCA GTGACCATGA GACAGAGTAG ATGGTTAAAA AGCCCGAAAG AAAAAGGCAT ACCCCCTCCT   
  
  
+ AATCTAACTA AACAGCGTCA CAGTGACCAA AACAAAGTTA GTTCGAGAGA TCCTTATTAA ATAATCCTTT   
  
  
+ TATGAACTTT TCTTCTCTTT CGTTCTTCTT TTACTTTTAG TTCCTAATGG TATCCTCTTA TCGTTCGTTT   
  
  
+ CCAGATACTT CTCTTCTACA TTGAGGTAAC CAGTGTGATT ATACATCGTA CCTCTTTCTT TCGACCTTCC   
  
  
+ ATTTCCGTTT AATATTATCG TATAGGTGTT CACTATATAG GTGAACACCA ACCTGAGACT TAGTTCGTCG   
  
  
+ ACGAAGACGA CCACACAACT TGAGGAGGGT CCGAAGTTAG GTTCACTGGT ACCGTAGACG TTCGAAGAGT   
  
  
+ TTCATTCCTA GTTATACTGG GTTAAAGAGA AACTGGACGG ACCTCTCCCG TTCCCTCGTG AAGTCCCGAC   
  
  
+ AACACGTCAG AAAACCGTCC TGTTGTCTAA CCTTGTTACC TTAGTCGACA AGGACGGTCA TATCTGGGAA   
  
  
+ CTTAAGGAAG AAGTTCAATC TTTTGCCCA  

- TATTGGAGCT AACAATCTAA ACCGAGCTTT TATTTGTTTC CTGTATGAGG AAGAACAAGA CAAAGTTGAA   
  
  
- AAGAGGAGAG GAGCAGGCTA GCTTATTATG CTAATAGAAA TGGCATAACA TGGGTAGAAA AGGGTGGGGG   
  
  
- TTAATGGAAA GGCGTCCAAC TTAACTGACT TGACAGAATT GACCCATGTA AAGAGTGATT TCCATGGATG   
  
  
- CATCCCCTCA CAAAGATTAA GAGTATGTGC CGTAAACGGC ACGCAAATTG ACAAACAGAA GGATGGTTTA   
  
  
- GTTTCTTGTC GAAAAGGTTG CCAAGAATAA ATGATTTTAA GCCCATAAGT TATGAAAGTG GACGAGCATG   
  
  
- GAAGTTGAAC TGATGAAGAA GCCATGCCAA CCAGCTTAAT CATAGAATTA CTTCTTGTGT TATATCTGAA   
  
  
- ATATAGATGT AACATCATGG ACTTCCATGG ATGACTAGCT CTGGTTGTAG CTTAGAGCTT GCTTAACGTA   
  
  
- CTTAAACTAC GAAGGAAAAG GAGTATGCTT GCTGTTTATT CTCACATAGA AGCCATCTTC AGCCAATCAG   
  
  
- CCAATAGCAT CACTTAAACC GGTAGGTGAA TTTCATGCCT CGAGGTCAAA AAGCAGATAT TTAAGTTTCA   
  
  
- ACTAATCAAT TGAGTGCACA CTCCCCACTT TCTACATGTA CAGTTCATCG CATGAGCTTT AACGCTAAAT   
  
  
- GACTTGGGCA TACATAAGAG GAATTTTCGC TGGGTTTTCC TCGGAGCTTC AAGGAAGATC AATATCTCAG   
  
  
- AAAAACACTA CCTGCTGTAG TGCATGGGAC ATGTCGGCAT GGTTTTATGG GGATATGAAG TCCTATTGCT   
  
  
- GTCTGTGGTT AATTATAATG ATTAATGATG GTATTAAGTA AATACATTCT CAAATCATTT GGACTTCTTT   
  
  
- CAAGAGGGGT CACTGGTACT CTGTCTCATC TACCAATTTT TCGGGCTTTC TTTTTCCGTA TGGGGGAGGA   
  
  
- TTAGATTGAT TTGTCGCAGT GTCACTGGTT TTGTTTCAAT CAAGCTCTCT AGGAATAATT TATTAGGAAA   
  
  
- ATACTTGAAA AGAAGAGAAA GCAAGAAGAA AATGAAAATC AAGGATTACC ATAGGAGAAT AGCAAGCAAA   
  
  
- GGTCTATGAA GAGAAGATGT AACTCCATTG GTCACACTAA TATGTAGCAT GGAGAAAGAA AGCTGGAAGG   
  
  
- TAAAGGCAAA TTATAATAGC ATATCCACAA GTGATATATC CACTTGTGGT TGGACTCTGA ATCAAGCAGC   
  
  
- TGCTTCTGCT GGTGTGTTGA ACTCCTCCCA GGCTTCAATC CAAGTGACCA TGGCATCTGC AAGCTTCTCA   
  
  
- AAGTAAGGAT CAATATGACC CAATTTCTCT TTGACCTGCC TGGAGAGGGC AAGGGAGCAC TTCAGGGCTG   
  
  
- TTGTGCAGTC TTTTGGCAGG ACAACAGATT GGAACAATGG AATCAGCTGT TCCTGCCAGT ATAGACCCTT   
  
  
- GAATTCCTTC TTCAAGTTAG AAAACGGGT

+     A-box

| Site Name | Organism | Position | Strand | Matrix score. | sequence | function |
| --- | --- | --- | --- | --- | --- | --- |
| A-box | Petroselinum crispum | 1415 | + | 6 | CCGTCC | cis-acting regulatory element |
| A-box | Petroselinum crispum | 1452 | - | 6 | CCGTCC | cis-acting regulatory element |
| A-box | Petroselinum crispum | 1365 | - | 6 | CCGTCC | cis-acting regulatory element |

> 2018/04/13 10:10:12  
+ ATAACCTCGA TTGTTAGATT TGGCTCGAAA ATAAACAAAG GACATACTCC TTCTTGTTCT GTTTCAACTT   
  
  
+ TTCTCCTCTC CTCGTCCGAT CGAATAATAC GATTATCTTT ACCGTATTGT ACCCATCTTT TCCCACCCCC   
  
  
+ AATTACCTTT CCGCAGGTTG AATTGACTGA ACTGTCTTAA CTGGGTACAT TTCTCACTAA AGGTACCTAC   
  
  
+ GTAGGGGAGT GTTTCTAATT CTCATACACG GCATTTGCCG TGCGTTTAAC TGTTTGTCTT CCTACCAAAT   
  
  
+ CAAAGAACAG CTTTTCCAAC GGTTCTTATT TACTAAAATT CGGGTATTCA ATACTTTCAC CTGCTCGTAC   
  
  
+ CTTCAACTTG ACTACTTCTT CGGTACGGTT GGTCGAATTA GTATCTTAAT GAAGAACACA ATATAGACTT   
  
  
+ TATATCTACA TTGTAGTACC TGAAGGTACC TACTGATCGA GACCAACATC GAATCTCGAA CGAATTGCAT   
  
  
+ GAATTTGATG CTTCCTTTTC CTCATACGAA CGACAAATAA GAGTGTATCT TCGGTAGAAG TCGGTTAGTC   
  
  
+ GGTTATCGTA GTGAATTTGG CCATCCACTT AAAGTACGGA GCTCCAGTTT TTCGTCTATA AATTCAAAGT   
  
  
+ TGATTAGTTA ACTCACGTGT GAGGGGTGAA AGATGTACAT GTCAAGTAGC GTACTCGAAA TTGCGATTTA   
  
  
+ CTGAACCCGT ATGTATTCTC CTTAAAAGCG ACCCAAAAGG AGCCTCGAAG TTCCTTCTAG TTATAGAGTC   
  
  
+ TTTTTGTGAT GGACGACATC ACGTACCCTG TACAGCCGTA CCAAAATACC CCTATACTTC AGGATAACGA   
  
  
+ CAGACACCAA TTAATATTAC TAATTACTAC CATAATTCAT TTATGTAAGA GTTTAGTAAA CCTGAAGAAA   
  
  
+ GTTCTCCCCA GTGACCATGA GACAGAGTAG ATGGTTAAAA AGCCCGAAAG AAAAAGGCAT ACCCCCTCCT   
  
  
+ AATCTAACTA AACAGCGTCA CAGTGACCAA AACAAAGTTA GTTCGAGAGA TCCTTATTAA ATAATCCTTT   
  
  
+ TATGAACTTT TCTTCTCTTT CGTTCTTCTT TTACTTTTAG TTCCTAATGG TATCCTCTTA TCGTTCGTTT   
  
  
+ CCAGATACTT CTCTTCTACA TTGAGGTAAC CAGTGTGATT ATACATCGTA CCTCTTTCTT TCGACCTTCC   
  
  
+ ATTTCCGTTT AATATTATCG TATAGGTGTT CACTATATAG GTGAACACCA ACCTGAGACT TAGTTCGTCG   
  
  
+ ACGAAGACGA CCACACAACT TGAGGAGGGT CCGAAGTTAG GTTCACTGGT ACCGTAGACG TTCGAAGAGT   
  
  
+ TTCATTCCTA GTTATACTGG GTTAAAGAGA AACTGGACGG ACCTCTCCCG TTCCCTCGTG AAGTCCCGAC   
  
  
+ AACACGTCAG AAAACCGTCC TGTTGTCTAA CCTTGTTACC TTAGTCGACA AGGACGGTCA TATCTGGGAA   
  
  
+ CTTAAGGAAG AAGTTCAATC TTTTGCCCA  

- TATTGGAGCT AACAATCTAA ACCGAGCTTT TATTTGTTTC CTGTATGAGG AAGAACAAGA CAAAGTTGAA   
  
  
- AAGAGGAGAG GAGCAGGCTA GCTTATTATG CTAATAGAAA TGGCATAACA TGGGTAGAAA AGGGTGGGGG   
  
  
- TTAATGGAAA GGCGTCCAAC TTAACTGACT TGACAGAATT GACCCATGTA AAGAGTGATT TCCATGGATG   
  
  
- CATCCCCTCA CAAAGATTAA GAGTATGTGC CGTAAACGGC ACGCAAATTG ACAAACAGAA GGATGGTTTA   
  
  
- GTTTCTTGTC GAAAAGGTTG CCAAGAATAA ATGATTTTAA GCCCATAAGT TATGAAAGTG GACGAGCATG   
  
  
- GAAGTTGAAC TGATGAAGAA GCCATGCCAA CCAGCTTAAT CATAGAATTA CTTCTTGTGT TATATCTGAA   
  
  
- ATATAGATGT AACATCATGG ACTTCCATGG ATGACTAGCT CTGGTTGTAG CTTAGAGCTT GCTTAACGTA   
  
  
- CTTAAACTAC GAAGGAAAAG GAGTATGCTT GCTGTTTATT CTCACATAGA AGCCATCTTC AGCCAATCAG   
  
  
- CCAATAGCAT CACTTAAACC GGTAGGTGAA TTTCATGCCT CGAGGTCAAA AAGCAGATAT TTAAGTTTCA   
  
  
- ACTAATCAAT TGAGTGCACA CTCCCCACTT TCTACATGTA CAGTTCATCG CATGAGCTTT AACGCTAAAT   
  
  
- GACTTGGGCA TACATAAGAG GAATTTTCGC TGGGTTTTCC TCGGAGCTTC AAGGAAGATC AATATCTCAG   
  
  
- AAAAACACTA CCTGCTGTAG TGCATGGGAC ATGTCGGCAT GGTTTTATGG GGATATGAAG TCCTATTGCT   
  
  
- GTCTGTGGTT AATTATAATG ATTAATGATG GTATTAAGTA AATACATTCT CAAATCATTT GGACTTCTTT   
  
  
- CAAGAGGGGT CACTGGTACT CTGTCTCATC TACCAATTTT TCGGGCTTTC TTTTTCCGTA TGGGGGAGGA   
  
  
- TTAGATTGAT TTGTCGCAGT GTCACTGGTT TTGTTTCAAT CAAGCTCTCT AGGAATAATT TATTAGGAAA   
  
  
- ATACTTGAAA AGAAGAGAAA GCAAGAAGAA AATGAAAATC AAGGATTACC ATAGGAGAAT AGCAAGCAAA   
  
  
- GGTCTATGAA GAGAAGATGT AACTCCATTG GTCACACTAA TATGTAGCAT GGAGAAAGAA AGCTGGAAGG   
  
  
- TAAAGGCAAA TTATAATAGC ATATCCACAA GTGATATATC CACTTGTGGT TGGACTCTGA ATCAAGCAGC   
  
  
- TGCTTCTGCT GGTGTGTTGA ACTCCTCCCA GGCTTCAATC CAAGTGACCA TGGCATCTGC AAGCTTCTCA   
  
  
- AAGTAAGGAT CAATATGACC CAATTTCTCT TTGACCTGCC TGGAGAGGGC AAGGGAGCAC TTCAGGGCTG   
  
  
- TTGTGCAGTC TTTTGGCAGG ACAACAGATT GGAACAATGG AATCAGCTGT TCCTGCCAGT ATAGACCCTT   
  
  
- GAATTCCTTC TTCAAGTTAG AAAACGGGT

+     AAGAA-motif

| Site Name | Organism | Position | Strand | Matrix score. | sequence | function |
| --- | --- | --- | --- | --- | --- | --- |
| AAGAA-motif | Avena sativa | 1176 | - | 7 | GAAAGAA |  |
| AAGAA-motif | Avena sativa | 104 | - | 9 | gGTAAAGAAA |  |
| AAGAA-motif | Avena sativa | 956 | + | 7 | GAAAGAA |  |

> 2018/04/13 10:10:12  
+ ATAACCTCGA TTGTTAGATT TGGCTCGAAA ATAAACAAAG GACATACTCC TTCTTGTTCT GTTTCAACTT   
  
  
+ TTCTCCTCTC CTCGTCCGAT CGAATAATAC GATTATCTTT ACCGTATTGT ACCCATCTTT TCCCACCCCC   
  
  
+ AATTACCTTT CCGCAGGTTG AATTGACTGA ACTGTCTTAA CTGGGTACAT TTCTCACTAA AGGTACCTAC   
  
  
+ GTAGGGGAGT GTTTCTAATT CTCATACACG GCATTTGCCG TGCGTTTAAC TGTTTGTCTT CCTACCAAAT   
  
  
+ CAAAGAACAG CTTTTCCAAC GGTTCTTATT TACTAAAATT CGGGTATTCA ATACTTTCAC CTGCTCGTAC   
  
  
+ CTTCAACTTG ACTACTTCTT CGGTACGGTT GGTCGAATTA GTATCTTAAT GAAGAACACA ATATAGACTT   
  
  
+ TATATCTACA TTGTAGTACC TGAAGGTACC TACTGATCGA GACCAACATC GAATCTCGAA CGAATTGCAT   
  
  
+ GAATTTGATG CTTCCTTTTC CTCATACGAA CGACAAATAA GAGTGTATCT TCGGTAGAAG TCGGTTAGTC   
  
  
+ GGTTATCGTA GTGAATTTGG CCATCCACTT AAAGTACGGA GCTCCAGTTT TTCGTCTATA AATTCAAAGT   
  
  
+ TGATTAGTTA ACTCACGTGT GAGGGGTGAA AGATGTACAT GTCAAGTAGC GTACTCGAAA TTGCGATTTA   
  
  
+ CTGAACCCGT ATGTATTCTC CTTAAAAGCG ACCCAAAAGG AGCCTCGAAG TTCCTTCTAG TTATAGAGTC   
  
  
+ TTTTTGTGAT GGACGACATC ACGTACCCTG TACAGCCGTA CCAAAATACC CCTATACTTC AGGATAACGA   
  
  
+ CAGACACCAA TTAATATTAC TAATTACTAC CATAATTCAT TTATGTAAGA GTTTAGTAAA CCTGAAGAAA   
  
  
+ GTTCTCCCCA GTGACCATGA GACAGAGTAG ATGGTTAAAA AGCCCGAAAG AAAAAGGCAT ACCCCCTCCT   
  
  
+ AATCTAACTA AACAGCGTCA CAGTGACCAA AACAAAGTTA GTTCGAGAGA TCCTTATTAA ATAATCCTTT   
  
  
+ TATGAACTTT TCTTCTCTTT CGTTCTTCTT TTACTTTTAG TTCCTAATGG TATCCTCTTA TCGTTCGTTT   
  
  
+ CCAGATACTT CTCTTCTACA TTGAGGTAAC CAGTGTGATT ATACATCGTA CCTCTTTCTT TCGACCTTCC   
  
  
+ ATTTCCGTTT AATATTATCG TATAGGTGTT CACTATATAG GTGAACACCA ACCTGAGACT TAGTTCGTCG   
  
  
+ ACGAAGACGA CCACACAACT TGAGGAGGGT CCGAAGTTAG GTTCACTGGT ACCGTAGACG TTCGAAGAGT   
  
  
+ TTCATTCCTA GTTATACTGG GTTAAAGAGA AACTGGACGG ACCTCTCCCG TTCCCTCGTG AAGTCCCGAC   
  
  
+ AACACGTCAG AAAACCGTCC TGTTGTCTAA CCTTGTTACC TTAGTCGACA AGGACGGTCA TATCTGGGAA   
  
  
+ CTTAAGGAAG AAGTTCAATC TTTTGCCCA  

- TATTGGAGCT AACAATCTAA ACCGAGCTTT TATTTGTTTC CTGTATGAGG AAGAACAAGA CAAAGTTGAA   
  
  
- AAGAGGAGAG GAGCAGGCTA GCTTATTATG CTAATAGAAA TGGCATAACA TGGGTAGAAA AGGGTGGGGG   
  
  
- TTAATGGAAA GGCGTCCAAC TTAACTGACT TGACAGAATT GACCCATGTA AAGAGTGATT TCCATGGATG   
  
  
- CATCCCCTCA CAAAGATTAA GAGTATGTGC CGTAAACGGC ACGCAAATTG ACAAACAGAA GGATGGTTTA   
  
  
- GTTTCTTGTC GAAAAGGTTG CCAAGAATAA ATGATTTTAA GCCCATAAGT TATGAAAGTG GACGAGCATG   
  
  
- GAAGTTGAAC TGATGAAGAA GCCATGCCAA CCAGCTTAAT CATAGAATTA CTTCTTGTGT TATATCTGAA   
  
  
- ATATAGATGT AACATCATGG ACTTCCATGG ATGACTAGCT CTGGTTGTAG CTTAGAGCTT GCTTAACGTA   
  
  
- CTTAAACTAC GAAGGAAAAG GAGTATGCTT GCTGTTTATT CTCACATAGA AGCCATCTTC AGCCAATCAG   
  
  
- CCAATAGCAT CACTTAAACC GGTAGGTGAA TTTCATGCCT CGAGGTCAAA AAGCAGATAT TTAAGTTTCA   
  
  
- ACTAATCAAT TGAGTGCACA CTCCCCACTT TCTACATGTA CAGTTCATCG CATGAGCTTT AACGCTAAAT   
  
  
- GACTTGGGCA TACATAAGAG GAATTTTCGC TGGGTTTTCC TCGGAGCTTC AAGGAAGATC AATATCTCAG   
  
  
- AAAAACACTA CCTGCTGTAG TGCATGGGAC ATGTCGGCAT GGTTTTATGG GGATATGAAG TCCTATTGCT   
  
  
- GTCTGTGGTT AATTATAATG ATTAATGATG GTATTAAGTA AATACATTCT CAAATCATTT GGACTTCTTT   
  
  
- CAAGAGGGGT CACTGGTACT CTGTCTCATC TACCAATTTT TCGGGCTTTC TTTTTCCGTA TGGGGGAGGA   
  
  
- TTAGATTGAT TTGTCGCAGT GTCACTGGTT TTGTTTCAAT CAAGCTCTCT AGGAATAATT TATTAGGAAA   
  
  
- ATACTTGAAA AGAAGAGAAA GCAAGAAGAA AATGAAAATC AAGGATTACC ATAGGAGAAT AGCAAGCAAA   
  
  
- GGTCTATGAA GAGAAGATGT AACTCCATTG GTCACACTAA TATGTAGCAT GGAGAAAGAA AGCTGGAAGG   
  
  
- TAAAGGCAAA TTATAATAGC ATATCCACAA GTGATATATC CACTTGTGGT TGGACTCTGA ATCAAGCAGC   
  
  
- TGCTTCTGCT GGTGTGTTGA ACTCCTCCCA GGCTTCAATC CAAGTGACCA TGGCATCTGC AAGCTTCTCA   
  
  
- AAGTAAGGAT CAATATGACC CAATTTCTCT TTGACCTGCC TGGAGAGGGC AAGGGAGCAC TTCAGGGCTG   
  
  
- TTGTGCAGTC TTTTGGCAGG ACAACAGATT GGAACAATGG AATCAGCTGT TCCTGCCAGT ATAGACCCTT   
  
  
- GAATTCCTTC TTCAAGTTAG AAAACGGGT

+     ABRE

| Site Name | Organism | Position | Strand | Matrix score. | sequence | function |
| --- | --- | --- | --- | --- | --- | --- |
| ABRE | Arabidopsis thaliana | 790 | - | 6 | TACGTG | cis-acting element involved in the abscisic acid responsiveness |
| ABRE | Arabidopsis thaliana | 644 | + | 6 | CACGTG | cis-acting element involved in the abscisic acid responsiveness |

> 2018/04/13 10:10:12  
+ ATAACCTCGA TTGTTAGATT TGGCTCGAAA ATAAACAAAG GACATACTCC TTCTTGTTCT GTTTCAACTT   
  
  
+ TTCTCCTCTC CTCGTCCGAT CGAATAATAC GATTATCTTT ACCGTATTGT ACCCATCTTT TCCCACCCCC   
  
  
+ AATTACCTTT CCGCAGGTTG AATTGACTGA ACTGTCTTAA CTGGGTACAT TTCTCACTAA AGGTACCTAC   
  
  
+ GTAGGGGAGT GTTTCTAATT CTCATACACG GCATTTGCCG TGCGTTTAAC TGTTTGTCTT CCTACCAAAT   
  
  
+ CAAAGAACAG CTTTTCCAAC GGTTCTTATT TACTAAAATT CGGGTATTCA ATACTTTCAC CTGCTCGTAC   
  
  
+ CTTCAACTTG ACTACTTCTT CGGTACGGTT GGTCGAATTA GTATCTTAAT GAAGAACACA ATATAGACTT   
  
  
+ TATATCTACA TTGTAGTACC TGAAGGTACC TACTGATCGA GACCAACATC GAATCTCGAA CGAATTGCAT   
  
  
+ GAATTTGATG CTTCCTTTTC CTCATACGAA CGACAAATAA GAGTGTATCT TCGGTAGAAG TCGGTTAGTC   
  
  
+ GGTTATCGTA GTGAATTTGG CCATCCACTT AAAGTACGGA GCTCCAGTTT TTCGTCTATA AATTCAAAGT   
  
  
+ TGATTAGTTA ACTCACGTGT GAGGGGTGAA AGATGTACAT GTCAAGTAGC GTACTCGAAA TTGCGATTTA   
  
  
+ CTGAACCCGT ATGTATTCTC CTTAAAAGCG ACCCAAAAGG AGCCTCGAAG TTCCTTCTAG TTATAGAGTC   
  
  
+ TTTTTGTGAT GGACGACATC ACGTACCCTG TACAGCCGTA CCAAAATACC CCTATACTTC AGGATAACGA   
  
  
+ CAGACACCAA TTAATATTAC TAATTACTAC CATAATTCAT TTATGTAAGA GTTTAGTAAA CCTGAAGAAA   
  
  
+ GTTCTCCCCA GTGACCATGA GACAGAGTAG ATGGTTAAAA AGCCCGAAAG AAAAAGGCAT ACCCCCTCCT   
  
  
+ AATCTAACTA AACAGCGTCA CAGTGACCAA AACAAAGTTA GTTCGAGAGA TCCTTATTAA ATAATCCTTT   
  
  
+ TATGAACTTT TCTTCTCTTT CGTTCTTCTT TTACTTTTAG TTCCTAATGG TATCCTCTTA TCGTTCGTTT   
  
  
+ CCAGATACTT CTCTTCTACA TTGAGGTAAC CAGTGTGATT ATACATCGTA CCTCTTTCTT TCGACCTTCC   
  
  
+ ATTTCCGTTT AATATTATCG TATAGGTGTT CACTATATAG GTGAACACCA ACCTGAGACT TAGTTCGTCG   
  
  
+ ACGAAGACGA CCACACAACT TGAGGAGGGT CCGAAGTTAG GTTCACTGGT ACCGTAGACG TTCGAAGAGT   
  
  
+ TTCATTCCTA GTTATACTGG GTTAAAGAGA AACTGGACGG ACCTCTCCCG TTCCCTCGTG AAGTCCCGAC   
  
  
+ AACACGTCAG AAAACCGTCC TGTTGTCTAA CCTTGTTACC TTAGTCGACA AGGACGGTCA TATCTGGGAA   
  
  
+ CTTAAGGAAG AAGTTCAATC TTTTGCCCA  

- TATTGGAGCT AACAATCTAA ACCGAGCTTT TATTTGTTTC CTGTATGAGG AAGAACAAGA CAAAGTTGAA   
  
  
- AAGAGGAGAG GAGCAGGCTA GCTTATTATG CTAATAGAAA TGGCATAACA TGGGTAGAAA AGGGTGGGGG   
  
  
- TTAATGGAAA GGCGTCCAAC TTAACTGACT TGACAGAATT GACCCATGTA AAGAGTGATT TCCATGGATG   
  
  
- CATCCCCTCA CAAAGATTAA GAGTATGTGC CGTAAACGGC ACGCAAATTG ACAAACAGAA GGATGGTTTA   
  
  
- GTTTCTTGTC GAAAAGGTTG CCAAGAATAA ATGATTTTAA GCCCATAAGT TATGAAAGTG GACGAGCATG   
  
  
- GAAGTTGAAC TGATGAAGAA GCCATGCCAA CCAGCTTAAT CATAGAATTA CTTCTTGTGT TATATCTGAA   
  
  
- ATATAGATGT AACATCATGG ACTTCCATGG ATGACTAGCT CTGGTTGTAG CTTAGAGCTT GCTTAACGTA   
  
  
- CTTAAACTAC GAAGGAAAAG GAGTATGCTT GCTGTTTATT CTCACATAGA AGCCATCTTC AGCCAATCAG   
  
  
- CCAATAGCAT CACTTAAACC GGTAGGTGAA TTTCATGCCT CGAGGTCAAA AAGCAGATAT TTAAGTTTCA   
  
  
- ACTAATCAAT TGAGTGCACA CTCCCCACTT TCTACATGTA CAGTTCATCG CATGAGCTTT AACGCTAAAT   
  
  
- GACTTGGGCA TACATAAGAG GAATTTTCGC TGGGTTTTCC TCGGAGCTTC AAGGAAGATC AATATCTCAG   
  
  
- AAAAACACTA CCTGCTGTAG TGCATGGGAC ATGTCGGCAT GGTTTTATGG GGATATGAAG TCCTATTGCT   
  
  
- GTCTGTGGTT AATTATAATG ATTAATGATG GTATTAAGTA AATACATTCT CAAATCATTT GGACTTCTTT   
  
  
- CAAGAGGGGT CACTGGTACT CTGTCTCATC TACCAATTTT TCGGGCTTTC TTTTTCCGTA TGGGGGAGGA   
  
  
- TTAGATTGAT TTGTCGCAGT GTCACTGGTT TTGTTTCAAT CAAGCTCTCT AGGAATAATT TATTAGGAAA   
  
  
- ATACTTGAAA AGAAGAGAAA GCAAGAAGAA AATGAAAATC AAGGATTACC ATAGGAGAAT AGCAAGCAAA   
  
  
- GGTCTATGAA GAGAAGATGT AACTCCATTG GTCACACTAA TATGTAGCAT GGAGAAAGAA AGCTGGAAGG   
  
  
- TAAAGGCAAA TTATAATAGC ATATCCACAA GTGATATATC CACTTGTGGT TGGACTCTGA ATCAAGCAGC   
  
  
- TGCTTCTGCT GGTGTGTTGA ACTCCTCCCA GGCTTCAATC CAAGTGACCA TGGCATCTGC AAGCTTCTCA   
  
  
- AAGTAAGGAT CAATATGACC CAATTTCTCT TTGACCTGCC TGGAGAGGGC AAGGGAGCAC TTCAGGGCTG   
  
  
- TTGTGCAGTC TTTTGGCAGG ACAACAGATT GGAACAATGG AATCAGCTGT TCCTGCCAGT ATAGACCCTT   
  
  
- GAATTCCTTC TTCAAGTTAG AAAACGGGT

+     AC-II

| Site Name | Organism | Position | Strand | Matrix score. | sequence | function |
| --- | --- | --- | --- | --- | --- | --- |
| AC-II | Phaseolus vulgaris | 127 | + | 9 | (C/T)T(T/C)(C/T)(A/C)(A/C)C(A/C)A(A/C)C(C/A)(C/A)C |  |

> 2018/04/13 10:10:12  
+ ATAACCTCGA TTGTTAGATT TGGCTCGAAA ATAAACAAAG GACATACTCC TTCTTGTTCT GTTTCAACTT   
  
  
+ TTCTCCTCTC CTCGTCCGAT CGAATAATAC GATTATCTTT ACCGTATTGT ACCCATCTTT TCCCACCCCC   
  
  
+ AATTACCTTT CCGCAGGTTG AATTGACTGA ACTGTCTTAA CTGGGTACAT TTCTCACTAA AGGTACCTAC   
  
  
+ GTAGGGGAGT GTTTCTAATT CTCATACACG GCATTTGCCG TGCGTTTAAC TGTTTGTCTT CCTACCAAAT   
  
  
+ CAAAGAACAG CTTTTCCAAC GGTTCTTATT TACTAAAATT CGGGTATTCA ATACTTTCAC CTGCTCGTAC   
  
  
+ CTTCAACTTG ACTACTTCTT CGGTACGGTT GGTCGAATTA GTATCTTAAT GAAGAACACA ATATAGACTT   
  
  
+ TATATCTACA TTGTAGTACC TGAAGGTACC TACTGATCGA GACCAACATC GAATCTCGAA CGAATTGCAT   
  
  
+ GAATTTGATG CTTCCTTTTC CTCATACGAA CGACAAATAA GAGTGTATCT TCGGTAGAAG TCGGTTAGTC   
  
  
+ GGTTATCGTA GTGAATTTGG CCATCCACTT AAAGTACGGA GCTCCAGTTT TTCGTCTATA AATTCAAAGT   
  
  
+ TGATTAGTTA ACTCACGTGT GAGGGGTGAA AGATGTACAT GTCAAGTAGC GTACTCGAAA TTGCGATTTA   
  
  
+ CTGAACCCGT ATGTATTCTC CTTAAAAGCG ACCCAAAAGG AGCCTCGAAG TTCCTTCTAG TTATAGAGTC   
  
  
+ TTTTTGTGAT GGACGACATC ACGTACCCTG TACAGCCGTA CCAAAATACC CCTATACTTC AGGATAACGA   
  
  
+ CAGACACCAA TTAATATTAC TAATTACTAC CATAATTCAT TTATGTAAGA GTTTAGTAAA CCTGAAGAAA   
  
  
+ GTTCTCCCCA GTGACCATGA GACAGAGTAG ATGGTTAAAA AGCCCGAAAG AAAAAGGCAT ACCCCCTCCT   
  
  
+ AATCTAACTA AACAGCGTCA CAGTGACCAA AACAAAGTTA GTTCGAGAGA TCCTTATTAA ATAATCCTTT   
  
  
+ TATGAACTTT TCTTCTCTTT CGTTCTTCTT TTACTTTTAG TTCCTAATGG TATCCTCTTA TCGTTCGTTT   
  
  
+ CCAGATACTT CTCTTCTACA TTGAGGTAAC CAGTGTGATT ATACATCGTA CCTCTTTCTT TCGACCTTCC   
  
  
+ ATTTCCGTTT AATATTATCG TATAGGTGTT CACTATATAG GTGAACACCA ACCTGAGACT TAGTTCGTCG   
  
  
+ ACGAAGACGA CCACACAACT TGAGGAGGGT CCGAAGTTAG GTTCACTGGT ACCGTAGACG TTCGAAGAGT   
  
  
+ TTCATTCCTA GTTATACTGG GTTAAAGAGA AACTGGACGG ACCTCTCCCG TTCCCTCGTG AAGTCCCGAC   
  
  
+ AACACGTCAG AAAACCGTCC TGTTGTCTAA CCTTGTTACC TTAGTCGACA AGGACGGTCA TATCTGGGAA   
  
  
+ CTTAAGGAAG AAGTTCAATC TTTTGCCCA  

- TATTGGAGCT AACAATCTAA ACCGAGCTTT TATTTGTTTC CTGTATGAGG AAGAACAAGA CAAAGTTGAA   
  
  
- AAGAGGAGAG GAGCAGGCTA GCTTATTATG CTAATAGAAA TGGCATAACA TGGGTAGAAA AGGGTGGGGG   
  
  
- TTAATGGAAA GGCGTCCAAC TTAACTGACT TGACAGAATT GACCCATGTA AAGAGTGATT TCCATGGATG   
  
  
- CATCCCCTCA CAAAGATTAA GAGTATGTGC CGTAAACGGC ACGCAAATTG ACAAACAGAA GGATGGTTTA   
  
  
- GTTTCTTGTC GAAAAGGTTG CCAAGAATAA ATGATTTTAA GCCCATAAGT TATGAAAGTG GACGAGCATG   
  
  
- GAAGTTGAAC TGATGAAGAA GCCATGCCAA CCAGCTTAAT CATAGAATTA CTTCTTGTGT TATATCTGAA   
  
  
- ATATAGATGT AACATCATGG ACTTCCATGG ATGACTAGCT CTGGTTGTAG CTTAGAGCTT GCTTAACGTA   
  
  
- CTTAAACTAC GAAGGAAAAG GAGTATGCTT GCTGTTTATT CTCACATAGA AGCCATCTTC AGCCAATCAG   
  
  
- CCAATAGCAT CACTTAAACC GGTAGGTGAA TTTCATGCCT CGAGGTCAAA AAGCAGATAT TTAAGTTTCA   
  
  
- ACTAATCAAT TGAGTGCACA CTCCCCACTT TCTACATGTA CAGTTCATCG CATGAGCTTT AACGCTAAAT   
  
  
- GACTTGGGCA TACATAAGAG GAATTTTCGC TGGGTTTTCC TCGGAGCTTC AAGGAAGATC AATATCTCAG   
  
  
- AAAAACACTA CCTGCTGTAG TGCATGGGAC ATGTCGGCAT GGTTTTATGG GGATATGAAG TCCTATTGCT   
  
  
- GTCTGTGGTT AATTATAATG ATTAATGATG GTATTAAGTA AATACATTCT CAAATCATTT GGACTTCTTT   
  
  
- CAAGAGGGGT CACTGGTACT CTGTCTCATC TACCAATTTT TCGGGCTTTC TTTTTCCGTA TGGGGGAGGA   
  
  
- TTAGATTGAT TTGTCGCAGT GTCACTGGTT TTGTTTCAAT CAAGCTCTCT AGGAATAATT TATTAGGAAA   
  
  
- ATACTTGAAA AGAAGAGAAA GCAAGAAGAA AATGAAAATC AAGGATTACC ATAGGAGAAT AGCAAGCAAA   
  
  
- GGTCTATGAA GAGAAGATGT AACTCCATTG GTCACACTAA TATGTAGCAT GGAGAAAGAA AGCTGGAAGG   
  
  
- TAAAGGCAAA TTATAATAGC ATATCCACAA GTGATATATC CACTTGTGGT TGGACTCTGA ATCAAGCAGC   
  
  
- TGCTTCTGCT GGTGTGTTGA ACTCCTCCCA GGCTTCAATC CAAGTGACCA TGGCATCTGC AAGCTTCTCA   
  
  
- AAGTAAGGAT CAATATGACC CAATTTCTCT TTGACCTGCC TGGAGAGGGC AAGGGAGCAC TTCAGGGCTG   
  
  
- TTGTGCAGTC TTTTGGCAGG ACAACAGATT GGAACAATGG AATCAGCTGT TCCTGCCAGT ATAGACCCTT   
  
  
- GAATTCCTTC TTCAAGTTAG AAAACGGGT

+     Box 4

| Site Name | Organism | Position | Strand | Matrix score. | sequence | function |
| --- | --- | --- | --- | --- | --- | --- |
| Box 4 | Petroselinum crispum | 850 | - | 6 | ATTAAT | part of a conserved DNA module involved in light responsiveness |

> 2018/04/13 10:10:12  
+ ATAACCTCGA TTGTTAGATT TGGCTCGAAA ATAAACAAAG GACATACTCC TTCTTGTTCT GTTTCAACTT   
  
  
+ TTCTCCTCTC CTCGTCCGAT CGAATAATAC GATTATCTTT ACCGTATTGT ACCCATCTTT TCCCACCCCC   
  
  
+ AATTACCTTT CCGCAGGTTG AATTGACTGA ACTGTCTTAA CTGGGTACAT TTCTCACTAA AGGTACCTAC   
  
  
+ GTAGGGGAGT GTTTCTAATT CTCATACACG GCATTTGCCG TGCGTTTAAC TGTTTGTCTT CCTACCAAAT   
  
  
+ CAAAGAACAG CTTTTCCAAC GGTTCTTATT TACTAAAATT CGGGTATTCA ATACTTTCAC CTGCTCGTAC   
  
  
+ CTTCAACTTG ACTACTTCTT CGGTACGGTT GGTCGAATTA GTATCTTAAT GAAGAACACA ATATAGACTT   
  
  
+ TATATCTACA TTGTAGTACC TGAAGGTACC TACTGATCGA GACCAACATC GAATCTCGAA CGAATTGCAT   
  
  
+ GAATTTGATG CTTCCTTTTC CTCATACGAA CGACAAATAA GAGTGTATCT TCGGTAGAAG TCGGTTAGTC   
  
  
+ GGTTATCGTA GTGAATTTGG CCATCCACTT AAAGTACGGA GCTCCAGTTT TTCGTCTATA AATTCAAAGT   
  
  
+ TGATTAGTTA ACTCACGTGT GAGGGGTGAA AGATGTACAT GTCAAGTAGC GTACTCGAAA TTGCGATTTA   
  
  
+ CTGAACCCGT ATGTATTCTC CTTAAAAGCG ACCCAAAAGG AGCCTCGAAG TTCCTTCTAG TTATAGAGTC   
  
  
+ TTTTTGTGAT GGACGACATC ACGTACCCTG TACAGCCGTA CCAAAATACC CCTATACTTC AGGATAACGA   
  
  
+ CAGACACCAA TTAATATTAC TAATTACTAC CATAATTCAT TTATGTAAGA GTTTAGTAAA CCTGAAGAAA   
  
  
+ GTTCTCCCCA GTGACCATGA GACAGAGTAG ATGGTTAAAA AGCCCGAAAG AAAAAGGCAT ACCCCCTCCT   
  
  
+ AATCTAACTA AACAGCGTCA CAGTGACCAA AACAAAGTTA GTTCGAGAGA TCCTTATTAA ATAATCCTTT   
  
  
+ TATGAACTTT TCTTCTCTTT CGTTCTTCTT TTACTTTTAG TTCCTAATGG TATCCTCTTA TCGTTCGTTT   
  
  
+ CCAGATACTT CTCTTCTACA TTGAGGTAAC CAGTGTGATT ATACATCGTA CCTCTTTCTT TCGACCTTCC   
  
  
+ ATTTCCGTTT AATATTATCG TATAGGTGTT CACTATATAG GTGAACACCA ACCTGAGACT TAGTTCGTCG   
  
  
+ ACGAAGACGA CCACACAACT TGAGGAGGGT CCGAAGTTAG GTTCACTGGT ACCGTAGACG TTCGAAGAGT   
  
  
+ TTCATTCCTA GTTATACTGG GTTAAAGAGA AACTGGACGG ACCTCTCCCG TTCCCTCGTG AAGTCCCGAC   
  
  
+ AACACGTCAG AAAACCGTCC TGTTGTCTAA CCTTGTTACC TTAGTCGACA AGGACGGTCA TATCTGGGAA   
  
  
+ CTTAAGGAAG AAGTTCAATC TTTTGCCCA  

- TATTGGAGCT AACAATCTAA ACCGAGCTTT TATTTGTTTC CTGTATGAGG AAGAACAAGA CAAAGTTGAA   
  
  
- AAGAGGAGAG GAGCAGGCTA GCTTATTATG CTAATAGAAA TGGCATAACA TGGGTAGAAA AGGGTGGGGG   
  
  
- TTAATGGAAA GGCGTCCAAC TTAACTGACT TGACAGAATT GACCCATGTA AAGAGTGATT TCCATGGATG   
  
  
- CATCCCCTCA CAAAGATTAA GAGTATGTGC CGTAAACGGC ACGCAAATTG ACAAACAGAA GGATGGTTTA   
  
  
- GTTTCTTGTC GAAAAGGTTG CCAAGAATAA ATGATTTTAA GCCCATAAGT TATGAAAGTG GACGAGCATG   
  
  
- GAAGTTGAAC TGATGAAGAA GCCATGCCAA CCAGCTTAAT CATAGAATTA CTTCTTGTGT TATATCTGAA   
  
  
- ATATAGATGT AACATCATGG ACTTCCATGG ATGACTAGCT CTGGTTGTAG CTTAGAGCTT GCTTAACGTA   
  
  
- CTTAAACTAC GAAGGAAAAG GAGTATGCTT GCTGTTTATT CTCACATAGA AGCCATCTTC AGCCAATCAG   
  
  
- CCAATAGCAT CACTTAAACC GGTAGGTGAA TTTCATGCCT CGAGGTCAAA AAGCAGATAT TTAAGTTTCA   
  
  
- ACTAATCAAT TGAGTGCACA CTCCCCACTT TCTACATGTA CAGTTCATCG CATGAGCTTT AACGCTAAAT   
  
  
- GACTTGGGCA TACATAAGAG GAATTTTCGC TGGGTTTTCC TCGGAGCTTC AAGGAAGATC AATATCTCAG   
  
  
- AAAAACACTA CCTGCTGTAG TGCATGGGAC ATGTCGGCAT GGTTTTATGG GGATATGAAG TCCTATTGCT   
  
  
- GTCTGTGGTT AATTATAATG ATTAATGATG GTATTAAGTA AATACATTCT CAAATCATTT GGACTTCTTT   
  
  
- CAAGAGGGGT CACTGGTACT CTGTCTCATC TACCAATTTT TCGGGCTTTC TTTTTCCGTA TGGGGGAGGA   
  
  
- TTAGATTGAT TTGTCGCAGT GTCACTGGTT TTGTTTCAAT CAAGCTCTCT AGGAATAATT TATTAGGAAA   
  
  
- ATACTTGAAA AGAAGAGAAA GCAAGAAGAA AATGAAAATC AAGGATTACC ATAGGAGAAT AGCAAGCAAA   
  
  
- GGTCTATGAA GAGAAGATGT AACTCCATTG GTCACACTAA TATGTAGCAT GGAGAAAGAA AGCTGGAAGG   
  
  
- TAAAGGCAAA TTATAATAGC ATATCCACAA GTGATATATC CACTTGTGGT TGGACTCTGA ATCAAGCAGC   
  
  
- TGCTTCTGCT GGTGTGTTGA ACTCCTCCCA GGCTTCAATC CAAGTGACCA TGGCATCTGC AAGCTTCTCA   
  
  
- AAGTAAGGAT CAATATGACC CAATTTCTCT TTGACCTGCC TGGAGAGGGC AAGGGAGCAC TTCAGGGCTG   
  
  
- TTGTGCAGTC TTTTGGCAGG ACAACAGATT GGAACAATGG AATCAGCTGT TCCTGCCAGT ATAGACCCTT   
  
  
- GAATTCCTTC TTCAAGTTAG AAAACGGGT

+     CAAT-box

| Site Name | Organism | Position | Strand | Matrix score. | sequence | function |
| --- | --- | --- | --- | --- | --- | --- |
| CAAT-box | Arabidopsis thaliana | 847 | + | 5 | CCAAT | common cis-acting element in promoter and enhancer regions |
| CAAT-box | Hordeum vulgare | 1486 | + | 4 | CAAT | common cis-acting element in promoter and enhancer regions |
| CAAT-box | Brassica rapa | 524 | + | 5 | CAAAT | common cis-acting element in promoter and enhancer regions |
| CAAT-box | Glycine max | 848 | + | 5 | CAATT | common cis-acting element in promoter and enhancer regions |
| CAAT-box | Glycine max | 161 | - | 5 | CAATT | common cis-acting element in promoter and enhancer regions |
| CAAT-box | Hordeum vulgare | 116 | - | 4 | CAAT | common cis-acting element in promoter and enhancer regions |
| CAAT-box | Hordeum vulgare | 690 | - | 4 | CAAT | common cis-acting element in promoter and enhancer regions |
| CAAT-box | Hordeum vulgare | 484 | - | 4 | CAAT | common cis-acting element in promoter and enhancer regions |
| CAAT-box | Hordeum vulgare | 162 | - | 4 | CAAT | common cis-acting element in promoter and enhancer regions |
| CAAT-box | Hordeum vulgare | 10 | - | 4 | CAAT | common cis-acting element in promoter and enhancer regions |
| CAAT-box | Glycine max | 140 | + | 5 | CAATT | common cis-acting element in promoter and enhancer regions |
| CAAT-box | Brassica rapa | 575 | - | 5 | CAAAT | common cis-acting element in promoter and enhancer regions |
| CAAT-box | Hordeum vulgare | 409 | + | 4 | CAAT | common cis-acting element in promoter and enhancer regions |
| CAAT-box | Glycine max | 689 | - | 5 | CAATT | common cis-acting element in promoter and enhancer regions |
| CAAT-box | Glycine max | 483 | - | 5 | CAATT | common cis-acting element in promoter and enhancer regions |
| CAAT-box | Arabidopsis thaliana | 139 | + | 5 | CCAAT | common cis-acting element in promoter and enhancer regions |
| CAAT-box | Hordeum vulgare | 329 | + | 4 | CAAT | common cis-acting element in promoter and enhancer regions |
| CAAT-box | Hordeum vulgare | 1140 | - | 4 | CAAT | common cis-acting element in promoter and enhancer regions |
| CAAT-box | Hordeum vulgare | 430 | - | 4 | CAAT | common cis-acting element in promoter and enhancer regions |
| CAAT-box | Brassica rapa | 493 | - | 5 | CAAAT | common cis-acting element in promoter and enhancer regions |
| CAAT-box | Brassica rapa | 18 | - | 5 | CAAAT | common cis-acting element in promoter and enhancer regions |
| CAAT-box | Brassica rapa | 243 | - | 5 | CAAAT | common cis-acting element in promoter and enhancer regions |
| CAAT-box | Brassica rapa | 276 | + | 5 | CAAAT | common cis-acting element in promoter and enhancer regions |

> 2018/04/13 10:10:12  
+ ATAACCTCGA TTGTTAGATT TGGCTCGAAA ATAAACAAAG GACATACTCC TTCTTGTTCT GTTTCAACTT   
  
  
+ TTCTCCTCTC CTCGTCCGAT CGAATAATAC GATTATCTTT ACCGTATTGT ACCCATCTTT TCCCACCCCC   
  
  
+ AATTACCTTT CCGCAGGTTG AATTGACTGA ACTGTCTTAA CTGGGTACAT TTCTCACTAA AGGTACCTAC   
  
  
+ GTAGGGGAGT GTTTCTAATT CTCATACACG GCATTTGCCG TGCGTTTAAC TGTTTGTCTT CCTACCAAAT   
  
  
+ CAAAGAACAG CTTTTCCAAC GGTTCTTATT TACTAAAATT CGGGTATTCA ATACTTTCAC CTGCTCGTAC   
  
  
+ CTTCAACTTG ACTACTTCTT CGGTACGGTT GGTCGAATTA GTATCTTAAT GAAGAACACA ATATAGACTT   
  
  
+ TATATCTACA TTGTAGTACC TGAAGGTACC TACTGATCGA GACCAACATC GAATCTCGAA CGAATTGCAT   
  
  
+ GAATTTGATG CTTCCTTTTC CTCATACGAA CGACAAATAA GAGTGTATCT TCGGTAGAAG TCGGTTAGTC   
  
  
+ GGTTATCGTA GTGAATTTGG CCATCCACTT AAAGTACGGA GCTCCAGTTT TTCGTCTATA AATTCAAAGT   
  
  
+ TGATTAGTTA ACTCACGTGT GAGGGGTGAA AGATGTACAT GTCAAGTAGC GTACTCGAAA TTGCGATTTA   
  
  
+ CTGAACCCGT ATGTATTCTC CTTAAAAGCG ACCCAAAAGG AGCCTCGAAG TTCCTTCTAG TTATAGAGTC   
  
  
+ TTTTTGTGAT GGACGACATC ACGTACCCTG TACAGCCGTA CCAAAATACC CCTATACTTC AGGATAACGA   
  
  
+ CAGACACCAA TTAATATTAC TAATTACTAC CATAATTCAT TTATGTAAGA GTTTAGTAAA CCTGAAGAAA   
  
  
+ GTTCTCCCCA GTGACCATGA GACAGAGTAG ATGGTTAAAA AGCCCGAAAG AAAAAGGCAT ACCCCCTCCT   
  
  
+ AATCTAACTA AACAGCGTCA CAGTGACCAA AACAAAGTTA GTTCGAGAGA TCCTTATTAA ATAATCCTTT   
  
  
+ TATGAACTTT TCTTCTCTTT CGTTCTTCTT TTACTTTTAG TTCCTAATGG TATCCTCTTA TCGTTCGTTT   
  
  
+ CCAGATACTT CTCTTCTACA TTGAGGTAAC CAGTGTGATT ATACATCGTA CCTCTTTCTT TCGACCTTCC   
  
  
+ ATTTCCGTTT AATATTATCG TATAGGTGTT CACTATATAG GTGAACACCA ACCTGAGACT TAGTTCGTCG   
  
  
+ ACGAAGACGA CCACACAACT TGAGGAGGGT CCGAAGTTAG GTTCACTGGT ACCGTAGACG TTCGAAGAGT   
  
  
+ TTCATTCCTA GTTATACTGG GTTAAAGAGA AACTGGACGG ACCTCTCCCG TTCCCTCGTG AAGTCCCGAC   
  
  
+ AACACGTCAG AAAACCGTCC TGTTGTCTAA CCTTGTTACC TTAGTCGACA AGGACGGTCA TATCTGGGAA   
  
  
+ CTTAAGGAAG AAGTTCAATC TTTTGCCCA  

- TATTGGAGCT AACAATCTAA ACCGAGCTTT TATTTGTTTC CTGTATGAGG AAGAACAAGA CAAAGTTGAA   
  
  
- AAGAGGAGAG GAGCAGGCTA GCTTATTATG CTAATAGAAA TGGCATAACA TGGGTAGAAA AGGGTGGGGG   
  
  
- TTAATGGAAA GGCGTCCAAC TTAACTGACT TGACAGAATT GACCCATGTA AAGAGTGATT TCCATGGATG   
  
  
- CATCCCCTCA CAAAGATTAA GAGTATGTGC CGTAAACGGC ACGCAAATTG ACAAACAGAA GGATGGTTTA   
  
  
- GTTTCTTGTC GAAAAGGTTG CCAAGAATAA ATGATTTTAA GCCCATAAGT TATGAAAGTG GACGAGCATG   
  
  
- GAAGTTGAAC TGATGAAGAA GCCATGCCAA CCAGCTTAAT CATAGAATTA CTTCTTGTGT TATATCTGAA   
  
  
- ATATAGATGT AACATCATGG ACTTCCATGG ATGACTAGCT CTGGTTGTAG CTTAGAGCTT GCTTAACGTA   
  
  
- CTTAAACTAC GAAGGAAAAG GAGTATGCTT GCTGTTTATT CTCACATAGA AGCCATCTTC AGCCAATCAG   
  
  
- CCAATAGCAT CACTTAAACC GGTAGGTGAA TTTCATGCCT CGAGGTCAAA AAGCAGATAT TTAAGTTTCA   
  
  
- ACTAATCAAT TGAGTGCACA CTCCCCACTT TCTACATGTA CAGTTCATCG CATGAGCTTT AACGCTAAAT   
  
  
- GACTTGGGCA TACATAAGAG GAATTTTCGC TGGGTTTTCC TCGGAGCTTC AAGGAAGATC AATATCTCAG   
  
  
- AAAAACACTA CCTGCTGTAG TGCATGGGAC ATGTCGGCAT GGTTTTATGG GGATATGAAG TCCTATTGCT   
  
  
- GTCTGTGGTT AATTATAATG ATTAATGATG GTATTAAGTA AATACATTCT CAAATCATTT GGACTTCTTT   
  
  
- CAAGAGGGGT CACTGGTACT CTGTCTCATC TACCAATTTT TCGGGCTTTC TTTTTCCGTA TGGGGGAGGA   
  
  
- TTAGATTGAT TTGTCGCAGT GTCACTGGTT TTGTTTCAAT CAAGCTCTCT AGGAATAATT TATTAGGAAA   
  
  
- ATACTTGAAA AGAAGAGAAA GCAAGAAGAA AATGAAAATC AAGGATTACC ATAGGAGAAT AGCAAGCAAA   
  
  
- GGTCTATGAA GAGAAGATGT AACTCCATTG GTCACACTAA TATGTAGCAT GGAGAAAGAA AGCTGGAAGG   
  
  
- TAAAGGCAAA TTATAATAGC ATATCCACAA GTGATATATC CACTTGTGGT TGGACTCTGA ATCAAGCAGC   
  
  
- TGCTTCTGCT GGTGTGTTGA ACTCCTCCCA GGCTTCAATC CAAGTGACCA TGGCATCTGC AAGCTTCTCA   
  
  
- AAGTAAGGAT CAATATGACC CAATTTCTCT TTGACCTGCC TGGAGAGGGC AAGGGAGCAC TTCAGGGCTG   
  
  
- TTGTGCAGTC TTTTGGCAGG ACAACAGATT GGAACAATGG AATCAGCTGT TCCTGCCAGT ATAGACCCTT   
  
  
- GAATTCCTTC TTCAAGTTAG AAAACGGGT

+     CCAAT-box

| Site Name | Organism | Position | Strand | Matrix score. | sequence | function |
| --- | --- | --- | --- | --- | --- | --- |
| CCAAT-box | Hordeum vulgare | 297 | + | 6 | CAACGG | MYBHv1 binding site |

> 2018/04/13 10:10:12  
+ ATAACCTCGA TTGTTAGATT TGGCTCGAAA ATAAACAAAG GACATACTCC TTCTTGTTCT GTTTCAACTT   
  
  
+ TTCTCCTCTC CTCGTCCGAT CGAATAATAC GATTATCTTT ACCGTATTGT ACCCATCTTT TCCCACCCCC   
  
  
+ AATTACCTTT CCGCAGGTTG AATTGACTGA ACTGTCTTAA CTGGGTACAT TTCTCACTAA AGGTACCTAC   
  
  
+ GTAGGGGAGT GTTTCTAATT CTCATACACG GCATTTGCCG TGCGTTTAAC TGTTTGTCTT CCTACCAAAT   
  
  
+ CAAAGAACAG CTTTTCCAAC GGTTCTTATT TACTAAAATT CGGGTATTCA ATACTTTCAC CTGCTCGTAC   
  
  
+ CTTCAACTTG ACTACTTCTT CGGTACGGTT GGTCGAATTA GTATCTTAAT GAAGAACACA ATATAGACTT   
  
  
+ TATATCTACA TTGTAGTACC TGAAGGTACC TACTGATCGA GACCAACATC GAATCTCGAA CGAATTGCAT   
  
  
+ GAATTTGATG CTTCCTTTTC CTCATACGAA CGACAAATAA GAGTGTATCT TCGGTAGAAG TCGGTTAGTC   
  
  
+ GGTTATCGTA GTGAATTTGG CCATCCACTT AAAGTACGGA GCTCCAGTTT TTCGTCTATA AATTCAAAGT   
  
  
+ TGATTAGTTA ACTCACGTGT GAGGGGTGAA AGATGTACAT GTCAAGTAGC GTACTCGAAA TTGCGATTTA   
  
  
+ CTGAACCCGT ATGTATTCTC CTTAAAAGCG ACCCAAAAGG AGCCTCGAAG TTCCTTCTAG TTATAGAGTC   
  
  
+ TTTTTGTGAT GGACGACATC ACGTACCCTG TACAGCCGTA CCAAAATACC CCTATACTTC AGGATAACGA   
  
  
+ CAGACACCAA TTAATATTAC TAATTACTAC CATAATTCAT TTATGTAAGA GTTTAGTAAA CCTGAAGAAA   
  
  
+ GTTCTCCCCA GTGACCATGA GACAGAGTAG ATGGTTAAAA AGCCCGAAAG AAAAAGGCAT ACCCCCTCCT   
  
  
+ AATCTAACTA AACAGCGTCA CAGTGACCAA AACAAAGTTA GTTCGAGAGA TCCTTATTAA ATAATCCTTT   
  
  
+ TATGAACTTT TCTTCTCTTT CGTTCTTCTT TTACTTTTAG TTCCTAATGG TATCCTCTTA TCGTTCGTTT   
  
  
+ CCAGATACTT CTCTTCTACA TTGAGGTAAC CAGTGTGATT ATACATCGTA CCTCTTTCTT TCGACCTTCC   
  
  
+ ATTTCCGTTT AATATTATCG TATAGGTGTT CACTATATAG GTGAACACCA ACCTGAGACT TAGTTCGTCG   
  
  
+ ACGAAGACGA CCACACAACT TGAGGAGGGT CCGAAGTTAG GTTCACTGGT ACCGTAGACG TTCGAAGAGT   
  
  
+ TTCATTCCTA GTTATACTGG GTTAAAGAGA AACTGGACGG ACCTCTCCCG TTCCCTCGTG AAGTCCCGAC   
  
  
+ AACACGTCAG AAAACCGTCC TGTTGTCTAA CCTTGTTACC TTAGTCGACA AGGACGGTCA TATCTGGGAA   
  
  
+ CTTAAGGAAG AAGTTCAATC TTTTGCCCA  

- TATTGGAGCT AACAATCTAA ACCGAGCTTT TATTTGTTTC CTGTATGAGG AAGAACAAGA CAAAGTTGAA   
  
  
- AAGAGGAGAG GAGCAGGCTA GCTTATTATG CTAATAGAAA TGGCATAACA TGGGTAGAAA AGGGTGGGGG   
  
  
- TTAATGGAAA GGCGTCCAAC TTAACTGACT TGACAGAATT GACCCATGTA AAGAGTGATT TCCATGGATG   
  
  
- CATCCCCTCA CAAAGATTAA GAGTATGTGC CGTAAACGGC ACGCAAATTG ACAAACAGAA GGATGGTTTA   
  
  
- GTTTCTTGTC GAAAAGGTTG CCAAGAATAA ATGATTTTAA GCCCATAAGT TATGAAAGTG GACGAGCATG   
  
  
- GAAGTTGAAC TGATGAAGAA GCCATGCCAA CCAGCTTAAT CATAGAATTA CTTCTTGTGT TATATCTGAA   
  
  
- ATATAGATGT AACATCATGG ACTTCCATGG ATGACTAGCT CTGGTTGTAG CTTAGAGCTT GCTTAACGTA   
  
  
- CTTAAACTAC GAAGGAAAAG GAGTATGCTT GCTGTTTATT CTCACATAGA AGCCATCTTC AGCCAATCAG   
  
  
- CCAATAGCAT CACTTAAACC GGTAGGTGAA TTTCATGCCT CGAGGTCAAA AAGCAGATAT TTAAGTTTCA   
  
  
- ACTAATCAAT TGAGTGCACA CTCCCCACTT TCTACATGTA CAGTTCATCG CATGAGCTTT AACGCTAAAT   
  
  
- GACTTGGGCA TACATAAGAG GAATTTTCGC TGGGTTTTCC TCGGAGCTTC AAGGAAGATC AATATCTCAG   
  
  
- AAAAACACTA CCTGCTGTAG TGCATGGGAC ATGTCGGCAT GGTTTTATGG GGATATGAAG TCCTATTGCT   
  
  
- GTCTGTGGTT AATTATAATG ATTAATGATG GTATTAAGTA AATACATTCT CAAATCATTT GGACTTCTTT   
  
  
- CAAGAGGGGT CACTGGTACT CTGTCTCATC TACCAATTTT TCGGGCTTTC TTTTTCCGTA TGGGGGAGGA   
  
  
- TTAGATTGAT TTGTCGCAGT GTCACTGGTT TTGTTTCAAT CAAGCTCTCT AGGAATAATT TATTAGGAAA   
  
  
- ATACTTGAAA AGAAGAGAAA GCAAGAAGAA AATGAAAATC AAGGATTACC ATAGGAGAAT AGCAAGCAAA   
  
  
- GGTCTATGAA GAGAAGATGT AACTCCATTG GTCACACTAA TATGTAGCAT GGAGAAAGAA AGCTGGAAGG   
  
  
- TAAAGGCAAA TTATAATAGC ATATCCACAA GTGATATATC CACTTGTGGT TGGACTCTGA ATCAAGCAGC   
  
  
- TGCTTCTGCT GGTGTGTTGA ACTCCTCCCA GGCTTCAATC CAAGTGACCA TGGCATCTGC AAGCTTCTCA   
  
  
- AAGTAAGGAT CAATATGACC CAATTTCTCT TTGACCTGCC TGGAGAGGGC AAGGGAGCAC TTCAGGGCTG   
  
  
- TTGTGCAGTC TTTTGGCAGG ACAACAGATT GGAACAATGG AATCAGCTGT TCCTGCCAGT ATAGACCCTT   
  
  
- GAATTCCTTC TTCAAGTTAG AAAACGGGT

+     CCGTCC-box

| Site Name | Organism | Position | Strand | Matrix score. | sequence | function |
| --- | --- | --- | --- | --- | --- | --- |
| CCGTCC-box | Arabidopsis thaliana | 1415 | + | 6 | CCGTCC | cis-acting regulatory element related to meristem specific activation |
| CCGTCC-box | Arabidopsis thaliana | 1365 | - | 6 | CCGTCC | cis-acting regulatory element related to meristem specific activation |
| CCGTCC-box | Arabidopsis thaliana | 1452 | - | 6 | CCGTCC | cis-acting regulatory element related to meristem specific activation |

> 2018/04/13 10:10:12  
+ ATAACCTCGA TTGTTAGATT TGGCTCGAAA ATAAACAAAG GACATACTCC TTCTTGTTCT GTTTCAACTT   
  
  
+ TTCTCCTCTC CTCGTCCGAT CGAATAATAC GATTATCTTT ACCGTATTGT ACCCATCTTT TCCCACCCCC   
  
  
+ AATTACCTTT CCGCAGGTTG AATTGACTGA ACTGTCTTAA CTGGGTACAT TTCTCACTAA AGGTACCTAC   
  
  
+ GTAGGGGAGT GTTTCTAATT CTCATACACG GCATTTGCCG TGCGTTTAAC TGTTTGTCTT CCTACCAAAT   
  
  
+ CAAAGAACAG CTTTTCCAAC GGTTCTTATT TACTAAAATT CGGGTATTCA ATACTTTCAC CTGCTCGTAC   
  
  
+ CTTCAACTTG ACTACTTCTT CGGTACGGTT GGTCGAATTA GTATCTTAAT GAAGAACACA ATATAGACTT   
  
  
+ TATATCTACA TTGTAGTACC TGAAGGTACC TACTGATCGA GACCAACATC GAATCTCGAA CGAATTGCAT   
  
  
+ GAATTTGATG CTTCCTTTTC CTCATACGAA CGACAAATAA GAGTGTATCT TCGGTAGAAG TCGGTTAGTC   
  
  
+ GGTTATCGTA GTGAATTTGG CCATCCACTT AAAGTACGGA GCTCCAGTTT TTCGTCTATA AATTCAAAGT   
  
  
+ TGATTAGTTA ACTCACGTGT GAGGGGTGAA AGATGTACAT GTCAAGTAGC GTACTCGAAA TTGCGATTTA   
  
  
+ CTGAACCCGT ATGTATTCTC CTTAAAAGCG ACCCAAAAGG AGCCTCGAAG TTCCTTCTAG TTATAGAGTC   
  
  
+ TTTTTGTGAT GGACGACATC ACGTACCCTG TACAGCCGTA CCAAAATACC CCTATACTTC AGGATAACGA   
  
  
+ CAGACACCAA TTAATATTAC TAATTACTAC CATAATTCAT TTATGTAAGA GTTTAGTAAA CCTGAAGAAA   
  
  
+ GTTCTCCCCA GTGACCATGA GACAGAGTAG ATGGTTAAAA AGCCCGAAAG AAAAAGGCAT ACCCCCTCCT   
  
  
+ AATCTAACTA AACAGCGTCA CAGTGACCAA AACAAAGTTA GTTCGAGAGA TCCTTATTAA ATAATCCTTT   
  
  
+ TATGAACTTT TCTTCTCTTT CGTTCTTCTT TTACTTTTAG TTCCTAATGG TATCCTCTTA TCGTTCGTTT   
  
  
+ CCAGATACTT CTCTTCTACA TTGAGGTAAC CAGTGTGATT ATACATCGTA CCTCTTTCTT TCGACCTTCC   
  
  
+ ATTTCCGTTT AATATTATCG TATAGGTGTT CACTATATAG GTGAACACCA ACCTGAGACT TAGTTCGTCG   
  
  
+ ACGAAGACGA CCACACAACT TGAGGAGGGT CCGAAGTTAG GTTCACTGGT ACCGTAGACG TTCGAAGAGT   
  
  
+ TTCATTCCTA GTTATACTGG GTTAAAGAGA AACTGGACGG ACCTCTCCCG TTCCCTCGTG AAGTCCCGAC   
  
  
+ AACACGTCAG AAAACCGTCC TGTTGTCTAA CCTTGTTACC TTAGTCGACA AGGACGGTCA TATCTGGGAA   
  
  
+ CTTAAGGAAG AAGTTCAATC TTTTGCCCA  

- TATTGGAGCT AACAATCTAA ACCGAGCTTT TATTTGTTTC CTGTATGAGG AAGAACAAGA CAAAGTTGAA   
  
  
- AAGAGGAGAG GAGCAGGCTA GCTTATTATG CTAATAGAAA TGGCATAACA TGGGTAGAAA AGGGTGGGGG   
  
  
- TTAATGGAAA GGCGTCCAAC TTAACTGACT TGACAGAATT GACCCATGTA AAGAGTGATT TCCATGGATG   
  
  
- CATCCCCTCA CAAAGATTAA GAGTATGTGC CGTAAACGGC ACGCAAATTG ACAAACAGAA GGATGGTTTA   
  
  
- GTTTCTTGTC GAAAAGGTTG CCAAGAATAA ATGATTTTAA GCCCATAAGT TATGAAAGTG GACGAGCATG   
  
  
- GAAGTTGAAC TGATGAAGAA GCCATGCCAA CCAGCTTAAT CATAGAATTA CTTCTTGTGT TATATCTGAA   
  
  
- ATATAGATGT AACATCATGG ACTTCCATGG ATGACTAGCT CTGGTTGTAG CTTAGAGCTT GCTTAACGTA   
  
  
- CTTAAACTAC GAAGGAAAAG GAGTATGCTT GCTGTTTATT CTCACATAGA AGCCATCTTC AGCCAATCAG   
  
  
- CCAATAGCAT CACTTAAACC GGTAGGTGAA TTTCATGCCT CGAGGTCAAA AAGCAGATAT TTAAGTTTCA   
  
  
- ACTAATCAAT TGAGTGCACA CTCCCCACTT TCTACATGTA CAGTTCATCG CATGAGCTTT AACGCTAAAT   
  
  
- GACTTGGGCA TACATAAGAG GAATTTTCGC TGGGTTTTCC TCGGAGCTTC AAGGAAGATC AATATCTCAG   
  
  
- AAAAACACTA CCTGCTGTAG TGCATGGGAC ATGTCGGCAT GGTTTTATGG GGATATGAAG TCCTATTGCT   
  
  
- GTCTGTGGTT AATTATAATG ATTAATGATG GTATTAAGTA AATACATTCT CAAATCATTT GGACTTCTTT   
  
  
- CAAGAGGGGT CACTGGTACT CTGTCTCATC TACCAATTTT TCGGGCTTTC TTTTTCCGTA TGGGGGAGGA   
  
  
- TTAGATTGAT TTGTCGCAGT GTCACTGGTT TTGTTTCAAT CAAGCTCTCT AGGAATAATT TATTAGGAAA   
  
  
- ATACTTGAAA AGAAGAGAAA GCAAGAAGAA AATGAAAATC AAGGATTACC ATAGGAGAAT AGCAAGCAAA   
  
  
- GGTCTATGAA GAGAAGATGT AACTCCATTG GTCACACTAA TATGTAGCAT GGAGAAAGAA AGCTGGAAGG   
  
  
- TAAAGGCAAA TTATAATAGC ATATCCACAA GTGATATATC CACTTGTGGT TGGACTCTGA ATCAAGCAGC   
  
  
- TGCTTCTGCT GGTGTGTTGA ACTCCTCCCA GGCTTCAATC CAAGTGACCA TGGCATCTGC AAGCTTCTCA   
  
  
- AAGTAAGGAT CAATATGACC CAATTTCTCT TTGACCTGCC TGGAGAGGGC AAGGGAGCAC TTCAGGGCTG   
  
  
- TTGTGCAGTC TTTTGGCAGG ACAACAGATT GGAACAATGG AATCAGCTGT TCCTGCCAGT ATAGACCCTT   
  
  
- GAATTCCTTC TTCAAGTTAG AAAACGGGT

+     CGTCA-motif

| Site Name | Organism | Position | Strand | Matrix score. | sequence | function |
| --- | --- | --- | --- | --- | --- | --- |
| CGTCA-motif | Hordeum vulgare | 1405 | + | 5 | CGTCA | cis-acting regulatory element involved in the MeJA-responsiveness |
| CGTCA-motif | Hordeum vulgare | 996 | + | 5 | CGTCA | cis-acting regulatory element involved in the MeJA-responsiveness |

> 2018/04/13 10:10:12  
+ ATAACCTCGA TTGTTAGATT TGGCTCGAAA ATAAACAAAG GACATACTCC TTCTTGTTCT GTTTCAACTT   
  
  
+ TTCTCCTCTC CTCGTCCGAT CGAATAATAC GATTATCTTT ACCGTATTGT ACCCATCTTT TCCCACCCCC   
  
  
+ AATTACCTTT CCGCAGGTTG AATTGACTGA ACTGTCTTAA CTGGGTACAT TTCTCACTAA AGGTACCTAC   
  
  
+ GTAGGGGAGT GTTTCTAATT CTCATACACG GCATTTGCCG TGCGTTTAAC TGTTTGTCTT CCTACCAAAT   
  
  
+ CAAAGAACAG CTTTTCCAAC GGTTCTTATT TACTAAAATT CGGGTATTCA ATACTTTCAC CTGCTCGTAC   
  
  
+ CTTCAACTTG ACTACTTCTT CGGTACGGTT GGTCGAATTA GTATCTTAAT GAAGAACACA ATATAGACTT   
  
  
+ TATATCTACA TTGTAGTACC TGAAGGTACC TACTGATCGA GACCAACATC GAATCTCGAA CGAATTGCAT   
  
  
+ GAATTTGATG CTTCCTTTTC CTCATACGAA CGACAAATAA GAGTGTATCT TCGGTAGAAG TCGGTTAGTC   
  
  
+ GGTTATCGTA GTGAATTTGG CCATCCACTT AAAGTACGGA GCTCCAGTTT TTCGTCTATA AATTCAAAGT   
  
  
+ TGATTAGTTA ACTCACGTGT GAGGGGTGAA AGATGTACAT GTCAAGTAGC GTACTCGAAA TTGCGATTTA   
  
  
+ CTGAACCCGT ATGTATTCTC CTTAAAAGCG ACCCAAAAGG AGCCTCGAAG TTCCTTCTAG TTATAGAGTC   
  
  
+ TTTTTGTGAT GGACGACATC ACGTACCCTG TACAGCCGTA CCAAAATACC CCTATACTTC AGGATAACGA   
  
  
+ CAGACACCAA TTAATATTAC TAATTACTAC CATAATTCAT TTATGTAAGA GTTTAGTAAA CCTGAAGAAA   
  
  
+ GTTCTCCCCA GTGACCATGA GACAGAGTAG ATGGTTAAAA AGCCCGAAAG AAAAAGGCAT ACCCCCTCCT   
  
  
+ AATCTAACTA AACAGCGTCA CAGTGACCAA AACAAAGTTA GTTCGAGAGA TCCTTATTAA ATAATCCTTT   
  
  
+ TATGAACTTT TCTTCTCTTT CGTTCTTCTT TTACTTTTAG TTCCTAATGG TATCCTCTTA TCGTTCGTTT   
  
  
+ CCAGATACTT CTCTTCTACA TTGAGGTAAC CAGTGTGATT ATACATCGTA CCTCTTTCTT TCGACCTTCC   
  
  
+ ATTTCCGTTT AATATTATCG TATAGGTGTT CACTATATAG GTGAACACCA ACCTGAGACT TAGTTCGTCG   
  
  
+ ACGAAGACGA CCACACAACT TGAGGAGGGT CCGAAGTTAG GTTCACTGGT ACCGTAGACG TTCGAAGAGT   
  
  
+ TTCATTCCTA GTTATACTGG GTTAAAGAGA AACTGGACGG ACCTCTCCCG TTCCCTCGTG AAGTCCCGAC   
  
  
+ AACACGTCAG AAAACCGTCC TGTTGTCTAA CCTTGTTACC TTAGTCGACA AGGACGGTCA TATCTGGGAA   
  
  
+ CTTAAGGAAG AAGTTCAATC TTTTGCCCA  

- TATTGGAGCT AACAATCTAA ACCGAGCTTT TATTTGTTTC CTGTATGAGG AAGAACAAGA CAAAGTTGAA   
  
  
- AAGAGGAGAG GAGCAGGCTA GCTTATTATG CTAATAGAAA TGGCATAACA TGGGTAGAAA AGGGTGGGGG   
  
  
- TTAATGGAAA GGCGTCCAAC TTAACTGACT TGACAGAATT GACCCATGTA AAGAGTGATT TCCATGGATG   
  
  
- CATCCCCTCA CAAAGATTAA GAGTATGTGC CGTAAACGGC ACGCAAATTG ACAAACAGAA GGATGGTTTA   
  
  
- GTTTCTTGTC GAAAAGGTTG CCAAGAATAA ATGATTTTAA GCCCATAAGT TATGAAAGTG GACGAGCATG   
  
  
- GAAGTTGAAC TGATGAAGAA GCCATGCCAA CCAGCTTAAT CATAGAATTA CTTCTTGTGT TATATCTGAA   
  
  
- ATATAGATGT AACATCATGG ACTTCCATGG ATGACTAGCT CTGGTTGTAG CTTAGAGCTT GCTTAACGTA   
  
  
- CTTAAACTAC GAAGGAAAAG GAGTATGCTT GCTGTTTATT CTCACATAGA AGCCATCTTC AGCCAATCAG   
  
  
- CCAATAGCAT CACTTAAACC GGTAGGTGAA TTTCATGCCT CGAGGTCAAA AAGCAGATAT TTAAGTTTCA   
  
  
- ACTAATCAAT TGAGTGCACA CTCCCCACTT TCTACATGTA CAGTTCATCG CATGAGCTTT AACGCTAAAT   
  
  
- GACTTGGGCA TACATAAGAG GAATTTTCGC TGGGTTTTCC TCGGAGCTTC AAGGAAGATC AATATCTCAG   
  
  
- AAAAACACTA CCTGCTGTAG TGCATGGGAC ATGTCGGCAT GGTTTTATGG GGATATGAAG TCCTATTGCT   
  
  
- GTCTGTGGTT AATTATAATG ATTAATGATG GTATTAAGTA AATACATTCT CAAATCATTT GGACTTCTTT   
  
  
- CAAGAGGGGT CACTGGTACT CTGTCTCATC TACCAATTTT TCGGGCTTTC TTTTTCCGTA TGGGGGAGGA   
  
  
- TTAGATTGAT TTGTCGCAGT GTCACTGGTT TTGTTTCAAT CAAGCTCTCT AGGAATAATT TATTAGGAAA   
  
  
- ATACTTGAAA AGAAGAGAAA GCAAGAAGAA AATGAAAATC AAGGATTACC ATAGGAGAAT AGCAAGCAAA   
  
  
- GGTCTATGAA GAGAAGATGT AACTCCATTG GTCACACTAA TATGTAGCAT GGAGAAAGAA AGCTGGAAGG   
  
  
- TAAAGGCAAA TTATAATAGC ATATCCACAA GTGATATATC CACTTGTGGT TGGACTCTGA ATCAAGCAGC   
  
  
- TGCTTCTGCT GGTGTGTTGA ACTCCTCCCA GGCTTCAATC CAAGTGACCA TGGCATCTGC AAGCTTCTCA   
  
  
- AAGTAAGGAT CAATATGACC CAATTTCTCT TTGACCTGCC TGGAGAGGGC AAGGGAGCAC TTCAGGGCTG   
  
  
- TTGTGCAGTC TTTTGGCAGG ACAACAGATT GGAACAATGG AATCAGCTGT TCCTGCCAGT ATAGACCCTT   
  
  
- GAATTCCTTC TTCAAGTTAG AAAACGGGT

+     EIRE

| Site Name | Organism | Position | Strand | Matrix score. | sequence | function |
| --- | --- | --- | --- | --- | --- | --- |
| EIRE | Nicotiana tabacum | 1180 | + | 7 | TTCGACC | elicitor-responsive element |
| EIRE | Nicotiana tabacum | 381 | - | 7 | TTCGACC | elicitor-responsive element |

> 2018/04/13 10:10:12  
+ ATAACCTCGA TTGTTAGATT TGGCTCGAAA ATAAACAAAG GACATACTCC TTCTTGTTCT GTTTCAACTT   
  
  
+ TTCTCCTCTC CTCGTCCGAT CGAATAATAC GATTATCTTT ACCGTATTGT ACCCATCTTT TCCCACCCCC   
  
  
+ AATTACCTTT CCGCAGGTTG AATTGACTGA ACTGTCTTAA CTGGGTACAT TTCTCACTAA AGGTACCTAC   
  
  
+ GTAGGGGAGT GTTTCTAATT CTCATACACG GCATTTGCCG TGCGTTTAAC TGTTTGTCTT CCTACCAAAT   
  
  
+ CAAAGAACAG CTTTTCCAAC GGTTCTTATT TACTAAAATT CGGGTATTCA ATACTTTCAC CTGCTCGTAC   
  
  
+ CTTCAACTTG ACTACTTCTT CGGTACGGTT GGTCGAATTA GTATCTTAAT GAAGAACACA ATATAGACTT   
  
  
+ TATATCTACA TTGTAGTACC TGAAGGTACC TACTGATCGA GACCAACATC GAATCTCGAA CGAATTGCAT   
  
  
+ GAATTTGATG CTTCCTTTTC CTCATACGAA CGACAAATAA GAGTGTATCT TCGGTAGAAG TCGGTTAGTC   
  
  
+ GGTTATCGTA GTGAATTTGG CCATCCACTT AAAGTACGGA GCTCCAGTTT TTCGTCTATA AATTCAAAGT   
  
  
+ TGATTAGTTA ACTCACGTGT GAGGGGTGAA AGATGTACAT GTCAAGTAGC GTACTCGAAA TTGCGATTTA   
  
  
+ CTGAACCCGT ATGTATTCTC CTTAAAAGCG ACCCAAAAGG AGCCTCGAAG TTCCTTCTAG TTATAGAGTC   
  
  
+ TTTTTGTGAT GGACGACATC ACGTACCCTG TACAGCCGTA CCAAAATACC CCTATACTTC AGGATAACGA   
  
  
+ CAGACACCAA TTAATATTAC TAATTACTAC CATAATTCAT TTATGTAAGA GTTTAGTAAA CCTGAAGAAA   
  
  
+ GTTCTCCCCA GTGACCATGA GACAGAGTAG ATGGTTAAAA AGCCCGAAAG AAAAAGGCAT ACCCCCTCCT   
  
  
+ AATCTAACTA AACAGCGTCA CAGTGACCAA AACAAAGTTA GTTCGAGAGA TCCTTATTAA ATAATCCTTT   
  
  
+ TATGAACTTT TCTTCTCTTT CGTTCTTCTT TTACTTTTAG TTCCTAATGG TATCCTCTTA TCGTTCGTTT   
  
  
+ CCAGATACTT CTCTTCTACA TTGAGGTAAC CAGTGTGATT ATACATCGTA CCTCTTTCTT TCGACCTTCC   
  
  
+ ATTTCCGTTT AATATTATCG TATAGGTGTT CACTATATAG GTGAACACCA ACCTGAGACT TAGTTCGTCG   
  
  
+ ACGAAGACGA CCACACAACT TGAGGAGGGT CCGAAGTTAG GTTCACTGGT ACCGTAGACG TTCGAAGAGT   
  
  
+ TTCATTCCTA GTTATACTGG GTTAAAGAGA AACTGGACGG ACCTCTCCCG TTCCCTCGTG AAGTCCCGAC   
  
  
+ AACACGTCAG AAAACCGTCC TGTTGTCTAA CCTTGTTACC TTAGTCGACA AGGACGGTCA TATCTGGGAA   
  
  
+ CTTAAGGAAG AAGTTCAATC TTTTGCCCA  

- TATTGGAGCT AACAATCTAA ACCGAGCTTT TATTTGTTTC CTGTATGAGG AAGAACAAGA CAAAGTTGAA   
  
  
- AAGAGGAGAG GAGCAGGCTA GCTTATTATG CTAATAGAAA TGGCATAACA TGGGTAGAAA AGGGTGGGGG   
  
  
- TTAATGGAAA GGCGTCCAAC TTAACTGACT TGACAGAATT GACCCATGTA AAGAGTGATT TCCATGGATG   
  
  
- CATCCCCTCA CAAAGATTAA GAGTATGTGC CGTAAACGGC ACGCAAATTG ACAAACAGAA GGATGGTTTA   
  
  
- GTTTCTTGTC GAAAAGGTTG CCAAGAATAA ATGATTTTAA GCCCATAAGT TATGAAAGTG GACGAGCATG   
  
  
- GAAGTTGAAC TGATGAAGAA GCCATGCCAA CCAGCTTAAT CATAGAATTA CTTCTTGTGT TATATCTGAA   
  
  
- ATATAGATGT AACATCATGG ACTTCCATGG ATGACTAGCT CTGGTTGTAG CTTAGAGCTT GCTTAACGTA   
  
  
- CTTAAACTAC GAAGGAAAAG GAGTATGCTT GCTGTTTATT CTCACATAGA AGCCATCTTC AGCCAATCAG   
  
  
- CCAATAGCAT CACTTAAACC GGTAGGTGAA TTTCATGCCT CGAGGTCAAA AAGCAGATAT TTAAGTTTCA   
  
  
- ACTAATCAAT TGAGTGCACA CTCCCCACTT TCTACATGTA CAGTTCATCG CATGAGCTTT AACGCTAAAT   
  
  
- GACTTGGGCA TACATAAGAG GAATTTTCGC TGGGTTTTCC TCGGAGCTTC AAGGAAGATC AATATCTCAG   
  
  
- AAAAACACTA CCTGCTGTAG TGCATGGGAC ATGTCGGCAT GGTTTTATGG GGATATGAAG TCCTATTGCT   
  
  
- GTCTGTGGTT AATTATAATG ATTAATGATG GTATTAAGTA AATACATTCT CAAATCATTT GGACTTCTTT   
  
  
- CAAGAGGGGT CACTGGTACT CTGTCTCATC TACCAATTTT TCGGGCTTTC TTTTTCCGTA TGGGGGAGGA   
  
  
- TTAGATTGAT TTGTCGCAGT GTCACTGGTT TTGTTTCAAT CAAGCTCTCT AGGAATAATT TATTAGGAAA   
  
  
- ATACTTGAAA AGAAGAGAAA GCAAGAAGAA AATGAAAATC AAGGATTACC ATAGGAGAAT AGCAAGCAAA   
  
  
- GGTCTATGAA GAGAAGATGT AACTCCATTG GTCACACTAA TATGTAGCAT GGAGAAAGAA AGCTGGAAGG   
  
  
- TAAAGGCAAA TTATAATAGC ATATCCACAA GTGATATATC CACTTGTGGT TGGACTCTGA ATCAAGCAGC   
  
  
- TGCTTCTGCT GGTGTGTTGA ACTCCTCCCA GGCTTCAATC CAAGTGACCA TGGCATCTGC AAGCTTCTCA   
  
  
- AAGTAAGGAT CAATATGACC CAATTTCTCT TTGACCTGCC TGGAGAGGGC AAGGGAGCAC TTCAGGGCTG   
  
  
- TTGTGCAGTC TTTTGGCAGG ACAACAGATT GGAACAATGG AATCAGCTGT TCCTGCCAGT ATAGACCCTT   
  
  
- GAATTCCTTC TTCAAGTTAG AAAACGGGT

+     G-Box

| Site Name | Organism | Position | Strand | Matrix score. | sequence | function |
| --- | --- | --- | --- | --- | --- | --- |
| G-Box | Antirrhinum majus | 790 | + | 6 | CACGTA | cis-acting regulatory element involved in light responsiveness |
| G-Box | Pisum sativum | 644 | + | 6 | CACGTG | cis-acting regulatory element involved in light responsiveness |

> 2018/04/13 10:10:12  
+ ATAACCTCGA TTGTTAGATT TGGCTCGAAA ATAAACAAAG GACATACTCC TTCTTGTTCT GTTTCAACTT   
  
  
+ TTCTCCTCTC CTCGTCCGAT CGAATAATAC GATTATCTTT ACCGTATTGT ACCCATCTTT TCCCACCCCC   
  
  
+ AATTACCTTT CCGCAGGTTG AATTGACTGA ACTGTCTTAA CTGGGTACAT TTCTCACTAA AGGTACCTAC   
  
  
+ GTAGGGGAGT GTTTCTAATT CTCATACACG GCATTTGCCG TGCGTTTAAC TGTTTGTCTT CCTACCAAAT   
  
  
+ CAAAGAACAG CTTTTCCAAC GGTTCTTATT TACTAAAATT CGGGTATTCA ATACTTTCAC CTGCTCGTAC   
  
  
+ CTTCAACTTG ACTACTTCTT CGGTACGGTT GGTCGAATTA GTATCTTAAT GAAGAACACA ATATAGACTT   
  
  
+ TATATCTACA TTGTAGTACC TGAAGGTACC TACTGATCGA GACCAACATC GAATCTCGAA CGAATTGCAT   
  
  
+ GAATTTGATG CTTCCTTTTC CTCATACGAA CGACAAATAA GAGTGTATCT TCGGTAGAAG TCGGTTAGTC   
  
  
+ GGTTATCGTA GTGAATTTGG CCATCCACTT AAAGTACGGA GCTCCAGTTT TTCGTCTATA AATTCAAAGT   
  
  
+ TGATTAGTTA ACTCACGTGT GAGGGGTGAA AGATGTACAT GTCAAGTAGC GTACTCGAAA TTGCGATTTA   
  
  
+ CTGAACCCGT ATGTATTCTC CTTAAAAGCG ACCCAAAAGG AGCCTCGAAG TTCCTTCTAG TTATAGAGTC   
  
  
+ TTTTTGTGAT GGACGACATC ACGTACCCTG TACAGCCGTA CCAAAATACC CCTATACTTC AGGATAACGA   
  
  
+ CAGACACCAA TTAATATTAC TAATTACTAC CATAATTCAT TTATGTAAGA GTTTAGTAAA CCTGAAGAAA   
  
  
+ GTTCTCCCCA GTGACCATGA GACAGAGTAG ATGGTTAAAA AGCCCGAAAG AAAAAGGCAT ACCCCCTCCT   
  
  
+ AATCTAACTA AACAGCGTCA CAGTGACCAA AACAAAGTTA GTTCGAGAGA TCCTTATTAA ATAATCCTTT   
  
  
+ TATGAACTTT TCTTCTCTTT CGTTCTTCTT TTACTTTTAG TTCCTAATGG TATCCTCTTA TCGTTCGTTT   
  
  
+ CCAGATACTT CTCTTCTACA TTGAGGTAAC CAGTGTGATT ATACATCGTA CCTCTTTCTT TCGACCTTCC   
  
  
+ ATTTCCGTTT AATATTATCG TATAGGTGTT CACTATATAG GTGAACACCA ACCTGAGACT TAGTTCGTCG   
  
  
+ ACGAAGACGA CCACACAACT TGAGGAGGGT CCGAAGTTAG GTTCACTGGT ACCGTAGACG TTCGAAGAGT   
  
  
+ TTCATTCCTA GTTATACTGG GTTAAAGAGA AACTGGACGG ACCTCTCCCG TTCCCTCGTG AAGTCCCGAC   
  
  
+ AACACGTCAG AAAACCGTCC TGTTGTCTAA CCTTGTTACC TTAGTCGACA AGGACGGTCA TATCTGGGAA   
  
  
+ CTTAAGGAAG AAGTTCAATC TTTTGCCCA  

- TATTGGAGCT AACAATCTAA ACCGAGCTTT TATTTGTTTC CTGTATGAGG AAGAACAAGA CAAAGTTGAA   
  
  
- AAGAGGAGAG GAGCAGGCTA GCTTATTATG CTAATAGAAA TGGCATAACA TGGGTAGAAA AGGGTGGGGG   
  
  
- TTAATGGAAA GGCGTCCAAC TTAACTGACT TGACAGAATT GACCCATGTA AAGAGTGATT TCCATGGATG   
  
  
- CATCCCCTCA CAAAGATTAA GAGTATGTGC CGTAAACGGC ACGCAAATTG ACAAACAGAA GGATGGTTTA   
  
  
- GTTTCTTGTC GAAAAGGTTG CCAAGAATAA ATGATTTTAA GCCCATAAGT TATGAAAGTG GACGAGCATG   
  
  
- GAAGTTGAAC TGATGAAGAA GCCATGCCAA CCAGCTTAAT CATAGAATTA CTTCTTGTGT TATATCTGAA   
  
  
- ATATAGATGT AACATCATGG ACTTCCATGG ATGACTAGCT CTGGTTGTAG CTTAGAGCTT GCTTAACGTA   
  
  
- CTTAAACTAC GAAGGAAAAG GAGTATGCTT GCTGTTTATT CTCACATAGA AGCCATCTTC AGCCAATCAG   
  
  
- CCAATAGCAT CACTTAAACC GGTAGGTGAA TTTCATGCCT CGAGGTCAAA AAGCAGATAT TTAAGTTTCA   
  
  
- ACTAATCAAT TGAGTGCACA CTCCCCACTT TCTACATGTA CAGTTCATCG CATGAGCTTT AACGCTAAAT   
  
  
- GACTTGGGCA TACATAAGAG GAATTTTCGC TGGGTTTTCC TCGGAGCTTC AAGGAAGATC AATATCTCAG   
  
  
- AAAAACACTA CCTGCTGTAG TGCATGGGAC ATGTCGGCAT GGTTTTATGG GGATATGAAG TCCTATTGCT   
  
  
- GTCTGTGGTT AATTATAATG ATTAATGATG GTATTAAGTA AATACATTCT CAAATCATTT GGACTTCTTT   
  
  
- CAAGAGGGGT CACTGGTACT CTGTCTCATC TACCAATTTT TCGGGCTTTC TTTTTCCGTA TGGGGGAGGA   
  
  
- TTAGATTGAT TTGTCGCAGT GTCACTGGTT TTGTTTCAAT CAAGCTCTCT AGGAATAATT TATTAGGAAA   
  
  
- ATACTTGAAA AGAAGAGAAA GCAAGAAGAA AATGAAAATC AAGGATTACC ATAGGAGAAT AGCAAGCAAA   
  
  
- GGTCTATGAA GAGAAGATGT AACTCCATTG GTCACACTAA TATGTAGCAT GGAGAAAGAA AGCTGGAAGG   
  
  
- TAAAGGCAAA TTATAATAGC ATATCCACAA GTGATATATC CACTTGTGGT TGGACTCTGA ATCAAGCAGC   
  
  
- TGCTTCTGCT GGTGTGTTGA ACTCCTCCCA GGCTTCAATC CAAGTGACCA TGGCATCTGC AAGCTTCTCA   
  
  
- AAGTAAGGAT CAATATGACC CAATTTCTCT TTGACCTGCC TGGAGAGGGC AAGGGAGCAC TTCAGGGCTG   
  
  
- TTGTGCAGTC TTTTGGCAGG ACAACAGATT GGAACAATGG AATCAGCTGT TCCTGCCAGT ATAGACCCTT   
  
  
- GAATTCCTTC TTCAAGTTAG AAAACGGGT

+     G-box

| Site Name | Organism | Position | Strand | Matrix score. | sequence | function |
| --- | --- | --- | --- | --- | --- | --- |
| G-box | Zea mays | 1403 | + | 6 | CACGTC | cis-acting regulatory element involved in light responsiveness |
| G-box | Daucus carota | 790 | - | 6 | TACGTG | cis-acting regulatory element involved in light responsiveness |
| G-box | Arabidopsis thaliana | 644 | + | 6 | CACGTG | cis-acting regulatory element involved in light responsiveness |

> 2018/04/13 10:10:12  
+ ATAACCTCGA TTGTTAGATT TGGCTCGAAA ATAAACAAAG GACATACTCC TTCTTGTTCT GTTTCAACTT   
  
  
+ TTCTCCTCTC CTCGTCCGAT CGAATAATAC GATTATCTTT ACCGTATTGT ACCCATCTTT TCCCACCCCC   
  
  
+ AATTACCTTT CCGCAGGTTG AATTGACTGA ACTGTCTTAA CTGGGTACAT TTCTCACTAA AGGTACCTAC   
  
  
+ GTAGGGGAGT GTTTCTAATT CTCATACACG GCATTTGCCG TGCGTTTAAC TGTTTGTCTT CCTACCAAAT   
  
  
+ CAAAGAACAG CTTTTCCAAC GGTTCTTATT TACTAAAATT CGGGTATTCA ATACTTTCAC CTGCTCGTAC   
  
  
+ CTTCAACTTG ACTACTTCTT CGGTACGGTT GGTCGAATTA GTATCTTAAT GAAGAACACA ATATAGACTT   
  
  
+ TATATCTACA TTGTAGTACC TGAAGGTACC TACTGATCGA GACCAACATC GAATCTCGAA CGAATTGCAT   
  
  
+ GAATTTGATG CTTCCTTTTC CTCATACGAA CGACAAATAA GAGTGTATCT TCGGTAGAAG TCGGTTAGTC   
  
  
+ GGTTATCGTA GTGAATTTGG CCATCCACTT AAAGTACGGA GCTCCAGTTT TTCGTCTATA AATTCAAAGT   
  
  
+ TGATTAGTTA ACTCACGTGT GAGGGGTGAA AGATGTACAT GTCAAGTAGC GTACTCGAAA TTGCGATTTA   
  
  
+ CTGAACCCGT ATGTATTCTC CTTAAAAGCG ACCCAAAAGG AGCCTCGAAG TTCCTTCTAG TTATAGAGTC   
  
  
+ TTTTTGTGAT GGACGACATC ACGTACCCTG TACAGCCGTA CCAAAATACC CCTATACTTC AGGATAACGA   
  
  
+ CAGACACCAA TTAATATTAC TAATTACTAC CATAATTCAT TTATGTAAGA GTTTAGTAAA CCTGAAGAAA   
  
  
+ GTTCTCCCCA GTGACCATGA GACAGAGTAG ATGGTTAAAA AGCCCGAAAG AAAAAGGCAT ACCCCCTCCT   
  
  
+ AATCTAACTA AACAGCGTCA CAGTGACCAA AACAAAGTTA GTTCGAGAGA TCCTTATTAA ATAATCCTTT   
  
  
+ TATGAACTTT TCTTCTCTTT CGTTCTTCTT TTACTTTTAG TTCCTAATGG TATCCTCTTA TCGTTCGTTT   
  
  
+ CCAGATACTT CTCTTCTACA TTGAGGTAAC CAGTGTGATT ATACATCGTA CCTCTTTCTT TCGACCTTCC   
  
  
+ ATTTCCGTTT AATATTATCG TATAGGTGTT CACTATATAG GTGAACACCA ACCTGAGACT TAGTTCGTCG   
  
  
+ ACGAAGACGA CCACACAACT TGAGGAGGGT CCGAAGTTAG GTTCACTGGT ACCGTAGACG TTCGAAGAGT   
  
  
+ TTCATTCCTA GTTATACTGG GTTAAAGAGA AACTGGACGG ACCTCTCCCG TTCCCTCGTG AAGTCCCGAC   
  
  
+ AACACGTCAG AAAACCGTCC TGTTGTCTAA CCTTGTTACC TTAGTCGACA AGGACGGTCA TATCTGGGAA   
  
  
+ CTTAAGGAAG AAGTTCAATC TTTTGCCCA  

- TATTGGAGCT AACAATCTAA ACCGAGCTTT TATTTGTTTC CTGTATGAGG AAGAACAAGA CAAAGTTGAA   
  
  
- AAGAGGAGAG GAGCAGGCTA GCTTATTATG CTAATAGAAA TGGCATAACA TGGGTAGAAA AGGGTGGGGG   
  
  
- TTAATGGAAA GGCGTCCAAC TTAACTGACT TGACAGAATT GACCCATGTA AAGAGTGATT TCCATGGATG   
  
  
- CATCCCCTCA CAAAGATTAA GAGTATGTGC CGTAAACGGC ACGCAAATTG ACAAACAGAA GGATGGTTTA   
  
  
- GTTTCTTGTC GAAAAGGTTG CCAAGAATAA ATGATTTTAA GCCCATAAGT TATGAAAGTG GACGAGCATG   
  
  
- GAAGTTGAAC TGATGAAGAA GCCATGCCAA CCAGCTTAAT CATAGAATTA CTTCTTGTGT TATATCTGAA   
  
  
- ATATAGATGT AACATCATGG ACTTCCATGG ATGACTAGCT CTGGTTGTAG CTTAGAGCTT GCTTAACGTA   
  
  
- CTTAAACTAC GAAGGAAAAG GAGTATGCTT GCTGTTTATT CTCACATAGA AGCCATCTTC AGCCAATCAG   
  
  
- CCAATAGCAT CACTTAAACC GGTAGGTGAA TTTCATGCCT CGAGGTCAAA AAGCAGATAT TTAAGTTTCA   
  
  
- ACTAATCAAT TGAGTGCACA CTCCCCACTT TCTACATGTA CAGTTCATCG CATGAGCTTT AACGCTAAAT   
  
  
- GACTTGGGCA TACATAAGAG GAATTTTCGC TGGGTTTTCC TCGGAGCTTC AAGGAAGATC AATATCTCAG   
  
  
- AAAAACACTA CCTGCTGTAG TGCATGGGAC ATGTCGGCAT GGTTTTATGG GGATATGAAG TCCTATTGCT   
  
  
- GTCTGTGGTT AATTATAATG ATTAATGATG GTATTAAGTA AATACATTCT CAAATCATTT GGACTTCTTT   
  
  
- CAAGAGGGGT CACTGGTACT CTGTCTCATC TACCAATTTT TCGGGCTTTC TTTTTCCGTA TGGGGGAGGA   
  
  
- TTAGATTGAT TTGTCGCAGT GTCACTGGTT TTGTTTCAAT CAAGCTCTCT AGGAATAATT TATTAGGAAA   
  
  
- ATACTTGAAA AGAAGAGAAA GCAAGAAGAA AATGAAAATC AAGGATTACC ATAGGAGAAT AGCAAGCAAA   
  
  
- GGTCTATGAA GAGAAGATGT AACTCCATTG GTCACACTAA TATGTAGCAT GGAGAAAGAA AGCTGGAAGG   
  
  
- TAAAGGCAAA TTATAATAGC ATATCCACAA GTGATATATC CACTTGTGGT TGGACTCTGA ATCAAGCAGC   
  
  
- TGCTTCTGCT GGTGTGTTGA ACTCCTCCCA GGCTTCAATC CAAGTGACCA TGGCATCTGC AAGCTTCTCA   
  
  
- AAGTAAGGAT CAATATGACC CAATTTCTCT TTGACCTGCC TGGAGAGGGC AAGGGAGCAC TTCAGGGCTG   
  
  
- TTGTGCAGTC TTTTGGCAGG ACAACAGATT GGAACAATGG AATCAGCTGT TCCTGCCAGT ATAGACCCTT   
  
  
- GAATTCCTTC TTCAAGTTAG AAAACGGGT

+     GA-motif

| Site Name | Organism | Position | Strand | Matrix score. | sequence | function |
| --- | --- | --- | --- | --- | --- | --- |
| GA-motif | Glycine max | 1474 | + | 8 | AAGGAAGA | part of a light responsive element |

> 2018/04/13 10:10:12  
+ ATAACCTCGA TTGTTAGATT TGGCTCGAAA ATAAACAAAG GACATACTCC TTCTTGTTCT GTTTCAACTT   
  
  
+ TTCTCCTCTC CTCGTCCGAT CGAATAATAC GATTATCTTT ACCGTATTGT ACCCATCTTT TCCCACCCCC   
  
  
+ AATTACCTTT CCGCAGGTTG AATTGACTGA ACTGTCTTAA CTGGGTACAT TTCTCACTAA AGGTACCTAC   
  
  
+ GTAGGGGAGT GTTTCTAATT CTCATACACG GCATTTGCCG TGCGTTTAAC TGTTTGTCTT CCTACCAAAT   
  
  
+ CAAAGAACAG CTTTTCCAAC GGTTCTTATT TACTAAAATT CGGGTATTCA ATACTTTCAC CTGCTCGTAC   
  
  
+ CTTCAACTTG ACTACTTCTT CGGTACGGTT GGTCGAATTA GTATCTTAAT GAAGAACACA ATATAGACTT   
  
  
+ TATATCTACA TTGTAGTACC TGAAGGTACC TACTGATCGA GACCAACATC GAATCTCGAA CGAATTGCAT   
  
  
+ GAATTTGATG CTTCCTTTTC CTCATACGAA CGACAAATAA GAGTGTATCT TCGGTAGAAG TCGGTTAGTC   
  
  
+ GGTTATCGTA GTGAATTTGG CCATCCACTT AAAGTACGGA GCTCCAGTTT TTCGTCTATA AATTCAAAGT   
  
  
+ TGATTAGTTA ACTCACGTGT GAGGGGTGAA AGATGTACAT GTCAAGTAGC GTACTCGAAA TTGCGATTTA   
  
  
+ CTGAACCCGT ATGTATTCTC CTTAAAAGCG ACCCAAAAGG AGCCTCGAAG TTCCTTCTAG TTATAGAGTC   
  
  
+ TTTTTGTGAT GGACGACATC ACGTACCCTG TACAGCCGTA CCAAAATACC CCTATACTTC AGGATAACGA   
  
  
+ CAGACACCAA TTAATATTAC TAATTACTAC CATAATTCAT TTATGTAAGA GTTTAGTAAA CCTGAAGAAA   
  
  
+ GTTCTCCCCA GTGACCATGA GACAGAGTAG ATGGTTAAAA AGCCCGAAAG AAAAAGGCAT ACCCCCTCCT   
  
  
+ AATCTAACTA AACAGCGTCA CAGTGACCAA AACAAAGTTA GTTCGAGAGA TCCTTATTAA ATAATCCTTT   
  
  
+ TATGAACTTT TCTTCTCTTT CGTTCTTCTT TTACTTTTAG TTCCTAATGG TATCCTCTTA TCGTTCGTTT   
  
  
+ CCAGATACTT CTCTTCTACA TTGAGGTAAC CAGTGTGATT ATACATCGTA CCTCTTTCTT TCGACCTTCC   
  
  
+ ATTTCCGTTT AATATTATCG TATAGGTGTT CACTATATAG GTGAACACCA ACCTGAGACT TAGTTCGTCG   
  
  
+ ACGAAGACGA CCACACAACT TGAGGAGGGT CCGAAGTTAG GTTCACTGGT ACCGTAGACG TTCGAAGAGT   
  
  
+ TTCATTCCTA GTTATACTGG GTTAAAGAGA AACTGGACGG ACCTCTCCCG TTCCCTCGTG AAGTCCCGAC   
  
  
+ AACACGTCAG AAAACCGTCC TGTTGTCTAA CCTTGTTACC TTAGTCGACA AGGACGGTCA TATCTGGGAA   
  
  
+ CTTAAGGAAG AAGTTCAATC TTTTGCCCA  

- TATTGGAGCT AACAATCTAA ACCGAGCTTT TATTTGTTTC CTGTATGAGG AAGAACAAGA CAAAGTTGAA   
  
  
- AAGAGGAGAG GAGCAGGCTA GCTTATTATG CTAATAGAAA TGGCATAACA TGGGTAGAAA AGGGTGGGGG   
  
  
- TTAATGGAAA GGCGTCCAAC TTAACTGACT TGACAGAATT GACCCATGTA AAGAGTGATT TCCATGGATG   
  
  
- CATCCCCTCA CAAAGATTAA GAGTATGTGC CGTAAACGGC ACGCAAATTG ACAAACAGAA GGATGGTTTA   
  
  
- GTTTCTTGTC GAAAAGGTTG CCAAGAATAA ATGATTTTAA GCCCATAAGT TATGAAAGTG GACGAGCATG   
  
  
- GAAGTTGAAC TGATGAAGAA GCCATGCCAA CCAGCTTAAT CATAGAATTA CTTCTTGTGT TATATCTGAA   
  
  
- ATATAGATGT AACATCATGG ACTTCCATGG ATGACTAGCT CTGGTTGTAG CTTAGAGCTT GCTTAACGTA   
  
  
- CTTAAACTAC GAAGGAAAAG GAGTATGCTT GCTGTTTATT CTCACATAGA AGCCATCTTC AGCCAATCAG   
  
  
- CCAATAGCAT CACTTAAACC GGTAGGTGAA TTTCATGCCT CGAGGTCAAA AAGCAGATAT TTAAGTTTCA   
  
  
- ACTAATCAAT TGAGTGCACA CTCCCCACTT TCTACATGTA CAGTTCATCG CATGAGCTTT AACGCTAAAT   
  
  
- GACTTGGGCA TACATAAGAG GAATTTTCGC TGGGTTTTCC TCGGAGCTTC AAGGAAGATC AATATCTCAG   
  
  
- AAAAACACTA CCTGCTGTAG TGCATGGGAC ATGTCGGCAT GGTTTTATGG GGATATGAAG TCCTATTGCT   
  
  
- GTCTGTGGTT AATTATAATG ATTAATGATG GTATTAAGTA AATACATTCT CAAATCATTT GGACTTCTTT   
  
  
- CAAGAGGGGT CACTGGTACT CTGTCTCATC TACCAATTTT TCGGGCTTTC TTTTTCCGTA TGGGGGAGGA   
  
  
- TTAGATTGAT TTGTCGCAGT GTCACTGGTT TTGTTTCAAT CAAGCTCTCT AGGAATAATT TATTAGGAAA   
  
  
- ATACTTGAAA AGAAGAGAAA GCAAGAAGAA AATGAAAATC AAGGATTACC ATAGGAGAAT AGCAAGCAAA   
  
  
- GGTCTATGAA GAGAAGATGT AACTCCATTG GTCACACTAA TATGTAGCAT GGAGAAAGAA AGCTGGAAGG   
  
  
- TAAAGGCAAA TTATAATAGC ATATCCACAA GTGATATATC CACTTGTGGT TGGACTCTGA ATCAAGCAGC   
  
  
- TGCTTCTGCT GGTGTGTTGA ACTCCTCCCA GGCTTCAATC CAAGTGACCA TGGCATCTGC AAGCTTCTCA   
  
  
- AAGTAAGGAT CAATATGACC CAATTTCTCT TTGACCTGCC TGGAGAGGGC AAGGGAGCAC TTCAGGGCTG   
  
  
- TTGTGCAGTC TTTTGGCAGG ACAACAGATT GGAACAATGG AATCAGCTGT TCCTGCCAGT ATAGACCCTT   
  
  
- GAATTCCTTC TTCAAGTTAG AAAACGGGT

+     GARE-motif

| Site Name | Organism | Position | Strand | Matrix score. | sequence | function |
| --- | --- | --- | --- | --- | --- | --- |
| GARE-motif | Brassica oleracea | 58 | - | 7 | AAACAGA | gibberellin-responsive element |

> 2018/04/13 10:10:12  
+ ATAACCTCGA TTGTTAGATT TGGCTCGAAA ATAAACAAAG GACATACTCC TTCTTGTTCT GTTTCAACTT   
  
  
+ TTCTCCTCTC CTCGTCCGAT CGAATAATAC GATTATCTTT ACCGTATTGT ACCCATCTTT TCCCACCCCC   
  
  
+ AATTACCTTT CCGCAGGTTG AATTGACTGA ACTGTCTTAA CTGGGTACAT TTCTCACTAA AGGTACCTAC   
  
  
+ GTAGGGGAGT GTTTCTAATT CTCATACACG GCATTTGCCG TGCGTTTAAC TGTTTGTCTT CCTACCAAAT   
  
  
+ CAAAGAACAG CTTTTCCAAC GGTTCTTATT TACTAAAATT CGGGTATTCA ATACTTTCAC CTGCTCGTAC   
  
  
+ CTTCAACTTG ACTACTTCTT CGGTACGGTT GGTCGAATTA GTATCTTAAT GAAGAACACA ATATAGACTT   
  
  
+ TATATCTACA TTGTAGTACC TGAAGGTACC TACTGATCGA GACCAACATC GAATCTCGAA CGAATTGCAT   
  
  
+ GAATTTGATG CTTCCTTTTC CTCATACGAA CGACAAATAA GAGTGTATCT TCGGTAGAAG TCGGTTAGTC   
  
  
+ GGTTATCGTA GTGAATTTGG CCATCCACTT AAAGTACGGA GCTCCAGTTT TTCGTCTATA AATTCAAAGT   
  
  
+ TGATTAGTTA ACTCACGTGT GAGGGGTGAA AGATGTACAT GTCAAGTAGC GTACTCGAAA TTGCGATTTA   
  
  
+ CTGAACCCGT ATGTATTCTC CTTAAAAGCG ACCCAAAAGG AGCCTCGAAG TTCCTTCTAG TTATAGAGTC   
  
  
+ TTTTTGTGAT GGACGACATC ACGTACCCTG TACAGCCGTA CCAAAATACC CCTATACTTC AGGATAACGA   
  
  
+ CAGACACCAA TTAATATTAC TAATTACTAC CATAATTCAT TTATGTAAGA GTTTAGTAAA CCTGAAGAAA   
  
  
+ GTTCTCCCCA GTGACCATGA GACAGAGTAG ATGGTTAAAA AGCCCGAAAG AAAAAGGCAT ACCCCCTCCT   
  
  
+ AATCTAACTA AACAGCGTCA CAGTGACCAA AACAAAGTTA GTTCGAGAGA TCCTTATTAA ATAATCCTTT   
  
  
+ TATGAACTTT TCTTCTCTTT CGTTCTTCTT TTACTTTTAG TTCCTAATGG TATCCTCTTA TCGTTCGTTT   
  
  
+ CCAGATACTT CTCTTCTACA TTGAGGTAAC CAGTGTGATT ATACATCGTA CCTCTTTCTT TCGACCTTCC   
  
  
+ ATTTCCGTTT AATATTATCG TATAGGTGTT CACTATATAG GTGAACACCA ACCTGAGACT TAGTTCGTCG   
  
  
+ ACGAAGACGA CCACACAACT TGAGGAGGGT CCGAAGTTAG GTTCACTGGT ACCGTAGACG TTCGAAGAGT   
  
  
+ TTCATTCCTA GTTATACTGG GTTAAAGAGA AACTGGACGG ACCTCTCCCG TTCCCTCGTG AAGTCCCGAC   
  
  
+ AACACGTCAG AAAACCGTCC TGTTGTCTAA CCTTGTTACC TTAGTCGACA AGGACGGTCA TATCTGGGAA   
  
  
+ CTTAAGGAAG AAGTTCAATC TTTTGCCCA  

- TATTGGAGCT AACAATCTAA ACCGAGCTTT TATTTGTTTC CTGTATGAGG AAGAACAAGA CAAAGTTGAA   
  
  
- AAGAGGAGAG GAGCAGGCTA GCTTATTATG CTAATAGAAA TGGCATAACA TGGGTAGAAA AGGGTGGGGG   
  
  
- TTAATGGAAA GGCGTCCAAC TTAACTGACT TGACAGAATT GACCCATGTA AAGAGTGATT TCCATGGATG   
  
  
- CATCCCCTCA CAAAGATTAA GAGTATGTGC CGTAAACGGC ACGCAAATTG ACAAACAGAA GGATGGTTTA   
  
  
- GTTTCTTGTC GAAAAGGTTG CCAAGAATAA ATGATTTTAA GCCCATAAGT TATGAAAGTG GACGAGCATG   
  
  
- GAAGTTGAAC TGATGAAGAA GCCATGCCAA CCAGCTTAAT CATAGAATTA CTTCTTGTGT TATATCTGAA   
  
  
- ATATAGATGT AACATCATGG ACTTCCATGG ATGACTAGCT CTGGTTGTAG CTTAGAGCTT GCTTAACGTA   
  
  
- CTTAAACTAC GAAGGAAAAG GAGTATGCTT GCTGTTTATT CTCACATAGA AGCCATCTTC AGCCAATCAG   
  
  
- CCAATAGCAT CACTTAAACC GGTAGGTGAA TTTCATGCCT CGAGGTCAAA AAGCAGATAT TTAAGTTTCA   
  
  
- ACTAATCAAT TGAGTGCACA CTCCCCACTT TCTACATGTA CAGTTCATCG CATGAGCTTT AACGCTAAAT   
  
  
- GACTTGGGCA TACATAAGAG GAATTTTCGC TGGGTTTTCC TCGGAGCTTC AAGGAAGATC AATATCTCAG   
  
  
- AAAAACACTA CCTGCTGTAG TGCATGGGAC ATGTCGGCAT GGTTTTATGG GGATATGAAG TCCTATTGCT   
  
  
- GTCTGTGGTT AATTATAATG ATTAATGATG GTATTAAGTA AATACATTCT CAAATCATTT GGACTTCTTT   
  
  
- CAAGAGGGGT CACTGGTACT CTGTCTCATC TACCAATTTT TCGGGCTTTC TTTTTCCGTA TGGGGGAGGA   
  
  
- TTAGATTGAT TTGTCGCAGT GTCACTGGTT TTGTTTCAAT CAAGCTCTCT AGGAATAATT TATTAGGAAA   
  
  
- ATACTTGAAA AGAAGAGAAA GCAAGAAGAA AATGAAAATC AAGGATTACC ATAGGAGAAT AGCAAGCAAA   
  
  
- GGTCTATGAA GAGAAGATGT AACTCCATTG GTCACACTAA TATGTAGCAT GGAGAAAGAA AGCTGGAAGG   
  
  
- TAAAGGCAAA TTATAATAGC ATATCCACAA GTGATATATC CACTTGTGGT TGGACTCTGA ATCAAGCAGC   
  
  
- TGCTTCTGCT GGTGTGTTGA ACTCCTCCCA GGCTTCAATC CAAGTGACCA TGGCATCTGC AAGCTTCTCA   
  
  
- AAGTAAGGAT CAATATGACC CAATTTCTCT TTGACCTGCC TGGAGAGGGC AAGGGAGCAC TTCAGGGCTG   
  
  
- TTGTGCAGTC TTTTGGCAGG ACAACAGATT GGAACAATGG AATCAGCTGT TCCTGCCAGT ATAGACCCTT   
  
  
- GAATTCCTTC TTCAAGTTAG AAAACGGGT

+     GT1-motif

| Site Name | Organism | Position | Strand | Matrix score. | sequence | function |
| --- | --- | --- | --- | --- | --- | --- |
| GT1-motif | Arabidopsis thaliana | 943 | + | 6 | GGTTAA | light responsive element |
| GT1-motif | Arabidopsis thaliana | 1350 | + | 6 | GGTTAA | light responsive element |

> 2018/04/13 10:10:12  
+ ATAACCTCGA TTGTTAGATT TGGCTCGAAA ATAAACAAAG GACATACTCC TTCTTGTTCT GTTTCAACTT   
  
  
+ TTCTCCTCTC CTCGTCCGAT CGAATAATAC GATTATCTTT ACCGTATTGT ACCCATCTTT TCCCACCCCC   
  
  
+ AATTACCTTT CCGCAGGTTG AATTGACTGA ACTGTCTTAA CTGGGTACAT TTCTCACTAA AGGTACCTAC   
  
  
+ GTAGGGGAGT GTTTCTAATT CTCATACACG GCATTTGCCG TGCGTTTAAC TGTTTGTCTT CCTACCAAAT   
  
  
+ CAAAGAACAG CTTTTCCAAC GGTTCTTATT TACTAAAATT CGGGTATTCA ATACTTTCAC CTGCTCGTAC   
  
  
+ CTTCAACTTG ACTACTTCTT CGGTACGGTT GGTCGAATTA GTATCTTAAT GAAGAACACA ATATAGACTT   
  
  
+ TATATCTACA TTGTAGTACC TGAAGGTACC TACTGATCGA GACCAACATC GAATCTCGAA CGAATTGCAT   
  
  
+ GAATTTGATG CTTCCTTTTC CTCATACGAA CGACAAATAA GAGTGTATCT TCGGTAGAAG TCGGTTAGTC   
  
  
+ GGTTATCGTA GTGAATTTGG CCATCCACTT AAAGTACGGA GCTCCAGTTT TTCGTCTATA AATTCAAAGT   
  
  
+ TGATTAGTTA ACTCACGTGT GAGGGGTGAA AGATGTACAT GTCAAGTAGC GTACTCGAAA TTGCGATTTA   
  
  
+ CTGAACCCGT ATGTATTCTC CTTAAAAGCG ACCCAAAAGG AGCCTCGAAG TTCCTTCTAG TTATAGAGTC   
  
  
+ TTTTTGTGAT GGACGACATC ACGTACCCTG TACAGCCGTA CCAAAATACC CCTATACTTC AGGATAACGA   
  
  
+ CAGACACCAA TTAATATTAC TAATTACTAC CATAATTCAT TTATGTAAGA GTTTAGTAAA CCTGAAGAAA   
  
  
+ GTTCTCCCCA GTGACCATGA GACAGAGTAG ATGGTTAAAA AGCCCGAAAG AAAAAGGCAT ACCCCCTCCT   
  
  
+ AATCTAACTA AACAGCGTCA CAGTGACCAA AACAAAGTTA GTTCGAGAGA TCCTTATTAA ATAATCCTTT   
  
  
+ TATGAACTTT TCTTCTCTTT CGTTCTTCTT TTACTTTTAG TTCCTAATGG TATCCTCTTA TCGTTCGTTT   
  
  
+ CCAGATACTT CTCTTCTACA TTGAGGTAAC CAGTGTGATT ATACATCGTA CCTCTTTCTT TCGACCTTCC   
  
  
+ ATTTCCGTTT AATATTATCG TATAGGTGTT CACTATATAG GTGAACACCA ACCTGAGACT TAGTTCGTCG   
  
  
+ ACGAAGACGA CCACACAACT TGAGGAGGGT CCGAAGTTAG GTTCACTGGT ACCGTAGACG TTCGAAGAGT   
  
  
+ TTCATTCCTA GTTATACTGG GTTAAAGAGA AACTGGACGG ACCTCTCCCG TTCCCTCGTG AAGTCCCGAC   
  
  
+ AACACGTCAG AAAACCGTCC TGTTGTCTAA CCTTGTTACC TTAGTCGACA AGGACGGTCA TATCTGGGAA   
  
  
+ CTTAAGGAAG AAGTTCAATC TTTTGCCCA  

- TATTGGAGCT AACAATCTAA ACCGAGCTTT TATTTGTTTC CTGTATGAGG AAGAACAAGA CAAAGTTGAA   
  
  
- AAGAGGAGAG GAGCAGGCTA GCTTATTATG CTAATAGAAA TGGCATAACA TGGGTAGAAA AGGGTGGGGG   
  
  
- TTAATGGAAA GGCGTCCAAC TTAACTGACT TGACAGAATT GACCCATGTA AAGAGTGATT TCCATGGATG   
  
  
- CATCCCCTCA CAAAGATTAA GAGTATGTGC CGTAAACGGC ACGCAAATTG ACAAACAGAA GGATGGTTTA   
  
  
- GTTTCTTGTC GAAAAGGTTG CCAAGAATAA ATGATTTTAA GCCCATAAGT TATGAAAGTG GACGAGCATG   
  
  
- GAAGTTGAAC TGATGAAGAA GCCATGCCAA CCAGCTTAAT CATAGAATTA CTTCTTGTGT TATATCTGAA   
  
  
- ATATAGATGT AACATCATGG ACTTCCATGG ATGACTAGCT CTGGTTGTAG CTTAGAGCTT GCTTAACGTA   
  
  
- CTTAAACTAC GAAGGAAAAG GAGTATGCTT GCTGTTTATT CTCACATAGA AGCCATCTTC AGCCAATCAG   
  
  
- CCAATAGCAT CACTTAAACC GGTAGGTGAA TTTCATGCCT CGAGGTCAAA AAGCAGATAT TTAAGTTTCA   
  
  
- ACTAATCAAT TGAGTGCACA CTCCCCACTT TCTACATGTA CAGTTCATCG CATGAGCTTT AACGCTAAAT   
  
  
- GACTTGGGCA TACATAAGAG GAATTTTCGC TGGGTTTTCC TCGGAGCTTC AAGGAAGATC AATATCTCAG   
  
  
- AAAAACACTA CCTGCTGTAG TGCATGGGAC ATGTCGGCAT GGTTTTATGG GGATATGAAG TCCTATTGCT   
  
  
- GTCTGTGGTT AATTATAATG ATTAATGATG GTATTAAGTA AATACATTCT CAAATCATTT GGACTTCTTT   
  
  
- CAAGAGGGGT CACTGGTACT CTGTCTCATC TACCAATTTT TCGGGCTTTC TTTTTCCGTA TGGGGGAGGA   
  
  
- TTAGATTGAT TTGTCGCAGT GTCACTGGTT TTGTTTCAAT CAAGCTCTCT AGGAATAATT TATTAGGAAA   
  
  
- ATACTTGAAA AGAAGAGAAA GCAAGAAGAA AATGAAAATC AAGGATTACC ATAGGAGAAT AGCAAGCAAA   
  
  
- GGTCTATGAA GAGAAGATGT AACTCCATTG GTCACACTAA TATGTAGCAT GGAGAAAGAA AGCTGGAAGG   
  
  
- TAAAGGCAAA TTATAATAGC ATATCCACAA GTGATATATC CACTTGTGGT TGGACTCTGA ATCAAGCAGC   
  
  
- TGCTTCTGCT GGTGTGTTGA ACTCCTCCCA GGCTTCAATC CAAGTGACCA TGGCATCTGC AAGCTTCTCA   
  
  
- AAGTAAGGAT CAATATGACC CAATTTCTCT TTGACCTGCC TGGAGAGGGC AAGGGAGCAC TTCAGGGCTG   
  
  
- TTGTGCAGTC TTTTGGCAGG ACAACAGATT GGAACAATGG AATCAGCTGT TCCTGCCAGT ATAGACCCTT   
  
  
- GAATTCCTTC TTCAAGTTAG AAAACGGGT

+     LTR

| Site Name | Organism | Position | Strand | Matrix score. | sequence | function |
| --- | --- | --- | --- | --- | --- | --- |
| LTR | Hordeum vulgare | 954 | + | 6 | CCGAAA | cis-acting element involved in low-temperature responsiveness |

> 2018/04/13 10:10:12  
+ ATAACCTCGA TTGTTAGATT TGGCTCGAAA ATAAACAAAG GACATACTCC TTCTTGTTCT GTTTCAACTT   
  
  
+ TTCTCCTCTC CTCGTCCGAT CGAATAATAC GATTATCTTT ACCGTATTGT ACCCATCTTT TCCCACCCCC   
  
  
+ AATTACCTTT CCGCAGGTTG AATTGACTGA ACTGTCTTAA CTGGGTACAT TTCTCACTAA AGGTACCTAC   
  
  
+ GTAGGGGAGT GTTTCTAATT CTCATACACG GCATTTGCCG TGCGTTTAAC TGTTTGTCTT CCTACCAAAT   
  
  
+ CAAAGAACAG CTTTTCCAAC GGTTCTTATT TACTAAAATT CGGGTATTCA ATACTTTCAC CTGCTCGTAC   
  
  
+ CTTCAACTTG ACTACTTCTT CGGTACGGTT GGTCGAATTA GTATCTTAAT GAAGAACACA ATATAGACTT   
  
  
+ TATATCTACA TTGTAGTACC TGAAGGTACC TACTGATCGA GACCAACATC GAATCTCGAA CGAATTGCAT   
  
  
+ GAATTTGATG CTTCCTTTTC CTCATACGAA CGACAAATAA GAGTGTATCT TCGGTAGAAG TCGGTTAGTC   
  
  
+ GGTTATCGTA GTGAATTTGG CCATCCACTT AAAGTACGGA GCTCCAGTTT TTCGTCTATA AATTCAAAGT   
  
  
+ TGATTAGTTA ACTCACGTGT GAGGGGTGAA AGATGTACAT GTCAAGTAGC GTACTCGAAA TTGCGATTTA   
  
  
+ CTGAACCCGT ATGTATTCTC CTTAAAAGCG ACCCAAAAGG AGCCTCGAAG TTCCTTCTAG TTATAGAGTC   
  
  
+ TTTTTGTGAT GGACGACATC ACGTACCCTG TACAGCCGTA CCAAAATACC CCTATACTTC AGGATAACGA   
  
  
+ CAGACACCAA TTAATATTAC TAATTACTAC CATAATTCAT TTATGTAAGA GTTTAGTAAA CCTGAAGAAA   
  
  
+ GTTCTCCCCA GTGACCATGA GACAGAGTAG ATGGTTAAAA AGCCCGAAAG AAAAAGGCAT ACCCCCTCCT   
  
  
+ AATCTAACTA AACAGCGTCA CAGTGACCAA AACAAAGTTA GTTCGAGAGA TCCTTATTAA ATAATCCTTT   
  
  
+ TATGAACTTT TCTTCTCTTT CGTTCTTCTT TTACTTTTAG TTCCTAATGG TATCCTCTTA TCGTTCGTTT   
  
  
+ CCAGATACTT CTCTTCTACA TTGAGGTAAC CAGTGTGATT ATACATCGTA CCTCTTTCTT TCGACCTTCC   
  
  
+ ATTTCCGTTT AATATTATCG TATAGGTGTT CACTATATAG GTGAACACCA ACCTGAGACT TAGTTCGTCG   
  
  
+ ACGAAGACGA CCACACAACT TGAGGAGGGT CCGAAGTTAG GTTCACTGGT ACCGTAGACG TTCGAAGAGT   
  
  
+ TTCATTCCTA GTTATACTGG GTTAAAGAGA AACTGGACGG ACCTCTCCCG TTCCCTCGTG AAGTCCCGAC   
  
  
+ AACACGTCAG AAAACCGTCC TGTTGTCTAA CCTTGTTACC TTAGTCGACA AGGACGGTCA TATCTGGGAA   
  
  
+ CTTAAGGAAG AAGTTCAATC TTTTGCCCA  

- TATTGGAGCT AACAATCTAA ACCGAGCTTT TATTTGTTTC CTGTATGAGG AAGAACAAGA CAAAGTTGAA   
  
  
- AAGAGGAGAG GAGCAGGCTA GCTTATTATG CTAATAGAAA TGGCATAACA TGGGTAGAAA AGGGTGGGGG   
  
  
- TTAATGGAAA GGCGTCCAAC TTAACTGACT TGACAGAATT GACCCATGTA AAGAGTGATT TCCATGGATG   
  
  
- CATCCCCTCA CAAAGATTAA GAGTATGTGC CGTAAACGGC ACGCAAATTG ACAAACAGAA GGATGGTTTA   
  
  
- GTTTCTTGTC GAAAAGGTTG CCAAGAATAA ATGATTTTAA GCCCATAAGT TATGAAAGTG GACGAGCATG   
  
  
- GAAGTTGAAC TGATGAAGAA GCCATGCCAA CCAGCTTAAT CATAGAATTA CTTCTTGTGT TATATCTGAA   
  
  
- ATATAGATGT AACATCATGG ACTTCCATGG ATGACTAGCT CTGGTTGTAG CTTAGAGCTT GCTTAACGTA   
  
  
- CTTAAACTAC GAAGGAAAAG GAGTATGCTT GCTGTTTATT CTCACATAGA AGCCATCTTC AGCCAATCAG   
  
  
- CCAATAGCAT CACTTAAACC GGTAGGTGAA TTTCATGCCT CGAGGTCAAA AAGCAGATAT TTAAGTTTCA   
  
  
- ACTAATCAAT TGAGTGCACA CTCCCCACTT TCTACATGTA CAGTTCATCG CATGAGCTTT AACGCTAAAT   
  
  
- GACTTGGGCA TACATAAGAG GAATTTTCGC TGGGTTTTCC TCGGAGCTTC AAGGAAGATC AATATCTCAG   
  
  
- AAAAACACTA CCTGCTGTAG TGCATGGGAC ATGTCGGCAT GGTTTTATGG GGATATGAAG TCCTATTGCT   
  
  
- GTCTGTGGTT AATTATAATG ATTAATGATG GTATTAAGTA AATACATTCT CAAATCATTT GGACTTCTTT   
  
  
- CAAGAGGGGT CACTGGTACT CTGTCTCATC TACCAATTTT TCGGGCTTTC TTTTTCCGTA TGGGGGAGGA   
  
  
- TTAGATTGAT TTGTCGCAGT GTCACTGGTT TTGTTTCAAT CAAGCTCTCT AGGAATAATT TATTAGGAAA   
  
  
- ATACTTGAAA AGAAGAGAAA GCAAGAAGAA AATGAAAATC AAGGATTACC ATAGGAGAAT AGCAAGCAAA   
  
  
- GGTCTATGAA GAGAAGATGT AACTCCATTG GTCACACTAA TATGTAGCAT GGAGAAAGAA AGCTGGAAGG   
  
  
- TAAAGGCAAA TTATAATAGC ATATCCACAA GTGATATATC CACTTGTGGT TGGACTCTGA ATCAAGCAGC   
  
  
- TGCTTCTGCT GGTGTGTTGA ACTCCTCCCA GGCTTCAATC CAAGTGACCA TGGCATCTGC AAGCTTCTCA   
  
  
- AAGTAAGGAT CAATATGACC CAATTTCTCT TTGACCTGCC TGGAGAGGGC AAGGGAGCAC TTCAGGGCTG   
  
  
- TTGTGCAGTC TTTTGGCAGG ACAACAGATT GGAACAATGG AATCAGCTGT TCCTGCCAGT ATAGACCCTT   
  
  
- GAATTCCTTC TTCAAGTTAG AAAACGGGT

+     MBS

| Site Name | Organism | Position | Strand | Matrix score. | sequence | function |
| --- | --- | --- | --- | --- | --- | --- |
| MBS | Arabidopsis thaliana | 257 | + | 6 | TAACTG | MYB binding site involved in drought-inducibility |
| MBS | Zea mays | 1455 | + | 6 | CGGTCA | MYB Binding Site |
| MBS | Arabidopsis thaliana | 178 | + | 6 | TAACTG | MYB binding site involved in drought-inducibility |

> 2018/04/13 10:10:12  
+ ATAACCTCGA TTGTTAGATT TGGCTCGAAA ATAAACAAAG GACATACTCC TTCTTGTTCT GTTTCAACTT   
  
  
+ TTCTCCTCTC CTCGTCCGAT CGAATAATAC GATTATCTTT ACCGTATTGT ACCCATCTTT TCCCACCCCC   
  
  
+ AATTACCTTT CCGCAGGTTG AATTGACTGA ACTGTCTTAA CTGGGTACAT TTCTCACTAA AGGTACCTAC   
  
  
+ GTAGGGGAGT GTTTCTAATT CTCATACACG GCATTTGCCG TGCGTTTAAC TGTTTGTCTT CCTACCAAAT   
  
  
+ CAAAGAACAG CTTTTCCAAC GGTTCTTATT TACTAAAATT CGGGTATTCA ATACTTTCAC CTGCTCGTAC   
  
  
+ CTTCAACTTG ACTACTTCTT CGGTACGGTT GGTCGAATTA GTATCTTAAT GAAGAACACA ATATAGACTT   
  
  
+ TATATCTACA TTGTAGTACC TGAAGGTACC TACTGATCGA GACCAACATC GAATCTCGAA CGAATTGCAT   
  
  
+ GAATTTGATG CTTCCTTTTC CTCATACGAA CGACAAATAA GAGTGTATCT TCGGTAGAAG TCGGTTAGTC   
  
  
+ GGTTATCGTA GTGAATTTGG CCATCCACTT AAAGTACGGA GCTCCAGTTT TTCGTCTATA AATTCAAAGT   
  
  
+ TGATTAGTTA ACTCACGTGT GAGGGGTGAA AGATGTACAT GTCAAGTAGC GTACTCGAAA TTGCGATTTA   
  
  
+ CTGAACCCGT ATGTATTCTC CTTAAAAGCG ACCCAAAAGG AGCCTCGAAG TTCCTTCTAG TTATAGAGTC   
  
  
+ TTTTTGTGAT GGACGACATC ACGTACCCTG TACAGCCGTA CCAAAATACC CCTATACTTC AGGATAACGA   
  
  
+ CAGACACCAA TTAATATTAC TAATTACTAC CATAATTCAT TTATGTAAGA GTTTAGTAAA CCTGAAGAAA   
  
  
+ GTTCTCCCCA GTGACCATGA GACAGAGTAG ATGGTTAAAA AGCCCGAAAG AAAAAGGCAT ACCCCCTCCT   
  
  
+ AATCTAACTA AACAGCGTCA CAGTGACCAA AACAAAGTTA GTTCGAGAGA TCCTTATTAA ATAATCCTTT   
  
  
+ TATGAACTTT TCTTCTCTTT CGTTCTTCTT TTACTTTTAG TTCCTAATGG TATCCTCTTA TCGTTCGTTT   
  
  
+ CCAGATACTT CTCTTCTACA TTGAGGTAAC CAGTGTGATT ATACATCGTA CCTCTTTCTT TCGACCTTCC   
  
  
+ ATTTCCGTTT AATATTATCG TATAGGTGTT CACTATATAG GTGAACACCA ACCTGAGACT TAGTTCGTCG   
  
  
+ ACGAAGACGA CCACACAACT TGAGGAGGGT CCGAAGTTAG GTTCACTGGT ACCGTAGACG TTCGAAGAGT   
  
  
+ TTCATTCCTA GTTATACTGG GTTAAAGAGA AACTGGACGG ACCTCTCCCG TTCCCTCGTG AAGTCCCGAC   
  
  
+ AACACGTCAG AAAACCGTCC TGTTGTCTAA CCTTGTTACC TTAGTCGACA AGGACGGTCA TATCTGGGAA   
  
  
+ CTTAAGGAAG AAGTTCAATC TTTTGCCCA  

- TATTGGAGCT AACAATCTAA ACCGAGCTTT TATTTGTTTC CTGTATGAGG AAGAACAAGA CAAAGTTGAA   
  
  
- AAGAGGAGAG GAGCAGGCTA GCTTATTATG CTAATAGAAA TGGCATAACA TGGGTAGAAA AGGGTGGGGG   
  
  
- TTAATGGAAA GGCGTCCAAC TTAACTGACT TGACAGAATT GACCCATGTA AAGAGTGATT TCCATGGATG   
  
  
- CATCCCCTCA CAAAGATTAA GAGTATGTGC CGTAAACGGC ACGCAAATTG ACAAACAGAA GGATGGTTTA   
  
  
- GTTTCTTGTC GAAAAGGTTG CCAAGAATAA ATGATTTTAA GCCCATAAGT TATGAAAGTG GACGAGCATG   
  
  
- GAAGTTGAAC TGATGAAGAA GCCATGCCAA CCAGCTTAAT CATAGAATTA CTTCTTGTGT TATATCTGAA   
  
  
- ATATAGATGT AACATCATGG ACTTCCATGG ATGACTAGCT CTGGTTGTAG CTTAGAGCTT GCTTAACGTA   
  
  
- CTTAAACTAC GAAGGAAAAG GAGTATGCTT GCTGTTTATT CTCACATAGA AGCCATCTTC AGCCAATCAG   
  
  
- CCAATAGCAT CACTTAAACC GGTAGGTGAA TTTCATGCCT CGAGGTCAAA AAGCAGATAT TTAAGTTTCA   
  
  
- ACTAATCAAT TGAGTGCACA CTCCCCACTT TCTACATGTA CAGTTCATCG CATGAGCTTT AACGCTAAAT   
  
  
- GACTTGGGCA TACATAAGAG GAATTTTCGC TGGGTTTTCC TCGGAGCTTC AAGGAAGATC AATATCTCAG   
  
  
- AAAAACACTA CCTGCTGTAG TGCATGGGAC ATGTCGGCAT GGTTTTATGG GGATATGAAG TCCTATTGCT   
  
  
- GTCTGTGGTT AATTATAATG ATTAATGATG GTATTAAGTA AATACATTCT CAAATCATTT GGACTTCTTT   
  
  
- CAAGAGGGGT CACTGGTACT CTGTCTCATC TACCAATTTT TCGGGCTTTC TTTTTCCGTA TGGGGGAGGA   
  
  
- TTAGATTGAT TTGTCGCAGT GTCACTGGTT TTGTTTCAAT CAAGCTCTCT AGGAATAATT TATTAGGAAA   
  
  
- ATACTTGAAA AGAAGAGAAA GCAAGAAGAA AATGAAAATC AAGGATTACC ATAGGAGAAT AGCAAGCAAA   
  
  
- GGTCTATGAA GAGAAGATGT AACTCCATTG GTCACACTAA TATGTAGCAT GGAGAAAGAA AGCTGGAAGG   
  
  
- TAAAGGCAAA TTATAATAGC ATATCCACAA GTGATATATC CACTTGTGGT TGGACTCTGA ATCAAGCAGC   
  
  
- TGCTTCTGCT GGTGTGTTGA ACTCCTCCCA GGCTTCAATC CAAGTGACCA TGGCATCTGC AAGCTTCTCA   
  
  
- AAGTAAGGAT CAATATGACC CAATTTCTCT TTGACCTGCC TGGAGAGGGC AAGGGAGCAC TTCAGGGCTG   
  
  
- TTGTGCAGTC TTTTGGCAGG ACAACAGATT GGAACAATGG AATCAGCTGT TCCTGCCAGT ATAGACCCTT   
  
  
- GAATTCCTTC TTCAAGTTAG AAAACGGGT

+     MRE

| Site Name | Organism | Position | Strand | Matrix score. | sequence | function |
| --- | --- | --- | --- | --- | --- | --- |
| MRE | Petroselinum crispum | 1297 | - | 7 | AACCTAA | MYB binding site involved in light responsiveness |

> 2018/04/13 10:10:12  
+ ATAACCTCGA TTGTTAGATT TGGCTCGAAA ATAAACAAAG GACATACTCC TTCTTGTTCT GTTTCAACTT   
  
  
+ TTCTCCTCTC CTCGTCCGAT CGAATAATAC GATTATCTTT ACCGTATTGT ACCCATCTTT TCCCACCCCC   
  
  
+ AATTACCTTT CCGCAGGTTG AATTGACTGA ACTGTCTTAA CTGGGTACAT TTCTCACTAA AGGTACCTAC   
  
  
+ GTAGGGGAGT GTTTCTAATT CTCATACACG GCATTTGCCG TGCGTTTAAC TGTTTGTCTT CCTACCAAAT   
  
  
+ CAAAGAACAG CTTTTCCAAC GGTTCTTATT TACTAAAATT CGGGTATTCA ATACTTTCAC CTGCTCGTAC   
  
  
+ CTTCAACTTG ACTACTTCTT CGGTACGGTT GGTCGAATTA GTATCTTAAT GAAGAACACA ATATAGACTT   
  
  
+ TATATCTACA TTGTAGTACC TGAAGGTACC TACTGATCGA GACCAACATC GAATCTCGAA CGAATTGCAT   
  
  
+ GAATTTGATG CTTCCTTTTC CTCATACGAA CGACAAATAA GAGTGTATCT TCGGTAGAAG TCGGTTAGTC   
  
  
+ GGTTATCGTA GTGAATTTGG CCATCCACTT AAAGTACGGA GCTCCAGTTT TTCGTCTATA AATTCAAAGT   
  
  
+ TGATTAGTTA ACTCACGTGT GAGGGGTGAA AGATGTACAT GTCAAGTAGC GTACTCGAAA TTGCGATTTA   
  
  
+ CTGAACCCGT ATGTATTCTC CTTAAAAGCG ACCCAAAAGG AGCCTCGAAG TTCCTTCTAG TTATAGAGTC   
  
  
+ TTTTTGTGAT GGACGACATC ACGTACCCTG TACAGCCGTA CCAAAATACC CCTATACTTC AGGATAACGA   
  
  
+ CAGACACCAA TTAATATTAC TAATTACTAC CATAATTCAT TTATGTAAGA GTTTAGTAAA CCTGAAGAAA   
  
  
+ GTTCTCCCCA GTGACCATGA GACAGAGTAG ATGGTTAAAA AGCCCGAAAG AAAAAGGCAT ACCCCCTCCT   
  
  
+ AATCTAACTA AACAGCGTCA CAGTGACCAA AACAAAGTTA GTTCGAGAGA TCCTTATTAA ATAATCCTTT   
  
  
+ TATGAACTTT TCTTCTCTTT CGTTCTTCTT TTACTTTTAG TTCCTAATGG TATCCTCTTA TCGTTCGTTT   
  
  
+ CCAGATACTT CTCTTCTACA TTGAGGTAAC CAGTGTGATT ATACATCGTA CCTCTTTCTT TCGACCTTCC   
  
  
+ ATTTCCGTTT AATATTATCG TATAGGTGTT CACTATATAG GTGAACACCA ACCTGAGACT TAGTTCGTCG   
  
  
+ ACGAAGACGA CCACACAACT TGAGGAGGGT CCGAAGTTAG GTTCACTGGT ACCGTAGACG TTCGAAGAGT   
  
  
+ TTCATTCCTA GTTATACTGG GTTAAAGAGA AACTGGACGG ACCTCTCCCG TTCCCTCGTG AAGTCCCGAC   
  
  
+ AACACGTCAG AAAACCGTCC TGTTGTCTAA CCTTGTTACC TTAGTCGACA AGGACGGTCA TATCTGGGAA   
  
  
+ CTTAAGGAAG AAGTTCAATC TTTTGCCCA  

- TATTGGAGCT AACAATCTAA ACCGAGCTTT TATTTGTTTC CTGTATGAGG AAGAACAAGA CAAAGTTGAA   
  
  
- AAGAGGAGAG GAGCAGGCTA GCTTATTATG CTAATAGAAA TGGCATAACA TGGGTAGAAA AGGGTGGGGG   
  
  
- TTAATGGAAA GGCGTCCAAC TTAACTGACT TGACAGAATT GACCCATGTA AAGAGTGATT TCCATGGATG   
  
  
- CATCCCCTCA CAAAGATTAA GAGTATGTGC CGTAAACGGC ACGCAAATTG ACAAACAGAA GGATGGTTTA   
  
  
- GTTTCTTGTC GAAAAGGTTG CCAAGAATAA ATGATTTTAA GCCCATAAGT TATGAAAGTG GACGAGCATG   
  
  
- GAAGTTGAAC TGATGAAGAA GCCATGCCAA CCAGCTTAAT CATAGAATTA CTTCTTGTGT TATATCTGAA   
  
  
- ATATAGATGT AACATCATGG ACTTCCATGG ATGACTAGCT CTGGTTGTAG CTTAGAGCTT GCTTAACGTA   
  
  
- CTTAAACTAC GAAGGAAAAG GAGTATGCTT GCTGTTTATT CTCACATAGA AGCCATCTTC AGCCAATCAG   
  
  
- CCAATAGCAT CACTTAAACC GGTAGGTGAA TTTCATGCCT CGAGGTCAAA AAGCAGATAT TTAAGTTTCA   
  
  
- ACTAATCAAT TGAGTGCACA CTCCCCACTT TCTACATGTA CAGTTCATCG CATGAGCTTT AACGCTAAAT   
  
  
- GACTTGGGCA TACATAAGAG GAATTTTCGC TGGGTTTTCC TCGGAGCTTC AAGGAAGATC AATATCTCAG   
  
  
- AAAAACACTA CCTGCTGTAG TGCATGGGAC ATGTCGGCAT GGTTTTATGG GGATATGAAG TCCTATTGCT   
  
  
- GTCTGTGGTT AATTATAATG ATTAATGATG GTATTAAGTA AATACATTCT CAAATCATTT GGACTTCTTT   
  
  
- CAAGAGGGGT CACTGGTACT CTGTCTCATC TACCAATTTT TCGGGCTTTC TTTTTCCGTA TGGGGGAGGA   
  
  
- TTAGATTGAT TTGTCGCAGT GTCACTGGTT TTGTTTCAAT CAAGCTCTCT AGGAATAATT TATTAGGAAA   
  
  
- ATACTTGAAA AGAAGAGAAA GCAAGAAGAA AATGAAAATC AAGGATTACC ATAGGAGAAT AGCAAGCAAA   
  
  
- GGTCTATGAA GAGAAGATGT AACTCCATTG GTCACACTAA TATGTAGCAT GGAGAAAGAA AGCTGGAAGG   
  
  
- TAAAGGCAAA TTATAATAGC ATATCCACAA GTGATATATC CACTTGTGGT TGGACTCTGA ATCAAGCAGC   
  
  
- TGCTTCTGCT GGTGTGTTGA ACTCCTCCCA GGCTTCAATC CAAGTGACCA TGGCATCTGC AAGCTTCTCA   
  
  
- AAGTAAGGAT CAATATGACC CAATTTCTCT TTGACCTGCC TGGAGAGGGC AAGGGAGCAC TTCAGGGCTG   
  
  
- TTGTGCAGTC TTTTGGCAGG ACAACAGATT GGAACAATGG AATCAGCTGT TCCTGCCAGT ATAGACCCTT   
  
  
- GAATTCCTTC TTCAAGTTAG AAAACGGGT

+     MSA-like

| Site Name | Organism | Position | Strand | Matrix score. | sequence | function |
| --- | --- | --- | --- | --- | --- | --- |
| MSA-like | Catharanthus roseus | 295 | + | 9 | TCCAACGGT | cis-acting element involved in cell cycle regulation |

> 2018/04/13 10:10:12  
+ ATAACCTCGA TTGTTAGATT TGGCTCGAAA ATAAACAAAG GACATACTCC TTCTTGTTCT GTTTCAACTT   
  
  
+ TTCTCCTCTC CTCGTCCGAT CGAATAATAC GATTATCTTT ACCGTATTGT ACCCATCTTT TCCCACCCCC   
  
  
+ AATTACCTTT CCGCAGGTTG AATTGACTGA ACTGTCTTAA CTGGGTACAT TTCTCACTAA AGGTACCTAC   
  
  
+ GTAGGGGAGT GTTTCTAATT CTCATACACG GCATTTGCCG TGCGTTTAAC TGTTTGTCTT CCTACCAAAT   
  
  
+ CAAAGAACAG CTTTTCCAAC GGTTCTTATT TACTAAAATT CGGGTATTCA ATACTTTCAC CTGCTCGTAC   
  
  
+ CTTCAACTTG ACTACTTCTT CGGTACGGTT GGTCGAATTA GTATCTTAAT GAAGAACACA ATATAGACTT   
  
  
+ TATATCTACA TTGTAGTACC TGAAGGTACC TACTGATCGA GACCAACATC GAATCTCGAA CGAATTGCAT   
  
  
+ GAATTTGATG CTTCCTTTTC CTCATACGAA CGACAAATAA GAGTGTATCT TCGGTAGAAG TCGGTTAGTC   
  
  
+ GGTTATCGTA GTGAATTTGG CCATCCACTT AAAGTACGGA GCTCCAGTTT TTCGTCTATA AATTCAAAGT   
  
  
+ TGATTAGTTA ACTCACGTGT GAGGGGTGAA AGATGTACAT GTCAAGTAGC GTACTCGAAA TTGCGATTTA   
  
  
+ CTGAACCCGT ATGTATTCTC CTTAAAAGCG ACCCAAAAGG AGCCTCGAAG TTCCTTCTAG TTATAGAGTC   
  
  
+ TTTTTGTGAT GGACGACATC ACGTACCCTG TACAGCCGTA CCAAAATACC CCTATACTTC AGGATAACGA   
  
  
+ CAGACACCAA TTAATATTAC TAATTACTAC CATAATTCAT TTATGTAAGA GTTTAGTAAA CCTGAAGAAA   
  
  
+ GTTCTCCCCA GTGACCATGA GACAGAGTAG ATGGTTAAAA AGCCCGAAAG AAAAAGGCAT ACCCCCTCCT   
  
  
+ AATCTAACTA AACAGCGTCA CAGTGACCAA AACAAAGTTA GTTCGAGAGA TCCTTATTAA ATAATCCTTT   
  
  
+ TATGAACTTT TCTTCTCTTT CGTTCTTCTT TTACTTTTAG TTCCTAATGG TATCCTCTTA TCGTTCGTTT   
  
  
+ CCAGATACTT CTCTTCTACA TTGAGGTAAC CAGTGTGATT ATACATCGTA CCTCTTTCTT TCGACCTTCC   
  
  
+ ATTTCCGTTT AATATTATCG TATAGGTGTT CACTATATAG GTGAACACCA ACCTGAGACT TAGTTCGTCG   
  
  
+ ACGAAGACGA CCACACAACT TGAGGAGGGT CCGAAGTTAG GTTCACTGGT ACCGTAGACG TTCGAAGAGT   
  
  
+ TTCATTCCTA GTTATACTGG GTTAAAGAGA AACTGGACGG ACCTCTCCCG TTCCCTCGTG AAGTCCCGAC   
  
  
+ AACACGTCAG AAAACCGTCC TGTTGTCTAA CCTTGTTACC TTAGTCGACA AGGACGGTCA TATCTGGGAA   
  
  
+ CTTAAGGAAG AAGTTCAATC TTTTGCCCA  

- TATTGGAGCT AACAATCTAA ACCGAGCTTT TATTTGTTTC CTGTATGAGG AAGAACAAGA CAAAGTTGAA   
  
  
- AAGAGGAGAG GAGCAGGCTA GCTTATTATG CTAATAGAAA TGGCATAACA TGGGTAGAAA AGGGTGGGGG   
  
  
- TTAATGGAAA GGCGTCCAAC TTAACTGACT TGACAGAATT GACCCATGTA AAGAGTGATT TCCATGGATG   
  
  
- CATCCCCTCA CAAAGATTAA GAGTATGTGC CGTAAACGGC ACGCAAATTG ACAAACAGAA GGATGGTTTA   
  
  
- GTTTCTTGTC GAAAAGGTTG CCAAGAATAA ATGATTTTAA GCCCATAAGT TATGAAAGTG GACGAGCATG   
  
  
- GAAGTTGAAC TGATGAAGAA GCCATGCCAA CCAGCTTAAT CATAGAATTA CTTCTTGTGT TATATCTGAA   
  
  
- ATATAGATGT AACATCATGG ACTTCCATGG ATGACTAGCT CTGGTTGTAG CTTAGAGCTT GCTTAACGTA   
  
  
- CTTAAACTAC GAAGGAAAAG GAGTATGCTT GCTGTTTATT CTCACATAGA AGCCATCTTC AGCCAATCAG   
  
  
- CCAATAGCAT CACTTAAACC GGTAGGTGAA TTTCATGCCT CGAGGTCAAA AAGCAGATAT TTAAGTTTCA   
  
  
- ACTAATCAAT TGAGTGCACA CTCCCCACTT TCTACATGTA CAGTTCATCG CATGAGCTTT AACGCTAAAT   
  
  
- GACTTGGGCA TACATAAGAG GAATTTTCGC TGGGTTTTCC TCGGAGCTTC AAGGAAGATC AATATCTCAG   
  
  
- AAAAACACTA CCTGCTGTAG TGCATGGGAC ATGTCGGCAT GGTTTTATGG GGATATGAAG TCCTATTGCT   
  
  
- GTCTGTGGTT AATTATAATG ATTAATGATG GTATTAAGTA AATACATTCT CAAATCATTT GGACTTCTTT   
  
  
- CAAGAGGGGT CACTGGTACT CTGTCTCATC TACCAATTTT TCGGGCTTTC TTTTTCCGTA TGGGGGAGGA   
  
  
- TTAGATTGAT TTGTCGCAGT GTCACTGGTT TTGTTTCAAT CAAGCTCTCT AGGAATAATT TATTAGGAAA   
  
  
- ATACTTGAAA AGAAGAGAAA GCAAGAAGAA AATGAAAATC AAGGATTACC ATAGGAGAAT AGCAAGCAAA   
  
  
- GGTCTATGAA GAGAAGATGT AACTCCATTG GTCACACTAA TATGTAGCAT GGAGAAAGAA AGCTGGAAGG   
  
  
- TAAAGGCAAA TTATAATAGC ATATCCACAA GTGATATATC CACTTGTGGT TGGACTCTGA ATCAAGCAGC   
  
  
- TGCTTCTGCT GGTGTGTTGA ACTCCTCCCA GGCTTCAATC CAAGTGACCA TGGCATCTGC AAGCTTCTCA   
  
  
- AAGTAAGGAT CAATATGACC CAATTTCTCT TTGACCTGCC TGGAGAGGGC AAGGGAGCAC TTCAGGGCTG   
  
  
- TTGTGCAGTC TTTTGGCAGG ACAACAGATT GGAACAATGG AATCAGCTGT TCCTGCCAGT ATAGACCCTT   
  
  
- GAATTCCTTC TTCAAGTTAG AAAACGGGT

+     P-box

| Site Name | Organism | Position | Strand | Matrix score. | sequence | function |
| --- | --- | --- | --- | --- | --- | --- |
| P-box | Oryza sativa | 734 | - | 7 | CCTTTTG | gibberellin-responsive element |

> 2018/04/13 10:10:12  
+ ATAACCTCGA TTGTTAGATT TGGCTCGAAA ATAAACAAAG GACATACTCC TTCTTGTTCT GTTTCAACTT   
  
  
+ TTCTCCTCTC CTCGTCCGAT CGAATAATAC GATTATCTTT ACCGTATTGT ACCCATCTTT TCCCACCCCC   
  
  
+ AATTACCTTT CCGCAGGTTG AATTGACTGA ACTGTCTTAA CTGGGTACAT TTCTCACTAA AGGTACCTAC   
  
  
+ GTAGGGGAGT GTTTCTAATT CTCATACACG GCATTTGCCG TGCGTTTAAC TGTTTGTCTT CCTACCAAAT   
  
  
+ CAAAGAACAG CTTTTCCAAC GGTTCTTATT TACTAAAATT CGGGTATTCA ATACTTTCAC CTGCTCGTAC   
  
  
+ CTTCAACTTG ACTACTTCTT CGGTACGGTT GGTCGAATTA GTATCTTAAT GAAGAACACA ATATAGACTT   
  
  
+ TATATCTACA TTGTAGTACC TGAAGGTACC TACTGATCGA GACCAACATC GAATCTCGAA CGAATTGCAT   
  
  
+ GAATTTGATG CTTCCTTTTC CTCATACGAA CGACAAATAA GAGTGTATCT TCGGTAGAAG TCGGTTAGTC   
  
  
+ GGTTATCGTA GTGAATTTGG CCATCCACTT AAAGTACGGA GCTCCAGTTT TTCGTCTATA AATTCAAAGT   
  
  
+ TGATTAGTTA ACTCACGTGT GAGGGGTGAA AGATGTACAT GTCAAGTAGC GTACTCGAAA TTGCGATTTA   
  
  
+ CTGAACCCGT ATGTATTCTC CTTAAAAGCG ACCCAAAAGG AGCCTCGAAG TTCCTTCTAG TTATAGAGTC   
  
  
+ TTTTTGTGAT GGACGACATC ACGTACCCTG TACAGCCGTA CCAAAATACC CCTATACTTC AGGATAACGA   
  
  
+ CAGACACCAA TTAATATTAC TAATTACTAC CATAATTCAT TTATGTAAGA GTTTAGTAAA CCTGAAGAAA   
  
  
+ GTTCTCCCCA GTGACCATGA GACAGAGTAG ATGGTTAAAA AGCCCGAAAG AAAAAGGCAT ACCCCCTCCT   
  
  
+ AATCTAACTA AACAGCGTCA CAGTGACCAA AACAAAGTTA GTTCGAGAGA TCCTTATTAA ATAATCCTTT   
  
  
+ TATGAACTTT TCTTCTCTTT CGTTCTTCTT TTACTTTTAG TTCCTAATGG TATCCTCTTA TCGTTCGTTT   
  
  
+ CCAGATACTT CTCTTCTACA TTGAGGTAAC CAGTGTGATT ATACATCGTA CCTCTTTCTT TCGACCTTCC   
  
  
+ ATTTCCGTTT AATATTATCG TATAGGTGTT CACTATATAG GTGAACACCA ACCTGAGACT TAGTTCGTCG   
  
  
+ ACGAAGACGA CCACACAACT TGAGGAGGGT CCGAAGTTAG GTTCACTGGT ACCGTAGACG TTCGAAGAGT   
  
  
+ TTCATTCCTA GTTATACTGG GTTAAAGAGA AACTGGACGG ACCTCTCCCG TTCCCTCGTG AAGTCCCGAC   
  
  
+ AACACGTCAG AAAACCGTCC TGTTGTCTAA CCTTGTTACC TTAGTCGACA AGGACGGTCA TATCTGGGAA   
  
  
+ CTTAAGGAAG AAGTTCAATC TTTTGCCCA  

- TATTGGAGCT AACAATCTAA ACCGAGCTTT TATTTGTTTC CTGTATGAGG AAGAACAAGA CAAAGTTGAA   
  
  
- AAGAGGAGAG GAGCAGGCTA GCTTATTATG CTAATAGAAA TGGCATAACA TGGGTAGAAA AGGGTGGGGG   
  
  
- TTAATGGAAA GGCGTCCAAC TTAACTGACT TGACAGAATT GACCCATGTA AAGAGTGATT TCCATGGATG   
  
  
- CATCCCCTCA CAAAGATTAA GAGTATGTGC CGTAAACGGC ACGCAAATTG ACAAACAGAA GGATGGTTTA   
  
  
- GTTTCTTGTC GAAAAGGTTG CCAAGAATAA ATGATTTTAA GCCCATAAGT TATGAAAGTG GACGAGCATG   
  
  
- GAAGTTGAAC TGATGAAGAA GCCATGCCAA CCAGCTTAAT CATAGAATTA CTTCTTGTGT TATATCTGAA   
  
  
- ATATAGATGT AACATCATGG ACTTCCATGG ATGACTAGCT CTGGTTGTAG CTTAGAGCTT GCTTAACGTA   
  
  
- CTTAAACTAC GAAGGAAAAG GAGTATGCTT GCTGTTTATT CTCACATAGA AGCCATCTTC AGCCAATCAG   
  
  
- CCAATAGCAT CACTTAAACC GGTAGGTGAA TTTCATGCCT CGAGGTCAAA AAGCAGATAT TTAAGTTTCA   
  
  
- ACTAATCAAT TGAGTGCACA CTCCCCACTT TCTACATGTA CAGTTCATCG CATGAGCTTT AACGCTAAAT   
  
  
- GACTTGGGCA TACATAAGAG GAATTTTCGC TGGGTTTTCC TCGGAGCTTC AAGGAAGATC AATATCTCAG   
  
  
- AAAAACACTA CCTGCTGTAG TGCATGGGAC ATGTCGGCAT GGTTTTATGG GGATATGAAG TCCTATTGCT   
  
  
- GTCTGTGGTT AATTATAATG ATTAATGATG GTATTAAGTA AATACATTCT CAAATCATTT GGACTTCTTT   
  
  
- CAAGAGGGGT CACTGGTACT CTGTCTCATC TACCAATTTT TCGGGCTTTC TTTTTCCGTA TGGGGGAGGA   
  
  
- TTAGATTGAT TTGTCGCAGT GTCACTGGTT TTGTTTCAAT CAAGCTCTCT AGGAATAATT TATTAGGAAA   
  
  
- ATACTTGAAA AGAAGAGAAA GCAAGAAGAA AATGAAAATC AAGGATTACC ATAGGAGAAT AGCAAGCAAA   
  
  
- GGTCTATGAA GAGAAGATGT AACTCCATTG GTCACACTAA TATGTAGCAT GGAGAAAGAA AGCTGGAAGG   
  
  
- TAAAGGCAAA TTATAATAGC ATATCCACAA GTGATATATC CACTTGTGGT TGGACTCTGA ATCAAGCAGC   
  
  
- TGCTTCTGCT GGTGTGTTGA ACTCCTCCCA GGCTTCAATC CAAGTGACCA TGGCATCTGC AAGCTTCTCA   
  
  
- AAGTAAGGAT CAATATGACC CAATTTCTCT TTGACCTGCC TGGAGAGGGC AAGGGAGCAC TTCAGGGCTG   
  
  
- TTGTGCAGTC TTTTGGCAGG ACAACAGATT GGAACAATGG AATCAGCTGT TCCTGCCAGT ATAGACCCTT   
  
  
- GAATTCCTTC TTCAAGTTAG AAAACGGGT

+     Skn-1\_motif

| Site Name | Organism | Position | Strand | Matrix score. | sequence | function |
| --- | --- | --- | --- | --- | --- | --- |
| Skn-1\_motif | Oryza sativa | 1457 | + | 5 | GTCAT | cis-acting regulatory element required for endosperm expression |

> 2018/04/13 10:10:12  
+ ATAACCTCGA TTGTTAGATT TGGCTCGAAA ATAAACAAAG GACATACTCC TTCTTGTTCT GTTTCAACTT   
  
  
+ TTCTCCTCTC CTCGTCCGAT CGAATAATAC GATTATCTTT ACCGTATTGT ACCCATCTTT TCCCACCCCC   
  
  
+ AATTACCTTT CCGCAGGTTG AATTGACTGA ACTGTCTTAA CTGGGTACAT TTCTCACTAA AGGTACCTAC   
  
  
+ GTAGGGGAGT GTTTCTAATT CTCATACACG GCATTTGCCG TGCGTTTAAC TGTTTGTCTT CCTACCAAAT   
  
  
+ CAAAGAACAG CTTTTCCAAC GGTTCTTATT TACTAAAATT CGGGTATTCA ATACTTTCAC CTGCTCGTAC   
  
  
+ CTTCAACTTG ACTACTTCTT CGGTACGGTT GGTCGAATTA GTATCTTAAT GAAGAACACA ATATAGACTT   
  
  
+ TATATCTACA TTGTAGTACC TGAAGGTACC TACTGATCGA GACCAACATC GAATCTCGAA CGAATTGCAT   
  
  
+ GAATTTGATG CTTCCTTTTC CTCATACGAA CGACAAATAA GAGTGTATCT TCGGTAGAAG TCGGTTAGTC   
  
  
+ GGTTATCGTA GTGAATTTGG CCATCCACTT AAAGTACGGA GCTCCAGTTT TTCGTCTATA AATTCAAAGT   
  
  
+ TGATTAGTTA ACTCACGTGT GAGGGGTGAA AGATGTACAT GTCAAGTAGC GTACTCGAAA TTGCGATTTA   
  
  
+ CTGAACCCGT ATGTATTCTC CTTAAAAGCG ACCCAAAAGG AGCCTCGAAG TTCCTTCTAG TTATAGAGTC   
  
  
+ TTTTTGTGAT GGACGACATC ACGTACCCTG TACAGCCGTA CCAAAATACC CCTATACTTC AGGATAACGA   
  
  
+ CAGACACCAA TTAATATTAC TAATTACTAC CATAATTCAT TTATGTAAGA GTTTAGTAAA CCTGAAGAAA   
  
  
+ GTTCTCCCCA GTGACCATGA GACAGAGTAG ATGGTTAAAA AGCCCGAAAG AAAAAGGCAT ACCCCCTCCT   
  
  
+ AATCTAACTA AACAGCGTCA CAGTGACCAA AACAAAGTTA GTTCGAGAGA TCCTTATTAA ATAATCCTTT   
  
  
+ TATGAACTTT TCTTCTCTTT CGTTCTTCTT TTACTTTTAG TTCCTAATGG TATCCTCTTA TCGTTCGTTT   
  
  
+ CCAGATACTT CTCTTCTACA TTGAGGTAAC CAGTGTGATT ATACATCGTA CCTCTTTCTT TCGACCTTCC   
  
  
+ ATTTCCGTTT AATATTATCG TATAGGTGTT CACTATATAG GTGAACACCA ACCTGAGACT TAGTTCGTCG   
  
  
+ ACGAAGACGA CCACACAACT TGAGGAGGGT CCGAAGTTAG GTTCACTGGT ACCGTAGACG TTCGAAGAGT   
  
  
+ TTCATTCCTA GTTATACTGG GTTAAAGAGA AACTGGACGG ACCTCTCCCG TTCCCTCGTG AAGTCCCGAC   
  
  
+ AACACGTCAG AAAACCGTCC TGTTGTCTAA CCTTGTTACC TTAGTCGACA AGGACGGTCA TATCTGGGAA   
  
  
+ CTTAAGGAAG AAGTTCAATC TTTTGCCCA  

- TATTGGAGCT AACAATCTAA ACCGAGCTTT TATTTGTTTC CTGTATGAGG AAGAACAAGA CAAAGTTGAA   
  
  
- AAGAGGAGAG GAGCAGGCTA GCTTATTATG CTAATAGAAA TGGCATAACA TGGGTAGAAA AGGGTGGGGG   
  
  
- TTAATGGAAA GGCGTCCAAC TTAACTGACT TGACAGAATT GACCCATGTA AAGAGTGATT TCCATGGATG   
  
  
- CATCCCCTCA CAAAGATTAA GAGTATGTGC CGTAAACGGC ACGCAAATTG ACAAACAGAA GGATGGTTTA   
  
  
- GTTTCTTGTC GAAAAGGTTG CCAAGAATAA ATGATTTTAA GCCCATAAGT TATGAAAGTG GACGAGCATG   
  
  
- GAAGTTGAAC TGATGAAGAA GCCATGCCAA CCAGCTTAAT CATAGAATTA CTTCTTGTGT TATATCTGAA   
  
  
- ATATAGATGT AACATCATGG ACTTCCATGG ATGACTAGCT CTGGTTGTAG CTTAGAGCTT GCTTAACGTA   
  
  
- CTTAAACTAC GAAGGAAAAG GAGTATGCTT GCTGTTTATT CTCACATAGA AGCCATCTTC AGCCAATCAG   
  
  
- CCAATAGCAT CACTTAAACC GGTAGGTGAA TTTCATGCCT CGAGGTCAAA AAGCAGATAT TTAAGTTTCA   
  
  
- ACTAATCAAT TGAGTGCACA CTCCCCACTT TCTACATGTA CAGTTCATCG CATGAGCTTT AACGCTAAAT   
  
  
- GACTTGGGCA TACATAAGAG GAATTTTCGC TGGGTTTTCC TCGGAGCTTC AAGGAAGATC AATATCTCAG   
  
  
- AAAAACACTA CCTGCTGTAG TGCATGGGAC ATGTCGGCAT GGTTTTATGG GGATATGAAG TCCTATTGCT   
  
  
- GTCTGTGGTT AATTATAATG ATTAATGATG GTATTAAGTA AATACATTCT CAAATCATTT GGACTTCTTT   
  
  
- CAAGAGGGGT CACTGGTACT CTGTCTCATC TACCAATTTT TCGGGCTTTC TTTTTCCGTA TGGGGGAGGA   
  
  
- TTAGATTGAT TTGTCGCAGT GTCACTGGTT TTGTTTCAAT CAAGCTCTCT AGGAATAATT TATTAGGAAA   
  
  
- ATACTTGAAA AGAAGAGAAA GCAAGAAGAA AATGAAAATC AAGGATTACC ATAGGAGAAT AGCAAGCAAA   
  
  
- GGTCTATGAA GAGAAGATGT AACTCCATTG GTCACACTAA TATGTAGCAT GGAGAAAGAA AGCTGGAAGG   
  
  
- TAAAGGCAAA TTATAATAGC ATATCCACAA GTGATATATC CACTTGTGGT TGGACTCTGA ATCAAGCAGC   
  
  
- TGCTTCTGCT GGTGTGTTGA ACTCCTCCCA GGCTTCAATC CAAGTGACCA TGGCATCTGC AAGCTTCTCA   
  
  
- AAGTAAGGAT CAATATGACC CAATTTCTCT TTGACCTGCC TGGAGAGGGC AAGGGAGCAC TTCAGGGCTG   
  
  
- TTGTGCAGTC TTTTGGCAGG ACAACAGATT GGAACAATGG AATCAGCTGT TCCTGCCAGT ATAGACCCTT   
  
  
- GAATTCCTTC TTCAAGTTAG AAAACGGGT

+     Sp1

| Site Name | Organism | Position | Strand | Matrix score. | sequence | function |
| --- | --- | --- | --- | --- | --- | --- |
| Sp1 | Zea mays | 133 | + | 5.5 | CC(G/A)CCC | light responsive element |

> 2018/04/13 10:10:12  
+ ATAACCTCGA TTGTTAGATT TGGCTCGAAA ATAAACAAAG GACATACTCC TTCTTGTTCT GTTTCAACTT   
  
  
+ TTCTCCTCTC CTCGTCCGAT CGAATAATAC GATTATCTTT ACCGTATTGT ACCCATCTTT TCCCACCCCC   
  
  
+ AATTACCTTT CCGCAGGTTG AATTGACTGA ACTGTCTTAA CTGGGTACAT TTCTCACTAA AGGTACCTAC   
  
  
+ GTAGGGGAGT GTTTCTAATT CTCATACACG GCATTTGCCG TGCGTTTAAC TGTTTGTCTT CCTACCAAAT   
  
  
+ CAAAGAACAG CTTTTCCAAC GGTTCTTATT TACTAAAATT CGGGTATTCA ATACTTTCAC CTGCTCGTAC   
  
  
+ CTTCAACTTG ACTACTTCTT CGGTACGGTT GGTCGAATTA GTATCTTAAT GAAGAACACA ATATAGACTT   
  
  
+ TATATCTACA TTGTAGTACC TGAAGGTACC TACTGATCGA GACCAACATC GAATCTCGAA CGAATTGCAT   
  
  
+ GAATTTGATG CTTCCTTTTC CTCATACGAA CGACAAATAA GAGTGTATCT TCGGTAGAAG TCGGTTAGTC   
  
  
+ GGTTATCGTA GTGAATTTGG CCATCCACTT AAAGTACGGA GCTCCAGTTT TTCGTCTATA AATTCAAAGT   
  
  
+ TGATTAGTTA ACTCACGTGT GAGGGGTGAA AGATGTACAT GTCAAGTAGC GTACTCGAAA TTGCGATTTA   
  
  
+ CTGAACCCGT ATGTATTCTC CTTAAAAGCG ACCCAAAAGG AGCCTCGAAG TTCCTTCTAG TTATAGAGTC   
  
  
+ TTTTTGTGAT GGACGACATC ACGTACCCTG TACAGCCGTA CCAAAATACC CCTATACTTC AGGATAACGA   
  
  
+ CAGACACCAA TTAATATTAC TAATTACTAC CATAATTCAT TTATGTAAGA GTTTAGTAAA CCTGAAGAAA   
  
  
+ GTTCTCCCCA GTGACCATGA GACAGAGTAG ATGGTTAAAA AGCCCGAAAG AAAAAGGCAT ACCCCCTCCT   
  
  
+ AATCTAACTA AACAGCGTCA CAGTGACCAA AACAAAGTTA GTTCGAGAGA TCCTTATTAA ATAATCCTTT   
  
  
+ TATGAACTTT TCTTCTCTTT CGTTCTTCTT TTACTTTTAG TTCCTAATGG TATCCTCTTA TCGTTCGTTT   
  
  
+ CCAGATACTT CTCTTCTACA TTGAGGTAAC CAGTGTGATT ATACATCGTA CCTCTTTCTT TCGACCTTCC   
  
  
+ ATTTCCGTTT AATATTATCG TATAGGTGTT CACTATATAG GTGAACACCA ACCTGAGACT TAGTTCGTCG   
  
  
+ ACGAAGACGA CCACACAACT TGAGGAGGGT CCGAAGTTAG GTTCACTGGT ACCGTAGACG TTCGAAGAGT   
  
  
+ TTCATTCCTA GTTATACTGG GTTAAAGAGA AACTGGACGG ACCTCTCCCG TTCCCTCGTG AAGTCCCGAC   
  
  
+ AACACGTCAG AAAACCGTCC TGTTGTCTAA CCTTGTTACC TTAGTCGACA AGGACGGTCA TATCTGGGAA   
  
  
+ CTTAAGGAAG AAGTTCAATC TTTTGCCCA  

- TATTGGAGCT AACAATCTAA ACCGAGCTTT TATTTGTTTC CTGTATGAGG AAGAACAAGA CAAAGTTGAA   
  
  
- AAGAGGAGAG GAGCAGGCTA GCTTATTATG CTAATAGAAA TGGCATAACA TGGGTAGAAA AGGGTGGGGG   
  
  
- TTAATGGAAA GGCGTCCAAC TTAACTGACT TGACAGAATT GACCCATGTA AAGAGTGATT TCCATGGATG   
  
  
- CATCCCCTCA CAAAGATTAA GAGTATGTGC CGTAAACGGC ACGCAAATTG ACAAACAGAA GGATGGTTTA   
  
  
- GTTTCTTGTC GAAAAGGTTG CCAAGAATAA ATGATTTTAA GCCCATAAGT TATGAAAGTG GACGAGCATG   
  
  
- GAAGTTGAAC TGATGAAGAA GCCATGCCAA CCAGCTTAAT CATAGAATTA CTTCTTGTGT TATATCTGAA   
  
  
- ATATAGATGT AACATCATGG ACTTCCATGG ATGACTAGCT CTGGTTGTAG CTTAGAGCTT GCTTAACGTA   
  
  
- CTTAAACTAC GAAGGAAAAG GAGTATGCTT GCTGTTTATT CTCACATAGA AGCCATCTTC AGCCAATCAG   
  
  
- CCAATAGCAT CACTTAAACC GGTAGGTGAA TTTCATGCCT CGAGGTCAAA AAGCAGATAT TTAAGTTTCA   
  
  
- ACTAATCAAT TGAGTGCACA CTCCCCACTT TCTACATGTA CAGTTCATCG CATGAGCTTT AACGCTAAAT   
  
  
- GACTTGGGCA TACATAAGAG GAATTTTCGC TGGGTTTTCC TCGGAGCTTC AAGGAAGATC AATATCTCAG   
  
  
- AAAAACACTA CCTGCTGTAG TGCATGGGAC ATGTCGGCAT GGTTTTATGG GGATATGAAG TCCTATTGCT   
  
  
- GTCTGTGGTT AATTATAATG ATTAATGATG GTATTAAGTA AATACATTCT CAAATCATTT GGACTTCTTT   
  
  
- CAAGAGGGGT CACTGGTACT CTGTCTCATC TACCAATTTT TCGGGCTTTC TTTTTCCGTA TGGGGGAGGA   
  
  
- TTAGATTGAT TTGTCGCAGT GTCACTGGTT TTGTTTCAAT CAAGCTCTCT AGGAATAATT TATTAGGAAA   
  
  
- ATACTTGAAA AGAAGAGAAA GCAAGAAGAA AATGAAAATC AAGGATTACC ATAGGAGAAT AGCAAGCAAA   
  
  
- GGTCTATGAA GAGAAGATGT AACTCCATTG GTCACACTAA TATGTAGCAT GGAGAAAGAA AGCTGGAAGG   
  
  
- TAAAGGCAAA TTATAATAGC ATATCCACAA GTGATATATC CACTTGTGGT TGGACTCTGA ATCAAGCAGC   
  
  
- TGCTTCTGCT GGTGTGTTGA ACTCCTCCCA GGCTTCAATC CAAGTGACCA TGGCATCTGC AAGCTTCTCA   
  
  
- AAGTAAGGAT CAATATGACC CAATTTCTCT TTGACCTGCC TGGAGAGGGC AAGGGAGCAC TTCAGGGCTG   
  
  
- TTGTGCAGTC TTTTGGCAGG ACAACAGATT GGAACAATGG AATCAGCTGT TCCTGCCAGT ATAGACCCTT   
  
  
- GAATTCCTTC TTCAAGTTAG AAAACGGGT

+     TATA-box

| Site Name | Organism | Position | Strand | Matrix score. | sequence | function |
| --- | --- | --- | --- | --- | --- | --- |
| TATA-box | Arabidopsis thaliana | 1224 | - | 4 | TATA | core promoter element around -30 of transcription start |
| TATA-box | Lycopersicon esculentum | 1079 | + | 5 | TTTTA | core promoter element around -30 of transcription start |
| TATA-box | Brassica napus | 1158 | + | 6 | ATTATA | core promoter element around -30 of transcription start |
| TATA-box | Arabidopsis thaliana | 762 | - | 4 | TATA | core promoter element around -30 of transcription start |
| TATA-box | Lycopersicon esculentum | 1085 | + | 5 | TTTTA | core promoter element around -30 of transcription start |
| TATA-box | Arabidopsis thaliana | 761 | - | 5 | TATAA | core promoter element around -30 of transcription start |
| TATA-box | Glycine max | 1203 | - | 5 | TAATA | core promoter element around -30 of transcription start |
| TATA-box | Arabidopsis thaliana | 1160 | - | 4 | TATA | core promoter element around -30 of transcription start |
| TATA-box | Arabidopsis thaliana | 1343 | - | 4 | TATA | core promoter element around -30 of transcription start |
| TATA-box | Glycine max | 1200 | + | 5 | TAATA | core promoter element around -30 of transcription start |
| TATA-box | Ac | 617 | + | 7 | TATAAAT | core promoter element around -30 of transcription start |
| TATA-box | Arabidopsis thaliana | 419 | - | 6 | TATAAA | core promoter element around -30 of transcription start |
| TATA-box | Lycopersicon esculentum | 946 | - | 5 | TTTTA | core promoter element around -30 of transcription start |
| TATA-box | Arabidopsis thaliana | 823 | - | 4 | TATA | core promoter element around -30 of transcription start |
| TATA-box | Arabidopsis thaliana | 412 | + | 4 | TATA | core promoter element around -30 of transcription start |
| TATA-box | Glycine max | 1035 | - | 5 | TAATA | core promoter element around -30 of transcription start |
| TATA-box | Glycine max | 855 | - | 5 | TAATA | core promoter element around -30 of transcription start |
| TATA-box | Arabidopsis thaliana | 1211 | - | 4 | TATA | core promoter element around -30 of transcription start |
| TATA-box | Arabidopsis thaliana | 615 | + | 9 | tcTATATAtt | core promoter element around -30 of transcription start |
| TATA-box | Arabidopsis thaliana | 1159 | - | 5 | TATAA | core promoter element around -30 of transcription start |
| TATA-box | Arabidopsis thaliana | 1226 | - | 4 | TATA | core promoter element around -30 of transcription start |
| TATA-box | Glycine max | 852 | + | 5 | TAATA | core promoter element around -30 of transcription start |
| TATA-box | Lycopersicon esculentum | 314 | - | 5 | TTTTA | core promoter element around -30 of transcription start |
| TATA-box | Oryza sativa | 880 | - | 8 | TACATAAA | core promoter element around -30 of transcription start |
| TATA-box | Arabidopsis thaliana | 421 | + | 4 | TATA | core promoter element around -30 of transcription start |
| TATA-box | Lycopersicon esculentum | 723 | - | 5 | TTTTA | core promoter element around -30 of transcription start |
| TATA-box | Nicotiana tabacum | 758 | - | 9 | tcTATAAAta | core promoter element around -30 of transcription start |
| TATA-box | Arabidopsis thaliana | 420 | - | 5 | TATAA | core promoter element around -30 of transcription start |
| TATA-box | Glycine max | 95 | + | 5 | TAATA | core promoter element around -30 of transcription start |
| TATA-box | Lycopersicon esculentum | 1048 | + | 5 | TTTTA | core promoter element around -30 of transcription start |
| TATA-box | Arabidopsis thaliana | 1342 | - | 5 | TATAA | core promoter element around -30 of transcription start |

> 2018/04/13 10:10:12  
+ ATAACCTCGA TTGTTAGATT TGGCTCGAAA ATAAACAAAG GACATACTCC TTCTTGTTCT GTTTCAACTT   
  
  
+ TTCTCCTCTC CTCGTCCGAT CGAATAATAC GATTATCTTT ACCGTATTGT ACCCATCTTT TCCCACCCCC   
  
  
+ AATTACCTTT CCGCAGGTTG AATTGACTGA ACTGTCTTAA CTGGGTACAT TTCTCACTAA AGGTACCTAC   
  
  
+ GTAGGGGAGT GTTTCTAATT CTCATACACG GCATTTGCCG TGCGTTTAAC TGTTTGTCTT CCTACCAAAT   
  
  
+ CAAAGAACAG CTTTTCCAAC GGTTCTTATT TACTAAAATT CGGGTATTCA ATACTTTCAC CTGCTCGTAC   
  
  
+ CTTCAACTTG ACTACTTCTT CGGTACGGTT GGTCGAATTA GTATCTTAAT GAAGAACACA ATATAGACTT   
  
  
+ TATATCTACA TTGTAGTACC TGAAGGTACC TACTGATCGA GACCAACATC GAATCTCGAA CGAATTGCAT   
  
  
+ GAATTTGATG CTTCCTTTTC CTCATACGAA CGACAAATAA GAGTGTATCT TCGGTAGAAG TCGGTTAGTC   
  
  
+ GGTTATCGTA GTGAATTTGG CCATCCACTT AAAGTACGGA GCTCCAGTTT TTCGTCTATA AATTCAAAGT   
  
  
+ TGATTAGTTA ACTCACGTGT GAGGGGTGAA AGATGTACAT GTCAAGTAGC GTACTCGAAA TTGCGATTTA   
  
  
+ CTGAACCCGT ATGTATTCTC CTTAAAAGCG ACCCAAAAGG AGCCTCGAAG TTCCTTCTAG TTATAGAGTC   
  
  
+ TTTTTGTGAT GGACGACATC ACGTACCCTG TACAGCCGTA CCAAAATACC CCTATACTTC AGGATAACGA   
  
  
+ CAGACACCAA TTAATATTAC TAATTACTAC CATAATTCAT TTATGTAAGA GTTTAGTAAA CCTGAAGAAA   
  
  
+ GTTCTCCCCA GTGACCATGA GACAGAGTAG ATGGTTAAAA AGCCCGAAAG AAAAAGGCAT ACCCCCTCCT   
  
  
+ AATCTAACTA AACAGCGTCA CAGTGACCAA AACAAAGTTA GTTCGAGAGA TCCTTATTAA ATAATCCTTT   
  
  
+ TATGAACTTT TCTTCTCTTT CGTTCTTCTT TTACTTTTAG TTCCTAATGG TATCCTCTTA TCGTTCGTTT   
  
  
+ CCAGATACTT CTCTTCTACA TTGAGGTAAC CAGTGTGATT ATACATCGTA CCTCTTTCTT TCGACCTTCC   
  
  
+ ATTTCCGTTT AATATTATCG TATAGGTGTT CACTATATAG GTGAACACCA ACCTGAGACT TAGTTCGTCG   
  
  
+ ACGAAGACGA CCACACAACT TGAGGAGGGT CCGAAGTTAG GTTCACTGGT ACCGTAGACG TTCGAAGAGT   
  
  
+ TTCATTCCTA GTTATACTGG GTTAAAGAGA AACTGGACGG ACCTCTCCCG TTCCCTCGTG AAGTCCCGAC   
  
  
+ AACACGTCAG AAAACCGTCC TGTTGTCTAA CCTTGTTACC TTAGTCGACA AGGACGGTCA TATCTGGGAA   
  
  
+ CTTAAGGAAG AAGTTCAATC TTTTGCCCA  

- TATTGGAGCT AACAATCTAA ACCGAGCTTT TATTTGTTTC CTGTATGAGG AAGAACAAGA CAAAGTTGAA   
  
  
- AAGAGGAGAG GAGCAGGCTA GCTTATTATG CTAATAGAAA TGGCATAACA TGGGTAGAAA AGGGTGGGGG   
  
  
- TTAATGGAAA GGCGTCCAAC TTAACTGACT TGACAGAATT GACCCATGTA AAGAGTGATT TCCATGGATG   
  
  
- CATCCCCTCA CAAAGATTAA GAGTATGTGC CGTAAACGGC ACGCAAATTG ACAAACAGAA GGATGGTTTA   
  
  
- GTTTCTTGTC GAAAAGGTTG CCAAGAATAA ATGATTTTAA GCCCATAAGT TATGAAAGTG GACGAGCATG   
  
  
- GAAGTTGAAC TGATGAAGAA GCCATGCCAA CCAGCTTAAT CATAGAATTA CTTCTTGTGT TATATCTGAA   
  
  
- ATATAGATGT AACATCATGG ACTTCCATGG ATGACTAGCT CTGGTTGTAG CTTAGAGCTT GCTTAACGTA   
  
  
- CTTAAACTAC GAAGGAAAAG GAGTATGCTT GCTGTTTATT CTCACATAGA AGCCATCTTC AGCCAATCAG   
  
  
- CCAATAGCAT CACTTAAACC GGTAGGTGAA TTTCATGCCT CGAGGTCAAA AAGCAGATAT TTAAGTTTCA   
  
  
- ACTAATCAAT TGAGTGCACA CTCCCCACTT TCTACATGTA CAGTTCATCG CATGAGCTTT AACGCTAAAT   
  
  
- GACTTGGGCA TACATAAGAG GAATTTTCGC TGGGTTTTCC TCGGAGCTTC AAGGAAGATC AATATCTCAG   
  
  
- AAAAACACTA CCTGCTGTAG TGCATGGGAC ATGTCGGCAT GGTTTTATGG GGATATGAAG TCCTATTGCT   
  
  
- GTCTGTGGTT AATTATAATG ATTAATGATG GTATTAAGTA AATACATTCT CAAATCATTT GGACTTCTTT   
  
  
- CAAGAGGGGT CACTGGTACT CTGTCTCATC TACCAATTTT TCGGGCTTTC TTTTTCCGTA TGGGGGAGGA   
  
  
- TTAGATTGAT TTGTCGCAGT GTCACTGGTT TTGTTTCAAT CAAGCTCTCT AGGAATAATT TATTAGGAAA   
  
  
- ATACTTGAAA AGAAGAGAAA GCAAGAAGAA AATGAAAATC AAGGATTACC ATAGGAGAAT AGCAAGCAAA   
  
  
- GGTCTATGAA GAGAAGATGT AACTCCATTG GTCACACTAA TATGTAGCAT GGAGAAAGAA AGCTGGAAGG   
  
  
- TAAAGGCAAA TTATAATAGC ATATCCACAA GTGATATATC CACTTGTGGT TGGACTCTGA ATCAAGCAGC   
  
  
- TGCTTCTGCT GGTGTGTTGA ACTCCTCCCA GGCTTCAATC CAAGTGACCA TGGCATCTGC AAGCTTCTCA   
  
  
- AAGTAAGGAT CAATATGACC CAATTTCTCT TTGACCTGCC TGGAGAGGGC AAGGGAGCAC TTCAGGGCTG   
  
  
- TTGTGCAGTC TTTTGGCAGG ACAACAGATT GGAACAATGG AATCAGCTGT TCCTGCCAGT ATAGACCCTT   
  
  
- GAATTCCTTC TTCAAGTTAG AAAACGGGT

+     TC-rich repeats

| Site Name | Organism | Position | Strand | Matrix score. | sequence | function |
| --- | --- | --- | --- | --- | --- | --- |
| TC-rich repeats | Nicotiana tabacum | 1406 | - | 9 | GTTTTCTTAC | cis-acting element involved in defense and stress responsiveness |
| TC-rich repeats | Nicotiana tabacum | 903 | - | 9 | ATTTTCTTCA | cis-acting element involved in defense and stress responsiveness |

> 2018/04/13 10:10:12  
+ ATAACCTCGA TTGTTAGATT TGGCTCGAAA ATAAACAAAG GACATACTCC TTCTTGTTCT GTTTCAACTT   
  
  
+ TTCTCCTCTC CTCGTCCGAT CGAATAATAC GATTATCTTT ACCGTATTGT ACCCATCTTT TCCCACCCCC   
  
  
+ AATTACCTTT CCGCAGGTTG AATTGACTGA ACTGTCTTAA CTGGGTACAT TTCTCACTAA AGGTACCTAC   
  
  
+ GTAGGGGAGT GTTTCTAATT CTCATACACG GCATTTGCCG TGCGTTTAAC TGTTTGTCTT CCTACCAAAT   
  
  
+ CAAAGAACAG CTTTTCCAAC GGTTCTTATT TACTAAAATT CGGGTATTCA ATACTTTCAC CTGCTCGTAC   
  
  
+ CTTCAACTTG ACTACTTCTT CGGTACGGTT GGTCGAATTA GTATCTTAAT GAAGAACACA ATATAGACTT   
  
  
+ TATATCTACA TTGTAGTACC TGAAGGTACC TACTGATCGA GACCAACATC GAATCTCGAA CGAATTGCAT   
  
  
+ GAATTTGATG CTTCCTTTTC CTCATACGAA CGACAAATAA GAGTGTATCT TCGGTAGAAG TCGGTTAGTC   
  
  
+ GGTTATCGTA GTGAATTTGG CCATCCACTT AAAGTACGGA GCTCCAGTTT TTCGTCTATA AATTCAAAGT   
  
  
+ TGATTAGTTA ACTCACGTGT GAGGGGTGAA AGATGTACAT GTCAAGTAGC GTACTCGAAA TTGCGATTTA   
  
  
+ CTGAACCCGT ATGTATTCTC CTTAAAAGCG ACCCAAAAGG AGCCTCGAAG TTCCTTCTAG TTATAGAGTC   
  
  
+ TTTTTGTGAT GGACGACATC ACGTACCCTG TACAGCCGTA CCAAAATACC CCTATACTTC AGGATAACGA   
  
  
+ CAGACACCAA TTAATATTAC TAATTACTAC CATAATTCAT TTATGTAAGA GTTTAGTAAA CCTGAAGAAA   
  
  
+ GTTCTCCCCA GTGACCATGA GACAGAGTAG ATGGTTAAAA AGCCCGAAAG AAAAAGGCAT ACCCCCTCCT   
  
  
+ AATCTAACTA AACAGCGTCA CAGTGACCAA AACAAAGTTA GTTCGAGAGA TCCTTATTAA ATAATCCTTT   
  
  
+ TATGAACTTT TCTTCTCTTT CGTTCTTCTT TTACTTTTAG TTCCTAATGG TATCCTCTTA TCGTTCGTTT   
  
  
+ CCAGATACTT CTCTTCTACA TTGAGGTAAC CAGTGTGATT ATACATCGTA CCTCTTTCTT TCGACCTTCC   
  
  
+ ATTTCCGTTT AATATTATCG TATAGGTGTT CACTATATAG GTGAACACCA ACCTGAGACT TAGTTCGTCG   
  
  
+ ACGAAGACGA CCACACAACT TGAGGAGGGT CCGAAGTTAG GTTCACTGGT ACCGTAGACG TTCGAAGAGT   
  
  
+ TTCATTCCTA GTTATACTGG GTTAAAGAGA AACTGGACGG ACCTCTCCCG TTCCCTCGTG AAGTCCCGAC   
  
  
+ AACACGTCAG AAAACCGTCC TGTTGTCTAA CCTTGTTACC TTAGTCGACA AGGACGGTCA TATCTGGGAA   
  
  
+ CTTAAGGAAG AAGTTCAATC TTTTGCCCA  

- TATTGGAGCT AACAATCTAA ACCGAGCTTT TATTTGTTTC CTGTATGAGG AAGAACAAGA CAAAGTTGAA   
  
  
- AAGAGGAGAG GAGCAGGCTA GCTTATTATG CTAATAGAAA TGGCATAACA TGGGTAGAAA AGGGTGGGGG   
  
  
- TTAATGGAAA GGCGTCCAAC TTAACTGACT TGACAGAATT GACCCATGTA AAGAGTGATT TCCATGGATG   
  
  
- CATCCCCTCA CAAAGATTAA GAGTATGTGC CGTAAACGGC ACGCAAATTG ACAAACAGAA GGATGGTTTA   
  
  
- GTTTCTTGTC GAAAAGGTTG CCAAGAATAA ATGATTTTAA GCCCATAAGT TATGAAAGTG GACGAGCATG   
  
  
- GAAGTTGAAC TGATGAAGAA GCCATGCCAA CCAGCTTAAT CATAGAATTA CTTCTTGTGT TATATCTGAA   
  
  
- ATATAGATGT AACATCATGG ACTTCCATGG ATGACTAGCT CTGGTTGTAG CTTAGAGCTT GCTTAACGTA   
  
  
- CTTAAACTAC GAAGGAAAAG GAGTATGCTT GCTGTTTATT CTCACATAGA AGCCATCTTC AGCCAATCAG   
  
  
- CCAATAGCAT CACTTAAACC GGTAGGTGAA TTTCATGCCT CGAGGTCAAA AAGCAGATAT TTAAGTTTCA   
  
  
- ACTAATCAAT TGAGTGCACA CTCCCCACTT TCTACATGTA CAGTTCATCG CATGAGCTTT AACGCTAAAT   
  
  
- GACTTGGGCA TACATAAGAG GAATTTTCGC TGGGTTTTCC TCGGAGCTTC AAGGAAGATC AATATCTCAG   
  
  
- AAAAACACTA CCTGCTGTAG TGCATGGGAC ATGTCGGCAT GGTTTTATGG GGATATGAAG TCCTATTGCT   
  
  
- GTCTGTGGTT AATTATAATG ATTAATGATG GTATTAAGTA AATACATTCT CAAATCATTT GGACTTCTTT   
  
  
- CAAGAGGGGT CACTGGTACT CTGTCTCATC TACCAATTTT TCGGGCTTTC TTTTTCCGTA TGGGGGAGGA   
  
  
- TTAGATTGAT TTGTCGCAGT GTCACTGGTT TTGTTTCAAT CAAGCTCTCT AGGAATAATT TATTAGGAAA   
  
  
- ATACTTGAAA AGAAGAGAAA GCAAGAAGAA AATGAAAATC AAGGATTACC ATAGGAGAAT AGCAAGCAAA   
  
  
- GGTCTATGAA GAGAAGATGT AACTCCATTG GTCACACTAA TATGTAGCAT GGAGAAAGAA AGCTGGAAGG   
  
  
- TAAAGGCAAA TTATAATAGC ATATCCACAA GTGATATATC CACTTGTGGT TGGACTCTGA ATCAAGCAGC   
  
  
- TGCTTCTGCT GGTGTGTTGA ACTCCTCCCA GGCTTCAATC CAAGTGACCA TGGCATCTGC AAGCTTCTCA   
  
  
- AAGTAAGGAT CAATATGACC CAATTTCTCT TTGACCTGCC TGGAGAGGGC AAGGGAGCAC TTCAGGGCTG   
  
  
- TTGTGCAGTC TTTTGGCAGG ACAACAGATT GGAACAATGG AATCAGCTGT TCCTGCCAGT ATAGACCCTT   
  
  
- GAATTCCTTC TTCAAGTTAG AAAACGGGT

+     TCA-element

| Site Name | Organism | Position | Strand | Matrix score. | sequence | function |
| --- | --- | --- | --- | --- | --- | --- |
| TCA-element | Nicotiana tabacum | 123 | + | 9 | CCATCTTTTT | cis-acting element involved in salicylic acid responsiveness |
| TCA-element | Brassica oleracea | 1045 | - | 9 | CAGAAAAGGA | cis-acting element involved in salicylic acid responsiveness |
| TCA-element | Brassica oleracea | 1058 | - | 9 | GAGAAGAATA | cis-acting element involved in salicylic acid responsiveness |

> 2018/04/13 10:10:12  
+ ATAACCTCGA TTGTTAGATT TGGCTCGAAA ATAAACAAAG GACATACTCC TTCTTGTTCT GTTTCAACTT   
  
  
+ TTCTCCTCTC CTCGTCCGAT CGAATAATAC GATTATCTTT ACCGTATTGT ACCCATCTTT TCCCACCCCC   
  
  
+ AATTACCTTT CCGCAGGTTG AATTGACTGA ACTGTCTTAA CTGGGTACAT TTCTCACTAA AGGTACCTAC   
  
  
+ GTAGGGGAGT GTTTCTAATT CTCATACACG GCATTTGCCG TGCGTTTAAC TGTTTGTCTT CCTACCAAAT   
  
  
+ CAAAGAACAG CTTTTCCAAC GGTTCTTATT TACTAAAATT CGGGTATTCA ATACTTTCAC CTGCTCGTAC   
  
  
+ CTTCAACTTG ACTACTTCTT CGGTACGGTT GGTCGAATTA GTATCTTAAT GAAGAACACA ATATAGACTT   
  
  
+ TATATCTACA TTGTAGTACC TGAAGGTACC TACTGATCGA GACCAACATC GAATCTCGAA CGAATTGCAT   
  
  
+ GAATTTGATG CTTCCTTTTC CTCATACGAA CGACAAATAA GAGTGTATCT TCGGTAGAAG TCGGTTAGTC   
  
  
+ GGTTATCGTA GTGAATTTGG CCATCCACTT AAAGTACGGA GCTCCAGTTT TTCGTCTATA AATTCAAAGT   
  
  
+ TGATTAGTTA ACTCACGTGT GAGGGGTGAA AGATGTACAT GTCAAGTAGC GTACTCGAAA TTGCGATTTA   
  
  
+ CTGAACCCGT ATGTATTCTC CTTAAAAGCG ACCCAAAAGG AGCCTCGAAG TTCCTTCTAG TTATAGAGTC   
  
  
+ TTTTTGTGAT GGACGACATC ACGTACCCTG TACAGCCGTA CCAAAATACC CCTATACTTC AGGATAACGA   
  
  
+ CAGACACCAA TTAATATTAC TAATTACTAC CATAATTCAT TTATGTAAGA GTTTAGTAAA CCTGAAGAAA   
  
  
+ GTTCTCCCCA GTGACCATGA GACAGAGTAG ATGGTTAAAA AGCCCGAAAG AAAAAGGCAT ACCCCCTCCT   
  
  
+ AATCTAACTA AACAGCGTCA CAGTGACCAA AACAAAGTTA GTTCGAGAGA TCCTTATTAA ATAATCCTTT   
  
  
+ TATGAACTTT TCTTCTCTTT CGTTCTTCTT TTACTTTTAG TTCCTAATGG TATCCTCTTA TCGTTCGTTT   
  
  
+ CCAGATACTT CTCTTCTACA TTGAGGTAAC CAGTGTGATT ATACATCGTA CCTCTTTCTT TCGACCTTCC   
  
  
+ ATTTCCGTTT AATATTATCG TATAGGTGTT CACTATATAG GTGAACACCA ACCTGAGACT TAGTTCGTCG   
  
  
+ ACGAAGACGA CCACACAACT TGAGGAGGGT CCGAAGTTAG GTTCACTGGT ACCGTAGACG TTCGAAGAGT   
  
  
+ TTCATTCCTA GTTATACTGG GTTAAAGAGA AACTGGACGG ACCTCTCCCG TTCCCTCGTG AAGTCCCGAC   
  
  
+ AACACGTCAG AAAACCGTCC TGTTGTCTAA CCTTGTTACC TTAGTCGACA AGGACGGTCA TATCTGGGAA   
  
  
+ CTTAAGGAAG AAGTTCAATC TTTTGCCCA  

- TATTGGAGCT AACAATCTAA ACCGAGCTTT TATTTGTTTC CTGTATGAGG AAGAACAAGA CAAAGTTGAA   
  
  
- AAGAGGAGAG GAGCAGGCTA GCTTATTATG CTAATAGAAA TGGCATAACA TGGGTAGAAA AGGGTGGGGG   
  
  
- TTAATGGAAA GGCGTCCAAC TTAACTGACT TGACAGAATT GACCCATGTA AAGAGTGATT TCCATGGATG   
  
  
- CATCCCCTCA CAAAGATTAA GAGTATGTGC CGTAAACGGC ACGCAAATTG ACAAACAGAA GGATGGTTTA   
  
  
- GTTTCTTGTC GAAAAGGTTG CCAAGAATAA ATGATTTTAA GCCCATAAGT TATGAAAGTG GACGAGCATG   
  
  
- GAAGTTGAAC TGATGAAGAA GCCATGCCAA CCAGCTTAAT CATAGAATTA CTTCTTGTGT TATATCTGAA   
  
  
- ATATAGATGT AACATCATGG ACTTCCATGG ATGACTAGCT CTGGTTGTAG CTTAGAGCTT GCTTAACGTA   
  
  
- CTTAAACTAC GAAGGAAAAG GAGTATGCTT GCTGTTTATT CTCACATAGA AGCCATCTTC AGCCAATCAG   
  
  
- CCAATAGCAT CACTTAAACC GGTAGGTGAA TTTCATGCCT CGAGGTCAAA AAGCAGATAT TTAAGTTTCA   
  
  
- ACTAATCAAT TGAGTGCACA CTCCCCACTT TCTACATGTA CAGTTCATCG CATGAGCTTT AACGCTAAAT   
  
  
- GACTTGGGCA TACATAAGAG GAATTTTCGC TGGGTTTTCC TCGGAGCTTC AAGGAAGATC AATATCTCAG   
  
  
- AAAAACACTA CCTGCTGTAG TGCATGGGAC ATGTCGGCAT GGTTTTATGG GGATATGAAG TCCTATTGCT   
  
  
- GTCTGTGGTT AATTATAATG ATTAATGATG GTATTAAGTA AATACATTCT CAAATCATTT GGACTTCTTT   
  
  
- CAAGAGGGGT CACTGGTACT CTGTCTCATC TACCAATTTT TCGGGCTTTC TTTTTCCGTA TGGGGGAGGA   
  
  
- TTAGATTGAT TTGTCGCAGT GTCACTGGTT TTGTTTCAAT CAAGCTCTCT AGGAATAATT TATTAGGAAA   
  
  
- ATACTTGAAA AGAAGAGAAA GCAAGAAGAA AATGAAAATC AAGGATTACC ATAGGAGAAT AGCAAGCAAA   
  
  
- GGTCTATGAA GAGAAGATGT AACTCCATTG GTCACACTAA TATGTAGCAT GGAGAAAGAA AGCTGGAAGG   
  
  
- TAAAGGCAAA TTATAATAGC ATATCCACAA GTGATATATC CACTTGTGGT TGGACTCTGA ATCAAGCAGC   
  
  
- TGCTTCTGCT GGTGTGTTGA ACTCCTCCCA GGCTTCAATC CAAGTGACCA TGGCATCTGC AAGCTTCTCA   
  
  
- AAGTAAGGAT CAATATGACC CAATTTCTCT TTGACCTGCC TGGAGAGGGC AAGGGAGCAC TTCAGGGCTG   
  
  
- TTGTGCAGTC TTTTGGCAGG ACAACAGATT GGAACAATGG AATCAGCTGT TCCTGCCAGT ATAGACCCTT   
  
  
- GAATTCCTTC TTCAAGTTAG AAAACGGGT

+     TCT-motif

| Site Name | Organism | Position | Strand | Matrix score. | sequence | function |
| --- | --- | --- | --- | --- | --- | --- |
| TCT-motif | Arabidopsis thaliana | 885 | - | 6 | TCTTAC | part of a light responsive element |

> 2018/04/13 10:10:12  
+ ATAACCTCGA TTGTTAGATT TGGCTCGAAA ATAAACAAAG GACATACTCC TTCTTGTTCT GTTTCAACTT   
  
  
+ TTCTCCTCTC CTCGTCCGAT CGAATAATAC GATTATCTTT ACCGTATTGT ACCCATCTTT TCCCACCCCC   
  
  
+ AATTACCTTT CCGCAGGTTG AATTGACTGA ACTGTCTTAA CTGGGTACAT TTCTCACTAA AGGTACCTAC   
  
  
+ GTAGGGGAGT GTTTCTAATT CTCATACACG GCATTTGCCG TGCGTTTAAC TGTTTGTCTT CCTACCAAAT   
  
  
+ CAAAGAACAG CTTTTCCAAC GGTTCTTATT TACTAAAATT CGGGTATTCA ATACTTTCAC CTGCTCGTAC   
  
  
+ CTTCAACTTG ACTACTTCTT CGGTACGGTT GGTCGAATTA GTATCTTAAT GAAGAACACA ATATAGACTT   
  
  
+ TATATCTACA TTGTAGTACC TGAAGGTACC TACTGATCGA GACCAACATC GAATCTCGAA CGAATTGCAT   
  
  
+ GAATTTGATG CTTCCTTTTC CTCATACGAA CGACAAATAA GAGTGTATCT TCGGTAGAAG TCGGTTAGTC   
  
  
+ GGTTATCGTA GTGAATTTGG CCATCCACTT AAAGTACGGA GCTCCAGTTT TTCGTCTATA AATTCAAAGT   
  
  
+ TGATTAGTTA ACTCACGTGT GAGGGGTGAA AGATGTACAT GTCAAGTAGC GTACTCGAAA TTGCGATTTA   
  
  
+ CTGAACCCGT ATGTATTCTC CTTAAAAGCG ACCCAAAAGG AGCCTCGAAG TTCCTTCTAG TTATAGAGTC   
  
  
+ TTTTTGTGAT GGACGACATC ACGTACCCTG TACAGCCGTA CCAAAATACC CCTATACTTC AGGATAACGA   
  
  
+ CAGACACCAA TTAATATTAC TAATTACTAC CATAATTCAT TTATGTAAGA GTTTAGTAAA CCTGAAGAAA   
  
  
+ GTTCTCCCCA GTGACCATGA GACAGAGTAG ATGGTTAAAA AGCCCGAAAG AAAAAGGCAT ACCCCCTCCT   
  
  
+ AATCTAACTA AACAGCGTCA CAGTGACCAA AACAAAGTTA GTTCGAGAGA TCCTTATTAA ATAATCCTTT   
  
  
+ TATGAACTTT TCTTCTCTTT CGTTCTTCTT TTACTTTTAG TTCCTAATGG TATCCTCTTA TCGTTCGTTT   
  
  
+ CCAGATACTT CTCTTCTACA TTGAGGTAAC CAGTGTGATT ATACATCGTA CCTCTTTCTT TCGACCTTCC   
  
  
+ ATTTCCGTTT AATATTATCG TATAGGTGTT CACTATATAG GTGAACACCA ACCTGAGACT TAGTTCGTCG   
  
  
+ ACGAAGACGA CCACACAACT TGAGGAGGGT CCGAAGTTAG GTTCACTGGT ACCGTAGACG TTCGAAGAGT   
  
  
+ TTCATTCCTA GTTATACTGG GTTAAAGAGA AACTGGACGG ACCTCTCCCG TTCCCTCGTG AAGTCCCGAC   
  
  
+ AACACGTCAG AAAACCGTCC TGTTGTCTAA CCTTGTTACC TTAGTCGACA AGGACGGTCA TATCTGGGAA   
  
  
+ CTTAAGGAAG AAGTTCAATC TTTTGCCCA  

- TATTGGAGCT AACAATCTAA ACCGAGCTTT TATTTGTTTC CTGTATGAGG AAGAACAAGA CAAAGTTGAA   
  
  
- AAGAGGAGAG GAGCAGGCTA GCTTATTATG CTAATAGAAA TGGCATAACA TGGGTAGAAA AGGGTGGGGG   
  
  
- TTAATGGAAA GGCGTCCAAC TTAACTGACT TGACAGAATT GACCCATGTA AAGAGTGATT TCCATGGATG   
  
  
- CATCCCCTCA CAAAGATTAA GAGTATGTGC CGTAAACGGC ACGCAAATTG ACAAACAGAA GGATGGTTTA   
  
  
- GTTTCTTGTC GAAAAGGTTG CCAAGAATAA ATGATTTTAA GCCCATAAGT TATGAAAGTG GACGAGCATG   
  
  
- GAAGTTGAAC TGATGAAGAA GCCATGCCAA CCAGCTTAAT CATAGAATTA CTTCTTGTGT TATATCTGAA   
  
  
- ATATAGATGT AACATCATGG ACTTCCATGG ATGACTAGCT CTGGTTGTAG CTTAGAGCTT GCTTAACGTA   
  
  
- CTTAAACTAC GAAGGAAAAG GAGTATGCTT GCTGTTTATT CTCACATAGA AGCCATCTTC AGCCAATCAG   
  
  
- CCAATAGCAT CACTTAAACC GGTAGGTGAA TTTCATGCCT CGAGGTCAAA AAGCAGATAT TTAAGTTTCA   
  
  
- ACTAATCAAT TGAGTGCACA CTCCCCACTT TCTACATGTA CAGTTCATCG CATGAGCTTT AACGCTAAAT   
  
  
- GACTTGGGCA TACATAAGAG GAATTTTCGC TGGGTTTTCC TCGGAGCTTC AAGGAAGATC AATATCTCAG   
  
  
- AAAAACACTA CCTGCTGTAG TGCATGGGAC ATGTCGGCAT GGTTTTATGG GGATATGAAG TCCTATTGCT   
  
  
- GTCTGTGGTT AATTATAATG ATTAATGATG GTATTAAGTA AATACATTCT CAAATCATTT GGACTTCTTT   
  
  
- CAAGAGGGGT CACTGGTACT CTGTCTCATC TACCAATTTT TCGGGCTTTC TTTTTCCGTA TGGGGGAGGA   
  
  
- TTAGATTGAT TTGTCGCAGT GTCACTGGTT TTGTTTCAAT CAAGCTCTCT AGGAATAATT TATTAGGAAA   
  
  
- ATACTTGAAA AGAAGAGAAA GCAAGAAGAA AATGAAAATC AAGGATTACC ATAGGAGAAT AGCAAGCAAA   
  
  
- GGTCTATGAA GAGAAGATGT AACTCCATTG GTCACACTAA TATGTAGCAT GGAGAAAGAA AGCTGGAAGG   
  
  
- TAAAGGCAAA TTATAATAGC ATATCCACAA GTGATATATC CACTTGTGGT TGGACTCTGA ATCAAGCAGC   
  
  
- TGCTTCTGCT GGTGTGTTGA ACTCCTCCCA GGCTTCAATC CAAGTGACCA TGGCATCTGC AAGCTTCTCA   
  
  
- AAGTAAGGAT CAATATGACC CAATTTCTCT TTGACCTGCC TGGAGAGGGC AAGGGAGCAC TTCAGGGCTG   
  
  
- TTGTGCAGTC TTTTGGCAGG ACAACAGATT GGAACAATGG AATCAGCTGT TCCTGCCAGT ATAGACCCTT   
  
  
- GAATTCCTTC TTCAAGTTAG AAAACGGGT

+     TGA-element

| Site Name | Organism | Position | Strand | Matrix score. | sequence | function |
| --- | --- | --- | --- | --- | --- | --- |
| TGA-element | Brassica oleracea | 519 | + | 6 | AACGAC | auxin-responsive element |
| TGA-element | Brassica oleracea | 836 | + | 6 | AACGAC | auxin-responsive element |

> 2018/04/13 10:10:12  
+ ATAACCTCGA TTGTTAGATT TGGCTCGAAA ATAAACAAAG GACATACTCC TTCTTGTTCT GTTTCAACTT   
  
  
+ TTCTCCTCTC CTCGTCCGAT CGAATAATAC GATTATCTTT ACCGTATTGT ACCCATCTTT TCCCACCCCC   
  
  
+ AATTACCTTT CCGCAGGTTG AATTGACTGA ACTGTCTTAA CTGGGTACAT TTCTCACTAA AGGTACCTAC   
  
  
+ GTAGGGGAGT GTTTCTAATT CTCATACACG GCATTTGCCG TGCGTTTAAC TGTTTGTCTT CCTACCAAAT   
  
  
+ CAAAGAACAG CTTTTCCAAC GGTTCTTATT TACTAAAATT CGGGTATTCA ATACTTTCAC CTGCTCGTAC   
  
  
+ CTTCAACTTG ACTACTTCTT CGGTACGGTT GGTCGAATTA GTATCTTAAT GAAGAACACA ATATAGACTT   
  
  
+ TATATCTACA TTGTAGTACC TGAAGGTACC TACTGATCGA GACCAACATC GAATCTCGAA CGAATTGCAT   
  
  
+ GAATTTGATG CTTCCTTTTC CTCATACGAA CGACAAATAA GAGTGTATCT TCGGTAGAAG TCGGTTAGTC   
  
  
+ GGTTATCGTA GTGAATTTGG CCATCCACTT AAAGTACGGA GCTCCAGTTT TTCGTCTATA AATTCAAAGT   
  
  
+ TGATTAGTTA ACTCACGTGT GAGGGGTGAA AGATGTACAT GTCAAGTAGC GTACTCGAAA TTGCGATTTA   
  
  
+ CTGAACCCGT ATGTATTCTC CTTAAAAGCG ACCCAAAAGG AGCCTCGAAG TTCCTTCTAG TTATAGAGTC   
  
  
+ TTTTTGTGAT GGACGACATC ACGTACCCTG TACAGCCGTA CCAAAATACC CCTATACTTC AGGATAACGA   
  
  
+ CAGACACCAA TTAATATTAC TAATTACTAC CATAATTCAT TTATGTAAGA GTTTAGTAAA CCTGAAGAAA   
  
  
+ GTTCTCCCCA GTGACCATGA GACAGAGTAG ATGGTTAAAA AGCCCGAAAG AAAAAGGCAT ACCCCCTCCT   
  
  
+ AATCTAACTA AACAGCGTCA CAGTGACCAA AACAAAGTTA GTTCGAGAGA TCCTTATTAA ATAATCCTTT   
  
  
+ TATGAACTTT TCTTCTCTTT CGTTCTTCTT TTACTTTTAG TTCCTAATGG TATCCTCTTA TCGTTCGTTT   
  
  
+ CCAGATACTT CTCTTCTACA TTGAGGTAAC CAGTGTGATT ATACATCGTA CCTCTTTCTT TCGACCTTCC   
  
  
+ ATTTCCGTTT AATATTATCG TATAGGTGTT CACTATATAG GTGAACACCA ACCTGAGACT TAGTTCGTCG   
  
  
+ ACGAAGACGA CCACACAACT TGAGGAGGGT CCGAAGTTAG GTTCACTGGT ACCGTAGACG TTCGAAGAGT   
  
  
+ TTCATTCCTA GTTATACTGG GTTAAAGAGA AACTGGACGG ACCTCTCCCG TTCCCTCGTG AAGTCCCGAC   
  
  
+ AACACGTCAG AAAACCGTCC TGTTGTCTAA CCTTGTTACC TTAGTCGACA AGGACGGTCA TATCTGGGAA   
  
  
+ CTTAAGGAAG AAGTTCAATC TTTTGCCCA  

- TATTGGAGCT AACAATCTAA ACCGAGCTTT TATTTGTTTC CTGTATGAGG AAGAACAAGA CAAAGTTGAA   
  
  
- AAGAGGAGAG GAGCAGGCTA GCTTATTATG CTAATAGAAA TGGCATAACA TGGGTAGAAA AGGGTGGGGG   
  
  
- TTAATGGAAA GGCGTCCAAC TTAACTGACT TGACAGAATT GACCCATGTA AAGAGTGATT TCCATGGATG   
  
  
- CATCCCCTCA CAAAGATTAA GAGTATGTGC CGTAAACGGC ACGCAAATTG ACAAACAGAA GGATGGTTTA   
  
  
- GTTTCTTGTC GAAAAGGTTG CCAAGAATAA ATGATTTTAA GCCCATAAGT TATGAAAGTG GACGAGCATG   
  
  
- GAAGTTGAAC TGATGAAGAA GCCATGCCAA CCAGCTTAAT CATAGAATTA CTTCTTGTGT TATATCTGAA   
  
  
- ATATAGATGT AACATCATGG ACTTCCATGG ATGACTAGCT CTGGTTGTAG CTTAGAGCTT GCTTAACGTA   
  
  
- CTTAAACTAC GAAGGAAAAG GAGTATGCTT GCTGTTTATT CTCACATAGA AGCCATCTTC AGCCAATCAG   
  
  
- CCAATAGCAT CACTTAAACC GGTAGGTGAA TTTCATGCCT CGAGGTCAAA AAGCAGATAT TTAAGTTTCA   
  
  
- ACTAATCAAT TGAGTGCACA CTCCCCACTT TCTACATGTA CAGTTCATCG CATGAGCTTT AACGCTAAAT   
  
  
- GACTTGGGCA TACATAAGAG GAATTTTCGC TGGGTTTTCC TCGGAGCTTC AAGGAAGATC AATATCTCAG   
  
  
- AAAAACACTA CCTGCTGTAG TGCATGGGAC ATGTCGGCAT GGTTTTATGG GGATATGAAG TCCTATTGCT   
  
  
- GTCTGTGGTT AATTATAATG ATTAATGATG GTATTAAGTA AATACATTCT CAAATCATTT GGACTTCTTT   
  
  
- CAAGAGGGGT CACTGGTACT CTGTCTCATC TACCAATTTT TCGGGCTTTC TTTTTCCGTA TGGGGGAGGA   
  
  
- TTAGATTGAT TTGTCGCAGT GTCACTGGTT TTGTTTCAAT CAAGCTCTCT AGGAATAATT TATTAGGAAA   
  
  
- ATACTTGAAA AGAAGAGAAA GCAAGAAGAA AATGAAAATC AAGGATTACC ATAGGAGAAT AGCAAGCAAA   
  
  
- GGTCTATGAA GAGAAGATGT AACTCCATTG GTCACACTAA TATGTAGCAT GGAGAAAGAA AGCTGGAAGG   
  
  
- TAAAGGCAAA TTATAATAGC ATATCCACAA GTGATATATC CACTTGTGGT TGGACTCTGA ATCAAGCAGC   
  
  
- TGCTTCTGCT GGTGTGTTGA ACTCCTCCCA GGCTTCAATC CAAGTGACCA TGGCATCTGC AAGCTTCTCA   
  
  
- AAGTAAGGAT CAATATGACC CAATTTCTCT TTGACCTGCC TGGAGAGGGC AAGGGAGCAC TTCAGGGCTG   
  
  
- TTGTGCAGTC TTTTGGCAGG ACAACAGATT GGAACAATGG AATCAGCTGT TCCTGCCAGT ATAGACCCTT   
  
  
- GAATTCCTTC TTCAAGTTAG AAAACGGGT

+     TGACG-motif

| Site Name | Organism | Position | Strand | Matrix score. | sequence | function |
| --- | --- | --- | --- | --- | --- | --- |
| TGACG-motif | Hordeum vulgare | 1405 | - | 5 | TGACG | cis-acting regulatory element involved in the MeJA-responsiveness |
| TGACG-motif | Hordeum vulgare | 996 | - | 5 | TGACG | cis-acting regulatory element involved in the MeJA-responsiveness |

> 2018/04/13 10:10:12  
+ ATAACCTCGA TTGTTAGATT TGGCTCGAAA ATAAACAAAG GACATACTCC TTCTTGTTCT GTTTCAACTT   
  
  
+ TTCTCCTCTC CTCGTCCGAT CGAATAATAC GATTATCTTT ACCGTATTGT ACCCATCTTT TCCCACCCCC   
  
  
+ AATTACCTTT CCGCAGGTTG AATTGACTGA ACTGTCTTAA CTGGGTACAT TTCTCACTAA AGGTACCTAC   
  
  
+ GTAGGGGAGT GTTTCTAATT CTCATACACG GCATTTGCCG TGCGTTTAAC TGTTTGTCTT CCTACCAAAT   
  
  
+ CAAAGAACAG CTTTTCCAAC GGTTCTTATT TACTAAAATT CGGGTATTCA ATACTTTCAC CTGCTCGTAC   
  
  
+ CTTCAACTTG ACTACTTCTT CGGTACGGTT GGTCGAATTA GTATCTTAAT GAAGAACACA ATATAGACTT   
  
  
+ TATATCTACA TTGTAGTACC TGAAGGTACC TACTGATCGA GACCAACATC GAATCTCGAA CGAATTGCAT   
  
  
+ GAATTTGATG CTTCCTTTTC CTCATACGAA CGACAAATAA GAGTGTATCT TCGGTAGAAG TCGGTTAGTC   
  
  
+ GGTTATCGTA GTGAATTTGG CCATCCACTT AAAGTACGGA GCTCCAGTTT TTCGTCTATA AATTCAAAGT   
  
  
+ TGATTAGTTA ACTCACGTGT GAGGGGTGAA AGATGTACAT GTCAAGTAGC GTACTCGAAA TTGCGATTTA   
  
  
+ CTGAACCCGT ATGTATTCTC CTTAAAAGCG ACCCAAAAGG AGCCTCGAAG TTCCTTCTAG TTATAGAGTC   
  
  
+ TTTTTGTGAT GGACGACATC ACGTACCCTG TACAGCCGTA CCAAAATACC CCTATACTTC AGGATAACGA   
  
  
+ CAGACACCAA TTAATATTAC TAATTACTAC CATAATTCAT TTATGTAAGA GTTTAGTAAA CCTGAAGAAA   
  
  
+ GTTCTCCCCA GTGACCATGA GACAGAGTAG ATGGTTAAAA AGCCCGAAAG AAAAAGGCAT ACCCCCTCCT   
  
  
+ AATCTAACTA AACAGCGTCA CAGTGACCAA AACAAAGTTA GTTCGAGAGA TCCTTATTAA ATAATCCTTT   
  
  
+ TATGAACTTT TCTTCTCTTT CGTTCTTCTT TTACTTTTAG TTCCTAATGG TATCCTCTTA TCGTTCGTTT   
  
  
+ CCAGATACTT CTCTTCTACA TTGAGGTAAC CAGTGTGATT ATACATCGTA CCTCTTTCTT TCGACCTTCC   
  
  
+ ATTTCCGTTT AATATTATCG TATAGGTGTT CACTATATAG GTGAACACCA ACCTGAGACT TAGTTCGTCG   
  
  
+ ACGAAGACGA CCACACAACT TGAGGAGGGT CCGAAGTTAG GTTCACTGGT ACCGTAGACG TTCGAAGAGT   
  
  
+ TTCATTCCTA GTTATACTGG GTTAAAGAGA AACTGGACGG ACCTCTCCCG TTCCCTCGTG AAGTCCCGAC   
  
  
+ AACACGTCAG AAAACCGTCC TGTTGTCTAA CCTTGTTACC TTAGTCGACA AGGACGGTCA TATCTGGGAA   
  
  
+ CTTAAGGAAG AAGTTCAATC TTTTGCCCA  

- TATTGGAGCT AACAATCTAA ACCGAGCTTT TATTTGTTTC CTGTATGAGG AAGAACAAGA CAAAGTTGAA   
  
  
- AAGAGGAGAG GAGCAGGCTA GCTTATTATG CTAATAGAAA TGGCATAACA TGGGTAGAAA AGGGTGGGGG   
  
  
- TTAATGGAAA GGCGTCCAAC TTAACTGACT TGACAGAATT GACCCATGTA AAGAGTGATT TCCATGGATG   
  
  
- CATCCCCTCA CAAAGATTAA GAGTATGTGC CGTAAACGGC ACGCAAATTG ACAAACAGAA GGATGGTTTA   
  
  
- GTTTCTTGTC GAAAAGGTTG CCAAGAATAA ATGATTTTAA GCCCATAAGT TATGAAAGTG GACGAGCATG   
  
  
- GAAGTTGAAC TGATGAAGAA GCCATGCCAA CCAGCTTAAT CATAGAATTA CTTCTTGTGT TATATCTGAA   
  
  
- ATATAGATGT AACATCATGG ACTTCCATGG ATGACTAGCT CTGGTTGTAG CTTAGAGCTT GCTTAACGTA   
  
  
- CTTAAACTAC GAAGGAAAAG GAGTATGCTT GCTGTTTATT CTCACATAGA AGCCATCTTC AGCCAATCAG   
  
  
- CCAATAGCAT CACTTAAACC GGTAGGTGAA TTTCATGCCT CGAGGTCAAA AAGCAGATAT TTAAGTTTCA   
  
  
- ACTAATCAAT TGAGTGCACA CTCCCCACTT TCTACATGTA CAGTTCATCG CATGAGCTTT AACGCTAAAT   
  
  
- GACTTGGGCA TACATAAGAG GAATTTTCGC TGGGTTTTCC TCGGAGCTTC AAGGAAGATC AATATCTCAG   
  
  
- AAAAACACTA CCTGCTGTAG TGCATGGGAC ATGTCGGCAT GGTTTTATGG GGATATGAAG TCCTATTGCT   
  
  
- GTCTGTGGTT AATTATAATG ATTAATGATG GTATTAAGTA AATACATTCT CAAATCATTT GGACTTCTTT   
  
  
- CAAGAGGGGT CACTGGTACT CTGTCTCATC TACCAATTTT TCGGGCTTTC TTTTTCCGTA TGGGGGAGGA   
  
  
- TTAGATTGAT TTGTCGCAGT GTCACTGGTT TTGTTTCAAT CAAGCTCTCT AGGAATAATT TATTAGGAAA   
  
  
- ATACTTGAAA AGAAGAGAAA GCAAGAAGAA AATGAAAATC AAGGATTACC ATAGGAGAAT AGCAAGCAAA   
  
  
- GGTCTATGAA GAGAAGATGT AACTCCATTG GTCACACTAA TATGTAGCAT GGAGAAAGAA AGCTGGAAGG   
  
  
- TAAAGGCAAA TTATAATAGC ATATCCACAA GTGATATATC CACTTGTGGT TGGACTCTGA ATCAAGCAGC   
  
  
- TGCTTCTGCT GGTGTGTTGA ACTCCTCCCA GGCTTCAATC CAAGTGACCA TGGCATCTGC AAGCTTCTCA   
  
  
- AAGTAAGGAT CAATATGACC CAATTTCTCT TTGACCTGCC TGGAGAGGGC AAGGGAGCAC TTCAGGGCTG   
  
  
- TTGTGCAGTC TTTTGGCAGG ACAACAGATT GGAACAATGG AATCAGCTGT TCCTGCCAGT ATAGACCCTT   
  
  
- GAATTCCTTC TTCAAGTTAG AAAACGGGT

+     Unnamed\_\_15

| Site Name | Organism | Position | Strand | Matrix score. | sequence | function |
| --- | --- | --- | --- | --- | --- | --- |
| Unnamed\_\_15 | Zea mays | 1372 | + | 10 | CCTCTCCCGTC |  |

> 2018/04/13 10:10:12  
+ ATAACCTCGA TTGTTAGATT TGGCTCGAAA ATAAACAAAG GACATACTCC TTCTTGTTCT GTTTCAACTT   
  
  
+ TTCTCCTCTC CTCGTCCGAT CGAATAATAC GATTATCTTT ACCGTATTGT ACCCATCTTT TCCCACCCCC   
  
  
+ AATTACCTTT CCGCAGGTTG AATTGACTGA ACTGTCTTAA CTGGGTACAT TTCTCACTAA AGGTACCTAC   
  
  
+ GTAGGGGAGT GTTTCTAATT CTCATACACG GCATTTGCCG TGCGTTTAAC TGTTTGTCTT CCTACCAAAT   
  
  
+ CAAAGAACAG CTTTTCCAAC GGTTCTTATT TACTAAAATT CGGGTATTCA ATACTTTCAC CTGCTCGTAC   
  
  
+ CTTCAACTTG ACTACTTCTT CGGTACGGTT GGTCGAATTA GTATCTTAAT GAAGAACACA ATATAGACTT   
  
  
+ TATATCTACA TTGTAGTACC TGAAGGTACC TACTGATCGA GACCAACATC GAATCTCGAA CGAATTGCAT   
  
  
+ GAATTTGATG CTTCCTTTTC CTCATACGAA CGACAAATAA GAGTGTATCT TCGGTAGAAG TCGGTTAGTC   
  
  
+ GGTTATCGTA GTGAATTTGG CCATCCACTT AAAGTACGGA GCTCCAGTTT TTCGTCTATA AATTCAAAGT   
  
  
+ TGATTAGTTA ACTCACGTGT GAGGGGTGAA AGATGTACAT GTCAAGTAGC GTACTCGAAA TTGCGATTTA   
  
  
+ CTGAACCCGT ATGTATTCTC CTTAAAAGCG ACCCAAAAGG AGCCTCGAAG TTCCTTCTAG TTATAGAGTC   
  
  
+ TTTTTGTGAT GGACGACATC ACGTACCCTG TACAGCCGTA CCAAAATACC CCTATACTTC AGGATAACGA   
  
  
+ CAGACACCAA TTAATATTAC TAATTACTAC CATAATTCAT TTATGTAAGA GTTTAGTAAA CCTGAAGAAA   
  
  
+ GTTCTCCCCA GTGACCATGA GACAGAGTAG ATGGTTAAAA AGCCCGAAAG AAAAAGGCAT ACCCCCTCCT   
  
  
+ AATCTAACTA AACAGCGTCA CAGTGACCAA AACAAAGTTA GTTCGAGAGA TCCTTATTAA ATAATCCTTT   
  
  
+ TATGAACTTT TCTTCTCTTT CGTTCTTCTT TTACTTTTAG TTCCTAATGG TATCCTCTTA TCGTTCGTTT   
  
  
+ CCAGATACTT CTCTTCTACA TTGAGGTAAC CAGTGTGATT ATACATCGTA CCTCTTTCTT TCGACCTTCC   
  
  
+ ATTTCCGTTT AATATTATCG TATAGGTGTT CACTATATAG GTGAACACCA ACCTGAGACT TAGTTCGTCG   
  
  
+ ACGAAGACGA CCACACAACT TGAGGAGGGT CCGAAGTTAG GTTCACTGGT ACCGTAGACG TTCGAAGAGT   
  
  
+ TTCATTCCTA GTTATACTGG GTTAAAGAGA AACTGGACGG ACCTCTCCCG TTCCCTCGTG AAGTCCCGAC   
  
  
+ AACACGTCAG AAAACCGTCC TGTTGTCTAA CCTTGTTACC TTAGTCGACA AGGACGGTCA TATCTGGGAA   
  
  
+ CTTAAGGAAG AAGTTCAATC TTTTGCCCA  

- TATTGGAGCT AACAATCTAA ACCGAGCTTT TATTTGTTTC CTGTATGAGG AAGAACAAGA CAAAGTTGAA   
  
  
- AAGAGGAGAG GAGCAGGCTA GCTTATTATG CTAATAGAAA TGGCATAACA TGGGTAGAAA AGGGTGGGGG   
  
  
- TTAATGGAAA GGCGTCCAAC TTAACTGACT TGACAGAATT GACCCATGTA AAGAGTGATT TCCATGGATG   
  
  
- CATCCCCTCA CAAAGATTAA GAGTATGTGC CGTAAACGGC ACGCAAATTG ACAAACAGAA GGATGGTTTA   
  
  
- GTTTCTTGTC GAAAAGGTTG CCAAGAATAA ATGATTTTAA GCCCATAAGT TATGAAAGTG GACGAGCATG   
  
  
- GAAGTTGAAC TGATGAAGAA GCCATGCCAA CCAGCTTAAT CATAGAATTA CTTCTTGTGT TATATCTGAA   
  
  
- ATATAGATGT AACATCATGG ACTTCCATGG ATGACTAGCT CTGGTTGTAG CTTAGAGCTT GCTTAACGTA   
  
  
- CTTAAACTAC GAAGGAAAAG GAGTATGCTT GCTGTTTATT CTCACATAGA AGCCATCTTC AGCCAATCAG   
  
  
- CCAATAGCAT CACTTAAACC GGTAGGTGAA TTTCATGCCT CGAGGTCAAA AAGCAGATAT TTAAGTTTCA   
  
  
- ACTAATCAAT TGAGTGCACA CTCCCCACTT TCTACATGTA CAGTTCATCG CATGAGCTTT AACGCTAAAT   
  
  
- GACTTGGGCA TACATAAGAG GAATTTTCGC TGGGTTTTCC TCGGAGCTTC AAGGAAGATC AATATCTCAG   
  
  
- AAAAACACTA CCTGCTGTAG TGCATGGGAC ATGTCGGCAT GGTTTTATGG GGATATGAAG TCCTATTGCT   
  
  
- GTCTGTGGTT AATTATAATG ATTAATGATG GTATTAAGTA AATACATTCT CAAATCATTT GGACTTCTTT   
  
  
- CAAGAGGGGT CACTGGTACT CTGTCTCATC TACCAATTTT TCGGGCTTTC TTTTTCCGTA TGGGGGAGGA   
  
  
- TTAGATTGAT TTGTCGCAGT GTCACTGGTT TTGTTTCAAT CAAGCTCTCT AGGAATAATT TATTAGGAAA   
  
  
- ATACTTGAAA AGAAGAGAAA GCAAGAAGAA AATGAAAATC AAGGATTACC ATAGGAGAAT AGCAAGCAAA   
  
  
- GGTCTATGAA GAGAAGATGT AACTCCATTG GTCACACTAA TATGTAGCAT GGAGAAAGAA AGCTGGAAGG   
  
  
- TAAAGGCAAA TTATAATAGC ATATCCACAA GTGATATATC CACTTGTGGT TGGACTCTGA ATCAAGCAGC   
  
  
- TGCTTCTGCT GGTGTGTTGA ACTCCTCCCA GGCTTCAATC CAAGTGACCA TGGCATCTGC AAGCTTCTCA   
  
  
- AAGTAAGGAT CAATATGACC CAATTTCTCT TTGACCTGCC TGGAGAGGGC AAGGGAGCAC TTCAGGGCTG   
  
  
- TTGTGCAGTC TTTTGGCAGG ACAACAGATT GGAACAATGG AATCAGCTGT TCCTGCCAGT ATAGACCCTT   
  
  
- GAATTCCTTC TTCAAGTTAG AAAACGGGT

+     Unnamed\_\_2

| Site Name | Organism | Position | Strand | Matrix score. | sequence | function |
| --- | --- | --- | --- | --- | --- | --- |
| Unnamed\_\_2 | Petroselinum hortense | 1294 | - | 9 | AACCTAACCT |  |

> 2018/04/13 10:10:12  
+ ATAACCTCGA TTGTTAGATT TGGCTCGAAA ATAAACAAAG GACATACTCC TTCTTGTTCT GTTTCAACTT   
  
  
+ TTCTCCTCTC CTCGTCCGAT CGAATAATAC GATTATCTTT ACCGTATTGT ACCCATCTTT TCCCACCCCC   
  
  
+ AATTACCTTT CCGCAGGTTG AATTGACTGA ACTGTCTTAA CTGGGTACAT TTCTCACTAA AGGTACCTAC   
  
  
+ GTAGGGGAGT GTTTCTAATT CTCATACACG GCATTTGCCG TGCGTTTAAC TGTTTGTCTT CCTACCAAAT   
  
  
+ CAAAGAACAG CTTTTCCAAC GGTTCTTATT TACTAAAATT CGGGTATTCA ATACTTTCAC CTGCTCGTAC   
  
  
+ CTTCAACTTG ACTACTTCTT CGGTACGGTT GGTCGAATTA GTATCTTAAT GAAGAACACA ATATAGACTT   
  
  
+ TATATCTACA TTGTAGTACC TGAAGGTACC TACTGATCGA GACCAACATC GAATCTCGAA CGAATTGCAT   
  
  
+ GAATTTGATG CTTCCTTTTC CTCATACGAA CGACAAATAA GAGTGTATCT TCGGTAGAAG TCGGTTAGTC   
  
  
+ GGTTATCGTA GTGAATTTGG CCATCCACTT AAAGTACGGA GCTCCAGTTT TTCGTCTATA AATTCAAAGT   
  
  
+ TGATTAGTTA ACTCACGTGT GAGGGGTGAA AGATGTACAT GTCAAGTAGC GTACTCGAAA TTGCGATTTA   
  
  
+ CTGAACCCGT ATGTATTCTC CTTAAAAGCG ACCCAAAAGG AGCCTCGAAG TTCCTTCTAG TTATAGAGTC   
  
  
+ TTTTTGTGAT GGACGACATC ACGTACCCTG TACAGCCGTA CCAAAATACC CCTATACTTC AGGATAACGA   
  
  
+ CAGACACCAA TTAATATTAC TAATTACTAC CATAATTCAT TTATGTAAGA GTTTAGTAAA CCTGAAGAAA   
  
  
+ GTTCTCCCCA GTGACCATGA GACAGAGTAG ATGGTTAAAA AGCCCGAAAG AAAAAGGCAT ACCCCCTCCT   
  
  
+ AATCTAACTA AACAGCGTCA CAGTGACCAA AACAAAGTTA GTTCGAGAGA TCCTTATTAA ATAATCCTTT   
  
  
+ TATGAACTTT TCTTCTCTTT CGTTCTTCTT TTACTTTTAG TTCCTAATGG TATCCTCTTA TCGTTCGTTT   
  
  
+ CCAGATACTT CTCTTCTACA TTGAGGTAAC CAGTGTGATT ATACATCGTA CCTCTTTCTT TCGACCTTCC   
  
  
+ ATTTCCGTTT AATATTATCG TATAGGTGTT CACTATATAG GTGAACACCA ACCTGAGACT TAGTTCGTCG   
  
  
+ ACGAAGACGA CCACACAACT TGAGGAGGGT CCGAAGTTAG GTTCACTGGT ACCGTAGACG TTCGAAGAGT   
  
  
+ TTCATTCCTA GTTATACTGG GTTAAAGAGA AACTGGACGG ACCTCTCCCG TTCCCTCGTG AAGTCCCGAC   
  
  
+ AACACGTCAG AAAACCGTCC TGTTGTCTAA CCTTGTTACC TTAGTCGACA AGGACGGTCA TATCTGGGAA   
  
  
+ CTTAAGGAAG AAGTTCAATC TTTTGCCCA  

- TATTGGAGCT AACAATCTAA ACCGAGCTTT TATTTGTTTC CTGTATGAGG AAGAACAAGA CAAAGTTGAA   
  
  
- AAGAGGAGAG GAGCAGGCTA GCTTATTATG CTAATAGAAA TGGCATAACA TGGGTAGAAA AGGGTGGGGG   
  
  
- TTAATGGAAA GGCGTCCAAC TTAACTGACT TGACAGAATT GACCCATGTA AAGAGTGATT TCCATGGATG   
  
  
- CATCCCCTCA CAAAGATTAA GAGTATGTGC CGTAAACGGC ACGCAAATTG ACAAACAGAA GGATGGTTTA   
  
  
- GTTTCTTGTC GAAAAGGTTG CCAAGAATAA ATGATTTTAA GCCCATAAGT TATGAAAGTG GACGAGCATG   
  
  
- GAAGTTGAAC TGATGAAGAA GCCATGCCAA CCAGCTTAAT CATAGAATTA CTTCTTGTGT TATATCTGAA   
  
  
- ATATAGATGT AACATCATGG ACTTCCATGG ATGACTAGCT CTGGTTGTAG CTTAGAGCTT GCTTAACGTA   
  
  
- CTTAAACTAC GAAGGAAAAG GAGTATGCTT GCTGTTTATT CTCACATAGA AGCCATCTTC AGCCAATCAG   
  
  
- CCAATAGCAT CACTTAAACC GGTAGGTGAA TTTCATGCCT CGAGGTCAAA AAGCAGATAT TTAAGTTTCA   
  
  
- ACTAATCAAT TGAGTGCACA CTCCCCACTT TCTACATGTA CAGTTCATCG CATGAGCTTT AACGCTAAAT   
  
  
- GACTTGGGCA TACATAAGAG GAATTTTCGC TGGGTTTTCC TCGGAGCTTC AAGGAAGATC AATATCTCAG   
  
  
- AAAAACACTA CCTGCTGTAG TGCATGGGAC ATGTCGGCAT GGTTTTATGG GGATATGAAG TCCTATTGCT   
  
  
- GTCTGTGGTT AATTATAATG ATTAATGATG GTATTAAGTA AATACATTCT CAAATCATTT GGACTTCTTT   
  
  
- CAAGAGGGGT CACTGGTACT CTGTCTCATC TACCAATTTT TCGGGCTTTC TTTTTCCGTA TGGGGGAGGA   
  
  
- TTAGATTGAT TTGTCGCAGT GTCACTGGTT TTGTTTCAAT CAAGCTCTCT AGGAATAATT TATTAGGAAA   
  
  
- ATACTTGAAA AGAAGAGAAA GCAAGAAGAA AATGAAAATC AAGGATTACC ATAGGAGAAT AGCAAGCAAA   
  
  
- GGTCTATGAA GAGAAGATGT AACTCCATTG GTCACACTAA TATGTAGCAT GGAGAAAGAA AGCTGGAAGG   
  
  
- TAAAGGCAAA TTATAATAGC ATATCCACAA GTGATATATC CACTTGTGGT TGGACTCTGA ATCAAGCAGC   
  
  
- TGCTTCTGCT GGTGTGTTGA ACTCCTCCCA GGCTTCAATC CAAGTGACCA TGGCATCTGC AAGCTTCTCA   
  
  
- AAGTAAGGAT CAATATGACC CAATTTCTCT TTGACCTGCC TGGAGAGGGC AAGGGAGCAC TTCAGGGCTG   
  
  
- TTGTGCAGTC TTTTGGCAGG ACAACAGATT GGAACAATGG AATCAGCTGT TCCTGCCAGT ATAGACCCTT   
  
  
- GAATTCCTTC TTCAAGTTAG AAAACGGGT

+     Unnamed\_\_4

| Site Name | Organism | Position | Strand | Matrix score. | sequence | function |
| --- | --- | --- | --- | --- | --- | --- |
| Unnamed\_\_4 | Petroselinum hortense | 976 | + | 4 | CTCC |  |
| Unnamed\_\_4 | Petroselinum hortense | 602 | + | 4 | CTCC |  |
| Unnamed\_\_4 | Petroselinum hortense | 598 | - | 4 | CTCC |  |
| Unnamed\_\_4 | Petroselinum hortense | 78 | + | 4 | CTCC |  |
| Unnamed\_\_4 | Petroselinum hortense | 216 | - | 4 | CTCC |  |
| Unnamed\_\_4 | Petroselinum hortense | 73 | + | 4 | CTCC |  |
| Unnamed\_\_4 | Petroselinum hortense | 47 | + | 4 | CTCC |  |
| Unnamed\_\_4 | Petroselinum hortense | 914 | + | 4 | CTCC |  |
| Unnamed\_\_4 | Petroselinum hortense | 739 | - | 4 | CTCC |  |
| Unnamed\_\_4 | Petroselinum hortense | 1375 | + | 4 | CTCC |  |
| Unnamed\_\_4 | Petroselinum hortense | 718 | + | 4 | CTCC |  |
| Unnamed\_\_4 | Petroselinum hortense | 1284 | - | 4 | CTCC |  |

> 2018/04/13 10:10:12  
+ ATAACCTCGA TTGTTAGATT TGGCTCGAAA ATAAACAAAG GACATACTCC TTCTTGTTCT GTTTCAACTT   
  
  
+ TTCTCCTCTC CTCGTCCGAT CGAATAATAC GATTATCTTT ACCGTATTGT ACCCATCTTT TCCCACCCCC   
  
  
+ AATTACCTTT CCGCAGGTTG AATTGACTGA ACTGTCTTAA CTGGGTACAT TTCTCACTAA AGGTACCTAC   
  
  
+ GTAGGGGAGT GTTTCTAATT CTCATACACG GCATTTGCCG TGCGTTTAAC TGTTTGTCTT CCTACCAAAT   
  
  
+ CAAAGAACAG CTTTTCCAAC GGTTCTTATT TACTAAAATT CGGGTATTCA ATACTTTCAC CTGCTCGTAC   
  
  
+ CTTCAACTTG ACTACTTCTT CGGTACGGTT GGTCGAATTA GTATCTTAAT GAAGAACACA ATATAGACTT   
  
  
+ TATATCTACA TTGTAGTACC TGAAGGTACC TACTGATCGA GACCAACATC GAATCTCGAA CGAATTGCAT   
  
  
+ GAATTTGATG CTTCCTTTTC CTCATACGAA CGACAAATAA GAGTGTATCT TCGGTAGAAG TCGGTTAGTC   
  
  
+ GGTTATCGTA GTGAATTTGG CCATCCACTT AAAGTACGGA GCTCCAGTTT TTCGTCTATA AATTCAAAGT   
  
  
+ TGATTAGTTA ACTCACGTGT GAGGGGTGAA AGATGTACAT GTCAAGTAGC GTACTCGAAA TTGCGATTTA   
  
  
+ CTGAACCCGT ATGTATTCTC CTTAAAAGCG ACCCAAAAGG AGCCTCGAAG TTCCTTCTAG TTATAGAGTC   
  
  
+ TTTTTGTGAT GGACGACATC ACGTACCCTG TACAGCCGTA CCAAAATACC CCTATACTTC AGGATAACGA   
  
  
+ CAGACACCAA TTAATATTAC TAATTACTAC CATAATTCAT TTATGTAAGA GTTTAGTAAA CCTGAAGAAA   
  
  
+ GTTCTCCCCA GTGACCATGA GACAGAGTAG ATGGTTAAAA AGCCCGAAAG AAAAAGGCAT ACCCCCTCCT   
  
  
+ AATCTAACTA AACAGCGTCA CAGTGACCAA AACAAAGTTA GTTCGAGAGA TCCTTATTAA ATAATCCTTT   
  
  
+ TATGAACTTT TCTTCTCTTT CGTTCTTCTT TTACTTTTAG TTCCTAATGG TATCCTCTTA TCGTTCGTTT   
  
  
+ CCAGATACTT CTCTTCTACA TTGAGGTAAC CAGTGTGATT ATACATCGTA CCTCTTTCTT TCGACCTTCC   
  
  
+ ATTTCCGTTT AATATTATCG TATAGGTGTT CACTATATAG GTGAACACCA ACCTGAGACT TAGTTCGTCG   
  
  
+ ACGAAGACGA CCACACAACT TGAGGAGGGT CCGAAGTTAG GTTCACTGGT ACCGTAGACG TTCGAAGAGT   
  
  
+ TTCATTCCTA GTTATACTGG GTTAAAGAGA AACTGGACGG ACCTCTCCCG TTCCCTCGTG AAGTCCCGAC   
  
  
+ AACACGTCAG AAAACCGTCC TGTTGTCTAA CCTTGTTACC TTAGTCGACA AGGACGGTCA TATCTGGGAA   
  
  
+ CTTAAGGAAG AAGTTCAATC TTTTGCCCA  

- TATTGGAGCT AACAATCTAA ACCGAGCTTT TATTTGTTTC CTGTATGAGG AAGAACAAGA CAAAGTTGAA   
  
  
- AAGAGGAGAG GAGCAGGCTA GCTTATTATG CTAATAGAAA TGGCATAACA TGGGTAGAAA AGGGTGGGGG   
  
  
- TTAATGGAAA GGCGTCCAAC TTAACTGACT TGACAGAATT GACCCATGTA AAGAGTGATT TCCATGGATG   
  
  
- CATCCCCTCA CAAAGATTAA GAGTATGTGC CGTAAACGGC ACGCAAATTG ACAAACAGAA GGATGGTTTA   
  
  
- GTTTCTTGTC GAAAAGGTTG CCAAGAATAA ATGATTTTAA GCCCATAAGT TATGAAAGTG GACGAGCATG   
  
  
- GAAGTTGAAC TGATGAAGAA GCCATGCCAA CCAGCTTAAT CATAGAATTA CTTCTTGTGT TATATCTGAA   
  
  
- ATATAGATGT AACATCATGG ACTTCCATGG ATGACTAGCT CTGGTTGTAG CTTAGAGCTT GCTTAACGTA   
  
  
- CTTAAACTAC GAAGGAAAAG GAGTATGCTT GCTGTTTATT CTCACATAGA AGCCATCTTC AGCCAATCAG   
  
  
- CCAATAGCAT CACTTAAACC GGTAGGTGAA TTTCATGCCT CGAGGTCAAA AAGCAGATAT TTAAGTTTCA   
  
  
- ACTAATCAAT TGAGTGCACA CTCCCCACTT TCTACATGTA CAGTTCATCG CATGAGCTTT AACGCTAAAT   
  
  
- GACTTGGGCA TACATAAGAG GAATTTTCGC TGGGTTTTCC TCGGAGCTTC AAGGAAGATC AATATCTCAG   
  
  
- AAAAACACTA CCTGCTGTAG TGCATGGGAC ATGTCGGCAT GGTTTTATGG GGATATGAAG TCCTATTGCT   
  
  
- GTCTGTGGTT AATTATAATG ATTAATGATG GTATTAAGTA AATACATTCT CAAATCATTT GGACTTCTTT   
  
  
- CAAGAGGGGT CACTGGTACT CTGTCTCATC TACCAATTTT TCGGGCTTTC TTTTTCCGTA TGGGGGAGGA   
  
  
- TTAGATTGAT TTGTCGCAGT GTCACTGGTT TTGTTTCAAT CAAGCTCTCT AGGAATAATT TATTAGGAAA   
  
  
- ATACTTGAAA AGAAGAGAAA GCAAGAAGAA AATGAAAATC AAGGATTACC ATAGGAGAAT AGCAAGCAAA   
  
  
- GGTCTATGAA GAGAAGATGT AACTCCATTG GTCACACTAA TATGTAGCAT GGAGAAAGAA AGCTGGAAGG   
  
  
- TAAAGGCAAA TTATAATAGC ATATCCACAA GTGATATATC CACTTGTGGT TGGACTCTGA ATCAAGCAGC   
  
  
- TGCTTCTGCT GGTGTGTTGA ACTCCTCCCA GGCTTCAATC CAAGTGACCA TGGCATCTGC AAGCTTCTCA   
  
  
- AAGTAAGGAT CAATATGACC CAATTTCTCT TTGACCTGCC TGGAGAGGGC AAGGGAGCAC TTCAGGGCTG   
  
  
- TTGTGCAGTC TTTTGGCAGG ACAACAGATT GGAACAATGG AATCAGCTGT TCCTGCCAGT ATAGACCCTT   
  
  
- GAATTCCTTC TTCAAGTTAG AAAACGGGT

+     rbcS-CMA7a

| Site Name | Organism | Position | Strand | Matrix score. | sequence | function |
| --- | --- | --- | --- | --- | --- | --- |
| rbcS-CMA7a | Lemna gibba | 1444 | + | 9 | GTCGATAAGG | part of a light responsive element |

> 2018/04/13 10:10:12  
+ ATAACCTCGA TTGTTAGATT TGGCTCGAAA ATAAACAAAG GACATACTCC TTCTTGTTCT GTTTCAACTT   
  
  
+ TTCTCCTCTC CTCGTCCGAT CGAATAATAC GATTATCTTT ACCGTATTGT ACCCATCTTT TCCCACCCCC   
  
  
+ AATTACCTTT CCGCAGGTTG AATTGACTGA ACTGTCTTAA CTGGGTACAT TTCTCACTAA AGGTACCTAC   
  
  
+ GTAGGGGAGT GTTTCTAATT CTCATACACG GCATTTGCCG TGCGTTTAAC TGTTTGTCTT CCTACCAAAT   
  
  
+ CAAAGAACAG CTTTTCCAAC GGTTCTTATT TACTAAAATT CGGGTATTCA ATACTTTCAC CTGCTCGTAC   
  
  
+ CTTCAACTTG ACTACTTCTT CGGTACGGTT GGTCGAATTA GTATCTTAAT GAAGAACACA ATATAGACTT   
  
  
+ TATATCTACA TTGTAGTACC TGAAGGTACC TACTGATCGA GACCAACATC GAATCTCGAA CGAATTGCAT   
  
  
+ GAATTTGATG CTTCCTTTTC CTCATACGAA CGACAAATAA GAGTGTATCT TCGGTAGAAG TCGGTTAGTC   
  
  
+ GGTTATCGTA GTGAATTTGG CCATCCACTT AAAGTACGGA GCTCCAGTTT TTCGTCTATA AATTCAAAGT   
  
  
+ TGATTAGTTA ACTCACGTGT GAGGGGTGAA AGATGTACAT GTCAAGTAGC GTACTCGAAA TTGCGATTTA   
  
  
+ CTGAACCCGT ATGTATTCTC CTTAAAAGCG ACCCAAAAGG AGCCTCGAAG TTCCTTCTAG TTATAGAGTC   
  
  
+ TTTTTGTGAT GGACGACATC ACGTACCCTG TACAGCCGTA CCAAAATACC CCTATACTTC AGGATAACGA   
  
  
+ CAGACACCAA TTAATATTAC TAATTACTAC CATAATTCAT TTATGTAAGA GTTTAGTAAA CCTGAAGAAA   
  
  
+ GTTCTCCCCA GTGACCATGA GACAGAGTAG ATGGTTAAAA AGCCCGAAAG AAAAAGGCAT ACCCCCTCCT   
  
  
+ AATCTAACTA AACAGCGTCA CAGTGACCAA AACAAAGTTA GTTCGAGAGA TCCTTATTAA ATAATCCTTT   
  
  
+ TATGAACTTT TCTTCTCTTT CGTTCTTCTT TTACTTTTAG TTCCTAATGG TATCCTCTTA TCGTTCGTTT   
  
  
+ CCAGATACTT CTCTTCTACA TTGAGGTAAC CAGTGTGATT ATACATCGTA CCTCTTTCTT TCGACCTTCC   
  
  
+ ATTTCCGTTT AATATTATCG TATAGGTGTT CACTATATAG GTGAACACCA ACCTGAGACT TAGTTCGTCG   
  
  
+ ACGAAGACGA CCACACAACT TGAGGAGGGT CCGAAGTTAG GTTCACTGGT ACCGTAGACG TTCGAAGAGT   
  
  
+ TTCATTCCTA GTTATACTGG GTTAAAGAGA AACTGGACGG ACCTCTCCCG TTCCCTCGTG AAGTCCCGAC   
  
  
+ AACACGTCAG AAAACCGTCC TGTTGTCTAA CCTTGTTACC TTAGTCGACA AGGACGGTCA TATCTGGGAA   
  
  
+ CTTAAGGAAG AAGTTCAATC TTTTGCCCA  

- TATTGGAGCT AACAATCTAA ACCGAGCTTT TATTTGTTTC CTGTATGAGG AAGAACAAGA CAAAGTTGAA   
  
  
- AAGAGGAGAG GAGCAGGCTA GCTTATTATG CTAATAGAAA TGGCATAACA TGGGTAGAAA AGGGTGGGGG   
  
  
- TTAATGGAAA GGCGTCCAAC TTAACTGACT TGACAGAATT GACCCATGTA AAGAGTGATT TCCATGGATG   
  
  
- CATCCCCTCA CAAAGATTAA GAGTATGTGC CGTAAACGGC ACGCAAATTG ACAAACAGAA GGATGGTTTA   
  
  
- GTTTCTTGTC GAAAAGGTTG CCAAGAATAA ATGATTTTAA GCCCATAAGT TATGAAAGTG GACGAGCATG   
  
  
- GAAGTTGAAC TGATGAAGAA GCCATGCCAA CCAGCTTAAT CATAGAATTA CTTCTTGTGT TATATCTGAA   
  
  
- ATATAGATGT AACATCATGG ACTTCCATGG ATGACTAGCT CTGGTTGTAG CTTAGAGCTT GCTTAACGTA   
  
  
- CTTAAACTAC GAAGGAAAAG GAGTATGCTT GCTGTTTATT CTCACATAGA AGCCATCTTC AGCCAATCAG   
  
  
- CCAATAGCAT CACTTAAACC GGTAGGTGAA TTTCATGCCT CGAGGTCAAA AAGCAGATAT TTAAGTTTCA   
  
  
- ACTAATCAAT TGAGTGCACA CTCCCCACTT TCTACATGTA CAGTTCATCG CATGAGCTTT AACGCTAAAT   
  
  
- GACTTGGGCA TACATAAGAG GAATTTTCGC TGGGTTTTCC TCGGAGCTTC AAGGAAGATC AATATCTCAG   
  
  
- AAAAACACTA CCTGCTGTAG TGCATGGGAC ATGTCGGCAT GGTTTTATGG GGATATGAAG TCCTATTGCT   
  
  
- GTCTGTGGTT AATTATAATG ATTAATGATG GTATTAAGTA AATACATTCT CAAATCATTT GGACTTCTTT   
  
  
- CAAGAGGGGT CACTGGTACT CTGTCTCATC TACCAATTTT TCGGGCTTTC TTTTTCCGTA TGGGGGAGGA   
  
  
- TTAGATTGAT TTGTCGCAGT GTCACTGGTT TTGTTTCAAT CAAGCTCTCT AGGAATAATT TATTAGGAAA   
  
  
- ATACTTGAAA AGAAGAGAAA GCAAGAAGAA AATGAAAATC AAGGATTACC ATAGGAGAAT AGCAAGCAAA   
  
  
- GGTCTATGAA GAGAAGATGT AACTCCATTG GTCACACTAA TATGTAGCAT GGAGAAAGAA AGCTGGAAGG   
  
  
- TAAAGGCAAA TTATAATAGC ATATCCACAA GTGATATATC CACTTGTGGT TGGACTCTGA ATCAAGCAGC   
  
  
- TGCTTCTGCT GGTGTGTTGA ACTCCTCCCA GGCTTCAATC CAAGTGACCA TGGCATCTGC AAGCTTCTCA   
  
  
- AAGTAAGGAT CAATATGACC CAATTTCTCT TTGACCTGCC TGGAGAGGGC AAGGGAGCAC TTCAGGGCTG   
  
  
- TTGTGCAGTC TTTTGGCAGG ACAACAGATT GGAACAATGG AATCAGCTGT TCCTGCCAGT ATAGACCCTT   
  
  
- GAATTCCTTC TTCAAGTTAG AAAACGGGT
