# Supplementary material for: Genome-wide analysis of the WRKY gene family in drumstick (Moringa oleifera Lam.)
Source: PeerJ. 2019 Jun 10;7:e7063. doi: 10.7717/peerj.7063 (PMC6563795; doi:10.7717/peerj.7063)
Supplement: Supplemental Information 1 [file peerj-07-7063-s003.gz › MoWRKY12_plantcare.html]

Content-Type: text/html; charset=ISO-8859-1


CallMat\_Firefox


Webmaster Firefox specific output  
To save the result:
click on the frame with the right mouse button and save the source code as a text file with extension .html  
REFERENCE:PlantCARE: a database of plant cis-acting regulatory elements and a portal to tools for in silico analysis of promoter sequences.  
Lescot, M., Déhais, P., Moreau, Y., De Moor, B., Rouzé ,P.,and Rombauts, S.  
Nucleic Acids Res., Database issue(2002), 30(1):325-327.   


---

> 2018/04/13 10:10:12  
+ GCTTCTTATG ATTCTTTCTT CTTCTTTACT TAGCCTTTCG TGGCTACTTT CAATCTTTCT CTAATCTCTC   
  
  
+ GAAGCCGATT CTAGAAACAA AAGGAGATCA CTCTTTCTCA AAACAAAGCT ACCCCAAGAA CAAAGCGAAA   
  
  
+ AGAAAGAGAG ACTCTCTCTC TCTCTCTTGG TCTTTTCCCC ATCCAAACCG AGGAGAGGTG AGGAAATATA   
  
  
+ AATGAAAAAA AAAAAAAGGG AAGCAATCAC CGGTTTTATA AGAAGTTTGT TGGGTTTTTA ACTCGAGACT   
  
  
+ AAACAATTTA AACTAATTTT TTATTTTTTA TAGAAGATAT TAAACACTGA AAAAAATGAA ATAAAATTTT   
  
  
+ GATCTGTACC AATATGAAGT ATTTTCTGTT AATTTTTAAT TTTTTTTGTT TTTTGTCAAA TTCGGGATCG   
  
  
+ CTACTTCCAT TTCTGTCCCT GTATGGGAAA AAAGAAGTAT AATTCTTTTT ATTTTTATTA ATTATTTTTT   
  
  
+ ATTAATCGAG AAATTAACGT ACTATTCAAA GACAAATTTA GGCTTAACCG ATTTTCTTAA CCCACTTTTG   
  
  
+ TCCCGTACCG TACTTGGTAC TCGCTCTTCC GAACCAGGCC AGGTAGCCAG CGTCCGTCAG TGGCAGTGCA   
  
  
+ CCTGTTCCGC GGTATTCTTT CCCCAACCAG TTACAAAAGT TGAGATGCCA ATAATAAGAG AAAAACCTCC   
  
  
+ TAATCCGATT GTGCACAGCA CCCAGCCCGT GGCAGTAAAC TGGCTTGCAC CGTGCACCCT CGGGTAGAGG   
  
  
+ ACAACCAAAA GGTGCCCGTT AATAACACCT GCAATCGCAT GTTTATAGGT GTAAACTGAG AAGACAGACA   
  
  
+ TGTAAAGATG TACGCAACGA CTGAGTCGAA AGCGCAGTAG GGTAGATCAA GGGCAATGAG AATTCAAACC   
  
  
+ ACCATGTGAG TTTTTTATTA TTATTATTAT TAATAATAAT TTAATTAGGC ATGTATTTAT CCGTTCTAGC   
  
  
+ ACTAATAAAA TATTATCGTA AAATAAACGA TAAGTTATTT CATTGCTACT ATTTACGAAT CACACTTTGT   
  
  
+ AAATATTACT ACTAGGGACT TGGTTGTATT AGTATGACTC TACTAATATA AAATACATAT TATTTGTAGT   
  
  
+ TTTATTAAAA ATACAATTAA CACAAAAAAT AATTTAAAAA ATAATAAAAA TCTACTTAAA ATATGTATTT   
  
  
+ TATAATTTAA TCAAGTTTAA TAATAACAAA AATAAAAAAA TTTTTTAATA TTAAGTTTAA ATTAATAACA   
  
  
+ AAAATAAAAA ATTTTTTTAA TATTAAGTTT AAATTCTAAA AAATATTATT TAAAAAATTA AAATTAAAAA   
  
  
+ AATGTTTCTA TTTCTTATAA GTTTAATATT ACTGTTTTTT TAGTTTAAAA AATTTACGAA TTTCACGAAA   
  
  
+ AATATTTTAA AATGCAATAA TTTTTAAATT TTAATTAAGT ATTTTTTATA AAAATCGTCA TTTTATTAAA   
  
  
+ ACATAAAAAA AGCAAACTTA TAGAAAAAT  

- CGAAGAATAC TAAGAAAGAA GAAGAAATGA ATCGGAAAGC ACCGATGAAA GTTAGAAAGA GATTAGAGAG   
  
  
- CTTCGGCTAA GATCTTTGTT TTCCTCTAGT GAGAAAGAGT TTTGTTTCGA TGGGGTTCTT GTTTCGCTTT   
  
  
- TCTTTCTCTC TGAGAGAGAG AGAGAGAACC AGAAAAGGGG TAGGTTTGGC TCCTCTCCAC TCCTTTATAT   
  
  
- TTACTTTTTT TTTTTTTCCC TTCGTTAGTG GCCAAAATAT TCTTCAAACA ACCCAAAAAT TGAGCTCTGA   
  
  
- TTTGTTAAAT TTGATTAAAA AATAAAAAAT ATCTTCTATA ATTTGTGACT TTTTTTACTT TATTTTAAAA   
  
  
- CTAGACATGG TTATACTTCA TAAAAGACAA TTAAAAATTA AAAAAAACAA AAAACAGTTT AAGCCCTAGC   
  
  
- GATGAAGGTA AAGACAGGGA CATACCCTTT TTTCTTCATA TTAAGAAAAA TAAAAATAAT TAATAAAAAA   
  
  
- TAATTAGCTC TTTAATTGCA TGATAAGTTT CTGTTTAAAT CCGAATTGGC TAAAAGAATT GGGTGAAAAC   
  
  
- AGGGCATGGC ATGAACCATG AGCGAGAAGG CTTGGTCCGG TCCATCGGTC GCAGGCAGTC ACCGTCACGT   
  
  
- GGACAAGGCG CCATAAGAAA GGGGTTGGTC AATGTTTTCA ACTCTACGGT TATTATTCTC TTTTTGGAGG   
  
  
- ATTAGGCTAA CACGTGTCGT GGGTCGGGCA CCGTCATTTG ACCGAACGTG GCACGTGGGA GCCCATCTCC   
  
  
- TGTTGGTTTT CCACGGGCAA TTATTGTGGA CGTTAGCGTA CAAATATCCA CATTTGACTC TTCTGTCTGT   
  
  
- ACATTTCTAC ATGCGTTGCT GACTCAGCTT TCGCGTCATC CCATCTAGTT CCCGTTACTC TTAAGTTTGG   
  
  
- TGGTACACTC AAAAAATAAT AATAATAATA ATTATTATTA AATTAATCCG TACATAAATA GGCAAGATCG   
  
  
- TGATTATTTT ATAATAGCAT TTTATTTGCT ATTCAATAAA GTAACGATGA TAAATGCTTA GTGTGAAACA   
  
  
- TTTATAATGA TGATCCCTGA ACCAACATAA TCATACTGAG ATGATTATAT TTTATGTATA ATAAACATCA   
  
  
- AAATAATTTT TATGTTAATT GTGTTTTTTA TTAAATTTTT TATTATTTTT AGATGAATTT TATACATAAA   
  
  
- ATATTAAATT AGTTCAAATT ATTATTGTTT TTATTTTTTT AAAAAATTAT AATTCAAATT TAATTATTGT   
  
  
- TTTTATTTTT TAAAAAAATT ATAATTCAAA TTTAAGATTT TTTATAATAA ATTTTTTAAT TTTAATTTTT   
  
  
- TTACAAAGAT AAAGAATATT CAAATTATAA TGACAAAAAA ATCAAATTTT TTAAATGCTT AAAGTGCTTT   
  
  
- TTATAAAATT TTACGTTATT AAAAATTTAA AATTAATTCA TAAAAAATAT TTTTAGCAGT AAAATAATTT   
  
  
- TGTATTTTTT TCGTTTGAAT ATCTTTTTA

  
  
Motifs Found  

+     5UTR Py-rich stretch

| Site Name | Organism | Position | Strand | Matrix score. | sequence | function |
| --- | --- | --- | --- | --- | --- | --- |
| 5UTR Py-rich stretch | Lycopersicon esculentum | 153 | + | 13 | TTTCTCTCTCTCTC | cis-acting element conferring high transcription levels |

> 2018/04/13 10:10:12  
+ GCTTCTTATG ATTCTTTCTT CTTCTTTACT TAGCCTTTCG TGGCTACTTT CAATCTTTCT CTAATCTCTC   
  
  
+ GAAGCCGATT CTAGAAACAA AAGGAGATCA CTCTTTCTCA AAACAAAGCT ACCCCAAGAA CAAAGCGAAA   
  
  
+ AGAAAGAGAG ACTCTCTCTC TCTCTCTTGG TCTTTTCCCC ATCCAAACCG AGGAGAGGTG AGGAAATATA   
  
  
+ AATGAAAAAA AAAAAAAGGG AAGCAATCAC CGGTTTTATA AGAAGTTTGT TGGGTTTTTA ACTCGAGACT   
  
  
+ AAACAATTTA AACTAATTTT TTATTTTTTA TAGAAGATAT TAAACACTGA AAAAAATGAA ATAAAATTTT   
  
  
+ GATCTGTACC AATATGAAGT ATTTTCTGTT AATTTTTAAT TTTTTTTGTT TTTTGTCAAA TTCGGGATCG   
  
  
+ CTACTTCCAT TTCTGTCCCT GTATGGGAAA AAAGAAGTAT AATTCTTTTT ATTTTTATTA ATTATTTTTT   
  
  
+ ATTAATCGAG AAATTAACGT ACTATTCAAA GACAAATTTA GGCTTAACCG ATTTTCTTAA CCCACTTTTG   
  
  
+ TCCCGTACCG TACTTGGTAC TCGCTCTTCC GAACCAGGCC AGGTAGCCAG CGTCCGTCAG TGGCAGTGCA   
  
  
+ CCTGTTCCGC GGTATTCTTT CCCCAACCAG TTACAAAAGT TGAGATGCCA ATAATAAGAG AAAAACCTCC   
  
  
+ TAATCCGATT GTGCACAGCA CCCAGCCCGT GGCAGTAAAC TGGCTTGCAC CGTGCACCCT CGGGTAGAGG   
  
  
+ ACAACCAAAA GGTGCCCGTT AATAACACCT GCAATCGCAT GTTTATAGGT GTAAACTGAG AAGACAGACA   
  
  
+ TGTAAAGATG TACGCAACGA CTGAGTCGAA AGCGCAGTAG GGTAGATCAA GGGCAATGAG AATTCAAACC   
  
  
+ ACCATGTGAG TTTTTTATTA TTATTATTAT TAATAATAAT TTAATTAGGC ATGTATTTAT CCGTTCTAGC   
  
  
+ ACTAATAAAA TATTATCGTA AAATAAACGA TAAGTTATTT CATTGCTACT ATTTACGAAT CACACTTTGT   
  
  
+ AAATATTACT ACTAGGGACT TGGTTGTATT AGTATGACTC TACTAATATA AAATACATAT TATTTGTAGT   
  
  
+ TTTATTAAAA ATACAATTAA CACAAAAAAT AATTTAAAAA ATAATAAAAA TCTACTTAAA ATATGTATTT   
  
  
+ TATAATTTAA TCAAGTTTAA TAATAACAAA AATAAAAAAA TTTTTTAATA TTAAGTTTAA ATTAATAACA   
  
  
+ AAAATAAAAA ATTTTTTTAA TATTAAGTTT AAATTCTAAA AAATATTATT TAAAAAATTA AAATTAAAAA   
  
  
+ AATGTTTCTA TTTCTTATAA GTTTAATATT ACTGTTTTTT TAGTTTAAAA AATTTACGAA TTTCACGAAA   
  
  
+ AATATTTTAA AATGCAATAA TTTTTAAATT TTAATTAAGT ATTTTTTATA AAAATCGTCA TTTTATTAAA   
  
  
+ ACATAAAAAA AGCAAACTTA TAGAAAAAT  

- CGAAGAATAC TAAGAAAGAA GAAGAAATGA ATCGGAAAGC ACCGATGAAA GTTAGAAAGA GATTAGAGAG   
  
  
- CTTCGGCTAA GATCTTTGTT TTCCTCTAGT GAGAAAGAGT TTTGTTTCGA TGGGGTTCTT GTTTCGCTTT   
  
  
- TCTTTCTCTC TGAGAGAGAG AGAGAGAACC AGAAAAGGGG TAGGTTTGGC TCCTCTCCAC TCCTTTATAT   
  
  
- TTACTTTTTT TTTTTTTCCC TTCGTTAGTG GCCAAAATAT TCTTCAAACA ACCCAAAAAT TGAGCTCTGA   
  
  
- TTTGTTAAAT TTGATTAAAA AATAAAAAAT ATCTTCTATA ATTTGTGACT TTTTTTACTT TATTTTAAAA   
  
  
- CTAGACATGG TTATACTTCA TAAAAGACAA TTAAAAATTA AAAAAAACAA AAAACAGTTT AAGCCCTAGC   
  
  
- GATGAAGGTA AAGACAGGGA CATACCCTTT TTTCTTCATA TTAAGAAAAA TAAAAATAAT TAATAAAAAA   
  
  
- TAATTAGCTC TTTAATTGCA TGATAAGTTT CTGTTTAAAT CCGAATTGGC TAAAAGAATT GGGTGAAAAC   
  
  
- AGGGCATGGC ATGAACCATG AGCGAGAAGG CTTGGTCCGG TCCATCGGTC GCAGGCAGTC ACCGTCACGT   
  
  
- GGACAAGGCG CCATAAGAAA GGGGTTGGTC AATGTTTTCA ACTCTACGGT TATTATTCTC TTTTTGGAGG   
  
  
- ATTAGGCTAA CACGTGTCGT GGGTCGGGCA CCGTCATTTG ACCGAACGTG GCACGTGGGA GCCCATCTCC   
  
  
- TGTTGGTTTT CCACGGGCAA TTATTGTGGA CGTTAGCGTA CAAATATCCA CATTTGACTC TTCTGTCTGT   
  
  
- ACATTTCTAC ATGCGTTGCT GACTCAGCTT TCGCGTCATC CCATCTAGTT CCCGTTACTC TTAAGTTTGG   
  
  
- TGGTACACTC AAAAAATAAT AATAATAATA ATTATTATTA AATTAATCCG TACATAAATA GGCAAGATCG   
  
  
- TGATTATTTT ATAATAGCAT TTTATTTGCT ATTCAATAAA GTAACGATGA TAAATGCTTA GTGTGAAACA   
  
  
- TTTATAATGA TGATCCCTGA ACCAACATAA TCATACTGAG ATGATTATAT TTTATGTATA ATAAACATCA   
  
  
- AAATAATTTT TATGTTAATT GTGTTTTTTA TTAAATTTTT TATTATTTTT AGATGAATTT TATACATAAA   
  
  
- ATATTAAATT AGTTCAAATT ATTATTGTTT TTATTTTTTT AAAAAATTAT AATTCAAATT TAATTATTGT   
  
  
- TTTTATTTTT TAAAAAAATT ATAATTCAAA TTTAAGATTT TTTATAATAA ATTTTTTAAT TTTAATTTTT   
  
  
- TTACAAAGAT AAAGAATATT CAAATTATAA TGACAAAAAA ATCAAATTTT TTAAATGCTT AAAGTGCTTT   
  
  
- TTATAAAATT TTACGTTATT AAAAATTTAA AATTAATTCA TAAAAAATAT TTTTAGCAGT AAAATAATTT   
  
  
- TGTATTTTTT TCGTTTGAAT ATCTTTTTA

+     AAGAA-motif

| Site Name | Organism | Position | Strand | Matrix score. | sequence | function |
| --- | --- | --- | --- | --- | --- | --- |
| AAGAA-motif | Avena sativa | 645 | - | 7 | GAAAGAA |  |
| AAGAA-motif | Avena sativa | 543 | - | 9 | gGTAAAGAAA |  |
| AAGAA-motif | Avena sativa | 12 | - | 7 | GAAAGAA |  |

> 2018/04/13 10:10:12  
+ GCTTCTTATG ATTCTTTCTT CTTCTTTACT TAGCCTTTCG TGGCTACTTT CAATCTTTCT CTAATCTCTC   
  
  
+ GAAGCCGATT CTAGAAACAA AAGGAGATCA CTCTTTCTCA AAACAAAGCT ACCCCAAGAA CAAAGCGAAA   
  
  
+ AGAAAGAGAG ACTCTCTCTC TCTCTCTTGG TCTTTTCCCC ATCCAAACCG AGGAGAGGTG AGGAAATATA   
  
  
+ AATGAAAAAA AAAAAAAGGG AAGCAATCAC CGGTTTTATA AGAAGTTTGT TGGGTTTTTA ACTCGAGACT   
  
  
+ AAACAATTTA AACTAATTTT TTATTTTTTA TAGAAGATAT TAAACACTGA AAAAAATGAA ATAAAATTTT   
  
  
+ GATCTGTACC AATATGAAGT ATTTTCTGTT AATTTTTAAT TTTTTTTGTT TTTTGTCAAA TTCGGGATCG   
  
  
+ CTACTTCCAT TTCTGTCCCT GTATGGGAAA AAAGAAGTAT AATTCTTTTT ATTTTTATTA ATTATTTTTT   
  
  
+ ATTAATCGAG AAATTAACGT ACTATTCAAA GACAAATTTA GGCTTAACCG ATTTTCTTAA CCCACTTTTG   
  
  
+ TCCCGTACCG TACTTGGTAC TCGCTCTTCC GAACCAGGCC AGGTAGCCAG CGTCCGTCAG TGGCAGTGCA   
  
  
+ CCTGTTCCGC GGTATTCTTT CCCCAACCAG TTACAAAAGT TGAGATGCCA ATAATAAGAG AAAAACCTCC   
  
  
+ TAATCCGATT GTGCACAGCA CCCAGCCCGT GGCAGTAAAC TGGCTTGCAC CGTGCACCCT CGGGTAGAGG   
  
  
+ ACAACCAAAA GGTGCCCGTT AATAACACCT GCAATCGCAT GTTTATAGGT GTAAACTGAG AAGACAGACA   
  
  
+ TGTAAAGATG TACGCAACGA CTGAGTCGAA AGCGCAGTAG GGTAGATCAA GGGCAATGAG AATTCAAACC   
  
  
+ ACCATGTGAG TTTTTTATTA TTATTATTAT TAATAATAAT TTAATTAGGC ATGTATTTAT CCGTTCTAGC   
  
  
+ ACTAATAAAA TATTATCGTA AAATAAACGA TAAGTTATTT CATTGCTACT ATTTACGAAT CACACTTTGT   
  
  
+ AAATATTACT ACTAGGGACT TGGTTGTATT AGTATGACTC TACTAATATA AAATACATAT TATTTGTAGT   
  
  
+ TTTATTAAAA ATACAATTAA CACAAAAAAT AATTTAAAAA ATAATAAAAA TCTACTTAAA ATATGTATTT   
  
  
+ TATAATTTAA TCAAGTTTAA TAATAACAAA AATAAAAAAA TTTTTTAATA TTAAGTTTAA ATTAATAACA   
  
  
+ AAAATAAAAA ATTTTTTTAA TATTAAGTTT AAATTCTAAA AAATATTATT TAAAAAATTA AAATTAAAAA   
  
  
+ AATGTTTCTA TTTCTTATAA GTTTAATATT ACTGTTTTTT TAGTTTAAAA AATTTACGAA TTTCACGAAA   
  
  
+ AATATTTTAA AATGCAATAA TTTTTAAATT TTAATTAAGT ATTTTTTATA AAAATCGTCA TTTTATTAAA   
  
  
+ ACATAAAAAA AGCAAACTTA TAGAAAAAT  

- CGAAGAATAC TAAGAAAGAA GAAGAAATGA ATCGGAAAGC ACCGATGAAA GTTAGAAAGA GATTAGAGAG   
  
  
- CTTCGGCTAA GATCTTTGTT TTCCTCTAGT GAGAAAGAGT TTTGTTTCGA TGGGGTTCTT GTTTCGCTTT   
  
  
- TCTTTCTCTC TGAGAGAGAG AGAGAGAACC AGAAAAGGGG TAGGTTTGGC TCCTCTCCAC TCCTTTATAT   
  
  
- TTACTTTTTT TTTTTTTCCC TTCGTTAGTG GCCAAAATAT TCTTCAAACA ACCCAAAAAT TGAGCTCTGA   
  
  
- TTTGTTAAAT TTGATTAAAA AATAAAAAAT ATCTTCTATA ATTTGTGACT TTTTTTACTT TATTTTAAAA   
  
  
- CTAGACATGG TTATACTTCA TAAAAGACAA TTAAAAATTA AAAAAAACAA AAAACAGTTT AAGCCCTAGC   
  
  
- GATGAAGGTA AAGACAGGGA CATACCCTTT TTTCTTCATA TTAAGAAAAA TAAAAATAAT TAATAAAAAA   
  
  
- TAATTAGCTC TTTAATTGCA TGATAAGTTT CTGTTTAAAT CCGAATTGGC TAAAAGAATT GGGTGAAAAC   
  
  
- AGGGCATGGC ATGAACCATG AGCGAGAAGG CTTGGTCCGG TCCATCGGTC GCAGGCAGTC ACCGTCACGT   
  
  
- GGACAAGGCG CCATAAGAAA GGGGTTGGTC AATGTTTTCA ACTCTACGGT TATTATTCTC TTTTTGGAGG   
  
  
- ATTAGGCTAA CACGTGTCGT GGGTCGGGCA CCGTCATTTG ACCGAACGTG GCACGTGGGA GCCCATCTCC   
  
  
- TGTTGGTTTT CCACGGGCAA TTATTGTGGA CGTTAGCGTA CAAATATCCA CATTTGACTC TTCTGTCTGT   
  
  
- ACATTTCTAC ATGCGTTGCT GACTCAGCTT TCGCGTCATC CCATCTAGTT CCCGTTACTC TTAAGTTTGG   
  
  
- TGGTACACTC AAAAAATAAT AATAATAATA ATTATTATTA AATTAATCCG TACATAAATA GGCAAGATCG   
  
  
- TGATTATTTT ATAATAGCAT TTTATTTGCT ATTCAATAAA GTAACGATGA TAAATGCTTA GTGTGAAACA   
  
  
- TTTATAATGA TGATCCCTGA ACCAACATAA TCATACTGAG ATGATTATAT TTTATGTATA ATAAACATCA   
  
  
- AAATAATTTT TATGTTAATT GTGTTTTTTA TTAAATTTTT TATTATTTTT AGATGAATTT TATACATAAA   
  
  
- ATATTAAATT AGTTCAAATT ATTATTGTTT TTATTTTTTT AAAAAATTAT AATTCAAATT TAATTATTGT   
  
  
- TTTTATTTTT TAAAAAAATT ATAATTCAAA TTTAAGATTT TTTATAATAA ATTTTTTAAT TTTAATTTTT   
  
  
- TTACAAAGAT AAAGAATATT CAAATTATAA TGACAAAAAA ATCAAATTTT TTAAATGCTT AAAGTGCTTT   
  
  
- TTATAAAATT TTACGTTATT AAAAATTTAA AATTAATTCA TAAAAAATAT TTTTAGCAGT AAAATAATTT   
  
  
- TGTATTTTTT TCGTTTGAAT ATCTTTTTA

+     AE-box

| Site Name | Organism | Position | Strand | Matrix score. | sequence | function |
| --- | --- | --- | --- | --- | --- | --- |
| AE-box | Arabidopsis thaliana | 1332 | - | 8 | AGAAACAT | part of a module for light response |
| AE-box | Arabidopsis thaliana | 83 | + | 8 | AGAAACAA | part of a module for light response |

> 2018/04/13 10:10:12  
+ GCTTCTTATG ATTCTTTCTT CTTCTTTACT TAGCCTTTCG TGGCTACTTT CAATCTTTCT CTAATCTCTC   
  
  
+ GAAGCCGATT CTAGAAACAA AAGGAGATCA CTCTTTCTCA AAACAAAGCT ACCCCAAGAA CAAAGCGAAA   
  
  
+ AGAAAGAGAG ACTCTCTCTC TCTCTCTTGG TCTTTTCCCC ATCCAAACCG AGGAGAGGTG AGGAAATATA   
  
  
+ AATGAAAAAA AAAAAAAGGG AAGCAATCAC CGGTTTTATA AGAAGTTTGT TGGGTTTTTA ACTCGAGACT   
  
  
+ AAACAATTTA AACTAATTTT TTATTTTTTA TAGAAGATAT TAAACACTGA AAAAAATGAA ATAAAATTTT   
  
  
+ GATCTGTACC AATATGAAGT ATTTTCTGTT AATTTTTAAT TTTTTTTGTT TTTTGTCAAA TTCGGGATCG   
  
  
+ CTACTTCCAT TTCTGTCCCT GTATGGGAAA AAAGAAGTAT AATTCTTTTT ATTTTTATTA ATTATTTTTT   
  
  
+ ATTAATCGAG AAATTAACGT ACTATTCAAA GACAAATTTA GGCTTAACCG ATTTTCTTAA CCCACTTTTG   
  
  
+ TCCCGTACCG TACTTGGTAC TCGCTCTTCC GAACCAGGCC AGGTAGCCAG CGTCCGTCAG TGGCAGTGCA   
  
  
+ CCTGTTCCGC GGTATTCTTT CCCCAACCAG TTACAAAAGT TGAGATGCCA ATAATAAGAG AAAAACCTCC   
  
  
+ TAATCCGATT GTGCACAGCA CCCAGCCCGT GGCAGTAAAC TGGCTTGCAC CGTGCACCCT CGGGTAGAGG   
  
  
+ ACAACCAAAA GGTGCCCGTT AATAACACCT GCAATCGCAT GTTTATAGGT GTAAACTGAG AAGACAGACA   
  
  
+ TGTAAAGATG TACGCAACGA CTGAGTCGAA AGCGCAGTAG GGTAGATCAA GGGCAATGAG AATTCAAACC   
  
  
+ ACCATGTGAG TTTTTTATTA TTATTATTAT TAATAATAAT TTAATTAGGC ATGTATTTAT CCGTTCTAGC   
  
  
+ ACTAATAAAA TATTATCGTA AAATAAACGA TAAGTTATTT CATTGCTACT ATTTACGAAT CACACTTTGT   
  
  
+ AAATATTACT ACTAGGGACT TGGTTGTATT AGTATGACTC TACTAATATA AAATACATAT TATTTGTAGT   
  
  
+ TTTATTAAAA ATACAATTAA CACAAAAAAT AATTTAAAAA ATAATAAAAA TCTACTTAAA ATATGTATTT   
  
  
+ TATAATTTAA TCAAGTTTAA TAATAACAAA AATAAAAAAA TTTTTTAATA TTAAGTTTAA ATTAATAACA   
  
  
+ AAAATAAAAA ATTTTTTTAA TATTAAGTTT AAATTCTAAA AAATATTATT TAAAAAATTA AAATTAAAAA   
  
  
+ AATGTTTCTA TTTCTTATAA GTTTAATATT ACTGTTTTTT TAGTTTAAAA AATTTACGAA TTTCACGAAA   
  
  
+ AATATTTTAA AATGCAATAA TTTTTAAATT TTAATTAAGT ATTTTTTATA AAAATCGTCA TTTTATTAAA   
  
  
+ ACATAAAAAA AGCAAACTTA TAGAAAAAT  

- CGAAGAATAC TAAGAAAGAA GAAGAAATGA ATCGGAAAGC ACCGATGAAA GTTAGAAAGA GATTAGAGAG   
  
  
- CTTCGGCTAA GATCTTTGTT TTCCTCTAGT GAGAAAGAGT TTTGTTTCGA TGGGGTTCTT GTTTCGCTTT   
  
  
- TCTTTCTCTC TGAGAGAGAG AGAGAGAACC AGAAAAGGGG TAGGTTTGGC TCCTCTCCAC TCCTTTATAT   
  
  
- TTACTTTTTT TTTTTTTCCC TTCGTTAGTG GCCAAAATAT TCTTCAAACA ACCCAAAAAT TGAGCTCTGA   
  
  
- TTTGTTAAAT TTGATTAAAA AATAAAAAAT ATCTTCTATA ATTTGTGACT TTTTTTACTT TATTTTAAAA   
  
  
- CTAGACATGG TTATACTTCA TAAAAGACAA TTAAAAATTA AAAAAAACAA AAAACAGTTT AAGCCCTAGC   
  
  
- GATGAAGGTA AAGACAGGGA CATACCCTTT TTTCTTCATA TTAAGAAAAA TAAAAATAAT TAATAAAAAA   
  
  
- TAATTAGCTC TTTAATTGCA TGATAAGTTT CTGTTTAAAT CCGAATTGGC TAAAAGAATT GGGTGAAAAC   
  
  
- AGGGCATGGC ATGAACCATG AGCGAGAAGG CTTGGTCCGG TCCATCGGTC GCAGGCAGTC ACCGTCACGT   
  
  
- GGACAAGGCG CCATAAGAAA GGGGTTGGTC AATGTTTTCA ACTCTACGGT TATTATTCTC TTTTTGGAGG   
  
  
- ATTAGGCTAA CACGTGTCGT GGGTCGGGCA CCGTCATTTG ACCGAACGTG GCACGTGGGA GCCCATCTCC   
  
  
- TGTTGGTTTT CCACGGGCAA TTATTGTGGA CGTTAGCGTA CAAATATCCA CATTTGACTC TTCTGTCTGT   
  
  
- ACATTTCTAC ATGCGTTGCT GACTCAGCTT TCGCGTCATC CCATCTAGTT CCCGTTACTC TTAAGTTTGG   
  
  
- TGGTACACTC AAAAAATAAT AATAATAATA ATTATTATTA AATTAATCCG TACATAAATA GGCAAGATCG   
  
  
- TGATTATTTT ATAATAGCAT TTTATTTGCT ATTCAATAAA GTAACGATGA TAAATGCTTA GTGTGAAACA   
  
  
- TTTATAATGA TGATCCCTGA ACCAACATAA TCATACTGAG ATGATTATAT TTTATGTATA ATAAACATCA   
  
  
- AAATAATTTT TATGTTAATT GTGTTTTTTA TTAAATTTTT TATTATTTTT AGATGAATTT TATACATAAA   
  
  
- ATATTAAATT AGTTCAAATT ATTATTGTTT TTATTTTTTT AAAAAATTAT AATTCAAATT TAATTATTGT   
  
  
- TTTTATTTTT TAAAAAAATT ATAATTCAAA TTTAAGATTT TTTATAATAA ATTTTTTAAT TTTAATTTTT   
  
  
- TTACAAAGAT AAAGAATATT CAAATTATAA TGACAAAAAA ATCAAATTTT TTAAATGCTT AAAGTGCTTT   
  
  
- TTATAAAATT TTACGTTATT AAAAATTTAA AATTAATTCA TAAAAAATAT TTTTAGCAGT AAAATAATTT   
  
  
- TGTATTTTTT TCGTTTGAAT ATCTTTTTA

+     ARE

| Site Name | Organism | Position | Strand | Matrix score. | sequence | function |
| --- | --- | --- | --- | --- | --- | --- |
| ARE | Zea mays | 906 | - | 6 | TGGTTT | cis-acting regulatory element essential for the anaerobic induction |

> 2018/04/13 10:10:12  
+ GCTTCTTATG ATTCTTTCTT CTTCTTTACT TAGCCTTTCG TGGCTACTTT CAATCTTTCT CTAATCTCTC   
  
  
+ GAAGCCGATT CTAGAAACAA AAGGAGATCA CTCTTTCTCA AAACAAAGCT ACCCCAAGAA CAAAGCGAAA   
  
  
+ AGAAAGAGAG ACTCTCTCTC TCTCTCTTGG TCTTTTCCCC ATCCAAACCG AGGAGAGGTG AGGAAATATA   
  
  
+ AATGAAAAAA AAAAAAAGGG AAGCAATCAC CGGTTTTATA AGAAGTTTGT TGGGTTTTTA ACTCGAGACT   
  
  
+ AAACAATTTA AACTAATTTT TTATTTTTTA TAGAAGATAT TAAACACTGA AAAAAATGAA ATAAAATTTT   
  
  
+ GATCTGTACC AATATGAAGT ATTTTCTGTT AATTTTTAAT TTTTTTTGTT TTTTGTCAAA TTCGGGATCG   
  
  
+ CTACTTCCAT TTCTGTCCCT GTATGGGAAA AAAGAAGTAT AATTCTTTTT ATTTTTATTA ATTATTTTTT   
  
  
+ ATTAATCGAG AAATTAACGT ACTATTCAAA GACAAATTTA GGCTTAACCG ATTTTCTTAA CCCACTTTTG   
  
  
+ TCCCGTACCG TACTTGGTAC TCGCTCTTCC GAACCAGGCC AGGTAGCCAG CGTCCGTCAG TGGCAGTGCA   
  
  
+ CCTGTTCCGC GGTATTCTTT CCCCAACCAG TTACAAAAGT TGAGATGCCA ATAATAAGAG AAAAACCTCC   
  
  
+ TAATCCGATT GTGCACAGCA CCCAGCCCGT GGCAGTAAAC TGGCTTGCAC CGTGCACCCT CGGGTAGAGG   
  
  
+ ACAACCAAAA GGTGCCCGTT AATAACACCT GCAATCGCAT GTTTATAGGT GTAAACTGAG AAGACAGACA   
  
  
+ TGTAAAGATG TACGCAACGA CTGAGTCGAA AGCGCAGTAG GGTAGATCAA GGGCAATGAG AATTCAAACC   
  
  
+ ACCATGTGAG TTTTTTATTA TTATTATTAT TAATAATAAT TTAATTAGGC ATGTATTTAT CCGTTCTAGC   
  
  
+ ACTAATAAAA TATTATCGTA AAATAAACGA TAAGTTATTT CATTGCTACT ATTTACGAAT CACACTTTGT   
  
  
+ AAATATTACT ACTAGGGACT TGGTTGTATT AGTATGACTC TACTAATATA AAATACATAT TATTTGTAGT   
  
  
+ TTTATTAAAA ATACAATTAA CACAAAAAAT AATTTAAAAA ATAATAAAAA TCTACTTAAA ATATGTATTT   
  
  
+ TATAATTTAA TCAAGTTTAA TAATAACAAA AATAAAAAAA TTTTTTAATA TTAAGTTTAA ATTAATAACA   
  
  
+ AAAATAAAAA ATTTTTTTAA TATTAAGTTT AAATTCTAAA AAATATTATT TAAAAAATTA AAATTAAAAA   
  
  
+ AATGTTTCTA TTTCTTATAA GTTTAATATT ACTGTTTTTT TAGTTTAAAA AATTTACGAA TTTCACGAAA   
  
  
+ AATATTTTAA AATGCAATAA TTTTTAAATT TTAATTAAGT ATTTTTTATA AAAATCGTCA TTTTATTAAA   
  
  
+ ACATAAAAAA AGCAAACTTA TAGAAAAAT  

- CGAAGAATAC TAAGAAAGAA GAAGAAATGA ATCGGAAAGC ACCGATGAAA GTTAGAAAGA GATTAGAGAG   
  
  
- CTTCGGCTAA GATCTTTGTT TTCCTCTAGT GAGAAAGAGT TTTGTTTCGA TGGGGTTCTT GTTTCGCTTT   
  
  
- TCTTTCTCTC TGAGAGAGAG AGAGAGAACC AGAAAAGGGG TAGGTTTGGC TCCTCTCCAC TCCTTTATAT   
  
  
- TTACTTTTTT TTTTTTTCCC TTCGTTAGTG GCCAAAATAT TCTTCAAACA ACCCAAAAAT TGAGCTCTGA   
  
  
- TTTGTTAAAT TTGATTAAAA AATAAAAAAT ATCTTCTATA ATTTGTGACT TTTTTTACTT TATTTTAAAA   
  
  
- CTAGACATGG TTATACTTCA TAAAAGACAA TTAAAAATTA AAAAAAACAA AAAACAGTTT AAGCCCTAGC   
  
  
- GATGAAGGTA AAGACAGGGA CATACCCTTT TTTCTTCATA TTAAGAAAAA TAAAAATAAT TAATAAAAAA   
  
  
- TAATTAGCTC TTTAATTGCA TGATAAGTTT CTGTTTAAAT CCGAATTGGC TAAAAGAATT GGGTGAAAAC   
  
  
- AGGGCATGGC ATGAACCATG AGCGAGAAGG CTTGGTCCGG TCCATCGGTC GCAGGCAGTC ACCGTCACGT   
  
  
- GGACAAGGCG CCATAAGAAA GGGGTTGGTC AATGTTTTCA ACTCTACGGT TATTATTCTC TTTTTGGAGG   
  
  
- ATTAGGCTAA CACGTGTCGT GGGTCGGGCA CCGTCATTTG ACCGAACGTG GCACGTGGGA GCCCATCTCC   
  
  
- TGTTGGTTTT CCACGGGCAA TTATTGTGGA CGTTAGCGTA CAAATATCCA CATTTGACTC TTCTGTCTGT   
  
  
- ACATTTCTAC ATGCGTTGCT GACTCAGCTT TCGCGTCATC CCATCTAGTT CCCGTTACTC TTAAGTTTGG   
  
  
- TGGTACACTC AAAAAATAAT AATAATAATA ATTATTATTA AATTAATCCG TACATAAATA GGCAAGATCG   
  
  
- TGATTATTTT ATAATAGCAT TTTATTTGCT ATTCAATAAA GTAACGATGA TAAATGCTTA GTGTGAAACA   
  
  
- TTTATAATGA TGATCCCTGA ACCAACATAA TCATACTGAG ATGATTATAT TTTATGTATA ATAAACATCA   
  
  
- AAATAATTTT TATGTTAATT GTGTTTTTTA TTAAATTTTT TATTATTTTT AGATGAATTT TATACATAAA   
  
  
- ATATTAAATT AGTTCAAATT ATTATTGTTT TTATTTTTTT AAAAAATTAT AATTCAAATT TAATTATTGT   
  
  
- TTTTATTTTT TAAAAAAATT ATAATTCAAA TTTAAGATTT TTTATAATAA ATTTTTTAAT TTTAATTTTT   
  
  
- TTACAAAGAT AAAGAATATT CAAATTATAA TGACAAAAAA ATCAAATTTT TTAAATGCTT AAAGTGCTTT   
  
  
- TTATAAAATT TTACGTTATT AAAAATTTAA AATTAATTCA TAAAAAATAT TTTTAGCAGT AAAATAATTT   
  
  
- TGTATTTTTT TCGTTTGAAT ATCTTTTTA

+     AT1-motif

| Site Name | Organism | Position | Strand | Matrix score. | sequence | function |
| --- | --- | --- | --- | --- | --- | --- |
| AT1-motif | Solanum tuberosum | 480 | + | 14 | AATTATTTTTTATT | part of a light responsive module |

> 2018/04/13 10:10:12  
+ GCTTCTTATG ATTCTTTCTT CTTCTTTACT TAGCCTTTCG TGGCTACTTT CAATCTTTCT CTAATCTCTC   
  
  
+ GAAGCCGATT CTAGAAACAA AAGGAGATCA CTCTTTCTCA AAACAAAGCT ACCCCAAGAA CAAAGCGAAA   
  
  
+ AGAAAGAGAG ACTCTCTCTC TCTCTCTTGG TCTTTTCCCC ATCCAAACCG AGGAGAGGTG AGGAAATATA   
  
  
+ AATGAAAAAA AAAAAAAGGG AAGCAATCAC CGGTTTTATA AGAAGTTTGT TGGGTTTTTA ACTCGAGACT   
  
  
+ AAACAATTTA AACTAATTTT TTATTTTTTA TAGAAGATAT TAAACACTGA AAAAAATGAA ATAAAATTTT   
  
  
+ GATCTGTACC AATATGAAGT ATTTTCTGTT AATTTTTAAT TTTTTTTGTT TTTTGTCAAA TTCGGGATCG   
  
  
+ CTACTTCCAT TTCTGTCCCT GTATGGGAAA AAAGAAGTAT AATTCTTTTT ATTTTTATTA ATTATTTTTT   
  
  
+ ATTAATCGAG AAATTAACGT ACTATTCAAA GACAAATTTA GGCTTAACCG ATTTTCTTAA CCCACTTTTG   
  
  
+ TCCCGTACCG TACTTGGTAC TCGCTCTTCC GAACCAGGCC AGGTAGCCAG CGTCCGTCAG TGGCAGTGCA   
  
  
+ CCTGTTCCGC GGTATTCTTT CCCCAACCAG TTACAAAAGT TGAGATGCCA ATAATAAGAG AAAAACCTCC   
  
  
+ TAATCCGATT GTGCACAGCA CCCAGCCCGT GGCAGTAAAC TGGCTTGCAC CGTGCACCCT CGGGTAGAGG   
  
  
+ ACAACCAAAA GGTGCCCGTT AATAACACCT GCAATCGCAT GTTTATAGGT GTAAACTGAG AAGACAGACA   
  
  
+ TGTAAAGATG TACGCAACGA CTGAGTCGAA AGCGCAGTAG GGTAGATCAA GGGCAATGAG AATTCAAACC   
  
  
+ ACCATGTGAG TTTTTTATTA TTATTATTAT TAATAATAAT TTAATTAGGC ATGTATTTAT CCGTTCTAGC   
  
  
+ ACTAATAAAA TATTATCGTA AAATAAACGA TAAGTTATTT CATTGCTACT ATTTACGAAT CACACTTTGT   
  
  
+ AAATATTACT ACTAGGGACT TGGTTGTATT AGTATGACTC TACTAATATA AAATACATAT TATTTGTAGT   
  
  
+ TTTATTAAAA ATACAATTAA CACAAAAAAT AATTTAAAAA ATAATAAAAA TCTACTTAAA ATATGTATTT   
  
  
+ TATAATTTAA TCAAGTTTAA TAATAACAAA AATAAAAAAA TTTTTTAATA TTAAGTTTAA ATTAATAACA   
  
  
+ AAAATAAAAA ATTTTTTTAA TATTAAGTTT AAATTCTAAA AAATATTATT TAAAAAATTA AAATTAAAAA   
  
  
+ AATGTTTCTA TTTCTTATAA GTTTAATATT ACTGTTTTTT TAGTTTAAAA AATTTACGAA TTTCACGAAA   
  
  
+ AATATTTTAA AATGCAATAA TTTTTAAATT TTAATTAAGT ATTTTTTATA AAAATCGTCA TTTTATTAAA   
  
  
+ ACATAAAAAA AGCAAACTTA TAGAAAAAT  

- CGAAGAATAC TAAGAAAGAA GAAGAAATGA ATCGGAAAGC ACCGATGAAA GTTAGAAAGA GATTAGAGAG   
  
  
- CTTCGGCTAA GATCTTTGTT TTCCTCTAGT GAGAAAGAGT TTTGTTTCGA TGGGGTTCTT GTTTCGCTTT   
  
  
- TCTTTCTCTC TGAGAGAGAG AGAGAGAACC AGAAAAGGGG TAGGTTTGGC TCCTCTCCAC TCCTTTATAT   
  
  
- TTACTTTTTT TTTTTTTCCC TTCGTTAGTG GCCAAAATAT TCTTCAAACA ACCCAAAAAT TGAGCTCTGA   
  
  
- TTTGTTAAAT TTGATTAAAA AATAAAAAAT ATCTTCTATA ATTTGTGACT TTTTTTACTT TATTTTAAAA   
  
  
- CTAGACATGG TTATACTTCA TAAAAGACAA TTAAAAATTA AAAAAAACAA AAAACAGTTT AAGCCCTAGC   
  
  
- GATGAAGGTA AAGACAGGGA CATACCCTTT TTTCTTCATA TTAAGAAAAA TAAAAATAAT TAATAAAAAA   
  
  
- TAATTAGCTC TTTAATTGCA TGATAAGTTT CTGTTTAAAT CCGAATTGGC TAAAAGAATT GGGTGAAAAC   
  
  
- AGGGCATGGC ATGAACCATG AGCGAGAAGG CTTGGTCCGG TCCATCGGTC GCAGGCAGTC ACCGTCACGT   
  
  
- GGACAAGGCG CCATAAGAAA GGGGTTGGTC AATGTTTTCA ACTCTACGGT TATTATTCTC TTTTTGGAGG   
  
  
- ATTAGGCTAA CACGTGTCGT GGGTCGGGCA CCGTCATTTG ACCGAACGTG GCACGTGGGA GCCCATCTCC   
  
  
- TGTTGGTTTT CCACGGGCAA TTATTGTGGA CGTTAGCGTA CAAATATCCA CATTTGACTC TTCTGTCTGT   
  
  
- ACATTTCTAC ATGCGTTGCT GACTCAGCTT TCGCGTCATC CCATCTAGTT CCCGTTACTC TTAAGTTTGG   
  
  
- TGGTACACTC AAAAAATAAT AATAATAATA ATTATTATTA AATTAATCCG TACATAAATA GGCAAGATCG   
  
  
- TGATTATTTT ATAATAGCAT TTTATTTGCT ATTCAATAAA GTAACGATGA TAAATGCTTA GTGTGAAACA   
  
  
- TTTATAATGA TGATCCCTGA ACCAACATAA TCATACTGAG ATGATTATAT TTTATGTATA ATAAACATCA   
  
  
- AAATAATTTT TATGTTAATT GTGTTTTTTA TTAAATTTTT TATTATTTTT AGATGAATTT TATACATAAA   
  
  
- ATATTAAATT AGTTCAAATT ATTATTGTTT TTATTTTTTT AAAAAATTAT AATTCAAATT TAATTATTGT   
  
  
- TTTTATTTTT TAAAAAAATT ATAATTCAAA TTTAAGATTT TTTATAATAA ATTTTTTAAT TTTAATTTTT   
  
  
- TTACAAAGAT AAAGAATATT CAAATTATAA TGACAAAAAA ATCAAATTTT TTAAATGCTT AAAGTGCTTT   
  
  
- TTATAAAATT TTACGTTATT AAAAATTTAA AATTAATTCA TAAAAAATAT TTTTAGCAGT AAAATAATTT   
  
  
- TGTATTTTTT TCGTTTGAAT ATCTTTTTA

+     Box 4

| Site Name | Organism | Position | Strand | Matrix score. | sequence | function |
| --- | --- | --- | --- | --- | --- | --- |
| Box 4 | Petroselinum crispum | 477 | + | 6 | ATTAAT | part of a conserved DNA module involved in light responsiveness |
| Box 4 | Petroselinum crispum | 1251 | - | 6 | ATTAAT | part of a conserved DNA module involved in light responsiveness |
| Box 4 | Petroselinum crispum | 939 | - | 6 | ATTAAT | part of a conserved DNA module involved in light responsiveness |
| Box 4 | Petroselinum crispum | 491 | + | 6 | ATTAAT | part of a conserved DNA module involved in light responsiveness |

> 2018/04/13 10:10:12  
+ GCTTCTTATG ATTCTTTCTT CTTCTTTACT TAGCCTTTCG TGGCTACTTT CAATCTTTCT CTAATCTCTC   
  
  
+ GAAGCCGATT CTAGAAACAA AAGGAGATCA CTCTTTCTCA AAACAAAGCT ACCCCAAGAA CAAAGCGAAA   
  
  
+ AGAAAGAGAG ACTCTCTCTC TCTCTCTTGG TCTTTTCCCC ATCCAAACCG AGGAGAGGTG AGGAAATATA   
  
  
+ AATGAAAAAA AAAAAAAGGG AAGCAATCAC CGGTTTTATA AGAAGTTTGT TGGGTTTTTA ACTCGAGACT   
  
  
+ AAACAATTTA AACTAATTTT TTATTTTTTA TAGAAGATAT TAAACACTGA AAAAAATGAA ATAAAATTTT   
  
  
+ GATCTGTACC AATATGAAGT ATTTTCTGTT AATTTTTAAT TTTTTTTGTT TTTTGTCAAA TTCGGGATCG   
  
  
+ CTACTTCCAT TTCTGTCCCT GTATGGGAAA AAAGAAGTAT AATTCTTTTT ATTTTTATTA ATTATTTTTT   
  
  
+ ATTAATCGAG AAATTAACGT ACTATTCAAA GACAAATTTA GGCTTAACCG ATTTTCTTAA CCCACTTTTG   
  
  
+ TCCCGTACCG TACTTGGTAC TCGCTCTTCC GAACCAGGCC AGGTAGCCAG CGTCCGTCAG TGGCAGTGCA   
  
  
+ CCTGTTCCGC GGTATTCTTT CCCCAACCAG TTACAAAAGT TGAGATGCCA ATAATAAGAG AAAAACCTCC   
  
  
+ TAATCCGATT GTGCACAGCA CCCAGCCCGT GGCAGTAAAC TGGCTTGCAC CGTGCACCCT CGGGTAGAGG   
  
  
+ ACAACCAAAA GGTGCCCGTT AATAACACCT GCAATCGCAT GTTTATAGGT GTAAACTGAG AAGACAGACA   
  
  
+ TGTAAAGATG TACGCAACGA CTGAGTCGAA AGCGCAGTAG GGTAGATCAA GGGCAATGAG AATTCAAACC   
  
  
+ ACCATGTGAG TTTTTTATTA TTATTATTAT TAATAATAAT TTAATTAGGC ATGTATTTAT CCGTTCTAGC   
  
  
+ ACTAATAAAA TATTATCGTA AAATAAACGA TAAGTTATTT CATTGCTACT ATTTACGAAT CACACTTTGT   
  
  
+ AAATATTACT ACTAGGGACT TGGTTGTATT AGTATGACTC TACTAATATA AAATACATAT TATTTGTAGT   
  
  
+ TTTATTAAAA ATACAATTAA CACAAAAAAT AATTTAAAAA ATAATAAAAA TCTACTTAAA ATATGTATTT   
  
  
+ TATAATTTAA TCAAGTTTAA TAATAACAAA AATAAAAAAA TTTTTTAATA TTAAGTTTAA ATTAATAACA   
  
  
+ AAAATAAAAA ATTTTTTTAA TATTAAGTTT AAATTCTAAA AAATATTATT TAAAAAATTA AAATTAAAAA   
  
  
+ AATGTTTCTA TTTCTTATAA GTTTAATATT ACTGTTTTTT TAGTTTAAAA AATTTACGAA TTTCACGAAA   
  
  
+ AATATTTTAA AATGCAATAA TTTTTAAATT TTAATTAAGT ATTTTTTATA AAAATCGTCA TTTTATTAAA   
  
  
+ ACATAAAAAA AGCAAACTTA TAGAAAAAT  

- CGAAGAATAC TAAGAAAGAA GAAGAAATGA ATCGGAAAGC ACCGATGAAA GTTAGAAAGA GATTAGAGAG   
  
  
- CTTCGGCTAA GATCTTTGTT TTCCTCTAGT GAGAAAGAGT TTTGTTTCGA TGGGGTTCTT GTTTCGCTTT   
  
  
- TCTTTCTCTC TGAGAGAGAG AGAGAGAACC AGAAAAGGGG TAGGTTTGGC TCCTCTCCAC TCCTTTATAT   
  
  
- TTACTTTTTT TTTTTTTCCC TTCGTTAGTG GCCAAAATAT TCTTCAAACA ACCCAAAAAT TGAGCTCTGA   
  
  
- TTTGTTAAAT TTGATTAAAA AATAAAAAAT ATCTTCTATA ATTTGTGACT TTTTTTACTT TATTTTAAAA   
  
  
- CTAGACATGG TTATACTTCA TAAAAGACAA TTAAAAATTA AAAAAAACAA AAAACAGTTT AAGCCCTAGC   
  
  
- GATGAAGGTA AAGACAGGGA CATACCCTTT TTTCTTCATA TTAAGAAAAA TAAAAATAAT TAATAAAAAA   
  
  
- TAATTAGCTC TTTAATTGCA TGATAAGTTT CTGTTTAAAT CCGAATTGGC TAAAAGAATT GGGTGAAAAC   
  
  
- AGGGCATGGC ATGAACCATG AGCGAGAAGG CTTGGTCCGG TCCATCGGTC GCAGGCAGTC ACCGTCACGT   
  
  
- GGACAAGGCG CCATAAGAAA GGGGTTGGTC AATGTTTTCA ACTCTACGGT TATTATTCTC TTTTTGGAGG   
  
  
- ATTAGGCTAA CACGTGTCGT GGGTCGGGCA CCGTCATTTG ACCGAACGTG GCACGTGGGA GCCCATCTCC   
  
  
- TGTTGGTTTT CCACGGGCAA TTATTGTGGA CGTTAGCGTA CAAATATCCA CATTTGACTC TTCTGTCTGT   
  
  
- ACATTTCTAC ATGCGTTGCT GACTCAGCTT TCGCGTCATC CCATCTAGTT CCCGTTACTC TTAAGTTTGG   
  
  
- TGGTACACTC AAAAAATAAT AATAATAATA ATTATTATTA AATTAATCCG TACATAAATA GGCAAGATCG   
  
  
- TGATTATTTT ATAATAGCAT TTTATTTGCT ATTCAATAAA GTAACGATGA TAAATGCTTA GTGTGAAACA   
  
  
- TTTATAATGA TGATCCCTGA ACCAACATAA TCATACTGAG ATGATTATAT TTTATGTATA ATAAACATCA   
  
  
- AAATAATTTT TATGTTAATT GTGTTTTTTA TTAAATTTTT TATTATTTTT AGATGAATTT TATACATAAA   
  
  
- ATATTAAATT AGTTCAAATT ATTATTGTTT TTATTTTTTT AAAAAATTAT AATTCAAATT TAATTATTGT   
  
  
- TTTTATTTTT TAAAAAAATT ATAATTCAAA TTTAAGATTT TTTATAATAA ATTTTTTAAT TTTAATTTTT   
  
  
- TTACAAAGAT AAAGAATATT CAAATTATAA TGACAAAAAA ATCAAATTTT TTAAATGCTT AAAGTGCTTT   
  
  
- TTATAAAATT TTACGTTATT AAAAATTTAA AATTAATTCA TAAAAAATAT TTTTAGCAGT AAAATAATTT   
  
  
- TGTATTTTTT TCGTTTGAAT ATCTTTTTA

+     Box II

| Site Name | Organism | Position | Strand | Matrix score. | sequence | function |
| --- | --- | --- | --- | --- | --- | --- |
| Box II | Pisum sativum | 198 | + | 11 | GTGAGGTAATAT | part of a light responsive element |

> 2018/04/13 10:10:12  
+ GCTTCTTATG ATTCTTTCTT CTTCTTTACT TAGCCTTTCG TGGCTACTTT CAATCTTTCT CTAATCTCTC   
  
  
+ GAAGCCGATT CTAGAAACAA AAGGAGATCA CTCTTTCTCA AAACAAAGCT ACCCCAAGAA CAAAGCGAAA   
  
  
+ AGAAAGAGAG ACTCTCTCTC TCTCTCTTGG TCTTTTCCCC ATCCAAACCG AGGAGAGGTG AGGAAATATA   
  
  
+ AATGAAAAAA AAAAAAAGGG AAGCAATCAC CGGTTTTATA AGAAGTTTGT TGGGTTTTTA ACTCGAGACT   
  
  
+ AAACAATTTA AACTAATTTT TTATTTTTTA TAGAAGATAT TAAACACTGA AAAAAATGAA ATAAAATTTT   
  
  
+ GATCTGTACC AATATGAAGT ATTTTCTGTT AATTTTTAAT TTTTTTTGTT TTTTGTCAAA TTCGGGATCG   
  
  
+ CTACTTCCAT TTCTGTCCCT GTATGGGAAA AAAGAAGTAT AATTCTTTTT ATTTTTATTA ATTATTTTTT   
  
  
+ ATTAATCGAG AAATTAACGT ACTATTCAAA GACAAATTTA GGCTTAACCG ATTTTCTTAA CCCACTTTTG   
  
  
+ TCCCGTACCG TACTTGGTAC TCGCTCTTCC GAACCAGGCC AGGTAGCCAG CGTCCGTCAG TGGCAGTGCA   
  
  
+ CCTGTTCCGC GGTATTCTTT CCCCAACCAG TTACAAAAGT TGAGATGCCA ATAATAAGAG AAAAACCTCC   
  
  
+ TAATCCGATT GTGCACAGCA CCCAGCCCGT GGCAGTAAAC TGGCTTGCAC CGTGCACCCT CGGGTAGAGG   
  
  
+ ACAACCAAAA GGTGCCCGTT AATAACACCT GCAATCGCAT GTTTATAGGT GTAAACTGAG AAGACAGACA   
  
  
+ TGTAAAGATG TACGCAACGA CTGAGTCGAA AGCGCAGTAG GGTAGATCAA GGGCAATGAG AATTCAAACC   
  
  
+ ACCATGTGAG TTTTTTATTA TTATTATTAT TAATAATAAT TTAATTAGGC ATGTATTTAT CCGTTCTAGC   
  
  
+ ACTAATAAAA TATTATCGTA AAATAAACGA TAAGTTATTT CATTGCTACT ATTTACGAAT CACACTTTGT   
  
  
+ AAATATTACT ACTAGGGACT TGGTTGTATT AGTATGACTC TACTAATATA AAATACATAT TATTTGTAGT   
  
  
+ TTTATTAAAA ATACAATTAA CACAAAAAAT AATTTAAAAA ATAATAAAAA TCTACTTAAA ATATGTATTT   
  
  
+ TATAATTTAA TCAAGTTTAA TAATAACAAA AATAAAAAAA TTTTTTAATA TTAAGTTTAA ATTAATAACA   
  
  
+ AAAATAAAAA ATTTTTTTAA TATTAAGTTT AAATTCTAAA AAATATTATT TAAAAAATTA AAATTAAAAA   
  
  
+ AATGTTTCTA TTTCTTATAA GTTTAATATT ACTGTTTTTT TAGTTTAAAA AATTTACGAA TTTCACGAAA   
  
  
+ AATATTTTAA AATGCAATAA TTTTTAAATT TTAATTAAGT ATTTTTTATA AAAATCGTCA TTTTATTAAA   
  
  
+ ACATAAAAAA AGCAAACTTA TAGAAAAAT  

- CGAAGAATAC TAAGAAAGAA GAAGAAATGA ATCGGAAAGC ACCGATGAAA GTTAGAAAGA GATTAGAGAG   
  
  
- CTTCGGCTAA GATCTTTGTT TTCCTCTAGT GAGAAAGAGT TTTGTTTCGA TGGGGTTCTT GTTTCGCTTT   
  
  
- TCTTTCTCTC TGAGAGAGAG AGAGAGAACC AGAAAAGGGG TAGGTTTGGC TCCTCTCCAC TCCTTTATAT   
  
  
- TTACTTTTTT TTTTTTTCCC TTCGTTAGTG GCCAAAATAT TCTTCAAACA ACCCAAAAAT TGAGCTCTGA   
  
  
- TTTGTTAAAT TTGATTAAAA AATAAAAAAT ATCTTCTATA ATTTGTGACT TTTTTTACTT TATTTTAAAA   
  
  
- CTAGACATGG TTATACTTCA TAAAAGACAA TTAAAAATTA AAAAAAACAA AAAACAGTTT AAGCCCTAGC   
  
  
- GATGAAGGTA AAGACAGGGA CATACCCTTT TTTCTTCATA TTAAGAAAAA TAAAAATAAT TAATAAAAAA   
  
  
- TAATTAGCTC TTTAATTGCA TGATAAGTTT CTGTTTAAAT CCGAATTGGC TAAAAGAATT GGGTGAAAAC   
  
  
- AGGGCATGGC ATGAACCATG AGCGAGAAGG CTTGGTCCGG TCCATCGGTC GCAGGCAGTC ACCGTCACGT   
  
  
- GGACAAGGCG CCATAAGAAA GGGGTTGGTC AATGTTTTCA ACTCTACGGT TATTATTCTC TTTTTGGAGG   
  
  
- ATTAGGCTAA CACGTGTCGT GGGTCGGGCA CCGTCATTTG ACCGAACGTG GCACGTGGGA GCCCATCTCC   
  
  
- TGTTGGTTTT CCACGGGCAA TTATTGTGGA CGTTAGCGTA CAAATATCCA CATTTGACTC TTCTGTCTGT   
  
  
- ACATTTCTAC ATGCGTTGCT GACTCAGCTT TCGCGTCATC CCATCTAGTT CCCGTTACTC TTAAGTTTGG   
  
  
- TGGTACACTC AAAAAATAAT AATAATAATA ATTATTATTA AATTAATCCG TACATAAATA GGCAAGATCG   
  
  
- TGATTATTTT ATAATAGCAT TTTATTTGCT ATTCAATAAA GTAACGATGA TAAATGCTTA GTGTGAAACA   
  
  
- TTTATAATGA TGATCCCTGA ACCAACATAA TCATACTGAG ATGATTATAT TTTATGTATA ATAAACATCA   
  
  
- AAATAATTTT TATGTTAATT GTGTTTTTTA TTAAATTTTT TATTATTTTT AGATGAATTT TATACATAAA   
  
  
- ATATTAAATT AGTTCAAATT ATTATTGTTT TTATTTTTTT AAAAAATTAT AATTCAAATT TAATTATTGT   
  
  
- TTTTATTTTT TAAAAAAATT ATAATTCAAA TTTAAGATTT TTTATAATAA ATTTTTTAAT TTTAATTTTT   
  
  
- TTACAAAGAT AAAGAATATT CAAATTATAA TGACAAAAAA ATCAAATTTT TTAAATGCTT AAAGTGCTTT   
  
  
- TTATAAAATT TTACGTTATT AAAAATTTAA AATTAATTCA TAAAAAATAT TTTTAGCAGT AAAATAATTT   
  
  
- TGTATTTTTT TCGTTTGAAT ATCTTTTTA

+     CAAT-box

| Site Name | Organism | Position | Strand | Matrix score. | sequence | function |
| --- | --- | --- | --- | --- | --- | --- |
| CAAT-box | Glycine max | 1134 | + | 5 | CAATT | common cis-acting element in promoter and enhancer regions |
| CAAT-box | Hordeum vulgare | 679 | + | 4 | CAAT | common cis-acting element in promoter and enhancer regions |
| CAAT-box | Arabidopsis thaliana | 359 | + | 5 | CCAAT | common cis-acting element in promoter and enhancer regions |
| CAAT-box | Glycine max | 284 | + | 5 | CAATT | common cis-acting element in promoter and enhancer regions |
| CAAT-box | Hordeum vulgare | 1415 | + | 4 | CAAT | common cis-acting element in promoter and enhancer regions |
| CAAT-box | Brassica rapa | 1112 | - | 5 | CAAAT | common cis-acting element in promoter and enhancer regions |
| CAAT-box | Arabidopsis thaliana | 1066 | - | 10 | CAACCAACTCC | common cis-acting element in promoter and enhancer regions |
| CAAT-box | Hordeum vulgare | 802 | + | 4 | CAAT | common cis-acting element in promoter and enhancer regions |
| CAAT-box | Brassica rapa | 523 | + | 5 | CAAAT | common cis-acting element in promoter and enhancer regions |
| CAAT-box | Brassica rapa | 407 | + | 5 | CAAAT | common cis-acting element in promoter and enhancer regions |
| CAAT-box | Hordeum vulgare | 894 | + | 4 | CAAT | common cis-acting element in promoter and enhancer regions |
| CAAT-box | Arabidopsis thaliana | 892 | + | 6 | gGCAAT | common cis-acting element in promoter and enhancer regions |
| CAAT-box | Arabidopsis thaliana | 678 | + | 5 | CCAAT | common cis-acting element in promoter and enhancer regions |
| CAAT-box | Hordeum vulgare | 51 | + | 4 | CAAT | common cis-acting element in promoter and enhancer regions |
| CAAT-box | Hordeum vulgare | 234 | + | 4 | CAAT | common cis-acting element in promoter and enhancer regions |
| CAAT-box | Hordeum vulgare | 1022 | - | 4 | CAAT | common cis-acting element in promoter and enhancer regions |
| CAAT-box | Hordeum vulgare | 360 | + | 4 | CAAT | common cis-acting element in promoter and enhancer regions |
| CAAT-box | Hordeum vulgare | 708 | - | 4 | CAAT | common cis-acting element in promoter and enhancer regions |

> 2018/04/13 10:10:12  
+ GCTTCTTATG ATTCTTTCTT CTTCTTTACT TAGCCTTTCG TGGCTACTTT CAATCTTTCT CTAATCTCTC   
  
  
+ GAAGCCGATT CTAGAAACAA AAGGAGATCA CTCTTTCTCA AAACAAAGCT ACCCCAAGAA CAAAGCGAAA   
  
  
+ AGAAAGAGAG ACTCTCTCTC TCTCTCTTGG TCTTTTCCCC ATCCAAACCG AGGAGAGGTG AGGAAATATA   
  
  
+ AATGAAAAAA AAAAAAAGGG AAGCAATCAC CGGTTTTATA AGAAGTTTGT TGGGTTTTTA ACTCGAGACT   
  
  
+ AAACAATTTA AACTAATTTT TTATTTTTTA TAGAAGATAT TAAACACTGA AAAAAATGAA ATAAAATTTT   
  
  
+ GATCTGTACC AATATGAAGT ATTTTCTGTT AATTTTTAAT TTTTTTTGTT TTTTGTCAAA TTCGGGATCG   
  
  
+ CTACTTCCAT TTCTGTCCCT GTATGGGAAA AAAGAAGTAT AATTCTTTTT ATTTTTATTA ATTATTTTTT   
  
  
+ ATTAATCGAG AAATTAACGT ACTATTCAAA GACAAATTTA GGCTTAACCG ATTTTCTTAA CCCACTTTTG   
  
  
+ TCCCGTACCG TACTTGGTAC TCGCTCTTCC GAACCAGGCC AGGTAGCCAG CGTCCGTCAG TGGCAGTGCA   
  
  
+ CCTGTTCCGC GGTATTCTTT CCCCAACCAG TTACAAAAGT TGAGATGCCA ATAATAAGAG AAAAACCTCC   
  
  
+ TAATCCGATT GTGCACAGCA CCCAGCCCGT GGCAGTAAAC TGGCTTGCAC CGTGCACCCT CGGGTAGAGG   
  
  
+ ACAACCAAAA GGTGCCCGTT AATAACACCT GCAATCGCAT GTTTATAGGT GTAAACTGAG AAGACAGACA   
  
  
+ TGTAAAGATG TACGCAACGA CTGAGTCGAA AGCGCAGTAG GGTAGATCAA GGGCAATGAG AATTCAAACC   
  
  
+ ACCATGTGAG TTTTTTATTA TTATTATTAT TAATAATAAT TTAATTAGGC ATGTATTTAT CCGTTCTAGC   
  
  
+ ACTAATAAAA TATTATCGTA AAATAAACGA TAAGTTATTT CATTGCTACT ATTTACGAAT CACACTTTGT   
  
  
+ AAATATTACT ACTAGGGACT TGGTTGTATT AGTATGACTC TACTAATATA AAATACATAT TATTTGTAGT   
  
  
+ TTTATTAAAA ATACAATTAA CACAAAAAAT AATTTAAAAA ATAATAAAAA TCTACTTAAA ATATGTATTT   
  
  
+ TATAATTTAA TCAAGTTTAA TAATAACAAA AATAAAAAAA TTTTTTAATA TTAAGTTTAA ATTAATAACA   
  
  
+ AAAATAAAAA ATTTTTTTAA TATTAAGTTT AAATTCTAAA AAATATTATT TAAAAAATTA AAATTAAAAA   
  
  
+ AATGTTTCTA TTTCTTATAA GTTTAATATT ACTGTTTTTT TAGTTTAAAA AATTTACGAA TTTCACGAAA   
  
  
+ AATATTTTAA AATGCAATAA TTTTTAAATT TTAATTAAGT ATTTTTTATA AAAATCGTCA TTTTATTAAA   
  
  
+ ACATAAAAAA AGCAAACTTA TAGAAAAAT  

- CGAAGAATAC TAAGAAAGAA GAAGAAATGA ATCGGAAAGC ACCGATGAAA GTTAGAAAGA GATTAGAGAG   
  
  
- CTTCGGCTAA GATCTTTGTT TTCCTCTAGT GAGAAAGAGT TTTGTTTCGA TGGGGTTCTT GTTTCGCTTT   
  
  
- TCTTTCTCTC TGAGAGAGAG AGAGAGAACC AGAAAAGGGG TAGGTTTGGC TCCTCTCCAC TCCTTTATAT   
  
  
- TTACTTTTTT TTTTTTTCCC TTCGTTAGTG GCCAAAATAT TCTTCAAACA ACCCAAAAAT TGAGCTCTGA   
  
  
- TTTGTTAAAT TTGATTAAAA AATAAAAAAT ATCTTCTATA ATTTGTGACT TTTTTTACTT TATTTTAAAA   
  
  
- CTAGACATGG TTATACTTCA TAAAAGACAA TTAAAAATTA AAAAAAACAA AAAACAGTTT AAGCCCTAGC   
  
  
- GATGAAGGTA AAGACAGGGA CATACCCTTT TTTCTTCATA TTAAGAAAAA TAAAAATAAT TAATAAAAAA   
  
  
- TAATTAGCTC TTTAATTGCA TGATAAGTTT CTGTTTAAAT CCGAATTGGC TAAAAGAATT GGGTGAAAAC   
  
  
- AGGGCATGGC ATGAACCATG AGCGAGAAGG CTTGGTCCGG TCCATCGGTC GCAGGCAGTC ACCGTCACGT   
  
  
- GGACAAGGCG CCATAAGAAA GGGGTTGGTC AATGTTTTCA ACTCTACGGT TATTATTCTC TTTTTGGAGG   
  
  
- ATTAGGCTAA CACGTGTCGT GGGTCGGGCA CCGTCATTTG ACCGAACGTG GCACGTGGGA GCCCATCTCC   
  
  
- TGTTGGTTTT CCACGGGCAA TTATTGTGGA CGTTAGCGTA CAAATATCCA CATTTGACTC TTCTGTCTGT   
  
  
- ACATTTCTAC ATGCGTTGCT GACTCAGCTT TCGCGTCATC CCATCTAGTT CCCGTTACTC TTAAGTTTGG   
  
  
- TGGTACACTC AAAAAATAAT AATAATAATA ATTATTATTA AATTAATCCG TACATAAATA GGCAAGATCG   
  
  
- TGATTATTTT ATAATAGCAT TTTATTTGCT ATTCAATAAA GTAACGATGA TAAATGCTTA GTGTGAAACA   
  
  
- TTTATAATGA TGATCCCTGA ACCAACATAA TCATACTGAG ATGATTATAT TTTATGTATA ATAAACATCA   
  
  
- AAATAATTTT TATGTTAATT GTGTTTTTTA TTAAATTTTT TATTATTTTT AGATGAATTT TATACATAAA   
  
  
- ATATTAAATT AGTTCAAATT ATTATTGTTT TTATTTTTTT AAAAAATTAT AATTCAAATT TAATTATTGT   
  
  
- TTTTATTTTT TAAAAAAATT ATAATTCAAA TTTAAGATTT TTTATAATAA ATTTTTTAAT TTTAATTTTT   
  
  
- TTACAAAGAT AAAGAATATT CAAATTATAA TGACAAAAAA ATCAAATTTT TTAAATGCTT AAAGTGCTTT   
  
  
- TTATAAAATT TTACGTTATT AAAAATTTAA AATTAATTCA TAAAAAATAT TTTTAGCAGT AAAATAATTT   
  
  
- TGTATTTTTT TCGTTTGAAT ATCTTTTTA

+     CAT-box

| Site Name | Organism | Position | Strand | Matrix score. | sequence | function |
| --- | --- | --- | --- | --- | --- | --- |
| CAT-box | Arabidopsis thaliana | 619 | - | 6 | GCCACT | cis-acting regulatory element related to meristem expression |

> 2018/04/13 10:10:12  
+ GCTTCTTATG ATTCTTTCTT CTTCTTTACT TAGCCTTTCG TGGCTACTTT CAATCTTTCT CTAATCTCTC   
  
  
+ GAAGCCGATT CTAGAAACAA AAGGAGATCA CTCTTTCTCA AAACAAAGCT ACCCCAAGAA CAAAGCGAAA   
  
  
+ AGAAAGAGAG ACTCTCTCTC TCTCTCTTGG TCTTTTCCCC ATCCAAACCG AGGAGAGGTG AGGAAATATA   
  
  
+ AATGAAAAAA AAAAAAAGGG AAGCAATCAC CGGTTTTATA AGAAGTTTGT TGGGTTTTTA ACTCGAGACT   
  
  
+ AAACAATTTA AACTAATTTT TTATTTTTTA TAGAAGATAT TAAACACTGA AAAAAATGAA ATAAAATTTT   
  
  
+ GATCTGTACC AATATGAAGT ATTTTCTGTT AATTTTTAAT TTTTTTTGTT TTTTGTCAAA TTCGGGATCG   
  
  
+ CTACTTCCAT TTCTGTCCCT GTATGGGAAA AAAGAAGTAT AATTCTTTTT ATTTTTATTA ATTATTTTTT   
  
  
+ ATTAATCGAG AAATTAACGT ACTATTCAAA GACAAATTTA GGCTTAACCG ATTTTCTTAA CCCACTTTTG   
  
  
+ TCCCGTACCG TACTTGGTAC TCGCTCTTCC GAACCAGGCC AGGTAGCCAG CGTCCGTCAG TGGCAGTGCA   
  
  
+ CCTGTTCCGC GGTATTCTTT CCCCAACCAG TTACAAAAGT TGAGATGCCA ATAATAAGAG AAAAACCTCC   
  
  
+ TAATCCGATT GTGCACAGCA CCCAGCCCGT GGCAGTAAAC TGGCTTGCAC CGTGCACCCT CGGGTAGAGG   
  
  
+ ACAACCAAAA GGTGCCCGTT AATAACACCT GCAATCGCAT GTTTATAGGT GTAAACTGAG AAGACAGACA   
  
  
+ TGTAAAGATG TACGCAACGA CTGAGTCGAA AGCGCAGTAG GGTAGATCAA GGGCAATGAG AATTCAAACC   
  
  
+ ACCATGTGAG TTTTTTATTA TTATTATTAT TAATAATAAT TTAATTAGGC ATGTATTTAT CCGTTCTAGC   
  
  
+ ACTAATAAAA TATTATCGTA AAATAAACGA TAAGTTATTT CATTGCTACT ATTTACGAAT CACACTTTGT   
  
  
+ AAATATTACT ACTAGGGACT TGGTTGTATT AGTATGACTC TACTAATATA AAATACATAT TATTTGTAGT   
  
  
+ TTTATTAAAA ATACAATTAA CACAAAAAAT AATTTAAAAA ATAATAAAAA TCTACTTAAA ATATGTATTT   
  
  
+ TATAATTTAA TCAAGTTTAA TAATAACAAA AATAAAAAAA TTTTTTAATA TTAAGTTTAA ATTAATAACA   
  
  
+ AAAATAAAAA ATTTTTTTAA TATTAAGTTT AAATTCTAAA AAATATTATT TAAAAAATTA AAATTAAAAA   
  
  
+ AATGTTTCTA TTTCTTATAA GTTTAATATT ACTGTTTTTT TAGTTTAAAA AATTTACGAA TTTCACGAAA   
  
  
+ AATATTTTAA AATGCAATAA TTTTTAAATT TTAATTAAGT ATTTTTTATA AAAATCGTCA TTTTATTAAA   
  
  
+ ACATAAAAAA AGCAAACTTA TAGAAAAAT  

- CGAAGAATAC TAAGAAAGAA GAAGAAATGA ATCGGAAAGC ACCGATGAAA GTTAGAAAGA GATTAGAGAG   
  
  
- CTTCGGCTAA GATCTTTGTT TTCCTCTAGT GAGAAAGAGT TTTGTTTCGA TGGGGTTCTT GTTTCGCTTT   
  
  
- TCTTTCTCTC TGAGAGAGAG AGAGAGAACC AGAAAAGGGG TAGGTTTGGC TCCTCTCCAC TCCTTTATAT   
  
  
- TTACTTTTTT TTTTTTTCCC TTCGTTAGTG GCCAAAATAT TCTTCAAACA ACCCAAAAAT TGAGCTCTGA   
  
  
- TTTGTTAAAT TTGATTAAAA AATAAAAAAT ATCTTCTATA ATTTGTGACT TTTTTTACTT TATTTTAAAA   
  
  
- CTAGACATGG TTATACTTCA TAAAAGACAA TTAAAAATTA AAAAAAACAA AAAACAGTTT AAGCCCTAGC   
  
  
- GATGAAGGTA AAGACAGGGA CATACCCTTT TTTCTTCATA TTAAGAAAAA TAAAAATAAT TAATAAAAAA   
  
  
- TAATTAGCTC TTTAATTGCA TGATAAGTTT CTGTTTAAAT CCGAATTGGC TAAAAGAATT GGGTGAAAAC   
  
  
- AGGGCATGGC ATGAACCATG AGCGAGAAGG CTTGGTCCGG TCCATCGGTC GCAGGCAGTC ACCGTCACGT   
  
  
- GGACAAGGCG CCATAAGAAA GGGGTTGGTC AATGTTTTCA ACTCTACGGT TATTATTCTC TTTTTGGAGG   
  
  
- ATTAGGCTAA CACGTGTCGT GGGTCGGGCA CCGTCATTTG ACCGAACGTG GCACGTGGGA GCCCATCTCC   
  
  
- TGTTGGTTTT CCACGGGCAA TTATTGTGGA CGTTAGCGTA CAAATATCCA CATTTGACTC TTCTGTCTGT   
  
  
- ACATTTCTAC ATGCGTTGCT GACTCAGCTT TCGCGTCATC CCATCTAGTT CCCGTTACTC TTAAGTTTGG   
  
  
- TGGTACACTC AAAAAATAAT AATAATAATA ATTATTATTA AATTAATCCG TACATAAATA GGCAAGATCG   
  
  
- TGATTATTTT ATAATAGCAT TTTATTTGCT ATTCAATAAA GTAACGATGA TAAATGCTTA GTGTGAAACA   
  
  
- TTTATAATGA TGATCCCTGA ACCAACATAA TCATACTGAG ATGATTATAT TTTATGTATA ATAAACATCA   
  
  
- AAATAATTTT TATGTTAATT GTGTTTTTTA TTAAATTTTT TATTATTTTT AGATGAATTT TATACATAAA   
  
  
- ATATTAAATT AGTTCAAATT ATTATTGTTT TTATTTTTTT AAAAAATTAT AATTCAAATT TAATTATTGT   
  
  
- TTTTATTTTT TAAAAAAATT ATAATTCAAA TTTAAGATTT TTTATAATAA ATTTTTTAAT TTTAATTTTT   
  
  
- TTACAAAGAT AAAGAATATT CAAATTATAA TGACAAAAAA ATCAAATTTT TTAAATGCTT AAAGTGCTTT   
  
  
- TTATAAAATT TTACGTTATT AAAAATTTAA AATTAATTCA TAAAAAATAT TTTTAGCAGT AAAATAATTT   
  
  
- TGTATTTTTT TCGTTTGAAT ATCTTTTTA

+     CGTCA-motif

| Site Name | Organism | Position | Strand | Matrix score. | sequence | function |
| --- | --- | --- | --- | --- | --- | --- |
| CGTCA-motif | Hordeum vulgare | 1456 | + | 5 | CGTCA | cis-acting regulatory element involved in the MeJA-responsiveness |
| CGTCA-motif | Hordeum vulgare | 615 | + | 5 | CGTCA | cis-acting regulatory element involved in the MeJA-responsiveness |

> 2018/04/13 10:10:12  
+ GCTTCTTATG ATTCTTTCTT CTTCTTTACT TAGCCTTTCG TGGCTACTTT CAATCTTTCT CTAATCTCTC   
  
  
+ GAAGCCGATT CTAGAAACAA AAGGAGATCA CTCTTTCTCA AAACAAAGCT ACCCCAAGAA CAAAGCGAAA   
  
  
+ AGAAAGAGAG ACTCTCTCTC TCTCTCTTGG TCTTTTCCCC ATCCAAACCG AGGAGAGGTG AGGAAATATA   
  
  
+ AATGAAAAAA AAAAAAAGGG AAGCAATCAC CGGTTTTATA AGAAGTTTGT TGGGTTTTTA ACTCGAGACT   
  
  
+ AAACAATTTA AACTAATTTT TTATTTTTTA TAGAAGATAT TAAACACTGA AAAAAATGAA ATAAAATTTT   
  
  
+ GATCTGTACC AATATGAAGT ATTTTCTGTT AATTTTTAAT TTTTTTTGTT TTTTGTCAAA TTCGGGATCG   
  
  
+ CTACTTCCAT TTCTGTCCCT GTATGGGAAA AAAGAAGTAT AATTCTTTTT ATTTTTATTA ATTATTTTTT   
  
  
+ ATTAATCGAG AAATTAACGT ACTATTCAAA GACAAATTTA GGCTTAACCG ATTTTCTTAA CCCACTTTTG   
  
  
+ TCCCGTACCG TACTTGGTAC TCGCTCTTCC GAACCAGGCC AGGTAGCCAG CGTCCGTCAG TGGCAGTGCA   
  
  
+ CCTGTTCCGC GGTATTCTTT CCCCAACCAG TTACAAAAGT TGAGATGCCA ATAATAAGAG AAAAACCTCC   
  
  
+ TAATCCGATT GTGCACAGCA CCCAGCCCGT GGCAGTAAAC TGGCTTGCAC CGTGCACCCT CGGGTAGAGG   
  
  
+ ACAACCAAAA GGTGCCCGTT AATAACACCT GCAATCGCAT GTTTATAGGT GTAAACTGAG AAGACAGACA   
  
  
+ TGTAAAGATG TACGCAACGA CTGAGTCGAA AGCGCAGTAG GGTAGATCAA GGGCAATGAG AATTCAAACC   
  
  
+ ACCATGTGAG TTTTTTATTA TTATTATTAT TAATAATAAT TTAATTAGGC ATGTATTTAT CCGTTCTAGC   
  
  
+ ACTAATAAAA TATTATCGTA AAATAAACGA TAAGTTATTT CATTGCTACT ATTTACGAAT CACACTTTGT   
  
  
+ AAATATTACT ACTAGGGACT TGGTTGTATT AGTATGACTC TACTAATATA AAATACATAT TATTTGTAGT   
  
  
+ TTTATTAAAA ATACAATTAA CACAAAAAAT AATTTAAAAA ATAATAAAAA TCTACTTAAA ATATGTATTT   
  
  
+ TATAATTTAA TCAAGTTTAA TAATAACAAA AATAAAAAAA TTTTTTAATA TTAAGTTTAA ATTAATAACA   
  
  
+ AAAATAAAAA ATTTTTTTAA TATTAAGTTT AAATTCTAAA AAATATTATT TAAAAAATTA AAATTAAAAA   
  
  
+ AATGTTTCTA TTTCTTATAA GTTTAATATT ACTGTTTTTT TAGTTTAAAA AATTTACGAA TTTCACGAAA   
  
  
+ AATATTTTAA AATGCAATAA TTTTTAAATT TTAATTAAGT ATTTTTTATA AAAATCGTCA TTTTATTAAA   
  
  
+ ACATAAAAAA AGCAAACTTA TAGAAAAAT  

- CGAAGAATAC TAAGAAAGAA GAAGAAATGA ATCGGAAAGC ACCGATGAAA GTTAGAAAGA GATTAGAGAG   
  
  
- CTTCGGCTAA GATCTTTGTT TTCCTCTAGT GAGAAAGAGT TTTGTTTCGA TGGGGTTCTT GTTTCGCTTT   
  
  
- TCTTTCTCTC TGAGAGAGAG AGAGAGAACC AGAAAAGGGG TAGGTTTGGC TCCTCTCCAC TCCTTTATAT   
  
  
- TTACTTTTTT TTTTTTTCCC TTCGTTAGTG GCCAAAATAT TCTTCAAACA ACCCAAAAAT TGAGCTCTGA   
  
  
- TTTGTTAAAT TTGATTAAAA AATAAAAAAT ATCTTCTATA ATTTGTGACT TTTTTTACTT TATTTTAAAA   
  
  
- CTAGACATGG TTATACTTCA TAAAAGACAA TTAAAAATTA AAAAAAACAA AAAACAGTTT AAGCCCTAGC   
  
  
- GATGAAGGTA AAGACAGGGA CATACCCTTT TTTCTTCATA TTAAGAAAAA TAAAAATAAT TAATAAAAAA   
  
  
- TAATTAGCTC TTTAATTGCA TGATAAGTTT CTGTTTAAAT CCGAATTGGC TAAAAGAATT GGGTGAAAAC   
  
  
- AGGGCATGGC ATGAACCATG AGCGAGAAGG CTTGGTCCGG TCCATCGGTC GCAGGCAGTC ACCGTCACGT   
  
  
- GGACAAGGCG CCATAAGAAA GGGGTTGGTC AATGTTTTCA ACTCTACGGT TATTATTCTC TTTTTGGAGG   
  
  
- ATTAGGCTAA CACGTGTCGT GGGTCGGGCA CCGTCATTTG ACCGAACGTG GCACGTGGGA GCCCATCTCC   
  
  
- TGTTGGTTTT CCACGGGCAA TTATTGTGGA CGTTAGCGTA CAAATATCCA CATTTGACTC TTCTGTCTGT   
  
  
- ACATTTCTAC ATGCGTTGCT GACTCAGCTT TCGCGTCATC CCATCTAGTT CCCGTTACTC TTAAGTTTGG   
  
  
- TGGTACACTC AAAAAATAAT AATAATAATA ATTATTATTA AATTAATCCG TACATAAATA GGCAAGATCG   
  
  
- TGATTATTTT ATAATAGCAT TTTATTTGCT ATTCAATAAA GTAACGATGA TAAATGCTTA GTGTGAAACA   
  
  
- TTTATAATGA TGATCCCTGA ACCAACATAA TCATACTGAG ATGATTATAT TTTATGTATA ATAAACATCA   
  
  
- AAATAATTTT TATGTTAATT GTGTTTTTTA TTAAATTTTT TATTATTTTT AGATGAATTT TATACATAAA   
  
  
- ATATTAAATT AGTTCAAATT ATTATTGTTT TTATTTTTTT AAAAAATTAT AATTCAAATT TAATTATTGT   
  
  
- TTTTATTTTT TAAAAAAATT ATAATTCAAA TTTAAGATTT TTTATAATAA ATTTTTTAAT TTTAATTTTT   
  
  
- TTACAAAGAT AAAGAATATT CAAATTATAA TGACAAAAAA ATCAAATTTT TTAAATGCTT AAAGTGCTTT   
  
  
- TTATAAAATT TTACGTTATT AAAAATTTAA AATTAATTCA TAAAAAATAT TTTTAGCAGT AAAATAATTT   
  
  
- TGTATTTTTT TCGTTTGAAT ATCTTTTTA

+     G-box

| Site Name | Organism | Position | Strand | Matrix score. | sequence | function |
| --- | --- | --- | --- | --- | --- | --- |
| G-box | Solanum tuberosum | 912 | - | 7 | CACATGG | cis-acting regulatory element involved in light responsiveness |

> 2018/04/13 10:10:12  
+ GCTTCTTATG ATTCTTTCTT CTTCTTTACT TAGCCTTTCG TGGCTACTTT CAATCTTTCT CTAATCTCTC   
  
  
+ GAAGCCGATT CTAGAAACAA AAGGAGATCA CTCTTTCTCA AAACAAAGCT ACCCCAAGAA CAAAGCGAAA   
  
  
+ AGAAAGAGAG ACTCTCTCTC TCTCTCTTGG TCTTTTCCCC ATCCAAACCG AGGAGAGGTG AGGAAATATA   
  
  
+ AATGAAAAAA AAAAAAAGGG AAGCAATCAC CGGTTTTATA AGAAGTTTGT TGGGTTTTTA ACTCGAGACT   
  
  
+ AAACAATTTA AACTAATTTT TTATTTTTTA TAGAAGATAT TAAACACTGA AAAAAATGAA ATAAAATTTT   
  
  
+ GATCTGTACC AATATGAAGT ATTTTCTGTT AATTTTTAAT TTTTTTTGTT TTTTGTCAAA TTCGGGATCG   
  
  
+ CTACTTCCAT TTCTGTCCCT GTATGGGAAA AAAGAAGTAT AATTCTTTTT ATTTTTATTA ATTATTTTTT   
  
  
+ ATTAATCGAG AAATTAACGT ACTATTCAAA GACAAATTTA GGCTTAACCG ATTTTCTTAA CCCACTTTTG   
  
  
+ TCCCGTACCG TACTTGGTAC TCGCTCTTCC GAACCAGGCC AGGTAGCCAG CGTCCGTCAG TGGCAGTGCA   
  
  
+ CCTGTTCCGC GGTATTCTTT CCCCAACCAG TTACAAAAGT TGAGATGCCA ATAATAAGAG AAAAACCTCC   
  
  
+ TAATCCGATT GTGCACAGCA CCCAGCCCGT GGCAGTAAAC TGGCTTGCAC CGTGCACCCT CGGGTAGAGG   
  
  
+ ACAACCAAAA GGTGCCCGTT AATAACACCT GCAATCGCAT GTTTATAGGT GTAAACTGAG AAGACAGACA   
  
  
+ TGTAAAGATG TACGCAACGA CTGAGTCGAA AGCGCAGTAG GGTAGATCAA GGGCAATGAG AATTCAAACC   
  
  
+ ACCATGTGAG TTTTTTATTA TTATTATTAT TAATAATAAT TTAATTAGGC ATGTATTTAT CCGTTCTAGC   
  
  
+ ACTAATAAAA TATTATCGTA AAATAAACGA TAAGTTATTT CATTGCTACT ATTTACGAAT CACACTTTGT   
  
  
+ AAATATTACT ACTAGGGACT TGGTTGTATT AGTATGACTC TACTAATATA AAATACATAT TATTTGTAGT   
  
  
+ TTTATTAAAA ATACAATTAA CACAAAAAAT AATTTAAAAA ATAATAAAAA TCTACTTAAA ATATGTATTT   
  
  
+ TATAATTTAA TCAAGTTTAA TAATAACAAA AATAAAAAAA TTTTTTAATA TTAAGTTTAA ATTAATAACA   
  
  
+ AAAATAAAAA ATTTTTTTAA TATTAAGTTT AAATTCTAAA AAATATTATT TAAAAAATTA AAATTAAAAA   
  
  
+ AATGTTTCTA TTTCTTATAA GTTTAATATT ACTGTTTTTT TAGTTTAAAA AATTTACGAA TTTCACGAAA   
  
  
+ AATATTTTAA AATGCAATAA TTTTTAAATT TTAATTAAGT ATTTTTTATA AAAATCGTCA TTTTATTAAA   
  
  
+ ACATAAAAAA AGCAAACTTA TAGAAAAAT  

- CGAAGAATAC TAAGAAAGAA GAAGAAATGA ATCGGAAAGC ACCGATGAAA GTTAGAAAGA GATTAGAGAG   
  
  
- CTTCGGCTAA GATCTTTGTT TTCCTCTAGT GAGAAAGAGT TTTGTTTCGA TGGGGTTCTT GTTTCGCTTT   
  
  
- TCTTTCTCTC TGAGAGAGAG AGAGAGAACC AGAAAAGGGG TAGGTTTGGC TCCTCTCCAC TCCTTTATAT   
  
  
- TTACTTTTTT TTTTTTTCCC TTCGTTAGTG GCCAAAATAT TCTTCAAACA ACCCAAAAAT TGAGCTCTGA   
  
  
- TTTGTTAAAT TTGATTAAAA AATAAAAAAT ATCTTCTATA ATTTGTGACT TTTTTTACTT TATTTTAAAA   
  
  
- CTAGACATGG TTATACTTCA TAAAAGACAA TTAAAAATTA AAAAAAACAA AAAACAGTTT AAGCCCTAGC   
  
  
- GATGAAGGTA AAGACAGGGA CATACCCTTT TTTCTTCATA TTAAGAAAAA TAAAAATAAT TAATAAAAAA   
  
  
- TAATTAGCTC TTTAATTGCA TGATAAGTTT CTGTTTAAAT CCGAATTGGC TAAAAGAATT GGGTGAAAAC   
  
  
- AGGGCATGGC ATGAACCATG AGCGAGAAGG CTTGGTCCGG TCCATCGGTC GCAGGCAGTC ACCGTCACGT   
  
  
- GGACAAGGCG CCATAAGAAA GGGGTTGGTC AATGTTTTCA ACTCTACGGT TATTATTCTC TTTTTGGAGG   
  
  
- ATTAGGCTAA CACGTGTCGT GGGTCGGGCA CCGTCATTTG ACCGAACGTG GCACGTGGGA GCCCATCTCC   
  
  
- TGTTGGTTTT CCACGGGCAA TTATTGTGGA CGTTAGCGTA CAAATATCCA CATTTGACTC TTCTGTCTGT   
  
  
- ACATTTCTAC ATGCGTTGCT GACTCAGCTT TCGCGTCATC CCATCTAGTT CCCGTTACTC TTAAGTTTGG   
  
  
- TGGTACACTC AAAAAATAAT AATAATAATA ATTATTATTA AATTAATCCG TACATAAATA GGCAAGATCG   
  
  
- TGATTATTTT ATAATAGCAT TTTATTTGCT ATTCAATAAA GTAACGATGA TAAATGCTTA GTGTGAAACA   
  
  
- TTTATAATGA TGATCCCTGA ACCAACATAA TCATACTGAG ATGATTATAT TTTATGTATA ATAAACATCA   
  
  
- AAATAATTTT TATGTTAATT GTGTTTTTTA TTAAATTTTT TATTATTTTT AGATGAATTT TATACATAAA   
  
  
- ATATTAAATT AGTTCAAATT ATTATTGTTT TTATTTTTTT AAAAAATTAT AATTCAAATT TAATTATTGT   
  
  
- TTTTATTTTT TAAAAAAATT ATAATTCAAA TTTAAGATTT TTTATAATAA ATTTTTTAAT TTTAATTTTT   
  
  
- TTACAAAGAT AAAGAATATT CAAATTATAA TGACAAAAAA ATCAAATTTT TTAAATGCTT AAAGTGCTTT   
  
  
- TTATAAAATT TTACGTTATT AAAAATTTAA AATTAATTCA TAAAAAATAT TTTTAGCAGT AAAATAATTT   
  
  
- TGTATTTTTT TCGTTTGAAT ATCTTTTTA

+     GAG-motif

| Site Name | Organism | Position | Strand | Matrix score. | sequence | function |
| --- | --- | --- | --- | --- | --- | --- |
| GAG-motif | Arabidopsis thaliana | 151 | - | 7 | AGAGAGT | part of a light responsive element |

> 2018/04/13 10:10:12  
+ GCTTCTTATG ATTCTTTCTT CTTCTTTACT TAGCCTTTCG TGGCTACTTT CAATCTTTCT CTAATCTCTC   
  
  
+ GAAGCCGATT CTAGAAACAA AAGGAGATCA CTCTTTCTCA AAACAAAGCT ACCCCAAGAA CAAAGCGAAA   
  
  
+ AGAAAGAGAG ACTCTCTCTC TCTCTCTTGG TCTTTTCCCC ATCCAAACCG AGGAGAGGTG AGGAAATATA   
  
  
+ AATGAAAAAA AAAAAAAGGG AAGCAATCAC CGGTTTTATA AGAAGTTTGT TGGGTTTTTA ACTCGAGACT   
  
  
+ AAACAATTTA AACTAATTTT TTATTTTTTA TAGAAGATAT TAAACACTGA AAAAAATGAA ATAAAATTTT   
  
  
+ GATCTGTACC AATATGAAGT ATTTTCTGTT AATTTTTAAT TTTTTTTGTT TTTTGTCAAA TTCGGGATCG   
  
  
+ CTACTTCCAT TTCTGTCCCT GTATGGGAAA AAAGAAGTAT AATTCTTTTT ATTTTTATTA ATTATTTTTT   
  
  
+ ATTAATCGAG AAATTAACGT ACTATTCAAA GACAAATTTA GGCTTAACCG ATTTTCTTAA CCCACTTTTG   
  
  
+ TCCCGTACCG TACTTGGTAC TCGCTCTTCC GAACCAGGCC AGGTAGCCAG CGTCCGTCAG TGGCAGTGCA   
  
  
+ CCTGTTCCGC GGTATTCTTT CCCCAACCAG TTACAAAAGT TGAGATGCCA ATAATAAGAG AAAAACCTCC   
  
  
+ TAATCCGATT GTGCACAGCA CCCAGCCCGT GGCAGTAAAC TGGCTTGCAC CGTGCACCCT CGGGTAGAGG   
  
  
+ ACAACCAAAA GGTGCCCGTT AATAACACCT GCAATCGCAT GTTTATAGGT GTAAACTGAG AAGACAGACA   
  
  
+ TGTAAAGATG TACGCAACGA CTGAGTCGAA AGCGCAGTAG GGTAGATCAA GGGCAATGAG AATTCAAACC   
  
  
+ ACCATGTGAG TTTTTTATTA TTATTATTAT TAATAATAAT TTAATTAGGC ATGTATTTAT CCGTTCTAGC   
  
  
+ ACTAATAAAA TATTATCGTA AAATAAACGA TAAGTTATTT CATTGCTACT ATTTACGAAT CACACTTTGT   
  
  
+ AAATATTACT ACTAGGGACT TGGTTGTATT AGTATGACTC TACTAATATA AAATACATAT TATTTGTAGT   
  
  
+ TTTATTAAAA ATACAATTAA CACAAAAAAT AATTTAAAAA ATAATAAAAA TCTACTTAAA ATATGTATTT   
  
  
+ TATAATTTAA TCAAGTTTAA TAATAACAAA AATAAAAAAA TTTTTTAATA TTAAGTTTAA ATTAATAACA   
  
  
+ AAAATAAAAA ATTTTTTTAA TATTAAGTTT AAATTCTAAA AAATATTATT TAAAAAATTA AAATTAAAAA   
  
  
+ AATGTTTCTA TTTCTTATAA GTTTAATATT ACTGTTTTTT TAGTTTAAAA AATTTACGAA TTTCACGAAA   
  
  
+ AATATTTTAA AATGCAATAA TTTTTAAATT TTAATTAAGT ATTTTTTATA AAAATCGTCA TTTTATTAAA   
  
  
+ ACATAAAAAA AGCAAACTTA TAGAAAAAT  

- CGAAGAATAC TAAGAAAGAA GAAGAAATGA ATCGGAAAGC ACCGATGAAA GTTAGAAAGA GATTAGAGAG   
  
  
- CTTCGGCTAA GATCTTTGTT TTCCTCTAGT GAGAAAGAGT TTTGTTTCGA TGGGGTTCTT GTTTCGCTTT   
  
  
- TCTTTCTCTC TGAGAGAGAG AGAGAGAACC AGAAAAGGGG TAGGTTTGGC TCCTCTCCAC TCCTTTATAT   
  
  
- TTACTTTTTT TTTTTTTCCC TTCGTTAGTG GCCAAAATAT TCTTCAAACA ACCCAAAAAT TGAGCTCTGA   
  
  
- TTTGTTAAAT TTGATTAAAA AATAAAAAAT ATCTTCTATA ATTTGTGACT TTTTTTACTT TATTTTAAAA   
  
  
- CTAGACATGG TTATACTTCA TAAAAGACAA TTAAAAATTA AAAAAAACAA AAAACAGTTT AAGCCCTAGC   
  
  
- GATGAAGGTA AAGACAGGGA CATACCCTTT TTTCTTCATA TTAAGAAAAA TAAAAATAAT TAATAAAAAA   
  
  
- TAATTAGCTC TTTAATTGCA TGATAAGTTT CTGTTTAAAT CCGAATTGGC TAAAAGAATT GGGTGAAAAC   
  
  
- AGGGCATGGC ATGAACCATG AGCGAGAAGG CTTGGTCCGG TCCATCGGTC GCAGGCAGTC ACCGTCACGT   
  
  
- GGACAAGGCG CCATAAGAAA GGGGTTGGTC AATGTTTTCA ACTCTACGGT TATTATTCTC TTTTTGGAGG   
  
  
- ATTAGGCTAA CACGTGTCGT GGGTCGGGCA CCGTCATTTG ACCGAACGTG GCACGTGGGA GCCCATCTCC   
  
  
- TGTTGGTTTT CCACGGGCAA TTATTGTGGA CGTTAGCGTA CAAATATCCA CATTTGACTC TTCTGTCTGT   
  
  
- ACATTTCTAC ATGCGTTGCT GACTCAGCTT TCGCGTCATC CCATCTAGTT CCCGTTACTC TTAAGTTTGG   
  
  
- TGGTACACTC AAAAAATAAT AATAATAATA ATTATTATTA AATTAATCCG TACATAAATA GGCAAGATCG   
  
  
- TGATTATTTT ATAATAGCAT TTTATTTGCT ATTCAATAAA GTAACGATGA TAAATGCTTA GTGTGAAACA   
  
  
- TTTATAATGA TGATCCCTGA ACCAACATAA TCATACTGAG ATGATTATAT TTTATGTATA ATAAACATCA   
  
  
- AAATAATTTT TATGTTAATT GTGTTTTTTA TTAAATTTTT TATTATTTTT AGATGAATTT TATACATAAA   
  
  
- ATATTAAATT AGTTCAAATT ATTATTGTTT TTATTTTTTT AAAAAATTAT AATTCAAATT TAATTATTGT   
  
  
- TTTTATTTTT TAAAAAAATT ATAATTCAAA TTTAAGATTT TTTATAATAA ATTTTTTAAT TTTAATTTTT   
  
  
- TTACAAAGAT AAAGAATATT CAAATTATAA TGACAAAAAA ATCAAATTTT TTAAATGCTT AAAGTGCTTT   
  
  
- TTATAAAATT TTACGTTATT AAAAATTTAA AATTAATTCA TAAAAAATAT TTTTAGCAGT AAAATAATTT   
  
  
- TGTATTTTTT TCGTTTGAAT ATCTTTTTA

+     GCN4\_motif

| Site Name | Organism | Position | Strand | Matrix score. | sequence | function |
| --- | --- | --- | --- | --- | --- | --- |
| GCN4\_motif | Oryza sativa | 741 | - | 7 | CAAGCCA | cis-regulatory element involved in endosperm expression |

> 2018/04/13 10:10:12  
+ GCTTCTTATG ATTCTTTCTT CTTCTTTACT TAGCCTTTCG TGGCTACTTT CAATCTTTCT CTAATCTCTC   
  
  
+ GAAGCCGATT CTAGAAACAA AAGGAGATCA CTCTTTCTCA AAACAAAGCT ACCCCAAGAA CAAAGCGAAA   
  
  
+ AGAAAGAGAG ACTCTCTCTC TCTCTCTTGG TCTTTTCCCC ATCCAAACCG AGGAGAGGTG AGGAAATATA   
  
  
+ AATGAAAAAA AAAAAAAGGG AAGCAATCAC CGGTTTTATA AGAAGTTTGT TGGGTTTTTA ACTCGAGACT   
  
  
+ AAACAATTTA AACTAATTTT TTATTTTTTA TAGAAGATAT TAAACACTGA AAAAAATGAA ATAAAATTTT   
  
  
+ GATCTGTACC AATATGAAGT ATTTTCTGTT AATTTTTAAT TTTTTTTGTT TTTTGTCAAA TTCGGGATCG   
  
  
+ CTACTTCCAT TTCTGTCCCT GTATGGGAAA AAAGAAGTAT AATTCTTTTT ATTTTTATTA ATTATTTTTT   
  
  
+ ATTAATCGAG AAATTAACGT ACTATTCAAA GACAAATTTA GGCTTAACCG ATTTTCTTAA CCCACTTTTG   
  
  
+ TCCCGTACCG TACTTGGTAC TCGCTCTTCC GAACCAGGCC AGGTAGCCAG CGTCCGTCAG TGGCAGTGCA   
  
  
+ CCTGTTCCGC GGTATTCTTT CCCCAACCAG TTACAAAAGT TGAGATGCCA ATAATAAGAG AAAAACCTCC   
  
  
+ TAATCCGATT GTGCACAGCA CCCAGCCCGT GGCAGTAAAC TGGCTTGCAC CGTGCACCCT CGGGTAGAGG   
  
  
+ ACAACCAAAA GGTGCCCGTT AATAACACCT GCAATCGCAT GTTTATAGGT GTAAACTGAG AAGACAGACA   
  
  
+ TGTAAAGATG TACGCAACGA CTGAGTCGAA AGCGCAGTAG GGTAGATCAA GGGCAATGAG AATTCAAACC   
  
  
+ ACCATGTGAG TTTTTTATTA TTATTATTAT TAATAATAAT TTAATTAGGC ATGTATTTAT CCGTTCTAGC   
  
  
+ ACTAATAAAA TATTATCGTA AAATAAACGA TAAGTTATTT CATTGCTACT ATTTACGAAT CACACTTTGT   
  
  
+ AAATATTACT ACTAGGGACT TGGTTGTATT AGTATGACTC TACTAATATA AAATACATAT TATTTGTAGT   
  
  
+ TTTATTAAAA ATACAATTAA CACAAAAAAT AATTTAAAAA ATAATAAAAA TCTACTTAAA ATATGTATTT   
  
  
+ TATAATTTAA TCAAGTTTAA TAATAACAAA AATAAAAAAA TTTTTTAATA TTAAGTTTAA ATTAATAACA   
  
  
+ AAAATAAAAA ATTTTTTTAA TATTAAGTTT AAATTCTAAA AAATATTATT TAAAAAATTA AAATTAAAAA   
  
  
+ AATGTTTCTA TTTCTTATAA GTTTAATATT ACTGTTTTTT TAGTTTAAAA AATTTACGAA TTTCACGAAA   
  
  
+ AATATTTTAA AATGCAATAA TTTTTAAATT TTAATTAAGT ATTTTTTATA AAAATCGTCA TTTTATTAAA   
  
  
+ ACATAAAAAA AGCAAACTTA TAGAAAAAT  

- CGAAGAATAC TAAGAAAGAA GAAGAAATGA ATCGGAAAGC ACCGATGAAA GTTAGAAAGA GATTAGAGAG   
  
  
- CTTCGGCTAA GATCTTTGTT TTCCTCTAGT GAGAAAGAGT TTTGTTTCGA TGGGGTTCTT GTTTCGCTTT   
  
  
- TCTTTCTCTC TGAGAGAGAG AGAGAGAACC AGAAAAGGGG TAGGTTTGGC TCCTCTCCAC TCCTTTATAT   
  
  
- TTACTTTTTT TTTTTTTCCC TTCGTTAGTG GCCAAAATAT TCTTCAAACA ACCCAAAAAT TGAGCTCTGA   
  
  
- TTTGTTAAAT TTGATTAAAA AATAAAAAAT ATCTTCTATA ATTTGTGACT TTTTTTACTT TATTTTAAAA   
  
  
- CTAGACATGG TTATACTTCA TAAAAGACAA TTAAAAATTA AAAAAAACAA AAAACAGTTT AAGCCCTAGC   
  
  
- GATGAAGGTA AAGACAGGGA CATACCCTTT TTTCTTCATA TTAAGAAAAA TAAAAATAAT TAATAAAAAA   
  
  
- TAATTAGCTC TTTAATTGCA TGATAAGTTT CTGTTTAAAT CCGAATTGGC TAAAAGAATT GGGTGAAAAC   
  
  
- AGGGCATGGC ATGAACCATG AGCGAGAAGG CTTGGTCCGG TCCATCGGTC GCAGGCAGTC ACCGTCACGT   
  
  
- GGACAAGGCG CCATAAGAAA GGGGTTGGTC AATGTTTTCA ACTCTACGGT TATTATTCTC TTTTTGGAGG   
  
  
- ATTAGGCTAA CACGTGTCGT GGGTCGGGCA CCGTCATTTG ACCGAACGTG GCACGTGGGA GCCCATCTCC   
  
  
- TGTTGGTTTT CCACGGGCAA TTATTGTGGA CGTTAGCGTA CAAATATCCA CATTTGACTC TTCTGTCTGT   
  
  
- ACATTTCTAC ATGCGTTGCT GACTCAGCTT TCGCGTCATC CCATCTAGTT CCCGTTACTC TTAAGTTTGG   
  
  
- TGGTACACTC AAAAAATAAT AATAATAATA ATTATTATTA AATTAATCCG TACATAAATA GGCAAGATCG   
  
  
- TGATTATTTT ATAATAGCAT TTTATTTGCT ATTCAATAAA GTAACGATGA TAAATGCTTA GTGTGAAACA   
  
  
- TTTATAATGA TGATCCCTGA ACCAACATAA TCATACTGAG ATGATTATAT TTTATGTATA ATAAACATCA   
  
  
- AAATAATTTT TATGTTAATT GTGTTTTTTA TTAAATTTTT TATTATTTTT AGATGAATTT TATACATAAA   
  
  
- ATATTAAATT AGTTCAAATT ATTATTGTTT TTATTTTTTT AAAAAATTAT AATTCAAATT TAATTATTGT   
  
  
- TTTTATTTTT TAAAAAAATT ATAATTCAAA TTTAAGATTT TTTATAATAA ATTTTTTAAT TTTAATTTTT   
  
  
- TTACAAAGAT AAAGAATATT CAAATTATAA TGACAAAAAA ATCAAATTTT TTAAATGCTT AAAGTGCTTT   
  
  
- TTATAAAATT TTACGTTATT AAAAATTTAA AATTAATTCA TAAAAAATAT TTTTAGCAGT AAAATAATTT   
  
  
- TGTATTTTTT TCGTTTGAAT ATCTTTTTA

+     GT1-motif

| Site Name | Organism | Position | Strand | Matrix score. | sequence | function |
| --- | --- | --- | --- | --- | --- | --- |
| GT1-motif | Solanum tuberosum | 905 | - | 10 | ATGGTGGTTGG | light responsive element |
| GT1-motif | Arabidopsis thaliana | 534 | - | 6 | GGTTAA | light responsive element |
| GT1-motif | Arabidopsis thaliana | 547 | - | 6 | GGTTAA | light responsive element |

> 2018/04/13 10:10:12  
+ GCTTCTTATG ATTCTTTCTT CTTCTTTACT TAGCCTTTCG TGGCTACTTT CAATCTTTCT CTAATCTCTC   
  
  
+ GAAGCCGATT CTAGAAACAA AAGGAGATCA CTCTTTCTCA AAACAAAGCT ACCCCAAGAA CAAAGCGAAA   
  
  
+ AGAAAGAGAG ACTCTCTCTC TCTCTCTTGG TCTTTTCCCC ATCCAAACCG AGGAGAGGTG AGGAAATATA   
  
  
+ AATGAAAAAA AAAAAAAGGG AAGCAATCAC CGGTTTTATA AGAAGTTTGT TGGGTTTTTA ACTCGAGACT   
  
  
+ AAACAATTTA AACTAATTTT TTATTTTTTA TAGAAGATAT TAAACACTGA AAAAAATGAA ATAAAATTTT   
  
  
+ GATCTGTACC AATATGAAGT ATTTTCTGTT AATTTTTAAT TTTTTTTGTT TTTTGTCAAA TTCGGGATCG   
  
  
+ CTACTTCCAT TTCTGTCCCT GTATGGGAAA AAAGAAGTAT AATTCTTTTT ATTTTTATTA ATTATTTTTT   
  
  
+ ATTAATCGAG AAATTAACGT ACTATTCAAA GACAAATTTA GGCTTAACCG ATTTTCTTAA CCCACTTTTG   
  
  
+ TCCCGTACCG TACTTGGTAC TCGCTCTTCC GAACCAGGCC AGGTAGCCAG CGTCCGTCAG TGGCAGTGCA   
  
  
+ CCTGTTCCGC GGTATTCTTT CCCCAACCAG TTACAAAAGT TGAGATGCCA ATAATAAGAG AAAAACCTCC   
  
  
+ TAATCCGATT GTGCACAGCA CCCAGCCCGT GGCAGTAAAC TGGCTTGCAC CGTGCACCCT CGGGTAGAGG   
  
  
+ ACAACCAAAA GGTGCCCGTT AATAACACCT GCAATCGCAT GTTTATAGGT GTAAACTGAG AAGACAGACA   
  
  
+ TGTAAAGATG TACGCAACGA CTGAGTCGAA AGCGCAGTAG GGTAGATCAA GGGCAATGAG AATTCAAACC   
  
  
+ ACCATGTGAG TTTTTTATTA TTATTATTAT TAATAATAAT TTAATTAGGC ATGTATTTAT CCGTTCTAGC   
  
  
+ ACTAATAAAA TATTATCGTA AAATAAACGA TAAGTTATTT CATTGCTACT ATTTACGAAT CACACTTTGT   
  
  
+ AAATATTACT ACTAGGGACT TGGTTGTATT AGTATGACTC TACTAATATA AAATACATAT TATTTGTAGT   
  
  
+ TTTATTAAAA ATACAATTAA CACAAAAAAT AATTTAAAAA ATAATAAAAA TCTACTTAAA ATATGTATTT   
  
  
+ TATAATTTAA TCAAGTTTAA TAATAACAAA AATAAAAAAA TTTTTTAATA TTAAGTTTAA ATTAATAACA   
  
  
+ AAAATAAAAA ATTTTTTTAA TATTAAGTTT AAATTCTAAA AAATATTATT TAAAAAATTA AAATTAAAAA   
  
  
+ AATGTTTCTA TTTCTTATAA GTTTAATATT ACTGTTTTTT TAGTTTAAAA AATTTACGAA TTTCACGAAA   
  
  
+ AATATTTTAA AATGCAATAA TTTTTAAATT TTAATTAAGT ATTTTTTATA AAAATCGTCA TTTTATTAAA   
  
  
+ ACATAAAAAA AGCAAACTTA TAGAAAAAT  

- CGAAGAATAC TAAGAAAGAA GAAGAAATGA ATCGGAAAGC ACCGATGAAA GTTAGAAAGA GATTAGAGAG   
  
  
- CTTCGGCTAA GATCTTTGTT TTCCTCTAGT GAGAAAGAGT TTTGTTTCGA TGGGGTTCTT GTTTCGCTTT   
  
  
- TCTTTCTCTC TGAGAGAGAG AGAGAGAACC AGAAAAGGGG TAGGTTTGGC TCCTCTCCAC TCCTTTATAT   
  
  
- TTACTTTTTT TTTTTTTCCC TTCGTTAGTG GCCAAAATAT TCTTCAAACA ACCCAAAAAT TGAGCTCTGA   
  
  
- TTTGTTAAAT TTGATTAAAA AATAAAAAAT ATCTTCTATA ATTTGTGACT TTTTTTACTT TATTTTAAAA   
  
  
- CTAGACATGG TTATACTTCA TAAAAGACAA TTAAAAATTA AAAAAAACAA AAAACAGTTT AAGCCCTAGC   
  
  
- GATGAAGGTA AAGACAGGGA CATACCCTTT TTTCTTCATA TTAAGAAAAA TAAAAATAAT TAATAAAAAA   
  
  
- TAATTAGCTC TTTAATTGCA TGATAAGTTT CTGTTTAAAT CCGAATTGGC TAAAAGAATT GGGTGAAAAC   
  
  
- AGGGCATGGC ATGAACCATG AGCGAGAAGG CTTGGTCCGG TCCATCGGTC GCAGGCAGTC ACCGTCACGT   
  
  
- GGACAAGGCG CCATAAGAAA GGGGTTGGTC AATGTTTTCA ACTCTACGGT TATTATTCTC TTTTTGGAGG   
  
  
- ATTAGGCTAA CACGTGTCGT GGGTCGGGCA CCGTCATTTG ACCGAACGTG GCACGTGGGA GCCCATCTCC   
  
  
- TGTTGGTTTT CCACGGGCAA TTATTGTGGA CGTTAGCGTA CAAATATCCA CATTTGACTC TTCTGTCTGT   
  
  
- ACATTTCTAC ATGCGTTGCT GACTCAGCTT TCGCGTCATC CCATCTAGTT CCCGTTACTC TTAAGTTTGG   
  
  
- TGGTACACTC AAAAAATAAT AATAATAATA ATTATTATTA AATTAATCCG TACATAAATA GGCAAGATCG   
  
  
- TGATTATTTT ATAATAGCAT TTTATTTGCT ATTCAATAAA GTAACGATGA TAAATGCTTA GTGTGAAACA   
  
  
- TTTATAATGA TGATCCCTGA ACCAACATAA TCATACTGAG ATGATTATAT TTTATGTATA ATAAACATCA   
  
  
- AAATAATTTT TATGTTAATT GTGTTTTTTA TTAAATTTTT TATTATTTTT AGATGAATTT TATACATAAA   
  
  
- ATATTAAATT AGTTCAAATT ATTATTGTTT TTATTTTTTT AAAAAATTAT AATTCAAATT TAATTATTGT   
  
  
- TTTTATTTTT TAAAAAAATT ATAATTCAAA TTTAAGATTT TTTATAATAA ATTTTTTAAT TTTAATTTTT   
  
  
- TTACAAAGAT AAAGAATATT CAAATTATAA TGACAAAAAA ATCAAATTTT TTAAATGCTT AAAGTGCTTT   
  
  
- TTATAAAATT TTACGTTATT AAAAATTTAA AATTAATTCA TAAAAAATAT TTTTAGCAGT AAAATAATTT   
  
  
- TGTATTTTTT TCGTTTGAAT ATCTTTTTA

+     HSE

| Site Name | Organism | Position | Strand | Matrix score. | sequence | function |
| --- | --- | --- | --- | --- | --- | --- |
| HSE | Brassica oleracea | 1268 | - | 9 | AAAAAATTTC | cis-acting element involved in heat stress responsiveness |
| HSE | Brassica oleracea | 1377 | + | 9 | AAAAAATTTC | cis-acting element involved in heat stress responsiveness |
| HSE | Brassica oleracea | 1225 | + | 9 | AAAAAATTTC | cis-acting element involved in heat stress responsiveness |
| HSE | Brassica oleracea | 1266 | + | 9 | AAAAAATTTC | cis-acting element involved in heat stress responsiveness |
| HSE | Brassica oleracea | 1227 | - | 9 | AAAAAATTTC | cis-acting element involved in heat stress responsiveness |

> 2018/04/13 10:10:12  
+ GCTTCTTATG ATTCTTTCTT CTTCTTTACT TAGCCTTTCG TGGCTACTTT CAATCTTTCT CTAATCTCTC   
  
  
+ GAAGCCGATT CTAGAAACAA AAGGAGATCA CTCTTTCTCA AAACAAAGCT ACCCCAAGAA CAAAGCGAAA   
  
  
+ AGAAAGAGAG ACTCTCTCTC TCTCTCTTGG TCTTTTCCCC ATCCAAACCG AGGAGAGGTG AGGAAATATA   
  
  
+ AATGAAAAAA AAAAAAAGGG AAGCAATCAC CGGTTTTATA AGAAGTTTGT TGGGTTTTTA ACTCGAGACT   
  
  
+ AAACAATTTA AACTAATTTT TTATTTTTTA TAGAAGATAT TAAACACTGA AAAAAATGAA ATAAAATTTT   
  
  
+ GATCTGTACC AATATGAAGT ATTTTCTGTT AATTTTTAAT TTTTTTTGTT TTTTGTCAAA TTCGGGATCG   
  
  
+ CTACTTCCAT TTCTGTCCCT GTATGGGAAA AAAGAAGTAT AATTCTTTTT ATTTTTATTA ATTATTTTTT   
  
  
+ ATTAATCGAG AAATTAACGT ACTATTCAAA GACAAATTTA GGCTTAACCG ATTTTCTTAA CCCACTTTTG   
  
  
+ TCCCGTACCG TACTTGGTAC TCGCTCTTCC GAACCAGGCC AGGTAGCCAG CGTCCGTCAG TGGCAGTGCA   
  
  
+ CCTGTTCCGC GGTATTCTTT CCCCAACCAG TTACAAAAGT TGAGATGCCA ATAATAAGAG AAAAACCTCC   
  
  
+ TAATCCGATT GTGCACAGCA CCCAGCCCGT GGCAGTAAAC TGGCTTGCAC CGTGCACCCT CGGGTAGAGG   
  
  
+ ACAACCAAAA GGTGCCCGTT AATAACACCT GCAATCGCAT GTTTATAGGT GTAAACTGAG AAGACAGACA   
  
  
+ TGTAAAGATG TACGCAACGA CTGAGTCGAA AGCGCAGTAG GGTAGATCAA GGGCAATGAG AATTCAAACC   
  
  
+ ACCATGTGAG TTTTTTATTA TTATTATTAT TAATAATAAT TTAATTAGGC ATGTATTTAT CCGTTCTAGC   
  
  
+ ACTAATAAAA TATTATCGTA AAATAAACGA TAAGTTATTT CATTGCTACT ATTTACGAAT CACACTTTGT   
  
  
+ AAATATTACT ACTAGGGACT TGGTTGTATT AGTATGACTC TACTAATATA AAATACATAT TATTTGTAGT   
  
  
+ TTTATTAAAA ATACAATTAA CACAAAAAAT AATTTAAAAA ATAATAAAAA TCTACTTAAA ATATGTATTT   
  
  
+ TATAATTTAA TCAAGTTTAA TAATAACAAA AATAAAAAAA TTTTTTAATA TTAAGTTTAA ATTAATAACA   
  
  
+ AAAATAAAAA ATTTTTTTAA TATTAAGTTT AAATTCTAAA AAATATTATT TAAAAAATTA AAATTAAAAA   
  
  
+ AATGTTTCTA TTTCTTATAA GTTTAATATT ACTGTTTTTT TAGTTTAAAA AATTTACGAA TTTCACGAAA   
  
  
+ AATATTTTAA AATGCAATAA TTTTTAAATT TTAATTAAGT ATTTTTTATA AAAATCGTCA TTTTATTAAA   
  
  
+ ACATAAAAAA AGCAAACTTA TAGAAAAAT  

- CGAAGAATAC TAAGAAAGAA GAAGAAATGA ATCGGAAAGC ACCGATGAAA GTTAGAAAGA GATTAGAGAG   
  
  
- CTTCGGCTAA GATCTTTGTT TTCCTCTAGT GAGAAAGAGT TTTGTTTCGA TGGGGTTCTT GTTTCGCTTT   
  
  
- TCTTTCTCTC TGAGAGAGAG AGAGAGAACC AGAAAAGGGG TAGGTTTGGC TCCTCTCCAC TCCTTTATAT   
  
  
- TTACTTTTTT TTTTTTTCCC TTCGTTAGTG GCCAAAATAT TCTTCAAACA ACCCAAAAAT TGAGCTCTGA   
  
  
- TTTGTTAAAT TTGATTAAAA AATAAAAAAT ATCTTCTATA ATTTGTGACT TTTTTTACTT TATTTTAAAA   
  
  
- CTAGACATGG TTATACTTCA TAAAAGACAA TTAAAAATTA AAAAAAACAA AAAACAGTTT AAGCCCTAGC   
  
  
- GATGAAGGTA AAGACAGGGA CATACCCTTT TTTCTTCATA TTAAGAAAAA TAAAAATAAT TAATAAAAAA   
  
  
- TAATTAGCTC TTTAATTGCA TGATAAGTTT CTGTTTAAAT CCGAATTGGC TAAAAGAATT GGGTGAAAAC   
  
  
- AGGGCATGGC ATGAACCATG AGCGAGAAGG CTTGGTCCGG TCCATCGGTC GCAGGCAGTC ACCGTCACGT   
  
  
- GGACAAGGCG CCATAAGAAA GGGGTTGGTC AATGTTTTCA ACTCTACGGT TATTATTCTC TTTTTGGAGG   
  
  
- ATTAGGCTAA CACGTGTCGT GGGTCGGGCA CCGTCATTTG ACCGAACGTG GCACGTGGGA GCCCATCTCC   
  
  
- TGTTGGTTTT CCACGGGCAA TTATTGTGGA CGTTAGCGTA CAAATATCCA CATTTGACTC TTCTGTCTGT   
  
  
- ACATTTCTAC ATGCGTTGCT GACTCAGCTT TCGCGTCATC CCATCTAGTT CCCGTTACTC TTAAGTTTGG   
  
  
- TGGTACACTC AAAAAATAAT AATAATAATA ATTATTATTA AATTAATCCG TACATAAATA GGCAAGATCG   
  
  
- TGATTATTTT ATAATAGCAT TTTATTTGCT ATTCAATAAA GTAACGATGA TAAATGCTTA GTGTGAAACA   
  
  
- TTTATAATGA TGATCCCTGA ACCAACATAA TCATACTGAG ATGATTATAT TTTATGTATA ATAAACATCA   
  
  
- AAATAATTTT TATGTTAATT GTGTTTTTTA TTAAATTTTT TATTATTTTT AGATGAATTT TATACATAAA   
  
  
- ATATTAAATT AGTTCAAATT ATTATTGTTT TTATTTTTTT AAAAAATTAT AATTCAAATT TAATTATTGT   
  
  
- TTTTATTTTT TAAAAAAATT ATAATTCAAA TTTAAGATTT TTTATAATAA ATTTTTTAAT TTTAATTTTT   
  
  
- TTACAAAGAT AAAGAATATT CAAATTATAA TGACAAAAAA ATCAAATTTT TTAAATGCTT AAAGTGCTTT   
  
  
- TTATAAAATT TTACGTTATT AAAAATTTAA AATTAATTCA TAAAAAATAT TTTTAGCAGT AAAATAATTT   
  
  
- TGTATTTTTT TCGTTTGAAT ATCTTTTTA

+     MBS

| Site Name | Organism | Position | Strand | Matrix score. | sequence | function |
| --- | --- | --- | --- | --- | --- | --- |
| MBS | Arabidopsis thaliana | 658 | - | 6 | TAACTG | MYB binding site involved in drought-inducibility |

> 2018/04/13 10:10:12  
+ GCTTCTTATG ATTCTTTCTT CTTCTTTACT TAGCCTTTCG TGGCTACTTT CAATCTTTCT CTAATCTCTC   
  
  
+ GAAGCCGATT CTAGAAACAA AAGGAGATCA CTCTTTCTCA AAACAAAGCT ACCCCAAGAA CAAAGCGAAA   
  
  
+ AGAAAGAGAG ACTCTCTCTC TCTCTCTTGG TCTTTTCCCC ATCCAAACCG AGGAGAGGTG AGGAAATATA   
  
  
+ AATGAAAAAA AAAAAAAGGG AAGCAATCAC CGGTTTTATA AGAAGTTTGT TGGGTTTTTA ACTCGAGACT   
  
  
+ AAACAATTTA AACTAATTTT TTATTTTTTA TAGAAGATAT TAAACACTGA AAAAAATGAA ATAAAATTTT   
  
  
+ GATCTGTACC AATATGAAGT ATTTTCTGTT AATTTTTAAT TTTTTTTGTT TTTTGTCAAA TTCGGGATCG   
  
  
+ CTACTTCCAT TTCTGTCCCT GTATGGGAAA AAAGAAGTAT AATTCTTTTT ATTTTTATTA ATTATTTTTT   
  
  
+ ATTAATCGAG AAATTAACGT ACTATTCAAA GACAAATTTA GGCTTAACCG ATTTTCTTAA CCCACTTTTG   
  
  
+ TCCCGTACCG TACTTGGTAC TCGCTCTTCC GAACCAGGCC AGGTAGCCAG CGTCCGTCAG TGGCAGTGCA   
  
  
+ CCTGTTCCGC GGTATTCTTT CCCCAACCAG TTACAAAAGT TGAGATGCCA ATAATAAGAG AAAAACCTCC   
  
  
+ TAATCCGATT GTGCACAGCA CCCAGCCCGT GGCAGTAAAC TGGCTTGCAC CGTGCACCCT CGGGTAGAGG   
  
  
+ ACAACCAAAA GGTGCCCGTT AATAACACCT GCAATCGCAT GTTTATAGGT GTAAACTGAG AAGACAGACA   
  
  
+ TGTAAAGATG TACGCAACGA CTGAGTCGAA AGCGCAGTAG GGTAGATCAA GGGCAATGAG AATTCAAACC   
  
  
+ ACCATGTGAG TTTTTTATTA TTATTATTAT TAATAATAAT TTAATTAGGC ATGTATTTAT CCGTTCTAGC   
  
  
+ ACTAATAAAA TATTATCGTA AAATAAACGA TAAGTTATTT CATTGCTACT ATTTACGAAT CACACTTTGT   
  
  
+ AAATATTACT ACTAGGGACT TGGTTGTATT AGTATGACTC TACTAATATA AAATACATAT TATTTGTAGT   
  
  
+ TTTATTAAAA ATACAATTAA CACAAAAAAT AATTTAAAAA ATAATAAAAA TCTACTTAAA ATATGTATTT   
  
  
+ TATAATTTAA TCAAGTTTAA TAATAACAAA AATAAAAAAA TTTTTTAATA TTAAGTTTAA ATTAATAACA   
  
  
+ AAAATAAAAA ATTTTTTTAA TATTAAGTTT AAATTCTAAA AAATATTATT TAAAAAATTA AAATTAAAAA   
  
  
+ AATGTTTCTA TTTCTTATAA GTTTAATATT ACTGTTTTTT TAGTTTAAAA AATTTACGAA TTTCACGAAA   
  
  
+ AATATTTTAA AATGCAATAA TTTTTAAATT TTAATTAAGT ATTTTTTATA AAAATCGTCA TTTTATTAAA   
  
  
+ ACATAAAAAA AGCAAACTTA TAGAAAAAT  

- CGAAGAATAC TAAGAAAGAA GAAGAAATGA ATCGGAAAGC ACCGATGAAA GTTAGAAAGA GATTAGAGAG   
  
  
- CTTCGGCTAA GATCTTTGTT TTCCTCTAGT GAGAAAGAGT TTTGTTTCGA TGGGGTTCTT GTTTCGCTTT   
  
  
- TCTTTCTCTC TGAGAGAGAG AGAGAGAACC AGAAAAGGGG TAGGTTTGGC TCCTCTCCAC TCCTTTATAT   
  
  
- TTACTTTTTT TTTTTTTCCC TTCGTTAGTG GCCAAAATAT TCTTCAAACA ACCCAAAAAT TGAGCTCTGA   
  
  
- TTTGTTAAAT TTGATTAAAA AATAAAAAAT ATCTTCTATA ATTTGTGACT TTTTTTACTT TATTTTAAAA   
  
  
- CTAGACATGG TTATACTTCA TAAAAGACAA TTAAAAATTA AAAAAAACAA AAAACAGTTT AAGCCCTAGC   
  
  
- GATGAAGGTA AAGACAGGGA CATACCCTTT TTTCTTCATA TTAAGAAAAA TAAAAATAAT TAATAAAAAA   
  
  
- TAATTAGCTC TTTAATTGCA TGATAAGTTT CTGTTTAAAT CCGAATTGGC TAAAAGAATT GGGTGAAAAC   
  
  
- AGGGCATGGC ATGAACCATG AGCGAGAAGG CTTGGTCCGG TCCATCGGTC GCAGGCAGTC ACCGTCACGT   
  
  
- GGACAAGGCG CCATAAGAAA GGGGTTGGTC AATGTTTTCA ACTCTACGGT TATTATTCTC TTTTTGGAGG   
  
  
- ATTAGGCTAA CACGTGTCGT GGGTCGGGCA CCGTCATTTG ACCGAACGTG GCACGTGGGA GCCCATCTCC   
  
  
- TGTTGGTTTT CCACGGGCAA TTATTGTGGA CGTTAGCGTA CAAATATCCA CATTTGACTC TTCTGTCTGT   
  
  
- ACATTTCTAC ATGCGTTGCT GACTCAGCTT TCGCGTCATC CCATCTAGTT CCCGTTACTC TTAAGTTTGG   
  
  
- TGGTACACTC AAAAAATAAT AATAATAATA ATTATTATTA AATTAATCCG TACATAAATA GGCAAGATCG   
  
  
- TGATTATTTT ATAATAGCAT TTTATTTGCT ATTCAATAAA GTAACGATGA TAAATGCTTA GTGTGAAACA   
  
  
- TTTATAATGA TGATCCCTGA ACCAACATAA TCATACTGAG ATGATTATAT TTTATGTATA ATAAACATCA   
  
  
- AAATAATTTT TATGTTAATT GTGTTTTTTA TTAAATTTTT TATTATTTTT AGATGAATTT TATACATAAA   
  
  
- ATATTAAATT AGTTCAAATT ATTATTGTTT TTATTTTTTT AAAAAATTAT AATTCAAATT TAATTATTGT   
  
  
- TTTTATTTTT TAAAAAAATT ATAATTCAAA TTTAAGATTT TTTATAATAA ATTTTTTAAT TTTAATTTTT   
  
  
- TTACAAAGAT AAAGAATATT CAAATTATAA TGACAAAAAA ATCAAATTTT TTAAATGCTT AAAGTGCTTT   
  
  
- TTATAAAATT TTACGTTATT AAAAATTTAA AATTAATTCA TAAAAAATAT TTTTAGCAGT AAAATAATTT   
  
  
- TGTATTTTTT TCGTTTGAAT ATCTTTTTA

+     MNF1

| Site Name | Organism | Position | Strand | Matrix score. | sequence | function |
| --- | --- | --- | --- | --- | --- | --- |
| MNF1 | Zea mays | 782 | + | 6.5 | GTGCCC(A/T)(A/T) | light responsive element |

> 2018/04/13 10:10:12  
+ GCTTCTTATG ATTCTTTCTT CTTCTTTACT TAGCCTTTCG TGGCTACTTT CAATCTTTCT CTAATCTCTC   
  
  
+ GAAGCCGATT CTAGAAACAA AAGGAGATCA CTCTTTCTCA AAACAAAGCT ACCCCAAGAA CAAAGCGAAA   
  
  
+ AGAAAGAGAG ACTCTCTCTC TCTCTCTTGG TCTTTTCCCC ATCCAAACCG AGGAGAGGTG AGGAAATATA   
  
  
+ AATGAAAAAA AAAAAAAGGG AAGCAATCAC CGGTTTTATA AGAAGTTTGT TGGGTTTTTA ACTCGAGACT   
  
  
+ AAACAATTTA AACTAATTTT TTATTTTTTA TAGAAGATAT TAAACACTGA AAAAAATGAA ATAAAATTTT   
  
  
+ GATCTGTACC AATATGAAGT ATTTTCTGTT AATTTTTAAT TTTTTTTGTT TTTTGTCAAA TTCGGGATCG   
  
  
+ CTACTTCCAT TTCTGTCCCT GTATGGGAAA AAAGAAGTAT AATTCTTTTT ATTTTTATTA ATTATTTTTT   
  
  
+ ATTAATCGAG AAATTAACGT ACTATTCAAA GACAAATTTA GGCTTAACCG ATTTTCTTAA CCCACTTTTG   
  
  
+ TCCCGTACCG TACTTGGTAC TCGCTCTTCC GAACCAGGCC AGGTAGCCAG CGTCCGTCAG TGGCAGTGCA   
  
  
+ CCTGTTCCGC GGTATTCTTT CCCCAACCAG TTACAAAAGT TGAGATGCCA ATAATAAGAG AAAAACCTCC   
  
  
+ TAATCCGATT GTGCACAGCA CCCAGCCCGT GGCAGTAAAC TGGCTTGCAC CGTGCACCCT CGGGTAGAGG   
  
  
+ ACAACCAAAA GGTGCCCGTT AATAACACCT GCAATCGCAT GTTTATAGGT GTAAACTGAG AAGACAGACA   
  
  
+ TGTAAAGATG TACGCAACGA CTGAGTCGAA AGCGCAGTAG GGTAGATCAA GGGCAATGAG AATTCAAACC   
  
  
+ ACCATGTGAG TTTTTTATTA TTATTATTAT TAATAATAAT TTAATTAGGC ATGTATTTAT CCGTTCTAGC   
  
  
+ ACTAATAAAA TATTATCGTA AAATAAACGA TAAGTTATTT CATTGCTACT ATTTACGAAT CACACTTTGT   
  
  
+ AAATATTACT ACTAGGGACT TGGTTGTATT AGTATGACTC TACTAATATA AAATACATAT TATTTGTAGT   
  
  
+ TTTATTAAAA ATACAATTAA CACAAAAAAT AATTTAAAAA ATAATAAAAA TCTACTTAAA ATATGTATTT   
  
  
+ TATAATTTAA TCAAGTTTAA TAATAACAAA AATAAAAAAA TTTTTTAATA TTAAGTTTAA ATTAATAACA   
  
  
+ AAAATAAAAA ATTTTTTTAA TATTAAGTTT AAATTCTAAA AAATATTATT TAAAAAATTA AAATTAAAAA   
  
  
+ AATGTTTCTA TTTCTTATAA GTTTAATATT ACTGTTTTTT TAGTTTAAAA AATTTACGAA TTTCACGAAA   
  
  
+ AATATTTTAA AATGCAATAA TTTTTAAATT TTAATTAAGT ATTTTTTATA AAAATCGTCA TTTTATTAAA   
  
  
+ ACATAAAAAA AGCAAACTTA TAGAAAAAT  

- CGAAGAATAC TAAGAAAGAA GAAGAAATGA ATCGGAAAGC ACCGATGAAA GTTAGAAAGA GATTAGAGAG   
  
  
- CTTCGGCTAA GATCTTTGTT TTCCTCTAGT GAGAAAGAGT TTTGTTTCGA TGGGGTTCTT GTTTCGCTTT   
  
  
- TCTTTCTCTC TGAGAGAGAG AGAGAGAACC AGAAAAGGGG TAGGTTTGGC TCCTCTCCAC TCCTTTATAT   
  
  
- TTACTTTTTT TTTTTTTCCC TTCGTTAGTG GCCAAAATAT TCTTCAAACA ACCCAAAAAT TGAGCTCTGA   
  
  
- TTTGTTAAAT TTGATTAAAA AATAAAAAAT ATCTTCTATA ATTTGTGACT TTTTTTACTT TATTTTAAAA   
  
  
- CTAGACATGG TTATACTTCA TAAAAGACAA TTAAAAATTA AAAAAAACAA AAAACAGTTT AAGCCCTAGC   
  
  
- GATGAAGGTA AAGACAGGGA CATACCCTTT TTTCTTCATA TTAAGAAAAA TAAAAATAAT TAATAAAAAA   
  
  
- TAATTAGCTC TTTAATTGCA TGATAAGTTT CTGTTTAAAT CCGAATTGGC TAAAAGAATT GGGTGAAAAC   
  
  
- AGGGCATGGC ATGAACCATG AGCGAGAAGG CTTGGTCCGG TCCATCGGTC GCAGGCAGTC ACCGTCACGT   
  
  
- GGACAAGGCG CCATAAGAAA GGGGTTGGTC AATGTTTTCA ACTCTACGGT TATTATTCTC TTTTTGGAGG   
  
  
- ATTAGGCTAA CACGTGTCGT GGGTCGGGCA CCGTCATTTG ACCGAACGTG GCACGTGGGA GCCCATCTCC   
  
  
- TGTTGGTTTT CCACGGGCAA TTATTGTGGA CGTTAGCGTA CAAATATCCA CATTTGACTC TTCTGTCTGT   
  
  
- ACATTTCTAC ATGCGTTGCT GACTCAGCTT TCGCGTCATC CCATCTAGTT CCCGTTACTC TTAAGTTTGG   
  
  
- TGGTACACTC AAAAAATAAT AATAATAATA ATTATTATTA AATTAATCCG TACATAAATA GGCAAGATCG   
  
  
- TGATTATTTT ATAATAGCAT TTTATTTGCT ATTCAATAAA GTAACGATGA TAAATGCTTA GTGTGAAACA   
  
  
- TTTATAATGA TGATCCCTGA ACCAACATAA TCATACTGAG ATGATTATAT TTTATGTATA ATAAACATCA   
  
  
- AAATAATTTT TATGTTAATT GTGTTTTTTA TTAAATTTTT TATTATTTTT AGATGAATTT TATACATAAA   
  
  
- ATATTAAATT AGTTCAAATT ATTATTGTTT TTATTTTTTT AAAAAATTAT AATTCAAATT TAATTATTGT   
  
  
- TTTTATTTTT TAAAAAAATT ATAATTCAAA TTTAAGATTT TTTATAATAA ATTTTTTAAT TTTAATTTTT   
  
  
- TTACAAAGAT AAAGAATATT CAAATTATAA TGACAAAAAA ATCAAATTTT TTAAATGCTT AAAGTGCTTT   
  
  
- TTATAAAATT TTACGTTATT AAAAATTTAA AATTAATTCA TAAAAAATAT TTTTAGCAGT AAAATAATTT   
  
  
- TGTATTTTTT TCGTTTGAAT ATCTTTTTA

+     P-box

| Site Name | Organism | Position | Strand | Matrix score. | sequence | function |
| --- | --- | --- | --- | --- | --- | --- |
| P-box | Oryza sativa | 88 | - | 7 | CCTTTTG | gibberellin-responsive element |
| P-box | Oryza sativa | 776 | - | 7 | CCTTTTG | gibberellin-responsive element |

> 2018/04/13 10:10:12  
+ GCTTCTTATG ATTCTTTCTT CTTCTTTACT TAGCCTTTCG TGGCTACTTT CAATCTTTCT CTAATCTCTC   
  
  
+ GAAGCCGATT CTAGAAACAA AAGGAGATCA CTCTTTCTCA AAACAAAGCT ACCCCAAGAA CAAAGCGAAA   
  
  
+ AGAAAGAGAG ACTCTCTCTC TCTCTCTTGG TCTTTTCCCC ATCCAAACCG AGGAGAGGTG AGGAAATATA   
  
  
+ AATGAAAAAA AAAAAAAGGG AAGCAATCAC CGGTTTTATA AGAAGTTTGT TGGGTTTTTA ACTCGAGACT   
  
  
+ AAACAATTTA AACTAATTTT TTATTTTTTA TAGAAGATAT TAAACACTGA AAAAAATGAA ATAAAATTTT   
  
  
+ GATCTGTACC AATATGAAGT ATTTTCTGTT AATTTTTAAT TTTTTTTGTT TTTTGTCAAA TTCGGGATCG   
  
  
+ CTACTTCCAT TTCTGTCCCT GTATGGGAAA AAAGAAGTAT AATTCTTTTT ATTTTTATTA ATTATTTTTT   
  
  
+ ATTAATCGAG AAATTAACGT ACTATTCAAA GACAAATTTA GGCTTAACCG ATTTTCTTAA CCCACTTTTG   
  
  
+ TCCCGTACCG TACTTGGTAC TCGCTCTTCC GAACCAGGCC AGGTAGCCAG CGTCCGTCAG TGGCAGTGCA   
  
  
+ CCTGTTCCGC GGTATTCTTT CCCCAACCAG TTACAAAAGT TGAGATGCCA ATAATAAGAG AAAAACCTCC   
  
  
+ TAATCCGATT GTGCACAGCA CCCAGCCCGT GGCAGTAAAC TGGCTTGCAC CGTGCACCCT CGGGTAGAGG   
  
  
+ ACAACCAAAA GGTGCCCGTT AATAACACCT GCAATCGCAT GTTTATAGGT GTAAACTGAG AAGACAGACA   
  
  
+ TGTAAAGATG TACGCAACGA CTGAGTCGAA AGCGCAGTAG GGTAGATCAA GGGCAATGAG AATTCAAACC   
  
  
+ ACCATGTGAG TTTTTTATTA TTATTATTAT TAATAATAAT TTAATTAGGC ATGTATTTAT CCGTTCTAGC   
  
  
+ ACTAATAAAA TATTATCGTA AAATAAACGA TAAGTTATTT CATTGCTACT ATTTACGAAT CACACTTTGT   
  
  
+ AAATATTACT ACTAGGGACT TGGTTGTATT AGTATGACTC TACTAATATA AAATACATAT TATTTGTAGT   
  
  
+ TTTATTAAAA ATACAATTAA CACAAAAAAT AATTTAAAAA ATAATAAAAA TCTACTTAAA ATATGTATTT   
  
  
+ TATAATTTAA TCAAGTTTAA TAATAACAAA AATAAAAAAA TTTTTTAATA TTAAGTTTAA ATTAATAACA   
  
  
+ AAAATAAAAA ATTTTTTTAA TATTAAGTTT AAATTCTAAA AAATATTATT TAAAAAATTA AAATTAAAAA   
  
  
+ AATGTTTCTA TTTCTTATAA GTTTAATATT ACTGTTTTTT TAGTTTAAAA AATTTACGAA TTTCACGAAA   
  
  
+ AATATTTTAA AATGCAATAA TTTTTAAATT TTAATTAAGT ATTTTTTATA AAAATCGTCA TTTTATTAAA   
  
  
+ ACATAAAAAA AGCAAACTTA TAGAAAAAT  

- CGAAGAATAC TAAGAAAGAA GAAGAAATGA ATCGGAAAGC ACCGATGAAA GTTAGAAAGA GATTAGAGAG   
  
  
- CTTCGGCTAA GATCTTTGTT TTCCTCTAGT GAGAAAGAGT TTTGTTTCGA TGGGGTTCTT GTTTCGCTTT   
  
  
- TCTTTCTCTC TGAGAGAGAG AGAGAGAACC AGAAAAGGGG TAGGTTTGGC TCCTCTCCAC TCCTTTATAT   
  
  
- TTACTTTTTT TTTTTTTCCC TTCGTTAGTG GCCAAAATAT TCTTCAAACA ACCCAAAAAT TGAGCTCTGA   
  
  
- TTTGTTAAAT TTGATTAAAA AATAAAAAAT ATCTTCTATA ATTTGTGACT TTTTTTACTT TATTTTAAAA   
  
  
- CTAGACATGG TTATACTTCA TAAAAGACAA TTAAAAATTA AAAAAAACAA AAAACAGTTT AAGCCCTAGC   
  
  
- GATGAAGGTA AAGACAGGGA CATACCCTTT TTTCTTCATA TTAAGAAAAA TAAAAATAAT TAATAAAAAA   
  
  
- TAATTAGCTC TTTAATTGCA TGATAAGTTT CTGTTTAAAT CCGAATTGGC TAAAAGAATT GGGTGAAAAC   
  
  
- AGGGCATGGC ATGAACCATG AGCGAGAAGG CTTGGTCCGG TCCATCGGTC GCAGGCAGTC ACCGTCACGT   
  
  
- GGACAAGGCG CCATAAGAAA GGGGTTGGTC AATGTTTTCA ACTCTACGGT TATTATTCTC TTTTTGGAGG   
  
  
- ATTAGGCTAA CACGTGTCGT GGGTCGGGCA CCGTCATTTG ACCGAACGTG GCACGTGGGA GCCCATCTCC   
  
  
- TGTTGGTTTT CCACGGGCAA TTATTGTGGA CGTTAGCGTA CAAATATCCA CATTTGACTC TTCTGTCTGT   
  
  
- ACATTTCTAC ATGCGTTGCT GACTCAGCTT TCGCGTCATC CCATCTAGTT CCCGTTACTC TTAAGTTTGG   
  
  
- TGGTACACTC AAAAAATAAT AATAATAATA ATTATTATTA AATTAATCCG TACATAAATA GGCAAGATCG   
  
  
- TGATTATTTT ATAATAGCAT TTTATTTGCT ATTCAATAAA GTAACGATGA TAAATGCTTA GTGTGAAACA   
  
  
- TTTATAATGA TGATCCCTGA ACCAACATAA TCATACTGAG ATGATTATAT TTTATGTATA ATAAACATCA   
  
  
- AAATAATTTT TATGTTAATT GTGTTTTTTA TTAAATTTTT TATTATTTTT AGATGAATTT TATACATAAA   
  
  
- ATATTAAATT AGTTCAAATT ATTATTGTTT TTATTTTTTT AAAAAATTAT AATTCAAATT TAATTATTGT   
  
  
- TTTTATTTTT TAAAAAAATT ATAATTCAAA TTTAAGATTT TTTATAATAA ATTTTTTAAT TTTAATTTTT   
  
  
- TTACAAAGAT AAAGAATATT CAAATTATAA TGACAAAAAA ATCAAATTTT TTAAATGCTT AAAGTGCTTT   
  
  
- TTATAAAATT TTACGTTATT AAAAATTTAA AATTAATTCA TAAAAAATAT TTTTAGCAGT AAAATAATTT   
  
  
- TGTATTTTTT TCGTTTGAAT ATCTTTTTA

+     Skn-1\_motif

| Site Name | Organism | Position | Strand | Matrix score. | sequence | function |
| --- | --- | --- | --- | --- | --- | --- |
| Skn-1\_motif | Oryza sativa | 1084 | - | 5 | GTCAT | cis-acting regulatory element required for endosperm expression |
| Skn-1\_motif | Oryza sativa | 1457 | + | 5 | GTCAT | cis-acting regulatory element required for endosperm expression |

> 2018/04/13 10:10:12  
+ GCTTCTTATG ATTCTTTCTT CTTCTTTACT TAGCCTTTCG TGGCTACTTT CAATCTTTCT CTAATCTCTC   
  
  
+ GAAGCCGATT CTAGAAACAA AAGGAGATCA CTCTTTCTCA AAACAAAGCT ACCCCAAGAA CAAAGCGAAA   
  
  
+ AGAAAGAGAG ACTCTCTCTC TCTCTCTTGG TCTTTTCCCC ATCCAAACCG AGGAGAGGTG AGGAAATATA   
  
  
+ AATGAAAAAA AAAAAAAGGG AAGCAATCAC CGGTTTTATA AGAAGTTTGT TGGGTTTTTA ACTCGAGACT   
  
  
+ AAACAATTTA AACTAATTTT TTATTTTTTA TAGAAGATAT TAAACACTGA AAAAAATGAA ATAAAATTTT   
  
  
+ GATCTGTACC AATATGAAGT ATTTTCTGTT AATTTTTAAT TTTTTTTGTT TTTTGTCAAA TTCGGGATCG   
  
  
+ CTACTTCCAT TTCTGTCCCT GTATGGGAAA AAAGAAGTAT AATTCTTTTT ATTTTTATTA ATTATTTTTT   
  
  
+ ATTAATCGAG AAATTAACGT ACTATTCAAA GACAAATTTA GGCTTAACCG ATTTTCTTAA CCCACTTTTG   
  
  
+ TCCCGTACCG TACTTGGTAC TCGCTCTTCC GAACCAGGCC AGGTAGCCAG CGTCCGTCAG TGGCAGTGCA   
  
  
+ CCTGTTCCGC GGTATTCTTT CCCCAACCAG TTACAAAAGT TGAGATGCCA ATAATAAGAG AAAAACCTCC   
  
  
+ TAATCCGATT GTGCACAGCA CCCAGCCCGT GGCAGTAAAC TGGCTTGCAC CGTGCACCCT CGGGTAGAGG   
  
  
+ ACAACCAAAA GGTGCCCGTT AATAACACCT GCAATCGCAT GTTTATAGGT GTAAACTGAG AAGACAGACA   
  
  
+ TGTAAAGATG TACGCAACGA CTGAGTCGAA AGCGCAGTAG GGTAGATCAA GGGCAATGAG AATTCAAACC   
  
  
+ ACCATGTGAG TTTTTTATTA TTATTATTAT TAATAATAAT TTAATTAGGC ATGTATTTAT CCGTTCTAGC   
  
  
+ ACTAATAAAA TATTATCGTA AAATAAACGA TAAGTTATTT CATTGCTACT ATTTACGAAT CACACTTTGT   
  
  
+ AAATATTACT ACTAGGGACT TGGTTGTATT AGTATGACTC TACTAATATA AAATACATAT TATTTGTAGT   
  
  
+ TTTATTAAAA ATACAATTAA CACAAAAAAT AATTTAAAAA ATAATAAAAA TCTACTTAAA ATATGTATTT   
  
  
+ TATAATTTAA TCAAGTTTAA TAATAACAAA AATAAAAAAA TTTTTTAATA TTAAGTTTAA ATTAATAACA   
  
  
+ AAAATAAAAA ATTTTTTTAA TATTAAGTTT AAATTCTAAA AAATATTATT TAAAAAATTA AAATTAAAAA   
  
  
+ AATGTTTCTA TTTCTTATAA GTTTAATATT ACTGTTTTTT TAGTTTAAAA AATTTACGAA TTTCACGAAA   
  
  
+ AATATTTTAA AATGCAATAA TTTTTAAATT TTAATTAAGT ATTTTTTATA AAAATCGTCA TTTTATTAAA   
  
  
+ ACATAAAAAA AGCAAACTTA TAGAAAAAT  

- CGAAGAATAC TAAGAAAGAA GAAGAAATGA ATCGGAAAGC ACCGATGAAA GTTAGAAAGA GATTAGAGAG   
  
  
- CTTCGGCTAA GATCTTTGTT TTCCTCTAGT GAGAAAGAGT TTTGTTTCGA TGGGGTTCTT GTTTCGCTTT   
  
  
- TCTTTCTCTC TGAGAGAGAG AGAGAGAACC AGAAAAGGGG TAGGTTTGGC TCCTCTCCAC TCCTTTATAT   
  
  
- TTACTTTTTT TTTTTTTCCC TTCGTTAGTG GCCAAAATAT TCTTCAAACA ACCCAAAAAT TGAGCTCTGA   
  
  
- TTTGTTAAAT TTGATTAAAA AATAAAAAAT ATCTTCTATA ATTTGTGACT TTTTTTACTT TATTTTAAAA   
  
  
- CTAGACATGG TTATACTTCA TAAAAGACAA TTAAAAATTA AAAAAAACAA AAAACAGTTT AAGCCCTAGC   
  
  
- GATGAAGGTA AAGACAGGGA CATACCCTTT TTTCTTCATA TTAAGAAAAA TAAAAATAAT TAATAAAAAA   
  
  
- TAATTAGCTC TTTAATTGCA TGATAAGTTT CTGTTTAAAT CCGAATTGGC TAAAAGAATT GGGTGAAAAC   
  
  
- AGGGCATGGC ATGAACCATG AGCGAGAAGG CTTGGTCCGG TCCATCGGTC GCAGGCAGTC ACCGTCACGT   
  
  
- GGACAAGGCG CCATAAGAAA GGGGTTGGTC AATGTTTTCA ACTCTACGGT TATTATTCTC TTTTTGGAGG   
  
  
- ATTAGGCTAA CACGTGTCGT GGGTCGGGCA CCGTCATTTG ACCGAACGTG GCACGTGGGA GCCCATCTCC   
  
  
- TGTTGGTTTT CCACGGGCAA TTATTGTGGA CGTTAGCGTA CAAATATCCA CATTTGACTC TTCTGTCTGT   
  
  
- ACATTTCTAC ATGCGTTGCT GACTCAGCTT TCGCGTCATC CCATCTAGTT CCCGTTACTC TTAAGTTTGG   
  
  
- TGGTACACTC AAAAAATAAT AATAATAATA ATTATTATTA AATTAATCCG TACATAAATA GGCAAGATCG   
  
  
- TGATTATTTT ATAATAGCAT TTTATTTGCT ATTCAATAAA GTAACGATGA TAAATGCTTA GTGTGAAACA   
  
  
- TTTATAATGA TGATCCCTGA ACCAACATAA TCATACTGAG ATGATTATAT TTTATGTATA ATAAACATCA   
  
  
- AAATAATTTT TATGTTAATT GTGTTTTTTA TTAAATTTTT TATTATTTTT AGATGAATTT TATACATAAA   
  
  
- ATATTAAATT AGTTCAAATT ATTATTGTTT TTATTTTTTT AAAAAATTAT AATTCAAATT TAATTATTGT   
  
  
- TTTTATTTTT TAAAAAAATT ATAATTCAAA TTTAAGATTT TTTATAATAA ATTTTTTAAT TTTAATTTTT   
  
  
- TTACAAAGAT AAAGAATATT CAAATTATAA TGACAAAAAA ATCAAATTTT TTAAATGCTT AAAGTGCTTT   
  
  
- TTATAAAATT TTACGTTATT AAAAATTTAA AATTAATTCA TAAAAAATAT TTTTAGCAGT AAAATAATTT   
  
  
- TGTATTTTTT TCGTTTGAAT ATCTTTTTA

+     TATA-box

| Site Name | Organism | Position | Strand | Matrix score. | sequence | function |
| --- | --- | --- | --- | --- | --- | --- |
| TATA-box | Lycopersicon esculentum | 999 | - | 5 | TTTTA | core promoter element around -30 of transcription start |
| TATA-box | Arabidopsis thaliana | 1345 | - | 5 | TATAA | core promoter element around -30 of transcription start |
| TATA-box | Oryza sativa | 1342 | - | 8 | TATAAGAA | core promoter element around -30 of transcription start |
| TATA-box | Lycopersicon esculentum | 1311 | - | 5 | TTTTA | core promoter element around -30 of transcription start |
| TATA-box | Arabidopsis thaliana | 1189 | - | 6 | TATAAA | core promoter element around -30 of transcription start |
| TATA-box | Arabidopsis thaliana | 1188 | - | 7 | TATAAAA | core promoter element around -30 of transcription start |
| TATA-box | Lycopersicon esculentum | 1165 | - | 5 | TTTTA | core promoter element around -30 of transcription start |
| TATA-box | Arabidopsis thaliana | 1488 | - | 5 | TATAA | core promoter element around -30 of transcription start |
| TATA-box | Arabidopsis thaliana | 1307 | + | 8 | TATTTAAA | core promoter element around -30 of transcription start |
| TATA-box | Lycopersicon esculentum | 1275 | + | 5 | TTTTA | core promoter element around -30 of transcription start |
| TATA-box | Glycine max | 941 | + | 5 | TAATA | core promoter element around -30 of transcription start |
| TATA-box | Lycopersicon esculentum | 1265 | - | 5 | TTTTA | core promoter element around -30 of transcription start |
| TATA-box | Lycopersicon esculentum | 1461 | + | 5 | TTTTA | core promoter element around -30 of transcription start |
| TATA-box | Glycine max | 1123 | - | 5 | TAATA | core promoter element around -30 of transcription start |
| TATA-box | Lycopersicon esculentum | 1126 | - | 5 | TTTTA | core promoter element around -30 of transcription start |
| TATA-box | Lycopersicon esculentum | 923 | + | 5 | TTTTA | core promoter element around -30 of transcription start |
| TATA-box | Arabidopsis thaliana | 469 | - | 9 | TAAAAATAA | core promoter element around -30 of transcription start |
| TATA-box | Lycopersicon esculentum | 1120 | + | 5 | TTTTA | core promoter element around -30 of transcription start |
| TATA-box | Arabidopsis thaliana | 812 | - | 6 | TATAAA | core promoter element around -30 of transcription start |
| TATA-box | Arabidopsis thaliana | 1489 | - | 4 | TATA | core promoter element around -30 of transcription start |
| TATA-box | Glycine max | 1108 | - | 5 | TAATA | core promoter element around -30 of transcription start |
| TATA-box | Lycopersicon esculentum | 1099 | - | 5 | TTTTA | core promoter element around -30 of transcription start |
| TATA-box | Lycopersicon esculentum | 473 | + | 5 | TTTTA | core promoter element around -30 of transcription start |
| TATA-box | Lycopersicon esculentum | 1325 | - | 5 | TTTTA | core promoter element around -30 of transcription start |
| TATA-box | Lycopersicon esculentum | 1449 | - | 5 | TTTTA | core promoter element around -30 of transcription start |
| TATA-box | Arabidopsis thaliana | 1447 | + | 6 | TATAAA | core promoter element around -30 of transcription start |
| TATA-box | Lycopersicon esculentum | 1429 | + | 5 | TTTTA | core promoter element around -30 of transcription start |
| TATA-box | Lycopersicon esculentum | 1233 | + | 5 | TTTTA | core promoter element around -30 of transcription start |
| TATA-box | Glycine max | 1077 | - | 5 | TAATA | core promoter element around -30 of transcription start |
| TATA-box | Arabidopsis thaliana | 1444 | - | 7 | TATAAAA | core promoter element around -30 of transcription start |
| TATA-box | Arabidopsis thaliana | 1445 | - | 6 | TATAAA | core promoter element around -30 of transcription start |
| TATA-box | Glycine max | 1253 | + | 5 | TAATA | core promoter element around -30 of transcription start |
| TATA-box | Glycine max | 926 | - | 5 | TAATA | core promoter element around -30 of transcription start |
| TATA-box | Arabidopsis thaliana | 1446 | - | 5 | TATAA | core promoter element around -30 of transcription start |
| TATA-box | Zea mays | 1421 | - | 8 | TTTAAAAA | core promoter element around -30 of transcription start |
| TATA-box | Zea mays | 1309 | + | 8 | TTTAAAAA | core promoter element around -30 of transcription start |
| TATA-box | Lycopersicon esculentum | 1297 | - | 5 | TTTTA | core promoter element around -30 of transcription start |
| TATA-box | Glycine max | 1281 | - | 5 | TAATA | core promoter element around -30 of transcription start |
| TATA-box | Glycine max | 476 | - | 5 | TAATA | core promoter element around -30 of transcription start |
| TATA-box | Lycopersicon esculentum | 299 | + | 5 | TTTTA | core promoter element around -30 of transcription start |
| TATA-box | Arabidopsis thaliana | 245 | - | 6 | TATAAA | core promoter element around -30 of transcription start |
| TATA-box | Arabidopsis thaliana | 1346 | - | 4 | TATA | core promoter element around -30 of transcription start |
| TATA-box | Glycine max | 944 | + | 5 | TAATA | core promoter element around -30 of transcription start |
| TATA-box | Arabidopsis thaliana | 247 | + | 4 | TATA | core promoter element around -30 of transcription start |
| TATA-box | Lycopersicon esculentum | 986 | - | 5 | TTTTA | core promoter element around -30 of transcription start |
| TATA-box | Arabidopsis thaliana | 1190 | - | 5 | TATAA | core promoter element around -30 of transcription start |
| TATA-box | Arabidopsis thaliana | 1191 | - | 4 | TATA | core promoter element around -30 of transcription start |
| TATA-box | Lycopersicon esculentum | 1319 | - | 5 | TTTTA | core promoter element around -30 of transcription start |
| TATA-box | Lycopersicon esculentum | 384 | + | 5 | TTTTA | core promoter element around -30 of transcription start |
| TATA-box | Arabidopsis thaliana | 813 | - | 5 | TATAA | core promoter element around -30 of transcription start |
| TATA-box | Arabidopsis thaliana | 246 | - | 5 | TATAA | core promoter element around -30 of transcription start |
| TATA-box | Lycopersicon esculentum | 1467 | - | 5 | TTTTA | core promoter element around -30 of transcription start |
| TATA-box | Glycine max | 682 | + | 5 | TAATA | core promoter element around -30 of transcription start |
| TATA-box | Glycine max | 1162 | + | 5 | TAATA | core promoter element around -30 of transcription start |
| TATA-box | Glycine max | 790 | + | 5 | TAATA | core promoter element around -30 of transcription start |
| TATA-box | Arabidopsis thaliana | 814 | - | 4 | TATA | core promoter element around -30 of transcription start |
| TATA-box | Glycine max | 1278 | + | 5 | TAATA | core promoter element around -30 of transcription start |
| TATA-box | Glycine max | 1239 | - | 5 | TAATA | core promoter element around -30 of transcription start |
| TATA-box | Glycine max | 1236 | + | 5 | TAATA | core promoter element around -30 of transcription start |
| TATA-box | Lycopersicon esculentum | 1368 | + | 5 | TTTTA | core promoter element around -30 of transcription start |
| TATA-box | Glycine max | 1357 | - | 5 | TAATA | core promoter element around -30 of transcription start |
| TATA-box | Arabidopsis thaliana | 306 | - | 7 | TATAAAA | core promoter element around -30 of transcription start |
| TATA-box | Glycine max | 1094 | + | 5 | TAATA | core promoter element around -30 of transcription start |
| TATA-box | Lycopersicon esculentum | 1177 | - | 5 | TTTTA | core promoter element around -30 of transcription start |
| TATA-box | Arabidopsis thaliana | 244 | - | 7 | TATAAAA | core promoter element around -30 of transcription start |
| TATA-box | Glycine max | 1464 | - | 5 | TAATA | core promoter element around -30 of transcription start |
| TATA-box | Arabidopsis thaliana | 1097 | + | 6 | TATAAA | core promoter element around -30 of transcription start |
| TATA-box | Arabidopsis thaliana | 305 | - | 9 | ccTATAAAaa | core promoter element around -30 of transcription start |
| TATA-box | Zea mays | 1374 | + | 8 | TTTAAAAA | core promoter element around -30 of transcription start |
| TATA-box | Lycopersicon esculentum | 342 | - | 5 | TTTTA | core promoter element around -30 of transcription start |
| TATA-box | Lycopersicon esculentum | 1223 | - | 5 | TTTTA | core promoter element around -30 of transcription start |
| TATA-box | Glycine max | 1054 | - | 5 | TAATA | core promoter element around -30 of transcription start |
| TATA-box | Lycopersicon esculentum | 1422 | + | 5 | TTTTA | core promoter element around -30 of transcription start |
| TATA-box | Glycine max | 991 | - | 5 | TAATA | core promoter element around -30 of transcription start |
| TATA-box | Glycine max | 1304 | - | 5 | TAATA | core promoter element around -30 of transcription start |
| TATA-box | Glycine max | 1211 | + | 5 | TAATA | core promoter element around -30 of transcription start |
| TATA-box | Ac | 207 | + | 7 | TATAAAT | core promoter element around -30 of transcription start |
| TATA-box | Brassica oleracea | 1096 | + | 6 | ATATAA | core promoter element around -30 of transcription start |
| TATA-box | Lycopersicon esculentum | 1474 | - | 5 | TTTTA | core promoter element around -30 of transcription start |
| TATA-box | Lycopersicon esculentum | 1155 | - | 5 | TTTTA | core promoter element around -30 of transcription start |
| TATA-box | Arabidopsis thaliana | 458 | + | 4 | TATA | core promoter element around -30 of transcription start |
| TATA-box | Arabidopsis thaliana | 309 | + | 4 | TATA | core promoter element around -30 of transcription start |
| TATA-box | Glycine max | 1354 | + | 5 | TAATA | core promoter element around -30 of transcription start |
| TATA-box | Glycine max | 983 | + | 5 | TAATA | core promoter element around -30 of transcription start |
| TATA-box | Pisum sativum | 1187 | - | 8 | TATAAAAT | core promoter element around -30 of transcription start |
| TATA-box | Lycopersicon esculentum | 1405 | + | 5 | TTTTA | core promoter element around -30 of transcription start |
| TATA-box | Glycine max | 929 | - | 5 | TAATA | core promoter element around -30 of transcription start |
| TATA-box | Lycopersicon esculentum | 487 | + | 5 | TTTTA | core promoter element around -30 of transcription start |
| TATA-box | Oryza sativa | 662 | + | 7 | TACAAAA | core promoter element around -30 of transcription start |
| TATA-box | Arabidopsis thaliana | 307 | - | 6 | TATAAA | core promoter element around -30 of transcription start |
| TATA-box | Glycine max | 1208 | + | 5 | TAATA | core promoter element around -30 of transcription start |
| TATA-box | Lycopersicon esculentum | 467 | + | 5 | TTTTA | core promoter element around -30 of transcription start |
| TATA-box | Arabidopsis thaliana | 308 | - | 5 | TATAA | core promoter element around -30 of transcription start |
| TATA-box | Lycopersicon esculentum | 1376 | - | 5 | TTTTA | core promoter element around -30 of transcription start |
| TATA-box | Glycine max | 935 | - | 5 | TAATA | core promoter element around -30 of transcription start |
| TATA-box | Zea mays | 1153 | + | 8 | TTTAAAAA | core promoter element around -30 of transcription start |
| TATA-box | Glycine max | 490 | - | 5 | TAATA | core promoter element around -30 of transcription start |
| TATA-box | Glycine max | 938 | - | 5 | TAATA | core promoter element around -30 of transcription start |
| TATA-box | Lycopersicon esculentum | 1408 | - | 5 | TTTTA | core promoter element around -30 of transcription start |
| TATA-box | Lycopersicon esculentum | 266 | + | 5 | TTTTA | core promoter element around -30 of transcription start |
| TATA-box | Brassica oleracea | 206 | + | 6 | ATATAA | core promoter element around -30 of transcription start |
| TATA-box | Glycine max | 318 | - | 5 | TAATA | core promoter element around -30 of transcription start |
| TATA-box | Arabidopsis thaliana | 810 | - | 9 | ccTATAAAaa | core promoter element around -30 of transcription start |
| TATA-box | Glycine max | 932 | - | 5 | TAATA | core promoter element around -30 of transcription start |

> 2018/04/13 10:10:12  
+ GCTTCTTATG ATTCTTTCTT CTTCTTTACT TAGCCTTTCG TGGCTACTTT CAATCTTTCT CTAATCTCTC   
  
  
+ GAAGCCGATT CTAGAAACAA AAGGAGATCA CTCTTTCTCA AAACAAAGCT ACCCCAAGAA CAAAGCGAAA   
  
  
+ AGAAAGAGAG ACTCTCTCTC TCTCTCTTGG TCTTTTCCCC ATCCAAACCG AGGAGAGGTG AGGAAATATA   
  
  
+ AATGAAAAAA AAAAAAAGGG AAGCAATCAC CGGTTTTATA AGAAGTTTGT TGGGTTTTTA ACTCGAGACT   
  
  
+ AAACAATTTA AACTAATTTT TTATTTTTTA TAGAAGATAT TAAACACTGA AAAAAATGAA ATAAAATTTT   
  
  
+ GATCTGTACC AATATGAAGT ATTTTCTGTT AATTTTTAAT TTTTTTTGTT TTTTGTCAAA TTCGGGATCG   
  
  
+ CTACTTCCAT TTCTGTCCCT GTATGGGAAA AAAGAAGTAT AATTCTTTTT ATTTTTATTA ATTATTTTTT   
  
  
+ ATTAATCGAG AAATTAACGT ACTATTCAAA GACAAATTTA GGCTTAACCG ATTTTCTTAA CCCACTTTTG   
  
  
+ TCCCGTACCG TACTTGGTAC TCGCTCTTCC GAACCAGGCC AGGTAGCCAG CGTCCGTCAG TGGCAGTGCA   
  
  
+ CCTGTTCCGC GGTATTCTTT CCCCAACCAG TTACAAAAGT TGAGATGCCA ATAATAAGAG AAAAACCTCC   
  
  
+ TAATCCGATT GTGCACAGCA CCCAGCCCGT GGCAGTAAAC TGGCTTGCAC CGTGCACCCT CGGGTAGAGG   
  
  
+ ACAACCAAAA GGTGCCCGTT AATAACACCT GCAATCGCAT GTTTATAGGT GTAAACTGAG AAGACAGACA   
  
  
+ TGTAAAGATG TACGCAACGA CTGAGTCGAA AGCGCAGTAG GGTAGATCAA GGGCAATGAG AATTCAAACC   
  
  
+ ACCATGTGAG TTTTTTATTA TTATTATTAT TAATAATAAT TTAATTAGGC ATGTATTTAT CCGTTCTAGC   
  
  
+ ACTAATAAAA TATTATCGTA AAATAAACGA TAAGTTATTT CATTGCTACT ATTTACGAAT CACACTTTGT   
  
  
+ AAATATTACT ACTAGGGACT TGGTTGTATT AGTATGACTC TACTAATATA AAATACATAT TATTTGTAGT   
  
  
+ TTTATTAAAA ATACAATTAA CACAAAAAAT AATTTAAAAA ATAATAAAAA TCTACTTAAA ATATGTATTT   
  
  
+ TATAATTTAA TCAAGTTTAA TAATAACAAA AATAAAAAAA TTTTTTAATA TTAAGTTTAA ATTAATAACA   
  
  
+ AAAATAAAAA ATTTTTTTAA TATTAAGTTT AAATTCTAAA AAATATTATT TAAAAAATTA AAATTAAAAA   
  
  
+ AATGTTTCTA TTTCTTATAA GTTTAATATT ACTGTTTTTT TAGTTTAAAA AATTTACGAA TTTCACGAAA   
  
  
+ AATATTTTAA AATGCAATAA TTTTTAAATT TTAATTAAGT ATTTTTTATA AAAATCGTCA TTTTATTAAA   
  
  
+ ACATAAAAAA AGCAAACTTA TAGAAAAAT  

- CGAAGAATAC TAAGAAAGAA GAAGAAATGA ATCGGAAAGC ACCGATGAAA GTTAGAAAGA GATTAGAGAG   
  
  
- CTTCGGCTAA GATCTTTGTT TTCCTCTAGT GAGAAAGAGT TTTGTTTCGA TGGGGTTCTT GTTTCGCTTT   
  
  
- TCTTTCTCTC TGAGAGAGAG AGAGAGAACC AGAAAAGGGG TAGGTTTGGC TCCTCTCCAC TCCTTTATAT   
  
  
- TTACTTTTTT TTTTTTTCCC TTCGTTAGTG GCCAAAATAT TCTTCAAACA ACCCAAAAAT TGAGCTCTGA   
  
  
- TTTGTTAAAT TTGATTAAAA AATAAAAAAT ATCTTCTATA ATTTGTGACT TTTTTTACTT TATTTTAAAA   
  
  
- CTAGACATGG TTATACTTCA TAAAAGACAA TTAAAAATTA AAAAAAACAA AAAACAGTTT AAGCCCTAGC   
  
  
- GATGAAGGTA AAGACAGGGA CATACCCTTT TTTCTTCATA TTAAGAAAAA TAAAAATAAT TAATAAAAAA   
  
  
- TAATTAGCTC TTTAATTGCA TGATAAGTTT CTGTTTAAAT CCGAATTGGC TAAAAGAATT GGGTGAAAAC   
  
  
- AGGGCATGGC ATGAACCATG AGCGAGAAGG CTTGGTCCGG TCCATCGGTC GCAGGCAGTC ACCGTCACGT   
  
  
- GGACAAGGCG CCATAAGAAA GGGGTTGGTC AATGTTTTCA ACTCTACGGT TATTATTCTC TTTTTGGAGG   
  
  
- ATTAGGCTAA CACGTGTCGT GGGTCGGGCA CCGTCATTTG ACCGAACGTG GCACGTGGGA GCCCATCTCC   
  
  
- TGTTGGTTTT CCACGGGCAA TTATTGTGGA CGTTAGCGTA CAAATATCCA CATTTGACTC TTCTGTCTGT   
  
  
- ACATTTCTAC ATGCGTTGCT GACTCAGCTT TCGCGTCATC CCATCTAGTT CCCGTTACTC TTAAGTTTGG   
  
  
- TGGTACACTC AAAAAATAAT AATAATAATA ATTATTATTA AATTAATCCG TACATAAATA GGCAAGATCG   
  
  
- TGATTATTTT ATAATAGCAT TTTATTTGCT ATTCAATAAA GTAACGATGA TAAATGCTTA GTGTGAAACA   
  
  
- TTTATAATGA TGATCCCTGA ACCAACATAA TCATACTGAG ATGATTATAT TTTATGTATA ATAAACATCA   
  
  
- AAATAATTTT TATGTTAATT GTGTTTTTTA TTAAATTTTT TATTATTTTT AGATGAATTT TATACATAAA   
  
  
- ATATTAAATT AGTTCAAATT ATTATTGTTT TTATTTTTTT AAAAAATTAT AATTCAAATT TAATTATTGT   
  
  
- TTTTATTTTT TAAAAAAATT ATAATTCAAA TTTAAGATTT TTTATAATAA ATTTTTTAAT TTTAATTTTT   
  
  
- TTACAAAGAT AAAGAATATT CAAATTATAA TGACAAAAAA ATCAAATTTT TTAAATGCTT AAAGTGCTTT   
  
  
- TTATAAAATT TTACGTTATT AAAAATTTAA AATTAATTCA TAAAAAATAT TTTTAGCAGT AAAATAATTT   
  
  
- TGTATTTTTT TCGTTTGAAT ATCTTTTTA

+     TC-rich repeats

| Site Name | Organism | Position | Strand | Matrix score. | sequence | function |
| --- | --- | --- | --- | --- | --- | --- |
| TC-rich repeats | Nicotiana tabacum | 328 | - | 9 | ATTTTCTTCA | cis-acting element involved in defense and stress responsiveness |
| TC-rich repeats | Nicotiana tabacum | 541 | + | 9 | ATTTTCTTCA | cis-acting element involved in defense and stress responsiveness |

> 2018/04/13 10:10:12  
+ GCTTCTTATG ATTCTTTCTT CTTCTTTACT TAGCCTTTCG TGGCTACTTT CAATCTTTCT CTAATCTCTC   
  
  
+ GAAGCCGATT CTAGAAACAA AAGGAGATCA CTCTTTCTCA AAACAAAGCT ACCCCAAGAA CAAAGCGAAA   
  
  
+ AGAAAGAGAG ACTCTCTCTC TCTCTCTTGG TCTTTTCCCC ATCCAAACCG AGGAGAGGTG AGGAAATATA   
  
  
+ AATGAAAAAA AAAAAAAGGG AAGCAATCAC CGGTTTTATA AGAAGTTTGT TGGGTTTTTA ACTCGAGACT   
  
  
+ AAACAATTTA AACTAATTTT TTATTTTTTA TAGAAGATAT TAAACACTGA AAAAAATGAA ATAAAATTTT   
  
  
+ GATCTGTACC AATATGAAGT ATTTTCTGTT AATTTTTAAT TTTTTTTGTT TTTTGTCAAA TTCGGGATCG   
  
  
+ CTACTTCCAT TTCTGTCCCT GTATGGGAAA AAAGAAGTAT AATTCTTTTT ATTTTTATTA ATTATTTTTT   
  
  
+ ATTAATCGAG AAATTAACGT ACTATTCAAA GACAAATTTA GGCTTAACCG ATTTTCTTAA CCCACTTTTG   
  
  
+ TCCCGTACCG TACTTGGTAC TCGCTCTTCC GAACCAGGCC AGGTAGCCAG CGTCCGTCAG TGGCAGTGCA   
  
  
+ CCTGTTCCGC GGTATTCTTT CCCCAACCAG TTACAAAAGT TGAGATGCCA ATAATAAGAG AAAAACCTCC   
  
  
+ TAATCCGATT GTGCACAGCA CCCAGCCCGT GGCAGTAAAC TGGCTTGCAC CGTGCACCCT CGGGTAGAGG   
  
  
+ ACAACCAAAA GGTGCCCGTT AATAACACCT GCAATCGCAT GTTTATAGGT GTAAACTGAG AAGACAGACA   
  
  
+ TGTAAAGATG TACGCAACGA CTGAGTCGAA AGCGCAGTAG GGTAGATCAA GGGCAATGAG AATTCAAACC   
  
  
+ ACCATGTGAG TTTTTTATTA TTATTATTAT TAATAATAAT TTAATTAGGC ATGTATTTAT CCGTTCTAGC   
  
  
+ ACTAATAAAA TATTATCGTA AAATAAACGA TAAGTTATTT CATTGCTACT ATTTACGAAT CACACTTTGT   
  
  
+ AAATATTACT ACTAGGGACT TGGTTGTATT AGTATGACTC TACTAATATA AAATACATAT TATTTGTAGT   
  
  
+ TTTATTAAAA ATACAATTAA CACAAAAAAT AATTTAAAAA ATAATAAAAA TCTACTTAAA ATATGTATTT   
  
  
+ TATAATTTAA TCAAGTTTAA TAATAACAAA AATAAAAAAA TTTTTTAATA TTAAGTTTAA ATTAATAACA   
  
  
+ AAAATAAAAA ATTTTTTTAA TATTAAGTTT AAATTCTAAA AAATATTATT TAAAAAATTA AAATTAAAAA   
  
  
+ AATGTTTCTA TTTCTTATAA GTTTAATATT ACTGTTTTTT TAGTTTAAAA AATTTACGAA TTTCACGAAA   
  
  
+ AATATTTTAA AATGCAATAA TTTTTAAATT TTAATTAAGT ATTTTTTATA AAAATCGTCA TTTTATTAAA   
  
  
+ ACATAAAAAA AGCAAACTTA TAGAAAAAT  

- CGAAGAATAC TAAGAAAGAA GAAGAAATGA ATCGGAAAGC ACCGATGAAA GTTAGAAAGA GATTAGAGAG   
  
  
- CTTCGGCTAA GATCTTTGTT TTCCTCTAGT GAGAAAGAGT TTTGTTTCGA TGGGGTTCTT GTTTCGCTTT   
  
  
- TCTTTCTCTC TGAGAGAGAG AGAGAGAACC AGAAAAGGGG TAGGTTTGGC TCCTCTCCAC TCCTTTATAT   
  
  
- TTACTTTTTT TTTTTTTCCC TTCGTTAGTG GCCAAAATAT TCTTCAAACA ACCCAAAAAT TGAGCTCTGA   
  
  
- TTTGTTAAAT TTGATTAAAA AATAAAAAAT ATCTTCTATA ATTTGTGACT TTTTTTACTT TATTTTAAAA   
  
  
- CTAGACATGG TTATACTTCA TAAAAGACAA TTAAAAATTA AAAAAAACAA AAAACAGTTT AAGCCCTAGC   
  
  
- GATGAAGGTA AAGACAGGGA CATACCCTTT TTTCTTCATA TTAAGAAAAA TAAAAATAAT TAATAAAAAA   
  
  
- TAATTAGCTC TTTAATTGCA TGATAAGTTT CTGTTTAAAT CCGAATTGGC TAAAAGAATT GGGTGAAAAC   
  
  
- AGGGCATGGC ATGAACCATG AGCGAGAAGG CTTGGTCCGG TCCATCGGTC GCAGGCAGTC ACCGTCACGT   
  
  
- GGACAAGGCG CCATAAGAAA GGGGTTGGTC AATGTTTTCA ACTCTACGGT TATTATTCTC TTTTTGGAGG   
  
  
- ATTAGGCTAA CACGTGTCGT GGGTCGGGCA CCGTCATTTG ACCGAACGTG GCACGTGGGA GCCCATCTCC   
  
  
- TGTTGGTTTT CCACGGGCAA TTATTGTGGA CGTTAGCGTA CAAATATCCA CATTTGACTC TTCTGTCTGT   
  
  
- ACATTTCTAC ATGCGTTGCT GACTCAGCTT TCGCGTCATC CCATCTAGTT CCCGTTACTC TTAAGTTTGG   
  
  
- TGGTACACTC AAAAAATAAT AATAATAATA ATTATTATTA AATTAATCCG TACATAAATA GGCAAGATCG   
  
  
- TGATTATTTT ATAATAGCAT TTTATTTGCT ATTCAATAAA GTAACGATGA TAAATGCTTA GTGTGAAACA   
  
  
- TTTATAATGA TGATCCCTGA ACCAACATAA TCATACTGAG ATGATTATAT TTTATGTATA ATAAACATCA   
  
  
- AAATAATTTT TATGTTAATT GTGTTTTTTA TTAAATTTTT TATTATTTTT AGATGAATTT TATACATAAA   
  
  
- ATATTAAATT AGTTCAAATT ATTATTGTTT TTATTTTTTT AAAAAATTAT AATTCAAATT TAATTATTGT   
  
  
- TTTTATTTTT TAAAAAAATT ATAATTCAAA TTTAAGATTT TTTATAATAA ATTTTTTAAT TTTAATTTTT   
  
  
- TTACAAAGAT AAAGAATATT CAAATTATAA TGACAAAAAA ATCAAATTTT TTAAATGCTT AAAGTGCTTT   
  
  
- TTATAAAATT TTACGTTATT AAAAATTTAA AATTAATTCA TAAAAAATAT TTTTAGCAGT AAAATAATTT   
  
  
- TGTATTTTTT TCGTTTGAAT ATCTTTTTA

+     TCA-element

| Site Name | Organism | Position | Strand | Matrix score. | sequence | function |
| --- | --- | --- | --- | --- | --- | --- |
| TCA-element | Brassica oleracea | 426 | - | 9 | CAGAAAAGGA | cis-acting element involved in salicylic acid responsiveness |

> 2018/04/13 10:10:12  
+ GCTTCTTATG ATTCTTTCTT CTTCTTTACT TAGCCTTTCG TGGCTACTTT CAATCTTTCT CTAATCTCTC   
  
  
+ GAAGCCGATT CTAGAAACAA AAGGAGATCA CTCTTTCTCA AAACAAAGCT ACCCCAAGAA CAAAGCGAAA   
  
  
+ AGAAAGAGAG ACTCTCTCTC TCTCTCTTGG TCTTTTCCCC ATCCAAACCG AGGAGAGGTG AGGAAATATA   
  
  
+ AATGAAAAAA AAAAAAAGGG AAGCAATCAC CGGTTTTATA AGAAGTTTGT TGGGTTTTTA ACTCGAGACT   
  
  
+ AAACAATTTA AACTAATTTT TTATTTTTTA TAGAAGATAT TAAACACTGA AAAAAATGAA ATAAAATTTT   
  
  
+ GATCTGTACC AATATGAAGT ATTTTCTGTT AATTTTTAAT TTTTTTTGTT TTTTGTCAAA TTCGGGATCG   
  
  
+ CTACTTCCAT TTCTGTCCCT GTATGGGAAA AAAGAAGTAT AATTCTTTTT ATTTTTATTA ATTATTTTTT   
  
  
+ ATTAATCGAG AAATTAACGT ACTATTCAAA GACAAATTTA GGCTTAACCG ATTTTCTTAA CCCACTTTTG   
  
  
+ TCCCGTACCG TACTTGGTAC TCGCTCTTCC GAACCAGGCC AGGTAGCCAG CGTCCGTCAG TGGCAGTGCA   
  
  
+ CCTGTTCCGC GGTATTCTTT CCCCAACCAG TTACAAAAGT TGAGATGCCA ATAATAAGAG AAAAACCTCC   
  
  
+ TAATCCGATT GTGCACAGCA CCCAGCCCGT GGCAGTAAAC TGGCTTGCAC CGTGCACCCT CGGGTAGAGG   
  
  
+ ACAACCAAAA GGTGCCCGTT AATAACACCT GCAATCGCAT GTTTATAGGT GTAAACTGAG AAGACAGACA   
  
  
+ TGTAAAGATG TACGCAACGA CTGAGTCGAA AGCGCAGTAG GGTAGATCAA GGGCAATGAG AATTCAAACC   
  
  
+ ACCATGTGAG TTTTTTATTA TTATTATTAT TAATAATAAT TTAATTAGGC ATGTATTTAT CCGTTCTAGC   
  
  
+ ACTAATAAAA TATTATCGTA AAATAAACGA TAAGTTATTT CATTGCTACT ATTTACGAAT CACACTTTGT   
  
  
+ AAATATTACT ACTAGGGACT TGGTTGTATT AGTATGACTC TACTAATATA AAATACATAT TATTTGTAGT   
  
  
+ TTTATTAAAA ATACAATTAA CACAAAAAAT AATTTAAAAA ATAATAAAAA TCTACTTAAA ATATGTATTT   
  
  
+ TATAATTTAA TCAAGTTTAA TAATAACAAA AATAAAAAAA TTTTTTAATA TTAAGTTTAA ATTAATAACA   
  
  
+ AAAATAAAAA ATTTTTTTAA TATTAAGTTT AAATTCTAAA AAATATTATT TAAAAAATTA AAATTAAAAA   
  
  
+ AATGTTTCTA TTTCTTATAA GTTTAATATT ACTGTTTTTT TAGTTTAAAA AATTTACGAA TTTCACGAAA   
  
  
+ AATATTTTAA AATGCAATAA TTTTTAAATT TTAATTAAGT ATTTTTTATA AAAATCGTCA TTTTATTAAA   
  
  
+ ACATAAAAAA AGCAAACTTA TAGAAAAAT  

- CGAAGAATAC TAAGAAAGAA GAAGAAATGA ATCGGAAAGC ACCGATGAAA GTTAGAAAGA GATTAGAGAG   
  
  
- CTTCGGCTAA GATCTTTGTT TTCCTCTAGT GAGAAAGAGT TTTGTTTCGA TGGGGTTCTT GTTTCGCTTT   
  
  
- TCTTTCTCTC TGAGAGAGAG AGAGAGAACC AGAAAAGGGG TAGGTTTGGC TCCTCTCCAC TCCTTTATAT   
  
  
- TTACTTTTTT TTTTTTTCCC TTCGTTAGTG GCCAAAATAT TCTTCAAACA ACCCAAAAAT TGAGCTCTGA   
  
  
- TTTGTTAAAT TTGATTAAAA AATAAAAAAT ATCTTCTATA ATTTGTGACT TTTTTTACTT TATTTTAAAA   
  
  
- CTAGACATGG TTATACTTCA TAAAAGACAA TTAAAAATTA AAAAAAACAA AAAACAGTTT AAGCCCTAGC   
  
  
- GATGAAGGTA AAGACAGGGA CATACCCTTT TTTCTTCATA TTAAGAAAAA TAAAAATAAT TAATAAAAAA   
  
  
- TAATTAGCTC TTTAATTGCA TGATAAGTTT CTGTTTAAAT CCGAATTGGC TAAAAGAATT GGGTGAAAAC   
  
  
- AGGGCATGGC ATGAACCATG AGCGAGAAGG CTTGGTCCGG TCCATCGGTC GCAGGCAGTC ACCGTCACGT   
  
  
- GGACAAGGCG CCATAAGAAA GGGGTTGGTC AATGTTTTCA ACTCTACGGT TATTATTCTC TTTTTGGAGG   
  
  
- ATTAGGCTAA CACGTGTCGT GGGTCGGGCA CCGTCATTTG ACCGAACGTG GCACGTGGGA GCCCATCTCC   
  
  
- TGTTGGTTTT CCACGGGCAA TTATTGTGGA CGTTAGCGTA CAAATATCCA CATTTGACTC TTCTGTCTGT   
  
  
- ACATTTCTAC ATGCGTTGCT GACTCAGCTT TCGCGTCATC CCATCTAGTT CCCGTTACTC TTAAGTTTGG   
  
  
- TGGTACACTC AAAAAATAAT AATAATAATA ATTATTATTA AATTAATCCG TACATAAATA GGCAAGATCG   
  
  
- TGATTATTTT ATAATAGCAT TTTATTTGCT ATTCAATAAA GTAACGATGA TAAATGCTTA GTGTGAAACA   
  
  
- TTTATAATGA TGATCCCTGA ACCAACATAA TCATACTGAG ATGATTATAT TTTATGTATA ATAAACATCA   
  
  
- AAATAATTTT TATGTTAATT GTGTTTTTTA TTAAATTTTT TATTATTTTT AGATGAATTT TATACATAAA   
  
  
- ATATTAAATT AGTTCAAATT ATTATTGTTT TTATTTTTTT AAAAAATTAT AATTCAAATT TAATTATTGT   
  
  
- TTTTATTTTT TAAAAAAATT ATAATTCAAA TTTAAGATTT TTTATAATAA ATTTTTTAAT TTTAATTTTT   
  
  
- TTACAAAGAT AAAGAATATT CAAATTATAA TGACAAAAAA ATCAAATTTT TTAAATGCTT AAAGTGCTTT   
  
  
- TTATAAAATT TTACGTTATT AAAAATTTAA AATTAATTCA TAAAAAATAT TTTTAGCAGT AAAATAATTT   
  
  
- TGTATTTTTT TCGTTTGAAT ATCTTTTTA

+     TGA-element

| Site Name | Organism | Position | Strand | Matrix score. | sequence | function |
| --- | --- | --- | --- | --- | --- | --- |
| TGA-element | Brassica oleracea | 856 | + | 6 | AACGAC | auxin-responsive element |

> 2018/04/13 10:10:12  
+ GCTTCTTATG ATTCTTTCTT CTTCTTTACT TAGCCTTTCG TGGCTACTTT CAATCTTTCT CTAATCTCTC   
  
  
+ GAAGCCGATT CTAGAAACAA AAGGAGATCA CTCTTTCTCA AAACAAAGCT ACCCCAAGAA CAAAGCGAAA   
  
  
+ AGAAAGAGAG ACTCTCTCTC TCTCTCTTGG TCTTTTCCCC ATCCAAACCG AGGAGAGGTG AGGAAATATA   
  
  
+ AATGAAAAAA AAAAAAAGGG AAGCAATCAC CGGTTTTATA AGAAGTTTGT TGGGTTTTTA ACTCGAGACT   
  
  
+ AAACAATTTA AACTAATTTT TTATTTTTTA TAGAAGATAT TAAACACTGA AAAAAATGAA ATAAAATTTT   
  
  
+ GATCTGTACC AATATGAAGT ATTTTCTGTT AATTTTTAAT TTTTTTTGTT TTTTGTCAAA TTCGGGATCG   
  
  
+ CTACTTCCAT TTCTGTCCCT GTATGGGAAA AAAGAAGTAT AATTCTTTTT ATTTTTATTA ATTATTTTTT   
  
  
+ ATTAATCGAG AAATTAACGT ACTATTCAAA GACAAATTTA GGCTTAACCG ATTTTCTTAA CCCACTTTTG   
  
  
+ TCCCGTACCG TACTTGGTAC TCGCTCTTCC GAACCAGGCC AGGTAGCCAG CGTCCGTCAG TGGCAGTGCA   
  
  
+ CCTGTTCCGC GGTATTCTTT CCCCAACCAG TTACAAAAGT TGAGATGCCA ATAATAAGAG AAAAACCTCC   
  
  
+ TAATCCGATT GTGCACAGCA CCCAGCCCGT GGCAGTAAAC TGGCTTGCAC CGTGCACCCT CGGGTAGAGG   
  
  
+ ACAACCAAAA GGTGCCCGTT AATAACACCT GCAATCGCAT GTTTATAGGT GTAAACTGAG AAGACAGACA   
  
  
+ TGTAAAGATG TACGCAACGA CTGAGTCGAA AGCGCAGTAG GGTAGATCAA GGGCAATGAG AATTCAAACC   
  
  
+ ACCATGTGAG TTTTTTATTA TTATTATTAT TAATAATAAT TTAATTAGGC ATGTATTTAT CCGTTCTAGC   
  
  
+ ACTAATAAAA TATTATCGTA AAATAAACGA TAAGTTATTT CATTGCTACT ATTTACGAAT CACACTTTGT   
  
  
+ AAATATTACT ACTAGGGACT TGGTTGTATT AGTATGACTC TACTAATATA AAATACATAT TATTTGTAGT   
  
  
+ TTTATTAAAA ATACAATTAA CACAAAAAAT AATTTAAAAA ATAATAAAAA TCTACTTAAA ATATGTATTT   
  
  
+ TATAATTTAA TCAAGTTTAA TAATAACAAA AATAAAAAAA TTTTTTAATA TTAAGTTTAA ATTAATAACA   
  
  
+ AAAATAAAAA ATTTTTTTAA TATTAAGTTT AAATTCTAAA AAATATTATT TAAAAAATTA AAATTAAAAA   
  
  
+ AATGTTTCTA TTTCTTATAA GTTTAATATT ACTGTTTTTT TAGTTTAAAA AATTTACGAA TTTCACGAAA   
  
  
+ AATATTTTAA AATGCAATAA TTTTTAAATT TTAATTAAGT ATTTTTTATA AAAATCGTCA TTTTATTAAA   
  
  
+ ACATAAAAAA AGCAAACTTA TAGAAAAAT  

- CGAAGAATAC TAAGAAAGAA GAAGAAATGA ATCGGAAAGC ACCGATGAAA GTTAGAAAGA GATTAGAGAG   
  
  
- CTTCGGCTAA GATCTTTGTT TTCCTCTAGT GAGAAAGAGT TTTGTTTCGA TGGGGTTCTT GTTTCGCTTT   
  
  
- TCTTTCTCTC TGAGAGAGAG AGAGAGAACC AGAAAAGGGG TAGGTTTGGC TCCTCTCCAC TCCTTTATAT   
  
  
- TTACTTTTTT TTTTTTTCCC TTCGTTAGTG GCCAAAATAT TCTTCAAACA ACCCAAAAAT TGAGCTCTGA   
  
  
- TTTGTTAAAT TTGATTAAAA AATAAAAAAT ATCTTCTATA ATTTGTGACT TTTTTTACTT TATTTTAAAA   
  
  
- CTAGACATGG TTATACTTCA TAAAAGACAA TTAAAAATTA AAAAAAACAA AAAACAGTTT AAGCCCTAGC   
  
  
- GATGAAGGTA AAGACAGGGA CATACCCTTT TTTCTTCATA TTAAGAAAAA TAAAAATAAT TAATAAAAAA   
  
  
- TAATTAGCTC TTTAATTGCA TGATAAGTTT CTGTTTAAAT CCGAATTGGC TAAAAGAATT GGGTGAAAAC   
  
  
- AGGGCATGGC ATGAACCATG AGCGAGAAGG CTTGGTCCGG TCCATCGGTC GCAGGCAGTC ACCGTCACGT   
  
  
- GGACAAGGCG CCATAAGAAA GGGGTTGGTC AATGTTTTCA ACTCTACGGT TATTATTCTC TTTTTGGAGG   
  
  
- ATTAGGCTAA CACGTGTCGT GGGTCGGGCA CCGTCATTTG ACCGAACGTG GCACGTGGGA GCCCATCTCC   
  
  
- TGTTGGTTTT CCACGGGCAA TTATTGTGGA CGTTAGCGTA CAAATATCCA CATTTGACTC TTCTGTCTGT   
  
  
- ACATTTCTAC ATGCGTTGCT GACTCAGCTT TCGCGTCATC CCATCTAGTT CCCGTTACTC TTAAGTTTGG   
  
  
- TGGTACACTC AAAAAATAAT AATAATAATA ATTATTATTA AATTAATCCG TACATAAATA GGCAAGATCG   
  
  
- TGATTATTTT ATAATAGCAT TTTATTTGCT ATTCAATAAA GTAACGATGA TAAATGCTTA GTGTGAAACA   
  
  
- TTTATAATGA TGATCCCTGA ACCAACATAA TCATACTGAG ATGATTATAT TTTATGTATA ATAAACATCA   
  
  
- AAATAATTTT TATGTTAATT GTGTTTTTTA TTAAATTTTT TATTATTTTT AGATGAATTT TATACATAAA   
  
  
- ATATTAAATT AGTTCAAATT ATTATTGTTT TTATTTTTTT AAAAAATTAT AATTCAAATT TAATTATTGT   
  
  
- TTTTATTTTT TAAAAAAATT ATAATTCAAA TTTAAGATTT TTTATAATAA ATTTTTTAAT TTTAATTTTT   
  
  
- TTACAAAGAT AAAGAATATT CAAATTATAA TGACAAAAAA ATCAAATTTT TTAAATGCTT AAAGTGCTTT   
  
  
- TTATAAAATT TTACGTTATT AAAAATTTAA AATTAATTCA TAAAAAATAT TTTTAGCAGT AAAATAATTT   
  
  
- TGTATTTTTT TCGTTTGAAT ATCTTTTTA

+     TGACG-motif

| Site Name | Organism | Position | Strand | Matrix score. | sequence | function |
| --- | --- | --- | --- | --- | --- | --- |
| TGACG-motif | Hordeum vulgare | 1456 | - | 5 | TGACG | cis-acting regulatory element involved in the MeJA-responsiveness |
| TGACG-motif | Hordeum vulgare | 615 | - | 5 | TGACG | cis-acting regulatory element involved in the MeJA-responsiveness |

> 2018/04/13 10:10:12  
+ GCTTCTTATG ATTCTTTCTT CTTCTTTACT TAGCCTTTCG TGGCTACTTT CAATCTTTCT CTAATCTCTC   
  
  
+ GAAGCCGATT CTAGAAACAA AAGGAGATCA CTCTTTCTCA AAACAAAGCT ACCCCAAGAA CAAAGCGAAA   
  
  
+ AGAAAGAGAG ACTCTCTCTC TCTCTCTTGG TCTTTTCCCC ATCCAAACCG AGGAGAGGTG AGGAAATATA   
  
  
+ AATGAAAAAA AAAAAAAGGG AAGCAATCAC CGGTTTTATA AGAAGTTTGT TGGGTTTTTA ACTCGAGACT   
  
  
+ AAACAATTTA AACTAATTTT TTATTTTTTA TAGAAGATAT TAAACACTGA AAAAAATGAA ATAAAATTTT   
  
  
+ GATCTGTACC AATATGAAGT ATTTTCTGTT AATTTTTAAT TTTTTTTGTT TTTTGTCAAA TTCGGGATCG   
  
  
+ CTACTTCCAT TTCTGTCCCT GTATGGGAAA AAAGAAGTAT AATTCTTTTT ATTTTTATTA ATTATTTTTT   
  
  
+ ATTAATCGAG AAATTAACGT ACTATTCAAA GACAAATTTA GGCTTAACCG ATTTTCTTAA CCCACTTTTG   
  
  
+ TCCCGTACCG TACTTGGTAC TCGCTCTTCC GAACCAGGCC AGGTAGCCAG CGTCCGTCAG TGGCAGTGCA   
  
  
+ CCTGTTCCGC GGTATTCTTT CCCCAACCAG TTACAAAAGT TGAGATGCCA ATAATAAGAG AAAAACCTCC   
  
  
+ TAATCCGATT GTGCACAGCA CCCAGCCCGT GGCAGTAAAC TGGCTTGCAC CGTGCACCCT CGGGTAGAGG   
  
  
+ ACAACCAAAA GGTGCCCGTT AATAACACCT GCAATCGCAT GTTTATAGGT GTAAACTGAG AAGACAGACA   
  
  
+ TGTAAAGATG TACGCAACGA CTGAGTCGAA AGCGCAGTAG GGTAGATCAA GGGCAATGAG AATTCAAACC   
  
  
+ ACCATGTGAG TTTTTTATTA TTATTATTAT TAATAATAAT TTAATTAGGC ATGTATTTAT CCGTTCTAGC   
  
  
+ ACTAATAAAA TATTATCGTA AAATAAACGA TAAGTTATTT CATTGCTACT ATTTACGAAT CACACTTTGT   
  
  
+ AAATATTACT ACTAGGGACT TGGTTGTATT AGTATGACTC TACTAATATA AAATACATAT TATTTGTAGT   
  
  
+ TTTATTAAAA ATACAATTAA CACAAAAAAT AATTTAAAAA ATAATAAAAA TCTACTTAAA ATATGTATTT   
  
  
+ TATAATTTAA TCAAGTTTAA TAATAACAAA AATAAAAAAA TTTTTTAATA TTAAGTTTAA ATTAATAACA   
  
  
+ AAAATAAAAA ATTTTTTTAA TATTAAGTTT AAATTCTAAA AAATATTATT TAAAAAATTA AAATTAAAAA   
  
  
+ AATGTTTCTA TTTCTTATAA GTTTAATATT ACTGTTTTTT TAGTTTAAAA AATTTACGAA TTTCACGAAA   
  
  
+ AATATTTTAA AATGCAATAA TTTTTAAATT TTAATTAAGT ATTTTTTATA AAAATCGTCA TTTTATTAAA   
  
  
+ ACATAAAAAA AGCAAACTTA TAGAAAAAT  

- CGAAGAATAC TAAGAAAGAA GAAGAAATGA ATCGGAAAGC ACCGATGAAA GTTAGAAAGA GATTAGAGAG   
  
  
- CTTCGGCTAA GATCTTTGTT TTCCTCTAGT GAGAAAGAGT TTTGTTTCGA TGGGGTTCTT GTTTCGCTTT   
  
  
- TCTTTCTCTC TGAGAGAGAG AGAGAGAACC AGAAAAGGGG TAGGTTTGGC TCCTCTCCAC TCCTTTATAT   
  
  
- TTACTTTTTT TTTTTTTCCC TTCGTTAGTG GCCAAAATAT TCTTCAAACA ACCCAAAAAT TGAGCTCTGA   
  
  
- TTTGTTAAAT TTGATTAAAA AATAAAAAAT ATCTTCTATA ATTTGTGACT TTTTTTACTT TATTTTAAAA   
  
  
- CTAGACATGG TTATACTTCA TAAAAGACAA TTAAAAATTA AAAAAAACAA AAAACAGTTT AAGCCCTAGC   
  
  
- GATGAAGGTA AAGACAGGGA CATACCCTTT TTTCTTCATA TTAAGAAAAA TAAAAATAAT TAATAAAAAA   
  
  
- TAATTAGCTC TTTAATTGCA TGATAAGTTT CTGTTTAAAT CCGAATTGGC TAAAAGAATT GGGTGAAAAC   
  
  
- AGGGCATGGC ATGAACCATG AGCGAGAAGG CTTGGTCCGG TCCATCGGTC GCAGGCAGTC ACCGTCACGT   
  
  
- GGACAAGGCG CCATAAGAAA GGGGTTGGTC AATGTTTTCA ACTCTACGGT TATTATTCTC TTTTTGGAGG   
  
  
- ATTAGGCTAA CACGTGTCGT GGGTCGGGCA CCGTCATTTG ACCGAACGTG GCACGTGGGA GCCCATCTCC   
  
  
- TGTTGGTTTT CCACGGGCAA TTATTGTGGA CGTTAGCGTA CAAATATCCA CATTTGACTC TTCTGTCTGT   
  
  
- ACATTTCTAC ATGCGTTGCT GACTCAGCTT TCGCGTCATC CCATCTAGTT CCCGTTACTC TTAAGTTTGG   
  
  
- TGGTACACTC AAAAAATAAT AATAATAATA ATTATTATTA AATTAATCCG TACATAAATA GGCAAGATCG   
  
  
- TGATTATTTT ATAATAGCAT TTTATTTGCT ATTCAATAAA GTAACGATGA TAAATGCTTA GTGTGAAACA   
  
  
- TTTATAATGA TGATCCCTGA ACCAACATAA TCATACTGAG ATGATTATAT TTTATGTATA ATAAACATCA   
  
  
- AAATAATTTT TATGTTAATT GTGTTTTTTA TTAAATTTTT TATTATTTTT AGATGAATTT TATACATAAA   
  
  
- ATATTAAATT AGTTCAAATT ATTATTGTTT TTATTTTTTT AAAAAATTAT AATTCAAATT TAATTATTGT   
  
  
- TTTTATTTTT TAAAAAAATT ATAATTCAAA TTTAAGATTT TTTATAATAA ATTTTTTAAT TTTAATTTTT   
  
  
- TTACAAAGAT AAAGAATATT CAAATTATAA TGACAAAAAA ATCAAATTTT TTAAATGCTT AAAGTGCTTT   
  
  
- TTATAAAATT TTACGTTATT AAAAATTTAA AATTAATTCA TAAAAAATAT TTTTAGCAGT AAAATAATTT   
  
  
- TGTATTTTTT TCGTTTGAAT ATCTTTTTA

+     Unnamed\_\_1

| Site Name | Organism | Position | Strand | Matrix score. | sequence | function |
| --- | --- | --- | --- | --- | --- | --- |
| Unnamed\_\_1 | Zea mays | 39 | + | 5 | CGTGG |  |
| Unnamed\_\_1 | Zea mays | 728 | + | 5 | CGTGG |  |

> 2018/04/13 10:10:12  
+ GCTTCTTATG ATTCTTTCTT CTTCTTTACT TAGCCTTTCG TGGCTACTTT CAATCTTTCT CTAATCTCTC   
  
  
+ GAAGCCGATT CTAGAAACAA AAGGAGATCA CTCTTTCTCA AAACAAAGCT ACCCCAAGAA CAAAGCGAAA   
  
  
+ AGAAAGAGAG ACTCTCTCTC TCTCTCTTGG TCTTTTCCCC ATCCAAACCG AGGAGAGGTG AGGAAATATA   
  
  
+ AATGAAAAAA AAAAAAAGGG AAGCAATCAC CGGTTTTATA AGAAGTTTGT TGGGTTTTTA ACTCGAGACT   
  
  
+ AAACAATTTA AACTAATTTT TTATTTTTTA TAGAAGATAT TAAACACTGA AAAAAATGAA ATAAAATTTT   
  
  
+ GATCTGTACC AATATGAAGT ATTTTCTGTT AATTTTTAAT TTTTTTTGTT TTTTGTCAAA TTCGGGATCG   
  
  
+ CTACTTCCAT TTCTGTCCCT GTATGGGAAA AAAGAAGTAT AATTCTTTTT ATTTTTATTA ATTATTTTTT   
  
  
+ ATTAATCGAG AAATTAACGT ACTATTCAAA GACAAATTTA GGCTTAACCG ATTTTCTTAA CCCACTTTTG   
  
  
+ TCCCGTACCG TACTTGGTAC TCGCTCTTCC GAACCAGGCC AGGTAGCCAG CGTCCGTCAG TGGCAGTGCA   
  
  
+ CCTGTTCCGC GGTATTCTTT CCCCAACCAG TTACAAAAGT TGAGATGCCA ATAATAAGAG AAAAACCTCC   
  
  
+ TAATCCGATT GTGCACAGCA CCCAGCCCGT GGCAGTAAAC TGGCTTGCAC CGTGCACCCT CGGGTAGAGG   
  
  
+ ACAACCAAAA GGTGCCCGTT AATAACACCT GCAATCGCAT GTTTATAGGT GTAAACTGAG AAGACAGACA   
  
  
+ TGTAAAGATG TACGCAACGA CTGAGTCGAA AGCGCAGTAG GGTAGATCAA GGGCAATGAG AATTCAAACC   
  
  
+ ACCATGTGAG TTTTTTATTA TTATTATTAT TAATAATAAT TTAATTAGGC ATGTATTTAT CCGTTCTAGC   
  
  
+ ACTAATAAAA TATTATCGTA AAATAAACGA TAAGTTATTT CATTGCTACT ATTTACGAAT CACACTTTGT   
  
  
+ AAATATTACT ACTAGGGACT TGGTTGTATT AGTATGACTC TACTAATATA AAATACATAT TATTTGTAGT   
  
  
+ TTTATTAAAA ATACAATTAA CACAAAAAAT AATTTAAAAA ATAATAAAAA TCTACTTAAA ATATGTATTT   
  
  
+ TATAATTTAA TCAAGTTTAA TAATAACAAA AATAAAAAAA TTTTTTAATA TTAAGTTTAA ATTAATAACA   
  
  
+ AAAATAAAAA ATTTTTTTAA TATTAAGTTT AAATTCTAAA AAATATTATT TAAAAAATTA AAATTAAAAA   
  
  
+ AATGTTTCTA TTTCTTATAA GTTTAATATT ACTGTTTTTT TAGTTTAAAA AATTTACGAA TTTCACGAAA   
  
  
+ AATATTTTAA AATGCAATAA TTTTTAAATT TTAATTAAGT ATTTTTTATA AAAATCGTCA TTTTATTAAA   
  
  
+ ACATAAAAAA AGCAAACTTA TAGAAAAAT  

- CGAAGAATAC TAAGAAAGAA GAAGAAATGA ATCGGAAAGC ACCGATGAAA GTTAGAAAGA GATTAGAGAG   
  
  
- CTTCGGCTAA GATCTTTGTT TTCCTCTAGT GAGAAAGAGT TTTGTTTCGA TGGGGTTCTT GTTTCGCTTT   
  
  
- TCTTTCTCTC TGAGAGAGAG AGAGAGAACC AGAAAAGGGG TAGGTTTGGC TCCTCTCCAC TCCTTTATAT   
  
  
- TTACTTTTTT TTTTTTTCCC TTCGTTAGTG GCCAAAATAT TCTTCAAACA ACCCAAAAAT TGAGCTCTGA   
  
  
- TTTGTTAAAT TTGATTAAAA AATAAAAAAT ATCTTCTATA ATTTGTGACT TTTTTTACTT TATTTTAAAA   
  
  
- CTAGACATGG TTATACTTCA TAAAAGACAA TTAAAAATTA AAAAAAACAA AAAACAGTTT AAGCCCTAGC   
  
  
- GATGAAGGTA AAGACAGGGA CATACCCTTT TTTCTTCATA TTAAGAAAAA TAAAAATAAT TAATAAAAAA   
  
  
- TAATTAGCTC TTTAATTGCA TGATAAGTTT CTGTTTAAAT CCGAATTGGC TAAAAGAATT GGGTGAAAAC   
  
  
- AGGGCATGGC ATGAACCATG AGCGAGAAGG CTTGGTCCGG TCCATCGGTC GCAGGCAGTC ACCGTCACGT   
  
  
- GGACAAGGCG CCATAAGAAA GGGGTTGGTC AATGTTTTCA ACTCTACGGT TATTATTCTC TTTTTGGAGG   
  
  
- ATTAGGCTAA CACGTGTCGT GGGTCGGGCA CCGTCATTTG ACCGAACGTG GCACGTGGGA GCCCATCTCC   
  
  
- TGTTGGTTTT CCACGGGCAA TTATTGTGGA CGTTAGCGTA CAAATATCCA CATTTGACTC TTCTGTCTGT   
  
  
- ACATTTCTAC ATGCGTTGCT GACTCAGCTT TCGCGTCATC CCATCTAGTT CCCGTTACTC TTAAGTTTGG   
  
  
- TGGTACACTC AAAAAATAAT AATAATAATA ATTATTATTA AATTAATCCG TACATAAATA GGCAAGATCG   
  
  
- TGATTATTTT ATAATAGCAT TTTATTTGCT ATTCAATAAA GTAACGATGA TAAATGCTTA GTGTGAAACA   
  
  
- TTTATAATGA TGATCCCTGA ACCAACATAA TCATACTGAG ATGATTATAT TTTATGTATA ATAAACATCA   
  
  
- AAATAATTTT TATGTTAATT GTGTTTTTTA TTAAATTTTT TATTATTTTT AGATGAATTT TATACATAAA   
  
  
- ATATTAAATT AGTTCAAATT ATTATTGTTT TTATTTTTTT AAAAAATTAT AATTCAAATT TAATTATTGT   
  
  
- TTTTATTTTT TAAAAAAATT ATAATTCAAA TTTAAGATTT TTTATAATAA ATTTTTTAAT TTTAATTTTT   
  
  
- TTACAAAGAT AAAGAATATT CAAATTATAA TGACAAAAAA ATCAAATTTT TTAAATGCTT AAAGTGCTTT   
  
  
- TTATAAAATT TTACGTTATT AAAAATTTAA AATTAATTCA TAAAAAATAT TTTTAGCAGT AAAATAATTT   
  
  
- TGTATTTTTT TCGTTTGAAT ATCTTTTTA

+     Unnamed\_\_3

| Site Name | Organism | Position | Strand | Matrix score. | sequence | function |
| --- | --- | --- | --- | --- | --- | --- |
| Unnamed\_\_3 | Zea mays | 39 | + | 5 | CGTGG |  |
| Unnamed\_\_3 | Zea mays | 728 | + | 5 | CGTGG |  |

> 2018/04/13 10:10:12  
+ GCTTCTTATG ATTCTTTCTT CTTCTTTACT TAGCCTTTCG TGGCTACTTT CAATCTTTCT CTAATCTCTC   
  
  
+ GAAGCCGATT CTAGAAACAA AAGGAGATCA CTCTTTCTCA AAACAAAGCT ACCCCAAGAA CAAAGCGAAA   
  
  
+ AGAAAGAGAG ACTCTCTCTC TCTCTCTTGG TCTTTTCCCC ATCCAAACCG AGGAGAGGTG AGGAAATATA   
  
  
+ AATGAAAAAA AAAAAAAGGG AAGCAATCAC CGGTTTTATA AGAAGTTTGT TGGGTTTTTA ACTCGAGACT   
  
  
+ AAACAATTTA AACTAATTTT TTATTTTTTA TAGAAGATAT TAAACACTGA AAAAAATGAA ATAAAATTTT   
  
  
+ GATCTGTACC AATATGAAGT ATTTTCTGTT AATTTTTAAT TTTTTTTGTT TTTTGTCAAA TTCGGGATCG   
  
  
+ CTACTTCCAT TTCTGTCCCT GTATGGGAAA AAAGAAGTAT AATTCTTTTT ATTTTTATTA ATTATTTTTT   
  
  
+ ATTAATCGAG AAATTAACGT ACTATTCAAA GACAAATTTA GGCTTAACCG ATTTTCTTAA CCCACTTTTG   
  
  
+ TCCCGTACCG TACTTGGTAC TCGCTCTTCC GAACCAGGCC AGGTAGCCAG CGTCCGTCAG TGGCAGTGCA   
  
  
+ CCTGTTCCGC GGTATTCTTT CCCCAACCAG TTACAAAAGT TGAGATGCCA ATAATAAGAG AAAAACCTCC   
  
  
+ TAATCCGATT GTGCACAGCA CCCAGCCCGT GGCAGTAAAC TGGCTTGCAC CGTGCACCCT CGGGTAGAGG   
  
  
+ ACAACCAAAA GGTGCCCGTT AATAACACCT GCAATCGCAT GTTTATAGGT GTAAACTGAG AAGACAGACA   
  
  
+ TGTAAAGATG TACGCAACGA CTGAGTCGAA AGCGCAGTAG GGTAGATCAA GGGCAATGAG AATTCAAACC   
  
  
+ ACCATGTGAG TTTTTTATTA TTATTATTAT TAATAATAAT TTAATTAGGC ATGTATTTAT CCGTTCTAGC   
  
  
+ ACTAATAAAA TATTATCGTA AAATAAACGA TAAGTTATTT CATTGCTACT ATTTACGAAT CACACTTTGT   
  
  
+ AAATATTACT ACTAGGGACT TGGTTGTATT AGTATGACTC TACTAATATA AAATACATAT TATTTGTAGT   
  
  
+ TTTATTAAAA ATACAATTAA CACAAAAAAT AATTTAAAAA ATAATAAAAA TCTACTTAAA ATATGTATTT   
  
  
+ TATAATTTAA TCAAGTTTAA TAATAACAAA AATAAAAAAA TTTTTTAATA TTAAGTTTAA ATTAATAACA   
  
  
+ AAAATAAAAA ATTTTTTTAA TATTAAGTTT AAATTCTAAA AAATATTATT TAAAAAATTA AAATTAAAAA   
  
  
+ AATGTTTCTA TTTCTTATAA GTTTAATATT ACTGTTTTTT TAGTTTAAAA AATTTACGAA TTTCACGAAA   
  
  
+ AATATTTTAA AATGCAATAA TTTTTAAATT TTAATTAAGT ATTTTTTATA AAAATCGTCA TTTTATTAAA   
  
  
+ ACATAAAAAA AGCAAACTTA TAGAAAAAT  

- CGAAGAATAC TAAGAAAGAA GAAGAAATGA ATCGGAAAGC ACCGATGAAA GTTAGAAAGA GATTAGAGAG   
  
  
- CTTCGGCTAA GATCTTTGTT TTCCTCTAGT GAGAAAGAGT TTTGTTTCGA TGGGGTTCTT GTTTCGCTTT   
  
  
- TCTTTCTCTC TGAGAGAGAG AGAGAGAACC AGAAAAGGGG TAGGTTTGGC TCCTCTCCAC TCCTTTATAT   
  
  
- TTACTTTTTT TTTTTTTCCC TTCGTTAGTG GCCAAAATAT TCTTCAAACA ACCCAAAAAT TGAGCTCTGA   
  
  
- TTTGTTAAAT TTGATTAAAA AATAAAAAAT ATCTTCTATA ATTTGTGACT TTTTTTACTT TATTTTAAAA   
  
  
- CTAGACATGG TTATACTTCA TAAAAGACAA TTAAAAATTA AAAAAAACAA AAAACAGTTT AAGCCCTAGC   
  
  
- GATGAAGGTA AAGACAGGGA CATACCCTTT TTTCTTCATA TTAAGAAAAA TAAAAATAAT TAATAAAAAA   
  
  
- TAATTAGCTC TTTAATTGCA TGATAAGTTT CTGTTTAAAT CCGAATTGGC TAAAAGAATT GGGTGAAAAC   
  
  
- AGGGCATGGC ATGAACCATG AGCGAGAAGG CTTGGTCCGG TCCATCGGTC GCAGGCAGTC ACCGTCACGT   
  
  
- GGACAAGGCG CCATAAGAAA GGGGTTGGTC AATGTTTTCA ACTCTACGGT TATTATTCTC TTTTTGGAGG   
  
  
- ATTAGGCTAA CACGTGTCGT GGGTCGGGCA CCGTCATTTG ACCGAACGTG GCACGTGGGA GCCCATCTCC   
  
  
- TGTTGGTTTT CCACGGGCAA TTATTGTGGA CGTTAGCGTA CAAATATCCA CATTTGACTC TTCTGTCTGT   
  
  
- ACATTTCTAC ATGCGTTGCT GACTCAGCTT TCGCGTCATC CCATCTAGTT CCCGTTACTC TTAAGTTTGG   
  
  
- TGGTACACTC AAAAAATAAT AATAATAATA ATTATTATTA AATTAATCCG TACATAAATA GGCAAGATCG   
  
  
- TGATTATTTT ATAATAGCAT TTTATTTGCT ATTCAATAAA GTAACGATGA TAAATGCTTA GTGTGAAACA   
  
  
- TTTATAATGA TGATCCCTGA ACCAACATAA TCATACTGAG ATGATTATAT TTTATGTATA ATAAACATCA   
  
  
- AAATAATTTT TATGTTAATT GTGTTTTTTA TTAAATTTTT TATTATTTTT AGATGAATTT TATACATAAA   
  
  
- ATATTAAATT AGTTCAAATT ATTATTGTTT TTATTTTTTT AAAAAATTAT AATTCAAATT TAATTATTGT   
  
  
- TTTTATTTTT TAAAAAAATT ATAATTCAAA TTTAAGATTT TTTATAATAA ATTTTTTAAT TTTAATTTTT   
  
  
- TTACAAAGAT AAAGAATATT CAAATTATAA TGACAAAAAA ATCAAATTTT TTAAATGCTT AAAGTGCTTT   
  
  
- TTATAAAATT TTACGTTATT AAAAATTTAA AATTAATTCA TAAAAAATAT TTTTAGCAGT AAAATAATTT   
  
  
- TGTATTTTTT TCGTTTGAAT ATCTTTTTA

+     Unnamed\_\_4

| Site Name | Organism | Position | Strand | Matrix score. | sequence | function |
| --- | --- | --- | --- | --- | --- | --- |
| Unnamed\_\_4 | Petroselinum hortense | 192 | - | 4 | CTCC |  |
| Unnamed\_\_4 | Petroselinum hortense | 93 | - | 4 | CTCC |  |
| Unnamed\_\_4 | Petroselinum hortense | 697 | + | 4 | CTCC |  |

> 2018/04/13 10:10:12  
+ GCTTCTTATG ATTCTTTCTT CTTCTTTACT TAGCCTTTCG TGGCTACTTT CAATCTTTCT CTAATCTCTC   
  
  
+ GAAGCCGATT CTAGAAACAA AAGGAGATCA CTCTTTCTCA AAACAAAGCT ACCCCAAGAA CAAAGCGAAA   
  
  
+ AGAAAGAGAG ACTCTCTCTC TCTCTCTTGG TCTTTTCCCC ATCCAAACCG AGGAGAGGTG AGGAAATATA   
  
  
+ AATGAAAAAA AAAAAAAGGG AAGCAATCAC CGGTTTTATA AGAAGTTTGT TGGGTTTTTA ACTCGAGACT   
  
  
+ AAACAATTTA AACTAATTTT TTATTTTTTA TAGAAGATAT TAAACACTGA AAAAAATGAA ATAAAATTTT   
  
  
+ GATCTGTACC AATATGAAGT ATTTTCTGTT AATTTTTAAT TTTTTTTGTT TTTTGTCAAA TTCGGGATCG   
  
  
+ CTACTTCCAT TTCTGTCCCT GTATGGGAAA AAAGAAGTAT AATTCTTTTT ATTTTTATTA ATTATTTTTT   
  
  
+ ATTAATCGAG AAATTAACGT ACTATTCAAA GACAAATTTA GGCTTAACCG ATTTTCTTAA CCCACTTTTG   
  
  
+ TCCCGTACCG TACTTGGTAC TCGCTCTTCC GAACCAGGCC AGGTAGCCAG CGTCCGTCAG TGGCAGTGCA   
  
  
+ CCTGTTCCGC GGTATTCTTT CCCCAACCAG TTACAAAAGT TGAGATGCCA ATAATAAGAG AAAAACCTCC   
  
  
+ TAATCCGATT GTGCACAGCA CCCAGCCCGT GGCAGTAAAC TGGCTTGCAC CGTGCACCCT CGGGTAGAGG   
  
  
+ ACAACCAAAA GGTGCCCGTT AATAACACCT GCAATCGCAT GTTTATAGGT GTAAACTGAG AAGACAGACA   
  
  
+ TGTAAAGATG TACGCAACGA CTGAGTCGAA AGCGCAGTAG GGTAGATCAA GGGCAATGAG AATTCAAACC   
  
  
+ ACCATGTGAG TTTTTTATTA TTATTATTAT TAATAATAAT TTAATTAGGC ATGTATTTAT CCGTTCTAGC   
  
  
+ ACTAATAAAA TATTATCGTA AAATAAACGA TAAGTTATTT CATTGCTACT ATTTACGAAT CACACTTTGT   
  
  
+ AAATATTACT ACTAGGGACT TGGTTGTATT AGTATGACTC TACTAATATA AAATACATAT TATTTGTAGT   
  
  
+ TTTATTAAAA ATACAATTAA CACAAAAAAT AATTTAAAAA ATAATAAAAA TCTACTTAAA ATATGTATTT   
  
  
+ TATAATTTAA TCAAGTTTAA TAATAACAAA AATAAAAAAA TTTTTTAATA TTAAGTTTAA ATTAATAACA   
  
  
+ AAAATAAAAA ATTTTTTTAA TATTAAGTTT AAATTCTAAA AAATATTATT TAAAAAATTA AAATTAAAAA   
  
  
+ AATGTTTCTA TTTCTTATAA GTTTAATATT ACTGTTTTTT TAGTTTAAAA AATTTACGAA TTTCACGAAA   
  
  
+ AATATTTTAA AATGCAATAA TTTTTAAATT TTAATTAAGT ATTTTTTATA AAAATCGTCA TTTTATTAAA   
  
  
+ ACATAAAAAA AGCAAACTTA TAGAAAAAT  

- CGAAGAATAC TAAGAAAGAA GAAGAAATGA ATCGGAAAGC ACCGATGAAA GTTAGAAAGA GATTAGAGAG   
  
  
- CTTCGGCTAA GATCTTTGTT TTCCTCTAGT GAGAAAGAGT TTTGTTTCGA TGGGGTTCTT GTTTCGCTTT   
  
  
- TCTTTCTCTC TGAGAGAGAG AGAGAGAACC AGAAAAGGGG TAGGTTTGGC TCCTCTCCAC TCCTTTATAT   
  
  
- TTACTTTTTT TTTTTTTCCC TTCGTTAGTG GCCAAAATAT TCTTCAAACA ACCCAAAAAT TGAGCTCTGA   
  
  
- TTTGTTAAAT TTGATTAAAA AATAAAAAAT ATCTTCTATA ATTTGTGACT TTTTTTACTT TATTTTAAAA   
  
  
- CTAGACATGG TTATACTTCA TAAAAGACAA TTAAAAATTA AAAAAAACAA AAAACAGTTT AAGCCCTAGC   
  
  
- GATGAAGGTA AAGACAGGGA CATACCCTTT TTTCTTCATA TTAAGAAAAA TAAAAATAAT TAATAAAAAA   
  
  
- TAATTAGCTC TTTAATTGCA TGATAAGTTT CTGTTTAAAT CCGAATTGGC TAAAAGAATT GGGTGAAAAC   
  
  
- AGGGCATGGC ATGAACCATG AGCGAGAAGG CTTGGTCCGG TCCATCGGTC GCAGGCAGTC ACCGTCACGT   
  
  
- GGACAAGGCG CCATAAGAAA GGGGTTGGTC AATGTTTTCA ACTCTACGGT TATTATTCTC TTTTTGGAGG   
  
  
- ATTAGGCTAA CACGTGTCGT GGGTCGGGCA CCGTCATTTG ACCGAACGTG GCACGTGGGA GCCCATCTCC   
  
  
- TGTTGGTTTT CCACGGGCAA TTATTGTGGA CGTTAGCGTA CAAATATCCA CATTTGACTC TTCTGTCTGT   
  
  
- ACATTTCTAC ATGCGTTGCT GACTCAGCTT TCGCGTCATC CCATCTAGTT CCCGTTACTC TTAAGTTTGG   
  
  
- TGGTACACTC AAAAAATAAT AATAATAATA ATTATTATTA AATTAATCCG TACATAAATA GGCAAGATCG   
  
  
- TGATTATTTT ATAATAGCAT TTTATTTGCT ATTCAATAAA GTAACGATGA TAAATGCTTA GTGTGAAACA   
  
  
- TTTATAATGA TGATCCCTGA ACCAACATAA TCATACTGAG ATGATTATAT TTTATGTATA ATAAACATCA   
  
  
- AAATAATTTT TATGTTAATT GTGTTTTTTA TTAAATTTTT TATTATTTTT AGATGAATTT TATACATAAA   
  
  
- ATATTAAATT AGTTCAAATT ATTATTGTTT TTATTTTTTT AAAAAATTAT AATTCAAATT TAATTATTGT   
  
  
- TTTTATTTTT TAAAAAAATT ATAATTCAAA TTTAAGATTT TTTATAATAA ATTTTTTAAT TTTAATTTTT   
  
  
- TTACAAAGAT AAAGAATATT CAAATTATAA TGACAAAAAA ATCAAATTTT TTAAATGCTT AAAGTGCTTT   
  
  
- TTATAAAATT TTACGTTATT AAAAATTTAA AATTAATTCA TAAAAAATAT TTTTAGCAGT AAAATAATTT   
  
  
- TGTATTTTTT TCGTTTGAAT ATCTTTTTA
