# Supplementary material for: Genome-wide analysis of the WRKY gene family in drumstick (Moringa oleifera Lam.)
Source: PeerJ. 2019 Jun 10;7:e7063. doi: 10.7717/peerj.7063 (PMC6563795; doi:10.7717/peerj.7063)
Supplement: Supplemental Information 1 [file peerj-07-7063-s003.gz › MoWRKY13_plantcare.html]

Content-Type: text/html; charset=ISO-8859-1


CallMat\_Firefox


Webmaster Firefox specific output  
To save the result:
click on the frame with the right mouse button and save the source code as a text file with extension .html  
REFERENCE:PlantCARE: a database of plant cis-acting regulatory elements and a portal to tools for in silico analysis of promoter sequences.  
Lescot, M., Déhais, P., Moreau, Y., De Moor, B., Rouzé ,P.,and Rombauts, S.  
Nucleic Acids Res., Database issue(2002), 30(1):325-327.   


---

> 2018/04/13 10:10:12  
+ CCTTCTTCTA CCTCTCGGAC GTCTTGTTCT TCTTCTTTTT CTTCTCTCTT TCTCTCTCGA ATATCTATAA   
  
  
+ TCGAATTAAA CACAACCTGA AAATCGAAGT GTATAGAGAG GAAAATACAT CAAACTCAAA CTATATATTT   
  
  
+ TTATTTTTCT CTTAATTTCT CTTTTTTCTT TTTTCTTTCT CACAAAAACA TCGAAACTTA CTCTGAAAAC   
  
  
+ AACGATCGTG AAATATATTT CTATCCAACT GTTTTTGTGG AAGGTTAATC TTCTCGTATT TCACGGTAGA   
  
  
+ TCATACCTTT GTAAAGTTTA GGTACGATGA TTAGGGTAGA TGGTTCATTG TTGTGAGTGA AAATCAAAGG   
  
  
+ AGAAGGGGTA ATTTATGGGG AGTACGTAAA GTAGAGGATT GTATTTGTTT TGTTTCTAGT TTCGAATGTA   
  
  
+ GTTTTAGAAA AATAGCTAGA CTTGAAGTAC ACAATTTGCA AACTTTTCTG CTATTATTAA ATTACTTTTT   
  
  
+ TTACTAATTC TAAAAAATAT TATTATTTGA CTTAATATTT TTATGAAATA TAAAACTAAT TAATTTTTGA   
  
  
+ GCTAGTCGTA TATTTAGTCT AGTTATCTTC TAGAGTTTAT ACAATTTTCT ATTAAATAAA AAATAATAAT   
  
  
+ GATTAATAGG AAAAATAACA TTAATCTAAA CACATATACT ACGAATCCCG AAATTCCGAA ACAAAAACAC   
  
  
+ ACAAATACAA TCACCCAACT TATCTATCTC TTTCCCTCAT CACACAAATC CACCGTTAAT GAATGATTAT   
  
  
+ TATTGTACAC TTACTCACAA CACACGTGCG AGACCCTACC TCGCACCCCG CCCCTAGTGT TCAGTCGCTT   
  
  
+ CTGATGTCCA TTTCCTACCT TCACTCTCGC CAGTTTCTAA GTTTGGGTTT TTTTTCCTTT TATTTTTATT   
  
  
+ TTTTTCGTTT CTTTTTATTT TTCCTCTAAA CCTTGTAATC CGAGTGTGCA CCGCCTTGTC GGTGGCAGTA   
  
  
+ AACTGGTATC GGCTGTGTAC TGTGCCGTGC ACCCTATGGG TAGAGCAGGG GGACGGCGAT AGGCAGCGCT   
  
  
+ CGGACAGGAC GACTACCCCG TGGTCGCAGT CGCAGGGAAA CCTTAAAAAG AGAATTAAAG AACACGAGTG   
  
  
+ CCTTTGGACT TAAATTATTT AGGAAATAAA TAAATAATAA AATAAATAAT AAAATAAATA AAAAGTACCG   
  
  
+ GTGAAAAATA AAAAATTAAA TTAAAAGCAA TGGTTCATTC TTCTAGTAAT TAATATAGGT GTGTGGGTTA   
  
  
+ ATTATATAAA TAATAAAATA AATTATTTAT TAATAATTAA TTCTAAAAAT AAACGAATTT TTATTTGTTT   
  
  
+ AGAGACTATT TTGTAACCTG CTCCTCAACT TAGTTAAAAT TACAAAAATT TTAAATAATT TATTATAAAA   
  
  
+ ATTAATTTAC GTTTTATAAA ACTACACATT ATGATTTAGT AAAGTTCACA AATTTTATAA ATCTATATAA   
  
  
+ TTACGACCTT CACTTTTAGA GAACAAGTA  

- GGAAGAAGAT GGAGAGCCTG CAGAACAAGA AGAAGAAAAA GAAGAGAGAA AGAGAGAGCT TATAGATATT   
  
  
- AGCTTAATTT GTGTTGGACT TTTAGCTTCA CATATCTCTC CTTTTATGTA GTTTGAGTTT GATATATAAA   
  
  
- AATAAAAAGA GAATTAAAGA GAAAAAAGAA AAAAGAAAGA GTGTTTTTGT AGCTTTGAAT GAGACTTTTG   
  
  
- TTGCTAGCAC TTTATATAAA GATAGGTTGA CAAAAACACC TTCCAATTAG AAGAGCATAA AGTGCCATCT   
  
  
- AGTATGGAAA CATTTCAAAT CCATGCTACT AATCCCATCT ACCAAGTAAC AACACTCACT TTTAGTTTCC   
  
  
- TCTTCCCCAT TAAATACCCC TCATGCATTT CATCTCCTAA CATAAACAAA ACAAAGATCA AAGCTTACAT   
  
  
- CAAAATCTTT TTATCGATCT GAACTTCATG TGTTAAACGT TTGAAAAGAC GATAATAATT TAATGAAAAA   
  
  
- AATGATTAAG ATTTTTTATA ATAATAAACT GAATTATAAA AATACTTTAT ATTTTGATTA ATTAAAAACT   
  
  
- CGATCAGCAT ATAAATCAGA TCAATAGAAG ATCTCAAATA TGTTAAAAGA TAATTTATTT TTTATTATTA   
  
  
- CTAATTATCC TTTTTATTGT AATTAGATTT GTGTATATGA TGCTTAGGGC TTTAAGGCTT TGTTTTTGTG   
  
  
- TGTTTATGTT AGTGGGTTGA ATAGATAGAG AAAGGGAGTA GTGTGTTTAG GTGGCAATTA CTTACTAATA   
  
  
- ATAACATGTG AATGAGTGTT GTGTGCACGC TCTGGGATGG AGCGTGGGGC GGGGATCACA AGTCAGCGAA   
  
  
- GACTACAGGT AAAGGATGGA AGTGAGAGCG GTCAAAGATT CAAACCCAAA AAAAAGGAAA ATAAAAATAA   
  
  
- AAAAAGCAAA GAAAAATAAA AAGGAGATTT GGAACATTAG GCTCACACGT GGCGGAACAG CCACCGTCAT   
  
  
- TTGACCATAG CCGACACATG ACACGGCACG TGGGATACCC ATCTCGTCCC CCTGCCGCTA TCCGTCGCGA   
  
  
- GCCTGTCCTG CTGATGGGGC ACCAGCGTCA GCGTCCCTTT GGAATTTTTC TCTTAATTTC TTGTGCTCAC   
  
  
- GGAAACCTGA ATTTAATAAA TCCTTTATTT ATTTATTATT TTATTTATTA TTTTATTTAT TTTTCATGGC   
  
  
- CACTTTTTAT TTTTTAATTT AATTTTCGTT ACCAAGTAAG AAGATCATTA ATTATATCCA CACACCCAAT   
  
  
- TAATATATTT ATTATTTTAT TTAATAAATA ATTATTAATT AAGATTTTTA TTTGCTTAAA AATAAACAAA   
  
  
- TCTCTGATAA AACATTGGAC GAGGAGTTGA ATCAATTTTA ATGTTTTTAA AATTTATTAA ATAATATTTT   
  
  
- TAATTAAATG CAAAATATTT TGATGTGTAA TACTAAATCA TTTCAAGTGT TTAAAATATT TAGATATATT   
  
  
- AATGCTGGAA GTGAAAATCT CTTGTTCAT

  
  
Motifs Found  

+     5UTR Py-rich stretch

| Site Name | Organism | Position | Strand | Matrix score. | sequence | function |
| --- | --- | --- | --- | --- | --- | --- |
| 5UTR Py-rich stretch | Lycopersicon esculentum | 105 | - | 9 | TTTCTTCTCT | cis-acting element conferring high transcription levels |
| 5UTR Py-rich stretch | Lycopersicon esculentum | 38 | + | 10 | TTTCTTCTCT | cis-acting element conferring high transcription levels |

> 2018/04/13 10:10:12  
+ CCTTCTTCTA CCTCTCGGAC GTCTTGTTCT TCTTCTTTTT CTTCTCTCTT TCTCTCTCGA ATATCTATAA   
  
  
+ TCGAATTAAA CACAACCTGA AAATCGAAGT GTATAGAGAG GAAAATACAT CAAACTCAAA CTATATATTT   
  
  
+ TTATTTTTCT CTTAATTTCT CTTTTTTCTT TTTTCTTTCT CACAAAAACA TCGAAACTTA CTCTGAAAAC   
  
  
+ AACGATCGTG AAATATATTT CTATCCAACT GTTTTTGTGG AAGGTTAATC TTCTCGTATT TCACGGTAGA   
  
  
+ TCATACCTTT GTAAAGTTTA GGTACGATGA TTAGGGTAGA TGGTTCATTG TTGTGAGTGA AAATCAAAGG   
  
  
+ AGAAGGGGTA ATTTATGGGG AGTACGTAAA GTAGAGGATT GTATTTGTTT TGTTTCTAGT TTCGAATGTA   
  
  
+ GTTTTAGAAA AATAGCTAGA CTTGAAGTAC ACAATTTGCA AACTTTTCTG CTATTATTAA ATTACTTTTT   
  
  
+ TTACTAATTC TAAAAAATAT TATTATTTGA CTTAATATTT TTATGAAATA TAAAACTAAT TAATTTTTGA   
  
  
+ GCTAGTCGTA TATTTAGTCT AGTTATCTTC TAGAGTTTAT ACAATTTTCT ATTAAATAAA AAATAATAAT   
  
  
+ GATTAATAGG AAAAATAACA TTAATCTAAA CACATATACT ACGAATCCCG AAATTCCGAA ACAAAAACAC   
  
  
+ ACAAATACAA TCACCCAACT TATCTATCTC TTTCCCTCAT CACACAAATC CACCGTTAAT GAATGATTAT   
  
  
+ TATTGTACAC TTACTCACAA CACACGTGCG AGACCCTACC TCGCACCCCG CCCCTAGTGT TCAGTCGCTT   
  
  
+ CTGATGTCCA TTTCCTACCT TCACTCTCGC CAGTTTCTAA GTTTGGGTTT TTTTTCCTTT TATTTTTATT   
  
  
+ TTTTTCGTTT CTTTTTATTT TTCCTCTAAA CCTTGTAATC CGAGTGTGCA CCGCCTTGTC GGTGGCAGTA   
  
  
+ AACTGGTATC GGCTGTGTAC TGTGCCGTGC ACCCTATGGG TAGAGCAGGG GGACGGCGAT AGGCAGCGCT   
  
  
+ CGGACAGGAC GACTACCCCG TGGTCGCAGT CGCAGGGAAA CCTTAAAAAG AGAATTAAAG AACACGAGTG   
  
  
+ CCTTTGGACT TAAATTATTT AGGAAATAAA TAAATAATAA AATAAATAAT AAAATAAATA AAAAGTACCG   
  
  
+ GTGAAAAATA AAAAATTAAA TTAAAAGCAA TGGTTCATTC TTCTAGTAAT TAATATAGGT GTGTGGGTTA   
  
  
+ ATTATATAAA TAATAAAATA AATTATTTAT TAATAATTAA TTCTAAAAAT AAACGAATTT TTATTTGTTT   
  
  
+ AGAGACTATT TTGTAACCTG CTCCTCAACT TAGTTAAAAT TACAAAAATT TTAAATAATT TATTATAAAA   
  
  
+ ATTAATTTAC GTTTTATAAA ACTACACATT ATGATTTAGT AAAGTTCACA AATTTTATAA ATCTATATAA   
  
  
+ TTACGACCTT CACTTTTAGA GAACAAGTA  

- GGAAGAAGAT GGAGAGCCTG CAGAACAAGA AGAAGAAAAA GAAGAGAGAA AGAGAGAGCT TATAGATATT   
  
  
- AGCTTAATTT GTGTTGGACT TTTAGCTTCA CATATCTCTC CTTTTATGTA GTTTGAGTTT GATATATAAA   
  
  
- AATAAAAAGA GAATTAAAGA GAAAAAAGAA AAAAGAAAGA GTGTTTTTGT AGCTTTGAAT GAGACTTTTG   
  
  
- TTGCTAGCAC TTTATATAAA GATAGGTTGA CAAAAACACC TTCCAATTAG AAGAGCATAA AGTGCCATCT   
  
  
- AGTATGGAAA CATTTCAAAT CCATGCTACT AATCCCATCT ACCAAGTAAC AACACTCACT TTTAGTTTCC   
  
  
- TCTTCCCCAT TAAATACCCC TCATGCATTT CATCTCCTAA CATAAACAAA ACAAAGATCA AAGCTTACAT   
  
  
- CAAAATCTTT TTATCGATCT GAACTTCATG TGTTAAACGT TTGAAAAGAC GATAATAATT TAATGAAAAA   
  
  
- AATGATTAAG ATTTTTTATA ATAATAAACT GAATTATAAA AATACTTTAT ATTTTGATTA ATTAAAAACT   
  
  
- CGATCAGCAT ATAAATCAGA TCAATAGAAG ATCTCAAATA TGTTAAAAGA TAATTTATTT TTTATTATTA   
  
  
- CTAATTATCC TTTTTATTGT AATTAGATTT GTGTATATGA TGCTTAGGGC TTTAAGGCTT TGTTTTTGTG   
  
  
- TGTTTATGTT AGTGGGTTGA ATAGATAGAG AAAGGGAGTA GTGTGTTTAG GTGGCAATTA CTTACTAATA   
  
  
- ATAACATGTG AATGAGTGTT GTGTGCACGC TCTGGGATGG AGCGTGGGGC GGGGATCACA AGTCAGCGAA   
  
  
- GACTACAGGT AAAGGATGGA AGTGAGAGCG GTCAAAGATT CAAACCCAAA AAAAAGGAAA ATAAAAATAA   
  
  
- AAAAAGCAAA GAAAAATAAA AAGGAGATTT GGAACATTAG GCTCACACGT GGCGGAACAG CCACCGTCAT   
  
  
- TTGACCATAG CCGACACATG ACACGGCACG TGGGATACCC ATCTCGTCCC CCTGCCGCTA TCCGTCGCGA   
  
  
- GCCTGTCCTG CTGATGGGGC ACCAGCGTCA GCGTCCCTTT GGAATTTTTC TCTTAATTTC TTGTGCTCAC   
  
  
- GGAAACCTGA ATTTAATAAA TCCTTTATTT ATTTATTATT TTATTTATTA TTTTATTTAT TTTTCATGGC   
  
  
- CACTTTTTAT TTTTTAATTT AATTTTCGTT ACCAAGTAAG AAGATCATTA ATTATATCCA CACACCCAAT   
  
  
- TAATATATTT ATTATTTTAT TTAATAAATA ATTATTAATT AAGATTTTTA TTTGCTTAAA AATAAACAAA   
  
  
- TCTCTGATAA AACATTGGAC GAGGAGTTGA ATCAATTTTA ATGTTTTTAA AATTTATTAA ATAATATTTT   
  
  
- TAATTAAATG CAAAATATTT TGATGTGTAA TACTAAATCA TTTCAAGTGT TTAAAATATT TAGATATATT   
  
  
- AATGCTGGAA GTGAAAATCT CTTGTTCAT

+     A-box

| Site Name | Organism | Position | Strand | Matrix score. | sequence | function |
| --- | --- | --- | --- | --- | --- | --- |
| A-box | Petroselinum crispum | 1031 | - | 6 | CCGTCC | cis-acting regulatory element |

> 2018/04/13 10:10:12  
+ CCTTCTTCTA CCTCTCGGAC GTCTTGTTCT TCTTCTTTTT CTTCTCTCTT TCTCTCTCGA ATATCTATAA   
  
  
+ TCGAATTAAA CACAACCTGA AAATCGAAGT GTATAGAGAG GAAAATACAT CAAACTCAAA CTATATATTT   
  
  
+ TTATTTTTCT CTTAATTTCT CTTTTTTCTT TTTTCTTTCT CACAAAAACA TCGAAACTTA CTCTGAAAAC   
  
  
+ AACGATCGTG AAATATATTT CTATCCAACT GTTTTTGTGG AAGGTTAATC TTCTCGTATT TCACGGTAGA   
  
  
+ TCATACCTTT GTAAAGTTTA GGTACGATGA TTAGGGTAGA TGGTTCATTG TTGTGAGTGA AAATCAAAGG   
  
  
+ AGAAGGGGTA ATTTATGGGG AGTACGTAAA GTAGAGGATT GTATTTGTTT TGTTTCTAGT TTCGAATGTA   
  
  
+ GTTTTAGAAA AATAGCTAGA CTTGAAGTAC ACAATTTGCA AACTTTTCTG CTATTATTAA ATTACTTTTT   
  
  
+ TTACTAATTC TAAAAAATAT TATTATTTGA CTTAATATTT TTATGAAATA TAAAACTAAT TAATTTTTGA   
  
  
+ GCTAGTCGTA TATTTAGTCT AGTTATCTTC TAGAGTTTAT ACAATTTTCT ATTAAATAAA AAATAATAAT   
  
  
+ GATTAATAGG AAAAATAACA TTAATCTAAA CACATATACT ACGAATCCCG AAATTCCGAA ACAAAAACAC   
  
  
+ ACAAATACAA TCACCCAACT TATCTATCTC TTTCCCTCAT CACACAAATC CACCGTTAAT GAATGATTAT   
  
  
+ TATTGTACAC TTACTCACAA CACACGTGCG AGACCCTACC TCGCACCCCG CCCCTAGTGT TCAGTCGCTT   
  
  
+ CTGATGTCCA TTTCCTACCT TCACTCTCGC CAGTTTCTAA GTTTGGGTTT TTTTTCCTTT TATTTTTATT   
  
  
+ TTTTTCGTTT CTTTTTATTT TTCCTCTAAA CCTTGTAATC CGAGTGTGCA CCGCCTTGTC GGTGGCAGTA   
  
  
+ AACTGGTATC GGCTGTGTAC TGTGCCGTGC ACCCTATGGG TAGAGCAGGG GGACGGCGAT AGGCAGCGCT   
  
  
+ CGGACAGGAC GACTACCCCG TGGTCGCAGT CGCAGGGAAA CCTTAAAAAG AGAATTAAAG AACACGAGTG   
  
  
+ CCTTTGGACT TAAATTATTT AGGAAATAAA TAAATAATAA AATAAATAAT AAAATAAATA AAAAGTACCG   
  
  
+ GTGAAAAATA AAAAATTAAA TTAAAAGCAA TGGTTCATTC TTCTAGTAAT TAATATAGGT GTGTGGGTTA   
  
  
+ ATTATATAAA TAATAAAATA AATTATTTAT TAATAATTAA TTCTAAAAAT AAACGAATTT TTATTTGTTT   
  
  
+ AGAGACTATT TTGTAACCTG CTCCTCAACT TAGTTAAAAT TACAAAAATT TTAAATAATT TATTATAAAA   
  
  
+ ATTAATTTAC GTTTTATAAA ACTACACATT ATGATTTAGT AAAGTTCACA AATTTTATAA ATCTATATAA   
  
  
+ TTACGACCTT CACTTTTAGA GAACAAGTA  

- GGAAGAAGAT GGAGAGCCTG CAGAACAAGA AGAAGAAAAA GAAGAGAGAA AGAGAGAGCT TATAGATATT   
  
  
- AGCTTAATTT GTGTTGGACT TTTAGCTTCA CATATCTCTC CTTTTATGTA GTTTGAGTTT GATATATAAA   
  
  
- AATAAAAAGA GAATTAAAGA GAAAAAAGAA AAAAGAAAGA GTGTTTTTGT AGCTTTGAAT GAGACTTTTG   
  
  
- TTGCTAGCAC TTTATATAAA GATAGGTTGA CAAAAACACC TTCCAATTAG AAGAGCATAA AGTGCCATCT   
  
  
- AGTATGGAAA CATTTCAAAT CCATGCTACT AATCCCATCT ACCAAGTAAC AACACTCACT TTTAGTTTCC   
  
  
- TCTTCCCCAT TAAATACCCC TCATGCATTT CATCTCCTAA CATAAACAAA ACAAAGATCA AAGCTTACAT   
  
  
- CAAAATCTTT TTATCGATCT GAACTTCATG TGTTAAACGT TTGAAAAGAC GATAATAATT TAATGAAAAA   
  
  
- AATGATTAAG ATTTTTTATA ATAATAAACT GAATTATAAA AATACTTTAT ATTTTGATTA ATTAAAAACT   
  
  
- CGATCAGCAT ATAAATCAGA TCAATAGAAG ATCTCAAATA TGTTAAAAGA TAATTTATTT TTTATTATTA   
  
  
- CTAATTATCC TTTTTATTGT AATTAGATTT GTGTATATGA TGCTTAGGGC TTTAAGGCTT TGTTTTTGTG   
  
  
- TGTTTATGTT AGTGGGTTGA ATAGATAGAG AAAGGGAGTA GTGTGTTTAG GTGGCAATTA CTTACTAATA   
  
  
- ATAACATGTG AATGAGTGTT GTGTGCACGC TCTGGGATGG AGCGTGGGGC GGGGATCACA AGTCAGCGAA   
  
  
- GACTACAGGT AAAGGATGGA AGTGAGAGCG GTCAAAGATT CAAACCCAAA AAAAAGGAAA ATAAAAATAA   
  
  
- AAAAAGCAAA GAAAAATAAA AAGGAGATTT GGAACATTAG GCTCACACGT GGCGGAACAG CCACCGTCAT   
  
  
- TTGACCATAG CCGACACATG ACACGGCACG TGGGATACCC ATCTCGTCCC CCTGCCGCTA TCCGTCGCGA   
  
  
- GCCTGTCCTG CTGATGGGGC ACCAGCGTCA GCGTCCCTTT GGAATTTTTC TCTTAATTTC TTGTGCTCAC   
  
  
- GGAAACCTGA ATTTAATAAA TCCTTTATTT ATTTATTATT TTATTTATTA TTTTATTTAT TTTTCATGGC   
  
  
- CACTTTTTAT TTTTTAATTT AATTTTCGTT ACCAAGTAAG AAGATCATTA ATTATATCCA CACACCCAAT   
  
  
- TAATATATTT ATTATTTTAT TTAATAAATA ATTATTAATT AAGATTTTTA TTTGCTTAAA AATAAACAAA   
  
  
- TCTCTGATAA AACATTGGAC GAGGAGTTGA ATCAATTTTA ATGTTTTTAA AATTTATTAA ATAATATTTT   
  
  
- TAATTAAATG CAAAATATTT TGATGTGTAA TACTAAATCA TTTCAAGTGT TTAAAATATT TAGATATATT   
  
  
- AATGCTGGAA GTGAAAATCT CTTGTTCAT

+     AAGAA-motif

| Site Name | Organism | Position | Strand | Matrix score. | sequence | function |
| --- | --- | --- | --- | --- | --- | --- |
| AAGAA-motif | Avena sativa | 173 | - | 7 | GAAAGAA |  |

> 2018/04/13 10:10:12  
+ CCTTCTTCTA CCTCTCGGAC GTCTTGTTCT TCTTCTTTTT CTTCTCTCTT TCTCTCTCGA ATATCTATAA   
  
  
+ TCGAATTAAA CACAACCTGA AAATCGAAGT GTATAGAGAG GAAAATACAT CAAACTCAAA CTATATATTT   
  
  
+ TTATTTTTCT CTTAATTTCT CTTTTTTCTT TTTTCTTTCT CACAAAAACA TCGAAACTTA CTCTGAAAAC   
  
  
+ AACGATCGTG AAATATATTT CTATCCAACT GTTTTTGTGG AAGGTTAATC TTCTCGTATT TCACGGTAGA   
  
  
+ TCATACCTTT GTAAAGTTTA GGTACGATGA TTAGGGTAGA TGGTTCATTG TTGTGAGTGA AAATCAAAGG   
  
  
+ AGAAGGGGTA ATTTATGGGG AGTACGTAAA GTAGAGGATT GTATTTGTTT TGTTTCTAGT TTCGAATGTA   
  
  
+ GTTTTAGAAA AATAGCTAGA CTTGAAGTAC ACAATTTGCA AACTTTTCTG CTATTATTAA ATTACTTTTT   
  
  
+ TTACTAATTC TAAAAAATAT TATTATTTGA CTTAATATTT TTATGAAATA TAAAACTAAT TAATTTTTGA   
  
  
+ GCTAGTCGTA TATTTAGTCT AGTTATCTTC TAGAGTTTAT ACAATTTTCT ATTAAATAAA AAATAATAAT   
  
  
+ GATTAATAGG AAAAATAACA TTAATCTAAA CACATATACT ACGAATCCCG AAATTCCGAA ACAAAAACAC   
  
  
+ ACAAATACAA TCACCCAACT TATCTATCTC TTTCCCTCAT CACACAAATC CACCGTTAAT GAATGATTAT   
  
  
+ TATTGTACAC TTACTCACAA CACACGTGCG AGACCCTACC TCGCACCCCG CCCCTAGTGT TCAGTCGCTT   
  
  
+ CTGATGTCCA TTTCCTACCT TCACTCTCGC CAGTTTCTAA GTTTGGGTTT TTTTTCCTTT TATTTTTATT   
  
  
+ TTTTTCGTTT CTTTTTATTT TTCCTCTAAA CCTTGTAATC CGAGTGTGCA CCGCCTTGTC GGTGGCAGTA   
  
  
+ AACTGGTATC GGCTGTGTAC TGTGCCGTGC ACCCTATGGG TAGAGCAGGG GGACGGCGAT AGGCAGCGCT   
  
  
+ CGGACAGGAC GACTACCCCG TGGTCGCAGT CGCAGGGAAA CCTTAAAAAG AGAATTAAAG AACACGAGTG   
  
  
+ CCTTTGGACT TAAATTATTT AGGAAATAAA TAAATAATAA AATAAATAAT AAAATAAATA AAAAGTACCG   
  
  
+ GTGAAAAATA AAAAATTAAA TTAAAAGCAA TGGTTCATTC TTCTAGTAAT TAATATAGGT GTGTGGGTTA   
  
  
+ ATTATATAAA TAATAAAATA AATTATTTAT TAATAATTAA TTCTAAAAAT AAACGAATTT TTATTTGTTT   
  
  
+ AGAGACTATT TTGTAACCTG CTCCTCAACT TAGTTAAAAT TACAAAAATT TTAAATAATT TATTATAAAA   
  
  
+ ATTAATTTAC GTTTTATAAA ACTACACATT ATGATTTAGT AAAGTTCACA AATTTTATAA ATCTATATAA   
  
  
+ TTACGACCTT CACTTTTAGA GAACAAGTA  

- GGAAGAAGAT GGAGAGCCTG CAGAACAAGA AGAAGAAAAA GAAGAGAGAA AGAGAGAGCT TATAGATATT   
  
  
- AGCTTAATTT GTGTTGGACT TTTAGCTTCA CATATCTCTC CTTTTATGTA GTTTGAGTTT GATATATAAA   
  
  
- AATAAAAAGA GAATTAAAGA GAAAAAAGAA AAAAGAAAGA GTGTTTTTGT AGCTTTGAAT GAGACTTTTG   
  
  
- TTGCTAGCAC TTTATATAAA GATAGGTTGA CAAAAACACC TTCCAATTAG AAGAGCATAA AGTGCCATCT   
  
  
- AGTATGGAAA CATTTCAAAT CCATGCTACT AATCCCATCT ACCAAGTAAC AACACTCACT TTTAGTTTCC   
  
  
- TCTTCCCCAT TAAATACCCC TCATGCATTT CATCTCCTAA CATAAACAAA ACAAAGATCA AAGCTTACAT   
  
  
- CAAAATCTTT TTATCGATCT GAACTTCATG TGTTAAACGT TTGAAAAGAC GATAATAATT TAATGAAAAA   
  
  
- AATGATTAAG ATTTTTTATA ATAATAAACT GAATTATAAA AATACTTTAT ATTTTGATTA ATTAAAAACT   
  
  
- CGATCAGCAT ATAAATCAGA TCAATAGAAG ATCTCAAATA TGTTAAAAGA TAATTTATTT TTTATTATTA   
  
  
- CTAATTATCC TTTTTATTGT AATTAGATTT GTGTATATGA TGCTTAGGGC TTTAAGGCTT TGTTTTTGTG   
  
  
- TGTTTATGTT AGTGGGTTGA ATAGATAGAG AAAGGGAGTA GTGTGTTTAG GTGGCAATTA CTTACTAATA   
  
  
- ATAACATGTG AATGAGTGTT GTGTGCACGC TCTGGGATGG AGCGTGGGGC GGGGATCACA AGTCAGCGAA   
  
  
- GACTACAGGT AAAGGATGGA AGTGAGAGCG GTCAAAGATT CAAACCCAAA AAAAAGGAAA ATAAAAATAA   
  
  
- AAAAAGCAAA GAAAAATAAA AAGGAGATTT GGAACATTAG GCTCACACGT GGCGGAACAG CCACCGTCAT   
  
  
- TTGACCATAG CCGACACATG ACACGGCACG TGGGATACCC ATCTCGTCCC CCTGCCGCTA TCCGTCGCGA   
  
  
- GCCTGTCCTG CTGATGGGGC ACCAGCGTCA GCGTCCCTTT GGAATTTTTC TCTTAATTTC TTGTGCTCAC   
  
  
- GGAAACCTGA ATTTAATAAA TCCTTTATTT ATTTATTATT TTATTTATTA TTTTATTTAT TTTTCATGGC   
  
  
- CACTTTTTAT TTTTTAATTT AATTTTCGTT ACCAAGTAAG AAGATCATTA ATTATATCCA CACACCCAAT   
  
  
- TAATATATTT ATTATTTTAT TTAATAAATA ATTATTAATT AAGATTTTTA TTTGCTTAAA AATAAACAAA   
  
  
- TCTCTGATAA AACATTGGAC GAGGAGTTGA ATCAATTTTA ATGTTTTTAA AATTTATTAA ATAATATTTT   
  
  
- TAATTAAATG CAAAATATTT TGATGTGTAA TACTAAATCA TTTCAAGTGT TTAAAATATT TAGATATATT   
  
  
- AATGCTGGAA GTGAAAATCT CTTGTTCAT

+     ABRE

| Site Name | Organism | Position | Strand | Matrix score. | sequence | function |
| --- | --- | --- | --- | --- | --- | --- |
| ABRE | Hordeum vulgare | 791 | - | 9 | CGCACGTGTC | cis-acting element involved in the abscisic acid responsiveness |
| ABRE | Arabidopsis thaliana | 793 | - | 6 | CACGTG | cis-acting element involved in the abscisic acid responsiveness |

> 2018/04/13 10:10:12  
+ CCTTCTTCTA CCTCTCGGAC GTCTTGTTCT TCTTCTTTTT CTTCTCTCTT TCTCTCTCGA ATATCTATAA   
  
  
+ TCGAATTAAA CACAACCTGA AAATCGAAGT GTATAGAGAG GAAAATACAT CAAACTCAAA CTATATATTT   
  
  
+ TTATTTTTCT CTTAATTTCT CTTTTTTCTT TTTTCTTTCT CACAAAAACA TCGAAACTTA CTCTGAAAAC   
  
  
+ AACGATCGTG AAATATATTT CTATCCAACT GTTTTTGTGG AAGGTTAATC TTCTCGTATT TCACGGTAGA   
  
  
+ TCATACCTTT GTAAAGTTTA GGTACGATGA TTAGGGTAGA TGGTTCATTG TTGTGAGTGA AAATCAAAGG   
  
  
+ AGAAGGGGTA ATTTATGGGG AGTACGTAAA GTAGAGGATT GTATTTGTTT TGTTTCTAGT TTCGAATGTA   
  
  
+ GTTTTAGAAA AATAGCTAGA CTTGAAGTAC ACAATTTGCA AACTTTTCTG CTATTATTAA ATTACTTTTT   
  
  
+ TTACTAATTC TAAAAAATAT TATTATTTGA CTTAATATTT TTATGAAATA TAAAACTAAT TAATTTTTGA   
  
  
+ GCTAGTCGTA TATTTAGTCT AGTTATCTTC TAGAGTTTAT ACAATTTTCT ATTAAATAAA AAATAATAAT   
  
  
+ GATTAATAGG AAAAATAACA TTAATCTAAA CACATATACT ACGAATCCCG AAATTCCGAA ACAAAAACAC   
  
  
+ ACAAATACAA TCACCCAACT TATCTATCTC TTTCCCTCAT CACACAAATC CACCGTTAAT GAATGATTAT   
  
  
+ TATTGTACAC TTACTCACAA CACACGTGCG AGACCCTACC TCGCACCCCG CCCCTAGTGT TCAGTCGCTT   
  
  
+ CTGATGTCCA TTTCCTACCT TCACTCTCGC CAGTTTCTAA GTTTGGGTTT TTTTTCCTTT TATTTTTATT   
  
  
+ TTTTTCGTTT CTTTTTATTT TTCCTCTAAA CCTTGTAATC CGAGTGTGCA CCGCCTTGTC GGTGGCAGTA   
  
  
+ AACTGGTATC GGCTGTGTAC TGTGCCGTGC ACCCTATGGG TAGAGCAGGG GGACGGCGAT AGGCAGCGCT   
  
  
+ CGGACAGGAC GACTACCCCG TGGTCGCAGT CGCAGGGAAA CCTTAAAAAG AGAATTAAAG AACACGAGTG   
  
  
+ CCTTTGGACT TAAATTATTT AGGAAATAAA TAAATAATAA AATAAATAAT AAAATAAATA AAAAGTACCG   
  
  
+ GTGAAAAATA AAAAATTAAA TTAAAAGCAA TGGTTCATTC TTCTAGTAAT TAATATAGGT GTGTGGGTTA   
  
  
+ ATTATATAAA TAATAAAATA AATTATTTAT TAATAATTAA TTCTAAAAAT AAACGAATTT TTATTTGTTT   
  
  
+ AGAGACTATT TTGTAACCTG CTCCTCAACT TAGTTAAAAT TACAAAAATT TTAAATAATT TATTATAAAA   
  
  
+ ATTAATTTAC GTTTTATAAA ACTACACATT ATGATTTAGT AAAGTTCACA AATTTTATAA ATCTATATAA   
  
  
+ TTACGACCTT CACTTTTAGA GAACAAGTA  

- GGAAGAAGAT GGAGAGCCTG CAGAACAAGA AGAAGAAAAA GAAGAGAGAA AGAGAGAGCT TATAGATATT   
  
  
- AGCTTAATTT GTGTTGGACT TTTAGCTTCA CATATCTCTC CTTTTATGTA GTTTGAGTTT GATATATAAA   
  
  
- AATAAAAAGA GAATTAAAGA GAAAAAAGAA AAAAGAAAGA GTGTTTTTGT AGCTTTGAAT GAGACTTTTG   
  
  
- TTGCTAGCAC TTTATATAAA GATAGGTTGA CAAAAACACC TTCCAATTAG AAGAGCATAA AGTGCCATCT   
  
  
- AGTATGGAAA CATTTCAAAT CCATGCTACT AATCCCATCT ACCAAGTAAC AACACTCACT TTTAGTTTCC   
  
  
- TCTTCCCCAT TAAATACCCC TCATGCATTT CATCTCCTAA CATAAACAAA ACAAAGATCA AAGCTTACAT   
  
  
- CAAAATCTTT TTATCGATCT GAACTTCATG TGTTAAACGT TTGAAAAGAC GATAATAATT TAATGAAAAA   
  
  
- AATGATTAAG ATTTTTTATA ATAATAAACT GAATTATAAA AATACTTTAT ATTTTGATTA ATTAAAAACT   
  
  
- CGATCAGCAT ATAAATCAGA TCAATAGAAG ATCTCAAATA TGTTAAAAGA TAATTTATTT TTTATTATTA   
  
  
- CTAATTATCC TTTTTATTGT AATTAGATTT GTGTATATGA TGCTTAGGGC TTTAAGGCTT TGTTTTTGTG   
  
  
- TGTTTATGTT AGTGGGTTGA ATAGATAGAG AAAGGGAGTA GTGTGTTTAG GTGGCAATTA CTTACTAATA   
  
  
- ATAACATGTG AATGAGTGTT GTGTGCACGC TCTGGGATGG AGCGTGGGGC GGGGATCACA AGTCAGCGAA   
  
  
- GACTACAGGT AAAGGATGGA AGTGAGAGCG GTCAAAGATT CAAACCCAAA AAAAAGGAAA ATAAAAATAA   
  
  
- AAAAAGCAAA GAAAAATAAA AAGGAGATTT GGAACATTAG GCTCACACGT GGCGGAACAG CCACCGTCAT   
  
  
- TTGACCATAG CCGACACATG ACACGGCACG TGGGATACCC ATCTCGTCCC CCTGCCGCTA TCCGTCGCGA   
  
  
- GCCTGTCCTG CTGATGGGGC ACCAGCGTCA GCGTCCCTTT GGAATTTTTC TCTTAATTTC TTGTGCTCAC   
  
  
- GGAAACCTGA ATTTAATAAA TCCTTTATTT ATTTATTATT TTATTTATTA TTTTATTTAT TTTTCATGGC   
  
  
- CACTTTTTAT TTTTTAATTT AATTTTCGTT ACCAAGTAAG AAGATCATTA ATTATATCCA CACACCCAAT   
  
  
- TAATATATTT ATTATTTTAT TTAATAAATA ATTATTAATT AAGATTTTTA TTTGCTTAAA AATAAACAAA   
  
  
- TCTCTGATAA AACATTGGAC GAGGAGTTGA ATCAATTTTA ATGTTTTTAA AATTTATTAA ATAATATTTT   
  
  
- TAATTAAATG CAAAATATTT TGATGTGTAA TACTAAATCA TTTCAAGTGT TTAAAATATT TAGATATATT   
  
  
- AATGCTGGAA GTGAAAATCT CTTGTTCAT

+     AE-box

| Site Name | Organism | Position | Strand | Matrix score. | sequence | function |
| --- | --- | --- | --- | --- | --- | --- |
| AE-box | Arabidopsis thaliana | 400 | - | 8 | AGAAACAA | part of a module for light response |

> 2018/04/13 10:10:12  
+ CCTTCTTCTA CCTCTCGGAC GTCTTGTTCT TCTTCTTTTT CTTCTCTCTT TCTCTCTCGA ATATCTATAA   
  
  
+ TCGAATTAAA CACAACCTGA AAATCGAAGT GTATAGAGAG GAAAATACAT CAAACTCAAA CTATATATTT   
  
  
+ TTATTTTTCT CTTAATTTCT CTTTTTTCTT TTTTCTTTCT CACAAAAACA TCGAAACTTA CTCTGAAAAC   
  
  
+ AACGATCGTG AAATATATTT CTATCCAACT GTTTTTGTGG AAGGTTAATC TTCTCGTATT TCACGGTAGA   
  
  
+ TCATACCTTT GTAAAGTTTA GGTACGATGA TTAGGGTAGA TGGTTCATTG TTGTGAGTGA AAATCAAAGG   
  
  
+ AGAAGGGGTA ATTTATGGGG AGTACGTAAA GTAGAGGATT GTATTTGTTT TGTTTCTAGT TTCGAATGTA   
  
  
+ GTTTTAGAAA AATAGCTAGA CTTGAAGTAC ACAATTTGCA AACTTTTCTG CTATTATTAA ATTACTTTTT   
  
  
+ TTACTAATTC TAAAAAATAT TATTATTTGA CTTAATATTT TTATGAAATA TAAAACTAAT TAATTTTTGA   
  
  
+ GCTAGTCGTA TATTTAGTCT AGTTATCTTC TAGAGTTTAT ACAATTTTCT ATTAAATAAA AAATAATAAT   
  
  
+ GATTAATAGG AAAAATAACA TTAATCTAAA CACATATACT ACGAATCCCG AAATTCCGAA ACAAAAACAC   
  
  
+ ACAAATACAA TCACCCAACT TATCTATCTC TTTCCCTCAT CACACAAATC CACCGTTAAT GAATGATTAT   
  
  
+ TATTGTACAC TTACTCACAA CACACGTGCG AGACCCTACC TCGCACCCCG CCCCTAGTGT TCAGTCGCTT   
  
  
+ CTGATGTCCA TTTCCTACCT TCACTCTCGC CAGTTTCTAA GTTTGGGTTT TTTTTCCTTT TATTTTTATT   
  
  
+ TTTTTCGTTT CTTTTTATTT TTCCTCTAAA CCTTGTAATC CGAGTGTGCA CCGCCTTGTC GGTGGCAGTA   
  
  
+ AACTGGTATC GGCTGTGTAC TGTGCCGTGC ACCCTATGGG TAGAGCAGGG GGACGGCGAT AGGCAGCGCT   
  
  
+ CGGACAGGAC GACTACCCCG TGGTCGCAGT CGCAGGGAAA CCTTAAAAAG AGAATTAAAG AACACGAGTG   
  
  
+ CCTTTGGACT TAAATTATTT AGGAAATAAA TAAATAATAA AATAAATAAT AAAATAAATA AAAAGTACCG   
  
  
+ GTGAAAAATA AAAAATTAAA TTAAAAGCAA TGGTTCATTC TTCTAGTAAT TAATATAGGT GTGTGGGTTA   
  
  
+ ATTATATAAA TAATAAAATA AATTATTTAT TAATAATTAA TTCTAAAAAT AAACGAATTT TTATTTGTTT   
  
  
+ AGAGACTATT TTGTAACCTG CTCCTCAACT TAGTTAAAAT TACAAAAATT TTAAATAATT TATTATAAAA   
  
  
+ ATTAATTTAC GTTTTATAAA ACTACACATT ATGATTTAGT AAAGTTCACA AATTTTATAA ATCTATATAA   
  
  
+ TTACGACCTT CACTTTTAGA GAACAAGTA  

- GGAAGAAGAT GGAGAGCCTG CAGAACAAGA AGAAGAAAAA GAAGAGAGAA AGAGAGAGCT TATAGATATT   
  
  
- AGCTTAATTT GTGTTGGACT TTTAGCTTCA CATATCTCTC CTTTTATGTA GTTTGAGTTT GATATATAAA   
  
  
- AATAAAAAGA GAATTAAAGA GAAAAAAGAA AAAAGAAAGA GTGTTTTTGT AGCTTTGAAT GAGACTTTTG   
  
  
- TTGCTAGCAC TTTATATAAA GATAGGTTGA CAAAAACACC TTCCAATTAG AAGAGCATAA AGTGCCATCT   
  
  
- AGTATGGAAA CATTTCAAAT CCATGCTACT AATCCCATCT ACCAAGTAAC AACACTCACT TTTAGTTTCC   
  
  
- TCTTCCCCAT TAAATACCCC TCATGCATTT CATCTCCTAA CATAAACAAA ACAAAGATCA AAGCTTACAT   
  
  
- CAAAATCTTT TTATCGATCT GAACTTCATG TGTTAAACGT TTGAAAAGAC GATAATAATT TAATGAAAAA   
  
  
- AATGATTAAG ATTTTTTATA ATAATAAACT GAATTATAAA AATACTTTAT ATTTTGATTA ATTAAAAACT   
  
  
- CGATCAGCAT ATAAATCAGA TCAATAGAAG ATCTCAAATA TGTTAAAAGA TAATTTATTT TTTATTATTA   
  
  
- CTAATTATCC TTTTTATTGT AATTAGATTT GTGTATATGA TGCTTAGGGC TTTAAGGCTT TGTTTTTGTG   
  
  
- TGTTTATGTT AGTGGGTTGA ATAGATAGAG AAAGGGAGTA GTGTGTTTAG GTGGCAATTA CTTACTAATA   
  
  
- ATAACATGTG AATGAGTGTT GTGTGCACGC TCTGGGATGG AGCGTGGGGC GGGGATCACA AGTCAGCGAA   
  
  
- GACTACAGGT AAAGGATGGA AGTGAGAGCG GTCAAAGATT CAAACCCAAA AAAAAGGAAA ATAAAAATAA   
  
  
- AAAAAGCAAA GAAAAATAAA AAGGAGATTT GGAACATTAG GCTCACACGT GGCGGAACAG CCACCGTCAT   
  
  
- TTGACCATAG CCGACACATG ACACGGCACG TGGGATACCC ATCTCGTCCC CCTGCCGCTA TCCGTCGCGA   
  
  
- GCCTGTCCTG CTGATGGGGC ACCAGCGTCA GCGTCCCTTT GGAATTTTTC TCTTAATTTC TTGTGCTCAC   
  
  
- GGAAACCTGA ATTTAATAAA TCCTTTATTT ATTTATTATT TTATTTATTA TTTTATTTAT TTTTCATGGC   
  
  
- CACTTTTTAT TTTTTAATTT AATTTTCGTT ACCAAGTAAG AAGATCATTA ATTATATCCA CACACCCAAT   
  
  
- TAATATATTT ATTATTTTAT TTAATAAATA ATTATTAATT AAGATTTTTA TTTGCTTAAA AATAAACAAA   
  
  
- TCTCTGATAA AACATTGGAC GAGGAGTTGA ATCAATTTTA ATGTTTTTAA AATTTATTAA ATAATATTTT   
  
  
- TAATTAAATG CAAAATATTT TGATGTGTAA TACTAAATCA TTTCAAGTGT TTAAAATATT TAGATATATT   
  
  
- AATGCTGGAA GTGAAAATCT CTTGTTCAT

+     AT1-motif

| Site Name | Organism | Position | Strand | Matrix score. | sequence | function |
| --- | --- | --- | --- | --- | --- | --- |
| AT1-motif | Solanum tuberosum | 615 | - | 13 | AATTATTTTTTATT | part of a light responsive module |

> 2018/04/13 10:10:12  
+ CCTTCTTCTA CCTCTCGGAC GTCTTGTTCT TCTTCTTTTT CTTCTCTCTT TCTCTCTCGA ATATCTATAA   
  
  
+ TCGAATTAAA CACAACCTGA AAATCGAAGT GTATAGAGAG GAAAATACAT CAAACTCAAA CTATATATTT   
  
  
+ TTATTTTTCT CTTAATTTCT CTTTTTTCTT TTTTCTTTCT CACAAAAACA TCGAAACTTA CTCTGAAAAC   
  
  
+ AACGATCGTG AAATATATTT CTATCCAACT GTTTTTGTGG AAGGTTAATC TTCTCGTATT TCACGGTAGA   
  
  
+ TCATACCTTT GTAAAGTTTA GGTACGATGA TTAGGGTAGA TGGTTCATTG TTGTGAGTGA AAATCAAAGG   
  
  
+ AGAAGGGGTA ATTTATGGGG AGTACGTAAA GTAGAGGATT GTATTTGTTT TGTTTCTAGT TTCGAATGTA   
  
  
+ GTTTTAGAAA AATAGCTAGA CTTGAAGTAC ACAATTTGCA AACTTTTCTG CTATTATTAA ATTACTTTTT   
  
  
+ TTACTAATTC TAAAAAATAT TATTATTTGA CTTAATATTT TTATGAAATA TAAAACTAAT TAATTTTTGA   
  
  
+ GCTAGTCGTA TATTTAGTCT AGTTATCTTC TAGAGTTTAT ACAATTTTCT ATTAAATAAA AAATAATAAT   
  
  
+ GATTAATAGG AAAAATAACA TTAATCTAAA CACATATACT ACGAATCCCG AAATTCCGAA ACAAAAACAC   
  
  
+ ACAAATACAA TCACCCAACT TATCTATCTC TTTCCCTCAT CACACAAATC CACCGTTAAT GAATGATTAT   
  
  
+ TATTGTACAC TTACTCACAA CACACGTGCG AGACCCTACC TCGCACCCCG CCCCTAGTGT TCAGTCGCTT   
  
  
+ CTGATGTCCA TTTCCTACCT TCACTCTCGC CAGTTTCTAA GTTTGGGTTT TTTTTCCTTT TATTTTTATT   
  
  
+ TTTTTCGTTT CTTTTTATTT TTCCTCTAAA CCTTGTAATC CGAGTGTGCA CCGCCTTGTC GGTGGCAGTA   
  
  
+ AACTGGTATC GGCTGTGTAC TGTGCCGTGC ACCCTATGGG TAGAGCAGGG GGACGGCGAT AGGCAGCGCT   
  
  
+ CGGACAGGAC GACTACCCCG TGGTCGCAGT CGCAGGGAAA CCTTAAAAAG AGAATTAAAG AACACGAGTG   
  
  
+ CCTTTGGACT TAAATTATTT AGGAAATAAA TAAATAATAA AATAAATAAT AAAATAAATA AAAAGTACCG   
  
  
+ GTGAAAAATA AAAAATTAAA TTAAAAGCAA TGGTTCATTC TTCTAGTAAT TAATATAGGT GTGTGGGTTA   
  
  
+ ATTATATAAA TAATAAAATA AATTATTTAT TAATAATTAA TTCTAAAAAT AAACGAATTT TTATTTGTTT   
  
  
+ AGAGACTATT TTGTAACCTG CTCCTCAACT TAGTTAAAAT TACAAAAATT TTAAATAATT TATTATAAAA   
  
  
+ ATTAATTTAC GTTTTATAAA ACTACACATT ATGATTTAGT AAAGTTCACA AATTTTATAA ATCTATATAA   
  
  
+ TTACGACCTT CACTTTTAGA GAACAAGTA  

- GGAAGAAGAT GGAGAGCCTG CAGAACAAGA AGAAGAAAAA GAAGAGAGAA AGAGAGAGCT TATAGATATT   
  
  
- AGCTTAATTT GTGTTGGACT TTTAGCTTCA CATATCTCTC CTTTTATGTA GTTTGAGTTT GATATATAAA   
  
  
- AATAAAAAGA GAATTAAAGA GAAAAAAGAA AAAAGAAAGA GTGTTTTTGT AGCTTTGAAT GAGACTTTTG   
  
  
- TTGCTAGCAC TTTATATAAA GATAGGTTGA CAAAAACACC TTCCAATTAG AAGAGCATAA AGTGCCATCT   
  
  
- AGTATGGAAA CATTTCAAAT CCATGCTACT AATCCCATCT ACCAAGTAAC AACACTCACT TTTAGTTTCC   
  
  
- TCTTCCCCAT TAAATACCCC TCATGCATTT CATCTCCTAA CATAAACAAA ACAAAGATCA AAGCTTACAT   
  
  
- CAAAATCTTT TTATCGATCT GAACTTCATG TGTTAAACGT TTGAAAAGAC GATAATAATT TAATGAAAAA   
  
  
- AATGATTAAG ATTTTTTATA ATAATAAACT GAATTATAAA AATACTTTAT ATTTTGATTA ATTAAAAACT   
  
  
- CGATCAGCAT ATAAATCAGA TCAATAGAAG ATCTCAAATA TGTTAAAAGA TAATTTATTT TTTATTATTA   
  
  
- CTAATTATCC TTTTTATTGT AATTAGATTT GTGTATATGA TGCTTAGGGC TTTAAGGCTT TGTTTTTGTG   
  
  
- TGTTTATGTT AGTGGGTTGA ATAGATAGAG AAAGGGAGTA GTGTGTTTAG GTGGCAATTA CTTACTAATA   
  
  
- ATAACATGTG AATGAGTGTT GTGTGCACGC TCTGGGATGG AGCGTGGGGC GGGGATCACA AGTCAGCGAA   
  
  
- GACTACAGGT AAAGGATGGA AGTGAGAGCG GTCAAAGATT CAAACCCAAA AAAAAGGAAA ATAAAAATAA   
  
  
- AAAAAGCAAA GAAAAATAAA AAGGAGATTT GGAACATTAG GCTCACACGT GGCGGAACAG CCACCGTCAT   
  
  
- TTGACCATAG CCGACACATG ACACGGCACG TGGGATACCC ATCTCGTCCC CCTGCCGCTA TCCGTCGCGA   
  
  
- GCCTGTCCTG CTGATGGGGC ACCAGCGTCA GCGTCCCTTT GGAATTTTTC TCTTAATTTC TTGTGCTCAC   
  
  
- GGAAACCTGA ATTTAATAAA TCCTTTATTT ATTTATTATT TTATTTATTA TTTTATTTAT TTTTCATGGC   
  
  
- CACTTTTTAT TTTTTAATTT AATTTTCGTT ACCAAGTAAG AAGATCATTA ATTATATCCA CACACCCAAT   
  
  
- TAATATATTT ATTATTTTAT TTAATAAATA ATTATTAATT AAGATTTTTA TTTGCTTAAA AATAAACAAA   
  
  
- TCTCTGATAA AACATTGGAC GAGGAGTTGA ATCAATTTTA ATGTTTTTAA AATTTATTAA ATAATATTTT   
  
  
- TAATTAAATG CAAAATATTT TGATGTGTAA TACTAAATCA TTTCAAGTGT TTAAAATATT TAGATATATT   
  
  
- AATGCTGGAA GTGAAAATCT CTTGTTCAT

+     ATCC-motif

| Site Name | Organism | Position | Strand | Matrix score. | sequence | function |
| --- | --- | --- | --- | --- | --- | --- |
| ATCC-motif | Pisum sativum | 384 | - | 8 | CAATCCTC | part of a conserved DNA module involved in light responsiveness |

> 2018/04/13 10:10:12  
+ CCTTCTTCTA CCTCTCGGAC GTCTTGTTCT TCTTCTTTTT CTTCTCTCTT TCTCTCTCGA ATATCTATAA   
  
  
+ TCGAATTAAA CACAACCTGA AAATCGAAGT GTATAGAGAG GAAAATACAT CAAACTCAAA CTATATATTT   
  
  
+ TTATTTTTCT CTTAATTTCT CTTTTTTCTT TTTTCTTTCT CACAAAAACA TCGAAACTTA CTCTGAAAAC   
  
  
+ AACGATCGTG AAATATATTT CTATCCAACT GTTTTTGTGG AAGGTTAATC TTCTCGTATT TCACGGTAGA   
  
  
+ TCATACCTTT GTAAAGTTTA GGTACGATGA TTAGGGTAGA TGGTTCATTG TTGTGAGTGA AAATCAAAGG   
  
  
+ AGAAGGGGTA ATTTATGGGG AGTACGTAAA GTAGAGGATT GTATTTGTTT TGTTTCTAGT TTCGAATGTA   
  
  
+ GTTTTAGAAA AATAGCTAGA CTTGAAGTAC ACAATTTGCA AACTTTTCTG CTATTATTAA ATTACTTTTT   
  
  
+ TTACTAATTC TAAAAAATAT TATTATTTGA CTTAATATTT TTATGAAATA TAAAACTAAT TAATTTTTGA   
  
  
+ GCTAGTCGTA TATTTAGTCT AGTTATCTTC TAGAGTTTAT ACAATTTTCT ATTAAATAAA AAATAATAAT   
  
  
+ GATTAATAGG AAAAATAACA TTAATCTAAA CACATATACT ACGAATCCCG AAATTCCGAA ACAAAAACAC   
  
  
+ ACAAATACAA TCACCCAACT TATCTATCTC TTTCCCTCAT CACACAAATC CACCGTTAAT GAATGATTAT   
  
  
+ TATTGTACAC TTACTCACAA CACACGTGCG AGACCCTACC TCGCACCCCG CCCCTAGTGT TCAGTCGCTT   
  
  
+ CTGATGTCCA TTTCCTACCT TCACTCTCGC CAGTTTCTAA GTTTGGGTTT TTTTTCCTTT TATTTTTATT   
  
  
+ TTTTTCGTTT CTTTTTATTT TTCCTCTAAA CCTTGTAATC CGAGTGTGCA CCGCCTTGTC GGTGGCAGTA   
  
  
+ AACTGGTATC GGCTGTGTAC TGTGCCGTGC ACCCTATGGG TAGAGCAGGG GGACGGCGAT AGGCAGCGCT   
  
  
+ CGGACAGGAC GACTACCCCG TGGTCGCAGT CGCAGGGAAA CCTTAAAAAG AGAATTAAAG AACACGAGTG   
  
  
+ CCTTTGGACT TAAATTATTT AGGAAATAAA TAAATAATAA AATAAATAAT AAAATAAATA AAAAGTACCG   
  
  
+ GTGAAAAATA AAAAATTAAA TTAAAAGCAA TGGTTCATTC TTCTAGTAAT TAATATAGGT GTGTGGGTTA   
  
  
+ ATTATATAAA TAATAAAATA AATTATTTAT TAATAATTAA TTCTAAAAAT AAACGAATTT TTATTTGTTT   
  
  
+ AGAGACTATT TTGTAACCTG CTCCTCAACT TAGTTAAAAT TACAAAAATT TTAAATAATT TATTATAAAA   
  
  
+ ATTAATTTAC GTTTTATAAA ACTACACATT ATGATTTAGT AAAGTTCACA AATTTTATAA ATCTATATAA   
  
  
+ TTACGACCTT CACTTTTAGA GAACAAGTA  

- GGAAGAAGAT GGAGAGCCTG CAGAACAAGA AGAAGAAAAA GAAGAGAGAA AGAGAGAGCT TATAGATATT   
  
  
- AGCTTAATTT GTGTTGGACT TTTAGCTTCA CATATCTCTC CTTTTATGTA GTTTGAGTTT GATATATAAA   
  
  
- AATAAAAAGA GAATTAAAGA GAAAAAAGAA AAAAGAAAGA GTGTTTTTGT AGCTTTGAAT GAGACTTTTG   
  
  
- TTGCTAGCAC TTTATATAAA GATAGGTTGA CAAAAACACC TTCCAATTAG AAGAGCATAA AGTGCCATCT   
  
  
- AGTATGGAAA CATTTCAAAT CCATGCTACT AATCCCATCT ACCAAGTAAC AACACTCACT TTTAGTTTCC   
  
  
- TCTTCCCCAT TAAATACCCC TCATGCATTT CATCTCCTAA CATAAACAAA ACAAAGATCA AAGCTTACAT   
  
  
- CAAAATCTTT TTATCGATCT GAACTTCATG TGTTAAACGT TTGAAAAGAC GATAATAATT TAATGAAAAA   
  
  
- AATGATTAAG ATTTTTTATA ATAATAAACT GAATTATAAA AATACTTTAT ATTTTGATTA ATTAAAAACT   
  
  
- CGATCAGCAT ATAAATCAGA TCAATAGAAG ATCTCAAATA TGTTAAAAGA TAATTTATTT TTTATTATTA   
  
  
- CTAATTATCC TTTTTATTGT AATTAGATTT GTGTATATGA TGCTTAGGGC TTTAAGGCTT TGTTTTTGTG   
  
  
- TGTTTATGTT AGTGGGTTGA ATAGATAGAG AAAGGGAGTA GTGTGTTTAG GTGGCAATTA CTTACTAATA   
  
  
- ATAACATGTG AATGAGTGTT GTGTGCACGC TCTGGGATGG AGCGTGGGGC GGGGATCACA AGTCAGCGAA   
  
  
- GACTACAGGT AAAGGATGGA AGTGAGAGCG GTCAAAGATT CAAACCCAAA AAAAAGGAAA ATAAAAATAA   
  
  
- AAAAAGCAAA GAAAAATAAA AAGGAGATTT GGAACATTAG GCTCACACGT GGCGGAACAG CCACCGTCAT   
  
  
- TTGACCATAG CCGACACATG ACACGGCACG TGGGATACCC ATCTCGTCCC CCTGCCGCTA TCCGTCGCGA   
  
  
- GCCTGTCCTG CTGATGGGGC ACCAGCGTCA GCGTCCCTTT GGAATTTTTC TCTTAATTTC TTGTGCTCAC   
  
  
- GGAAACCTGA ATTTAATAAA TCCTTTATTT ATTTATTATT TTATTTATTA TTTTATTTAT TTTTCATGGC   
  
  
- CACTTTTTAT TTTTTAATTT AATTTTCGTT ACCAAGTAAG AAGATCATTA ATTATATCCA CACACCCAAT   
  
  
- TAATATATTT ATTATTTTAT TTAATAAATA ATTATTAATT AAGATTTTTA TTTGCTTAAA AATAAACAAA   
  
  
- TCTCTGATAA AACATTGGAC GAGGAGTTGA ATCAATTTTA ATGTTTTTAA AATTTATTAA ATAATATTTT   
  
  
- TAATTAAATG CAAAATATTT TGATGTGTAA TACTAAATCA TTTCAAGTGT TTAAAATATT TAGATATATT   
  
  
- AATGCTGGAA GTGAAAATCT CTTGTTCAT

+     Box 4

| Site Name | Organism | Position | Strand | Matrix score. | sequence | function |
| --- | --- | --- | --- | --- | --- | --- |
| Box 4 | Petroselinum crispum | 1239 | - | 6 | ATTAAT | part of a conserved DNA module involved in light responsiveness |
| Box 4 | Petroselinum crispum | 650 | + | 6 | ATTAAT | part of a conserved DNA module involved in light responsiveness |
| Box 4 | Petroselinum crispum | 632 | + | 6 | ATTAAT | part of a conserved DNA module involved in light responsiveness |
| Box 4 | Petroselinum crispum | 1401 | - | 6 | ATTAAT | part of a conserved DNA module involved in light responsiveness |
| Box 4 | Petroselinum crispum | 1296 | - | 6 | ATTAAT | part of a conserved DNA module involved in light responsiveness |
| Box 4 | Petroselinum crispum | 1289 | - | 6 | ATTAAT | part of a conserved DNA module involved in light responsiveness |
| Box 4 | Petroselinum crispum | 549 | + | 6 | ATTAAT | part of a conserved DNA module involved in light responsiveness |

> 2018/04/13 10:10:12  
+ CCTTCTTCTA CCTCTCGGAC GTCTTGTTCT TCTTCTTTTT CTTCTCTCTT TCTCTCTCGA ATATCTATAA   
  
  
+ TCGAATTAAA CACAACCTGA AAATCGAAGT GTATAGAGAG GAAAATACAT CAAACTCAAA CTATATATTT   
  
  
+ TTATTTTTCT CTTAATTTCT CTTTTTTCTT TTTTCTTTCT CACAAAAACA TCGAAACTTA CTCTGAAAAC   
  
  
+ AACGATCGTG AAATATATTT CTATCCAACT GTTTTTGTGG AAGGTTAATC TTCTCGTATT TCACGGTAGA   
  
  
+ TCATACCTTT GTAAAGTTTA GGTACGATGA TTAGGGTAGA TGGTTCATTG TTGTGAGTGA AAATCAAAGG   
  
  
+ AGAAGGGGTA ATTTATGGGG AGTACGTAAA GTAGAGGATT GTATTTGTTT TGTTTCTAGT TTCGAATGTA   
  
  
+ GTTTTAGAAA AATAGCTAGA CTTGAAGTAC ACAATTTGCA AACTTTTCTG CTATTATTAA ATTACTTTTT   
  
  
+ TTACTAATTC TAAAAAATAT TATTATTTGA CTTAATATTT TTATGAAATA TAAAACTAAT TAATTTTTGA   
  
  
+ GCTAGTCGTA TATTTAGTCT AGTTATCTTC TAGAGTTTAT ACAATTTTCT ATTAAATAAA AAATAATAAT   
  
  
+ GATTAATAGG AAAAATAACA TTAATCTAAA CACATATACT ACGAATCCCG AAATTCCGAA ACAAAAACAC   
  
  
+ ACAAATACAA TCACCCAACT TATCTATCTC TTTCCCTCAT CACACAAATC CACCGTTAAT GAATGATTAT   
  
  
+ TATTGTACAC TTACTCACAA CACACGTGCG AGACCCTACC TCGCACCCCG CCCCTAGTGT TCAGTCGCTT   
  
  
+ CTGATGTCCA TTTCCTACCT TCACTCTCGC CAGTTTCTAA GTTTGGGTTT TTTTTCCTTT TATTTTTATT   
  
  
+ TTTTTCGTTT CTTTTTATTT TTCCTCTAAA CCTTGTAATC CGAGTGTGCA CCGCCTTGTC GGTGGCAGTA   
  
  
+ AACTGGTATC GGCTGTGTAC TGTGCCGTGC ACCCTATGGG TAGAGCAGGG GGACGGCGAT AGGCAGCGCT   
  
  
+ CGGACAGGAC GACTACCCCG TGGTCGCAGT CGCAGGGAAA CCTTAAAAAG AGAATTAAAG AACACGAGTG   
  
  
+ CCTTTGGACT TAAATTATTT AGGAAATAAA TAAATAATAA AATAAATAAT AAAATAAATA AAAAGTACCG   
  
  
+ GTGAAAAATA AAAAATTAAA TTAAAAGCAA TGGTTCATTC TTCTAGTAAT TAATATAGGT GTGTGGGTTA   
  
  
+ ATTATATAAA TAATAAAATA AATTATTTAT TAATAATTAA TTCTAAAAAT AAACGAATTT TTATTTGTTT   
  
  
+ AGAGACTATT TTGTAACCTG CTCCTCAACT TAGTTAAAAT TACAAAAATT TTAAATAATT TATTATAAAA   
  
  
+ ATTAATTTAC GTTTTATAAA ACTACACATT ATGATTTAGT AAAGTTCACA AATTTTATAA ATCTATATAA   
  
  
+ TTACGACCTT CACTTTTAGA GAACAAGTA  

- GGAAGAAGAT GGAGAGCCTG CAGAACAAGA AGAAGAAAAA GAAGAGAGAA AGAGAGAGCT TATAGATATT   
  
  
- AGCTTAATTT GTGTTGGACT TTTAGCTTCA CATATCTCTC CTTTTATGTA GTTTGAGTTT GATATATAAA   
  
  
- AATAAAAAGA GAATTAAAGA GAAAAAAGAA AAAAGAAAGA GTGTTTTTGT AGCTTTGAAT GAGACTTTTG   
  
  
- TTGCTAGCAC TTTATATAAA GATAGGTTGA CAAAAACACC TTCCAATTAG AAGAGCATAA AGTGCCATCT   
  
  
- AGTATGGAAA CATTTCAAAT CCATGCTACT AATCCCATCT ACCAAGTAAC AACACTCACT TTTAGTTTCC   
  
  
- TCTTCCCCAT TAAATACCCC TCATGCATTT CATCTCCTAA CATAAACAAA ACAAAGATCA AAGCTTACAT   
  
  
- CAAAATCTTT TTATCGATCT GAACTTCATG TGTTAAACGT TTGAAAAGAC GATAATAATT TAATGAAAAA   
  
  
- AATGATTAAG ATTTTTTATA ATAATAAACT GAATTATAAA AATACTTTAT ATTTTGATTA ATTAAAAACT   
  
  
- CGATCAGCAT ATAAATCAGA TCAATAGAAG ATCTCAAATA TGTTAAAAGA TAATTTATTT TTTATTATTA   
  
  
- CTAATTATCC TTTTTATTGT AATTAGATTT GTGTATATGA TGCTTAGGGC TTTAAGGCTT TGTTTTTGTG   
  
  
- TGTTTATGTT AGTGGGTTGA ATAGATAGAG AAAGGGAGTA GTGTGTTTAG GTGGCAATTA CTTACTAATA   
  
  
- ATAACATGTG AATGAGTGTT GTGTGCACGC TCTGGGATGG AGCGTGGGGC GGGGATCACA AGTCAGCGAA   
  
  
- GACTACAGGT AAAGGATGGA AGTGAGAGCG GTCAAAGATT CAAACCCAAA AAAAAGGAAA ATAAAAATAA   
  
  
- AAAAAGCAAA GAAAAATAAA AAGGAGATTT GGAACATTAG GCTCACACGT GGCGGAACAG CCACCGTCAT   
  
  
- TTGACCATAG CCGACACATG ACACGGCACG TGGGATACCC ATCTCGTCCC CCTGCCGCTA TCCGTCGCGA   
  
  
- GCCTGTCCTG CTGATGGGGC ACCAGCGTCA GCGTCCCTTT GGAATTTTTC TCTTAATTTC TTGTGCTCAC   
  
  
- GGAAACCTGA ATTTAATAAA TCCTTTATTT ATTTATTATT TTATTTATTA TTTTATTTAT TTTTCATGGC   
  
  
- CACTTTTTAT TTTTTAATTT AATTTTCGTT ACCAAGTAAG AAGATCATTA ATTATATCCA CACACCCAAT   
  
  
- TAATATATTT ATTATTTTAT TTAATAAATA ATTATTAATT AAGATTTTTA TTTGCTTAAA AATAAACAAA   
  
  
- TCTCTGATAA AACATTGGAC GAGGAGTTGA ATCAATTTTA ATGTTTTTAA AATTTATTAA ATAATATTTT   
  
  
- TAATTAAATG CAAAATATTT TGATGTGTAA TACTAAATCA TTTCAAGTGT TTAAAATATT TAGATATATT   
  
  
- AATGCTGGAA GTGAAAATCT CTTGTTCAT

+     CAAT-box

| Site Name | Organism | Position | Strand | Matrix score. | sequence | function |
| --- | --- | --- | --- | --- | --- | --- |
| CAAT-box | Hordeum vulgare | 388 | - | 4 | CAAT | common cis-acting element in promoter and enhancer regions |
| CAAT-box | Brassica rapa | 1323 | - | 5 | CAAAT | common cis-acting element in promoter and enhancer regions |
| CAAT-box | Brassica rapa | 515 | - | 5 | CAAAT | common cis-acting element in promoter and enhancer regions |
| CAAT-box | Brassica rapa | 1449 | + | 5 | CAAAT | common cis-acting element in promoter and enhancer regions |
| CAAT-box | Hordeum vulgare | 1218 | + | 4 | CAAT | common cis-acting element in promoter and enhancer regions |
| CAAT-box | Hordeum vulgare | 327 | - | 4 | CAAT | common cis-acting element in promoter and enhancer regions |
| CAAT-box | Brassica rapa | 393 | - | 5 | CAAAT | common cis-acting element in promoter and enhancer regions |
| CAAT-box | Glycine max | 452 | + | 5 | CAATT | common cis-acting element in promoter and enhancer regions |
| CAAT-box | Brassica rapa | 745 | + | 5 | CAAAT | common cis-acting element in promoter and enhancer regions |
| CAAT-box | Hordeum vulgare | 708 | + | 4 | CAAT | common cis-acting element in promoter and enhancer regions |
| CAAT-box | Brassica rapa | 702 | + | 5 | CAAAT | common cis-acting element in promoter and enhancer regions |
| CAAT-box | Glycine max | 602 | + | 5 | CAATT | common cis-acting element in promoter and enhancer regions |
| CAAT-box | Hordeum vulgare | 772 | - | 4 | CAAT | common cis-acting element in promoter and enhancer regions |
| CAAT-box | Brassica rapa | 454 | - | 5 | CAAAT | common cis-acting element in promoter and enhancer regions |

> 2018/04/13 10:10:12  
+ CCTTCTTCTA CCTCTCGGAC GTCTTGTTCT TCTTCTTTTT CTTCTCTCTT TCTCTCTCGA ATATCTATAA   
  
  
+ TCGAATTAAA CACAACCTGA AAATCGAAGT GTATAGAGAG GAAAATACAT CAAACTCAAA CTATATATTT   
  
  
+ TTATTTTTCT CTTAATTTCT CTTTTTTCTT TTTTCTTTCT CACAAAAACA TCGAAACTTA CTCTGAAAAC   
  
  
+ AACGATCGTG AAATATATTT CTATCCAACT GTTTTTGTGG AAGGTTAATC TTCTCGTATT TCACGGTAGA   
  
  
+ TCATACCTTT GTAAAGTTTA GGTACGATGA TTAGGGTAGA TGGTTCATTG TTGTGAGTGA AAATCAAAGG   
  
  
+ AGAAGGGGTA ATTTATGGGG AGTACGTAAA GTAGAGGATT GTATTTGTTT TGTTTCTAGT TTCGAATGTA   
  
  
+ GTTTTAGAAA AATAGCTAGA CTTGAAGTAC ACAATTTGCA AACTTTTCTG CTATTATTAA ATTACTTTTT   
  
  
+ TTACTAATTC TAAAAAATAT TATTATTTGA CTTAATATTT TTATGAAATA TAAAACTAAT TAATTTTTGA   
  
  
+ GCTAGTCGTA TATTTAGTCT AGTTATCTTC TAGAGTTTAT ACAATTTTCT ATTAAATAAA AAATAATAAT   
  
  
+ GATTAATAGG AAAAATAACA TTAATCTAAA CACATATACT ACGAATCCCG AAATTCCGAA ACAAAAACAC   
  
  
+ ACAAATACAA TCACCCAACT TATCTATCTC TTTCCCTCAT CACACAAATC CACCGTTAAT GAATGATTAT   
  
  
+ TATTGTACAC TTACTCACAA CACACGTGCG AGACCCTACC TCGCACCCCG CCCCTAGTGT TCAGTCGCTT   
  
  
+ CTGATGTCCA TTTCCTACCT TCACTCTCGC CAGTTTCTAA GTTTGGGTTT TTTTTCCTTT TATTTTTATT   
  
  
+ TTTTTCGTTT CTTTTTATTT TTCCTCTAAA CCTTGTAATC CGAGTGTGCA CCGCCTTGTC GGTGGCAGTA   
  
  
+ AACTGGTATC GGCTGTGTAC TGTGCCGTGC ACCCTATGGG TAGAGCAGGG GGACGGCGAT AGGCAGCGCT   
  
  
+ CGGACAGGAC GACTACCCCG TGGTCGCAGT CGCAGGGAAA CCTTAAAAAG AGAATTAAAG AACACGAGTG   
  
  
+ CCTTTGGACT TAAATTATTT AGGAAATAAA TAAATAATAA AATAAATAAT AAAATAAATA AAAAGTACCG   
  
  
+ GTGAAAAATA AAAAATTAAA TTAAAAGCAA TGGTTCATTC TTCTAGTAAT TAATATAGGT GTGTGGGTTA   
  
  
+ ATTATATAAA TAATAAAATA AATTATTTAT TAATAATTAA TTCTAAAAAT AAACGAATTT TTATTTGTTT   
  
  
+ AGAGACTATT TTGTAACCTG CTCCTCAACT TAGTTAAAAT TACAAAAATT TTAAATAATT TATTATAAAA   
  
  
+ ATTAATTTAC GTTTTATAAA ACTACACATT ATGATTTAGT AAAGTTCACA AATTTTATAA ATCTATATAA   
  
  
+ TTACGACCTT CACTTTTAGA GAACAAGTA  

- GGAAGAAGAT GGAGAGCCTG CAGAACAAGA AGAAGAAAAA GAAGAGAGAA AGAGAGAGCT TATAGATATT   
  
  
- AGCTTAATTT GTGTTGGACT TTTAGCTTCA CATATCTCTC CTTTTATGTA GTTTGAGTTT GATATATAAA   
  
  
- AATAAAAAGA GAATTAAAGA GAAAAAAGAA AAAAGAAAGA GTGTTTTTGT AGCTTTGAAT GAGACTTTTG   
  
  
- TTGCTAGCAC TTTATATAAA GATAGGTTGA CAAAAACACC TTCCAATTAG AAGAGCATAA AGTGCCATCT   
  
  
- AGTATGGAAA CATTTCAAAT CCATGCTACT AATCCCATCT ACCAAGTAAC AACACTCACT TTTAGTTTCC   
  
  
- TCTTCCCCAT TAAATACCCC TCATGCATTT CATCTCCTAA CATAAACAAA ACAAAGATCA AAGCTTACAT   
  
  
- CAAAATCTTT TTATCGATCT GAACTTCATG TGTTAAACGT TTGAAAAGAC GATAATAATT TAATGAAAAA   
  
  
- AATGATTAAG ATTTTTTATA ATAATAAACT GAATTATAAA AATACTTTAT ATTTTGATTA ATTAAAAACT   
  
  
- CGATCAGCAT ATAAATCAGA TCAATAGAAG ATCTCAAATA TGTTAAAAGA TAATTTATTT TTTATTATTA   
  
  
- CTAATTATCC TTTTTATTGT AATTAGATTT GTGTATATGA TGCTTAGGGC TTTAAGGCTT TGTTTTTGTG   
  
  
- TGTTTATGTT AGTGGGTTGA ATAGATAGAG AAAGGGAGTA GTGTGTTTAG GTGGCAATTA CTTACTAATA   
  
  
- ATAACATGTG AATGAGTGTT GTGTGCACGC TCTGGGATGG AGCGTGGGGC GGGGATCACA AGTCAGCGAA   
  
  
- GACTACAGGT AAAGGATGGA AGTGAGAGCG GTCAAAGATT CAAACCCAAA AAAAAGGAAA ATAAAAATAA   
  
  
- AAAAAGCAAA GAAAAATAAA AAGGAGATTT GGAACATTAG GCTCACACGT GGCGGAACAG CCACCGTCAT   
  
  
- TTGACCATAG CCGACACATG ACACGGCACG TGGGATACCC ATCTCGTCCC CCTGCCGCTA TCCGTCGCGA   
  
  
- GCCTGTCCTG CTGATGGGGC ACCAGCGTCA GCGTCCCTTT GGAATTTTTC TCTTAATTTC TTGTGCTCAC   
  
  
- GGAAACCTGA ATTTAATAAA TCCTTTATTT ATTTATTATT TTATTTATTA TTTTATTTAT TTTTCATGGC   
  
  
- CACTTTTTAT TTTTTAATTT AATTTTCGTT ACCAAGTAAG AAGATCATTA ATTATATCCA CACACCCAAT   
  
  
- TAATATATTT ATTATTTTAT TTAATAAATA ATTATTAATT AAGATTTTTA TTTGCTTAAA AATAAACAAA   
  
  
- TCTCTGATAA AACATTGGAC GAGGAGTTGA ATCAATTTTA ATGTTTTTAA AATTTATTAA ATAATATTTT   
  
  
- TAATTAAATG CAAAATATTT TGATGTGTAA TACTAAATCA TTTCAAGTGT TTAAAATATT TAGATATATT   
  
  
- AATGCTGGAA GTGAAAATCT CTTGTTCAT

+     CCGTCC-box

| Site Name | Organism | Position | Strand | Matrix score. | sequence | function |
| --- | --- | --- | --- | --- | --- | --- |
| CCGTCC-box | Arabidopsis thaliana | 1031 | - | 6 | CCGTCC | cis-acting regulatory element related to meristem specific activation |

> 2018/04/13 10:10:12  
+ CCTTCTTCTA CCTCTCGGAC GTCTTGTTCT TCTTCTTTTT CTTCTCTCTT TCTCTCTCGA ATATCTATAA   
  
  
+ TCGAATTAAA CACAACCTGA AAATCGAAGT GTATAGAGAG GAAAATACAT CAAACTCAAA CTATATATTT   
  
  
+ TTATTTTTCT CTTAATTTCT CTTTTTTCTT TTTTCTTTCT CACAAAAACA TCGAAACTTA CTCTGAAAAC   
  
  
+ AACGATCGTG AAATATATTT CTATCCAACT GTTTTTGTGG AAGGTTAATC TTCTCGTATT TCACGGTAGA   
  
  
+ TCATACCTTT GTAAAGTTTA GGTACGATGA TTAGGGTAGA TGGTTCATTG TTGTGAGTGA AAATCAAAGG   
  
  
+ AGAAGGGGTA ATTTATGGGG AGTACGTAAA GTAGAGGATT GTATTTGTTT TGTTTCTAGT TTCGAATGTA   
  
  
+ GTTTTAGAAA AATAGCTAGA CTTGAAGTAC ACAATTTGCA AACTTTTCTG CTATTATTAA ATTACTTTTT   
  
  
+ TTACTAATTC TAAAAAATAT TATTATTTGA CTTAATATTT TTATGAAATA TAAAACTAAT TAATTTTTGA   
  
  
+ GCTAGTCGTA TATTTAGTCT AGTTATCTTC TAGAGTTTAT ACAATTTTCT ATTAAATAAA AAATAATAAT   
  
  
+ GATTAATAGG AAAAATAACA TTAATCTAAA CACATATACT ACGAATCCCG AAATTCCGAA ACAAAAACAC   
  
  
+ ACAAATACAA TCACCCAACT TATCTATCTC TTTCCCTCAT CACACAAATC CACCGTTAAT GAATGATTAT   
  
  
+ TATTGTACAC TTACTCACAA CACACGTGCG AGACCCTACC TCGCACCCCG CCCCTAGTGT TCAGTCGCTT   
  
  
+ CTGATGTCCA TTTCCTACCT TCACTCTCGC CAGTTTCTAA GTTTGGGTTT TTTTTCCTTT TATTTTTATT   
  
  
+ TTTTTCGTTT CTTTTTATTT TTCCTCTAAA CCTTGTAATC CGAGTGTGCA CCGCCTTGTC GGTGGCAGTA   
  
  
+ AACTGGTATC GGCTGTGTAC TGTGCCGTGC ACCCTATGGG TAGAGCAGGG GGACGGCGAT AGGCAGCGCT   
  
  
+ CGGACAGGAC GACTACCCCG TGGTCGCAGT CGCAGGGAAA CCTTAAAAAG AGAATTAAAG AACACGAGTG   
  
  
+ CCTTTGGACT TAAATTATTT AGGAAATAAA TAAATAATAA AATAAATAAT AAAATAAATA AAAAGTACCG   
  
  
+ GTGAAAAATA AAAAATTAAA TTAAAAGCAA TGGTTCATTC TTCTAGTAAT TAATATAGGT GTGTGGGTTA   
  
  
+ ATTATATAAA TAATAAAATA AATTATTTAT TAATAATTAA TTCTAAAAAT AAACGAATTT TTATTTGTTT   
  
  
+ AGAGACTATT TTGTAACCTG CTCCTCAACT TAGTTAAAAT TACAAAAATT TTAAATAATT TATTATAAAA   
  
  
+ ATTAATTTAC GTTTTATAAA ACTACACATT ATGATTTAGT AAAGTTCACA AATTTTATAA ATCTATATAA   
  
  
+ TTACGACCTT CACTTTTAGA GAACAAGTA  

- GGAAGAAGAT GGAGAGCCTG CAGAACAAGA AGAAGAAAAA GAAGAGAGAA AGAGAGAGCT TATAGATATT   
  
  
- AGCTTAATTT GTGTTGGACT TTTAGCTTCA CATATCTCTC CTTTTATGTA GTTTGAGTTT GATATATAAA   
  
  
- AATAAAAAGA GAATTAAAGA GAAAAAAGAA AAAAGAAAGA GTGTTTTTGT AGCTTTGAAT GAGACTTTTG   
  
  
- TTGCTAGCAC TTTATATAAA GATAGGTTGA CAAAAACACC TTCCAATTAG AAGAGCATAA AGTGCCATCT   
  
  
- AGTATGGAAA CATTTCAAAT CCATGCTACT AATCCCATCT ACCAAGTAAC AACACTCACT TTTAGTTTCC   
  
  
- TCTTCCCCAT TAAATACCCC TCATGCATTT CATCTCCTAA CATAAACAAA ACAAAGATCA AAGCTTACAT   
  
  
- CAAAATCTTT TTATCGATCT GAACTTCATG TGTTAAACGT TTGAAAAGAC GATAATAATT TAATGAAAAA   
  
  
- AATGATTAAG ATTTTTTATA ATAATAAACT GAATTATAAA AATACTTTAT ATTTTGATTA ATTAAAAACT   
  
  
- CGATCAGCAT ATAAATCAGA TCAATAGAAG ATCTCAAATA TGTTAAAAGA TAATTTATTT TTTATTATTA   
  
  
- CTAATTATCC TTTTTATTGT AATTAGATTT GTGTATATGA TGCTTAGGGC TTTAAGGCTT TGTTTTTGTG   
  
  
- TGTTTATGTT AGTGGGTTGA ATAGATAGAG AAAGGGAGTA GTGTGTTTAG GTGGCAATTA CTTACTAATA   
  
  
- ATAACATGTG AATGAGTGTT GTGTGCACGC TCTGGGATGG AGCGTGGGGC GGGGATCACA AGTCAGCGAA   
  
  
- GACTACAGGT AAAGGATGGA AGTGAGAGCG GTCAAAGATT CAAACCCAAA AAAAAGGAAA ATAAAAATAA   
  
  
- AAAAAGCAAA GAAAAATAAA AAGGAGATTT GGAACATTAG GCTCACACGT GGCGGAACAG CCACCGTCAT   
  
  
- TTGACCATAG CCGACACATG ACACGGCACG TGGGATACCC ATCTCGTCCC CCTGCCGCTA TCCGTCGCGA   
  
  
- GCCTGTCCTG CTGATGGGGC ACCAGCGTCA GCGTCCCTTT GGAATTTTTC TCTTAATTTC TTGTGCTCAC   
  
  
- GGAAACCTGA ATTTAATAAA TCCTTTATTT ATTTATTATT TTATTTATTA TTTTATTTAT TTTTCATGGC   
  
  
- CACTTTTTAT TTTTTAATTT AATTTTCGTT ACCAAGTAAG AAGATCATTA ATTATATCCA CACACCCAAT   
  
  
- TAATATATTT ATTATTTTAT TTAATAAATA ATTATTAATT AAGATTTTTA TTTGCTTAAA AATAAACAAA   
  
  
- TCTCTGATAA AACATTGGAC GAGGAGTTGA ATCAATTTTA ATGTTTTTAA AATTTATTAA ATAATATTTT   
  
  
- TAATTAAATG CAAAATATTT TGATGTGTAA TACTAAATCA TTTCAAGTGT TTAAAATATT TAGATATATT   
  
  
- AATGCTGGAA GTGAAAATCT CTTGTTCAT

+     CTAG-motif

| Site Name | Organism | Position | Strand | Matrix score. | sequence | function |
| --- | --- | --- | --- | --- | --- | --- |
| CTAG-motif | Avena sativa | 1228 | - | 9 | ACTAGCAGAA |  |
| CTAG-motif | Avena sativa | 466 | - | 9 | ACTAGCAGAA |  |

> 2018/04/13 10:10:12  
+ CCTTCTTCTA CCTCTCGGAC GTCTTGTTCT TCTTCTTTTT CTTCTCTCTT TCTCTCTCGA ATATCTATAA   
  
  
+ TCGAATTAAA CACAACCTGA AAATCGAAGT GTATAGAGAG GAAAATACAT CAAACTCAAA CTATATATTT   
  
  
+ TTATTTTTCT CTTAATTTCT CTTTTTTCTT TTTTCTTTCT CACAAAAACA TCGAAACTTA CTCTGAAAAC   
  
  
+ AACGATCGTG AAATATATTT CTATCCAACT GTTTTTGTGG AAGGTTAATC TTCTCGTATT TCACGGTAGA   
  
  
+ TCATACCTTT GTAAAGTTTA GGTACGATGA TTAGGGTAGA TGGTTCATTG TTGTGAGTGA AAATCAAAGG   
  
  
+ AGAAGGGGTA ATTTATGGGG AGTACGTAAA GTAGAGGATT GTATTTGTTT TGTTTCTAGT TTCGAATGTA   
  
  
+ GTTTTAGAAA AATAGCTAGA CTTGAAGTAC ACAATTTGCA AACTTTTCTG CTATTATTAA ATTACTTTTT   
  
  
+ TTACTAATTC TAAAAAATAT TATTATTTGA CTTAATATTT TTATGAAATA TAAAACTAAT TAATTTTTGA   
  
  
+ GCTAGTCGTA TATTTAGTCT AGTTATCTTC TAGAGTTTAT ACAATTTTCT ATTAAATAAA AAATAATAAT   
  
  
+ GATTAATAGG AAAAATAACA TTAATCTAAA CACATATACT ACGAATCCCG AAATTCCGAA ACAAAAACAC   
  
  
+ ACAAATACAA TCACCCAACT TATCTATCTC TTTCCCTCAT CACACAAATC CACCGTTAAT GAATGATTAT   
  
  
+ TATTGTACAC TTACTCACAA CACACGTGCG AGACCCTACC TCGCACCCCG CCCCTAGTGT TCAGTCGCTT   
  
  
+ CTGATGTCCA TTTCCTACCT TCACTCTCGC CAGTTTCTAA GTTTGGGTTT TTTTTCCTTT TATTTTTATT   
  
  
+ TTTTTCGTTT CTTTTTATTT TTCCTCTAAA CCTTGTAATC CGAGTGTGCA CCGCCTTGTC GGTGGCAGTA   
  
  
+ AACTGGTATC GGCTGTGTAC TGTGCCGTGC ACCCTATGGG TAGAGCAGGG GGACGGCGAT AGGCAGCGCT   
  
  
+ CGGACAGGAC GACTACCCCG TGGTCGCAGT CGCAGGGAAA CCTTAAAAAG AGAATTAAAG AACACGAGTG   
  
  
+ CCTTTGGACT TAAATTATTT AGGAAATAAA TAAATAATAA AATAAATAAT AAAATAAATA AAAAGTACCG   
  
  
+ GTGAAAAATA AAAAATTAAA TTAAAAGCAA TGGTTCATTC TTCTAGTAAT TAATATAGGT GTGTGGGTTA   
  
  
+ ATTATATAAA TAATAAAATA AATTATTTAT TAATAATTAA TTCTAAAAAT AAACGAATTT TTATTTGTTT   
  
  
+ AGAGACTATT TTGTAACCTG CTCCTCAACT TAGTTAAAAT TACAAAAATT TTAAATAATT TATTATAAAA   
  
  
+ ATTAATTTAC GTTTTATAAA ACTACACATT ATGATTTAGT AAAGTTCACA AATTTTATAA ATCTATATAA   
  
  
+ TTACGACCTT CACTTTTAGA GAACAAGTA  

- GGAAGAAGAT GGAGAGCCTG CAGAACAAGA AGAAGAAAAA GAAGAGAGAA AGAGAGAGCT TATAGATATT   
  
  
- AGCTTAATTT GTGTTGGACT TTTAGCTTCA CATATCTCTC CTTTTATGTA GTTTGAGTTT GATATATAAA   
  
  
- AATAAAAAGA GAATTAAAGA GAAAAAAGAA AAAAGAAAGA GTGTTTTTGT AGCTTTGAAT GAGACTTTTG   
  
  
- TTGCTAGCAC TTTATATAAA GATAGGTTGA CAAAAACACC TTCCAATTAG AAGAGCATAA AGTGCCATCT   
  
  
- AGTATGGAAA CATTTCAAAT CCATGCTACT AATCCCATCT ACCAAGTAAC AACACTCACT TTTAGTTTCC   
  
  
- TCTTCCCCAT TAAATACCCC TCATGCATTT CATCTCCTAA CATAAACAAA ACAAAGATCA AAGCTTACAT   
  
  
- CAAAATCTTT TTATCGATCT GAACTTCATG TGTTAAACGT TTGAAAAGAC GATAATAATT TAATGAAAAA   
  
  
- AATGATTAAG ATTTTTTATA ATAATAAACT GAATTATAAA AATACTTTAT ATTTTGATTA ATTAAAAACT   
  
  
- CGATCAGCAT ATAAATCAGA TCAATAGAAG ATCTCAAATA TGTTAAAAGA TAATTTATTT TTTATTATTA   
  
  
- CTAATTATCC TTTTTATTGT AATTAGATTT GTGTATATGA TGCTTAGGGC TTTAAGGCTT TGTTTTTGTG   
  
  
- TGTTTATGTT AGTGGGTTGA ATAGATAGAG AAAGGGAGTA GTGTGTTTAG GTGGCAATTA CTTACTAATA   
  
  
- ATAACATGTG AATGAGTGTT GTGTGCACGC TCTGGGATGG AGCGTGGGGC GGGGATCACA AGTCAGCGAA   
  
  
- GACTACAGGT AAAGGATGGA AGTGAGAGCG GTCAAAGATT CAAACCCAAA AAAAAGGAAA ATAAAAATAA   
  
  
- AAAAAGCAAA GAAAAATAAA AAGGAGATTT GGAACATTAG GCTCACACGT GGCGGAACAG CCACCGTCAT   
  
  
- TTGACCATAG CCGACACATG ACACGGCACG TGGGATACCC ATCTCGTCCC CCTGCCGCTA TCCGTCGCGA   
  
  
- GCCTGTCCTG CTGATGGGGC ACCAGCGTCA GCGTCCCTTT GGAATTTTTC TCTTAATTTC TTGTGCTCAC   
  
  
- GGAAACCTGA ATTTAATAAA TCCTTTATTT ATTTATTATT TTATTTATTA TTTTATTTAT TTTTCATGGC   
  
  
- CACTTTTTAT TTTTTAATTT AATTTTCGTT ACCAAGTAAG AAGATCATTA ATTATATCCA CACACCCAAT   
  
  
- TAATATATTT ATTATTTTAT TTAATAAATA ATTATTAATT AAGATTTTTA TTTGCTTAAA AATAAACAAA   
  
  
- TCTCTGATAA AACATTGGAC GAGGAGTTGA ATCAATTTTA ATGTTTTTAA AATTTATTAA ATAATATTTT   
  
  
- TAATTAAATG CAAAATATTT TGATGTGTAA TACTAAATCA TTTCAAGTGT TTAAAATATT TAGATATATT   
  
  
- AATGCTGGAA GTGAAAATCT CTTGTTCAT

+     G-Box

| Site Name | Organism | Position | Strand | Matrix score. | sequence | function |
| --- | --- | --- | --- | --- | --- | --- |
| G-Box | Pisum sativum | 793 | - | 6 | CACGTG | cis-acting regulatory element involved in light responsiveness |

> 2018/04/13 10:10:12  
+ CCTTCTTCTA CCTCTCGGAC GTCTTGTTCT TCTTCTTTTT CTTCTCTCTT TCTCTCTCGA ATATCTATAA   
  
  
+ TCGAATTAAA CACAACCTGA AAATCGAAGT GTATAGAGAG GAAAATACAT CAAACTCAAA CTATATATTT   
  
  
+ TTATTTTTCT CTTAATTTCT CTTTTTTCTT TTTTCTTTCT CACAAAAACA TCGAAACTTA CTCTGAAAAC   
  
  
+ AACGATCGTG AAATATATTT CTATCCAACT GTTTTTGTGG AAGGTTAATC TTCTCGTATT TCACGGTAGA   
  
  
+ TCATACCTTT GTAAAGTTTA GGTACGATGA TTAGGGTAGA TGGTTCATTG TTGTGAGTGA AAATCAAAGG   
  
  
+ AGAAGGGGTA ATTTATGGGG AGTACGTAAA GTAGAGGATT GTATTTGTTT TGTTTCTAGT TTCGAATGTA   
  
  
+ GTTTTAGAAA AATAGCTAGA CTTGAAGTAC ACAATTTGCA AACTTTTCTG CTATTATTAA ATTACTTTTT   
  
  
+ TTACTAATTC TAAAAAATAT TATTATTTGA CTTAATATTT TTATGAAATA TAAAACTAAT TAATTTTTGA   
  
  
+ GCTAGTCGTA TATTTAGTCT AGTTATCTTC TAGAGTTTAT ACAATTTTCT ATTAAATAAA AAATAATAAT   
  
  
+ GATTAATAGG AAAAATAACA TTAATCTAAA CACATATACT ACGAATCCCG AAATTCCGAA ACAAAAACAC   
  
  
+ ACAAATACAA TCACCCAACT TATCTATCTC TTTCCCTCAT CACACAAATC CACCGTTAAT GAATGATTAT   
  
  
+ TATTGTACAC TTACTCACAA CACACGTGCG AGACCCTACC TCGCACCCCG CCCCTAGTGT TCAGTCGCTT   
  
  
+ CTGATGTCCA TTTCCTACCT TCACTCTCGC CAGTTTCTAA GTTTGGGTTT TTTTTCCTTT TATTTTTATT   
  
  
+ TTTTTCGTTT CTTTTTATTT TTCCTCTAAA CCTTGTAATC CGAGTGTGCA CCGCCTTGTC GGTGGCAGTA   
  
  
+ AACTGGTATC GGCTGTGTAC TGTGCCGTGC ACCCTATGGG TAGAGCAGGG GGACGGCGAT AGGCAGCGCT   
  
  
+ CGGACAGGAC GACTACCCCG TGGTCGCAGT CGCAGGGAAA CCTTAAAAAG AGAATTAAAG AACACGAGTG   
  
  
+ CCTTTGGACT TAAATTATTT AGGAAATAAA TAAATAATAA AATAAATAAT AAAATAAATA AAAAGTACCG   
  
  
+ GTGAAAAATA AAAAATTAAA TTAAAAGCAA TGGTTCATTC TTCTAGTAAT TAATATAGGT GTGTGGGTTA   
  
  
+ ATTATATAAA TAATAAAATA AATTATTTAT TAATAATTAA TTCTAAAAAT AAACGAATTT TTATTTGTTT   
  
  
+ AGAGACTATT TTGTAACCTG CTCCTCAACT TAGTTAAAAT TACAAAAATT TTAAATAATT TATTATAAAA   
  
  
+ ATTAATTTAC GTTTTATAAA ACTACACATT ATGATTTAGT AAAGTTCACA AATTTTATAA ATCTATATAA   
  
  
+ TTACGACCTT CACTTTTAGA GAACAAGTA  

- GGAAGAAGAT GGAGAGCCTG CAGAACAAGA AGAAGAAAAA GAAGAGAGAA AGAGAGAGCT TATAGATATT   
  
  
- AGCTTAATTT GTGTTGGACT TTTAGCTTCA CATATCTCTC CTTTTATGTA GTTTGAGTTT GATATATAAA   
  
  
- AATAAAAAGA GAATTAAAGA GAAAAAAGAA AAAAGAAAGA GTGTTTTTGT AGCTTTGAAT GAGACTTTTG   
  
  
- TTGCTAGCAC TTTATATAAA GATAGGTTGA CAAAAACACC TTCCAATTAG AAGAGCATAA AGTGCCATCT   
  
  
- AGTATGGAAA CATTTCAAAT CCATGCTACT AATCCCATCT ACCAAGTAAC AACACTCACT TTTAGTTTCC   
  
  
- TCTTCCCCAT TAAATACCCC TCATGCATTT CATCTCCTAA CATAAACAAA ACAAAGATCA AAGCTTACAT   
  
  
- CAAAATCTTT TTATCGATCT GAACTTCATG TGTTAAACGT TTGAAAAGAC GATAATAATT TAATGAAAAA   
  
  
- AATGATTAAG ATTTTTTATA ATAATAAACT GAATTATAAA AATACTTTAT ATTTTGATTA ATTAAAAACT   
  
  
- CGATCAGCAT ATAAATCAGA TCAATAGAAG ATCTCAAATA TGTTAAAAGA TAATTTATTT TTTATTATTA   
  
  
- CTAATTATCC TTTTTATTGT AATTAGATTT GTGTATATGA TGCTTAGGGC TTTAAGGCTT TGTTTTTGTG   
  
  
- TGTTTATGTT AGTGGGTTGA ATAGATAGAG AAAGGGAGTA GTGTGTTTAG GTGGCAATTA CTTACTAATA   
  
  
- ATAACATGTG AATGAGTGTT GTGTGCACGC TCTGGGATGG AGCGTGGGGC GGGGATCACA AGTCAGCGAA   
  
  
- GACTACAGGT AAAGGATGGA AGTGAGAGCG GTCAAAGATT CAAACCCAAA AAAAAGGAAA ATAAAAATAA   
  
  
- AAAAAGCAAA GAAAAATAAA AAGGAGATTT GGAACATTAG GCTCACACGT GGCGGAACAG CCACCGTCAT   
  
  
- TTGACCATAG CCGACACATG ACACGGCACG TGGGATACCC ATCTCGTCCC CCTGCCGCTA TCCGTCGCGA   
  
  
- GCCTGTCCTG CTGATGGGGC ACCAGCGTCA GCGTCCCTTT GGAATTTTTC TCTTAATTTC TTGTGCTCAC   
  
  
- GGAAACCTGA ATTTAATAAA TCCTTTATTT ATTTATTATT TTATTTATTA TTTTATTTAT TTTTCATGGC   
  
  
- CACTTTTTAT TTTTTAATTT AATTTTCGTT ACCAAGTAAG AAGATCATTA ATTATATCCA CACACCCAAT   
  
  
- TAATATATTT ATTATTTTAT TTAATAAATA ATTATTAATT AAGATTTTTA TTTGCTTAAA AATAAACAAA   
  
  
- TCTCTGATAA AACATTGGAC GAGGAGTTGA ATCAATTTTA ATGTTTTTAA AATTTATTAA ATAATATTTT   
  
  
- TAATTAAATG CAAAATATTT TGATGTGTAA TACTAAATCA TTTCAAGTGT TTAAAATATT TAGATATATT   
  
  
- AATGCTGGAA GTGAAAATCT CTTGTTCAT

+     G-box

| Site Name | Organism | Position | Strand | Matrix score. | sequence | function |
| --- | --- | --- | --- | --- | --- | --- |
| G-box | Arabidopsis thaliana | 793 | - | 6 | CACGTG | cis-acting regulatory element involved in light responsiveness |

> 2018/04/13 10:10:12  
+ CCTTCTTCTA CCTCTCGGAC GTCTTGTTCT TCTTCTTTTT CTTCTCTCTT TCTCTCTCGA ATATCTATAA   
  
  
+ TCGAATTAAA CACAACCTGA AAATCGAAGT GTATAGAGAG GAAAATACAT CAAACTCAAA CTATATATTT   
  
  
+ TTATTTTTCT CTTAATTTCT CTTTTTTCTT TTTTCTTTCT CACAAAAACA TCGAAACTTA CTCTGAAAAC   
  
  
+ AACGATCGTG AAATATATTT CTATCCAACT GTTTTTGTGG AAGGTTAATC TTCTCGTATT TCACGGTAGA   
  
  
+ TCATACCTTT GTAAAGTTTA GGTACGATGA TTAGGGTAGA TGGTTCATTG TTGTGAGTGA AAATCAAAGG   
  
  
+ AGAAGGGGTA ATTTATGGGG AGTACGTAAA GTAGAGGATT GTATTTGTTT TGTTTCTAGT TTCGAATGTA   
  
  
+ GTTTTAGAAA AATAGCTAGA CTTGAAGTAC ACAATTTGCA AACTTTTCTG CTATTATTAA ATTACTTTTT   
  
  
+ TTACTAATTC TAAAAAATAT TATTATTTGA CTTAATATTT TTATGAAATA TAAAACTAAT TAATTTTTGA   
  
  
+ GCTAGTCGTA TATTTAGTCT AGTTATCTTC TAGAGTTTAT ACAATTTTCT ATTAAATAAA AAATAATAAT   
  
  
+ GATTAATAGG AAAAATAACA TTAATCTAAA CACATATACT ACGAATCCCG AAATTCCGAA ACAAAAACAC   
  
  
+ ACAAATACAA TCACCCAACT TATCTATCTC TTTCCCTCAT CACACAAATC CACCGTTAAT GAATGATTAT   
  
  
+ TATTGTACAC TTACTCACAA CACACGTGCG AGACCCTACC TCGCACCCCG CCCCTAGTGT TCAGTCGCTT   
  
  
+ CTGATGTCCA TTTCCTACCT TCACTCTCGC CAGTTTCTAA GTTTGGGTTT TTTTTCCTTT TATTTTTATT   
  
  
+ TTTTTCGTTT CTTTTTATTT TTCCTCTAAA CCTTGTAATC CGAGTGTGCA CCGCCTTGTC GGTGGCAGTA   
  
  
+ AACTGGTATC GGCTGTGTAC TGTGCCGTGC ACCCTATGGG TAGAGCAGGG GGACGGCGAT AGGCAGCGCT   
  
  
+ CGGACAGGAC GACTACCCCG TGGTCGCAGT CGCAGGGAAA CCTTAAAAAG AGAATTAAAG AACACGAGTG   
  
  
+ CCTTTGGACT TAAATTATTT AGGAAATAAA TAAATAATAA AATAAATAAT AAAATAAATA AAAAGTACCG   
  
  
+ GTGAAAAATA AAAAATTAAA TTAAAAGCAA TGGTTCATTC TTCTAGTAAT TAATATAGGT GTGTGGGTTA   
  
  
+ ATTATATAAA TAATAAAATA AATTATTTAT TAATAATTAA TTCTAAAAAT AAACGAATTT TTATTTGTTT   
  
  
+ AGAGACTATT TTGTAACCTG CTCCTCAACT TAGTTAAAAT TACAAAAATT TTAAATAATT TATTATAAAA   
  
  
+ ATTAATTTAC GTTTTATAAA ACTACACATT ATGATTTAGT AAAGTTCACA AATTTTATAA ATCTATATAA   
  
  
+ TTACGACCTT CACTTTTAGA GAACAAGTA  

- GGAAGAAGAT GGAGAGCCTG CAGAACAAGA AGAAGAAAAA GAAGAGAGAA AGAGAGAGCT TATAGATATT   
  
  
- AGCTTAATTT GTGTTGGACT TTTAGCTTCA CATATCTCTC CTTTTATGTA GTTTGAGTTT GATATATAAA   
  
  
- AATAAAAAGA GAATTAAAGA GAAAAAAGAA AAAAGAAAGA GTGTTTTTGT AGCTTTGAAT GAGACTTTTG   
  
  
- TTGCTAGCAC TTTATATAAA GATAGGTTGA CAAAAACACC TTCCAATTAG AAGAGCATAA AGTGCCATCT   
  
  
- AGTATGGAAA CATTTCAAAT CCATGCTACT AATCCCATCT ACCAAGTAAC AACACTCACT TTTAGTTTCC   
  
  
- TCTTCCCCAT TAAATACCCC TCATGCATTT CATCTCCTAA CATAAACAAA ACAAAGATCA AAGCTTACAT   
  
  
- CAAAATCTTT TTATCGATCT GAACTTCATG TGTTAAACGT TTGAAAAGAC GATAATAATT TAATGAAAAA   
  
  
- AATGATTAAG ATTTTTTATA ATAATAAACT GAATTATAAA AATACTTTAT ATTTTGATTA ATTAAAAACT   
  
  
- CGATCAGCAT ATAAATCAGA TCAATAGAAG ATCTCAAATA TGTTAAAAGA TAATTTATTT TTTATTATTA   
  
  
- CTAATTATCC TTTTTATTGT AATTAGATTT GTGTATATGA TGCTTAGGGC TTTAAGGCTT TGTTTTTGTG   
  
  
- TGTTTATGTT AGTGGGTTGA ATAGATAGAG AAAGGGAGTA GTGTGTTTAG GTGGCAATTA CTTACTAATA   
  
  
- ATAACATGTG AATGAGTGTT GTGTGCACGC TCTGGGATGG AGCGTGGGGC GGGGATCACA AGTCAGCGAA   
  
  
- GACTACAGGT AAAGGATGGA AGTGAGAGCG GTCAAAGATT CAAACCCAAA AAAAAGGAAA ATAAAAATAA   
  
  
- AAAAAGCAAA GAAAAATAAA AAGGAGATTT GGAACATTAG GCTCACACGT GGCGGAACAG CCACCGTCAT   
  
  
- TTGACCATAG CCGACACATG ACACGGCACG TGGGATACCC ATCTCGTCCC CCTGCCGCTA TCCGTCGCGA   
  
  
- GCCTGTCCTG CTGATGGGGC ACCAGCGTCA GCGTCCCTTT GGAATTTTTC TCTTAATTTC TTGTGCTCAC   
  
  
- GGAAACCTGA ATTTAATAAA TCCTTTATTT ATTTATTATT TTATTTATTA TTTTATTTAT TTTTCATGGC   
  
  
- CACTTTTTAT TTTTTAATTT AATTTTCGTT ACCAAGTAAG AAGATCATTA ATTATATCCA CACACCCAAT   
  
  
- TAATATATTT ATTATTTTAT TTAATAAATA ATTATTAATT AAGATTTTTA TTTGCTTAAA AATAAACAAA   
  
  
- TCTCTGATAA AACATTGGAC GAGGAGTTGA ATCAATTTTA ATGTTTTTAA AATTTATTAA ATAATATTTT   
  
  
- TAATTAAATG CAAAATATTT TGATGTGTAA TACTAAATCA TTTCAAGTGT TTAAAATATT TAGATATATT   
  
  
- AATGCTGGAA GTGAAAATCT CTTGTTCAT

+     GA-motif

| Site Name | Organism | Position | Strand | Matrix score. | sequence | function |
| --- | --- | --- | --- | --- | --- | --- |
| GA-motif | Arabidopsis thaliana | 720 | - | 8 | ATAGATAA | part of a light responsive element |

> 2018/04/13 10:10:12  
+ CCTTCTTCTA CCTCTCGGAC GTCTTGTTCT TCTTCTTTTT CTTCTCTCTT TCTCTCTCGA ATATCTATAA   
  
  
+ TCGAATTAAA CACAACCTGA AAATCGAAGT GTATAGAGAG GAAAATACAT CAAACTCAAA CTATATATTT   
  
  
+ TTATTTTTCT CTTAATTTCT CTTTTTTCTT TTTTCTTTCT CACAAAAACA TCGAAACTTA CTCTGAAAAC   
  
  
+ AACGATCGTG AAATATATTT CTATCCAACT GTTTTTGTGG AAGGTTAATC TTCTCGTATT TCACGGTAGA   
  
  
+ TCATACCTTT GTAAAGTTTA GGTACGATGA TTAGGGTAGA TGGTTCATTG TTGTGAGTGA AAATCAAAGG   
  
  
+ AGAAGGGGTA ATTTATGGGG AGTACGTAAA GTAGAGGATT GTATTTGTTT TGTTTCTAGT TTCGAATGTA   
  
  
+ GTTTTAGAAA AATAGCTAGA CTTGAAGTAC ACAATTTGCA AACTTTTCTG CTATTATTAA ATTACTTTTT   
  
  
+ TTACTAATTC TAAAAAATAT TATTATTTGA CTTAATATTT TTATGAAATA TAAAACTAAT TAATTTTTGA   
  
  
+ GCTAGTCGTA TATTTAGTCT AGTTATCTTC TAGAGTTTAT ACAATTTTCT ATTAAATAAA AAATAATAAT   
  
  
+ GATTAATAGG AAAAATAACA TTAATCTAAA CACATATACT ACGAATCCCG AAATTCCGAA ACAAAAACAC   
  
  
+ ACAAATACAA TCACCCAACT TATCTATCTC TTTCCCTCAT CACACAAATC CACCGTTAAT GAATGATTAT   
  
  
+ TATTGTACAC TTACTCACAA CACACGTGCG AGACCCTACC TCGCACCCCG CCCCTAGTGT TCAGTCGCTT   
  
  
+ CTGATGTCCA TTTCCTACCT TCACTCTCGC CAGTTTCTAA GTTTGGGTTT TTTTTCCTTT TATTTTTATT   
  
  
+ TTTTTCGTTT CTTTTTATTT TTCCTCTAAA CCTTGTAATC CGAGTGTGCA CCGCCTTGTC GGTGGCAGTA   
  
  
+ AACTGGTATC GGCTGTGTAC TGTGCCGTGC ACCCTATGGG TAGAGCAGGG GGACGGCGAT AGGCAGCGCT   
  
  
+ CGGACAGGAC GACTACCCCG TGGTCGCAGT CGCAGGGAAA CCTTAAAAAG AGAATTAAAG AACACGAGTG   
  
  
+ CCTTTGGACT TAAATTATTT AGGAAATAAA TAAATAATAA AATAAATAAT AAAATAAATA AAAAGTACCG   
  
  
+ GTGAAAAATA AAAAATTAAA TTAAAAGCAA TGGTTCATTC TTCTAGTAAT TAATATAGGT GTGTGGGTTA   
  
  
+ ATTATATAAA TAATAAAATA AATTATTTAT TAATAATTAA TTCTAAAAAT AAACGAATTT TTATTTGTTT   
  
  
+ AGAGACTATT TTGTAACCTG CTCCTCAACT TAGTTAAAAT TACAAAAATT TTAAATAATT TATTATAAAA   
  
  
+ ATTAATTTAC GTTTTATAAA ACTACACATT ATGATTTAGT AAAGTTCACA AATTTTATAA ATCTATATAA   
  
  
+ TTACGACCTT CACTTTTAGA GAACAAGTA  

- GGAAGAAGAT GGAGAGCCTG CAGAACAAGA AGAAGAAAAA GAAGAGAGAA AGAGAGAGCT TATAGATATT   
  
  
- AGCTTAATTT GTGTTGGACT TTTAGCTTCA CATATCTCTC CTTTTATGTA GTTTGAGTTT GATATATAAA   
  
  
- AATAAAAAGA GAATTAAAGA GAAAAAAGAA AAAAGAAAGA GTGTTTTTGT AGCTTTGAAT GAGACTTTTG   
  
  
- TTGCTAGCAC TTTATATAAA GATAGGTTGA CAAAAACACC TTCCAATTAG AAGAGCATAA AGTGCCATCT   
  
  
- AGTATGGAAA CATTTCAAAT CCATGCTACT AATCCCATCT ACCAAGTAAC AACACTCACT TTTAGTTTCC   
  
  
- TCTTCCCCAT TAAATACCCC TCATGCATTT CATCTCCTAA CATAAACAAA ACAAAGATCA AAGCTTACAT   
  
  
- CAAAATCTTT TTATCGATCT GAACTTCATG TGTTAAACGT TTGAAAAGAC GATAATAATT TAATGAAAAA   
  
  
- AATGATTAAG ATTTTTTATA ATAATAAACT GAATTATAAA AATACTTTAT ATTTTGATTA ATTAAAAACT   
  
  
- CGATCAGCAT ATAAATCAGA TCAATAGAAG ATCTCAAATA TGTTAAAAGA TAATTTATTT TTTATTATTA   
  
  
- CTAATTATCC TTTTTATTGT AATTAGATTT GTGTATATGA TGCTTAGGGC TTTAAGGCTT TGTTTTTGTG   
  
  
- TGTTTATGTT AGTGGGTTGA ATAGATAGAG AAAGGGAGTA GTGTGTTTAG GTGGCAATTA CTTACTAATA   
  
  
- ATAACATGTG AATGAGTGTT GTGTGCACGC TCTGGGATGG AGCGTGGGGC GGGGATCACA AGTCAGCGAA   
  
  
- GACTACAGGT AAAGGATGGA AGTGAGAGCG GTCAAAGATT CAAACCCAAA AAAAAGGAAA ATAAAAATAA   
  
  
- AAAAAGCAAA GAAAAATAAA AAGGAGATTT GGAACATTAG GCTCACACGT GGCGGAACAG CCACCGTCAT   
  
  
- TTGACCATAG CCGACACATG ACACGGCACG TGGGATACCC ATCTCGTCCC CCTGCCGCTA TCCGTCGCGA   
  
  
- GCCTGTCCTG CTGATGGGGC ACCAGCGTCA GCGTCCCTTT GGAATTTTTC TCTTAATTTC TTGTGCTCAC   
  
  
- GGAAACCTGA ATTTAATAAA TCCTTTATTT ATTTATTATT TTATTTATTA TTTTATTTAT TTTTCATGGC   
  
  
- CACTTTTTAT TTTTTAATTT AATTTTCGTT ACCAAGTAAG AAGATCATTA ATTATATCCA CACACCCAAT   
  
  
- TAATATATTT ATTATTTTAT TTAATAAATA ATTATTAATT AAGATTTTTA TTTGCTTAAA AATAAACAAA   
  
  
- TCTCTGATAA AACATTGGAC GAGGAGTTGA ATCAATTTTA ATGTTTTTAA AATTTATTAA ATAATATTTT   
  
  
- TAATTAAATG CAAAATATTT TGATGTGTAA TACTAAATCA TTTCAAGTGT TTAAAATATT TAGATATATT   
  
  
- AATGCTGGAA GTGAAAATCT CTTGTTCAT

+     GATA-motif

| Site Name | Organism | Position | Strand | Matrix score. | sequence | function |
| --- | --- | --- | --- | --- | --- | --- |
| GATA-motif | Solanum tuberosum | 347 | + | 9 | AAGGATAAGG | part of a light responsive element |

> 2018/04/13 10:10:12  
+ CCTTCTTCTA CCTCTCGGAC GTCTTGTTCT TCTTCTTTTT CTTCTCTCTT TCTCTCTCGA ATATCTATAA   
  
  
+ TCGAATTAAA CACAACCTGA AAATCGAAGT GTATAGAGAG GAAAATACAT CAAACTCAAA CTATATATTT   
  
  
+ TTATTTTTCT CTTAATTTCT CTTTTTTCTT TTTTCTTTCT CACAAAAACA TCGAAACTTA CTCTGAAAAC   
  
  
+ AACGATCGTG AAATATATTT CTATCCAACT GTTTTTGTGG AAGGTTAATC TTCTCGTATT TCACGGTAGA   
  
  
+ TCATACCTTT GTAAAGTTTA GGTACGATGA TTAGGGTAGA TGGTTCATTG TTGTGAGTGA AAATCAAAGG   
  
  
+ AGAAGGGGTA ATTTATGGGG AGTACGTAAA GTAGAGGATT GTATTTGTTT TGTTTCTAGT TTCGAATGTA   
  
  
+ GTTTTAGAAA AATAGCTAGA CTTGAAGTAC ACAATTTGCA AACTTTTCTG CTATTATTAA ATTACTTTTT   
  
  
+ TTACTAATTC TAAAAAATAT TATTATTTGA CTTAATATTT TTATGAAATA TAAAACTAAT TAATTTTTGA   
  
  
+ GCTAGTCGTA TATTTAGTCT AGTTATCTTC TAGAGTTTAT ACAATTTTCT ATTAAATAAA AAATAATAAT   
  
  
+ GATTAATAGG AAAAATAACA TTAATCTAAA CACATATACT ACGAATCCCG AAATTCCGAA ACAAAAACAC   
  
  
+ ACAAATACAA TCACCCAACT TATCTATCTC TTTCCCTCAT CACACAAATC CACCGTTAAT GAATGATTAT   
  
  
+ TATTGTACAC TTACTCACAA CACACGTGCG AGACCCTACC TCGCACCCCG CCCCTAGTGT TCAGTCGCTT   
  
  
+ CTGATGTCCA TTTCCTACCT TCACTCTCGC CAGTTTCTAA GTTTGGGTTT TTTTTCCTTT TATTTTTATT   
  
  
+ TTTTTCGTTT CTTTTTATTT TTCCTCTAAA CCTTGTAATC CGAGTGTGCA CCGCCTTGTC GGTGGCAGTA   
  
  
+ AACTGGTATC GGCTGTGTAC TGTGCCGTGC ACCCTATGGG TAGAGCAGGG GGACGGCGAT AGGCAGCGCT   
  
  
+ CGGACAGGAC GACTACCCCG TGGTCGCAGT CGCAGGGAAA CCTTAAAAAG AGAATTAAAG AACACGAGTG   
  
  
+ CCTTTGGACT TAAATTATTT AGGAAATAAA TAAATAATAA AATAAATAAT AAAATAAATA AAAAGTACCG   
  
  
+ GTGAAAAATA AAAAATTAAA TTAAAAGCAA TGGTTCATTC TTCTAGTAAT TAATATAGGT GTGTGGGTTA   
  
  
+ ATTATATAAA TAATAAAATA AATTATTTAT TAATAATTAA TTCTAAAAAT AAACGAATTT TTATTTGTTT   
  
  
+ AGAGACTATT TTGTAACCTG CTCCTCAACT TAGTTAAAAT TACAAAAATT TTAAATAATT TATTATAAAA   
  
  
+ ATTAATTTAC GTTTTATAAA ACTACACATT ATGATTTAGT AAAGTTCACA AATTTTATAA ATCTATATAA   
  
  
+ TTACGACCTT CACTTTTAGA GAACAAGTA  

- GGAAGAAGAT GGAGAGCCTG CAGAACAAGA AGAAGAAAAA GAAGAGAGAA AGAGAGAGCT TATAGATATT   
  
  
- AGCTTAATTT GTGTTGGACT TTTAGCTTCA CATATCTCTC CTTTTATGTA GTTTGAGTTT GATATATAAA   
  
  
- AATAAAAAGA GAATTAAAGA GAAAAAAGAA AAAAGAAAGA GTGTTTTTGT AGCTTTGAAT GAGACTTTTG   
  
  
- TTGCTAGCAC TTTATATAAA GATAGGTTGA CAAAAACACC TTCCAATTAG AAGAGCATAA AGTGCCATCT   
  
  
- AGTATGGAAA CATTTCAAAT CCATGCTACT AATCCCATCT ACCAAGTAAC AACACTCACT TTTAGTTTCC   
  
  
- TCTTCCCCAT TAAATACCCC TCATGCATTT CATCTCCTAA CATAAACAAA ACAAAGATCA AAGCTTACAT   
  
  
- CAAAATCTTT TTATCGATCT GAACTTCATG TGTTAAACGT TTGAAAAGAC GATAATAATT TAATGAAAAA   
  
  
- AATGATTAAG ATTTTTTATA ATAATAAACT GAATTATAAA AATACTTTAT ATTTTGATTA ATTAAAAACT   
  
  
- CGATCAGCAT ATAAATCAGA TCAATAGAAG ATCTCAAATA TGTTAAAAGA TAATTTATTT TTTATTATTA   
  
  
- CTAATTATCC TTTTTATTGT AATTAGATTT GTGTATATGA TGCTTAGGGC TTTAAGGCTT TGTTTTTGTG   
  
  
- TGTTTATGTT AGTGGGTTGA ATAGATAGAG AAAGGGAGTA GTGTGTTTAG GTGGCAATTA CTTACTAATA   
  
  
- ATAACATGTG AATGAGTGTT GTGTGCACGC TCTGGGATGG AGCGTGGGGC GGGGATCACA AGTCAGCGAA   
  
  
- GACTACAGGT AAAGGATGGA AGTGAGAGCG GTCAAAGATT CAAACCCAAA AAAAAGGAAA ATAAAAATAA   
  
  
- AAAAAGCAAA GAAAAATAAA AAGGAGATTT GGAACATTAG GCTCACACGT GGCGGAACAG CCACCGTCAT   
  
  
- TTGACCATAG CCGACACATG ACACGGCACG TGGGATACCC ATCTCGTCCC CCTGCCGCTA TCCGTCGCGA   
  
  
- GCCTGTCCTG CTGATGGGGC ACCAGCGTCA GCGTCCCTTT GGAATTTTTC TCTTAATTTC TTGTGCTCAC   
  
  
- GGAAACCTGA ATTTAATAAA TCCTTTATTT ATTTATTATT TTATTTATTA TTTTATTTAT TTTTCATGGC   
  
  
- CACTTTTTAT TTTTTAATTT AATTTTCGTT ACCAAGTAAG AAGATCATTA ATTATATCCA CACACCCAAT   
  
  
- TAATATATTT ATTATTTTAT TTAATAAATA ATTATTAATT AAGATTTTTA TTTGCTTAAA AATAAACAAA   
  
  
- TCTCTGATAA AACATTGGAC GAGGAGTTGA ATCAATTTTA ATGTTTTTAA AATTTATTAA ATAATATTTT   
  
  
- TAATTAAATG CAAAATATTT TGATGTGTAA TACTAAATCA TTTCAAGTGT TTAAAATATT TAGATATATT   
  
  
- AATGCTGGAA GTGAAAATCT CTTGTTCAT

+     GC-motif

| Site Name | Organism | Position | Strand | Matrix score. | sequence | function |
| --- | --- | --- | --- | --- | --- | --- |
| GC-motif | Zea mays | 1066 | - | 8 | CCACGGGG | enhancer-like element involved in anoxic specific inducibility |

> 2018/04/13 10:10:12  
+ CCTTCTTCTA CCTCTCGGAC GTCTTGTTCT TCTTCTTTTT CTTCTCTCTT TCTCTCTCGA ATATCTATAA   
  
  
+ TCGAATTAAA CACAACCTGA AAATCGAAGT GTATAGAGAG GAAAATACAT CAAACTCAAA CTATATATTT   
  
  
+ TTATTTTTCT CTTAATTTCT CTTTTTTCTT TTTTCTTTCT CACAAAAACA TCGAAACTTA CTCTGAAAAC   
  
  
+ AACGATCGTG AAATATATTT CTATCCAACT GTTTTTGTGG AAGGTTAATC TTCTCGTATT TCACGGTAGA   
  
  
+ TCATACCTTT GTAAAGTTTA GGTACGATGA TTAGGGTAGA TGGTTCATTG TTGTGAGTGA AAATCAAAGG   
  
  
+ AGAAGGGGTA ATTTATGGGG AGTACGTAAA GTAGAGGATT GTATTTGTTT TGTTTCTAGT TTCGAATGTA   
  
  
+ GTTTTAGAAA AATAGCTAGA CTTGAAGTAC ACAATTTGCA AACTTTTCTG CTATTATTAA ATTACTTTTT   
  
  
+ TTACTAATTC TAAAAAATAT TATTATTTGA CTTAATATTT TTATGAAATA TAAAACTAAT TAATTTTTGA   
  
  
+ GCTAGTCGTA TATTTAGTCT AGTTATCTTC TAGAGTTTAT ACAATTTTCT ATTAAATAAA AAATAATAAT   
  
  
+ GATTAATAGG AAAAATAACA TTAATCTAAA CACATATACT ACGAATCCCG AAATTCCGAA ACAAAAACAC   
  
  
+ ACAAATACAA TCACCCAACT TATCTATCTC TTTCCCTCAT CACACAAATC CACCGTTAAT GAATGATTAT   
  
  
+ TATTGTACAC TTACTCACAA CACACGTGCG AGACCCTACC TCGCACCCCG CCCCTAGTGT TCAGTCGCTT   
  
  
+ CTGATGTCCA TTTCCTACCT TCACTCTCGC CAGTTTCTAA GTTTGGGTTT TTTTTCCTTT TATTTTTATT   
  
  
+ TTTTTCGTTT CTTTTTATTT TTCCTCTAAA CCTTGTAATC CGAGTGTGCA CCGCCTTGTC GGTGGCAGTA   
  
  
+ AACTGGTATC GGCTGTGTAC TGTGCCGTGC ACCCTATGGG TAGAGCAGGG GGACGGCGAT AGGCAGCGCT   
  
  
+ CGGACAGGAC GACTACCCCG TGGTCGCAGT CGCAGGGAAA CCTTAAAAAG AGAATTAAAG AACACGAGTG   
  
  
+ CCTTTGGACT TAAATTATTT AGGAAATAAA TAAATAATAA AATAAATAAT AAAATAAATA AAAAGTACCG   
  
  
+ GTGAAAAATA AAAAATTAAA TTAAAAGCAA TGGTTCATTC TTCTAGTAAT TAATATAGGT GTGTGGGTTA   
  
  
+ ATTATATAAA TAATAAAATA AATTATTTAT TAATAATTAA TTCTAAAAAT AAACGAATTT TTATTTGTTT   
  
  
+ AGAGACTATT TTGTAACCTG CTCCTCAACT TAGTTAAAAT TACAAAAATT TTAAATAATT TATTATAAAA   
  
  
+ ATTAATTTAC GTTTTATAAA ACTACACATT ATGATTTAGT AAAGTTCACA AATTTTATAA ATCTATATAA   
  
  
+ TTACGACCTT CACTTTTAGA GAACAAGTA  

- GGAAGAAGAT GGAGAGCCTG CAGAACAAGA AGAAGAAAAA GAAGAGAGAA AGAGAGAGCT TATAGATATT   
  
  
- AGCTTAATTT GTGTTGGACT TTTAGCTTCA CATATCTCTC CTTTTATGTA GTTTGAGTTT GATATATAAA   
  
  
- AATAAAAAGA GAATTAAAGA GAAAAAAGAA AAAAGAAAGA GTGTTTTTGT AGCTTTGAAT GAGACTTTTG   
  
  
- TTGCTAGCAC TTTATATAAA GATAGGTTGA CAAAAACACC TTCCAATTAG AAGAGCATAA AGTGCCATCT   
  
  
- AGTATGGAAA CATTTCAAAT CCATGCTACT AATCCCATCT ACCAAGTAAC AACACTCACT TTTAGTTTCC   
  
  
- TCTTCCCCAT TAAATACCCC TCATGCATTT CATCTCCTAA CATAAACAAA ACAAAGATCA AAGCTTACAT   
  
  
- CAAAATCTTT TTATCGATCT GAACTTCATG TGTTAAACGT TTGAAAAGAC GATAATAATT TAATGAAAAA   
  
  
- AATGATTAAG ATTTTTTATA ATAATAAACT GAATTATAAA AATACTTTAT ATTTTGATTA ATTAAAAACT   
  
  
- CGATCAGCAT ATAAATCAGA TCAATAGAAG ATCTCAAATA TGTTAAAAGA TAATTTATTT TTTATTATTA   
  
  
- CTAATTATCC TTTTTATTGT AATTAGATTT GTGTATATGA TGCTTAGGGC TTTAAGGCTT TGTTTTTGTG   
  
  
- TGTTTATGTT AGTGGGTTGA ATAGATAGAG AAAGGGAGTA GTGTGTTTAG GTGGCAATTA CTTACTAATA   
  
  
- ATAACATGTG AATGAGTGTT GTGTGCACGC TCTGGGATGG AGCGTGGGGC GGGGATCACA AGTCAGCGAA   
  
  
- GACTACAGGT AAAGGATGGA AGTGAGAGCG GTCAAAGATT CAAACCCAAA AAAAAGGAAA ATAAAAATAA   
  
  
- AAAAAGCAAA GAAAAATAAA AAGGAGATTT GGAACATTAG GCTCACACGT GGCGGAACAG CCACCGTCAT   
  
  
- TTGACCATAG CCGACACATG ACACGGCACG TGGGATACCC ATCTCGTCCC CCTGCCGCTA TCCGTCGCGA   
  
  
- GCCTGTCCTG CTGATGGGGC ACCAGCGTCA GCGTCCCTTT GGAATTTTTC TCTTAATTTC TTGTGCTCAC   
  
  
- GGAAACCTGA ATTTAATAAA TCCTTTATTT ATTTATTATT TTATTTATTA TTTTATTTAT TTTTCATGGC   
  
  
- CACTTTTTAT TTTTTAATTT AATTTTCGTT ACCAAGTAAG AAGATCATTA ATTATATCCA CACACCCAAT   
  
  
- TAATATATTT ATTATTTTAT TTAATAAATA ATTATTAATT AAGATTTTTA TTTGCTTAAA AATAAACAAA   
  
  
- TCTCTGATAA AACATTGGAC GAGGAGTTGA ATCAATTTTA ATGTTTTTAA AATTTATTAA ATAATATTTT   
  
  
- TAATTAAATG CAAAATATTT TGATGTGTAA TACTAAATCA TTTCAAGTGT TTAAAATATT TAGATATATT   
  
  
- AATGCTGGAA GTGAAAATCT CTTGTTCAT

+     GT1-motif

| Site Name | Organism | Position | Strand | Matrix score. | sequence | function |
| --- | --- | --- | --- | --- | --- | --- |
| GT1-motif | Avena sativa | 253 | + | 7 | GGTTAAT | light responsive element |
| GT1-motif | Avena sativa | 1256 | + | 7 | GGTTAAT | light responsive element |

> 2018/04/13 10:10:12  
+ CCTTCTTCTA CCTCTCGGAC GTCTTGTTCT TCTTCTTTTT CTTCTCTCTT TCTCTCTCGA ATATCTATAA   
  
  
+ TCGAATTAAA CACAACCTGA AAATCGAAGT GTATAGAGAG GAAAATACAT CAAACTCAAA CTATATATTT   
  
  
+ TTATTTTTCT CTTAATTTCT CTTTTTTCTT TTTTCTTTCT CACAAAAACA TCGAAACTTA CTCTGAAAAC   
  
  
+ AACGATCGTG AAATATATTT CTATCCAACT GTTTTTGTGG AAGGTTAATC TTCTCGTATT TCACGGTAGA   
  
  
+ TCATACCTTT GTAAAGTTTA GGTACGATGA TTAGGGTAGA TGGTTCATTG TTGTGAGTGA AAATCAAAGG   
  
  
+ AGAAGGGGTA ATTTATGGGG AGTACGTAAA GTAGAGGATT GTATTTGTTT TGTTTCTAGT TTCGAATGTA   
  
  
+ GTTTTAGAAA AATAGCTAGA CTTGAAGTAC ACAATTTGCA AACTTTTCTG CTATTATTAA ATTACTTTTT   
  
  
+ TTACTAATTC TAAAAAATAT TATTATTTGA CTTAATATTT TTATGAAATA TAAAACTAAT TAATTTTTGA   
  
  
+ GCTAGTCGTA TATTTAGTCT AGTTATCTTC TAGAGTTTAT ACAATTTTCT ATTAAATAAA AAATAATAAT   
  
  
+ GATTAATAGG AAAAATAACA TTAATCTAAA CACATATACT ACGAATCCCG AAATTCCGAA ACAAAAACAC   
  
  
+ ACAAATACAA TCACCCAACT TATCTATCTC TTTCCCTCAT CACACAAATC CACCGTTAAT GAATGATTAT   
  
  
+ TATTGTACAC TTACTCACAA CACACGTGCG AGACCCTACC TCGCACCCCG CCCCTAGTGT TCAGTCGCTT   
  
  
+ CTGATGTCCA TTTCCTACCT TCACTCTCGC CAGTTTCTAA GTTTGGGTTT TTTTTCCTTT TATTTTTATT   
  
  
+ TTTTTCGTTT CTTTTTATTT TTCCTCTAAA CCTTGTAATC CGAGTGTGCA CCGCCTTGTC GGTGGCAGTA   
  
  
+ AACTGGTATC GGCTGTGTAC TGTGCCGTGC ACCCTATGGG TAGAGCAGGG GGACGGCGAT AGGCAGCGCT   
  
  
+ CGGACAGGAC GACTACCCCG TGGTCGCAGT CGCAGGGAAA CCTTAAAAAG AGAATTAAAG AACACGAGTG   
  
  
+ CCTTTGGACT TAAATTATTT AGGAAATAAA TAAATAATAA AATAAATAAT AAAATAAATA AAAAGTACCG   
  
  
+ GTGAAAAATA AAAAATTAAA TTAAAAGCAA TGGTTCATTC TTCTAGTAAT TAATATAGGT GTGTGGGTTA   
  
  
+ ATTATATAAA TAATAAAATA AATTATTTAT TAATAATTAA TTCTAAAAAT AAACGAATTT TTATTTGTTT   
  
  
+ AGAGACTATT TTGTAACCTG CTCCTCAACT TAGTTAAAAT TACAAAAATT TTAAATAATT TATTATAAAA   
  
  
+ ATTAATTTAC GTTTTATAAA ACTACACATT ATGATTTAGT AAAGTTCACA AATTTTATAA ATCTATATAA   
  
  
+ TTACGACCTT CACTTTTAGA GAACAAGTA  

- GGAAGAAGAT GGAGAGCCTG CAGAACAAGA AGAAGAAAAA GAAGAGAGAA AGAGAGAGCT TATAGATATT   
  
  
- AGCTTAATTT GTGTTGGACT TTTAGCTTCA CATATCTCTC CTTTTATGTA GTTTGAGTTT GATATATAAA   
  
  
- AATAAAAAGA GAATTAAAGA GAAAAAAGAA AAAAGAAAGA GTGTTTTTGT AGCTTTGAAT GAGACTTTTG   
  
  
- TTGCTAGCAC TTTATATAAA GATAGGTTGA CAAAAACACC TTCCAATTAG AAGAGCATAA AGTGCCATCT   
  
  
- AGTATGGAAA CATTTCAAAT CCATGCTACT AATCCCATCT ACCAAGTAAC AACACTCACT TTTAGTTTCC   
  
  
- TCTTCCCCAT TAAATACCCC TCATGCATTT CATCTCCTAA CATAAACAAA ACAAAGATCA AAGCTTACAT   
  
  
- CAAAATCTTT TTATCGATCT GAACTTCATG TGTTAAACGT TTGAAAAGAC GATAATAATT TAATGAAAAA   
  
  
- AATGATTAAG ATTTTTTATA ATAATAAACT GAATTATAAA AATACTTTAT ATTTTGATTA ATTAAAAACT   
  
  
- CGATCAGCAT ATAAATCAGA TCAATAGAAG ATCTCAAATA TGTTAAAAGA TAATTTATTT TTTATTATTA   
  
  
- CTAATTATCC TTTTTATTGT AATTAGATTT GTGTATATGA TGCTTAGGGC TTTAAGGCTT TGTTTTTGTG   
  
  
- TGTTTATGTT AGTGGGTTGA ATAGATAGAG AAAGGGAGTA GTGTGTTTAG GTGGCAATTA CTTACTAATA   
  
  
- ATAACATGTG AATGAGTGTT GTGTGCACGC TCTGGGATGG AGCGTGGGGC GGGGATCACA AGTCAGCGAA   
  
  
- GACTACAGGT AAAGGATGGA AGTGAGAGCG GTCAAAGATT CAAACCCAAA AAAAAGGAAA ATAAAAATAA   
  
  
- AAAAAGCAAA GAAAAATAAA AAGGAGATTT GGAACATTAG GCTCACACGT GGCGGAACAG CCACCGTCAT   
  
  
- TTGACCATAG CCGACACATG ACACGGCACG TGGGATACCC ATCTCGTCCC CCTGCCGCTA TCCGTCGCGA   
  
  
- GCCTGTCCTG CTGATGGGGC ACCAGCGTCA GCGTCCCTTT GGAATTTTTC TCTTAATTTC TTGTGCTCAC   
  
  
- GGAAACCTGA ATTTAATAAA TCCTTTATTT ATTTATTATT TTATTTATTA TTTTATTTAT TTTTCATGGC   
  
  
- CACTTTTTAT TTTTTAATTT AATTTTCGTT ACCAAGTAAG AAGATCATTA ATTATATCCA CACACCCAAT   
  
  
- TAATATATTT ATTATTTTAT TTAATAAATA ATTATTAATT AAGATTTTTA TTTGCTTAAA AATAAACAAA   
  
  
- TCTCTGATAA AACATTGGAC GAGGAGTTGA ATCAATTTTA ATGTTTTTAA AATTTATTAA ATAATATTTT   
  
  
- TAATTAAATG CAAAATATTT TGATGTGTAA TACTAAATCA TTTCAAGTGT TTAAAATATT TAGATATATT   
  
  
- AATGCTGGAA GTGAAAATCT CTTGTTCAT

+     I-box

| Site Name | Organism | Position | Strand | Matrix score. | sequence | function |
| --- | --- | --- | --- | --- | --- | --- |
| I-box | Zea mays | 962 | - | 9 | cGATAAGGCG | part of a light responsive element |

> 2018/04/13 10:10:12  
+ CCTTCTTCTA CCTCTCGGAC GTCTTGTTCT TCTTCTTTTT CTTCTCTCTT TCTCTCTCGA ATATCTATAA   
  
  
+ TCGAATTAAA CACAACCTGA AAATCGAAGT GTATAGAGAG GAAAATACAT CAAACTCAAA CTATATATTT   
  
  
+ TTATTTTTCT CTTAATTTCT CTTTTTTCTT TTTTCTTTCT CACAAAAACA TCGAAACTTA CTCTGAAAAC   
  
  
+ AACGATCGTG AAATATATTT CTATCCAACT GTTTTTGTGG AAGGTTAATC TTCTCGTATT TCACGGTAGA   
  
  
+ TCATACCTTT GTAAAGTTTA GGTACGATGA TTAGGGTAGA TGGTTCATTG TTGTGAGTGA AAATCAAAGG   
  
  
+ AGAAGGGGTA ATTTATGGGG AGTACGTAAA GTAGAGGATT GTATTTGTTT TGTTTCTAGT TTCGAATGTA   
  
  
+ GTTTTAGAAA AATAGCTAGA CTTGAAGTAC ACAATTTGCA AACTTTTCTG CTATTATTAA ATTACTTTTT   
  
  
+ TTACTAATTC TAAAAAATAT TATTATTTGA CTTAATATTT TTATGAAATA TAAAACTAAT TAATTTTTGA   
  
  
+ GCTAGTCGTA TATTTAGTCT AGTTATCTTC TAGAGTTTAT ACAATTTTCT ATTAAATAAA AAATAATAAT   
  
  
+ GATTAATAGG AAAAATAACA TTAATCTAAA CACATATACT ACGAATCCCG AAATTCCGAA ACAAAAACAC   
  
  
+ ACAAATACAA TCACCCAACT TATCTATCTC TTTCCCTCAT CACACAAATC CACCGTTAAT GAATGATTAT   
  
  
+ TATTGTACAC TTACTCACAA CACACGTGCG AGACCCTACC TCGCACCCCG CCCCTAGTGT TCAGTCGCTT   
  
  
+ CTGATGTCCA TTTCCTACCT TCACTCTCGC CAGTTTCTAA GTTTGGGTTT TTTTTCCTTT TATTTTTATT   
  
  
+ TTTTTCGTTT CTTTTTATTT TTCCTCTAAA CCTTGTAATC CGAGTGTGCA CCGCCTTGTC GGTGGCAGTA   
  
  
+ AACTGGTATC GGCTGTGTAC TGTGCCGTGC ACCCTATGGG TAGAGCAGGG GGACGGCGAT AGGCAGCGCT   
  
  
+ CGGACAGGAC GACTACCCCG TGGTCGCAGT CGCAGGGAAA CCTTAAAAAG AGAATTAAAG AACACGAGTG   
  
  
+ CCTTTGGACT TAAATTATTT AGGAAATAAA TAAATAATAA AATAAATAAT AAAATAAATA AAAAGTACCG   
  
  
+ GTGAAAAATA AAAAATTAAA TTAAAAGCAA TGGTTCATTC TTCTAGTAAT TAATATAGGT GTGTGGGTTA   
  
  
+ ATTATATAAA TAATAAAATA AATTATTTAT TAATAATTAA TTCTAAAAAT AAACGAATTT TTATTTGTTT   
  
  
+ AGAGACTATT TTGTAACCTG CTCCTCAACT TAGTTAAAAT TACAAAAATT TTAAATAATT TATTATAAAA   
  
  
+ ATTAATTTAC GTTTTATAAA ACTACACATT ATGATTTAGT AAAGTTCACA AATTTTATAA ATCTATATAA   
  
  
+ TTACGACCTT CACTTTTAGA GAACAAGTA  

- GGAAGAAGAT GGAGAGCCTG CAGAACAAGA AGAAGAAAAA GAAGAGAGAA AGAGAGAGCT TATAGATATT   
  
  
- AGCTTAATTT GTGTTGGACT TTTAGCTTCA CATATCTCTC CTTTTATGTA GTTTGAGTTT GATATATAAA   
  
  
- AATAAAAAGA GAATTAAAGA GAAAAAAGAA AAAAGAAAGA GTGTTTTTGT AGCTTTGAAT GAGACTTTTG   
  
  
- TTGCTAGCAC TTTATATAAA GATAGGTTGA CAAAAACACC TTCCAATTAG AAGAGCATAA AGTGCCATCT   
  
  
- AGTATGGAAA CATTTCAAAT CCATGCTACT AATCCCATCT ACCAAGTAAC AACACTCACT TTTAGTTTCC   
  
  
- TCTTCCCCAT TAAATACCCC TCATGCATTT CATCTCCTAA CATAAACAAA ACAAAGATCA AAGCTTACAT   
  
  
- CAAAATCTTT TTATCGATCT GAACTTCATG TGTTAAACGT TTGAAAAGAC GATAATAATT TAATGAAAAA   
  
  
- AATGATTAAG ATTTTTTATA ATAATAAACT GAATTATAAA AATACTTTAT ATTTTGATTA ATTAAAAACT   
  
  
- CGATCAGCAT ATAAATCAGA TCAATAGAAG ATCTCAAATA TGTTAAAAGA TAATTTATTT TTTATTATTA   
  
  
- CTAATTATCC TTTTTATTGT AATTAGATTT GTGTATATGA TGCTTAGGGC TTTAAGGCTT TGTTTTTGTG   
  
  
- TGTTTATGTT AGTGGGTTGA ATAGATAGAG AAAGGGAGTA GTGTGTTTAG GTGGCAATTA CTTACTAATA   
  
  
- ATAACATGTG AATGAGTGTT GTGTGCACGC TCTGGGATGG AGCGTGGGGC GGGGATCACA AGTCAGCGAA   
  
  
- GACTACAGGT AAAGGATGGA AGTGAGAGCG GTCAAAGATT CAAACCCAAA AAAAAGGAAA ATAAAAATAA   
  
  
- AAAAAGCAAA GAAAAATAAA AAGGAGATTT GGAACATTAG GCTCACACGT GGCGGAACAG CCACCGTCAT   
  
  
- TTGACCATAG CCGACACATG ACACGGCACG TGGGATACCC ATCTCGTCCC CCTGCCGCTA TCCGTCGCGA   
  
  
- GCCTGTCCTG CTGATGGGGC ACCAGCGTCA GCGTCCCTTT GGAATTTTTC TCTTAATTTC TTGTGCTCAC   
  
  
- GGAAACCTGA ATTTAATAAA TCCTTTATTT ATTTATTATT TTATTTATTA TTTTATTTAT TTTTCATGGC   
  
  
- CACTTTTTAT TTTTTAATTT AATTTTCGTT ACCAAGTAAG AAGATCATTA ATTATATCCA CACACCCAAT   
  
  
- TAATATATTT ATTATTTTAT TTAATAAATA ATTATTAATT AAGATTTTTA TTTGCTTAAA AATAAACAAA   
  
  
- TCTCTGATAA AACATTGGAC GAGGAGTTGA ATCAATTTTA ATGTTTTTAA AATTTATTAA ATAATATTTT   
  
  
- TAATTAAATG CAAAATATTT TGATGTGTAA TACTAAATCA TTTCAAGTGT TTAAAATATT TAGATATATT   
  
  
- AATGCTGGAA GTGAAAATCT CTTGTTCAT

+     LTR

| Site Name | Organism | Position | Strand | Matrix score. | sequence | function |
| --- | --- | --- | --- | --- | --- | --- |
| LTR | Hordeum vulgare | 686 | + | 6 | CCGAAA | cis-acting element involved in low-temperature responsiveness |
| LTR | Hordeum vulgare | 678 | + | 6 | CCGAAA | cis-acting element involved in low-temperature responsiveness |

> 2018/04/13 10:10:12  
+ CCTTCTTCTA CCTCTCGGAC GTCTTGTTCT TCTTCTTTTT CTTCTCTCTT TCTCTCTCGA ATATCTATAA   
  
  
+ TCGAATTAAA CACAACCTGA AAATCGAAGT GTATAGAGAG GAAAATACAT CAAACTCAAA CTATATATTT   
  
  
+ TTATTTTTCT CTTAATTTCT CTTTTTTCTT TTTTCTTTCT CACAAAAACA TCGAAACTTA CTCTGAAAAC   
  
  
+ AACGATCGTG AAATATATTT CTATCCAACT GTTTTTGTGG AAGGTTAATC TTCTCGTATT TCACGGTAGA   
  
  
+ TCATACCTTT GTAAAGTTTA GGTACGATGA TTAGGGTAGA TGGTTCATTG TTGTGAGTGA AAATCAAAGG   
  
  
+ AGAAGGGGTA ATTTATGGGG AGTACGTAAA GTAGAGGATT GTATTTGTTT TGTTTCTAGT TTCGAATGTA   
  
  
+ GTTTTAGAAA AATAGCTAGA CTTGAAGTAC ACAATTTGCA AACTTTTCTG CTATTATTAA ATTACTTTTT   
  
  
+ TTACTAATTC TAAAAAATAT TATTATTTGA CTTAATATTT TTATGAAATA TAAAACTAAT TAATTTTTGA   
  
  
+ GCTAGTCGTA TATTTAGTCT AGTTATCTTC TAGAGTTTAT ACAATTTTCT ATTAAATAAA AAATAATAAT   
  
  
+ GATTAATAGG AAAAATAACA TTAATCTAAA CACATATACT ACGAATCCCG AAATTCCGAA ACAAAAACAC   
  
  
+ ACAAATACAA TCACCCAACT TATCTATCTC TTTCCCTCAT CACACAAATC CACCGTTAAT GAATGATTAT   
  
  
+ TATTGTACAC TTACTCACAA CACACGTGCG AGACCCTACC TCGCACCCCG CCCCTAGTGT TCAGTCGCTT   
  
  
+ CTGATGTCCA TTTCCTACCT TCACTCTCGC CAGTTTCTAA GTTTGGGTTT TTTTTCCTTT TATTTTTATT   
  
  
+ TTTTTCGTTT CTTTTTATTT TTCCTCTAAA CCTTGTAATC CGAGTGTGCA CCGCCTTGTC GGTGGCAGTA   
  
  
+ AACTGGTATC GGCTGTGTAC TGTGCCGTGC ACCCTATGGG TAGAGCAGGG GGACGGCGAT AGGCAGCGCT   
  
  
+ CGGACAGGAC GACTACCCCG TGGTCGCAGT CGCAGGGAAA CCTTAAAAAG AGAATTAAAG AACACGAGTG   
  
  
+ CCTTTGGACT TAAATTATTT AGGAAATAAA TAAATAATAA AATAAATAAT AAAATAAATA AAAAGTACCG   
  
  
+ GTGAAAAATA AAAAATTAAA TTAAAAGCAA TGGTTCATTC TTCTAGTAAT TAATATAGGT GTGTGGGTTA   
  
  
+ ATTATATAAA TAATAAAATA AATTATTTAT TAATAATTAA TTCTAAAAAT AAACGAATTT TTATTTGTTT   
  
  
+ AGAGACTATT TTGTAACCTG CTCCTCAACT TAGTTAAAAT TACAAAAATT TTAAATAATT TATTATAAAA   
  
  
+ ATTAATTTAC GTTTTATAAA ACTACACATT ATGATTTAGT AAAGTTCACA AATTTTATAA ATCTATATAA   
  
  
+ TTACGACCTT CACTTTTAGA GAACAAGTA  

- GGAAGAAGAT GGAGAGCCTG CAGAACAAGA AGAAGAAAAA GAAGAGAGAA AGAGAGAGCT TATAGATATT   
  
  
- AGCTTAATTT GTGTTGGACT TTTAGCTTCA CATATCTCTC CTTTTATGTA GTTTGAGTTT GATATATAAA   
  
  
- AATAAAAAGA GAATTAAAGA GAAAAAAGAA AAAAGAAAGA GTGTTTTTGT AGCTTTGAAT GAGACTTTTG   
  
  
- TTGCTAGCAC TTTATATAAA GATAGGTTGA CAAAAACACC TTCCAATTAG AAGAGCATAA AGTGCCATCT   
  
  
- AGTATGGAAA CATTTCAAAT CCATGCTACT AATCCCATCT ACCAAGTAAC AACACTCACT TTTAGTTTCC   
  
  
- TCTTCCCCAT TAAATACCCC TCATGCATTT CATCTCCTAA CATAAACAAA ACAAAGATCA AAGCTTACAT   
  
  
- CAAAATCTTT TTATCGATCT GAACTTCATG TGTTAAACGT TTGAAAAGAC GATAATAATT TAATGAAAAA   
  
  
- AATGATTAAG ATTTTTTATA ATAATAAACT GAATTATAAA AATACTTTAT ATTTTGATTA ATTAAAAACT   
  
  
- CGATCAGCAT ATAAATCAGA TCAATAGAAG ATCTCAAATA TGTTAAAAGA TAATTTATTT TTTATTATTA   
  
  
- CTAATTATCC TTTTTATTGT AATTAGATTT GTGTATATGA TGCTTAGGGC TTTAAGGCTT TGTTTTTGTG   
  
  
- TGTTTATGTT AGTGGGTTGA ATAGATAGAG AAAGGGAGTA GTGTGTTTAG GTGGCAATTA CTTACTAATA   
  
  
- ATAACATGTG AATGAGTGTT GTGTGCACGC TCTGGGATGG AGCGTGGGGC GGGGATCACA AGTCAGCGAA   
  
  
- GACTACAGGT AAAGGATGGA AGTGAGAGCG GTCAAAGATT CAAACCCAAA AAAAAGGAAA ATAAAAATAA   
  
  
- AAAAAGCAAA GAAAAATAAA AAGGAGATTT GGAACATTAG GCTCACACGT GGCGGAACAG CCACCGTCAT   
  
  
- TTGACCATAG CCGACACATG ACACGGCACG TGGGATACCC ATCTCGTCCC CCTGCCGCTA TCCGTCGCGA   
  
  
- GCCTGTCCTG CTGATGGGGC ACCAGCGTCA GCGTCCCTTT GGAATTTTTC TCTTAATTTC TTGTGCTCAC   
  
  
- GGAAACCTGA ATTTAATAAA TCCTTTATTT ATTTATTATT TTATTTATTA TTTTATTTAT TTTTCATGGC   
  
  
- CACTTTTTAT TTTTTAATTT AATTTTCGTT ACCAAGTAAG AAGATCATTA ATTATATCCA CACACCCAAT   
  
  
- TAATATATTT ATTATTTTAT TTAATAAATA ATTATTAATT AAGATTTTTA TTTGCTTAAA AATAAACAAA   
  
  
- TCTCTGATAA AACATTGGAC GAGGAGTTGA ATCAATTTTA ATGTTTTTAA AATTTATTAA ATAATATTTT   
  
  
- TAATTAAATG CAAAATATTT TGATGTGTAA TACTAAATCA TTTCAAGTGT TTAAAATATT TAGATATATT   
  
  
- AATGCTGGAA GTGAAAATCT CTTGTTCAT

+     MBS

| Site Name | Organism | Position | Strand | Matrix score. | sequence | function |
| --- | --- | --- | --- | --- | --- | --- |
| MBS | Arabidopsis thaliana | 236 | + | 6 | CAACTG | MYB binding site involved in drought-inducibility |

> 2018/04/13 10:10:12  
+ CCTTCTTCTA CCTCTCGGAC GTCTTGTTCT TCTTCTTTTT CTTCTCTCTT TCTCTCTCGA ATATCTATAA   
  
  
+ TCGAATTAAA CACAACCTGA AAATCGAAGT GTATAGAGAG GAAAATACAT CAAACTCAAA CTATATATTT   
  
  
+ TTATTTTTCT CTTAATTTCT CTTTTTTCTT TTTTCTTTCT CACAAAAACA TCGAAACTTA CTCTGAAAAC   
  
  
+ AACGATCGTG AAATATATTT CTATCCAACT GTTTTTGTGG AAGGTTAATC TTCTCGTATT TCACGGTAGA   
  
  
+ TCATACCTTT GTAAAGTTTA GGTACGATGA TTAGGGTAGA TGGTTCATTG TTGTGAGTGA AAATCAAAGG   
  
  
+ AGAAGGGGTA ATTTATGGGG AGTACGTAAA GTAGAGGATT GTATTTGTTT TGTTTCTAGT TTCGAATGTA   
  
  
+ GTTTTAGAAA AATAGCTAGA CTTGAAGTAC ACAATTTGCA AACTTTTCTG CTATTATTAA ATTACTTTTT   
  
  
+ TTACTAATTC TAAAAAATAT TATTATTTGA CTTAATATTT TTATGAAATA TAAAACTAAT TAATTTTTGA   
  
  
+ GCTAGTCGTA TATTTAGTCT AGTTATCTTC TAGAGTTTAT ACAATTTTCT ATTAAATAAA AAATAATAAT   
  
  
+ GATTAATAGG AAAAATAACA TTAATCTAAA CACATATACT ACGAATCCCG AAATTCCGAA ACAAAAACAC   
  
  
+ ACAAATACAA TCACCCAACT TATCTATCTC TTTCCCTCAT CACACAAATC CACCGTTAAT GAATGATTAT   
  
  
+ TATTGTACAC TTACTCACAA CACACGTGCG AGACCCTACC TCGCACCCCG CCCCTAGTGT TCAGTCGCTT   
  
  
+ CTGATGTCCA TTTCCTACCT TCACTCTCGC CAGTTTCTAA GTTTGGGTTT TTTTTCCTTT TATTTTTATT   
  
  
+ TTTTTCGTTT CTTTTTATTT TTCCTCTAAA CCTTGTAATC CGAGTGTGCA CCGCCTTGTC GGTGGCAGTA   
  
  
+ AACTGGTATC GGCTGTGTAC TGTGCCGTGC ACCCTATGGG TAGAGCAGGG GGACGGCGAT AGGCAGCGCT   
  
  
+ CGGACAGGAC GACTACCCCG TGGTCGCAGT CGCAGGGAAA CCTTAAAAAG AGAATTAAAG AACACGAGTG   
  
  
+ CCTTTGGACT TAAATTATTT AGGAAATAAA TAAATAATAA AATAAATAAT AAAATAAATA AAAAGTACCG   
  
  
+ GTGAAAAATA AAAAATTAAA TTAAAAGCAA TGGTTCATTC TTCTAGTAAT TAATATAGGT GTGTGGGTTA   
  
  
+ ATTATATAAA TAATAAAATA AATTATTTAT TAATAATTAA TTCTAAAAAT AAACGAATTT TTATTTGTTT   
  
  
+ AGAGACTATT TTGTAACCTG CTCCTCAACT TAGTTAAAAT TACAAAAATT TTAAATAATT TATTATAAAA   
  
  
+ ATTAATTTAC GTTTTATAAA ACTACACATT ATGATTTAGT AAAGTTCACA AATTTTATAA ATCTATATAA   
  
  
+ TTACGACCTT CACTTTTAGA GAACAAGTA  

- GGAAGAAGAT GGAGAGCCTG CAGAACAAGA AGAAGAAAAA GAAGAGAGAA AGAGAGAGCT TATAGATATT   
  
  
- AGCTTAATTT GTGTTGGACT TTTAGCTTCA CATATCTCTC CTTTTATGTA GTTTGAGTTT GATATATAAA   
  
  
- AATAAAAAGA GAATTAAAGA GAAAAAAGAA AAAAGAAAGA GTGTTTTTGT AGCTTTGAAT GAGACTTTTG   
  
  
- TTGCTAGCAC TTTATATAAA GATAGGTTGA CAAAAACACC TTCCAATTAG AAGAGCATAA AGTGCCATCT   
  
  
- AGTATGGAAA CATTTCAAAT CCATGCTACT AATCCCATCT ACCAAGTAAC AACACTCACT TTTAGTTTCC   
  
  
- TCTTCCCCAT TAAATACCCC TCATGCATTT CATCTCCTAA CATAAACAAA ACAAAGATCA AAGCTTACAT   
  
  
- CAAAATCTTT TTATCGATCT GAACTTCATG TGTTAAACGT TTGAAAAGAC GATAATAATT TAATGAAAAA   
  
  
- AATGATTAAG ATTTTTTATA ATAATAAACT GAATTATAAA AATACTTTAT ATTTTGATTA ATTAAAAACT   
  
  
- CGATCAGCAT ATAAATCAGA TCAATAGAAG ATCTCAAATA TGTTAAAAGA TAATTTATTT TTTATTATTA   
  
  
- CTAATTATCC TTTTTATTGT AATTAGATTT GTGTATATGA TGCTTAGGGC TTTAAGGCTT TGTTTTTGTG   
  
  
- TGTTTATGTT AGTGGGTTGA ATAGATAGAG AAAGGGAGTA GTGTGTTTAG GTGGCAATTA CTTACTAATA   
  
  
- ATAACATGTG AATGAGTGTT GTGTGCACGC TCTGGGATGG AGCGTGGGGC GGGGATCACA AGTCAGCGAA   
  
  
- GACTACAGGT AAAGGATGGA AGTGAGAGCG GTCAAAGATT CAAACCCAAA AAAAAGGAAA ATAAAAATAA   
  
  
- AAAAAGCAAA GAAAAATAAA AAGGAGATTT GGAACATTAG GCTCACACGT GGCGGAACAG CCACCGTCAT   
  
  
- TTGACCATAG CCGACACATG ACACGGCACG TGGGATACCC ATCTCGTCCC CCTGCCGCTA TCCGTCGCGA   
  
  
- GCCTGTCCTG CTGATGGGGC ACCAGCGTCA GCGTCCCTTT GGAATTTTTC TCTTAATTTC TTGTGCTCAC   
  
  
- GGAAACCTGA ATTTAATAAA TCCTTTATTT ATTTATTATT TTATTTATTA TTTTATTTAT TTTTCATGGC   
  
  
- CACTTTTTAT TTTTTAATTT AATTTTCGTT ACCAAGTAAG AAGATCATTA ATTATATCCA CACACCCAAT   
  
  
- TAATATATTT ATTATTTTAT TTAATAAATA ATTATTAATT AAGATTTTTA TTTGCTTAAA AATAAACAAA   
  
  
- TCTCTGATAA AACATTGGAC GAGGAGTTGA ATCAATTTTA ATGTTTTTAA AATTTATTAA ATAATATTTT   
  
  
- TAATTAAATG CAAAATATTT TGATGTGTAA TACTAAATCA TTTCAAGTGT TTAAAATATT TAGATATATT   
  
  
- AATGCTGGAA GTGAAAATCT CTTGTTCAT

+     Sp1

| Site Name | Organism | Position | Strand | Matrix score. | sequence | function |
| --- | --- | --- | --- | --- | --- | --- |
| Sp1 | Oryza sativa | 818 | - | 6 | GGGCGG | light responsive element |

> 2018/04/13 10:10:12  
+ CCTTCTTCTA CCTCTCGGAC GTCTTGTTCT TCTTCTTTTT CTTCTCTCTT TCTCTCTCGA ATATCTATAA   
  
  
+ TCGAATTAAA CACAACCTGA AAATCGAAGT GTATAGAGAG GAAAATACAT CAAACTCAAA CTATATATTT   
  
  
+ TTATTTTTCT CTTAATTTCT CTTTTTTCTT TTTTCTTTCT CACAAAAACA TCGAAACTTA CTCTGAAAAC   
  
  
+ AACGATCGTG AAATATATTT CTATCCAACT GTTTTTGTGG AAGGTTAATC TTCTCGTATT TCACGGTAGA   
  
  
+ TCATACCTTT GTAAAGTTTA GGTACGATGA TTAGGGTAGA TGGTTCATTG TTGTGAGTGA AAATCAAAGG   
  
  
+ AGAAGGGGTA ATTTATGGGG AGTACGTAAA GTAGAGGATT GTATTTGTTT TGTTTCTAGT TTCGAATGTA   
  
  
+ GTTTTAGAAA AATAGCTAGA CTTGAAGTAC ACAATTTGCA AACTTTTCTG CTATTATTAA ATTACTTTTT   
  
  
+ TTACTAATTC TAAAAAATAT TATTATTTGA CTTAATATTT TTATGAAATA TAAAACTAAT TAATTTTTGA   
  
  
+ GCTAGTCGTA TATTTAGTCT AGTTATCTTC TAGAGTTTAT ACAATTTTCT ATTAAATAAA AAATAATAAT   
  
  
+ GATTAATAGG AAAAATAACA TTAATCTAAA CACATATACT ACGAATCCCG AAATTCCGAA ACAAAAACAC   
  
  
+ ACAAATACAA TCACCCAACT TATCTATCTC TTTCCCTCAT CACACAAATC CACCGTTAAT GAATGATTAT   
  
  
+ TATTGTACAC TTACTCACAA CACACGTGCG AGACCCTACC TCGCACCCCG CCCCTAGTGT TCAGTCGCTT   
  
  
+ CTGATGTCCA TTTCCTACCT TCACTCTCGC CAGTTTCTAA GTTTGGGTTT TTTTTCCTTT TATTTTTATT   
  
  
+ TTTTTCGTTT CTTTTTATTT TTCCTCTAAA CCTTGTAATC CGAGTGTGCA CCGCCTTGTC GGTGGCAGTA   
  
  
+ AACTGGTATC GGCTGTGTAC TGTGCCGTGC ACCCTATGGG TAGAGCAGGG GGACGGCGAT AGGCAGCGCT   
  
  
+ CGGACAGGAC GACTACCCCG TGGTCGCAGT CGCAGGGAAA CCTTAAAAAG AGAATTAAAG AACACGAGTG   
  
  
+ CCTTTGGACT TAAATTATTT AGGAAATAAA TAAATAATAA AATAAATAAT AAAATAAATA AAAAGTACCG   
  
  
+ GTGAAAAATA AAAAATTAAA TTAAAAGCAA TGGTTCATTC TTCTAGTAAT TAATATAGGT GTGTGGGTTA   
  
  
+ ATTATATAAA TAATAAAATA AATTATTTAT TAATAATTAA TTCTAAAAAT AAACGAATTT TTATTTGTTT   
  
  
+ AGAGACTATT TTGTAACCTG CTCCTCAACT TAGTTAAAAT TACAAAAATT TTAAATAATT TATTATAAAA   
  
  
+ ATTAATTTAC GTTTTATAAA ACTACACATT ATGATTTAGT AAAGTTCACA AATTTTATAA ATCTATATAA   
  
  
+ TTACGACCTT CACTTTTAGA GAACAAGTA  

- GGAAGAAGAT GGAGAGCCTG CAGAACAAGA AGAAGAAAAA GAAGAGAGAA AGAGAGAGCT TATAGATATT   
  
  
- AGCTTAATTT GTGTTGGACT TTTAGCTTCA CATATCTCTC CTTTTATGTA GTTTGAGTTT GATATATAAA   
  
  
- AATAAAAAGA GAATTAAAGA GAAAAAAGAA AAAAGAAAGA GTGTTTTTGT AGCTTTGAAT GAGACTTTTG   
  
  
- TTGCTAGCAC TTTATATAAA GATAGGTTGA CAAAAACACC TTCCAATTAG AAGAGCATAA AGTGCCATCT   
  
  
- AGTATGGAAA CATTTCAAAT CCATGCTACT AATCCCATCT ACCAAGTAAC AACACTCACT TTTAGTTTCC   
  
  
- TCTTCCCCAT TAAATACCCC TCATGCATTT CATCTCCTAA CATAAACAAA ACAAAGATCA AAGCTTACAT   
  
  
- CAAAATCTTT TTATCGATCT GAACTTCATG TGTTAAACGT TTGAAAAGAC GATAATAATT TAATGAAAAA   
  
  
- AATGATTAAG ATTTTTTATA ATAATAAACT GAATTATAAA AATACTTTAT ATTTTGATTA ATTAAAAACT   
  
  
- CGATCAGCAT ATAAATCAGA TCAATAGAAG ATCTCAAATA TGTTAAAAGA TAATTTATTT TTTATTATTA   
  
  
- CTAATTATCC TTTTTATTGT AATTAGATTT GTGTATATGA TGCTTAGGGC TTTAAGGCTT TGTTTTTGTG   
  
  
- TGTTTATGTT AGTGGGTTGA ATAGATAGAG AAAGGGAGTA GTGTGTTTAG GTGGCAATTA CTTACTAATA   
  
  
- ATAACATGTG AATGAGTGTT GTGTGCACGC TCTGGGATGG AGCGTGGGGC GGGGATCACA AGTCAGCGAA   
  
  
- GACTACAGGT AAAGGATGGA AGTGAGAGCG GTCAAAGATT CAAACCCAAA AAAAAGGAAA ATAAAAATAA   
  
  
- AAAAAGCAAA GAAAAATAAA AAGGAGATTT GGAACATTAG GCTCACACGT GGCGGAACAG CCACCGTCAT   
  
  
- TTGACCATAG CCGACACATG ACACGGCACG TGGGATACCC ATCTCGTCCC CCTGCCGCTA TCCGTCGCGA   
  
  
- GCCTGTCCTG CTGATGGGGC ACCAGCGTCA GCGTCCCTTT GGAATTTTTC TCTTAATTTC TTGTGCTCAC   
  
  
- GGAAACCTGA ATTTAATAAA TCCTTTATTT ATTTATTATT TTATTTATTA TTTTATTTAT TTTTCATGGC   
  
  
- CACTTTTTAT TTTTTAATTT AATTTTCGTT ACCAAGTAAG AAGATCATTA ATTATATCCA CACACCCAAT   
  
  
- TAATATATTT ATTATTTTAT TTAATAAATA ATTATTAATT AAGATTTTTA TTTGCTTAAA AATAAACAAA   
  
  
- TCTCTGATAA AACATTGGAC GAGGAGTTGA ATCAATTTTA ATGTTTTTAA AATTTATTAA ATAATATTTT   
  
  
- TAATTAAATG CAAAATATTT TGATGTGTAA TACTAAATCA TTTCAAGTGT TTAAAATATT TAGATATATT   
  
  
- AATGCTGGAA GTGAAAATCT CTTGTTCAT

+     TATA-box

| Site Name | Organism | Position | Strand | Matrix score. | sequence | function |
| --- | --- | --- | --- | --- | --- | --- |
| TATA-box | Lycopersicon esculentum | 1170 | - | 5 | TTTTA | core promoter element around -30 of transcription start |
| TATA-box | Lycopersicon esculentum | 501 | - | 5 | TTTTA | core promoter element around -30 of transcription start |
| TATA-box | Brassica napus | 133 | + | 6 | ATATAT | core promoter element around -30 of transcription start |
| TATA-box | Arabidopsis thaliana | 132 | + | 4 | TATA | core promoter element around -30 of transcription start |
| TATA-box | Lycopersicon esculentum | 1274 | - | 5 | TTTTA | core promoter element around -30 of transcription start |
| TATA-box | Glycine max | 1288 | - | 5 | TAATA | core promoter element around -30 of transcription start |
| TATA-box | Brassica oleracea | 538 | + | 6 | ATATAA | core promoter element around -30 of transcription start |
| TATA-box | Arabidopsis thaliana | 598 | + | 4 | TATA | core promoter element around -30 of transcription start |
| TATA-box | Arabidopsis thaliana | 665 | + | 4 | TATA | core promoter element around -30 of transcription start |
| TATA-box | Lycopersicon esculentum | 1199 | - | 5 | TTTTA | core promoter element around -30 of transcription start |
| TATA-box | Glycine max | 508 | - | 5 | TAATA | core promoter element around -30 of transcription start |
| TATA-box | Oryza sativa | 1371 | + | 7 | TACAAAA | core promoter element around -30 of transcription start |
| TATA-box | Brassica oleracea | 1465 | + | 7 | ATATAAT | core promoter element around -30 of transcription start |
| TATA-box | Lycopersicon esculentum | 1417 | - | 5 | TTTTA | core promoter element around -30 of transcription start |
| TATA-box | Arabidopsis thaliana | 1262 | - | 7 | TATATAA | core promoter element around -30 of transcription start |
| TATA-box | Brassica napus | 1261 | + | 6 | ATTATA | core promoter element around -30 of transcription start |
| TATA-box | Arabidopsis thaliana | 1454 | - | 6 | TATAAA | core promoter element around -30 of transcription start |
| TATA-box | Lycopersicon esculentum | 1396 | - | 5 | TTTTA | core promoter element around -30 of transcription start |
| TATA-box | Lycopersicon esculentum | 422 | + | 5 | TTTTA | core promoter element around -30 of transcription start |
| TATA-box | Ac | 1265 | + | 7 | TATAAAT | core promoter element around -30 of transcription start |
| TATA-box | Pisum sativum | 1452 | - | 8 | TATAAAAT | core promoter element around -30 of transcription start |
| TATA-box | Arabidopsis thaliana | 1393 | - | 5 | TATAA | core promoter element around -30 of transcription start |
| TATA-box | Glycine max | 1271 | + | 5 | TAATA | core promoter element around -30 of transcription start |
| TATA-box | Lycopersicon esculentum | 1379 | + | 5 | TTTTA | core promoter element around -30 of transcription start |
| TATA-box | Glycine max | 1291 | + | 5 | TAATA | core promoter element around -30 of transcription start |
| TATA-box | Glycine max | 523 | + | 5 | TAATA | core promoter element around -30 of transcription start |
| TATA-box | Brassica napus | 223 | + | 6 | ATATAT | core promoter element around -30 of transcription start |
| TATA-box | Arabidopsis thaliana | 1462 | + | 9 | tcTATATAtt | core promoter element around -30 of transcription start |
| TATA-box | Lycopersicon esculentum | 1094 | - | 5 | TTTTA | core promoter element around -30 of transcription start |
| TATA-box | Arabidopsis thaliana | 224 | + | 4 | TATA | core promoter element around -30 of transcription start |
| TATA-box | Lycopersicon esculentum | 139 | + | 5 | TTTTA | core promoter element around -30 of transcription start |
| TATA-box | Avena sativa | 134 | + | 12 | TATATTTATATTT | core promoter element around -30 of transcription start |
| TATA-box | Glycine max | 511 | - | 5 | TAATA | core promoter element around -30 of transcription start |
| TATA-box | Arabidopsis thaliana | 1263 | + | 9 | taTATAAAtc | core promoter element around -30 of transcription start |
| TATA-box | Oryza sativa | 1339 | - | 7 | TACAAAA | core promoter element around -30 of transcription start |
| TATA-box | Arabidopsis thaliana | 1464 | - | 4 | TATA | core promoter element around -30 of transcription start |
| TATA-box | Glycine max | 1155 | + | 5 | TAATA | core promoter element around -30 of transcription start |
| TATA-box | Arabidopsis thaliana | 66 | + | 4 | TATA | core promoter element around -30 of transcription start |
| TATA-box | Lycopersicon esculentum | 923 | + | 5 | TTTTA | core promoter element around -30 of transcription start |
| TATA-box | Arabidopsis thaliana | 900 | - | 9 | TAAAAATAA | core promoter element around -30 of transcription start |
| TATA-box | Arabidopsis thaliana | 1244 | - | 4 | TATA | core promoter element around -30 of transcription start |
| TATA-box | Arabidopsis thaliana | 1415 | + | 6 | TATAAA | core promoter element around -30 of transcription start |
| TATA-box | Lycopersicon esculentum | 529 | + | 5 | TTTTA | core promoter element around -30 of transcription start |
| TATA-box | Lycopersicon esculentum | 898 | + | 5 | TTTTA | core promoter element around -30 of transcription start |
| TATA-box | Arabidopsis thaliana | 1414 | - | 5 | TATAA | core promoter element around -30 of transcription start |
| TATA-box | Arabidopsis thaliana | 1304 | + | 9 | TAAAAATAA | core promoter element around -30 of transcription start |
| TATA-box | Daucus carota | 360 | - | 9 | ccTATAAATT | core promoter element around -30 of transcription start |
| TATA-box | Arabidopsis thaliana | 596 | - | 6 | TATAAA | core promoter element around -30 of transcription start |
| TATA-box | Lycopersicon esculentum | 1484 | + | 5 | TTTTA | core promoter element around -30 of transcription start |
| TATA-box | Arabidopsis thaliana | 1413 | - | 6 | TATAAA | core promoter element around -30 of transcription start |
| TATA-box | Arabidopsis thaliana | 539 | + | 6 | TATAAA | core promoter element around -30 of transcription start |
| TATA-box | Helianthus annuus | 100 | - | 6 | TATACA | core promoter element around -30 of transcription start |
| TATA-box | Glycine max | 1241 | + | 5 | TAATA | core promoter element around -30 of transcription start |
| TATA-box | Glycine max | 610 | - | 5 | TAATA | core promoter element around -30 of transcription start |
| TATA-box | Arabidopsis thaliana | 569 | + | 4 | TATA | core promoter element around -30 of transcription start |
| TATA-box | Arabidopsis thaliana | 1412 | - | 7 | TATAAAA | core promoter element around -30 of transcription start |
| TATA-box | Lycopersicon esculentum | 489 | + | 5 | TTTTA | core promoter element around -30 of transcription start |
| TATA-box | Glycine max | 475 | - | 5 | TAATA | core promoter element around -30 of transcription start |
| TATA-box | Brassica oleracea | 1264 | + | 6 | ATATAA | core promoter element around -30 of transcription start |
| TATA-box | Lycopersicon esculentum | 1179 | - | 5 | TTTTA | core promoter element around -30 of transcription start |
| TATA-box | Arabidopsis thaliana | 1380 | - | 8 | TATTTAAA | core promoter element around -30 of transcription start |
| TATA-box | Arabidopsis thaliana | 1453 | - | 7 | TATAAAA | core promoter element around -30 of transcription start |
| TATA-box | Arabidopsis thaliana | 102 | + | 4 | TATA | core promoter element around -30 of transcription start |
| TATA-box | Lycopersicon esculentum | 541 | - | 5 | TTTTA | core promoter element around -30 of transcription start |
| TATA-box | Arabidopsis thaliana | 597 | - | 5 | TATAA | core promoter element around -30 of transcription start |
| TATA-box | Lycopersicon esculentum | 1212 | - | 5 | TTTTA | core promoter element around -30 of transcription start |
| TATA-box | Brassica napus | 1392 | + | 6 | ATTATA | core promoter element around -30 of transcription start |
| TATA-box | Glycine max | 472 | - | 5 | TAATA | core promoter element around -30 of transcription start |
| TATA-box | Arabidopsis thaliana | 130 | + | 9 | tcTATATAtt | core promoter element around -30 of transcription start |
| TATA-box | Glycine max | 768 | - | 5 | TAATA | core promoter element around -30 of transcription start |
| TATA-box | Pisum sativum | 662 | - | 7 | TATATGT | core promoter element around -30 of transcription start |
| TATA-box | Glycine max | 624 | + | 5 | TAATA | core promoter element around -30 of transcription start |
| TATA-box | Lycopersicon esculentum | 617 | - | 5 | TTTTA | core promoter element around -30 of transcription start |
| TATA-box | Lycopersicon esculentum | 1365 | - | 5 | TTTTA | core promoter element around -30 of transcription start |
| TATA-box | Glycine max | 634 | + | 5 | TAATA | core promoter element around -30 of transcription start |
| TATA-box | Glycine max | 1167 | + | 5 | TAATA | core promoter element around -30 of transcription start |
| TATA-box | Arabidopsis thaliana | 1466 | - | 4 | TATA | core promoter element around -30 of transcription start |
| TATA-box | Lycopersicon esculentum | 1158 | - | 5 | TTTTA | core promoter element around -30 of transcription start |
| TATA-box | Glycine max | 1391 | - | 5 | TAATA | core promoter element around -30 of transcription start |
| TATA-box | Lycopersicon esculentum | 1319 | + | 5 | TTTTA | core promoter element around -30 of transcription start |
| TATA-box | Lycopersicon esculentum | 904 | + | 5 | TTTTA | core promoter element around -30 of transcription start |
| TATA-box | Arabidopsis thaliana | 1394 | + | 6 | TATAAA | core promoter element around -30 of transcription start |
| TATA-box | Nicotiana tabacum | 1302 | + | 9 | tcTATAAAta | core promoter element around -30 of transcription start |
| TATA-box | Arabidopsis thaliana | 1455 | - | 5 | TATAA | core promoter element around -30 of transcription start |
| TATA-box | Ac | 1456 | + | 7 | TATAAAT | core promoter element around -30 of transcription start |

> 2018/04/13 10:10:12  
+ CCTTCTTCTA CCTCTCGGAC GTCTTGTTCT TCTTCTTTTT CTTCTCTCTT TCTCTCTCGA ATATCTATAA   
  
  
+ TCGAATTAAA CACAACCTGA AAATCGAAGT GTATAGAGAG GAAAATACAT CAAACTCAAA CTATATATTT   
  
  
+ TTATTTTTCT CTTAATTTCT CTTTTTTCTT TTTTCTTTCT CACAAAAACA TCGAAACTTA CTCTGAAAAC   
  
  
+ AACGATCGTG AAATATATTT CTATCCAACT GTTTTTGTGG AAGGTTAATC TTCTCGTATT TCACGGTAGA   
  
  
+ TCATACCTTT GTAAAGTTTA GGTACGATGA TTAGGGTAGA TGGTTCATTG TTGTGAGTGA AAATCAAAGG   
  
  
+ AGAAGGGGTA ATTTATGGGG AGTACGTAAA GTAGAGGATT GTATTTGTTT TGTTTCTAGT TTCGAATGTA   
  
  
+ GTTTTAGAAA AATAGCTAGA CTTGAAGTAC ACAATTTGCA AACTTTTCTG CTATTATTAA ATTACTTTTT   
  
  
+ TTACTAATTC TAAAAAATAT TATTATTTGA CTTAATATTT TTATGAAATA TAAAACTAAT TAATTTTTGA   
  
  
+ GCTAGTCGTA TATTTAGTCT AGTTATCTTC TAGAGTTTAT ACAATTTTCT ATTAAATAAA AAATAATAAT   
  
  
+ GATTAATAGG AAAAATAACA TTAATCTAAA CACATATACT ACGAATCCCG AAATTCCGAA ACAAAAACAC   
  
  
+ ACAAATACAA TCACCCAACT TATCTATCTC TTTCCCTCAT CACACAAATC CACCGTTAAT GAATGATTAT   
  
  
+ TATTGTACAC TTACTCACAA CACACGTGCG AGACCCTACC TCGCACCCCG CCCCTAGTGT TCAGTCGCTT   
  
  
+ CTGATGTCCA TTTCCTACCT TCACTCTCGC CAGTTTCTAA GTTTGGGTTT TTTTTCCTTT TATTTTTATT   
  
  
+ TTTTTCGTTT CTTTTTATTT TTCCTCTAAA CCTTGTAATC CGAGTGTGCA CCGCCTTGTC GGTGGCAGTA   
  
  
+ AACTGGTATC GGCTGTGTAC TGTGCCGTGC ACCCTATGGG TAGAGCAGGG GGACGGCGAT AGGCAGCGCT   
  
  
+ CGGACAGGAC GACTACCCCG TGGTCGCAGT CGCAGGGAAA CCTTAAAAAG AGAATTAAAG AACACGAGTG   
  
  
+ CCTTTGGACT TAAATTATTT AGGAAATAAA TAAATAATAA AATAAATAAT AAAATAAATA AAAAGTACCG   
  
  
+ GTGAAAAATA AAAAATTAAA TTAAAAGCAA TGGTTCATTC TTCTAGTAAT TAATATAGGT GTGTGGGTTA   
  
  
+ ATTATATAAA TAATAAAATA AATTATTTAT TAATAATTAA TTCTAAAAAT AAACGAATTT TTATTTGTTT   
  
  
+ AGAGACTATT TTGTAACCTG CTCCTCAACT TAGTTAAAAT TACAAAAATT TTAAATAATT TATTATAAAA   
  
  
+ ATTAATTTAC GTTTTATAAA ACTACACATT ATGATTTAGT AAAGTTCACA AATTTTATAA ATCTATATAA   
  
  
+ TTACGACCTT CACTTTTAGA GAACAAGTA  

- GGAAGAAGAT GGAGAGCCTG CAGAACAAGA AGAAGAAAAA GAAGAGAGAA AGAGAGAGCT TATAGATATT   
  
  
- AGCTTAATTT GTGTTGGACT TTTAGCTTCA CATATCTCTC CTTTTATGTA GTTTGAGTTT GATATATAAA   
  
  
- AATAAAAAGA GAATTAAAGA GAAAAAAGAA AAAAGAAAGA GTGTTTTTGT AGCTTTGAAT GAGACTTTTG   
  
  
- TTGCTAGCAC TTTATATAAA GATAGGTTGA CAAAAACACC TTCCAATTAG AAGAGCATAA AGTGCCATCT   
  
  
- AGTATGGAAA CATTTCAAAT CCATGCTACT AATCCCATCT ACCAAGTAAC AACACTCACT TTTAGTTTCC   
  
  
- TCTTCCCCAT TAAATACCCC TCATGCATTT CATCTCCTAA CATAAACAAA ACAAAGATCA AAGCTTACAT   
  
  
- CAAAATCTTT TTATCGATCT GAACTTCATG TGTTAAACGT TTGAAAAGAC GATAATAATT TAATGAAAAA   
  
  
- AATGATTAAG ATTTTTTATA ATAATAAACT GAATTATAAA AATACTTTAT ATTTTGATTA ATTAAAAACT   
  
  
- CGATCAGCAT ATAAATCAGA TCAATAGAAG ATCTCAAATA TGTTAAAAGA TAATTTATTT TTTATTATTA   
  
  
- CTAATTATCC TTTTTATTGT AATTAGATTT GTGTATATGA TGCTTAGGGC TTTAAGGCTT TGTTTTTGTG   
  
  
- TGTTTATGTT AGTGGGTTGA ATAGATAGAG AAAGGGAGTA GTGTGTTTAG GTGGCAATTA CTTACTAATA   
  
  
- ATAACATGTG AATGAGTGTT GTGTGCACGC TCTGGGATGG AGCGTGGGGC GGGGATCACA AGTCAGCGAA   
  
  
- GACTACAGGT AAAGGATGGA AGTGAGAGCG GTCAAAGATT CAAACCCAAA AAAAAGGAAA ATAAAAATAA   
  
  
- AAAAAGCAAA GAAAAATAAA AAGGAGATTT GGAACATTAG GCTCACACGT GGCGGAACAG CCACCGTCAT   
  
  
- TTGACCATAG CCGACACATG ACACGGCACG TGGGATACCC ATCTCGTCCC CCTGCCGCTA TCCGTCGCGA   
  
  
- GCCTGTCCTG CTGATGGGGC ACCAGCGTCA GCGTCCCTTT GGAATTTTTC TCTTAATTTC TTGTGCTCAC   
  
  
- GGAAACCTGA ATTTAATAAA TCCTTTATTT ATTTATTATT TTATTTATTA TTTTATTTAT TTTTCATGGC   
  
  
- CACTTTTTAT TTTTTAATTT AATTTTCGTT ACCAAGTAAG AAGATCATTA ATTATATCCA CACACCCAAT   
  
  
- TAATATATTT ATTATTTTAT TTAATAAATA ATTATTAATT AAGATTTTTA TTTGCTTAAA AATAAACAAA   
  
  
- TCTCTGATAA AACATTGGAC GAGGAGTTGA ATCAATTTTA ATGTTTTTAA AATTTATTAA ATAATATTTT   
  
  
- TAATTAAATG CAAAATATTT TGATGTGTAA TACTAAATCA TTTCAAGTGT TTAAAATATT TAGATATATT   
  
  
- AATGCTGGAA GTGAAAATCT CTTGTTCAT

+     TCA-element

| Site Name | Organism | Position | Strand | Matrix score. | sequence | function |
| --- | --- | --- | --- | --- | --- | --- |
| TCA-element | Brassica oleracea | 37 | - | 9 | GAGAAGAATA | cis-acting element involved in salicylic acid responsiveness |
| TCA-element | Brassica oleracea | 256 | - | 9 | GAGAAGAATA | cis-acting element involved in salicylic acid responsiveness |
| TCA-element | Brassica oleracea | 142 | - | 9 | GAGAAGAATA | cis-acting element involved in salicylic acid responsiveness |

> 2018/04/13 10:10:12  
+ CCTTCTTCTA CCTCTCGGAC GTCTTGTTCT TCTTCTTTTT CTTCTCTCTT TCTCTCTCGA ATATCTATAA   
  
  
+ TCGAATTAAA CACAACCTGA AAATCGAAGT GTATAGAGAG GAAAATACAT CAAACTCAAA CTATATATTT   
  
  
+ TTATTTTTCT CTTAATTTCT CTTTTTTCTT TTTTCTTTCT CACAAAAACA TCGAAACTTA CTCTGAAAAC   
  
  
+ AACGATCGTG AAATATATTT CTATCCAACT GTTTTTGTGG AAGGTTAATC TTCTCGTATT TCACGGTAGA   
  
  
+ TCATACCTTT GTAAAGTTTA GGTACGATGA TTAGGGTAGA TGGTTCATTG TTGTGAGTGA AAATCAAAGG   
  
  
+ AGAAGGGGTA ATTTATGGGG AGTACGTAAA GTAGAGGATT GTATTTGTTT TGTTTCTAGT TTCGAATGTA   
  
  
+ GTTTTAGAAA AATAGCTAGA CTTGAAGTAC ACAATTTGCA AACTTTTCTG CTATTATTAA ATTACTTTTT   
  
  
+ TTACTAATTC TAAAAAATAT TATTATTTGA CTTAATATTT TTATGAAATA TAAAACTAAT TAATTTTTGA   
  
  
+ GCTAGTCGTA TATTTAGTCT AGTTATCTTC TAGAGTTTAT ACAATTTTCT ATTAAATAAA AAATAATAAT   
  
  
+ GATTAATAGG AAAAATAACA TTAATCTAAA CACATATACT ACGAATCCCG AAATTCCGAA ACAAAAACAC   
  
  
+ ACAAATACAA TCACCCAACT TATCTATCTC TTTCCCTCAT CACACAAATC CACCGTTAAT GAATGATTAT   
  
  
+ TATTGTACAC TTACTCACAA CACACGTGCG AGACCCTACC TCGCACCCCG CCCCTAGTGT TCAGTCGCTT   
  
  
+ CTGATGTCCA TTTCCTACCT TCACTCTCGC CAGTTTCTAA GTTTGGGTTT TTTTTCCTTT TATTTTTATT   
  
  
+ TTTTTCGTTT CTTTTTATTT TTCCTCTAAA CCTTGTAATC CGAGTGTGCA CCGCCTTGTC GGTGGCAGTA   
  
  
+ AACTGGTATC GGCTGTGTAC TGTGCCGTGC ACCCTATGGG TAGAGCAGGG GGACGGCGAT AGGCAGCGCT   
  
  
+ CGGACAGGAC GACTACCCCG TGGTCGCAGT CGCAGGGAAA CCTTAAAAAG AGAATTAAAG AACACGAGTG   
  
  
+ CCTTTGGACT TAAATTATTT AGGAAATAAA TAAATAATAA AATAAATAAT AAAATAAATA AAAAGTACCG   
  
  
+ GTGAAAAATA AAAAATTAAA TTAAAAGCAA TGGTTCATTC TTCTAGTAAT TAATATAGGT GTGTGGGTTA   
  
  
+ ATTATATAAA TAATAAAATA AATTATTTAT TAATAATTAA TTCTAAAAAT AAACGAATTT TTATTTGTTT   
  
  
+ AGAGACTATT TTGTAACCTG CTCCTCAACT TAGTTAAAAT TACAAAAATT TTAAATAATT TATTATAAAA   
  
  
+ ATTAATTTAC GTTTTATAAA ACTACACATT ATGATTTAGT AAAGTTCACA AATTTTATAA ATCTATATAA   
  
  
+ TTACGACCTT CACTTTTAGA GAACAAGTA  

- GGAAGAAGAT GGAGAGCCTG CAGAACAAGA AGAAGAAAAA GAAGAGAGAA AGAGAGAGCT TATAGATATT   
  
  
- AGCTTAATTT GTGTTGGACT TTTAGCTTCA CATATCTCTC CTTTTATGTA GTTTGAGTTT GATATATAAA   
  
  
- AATAAAAAGA GAATTAAAGA GAAAAAAGAA AAAAGAAAGA GTGTTTTTGT AGCTTTGAAT GAGACTTTTG   
  
  
- TTGCTAGCAC TTTATATAAA GATAGGTTGA CAAAAACACC TTCCAATTAG AAGAGCATAA AGTGCCATCT   
  
  
- AGTATGGAAA CATTTCAAAT CCATGCTACT AATCCCATCT ACCAAGTAAC AACACTCACT TTTAGTTTCC   
  
  
- TCTTCCCCAT TAAATACCCC TCATGCATTT CATCTCCTAA CATAAACAAA ACAAAGATCA AAGCTTACAT   
  
  
- CAAAATCTTT TTATCGATCT GAACTTCATG TGTTAAACGT TTGAAAAGAC GATAATAATT TAATGAAAAA   
  
  
- AATGATTAAG ATTTTTTATA ATAATAAACT GAATTATAAA AATACTTTAT ATTTTGATTA ATTAAAAACT   
  
  
- CGATCAGCAT ATAAATCAGA TCAATAGAAG ATCTCAAATA TGTTAAAAGA TAATTTATTT TTTATTATTA   
  
  
- CTAATTATCC TTTTTATTGT AATTAGATTT GTGTATATGA TGCTTAGGGC TTTAAGGCTT TGTTTTTGTG   
  
  
- TGTTTATGTT AGTGGGTTGA ATAGATAGAG AAAGGGAGTA GTGTGTTTAG GTGGCAATTA CTTACTAATA   
  
  
- ATAACATGTG AATGAGTGTT GTGTGCACGC TCTGGGATGG AGCGTGGGGC GGGGATCACA AGTCAGCGAA   
  
  
- GACTACAGGT AAAGGATGGA AGTGAGAGCG GTCAAAGATT CAAACCCAAA AAAAAGGAAA ATAAAAATAA   
  
  
- AAAAAGCAAA GAAAAATAAA AAGGAGATTT GGAACATTAG GCTCACACGT GGCGGAACAG CCACCGTCAT   
  
  
- TTGACCATAG CCGACACATG ACACGGCACG TGGGATACCC ATCTCGTCCC CCTGCCGCTA TCCGTCGCGA   
  
  
- GCCTGTCCTG CTGATGGGGC ACCAGCGTCA GCGTCCCTTT GGAATTTTTC TCTTAATTTC TTGTGCTCAC   
  
  
- GGAAACCTGA ATTTAATAAA TCCTTTATTT ATTTATTATT TTATTTATTA TTTTATTTAT TTTTCATGGC   
  
  
- CACTTTTTAT TTTTTAATTT AATTTTCGTT ACCAAGTAAG AAGATCATTA ATTATATCCA CACACCCAAT   
  
  
- TAATATATTT ATTATTTTAT TTAATAAATA ATTATTAATT AAGATTTTTA TTTGCTTAAA AATAAACAAA   
  
  
- TCTCTGATAA AACATTGGAC GAGGAGTTGA ATCAATTTTA ATGTTTTTAA AATTTATTAA ATAATATTTT   
  
  
- TAATTAAATG CAAAATATTT TGATGTGTAA TACTAAATCA TTTCAAGTGT TTAAAATATT TAGATATATT   
  
  
- AATGCTGGAA GTGAAAATCT CTTGTTCAT

+     Unnamed\_\_1

| Site Name | Organism | Position | Strand | Matrix score. | sequence | function |
| --- | --- | --- | --- | --- | --- | --- |
| Unnamed\_\_1 | Zea mays | 1069 | + | 5 | CGTGG |  |

> 2018/04/13 10:10:12  
+ CCTTCTTCTA CCTCTCGGAC GTCTTGTTCT TCTTCTTTTT CTTCTCTCTT TCTCTCTCGA ATATCTATAA   
  
  
+ TCGAATTAAA CACAACCTGA AAATCGAAGT GTATAGAGAG GAAAATACAT CAAACTCAAA CTATATATTT   
  
  
+ TTATTTTTCT CTTAATTTCT CTTTTTTCTT TTTTCTTTCT CACAAAAACA TCGAAACTTA CTCTGAAAAC   
  
  
+ AACGATCGTG AAATATATTT CTATCCAACT GTTTTTGTGG AAGGTTAATC TTCTCGTATT TCACGGTAGA   
  
  
+ TCATACCTTT GTAAAGTTTA GGTACGATGA TTAGGGTAGA TGGTTCATTG TTGTGAGTGA AAATCAAAGG   
  
  
+ AGAAGGGGTA ATTTATGGGG AGTACGTAAA GTAGAGGATT GTATTTGTTT TGTTTCTAGT TTCGAATGTA   
  
  
+ GTTTTAGAAA AATAGCTAGA CTTGAAGTAC ACAATTTGCA AACTTTTCTG CTATTATTAA ATTACTTTTT   
  
  
+ TTACTAATTC TAAAAAATAT TATTATTTGA CTTAATATTT TTATGAAATA TAAAACTAAT TAATTTTTGA   
  
  
+ GCTAGTCGTA TATTTAGTCT AGTTATCTTC TAGAGTTTAT ACAATTTTCT ATTAAATAAA AAATAATAAT   
  
  
+ GATTAATAGG AAAAATAACA TTAATCTAAA CACATATACT ACGAATCCCG AAATTCCGAA ACAAAAACAC   
  
  
+ ACAAATACAA TCACCCAACT TATCTATCTC TTTCCCTCAT CACACAAATC CACCGTTAAT GAATGATTAT   
  
  
+ TATTGTACAC TTACTCACAA CACACGTGCG AGACCCTACC TCGCACCCCG CCCCTAGTGT TCAGTCGCTT   
  
  
+ CTGATGTCCA TTTCCTACCT TCACTCTCGC CAGTTTCTAA GTTTGGGTTT TTTTTCCTTT TATTTTTATT   
  
  
+ TTTTTCGTTT CTTTTTATTT TTCCTCTAAA CCTTGTAATC CGAGTGTGCA CCGCCTTGTC GGTGGCAGTA   
  
  
+ AACTGGTATC GGCTGTGTAC TGTGCCGTGC ACCCTATGGG TAGAGCAGGG GGACGGCGAT AGGCAGCGCT   
  
  
+ CGGACAGGAC GACTACCCCG TGGTCGCAGT CGCAGGGAAA CCTTAAAAAG AGAATTAAAG AACACGAGTG   
  
  
+ CCTTTGGACT TAAATTATTT AGGAAATAAA TAAATAATAA AATAAATAAT AAAATAAATA AAAAGTACCG   
  
  
+ GTGAAAAATA AAAAATTAAA TTAAAAGCAA TGGTTCATTC TTCTAGTAAT TAATATAGGT GTGTGGGTTA   
  
  
+ ATTATATAAA TAATAAAATA AATTATTTAT TAATAATTAA TTCTAAAAAT AAACGAATTT TTATTTGTTT   
  
  
+ AGAGACTATT TTGTAACCTG CTCCTCAACT TAGTTAAAAT TACAAAAATT TTAAATAATT TATTATAAAA   
  
  
+ ATTAATTTAC GTTTTATAAA ACTACACATT ATGATTTAGT AAAGTTCACA AATTTTATAA ATCTATATAA   
  
  
+ TTACGACCTT CACTTTTAGA GAACAAGTA  

- GGAAGAAGAT GGAGAGCCTG CAGAACAAGA AGAAGAAAAA GAAGAGAGAA AGAGAGAGCT TATAGATATT   
  
  
- AGCTTAATTT GTGTTGGACT TTTAGCTTCA CATATCTCTC CTTTTATGTA GTTTGAGTTT GATATATAAA   
  
  
- AATAAAAAGA GAATTAAAGA GAAAAAAGAA AAAAGAAAGA GTGTTTTTGT AGCTTTGAAT GAGACTTTTG   
  
  
- TTGCTAGCAC TTTATATAAA GATAGGTTGA CAAAAACACC TTCCAATTAG AAGAGCATAA AGTGCCATCT   
  
  
- AGTATGGAAA CATTTCAAAT CCATGCTACT AATCCCATCT ACCAAGTAAC AACACTCACT TTTAGTTTCC   
  
  
- TCTTCCCCAT TAAATACCCC TCATGCATTT CATCTCCTAA CATAAACAAA ACAAAGATCA AAGCTTACAT   
  
  
- CAAAATCTTT TTATCGATCT GAACTTCATG TGTTAAACGT TTGAAAAGAC GATAATAATT TAATGAAAAA   
  
  
- AATGATTAAG ATTTTTTATA ATAATAAACT GAATTATAAA AATACTTTAT ATTTTGATTA ATTAAAAACT   
  
  
- CGATCAGCAT ATAAATCAGA TCAATAGAAG ATCTCAAATA TGTTAAAAGA TAATTTATTT TTTATTATTA   
  
  
- CTAATTATCC TTTTTATTGT AATTAGATTT GTGTATATGA TGCTTAGGGC TTTAAGGCTT TGTTTTTGTG   
  
  
- TGTTTATGTT AGTGGGTTGA ATAGATAGAG AAAGGGAGTA GTGTGTTTAG GTGGCAATTA CTTACTAATA   
  
  
- ATAACATGTG AATGAGTGTT GTGTGCACGC TCTGGGATGG AGCGTGGGGC GGGGATCACA AGTCAGCGAA   
  
  
- GACTACAGGT AAAGGATGGA AGTGAGAGCG GTCAAAGATT CAAACCCAAA AAAAAGGAAA ATAAAAATAA   
  
  
- AAAAAGCAAA GAAAAATAAA AAGGAGATTT GGAACATTAG GCTCACACGT GGCGGAACAG CCACCGTCAT   
  
  
- TTGACCATAG CCGACACATG ACACGGCACG TGGGATACCC ATCTCGTCCC CCTGCCGCTA TCCGTCGCGA   
  
  
- GCCTGTCCTG CTGATGGGGC ACCAGCGTCA GCGTCCCTTT GGAATTTTTC TCTTAATTTC TTGTGCTCAC   
  
  
- GGAAACCTGA ATTTAATAAA TCCTTTATTT ATTTATTATT TTATTTATTA TTTTATTTAT TTTTCATGGC   
  
  
- CACTTTTTAT TTTTTAATTT AATTTTCGTT ACCAAGTAAG AAGATCATTA ATTATATCCA CACACCCAAT   
  
  
- TAATATATTT ATTATTTTAT TTAATAAATA ATTATTAATT AAGATTTTTA TTTGCTTAAA AATAAACAAA   
  
  
- TCTCTGATAA AACATTGGAC GAGGAGTTGA ATCAATTTTA ATGTTTTTAA AATTTATTAA ATAATATTTT   
  
  
- TAATTAAATG CAAAATATTT TGATGTGTAA TACTAAATCA TTTCAAGTGT TTAAAATATT TAGATATATT   
  
  
- AATGCTGGAA GTGAAAATCT CTTGTTCAT

+     Unnamed\_\_3

| Site Name | Organism | Position | Strand | Matrix score. | sequence | function |
| --- | --- | --- | --- | --- | --- | --- |
| Unnamed\_\_3 | Zea mays | 1069 | + | 5 | CGTGG |  |

> 2018/04/13 10:10:12  
+ CCTTCTTCTA CCTCTCGGAC GTCTTGTTCT TCTTCTTTTT CTTCTCTCTT TCTCTCTCGA ATATCTATAA   
  
  
+ TCGAATTAAA CACAACCTGA AAATCGAAGT GTATAGAGAG GAAAATACAT CAAACTCAAA CTATATATTT   
  
  
+ TTATTTTTCT CTTAATTTCT CTTTTTTCTT TTTTCTTTCT CACAAAAACA TCGAAACTTA CTCTGAAAAC   
  
  
+ AACGATCGTG AAATATATTT CTATCCAACT GTTTTTGTGG AAGGTTAATC TTCTCGTATT TCACGGTAGA   
  
  
+ TCATACCTTT GTAAAGTTTA GGTACGATGA TTAGGGTAGA TGGTTCATTG TTGTGAGTGA AAATCAAAGG   
  
  
+ AGAAGGGGTA ATTTATGGGG AGTACGTAAA GTAGAGGATT GTATTTGTTT TGTTTCTAGT TTCGAATGTA   
  
  
+ GTTTTAGAAA AATAGCTAGA CTTGAAGTAC ACAATTTGCA AACTTTTCTG CTATTATTAA ATTACTTTTT   
  
  
+ TTACTAATTC TAAAAAATAT TATTATTTGA CTTAATATTT TTATGAAATA TAAAACTAAT TAATTTTTGA   
  
  
+ GCTAGTCGTA TATTTAGTCT AGTTATCTTC TAGAGTTTAT ACAATTTTCT ATTAAATAAA AAATAATAAT   
  
  
+ GATTAATAGG AAAAATAACA TTAATCTAAA CACATATACT ACGAATCCCG AAATTCCGAA ACAAAAACAC   
  
  
+ ACAAATACAA TCACCCAACT TATCTATCTC TTTCCCTCAT CACACAAATC CACCGTTAAT GAATGATTAT   
  
  
+ TATTGTACAC TTACTCACAA CACACGTGCG AGACCCTACC TCGCACCCCG CCCCTAGTGT TCAGTCGCTT   
  
  
+ CTGATGTCCA TTTCCTACCT TCACTCTCGC CAGTTTCTAA GTTTGGGTTT TTTTTCCTTT TATTTTTATT   
  
  
+ TTTTTCGTTT CTTTTTATTT TTCCTCTAAA CCTTGTAATC CGAGTGTGCA CCGCCTTGTC GGTGGCAGTA   
  
  
+ AACTGGTATC GGCTGTGTAC TGTGCCGTGC ACCCTATGGG TAGAGCAGGG GGACGGCGAT AGGCAGCGCT   
  
  
+ CGGACAGGAC GACTACCCCG TGGTCGCAGT CGCAGGGAAA CCTTAAAAAG AGAATTAAAG AACACGAGTG   
  
  
+ CCTTTGGACT TAAATTATTT AGGAAATAAA TAAATAATAA AATAAATAAT AAAATAAATA AAAAGTACCG   
  
  
+ GTGAAAAATA AAAAATTAAA TTAAAAGCAA TGGTTCATTC TTCTAGTAAT TAATATAGGT GTGTGGGTTA   
  
  
+ ATTATATAAA TAATAAAATA AATTATTTAT TAATAATTAA TTCTAAAAAT AAACGAATTT TTATTTGTTT   
  
  
+ AGAGACTATT TTGTAACCTG CTCCTCAACT TAGTTAAAAT TACAAAAATT TTAAATAATT TATTATAAAA   
  
  
+ ATTAATTTAC GTTTTATAAA ACTACACATT ATGATTTAGT AAAGTTCACA AATTTTATAA ATCTATATAA   
  
  
+ TTACGACCTT CACTTTTAGA GAACAAGTA  

- GGAAGAAGAT GGAGAGCCTG CAGAACAAGA AGAAGAAAAA GAAGAGAGAA AGAGAGAGCT TATAGATATT   
  
  
- AGCTTAATTT GTGTTGGACT TTTAGCTTCA CATATCTCTC CTTTTATGTA GTTTGAGTTT GATATATAAA   
  
  
- AATAAAAAGA GAATTAAAGA GAAAAAAGAA AAAAGAAAGA GTGTTTTTGT AGCTTTGAAT GAGACTTTTG   
  
  
- TTGCTAGCAC TTTATATAAA GATAGGTTGA CAAAAACACC TTCCAATTAG AAGAGCATAA AGTGCCATCT   
  
  
- AGTATGGAAA CATTTCAAAT CCATGCTACT AATCCCATCT ACCAAGTAAC AACACTCACT TTTAGTTTCC   
  
  
- TCTTCCCCAT TAAATACCCC TCATGCATTT CATCTCCTAA CATAAACAAA ACAAAGATCA AAGCTTACAT   
  
  
- CAAAATCTTT TTATCGATCT GAACTTCATG TGTTAAACGT TTGAAAAGAC GATAATAATT TAATGAAAAA   
  
  
- AATGATTAAG ATTTTTTATA ATAATAAACT GAATTATAAA AATACTTTAT ATTTTGATTA ATTAAAAACT   
  
  
- CGATCAGCAT ATAAATCAGA TCAATAGAAG ATCTCAAATA TGTTAAAAGA TAATTTATTT TTTATTATTA   
  
  
- CTAATTATCC TTTTTATTGT AATTAGATTT GTGTATATGA TGCTTAGGGC TTTAAGGCTT TGTTTTTGTG   
  
  
- TGTTTATGTT AGTGGGTTGA ATAGATAGAG AAAGGGAGTA GTGTGTTTAG GTGGCAATTA CTTACTAATA   
  
  
- ATAACATGTG AATGAGTGTT GTGTGCACGC TCTGGGATGG AGCGTGGGGC GGGGATCACA AGTCAGCGAA   
  
  
- GACTACAGGT AAAGGATGGA AGTGAGAGCG GTCAAAGATT CAAACCCAAA AAAAAGGAAA ATAAAAATAA   
  
  
- AAAAAGCAAA GAAAAATAAA AAGGAGATTT GGAACATTAG GCTCACACGT GGCGGAACAG CCACCGTCAT   
  
  
- TTGACCATAG CCGACACATG ACACGGCACG TGGGATACCC ATCTCGTCCC CCTGCCGCTA TCCGTCGCGA   
  
  
- GCCTGTCCTG CTGATGGGGC ACCAGCGTCA GCGTCCCTTT GGAATTTTTC TCTTAATTTC TTGTGCTCAC   
  
  
- GGAAACCTGA ATTTAATAAA TCCTTTATTT ATTTATTATT TTATTTATTA TTTTATTTAT TTTTCATGGC   
  
  
- CACTTTTTAT TTTTTAATTT AATTTTCGTT ACCAAGTAAG AAGATCATTA ATTATATCCA CACACCCAAT   
  
  
- TAATATATTT ATTATTTTAT TTAATAAATA ATTATTAATT AAGATTTTTA TTTGCTTAAA AATAAACAAA   
  
  
- TCTCTGATAA AACATTGGAC GAGGAGTTGA ATCAATTTTA ATGTTTTTAA AATTTATTAA ATAATATTTT   
  
  
- TAATTAAATG CAAAATATTT TGATGTGTAA TACTAAATCA TTTCAAGTGT TTAAAATATT TAGATATATT   
  
  
- AATGCTGGAA GTGAAAATCT CTTGTTCAT

+     Unnamed\_\_4

| Site Name | Organism | Position | Strand | Matrix score. | sequence | function |
| --- | --- | --- | --- | --- | --- | --- |
| Unnamed\_\_4 | Petroselinum hortense | 1351 | + | 4 | CTCC |  |
| Unnamed\_\_4 | Petroselinum hortense | 369 | - | 4 | CTCC |  |
| Unnamed\_\_4 | Petroselinum hortense | 349 | - | 4 | CTCC |  |

> 2018/04/13 10:10:12  
+ CCTTCTTCTA CCTCTCGGAC GTCTTGTTCT TCTTCTTTTT CTTCTCTCTT TCTCTCTCGA ATATCTATAA   
  
  
+ TCGAATTAAA CACAACCTGA AAATCGAAGT GTATAGAGAG GAAAATACAT CAAACTCAAA CTATATATTT   
  
  
+ TTATTTTTCT CTTAATTTCT CTTTTTTCTT TTTTCTTTCT CACAAAAACA TCGAAACTTA CTCTGAAAAC   
  
  
+ AACGATCGTG AAATATATTT CTATCCAACT GTTTTTGTGG AAGGTTAATC TTCTCGTATT TCACGGTAGA   
  
  
+ TCATACCTTT GTAAAGTTTA GGTACGATGA TTAGGGTAGA TGGTTCATTG TTGTGAGTGA AAATCAAAGG   
  
  
+ AGAAGGGGTA ATTTATGGGG AGTACGTAAA GTAGAGGATT GTATTTGTTT TGTTTCTAGT TTCGAATGTA   
  
  
+ GTTTTAGAAA AATAGCTAGA CTTGAAGTAC ACAATTTGCA AACTTTTCTG CTATTATTAA ATTACTTTTT   
  
  
+ TTACTAATTC TAAAAAATAT TATTATTTGA CTTAATATTT TTATGAAATA TAAAACTAAT TAATTTTTGA   
  
  
+ GCTAGTCGTA TATTTAGTCT AGTTATCTTC TAGAGTTTAT ACAATTTTCT ATTAAATAAA AAATAATAAT   
  
  
+ GATTAATAGG AAAAATAACA TTAATCTAAA CACATATACT ACGAATCCCG AAATTCCGAA ACAAAAACAC   
  
  
+ ACAAATACAA TCACCCAACT TATCTATCTC TTTCCCTCAT CACACAAATC CACCGTTAAT GAATGATTAT   
  
  
+ TATTGTACAC TTACTCACAA CACACGTGCG AGACCCTACC TCGCACCCCG CCCCTAGTGT TCAGTCGCTT   
  
  
+ CTGATGTCCA TTTCCTACCT TCACTCTCGC CAGTTTCTAA GTTTGGGTTT TTTTTCCTTT TATTTTTATT   
  
  
+ TTTTTCGTTT CTTTTTATTT TTCCTCTAAA CCTTGTAATC CGAGTGTGCA CCGCCTTGTC GGTGGCAGTA   
  
  
+ AACTGGTATC GGCTGTGTAC TGTGCCGTGC ACCCTATGGG TAGAGCAGGG GGACGGCGAT AGGCAGCGCT   
  
  
+ CGGACAGGAC GACTACCCCG TGGTCGCAGT CGCAGGGAAA CCTTAAAAAG AGAATTAAAG AACACGAGTG   
  
  
+ CCTTTGGACT TAAATTATTT AGGAAATAAA TAAATAATAA AATAAATAAT AAAATAAATA AAAAGTACCG   
  
  
+ GTGAAAAATA AAAAATTAAA TTAAAAGCAA TGGTTCATTC TTCTAGTAAT TAATATAGGT GTGTGGGTTA   
  
  
+ ATTATATAAA TAATAAAATA AATTATTTAT TAATAATTAA TTCTAAAAAT AAACGAATTT TTATTTGTTT   
  
  
+ AGAGACTATT TTGTAACCTG CTCCTCAACT TAGTTAAAAT TACAAAAATT TTAAATAATT TATTATAAAA   
  
  
+ ATTAATTTAC GTTTTATAAA ACTACACATT ATGATTTAGT AAAGTTCACA AATTTTATAA ATCTATATAA   
  
  
+ TTACGACCTT CACTTTTAGA GAACAAGTA  

- GGAAGAAGAT GGAGAGCCTG CAGAACAAGA AGAAGAAAAA GAAGAGAGAA AGAGAGAGCT TATAGATATT   
  
  
- AGCTTAATTT GTGTTGGACT TTTAGCTTCA CATATCTCTC CTTTTATGTA GTTTGAGTTT GATATATAAA   
  
  
- AATAAAAAGA GAATTAAAGA GAAAAAAGAA AAAAGAAAGA GTGTTTTTGT AGCTTTGAAT GAGACTTTTG   
  
  
- TTGCTAGCAC TTTATATAAA GATAGGTTGA CAAAAACACC TTCCAATTAG AAGAGCATAA AGTGCCATCT   
  
  
- AGTATGGAAA CATTTCAAAT CCATGCTACT AATCCCATCT ACCAAGTAAC AACACTCACT TTTAGTTTCC   
  
  
- TCTTCCCCAT TAAATACCCC TCATGCATTT CATCTCCTAA CATAAACAAA ACAAAGATCA AAGCTTACAT   
  
  
- CAAAATCTTT TTATCGATCT GAACTTCATG TGTTAAACGT TTGAAAAGAC GATAATAATT TAATGAAAAA   
  
  
- AATGATTAAG ATTTTTTATA ATAATAAACT GAATTATAAA AATACTTTAT ATTTTGATTA ATTAAAAACT   
  
  
- CGATCAGCAT ATAAATCAGA TCAATAGAAG ATCTCAAATA TGTTAAAAGA TAATTTATTT TTTATTATTA   
  
  
- CTAATTATCC TTTTTATTGT AATTAGATTT GTGTATATGA TGCTTAGGGC TTTAAGGCTT TGTTTTTGTG   
  
  
- TGTTTATGTT AGTGGGTTGA ATAGATAGAG AAAGGGAGTA GTGTGTTTAG GTGGCAATTA CTTACTAATA   
  
  
- ATAACATGTG AATGAGTGTT GTGTGCACGC TCTGGGATGG AGCGTGGGGC GGGGATCACA AGTCAGCGAA   
  
  
- GACTACAGGT AAAGGATGGA AGTGAGAGCG GTCAAAGATT CAAACCCAAA AAAAAGGAAA ATAAAAATAA   
  
  
- AAAAAGCAAA GAAAAATAAA AAGGAGATTT GGAACATTAG GCTCACACGT GGCGGAACAG CCACCGTCAT   
  
  
- TTGACCATAG CCGACACATG ACACGGCACG TGGGATACCC ATCTCGTCCC CCTGCCGCTA TCCGTCGCGA   
  
  
- GCCTGTCCTG CTGATGGGGC ACCAGCGTCA GCGTCCCTTT GGAATTTTTC TCTTAATTTC TTGTGCTCAC   
  
  
- GGAAACCTGA ATTTAATAAA TCCTTTATTT ATTTATTATT TTATTTATTA TTTTATTTAT TTTTCATGGC   
  
  
- CACTTTTTAT TTTTTAATTT AATTTTCGTT ACCAAGTAAG AAGATCATTA ATTATATCCA CACACCCAAT   
  
  
- TAATATATTT ATTATTTTAT TTAATAAATA ATTATTAATT AAGATTTTTA TTTGCTTAAA AATAAACAAA   
  
  
- TCTCTGATAA AACATTGGAC GAGGAGTTGA ATCAATTTTA ATGTTTTTAA AATTTATTAA ATAATATTTT   
  
  
- TAATTAAATG CAAAATATTT TGATGTGTAA TACTAAATCA TTTCAAGTGT TTAAAATATT TAGATATATT   
  
  
- AATGCTGGAA GTGAAAATCT CTTGTTCAT

+     circadian

| Site Name | Organism | Position | Strand | Matrix score. | sequence | function |
| --- | --- | --- | --- | --- | --- | --- |
| circadian | Lycopersicon esculentum | 183 | + | 6 | CAANNNNATC | cis-acting regulatory element involved in circadian control |

> 2018/04/13 10:10:12  
+ CCTTCTTCTA CCTCTCGGAC GTCTTGTTCT TCTTCTTTTT CTTCTCTCTT TCTCTCTCGA ATATCTATAA   
  
  
+ TCGAATTAAA CACAACCTGA AAATCGAAGT GTATAGAGAG GAAAATACAT CAAACTCAAA CTATATATTT   
  
  
+ TTATTTTTCT CTTAATTTCT CTTTTTTCTT TTTTCTTTCT CACAAAAACA TCGAAACTTA CTCTGAAAAC   
  
  
+ AACGATCGTG AAATATATTT CTATCCAACT GTTTTTGTGG AAGGTTAATC TTCTCGTATT TCACGGTAGA   
  
  
+ TCATACCTTT GTAAAGTTTA GGTACGATGA TTAGGGTAGA TGGTTCATTG TTGTGAGTGA AAATCAAAGG   
  
  
+ AGAAGGGGTA ATTTATGGGG AGTACGTAAA GTAGAGGATT GTATTTGTTT TGTTTCTAGT TTCGAATGTA   
  
  
+ GTTTTAGAAA AATAGCTAGA CTTGAAGTAC ACAATTTGCA AACTTTTCTG CTATTATTAA ATTACTTTTT   
  
  
+ TTACTAATTC TAAAAAATAT TATTATTTGA CTTAATATTT TTATGAAATA TAAAACTAAT TAATTTTTGA   
  
  
+ GCTAGTCGTA TATTTAGTCT AGTTATCTTC TAGAGTTTAT ACAATTTTCT ATTAAATAAA AAATAATAAT   
  
  
+ GATTAATAGG AAAAATAACA TTAATCTAAA CACATATACT ACGAATCCCG AAATTCCGAA ACAAAAACAC   
  
  
+ ACAAATACAA TCACCCAACT TATCTATCTC TTTCCCTCAT CACACAAATC CACCGTTAAT GAATGATTAT   
  
  
+ TATTGTACAC TTACTCACAA CACACGTGCG AGACCCTACC TCGCACCCCG CCCCTAGTGT TCAGTCGCTT   
  
  
+ CTGATGTCCA TTTCCTACCT TCACTCTCGC CAGTTTCTAA GTTTGGGTTT TTTTTCCTTT TATTTTTATT   
  
  
+ TTTTTCGTTT CTTTTTATTT TTCCTCTAAA CCTTGTAATC CGAGTGTGCA CCGCCTTGTC GGTGGCAGTA   
  
  
+ AACTGGTATC GGCTGTGTAC TGTGCCGTGC ACCCTATGGG TAGAGCAGGG GGACGGCGAT AGGCAGCGCT   
  
  
+ CGGACAGGAC GACTACCCCG TGGTCGCAGT CGCAGGGAAA CCTTAAAAAG AGAATTAAAG AACACGAGTG   
  
  
+ CCTTTGGACT TAAATTATTT AGGAAATAAA TAAATAATAA AATAAATAAT AAAATAAATA AAAAGTACCG   
  
  
+ GTGAAAAATA AAAAATTAAA TTAAAAGCAA TGGTTCATTC TTCTAGTAAT TAATATAGGT GTGTGGGTTA   
  
  
+ ATTATATAAA TAATAAAATA AATTATTTAT TAATAATTAA TTCTAAAAAT AAACGAATTT TTATTTGTTT   
  
  
+ AGAGACTATT TTGTAACCTG CTCCTCAACT TAGTTAAAAT TACAAAAATT TTAAATAATT TATTATAAAA   
  
  
+ ATTAATTTAC GTTTTATAAA ACTACACATT ATGATTTAGT AAAGTTCACA AATTTTATAA ATCTATATAA   
  
  
+ TTACGACCTT CACTTTTAGA GAACAAGTA  

- GGAAGAAGAT GGAGAGCCTG CAGAACAAGA AGAAGAAAAA GAAGAGAGAA AGAGAGAGCT TATAGATATT   
  
  
- AGCTTAATTT GTGTTGGACT TTTAGCTTCA CATATCTCTC CTTTTATGTA GTTTGAGTTT GATATATAAA   
  
  
- AATAAAAAGA GAATTAAAGA GAAAAAAGAA AAAAGAAAGA GTGTTTTTGT AGCTTTGAAT GAGACTTTTG   
  
  
- TTGCTAGCAC TTTATATAAA GATAGGTTGA CAAAAACACC TTCCAATTAG AAGAGCATAA AGTGCCATCT   
  
  
- AGTATGGAAA CATTTCAAAT CCATGCTACT AATCCCATCT ACCAAGTAAC AACACTCACT TTTAGTTTCC   
  
  
- TCTTCCCCAT TAAATACCCC TCATGCATTT CATCTCCTAA CATAAACAAA ACAAAGATCA AAGCTTACAT   
  
  
- CAAAATCTTT TTATCGATCT GAACTTCATG TGTTAAACGT TTGAAAAGAC GATAATAATT TAATGAAAAA   
  
  
- AATGATTAAG ATTTTTTATA ATAATAAACT GAATTATAAA AATACTTTAT ATTTTGATTA ATTAAAAACT   
  
  
- CGATCAGCAT ATAAATCAGA TCAATAGAAG ATCTCAAATA TGTTAAAAGA TAATTTATTT TTTATTATTA   
  
  
- CTAATTATCC TTTTTATTGT AATTAGATTT GTGTATATGA TGCTTAGGGC TTTAAGGCTT TGTTTTTGTG   
  
  
- TGTTTATGTT AGTGGGTTGA ATAGATAGAG AAAGGGAGTA GTGTGTTTAG GTGGCAATTA CTTACTAATA   
  
  
- ATAACATGTG AATGAGTGTT GTGTGCACGC TCTGGGATGG AGCGTGGGGC GGGGATCACA AGTCAGCGAA   
  
  
- GACTACAGGT AAAGGATGGA AGTGAGAGCG GTCAAAGATT CAAACCCAAA AAAAAGGAAA ATAAAAATAA   
  
  
- AAAAAGCAAA GAAAAATAAA AAGGAGATTT GGAACATTAG GCTCACACGT GGCGGAACAG CCACCGTCAT   
  
  
- TTGACCATAG CCGACACATG ACACGGCACG TGGGATACCC ATCTCGTCCC CCTGCCGCTA TCCGTCGCGA   
  
  
- GCCTGTCCTG CTGATGGGGC ACCAGCGTCA GCGTCCCTTT GGAATTTTTC TCTTAATTTC TTGTGCTCAC   
  
  
- GGAAACCTGA ATTTAATAAA TCCTTTATTT ATTTATTATT TTATTTATTA TTTTATTTAT TTTTCATGGC   
  
  
- CACTTTTTAT TTTTTAATTT AATTTTCGTT ACCAAGTAAG AAGATCATTA ATTATATCCA CACACCCAAT   
  
  
- TAATATATTT ATTATTTTAT TTAATAAATA ATTATTAATT AAGATTTTTA TTTGCTTAAA AATAAACAAA   
  
  
- TCTCTGATAA AACATTGGAC GAGGAGTTGA ATCAATTTTA ATGTTTTTAA AATTTATTAA ATAATATTTT   
  
  
- TAATTAAATG CAAAATATTT TGATGTGTAA TACTAAATCA TTTCAAGTGT TTAAAATATT TAGATATATT   
  
  
- AATGCTGGAA GTGAAAATCT CTTGTTCAT
