# Supplementary material for: Genome-wide analysis of the WRKY gene family in drumstick (Moringa oleifera Lam.)
Source: PeerJ. 2019 Jun 10;7:e7063. doi: 10.7717/peerj.7063 (PMC6563795; doi:10.7717/peerj.7063)
Supplement: Supplemental Information 1 [file peerj-07-7063-s003.gz › MoWRKY14_plantcare.html]

Content-Type: text/html; charset=ISO-8859-1


CallMat\_Firefox


Webmaster Firefox specific output  
To save the result:
click on the frame with the right mouse button and save the source code as a text file with extension .html  
REFERENCE:PlantCARE: a database of plant cis-acting regulatory elements and a portal to tools for in silico analysis of promoter sequences.  
Lescot, M., Déhais, P., Moreau, Y., De Moor, B., Rouzé ,P.,and Rombauts, S.  
Nucleic Acids Res., Database issue(2002), 30(1):325-327.   


---

> 2018/04/13 10:10:12  
+ TTAATTTTCA GGTGCTCTCA TTCGTTTTCA CCTTCGCCAA TTGTGAAGTT GAAGCCACAT TTTTCTAGGG   
  
  
+ GCTACGATCT TGAAATAATG TCGATTTGAT TTTGTAGATA CCTTTTAATC ATTCTTCTAA TAGAATCATC   
  
  
+ TTTTTTTTTT TTTGGGTTGA TCTCATTTTG TTAAATTGAA TAAATTTAAT TGAATGAATT GAAAATTCTT   
  
  
+ TTTTTTTAAG GATATAAATT TTGTTGGTAT TTTAAAAAAA TATTTTTTAT TAAATACTTT TTAAAAAGAT   
  
  
+ GTTGATATAT ATATATATAT GTTAGAAAAT AAAGGATATA AATTTTGTTA GTATTTTAAA AAAATATTTT   
  
  
+ TTATTAAATA CTTTTTAAAA AGATGTTGAT ATATATATAT ATGTTAGAAA ATACAATAAA ATTTATTGCA   
  
  
+ TTTGTTTTCT TGATTAATGT AACCAATTAT AAAAATAGAA TTTTAAAAAA AAGATTAATA AGAATCATAG   
  
  
+ TAACAGATGA TTTTTTGATT CAAAGGAAAC ATTTCTTCAA TATTTTAAAG ATTCATTATT AAAAATGATT   
  
  
+ AGAAATTATG GTAAAATCTA TGAAATTTTT AAAAAATATA AATAGTATTG AAGTAAGAAG GGTAGTTATA   
  
  
+ TTTTTATAAC TTAAATAATA ATATAAAATA AAAAATGTAA CCATTAATTT TAATTAAATT ACCATGTATC   
  
  
+ TTAATTTATT TAAGTCGAAA ATTTGATCCT TTCTTAATTT TATTTATTAA GACACGTGAA AATACCTTTT   
  
  
+ TGCTTAATTA ATTGAATAAG AGCCACGCAT AGCATAACAA CATTATCTAT TTGCCCATTT CATGAGCAAT   
  
  
+ AAAGAATTCC ACTGACTGAA AAGAATACAA GACCCCCCAA AAAAAAAAGA AAAAAAGAGA AAAGAGAAAA   
  
  
+ AAAAAGATGA AAAAAATGAT TAAAGGGAAA AGAAGAAAAG GGAATATCTT AGGAGGATTC AGTTGGTGAG   
  
  
+ CTTGAATTGA GTTCACACAG AGGAGAGTTC ACATGCAAGA CGACGGAGCT TGGGGCTGTA ATGGGTAAGA   
  
  
+ ATCTCACGTG CAGAGAAACC CCGTTCTCAC TGGGGGAACC TGTTAACTGT TGAGCCGGCC AACGAAGCGT   
  
  
+ ATGAAAACGT CGGCCAACCA CGACAGAATA CTCGGCCGCA TGCATTGAAT TTGCCACCGG CACTGTGGGT   
  
  
+ GTCCGTACGA AATGTTGACC TACGATCTCC GGTCAATCTT TGTTGCCATT TGTCTACTCT CGAGCAGAAT   
  
  
+ AGAAAAGTAA CTCACCTTCT CTTCTCTTTG GCCAAATCTC ACTTTCTTCT ATACCTAAAG CTCTCTCTCT   
  
  
+ CTCTCTCTCC CCCTCCCACC TACCTGATAA ACGCAATCAG GCCTAGCTTT CTCTCTCTCT CTCTCTCTCT   
  
  
+ CTTTCTTTCT CAGTAAGCAA ACATCACTCC CGCTGTGTGT CACTCTCTGT TAAGTTACAA GCTTTTATAT   
  
  
+ GAGGATTTTA TAGGCCTGAT TGATAGAAA  

- AATTAAAAGT CCACGAGAGT AAGCAAAAGT GGAAGCGGTT AACACTTCAA CTTCGGTGTA AAAAGATCCC   
  
  
- CGATGCTAGA ACTTTATTAC AGCTAAACTA AAACATCTAT GGAAAATTAG TAAGAAGATT ATCTTAGTAG   
  
  
- AAAAAAAAAA AAACCCAACT AGAGTAAAAC AATTTAACTT ATTTAAATTA ACTTACTTAA CTTTTAAGAA   
  
  
- AAAAAAATTC CTATATTTAA AACAACCATA AAATTTTTTT ATAAAAAATA ATTTATGAAA AATTTTTCTA   
  
  
- CAACTATATA TATATATATA CAATCTTTTA TTTCCTATAT TTAAAACAAT CATAAAATTT TTTTATAAAA   
  
  
- AATAATTTAT GAAAAATTTT TCTACAACTA TATATATATA TACAATCTTT TATGTTATTT TAAATAACGT   
  
  
- AAACAAAAGA ACTAATTACA TTGGTTAATA TTTTTATCTT AAAATTTTTT TTCTAATTAT TCTTAGTATC   
  
  
- ATTGTCTACT AAAAAACTAA GTTTCCTTTG TAAAGAAGTT ATAAAATTTC TAAGTAATAA TTTTTACTAA   
  
  
- TCTTTAATAC CATTTTAGAT ACTTTAAAAA TTTTTTATAT TTATCATAAC TTCATTCTTC CCATCAATAT   
  
  
- AAAAATATTG AATTTATTAT TATATTTTAT TTTTTACATT GGTAATTAAA ATTAATTTAA TGGTACATAG   
  
  
- AATTAAATAA ATTCAGCTTT TAAACTAGGA AAGAATTAAA ATAAATAATT CTGTGCACTT TTATGGAAAA   
  
  
- ACGAATTAAT TAACTTATTC TCGGTGCGTA TCGTATTGTT GTAATAGATA AACGGGTAAA GTACTCGTTA   
  
  
- TTTCTTAAGG TGACTGACTT TTCTTATGTT CTGGGGGGTT TTTTTTTTCT TTTTTTCTCT TTTCTCTTTT   
  
  
- TTTTTCTACT TTTTTTACTA ATTTCCCTTT TCTTCTTTTC CCTTATAGAA TCCTCCTAAG TCAACCACTC   
  
  
- GAACTTAACT CAAGTGTGTC TCCTCTCAAG TGTACGTTCT GCTGCCTCGA ACCCCGACAT TACCCATTCT   
  
  
- TAGAGTGCAC GTCTCTTTGG GGCAAGAGTG ACCCCCTTGG ACAATTGACA ACTCGGCCGG TTGCTTCGCA   
  
  
- TACTTTTGCA GCCGGTTGGT GCTGTCTTAT GAGCCGGCGT ACGTAACTTA AACGGTGGCC GTGACACCCA   
  
  
- CAGGCATGCT TTACAACTGG ATGCTAGAGG CCAGTTAGAA ACAACGGTAA ACAGATGAGA GCTCGTCTTA   
  
  
- TCTTTTCATT GAGTGGAAGA GAAGAGAAAC CGGTTTAGAG TGAAAGAAGA TATGGATTTC GAGAGAGAGA   
  
  
- GAGAGAGAGG GGGAGGGTGG ATGGACTATT TGCGTTAGTC CGGATCGAAA GAGAGAGAGA GAGAGAGAGA   
  
  
- GAAAGAAAGA GTCATTCGTT TGTAGTGAGG GCGACACACA GTGAGAGACA ATTCAATGTT CGAAAATATA   
  
  
- CTCCTAAAAT ATCCGGACTA ACTATCTTT

  
  
Motifs Found  

+     5UTR Py-rich stretch

| Site Name | Organism | Position | Strand | Matrix score. | sequence | function |
| --- | --- | --- | --- | --- | --- | --- |
| 5UTR Py-rich stretch | Lycopersicon esculentum | 903 | - | 9 | TTTCTTCTCT | cis-acting element conferring high transcription levels |
| 5UTR Py-rich stretch | Lycopersicon esculentum | 1380 | + | 13 | TTTCTCTCTCTCTC | cis-acting element conferring high transcription levels |
| 5UTR Py-rich stretch | Lycopersicon esculentum | 1322 | + | 13 | TTTCTCTCTCTCTC | cis-acting element conferring high transcription levels |
| 5UTR Py-rich stretch | Lycopersicon esculentum | 1388 | + | 13 | TTTCTCTCTCTCTC | cis-acting element conferring high transcription levels |
| 5UTR Py-rich stretch | Lycopersicon esculentum | 1278 | + | 9 | TTTCTTCTCT | cis-acting element conferring high transcription levels |
| 5UTR Py-rich stretch | Lycopersicon esculentum | 1384 | + | 13 | TTTCTCTCTCTCTC | cis-acting element conferring high transcription levels |
| 5UTR Py-rich stretch | Lycopersicon esculentum | 1326 | + | 13 | TTTCTCTCTCTCTC | cis-acting element conferring high transcription levels |
| 5UTR Py-rich stretch | Lycopersicon esculentum | 1382 | + | 13 | TTTCTCTCTCTCTC | cis-acting element conferring high transcription levels |
| 5UTR Py-rich stretch | Lycopersicon esculentum | 939 | - | 9 | TTTCTTCTCT | cis-acting element conferring high transcription levels |
| 5UTR Py-rich stretch | Lycopersicon esculentum | 1386 | + | 13 | TTTCTCTCTCTCTC | cis-acting element conferring high transcription levels |
| 5UTR Py-rich stretch | Lycopersicon esculentum | 1324 | + | 13 | TTTCTCTCTCTCTC | cis-acting element conferring high transcription levels |
| 5UTR Py-rich stretch | Lycopersicon esculentum | 1303 | + | 9 | TTTCTTCTCT | cis-acting element conferring high transcription levels |
| 5UTR Py-rich stretch | Lycopersicon esculentum | 1378 | + | 14 | TTTCTCTCTCTCTC | cis-acting element conferring high transcription levels |

> 2018/04/13 10:10:12  
+ TTAATTTTCA GGTGCTCTCA TTCGTTTTCA CCTTCGCCAA TTGTGAAGTT GAAGCCACAT TTTTCTAGGG   
  
  
+ GCTACGATCT TGAAATAATG TCGATTTGAT TTTGTAGATA CCTTTTAATC ATTCTTCTAA TAGAATCATC   
  
  
+ TTTTTTTTTT TTTGGGTTGA TCTCATTTTG TTAAATTGAA TAAATTTAAT TGAATGAATT GAAAATTCTT   
  
  
+ TTTTTTTAAG GATATAAATT TTGTTGGTAT TTTAAAAAAA TATTTTTTAT TAAATACTTT TTAAAAAGAT   
  
  
+ GTTGATATAT ATATATATAT GTTAGAAAAT AAAGGATATA AATTTTGTTA GTATTTTAAA AAAATATTTT   
  
  
+ TTATTAAATA CTTTTTAAAA AGATGTTGAT ATATATATAT ATGTTAGAAA ATACAATAAA ATTTATTGCA   
  
  
+ TTTGTTTTCT TGATTAATGT AACCAATTAT AAAAATAGAA TTTTAAAAAA AAGATTAATA AGAATCATAG   
  
  
+ TAACAGATGA TTTTTTGATT CAAAGGAAAC ATTTCTTCAA TATTTTAAAG ATTCATTATT AAAAATGATT   
  
  
+ AGAAATTATG GTAAAATCTA TGAAATTTTT AAAAAATATA AATAGTATTG AAGTAAGAAG GGTAGTTATA   
  
  
+ TTTTTATAAC TTAAATAATA ATATAAAATA AAAAATGTAA CCATTAATTT TAATTAAATT ACCATGTATC   
  
  
+ TTAATTTATT TAAGTCGAAA ATTTGATCCT TTCTTAATTT TATTTATTAA GACACGTGAA AATACCTTTT   
  
  
+ TGCTTAATTA ATTGAATAAG AGCCACGCAT AGCATAACAA CATTATCTAT TTGCCCATTT CATGAGCAAT   
  
  
+ AAAGAATTCC ACTGACTGAA AAGAATACAA GACCCCCCAA AAAAAAAAGA AAAAAAGAGA AAAGAGAAAA   
  
  
+ AAAAAGATGA AAAAAATGAT TAAAGGGAAA AGAAGAAAAG GGAATATCTT AGGAGGATTC AGTTGGTGAG   
  
  
+ CTTGAATTGA GTTCACACAG AGGAGAGTTC ACATGCAAGA CGACGGAGCT TGGGGCTGTA ATGGGTAAGA   
  
  
+ ATCTCACGTG CAGAGAAACC CCGTTCTCAC TGGGGGAACC TGTTAACTGT TGAGCCGGCC AACGAAGCGT   
  
  
+ ATGAAAACGT CGGCCAACCA CGACAGAATA CTCGGCCGCA TGCATTGAAT TTGCCACCGG CACTGTGGGT   
  
  
+ GTCCGTACGA AATGTTGACC TACGATCTCC GGTCAATCTT TGTTGCCATT TGTCTACTCT CGAGCAGAAT   
  
  
+ AGAAAAGTAA CTCACCTTCT CTTCTCTTTG GCCAAATCTC ACTTTCTTCT ATACCTAAAG CTCTCTCTCT   
  
  
+ CTCTCTCTCC CCCTCCCACC TACCTGATAA ACGCAATCAG GCCTAGCTTT CTCTCTCTCT CTCTCTCTCT   
  
  
+ CTTTCTTTCT CAGTAAGCAA ACATCACTCC CGCTGTGTGT CACTCTCTGT TAAGTTACAA GCTTTTATAT   
  
  
+ GAGGATTTTA TAGGCCTGAT TGATAGAAA  

- AATTAAAAGT CCACGAGAGT AAGCAAAAGT GGAAGCGGTT AACACTTCAA CTTCGGTGTA AAAAGATCCC   
  
  
- CGATGCTAGA ACTTTATTAC AGCTAAACTA AAACATCTAT GGAAAATTAG TAAGAAGATT ATCTTAGTAG   
  
  
- AAAAAAAAAA AAACCCAACT AGAGTAAAAC AATTTAACTT ATTTAAATTA ACTTACTTAA CTTTTAAGAA   
  
  
- AAAAAAATTC CTATATTTAA AACAACCATA AAATTTTTTT ATAAAAAATA ATTTATGAAA AATTTTTCTA   
  
  
- CAACTATATA TATATATATA CAATCTTTTA TTTCCTATAT TTAAAACAAT CATAAAATTT TTTTATAAAA   
  
  
- AATAATTTAT GAAAAATTTT TCTACAACTA TATATATATA TACAATCTTT TATGTTATTT TAAATAACGT   
  
  
- AAACAAAAGA ACTAATTACA TTGGTTAATA TTTTTATCTT AAAATTTTTT TTCTAATTAT TCTTAGTATC   
  
  
- ATTGTCTACT AAAAAACTAA GTTTCCTTTG TAAAGAAGTT ATAAAATTTC TAAGTAATAA TTTTTACTAA   
  
  
- TCTTTAATAC CATTTTAGAT ACTTTAAAAA TTTTTTATAT TTATCATAAC TTCATTCTTC CCATCAATAT   
  
  
- AAAAATATTG AATTTATTAT TATATTTTAT TTTTTACATT GGTAATTAAA ATTAATTTAA TGGTACATAG   
  
  
- AATTAAATAA ATTCAGCTTT TAAACTAGGA AAGAATTAAA ATAAATAATT CTGTGCACTT TTATGGAAAA   
  
  
- ACGAATTAAT TAACTTATTC TCGGTGCGTA TCGTATTGTT GTAATAGATA AACGGGTAAA GTACTCGTTA   
  
  
- TTTCTTAAGG TGACTGACTT TTCTTATGTT CTGGGGGGTT TTTTTTTTCT TTTTTTCTCT TTTCTCTTTT   
  
  
- TTTTTCTACT TTTTTTACTA ATTTCCCTTT TCTTCTTTTC CCTTATAGAA TCCTCCTAAG TCAACCACTC   
  
  
- GAACTTAACT CAAGTGTGTC TCCTCTCAAG TGTACGTTCT GCTGCCTCGA ACCCCGACAT TACCCATTCT   
  
  
- TAGAGTGCAC GTCTCTTTGG GGCAAGAGTG ACCCCCTTGG ACAATTGACA ACTCGGCCGG TTGCTTCGCA   
  
  
- TACTTTTGCA GCCGGTTGGT GCTGTCTTAT GAGCCGGCGT ACGTAACTTA AACGGTGGCC GTGACACCCA   
  
  
- CAGGCATGCT TTACAACTGG ATGCTAGAGG CCAGTTAGAA ACAACGGTAA ACAGATGAGA GCTCGTCTTA   
  
  
- TCTTTTCATT GAGTGGAAGA GAAGAGAAAC CGGTTTAGAG TGAAAGAAGA TATGGATTTC GAGAGAGAGA   
  
  
- GAGAGAGAGG GGGAGGGTGG ATGGACTATT TGCGTTAGTC CGGATCGAAA GAGAGAGAGA GAGAGAGAGA   
  
  
- GAAAGAAAGA GTCATTCGTT TGTAGTGAGG GCGACACACA GTGAGAGACA ATTCAATGTT CGAAAATATA   
  
  
- CTCCTAAAAT ATCCGGACTA ACTATCTTT

+     AAGAA-motif

| Site Name | Organism | Position | Strand | Matrix score. | sequence | function |
| --- | --- | --- | --- | --- | --- | --- |
| AAGAA-motif | Avena sativa | 1044 | + | 8 | gGTAAGAA |  |
| AAGAA-motif | Avena sativa | 1403 | - | 7 | GAAAGAA |  |

> 2018/04/13 10:10:12  
+ TTAATTTTCA GGTGCTCTCA TTCGTTTTCA CCTTCGCCAA TTGTGAAGTT GAAGCCACAT TTTTCTAGGG   
  
  
+ GCTACGATCT TGAAATAATG TCGATTTGAT TTTGTAGATA CCTTTTAATC ATTCTTCTAA TAGAATCATC   
  
  
+ TTTTTTTTTT TTTGGGTTGA TCTCATTTTG TTAAATTGAA TAAATTTAAT TGAATGAATT GAAAATTCTT   
  
  
+ TTTTTTTAAG GATATAAATT TTGTTGGTAT TTTAAAAAAA TATTTTTTAT TAAATACTTT TTAAAAAGAT   
  
  
+ GTTGATATAT ATATATATAT GTTAGAAAAT AAAGGATATA AATTTTGTTA GTATTTTAAA AAAATATTTT   
  
  
+ TTATTAAATA CTTTTTAAAA AGATGTTGAT ATATATATAT ATGTTAGAAA ATACAATAAA ATTTATTGCA   
  
  
+ TTTGTTTTCT TGATTAATGT AACCAATTAT AAAAATAGAA TTTTAAAAAA AAGATTAATA AGAATCATAG   
  
  
+ TAACAGATGA TTTTTTGATT CAAAGGAAAC ATTTCTTCAA TATTTTAAAG ATTCATTATT AAAAATGATT   
  
  
+ AGAAATTATG GTAAAATCTA TGAAATTTTT AAAAAATATA AATAGTATTG AAGTAAGAAG GGTAGTTATA   
  
  
+ TTTTTATAAC TTAAATAATA ATATAAAATA AAAAATGTAA CCATTAATTT TAATTAAATT ACCATGTATC   
  
  
+ TTAATTTATT TAAGTCGAAA ATTTGATCCT TTCTTAATTT TATTTATTAA GACACGTGAA AATACCTTTT   
  
  
+ TGCTTAATTA ATTGAATAAG AGCCACGCAT AGCATAACAA CATTATCTAT TTGCCCATTT CATGAGCAAT   
  
  
+ AAAGAATTCC ACTGACTGAA AAGAATACAA GACCCCCCAA AAAAAAAAGA AAAAAAGAGA AAAGAGAAAA   
  
  
+ AAAAAGATGA AAAAAATGAT TAAAGGGAAA AGAAGAAAAG GGAATATCTT AGGAGGATTC AGTTGGTGAG   
  
  
+ CTTGAATTGA GTTCACACAG AGGAGAGTTC ACATGCAAGA CGACGGAGCT TGGGGCTGTA ATGGGTAAGA   
  
  
+ ATCTCACGTG CAGAGAAACC CCGTTCTCAC TGGGGGAACC TGTTAACTGT TGAGCCGGCC AACGAAGCGT   
  
  
+ ATGAAAACGT CGGCCAACCA CGACAGAATA CTCGGCCGCA TGCATTGAAT TTGCCACCGG CACTGTGGGT   
  
  
+ GTCCGTACGA AATGTTGACC TACGATCTCC GGTCAATCTT TGTTGCCATT TGTCTACTCT CGAGCAGAAT   
  
  
+ AGAAAAGTAA CTCACCTTCT CTTCTCTTTG GCCAAATCTC ACTTTCTTCT ATACCTAAAG CTCTCTCTCT   
  
  
+ CTCTCTCTCC CCCTCCCACC TACCTGATAA ACGCAATCAG GCCTAGCTTT CTCTCTCTCT CTCTCTCTCT   
  
  
+ CTTTCTTTCT CAGTAAGCAA ACATCACTCC CGCTGTGTGT CACTCTCTGT TAAGTTACAA GCTTTTATAT   
  
  
+ GAGGATTTTA TAGGCCTGAT TGATAGAAA  

- AATTAAAAGT CCACGAGAGT AAGCAAAAGT GGAAGCGGTT AACACTTCAA CTTCGGTGTA AAAAGATCCC   
  
  
- CGATGCTAGA ACTTTATTAC AGCTAAACTA AAACATCTAT GGAAAATTAG TAAGAAGATT ATCTTAGTAG   
  
  
- AAAAAAAAAA AAACCCAACT AGAGTAAAAC AATTTAACTT ATTTAAATTA ACTTACTTAA CTTTTAAGAA   
  
  
- AAAAAAATTC CTATATTTAA AACAACCATA AAATTTTTTT ATAAAAAATA ATTTATGAAA AATTTTTCTA   
  
  
- CAACTATATA TATATATATA CAATCTTTTA TTTCCTATAT TTAAAACAAT CATAAAATTT TTTTATAAAA   
  
  
- AATAATTTAT GAAAAATTTT TCTACAACTA TATATATATA TACAATCTTT TATGTTATTT TAAATAACGT   
  
  
- AAACAAAAGA ACTAATTACA TTGGTTAATA TTTTTATCTT AAAATTTTTT TTCTAATTAT TCTTAGTATC   
  
  
- ATTGTCTACT AAAAAACTAA GTTTCCTTTG TAAAGAAGTT ATAAAATTTC TAAGTAATAA TTTTTACTAA   
  
  
- TCTTTAATAC CATTTTAGAT ACTTTAAAAA TTTTTTATAT TTATCATAAC TTCATTCTTC CCATCAATAT   
  
  
- AAAAATATTG AATTTATTAT TATATTTTAT TTTTTACATT GGTAATTAAA ATTAATTTAA TGGTACATAG   
  
  
- AATTAAATAA ATTCAGCTTT TAAACTAGGA AAGAATTAAA ATAAATAATT CTGTGCACTT TTATGGAAAA   
  
  
- ACGAATTAAT TAACTTATTC TCGGTGCGTA TCGTATTGTT GTAATAGATA AACGGGTAAA GTACTCGTTA   
  
  
- TTTCTTAAGG TGACTGACTT TTCTTATGTT CTGGGGGGTT TTTTTTTTCT TTTTTTCTCT TTTCTCTTTT   
  
  
- TTTTTCTACT TTTTTTACTA ATTTCCCTTT TCTTCTTTTC CCTTATAGAA TCCTCCTAAG TCAACCACTC   
  
  
- GAACTTAACT CAAGTGTGTC TCCTCTCAAG TGTACGTTCT GCTGCCTCGA ACCCCGACAT TACCCATTCT   
  
  
- TAGAGTGCAC GTCTCTTTGG GGCAAGAGTG ACCCCCTTGG ACAATTGACA ACTCGGCCGG TTGCTTCGCA   
  
  
- TACTTTTGCA GCCGGTTGGT GCTGTCTTAT GAGCCGGCGT ACGTAACTTA AACGGTGGCC GTGACACCCA   
  
  
- CAGGCATGCT TTACAACTGG ATGCTAGAGG CCAGTTAGAA ACAACGGTAA ACAGATGAGA GCTCGTCTTA   
  
  
- TCTTTTCATT GAGTGGAAGA GAAGAGAAAC CGGTTTAGAG TGAAAGAAGA TATGGATTTC GAGAGAGAGA   
  
  
- GAGAGAGAGG GGGAGGGTGG ATGGACTATT TGCGTTAGTC CGGATCGAAA GAGAGAGAGA GAGAGAGAGA   
  
  
- GAAAGAAAGA GTCATTCGTT TGTAGTGAGG GCGACACACA GTGAGAGACA ATTCAATGTT CGAAAATATA   
  
  
- CTCCTAAAAT ATCCGGACTA ACTATCTTT

+     ABRE

| Site Name | Organism | Position | Strand | Matrix score. | sequence | function |
| --- | --- | --- | --- | --- | --- | --- |
| ABRE | Arabidopsis thaliana | 1055 | - | 6 | CACGTG | cis-acting element involved in the abscisic acid responsiveness |
| ABRE | Arabidopsis thaliana | 753 | - | 6 | CACGTG | cis-acting element involved in the abscisic acid responsiveness |

> 2018/04/13 10:10:12  
+ TTAATTTTCA GGTGCTCTCA TTCGTTTTCA CCTTCGCCAA TTGTGAAGTT GAAGCCACAT TTTTCTAGGG   
  
  
+ GCTACGATCT TGAAATAATG TCGATTTGAT TTTGTAGATA CCTTTTAATC ATTCTTCTAA TAGAATCATC   
  
  
+ TTTTTTTTTT TTTGGGTTGA TCTCATTTTG TTAAATTGAA TAAATTTAAT TGAATGAATT GAAAATTCTT   
  
  
+ TTTTTTTAAG GATATAAATT TTGTTGGTAT TTTAAAAAAA TATTTTTTAT TAAATACTTT TTAAAAAGAT   
  
  
+ GTTGATATAT ATATATATAT GTTAGAAAAT AAAGGATATA AATTTTGTTA GTATTTTAAA AAAATATTTT   
  
  
+ TTATTAAATA CTTTTTAAAA AGATGTTGAT ATATATATAT ATGTTAGAAA ATACAATAAA ATTTATTGCA   
  
  
+ TTTGTTTTCT TGATTAATGT AACCAATTAT AAAAATAGAA TTTTAAAAAA AAGATTAATA AGAATCATAG   
  
  
+ TAACAGATGA TTTTTTGATT CAAAGGAAAC ATTTCTTCAA TATTTTAAAG ATTCATTATT AAAAATGATT   
  
  
+ AGAAATTATG GTAAAATCTA TGAAATTTTT AAAAAATATA AATAGTATTG AAGTAAGAAG GGTAGTTATA   
  
  
+ TTTTTATAAC TTAAATAATA ATATAAAATA AAAAATGTAA CCATTAATTT TAATTAAATT ACCATGTATC   
  
  
+ TTAATTTATT TAAGTCGAAA ATTTGATCCT TTCTTAATTT TATTTATTAA GACACGTGAA AATACCTTTT   
  
  
+ TGCTTAATTA ATTGAATAAG AGCCACGCAT AGCATAACAA CATTATCTAT TTGCCCATTT CATGAGCAAT   
  
  
+ AAAGAATTCC ACTGACTGAA AAGAATACAA GACCCCCCAA AAAAAAAAGA AAAAAAGAGA AAAGAGAAAA   
  
  
+ AAAAAGATGA AAAAAATGAT TAAAGGGAAA AGAAGAAAAG GGAATATCTT AGGAGGATTC AGTTGGTGAG   
  
  
+ CTTGAATTGA GTTCACACAG AGGAGAGTTC ACATGCAAGA CGACGGAGCT TGGGGCTGTA ATGGGTAAGA   
  
  
+ ATCTCACGTG CAGAGAAACC CCGTTCTCAC TGGGGGAACC TGTTAACTGT TGAGCCGGCC AACGAAGCGT   
  
  
+ ATGAAAACGT CGGCCAACCA CGACAGAATA CTCGGCCGCA TGCATTGAAT TTGCCACCGG CACTGTGGGT   
  
  
+ GTCCGTACGA AATGTTGACC TACGATCTCC GGTCAATCTT TGTTGCCATT TGTCTACTCT CGAGCAGAAT   
  
  
+ AGAAAAGTAA CTCACCTTCT CTTCTCTTTG GCCAAATCTC ACTTTCTTCT ATACCTAAAG CTCTCTCTCT   
  
  
+ CTCTCTCTCC CCCTCCCACC TACCTGATAA ACGCAATCAG GCCTAGCTTT CTCTCTCTCT CTCTCTCTCT   
  
  
+ CTTTCTTTCT CAGTAAGCAA ACATCACTCC CGCTGTGTGT CACTCTCTGT TAAGTTACAA GCTTTTATAT   
  
  
+ GAGGATTTTA TAGGCCTGAT TGATAGAAA  

- AATTAAAAGT CCACGAGAGT AAGCAAAAGT GGAAGCGGTT AACACTTCAA CTTCGGTGTA AAAAGATCCC   
  
  
- CGATGCTAGA ACTTTATTAC AGCTAAACTA AAACATCTAT GGAAAATTAG TAAGAAGATT ATCTTAGTAG   
  
  
- AAAAAAAAAA AAACCCAACT AGAGTAAAAC AATTTAACTT ATTTAAATTA ACTTACTTAA CTTTTAAGAA   
  
  
- AAAAAAATTC CTATATTTAA AACAACCATA AAATTTTTTT ATAAAAAATA ATTTATGAAA AATTTTTCTA   
  
  
- CAACTATATA TATATATATA CAATCTTTTA TTTCCTATAT TTAAAACAAT CATAAAATTT TTTTATAAAA   
  
  
- AATAATTTAT GAAAAATTTT TCTACAACTA TATATATATA TACAATCTTT TATGTTATTT TAAATAACGT   
  
  
- AAACAAAAGA ACTAATTACA TTGGTTAATA TTTTTATCTT AAAATTTTTT TTCTAATTAT TCTTAGTATC   
  
  
- ATTGTCTACT AAAAAACTAA GTTTCCTTTG TAAAGAAGTT ATAAAATTTC TAAGTAATAA TTTTTACTAA   
  
  
- TCTTTAATAC CATTTTAGAT ACTTTAAAAA TTTTTTATAT TTATCATAAC TTCATTCTTC CCATCAATAT   
  
  
- AAAAATATTG AATTTATTAT TATATTTTAT TTTTTACATT GGTAATTAAA ATTAATTTAA TGGTACATAG   
  
  
- AATTAAATAA ATTCAGCTTT TAAACTAGGA AAGAATTAAA ATAAATAATT CTGTGCACTT TTATGGAAAA   
  
  
- ACGAATTAAT TAACTTATTC TCGGTGCGTA TCGTATTGTT GTAATAGATA AACGGGTAAA GTACTCGTTA   
  
  
- TTTCTTAAGG TGACTGACTT TTCTTATGTT CTGGGGGGTT TTTTTTTTCT TTTTTTCTCT TTTCTCTTTT   
  
  
- TTTTTCTACT TTTTTTACTA ATTTCCCTTT TCTTCTTTTC CCTTATAGAA TCCTCCTAAG TCAACCACTC   
  
  
- GAACTTAACT CAAGTGTGTC TCCTCTCAAG TGTACGTTCT GCTGCCTCGA ACCCCGACAT TACCCATTCT   
  
  
- TAGAGTGCAC GTCTCTTTGG GGCAAGAGTG ACCCCCTTGG ACAATTGACA ACTCGGCCGG TTGCTTCGCA   
  
  
- TACTTTTGCA GCCGGTTGGT GCTGTCTTAT GAGCCGGCGT ACGTAACTTA AACGGTGGCC GTGACACCCA   
  
  
- CAGGCATGCT TTACAACTGG ATGCTAGAGG CCAGTTAGAA ACAACGGTAA ACAGATGAGA GCTCGTCTTA   
  
  
- TCTTTTCATT GAGTGGAAGA GAAGAGAAAC CGGTTTAGAG TGAAAGAAGA TATGGATTTC GAGAGAGAGA   
  
  
- GAGAGAGAGG GGGAGGGTGG ATGGACTATT TGCGTTAGTC CGGATCGAAA GAGAGAGAGA GAGAGAGAGA   
  
  
- GAAAGAAAGA GTCATTCGTT TGTAGTGAGG GCGACACACA GTGAGAGACA ATTCAATGTT CGAAAATATA   
  
  
- CTCCTAAAAT ATCCGGACTA ACTATCTTT

+     AC-I

| Site Name | Organism | Position | Strand | Matrix score. | sequence | function |
| --- | --- | --- | --- | --- | --- | --- |
| AC-I | Phaseolus vulgaris | 1344 | + | 9 | (T/C)C(T/C)(C/T)ACC(T/C)ACC |  |
| AC-I | Phaseolus vulgaris | 1345 | + | 10 | CCCACCTACC |  |

> 2018/04/13 10:10:12  
+ TTAATTTTCA GGTGCTCTCA TTCGTTTTCA CCTTCGCCAA TTGTGAAGTT GAAGCCACAT TTTTCTAGGG   
  
  
+ GCTACGATCT TGAAATAATG TCGATTTGAT TTTGTAGATA CCTTTTAATC ATTCTTCTAA TAGAATCATC   
  
  
+ TTTTTTTTTT TTTGGGTTGA TCTCATTTTG TTAAATTGAA TAAATTTAAT TGAATGAATT GAAAATTCTT   
  
  
+ TTTTTTTAAG GATATAAATT TTGTTGGTAT TTTAAAAAAA TATTTTTTAT TAAATACTTT TTAAAAAGAT   
  
  
+ GTTGATATAT ATATATATAT GTTAGAAAAT AAAGGATATA AATTTTGTTA GTATTTTAAA AAAATATTTT   
  
  
+ TTATTAAATA CTTTTTAAAA AGATGTTGAT ATATATATAT ATGTTAGAAA ATACAATAAA ATTTATTGCA   
  
  
+ TTTGTTTTCT TGATTAATGT AACCAATTAT AAAAATAGAA TTTTAAAAAA AAGATTAATA AGAATCATAG   
  
  
+ TAACAGATGA TTTTTTGATT CAAAGGAAAC ATTTCTTCAA TATTTTAAAG ATTCATTATT AAAAATGATT   
  
  
+ AGAAATTATG GTAAAATCTA TGAAATTTTT AAAAAATATA AATAGTATTG AAGTAAGAAG GGTAGTTATA   
  
  
+ TTTTTATAAC TTAAATAATA ATATAAAATA AAAAATGTAA CCATTAATTT TAATTAAATT ACCATGTATC   
  
  
+ TTAATTTATT TAAGTCGAAA ATTTGATCCT TTCTTAATTT TATTTATTAA GACACGTGAA AATACCTTTT   
  
  
+ TGCTTAATTA ATTGAATAAG AGCCACGCAT AGCATAACAA CATTATCTAT TTGCCCATTT CATGAGCAAT   
  
  
+ AAAGAATTCC ACTGACTGAA AAGAATACAA GACCCCCCAA AAAAAAAAGA AAAAAAGAGA AAAGAGAAAA   
  
  
+ AAAAAGATGA AAAAAATGAT TAAAGGGAAA AGAAGAAAAG GGAATATCTT AGGAGGATTC AGTTGGTGAG   
  
  
+ CTTGAATTGA GTTCACACAG AGGAGAGTTC ACATGCAAGA CGACGGAGCT TGGGGCTGTA ATGGGTAAGA   
  
  
+ ATCTCACGTG CAGAGAAACC CCGTTCTCAC TGGGGGAACC TGTTAACTGT TGAGCCGGCC AACGAAGCGT   
  
  
+ ATGAAAACGT CGGCCAACCA CGACAGAATA CTCGGCCGCA TGCATTGAAT TTGCCACCGG CACTGTGGGT   
  
  
+ GTCCGTACGA AATGTTGACC TACGATCTCC GGTCAATCTT TGTTGCCATT TGTCTACTCT CGAGCAGAAT   
  
  
+ AGAAAAGTAA CTCACCTTCT CTTCTCTTTG GCCAAATCTC ACTTTCTTCT ATACCTAAAG CTCTCTCTCT   
  
  
+ CTCTCTCTCC CCCTCCCACC TACCTGATAA ACGCAATCAG GCCTAGCTTT CTCTCTCTCT CTCTCTCTCT   
  
  
+ CTTTCTTTCT CAGTAAGCAA ACATCACTCC CGCTGTGTGT CACTCTCTGT TAAGTTACAA GCTTTTATAT   
  
  
+ GAGGATTTTA TAGGCCTGAT TGATAGAAA  

- AATTAAAAGT CCACGAGAGT AAGCAAAAGT GGAAGCGGTT AACACTTCAA CTTCGGTGTA AAAAGATCCC   
  
  
- CGATGCTAGA ACTTTATTAC AGCTAAACTA AAACATCTAT GGAAAATTAG TAAGAAGATT ATCTTAGTAG   
  
  
- AAAAAAAAAA AAACCCAACT AGAGTAAAAC AATTTAACTT ATTTAAATTA ACTTACTTAA CTTTTAAGAA   
  
  
- AAAAAAATTC CTATATTTAA AACAACCATA AAATTTTTTT ATAAAAAATA ATTTATGAAA AATTTTTCTA   
  
  
- CAACTATATA TATATATATA CAATCTTTTA TTTCCTATAT TTAAAACAAT CATAAAATTT TTTTATAAAA   
  
  
- AATAATTTAT GAAAAATTTT TCTACAACTA TATATATATA TACAATCTTT TATGTTATTT TAAATAACGT   
  
  
- AAACAAAAGA ACTAATTACA TTGGTTAATA TTTTTATCTT AAAATTTTTT TTCTAATTAT TCTTAGTATC   
  
  
- ATTGTCTACT AAAAAACTAA GTTTCCTTTG TAAAGAAGTT ATAAAATTTC TAAGTAATAA TTTTTACTAA   
  
  
- TCTTTAATAC CATTTTAGAT ACTTTAAAAA TTTTTTATAT TTATCATAAC TTCATTCTTC CCATCAATAT   
  
  
- AAAAATATTG AATTTATTAT TATATTTTAT TTTTTACATT GGTAATTAAA ATTAATTTAA TGGTACATAG   
  
  
- AATTAAATAA ATTCAGCTTT TAAACTAGGA AAGAATTAAA ATAAATAATT CTGTGCACTT TTATGGAAAA   
  
  
- ACGAATTAAT TAACTTATTC TCGGTGCGTA TCGTATTGTT GTAATAGATA AACGGGTAAA GTACTCGTTA   
  
  
- TTTCTTAAGG TGACTGACTT TTCTTATGTT CTGGGGGGTT TTTTTTTTCT TTTTTTCTCT TTTCTCTTTT   
  
  
- TTTTTCTACT TTTTTTACTA ATTTCCCTTT TCTTCTTTTC CCTTATAGAA TCCTCCTAAG TCAACCACTC   
  
  
- GAACTTAACT CAAGTGTGTC TCCTCTCAAG TGTACGTTCT GCTGCCTCGA ACCCCGACAT TACCCATTCT   
  
  
- TAGAGTGCAC GTCTCTTTGG GGCAAGAGTG ACCCCCTTGG ACAATTGACA ACTCGGCCGG TTGCTTCGCA   
  
  
- TACTTTTGCA GCCGGTTGGT GCTGTCTTAT GAGCCGGCGT ACGTAACTTA AACGGTGGCC GTGACACCCA   
  
  
- CAGGCATGCT TTACAACTGG ATGCTAGAGG CCAGTTAGAA ACAACGGTAA ACAGATGAGA GCTCGTCTTA   
  
  
- TCTTTTCATT GAGTGGAAGA GAAGAGAAAC CGGTTTAGAG TGAAAGAAGA TATGGATTTC GAGAGAGAGA   
  
  
- GAGAGAGAGG GGGAGGGTGG ATGGACTATT TGCGTTAGTC CGGATCGAAA GAGAGAGAGA GAGAGAGAGA   
  
  
- GAAAGAAAGA GTCATTCGTT TGTAGTGAGG GCGACACACA GTGAGAGACA ATTCAATGTT CGAAAATATA   
  
  
- CTCCTAAAAT ATCCGGACTA ACTATCTTT

+     AT-rich sequence

| Site Name | Organism | Position | Strand | Matrix score. | sequence | function |
| --- | --- | --- | --- | --- | --- | --- |
| AT-rich sequence | Pisum sativum | 330 | - | 9 | TAAAATACT | element for maximal elicitor-mediated activation (2copies) |

> 2018/04/13 10:10:12  
+ TTAATTTTCA GGTGCTCTCA TTCGTTTTCA CCTTCGCCAA TTGTGAAGTT GAAGCCACAT TTTTCTAGGG   
  
  
+ GCTACGATCT TGAAATAATG TCGATTTGAT TTTGTAGATA CCTTTTAATC ATTCTTCTAA TAGAATCATC   
  
  
+ TTTTTTTTTT TTTGGGTTGA TCTCATTTTG TTAAATTGAA TAAATTTAAT TGAATGAATT GAAAATTCTT   
  
  
+ TTTTTTTAAG GATATAAATT TTGTTGGTAT TTTAAAAAAA TATTTTTTAT TAAATACTTT TTAAAAAGAT   
  
  
+ GTTGATATAT ATATATATAT GTTAGAAAAT AAAGGATATA AATTTTGTTA GTATTTTAAA AAAATATTTT   
  
  
+ TTATTAAATA CTTTTTAAAA AGATGTTGAT ATATATATAT ATGTTAGAAA ATACAATAAA ATTTATTGCA   
  
  
+ TTTGTTTTCT TGATTAATGT AACCAATTAT AAAAATAGAA TTTTAAAAAA AAGATTAATA AGAATCATAG   
  
  
+ TAACAGATGA TTTTTTGATT CAAAGGAAAC ATTTCTTCAA TATTTTAAAG ATTCATTATT AAAAATGATT   
  
  
+ AGAAATTATG GTAAAATCTA TGAAATTTTT AAAAAATATA AATAGTATTG AAGTAAGAAG GGTAGTTATA   
  
  
+ TTTTTATAAC TTAAATAATA ATATAAAATA AAAAATGTAA CCATTAATTT TAATTAAATT ACCATGTATC   
  
  
+ TTAATTTATT TAAGTCGAAA ATTTGATCCT TTCTTAATTT TATTTATTAA GACACGTGAA AATACCTTTT   
  
  
+ TGCTTAATTA ATTGAATAAG AGCCACGCAT AGCATAACAA CATTATCTAT TTGCCCATTT CATGAGCAAT   
  
  
+ AAAGAATTCC ACTGACTGAA AAGAATACAA GACCCCCCAA AAAAAAAAGA AAAAAAGAGA AAAGAGAAAA   
  
  
+ AAAAAGATGA AAAAAATGAT TAAAGGGAAA AGAAGAAAAG GGAATATCTT AGGAGGATTC AGTTGGTGAG   
  
  
+ CTTGAATTGA GTTCACACAG AGGAGAGTTC ACATGCAAGA CGACGGAGCT TGGGGCTGTA ATGGGTAAGA   
  
  
+ ATCTCACGTG CAGAGAAACC CCGTTCTCAC TGGGGGAACC TGTTAACTGT TGAGCCGGCC AACGAAGCGT   
  
  
+ ATGAAAACGT CGGCCAACCA CGACAGAATA CTCGGCCGCA TGCATTGAAT TTGCCACCGG CACTGTGGGT   
  
  
+ GTCCGTACGA AATGTTGACC TACGATCTCC GGTCAATCTT TGTTGCCATT TGTCTACTCT CGAGCAGAAT   
  
  
+ AGAAAAGTAA CTCACCTTCT CTTCTCTTTG GCCAAATCTC ACTTTCTTCT ATACCTAAAG CTCTCTCTCT   
  
  
+ CTCTCTCTCC CCCTCCCACC TACCTGATAA ACGCAATCAG GCCTAGCTTT CTCTCTCTCT CTCTCTCTCT   
  
  
+ CTTTCTTTCT CAGTAAGCAA ACATCACTCC CGCTGTGTGT CACTCTCTGT TAAGTTACAA GCTTTTATAT   
  
  
+ GAGGATTTTA TAGGCCTGAT TGATAGAAA  

- AATTAAAAGT CCACGAGAGT AAGCAAAAGT GGAAGCGGTT AACACTTCAA CTTCGGTGTA AAAAGATCCC   
  
  
- CGATGCTAGA ACTTTATTAC AGCTAAACTA AAACATCTAT GGAAAATTAG TAAGAAGATT ATCTTAGTAG   
  
  
- AAAAAAAAAA AAACCCAACT AGAGTAAAAC AATTTAACTT ATTTAAATTA ACTTACTTAA CTTTTAAGAA   
  
  
- AAAAAAATTC CTATATTTAA AACAACCATA AAATTTTTTT ATAAAAAATA ATTTATGAAA AATTTTTCTA   
  
  
- CAACTATATA TATATATATA CAATCTTTTA TTTCCTATAT TTAAAACAAT CATAAAATTT TTTTATAAAA   
  
  
- AATAATTTAT GAAAAATTTT TCTACAACTA TATATATATA TACAATCTTT TATGTTATTT TAAATAACGT   
  
  
- AAACAAAAGA ACTAATTACA TTGGTTAATA TTTTTATCTT AAAATTTTTT TTCTAATTAT TCTTAGTATC   
  
  
- ATTGTCTACT AAAAAACTAA GTTTCCTTTG TAAAGAAGTT ATAAAATTTC TAAGTAATAA TTTTTACTAA   
  
  
- TCTTTAATAC CATTTTAGAT ACTTTAAAAA TTTTTTATAT TTATCATAAC TTCATTCTTC CCATCAATAT   
  
  
- AAAAATATTG AATTTATTAT TATATTTTAT TTTTTACATT GGTAATTAAA ATTAATTTAA TGGTACATAG   
  
  
- AATTAAATAA ATTCAGCTTT TAAACTAGGA AAGAATTAAA ATAAATAATT CTGTGCACTT TTATGGAAAA   
  
  
- ACGAATTAAT TAACTTATTC TCGGTGCGTA TCGTATTGTT GTAATAGATA AACGGGTAAA GTACTCGTTA   
  
  
- TTTCTTAAGG TGACTGACTT TTCTTATGTT CTGGGGGGTT TTTTTTTTCT TTTTTTCTCT TTTCTCTTTT   
  
  
- TTTTTCTACT TTTTTTACTA ATTTCCCTTT TCTTCTTTTC CCTTATAGAA TCCTCCTAAG TCAACCACTC   
  
  
- GAACTTAACT CAAGTGTGTC TCCTCTCAAG TGTACGTTCT GCTGCCTCGA ACCCCGACAT TACCCATTCT   
  
  
- TAGAGTGCAC GTCTCTTTGG GGCAAGAGTG ACCCCCTTGG ACAATTGACA ACTCGGCCGG TTGCTTCGCA   
  
  
- TACTTTTGCA GCCGGTTGGT GCTGTCTTAT GAGCCGGCGT ACGTAACTTA AACGGTGGCC GTGACACCCA   
  
  
- CAGGCATGCT TTACAACTGG ATGCTAGAGG CCAGTTAGAA ACAACGGTAA ACAGATGAGA GCTCGTCTTA   
  
  
- TCTTTTCATT GAGTGGAAGA GAAGAGAAAC CGGTTTAGAG TGAAAGAAGA TATGGATTTC GAGAGAGAGA   
  
  
- GAGAGAGAGG GGGAGGGTGG ATGGACTATT TGCGTTAGTC CGGATCGAAA GAGAGAGAGA GAGAGAGAGA   
  
  
- GAAAGAAAGA GTCATTCGTT TGTAGTGAGG GCGACACACA GTGAGAGACA ATTCAATGTT CGAAAATATA   
  
  
- CTCCTAAAAT ATCCGGACTA ACTATCTTT

+     AT1-motif

| Site Name | Organism | Position | Strand | Matrix score. | sequence | function |
| --- | --- | --- | --- | --- | --- | --- |
| AT1-motif | Solanum tuberosum | 342 | + | 13 | AATTATTTTTTATT | part of a light responsive module |
| AT1-motif | Solanum tuberosum | 248 | + | 13 | AATTATTTTTTATT | part of a light responsive module |

> 2018/04/13 10:10:12  
+ TTAATTTTCA GGTGCTCTCA TTCGTTTTCA CCTTCGCCAA TTGTGAAGTT GAAGCCACAT TTTTCTAGGG   
  
  
+ GCTACGATCT TGAAATAATG TCGATTTGAT TTTGTAGATA CCTTTTAATC ATTCTTCTAA TAGAATCATC   
  
  
+ TTTTTTTTTT TTTGGGTTGA TCTCATTTTG TTAAATTGAA TAAATTTAAT TGAATGAATT GAAAATTCTT   
  
  
+ TTTTTTTAAG GATATAAATT TTGTTGGTAT TTTAAAAAAA TATTTTTTAT TAAATACTTT TTAAAAAGAT   
  
  
+ GTTGATATAT ATATATATAT GTTAGAAAAT AAAGGATATA AATTTTGTTA GTATTTTAAA AAAATATTTT   
  
  
+ TTATTAAATA CTTTTTAAAA AGATGTTGAT ATATATATAT ATGTTAGAAA ATACAATAAA ATTTATTGCA   
  
  
+ TTTGTTTTCT TGATTAATGT AACCAATTAT AAAAATAGAA TTTTAAAAAA AAGATTAATA AGAATCATAG   
  
  
+ TAACAGATGA TTTTTTGATT CAAAGGAAAC ATTTCTTCAA TATTTTAAAG ATTCATTATT AAAAATGATT   
  
  
+ AGAAATTATG GTAAAATCTA TGAAATTTTT AAAAAATATA AATAGTATTG AAGTAAGAAG GGTAGTTATA   
  
  
+ TTTTTATAAC TTAAATAATA ATATAAAATA AAAAATGTAA CCATTAATTT TAATTAAATT ACCATGTATC   
  
  
+ TTAATTTATT TAAGTCGAAA ATTTGATCCT TTCTTAATTT TATTTATTAA GACACGTGAA AATACCTTTT   
  
  
+ TGCTTAATTA ATTGAATAAG AGCCACGCAT AGCATAACAA CATTATCTAT TTGCCCATTT CATGAGCAAT   
  
  
+ AAAGAATTCC ACTGACTGAA AAGAATACAA GACCCCCCAA AAAAAAAAGA AAAAAAGAGA AAAGAGAAAA   
  
  
+ AAAAAGATGA AAAAAATGAT TAAAGGGAAA AGAAGAAAAG GGAATATCTT AGGAGGATTC AGTTGGTGAG   
  
  
+ CTTGAATTGA GTTCACACAG AGGAGAGTTC ACATGCAAGA CGACGGAGCT TGGGGCTGTA ATGGGTAAGA   
  
  
+ ATCTCACGTG CAGAGAAACC CCGTTCTCAC TGGGGGAACC TGTTAACTGT TGAGCCGGCC AACGAAGCGT   
  
  
+ ATGAAAACGT CGGCCAACCA CGACAGAATA CTCGGCCGCA TGCATTGAAT TTGCCACCGG CACTGTGGGT   
  
  
+ GTCCGTACGA AATGTTGACC TACGATCTCC GGTCAATCTT TGTTGCCATT TGTCTACTCT CGAGCAGAAT   
  
  
+ AGAAAAGTAA CTCACCTTCT CTTCTCTTTG GCCAAATCTC ACTTTCTTCT ATACCTAAAG CTCTCTCTCT   
  
  
+ CTCTCTCTCC CCCTCCCACC TACCTGATAA ACGCAATCAG GCCTAGCTTT CTCTCTCTCT CTCTCTCTCT   
  
  
+ CTTTCTTTCT CAGTAAGCAA ACATCACTCC CGCTGTGTGT CACTCTCTGT TAAGTTACAA GCTTTTATAT   
  
  
+ GAGGATTTTA TAGGCCTGAT TGATAGAAA  

- AATTAAAAGT CCACGAGAGT AAGCAAAAGT GGAAGCGGTT AACACTTCAA CTTCGGTGTA AAAAGATCCC   
  
  
- CGATGCTAGA ACTTTATTAC AGCTAAACTA AAACATCTAT GGAAAATTAG TAAGAAGATT ATCTTAGTAG   
  
  
- AAAAAAAAAA AAACCCAACT AGAGTAAAAC AATTTAACTT ATTTAAATTA ACTTACTTAA CTTTTAAGAA   
  
  
- AAAAAAATTC CTATATTTAA AACAACCATA AAATTTTTTT ATAAAAAATA ATTTATGAAA AATTTTTCTA   
  
  
- CAACTATATA TATATATATA CAATCTTTTA TTTCCTATAT TTAAAACAAT CATAAAATTT TTTTATAAAA   
  
  
- AATAATTTAT GAAAAATTTT TCTACAACTA TATATATATA TACAATCTTT TATGTTATTT TAAATAACGT   
  
  
- AAACAAAAGA ACTAATTACA TTGGTTAATA TTTTTATCTT AAAATTTTTT TTCTAATTAT TCTTAGTATC   
  
  
- ATTGTCTACT AAAAAACTAA GTTTCCTTTG TAAAGAAGTT ATAAAATTTC TAAGTAATAA TTTTTACTAA   
  
  
- TCTTTAATAC CATTTTAGAT ACTTTAAAAA TTTTTTATAT TTATCATAAC TTCATTCTTC CCATCAATAT   
  
  
- AAAAATATTG AATTTATTAT TATATTTTAT TTTTTACATT GGTAATTAAA ATTAATTTAA TGGTACATAG   
  
  
- AATTAAATAA ATTCAGCTTT TAAACTAGGA AAGAATTAAA ATAAATAATT CTGTGCACTT TTATGGAAAA   
  
  
- ACGAATTAAT TAACTTATTC TCGGTGCGTA TCGTATTGTT GTAATAGATA AACGGGTAAA GTACTCGTTA   
  
  
- TTTCTTAAGG TGACTGACTT TTCTTATGTT CTGGGGGGTT TTTTTTTTCT TTTTTTCTCT TTTCTCTTTT   
  
  
- TTTTTCTACT TTTTTTACTA ATTTCCCTTT TCTTCTTTTC CCTTATAGAA TCCTCCTAAG TCAACCACTC   
  
  
- GAACTTAACT CAAGTGTGTC TCCTCTCAAG TGTACGTTCT GCTGCCTCGA ACCCCGACAT TACCCATTCT   
  
  
- TAGAGTGCAC GTCTCTTTGG GGCAAGAGTG ACCCCCTTGG ACAATTGACA ACTCGGCCGG TTGCTTCGCA   
  
  
- TACTTTTGCA GCCGGTTGGT GCTGTCTTAT GAGCCGGCGT ACGTAACTTA AACGGTGGCC GTGACACCCA   
  
  
- CAGGCATGCT TTACAACTGG ATGCTAGAGG CCAGTTAGAA ACAACGGTAA ACAGATGAGA GCTCGTCTTA   
  
  
- TCTTTTCATT GAGTGGAAGA GAAGAGAAAC CGGTTTAGAG TGAAAGAAGA TATGGATTTC GAGAGAGAGA   
  
  
- GAGAGAGAGG GGGAGGGTGG ATGGACTATT TGCGTTAGTC CGGATCGAAA GAGAGAGAGA GAGAGAGAGA   
  
  
- GAAAGAAAGA GTCATTCGTT TGTAGTGAGG GCGACACACA GTGAGAGACA ATTCAATGTT CGAAAATATA   
  
  
- CTCCTAAAAT ATCCGGACTA ACTATCTTT

+     Box 4

| Site Name | Organism | Position | Strand | Matrix score. | sequence | function |
| --- | --- | --- | --- | --- | --- | --- |
| Box 4 | Petroselinum crispum | 433 | + | 6 | ATTAAT | part of a conserved DNA module involved in light responsiveness |
| Box 4 | Petroselinum crispum | 673 | + | 6 | ATTAAT | part of a conserved DNA module involved in light responsiveness |
| Box 4 | Petroselinum crispum | 777 | - | 6 | ATTAAT | part of a conserved DNA module involved in light responsiveness |
| Box 4 | Petroselinum crispum | 474 | + | 6 | ATTAAT | part of a conserved DNA module involved in light responsiveness |

> 2018/04/13 10:10:12  
+ TTAATTTTCA GGTGCTCTCA TTCGTTTTCA CCTTCGCCAA TTGTGAAGTT GAAGCCACAT TTTTCTAGGG   
  
  
+ GCTACGATCT TGAAATAATG TCGATTTGAT TTTGTAGATA CCTTTTAATC ATTCTTCTAA TAGAATCATC   
  
  
+ TTTTTTTTTT TTTGGGTTGA TCTCATTTTG TTAAATTGAA TAAATTTAAT TGAATGAATT GAAAATTCTT   
  
  
+ TTTTTTTAAG GATATAAATT TTGTTGGTAT TTTAAAAAAA TATTTTTTAT TAAATACTTT TTAAAAAGAT   
  
  
+ GTTGATATAT ATATATATAT GTTAGAAAAT AAAGGATATA AATTTTGTTA GTATTTTAAA AAAATATTTT   
  
  
+ TTATTAAATA CTTTTTAAAA AGATGTTGAT ATATATATAT ATGTTAGAAA ATACAATAAA ATTTATTGCA   
  
  
+ TTTGTTTTCT TGATTAATGT AACCAATTAT AAAAATAGAA TTTTAAAAAA AAGATTAATA AGAATCATAG   
  
  
+ TAACAGATGA TTTTTTGATT CAAAGGAAAC ATTTCTTCAA TATTTTAAAG ATTCATTATT AAAAATGATT   
  
  
+ AGAAATTATG GTAAAATCTA TGAAATTTTT AAAAAATATA AATAGTATTG AAGTAAGAAG GGTAGTTATA   
  
  
+ TTTTTATAAC TTAAATAATA ATATAAAATA AAAAATGTAA CCATTAATTT TAATTAAATT ACCATGTATC   
  
  
+ TTAATTTATT TAAGTCGAAA ATTTGATCCT TTCTTAATTT TATTTATTAA GACACGTGAA AATACCTTTT   
  
  
+ TGCTTAATTA ATTGAATAAG AGCCACGCAT AGCATAACAA CATTATCTAT TTGCCCATTT CATGAGCAAT   
  
  
+ AAAGAATTCC ACTGACTGAA AAGAATACAA GACCCCCCAA AAAAAAAAGA AAAAAAGAGA AAAGAGAAAA   
  
  
+ AAAAAGATGA AAAAAATGAT TAAAGGGAAA AGAAGAAAAG GGAATATCTT AGGAGGATTC AGTTGGTGAG   
  
  
+ CTTGAATTGA GTTCACACAG AGGAGAGTTC ACATGCAAGA CGACGGAGCT TGGGGCTGTA ATGGGTAAGA   
  
  
+ ATCTCACGTG CAGAGAAACC CCGTTCTCAC TGGGGGAACC TGTTAACTGT TGAGCCGGCC AACGAAGCGT   
  
  
+ ATGAAAACGT CGGCCAACCA CGACAGAATA CTCGGCCGCA TGCATTGAAT TTGCCACCGG CACTGTGGGT   
  
  
+ GTCCGTACGA AATGTTGACC TACGATCTCC GGTCAATCTT TGTTGCCATT TGTCTACTCT CGAGCAGAAT   
  
  
+ AGAAAAGTAA CTCACCTTCT CTTCTCTTTG GCCAAATCTC ACTTTCTTCT ATACCTAAAG CTCTCTCTCT   
  
  
+ CTCTCTCTCC CCCTCCCACC TACCTGATAA ACGCAATCAG GCCTAGCTTT CTCTCTCTCT CTCTCTCTCT   
  
  
+ CTTTCTTTCT CAGTAAGCAA ACATCACTCC CGCTGTGTGT CACTCTCTGT TAAGTTACAA GCTTTTATAT   
  
  
+ GAGGATTTTA TAGGCCTGAT TGATAGAAA  

- AATTAAAAGT CCACGAGAGT AAGCAAAAGT GGAAGCGGTT AACACTTCAA CTTCGGTGTA AAAAGATCCC   
  
  
- CGATGCTAGA ACTTTATTAC AGCTAAACTA AAACATCTAT GGAAAATTAG TAAGAAGATT ATCTTAGTAG   
  
  
- AAAAAAAAAA AAACCCAACT AGAGTAAAAC AATTTAACTT ATTTAAATTA ACTTACTTAA CTTTTAAGAA   
  
  
- AAAAAAATTC CTATATTTAA AACAACCATA AAATTTTTTT ATAAAAAATA ATTTATGAAA AATTTTTCTA   
  
  
- CAACTATATA TATATATATA CAATCTTTTA TTTCCTATAT TTAAAACAAT CATAAAATTT TTTTATAAAA   
  
  
- AATAATTTAT GAAAAATTTT TCTACAACTA TATATATATA TACAATCTTT TATGTTATTT TAAATAACGT   
  
  
- AAACAAAAGA ACTAATTACA TTGGTTAATA TTTTTATCTT AAAATTTTTT TTCTAATTAT TCTTAGTATC   
  
  
- ATTGTCTACT AAAAAACTAA GTTTCCTTTG TAAAGAAGTT ATAAAATTTC TAAGTAATAA TTTTTACTAA   
  
  
- TCTTTAATAC CATTTTAGAT ACTTTAAAAA TTTTTTATAT TTATCATAAC TTCATTCTTC CCATCAATAT   
  
  
- AAAAATATTG AATTTATTAT TATATTTTAT TTTTTACATT GGTAATTAAA ATTAATTTAA TGGTACATAG   
  
  
- AATTAAATAA ATTCAGCTTT TAAACTAGGA AAGAATTAAA ATAAATAATT CTGTGCACTT TTATGGAAAA   
  
  
- ACGAATTAAT TAACTTATTC TCGGTGCGTA TCGTATTGTT GTAATAGATA AACGGGTAAA GTACTCGTTA   
  
  
- TTTCTTAAGG TGACTGACTT TTCTTATGTT CTGGGGGGTT TTTTTTTTCT TTTTTTCTCT TTTCTCTTTT   
  
  
- TTTTTCTACT TTTTTTACTA ATTTCCCTTT TCTTCTTTTC CCTTATAGAA TCCTCCTAAG TCAACCACTC   
  
  
- GAACTTAACT CAAGTGTGTC TCCTCTCAAG TGTACGTTCT GCTGCCTCGA ACCCCGACAT TACCCATTCT   
  
  
- TAGAGTGCAC GTCTCTTTGG GGCAAGAGTG ACCCCCTTGG ACAATTGACA ACTCGGCCGG TTGCTTCGCA   
  
  
- TACTTTTGCA GCCGGTTGGT GCTGTCTTAT GAGCCGGCGT ACGTAACTTA AACGGTGGCC GTGACACCCA   
  
  
- CAGGCATGCT TTACAACTGG ATGCTAGAGG CCAGTTAGAA ACAACGGTAA ACAGATGAGA GCTCGTCTTA   
  
  
- TCTTTTCATT GAGTGGAAGA GAAGAGAAAC CGGTTTAGAG TGAAAGAAGA TATGGATTTC GAGAGAGAGA   
  
  
- GAGAGAGAGG GGGAGGGTGG ATGGACTATT TGCGTTAGTC CGGATCGAAA GAGAGAGAGA GAGAGAGAGA   
  
  
- GAAAGAAAGA GTCATTCGTT TGTAGTGAGG GCGACACACA GTGAGAGACA ATTCAATGTT CGAAAATATA   
  
  
- CTCCTAAAAT ATCCGGACTA ACTATCTTT

+     Box-W1

| Site Name | Organism | Position | Strand | Matrix score. | sequence | function |
| --- | --- | --- | --- | --- | --- | --- |
| Box-W1 | Petroselinum crispum | 1205 | + | 6 | TTGACC | fungal elicitor responsive element |
| Box-W1 | Petroselinum crispum | 1221 | - | 6 | TTGACC | fungal elicitor responsive element |

> 2018/04/13 10:10:12  
+ TTAATTTTCA GGTGCTCTCA TTCGTTTTCA CCTTCGCCAA TTGTGAAGTT GAAGCCACAT TTTTCTAGGG   
  
  
+ GCTACGATCT TGAAATAATG TCGATTTGAT TTTGTAGATA CCTTTTAATC ATTCTTCTAA TAGAATCATC   
  
  
+ TTTTTTTTTT TTTGGGTTGA TCTCATTTTG TTAAATTGAA TAAATTTAAT TGAATGAATT GAAAATTCTT   
  
  
+ TTTTTTTAAG GATATAAATT TTGTTGGTAT TTTAAAAAAA TATTTTTTAT TAAATACTTT TTAAAAAGAT   
  
  
+ GTTGATATAT ATATATATAT GTTAGAAAAT AAAGGATATA AATTTTGTTA GTATTTTAAA AAAATATTTT   
  
  
+ TTATTAAATA CTTTTTAAAA AGATGTTGAT ATATATATAT ATGTTAGAAA ATACAATAAA ATTTATTGCA   
  
  
+ TTTGTTTTCT TGATTAATGT AACCAATTAT AAAAATAGAA TTTTAAAAAA AAGATTAATA AGAATCATAG   
  
  
+ TAACAGATGA TTTTTTGATT CAAAGGAAAC ATTTCTTCAA TATTTTAAAG ATTCATTATT AAAAATGATT   
  
  
+ AGAAATTATG GTAAAATCTA TGAAATTTTT AAAAAATATA AATAGTATTG AAGTAAGAAG GGTAGTTATA   
  
  
+ TTTTTATAAC TTAAATAATA ATATAAAATA AAAAATGTAA CCATTAATTT TAATTAAATT ACCATGTATC   
  
  
+ TTAATTTATT TAAGTCGAAA ATTTGATCCT TTCTTAATTT TATTTATTAA GACACGTGAA AATACCTTTT   
  
  
+ TGCTTAATTA ATTGAATAAG AGCCACGCAT AGCATAACAA CATTATCTAT TTGCCCATTT CATGAGCAAT   
  
  
+ AAAGAATTCC ACTGACTGAA AAGAATACAA GACCCCCCAA AAAAAAAAGA AAAAAAGAGA AAAGAGAAAA   
  
  
+ AAAAAGATGA AAAAAATGAT TAAAGGGAAA AGAAGAAAAG GGAATATCTT AGGAGGATTC AGTTGGTGAG   
  
  
+ CTTGAATTGA GTTCACACAG AGGAGAGTTC ACATGCAAGA CGACGGAGCT TGGGGCTGTA ATGGGTAAGA   
  
  
+ ATCTCACGTG CAGAGAAACC CCGTTCTCAC TGGGGGAACC TGTTAACTGT TGAGCCGGCC AACGAAGCGT   
  
  
+ ATGAAAACGT CGGCCAACCA CGACAGAATA CTCGGCCGCA TGCATTGAAT TTGCCACCGG CACTGTGGGT   
  
  
+ GTCCGTACGA AATGTTGACC TACGATCTCC GGTCAATCTT TGTTGCCATT TGTCTACTCT CGAGCAGAAT   
  
  
+ AGAAAAGTAA CTCACCTTCT CTTCTCTTTG GCCAAATCTC ACTTTCTTCT ATACCTAAAG CTCTCTCTCT   
  
  
+ CTCTCTCTCC CCCTCCCACC TACCTGATAA ACGCAATCAG GCCTAGCTTT CTCTCTCTCT CTCTCTCTCT   
  
  
+ CTTTCTTTCT CAGTAAGCAA ACATCACTCC CGCTGTGTGT CACTCTCTGT TAAGTTACAA GCTTTTATAT   
  
  
+ GAGGATTTTA TAGGCCTGAT TGATAGAAA  

- AATTAAAAGT CCACGAGAGT AAGCAAAAGT GGAAGCGGTT AACACTTCAA CTTCGGTGTA AAAAGATCCC   
  
  
- CGATGCTAGA ACTTTATTAC AGCTAAACTA AAACATCTAT GGAAAATTAG TAAGAAGATT ATCTTAGTAG   
  
  
- AAAAAAAAAA AAACCCAACT AGAGTAAAAC AATTTAACTT ATTTAAATTA ACTTACTTAA CTTTTAAGAA   
  
  
- AAAAAAATTC CTATATTTAA AACAACCATA AAATTTTTTT ATAAAAAATA ATTTATGAAA AATTTTTCTA   
  
  
- CAACTATATA TATATATATA CAATCTTTTA TTTCCTATAT TTAAAACAAT CATAAAATTT TTTTATAAAA   
  
  
- AATAATTTAT GAAAAATTTT TCTACAACTA TATATATATA TACAATCTTT TATGTTATTT TAAATAACGT   
  
  
- AAACAAAAGA ACTAATTACA TTGGTTAATA TTTTTATCTT AAAATTTTTT TTCTAATTAT TCTTAGTATC   
  
  
- ATTGTCTACT AAAAAACTAA GTTTCCTTTG TAAAGAAGTT ATAAAATTTC TAAGTAATAA TTTTTACTAA   
  
  
- TCTTTAATAC CATTTTAGAT ACTTTAAAAA TTTTTTATAT TTATCATAAC TTCATTCTTC CCATCAATAT   
  
  
- AAAAATATTG AATTTATTAT TATATTTTAT TTTTTACATT GGTAATTAAA ATTAATTTAA TGGTACATAG   
  
  
- AATTAAATAA ATTCAGCTTT TAAACTAGGA AAGAATTAAA ATAAATAATT CTGTGCACTT TTATGGAAAA   
  
  
- ACGAATTAAT TAACTTATTC TCGGTGCGTA TCGTATTGTT GTAATAGATA AACGGGTAAA GTACTCGTTA   
  
  
- TTTCTTAAGG TGACTGACTT TTCTTATGTT CTGGGGGGTT TTTTTTTTCT TTTTTTCTCT TTTCTCTTTT   
  
  
- TTTTTCTACT TTTTTTACTA ATTTCCCTTT TCTTCTTTTC CCTTATAGAA TCCTCCTAAG TCAACCACTC   
  
  
- GAACTTAACT CAAGTGTGTC TCCTCTCAAG TGTACGTTCT GCTGCCTCGA ACCCCGACAT TACCCATTCT   
  
  
- TAGAGTGCAC GTCTCTTTGG GGCAAGAGTG ACCCCCTTGG ACAATTGACA ACTCGGCCGG TTGCTTCGCA   
  
  
- TACTTTTGCA GCCGGTTGGT GCTGTCTTAT GAGCCGGCGT ACGTAACTTA AACGGTGGCC GTGACACCCA   
  
  
- CAGGCATGCT TTACAACTGG ATGCTAGAGG CCAGTTAGAA ACAACGGTAA ACAGATGAGA GCTCGTCTTA   
  
  
- TCTTTTCATT GAGTGGAAGA GAAGAGAAAC CGGTTTAGAG TGAAAGAAGA TATGGATTTC GAGAGAGAGA   
  
  
- GAGAGAGAGG GGGAGGGTGG ATGGACTATT TGCGTTAGTC CGGATCGAAA GAGAGAGAGA GAGAGAGAGA   
  
  
- GAAAGAAAGA GTCATTCGTT TGTAGTGAGG GCGACACACA GTGAGAGACA ATTCAATGTT CGAAAATATA   
  
  
- CTCCTAAAAT ATCCGGACTA ACTATCTTT

+     C-repeat/DRE

| Site Name | Organism | Position | Strand | Matrix score. | sequence | function |
| --- | --- | --- | --- | --- | --- | --- |
| C-repeat/DRE | Arabidopsis thaliana | 1129 | - | 8 | TGGCCGAC | regulatory element involved in cold- and dehydration-responsiveness |

> 2018/04/13 10:10:12  
+ TTAATTTTCA GGTGCTCTCA TTCGTTTTCA CCTTCGCCAA TTGTGAAGTT GAAGCCACAT TTTTCTAGGG   
  
  
+ GCTACGATCT TGAAATAATG TCGATTTGAT TTTGTAGATA CCTTTTAATC ATTCTTCTAA TAGAATCATC   
  
  
+ TTTTTTTTTT TTTGGGTTGA TCTCATTTTG TTAAATTGAA TAAATTTAAT TGAATGAATT GAAAATTCTT   
  
  
+ TTTTTTTAAG GATATAAATT TTGTTGGTAT TTTAAAAAAA TATTTTTTAT TAAATACTTT TTAAAAAGAT   
  
  
+ GTTGATATAT ATATATATAT GTTAGAAAAT AAAGGATATA AATTTTGTTA GTATTTTAAA AAAATATTTT   
  
  
+ TTATTAAATA CTTTTTAAAA AGATGTTGAT ATATATATAT ATGTTAGAAA ATACAATAAA ATTTATTGCA   
  
  
+ TTTGTTTTCT TGATTAATGT AACCAATTAT AAAAATAGAA TTTTAAAAAA AAGATTAATA AGAATCATAG   
  
  
+ TAACAGATGA TTTTTTGATT CAAAGGAAAC ATTTCTTCAA TATTTTAAAG ATTCATTATT AAAAATGATT   
  
  
+ AGAAATTATG GTAAAATCTA TGAAATTTTT AAAAAATATA AATAGTATTG AAGTAAGAAG GGTAGTTATA   
  
  
+ TTTTTATAAC TTAAATAATA ATATAAAATA AAAAATGTAA CCATTAATTT TAATTAAATT ACCATGTATC   
  
  
+ TTAATTTATT TAAGTCGAAA ATTTGATCCT TTCTTAATTT TATTTATTAA GACACGTGAA AATACCTTTT   
  
  
+ TGCTTAATTA ATTGAATAAG AGCCACGCAT AGCATAACAA CATTATCTAT TTGCCCATTT CATGAGCAAT   
  
  
+ AAAGAATTCC ACTGACTGAA AAGAATACAA GACCCCCCAA AAAAAAAAGA AAAAAAGAGA AAAGAGAAAA   
  
  
+ AAAAAGATGA AAAAAATGAT TAAAGGGAAA AGAAGAAAAG GGAATATCTT AGGAGGATTC AGTTGGTGAG   
  
  
+ CTTGAATTGA GTTCACACAG AGGAGAGTTC ACATGCAAGA CGACGGAGCT TGGGGCTGTA ATGGGTAAGA   
  
  
+ ATCTCACGTG CAGAGAAACC CCGTTCTCAC TGGGGGAACC TGTTAACTGT TGAGCCGGCC AACGAAGCGT   
  
  
+ ATGAAAACGT CGGCCAACCA CGACAGAATA CTCGGCCGCA TGCATTGAAT TTGCCACCGG CACTGTGGGT   
  
  
+ GTCCGTACGA AATGTTGACC TACGATCTCC GGTCAATCTT TGTTGCCATT TGTCTACTCT CGAGCAGAAT   
  
  
+ AGAAAAGTAA CTCACCTTCT CTTCTCTTTG GCCAAATCTC ACTTTCTTCT ATACCTAAAG CTCTCTCTCT   
  
  
+ CTCTCTCTCC CCCTCCCACC TACCTGATAA ACGCAATCAG GCCTAGCTTT CTCTCTCTCT CTCTCTCTCT   
  
  
+ CTTTCTTTCT CAGTAAGCAA ACATCACTCC CGCTGTGTGT CACTCTCTGT TAAGTTACAA GCTTTTATAT   
  
  
+ GAGGATTTTA TAGGCCTGAT TGATAGAAA  

- AATTAAAAGT CCACGAGAGT AAGCAAAAGT GGAAGCGGTT AACACTTCAA CTTCGGTGTA AAAAGATCCC   
  
  
- CGATGCTAGA ACTTTATTAC AGCTAAACTA AAACATCTAT GGAAAATTAG TAAGAAGATT ATCTTAGTAG   
  
  
- AAAAAAAAAA AAACCCAACT AGAGTAAAAC AATTTAACTT ATTTAAATTA ACTTACTTAA CTTTTAAGAA   
  
  
- AAAAAAATTC CTATATTTAA AACAACCATA AAATTTTTTT ATAAAAAATA ATTTATGAAA AATTTTTCTA   
  
  
- CAACTATATA TATATATATA CAATCTTTTA TTTCCTATAT TTAAAACAAT CATAAAATTT TTTTATAAAA   
  
  
- AATAATTTAT GAAAAATTTT TCTACAACTA TATATATATA TACAATCTTT TATGTTATTT TAAATAACGT   
  
  
- AAACAAAAGA ACTAATTACA TTGGTTAATA TTTTTATCTT AAAATTTTTT TTCTAATTAT TCTTAGTATC   
  
  
- ATTGTCTACT AAAAAACTAA GTTTCCTTTG TAAAGAAGTT ATAAAATTTC TAAGTAATAA TTTTTACTAA   
  
  
- TCTTTAATAC CATTTTAGAT ACTTTAAAAA TTTTTTATAT TTATCATAAC TTCATTCTTC CCATCAATAT   
  
  
- AAAAATATTG AATTTATTAT TATATTTTAT TTTTTACATT GGTAATTAAA ATTAATTTAA TGGTACATAG   
  
  
- AATTAAATAA ATTCAGCTTT TAAACTAGGA AAGAATTAAA ATAAATAATT CTGTGCACTT TTATGGAAAA   
  
  
- ACGAATTAAT TAACTTATTC TCGGTGCGTA TCGTATTGTT GTAATAGATA AACGGGTAAA GTACTCGTTA   
  
  
- TTTCTTAAGG TGACTGACTT TTCTTATGTT CTGGGGGGTT TTTTTTTTCT TTTTTTCTCT TTTCTCTTTT   
  
  
- TTTTTCTACT TTTTTTACTA ATTTCCCTTT TCTTCTTTTC CCTTATAGAA TCCTCCTAAG TCAACCACTC   
  
  
- GAACTTAACT CAAGTGTGTC TCCTCTCAAG TGTACGTTCT GCTGCCTCGA ACCCCGACAT TACCCATTCT   
  
  
- TAGAGTGCAC GTCTCTTTGG GGCAAGAGTG ACCCCCTTGG ACAATTGACA ACTCGGCCGG TTGCTTCGCA   
  
  
- TACTTTTGCA GCCGGTTGGT GCTGTCTTAT GAGCCGGCGT ACGTAACTTA AACGGTGGCC GTGACACCCA   
  
  
- CAGGCATGCT TTACAACTGG ATGCTAGAGG CCAGTTAGAA ACAACGGTAA ACAGATGAGA GCTCGTCTTA   
  
  
- TCTTTTCATT GAGTGGAAGA GAAGAGAAAC CGGTTTAGAG TGAAAGAAGA TATGGATTTC GAGAGAGAGA   
  
  
- GAGAGAGAGG GGGAGGGTGG ATGGACTATT TGCGTTAGTC CGGATCGAAA GAGAGAGAGA GAGAGAGAGA   
  
  
- GAAAGAAAGA GTCATTCGTT TGTAGTGAGG GCGACACACA GTGAGAGACA ATTCAATGTT CGAAAATATA   
  
  
- CTCCTAAAAT ATCCGGACTA ACTATCTTT

+     CAAT-box

| Site Name | Organism | Position | Strand | Matrix score. | sequence | function |
| --- | --- | --- | --- | --- | --- | --- |
| CAAT-box | Brassica rapa | 1293 | + | 5 | CAAAT | common cis-acting element in promoter and enhancer regions |
| CAAT-box | Brassica rapa | 1238 | - | 5 | CAAAT | common cis-acting element in promoter and enhancer regions |
| CAAT-box | Glycine max | 444 | + | 5 | CAATT | common cis-acting element in promoter and enhancer regions |
| CAAT-box | Hordeum vulgare | 1224 | + | 4 | CAAT | common cis-acting element in promoter and enhancer regions |
| CAAT-box | Brassica rapa | 1169 | - | 5 | CAAAT | common cis-acting element in promoter and enhancer regions |
| CAAT-box | Glycine max | 985 | - | 5 | CAATT | common cis-acting element in promoter and enhancer regions |
| CAAT-box | Arabidopsis thaliana | 443 | + | 5 | CCAAT | common cis-acting element in promoter and enhancer regions |
| CAAT-box | Hordeum vulgare | 1164 | - | 4 | CAAT | common cis-acting element in promoter and enhancer regions |
| CAAT-box | Glycine max | 780 | - | 5 | CAATT | common cis-acting element in promoter and enhancer regions |
| CAAT-box | Hordeum vulgare | 837 | + | 4 | CAAT | common cis-acting element in promoter and enhancer regions |
| CAAT-box | Brassica rapa | 819 | - | 5 | CAAAT | common cis-acting element in promoter and enhancer regions |
| CAAT-box | Hordeum vulgare | 781 | - | 4 | CAAT | common cis-acting element in promoter and enhancer regions |
| CAAT-box | Hordeum vulgare | 607 | - | 4 | CAAT | common cis-acting element in promoter and enhancer regions |
| CAAT-box | Hordeum vulgare | 1364 | + | 4 | CAAT | common cis-acting element in promoter and enhancer regions |
| CAAT-box | Brassica rapa | 721 | - | 5 | CAAAT | common cis-acting element in promoter and enhancer regions |
| CAAT-box | Brassica rapa | 420 | - | 5 | CAAAT | common cis-acting element in promoter and enhancer regions |
| CAAT-box | Hordeum vulgare | 986 | - | 4 | CAAT | common cis-acting element in promoter and enhancer regions |
| CAAT-box | Hordeum vulgare | 528 | + | 4 | CAAT | common cis-acting element in promoter and enhancer regions |
| CAAT-box | Glycine max | 188 | - | 5 | CAATT | common cis-acting element in promoter and enhancer regions |
| CAAT-box | Glycine max | 39 | - | 5 | CAATT | common cis-acting element in promoter and enhancer regions |
| CAAT-box | Arabidopsis thaliana | 37 | + | 5 | CCAAT | common cis-acting element in promoter and enhancer regions |
| CAAT-box | Hordeum vulgare | 1489 | - | 4 | CAAT | common cis-acting element in promoter and enhancer regions |
| CAAT-box | Hordeum vulgare | 175 | - | 4 | CAAT | common cis-acting element in promoter and enhancer regions |
| CAAT-box | Hordeum vulgare | 415 | - | 4 | CAAT | common cis-acting element in promoter and enhancer regions |
| CAAT-box | Hordeum vulgare | 404 | + | 4 | CAAT | common cis-acting element in promoter and enhancer regions |
| CAAT-box | Glycine max | 38 | + | 5 | CAATT | common cis-acting element in promoter and enhancer regions |
| CAAT-box | Brassica rapa | 94 | - | 5 | CAAAT | common cis-acting element in promoter and enhancer regions |
| CAAT-box | Glycine max | 174 | - | 5 | CAATT | common cis-acting element in promoter and enhancer regions |
| CAAT-box | Hordeum vulgare | 40 | - | 4 | CAAT | common cis-acting element in promoter and enhancer regions |
| CAAT-box | Hordeum vulgare | 189 | - | 4 | CAAT | common cis-acting element in promoter and enhancer regions |
| CAAT-box | Glycine max | 197 | - | 5 | CAATT | common cis-acting element in promoter and enhancer regions |
| CAAT-box | Hordeum vulgare | 198 | - | 4 | CAAT | common cis-acting element in promoter and enhancer regions |

> 2018/04/13 10:10:12  
+ TTAATTTTCA GGTGCTCTCA TTCGTTTTCA CCTTCGCCAA TTGTGAAGTT GAAGCCACAT TTTTCTAGGG   
  
  
+ GCTACGATCT TGAAATAATG TCGATTTGAT TTTGTAGATA CCTTTTAATC ATTCTTCTAA TAGAATCATC   
  
  
+ TTTTTTTTTT TTTGGGTTGA TCTCATTTTG TTAAATTGAA TAAATTTAAT TGAATGAATT GAAAATTCTT   
  
  
+ TTTTTTTAAG GATATAAATT TTGTTGGTAT TTTAAAAAAA TATTTTTTAT TAAATACTTT TTAAAAAGAT   
  
  
+ GTTGATATAT ATATATATAT GTTAGAAAAT AAAGGATATA AATTTTGTTA GTATTTTAAA AAAATATTTT   
  
  
+ TTATTAAATA CTTTTTAAAA AGATGTTGAT ATATATATAT ATGTTAGAAA ATACAATAAA ATTTATTGCA   
  
  
+ TTTGTTTTCT TGATTAATGT AACCAATTAT AAAAATAGAA TTTTAAAAAA AAGATTAATA AGAATCATAG   
  
  
+ TAACAGATGA TTTTTTGATT CAAAGGAAAC ATTTCTTCAA TATTTTAAAG ATTCATTATT AAAAATGATT   
  
  
+ AGAAATTATG GTAAAATCTA TGAAATTTTT AAAAAATATA AATAGTATTG AAGTAAGAAG GGTAGTTATA   
  
  
+ TTTTTATAAC TTAAATAATA ATATAAAATA AAAAATGTAA CCATTAATTT TAATTAAATT ACCATGTATC   
  
  
+ TTAATTTATT TAAGTCGAAA ATTTGATCCT TTCTTAATTT TATTTATTAA GACACGTGAA AATACCTTTT   
  
  
+ TGCTTAATTA ATTGAATAAG AGCCACGCAT AGCATAACAA CATTATCTAT TTGCCCATTT CATGAGCAAT   
  
  
+ AAAGAATTCC ACTGACTGAA AAGAATACAA GACCCCCCAA AAAAAAAAGA AAAAAAGAGA AAAGAGAAAA   
  
  
+ AAAAAGATGA AAAAAATGAT TAAAGGGAAA AGAAGAAAAG GGAATATCTT AGGAGGATTC AGTTGGTGAG   
  
  
+ CTTGAATTGA GTTCACACAG AGGAGAGTTC ACATGCAAGA CGACGGAGCT TGGGGCTGTA ATGGGTAAGA   
  
  
+ ATCTCACGTG CAGAGAAACC CCGTTCTCAC TGGGGGAACC TGTTAACTGT TGAGCCGGCC AACGAAGCGT   
  
  
+ ATGAAAACGT CGGCCAACCA CGACAGAATA CTCGGCCGCA TGCATTGAAT TTGCCACCGG CACTGTGGGT   
  
  
+ GTCCGTACGA AATGTTGACC TACGATCTCC GGTCAATCTT TGTTGCCATT TGTCTACTCT CGAGCAGAAT   
  
  
+ AGAAAAGTAA CTCACCTTCT CTTCTCTTTG GCCAAATCTC ACTTTCTTCT ATACCTAAAG CTCTCTCTCT   
  
  
+ CTCTCTCTCC CCCTCCCACC TACCTGATAA ACGCAATCAG GCCTAGCTTT CTCTCTCTCT CTCTCTCTCT   
  
  
+ CTTTCTTTCT CAGTAAGCAA ACATCACTCC CGCTGTGTGT CACTCTCTGT TAAGTTACAA GCTTTTATAT   
  
  
+ GAGGATTTTA TAGGCCTGAT TGATAGAAA  

- AATTAAAAGT CCACGAGAGT AAGCAAAAGT GGAAGCGGTT AACACTTCAA CTTCGGTGTA AAAAGATCCC   
  
  
- CGATGCTAGA ACTTTATTAC AGCTAAACTA AAACATCTAT GGAAAATTAG TAAGAAGATT ATCTTAGTAG   
  
  
- AAAAAAAAAA AAACCCAACT AGAGTAAAAC AATTTAACTT ATTTAAATTA ACTTACTTAA CTTTTAAGAA   
  
  
- AAAAAAATTC CTATATTTAA AACAACCATA AAATTTTTTT ATAAAAAATA ATTTATGAAA AATTTTTCTA   
  
  
- CAACTATATA TATATATATA CAATCTTTTA TTTCCTATAT TTAAAACAAT CATAAAATTT TTTTATAAAA   
  
  
- AATAATTTAT GAAAAATTTT TCTACAACTA TATATATATA TACAATCTTT TATGTTATTT TAAATAACGT   
  
  
- AAACAAAAGA ACTAATTACA TTGGTTAATA TTTTTATCTT AAAATTTTTT TTCTAATTAT TCTTAGTATC   
  
  
- ATTGTCTACT AAAAAACTAA GTTTCCTTTG TAAAGAAGTT ATAAAATTTC TAAGTAATAA TTTTTACTAA   
  
  
- TCTTTAATAC CATTTTAGAT ACTTTAAAAA TTTTTTATAT TTATCATAAC TTCATTCTTC CCATCAATAT   
  
  
- AAAAATATTG AATTTATTAT TATATTTTAT TTTTTACATT GGTAATTAAA ATTAATTTAA TGGTACATAG   
  
  
- AATTAAATAA ATTCAGCTTT TAAACTAGGA AAGAATTAAA ATAAATAATT CTGTGCACTT TTATGGAAAA   
  
  
- ACGAATTAAT TAACTTATTC TCGGTGCGTA TCGTATTGTT GTAATAGATA AACGGGTAAA GTACTCGTTA   
  
  
- TTTCTTAAGG TGACTGACTT TTCTTATGTT CTGGGGGGTT TTTTTTTTCT TTTTTTCTCT TTTCTCTTTT   
  
  
- TTTTTCTACT TTTTTTACTA ATTTCCCTTT TCTTCTTTTC CCTTATAGAA TCCTCCTAAG TCAACCACTC   
  
  
- GAACTTAACT CAAGTGTGTC TCCTCTCAAG TGTACGTTCT GCTGCCTCGA ACCCCGACAT TACCCATTCT   
  
  
- TAGAGTGCAC GTCTCTTTGG GGCAAGAGTG ACCCCCTTGG ACAATTGACA ACTCGGCCGG TTGCTTCGCA   
  
  
- TACTTTTGCA GCCGGTTGGT GCTGTCTTAT GAGCCGGCGT ACGTAACTTA AACGGTGGCC GTGACACCCA   
  
  
- CAGGCATGCT TTACAACTGG ATGCTAGAGG CCAGTTAGAA ACAACGGTAA ACAGATGAGA GCTCGTCTTA   
  
  
- TCTTTTCATT GAGTGGAAGA GAAGAGAAAC CGGTTTAGAG TGAAAGAAGA TATGGATTTC GAGAGAGAGA   
  
  
- GAGAGAGAGG GGGAGGGTGG ATGGACTATT TGCGTTAGTC CGGATCGAAA GAGAGAGAGA GAGAGAGAGA   
  
  
- GAAAGAAAGA GTCATTCGTT TGTAGTGAGG GCGACACACA GTGAGAGACA ATTCAATGTT CGAAAATATA   
  
  
- CTCCTAAAAT ATCCGGACTA ACTATCTTT

+     CE1

| Site Name | Organism | Position | Strand | Matrix score. | sequence | function |
| --- | --- | --- | --- | --- | --- | --- |
| CE1 | Hordeum vulgare | 1172 | + | 9 | TGCCACCGG | "cis-acting element associated to ABRE, involved in ABA responsiveness |

> 2018/04/13 10:10:12  
+ TTAATTTTCA GGTGCTCTCA TTCGTTTTCA CCTTCGCCAA TTGTGAAGTT GAAGCCACAT TTTTCTAGGG   
  
  
+ GCTACGATCT TGAAATAATG TCGATTTGAT TTTGTAGATA CCTTTTAATC ATTCTTCTAA TAGAATCATC   
  
  
+ TTTTTTTTTT TTTGGGTTGA TCTCATTTTG TTAAATTGAA TAAATTTAAT TGAATGAATT GAAAATTCTT   
  
  
+ TTTTTTTAAG GATATAAATT TTGTTGGTAT TTTAAAAAAA TATTTTTTAT TAAATACTTT TTAAAAAGAT   
  
  
+ GTTGATATAT ATATATATAT GTTAGAAAAT AAAGGATATA AATTTTGTTA GTATTTTAAA AAAATATTTT   
  
  
+ TTATTAAATA CTTTTTAAAA AGATGTTGAT ATATATATAT ATGTTAGAAA ATACAATAAA ATTTATTGCA   
  
  
+ TTTGTTTTCT TGATTAATGT AACCAATTAT AAAAATAGAA TTTTAAAAAA AAGATTAATA AGAATCATAG   
  
  
+ TAACAGATGA TTTTTTGATT CAAAGGAAAC ATTTCTTCAA TATTTTAAAG ATTCATTATT AAAAATGATT   
  
  
+ AGAAATTATG GTAAAATCTA TGAAATTTTT AAAAAATATA AATAGTATTG AAGTAAGAAG GGTAGTTATA   
  
  
+ TTTTTATAAC TTAAATAATA ATATAAAATA AAAAATGTAA CCATTAATTT TAATTAAATT ACCATGTATC   
  
  
+ TTAATTTATT TAAGTCGAAA ATTTGATCCT TTCTTAATTT TATTTATTAA GACACGTGAA AATACCTTTT   
  
  
+ TGCTTAATTA ATTGAATAAG AGCCACGCAT AGCATAACAA CATTATCTAT TTGCCCATTT CATGAGCAAT   
  
  
+ AAAGAATTCC ACTGACTGAA AAGAATACAA GACCCCCCAA AAAAAAAAGA AAAAAAGAGA AAAGAGAAAA   
  
  
+ AAAAAGATGA AAAAAATGAT TAAAGGGAAA AGAAGAAAAG GGAATATCTT AGGAGGATTC AGTTGGTGAG   
  
  
+ CTTGAATTGA GTTCACACAG AGGAGAGTTC ACATGCAAGA CGACGGAGCT TGGGGCTGTA ATGGGTAAGA   
  
  
+ ATCTCACGTG CAGAGAAACC CCGTTCTCAC TGGGGGAACC TGTTAACTGT TGAGCCGGCC AACGAAGCGT   
  
  
+ ATGAAAACGT CGGCCAACCA CGACAGAATA CTCGGCCGCA TGCATTGAAT TTGCCACCGG CACTGTGGGT   
  
  
+ GTCCGTACGA AATGTTGACC TACGATCTCC GGTCAATCTT TGTTGCCATT TGTCTACTCT CGAGCAGAAT   
  
  
+ AGAAAAGTAA CTCACCTTCT CTTCTCTTTG GCCAAATCTC ACTTTCTTCT ATACCTAAAG CTCTCTCTCT   
  
  
+ CTCTCTCTCC CCCTCCCACC TACCTGATAA ACGCAATCAG GCCTAGCTTT CTCTCTCTCT CTCTCTCTCT   
  
  
+ CTTTCTTTCT CAGTAAGCAA ACATCACTCC CGCTGTGTGT CACTCTCTGT TAAGTTACAA GCTTTTATAT   
  
  
+ GAGGATTTTA TAGGCCTGAT TGATAGAAA  

- AATTAAAAGT CCACGAGAGT AAGCAAAAGT GGAAGCGGTT AACACTTCAA CTTCGGTGTA AAAAGATCCC   
  
  
- CGATGCTAGA ACTTTATTAC AGCTAAACTA AAACATCTAT GGAAAATTAG TAAGAAGATT ATCTTAGTAG   
  
  
- AAAAAAAAAA AAACCCAACT AGAGTAAAAC AATTTAACTT ATTTAAATTA ACTTACTTAA CTTTTAAGAA   
  
  
- AAAAAAATTC CTATATTTAA AACAACCATA AAATTTTTTT ATAAAAAATA ATTTATGAAA AATTTTTCTA   
  
  
- CAACTATATA TATATATATA CAATCTTTTA TTTCCTATAT TTAAAACAAT CATAAAATTT TTTTATAAAA   
  
  
- AATAATTTAT GAAAAATTTT TCTACAACTA TATATATATA TACAATCTTT TATGTTATTT TAAATAACGT   
  
  
- AAACAAAAGA ACTAATTACA TTGGTTAATA TTTTTATCTT AAAATTTTTT TTCTAATTAT TCTTAGTATC   
  
  
- ATTGTCTACT AAAAAACTAA GTTTCCTTTG TAAAGAAGTT ATAAAATTTC TAAGTAATAA TTTTTACTAA   
  
  
- TCTTTAATAC CATTTTAGAT ACTTTAAAAA TTTTTTATAT TTATCATAAC TTCATTCTTC CCATCAATAT   
  
  
- AAAAATATTG AATTTATTAT TATATTTTAT TTTTTACATT GGTAATTAAA ATTAATTTAA TGGTACATAG   
  
  
- AATTAAATAA ATTCAGCTTT TAAACTAGGA AAGAATTAAA ATAAATAATT CTGTGCACTT TTATGGAAAA   
  
  
- ACGAATTAAT TAACTTATTC TCGGTGCGTA TCGTATTGTT GTAATAGATA AACGGGTAAA GTACTCGTTA   
  
  
- TTTCTTAAGG TGACTGACTT TTCTTATGTT CTGGGGGGTT TTTTTTTTCT TTTTTTCTCT TTTCTCTTTT   
  
  
- TTTTTCTACT TTTTTTACTA ATTTCCCTTT TCTTCTTTTC CCTTATAGAA TCCTCCTAAG TCAACCACTC   
  
  
- GAACTTAACT CAAGTGTGTC TCCTCTCAAG TGTACGTTCT GCTGCCTCGA ACCCCGACAT TACCCATTCT   
  
  
- TAGAGTGCAC GTCTCTTTGG GGCAAGAGTG ACCCCCTTGG ACAATTGACA ACTCGGCCGG TTGCTTCGCA   
  
  
- TACTTTTGCA GCCGGTTGGT GCTGTCTTAT GAGCCGGCGT ACGTAACTTA AACGGTGGCC GTGACACCCA   
  
  
- CAGGCATGCT TTACAACTGG ATGCTAGAGG CCAGTTAGAA ACAACGGTAA ACAGATGAGA GCTCGTCTTA   
  
  
- TCTTTTCATT GAGTGGAAGA GAAGAGAAAC CGGTTTAGAG TGAAAGAAGA TATGGATTTC GAGAGAGAGA   
  
  
- GAGAGAGAGG GGGAGGGTGG ATGGACTATT TGCGTTAGTC CGGATCGAAA GAGAGAGAGA GAGAGAGAGA   
  
  
- GAAAGAAAGA GTCATTCGTT TGTAGTGAGG GCGACACACA GTGAGAGACA ATTCAATGTT CGAAAATATA   
  
  
- CTCCTAAAAT ATCCGGACTA ACTATCTTT

+     G-Box

| Site Name | Organism | Position | Strand | Matrix score. | sequence | function |
| --- | --- | --- | --- | --- | --- | --- |
| G-Box | Pisum sativum | 753 | - | 6 | CACGTG | cis-acting regulatory element involved in light responsiveness |
| G-Box | Pisum sativum | 1055 | - | 6 | CACGTG | cis-acting regulatory element involved in light responsiveness |

> 2018/04/13 10:10:12  
+ TTAATTTTCA GGTGCTCTCA TTCGTTTTCA CCTTCGCCAA TTGTGAAGTT GAAGCCACAT TTTTCTAGGG   
  
  
+ GCTACGATCT TGAAATAATG TCGATTTGAT TTTGTAGATA CCTTTTAATC ATTCTTCTAA TAGAATCATC   
  
  
+ TTTTTTTTTT TTTGGGTTGA TCTCATTTTG TTAAATTGAA TAAATTTAAT TGAATGAATT GAAAATTCTT   
  
  
+ TTTTTTTAAG GATATAAATT TTGTTGGTAT TTTAAAAAAA TATTTTTTAT TAAATACTTT TTAAAAAGAT   
  
  
+ GTTGATATAT ATATATATAT GTTAGAAAAT AAAGGATATA AATTTTGTTA GTATTTTAAA AAAATATTTT   
  
  
+ TTATTAAATA CTTTTTAAAA AGATGTTGAT ATATATATAT ATGTTAGAAA ATACAATAAA ATTTATTGCA   
  
  
+ TTTGTTTTCT TGATTAATGT AACCAATTAT AAAAATAGAA TTTTAAAAAA AAGATTAATA AGAATCATAG   
  
  
+ TAACAGATGA TTTTTTGATT CAAAGGAAAC ATTTCTTCAA TATTTTAAAG ATTCATTATT AAAAATGATT   
  
  
+ AGAAATTATG GTAAAATCTA TGAAATTTTT AAAAAATATA AATAGTATTG AAGTAAGAAG GGTAGTTATA   
  
  
+ TTTTTATAAC TTAAATAATA ATATAAAATA AAAAATGTAA CCATTAATTT TAATTAAATT ACCATGTATC   
  
  
+ TTAATTTATT TAAGTCGAAA ATTTGATCCT TTCTTAATTT TATTTATTAA GACACGTGAA AATACCTTTT   
  
  
+ TGCTTAATTA ATTGAATAAG AGCCACGCAT AGCATAACAA CATTATCTAT TTGCCCATTT CATGAGCAAT   
  
  
+ AAAGAATTCC ACTGACTGAA AAGAATACAA GACCCCCCAA AAAAAAAAGA AAAAAAGAGA AAAGAGAAAA   
  
  
+ AAAAAGATGA AAAAAATGAT TAAAGGGAAA AGAAGAAAAG GGAATATCTT AGGAGGATTC AGTTGGTGAG   
  
  
+ CTTGAATTGA GTTCACACAG AGGAGAGTTC ACATGCAAGA CGACGGAGCT TGGGGCTGTA ATGGGTAAGA   
  
  
+ ATCTCACGTG CAGAGAAACC CCGTTCTCAC TGGGGGAACC TGTTAACTGT TGAGCCGGCC AACGAAGCGT   
  
  
+ ATGAAAACGT CGGCCAACCA CGACAGAATA CTCGGCCGCA TGCATTGAAT TTGCCACCGG CACTGTGGGT   
  
  
+ GTCCGTACGA AATGTTGACC TACGATCTCC GGTCAATCTT TGTTGCCATT TGTCTACTCT CGAGCAGAAT   
  
  
+ AGAAAAGTAA CTCACCTTCT CTTCTCTTTG GCCAAATCTC ACTTTCTTCT ATACCTAAAG CTCTCTCTCT   
  
  
+ CTCTCTCTCC CCCTCCCACC TACCTGATAA ACGCAATCAG GCCTAGCTTT CTCTCTCTCT CTCTCTCTCT   
  
  
+ CTTTCTTTCT CAGTAAGCAA ACATCACTCC CGCTGTGTGT CACTCTCTGT TAAGTTACAA GCTTTTATAT   
  
  
+ GAGGATTTTA TAGGCCTGAT TGATAGAAA  

- AATTAAAAGT CCACGAGAGT AAGCAAAAGT GGAAGCGGTT AACACTTCAA CTTCGGTGTA AAAAGATCCC   
  
  
- CGATGCTAGA ACTTTATTAC AGCTAAACTA AAACATCTAT GGAAAATTAG TAAGAAGATT ATCTTAGTAG   
  
  
- AAAAAAAAAA AAACCCAACT AGAGTAAAAC AATTTAACTT ATTTAAATTA ACTTACTTAA CTTTTAAGAA   
  
  
- AAAAAAATTC CTATATTTAA AACAACCATA AAATTTTTTT ATAAAAAATA ATTTATGAAA AATTTTTCTA   
  
  
- CAACTATATA TATATATATA CAATCTTTTA TTTCCTATAT TTAAAACAAT CATAAAATTT TTTTATAAAA   
  
  
- AATAATTTAT GAAAAATTTT TCTACAACTA TATATATATA TACAATCTTT TATGTTATTT TAAATAACGT   
  
  
- AAACAAAAGA ACTAATTACA TTGGTTAATA TTTTTATCTT AAAATTTTTT TTCTAATTAT TCTTAGTATC   
  
  
- ATTGTCTACT AAAAAACTAA GTTTCCTTTG TAAAGAAGTT ATAAAATTTC TAAGTAATAA TTTTTACTAA   
  
  
- TCTTTAATAC CATTTTAGAT ACTTTAAAAA TTTTTTATAT TTATCATAAC TTCATTCTTC CCATCAATAT   
  
  
- AAAAATATTG AATTTATTAT TATATTTTAT TTTTTACATT GGTAATTAAA ATTAATTTAA TGGTACATAG   
  
  
- AATTAAATAA ATTCAGCTTT TAAACTAGGA AAGAATTAAA ATAAATAATT CTGTGCACTT TTATGGAAAA   
  
  
- ACGAATTAAT TAACTTATTC TCGGTGCGTA TCGTATTGTT GTAATAGATA AACGGGTAAA GTACTCGTTA   
  
  
- TTTCTTAAGG TGACTGACTT TTCTTATGTT CTGGGGGGTT TTTTTTTTCT TTTTTTCTCT TTTCTCTTTT   
  
  
- TTTTTCTACT TTTTTTACTA ATTTCCCTTT TCTTCTTTTC CCTTATAGAA TCCTCCTAAG TCAACCACTC   
  
  
- GAACTTAACT CAAGTGTGTC TCCTCTCAAG TGTACGTTCT GCTGCCTCGA ACCCCGACAT TACCCATTCT   
  
  
- TAGAGTGCAC GTCTCTTTGG GGCAAGAGTG ACCCCCTTGG ACAATTGACA ACTCGGCCGG TTGCTTCGCA   
  
  
- TACTTTTGCA GCCGGTTGGT GCTGTCTTAT GAGCCGGCGT ACGTAACTTA AACGGTGGCC GTGACACCCA   
  
  
- CAGGCATGCT TTACAACTGG ATGCTAGAGG CCAGTTAGAA ACAACGGTAA ACAGATGAGA GCTCGTCTTA   
  
  
- TCTTTTCATT GAGTGGAAGA GAAGAGAAAC CGGTTTAGAG TGAAAGAAGA TATGGATTTC GAGAGAGAGA   
  
  
- GAGAGAGAGG GGGAGGGTGG ATGGACTATT TGCGTTAGTC CGGATCGAAA GAGAGAGAGA GAGAGAGAGA   
  
  
- GAAAGAAAGA GTCATTCGTT TGTAGTGAGG GCGACACACA GTGAGAGACA ATTCAATGTT CGAAAATATA   
  
  
- CTCCTAAAAT ATCCGGACTA ACTATCTTT

+     G-box

| Site Name | Organism | Position | Strand | Matrix score. | sequence | function |
| --- | --- | --- | --- | --- | --- | --- |
| G-box | Zea mays | 1139 | + | 6 | CACGAC | cis-acting regulatory element involved in light responsiveness |
| G-box | Arabidopsis thaliana | 1055 | - | 6 | CACGTG | cis-acting regulatory element involved in light responsiveness |
| G-box | Arabidopsis thaliana | 753 | - | 6 | CACGTG | cis-acting regulatory element involved in light responsiveness |

> 2018/04/13 10:10:12  
+ TTAATTTTCA GGTGCTCTCA TTCGTTTTCA CCTTCGCCAA TTGTGAAGTT GAAGCCACAT TTTTCTAGGG   
  
  
+ GCTACGATCT TGAAATAATG TCGATTTGAT TTTGTAGATA CCTTTTAATC ATTCTTCTAA TAGAATCATC   
  
  
+ TTTTTTTTTT TTTGGGTTGA TCTCATTTTG TTAAATTGAA TAAATTTAAT TGAATGAATT GAAAATTCTT   
  
  
+ TTTTTTTAAG GATATAAATT TTGTTGGTAT TTTAAAAAAA TATTTTTTAT TAAATACTTT TTAAAAAGAT   
  
  
+ GTTGATATAT ATATATATAT GTTAGAAAAT AAAGGATATA AATTTTGTTA GTATTTTAAA AAAATATTTT   
  
  
+ TTATTAAATA CTTTTTAAAA AGATGTTGAT ATATATATAT ATGTTAGAAA ATACAATAAA ATTTATTGCA   
  
  
+ TTTGTTTTCT TGATTAATGT AACCAATTAT AAAAATAGAA TTTTAAAAAA AAGATTAATA AGAATCATAG   
  
  
+ TAACAGATGA TTTTTTGATT CAAAGGAAAC ATTTCTTCAA TATTTTAAAG ATTCATTATT AAAAATGATT   
  
  
+ AGAAATTATG GTAAAATCTA TGAAATTTTT AAAAAATATA AATAGTATTG AAGTAAGAAG GGTAGTTATA   
  
  
+ TTTTTATAAC TTAAATAATA ATATAAAATA AAAAATGTAA CCATTAATTT TAATTAAATT ACCATGTATC   
  
  
+ TTAATTTATT TAAGTCGAAA ATTTGATCCT TTCTTAATTT TATTTATTAA GACACGTGAA AATACCTTTT   
  
  
+ TGCTTAATTA ATTGAATAAG AGCCACGCAT AGCATAACAA CATTATCTAT TTGCCCATTT CATGAGCAAT   
  
  
+ AAAGAATTCC ACTGACTGAA AAGAATACAA GACCCCCCAA AAAAAAAAGA AAAAAAGAGA AAAGAGAAAA   
  
  
+ AAAAAGATGA AAAAAATGAT TAAAGGGAAA AGAAGAAAAG GGAATATCTT AGGAGGATTC AGTTGGTGAG   
  
  
+ CTTGAATTGA GTTCACACAG AGGAGAGTTC ACATGCAAGA CGACGGAGCT TGGGGCTGTA ATGGGTAAGA   
  
  
+ ATCTCACGTG CAGAGAAACC CCGTTCTCAC TGGGGGAACC TGTTAACTGT TGAGCCGGCC AACGAAGCGT   
  
  
+ ATGAAAACGT CGGCCAACCA CGACAGAATA CTCGGCCGCA TGCATTGAAT TTGCCACCGG CACTGTGGGT   
  
  
+ GTCCGTACGA AATGTTGACC TACGATCTCC GGTCAATCTT TGTTGCCATT TGTCTACTCT CGAGCAGAAT   
  
  
+ AGAAAAGTAA CTCACCTTCT CTTCTCTTTG GCCAAATCTC ACTTTCTTCT ATACCTAAAG CTCTCTCTCT   
  
  
+ CTCTCTCTCC CCCTCCCACC TACCTGATAA ACGCAATCAG GCCTAGCTTT CTCTCTCTCT CTCTCTCTCT   
  
  
+ CTTTCTTTCT CAGTAAGCAA ACATCACTCC CGCTGTGTGT CACTCTCTGT TAAGTTACAA GCTTTTATAT   
  
  
+ GAGGATTTTA TAGGCCTGAT TGATAGAAA  

- AATTAAAAGT CCACGAGAGT AAGCAAAAGT GGAAGCGGTT AACACTTCAA CTTCGGTGTA AAAAGATCCC   
  
  
- CGATGCTAGA ACTTTATTAC AGCTAAACTA AAACATCTAT GGAAAATTAG TAAGAAGATT ATCTTAGTAG   
  
  
- AAAAAAAAAA AAACCCAACT AGAGTAAAAC AATTTAACTT ATTTAAATTA ACTTACTTAA CTTTTAAGAA   
  
  
- AAAAAAATTC CTATATTTAA AACAACCATA AAATTTTTTT ATAAAAAATA ATTTATGAAA AATTTTTCTA   
  
  
- CAACTATATA TATATATATA CAATCTTTTA TTTCCTATAT TTAAAACAAT CATAAAATTT TTTTATAAAA   
  
  
- AATAATTTAT GAAAAATTTT TCTACAACTA TATATATATA TACAATCTTT TATGTTATTT TAAATAACGT   
  
  
- AAACAAAAGA ACTAATTACA TTGGTTAATA TTTTTATCTT AAAATTTTTT TTCTAATTAT TCTTAGTATC   
  
  
- ATTGTCTACT AAAAAACTAA GTTTCCTTTG TAAAGAAGTT ATAAAATTTC TAAGTAATAA TTTTTACTAA   
  
  
- TCTTTAATAC CATTTTAGAT ACTTTAAAAA TTTTTTATAT TTATCATAAC TTCATTCTTC CCATCAATAT   
  
  
- AAAAATATTG AATTTATTAT TATATTTTAT TTTTTACATT GGTAATTAAA ATTAATTTAA TGGTACATAG   
  
  
- AATTAAATAA ATTCAGCTTT TAAACTAGGA AAGAATTAAA ATAAATAATT CTGTGCACTT TTATGGAAAA   
  
  
- ACGAATTAAT TAACTTATTC TCGGTGCGTA TCGTATTGTT GTAATAGATA AACGGGTAAA GTACTCGTTA   
  
  
- TTTCTTAAGG TGACTGACTT TTCTTATGTT CTGGGGGGTT TTTTTTTTCT TTTTTTCTCT TTTCTCTTTT   
  
  
- TTTTTCTACT TTTTTTACTA ATTTCCCTTT TCTTCTTTTC CCTTATAGAA TCCTCCTAAG TCAACCACTC   
  
  
- GAACTTAACT CAAGTGTGTC TCCTCTCAAG TGTACGTTCT GCTGCCTCGA ACCCCGACAT TACCCATTCT   
  
  
- TAGAGTGCAC GTCTCTTTGG GGCAAGAGTG ACCCCCTTGG ACAATTGACA ACTCGGCCGG TTGCTTCGCA   
  
  
- TACTTTTGCA GCCGGTTGGT GCTGTCTTAT GAGCCGGCGT ACGTAACTTA AACGGTGGCC GTGACACCCA   
  
  
- CAGGCATGCT TTACAACTGG ATGCTAGAGG CCAGTTAGAA ACAACGGTAA ACAGATGAGA GCTCGTCTTA   
  
  
- TCTTTTCATT GAGTGGAAGA GAAGAGAAAC CGGTTTAGAG TGAAAGAAGA TATGGATTTC GAGAGAGAGA   
  
  
- GAGAGAGAGG GGGAGGGTGG ATGGACTATT TGCGTTAGTC CGGATCGAAA GAGAGAGAGA GAGAGAGAGA   
  
  
- GAAAGAAAGA GTCATTCGTT TGTAGTGAGG GCGACACACA GTGAGAGACA ATTCAATGTT CGAAAATATA   
  
  
- CTCCTAAAAT ATCCGGACTA ACTATCTTT

+     GA-motif

| Site Name | Organism | Position | Strand | Matrix score. | sequence | function |
| --- | --- | --- | --- | --- | --- | --- |
| GA-motif | Arabidopsis thaliana | 813 | - | 8 | ATAGATAA | part of a light responsive element |
| GA-motif | Helianthus annuus | 913 | + | 8 | AAAGATGA | part of a light responsive element |
| GA-motif | Helianthus annuus | 136 | - | 8 | AAAGATGA | part of a light responsive element |

> 2018/04/13 10:10:12  
+ TTAATTTTCA GGTGCTCTCA TTCGTTTTCA CCTTCGCCAA TTGTGAAGTT GAAGCCACAT TTTTCTAGGG   
  
  
+ GCTACGATCT TGAAATAATG TCGATTTGAT TTTGTAGATA CCTTTTAATC ATTCTTCTAA TAGAATCATC   
  
  
+ TTTTTTTTTT TTTGGGTTGA TCTCATTTTG TTAAATTGAA TAAATTTAAT TGAATGAATT GAAAATTCTT   
  
  
+ TTTTTTTAAG GATATAAATT TTGTTGGTAT TTTAAAAAAA TATTTTTTAT TAAATACTTT TTAAAAAGAT   
  
  
+ GTTGATATAT ATATATATAT GTTAGAAAAT AAAGGATATA AATTTTGTTA GTATTTTAAA AAAATATTTT   
  
  
+ TTATTAAATA CTTTTTAAAA AGATGTTGAT ATATATATAT ATGTTAGAAA ATACAATAAA ATTTATTGCA   
  
  
+ TTTGTTTTCT TGATTAATGT AACCAATTAT AAAAATAGAA TTTTAAAAAA AAGATTAATA AGAATCATAG   
  
  
+ TAACAGATGA TTTTTTGATT CAAAGGAAAC ATTTCTTCAA TATTTTAAAG ATTCATTATT AAAAATGATT   
  
  
+ AGAAATTATG GTAAAATCTA TGAAATTTTT AAAAAATATA AATAGTATTG AAGTAAGAAG GGTAGTTATA   
  
  
+ TTTTTATAAC TTAAATAATA ATATAAAATA AAAAATGTAA CCATTAATTT TAATTAAATT ACCATGTATC   
  
  
+ TTAATTTATT TAAGTCGAAA ATTTGATCCT TTCTTAATTT TATTTATTAA GACACGTGAA AATACCTTTT   
  
  
+ TGCTTAATTA ATTGAATAAG AGCCACGCAT AGCATAACAA CATTATCTAT TTGCCCATTT CATGAGCAAT   
  
  
+ AAAGAATTCC ACTGACTGAA AAGAATACAA GACCCCCCAA AAAAAAAAGA AAAAAAGAGA AAAGAGAAAA   
  
  
+ AAAAAGATGA AAAAAATGAT TAAAGGGAAA AGAAGAAAAG GGAATATCTT AGGAGGATTC AGTTGGTGAG   
  
  
+ CTTGAATTGA GTTCACACAG AGGAGAGTTC ACATGCAAGA CGACGGAGCT TGGGGCTGTA ATGGGTAAGA   
  
  
+ ATCTCACGTG CAGAGAAACC CCGTTCTCAC TGGGGGAACC TGTTAACTGT TGAGCCGGCC AACGAAGCGT   
  
  
+ ATGAAAACGT CGGCCAACCA CGACAGAATA CTCGGCCGCA TGCATTGAAT TTGCCACCGG CACTGTGGGT   
  
  
+ GTCCGTACGA AATGTTGACC TACGATCTCC GGTCAATCTT TGTTGCCATT TGTCTACTCT CGAGCAGAAT   
  
  
+ AGAAAAGTAA CTCACCTTCT CTTCTCTTTG GCCAAATCTC ACTTTCTTCT ATACCTAAAG CTCTCTCTCT   
  
  
+ CTCTCTCTCC CCCTCCCACC TACCTGATAA ACGCAATCAG GCCTAGCTTT CTCTCTCTCT CTCTCTCTCT   
  
  
+ CTTTCTTTCT CAGTAAGCAA ACATCACTCC CGCTGTGTGT CACTCTCTGT TAAGTTACAA GCTTTTATAT   
  
  
+ GAGGATTTTA TAGGCCTGAT TGATAGAAA  

- AATTAAAAGT CCACGAGAGT AAGCAAAAGT GGAAGCGGTT AACACTTCAA CTTCGGTGTA AAAAGATCCC   
  
  
- CGATGCTAGA ACTTTATTAC AGCTAAACTA AAACATCTAT GGAAAATTAG TAAGAAGATT ATCTTAGTAG   
  
  
- AAAAAAAAAA AAACCCAACT AGAGTAAAAC AATTTAACTT ATTTAAATTA ACTTACTTAA CTTTTAAGAA   
  
  
- AAAAAAATTC CTATATTTAA AACAACCATA AAATTTTTTT ATAAAAAATA ATTTATGAAA AATTTTTCTA   
  
  
- CAACTATATA TATATATATA CAATCTTTTA TTTCCTATAT TTAAAACAAT CATAAAATTT TTTTATAAAA   
  
  
- AATAATTTAT GAAAAATTTT TCTACAACTA TATATATATA TACAATCTTT TATGTTATTT TAAATAACGT   
  
  
- AAACAAAAGA ACTAATTACA TTGGTTAATA TTTTTATCTT AAAATTTTTT TTCTAATTAT TCTTAGTATC   
  
  
- ATTGTCTACT AAAAAACTAA GTTTCCTTTG TAAAGAAGTT ATAAAATTTC TAAGTAATAA TTTTTACTAA   
  
  
- TCTTTAATAC CATTTTAGAT ACTTTAAAAA TTTTTTATAT TTATCATAAC TTCATTCTTC CCATCAATAT   
  
  
- AAAAATATTG AATTTATTAT TATATTTTAT TTTTTACATT GGTAATTAAA ATTAATTTAA TGGTACATAG   
  
  
- AATTAAATAA ATTCAGCTTT TAAACTAGGA AAGAATTAAA ATAAATAATT CTGTGCACTT TTATGGAAAA   
  
  
- ACGAATTAAT TAACTTATTC TCGGTGCGTA TCGTATTGTT GTAATAGATA AACGGGTAAA GTACTCGTTA   
  
  
- TTTCTTAAGG TGACTGACTT TTCTTATGTT CTGGGGGGTT TTTTTTTTCT TTTTTTCTCT TTTCTCTTTT   
  
  
- TTTTTCTACT TTTTTTACTA ATTTCCCTTT TCTTCTTTTC CCTTATAGAA TCCTCCTAAG TCAACCACTC   
  
  
- GAACTTAACT CAAGTGTGTC TCCTCTCAAG TGTACGTTCT GCTGCCTCGA ACCCCGACAT TACCCATTCT   
  
  
- TAGAGTGCAC GTCTCTTTGG GGCAAGAGTG ACCCCCTTGG ACAATTGACA ACTCGGCCGG TTGCTTCGCA   
  
  
- TACTTTTGCA GCCGGTTGGT GCTGTCTTAT GAGCCGGCGT ACGTAACTTA AACGGTGGCC GTGACACCCA   
  
  
- CAGGCATGCT TTACAACTGG ATGCTAGAGG CCAGTTAGAA ACAACGGTAA ACAGATGAGA GCTCGTCTTA   
  
  
- TCTTTTCATT GAGTGGAAGA GAAGAGAAAC CGGTTTAGAG TGAAAGAAGA TATGGATTTC GAGAGAGAGA   
  
  
- GAGAGAGAGG GGGAGGGTGG ATGGACTATT TGCGTTAGTC CGGATCGAAA GAGAGAGAGA GAGAGAGAGA   
  
  
- GAAAGAAAGA GTCATTCGTT TGTAGTGAGG GCGACACACA GTGAGAGACA ATTCAATGTT CGAAAATATA   
  
  
- CTCCTAAAAT ATCCGGACTA ACTATCTTT

+     GAG-motif

| Site Name | Organism | Position | Strand | Matrix score. | sequence | function |
| --- | --- | --- | --- | --- | --- | --- |
| GAG-motif | Arabidopsis thaliana | 1442 | - | 7 | AGAGAGT | part of a light responsive element |

> 2018/04/13 10:10:12  
+ TTAATTTTCA GGTGCTCTCA TTCGTTTTCA CCTTCGCCAA TTGTGAAGTT GAAGCCACAT TTTTCTAGGG   
  
  
+ GCTACGATCT TGAAATAATG TCGATTTGAT TTTGTAGATA CCTTTTAATC ATTCTTCTAA TAGAATCATC   
  
  
+ TTTTTTTTTT TTTGGGTTGA TCTCATTTTG TTAAATTGAA TAAATTTAAT TGAATGAATT GAAAATTCTT   
  
  
+ TTTTTTTAAG GATATAAATT TTGTTGGTAT TTTAAAAAAA TATTTTTTAT TAAATACTTT TTAAAAAGAT   
  
  
+ GTTGATATAT ATATATATAT GTTAGAAAAT AAAGGATATA AATTTTGTTA GTATTTTAAA AAAATATTTT   
  
  
+ TTATTAAATA CTTTTTAAAA AGATGTTGAT ATATATATAT ATGTTAGAAA ATACAATAAA ATTTATTGCA   
  
  
+ TTTGTTTTCT TGATTAATGT AACCAATTAT AAAAATAGAA TTTTAAAAAA AAGATTAATA AGAATCATAG   
  
  
+ TAACAGATGA TTTTTTGATT CAAAGGAAAC ATTTCTTCAA TATTTTAAAG ATTCATTATT AAAAATGATT   
  
  
+ AGAAATTATG GTAAAATCTA TGAAATTTTT AAAAAATATA AATAGTATTG AAGTAAGAAG GGTAGTTATA   
  
  
+ TTTTTATAAC TTAAATAATA ATATAAAATA AAAAATGTAA CCATTAATTT TAATTAAATT ACCATGTATC   
  
  
+ TTAATTTATT TAAGTCGAAA ATTTGATCCT TTCTTAATTT TATTTATTAA GACACGTGAA AATACCTTTT   
  
  
+ TGCTTAATTA ATTGAATAAG AGCCACGCAT AGCATAACAA CATTATCTAT TTGCCCATTT CATGAGCAAT   
  
  
+ AAAGAATTCC ACTGACTGAA AAGAATACAA GACCCCCCAA AAAAAAAAGA AAAAAAGAGA AAAGAGAAAA   
  
  
+ AAAAAGATGA AAAAAATGAT TAAAGGGAAA AGAAGAAAAG GGAATATCTT AGGAGGATTC AGTTGGTGAG   
  
  
+ CTTGAATTGA GTTCACACAG AGGAGAGTTC ACATGCAAGA CGACGGAGCT TGGGGCTGTA ATGGGTAAGA   
  
  
+ ATCTCACGTG CAGAGAAACC CCGTTCTCAC TGGGGGAACC TGTTAACTGT TGAGCCGGCC AACGAAGCGT   
  
  
+ ATGAAAACGT CGGCCAACCA CGACAGAATA CTCGGCCGCA TGCATTGAAT TTGCCACCGG CACTGTGGGT   
  
  
+ GTCCGTACGA AATGTTGACC TACGATCTCC GGTCAATCTT TGTTGCCATT TGTCTACTCT CGAGCAGAAT   
  
  
+ AGAAAAGTAA CTCACCTTCT CTTCTCTTTG GCCAAATCTC ACTTTCTTCT ATACCTAAAG CTCTCTCTCT   
  
  
+ CTCTCTCTCC CCCTCCCACC TACCTGATAA ACGCAATCAG GCCTAGCTTT CTCTCTCTCT CTCTCTCTCT   
  
  
+ CTTTCTTTCT CAGTAAGCAA ACATCACTCC CGCTGTGTGT CACTCTCTGT TAAGTTACAA GCTTTTATAT   
  
  
+ GAGGATTTTA TAGGCCTGAT TGATAGAAA  

- AATTAAAAGT CCACGAGAGT AAGCAAAAGT GGAAGCGGTT AACACTTCAA CTTCGGTGTA AAAAGATCCC   
  
  
- CGATGCTAGA ACTTTATTAC AGCTAAACTA AAACATCTAT GGAAAATTAG TAAGAAGATT ATCTTAGTAG   
  
  
- AAAAAAAAAA AAACCCAACT AGAGTAAAAC AATTTAACTT ATTTAAATTA ACTTACTTAA CTTTTAAGAA   
  
  
- AAAAAAATTC CTATATTTAA AACAACCATA AAATTTTTTT ATAAAAAATA ATTTATGAAA AATTTTTCTA   
  
  
- CAACTATATA TATATATATA CAATCTTTTA TTTCCTATAT TTAAAACAAT CATAAAATTT TTTTATAAAA   
  
  
- AATAATTTAT GAAAAATTTT TCTACAACTA TATATATATA TACAATCTTT TATGTTATTT TAAATAACGT   
  
  
- AAACAAAAGA ACTAATTACA TTGGTTAATA TTTTTATCTT AAAATTTTTT TTCTAATTAT TCTTAGTATC   
  
  
- ATTGTCTACT AAAAAACTAA GTTTCCTTTG TAAAGAAGTT ATAAAATTTC TAAGTAATAA TTTTTACTAA   
  
  
- TCTTTAATAC CATTTTAGAT ACTTTAAAAA TTTTTTATAT TTATCATAAC TTCATTCTTC CCATCAATAT   
  
  
- AAAAATATTG AATTTATTAT TATATTTTAT TTTTTACATT GGTAATTAAA ATTAATTTAA TGGTACATAG   
  
  
- AATTAAATAA ATTCAGCTTT TAAACTAGGA AAGAATTAAA ATAAATAATT CTGTGCACTT TTATGGAAAA   
  
  
- ACGAATTAAT TAACTTATTC TCGGTGCGTA TCGTATTGTT GTAATAGATA AACGGGTAAA GTACTCGTTA   
  
  
- TTTCTTAAGG TGACTGACTT TTCTTATGTT CTGGGGGGTT TTTTTTTTCT TTTTTTCTCT TTTCTCTTTT   
  
  
- TTTTTCTACT TTTTTTACTA ATTTCCCTTT TCTTCTTTTC CCTTATAGAA TCCTCCTAAG TCAACCACTC   
  
  
- GAACTTAACT CAAGTGTGTC TCCTCTCAAG TGTACGTTCT GCTGCCTCGA ACCCCGACAT TACCCATTCT   
  
  
- TAGAGTGCAC GTCTCTTTGG GGCAAGAGTG ACCCCCTTGG ACAATTGACA ACTCGGCCGG TTGCTTCGCA   
  
  
- TACTTTTGCA GCCGGTTGGT GCTGTCTTAT GAGCCGGCGT ACGTAACTTA AACGGTGGCC GTGACACCCA   
  
  
- CAGGCATGCT TTACAACTGG ATGCTAGAGG CCAGTTAGAA ACAACGGTAA ACAGATGAGA GCTCGTCTTA   
  
  
- TCTTTTCATT GAGTGGAAGA GAAGAGAAAC CGGTTTAGAG TGAAAGAAGA TATGGATTTC GAGAGAGAGA   
  
  
- GAGAGAGAGG GGGAGGGTGG ATGGACTATT TGCGTTAGTC CGGATCGAAA GAGAGAGAGA GAGAGAGAGA   
  
  
- GAAAGAAAGA GTCATTCGTT TGTAGTGAGG GCGACACACA GTGAGAGACA ATTCAATGTT CGAAAATATA   
  
  
- CTCCTAAAAT ATCCGGACTA ACTATCTTT

+     GCN4\_motif

| Site Name | Organism | Position | Strand | Matrix score. | sequence | function |
| --- | --- | --- | --- | --- | --- | --- |
| GCN4\_motif | Oryza sativa | 1436 | + | 7 | TGTGTCA | cis-regulatory element involved in endosperm expression |

> 2018/04/13 10:10:12  
+ TTAATTTTCA GGTGCTCTCA TTCGTTTTCA CCTTCGCCAA TTGTGAAGTT GAAGCCACAT TTTTCTAGGG   
  
  
+ GCTACGATCT TGAAATAATG TCGATTTGAT TTTGTAGATA CCTTTTAATC ATTCTTCTAA TAGAATCATC   
  
  
+ TTTTTTTTTT TTTGGGTTGA TCTCATTTTG TTAAATTGAA TAAATTTAAT TGAATGAATT GAAAATTCTT   
  
  
+ TTTTTTTAAG GATATAAATT TTGTTGGTAT TTTAAAAAAA TATTTTTTAT TAAATACTTT TTAAAAAGAT   
  
  
+ GTTGATATAT ATATATATAT GTTAGAAAAT AAAGGATATA AATTTTGTTA GTATTTTAAA AAAATATTTT   
  
  
+ TTATTAAATA CTTTTTAAAA AGATGTTGAT ATATATATAT ATGTTAGAAA ATACAATAAA ATTTATTGCA   
  
  
+ TTTGTTTTCT TGATTAATGT AACCAATTAT AAAAATAGAA TTTTAAAAAA AAGATTAATA AGAATCATAG   
  
  
+ TAACAGATGA TTTTTTGATT CAAAGGAAAC ATTTCTTCAA TATTTTAAAG ATTCATTATT AAAAATGATT   
  
  
+ AGAAATTATG GTAAAATCTA TGAAATTTTT AAAAAATATA AATAGTATTG AAGTAAGAAG GGTAGTTATA   
  
  
+ TTTTTATAAC TTAAATAATA ATATAAAATA AAAAATGTAA CCATTAATTT TAATTAAATT ACCATGTATC   
  
  
+ TTAATTTATT TAAGTCGAAA ATTTGATCCT TTCTTAATTT TATTTATTAA GACACGTGAA AATACCTTTT   
  
  
+ TGCTTAATTA ATTGAATAAG AGCCACGCAT AGCATAACAA CATTATCTAT TTGCCCATTT CATGAGCAAT   
  
  
+ AAAGAATTCC ACTGACTGAA AAGAATACAA GACCCCCCAA AAAAAAAAGA AAAAAAGAGA AAAGAGAAAA   
  
  
+ AAAAAGATGA AAAAAATGAT TAAAGGGAAA AGAAGAAAAG GGAATATCTT AGGAGGATTC AGTTGGTGAG   
  
  
+ CTTGAATTGA GTTCACACAG AGGAGAGTTC ACATGCAAGA CGACGGAGCT TGGGGCTGTA ATGGGTAAGA   
  
  
+ ATCTCACGTG CAGAGAAACC CCGTTCTCAC TGGGGGAACC TGTTAACTGT TGAGCCGGCC AACGAAGCGT   
  
  
+ ATGAAAACGT CGGCCAACCA CGACAGAATA CTCGGCCGCA TGCATTGAAT TTGCCACCGG CACTGTGGGT   
  
  
+ GTCCGTACGA AATGTTGACC TACGATCTCC GGTCAATCTT TGTTGCCATT TGTCTACTCT CGAGCAGAAT   
  
  
+ AGAAAAGTAA CTCACCTTCT CTTCTCTTTG GCCAAATCTC ACTTTCTTCT ATACCTAAAG CTCTCTCTCT   
  
  
+ CTCTCTCTCC CCCTCCCACC TACCTGATAA ACGCAATCAG GCCTAGCTTT CTCTCTCTCT CTCTCTCTCT   
  
  
+ CTTTCTTTCT CAGTAAGCAA ACATCACTCC CGCTGTGTGT CACTCTCTGT TAAGTTACAA GCTTTTATAT   
  
  
+ GAGGATTTTA TAGGCCTGAT TGATAGAAA  

- AATTAAAAGT CCACGAGAGT AAGCAAAAGT GGAAGCGGTT AACACTTCAA CTTCGGTGTA AAAAGATCCC   
  
  
- CGATGCTAGA ACTTTATTAC AGCTAAACTA AAACATCTAT GGAAAATTAG TAAGAAGATT ATCTTAGTAG   
  
  
- AAAAAAAAAA AAACCCAACT AGAGTAAAAC AATTTAACTT ATTTAAATTA ACTTACTTAA CTTTTAAGAA   
  
  
- AAAAAAATTC CTATATTTAA AACAACCATA AAATTTTTTT ATAAAAAATA ATTTATGAAA AATTTTTCTA   
  
  
- CAACTATATA TATATATATA CAATCTTTTA TTTCCTATAT TTAAAACAAT CATAAAATTT TTTTATAAAA   
  
  
- AATAATTTAT GAAAAATTTT TCTACAACTA TATATATATA TACAATCTTT TATGTTATTT TAAATAACGT   
  
  
- AAACAAAAGA ACTAATTACA TTGGTTAATA TTTTTATCTT AAAATTTTTT TTCTAATTAT TCTTAGTATC   
  
  
- ATTGTCTACT AAAAAACTAA GTTTCCTTTG TAAAGAAGTT ATAAAATTTC TAAGTAATAA TTTTTACTAA   
  
  
- TCTTTAATAC CATTTTAGAT ACTTTAAAAA TTTTTTATAT TTATCATAAC TTCATTCTTC CCATCAATAT   
  
  
- AAAAATATTG AATTTATTAT TATATTTTAT TTTTTACATT GGTAATTAAA ATTAATTTAA TGGTACATAG   
  
  
- AATTAAATAA ATTCAGCTTT TAAACTAGGA AAGAATTAAA ATAAATAATT CTGTGCACTT TTATGGAAAA   
  
  
- ACGAATTAAT TAACTTATTC TCGGTGCGTA TCGTATTGTT GTAATAGATA AACGGGTAAA GTACTCGTTA   
  
  
- TTTCTTAAGG TGACTGACTT TTCTTATGTT CTGGGGGGTT TTTTTTTTCT TTTTTTCTCT TTTCTCTTTT   
  
  
- TTTTTCTACT TTTTTTACTA ATTTCCCTTT TCTTCTTTTC CCTTATAGAA TCCTCCTAAG TCAACCACTC   
  
  
- GAACTTAACT CAAGTGTGTC TCCTCTCAAG TGTACGTTCT GCTGCCTCGA ACCCCGACAT TACCCATTCT   
  
  
- TAGAGTGCAC GTCTCTTTGG GGCAAGAGTG ACCCCCTTGG ACAATTGACA ACTCGGCCGG TTGCTTCGCA   
  
  
- TACTTTTGCA GCCGGTTGGT GCTGTCTTAT GAGCCGGCGT ACGTAACTTA AACGGTGGCC GTGACACCCA   
  
  
- CAGGCATGCT TTACAACTGG ATGCTAGAGG CCAGTTAGAA ACAACGGTAA ACAGATGAGA GCTCGTCTTA   
  
  
- TCTTTTCATT GAGTGGAAGA GAAGAGAAAC CGGTTTAGAG TGAAAGAAGA TATGGATTTC GAGAGAGAGA   
  
  
- GAGAGAGAGG GGGAGGGTGG ATGGACTATT TGCGTTAGTC CGGATCGAAA GAGAGAGAGA GAGAGAGAGA   
  
  
- GAAAGAAAGA GTCATTCGTT TGTAGTGAGG GCGACACACA GTGAGAGACA ATTCAATGTT CGAAAATATA   
  
  
- CTCCTAAAAT ATCCGGACTA ACTATCTTT

+     HSE

| Site Name | Organism | Position | Strand | Matrix score. | sequence | function |
| --- | --- | --- | --- | --- | --- | --- |
| HSE | Brassica oleracea | 582 | - | 9 | AAAAAATTTC | cis-acting element involved in heat stress responsiveness |

> 2018/04/13 10:10:12  
+ TTAATTTTCA GGTGCTCTCA TTCGTTTTCA CCTTCGCCAA TTGTGAAGTT GAAGCCACAT TTTTCTAGGG   
  
  
+ GCTACGATCT TGAAATAATG TCGATTTGAT TTTGTAGATA CCTTTTAATC ATTCTTCTAA TAGAATCATC   
  
  
+ TTTTTTTTTT TTTGGGTTGA TCTCATTTTG TTAAATTGAA TAAATTTAAT TGAATGAATT GAAAATTCTT   
  
  
+ TTTTTTTAAG GATATAAATT TTGTTGGTAT TTTAAAAAAA TATTTTTTAT TAAATACTTT TTAAAAAGAT   
  
  
+ GTTGATATAT ATATATATAT GTTAGAAAAT AAAGGATATA AATTTTGTTA GTATTTTAAA AAAATATTTT   
  
  
+ TTATTAAATA CTTTTTAAAA AGATGTTGAT ATATATATAT ATGTTAGAAA ATACAATAAA ATTTATTGCA   
  
  
+ TTTGTTTTCT TGATTAATGT AACCAATTAT AAAAATAGAA TTTTAAAAAA AAGATTAATA AGAATCATAG   
  
  
+ TAACAGATGA TTTTTTGATT CAAAGGAAAC ATTTCTTCAA TATTTTAAAG ATTCATTATT AAAAATGATT   
  
  
+ AGAAATTATG GTAAAATCTA TGAAATTTTT AAAAAATATA AATAGTATTG AAGTAAGAAG GGTAGTTATA   
  
  
+ TTTTTATAAC TTAAATAATA ATATAAAATA AAAAATGTAA CCATTAATTT TAATTAAATT ACCATGTATC   
  
  
+ TTAATTTATT TAAGTCGAAA ATTTGATCCT TTCTTAATTT TATTTATTAA GACACGTGAA AATACCTTTT   
  
  
+ TGCTTAATTA ATTGAATAAG AGCCACGCAT AGCATAACAA CATTATCTAT TTGCCCATTT CATGAGCAAT   
  
  
+ AAAGAATTCC ACTGACTGAA AAGAATACAA GACCCCCCAA AAAAAAAAGA AAAAAAGAGA AAAGAGAAAA   
  
  
+ AAAAAGATGA AAAAAATGAT TAAAGGGAAA AGAAGAAAAG GGAATATCTT AGGAGGATTC AGTTGGTGAG   
  
  
+ CTTGAATTGA GTTCACACAG AGGAGAGTTC ACATGCAAGA CGACGGAGCT TGGGGCTGTA ATGGGTAAGA   
  
  
+ ATCTCACGTG CAGAGAAACC CCGTTCTCAC TGGGGGAACC TGTTAACTGT TGAGCCGGCC AACGAAGCGT   
  
  
+ ATGAAAACGT CGGCCAACCA CGACAGAATA CTCGGCCGCA TGCATTGAAT TTGCCACCGG CACTGTGGGT   
  
  
+ GTCCGTACGA AATGTTGACC TACGATCTCC GGTCAATCTT TGTTGCCATT TGTCTACTCT CGAGCAGAAT   
  
  
+ AGAAAAGTAA CTCACCTTCT CTTCTCTTTG GCCAAATCTC ACTTTCTTCT ATACCTAAAG CTCTCTCTCT   
  
  
+ CTCTCTCTCC CCCTCCCACC TACCTGATAA ACGCAATCAG GCCTAGCTTT CTCTCTCTCT CTCTCTCTCT   
  
  
+ CTTTCTTTCT CAGTAAGCAA ACATCACTCC CGCTGTGTGT CACTCTCTGT TAAGTTACAA GCTTTTATAT   
  
  
+ GAGGATTTTA TAGGCCTGAT TGATAGAAA  

- AATTAAAAGT CCACGAGAGT AAGCAAAAGT GGAAGCGGTT AACACTTCAA CTTCGGTGTA AAAAGATCCC   
  
  
- CGATGCTAGA ACTTTATTAC AGCTAAACTA AAACATCTAT GGAAAATTAG TAAGAAGATT ATCTTAGTAG   
  
  
- AAAAAAAAAA AAACCCAACT AGAGTAAAAC AATTTAACTT ATTTAAATTA ACTTACTTAA CTTTTAAGAA   
  
  
- AAAAAAATTC CTATATTTAA AACAACCATA AAATTTTTTT ATAAAAAATA ATTTATGAAA AATTTTTCTA   
  
  
- CAACTATATA TATATATATA CAATCTTTTA TTTCCTATAT TTAAAACAAT CATAAAATTT TTTTATAAAA   
  
  
- AATAATTTAT GAAAAATTTT TCTACAACTA TATATATATA TACAATCTTT TATGTTATTT TAAATAACGT   
  
  
- AAACAAAAGA ACTAATTACA TTGGTTAATA TTTTTATCTT AAAATTTTTT TTCTAATTAT TCTTAGTATC   
  
  
- ATTGTCTACT AAAAAACTAA GTTTCCTTTG TAAAGAAGTT ATAAAATTTC TAAGTAATAA TTTTTACTAA   
  
  
- TCTTTAATAC CATTTTAGAT ACTTTAAAAA TTTTTTATAT TTATCATAAC TTCATTCTTC CCATCAATAT   
  
  
- AAAAATATTG AATTTATTAT TATATTTTAT TTTTTACATT GGTAATTAAA ATTAATTTAA TGGTACATAG   
  
  
- AATTAAATAA ATTCAGCTTT TAAACTAGGA AAGAATTAAA ATAAATAATT CTGTGCACTT TTATGGAAAA   
  
  
- ACGAATTAAT TAACTTATTC TCGGTGCGTA TCGTATTGTT GTAATAGATA AACGGGTAAA GTACTCGTTA   
  
  
- TTTCTTAAGG TGACTGACTT TTCTTATGTT CTGGGGGGTT TTTTTTTTCT TTTTTTCTCT TTTCTCTTTT   
  
  
- TTTTTCTACT TTTTTTACTA ATTTCCCTTT TCTTCTTTTC CCTTATAGAA TCCTCCTAAG TCAACCACTC   
  
  
- GAACTTAACT CAAGTGTGTC TCCTCTCAAG TGTACGTTCT GCTGCCTCGA ACCCCGACAT TACCCATTCT   
  
  
- TAGAGTGCAC GTCTCTTTGG GGCAAGAGTG ACCCCCTTGG ACAATTGACA ACTCGGCCGG TTGCTTCGCA   
  
  
- TACTTTTGCA GCCGGTTGGT GCTGTCTTAT GAGCCGGCGT ACGTAACTTA AACGGTGGCC GTGACACCCA   
  
  
- CAGGCATGCT TTACAACTGG ATGCTAGAGG CCAGTTAGAA ACAACGGTAA ACAGATGAGA GCTCGTCTTA   
  
  
- TCTTTTCATT GAGTGGAAGA GAAGAGAAAC CGGTTTAGAG TGAAAGAAGA TATGGATTTC GAGAGAGAGA   
  
  
- GAGAGAGAGG GGGAGGGTGG ATGGACTATT TGCGTTAGTC CGGATCGAAA GAGAGAGAGA GAGAGAGAGA   
  
  
- GAAAGAAAGA GTCATTCGTT TGTAGTGAGG GCGACACACA GTGAGAGACA ATTCAATGTT CGAAAATATA   
  
  
- CTCCTAAAAT ATCCGGACTA ACTATCTTT

+     L-box

| Site Name | Organism | Position | Strand | Matrix score. | sequence | function |
| --- | --- | --- | --- | --- | --- | --- |
| L-box | Petroselinum crispum | 1344 | + | 10 | TCTCACCTACC | part of a light responsive element |
| L-box | Petroselinum crispum | 1343 | + | 10 | ATCCCACCTAC | part of a light responsive element |

> 2018/04/13 10:10:12  
+ TTAATTTTCA GGTGCTCTCA TTCGTTTTCA CCTTCGCCAA TTGTGAAGTT GAAGCCACAT TTTTCTAGGG   
  
  
+ GCTACGATCT TGAAATAATG TCGATTTGAT TTTGTAGATA CCTTTTAATC ATTCTTCTAA TAGAATCATC   
  
  
+ TTTTTTTTTT TTTGGGTTGA TCTCATTTTG TTAAATTGAA TAAATTTAAT TGAATGAATT GAAAATTCTT   
  
  
+ TTTTTTTAAG GATATAAATT TTGTTGGTAT TTTAAAAAAA TATTTTTTAT TAAATACTTT TTAAAAAGAT   
  
  
+ GTTGATATAT ATATATATAT GTTAGAAAAT AAAGGATATA AATTTTGTTA GTATTTTAAA AAAATATTTT   
  
  
+ TTATTAAATA CTTTTTAAAA AGATGTTGAT ATATATATAT ATGTTAGAAA ATACAATAAA ATTTATTGCA   
  
  
+ TTTGTTTTCT TGATTAATGT AACCAATTAT AAAAATAGAA TTTTAAAAAA AAGATTAATA AGAATCATAG   
  
  
+ TAACAGATGA TTTTTTGATT CAAAGGAAAC ATTTCTTCAA TATTTTAAAG ATTCATTATT AAAAATGATT   
  
  
+ AGAAATTATG GTAAAATCTA TGAAATTTTT AAAAAATATA AATAGTATTG AAGTAAGAAG GGTAGTTATA   
  
  
+ TTTTTATAAC TTAAATAATA ATATAAAATA AAAAATGTAA CCATTAATTT TAATTAAATT ACCATGTATC   
  
  
+ TTAATTTATT TAAGTCGAAA ATTTGATCCT TTCTTAATTT TATTTATTAA GACACGTGAA AATACCTTTT   
  
  
+ TGCTTAATTA ATTGAATAAG AGCCACGCAT AGCATAACAA CATTATCTAT TTGCCCATTT CATGAGCAAT   
  
  
+ AAAGAATTCC ACTGACTGAA AAGAATACAA GACCCCCCAA AAAAAAAAGA AAAAAAGAGA AAAGAGAAAA   
  
  
+ AAAAAGATGA AAAAAATGAT TAAAGGGAAA AGAAGAAAAG GGAATATCTT AGGAGGATTC AGTTGGTGAG   
  
  
+ CTTGAATTGA GTTCACACAG AGGAGAGTTC ACATGCAAGA CGACGGAGCT TGGGGCTGTA ATGGGTAAGA   
  
  
+ ATCTCACGTG CAGAGAAACC CCGTTCTCAC TGGGGGAACC TGTTAACTGT TGAGCCGGCC AACGAAGCGT   
  
  
+ ATGAAAACGT CGGCCAACCA CGACAGAATA CTCGGCCGCA TGCATTGAAT TTGCCACCGG CACTGTGGGT   
  
  
+ GTCCGTACGA AATGTTGACC TACGATCTCC GGTCAATCTT TGTTGCCATT TGTCTACTCT CGAGCAGAAT   
  
  
+ AGAAAAGTAA CTCACCTTCT CTTCTCTTTG GCCAAATCTC ACTTTCTTCT ATACCTAAAG CTCTCTCTCT   
  
  
+ CTCTCTCTCC CCCTCCCACC TACCTGATAA ACGCAATCAG GCCTAGCTTT CTCTCTCTCT CTCTCTCTCT   
  
  
+ CTTTCTTTCT CAGTAAGCAA ACATCACTCC CGCTGTGTGT CACTCTCTGT TAAGTTACAA GCTTTTATAT   
  
  
+ GAGGATTTTA TAGGCCTGAT TGATAGAAA  

- AATTAAAAGT CCACGAGAGT AAGCAAAAGT GGAAGCGGTT AACACTTCAA CTTCGGTGTA AAAAGATCCC   
  
  
- CGATGCTAGA ACTTTATTAC AGCTAAACTA AAACATCTAT GGAAAATTAG TAAGAAGATT ATCTTAGTAG   
  
  
- AAAAAAAAAA AAACCCAACT AGAGTAAAAC AATTTAACTT ATTTAAATTA ACTTACTTAA CTTTTAAGAA   
  
  
- AAAAAAATTC CTATATTTAA AACAACCATA AAATTTTTTT ATAAAAAATA ATTTATGAAA AATTTTTCTA   
  
  
- CAACTATATA TATATATATA CAATCTTTTA TTTCCTATAT TTAAAACAAT CATAAAATTT TTTTATAAAA   
  
  
- AATAATTTAT GAAAAATTTT TCTACAACTA TATATATATA TACAATCTTT TATGTTATTT TAAATAACGT   
  
  
- AAACAAAAGA ACTAATTACA TTGGTTAATA TTTTTATCTT AAAATTTTTT TTCTAATTAT TCTTAGTATC   
  
  
- ATTGTCTACT AAAAAACTAA GTTTCCTTTG TAAAGAAGTT ATAAAATTTC TAAGTAATAA TTTTTACTAA   
  
  
- TCTTTAATAC CATTTTAGAT ACTTTAAAAA TTTTTTATAT TTATCATAAC TTCATTCTTC CCATCAATAT   
  
  
- AAAAATATTG AATTTATTAT TATATTTTAT TTTTTACATT GGTAATTAAA ATTAATTTAA TGGTACATAG   
  
  
- AATTAAATAA ATTCAGCTTT TAAACTAGGA AAGAATTAAA ATAAATAATT CTGTGCACTT TTATGGAAAA   
  
  
- ACGAATTAAT TAACTTATTC TCGGTGCGTA TCGTATTGTT GTAATAGATA AACGGGTAAA GTACTCGTTA   
  
  
- TTTCTTAAGG TGACTGACTT TTCTTATGTT CTGGGGGGTT TTTTTTTTCT TTTTTTCTCT TTTCTCTTTT   
  
  
- TTTTTCTACT TTTTTTACTA ATTTCCCTTT TCTTCTTTTC CCTTATAGAA TCCTCCTAAG TCAACCACTC   
  
  
- GAACTTAACT CAAGTGTGTC TCCTCTCAAG TGTACGTTCT GCTGCCTCGA ACCCCGACAT TACCCATTCT   
  
  
- TAGAGTGCAC GTCTCTTTGG GGCAAGAGTG ACCCCCTTGG ACAATTGACA ACTCGGCCGG TTGCTTCGCA   
  
  
- TACTTTTGCA GCCGGTTGGT GCTGTCTTAT GAGCCGGCGT ACGTAACTTA AACGGTGGCC GTGACACCCA   
  
  
- CAGGCATGCT TTACAACTGG ATGCTAGAGG CCAGTTAGAA ACAACGGTAA ACAGATGAGA GCTCGTCTTA   
  
  
- TCTTTTCATT GAGTGGAAGA GAAGAGAAAC CGGTTTAGAG TGAAAGAAGA TATGGATTTC GAGAGAGAGA   
  
  
- GAGAGAGAGG GGGAGGGTGG ATGGACTATT TGCGTTAGTC CGGATCGAAA GAGAGAGAGA GAGAGAGAGA   
  
  
- GAAAGAAAGA GTCATTCGTT TGTAGTGAGG GCGACACACA GTGAGAGACA ATTCAATGTT CGAAAATATA   
  
  
- CTCCTAAAAT ATCCGGACTA ACTATCTTT

+     MBS

| Site Name | Organism | Position | Strand | Matrix score. | sequence | function |
| --- | --- | --- | --- | --- | --- | --- |
| MBS | Arabidopsis thaliana | 1094 | + | 6 | TAACTG | MYB binding site involved in drought-inducibility |
| MBS | Arabidopsis thaliana | 970 | - | 6 | CAACTG | MYB binding site involved in drought-inducibility |
| MBS | Zea mays | 1220 | + | 6 | CGGTCA | MYB Binding Site |

> 2018/04/13 10:10:12  
+ TTAATTTTCA GGTGCTCTCA TTCGTTTTCA CCTTCGCCAA TTGTGAAGTT GAAGCCACAT TTTTCTAGGG   
  
  
+ GCTACGATCT TGAAATAATG TCGATTTGAT TTTGTAGATA CCTTTTAATC ATTCTTCTAA TAGAATCATC   
  
  
+ TTTTTTTTTT TTTGGGTTGA TCTCATTTTG TTAAATTGAA TAAATTTAAT TGAATGAATT GAAAATTCTT   
  
  
+ TTTTTTTAAG GATATAAATT TTGTTGGTAT TTTAAAAAAA TATTTTTTAT TAAATACTTT TTAAAAAGAT   
  
  
+ GTTGATATAT ATATATATAT GTTAGAAAAT AAAGGATATA AATTTTGTTA GTATTTTAAA AAAATATTTT   
  
  
+ TTATTAAATA CTTTTTAAAA AGATGTTGAT ATATATATAT ATGTTAGAAA ATACAATAAA ATTTATTGCA   
  
  
+ TTTGTTTTCT TGATTAATGT AACCAATTAT AAAAATAGAA TTTTAAAAAA AAGATTAATA AGAATCATAG   
  
  
+ TAACAGATGA TTTTTTGATT CAAAGGAAAC ATTTCTTCAA TATTTTAAAG ATTCATTATT AAAAATGATT   
  
  
+ AGAAATTATG GTAAAATCTA TGAAATTTTT AAAAAATATA AATAGTATTG AAGTAAGAAG GGTAGTTATA   
  
  
+ TTTTTATAAC TTAAATAATA ATATAAAATA AAAAATGTAA CCATTAATTT TAATTAAATT ACCATGTATC   
  
  
+ TTAATTTATT TAAGTCGAAA ATTTGATCCT TTCTTAATTT TATTTATTAA GACACGTGAA AATACCTTTT   
  
  
+ TGCTTAATTA ATTGAATAAG AGCCACGCAT AGCATAACAA CATTATCTAT TTGCCCATTT CATGAGCAAT   
  
  
+ AAAGAATTCC ACTGACTGAA AAGAATACAA GACCCCCCAA AAAAAAAAGA AAAAAAGAGA AAAGAGAAAA   
  
  
+ AAAAAGATGA AAAAAATGAT TAAAGGGAAA AGAAGAAAAG GGAATATCTT AGGAGGATTC AGTTGGTGAG   
  
  
+ CTTGAATTGA GTTCACACAG AGGAGAGTTC ACATGCAAGA CGACGGAGCT TGGGGCTGTA ATGGGTAAGA   
  
  
+ ATCTCACGTG CAGAGAAACC CCGTTCTCAC TGGGGGAACC TGTTAACTGT TGAGCCGGCC AACGAAGCGT   
  
  
+ ATGAAAACGT CGGCCAACCA CGACAGAATA CTCGGCCGCA TGCATTGAAT TTGCCACCGG CACTGTGGGT   
  
  
+ GTCCGTACGA AATGTTGACC TACGATCTCC GGTCAATCTT TGTTGCCATT TGTCTACTCT CGAGCAGAAT   
  
  
+ AGAAAAGTAA CTCACCTTCT CTTCTCTTTG GCCAAATCTC ACTTTCTTCT ATACCTAAAG CTCTCTCTCT   
  
  
+ CTCTCTCTCC CCCTCCCACC TACCTGATAA ACGCAATCAG GCCTAGCTTT CTCTCTCTCT CTCTCTCTCT   
  
  
+ CTTTCTTTCT CAGTAAGCAA ACATCACTCC CGCTGTGTGT CACTCTCTGT TAAGTTACAA GCTTTTATAT   
  
  
+ GAGGATTTTA TAGGCCTGAT TGATAGAAA  

- AATTAAAAGT CCACGAGAGT AAGCAAAAGT GGAAGCGGTT AACACTTCAA CTTCGGTGTA AAAAGATCCC   
  
  
- CGATGCTAGA ACTTTATTAC AGCTAAACTA AAACATCTAT GGAAAATTAG TAAGAAGATT ATCTTAGTAG   
  
  
- AAAAAAAAAA AAACCCAACT AGAGTAAAAC AATTTAACTT ATTTAAATTA ACTTACTTAA CTTTTAAGAA   
  
  
- AAAAAAATTC CTATATTTAA AACAACCATA AAATTTTTTT ATAAAAAATA ATTTATGAAA AATTTTTCTA   
  
  
- CAACTATATA TATATATATA CAATCTTTTA TTTCCTATAT TTAAAACAAT CATAAAATTT TTTTATAAAA   
  
  
- AATAATTTAT GAAAAATTTT TCTACAACTA TATATATATA TACAATCTTT TATGTTATTT TAAATAACGT   
  
  
- AAACAAAAGA ACTAATTACA TTGGTTAATA TTTTTATCTT AAAATTTTTT TTCTAATTAT TCTTAGTATC   
  
  
- ATTGTCTACT AAAAAACTAA GTTTCCTTTG TAAAGAAGTT ATAAAATTTC TAAGTAATAA TTTTTACTAA   
  
  
- TCTTTAATAC CATTTTAGAT ACTTTAAAAA TTTTTTATAT TTATCATAAC TTCATTCTTC CCATCAATAT   
  
  
- AAAAATATTG AATTTATTAT TATATTTTAT TTTTTACATT GGTAATTAAA ATTAATTTAA TGGTACATAG   
  
  
- AATTAAATAA ATTCAGCTTT TAAACTAGGA AAGAATTAAA ATAAATAATT CTGTGCACTT TTATGGAAAA   
  
  
- ACGAATTAAT TAACTTATTC TCGGTGCGTA TCGTATTGTT GTAATAGATA AACGGGTAAA GTACTCGTTA   
  
  
- TTTCTTAAGG TGACTGACTT TTCTTATGTT CTGGGGGGTT TTTTTTTTCT TTTTTTCTCT TTTCTCTTTT   
  
  
- TTTTTCTACT TTTTTTACTA ATTTCCCTTT TCTTCTTTTC CCTTATAGAA TCCTCCTAAG TCAACCACTC   
  
  
- GAACTTAACT CAAGTGTGTC TCCTCTCAAG TGTACGTTCT GCTGCCTCGA ACCCCGACAT TACCCATTCT   
  
  
- TAGAGTGCAC GTCTCTTTGG GGCAAGAGTG ACCCCCTTGG ACAATTGACA ACTCGGCCGG TTGCTTCGCA   
  
  
- TACTTTTGCA GCCGGTTGGT GCTGTCTTAT GAGCCGGCGT ACGTAACTTA AACGGTGGCC GTGACACCCA   
  
  
- CAGGCATGCT TTACAACTGG ATGCTAGAGG CCAGTTAGAA ACAACGGTAA ACAGATGAGA GCTCGTCTTA   
  
  
- TCTTTTCATT GAGTGGAAGA GAAGAGAAAC CGGTTTAGAG TGAAAGAAGA TATGGATTTC GAGAGAGAGA   
  
  
- GAGAGAGAGG GGGAGGGTGG ATGGACTATT TGCGTTAGTC CGGATCGAAA GAGAGAGAGA GAGAGAGAGA   
  
  
- GAAAGAAAGA GTCATTCGTT TGTAGTGAGG GCGACACACA GTGAGAGACA ATTCAATGTT CGAAAATATA   
  
  
- CTCCTAAAAT ATCCGGACTA ACTATCTTT

+     Sp1

| Site Name | Organism | Position | Strand | Matrix score. | sequence | function |
| --- | --- | --- | --- | --- | --- | --- |
| Sp1 | Zea mays | 1342 | + | 5 | CC(G/A)CCC | light responsive element |
| Sp1 | Zea mays | 873 | + | 5 | CC(G/A)CCC | light responsive element |

> 2018/04/13 10:10:12  
+ TTAATTTTCA GGTGCTCTCA TTCGTTTTCA CCTTCGCCAA TTGTGAAGTT GAAGCCACAT TTTTCTAGGG   
  
  
+ GCTACGATCT TGAAATAATG TCGATTTGAT TTTGTAGATA CCTTTTAATC ATTCTTCTAA TAGAATCATC   
  
  
+ TTTTTTTTTT TTTGGGTTGA TCTCATTTTG TTAAATTGAA TAAATTTAAT TGAATGAATT GAAAATTCTT   
  
  
+ TTTTTTTAAG GATATAAATT TTGTTGGTAT TTTAAAAAAA TATTTTTTAT TAAATACTTT TTAAAAAGAT   
  
  
+ GTTGATATAT ATATATATAT GTTAGAAAAT AAAGGATATA AATTTTGTTA GTATTTTAAA AAAATATTTT   
  
  
+ TTATTAAATA CTTTTTAAAA AGATGTTGAT ATATATATAT ATGTTAGAAA ATACAATAAA ATTTATTGCA   
  
  
+ TTTGTTTTCT TGATTAATGT AACCAATTAT AAAAATAGAA TTTTAAAAAA AAGATTAATA AGAATCATAG   
  
  
+ TAACAGATGA TTTTTTGATT CAAAGGAAAC ATTTCTTCAA TATTTTAAAG ATTCATTATT AAAAATGATT   
  
  
+ AGAAATTATG GTAAAATCTA TGAAATTTTT AAAAAATATA AATAGTATTG AAGTAAGAAG GGTAGTTATA   
  
  
+ TTTTTATAAC TTAAATAATA ATATAAAATA AAAAATGTAA CCATTAATTT TAATTAAATT ACCATGTATC   
  
  
+ TTAATTTATT TAAGTCGAAA ATTTGATCCT TTCTTAATTT TATTTATTAA GACACGTGAA AATACCTTTT   
  
  
+ TGCTTAATTA ATTGAATAAG AGCCACGCAT AGCATAACAA CATTATCTAT TTGCCCATTT CATGAGCAAT   
  
  
+ AAAGAATTCC ACTGACTGAA AAGAATACAA GACCCCCCAA AAAAAAAAGA AAAAAAGAGA AAAGAGAAAA   
  
  
+ AAAAAGATGA AAAAAATGAT TAAAGGGAAA AGAAGAAAAG GGAATATCTT AGGAGGATTC AGTTGGTGAG   
  
  
+ CTTGAATTGA GTTCACACAG AGGAGAGTTC ACATGCAAGA CGACGGAGCT TGGGGCTGTA ATGGGTAAGA   
  
  
+ ATCTCACGTG CAGAGAAACC CCGTTCTCAC TGGGGGAACC TGTTAACTGT TGAGCCGGCC AACGAAGCGT   
  
  
+ ATGAAAACGT CGGCCAACCA CGACAGAATA CTCGGCCGCA TGCATTGAAT TTGCCACCGG CACTGTGGGT   
  
  
+ GTCCGTACGA AATGTTGACC TACGATCTCC GGTCAATCTT TGTTGCCATT TGTCTACTCT CGAGCAGAAT   
  
  
+ AGAAAAGTAA CTCACCTTCT CTTCTCTTTG GCCAAATCTC ACTTTCTTCT ATACCTAAAG CTCTCTCTCT   
  
  
+ CTCTCTCTCC CCCTCCCACC TACCTGATAA ACGCAATCAG GCCTAGCTTT CTCTCTCTCT CTCTCTCTCT   
  
  
+ CTTTCTTTCT CAGTAAGCAA ACATCACTCC CGCTGTGTGT CACTCTCTGT TAAGTTACAA GCTTTTATAT   
  
  
+ GAGGATTTTA TAGGCCTGAT TGATAGAAA  

- AATTAAAAGT CCACGAGAGT AAGCAAAAGT GGAAGCGGTT AACACTTCAA CTTCGGTGTA AAAAGATCCC   
  
  
- CGATGCTAGA ACTTTATTAC AGCTAAACTA AAACATCTAT GGAAAATTAG TAAGAAGATT ATCTTAGTAG   
  
  
- AAAAAAAAAA AAACCCAACT AGAGTAAAAC AATTTAACTT ATTTAAATTA ACTTACTTAA CTTTTAAGAA   
  
  
- AAAAAAATTC CTATATTTAA AACAACCATA AAATTTTTTT ATAAAAAATA ATTTATGAAA AATTTTTCTA   
  
  
- CAACTATATA TATATATATA CAATCTTTTA TTTCCTATAT TTAAAACAAT CATAAAATTT TTTTATAAAA   
  
  
- AATAATTTAT GAAAAATTTT TCTACAACTA TATATATATA TACAATCTTT TATGTTATTT TAAATAACGT   
  
  
- AAACAAAAGA ACTAATTACA TTGGTTAATA TTTTTATCTT AAAATTTTTT TTCTAATTAT TCTTAGTATC   
  
  
- ATTGTCTACT AAAAAACTAA GTTTCCTTTG TAAAGAAGTT ATAAAATTTC TAAGTAATAA TTTTTACTAA   
  
  
- TCTTTAATAC CATTTTAGAT ACTTTAAAAA TTTTTTATAT TTATCATAAC TTCATTCTTC CCATCAATAT   
  
  
- AAAAATATTG AATTTATTAT TATATTTTAT TTTTTACATT GGTAATTAAA ATTAATTTAA TGGTACATAG   
  
  
- AATTAAATAA ATTCAGCTTT TAAACTAGGA AAGAATTAAA ATAAATAATT CTGTGCACTT TTATGGAAAA   
  
  
- ACGAATTAAT TAACTTATTC TCGGTGCGTA TCGTATTGTT GTAATAGATA AACGGGTAAA GTACTCGTTA   
  
  
- TTTCTTAAGG TGACTGACTT TTCTTATGTT CTGGGGGGTT TTTTTTTTCT TTTTTTCTCT TTTCTCTTTT   
  
  
- TTTTTCTACT TTTTTTACTA ATTTCCCTTT TCTTCTTTTC CCTTATAGAA TCCTCCTAAG TCAACCACTC   
  
  
- GAACTTAACT CAAGTGTGTC TCCTCTCAAG TGTACGTTCT GCTGCCTCGA ACCCCGACAT TACCCATTCT   
  
  
- TAGAGTGCAC GTCTCTTTGG GGCAAGAGTG ACCCCCTTGG ACAATTGACA ACTCGGCCGG TTGCTTCGCA   
  
  
- TACTTTTGCA GCCGGTTGGT GCTGTCTTAT GAGCCGGCGT ACGTAACTTA AACGGTGGCC GTGACACCCA   
  
  
- CAGGCATGCT TTACAACTGG ATGCTAGAGG CCAGTTAGAA ACAACGGTAA ACAGATGAGA GCTCGTCTTA   
  
  
- TCTTTTCATT GAGTGGAAGA GAAGAGAAAC CGGTTTAGAG TGAAAGAAGA TATGGATTTC GAGAGAGAGA   
  
  
- GAGAGAGAGG GGGAGGGTGG ATGGACTATT TGCGTTAGTC CGGATCGAAA GAGAGAGAGA GAGAGAGAGA   
  
  
- GAAAGAAAGA GTCATTCGTT TGTAGTGAGG GCGACACACA GTGAGAGACA ATTCAATGTT CGAAAATATA   
  
  
- CTCCTAAAAT ATCCGGACTA ACTATCTTT

+     TATA-box

| Site Name | Organism | Position | Strand | Matrix score. | sequence | function |
| --- | --- | --- | --- | --- | --- | --- |
| TATA-box | Arabidopsis thaliana | 1477 | - | 6 | TATAAA | core promoter element around -30 of transcription start |
| TATA-box | Lycopersicon esculentum | 659 | - | 5 | TTTTA | core promoter element around -30 of transcription start |
| TATA-box | Lycopersicon esculentum | 366 | - | 5 | TTTTA | core promoter element around -30 of transcription start |
| TATA-box | Brassica oleracea | 651 | + | 6 | ATATAA | core promoter element around -30 of transcription start |
| TATA-box | Lycopersicon esculentum | 654 | - | 5 | TTTTA | core promoter element around -30 of transcription start |
| TATA-box | Arabidopsis thaliana | 652 | + | 6 | TATAAA | core promoter element around -30 of transcription start |
| TATA-box | Arabidopsis thaliana | 626 | - | 5 | TATAA | core promoter element around -30 of transcription start |
| TATA-box | Arabidopsis thaliana | 380 | + | 8 | TATATATA | core promoter element around -30 of transcription start |
| TATA-box | Glycine max | 258 | - | 5 | TAATA | core promoter element around -30 of transcription start |
| TATA-box | Arabidopsis thaliana | 448 | + | 6 | TATAAA | core promoter element around -30 of transcription start |
| TATA-box | Zea mays | 586 | - | 8 | TTTAAAAA | core promoter element around -30 of transcription start |
| TATA-box | Zea mays | 268 | - | 8 | TTTAAAAA | core promoter element around -30 of transcription start |
| TATA-box | Arabidopsis thaliana | 284 | - | 9 | taTATAAAtc | core promoter element around -30 of transcription start |
| TATA-box | Arabidopsis thaliana | 1478 | - | 5 | TATAA | core promoter element around -30 of transcription start |
| TATA-box | Arabidopsis thaliana | 1463 | - | 7 | TATAAAA | core promoter element around -30 of transcription start |
| TATA-box | Arabidopsis thaliana | 296 | + | 4 | TATA | core promoter element around -30 of transcription start |
| TATA-box | Brassica oleracea | 316 | + | 6 | ATATAA | core promoter element around -30 of transcription start |
| TATA-box | Zea mays | 241 | + | 8 | TTTAAAAA | core promoter element around -30 of transcription start |
| TATA-box | Lycopersicon esculentum | 214 | + | 5 | TTTTA | core promoter element around -30 of transcription start |
| TATA-box | Glycine max | 128 | + | 5 | TAATA | core promoter element around -30 of transcription start |
| TATA-box | Arabidopsis thaliana | 1479 | - | 4 | TATA | core promoter element around -30 of transcription start |
| TATA-box | Arabidopsis thaliana | 1310 | - | 4 | TATA | core promoter element around -30 of transcription start |
| TATA-box | Ac | 597 | + | 7 | TATAAAT | core promoter element around -30 of transcription start |
| TATA-box | Brassica oleracea | 596 | + | 6 | ATATAA | core promoter element around -30 of transcription start |
| TATA-box | Lycopersicon esculentum | 590 | - | 5 | TTTTA | core promoter element around -30 of transcription start |
| TATA-box | Zea mays | 364 | + | 8 | TTTAAAAA | core promoter element around -30 of transcription start |
| TATA-box | Brassica napus | 287 | + | 6 | ATATAT | core promoter element around -30 of transcription start |
| TATA-box | Arabidopsis thaliana | 632 | - | 7 | TATAAAA | core promoter element around -30 of transcription start |
| TATA-box | Arabidopsis thaliana | 1466 | - | 4 | TATA | core promoter element around -30 of transcription start |
| TATA-box | Lycopersicon esculentum | 587 | + | 5 | TTTTA | core promoter element around -30 of transcription start |
| TATA-box | Arabidopsis thaliana | 290 | + | 8 | TATATATA | core promoter element around -30 of transcription start |
| TATA-box | Zea mays | 270 | + | 8 | TTTAAAAA | core promoter element around -30 of transcription start |
| TATA-box | Zea mays | 462 | + | 8 | TTTAAAAA | core promoter element around -30 of transcription start |
| TATA-box | Lycopersicon esculentum | 572 | - | 5 | TTTTA | core promoter element around -30 of transcription start |
| TATA-box | Glycine max | 547 | - | 5 | TAATA | core promoter element around -30 of transcription start |
| TATA-box | Lycopersicon esculentum | 243 | - | 5 | TTTTA | core promoter element around -30 of transcription start |
| TATA-box | Arabidopsis thaliana | 627 | + | 4 | TATA | core promoter element around -30 of transcription start |
| TATA-box | Arabidopsis thaliana | 1476 | - | 7 | TATAAAA | core promoter element around -30 of transcription start |
| TATA-box | Glycine max | 745 | - | 5 | TAATA | core promoter element around -30 of transcription start |
| TATA-box | Arabidopsis thaliana | 1475 | - | 9 | ccTATAAAaa | core promoter element around -30 of transcription start |
| TATA-box | Lycopersicon esculentum | 738 | + | 5 | TTTTA | core promoter element around -30 of transcription start |
| TATA-box | Lycopersicon esculentum | 240 | + | 5 | TTTTA | core promoter element around -30 of transcription start |
| TATA-box | Lycopersicon esculentum | 464 | - | 5 | TTTTA | core promoter element around -30 of transcription start |
| TATA-box | Brassica napus | 379 | + | 6 | ATATAT | core promoter element around -30 of transcription start |
| TATA-box | Arabidopsis thaliana | 1464 | - | 6 | TATAAA | core promoter element around -30 of transcription start |
| TATA-box | Lycopersicon esculentum | 349 | + | 5 | TTTTA | core promoter element around -30 of transcription start |
| TATA-box | Glycine max | 352 | - | 5 | TAATA | core promoter element around -30 of transcription start |
| TATA-box | Arabidopsis thaliana | 633 | - | 6 | TATAAA | core promoter element around -30 of transcription start |
| TATA-box | Glycine max | 476 | + | 5 | TAATA | core promoter element around -30 of transcription start |
| TATA-box | Zea mays | 588 | + | 8 | TTTAAAAA | core promoter element around -30 of transcription start |
| TATA-box | Ac | 223 | + | 7 | TATAAAT | core promoter element around -30 of transcription start |
| TATA-box | Lycopersicon esculentum | 334 | + | 5 | TTTTA | core promoter element around -30 of transcription start |
| TATA-box | Lycopersicon esculentum | 678 | + | 5 | TTTTA | core promoter element around -30 of transcription start |
| TATA-box | Lycopersicon esculentum | 533 | + | 5 | TTTTA | core promoter element around -30 of transcription start |
| TATA-box | Brassica napus | 293 | + | 6 | ATATAT | core promoter element around -30 of transcription start |
| TATA-box | Arabidopsis thaliana | 292 | + | 8 | TATATATA | core promoter element around -30 of transcription start |
| TATA-box | Lycopersicon esculentum | 113 | + | 5 | TTTTA | core promoter element around -30 of transcription start |
| TATA-box | Lycopersicon esculentum | 255 | + | 5 | TTTTA | core promoter element around -30 of transcription start |
| TATA-box | Arabidopsis thaliana | 1465 | - | 5 | TATAA | core promoter element around -30 of transcription start |
| TATA-box | Arabidopsis thaliana | 384 | + | 8 | TATATATA | core promoter element around -30 of transcription start |
| TATA-box | Brassica napus | 446 | + | 6 | ATTATA | core promoter element around -30 of transcription start |
| TATA-box | Brassica napus | 285 | + | 6 | ATATAT | core promoter element around -30 of transcription start |
| TATA-box | Oryza sativa | 100 | - | 7 | TACAAAA | core promoter element around -30 of transcription start |
| TATA-box | Arabidopsis thaliana | 635 | + | 4 | TATA | core promoter element around -30 of transcription start |
| TATA-box | Glycine max | 646 | + | 5 | TAATA | core promoter element around -30 of transcription start |
| TATA-box | Arabidopsis thaliana | 536 | + | 8 | TAAAGATT | core promoter element around -30 of transcription start |
| TATA-box | Zea mays | 335 | + | 8 | TTTAAAAA | core promoter element around -30 of transcription start |
| TATA-box | Ac | 317 | + | 7 | TATAAAT | core promoter element around -30 of transcription start |
| TATA-box | Lycopersicon esculentum | 450 | - | 5 | TTTTA | core promoter element around -30 of transcription start |
| TATA-box | Arabidopsis thaliana | 286 | + | 8 | TATATATA | core promoter element around -30 of transcription start |
| TATA-box | Glycine max | 649 | + | 5 | TAATA | core promoter element around -30 of transcription start |
| TATA-box | Arabidopsis thaliana | 382 | + | 8 | TATATATA | core promoter element around -30 of transcription start |
| TATA-box | Lycopersicon esculentum | 461 | + | 5 | TTTTA | core promoter element around -30 of transcription start |
| TATA-box | Arabidopsis thaliana | 447 | - | 5 | TATAA | core promoter element around -30 of transcription start |
| TATA-box | Arabidopsis thaliana | 388 | + | 4 | TATA | core promoter element around -30 of transcription start |
| TATA-box | Arabidopsis thaliana | 60 | - | 9 | ccTATAAAaa | core promoter element around -30 of transcription start |
| TATA-box | Brassica oleracea | 222 | + | 6 | ATATAA | core promoter element around -30 of transcription start |
| TATA-box | Brassica napus | 295 | + | 6 | ATATAT | core promoter element around -30 of transcription start |
| TATA-box | Arabidopsis thaliana | 634 | - | 5 | TATAA | core promoter element around -30 of transcription start |
| TATA-box | Brassica napus | 291 | + | 6 | ATATAT | core promoter element around -30 of transcription start |
| TATA-box | Lycopersicon esculentum | 550 | - | 5 | TTTTA | core promoter element around -30 of transcription start |
| TATA-box | Zea mays | 362 | - | 8 | TTTAAAAA | core promoter element around -30 of transcription start |
| TATA-box | Arabidopsis thaliana | 294 | + | 4 | TATA | core promoter element around -30 of transcription start |
| TATA-box | Lycopersicon esculentum | 407 | - | 5 | TTTTA | core promoter element around -30 of transcription start |
| TATA-box | Arabidopsis thaliana | 386 | + | 4 | TATA | core promoter element around -30 of transcription start |
| TATA-box | Lycopersicon esculentum | 269 | + | 5 | TTTTA | core promoter element around -30 of transcription start |
| TATA-box | Lycopersicon esculentum | 272 | - | 5 | TTTTA | core promoter element around -30 of transcription start |
| TATA-box | Brassica napus | 387 | + | 6 | ATATAT | core promoter element around -30 of transcription start |
| TATA-box | Brassica napus | 385 | + | 6 | ATATAT | core promoter element around -30 of transcription start |
| TATA-box | Arabidopsis thaliana | 288 | + | 8 | TATATATA | core promoter element around -30 of transcription start |
| TATA-box | Brassica napus | 289 | + | 6 | ATATAT | core promoter element around -30 of transcription start |
| TATA-box | Lycopersicon esculentum | 337 | - | 5 | TTTTA | core promoter element around -30 of transcription start |
| TATA-box | Arabidopsis thaliana | 378 | - | 9 | taTATAAAtc | core promoter element around -30 of transcription start |
| TATA-box | Lycopersicon esculentum | 363 | + | 5 | TTTTA | core promoter element around -30 of transcription start |
| TATA-box | Brassica napus | 381 | + | 6 | ATATAT | core promoter element around -30 of transcription start |
| TATA-box | Brassica napus | 383 | + | 6 | ATATAT | core promoter element around -30 of transcription start |

> 2018/04/13 10:10:12  
+ TTAATTTTCA GGTGCTCTCA TTCGTTTTCA CCTTCGCCAA TTGTGAAGTT GAAGCCACAT TTTTCTAGGG   
  
  
+ GCTACGATCT TGAAATAATG TCGATTTGAT TTTGTAGATA CCTTTTAATC ATTCTTCTAA TAGAATCATC   
  
  
+ TTTTTTTTTT TTTGGGTTGA TCTCATTTTG TTAAATTGAA TAAATTTAAT TGAATGAATT GAAAATTCTT   
  
  
+ TTTTTTTAAG GATATAAATT TTGTTGGTAT TTTAAAAAAA TATTTTTTAT TAAATACTTT TTAAAAAGAT   
  
  
+ GTTGATATAT ATATATATAT GTTAGAAAAT AAAGGATATA AATTTTGTTA GTATTTTAAA AAAATATTTT   
  
  
+ TTATTAAATA CTTTTTAAAA AGATGTTGAT ATATATATAT ATGTTAGAAA ATACAATAAA ATTTATTGCA   
  
  
+ TTTGTTTTCT TGATTAATGT AACCAATTAT AAAAATAGAA TTTTAAAAAA AAGATTAATA AGAATCATAG   
  
  
+ TAACAGATGA TTTTTTGATT CAAAGGAAAC ATTTCTTCAA TATTTTAAAG ATTCATTATT AAAAATGATT   
  
  
+ AGAAATTATG GTAAAATCTA TGAAATTTTT AAAAAATATA AATAGTATTG AAGTAAGAAG GGTAGTTATA   
  
  
+ TTTTTATAAC TTAAATAATA ATATAAAATA AAAAATGTAA CCATTAATTT TAATTAAATT ACCATGTATC   
  
  
+ TTAATTTATT TAAGTCGAAA ATTTGATCCT TTCTTAATTT TATTTATTAA GACACGTGAA AATACCTTTT   
  
  
+ TGCTTAATTA ATTGAATAAG AGCCACGCAT AGCATAACAA CATTATCTAT TTGCCCATTT CATGAGCAAT   
  
  
+ AAAGAATTCC ACTGACTGAA AAGAATACAA GACCCCCCAA AAAAAAAAGA AAAAAAGAGA AAAGAGAAAA   
  
  
+ AAAAAGATGA AAAAAATGAT TAAAGGGAAA AGAAGAAAAG GGAATATCTT AGGAGGATTC AGTTGGTGAG   
  
  
+ CTTGAATTGA GTTCACACAG AGGAGAGTTC ACATGCAAGA CGACGGAGCT TGGGGCTGTA ATGGGTAAGA   
  
  
+ ATCTCACGTG CAGAGAAACC CCGTTCTCAC TGGGGGAACC TGTTAACTGT TGAGCCGGCC AACGAAGCGT   
  
  
+ ATGAAAACGT CGGCCAACCA CGACAGAATA CTCGGCCGCA TGCATTGAAT TTGCCACCGG CACTGTGGGT   
  
  
+ GTCCGTACGA AATGTTGACC TACGATCTCC GGTCAATCTT TGTTGCCATT TGTCTACTCT CGAGCAGAAT   
  
  
+ AGAAAAGTAA CTCACCTTCT CTTCTCTTTG GCCAAATCTC ACTTTCTTCT ATACCTAAAG CTCTCTCTCT   
  
  
+ CTCTCTCTCC CCCTCCCACC TACCTGATAA ACGCAATCAG GCCTAGCTTT CTCTCTCTCT CTCTCTCTCT   
  
  
+ CTTTCTTTCT CAGTAAGCAA ACATCACTCC CGCTGTGTGT CACTCTCTGT TAAGTTACAA GCTTTTATAT   
  
  
+ GAGGATTTTA TAGGCCTGAT TGATAGAAA  

- AATTAAAAGT CCACGAGAGT AAGCAAAAGT GGAAGCGGTT AACACTTCAA CTTCGGTGTA AAAAGATCCC   
  
  
- CGATGCTAGA ACTTTATTAC AGCTAAACTA AAACATCTAT GGAAAATTAG TAAGAAGATT ATCTTAGTAG   
  
  
- AAAAAAAAAA AAACCCAACT AGAGTAAAAC AATTTAACTT ATTTAAATTA ACTTACTTAA CTTTTAAGAA   
  
  
- AAAAAAATTC CTATATTTAA AACAACCATA AAATTTTTTT ATAAAAAATA ATTTATGAAA AATTTTTCTA   
  
  
- CAACTATATA TATATATATA CAATCTTTTA TTTCCTATAT TTAAAACAAT CATAAAATTT TTTTATAAAA   
  
  
- AATAATTTAT GAAAAATTTT TCTACAACTA TATATATATA TACAATCTTT TATGTTATTT TAAATAACGT   
  
  
- AAACAAAAGA ACTAATTACA TTGGTTAATA TTTTTATCTT AAAATTTTTT TTCTAATTAT TCTTAGTATC   
  
  
- ATTGTCTACT AAAAAACTAA GTTTCCTTTG TAAAGAAGTT ATAAAATTTC TAAGTAATAA TTTTTACTAA   
  
  
- TCTTTAATAC CATTTTAGAT ACTTTAAAAA TTTTTTATAT TTATCATAAC TTCATTCTTC CCATCAATAT   
  
  
- AAAAATATTG AATTTATTAT TATATTTTAT TTTTTACATT GGTAATTAAA ATTAATTTAA TGGTACATAG   
  
  
- AATTAAATAA ATTCAGCTTT TAAACTAGGA AAGAATTAAA ATAAATAATT CTGTGCACTT TTATGGAAAA   
  
  
- ACGAATTAAT TAACTTATTC TCGGTGCGTA TCGTATTGTT GTAATAGATA AACGGGTAAA GTACTCGTTA   
  
  
- TTTCTTAAGG TGACTGACTT TTCTTATGTT CTGGGGGGTT TTTTTTTTCT TTTTTTCTCT TTTCTCTTTT   
  
  
- TTTTTCTACT TTTTTTACTA ATTTCCCTTT TCTTCTTTTC CCTTATAGAA TCCTCCTAAG TCAACCACTC   
  
  
- GAACTTAACT CAAGTGTGTC TCCTCTCAAG TGTACGTTCT GCTGCCTCGA ACCCCGACAT TACCCATTCT   
  
  
- TAGAGTGCAC GTCTCTTTGG GGCAAGAGTG ACCCCCTTGG ACAATTGACA ACTCGGCCGG TTGCTTCGCA   
  
  
- TACTTTTGCA GCCGGTTGGT GCTGTCTTAT GAGCCGGCGT ACGTAACTTA AACGGTGGCC GTGACACCCA   
  
  
- CAGGCATGCT TTACAACTGG ATGCTAGAGG CCAGTTAGAA ACAACGGTAA ACAGATGAGA GCTCGTCTTA   
  
  
- TCTTTTCATT GAGTGGAAGA GAAGAGAAAC CGGTTTAGAG TGAAAGAAGA TATGGATTTC GAGAGAGAGA   
  
  
- GAGAGAGAGG GGGAGGGTGG ATGGACTATT TGCGTTAGTC CGGATCGAAA GAGAGAGAGA GAGAGAGAGA   
  
  
- GAAAGAAAGA GTCATTCGTT TGTAGTGAGG GCGACACACA GTGAGAGACA ATTCAATGTT CGAAAATATA   
  
  
- CTCCTAAAAT ATCCGGACTA ACTATCTTT

+     TC-rich repeats

| Site Name | Organism | Position | Strand | Matrix score. | sequence | function |
| --- | --- | --- | --- | --- | --- | --- |
| TC-rich repeats | Nicotiana tabacum | 301 | - | 9 | ATTCTCTAAC | cis-acting element involved in defense and stress responsiveness |
| TC-rich repeats | Nicotiana tabacum | 1413 | - | 9 | GTTTTCTTAC | cis-acting element involved in defense and stress responsiveness |
| TC-rich repeats | Nicotiana tabacum | 918 | - | 9 | ATTTTCTTCA | cis-acting element involved in defense and stress responsiveness |
| TC-rich repeats | Nicotiana tabacum | 393 | - | 9 | ATTCTCTAAC | cis-acting element involved in defense and stress responsiveness |
| TC-rich repeats | Nicotiana tabacum | 1119 | - | 9 | GTTTTCTTAC | cis-acting element involved in defense and stress responsiveness |

> 2018/04/13 10:10:12  
+ TTAATTTTCA GGTGCTCTCA TTCGTTTTCA CCTTCGCCAA TTGTGAAGTT GAAGCCACAT TTTTCTAGGG   
  
  
+ GCTACGATCT TGAAATAATG TCGATTTGAT TTTGTAGATA CCTTTTAATC ATTCTTCTAA TAGAATCATC   
  
  
+ TTTTTTTTTT TTTGGGTTGA TCTCATTTTG TTAAATTGAA TAAATTTAAT TGAATGAATT GAAAATTCTT   
  
  
+ TTTTTTTAAG GATATAAATT TTGTTGGTAT TTTAAAAAAA TATTTTTTAT TAAATACTTT TTAAAAAGAT   
  
  
+ GTTGATATAT ATATATATAT GTTAGAAAAT AAAGGATATA AATTTTGTTA GTATTTTAAA AAAATATTTT   
  
  
+ TTATTAAATA CTTTTTAAAA AGATGTTGAT ATATATATAT ATGTTAGAAA ATACAATAAA ATTTATTGCA   
  
  
+ TTTGTTTTCT TGATTAATGT AACCAATTAT AAAAATAGAA TTTTAAAAAA AAGATTAATA AGAATCATAG   
  
  
+ TAACAGATGA TTTTTTGATT CAAAGGAAAC ATTTCTTCAA TATTTTAAAG ATTCATTATT AAAAATGATT   
  
  
+ AGAAATTATG GTAAAATCTA TGAAATTTTT AAAAAATATA AATAGTATTG AAGTAAGAAG GGTAGTTATA   
  
  
+ TTTTTATAAC TTAAATAATA ATATAAAATA AAAAATGTAA CCATTAATTT TAATTAAATT ACCATGTATC   
  
  
+ TTAATTTATT TAAGTCGAAA ATTTGATCCT TTCTTAATTT TATTTATTAA GACACGTGAA AATACCTTTT   
  
  
+ TGCTTAATTA ATTGAATAAG AGCCACGCAT AGCATAACAA CATTATCTAT TTGCCCATTT CATGAGCAAT   
  
  
+ AAAGAATTCC ACTGACTGAA AAGAATACAA GACCCCCCAA AAAAAAAAGA AAAAAAGAGA AAAGAGAAAA   
  
  
+ AAAAAGATGA AAAAAATGAT TAAAGGGAAA AGAAGAAAAG GGAATATCTT AGGAGGATTC AGTTGGTGAG   
  
  
+ CTTGAATTGA GTTCACACAG AGGAGAGTTC ACATGCAAGA CGACGGAGCT TGGGGCTGTA ATGGGTAAGA   
  
  
+ ATCTCACGTG CAGAGAAACC CCGTTCTCAC TGGGGGAACC TGTTAACTGT TGAGCCGGCC AACGAAGCGT   
  
  
+ ATGAAAACGT CGGCCAACCA CGACAGAATA CTCGGCCGCA TGCATTGAAT TTGCCACCGG CACTGTGGGT   
  
  
+ GTCCGTACGA AATGTTGACC TACGATCTCC GGTCAATCTT TGTTGCCATT TGTCTACTCT CGAGCAGAAT   
  
  
+ AGAAAAGTAA CTCACCTTCT CTTCTCTTTG GCCAAATCTC ACTTTCTTCT ATACCTAAAG CTCTCTCTCT   
  
  
+ CTCTCTCTCC CCCTCCCACC TACCTGATAA ACGCAATCAG GCCTAGCTTT CTCTCTCTCT CTCTCTCTCT   
  
  
+ CTTTCTTTCT CAGTAAGCAA ACATCACTCC CGCTGTGTGT CACTCTCTGT TAAGTTACAA GCTTTTATAT   
  
  
+ GAGGATTTTA TAGGCCTGAT TGATAGAAA  

- AATTAAAAGT CCACGAGAGT AAGCAAAAGT GGAAGCGGTT AACACTTCAA CTTCGGTGTA AAAAGATCCC   
  
  
- CGATGCTAGA ACTTTATTAC AGCTAAACTA AAACATCTAT GGAAAATTAG TAAGAAGATT ATCTTAGTAG   
  
  
- AAAAAAAAAA AAACCCAACT AGAGTAAAAC AATTTAACTT ATTTAAATTA ACTTACTTAA CTTTTAAGAA   
  
  
- AAAAAAATTC CTATATTTAA AACAACCATA AAATTTTTTT ATAAAAAATA ATTTATGAAA AATTTTTCTA   
  
  
- CAACTATATA TATATATATA CAATCTTTTA TTTCCTATAT TTAAAACAAT CATAAAATTT TTTTATAAAA   
  
  
- AATAATTTAT GAAAAATTTT TCTACAACTA TATATATATA TACAATCTTT TATGTTATTT TAAATAACGT   
  
  
- AAACAAAAGA ACTAATTACA TTGGTTAATA TTTTTATCTT AAAATTTTTT TTCTAATTAT TCTTAGTATC   
  
  
- ATTGTCTACT AAAAAACTAA GTTTCCTTTG TAAAGAAGTT ATAAAATTTC TAAGTAATAA TTTTTACTAA   
  
  
- TCTTTAATAC CATTTTAGAT ACTTTAAAAA TTTTTTATAT TTATCATAAC TTCATTCTTC CCATCAATAT   
  
  
- AAAAATATTG AATTTATTAT TATATTTTAT TTTTTACATT GGTAATTAAA ATTAATTTAA TGGTACATAG   
  
  
- AATTAAATAA ATTCAGCTTT TAAACTAGGA AAGAATTAAA ATAAATAATT CTGTGCACTT TTATGGAAAA   
  
  
- ACGAATTAAT TAACTTATTC TCGGTGCGTA TCGTATTGTT GTAATAGATA AACGGGTAAA GTACTCGTTA   
  
  
- TTTCTTAAGG TGACTGACTT TTCTTATGTT CTGGGGGGTT TTTTTTTTCT TTTTTTCTCT TTTCTCTTTT   
  
  
- TTTTTCTACT TTTTTTACTA ATTTCCCTTT TCTTCTTTTC CCTTATAGAA TCCTCCTAAG TCAACCACTC   
  
  
- GAACTTAACT CAAGTGTGTC TCCTCTCAAG TGTACGTTCT GCTGCCTCGA ACCCCGACAT TACCCATTCT   
  
  
- TAGAGTGCAC GTCTCTTTGG GGCAAGAGTG ACCCCCTTGG ACAATTGACA ACTCGGCCGG TTGCTTCGCA   
  
  
- TACTTTTGCA GCCGGTTGGT GCTGTCTTAT GAGCCGGCGT ACGTAACTTA AACGGTGGCC GTGACACCCA   
  
  
- CAGGCATGCT TTACAACTGG ATGCTAGAGG CCAGTTAGAA ACAACGGTAA ACAGATGAGA GCTCGTCTTA   
  
  
- TCTTTTCATT GAGTGGAAGA GAAGAGAAAC CGGTTTAGAG TGAAAGAAGA TATGGATTTC GAGAGAGAGA   
  
  
- GAGAGAGAGG GGGAGGGTGG ATGGACTATT TGCGTTAGTC CGGATCGAAA GAGAGAGAGA GAGAGAGAGA   
  
  
- GAAAGAAAGA GTCATTCGTT TGTAGTGAGG GCGACACACA GTGAGAGACA ATTCAATGTT CGAAAATATA   
  
  
- CTCCTAAAAT ATCCGGACTA ACTATCTTT

+     TCA-element

| Site Name | Organism | Position | Strand | Matrix score. | sequence | function |
| --- | --- | --- | --- | --- | --- | --- |
| TCA-element | Nicotiana tabacum | 136 | + | 9 | CCATCTTTTT | cis-acting element involved in salicylic acid responsiveness |
| TCA-element | Brassica oleracea | 1252 | + | 9 | GAGAAGAATA | cis-acting element involved in salicylic acid responsiveness |
| TCA-element | Nicotiana tabacum | 367 | - | 9 | CCATCTTTTT | cis-acting element involved in salicylic acid responsiveness |
| TCA-element | Brassica oleracea | 858 | + | 9 | GAGAAGAATA | cis-acting element involved in salicylic acid responsiveness |
| TCA-element | Nicotiana tabacum | 911 | - | 9 | CCATCTTTTT | cis-acting element involved in salicylic acid responsiveness |
| TCA-element | Nicotiana tabacum | 273 | - | 9 | CCATCTTTTT | cis-acting element involved in salicylic acid responsiveness |

> 2018/04/13 10:10:12  
+ TTAATTTTCA GGTGCTCTCA TTCGTTTTCA CCTTCGCCAA TTGTGAAGTT GAAGCCACAT TTTTCTAGGG   
  
  
+ GCTACGATCT TGAAATAATG TCGATTTGAT TTTGTAGATA CCTTTTAATC ATTCTTCTAA TAGAATCATC   
  
  
+ TTTTTTTTTT TTTGGGTTGA TCTCATTTTG TTAAATTGAA TAAATTTAAT TGAATGAATT GAAAATTCTT   
  
  
+ TTTTTTTAAG GATATAAATT TTGTTGGTAT TTTAAAAAAA TATTTTTTAT TAAATACTTT TTAAAAAGAT   
  
  
+ GTTGATATAT ATATATATAT GTTAGAAAAT AAAGGATATA AATTTTGTTA GTATTTTAAA AAAATATTTT   
  
  
+ TTATTAAATA CTTTTTAAAA AGATGTTGAT ATATATATAT ATGTTAGAAA ATACAATAAA ATTTATTGCA   
  
  
+ TTTGTTTTCT TGATTAATGT AACCAATTAT AAAAATAGAA TTTTAAAAAA AAGATTAATA AGAATCATAG   
  
  
+ TAACAGATGA TTTTTTGATT CAAAGGAAAC ATTTCTTCAA TATTTTAAAG ATTCATTATT AAAAATGATT   
  
  
+ AGAAATTATG GTAAAATCTA TGAAATTTTT AAAAAATATA AATAGTATTG AAGTAAGAAG GGTAGTTATA   
  
  
+ TTTTTATAAC TTAAATAATA ATATAAAATA AAAAATGTAA CCATTAATTT TAATTAAATT ACCATGTATC   
  
  
+ TTAATTTATT TAAGTCGAAA ATTTGATCCT TTCTTAATTT TATTTATTAA GACACGTGAA AATACCTTTT   
  
  
+ TGCTTAATTA ATTGAATAAG AGCCACGCAT AGCATAACAA CATTATCTAT TTGCCCATTT CATGAGCAAT   
  
  
+ AAAGAATTCC ACTGACTGAA AAGAATACAA GACCCCCCAA AAAAAAAAGA AAAAAAGAGA AAAGAGAAAA   
  
  
+ AAAAAGATGA AAAAAATGAT TAAAGGGAAA AGAAGAAAAG GGAATATCTT AGGAGGATTC AGTTGGTGAG   
  
  
+ CTTGAATTGA GTTCACACAG AGGAGAGTTC ACATGCAAGA CGACGGAGCT TGGGGCTGTA ATGGGTAAGA   
  
  
+ ATCTCACGTG CAGAGAAACC CCGTTCTCAC TGGGGGAACC TGTTAACTGT TGAGCCGGCC AACGAAGCGT   
  
  
+ ATGAAAACGT CGGCCAACCA CGACAGAATA CTCGGCCGCA TGCATTGAAT TTGCCACCGG CACTGTGGGT   
  
  
+ GTCCGTACGA AATGTTGACC TACGATCTCC GGTCAATCTT TGTTGCCATT TGTCTACTCT CGAGCAGAAT   
  
  
+ AGAAAAGTAA CTCACCTTCT CTTCTCTTTG GCCAAATCTC ACTTTCTTCT ATACCTAAAG CTCTCTCTCT   
  
  
+ CTCTCTCTCC CCCTCCCACC TACCTGATAA ACGCAATCAG GCCTAGCTTT CTCTCTCTCT CTCTCTCTCT   
  
  
+ CTTTCTTTCT CAGTAAGCAA ACATCACTCC CGCTGTGTGT CACTCTCTGT TAAGTTACAA GCTTTTATAT   
  
  
+ GAGGATTTTA TAGGCCTGAT TGATAGAAA  

- AATTAAAAGT CCACGAGAGT AAGCAAAAGT GGAAGCGGTT AACACTTCAA CTTCGGTGTA AAAAGATCCC   
  
  
- CGATGCTAGA ACTTTATTAC AGCTAAACTA AAACATCTAT GGAAAATTAG TAAGAAGATT ATCTTAGTAG   
  
  
- AAAAAAAAAA AAACCCAACT AGAGTAAAAC AATTTAACTT ATTTAAATTA ACTTACTTAA CTTTTAAGAA   
  
  
- AAAAAAATTC CTATATTTAA AACAACCATA AAATTTTTTT ATAAAAAATA ATTTATGAAA AATTTTTCTA   
  
  
- CAACTATATA TATATATATA CAATCTTTTA TTTCCTATAT TTAAAACAAT CATAAAATTT TTTTATAAAA   
  
  
- AATAATTTAT GAAAAATTTT TCTACAACTA TATATATATA TACAATCTTT TATGTTATTT TAAATAACGT   
  
  
- AAACAAAAGA ACTAATTACA TTGGTTAATA TTTTTATCTT AAAATTTTTT TTCTAATTAT TCTTAGTATC   
  
  
- ATTGTCTACT AAAAAACTAA GTTTCCTTTG TAAAGAAGTT ATAAAATTTC TAAGTAATAA TTTTTACTAA   
  
  
- TCTTTAATAC CATTTTAGAT ACTTTAAAAA TTTTTTATAT TTATCATAAC TTCATTCTTC CCATCAATAT   
  
  
- AAAAATATTG AATTTATTAT TATATTTTAT TTTTTACATT GGTAATTAAA ATTAATTTAA TGGTACATAG   
  
  
- AATTAAATAA ATTCAGCTTT TAAACTAGGA AAGAATTAAA ATAAATAATT CTGTGCACTT TTATGGAAAA   
  
  
- ACGAATTAAT TAACTTATTC TCGGTGCGTA TCGTATTGTT GTAATAGATA AACGGGTAAA GTACTCGTTA   
  
  
- TTTCTTAAGG TGACTGACTT TTCTTATGTT CTGGGGGGTT TTTTTTTTCT TTTTTTCTCT TTTCTCTTTT   
  
  
- TTTTTCTACT TTTTTTACTA ATTTCCCTTT TCTTCTTTTC CCTTATAGAA TCCTCCTAAG TCAACCACTC   
  
  
- GAACTTAACT CAAGTGTGTC TCCTCTCAAG TGTACGTTCT GCTGCCTCGA ACCCCGACAT TACCCATTCT   
  
  
- TAGAGTGCAC GTCTCTTTGG GGCAAGAGTG ACCCCCTTGG ACAATTGACA ACTCGGCCGG TTGCTTCGCA   
  
  
- TACTTTTGCA GCCGGTTGGT GCTGTCTTAT GAGCCGGCGT ACGTAACTTA AACGGTGGCC GTGACACCCA   
  
  
- CAGGCATGCT TTACAACTGG ATGCTAGAGG CCAGTTAGAA ACAACGGTAA ACAGATGAGA GCTCGTCTTA   
  
  
- TCTTTTCATT GAGTGGAAGA GAAGAGAAAC CGGTTTAGAG TGAAAGAAGA TATGGATTTC GAGAGAGAGA   
  
  
- GAGAGAGAGG GGGAGGGTGG ATGGACTATT TGCGTTAGTC CGGATCGAAA GAGAGAGAGA GAGAGAGAGA   
  
  
- GAAAGAAAGA GTCATTCGTT TGTAGTGAGG GCGACACACA GTGAGAGACA ATTCAATGTT CGAAAATATA   
  
  
- CTCCTAAAAT ATCCGGACTA ACTATCTTT

+     TCT-motif

| Site Name | Organism | Position | Strand | Matrix score. | sequence | function |
| --- | --- | --- | --- | --- | --- | --- |
| TCT-motif | Arabidopsis thaliana | 613 | - | 6 | TCTTAC | part of a light responsive element |
| TCT-motif | Arabidopsis thaliana | 1045 | - | 6 | TCTTAC | part of a light responsive element |

> 2018/04/13 10:10:12  
+ TTAATTTTCA GGTGCTCTCA TTCGTTTTCA CCTTCGCCAA TTGTGAAGTT GAAGCCACAT TTTTCTAGGG   
  
  
+ GCTACGATCT TGAAATAATG TCGATTTGAT TTTGTAGATA CCTTTTAATC ATTCTTCTAA TAGAATCATC   
  
  
+ TTTTTTTTTT TTTGGGTTGA TCTCATTTTG TTAAATTGAA TAAATTTAAT TGAATGAATT GAAAATTCTT   
  
  
+ TTTTTTTAAG GATATAAATT TTGTTGGTAT TTTAAAAAAA TATTTTTTAT TAAATACTTT TTAAAAAGAT   
  
  
+ GTTGATATAT ATATATATAT GTTAGAAAAT AAAGGATATA AATTTTGTTA GTATTTTAAA AAAATATTTT   
  
  
+ TTATTAAATA CTTTTTAAAA AGATGTTGAT ATATATATAT ATGTTAGAAA ATACAATAAA ATTTATTGCA   
  
  
+ TTTGTTTTCT TGATTAATGT AACCAATTAT AAAAATAGAA TTTTAAAAAA AAGATTAATA AGAATCATAG   
  
  
+ TAACAGATGA TTTTTTGATT CAAAGGAAAC ATTTCTTCAA TATTTTAAAG ATTCATTATT AAAAATGATT   
  
  
+ AGAAATTATG GTAAAATCTA TGAAATTTTT AAAAAATATA AATAGTATTG AAGTAAGAAG GGTAGTTATA   
  
  
+ TTTTTATAAC TTAAATAATA ATATAAAATA AAAAATGTAA CCATTAATTT TAATTAAATT ACCATGTATC   
  
  
+ TTAATTTATT TAAGTCGAAA ATTTGATCCT TTCTTAATTT TATTTATTAA GACACGTGAA AATACCTTTT   
  
  
+ TGCTTAATTA ATTGAATAAG AGCCACGCAT AGCATAACAA CATTATCTAT TTGCCCATTT CATGAGCAAT   
  
  
+ AAAGAATTCC ACTGACTGAA AAGAATACAA GACCCCCCAA AAAAAAAAGA AAAAAAGAGA AAAGAGAAAA   
  
  
+ AAAAAGATGA AAAAAATGAT TAAAGGGAAA AGAAGAAAAG GGAATATCTT AGGAGGATTC AGTTGGTGAG   
  
  
+ CTTGAATTGA GTTCACACAG AGGAGAGTTC ACATGCAAGA CGACGGAGCT TGGGGCTGTA ATGGGTAAGA   
  
  
+ ATCTCACGTG CAGAGAAACC CCGTTCTCAC TGGGGGAACC TGTTAACTGT TGAGCCGGCC AACGAAGCGT   
  
  
+ ATGAAAACGT CGGCCAACCA CGACAGAATA CTCGGCCGCA TGCATTGAAT TTGCCACCGG CACTGTGGGT   
  
  
+ GTCCGTACGA AATGTTGACC TACGATCTCC GGTCAATCTT TGTTGCCATT TGTCTACTCT CGAGCAGAAT   
  
  
+ AGAAAAGTAA CTCACCTTCT CTTCTCTTTG GCCAAATCTC ACTTTCTTCT ATACCTAAAG CTCTCTCTCT   
  
  
+ CTCTCTCTCC CCCTCCCACC TACCTGATAA ACGCAATCAG GCCTAGCTTT CTCTCTCTCT CTCTCTCTCT   
  
  
+ CTTTCTTTCT CAGTAAGCAA ACATCACTCC CGCTGTGTGT CACTCTCTGT TAAGTTACAA GCTTTTATAT   
  
  
+ GAGGATTTTA TAGGCCTGAT TGATAGAAA  

- AATTAAAAGT CCACGAGAGT AAGCAAAAGT GGAAGCGGTT AACACTTCAA CTTCGGTGTA AAAAGATCCC   
  
  
- CGATGCTAGA ACTTTATTAC AGCTAAACTA AAACATCTAT GGAAAATTAG TAAGAAGATT ATCTTAGTAG   
  
  
- AAAAAAAAAA AAACCCAACT AGAGTAAAAC AATTTAACTT ATTTAAATTA ACTTACTTAA CTTTTAAGAA   
  
  
- AAAAAAATTC CTATATTTAA AACAACCATA AAATTTTTTT ATAAAAAATA ATTTATGAAA AATTTTTCTA   
  
  
- CAACTATATA TATATATATA CAATCTTTTA TTTCCTATAT TTAAAACAAT CATAAAATTT TTTTATAAAA   
  
  
- AATAATTTAT GAAAAATTTT TCTACAACTA TATATATATA TACAATCTTT TATGTTATTT TAAATAACGT   
  
  
- AAACAAAAGA ACTAATTACA TTGGTTAATA TTTTTATCTT AAAATTTTTT TTCTAATTAT TCTTAGTATC   
  
  
- ATTGTCTACT AAAAAACTAA GTTTCCTTTG TAAAGAAGTT ATAAAATTTC TAAGTAATAA TTTTTACTAA   
  
  
- TCTTTAATAC CATTTTAGAT ACTTTAAAAA TTTTTTATAT TTATCATAAC TTCATTCTTC CCATCAATAT   
  
  
- AAAAATATTG AATTTATTAT TATATTTTAT TTTTTACATT GGTAATTAAA ATTAATTTAA TGGTACATAG   
  
  
- AATTAAATAA ATTCAGCTTT TAAACTAGGA AAGAATTAAA ATAAATAATT CTGTGCACTT TTATGGAAAA   
  
  
- ACGAATTAAT TAACTTATTC TCGGTGCGTA TCGTATTGTT GTAATAGATA AACGGGTAAA GTACTCGTTA   
  
  
- TTTCTTAAGG TGACTGACTT TTCTTATGTT CTGGGGGGTT TTTTTTTTCT TTTTTTCTCT TTTCTCTTTT   
  
  
- TTTTTCTACT TTTTTTACTA ATTTCCCTTT TCTTCTTTTC CCTTATAGAA TCCTCCTAAG TCAACCACTC   
  
  
- GAACTTAACT CAAGTGTGTC TCCTCTCAAG TGTACGTTCT GCTGCCTCGA ACCCCGACAT TACCCATTCT   
  
  
- TAGAGTGCAC GTCTCTTTGG GGCAAGAGTG ACCCCCTTGG ACAATTGACA ACTCGGCCGG TTGCTTCGCA   
  
  
- TACTTTTGCA GCCGGTTGGT GCTGTCTTAT GAGCCGGCGT ACGTAACTTA AACGGTGGCC GTGACACCCA   
  
  
- CAGGCATGCT TTACAACTGG ATGCTAGAGG CCAGTTAGAA ACAACGGTAA ACAGATGAGA GCTCGTCTTA   
  
  
- TCTTTTCATT GAGTGGAAGA GAAGAGAAAC CGGTTTAGAG TGAAAGAAGA TATGGATTTC GAGAGAGAGA   
  
  
- GAGAGAGAGG GGGAGGGTGG ATGGACTATT TGCGTTAGTC CGGATCGAAA GAGAGAGAGA GAGAGAGAGA   
  
  
- GAAAGAAAGA GTCATTCGTT TGTAGTGAGG GCGACACACA GTGAGAGACA ATTCAATGTT CGAAAATATA   
  
  
- CTCCTAAAAT ATCCGGACTA ACTATCTTT

+     Unnamed\_\_1

| Site Name | Organism | Position | Strand | Matrix score. | sequence | function |
| --- | --- | --- | --- | --- | --- | --- |
| Unnamed\_\_1 | Zea mays | 793 | - | 5 | CGTGG |  |
| Unnamed\_\_1 | Zea mays | 1138 | - | 5 | CGTGG |  |
| Unnamed\_\_1 | Glycine max | 680 | - | 11 | GAATTTAATTAA | 60K protein binding site |

> 2018/04/13 10:10:12  
+ TTAATTTTCA GGTGCTCTCA TTCGTTTTCA CCTTCGCCAA TTGTGAAGTT GAAGCCACAT TTTTCTAGGG   
  
  
+ GCTACGATCT TGAAATAATG TCGATTTGAT TTTGTAGATA CCTTTTAATC ATTCTTCTAA TAGAATCATC   
  
  
+ TTTTTTTTTT TTTGGGTTGA TCTCATTTTG TTAAATTGAA TAAATTTAAT TGAATGAATT GAAAATTCTT   
  
  
+ TTTTTTTAAG GATATAAATT TTGTTGGTAT TTTAAAAAAA TATTTTTTAT TAAATACTTT TTAAAAAGAT   
  
  
+ GTTGATATAT ATATATATAT GTTAGAAAAT AAAGGATATA AATTTTGTTA GTATTTTAAA AAAATATTTT   
  
  
+ TTATTAAATA CTTTTTAAAA AGATGTTGAT ATATATATAT ATGTTAGAAA ATACAATAAA ATTTATTGCA   
  
  
+ TTTGTTTTCT TGATTAATGT AACCAATTAT AAAAATAGAA TTTTAAAAAA AAGATTAATA AGAATCATAG   
  
  
+ TAACAGATGA TTTTTTGATT CAAAGGAAAC ATTTCTTCAA TATTTTAAAG ATTCATTATT AAAAATGATT   
  
  
+ AGAAATTATG GTAAAATCTA TGAAATTTTT AAAAAATATA AATAGTATTG AAGTAAGAAG GGTAGTTATA   
  
  
+ TTTTTATAAC TTAAATAATA ATATAAAATA AAAAATGTAA CCATTAATTT TAATTAAATT ACCATGTATC   
  
  
+ TTAATTTATT TAAGTCGAAA ATTTGATCCT TTCTTAATTT TATTTATTAA GACACGTGAA AATACCTTTT   
  
  
+ TGCTTAATTA ATTGAATAAG AGCCACGCAT AGCATAACAA CATTATCTAT TTGCCCATTT CATGAGCAAT   
  
  
+ AAAGAATTCC ACTGACTGAA AAGAATACAA GACCCCCCAA AAAAAAAAGA AAAAAAGAGA AAAGAGAAAA   
  
  
+ AAAAAGATGA AAAAAATGAT TAAAGGGAAA AGAAGAAAAG GGAATATCTT AGGAGGATTC AGTTGGTGAG   
  
  
+ CTTGAATTGA GTTCACACAG AGGAGAGTTC ACATGCAAGA CGACGGAGCT TGGGGCTGTA ATGGGTAAGA   
  
  
+ ATCTCACGTG CAGAGAAACC CCGTTCTCAC TGGGGGAACC TGTTAACTGT TGAGCCGGCC AACGAAGCGT   
  
  
+ ATGAAAACGT CGGCCAACCA CGACAGAATA CTCGGCCGCA TGCATTGAAT TTGCCACCGG CACTGTGGGT   
  
  
+ GTCCGTACGA AATGTTGACC TACGATCTCC GGTCAATCTT TGTTGCCATT TGTCTACTCT CGAGCAGAAT   
  
  
+ AGAAAAGTAA CTCACCTTCT CTTCTCTTTG GCCAAATCTC ACTTTCTTCT ATACCTAAAG CTCTCTCTCT   
  
  
+ CTCTCTCTCC CCCTCCCACC TACCTGATAA ACGCAATCAG GCCTAGCTTT CTCTCTCTCT CTCTCTCTCT   
  
  
+ CTTTCTTTCT CAGTAAGCAA ACATCACTCC CGCTGTGTGT CACTCTCTGT TAAGTTACAA GCTTTTATAT   
  
  
+ GAGGATTTTA TAGGCCTGAT TGATAGAAA  

- AATTAAAAGT CCACGAGAGT AAGCAAAAGT GGAAGCGGTT AACACTTCAA CTTCGGTGTA AAAAGATCCC   
  
  
- CGATGCTAGA ACTTTATTAC AGCTAAACTA AAACATCTAT GGAAAATTAG TAAGAAGATT ATCTTAGTAG   
  
  
- AAAAAAAAAA AAACCCAACT AGAGTAAAAC AATTTAACTT ATTTAAATTA ACTTACTTAA CTTTTAAGAA   
  
  
- AAAAAAATTC CTATATTTAA AACAACCATA AAATTTTTTT ATAAAAAATA ATTTATGAAA AATTTTTCTA   
  
  
- CAACTATATA TATATATATA CAATCTTTTA TTTCCTATAT TTAAAACAAT CATAAAATTT TTTTATAAAA   
  
  
- AATAATTTAT GAAAAATTTT TCTACAACTA TATATATATA TACAATCTTT TATGTTATTT TAAATAACGT   
  
  
- AAACAAAAGA ACTAATTACA TTGGTTAATA TTTTTATCTT AAAATTTTTT TTCTAATTAT TCTTAGTATC   
  
  
- ATTGTCTACT AAAAAACTAA GTTTCCTTTG TAAAGAAGTT ATAAAATTTC TAAGTAATAA TTTTTACTAA   
  
  
- TCTTTAATAC CATTTTAGAT ACTTTAAAAA TTTTTTATAT TTATCATAAC TTCATTCTTC CCATCAATAT   
  
  
- AAAAATATTG AATTTATTAT TATATTTTAT TTTTTACATT GGTAATTAAA ATTAATTTAA TGGTACATAG   
  
  
- AATTAAATAA ATTCAGCTTT TAAACTAGGA AAGAATTAAA ATAAATAATT CTGTGCACTT TTATGGAAAA   
  
  
- ACGAATTAAT TAACTTATTC TCGGTGCGTA TCGTATTGTT GTAATAGATA AACGGGTAAA GTACTCGTTA   
  
  
- TTTCTTAAGG TGACTGACTT TTCTTATGTT CTGGGGGGTT TTTTTTTTCT TTTTTTCTCT TTTCTCTTTT   
  
  
- TTTTTCTACT TTTTTTACTA ATTTCCCTTT TCTTCTTTTC CCTTATAGAA TCCTCCTAAG TCAACCACTC   
  
  
- GAACTTAACT CAAGTGTGTC TCCTCTCAAG TGTACGTTCT GCTGCCTCGA ACCCCGACAT TACCCATTCT   
  
  
- TAGAGTGCAC GTCTCTTTGG GGCAAGAGTG ACCCCCTTGG ACAATTGACA ACTCGGCCGG TTGCTTCGCA   
  
  
- TACTTTTGCA GCCGGTTGGT GCTGTCTTAT GAGCCGGCGT ACGTAACTTA AACGGTGGCC GTGACACCCA   
  
  
- CAGGCATGCT TTACAACTGG ATGCTAGAGG CCAGTTAGAA ACAACGGTAA ACAGATGAGA GCTCGTCTTA   
  
  
- TCTTTTCATT GAGTGGAAGA GAAGAGAAAC CGGTTTAGAG TGAAAGAAGA TATGGATTTC GAGAGAGAGA   
  
  
- GAGAGAGAGG GGGAGGGTGG ATGGACTATT TGCGTTAGTC CGGATCGAAA GAGAGAGAGA GAGAGAGAGA   
  
  
- GAAAGAAAGA GTCATTCGTT TGTAGTGAGG GCGACACACA GTGAGAGACA ATTCAATGTT CGAAAATATA   
  
  
- CTCCTAAAAT ATCCGGACTA ACTATCTTT

+     Unnamed\_\_3

| Site Name | Organism | Position | Strand | Matrix score. | sequence | function |
| --- | --- | --- | --- | --- | --- | --- |
| Unnamed\_\_3 | Zea mays | 1138 | - | 5 | CGTGG |  |
| Unnamed\_\_3 | Zea mays | 793 | - | 5 | CGTGG |  |

> 2018/04/13 10:10:12  
+ TTAATTTTCA GGTGCTCTCA TTCGTTTTCA CCTTCGCCAA TTGTGAAGTT GAAGCCACAT TTTTCTAGGG   
  
  
+ GCTACGATCT TGAAATAATG TCGATTTGAT TTTGTAGATA CCTTTTAATC ATTCTTCTAA TAGAATCATC   
  
  
+ TTTTTTTTTT TTTGGGTTGA TCTCATTTTG TTAAATTGAA TAAATTTAAT TGAATGAATT GAAAATTCTT   
  
  
+ TTTTTTTAAG GATATAAATT TTGTTGGTAT TTTAAAAAAA TATTTTTTAT TAAATACTTT TTAAAAAGAT   
  
  
+ GTTGATATAT ATATATATAT GTTAGAAAAT AAAGGATATA AATTTTGTTA GTATTTTAAA AAAATATTTT   
  
  
+ TTATTAAATA CTTTTTAAAA AGATGTTGAT ATATATATAT ATGTTAGAAA ATACAATAAA ATTTATTGCA   
  
  
+ TTTGTTTTCT TGATTAATGT AACCAATTAT AAAAATAGAA TTTTAAAAAA AAGATTAATA AGAATCATAG   
  
  
+ TAACAGATGA TTTTTTGATT CAAAGGAAAC ATTTCTTCAA TATTTTAAAG ATTCATTATT AAAAATGATT   
  
  
+ AGAAATTATG GTAAAATCTA TGAAATTTTT AAAAAATATA AATAGTATTG AAGTAAGAAG GGTAGTTATA   
  
  
+ TTTTTATAAC TTAAATAATA ATATAAAATA AAAAATGTAA CCATTAATTT TAATTAAATT ACCATGTATC   
  
  
+ TTAATTTATT TAAGTCGAAA ATTTGATCCT TTCTTAATTT TATTTATTAA GACACGTGAA AATACCTTTT   
  
  
+ TGCTTAATTA ATTGAATAAG AGCCACGCAT AGCATAACAA CATTATCTAT TTGCCCATTT CATGAGCAAT   
  
  
+ AAAGAATTCC ACTGACTGAA AAGAATACAA GACCCCCCAA AAAAAAAAGA AAAAAAGAGA AAAGAGAAAA   
  
  
+ AAAAAGATGA AAAAAATGAT TAAAGGGAAA AGAAGAAAAG GGAATATCTT AGGAGGATTC AGTTGGTGAG   
  
  
+ CTTGAATTGA GTTCACACAG AGGAGAGTTC ACATGCAAGA CGACGGAGCT TGGGGCTGTA ATGGGTAAGA   
  
  
+ ATCTCACGTG CAGAGAAACC CCGTTCTCAC TGGGGGAACC TGTTAACTGT TGAGCCGGCC AACGAAGCGT   
  
  
+ ATGAAAACGT CGGCCAACCA CGACAGAATA CTCGGCCGCA TGCATTGAAT TTGCCACCGG CACTGTGGGT   
  
  
+ GTCCGTACGA AATGTTGACC TACGATCTCC GGTCAATCTT TGTTGCCATT TGTCTACTCT CGAGCAGAAT   
  
  
+ AGAAAAGTAA CTCACCTTCT CTTCTCTTTG GCCAAATCTC ACTTTCTTCT ATACCTAAAG CTCTCTCTCT   
  
  
+ CTCTCTCTCC CCCTCCCACC TACCTGATAA ACGCAATCAG GCCTAGCTTT CTCTCTCTCT CTCTCTCTCT   
  
  
+ CTTTCTTTCT CAGTAAGCAA ACATCACTCC CGCTGTGTGT CACTCTCTGT TAAGTTACAA GCTTTTATAT   
  
  
+ GAGGATTTTA TAGGCCTGAT TGATAGAAA  

- AATTAAAAGT CCACGAGAGT AAGCAAAAGT GGAAGCGGTT AACACTTCAA CTTCGGTGTA AAAAGATCCC   
  
  
- CGATGCTAGA ACTTTATTAC AGCTAAACTA AAACATCTAT GGAAAATTAG TAAGAAGATT ATCTTAGTAG   
  
  
- AAAAAAAAAA AAACCCAACT AGAGTAAAAC AATTTAACTT ATTTAAATTA ACTTACTTAA CTTTTAAGAA   
  
  
- AAAAAAATTC CTATATTTAA AACAACCATA AAATTTTTTT ATAAAAAATA ATTTATGAAA AATTTTTCTA   
  
  
- CAACTATATA TATATATATA CAATCTTTTA TTTCCTATAT TTAAAACAAT CATAAAATTT TTTTATAAAA   
  
  
- AATAATTTAT GAAAAATTTT TCTACAACTA TATATATATA TACAATCTTT TATGTTATTT TAAATAACGT   
  
  
- AAACAAAAGA ACTAATTACA TTGGTTAATA TTTTTATCTT AAAATTTTTT TTCTAATTAT TCTTAGTATC   
  
  
- ATTGTCTACT AAAAAACTAA GTTTCCTTTG TAAAGAAGTT ATAAAATTTC TAAGTAATAA TTTTTACTAA   
  
  
- TCTTTAATAC CATTTTAGAT ACTTTAAAAA TTTTTTATAT TTATCATAAC TTCATTCTTC CCATCAATAT   
  
  
- AAAAATATTG AATTTATTAT TATATTTTAT TTTTTACATT GGTAATTAAA ATTAATTTAA TGGTACATAG   
  
  
- AATTAAATAA ATTCAGCTTT TAAACTAGGA AAGAATTAAA ATAAATAATT CTGTGCACTT TTATGGAAAA   
  
  
- ACGAATTAAT TAACTTATTC TCGGTGCGTA TCGTATTGTT GTAATAGATA AACGGGTAAA GTACTCGTTA   
  
  
- TTTCTTAAGG TGACTGACTT TTCTTATGTT CTGGGGGGTT TTTTTTTTCT TTTTTTCTCT TTTCTCTTTT   
  
  
- TTTTTCTACT TTTTTTACTA ATTTCCCTTT TCTTCTTTTC CCTTATAGAA TCCTCCTAAG TCAACCACTC   
  
  
- GAACTTAACT CAAGTGTGTC TCCTCTCAAG TGTACGTTCT GCTGCCTCGA ACCCCGACAT TACCCATTCT   
  
  
- TAGAGTGCAC GTCTCTTTGG GGCAAGAGTG ACCCCCTTGG ACAATTGACA ACTCGGCCGG TTGCTTCGCA   
  
  
- TACTTTTGCA GCCGGTTGGT GCTGTCTTAT GAGCCGGCGT ACGTAACTTA AACGGTGGCC GTGACACCCA   
  
  
- CAGGCATGCT TTACAACTGG ATGCTAGAGG CCAGTTAGAA ACAACGGTAA ACAGATGAGA GCTCGTCTTA   
  
  
- TCTTTTCATT GAGTGGAAGA GAAGAGAAAC CGGTTTAGAG TGAAAGAAGA TATGGATTTC GAGAGAGAGA   
  
  
- GAGAGAGAGG GGGAGGGTGG ATGGACTATT TGCGTTAGTC CGGATCGAAA GAGAGAGAGA GAGAGAGAGA   
  
  
- GAAAGAAAGA GTCATTCGTT TGTAGTGAGG GCGACACACA GTGAGAGACA ATTCAATGTT CGAAAATATA   
  
  
- CTCCTAAAAT ATCCGGACTA ACTATCTTT

+     Unnamed\_\_4

| Site Name | Organism | Position | Strand | Matrix score. | sequence | function |
| --- | --- | --- | --- | --- | --- | --- |
| Unnamed\_\_4 | Petroselinum hortense | 1427 | + | 4 | CTCC |  |
| Unnamed\_\_4 | Petroselinum hortense | 1025 | - | 4 | CTCC |  |
| Unnamed\_\_4 | Petroselinum hortense | 1002 | - | 4 | CTCC |  |
| Unnamed\_\_4 | Petroselinum hortense | 1337 | + | 4 | CTCC |  |
| Unnamed\_\_4 | Petroselinum hortense | 1343 | + | 4 | CTCC |  |
| Unnamed\_\_4 | Petroselinum hortense | 1217 | + | 4 | CTCC |  |
| Unnamed\_\_4 | Petroselinum hortense | 962 | - | 4 | CTCC |  |

> 2018/04/13 10:10:12  
+ TTAATTTTCA GGTGCTCTCA TTCGTTTTCA CCTTCGCCAA TTGTGAAGTT GAAGCCACAT TTTTCTAGGG   
  
  
+ GCTACGATCT TGAAATAATG TCGATTTGAT TTTGTAGATA CCTTTTAATC ATTCTTCTAA TAGAATCATC   
  
  
+ TTTTTTTTTT TTTGGGTTGA TCTCATTTTG TTAAATTGAA TAAATTTAAT TGAATGAATT GAAAATTCTT   
  
  
+ TTTTTTTAAG GATATAAATT TTGTTGGTAT TTTAAAAAAA TATTTTTTAT TAAATACTTT TTAAAAAGAT   
  
  
+ GTTGATATAT ATATATATAT GTTAGAAAAT AAAGGATATA AATTTTGTTA GTATTTTAAA AAAATATTTT   
  
  
+ TTATTAAATA CTTTTTAAAA AGATGTTGAT ATATATATAT ATGTTAGAAA ATACAATAAA ATTTATTGCA   
  
  
+ TTTGTTTTCT TGATTAATGT AACCAATTAT AAAAATAGAA TTTTAAAAAA AAGATTAATA AGAATCATAG   
  
  
+ TAACAGATGA TTTTTTGATT CAAAGGAAAC ATTTCTTCAA TATTTTAAAG ATTCATTATT AAAAATGATT   
  
  
+ AGAAATTATG GTAAAATCTA TGAAATTTTT AAAAAATATA AATAGTATTG AAGTAAGAAG GGTAGTTATA   
  
  
+ TTTTTATAAC TTAAATAATA ATATAAAATA AAAAATGTAA CCATTAATTT TAATTAAATT ACCATGTATC   
  
  
+ TTAATTTATT TAAGTCGAAA ATTTGATCCT TTCTTAATTT TATTTATTAA GACACGTGAA AATACCTTTT   
  
  
+ TGCTTAATTA ATTGAATAAG AGCCACGCAT AGCATAACAA CATTATCTAT TTGCCCATTT CATGAGCAAT   
  
  
+ AAAGAATTCC ACTGACTGAA AAGAATACAA GACCCCCCAA AAAAAAAAGA AAAAAAGAGA AAAGAGAAAA   
  
  
+ AAAAAGATGA AAAAAATGAT TAAAGGGAAA AGAAGAAAAG GGAATATCTT AGGAGGATTC AGTTGGTGAG   
  
  
+ CTTGAATTGA GTTCACACAG AGGAGAGTTC ACATGCAAGA CGACGGAGCT TGGGGCTGTA ATGGGTAAGA   
  
  
+ ATCTCACGTG CAGAGAAACC CCGTTCTCAC TGGGGGAACC TGTTAACTGT TGAGCCGGCC AACGAAGCGT   
  
  
+ ATGAAAACGT CGGCCAACCA CGACAGAATA CTCGGCCGCA TGCATTGAAT TTGCCACCGG CACTGTGGGT   
  
  
+ GTCCGTACGA AATGTTGACC TACGATCTCC GGTCAATCTT TGTTGCCATT TGTCTACTCT CGAGCAGAAT   
  
  
+ AGAAAAGTAA CTCACCTTCT CTTCTCTTTG GCCAAATCTC ACTTTCTTCT ATACCTAAAG CTCTCTCTCT   
  
  
+ CTCTCTCTCC CCCTCCCACC TACCTGATAA ACGCAATCAG GCCTAGCTTT CTCTCTCTCT CTCTCTCTCT   
  
  
+ CTTTCTTTCT CAGTAAGCAA ACATCACTCC CGCTGTGTGT CACTCTCTGT TAAGTTACAA GCTTTTATAT   
  
  
+ GAGGATTTTA TAGGCCTGAT TGATAGAAA  

- AATTAAAAGT CCACGAGAGT AAGCAAAAGT GGAAGCGGTT AACACTTCAA CTTCGGTGTA AAAAGATCCC   
  
  
- CGATGCTAGA ACTTTATTAC AGCTAAACTA AAACATCTAT GGAAAATTAG TAAGAAGATT ATCTTAGTAG   
  
  
- AAAAAAAAAA AAACCCAACT AGAGTAAAAC AATTTAACTT ATTTAAATTA ACTTACTTAA CTTTTAAGAA   
  
  
- AAAAAAATTC CTATATTTAA AACAACCATA AAATTTTTTT ATAAAAAATA ATTTATGAAA AATTTTTCTA   
  
  
- CAACTATATA TATATATATA CAATCTTTTA TTTCCTATAT TTAAAACAAT CATAAAATTT TTTTATAAAA   
  
  
- AATAATTTAT GAAAAATTTT TCTACAACTA TATATATATA TACAATCTTT TATGTTATTT TAAATAACGT   
  
  
- AAACAAAAGA ACTAATTACA TTGGTTAATA TTTTTATCTT AAAATTTTTT TTCTAATTAT TCTTAGTATC   
  
  
- ATTGTCTACT AAAAAACTAA GTTTCCTTTG TAAAGAAGTT ATAAAATTTC TAAGTAATAA TTTTTACTAA   
  
  
- TCTTTAATAC CATTTTAGAT ACTTTAAAAA TTTTTTATAT TTATCATAAC TTCATTCTTC CCATCAATAT   
  
  
- AAAAATATTG AATTTATTAT TATATTTTAT TTTTTACATT GGTAATTAAA ATTAATTTAA TGGTACATAG   
  
  
- AATTAAATAA ATTCAGCTTT TAAACTAGGA AAGAATTAAA ATAAATAATT CTGTGCACTT TTATGGAAAA   
  
  
- ACGAATTAAT TAACTTATTC TCGGTGCGTA TCGTATTGTT GTAATAGATA AACGGGTAAA GTACTCGTTA   
  
  
- TTTCTTAAGG TGACTGACTT TTCTTATGTT CTGGGGGGTT TTTTTTTTCT TTTTTTCTCT TTTCTCTTTT   
  
  
- TTTTTCTACT TTTTTTACTA ATTTCCCTTT TCTTCTTTTC CCTTATAGAA TCCTCCTAAG TCAACCACTC   
  
  
- GAACTTAACT CAAGTGTGTC TCCTCTCAAG TGTACGTTCT GCTGCCTCGA ACCCCGACAT TACCCATTCT   
  
  
- TAGAGTGCAC GTCTCTTTGG GGCAAGAGTG ACCCCCTTGG ACAATTGACA ACTCGGCCGG TTGCTTCGCA   
  
  
- TACTTTTGCA GCCGGTTGGT GCTGTCTTAT GAGCCGGCGT ACGTAACTTA AACGGTGGCC GTGACACCCA   
  
  
- CAGGCATGCT TTACAACTGG ATGCTAGAGG CCAGTTAGAA ACAACGGTAA ACAGATGAGA GCTCGTCTTA   
  
  
- TCTTTTCATT GAGTGGAAGA GAAGAGAAAC CGGTTTAGAG TGAAAGAAGA TATGGATTTC GAGAGAGAGA   
  
  
- GAGAGAGAGG GGGAGGGTGG ATGGACTATT TGCGTTAGTC CGGATCGAAA GAGAGAGAGA GAGAGAGAGA   
  
  
- GAAAGAAAGA GTCATTCGTT TGTAGTGAGG GCGACACACA GTGAGAGACA ATTCAATGTT CGAAAATATA   
  
  
- CTCCTAAAAT ATCCGGACTA ACTATCTTT

+     W box

| Site Name | Organism | Position | Strand | Matrix score. | sequence | function |
| --- | --- | --- | --- | --- | --- | --- |
| W box | Arabidopsis thaliana | 1221 | - | 6 | TTGACC |  |
| W box | Arabidopsis thaliana | 1205 | + | 6 | TTGACC |  |

> 2018/04/13 10:10:12  
+ TTAATTTTCA GGTGCTCTCA TTCGTTTTCA CCTTCGCCAA TTGTGAAGTT GAAGCCACAT TTTTCTAGGG   
  
  
+ GCTACGATCT TGAAATAATG TCGATTTGAT TTTGTAGATA CCTTTTAATC ATTCTTCTAA TAGAATCATC   
  
  
+ TTTTTTTTTT TTTGGGTTGA TCTCATTTTG TTAAATTGAA TAAATTTAAT TGAATGAATT GAAAATTCTT   
  
  
+ TTTTTTTAAG GATATAAATT TTGTTGGTAT TTTAAAAAAA TATTTTTTAT TAAATACTTT TTAAAAAGAT   
  
  
+ GTTGATATAT ATATATATAT GTTAGAAAAT AAAGGATATA AATTTTGTTA GTATTTTAAA AAAATATTTT   
  
  
+ TTATTAAATA CTTTTTAAAA AGATGTTGAT ATATATATAT ATGTTAGAAA ATACAATAAA ATTTATTGCA   
  
  
+ TTTGTTTTCT TGATTAATGT AACCAATTAT AAAAATAGAA TTTTAAAAAA AAGATTAATA AGAATCATAG   
  
  
+ TAACAGATGA TTTTTTGATT CAAAGGAAAC ATTTCTTCAA TATTTTAAAG ATTCATTATT AAAAATGATT   
  
  
+ AGAAATTATG GTAAAATCTA TGAAATTTTT AAAAAATATA AATAGTATTG AAGTAAGAAG GGTAGTTATA   
  
  
+ TTTTTATAAC TTAAATAATA ATATAAAATA AAAAATGTAA CCATTAATTT TAATTAAATT ACCATGTATC   
  
  
+ TTAATTTATT TAAGTCGAAA ATTTGATCCT TTCTTAATTT TATTTATTAA GACACGTGAA AATACCTTTT   
  
  
+ TGCTTAATTA ATTGAATAAG AGCCACGCAT AGCATAACAA CATTATCTAT TTGCCCATTT CATGAGCAAT   
  
  
+ AAAGAATTCC ACTGACTGAA AAGAATACAA GACCCCCCAA AAAAAAAAGA AAAAAAGAGA AAAGAGAAAA   
  
  
+ AAAAAGATGA AAAAAATGAT TAAAGGGAAA AGAAGAAAAG GGAATATCTT AGGAGGATTC AGTTGGTGAG   
  
  
+ CTTGAATTGA GTTCACACAG AGGAGAGTTC ACATGCAAGA CGACGGAGCT TGGGGCTGTA ATGGGTAAGA   
  
  
+ ATCTCACGTG CAGAGAAACC CCGTTCTCAC TGGGGGAACC TGTTAACTGT TGAGCCGGCC AACGAAGCGT   
  
  
+ ATGAAAACGT CGGCCAACCA CGACAGAATA CTCGGCCGCA TGCATTGAAT TTGCCACCGG CACTGTGGGT   
  
  
+ GTCCGTACGA AATGTTGACC TACGATCTCC GGTCAATCTT TGTTGCCATT TGTCTACTCT CGAGCAGAAT   
  
  
+ AGAAAAGTAA CTCACCTTCT CTTCTCTTTG GCCAAATCTC ACTTTCTTCT ATACCTAAAG CTCTCTCTCT   
  
  
+ CTCTCTCTCC CCCTCCCACC TACCTGATAA ACGCAATCAG GCCTAGCTTT CTCTCTCTCT CTCTCTCTCT   
  
  
+ CTTTCTTTCT CAGTAAGCAA ACATCACTCC CGCTGTGTGT CACTCTCTGT TAAGTTACAA GCTTTTATAT   
  
  
+ GAGGATTTTA TAGGCCTGAT TGATAGAAA  

- AATTAAAAGT CCACGAGAGT AAGCAAAAGT GGAAGCGGTT AACACTTCAA CTTCGGTGTA AAAAGATCCC   
  
  
- CGATGCTAGA ACTTTATTAC AGCTAAACTA AAACATCTAT GGAAAATTAG TAAGAAGATT ATCTTAGTAG   
  
  
- AAAAAAAAAA AAACCCAACT AGAGTAAAAC AATTTAACTT ATTTAAATTA ACTTACTTAA CTTTTAAGAA   
  
  
- AAAAAAATTC CTATATTTAA AACAACCATA AAATTTTTTT ATAAAAAATA ATTTATGAAA AATTTTTCTA   
  
  
- CAACTATATA TATATATATA CAATCTTTTA TTTCCTATAT TTAAAACAAT CATAAAATTT TTTTATAAAA   
  
  
- AATAATTTAT GAAAAATTTT TCTACAACTA TATATATATA TACAATCTTT TATGTTATTT TAAATAACGT   
  
  
- AAACAAAAGA ACTAATTACA TTGGTTAATA TTTTTATCTT AAAATTTTTT TTCTAATTAT TCTTAGTATC   
  
  
- ATTGTCTACT AAAAAACTAA GTTTCCTTTG TAAAGAAGTT ATAAAATTTC TAAGTAATAA TTTTTACTAA   
  
  
- TCTTTAATAC CATTTTAGAT ACTTTAAAAA TTTTTTATAT TTATCATAAC TTCATTCTTC CCATCAATAT   
  
  
- AAAAATATTG AATTTATTAT TATATTTTAT TTTTTACATT GGTAATTAAA ATTAATTTAA TGGTACATAG   
  
  
- AATTAAATAA ATTCAGCTTT TAAACTAGGA AAGAATTAAA ATAAATAATT CTGTGCACTT TTATGGAAAA   
  
  
- ACGAATTAAT TAACTTATTC TCGGTGCGTA TCGTATTGTT GTAATAGATA AACGGGTAAA GTACTCGTTA   
  
  
- TTTCTTAAGG TGACTGACTT TTCTTATGTT CTGGGGGGTT TTTTTTTTCT TTTTTTCTCT TTTCTCTTTT   
  
  
- TTTTTCTACT TTTTTTACTA ATTTCCCTTT TCTTCTTTTC CCTTATAGAA TCCTCCTAAG TCAACCACTC   
  
  
- GAACTTAACT CAAGTGTGTC TCCTCTCAAG TGTACGTTCT GCTGCCTCGA ACCCCGACAT TACCCATTCT   
  
  
- TAGAGTGCAC GTCTCTTTGG GGCAAGAGTG ACCCCCTTGG ACAATTGACA ACTCGGCCGG TTGCTTCGCA   
  
  
- TACTTTTGCA GCCGGTTGGT GCTGTCTTAT GAGCCGGCGT ACGTAACTTA AACGGTGGCC GTGACACCCA   
  
  
- CAGGCATGCT TTACAACTGG ATGCTAGAGG CCAGTTAGAA ACAACGGTAA ACAGATGAGA GCTCGTCTTA   
  
  
- TCTTTTCATT GAGTGGAAGA GAAGAGAAAC CGGTTTAGAG TGAAAGAAGA TATGGATTTC GAGAGAGAGA   
  
  
- GAGAGAGAGG GGGAGGGTGG ATGGACTATT TGCGTTAGTC CGGATCGAAA GAGAGAGAGA GAGAGAGAGA   
  
  
- GAAAGAAAGA GTCATTCGTT TGTAGTGAGG GCGACACACA GTGAGAGACA ATTCAATGTT CGAAAATATA   
  
  
- CTCCTAAAAT ATCCGGACTA ACTATCTTT

+     as-2-box

| Site Name | Organism | Position | Strand | Matrix score. | sequence | function |
| --- | --- | --- | --- | --- | --- | --- |
| as-2-box | Nicotiana tabacum | 808 | - | 9 | GATAatGATG | involved in shoot-specific expression and light responsiveness |

> 2018/04/13 10:10:12  
+ TTAATTTTCA GGTGCTCTCA TTCGTTTTCA CCTTCGCCAA TTGTGAAGTT GAAGCCACAT TTTTCTAGGG   
  
  
+ GCTACGATCT TGAAATAATG TCGATTTGAT TTTGTAGATA CCTTTTAATC ATTCTTCTAA TAGAATCATC   
  
  
+ TTTTTTTTTT TTTGGGTTGA TCTCATTTTG TTAAATTGAA TAAATTTAAT TGAATGAATT GAAAATTCTT   
  
  
+ TTTTTTTAAG GATATAAATT TTGTTGGTAT TTTAAAAAAA TATTTTTTAT TAAATACTTT TTAAAAAGAT   
  
  
+ GTTGATATAT ATATATATAT GTTAGAAAAT AAAGGATATA AATTTTGTTA GTATTTTAAA AAAATATTTT   
  
  
+ TTATTAAATA CTTTTTAAAA AGATGTTGAT ATATATATAT ATGTTAGAAA ATACAATAAA ATTTATTGCA   
  
  
+ TTTGTTTTCT TGATTAATGT AACCAATTAT AAAAATAGAA TTTTAAAAAA AAGATTAATA AGAATCATAG   
  
  
+ TAACAGATGA TTTTTTGATT CAAAGGAAAC ATTTCTTCAA TATTTTAAAG ATTCATTATT AAAAATGATT   
  
  
+ AGAAATTATG GTAAAATCTA TGAAATTTTT AAAAAATATA AATAGTATTG AAGTAAGAAG GGTAGTTATA   
  
  
+ TTTTTATAAC TTAAATAATA ATATAAAATA AAAAATGTAA CCATTAATTT TAATTAAATT ACCATGTATC   
  
  
+ TTAATTTATT TAAGTCGAAA ATTTGATCCT TTCTTAATTT TATTTATTAA GACACGTGAA AATACCTTTT   
  
  
+ TGCTTAATTA ATTGAATAAG AGCCACGCAT AGCATAACAA CATTATCTAT TTGCCCATTT CATGAGCAAT   
  
  
+ AAAGAATTCC ACTGACTGAA AAGAATACAA GACCCCCCAA AAAAAAAAGA AAAAAAGAGA AAAGAGAAAA   
  
  
+ AAAAAGATGA AAAAAATGAT TAAAGGGAAA AGAAGAAAAG GGAATATCTT AGGAGGATTC AGTTGGTGAG   
  
  
+ CTTGAATTGA GTTCACACAG AGGAGAGTTC ACATGCAAGA CGACGGAGCT TGGGGCTGTA ATGGGTAAGA   
  
  
+ ATCTCACGTG CAGAGAAACC CCGTTCTCAC TGGGGGAACC TGTTAACTGT TGAGCCGGCC AACGAAGCGT   
  
  
+ ATGAAAACGT CGGCCAACCA CGACAGAATA CTCGGCCGCA TGCATTGAAT TTGCCACCGG CACTGTGGGT   
  
  
+ GTCCGTACGA AATGTTGACC TACGATCTCC GGTCAATCTT TGTTGCCATT TGTCTACTCT CGAGCAGAAT   
  
  
+ AGAAAAGTAA CTCACCTTCT CTTCTCTTTG GCCAAATCTC ACTTTCTTCT ATACCTAAAG CTCTCTCTCT   
  
  
+ CTCTCTCTCC CCCTCCCACC TACCTGATAA ACGCAATCAG GCCTAGCTTT CTCTCTCTCT CTCTCTCTCT   
  
  
+ CTTTCTTTCT CAGTAAGCAA ACATCACTCC CGCTGTGTGT CACTCTCTGT TAAGTTACAA GCTTTTATAT   
  
  
+ GAGGATTTTA TAGGCCTGAT TGATAGAAA  

- AATTAAAAGT CCACGAGAGT AAGCAAAAGT GGAAGCGGTT AACACTTCAA CTTCGGTGTA AAAAGATCCC   
  
  
- CGATGCTAGA ACTTTATTAC AGCTAAACTA AAACATCTAT GGAAAATTAG TAAGAAGATT ATCTTAGTAG   
  
  
- AAAAAAAAAA AAACCCAACT AGAGTAAAAC AATTTAACTT ATTTAAATTA ACTTACTTAA CTTTTAAGAA   
  
  
- AAAAAAATTC CTATATTTAA AACAACCATA AAATTTTTTT ATAAAAAATA ATTTATGAAA AATTTTTCTA   
  
  
- CAACTATATA TATATATATA CAATCTTTTA TTTCCTATAT TTAAAACAAT CATAAAATTT TTTTATAAAA   
  
  
- AATAATTTAT GAAAAATTTT TCTACAACTA TATATATATA TACAATCTTT TATGTTATTT TAAATAACGT   
  
  
- AAACAAAAGA ACTAATTACA TTGGTTAATA TTTTTATCTT AAAATTTTTT TTCTAATTAT TCTTAGTATC   
  
  
- ATTGTCTACT AAAAAACTAA GTTTCCTTTG TAAAGAAGTT ATAAAATTTC TAAGTAATAA TTTTTACTAA   
  
  
- TCTTTAATAC CATTTTAGAT ACTTTAAAAA TTTTTTATAT TTATCATAAC TTCATTCTTC CCATCAATAT   
  
  
- AAAAATATTG AATTTATTAT TATATTTTAT TTTTTACATT GGTAATTAAA ATTAATTTAA TGGTACATAG   
  
  
- AATTAAATAA ATTCAGCTTT TAAACTAGGA AAGAATTAAA ATAAATAATT CTGTGCACTT TTATGGAAAA   
  
  
- ACGAATTAAT TAACTTATTC TCGGTGCGTA TCGTATTGTT GTAATAGATA AACGGGTAAA GTACTCGTTA   
  
  
- TTTCTTAAGG TGACTGACTT TTCTTATGTT CTGGGGGGTT TTTTTTTTCT TTTTTTCTCT TTTCTCTTTT   
  
  
- TTTTTCTACT TTTTTTACTA ATTTCCCTTT TCTTCTTTTC CCTTATAGAA TCCTCCTAAG TCAACCACTC   
  
  
- GAACTTAACT CAAGTGTGTC TCCTCTCAAG TGTACGTTCT GCTGCCTCGA ACCCCGACAT TACCCATTCT   
  
  
- TAGAGTGCAC GTCTCTTTGG GGCAAGAGTG ACCCCCTTGG ACAATTGACA ACTCGGCCGG TTGCTTCGCA   
  
  
- TACTTTTGCA GCCGGTTGGT GCTGTCTTAT GAGCCGGCGT ACGTAACTTA AACGGTGGCC GTGACACCCA   
  
  
- CAGGCATGCT TTACAACTGG ATGCTAGAGG CCAGTTAGAA ACAACGGTAA ACAGATGAGA GCTCGTCTTA   
  
  
- TCTTTTCATT GAGTGGAAGA GAAGAGAAAC CGGTTTAGAG TGAAAGAAGA TATGGATTTC GAGAGAGAGA   
  
  
- GAGAGAGAGG GGGAGGGTGG ATGGACTATT TGCGTTAGTC CGGATCGAAA GAGAGAGAGA GAGAGAGAGA   
  
  
- GAAAGAAAGA GTCATTCGTT TGTAGTGAGG GCGACACACA GTGAGAGACA ATTCAATGTT CGAAAATATA   
  
  
- CTCCTAAAAT ATCCGGACTA ACTATCTTT

+     circadian

| Site Name | Organism | Position | Strand | Matrix score. | sequence | function |
| --- | --- | --- | --- | --- | --- | --- |
| circadian | Lycopersicon esculentum | 966 | - | 6 | CAANNNNATC | cis-acting regulatory element involved in circadian control |
| circadian | Lycopersicon esculentum | 808 | + | 6 | CAANNNNATC | cis-acting regulatory element involved in circadian control |

> 2018/04/13 10:10:12  
+ TTAATTTTCA GGTGCTCTCA TTCGTTTTCA CCTTCGCCAA TTGTGAAGTT GAAGCCACAT TTTTCTAGGG   
  
  
+ GCTACGATCT TGAAATAATG TCGATTTGAT TTTGTAGATA CCTTTTAATC ATTCTTCTAA TAGAATCATC   
  
  
+ TTTTTTTTTT TTTGGGTTGA TCTCATTTTG TTAAATTGAA TAAATTTAAT TGAATGAATT GAAAATTCTT   
  
  
+ TTTTTTTAAG GATATAAATT TTGTTGGTAT TTTAAAAAAA TATTTTTTAT TAAATACTTT TTAAAAAGAT   
  
  
+ GTTGATATAT ATATATATAT GTTAGAAAAT AAAGGATATA AATTTTGTTA GTATTTTAAA AAAATATTTT   
  
  
+ TTATTAAATA CTTTTTAAAA AGATGTTGAT ATATATATAT ATGTTAGAAA ATACAATAAA ATTTATTGCA   
  
  
+ TTTGTTTTCT TGATTAATGT AACCAATTAT AAAAATAGAA TTTTAAAAAA AAGATTAATA AGAATCATAG   
  
  
+ TAACAGATGA TTTTTTGATT CAAAGGAAAC ATTTCTTCAA TATTTTAAAG ATTCATTATT AAAAATGATT   
  
  
+ AGAAATTATG GTAAAATCTA TGAAATTTTT AAAAAATATA AATAGTATTG AAGTAAGAAG GGTAGTTATA   
  
  
+ TTTTTATAAC TTAAATAATA ATATAAAATA AAAAATGTAA CCATTAATTT TAATTAAATT ACCATGTATC   
  
  
+ TTAATTTATT TAAGTCGAAA ATTTGATCCT TTCTTAATTT TATTTATTAA GACACGTGAA AATACCTTTT   
  
  
+ TGCTTAATTA ATTGAATAAG AGCCACGCAT AGCATAACAA CATTATCTAT TTGCCCATTT CATGAGCAAT   
  
  
+ AAAGAATTCC ACTGACTGAA AAGAATACAA GACCCCCCAA AAAAAAAAGA AAAAAAGAGA AAAGAGAAAA   
  
  
+ AAAAAGATGA AAAAAATGAT TAAAGGGAAA AGAAGAAAAG GGAATATCTT AGGAGGATTC AGTTGGTGAG   
  
  
+ CTTGAATTGA GTTCACACAG AGGAGAGTTC ACATGCAAGA CGACGGAGCT TGGGGCTGTA ATGGGTAAGA   
  
  
+ ATCTCACGTG CAGAGAAACC CCGTTCTCAC TGGGGGAACC TGTTAACTGT TGAGCCGGCC AACGAAGCGT   
  
  
+ ATGAAAACGT CGGCCAACCA CGACAGAATA CTCGGCCGCA TGCATTGAAT TTGCCACCGG CACTGTGGGT   
  
  
+ GTCCGTACGA AATGTTGACC TACGATCTCC GGTCAATCTT TGTTGCCATT TGTCTACTCT CGAGCAGAAT   
  
  
+ AGAAAAGTAA CTCACCTTCT CTTCTCTTTG GCCAAATCTC ACTTTCTTCT ATACCTAAAG CTCTCTCTCT   
  
  
+ CTCTCTCTCC CCCTCCCACC TACCTGATAA ACGCAATCAG GCCTAGCTTT CTCTCTCTCT CTCTCTCTCT   
  
  
+ CTTTCTTTCT CAGTAAGCAA ACATCACTCC CGCTGTGTGT CACTCTCTGT TAAGTTACAA GCTTTTATAT   
  
  
+ GAGGATTTTA TAGGCCTGAT TGATAGAAA  

- AATTAAAAGT CCACGAGAGT AAGCAAAAGT GGAAGCGGTT AACACTTCAA CTTCGGTGTA AAAAGATCCC   
  
  
- CGATGCTAGA ACTTTATTAC AGCTAAACTA AAACATCTAT GGAAAATTAG TAAGAAGATT ATCTTAGTAG   
  
  
- AAAAAAAAAA AAACCCAACT AGAGTAAAAC AATTTAACTT ATTTAAATTA ACTTACTTAA CTTTTAAGAA   
  
  
- AAAAAAATTC CTATATTTAA AACAACCATA AAATTTTTTT ATAAAAAATA ATTTATGAAA AATTTTTCTA   
  
  
- CAACTATATA TATATATATA CAATCTTTTA TTTCCTATAT TTAAAACAAT CATAAAATTT TTTTATAAAA   
  
  
- AATAATTTAT GAAAAATTTT TCTACAACTA TATATATATA TACAATCTTT TATGTTATTT TAAATAACGT   
  
  
- AAACAAAAGA ACTAATTACA TTGGTTAATA TTTTTATCTT AAAATTTTTT TTCTAATTAT TCTTAGTATC   
  
  
- ATTGTCTACT AAAAAACTAA GTTTCCTTTG TAAAGAAGTT ATAAAATTTC TAAGTAATAA TTTTTACTAA   
  
  
- TCTTTAATAC CATTTTAGAT ACTTTAAAAA TTTTTTATAT TTATCATAAC TTCATTCTTC CCATCAATAT   
  
  
- AAAAATATTG AATTTATTAT TATATTTTAT TTTTTACATT GGTAATTAAA ATTAATTTAA TGGTACATAG   
  
  
- AATTAAATAA ATTCAGCTTT TAAACTAGGA AAGAATTAAA ATAAATAATT CTGTGCACTT TTATGGAAAA   
  
  
- ACGAATTAAT TAACTTATTC TCGGTGCGTA TCGTATTGTT GTAATAGATA AACGGGTAAA GTACTCGTTA   
  
  
- TTTCTTAAGG TGACTGACTT TTCTTATGTT CTGGGGGGTT TTTTTTTTCT TTTTTTCTCT TTTCTCTTTT   
  
  
- TTTTTCTACT TTTTTTACTA ATTTCCCTTT TCTTCTTTTC CCTTATAGAA TCCTCCTAAG TCAACCACTC   
  
  
- GAACTTAACT CAAGTGTGTC TCCTCTCAAG TGTACGTTCT GCTGCCTCGA ACCCCGACAT TACCCATTCT   
  
  
- TAGAGTGCAC GTCTCTTTGG GGCAAGAGTG ACCCCCTTGG ACAATTGACA ACTCGGCCGG TTGCTTCGCA   
  
  
- TACTTTTGCA GCCGGTTGGT GCTGTCTTAT GAGCCGGCGT ACGTAACTTA AACGGTGGCC GTGACACCCA   
  
  
- CAGGCATGCT TTACAACTGG ATGCTAGAGG CCAGTTAGAA ACAACGGTAA ACAGATGAGA GCTCGTCTTA   
  
  
- TCTTTTCATT GAGTGGAAGA GAAGAGAAAC CGGTTTAGAG TGAAAGAAGA TATGGATTTC GAGAGAGAGA   
  
  
- GAGAGAGAGG GGGAGGGTGG ATGGACTATT TGCGTTAGTC CGGATCGAAA GAGAGAGAGA GAGAGAGAGA   
  
  
- GAAAGAAAGA GTCATTCGTT TGTAGTGAGG GCGACACACA GTGAGAGACA ATTCAATGTT CGAAAATATA   
  
  
- CTCCTAAAAT ATCCGGACTA ACTATCTTT
