# Supplementary material for: Genome-wide analysis of the WRKY gene family in drumstick (Moringa oleifera Lam.)
Source: PeerJ. 2019 Jun 10;7:e7063. doi: 10.7717/peerj.7063 (PMC6563795; doi:10.7717/peerj.7063)
Supplement: Supplemental Information 1 [file peerj-07-7063-s003.gz › MoWRKY15_plantcare.html]

Content-Type: text/html; charset=ISO-8859-1


CallMat\_Firefox


Webmaster Firefox specific output  
To save the result:
click on the frame with the right mouse button and save the source code as a text file with extension .html  
REFERENCE:PlantCARE: a database of plant cis-acting regulatory elements and a portal to tools for in silico analysis of promoter sequences.  
Lescot, M., Déhais, P., Moreau, Y., De Moor, B., Rouzé ,P.,and Rombauts, S.  
Nucleic Acids Res., Database issue(2002), 30(1):325-327.   


---

> 2018/04/13 10:10:12  
+ CCCCCTTCTC GAGTTTCTCA ACTCTCTCCT TGACTCTTAC CTTCTCTTCC TTCCAATTAT ACCCAGTTCC   
  
  
+ CTTCACTATA GTACCTTCCA CTCTTCTCCC ACCTATCTGG GTGCCTATAT AAATGTCTCT TTCTCTCTCT   
  
  
+ CTTTTATACC ACGAAACCGA ACACTACACC TCTACCTCCT ACCTCTCTGA TCAGACTCAA AGTACCCATT   
  
  
+ TGGGTTGATT TAGTATGGAT GAAGACAAGT GGACGGGAAG TCACTTTTCA CATGCAGGAT ATATACGTAG   
  
  
+ CAATAGTGGC TACAAAGTGT AAGAATAAAC GGTTTTCTCT TGGTAAGTAA ATCCTGTACG ATGCAAAGTC   
  
  
+ ATTGGGGTTA CAGAAGAAAA CATATACGAT ATGACAAATG GTTGGTTCGT GACCACAAAA GAAAACTGGT   
  
  
+ TAATCTAGTT GACGTCGATG AAATTAAAGG ACATGCTTTA TAAGTCGGCA TTTGGTTCAA GTAGTTCTTT   
  
  
+ TTCTTCAGTC GTAAAACTAC TGATACGGAC AGGGGAAGCT AGAGTTAATG TCAAATTTGT TTTATTTCCC   
  
  
+ TAAATACACT TATATTTTCT GTATTATATA CAGTATTTTT GAAGTGGTAA TAACTTCTTT CCTAAACCCC   
  
  
+ ACGAGACCTA CTTGGAAGCA AAGGATGAGT ACGTTCCGTT CGGTTTCACG TACCTGTGTA CTATCGTTTG   
  
  
+ TTCGAACCTT TCCCTCTCAG GACTAATCCA GTGGACATCA CTGAAGGGTT CTGAAAAGAT CGAGGACTAA   
  
  
+ CCATTGTACG GTAAAGTATT ATGTGGGTTA GATGGTTCCT TCTTATGATG TATGGTGTTA GTTGTAGTAT   
  
  
+ TCATTGGTAT ATCCAGTAGT TCTACTTAGC CGACTCATCG TCCAAGTATA TCTAAAAAAA CGGTTCTTTT   
  
  
+ GGTTTGTATA TTTTGAACGG TTACACCTCT AATGACCTTC TTGTCTTTCT TTGTCTACGT ATAGTCCTGT   
  
  
+ TCAAGGTACG TGGTATATAG AGGTTATCTT ATAGGTTTTA AAATTGATGT TTCGAATACA GCTTGGTCTT   
  
  
+ TTTGAACAAT GTCCCCCAAG GTTTGAGACT TAATGCCGTG GAACTCCGGA GTTAAATAGG TTATAGTTCA   
  
  
+ TCTTAGTTTA GAGAGTCCGT ACCCCAACTT CAAAGTTCTT AAACTGACGT CTTGTTTATG TGAAGTTGGT   
  
  
+ ATAATTACTA AAAAAAAAGT ATATTCTTTG GTGTTAATAC TAAGACTGTT CTGATTTTAC ACCGAGTTCC   
  
  
+ AGTTTAGTCC CTGAGGTGTA GTCGACTACT AGTAGAAAGA TTTCTTAAGA CGAACGAGTC CCTTTATGAG   
  
  
+ GTTGTCGAGT AGGAAGCATT GGGTTCGTTT TAGGAGTGTG AAGGGTTACA ATTTAAACAA CCAACGTTAA   
  
  
+ TGAACCCTAA ATTTCTTCTT AACGACTGTT TTTTTTCTTT TTTTTAGTTC GTGCCTTTAA TTTTTTTTGT   
  
  
+ ATTAAATCGG ATTTGTTCTT TCCGTGTAA  

- GGGGGAAGAG CTCAAAGAGT TGAGAGAGGA ACTGAGAATG GAAGAGAAGG AAGGTTAATA TGGGTCAAGG   
  
  
- GAAGTGATAT CATGGAAGGT GAGAAGAGGG TGGATAGACC CACGGATATA TTTACAGAGA AAGAGAGAGA   
  
  
- GAAAATATGG TGCTTTGGCT TGTGATGTGG AGATGGAGGA TGGAGAGACT AGTCTGAGTT TCATGGGTAA   
  
  
- ACCCAACTAA ATCATACCTA CTTCTGTTCA CCTGCCCTTC AGTGAAAAGT GTACGTCCTA TATATGCATC   
  
  
- GTTATCACCG ATGTTTCACA TTCTTATTTG CCAAAAGAGA ACCATTCATT TAGGACATGC TACGTTTCAG   
  
  
- TAACCCCAAT GTCTTCTTTT GTATATGCTA TACTGTTTAC CAACCAAGCA CTGGTGTTTT CTTTTGACCA   
  
  
- ATTAGATCAA CTGCAGCTAC TTTAATTTCC TGTACGAAAT ATTCAGCCGT AAACCAAGTT CATCAAGAAA   
  
  
- AAGAAGTCAG CATTTTGATG ACTATGCCTG TCCCCTTCGA TCTCAATTAC AGTTTAAACA AAATAAAGGG   
  
  
- ATTTATGTGA ATATAAAAGA CATAATATAT GTCATAAAAA CTTCACCATT ATTGAAGAAA GGATTTGGGG   
  
  
- TGCTCTGGAT GAACCTTCGT TTCCTACTCA TGCAAGGCAA GCCAAAGTGC ATGGACACAT GATAGCAAAC   
  
  
- AAGCTTGGAA AGGGAGAGTC CTGATTAGGT CACCTGTAGT GACTTCCCAA GACTTTTCTA GCTCCTGATT   
  
  
- GGTAACATGC CATTTCATAA TACACCCAAT CTACCAAGGA AGAATACTAC ATACCACAAT CAACATCATA   
  
  
- AGTAACCATA TAGGTCATCA AGATGAATCG GCTGAGTAGC AGGTTCATAT AGATTTTTTT GCCAAGAAAA   
  
  
- CCAAACATAT AAAACTTGCC AATGTGGAGA TTACTGGAAG AACAGAAAGA AACAGATGCA TATCAGGACA   
  
  
- AGTTCCATGC ACCATATATC TCCAATAGAA TATCCAAAAT TTTAACTACA AAGCTTATGT CGAACCAGAA   
  
  
- AAACTTGTTA CAGGGGGTTC CAAACTCTGA ATTACGGCAC CTTGAGGCCT CAATTTATCC AATATCAAGT   
  
  
- AGAATCAAAT CTCTCAGGCA TGGGGTTGAA GTTTCAAGAA TTTGACTGCA GAACAAATAC ACTTCAACCA   
  
  
- TATTAATGAT TTTTTTTTCA TATAAGAAAC CACAATTATG ATTCTGACAA GACTAAAATG TGGCTCAAGG   
  
  
- TCAAATCAGG GACTCCACAT CAGCTGATGA TCATCTTTCT AAAGAATTCT GCTTGCTCAG GGAAATACTC   
  
  
- CAACAGCTCA TCCTTCGTAA CCCAAGCAAA ATCCTCACAC TTCCCAATGT TAAATTTGTT GGTTGCAATT   
  
  
- ACTTGGGATT TAAAGAAGAA TTGCTGACAA AAAAAAGAAA AAAAATCAAG CACGGAAATT AAAAAAAACA   
  
  
- TAATTTAGCC TAAACAAGAA AGGCACATT

  
  
Motifs Found  

+     3-AF1 binding site

| Site Name | Organism | Position | Strand | Matrix score. | sequence | function |
| --- | --- | --- | --- | --- | --- | --- |
| 3-AF1 binding site | Solanum tuberosum | 121 | - | 10 | AAGAGATATTT | light responsive element |

> 2018/04/13 10:10:12  
+ CCCCCTTCTC GAGTTTCTCA ACTCTCTCCT TGACTCTTAC CTTCTCTTCC TTCCAATTAT ACCCAGTTCC   
  
  
+ CTTCACTATA GTACCTTCCA CTCTTCTCCC ACCTATCTGG GTGCCTATAT AAATGTCTCT TTCTCTCTCT   
  
  
+ CTTTTATACC ACGAAACCGA ACACTACACC TCTACCTCCT ACCTCTCTGA TCAGACTCAA AGTACCCATT   
  
  
+ TGGGTTGATT TAGTATGGAT GAAGACAAGT GGACGGGAAG TCACTTTTCA CATGCAGGAT ATATACGTAG   
  
  
+ CAATAGTGGC TACAAAGTGT AAGAATAAAC GGTTTTCTCT TGGTAAGTAA ATCCTGTACG ATGCAAAGTC   
  
  
+ ATTGGGGTTA CAGAAGAAAA CATATACGAT ATGACAAATG GTTGGTTCGT GACCACAAAA GAAAACTGGT   
  
  
+ TAATCTAGTT GACGTCGATG AAATTAAAGG ACATGCTTTA TAAGTCGGCA TTTGGTTCAA GTAGTTCTTT   
  
  
+ TTCTTCAGTC GTAAAACTAC TGATACGGAC AGGGGAAGCT AGAGTTAATG TCAAATTTGT TTTATTTCCC   
  
  
+ TAAATACACT TATATTTTCT GTATTATATA CAGTATTTTT GAAGTGGTAA TAACTTCTTT CCTAAACCCC   
  
  
+ ACGAGACCTA CTTGGAAGCA AAGGATGAGT ACGTTCCGTT CGGTTTCACG TACCTGTGTA CTATCGTTTG   
  
  
+ TTCGAACCTT TCCCTCTCAG GACTAATCCA GTGGACATCA CTGAAGGGTT CTGAAAAGAT CGAGGACTAA   
  
  
+ CCATTGTACG GTAAAGTATT ATGTGGGTTA GATGGTTCCT TCTTATGATG TATGGTGTTA GTTGTAGTAT   
  
  
+ TCATTGGTAT ATCCAGTAGT TCTACTTAGC CGACTCATCG TCCAAGTATA TCTAAAAAAA CGGTTCTTTT   
  
  
+ GGTTTGTATA TTTTGAACGG TTACACCTCT AATGACCTTC TTGTCTTTCT TTGTCTACGT ATAGTCCTGT   
  
  
+ TCAAGGTACG TGGTATATAG AGGTTATCTT ATAGGTTTTA AAATTGATGT TTCGAATACA GCTTGGTCTT   
  
  
+ TTTGAACAAT GTCCCCCAAG GTTTGAGACT TAATGCCGTG GAACTCCGGA GTTAAATAGG TTATAGTTCA   
  
  
+ TCTTAGTTTA GAGAGTCCGT ACCCCAACTT CAAAGTTCTT AAACTGACGT CTTGTTTATG TGAAGTTGGT   
  
  
+ ATAATTACTA AAAAAAAAGT ATATTCTTTG GTGTTAATAC TAAGACTGTT CTGATTTTAC ACCGAGTTCC   
  
  
+ AGTTTAGTCC CTGAGGTGTA GTCGACTACT AGTAGAAAGA TTTCTTAAGA CGAACGAGTC CCTTTATGAG   
  
  
+ GTTGTCGAGT AGGAAGCATT GGGTTCGTTT TAGGAGTGTG AAGGGTTACA ATTTAAACAA CCAACGTTAA   
  
  
+ TGAACCCTAA ATTTCTTCTT AACGACTGTT TTTTTTCTTT TTTTTAGTTC GTGCCTTTAA TTTTTTTTGT   
  
  
+ ATTAAATCGG ATTTGTTCTT TCCGTGTAA  

- GGGGGAAGAG CTCAAAGAGT TGAGAGAGGA ACTGAGAATG GAAGAGAAGG AAGGTTAATA TGGGTCAAGG   
  
  
- GAAGTGATAT CATGGAAGGT GAGAAGAGGG TGGATAGACC CACGGATATA TTTACAGAGA AAGAGAGAGA   
  
  
- GAAAATATGG TGCTTTGGCT TGTGATGTGG AGATGGAGGA TGGAGAGACT AGTCTGAGTT TCATGGGTAA   
  
  
- ACCCAACTAA ATCATACCTA CTTCTGTTCA CCTGCCCTTC AGTGAAAAGT GTACGTCCTA TATATGCATC   
  
  
- GTTATCACCG ATGTTTCACA TTCTTATTTG CCAAAAGAGA ACCATTCATT TAGGACATGC TACGTTTCAG   
  
  
- TAACCCCAAT GTCTTCTTTT GTATATGCTA TACTGTTTAC CAACCAAGCA CTGGTGTTTT CTTTTGACCA   
  
  
- ATTAGATCAA CTGCAGCTAC TTTAATTTCC TGTACGAAAT ATTCAGCCGT AAACCAAGTT CATCAAGAAA   
  
  
- AAGAAGTCAG CATTTTGATG ACTATGCCTG TCCCCTTCGA TCTCAATTAC AGTTTAAACA AAATAAAGGG   
  
  
- ATTTATGTGA ATATAAAAGA CATAATATAT GTCATAAAAA CTTCACCATT ATTGAAGAAA GGATTTGGGG   
  
  
- TGCTCTGGAT GAACCTTCGT TTCCTACTCA TGCAAGGCAA GCCAAAGTGC ATGGACACAT GATAGCAAAC   
  
  
- AAGCTTGGAA AGGGAGAGTC CTGATTAGGT CACCTGTAGT GACTTCCCAA GACTTTTCTA GCTCCTGATT   
  
  
- GGTAACATGC CATTTCATAA TACACCCAAT CTACCAAGGA AGAATACTAC ATACCACAAT CAACATCATA   
  
  
- AGTAACCATA TAGGTCATCA AGATGAATCG GCTGAGTAGC AGGTTCATAT AGATTTTTTT GCCAAGAAAA   
  
  
- CCAAACATAT AAAACTTGCC AATGTGGAGA TTACTGGAAG AACAGAAAGA AACAGATGCA TATCAGGACA   
  
  
- AGTTCCATGC ACCATATATC TCCAATAGAA TATCCAAAAT TTTAACTACA AAGCTTATGT CGAACCAGAA   
  
  
- AAACTTGTTA CAGGGGGTTC CAAACTCTGA ATTACGGCAC CTTGAGGCCT CAATTTATCC AATATCAAGT   
  
  
- AGAATCAAAT CTCTCAGGCA TGGGGTTGAA GTTTCAAGAA TTTGACTGCA GAACAAATAC ACTTCAACCA   
  
  
- TATTAATGAT TTTTTTTTCA TATAAGAAAC CACAATTATG ATTCTGACAA GACTAAAATG TGGCTCAAGG   
  
  
- TCAAATCAGG GACTCCACAT CAGCTGATGA TCATCTTTCT AAAGAATTCT GCTTGCTCAG GGAAATACTC   
  
  
- CAACAGCTCA TCCTTCGTAA CCCAAGCAAA ATCCTCACAC TTCCCAATGT TAAATTTGTT GGTTGCAATT   
  
  
- ACTTGGGATT TAAAGAAGAA TTGCTGACAA AAAAAAGAAA AAAAATCAAG CACGGAAATT AAAAAAAACA   
  
  
- TAATTTAGCC TAAACAAGAA AGGCACATT

+     5UTR Py-rich stretch

| Site Name | Organism | Position | Strand | Matrix score. | sequence | function |
| --- | --- | --- | --- | --- | --- | --- |
| 5UTR Py-rich stretch | Lycopersicon esculentum | 360 | - | 9 | TTTCTTCTCT | cis-acting element conferring high transcription levels |
| 5UTR Py-rich stretch | Lycopersicon esculentum | 130 | + | 13 | TTTCTCTCTCTCTC | cis-acting element conferring high transcription levels |

> 2018/04/13 10:10:12  
+ CCCCCTTCTC GAGTTTCTCA ACTCTCTCCT TGACTCTTAC CTTCTCTTCC TTCCAATTAT ACCCAGTTCC   
  
  
+ CTTCACTATA GTACCTTCCA CTCTTCTCCC ACCTATCTGG GTGCCTATAT AAATGTCTCT TTCTCTCTCT   
  
  
+ CTTTTATACC ACGAAACCGA ACACTACACC TCTACCTCCT ACCTCTCTGA TCAGACTCAA AGTACCCATT   
  
  
+ TGGGTTGATT TAGTATGGAT GAAGACAAGT GGACGGGAAG TCACTTTTCA CATGCAGGAT ATATACGTAG   
  
  
+ CAATAGTGGC TACAAAGTGT AAGAATAAAC GGTTTTCTCT TGGTAAGTAA ATCCTGTACG ATGCAAAGTC   
  
  
+ ATTGGGGTTA CAGAAGAAAA CATATACGAT ATGACAAATG GTTGGTTCGT GACCACAAAA GAAAACTGGT   
  
  
+ TAATCTAGTT GACGTCGATG AAATTAAAGG ACATGCTTTA TAAGTCGGCA TTTGGTTCAA GTAGTTCTTT   
  
  
+ TTCTTCAGTC GTAAAACTAC TGATACGGAC AGGGGAAGCT AGAGTTAATG TCAAATTTGT TTTATTTCCC   
  
  
+ TAAATACACT TATATTTTCT GTATTATATA CAGTATTTTT GAAGTGGTAA TAACTTCTTT CCTAAACCCC   
  
  
+ ACGAGACCTA CTTGGAAGCA AAGGATGAGT ACGTTCCGTT CGGTTTCACG TACCTGTGTA CTATCGTTTG   
  
  
+ TTCGAACCTT TCCCTCTCAG GACTAATCCA GTGGACATCA CTGAAGGGTT CTGAAAAGAT CGAGGACTAA   
  
  
+ CCATTGTACG GTAAAGTATT ATGTGGGTTA GATGGTTCCT TCTTATGATG TATGGTGTTA GTTGTAGTAT   
  
  
+ TCATTGGTAT ATCCAGTAGT TCTACTTAGC CGACTCATCG TCCAAGTATA TCTAAAAAAA CGGTTCTTTT   
  
  
+ GGTTTGTATA TTTTGAACGG TTACACCTCT AATGACCTTC TTGTCTTTCT TTGTCTACGT ATAGTCCTGT   
  
  
+ TCAAGGTACG TGGTATATAG AGGTTATCTT ATAGGTTTTA AAATTGATGT TTCGAATACA GCTTGGTCTT   
  
  
+ TTTGAACAAT GTCCCCCAAG GTTTGAGACT TAATGCCGTG GAACTCCGGA GTTAAATAGG TTATAGTTCA   
  
  
+ TCTTAGTTTA GAGAGTCCGT ACCCCAACTT CAAAGTTCTT AAACTGACGT CTTGTTTATG TGAAGTTGGT   
  
  
+ ATAATTACTA AAAAAAAAGT ATATTCTTTG GTGTTAATAC TAAGACTGTT CTGATTTTAC ACCGAGTTCC   
  
  
+ AGTTTAGTCC CTGAGGTGTA GTCGACTACT AGTAGAAAGA TTTCTTAAGA CGAACGAGTC CCTTTATGAG   
  
  
+ GTTGTCGAGT AGGAAGCATT GGGTTCGTTT TAGGAGTGTG AAGGGTTACA ATTTAAACAA CCAACGTTAA   
  
  
+ TGAACCCTAA ATTTCTTCTT AACGACTGTT TTTTTTCTTT TTTTTAGTTC GTGCCTTTAA TTTTTTTTGT   
  
  
+ ATTAAATCGG ATTTGTTCTT TCCGTGTAA  

- GGGGGAAGAG CTCAAAGAGT TGAGAGAGGA ACTGAGAATG GAAGAGAAGG AAGGTTAATA TGGGTCAAGG   
  
  
- GAAGTGATAT CATGGAAGGT GAGAAGAGGG TGGATAGACC CACGGATATA TTTACAGAGA AAGAGAGAGA   
  
  
- GAAAATATGG TGCTTTGGCT TGTGATGTGG AGATGGAGGA TGGAGAGACT AGTCTGAGTT TCATGGGTAA   
  
  
- ACCCAACTAA ATCATACCTA CTTCTGTTCA CCTGCCCTTC AGTGAAAAGT GTACGTCCTA TATATGCATC   
  
  
- GTTATCACCG ATGTTTCACA TTCTTATTTG CCAAAAGAGA ACCATTCATT TAGGACATGC TACGTTTCAG   
  
  
- TAACCCCAAT GTCTTCTTTT GTATATGCTA TACTGTTTAC CAACCAAGCA CTGGTGTTTT CTTTTGACCA   
  
  
- ATTAGATCAA CTGCAGCTAC TTTAATTTCC TGTACGAAAT ATTCAGCCGT AAACCAAGTT CATCAAGAAA   
  
  
- AAGAAGTCAG CATTTTGATG ACTATGCCTG TCCCCTTCGA TCTCAATTAC AGTTTAAACA AAATAAAGGG   
  
  
- ATTTATGTGA ATATAAAAGA CATAATATAT GTCATAAAAA CTTCACCATT ATTGAAGAAA GGATTTGGGG   
  
  
- TGCTCTGGAT GAACCTTCGT TTCCTACTCA TGCAAGGCAA GCCAAAGTGC ATGGACACAT GATAGCAAAC   
  
  
- AAGCTTGGAA AGGGAGAGTC CTGATTAGGT CACCTGTAGT GACTTCCCAA GACTTTTCTA GCTCCTGATT   
  
  
- GGTAACATGC CATTTCATAA TACACCCAAT CTACCAAGGA AGAATACTAC ATACCACAAT CAACATCATA   
  
  
- AGTAACCATA TAGGTCATCA AGATGAATCG GCTGAGTAGC AGGTTCATAT AGATTTTTTT GCCAAGAAAA   
  
  
- CCAAACATAT AAAACTTGCC AATGTGGAGA TTACTGGAAG AACAGAAAGA AACAGATGCA TATCAGGACA   
  
  
- AGTTCCATGC ACCATATATC TCCAATAGAA TATCCAAAAT TTTAACTACA AAGCTTATGT CGAACCAGAA   
  
  
- AAACTTGTTA CAGGGGGTTC CAAACTCTGA ATTACGGCAC CTTGAGGCCT CAATTTATCC AATATCAAGT   
  
  
- AGAATCAAAT CTCTCAGGCA TGGGGTTGAA GTTTCAAGAA TTTGACTGCA GAACAAATAC ACTTCAACCA   
  
  
- TATTAATGAT TTTTTTTTCA TATAAGAAAC CACAATTATG ATTCTGACAA GACTAAAATG TGGCTCAAGG   
  
  
- TCAAATCAGG GACTCCACAT CAGCTGATGA TCATCTTTCT AAAGAATTCT GCTTGCTCAG GGAAATACTC   
  
  
- CAACAGCTCA TCCTTCGTAA CCCAAGCAAA ATCCTCACAC TTCCCAATGT TAAATTTGTT GGTTGCAATT   
  
  
- ACTTGGGATT TAAAGAAGAA TTGCTGACAA AAAAAAGAAA AAAAATCAAG CACGGAAATT AAAAAAAACA   
  
  
- TAATTTAGCC TAAACAAGAA AGGCACATT

+     A-box

| Site Name | Organism | Position | Strand | Matrix score. | sequence | function |
| --- | --- | --- | --- | --- | --- | --- |
| A-box | Petroselinum crispum | 241 | - | 6 | CCGTCC | cis-acting regulatory element |

> 2018/04/13 10:10:12  
+ CCCCCTTCTC GAGTTTCTCA ACTCTCTCCT TGACTCTTAC CTTCTCTTCC TTCCAATTAT ACCCAGTTCC   
  
  
+ CTTCACTATA GTACCTTCCA CTCTTCTCCC ACCTATCTGG GTGCCTATAT AAATGTCTCT TTCTCTCTCT   
  
  
+ CTTTTATACC ACGAAACCGA ACACTACACC TCTACCTCCT ACCTCTCTGA TCAGACTCAA AGTACCCATT   
  
  
+ TGGGTTGATT TAGTATGGAT GAAGACAAGT GGACGGGAAG TCACTTTTCA CATGCAGGAT ATATACGTAG   
  
  
+ CAATAGTGGC TACAAAGTGT AAGAATAAAC GGTTTTCTCT TGGTAAGTAA ATCCTGTACG ATGCAAAGTC   
  
  
+ ATTGGGGTTA CAGAAGAAAA CATATACGAT ATGACAAATG GTTGGTTCGT GACCACAAAA GAAAACTGGT   
  
  
+ TAATCTAGTT GACGTCGATG AAATTAAAGG ACATGCTTTA TAAGTCGGCA TTTGGTTCAA GTAGTTCTTT   
  
  
+ TTCTTCAGTC GTAAAACTAC TGATACGGAC AGGGGAAGCT AGAGTTAATG TCAAATTTGT TTTATTTCCC   
  
  
+ TAAATACACT TATATTTTCT GTATTATATA CAGTATTTTT GAAGTGGTAA TAACTTCTTT CCTAAACCCC   
  
  
+ ACGAGACCTA CTTGGAAGCA AAGGATGAGT ACGTTCCGTT CGGTTTCACG TACCTGTGTA CTATCGTTTG   
  
  
+ TTCGAACCTT TCCCTCTCAG GACTAATCCA GTGGACATCA CTGAAGGGTT CTGAAAAGAT CGAGGACTAA   
  
  
+ CCATTGTACG GTAAAGTATT ATGTGGGTTA GATGGTTCCT TCTTATGATG TATGGTGTTA GTTGTAGTAT   
  
  
+ TCATTGGTAT ATCCAGTAGT TCTACTTAGC CGACTCATCG TCCAAGTATA TCTAAAAAAA CGGTTCTTTT   
  
  
+ GGTTTGTATA TTTTGAACGG TTACACCTCT AATGACCTTC TTGTCTTTCT TTGTCTACGT ATAGTCCTGT   
  
  
+ TCAAGGTACG TGGTATATAG AGGTTATCTT ATAGGTTTTA AAATTGATGT TTCGAATACA GCTTGGTCTT   
  
  
+ TTTGAACAAT GTCCCCCAAG GTTTGAGACT TAATGCCGTG GAACTCCGGA GTTAAATAGG TTATAGTTCA   
  
  
+ TCTTAGTTTA GAGAGTCCGT ACCCCAACTT CAAAGTTCTT AAACTGACGT CTTGTTTATG TGAAGTTGGT   
  
  
+ ATAATTACTA AAAAAAAAGT ATATTCTTTG GTGTTAATAC TAAGACTGTT CTGATTTTAC ACCGAGTTCC   
  
  
+ AGTTTAGTCC CTGAGGTGTA GTCGACTACT AGTAGAAAGA TTTCTTAAGA CGAACGAGTC CCTTTATGAG   
  
  
+ GTTGTCGAGT AGGAAGCATT GGGTTCGTTT TAGGAGTGTG AAGGGTTACA ATTTAAACAA CCAACGTTAA   
  
  
+ TGAACCCTAA ATTTCTTCTT AACGACTGTT TTTTTTCTTT TTTTTAGTTC GTGCCTTTAA TTTTTTTTGT   
  
  
+ ATTAAATCGG ATTTGTTCTT TCCGTGTAA  

- GGGGGAAGAG CTCAAAGAGT TGAGAGAGGA ACTGAGAATG GAAGAGAAGG AAGGTTAATA TGGGTCAAGG   
  
  
- GAAGTGATAT CATGGAAGGT GAGAAGAGGG TGGATAGACC CACGGATATA TTTACAGAGA AAGAGAGAGA   
  
  
- GAAAATATGG TGCTTTGGCT TGTGATGTGG AGATGGAGGA TGGAGAGACT AGTCTGAGTT TCATGGGTAA   
  
  
- ACCCAACTAA ATCATACCTA CTTCTGTTCA CCTGCCCTTC AGTGAAAAGT GTACGTCCTA TATATGCATC   
  
  
- GTTATCACCG ATGTTTCACA TTCTTATTTG CCAAAAGAGA ACCATTCATT TAGGACATGC TACGTTTCAG   
  
  
- TAACCCCAAT GTCTTCTTTT GTATATGCTA TACTGTTTAC CAACCAAGCA CTGGTGTTTT CTTTTGACCA   
  
  
- ATTAGATCAA CTGCAGCTAC TTTAATTTCC TGTACGAAAT ATTCAGCCGT AAACCAAGTT CATCAAGAAA   
  
  
- AAGAAGTCAG CATTTTGATG ACTATGCCTG TCCCCTTCGA TCTCAATTAC AGTTTAAACA AAATAAAGGG   
  
  
- ATTTATGTGA ATATAAAAGA CATAATATAT GTCATAAAAA CTTCACCATT ATTGAAGAAA GGATTTGGGG   
  
  
- TGCTCTGGAT GAACCTTCGT TTCCTACTCA TGCAAGGCAA GCCAAAGTGC ATGGACACAT GATAGCAAAC   
  
  
- AAGCTTGGAA AGGGAGAGTC CTGATTAGGT CACCTGTAGT GACTTCCCAA GACTTTTCTA GCTCCTGATT   
  
  
- GGTAACATGC CATTTCATAA TACACCCAAT CTACCAAGGA AGAATACTAC ATACCACAAT CAACATCATA   
  
  
- AGTAACCATA TAGGTCATCA AGATGAATCG GCTGAGTAGC AGGTTCATAT AGATTTTTTT GCCAAGAAAA   
  
  
- CCAAACATAT AAAACTTGCC AATGTGGAGA TTACTGGAAG AACAGAAAGA AACAGATGCA TATCAGGACA   
  
  
- AGTTCCATGC ACCATATATC TCCAATAGAA TATCCAAAAT TTTAACTACA AAGCTTATGT CGAACCAGAA   
  
  
- AAACTTGTTA CAGGGGGTTC CAAACTCTGA ATTACGGCAC CTTGAGGCCT CAATTTATCC AATATCAAGT   
  
  
- AGAATCAAAT CTCTCAGGCA TGGGGTTGAA GTTTCAAGAA TTTGACTGCA GAACAAATAC ACTTCAACCA   
  
  
- TATTAATGAT TTTTTTTTCA TATAAGAAAC CACAATTATG ATTCTGACAA GACTAAAATG TGGCTCAAGG   
  
  
- TCAAATCAGG GACTCCACAT CAGCTGATGA TCATCTTTCT AAAGAATTCT GCTTGCTCAG GGAAATACTC   
  
  
- CAACAGCTCA TCCTTCGTAA CCCAAGCAAA ATCCTCACAC TTCCCAATGT TAAATTTGTT GGTTGCAATT   
  
  
- ACTTGGGATT TAAAGAAGAA TTGCTGACAA AAAAAAGAAA AAAAATCAAG CACGGAAATT AAAAAAAACA   
  
  
- TAATTTAGCC TAAACAAGAA AGGCACATT

+     AAGAA-motif

| Site Name | Organism | Position | Strand | Matrix score. | sequence | function |
| --- | --- | --- | --- | --- | --- | --- |
| AAGAA-motif | Avena sativa | 1486 | - | 7 | GAAAGAA |  |
| AAGAA-motif | Avena sativa | 615 | - | 7 | GAAAGAA |  |

> 2018/04/13 10:10:12  
+ CCCCCTTCTC GAGTTTCTCA ACTCTCTCCT TGACTCTTAC CTTCTCTTCC TTCCAATTAT ACCCAGTTCC   
  
  
+ CTTCACTATA GTACCTTCCA CTCTTCTCCC ACCTATCTGG GTGCCTATAT AAATGTCTCT TTCTCTCTCT   
  
  
+ CTTTTATACC ACGAAACCGA ACACTACACC TCTACCTCCT ACCTCTCTGA TCAGACTCAA AGTACCCATT   
  
  
+ TGGGTTGATT TAGTATGGAT GAAGACAAGT GGACGGGAAG TCACTTTTCA CATGCAGGAT ATATACGTAG   
  
  
+ CAATAGTGGC TACAAAGTGT AAGAATAAAC GGTTTTCTCT TGGTAAGTAA ATCCTGTACG ATGCAAAGTC   
  
  
+ ATTGGGGTTA CAGAAGAAAA CATATACGAT ATGACAAATG GTTGGTTCGT GACCACAAAA GAAAACTGGT   
  
  
+ TAATCTAGTT GACGTCGATG AAATTAAAGG ACATGCTTTA TAAGTCGGCA TTTGGTTCAA GTAGTTCTTT   
  
  
+ TTCTTCAGTC GTAAAACTAC TGATACGGAC AGGGGAAGCT AGAGTTAATG TCAAATTTGT TTTATTTCCC   
  
  
+ TAAATACACT TATATTTTCT GTATTATATA CAGTATTTTT GAAGTGGTAA TAACTTCTTT CCTAAACCCC   
  
  
+ ACGAGACCTA CTTGGAAGCA AAGGATGAGT ACGTTCCGTT CGGTTTCACG TACCTGTGTA CTATCGTTTG   
  
  
+ TTCGAACCTT TCCCTCTCAG GACTAATCCA GTGGACATCA CTGAAGGGTT CTGAAAAGAT CGAGGACTAA   
  
  
+ CCATTGTACG GTAAAGTATT ATGTGGGTTA GATGGTTCCT TCTTATGATG TATGGTGTTA GTTGTAGTAT   
  
  
+ TCATTGGTAT ATCCAGTAGT TCTACTTAGC CGACTCATCG TCCAAGTATA TCTAAAAAAA CGGTTCTTTT   
  
  
+ GGTTTGTATA TTTTGAACGG TTACACCTCT AATGACCTTC TTGTCTTTCT TTGTCTACGT ATAGTCCTGT   
  
  
+ TCAAGGTACG TGGTATATAG AGGTTATCTT ATAGGTTTTA AAATTGATGT TTCGAATACA GCTTGGTCTT   
  
  
+ TTTGAACAAT GTCCCCCAAG GTTTGAGACT TAATGCCGTG GAACTCCGGA GTTAAATAGG TTATAGTTCA   
  
  
+ TCTTAGTTTA GAGAGTCCGT ACCCCAACTT CAAAGTTCTT AAACTGACGT CTTGTTTATG TGAAGTTGGT   
  
  
+ ATAATTACTA AAAAAAAAGT ATATTCTTTG GTGTTAATAC TAAGACTGTT CTGATTTTAC ACCGAGTTCC   
  
  
+ AGTTTAGTCC CTGAGGTGTA GTCGACTACT AGTAGAAAGA TTTCTTAAGA CGAACGAGTC CCTTTATGAG   
  
  
+ GTTGTCGAGT AGGAAGCATT GGGTTCGTTT TAGGAGTGTG AAGGGTTACA ATTTAAACAA CCAACGTTAA   
  
  
+ TGAACCCTAA ATTTCTTCTT AACGACTGTT TTTTTTCTTT TTTTTAGTTC GTGCCTTTAA TTTTTTTTGT   
  
  
+ ATTAAATCGG ATTTGTTCTT TCCGTGTAA  

- GGGGGAAGAG CTCAAAGAGT TGAGAGAGGA ACTGAGAATG GAAGAGAAGG AAGGTTAATA TGGGTCAAGG   
  
  
- GAAGTGATAT CATGGAAGGT GAGAAGAGGG TGGATAGACC CACGGATATA TTTACAGAGA AAGAGAGAGA   
  
  
- GAAAATATGG TGCTTTGGCT TGTGATGTGG AGATGGAGGA TGGAGAGACT AGTCTGAGTT TCATGGGTAA   
  
  
- ACCCAACTAA ATCATACCTA CTTCTGTTCA CCTGCCCTTC AGTGAAAAGT GTACGTCCTA TATATGCATC   
  
  
- GTTATCACCG ATGTTTCACA TTCTTATTTG CCAAAAGAGA ACCATTCATT TAGGACATGC TACGTTTCAG   
  
  
- TAACCCCAAT GTCTTCTTTT GTATATGCTA TACTGTTTAC CAACCAAGCA CTGGTGTTTT CTTTTGACCA   
  
  
- ATTAGATCAA CTGCAGCTAC TTTAATTTCC TGTACGAAAT ATTCAGCCGT AAACCAAGTT CATCAAGAAA   
  
  
- AAGAAGTCAG CATTTTGATG ACTATGCCTG TCCCCTTCGA TCTCAATTAC AGTTTAAACA AAATAAAGGG   
  
  
- ATTTATGTGA ATATAAAAGA CATAATATAT GTCATAAAAA CTTCACCATT ATTGAAGAAA GGATTTGGGG   
  
  
- TGCTCTGGAT GAACCTTCGT TTCCTACTCA TGCAAGGCAA GCCAAAGTGC ATGGACACAT GATAGCAAAC   
  
  
- AAGCTTGGAA AGGGAGAGTC CTGATTAGGT CACCTGTAGT GACTTCCCAA GACTTTTCTA GCTCCTGATT   
  
  
- GGTAACATGC CATTTCATAA TACACCCAAT CTACCAAGGA AGAATACTAC ATACCACAAT CAACATCATA   
  
  
- AGTAACCATA TAGGTCATCA AGATGAATCG GCTGAGTAGC AGGTTCATAT AGATTTTTTT GCCAAGAAAA   
  
  
- CCAAACATAT AAAACTTGCC AATGTGGAGA TTACTGGAAG AACAGAAAGA AACAGATGCA TATCAGGACA   
  
  
- AGTTCCATGC ACCATATATC TCCAATAGAA TATCCAAAAT TTTAACTACA AAGCTTATGT CGAACCAGAA   
  
  
- AAACTTGTTA CAGGGGGTTC CAAACTCTGA ATTACGGCAC CTTGAGGCCT CAATTTATCC AATATCAAGT   
  
  
- AGAATCAAAT CTCTCAGGCA TGGGGTTGAA GTTTCAAGAA TTTGACTGCA GAACAAATAC ACTTCAACCA   
  
  
- TATTAATGAT TTTTTTTTCA TATAAGAAAC CACAATTATG ATTCTGACAA GACTAAAATG TGGCTCAAGG   
  
  
- TCAAATCAGG GACTCCACAT CAGCTGATGA TCATCTTTCT AAAGAATTCT GCTTGCTCAG GGAAATACTC   
  
  
- CAACAGCTCA TCCTTCGTAA CCCAAGCAAA ATCCTCACAC TTCCCAATGT TAAATTTGTT GGTTGCAATT   
  
  
- ACTTGGGATT TAAAGAAGAA TTGCTGACAA AAAAAAGAAA AAAAATCAAG CACGGAAATT AAAAAAAACA   
  
  
- TAATTTAGCC TAAACAAGAA AGGCACATT

+     ABRE

| Site Name | Organism | Position | Strand | Matrix score. | sequence | function |
| --- | --- | --- | --- | --- | --- | --- |
| ABRE | Arabidopsis thaliana | 987 | + | 6 | TACGTG | cis-acting element involved in the abscisic acid responsiveness |
| ABRE | Arabidopsis thaliana | 677 | - | 6 | TACGTG | cis-acting element involved in the abscisic acid responsiveness |

> 2018/04/13 10:10:12  
+ CCCCCTTCTC GAGTTTCTCA ACTCTCTCCT TGACTCTTAC CTTCTCTTCC TTCCAATTAT ACCCAGTTCC   
  
  
+ CTTCACTATA GTACCTTCCA CTCTTCTCCC ACCTATCTGG GTGCCTATAT AAATGTCTCT TTCTCTCTCT   
  
  
+ CTTTTATACC ACGAAACCGA ACACTACACC TCTACCTCCT ACCTCTCTGA TCAGACTCAA AGTACCCATT   
  
  
+ TGGGTTGATT TAGTATGGAT GAAGACAAGT GGACGGGAAG TCACTTTTCA CATGCAGGAT ATATACGTAG   
  
  
+ CAATAGTGGC TACAAAGTGT AAGAATAAAC GGTTTTCTCT TGGTAAGTAA ATCCTGTACG ATGCAAAGTC   
  
  
+ ATTGGGGTTA CAGAAGAAAA CATATACGAT ATGACAAATG GTTGGTTCGT GACCACAAAA GAAAACTGGT   
  
  
+ TAATCTAGTT GACGTCGATG AAATTAAAGG ACATGCTTTA TAAGTCGGCA TTTGGTTCAA GTAGTTCTTT   
  
  
+ TTCTTCAGTC GTAAAACTAC TGATACGGAC AGGGGAAGCT AGAGTTAATG TCAAATTTGT TTTATTTCCC   
  
  
+ TAAATACACT TATATTTTCT GTATTATATA CAGTATTTTT GAAGTGGTAA TAACTTCTTT CCTAAACCCC   
  
  
+ ACGAGACCTA CTTGGAAGCA AAGGATGAGT ACGTTCCGTT CGGTTTCACG TACCTGTGTA CTATCGTTTG   
  
  
+ TTCGAACCTT TCCCTCTCAG GACTAATCCA GTGGACATCA CTGAAGGGTT CTGAAAAGAT CGAGGACTAA   
  
  
+ CCATTGTACG GTAAAGTATT ATGTGGGTTA GATGGTTCCT TCTTATGATG TATGGTGTTA GTTGTAGTAT   
  
  
+ TCATTGGTAT ATCCAGTAGT TCTACTTAGC CGACTCATCG TCCAAGTATA TCTAAAAAAA CGGTTCTTTT   
  
  
+ GGTTTGTATA TTTTGAACGG TTACACCTCT AATGACCTTC TTGTCTTTCT TTGTCTACGT ATAGTCCTGT   
  
  
+ TCAAGGTACG TGGTATATAG AGGTTATCTT ATAGGTTTTA AAATTGATGT TTCGAATACA GCTTGGTCTT   
  
  
+ TTTGAACAAT GTCCCCCAAG GTTTGAGACT TAATGCCGTG GAACTCCGGA GTTAAATAGG TTATAGTTCA   
  
  
+ TCTTAGTTTA GAGAGTCCGT ACCCCAACTT CAAAGTTCTT AAACTGACGT CTTGTTTATG TGAAGTTGGT   
  
  
+ ATAATTACTA AAAAAAAAGT ATATTCTTTG GTGTTAATAC TAAGACTGTT CTGATTTTAC ACCGAGTTCC   
  
  
+ AGTTTAGTCC CTGAGGTGTA GTCGACTACT AGTAGAAAGA TTTCTTAAGA CGAACGAGTC CCTTTATGAG   
  
  
+ GTTGTCGAGT AGGAAGCATT GGGTTCGTTT TAGGAGTGTG AAGGGTTACA ATTTAAACAA CCAACGTTAA   
  
  
+ TGAACCCTAA ATTTCTTCTT AACGACTGTT TTTTTTCTTT TTTTTAGTTC GTGCCTTTAA TTTTTTTTGT   
  
  
+ ATTAAATCGG ATTTGTTCTT TCCGTGTAA  

- GGGGGAAGAG CTCAAAGAGT TGAGAGAGGA ACTGAGAATG GAAGAGAAGG AAGGTTAATA TGGGTCAAGG   
  
  
- GAAGTGATAT CATGGAAGGT GAGAAGAGGG TGGATAGACC CACGGATATA TTTACAGAGA AAGAGAGAGA   
  
  
- GAAAATATGG TGCTTTGGCT TGTGATGTGG AGATGGAGGA TGGAGAGACT AGTCTGAGTT TCATGGGTAA   
  
  
- ACCCAACTAA ATCATACCTA CTTCTGTTCA CCTGCCCTTC AGTGAAAAGT GTACGTCCTA TATATGCATC   
  
  
- GTTATCACCG ATGTTTCACA TTCTTATTTG CCAAAAGAGA ACCATTCATT TAGGACATGC TACGTTTCAG   
  
  
- TAACCCCAAT GTCTTCTTTT GTATATGCTA TACTGTTTAC CAACCAAGCA CTGGTGTTTT CTTTTGACCA   
  
  
- ATTAGATCAA CTGCAGCTAC TTTAATTTCC TGTACGAAAT ATTCAGCCGT AAACCAAGTT CATCAAGAAA   
  
  
- AAGAAGTCAG CATTTTGATG ACTATGCCTG TCCCCTTCGA TCTCAATTAC AGTTTAAACA AAATAAAGGG   
  
  
- ATTTATGTGA ATATAAAAGA CATAATATAT GTCATAAAAA CTTCACCATT ATTGAAGAAA GGATTTGGGG   
  
  
- TGCTCTGGAT GAACCTTCGT TTCCTACTCA TGCAAGGCAA GCCAAAGTGC ATGGACACAT GATAGCAAAC   
  
  
- AAGCTTGGAA AGGGAGAGTC CTGATTAGGT CACCTGTAGT GACTTCCCAA GACTTTTCTA GCTCCTGATT   
  
  
- GGTAACATGC CATTTCATAA TACACCCAAT CTACCAAGGA AGAATACTAC ATACCACAAT CAACATCATA   
  
  
- AGTAACCATA TAGGTCATCA AGATGAATCG GCTGAGTAGC AGGTTCATAT AGATTTTTTT GCCAAGAAAA   
  
  
- CCAAACATAT AAAACTTGCC AATGTGGAGA TTACTGGAAG AACAGAAAGA AACAGATGCA TATCAGGACA   
  
  
- AGTTCCATGC ACCATATATC TCCAATAGAA TATCCAAAAT TTTAACTACA AAGCTTATGT CGAACCAGAA   
  
  
- AAACTTGTTA CAGGGGGTTC CAAACTCTGA ATTACGGCAC CTTGAGGCCT CAATTTATCC AATATCAAGT   
  
  
- AGAATCAAAT CTCTCAGGCA TGGGGTTGAA GTTTCAAGAA TTTGACTGCA GAACAAATAC ACTTCAACCA   
  
  
- TATTAATGAT TTTTTTTTCA TATAAGAAAC CACAATTATG ATTCTGACAA GACTAAAATG TGGCTCAAGG   
  
  
- TCAAATCAGG GACTCCACAT CAGCTGATGA TCATCTTTCT AAAGAATTCT GCTTGCTCAG GGAAATACTC   
  
  
- CAACAGCTCA TCCTTCGTAA CCCAAGCAAA ATCCTCACAC TTCCCAATGT TAAATTTGTT GGTTGCAATT   
  
  
- ACTTGGGATT TAAAGAAGAA TTGCTGACAA AAAAAAGAAA AAAAATCAAG CACGGAAATT AAAAAAAACA   
  
  
- TAATTTAGCC TAAACAAGAA AGGCACATT

+     AC-I

| Site Name | Organism | Position | Strand | Matrix score. | sequence | function |
| --- | --- | --- | --- | --- | --- | --- |
| AC-I | Phaseolus vulgaris | 98 | + | 9 | CCCACCTACC |  |

> 2018/04/13 10:10:12  
+ CCCCCTTCTC GAGTTTCTCA ACTCTCTCCT TGACTCTTAC CTTCTCTTCC TTCCAATTAT ACCCAGTTCC   
  
  
+ CTTCACTATA GTACCTTCCA CTCTTCTCCC ACCTATCTGG GTGCCTATAT AAATGTCTCT TTCTCTCTCT   
  
  
+ CTTTTATACC ACGAAACCGA ACACTACACC TCTACCTCCT ACCTCTCTGA TCAGACTCAA AGTACCCATT   
  
  
+ TGGGTTGATT TAGTATGGAT GAAGACAAGT GGACGGGAAG TCACTTTTCA CATGCAGGAT ATATACGTAG   
  
  
+ CAATAGTGGC TACAAAGTGT AAGAATAAAC GGTTTTCTCT TGGTAAGTAA ATCCTGTACG ATGCAAAGTC   
  
  
+ ATTGGGGTTA CAGAAGAAAA CATATACGAT ATGACAAATG GTTGGTTCGT GACCACAAAA GAAAACTGGT   
  
  
+ TAATCTAGTT GACGTCGATG AAATTAAAGG ACATGCTTTA TAAGTCGGCA TTTGGTTCAA GTAGTTCTTT   
  
  
+ TTCTTCAGTC GTAAAACTAC TGATACGGAC AGGGGAAGCT AGAGTTAATG TCAAATTTGT TTTATTTCCC   
  
  
+ TAAATACACT TATATTTTCT GTATTATATA CAGTATTTTT GAAGTGGTAA TAACTTCTTT CCTAAACCCC   
  
  
+ ACGAGACCTA CTTGGAAGCA AAGGATGAGT ACGTTCCGTT CGGTTTCACG TACCTGTGTA CTATCGTTTG   
  
  
+ TTCGAACCTT TCCCTCTCAG GACTAATCCA GTGGACATCA CTGAAGGGTT CTGAAAAGAT CGAGGACTAA   
  
  
+ CCATTGTACG GTAAAGTATT ATGTGGGTTA GATGGTTCCT TCTTATGATG TATGGTGTTA GTTGTAGTAT   
  
  
+ TCATTGGTAT ATCCAGTAGT TCTACTTAGC CGACTCATCG TCCAAGTATA TCTAAAAAAA CGGTTCTTTT   
  
  
+ GGTTTGTATA TTTTGAACGG TTACACCTCT AATGACCTTC TTGTCTTTCT TTGTCTACGT ATAGTCCTGT   
  
  
+ TCAAGGTACG TGGTATATAG AGGTTATCTT ATAGGTTTTA AAATTGATGT TTCGAATACA GCTTGGTCTT   
  
  
+ TTTGAACAAT GTCCCCCAAG GTTTGAGACT TAATGCCGTG GAACTCCGGA GTTAAATAGG TTATAGTTCA   
  
  
+ TCTTAGTTTA GAGAGTCCGT ACCCCAACTT CAAAGTTCTT AAACTGACGT CTTGTTTATG TGAAGTTGGT   
  
  
+ ATAATTACTA AAAAAAAAGT ATATTCTTTG GTGTTAATAC TAAGACTGTT CTGATTTTAC ACCGAGTTCC   
  
  
+ AGTTTAGTCC CTGAGGTGTA GTCGACTACT AGTAGAAAGA TTTCTTAAGA CGAACGAGTC CCTTTATGAG   
  
  
+ GTTGTCGAGT AGGAAGCATT GGGTTCGTTT TAGGAGTGTG AAGGGTTACA ATTTAAACAA CCAACGTTAA   
  
  
+ TGAACCCTAA ATTTCTTCTT AACGACTGTT TTTTTTCTTT TTTTTAGTTC GTGCCTTTAA TTTTTTTTGT   
  
  
+ ATTAAATCGG ATTTGTTCTT TCCGTGTAA  

- GGGGGAAGAG CTCAAAGAGT TGAGAGAGGA ACTGAGAATG GAAGAGAAGG AAGGTTAATA TGGGTCAAGG   
  
  
- GAAGTGATAT CATGGAAGGT GAGAAGAGGG TGGATAGACC CACGGATATA TTTACAGAGA AAGAGAGAGA   
  
  
- GAAAATATGG TGCTTTGGCT TGTGATGTGG AGATGGAGGA TGGAGAGACT AGTCTGAGTT TCATGGGTAA   
  
  
- ACCCAACTAA ATCATACCTA CTTCTGTTCA CCTGCCCTTC AGTGAAAAGT GTACGTCCTA TATATGCATC   
  
  
- GTTATCACCG ATGTTTCACA TTCTTATTTG CCAAAAGAGA ACCATTCATT TAGGACATGC TACGTTTCAG   
  
  
- TAACCCCAAT GTCTTCTTTT GTATATGCTA TACTGTTTAC CAACCAAGCA CTGGTGTTTT CTTTTGACCA   
  
  
- ATTAGATCAA CTGCAGCTAC TTTAATTTCC TGTACGAAAT ATTCAGCCGT AAACCAAGTT CATCAAGAAA   
  
  
- AAGAAGTCAG CATTTTGATG ACTATGCCTG TCCCCTTCGA TCTCAATTAC AGTTTAAACA AAATAAAGGG   
  
  
- ATTTATGTGA ATATAAAAGA CATAATATAT GTCATAAAAA CTTCACCATT ATTGAAGAAA GGATTTGGGG   
  
  
- TGCTCTGGAT GAACCTTCGT TTCCTACTCA TGCAAGGCAA GCCAAAGTGC ATGGACACAT GATAGCAAAC   
  
  
- AAGCTTGGAA AGGGAGAGTC CTGATTAGGT CACCTGTAGT GACTTCCCAA GACTTTTCTA GCTCCTGATT   
  
  
- GGTAACATGC CATTTCATAA TACACCCAAT CTACCAAGGA AGAATACTAC ATACCACAAT CAACATCATA   
  
  
- AGTAACCATA TAGGTCATCA AGATGAATCG GCTGAGTAGC AGGTTCATAT AGATTTTTTT GCCAAGAAAA   
  
  
- CCAAACATAT AAAACTTGCC AATGTGGAGA TTACTGGAAG AACAGAAAGA AACAGATGCA TATCAGGACA   
  
  
- AGTTCCATGC ACCATATATC TCCAATAGAA TATCCAAAAT TTTAACTACA AAGCTTATGT CGAACCAGAA   
  
  
- AAACTTGTTA CAGGGGGTTC CAAACTCTGA ATTACGGCAC CTTGAGGCCT CAATTTATCC AATATCAAGT   
  
  
- AGAATCAAAT CTCTCAGGCA TGGGGTTGAA GTTTCAAGAA TTTGACTGCA GAACAAATAC ACTTCAACCA   
  
  
- TATTAATGAT TTTTTTTTCA TATAAGAAAC CACAATTATG ATTCTGACAA GACTAAAATG TGGCTCAAGG   
  
  
- TCAAATCAGG GACTCCACAT CAGCTGATGA TCATCTTTCT AAAGAATTCT GCTTGCTCAG GGAAATACTC   
  
  
- CAACAGCTCA TCCTTCGTAA CCCAAGCAAA ATCCTCACAC TTCCCAATGT TAAATTTGTT GGTTGCAATT   
  
  
- ACTTGGGATT TAAAGAAGAA TTGCTGACAA AAAAAAGAAA AAAAATCAAG CACGGAAATT AAAAAAAACA   
  
  
- TAATTTAGCC TAAACAAGAA AGGCACATT

+     AC-II

| Site Name | Organism | Position | Strand | Matrix score. | sequence | function |
| --- | --- | --- | --- | --- | --- | --- |
| AC-II | Phaseolus vulgaris | 1382 | + | 9 | (C/T)T(T/C)(C/T)(A/C)(A/C)C(A/C)A(A/C)C(C/A)(C/A)C |  |

> 2018/04/13 10:10:12  
+ CCCCCTTCTC GAGTTTCTCA ACTCTCTCCT TGACTCTTAC CTTCTCTTCC TTCCAATTAT ACCCAGTTCC   
  
  
+ CTTCACTATA GTACCTTCCA CTCTTCTCCC ACCTATCTGG GTGCCTATAT AAATGTCTCT TTCTCTCTCT   
  
  
+ CTTTTATACC ACGAAACCGA ACACTACACC TCTACCTCCT ACCTCTCTGA TCAGACTCAA AGTACCCATT   
  
  
+ TGGGTTGATT TAGTATGGAT GAAGACAAGT GGACGGGAAG TCACTTTTCA CATGCAGGAT ATATACGTAG   
  
  
+ CAATAGTGGC TACAAAGTGT AAGAATAAAC GGTTTTCTCT TGGTAAGTAA ATCCTGTACG ATGCAAAGTC   
  
  
+ ATTGGGGTTA CAGAAGAAAA CATATACGAT ATGACAAATG GTTGGTTCGT GACCACAAAA GAAAACTGGT   
  
  
+ TAATCTAGTT GACGTCGATG AAATTAAAGG ACATGCTTTA TAAGTCGGCA TTTGGTTCAA GTAGTTCTTT   
  
  
+ TTCTTCAGTC GTAAAACTAC TGATACGGAC AGGGGAAGCT AGAGTTAATG TCAAATTTGT TTTATTTCCC   
  
  
+ TAAATACACT TATATTTTCT GTATTATATA CAGTATTTTT GAAGTGGTAA TAACTTCTTT CCTAAACCCC   
  
  
+ ACGAGACCTA CTTGGAAGCA AAGGATGAGT ACGTTCCGTT CGGTTTCACG TACCTGTGTA CTATCGTTTG   
  
  
+ TTCGAACCTT TCCCTCTCAG GACTAATCCA GTGGACATCA CTGAAGGGTT CTGAAAAGAT CGAGGACTAA   
  
  
+ CCATTGTACG GTAAAGTATT ATGTGGGTTA GATGGTTCCT TCTTATGATG TATGGTGTTA GTTGTAGTAT   
  
  
+ TCATTGGTAT ATCCAGTAGT TCTACTTAGC CGACTCATCG TCCAAGTATA TCTAAAAAAA CGGTTCTTTT   
  
  
+ GGTTTGTATA TTTTGAACGG TTACACCTCT AATGACCTTC TTGTCTTTCT TTGTCTACGT ATAGTCCTGT   
  
  
+ TCAAGGTACG TGGTATATAG AGGTTATCTT ATAGGTTTTA AAATTGATGT TTCGAATACA GCTTGGTCTT   
  
  
+ TTTGAACAAT GTCCCCCAAG GTTTGAGACT TAATGCCGTG GAACTCCGGA GTTAAATAGG TTATAGTTCA   
  
  
+ TCTTAGTTTA GAGAGTCCGT ACCCCAACTT CAAAGTTCTT AAACTGACGT CTTGTTTATG TGAAGTTGGT   
  
  
+ ATAATTACTA AAAAAAAAGT ATATTCTTTG GTGTTAATAC TAAGACTGTT CTGATTTTAC ACCGAGTTCC   
  
  
+ AGTTTAGTCC CTGAGGTGTA GTCGACTACT AGTAGAAAGA TTTCTTAAGA CGAACGAGTC CCTTTATGAG   
  
  
+ GTTGTCGAGT AGGAAGCATT GGGTTCGTTT TAGGAGTGTG AAGGGTTACA ATTTAAACAA CCAACGTTAA   
  
  
+ TGAACCCTAA ATTTCTTCTT AACGACTGTT TTTTTTCTTT TTTTTAGTTC GTGCCTTTAA TTTTTTTTGT   
  
  
+ ATTAAATCGG ATTTGTTCTT TCCGTGTAA  

- GGGGGAAGAG CTCAAAGAGT TGAGAGAGGA ACTGAGAATG GAAGAGAAGG AAGGTTAATA TGGGTCAAGG   
  
  
- GAAGTGATAT CATGGAAGGT GAGAAGAGGG TGGATAGACC CACGGATATA TTTACAGAGA AAGAGAGAGA   
  
  
- GAAAATATGG TGCTTTGGCT TGTGATGTGG AGATGGAGGA TGGAGAGACT AGTCTGAGTT TCATGGGTAA   
  
  
- ACCCAACTAA ATCATACCTA CTTCTGTTCA CCTGCCCTTC AGTGAAAAGT GTACGTCCTA TATATGCATC   
  
  
- GTTATCACCG ATGTTTCACA TTCTTATTTG CCAAAAGAGA ACCATTCATT TAGGACATGC TACGTTTCAG   
  
  
- TAACCCCAAT GTCTTCTTTT GTATATGCTA TACTGTTTAC CAACCAAGCA CTGGTGTTTT CTTTTGACCA   
  
  
- ATTAGATCAA CTGCAGCTAC TTTAATTTCC TGTACGAAAT ATTCAGCCGT AAACCAAGTT CATCAAGAAA   
  
  
- AAGAAGTCAG CATTTTGATG ACTATGCCTG TCCCCTTCGA TCTCAATTAC AGTTTAAACA AAATAAAGGG   
  
  
- ATTTATGTGA ATATAAAAGA CATAATATAT GTCATAAAAA CTTCACCATT ATTGAAGAAA GGATTTGGGG   
  
  
- TGCTCTGGAT GAACCTTCGT TTCCTACTCA TGCAAGGCAA GCCAAAGTGC ATGGACACAT GATAGCAAAC   
  
  
- AAGCTTGGAA AGGGAGAGTC CTGATTAGGT CACCTGTAGT GACTTCCCAA GACTTTTCTA GCTCCTGATT   
  
  
- GGTAACATGC CATTTCATAA TACACCCAAT CTACCAAGGA AGAATACTAC ATACCACAAT CAACATCATA   
  
  
- AGTAACCATA TAGGTCATCA AGATGAATCG GCTGAGTAGC AGGTTCATAT AGATTTTTTT GCCAAGAAAA   
  
  
- CCAAACATAT AAAACTTGCC AATGTGGAGA TTACTGGAAG AACAGAAAGA AACAGATGCA TATCAGGACA   
  
  
- AGTTCCATGC ACCATATATC TCCAATAGAA TATCCAAAAT TTTAACTACA AAGCTTATGT CGAACCAGAA   
  
  
- AAACTTGTTA CAGGGGGTTC CAAACTCTGA ATTACGGCAC CTTGAGGCCT CAATTTATCC AATATCAAGT   
  
  
- AGAATCAAAT CTCTCAGGCA TGGGGTTGAA GTTTCAAGAA TTTGACTGCA GAACAAATAC ACTTCAACCA   
  
  
- TATTAATGAT TTTTTTTTCA TATAAGAAAC CACAATTATG ATTCTGACAA GACTAAAATG TGGCTCAAGG   
  
  
- TCAAATCAGG GACTCCACAT CAGCTGATGA TCATCTTTCT AAAGAATTCT GCTTGCTCAG GGAAATACTC   
  
  
- CAACAGCTCA TCCTTCGTAA CCCAAGCAAA ATCCTCACAC TTCCCAATGT TAAATTTGTT GGTTGCAATT   
  
  
- ACTTGGGATT TAAAGAAGAA TTGCTGACAA AAAAAAGAAA AAAAATCAAG CACGGAAATT AAAAAAAACA   
  
  
- TAATTTAGCC TAAACAAGAA AGGCACATT

+     ACE

| Site Name | Organism | Position | Strand | Matrix score. | sequence | function |
| --- | --- | --- | --- | --- | --- | --- |
| ACE | Petroselinum crispum | 306 | - | 9 | AAAACGTTTA | cis-acting element involved in light responsiveness |

> 2018/04/13 10:10:12  
+ CCCCCTTCTC GAGTTTCTCA ACTCTCTCCT TGACTCTTAC CTTCTCTTCC TTCCAATTAT ACCCAGTTCC   
  
  
+ CTTCACTATA GTACCTTCCA CTCTTCTCCC ACCTATCTGG GTGCCTATAT AAATGTCTCT TTCTCTCTCT   
  
  
+ CTTTTATACC ACGAAACCGA ACACTACACC TCTACCTCCT ACCTCTCTGA TCAGACTCAA AGTACCCATT   
  
  
+ TGGGTTGATT TAGTATGGAT GAAGACAAGT GGACGGGAAG TCACTTTTCA CATGCAGGAT ATATACGTAG   
  
  
+ CAATAGTGGC TACAAAGTGT AAGAATAAAC GGTTTTCTCT TGGTAAGTAA ATCCTGTACG ATGCAAAGTC   
  
  
+ ATTGGGGTTA CAGAAGAAAA CATATACGAT ATGACAAATG GTTGGTTCGT GACCACAAAA GAAAACTGGT   
  
  
+ TAATCTAGTT GACGTCGATG AAATTAAAGG ACATGCTTTA TAAGTCGGCA TTTGGTTCAA GTAGTTCTTT   
  
  
+ TTCTTCAGTC GTAAAACTAC TGATACGGAC AGGGGAAGCT AGAGTTAATG TCAAATTTGT TTTATTTCCC   
  
  
+ TAAATACACT TATATTTTCT GTATTATATA CAGTATTTTT GAAGTGGTAA TAACTTCTTT CCTAAACCCC   
  
  
+ ACGAGACCTA CTTGGAAGCA AAGGATGAGT ACGTTCCGTT CGGTTTCACG TACCTGTGTA CTATCGTTTG   
  
  
+ TTCGAACCTT TCCCTCTCAG GACTAATCCA GTGGACATCA CTGAAGGGTT CTGAAAAGAT CGAGGACTAA   
  
  
+ CCATTGTACG GTAAAGTATT ATGTGGGTTA GATGGTTCCT TCTTATGATG TATGGTGTTA GTTGTAGTAT   
  
  
+ TCATTGGTAT ATCCAGTAGT TCTACTTAGC CGACTCATCG TCCAAGTATA TCTAAAAAAA CGGTTCTTTT   
  
  
+ GGTTTGTATA TTTTGAACGG TTACACCTCT AATGACCTTC TTGTCTTTCT TTGTCTACGT ATAGTCCTGT   
  
  
+ TCAAGGTACG TGGTATATAG AGGTTATCTT ATAGGTTTTA AAATTGATGT TTCGAATACA GCTTGGTCTT   
  
  
+ TTTGAACAAT GTCCCCCAAG GTTTGAGACT TAATGCCGTG GAACTCCGGA GTTAAATAGG TTATAGTTCA   
  
  
+ TCTTAGTTTA GAGAGTCCGT ACCCCAACTT CAAAGTTCTT AAACTGACGT CTTGTTTATG TGAAGTTGGT   
  
  
+ ATAATTACTA AAAAAAAAGT ATATTCTTTG GTGTTAATAC TAAGACTGTT CTGATTTTAC ACCGAGTTCC   
  
  
+ AGTTTAGTCC CTGAGGTGTA GTCGACTACT AGTAGAAAGA TTTCTTAAGA CGAACGAGTC CCTTTATGAG   
  
  
+ GTTGTCGAGT AGGAAGCATT GGGTTCGTTT TAGGAGTGTG AAGGGTTACA ATTTAAACAA CCAACGTTAA   
  
  
+ TGAACCCTAA ATTTCTTCTT AACGACTGTT TTTTTTCTTT TTTTTAGTTC GTGCCTTTAA TTTTTTTTGT   
  
  
+ ATTAAATCGG ATTTGTTCTT TCCGTGTAA  

- GGGGGAAGAG CTCAAAGAGT TGAGAGAGGA ACTGAGAATG GAAGAGAAGG AAGGTTAATA TGGGTCAAGG   
  
  
- GAAGTGATAT CATGGAAGGT GAGAAGAGGG TGGATAGACC CACGGATATA TTTACAGAGA AAGAGAGAGA   
  
  
- GAAAATATGG TGCTTTGGCT TGTGATGTGG AGATGGAGGA TGGAGAGACT AGTCTGAGTT TCATGGGTAA   
  
  
- ACCCAACTAA ATCATACCTA CTTCTGTTCA CCTGCCCTTC AGTGAAAAGT GTACGTCCTA TATATGCATC   
  
  
- GTTATCACCG ATGTTTCACA TTCTTATTTG CCAAAAGAGA ACCATTCATT TAGGACATGC TACGTTTCAG   
  
  
- TAACCCCAAT GTCTTCTTTT GTATATGCTA TACTGTTTAC CAACCAAGCA CTGGTGTTTT CTTTTGACCA   
  
  
- ATTAGATCAA CTGCAGCTAC TTTAATTTCC TGTACGAAAT ATTCAGCCGT AAACCAAGTT CATCAAGAAA   
  
  
- AAGAAGTCAG CATTTTGATG ACTATGCCTG TCCCCTTCGA TCTCAATTAC AGTTTAAACA AAATAAAGGG   
  
  
- ATTTATGTGA ATATAAAAGA CATAATATAT GTCATAAAAA CTTCACCATT ATTGAAGAAA GGATTTGGGG   
  
  
- TGCTCTGGAT GAACCTTCGT TTCCTACTCA TGCAAGGCAA GCCAAAGTGC ATGGACACAT GATAGCAAAC   
  
  
- AAGCTTGGAA AGGGAGAGTC CTGATTAGGT CACCTGTAGT GACTTCCCAA GACTTTTCTA GCTCCTGATT   
  
  
- GGTAACATGC CATTTCATAA TACACCCAAT CTACCAAGGA AGAATACTAC ATACCACAAT CAACATCATA   
  
  
- AGTAACCATA TAGGTCATCA AGATGAATCG GCTGAGTAGC AGGTTCATAT AGATTTTTTT GCCAAGAAAA   
  
  
- CCAAACATAT AAAACTTGCC AATGTGGAGA TTACTGGAAG AACAGAAAGA AACAGATGCA TATCAGGACA   
  
  
- AGTTCCATGC ACCATATATC TCCAATAGAA TATCCAAAAT TTTAACTACA AAGCTTATGT CGAACCAGAA   
  
  
- AAACTTGTTA CAGGGGGTTC CAAACTCTGA ATTACGGCAC CTTGAGGCCT CAATTTATCC AATATCAAGT   
  
  
- AGAATCAAAT CTCTCAGGCA TGGGGTTGAA GTTTCAAGAA TTTGACTGCA GAACAAATAC ACTTCAACCA   
  
  
- TATTAATGAT TTTTTTTTCA TATAAGAAAC CACAATTATG ATTCTGACAA GACTAAAATG TGGCTCAAGG   
  
  
- TCAAATCAGG GACTCCACAT CAGCTGATGA TCATCTTTCT AAAGAATTCT GCTTGCTCAG GGAAATACTC   
  
  
- CAACAGCTCA TCCTTCGTAA CCCAAGCAAA ATCCTCACAC TTCCCAATGT TAAATTTGTT GGTTGCAATT   
  
  
- ACTTGGGATT TAAAGAAGAA TTGCTGACAA AAAAAAGAAA AAAAATCAAG CACGGAAATT AAAAAAAACA   
  
  
- TAATTTAGCC TAAACAAGAA AGGCACATT

+     ARE

| Site Name | Organism | Position | Strand | Matrix score. | sequence | function |
| --- | --- | --- | --- | --- | --- | --- |
| ARE | Zea mays | 910 | + | 6 | TGGTTT | cis-acting regulatory element essential for the anaerobic induction |

> 2018/04/13 10:10:12  
+ CCCCCTTCTC GAGTTTCTCA ACTCTCTCCT TGACTCTTAC CTTCTCTTCC TTCCAATTAT ACCCAGTTCC   
  
  
+ CTTCACTATA GTACCTTCCA CTCTTCTCCC ACCTATCTGG GTGCCTATAT AAATGTCTCT TTCTCTCTCT   
  
  
+ CTTTTATACC ACGAAACCGA ACACTACACC TCTACCTCCT ACCTCTCTGA TCAGACTCAA AGTACCCATT   
  
  
+ TGGGTTGATT TAGTATGGAT GAAGACAAGT GGACGGGAAG TCACTTTTCA CATGCAGGAT ATATACGTAG   
  
  
+ CAATAGTGGC TACAAAGTGT AAGAATAAAC GGTTTTCTCT TGGTAAGTAA ATCCTGTACG ATGCAAAGTC   
  
  
+ ATTGGGGTTA CAGAAGAAAA CATATACGAT ATGACAAATG GTTGGTTCGT GACCACAAAA GAAAACTGGT   
  
  
+ TAATCTAGTT GACGTCGATG AAATTAAAGG ACATGCTTTA TAAGTCGGCA TTTGGTTCAA GTAGTTCTTT   
  
  
+ TTCTTCAGTC GTAAAACTAC TGATACGGAC AGGGGAAGCT AGAGTTAATG TCAAATTTGT TTTATTTCCC   
  
  
+ TAAATACACT TATATTTTCT GTATTATATA CAGTATTTTT GAAGTGGTAA TAACTTCTTT CCTAAACCCC   
  
  
+ ACGAGACCTA CTTGGAAGCA AAGGATGAGT ACGTTCCGTT CGGTTTCACG TACCTGTGTA CTATCGTTTG   
  
  
+ TTCGAACCTT TCCCTCTCAG GACTAATCCA GTGGACATCA CTGAAGGGTT CTGAAAAGAT CGAGGACTAA   
  
  
+ CCATTGTACG GTAAAGTATT ATGTGGGTTA GATGGTTCCT TCTTATGATG TATGGTGTTA GTTGTAGTAT   
  
  
+ TCATTGGTAT ATCCAGTAGT TCTACTTAGC CGACTCATCG TCCAAGTATA TCTAAAAAAA CGGTTCTTTT   
  
  
+ GGTTTGTATA TTTTGAACGG TTACACCTCT AATGACCTTC TTGTCTTTCT TTGTCTACGT ATAGTCCTGT   
  
  
+ TCAAGGTACG TGGTATATAG AGGTTATCTT ATAGGTTTTA AAATTGATGT TTCGAATACA GCTTGGTCTT   
  
  
+ TTTGAACAAT GTCCCCCAAG GTTTGAGACT TAATGCCGTG GAACTCCGGA GTTAAATAGG TTATAGTTCA   
  
  
+ TCTTAGTTTA GAGAGTCCGT ACCCCAACTT CAAAGTTCTT AAACTGACGT CTTGTTTATG TGAAGTTGGT   
  
  
+ ATAATTACTA AAAAAAAAGT ATATTCTTTG GTGTTAATAC TAAGACTGTT CTGATTTTAC ACCGAGTTCC   
  
  
+ AGTTTAGTCC CTGAGGTGTA GTCGACTACT AGTAGAAAGA TTTCTTAAGA CGAACGAGTC CCTTTATGAG   
  
  
+ GTTGTCGAGT AGGAAGCATT GGGTTCGTTT TAGGAGTGTG AAGGGTTACA ATTTAAACAA CCAACGTTAA   
  
  
+ TGAACCCTAA ATTTCTTCTT AACGACTGTT TTTTTTCTTT TTTTTAGTTC GTGCCTTTAA TTTTTTTTGT   
  
  
+ ATTAAATCGG ATTTGTTCTT TCCGTGTAA  

- GGGGGAAGAG CTCAAAGAGT TGAGAGAGGA ACTGAGAATG GAAGAGAAGG AAGGTTAATA TGGGTCAAGG   
  
  
- GAAGTGATAT CATGGAAGGT GAGAAGAGGG TGGATAGACC CACGGATATA TTTACAGAGA AAGAGAGAGA   
  
  
- GAAAATATGG TGCTTTGGCT TGTGATGTGG AGATGGAGGA TGGAGAGACT AGTCTGAGTT TCATGGGTAA   
  
  
- ACCCAACTAA ATCATACCTA CTTCTGTTCA CCTGCCCTTC AGTGAAAAGT GTACGTCCTA TATATGCATC   
  
  
- GTTATCACCG ATGTTTCACA TTCTTATTTG CCAAAAGAGA ACCATTCATT TAGGACATGC TACGTTTCAG   
  
  
- TAACCCCAAT GTCTTCTTTT GTATATGCTA TACTGTTTAC CAACCAAGCA CTGGTGTTTT CTTTTGACCA   
  
  
- ATTAGATCAA CTGCAGCTAC TTTAATTTCC TGTACGAAAT ATTCAGCCGT AAACCAAGTT CATCAAGAAA   
  
  
- AAGAAGTCAG CATTTTGATG ACTATGCCTG TCCCCTTCGA TCTCAATTAC AGTTTAAACA AAATAAAGGG   
  
  
- ATTTATGTGA ATATAAAAGA CATAATATAT GTCATAAAAA CTTCACCATT ATTGAAGAAA GGATTTGGGG   
  
  
- TGCTCTGGAT GAACCTTCGT TTCCTACTCA TGCAAGGCAA GCCAAAGTGC ATGGACACAT GATAGCAAAC   
  
  
- AAGCTTGGAA AGGGAGAGTC CTGATTAGGT CACCTGTAGT GACTTCCCAA GACTTTTCTA GCTCCTGATT   
  
  
- GGTAACATGC CATTTCATAA TACACCCAAT CTACCAAGGA AGAATACTAC ATACCACAAT CAACATCATA   
  
  
- AGTAACCATA TAGGTCATCA AGATGAATCG GCTGAGTAGC AGGTTCATAT AGATTTTTTT GCCAAGAAAA   
  
  
- CCAAACATAT AAAACTTGCC AATGTGGAGA TTACTGGAAG AACAGAAAGA AACAGATGCA TATCAGGACA   
  
  
- AGTTCCATGC ACCATATATC TCCAATAGAA TATCCAAAAT TTTAACTACA AAGCTTATGT CGAACCAGAA   
  
  
- AAACTTGTTA CAGGGGGTTC CAAACTCTGA ATTACGGCAC CTTGAGGCCT CAATTTATCC AATATCAAGT   
  
  
- AGAATCAAAT CTCTCAGGCA TGGGGTTGAA GTTTCAAGAA TTTGACTGCA GAACAAATAC ACTTCAACCA   
  
  
- TATTAATGAT TTTTTTTTCA TATAAGAAAC CACAATTATG ATTCTGACAA GACTAAAATG TGGCTCAAGG   
  
  
- TCAAATCAGG GACTCCACAT CAGCTGATGA TCATCTTTCT AAAGAATTCT GCTTGCTCAG GGAAATACTC   
  
  
- CAACAGCTCA TCCTTCGTAA CCCAAGCAAA ATCCTCACAC TTCCCAATGT TAAATTTGTT GGTTGCAATT   
  
  
- ACTTGGGATT TAAAGAAGAA TTGCTGACAA AAAAAAGAAA AAAAATCAAG CACGGAAATT AAAAAAAACA   
  
  
- TAATTTAGCC TAAACAAGAA AGGCACATT

+     Box II

| Site Name | Organism | Position | Strand | Matrix score. | sequence | function |
| --- | --- | --- | --- | --- | --- | --- |
| Box II | Solanum tuberosum | 605 | + | 9 | TGGTAATAA | part of a light responsive element |

> 2018/04/13 10:10:12  
+ CCCCCTTCTC GAGTTTCTCA ACTCTCTCCT TGACTCTTAC CTTCTCTTCC TTCCAATTAT ACCCAGTTCC   
  
  
+ CTTCACTATA GTACCTTCCA CTCTTCTCCC ACCTATCTGG GTGCCTATAT AAATGTCTCT TTCTCTCTCT   
  
  
+ CTTTTATACC ACGAAACCGA ACACTACACC TCTACCTCCT ACCTCTCTGA TCAGACTCAA AGTACCCATT   
  
  
+ TGGGTTGATT TAGTATGGAT GAAGACAAGT GGACGGGAAG TCACTTTTCA CATGCAGGAT ATATACGTAG   
  
  
+ CAATAGTGGC TACAAAGTGT AAGAATAAAC GGTTTTCTCT TGGTAAGTAA ATCCTGTACG ATGCAAAGTC   
  
  
+ ATTGGGGTTA CAGAAGAAAA CATATACGAT ATGACAAATG GTTGGTTCGT GACCACAAAA GAAAACTGGT   
  
  
+ TAATCTAGTT GACGTCGATG AAATTAAAGG ACATGCTTTA TAAGTCGGCA TTTGGTTCAA GTAGTTCTTT   
  
  
+ TTCTTCAGTC GTAAAACTAC TGATACGGAC AGGGGAAGCT AGAGTTAATG TCAAATTTGT TTTATTTCCC   
  
  
+ TAAATACACT TATATTTTCT GTATTATATA CAGTATTTTT GAAGTGGTAA TAACTTCTTT CCTAAACCCC   
  
  
+ ACGAGACCTA CTTGGAAGCA AAGGATGAGT ACGTTCCGTT CGGTTTCACG TACCTGTGTA CTATCGTTTG   
  
  
+ TTCGAACCTT TCCCTCTCAG GACTAATCCA GTGGACATCA CTGAAGGGTT CTGAAAAGAT CGAGGACTAA   
  
  
+ CCATTGTACG GTAAAGTATT ATGTGGGTTA GATGGTTCCT TCTTATGATG TATGGTGTTA GTTGTAGTAT   
  
  
+ TCATTGGTAT ATCCAGTAGT TCTACTTAGC CGACTCATCG TCCAAGTATA TCTAAAAAAA CGGTTCTTTT   
  
  
+ GGTTTGTATA TTTTGAACGG TTACACCTCT AATGACCTTC TTGTCTTTCT TTGTCTACGT ATAGTCCTGT   
  
  
+ TCAAGGTACG TGGTATATAG AGGTTATCTT ATAGGTTTTA AAATTGATGT TTCGAATACA GCTTGGTCTT   
  
  
+ TTTGAACAAT GTCCCCCAAG GTTTGAGACT TAATGCCGTG GAACTCCGGA GTTAAATAGG TTATAGTTCA   
  
  
+ TCTTAGTTTA GAGAGTCCGT ACCCCAACTT CAAAGTTCTT AAACTGACGT CTTGTTTATG TGAAGTTGGT   
  
  
+ ATAATTACTA AAAAAAAAGT ATATTCTTTG GTGTTAATAC TAAGACTGTT CTGATTTTAC ACCGAGTTCC   
  
  
+ AGTTTAGTCC CTGAGGTGTA GTCGACTACT AGTAGAAAGA TTTCTTAAGA CGAACGAGTC CCTTTATGAG   
  
  
+ GTTGTCGAGT AGGAAGCATT GGGTTCGTTT TAGGAGTGTG AAGGGTTACA ATTTAAACAA CCAACGTTAA   
  
  
+ TGAACCCTAA ATTTCTTCTT AACGACTGTT TTTTTTCTTT TTTTTAGTTC GTGCCTTTAA TTTTTTTTGT   
  
  
+ ATTAAATCGG ATTTGTTCTT TCCGTGTAA  

- GGGGGAAGAG CTCAAAGAGT TGAGAGAGGA ACTGAGAATG GAAGAGAAGG AAGGTTAATA TGGGTCAAGG   
  
  
- GAAGTGATAT CATGGAAGGT GAGAAGAGGG TGGATAGACC CACGGATATA TTTACAGAGA AAGAGAGAGA   
  
  
- GAAAATATGG TGCTTTGGCT TGTGATGTGG AGATGGAGGA TGGAGAGACT AGTCTGAGTT TCATGGGTAA   
  
  
- ACCCAACTAA ATCATACCTA CTTCTGTTCA CCTGCCCTTC AGTGAAAAGT GTACGTCCTA TATATGCATC   
  
  
- GTTATCACCG ATGTTTCACA TTCTTATTTG CCAAAAGAGA ACCATTCATT TAGGACATGC TACGTTTCAG   
  
  
- TAACCCCAAT GTCTTCTTTT GTATATGCTA TACTGTTTAC CAACCAAGCA CTGGTGTTTT CTTTTGACCA   
  
  
- ATTAGATCAA CTGCAGCTAC TTTAATTTCC TGTACGAAAT ATTCAGCCGT AAACCAAGTT CATCAAGAAA   
  
  
- AAGAAGTCAG CATTTTGATG ACTATGCCTG TCCCCTTCGA TCTCAATTAC AGTTTAAACA AAATAAAGGG   
  
  
- ATTTATGTGA ATATAAAAGA CATAATATAT GTCATAAAAA CTTCACCATT ATTGAAGAAA GGATTTGGGG   
  
  
- TGCTCTGGAT GAACCTTCGT TTCCTACTCA TGCAAGGCAA GCCAAAGTGC ATGGACACAT GATAGCAAAC   
  
  
- AAGCTTGGAA AGGGAGAGTC CTGATTAGGT CACCTGTAGT GACTTCCCAA GACTTTTCTA GCTCCTGATT   
  
  
- GGTAACATGC CATTTCATAA TACACCCAAT CTACCAAGGA AGAATACTAC ATACCACAAT CAACATCATA   
  
  
- AGTAACCATA TAGGTCATCA AGATGAATCG GCTGAGTAGC AGGTTCATAT AGATTTTTTT GCCAAGAAAA   
  
  
- CCAAACATAT AAAACTTGCC AATGTGGAGA TTACTGGAAG AACAGAAAGA AACAGATGCA TATCAGGACA   
  
  
- AGTTCCATGC ACCATATATC TCCAATAGAA TATCCAAAAT TTTAACTACA AAGCTTATGT CGAACCAGAA   
  
  
- AAACTTGTTA CAGGGGGTTC CAAACTCTGA ATTACGGCAC CTTGAGGCCT CAATTTATCC AATATCAAGT   
  
  
- AGAATCAAAT CTCTCAGGCA TGGGGTTGAA GTTTCAAGAA TTTGACTGCA GAACAAATAC ACTTCAACCA   
  
  
- TATTAATGAT TTTTTTTTCA TATAAGAAAC CACAATTATG ATTCTGACAA GACTAAAATG TGGCTCAAGG   
  
  
- TCAAATCAGG GACTCCACAT CAGCTGATGA TCATCTTTCT AAAGAATTCT GCTTGCTCAG GGAAATACTC   
  
  
- CAACAGCTCA TCCTTCGTAA CCCAAGCAAA ATCCTCACAC TTCCCAATGT TAAATTTGTT GGTTGCAATT   
  
  
- ACTTGGGATT TAAAGAAGAA TTGCTGACAA AAAAAAGAAA AAAAATCAAG CACGGAAATT AAAAAAAACA   
  
  
- TAATTTAGCC TAAACAAGAA AGGCACATT

+     CAAT-box

| Site Name | Organism | Position | Strand | Matrix score. | sequence | function |
| --- | --- | --- | --- | --- | --- | --- |
| CAAT-box | Brassica rapa | 545 | - | 5 | CAAAT | common cis-acting element in promoter and enhancer regions |
| CAAT-box | Hordeum vulgare | 1023 | - | 4 | CAAT | common cis-acting element in promoter and enhancer regions |
| CAAT-box | Glycine max | 1022 | - | 5 | CAATT | common cis-acting element in promoter and enhancer regions |
| CAAT-box | Hordeum vulgare | 1057 | + | 4 | CAAT | common cis-acting element in promoter and enhancer regions |
| CAAT-box | Arabidopsis thaliana | 53 | + | 5 | CCAAT | common cis-acting element in promoter and enhancer regions |
| CAAT-box | Arabidopsis thaliana | 351 | - | 5 | CCAAT | common cis-acting element in promoter and enhancer regions |
| CAAT-box | Arabidopsis thaliana | 843 | - | 5 | CCAAT | common cis-acting element in promoter and enhancer regions |
| CAAT-box | Brassica rapa | 1481 | - | 5 | CAAAT | common cis-acting element in promoter and enhancer regions |
| CAAT-box | Hordeum vulgare | 281 | + | 4 | CAAT | common cis-acting element in promoter and enhancer regions |
| CAAT-box | Brassica rapa | 385 | + | 5 | CAAAT | common cis-acting element in promoter and enhancer regions |
| CAAT-box | Arabidopsis thaliana | 1348 | - | 5 | CCAAT | common cis-acting element in promoter and enhancer regions |
| CAAT-box | Glycine max | 54 | + | 5 | CAATT | common cis-acting element in promoter and enhancer regions |
| CAAT-box | Hordeum vulgare | 773 | - | 4 | CAAT | common cis-acting element in promoter and enhancer regions |
| CAAT-box | Glycine max | 1379 | + | 5 | CAATT | common cis-acting element in promoter and enhancer regions |
| CAAT-box | Brassica rapa | 208 | - | 5 | CAAAT | common cis-acting element in promoter and enhancer regions |
| CAAT-box | Brassica rapa | 542 | + | 5 | CAAAT | common cis-acting element in promoter and enhancer regions |
| CAAT-box | Brassica rapa | 470 | - | 5 | CAAAT | common cis-acting element in promoter and enhancer regions |

> 2018/04/13 10:10:12  
+ CCCCCTTCTC GAGTTTCTCA ACTCTCTCCT TGACTCTTAC CTTCTCTTCC TTCCAATTAT ACCCAGTTCC   
  
  
+ CTTCACTATA GTACCTTCCA CTCTTCTCCC ACCTATCTGG GTGCCTATAT AAATGTCTCT TTCTCTCTCT   
  
  
+ CTTTTATACC ACGAAACCGA ACACTACACC TCTACCTCCT ACCTCTCTGA TCAGACTCAA AGTACCCATT   
  
  
+ TGGGTTGATT TAGTATGGAT GAAGACAAGT GGACGGGAAG TCACTTTTCA CATGCAGGAT ATATACGTAG   
  
  
+ CAATAGTGGC TACAAAGTGT AAGAATAAAC GGTTTTCTCT TGGTAAGTAA ATCCTGTACG ATGCAAAGTC   
  
  
+ ATTGGGGTTA CAGAAGAAAA CATATACGAT ATGACAAATG GTTGGTTCGT GACCACAAAA GAAAACTGGT   
  
  
+ TAATCTAGTT GACGTCGATG AAATTAAAGG ACATGCTTTA TAAGTCGGCA TTTGGTTCAA GTAGTTCTTT   
  
  
+ TTCTTCAGTC GTAAAACTAC TGATACGGAC AGGGGAAGCT AGAGTTAATG TCAAATTTGT TTTATTTCCC   
  
  
+ TAAATACACT TATATTTTCT GTATTATATA CAGTATTTTT GAAGTGGTAA TAACTTCTTT CCTAAACCCC   
  
  
+ ACGAGACCTA CTTGGAAGCA AAGGATGAGT ACGTTCCGTT CGGTTTCACG TACCTGTGTA CTATCGTTTG   
  
  
+ TTCGAACCTT TCCCTCTCAG GACTAATCCA GTGGACATCA CTGAAGGGTT CTGAAAAGAT CGAGGACTAA   
  
  
+ CCATTGTACG GTAAAGTATT ATGTGGGTTA GATGGTTCCT TCTTATGATG TATGGTGTTA GTTGTAGTAT   
  
  
+ TCATTGGTAT ATCCAGTAGT TCTACTTAGC CGACTCATCG TCCAAGTATA TCTAAAAAAA CGGTTCTTTT   
  
  
+ GGTTTGTATA TTTTGAACGG TTACACCTCT AATGACCTTC TTGTCTTTCT TTGTCTACGT ATAGTCCTGT   
  
  
+ TCAAGGTACG TGGTATATAG AGGTTATCTT ATAGGTTTTA AAATTGATGT TTCGAATACA GCTTGGTCTT   
  
  
+ TTTGAACAAT GTCCCCCAAG GTTTGAGACT TAATGCCGTG GAACTCCGGA GTTAAATAGG TTATAGTTCA   
  
  
+ TCTTAGTTTA GAGAGTCCGT ACCCCAACTT CAAAGTTCTT AAACTGACGT CTTGTTTATG TGAAGTTGGT   
  
  
+ ATAATTACTA AAAAAAAAGT ATATTCTTTG GTGTTAATAC TAAGACTGTT CTGATTTTAC ACCGAGTTCC   
  
  
+ AGTTTAGTCC CTGAGGTGTA GTCGACTACT AGTAGAAAGA TTTCTTAAGA CGAACGAGTC CCTTTATGAG   
  
  
+ GTTGTCGAGT AGGAAGCATT GGGTTCGTTT TAGGAGTGTG AAGGGTTACA ATTTAAACAA CCAACGTTAA   
  
  
+ TGAACCCTAA ATTTCTTCTT AACGACTGTT TTTTTTCTTT TTTTTAGTTC GTGCCTTTAA TTTTTTTTGT   
  
  
+ ATTAAATCGG ATTTGTTCTT TCCGTGTAA  

- GGGGGAAGAG CTCAAAGAGT TGAGAGAGGA ACTGAGAATG GAAGAGAAGG AAGGTTAATA TGGGTCAAGG   
  
  
- GAAGTGATAT CATGGAAGGT GAGAAGAGGG TGGATAGACC CACGGATATA TTTACAGAGA AAGAGAGAGA   
  
  
- GAAAATATGG TGCTTTGGCT TGTGATGTGG AGATGGAGGA TGGAGAGACT AGTCTGAGTT TCATGGGTAA   
  
  
- ACCCAACTAA ATCATACCTA CTTCTGTTCA CCTGCCCTTC AGTGAAAAGT GTACGTCCTA TATATGCATC   
  
  
- GTTATCACCG ATGTTTCACA TTCTTATTTG CCAAAAGAGA ACCATTCATT TAGGACATGC TACGTTTCAG   
  
  
- TAACCCCAAT GTCTTCTTTT GTATATGCTA TACTGTTTAC CAACCAAGCA CTGGTGTTTT CTTTTGACCA   
  
  
- ATTAGATCAA CTGCAGCTAC TTTAATTTCC TGTACGAAAT ATTCAGCCGT AAACCAAGTT CATCAAGAAA   
  
  
- AAGAAGTCAG CATTTTGATG ACTATGCCTG TCCCCTTCGA TCTCAATTAC AGTTTAAACA AAATAAAGGG   
  
  
- ATTTATGTGA ATATAAAAGA CATAATATAT GTCATAAAAA CTTCACCATT ATTGAAGAAA GGATTTGGGG   
  
  
- TGCTCTGGAT GAACCTTCGT TTCCTACTCA TGCAAGGCAA GCCAAAGTGC ATGGACACAT GATAGCAAAC   
  
  
- AAGCTTGGAA AGGGAGAGTC CTGATTAGGT CACCTGTAGT GACTTCCCAA GACTTTTCTA GCTCCTGATT   
  
  
- GGTAACATGC CATTTCATAA TACACCCAAT CTACCAAGGA AGAATACTAC ATACCACAAT CAACATCATA   
  
  
- AGTAACCATA TAGGTCATCA AGATGAATCG GCTGAGTAGC AGGTTCATAT AGATTTTTTT GCCAAGAAAA   
  
  
- CCAAACATAT AAAACTTGCC AATGTGGAGA TTACTGGAAG AACAGAAAGA AACAGATGCA TATCAGGACA   
  
  
- AGTTCCATGC ACCATATATC TCCAATAGAA TATCCAAAAT TTTAACTACA AAGCTTATGT CGAACCAGAA   
  
  
- AAACTTGTTA CAGGGGGTTC CAAACTCTGA ATTACGGCAC CTTGAGGCCT CAATTTATCC AATATCAAGT   
  
  
- AGAATCAAAT CTCTCAGGCA TGGGGTTGAA GTTTCAAGAA TTTGACTGCA GAACAAATAC ACTTCAACCA   
  
  
- TATTAATGAT TTTTTTTTCA TATAAGAAAC CACAATTATG ATTCTGACAA GACTAAAATG TGGCTCAAGG   
  
  
- TCAAATCAGG GACTCCACAT CAGCTGATGA TCATCTTTCT AAAGAATTCT GCTTGCTCAG GGAAATACTC   
  
  
- CAACAGCTCA TCCTTCGTAA CCCAAGCAAA ATCCTCACAC TTCCCAATGT TAAATTTGTT GGTTGCAATT   
  
  
- ACTTGGGATT TAAAGAAGAA TTGCTGACAA AAAAAAGAAA AAAAATCAAG CACGGAAATT AAAAAAAACA   
  
  
- TAATTTAGCC TAAACAAGAA AGGCACATT

+     CAT-box

| Site Name | Organism | Position | Strand | Matrix score. | sequence | function |
| --- | --- | --- | --- | --- | --- | --- |
| CAT-box | Arabidopsis thaliana | 285 | - | 6 | GCCACT | cis-acting regulatory element related to meristem expression |

> 2018/04/13 10:10:12  
+ CCCCCTTCTC GAGTTTCTCA ACTCTCTCCT TGACTCTTAC CTTCTCTTCC TTCCAATTAT ACCCAGTTCC   
  
  
+ CTTCACTATA GTACCTTCCA CTCTTCTCCC ACCTATCTGG GTGCCTATAT AAATGTCTCT TTCTCTCTCT   
  
  
+ CTTTTATACC ACGAAACCGA ACACTACACC TCTACCTCCT ACCTCTCTGA TCAGACTCAA AGTACCCATT   
  
  
+ TGGGTTGATT TAGTATGGAT GAAGACAAGT GGACGGGAAG TCACTTTTCA CATGCAGGAT ATATACGTAG   
  
  
+ CAATAGTGGC TACAAAGTGT AAGAATAAAC GGTTTTCTCT TGGTAAGTAA ATCCTGTACG ATGCAAAGTC   
  
  
+ ATTGGGGTTA CAGAAGAAAA CATATACGAT ATGACAAATG GTTGGTTCGT GACCACAAAA GAAAACTGGT   
  
  
+ TAATCTAGTT GACGTCGATG AAATTAAAGG ACATGCTTTA TAAGTCGGCA TTTGGTTCAA GTAGTTCTTT   
  
  
+ TTCTTCAGTC GTAAAACTAC TGATACGGAC AGGGGAAGCT AGAGTTAATG TCAAATTTGT TTTATTTCCC   
  
  
+ TAAATACACT TATATTTTCT GTATTATATA CAGTATTTTT GAAGTGGTAA TAACTTCTTT CCTAAACCCC   
  
  
+ ACGAGACCTA CTTGGAAGCA AAGGATGAGT ACGTTCCGTT CGGTTTCACG TACCTGTGTA CTATCGTTTG   
  
  
+ TTCGAACCTT TCCCTCTCAG GACTAATCCA GTGGACATCA CTGAAGGGTT CTGAAAAGAT CGAGGACTAA   
  
  
+ CCATTGTACG GTAAAGTATT ATGTGGGTTA GATGGTTCCT TCTTATGATG TATGGTGTTA GTTGTAGTAT   
  
  
+ TCATTGGTAT ATCCAGTAGT TCTACTTAGC CGACTCATCG TCCAAGTATA TCTAAAAAAA CGGTTCTTTT   
  
  
+ GGTTTGTATA TTTTGAACGG TTACACCTCT AATGACCTTC TTGTCTTTCT TTGTCTACGT ATAGTCCTGT   
  
  
+ TCAAGGTACG TGGTATATAG AGGTTATCTT ATAGGTTTTA AAATTGATGT TTCGAATACA GCTTGGTCTT   
  
  
+ TTTGAACAAT GTCCCCCAAG GTTTGAGACT TAATGCCGTG GAACTCCGGA GTTAAATAGG TTATAGTTCA   
  
  
+ TCTTAGTTTA GAGAGTCCGT ACCCCAACTT CAAAGTTCTT AAACTGACGT CTTGTTTATG TGAAGTTGGT   
  
  
+ ATAATTACTA AAAAAAAAGT ATATTCTTTG GTGTTAATAC TAAGACTGTT CTGATTTTAC ACCGAGTTCC   
  
  
+ AGTTTAGTCC CTGAGGTGTA GTCGACTACT AGTAGAAAGA TTTCTTAAGA CGAACGAGTC CCTTTATGAG   
  
  
+ GTTGTCGAGT AGGAAGCATT GGGTTCGTTT TAGGAGTGTG AAGGGTTACA ATTTAAACAA CCAACGTTAA   
  
  
+ TGAACCCTAA ATTTCTTCTT AACGACTGTT TTTTTTCTTT TTTTTAGTTC GTGCCTTTAA TTTTTTTTGT   
  
  
+ ATTAAATCGG ATTTGTTCTT TCCGTGTAA  

- GGGGGAAGAG CTCAAAGAGT TGAGAGAGGA ACTGAGAATG GAAGAGAAGG AAGGTTAATA TGGGTCAAGG   
  
  
- GAAGTGATAT CATGGAAGGT GAGAAGAGGG TGGATAGACC CACGGATATA TTTACAGAGA AAGAGAGAGA   
  
  
- GAAAATATGG TGCTTTGGCT TGTGATGTGG AGATGGAGGA TGGAGAGACT AGTCTGAGTT TCATGGGTAA   
  
  
- ACCCAACTAA ATCATACCTA CTTCTGTTCA CCTGCCCTTC AGTGAAAAGT GTACGTCCTA TATATGCATC   
  
  
- GTTATCACCG ATGTTTCACA TTCTTATTTG CCAAAAGAGA ACCATTCATT TAGGACATGC TACGTTTCAG   
  
  
- TAACCCCAAT GTCTTCTTTT GTATATGCTA TACTGTTTAC CAACCAAGCA CTGGTGTTTT CTTTTGACCA   
  
  
- ATTAGATCAA CTGCAGCTAC TTTAATTTCC TGTACGAAAT ATTCAGCCGT AAACCAAGTT CATCAAGAAA   
  
  
- AAGAAGTCAG CATTTTGATG ACTATGCCTG TCCCCTTCGA TCTCAATTAC AGTTTAAACA AAATAAAGGG   
  
  
- ATTTATGTGA ATATAAAAGA CATAATATAT GTCATAAAAA CTTCACCATT ATTGAAGAAA GGATTTGGGG   
  
  
- TGCTCTGGAT GAACCTTCGT TTCCTACTCA TGCAAGGCAA GCCAAAGTGC ATGGACACAT GATAGCAAAC   
  
  
- AAGCTTGGAA AGGGAGAGTC CTGATTAGGT CACCTGTAGT GACTTCCCAA GACTTTTCTA GCTCCTGATT   
  
  
- GGTAACATGC CATTTCATAA TACACCCAAT CTACCAAGGA AGAATACTAC ATACCACAAT CAACATCATA   
  
  
- AGTAACCATA TAGGTCATCA AGATGAATCG GCTGAGTAGC AGGTTCATAT AGATTTTTTT GCCAAGAAAA   
  
  
- CCAAACATAT AAAACTTGCC AATGTGGAGA TTACTGGAAG AACAGAAAGA AACAGATGCA TATCAGGACA   
  
  
- AGTTCCATGC ACCATATATC TCCAATAGAA TATCCAAAAT TTTAACTACA AAGCTTATGT CGAACCAGAA   
  
  
- AAACTTGTTA CAGGGGGTTC CAAACTCTGA ATTACGGCAC CTTGAGGCCT CAATTTATCC AATATCAAGT   
  
  
- AGAATCAAAT CTCTCAGGCA TGGGGTTGAA GTTTCAAGAA TTTGACTGCA GAACAAATAC ACTTCAACCA   
  
  
- TATTAATGAT TTTTTTTTCA TATAAGAAAC CACAATTATG ATTCTGACAA GACTAAAATG TGGCTCAAGG   
  
  
- TCAAATCAGG GACTCCACAT CAGCTGATGA TCATCTTTCT AAAGAATTCT GCTTGCTCAG GGAAATACTC   
  
  
- CAACAGCTCA TCCTTCGTAA CCCAAGCAAA ATCCTCACAC TTCCCAATGT TAAATTTGTT GGTTGCAATT   
  
  
- ACTTGGGATT TAAAGAAGAA TTGCTGACAA AAAAAAGAAA AAAAATCAAG CACGGAAATT AAAAAAAACA   
  
  
- TAATTTAGCC TAAACAAGAA AGGCACATT

+     CCGTCC-box

| Site Name | Organism | Position | Strand | Matrix score. | sequence | function |
| --- | --- | --- | --- | --- | --- | --- |
| CCGTCC-box | Arabidopsis thaliana | 241 | - | 6 | CCGTCC | cis-acting regulatory element related to meristem specific activation |

> 2018/04/13 10:10:12  
+ CCCCCTTCTC GAGTTTCTCA ACTCTCTCCT TGACTCTTAC CTTCTCTTCC TTCCAATTAT ACCCAGTTCC   
  
  
+ CTTCACTATA GTACCTTCCA CTCTTCTCCC ACCTATCTGG GTGCCTATAT AAATGTCTCT TTCTCTCTCT   
  
  
+ CTTTTATACC ACGAAACCGA ACACTACACC TCTACCTCCT ACCTCTCTGA TCAGACTCAA AGTACCCATT   
  
  
+ TGGGTTGATT TAGTATGGAT GAAGACAAGT GGACGGGAAG TCACTTTTCA CATGCAGGAT ATATACGTAG   
  
  
+ CAATAGTGGC TACAAAGTGT AAGAATAAAC GGTTTTCTCT TGGTAAGTAA ATCCTGTACG ATGCAAAGTC   
  
  
+ ATTGGGGTTA CAGAAGAAAA CATATACGAT ATGACAAATG GTTGGTTCGT GACCACAAAA GAAAACTGGT   
  
  
+ TAATCTAGTT GACGTCGATG AAATTAAAGG ACATGCTTTA TAAGTCGGCA TTTGGTTCAA GTAGTTCTTT   
  
  
+ TTCTTCAGTC GTAAAACTAC TGATACGGAC AGGGGAAGCT AGAGTTAATG TCAAATTTGT TTTATTTCCC   
  
  
+ TAAATACACT TATATTTTCT GTATTATATA CAGTATTTTT GAAGTGGTAA TAACTTCTTT CCTAAACCCC   
  
  
+ ACGAGACCTA CTTGGAAGCA AAGGATGAGT ACGTTCCGTT CGGTTTCACG TACCTGTGTA CTATCGTTTG   
  
  
+ TTCGAACCTT TCCCTCTCAG GACTAATCCA GTGGACATCA CTGAAGGGTT CTGAAAAGAT CGAGGACTAA   
  
  
+ CCATTGTACG GTAAAGTATT ATGTGGGTTA GATGGTTCCT TCTTATGATG TATGGTGTTA GTTGTAGTAT   
  
  
+ TCATTGGTAT ATCCAGTAGT TCTACTTAGC CGACTCATCG TCCAAGTATA TCTAAAAAAA CGGTTCTTTT   
  
  
+ GGTTTGTATA TTTTGAACGG TTACACCTCT AATGACCTTC TTGTCTTTCT TTGTCTACGT ATAGTCCTGT   
  
  
+ TCAAGGTACG TGGTATATAG AGGTTATCTT ATAGGTTTTA AAATTGATGT TTCGAATACA GCTTGGTCTT   
  
  
+ TTTGAACAAT GTCCCCCAAG GTTTGAGACT TAATGCCGTG GAACTCCGGA GTTAAATAGG TTATAGTTCA   
  
  
+ TCTTAGTTTA GAGAGTCCGT ACCCCAACTT CAAAGTTCTT AAACTGACGT CTTGTTTATG TGAAGTTGGT   
  
  
+ ATAATTACTA AAAAAAAAGT ATATTCTTTG GTGTTAATAC TAAGACTGTT CTGATTTTAC ACCGAGTTCC   
  
  
+ AGTTTAGTCC CTGAGGTGTA GTCGACTACT AGTAGAAAGA TTTCTTAAGA CGAACGAGTC CCTTTATGAG   
  
  
+ GTTGTCGAGT AGGAAGCATT GGGTTCGTTT TAGGAGTGTG AAGGGTTACA ATTTAAACAA CCAACGTTAA   
  
  
+ TGAACCCTAA ATTTCTTCTT AACGACTGTT TTTTTTCTTT TTTTTAGTTC GTGCCTTTAA TTTTTTTTGT   
  
  
+ ATTAAATCGG ATTTGTTCTT TCCGTGTAA  

- GGGGGAAGAG CTCAAAGAGT TGAGAGAGGA ACTGAGAATG GAAGAGAAGG AAGGTTAATA TGGGTCAAGG   
  
  
- GAAGTGATAT CATGGAAGGT GAGAAGAGGG TGGATAGACC CACGGATATA TTTACAGAGA AAGAGAGAGA   
  
  
- GAAAATATGG TGCTTTGGCT TGTGATGTGG AGATGGAGGA TGGAGAGACT AGTCTGAGTT TCATGGGTAA   
  
  
- ACCCAACTAA ATCATACCTA CTTCTGTTCA CCTGCCCTTC AGTGAAAAGT GTACGTCCTA TATATGCATC   
  
  
- GTTATCACCG ATGTTTCACA TTCTTATTTG CCAAAAGAGA ACCATTCATT TAGGACATGC TACGTTTCAG   
  
  
- TAACCCCAAT GTCTTCTTTT GTATATGCTA TACTGTTTAC CAACCAAGCA CTGGTGTTTT CTTTTGACCA   
  
  
- ATTAGATCAA CTGCAGCTAC TTTAATTTCC TGTACGAAAT ATTCAGCCGT AAACCAAGTT CATCAAGAAA   
  
  
- AAGAAGTCAG CATTTTGATG ACTATGCCTG TCCCCTTCGA TCTCAATTAC AGTTTAAACA AAATAAAGGG   
  
  
- ATTTATGTGA ATATAAAAGA CATAATATAT GTCATAAAAA CTTCACCATT ATTGAAGAAA GGATTTGGGG   
  
  
- TGCTCTGGAT GAACCTTCGT TTCCTACTCA TGCAAGGCAA GCCAAAGTGC ATGGACACAT GATAGCAAAC   
  
  
- AAGCTTGGAA AGGGAGAGTC CTGATTAGGT CACCTGTAGT GACTTCCCAA GACTTTTCTA GCTCCTGATT   
  
  
- GGTAACATGC CATTTCATAA TACACCCAAT CTACCAAGGA AGAATACTAC ATACCACAAT CAACATCATA   
  
  
- AGTAACCATA TAGGTCATCA AGATGAATCG GCTGAGTAGC AGGTTCATAT AGATTTTTTT GCCAAGAAAA   
  
  
- CCAAACATAT AAAACTTGCC AATGTGGAGA TTACTGGAAG AACAGAAAGA AACAGATGCA TATCAGGACA   
  
  
- AGTTCCATGC ACCATATATC TCCAATAGAA TATCCAAAAT TTTAACTACA AAGCTTATGT CGAACCAGAA   
  
  
- AAACTTGTTA CAGGGGGTTC CAAACTCTGA ATTACGGCAC CTTGAGGCCT CAATTTATCC AATATCAAGT   
  
  
- AGAATCAAAT CTCTCAGGCA TGGGGTTGAA GTTTCAAGAA TTTGACTGCA GAACAAATAC ACTTCAACCA   
  
  
- TATTAATGAT TTTTTTTTCA TATAAGAAAC CACAATTATG ATTCTGACAA GACTAAAATG TGGCTCAAGG   
  
  
- TCAAATCAGG GACTCCACAT CAGCTGATGA TCATCTTTCT AAAGAATTCT GCTTGCTCAG GGAAATACTC   
  
  
- CAACAGCTCA TCCTTCGTAA CCCAAGCAAA ATCCTCACAC TTCCCAATGT TAAATTTGTT GGTTGCAATT   
  
  
- ACTTGGGATT TAAAGAAGAA TTGCTGACAA AAAAAAGAAA AAAAATCAAG CACGGAAATT AAAAAAAACA   
  
  
- TAATTTAGCC TAAACAAGAA AGGCACATT

+     CGTCA-motif

| Site Name | Organism | Position | Strand | Matrix score. | sequence | function |
| --- | --- | --- | --- | --- | --- | --- |
| CGTCA-motif | Hordeum vulgare | 1165 | - | 5 | CGTCA | cis-acting regulatory element involved in the MeJA-responsiveness |
| CGTCA-motif | Hordeum vulgare | 430 | - | 5 | CGTCA | cis-acting regulatory element involved in the MeJA-responsiveness |

> 2018/04/13 10:10:12  
+ CCCCCTTCTC GAGTTTCTCA ACTCTCTCCT TGACTCTTAC CTTCTCTTCC TTCCAATTAT ACCCAGTTCC   
  
  
+ CTTCACTATA GTACCTTCCA CTCTTCTCCC ACCTATCTGG GTGCCTATAT AAATGTCTCT TTCTCTCTCT   
  
  
+ CTTTTATACC ACGAAACCGA ACACTACACC TCTACCTCCT ACCTCTCTGA TCAGACTCAA AGTACCCATT   
  
  
+ TGGGTTGATT TAGTATGGAT GAAGACAAGT GGACGGGAAG TCACTTTTCA CATGCAGGAT ATATACGTAG   
  
  
+ CAATAGTGGC TACAAAGTGT AAGAATAAAC GGTTTTCTCT TGGTAAGTAA ATCCTGTACG ATGCAAAGTC   
  
  
+ ATTGGGGTTA CAGAAGAAAA CATATACGAT ATGACAAATG GTTGGTTCGT GACCACAAAA GAAAACTGGT   
  
  
+ TAATCTAGTT GACGTCGATG AAATTAAAGG ACATGCTTTA TAAGTCGGCA TTTGGTTCAA GTAGTTCTTT   
  
  
+ TTCTTCAGTC GTAAAACTAC TGATACGGAC AGGGGAAGCT AGAGTTAATG TCAAATTTGT TTTATTTCCC   
  
  
+ TAAATACACT TATATTTTCT GTATTATATA CAGTATTTTT GAAGTGGTAA TAACTTCTTT CCTAAACCCC   
  
  
+ ACGAGACCTA CTTGGAAGCA AAGGATGAGT ACGTTCCGTT CGGTTTCACG TACCTGTGTA CTATCGTTTG   
  
  
+ TTCGAACCTT TCCCTCTCAG GACTAATCCA GTGGACATCA CTGAAGGGTT CTGAAAAGAT CGAGGACTAA   
  
  
+ CCATTGTACG GTAAAGTATT ATGTGGGTTA GATGGTTCCT TCTTATGATG TATGGTGTTA GTTGTAGTAT   
  
  
+ TCATTGGTAT ATCCAGTAGT TCTACTTAGC CGACTCATCG TCCAAGTATA TCTAAAAAAA CGGTTCTTTT   
  
  
+ GGTTTGTATA TTTTGAACGG TTACACCTCT AATGACCTTC TTGTCTTTCT TTGTCTACGT ATAGTCCTGT   
  
  
+ TCAAGGTACG TGGTATATAG AGGTTATCTT ATAGGTTTTA AAATTGATGT TTCGAATACA GCTTGGTCTT   
  
  
+ TTTGAACAAT GTCCCCCAAG GTTTGAGACT TAATGCCGTG GAACTCCGGA GTTAAATAGG TTATAGTTCA   
  
  
+ TCTTAGTTTA GAGAGTCCGT ACCCCAACTT CAAAGTTCTT AAACTGACGT CTTGTTTATG TGAAGTTGGT   
  
  
+ ATAATTACTA AAAAAAAAGT ATATTCTTTG GTGTTAATAC TAAGACTGTT CTGATTTTAC ACCGAGTTCC   
  
  
+ AGTTTAGTCC CTGAGGTGTA GTCGACTACT AGTAGAAAGA TTTCTTAAGA CGAACGAGTC CCTTTATGAG   
  
  
+ GTTGTCGAGT AGGAAGCATT GGGTTCGTTT TAGGAGTGTG AAGGGTTACA ATTTAAACAA CCAACGTTAA   
  
  
+ TGAACCCTAA ATTTCTTCTT AACGACTGTT TTTTTTCTTT TTTTTAGTTC GTGCCTTTAA TTTTTTTTGT   
  
  
+ ATTAAATCGG ATTTGTTCTT TCCGTGTAA  

- GGGGGAAGAG CTCAAAGAGT TGAGAGAGGA ACTGAGAATG GAAGAGAAGG AAGGTTAATA TGGGTCAAGG   
  
  
- GAAGTGATAT CATGGAAGGT GAGAAGAGGG TGGATAGACC CACGGATATA TTTACAGAGA AAGAGAGAGA   
  
  
- GAAAATATGG TGCTTTGGCT TGTGATGTGG AGATGGAGGA TGGAGAGACT AGTCTGAGTT TCATGGGTAA   
  
  
- ACCCAACTAA ATCATACCTA CTTCTGTTCA CCTGCCCTTC AGTGAAAAGT GTACGTCCTA TATATGCATC   
  
  
- GTTATCACCG ATGTTTCACA TTCTTATTTG CCAAAAGAGA ACCATTCATT TAGGACATGC TACGTTTCAG   
  
  
- TAACCCCAAT GTCTTCTTTT GTATATGCTA TACTGTTTAC CAACCAAGCA CTGGTGTTTT CTTTTGACCA   
  
  
- ATTAGATCAA CTGCAGCTAC TTTAATTTCC TGTACGAAAT ATTCAGCCGT AAACCAAGTT CATCAAGAAA   
  
  
- AAGAAGTCAG CATTTTGATG ACTATGCCTG TCCCCTTCGA TCTCAATTAC AGTTTAAACA AAATAAAGGG   
  
  
- ATTTATGTGA ATATAAAAGA CATAATATAT GTCATAAAAA CTTCACCATT ATTGAAGAAA GGATTTGGGG   
  
  
- TGCTCTGGAT GAACCTTCGT TTCCTACTCA TGCAAGGCAA GCCAAAGTGC ATGGACACAT GATAGCAAAC   
  
  
- AAGCTTGGAA AGGGAGAGTC CTGATTAGGT CACCTGTAGT GACTTCCCAA GACTTTTCTA GCTCCTGATT   
  
  
- GGTAACATGC CATTTCATAA TACACCCAAT CTACCAAGGA AGAATACTAC ATACCACAAT CAACATCATA   
  
  
- AGTAACCATA TAGGTCATCA AGATGAATCG GCTGAGTAGC AGGTTCATAT AGATTTTTTT GCCAAGAAAA   
  
  
- CCAAACATAT AAAACTTGCC AATGTGGAGA TTACTGGAAG AACAGAAAGA AACAGATGCA TATCAGGACA   
  
  
- AGTTCCATGC ACCATATATC TCCAATAGAA TATCCAAAAT TTTAACTACA AAGCTTATGT CGAACCAGAA   
  
  
- AAACTTGTTA CAGGGGGTTC CAAACTCTGA ATTACGGCAC CTTGAGGCCT CAATTTATCC AATATCAAGT   
  
  
- AGAATCAAAT CTCTCAGGCA TGGGGTTGAA GTTTCAAGAA TTTGACTGCA GAACAAATAC ACTTCAACCA   
  
  
- TATTAATGAT TTTTTTTTCA TATAAGAAAC CACAATTATG ATTCTGACAA GACTAAAATG TGGCTCAAGG   
  
  
- TCAAATCAGG GACTCCACAT CAGCTGATGA TCATCTTTCT AAAGAATTCT GCTTGCTCAG GGAAATACTC   
  
  
- CAACAGCTCA TCCTTCGTAA CCCAAGCAAA ATCCTCACAC TTCCCAATGT TAAATTTGTT GGTTGCAATT   
  
  
- ACTTGGGATT TAAAGAAGAA TTGCTGACAA AAAAAAGAAA AAAAATCAAG CACGGAAATT AAAAAAAACA   
  
  
- TAATTTAGCC TAAACAAGAA AGGCACATT

+     CTAG-motif

| Site Name | Organism | Position | Strand | Matrix score. | sequence | function |
| --- | --- | --- | --- | --- | --- | --- |
| CTAG-motif | Avena sativa | 1288 | + | 9 | ACTAGCAGAA |  |

> 2018/04/13 10:10:12  
+ CCCCCTTCTC GAGTTTCTCA ACTCTCTCCT TGACTCTTAC CTTCTCTTCC TTCCAATTAT ACCCAGTTCC   
  
  
+ CTTCACTATA GTACCTTCCA CTCTTCTCCC ACCTATCTGG GTGCCTATAT AAATGTCTCT TTCTCTCTCT   
  
  
+ CTTTTATACC ACGAAACCGA ACACTACACC TCTACCTCCT ACCTCTCTGA TCAGACTCAA AGTACCCATT   
  
  
+ TGGGTTGATT TAGTATGGAT GAAGACAAGT GGACGGGAAG TCACTTTTCA CATGCAGGAT ATATACGTAG   
  
  
+ CAATAGTGGC TACAAAGTGT AAGAATAAAC GGTTTTCTCT TGGTAAGTAA ATCCTGTACG ATGCAAAGTC   
  
  
+ ATTGGGGTTA CAGAAGAAAA CATATACGAT ATGACAAATG GTTGGTTCGT GACCACAAAA GAAAACTGGT   
  
  
+ TAATCTAGTT GACGTCGATG AAATTAAAGG ACATGCTTTA TAAGTCGGCA TTTGGTTCAA GTAGTTCTTT   
  
  
+ TTCTTCAGTC GTAAAACTAC TGATACGGAC AGGGGAAGCT AGAGTTAATG TCAAATTTGT TTTATTTCCC   
  
  
+ TAAATACACT TATATTTTCT GTATTATATA CAGTATTTTT GAAGTGGTAA TAACTTCTTT CCTAAACCCC   
  
  
+ ACGAGACCTA CTTGGAAGCA AAGGATGAGT ACGTTCCGTT CGGTTTCACG TACCTGTGTA CTATCGTTTG   
  
  
+ TTCGAACCTT TCCCTCTCAG GACTAATCCA GTGGACATCA CTGAAGGGTT CTGAAAAGAT CGAGGACTAA   
  
  
+ CCATTGTACG GTAAAGTATT ATGTGGGTTA GATGGTTCCT TCTTATGATG TATGGTGTTA GTTGTAGTAT   
  
  
+ TCATTGGTAT ATCCAGTAGT TCTACTTAGC CGACTCATCG TCCAAGTATA TCTAAAAAAA CGGTTCTTTT   
  
  
+ GGTTTGTATA TTTTGAACGG TTACACCTCT AATGACCTTC TTGTCTTTCT TTGTCTACGT ATAGTCCTGT   
  
  
+ TCAAGGTACG TGGTATATAG AGGTTATCTT ATAGGTTTTA AAATTGATGT TTCGAATACA GCTTGGTCTT   
  
  
+ TTTGAACAAT GTCCCCCAAG GTTTGAGACT TAATGCCGTG GAACTCCGGA GTTAAATAGG TTATAGTTCA   
  
  
+ TCTTAGTTTA GAGAGTCCGT ACCCCAACTT CAAAGTTCTT AAACTGACGT CTTGTTTATG TGAAGTTGGT   
  
  
+ ATAATTACTA AAAAAAAAGT ATATTCTTTG GTGTTAATAC TAAGACTGTT CTGATTTTAC ACCGAGTTCC   
  
  
+ AGTTTAGTCC CTGAGGTGTA GTCGACTACT AGTAGAAAGA TTTCTTAAGA CGAACGAGTC CCTTTATGAG   
  
  
+ GTTGTCGAGT AGGAAGCATT GGGTTCGTTT TAGGAGTGTG AAGGGTTACA ATTTAAACAA CCAACGTTAA   
  
  
+ TGAACCCTAA ATTTCTTCTT AACGACTGTT TTTTTTCTTT TTTTTAGTTC GTGCCTTTAA TTTTTTTTGT   
  
  
+ ATTAAATCGG ATTTGTTCTT TCCGTGTAA  

- GGGGGAAGAG CTCAAAGAGT TGAGAGAGGA ACTGAGAATG GAAGAGAAGG AAGGTTAATA TGGGTCAAGG   
  
  
- GAAGTGATAT CATGGAAGGT GAGAAGAGGG TGGATAGACC CACGGATATA TTTACAGAGA AAGAGAGAGA   
  
  
- GAAAATATGG TGCTTTGGCT TGTGATGTGG AGATGGAGGA TGGAGAGACT AGTCTGAGTT TCATGGGTAA   
  
  
- ACCCAACTAA ATCATACCTA CTTCTGTTCA CCTGCCCTTC AGTGAAAAGT GTACGTCCTA TATATGCATC   
  
  
- GTTATCACCG ATGTTTCACA TTCTTATTTG CCAAAAGAGA ACCATTCATT TAGGACATGC TACGTTTCAG   
  
  
- TAACCCCAAT GTCTTCTTTT GTATATGCTA TACTGTTTAC CAACCAAGCA CTGGTGTTTT CTTTTGACCA   
  
  
- ATTAGATCAA CTGCAGCTAC TTTAATTTCC TGTACGAAAT ATTCAGCCGT AAACCAAGTT CATCAAGAAA   
  
  
- AAGAAGTCAG CATTTTGATG ACTATGCCTG TCCCCTTCGA TCTCAATTAC AGTTTAAACA AAATAAAGGG   
  
  
- ATTTATGTGA ATATAAAAGA CATAATATAT GTCATAAAAA CTTCACCATT ATTGAAGAAA GGATTTGGGG   
  
  
- TGCTCTGGAT GAACCTTCGT TTCCTACTCA TGCAAGGCAA GCCAAAGTGC ATGGACACAT GATAGCAAAC   
  
  
- AAGCTTGGAA AGGGAGAGTC CTGATTAGGT CACCTGTAGT GACTTCCCAA GACTTTTCTA GCTCCTGATT   
  
  
- GGTAACATGC CATTTCATAA TACACCCAAT CTACCAAGGA AGAATACTAC ATACCACAAT CAACATCATA   
  
  
- AGTAACCATA TAGGTCATCA AGATGAATCG GCTGAGTAGC AGGTTCATAT AGATTTTTTT GCCAAGAAAA   
  
  
- CCAAACATAT AAAACTTGCC AATGTGGAGA TTACTGGAAG AACAGAAAGA AACAGATGCA TATCAGGACA   
  
  
- AGTTCCATGC ACCATATATC TCCAATAGAA TATCCAAAAT TTTAACTACA AAGCTTATGT CGAACCAGAA   
  
  
- AAACTTGTTA CAGGGGGTTC CAAACTCTGA ATTACGGCAC CTTGAGGCCT CAATTTATCC AATATCAAGT   
  
  
- AGAATCAAAT CTCTCAGGCA TGGGGTTGAA GTTTCAAGAA TTTGACTGCA GAACAAATAC ACTTCAACCA   
  
  
- TATTAATGAT TTTTTTTTCA TATAAGAAAC CACAATTATG ATTCTGACAA GACTAAAATG TGGCTCAAGG   
  
  
- TCAAATCAGG GACTCCACAT CAGCTGATGA TCATCTTTCT AAAGAATTCT GCTTGCTCAG GGAAATACTC   
  
  
- CAACAGCTCA TCCTTCGTAA CCCAAGCAAA ATCCTCACAC TTCCCAATGT TAAATTTGTT GGTTGCAATT   
  
  
- ACTTGGGATT TAAAGAAGAA TTGCTGACAA AAAAAAGAAA AAAAATCAAG CACGGAAATT AAAAAAAACA   
  
  
- TAATTTAGCC TAAACAAGAA AGGCACATT

+     G-Box

| Site Name | Organism | Position | Strand | Matrix score. | sequence | function |
| --- | --- | --- | --- | --- | --- | --- |
| G-Box | Antirrhinum majus | 987 | - | 6 | CACGTA | cis-acting regulatory element involved in light responsiveness |
| G-Box | Antirrhinum majus | 677 | + | 6 | CACGTA | cis-acting regulatory element involved in light responsiveness |

> 2018/04/13 10:10:12  
+ CCCCCTTCTC GAGTTTCTCA ACTCTCTCCT TGACTCTTAC CTTCTCTTCC TTCCAATTAT ACCCAGTTCC   
  
  
+ CTTCACTATA GTACCTTCCA CTCTTCTCCC ACCTATCTGG GTGCCTATAT AAATGTCTCT TTCTCTCTCT   
  
  
+ CTTTTATACC ACGAAACCGA ACACTACACC TCTACCTCCT ACCTCTCTGA TCAGACTCAA AGTACCCATT   
  
  
+ TGGGTTGATT TAGTATGGAT GAAGACAAGT GGACGGGAAG TCACTTTTCA CATGCAGGAT ATATACGTAG   
  
  
+ CAATAGTGGC TACAAAGTGT AAGAATAAAC GGTTTTCTCT TGGTAAGTAA ATCCTGTACG ATGCAAAGTC   
  
  
+ ATTGGGGTTA CAGAAGAAAA CATATACGAT ATGACAAATG GTTGGTTCGT GACCACAAAA GAAAACTGGT   
  
  
+ TAATCTAGTT GACGTCGATG AAATTAAAGG ACATGCTTTA TAAGTCGGCA TTTGGTTCAA GTAGTTCTTT   
  
  
+ TTCTTCAGTC GTAAAACTAC TGATACGGAC AGGGGAAGCT AGAGTTAATG TCAAATTTGT TTTATTTCCC   
  
  
+ TAAATACACT TATATTTTCT GTATTATATA CAGTATTTTT GAAGTGGTAA TAACTTCTTT CCTAAACCCC   
  
  
+ ACGAGACCTA CTTGGAAGCA AAGGATGAGT ACGTTCCGTT CGGTTTCACG TACCTGTGTA CTATCGTTTG   
  
  
+ TTCGAACCTT TCCCTCTCAG GACTAATCCA GTGGACATCA CTGAAGGGTT CTGAAAAGAT CGAGGACTAA   
  
  
+ CCATTGTACG GTAAAGTATT ATGTGGGTTA GATGGTTCCT TCTTATGATG TATGGTGTTA GTTGTAGTAT   
  
  
+ TCATTGGTAT ATCCAGTAGT TCTACTTAGC CGACTCATCG TCCAAGTATA TCTAAAAAAA CGGTTCTTTT   
  
  
+ GGTTTGTATA TTTTGAACGG TTACACCTCT AATGACCTTC TTGTCTTTCT TTGTCTACGT ATAGTCCTGT   
  
  
+ TCAAGGTACG TGGTATATAG AGGTTATCTT ATAGGTTTTA AAATTGATGT TTCGAATACA GCTTGGTCTT   
  
  
+ TTTGAACAAT GTCCCCCAAG GTTTGAGACT TAATGCCGTG GAACTCCGGA GTTAAATAGG TTATAGTTCA   
  
  
+ TCTTAGTTTA GAGAGTCCGT ACCCCAACTT CAAAGTTCTT AAACTGACGT CTTGTTTATG TGAAGTTGGT   
  
  
+ ATAATTACTA AAAAAAAAGT ATATTCTTTG GTGTTAATAC TAAGACTGTT CTGATTTTAC ACCGAGTTCC   
  
  
+ AGTTTAGTCC CTGAGGTGTA GTCGACTACT AGTAGAAAGA TTTCTTAAGA CGAACGAGTC CCTTTATGAG   
  
  
+ GTTGTCGAGT AGGAAGCATT GGGTTCGTTT TAGGAGTGTG AAGGGTTACA ATTTAAACAA CCAACGTTAA   
  
  
+ TGAACCCTAA ATTTCTTCTT AACGACTGTT TTTTTTCTTT TTTTTAGTTC GTGCCTTTAA TTTTTTTTGT   
  
  
+ ATTAAATCGG ATTTGTTCTT TCCGTGTAA  

- GGGGGAAGAG CTCAAAGAGT TGAGAGAGGA ACTGAGAATG GAAGAGAAGG AAGGTTAATA TGGGTCAAGG   
  
  
- GAAGTGATAT CATGGAAGGT GAGAAGAGGG TGGATAGACC CACGGATATA TTTACAGAGA AAGAGAGAGA   
  
  
- GAAAATATGG TGCTTTGGCT TGTGATGTGG AGATGGAGGA TGGAGAGACT AGTCTGAGTT TCATGGGTAA   
  
  
- ACCCAACTAA ATCATACCTA CTTCTGTTCA CCTGCCCTTC AGTGAAAAGT GTACGTCCTA TATATGCATC   
  
  
- GTTATCACCG ATGTTTCACA TTCTTATTTG CCAAAAGAGA ACCATTCATT TAGGACATGC TACGTTTCAG   
  
  
- TAACCCCAAT GTCTTCTTTT GTATATGCTA TACTGTTTAC CAACCAAGCA CTGGTGTTTT CTTTTGACCA   
  
  
- ATTAGATCAA CTGCAGCTAC TTTAATTTCC TGTACGAAAT ATTCAGCCGT AAACCAAGTT CATCAAGAAA   
  
  
- AAGAAGTCAG CATTTTGATG ACTATGCCTG TCCCCTTCGA TCTCAATTAC AGTTTAAACA AAATAAAGGG   
  
  
- ATTTATGTGA ATATAAAAGA CATAATATAT GTCATAAAAA CTTCACCATT ATTGAAGAAA GGATTTGGGG   
  
  
- TGCTCTGGAT GAACCTTCGT TTCCTACTCA TGCAAGGCAA GCCAAAGTGC ATGGACACAT GATAGCAAAC   
  
  
- AAGCTTGGAA AGGGAGAGTC CTGATTAGGT CACCTGTAGT GACTTCCCAA GACTTTTCTA GCTCCTGATT   
  
  
- GGTAACATGC CATTTCATAA TACACCCAAT CTACCAAGGA AGAATACTAC ATACCACAAT CAACATCATA   
  
  
- AGTAACCATA TAGGTCATCA AGATGAATCG GCTGAGTAGC AGGTTCATAT AGATTTTTTT GCCAAGAAAA   
  
  
- CCAAACATAT AAAACTTGCC AATGTGGAGA TTACTGGAAG AACAGAAAGA AACAGATGCA TATCAGGACA   
  
  
- AGTTCCATGC ACCATATATC TCCAATAGAA TATCCAAAAT TTTAACTACA AAGCTTATGT CGAACCAGAA   
  
  
- AAACTTGTTA CAGGGGGTTC CAAACTCTGA ATTACGGCAC CTTGAGGCCT CAATTTATCC AATATCAAGT   
  
  
- AGAATCAAAT CTCTCAGGCA TGGGGTTGAA GTTTCAAGAA TTTGACTGCA GAACAAATAC ACTTCAACCA   
  
  
- TATTAATGAT TTTTTTTTCA TATAAGAAAC CACAATTATG ATTCTGACAA GACTAAAATG TGGCTCAAGG   
  
  
- TCAAATCAGG GACTCCACAT CAGCTGATGA TCATCTTTCT AAAGAATTCT GCTTGCTCAG GGAAATACTC   
  
  
- CAACAGCTCA TCCTTCGTAA CCCAAGCAAA ATCCTCACAC TTCCCAATGT TAAATTTGTT GGTTGCAATT   
  
  
- ACTTGGGATT TAAAGAAGAA TTGCTGACAA AAAAAAGAAA AAAAATCAAG CACGGAAATT AAAAAAAACA   
  
  
- TAATTTAGCC TAAACAAGAA AGGCACATT

+     G-box

| Site Name | Organism | Position | Strand | Matrix score. | sequence | function |
| --- | --- | --- | --- | --- | --- | --- |
| G-box | Oryza sativa | 986 | + | 7 | GTACGTG | cis-acting regulatory element involved in light responsiveness |
| G-box | Daucus carota | 677 | - | 6 | TACGTG | cis-acting regulatory element involved in light responsiveness |
| G-box | Daucus carota | 987 | + | 6 | TACGTG | cis-acting regulatory element involved in light responsiveness |

> 2018/04/13 10:10:12  
+ CCCCCTTCTC GAGTTTCTCA ACTCTCTCCT TGACTCTTAC CTTCTCTTCC TTCCAATTAT ACCCAGTTCC   
  
  
+ CTTCACTATA GTACCTTCCA CTCTTCTCCC ACCTATCTGG GTGCCTATAT AAATGTCTCT TTCTCTCTCT   
  
  
+ CTTTTATACC ACGAAACCGA ACACTACACC TCTACCTCCT ACCTCTCTGA TCAGACTCAA AGTACCCATT   
  
  
+ TGGGTTGATT TAGTATGGAT GAAGACAAGT GGACGGGAAG TCACTTTTCA CATGCAGGAT ATATACGTAG   
  
  
+ CAATAGTGGC TACAAAGTGT AAGAATAAAC GGTTTTCTCT TGGTAAGTAA ATCCTGTACG ATGCAAAGTC   
  
  
+ ATTGGGGTTA CAGAAGAAAA CATATACGAT ATGACAAATG GTTGGTTCGT GACCACAAAA GAAAACTGGT   
  
  
+ TAATCTAGTT GACGTCGATG AAATTAAAGG ACATGCTTTA TAAGTCGGCA TTTGGTTCAA GTAGTTCTTT   
  
  
+ TTCTTCAGTC GTAAAACTAC TGATACGGAC AGGGGAAGCT AGAGTTAATG TCAAATTTGT TTTATTTCCC   
  
  
+ TAAATACACT TATATTTTCT GTATTATATA CAGTATTTTT GAAGTGGTAA TAACTTCTTT CCTAAACCCC   
  
  
+ ACGAGACCTA CTTGGAAGCA AAGGATGAGT ACGTTCCGTT CGGTTTCACG TACCTGTGTA CTATCGTTTG   
  
  
+ TTCGAACCTT TCCCTCTCAG GACTAATCCA GTGGACATCA CTGAAGGGTT CTGAAAAGAT CGAGGACTAA   
  
  
+ CCATTGTACG GTAAAGTATT ATGTGGGTTA GATGGTTCCT TCTTATGATG TATGGTGTTA GTTGTAGTAT   
  
  
+ TCATTGGTAT ATCCAGTAGT TCTACTTAGC CGACTCATCG TCCAAGTATA TCTAAAAAAA CGGTTCTTTT   
  
  
+ GGTTTGTATA TTTTGAACGG TTACACCTCT AATGACCTTC TTGTCTTTCT TTGTCTACGT ATAGTCCTGT   
  
  
+ TCAAGGTACG TGGTATATAG AGGTTATCTT ATAGGTTTTA AAATTGATGT TTCGAATACA GCTTGGTCTT   
  
  
+ TTTGAACAAT GTCCCCCAAG GTTTGAGACT TAATGCCGTG GAACTCCGGA GTTAAATAGG TTATAGTTCA   
  
  
+ TCTTAGTTTA GAGAGTCCGT ACCCCAACTT CAAAGTTCTT AAACTGACGT CTTGTTTATG TGAAGTTGGT   
  
  
+ ATAATTACTA AAAAAAAAGT ATATTCTTTG GTGTTAATAC TAAGACTGTT CTGATTTTAC ACCGAGTTCC   
  
  
+ AGTTTAGTCC CTGAGGTGTA GTCGACTACT AGTAGAAAGA TTTCTTAAGA CGAACGAGTC CCTTTATGAG   
  
  
+ GTTGTCGAGT AGGAAGCATT GGGTTCGTTT TAGGAGTGTG AAGGGTTACA ATTTAAACAA CCAACGTTAA   
  
  
+ TGAACCCTAA ATTTCTTCTT AACGACTGTT TTTTTTCTTT TTTTTAGTTC GTGCCTTTAA TTTTTTTTGT   
  
  
+ ATTAAATCGG ATTTGTTCTT TCCGTGTAA  

- GGGGGAAGAG CTCAAAGAGT TGAGAGAGGA ACTGAGAATG GAAGAGAAGG AAGGTTAATA TGGGTCAAGG   
  
  
- GAAGTGATAT CATGGAAGGT GAGAAGAGGG TGGATAGACC CACGGATATA TTTACAGAGA AAGAGAGAGA   
  
  
- GAAAATATGG TGCTTTGGCT TGTGATGTGG AGATGGAGGA TGGAGAGACT AGTCTGAGTT TCATGGGTAA   
  
  
- ACCCAACTAA ATCATACCTA CTTCTGTTCA CCTGCCCTTC AGTGAAAAGT GTACGTCCTA TATATGCATC   
  
  
- GTTATCACCG ATGTTTCACA TTCTTATTTG CCAAAAGAGA ACCATTCATT TAGGACATGC TACGTTTCAG   
  
  
- TAACCCCAAT GTCTTCTTTT GTATATGCTA TACTGTTTAC CAACCAAGCA CTGGTGTTTT CTTTTGACCA   
  
  
- ATTAGATCAA CTGCAGCTAC TTTAATTTCC TGTACGAAAT ATTCAGCCGT AAACCAAGTT CATCAAGAAA   
  
  
- AAGAAGTCAG CATTTTGATG ACTATGCCTG TCCCCTTCGA TCTCAATTAC AGTTTAAACA AAATAAAGGG   
  
  
- ATTTATGTGA ATATAAAAGA CATAATATAT GTCATAAAAA CTTCACCATT ATTGAAGAAA GGATTTGGGG   
  
  
- TGCTCTGGAT GAACCTTCGT TTCCTACTCA TGCAAGGCAA GCCAAAGTGC ATGGACACAT GATAGCAAAC   
  
  
- AAGCTTGGAA AGGGAGAGTC CTGATTAGGT CACCTGTAGT GACTTCCCAA GACTTTTCTA GCTCCTGATT   
  
  
- GGTAACATGC CATTTCATAA TACACCCAAT CTACCAAGGA AGAATACTAC ATACCACAAT CAACATCATA   
  
  
- AGTAACCATA TAGGTCATCA AGATGAATCG GCTGAGTAGC AGGTTCATAT AGATTTTTTT GCCAAGAAAA   
  
  
- CCAAACATAT AAAACTTGCC AATGTGGAGA TTACTGGAAG AACAGAAAGA AACAGATGCA TATCAGGACA   
  
  
- AGTTCCATGC ACCATATATC TCCAATAGAA TATCCAAAAT TTTAACTACA AAGCTTATGT CGAACCAGAA   
  
  
- AAACTTGTTA CAGGGGGTTC CAAACTCTGA ATTACGGCAC CTTGAGGCCT CAATTTATCC AATATCAAGT   
  
  
- AGAATCAAAT CTCTCAGGCA TGGGGTTGAA GTTTCAAGAA TTTGACTGCA GAACAAATAC ACTTCAACCA   
  
  
- TATTAATGAT TTTTTTTTCA TATAAGAAAC CACAATTATG ATTCTGACAA GACTAAAATG TGGCTCAAGG   
  
  
- TCAAATCAGG GACTCCACAT CAGCTGATGA TCATCTTTCT AAAGAATTCT GCTTGCTCAG GGAAATACTC   
  
  
- CAACAGCTCA TCCTTCGTAA CCCAAGCAAA ATCCTCACAC TTCCCAATGT TAAATTTGTT GGTTGCAATT   
  
  
- ACTTGGGATT TAAAGAAGAA TTGCTGACAA AAAAAAGAAA AAAAATCAAG CACGGAAATT AAAAAAAACA   
  
  
- TAATTTAGCC TAAACAAGAA AGGCACATT

+     GA-motif

| Site Name | Organism | Position | Strand | Matrix score. | sequence | function |
| --- | --- | --- | --- | --- | --- | --- |
| GA-motif | Glycine max | 45 | - | 8 | AAGGAAGA | part of a light responsive element |

> 2018/04/13 10:10:12  
+ CCCCCTTCTC GAGTTTCTCA ACTCTCTCCT TGACTCTTAC CTTCTCTTCC TTCCAATTAT ACCCAGTTCC   
  
  
+ CTTCACTATA GTACCTTCCA CTCTTCTCCC ACCTATCTGG GTGCCTATAT AAATGTCTCT TTCTCTCTCT   
  
  
+ CTTTTATACC ACGAAACCGA ACACTACACC TCTACCTCCT ACCTCTCTGA TCAGACTCAA AGTACCCATT   
  
  
+ TGGGTTGATT TAGTATGGAT GAAGACAAGT GGACGGGAAG TCACTTTTCA CATGCAGGAT ATATACGTAG   
  
  
+ CAATAGTGGC TACAAAGTGT AAGAATAAAC GGTTTTCTCT TGGTAAGTAA ATCCTGTACG ATGCAAAGTC   
  
  
+ ATTGGGGTTA CAGAAGAAAA CATATACGAT ATGACAAATG GTTGGTTCGT GACCACAAAA GAAAACTGGT   
  
  
+ TAATCTAGTT GACGTCGATG AAATTAAAGG ACATGCTTTA TAAGTCGGCA TTTGGTTCAA GTAGTTCTTT   
  
  
+ TTCTTCAGTC GTAAAACTAC TGATACGGAC AGGGGAAGCT AGAGTTAATG TCAAATTTGT TTTATTTCCC   
  
  
+ TAAATACACT TATATTTTCT GTATTATATA CAGTATTTTT GAAGTGGTAA TAACTTCTTT CCTAAACCCC   
  
  
+ ACGAGACCTA CTTGGAAGCA AAGGATGAGT ACGTTCCGTT CGGTTTCACG TACCTGTGTA CTATCGTTTG   
  
  
+ TTCGAACCTT TCCCTCTCAG GACTAATCCA GTGGACATCA CTGAAGGGTT CTGAAAAGAT CGAGGACTAA   
  
  
+ CCATTGTACG GTAAAGTATT ATGTGGGTTA GATGGTTCCT TCTTATGATG TATGGTGTTA GTTGTAGTAT   
  
  
+ TCATTGGTAT ATCCAGTAGT TCTACTTAGC CGACTCATCG TCCAAGTATA TCTAAAAAAA CGGTTCTTTT   
  
  
+ GGTTTGTATA TTTTGAACGG TTACACCTCT AATGACCTTC TTGTCTTTCT TTGTCTACGT ATAGTCCTGT   
  
  
+ TCAAGGTACG TGGTATATAG AGGTTATCTT ATAGGTTTTA AAATTGATGT TTCGAATACA GCTTGGTCTT   
  
  
+ TTTGAACAAT GTCCCCCAAG GTTTGAGACT TAATGCCGTG GAACTCCGGA GTTAAATAGG TTATAGTTCA   
  
  
+ TCTTAGTTTA GAGAGTCCGT ACCCCAACTT CAAAGTTCTT AAACTGACGT CTTGTTTATG TGAAGTTGGT   
  
  
+ ATAATTACTA AAAAAAAAGT ATATTCTTTG GTGTTAATAC TAAGACTGTT CTGATTTTAC ACCGAGTTCC   
  
  
+ AGTTTAGTCC CTGAGGTGTA GTCGACTACT AGTAGAAAGA TTTCTTAAGA CGAACGAGTC CCTTTATGAG   
  
  
+ GTTGTCGAGT AGGAAGCATT GGGTTCGTTT TAGGAGTGTG AAGGGTTACA ATTTAAACAA CCAACGTTAA   
  
  
+ TGAACCCTAA ATTTCTTCTT AACGACTGTT TTTTTTCTTT TTTTTAGTTC GTGCCTTTAA TTTTTTTTGT   
  
  
+ ATTAAATCGG ATTTGTTCTT TCCGTGTAA  

- GGGGGAAGAG CTCAAAGAGT TGAGAGAGGA ACTGAGAATG GAAGAGAAGG AAGGTTAATA TGGGTCAAGG   
  
  
- GAAGTGATAT CATGGAAGGT GAGAAGAGGG TGGATAGACC CACGGATATA TTTACAGAGA AAGAGAGAGA   
  
  
- GAAAATATGG TGCTTTGGCT TGTGATGTGG AGATGGAGGA TGGAGAGACT AGTCTGAGTT TCATGGGTAA   
  
  
- ACCCAACTAA ATCATACCTA CTTCTGTTCA CCTGCCCTTC AGTGAAAAGT GTACGTCCTA TATATGCATC   
  
  
- GTTATCACCG ATGTTTCACA TTCTTATTTG CCAAAAGAGA ACCATTCATT TAGGACATGC TACGTTTCAG   
  
  
- TAACCCCAAT GTCTTCTTTT GTATATGCTA TACTGTTTAC CAACCAAGCA CTGGTGTTTT CTTTTGACCA   
  
  
- ATTAGATCAA CTGCAGCTAC TTTAATTTCC TGTACGAAAT ATTCAGCCGT AAACCAAGTT CATCAAGAAA   
  
  
- AAGAAGTCAG CATTTTGATG ACTATGCCTG TCCCCTTCGA TCTCAATTAC AGTTTAAACA AAATAAAGGG   
  
  
- ATTTATGTGA ATATAAAAGA CATAATATAT GTCATAAAAA CTTCACCATT ATTGAAGAAA GGATTTGGGG   
  
  
- TGCTCTGGAT GAACCTTCGT TTCCTACTCA TGCAAGGCAA GCCAAAGTGC ATGGACACAT GATAGCAAAC   
  
  
- AAGCTTGGAA AGGGAGAGTC CTGATTAGGT CACCTGTAGT GACTTCCCAA GACTTTTCTA GCTCCTGATT   
  
  
- GGTAACATGC CATTTCATAA TACACCCAAT CTACCAAGGA AGAATACTAC ATACCACAAT CAACATCATA   
  
  
- AGTAACCATA TAGGTCATCA AGATGAATCG GCTGAGTAGC AGGTTCATAT AGATTTTTTT GCCAAGAAAA   
  
  
- CCAAACATAT AAAACTTGCC AATGTGGAGA TTACTGGAAG AACAGAAAGA AACAGATGCA TATCAGGACA   
  
  
- AGTTCCATGC ACCATATATC TCCAATAGAA TATCCAAAAT TTTAACTACA AAGCTTATGT CGAACCAGAA   
  
  
- AAACTTGTTA CAGGGGGTTC CAAACTCTGA ATTACGGCAC CTTGAGGCCT CAATTTATCC AATATCAAGT   
  
  
- AGAATCAAAT CTCTCAGGCA TGGGGTTGAA GTTTCAAGAA TTTGACTGCA GAACAAATAC ACTTCAACCA   
  
  
- TATTAATGAT TTTTTTTTCA TATAAGAAAC CACAATTATG ATTCTGACAA GACTAAAATG TGGCTCAAGG   
  
  
- TCAAATCAGG GACTCCACAT CAGCTGATGA TCATCTTTCT AAAGAATTCT GCTTGCTCAG GGAAATACTC   
  
  
- CAACAGCTCA TCCTTCGTAA CCCAAGCAAA ATCCTCACAC TTCCCAATGT TAAATTTGTT GGTTGCAATT   
  
  
- ACTTGGGATT TAAAGAAGAA TTGCTGACAA AAAAAAGAAA AAAAATCAAG CACGGAAATT AAAAAAAACA   
  
  
- TAATTTAGCC TAAACAAGAA AGGCACATT

+     GAG-motif

| Site Name | Organism | Position | Strand | Matrix score. | sequence | function |
| --- | --- | --- | --- | --- | --- | --- |
| GAG-motif | Arabidopsis thaliana | 21 | - | 7 | AGAGAGT | part of a light responsive element |
| GAG-motif | Arabidopsis thaliana | 1130 | + | 7 | AGAGAGT | part of a light responsive element |

> 2018/04/13 10:10:12  
+ CCCCCTTCTC GAGTTTCTCA ACTCTCTCCT TGACTCTTAC CTTCTCTTCC TTCCAATTAT ACCCAGTTCC   
  
  
+ CTTCACTATA GTACCTTCCA CTCTTCTCCC ACCTATCTGG GTGCCTATAT AAATGTCTCT TTCTCTCTCT   
  
  
+ CTTTTATACC ACGAAACCGA ACACTACACC TCTACCTCCT ACCTCTCTGA TCAGACTCAA AGTACCCATT   
  
  
+ TGGGTTGATT TAGTATGGAT GAAGACAAGT GGACGGGAAG TCACTTTTCA CATGCAGGAT ATATACGTAG   
  
  
+ CAATAGTGGC TACAAAGTGT AAGAATAAAC GGTTTTCTCT TGGTAAGTAA ATCCTGTACG ATGCAAAGTC   
  
  
+ ATTGGGGTTA CAGAAGAAAA CATATACGAT ATGACAAATG GTTGGTTCGT GACCACAAAA GAAAACTGGT   
  
  
+ TAATCTAGTT GACGTCGATG AAATTAAAGG ACATGCTTTA TAAGTCGGCA TTTGGTTCAA GTAGTTCTTT   
  
  
+ TTCTTCAGTC GTAAAACTAC TGATACGGAC AGGGGAAGCT AGAGTTAATG TCAAATTTGT TTTATTTCCC   
  
  
+ TAAATACACT TATATTTTCT GTATTATATA CAGTATTTTT GAAGTGGTAA TAACTTCTTT CCTAAACCCC   
  
  
+ ACGAGACCTA CTTGGAAGCA AAGGATGAGT ACGTTCCGTT CGGTTTCACG TACCTGTGTA CTATCGTTTG   
  
  
+ TTCGAACCTT TCCCTCTCAG GACTAATCCA GTGGACATCA CTGAAGGGTT CTGAAAAGAT CGAGGACTAA   
  
  
+ CCATTGTACG GTAAAGTATT ATGTGGGTTA GATGGTTCCT TCTTATGATG TATGGTGTTA GTTGTAGTAT   
  
  
+ TCATTGGTAT ATCCAGTAGT TCTACTTAGC CGACTCATCG TCCAAGTATA TCTAAAAAAA CGGTTCTTTT   
  
  
+ GGTTTGTATA TTTTGAACGG TTACACCTCT AATGACCTTC TTGTCTTTCT TTGTCTACGT ATAGTCCTGT   
  
  
+ TCAAGGTACG TGGTATATAG AGGTTATCTT ATAGGTTTTA AAATTGATGT TTCGAATACA GCTTGGTCTT   
  
  
+ TTTGAACAAT GTCCCCCAAG GTTTGAGACT TAATGCCGTG GAACTCCGGA GTTAAATAGG TTATAGTTCA   
  
  
+ TCTTAGTTTA GAGAGTCCGT ACCCCAACTT CAAAGTTCTT AAACTGACGT CTTGTTTATG TGAAGTTGGT   
  
  
+ ATAATTACTA AAAAAAAAGT ATATTCTTTG GTGTTAATAC TAAGACTGTT CTGATTTTAC ACCGAGTTCC   
  
  
+ AGTTTAGTCC CTGAGGTGTA GTCGACTACT AGTAGAAAGA TTTCTTAAGA CGAACGAGTC CCTTTATGAG   
  
  
+ GTTGTCGAGT AGGAAGCATT GGGTTCGTTT TAGGAGTGTG AAGGGTTACA ATTTAAACAA CCAACGTTAA   
  
  
+ TGAACCCTAA ATTTCTTCTT AACGACTGTT TTTTTTCTTT TTTTTAGTTC GTGCCTTTAA TTTTTTTTGT   
  
  
+ ATTAAATCGG ATTTGTTCTT TCCGTGTAA  

- GGGGGAAGAG CTCAAAGAGT TGAGAGAGGA ACTGAGAATG GAAGAGAAGG AAGGTTAATA TGGGTCAAGG   
  
  
- GAAGTGATAT CATGGAAGGT GAGAAGAGGG TGGATAGACC CACGGATATA TTTACAGAGA AAGAGAGAGA   
  
  
- GAAAATATGG TGCTTTGGCT TGTGATGTGG AGATGGAGGA TGGAGAGACT AGTCTGAGTT TCATGGGTAA   
  
  
- ACCCAACTAA ATCATACCTA CTTCTGTTCA CCTGCCCTTC AGTGAAAAGT GTACGTCCTA TATATGCATC   
  
  
- GTTATCACCG ATGTTTCACA TTCTTATTTG CCAAAAGAGA ACCATTCATT TAGGACATGC TACGTTTCAG   
  
  
- TAACCCCAAT GTCTTCTTTT GTATATGCTA TACTGTTTAC CAACCAAGCA CTGGTGTTTT CTTTTGACCA   
  
  
- ATTAGATCAA CTGCAGCTAC TTTAATTTCC TGTACGAAAT ATTCAGCCGT AAACCAAGTT CATCAAGAAA   
  
  
- AAGAAGTCAG CATTTTGATG ACTATGCCTG TCCCCTTCGA TCTCAATTAC AGTTTAAACA AAATAAAGGG   
  
  
- ATTTATGTGA ATATAAAAGA CATAATATAT GTCATAAAAA CTTCACCATT ATTGAAGAAA GGATTTGGGG   
  
  
- TGCTCTGGAT GAACCTTCGT TTCCTACTCA TGCAAGGCAA GCCAAAGTGC ATGGACACAT GATAGCAAAC   
  
  
- AAGCTTGGAA AGGGAGAGTC CTGATTAGGT CACCTGTAGT GACTTCCCAA GACTTTTCTA GCTCCTGATT   
  
  
- GGTAACATGC CATTTCATAA TACACCCAAT CTACCAAGGA AGAATACTAC ATACCACAAT CAACATCATA   
  
  
- AGTAACCATA TAGGTCATCA AGATGAATCG GCTGAGTAGC AGGTTCATAT AGATTTTTTT GCCAAGAAAA   
  
  
- CCAAACATAT AAAACTTGCC AATGTGGAGA TTACTGGAAG AACAGAAAGA AACAGATGCA TATCAGGACA   
  
  
- AGTTCCATGC ACCATATATC TCCAATAGAA TATCCAAAAT TTTAACTACA AAGCTTATGT CGAACCAGAA   
  
  
- AAACTTGTTA CAGGGGGTTC CAAACTCTGA ATTACGGCAC CTTGAGGCCT CAATTTATCC AATATCAAGT   
  
  
- AGAATCAAAT CTCTCAGGCA TGGGGTTGAA GTTTCAAGAA TTTGACTGCA GAACAAATAC ACTTCAACCA   
  
  
- TATTAATGAT TTTTTTTTCA TATAAGAAAC CACAATTATG ATTCTGACAA GACTAAAATG TGGCTCAAGG   
  
  
- TCAAATCAGG GACTCCACAT CAGCTGATGA TCATCTTTCT AAAGAATTCT GCTTGCTCAG GGAAATACTC   
  
  
- CAACAGCTCA TCCTTCGTAA CCCAAGCAAA ATCCTCACAC TTCCCAATGT TAAATTTGTT GGTTGCAATT   
  
  
- ACTTGGGATT TAAAGAAGAA TTGCTGACAA AAAAAAGAAA AAAAATCAAG CACGGAAATT AAAAAAAACA   
  
  
- TAATTTAGCC TAAACAAGAA AGGCACATT

+     GT1-motif

| Site Name | Organism | Position | Strand | Matrix score. | sequence | function |
| --- | --- | --- | --- | --- | --- | --- |
| GT1-motif | Avena sativa | 418 | + | 7 | GGTTAAT | light responsive element |

> 2018/04/13 10:10:12  
+ CCCCCTTCTC GAGTTTCTCA ACTCTCTCCT TGACTCTTAC CTTCTCTTCC TTCCAATTAT ACCCAGTTCC   
  
  
+ CTTCACTATA GTACCTTCCA CTCTTCTCCC ACCTATCTGG GTGCCTATAT AAATGTCTCT TTCTCTCTCT   
  
  
+ CTTTTATACC ACGAAACCGA ACACTACACC TCTACCTCCT ACCTCTCTGA TCAGACTCAA AGTACCCATT   
  
  
+ TGGGTTGATT TAGTATGGAT GAAGACAAGT GGACGGGAAG TCACTTTTCA CATGCAGGAT ATATACGTAG   
  
  
+ CAATAGTGGC TACAAAGTGT AAGAATAAAC GGTTTTCTCT TGGTAAGTAA ATCCTGTACG ATGCAAAGTC   
  
  
+ ATTGGGGTTA CAGAAGAAAA CATATACGAT ATGACAAATG GTTGGTTCGT GACCACAAAA GAAAACTGGT   
  
  
+ TAATCTAGTT GACGTCGATG AAATTAAAGG ACATGCTTTA TAAGTCGGCA TTTGGTTCAA GTAGTTCTTT   
  
  
+ TTCTTCAGTC GTAAAACTAC TGATACGGAC AGGGGAAGCT AGAGTTAATG TCAAATTTGT TTTATTTCCC   
  
  
+ TAAATACACT TATATTTTCT GTATTATATA CAGTATTTTT GAAGTGGTAA TAACTTCTTT CCTAAACCCC   
  
  
+ ACGAGACCTA CTTGGAAGCA AAGGATGAGT ACGTTCCGTT CGGTTTCACG TACCTGTGTA CTATCGTTTG   
  
  
+ TTCGAACCTT TCCCTCTCAG GACTAATCCA GTGGACATCA CTGAAGGGTT CTGAAAAGAT CGAGGACTAA   
  
  
+ CCATTGTACG GTAAAGTATT ATGTGGGTTA GATGGTTCCT TCTTATGATG TATGGTGTTA GTTGTAGTAT   
  
  
+ TCATTGGTAT ATCCAGTAGT TCTACTTAGC CGACTCATCG TCCAAGTATA TCTAAAAAAA CGGTTCTTTT   
  
  
+ GGTTTGTATA TTTTGAACGG TTACACCTCT AATGACCTTC TTGTCTTTCT TTGTCTACGT ATAGTCCTGT   
  
  
+ TCAAGGTACG TGGTATATAG AGGTTATCTT ATAGGTTTTA AAATTGATGT TTCGAATACA GCTTGGTCTT   
  
  
+ TTTGAACAAT GTCCCCCAAG GTTTGAGACT TAATGCCGTG GAACTCCGGA GTTAAATAGG TTATAGTTCA   
  
  
+ TCTTAGTTTA GAGAGTCCGT ACCCCAACTT CAAAGTTCTT AAACTGACGT CTTGTTTATG TGAAGTTGGT   
  
  
+ ATAATTACTA AAAAAAAAGT ATATTCTTTG GTGTTAATAC TAAGACTGTT CTGATTTTAC ACCGAGTTCC   
  
  
+ AGTTTAGTCC CTGAGGTGTA GTCGACTACT AGTAGAAAGA TTTCTTAAGA CGAACGAGTC CCTTTATGAG   
  
  
+ GTTGTCGAGT AGGAAGCATT GGGTTCGTTT TAGGAGTGTG AAGGGTTACA ATTTAAACAA CCAACGTTAA   
  
  
+ TGAACCCTAA ATTTCTTCTT AACGACTGTT TTTTTTCTTT TTTTTAGTTC GTGCCTTTAA TTTTTTTTGT   
  
  
+ ATTAAATCGG ATTTGTTCTT TCCGTGTAA  

- GGGGGAAGAG CTCAAAGAGT TGAGAGAGGA ACTGAGAATG GAAGAGAAGG AAGGTTAATA TGGGTCAAGG   
  
  
- GAAGTGATAT CATGGAAGGT GAGAAGAGGG TGGATAGACC CACGGATATA TTTACAGAGA AAGAGAGAGA   
  
  
- GAAAATATGG TGCTTTGGCT TGTGATGTGG AGATGGAGGA TGGAGAGACT AGTCTGAGTT TCATGGGTAA   
  
  
- ACCCAACTAA ATCATACCTA CTTCTGTTCA CCTGCCCTTC AGTGAAAAGT GTACGTCCTA TATATGCATC   
  
  
- GTTATCACCG ATGTTTCACA TTCTTATTTG CCAAAAGAGA ACCATTCATT TAGGACATGC TACGTTTCAG   
  
  
- TAACCCCAAT GTCTTCTTTT GTATATGCTA TACTGTTTAC CAACCAAGCA CTGGTGTTTT CTTTTGACCA   
  
  
- ATTAGATCAA CTGCAGCTAC TTTAATTTCC TGTACGAAAT ATTCAGCCGT AAACCAAGTT CATCAAGAAA   
  
  
- AAGAAGTCAG CATTTTGATG ACTATGCCTG TCCCCTTCGA TCTCAATTAC AGTTTAAACA AAATAAAGGG   
  
  
- ATTTATGTGA ATATAAAAGA CATAATATAT GTCATAAAAA CTTCACCATT ATTGAAGAAA GGATTTGGGG   
  
  
- TGCTCTGGAT GAACCTTCGT TTCCTACTCA TGCAAGGCAA GCCAAAGTGC ATGGACACAT GATAGCAAAC   
  
  
- AAGCTTGGAA AGGGAGAGTC CTGATTAGGT CACCTGTAGT GACTTCCCAA GACTTTTCTA GCTCCTGATT   
  
  
- GGTAACATGC CATTTCATAA TACACCCAAT CTACCAAGGA AGAATACTAC ATACCACAAT CAACATCATA   
  
  
- AGTAACCATA TAGGTCATCA AGATGAATCG GCTGAGTAGC AGGTTCATAT AGATTTTTTT GCCAAGAAAA   
  
  
- CCAAACATAT AAAACTTGCC AATGTGGAGA TTACTGGAAG AACAGAAAGA AACAGATGCA TATCAGGACA   
  
  
- AGTTCCATGC ACCATATATC TCCAATAGAA TATCCAAAAT TTTAACTACA AAGCTTATGT CGAACCAGAA   
  
  
- AAACTTGTTA CAGGGGGTTC CAAACTCTGA ATTACGGCAC CTTGAGGCCT CAATTTATCC AATATCAAGT   
  
  
- AGAATCAAAT CTCTCAGGCA TGGGGTTGAA GTTTCAAGAA TTTGACTGCA GAACAAATAC ACTTCAACCA   
  
  
- TATTAATGAT TTTTTTTTCA TATAAGAAAC CACAATTATG ATTCTGACAA GACTAAAATG TGGCTCAAGG   
  
  
- TCAAATCAGG GACTCCACAT CAGCTGATGA TCATCTTTCT AAAGAATTCT GCTTGCTCAG GGAAATACTC   
  
  
- CAACAGCTCA TCCTTCGTAA CCCAAGCAAA ATCCTCACAC TTCCCAATGT TAAATTTGTT GGTTGCAATT   
  
  
- ACTTGGGATT TAAAGAAGAA TTGCTGACAA AAAAAAGAAA AAAAATCAAG CACGGAAATT AAAAAAAACA   
  
  
- TAATTTAGCC TAAACAAGAA AGGCACATT

+     MBSI

| Site Name | Organism | Position | Strand | Matrix score. | sequence | function |
| --- | --- | --- | --- | --- | --- | --- |
| MBSI | Petunia hybrida | 895 | + | 10.5 | aaaAaaC(G/C)GTTA | MYB binding site involved in flavonoid biosynthetic genes regulation |

> 2018/04/13 10:10:12  
+ CCCCCTTCTC GAGTTTCTCA ACTCTCTCCT TGACTCTTAC CTTCTCTTCC TTCCAATTAT ACCCAGTTCC   
  
  
+ CTTCACTATA GTACCTTCCA CTCTTCTCCC ACCTATCTGG GTGCCTATAT AAATGTCTCT TTCTCTCTCT   
  
  
+ CTTTTATACC ACGAAACCGA ACACTACACC TCTACCTCCT ACCTCTCTGA TCAGACTCAA AGTACCCATT   
  
  
+ TGGGTTGATT TAGTATGGAT GAAGACAAGT GGACGGGAAG TCACTTTTCA CATGCAGGAT ATATACGTAG   
  
  
+ CAATAGTGGC TACAAAGTGT AAGAATAAAC GGTTTTCTCT TGGTAAGTAA ATCCTGTACG ATGCAAAGTC   
  
  
+ ATTGGGGTTA CAGAAGAAAA CATATACGAT ATGACAAATG GTTGGTTCGT GACCACAAAA GAAAACTGGT   
  
  
+ TAATCTAGTT GACGTCGATG AAATTAAAGG ACATGCTTTA TAAGTCGGCA TTTGGTTCAA GTAGTTCTTT   
  
  
+ TTCTTCAGTC GTAAAACTAC TGATACGGAC AGGGGAAGCT AGAGTTAATG TCAAATTTGT TTTATTTCCC   
  
  
+ TAAATACACT TATATTTTCT GTATTATATA CAGTATTTTT GAAGTGGTAA TAACTTCTTT CCTAAACCCC   
  
  
+ ACGAGACCTA CTTGGAAGCA AAGGATGAGT ACGTTCCGTT CGGTTTCACG TACCTGTGTA CTATCGTTTG   
  
  
+ TTCGAACCTT TCCCTCTCAG GACTAATCCA GTGGACATCA CTGAAGGGTT CTGAAAAGAT CGAGGACTAA   
  
  
+ CCATTGTACG GTAAAGTATT ATGTGGGTTA GATGGTTCCT TCTTATGATG TATGGTGTTA GTTGTAGTAT   
  
  
+ TCATTGGTAT ATCCAGTAGT TCTACTTAGC CGACTCATCG TCCAAGTATA TCTAAAAAAA CGGTTCTTTT   
  
  
+ GGTTTGTATA TTTTGAACGG TTACACCTCT AATGACCTTC TTGTCTTTCT TTGTCTACGT ATAGTCCTGT   
  
  
+ TCAAGGTACG TGGTATATAG AGGTTATCTT ATAGGTTTTA AAATTGATGT TTCGAATACA GCTTGGTCTT   
  
  
+ TTTGAACAAT GTCCCCCAAG GTTTGAGACT TAATGCCGTG GAACTCCGGA GTTAAATAGG TTATAGTTCA   
  
  
+ TCTTAGTTTA GAGAGTCCGT ACCCCAACTT CAAAGTTCTT AAACTGACGT CTTGTTTATG TGAAGTTGGT   
  
  
+ ATAATTACTA AAAAAAAAGT ATATTCTTTG GTGTTAATAC TAAGACTGTT CTGATTTTAC ACCGAGTTCC   
  
  
+ AGTTTAGTCC CTGAGGTGTA GTCGACTACT AGTAGAAAGA TTTCTTAAGA CGAACGAGTC CCTTTATGAG   
  
  
+ GTTGTCGAGT AGGAAGCATT GGGTTCGTTT TAGGAGTGTG AAGGGTTACA ATTTAAACAA CCAACGTTAA   
  
  
+ TGAACCCTAA ATTTCTTCTT AACGACTGTT TTTTTTCTTT TTTTTAGTTC GTGCCTTTAA TTTTTTTTGT   
  
  
+ ATTAAATCGG ATTTGTTCTT TCCGTGTAA  

- GGGGGAAGAG CTCAAAGAGT TGAGAGAGGA ACTGAGAATG GAAGAGAAGG AAGGTTAATA TGGGTCAAGG   
  
  
- GAAGTGATAT CATGGAAGGT GAGAAGAGGG TGGATAGACC CACGGATATA TTTACAGAGA AAGAGAGAGA   
  
  
- GAAAATATGG TGCTTTGGCT TGTGATGTGG AGATGGAGGA TGGAGAGACT AGTCTGAGTT TCATGGGTAA   
  
  
- ACCCAACTAA ATCATACCTA CTTCTGTTCA CCTGCCCTTC AGTGAAAAGT GTACGTCCTA TATATGCATC   
  
  
- GTTATCACCG ATGTTTCACA TTCTTATTTG CCAAAAGAGA ACCATTCATT TAGGACATGC TACGTTTCAG   
  
  
- TAACCCCAAT GTCTTCTTTT GTATATGCTA TACTGTTTAC CAACCAAGCA CTGGTGTTTT CTTTTGACCA   
  
  
- ATTAGATCAA CTGCAGCTAC TTTAATTTCC TGTACGAAAT ATTCAGCCGT AAACCAAGTT CATCAAGAAA   
  
  
- AAGAAGTCAG CATTTTGATG ACTATGCCTG TCCCCTTCGA TCTCAATTAC AGTTTAAACA AAATAAAGGG   
  
  
- ATTTATGTGA ATATAAAAGA CATAATATAT GTCATAAAAA CTTCACCATT ATTGAAGAAA GGATTTGGGG   
  
  
- TGCTCTGGAT GAACCTTCGT TTCCTACTCA TGCAAGGCAA GCCAAAGTGC ATGGACACAT GATAGCAAAC   
  
  
- AAGCTTGGAA AGGGAGAGTC CTGATTAGGT CACCTGTAGT GACTTCCCAA GACTTTTCTA GCTCCTGATT   
  
  
- GGTAACATGC CATTTCATAA TACACCCAAT CTACCAAGGA AGAATACTAC ATACCACAAT CAACATCATA   
  
  
- AGTAACCATA TAGGTCATCA AGATGAATCG GCTGAGTAGC AGGTTCATAT AGATTTTTTT GCCAAGAAAA   
  
  
- CCAAACATAT AAAACTTGCC AATGTGGAGA TTACTGGAAG AACAGAAAGA AACAGATGCA TATCAGGACA   
  
  
- AGTTCCATGC ACCATATATC TCCAATAGAA TATCCAAAAT TTTAACTACA AAGCTTATGT CGAACCAGAA   
  
  
- AAACTTGTTA CAGGGGGTTC CAAACTCTGA ATTACGGCAC CTTGAGGCCT CAATTTATCC AATATCAAGT   
  
  
- AGAATCAAAT CTCTCAGGCA TGGGGTTGAA GTTTCAAGAA TTTGACTGCA GAACAAATAC ACTTCAACCA   
  
  
- TATTAATGAT TTTTTTTTCA TATAAGAAAC CACAATTATG ATTCTGACAA GACTAAAATG TGGCTCAAGG   
  
  
- TCAAATCAGG GACTCCACAT CAGCTGATGA TCATCTTTCT AAAGAATTCT GCTTGCTCAG GGAAATACTC   
  
  
- CAACAGCTCA TCCTTCGTAA CCCAAGCAAA ATCCTCACAC TTCCCAATGT TAAATTTGTT GGTTGCAATT   
  
  
- ACTTGGGATT TAAAGAAGAA TTGCTGACAA AAAAAAGAAA AAAAATCAAG CACGGAAATT AAAAAAAACA   
  
  
- TAATTTAGCC TAAACAAGAA AGGCACATT

+     Skn-1\_motif

| Site Name | Organism | Position | Strand | Matrix score. | sequence | function |
| --- | --- | --- | --- | --- | --- | --- |
| Skn-1\_motif | Oryza sativa | 942 | - | 5 | GTCAT | cis-acting regulatory element required for endosperm expression |
| Skn-1\_motif | Oryza sativa | 381 | - | 5 | GTCAT | cis-acting regulatory element required for endosperm expression |
| Skn-1\_motif | Oryza sativa | 348 | + | 5 | GTCAT | cis-acting regulatory element required for endosperm expression |

> 2018/04/13 10:10:12  
+ CCCCCTTCTC GAGTTTCTCA ACTCTCTCCT TGACTCTTAC CTTCTCTTCC TTCCAATTAT ACCCAGTTCC   
  
  
+ CTTCACTATA GTACCTTCCA CTCTTCTCCC ACCTATCTGG GTGCCTATAT AAATGTCTCT TTCTCTCTCT   
  
  
+ CTTTTATACC ACGAAACCGA ACACTACACC TCTACCTCCT ACCTCTCTGA TCAGACTCAA AGTACCCATT   
  
  
+ TGGGTTGATT TAGTATGGAT GAAGACAAGT GGACGGGAAG TCACTTTTCA CATGCAGGAT ATATACGTAG   
  
  
+ CAATAGTGGC TACAAAGTGT AAGAATAAAC GGTTTTCTCT TGGTAAGTAA ATCCTGTACG ATGCAAAGTC   
  
  
+ ATTGGGGTTA CAGAAGAAAA CATATACGAT ATGACAAATG GTTGGTTCGT GACCACAAAA GAAAACTGGT   
  
  
+ TAATCTAGTT GACGTCGATG AAATTAAAGG ACATGCTTTA TAAGTCGGCA TTTGGTTCAA GTAGTTCTTT   
  
  
+ TTCTTCAGTC GTAAAACTAC TGATACGGAC AGGGGAAGCT AGAGTTAATG TCAAATTTGT TTTATTTCCC   
  
  
+ TAAATACACT TATATTTTCT GTATTATATA CAGTATTTTT GAAGTGGTAA TAACTTCTTT CCTAAACCCC   
  
  
+ ACGAGACCTA CTTGGAAGCA AAGGATGAGT ACGTTCCGTT CGGTTTCACG TACCTGTGTA CTATCGTTTG   
  
  
+ TTCGAACCTT TCCCTCTCAG GACTAATCCA GTGGACATCA CTGAAGGGTT CTGAAAAGAT CGAGGACTAA   
  
  
+ CCATTGTACG GTAAAGTATT ATGTGGGTTA GATGGTTCCT TCTTATGATG TATGGTGTTA GTTGTAGTAT   
  
  
+ TCATTGGTAT ATCCAGTAGT TCTACTTAGC CGACTCATCG TCCAAGTATA TCTAAAAAAA CGGTTCTTTT   
  
  
+ GGTTTGTATA TTTTGAACGG TTACACCTCT AATGACCTTC TTGTCTTTCT TTGTCTACGT ATAGTCCTGT   
  
  
+ TCAAGGTACG TGGTATATAG AGGTTATCTT ATAGGTTTTA AAATTGATGT TTCGAATACA GCTTGGTCTT   
  
  
+ TTTGAACAAT GTCCCCCAAG GTTTGAGACT TAATGCCGTG GAACTCCGGA GTTAAATAGG TTATAGTTCA   
  
  
+ TCTTAGTTTA GAGAGTCCGT ACCCCAACTT CAAAGTTCTT AAACTGACGT CTTGTTTATG TGAAGTTGGT   
  
  
+ ATAATTACTA AAAAAAAAGT ATATTCTTTG GTGTTAATAC TAAGACTGTT CTGATTTTAC ACCGAGTTCC   
  
  
+ AGTTTAGTCC CTGAGGTGTA GTCGACTACT AGTAGAAAGA TTTCTTAAGA CGAACGAGTC CCTTTATGAG   
  
  
+ GTTGTCGAGT AGGAAGCATT GGGTTCGTTT TAGGAGTGTG AAGGGTTACA ATTTAAACAA CCAACGTTAA   
  
  
+ TGAACCCTAA ATTTCTTCTT AACGACTGTT TTTTTTCTTT TTTTTAGTTC GTGCCTTTAA TTTTTTTTGT   
  
  
+ ATTAAATCGG ATTTGTTCTT TCCGTGTAA  

- GGGGGAAGAG CTCAAAGAGT TGAGAGAGGA ACTGAGAATG GAAGAGAAGG AAGGTTAATA TGGGTCAAGG   
  
  
- GAAGTGATAT CATGGAAGGT GAGAAGAGGG TGGATAGACC CACGGATATA TTTACAGAGA AAGAGAGAGA   
  
  
- GAAAATATGG TGCTTTGGCT TGTGATGTGG AGATGGAGGA TGGAGAGACT AGTCTGAGTT TCATGGGTAA   
  
  
- ACCCAACTAA ATCATACCTA CTTCTGTTCA CCTGCCCTTC AGTGAAAAGT GTACGTCCTA TATATGCATC   
  
  
- GTTATCACCG ATGTTTCACA TTCTTATTTG CCAAAAGAGA ACCATTCATT TAGGACATGC TACGTTTCAG   
  
  
- TAACCCCAAT GTCTTCTTTT GTATATGCTA TACTGTTTAC CAACCAAGCA CTGGTGTTTT CTTTTGACCA   
  
  
- ATTAGATCAA CTGCAGCTAC TTTAATTTCC TGTACGAAAT ATTCAGCCGT AAACCAAGTT CATCAAGAAA   
  
  
- AAGAAGTCAG CATTTTGATG ACTATGCCTG TCCCCTTCGA TCTCAATTAC AGTTTAAACA AAATAAAGGG   
  
  
- ATTTATGTGA ATATAAAAGA CATAATATAT GTCATAAAAA CTTCACCATT ATTGAAGAAA GGATTTGGGG   
  
  
- TGCTCTGGAT GAACCTTCGT TTCCTACTCA TGCAAGGCAA GCCAAAGTGC ATGGACACAT GATAGCAAAC   
  
  
- AAGCTTGGAA AGGGAGAGTC CTGATTAGGT CACCTGTAGT GACTTCCCAA GACTTTTCTA GCTCCTGATT   
  
  
- GGTAACATGC CATTTCATAA TACACCCAAT CTACCAAGGA AGAATACTAC ATACCACAAT CAACATCATA   
  
  
- AGTAACCATA TAGGTCATCA AGATGAATCG GCTGAGTAGC AGGTTCATAT AGATTTTTTT GCCAAGAAAA   
  
  
- CCAAACATAT AAAACTTGCC AATGTGGAGA TTACTGGAAG AACAGAAAGA AACAGATGCA TATCAGGACA   
  
  
- AGTTCCATGC ACCATATATC TCCAATAGAA TATCCAAAAT TTTAACTACA AAGCTTATGT CGAACCAGAA   
  
  
- AAACTTGTTA CAGGGGGTTC CAAACTCTGA ATTACGGCAC CTTGAGGCCT CAATTTATCC AATATCAAGT   
  
  
- AGAATCAAAT CTCTCAGGCA TGGGGTTGAA GTTTCAAGAA TTTGACTGCA GAACAAATAC ACTTCAACCA   
  
  
- TATTAATGAT TTTTTTTTCA TATAAGAAAC CACAATTATG ATTCTGACAA GACTAAAATG TGGCTCAAGG   
  
  
- TCAAATCAGG GACTCCACAT CAGCTGATGA TCATCTTTCT AAAGAATTCT GCTTGCTCAG GGAAATACTC   
  
  
- CAACAGCTCA TCCTTCGTAA CCCAAGCAAA ATCCTCACAC TTCCCAATGT TAAATTTGTT GGTTGCAATT   
  
  
- ACTTGGGATT TAAAGAAGAA TTGCTGACAA AAAAAAGAAA AAAAATCAAG CACGGAAATT AAAAAAAACA   
  
  
- TAATTTAGCC TAAACAAGAA AGGCACATT

+     TATA-box

| Site Name | Organism | Position | Strand | Matrix score. | sequence | function |
| --- | --- | --- | --- | --- | --- | --- |
| TATA-box | Lycopersicon esculentum | 1358 | + | 5 | TTTTA | core promoter element around -30 of transcription start |
| TATA-box | Lycopersicon esculentum | 1016 | + | 5 | TTTTA | core promoter element around -30 of transcription start |
| TATA-box | Arabidopsis thaliana | 570 | - | 5 | TATAA | core promoter element around -30 of transcription start |
| TATA-box | Lycopersicon esculentum | 1442 | + | 5 | TTTTA | core promoter element around -30 of transcription start |
| TATA-box | Glycine max | 1225 | + | 5 | TAATA | core promoter element around -30 of transcription start |
| TATA-box | Arabidopsis thaliana | 1210 | - | 4 | TATA | core promoter element around -30 of transcription start |
| TATA-box | Arabidopsis thaliana | 1190 | - | 4 | TATA | core promoter element around -30 of transcription start |
| TATA-box | Arabidopsis thaliana | 1111 | - | 5 | TATAA | core promoter element around -30 of transcription start |
| TATA-box | Arabidopsis thaliana | 1010 | - | 4 | TATA | core promoter element around -30 of transcription start |
| TATA-box | Arabidopsis thaliana | 270 | + | 4 | TATA | core promoter element around -30 of transcription start |
| TATA-box | Arabidopsis thaliana | 996 | - | 4 | TATA | core promoter element around -30 of transcription start |
| TATA-box | Lycopersicon esculentum | 502 | - | 5 | TTTTA | core promoter element around -30 of transcription start |
| TATA-box | Lycopersicon esculentum | 550 | + | 5 | TTTTA | core promoter element around -30 of transcription start |
| TATA-box | Glycine max | 787 | - | 5 | TAATA | core promoter element around -30 of transcription start |
| TATA-box | Glycine max | 608 | + | 5 | TAATA | core promoter element around -30 of transcription start |
| TATA-box | Arabidopsis thaliana | 458 | - | 5 | TATAA | core promoter element around -30 of transcription start |
| TATA-box | Arabidopsis thaliana | 272 | + | 4 | TATA | core promoter element around -30 of transcription start |
| TATA-box | Oryza sativa | 1465 | - | 7 | TACAAAA | core promoter element around -30 of transcription start |
| TATA-box | Brassica napus | 56 | + | 6 | ATTATA | core promoter element around -30 of transcription start |
| TATA-box | Arabidopsis thaliana | 1009 | - | 5 | TATAA | core promoter element around -30 of transcription start |
| TATA-box | Arabidopsis thaliana | 57 | - | 5 | TATAA | core promoter element around -30 of transcription start |
| TATA-box | Lycopersicon esculentum | 1245 | + | 5 | TTTTA | core promoter element around -30 of transcription start |
| TATA-box | Arabidopsis thaliana | 994 | - | 4 | TATA | core promoter element around -30 of transcription start |
| TATA-box | Lycopersicon esculentum | 1199 | - | 5 | TTTTA | core promoter element around -30 of transcription start |
| TATA-box | Glycine max | 582 | - | 5 | TAATA | core promoter element around -30 of transcription start |
| TATA-box | Helianthus annuus | 915 | - | 6 | TATACA | core promoter element around -30 of transcription start |
| TATA-box | Lycopersicon esculentum | 893 | - | 5 | TTTTA | core promoter element around -30 of transcription start |
| TATA-box | Arabidopsis thaliana | 917 | - | 4 | TATA | core promoter element around -30 of transcription start |
| TATA-box | Arabidopsis thaliana | 1112 | - | 4 | TATA | core promoter element around -30 of transcription start |
| TATA-box | Brassica napus | 269 | + | 6 | ATATAT | core promoter element around -30 of transcription start |
| TATA-box | Brassica napus | 583 | + | 6 | ATTATA | core promoter element around -30 of transcription start |
| TATA-box | Arabidopsis thaliana | 848 | - | 4 | TATA | core promoter element around -30 of transcription start |
| TATA-box | Pisum sativum | 370 | - | 7 | TATATGT | core promoter element around -30 of transcription start |
| TATA-box | Glycine max | 1470 | - | 5 | TAATA | core promoter element around -30 of transcription start |
| TATA-box | Arabidopsis thaliana | 58 | + | 4 | TATA | core promoter element around -30 of transcription start |
| TATA-box | Arabidopsis thaliana | 145 | + | 4 | TATA | core promoter element around -30 of transcription start |
| TATA-box | Lycopersicon esculentum | 1019 | - | 5 | TTTTA | core promoter element around -30 of transcription start |
| TATA-box | Arabidopsis thaliana | 457 | - | 6 | TATAAA | core promoter element around -30 of transcription start |
| TATA-box | Arabidopsis thaliana | 970 | - | 4 | TATA | core promoter element around -30 of transcription start |
| TATA-box | Arabidopsis thaliana | 887 | - | 4 | TATA | core promoter element around -30 of transcription start |
| TATA-box | Arabidopsis thaliana | 144 | - | 5 | TATAA | core promoter element around -30 of transcription start |
| TATA-box | Arabidopsis thaliana | 584 | - | 7 | TATATAA | core promoter element around -30 of transcription start |
| TATA-box | Brassica oleracea | 117 | + | 6 | ATATAA | core promoter element around -30 of transcription start |
| TATA-box | Arabidopsis thaliana | 587 | + | 4 | TATA | core promoter element around -30 of transcription start |
| TATA-box | Arabidopsis thaliana | 459 | + | 4 | TATA | core promoter element around -30 of transcription start |
| TATA-box | Arabidopsis thaliana | 373 | + | 4 | TATA | core promoter element around -30 of transcription start |
| TATA-box | Arabidopsis thaliana | 77 | + | 4 | TATA | core promoter element around -30 of transcription start |
| TATA-box | Arabidopsis thaliana | 114 | + | 9 | ccTATAAAaa | core promoter element around -30 of transcription start |
| TATA-box | Arabidopsis thaliana | 571 | + | 4 | TATA | core promoter element around -30 of transcription start |
| TATA-box | Arabidopsis thaliana | 143 | - | 6 | TATAAA | core promoter element around -30 of transcription start |
| TATA-box | Arabidopsis thaliana | 585 | + | 4 | TATA | core promoter element around -30 of transcription start |
| TATA-box | Ac | 118 | + | 7 | TATAAAT | core promoter element around -30 of transcription start |
| TATA-box | Arabidopsis thaliana | 142 | - | 7 | TATAAAA | core promoter element around -30 of transcription start |
| TATA-box | Arabidopsis thaliana | 116 | + | 9 | taTATAAAtc | core promoter element around -30 of transcription start |

> 2018/04/13 10:10:12  
+ CCCCCTTCTC GAGTTTCTCA ACTCTCTCCT TGACTCTTAC CTTCTCTTCC TTCCAATTAT ACCCAGTTCC   
  
  
+ CTTCACTATA GTACCTTCCA CTCTTCTCCC ACCTATCTGG GTGCCTATAT AAATGTCTCT TTCTCTCTCT   
  
  
+ CTTTTATACC ACGAAACCGA ACACTACACC TCTACCTCCT ACCTCTCTGA TCAGACTCAA AGTACCCATT   
  
  
+ TGGGTTGATT TAGTATGGAT GAAGACAAGT GGACGGGAAG TCACTTTTCA CATGCAGGAT ATATACGTAG   
  
  
+ CAATAGTGGC TACAAAGTGT AAGAATAAAC GGTTTTCTCT TGGTAAGTAA ATCCTGTACG ATGCAAAGTC   
  
  
+ ATTGGGGTTA CAGAAGAAAA CATATACGAT ATGACAAATG GTTGGTTCGT GACCACAAAA GAAAACTGGT   
  
  
+ TAATCTAGTT GACGTCGATG AAATTAAAGG ACATGCTTTA TAAGTCGGCA TTTGGTTCAA GTAGTTCTTT   
  
  
+ TTCTTCAGTC GTAAAACTAC TGATACGGAC AGGGGAAGCT AGAGTTAATG TCAAATTTGT TTTATTTCCC   
  
  
+ TAAATACACT TATATTTTCT GTATTATATA CAGTATTTTT GAAGTGGTAA TAACTTCTTT CCTAAACCCC   
  
  
+ ACGAGACCTA CTTGGAAGCA AAGGATGAGT ACGTTCCGTT CGGTTTCACG TACCTGTGTA CTATCGTTTG   
  
  
+ TTCGAACCTT TCCCTCTCAG GACTAATCCA GTGGACATCA CTGAAGGGTT CTGAAAAGAT CGAGGACTAA   
  
  
+ CCATTGTACG GTAAAGTATT ATGTGGGTTA GATGGTTCCT TCTTATGATG TATGGTGTTA GTTGTAGTAT   
  
  
+ TCATTGGTAT ATCCAGTAGT TCTACTTAGC CGACTCATCG TCCAAGTATA TCTAAAAAAA CGGTTCTTTT   
  
  
+ GGTTTGTATA TTTTGAACGG TTACACCTCT AATGACCTTC TTGTCTTTCT TTGTCTACGT ATAGTCCTGT   
  
  
+ TCAAGGTACG TGGTATATAG AGGTTATCTT ATAGGTTTTA AAATTGATGT TTCGAATACA GCTTGGTCTT   
  
  
+ TTTGAACAAT GTCCCCCAAG GTTTGAGACT TAATGCCGTG GAACTCCGGA GTTAAATAGG TTATAGTTCA   
  
  
+ TCTTAGTTTA GAGAGTCCGT ACCCCAACTT CAAAGTTCTT AAACTGACGT CTTGTTTATG TGAAGTTGGT   
  
  
+ ATAATTACTA AAAAAAAAGT ATATTCTTTG GTGTTAATAC TAAGACTGTT CTGATTTTAC ACCGAGTTCC   
  
  
+ AGTTTAGTCC CTGAGGTGTA GTCGACTACT AGTAGAAAGA TTTCTTAAGA CGAACGAGTC CCTTTATGAG   
  
  
+ GTTGTCGAGT AGGAAGCATT GGGTTCGTTT TAGGAGTGTG AAGGGTTACA ATTTAAACAA CCAACGTTAA   
  
  
+ TGAACCCTAA ATTTCTTCTT AACGACTGTT TTTTTTCTTT TTTTTAGTTC GTGCCTTTAA TTTTTTTTGT   
  
  
+ ATTAAATCGG ATTTGTTCTT TCCGTGTAA  

- GGGGGAAGAG CTCAAAGAGT TGAGAGAGGA ACTGAGAATG GAAGAGAAGG AAGGTTAATA TGGGTCAAGG   
  
  
- GAAGTGATAT CATGGAAGGT GAGAAGAGGG TGGATAGACC CACGGATATA TTTACAGAGA AAGAGAGAGA   
  
  
- GAAAATATGG TGCTTTGGCT TGTGATGTGG AGATGGAGGA TGGAGAGACT AGTCTGAGTT TCATGGGTAA   
  
  
- ACCCAACTAA ATCATACCTA CTTCTGTTCA CCTGCCCTTC AGTGAAAAGT GTACGTCCTA TATATGCATC   
  
  
- GTTATCACCG ATGTTTCACA TTCTTATTTG CCAAAAGAGA ACCATTCATT TAGGACATGC TACGTTTCAG   
  
  
- TAACCCCAAT GTCTTCTTTT GTATATGCTA TACTGTTTAC CAACCAAGCA CTGGTGTTTT CTTTTGACCA   
  
  
- ATTAGATCAA CTGCAGCTAC TTTAATTTCC TGTACGAAAT ATTCAGCCGT AAACCAAGTT CATCAAGAAA   
  
  
- AAGAAGTCAG CATTTTGATG ACTATGCCTG TCCCCTTCGA TCTCAATTAC AGTTTAAACA AAATAAAGGG   
  
  
- ATTTATGTGA ATATAAAAGA CATAATATAT GTCATAAAAA CTTCACCATT ATTGAAGAAA GGATTTGGGG   
  
  
- TGCTCTGGAT GAACCTTCGT TTCCTACTCA TGCAAGGCAA GCCAAAGTGC ATGGACACAT GATAGCAAAC   
  
  
- AAGCTTGGAA AGGGAGAGTC CTGATTAGGT CACCTGTAGT GACTTCCCAA GACTTTTCTA GCTCCTGATT   
  
  
- GGTAACATGC CATTTCATAA TACACCCAAT CTACCAAGGA AGAATACTAC ATACCACAAT CAACATCATA   
  
  
- AGTAACCATA TAGGTCATCA AGATGAATCG GCTGAGTAGC AGGTTCATAT AGATTTTTTT GCCAAGAAAA   
  
  
- CCAAACATAT AAAACTTGCC AATGTGGAGA TTACTGGAAG AACAGAAAGA AACAGATGCA TATCAGGACA   
  
  
- AGTTCCATGC ACCATATATC TCCAATAGAA TATCCAAAAT TTTAACTACA AAGCTTATGT CGAACCAGAA   
  
  
- AAACTTGTTA CAGGGGGTTC CAAACTCTGA ATTACGGCAC CTTGAGGCCT CAATTTATCC AATATCAAGT   
  
  
- AGAATCAAAT CTCTCAGGCA TGGGGTTGAA GTTTCAAGAA TTTGACTGCA GAACAAATAC ACTTCAACCA   
  
  
- TATTAATGAT TTTTTTTTCA TATAAGAAAC CACAATTATG ATTCTGACAA GACTAAAATG TGGCTCAAGG   
  
  
- TCAAATCAGG GACTCCACAT CAGCTGATGA TCATCTTTCT AAAGAATTCT GCTTGCTCAG GGAAATACTC   
  
  
- CAACAGCTCA TCCTTCGTAA CCCAAGCAAA ATCCTCACAC TTCCCAATGT TAAATTTGTT GGTTGCAATT   
  
  
- ACTTGGGATT TAAAGAAGAA TTGCTGACAA AAAAAAGAAA AAAAATCAAG CACGGAAATT AAAAAAAACA   
  
  
- TAATTTAGCC TAAACAAGAA AGGCACATT

+     TC-rich repeats

| Site Name | Organism | Position | Strand | Matrix score. | sequence | function |
| --- | --- | --- | --- | --- | --- | --- |
| TC-rich repeats | Nicotiana tabacum | 488 | + | 9 | ATTTTCTTCA | cis-acting element involved in defense and stress responsiveness |

> 2018/04/13 10:10:12  
+ CCCCCTTCTC GAGTTTCTCA ACTCTCTCCT TGACTCTTAC CTTCTCTTCC TTCCAATTAT ACCCAGTTCC   
  
  
+ CTTCACTATA GTACCTTCCA CTCTTCTCCC ACCTATCTGG GTGCCTATAT AAATGTCTCT TTCTCTCTCT   
  
  
+ CTTTTATACC ACGAAACCGA ACACTACACC TCTACCTCCT ACCTCTCTGA TCAGACTCAA AGTACCCATT   
  
  
+ TGGGTTGATT TAGTATGGAT GAAGACAAGT GGACGGGAAG TCACTTTTCA CATGCAGGAT ATATACGTAG   
  
  
+ CAATAGTGGC TACAAAGTGT AAGAATAAAC GGTTTTCTCT TGGTAAGTAA ATCCTGTACG ATGCAAAGTC   
  
  
+ ATTGGGGTTA CAGAAGAAAA CATATACGAT ATGACAAATG GTTGGTTCGT GACCACAAAA GAAAACTGGT   
  
  
+ TAATCTAGTT GACGTCGATG AAATTAAAGG ACATGCTTTA TAAGTCGGCA TTTGGTTCAA GTAGTTCTTT   
  
  
+ TTCTTCAGTC GTAAAACTAC TGATACGGAC AGGGGAAGCT AGAGTTAATG TCAAATTTGT TTTATTTCCC   
  
  
+ TAAATACACT TATATTTTCT GTATTATATA CAGTATTTTT GAAGTGGTAA TAACTTCTTT CCTAAACCCC   
  
  
+ ACGAGACCTA CTTGGAAGCA AAGGATGAGT ACGTTCCGTT CGGTTTCACG TACCTGTGTA CTATCGTTTG   
  
  
+ TTCGAACCTT TCCCTCTCAG GACTAATCCA GTGGACATCA CTGAAGGGTT CTGAAAAGAT CGAGGACTAA   
  
  
+ CCATTGTACG GTAAAGTATT ATGTGGGTTA GATGGTTCCT TCTTATGATG TATGGTGTTA GTTGTAGTAT   
  
  
+ TCATTGGTAT ATCCAGTAGT TCTACTTAGC CGACTCATCG TCCAAGTATA TCTAAAAAAA CGGTTCTTTT   
  
  
+ GGTTTGTATA TTTTGAACGG TTACACCTCT AATGACCTTC TTGTCTTTCT TTGTCTACGT ATAGTCCTGT   
  
  
+ TCAAGGTACG TGGTATATAG AGGTTATCTT ATAGGTTTTA AAATTGATGT TTCGAATACA GCTTGGTCTT   
  
  
+ TTTGAACAAT GTCCCCCAAG GTTTGAGACT TAATGCCGTG GAACTCCGGA GTTAAATAGG TTATAGTTCA   
  
  
+ TCTTAGTTTA GAGAGTCCGT ACCCCAACTT CAAAGTTCTT AAACTGACGT CTTGTTTATG TGAAGTTGGT   
  
  
+ ATAATTACTA AAAAAAAAGT ATATTCTTTG GTGTTAATAC TAAGACTGTT CTGATTTTAC ACCGAGTTCC   
  
  
+ AGTTTAGTCC CTGAGGTGTA GTCGACTACT AGTAGAAAGA TTTCTTAAGA CGAACGAGTC CCTTTATGAG   
  
  
+ GTTGTCGAGT AGGAAGCATT GGGTTCGTTT TAGGAGTGTG AAGGGTTACA ATTTAAACAA CCAACGTTAA   
  
  
+ TGAACCCTAA ATTTCTTCTT AACGACTGTT TTTTTTCTTT TTTTTAGTTC GTGCCTTTAA TTTTTTTTGT   
  
  
+ ATTAAATCGG ATTTGTTCTT TCCGTGTAA  

- GGGGGAAGAG CTCAAAGAGT TGAGAGAGGA ACTGAGAATG GAAGAGAAGG AAGGTTAATA TGGGTCAAGG   
  
  
- GAAGTGATAT CATGGAAGGT GAGAAGAGGG TGGATAGACC CACGGATATA TTTACAGAGA AAGAGAGAGA   
  
  
- GAAAATATGG TGCTTTGGCT TGTGATGTGG AGATGGAGGA TGGAGAGACT AGTCTGAGTT TCATGGGTAA   
  
  
- ACCCAACTAA ATCATACCTA CTTCTGTTCA CCTGCCCTTC AGTGAAAAGT GTACGTCCTA TATATGCATC   
  
  
- GTTATCACCG ATGTTTCACA TTCTTATTTG CCAAAAGAGA ACCATTCATT TAGGACATGC TACGTTTCAG   
  
  
- TAACCCCAAT GTCTTCTTTT GTATATGCTA TACTGTTTAC CAACCAAGCA CTGGTGTTTT CTTTTGACCA   
  
  
- ATTAGATCAA CTGCAGCTAC TTTAATTTCC TGTACGAAAT ATTCAGCCGT AAACCAAGTT CATCAAGAAA   
  
  
- AAGAAGTCAG CATTTTGATG ACTATGCCTG TCCCCTTCGA TCTCAATTAC AGTTTAAACA AAATAAAGGG   
  
  
- ATTTATGTGA ATATAAAAGA CATAATATAT GTCATAAAAA CTTCACCATT ATTGAAGAAA GGATTTGGGG   
  
  
- TGCTCTGGAT GAACCTTCGT TTCCTACTCA TGCAAGGCAA GCCAAAGTGC ATGGACACAT GATAGCAAAC   
  
  
- AAGCTTGGAA AGGGAGAGTC CTGATTAGGT CACCTGTAGT GACTTCCCAA GACTTTTCTA GCTCCTGATT   
  
  
- GGTAACATGC CATTTCATAA TACACCCAAT CTACCAAGGA AGAATACTAC ATACCACAAT CAACATCATA   
  
  
- AGTAACCATA TAGGTCATCA AGATGAATCG GCTGAGTAGC AGGTTCATAT AGATTTTTTT GCCAAGAAAA   
  
  
- CCAAACATAT AAAACTTGCC AATGTGGAGA TTACTGGAAG AACAGAAAGA AACAGATGCA TATCAGGACA   
  
  
- AGTTCCATGC ACCATATATC TCCAATAGAA TATCCAAAAT TTTAACTACA AAGCTTATGT CGAACCAGAA   
  
  
- AAACTTGTTA CAGGGGGTTC CAAACTCTGA ATTACGGCAC CTTGAGGCCT CAATTTATCC AATATCAAGT   
  
  
- AGAATCAAAT CTCTCAGGCA TGGGGTTGAA GTTTCAAGAA TTTGACTGCA GAACAAATAC ACTTCAACCA   
  
  
- TATTAATGAT TTTTTTTTCA TATAAGAAAC CACAATTATG ATTCTGACAA GACTAAAATG TGGCTCAAGG   
  
  
- TCAAATCAGG GACTCCACAT CAGCTGATGA TCATCTTTCT AAAGAATTCT GCTTGCTCAG GGAAATACTC   
  
  
- CAACAGCTCA TCCTTCGTAA CCCAAGCAAA ATCCTCACAC TTCCCAATGT TAAATTTGTT GGTTGCAATT   
  
  
- ACTTGGGATT TAAAGAAGAA TTGCTGACAA AAAAAAGAAA AAAAATCAAG CACGGAAATT AAAAAAAACA   
  
  
- TAATTTAGCC TAAACAAGAA AGGCACATT

+     TCT-motif

| Site Name | Organism | Position | Strand | Matrix score. | sequence | function |
| --- | --- | --- | --- | --- | --- | --- |
| TCT-motif | Arabidopsis thaliana | 299 | - | 6 | TCTTAC | part of a light responsive element |
| TCT-motif | Arabidopsis thaliana | 35 | + | 6 | TCTTAC | part of a light responsive element |

> 2018/04/13 10:10:12  
+ CCCCCTTCTC GAGTTTCTCA ACTCTCTCCT TGACTCTTAC CTTCTCTTCC TTCCAATTAT ACCCAGTTCC   
  
  
+ CTTCACTATA GTACCTTCCA CTCTTCTCCC ACCTATCTGG GTGCCTATAT AAATGTCTCT TTCTCTCTCT   
  
  
+ CTTTTATACC ACGAAACCGA ACACTACACC TCTACCTCCT ACCTCTCTGA TCAGACTCAA AGTACCCATT   
  
  
+ TGGGTTGATT TAGTATGGAT GAAGACAAGT GGACGGGAAG TCACTTTTCA CATGCAGGAT ATATACGTAG   
  
  
+ CAATAGTGGC TACAAAGTGT AAGAATAAAC GGTTTTCTCT TGGTAAGTAA ATCCTGTACG ATGCAAAGTC   
  
  
+ ATTGGGGTTA CAGAAGAAAA CATATACGAT ATGACAAATG GTTGGTTCGT GACCACAAAA GAAAACTGGT   
  
  
+ TAATCTAGTT GACGTCGATG AAATTAAAGG ACATGCTTTA TAAGTCGGCA TTTGGTTCAA GTAGTTCTTT   
  
  
+ TTCTTCAGTC GTAAAACTAC TGATACGGAC AGGGGAAGCT AGAGTTAATG TCAAATTTGT TTTATTTCCC   
  
  
+ TAAATACACT TATATTTTCT GTATTATATA CAGTATTTTT GAAGTGGTAA TAACTTCTTT CCTAAACCCC   
  
  
+ ACGAGACCTA CTTGGAAGCA AAGGATGAGT ACGTTCCGTT CGGTTTCACG TACCTGTGTA CTATCGTTTG   
  
  
+ TTCGAACCTT TCCCTCTCAG GACTAATCCA GTGGACATCA CTGAAGGGTT CTGAAAAGAT CGAGGACTAA   
  
  
+ CCATTGTACG GTAAAGTATT ATGTGGGTTA GATGGTTCCT TCTTATGATG TATGGTGTTA GTTGTAGTAT   
  
  
+ TCATTGGTAT ATCCAGTAGT TCTACTTAGC CGACTCATCG TCCAAGTATA TCTAAAAAAA CGGTTCTTTT   
  
  
+ GGTTTGTATA TTTTGAACGG TTACACCTCT AATGACCTTC TTGTCTTTCT TTGTCTACGT ATAGTCCTGT   
  
  
+ TCAAGGTACG TGGTATATAG AGGTTATCTT ATAGGTTTTA AAATTGATGT TTCGAATACA GCTTGGTCTT   
  
  
+ TTTGAACAAT GTCCCCCAAG GTTTGAGACT TAATGCCGTG GAACTCCGGA GTTAAATAGG TTATAGTTCA   
  
  
+ TCTTAGTTTA GAGAGTCCGT ACCCCAACTT CAAAGTTCTT AAACTGACGT CTTGTTTATG TGAAGTTGGT   
  
  
+ ATAATTACTA AAAAAAAAGT ATATTCTTTG GTGTTAATAC TAAGACTGTT CTGATTTTAC ACCGAGTTCC   
  
  
+ AGTTTAGTCC CTGAGGTGTA GTCGACTACT AGTAGAAAGA TTTCTTAAGA CGAACGAGTC CCTTTATGAG   
  
  
+ GTTGTCGAGT AGGAAGCATT GGGTTCGTTT TAGGAGTGTG AAGGGTTACA ATTTAAACAA CCAACGTTAA   
  
  
+ TGAACCCTAA ATTTCTTCTT AACGACTGTT TTTTTTCTTT TTTTTAGTTC GTGCCTTTAA TTTTTTTTGT   
  
  
+ ATTAAATCGG ATTTGTTCTT TCCGTGTAA  

- GGGGGAAGAG CTCAAAGAGT TGAGAGAGGA ACTGAGAATG GAAGAGAAGG AAGGTTAATA TGGGTCAAGG   
  
  
- GAAGTGATAT CATGGAAGGT GAGAAGAGGG TGGATAGACC CACGGATATA TTTACAGAGA AAGAGAGAGA   
  
  
- GAAAATATGG TGCTTTGGCT TGTGATGTGG AGATGGAGGA TGGAGAGACT AGTCTGAGTT TCATGGGTAA   
  
  
- ACCCAACTAA ATCATACCTA CTTCTGTTCA CCTGCCCTTC AGTGAAAAGT GTACGTCCTA TATATGCATC   
  
  
- GTTATCACCG ATGTTTCACA TTCTTATTTG CCAAAAGAGA ACCATTCATT TAGGACATGC TACGTTTCAG   
  
  
- TAACCCCAAT GTCTTCTTTT GTATATGCTA TACTGTTTAC CAACCAAGCA CTGGTGTTTT CTTTTGACCA   
  
  
- ATTAGATCAA CTGCAGCTAC TTTAATTTCC TGTACGAAAT ATTCAGCCGT AAACCAAGTT CATCAAGAAA   
  
  
- AAGAAGTCAG CATTTTGATG ACTATGCCTG TCCCCTTCGA TCTCAATTAC AGTTTAAACA AAATAAAGGG   
  
  
- ATTTATGTGA ATATAAAAGA CATAATATAT GTCATAAAAA CTTCACCATT ATTGAAGAAA GGATTTGGGG   
  
  
- TGCTCTGGAT GAACCTTCGT TTCCTACTCA TGCAAGGCAA GCCAAAGTGC ATGGACACAT GATAGCAAAC   
  
  
- AAGCTTGGAA AGGGAGAGTC CTGATTAGGT CACCTGTAGT GACTTCCCAA GACTTTTCTA GCTCCTGATT   
  
  
- GGTAACATGC CATTTCATAA TACACCCAAT CTACCAAGGA AGAATACTAC ATACCACAAT CAACATCATA   
  
  
- AGTAACCATA TAGGTCATCA AGATGAATCG GCTGAGTAGC AGGTTCATAT AGATTTTTTT GCCAAGAAAA   
  
  
- CCAAACATAT AAAACTTGCC AATGTGGAGA TTACTGGAAG AACAGAAAGA AACAGATGCA TATCAGGACA   
  
  
- AGTTCCATGC ACCATATATC TCCAATAGAA TATCCAAAAT TTTAACTACA AAGCTTATGT CGAACCAGAA   
  
  
- AAACTTGTTA CAGGGGGTTC CAAACTCTGA ATTACGGCAC CTTGAGGCCT CAATTTATCC AATATCAAGT   
  
  
- AGAATCAAAT CTCTCAGGCA TGGGGTTGAA GTTTCAAGAA TTTGACTGCA GAACAAATAC ACTTCAACCA   
  
  
- TATTAATGAT TTTTTTTTCA TATAAGAAAC CACAATTATG ATTCTGACAA GACTAAAATG TGGCTCAAGG   
  
  
- TCAAATCAGG GACTCCACAT CAGCTGATGA TCATCTTTCT AAAGAATTCT GCTTGCTCAG GGAAATACTC   
  
  
- CAACAGCTCA TCCTTCGTAA CCCAAGCAAA ATCCTCACAC TTCCCAATGT TAAATTTGTT GGTTGCAATT   
  
  
- ACTTGGGATT TAAAGAAGAA TTGCTGACAA AAAAAAGAAA AAAAATCAAG CACGGAAATT AAAAAAAACA   
  
  
- TAATTTAGCC TAAACAAGAA AGGCACATT

+     TGA-element

| Site Name | Organism | Position | Strand | Matrix score. | sequence | function |
| --- | --- | --- | --- | --- | --- | --- |
| TGA-element | Brassica oleracea | 1421 | + | 6 | AACGAC | auxin-responsive element |

> 2018/04/13 10:10:12  
+ CCCCCTTCTC GAGTTTCTCA ACTCTCTCCT TGACTCTTAC CTTCTCTTCC TTCCAATTAT ACCCAGTTCC   
  
  
+ CTTCACTATA GTACCTTCCA CTCTTCTCCC ACCTATCTGG GTGCCTATAT AAATGTCTCT TTCTCTCTCT   
  
  
+ CTTTTATACC ACGAAACCGA ACACTACACC TCTACCTCCT ACCTCTCTGA TCAGACTCAA AGTACCCATT   
  
  
+ TGGGTTGATT TAGTATGGAT GAAGACAAGT GGACGGGAAG TCACTTTTCA CATGCAGGAT ATATACGTAG   
  
  
+ CAATAGTGGC TACAAAGTGT AAGAATAAAC GGTTTTCTCT TGGTAAGTAA ATCCTGTACG ATGCAAAGTC   
  
  
+ ATTGGGGTTA CAGAAGAAAA CATATACGAT ATGACAAATG GTTGGTTCGT GACCACAAAA GAAAACTGGT   
  
  
+ TAATCTAGTT GACGTCGATG AAATTAAAGG ACATGCTTTA TAAGTCGGCA TTTGGTTCAA GTAGTTCTTT   
  
  
+ TTCTTCAGTC GTAAAACTAC TGATACGGAC AGGGGAAGCT AGAGTTAATG TCAAATTTGT TTTATTTCCC   
  
  
+ TAAATACACT TATATTTTCT GTATTATATA CAGTATTTTT GAAGTGGTAA TAACTTCTTT CCTAAACCCC   
  
  
+ ACGAGACCTA CTTGGAAGCA AAGGATGAGT ACGTTCCGTT CGGTTTCACG TACCTGTGTA CTATCGTTTG   
  
  
+ TTCGAACCTT TCCCTCTCAG GACTAATCCA GTGGACATCA CTGAAGGGTT CTGAAAAGAT CGAGGACTAA   
  
  
+ CCATTGTACG GTAAAGTATT ATGTGGGTTA GATGGTTCCT TCTTATGATG TATGGTGTTA GTTGTAGTAT   
  
  
+ TCATTGGTAT ATCCAGTAGT TCTACTTAGC CGACTCATCG TCCAAGTATA TCTAAAAAAA CGGTTCTTTT   
  
  
+ GGTTTGTATA TTTTGAACGG TTACACCTCT AATGACCTTC TTGTCTTTCT TTGTCTACGT ATAGTCCTGT   
  
  
+ TCAAGGTACG TGGTATATAG AGGTTATCTT ATAGGTTTTA AAATTGATGT TTCGAATACA GCTTGGTCTT   
  
  
+ TTTGAACAAT GTCCCCCAAG GTTTGAGACT TAATGCCGTG GAACTCCGGA GTTAAATAGG TTATAGTTCA   
  
  
+ TCTTAGTTTA GAGAGTCCGT ACCCCAACTT CAAAGTTCTT AAACTGACGT CTTGTTTATG TGAAGTTGGT   
  
  
+ ATAATTACTA AAAAAAAAGT ATATTCTTTG GTGTTAATAC TAAGACTGTT CTGATTTTAC ACCGAGTTCC   
  
  
+ AGTTTAGTCC CTGAGGTGTA GTCGACTACT AGTAGAAAGA TTTCTTAAGA CGAACGAGTC CCTTTATGAG   
  
  
+ GTTGTCGAGT AGGAAGCATT GGGTTCGTTT TAGGAGTGTG AAGGGTTACA ATTTAAACAA CCAACGTTAA   
  
  
+ TGAACCCTAA ATTTCTTCTT AACGACTGTT TTTTTTCTTT TTTTTAGTTC GTGCCTTTAA TTTTTTTTGT   
  
  
+ ATTAAATCGG ATTTGTTCTT TCCGTGTAA  

- GGGGGAAGAG CTCAAAGAGT TGAGAGAGGA ACTGAGAATG GAAGAGAAGG AAGGTTAATA TGGGTCAAGG   
  
  
- GAAGTGATAT CATGGAAGGT GAGAAGAGGG TGGATAGACC CACGGATATA TTTACAGAGA AAGAGAGAGA   
  
  
- GAAAATATGG TGCTTTGGCT TGTGATGTGG AGATGGAGGA TGGAGAGACT AGTCTGAGTT TCATGGGTAA   
  
  
- ACCCAACTAA ATCATACCTA CTTCTGTTCA CCTGCCCTTC AGTGAAAAGT GTACGTCCTA TATATGCATC   
  
  
- GTTATCACCG ATGTTTCACA TTCTTATTTG CCAAAAGAGA ACCATTCATT TAGGACATGC TACGTTTCAG   
  
  
- TAACCCCAAT GTCTTCTTTT GTATATGCTA TACTGTTTAC CAACCAAGCA CTGGTGTTTT CTTTTGACCA   
  
  
- ATTAGATCAA CTGCAGCTAC TTTAATTTCC TGTACGAAAT ATTCAGCCGT AAACCAAGTT CATCAAGAAA   
  
  
- AAGAAGTCAG CATTTTGATG ACTATGCCTG TCCCCTTCGA TCTCAATTAC AGTTTAAACA AAATAAAGGG   
  
  
- ATTTATGTGA ATATAAAAGA CATAATATAT GTCATAAAAA CTTCACCATT ATTGAAGAAA GGATTTGGGG   
  
  
- TGCTCTGGAT GAACCTTCGT TTCCTACTCA TGCAAGGCAA GCCAAAGTGC ATGGACACAT GATAGCAAAC   
  
  
- AAGCTTGGAA AGGGAGAGTC CTGATTAGGT CACCTGTAGT GACTTCCCAA GACTTTTCTA GCTCCTGATT   
  
  
- GGTAACATGC CATTTCATAA TACACCCAAT CTACCAAGGA AGAATACTAC ATACCACAAT CAACATCATA   
  
  
- AGTAACCATA TAGGTCATCA AGATGAATCG GCTGAGTAGC AGGTTCATAT AGATTTTTTT GCCAAGAAAA   
  
  
- CCAAACATAT AAAACTTGCC AATGTGGAGA TTACTGGAAG AACAGAAAGA AACAGATGCA TATCAGGACA   
  
  
- AGTTCCATGC ACCATATATC TCCAATAGAA TATCCAAAAT TTTAACTACA AAGCTTATGT CGAACCAGAA   
  
  
- AAACTTGTTA CAGGGGGTTC CAAACTCTGA ATTACGGCAC CTTGAGGCCT CAATTTATCC AATATCAAGT   
  
  
- AGAATCAAAT CTCTCAGGCA TGGGGTTGAA GTTTCAAGAA TTTGACTGCA GAACAAATAC ACTTCAACCA   
  
  
- TATTAATGAT TTTTTTTTCA TATAAGAAAC CACAATTATG ATTCTGACAA GACTAAAATG TGGCTCAAGG   
  
  
- TCAAATCAGG GACTCCACAT CAGCTGATGA TCATCTTTCT AAAGAATTCT GCTTGCTCAG GGAAATACTC   
  
  
- CAACAGCTCA TCCTTCGTAA CCCAAGCAAA ATCCTCACAC TTCCCAATGT TAAATTTGTT GGTTGCAATT   
  
  
- ACTTGGGATT TAAAGAAGAA TTGCTGACAA AAAAAAGAAA AAAAATCAAG CACGGAAATT AAAAAAAACA   
  
  
- TAATTTAGCC TAAACAAGAA AGGCACATT

+     TGACG-motif

| Site Name | Organism | Position | Strand | Matrix score. | sequence | function |
| --- | --- | --- | --- | --- | --- | --- |
| TGACG-motif | Hordeum vulgare | 430 | + | 5 | TGACG | cis-acting regulatory element involved in the MeJA-responsiveness |
| TGACG-motif | Hordeum vulgare | 1165 | + | 5 | TGACG | cis-acting regulatory element involved in the MeJA-responsiveness |

> 2018/04/13 10:10:12  
+ CCCCCTTCTC GAGTTTCTCA ACTCTCTCCT TGACTCTTAC CTTCTCTTCC TTCCAATTAT ACCCAGTTCC   
  
  
+ CTTCACTATA GTACCTTCCA CTCTTCTCCC ACCTATCTGG GTGCCTATAT AAATGTCTCT TTCTCTCTCT   
  
  
+ CTTTTATACC ACGAAACCGA ACACTACACC TCTACCTCCT ACCTCTCTGA TCAGACTCAA AGTACCCATT   
  
  
+ TGGGTTGATT TAGTATGGAT GAAGACAAGT GGACGGGAAG TCACTTTTCA CATGCAGGAT ATATACGTAG   
  
  
+ CAATAGTGGC TACAAAGTGT AAGAATAAAC GGTTTTCTCT TGGTAAGTAA ATCCTGTACG ATGCAAAGTC   
  
  
+ ATTGGGGTTA CAGAAGAAAA CATATACGAT ATGACAAATG GTTGGTTCGT GACCACAAAA GAAAACTGGT   
  
  
+ TAATCTAGTT GACGTCGATG AAATTAAAGG ACATGCTTTA TAAGTCGGCA TTTGGTTCAA GTAGTTCTTT   
  
  
+ TTCTTCAGTC GTAAAACTAC TGATACGGAC AGGGGAAGCT AGAGTTAATG TCAAATTTGT TTTATTTCCC   
  
  
+ TAAATACACT TATATTTTCT GTATTATATA CAGTATTTTT GAAGTGGTAA TAACTTCTTT CCTAAACCCC   
  
  
+ ACGAGACCTA CTTGGAAGCA AAGGATGAGT ACGTTCCGTT CGGTTTCACG TACCTGTGTA CTATCGTTTG   
  
  
+ TTCGAACCTT TCCCTCTCAG GACTAATCCA GTGGACATCA CTGAAGGGTT CTGAAAAGAT CGAGGACTAA   
  
  
+ CCATTGTACG GTAAAGTATT ATGTGGGTTA GATGGTTCCT TCTTATGATG TATGGTGTTA GTTGTAGTAT   
  
  
+ TCATTGGTAT ATCCAGTAGT TCTACTTAGC CGACTCATCG TCCAAGTATA TCTAAAAAAA CGGTTCTTTT   
  
  
+ GGTTTGTATA TTTTGAACGG TTACACCTCT AATGACCTTC TTGTCTTTCT TTGTCTACGT ATAGTCCTGT   
  
  
+ TCAAGGTACG TGGTATATAG AGGTTATCTT ATAGGTTTTA AAATTGATGT TTCGAATACA GCTTGGTCTT   
  
  
+ TTTGAACAAT GTCCCCCAAG GTTTGAGACT TAATGCCGTG GAACTCCGGA GTTAAATAGG TTATAGTTCA   
  
  
+ TCTTAGTTTA GAGAGTCCGT ACCCCAACTT CAAAGTTCTT AAACTGACGT CTTGTTTATG TGAAGTTGGT   
  
  
+ ATAATTACTA AAAAAAAAGT ATATTCTTTG GTGTTAATAC TAAGACTGTT CTGATTTTAC ACCGAGTTCC   
  
  
+ AGTTTAGTCC CTGAGGTGTA GTCGACTACT AGTAGAAAGA TTTCTTAAGA CGAACGAGTC CCTTTATGAG   
  
  
+ GTTGTCGAGT AGGAAGCATT GGGTTCGTTT TAGGAGTGTG AAGGGTTACA ATTTAAACAA CCAACGTTAA   
  
  
+ TGAACCCTAA ATTTCTTCTT AACGACTGTT TTTTTTCTTT TTTTTAGTTC GTGCCTTTAA TTTTTTTTGT   
  
  
+ ATTAAATCGG ATTTGTTCTT TCCGTGTAA  

- GGGGGAAGAG CTCAAAGAGT TGAGAGAGGA ACTGAGAATG GAAGAGAAGG AAGGTTAATA TGGGTCAAGG   
  
  
- GAAGTGATAT CATGGAAGGT GAGAAGAGGG TGGATAGACC CACGGATATA TTTACAGAGA AAGAGAGAGA   
  
  
- GAAAATATGG TGCTTTGGCT TGTGATGTGG AGATGGAGGA TGGAGAGACT AGTCTGAGTT TCATGGGTAA   
  
  
- ACCCAACTAA ATCATACCTA CTTCTGTTCA CCTGCCCTTC AGTGAAAAGT GTACGTCCTA TATATGCATC   
  
  
- GTTATCACCG ATGTTTCACA TTCTTATTTG CCAAAAGAGA ACCATTCATT TAGGACATGC TACGTTTCAG   
  
  
- TAACCCCAAT GTCTTCTTTT GTATATGCTA TACTGTTTAC CAACCAAGCA CTGGTGTTTT CTTTTGACCA   
  
  
- ATTAGATCAA CTGCAGCTAC TTTAATTTCC TGTACGAAAT ATTCAGCCGT AAACCAAGTT CATCAAGAAA   
  
  
- AAGAAGTCAG CATTTTGATG ACTATGCCTG TCCCCTTCGA TCTCAATTAC AGTTTAAACA AAATAAAGGG   
  
  
- ATTTATGTGA ATATAAAAGA CATAATATAT GTCATAAAAA CTTCACCATT ATTGAAGAAA GGATTTGGGG   
  
  
- TGCTCTGGAT GAACCTTCGT TTCCTACTCA TGCAAGGCAA GCCAAAGTGC ATGGACACAT GATAGCAAAC   
  
  
- AAGCTTGGAA AGGGAGAGTC CTGATTAGGT CACCTGTAGT GACTTCCCAA GACTTTTCTA GCTCCTGATT   
  
  
- GGTAACATGC CATTTCATAA TACACCCAAT CTACCAAGGA AGAATACTAC ATACCACAAT CAACATCATA   
  
  
- AGTAACCATA TAGGTCATCA AGATGAATCG GCTGAGTAGC AGGTTCATAT AGATTTTTTT GCCAAGAAAA   
  
  
- CCAAACATAT AAAACTTGCC AATGTGGAGA TTACTGGAAG AACAGAAAGA AACAGATGCA TATCAGGACA   
  
  
- AGTTCCATGC ACCATATATC TCCAATAGAA TATCCAAAAT TTTAACTACA AAGCTTATGT CGAACCAGAA   
  
  
- AAACTTGTTA CAGGGGGTTC CAAACTCTGA ATTACGGCAC CTTGAGGCCT CAATTTATCC AATATCAAGT   
  
  
- AGAATCAAAT CTCTCAGGCA TGGGGTTGAA GTTTCAAGAA TTTGACTGCA GAACAAATAC ACTTCAACCA   
  
  
- TATTAATGAT TTTTTTTTCA TATAAGAAAC CACAATTATG ATTCTGACAA GACTAAAATG TGGCTCAAGG   
  
  
- TCAAATCAGG GACTCCACAT CAGCTGATGA TCATCTTTCT AAAGAATTCT GCTTGCTCAG GGAAATACTC   
  
  
- CAACAGCTCA TCCTTCGTAA CCCAAGCAAA ATCCTCACAC TTCCCAATGT TAAATTTGTT GGTTGCAATT   
  
  
- ACTTGGGATT TAAAGAAGAA TTGCTGACAA AAAAAAGAAA AAAAATCAAG CACGGAAATT AAAAAAAACA   
  
  
- TAATTTAGCC TAAACAAGAA AGGCACATT

+     Unnamed\_\_1

| Site Name | Organism | Position | Strand | Matrix score. | sequence | function |
| --- | --- | --- | --- | --- | --- | --- |
| Unnamed\_\_1 | Zea mays | 1087 | + | 5 | CGTGG |  |
| Unnamed\_\_1 | Zea mays | 149 | - | 5 | CGTGG |  |
| Unnamed\_\_1 | Zea mays | 629 | - | 5 | CGTGG |  |
| Unnamed\_\_1 | Zea mays | 989 | + | 5 | CGTGG |  |

> 2018/04/13 10:10:12  
+ CCCCCTTCTC GAGTTTCTCA ACTCTCTCCT TGACTCTTAC CTTCTCTTCC TTCCAATTAT ACCCAGTTCC   
  
  
+ CTTCACTATA GTACCTTCCA CTCTTCTCCC ACCTATCTGG GTGCCTATAT AAATGTCTCT TTCTCTCTCT   
  
  
+ CTTTTATACC ACGAAACCGA ACACTACACC TCTACCTCCT ACCTCTCTGA TCAGACTCAA AGTACCCATT   
  
  
+ TGGGTTGATT TAGTATGGAT GAAGACAAGT GGACGGGAAG TCACTTTTCA CATGCAGGAT ATATACGTAG   
  
  
+ CAATAGTGGC TACAAAGTGT AAGAATAAAC GGTTTTCTCT TGGTAAGTAA ATCCTGTACG ATGCAAAGTC   
  
  
+ ATTGGGGTTA CAGAAGAAAA CATATACGAT ATGACAAATG GTTGGTTCGT GACCACAAAA GAAAACTGGT   
  
  
+ TAATCTAGTT GACGTCGATG AAATTAAAGG ACATGCTTTA TAAGTCGGCA TTTGGTTCAA GTAGTTCTTT   
  
  
+ TTCTTCAGTC GTAAAACTAC TGATACGGAC AGGGGAAGCT AGAGTTAATG TCAAATTTGT TTTATTTCCC   
  
  
+ TAAATACACT TATATTTTCT GTATTATATA CAGTATTTTT GAAGTGGTAA TAACTTCTTT CCTAAACCCC   
  
  
+ ACGAGACCTA CTTGGAAGCA AAGGATGAGT ACGTTCCGTT CGGTTTCACG TACCTGTGTA CTATCGTTTG   
  
  
+ TTCGAACCTT TCCCTCTCAG GACTAATCCA GTGGACATCA CTGAAGGGTT CTGAAAAGAT CGAGGACTAA   
  
  
+ CCATTGTACG GTAAAGTATT ATGTGGGTTA GATGGTTCCT TCTTATGATG TATGGTGTTA GTTGTAGTAT   
  
  
+ TCATTGGTAT ATCCAGTAGT TCTACTTAGC CGACTCATCG TCCAAGTATA TCTAAAAAAA CGGTTCTTTT   
  
  
+ GGTTTGTATA TTTTGAACGG TTACACCTCT AATGACCTTC TTGTCTTTCT TTGTCTACGT ATAGTCCTGT   
  
  
+ TCAAGGTACG TGGTATATAG AGGTTATCTT ATAGGTTTTA AAATTGATGT TTCGAATACA GCTTGGTCTT   
  
  
+ TTTGAACAAT GTCCCCCAAG GTTTGAGACT TAATGCCGTG GAACTCCGGA GTTAAATAGG TTATAGTTCA   
  
  
+ TCTTAGTTTA GAGAGTCCGT ACCCCAACTT CAAAGTTCTT AAACTGACGT CTTGTTTATG TGAAGTTGGT   
  
  
+ ATAATTACTA AAAAAAAAGT ATATTCTTTG GTGTTAATAC TAAGACTGTT CTGATTTTAC ACCGAGTTCC   
  
  
+ AGTTTAGTCC CTGAGGTGTA GTCGACTACT AGTAGAAAGA TTTCTTAAGA CGAACGAGTC CCTTTATGAG   
  
  
+ GTTGTCGAGT AGGAAGCATT GGGTTCGTTT TAGGAGTGTG AAGGGTTACA ATTTAAACAA CCAACGTTAA   
  
  
+ TGAACCCTAA ATTTCTTCTT AACGACTGTT TTTTTTCTTT TTTTTAGTTC GTGCCTTTAA TTTTTTTTGT   
  
  
+ ATTAAATCGG ATTTGTTCTT TCCGTGTAA  

- GGGGGAAGAG CTCAAAGAGT TGAGAGAGGA ACTGAGAATG GAAGAGAAGG AAGGTTAATA TGGGTCAAGG   
  
  
- GAAGTGATAT CATGGAAGGT GAGAAGAGGG TGGATAGACC CACGGATATA TTTACAGAGA AAGAGAGAGA   
  
  
- GAAAATATGG TGCTTTGGCT TGTGATGTGG AGATGGAGGA TGGAGAGACT AGTCTGAGTT TCATGGGTAA   
  
  
- ACCCAACTAA ATCATACCTA CTTCTGTTCA CCTGCCCTTC AGTGAAAAGT GTACGTCCTA TATATGCATC   
  
  
- GTTATCACCG ATGTTTCACA TTCTTATTTG CCAAAAGAGA ACCATTCATT TAGGACATGC TACGTTTCAG   
  
  
- TAACCCCAAT GTCTTCTTTT GTATATGCTA TACTGTTTAC CAACCAAGCA CTGGTGTTTT CTTTTGACCA   
  
  
- ATTAGATCAA CTGCAGCTAC TTTAATTTCC TGTACGAAAT ATTCAGCCGT AAACCAAGTT CATCAAGAAA   
  
  
- AAGAAGTCAG CATTTTGATG ACTATGCCTG TCCCCTTCGA TCTCAATTAC AGTTTAAACA AAATAAAGGG   
  
  
- ATTTATGTGA ATATAAAAGA CATAATATAT GTCATAAAAA CTTCACCATT ATTGAAGAAA GGATTTGGGG   
  
  
- TGCTCTGGAT GAACCTTCGT TTCCTACTCA TGCAAGGCAA GCCAAAGTGC ATGGACACAT GATAGCAAAC   
  
  
- AAGCTTGGAA AGGGAGAGTC CTGATTAGGT CACCTGTAGT GACTTCCCAA GACTTTTCTA GCTCCTGATT   
  
  
- GGTAACATGC CATTTCATAA TACACCCAAT CTACCAAGGA AGAATACTAC ATACCACAAT CAACATCATA   
  
  
- AGTAACCATA TAGGTCATCA AGATGAATCG GCTGAGTAGC AGGTTCATAT AGATTTTTTT GCCAAGAAAA   
  
  
- CCAAACATAT AAAACTTGCC AATGTGGAGA TTACTGGAAG AACAGAAAGA AACAGATGCA TATCAGGACA   
  
  
- AGTTCCATGC ACCATATATC TCCAATAGAA TATCCAAAAT TTTAACTACA AAGCTTATGT CGAACCAGAA   
  
  
- AAACTTGTTA CAGGGGGTTC CAAACTCTGA ATTACGGCAC CTTGAGGCCT CAATTTATCC AATATCAAGT   
  
  
- AGAATCAAAT CTCTCAGGCA TGGGGTTGAA GTTTCAAGAA TTTGACTGCA GAACAAATAC ACTTCAACCA   
  
  
- TATTAATGAT TTTTTTTTCA TATAAGAAAC CACAATTATG ATTCTGACAA GACTAAAATG TGGCTCAAGG   
  
  
- TCAAATCAGG GACTCCACAT CAGCTGATGA TCATCTTTCT AAAGAATTCT GCTTGCTCAG GGAAATACTC   
  
  
- CAACAGCTCA TCCTTCGTAA CCCAAGCAAA ATCCTCACAC TTCCCAATGT TAAATTTGTT GGTTGCAATT   
  
  
- ACTTGGGATT TAAAGAAGAA TTGCTGACAA AAAAAAGAAA AAAAATCAAG CACGGAAATT AAAAAAAACA   
  
  
- TAATTTAGCC TAAACAAGAA AGGCACATT

+     Unnamed\_\_13

| Site Name | Organism | Position | Strand | Matrix score. | sequence | function |
| --- | --- | --- | --- | --- | --- | --- |
| Unnamed\_\_13 | Zea mays | 881 | + | 10 | TCCAAGTATA |  |
| Unnamed\_\_13 | Zea mays | 52 | + | 9 | TCCAAGTATA |  |

> 2018/04/13 10:10:12  
+ CCCCCTTCTC GAGTTTCTCA ACTCTCTCCT TGACTCTTAC CTTCTCTTCC TTCCAATTAT ACCCAGTTCC   
  
  
+ CTTCACTATA GTACCTTCCA CTCTTCTCCC ACCTATCTGG GTGCCTATAT AAATGTCTCT TTCTCTCTCT   
  
  
+ CTTTTATACC ACGAAACCGA ACACTACACC TCTACCTCCT ACCTCTCTGA TCAGACTCAA AGTACCCATT   
  
  
+ TGGGTTGATT TAGTATGGAT GAAGACAAGT GGACGGGAAG TCACTTTTCA CATGCAGGAT ATATACGTAG   
  
  
+ CAATAGTGGC TACAAAGTGT AAGAATAAAC GGTTTTCTCT TGGTAAGTAA ATCCTGTACG ATGCAAAGTC   
  
  
+ ATTGGGGTTA CAGAAGAAAA CATATACGAT ATGACAAATG GTTGGTTCGT GACCACAAAA GAAAACTGGT   
  
  
+ TAATCTAGTT GACGTCGATG AAATTAAAGG ACATGCTTTA TAAGTCGGCA TTTGGTTCAA GTAGTTCTTT   
  
  
+ TTCTTCAGTC GTAAAACTAC TGATACGGAC AGGGGAAGCT AGAGTTAATG TCAAATTTGT TTTATTTCCC   
  
  
+ TAAATACACT TATATTTTCT GTATTATATA CAGTATTTTT GAAGTGGTAA TAACTTCTTT CCTAAACCCC   
  
  
+ ACGAGACCTA CTTGGAAGCA AAGGATGAGT ACGTTCCGTT CGGTTTCACG TACCTGTGTA CTATCGTTTG   
  
  
+ TTCGAACCTT TCCCTCTCAG GACTAATCCA GTGGACATCA CTGAAGGGTT CTGAAAAGAT CGAGGACTAA   
  
  
+ CCATTGTACG GTAAAGTATT ATGTGGGTTA GATGGTTCCT TCTTATGATG TATGGTGTTA GTTGTAGTAT   
  
  
+ TCATTGGTAT ATCCAGTAGT TCTACTTAGC CGACTCATCG TCCAAGTATA TCTAAAAAAA CGGTTCTTTT   
  
  
+ GGTTTGTATA TTTTGAACGG TTACACCTCT AATGACCTTC TTGTCTTTCT TTGTCTACGT ATAGTCCTGT   
  
  
+ TCAAGGTACG TGGTATATAG AGGTTATCTT ATAGGTTTTA AAATTGATGT TTCGAATACA GCTTGGTCTT   
  
  
+ TTTGAACAAT GTCCCCCAAG GTTTGAGACT TAATGCCGTG GAACTCCGGA GTTAAATAGG TTATAGTTCA   
  
  
+ TCTTAGTTTA GAGAGTCCGT ACCCCAACTT CAAAGTTCTT AAACTGACGT CTTGTTTATG TGAAGTTGGT   
  
  
+ ATAATTACTA AAAAAAAAGT ATATTCTTTG GTGTTAATAC TAAGACTGTT CTGATTTTAC ACCGAGTTCC   
  
  
+ AGTTTAGTCC CTGAGGTGTA GTCGACTACT AGTAGAAAGA TTTCTTAAGA CGAACGAGTC CCTTTATGAG   
  
  
+ GTTGTCGAGT AGGAAGCATT GGGTTCGTTT TAGGAGTGTG AAGGGTTACA ATTTAAACAA CCAACGTTAA   
  
  
+ TGAACCCTAA ATTTCTTCTT AACGACTGTT TTTTTTCTTT TTTTTAGTTC GTGCCTTTAA TTTTTTTTGT   
  
  
+ ATTAAATCGG ATTTGTTCTT TCCGTGTAA  

- GGGGGAAGAG CTCAAAGAGT TGAGAGAGGA ACTGAGAATG GAAGAGAAGG AAGGTTAATA TGGGTCAAGG   
  
  
- GAAGTGATAT CATGGAAGGT GAGAAGAGGG TGGATAGACC CACGGATATA TTTACAGAGA AAGAGAGAGA   
  
  
- GAAAATATGG TGCTTTGGCT TGTGATGTGG AGATGGAGGA TGGAGAGACT AGTCTGAGTT TCATGGGTAA   
  
  
- ACCCAACTAA ATCATACCTA CTTCTGTTCA CCTGCCCTTC AGTGAAAAGT GTACGTCCTA TATATGCATC   
  
  
- GTTATCACCG ATGTTTCACA TTCTTATTTG CCAAAAGAGA ACCATTCATT TAGGACATGC TACGTTTCAG   
  
  
- TAACCCCAAT GTCTTCTTTT GTATATGCTA TACTGTTTAC CAACCAAGCA CTGGTGTTTT CTTTTGACCA   
  
  
- ATTAGATCAA CTGCAGCTAC TTTAATTTCC TGTACGAAAT ATTCAGCCGT AAACCAAGTT CATCAAGAAA   
  
  
- AAGAAGTCAG CATTTTGATG ACTATGCCTG TCCCCTTCGA TCTCAATTAC AGTTTAAACA AAATAAAGGG   
  
  
- ATTTATGTGA ATATAAAAGA CATAATATAT GTCATAAAAA CTTCACCATT ATTGAAGAAA GGATTTGGGG   
  
  
- TGCTCTGGAT GAACCTTCGT TTCCTACTCA TGCAAGGCAA GCCAAAGTGC ATGGACACAT GATAGCAAAC   
  
  
- AAGCTTGGAA AGGGAGAGTC CTGATTAGGT CACCTGTAGT GACTTCCCAA GACTTTTCTA GCTCCTGATT   
  
  
- GGTAACATGC CATTTCATAA TACACCCAAT CTACCAAGGA AGAATACTAC ATACCACAAT CAACATCATA   
  
  
- AGTAACCATA TAGGTCATCA AGATGAATCG GCTGAGTAGC AGGTTCATAT AGATTTTTTT GCCAAGAAAA   
  
  
- CCAAACATAT AAAACTTGCC AATGTGGAGA TTACTGGAAG AACAGAAAGA AACAGATGCA TATCAGGACA   
  
  
- AGTTCCATGC ACCATATATC TCCAATAGAA TATCCAAAAT TTTAACTACA AAGCTTATGT CGAACCAGAA   
  
  
- AAACTTGTTA CAGGGGGTTC CAAACTCTGA ATTACGGCAC CTTGAGGCCT CAATTTATCC AATATCAAGT   
  
  
- AGAATCAAAT CTCTCAGGCA TGGGGTTGAA GTTTCAAGAA TTTGACTGCA GAACAAATAC ACTTCAACCA   
  
  
- TATTAATGAT TTTTTTTTCA TATAAGAAAC CACAATTATG ATTCTGACAA GACTAAAATG TGGCTCAAGG   
  
  
- TCAAATCAGG GACTCCACAT CAGCTGATGA TCATCTTTCT AAAGAATTCT GCTTGCTCAG GGAAATACTC   
  
  
- CAACAGCTCA TCCTTCGTAA CCCAAGCAAA ATCCTCACAC TTCCCAATGT TAAATTTGTT GGTTGCAATT   
  
  
- ACTTGGGATT TAAAGAAGAA TTGCTGACAA AAAAAAGAAA AAAAATCAAG CACGGAAATT AAAAAAAACA   
  
  
- TAATTTAGCC TAAACAAGAA AGGCACATT

+     Unnamed\_\_3

| Site Name | Organism | Position | Strand | Matrix score. | sequence | function |
| --- | --- | --- | --- | --- | --- | --- |
| Unnamed\_\_3 | Zea mays | 1087 | + | 5 | CGTGG |  |
| Unnamed\_\_3 | Zea mays | 989 | + | 5 | CGTGG |  |
| Unnamed\_\_3 | Zea mays | 629 | - | 5 | CGTGG |  |
| Unnamed\_\_3 | Zea mays | 149 | - | 5 | CGTGG |  |

> 2018/04/13 10:10:12  
+ CCCCCTTCTC GAGTTTCTCA ACTCTCTCCT TGACTCTTAC CTTCTCTTCC TTCCAATTAT ACCCAGTTCC   
  
  
+ CTTCACTATA GTACCTTCCA CTCTTCTCCC ACCTATCTGG GTGCCTATAT AAATGTCTCT TTCTCTCTCT   
  
  
+ CTTTTATACC ACGAAACCGA ACACTACACC TCTACCTCCT ACCTCTCTGA TCAGACTCAA AGTACCCATT   
  
  
+ TGGGTTGATT TAGTATGGAT GAAGACAAGT GGACGGGAAG TCACTTTTCA CATGCAGGAT ATATACGTAG   
  
  
+ CAATAGTGGC TACAAAGTGT AAGAATAAAC GGTTTTCTCT TGGTAAGTAA ATCCTGTACG ATGCAAAGTC   
  
  
+ ATTGGGGTTA CAGAAGAAAA CATATACGAT ATGACAAATG GTTGGTTCGT GACCACAAAA GAAAACTGGT   
  
  
+ TAATCTAGTT GACGTCGATG AAATTAAAGG ACATGCTTTA TAAGTCGGCA TTTGGTTCAA GTAGTTCTTT   
  
  
+ TTCTTCAGTC GTAAAACTAC TGATACGGAC AGGGGAAGCT AGAGTTAATG TCAAATTTGT TTTATTTCCC   
  
  
+ TAAATACACT TATATTTTCT GTATTATATA CAGTATTTTT GAAGTGGTAA TAACTTCTTT CCTAAACCCC   
  
  
+ ACGAGACCTA CTTGGAAGCA AAGGATGAGT ACGTTCCGTT CGGTTTCACG TACCTGTGTA CTATCGTTTG   
  
  
+ TTCGAACCTT TCCCTCTCAG GACTAATCCA GTGGACATCA CTGAAGGGTT CTGAAAAGAT CGAGGACTAA   
  
  
+ CCATTGTACG GTAAAGTATT ATGTGGGTTA GATGGTTCCT TCTTATGATG TATGGTGTTA GTTGTAGTAT   
  
  
+ TCATTGGTAT ATCCAGTAGT TCTACTTAGC CGACTCATCG TCCAAGTATA TCTAAAAAAA CGGTTCTTTT   
  
  
+ GGTTTGTATA TTTTGAACGG TTACACCTCT AATGACCTTC TTGTCTTTCT TTGTCTACGT ATAGTCCTGT   
  
  
+ TCAAGGTACG TGGTATATAG AGGTTATCTT ATAGGTTTTA AAATTGATGT TTCGAATACA GCTTGGTCTT   
  
  
+ TTTGAACAAT GTCCCCCAAG GTTTGAGACT TAATGCCGTG GAACTCCGGA GTTAAATAGG TTATAGTTCA   
  
  
+ TCTTAGTTTA GAGAGTCCGT ACCCCAACTT CAAAGTTCTT AAACTGACGT CTTGTTTATG TGAAGTTGGT   
  
  
+ ATAATTACTA AAAAAAAAGT ATATTCTTTG GTGTTAATAC TAAGACTGTT CTGATTTTAC ACCGAGTTCC   
  
  
+ AGTTTAGTCC CTGAGGTGTA GTCGACTACT AGTAGAAAGA TTTCTTAAGA CGAACGAGTC CCTTTATGAG   
  
  
+ GTTGTCGAGT AGGAAGCATT GGGTTCGTTT TAGGAGTGTG AAGGGTTACA ATTTAAACAA CCAACGTTAA   
  
  
+ TGAACCCTAA ATTTCTTCTT AACGACTGTT TTTTTTCTTT TTTTTAGTTC GTGCCTTTAA TTTTTTTTGT   
  
  
+ ATTAAATCGG ATTTGTTCTT TCCGTGTAA  

- GGGGGAAGAG CTCAAAGAGT TGAGAGAGGA ACTGAGAATG GAAGAGAAGG AAGGTTAATA TGGGTCAAGG   
  
  
- GAAGTGATAT CATGGAAGGT GAGAAGAGGG TGGATAGACC CACGGATATA TTTACAGAGA AAGAGAGAGA   
  
  
- GAAAATATGG TGCTTTGGCT TGTGATGTGG AGATGGAGGA TGGAGAGACT AGTCTGAGTT TCATGGGTAA   
  
  
- ACCCAACTAA ATCATACCTA CTTCTGTTCA CCTGCCCTTC AGTGAAAAGT GTACGTCCTA TATATGCATC   
  
  
- GTTATCACCG ATGTTTCACA TTCTTATTTG CCAAAAGAGA ACCATTCATT TAGGACATGC TACGTTTCAG   
  
  
- TAACCCCAAT GTCTTCTTTT GTATATGCTA TACTGTTTAC CAACCAAGCA CTGGTGTTTT CTTTTGACCA   
  
  
- ATTAGATCAA CTGCAGCTAC TTTAATTTCC TGTACGAAAT ATTCAGCCGT AAACCAAGTT CATCAAGAAA   
  
  
- AAGAAGTCAG CATTTTGATG ACTATGCCTG TCCCCTTCGA TCTCAATTAC AGTTTAAACA AAATAAAGGG   
  
  
- ATTTATGTGA ATATAAAAGA CATAATATAT GTCATAAAAA CTTCACCATT ATTGAAGAAA GGATTTGGGG   
  
  
- TGCTCTGGAT GAACCTTCGT TTCCTACTCA TGCAAGGCAA GCCAAAGTGC ATGGACACAT GATAGCAAAC   
  
  
- AAGCTTGGAA AGGGAGAGTC CTGATTAGGT CACCTGTAGT GACTTCCCAA GACTTTTCTA GCTCCTGATT   
  
  
- GGTAACATGC CATTTCATAA TACACCCAAT CTACCAAGGA AGAATACTAC ATACCACAAT CAACATCATA   
  
  
- AGTAACCATA TAGGTCATCA AGATGAATCG GCTGAGTAGC AGGTTCATAT AGATTTTTTT GCCAAGAAAA   
  
  
- CCAAACATAT AAAACTTGCC AATGTGGAGA TTACTGGAAG AACAGAAAGA AACAGATGCA TATCAGGACA   
  
  
- AGTTCCATGC ACCATATATC TCCAATAGAA TATCCAAAAT TTTAACTACA AAGCTTATGT CGAACCAGAA   
  
  
- AAACTTGTTA CAGGGGGTTC CAAACTCTGA ATTACGGCAC CTTGAGGCCT CAATTTATCC AATATCAAGT   
  
  
- AGAATCAAAT CTCTCAGGCA TGGGGTTGAA GTTTCAAGAA TTTGACTGCA GAACAAATAC ACTTCAACCA   
  
  
- TATTAATGAT TTTTTTTTCA TATAAGAAAC CACAATTATG ATTCTGACAA GACTAAAATG TGGCTCAAGG   
  
  
- TCAAATCAGG GACTCCACAT CAGCTGATGA TCATCTTTCT AAAGAATTCT GCTTGCTCAG GGAAATACTC   
  
  
- CAACAGCTCA TCCTTCGTAA CCCAAGCAAA ATCCTCACAC TTCCCAATGT TAAATTTGTT GGTTGCAATT   
  
  
- ACTTGGGATT TAAAGAAGAA TTGCTGACAA AAAAAAGAAA AAAAATCAAG CACGGAAATT AAAAAAAACA   
  
  
- TAATTTAGCC TAAACAAGAA AGGCACATT

+     Unnamed\_\_4

| Site Name | Organism | Position | Strand | Matrix score. | sequence | function |
| --- | --- | --- | --- | --- | --- | --- |
| Unnamed\_\_4 | Petroselinum hortense | 96 | + | 4 | CTCC |  |
| Unnamed\_\_4 | Petroselinum hortense | 1363 | - | 4 | CTCC |  |
| Unnamed\_\_4 | Petroselinum hortense | 1098 | - | 4 | CTCC |  |
| Unnamed\_\_4 | Petroselinum hortense | 176 | + | 4 | CTCC |  |
| Unnamed\_\_4 | Petroselinum hortense | 26 | + | 4 | CTCC |  |
| Unnamed\_\_4 | Petroselinum hortense | 1094 | + | 4 | CTCC |  |

> 2018/04/13 10:10:12  
+ CCCCCTTCTC GAGTTTCTCA ACTCTCTCCT TGACTCTTAC CTTCTCTTCC TTCCAATTAT ACCCAGTTCC   
  
  
+ CTTCACTATA GTACCTTCCA CTCTTCTCCC ACCTATCTGG GTGCCTATAT AAATGTCTCT TTCTCTCTCT   
  
  
+ CTTTTATACC ACGAAACCGA ACACTACACC TCTACCTCCT ACCTCTCTGA TCAGACTCAA AGTACCCATT   
  
  
+ TGGGTTGATT TAGTATGGAT GAAGACAAGT GGACGGGAAG TCACTTTTCA CATGCAGGAT ATATACGTAG   
  
  
+ CAATAGTGGC TACAAAGTGT AAGAATAAAC GGTTTTCTCT TGGTAAGTAA ATCCTGTACG ATGCAAAGTC   
  
  
+ ATTGGGGTTA CAGAAGAAAA CATATACGAT ATGACAAATG GTTGGTTCGT GACCACAAAA GAAAACTGGT   
  
  
+ TAATCTAGTT GACGTCGATG AAATTAAAGG ACATGCTTTA TAAGTCGGCA TTTGGTTCAA GTAGTTCTTT   
  
  
+ TTCTTCAGTC GTAAAACTAC TGATACGGAC AGGGGAAGCT AGAGTTAATG TCAAATTTGT TTTATTTCCC   
  
  
+ TAAATACACT TATATTTTCT GTATTATATA CAGTATTTTT GAAGTGGTAA TAACTTCTTT CCTAAACCCC   
  
  
+ ACGAGACCTA CTTGGAAGCA AAGGATGAGT ACGTTCCGTT CGGTTTCACG TACCTGTGTA CTATCGTTTG   
  
  
+ TTCGAACCTT TCCCTCTCAG GACTAATCCA GTGGACATCA CTGAAGGGTT CTGAAAAGAT CGAGGACTAA   
  
  
+ CCATTGTACG GTAAAGTATT ATGTGGGTTA GATGGTTCCT TCTTATGATG TATGGTGTTA GTTGTAGTAT   
  
  
+ TCATTGGTAT ATCCAGTAGT TCTACTTAGC CGACTCATCG TCCAAGTATA TCTAAAAAAA CGGTTCTTTT   
  
  
+ GGTTTGTATA TTTTGAACGG TTACACCTCT AATGACCTTC TTGTCTTTCT TTGTCTACGT ATAGTCCTGT   
  
  
+ TCAAGGTACG TGGTATATAG AGGTTATCTT ATAGGTTTTA AAATTGATGT TTCGAATACA GCTTGGTCTT   
  
  
+ TTTGAACAAT GTCCCCCAAG GTTTGAGACT TAATGCCGTG GAACTCCGGA GTTAAATAGG TTATAGTTCA   
  
  
+ TCTTAGTTTA GAGAGTCCGT ACCCCAACTT CAAAGTTCTT AAACTGACGT CTTGTTTATG TGAAGTTGGT   
  
  
+ ATAATTACTA AAAAAAAAGT ATATTCTTTG GTGTTAATAC TAAGACTGTT CTGATTTTAC ACCGAGTTCC   
  
  
+ AGTTTAGTCC CTGAGGTGTA GTCGACTACT AGTAGAAAGA TTTCTTAAGA CGAACGAGTC CCTTTATGAG   
  
  
+ GTTGTCGAGT AGGAAGCATT GGGTTCGTTT TAGGAGTGTG AAGGGTTACA ATTTAAACAA CCAACGTTAA   
  
  
+ TGAACCCTAA ATTTCTTCTT AACGACTGTT TTTTTTCTTT TTTTTAGTTC GTGCCTTTAA TTTTTTTTGT   
  
  
+ ATTAAATCGG ATTTGTTCTT TCCGTGTAA  

- GGGGGAAGAG CTCAAAGAGT TGAGAGAGGA ACTGAGAATG GAAGAGAAGG AAGGTTAATA TGGGTCAAGG   
  
  
- GAAGTGATAT CATGGAAGGT GAGAAGAGGG TGGATAGACC CACGGATATA TTTACAGAGA AAGAGAGAGA   
  
  
- GAAAATATGG TGCTTTGGCT TGTGATGTGG AGATGGAGGA TGGAGAGACT AGTCTGAGTT TCATGGGTAA   
  
  
- ACCCAACTAA ATCATACCTA CTTCTGTTCA CCTGCCCTTC AGTGAAAAGT GTACGTCCTA TATATGCATC   
  
  
- GTTATCACCG ATGTTTCACA TTCTTATTTG CCAAAAGAGA ACCATTCATT TAGGACATGC TACGTTTCAG   
  
  
- TAACCCCAAT GTCTTCTTTT GTATATGCTA TACTGTTTAC CAACCAAGCA CTGGTGTTTT CTTTTGACCA   
  
  
- ATTAGATCAA CTGCAGCTAC TTTAATTTCC TGTACGAAAT ATTCAGCCGT AAACCAAGTT CATCAAGAAA   
  
  
- AAGAAGTCAG CATTTTGATG ACTATGCCTG TCCCCTTCGA TCTCAATTAC AGTTTAAACA AAATAAAGGG   
  
  
- ATTTATGTGA ATATAAAAGA CATAATATAT GTCATAAAAA CTTCACCATT ATTGAAGAAA GGATTTGGGG   
  
  
- TGCTCTGGAT GAACCTTCGT TTCCTACTCA TGCAAGGCAA GCCAAAGTGC ATGGACACAT GATAGCAAAC   
  
  
- AAGCTTGGAA AGGGAGAGTC CTGATTAGGT CACCTGTAGT GACTTCCCAA GACTTTTCTA GCTCCTGATT   
  
  
- GGTAACATGC CATTTCATAA TACACCCAAT CTACCAAGGA AGAATACTAC ATACCACAAT CAACATCATA   
  
  
- AGTAACCATA TAGGTCATCA AGATGAATCG GCTGAGTAGC AGGTTCATAT AGATTTTTTT GCCAAGAAAA   
  
  
- CCAAACATAT AAAACTTGCC AATGTGGAGA TTACTGGAAG AACAGAAAGA AACAGATGCA TATCAGGACA   
  
  
- AGTTCCATGC ACCATATATC TCCAATAGAA TATCCAAAAT TTTAACTACA AAGCTTATGT CGAACCAGAA   
  
  
- AAACTTGTTA CAGGGGGTTC CAAACTCTGA ATTACGGCAC CTTGAGGCCT CAATTTATCC AATATCAAGT   
  
  
- AGAATCAAAT CTCTCAGGCA TGGGGTTGAA GTTTCAAGAA TTTGACTGCA GAACAAATAC ACTTCAACCA   
  
  
- TATTAATGAT TTTTTTTTCA TATAAGAAAC CACAATTATG ATTCTGACAA GACTAAAATG TGGCTCAAGG   
  
  
- TCAAATCAGG GACTCCACAT CAGCTGATGA TCATCTTTCT AAAGAATTCT GCTTGCTCAG GGAAATACTC   
  
  
- CAACAGCTCA TCCTTCGTAA CCCAAGCAAA ATCCTCACAC TTCCCAATGT TAAATTTGTT GGTTGCAATT   
  
  
- ACTTGGGATT TAAAGAAGAA TTGCTGACAA AAAAAAGAAA AAAAATCAAG CACGGAAATT AAAAAAAACA   
  
  
- TAATTTAGCC TAAACAAGAA AGGCACATT

+     Unnamed\_\_6

| Site Name | Organism | Position | Strand | Matrix score. | sequence | function |
| --- | --- | --- | --- | --- | --- | --- |
| Unnamed\_\_6 | Zea mays | 118 | + | 10 | taTAAATATct |  |

> 2018/04/13 10:10:12  
+ CCCCCTTCTC GAGTTTCTCA ACTCTCTCCT TGACTCTTAC CTTCTCTTCC TTCCAATTAT ACCCAGTTCC   
  
  
+ CTTCACTATA GTACCTTCCA CTCTTCTCCC ACCTATCTGG GTGCCTATAT AAATGTCTCT TTCTCTCTCT   
  
  
+ CTTTTATACC ACGAAACCGA ACACTACACC TCTACCTCCT ACCTCTCTGA TCAGACTCAA AGTACCCATT   
  
  
+ TGGGTTGATT TAGTATGGAT GAAGACAAGT GGACGGGAAG TCACTTTTCA CATGCAGGAT ATATACGTAG   
  
  
+ CAATAGTGGC TACAAAGTGT AAGAATAAAC GGTTTTCTCT TGGTAAGTAA ATCCTGTACG ATGCAAAGTC   
  
  
+ ATTGGGGTTA CAGAAGAAAA CATATACGAT ATGACAAATG GTTGGTTCGT GACCACAAAA GAAAACTGGT   
  
  
+ TAATCTAGTT GACGTCGATG AAATTAAAGG ACATGCTTTA TAAGTCGGCA TTTGGTTCAA GTAGTTCTTT   
  
  
+ TTCTTCAGTC GTAAAACTAC TGATACGGAC AGGGGAAGCT AGAGTTAATG TCAAATTTGT TTTATTTCCC   
  
  
+ TAAATACACT TATATTTTCT GTATTATATA CAGTATTTTT GAAGTGGTAA TAACTTCTTT CCTAAACCCC   
  
  
+ ACGAGACCTA CTTGGAAGCA AAGGATGAGT ACGTTCCGTT CGGTTTCACG TACCTGTGTA CTATCGTTTG   
  
  
+ TTCGAACCTT TCCCTCTCAG GACTAATCCA GTGGACATCA CTGAAGGGTT CTGAAAAGAT CGAGGACTAA   
  
  
+ CCATTGTACG GTAAAGTATT ATGTGGGTTA GATGGTTCCT TCTTATGATG TATGGTGTTA GTTGTAGTAT   
  
  
+ TCATTGGTAT ATCCAGTAGT TCTACTTAGC CGACTCATCG TCCAAGTATA TCTAAAAAAA CGGTTCTTTT   
  
  
+ GGTTTGTATA TTTTGAACGG TTACACCTCT AATGACCTTC TTGTCTTTCT TTGTCTACGT ATAGTCCTGT   
  
  
+ TCAAGGTACG TGGTATATAG AGGTTATCTT ATAGGTTTTA AAATTGATGT TTCGAATACA GCTTGGTCTT   
  
  
+ TTTGAACAAT GTCCCCCAAG GTTTGAGACT TAATGCCGTG GAACTCCGGA GTTAAATAGG TTATAGTTCA   
  
  
+ TCTTAGTTTA GAGAGTCCGT ACCCCAACTT CAAAGTTCTT AAACTGACGT CTTGTTTATG TGAAGTTGGT   
  
  
+ ATAATTACTA AAAAAAAAGT ATATTCTTTG GTGTTAATAC TAAGACTGTT CTGATTTTAC ACCGAGTTCC   
  
  
+ AGTTTAGTCC CTGAGGTGTA GTCGACTACT AGTAGAAAGA TTTCTTAAGA CGAACGAGTC CCTTTATGAG   
  
  
+ GTTGTCGAGT AGGAAGCATT GGGTTCGTTT TAGGAGTGTG AAGGGTTACA ATTTAAACAA CCAACGTTAA   
  
  
+ TGAACCCTAA ATTTCTTCTT AACGACTGTT TTTTTTCTTT TTTTTAGTTC GTGCCTTTAA TTTTTTTTGT   
  
  
+ ATTAAATCGG ATTTGTTCTT TCCGTGTAA  

- GGGGGAAGAG CTCAAAGAGT TGAGAGAGGA ACTGAGAATG GAAGAGAAGG AAGGTTAATA TGGGTCAAGG   
  
  
- GAAGTGATAT CATGGAAGGT GAGAAGAGGG TGGATAGACC CACGGATATA TTTACAGAGA AAGAGAGAGA   
  
  
- GAAAATATGG TGCTTTGGCT TGTGATGTGG AGATGGAGGA TGGAGAGACT AGTCTGAGTT TCATGGGTAA   
  
  
- ACCCAACTAA ATCATACCTA CTTCTGTTCA CCTGCCCTTC AGTGAAAAGT GTACGTCCTA TATATGCATC   
  
  
- GTTATCACCG ATGTTTCACA TTCTTATTTG CCAAAAGAGA ACCATTCATT TAGGACATGC TACGTTTCAG   
  
  
- TAACCCCAAT GTCTTCTTTT GTATATGCTA TACTGTTTAC CAACCAAGCA CTGGTGTTTT CTTTTGACCA   
  
  
- ATTAGATCAA CTGCAGCTAC TTTAATTTCC TGTACGAAAT ATTCAGCCGT AAACCAAGTT CATCAAGAAA   
  
  
- AAGAAGTCAG CATTTTGATG ACTATGCCTG TCCCCTTCGA TCTCAATTAC AGTTTAAACA AAATAAAGGG   
  
  
- ATTTATGTGA ATATAAAAGA CATAATATAT GTCATAAAAA CTTCACCATT ATTGAAGAAA GGATTTGGGG   
  
  
- TGCTCTGGAT GAACCTTCGT TTCCTACTCA TGCAAGGCAA GCCAAAGTGC ATGGACACAT GATAGCAAAC   
  
  
- AAGCTTGGAA AGGGAGAGTC CTGATTAGGT CACCTGTAGT GACTTCCCAA GACTTTTCTA GCTCCTGATT   
  
  
- GGTAACATGC CATTTCATAA TACACCCAAT CTACCAAGGA AGAATACTAC ATACCACAAT CAACATCATA   
  
  
- AGTAACCATA TAGGTCATCA AGATGAATCG GCTGAGTAGC AGGTTCATAT AGATTTTTTT GCCAAGAAAA   
  
  
- CCAAACATAT AAAACTTGCC AATGTGGAGA TTACTGGAAG AACAGAAAGA AACAGATGCA TATCAGGACA   
  
  
- AGTTCCATGC ACCATATATC TCCAATAGAA TATCCAAAAT TTTAACTACA AAGCTTATGT CGAACCAGAA   
  
  
- AAACTTGTTA CAGGGGGTTC CAAACTCTGA ATTACGGCAC CTTGAGGCCT CAATTTATCC AATATCAAGT   
  
  
- AGAATCAAAT CTCTCAGGCA TGGGGTTGAA GTTTCAAGAA TTTGACTGCA GAACAAATAC ACTTCAACCA   
  
  
- TATTAATGAT TTTTTTTTCA TATAAGAAAC CACAATTATG ATTCTGACAA GACTAAAATG TGGCTCAAGG   
  
  
- TCAAATCAGG GACTCCACAT CAGCTGATGA TCATCTTTCT AAAGAATTCT GCTTGCTCAG GGAAATACTC   
  
  
- CAACAGCTCA TCCTTCGTAA CCCAAGCAAA ATCCTCACAC TTCCCAATGT TAAATTTGTT GGTTGCAATT   
  
  
- ACTTGGGATT TAAAGAAGAA TTGCTGACAA AAAAAAGAAA AAAAATCAAG CACGGAAATT AAAAAAAACA   
  
  
- TAATTTAGCC TAAACAAGAA AGGCACATT

+     circadian

| Site Name | Organism | Position | Strand | Matrix score. | sequence | function |
| --- | --- | --- | --- | --- | --- | --- |
| circadian | Lycopersicon esculentum | 883 | + | 6 | CAANNNNATC | cis-acting regulatory element involved in circadian control |

> 2018/04/13 10:10:12  
+ CCCCCTTCTC GAGTTTCTCA ACTCTCTCCT TGACTCTTAC CTTCTCTTCC TTCCAATTAT ACCCAGTTCC   
  
  
+ CTTCACTATA GTACCTTCCA CTCTTCTCCC ACCTATCTGG GTGCCTATAT AAATGTCTCT TTCTCTCTCT   
  
  
+ CTTTTATACC ACGAAACCGA ACACTACACC TCTACCTCCT ACCTCTCTGA TCAGACTCAA AGTACCCATT   
  
  
+ TGGGTTGATT TAGTATGGAT GAAGACAAGT GGACGGGAAG TCACTTTTCA CATGCAGGAT ATATACGTAG   
  
  
+ CAATAGTGGC TACAAAGTGT AAGAATAAAC GGTTTTCTCT TGGTAAGTAA ATCCTGTACG ATGCAAAGTC   
  
  
+ ATTGGGGTTA CAGAAGAAAA CATATACGAT ATGACAAATG GTTGGTTCGT GACCACAAAA GAAAACTGGT   
  
  
+ TAATCTAGTT GACGTCGATG AAATTAAAGG ACATGCTTTA TAAGTCGGCA TTTGGTTCAA GTAGTTCTTT   
  
  
+ TTCTTCAGTC GTAAAACTAC TGATACGGAC AGGGGAAGCT AGAGTTAATG TCAAATTTGT TTTATTTCCC   
  
  
+ TAAATACACT TATATTTTCT GTATTATATA CAGTATTTTT GAAGTGGTAA TAACTTCTTT CCTAAACCCC   
  
  
+ ACGAGACCTA CTTGGAAGCA AAGGATGAGT ACGTTCCGTT CGGTTTCACG TACCTGTGTA CTATCGTTTG   
  
  
+ TTCGAACCTT TCCCTCTCAG GACTAATCCA GTGGACATCA CTGAAGGGTT CTGAAAAGAT CGAGGACTAA   
  
  
+ CCATTGTACG GTAAAGTATT ATGTGGGTTA GATGGTTCCT TCTTATGATG TATGGTGTTA GTTGTAGTAT   
  
  
+ TCATTGGTAT ATCCAGTAGT TCTACTTAGC CGACTCATCG TCCAAGTATA TCTAAAAAAA CGGTTCTTTT   
  
  
+ GGTTTGTATA TTTTGAACGG TTACACCTCT AATGACCTTC TTGTCTTTCT TTGTCTACGT ATAGTCCTGT   
  
  
+ TCAAGGTACG TGGTATATAG AGGTTATCTT ATAGGTTTTA AAATTGATGT TTCGAATACA GCTTGGTCTT   
  
  
+ TTTGAACAAT GTCCCCCAAG GTTTGAGACT TAATGCCGTG GAACTCCGGA GTTAAATAGG TTATAGTTCA   
  
  
+ TCTTAGTTTA GAGAGTCCGT ACCCCAACTT CAAAGTTCTT AAACTGACGT CTTGTTTATG TGAAGTTGGT   
  
  
+ ATAATTACTA AAAAAAAAGT ATATTCTTTG GTGTTAATAC TAAGACTGTT CTGATTTTAC ACCGAGTTCC   
  
  
+ AGTTTAGTCC CTGAGGTGTA GTCGACTACT AGTAGAAAGA TTTCTTAAGA CGAACGAGTC CCTTTATGAG   
  
  
+ GTTGTCGAGT AGGAAGCATT GGGTTCGTTT TAGGAGTGTG AAGGGTTACA ATTTAAACAA CCAACGTTAA   
  
  
+ TGAACCCTAA ATTTCTTCTT AACGACTGTT TTTTTTCTTT TTTTTAGTTC GTGCCTTTAA TTTTTTTTGT   
  
  
+ ATTAAATCGG ATTTGTTCTT TCCGTGTAA  

- GGGGGAAGAG CTCAAAGAGT TGAGAGAGGA ACTGAGAATG GAAGAGAAGG AAGGTTAATA TGGGTCAAGG   
  
  
- GAAGTGATAT CATGGAAGGT GAGAAGAGGG TGGATAGACC CACGGATATA TTTACAGAGA AAGAGAGAGA   
  
  
- GAAAATATGG TGCTTTGGCT TGTGATGTGG AGATGGAGGA TGGAGAGACT AGTCTGAGTT TCATGGGTAA   
  
  
- ACCCAACTAA ATCATACCTA CTTCTGTTCA CCTGCCCTTC AGTGAAAAGT GTACGTCCTA TATATGCATC   
  
  
- GTTATCACCG ATGTTTCACA TTCTTATTTG CCAAAAGAGA ACCATTCATT TAGGACATGC TACGTTTCAG   
  
  
- TAACCCCAAT GTCTTCTTTT GTATATGCTA TACTGTTTAC CAACCAAGCA CTGGTGTTTT CTTTTGACCA   
  
  
- ATTAGATCAA CTGCAGCTAC TTTAATTTCC TGTACGAAAT ATTCAGCCGT AAACCAAGTT CATCAAGAAA   
  
  
- AAGAAGTCAG CATTTTGATG ACTATGCCTG TCCCCTTCGA TCTCAATTAC AGTTTAAACA AAATAAAGGG   
  
  
- ATTTATGTGA ATATAAAAGA CATAATATAT GTCATAAAAA CTTCACCATT ATTGAAGAAA GGATTTGGGG   
  
  
- TGCTCTGGAT GAACCTTCGT TTCCTACTCA TGCAAGGCAA GCCAAAGTGC ATGGACACAT GATAGCAAAC   
  
  
- AAGCTTGGAA AGGGAGAGTC CTGATTAGGT CACCTGTAGT GACTTCCCAA GACTTTTCTA GCTCCTGATT   
  
  
- GGTAACATGC CATTTCATAA TACACCCAAT CTACCAAGGA AGAATACTAC ATACCACAAT CAACATCATA   
  
  
- AGTAACCATA TAGGTCATCA AGATGAATCG GCTGAGTAGC AGGTTCATAT AGATTTTTTT GCCAAGAAAA   
  
  
- CCAAACATAT AAAACTTGCC AATGTGGAGA TTACTGGAAG AACAGAAAGA AACAGATGCA TATCAGGACA   
  
  
- AGTTCCATGC ACCATATATC TCCAATAGAA TATCCAAAAT TTTAACTACA AAGCTTATGT CGAACCAGAA   
  
  
- AAACTTGTTA CAGGGGGTTC CAAACTCTGA ATTACGGCAC CTTGAGGCCT CAATTTATCC AATATCAAGT   
  
  
- AGAATCAAAT CTCTCAGGCA TGGGGTTGAA GTTTCAAGAA TTTGACTGCA GAACAAATAC ACTTCAACCA   
  
  
- TATTAATGAT TTTTTTTTCA TATAAGAAAC CACAATTATG ATTCTGACAA GACTAAAATG TGGCTCAAGG   
  
  
- TCAAATCAGG GACTCCACAT CAGCTGATGA TCATCTTTCT AAAGAATTCT GCTTGCTCAG GGAAATACTC   
  
  
- CAACAGCTCA TCCTTCGTAA CCCAAGCAAA ATCCTCACAC TTCCCAATGT TAAATTTGTT GGTTGCAATT   
  
  
- ACTTGGGATT TAAAGAAGAA TTGCTGACAA AAAAAAGAAA AAAAATCAAG CACGGAAATT AAAAAAAACA   
  
  
- TAATTTAGCC TAAACAAGAA AGGCACATT
