# Supplementary material for: Genome-wide analysis of the WRKY gene family in drumstick (Moringa oleifera Lam.)
Source: PeerJ. 2019 Jun 10;7:e7063. doi: 10.7717/peerj.7063 (PMC6563795; doi:10.7717/peerj.7063)
Supplement: Supplemental Information 1 [file peerj-07-7063-s003.gz › MoWRKY16_plantcare.html]

Content-Type: text/html; charset=ISO-8859-1


CallMat\_Firefox


Webmaster Firefox specific output  
To save the result:
click on the frame with the right mouse button and save the source code as a text file with extension .html  
REFERENCE:PlantCARE: a database of plant cis-acting regulatory elements and a portal to tools for in silico analysis of promoter sequences.  
Lescot, M., Déhais, P., Moreau, Y., De Moor, B., Rouzé ,P.,and Rombauts, S.  
Nucleic Acids Res., Database issue(2002), 30(1):325-327.   


---

> 2018/04/13 10:10:12  
+ CTGACATTGT TACTAGGCAG AGTCTGTCTC TCGGACGGCT TTAAGACGCT TGCGCCTCTC TCTTTCACGC   
  
  
+ TCCGATTCTC CACGCAAAAT CTGACTCCTT TTCCCTCTTC CTACCTTTCT CTTTCCCTCT CTCGATCCCA   
  
  
+ AAACACTACC ATTCCTGCGC ATCAAAAACT TGAAAAAAGG ACACTTCGTC ACAGGATAGT TGTCGCTGCG   
  
  
+ CGCGAGAGAA GAGACAATAG AGAGATTTCT GTTTGTCAGT ATCTCTGTCT CTCTCTTTTC GTTTGTTTTT   
  
  
+ TGTGACTTTT CTTACTTTCT TTTCTTCTCT CTTCCCTTAT CTTTTCTCTA TTCAATCTTT TTAGCGGCAG   
  
  
+ TTGGCTTTTG TCATGCCCTC CGACAATAAA TCCCCTTGCG TTCTCACATC CAGGGTGCGC GTACTTTAAC   
  
  
+ TTTAAAAGGT CTCTTCTTTT TTCTATGAAA GGCTGTTCTT CCGCGTGGAA CAAAGAGAGG TCATGAATGG   
  
  
+ ACACGCATTT GGTAGGGGTT AAAGAAAGAA AAGAATCAAA ATGGGGGTTT TTTTTTTTTT ATTTCATTTT   
  
  
+ AATTTTAGAA GAAATGTTTC TATTGAATAA AAATTTAAGT GTAATGTATA ATATTTTATT TTTAATTTAG   
  
  
+ GTTTGAAAGG TGTAAAATTT TTAGAAATTT TATTCAATTT AAAGGTCCAG TTAGTACATT CTGTATAAAA   
  
  
+ AAATTTTACT TATTTTAGAA ATTTTTAATA GCAATAATTT TATCTATTGT AAAATGTAAT AATACTGTTT   
  
  
+ TTTAATGACA TTTATAGTGT TAAATTTAAC TATCCTATTT ACACATTATA ACTTTTTGAA AGAAAAAGTC   
  
  
+ ATCTTTTTTT AGAACCCCTC CTCTGGAAAC TAACCGTATA TATAATATAC CACTCATTAC TTTCTTCTCC   
  
  
+ TGCAAACCAC ACAAACAAAT TAATTTGTTA ATTAATTAAA TTACAGGTTT CGTCTACTTC GCTTCTCCAG   
  
  
+ TAGTCTTCTT CTTCGTATTT ATTTTATAAC CTTTTGTTCT CCACCTCCTC CTCTGTCTCC GAACCCTACG   
  
  
+ TCGTCACCAT TCATTGTTCA CCCGAGACAG CGAGAGAGAG ATAAACGACT CACTTATTAA GTCGTGTTGT   
  
  
+ CGTGTGCTTG TCCTCGACCA GTGACTAAAC ACTTGTAAGA ACCGTTTATG ATGGGTAGTC GGTAGGAGAA   
  
  
+ ATAATGAGGG AGATTGAGGA TGTGAGAAAC TGGGTTAGGT GGAAAGAGTA GGGAAGATAT AAATAAAAAA   
  
  
+ TAGGCAAGTA AAAAATAAAA AAATTTTTAC TTAATTTAAT AAATAAAAAA TTTTAATATT AATAGATAAA   
  
  
+ GAAAAAATTT TCTAATTATT TTTAGTTTGA CTATCTTTTC TACAAATCAA ATAGTTTTAT TAAAAAAAAT   
  
  
+ CTACGGGTAT ATATGATTAA AGTAGTTCAC CCGTACCAAT AACTTAACTA CGATAACCAC ACGGCTTCTT   
  
  
+ TCAATAAGAA GTGTAAACAG CTGCTCCTG  

- GACTGTAACA ATGATCCGTC TCAGACAGAG AGCCTGCCGA AATTCTGCGA ACGCGGAGAG AGAAAGTGCG   
  
  
- AGGCTAAGAG GTGCGTTTTA GACTGAGGAA AAGGGAGAAG GATGGAAAGA GAAAGGGAGA GAGCTAGGGT   
  
  
- TTTGTGATGG TAAGGACGCG TAGTTTTTGA ACTTTTTTCC TGTGAAGCAG TGTCCTATCA ACAGCGACGC   
  
  
- GCGCTCTCTT CTCTGTTATC TCTCTAAAGA CAAACAGTCA TAGAGACAGA GAGAGAAAAG CAAACAAAAA   
  
  
- ACACTGAAAA GAATGAAAGA AAAGAAGAGA GAAGGGAATA GAAAAGAGAT AAGTTAGAAA AATCGCCGTC   
  
  
- AACCGAAAAC AGTACGGGAG GCTGTTATTT AGGGGAACGC AAGAGTGTAG GTCCCACGCG CATGAAATTG   
  
  
- AAATTTTCCA GAGAAGAAAA AAGATACTTT CCGACAAGAA GGCGCACCTT GTTTCTCTCC AGTACTTACC   
  
  
- TGTGCGTAAA CCATCCCCAA TTTCTTTCTT TTCTTAGTTT TACCCCCAAA AAAAAAAAAA TAAAGTAAAA   
  
  
- TTAAAATCTT CTTTACAAAG ATAACTTATT TTTAAATTCA CATTACATAT TATAAAATAA AAATTAAATC   
  
  
- CAAACTTTCC ACATTTTAAA AATCTTTAAA ATAAGTTAAA TTTCCAGGTC AATCATGTAA GACATATTTT   
  
  
- TTTAAAATGA ATAAAATCTT TAAAAATTAT CGTTATTAAA ATAGATAACA TTTTACATTA TTATGACAAA   
  
  
- AAATTACTGT AAATATCACA ATTTAAATTG ATAGGATAAA TGTGTAATAT TGAAAAACTT TCTTTTTCAG   
  
  
- TAGAAAAAAA TCTTGGGGAG GAGACCTTTG ATTGGCATAT ATATTATATG GTGAGTAATG AAAGAAGAGG   
  
  
- ACGTTTGGTG TGTTTGTTTA ATTAAACAAT TAATTAATTT AATGTCCAAA GCAGATGAAG CGAAGAGGTC   
  
  
- ATCAGAAGAA GAAGCATAAA TAAAATATTG GAAAACAAGA GGTGGAGGAG GAGACAGAGG CTTGGGATGC   
  
  
- AGCAGTGGTA AGTAACAAGT GGGCTCTGTC GCTCTCTCTC TATTTGCTGA GTGAATAATT CAGCACAACA   
  
  
- GCACACGAAC AGGAGCTGGT CACTGATTTG TGAACATTCT TGGCAAATAC TACCCATCAG CCATCCTCTT   
  
  
- TATTACTCCC TCTAACTCCT ACACTCTTTG ACCCAATCCA CCTTTCTCAT CCCTTCTATA TTTATTTTTT   
  
  
- ATCCGTTCAT TTTTTATTTT TTTAAAAATG AATTAAATTA TTTATTTTTT AAAATTATAA TTATCTATTT   
  
  
- CTTTTTTAAA AGATTAATAA AAATCAAACT GATAGAAAAG ATGTTTAGTT TATCAAAATA ATTTTTTTTA   
  
  
- GATGCCCATA TATACTAATT TCATCAAGTG GGCATGGTTA TTGAATTGAT GCTATTGGTG TGCCGAAGAA   
  
  
- AGTTATTCTT CACATTTGTC GACGAGGAC

  
  
Motifs Found  

+     5UTR Py-rich stretch

| Site Name | Organism | Position | Strand | Matrix score. | sequence | function |
| --- | --- | --- | --- | --- | --- | --- |
| 5UTR Py-rich stretch | Lycopersicon esculentum | 1082 | - | 13 | TTTCTCTCTCTCTC | cis-acting element conferring high transcription levels |
| 5UTR Py-rich stretch | Lycopersicon esculentum | 301 | + | 10 | TTTCTTCTCT | cis-acting element conferring high transcription levels |
| 5UTR Py-rich stretch | Lycopersicon esculentum | 296 | + | 9 | TTTCTTCTCT | cis-acting element conferring high transcription levels |
| 5UTR Py-rich stretch | Lycopersicon esculentum | 215 | - | 9 | TTTCTTCTCT | cis-acting element conferring high transcription levels |
| 5UTR Py-rich stretch | Lycopersicon esculentum | 901 | + | 9 | TTTCTTCTCT | cis-acting element conferring high transcription levels |

> 2018/04/13 10:10:12  
+ CTGACATTGT TACTAGGCAG AGTCTGTCTC TCGGACGGCT TTAAGACGCT TGCGCCTCTC TCTTTCACGC   
  
  
+ TCCGATTCTC CACGCAAAAT CTGACTCCTT TTCCCTCTTC CTACCTTTCT CTTTCCCTCT CTCGATCCCA   
  
  
+ AAACACTACC ATTCCTGCGC ATCAAAAACT TGAAAAAAGG ACACTTCGTC ACAGGATAGT TGTCGCTGCG   
  
  
+ CGCGAGAGAA GAGACAATAG AGAGATTTCT GTTTGTCAGT ATCTCTGTCT CTCTCTTTTC GTTTGTTTTT   
  
  
+ TGTGACTTTT CTTACTTTCT TTTCTTCTCT CTTCCCTTAT CTTTTCTCTA TTCAATCTTT TTAGCGGCAG   
  
  
+ TTGGCTTTTG TCATGCCCTC CGACAATAAA TCCCCTTGCG TTCTCACATC CAGGGTGCGC GTACTTTAAC   
  
  
+ TTTAAAAGGT CTCTTCTTTT TTCTATGAAA GGCTGTTCTT CCGCGTGGAA CAAAGAGAGG TCATGAATGG   
  
  
+ ACACGCATTT GGTAGGGGTT AAAGAAAGAA AAGAATCAAA ATGGGGGTTT TTTTTTTTTT ATTTCATTTT   
  
  
+ AATTTTAGAA GAAATGTTTC TATTGAATAA AAATTTAAGT GTAATGTATA ATATTTTATT TTTAATTTAG   
  
  
+ GTTTGAAAGG TGTAAAATTT TTAGAAATTT TATTCAATTT AAAGGTCCAG TTAGTACATT CTGTATAAAA   
  
  
+ AAATTTTACT TATTTTAGAA ATTTTTAATA GCAATAATTT TATCTATTGT AAAATGTAAT AATACTGTTT   
  
  
+ TTTAATGACA TTTATAGTGT TAAATTTAAC TATCCTATTT ACACATTATA ACTTTTTGAA AGAAAAAGTC   
  
  
+ ATCTTTTTTT AGAACCCCTC CTCTGGAAAC TAACCGTATA TATAATATAC CACTCATTAC TTTCTTCTCC   
  
  
+ TGCAAACCAC ACAAACAAAT TAATTTGTTA ATTAATTAAA TTACAGGTTT CGTCTACTTC GCTTCTCCAG   
  
  
+ TAGTCTTCTT CTTCGTATTT ATTTTATAAC CTTTTGTTCT CCACCTCCTC CTCTGTCTCC GAACCCTACG   
  
  
+ TCGTCACCAT TCATTGTTCA CCCGAGACAG CGAGAGAGAG ATAAACGACT CACTTATTAA GTCGTGTTGT   
  
  
+ CGTGTGCTTG TCCTCGACCA GTGACTAAAC ACTTGTAAGA ACCGTTTATG ATGGGTAGTC GGTAGGAGAA   
  
  
+ ATAATGAGGG AGATTGAGGA TGTGAGAAAC TGGGTTAGGT GGAAAGAGTA GGGAAGATAT AAATAAAAAA   
  
  
+ TAGGCAAGTA AAAAATAAAA AAATTTTTAC TTAATTTAAT AAATAAAAAA TTTTAATATT AATAGATAAA   
  
  
+ GAAAAAATTT TCTAATTATT TTTAGTTTGA CTATCTTTTC TACAAATCAA ATAGTTTTAT TAAAAAAAAT   
  
  
+ CTACGGGTAT ATATGATTAA AGTAGTTCAC CCGTACCAAT AACTTAACTA CGATAACCAC ACGGCTTCTT   
  
  
+ TCAATAAGAA GTGTAAACAG CTGCTCCTG  

- GACTGTAACA ATGATCCGTC TCAGACAGAG AGCCTGCCGA AATTCTGCGA ACGCGGAGAG AGAAAGTGCG   
  
  
- AGGCTAAGAG GTGCGTTTTA GACTGAGGAA AAGGGAGAAG GATGGAAAGA GAAAGGGAGA GAGCTAGGGT   
  
  
- TTTGTGATGG TAAGGACGCG TAGTTTTTGA ACTTTTTTCC TGTGAAGCAG TGTCCTATCA ACAGCGACGC   
  
  
- GCGCTCTCTT CTCTGTTATC TCTCTAAAGA CAAACAGTCA TAGAGACAGA GAGAGAAAAG CAAACAAAAA   
  
  
- ACACTGAAAA GAATGAAAGA AAAGAAGAGA GAAGGGAATA GAAAAGAGAT AAGTTAGAAA AATCGCCGTC   
  
  
- AACCGAAAAC AGTACGGGAG GCTGTTATTT AGGGGAACGC AAGAGTGTAG GTCCCACGCG CATGAAATTG   
  
  
- AAATTTTCCA GAGAAGAAAA AAGATACTTT CCGACAAGAA GGCGCACCTT GTTTCTCTCC AGTACTTACC   
  
  
- TGTGCGTAAA CCATCCCCAA TTTCTTTCTT TTCTTAGTTT TACCCCCAAA AAAAAAAAAA TAAAGTAAAA   
  
  
- TTAAAATCTT CTTTACAAAG ATAACTTATT TTTAAATTCA CATTACATAT TATAAAATAA AAATTAAATC   
  
  
- CAAACTTTCC ACATTTTAAA AATCTTTAAA ATAAGTTAAA TTTCCAGGTC AATCATGTAA GACATATTTT   
  
  
- TTTAAAATGA ATAAAATCTT TAAAAATTAT CGTTATTAAA ATAGATAACA TTTTACATTA TTATGACAAA   
  
  
- AAATTACTGT AAATATCACA ATTTAAATTG ATAGGATAAA TGTGTAATAT TGAAAAACTT TCTTTTTCAG   
  
  
- TAGAAAAAAA TCTTGGGGAG GAGACCTTTG ATTGGCATAT ATATTATATG GTGAGTAATG AAAGAAGAGG   
  
  
- ACGTTTGGTG TGTTTGTTTA ATTAAACAAT TAATTAATTT AATGTCCAAA GCAGATGAAG CGAAGAGGTC   
  
  
- ATCAGAAGAA GAAGCATAAA TAAAATATTG GAAAACAAGA GGTGGAGGAG GAGACAGAGG CTTGGGATGC   
  
  
- AGCAGTGGTA AGTAACAAGT GGGCTCTGTC GCTCTCTCTC TATTTGCTGA GTGAATAATT CAGCACAACA   
  
  
- GCACACGAAC AGGAGCTGGT CACTGATTTG TGAACATTCT TGGCAAATAC TACCCATCAG CCATCCTCTT   
  
  
- TATTACTCCC TCTAACTCCT ACACTCTTTG ACCCAATCCA CCTTTCTCAT CCCTTCTATA TTTATTTTTT   
  
  
- ATCCGTTCAT TTTTTATTTT TTTAAAAATG AATTAAATTA TTTATTTTTT AAAATTATAA TTATCTATTT   
  
  
- CTTTTTTAAA AGATTAATAA AAATCAAACT GATAGAAAAG ATGTTTAGTT TATCAAAATA ATTTTTTTTA   
  
  
- GATGCCCATA TATACTAATT TCATCAAGTG GGCATGGTTA TTGAATTGAT GCTATTGGTG TGCCGAAGAA   
  
  
- AGTTATTCTT CACATTTGTC GACGAGGAC

+     A-box

| Site Name | Organism | Position | Strand | Matrix score. | sequence | function |
| --- | --- | --- | --- | --- | --- | --- |
| A-box | Petroselinum crispum | 33 | - | 6 | CCGTCC | cis-acting regulatory element |

> 2018/04/13 10:10:12  
+ CTGACATTGT TACTAGGCAG AGTCTGTCTC TCGGACGGCT TTAAGACGCT TGCGCCTCTC TCTTTCACGC   
  
  
+ TCCGATTCTC CACGCAAAAT CTGACTCCTT TTCCCTCTTC CTACCTTTCT CTTTCCCTCT CTCGATCCCA   
  
  
+ AAACACTACC ATTCCTGCGC ATCAAAAACT TGAAAAAAGG ACACTTCGTC ACAGGATAGT TGTCGCTGCG   
  
  
+ CGCGAGAGAA GAGACAATAG AGAGATTTCT GTTTGTCAGT ATCTCTGTCT CTCTCTTTTC GTTTGTTTTT   
  
  
+ TGTGACTTTT CTTACTTTCT TTTCTTCTCT CTTCCCTTAT CTTTTCTCTA TTCAATCTTT TTAGCGGCAG   
  
  
+ TTGGCTTTTG TCATGCCCTC CGACAATAAA TCCCCTTGCG TTCTCACATC CAGGGTGCGC GTACTTTAAC   
  
  
+ TTTAAAAGGT CTCTTCTTTT TTCTATGAAA GGCTGTTCTT CCGCGTGGAA CAAAGAGAGG TCATGAATGG   
  
  
+ ACACGCATTT GGTAGGGGTT AAAGAAAGAA AAGAATCAAA ATGGGGGTTT TTTTTTTTTT ATTTCATTTT   
  
  
+ AATTTTAGAA GAAATGTTTC TATTGAATAA AAATTTAAGT GTAATGTATA ATATTTTATT TTTAATTTAG   
  
  
+ GTTTGAAAGG TGTAAAATTT TTAGAAATTT TATTCAATTT AAAGGTCCAG TTAGTACATT CTGTATAAAA   
  
  
+ AAATTTTACT TATTTTAGAA ATTTTTAATA GCAATAATTT TATCTATTGT AAAATGTAAT AATACTGTTT   
  
  
+ TTTAATGACA TTTATAGTGT TAAATTTAAC TATCCTATTT ACACATTATA ACTTTTTGAA AGAAAAAGTC   
  
  
+ ATCTTTTTTT AGAACCCCTC CTCTGGAAAC TAACCGTATA TATAATATAC CACTCATTAC TTTCTTCTCC   
  
  
+ TGCAAACCAC ACAAACAAAT TAATTTGTTA ATTAATTAAA TTACAGGTTT CGTCTACTTC GCTTCTCCAG   
  
  
+ TAGTCTTCTT CTTCGTATTT ATTTTATAAC CTTTTGTTCT CCACCTCCTC CTCTGTCTCC GAACCCTACG   
  
  
+ TCGTCACCAT TCATTGTTCA CCCGAGACAG CGAGAGAGAG ATAAACGACT CACTTATTAA GTCGTGTTGT   
  
  
+ CGTGTGCTTG TCCTCGACCA GTGACTAAAC ACTTGTAAGA ACCGTTTATG ATGGGTAGTC GGTAGGAGAA   
  
  
+ ATAATGAGGG AGATTGAGGA TGTGAGAAAC TGGGTTAGGT GGAAAGAGTA GGGAAGATAT AAATAAAAAA   
  
  
+ TAGGCAAGTA AAAAATAAAA AAATTTTTAC TTAATTTAAT AAATAAAAAA TTTTAATATT AATAGATAAA   
  
  
+ GAAAAAATTT TCTAATTATT TTTAGTTTGA CTATCTTTTC TACAAATCAA ATAGTTTTAT TAAAAAAAAT   
  
  
+ CTACGGGTAT ATATGATTAA AGTAGTTCAC CCGTACCAAT AACTTAACTA CGATAACCAC ACGGCTTCTT   
  
  
+ TCAATAAGAA GTGTAAACAG CTGCTCCTG  

- GACTGTAACA ATGATCCGTC TCAGACAGAG AGCCTGCCGA AATTCTGCGA ACGCGGAGAG AGAAAGTGCG   
  
  
- AGGCTAAGAG GTGCGTTTTA GACTGAGGAA AAGGGAGAAG GATGGAAAGA GAAAGGGAGA GAGCTAGGGT   
  
  
- TTTGTGATGG TAAGGACGCG TAGTTTTTGA ACTTTTTTCC TGTGAAGCAG TGTCCTATCA ACAGCGACGC   
  
  
- GCGCTCTCTT CTCTGTTATC TCTCTAAAGA CAAACAGTCA TAGAGACAGA GAGAGAAAAG CAAACAAAAA   
  
  
- ACACTGAAAA GAATGAAAGA AAAGAAGAGA GAAGGGAATA GAAAAGAGAT AAGTTAGAAA AATCGCCGTC   
  
  
- AACCGAAAAC AGTACGGGAG GCTGTTATTT AGGGGAACGC AAGAGTGTAG GTCCCACGCG CATGAAATTG   
  
  
- AAATTTTCCA GAGAAGAAAA AAGATACTTT CCGACAAGAA GGCGCACCTT GTTTCTCTCC AGTACTTACC   
  
  
- TGTGCGTAAA CCATCCCCAA TTTCTTTCTT TTCTTAGTTT TACCCCCAAA AAAAAAAAAA TAAAGTAAAA   
  
  
- TTAAAATCTT CTTTACAAAG ATAACTTATT TTTAAATTCA CATTACATAT TATAAAATAA AAATTAAATC   
  
  
- CAAACTTTCC ACATTTTAAA AATCTTTAAA ATAAGTTAAA TTTCCAGGTC AATCATGTAA GACATATTTT   
  
  
- TTTAAAATGA ATAAAATCTT TAAAAATTAT CGTTATTAAA ATAGATAACA TTTTACATTA TTATGACAAA   
  
  
- AAATTACTGT AAATATCACA ATTTAAATTG ATAGGATAAA TGTGTAATAT TGAAAAACTT TCTTTTTCAG   
  
  
- TAGAAAAAAA TCTTGGGGAG GAGACCTTTG ATTGGCATAT ATATTATATG GTGAGTAATG AAAGAAGAGG   
  
  
- ACGTTTGGTG TGTTTGTTTA ATTAAACAAT TAATTAATTT AATGTCCAAA GCAGATGAAG CGAAGAGGTC   
  
  
- ATCAGAAGAA GAAGCATAAA TAAAATATTG GAAAACAAGA GGTGGAGGAG GAGACAGAGG CTTGGGATGC   
  
  
- AGCAGTGGTA AGTAACAAGT GGGCTCTGTC GCTCTCTCTC TATTTGCTGA GTGAATAATT CAGCACAACA   
  
  
- GCACACGAAC AGGAGCTGGT CACTGATTTG TGAACATTCT TGGCAAATAC TACCCATCAG CCATCCTCTT   
  
  
- TATTACTCCC TCTAACTCCT ACACTCTTTG ACCCAATCCA CCTTTCTCAT CCCTTCTATA TTTATTTTTT   
  
  
- ATCCGTTCAT TTTTTATTTT TTTAAAAATG AATTAAATTA TTTATTTTTT AAAATTATAA TTATCTATTT   
  
  
- CTTTTTTAAA AGATTAATAA AAATCAAACT GATAGAAAAG ATGTTTAGTT TATCAAAATA ATTTTTTTTA   
  
  
- GATGCCCATA TATACTAATT TCATCAAGTG GGCATGGTTA TTGAATTGAT GCTATTGGTG TGCCGAAGAA   
  
  
- AGTTATTCTT CACATTTGTC GACGAGGAC

+     AAGAA-motif

| Site Name | Organism | Position | Strand | Matrix score. | sequence | function |
| --- | --- | --- | --- | --- | --- | --- |
| AAGAA-motif | Avena sativa | 1466 | - | 7 | GAAAGAA |  |
| AAGAA-motif | Avena sativa | 1325 | + | 9 | gGTAAAGAAA |  |
| AAGAA-motif | Avena sativa | 828 | + | 7 | GAAAGAA |  |
| AAGAA-motif | Avena sativa | 508 | + | 9 | gGTAAAGAAA |  |
| AAGAA-motif | Avena sativa | 514 | + | 7 | GAAAGAA |  |

> 2018/04/13 10:10:12  
+ CTGACATTGT TACTAGGCAG AGTCTGTCTC TCGGACGGCT TTAAGACGCT TGCGCCTCTC TCTTTCACGC   
  
  
+ TCCGATTCTC CACGCAAAAT CTGACTCCTT TTCCCTCTTC CTACCTTTCT CTTTCCCTCT CTCGATCCCA   
  
  
+ AAACACTACC ATTCCTGCGC ATCAAAAACT TGAAAAAAGG ACACTTCGTC ACAGGATAGT TGTCGCTGCG   
  
  
+ CGCGAGAGAA GAGACAATAG AGAGATTTCT GTTTGTCAGT ATCTCTGTCT CTCTCTTTTC GTTTGTTTTT   
  
  
+ TGTGACTTTT CTTACTTTCT TTTCTTCTCT CTTCCCTTAT CTTTTCTCTA TTCAATCTTT TTAGCGGCAG   
  
  
+ TTGGCTTTTG TCATGCCCTC CGACAATAAA TCCCCTTGCG TTCTCACATC CAGGGTGCGC GTACTTTAAC   
  
  
+ TTTAAAAGGT CTCTTCTTTT TTCTATGAAA GGCTGTTCTT CCGCGTGGAA CAAAGAGAGG TCATGAATGG   
  
  
+ ACACGCATTT GGTAGGGGTT AAAGAAAGAA AAGAATCAAA ATGGGGGTTT TTTTTTTTTT ATTTCATTTT   
  
  
+ AATTTTAGAA GAAATGTTTC TATTGAATAA AAATTTAAGT GTAATGTATA ATATTTTATT TTTAATTTAG   
  
  
+ GTTTGAAAGG TGTAAAATTT TTAGAAATTT TATTCAATTT AAAGGTCCAG TTAGTACATT CTGTATAAAA   
  
  
+ AAATTTTACT TATTTTAGAA ATTTTTAATA GCAATAATTT TATCTATTGT AAAATGTAAT AATACTGTTT   
  
  
+ TTTAATGACA TTTATAGTGT TAAATTTAAC TATCCTATTT ACACATTATA ACTTTTTGAA AGAAAAAGTC   
  
  
+ ATCTTTTTTT AGAACCCCTC CTCTGGAAAC TAACCGTATA TATAATATAC CACTCATTAC TTTCTTCTCC   
  
  
+ TGCAAACCAC ACAAACAAAT TAATTTGTTA ATTAATTAAA TTACAGGTTT CGTCTACTTC GCTTCTCCAG   
  
  
+ TAGTCTTCTT CTTCGTATTT ATTTTATAAC CTTTTGTTCT CCACCTCCTC CTCTGTCTCC GAACCCTACG   
  
  
+ TCGTCACCAT TCATTGTTCA CCCGAGACAG CGAGAGAGAG ATAAACGACT CACTTATTAA GTCGTGTTGT   
  
  
+ CGTGTGCTTG TCCTCGACCA GTGACTAAAC ACTTGTAAGA ACCGTTTATG ATGGGTAGTC GGTAGGAGAA   
  
  
+ ATAATGAGGG AGATTGAGGA TGTGAGAAAC TGGGTTAGGT GGAAAGAGTA GGGAAGATAT AAATAAAAAA   
  
  
+ TAGGCAAGTA AAAAATAAAA AAATTTTTAC TTAATTTAAT AAATAAAAAA TTTTAATATT AATAGATAAA   
  
  
+ GAAAAAATTT TCTAATTATT TTTAGTTTGA CTATCTTTTC TACAAATCAA ATAGTTTTAT TAAAAAAAAT   
  
  
+ CTACGGGTAT ATATGATTAA AGTAGTTCAC CCGTACCAAT AACTTAACTA CGATAACCAC ACGGCTTCTT   
  
  
+ TCAATAAGAA GTGTAAACAG CTGCTCCTG  

- GACTGTAACA ATGATCCGTC TCAGACAGAG AGCCTGCCGA AATTCTGCGA ACGCGGAGAG AGAAAGTGCG   
  
  
- AGGCTAAGAG GTGCGTTTTA GACTGAGGAA AAGGGAGAAG GATGGAAAGA GAAAGGGAGA GAGCTAGGGT   
  
  
- TTTGTGATGG TAAGGACGCG TAGTTTTTGA ACTTTTTTCC TGTGAAGCAG TGTCCTATCA ACAGCGACGC   
  
  
- GCGCTCTCTT CTCTGTTATC TCTCTAAAGA CAAACAGTCA TAGAGACAGA GAGAGAAAAG CAAACAAAAA   
  
  
- ACACTGAAAA GAATGAAAGA AAAGAAGAGA GAAGGGAATA GAAAAGAGAT AAGTTAGAAA AATCGCCGTC   
  
  
- AACCGAAAAC AGTACGGGAG GCTGTTATTT AGGGGAACGC AAGAGTGTAG GTCCCACGCG CATGAAATTG   
  
  
- AAATTTTCCA GAGAAGAAAA AAGATACTTT CCGACAAGAA GGCGCACCTT GTTTCTCTCC AGTACTTACC   
  
  
- TGTGCGTAAA CCATCCCCAA TTTCTTTCTT TTCTTAGTTT TACCCCCAAA AAAAAAAAAA TAAAGTAAAA   
  
  
- TTAAAATCTT CTTTACAAAG ATAACTTATT TTTAAATTCA CATTACATAT TATAAAATAA AAATTAAATC   
  
  
- CAAACTTTCC ACATTTTAAA AATCTTTAAA ATAAGTTAAA TTTCCAGGTC AATCATGTAA GACATATTTT   
  
  
- TTTAAAATGA ATAAAATCTT TAAAAATTAT CGTTATTAAA ATAGATAACA TTTTACATTA TTATGACAAA   
  
  
- AAATTACTGT AAATATCACA ATTTAAATTG ATAGGATAAA TGTGTAATAT TGAAAAACTT TCTTTTTCAG   
  
  
- TAGAAAAAAA TCTTGGGGAG GAGACCTTTG ATTGGCATAT ATATTATATG GTGAGTAATG AAAGAAGAGG   
  
  
- ACGTTTGGTG TGTTTGTTTA ATTAAACAAT TAATTAATTT AATGTCCAAA GCAGATGAAG CGAAGAGGTC   
  
  
- ATCAGAAGAA GAAGCATAAA TAAAATATTG GAAAACAAGA GGTGGAGGAG GAGACAGAGG CTTGGGATGC   
  
  
- AGCAGTGGTA AGTAACAAGT GGGCTCTGTC GCTCTCTCTC TATTTGCTGA GTGAATAATT CAGCACAACA   
  
  
- GCACACGAAC AGGAGCTGGT CACTGATTTG TGAACATTCT TGGCAAATAC TACCCATCAG CCATCCTCTT   
  
  
- TATTACTCCC TCTAACTCCT ACACTCTTTG ACCCAATCCA CCTTTCTCAT CCCTTCTATA TTTATTTTTT   
  
  
- ATCCGTTCAT TTTTTATTTT TTTAAAAATG AATTAAATTA TTTATTTTTT AAAATTATAA TTATCTATTT   
  
  
- CTTTTTTAAA AGATTAATAA AAATCAAACT GATAGAAAAG ATGTTTAGTT TATCAAAATA ATTTTTTTTA   
  
  
- GATGCCCATA TATACTAATT TCATCAAGTG GGCATGGTTA TTGAATTGAT GCTATTGGTG TGCCGAAGAA   
  
  
- AGTTATTCTT CACATTTGTC GACGAGGAC

+     AE-box

| Site Name | Organism | Position | Strand | Matrix score. | sequence | function |
| --- | --- | --- | --- | --- | --- | --- |
| AE-box | Arabidopsis thaliana | 574 | - | 8 | AGAAACAT | part of a module for light response |

> 2018/04/13 10:10:12  
+ CTGACATTGT TACTAGGCAG AGTCTGTCTC TCGGACGGCT TTAAGACGCT TGCGCCTCTC TCTTTCACGC   
  
  
+ TCCGATTCTC CACGCAAAAT CTGACTCCTT TTCCCTCTTC CTACCTTTCT CTTTCCCTCT CTCGATCCCA   
  
  
+ AAACACTACC ATTCCTGCGC ATCAAAAACT TGAAAAAAGG ACACTTCGTC ACAGGATAGT TGTCGCTGCG   
  
  
+ CGCGAGAGAA GAGACAATAG AGAGATTTCT GTTTGTCAGT ATCTCTGTCT CTCTCTTTTC GTTTGTTTTT   
  
  
+ TGTGACTTTT CTTACTTTCT TTTCTTCTCT CTTCCCTTAT CTTTTCTCTA TTCAATCTTT TTAGCGGCAG   
  
  
+ TTGGCTTTTG TCATGCCCTC CGACAATAAA TCCCCTTGCG TTCTCACATC CAGGGTGCGC GTACTTTAAC   
  
  
+ TTTAAAAGGT CTCTTCTTTT TTCTATGAAA GGCTGTTCTT CCGCGTGGAA CAAAGAGAGG TCATGAATGG   
  
  
+ ACACGCATTT GGTAGGGGTT AAAGAAAGAA AAGAATCAAA ATGGGGGTTT TTTTTTTTTT ATTTCATTTT   
  
  
+ AATTTTAGAA GAAATGTTTC TATTGAATAA AAATTTAAGT GTAATGTATA ATATTTTATT TTTAATTTAG   
  
  
+ GTTTGAAAGG TGTAAAATTT TTAGAAATTT TATTCAATTT AAAGGTCCAG TTAGTACATT CTGTATAAAA   
  
  
+ AAATTTTACT TATTTTAGAA ATTTTTAATA GCAATAATTT TATCTATTGT AAAATGTAAT AATACTGTTT   
  
  
+ TTTAATGACA TTTATAGTGT TAAATTTAAC TATCCTATTT ACACATTATA ACTTTTTGAA AGAAAAAGTC   
  
  
+ ATCTTTTTTT AGAACCCCTC CTCTGGAAAC TAACCGTATA TATAATATAC CACTCATTAC TTTCTTCTCC   
  
  
+ TGCAAACCAC ACAAACAAAT TAATTTGTTA ATTAATTAAA TTACAGGTTT CGTCTACTTC GCTTCTCCAG   
  
  
+ TAGTCTTCTT CTTCGTATTT ATTTTATAAC CTTTTGTTCT CCACCTCCTC CTCTGTCTCC GAACCCTACG   
  
  
+ TCGTCACCAT TCATTGTTCA CCCGAGACAG CGAGAGAGAG ATAAACGACT CACTTATTAA GTCGTGTTGT   
  
  
+ CGTGTGCTTG TCCTCGACCA GTGACTAAAC ACTTGTAAGA ACCGTTTATG ATGGGTAGTC GGTAGGAGAA   
  
  
+ ATAATGAGGG AGATTGAGGA TGTGAGAAAC TGGGTTAGGT GGAAAGAGTA GGGAAGATAT AAATAAAAAA   
  
  
+ TAGGCAAGTA AAAAATAAAA AAATTTTTAC TTAATTTAAT AAATAAAAAA TTTTAATATT AATAGATAAA   
  
  
+ GAAAAAATTT TCTAATTATT TTTAGTTTGA CTATCTTTTC TACAAATCAA ATAGTTTTAT TAAAAAAAAT   
  
  
+ CTACGGGTAT ATATGATTAA AGTAGTTCAC CCGTACCAAT AACTTAACTA CGATAACCAC ACGGCTTCTT   
  
  
+ TCAATAAGAA GTGTAAACAG CTGCTCCTG  

- GACTGTAACA ATGATCCGTC TCAGACAGAG AGCCTGCCGA AATTCTGCGA ACGCGGAGAG AGAAAGTGCG   
  
  
- AGGCTAAGAG GTGCGTTTTA GACTGAGGAA AAGGGAGAAG GATGGAAAGA GAAAGGGAGA GAGCTAGGGT   
  
  
- TTTGTGATGG TAAGGACGCG TAGTTTTTGA ACTTTTTTCC TGTGAAGCAG TGTCCTATCA ACAGCGACGC   
  
  
- GCGCTCTCTT CTCTGTTATC TCTCTAAAGA CAAACAGTCA TAGAGACAGA GAGAGAAAAG CAAACAAAAA   
  
  
- ACACTGAAAA GAATGAAAGA AAAGAAGAGA GAAGGGAATA GAAAAGAGAT AAGTTAGAAA AATCGCCGTC   
  
  
- AACCGAAAAC AGTACGGGAG GCTGTTATTT AGGGGAACGC AAGAGTGTAG GTCCCACGCG CATGAAATTG   
  
  
- AAATTTTCCA GAGAAGAAAA AAGATACTTT CCGACAAGAA GGCGCACCTT GTTTCTCTCC AGTACTTACC   
  
  
- TGTGCGTAAA CCATCCCCAA TTTCTTTCTT TTCTTAGTTT TACCCCCAAA AAAAAAAAAA TAAAGTAAAA   
  
  
- TTAAAATCTT CTTTACAAAG ATAACTTATT TTTAAATTCA CATTACATAT TATAAAATAA AAATTAAATC   
  
  
- CAAACTTTCC ACATTTTAAA AATCTTTAAA ATAAGTTAAA TTTCCAGGTC AATCATGTAA GACATATTTT   
  
  
- TTTAAAATGA ATAAAATCTT TAAAAATTAT CGTTATTAAA ATAGATAACA TTTTACATTA TTATGACAAA   
  
  
- AAATTACTGT AAATATCACA ATTTAAATTG ATAGGATAAA TGTGTAATAT TGAAAAACTT TCTTTTTCAG   
  
  
- TAGAAAAAAA TCTTGGGGAG GAGACCTTTG ATTGGCATAT ATATTATATG GTGAGTAATG AAAGAAGAGG   
  
  
- ACGTTTGGTG TGTTTGTTTA ATTAAACAAT TAATTAATTT AATGTCCAAA GCAGATGAAG CGAAGAGGTC   
  
  
- ATCAGAAGAA GAAGCATAAA TAAAATATTG GAAAACAAGA GGTGGAGGAG GAGACAGAGG CTTGGGATGC   
  
  
- AGCAGTGGTA AGTAACAAGT GGGCTCTGTC GCTCTCTCTC TATTTGCTGA GTGAATAATT CAGCACAACA   
  
  
- GCACACGAAC AGGAGCTGGT CACTGATTTG TGAACATTCT TGGCAAATAC TACCCATCAG CCATCCTCTT   
  
  
- TATTACTCCC TCTAACTCCT ACACTCTTTG ACCCAATCCA CCTTTCTCAT CCCTTCTATA TTTATTTTTT   
  
  
- ATCCGTTCAT TTTTTATTTT TTTAAAAATG AATTAAATTA TTTATTTTTT AAAATTATAA TTATCTATTT   
  
  
- CTTTTTTAAA AGATTAATAA AAATCAAACT GATAGAAAAG ATGTTTAGTT TATCAAAATA ATTTTTTTTA   
  
  
- GATGCCCATA TATACTAATT TCATCAAGTG GGCATGGTTA TTGAATTGAT GCTATTGGTG TGCCGAAGAA   
  
  
- AGTTATTCTT CACATTTGTC GACGAGGAC

+     ARE

| Site Name | Organism | Position | Strand | Matrix score. | sequence | function |
| --- | --- | --- | --- | --- | --- | --- |
| ARE | Zea mays | 914 | - | 6 | TGGTTT | cis-acting regulatory element essential for the anaerobic induction |

> 2018/04/13 10:10:12  
+ CTGACATTGT TACTAGGCAG AGTCTGTCTC TCGGACGGCT TTAAGACGCT TGCGCCTCTC TCTTTCACGC   
  
  
+ TCCGATTCTC CACGCAAAAT CTGACTCCTT TTCCCTCTTC CTACCTTTCT CTTTCCCTCT CTCGATCCCA   
  
  
+ AAACACTACC ATTCCTGCGC ATCAAAAACT TGAAAAAAGG ACACTTCGTC ACAGGATAGT TGTCGCTGCG   
  
  
+ CGCGAGAGAA GAGACAATAG AGAGATTTCT GTTTGTCAGT ATCTCTGTCT CTCTCTTTTC GTTTGTTTTT   
  
  
+ TGTGACTTTT CTTACTTTCT TTTCTTCTCT CTTCCCTTAT CTTTTCTCTA TTCAATCTTT TTAGCGGCAG   
  
  
+ TTGGCTTTTG TCATGCCCTC CGACAATAAA TCCCCTTGCG TTCTCACATC CAGGGTGCGC GTACTTTAAC   
  
  
+ TTTAAAAGGT CTCTTCTTTT TTCTATGAAA GGCTGTTCTT CCGCGTGGAA CAAAGAGAGG TCATGAATGG   
  
  
+ ACACGCATTT GGTAGGGGTT AAAGAAAGAA AAGAATCAAA ATGGGGGTTT TTTTTTTTTT ATTTCATTTT   
  
  
+ AATTTTAGAA GAAATGTTTC TATTGAATAA AAATTTAAGT GTAATGTATA ATATTTTATT TTTAATTTAG   
  
  
+ GTTTGAAAGG TGTAAAATTT TTAGAAATTT TATTCAATTT AAAGGTCCAG TTAGTACATT CTGTATAAAA   
  
  
+ AAATTTTACT TATTTTAGAA ATTTTTAATA GCAATAATTT TATCTATTGT AAAATGTAAT AATACTGTTT   
  
  
+ TTTAATGACA TTTATAGTGT TAAATTTAAC TATCCTATTT ACACATTATA ACTTTTTGAA AGAAAAAGTC   
  
  
+ ATCTTTTTTT AGAACCCCTC CTCTGGAAAC TAACCGTATA TATAATATAC CACTCATTAC TTTCTTCTCC   
  
  
+ TGCAAACCAC ACAAACAAAT TAATTTGTTA ATTAATTAAA TTACAGGTTT CGTCTACTTC GCTTCTCCAG   
  
  
+ TAGTCTTCTT CTTCGTATTT ATTTTATAAC CTTTTGTTCT CCACCTCCTC CTCTGTCTCC GAACCCTACG   
  
  
+ TCGTCACCAT TCATTGTTCA CCCGAGACAG CGAGAGAGAG ATAAACGACT CACTTATTAA GTCGTGTTGT   
  
  
+ CGTGTGCTTG TCCTCGACCA GTGACTAAAC ACTTGTAAGA ACCGTTTATG ATGGGTAGTC GGTAGGAGAA   
  
  
+ ATAATGAGGG AGATTGAGGA TGTGAGAAAC TGGGTTAGGT GGAAAGAGTA GGGAAGATAT AAATAAAAAA   
  
  
+ TAGGCAAGTA AAAAATAAAA AAATTTTTAC TTAATTTAAT AAATAAAAAA TTTTAATATT AATAGATAAA   
  
  
+ GAAAAAATTT TCTAATTATT TTTAGTTTGA CTATCTTTTC TACAAATCAA ATAGTTTTAT TAAAAAAAAT   
  
  
+ CTACGGGTAT ATATGATTAA AGTAGTTCAC CCGTACCAAT AACTTAACTA CGATAACCAC ACGGCTTCTT   
  
  
+ TCAATAAGAA GTGTAAACAG CTGCTCCTG  

- GACTGTAACA ATGATCCGTC TCAGACAGAG AGCCTGCCGA AATTCTGCGA ACGCGGAGAG AGAAAGTGCG   
  
  
- AGGCTAAGAG GTGCGTTTTA GACTGAGGAA AAGGGAGAAG GATGGAAAGA GAAAGGGAGA GAGCTAGGGT   
  
  
- TTTGTGATGG TAAGGACGCG TAGTTTTTGA ACTTTTTTCC TGTGAAGCAG TGTCCTATCA ACAGCGACGC   
  
  
- GCGCTCTCTT CTCTGTTATC TCTCTAAAGA CAAACAGTCA TAGAGACAGA GAGAGAAAAG CAAACAAAAA   
  
  
- ACACTGAAAA GAATGAAAGA AAAGAAGAGA GAAGGGAATA GAAAAGAGAT AAGTTAGAAA AATCGCCGTC   
  
  
- AACCGAAAAC AGTACGGGAG GCTGTTATTT AGGGGAACGC AAGAGTGTAG GTCCCACGCG CATGAAATTG   
  
  
- AAATTTTCCA GAGAAGAAAA AAGATACTTT CCGACAAGAA GGCGCACCTT GTTTCTCTCC AGTACTTACC   
  
  
- TGTGCGTAAA CCATCCCCAA TTTCTTTCTT TTCTTAGTTT TACCCCCAAA AAAAAAAAAA TAAAGTAAAA   
  
  
- TTAAAATCTT CTTTACAAAG ATAACTTATT TTTAAATTCA CATTACATAT TATAAAATAA AAATTAAATC   
  
  
- CAAACTTTCC ACATTTTAAA AATCTTTAAA ATAAGTTAAA TTTCCAGGTC AATCATGTAA GACATATTTT   
  
  
- TTTAAAATGA ATAAAATCTT TAAAAATTAT CGTTATTAAA ATAGATAACA TTTTACATTA TTATGACAAA   
  
  
- AAATTACTGT AAATATCACA ATTTAAATTG ATAGGATAAA TGTGTAATAT TGAAAAACTT TCTTTTTCAG   
  
  
- TAGAAAAAAA TCTTGGGGAG GAGACCTTTG ATTGGCATAT ATATTATATG GTGAGTAATG AAAGAAGAGG   
  
  
- ACGTTTGGTG TGTTTGTTTA ATTAAACAAT TAATTAATTT AATGTCCAAA GCAGATGAAG CGAAGAGGTC   
  
  
- ATCAGAAGAA GAAGCATAAA TAAAATATTG GAAAACAAGA GGTGGAGGAG GAGACAGAGG CTTGGGATGC   
  
  
- AGCAGTGGTA AGTAACAAGT GGGCTCTGTC GCTCTCTCTC TATTTGCTGA GTGAATAATT CAGCACAACA   
  
  
- GCACACGAAC AGGAGCTGGT CACTGATTTG TGAACATTCT TGGCAAATAC TACCCATCAG CCATCCTCTT   
  
  
- TATTACTCCC TCTAACTCCT ACACTCTTTG ACCCAATCCA CCTTTCTCAT CCCTTCTATA TTTATTTTTT   
  
  
- ATCCGTTCAT TTTTTATTTT TTTAAAAATG AATTAAATTA TTTATTTTTT AAAATTATAA TTATCTATTT   
  
  
- CTTTTTTAAA AGATTAATAA AAATCAAACT GATAGAAAAG ATGTTTAGTT TATCAAAATA ATTTTTTTTA   
  
  
- GATGCCCATA TATACTAATT TCATCAAGTG GGCATGGTTA TTGAATTGAT GCTATTGGTG TGCCGAAGAA   
  
  
- AGTTATTCTT CACATTTGTC GACGAGGAC

+     Box 4

| Site Name | Organism | Position | Strand | Matrix score. | sequence | function |
| --- | --- | --- | --- | --- | --- | --- |
| Box 4 | Petroselinum crispum | 1318 | - | 6 | ATTAAT | part of a conserved DNA module involved in light responsiveness |
| Box 4 | Petroselinum crispum | 941 | - | 6 | ATTAAT | part of a conserved DNA module involved in light responsiveness |
| Box 4 | Petroselinum crispum | 929 | - | 6 | ATTAAT | part of a conserved DNA module involved in light responsiveness |

> 2018/04/13 10:10:12  
+ CTGACATTGT TACTAGGCAG AGTCTGTCTC TCGGACGGCT TTAAGACGCT TGCGCCTCTC TCTTTCACGC   
  
  
+ TCCGATTCTC CACGCAAAAT CTGACTCCTT TTCCCTCTTC CTACCTTTCT CTTTCCCTCT CTCGATCCCA   
  
  
+ AAACACTACC ATTCCTGCGC ATCAAAAACT TGAAAAAAGG ACACTTCGTC ACAGGATAGT TGTCGCTGCG   
  
  
+ CGCGAGAGAA GAGACAATAG AGAGATTTCT GTTTGTCAGT ATCTCTGTCT CTCTCTTTTC GTTTGTTTTT   
  
  
+ TGTGACTTTT CTTACTTTCT TTTCTTCTCT CTTCCCTTAT CTTTTCTCTA TTCAATCTTT TTAGCGGCAG   
  
  
+ TTGGCTTTTG TCATGCCCTC CGACAATAAA TCCCCTTGCG TTCTCACATC CAGGGTGCGC GTACTTTAAC   
  
  
+ TTTAAAAGGT CTCTTCTTTT TTCTATGAAA GGCTGTTCTT CCGCGTGGAA CAAAGAGAGG TCATGAATGG   
  
  
+ ACACGCATTT GGTAGGGGTT AAAGAAAGAA AAGAATCAAA ATGGGGGTTT TTTTTTTTTT ATTTCATTTT   
  
  
+ AATTTTAGAA GAAATGTTTC TATTGAATAA AAATTTAAGT GTAATGTATA ATATTTTATT TTTAATTTAG   
  
  
+ GTTTGAAAGG TGTAAAATTT TTAGAAATTT TATTCAATTT AAAGGTCCAG TTAGTACATT CTGTATAAAA   
  
  
+ AAATTTTACT TATTTTAGAA ATTTTTAATA GCAATAATTT TATCTATTGT AAAATGTAAT AATACTGTTT   
  
  
+ TTTAATGACA TTTATAGTGT TAAATTTAAC TATCCTATTT ACACATTATA ACTTTTTGAA AGAAAAAGTC   
  
  
+ ATCTTTTTTT AGAACCCCTC CTCTGGAAAC TAACCGTATA TATAATATAC CACTCATTAC TTTCTTCTCC   
  
  
+ TGCAAACCAC ACAAACAAAT TAATTTGTTA ATTAATTAAA TTACAGGTTT CGTCTACTTC GCTTCTCCAG   
  
  
+ TAGTCTTCTT CTTCGTATTT ATTTTATAAC CTTTTGTTCT CCACCTCCTC CTCTGTCTCC GAACCCTACG   
  
  
+ TCGTCACCAT TCATTGTTCA CCCGAGACAG CGAGAGAGAG ATAAACGACT CACTTATTAA GTCGTGTTGT   
  
  
+ CGTGTGCTTG TCCTCGACCA GTGACTAAAC ACTTGTAAGA ACCGTTTATG ATGGGTAGTC GGTAGGAGAA   
  
  
+ ATAATGAGGG AGATTGAGGA TGTGAGAAAC TGGGTTAGGT GGAAAGAGTA GGGAAGATAT AAATAAAAAA   
  
  
+ TAGGCAAGTA AAAAATAAAA AAATTTTTAC TTAATTTAAT AAATAAAAAA TTTTAATATT AATAGATAAA   
  
  
+ GAAAAAATTT TCTAATTATT TTTAGTTTGA CTATCTTTTC TACAAATCAA ATAGTTTTAT TAAAAAAAAT   
  
  
+ CTACGGGTAT ATATGATTAA AGTAGTTCAC CCGTACCAAT AACTTAACTA CGATAACCAC ACGGCTTCTT   
  
  
+ TCAATAAGAA GTGTAAACAG CTGCTCCTG  

- GACTGTAACA ATGATCCGTC TCAGACAGAG AGCCTGCCGA AATTCTGCGA ACGCGGAGAG AGAAAGTGCG   
  
  
- AGGCTAAGAG GTGCGTTTTA GACTGAGGAA AAGGGAGAAG GATGGAAAGA GAAAGGGAGA GAGCTAGGGT   
  
  
- TTTGTGATGG TAAGGACGCG TAGTTTTTGA ACTTTTTTCC TGTGAAGCAG TGTCCTATCA ACAGCGACGC   
  
  
- GCGCTCTCTT CTCTGTTATC TCTCTAAAGA CAAACAGTCA TAGAGACAGA GAGAGAAAAG CAAACAAAAA   
  
  
- ACACTGAAAA GAATGAAAGA AAAGAAGAGA GAAGGGAATA GAAAAGAGAT AAGTTAGAAA AATCGCCGTC   
  
  
- AACCGAAAAC AGTACGGGAG GCTGTTATTT AGGGGAACGC AAGAGTGTAG GTCCCACGCG CATGAAATTG   
  
  
- AAATTTTCCA GAGAAGAAAA AAGATACTTT CCGACAAGAA GGCGCACCTT GTTTCTCTCC AGTACTTACC   
  
  
- TGTGCGTAAA CCATCCCCAA TTTCTTTCTT TTCTTAGTTT TACCCCCAAA AAAAAAAAAA TAAAGTAAAA   
  
  
- TTAAAATCTT CTTTACAAAG ATAACTTATT TTTAAATTCA CATTACATAT TATAAAATAA AAATTAAATC   
  
  
- CAAACTTTCC ACATTTTAAA AATCTTTAAA ATAAGTTAAA TTTCCAGGTC AATCATGTAA GACATATTTT   
  
  
- TTTAAAATGA ATAAAATCTT TAAAAATTAT CGTTATTAAA ATAGATAACA TTTTACATTA TTATGACAAA   
  
  
- AAATTACTGT AAATATCACA ATTTAAATTG ATAGGATAAA TGTGTAATAT TGAAAAACTT TCTTTTTCAG   
  
  
- TAGAAAAAAA TCTTGGGGAG GAGACCTTTG ATTGGCATAT ATATTATATG GTGAGTAATG AAAGAAGAGG   
  
  
- ACGTTTGGTG TGTTTGTTTA ATTAAACAAT TAATTAATTT AATGTCCAAA GCAGATGAAG CGAAGAGGTC   
  
  
- ATCAGAAGAA GAAGCATAAA TAAAATATTG GAAAACAAGA GGTGGAGGAG GAGACAGAGG CTTGGGATGC   
  
  
- AGCAGTGGTA AGTAACAAGT GGGCTCTGTC GCTCTCTCTC TATTTGCTGA GTGAATAATT CAGCACAACA   
  
  
- GCACACGAAC AGGAGCTGGT CACTGATTTG TGAACATTCT TGGCAAATAC TACCCATCAG CCATCCTCTT   
  
  
- TATTACTCCC TCTAACTCCT ACACTCTTTG ACCCAATCCA CCTTTCTCAT CCCTTCTATA TTTATTTTTT   
  
  
- ATCCGTTCAT TTTTTATTTT TTTAAAAATG AATTAAATTA TTTATTTTTT AAAATTATAA TTATCTATTT   
  
  
- CTTTTTTAAA AGATTAATAA AAATCAAACT GATAGAAAAG ATGTTTAGTT TATCAAAATA ATTTTTTTTA   
  
  
- GATGCCCATA TATACTAATT TCATCAAGTG GGCATGGTTA TTGAATTGAT GCTATTGGTG TGCCGAAGAA   
  
  
- AGTTATTCTT CACATTTGTC GACGAGGAC

+     Box I

| Site Name | Organism | Position | Strand | Matrix score. | sequence | function |
| --- | --- | --- | --- | --- | --- | --- |
| Box I | Pisum sativum | 825 | - | 7 | TTTCAAA | light responsive element |
| Box I | Pisum sativum | 632 | - | 7 | TTTCAAA | light responsive element |

> 2018/04/13 10:10:12  
+ CTGACATTGT TACTAGGCAG AGTCTGTCTC TCGGACGGCT TTAAGACGCT TGCGCCTCTC TCTTTCACGC   
  
  
+ TCCGATTCTC CACGCAAAAT CTGACTCCTT TTCCCTCTTC CTACCTTTCT CTTTCCCTCT CTCGATCCCA   
  
  
+ AAACACTACC ATTCCTGCGC ATCAAAAACT TGAAAAAAGG ACACTTCGTC ACAGGATAGT TGTCGCTGCG   
  
  
+ CGCGAGAGAA GAGACAATAG AGAGATTTCT GTTTGTCAGT ATCTCTGTCT CTCTCTTTTC GTTTGTTTTT   
  
  
+ TGTGACTTTT CTTACTTTCT TTTCTTCTCT CTTCCCTTAT CTTTTCTCTA TTCAATCTTT TTAGCGGCAG   
  
  
+ TTGGCTTTTG TCATGCCCTC CGACAATAAA TCCCCTTGCG TTCTCACATC CAGGGTGCGC GTACTTTAAC   
  
  
+ TTTAAAAGGT CTCTTCTTTT TTCTATGAAA GGCTGTTCTT CCGCGTGGAA CAAAGAGAGG TCATGAATGG   
  
  
+ ACACGCATTT GGTAGGGGTT AAAGAAAGAA AAGAATCAAA ATGGGGGTTT TTTTTTTTTT ATTTCATTTT   
  
  
+ AATTTTAGAA GAAATGTTTC TATTGAATAA AAATTTAAGT GTAATGTATA ATATTTTATT TTTAATTTAG   
  
  
+ GTTTGAAAGG TGTAAAATTT TTAGAAATTT TATTCAATTT AAAGGTCCAG TTAGTACATT CTGTATAAAA   
  
  
+ AAATTTTACT TATTTTAGAA ATTTTTAATA GCAATAATTT TATCTATTGT AAAATGTAAT AATACTGTTT   
  
  
+ TTTAATGACA TTTATAGTGT TAAATTTAAC TATCCTATTT ACACATTATA ACTTTTTGAA AGAAAAAGTC   
  
  
+ ATCTTTTTTT AGAACCCCTC CTCTGGAAAC TAACCGTATA TATAATATAC CACTCATTAC TTTCTTCTCC   
  
  
+ TGCAAACCAC ACAAACAAAT TAATTTGTTA ATTAATTAAA TTACAGGTTT CGTCTACTTC GCTTCTCCAG   
  
  
+ TAGTCTTCTT CTTCGTATTT ATTTTATAAC CTTTTGTTCT CCACCTCCTC CTCTGTCTCC GAACCCTACG   
  
  
+ TCGTCACCAT TCATTGTTCA CCCGAGACAG CGAGAGAGAG ATAAACGACT CACTTATTAA GTCGTGTTGT   
  
  
+ CGTGTGCTTG TCCTCGACCA GTGACTAAAC ACTTGTAAGA ACCGTTTATG ATGGGTAGTC GGTAGGAGAA   
  
  
+ ATAATGAGGG AGATTGAGGA TGTGAGAAAC TGGGTTAGGT GGAAAGAGTA GGGAAGATAT AAATAAAAAA   
  
  
+ TAGGCAAGTA AAAAATAAAA AAATTTTTAC TTAATTTAAT AAATAAAAAA TTTTAATATT AATAGATAAA   
  
  
+ GAAAAAATTT TCTAATTATT TTTAGTTTGA CTATCTTTTC TACAAATCAA ATAGTTTTAT TAAAAAAAAT   
  
  
+ CTACGGGTAT ATATGATTAA AGTAGTTCAC CCGTACCAAT AACTTAACTA CGATAACCAC ACGGCTTCTT   
  
  
+ TCAATAAGAA GTGTAAACAG CTGCTCCTG  

- GACTGTAACA ATGATCCGTC TCAGACAGAG AGCCTGCCGA AATTCTGCGA ACGCGGAGAG AGAAAGTGCG   
  
  
- AGGCTAAGAG GTGCGTTTTA GACTGAGGAA AAGGGAGAAG GATGGAAAGA GAAAGGGAGA GAGCTAGGGT   
  
  
- TTTGTGATGG TAAGGACGCG TAGTTTTTGA ACTTTTTTCC TGTGAAGCAG TGTCCTATCA ACAGCGACGC   
  
  
- GCGCTCTCTT CTCTGTTATC TCTCTAAAGA CAAACAGTCA TAGAGACAGA GAGAGAAAAG CAAACAAAAA   
  
  
- ACACTGAAAA GAATGAAAGA AAAGAAGAGA GAAGGGAATA GAAAAGAGAT AAGTTAGAAA AATCGCCGTC   
  
  
- AACCGAAAAC AGTACGGGAG GCTGTTATTT AGGGGAACGC AAGAGTGTAG GTCCCACGCG CATGAAATTG   
  
  
- AAATTTTCCA GAGAAGAAAA AAGATACTTT CCGACAAGAA GGCGCACCTT GTTTCTCTCC AGTACTTACC   
  
  
- TGTGCGTAAA CCATCCCCAA TTTCTTTCTT TTCTTAGTTT TACCCCCAAA AAAAAAAAAA TAAAGTAAAA   
  
  
- TTAAAATCTT CTTTACAAAG ATAACTTATT TTTAAATTCA CATTACATAT TATAAAATAA AAATTAAATC   
  
  
- CAAACTTTCC ACATTTTAAA AATCTTTAAA ATAAGTTAAA TTTCCAGGTC AATCATGTAA GACATATTTT   
  
  
- TTTAAAATGA ATAAAATCTT TAAAAATTAT CGTTATTAAA ATAGATAACA TTTTACATTA TTATGACAAA   
  
  
- AAATTACTGT AAATATCACA ATTTAAATTG ATAGGATAAA TGTGTAATAT TGAAAAACTT TCTTTTTCAG   
  
  
- TAGAAAAAAA TCTTGGGGAG GAGACCTTTG ATTGGCATAT ATATTATATG GTGAGTAATG AAAGAAGAGG   
  
  
- ACGTTTGGTG TGTTTGTTTA ATTAAACAAT TAATTAATTT AATGTCCAAA GCAGATGAAG CGAAGAGGTC   
  
  
- ATCAGAAGAA GAAGCATAAA TAAAATATTG GAAAACAAGA GGTGGAGGAG GAGACAGAGG CTTGGGATGC   
  
  
- AGCAGTGGTA AGTAACAAGT GGGCTCTGTC GCTCTCTCTC TATTTGCTGA GTGAATAATT CAGCACAACA   
  
  
- GCACACGAAC AGGAGCTGGT CACTGATTTG TGAACATTCT TGGCAAATAC TACCCATCAG CCATCCTCTT   
  
  
- TATTACTCCC TCTAACTCCT ACACTCTTTG ACCCAATCCA CCTTTCTCAT CCCTTCTATA TTTATTTTTT   
  
  
- ATCCGTTCAT TTTTTATTTT TTTAAAAATG AATTAAATTA TTTATTTTTT AAAATTATAA TTATCTATTT   
  
  
- CTTTTTTAAA AGATTAATAA AAATCAAACT GATAGAAAAG ATGTTTAGTT TATCAAAATA ATTTTTTTTA   
  
  
- GATGCCCATA TATACTAATT TCATCAAGTG GGCATGGTTA TTGAATTGAT GCTATTGGTG TGCCGAAGAA   
  
  
- AGTTATTCTT CACATTTGTC GACGAGGAC

+     CAAT-box

| Site Name | Organism | Position | Strand | Matrix score. | sequence | function |
| --- | --- | --- | --- | --- | --- | --- |
| CAAT-box | Hordeum vulgare | 1472 | + | 4 | CAAT | common cis-acting element in promoter and enhancer regions |
| CAAT-box | Hordeum vulgare | 582 | - | 4 | CAAT | common cis-acting element in promoter and enhancer regions |
| CAAT-box | Brassica rapa | 1378 | + | 5 | CAAAT | common cis-acting element in promoter and enhancer regions |
| CAAT-box | Hordeum vulgare | 374 | + | 4 | CAAT | common cis-acting element in promoter and enhancer regions |
| CAAT-box | Brassica rapa | 1373 | + | 5 | CAAAT | common cis-acting element in promoter and enhancer regions |
| CAAT-box | Hordeum vulgare | 1203 | - | 4 | CAAT | common cis-acting element in promoter and enhancer regions |
| CAAT-box | Hordeum vulgare | 1063 | - | 4 | CAAT | common cis-acting element in promoter and enhancer regions |
| CAAT-box | Brassica rapa | 933 | - | 5 | CAAAT | common cis-acting element in promoter and enhancer regions |
| CAAT-box | Hordeum vulgare | 6 | - | 4 | CAAT | common cis-acting element in promoter and enhancer regions |
| CAAT-box | Glycine max | 665 | + | 5 | CAATT | common cis-acting element in promoter and enhancer regions |
| CAAT-box | Arabidopsis thaliana | 1436 | + | 5 | CCAAT | common cis-acting element in promoter and enhancer regions |
| CAAT-box | Hordeum vulgare | 746 | - | 4 | CAAT | common cis-acting element in promoter and enhancer regions |
| CAAT-box | Hordeum vulgare | 1437 | + | 4 | CAAT | common cis-acting element in promoter and enhancer regions |
| CAAT-box | Hordeum vulgare | 732 | + | 4 | CAAT | common cis-acting element in promoter and enhancer regions |
| CAAT-box | Brassica rapa | 497 | - | 5 | CAAAT | common cis-acting element in promoter and enhancer regions |
| CAAT-box | Brassica rapa | 926 | + | 5 | CAAAT | common cis-acting element in promoter and enhancer regions |
| CAAT-box | Hordeum vulgare | 333 | + | 4 | CAAT | common cis-acting element in promoter and enhancer regions |
| CAAT-box | Hordeum vulgare | 225 | + | 4 | CAAT | common cis-acting element in promoter and enhancer regions |

> 2018/04/13 10:10:12  
+ CTGACATTGT TACTAGGCAG AGTCTGTCTC TCGGACGGCT TTAAGACGCT TGCGCCTCTC TCTTTCACGC   
  
  
+ TCCGATTCTC CACGCAAAAT CTGACTCCTT TTCCCTCTTC CTACCTTTCT CTTTCCCTCT CTCGATCCCA   
  
  
+ AAACACTACC ATTCCTGCGC ATCAAAAACT TGAAAAAAGG ACACTTCGTC ACAGGATAGT TGTCGCTGCG   
  
  
+ CGCGAGAGAA GAGACAATAG AGAGATTTCT GTTTGTCAGT ATCTCTGTCT CTCTCTTTTC GTTTGTTTTT   
  
  
+ TGTGACTTTT CTTACTTTCT TTTCTTCTCT CTTCCCTTAT CTTTTCTCTA TTCAATCTTT TTAGCGGCAG   
  
  
+ TTGGCTTTTG TCATGCCCTC CGACAATAAA TCCCCTTGCG TTCTCACATC CAGGGTGCGC GTACTTTAAC   
  
  
+ TTTAAAAGGT CTCTTCTTTT TTCTATGAAA GGCTGTTCTT CCGCGTGGAA CAAAGAGAGG TCATGAATGG   
  
  
+ ACACGCATTT GGTAGGGGTT AAAGAAAGAA AAGAATCAAA ATGGGGGTTT TTTTTTTTTT ATTTCATTTT   
  
  
+ AATTTTAGAA GAAATGTTTC TATTGAATAA AAATTTAAGT GTAATGTATA ATATTTTATT TTTAATTTAG   
  
  
+ GTTTGAAAGG TGTAAAATTT TTAGAAATTT TATTCAATTT AAAGGTCCAG TTAGTACATT CTGTATAAAA   
  
  
+ AAATTTTACT TATTTTAGAA ATTTTTAATA GCAATAATTT TATCTATTGT AAAATGTAAT AATACTGTTT   
  
  
+ TTTAATGACA TTTATAGTGT TAAATTTAAC TATCCTATTT ACACATTATA ACTTTTTGAA AGAAAAAGTC   
  
  
+ ATCTTTTTTT AGAACCCCTC CTCTGGAAAC TAACCGTATA TATAATATAC CACTCATTAC TTTCTTCTCC   
  
  
+ TGCAAACCAC ACAAACAAAT TAATTTGTTA ATTAATTAAA TTACAGGTTT CGTCTACTTC GCTTCTCCAG   
  
  
+ TAGTCTTCTT CTTCGTATTT ATTTTATAAC CTTTTGTTCT CCACCTCCTC CTCTGTCTCC GAACCCTACG   
  
  
+ TCGTCACCAT TCATTGTTCA CCCGAGACAG CGAGAGAGAG ATAAACGACT CACTTATTAA GTCGTGTTGT   
  
  
+ CGTGTGCTTG TCCTCGACCA GTGACTAAAC ACTTGTAAGA ACCGTTTATG ATGGGTAGTC GGTAGGAGAA   
  
  
+ ATAATGAGGG AGATTGAGGA TGTGAGAAAC TGGGTTAGGT GGAAAGAGTA GGGAAGATAT AAATAAAAAA   
  
  
+ TAGGCAAGTA AAAAATAAAA AAATTTTTAC TTAATTTAAT AAATAAAAAA TTTTAATATT AATAGATAAA   
  
  
+ GAAAAAATTT TCTAATTATT TTTAGTTTGA CTATCTTTTC TACAAATCAA ATAGTTTTAT TAAAAAAAAT   
  
  
+ CTACGGGTAT ATATGATTAA AGTAGTTCAC CCGTACCAAT AACTTAACTA CGATAACCAC ACGGCTTCTT   
  
  
+ TCAATAAGAA GTGTAAACAG CTGCTCCTG  

- GACTGTAACA ATGATCCGTC TCAGACAGAG AGCCTGCCGA AATTCTGCGA ACGCGGAGAG AGAAAGTGCG   
  
  
- AGGCTAAGAG GTGCGTTTTA GACTGAGGAA AAGGGAGAAG GATGGAAAGA GAAAGGGAGA GAGCTAGGGT   
  
  
- TTTGTGATGG TAAGGACGCG TAGTTTTTGA ACTTTTTTCC TGTGAAGCAG TGTCCTATCA ACAGCGACGC   
  
  
- GCGCTCTCTT CTCTGTTATC TCTCTAAAGA CAAACAGTCA TAGAGACAGA GAGAGAAAAG CAAACAAAAA   
  
  
- ACACTGAAAA GAATGAAAGA AAAGAAGAGA GAAGGGAATA GAAAAGAGAT AAGTTAGAAA AATCGCCGTC   
  
  
- AACCGAAAAC AGTACGGGAG GCTGTTATTT AGGGGAACGC AAGAGTGTAG GTCCCACGCG CATGAAATTG   
  
  
- AAATTTTCCA GAGAAGAAAA AAGATACTTT CCGACAAGAA GGCGCACCTT GTTTCTCTCC AGTACTTACC   
  
  
- TGTGCGTAAA CCATCCCCAA TTTCTTTCTT TTCTTAGTTT TACCCCCAAA AAAAAAAAAA TAAAGTAAAA   
  
  
- TTAAAATCTT CTTTACAAAG ATAACTTATT TTTAAATTCA CATTACATAT TATAAAATAA AAATTAAATC   
  
  
- CAAACTTTCC ACATTTTAAA AATCTTTAAA ATAAGTTAAA TTTCCAGGTC AATCATGTAA GACATATTTT   
  
  
- TTTAAAATGA ATAAAATCTT TAAAAATTAT CGTTATTAAA ATAGATAACA TTTTACATTA TTATGACAAA   
  
  
- AAATTACTGT AAATATCACA ATTTAAATTG ATAGGATAAA TGTGTAATAT TGAAAAACTT TCTTTTTCAG   
  
  
- TAGAAAAAAA TCTTGGGGAG GAGACCTTTG ATTGGCATAT ATATTATATG GTGAGTAATG AAAGAAGAGG   
  
  
- ACGTTTGGTG TGTTTGTTTA ATTAAACAAT TAATTAATTT AATGTCCAAA GCAGATGAAG CGAAGAGGTC   
  
  
- ATCAGAAGAA GAAGCATAAA TAAAATATTG GAAAACAAGA GGTGGAGGAG GAGACAGAGG CTTGGGATGC   
  
  
- AGCAGTGGTA AGTAACAAGT GGGCTCTGTC GCTCTCTCTC TATTTGCTGA GTGAATAATT CAGCACAACA   
  
  
- GCACACGAAC AGGAGCTGGT CACTGATTTG TGAACATTCT TGGCAAATAC TACCCATCAG CCATCCTCTT   
  
  
- TATTACTCCC TCTAACTCCT ACACTCTTTG ACCCAATCCA CCTTTCTCAT CCCTTCTATA TTTATTTTTT   
  
  
- ATCCGTTCAT TTTTTATTTT TTTAAAAATG AATTAAATTA TTTATTTTTT AAAATTATAA TTATCTATTT   
  
  
- CTTTTTTAAA AGATTAATAA AAATCAAACT GATAGAAAAG ATGTTTAGTT TATCAAAATA ATTTTTTTTA   
  
  
- GATGCCCATA TATACTAATT TCATCAAGTG GGCATGGTTA TTGAATTGAT GCTATTGGTG TGCCGAAGAA   
  
  
- AGTTATTCTT CACATTTGTC GACGAGGAC

+     CCGTCC-box

| Site Name | Organism | Position | Strand | Matrix score. | sequence | function |
| --- | --- | --- | --- | --- | --- | --- |
| CCGTCC-box | Arabidopsis thaliana | 33 | - | 6 | CCGTCC | cis-acting regulatory element related to meristem specific activation |

> 2018/04/13 10:10:12  
+ CTGACATTGT TACTAGGCAG AGTCTGTCTC TCGGACGGCT TTAAGACGCT TGCGCCTCTC TCTTTCACGC   
  
  
+ TCCGATTCTC CACGCAAAAT CTGACTCCTT TTCCCTCTTC CTACCTTTCT CTTTCCCTCT CTCGATCCCA   
  
  
+ AAACACTACC ATTCCTGCGC ATCAAAAACT TGAAAAAAGG ACACTTCGTC ACAGGATAGT TGTCGCTGCG   
  
  
+ CGCGAGAGAA GAGACAATAG AGAGATTTCT GTTTGTCAGT ATCTCTGTCT CTCTCTTTTC GTTTGTTTTT   
  
  
+ TGTGACTTTT CTTACTTTCT TTTCTTCTCT CTTCCCTTAT CTTTTCTCTA TTCAATCTTT TTAGCGGCAG   
  
  
+ TTGGCTTTTG TCATGCCCTC CGACAATAAA TCCCCTTGCG TTCTCACATC CAGGGTGCGC GTACTTTAAC   
  
  
+ TTTAAAAGGT CTCTTCTTTT TTCTATGAAA GGCTGTTCTT CCGCGTGGAA CAAAGAGAGG TCATGAATGG   
  
  
+ ACACGCATTT GGTAGGGGTT AAAGAAAGAA AAGAATCAAA ATGGGGGTTT TTTTTTTTTT ATTTCATTTT   
  
  
+ AATTTTAGAA GAAATGTTTC TATTGAATAA AAATTTAAGT GTAATGTATA ATATTTTATT TTTAATTTAG   
  
  
+ GTTTGAAAGG TGTAAAATTT TTAGAAATTT TATTCAATTT AAAGGTCCAG TTAGTACATT CTGTATAAAA   
  
  
+ AAATTTTACT TATTTTAGAA ATTTTTAATA GCAATAATTT TATCTATTGT AAAATGTAAT AATACTGTTT   
  
  
+ TTTAATGACA TTTATAGTGT TAAATTTAAC TATCCTATTT ACACATTATA ACTTTTTGAA AGAAAAAGTC   
  
  
+ ATCTTTTTTT AGAACCCCTC CTCTGGAAAC TAACCGTATA TATAATATAC CACTCATTAC TTTCTTCTCC   
  
  
+ TGCAAACCAC ACAAACAAAT TAATTTGTTA ATTAATTAAA TTACAGGTTT CGTCTACTTC GCTTCTCCAG   
  
  
+ TAGTCTTCTT CTTCGTATTT ATTTTATAAC CTTTTGTTCT CCACCTCCTC CTCTGTCTCC GAACCCTACG   
  
  
+ TCGTCACCAT TCATTGTTCA CCCGAGACAG CGAGAGAGAG ATAAACGACT CACTTATTAA GTCGTGTTGT   
  
  
+ CGTGTGCTTG TCCTCGACCA GTGACTAAAC ACTTGTAAGA ACCGTTTATG ATGGGTAGTC GGTAGGAGAA   
  
  
+ ATAATGAGGG AGATTGAGGA TGTGAGAAAC TGGGTTAGGT GGAAAGAGTA GGGAAGATAT AAATAAAAAA   
  
  
+ TAGGCAAGTA AAAAATAAAA AAATTTTTAC TTAATTTAAT AAATAAAAAA TTTTAATATT AATAGATAAA   
  
  
+ GAAAAAATTT TCTAATTATT TTTAGTTTGA CTATCTTTTC TACAAATCAA ATAGTTTTAT TAAAAAAAAT   
  
  
+ CTACGGGTAT ATATGATTAA AGTAGTTCAC CCGTACCAAT AACTTAACTA CGATAACCAC ACGGCTTCTT   
  
  
+ TCAATAAGAA GTGTAAACAG CTGCTCCTG  

- GACTGTAACA ATGATCCGTC TCAGACAGAG AGCCTGCCGA AATTCTGCGA ACGCGGAGAG AGAAAGTGCG   
  
  
- AGGCTAAGAG GTGCGTTTTA GACTGAGGAA AAGGGAGAAG GATGGAAAGA GAAAGGGAGA GAGCTAGGGT   
  
  
- TTTGTGATGG TAAGGACGCG TAGTTTTTGA ACTTTTTTCC TGTGAAGCAG TGTCCTATCA ACAGCGACGC   
  
  
- GCGCTCTCTT CTCTGTTATC TCTCTAAAGA CAAACAGTCA TAGAGACAGA GAGAGAAAAG CAAACAAAAA   
  
  
- ACACTGAAAA GAATGAAAGA AAAGAAGAGA GAAGGGAATA GAAAAGAGAT AAGTTAGAAA AATCGCCGTC   
  
  
- AACCGAAAAC AGTACGGGAG GCTGTTATTT AGGGGAACGC AAGAGTGTAG GTCCCACGCG CATGAAATTG   
  
  
- AAATTTTCCA GAGAAGAAAA AAGATACTTT CCGACAAGAA GGCGCACCTT GTTTCTCTCC AGTACTTACC   
  
  
- TGTGCGTAAA CCATCCCCAA TTTCTTTCTT TTCTTAGTTT TACCCCCAAA AAAAAAAAAA TAAAGTAAAA   
  
  
- TTAAAATCTT CTTTACAAAG ATAACTTATT TTTAAATTCA CATTACATAT TATAAAATAA AAATTAAATC   
  
  
- CAAACTTTCC ACATTTTAAA AATCTTTAAA ATAAGTTAAA TTTCCAGGTC AATCATGTAA GACATATTTT   
  
  
- TTTAAAATGA ATAAAATCTT TAAAAATTAT CGTTATTAAA ATAGATAACA TTTTACATTA TTATGACAAA   
  
  
- AAATTACTGT AAATATCACA ATTTAAATTG ATAGGATAAA TGTGTAATAT TGAAAAACTT TCTTTTTCAG   
  
  
- TAGAAAAAAA TCTTGGGGAG GAGACCTTTG ATTGGCATAT ATATTATATG GTGAGTAATG AAAGAAGAGG   
  
  
- ACGTTTGGTG TGTTTGTTTA ATTAAACAAT TAATTAATTT AATGTCCAAA GCAGATGAAG CGAAGAGGTC   
  
  
- ATCAGAAGAA GAAGCATAAA TAAAATATTG GAAAACAAGA GGTGGAGGAG GAGACAGAGG CTTGGGATGC   
  
  
- AGCAGTGGTA AGTAACAAGT GGGCTCTGTC GCTCTCTCTC TATTTGCTGA GTGAATAATT CAGCACAACA   
  
  
- GCACACGAAC AGGAGCTGGT CACTGATTTG TGAACATTCT TGGCAAATAC TACCCATCAG CCATCCTCTT   
  
  
- TATTACTCCC TCTAACTCCT ACACTCTTTG ACCCAATCCA CCTTTCTCAT CCCTTCTATA TTTATTTTTT   
  
  
- ATCCGTTCAT TTTTTATTTT TTTAAAAATG AATTAAATTA TTTATTTTTT AAAATTATAA TTATCTATTT   
  
  
- CTTTTTTAAA AGATTAATAA AAATCAAACT GATAGAAAAG ATGTTTAGTT TATCAAAATA ATTTTTTTTA   
  
  
- GATGCCCATA TATACTAATT TCATCAAGTG GGCATGGTTA TTGAATTGAT GCTATTGGTG TGCCGAAGAA   
  
  
- AGTTATTCTT CACATTTGTC GACGAGGAC

+     CGTCA-motif

| Site Name | Organism | Position | Strand | Matrix score. | sequence | function |
| --- | --- | --- | --- | --- | --- | --- |
| CGTCA-motif | Hordeum vulgare | 187 | + | 5 | CGTCA | cis-acting regulatory element involved in the MeJA-responsiveness |
| CGTCA-motif | Hordeum vulgare | 1052 | + | 5 | CGTCA | cis-acting regulatory element involved in the MeJA-responsiveness |

> 2018/04/13 10:10:12  
+ CTGACATTGT TACTAGGCAG AGTCTGTCTC TCGGACGGCT TTAAGACGCT TGCGCCTCTC TCTTTCACGC   
  
  
+ TCCGATTCTC CACGCAAAAT CTGACTCCTT TTCCCTCTTC CTACCTTTCT CTTTCCCTCT CTCGATCCCA   
  
  
+ AAACACTACC ATTCCTGCGC ATCAAAAACT TGAAAAAAGG ACACTTCGTC ACAGGATAGT TGTCGCTGCG   
  
  
+ CGCGAGAGAA GAGACAATAG AGAGATTTCT GTTTGTCAGT ATCTCTGTCT CTCTCTTTTC GTTTGTTTTT   
  
  
+ TGTGACTTTT CTTACTTTCT TTTCTTCTCT CTTCCCTTAT CTTTTCTCTA TTCAATCTTT TTAGCGGCAG   
  
  
+ TTGGCTTTTG TCATGCCCTC CGACAATAAA TCCCCTTGCG TTCTCACATC CAGGGTGCGC GTACTTTAAC   
  
  
+ TTTAAAAGGT CTCTTCTTTT TTCTATGAAA GGCTGTTCTT CCGCGTGGAA CAAAGAGAGG TCATGAATGG   
  
  
+ ACACGCATTT GGTAGGGGTT AAAGAAAGAA AAGAATCAAA ATGGGGGTTT TTTTTTTTTT ATTTCATTTT   
  
  
+ AATTTTAGAA GAAATGTTTC TATTGAATAA AAATTTAAGT GTAATGTATA ATATTTTATT TTTAATTTAG   
  
  
+ GTTTGAAAGG TGTAAAATTT TTAGAAATTT TATTCAATTT AAAGGTCCAG TTAGTACATT CTGTATAAAA   
  
  
+ AAATTTTACT TATTTTAGAA ATTTTTAATA GCAATAATTT TATCTATTGT AAAATGTAAT AATACTGTTT   
  
  
+ TTTAATGACA TTTATAGTGT TAAATTTAAC TATCCTATTT ACACATTATA ACTTTTTGAA AGAAAAAGTC   
  
  
+ ATCTTTTTTT AGAACCCCTC CTCTGGAAAC TAACCGTATA TATAATATAC CACTCATTAC TTTCTTCTCC   
  
  
+ TGCAAACCAC ACAAACAAAT TAATTTGTTA ATTAATTAAA TTACAGGTTT CGTCTACTTC GCTTCTCCAG   
  
  
+ TAGTCTTCTT CTTCGTATTT ATTTTATAAC CTTTTGTTCT CCACCTCCTC CTCTGTCTCC GAACCCTACG   
  
  
+ TCGTCACCAT TCATTGTTCA CCCGAGACAG CGAGAGAGAG ATAAACGACT CACTTATTAA GTCGTGTTGT   
  
  
+ CGTGTGCTTG TCCTCGACCA GTGACTAAAC ACTTGTAAGA ACCGTTTATG ATGGGTAGTC GGTAGGAGAA   
  
  
+ ATAATGAGGG AGATTGAGGA TGTGAGAAAC TGGGTTAGGT GGAAAGAGTA GGGAAGATAT AAATAAAAAA   
  
  
+ TAGGCAAGTA AAAAATAAAA AAATTTTTAC TTAATTTAAT AAATAAAAAA TTTTAATATT AATAGATAAA   
  
  
+ GAAAAAATTT TCTAATTATT TTTAGTTTGA CTATCTTTTC TACAAATCAA ATAGTTTTAT TAAAAAAAAT   
  
  
+ CTACGGGTAT ATATGATTAA AGTAGTTCAC CCGTACCAAT AACTTAACTA CGATAACCAC ACGGCTTCTT   
  
  
+ TCAATAAGAA GTGTAAACAG CTGCTCCTG  

- GACTGTAACA ATGATCCGTC TCAGACAGAG AGCCTGCCGA AATTCTGCGA ACGCGGAGAG AGAAAGTGCG   
  
  
- AGGCTAAGAG GTGCGTTTTA GACTGAGGAA AAGGGAGAAG GATGGAAAGA GAAAGGGAGA GAGCTAGGGT   
  
  
- TTTGTGATGG TAAGGACGCG TAGTTTTTGA ACTTTTTTCC TGTGAAGCAG TGTCCTATCA ACAGCGACGC   
  
  
- GCGCTCTCTT CTCTGTTATC TCTCTAAAGA CAAACAGTCA TAGAGACAGA GAGAGAAAAG CAAACAAAAA   
  
  
- ACACTGAAAA GAATGAAAGA AAAGAAGAGA GAAGGGAATA GAAAAGAGAT AAGTTAGAAA AATCGCCGTC   
  
  
- AACCGAAAAC AGTACGGGAG GCTGTTATTT AGGGGAACGC AAGAGTGTAG GTCCCACGCG CATGAAATTG   
  
  
- AAATTTTCCA GAGAAGAAAA AAGATACTTT CCGACAAGAA GGCGCACCTT GTTTCTCTCC AGTACTTACC   
  
  
- TGTGCGTAAA CCATCCCCAA TTTCTTTCTT TTCTTAGTTT TACCCCCAAA AAAAAAAAAA TAAAGTAAAA   
  
  
- TTAAAATCTT CTTTACAAAG ATAACTTATT TTTAAATTCA CATTACATAT TATAAAATAA AAATTAAATC   
  
  
- CAAACTTTCC ACATTTTAAA AATCTTTAAA ATAAGTTAAA TTTCCAGGTC AATCATGTAA GACATATTTT   
  
  
- TTTAAAATGA ATAAAATCTT TAAAAATTAT CGTTATTAAA ATAGATAACA TTTTACATTA TTATGACAAA   
  
  
- AAATTACTGT AAATATCACA ATTTAAATTG ATAGGATAAA TGTGTAATAT TGAAAAACTT TCTTTTTCAG   
  
  
- TAGAAAAAAA TCTTGGGGAG GAGACCTTTG ATTGGCATAT ATATTATATG GTGAGTAATG AAAGAAGAGG   
  
  
- ACGTTTGGTG TGTTTGTTTA ATTAAACAAT TAATTAATTT AATGTCCAAA GCAGATGAAG CGAAGAGGTC   
  
  
- ATCAGAAGAA GAAGCATAAA TAAAATATTG GAAAACAAGA GGTGGAGGAG GAGACAGAGG CTTGGGATGC   
  
  
- AGCAGTGGTA AGTAACAAGT GGGCTCTGTC GCTCTCTCTC TATTTGCTGA GTGAATAATT CAGCACAACA   
  
  
- GCACACGAAC AGGAGCTGGT CACTGATTTG TGAACATTCT TGGCAAATAC TACCCATCAG CCATCCTCTT   
  
  
- TATTACTCCC TCTAACTCCT ACACTCTTTG ACCCAATCCA CCTTTCTCAT CCCTTCTATA TTTATTTTTT   
  
  
- ATCCGTTCAT TTTTTATTTT TTTAAAAATG AATTAAATTA TTTATTTTTT AAAATTATAA TTATCTATTT   
  
  
- CTTTTTTAAA AGATTAATAA AAATCAAACT GATAGAAAAG ATGTTTAGTT TATCAAAATA ATTTTTTTTA   
  
  
- GATGCCCATA TATACTAATT TCATCAAGTG GGCATGGTTA TTGAATTGAT GCTATTGGTG TGCCGAAGAA   
  
  
- AGTTATTCTT CACATTTGTC GACGAGGAC

+     G-box

| Site Name | Organism | Position | Strand | Matrix score. | sequence | function |
| --- | --- | --- | --- | --- | --- | --- |
| G-box | Zea mays | 1119 | - | 6 | CACGAC | cis-acting regulatory element involved in light responsiveness |
| G-box | Zea mays | 1111 | - | 6 | CACGAC | cis-acting regulatory element involved in light responsiveness |

> 2018/04/13 10:10:12  
+ CTGACATTGT TACTAGGCAG AGTCTGTCTC TCGGACGGCT TTAAGACGCT TGCGCCTCTC TCTTTCACGC   
  
  
+ TCCGATTCTC CACGCAAAAT CTGACTCCTT TTCCCTCTTC CTACCTTTCT CTTTCCCTCT CTCGATCCCA   
  
  
+ AAACACTACC ATTCCTGCGC ATCAAAAACT TGAAAAAAGG ACACTTCGTC ACAGGATAGT TGTCGCTGCG   
  
  
+ CGCGAGAGAA GAGACAATAG AGAGATTTCT GTTTGTCAGT ATCTCTGTCT CTCTCTTTTC GTTTGTTTTT   
  
  
+ TGTGACTTTT CTTACTTTCT TTTCTTCTCT CTTCCCTTAT CTTTTCTCTA TTCAATCTTT TTAGCGGCAG   
  
  
+ TTGGCTTTTG TCATGCCCTC CGACAATAAA TCCCCTTGCG TTCTCACATC CAGGGTGCGC GTACTTTAAC   
  
  
+ TTTAAAAGGT CTCTTCTTTT TTCTATGAAA GGCTGTTCTT CCGCGTGGAA CAAAGAGAGG TCATGAATGG   
  
  
+ ACACGCATTT GGTAGGGGTT AAAGAAAGAA AAGAATCAAA ATGGGGGTTT TTTTTTTTTT ATTTCATTTT   
  
  
+ AATTTTAGAA GAAATGTTTC TATTGAATAA AAATTTAAGT GTAATGTATA ATATTTTATT TTTAATTTAG   
  
  
+ GTTTGAAAGG TGTAAAATTT TTAGAAATTT TATTCAATTT AAAGGTCCAG TTAGTACATT CTGTATAAAA   
  
  
+ AAATTTTACT TATTTTAGAA ATTTTTAATA GCAATAATTT TATCTATTGT AAAATGTAAT AATACTGTTT   
  
  
+ TTTAATGACA TTTATAGTGT TAAATTTAAC TATCCTATTT ACACATTATA ACTTTTTGAA AGAAAAAGTC   
  
  
+ ATCTTTTTTT AGAACCCCTC CTCTGGAAAC TAACCGTATA TATAATATAC CACTCATTAC TTTCTTCTCC   
  
  
+ TGCAAACCAC ACAAACAAAT TAATTTGTTA ATTAATTAAA TTACAGGTTT CGTCTACTTC GCTTCTCCAG   
  
  
+ TAGTCTTCTT CTTCGTATTT ATTTTATAAC CTTTTGTTCT CCACCTCCTC CTCTGTCTCC GAACCCTACG   
  
  
+ TCGTCACCAT TCATTGTTCA CCCGAGACAG CGAGAGAGAG ATAAACGACT CACTTATTAA GTCGTGTTGT   
  
  
+ CGTGTGCTTG TCCTCGACCA GTGACTAAAC ACTTGTAAGA ACCGTTTATG ATGGGTAGTC GGTAGGAGAA   
  
  
+ ATAATGAGGG AGATTGAGGA TGTGAGAAAC TGGGTTAGGT GGAAAGAGTA GGGAAGATAT AAATAAAAAA   
  
  
+ TAGGCAAGTA AAAAATAAAA AAATTTTTAC TTAATTTAAT AAATAAAAAA TTTTAATATT AATAGATAAA   
  
  
+ GAAAAAATTT TCTAATTATT TTTAGTTTGA CTATCTTTTC TACAAATCAA ATAGTTTTAT TAAAAAAAAT   
  
  
+ CTACGGGTAT ATATGATTAA AGTAGTTCAC CCGTACCAAT AACTTAACTA CGATAACCAC ACGGCTTCTT   
  
  
+ TCAATAAGAA GTGTAAACAG CTGCTCCTG  

- GACTGTAACA ATGATCCGTC TCAGACAGAG AGCCTGCCGA AATTCTGCGA ACGCGGAGAG AGAAAGTGCG   
  
  
- AGGCTAAGAG GTGCGTTTTA GACTGAGGAA AAGGGAGAAG GATGGAAAGA GAAAGGGAGA GAGCTAGGGT   
  
  
- TTTGTGATGG TAAGGACGCG TAGTTTTTGA ACTTTTTTCC TGTGAAGCAG TGTCCTATCA ACAGCGACGC   
  
  
- GCGCTCTCTT CTCTGTTATC TCTCTAAAGA CAAACAGTCA TAGAGACAGA GAGAGAAAAG CAAACAAAAA   
  
  
- ACACTGAAAA GAATGAAAGA AAAGAAGAGA GAAGGGAATA GAAAAGAGAT AAGTTAGAAA AATCGCCGTC   
  
  
- AACCGAAAAC AGTACGGGAG GCTGTTATTT AGGGGAACGC AAGAGTGTAG GTCCCACGCG CATGAAATTG   
  
  
- AAATTTTCCA GAGAAGAAAA AAGATACTTT CCGACAAGAA GGCGCACCTT GTTTCTCTCC AGTACTTACC   
  
  
- TGTGCGTAAA CCATCCCCAA TTTCTTTCTT TTCTTAGTTT TACCCCCAAA AAAAAAAAAA TAAAGTAAAA   
  
  
- TTAAAATCTT CTTTACAAAG ATAACTTATT TTTAAATTCA CATTACATAT TATAAAATAA AAATTAAATC   
  
  
- CAAACTTTCC ACATTTTAAA AATCTTTAAA ATAAGTTAAA TTTCCAGGTC AATCATGTAA GACATATTTT   
  
  
- TTTAAAATGA ATAAAATCTT TAAAAATTAT CGTTATTAAA ATAGATAACA TTTTACATTA TTATGACAAA   
  
  
- AAATTACTGT AAATATCACA ATTTAAATTG ATAGGATAAA TGTGTAATAT TGAAAAACTT TCTTTTTCAG   
  
  
- TAGAAAAAAA TCTTGGGGAG GAGACCTTTG ATTGGCATAT ATATTATATG GTGAGTAATG AAAGAAGAGG   
  
  
- ACGTTTGGTG TGTTTGTTTA ATTAAACAAT TAATTAATTT AATGTCCAAA GCAGATGAAG CGAAGAGGTC   
  
  
- ATCAGAAGAA GAAGCATAAA TAAAATATTG GAAAACAAGA GGTGGAGGAG GAGACAGAGG CTTGGGATGC   
  
  
- AGCAGTGGTA AGTAACAAGT GGGCTCTGTC GCTCTCTCTC TATTTGCTGA GTGAATAATT CAGCACAACA   
  
  
- GCACACGAAC AGGAGCTGGT CACTGATTTG TGAACATTCT TGGCAAATAC TACCCATCAG CCATCCTCTT   
  
  
- TATTACTCCC TCTAACTCCT ACACTCTTTG ACCCAATCCA CCTTTCTCAT CCCTTCTATA TTTATTTTTT   
  
  
- ATCCGTTCAT TTTTTATTTT TTTAAAAATG AATTAAATTA TTTATTTTTT AAAATTATAA TTATCTATTT   
  
  
- CTTTTTTAAA AGATTAATAA AAATCAAACT GATAGAAAAG ATGTTTAGTT TATCAAAATA ATTTTTTTTA   
  
  
- GATGCCCATA TATACTAATT TCATCAAGTG GGCATGGTTA TTGAATTGAT GCTATTGGTG TGCCGAAGAA   
  
  
- AGTTATTCTT CACATTTGTC GACGAGGAC

+     GA-motif

| Site Name | Organism | Position | Strand | Matrix score. | sequence | function |
| --- | --- | --- | --- | --- | --- | --- |
| GA-motif | Arabidopsis thaliana | 1322 | + | 8 | ATAGATAA | part of a light responsive element |
| GA-motif | Helianthus annuus | 839 | - | 8 | AAAGATGA | part of a light responsive element |
| GA-motif | Arabidopsis thaliana | 740 | - | 8 | ATAGATAA | part of a light responsive element |

> 2018/04/13 10:10:12  
+ CTGACATTGT TACTAGGCAG AGTCTGTCTC TCGGACGGCT TTAAGACGCT TGCGCCTCTC TCTTTCACGC   
  
  
+ TCCGATTCTC CACGCAAAAT CTGACTCCTT TTCCCTCTTC CTACCTTTCT CTTTCCCTCT CTCGATCCCA   
  
  
+ AAACACTACC ATTCCTGCGC ATCAAAAACT TGAAAAAAGG ACACTTCGTC ACAGGATAGT TGTCGCTGCG   
  
  
+ CGCGAGAGAA GAGACAATAG AGAGATTTCT GTTTGTCAGT ATCTCTGTCT CTCTCTTTTC GTTTGTTTTT   
  
  
+ TGTGACTTTT CTTACTTTCT TTTCTTCTCT CTTCCCTTAT CTTTTCTCTA TTCAATCTTT TTAGCGGCAG   
  
  
+ TTGGCTTTTG TCATGCCCTC CGACAATAAA TCCCCTTGCG TTCTCACATC CAGGGTGCGC GTACTTTAAC   
  
  
+ TTTAAAAGGT CTCTTCTTTT TTCTATGAAA GGCTGTTCTT CCGCGTGGAA CAAAGAGAGG TCATGAATGG   
  
  
+ ACACGCATTT GGTAGGGGTT AAAGAAAGAA AAGAATCAAA ATGGGGGTTT TTTTTTTTTT ATTTCATTTT   
  
  
+ AATTTTAGAA GAAATGTTTC TATTGAATAA AAATTTAAGT GTAATGTATA ATATTTTATT TTTAATTTAG   
  
  
+ GTTTGAAAGG TGTAAAATTT TTAGAAATTT TATTCAATTT AAAGGTCCAG TTAGTACATT CTGTATAAAA   
  
  
+ AAATTTTACT TATTTTAGAA ATTTTTAATA GCAATAATTT TATCTATTGT AAAATGTAAT AATACTGTTT   
  
  
+ TTTAATGACA TTTATAGTGT TAAATTTAAC TATCCTATTT ACACATTATA ACTTTTTGAA AGAAAAAGTC   
  
  
+ ATCTTTTTTT AGAACCCCTC CTCTGGAAAC TAACCGTATA TATAATATAC CACTCATTAC TTTCTTCTCC   
  
  
+ TGCAAACCAC ACAAACAAAT TAATTTGTTA ATTAATTAAA TTACAGGTTT CGTCTACTTC GCTTCTCCAG   
  
  
+ TAGTCTTCTT CTTCGTATTT ATTTTATAAC CTTTTGTTCT CCACCTCCTC CTCTGTCTCC GAACCCTACG   
  
  
+ TCGTCACCAT TCATTGTTCA CCCGAGACAG CGAGAGAGAG ATAAACGACT CACTTATTAA GTCGTGTTGT   
  
  
+ CGTGTGCTTG TCCTCGACCA GTGACTAAAC ACTTGTAAGA ACCGTTTATG ATGGGTAGTC GGTAGGAGAA   
  
  
+ ATAATGAGGG AGATTGAGGA TGTGAGAAAC TGGGTTAGGT GGAAAGAGTA GGGAAGATAT AAATAAAAAA   
  
  
+ TAGGCAAGTA AAAAATAAAA AAATTTTTAC TTAATTTAAT AAATAAAAAA TTTTAATATT AATAGATAAA   
  
  
+ GAAAAAATTT TCTAATTATT TTTAGTTTGA CTATCTTTTC TACAAATCAA ATAGTTTTAT TAAAAAAAAT   
  
  
+ CTACGGGTAT ATATGATTAA AGTAGTTCAC CCGTACCAAT AACTTAACTA CGATAACCAC ACGGCTTCTT   
  
  
+ TCAATAAGAA GTGTAAACAG CTGCTCCTG  

- GACTGTAACA ATGATCCGTC TCAGACAGAG AGCCTGCCGA AATTCTGCGA ACGCGGAGAG AGAAAGTGCG   
  
  
- AGGCTAAGAG GTGCGTTTTA GACTGAGGAA AAGGGAGAAG GATGGAAAGA GAAAGGGAGA GAGCTAGGGT   
  
  
- TTTGTGATGG TAAGGACGCG TAGTTTTTGA ACTTTTTTCC TGTGAAGCAG TGTCCTATCA ACAGCGACGC   
  
  
- GCGCTCTCTT CTCTGTTATC TCTCTAAAGA CAAACAGTCA TAGAGACAGA GAGAGAAAAG CAAACAAAAA   
  
  
- ACACTGAAAA GAATGAAAGA AAAGAAGAGA GAAGGGAATA GAAAAGAGAT AAGTTAGAAA AATCGCCGTC   
  
  
- AACCGAAAAC AGTACGGGAG GCTGTTATTT AGGGGAACGC AAGAGTGTAG GTCCCACGCG CATGAAATTG   
  
  
- AAATTTTCCA GAGAAGAAAA AAGATACTTT CCGACAAGAA GGCGCACCTT GTTTCTCTCC AGTACTTACC   
  
  
- TGTGCGTAAA CCATCCCCAA TTTCTTTCTT TTCTTAGTTT TACCCCCAAA AAAAAAAAAA TAAAGTAAAA   
  
  
- TTAAAATCTT CTTTACAAAG ATAACTTATT TTTAAATTCA CATTACATAT TATAAAATAA AAATTAAATC   
  
  
- CAAACTTTCC ACATTTTAAA AATCTTTAAA ATAAGTTAAA TTTCCAGGTC AATCATGTAA GACATATTTT   
  
  
- TTTAAAATGA ATAAAATCTT TAAAAATTAT CGTTATTAAA ATAGATAACA TTTTACATTA TTATGACAAA   
  
  
- AAATTACTGT AAATATCACA ATTTAAATTG ATAGGATAAA TGTGTAATAT TGAAAAACTT TCTTTTTCAG   
  
  
- TAGAAAAAAA TCTTGGGGAG GAGACCTTTG ATTGGCATAT ATATTATATG GTGAGTAATG AAAGAAGAGG   
  
  
- ACGTTTGGTG TGTTTGTTTA ATTAAACAAT TAATTAATTT AATGTCCAAA GCAGATGAAG CGAAGAGGTC   
  
  
- ATCAGAAGAA GAAGCATAAA TAAAATATTG GAAAACAAGA GGTGGAGGAG GAGACAGAGG CTTGGGATGC   
  
  
- AGCAGTGGTA AGTAACAAGT GGGCTCTGTC GCTCTCTCTC TATTTGCTGA GTGAATAATT CAGCACAACA   
  
  
- GCACACGAAC AGGAGCTGGT CACTGATTTG TGAACATTCT TGGCAAATAC TACCCATCAG CCATCCTCTT   
  
  
- TATTACTCCC TCTAACTCCT ACACTCTTTG ACCCAATCCA CCTTTCTCAT CCCTTCTATA TTTATTTTTT   
  
  
- ATCCGTTCAT TTTTTATTTT TTTAAAAATG AATTAAATTA TTTATTTTTT AAAATTATAA TTATCTATTT   
  
  
- CTTTTTTAAA AGATTAATAA AAATCAAACT GATAGAAAAG ATGTTTAGTT TATCAAAATA ATTTTTTTTA   
  
  
- GATGCCCATA TATACTAATT TCATCAAGTG GGCATGGTTA TTGAATTGAT GCTATTGGTG TGCCGAAGAA   
  
  
- AGTTATTCTT CACATTTGTC GACGAGGAC

+     GARE-motif

| Site Name | Organism | Position | Strand | Matrix score. | sequence | function |
| --- | --- | --- | --- | --- | --- | --- |
| GARE-motif | Brassica oleracea | 238 | - | 7 | AAACAGA | gibberellin-responsive element |

> 2018/04/13 10:10:12  
+ CTGACATTGT TACTAGGCAG AGTCTGTCTC TCGGACGGCT TTAAGACGCT TGCGCCTCTC TCTTTCACGC   
  
  
+ TCCGATTCTC CACGCAAAAT CTGACTCCTT TTCCCTCTTC CTACCTTTCT CTTTCCCTCT CTCGATCCCA   
  
  
+ AAACACTACC ATTCCTGCGC ATCAAAAACT TGAAAAAAGG ACACTTCGTC ACAGGATAGT TGTCGCTGCG   
  
  
+ CGCGAGAGAA GAGACAATAG AGAGATTTCT GTTTGTCAGT ATCTCTGTCT CTCTCTTTTC GTTTGTTTTT   
  
  
+ TGTGACTTTT CTTACTTTCT TTTCTTCTCT CTTCCCTTAT CTTTTCTCTA TTCAATCTTT TTAGCGGCAG   
  
  
+ TTGGCTTTTG TCATGCCCTC CGACAATAAA TCCCCTTGCG TTCTCACATC CAGGGTGCGC GTACTTTAAC   
  
  
+ TTTAAAAGGT CTCTTCTTTT TTCTATGAAA GGCTGTTCTT CCGCGTGGAA CAAAGAGAGG TCATGAATGG   
  
  
+ ACACGCATTT GGTAGGGGTT AAAGAAAGAA AAGAATCAAA ATGGGGGTTT TTTTTTTTTT ATTTCATTTT   
  
  
+ AATTTTAGAA GAAATGTTTC TATTGAATAA AAATTTAAGT GTAATGTATA ATATTTTATT TTTAATTTAG   
  
  
+ GTTTGAAAGG TGTAAAATTT TTAGAAATTT TATTCAATTT AAAGGTCCAG TTAGTACATT CTGTATAAAA   
  
  
+ AAATTTTACT TATTTTAGAA ATTTTTAATA GCAATAATTT TATCTATTGT AAAATGTAAT AATACTGTTT   
  
  
+ TTTAATGACA TTTATAGTGT TAAATTTAAC TATCCTATTT ACACATTATA ACTTTTTGAA AGAAAAAGTC   
  
  
+ ATCTTTTTTT AGAACCCCTC CTCTGGAAAC TAACCGTATA TATAATATAC CACTCATTAC TTTCTTCTCC   
  
  
+ TGCAAACCAC ACAAACAAAT TAATTTGTTA ATTAATTAAA TTACAGGTTT CGTCTACTTC GCTTCTCCAG   
  
  
+ TAGTCTTCTT CTTCGTATTT ATTTTATAAC CTTTTGTTCT CCACCTCCTC CTCTGTCTCC GAACCCTACG   
  
  
+ TCGTCACCAT TCATTGTTCA CCCGAGACAG CGAGAGAGAG ATAAACGACT CACTTATTAA GTCGTGTTGT   
  
  
+ CGTGTGCTTG TCCTCGACCA GTGACTAAAC ACTTGTAAGA ACCGTTTATG ATGGGTAGTC GGTAGGAGAA   
  
  
+ ATAATGAGGG AGATTGAGGA TGTGAGAAAC TGGGTTAGGT GGAAAGAGTA GGGAAGATAT AAATAAAAAA   
  
  
+ TAGGCAAGTA AAAAATAAAA AAATTTTTAC TTAATTTAAT AAATAAAAAA TTTTAATATT AATAGATAAA   
  
  
+ GAAAAAATTT TCTAATTATT TTTAGTTTGA CTATCTTTTC TACAAATCAA ATAGTTTTAT TAAAAAAAAT   
  
  
+ CTACGGGTAT ATATGATTAA AGTAGTTCAC CCGTACCAAT AACTTAACTA CGATAACCAC ACGGCTTCTT   
  
  
+ TCAATAAGAA GTGTAAACAG CTGCTCCTG  

- GACTGTAACA ATGATCCGTC TCAGACAGAG AGCCTGCCGA AATTCTGCGA ACGCGGAGAG AGAAAGTGCG   
  
  
- AGGCTAAGAG GTGCGTTTTA GACTGAGGAA AAGGGAGAAG GATGGAAAGA GAAAGGGAGA GAGCTAGGGT   
  
  
- TTTGTGATGG TAAGGACGCG TAGTTTTTGA ACTTTTTTCC TGTGAAGCAG TGTCCTATCA ACAGCGACGC   
  
  
- GCGCTCTCTT CTCTGTTATC TCTCTAAAGA CAAACAGTCA TAGAGACAGA GAGAGAAAAG CAAACAAAAA   
  
  
- ACACTGAAAA GAATGAAAGA AAAGAAGAGA GAAGGGAATA GAAAAGAGAT AAGTTAGAAA AATCGCCGTC   
  
  
- AACCGAAAAC AGTACGGGAG GCTGTTATTT AGGGGAACGC AAGAGTGTAG GTCCCACGCG CATGAAATTG   
  
  
- AAATTTTCCA GAGAAGAAAA AAGATACTTT CCGACAAGAA GGCGCACCTT GTTTCTCTCC AGTACTTACC   
  
  
- TGTGCGTAAA CCATCCCCAA TTTCTTTCTT TTCTTAGTTT TACCCCCAAA AAAAAAAAAA TAAAGTAAAA   
  
  
- TTAAAATCTT CTTTACAAAG ATAACTTATT TTTAAATTCA CATTACATAT TATAAAATAA AAATTAAATC   
  
  
- CAAACTTTCC ACATTTTAAA AATCTTTAAA ATAAGTTAAA TTTCCAGGTC AATCATGTAA GACATATTTT   
  
  
- TTTAAAATGA ATAAAATCTT TAAAAATTAT CGTTATTAAA ATAGATAACA TTTTACATTA TTATGACAAA   
  
  
- AAATTACTGT AAATATCACA ATTTAAATTG ATAGGATAAA TGTGTAATAT TGAAAAACTT TCTTTTTCAG   
  
  
- TAGAAAAAAA TCTTGGGGAG GAGACCTTTG ATTGGCATAT ATATTATATG GTGAGTAATG AAAGAAGAGG   
  
  
- ACGTTTGGTG TGTTTGTTTA ATTAAACAAT TAATTAATTT AATGTCCAAA GCAGATGAAG CGAAGAGGTC   
  
  
- ATCAGAAGAA GAAGCATAAA TAAAATATTG GAAAACAAGA GGTGGAGGAG GAGACAGAGG CTTGGGATGC   
  
  
- AGCAGTGGTA AGTAACAAGT GGGCTCTGTC GCTCTCTCTC TATTTGCTGA GTGAATAATT CAGCACAACA   
  
  
- GCACACGAAC AGGAGCTGGT CACTGATTTG TGAACATTCT TGGCAAATAC TACCCATCAG CCATCCTCTT   
  
  
- TATTACTCCC TCTAACTCCT ACACTCTTTG ACCCAATCCA CCTTTCTCAT CCCTTCTATA TTTATTTTTT   
  
  
- ATCCGTTCAT TTTTTATTTT TTTAAAAATG AATTAAATTA TTTATTTTTT AAAATTATAA TTATCTATTT   
  
  
- CTTTTTTAAA AGATTAATAA AAATCAAACT GATAGAAAAG ATGTTTAGTT TATCAAAATA ATTTTTTTTA   
  
  
- GATGCCCATA TATACTAATT TCATCAAGTG GGCATGGTTA TTGAATTGAT GCTATTGGTG TGCCGAAGAA   
  
  
- AGTTATTCTT CACATTTGTC GACGAGGAC

+     GATA-motif

| Site Name | Organism | Position | Strand | Matrix score. | sequence | function |
| --- | --- | --- | --- | --- | --- | --- |
| GATA-motif | Solanum tuberosum | 315 | - | 9 | AAGGATAAGG | part of a light responsive element |

> 2018/04/13 10:10:12  
+ CTGACATTGT TACTAGGCAG AGTCTGTCTC TCGGACGGCT TTAAGACGCT TGCGCCTCTC TCTTTCACGC   
  
  
+ TCCGATTCTC CACGCAAAAT CTGACTCCTT TTCCCTCTTC CTACCTTTCT CTTTCCCTCT CTCGATCCCA   
  
  
+ AAACACTACC ATTCCTGCGC ATCAAAAACT TGAAAAAAGG ACACTTCGTC ACAGGATAGT TGTCGCTGCG   
  
  
+ CGCGAGAGAA GAGACAATAG AGAGATTTCT GTTTGTCAGT ATCTCTGTCT CTCTCTTTTC GTTTGTTTTT   
  
  
+ TGTGACTTTT CTTACTTTCT TTTCTTCTCT CTTCCCTTAT CTTTTCTCTA TTCAATCTTT TTAGCGGCAG   
  
  
+ TTGGCTTTTG TCATGCCCTC CGACAATAAA TCCCCTTGCG TTCTCACATC CAGGGTGCGC GTACTTTAAC   
  
  
+ TTTAAAAGGT CTCTTCTTTT TTCTATGAAA GGCTGTTCTT CCGCGTGGAA CAAAGAGAGG TCATGAATGG   
  
  
+ ACACGCATTT GGTAGGGGTT AAAGAAAGAA AAGAATCAAA ATGGGGGTTT TTTTTTTTTT ATTTCATTTT   
  
  
+ AATTTTAGAA GAAATGTTTC TATTGAATAA AAATTTAAGT GTAATGTATA ATATTTTATT TTTAATTTAG   
  
  
+ GTTTGAAAGG TGTAAAATTT TTAGAAATTT TATTCAATTT AAAGGTCCAG TTAGTACATT CTGTATAAAA   
  
  
+ AAATTTTACT TATTTTAGAA ATTTTTAATA GCAATAATTT TATCTATTGT AAAATGTAAT AATACTGTTT   
  
  
+ TTTAATGACA TTTATAGTGT TAAATTTAAC TATCCTATTT ACACATTATA ACTTTTTGAA AGAAAAAGTC   
  
  
+ ATCTTTTTTT AGAACCCCTC CTCTGGAAAC TAACCGTATA TATAATATAC CACTCATTAC TTTCTTCTCC   
  
  
+ TGCAAACCAC ACAAACAAAT TAATTTGTTA ATTAATTAAA TTACAGGTTT CGTCTACTTC GCTTCTCCAG   
  
  
+ TAGTCTTCTT CTTCGTATTT ATTTTATAAC CTTTTGTTCT CCACCTCCTC CTCTGTCTCC GAACCCTACG   
  
  
+ TCGTCACCAT TCATTGTTCA CCCGAGACAG CGAGAGAGAG ATAAACGACT CACTTATTAA GTCGTGTTGT   
  
  
+ CGTGTGCTTG TCCTCGACCA GTGACTAAAC ACTTGTAAGA ACCGTTTATG ATGGGTAGTC GGTAGGAGAA   
  
  
+ ATAATGAGGG AGATTGAGGA TGTGAGAAAC TGGGTTAGGT GGAAAGAGTA GGGAAGATAT AAATAAAAAA   
  
  
+ TAGGCAAGTA AAAAATAAAA AAATTTTTAC TTAATTTAAT AAATAAAAAA TTTTAATATT AATAGATAAA   
  
  
+ GAAAAAATTT TCTAATTATT TTTAGTTTGA CTATCTTTTC TACAAATCAA ATAGTTTTAT TAAAAAAAAT   
  
  
+ CTACGGGTAT ATATGATTAA AGTAGTTCAC CCGTACCAAT AACTTAACTA CGATAACCAC ACGGCTTCTT   
  
  
+ TCAATAAGAA GTGTAAACAG CTGCTCCTG  

- GACTGTAACA ATGATCCGTC TCAGACAGAG AGCCTGCCGA AATTCTGCGA ACGCGGAGAG AGAAAGTGCG   
  
  
- AGGCTAAGAG GTGCGTTTTA GACTGAGGAA AAGGGAGAAG GATGGAAAGA GAAAGGGAGA GAGCTAGGGT   
  
  
- TTTGTGATGG TAAGGACGCG TAGTTTTTGA ACTTTTTTCC TGTGAAGCAG TGTCCTATCA ACAGCGACGC   
  
  
- GCGCTCTCTT CTCTGTTATC TCTCTAAAGA CAAACAGTCA TAGAGACAGA GAGAGAAAAG CAAACAAAAA   
  
  
- ACACTGAAAA GAATGAAAGA AAAGAAGAGA GAAGGGAATA GAAAAGAGAT AAGTTAGAAA AATCGCCGTC   
  
  
- AACCGAAAAC AGTACGGGAG GCTGTTATTT AGGGGAACGC AAGAGTGTAG GTCCCACGCG CATGAAATTG   
  
  
- AAATTTTCCA GAGAAGAAAA AAGATACTTT CCGACAAGAA GGCGCACCTT GTTTCTCTCC AGTACTTACC   
  
  
- TGTGCGTAAA CCATCCCCAA TTTCTTTCTT TTCTTAGTTT TACCCCCAAA AAAAAAAAAA TAAAGTAAAA   
  
  
- TTAAAATCTT CTTTACAAAG ATAACTTATT TTTAAATTCA CATTACATAT TATAAAATAA AAATTAAATC   
  
  
- CAAACTTTCC ACATTTTAAA AATCTTTAAA ATAAGTTAAA TTTCCAGGTC AATCATGTAA GACATATTTT   
  
  
- TTTAAAATGA ATAAAATCTT TAAAAATTAT CGTTATTAAA ATAGATAACA TTTTACATTA TTATGACAAA   
  
  
- AAATTACTGT AAATATCACA ATTTAAATTG ATAGGATAAA TGTGTAATAT TGAAAAACTT TCTTTTTCAG   
  
  
- TAGAAAAAAA TCTTGGGGAG GAGACCTTTG ATTGGCATAT ATATTATATG GTGAGTAATG AAAGAAGAGG   
  
  
- ACGTTTGGTG TGTTTGTTTA ATTAAACAAT TAATTAATTT AATGTCCAAA GCAGATGAAG CGAAGAGGTC   
  
  
- ATCAGAAGAA GAAGCATAAA TAAAATATTG GAAAACAAGA GGTGGAGGAG GAGACAGAGG CTTGGGATGC   
  
  
- AGCAGTGGTA AGTAACAAGT GGGCTCTGTC GCTCTCTCTC TATTTGCTGA GTGAATAATT CAGCACAACA   
  
  
- GCACACGAAC AGGAGCTGGT CACTGATTTG TGAACATTCT TGGCAAATAC TACCCATCAG CCATCCTCTT   
  
  
- TATTACTCCC TCTAACTCCT ACACTCTTTG ACCCAATCCA CCTTTCTCAT CCCTTCTATA TTTATTTTTT   
  
  
- ATCCGTTCAT TTTTTATTTT TTTAAAAATG AATTAAATTA TTTATTTTTT AAAATTATAA TTATCTATTT   
  
  
- CTTTTTTAAA AGATTAATAA AAATCAAACT GATAGAAAAG ATGTTTAGTT TATCAAAATA ATTTTTTTTA   
  
  
- GATGCCCATA TATACTAATT TCATCAAGTG GGCATGGTTA TTGAATTGAT GCTATTGGTG TGCCGAAGAA   
  
  
- AGTTATTCTT CACATTTGTC GACGAGGAC

+     GT1-motif

| Site Name | Organism | Position | Strand | Matrix score. | sequence | function |
| --- | --- | --- | --- | --- | --- | --- |
| GT1-motif | Arabidopsis thaliana | 507 | + | 6 | GGTTAA | light responsive element |

> 2018/04/13 10:10:12  
+ CTGACATTGT TACTAGGCAG AGTCTGTCTC TCGGACGGCT TTAAGACGCT TGCGCCTCTC TCTTTCACGC   
  
  
+ TCCGATTCTC CACGCAAAAT CTGACTCCTT TTCCCTCTTC CTACCTTTCT CTTTCCCTCT CTCGATCCCA   
  
  
+ AAACACTACC ATTCCTGCGC ATCAAAAACT TGAAAAAAGG ACACTTCGTC ACAGGATAGT TGTCGCTGCG   
  
  
+ CGCGAGAGAA GAGACAATAG AGAGATTTCT GTTTGTCAGT ATCTCTGTCT CTCTCTTTTC GTTTGTTTTT   
  
  
+ TGTGACTTTT CTTACTTTCT TTTCTTCTCT CTTCCCTTAT CTTTTCTCTA TTCAATCTTT TTAGCGGCAG   
  
  
+ TTGGCTTTTG TCATGCCCTC CGACAATAAA TCCCCTTGCG TTCTCACATC CAGGGTGCGC GTACTTTAAC   
  
  
+ TTTAAAAGGT CTCTTCTTTT TTCTATGAAA GGCTGTTCTT CCGCGTGGAA CAAAGAGAGG TCATGAATGG   
  
  
+ ACACGCATTT GGTAGGGGTT AAAGAAAGAA AAGAATCAAA ATGGGGGTTT TTTTTTTTTT ATTTCATTTT   
  
  
+ AATTTTAGAA GAAATGTTTC TATTGAATAA AAATTTAAGT GTAATGTATA ATATTTTATT TTTAATTTAG   
  
  
+ GTTTGAAAGG TGTAAAATTT TTAGAAATTT TATTCAATTT AAAGGTCCAG TTAGTACATT CTGTATAAAA   
  
  
+ AAATTTTACT TATTTTAGAA ATTTTTAATA GCAATAATTT TATCTATTGT AAAATGTAAT AATACTGTTT   
  
  
+ TTTAATGACA TTTATAGTGT TAAATTTAAC TATCCTATTT ACACATTATA ACTTTTTGAA AGAAAAAGTC   
  
  
+ ATCTTTTTTT AGAACCCCTC CTCTGGAAAC TAACCGTATA TATAATATAC CACTCATTAC TTTCTTCTCC   
  
  
+ TGCAAACCAC ACAAACAAAT TAATTTGTTA ATTAATTAAA TTACAGGTTT CGTCTACTTC GCTTCTCCAG   
  
  
+ TAGTCTTCTT CTTCGTATTT ATTTTATAAC CTTTTGTTCT CCACCTCCTC CTCTGTCTCC GAACCCTACG   
  
  
+ TCGTCACCAT TCATTGTTCA CCCGAGACAG CGAGAGAGAG ATAAACGACT CACTTATTAA GTCGTGTTGT   
  
  
+ CGTGTGCTTG TCCTCGACCA GTGACTAAAC ACTTGTAAGA ACCGTTTATG ATGGGTAGTC GGTAGGAGAA   
  
  
+ ATAATGAGGG AGATTGAGGA TGTGAGAAAC TGGGTTAGGT GGAAAGAGTA GGGAAGATAT AAATAAAAAA   
  
  
+ TAGGCAAGTA AAAAATAAAA AAATTTTTAC TTAATTTAAT AAATAAAAAA TTTTAATATT AATAGATAAA   
  
  
+ GAAAAAATTT TCTAATTATT TTTAGTTTGA CTATCTTTTC TACAAATCAA ATAGTTTTAT TAAAAAAAAT   
  
  
+ CTACGGGTAT ATATGATTAA AGTAGTTCAC CCGTACCAAT AACTTAACTA CGATAACCAC ACGGCTTCTT   
  
  
+ TCAATAAGAA GTGTAAACAG CTGCTCCTG  

- GACTGTAACA ATGATCCGTC TCAGACAGAG AGCCTGCCGA AATTCTGCGA ACGCGGAGAG AGAAAGTGCG   
  
  
- AGGCTAAGAG GTGCGTTTTA GACTGAGGAA AAGGGAGAAG GATGGAAAGA GAAAGGGAGA GAGCTAGGGT   
  
  
- TTTGTGATGG TAAGGACGCG TAGTTTTTGA ACTTTTTTCC TGTGAAGCAG TGTCCTATCA ACAGCGACGC   
  
  
- GCGCTCTCTT CTCTGTTATC TCTCTAAAGA CAAACAGTCA TAGAGACAGA GAGAGAAAAG CAAACAAAAA   
  
  
- ACACTGAAAA GAATGAAAGA AAAGAAGAGA GAAGGGAATA GAAAAGAGAT AAGTTAGAAA AATCGCCGTC   
  
  
- AACCGAAAAC AGTACGGGAG GCTGTTATTT AGGGGAACGC AAGAGTGTAG GTCCCACGCG CATGAAATTG   
  
  
- AAATTTTCCA GAGAAGAAAA AAGATACTTT CCGACAAGAA GGCGCACCTT GTTTCTCTCC AGTACTTACC   
  
  
- TGTGCGTAAA CCATCCCCAA TTTCTTTCTT TTCTTAGTTT TACCCCCAAA AAAAAAAAAA TAAAGTAAAA   
  
  
- TTAAAATCTT CTTTACAAAG ATAACTTATT TTTAAATTCA CATTACATAT TATAAAATAA AAATTAAATC   
  
  
- CAAACTTTCC ACATTTTAAA AATCTTTAAA ATAAGTTAAA TTTCCAGGTC AATCATGTAA GACATATTTT   
  
  
- TTTAAAATGA ATAAAATCTT TAAAAATTAT CGTTATTAAA ATAGATAACA TTTTACATTA TTATGACAAA   
  
  
- AAATTACTGT AAATATCACA ATTTAAATTG ATAGGATAAA TGTGTAATAT TGAAAAACTT TCTTTTTCAG   
  
  
- TAGAAAAAAA TCTTGGGGAG GAGACCTTTG ATTGGCATAT ATATTATATG GTGAGTAATG AAAGAAGAGG   
  
  
- ACGTTTGGTG TGTTTGTTTA ATTAAACAAT TAATTAATTT AATGTCCAAA GCAGATGAAG CGAAGAGGTC   
  
  
- ATCAGAAGAA GAAGCATAAA TAAAATATTG GAAAACAAGA GGTGGAGGAG GAGACAGAGG CTTGGGATGC   
  
  
- AGCAGTGGTA AGTAACAAGT GGGCTCTGTC GCTCTCTCTC TATTTGCTGA GTGAATAATT CAGCACAACA   
  
  
- GCACACGAAC AGGAGCTGGT CACTGATTTG TGAACATTCT TGGCAAATAC TACCCATCAG CCATCCTCTT   
  
  
- TATTACTCCC TCTAACTCCT ACACTCTTTG ACCCAATCCA CCTTTCTCAT CCCTTCTATA TTTATTTTTT   
  
  
- ATCCGTTCAT TTTTTATTTT TTTAAAAATG AATTAAATTA TTTATTTTTT AAAATTATAA TTATCTATTT   
  
  
- CTTTTTTAAA AGATTAATAA AAATCAAACT GATAGAAAAG ATGTTTAGTT TATCAAAATA ATTTTTTTTA   
  
  
- GATGCCCATA TATACTAATT TCATCAAGTG GGCATGGTTA TTGAATTGAT GCTATTGGTG TGCCGAAGAA   
  
  
- AGTTATTCTT CACATTTGTC GACGAGGAC

+     HSE

| Site Name | Organism | Position | Strand | Matrix score. | sequence | function |
| --- | --- | --- | --- | --- | --- | --- |
| HSE | Brassica oleracea | 1333 | + | 9 | AAAAAATTTC | cis-acting element involved in heat stress responsiveness |
| HSE | Brassica oleracea | 1332 | + | 9 | AAAAAATTTC | cis-acting element involved in heat stress responsiveness |
| HSE | Brassica oleracea | 1305 | + | 9 | AAAAAATTTC | cis-acting element involved in heat stress responsiveness |
| HSE | Brassica oleracea | 718 | - | 9 | AAAAAATTTC | cis-acting element involved in heat stress responsiveness |
| HSE | Brassica oleracea | 1278 | + | 9 | AAAAAATTTC | cis-acting element involved in heat stress responsiveness |
| HSE | Brassica oleracea | 654 | - | 9 | AAAAAATTTC | cis-acting element involved in heat stress responsiveness |
| HSE | Brassica oleracea | 698 | + | 9 | AAAAAATTTC | cis-acting element involved in heat stress responsiveness |

> 2018/04/13 10:10:12  
+ CTGACATTGT TACTAGGCAG AGTCTGTCTC TCGGACGGCT TTAAGACGCT TGCGCCTCTC TCTTTCACGC   
  
  
+ TCCGATTCTC CACGCAAAAT CTGACTCCTT TTCCCTCTTC CTACCTTTCT CTTTCCCTCT CTCGATCCCA   
  
  
+ AAACACTACC ATTCCTGCGC ATCAAAAACT TGAAAAAAGG ACACTTCGTC ACAGGATAGT TGTCGCTGCG   
  
  
+ CGCGAGAGAA GAGACAATAG AGAGATTTCT GTTTGTCAGT ATCTCTGTCT CTCTCTTTTC GTTTGTTTTT   
  
  
+ TGTGACTTTT CTTACTTTCT TTTCTTCTCT CTTCCCTTAT CTTTTCTCTA TTCAATCTTT TTAGCGGCAG   
  
  
+ TTGGCTTTTG TCATGCCCTC CGACAATAAA TCCCCTTGCG TTCTCACATC CAGGGTGCGC GTACTTTAAC   
  
  
+ TTTAAAAGGT CTCTTCTTTT TTCTATGAAA GGCTGTTCTT CCGCGTGGAA CAAAGAGAGG TCATGAATGG   
  
  
+ ACACGCATTT GGTAGGGGTT AAAGAAAGAA AAGAATCAAA ATGGGGGTTT TTTTTTTTTT ATTTCATTTT   
  
  
+ AATTTTAGAA GAAATGTTTC TATTGAATAA AAATTTAAGT GTAATGTATA ATATTTTATT TTTAATTTAG   
  
  
+ GTTTGAAAGG TGTAAAATTT TTAGAAATTT TATTCAATTT AAAGGTCCAG TTAGTACATT CTGTATAAAA   
  
  
+ AAATTTTACT TATTTTAGAA ATTTTTAATA GCAATAATTT TATCTATTGT AAAATGTAAT AATACTGTTT   
  
  
+ TTTAATGACA TTTATAGTGT TAAATTTAAC TATCCTATTT ACACATTATA ACTTTTTGAA AGAAAAAGTC   
  
  
+ ATCTTTTTTT AGAACCCCTC CTCTGGAAAC TAACCGTATA TATAATATAC CACTCATTAC TTTCTTCTCC   
  
  
+ TGCAAACCAC ACAAACAAAT TAATTTGTTA ATTAATTAAA TTACAGGTTT CGTCTACTTC GCTTCTCCAG   
  
  
+ TAGTCTTCTT CTTCGTATTT ATTTTATAAC CTTTTGTTCT CCACCTCCTC CTCTGTCTCC GAACCCTACG   
  
  
+ TCGTCACCAT TCATTGTTCA CCCGAGACAG CGAGAGAGAG ATAAACGACT CACTTATTAA GTCGTGTTGT   
  
  
+ CGTGTGCTTG TCCTCGACCA GTGACTAAAC ACTTGTAAGA ACCGTTTATG ATGGGTAGTC GGTAGGAGAA   
  
  
+ ATAATGAGGG AGATTGAGGA TGTGAGAAAC TGGGTTAGGT GGAAAGAGTA GGGAAGATAT AAATAAAAAA   
  
  
+ TAGGCAAGTA AAAAATAAAA AAATTTTTAC TTAATTTAAT AAATAAAAAA TTTTAATATT AATAGATAAA   
  
  
+ GAAAAAATTT TCTAATTATT TTTAGTTTGA CTATCTTTTC TACAAATCAA ATAGTTTTAT TAAAAAAAAT   
  
  
+ CTACGGGTAT ATATGATTAA AGTAGTTCAC CCGTACCAAT AACTTAACTA CGATAACCAC ACGGCTTCTT   
  
  
+ TCAATAAGAA GTGTAAACAG CTGCTCCTG  

- GACTGTAACA ATGATCCGTC TCAGACAGAG AGCCTGCCGA AATTCTGCGA ACGCGGAGAG AGAAAGTGCG   
  
  
- AGGCTAAGAG GTGCGTTTTA GACTGAGGAA AAGGGAGAAG GATGGAAAGA GAAAGGGAGA GAGCTAGGGT   
  
  
- TTTGTGATGG TAAGGACGCG TAGTTTTTGA ACTTTTTTCC TGTGAAGCAG TGTCCTATCA ACAGCGACGC   
  
  
- GCGCTCTCTT CTCTGTTATC TCTCTAAAGA CAAACAGTCA TAGAGACAGA GAGAGAAAAG CAAACAAAAA   
  
  
- ACACTGAAAA GAATGAAAGA AAAGAAGAGA GAAGGGAATA GAAAAGAGAT AAGTTAGAAA AATCGCCGTC   
  
  
- AACCGAAAAC AGTACGGGAG GCTGTTATTT AGGGGAACGC AAGAGTGTAG GTCCCACGCG CATGAAATTG   
  
  
- AAATTTTCCA GAGAAGAAAA AAGATACTTT CCGACAAGAA GGCGCACCTT GTTTCTCTCC AGTACTTACC   
  
  
- TGTGCGTAAA CCATCCCCAA TTTCTTTCTT TTCTTAGTTT TACCCCCAAA AAAAAAAAAA TAAAGTAAAA   
  
  
- TTAAAATCTT CTTTACAAAG ATAACTTATT TTTAAATTCA CATTACATAT TATAAAATAA AAATTAAATC   
  
  
- CAAACTTTCC ACATTTTAAA AATCTTTAAA ATAAGTTAAA TTTCCAGGTC AATCATGTAA GACATATTTT   
  
  
- TTTAAAATGA ATAAAATCTT TAAAAATTAT CGTTATTAAA ATAGATAACA TTTTACATTA TTATGACAAA   
  
  
- AAATTACTGT AAATATCACA ATTTAAATTG ATAGGATAAA TGTGTAATAT TGAAAAACTT TCTTTTTCAG   
  
  
- TAGAAAAAAA TCTTGGGGAG GAGACCTTTG ATTGGCATAT ATATTATATG GTGAGTAATG AAAGAAGAGG   
  
  
- ACGTTTGGTG TGTTTGTTTA ATTAAACAAT TAATTAATTT AATGTCCAAA GCAGATGAAG CGAAGAGGTC   
  
  
- ATCAGAAGAA GAAGCATAAA TAAAATATTG GAAAACAAGA GGTGGAGGAG GAGACAGAGG CTTGGGATGC   
  
  
- AGCAGTGGTA AGTAACAAGT GGGCTCTGTC GCTCTCTCTC TATTTGCTGA GTGAATAATT CAGCACAACA   
  
  
- GCACACGAAC AGGAGCTGGT CACTGATTTG TGAACATTCT TGGCAAATAC TACCCATCAG CCATCCTCTT   
  
  
- TATTACTCCC TCTAACTCCT ACACTCTTTG ACCCAATCCA CCTTTCTCAT CCCTTCTATA TTTATTTTTT   
  
  
- ATCCGTTCAT TTTTTATTTT TTTAAAAATG AATTAAATTA TTTATTTTTT AAAATTATAA TTATCTATTT   
  
  
- CTTTTTTAAA AGATTAATAA AAATCAAACT GATAGAAAAG ATGTTTAGTT TATCAAAATA ATTTTTTTTA   
  
  
- GATGCCCATA TATACTAATT TCATCAAGTG GGCATGGTTA TTGAATTGAT GCTATTGGTG TGCCGAAGAA   
  
  
- AGTTATTCTT CACATTTGTC GACGAGGAC

+     I-box

| Site Name | Organism | Position | Strand | Matrix score. | sequence | function |
| --- | --- | --- | --- | --- | --- | --- |
| I-box | Triticum aestivum | 315 | - | 8 | AGATAAGG | part of a light responsive element |

> 2018/04/13 10:10:12  
+ CTGACATTGT TACTAGGCAG AGTCTGTCTC TCGGACGGCT TTAAGACGCT TGCGCCTCTC TCTTTCACGC   
  
  
+ TCCGATTCTC CACGCAAAAT CTGACTCCTT TTCCCTCTTC CTACCTTTCT CTTTCCCTCT CTCGATCCCA   
  
  
+ AAACACTACC ATTCCTGCGC ATCAAAAACT TGAAAAAAGG ACACTTCGTC ACAGGATAGT TGTCGCTGCG   
  
  
+ CGCGAGAGAA GAGACAATAG AGAGATTTCT GTTTGTCAGT ATCTCTGTCT CTCTCTTTTC GTTTGTTTTT   
  
  
+ TGTGACTTTT CTTACTTTCT TTTCTTCTCT CTTCCCTTAT CTTTTCTCTA TTCAATCTTT TTAGCGGCAG   
  
  
+ TTGGCTTTTG TCATGCCCTC CGACAATAAA TCCCCTTGCG TTCTCACATC CAGGGTGCGC GTACTTTAAC   
  
  
+ TTTAAAAGGT CTCTTCTTTT TTCTATGAAA GGCTGTTCTT CCGCGTGGAA CAAAGAGAGG TCATGAATGG   
  
  
+ ACACGCATTT GGTAGGGGTT AAAGAAAGAA AAGAATCAAA ATGGGGGTTT TTTTTTTTTT ATTTCATTTT   
  
  
+ AATTTTAGAA GAAATGTTTC TATTGAATAA AAATTTAAGT GTAATGTATA ATATTTTATT TTTAATTTAG   
  
  
+ GTTTGAAAGG TGTAAAATTT TTAGAAATTT TATTCAATTT AAAGGTCCAG TTAGTACATT CTGTATAAAA   
  
  
+ AAATTTTACT TATTTTAGAA ATTTTTAATA GCAATAATTT TATCTATTGT AAAATGTAAT AATACTGTTT   
  
  
+ TTTAATGACA TTTATAGTGT TAAATTTAAC TATCCTATTT ACACATTATA ACTTTTTGAA AGAAAAAGTC   
  
  
+ ATCTTTTTTT AGAACCCCTC CTCTGGAAAC TAACCGTATA TATAATATAC CACTCATTAC TTTCTTCTCC   
  
  
+ TGCAAACCAC ACAAACAAAT TAATTTGTTA ATTAATTAAA TTACAGGTTT CGTCTACTTC GCTTCTCCAG   
  
  
+ TAGTCTTCTT CTTCGTATTT ATTTTATAAC CTTTTGTTCT CCACCTCCTC CTCTGTCTCC GAACCCTACG   
  
  
+ TCGTCACCAT TCATTGTTCA CCCGAGACAG CGAGAGAGAG ATAAACGACT CACTTATTAA GTCGTGTTGT   
  
  
+ CGTGTGCTTG TCCTCGACCA GTGACTAAAC ACTTGTAAGA ACCGTTTATG ATGGGTAGTC GGTAGGAGAA   
  
  
+ ATAATGAGGG AGATTGAGGA TGTGAGAAAC TGGGTTAGGT GGAAAGAGTA GGGAAGATAT AAATAAAAAA   
  
  
+ TAGGCAAGTA AAAAATAAAA AAATTTTTAC TTAATTTAAT AAATAAAAAA TTTTAATATT AATAGATAAA   
  
  
+ GAAAAAATTT TCTAATTATT TTTAGTTTGA CTATCTTTTC TACAAATCAA ATAGTTTTAT TAAAAAAAAT   
  
  
+ CTACGGGTAT ATATGATTAA AGTAGTTCAC CCGTACCAAT AACTTAACTA CGATAACCAC ACGGCTTCTT   
  
  
+ TCAATAAGAA GTGTAAACAG CTGCTCCTG  

- GACTGTAACA ATGATCCGTC TCAGACAGAG AGCCTGCCGA AATTCTGCGA ACGCGGAGAG AGAAAGTGCG   
  
  
- AGGCTAAGAG GTGCGTTTTA GACTGAGGAA AAGGGAGAAG GATGGAAAGA GAAAGGGAGA GAGCTAGGGT   
  
  
- TTTGTGATGG TAAGGACGCG TAGTTTTTGA ACTTTTTTCC TGTGAAGCAG TGTCCTATCA ACAGCGACGC   
  
  
- GCGCTCTCTT CTCTGTTATC TCTCTAAAGA CAAACAGTCA TAGAGACAGA GAGAGAAAAG CAAACAAAAA   
  
  
- ACACTGAAAA GAATGAAAGA AAAGAAGAGA GAAGGGAATA GAAAAGAGAT AAGTTAGAAA AATCGCCGTC   
  
  
- AACCGAAAAC AGTACGGGAG GCTGTTATTT AGGGGAACGC AAGAGTGTAG GTCCCACGCG CATGAAATTG   
  
  
- AAATTTTCCA GAGAAGAAAA AAGATACTTT CCGACAAGAA GGCGCACCTT GTTTCTCTCC AGTACTTACC   
  
  
- TGTGCGTAAA CCATCCCCAA TTTCTTTCTT TTCTTAGTTT TACCCCCAAA AAAAAAAAAA TAAAGTAAAA   
  
  
- TTAAAATCTT CTTTACAAAG ATAACTTATT TTTAAATTCA CATTACATAT TATAAAATAA AAATTAAATC   
  
  
- CAAACTTTCC ACATTTTAAA AATCTTTAAA ATAAGTTAAA TTTCCAGGTC AATCATGTAA GACATATTTT   
  
  
- TTTAAAATGA ATAAAATCTT TAAAAATTAT CGTTATTAAA ATAGATAACA TTTTACATTA TTATGACAAA   
  
  
- AAATTACTGT AAATATCACA ATTTAAATTG ATAGGATAAA TGTGTAATAT TGAAAAACTT TCTTTTTCAG   
  
  
- TAGAAAAAAA TCTTGGGGAG GAGACCTTTG ATTGGCATAT ATATTATATG GTGAGTAATG AAAGAAGAGG   
  
  
- ACGTTTGGTG TGTTTGTTTA ATTAAACAAT TAATTAATTT AATGTCCAAA GCAGATGAAG CGAAGAGGTC   
  
  
- ATCAGAAGAA GAAGCATAAA TAAAATATTG GAAAACAAGA GGTGGAGGAG GAGACAGAGG CTTGGGATGC   
  
  
- AGCAGTGGTA AGTAACAAGT GGGCTCTGTC GCTCTCTCTC TATTTGCTGA GTGAATAATT CAGCACAACA   
  
  
- GCACACGAAC AGGAGCTGGT CACTGATTTG TGAACATTCT TGGCAAATAC TACCCATCAG CCATCCTCTT   
  
  
- TATTACTCCC TCTAACTCCT ACACTCTTTG ACCCAATCCA CCTTTCTCAT CCCTTCTATA TTTATTTTTT   
  
  
- ATCCGTTCAT TTTTTATTTT TTTAAAAATG AATTAAATTA TTTATTTTTT AAAATTATAA TTATCTATTT   
  
  
- CTTTTTTAAA AGATTAATAA AAATCAAACT GATAGAAAAG ATGTTTAGTT TATCAAAATA ATTTTTTTTA   
  
  
- GATGCCCATA TATACTAATT TCATCAAGTG GGCATGGTTA TTGAATTGAT GCTATTGGTG TGCCGAAGAA   
  
  
- AGTTATTCTT CACATTTGTC GACGAGGAC

+     MBS

| Site Name | Organism | Position | Strand | Matrix score. | sequence | function |
| --- | --- | --- | --- | --- | --- | --- |
| MBS | Arabidopsis thaliana | 348 | - | 6 | CAACTG | MYB binding site involved in drought-inducibility |
| MBS | Arabidopsis thaliana | 678 | - | 6 | TAACTG | MYB binding site involved in drought-inducibility |

> 2018/04/13 10:10:12  
+ CTGACATTGT TACTAGGCAG AGTCTGTCTC TCGGACGGCT TTAAGACGCT TGCGCCTCTC TCTTTCACGC   
  
  
+ TCCGATTCTC CACGCAAAAT CTGACTCCTT TTCCCTCTTC CTACCTTTCT CTTTCCCTCT CTCGATCCCA   
  
  
+ AAACACTACC ATTCCTGCGC ATCAAAAACT TGAAAAAAGG ACACTTCGTC ACAGGATAGT TGTCGCTGCG   
  
  
+ CGCGAGAGAA GAGACAATAG AGAGATTTCT GTTTGTCAGT ATCTCTGTCT CTCTCTTTTC GTTTGTTTTT   
  
  
+ TGTGACTTTT CTTACTTTCT TTTCTTCTCT CTTCCCTTAT CTTTTCTCTA TTCAATCTTT TTAGCGGCAG   
  
  
+ TTGGCTTTTG TCATGCCCTC CGACAATAAA TCCCCTTGCG TTCTCACATC CAGGGTGCGC GTACTTTAAC   
  
  
+ TTTAAAAGGT CTCTTCTTTT TTCTATGAAA GGCTGTTCTT CCGCGTGGAA CAAAGAGAGG TCATGAATGG   
  
  
+ ACACGCATTT GGTAGGGGTT AAAGAAAGAA AAGAATCAAA ATGGGGGTTT TTTTTTTTTT ATTTCATTTT   
  
  
+ AATTTTAGAA GAAATGTTTC TATTGAATAA AAATTTAAGT GTAATGTATA ATATTTTATT TTTAATTTAG   
  
  
+ GTTTGAAAGG TGTAAAATTT TTAGAAATTT TATTCAATTT AAAGGTCCAG TTAGTACATT CTGTATAAAA   
  
  
+ AAATTTTACT TATTTTAGAA ATTTTTAATA GCAATAATTT TATCTATTGT AAAATGTAAT AATACTGTTT   
  
  
+ TTTAATGACA TTTATAGTGT TAAATTTAAC TATCCTATTT ACACATTATA ACTTTTTGAA AGAAAAAGTC   
  
  
+ ATCTTTTTTT AGAACCCCTC CTCTGGAAAC TAACCGTATA TATAATATAC CACTCATTAC TTTCTTCTCC   
  
  
+ TGCAAACCAC ACAAACAAAT TAATTTGTTA ATTAATTAAA TTACAGGTTT CGTCTACTTC GCTTCTCCAG   
  
  
+ TAGTCTTCTT CTTCGTATTT ATTTTATAAC CTTTTGTTCT CCACCTCCTC CTCTGTCTCC GAACCCTACG   
  
  
+ TCGTCACCAT TCATTGTTCA CCCGAGACAG CGAGAGAGAG ATAAACGACT CACTTATTAA GTCGTGTTGT   
  
  
+ CGTGTGCTTG TCCTCGACCA GTGACTAAAC ACTTGTAAGA ACCGTTTATG ATGGGTAGTC GGTAGGAGAA   
  
  
+ ATAATGAGGG AGATTGAGGA TGTGAGAAAC TGGGTTAGGT GGAAAGAGTA GGGAAGATAT AAATAAAAAA   
  
  
+ TAGGCAAGTA AAAAATAAAA AAATTTTTAC TTAATTTAAT AAATAAAAAA TTTTAATATT AATAGATAAA   
  
  
+ GAAAAAATTT TCTAATTATT TTTAGTTTGA CTATCTTTTC TACAAATCAA ATAGTTTTAT TAAAAAAAAT   
  
  
+ CTACGGGTAT ATATGATTAA AGTAGTTCAC CCGTACCAAT AACTTAACTA CGATAACCAC ACGGCTTCTT   
  
  
+ TCAATAAGAA GTGTAAACAG CTGCTCCTG  

- GACTGTAACA ATGATCCGTC TCAGACAGAG AGCCTGCCGA AATTCTGCGA ACGCGGAGAG AGAAAGTGCG   
  
  
- AGGCTAAGAG GTGCGTTTTA GACTGAGGAA AAGGGAGAAG GATGGAAAGA GAAAGGGAGA GAGCTAGGGT   
  
  
- TTTGTGATGG TAAGGACGCG TAGTTTTTGA ACTTTTTTCC TGTGAAGCAG TGTCCTATCA ACAGCGACGC   
  
  
- GCGCTCTCTT CTCTGTTATC TCTCTAAAGA CAAACAGTCA TAGAGACAGA GAGAGAAAAG CAAACAAAAA   
  
  
- ACACTGAAAA GAATGAAAGA AAAGAAGAGA GAAGGGAATA GAAAAGAGAT AAGTTAGAAA AATCGCCGTC   
  
  
- AACCGAAAAC AGTACGGGAG GCTGTTATTT AGGGGAACGC AAGAGTGTAG GTCCCACGCG CATGAAATTG   
  
  
- AAATTTTCCA GAGAAGAAAA AAGATACTTT CCGACAAGAA GGCGCACCTT GTTTCTCTCC AGTACTTACC   
  
  
- TGTGCGTAAA CCATCCCCAA TTTCTTTCTT TTCTTAGTTT TACCCCCAAA AAAAAAAAAA TAAAGTAAAA   
  
  
- TTAAAATCTT CTTTACAAAG ATAACTTATT TTTAAATTCA CATTACATAT TATAAAATAA AAATTAAATC   
  
  
- CAAACTTTCC ACATTTTAAA AATCTTTAAA ATAAGTTAAA TTTCCAGGTC AATCATGTAA GACATATTTT   
  
  
- TTTAAAATGA ATAAAATCTT TAAAAATTAT CGTTATTAAA ATAGATAACA TTTTACATTA TTATGACAAA   
  
  
- AAATTACTGT AAATATCACA ATTTAAATTG ATAGGATAAA TGTGTAATAT TGAAAAACTT TCTTTTTCAG   
  
  
- TAGAAAAAAA TCTTGGGGAG GAGACCTTTG ATTGGCATAT ATATTATATG GTGAGTAATG AAAGAAGAGG   
  
  
- ACGTTTGGTG TGTTTGTTTA ATTAAACAAT TAATTAATTT AATGTCCAAA GCAGATGAAG CGAAGAGGTC   
  
  
- ATCAGAAGAA GAAGCATAAA TAAAATATTG GAAAACAAGA GGTGGAGGAG GAGACAGAGG CTTGGGATGC   
  
  
- AGCAGTGGTA AGTAACAAGT GGGCTCTGTC GCTCTCTCTC TATTTGCTGA GTGAATAATT CAGCACAACA   
  
  
- GCACACGAAC AGGAGCTGGT CACTGATTTG TGAACATTCT TGGCAAATAC TACCCATCAG CCATCCTCTT   
  
  
- TATTACTCCC TCTAACTCCT ACACTCTTTG ACCCAATCCA CCTTTCTCAT CCCTTCTATA TTTATTTTTT   
  
  
- ATCCGTTCAT TTTTTATTTT TTTAAAAATG AATTAAATTA TTTATTTTTT AAAATTATAA TTATCTATTT   
  
  
- CTTTTTTAAA AGATTAATAA AAATCAAACT GATAGAAAAG ATGTTTAGTT TATCAAAATA ATTTTTTTTA   
  
  
- GATGCCCATA TATACTAATT TCATCAAGTG GGCATGGTTA TTGAATTGAT GCTATTGGTG TGCCGAAGAA   
  
  
- AGTTATTCTT CACATTTGTC GACGAGGAC

+     MRE

| Site Name | Organism | Position | Strand | Matrix score. | sequence | function |
| --- | --- | --- | --- | --- | --- | --- |
| MRE | Petroselinum crispum | 627 | - | 7 | AACCTAA | MYB binding site involved in light responsiveness |

> 2018/04/13 10:10:12  
+ CTGACATTGT TACTAGGCAG AGTCTGTCTC TCGGACGGCT TTAAGACGCT TGCGCCTCTC TCTTTCACGC   
  
  
+ TCCGATTCTC CACGCAAAAT CTGACTCCTT TTCCCTCTTC CTACCTTTCT CTTTCCCTCT CTCGATCCCA   
  
  
+ AAACACTACC ATTCCTGCGC ATCAAAAACT TGAAAAAAGG ACACTTCGTC ACAGGATAGT TGTCGCTGCG   
  
  
+ CGCGAGAGAA GAGACAATAG AGAGATTTCT GTTTGTCAGT ATCTCTGTCT CTCTCTTTTC GTTTGTTTTT   
  
  
+ TGTGACTTTT CTTACTTTCT TTTCTTCTCT CTTCCCTTAT CTTTTCTCTA TTCAATCTTT TTAGCGGCAG   
  
  
+ TTGGCTTTTG TCATGCCCTC CGACAATAAA TCCCCTTGCG TTCTCACATC CAGGGTGCGC GTACTTTAAC   
  
  
+ TTTAAAAGGT CTCTTCTTTT TTCTATGAAA GGCTGTTCTT CCGCGTGGAA CAAAGAGAGG TCATGAATGG   
  
  
+ ACACGCATTT GGTAGGGGTT AAAGAAAGAA AAGAATCAAA ATGGGGGTTT TTTTTTTTTT ATTTCATTTT   
  
  
+ AATTTTAGAA GAAATGTTTC TATTGAATAA AAATTTAAGT GTAATGTATA ATATTTTATT TTTAATTTAG   
  
  
+ GTTTGAAAGG TGTAAAATTT TTAGAAATTT TATTCAATTT AAAGGTCCAG TTAGTACATT CTGTATAAAA   
  
  
+ AAATTTTACT TATTTTAGAA ATTTTTAATA GCAATAATTT TATCTATTGT AAAATGTAAT AATACTGTTT   
  
  
+ TTTAATGACA TTTATAGTGT TAAATTTAAC TATCCTATTT ACACATTATA ACTTTTTGAA AGAAAAAGTC   
  
  
+ ATCTTTTTTT AGAACCCCTC CTCTGGAAAC TAACCGTATA TATAATATAC CACTCATTAC TTTCTTCTCC   
  
  
+ TGCAAACCAC ACAAACAAAT TAATTTGTTA ATTAATTAAA TTACAGGTTT CGTCTACTTC GCTTCTCCAG   
  
  
+ TAGTCTTCTT CTTCGTATTT ATTTTATAAC CTTTTGTTCT CCACCTCCTC CTCTGTCTCC GAACCCTACG   
  
  
+ TCGTCACCAT TCATTGTTCA CCCGAGACAG CGAGAGAGAG ATAAACGACT CACTTATTAA GTCGTGTTGT   
  
  
+ CGTGTGCTTG TCCTCGACCA GTGACTAAAC ACTTGTAAGA ACCGTTTATG ATGGGTAGTC GGTAGGAGAA   
  
  
+ ATAATGAGGG AGATTGAGGA TGTGAGAAAC TGGGTTAGGT GGAAAGAGTA GGGAAGATAT AAATAAAAAA   
  
  
+ TAGGCAAGTA AAAAATAAAA AAATTTTTAC TTAATTTAAT AAATAAAAAA TTTTAATATT AATAGATAAA   
  
  
+ GAAAAAATTT TCTAATTATT TTTAGTTTGA CTATCTTTTC TACAAATCAA ATAGTTTTAT TAAAAAAAAT   
  
  
+ CTACGGGTAT ATATGATTAA AGTAGTTCAC CCGTACCAAT AACTTAACTA CGATAACCAC ACGGCTTCTT   
  
  
+ TCAATAAGAA GTGTAAACAG CTGCTCCTG  

- GACTGTAACA ATGATCCGTC TCAGACAGAG AGCCTGCCGA AATTCTGCGA ACGCGGAGAG AGAAAGTGCG   
  
  
- AGGCTAAGAG GTGCGTTTTA GACTGAGGAA AAGGGAGAAG GATGGAAAGA GAAAGGGAGA GAGCTAGGGT   
  
  
- TTTGTGATGG TAAGGACGCG TAGTTTTTGA ACTTTTTTCC TGTGAAGCAG TGTCCTATCA ACAGCGACGC   
  
  
- GCGCTCTCTT CTCTGTTATC TCTCTAAAGA CAAACAGTCA TAGAGACAGA GAGAGAAAAG CAAACAAAAA   
  
  
- ACACTGAAAA GAATGAAAGA AAAGAAGAGA GAAGGGAATA GAAAAGAGAT AAGTTAGAAA AATCGCCGTC   
  
  
- AACCGAAAAC AGTACGGGAG GCTGTTATTT AGGGGAACGC AAGAGTGTAG GTCCCACGCG CATGAAATTG   
  
  
- AAATTTTCCA GAGAAGAAAA AAGATACTTT CCGACAAGAA GGCGCACCTT GTTTCTCTCC AGTACTTACC   
  
  
- TGTGCGTAAA CCATCCCCAA TTTCTTTCTT TTCTTAGTTT TACCCCCAAA AAAAAAAAAA TAAAGTAAAA   
  
  
- TTAAAATCTT CTTTACAAAG ATAACTTATT TTTAAATTCA CATTACATAT TATAAAATAA AAATTAAATC   
  
  
- CAAACTTTCC ACATTTTAAA AATCTTTAAA ATAAGTTAAA TTTCCAGGTC AATCATGTAA GACATATTTT   
  
  
- TTTAAAATGA ATAAAATCTT TAAAAATTAT CGTTATTAAA ATAGATAACA TTTTACATTA TTATGACAAA   
  
  
- AAATTACTGT AAATATCACA ATTTAAATTG ATAGGATAAA TGTGTAATAT TGAAAAACTT TCTTTTTCAG   
  
  
- TAGAAAAAAA TCTTGGGGAG GAGACCTTTG ATTGGCATAT ATATTATATG GTGAGTAATG AAAGAAGAGG   
  
  
- ACGTTTGGTG TGTTTGTTTA ATTAAACAAT TAATTAATTT AATGTCCAAA GCAGATGAAG CGAAGAGGTC   
  
  
- ATCAGAAGAA GAAGCATAAA TAAAATATTG GAAAACAAGA GGTGGAGGAG GAGACAGAGG CTTGGGATGC   
  
  
- AGCAGTGGTA AGTAACAAGT GGGCTCTGTC GCTCTCTCTC TATTTGCTGA GTGAATAATT CAGCACAACA   
  
  
- GCACACGAAC AGGAGCTGGT CACTGATTTG TGAACATTCT TGGCAAATAC TACCCATCAG CCATCCTCTT   
  
  
- TATTACTCCC TCTAACTCCT ACACTCTTTG ACCCAATCCA CCTTTCTCAT CCCTTCTATA TTTATTTTTT   
  
  
- ATCCGTTCAT TTTTTATTTT TTTAAAAATG AATTAAATTA TTTATTTTTT AAAATTATAA TTATCTATTT   
  
  
- CTTTTTTAAA AGATTAATAA AAATCAAACT GATAGAAAAG ATGTTTAGTT TATCAAAATA ATTTTTTTTA   
  
  
- GATGCCCATA TATACTAATT TCATCAAGTG GGCATGGTTA TTGAATTGAT GCTATTGGTG TGCCGAAGAA   
  
  
- AGTTATTCTT CACATTTGTC GACGAGGAC

+     P-box

| Site Name | Organism | Position | Strand | Matrix score. | sequence | function |
| --- | --- | --- | --- | --- | --- | --- |
| P-box | Oryza sativa | 1010 | + | 7 | CCTTTTG | gibberellin-responsive element |

> 2018/04/13 10:10:12  
+ CTGACATTGT TACTAGGCAG AGTCTGTCTC TCGGACGGCT TTAAGACGCT TGCGCCTCTC TCTTTCACGC   
  
  
+ TCCGATTCTC CACGCAAAAT CTGACTCCTT TTCCCTCTTC CTACCTTTCT CTTTCCCTCT CTCGATCCCA   
  
  
+ AAACACTACC ATTCCTGCGC ATCAAAAACT TGAAAAAAGG ACACTTCGTC ACAGGATAGT TGTCGCTGCG   
  
  
+ CGCGAGAGAA GAGACAATAG AGAGATTTCT GTTTGTCAGT ATCTCTGTCT CTCTCTTTTC GTTTGTTTTT   
  
  
+ TGTGACTTTT CTTACTTTCT TTTCTTCTCT CTTCCCTTAT CTTTTCTCTA TTCAATCTTT TTAGCGGCAG   
  
  
+ TTGGCTTTTG TCATGCCCTC CGACAATAAA TCCCCTTGCG TTCTCACATC CAGGGTGCGC GTACTTTAAC   
  
  
+ TTTAAAAGGT CTCTTCTTTT TTCTATGAAA GGCTGTTCTT CCGCGTGGAA CAAAGAGAGG TCATGAATGG   
  
  
+ ACACGCATTT GGTAGGGGTT AAAGAAAGAA AAGAATCAAA ATGGGGGTTT TTTTTTTTTT ATTTCATTTT   
  
  
+ AATTTTAGAA GAAATGTTTC TATTGAATAA AAATTTAAGT GTAATGTATA ATATTTTATT TTTAATTTAG   
  
  
+ GTTTGAAAGG TGTAAAATTT TTAGAAATTT TATTCAATTT AAAGGTCCAG TTAGTACATT CTGTATAAAA   
  
  
+ AAATTTTACT TATTTTAGAA ATTTTTAATA GCAATAATTT TATCTATTGT AAAATGTAAT AATACTGTTT   
  
  
+ TTTAATGACA TTTATAGTGT TAAATTTAAC TATCCTATTT ACACATTATA ACTTTTTGAA AGAAAAAGTC   
  
  
+ ATCTTTTTTT AGAACCCCTC CTCTGGAAAC TAACCGTATA TATAATATAC CACTCATTAC TTTCTTCTCC   
  
  
+ TGCAAACCAC ACAAACAAAT TAATTTGTTA ATTAATTAAA TTACAGGTTT CGTCTACTTC GCTTCTCCAG   
  
  
+ TAGTCTTCTT CTTCGTATTT ATTTTATAAC CTTTTGTTCT CCACCTCCTC CTCTGTCTCC GAACCCTACG   
  
  
+ TCGTCACCAT TCATTGTTCA CCCGAGACAG CGAGAGAGAG ATAAACGACT CACTTATTAA GTCGTGTTGT   
  
  
+ CGTGTGCTTG TCCTCGACCA GTGACTAAAC ACTTGTAAGA ACCGTTTATG ATGGGTAGTC GGTAGGAGAA   
  
  
+ ATAATGAGGG AGATTGAGGA TGTGAGAAAC TGGGTTAGGT GGAAAGAGTA GGGAAGATAT AAATAAAAAA   
  
  
+ TAGGCAAGTA AAAAATAAAA AAATTTTTAC TTAATTTAAT AAATAAAAAA TTTTAATATT AATAGATAAA   
  
  
+ GAAAAAATTT TCTAATTATT TTTAGTTTGA CTATCTTTTC TACAAATCAA ATAGTTTTAT TAAAAAAAAT   
  
  
+ CTACGGGTAT ATATGATTAA AGTAGTTCAC CCGTACCAAT AACTTAACTA CGATAACCAC ACGGCTTCTT   
  
  
+ TCAATAAGAA GTGTAAACAG CTGCTCCTG  

- GACTGTAACA ATGATCCGTC TCAGACAGAG AGCCTGCCGA AATTCTGCGA ACGCGGAGAG AGAAAGTGCG   
  
  
- AGGCTAAGAG GTGCGTTTTA GACTGAGGAA AAGGGAGAAG GATGGAAAGA GAAAGGGAGA GAGCTAGGGT   
  
  
- TTTGTGATGG TAAGGACGCG TAGTTTTTGA ACTTTTTTCC TGTGAAGCAG TGTCCTATCA ACAGCGACGC   
  
  
- GCGCTCTCTT CTCTGTTATC TCTCTAAAGA CAAACAGTCA TAGAGACAGA GAGAGAAAAG CAAACAAAAA   
  
  
- ACACTGAAAA GAATGAAAGA AAAGAAGAGA GAAGGGAATA GAAAAGAGAT AAGTTAGAAA AATCGCCGTC   
  
  
- AACCGAAAAC AGTACGGGAG GCTGTTATTT AGGGGAACGC AAGAGTGTAG GTCCCACGCG CATGAAATTG   
  
  
- AAATTTTCCA GAGAAGAAAA AAGATACTTT CCGACAAGAA GGCGCACCTT GTTTCTCTCC AGTACTTACC   
  
  
- TGTGCGTAAA CCATCCCCAA TTTCTTTCTT TTCTTAGTTT TACCCCCAAA AAAAAAAAAA TAAAGTAAAA   
  
  
- TTAAAATCTT CTTTACAAAG ATAACTTATT TTTAAATTCA CATTACATAT TATAAAATAA AAATTAAATC   
  
  
- CAAACTTTCC ACATTTTAAA AATCTTTAAA ATAAGTTAAA TTTCCAGGTC AATCATGTAA GACATATTTT   
  
  
- TTTAAAATGA ATAAAATCTT TAAAAATTAT CGTTATTAAA ATAGATAACA TTTTACATTA TTATGACAAA   
  
  
- AAATTACTGT AAATATCACA ATTTAAATTG ATAGGATAAA TGTGTAATAT TGAAAAACTT TCTTTTTCAG   
  
  
- TAGAAAAAAA TCTTGGGGAG GAGACCTTTG ATTGGCATAT ATATTATATG GTGAGTAATG AAAGAAGAGG   
  
  
- ACGTTTGGTG TGTTTGTTTA ATTAAACAAT TAATTAATTT AATGTCCAAA GCAGATGAAG CGAAGAGGTC   
  
  
- ATCAGAAGAA GAAGCATAAA TAAAATATTG GAAAACAAGA GGTGGAGGAG GAGACAGAGG CTTGGGATGC   
  
  
- AGCAGTGGTA AGTAACAAGT GGGCTCTGTC GCTCTCTCTC TATTTGCTGA GTGAATAATT CAGCACAACA   
  
  
- GCACACGAAC AGGAGCTGGT CACTGATTTG TGAACATTCT TGGCAAATAC TACCCATCAG CCATCCTCTT   
  
  
- TATTACTCCC TCTAACTCCT ACACTCTTTG ACCCAATCCA CCTTTCTCAT CCCTTCTATA TTTATTTTTT   
  
  
- ATCCGTTCAT TTTTTATTTT TTTAAAAATG AATTAAATTA TTTATTTTTT AAAATTATAA TTATCTATTT   
  
  
- CTTTTTTAAA AGATTAATAA AAATCAAACT GATAGAAAAG ATGTTTAGTT TATCAAAATA ATTTTTTTTA   
  
  
- GATGCCCATA TATACTAATT TCATCAAGTG GGCATGGTTA TTGAATTGAT GCTATTGGTG TGCCGAAGAA   
  
  
- AGTTATTCTT CACATTTGTC GACGAGGAC

+     Skn-1\_motif

| Site Name | Organism | Position | Strand | Matrix score. | sequence | function |
| --- | --- | --- | --- | --- | --- | --- |
| Skn-1\_motif | Oryza sativa | 775 | - | 5 | GTCAT | cis-acting regulatory element required for endosperm expression |
| Skn-1\_motif | Oryza sativa | 360 | + | 5 | GTCAT | cis-acting regulatory element required for endosperm expression |
| Skn-1\_motif | Oryza sativa | 480 | + | 5 | GTCAT | cis-acting regulatory element required for endosperm expression |
| Skn-1\_motif | Oryza sativa | 838 | + | 5 | GTCAT | cis-acting regulatory element required for endosperm expression |

> 2018/04/13 10:10:12  
+ CTGACATTGT TACTAGGCAG AGTCTGTCTC TCGGACGGCT TTAAGACGCT TGCGCCTCTC TCTTTCACGC   
  
  
+ TCCGATTCTC CACGCAAAAT CTGACTCCTT TTCCCTCTTC CTACCTTTCT CTTTCCCTCT CTCGATCCCA   
  
  
+ AAACACTACC ATTCCTGCGC ATCAAAAACT TGAAAAAAGG ACACTTCGTC ACAGGATAGT TGTCGCTGCG   
  
  
+ CGCGAGAGAA GAGACAATAG AGAGATTTCT GTTTGTCAGT ATCTCTGTCT CTCTCTTTTC GTTTGTTTTT   
  
  
+ TGTGACTTTT CTTACTTTCT TTTCTTCTCT CTTCCCTTAT CTTTTCTCTA TTCAATCTTT TTAGCGGCAG   
  
  
+ TTGGCTTTTG TCATGCCCTC CGACAATAAA TCCCCTTGCG TTCTCACATC CAGGGTGCGC GTACTTTAAC   
  
  
+ TTTAAAAGGT CTCTTCTTTT TTCTATGAAA GGCTGTTCTT CCGCGTGGAA CAAAGAGAGG TCATGAATGG   
  
  
+ ACACGCATTT GGTAGGGGTT AAAGAAAGAA AAGAATCAAA ATGGGGGTTT TTTTTTTTTT ATTTCATTTT   
  
  
+ AATTTTAGAA GAAATGTTTC TATTGAATAA AAATTTAAGT GTAATGTATA ATATTTTATT TTTAATTTAG   
  
  
+ GTTTGAAAGG TGTAAAATTT TTAGAAATTT TATTCAATTT AAAGGTCCAG TTAGTACATT CTGTATAAAA   
  
  
+ AAATTTTACT TATTTTAGAA ATTTTTAATA GCAATAATTT TATCTATTGT AAAATGTAAT AATACTGTTT   
  
  
+ TTTAATGACA TTTATAGTGT TAAATTTAAC TATCCTATTT ACACATTATA ACTTTTTGAA AGAAAAAGTC   
  
  
+ ATCTTTTTTT AGAACCCCTC CTCTGGAAAC TAACCGTATA TATAATATAC CACTCATTAC TTTCTTCTCC   
  
  
+ TGCAAACCAC ACAAACAAAT TAATTTGTTA ATTAATTAAA TTACAGGTTT CGTCTACTTC GCTTCTCCAG   
  
  
+ TAGTCTTCTT CTTCGTATTT ATTTTATAAC CTTTTGTTCT CCACCTCCTC CTCTGTCTCC GAACCCTACG   
  
  
+ TCGTCACCAT TCATTGTTCA CCCGAGACAG CGAGAGAGAG ATAAACGACT CACTTATTAA GTCGTGTTGT   
  
  
+ CGTGTGCTTG TCCTCGACCA GTGACTAAAC ACTTGTAAGA ACCGTTTATG ATGGGTAGTC GGTAGGAGAA   
  
  
+ ATAATGAGGG AGATTGAGGA TGTGAGAAAC TGGGTTAGGT GGAAAGAGTA GGGAAGATAT AAATAAAAAA   
  
  
+ TAGGCAAGTA AAAAATAAAA AAATTTTTAC TTAATTTAAT AAATAAAAAA TTTTAATATT AATAGATAAA   
  
  
+ GAAAAAATTT TCTAATTATT TTTAGTTTGA CTATCTTTTC TACAAATCAA ATAGTTTTAT TAAAAAAAAT   
  
  
+ CTACGGGTAT ATATGATTAA AGTAGTTCAC CCGTACCAAT AACTTAACTA CGATAACCAC ACGGCTTCTT   
  
  
+ TCAATAAGAA GTGTAAACAG CTGCTCCTG  

- GACTGTAACA ATGATCCGTC TCAGACAGAG AGCCTGCCGA AATTCTGCGA ACGCGGAGAG AGAAAGTGCG   
  
  
- AGGCTAAGAG GTGCGTTTTA GACTGAGGAA AAGGGAGAAG GATGGAAAGA GAAAGGGAGA GAGCTAGGGT   
  
  
- TTTGTGATGG TAAGGACGCG TAGTTTTTGA ACTTTTTTCC TGTGAAGCAG TGTCCTATCA ACAGCGACGC   
  
  
- GCGCTCTCTT CTCTGTTATC TCTCTAAAGA CAAACAGTCA TAGAGACAGA GAGAGAAAAG CAAACAAAAA   
  
  
- ACACTGAAAA GAATGAAAGA AAAGAAGAGA GAAGGGAATA GAAAAGAGAT AAGTTAGAAA AATCGCCGTC   
  
  
- AACCGAAAAC AGTACGGGAG GCTGTTATTT AGGGGAACGC AAGAGTGTAG GTCCCACGCG CATGAAATTG   
  
  
- AAATTTTCCA GAGAAGAAAA AAGATACTTT CCGACAAGAA GGCGCACCTT GTTTCTCTCC AGTACTTACC   
  
  
- TGTGCGTAAA CCATCCCCAA TTTCTTTCTT TTCTTAGTTT TACCCCCAAA AAAAAAAAAA TAAAGTAAAA   
  
  
- TTAAAATCTT CTTTACAAAG ATAACTTATT TTTAAATTCA CATTACATAT TATAAAATAA AAATTAAATC   
  
  
- CAAACTTTCC ACATTTTAAA AATCTTTAAA ATAAGTTAAA TTTCCAGGTC AATCATGTAA GACATATTTT   
  
  
- TTTAAAATGA ATAAAATCTT TAAAAATTAT CGTTATTAAA ATAGATAACA TTTTACATTA TTATGACAAA   
  
  
- AAATTACTGT AAATATCACA ATTTAAATTG ATAGGATAAA TGTGTAATAT TGAAAAACTT TCTTTTTCAG   
  
  
- TAGAAAAAAA TCTTGGGGAG GAGACCTTTG ATTGGCATAT ATATTATATG GTGAGTAATG AAAGAAGAGG   
  
  
- ACGTTTGGTG TGTTTGTTTA ATTAAACAAT TAATTAATTT AATGTCCAAA GCAGATGAAG CGAAGAGGTC   
  
  
- ATCAGAAGAA GAAGCATAAA TAAAATATTG GAAAACAAGA GGTGGAGGAG GAGACAGAGG CTTGGGATGC   
  
  
- AGCAGTGGTA AGTAACAAGT GGGCTCTGTC GCTCTCTCTC TATTTGCTGA GTGAATAATT CAGCACAACA   
  
  
- GCACACGAAC AGGAGCTGGT CACTGATTTG TGAACATTCT TGGCAAATAC TACCCATCAG CCATCCTCTT   
  
  
- TATTACTCCC TCTAACTCCT ACACTCTTTG ACCCAATCCA CCTTTCTCAT CCCTTCTATA TTTATTTTTT   
  
  
- ATCCGTTCAT TTTTTATTTT TTTAAAAATG AATTAAATTA TTTATTTTTT AAAATTATAA TTATCTATTT   
  
  
- CTTTTTTAAA AGATTAATAA AAATCAAACT GATAGAAAAG ATGTTTAGTT TATCAAAATA ATTTTTTTTA   
  
  
- GATGCCCATA TATACTAATT TCATCAAGTG GGCATGGTTA TTGAATTGAT GCTATTGGTG TGCCGAAGAA   
  
  
- AGTTATTCTT CACATTTGTC GACGAGGAC

+     TATA-box

| Site Name | Organism | Position | Strand | Matrix score. | sequence | function |
| --- | --- | --- | --- | --- | --- | --- |
| TATA-box | Arabidopsis thaliana | 816 | - | 5 | TATAA | core promoter element around -30 of transcription start |
| TATA-box | Ac | 780 | - | 7 | TATAAAT | core promoter element around -30 of transcription start |
| TATA-box | Lycopersicon esculentum | 750 | - | 5 | TTTTA | core promoter element around -30 of transcription start |
| TATA-box | Arabidopsis thaliana | 607 | + | 4 | TATA | core promoter element around -30 of transcription start |
| TATA-box | Lycopersicon esculentum | 588 | - | 5 | TTTTA | core promoter element around -30 of transcription start |
| TATA-box | Lycopersicon esculentum | 649 | + | 5 | TTTTA | core promoter element around -30 of transcription start |
| TATA-box | Glycine max | 726 | + | 5 | TAATA | core promoter element around -30 of transcription start |
| TATA-box | Lycopersicon esculentum | 1350 | + | 5 | TTTTA | core promoter element around -30 of transcription start |
| TATA-box | Lycopersicon esculentum | 1385 | + | 5 | TTTTA | core promoter element around -30 of transcription start |
| TATA-box | Glycine max | 609 | + | 5 | TAATA | core promoter element around -30 of transcription start |
| TATA-box | Glycine max | 1388 | - | 5 | TAATA | core promoter element around -30 of transcription start |
| TATA-box | Arabidopsis thaliana | 1003 | - | 6 | TATAAA | core promoter element around -30 of transcription start |
| TATA-box | Arabidopsis thaliana | 1002 | - | 7 | TATAAAA | core promoter element around -30 of transcription start |
| TATA-box | Lycopersicon esculentum | 614 | + | 5 | TTTTA | core promoter element around -30 of transcription start |
| TATA-box | Lycopersicon esculentum | 738 | + | 5 | TTTTA | core promoter element around -30 of transcription start |
| TATA-box | Daucus carota | 666 | - | 9 | ccTATAAATT | core promoter element around -30 of transcription start |
| TATA-box | Lycopersicon esculentum | 658 | + | 5 | TTTTA | core promoter element around -30 of transcription start |
| TATA-box | Arabidopsis thaliana | 1005 | - | 4 | TATA | core promoter element around -30 of transcription start |
| TATA-box | Glycine max | 883 | + | 5 | TAATA | core promoter element around -30 of transcription start |
| TATA-box | Pisum sativum | 1001 | - | 8 | TATAAAAT | core promoter element around -30 of transcription start |
| TATA-box | Arabidopsis thaliana | 694 | + | 6 | TATAAA | core promoter element around -30 of transcription start |
| TATA-box | Arabidopsis thaliana | 881 | - | 4 | TATA | core promoter element around -30 of transcription start |
| TATA-box | Helianthus annuus | 692 | - | 6 | TATACA | core promoter element around -30 of transcription start |
| TATA-box | Lycopersicon esculentum | 423 | - | 5 | TTTTA | core promoter element around -30 of transcription start |
| TATA-box | Arabidopsis thaliana | 782 | - | 5 | TATAA | core promoter element around -30 of transcription start |
| TATA-box | Lycopersicon esculentum | 704 | + | 5 | TTTTA | core promoter element around -30 of transcription start |
| TATA-box | Arabidopsis thaliana | 886 | - | 4 | TATA | core promoter element around -30 of transcription start |
| TATA-box | Arabidopsis thaliana | 879 | - | 4 | TATA | core promoter element around -30 of transcription start |
| TATA-box | Lycopersicon esculentum | 547 | + | 5 | TTTTA | core promoter element around -30 of transcription start |
| TATA-box | Lycopersicon esculentum | 1276 | - | 5 | TTTTA | core promoter element around -30 of transcription start |
| TATA-box | Brassica napus | 1409 | - | 6 | ATATAT | core promoter element around -30 of transcription start |
| TATA-box | Lycopersicon esculentum | 620 | + | 5 | TTTTA | core promoter element around -30 of transcription start |
| TATA-box | Glycine max | 757 | + | 5 | TAATA | core promoter element around -30 of transcription start |
| TATA-box | Glycine max | 760 | + | 5 | TAATA | core promoter element around -30 of transcription start |
| TATA-box | Glycine max | 1317 | - | 5 | TAATA | core promoter element around -30 of transcription start |
| TATA-box | Helianthus annuus | 605 | - | 6 | TATACA | core promoter element around -30 of transcription start |
| TATA-box | Lycopersicon esculentum | 1391 | - | 5 | TTTTA | core promoter element around -30 of transcription start |
| TATA-box | Brassica oleracea | 880 | + | 7 | ATATAAT | core promoter element around -30 of transcription start |
| TATA-box | Lycopersicon esculentum | 1254 | - | 5 | TTTTA | core promoter element around -30 of transcription start |
| TATA-box | Brassica napus | 878 | - | 6 | ATATAT | core promoter element around -30 of transcription start |
| TATA-box | Glycine max | 1314 | + | 5 | TAATA | core promoter element around -30 of transcription start |
| TATA-box | Arabidopsis thaliana | 616 | - | 9 | TAAAAATAA | core promoter element around -30 of transcription start |
| TATA-box | Arabidopsis thaliana | 817 | - | 4 | TATA | core promoter element around -30 of transcription start |
| TATA-box | Brassica napus | 815 | + | 6 | ATTATA | core promoter element around -30 of transcription start |
| TATA-box | Lycopersicon esculentum | 713 | + | 5 | TTTTA | core promoter element around -30 of transcription start |
| TATA-box | Arabidopsis thaliana | 877 | - | 8 | TATATATA | core promoter element around -30 of transcription start |
| TATA-box | Lycopersicon esculentum | 563 | + | 5 | TTTTA | core promoter element around -30 of transcription start |
| TATA-box | Arabidopsis thaliana | 1004 | - | 5 | TATAA | core promoter element around -30 of transcription start |
| TATA-box | Brassica oleracea | 1247 | + | 6 | ATATAA | core promoter element around -30 of transcription start |
| TATA-box | Lycopersicon esculentum | 339 | + | 5 | TTTTA | core promoter element around -30 of transcription start |
| TATA-box | Arabidopsis thaliana | 781 | - | 6 | TATAAA | core promoter element around -30 of transcription start |
| TATA-box | Lycopersicon esculentum | 1269 | - | 5 | TTTTA | core promoter element around -30 of transcription start |
| TATA-box | Lycopersicon esculentum | 696 | - | 5 | TTTTA | core promoter element around -30 of transcription start |
| TATA-box | Arabidopsis thaliana | 1346 | - | 9 | TAAAAATAA | core promoter element around -30 of transcription start |
| TATA-box | Lycopersicon esculentum | 643 | - | 5 | TTTTA | core promoter element around -30 of transcription start |
| TATA-box | Arabidopsis thaliana | 1410 | - | 4 | TATA | core promoter element around -30 of transcription start |
| TATA-box | Lycopersicon esculentum | 557 | + | 5 | TTTTA | core promoter element around -30 of transcription start |
| TATA-box | Arabidopsis thaliana | 1408 | - | 4 | TATA | core promoter element around -30 of transcription start |
| TATA-box | Lycopersicon esculentum | 847 | + | 5 | TTTTA | core promoter element around -30 of transcription start |
| TATA-box | Glycine max | 1105 | - | 5 | TAATA | core promoter element around -30 of transcription start |
| TATA-box | Ac | 1248 | + | 7 | TATAAAT | core promoter element around -30 of transcription start |
| TATA-box | Lycopersicon esculentum | 723 | + | 5 | TTTTA | core promoter element around -30 of transcription start |
| TATA-box | Lycopersicon esculentum | 770 | + | 5 | TTTTA | core promoter element around -30 of transcription start |
| TATA-box | Lycopersicon esculentum | 1285 | + | 5 | TTTTA | core promoter element around -30 of transcription start |
| TATA-box | Glycine max | 1320 | + | 5 | TAATA | core promoter element around -30 of transcription start |
| TATA-box | Arabidopsis thaliana | 783 | - | 4 | TATA | core promoter element around -30 of transcription start |
| TATA-box | Lycopersicon esculentum | 1304 | - | 5 | TTTTA | core promoter element around -30 of transcription start |
| TATA-box | Glycine max | 1297 | + | 5 | TAATA | core promoter element around -30 of transcription start |
| TATA-box | Lycopersicon esculentum | 1311 | + | 5 | TTTTA | core promoter element around -30 of transcription start |

> 2018/04/13 10:10:12  
+ CTGACATTGT TACTAGGCAG AGTCTGTCTC TCGGACGGCT TTAAGACGCT TGCGCCTCTC TCTTTCACGC   
  
  
+ TCCGATTCTC CACGCAAAAT CTGACTCCTT TTCCCTCTTC CTACCTTTCT CTTTCCCTCT CTCGATCCCA   
  
  
+ AAACACTACC ATTCCTGCGC ATCAAAAACT TGAAAAAAGG ACACTTCGTC ACAGGATAGT TGTCGCTGCG   
  
  
+ CGCGAGAGAA GAGACAATAG AGAGATTTCT GTTTGTCAGT ATCTCTGTCT CTCTCTTTTC GTTTGTTTTT   
  
  
+ TGTGACTTTT CTTACTTTCT TTTCTTCTCT CTTCCCTTAT CTTTTCTCTA TTCAATCTTT TTAGCGGCAG   
  
  
+ TTGGCTTTTG TCATGCCCTC CGACAATAAA TCCCCTTGCG TTCTCACATC CAGGGTGCGC GTACTTTAAC   
  
  
+ TTTAAAAGGT CTCTTCTTTT TTCTATGAAA GGCTGTTCTT CCGCGTGGAA CAAAGAGAGG TCATGAATGG   
  
  
+ ACACGCATTT GGTAGGGGTT AAAGAAAGAA AAGAATCAAA ATGGGGGTTT TTTTTTTTTT ATTTCATTTT   
  
  
+ AATTTTAGAA GAAATGTTTC TATTGAATAA AAATTTAAGT GTAATGTATA ATATTTTATT TTTAATTTAG   
  
  
+ GTTTGAAAGG TGTAAAATTT TTAGAAATTT TATTCAATTT AAAGGTCCAG TTAGTACATT CTGTATAAAA   
  
  
+ AAATTTTACT TATTTTAGAA ATTTTTAATA GCAATAATTT TATCTATTGT AAAATGTAAT AATACTGTTT   
  
  
+ TTTAATGACA TTTATAGTGT TAAATTTAAC TATCCTATTT ACACATTATA ACTTTTTGAA AGAAAAAGTC   
  
  
+ ATCTTTTTTT AGAACCCCTC CTCTGGAAAC TAACCGTATA TATAATATAC CACTCATTAC TTTCTTCTCC   
  
  
+ TGCAAACCAC ACAAACAAAT TAATTTGTTA ATTAATTAAA TTACAGGTTT CGTCTACTTC GCTTCTCCAG   
  
  
+ TAGTCTTCTT CTTCGTATTT ATTTTATAAC CTTTTGTTCT CCACCTCCTC CTCTGTCTCC GAACCCTACG   
  
  
+ TCGTCACCAT TCATTGTTCA CCCGAGACAG CGAGAGAGAG ATAAACGACT CACTTATTAA GTCGTGTTGT   
  
  
+ CGTGTGCTTG TCCTCGACCA GTGACTAAAC ACTTGTAAGA ACCGTTTATG ATGGGTAGTC GGTAGGAGAA   
  
  
+ ATAATGAGGG AGATTGAGGA TGTGAGAAAC TGGGTTAGGT GGAAAGAGTA GGGAAGATAT AAATAAAAAA   
  
  
+ TAGGCAAGTA AAAAATAAAA AAATTTTTAC TTAATTTAAT AAATAAAAAA TTTTAATATT AATAGATAAA   
  
  
+ GAAAAAATTT TCTAATTATT TTTAGTTTGA CTATCTTTTC TACAAATCAA ATAGTTTTAT TAAAAAAAAT   
  
  
+ CTACGGGTAT ATATGATTAA AGTAGTTCAC CCGTACCAAT AACTTAACTA CGATAACCAC ACGGCTTCTT   
  
  
+ TCAATAAGAA GTGTAAACAG CTGCTCCTG  

- GACTGTAACA ATGATCCGTC TCAGACAGAG AGCCTGCCGA AATTCTGCGA ACGCGGAGAG AGAAAGTGCG   
  
  
- AGGCTAAGAG GTGCGTTTTA GACTGAGGAA AAGGGAGAAG GATGGAAAGA GAAAGGGAGA GAGCTAGGGT   
  
  
- TTTGTGATGG TAAGGACGCG TAGTTTTTGA ACTTTTTTCC TGTGAAGCAG TGTCCTATCA ACAGCGACGC   
  
  
- GCGCTCTCTT CTCTGTTATC TCTCTAAAGA CAAACAGTCA TAGAGACAGA GAGAGAAAAG CAAACAAAAA   
  
  
- ACACTGAAAA GAATGAAAGA AAAGAAGAGA GAAGGGAATA GAAAAGAGAT AAGTTAGAAA AATCGCCGTC   
  
  
- AACCGAAAAC AGTACGGGAG GCTGTTATTT AGGGGAACGC AAGAGTGTAG GTCCCACGCG CATGAAATTG   
  
  
- AAATTTTCCA GAGAAGAAAA AAGATACTTT CCGACAAGAA GGCGCACCTT GTTTCTCTCC AGTACTTACC   
  
  
- TGTGCGTAAA CCATCCCCAA TTTCTTTCTT TTCTTAGTTT TACCCCCAAA AAAAAAAAAA TAAAGTAAAA   
  
  
- TTAAAATCTT CTTTACAAAG ATAACTTATT TTTAAATTCA CATTACATAT TATAAAATAA AAATTAAATC   
  
  
- CAAACTTTCC ACATTTTAAA AATCTTTAAA ATAAGTTAAA TTTCCAGGTC AATCATGTAA GACATATTTT   
  
  
- TTTAAAATGA ATAAAATCTT TAAAAATTAT CGTTATTAAA ATAGATAACA TTTTACATTA TTATGACAAA   
  
  
- AAATTACTGT AAATATCACA ATTTAAATTG ATAGGATAAA TGTGTAATAT TGAAAAACTT TCTTTTTCAG   
  
  
- TAGAAAAAAA TCTTGGGGAG GAGACCTTTG ATTGGCATAT ATATTATATG GTGAGTAATG AAAGAAGAGG   
  
  
- ACGTTTGGTG TGTTTGTTTA ATTAAACAAT TAATTAATTT AATGTCCAAA GCAGATGAAG CGAAGAGGTC   
  
  
- ATCAGAAGAA GAAGCATAAA TAAAATATTG GAAAACAAGA GGTGGAGGAG GAGACAGAGG CTTGGGATGC   
  
  
- AGCAGTGGTA AGTAACAAGT GGGCTCTGTC GCTCTCTCTC TATTTGCTGA GTGAATAATT CAGCACAACA   
  
  
- GCACACGAAC AGGAGCTGGT CACTGATTTG TGAACATTCT TGGCAAATAC TACCCATCAG CCATCCTCTT   
  
  
- TATTACTCCC TCTAACTCCT ACACTCTTTG ACCCAATCCA CCTTTCTCAT CCCTTCTATA TTTATTTTTT   
  
  
- ATCCGTTCAT TTTTTATTTT TTTAAAAATG AATTAAATTA TTTATTTTTT AAAATTATAA TTATCTATTT   
  
  
- CTTTTTTAAA AGATTAATAA AAATCAAACT GATAGAAAAG ATGTTTAGTT TATCAAAATA ATTTTTTTTA   
  
  
- GATGCCCATA TATACTAATT TCATCAAGTG GGCATGGTTA TTGAATTGAT GCTATTGGTG TGCCGAAGAA   
  
  
- AGTTATTCTT CACATTTGTC GACGAGGAC

+     TC-rich repeats

| Site Name | Organism | Position | Strand | Matrix score. | sequence | function |
| --- | --- | --- | --- | --- | --- | --- |
| TC-rich repeats | Nicotiana tabacum | 657 | + | 9 | ATTTTCTTCA | cis-acting element involved in defense and stress responsiveness |
| TC-rich repeats | Nicotiana tabacum | 286 | + | 9 | GTTTTCTTAC | cis-acting element involved in defense and stress responsiveness |

> 2018/04/13 10:10:12  
+ CTGACATTGT TACTAGGCAG AGTCTGTCTC TCGGACGGCT TTAAGACGCT TGCGCCTCTC TCTTTCACGC   
  
  
+ TCCGATTCTC CACGCAAAAT CTGACTCCTT TTCCCTCTTC CTACCTTTCT CTTTCCCTCT CTCGATCCCA   
  
  
+ AAACACTACC ATTCCTGCGC ATCAAAAACT TGAAAAAAGG ACACTTCGTC ACAGGATAGT TGTCGCTGCG   
  
  
+ CGCGAGAGAA GAGACAATAG AGAGATTTCT GTTTGTCAGT ATCTCTGTCT CTCTCTTTTC GTTTGTTTTT   
  
  
+ TGTGACTTTT CTTACTTTCT TTTCTTCTCT CTTCCCTTAT CTTTTCTCTA TTCAATCTTT TTAGCGGCAG   
  
  
+ TTGGCTTTTG TCATGCCCTC CGACAATAAA TCCCCTTGCG TTCTCACATC CAGGGTGCGC GTACTTTAAC   
  
  
+ TTTAAAAGGT CTCTTCTTTT TTCTATGAAA GGCTGTTCTT CCGCGTGGAA CAAAGAGAGG TCATGAATGG   
  
  
+ ACACGCATTT GGTAGGGGTT AAAGAAAGAA AAGAATCAAA ATGGGGGTTT TTTTTTTTTT ATTTCATTTT   
  
  
+ AATTTTAGAA GAAATGTTTC TATTGAATAA AAATTTAAGT GTAATGTATA ATATTTTATT TTTAATTTAG   
  
  
+ GTTTGAAAGG TGTAAAATTT TTAGAAATTT TATTCAATTT AAAGGTCCAG TTAGTACATT CTGTATAAAA   
  
  
+ AAATTTTACT TATTTTAGAA ATTTTTAATA GCAATAATTT TATCTATTGT AAAATGTAAT AATACTGTTT   
  
  
+ TTTAATGACA TTTATAGTGT TAAATTTAAC TATCCTATTT ACACATTATA ACTTTTTGAA AGAAAAAGTC   
  
  
+ ATCTTTTTTT AGAACCCCTC CTCTGGAAAC TAACCGTATA TATAATATAC CACTCATTAC TTTCTTCTCC   
  
  
+ TGCAAACCAC ACAAACAAAT TAATTTGTTA ATTAATTAAA TTACAGGTTT CGTCTACTTC GCTTCTCCAG   
  
  
+ TAGTCTTCTT CTTCGTATTT ATTTTATAAC CTTTTGTTCT CCACCTCCTC CTCTGTCTCC GAACCCTACG   
  
  
+ TCGTCACCAT TCATTGTTCA CCCGAGACAG CGAGAGAGAG ATAAACGACT CACTTATTAA GTCGTGTTGT   
  
  
+ CGTGTGCTTG TCCTCGACCA GTGACTAAAC ACTTGTAAGA ACCGTTTATG ATGGGTAGTC GGTAGGAGAA   
  
  
+ ATAATGAGGG AGATTGAGGA TGTGAGAAAC TGGGTTAGGT GGAAAGAGTA GGGAAGATAT AAATAAAAAA   
  
  
+ TAGGCAAGTA AAAAATAAAA AAATTTTTAC TTAATTTAAT AAATAAAAAA TTTTAATATT AATAGATAAA   
  
  
+ GAAAAAATTT TCTAATTATT TTTAGTTTGA CTATCTTTTC TACAAATCAA ATAGTTTTAT TAAAAAAAAT   
  
  
+ CTACGGGTAT ATATGATTAA AGTAGTTCAC CCGTACCAAT AACTTAACTA CGATAACCAC ACGGCTTCTT   
  
  
+ TCAATAAGAA GTGTAAACAG CTGCTCCTG  

- GACTGTAACA ATGATCCGTC TCAGACAGAG AGCCTGCCGA AATTCTGCGA ACGCGGAGAG AGAAAGTGCG   
  
  
- AGGCTAAGAG GTGCGTTTTA GACTGAGGAA AAGGGAGAAG GATGGAAAGA GAAAGGGAGA GAGCTAGGGT   
  
  
- TTTGTGATGG TAAGGACGCG TAGTTTTTGA ACTTTTTTCC TGTGAAGCAG TGTCCTATCA ACAGCGACGC   
  
  
- GCGCTCTCTT CTCTGTTATC TCTCTAAAGA CAAACAGTCA TAGAGACAGA GAGAGAAAAG CAAACAAAAA   
  
  
- ACACTGAAAA GAATGAAAGA AAAGAAGAGA GAAGGGAATA GAAAAGAGAT AAGTTAGAAA AATCGCCGTC   
  
  
- AACCGAAAAC AGTACGGGAG GCTGTTATTT AGGGGAACGC AAGAGTGTAG GTCCCACGCG CATGAAATTG   
  
  
- AAATTTTCCA GAGAAGAAAA AAGATACTTT CCGACAAGAA GGCGCACCTT GTTTCTCTCC AGTACTTACC   
  
  
- TGTGCGTAAA CCATCCCCAA TTTCTTTCTT TTCTTAGTTT TACCCCCAAA AAAAAAAAAA TAAAGTAAAA   
  
  
- TTAAAATCTT CTTTACAAAG ATAACTTATT TTTAAATTCA CATTACATAT TATAAAATAA AAATTAAATC   
  
  
- CAAACTTTCC ACATTTTAAA AATCTTTAAA ATAAGTTAAA TTTCCAGGTC AATCATGTAA GACATATTTT   
  
  
- TTTAAAATGA ATAAAATCTT TAAAAATTAT CGTTATTAAA ATAGATAACA TTTTACATTA TTATGACAAA   
  
  
- AAATTACTGT AAATATCACA ATTTAAATTG ATAGGATAAA TGTGTAATAT TGAAAAACTT TCTTTTTCAG   
  
  
- TAGAAAAAAA TCTTGGGGAG GAGACCTTTG ATTGGCATAT ATATTATATG GTGAGTAATG AAAGAAGAGG   
  
  
- ACGTTTGGTG TGTTTGTTTA ATTAAACAAT TAATTAATTT AATGTCCAAA GCAGATGAAG CGAAGAGGTC   
  
  
- ATCAGAAGAA GAAGCATAAA TAAAATATTG GAAAACAAGA GGTGGAGGAG GAGACAGAGG CTTGGGATGC   
  
  
- AGCAGTGGTA AGTAACAAGT GGGCTCTGTC GCTCTCTCTC TATTTGCTGA GTGAATAATT CAGCACAACA   
  
  
- GCACACGAAC AGGAGCTGGT CACTGATTTG TGAACATTCT TGGCAAATAC TACCCATCAG CCATCCTCTT   
  
  
- TATTACTCCC TCTAACTCCT ACACTCTTTG ACCCAATCCA CCTTTCTCAT CCCTTCTATA TTTATTTTTT   
  
  
- ATCCGTTCAT TTTTTATTTT TTTAAAAATG AATTAAATTA TTTATTTTTT AAAATTATAA TTATCTATTT   
  
  
- CTTTTTTAAA AGATTAATAA AAATCAAACT GATAGAAAAG ATGTTTAGTT TATCAAAATA ATTTTTTTTA   
  
  
- GATGCCCATA TATACTAATT TCATCAAGTG GGCATGGTTA TTGAATTGAT GCTATTGGTG TGCCGAAGAA   
  
  
- AGTTATTCTT CACATTTGTC GACGAGGAC

+     TCA-element

| Site Name | Organism | Position | Strand | Matrix score. | sequence | function |
| --- | --- | --- | --- | --- | --- | --- |
| TCA-element | Nicotiana tabacum | 839 | + | 9 | CCATCTTTTT | cis-acting element involved in salicylic acid responsiveness |
| TCA-element | Brassica oleracea | 300 | - | 9 | GAGAAGAATA | cis-acting element involved in salicylic acid responsiveness |
| TCA-element | Nicotiana tabacum | 333 | + | 9 | CCATCTTTTT | cis-acting element involved in salicylic acid responsiveness |

> 2018/04/13 10:10:12  
+ CTGACATTGT TACTAGGCAG AGTCTGTCTC TCGGACGGCT TTAAGACGCT TGCGCCTCTC TCTTTCACGC   
  
  
+ TCCGATTCTC CACGCAAAAT CTGACTCCTT TTCCCTCTTC CTACCTTTCT CTTTCCCTCT CTCGATCCCA   
  
  
+ AAACACTACC ATTCCTGCGC ATCAAAAACT TGAAAAAAGG ACACTTCGTC ACAGGATAGT TGTCGCTGCG   
  
  
+ CGCGAGAGAA GAGACAATAG AGAGATTTCT GTTTGTCAGT ATCTCTGTCT CTCTCTTTTC GTTTGTTTTT   
  
  
+ TGTGACTTTT CTTACTTTCT TTTCTTCTCT CTTCCCTTAT CTTTTCTCTA TTCAATCTTT TTAGCGGCAG   
  
  
+ TTGGCTTTTG TCATGCCCTC CGACAATAAA TCCCCTTGCG TTCTCACATC CAGGGTGCGC GTACTTTAAC   
  
  
+ TTTAAAAGGT CTCTTCTTTT TTCTATGAAA GGCTGTTCTT CCGCGTGGAA CAAAGAGAGG TCATGAATGG   
  
  
+ ACACGCATTT GGTAGGGGTT AAAGAAAGAA AAGAATCAAA ATGGGGGTTT TTTTTTTTTT ATTTCATTTT   
  
  
+ AATTTTAGAA GAAATGTTTC TATTGAATAA AAATTTAAGT GTAATGTATA ATATTTTATT TTTAATTTAG   
  
  
+ GTTTGAAAGG TGTAAAATTT TTAGAAATTT TATTCAATTT AAAGGTCCAG TTAGTACATT CTGTATAAAA   
  
  
+ AAATTTTACT TATTTTAGAA ATTTTTAATA GCAATAATTT TATCTATTGT AAAATGTAAT AATACTGTTT   
  
  
+ TTTAATGACA TTTATAGTGT TAAATTTAAC TATCCTATTT ACACATTATA ACTTTTTGAA AGAAAAAGTC   
  
  
+ ATCTTTTTTT AGAACCCCTC CTCTGGAAAC TAACCGTATA TATAATATAC CACTCATTAC TTTCTTCTCC   
  
  
+ TGCAAACCAC ACAAACAAAT TAATTTGTTA ATTAATTAAA TTACAGGTTT CGTCTACTTC GCTTCTCCAG   
  
  
+ TAGTCTTCTT CTTCGTATTT ATTTTATAAC CTTTTGTTCT CCACCTCCTC CTCTGTCTCC GAACCCTACG   
  
  
+ TCGTCACCAT TCATTGTTCA CCCGAGACAG CGAGAGAGAG ATAAACGACT CACTTATTAA GTCGTGTTGT   
  
  
+ CGTGTGCTTG TCCTCGACCA GTGACTAAAC ACTTGTAAGA ACCGTTTATG ATGGGTAGTC GGTAGGAGAA   
  
  
+ ATAATGAGGG AGATTGAGGA TGTGAGAAAC TGGGTTAGGT GGAAAGAGTA GGGAAGATAT AAATAAAAAA   
  
  
+ TAGGCAAGTA AAAAATAAAA AAATTTTTAC TTAATTTAAT AAATAAAAAA TTTTAATATT AATAGATAAA   
  
  
+ GAAAAAATTT TCTAATTATT TTTAGTTTGA CTATCTTTTC TACAAATCAA ATAGTTTTAT TAAAAAAAAT   
  
  
+ CTACGGGTAT ATATGATTAA AGTAGTTCAC CCGTACCAAT AACTTAACTA CGATAACCAC ACGGCTTCTT   
  
  
+ TCAATAAGAA GTGTAAACAG CTGCTCCTG  

- GACTGTAACA ATGATCCGTC TCAGACAGAG AGCCTGCCGA AATTCTGCGA ACGCGGAGAG AGAAAGTGCG   
  
  
- AGGCTAAGAG GTGCGTTTTA GACTGAGGAA AAGGGAGAAG GATGGAAAGA GAAAGGGAGA GAGCTAGGGT   
  
  
- TTTGTGATGG TAAGGACGCG TAGTTTTTGA ACTTTTTTCC TGTGAAGCAG TGTCCTATCA ACAGCGACGC   
  
  
- GCGCTCTCTT CTCTGTTATC TCTCTAAAGA CAAACAGTCA TAGAGACAGA GAGAGAAAAG CAAACAAAAA   
  
  
- ACACTGAAAA GAATGAAAGA AAAGAAGAGA GAAGGGAATA GAAAAGAGAT AAGTTAGAAA AATCGCCGTC   
  
  
- AACCGAAAAC AGTACGGGAG GCTGTTATTT AGGGGAACGC AAGAGTGTAG GTCCCACGCG CATGAAATTG   
  
  
- AAATTTTCCA GAGAAGAAAA AAGATACTTT CCGACAAGAA GGCGCACCTT GTTTCTCTCC AGTACTTACC   
  
  
- TGTGCGTAAA CCATCCCCAA TTTCTTTCTT TTCTTAGTTT TACCCCCAAA AAAAAAAAAA TAAAGTAAAA   
  
  
- TTAAAATCTT CTTTACAAAG ATAACTTATT TTTAAATTCA CATTACATAT TATAAAATAA AAATTAAATC   
  
  
- CAAACTTTCC ACATTTTAAA AATCTTTAAA ATAAGTTAAA TTTCCAGGTC AATCATGTAA GACATATTTT   
  
  
- TTTAAAATGA ATAAAATCTT TAAAAATTAT CGTTATTAAA ATAGATAACA TTTTACATTA TTATGACAAA   
  
  
- AAATTACTGT AAATATCACA ATTTAAATTG ATAGGATAAA TGTGTAATAT TGAAAAACTT TCTTTTTCAG   
  
  
- TAGAAAAAAA TCTTGGGGAG GAGACCTTTG ATTGGCATAT ATATTATATG GTGAGTAATG AAAGAAGAGG   
  
  
- ACGTTTGGTG TGTTTGTTTA ATTAAACAAT TAATTAATTT AATGTCCAAA GCAGATGAAG CGAAGAGGTC   
  
  
- ATCAGAAGAA GAAGCATAAA TAAAATATTG GAAAACAAGA GGTGGAGGAG GAGACAGAGG CTTGGGATGC   
  
  
- AGCAGTGGTA AGTAACAAGT GGGCTCTGTC GCTCTCTCTC TATTTGCTGA GTGAATAATT CAGCACAACA   
  
  
- GCACACGAAC AGGAGCTGGT CACTGATTTG TGAACATTCT TGGCAAATAC TACCCATCAG CCATCCTCTT   
  
  
- TATTACTCCC TCTAACTCCT ACACTCTTTG ACCCAATCCA CCTTTCTCAT CCCTTCTATA TTTATTTTTT   
  
  
- ATCCGTTCAT TTTTTATTTT TTTAAAAATG AATTAAATTA TTTATTTTTT AAAATTATAA TTATCTATTT   
  
  
- CTTTTTTAAA AGATTAATAA AAATCAAACT GATAGAAAAG ATGTTTAGTT TATCAAAATA ATTTTTTTTA   
  
  
- GATGCCCATA TATACTAATT TCATCAAGTG GGCATGGTTA TTGAATTGAT GCTATTGGTG TGCCGAAGAA   
  
  
- AGTTATTCTT CACATTTGTC GACGAGGAC

+     TCCACCT-motif

| Site Name | Organism | Position | Strand | Matrix score. | sequence | function |
| --- | --- | --- | --- | --- | --- | --- |
| TCCACCT-motif | Petroselinum hortense | 1020 | + | 7 | TCCACCT |  |
| TCCACCT-motif | Petroselinum hortense | 1227 | - | 7 | TCCACCT |  |

> 2018/04/13 10:10:12  
+ CTGACATTGT TACTAGGCAG AGTCTGTCTC TCGGACGGCT TTAAGACGCT TGCGCCTCTC TCTTTCACGC   
  
  
+ TCCGATTCTC CACGCAAAAT CTGACTCCTT TTCCCTCTTC CTACCTTTCT CTTTCCCTCT CTCGATCCCA   
  
  
+ AAACACTACC ATTCCTGCGC ATCAAAAACT TGAAAAAAGG ACACTTCGTC ACAGGATAGT TGTCGCTGCG   
  
  
+ CGCGAGAGAA GAGACAATAG AGAGATTTCT GTTTGTCAGT ATCTCTGTCT CTCTCTTTTC GTTTGTTTTT   
  
  
+ TGTGACTTTT CTTACTTTCT TTTCTTCTCT CTTCCCTTAT CTTTTCTCTA TTCAATCTTT TTAGCGGCAG   
  
  
+ TTGGCTTTTG TCATGCCCTC CGACAATAAA TCCCCTTGCG TTCTCACATC CAGGGTGCGC GTACTTTAAC   
  
  
+ TTTAAAAGGT CTCTTCTTTT TTCTATGAAA GGCTGTTCTT CCGCGTGGAA CAAAGAGAGG TCATGAATGG   
  
  
+ ACACGCATTT GGTAGGGGTT AAAGAAAGAA AAGAATCAAA ATGGGGGTTT TTTTTTTTTT ATTTCATTTT   
  
  
+ AATTTTAGAA GAAATGTTTC TATTGAATAA AAATTTAAGT GTAATGTATA ATATTTTATT TTTAATTTAG   
  
  
+ GTTTGAAAGG TGTAAAATTT TTAGAAATTT TATTCAATTT AAAGGTCCAG TTAGTACATT CTGTATAAAA   
  
  
+ AAATTTTACT TATTTTAGAA ATTTTTAATA GCAATAATTT TATCTATTGT AAAATGTAAT AATACTGTTT   
  
  
+ TTTAATGACA TTTATAGTGT TAAATTTAAC TATCCTATTT ACACATTATA ACTTTTTGAA AGAAAAAGTC   
  
  
+ ATCTTTTTTT AGAACCCCTC CTCTGGAAAC TAACCGTATA TATAATATAC CACTCATTAC TTTCTTCTCC   
  
  
+ TGCAAACCAC ACAAACAAAT TAATTTGTTA ATTAATTAAA TTACAGGTTT CGTCTACTTC GCTTCTCCAG   
  
  
+ TAGTCTTCTT CTTCGTATTT ATTTTATAAC CTTTTGTTCT CCACCTCCTC CTCTGTCTCC GAACCCTACG   
  
  
+ TCGTCACCAT TCATTGTTCA CCCGAGACAG CGAGAGAGAG ATAAACGACT CACTTATTAA GTCGTGTTGT   
  
  
+ CGTGTGCTTG TCCTCGACCA GTGACTAAAC ACTTGTAAGA ACCGTTTATG ATGGGTAGTC GGTAGGAGAA   
  
  
+ ATAATGAGGG AGATTGAGGA TGTGAGAAAC TGGGTTAGGT GGAAAGAGTA GGGAAGATAT AAATAAAAAA   
  
  
+ TAGGCAAGTA AAAAATAAAA AAATTTTTAC TTAATTTAAT AAATAAAAAA TTTTAATATT AATAGATAAA   
  
  
+ GAAAAAATTT TCTAATTATT TTTAGTTTGA CTATCTTTTC TACAAATCAA ATAGTTTTAT TAAAAAAAAT   
  
  
+ CTACGGGTAT ATATGATTAA AGTAGTTCAC CCGTACCAAT AACTTAACTA CGATAACCAC ACGGCTTCTT   
  
  
+ TCAATAAGAA GTGTAAACAG CTGCTCCTG  

- GACTGTAACA ATGATCCGTC TCAGACAGAG AGCCTGCCGA AATTCTGCGA ACGCGGAGAG AGAAAGTGCG   
  
  
- AGGCTAAGAG GTGCGTTTTA GACTGAGGAA AAGGGAGAAG GATGGAAAGA GAAAGGGAGA GAGCTAGGGT   
  
  
- TTTGTGATGG TAAGGACGCG TAGTTTTTGA ACTTTTTTCC TGTGAAGCAG TGTCCTATCA ACAGCGACGC   
  
  
- GCGCTCTCTT CTCTGTTATC TCTCTAAAGA CAAACAGTCA TAGAGACAGA GAGAGAAAAG CAAACAAAAA   
  
  
- ACACTGAAAA GAATGAAAGA AAAGAAGAGA GAAGGGAATA GAAAAGAGAT AAGTTAGAAA AATCGCCGTC   
  
  
- AACCGAAAAC AGTACGGGAG GCTGTTATTT AGGGGAACGC AAGAGTGTAG GTCCCACGCG CATGAAATTG   
  
  
- AAATTTTCCA GAGAAGAAAA AAGATACTTT CCGACAAGAA GGCGCACCTT GTTTCTCTCC AGTACTTACC   
  
  
- TGTGCGTAAA CCATCCCCAA TTTCTTTCTT TTCTTAGTTT TACCCCCAAA AAAAAAAAAA TAAAGTAAAA   
  
  
- TTAAAATCTT CTTTACAAAG ATAACTTATT TTTAAATTCA CATTACATAT TATAAAATAA AAATTAAATC   
  
  
- CAAACTTTCC ACATTTTAAA AATCTTTAAA ATAAGTTAAA TTTCCAGGTC AATCATGTAA GACATATTTT   
  
  
- TTTAAAATGA ATAAAATCTT TAAAAATTAT CGTTATTAAA ATAGATAACA TTTTACATTA TTATGACAAA   
  
  
- AAATTACTGT AAATATCACA ATTTAAATTG ATAGGATAAA TGTGTAATAT TGAAAAACTT TCTTTTTCAG   
  
  
- TAGAAAAAAA TCTTGGGGAG GAGACCTTTG ATTGGCATAT ATATTATATG GTGAGTAATG AAAGAAGAGG   
  
  
- ACGTTTGGTG TGTTTGTTTA ATTAAACAAT TAATTAATTT AATGTCCAAA GCAGATGAAG CGAAGAGGTC   
  
  
- ATCAGAAGAA GAAGCATAAA TAAAATATTG GAAAACAAGA GGTGGAGGAG GAGACAGAGG CTTGGGATGC   
  
  
- AGCAGTGGTA AGTAACAAGT GGGCTCTGTC GCTCTCTCTC TATTTGCTGA GTGAATAATT CAGCACAACA   
  
  
- GCACACGAAC AGGAGCTGGT CACTGATTTG TGAACATTCT TGGCAAATAC TACCCATCAG CCATCCTCTT   
  
  
- TATTACTCCC TCTAACTCCT ACACTCTTTG ACCCAATCCA CCTTTCTCAT CCCTTCTATA TTTATTTTTT   
  
  
- ATCCGTTCAT TTTTTATTTT TTTAAAAATG AATTAAATTA TTTATTTTTT AAAATTATAA TTATCTATTT   
  
  
- CTTTTTTAAA AGATTAATAA AAATCAAACT GATAGAAAAG ATGTTTAGTT TATCAAAATA ATTTTTTTTA   
  
  
- GATGCCCATA TATACTAATT TCATCAAGTG GGCATGGTTA TTGAATTGAT GCTATTGGTG TGCCGAAGAA   
  
  
- AGTTATTCTT CACATTTGTC GACGAGGAC

+     TCCC-motif

| Site Name | Organism | Position | Strand | Matrix score. | sequence | function |
| --- | --- | --- | --- | --- | --- | --- |
| TCCC-motif | Spinacia oleracea | 1197 | - | 7 | TCTCCCT | part of a light responsive element |

> 2018/04/13 10:10:12  
+ CTGACATTGT TACTAGGCAG AGTCTGTCTC TCGGACGGCT TTAAGACGCT TGCGCCTCTC TCTTTCACGC   
  
  
+ TCCGATTCTC CACGCAAAAT CTGACTCCTT TTCCCTCTTC CTACCTTTCT CTTTCCCTCT CTCGATCCCA   
  
  
+ AAACACTACC ATTCCTGCGC ATCAAAAACT TGAAAAAAGG ACACTTCGTC ACAGGATAGT TGTCGCTGCG   
  
  
+ CGCGAGAGAA GAGACAATAG AGAGATTTCT GTTTGTCAGT ATCTCTGTCT CTCTCTTTTC GTTTGTTTTT   
  
  
+ TGTGACTTTT CTTACTTTCT TTTCTTCTCT CTTCCCTTAT CTTTTCTCTA TTCAATCTTT TTAGCGGCAG   
  
  
+ TTGGCTTTTG TCATGCCCTC CGACAATAAA TCCCCTTGCG TTCTCACATC CAGGGTGCGC GTACTTTAAC   
  
  
+ TTTAAAAGGT CTCTTCTTTT TTCTATGAAA GGCTGTTCTT CCGCGTGGAA CAAAGAGAGG TCATGAATGG   
  
  
+ ACACGCATTT GGTAGGGGTT AAAGAAAGAA AAGAATCAAA ATGGGGGTTT TTTTTTTTTT ATTTCATTTT   
  
  
+ AATTTTAGAA GAAATGTTTC TATTGAATAA AAATTTAAGT GTAATGTATA ATATTTTATT TTTAATTTAG   
  
  
+ GTTTGAAAGG TGTAAAATTT TTAGAAATTT TATTCAATTT AAAGGTCCAG TTAGTACATT CTGTATAAAA   
  
  
+ AAATTTTACT TATTTTAGAA ATTTTTAATA GCAATAATTT TATCTATTGT AAAATGTAAT AATACTGTTT   
  
  
+ TTTAATGACA TTTATAGTGT TAAATTTAAC TATCCTATTT ACACATTATA ACTTTTTGAA AGAAAAAGTC   
  
  
+ ATCTTTTTTT AGAACCCCTC CTCTGGAAAC TAACCGTATA TATAATATAC CACTCATTAC TTTCTTCTCC   
  
  
+ TGCAAACCAC ACAAACAAAT TAATTTGTTA ATTAATTAAA TTACAGGTTT CGTCTACTTC GCTTCTCCAG   
  
  
+ TAGTCTTCTT CTTCGTATTT ATTTTATAAC CTTTTGTTCT CCACCTCCTC CTCTGTCTCC GAACCCTACG   
  
  
+ TCGTCACCAT TCATTGTTCA CCCGAGACAG CGAGAGAGAG ATAAACGACT CACTTATTAA GTCGTGTTGT   
  
  
+ CGTGTGCTTG TCCTCGACCA GTGACTAAAC ACTTGTAAGA ACCGTTTATG ATGGGTAGTC GGTAGGAGAA   
  
  
+ ATAATGAGGG AGATTGAGGA TGTGAGAAAC TGGGTTAGGT GGAAAGAGTA GGGAAGATAT AAATAAAAAA   
  
  
+ TAGGCAAGTA AAAAATAAAA AAATTTTTAC TTAATTTAAT AAATAAAAAA TTTTAATATT AATAGATAAA   
  
  
+ GAAAAAATTT TCTAATTATT TTTAGTTTGA CTATCTTTTC TACAAATCAA ATAGTTTTAT TAAAAAAAAT   
  
  
+ CTACGGGTAT ATATGATTAA AGTAGTTCAC CCGTACCAAT AACTTAACTA CGATAACCAC ACGGCTTCTT   
  
  
+ TCAATAAGAA GTGTAAACAG CTGCTCCTG  

- GACTGTAACA ATGATCCGTC TCAGACAGAG AGCCTGCCGA AATTCTGCGA ACGCGGAGAG AGAAAGTGCG   
  
  
- AGGCTAAGAG GTGCGTTTTA GACTGAGGAA AAGGGAGAAG GATGGAAAGA GAAAGGGAGA GAGCTAGGGT   
  
  
- TTTGTGATGG TAAGGACGCG TAGTTTTTGA ACTTTTTTCC TGTGAAGCAG TGTCCTATCA ACAGCGACGC   
  
  
- GCGCTCTCTT CTCTGTTATC TCTCTAAAGA CAAACAGTCA TAGAGACAGA GAGAGAAAAG CAAACAAAAA   
  
  
- ACACTGAAAA GAATGAAAGA AAAGAAGAGA GAAGGGAATA GAAAAGAGAT AAGTTAGAAA AATCGCCGTC   
  
  
- AACCGAAAAC AGTACGGGAG GCTGTTATTT AGGGGAACGC AAGAGTGTAG GTCCCACGCG CATGAAATTG   
  
  
- AAATTTTCCA GAGAAGAAAA AAGATACTTT CCGACAAGAA GGCGCACCTT GTTTCTCTCC AGTACTTACC   
  
  
- TGTGCGTAAA CCATCCCCAA TTTCTTTCTT TTCTTAGTTT TACCCCCAAA AAAAAAAAAA TAAAGTAAAA   
  
  
- TTAAAATCTT CTTTACAAAG ATAACTTATT TTTAAATTCA CATTACATAT TATAAAATAA AAATTAAATC   
  
  
- CAAACTTTCC ACATTTTAAA AATCTTTAAA ATAAGTTAAA TTTCCAGGTC AATCATGTAA GACATATTTT   
  
  
- TTTAAAATGA ATAAAATCTT TAAAAATTAT CGTTATTAAA ATAGATAACA TTTTACATTA TTATGACAAA   
  
  
- AAATTACTGT AAATATCACA ATTTAAATTG ATAGGATAAA TGTGTAATAT TGAAAAACTT TCTTTTTCAG   
  
  
- TAGAAAAAAA TCTTGGGGAG GAGACCTTTG ATTGGCATAT ATATTATATG GTGAGTAATG AAAGAAGAGG   
  
  
- ACGTTTGGTG TGTTTGTTTA ATTAAACAAT TAATTAATTT AATGTCCAAA GCAGATGAAG CGAAGAGGTC   
  
  
- ATCAGAAGAA GAAGCATAAA TAAAATATTG GAAAACAAGA GGTGGAGGAG GAGACAGAGG CTTGGGATGC   
  
  
- AGCAGTGGTA AGTAACAAGT GGGCTCTGTC GCTCTCTCTC TATTTGCTGA GTGAATAATT CAGCACAACA   
  
  
- GCACACGAAC AGGAGCTGGT CACTGATTTG TGAACATTCT TGGCAAATAC TACCCATCAG CCATCCTCTT   
  
  
- TATTACTCCC TCTAACTCCT ACACTCTTTG ACCCAATCCA CCTTTCTCAT CCCTTCTATA TTTATTTTTT   
  
  
- ATCCGTTCAT TTTTTATTTT TTTAAAAATG AATTAAATTA TTTATTTTTT AAAATTATAA TTATCTATTT   
  
  
- CTTTTTTAAA AGATTAATAA AAATCAAACT GATAGAAAAG ATGTTTAGTT TATCAAAATA ATTTTTTTTA   
  
  
- GATGCCCATA TATACTAATT TCATCAAGTG GGCATGGTTA TTGAATTGAT GCTATTGGTG TGCCGAAGAA   
  
  
- AGTTATTCTT CACATTTGTC GACGAGGAC

+     TCT-motif

| Site Name | Organism | Position | Strand | Matrix score. | sequence | function |
| --- | --- | --- | --- | --- | --- | --- |
| TCT-motif | Arabidopsis thaliana | 1155 | - | 6 | TCTTAC | part of a light responsive element |
| TCT-motif | Arabidopsis thaliana | 290 | + | 6 | TCTTAC | part of a light responsive element |

> 2018/04/13 10:10:12  
+ CTGACATTGT TACTAGGCAG AGTCTGTCTC TCGGACGGCT TTAAGACGCT TGCGCCTCTC TCTTTCACGC   
  
  
+ TCCGATTCTC CACGCAAAAT CTGACTCCTT TTCCCTCTTC CTACCTTTCT CTTTCCCTCT CTCGATCCCA   
  
  
+ AAACACTACC ATTCCTGCGC ATCAAAAACT TGAAAAAAGG ACACTTCGTC ACAGGATAGT TGTCGCTGCG   
  
  
+ CGCGAGAGAA GAGACAATAG AGAGATTTCT GTTTGTCAGT ATCTCTGTCT CTCTCTTTTC GTTTGTTTTT   
  
  
+ TGTGACTTTT CTTACTTTCT TTTCTTCTCT CTTCCCTTAT CTTTTCTCTA TTCAATCTTT TTAGCGGCAG   
  
  
+ TTGGCTTTTG TCATGCCCTC CGACAATAAA TCCCCTTGCG TTCTCACATC CAGGGTGCGC GTACTTTAAC   
  
  
+ TTTAAAAGGT CTCTTCTTTT TTCTATGAAA GGCTGTTCTT CCGCGTGGAA CAAAGAGAGG TCATGAATGG   
  
  
+ ACACGCATTT GGTAGGGGTT AAAGAAAGAA AAGAATCAAA ATGGGGGTTT TTTTTTTTTT ATTTCATTTT   
  
  
+ AATTTTAGAA GAAATGTTTC TATTGAATAA AAATTTAAGT GTAATGTATA ATATTTTATT TTTAATTTAG   
  
  
+ GTTTGAAAGG TGTAAAATTT TTAGAAATTT TATTCAATTT AAAGGTCCAG TTAGTACATT CTGTATAAAA   
  
  
+ AAATTTTACT TATTTTAGAA ATTTTTAATA GCAATAATTT TATCTATTGT AAAATGTAAT AATACTGTTT   
  
  
+ TTTAATGACA TTTATAGTGT TAAATTTAAC TATCCTATTT ACACATTATA ACTTTTTGAA AGAAAAAGTC   
  
  
+ ATCTTTTTTT AGAACCCCTC CTCTGGAAAC TAACCGTATA TATAATATAC CACTCATTAC TTTCTTCTCC   
  
  
+ TGCAAACCAC ACAAACAAAT TAATTTGTTA ATTAATTAAA TTACAGGTTT CGTCTACTTC GCTTCTCCAG   
  
  
+ TAGTCTTCTT CTTCGTATTT ATTTTATAAC CTTTTGTTCT CCACCTCCTC CTCTGTCTCC GAACCCTACG   
  
  
+ TCGTCACCAT TCATTGTTCA CCCGAGACAG CGAGAGAGAG ATAAACGACT CACTTATTAA GTCGTGTTGT   
  
  
+ CGTGTGCTTG TCCTCGACCA GTGACTAAAC ACTTGTAAGA ACCGTTTATG ATGGGTAGTC GGTAGGAGAA   
  
  
+ ATAATGAGGG AGATTGAGGA TGTGAGAAAC TGGGTTAGGT GGAAAGAGTA GGGAAGATAT AAATAAAAAA   
  
  
+ TAGGCAAGTA AAAAATAAAA AAATTTTTAC TTAATTTAAT AAATAAAAAA TTTTAATATT AATAGATAAA   
  
  
+ GAAAAAATTT TCTAATTATT TTTAGTTTGA CTATCTTTTC TACAAATCAA ATAGTTTTAT TAAAAAAAAT   
  
  
+ CTACGGGTAT ATATGATTAA AGTAGTTCAC CCGTACCAAT AACTTAACTA CGATAACCAC ACGGCTTCTT   
  
  
+ TCAATAAGAA GTGTAAACAG CTGCTCCTG  

- GACTGTAACA ATGATCCGTC TCAGACAGAG AGCCTGCCGA AATTCTGCGA ACGCGGAGAG AGAAAGTGCG   
  
  
- AGGCTAAGAG GTGCGTTTTA GACTGAGGAA AAGGGAGAAG GATGGAAAGA GAAAGGGAGA GAGCTAGGGT   
  
  
- TTTGTGATGG TAAGGACGCG TAGTTTTTGA ACTTTTTTCC TGTGAAGCAG TGTCCTATCA ACAGCGACGC   
  
  
- GCGCTCTCTT CTCTGTTATC TCTCTAAAGA CAAACAGTCA TAGAGACAGA GAGAGAAAAG CAAACAAAAA   
  
  
- ACACTGAAAA GAATGAAAGA AAAGAAGAGA GAAGGGAATA GAAAAGAGAT AAGTTAGAAA AATCGCCGTC   
  
  
- AACCGAAAAC AGTACGGGAG GCTGTTATTT AGGGGAACGC AAGAGTGTAG GTCCCACGCG CATGAAATTG   
  
  
- AAATTTTCCA GAGAAGAAAA AAGATACTTT CCGACAAGAA GGCGCACCTT GTTTCTCTCC AGTACTTACC   
  
  
- TGTGCGTAAA CCATCCCCAA TTTCTTTCTT TTCTTAGTTT TACCCCCAAA AAAAAAAAAA TAAAGTAAAA   
  
  
- TTAAAATCTT CTTTACAAAG ATAACTTATT TTTAAATTCA CATTACATAT TATAAAATAA AAATTAAATC   
  
  
- CAAACTTTCC ACATTTTAAA AATCTTTAAA ATAAGTTAAA TTTCCAGGTC AATCATGTAA GACATATTTT   
  
  
- TTTAAAATGA ATAAAATCTT TAAAAATTAT CGTTATTAAA ATAGATAACA TTTTACATTA TTATGACAAA   
  
  
- AAATTACTGT AAATATCACA ATTTAAATTG ATAGGATAAA TGTGTAATAT TGAAAAACTT TCTTTTTCAG   
  
  
- TAGAAAAAAA TCTTGGGGAG GAGACCTTTG ATTGGCATAT ATATTATATG GTGAGTAATG AAAGAAGAGG   
  
  
- ACGTTTGGTG TGTTTGTTTA ATTAAACAAT TAATTAATTT AATGTCCAAA GCAGATGAAG CGAAGAGGTC   
  
  
- ATCAGAAGAA GAAGCATAAA TAAAATATTG GAAAACAAGA GGTGGAGGAG GAGACAGAGG CTTGGGATGC   
  
  
- AGCAGTGGTA AGTAACAAGT GGGCTCTGTC GCTCTCTCTC TATTTGCTGA GTGAATAATT CAGCACAACA   
  
  
- GCACACGAAC AGGAGCTGGT CACTGATTTG TGAACATTCT TGGCAAATAC TACCCATCAG CCATCCTCTT   
  
  
- TATTACTCCC TCTAACTCCT ACACTCTTTG ACCCAATCCA CCTTTCTCAT CCCTTCTATA TTTATTTTTT   
  
  
- ATCCGTTCAT TTTTTATTTT TTTAAAAATG AATTAAATTA TTTATTTTTT AAAATTATAA TTATCTATTT   
  
  
- CTTTTTTAAA AGATTAATAA AAATCAAACT GATAGAAAAG ATGTTTAGTT TATCAAAATA ATTTTTTTTA   
  
  
- GATGCCCATA TATACTAATT TCATCAAGTG GGCATGGTTA TTGAATTGAT GCTATTGGTG TGCCGAAGAA   
  
  
- AGTTATTCTT CACATTTGTC GACGAGGAC

+     TGA-element

| Site Name | Organism | Position | Strand | Matrix score. | sequence | function |
| --- | --- | --- | --- | --- | --- | --- |
| TGA-element | Brassica oleracea | 1094 | + | 6 | AACGAC | auxin-responsive element |

> 2018/04/13 10:10:12  
+ CTGACATTGT TACTAGGCAG AGTCTGTCTC TCGGACGGCT TTAAGACGCT TGCGCCTCTC TCTTTCACGC   
  
  
+ TCCGATTCTC CACGCAAAAT CTGACTCCTT TTCCCTCTTC CTACCTTTCT CTTTCCCTCT CTCGATCCCA   
  
  
+ AAACACTACC ATTCCTGCGC ATCAAAAACT TGAAAAAAGG ACACTTCGTC ACAGGATAGT TGTCGCTGCG   
  
  
+ CGCGAGAGAA GAGACAATAG AGAGATTTCT GTTTGTCAGT ATCTCTGTCT CTCTCTTTTC GTTTGTTTTT   
  
  
+ TGTGACTTTT CTTACTTTCT TTTCTTCTCT CTTCCCTTAT CTTTTCTCTA TTCAATCTTT TTAGCGGCAG   
  
  
+ TTGGCTTTTG TCATGCCCTC CGACAATAAA TCCCCTTGCG TTCTCACATC CAGGGTGCGC GTACTTTAAC   
  
  
+ TTTAAAAGGT CTCTTCTTTT TTCTATGAAA GGCTGTTCTT CCGCGTGGAA CAAAGAGAGG TCATGAATGG   
  
  
+ ACACGCATTT GGTAGGGGTT AAAGAAAGAA AAGAATCAAA ATGGGGGTTT TTTTTTTTTT ATTTCATTTT   
  
  
+ AATTTTAGAA GAAATGTTTC TATTGAATAA AAATTTAAGT GTAATGTATA ATATTTTATT TTTAATTTAG   
  
  
+ GTTTGAAAGG TGTAAAATTT TTAGAAATTT TATTCAATTT AAAGGTCCAG TTAGTACATT CTGTATAAAA   
  
  
+ AAATTTTACT TATTTTAGAA ATTTTTAATA GCAATAATTT TATCTATTGT AAAATGTAAT AATACTGTTT   
  
  
+ TTTAATGACA TTTATAGTGT TAAATTTAAC TATCCTATTT ACACATTATA ACTTTTTGAA AGAAAAAGTC   
  
  
+ ATCTTTTTTT AGAACCCCTC CTCTGGAAAC TAACCGTATA TATAATATAC CACTCATTAC TTTCTTCTCC   
  
  
+ TGCAAACCAC ACAAACAAAT TAATTTGTTA ATTAATTAAA TTACAGGTTT CGTCTACTTC GCTTCTCCAG   
  
  
+ TAGTCTTCTT CTTCGTATTT ATTTTATAAC CTTTTGTTCT CCACCTCCTC CTCTGTCTCC GAACCCTACG   
  
  
+ TCGTCACCAT TCATTGTTCA CCCGAGACAG CGAGAGAGAG ATAAACGACT CACTTATTAA GTCGTGTTGT   
  
  
+ CGTGTGCTTG TCCTCGACCA GTGACTAAAC ACTTGTAAGA ACCGTTTATG ATGGGTAGTC GGTAGGAGAA   
  
  
+ ATAATGAGGG AGATTGAGGA TGTGAGAAAC TGGGTTAGGT GGAAAGAGTA GGGAAGATAT AAATAAAAAA   
  
  
+ TAGGCAAGTA AAAAATAAAA AAATTTTTAC TTAATTTAAT AAATAAAAAA TTTTAATATT AATAGATAAA   
  
  
+ GAAAAAATTT TCTAATTATT TTTAGTTTGA CTATCTTTTC TACAAATCAA ATAGTTTTAT TAAAAAAAAT   
  
  
+ CTACGGGTAT ATATGATTAA AGTAGTTCAC CCGTACCAAT AACTTAACTA CGATAACCAC ACGGCTTCTT   
  
  
+ TCAATAAGAA GTGTAAACAG CTGCTCCTG  

- GACTGTAACA ATGATCCGTC TCAGACAGAG AGCCTGCCGA AATTCTGCGA ACGCGGAGAG AGAAAGTGCG   
  
  
- AGGCTAAGAG GTGCGTTTTA GACTGAGGAA AAGGGAGAAG GATGGAAAGA GAAAGGGAGA GAGCTAGGGT   
  
  
- TTTGTGATGG TAAGGACGCG TAGTTTTTGA ACTTTTTTCC TGTGAAGCAG TGTCCTATCA ACAGCGACGC   
  
  
- GCGCTCTCTT CTCTGTTATC TCTCTAAAGA CAAACAGTCA TAGAGACAGA GAGAGAAAAG CAAACAAAAA   
  
  
- ACACTGAAAA GAATGAAAGA AAAGAAGAGA GAAGGGAATA GAAAAGAGAT AAGTTAGAAA AATCGCCGTC   
  
  
- AACCGAAAAC AGTACGGGAG GCTGTTATTT AGGGGAACGC AAGAGTGTAG GTCCCACGCG CATGAAATTG   
  
  
- AAATTTTCCA GAGAAGAAAA AAGATACTTT CCGACAAGAA GGCGCACCTT GTTTCTCTCC AGTACTTACC   
  
  
- TGTGCGTAAA CCATCCCCAA TTTCTTTCTT TTCTTAGTTT TACCCCCAAA AAAAAAAAAA TAAAGTAAAA   
  
  
- TTAAAATCTT CTTTACAAAG ATAACTTATT TTTAAATTCA CATTACATAT TATAAAATAA AAATTAAATC   
  
  
- CAAACTTTCC ACATTTTAAA AATCTTTAAA ATAAGTTAAA TTTCCAGGTC AATCATGTAA GACATATTTT   
  
  
- TTTAAAATGA ATAAAATCTT TAAAAATTAT CGTTATTAAA ATAGATAACA TTTTACATTA TTATGACAAA   
  
  
- AAATTACTGT AAATATCACA ATTTAAATTG ATAGGATAAA TGTGTAATAT TGAAAAACTT TCTTTTTCAG   
  
  
- TAGAAAAAAA TCTTGGGGAG GAGACCTTTG ATTGGCATAT ATATTATATG GTGAGTAATG AAAGAAGAGG   
  
  
- ACGTTTGGTG TGTTTGTTTA ATTAAACAAT TAATTAATTT AATGTCCAAA GCAGATGAAG CGAAGAGGTC   
  
  
- ATCAGAAGAA GAAGCATAAA TAAAATATTG GAAAACAAGA GGTGGAGGAG GAGACAGAGG CTTGGGATGC   
  
  
- AGCAGTGGTA AGTAACAAGT GGGCTCTGTC GCTCTCTCTC TATTTGCTGA GTGAATAATT CAGCACAACA   
  
  
- GCACACGAAC AGGAGCTGGT CACTGATTTG TGAACATTCT TGGCAAATAC TACCCATCAG CCATCCTCTT   
  
  
- TATTACTCCC TCTAACTCCT ACACTCTTTG ACCCAATCCA CCTTTCTCAT CCCTTCTATA TTTATTTTTT   
  
  
- ATCCGTTCAT TTTTTATTTT TTTAAAAATG AATTAAATTA TTTATTTTTT AAAATTATAA TTATCTATTT   
  
  
- CTTTTTTAAA AGATTAATAA AAATCAAACT GATAGAAAAG ATGTTTAGTT TATCAAAATA ATTTTTTTTA   
  
  
- GATGCCCATA TATACTAATT TCATCAAGTG GGCATGGTTA TTGAATTGAT GCTATTGGTG TGCCGAAGAA   
  
  
- AGTTATTCTT CACATTTGTC GACGAGGAC

+     TGACG-motif

| Site Name | Organism | Position | Strand | Matrix score. | sequence | function |
| --- | --- | --- | --- | --- | --- | --- |
| TGACG-motif | Hordeum vulgare | 187 | - | 5 | TGACG | cis-acting regulatory element involved in the MeJA-responsiveness |
| TGACG-motif | Hordeum vulgare | 1052 | - | 5 | TGACG | cis-acting regulatory element involved in the MeJA-responsiveness |

> 2018/04/13 10:10:12  
+ CTGACATTGT TACTAGGCAG AGTCTGTCTC TCGGACGGCT TTAAGACGCT TGCGCCTCTC TCTTTCACGC   
  
  
+ TCCGATTCTC CACGCAAAAT CTGACTCCTT TTCCCTCTTC CTACCTTTCT CTTTCCCTCT CTCGATCCCA   
  
  
+ AAACACTACC ATTCCTGCGC ATCAAAAACT TGAAAAAAGG ACACTTCGTC ACAGGATAGT TGTCGCTGCG   
  
  
+ CGCGAGAGAA GAGACAATAG AGAGATTTCT GTTTGTCAGT ATCTCTGTCT CTCTCTTTTC GTTTGTTTTT   
  
  
+ TGTGACTTTT CTTACTTTCT TTTCTTCTCT CTTCCCTTAT CTTTTCTCTA TTCAATCTTT TTAGCGGCAG   
  
  
+ TTGGCTTTTG TCATGCCCTC CGACAATAAA TCCCCTTGCG TTCTCACATC CAGGGTGCGC GTACTTTAAC   
  
  
+ TTTAAAAGGT CTCTTCTTTT TTCTATGAAA GGCTGTTCTT CCGCGTGGAA CAAAGAGAGG TCATGAATGG   
  
  
+ ACACGCATTT GGTAGGGGTT AAAGAAAGAA AAGAATCAAA ATGGGGGTTT TTTTTTTTTT ATTTCATTTT   
  
  
+ AATTTTAGAA GAAATGTTTC TATTGAATAA AAATTTAAGT GTAATGTATA ATATTTTATT TTTAATTTAG   
  
  
+ GTTTGAAAGG TGTAAAATTT TTAGAAATTT TATTCAATTT AAAGGTCCAG TTAGTACATT CTGTATAAAA   
  
  
+ AAATTTTACT TATTTTAGAA ATTTTTAATA GCAATAATTT TATCTATTGT AAAATGTAAT AATACTGTTT   
  
  
+ TTTAATGACA TTTATAGTGT TAAATTTAAC TATCCTATTT ACACATTATA ACTTTTTGAA AGAAAAAGTC   
  
  
+ ATCTTTTTTT AGAACCCCTC CTCTGGAAAC TAACCGTATA TATAATATAC CACTCATTAC TTTCTTCTCC   
  
  
+ TGCAAACCAC ACAAACAAAT TAATTTGTTA ATTAATTAAA TTACAGGTTT CGTCTACTTC GCTTCTCCAG   
  
  
+ TAGTCTTCTT CTTCGTATTT ATTTTATAAC CTTTTGTTCT CCACCTCCTC CTCTGTCTCC GAACCCTACG   
  
  
+ TCGTCACCAT TCATTGTTCA CCCGAGACAG CGAGAGAGAG ATAAACGACT CACTTATTAA GTCGTGTTGT   
  
  
+ CGTGTGCTTG TCCTCGACCA GTGACTAAAC ACTTGTAAGA ACCGTTTATG ATGGGTAGTC GGTAGGAGAA   
  
  
+ ATAATGAGGG AGATTGAGGA TGTGAGAAAC TGGGTTAGGT GGAAAGAGTA GGGAAGATAT AAATAAAAAA   
  
  
+ TAGGCAAGTA AAAAATAAAA AAATTTTTAC TTAATTTAAT AAATAAAAAA TTTTAATATT AATAGATAAA   
  
  
+ GAAAAAATTT TCTAATTATT TTTAGTTTGA CTATCTTTTC TACAAATCAA ATAGTTTTAT TAAAAAAAAT   
  
  
+ CTACGGGTAT ATATGATTAA AGTAGTTCAC CCGTACCAAT AACTTAACTA CGATAACCAC ACGGCTTCTT   
  
  
+ TCAATAAGAA GTGTAAACAG CTGCTCCTG  

- GACTGTAACA ATGATCCGTC TCAGACAGAG AGCCTGCCGA AATTCTGCGA ACGCGGAGAG AGAAAGTGCG   
  
  
- AGGCTAAGAG GTGCGTTTTA GACTGAGGAA AAGGGAGAAG GATGGAAAGA GAAAGGGAGA GAGCTAGGGT   
  
  
- TTTGTGATGG TAAGGACGCG TAGTTTTTGA ACTTTTTTCC TGTGAAGCAG TGTCCTATCA ACAGCGACGC   
  
  
- GCGCTCTCTT CTCTGTTATC TCTCTAAAGA CAAACAGTCA TAGAGACAGA GAGAGAAAAG CAAACAAAAA   
  
  
- ACACTGAAAA GAATGAAAGA AAAGAAGAGA GAAGGGAATA GAAAAGAGAT AAGTTAGAAA AATCGCCGTC   
  
  
- AACCGAAAAC AGTACGGGAG GCTGTTATTT AGGGGAACGC AAGAGTGTAG GTCCCACGCG CATGAAATTG   
  
  
- AAATTTTCCA GAGAAGAAAA AAGATACTTT CCGACAAGAA GGCGCACCTT GTTTCTCTCC AGTACTTACC   
  
  
- TGTGCGTAAA CCATCCCCAA TTTCTTTCTT TTCTTAGTTT TACCCCCAAA AAAAAAAAAA TAAAGTAAAA   
  
  
- TTAAAATCTT CTTTACAAAG ATAACTTATT TTTAAATTCA CATTACATAT TATAAAATAA AAATTAAATC   
  
  
- CAAACTTTCC ACATTTTAAA AATCTTTAAA ATAAGTTAAA TTTCCAGGTC AATCATGTAA GACATATTTT   
  
  
- TTTAAAATGA ATAAAATCTT TAAAAATTAT CGTTATTAAA ATAGATAACA TTTTACATTA TTATGACAAA   
  
  
- AAATTACTGT AAATATCACA ATTTAAATTG ATAGGATAAA TGTGTAATAT TGAAAAACTT TCTTTTTCAG   
  
  
- TAGAAAAAAA TCTTGGGGAG GAGACCTTTG ATTGGCATAT ATATTATATG GTGAGTAATG AAAGAAGAGG   
  
  
- ACGTTTGGTG TGTTTGTTTA ATTAAACAAT TAATTAATTT AATGTCCAAA GCAGATGAAG CGAAGAGGTC   
  
  
- ATCAGAAGAA GAAGCATAAA TAAAATATTG GAAAACAAGA GGTGGAGGAG GAGACAGAGG CTTGGGATGC   
  
  
- AGCAGTGGTA AGTAACAAGT GGGCTCTGTC GCTCTCTCTC TATTTGCTGA GTGAATAATT CAGCACAACA   
  
  
- GCACACGAAC AGGAGCTGGT CACTGATTTG TGAACATTCT TGGCAAATAC TACCCATCAG CCATCCTCTT   
  
  
- TATTACTCCC TCTAACTCCT ACACTCTTTG ACCCAATCCA CCTTTCTCAT CCCTTCTATA TTTATTTTTT   
  
  
- ATCCGTTCAT TTTTTATTTT TTTAAAAATG AATTAAATTA TTTATTTTTT AAAATTATAA TTATCTATTT   
  
  
- CTTTTTTAAA AGATTAATAA AAATCAAACT GATAGAAAAG ATGTTTAGTT TATCAAAATA ATTTTTTTTA   
  
  
- GATGCCCATA TATACTAATT TCATCAAGTG GGCATGGTTA TTGAATTGAT GCTATTGGTG TGCCGAAGAA   
  
  
- AGTTATTCTT CACATTTGTC GACGAGGAC

+     Unnamed\_\_1

| Site Name | Organism | Position | Strand | Matrix score. | sequence | function |
| --- | --- | --- | --- | --- | --- | --- |
| Unnamed\_\_1 | Zea mays | 80 | - | 5 | CGTGG |  |
| Unnamed\_\_1 | Zea mays | 464 | + | 5 | CGTGG |  |
| Unnamed\_\_1 | Glycine max | 942 | - | 11 | GAATTTAATTAA | 60K protein binding site |

> 2018/04/13 10:10:12  
+ CTGACATTGT TACTAGGCAG AGTCTGTCTC TCGGACGGCT TTAAGACGCT TGCGCCTCTC TCTTTCACGC   
  
  
+ TCCGATTCTC CACGCAAAAT CTGACTCCTT TTCCCTCTTC CTACCTTTCT CTTTCCCTCT CTCGATCCCA   
  
  
+ AAACACTACC ATTCCTGCGC ATCAAAAACT TGAAAAAAGG ACACTTCGTC ACAGGATAGT TGTCGCTGCG   
  
  
+ CGCGAGAGAA GAGACAATAG AGAGATTTCT GTTTGTCAGT ATCTCTGTCT CTCTCTTTTC GTTTGTTTTT   
  
  
+ TGTGACTTTT CTTACTTTCT TTTCTTCTCT CTTCCCTTAT CTTTTCTCTA TTCAATCTTT TTAGCGGCAG   
  
  
+ TTGGCTTTTG TCATGCCCTC CGACAATAAA TCCCCTTGCG TTCTCACATC CAGGGTGCGC GTACTTTAAC   
  
  
+ TTTAAAAGGT CTCTTCTTTT TTCTATGAAA GGCTGTTCTT CCGCGTGGAA CAAAGAGAGG TCATGAATGG   
  
  
+ ACACGCATTT GGTAGGGGTT AAAGAAAGAA AAGAATCAAA ATGGGGGTTT TTTTTTTTTT ATTTCATTTT   
  
  
+ AATTTTAGAA GAAATGTTTC TATTGAATAA AAATTTAAGT GTAATGTATA ATATTTTATT TTTAATTTAG   
  
  
+ GTTTGAAAGG TGTAAAATTT TTAGAAATTT TATTCAATTT AAAGGTCCAG TTAGTACATT CTGTATAAAA   
  
  
+ AAATTTTACT TATTTTAGAA ATTTTTAATA GCAATAATTT TATCTATTGT AAAATGTAAT AATACTGTTT   
  
  
+ TTTAATGACA TTTATAGTGT TAAATTTAAC TATCCTATTT ACACATTATA ACTTTTTGAA AGAAAAAGTC   
  
  
+ ATCTTTTTTT AGAACCCCTC CTCTGGAAAC TAACCGTATA TATAATATAC CACTCATTAC TTTCTTCTCC   
  
  
+ TGCAAACCAC ACAAACAAAT TAATTTGTTA ATTAATTAAA TTACAGGTTT CGTCTACTTC GCTTCTCCAG   
  
  
+ TAGTCTTCTT CTTCGTATTT ATTTTATAAC CTTTTGTTCT CCACCTCCTC CTCTGTCTCC GAACCCTACG   
  
  
+ TCGTCACCAT TCATTGTTCA CCCGAGACAG CGAGAGAGAG ATAAACGACT CACTTATTAA GTCGTGTTGT   
  
  
+ CGTGTGCTTG TCCTCGACCA GTGACTAAAC ACTTGTAAGA ACCGTTTATG ATGGGTAGTC GGTAGGAGAA   
  
  
+ ATAATGAGGG AGATTGAGGA TGTGAGAAAC TGGGTTAGGT GGAAAGAGTA GGGAAGATAT AAATAAAAAA   
  
  
+ TAGGCAAGTA AAAAATAAAA AAATTTTTAC TTAATTTAAT AAATAAAAAA TTTTAATATT AATAGATAAA   
  
  
+ GAAAAAATTT TCTAATTATT TTTAGTTTGA CTATCTTTTC TACAAATCAA ATAGTTTTAT TAAAAAAAAT   
  
  
+ CTACGGGTAT ATATGATTAA AGTAGTTCAC CCGTACCAAT AACTTAACTA CGATAACCAC ACGGCTTCTT   
  
  
+ TCAATAAGAA GTGTAAACAG CTGCTCCTG  

- GACTGTAACA ATGATCCGTC TCAGACAGAG AGCCTGCCGA AATTCTGCGA ACGCGGAGAG AGAAAGTGCG   
  
  
- AGGCTAAGAG GTGCGTTTTA GACTGAGGAA AAGGGAGAAG GATGGAAAGA GAAAGGGAGA GAGCTAGGGT   
  
  
- TTTGTGATGG TAAGGACGCG TAGTTTTTGA ACTTTTTTCC TGTGAAGCAG TGTCCTATCA ACAGCGACGC   
  
  
- GCGCTCTCTT CTCTGTTATC TCTCTAAAGA CAAACAGTCA TAGAGACAGA GAGAGAAAAG CAAACAAAAA   
  
  
- ACACTGAAAA GAATGAAAGA AAAGAAGAGA GAAGGGAATA GAAAAGAGAT AAGTTAGAAA AATCGCCGTC   
  
  
- AACCGAAAAC AGTACGGGAG GCTGTTATTT AGGGGAACGC AAGAGTGTAG GTCCCACGCG CATGAAATTG   
  
  
- AAATTTTCCA GAGAAGAAAA AAGATACTTT CCGACAAGAA GGCGCACCTT GTTTCTCTCC AGTACTTACC   
  
  
- TGTGCGTAAA CCATCCCCAA TTTCTTTCTT TTCTTAGTTT TACCCCCAAA AAAAAAAAAA TAAAGTAAAA   
  
  
- TTAAAATCTT CTTTACAAAG ATAACTTATT TTTAAATTCA CATTACATAT TATAAAATAA AAATTAAATC   
  
  
- CAAACTTTCC ACATTTTAAA AATCTTTAAA ATAAGTTAAA TTTCCAGGTC AATCATGTAA GACATATTTT   
  
  
- TTTAAAATGA ATAAAATCTT TAAAAATTAT CGTTATTAAA ATAGATAACA TTTTACATTA TTATGACAAA   
  
  
- AAATTACTGT AAATATCACA ATTTAAATTG ATAGGATAAA TGTGTAATAT TGAAAAACTT TCTTTTTCAG   
  
  
- TAGAAAAAAA TCTTGGGGAG GAGACCTTTG ATTGGCATAT ATATTATATG GTGAGTAATG AAAGAAGAGG   
  
  
- ACGTTTGGTG TGTTTGTTTA ATTAAACAAT TAATTAATTT AATGTCCAAA GCAGATGAAG CGAAGAGGTC   
  
  
- ATCAGAAGAA GAAGCATAAA TAAAATATTG GAAAACAAGA GGTGGAGGAG GAGACAGAGG CTTGGGATGC   
  
  
- AGCAGTGGTA AGTAACAAGT GGGCTCTGTC GCTCTCTCTC TATTTGCTGA GTGAATAATT CAGCACAACA   
  
  
- GCACACGAAC AGGAGCTGGT CACTGATTTG TGAACATTCT TGGCAAATAC TACCCATCAG CCATCCTCTT   
  
  
- TATTACTCCC TCTAACTCCT ACACTCTTTG ACCCAATCCA CCTTTCTCAT CCCTTCTATA TTTATTTTTT   
  
  
- ATCCGTTCAT TTTTTATTTT TTTAAAAATG AATTAAATTA TTTATTTTTT AAAATTATAA TTATCTATTT   
  
  
- CTTTTTTAAA AGATTAATAA AAATCAAACT GATAGAAAAG ATGTTTAGTT TATCAAAATA ATTTTTTTTA   
  
  
- GATGCCCATA TATACTAATT TCATCAAGTG GGCATGGTTA TTGAATTGAT GCTATTGGTG TGCCGAAGAA   
  
  
- AGTTATTCTT CACATTTGTC GACGAGGAC

+     Unnamed\_\_17

| Site Name | Organism | Position | Strand | Matrix score. | sequence | function |
| --- | --- | --- | --- | --- | --- | --- |
| Unnamed\_\_17 | Zea mays | 1489 | - | 10 | TAGGAGCAGCT |  |

> 2018/04/13 10:10:12  
+ CTGACATTGT TACTAGGCAG AGTCTGTCTC TCGGACGGCT TTAAGACGCT TGCGCCTCTC TCTTTCACGC   
  
  
+ TCCGATTCTC CACGCAAAAT CTGACTCCTT TTCCCTCTTC CTACCTTTCT CTTTCCCTCT CTCGATCCCA   
  
  
+ AAACACTACC ATTCCTGCGC ATCAAAAACT TGAAAAAAGG ACACTTCGTC ACAGGATAGT TGTCGCTGCG   
  
  
+ CGCGAGAGAA GAGACAATAG AGAGATTTCT GTTTGTCAGT ATCTCTGTCT CTCTCTTTTC GTTTGTTTTT   
  
  
+ TGTGACTTTT CTTACTTTCT TTTCTTCTCT CTTCCCTTAT CTTTTCTCTA TTCAATCTTT TTAGCGGCAG   
  
  
+ TTGGCTTTTG TCATGCCCTC CGACAATAAA TCCCCTTGCG TTCTCACATC CAGGGTGCGC GTACTTTAAC   
  
  
+ TTTAAAAGGT CTCTTCTTTT TTCTATGAAA GGCTGTTCTT CCGCGTGGAA CAAAGAGAGG TCATGAATGG   
  
  
+ ACACGCATTT GGTAGGGGTT AAAGAAAGAA AAGAATCAAA ATGGGGGTTT TTTTTTTTTT ATTTCATTTT   
  
  
+ AATTTTAGAA GAAATGTTTC TATTGAATAA AAATTTAAGT GTAATGTATA ATATTTTATT TTTAATTTAG   
  
  
+ GTTTGAAAGG TGTAAAATTT TTAGAAATTT TATTCAATTT AAAGGTCCAG TTAGTACATT CTGTATAAAA   
  
  
+ AAATTTTACT TATTTTAGAA ATTTTTAATA GCAATAATTT TATCTATTGT AAAATGTAAT AATACTGTTT   
  
  
+ TTTAATGACA TTTATAGTGT TAAATTTAAC TATCCTATTT ACACATTATA ACTTTTTGAA AGAAAAAGTC   
  
  
+ ATCTTTTTTT AGAACCCCTC CTCTGGAAAC TAACCGTATA TATAATATAC CACTCATTAC TTTCTTCTCC   
  
  
+ TGCAAACCAC ACAAACAAAT TAATTTGTTA ATTAATTAAA TTACAGGTTT CGTCTACTTC GCTTCTCCAG   
  
  
+ TAGTCTTCTT CTTCGTATTT ATTTTATAAC CTTTTGTTCT CCACCTCCTC CTCTGTCTCC GAACCCTACG   
  
  
+ TCGTCACCAT TCATTGTTCA CCCGAGACAG CGAGAGAGAG ATAAACGACT CACTTATTAA GTCGTGTTGT   
  
  
+ CGTGTGCTTG TCCTCGACCA GTGACTAAAC ACTTGTAAGA ACCGTTTATG ATGGGTAGTC GGTAGGAGAA   
  
  
+ ATAATGAGGG AGATTGAGGA TGTGAGAAAC TGGGTTAGGT GGAAAGAGTA GGGAAGATAT AAATAAAAAA   
  
  
+ TAGGCAAGTA AAAAATAAAA AAATTTTTAC TTAATTTAAT AAATAAAAAA TTTTAATATT AATAGATAAA   
  
  
+ GAAAAAATTT TCTAATTATT TTTAGTTTGA CTATCTTTTC TACAAATCAA ATAGTTTTAT TAAAAAAAAT   
  
  
+ CTACGGGTAT ATATGATTAA AGTAGTTCAC CCGTACCAAT AACTTAACTA CGATAACCAC ACGGCTTCTT   
  
  
+ TCAATAAGAA GTGTAAACAG CTGCTCCTG  

- GACTGTAACA ATGATCCGTC TCAGACAGAG AGCCTGCCGA AATTCTGCGA ACGCGGAGAG AGAAAGTGCG   
  
  
- AGGCTAAGAG GTGCGTTTTA GACTGAGGAA AAGGGAGAAG GATGGAAAGA GAAAGGGAGA GAGCTAGGGT   
  
  
- TTTGTGATGG TAAGGACGCG TAGTTTTTGA ACTTTTTTCC TGTGAAGCAG TGTCCTATCA ACAGCGACGC   
  
  
- GCGCTCTCTT CTCTGTTATC TCTCTAAAGA CAAACAGTCA TAGAGACAGA GAGAGAAAAG CAAACAAAAA   
  
  
- ACACTGAAAA GAATGAAAGA AAAGAAGAGA GAAGGGAATA GAAAAGAGAT AAGTTAGAAA AATCGCCGTC   
  
  
- AACCGAAAAC AGTACGGGAG GCTGTTATTT AGGGGAACGC AAGAGTGTAG GTCCCACGCG CATGAAATTG   
  
  
- AAATTTTCCA GAGAAGAAAA AAGATACTTT CCGACAAGAA GGCGCACCTT GTTTCTCTCC AGTACTTACC   
  
  
- TGTGCGTAAA CCATCCCCAA TTTCTTTCTT TTCTTAGTTT TACCCCCAAA AAAAAAAAAA TAAAGTAAAA   
  
  
- TTAAAATCTT CTTTACAAAG ATAACTTATT TTTAAATTCA CATTACATAT TATAAAATAA AAATTAAATC   
  
  
- CAAACTTTCC ACATTTTAAA AATCTTTAAA ATAAGTTAAA TTTCCAGGTC AATCATGTAA GACATATTTT   
  
  
- TTTAAAATGA ATAAAATCTT TAAAAATTAT CGTTATTAAA ATAGATAACA TTTTACATTA TTATGACAAA   
  
  
- AAATTACTGT AAATATCACA ATTTAAATTG ATAGGATAAA TGTGTAATAT TGAAAAACTT TCTTTTTCAG   
  
  
- TAGAAAAAAA TCTTGGGGAG GAGACCTTTG ATTGGCATAT ATATTATATG GTGAGTAATG AAAGAAGAGG   
  
  
- ACGTTTGGTG TGTTTGTTTA ATTAAACAAT TAATTAATTT AATGTCCAAA GCAGATGAAG CGAAGAGGTC   
  
  
- ATCAGAAGAA GAAGCATAAA TAAAATATTG GAAAACAAGA GGTGGAGGAG GAGACAGAGG CTTGGGATGC   
  
  
- AGCAGTGGTA AGTAACAAGT GGGCTCTGTC GCTCTCTCTC TATTTGCTGA GTGAATAATT CAGCACAACA   
  
  
- GCACACGAAC AGGAGCTGGT CACTGATTTG TGAACATTCT TGGCAAATAC TACCCATCAG CCATCCTCTT   
  
  
- TATTACTCCC TCTAACTCCT ACACTCTTTG ACCCAATCCA CCTTTCTCAT CCCTTCTATA TTTATTTTTT   
  
  
- ATCCGTTCAT TTTTTATTTT TTTAAAAATG AATTAAATTA TTTATTTTTT AAAATTATAA TTATCTATTT   
  
  
- CTTTTTTAAA AGATTAATAA AAATCAAACT GATAGAAAAG ATGTTTAGTT TATCAAAATA ATTTTTTTTA   
  
  
- GATGCCCATA TATACTAATT TCATCAAGTG GGCATGGTTA TTGAATTGAT GCTATTGGTG TGCCGAAGAA   
  
  
- AGTTATTCTT CACATTTGTC GACGAGGAC

+     Unnamed\_\_3

| Site Name | Organism | Position | Strand | Matrix score. | sequence | function |
| --- | --- | --- | --- | --- | --- | --- |
| Unnamed\_\_3 | Zea mays | 80 | - | 5 | CGTGG |  |
| Unnamed\_\_3 | Zea mays | 464 | + | 5 | CGTGG |  |

> 2018/04/13 10:10:12  
+ CTGACATTGT TACTAGGCAG AGTCTGTCTC TCGGACGGCT TTAAGACGCT TGCGCCTCTC TCTTTCACGC   
  
  
+ TCCGATTCTC CACGCAAAAT CTGACTCCTT TTCCCTCTTC CTACCTTTCT CTTTCCCTCT CTCGATCCCA   
  
  
+ AAACACTACC ATTCCTGCGC ATCAAAAACT TGAAAAAAGG ACACTTCGTC ACAGGATAGT TGTCGCTGCG   
  
  
+ CGCGAGAGAA GAGACAATAG AGAGATTTCT GTTTGTCAGT ATCTCTGTCT CTCTCTTTTC GTTTGTTTTT   
  
  
+ TGTGACTTTT CTTACTTTCT TTTCTTCTCT CTTCCCTTAT CTTTTCTCTA TTCAATCTTT TTAGCGGCAG   
  
  
+ TTGGCTTTTG TCATGCCCTC CGACAATAAA TCCCCTTGCG TTCTCACATC CAGGGTGCGC GTACTTTAAC   
  
  
+ TTTAAAAGGT CTCTTCTTTT TTCTATGAAA GGCTGTTCTT CCGCGTGGAA CAAAGAGAGG TCATGAATGG   
  
  
+ ACACGCATTT GGTAGGGGTT AAAGAAAGAA AAGAATCAAA ATGGGGGTTT TTTTTTTTTT ATTTCATTTT   
  
  
+ AATTTTAGAA GAAATGTTTC TATTGAATAA AAATTTAAGT GTAATGTATA ATATTTTATT TTTAATTTAG   
  
  
+ GTTTGAAAGG TGTAAAATTT TTAGAAATTT TATTCAATTT AAAGGTCCAG TTAGTACATT CTGTATAAAA   
  
  
+ AAATTTTACT TATTTTAGAA ATTTTTAATA GCAATAATTT TATCTATTGT AAAATGTAAT AATACTGTTT   
  
  
+ TTTAATGACA TTTATAGTGT TAAATTTAAC TATCCTATTT ACACATTATA ACTTTTTGAA AGAAAAAGTC   
  
  
+ ATCTTTTTTT AGAACCCCTC CTCTGGAAAC TAACCGTATA TATAATATAC CACTCATTAC TTTCTTCTCC   
  
  
+ TGCAAACCAC ACAAACAAAT TAATTTGTTA ATTAATTAAA TTACAGGTTT CGTCTACTTC GCTTCTCCAG   
  
  
+ TAGTCTTCTT CTTCGTATTT ATTTTATAAC CTTTTGTTCT CCACCTCCTC CTCTGTCTCC GAACCCTACG   
  
  
+ TCGTCACCAT TCATTGTTCA CCCGAGACAG CGAGAGAGAG ATAAACGACT CACTTATTAA GTCGTGTTGT   
  
  
+ CGTGTGCTTG TCCTCGACCA GTGACTAAAC ACTTGTAAGA ACCGTTTATG ATGGGTAGTC GGTAGGAGAA   
  
  
+ ATAATGAGGG AGATTGAGGA TGTGAGAAAC TGGGTTAGGT GGAAAGAGTA GGGAAGATAT AAATAAAAAA   
  
  
+ TAGGCAAGTA AAAAATAAAA AAATTTTTAC TTAATTTAAT AAATAAAAAA TTTTAATATT AATAGATAAA   
  
  
+ GAAAAAATTT TCTAATTATT TTTAGTTTGA CTATCTTTTC TACAAATCAA ATAGTTTTAT TAAAAAAAAT   
  
  
+ CTACGGGTAT ATATGATTAA AGTAGTTCAC CCGTACCAAT AACTTAACTA CGATAACCAC ACGGCTTCTT   
  
  
+ TCAATAAGAA GTGTAAACAG CTGCTCCTG  

- GACTGTAACA ATGATCCGTC TCAGACAGAG AGCCTGCCGA AATTCTGCGA ACGCGGAGAG AGAAAGTGCG   
  
  
- AGGCTAAGAG GTGCGTTTTA GACTGAGGAA AAGGGAGAAG GATGGAAAGA GAAAGGGAGA GAGCTAGGGT   
  
  
- TTTGTGATGG TAAGGACGCG TAGTTTTTGA ACTTTTTTCC TGTGAAGCAG TGTCCTATCA ACAGCGACGC   
  
  
- GCGCTCTCTT CTCTGTTATC TCTCTAAAGA CAAACAGTCA TAGAGACAGA GAGAGAAAAG CAAACAAAAA   
  
  
- ACACTGAAAA GAATGAAAGA AAAGAAGAGA GAAGGGAATA GAAAAGAGAT AAGTTAGAAA AATCGCCGTC   
  
  
- AACCGAAAAC AGTACGGGAG GCTGTTATTT AGGGGAACGC AAGAGTGTAG GTCCCACGCG CATGAAATTG   
  
  
- AAATTTTCCA GAGAAGAAAA AAGATACTTT CCGACAAGAA GGCGCACCTT GTTTCTCTCC AGTACTTACC   
  
  
- TGTGCGTAAA CCATCCCCAA TTTCTTTCTT TTCTTAGTTT TACCCCCAAA AAAAAAAAAA TAAAGTAAAA   
  
  
- TTAAAATCTT CTTTACAAAG ATAACTTATT TTTAAATTCA CATTACATAT TATAAAATAA AAATTAAATC   
  
  
- CAAACTTTCC ACATTTTAAA AATCTTTAAA ATAAGTTAAA TTTCCAGGTC AATCATGTAA GACATATTTT   
  
  
- TTTAAAATGA ATAAAATCTT TAAAAATTAT CGTTATTAAA ATAGATAACA TTTTACATTA TTATGACAAA   
  
  
- AAATTACTGT AAATATCACA ATTTAAATTG ATAGGATAAA TGTGTAATAT TGAAAAACTT TCTTTTTCAG   
  
  
- TAGAAAAAAA TCTTGGGGAG GAGACCTTTG ATTGGCATAT ATATTATATG GTGAGTAATG AAAGAAGAGG   
  
  
- ACGTTTGGTG TGTTTGTTTA ATTAAACAAT TAATTAATTT AATGTCCAAA GCAGATGAAG CGAAGAGGTC   
  
  
- ATCAGAAGAA GAAGCATAAA TAAAATATTG GAAAACAAGA GGTGGAGGAG GAGACAGAGG CTTGGGATGC   
  
  
- AGCAGTGGTA AGTAACAAGT GGGCTCTGTC GCTCTCTCTC TATTTGCTGA GTGAATAATT CAGCACAACA   
  
  
- GCACACGAAC AGGAGCTGGT CACTGATTTG TGAACATTCT TGGCAAATAC TACCCATCAG CCATCCTCTT   
  
  
- TATTACTCCC TCTAACTCCT ACACTCTTTG ACCCAATCCA CCTTTCTCAT CCCTTCTATA TTTATTTTTT   
  
  
- ATCCGTTCAT TTTTTATTTT TTTAAAAATG AATTAAATTA TTTATTTTTT AAAATTATAA TTATCTATTT   
  
  
- CTTTTTTAAA AGATTAATAA AAATCAAACT GATAGAAAAG ATGTTTAGTT TATCAAAATA ATTTTTTTTA   
  
  
- GATGCCCATA TATACTAATT TCATCAAGTG GGCATGGTTA TTGAATTGAT GCTATTGGTG TGCCGAAGAA   
  
  
- AGTTATTCTT CACATTTGTC GACGAGGAC

+     Unnamed\_\_4

| Site Name | Organism | Position | Strand | Matrix score. | sequence | function |
| --- | --- | --- | --- | --- | --- | --- |
| Unnamed\_\_4 | Petroselinum hortense | 907 | + | 4 | CTCC |  |
| Unnamed\_\_4 | Petroselinum hortense | 858 | + | 4 | CTCC |  |
| Unnamed\_\_4 | Petroselinum hortense | 1494 | + | 4 | CTCC |  |
| Unnamed\_\_4 | Petroselinum hortense | 70 | + | 4 | CTCC |  |
| Unnamed\_\_4 | Petroselinum hortense | 1199 | - | 4 | CTCC |  |
| Unnamed\_\_4 | Petroselinum hortense | 1185 | - | 4 | CTCC |  |
| Unnamed\_\_4 | Petroselinum hortense | 1019 | + | 4 | CTCC |  |
| Unnamed\_\_4 | Petroselinum hortense | 975 | + | 4 | CTCC |  |
| Unnamed\_\_4 | Petroselinum hortense | 95 | + | 4 | CTCC |  |
| Unnamed\_\_4 | Petroselinum hortense | 1028 | + | 4 | CTCC |  |
| Unnamed\_\_4 | Petroselinum hortense | 78 | + | 4 | CTCC |  |
| Unnamed\_\_4 | Petroselinum hortense | 1037 | + | 4 | CTCC |  |
| Unnamed\_\_4 | Petroselinum hortense | 368 | + | 4 | CTCC |  |
| Unnamed\_\_4 | Petroselinum hortense | 1025 | + | 4 | CTCC |  |

> 2018/04/13 10:10:12  
+ CTGACATTGT TACTAGGCAG AGTCTGTCTC TCGGACGGCT TTAAGACGCT TGCGCCTCTC TCTTTCACGC   
  
  
+ TCCGATTCTC CACGCAAAAT CTGACTCCTT TTCCCTCTTC CTACCTTTCT CTTTCCCTCT CTCGATCCCA   
  
  
+ AAACACTACC ATTCCTGCGC ATCAAAAACT TGAAAAAAGG ACACTTCGTC ACAGGATAGT TGTCGCTGCG   
  
  
+ CGCGAGAGAA GAGACAATAG AGAGATTTCT GTTTGTCAGT ATCTCTGTCT CTCTCTTTTC GTTTGTTTTT   
  
  
+ TGTGACTTTT CTTACTTTCT TTTCTTCTCT CTTCCCTTAT CTTTTCTCTA TTCAATCTTT TTAGCGGCAG   
  
  
+ TTGGCTTTTG TCATGCCCTC CGACAATAAA TCCCCTTGCG TTCTCACATC CAGGGTGCGC GTACTTTAAC   
  
  
+ TTTAAAAGGT CTCTTCTTTT TTCTATGAAA GGCTGTTCTT CCGCGTGGAA CAAAGAGAGG TCATGAATGG   
  
  
+ ACACGCATTT GGTAGGGGTT AAAGAAAGAA AAGAATCAAA ATGGGGGTTT TTTTTTTTTT ATTTCATTTT   
  
  
+ AATTTTAGAA GAAATGTTTC TATTGAATAA AAATTTAAGT GTAATGTATA ATATTTTATT TTTAATTTAG   
  
  
+ GTTTGAAAGG TGTAAAATTT TTAGAAATTT TATTCAATTT AAAGGTCCAG TTAGTACATT CTGTATAAAA   
  
  
+ AAATTTTACT TATTTTAGAA ATTTTTAATA GCAATAATTT TATCTATTGT AAAATGTAAT AATACTGTTT   
  
  
+ TTTAATGACA TTTATAGTGT TAAATTTAAC TATCCTATTT ACACATTATA ACTTTTTGAA AGAAAAAGTC   
  
  
+ ATCTTTTTTT AGAACCCCTC CTCTGGAAAC TAACCGTATA TATAATATAC CACTCATTAC TTTCTTCTCC   
  
  
+ TGCAAACCAC ACAAACAAAT TAATTTGTTA ATTAATTAAA TTACAGGTTT CGTCTACTTC GCTTCTCCAG   
  
  
+ TAGTCTTCTT CTTCGTATTT ATTTTATAAC CTTTTGTTCT CCACCTCCTC CTCTGTCTCC GAACCCTACG   
  
  
+ TCGTCACCAT TCATTGTTCA CCCGAGACAG CGAGAGAGAG ATAAACGACT CACTTATTAA GTCGTGTTGT   
  
  
+ CGTGTGCTTG TCCTCGACCA GTGACTAAAC ACTTGTAAGA ACCGTTTATG ATGGGTAGTC GGTAGGAGAA   
  
  
+ ATAATGAGGG AGATTGAGGA TGTGAGAAAC TGGGTTAGGT GGAAAGAGTA GGGAAGATAT AAATAAAAAA   
  
  
+ TAGGCAAGTA AAAAATAAAA AAATTTTTAC TTAATTTAAT AAATAAAAAA TTTTAATATT AATAGATAAA   
  
  
+ GAAAAAATTT TCTAATTATT TTTAGTTTGA CTATCTTTTC TACAAATCAA ATAGTTTTAT TAAAAAAAAT   
  
  
+ CTACGGGTAT ATATGATTAA AGTAGTTCAC CCGTACCAAT AACTTAACTA CGATAACCAC ACGGCTTCTT   
  
  
+ TCAATAAGAA GTGTAAACAG CTGCTCCTG  

- GACTGTAACA ATGATCCGTC TCAGACAGAG AGCCTGCCGA AATTCTGCGA ACGCGGAGAG AGAAAGTGCG   
  
  
- AGGCTAAGAG GTGCGTTTTA GACTGAGGAA AAGGGAGAAG GATGGAAAGA GAAAGGGAGA GAGCTAGGGT   
  
  
- TTTGTGATGG TAAGGACGCG TAGTTTTTGA ACTTTTTTCC TGTGAAGCAG TGTCCTATCA ACAGCGACGC   
  
  
- GCGCTCTCTT CTCTGTTATC TCTCTAAAGA CAAACAGTCA TAGAGACAGA GAGAGAAAAG CAAACAAAAA   
  
  
- ACACTGAAAA GAATGAAAGA AAAGAAGAGA GAAGGGAATA GAAAAGAGAT AAGTTAGAAA AATCGCCGTC   
  
  
- AACCGAAAAC AGTACGGGAG GCTGTTATTT AGGGGAACGC AAGAGTGTAG GTCCCACGCG CATGAAATTG   
  
  
- AAATTTTCCA GAGAAGAAAA AAGATACTTT CCGACAAGAA GGCGCACCTT GTTTCTCTCC AGTACTTACC   
  
  
- TGTGCGTAAA CCATCCCCAA TTTCTTTCTT TTCTTAGTTT TACCCCCAAA AAAAAAAAAA TAAAGTAAAA   
  
  
- TTAAAATCTT CTTTACAAAG ATAACTTATT TTTAAATTCA CATTACATAT TATAAAATAA AAATTAAATC   
  
  
- CAAACTTTCC ACATTTTAAA AATCTTTAAA ATAAGTTAAA TTTCCAGGTC AATCATGTAA GACATATTTT   
  
  
- TTTAAAATGA ATAAAATCTT TAAAAATTAT CGTTATTAAA ATAGATAACA TTTTACATTA TTATGACAAA   
  
  
- AAATTACTGT AAATATCACA ATTTAAATTG ATAGGATAAA TGTGTAATAT TGAAAAACTT TCTTTTTCAG   
  
  
- TAGAAAAAAA TCTTGGGGAG GAGACCTTTG ATTGGCATAT ATATTATATG GTGAGTAATG AAAGAAGAGG   
  
  
- ACGTTTGGTG TGTTTGTTTA ATTAAACAAT TAATTAATTT AATGTCCAAA GCAGATGAAG CGAAGAGGTC   
  
  
- ATCAGAAGAA GAAGCATAAA TAAAATATTG GAAAACAAGA GGTGGAGGAG GAGACAGAGG CTTGGGATGC   
  
  
- AGCAGTGGTA AGTAACAAGT GGGCTCTGTC GCTCTCTCTC TATTTGCTGA GTGAATAATT CAGCACAACA   
  
  
- GCACACGAAC AGGAGCTGGT CACTGATTTG TGAACATTCT TGGCAAATAC TACCCATCAG CCATCCTCTT   
  
  
- TATTACTCCC TCTAACTCCT ACACTCTTTG ACCCAATCCA CCTTTCTCAT CCCTTCTATA TTTATTTTTT   
  
  
- ATCCGTTCAT TTTTTATTTT TTTAAAAATG AATTAAATTA TTTATTTTTT AAAATTATAA TTATCTATTT   
  
  
- CTTTTTTAAA AGATTAATAA AAATCAAACT GATAGAAAAG ATGTTTAGTT TATCAAAATA ATTTTTTTTA   
  
  
- GATGCCCATA TATACTAATT TCATCAAGTG GGCATGGTTA TTGAATTGAT GCTATTGGTG TGCCGAAGAA   
  
  
- AGTTATTCTT CACATTTGTC GACGAGGAC

+     chs-CMA1a

| Site Name | Organism | Position | Strand | Matrix score. | sequence | function |
| --- | --- | --- | --- | --- | --- | --- |
| chs-CMA1a | Daucus carota | 1287 | + | 8 | TTACTTAA | part of a light responsive element |

> 2018/04/13 10:10:12  
+ CTGACATTGT TACTAGGCAG AGTCTGTCTC TCGGACGGCT TTAAGACGCT TGCGCCTCTC TCTTTCACGC   
  
  
+ TCCGATTCTC CACGCAAAAT CTGACTCCTT TTCCCTCTTC CTACCTTTCT CTTTCCCTCT CTCGATCCCA   
  
  
+ AAACACTACC ATTCCTGCGC ATCAAAAACT TGAAAAAAGG ACACTTCGTC ACAGGATAGT TGTCGCTGCG   
  
  
+ CGCGAGAGAA GAGACAATAG AGAGATTTCT GTTTGTCAGT ATCTCTGTCT CTCTCTTTTC GTTTGTTTTT   
  
  
+ TGTGACTTTT CTTACTTTCT TTTCTTCTCT CTTCCCTTAT CTTTTCTCTA TTCAATCTTT TTAGCGGCAG   
  
  
+ TTGGCTTTTG TCATGCCCTC CGACAATAAA TCCCCTTGCG TTCTCACATC CAGGGTGCGC GTACTTTAAC   
  
  
+ TTTAAAAGGT CTCTTCTTTT TTCTATGAAA GGCTGTTCTT CCGCGTGGAA CAAAGAGAGG TCATGAATGG   
  
  
+ ACACGCATTT GGTAGGGGTT AAAGAAAGAA AAGAATCAAA ATGGGGGTTT TTTTTTTTTT ATTTCATTTT   
  
  
+ AATTTTAGAA GAAATGTTTC TATTGAATAA AAATTTAAGT GTAATGTATA ATATTTTATT TTTAATTTAG   
  
  
+ GTTTGAAAGG TGTAAAATTT TTAGAAATTT TATTCAATTT AAAGGTCCAG TTAGTACATT CTGTATAAAA   
  
  
+ AAATTTTACT TATTTTAGAA ATTTTTAATA GCAATAATTT TATCTATTGT AAAATGTAAT AATACTGTTT   
  
  
+ TTTAATGACA TTTATAGTGT TAAATTTAAC TATCCTATTT ACACATTATA ACTTTTTGAA AGAAAAAGTC   
  
  
+ ATCTTTTTTT AGAACCCCTC CTCTGGAAAC TAACCGTATA TATAATATAC CACTCATTAC TTTCTTCTCC   
  
  
+ TGCAAACCAC ACAAACAAAT TAATTTGTTA ATTAATTAAA TTACAGGTTT CGTCTACTTC GCTTCTCCAG   
  
  
+ TAGTCTTCTT CTTCGTATTT ATTTTATAAC CTTTTGTTCT CCACCTCCTC CTCTGTCTCC GAACCCTACG   
  
  
+ TCGTCACCAT TCATTGTTCA CCCGAGACAG CGAGAGAGAG ATAAACGACT CACTTATTAA GTCGTGTTGT   
  
  
+ CGTGTGCTTG TCCTCGACCA GTGACTAAAC ACTTGTAAGA ACCGTTTATG ATGGGTAGTC GGTAGGAGAA   
  
  
+ ATAATGAGGG AGATTGAGGA TGTGAGAAAC TGGGTTAGGT GGAAAGAGTA GGGAAGATAT AAATAAAAAA   
  
  
+ TAGGCAAGTA AAAAATAAAA AAATTTTTAC TTAATTTAAT AAATAAAAAA TTTTAATATT AATAGATAAA   
  
  
+ GAAAAAATTT TCTAATTATT TTTAGTTTGA CTATCTTTTC TACAAATCAA ATAGTTTTAT TAAAAAAAAT   
  
  
+ CTACGGGTAT ATATGATTAA AGTAGTTCAC CCGTACCAAT AACTTAACTA CGATAACCAC ACGGCTTCTT   
  
  
+ TCAATAAGAA GTGTAAACAG CTGCTCCTG  

- GACTGTAACA ATGATCCGTC TCAGACAGAG AGCCTGCCGA AATTCTGCGA ACGCGGAGAG AGAAAGTGCG   
  
  
- AGGCTAAGAG GTGCGTTTTA GACTGAGGAA AAGGGAGAAG GATGGAAAGA GAAAGGGAGA GAGCTAGGGT   
  
  
- TTTGTGATGG TAAGGACGCG TAGTTTTTGA ACTTTTTTCC TGTGAAGCAG TGTCCTATCA ACAGCGACGC   
  
  
- GCGCTCTCTT CTCTGTTATC TCTCTAAAGA CAAACAGTCA TAGAGACAGA GAGAGAAAAG CAAACAAAAA   
  
  
- ACACTGAAAA GAATGAAAGA AAAGAAGAGA GAAGGGAATA GAAAAGAGAT AAGTTAGAAA AATCGCCGTC   
  
  
- AACCGAAAAC AGTACGGGAG GCTGTTATTT AGGGGAACGC AAGAGTGTAG GTCCCACGCG CATGAAATTG   
  
  
- AAATTTTCCA GAGAAGAAAA AAGATACTTT CCGACAAGAA GGCGCACCTT GTTTCTCTCC AGTACTTACC   
  
  
- TGTGCGTAAA CCATCCCCAA TTTCTTTCTT TTCTTAGTTT TACCCCCAAA AAAAAAAAAA TAAAGTAAAA   
  
  
- TTAAAATCTT CTTTACAAAG ATAACTTATT TTTAAATTCA CATTACATAT TATAAAATAA AAATTAAATC   
  
  
- CAAACTTTCC ACATTTTAAA AATCTTTAAA ATAAGTTAAA TTTCCAGGTC AATCATGTAA GACATATTTT   
  
  
- TTTAAAATGA ATAAAATCTT TAAAAATTAT CGTTATTAAA ATAGATAACA TTTTACATTA TTATGACAAA   
  
  
- AAATTACTGT AAATATCACA ATTTAAATTG ATAGGATAAA TGTGTAATAT TGAAAAACTT TCTTTTTCAG   
  
  
- TAGAAAAAAA TCTTGGGGAG GAGACCTTTG ATTGGCATAT ATATTATATG GTGAGTAATG AAAGAAGAGG   
  
  
- ACGTTTGGTG TGTTTGTTTA ATTAAACAAT TAATTAATTT AATGTCCAAA GCAGATGAAG CGAAGAGGTC   
  
  
- ATCAGAAGAA GAAGCATAAA TAAAATATTG GAAAACAAGA GGTGGAGGAG GAGACAGAGG CTTGGGATGC   
  
  
- AGCAGTGGTA AGTAACAAGT GGGCTCTGTC GCTCTCTCTC TATTTGCTGA GTGAATAATT CAGCACAACA   
  
  
- GCACACGAAC AGGAGCTGGT CACTGATTTG TGAACATTCT TGGCAAATAC TACCCATCAG CCATCCTCTT   
  
  
- TATTACTCCC TCTAACTCCT ACACTCTTTG ACCCAATCCA CCTTTCTCAT CCCTTCTATA TTTATTTTTT   
  
  
- ATCCGTTCAT TTTTTATTTT TTTAAAAATG AATTAAATTA TTTATTTTTT AAAATTATAA TTATCTATTT   
  
  
- CTTTTTTAAA AGATTAATAA AAATCAAACT GATAGAAAAG ATGTTTAGTT TATCAAAATA ATTTTTTTTA   
  
  
- GATGCCCATA TATACTAATT TCATCAAGTG GGCATGGTTA TTGAATTGAT GCTATTGGTG TGCCGAAGAA   
  
  
- AGTTATTCTT CACATTTGTC GACGAGGAC

+     chs-Unit 1 m1

| Site Name | Organism | Position | Strand | Matrix score. | sequence | function |
| --- | --- | --- | --- | --- | --- | --- |
| chs-Unit 1 m1 | Zea mays | 1220 | - | 10 | ACCTAACCCGG | part of a light responsive element |

> 2018/04/13 10:10:12  
+ CTGACATTGT TACTAGGCAG AGTCTGTCTC TCGGACGGCT TTAAGACGCT TGCGCCTCTC TCTTTCACGC   
  
  
+ TCCGATTCTC CACGCAAAAT CTGACTCCTT TTCCCTCTTC CTACCTTTCT CTTTCCCTCT CTCGATCCCA   
  
  
+ AAACACTACC ATTCCTGCGC ATCAAAAACT TGAAAAAAGG ACACTTCGTC ACAGGATAGT TGTCGCTGCG   
  
  
+ CGCGAGAGAA GAGACAATAG AGAGATTTCT GTTTGTCAGT ATCTCTGTCT CTCTCTTTTC GTTTGTTTTT   
  
  
+ TGTGACTTTT CTTACTTTCT TTTCTTCTCT CTTCCCTTAT CTTTTCTCTA TTCAATCTTT TTAGCGGCAG   
  
  
+ TTGGCTTTTG TCATGCCCTC CGACAATAAA TCCCCTTGCG TTCTCACATC CAGGGTGCGC GTACTTTAAC   
  
  
+ TTTAAAAGGT CTCTTCTTTT TTCTATGAAA GGCTGTTCTT CCGCGTGGAA CAAAGAGAGG TCATGAATGG   
  
  
+ ACACGCATTT GGTAGGGGTT AAAGAAAGAA AAGAATCAAA ATGGGGGTTT TTTTTTTTTT ATTTCATTTT   
  
  
+ AATTTTAGAA GAAATGTTTC TATTGAATAA AAATTTAAGT GTAATGTATA ATATTTTATT TTTAATTTAG   
  
  
+ GTTTGAAAGG TGTAAAATTT TTAGAAATTT TATTCAATTT AAAGGTCCAG TTAGTACATT CTGTATAAAA   
  
  
+ AAATTTTACT TATTTTAGAA ATTTTTAATA GCAATAATTT TATCTATTGT AAAATGTAAT AATACTGTTT   
  
  
+ TTTAATGACA TTTATAGTGT TAAATTTAAC TATCCTATTT ACACATTATA ACTTTTTGAA AGAAAAAGTC   
  
  
+ ATCTTTTTTT AGAACCCCTC CTCTGGAAAC TAACCGTATA TATAATATAC CACTCATTAC TTTCTTCTCC   
  
  
+ TGCAAACCAC ACAAACAAAT TAATTTGTTA ATTAATTAAA TTACAGGTTT CGTCTACTTC GCTTCTCCAG   
  
  
+ TAGTCTTCTT CTTCGTATTT ATTTTATAAC CTTTTGTTCT CCACCTCCTC CTCTGTCTCC GAACCCTACG   
  
  
+ TCGTCACCAT TCATTGTTCA CCCGAGACAG CGAGAGAGAG ATAAACGACT CACTTATTAA GTCGTGTTGT   
  
  
+ CGTGTGCTTG TCCTCGACCA GTGACTAAAC ACTTGTAAGA ACCGTTTATG ATGGGTAGTC GGTAGGAGAA   
  
  
+ ATAATGAGGG AGATTGAGGA TGTGAGAAAC TGGGTTAGGT GGAAAGAGTA GGGAAGATAT AAATAAAAAA   
  
  
+ TAGGCAAGTA AAAAATAAAA AAATTTTTAC TTAATTTAAT AAATAAAAAA TTTTAATATT AATAGATAAA   
  
  
+ GAAAAAATTT TCTAATTATT TTTAGTTTGA CTATCTTTTC TACAAATCAA ATAGTTTTAT TAAAAAAAAT   
  
  
+ CTACGGGTAT ATATGATTAA AGTAGTTCAC CCGTACCAAT AACTTAACTA CGATAACCAC ACGGCTTCTT   
  
  
+ TCAATAAGAA GTGTAAACAG CTGCTCCTG  

- GACTGTAACA ATGATCCGTC TCAGACAGAG AGCCTGCCGA AATTCTGCGA ACGCGGAGAG AGAAAGTGCG   
  
  
- AGGCTAAGAG GTGCGTTTTA GACTGAGGAA AAGGGAGAAG GATGGAAAGA GAAAGGGAGA GAGCTAGGGT   
  
  
- TTTGTGATGG TAAGGACGCG TAGTTTTTGA ACTTTTTTCC TGTGAAGCAG TGTCCTATCA ACAGCGACGC   
  
  
- GCGCTCTCTT CTCTGTTATC TCTCTAAAGA CAAACAGTCA TAGAGACAGA GAGAGAAAAG CAAACAAAAA   
  
  
- ACACTGAAAA GAATGAAAGA AAAGAAGAGA GAAGGGAATA GAAAAGAGAT AAGTTAGAAA AATCGCCGTC   
  
  
- AACCGAAAAC AGTACGGGAG GCTGTTATTT AGGGGAACGC AAGAGTGTAG GTCCCACGCG CATGAAATTG   
  
  
- AAATTTTCCA GAGAAGAAAA AAGATACTTT CCGACAAGAA GGCGCACCTT GTTTCTCTCC AGTACTTACC   
  
  
- TGTGCGTAAA CCATCCCCAA TTTCTTTCTT TTCTTAGTTT TACCCCCAAA AAAAAAAAAA TAAAGTAAAA   
  
  
- TTAAAATCTT CTTTACAAAG ATAACTTATT TTTAAATTCA CATTACATAT TATAAAATAA AAATTAAATC   
  
  
- CAAACTTTCC ACATTTTAAA AATCTTTAAA ATAAGTTAAA TTTCCAGGTC AATCATGTAA GACATATTTT   
  
  
- TTTAAAATGA ATAAAATCTT TAAAAATTAT CGTTATTAAA ATAGATAACA TTTTACATTA TTATGACAAA   
  
  
- AAATTACTGT AAATATCACA ATTTAAATTG ATAGGATAAA TGTGTAATAT TGAAAAACTT TCTTTTTCAG   
  
  
- TAGAAAAAAA TCTTGGGGAG GAGACCTTTG ATTGGCATAT ATATTATATG GTGAGTAATG AAAGAAGAGG   
  
  
- ACGTTTGGTG TGTTTGTTTA ATTAAACAAT TAATTAATTT AATGTCCAAA GCAGATGAAG CGAAGAGGTC   
  
  
- ATCAGAAGAA GAAGCATAAA TAAAATATTG GAAAACAAGA GGTGGAGGAG GAGACAGAGG CTTGGGATGC   
  
  
- AGCAGTGGTA AGTAACAAGT GGGCTCTGTC GCTCTCTCTC TATTTGCTGA GTGAATAATT CAGCACAACA   
  
  
- GCACACGAAC AGGAGCTGGT CACTGATTTG TGAACATTCT TGGCAAATAC TACCCATCAG CCATCCTCTT   
  
  
- TATTACTCCC TCTAACTCCT ACACTCTTTG ACCCAATCCA CCTTTCTCAT CCCTTCTATA TTTATTTTTT   
  
  
- ATCCGTTCAT TTTTTATTTT TTTAAAAATG AATTAAATTA TTTATTTTTT AAAATTATAA TTATCTATTT   
  
  
- CTTTTTTAAA AGATTAATAA AAATCAAACT GATAGAAAAG ATGTTTAGTT TATCAAAATA ATTTTTTTTA   
  
  
- GATGCCCATA TATACTAATT TCATCAAGTG GGCATGGTTA TTGAATTGAT GCTATTGGTG TGCCGAAGAA   
  
  
- AGTTATTCTT CACATTTGTC GACGAGGAC
