# Supplementary material for: Genome-wide analysis of the WRKY gene family in drumstick (Moringa oleifera Lam.)
Source: PeerJ. 2019 Jun 10;7:e7063. doi: 10.7717/peerj.7063 (PMC6563795; doi:10.7717/peerj.7063)
Supplement: Supplemental Information 1 [file peerj-07-7063-s003.gz › MoWRKY17_plantcare.html]

Content-Type: text/html; charset=ISO-8859-1


CallMat\_Firefox


Webmaster Firefox specific output  
To save the result:
click on the frame with the right mouse button and save the source code as a text file with extension .html  
REFERENCE:PlantCARE: a database of plant cis-acting regulatory elements and a portal to tools for in silico analysis of promoter sequences.  
Lescot, M., Déhais, P., Moreau, Y., De Moor, B., Rouzé ,P.,and Rombauts, S.  
Nucleic Acids Res., Database issue(2002), 30(1):325-327.   


---

> 2018/04/13 10:10:12  
+ CGCGGGGATC CAGGGCCAGT AGGGGGTCCG ACAAGAAGGG GACAGCTCGA ACCGCTCCAT TTTCCGACAC   
  
  
+ GTGATCATCT ACACGAGTGG GGGCCTCGAG CATTTACACA TGTCACACGA TGACAGGCAG ATCGGGCCCA   
  
  
+ CTTTATTAAA GGGTTGACTT TTTAGGGGAG AAAACGTCCA GGAAGATTTG GTGCTGGGCT GCAACTGAAC   
  
  
+ CTAAGCCCAC TCTGACAGTC AACCCCACTT TTTGTCTTAA CGTGTGAAGG GTCACGTTGG GTTCTCAACG   
  
  
+ GGTCACGAAG TCAAAGCCGC ATATGTACAA AACTTCCCTC ACTTTGAAGC AAACCACTCA TGGGGATCAT   
  
  
+ ATCATCTTGT TGTGTTGCGT TGAAGATAAA GTTGTATATT AGCCTTGTGG TGCTTGTATC ATGAATTTAA   
  
  
+ TAGATAGGCC CAATTTAAGA GAACCGAGAC ATTGAAACTT TTCTTGTCTC CTTCTCTAGA GTACATAATT   
  
  
+ TCCCCAAATT TGACCACTTT TGCCTCATGG GTATAAGATG ATCAGATTTG AATCGATAAA AATTGAATCT   
  
  
+ TGGAAATTGC AAGAAGAAGA CTGTGGAACA GCTCATGAAC TTGGTTGCAT TAGAGGAATT GGGAAATGGG   
  
  
+ TAGATATGTT TCTTTGTTTT GTAAGTTGTT AATCTTGCAA TCAAGGGCAT TATGCACTTT TATCTACCTA   
  
  
+ ATGAGGCATA CCCAACTGCC TCCAAAGAAT TTAAAACAGA AGGATTCCAT TGAAACAAAG AGCTACAAGA   
  
  
+ GTAACAGGAC AATGGTTTTT TCAACTTTAA TTTCTATAAT TCTTTAGTAG TTGACTTTTA AGCTTTGGTT   
  
  
+ GAATGAAGCC ATCGGTGTTC TAGCTCCATC TGGTCCACCT CCGATGCATC TATCCCATCA AAACAATGTA   
  
  
+ ACAGGACAAA ACAGACTCTC AACCAAAGAT ACCAACGATG AGTAATTAAG ACACACTAGT ACTGTAAGAA   
  
  
+ AATCGATCTA TCTCTAAGAT TAATCTACAG AATCCGAAAC CCTGAAACTG TAACTTTCTT GGTACTGAGT   
  
  
+ CCTTCTGAAA TTAAAGTATA TACCCACATG CAATATTTGC TCAAAAACAC GAAATGTGGA TGAGAGAAGG   
  
  
+ CTCCATCAGA AGTAATGAGG TGGTGTCCCA GTGTTACAGT TTACCAAACC TGTAACCCTT GACGAGTGTG   
  
  
+ GAATTCAGAA CTAAAGTCGA AGTGAAAATA TCGTAGAATT GTAAGAACCA CAACTTGTCC CCCTGAATAA   
  
  
+ GATATCTTAA TTAGTTTAAA GAGTCAAGAG AGCACGCACG TAAACACACA GCTGCTTCCT TTTCATCTGC   
  
  
+ TCCACTCTCT CTCCTCTCTT TTTCATAGCC TTCTCGCTCT CTCTTTCTCT CTTTCTCCTT ATCGCTTAAT   
  
  
+ CTCCTTCTCC TCTTCTTCCC TCTCGAGTTC GTTTCCTCTT CAGTAATGCA AAGCTAAGGC TGTGTACGTC   
  
  
+ GCTTATCTTA TGAAAGGCTA GGTTATATT  

- GCGCCCCTAG GTCCCGGTCA TCCCCCAGGC TGTTCTTCCC CTGTCGAGCT TGGCGAGGTA AAAGGCTGTG   
  
  
- CACTAGTAGA TGTGCTCACC CCCGGAGCTC GTAAATGTGT ACAGTGTGCT ACTGTCCGTC TAGCCCGGGT   
  
  
- GAAATAATTT CCCAACTGAA AAATCCCCTC TTTTGCAGGT CCTTCTAAAC CACGACCCGA CGTTGACTTG   
  
  
- GATTCGGGTG AGACTGTCAG TTGGGGTGAA AAACAGAATT GCACACTTCC CAGTGCAACC CAAGAGTTGC   
  
  
- CCAGTGCTTC AGTTTCGGCG TATACATGTT TTGAAGGGAG TGAAACTTCG TTTGGTGAGT ACCCCTAGTA   
  
  
- TAGTAGAACA ACACAACGCA ACTTCTATTT CAACATATAA TCGGAACACC ACGAACATAG TACTTAAATT   
  
  
- ATCTATCCGG GTTAAATTCT CTTGGCTCTG TAACTTTGAA AAGAACAGAG GAAGAGATCT CATGTATTAA   
  
  
- AGGGGTTTAA ACTGGTGAAA ACGGAGTACC CATATTCTAC TAGTCTAAAC TTAGCTATTT TTAACTTAGA   
  
  
- ACCTTTAACG TTCTTCTTCT GACACCTTGT CGAGTACTTG AACCAACGTA ATCTCCTTAA CCCTTTACCC   
  
  
- ATCTATACAA AGAAACAAAA CATTCAACAA TTAGAACGTT AGTTCCCGTA ATACGTGAAA ATAGATGGAT   
  
  
- TACTCCGTAT GGGTTGACGG AGGTTTCTTA AATTTTGTCT TCCTAAGGTA ACTTTGTTTC TCGATGTTCT   
  
  
- CATTGTCCTG TTACCAAAAA AGTTGAAATT AAAGATATTA AGAAATCATC AACTGAAAAT TCGAAACCAA   
  
  
- CTTACTTCGG TAGCCACAAG ATCGAGGTAG ACCAGGTGGA GGCTACGTAG ATAGGGTAGT TTTGTTACAT   
  
  
- TGTCCTGTTT TGTCTGAGAG TTGGTTTCTA TGGTTGCTAC TCATTAATTC TGTGTGATCA TGACATTCTT   
  
  
- TTAGCTAGAT AGAGATTCTA ATTAGATGTC TTAGGCTTTG GGACTTTGAC ATTGAAAGAA CCATGACTCA   
  
  
- GGAAGACTTT AATTTCATAT ATGGGTGTAC GTTATAAACG AGTTTTTGTG CTTTACACCT ACTCTCTTCC   
  
  
- GAGGTAGTCT TCATTACTCC ACCACAGGGT CACAATGTCA AATGGTTTGG ACATTGGGAA CTGCTCACAC   
  
  
- CTTAAGTCTT GATTTCAGCT TCACTTTTAT AGCATCTTAA CATTCTTGGT GTTGAACAGG GGGACTTATT   
  
  
- CTATAGAATT AATCAAATTT CTCAGTTCTC TCGTGCGTGC ATTTGTGTGT CGACGAAGGA AAAGTAGACG   
  
  
- AGGTGAGAGA GAGGAGAGAA AAAGTATCGG AAGAGCGAGA GAGAAAGAGA GAAAGAGGAA TAGCGAATTA   
  
  
- GAGGAAGAGG AGAAGAAGGG AGAGCTCAAG CAAAGGAGAA GTCATTACGT TTCGATTCCG ACACATGCAG   
  
  
- CGAATAGAAT ACTTTCCGAT CCAATATAA

  
  
Motifs Found  

+     5UTR Py-rich stretch

| Site Name | Organism | Position | Strand | Matrix score. | sequence | function |
| --- | --- | --- | --- | --- | --- | --- |
| 5UTR Py-rich stretch | Lycopersicon esculentum | 460 | + | 9 | TTTCTTCTCT | cis-acting element conferring high transcription levels |
| 5UTR Py-rich stretch | Lycopersicon esculentum | 1374 | + | 13 | TTTCTCTCTCTCTC | cis-acting element conferring high transcription levels |

> 2018/04/13 10:10:12  
+ CGCGGGGATC CAGGGCCAGT AGGGGGTCCG ACAAGAAGGG GACAGCTCGA ACCGCTCCAT TTTCCGACAC   
  
  
+ GTGATCATCT ACACGAGTGG GGGCCTCGAG CATTTACACA TGTCACACGA TGACAGGCAG ATCGGGCCCA   
  
  
+ CTTTATTAAA GGGTTGACTT TTTAGGGGAG AAAACGTCCA GGAAGATTTG GTGCTGGGCT GCAACTGAAC   
  
  
+ CTAAGCCCAC TCTGACAGTC AACCCCACTT TTTGTCTTAA CGTGTGAAGG GTCACGTTGG GTTCTCAACG   
  
  
+ GGTCACGAAG TCAAAGCCGC ATATGTACAA AACTTCCCTC ACTTTGAAGC AAACCACTCA TGGGGATCAT   
  
  
+ ATCATCTTGT TGTGTTGCGT TGAAGATAAA GTTGTATATT AGCCTTGTGG TGCTTGTATC ATGAATTTAA   
  
  
+ TAGATAGGCC CAATTTAAGA GAACCGAGAC ATTGAAACTT TTCTTGTCTC CTTCTCTAGA GTACATAATT   
  
  
+ TCCCCAAATT TGACCACTTT TGCCTCATGG GTATAAGATG ATCAGATTTG AATCGATAAA AATTGAATCT   
  
  
+ TGGAAATTGC AAGAAGAAGA CTGTGGAACA GCTCATGAAC TTGGTTGCAT TAGAGGAATT GGGAAATGGG   
  
  
+ TAGATATGTT TCTTTGTTTT GTAAGTTGTT AATCTTGCAA TCAAGGGCAT TATGCACTTT TATCTACCTA   
  
  
+ ATGAGGCATA CCCAACTGCC TCCAAAGAAT TTAAAACAGA AGGATTCCAT TGAAACAAAG AGCTACAAGA   
  
  
+ GTAACAGGAC AATGGTTTTT TCAACTTTAA TTTCTATAAT TCTTTAGTAG TTGACTTTTA AGCTTTGGTT   
  
  
+ GAATGAAGCC ATCGGTGTTC TAGCTCCATC TGGTCCACCT CCGATGCATC TATCCCATCA AAACAATGTA   
  
  
+ ACAGGACAAA ACAGACTCTC AACCAAAGAT ACCAACGATG AGTAATTAAG ACACACTAGT ACTGTAAGAA   
  
  
+ AATCGATCTA TCTCTAAGAT TAATCTACAG AATCCGAAAC CCTGAAACTG TAACTTTCTT GGTACTGAGT   
  
  
+ CCTTCTGAAA TTAAAGTATA TACCCACATG CAATATTTGC TCAAAAACAC GAAATGTGGA TGAGAGAAGG   
  
  
+ CTCCATCAGA AGTAATGAGG TGGTGTCCCA GTGTTACAGT TTACCAAACC TGTAACCCTT GACGAGTGTG   
  
  
+ GAATTCAGAA CTAAAGTCGA AGTGAAAATA TCGTAGAATT GTAAGAACCA CAACTTGTCC CCCTGAATAA   
  
  
+ GATATCTTAA TTAGTTTAAA GAGTCAAGAG AGCACGCACG TAAACACACA GCTGCTTCCT TTTCATCTGC   
  
  
+ TCCACTCTCT CTCCTCTCTT TTTCATAGCC TTCTCGCTCT CTCTTTCTCT CTTTCTCCTT ATCGCTTAAT   
  
  
+ CTCCTTCTCC TCTTCTTCCC TCTCGAGTTC GTTTCCTCTT CAGTAATGCA AAGCTAAGGC TGTGTACGTC   
  
  
+ GCTTATCTTA TGAAAGGCTA GGTTATATT  

- GCGCCCCTAG GTCCCGGTCA TCCCCCAGGC TGTTCTTCCC CTGTCGAGCT TGGCGAGGTA AAAGGCTGTG   
  
  
- CACTAGTAGA TGTGCTCACC CCCGGAGCTC GTAAATGTGT ACAGTGTGCT ACTGTCCGTC TAGCCCGGGT   
  
  
- GAAATAATTT CCCAACTGAA AAATCCCCTC TTTTGCAGGT CCTTCTAAAC CACGACCCGA CGTTGACTTG   
  
  
- GATTCGGGTG AGACTGTCAG TTGGGGTGAA AAACAGAATT GCACACTTCC CAGTGCAACC CAAGAGTTGC   
  
  
- CCAGTGCTTC AGTTTCGGCG TATACATGTT TTGAAGGGAG TGAAACTTCG TTTGGTGAGT ACCCCTAGTA   
  
  
- TAGTAGAACA ACACAACGCA ACTTCTATTT CAACATATAA TCGGAACACC ACGAACATAG TACTTAAATT   
  
  
- ATCTATCCGG GTTAAATTCT CTTGGCTCTG TAACTTTGAA AAGAACAGAG GAAGAGATCT CATGTATTAA   
  
  
- AGGGGTTTAA ACTGGTGAAA ACGGAGTACC CATATTCTAC TAGTCTAAAC TTAGCTATTT TTAACTTAGA   
  
  
- ACCTTTAACG TTCTTCTTCT GACACCTTGT CGAGTACTTG AACCAACGTA ATCTCCTTAA CCCTTTACCC   
  
  
- ATCTATACAA AGAAACAAAA CATTCAACAA TTAGAACGTT AGTTCCCGTA ATACGTGAAA ATAGATGGAT   
  
  
- TACTCCGTAT GGGTTGACGG AGGTTTCTTA AATTTTGTCT TCCTAAGGTA ACTTTGTTTC TCGATGTTCT   
  
  
- CATTGTCCTG TTACCAAAAA AGTTGAAATT AAAGATATTA AGAAATCATC AACTGAAAAT TCGAAACCAA   
  
  
- CTTACTTCGG TAGCCACAAG ATCGAGGTAG ACCAGGTGGA GGCTACGTAG ATAGGGTAGT TTTGTTACAT   
  
  
- TGTCCTGTTT TGTCTGAGAG TTGGTTTCTA TGGTTGCTAC TCATTAATTC TGTGTGATCA TGACATTCTT   
  
  
- TTAGCTAGAT AGAGATTCTA ATTAGATGTC TTAGGCTTTG GGACTTTGAC ATTGAAAGAA CCATGACTCA   
  
  
- GGAAGACTTT AATTTCATAT ATGGGTGTAC GTTATAAACG AGTTTTTGTG CTTTACACCT ACTCTCTTCC   
  
  
- GAGGTAGTCT TCATTACTCC ACCACAGGGT CACAATGTCA AATGGTTTGG ACATTGGGAA CTGCTCACAC   
  
  
- CTTAAGTCTT GATTTCAGCT TCACTTTTAT AGCATCTTAA CATTCTTGGT GTTGAACAGG GGGACTTATT   
  
  
- CTATAGAATT AATCAAATTT CTCAGTTCTC TCGTGCGTGC ATTTGTGTGT CGACGAAGGA AAAGTAGACG   
  
  
- AGGTGAGAGA GAGGAGAGAA AAAGTATCGG AAGAGCGAGA GAGAAAGAGA GAAAGAGGAA TAGCGAATTA   
  
  
- GAGGAAGAGG AGAAGAAGGG AGAGCTCAAG CAAAGGAGAA GTCATTACGT TTCGATTCCG ACACATGCAG   
  
  
- CGAATAGAAT ACTTTCCGAT CCAATATAA

+     ABRE

| Site Name | Organism | Position | Strand | Matrix score. | sequence | function |
| --- | --- | --- | --- | --- | --- | --- |
| ABRE | Arabidopsis thaliana | 68 | + | 6 | CACGTG | cis-acting element involved in the abscisic acid responsiveness |
| ABRE | Hordeum vulgare | 1463 | - | 9 | GCCACGTACA | cis-acting element involved in the abscisic acid responsiveness |
| ABRE | Arabidopsis thaliana | 1297 | - | 6 | TACGTG | cis-acting element involved in the abscisic acid responsiveness |

> 2018/04/13 10:10:12  
+ CGCGGGGATC CAGGGCCAGT AGGGGGTCCG ACAAGAAGGG GACAGCTCGA ACCGCTCCAT TTTCCGACAC   
  
  
+ GTGATCATCT ACACGAGTGG GGGCCTCGAG CATTTACACA TGTCACACGA TGACAGGCAG ATCGGGCCCA   
  
  
+ CTTTATTAAA GGGTTGACTT TTTAGGGGAG AAAACGTCCA GGAAGATTTG GTGCTGGGCT GCAACTGAAC   
  
  
+ CTAAGCCCAC TCTGACAGTC AACCCCACTT TTTGTCTTAA CGTGTGAAGG GTCACGTTGG GTTCTCAACG   
  
  
+ GGTCACGAAG TCAAAGCCGC ATATGTACAA AACTTCCCTC ACTTTGAAGC AAACCACTCA TGGGGATCAT   
  
  
+ ATCATCTTGT TGTGTTGCGT TGAAGATAAA GTTGTATATT AGCCTTGTGG TGCTTGTATC ATGAATTTAA   
  
  
+ TAGATAGGCC CAATTTAAGA GAACCGAGAC ATTGAAACTT TTCTTGTCTC CTTCTCTAGA GTACATAATT   
  
  
+ TCCCCAAATT TGACCACTTT TGCCTCATGG GTATAAGATG ATCAGATTTG AATCGATAAA AATTGAATCT   
  
  
+ TGGAAATTGC AAGAAGAAGA CTGTGGAACA GCTCATGAAC TTGGTTGCAT TAGAGGAATT GGGAAATGGG   
  
  
+ TAGATATGTT TCTTTGTTTT GTAAGTTGTT AATCTTGCAA TCAAGGGCAT TATGCACTTT TATCTACCTA   
  
  
+ ATGAGGCATA CCCAACTGCC TCCAAAGAAT TTAAAACAGA AGGATTCCAT TGAAACAAAG AGCTACAAGA   
  
  
+ GTAACAGGAC AATGGTTTTT TCAACTTTAA TTTCTATAAT TCTTTAGTAG TTGACTTTTA AGCTTTGGTT   
  
  
+ GAATGAAGCC ATCGGTGTTC TAGCTCCATC TGGTCCACCT CCGATGCATC TATCCCATCA AAACAATGTA   
  
  
+ ACAGGACAAA ACAGACTCTC AACCAAAGAT ACCAACGATG AGTAATTAAG ACACACTAGT ACTGTAAGAA   
  
  
+ AATCGATCTA TCTCTAAGAT TAATCTACAG AATCCGAAAC CCTGAAACTG TAACTTTCTT GGTACTGAGT   
  
  
+ CCTTCTGAAA TTAAAGTATA TACCCACATG CAATATTTGC TCAAAAACAC GAAATGTGGA TGAGAGAAGG   
  
  
+ CTCCATCAGA AGTAATGAGG TGGTGTCCCA GTGTTACAGT TTACCAAACC TGTAACCCTT GACGAGTGTG   
  
  
+ GAATTCAGAA CTAAAGTCGA AGTGAAAATA TCGTAGAATT GTAAGAACCA CAACTTGTCC CCCTGAATAA   
  
  
+ GATATCTTAA TTAGTTTAAA GAGTCAAGAG AGCACGCACG TAAACACACA GCTGCTTCCT TTTCATCTGC   
  
  
+ TCCACTCTCT CTCCTCTCTT TTTCATAGCC TTCTCGCTCT CTCTTTCTCT CTTTCTCCTT ATCGCTTAAT   
  
  
+ CTCCTTCTCC TCTTCTTCCC TCTCGAGTTC GTTTCCTCTT CAGTAATGCA AAGCTAAGGC TGTGTACGTC   
  
  
+ GCTTATCTTA TGAAAGGCTA GGTTATATT  

- GCGCCCCTAG GTCCCGGTCA TCCCCCAGGC TGTTCTTCCC CTGTCGAGCT TGGCGAGGTA AAAGGCTGTG   
  
  
- CACTAGTAGA TGTGCTCACC CCCGGAGCTC GTAAATGTGT ACAGTGTGCT ACTGTCCGTC TAGCCCGGGT   
  
  
- GAAATAATTT CCCAACTGAA AAATCCCCTC TTTTGCAGGT CCTTCTAAAC CACGACCCGA CGTTGACTTG   
  
  
- GATTCGGGTG AGACTGTCAG TTGGGGTGAA AAACAGAATT GCACACTTCC CAGTGCAACC CAAGAGTTGC   
  
  
- CCAGTGCTTC AGTTTCGGCG TATACATGTT TTGAAGGGAG TGAAACTTCG TTTGGTGAGT ACCCCTAGTA   
  
  
- TAGTAGAACA ACACAACGCA ACTTCTATTT CAACATATAA TCGGAACACC ACGAACATAG TACTTAAATT   
  
  
- ATCTATCCGG GTTAAATTCT CTTGGCTCTG TAACTTTGAA AAGAACAGAG GAAGAGATCT CATGTATTAA   
  
  
- AGGGGTTTAA ACTGGTGAAA ACGGAGTACC CATATTCTAC TAGTCTAAAC TTAGCTATTT TTAACTTAGA   
  
  
- ACCTTTAACG TTCTTCTTCT GACACCTTGT CGAGTACTTG AACCAACGTA ATCTCCTTAA CCCTTTACCC   
  
  
- ATCTATACAA AGAAACAAAA CATTCAACAA TTAGAACGTT AGTTCCCGTA ATACGTGAAA ATAGATGGAT   
  
  
- TACTCCGTAT GGGTTGACGG AGGTTTCTTA AATTTTGTCT TCCTAAGGTA ACTTTGTTTC TCGATGTTCT   
  
  
- CATTGTCCTG TTACCAAAAA AGTTGAAATT AAAGATATTA AGAAATCATC AACTGAAAAT TCGAAACCAA   
  
  
- CTTACTTCGG TAGCCACAAG ATCGAGGTAG ACCAGGTGGA GGCTACGTAG ATAGGGTAGT TTTGTTACAT   
  
  
- TGTCCTGTTT TGTCTGAGAG TTGGTTTCTA TGGTTGCTAC TCATTAATTC TGTGTGATCA TGACATTCTT   
  
  
- TTAGCTAGAT AGAGATTCTA ATTAGATGTC TTAGGCTTTG GGACTTTGAC ATTGAAAGAA CCATGACTCA   
  
  
- GGAAGACTTT AATTTCATAT ATGGGTGTAC GTTATAAACG AGTTTTTGTG CTTTACACCT ACTCTCTTCC   
  
  
- GAGGTAGTCT TCATTACTCC ACCACAGGGT CACAATGTCA AATGGTTTGG ACATTGGGAA CTGCTCACAC   
  
  
- CTTAAGTCTT GATTTCAGCT TCACTTTTAT AGCATCTTAA CATTCTTGGT GTTGAACAGG GGGACTTATT   
  
  
- CTATAGAATT AATCAAATTT CTCAGTTCTC TCGTGCGTGC ATTTGTGTGT CGACGAAGGA AAAGTAGACG   
  
  
- AGGTGAGAGA GAGGAGAGAA AAAGTATCGG AAGAGCGAGA GAGAAAGAGA GAAAGAGGAA TAGCGAATTA   
  
  
- GAGGAAGAGG AGAAGAAGGG AGAGCTCAAG CAAAGGAGAA GTCATTACGT TTCGATTCCG ACACATGCAG   
  
  
- CGAATAGAAT ACTTTCCGAT CCAATATAA

+     ACE

| Site Name | Organism | Position | Strand | Matrix score. | sequence | function |
| --- | --- | --- | --- | --- | --- | --- |
| ACE | Petroselinum crispum | 1463 | - | 9 | GCGACGTACC | cis-acting element involved in light responsiveness |

> 2018/04/13 10:10:12  
+ CGCGGGGATC CAGGGCCAGT AGGGGGTCCG ACAAGAAGGG GACAGCTCGA ACCGCTCCAT TTTCCGACAC   
  
  
+ GTGATCATCT ACACGAGTGG GGGCCTCGAG CATTTACACA TGTCACACGA TGACAGGCAG ATCGGGCCCA   
  
  
+ CTTTATTAAA GGGTTGACTT TTTAGGGGAG AAAACGTCCA GGAAGATTTG GTGCTGGGCT GCAACTGAAC   
  
  
+ CTAAGCCCAC TCTGACAGTC AACCCCACTT TTTGTCTTAA CGTGTGAAGG GTCACGTTGG GTTCTCAACG   
  
  
+ GGTCACGAAG TCAAAGCCGC ATATGTACAA AACTTCCCTC ACTTTGAAGC AAACCACTCA TGGGGATCAT   
  
  
+ ATCATCTTGT TGTGTTGCGT TGAAGATAAA GTTGTATATT AGCCTTGTGG TGCTTGTATC ATGAATTTAA   
  
  
+ TAGATAGGCC CAATTTAAGA GAACCGAGAC ATTGAAACTT TTCTTGTCTC CTTCTCTAGA GTACATAATT   
  
  
+ TCCCCAAATT TGACCACTTT TGCCTCATGG GTATAAGATG ATCAGATTTG AATCGATAAA AATTGAATCT   
  
  
+ TGGAAATTGC AAGAAGAAGA CTGTGGAACA GCTCATGAAC TTGGTTGCAT TAGAGGAATT GGGAAATGGG   
  
  
+ TAGATATGTT TCTTTGTTTT GTAAGTTGTT AATCTTGCAA TCAAGGGCAT TATGCACTTT TATCTACCTA   
  
  
+ ATGAGGCATA CCCAACTGCC TCCAAAGAAT TTAAAACAGA AGGATTCCAT TGAAACAAAG AGCTACAAGA   
  
  
+ GTAACAGGAC AATGGTTTTT TCAACTTTAA TTTCTATAAT TCTTTAGTAG TTGACTTTTA AGCTTTGGTT   
  
  
+ GAATGAAGCC ATCGGTGTTC TAGCTCCATC TGGTCCACCT CCGATGCATC TATCCCATCA AAACAATGTA   
  
  
+ ACAGGACAAA ACAGACTCTC AACCAAAGAT ACCAACGATG AGTAATTAAG ACACACTAGT ACTGTAAGAA   
  
  
+ AATCGATCTA TCTCTAAGAT TAATCTACAG AATCCGAAAC CCTGAAACTG TAACTTTCTT GGTACTGAGT   
  
  
+ CCTTCTGAAA TTAAAGTATA TACCCACATG CAATATTTGC TCAAAAACAC GAAATGTGGA TGAGAGAAGG   
  
  
+ CTCCATCAGA AGTAATGAGG TGGTGTCCCA GTGTTACAGT TTACCAAACC TGTAACCCTT GACGAGTGTG   
  
  
+ GAATTCAGAA CTAAAGTCGA AGTGAAAATA TCGTAGAATT GTAAGAACCA CAACTTGTCC CCCTGAATAA   
  
  
+ GATATCTTAA TTAGTTTAAA GAGTCAAGAG AGCACGCACG TAAACACACA GCTGCTTCCT TTTCATCTGC   
  
  
+ TCCACTCTCT CTCCTCTCTT TTTCATAGCC TTCTCGCTCT CTCTTTCTCT CTTTCTCCTT ATCGCTTAAT   
  
  
+ CTCCTTCTCC TCTTCTTCCC TCTCGAGTTC GTTTCCTCTT CAGTAATGCA AAGCTAAGGC TGTGTACGTC   
  
  
+ GCTTATCTTA TGAAAGGCTA GGTTATATT  

- GCGCCCCTAG GTCCCGGTCA TCCCCCAGGC TGTTCTTCCC CTGTCGAGCT TGGCGAGGTA AAAGGCTGTG   
  
  
- CACTAGTAGA TGTGCTCACC CCCGGAGCTC GTAAATGTGT ACAGTGTGCT ACTGTCCGTC TAGCCCGGGT   
  
  
- GAAATAATTT CCCAACTGAA AAATCCCCTC TTTTGCAGGT CCTTCTAAAC CACGACCCGA CGTTGACTTG   
  
  
- GATTCGGGTG AGACTGTCAG TTGGGGTGAA AAACAGAATT GCACACTTCC CAGTGCAACC CAAGAGTTGC   
  
  
- CCAGTGCTTC AGTTTCGGCG TATACATGTT TTGAAGGGAG TGAAACTTCG TTTGGTGAGT ACCCCTAGTA   
  
  
- TAGTAGAACA ACACAACGCA ACTTCTATTT CAACATATAA TCGGAACACC ACGAACATAG TACTTAAATT   
  
  
- ATCTATCCGG GTTAAATTCT CTTGGCTCTG TAACTTTGAA AAGAACAGAG GAAGAGATCT CATGTATTAA   
  
  
- AGGGGTTTAA ACTGGTGAAA ACGGAGTACC CATATTCTAC TAGTCTAAAC TTAGCTATTT TTAACTTAGA   
  
  
- ACCTTTAACG TTCTTCTTCT GACACCTTGT CGAGTACTTG AACCAACGTA ATCTCCTTAA CCCTTTACCC   
  
  
- ATCTATACAA AGAAACAAAA CATTCAACAA TTAGAACGTT AGTTCCCGTA ATACGTGAAA ATAGATGGAT   
  
  
- TACTCCGTAT GGGTTGACGG AGGTTTCTTA AATTTTGTCT TCCTAAGGTA ACTTTGTTTC TCGATGTTCT   
  
  
- CATTGTCCTG TTACCAAAAA AGTTGAAATT AAAGATATTA AGAAATCATC AACTGAAAAT TCGAAACCAA   
  
  
- CTTACTTCGG TAGCCACAAG ATCGAGGTAG ACCAGGTGGA GGCTACGTAG ATAGGGTAGT TTTGTTACAT   
  
  
- TGTCCTGTTT TGTCTGAGAG TTGGTTTCTA TGGTTGCTAC TCATTAATTC TGTGTGATCA TGACATTCTT   
  
  
- TTAGCTAGAT AGAGATTCTA ATTAGATGTC TTAGGCTTTG GGACTTTGAC ATTGAAAGAA CCATGACTCA   
  
  
- GGAAGACTTT AATTTCATAT ATGGGTGTAC GTTATAAACG AGTTTTTGTG CTTTACACCT ACTCTCTTCC   
  
  
- GAGGTAGTCT TCATTACTCC ACCACAGGGT CACAATGTCA AATGGTTTGG ACATTGGGAA CTGCTCACAC   
  
  
- CTTAAGTCTT GATTTCAGCT TCACTTTTAT AGCATCTTAA CATTCTTGGT GTTGAACAGG GGGACTTATT   
  
  
- CTATAGAATT AATCAAATTT CTCAGTTCTC TCGTGCGTGC ATTTGTGTGT CGACGAAGGA AAAGTAGACG   
  
  
- AGGTGAGAGA GAGGAGAGAA AAAGTATCGG AAGAGCGAGA GAGAAAGAGA GAAAGAGGAA TAGCGAATTA   
  
  
- GAGGAAGAGG AGAAGAAGGG AGAGCTCAAG CAAAGGAGAA GTCATTACGT TTCGATTCCG ACACATGCAG   
  
  
- CGAATAGAAT ACTTTCCGAT CCAATATAA

+     AE-box

| Site Name | Organism | Position | Strand | Matrix score. | sequence | function |
| --- | --- | --- | --- | --- | --- | --- |
| AE-box | Arabidopsis thaliana | 636 | - | 8 | AGAAACAT | part of a module for light response |

> 2018/04/13 10:10:12  
+ CGCGGGGATC CAGGGCCAGT AGGGGGTCCG ACAAGAAGGG GACAGCTCGA ACCGCTCCAT TTTCCGACAC   
  
  
+ GTGATCATCT ACACGAGTGG GGGCCTCGAG CATTTACACA TGTCACACGA TGACAGGCAG ATCGGGCCCA   
  
  
+ CTTTATTAAA GGGTTGACTT TTTAGGGGAG AAAACGTCCA GGAAGATTTG GTGCTGGGCT GCAACTGAAC   
  
  
+ CTAAGCCCAC TCTGACAGTC AACCCCACTT TTTGTCTTAA CGTGTGAAGG GTCACGTTGG GTTCTCAACG   
  
  
+ GGTCACGAAG TCAAAGCCGC ATATGTACAA AACTTCCCTC ACTTTGAAGC AAACCACTCA TGGGGATCAT   
  
  
+ ATCATCTTGT TGTGTTGCGT TGAAGATAAA GTTGTATATT AGCCTTGTGG TGCTTGTATC ATGAATTTAA   
  
  
+ TAGATAGGCC CAATTTAAGA GAACCGAGAC ATTGAAACTT TTCTTGTCTC CTTCTCTAGA GTACATAATT   
  
  
+ TCCCCAAATT TGACCACTTT TGCCTCATGG GTATAAGATG ATCAGATTTG AATCGATAAA AATTGAATCT   
  
  
+ TGGAAATTGC AAGAAGAAGA CTGTGGAACA GCTCATGAAC TTGGTTGCAT TAGAGGAATT GGGAAATGGG   
  
  
+ TAGATATGTT TCTTTGTTTT GTAAGTTGTT AATCTTGCAA TCAAGGGCAT TATGCACTTT TATCTACCTA   
  
  
+ ATGAGGCATA CCCAACTGCC TCCAAAGAAT TTAAAACAGA AGGATTCCAT TGAAACAAAG AGCTACAAGA   
  
  
+ GTAACAGGAC AATGGTTTTT TCAACTTTAA TTTCTATAAT TCTTTAGTAG TTGACTTTTA AGCTTTGGTT   
  
  
+ GAATGAAGCC ATCGGTGTTC TAGCTCCATC TGGTCCACCT CCGATGCATC TATCCCATCA AAACAATGTA   
  
  
+ ACAGGACAAA ACAGACTCTC AACCAAAGAT ACCAACGATG AGTAATTAAG ACACACTAGT ACTGTAAGAA   
  
  
+ AATCGATCTA TCTCTAAGAT TAATCTACAG AATCCGAAAC CCTGAAACTG TAACTTTCTT GGTACTGAGT   
  
  
+ CCTTCTGAAA TTAAAGTATA TACCCACATG CAATATTTGC TCAAAAACAC GAAATGTGGA TGAGAGAAGG   
  
  
+ CTCCATCAGA AGTAATGAGG TGGTGTCCCA GTGTTACAGT TTACCAAACC TGTAACCCTT GACGAGTGTG   
  
  
+ GAATTCAGAA CTAAAGTCGA AGTGAAAATA TCGTAGAATT GTAAGAACCA CAACTTGTCC CCCTGAATAA   
  
  
+ GATATCTTAA TTAGTTTAAA GAGTCAAGAG AGCACGCACG TAAACACACA GCTGCTTCCT TTTCATCTGC   
  
  
+ TCCACTCTCT CTCCTCTCTT TTTCATAGCC TTCTCGCTCT CTCTTTCTCT CTTTCTCCTT ATCGCTTAAT   
  
  
+ CTCCTTCTCC TCTTCTTCCC TCTCGAGTTC GTTTCCTCTT CAGTAATGCA AAGCTAAGGC TGTGTACGTC   
  
  
+ GCTTATCTTA TGAAAGGCTA GGTTATATT  

- GCGCCCCTAG GTCCCGGTCA TCCCCCAGGC TGTTCTTCCC CTGTCGAGCT TGGCGAGGTA AAAGGCTGTG   
  
  
- CACTAGTAGA TGTGCTCACC CCCGGAGCTC GTAAATGTGT ACAGTGTGCT ACTGTCCGTC TAGCCCGGGT   
  
  
- GAAATAATTT CCCAACTGAA AAATCCCCTC TTTTGCAGGT CCTTCTAAAC CACGACCCGA CGTTGACTTG   
  
  
- GATTCGGGTG AGACTGTCAG TTGGGGTGAA AAACAGAATT GCACACTTCC CAGTGCAACC CAAGAGTTGC   
  
  
- CCAGTGCTTC AGTTTCGGCG TATACATGTT TTGAAGGGAG TGAAACTTCG TTTGGTGAGT ACCCCTAGTA   
  
  
- TAGTAGAACA ACACAACGCA ACTTCTATTT CAACATATAA TCGGAACACC ACGAACATAG TACTTAAATT   
  
  
- ATCTATCCGG GTTAAATTCT CTTGGCTCTG TAACTTTGAA AAGAACAGAG GAAGAGATCT CATGTATTAA   
  
  
- AGGGGTTTAA ACTGGTGAAA ACGGAGTACC CATATTCTAC TAGTCTAAAC TTAGCTATTT TTAACTTAGA   
  
  
- ACCTTTAACG TTCTTCTTCT GACACCTTGT CGAGTACTTG AACCAACGTA ATCTCCTTAA CCCTTTACCC   
  
  
- ATCTATACAA AGAAACAAAA CATTCAACAA TTAGAACGTT AGTTCCCGTA ATACGTGAAA ATAGATGGAT   
  
  
- TACTCCGTAT GGGTTGACGG AGGTTTCTTA AATTTTGTCT TCCTAAGGTA ACTTTGTTTC TCGATGTTCT   
  
  
- CATTGTCCTG TTACCAAAAA AGTTGAAATT AAAGATATTA AGAAATCATC AACTGAAAAT TCGAAACCAA   
  
  
- CTTACTTCGG TAGCCACAAG ATCGAGGTAG ACCAGGTGGA GGCTACGTAG ATAGGGTAGT TTTGTTACAT   
  
  
- TGTCCTGTTT TGTCTGAGAG TTGGTTTCTA TGGTTGCTAC TCATTAATTC TGTGTGATCA TGACATTCTT   
  
  
- TTAGCTAGAT AGAGATTCTA ATTAGATGTC TTAGGCTTTG GGACTTTGAC ATTGAAAGAA CCATGACTCA   
  
  
- GGAAGACTTT AATTTCATAT ATGGGTGTAC GTTATAAACG AGTTTTTGTG CTTTACACCT ACTCTCTTCC   
  
  
- GAGGTAGTCT TCATTACTCC ACCACAGGGT CACAATGTCA AATGGTTTGG ACATTGGGAA CTGCTCACAC   
  
  
- CTTAAGTCTT GATTTCAGCT TCACTTTTAT AGCATCTTAA CATTCTTGGT GTTGAACAGG GGGACTTATT   
  
  
- CTATAGAATT AATCAAATTT CTCAGTTCTC TCGTGCGTGC ATTTGTGTGT CGACGAAGGA AAAGTAGACG   
  
  
- AGGTGAGAGA GAGGAGAGAA AAAGTATCGG AAGAGCGAGA GAGAAAGAGA GAAAGAGGAA TAGCGAATTA   
  
  
- GAGGAAGAGG AGAAGAAGGG AGAGCTCAAG CAAAGGAGAA GTCATTACGT TTCGATTCCG ACACATGCAG   
  
  
- CGAATAGAAT ACTTTCCGAT CCAATATAA

+     ARE

| Site Name | Organism | Position | Strand | Matrix score. | sequence | function |
| --- | --- | --- | --- | --- | --- | --- |
| ARE | Zea mays | 783 | + | 6 | TGGTTT | cis-acting regulatory element essential for the anaerobic induction |
| ARE | Zea mays | 331 | - | 6 | TGGTTT | cis-acting regulatory element essential for the anaerobic induction |

> 2018/04/13 10:10:12  
+ CGCGGGGATC CAGGGCCAGT AGGGGGTCCG ACAAGAAGGG GACAGCTCGA ACCGCTCCAT TTTCCGACAC   
  
  
+ GTGATCATCT ACACGAGTGG GGGCCTCGAG CATTTACACA TGTCACACGA TGACAGGCAG ATCGGGCCCA   
  
  
+ CTTTATTAAA GGGTTGACTT TTTAGGGGAG AAAACGTCCA GGAAGATTTG GTGCTGGGCT GCAACTGAAC   
  
  
+ CTAAGCCCAC TCTGACAGTC AACCCCACTT TTTGTCTTAA CGTGTGAAGG GTCACGTTGG GTTCTCAACG   
  
  
+ GGTCACGAAG TCAAAGCCGC ATATGTACAA AACTTCCCTC ACTTTGAAGC AAACCACTCA TGGGGATCAT   
  
  
+ ATCATCTTGT TGTGTTGCGT TGAAGATAAA GTTGTATATT AGCCTTGTGG TGCTTGTATC ATGAATTTAA   
  
  
+ TAGATAGGCC CAATTTAAGA GAACCGAGAC ATTGAAACTT TTCTTGTCTC CTTCTCTAGA GTACATAATT   
  
  
+ TCCCCAAATT TGACCACTTT TGCCTCATGG GTATAAGATG ATCAGATTTG AATCGATAAA AATTGAATCT   
  
  
+ TGGAAATTGC AAGAAGAAGA CTGTGGAACA GCTCATGAAC TTGGTTGCAT TAGAGGAATT GGGAAATGGG   
  
  
+ TAGATATGTT TCTTTGTTTT GTAAGTTGTT AATCTTGCAA TCAAGGGCAT TATGCACTTT TATCTACCTA   
  
  
+ ATGAGGCATA CCCAACTGCC TCCAAAGAAT TTAAAACAGA AGGATTCCAT TGAAACAAAG AGCTACAAGA   
  
  
+ GTAACAGGAC AATGGTTTTT TCAACTTTAA TTTCTATAAT TCTTTAGTAG TTGACTTTTA AGCTTTGGTT   
  
  
+ GAATGAAGCC ATCGGTGTTC TAGCTCCATC TGGTCCACCT CCGATGCATC TATCCCATCA AAACAATGTA   
  
  
+ ACAGGACAAA ACAGACTCTC AACCAAAGAT ACCAACGATG AGTAATTAAG ACACACTAGT ACTGTAAGAA   
  
  
+ AATCGATCTA TCTCTAAGAT TAATCTACAG AATCCGAAAC CCTGAAACTG TAACTTTCTT GGTACTGAGT   
  
  
+ CCTTCTGAAA TTAAAGTATA TACCCACATG CAATATTTGC TCAAAAACAC GAAATGTGGA TGAGAGAAGG   
  
  
+ CTCCATCAGA AGTAATGAGG TGGTGTCCCA GTGTTACAGT TTACCAAACC TGTAACCCTT GACGAGTGTG   
  
  
+ GAATTCAGAA CTAAAGTCGA AGTGAAAATA TCGTAGAATT GTAAGAACCA CAACTTGTCC CCCTGAATAA   
  
  
+ GATATCTTAA TTAGTTTAAA GAGTCAAGAG AGCACGCACG TAAACACACA GCTGCTTCCT TTTCATCTGC   
  
  
+ TCCACTCTCT CTCCTCTCTT TTTCATAGCC TTCTCGCTCT CTCTTTCTCT CTTTCTCCTT ATCGCTTAAT   
  
  
+ CTCCTTCTCC TCTTCTTCCC TCTCGAGTTC GTTTCCTCTT CAGTAATGCA AAGCTAAGGC TGTGTACGTC   
  
  
+ GCTTATCTTA TGAAAGGCTA GGTTATATT  

- GCGCCCCTAG GTCCCGGTCA TCCCCCAGGC TGTTCTTCCC CTGTCGAGCT TGGCGAGGTA AAAGGCTGTG   
  
  
- CACTAGTAGA TGTGCTCACC CCCGGAGCTC GTAAATGTGT ACAGTGTGCT ACTGTCCGTC TAGCCCGGGT   
  
  
- GAAATAATTT CCCAACTGAA AAATCCCCTC TTTTGCAGGT CCTTCTAAAC CACGACCCGA CGTTGACTTG   
  
  
- GATTCGGGTG AGACTGTCAG TTGGGGTGAA AAACAGAATT GCACACTTCC CAGTGCAACC CAAGAGTTGC   
  
  
- CCAGTGCTTC AGTTTCGGCG TATACATGTT TTGAAGGGAG TGAAACTTCG TTTGGTGAGT ACCCCTAGTA   
  
  
- TAGTAGAACA ACACAACGCA ACTTCTATTT CAACATATAA TCGGAACACC ACGAACATAG TACTTAAATT   
  
  
- ATCTATCCGG GTTAAATTCT CTTGGCTCTG TAACTTTGAA AAGAACAGAG GAAGAGATCT CATGTATTAA   
  
  
- AGGGGTTTAA ACTGGTGAAA ACGGAGTACC CATATTCTAC TAGTCTAAAC TTAGCTATTT TTAACTTAGA   
  
  
- ACCTTTAACG TTCTTCTTCT GACACCTTGT CGAGTACTTG AACCAACGTA ATCTCCTTAA CCCTTTACCC   
  
  
- ATCTATACAA AGAAACAAAA CATTCAACAA TTAGAACGTT AGTTCCCGTA ATACGTGAAA ATAGATGGAT   
  
  
- TACTCCGTAT GGGTTGACGG AGGTTTCTTA AATTTTGTCT TCCTAAGGTA ACTTTGTTTC TCGATGTTCT   
  
  
- CATTGTCCTG TTACCAAAAA AGTTGAAATT AAAGATATTA AGAAATCATC AACTGAAAAT TCGAAACCAA   
  
  
- CTTACTTCGG TAGCCACAAG ATCGAGGTAG ACCAGGTGGA GGCTACGTAG ATAGGGTAGT TTTGTTACAT   
  
  
- TGTCCTGTTT TGTCTGAGAG TTGGTTTCTA TGGTTGCTAC TCATTAATTC TGTGTGATCA TGACATTCTT   
  
  
- TTAGCTAGAT AGAGATTCTA ATTAGATGTC TTAGGCTTTG GGACTTTGAC ATTGAAAGAA CCATGACTCA   
  
  
- GGAAGACTTT AATTTCATAT ATGGGTGTAC GTTATAAACG AGTTTTTGTG CTTTACACCT ACTCTCTTCC   
  
  
- GAGGTAGTCT TCATTACTCC ACCACAGGGT CACAATGTCA AATGGTTTGG ACATTGGGAA CTGCTCACAC   
  
  
- CTTAAGTCTT GATTTCAGCT TCACTTTTAT AGCATCTTAA CATTCTTGGT GTTGAACAGG GGGACTTATT   
  
  
- CTATAGAATT AATCAAATTT CTCAGTTCTC TCGTGCGTGC ATTTGTGTGT CGACGAAGGA AAAGTAGACG   
  
  
- AGGTGAGAGA GAGGAGAGAA AAAGTATCGG AAGAGCGAGA GAGAAAGAGA GAAAGAGGAA TAGCGAATTA   
  
  
- GAGGAAGAGG AGAAGAAGGG AGAGCTCAAG CAAAGGAGAA GTCATTACGT TTCGATTCCG ACACATGCAG   
  
  
- CGAATAGAAT ACTTTCCGAT CCAATATAA

+     AT-rich element

| Site Name | Organism | Position | Strand | Matrix score. | sequence | function |
| --- | --- | --- | --- | --- | --- | --- |
| AT-rich element | Glycine max | 797 | - | 10 | ATAGAAATCAA | binding site of AT-rich DNA binding protein (ATBP-1) |

> 2018/04/13 10:10:12  
+ CGCGGGGATC CAGGGCCAGT AGGGGGTCCG ACAAGAAGGG GACAGCTCGA ACCGCTCCAT TTTCCGACAC   
  
  
+ GTGATCATCT ACACGAGTGG GGGCCTCGAG CATTTACACA TGTCACACGA TGACAGGCAG ATCGGGCCCA   
  
  
+ CTTTATTAAA GGGTTGACTT TTTAGGGGAG AAAACGTCCA GGAAGATTTG GTGCTGGGCT GCAACTGAAC   
  
  
+ CTAAGCCCAC TCTGACAGTC AACCCCACTT TTTGTCTTAA CGTGTGAAGG GTCACGTTGG GTTCTCAACG   
  
  
+ GGTCACGAAG TCAAAGCCGC ATATGTACAA AACTTCCCTC ACTTTGAAGC AAACCACTCA TGGGGATCAT   
  
  
+ ATCATCTTGT TGTGTTGCGT TGAAGATAAA GTTGTATATT AGCCTTGTGG TGCTTGTATC ATGAATTTAA   
  
  
+ TAGATAGGCC CAATTTAAGA GAACCGAGAC ATTGAAACTT TTCTTGTCTC CTTCTCTAGA GTACATAATT   
  
  
+ TCCCCAAATT TGACCACTTT TGCCTCATGG GTATAAGATG ATCAGATTTG AATCGATAAA AATTGAATCT   
  
  
+ TGGAAATTGC AAGAAGAAGA CTGTGGAACA GCTCATGAAC TTGGTTGCAT TAGAGGAATT GGGAAATGGG   
  
  
+ TAGATATGTT TCTTTGTTTT GTAAGTTGTT AATCTTGCAA TCAAGGGCAT TATGCACTTT TATCTACCTA   
  
  
+ ATGAGGCATA CCCAACTGCC TCCAAAGAAT TTAAAACAGA AGGATTCCAT TGAAACAAAG AGCTACAAGA   
  
  
+ GTAACAGGAC AATGGTTTTT TCAACTTTAA TTTCTATAAT TCTTTAGTAG TTGACTTTTA AGCTTTGGTT   
  
  
+ GAATGAAGCC ATCGGTGTTC TAGCTCCATC TGGTCCACCT CCGATGCATC TATCCCATCA AAACAATGTA   
  
  
+ ACAGGACAAA ACAGACTCTC AACCAAAGAT ACCAACGATG AGTAATTAAG ACACACTAGT ACTGTAAGAA   
  
  
+ AATCGATCTA TCTCTAAGAT TAATCTACAG AATCCGAAAC CCTGAAACTG TAACTTTCTT GGTACTGAGT   
  
  
+ CCTTCTGAAA TTAAAGTATA TACCCACATG CAATATTTGC TCAAAAACAC GAAATGTGGA TGAGAGAAGG   
  
  
+ CTCCATCAGA AGTAATGAGG TGGTGTCCCA GTGTTACAGT TTACCAAACC TGTAACCCTT GACGAGTGTG   
  
  
+ GAATTCAGAA CTAAAGTCGA AGTGAAAATA TCGTAGAATT GTAAGAACCA CAACTTGTCC CCCTGAATAA   
  
  
+ GATATCTTAA TTAGTTTAAA GAGTCAAGAG AGCACGCACG TAAACACACA GCTGCTTCCT TTTCATCTGC   
  
  
+ TCCACTCTCT CTCCTCTCTT TTTCATAGCC TTCTCGCTCT CTCTTTCTCT CTTTCTCCTT ATCGCTTAAT   
  
  
+ CTCCTTCTCC TCTTCTTCCC TCTCGAGTTC GTTTCCTCTT CAGTAATGCA AAGCTAAGGC TGTGTACGTC   
  
  
+ GCTTATCTTA TGAAAGGCTA GGTTATATT  

- GCGCCCCTAG GTCCCGGTCA TCCCCCAGGC TGTTCTTCCC CTGTCGAGCT TGGCGAGGTA AAAGGCTGTG   
  
  
- CACTAGTAGA TGTGCTCACC CCCGGAGCTC GTAAATGTGT ACAGTGTGCT ACTGTCCGTC TAGCCCGGGT   
  
  
- GAAATAATTT CCCAACTGAA AAATCCCCTC TTTTGCAGGT CCTTCTAAAC CACGACCCGA CGTTGACTTG   
  
  
- GATTCGGGTG AGACTGTCAG TTGGGGTGAA AAACAGAATT GCACACTTCC CAGTGCAACC CAAGAGTTGC   
  
  
- CCAGTGCTTC AGTTTCGGCG TATACATGTT TTGAAGGGAG TGAAACTTCG TTTGGTGAGT ACCCCTAGTA   
  
  
- TAGTAGAACA ACACAACGCA ACTTCTATTT CAACATATAA TCGGAACACC ACGAACATAG TACTTAAATT   
  
  
- ATCTATCCGG GTTAAATTCT CTTGGCTCTG TAACTTTGAA AAGAACAGAG GAAGAGATCT CATGTATTAA   
  
  
- AGGGGTTTAA ACTGGTGAAA ACGGAGTACC CATATTCTAC TAGTCTAAAC TTAGCTATTT TTAACTTAGA   
  
  
- ACCTTTAACG TTCTTCTTCT GACACCTTGT CGAGTACTTG AACCAACGTA ATCTCCTTAA CCCTTTACCC   
  
  
- ATCTATACAA AGAAACAAAA CATTCAACAA TTAGAACGTT AGTTCCCGTA ATACGTGAAA ATAGATGGAT   
  
  
- TACTCCGTAT GGGTTGACGG AGGTTTCTTA AATTTTGTCT TCCTAAGGTA ACTTTGTTTC TCGATGTTCT   
  
  
- CATTGTCCTG TTACCAAAAA AGTTGAAATT AAAGATATTA AGAAATCATC AACTGAAAAT TCGAAACCAA   
  
  
- CTTACTTCGG TAGCCACAAG ATCGAGGTAG ACCAGGTGGA GGCTACGTAG ATAGGGTAGT TTTGTTACAT   
  
  
- TGTCCTGTTT TGTCTGAGAG TTGGTTTCTA TGGTTGCTAC TCATTAATTC TGTGTGATCA TGACATTCTT   
  
  
- TTAGCTAGAT AGAGATTCTA ATTAGATGTC TTAGGCTTTG GGACTTTGAC ATTGAAAGAA CCATGACTCA   
  
  
- GGAAGACTTT AATTTCATAT ATGGGTGTAC GTTATAAACG AGTTTTTGTG CTTTACACCT ACTCTCTTCC   
  
  
- GAGGTAGTCT TCATTACTCC ACCACAGGGT CACAATGTCA AATGGTTTGG ACATTGGGAA CTGCTCACAC   
  
  
- CTTAAGTCTT GATTTCAGCT TCACTTTTAT AGCATCTTAA CATTCTTGGT GTTGAACAGG GGGACTTATT   
  
  
- CTATAGAATT AATCAAATTT CTCAGTTCTC TCGTGCGTGC ATTTGTGTGT CGACGAAGGA AAAGTAGACG   
  
  
- AGGTGAGAGA GAGGAGAGAA AAAGTATCGG AAGAGCGAGA GAGAAAGAGA GAAAGAGGAA TAGCGAATTA   
  
  
- GAGGAAGAGG AGAAGAAGGG AGAGCTCAAG CAAAGGAGAA GTCATTACGT TTCGATTCCG ACACATGCAG   
  
  
- CGAATAGAAT ACTTTCCGAT CCAATATAA

+     ATCT-motif

| Site Name | Organism | Position | Strand | Matrix score. | sequence | function |
| --- | --- | --- | --- | --- | --- | --- |
| ATCT-motif | Zea mays | 529 | - | 9 | AATCTGATCG | part of a conserved DNA module involved in light responsiveness |

> 2018/04/13 10:10:12  
+ CGCGGGGATC CAGGGCCAGT AGGGGGTCCG ACAAGAAGGG GACAGCTCGA ACCGCTCCAT TTTCCGACAC   
  
  
+ GTGATCATCT ACACGAGTGG GGGCCTCGAG CATTTACACA TGTCACACGA TGACAGGCAG ATCGGGCCCA   
  
  
+ CTTTATTAAA GGGTTGACTT TTTAGGGGAG AAAACGTCCA GGAAGATTTG GTGCTGGGCT GCAACTGAAC   
  
  
+ CTAAGCCCAC TCTGACAGTC AACCCCACTT TTTGTCTTAA CGTGTGAAGG GTCACGTTGG GTTCTCAACG   
  
  
+ GGTCACGAAG TCAAAGCCGC ATATGTACAA AACTTCCCTC ACTTTGAAGC AAACCACTCA TGGGGATCAT   
  
  
+ ATCATCTTGT TGTGTTGCGT TGAAGATAAA GTTGTATATT AGCCTTGTGG TGCTTGTATC ATGAATTTAA   
  
  
+ TAGATAGGCC CAATTTAAGA GAACCGAGAC ATTGAAACTT TTCTTGTCTC CTTCTCTAGA GTACATAATT   
  
  
+ TCCCCAAATT TGACCACTTT TGCCTCATGG GTATAAGATG ATCAGATTTG AATCGATAAA AATTGAATCT   
  
  
+ TGGAAATTGC AAGAAGAAGA CTGTGGAACA GCTCATGAAC TTGGTTGCAT TAGAGGAATT GGGAAATGGG   
  
  
+ TAGATATGTT TCTTTGTTTT GTAAGTTGTT AATCTTGCAA TCAAGGGCAT TATGCACTTT TATCTACCTA   
  
  
+ ATGAGGCATA CCCAACTGCC TCCAAAGAAT TTAAAACAGA AGGATTCCAT TGAAACAAAG AGCTACAAGA   
  
  
+ GTAACAGGAC AATGGTTTTT TCAACTTTAA TTTCTATAAT TCTTTAGTAG TTGACTTTTA AGCTTTGGTT   
  
  
+ GAATGAAGCC ATCGGTGTTC TAGCTCCATC TGGTCCACCT CCGATGCATC TATCCCATCA AAACAATGTA   
  
  
+ ACAGGACAAA ACAGACTCTC AACCAAAGAT ACCAACGATG AGTAATTAAG ACACACTAGT ACTGTAAGAA   
  
  
+ AATCGATCTA TCTCTAAGAT TAATCTACAG AATCCGAAAC CCTGAAACTG TAACTTTCTT GGTACTGAGT   
  
  
+ CCTTCTGAAA TTAAAGTATA TACCCACATG CAATATTTGC TCAAAAACAC GAAATGTGGA TGAGAGAAGG   
  
  
+ CTCCATCAGA AGTAATGAGG TGGTGTCCCA GTGTTACAGT TTACCAAACC TGTAACCCTT GACGAGTGTG   
  
  
+ GAATTCAGAA CTAAAGTCGA AGTGAAAATA TCGTAGAATT GTAAGAACCA CAACTTGTCC CCCTGAATAA   
  
  
+ GATATCTTAA TTAGTTTAAA GAGTCAAGAG AGCACGCACG TAAACACACA GCTGCTTCCT TTTCATCTGC   
  
  
+ TCCACTCTCT CTCCTCTCTT TTTCATAGCC TTCTCGCTCT CTCTTTCTCT CTTTCTCCTT ATCGCTTAAT   
  
  
+ CTCCTTCTCC TCTTCTTCCC TCTCGAGTTC GTTTCCTCTT CAGTAATGCA AAGCTAAGGC TGTGTACGTC   
  
  
+ GCTTATCTTA TGAAAGGCTA GGTTATATT  

- GCGCCCCTAG GTCCCGGTCA TCCCCCAGGC TGTTCTTCCC CTGTCGAGCT TGGCGAGGTA AAAGGCTGTG   
  
  
- CACTAGTAGA TGTGCTCACC CCCGGAGCTC GTAAATGTGT ACAGTGTGCT ACTGTCCGTC TAGCCCGGGT   
  
  
- GAAATAATTT CCCAACTGAA AAATCCCCTC TTTTGCAGGT CCTTCTAAAC CACGACCCGA CGTTGACTTG   
  
  
- GATTCGGGTG AGACTGTCAG TTGGGGTGAA AAACAGAATT GCACACTTCC CAGTGCAACC CAAGAGTTGC   
  
  
- CCAGTGCTTC AGTTTCGGCG TATACATGTT TTGAAGGGAG TGAAACTTCG TTTGGTGAGT ACCCCTAGTA   
  
  
- TAGTAGAACA ACACAACGCA ACTTCTATTT CAACATATAA TCGGAACACC ACGAACATAG TACTTAAATT   
  
  
- ATCTATCCGG GTTAAATTCT CTTGGCTCTG TAACTTTGAA AAGAACAGAG GAAGAGATCT CATGTATTAA   
  
  
- AGGGGTTTAA ACTGGTGAAA ACGGAGTACC CATATTCTAC TAGTCTAAAC TTAGCTATTT TTAACTTAGA   
  
  
- ACCTTTAACG TTCTTCTTCT GACACCTTGT CGAGTACTTG AACCAACGTA ATCTCCTTAA CCCTTTACCC   
  
  
- ATCTATACAA AGAAACAAAA CATTCAACAA TTAGAACGTT AGTTCCCGTA ATACGTGAAA ATAGATGGAT   
  
  
- TACTCCGTAT GGGTTGACGG AGGTTTCTTA AATTTTGTCT TCCTAAGGTA ACTTTGTTTC TCGATGTTCT   
  
  
- CATTGTCCTG TTACCAAAAA AGTTGAAATT AAAGATATTA AGAAATCATC AACTGAAAAT TCGAAACCAA   
  
  
- CTTACTTCGG TAGCCACAAG ATCGAGGTAG ACCAGGTGGA GGCTACGTAG ATAGGGTAGT TTTGTTACAT   
  
  
- TGTCCTGTTT TGTCTGAGAG TTGGTTTCTA TGGTTGCTAC TCATTAATTC TGTGTGATCA TGACATTCTT   
  
  
- TTAGCTAGAT AGAGATTCTA ATTAGATGTC TTAGGCTTTG GGACTTTGAC ATTGAAAGAA CCATGACTCA   
  
  
- GGAAGACTTT AATTTCATAT ATGGGTGTAC GTTATAAACG AGTTTTTGTG CTTTACACCT ACTCTCTTCC   
  
  
- GAGGTAGTCT TCATTACTCC ACCACAGGGT CACAATGTCA AATGGTTTGG ACATTGGGAA CTGCTCACAC   
  
  
- CTTAAGTCTT GATTTCAGCT TCACTTTTAT AGCATCTTAA CATTCTTGGT GTTGAACAGG GGGACTTATT   
  
  
- CTATAGAATT AATCAAATTT CTCAGTTCTC TCGTGCGTGC ATTTGTGTGT CGACGAAGGA AAAGTAGACG   
  
  
- AGGTGAGAGA GAGGAGAGAA AAAGTATCGG AAGAGCGAGA GAGAAAGAGA GAAAGAGGAA TAGCGAATTA   
  
  
- GAGGAAGAGG AGAAGAAGGG AGAGCTCAAG CAAAGGAGAA GTCATTACGT TTCGATTCCG ACACATGCAG   
  
  
- CGAATAGAAT ACTTTCCGAT CCAATATAA

+     Box 4

| Site Name | Organism | Position | Strand | Matrix score. | sequence | function |
| --- | --- | --- | --- | --- | --- | --- |
| Box 4 | Petroselinum crispum | 999 | - | 6 | ATTAAT | part of a conserved DNA module involved in light responsiveness |

> 2018/04/13 10:10:12  
+ CGCGGGGATC CAGGGCCAGT AGGGGGTCCG ACAAGAAGGG GACAGCTCGA ACCGCTCCAT TTTCCGACAC   
  
  
+ GTGATCATCT ACACGAGTGG GGGCCTCGAG CATTTACACA TGTCACACGA TGACAGGCAG ATCGGGCCCA   
  
  
+ CTTTATTAAA GGGTTGACTT TTTAGGGGAG AAAACGTCCA GGAAGATTTG GTGCTGGGCT GCAACTGAAC   
  
  
+ CTAAGCCCAC TCTGACAGTC AACCCCACTT TTTGTCTTAA CGTGTGAAGG GTCACGTTGG GTTCTCAACG   
  
  
+ GGTCACGAAG TCAAAGCCGC ATATGTACAA AACTTCCCTC ACTTTGAAGC AAACCACTCA TGGGGATCAT   
  
  
+ ATCATCTTGT TGTGTTGCGT TGAAGATAAA GTTGTATATT AGCCTTGTGG TGCTTGTATC ATGAATTTAA   
  
  
+ TAGATAGGCC CAATTTAAGA GAACCGAGAC ATTGAAACTT TTCTTGTCTC CTTCTCTAGA GTACATAATT   
  
  
+ TCCCCAAATT TGACCACTTT TGCCTCATGG GTATAAGATG ATCAGATTTG AATCGATAAA AATTGAATCT   
  
  
+ TGGAAATTGC AAGAAGAAGA CTGTGGAACA GCTCATGAAC TTGGTTGCAT TAGAGGAATT GGGAAATGGG   
  
  
+ TAGATATGTT TCTTTGTTTT GTAAGTTGTT AATCTTGCAA TCAAGGGCAT TATGCACTTT TATCTACCTA   
  
  
+ ATGAGGCATA CCCAACTGCC TCCAAAGAAT TTAAAACAGA AGGATTCCAT TGAAACAAAG AGCTACAAGA   
  
  
+ GTAACAGGAC AATGGTTTTT TCAACTTTAA TTTCTATAAT TCTTTAGTAG TTGACTTTTA AGCTTTGGTT   
  
  
+ GAATGAAGCC ATCGGTGTTC TAGCTCCATC TGGTCCACCT CCGATGCATC TATCCCATCA AAACAATGTA   
  
  
+ ACAGGACAAA ACAGACTCTC AACCAAAGAT ACCAACGATG AGTAATTAAG ACACACTAGT ACTGTAAGAA   
  
  
+ AATCGATCTA TCTCTAAGAT TAATCTACAG AATCCGAAAC CCTGAAACTG TAACTTTCTT GGTACTGAGT   
  
  
+ CCTTCTGAAA TTAAAGTATA TACCCACATG CAATATTTGC TCAAAAACAC GAAATGTGGA TGAGAGAAGG   
  
  
+ CTCCATCAGA AGTAATGAGG TGGTGTCCCA GTGTTACAGT TTACCAAACC TGTAACCCTT GACGAGTGTG   
  
  
+ GAATTCAGAA CTAAAGTCGA AGTGAAAATA TCGTAGAATT GTAAGAACCA CAACTTGTCC CCCTGAATAA   
  
  
+ GATATCTTAA TTAGTTTAAA GAGTCAAGAG AGCACGCACG TAAACACACA GCTGCTTCCT TTTCATCTGC   
  
  
+ TCCACTCTCT CTCCTCTCTT TTTCATAGCC TTCTCGCTCT CTCTTTCTCT CTTTCTCCTT ATCGCTTAAT   
  
  
+ CTCCTTCTCC TCTTCTTCCC TCTCGAGTTC GTTTCCTCTT CAGTAATGCA AAGCTAAGGC TGTGTACGTC   
  
  
+ GCTTATCTTA TGAAAGGCTA GGTTATATT  

- GCGCCCCTAG GTCCCGGTCA TCCCCCAGGC TGTTCTTCCC CTGTCGAGCT TGGCGAGGTA AAAGGCTGTG   
  
  
- CACTAGTAGA TGTGCTCACC CCCGGAGCTC GTAAATGTGT ACAGTGTGCT ACTGTCCGTC TAGCCCGGGT   
  
  
- GAAATAATTT CCCAACTGAA AAATCCCCTC TTTTGCAGGT CCTTCTAAAC CACGACCCGA CGTTGACTTG   
  
  
- GATTCGGGTG AGACTGTCAG TTGGGGTGAA AAACAGAATT GCACACTTCC CAGTGCAACC CAAGAGTTGC   
  
  
- CCAGTGCTTC AGTTTCGGCG TATACATGTT TTGAAGGGAG TGAAACTTCG TTTGGTGAGT ACCCCTAGTA   
  
  
- TAGTAGAACA ACACAACGCA ACTTCTATTT CAACATATAA TCGGAACACC ACGAACATAG TACTTAAATT   
  
  
- ATCTATCCGG GTTAAATTCT CTTGGCTCTG TAACTTTGAA AAGAACAGAG GAAGAGATCT CATGTATTAA   
  
  
- AGGGGTTTAA ACTGGTGAAA ACGGAGTACC CATATTCTAC TAGTCTAAAC TTAGCTATTT TTAACTTAGA   
  
  
- ACCTTTAACG TTCTTCTTCT GACACCTTGT CGAGTACTTG AACCAACGTA ATCTCCTTAA CCCTTTACCC   
  
  
- ATCTATACAA AGAAACAAAA CATTCAACAA TTAGAACGTT AGTTCCCGTA ATACGTGAAA ATAGATGGAT   
  
  
- TACTCCGTAT GGGTTGACGG AGGTTTCTTA AATTTTGTCT TCCTAAGGTA ACTTTGTTTC TCGATGTTCT   
  
  
- CATTGTCCTG TTACCAAAAA AGTTGAAATT AAAGATATTA AGAAATCATC AACTGAAAAT TCGAAACCAA   
  
  
- CTTACTTCGG TAGCCACAAG ATCGAGGTAG ACCAGGTGGA GGCTACGTAG ATAGGGTAGT TTTGTTACAT   
  
  
- TGTCCTGTTT TGTCTGAGAG TTGGTTTCTA TGGTTGCTAC TCATTAATTC TGTGTGATCA TGACATTCTT   
  
  
- TTAGCTAGAT AGAGATTCTA ATTAGATGTC TTAGGCTTTG GGACTTTGAC ATTGAAAGAA CCATGACTCA   
  
  
- GGAAGACTTT AATTTCATAT ATGGGTGTAC GTTATAAACG AGTTTTTGTG CTTTACACCT ACTCTCTTCC   
  
  
- GAGGTAGTCT TCATTACTCC ACCACAGGGT CACAATGTCA AATGGTTTGG ACATTGGGAA CTGCTCACAC   
  
  
- CTTAAGTCTT GATTTCAGCT TCACTTTTAT AGCATCTTAA CATTCTTGGT GTTGAACAGG GGGACTTATT   
  
  
- CTATAGAATT AATCAAATTT CTCAGTTCTC TCGTGCGTGC ATTTGTGTGT CGACGAAGGA AAAGTAGACG   
  
  
- AGGTGAGAGA GAGGAGAGAA AAAGTATCGG AAGAGCGAGA GAGAAAGAGA GAAAGAGGAA TAGCGAATTA   
  
  
- GAGGAAGAGG AGAAGAAGGG AGAGCTCAAG CAAAGGAGAA GTCATTACGT TTCGATTCCG ACACATGCAG   
  
  
- CGAATAGAAT ACTTTCCGAT CCAATATAA

+     Box III

| Site Name | Organism | Position | Strand | Matrix score. | sequence | function |
| --- | --- | --- | --- | --- | --- | --- |
| Box III | Pisum sativum | 101 | + | 9 | CATTTACACT | protein binding site |

> 2018/04/13 10:10:12  
+ CGCGGGGATC CAGGGCCAGT AGGGGGTCCG ACAAGAAGGG GACAGCTCGA ACCGCTCCAT TTTCCGACAC   
  
  
+ GTGATCATCT ACACGAGTGG GGGCCTCGAG CATTTACACA TGTCACACGA TGACAGGCAG ATCGGGCCCA   
  
  
+ CTTTATTAAA GGGTTGACTT TTTAGGGGAG AAAACGTCCA GGAAGATTTG GTGCTGGGCT GCAACTGAAC   
  
  
+ CTAAGCCCAC TCTGACAGTC AACCCCACTT TTTGTCTTAA CGTGTGAAGG GTCACGTTGG GTTCTCAACG   
  
  
+ GGTCACGAAG TCAAAGCCGC ATATGTACAA AACTTCCCTC ACTTTGAAGC AAACCACTCA TGGGGATCAT   
  
  
+ ATCATCTTGT TGTGTTGCGT TGAAGATAAA GTTGTATATT AGCCTTGTGG TGCTTGTATC ATGAATTTAA   
  
  
+ TAGATAGGCC CAATTTAAGA GAACCGAGAC ATTGAAACTT TTCTTGTCTC CTTCTCTAGA GTACATAATT   
  
  
+ TCCCCAAATT TGACCACTTT TGCCTCATGG GTATAAGATG ATCAGATTTG AATCGATAAA AATTGAATCT   
  
  
+ TGGAAATTGC AAGAAGAAGA CTGTGGAACA GCTCATGAAC TTGGTTGCAT TAGAGGAATT GGGAAATGGG   
  
  
+ TAGATATGTT TCTTTGTTTT GTAAGTTGTT AATCTTGCAA TCAAGGGCAT TATGCACTTT TATCTACCTA   
  
  
+ ATGAGGCATA CCCAACTGCC TCCAAAGAAT TTAAAACAGA AGGATTCCAT TGAAACAAAG AGCTACAAGA   
  
  
+ GTAACAGGAC AATGGTTTTT TCAACTTTAA TTTCTATAAT TCTTTAGTAG TTGACTTTTA AGCTTTGGTT   
  
  
+ GAATGAAGCC ATCGGTGTTC TAGCTCCATC TGGTCCACCT CCGATGCATC TATCCCATCA AAACAATGTA   
  
  
+ ACAGGACAAA ACAGACTCTC AACCAAAGAT ACCAACGATG AGTAATTAAG ACACACTAGT ACTGTAAGAA   
  
  
+ AATCGATCTA TCTCTAAGAT TAATCTACAG AATCCGAAAC CCTGAAACTG TAACTTTCTT GGTACTGAGT   
  
  
+ CCTTCTGAAA TTAAAGTATA TACCCACATG CAATATTTGC TCAAAAACAC GAAATGTGGA TGAGAGAAGG   
  
  
+ CTCCATCAGA AGTAATGAGG TGGTGTCCCA GTGTTACAGT TTACCAAACC TGTAACCCTT GACGAGTGTG   
  
  
+ GAATTCAGAA CTAAAGTCGA AGTGAAAATA TCGTAGAATT GTAAGAACCA CAACTTGTCC CCCTGAATAA   
  
  
+ GATATCTTAA TTAGTTTAAA GAGTCAAGAG AGCACGCACG TAAACACACA GCTGCTTCCT TTTCATCTGC   
  
  
+ TCCACTCTCT CTCCTCTCTT TTTCATAGCC TTCTCGCTCT CTCTTTCTCT CTTTCTCCTT ATCGCTTAAT   
  
  
+ CTCCTTCTCC TCTTCTTCCC TCTCGAGTTC GTTTCCTCTT CAGTAATGCA AAGCTAAGGC TGTGTACGTC   
  
  
+ GCTTATCTTA TGAAAGGCTA GGTTATATT  

- GCGCCCCTAG GTCCCGGTCA TCCCCCAGGC TGTTCTTCCC CTGTCGAGCT TGGCGAGGTA AAAGGCTGTG   
  
  
- CACTAGTAGA TGTGCTCACC CCCGGAGCTC GTAAATGTGT ACAGTGTGCT ACTGTCCGTC TAGCCCGGGT   
  
  
- GAAATAATTT CCCAACTGAA AAATCCCCTC TTTTGCAGGT CCTTCTAAAC CACGACCCGA CGTTGACTTG   
  
  
- GATTCGGGTG AGACTGTCAG TTGGGGTGAA AAACAGAATT GCACACTTCC CAGTGCAACC CAAGAGTTGC   
  
  
- CCAGTGCTTC AGTTTCGGCG TATACATGTT TTGAAGGGAG TGAAACTTCG TTTGGTGAGT ACCCCTAGTA   
  
  
- TAGTAGAACA ACACAACGCA ACTTCTATTT CAACATATAA TCGGAACACC ACGAACATAG TACTTAAATT   
  
  
- ATCTATCCGG GTTAAATTCT CTTGGCTCTG TAACTTTGAA AAGAACAGAG GAAGAGATCT CATGTATTAA   
  
  
- AGGGGTTTAA ACTGGTGAAA ACGGAGTACC CATATTCTAC TAGTCTAAAC TTAGCTATTT TTAACTTAGA   
  
  
- ACCTTTAACG TTCTTCTTCT GACACCTTGT CGAGTACTTG AACCAACGTA ATCTCCTTAA CCCTTTACCC   
  
  
- ATCTATACAA AGAAACAAAA CATTCAACAA TTAGAACGTT AGTTCCCGTA ATACGTGAAA ATAGATGGAT   
  
  
- TACTCCGTAT GGGTTGACGG AGGTTTCTTA AATTTTGTCT TCCTAAGGTA ACTTTGTTTC TCGATGTTCT   
  
  
- CATTGTCCTG TTACCAAAAA AGTTGAAATT AAAGATATTA AGAAATCATC AACTGAAAAT TCGAAACCAA   
  
  
- CTTACTTCGG TAGCCACAAG ATCGAGGTAG ACCAGGTGGA GGCTACGTAG ATAGGGTAGT TTTGTTACAT   
  
  
- TGTCCTGTTT TGTCTGAGAG TTGGTTTCTA TGGTTGCTAC TCATTAATTC TGTGTGATCA TGACATTCTT   
  
  
- TTAGCTAGAT AGAGATTCTA ATTAGATGTC TTAGGCTTTG GGACTTTGAC ATTGAAAGAA CCATGACTCA   
  
  
- GGAAGACTTT AATTTCATAT ATGGGTGTAC GTTATAAACG AGTTTTTGTG CTTTACACCT ACTCTCTTCC   
  
  
- GAGGTAGTCT TCATTACTCC ACCACAGGGT CACAATGTCA AATGGTTTGG ACATTGGGAA CTGCTCACAC   
  
  
- CTTAAGTCTT GATTTCAGCT TCACTTTTAT AGCATCTTAA CATTCTTGGT GTTGAACAGG GGGACTTATT   
  
  
- CTATAGAATT AATCAAATTT CTCAGTTCTC TCGTGCGTGC ATTTGTGTGT CGACGAAGGA AAAGTAGACG   
  
  
- AGGTGAGAGA GAGGAGAGAA AAAGTATCGG AAGAGCGAGA GAGAAAGAGA GAAAGAGGAA TAGCGAATTA   
  
  
- GAGGAAGAGG AGAAGAAGGG AGAGCTCAAG CAAAGGAGAA GTCATTACGT TTCGATTCCG ACACATGCAG   
  
  
- CGAATAGAAT ACTTTCCGAT CCAATATAA

+     Box-W1

| Site Name | Organism | Position | Strand | Matrix score. | sequence | function |
| --- | --- | --- | --- | --- | --- | --- |
| Box-W1 | Petroselinum crispum | 500 | + | 6 | TTGACC | fungal elicitor responsive element |

> 2018/04/13 10:10:12  
+ CGCGGGGATC CAGGGCCAGT AGGGGGTCCG ACAAGAAGGG GACAGCTCGA ACCGCTCCAT TTTCCGACAC   
  
  
+ GTGATCATCT ACACGAGTGG GGGCCTCGAG CATTTACACA TGTCACACGA TGACAGGCAG ATCGGGCCCA   
  
  
+ CTTTATTAAA GGGTTGACTT TTTAGGGGAG AAAACGTCCA GGAAGATTTG GTGCTGGGCT GCAACTGAAC   
  
  
+ CTAAGCCCAC TCTGACAGTC AACCCCACTT TTTGTCTTAA CGTGTGAAGG GTCACGTTGG GTTCTCAACG   
  
  
+ GGTCACGAAG TCAAAGCCGC ATATGTACAA AACTTCCCTC ACTTTGAAGC AAACCACTCA TGGGGATCAT   
  
  
+ ATCATCTTGT TGTGTTGCGT TGAAGATAAA GTTGTATATT AGCCTTGTGG TGCTTGTATC ATGAATTTAA   
  
  
+ TAGATAGGCC CAATTTAAGA GAACCGAGAC ATTGAAACTT TTCTTGTCTC CTTCTCTAGA GTACATAATT   
  
  
+ TCCCCAAATT TGACCACTTT TGCCTCATGG GTATAAGATG ATCAGATTTG AATCGATAAA AATTGAATCT   
  
  
+ TGGAAATTGC AAGAAGAAGA CTGTGGAACA GCTCATGAAC TTGGTTGCAT TAGAGGAATT GGGAAATGGG   
  
  
+ TAGATATGTT TCTTTGTTTT GTAAGTTGTT AATCTTGCAA TCAAGGGCAT TATGCACTTT TATCTACCTA   
  
  
+ ATGAGGCATA CCCAACTGCC TCCAAAGAAT TTAAAACAGA AGGATTCCAT TGAAACAAAG AGCTACAAGA   
  
  
+ GTAACAGGAC AATGGTTTTT TCAACTTTAA TTTCTATAAT TCTTTAGTAG TTGACTTTTA AGCTTTGGTT   
  
  
+ GAATGAAGCC ATCGGTGTTC TAGCTCCATC TGGTCCACCT CCGATGCATC TATCCCATCA AAACAATGTA   
  
  
+ ACAGGACAAA ACAGACTCTC AACCAAAGAT ACCAACGATG AGTAATTAAG ACACACTAGT ACTGTAAGAA   
  
  
+ AATCGATCTA TCTCTAAGAT TAATCTACAG AATCCGAAAC CCTGAAACTG TAACTTTCTT GGTACTGAGT   
  
  
+ CCTTCTGAAA TTAAAGTATA TACCCACATG CAATATTTGC TCAAAAACAC GAAATGTGGA TGAGAGAAGG   
  
  
+ CTCCATCAGA AGTAATGAGG TGGTGTCCCA GTGTTACAGT TTACCAAACC TGTAACCCTT GACGAGTGTG   
  
  
+ GAATTCAGAA CTAAAGTCGA AGTGAAAATA TCGTAGAATT GTAAGAACCA CAACTTGTCC CCCTGAATAA   
  
  
+ GATATCTTAA TTAGTTTAAA GAGTCAAGAG AGCACGCACG TAAACACACA GCTGCTTCCT TTTCATCTGC   
  
  
+ TCCACTCTCT CTCCTCTCTT TTTCATAGCC TTCTCGCTCT CTCTTTCTCT CTTTCTCCTT ATCGCTTAAT   
  
  
+ CTCCTTCTCC TCTTCTTCCC TCTCGAGTTC GTTTCCTCTT CAGTAATGCA AAGCTAAGGC TGTGTACGTC   
  
  
+ GCTTATCTTA TGAAAGGCTA GGTTATATT  

- GCGCCCCTAG GTCCCGGTCA TCCCCCAGGC TGTTCTTCCC CTGTCGAGCT TGGCGAGGTA AAAGGCTGTG   
  
  
- CACTAGTAGA TGTGCTCACC CCCGGAGCTC GTAAATGTGT ACAGTGTGCT ACTGTCCGTC TAGCCCGGGT   
  
  
- GAAATAATTT CCCAACTGAA AAATCCCCTC TTTTGCAGGT CCTTCTAAAC CACGACCCGA CGTTGACTTG   
  
  
- GATTCGGGTG AGACTGTCAG TTGGGGTGAA AAACAGAATT GCACACTTCC CAGTGCAACC CAAGAGTTGC   
  
  
- CCAGTGCTTC AGTTTCGGCG TATACATGTT TTGAAGGGAG TGAAACTTCG TTTGGTGAGT ACCCCTAGTA   
  
  
- TAGTAGAACA ACACAACGCA ACTTCTATTT CAACATATAA TCGGAACACC ACGAACATAG TACTTAAATT   
  
  
- ATCTATCCGG GTTAAATTCT CTTGGCTCTG TAACTTTGAA AAGAACAGAG GAAGAGATCT CATGTATTAA   
  
  
- AGGGGTTTAA ACTGGTGAAA ACGGAGTACC CATATTCTAC TAGTCTAAAC TTAGCTATTT TTAACTTAGA   
  
  
- ACCTTTAACG TTCTTCTTCT GACACCTTGT CGAGTACTTG AACCAACGTA ATCTCCTTAA CCCTTTACCC   
  
  
- ATCTATACAA AGAAACAAAA CATTCAACAA TTAGAACGTT AGTTCCCGTA ATACGTGAAA ATAGATGGAT   
  
  
- TACTCCGTAT GGGTTGACGG AGGTTTCTTA AATTTTGTCT TCCTAAGGTA ACTTTGTTTC TCGATGTTCT   
  
  
- CATTGTCCTG TTACCAAAAA AGTTGAAATT AAAGATATTA AGAAATCATC AACTGAAAAT TCGAAACCAA   
  
  
- CTTACTTCGG TAGCCACAAG ATCGAGGTAG ACCAGGTGGA GGCTACGTAG ATAGGGTAGT TTTGTTACAT   
  
  
- TGTCCTGTTT TGTCTGAGAG TTGGTTTCTA TGGTTGCTAC TCATTAATTC TGTGTGATCA TGACATTCTT   
  
  
- TTAGCTAGAT AGAGATTCTA ATTAGATGTC TTAGGCTTTG GGACTTTGAC ATTGAAAGAA CCATGACTCA   
  
  
- GGAAGACTTT AATTTCATAT ATGGGTGTAC GTTATAAACG AGTTTTTGTG CTTTACACCT ACTCTCTTCC   
  
  
- GAGGTAGTCT TCATTACTCC ACCACAGGGT CACAATGTCA AATGGTTTGG ACATTGGGAA CTGCTCACAC   
  
  
- CTTAAGTCTT GATTTCAGCT TCACTTTTAT AGCATCTTAA CATTCTTGGT GTTGAACAGG GGGACTTATT   
  
  
- CTATAGAATT AATCAAATTT CTCAGTTCTC TCGTGCGTGC ATTTGTGTGT CGACGAAGGA AAAGTAGACG   
  
  
- AGGTGAGAGA GAGGAGAGAA AAAGTATCGG AAGAGCGAGA GAGAAAGAGA GAAAGAGGAA TAGCGAATTA   
  
  
- GAGGAAGAGG AGAAGAAGGG AGAGCTCAAG CAAAGGAGAA GTCATTACGT TTCGATTCCG ACACATGCAG   
  
  
- CGAATAGAAT ACTTTCCGAT CCAATATAA

+     CAAT-box

| Site Name | Organism | Position | Strand | Matrix score. | sequence | function |
| --- | --- | --- | --- | --- | --- | --- |
| CAAT-box | Glycine max | 1227 | - | 5 | CAATT | common cis-acting element in promoter and enhancer regions |
| CAAT-box | Brassica rapa | 498 | - | 5 | CAAAT | common cis-acting element in promoter and enhancer regions |
| CAAT-box | Brassica rapa | 1085 | - | 5 | CAAAT | common cis-acting element in promoter and enhancer regions |
| CAAT-box | Hordeum vulgare | 1081 | + | 4 | CAAT | common cis-acting element in promoter and enhancer regions |
| CAAT-box | Hordeum vulgare | 904 | + | 4 | CAAT | common cis-acting element in promoter and enhancer regions |
| CAAT-box | Hordeum vulgare | 780 | + | 4 | CAAT | common cis-acting element in promoter and enhancer regions |
| CAAT-box | Hordeum vulgare | 668 | + | 4 | CAAT | common cis-acting element in promoter and enhancer regions |
| CAAT-box | Glycine max | 431 | + | 5 | CAATT | common cis-acting element in promoter and enhancer regions |
| CAAT-box | Arabidopsis thaliana | 430 | + | 5 | CCAAT | common cis-acting element in promoter and enhancer regions |
| CAAT-box | Hordeum vulgare | 1228 | - | 4 | CAAT | common cis-acting element in promoter and enhancer regions |
| CAAT-box | Glycine max | 565 | - | 5 | CAATT | common cis-acting element in promoter and enhancer regions |
| CAAT-box | Arabidopsis thaliana | 618 | - | 5 | CCAAT | common cis-acting element in promoter and enhancer regions |
| CAAT-box | Glycine max | 617 | - | 5 | CAATT | common cis-acting element in promoter and enhancer regions |
| CAAT-box | Hordeum vulgare | 451 | - | 4 | CAAT | common cis-acting element in promoter and enhancer regions |
| CAAT-box | Glycine max | 551 | - | 5 | CAATT | common cis-acting element in promoter and enhancer regions |
| CAAT-box | Hordeum vulgare | 566 | - | 4 | CAAT | common cis-acting element in promoter and enhancer regions |
| CAAT-box | Hordeum vulgare | 749 | - | 4 | CAAT | common cis-acting element in promoter and enhancer regions |
| CAAT-box | Brassica rapa | 186 | - | 5 | CAAAT | common cis-acting element in promoter and enhancer regions |
| CAAT-box | Arabidopsis thaliana | 429 | + | 8 | CCCAATTT | common cis-acting element in promoter and enhancer regions |
| CAAT-box | Brassica rapa | 536 | - | 5 | CAAAT | common cis-acting element in promoter and enhancer regions |
| CAAT-box | Hordeum vulgare | 552 | - | 4 | CAAT | common cis-acting element in promoter and enhancer regions |
| CAAT-box | Brassica rapa | 495 | + | 5 | CAAAT | common cis-acting element in promoter and enhancer regions |

> 2018/04/13 10:10:12  
+ CGCGGGGATC CAGGGCCAGT AGGGGGTCCG ACAAGAAGGG GACAGCTCGA ACCGCTCCAT TTTCCGACAC   
  
  
+ GTGATCATCT ACACGAGTGG GGGCCTCGAG CATTTACACA TGTCACACGA TGACAGGCAG ATCGGGCCCA   
  
  
+ CTTTATTAAA GGGTTGACTT TTTAGGGGAG AAAACGTCCA GGAAGATTTG GTGCTGGGCT GCAACTGAAC   
  
  
+ CTAAGCCCAC TCTGACAGTC AACCCCACTT TTTGTCTTAA CGTGTGAAGG GTCACGTTGG GTTCTCAACG   
  
  
+ GGTCACGAAG TCAAAGCCGC ATATGTACAA AACTTCCCTC ACTTTGAAGC AAACCACTCA TGGGGATCAT   
  
  
+ ATCATCTTGT TGTGTTGCGT TGAAGATAAA GTTGTATATT AGCCTTGTGG TGCTTGTATC ATGAATTTAA   
  
  
+ TAGATAGGCC CAATTTAAGA GAACCGAGAC ATTGAAACTT TTCTTGTCTC CTTCTCTAGA GTACATAATT   
  
  
+ TCCCCAAATT TGACCACTTT TGCCTCATGG GTATAAGATG ATCAGATTTG AATCGATAAA AATTGAATCT   
  
  
+ TGGAAATTGC AAGAAGAAGA CTGTGGAACA GCTCATGAAC TTGGTTGCAT TAGAGGAATT GGGAAATGGG   
  
  
+ TAGATATGTT TCTTTGTTTT GTAAGTTGTT AATCTTGCAA TCAAGGGCAT TATGCACTTT TATCTACCTA   
  
  
+ ATGAGGCATA CCCAACTGCC TCCAAAGAAT TTAAAACAGA AGGATTCCAT TGAAACAAAG AGCTACAAGA   
  
  
+ GTAACAGGAC AATGGTTTTT TCAACTTTAA TTTCTATAAT TCTTTAGTAG TTGACTTTTA AGCTTTGGTT   
  
  
+ GAATGAAGCC ATCGGTGTTC TAGCTCCATC TGGTCCACCT CCGATGCATC TATCCCATCA AAACAATGTA   
  
  
+ ACAGGACAAA ACAGACTCTC AACCAAAGAT ACCAACGATG AGTAATTAAG ACACACTAGT ACTGTAAGAA   
  
  
+ AATCGATCTA TCTCTAAGAT TAATCTACAG AATCCGAAAC CCTGAAACTG TAACTTTCTT GGTACTGAGT   
  
  
+ CCTTCTGAAA TTAAAGTATA TACCCACATG CAATATTTGC TCAAAAACAC GAAATGTGGA TGAGAGAAGG   
  
  
+ CTCCATCAGA AGTAATGAGG TGGTGTCCCA GTGTTACAGT TTACCAAACC TGTAACCCTT GACGAGTGTG   
  
  
+ GAATTCAGAA CTAAAGTCGA AGTGAAAATA TCGTAGAATT GTAAGAACCA CAACTTGTCC CCCTGAATAA   
  
  
+ GATATCTTAA TTAGTTTAAA GAGTCAAGAG AGCACGCACG TAAACACACA GCTGCTTCCT TTTCATCTGC   
  
  
+ TCCACTCTCT CTCCTCTCTT TTTCATAGCC TTCTCGCTCT CTCTTTCTCT CTTTCTCCTT ATCGCTTAAT   
  
  
+ CTCCTTCTCC TCTTCTTCCC TCTCGAGTTC GTTTCCTCTT CAGTAATGCA AAGCTAAGGC TGTGTACGTC   
  
  
+ GCTTATCTTA TGAAAGGCTA GGTTATATT  

- GCGCCCCTAG GTCCCGGTCA TCCCCCAGGC TGTTCTTCCC CTGTCGAGCT TGGCGAGGTA AAAGGCTGTG   
  
  
- CACTAGTAGA TGTGCTCACC CCCGGAGCTC GTAAATGTGT ACAGTGTGCT ACTGTCCGTC TAGCCCGGGT   
  
  
- GAAATAATTT CCCAACTGAA AAATCCCCTC TTTTGCAGGT CCTTCTAAAC CACGACCCGA CGTTGACTTG   
  
  
- GATTCGGGTG AGACTGTCAG TTGGGGTGAA AAACAGAATT GCACACTTCC CAGTGCAACC CAAGAGTTGC   
  
  
- CCAGTGCTTC AGTTTCGGCG TATACATGTT TTGAAGGGAG TGAAACTTCG TTTGGTGAGT ACCCCTAGTA   
  
  
- TAGTAGAACA ACACAACGCA ACTTCTATTT CAACATATAA TCGGAACACC ACGAACATAG TACTTAAATT   
  
  
- ATCTATCCGG GTTAAATTCT CTTGGCTCTG TAACTTTGAA AAGAACAGAG GAAGAGATCT CATGTATTAA   
  
  
- AGGGGTTTAA ACTGGTGAAA ACGGAGTACC CATATTCTAC TAGTCTAAAC TTAGCTATTT TTAACTTAGA   
  
  
- ACCTTTAACG TTCTTCTTCT GACACCTTGT CGAGTACTTG AACCAACGTA ATCTCCTTAA CCCTTTACCC   
  
  
- ATCTATACAA AGAAACAAAA CATTCAACAA TTAGAACGTT AGTTCCCGTA ATACGTGAAA ATAGATGGAT   
  
  
- TACTCCGTAT GGGTTGACGG AGGTTTCTTA AATTTTGTCT TCCTAAGGTA ACTTTGTTTC TCGATGTTCT   
  
  
- CATTGTCCTG TTACCAAAAA AGTTGAAATT AAAGATATTA AGAAATCATC AACTGAAAAT TCGAAACCAA   
  
  
- CTTACTTCGG TAGCCACAAG ATCGAGGTAG ACCAGGTGGA GGCTACGTAG ATAGGGTAGT TTTGTTACAT   
  
  
- TGTCCTGTTT TGTCTGAGAG TTGGTTTCTA TGGTTGCTAC TCATTAATTC TGTGTGATCA TGACATTCTT   
  
  
- TTAGCTAGAT AGAGATTCTA ATTAGATGTC TTAGGCTTTG GGACTTTGAC ATTGAAAGAA CCATGACTCA   
  
  
- GGAAGACTTT AATTTCATAT ATGGGTGTAC GTTATAAACG AGTTTTTGTG CTTTACACCT ACTCTCTTCC   
  
  
- GAGGTAGTCT TCATTACTCC ACCACAGGGT CACAATGTCA AATGGTTTGG ACATTGGGAA CTGCTCACAC   
  
  
- CTTAAGTCTT GATTTCAGCT TCACTTTTAT AGCATCTTAA CATTCTTGGT GTTGAACAGG GGGACTTATT   
  
  
- CTATAGAATT AATCAAATTT CTCAGTTCTC TCGTGCGTGC ATTTGTGTGT CGACGAAGGA AAAGTAGACG   
  
  
- AGGTGAGAGA GAGGAGAGAA AAAGTATCGG AAGAGCGAGA GAGAAAGAGA GAAAGAGGAA TAGCGAATTA   
  
  
- GAGGAAGAGG AGAAGAAGGG AGAGCTCAAG CAAAGGAGAA GTCATTACGT TTCGATTCCG ACACATGCAG   
  
  
- CGAATAGAAT ACTTTCCGAT CCAATATAA

+     CCAAT-box

| Site Name | Organism | Position | Strand | Matrix score. | sequence | function |
| --- | --- | --- | --- | --- | --- | --- |
| CCAAT-box | Hordeum vulgare | 276 | + | 6 | CAACGG | MYBHv1 binding site |

> 2018/04/13 10:10:12  
+ CGCGGGGATC CAGGGCCAGT AGGGGGTCCG ACAAGAAGGG GACAGCTCGA ACCGCTCCAT TTTCCGACAC   
  
  
+ GTGATCATCT ACACGAGTGG GGGCCTCGAG CATTTACACA TGTCACACGA TGACAGGCAG ATCGGGCCCA   
  
  
+ CTTTATTAAA GGGTTGACTT TTTAGGGGAG AAAACGTCCA GGAAGATTTG GTGCTGGGCT GCAACTGAAC   
  
  
+ CTAAGCCCAC TCTGACAGTC AACCCCACTT TTTGTCTTAA CGTGTGAAGG GTCACGTTGG GTTCTCAACG   
  
  
+ GGTCACGAAG TCAAAGCCGC ATATGTACAA AACTTCCCTC ACTTTGAAGC AAACCACTCA TGGGGATCAT   
  
  
+ ATCATCTTGT TGTGTTGCGT TGAAGATAAA GTTGTATATT AGCCTTGTGG TGCTTGTATC ATGAATTTAA   
  
  
+ TAGATAGGCC CAATTTAAGA GAACCGAGAC ATTGAAACTT TTCTTGTCTC CTTCTCTAGA GTACATAATT   
  
  
+ TCCCCAAATT TGACCACTTT TGCCTCATGG GTATAAGATG ATCAGATTTG AATCGATAAA AATTGAATCT   
  
  
+ TGGAAATTGC AAGAAGAAGA CTGTGGAACA GCTCATGAAC TTGGTTGCAT TAGAGGAATT GGGAAATGGG   
  
  
+ TAGATATGTT TCTTTGTTTT GTAAGTTGTT AATCTTGCAA TCAAGGGCAT TATGCACTTT TATCTACCTA   
  
  
+ ATGAGGCATA CCCAACTGCC TCCAAAGAAT TTAAAACAGA AGGATTCCAT TGAAACAAAG AGCTACAAGA   
  
  
+ GTAACAGGAC AATGGTTTTT TCAACTTTAA TTTCTATAAT TCTTTAGTAG TTGACTTTTA AGCTTTGGTT   
  
  
+ GAATGAAGCC ATCGGTGTTC TAGCTCCATC TGGTCCACCT CCGATGCATC TATCCCATCA AAACAATGTA   
  
  
+ ACAGGACAAA ACAGACTCTC AACCAAAGAT ACCAACGATG AGTAATTAAG ACACACTAGT ACTGTAAGAA   
  
  
+ AATCGATCTA TCTCTAAGAT TAATCTACAG AATCCGAAAC CCTGAAACTG TAACTTTCTT GGTACTGAGT   
  
  
+ CCTTCTGAAA TTAAAGTATA TACCCACATG CAATATTTGC TCAAAAACAC GAAATGTGGA TGAGAGAAGG   
  
  
+ CTCCATCAGA AGTAATGAGG TGGTGTCCCA GTGTTACAGT TTACCAAACC TGTAACCCTT GACGAGTGTG   
  
  
+ GAATTCAGAA CTAAAGTCGA AGTGAAAATA TCGTAGAATT GTAAGAACCA CAACTTGTCC CCCTGAATAA   
  
  
+ GATATCTTAA TTAGTTTAAA GAGTCAAGAG AGCACGCACG TAAACACACA GCTGCTTCCT TTTCATCTGC   
  
  
+ TCCACTCTCT CTCCTCTCTT TTTCATAGCC TTCTCGCTCT CTCTTTCTCT CTTTCTCCTT ATCGCTTAAT   
  
  
+ CTCCTTCTCC TCTTCTTCCC TCTCGAGTTC GTTTCCTCTT CAGTAATGCA AAGCTAAGGC TGTGTACGTC   
  
  
+ GCTTATCTTA TGAAAGGCTA GGTTATATT  

- GCGCCCCTAG GTCCCGGTCA TCCCCCAGGC TGTTCTTCCC CTGTCGAGCT TGGCGAGGTA AAAGGCTGTG   
  
  
- CACTAGTAGA TGTGCTCACC CCCGGAGCTC GTAAATGTGT ACAGTGTGCT ACTGTCCGTC TAGCCCGGGT   
  
  
- GAAATAATTT CCCAACTGAA AAATCCCCTC TTTTGCAGGT CCTTCTAAAC CACGACCCGA CGTTGACTTG   
  
  
- GATTCGGGTG AGACTGTCAG TTGGGGTGAA AAACAGAATT GCACACTTCC CAGTGCAACC CAAGAGTTGC   
  
  
- CCAGTGCTTC AGTTTCGGCG TATACATGTT TTGAAGGGAG TGAAACTTCG TTTGGTGAGT ACCCCTAGTA   
  
  
- TAGTAGAACA ACACAACGCA ACTTCTATTT CAACATATAA TCGGAACACC ACGAACATAG TACTTAAATT   
  
  
- ATCTATCCGG GTTAAATTCT CTTGGCTCTG TAACTTTGAA AAGAACAGAG GAAGAGATCT CATGTATTAA   
  
  
- AGGGGTTTAA ACTGGTGAAA ACGGAGTACC CATATTCTAC TAGTCTAAAC TTAGCTATTT TTAACTTAGA   
  
  
- ACCTTTAACG TTCTTCTTCT GACACCTTGT CGAGTACTTG AACCAACGTA ATCTCCTTAA CCCTTTACCC   
  
  
- ATCTATACAA AGAAACAAAA CATTCAACAA TTAGAACGTT AGTTCCCGTA ATACGTGAAA ATAGATGGAT   
  
  
- TACTCCGTAT GGGTTGACGG AGGTTTCTTA AATTTTGTCT TCCTAAGGTA ACTTTGTTTC TCGATGTTCT   
  
  
- CATTGTCCTG TTACCAAAAA AGTTGAAATT AAAGATATTA AGAAATCATC AACTGAAAAT TCGAAACCAA   
  
  
- CTTACTTCGG TAGCCACAAG ATCGAGGTAG ACCAGGTGGA GGCTACGTAG ATAGGGTAGT TTTGTTACAT   
  
  
- TGTCCTGTTT TGTCTGAGAG TTGGTTTCTA TGGTTGCTAC TCATTAATTC TGTGTGATCA TGACATTCTT   
  
  
- TTAGCTAGAT AGAGATTCTA ATTAGATGTC TTAGGCTTTG GGACTTTGAC ATTGAAAGAA CCATGACTCA   
  
  
- GGAAGACTTT AATTTCATAT ATGGGTGTAC GTTATAAACG AGTTTTTGTG CTTTACACCT ACTCTCTTCC   
  
  
- GAGGTAGTCT TCATTACTCC ACCACAGGGT CACAATGTCA AATGGTTTGG ACATTGGGAA CTGCTCACAC   
  
  
- CTTAAGTCTT GATTTCAGCT TCACTTTTAT AGCATCTTAA CATTCTTGGT GTTGAACAGG GGGACTTATT   
  
  
- CTATAGAATT AATCAAATTT CTCAGTTCTC TCGTGCGTGC ATTTGTGTGT CGACGAAGGA AAAGTAGACG   
  
  
- AGGTGAGAGA GAGGAGAGAA AAAGTATCGG AAGAGCGAGA GAGAAAGAGA GAAAGAGGAA TAGCGAATTA   
  
  
- GAGGAAGAGG AGAAGAAGGG AGAGCTCAAG CAAAGGAGAA GTCATTACGT TTCGATTCCG ACACATGCAG   
  
  
- CGAATAGAAT ACTTTCCGAT CCAATATAA

+     CGTCA-motif

| Site Name | Organism | Position | Strand | Matrix score. | sequence | function |
| --- | --- | --- | --- | --- | --- | --- |
| CGTCA-motif | Hordeum vulgare | 1180 | - | 5 | CGTCA | cis-acting regulatory element involved in the MeJA-responsiveness |

> 2018/04/13 10:10:12  
+ CGCGGGGATC CAGGGCCAGT AGGGGGTCCG ACAAGAAGGG GACAGCTCGA ACCGCTCCAT TTTCCGACAC   
  
  
+ GTGATCATCT ACACGAGTGG GGGCCTCGAG CATTTACACA TGTCACACGA TGACAGGCAG ATCGGGCCCA   
  
  
+ CTTTATTAAA GGGTTGACTT TTTAGGGGAG AAAACGTCCA GGAAGATTTG GTGCTGGGCT GCAACTGAAC   
  
  
+ CTAAGCCCAC TCTGACAGTC AACCCCACTT TTTGTCTTAA CGTGTGAAGG GTCACGTTGG GTTCTCAACG   
  
  
+ GGTCACGAAG TCAAAGCCGC ATATGTACAA AACTTCCCTC ACTTTGAAGC AAACCACTCA TGGGGATCAT   
  
  
+ ATCATCTTGT TGTGTTGCGT TGAAGATAAA GTTGTATATT AGCCTTGTGG TGCTTGTATC ATGAATTTAA   
  
  
+ TAGATAGGCC CAATTTAAGA GAACCGAGAC ATTGAAACTT TTCTTGTCTC CTTCTCTAGA GTACATAATT   
  
  
+ TCCCCAAATT TGACCACTTT TGCCTCATGG GTATAAGATG ATCAGATTTG AATCGATAAA AATTGAATCT   
  
  
+ TGGAAATTGC AAGAAGAAGA CTGTGGAACA GCTCATGAAC TTGGTTGCAT TAGAGGAATT GGGAAATGGG   
  
  
+ TAGATATGTT TCTTTGTTTT GTAAGTTGTT AATCTTGCAA TCAAGGGCAT TATGCACTTT TATCTACCTA   
  
  
+ ATGAGGCATA CCCAACTGCC TCCAAAGAAT TTAAAACAGA AGGATTCCAT TGAAACAAAG AGCTACAAGA   
  
  
+ GTAACAGGAC AATGGTTTTT TCAACTTTAA TTTCTATAAT TCTTTAGTAG TTGACTTTTA AGCTTTGGTT   
  
  
+ GAATGAAGCC ATCGGTGTTC TAGCTCCATC TGGTCCACCT CCGATGCATC TATCCCATCA AAACAATGTA   
  
  
+ ACAGGACAAA ACAGACTCTC AACCAAAGAT ACCAACGATG AGTAATTAAG ACACACTAGT ACTGTAAGAA   
  
  
+ AATCGATCTA TCTCTAAGAT TAATCTACAG AATCCGAAAC CCTGAAACTG TAACTTTCTT GGTACTGAGT   
  
  
+ CCTTCTGAAA TTAAAGTATA TACCCACATG CAATATTTGC TCAAAAACAC GAAATGTGGA TGAGAGAAGG   
  
  
+ CTCCATCAGA AGTAATGAGG TGGTGTCCCA GTGTTACAGT TTACCAAACC TGTAACCCTT GACGAGTGTG   
  
  
+ GAATTCAGAA CTAAAGTCGA AGTGAAAATA TCGTAGAATT GTAAGAACCA CAACTTGTCC CCCTGAATAA   
  
  
+ GATATCTTAA TTAGTTTAAA GAGTCAAGAG AGCACGCACG TAAACACACA GCTGCTTCCT TTTCATCTGC   
  
  
+ TCCACTCTCT CTCCTCTCTT TTTCATAGCC TTCTCGCTCT CTCTTTCTCT CTTTCTCCTT ATCGCTTAAT   
  
  
+ CTCCTTCTCC TCTTCTTCCC TCTCGAGTTC GTTTCCTCTT CAGTAATGCA AAGCTAAGGC TGTGTACGTC   
  
  
+ GCTTATCTTA TGAAAGGCTA GGTTATATT  

- GCGCCCCTAG GTCCCGGTCA TCCCCCAGGC TGTTCTTCCC CTGTCGAGCT TGGCGAGGTA AAAGGCTGTG   
  
  
- CACTAGTAGA TGTGCTCACC CCCGGAGCTC GTAAATGTGT ACAGTGTGCT ACTGTCCGTC TAGCCCGGGT   
  
  
- GAAATAATTT CCCAACTGAA AAATCCCCTC TTTTGCAGGT CCTTCTAAAC CACGACCCGA CGTTGACTTG   
  
  
- GATTCGGGTG AGACTGTCAG TTGGGGTGAA AAACAGAATT GCACACTTCC CAGTGCAACC CAAGAGTTGC   
  
  
- CCAGTGCTTC AGTTTCGGCG TATACATGTT TTGAAGGGAG TGAAACTTCG TTTGGTGAGT ACCCCTAGTA   
  
  
- TAGTAGAACA ACACAACGCA ACTTCTATTT CAACATATAA TCGGAACACC ACGAACATAG TACTTAAATT   
  
  
- ATCTATCCGG GTTAAATTCT CTTGGCTCTG TAACTTTGAA AAGAACAGAG GAAGAGATCT CATGTATTAA   
  
  
- AGGGGTTTAA ACTGGTGAAA ACGGAGTACC CATATTCTAC TAGTCTAAAC TTAGCTATTT TTAACTTAGA   
  
  
- ACCTTTAACG TTCTTCTTCT GACACCTTGT CGAGTACTTG AACCAACGTA ATCTCCTTAA CCCTTTACCC   
  
  
- ATCTATACAA AGAAACAAAA CATTCAACAA TTAGAACGTT AGTTCCCGTA ATACGTGAAA ATAGATGGAT   
  
  
- TACTCCGTAT GGGTTGACGG AGGTTTCTTA AATTTTGTCT TCCTAAGGTA ACTTTGTTTC TCGATGTTCT   
  
  
- CATTGTCCTG TTACCAAAAA AGTTGAAATT AAAGATATTA AGAAATCATC AACTGAAAAT TCGAAACCAA   
  
  
- CTTACTTCGG TAGCCACAAG ATCGAGGTAG ACCAGGTGGA GGCTACGTAG ATAGGGTAGT TTTGTTACAT   
  
  
- TGTCCTGTTT TGTCTGAGAG TTGGTTTCTA TGGTTGCTAC TCATTAATTC TGTGTGATCA TGACATTCTT   
  
  
- TTAGCTAGAT AGAGATTCTA ATTAGATGTC TTAGGCTTTG GGACTTTGAC ATTGAAAGAA CCATGACTCA   
  
  
- GGAAGACTTT AATTTCATAT ATGGGTGTAC GTTATAAACG AGTTTTTGTG CTTTACACCT ACTCTCTTCC   
  
  
- GAGGTAGTCT TCATTACTCC ACCACAGGGT CACAATGTCA AATGGTTTGG ACATTGGGAA CTGCTCACAC   
  
  
- CTTAAGTCTT GATTTCAGCT TCACTTTTAT AGCATCTTAA CATTCTTGGT GTTGAACAGG GGGACTTATT   
  
  
- CTATAGAATT AATCAAATTT CTCAGTTCTC TCGTGCGTGC ATTTGTGTGT CGACGAAGGA AAAGTAGACG   
  
  
- AGGTGAGAGA GAGGAGAGAA AAAGTATCGG AAGAGCGAGA GAGAAAGAGA GAAAGAGGAA TAGCGAATTA   
  
  
- GAGGAAGAGG AGAAGAAGGG AGAGCTCAAG CAAAGGAGAA GTCATTACGT TTCGATTCCG ACACATGCAG   
  
  
- CGAATAGAAT ACTTTCCGAT CCAATATAA

+     G-Box

| Site Name | Organism | Position | Strand | Matrix score. | sequence | function |
| --- | --- | --- | --- | --- | --- | --- |
| G-Box | Pisum sativum | 249 | - | 6 | CACGTT | cis-acting regulatory element involved in light responsiveness |
| G-Box | Pisum sativum | 263 | + | 6 | CACGTT | cis-acting regulatory element involved in light responsiveness |
| G-Box | Antirrhinum majus | 1297 | + | 6 | CACGTA | cis-acting regulatory element involved in light responsiveness |
| G-Box | Pisum sativum | 68 | + | 6 | CACGTG | cis-acting regulatory element involved in light responsiveness |

> 2018/04/13 10:10:12  
+ CGCGGGGATC CAGGGCCAGT AGGGGGTCCG ACAAGAAGGG GACAGCTCGA ACCGCTCCAT TTTCCGACAC   
  
  
+ GTGATCATCT ACACGAGTGG GGGCCTCGAG CATTTACACA TGTCACACGA TGACAGGCAG ATCGGGCCCA   
  
  
+ CTTTATTAAA GGGTTGACTT TTTAGGGGAG AAAACGTCCA GGAAGATTTG GTGCTGGGCT GCAACTGAAC   
  
  
+ CTAAGCCCAC TCTGACAGTC AACCCCACTT TTTGTCTTAA CGTGTGAAGG GTCACGTTGG GTTCTCAACG   
  
  
+ GGTCACGAAG TCAAAGCCGC ATATGTACAA AACTTCCCTC ACTTTGAAGC AAACCACTCA TGGGGATCAT   
  
  
+ ATCATCTTGT TGTGTTGCGT TGAAGATAAA GTTGTATATT AGCCTTGTGG TGCTTGTATC ATGAATTTAA   
  
  
+ TAGATAGGCC CAATTTAAGA GAACCGAGAC ATTGAAACTT TTCTTGTCTC CTTCTCTAGA GTACATAATT   
  
  
+ TCCCCAAATT TGACCACTTT TGCCTCATGG GTATAAGATG ATCAGATTTG AATCGATAAA AATTGAATCT   
  
  
+ TGGAAATTGC AAGAAGAAGA CTGTGGAACA GCTCATGAAC TTGGTTGCAT TAGAGGAATT GGGAAATGGG   
  
  
+ TAGATATGTT TCTTTGTTTT GTAAGTTGTT AATCTTGCAA TCAAGGGCAT TATGCACTTT TATCTACCTA   
  
  
+ ATGAGGCATA CCCAACTGCC TCCAAAGAAT TTAAAACAGA AGGATTCCAT TGAAACAAAG AGCTACAAGA   
  
  
+ GTAACAGGAC AATGGTTTTT TCAACTTTAA TTTCTATAAT TCTTTAGTAG TTGACTTTTA AGCTTTGGTT   
  
  
+ GAATGAAGCC ATCGGTGTTC TAGCTCCATC TGGTCCACCT CCGATGCATC TATCCCATCA AAACAATGTA   
  
  
+ ACAGGACAAA ACAGACTCTC AACCAAAGAT ACCAACGATG AGTAATTAAG ACACACTAGT ACTGTAAGAA   
  
  
+ AATCGATCTA TCTCTAAGAT TAATCTACAG AATCCGAAAC CCTGAAACTG TAACTTTCTT GGTACTGAGT   
  
  
+ CCTTCTGAAA TTAAAGTATA TACCCACATG CAATATTTGC TCAAAAACAC GAAATGTGGA TGAGAGAAGG   
  
  
+ CTCCATCAGA AGTAATGAGG TGGTGTCCCA GTGTTACAGT TTACCAAACC TGTAACCCTT GACGAGTGTG   
  
  
+ GAATTCAGAA CTAAAGTCGA AGTGAAAATA TCGTAGAATT GTAAGAACCA CAACTTGTCC CCCTGAATAA   
  
  
+ GATATCTTAA TTAGTTTAAA GAGTCAAGAG AGCACGCACG TAAACACACA GCTGCTTCCT TTTCATCTGC   
  
  
+ TCCACTCTCT CTCCTCTCTT TTTCATAGCC TTCTCGCTCT CTCTTTCTCT CTTTCTCCTT ATCGCTTAAT   
  
  
+ CTCCTTCTCC TCTTCTTCCC TCTCGAGTTC GTTTCCTCTT CAGTAATGCA AAGCTAAGGC TGTGTACGTC   
  
  
+ GCTTATCTTA TGAAAGGCTA GGTTATATT  

- GCGCCCCTAG GTCCCGGTCA TCCCCCAGGC TGTTCTTCCC CTGTCGAGCT TGGCGAGGTA AAAGGCTGTG   
  
  
- CACTAGTAGA TGTGCTCACC CCCGGAGCTC GTAAATGTGT ACAGTGTGCT ACTGTCCGTC TAGCCCGGGT   
  
  
- GAAATAATTT CCCAACTGAA AAATCCCCTC TTTTGCAGGT CCTTCTAAAC CACGACCCGA CGTTGACTTG   
  
  
- GATTCGGGTG AGACTGTCAG TTGGGGTGAA AAACAGAATT GCACACTTCC CAGTGCAACC CAAGAGTTGC   
  
  
- CCAGTGCTTC AGTTTCGGCG TATACATGTT TTGAAGGGAG TGAAACTTCG TTTGGTGAGT ACCCCTAGTA   
  
  
- TAGTAGAACA ACACAACGCA ACTTCTATTT CAACATATAA TCGGAACACC ACGAACATAG TACTTAAATT   
  
  
- ATCTATCCGG GTTAAATTCT CTTGGCTCTG TAACTTTGAA AAGAACAGAG GAAGAGATCT CATGTATTAA   
  
  
- AGGGGTTTAA ACTGGTGAAA ACGGAGTACC CATATTCTAC TAGTCTAAAC TTAGCTATTT TTAACTTAGA   
  
  
- ACCTTTAACG TTCTTCTTCT GACACCTTGT CGAGTACTTG AACCAACGTA ATCTCCTTAA CCCTTTACCC   
  
  
- ATCTATACAA AGAAACAAAA CATTCAACAA TTAGAACGTT AGTTCCCGTA ATACGTGAAA ATAGATGGAT   
  
  
- TACTCCGTAT GGGTTGACGG AGGTTTCTTA AATTTTGTCT TCCTAAGGTA ACTTTGTTTC TCGATGTTCT   
  
  
- CATTGTCCTG TTACCAAAAA AGTTGAAATT AAAGATATTA AGAAATCATC AACTGAAAAT TCGAAACCAA   
  
  
- CTTACTTCGG TAGCCACAAG ATCGAGGTAG ACCAGGTGGA GGCTACGTAG ATAGGGTAGT TTTGTTACAT   
  
  
- TGTCCTGTTT TGTCTGAGAG TTGGTTTCTA TGGTTGCTAC TCATTAATTC TGTGTGATCA TGACATTCTT   
  
  
- TTAGCTAGAT AGAGATTCTA ATTAGATGTC TTAGGCTTTG GGACTTTGAC ATTGAAAGAA CCATGACTCA   
  
  
- GGAAGACTTT AATTTCATAT ATGGGTGTAC GTTATAAACG AGTTTTTGTG CTTTACACCT ACTCTCTTCC   
  
  
- GAGGTAGTCT TCATTACTCC ACCACAGGGT CACAATGTCA AATGGTTTGG ACATTGGGAA CTGCTCACAC   
  
  
- CTTAAGTCTT GATTTCAGCT TCACTTTTAT AGCATCTTAA CATTCTTGGT GTTGAACAGG GGGACTTATT   
  
  
- CTATAGAATT AATCAAATTT CTCAGTTCTC TCGTGCGTGC ATTTGTGTGT CGACGAAGGA AAAGTAGACG   
  
  
- AGGTGAGAGA GAGGAGAGAA AAAGTATCGG AAGAGCGAGA GAGAAAGAGA GAAAGAGGAA TAGCGAATTA   
  
  
- GAGGAAGAGG AGAAGAAGGG AGAGCTCAAG CAAAGGAGAA GTCATTACGT TTCGATTCCG ACACATGCAG   
  
  
- CGAATAGAAT ACTTTCCGAT CCAATATAA

+     G-box

| Site Name | Organism | Position | Strand | Matrix score. | sequence | function |
| --- | --- | --- | --- | --- | --- | --- |
| G-box | Zea mays | 263 | + | 6 | CACGTT | cis-acting regulatory element involved in light responsiveness |
| G-box | Arabidopsis thaliana | 68 | + | 6 | CACGTG | cis-acting regulatory element involved in light responsiveness |
| G-box | Zea mays | 249 | - | 6 | CACGTT | cis-acting regulatory element involved in light responsiveness |
| G-box | Daucus carota | 1297 | - | 6 | TACGTG | cis-acting regulatory element involved in light responsiveness |

> 2018/04/13 10:10:12  
+ CGCGGGGATC CAGGGCCAGT AGGGGGTCCG ACAAGAAGGG GACAGCTCGA ACCGCTCCAT TTTCCGACAC   
  
  
+ GTGATCATCT ACACGAGTGG GGGCCTCGAG CATTTACACA TGTCACACGA TGACAGGCAG ATCGGGCCCA   
  
  
+ CTTTATTAAA GGGTTGACTT TTTAGGGGAG AAAACGTCCA GGAAGATTTG GTGCTGGGCT GCAACTGAAC   
  
  
+ CTAAGCCCAC TCTGACAGTC AACCCCACTT TTTGTCTTAA CGTGTGAAGG GTCACGTTGG GTTCTCAACG   
  
  
+ GGTCACGAAG TCAAAGCCGC ATATGTACAA AACTTCCCTC ACTTTGAAGC AAACCACTCA TGGGGATCAT   
  
  
+ ATCATCTTGT TGTGTTGCGT TGAAGATAAA GTTGTATATT AGCCTTGTGG TGCTTGTATC ATGAATTTAA   
  
  
+ TAGATAGGCC CAATTTAAGA GAACCGAGAC ATTGAAACTT TTCTTGTCTC CTTCTCTAGA GTACATAATT   
  
  
+ TCCCCAAATT TGACCACTTT TGCCTCATGG GTATAAGATG ATCAGATTTG AATCGATAAA AATTGAATCT   
  
  
+ TGGAAATTGC AAGAAGAAGA CTGTGGAACA GCTCATGAAC TTGGTTGCAT TAGAGGAATT GGGAAATGGG   
  
  
+ TAGATATGTT TCTTTGTTTT GTAAGTTGTT AATCTTGCAA TCAAGGGCAT TATGCACTTT TATCTACCTA   
  
  
+ ATGAGGCATA CCCAACTGCC TCCAAAGAAT TTAAAACAGA AGGATTCCAT TGAAACAAAG AGCTACAAGA   
  
  
+ GTAACAGGAC AATGGTTTTT TCAACTTTAA TTTCTATAAT TCTTTAGTAG TTGACTTTTA AGCTTTGGTT   
  
  
+ GAATGAAGCC ATCGGTGTTC TAGCTCCATC TGGTCCACCT CCGATGCATC TATCCCATCA AAACAATGTA   
  
  
+ ACAGGACAAA ACAGACTCTC AACCAAAGAT ACCAACGATG AGTAATTAAG ACACACTAGT ACTGTAAGAA   
  
  
+ AATCGATCTA TCTCTAAGAT TAATCTACAG AATCCGAAAC CCTGAAACTG TAACTTTCTT GGTACTGAGT   
  
  
+ CCTTCTGAAA TTAAAGTATA TACCCACATG CAATATTTGC TCAAAAACAC GAAATGTGGA TGAGAGAAGG   
  
  
+ CTCCATCAGA AGTAATGAGG TGGTGTCCCA GTGTTACAGT TTACCAAACC TGTAACCCTT GACGAGTGTG   
  
  
+ GAATTCAGAA CTAAAGTCGA AGTGAAAATA TCGTAGAATT GTAAGAACCA CAACTTGTCC CCCTGAATAA   
  
  
+ GATATCTTAA TTAGTTTAAA GAGTCAAGAG AGCACGCACG TAAACACACA GCTGCTTCCT TTTCATCTGC   
  
  
+ TCCACTCTCT CTCCTCTCTT TTTCATAGCC TTCTCGCTCT CTCTTTCTCT CTTTCTCCTT ATCGCTTAAT   
  
  
+ CTCCTTCTCC TCTTCTTCCC TCTCGAGTTC GTTTCCTCTT CAGTAATGCA AAGCTAAGGC TGTGTACGTC   
  
  
+ GCTTATCTTA TGAAAGGCTA GGTTATATT  

- GCGCCCCTAG GTCCCGGTCA TCCCCCAGGC TGTTCTTCCC CTGTCGAGCT TGGCGAGGTA AAAGGCTGTG   
  
  
- CACTAGTAGA TGTGCTCACC CCCGGAGCTC GTAAATGTGT ACAGTGTGCT ACTGTCCGTC TAGCCCGGGT   
  
  
- GAAATAATTT CCCAACTGAA AAATCCCCTC TTTTGCAGGT CCTTCTAAAC CACGACCCGA CGTTGACTTG   
  
  
- GATTCGGGTG AGACTGTCAG TTGGGGTGAA AAACAGAATT GCACACTTCC CAGTGCAACC CAAGAGTTGC   
  
  
- CCAGTGCTTC AGTTTCGGCG TATACATGTT TTGAAGGGAG TGAAACTTCG TTTGGTGAGT ACCCCTAGTA   
  
  
- TAGTAGAACA ACACAACGCA ACTTCTATTT CAACATATAA TCGGAACACC ACGAACATAG TACTTAAATT   
  
  
- ATCTATCCGG GTTAAATTCT CTTGGCTCTG TAACTTTGAA AAGAACAGAG GAAGAGATCT CATGTATTAA   
  
  
- AGGGGTTTAA ACTGGTGAAA ACGGAGTACC CATATTCTAC TAGTCTAAAC TTAGCTATTT TTAACTTAGA   
  
  
- ACCTTTAACG TTCTTCTTCT GACACCTTGT CGAGTACTTG AACCAACGTA ATCTCCTTAA CCCTTTACCC   
  
  
- ATCTATACAA AGAAACAAAA CATTCAACAA TTAGAACGTT AGTTCCCGTA ATACGTGAAA ATAGATGGAT   
  
  
- TACTCCGTAT GGGTTGACGG AGGTTTCTTA AATTTTGTCT TCCTAAGGTA ACTTTGTTTC TCGATGTTCT   
  
  
- CATTGTCCTG TTACCAAAAA AGTTGAAATT AAAGATATTA AGAAATCATC AACTGAAAAT TCGAAACCAA   
  
  
- CTTACTTCGG TAGCCACAAG ATCGAGGTAG ACCAGGTGGA GGCTACGTAG ATAGGGTAGT TTTGTTACAT   
  
  
- TGTCCTGTTT TGTCTGAGAG TTGGTTTCTA TGGTTGCTAC TCATTAATTC TGTGTGATCA TGACATTCTT   
  
  
- TTAGCTAGAT AGAGATTCTA ATTAGATGTC TTAGGCTTTG GGACTTTGAC ATTGAAAGAA CCATGACTCA   
  
  
- GGAAGACTTT AATTTCATAT ATGGGTGTAC GTTATAAACG AGTTTTTGTG CTTTACACCT ACTCTCTTCC   
  
  
- GAGGTAGTCT TCATTACTCC ACCACAGGGT CACAATGTCA AATGGTTTGG ACATTGGGAA CTGCTCACAC   
  
  
- CTTAAGTCTT GATTTCAGCT TCACTTTTAT AGCATCTTAA CATTCTTGGT GTTGAACAGG GGGACTTATT   
  
  
- CTATAGAATT AATCAAATTT CTCAGTTCTC TCGTGCGTGC ATTTGTGTGT CGACGAAGGA AAAGTAGACG   
  
  
- AGGTGAGAGA GAGGAGAGAA AAAGTATCGG AAGAGCGAGA GAGAAAGAGA GAAAGAGGAA TAGCGAATTA   
  
  
- GAGGAAGAGG AGAAGAAGGG AGAGCTCAAG CAAAGGAGAA GTCATTACGT TTCGATTCCG ACACATGCAG   
  
  
- CGAATAGAAT ACTTTCCGAT CCAATATAA

+     GAG-motif

| Site Name | Organism | Position | Strand | Matrix score. | sequence | function |
| --- | --- | --- | --- | --- | --- | --- |
| GAG-motif | Arabidopsis thaliana | 1334 | - | 7 | AGAGAGT | part of a light responsive element |

> 2018/04/13 10:10:12  
+ CGCGGGGATC CAGGGCCAGT AGGGGGTCCG ACAAGAAGGG GACAGCTCGA ACCGCTCCAT TTTCCGACAC   
  
  
+ GTGATCATCT ACACGAGTGG GGGCCTCGAG CATTTACACA TGTCACACGA TGACAGGCAG ATCGGGCCCA   
  
  
+ CTTTATTAAA GGGTTGACTT TTTAGGGGAG AAAACGTCCA GGAAGATTTG GTGCTGGGCT GCAACTGAAC   
  
  
+ CTAAGCCCAC TCTGACAGTC AACCCCACTT TTTGTCTTAA CGTGTGAAGG GTCACGTTGG GTTCTCAACG   
  
  
+ GGTCACGAAG TCAAAGCCGC ATATGTACAA AACTTCCCTC ACTTTGAAGC AAACCACTCA TGGGGATCAT   
  
  
+ ATCATCTTGT TGTGTTGCGT TGAAGATAAA GTTGTATATT AGCCTTGTGG TGCTTGTATC ATGAATTTAA   
  
  
+ TAGATAGGCC CAATTTAAGA GAACCGAGAC ATTGAAACTT TTCTTGTCTC CTTCTCTAGA GTACATAATT   
  
  
+ TCCCCAAATT TGACCACTTT TGCCTCATGG GTATAAGATG ATCAGATTTG AATCGATAAA AATTGAATCT   
  
  
+ TGGAAATTGC AAGAAGAAGA CTGTGGAACA GCTCATGAAC TTGGTTGCAT TAGAGGAATT GGGAAATGGG   
  
  
+ TAGATATGTT TCTTTGTTTT GTAAGTTGTT AATCTTGCAA TCAAGGGCAT TATGCACTTT TATCTACCTA   
  
  
+ ATGAGGCATA CCCAACTGCC TCCAAAGAAT TTAAAACAGA AGGATTCCAT TGAAACAAAG AGCTACAAGA   
  
  
+ GTAACAGGAC AATGGTTTTT TCAACTTTAA TTTCTATAAT TCTTTAGTAG TTGACTTTTA AGCTTTGGTT   
  
  
+ GAATGAAGCC ATCGGTGTTC TAGCTCCATC TGGTCCACCT CCGATGCATC TATCCCATCA AAACAATGTA   
  
  
+ ACAGGACAAA ACAGACTCTC AACCAAAGAT ACCAACGATG AGTAATTAAG ACACACTAGT ACTGTAAGAA   
  
  
+ AATCGATCTA TCTCTAAGAT TAATCTACAG AATCCGAAAC CCTGAAACTG TAACTTTCTT GGTACTGAGT   
  
  
+ CCTTCTGAAA TTAAAGTATA TACCCACATG CAATATTTGC TCAAAAACAC GAAATGTGGA TGAGAGAAGG   
  
  
+ CTCCATCAGA AGTAATGAGG TGGTGTCCCA GTGTTACAGT TTACCAAACC TGTAACCCTT GACGAGTGTG   
  
  
+ GAATTCAGAA CTAAAGTCGA AGTGAAAATA TCGTAGAATT GTAAGAACCA CAACTTGTCC CCCTGAATAA   
  
  
+ GATATCTTAA TTAGTTTAAA GAGTCAAGAG AGCACGCACG TAAACACACA GCTGCTTCCT TTTCATCTGC   
  
  
+ TCCACTCTCT CTCCTCTCTT TTTCATAGCC TTCTCGCTCT CTCTTTCTCT CTTTCTCCTT ATCGCTTAAT   
  
  
+ CTCCTTCTCC TCTTCTTCCC TCTCGAGTTC GTTTCCTCTT CAGTAATGCA AAGCTAAGGC TGTGTACGTC   
  
  
+ GCTTATCTTA TGAAAGGCTA GGTTATATT  

- GCGCCCCTAG GTCCCGGTCA TCCCCCAGGC TGTTCTTCCC CTGTCGAGCT TGGCGAGGTA AAAGGCTGTG   
  
  
- CACTAGTAGA TGTGCTCACC CCCGGAGCTC GTAAATGTGT ACAGTGTGCT ACTGTCCGTC TAGCCCGGGT   
  
  
- GAAATAATTT CCCAACTGAA AAATCCCCTC TTTTGCAGGT CCTTCTAAAC CACGACCCGA CGTTGACTTG   
  
  
- GATTCGGGTG AGACTGTCAG TTGGGGTGAA AAACAGAATT GCACACTTCC CAGTGCAACC CAAGAGTTGC   
  
  
- CCAGTGCTTC AGTTTCGGCG TATACATGTT TTGAAGGGAG TGAAACTTCG TTTGGTGAGT ACCCCTAGTA   
  
  
- TAGTAGAACA ACACAACGCA ACTTCTATTT CAACATATAA TCGGAACACC ACGAACATAG TACTTAAATT   
  
  
- ATCTATCCGG GTTAAATTCT CTTGGCTCTG TAACTTTGAA AAGAACAGAG GAAGAGATCT CATGTATTAA   
  
  
- AGGGGTTTAA ACTGGTGAAA ACGGAGTACC CATATTCTAC TAGTCTAAAC TTAGCTATTT TTAACTTAGA   
  
  
- ACCTTTAACG TTCTTCTTCT GACACCTTGT CGAGTACTTG AACCAACGTA ATCTCCTTAA CCCTTTACCC   
  
  
- ATCTATACAA AGAAACAAAA CATTCAACAA TTAGAACGTT AGTTCCCGTA ATACGTGAAA ATAGATGGAT   
  
  
- TACTCCGTAT GGGTTGACGG AGGTTTCTTA AATTTTGTCT TCCTAAGGTA ACTTTGTTTC TCGATGTTCT   
  
  
- CATTGTCCTG TTACCAAAAA AGTTGAAATT AAAGATATTA AGAAATCATC AACTGAAAAT TCGAAACCAA   
  
  
- CTTACTTCGG TAGCCACAAG ATCGAGGTAG ACCAGGTGGA GGCTACGTAG ATAGGGTAGT TTTGTTACAT   
  
  
- TGTCCTGTTT TGTCTGAGAG TTGGTTTCTA TGGTTGCTAC TCATTAATTC TGTGTGATCA TGACATTCTT   
  
  
- TTAGCTAGAT AGAGATTCTA ATTAGATGTC TTAGGCTTTG GGACTTTGAC ATTGAAAGAA CCATGACTCA   
  
  
- GGAAGACTTT AATTTCATAT ATGGGTGTAC GTTATAAACG AGTTTTTGTG CTTTACACCT ACTCTCTTCC   
  
  
- GAGGTAGTCT TCATTACTCC ACCACAGGGT CACAATGTCA AATGGTTTGG ACATTGGGAA CTGCTCACAC   
  
  
- CTTAAGTCTT GATTTCAGCT TCACTTTTAT AGCATCTTAA CATTCTTGGT GTTGAACAGG GGGACTTATT   
  
  
- CTATAGAATT AATCAAATTT CTCAGTTCTC TCGTGCGTGC ATTTGTGTGT CGACGAAGGA AAAGTAGACG   
  
  
- AGGTGAGAGA GAGGAGAGAA AAAGTATCGG AAGAGCGAGA GAGAAAGAGA GAAAGAGGAA TAGCGAATTA   
  
  
- GAGGAAGAGG AGAAGAAGGG AGAGCTCAAG CAAAGGAGAA GTCATTACGT TTCGATTCCG ACACATGCAG   
  
  
- CGAATAGAAT ACTTTCCGAT CCAATATAA

+     GARE-motif

| Site Name | Organism | Position | Strand | Matrix score. | sequence | function |
| --- | --- | --- | --- | --- | --- | --- |
| GARE-motif | Brassica oleracea | 919 | + | 7 | AAACAGA | gibberellin-responsive element |
| GARE-motif | Brassica oleracea | 734 | + | 7 | AAACAGA | gibberellin-responsive element |

> 2018/04/13 10:10:12  
+ CGCGGGGATC CAGGGCCAGT AGGGGGTCCG ACAAGAAGGG GACAGCTCGA ACCGCTCCAT TTTCCGACAC   
  
  
+ GTGATCATCT ACACGAGTGG GGGCCTCGAG CATTTACACA TGTCACACGA TGACAGGCAG ATCGGGCCCA   
  
  
+ CTTTATTAAA GGGTTGACTT TTTAGGGGAG AAAACGTCCA GGAAGATTTG GTGCTGGGCT GCAACTGAAC   
  
  
+ CTAAGCCCAC TCTGACAGTC AACCCCACTT TTTGTCTTAA CGTGTGAAGG GTCACGTTGG GTTCTCAACG   
  
  
+ GGTCACGAAG TCAAAGCCGC ATATGTACAA AACTTCCCTC ACTTTGAAGC AAACCACTCA TGGGGATCAT   
  
  
+ ATCATCTTGT TGTGTTGCGT TGAAGATAAA GTTGTATATT AGCCTTGTGG TGCTTGTATC ATGAATTTAA   
  
  
+ TAGATAGGCC CAATTTAAGA GAACCGAGAC ATTGAAACTT TTCTTGTCTC CTTCTCTAGA GTACATAATT   
  
  
+ TCCCCAAATT TGACCACTTT TGCCTCATGG GTATAAGATG ATCAGATTTG AATCGATAAA AATTGAATCT   
  
  
+ TGGAAATTGC AAGAAGAAGA CTGTGGAACA GCTCATGAAC TTGGTTGCAT TAGAGGAATT GGGAAATGGG   
  
  
+ TAGATATGTT TCTTTGTTTT GTAAGTTGTT AATCTTGCAA TCAAGGGCAT TATGCACTTT TATCTACCTA   
  
  
+ ATGAGGCATA CCCAACTGCC TCCAAAGAAT TTAAAACAGA AGGATTCCAT TGAAACAAAG AGCTACAAGA   
  
  
+ GTAACAGGAC AATGGTTTTT TCAACTTTAA TTTCTATAAT TCTTTAGTAG TTGACTTTTA AGCTTTGGTT   
  
  
+ GAATGAAGCC ATCGGTGTTC TAGCTCCATC TGGTCCACCT CCGATGCATC TATCCCATCA AAACAATGTA   
  
  
+ ACAGGACAAA ACAGACTCTC AACCAAAGAT ACCAACGATG AGTAATTAAG ACACACTAGT ACTGTAAGAA   
  
  
+ AATCGATCTA TCTCTAAGAT TAATCTACAG AATCCGAAAC CCTGAAACTG TAACTTTCTT GGTACTGAGT   
  
  
+ CCTTCTGAAA TTAAAGTATA TACCCACATG CAATATTTGC TCAAAAACAC GAAATGTGGA TGAGAGAAGG   
  
  
+ CTCCATCAGA AGTAATGAGG TGGTGTCCCA GTGTTACAGT TTACCAAACC TGTAACCCTT GACGAGTGTG   
  
  
+ GAATTCAGAA CTAAAGTCGA AGTGAAAATA TCGTAGAATT GTAAGAACCA CAACTTGTCC CCCTGAATAA   
  
  
+ GATATCTTAA TTAGTTTAAA GAGTCAAGAG AGCACGCACG TAAACACACA GCTGCTTCCT TTTCATCTGC   
  
  
+ TCCACTCTCT CTCCTCTCTT TTTCATAGCC TTCTCGCTCT CTCTTTCTCT CTTTCTCCTT ATCGCTTAAT   
  
  
+ CTCCTTCTCC TCTTCTTCCC TCTCGAGTTC GTTTCCTCTT CAGTAATGCA AAGCTAAGGC TGTGTACGTC   
  
  
+ GCTTATCTTA TGAAAGGCTA GGTTATATT  

- GCGCCCCTAG GTCCCGGTCA TCCCCCAGGC TGTTCTTCCC CTGTCGAGCT TGGCGAGGTA AAAGGCTGTG   
  
  
- CACTAGTAGA TGTGCTCACC CCCGGAGCTC GTAAATGTGT ACAGTGTGCT ACTGTCCGTC TAGCCCGGGT   
  
  
- GAAATAATTT CCCAACTGAA AAATCCCCTC TTTTGCAGGT CCTTCTAAAC CACGACCCGA CGTTGACTTG   
  
  
- GATTCGGGTG AGACTGTCAG TTGGGGTGAA AAACAGAATT GCACACTTCC CAGTGCAACC CAAGAGTTGC   
  
  
- CCAGTGCTTC AGTTTCGGCG TATACATGTT TTGAAGGGAG TGAAACTTCG TTTGGTGAGT ACCCCTAGTA   
  
  
- TAGTAGAACA ACACAACGCA ACTTCTATTT CAACATATAA TCGGAACACC ACGAACATAG TACTTAAATT   
  
  
- ATCTATCCGG GTTAAATTCT CTTGGCTCTG TAACTTTGAA AAGAACAGAG GAAGAGATCT CATGTATTAA   
  
  
- AGGGGTTTAA ACTGGTGAAA ACGGAGTACC CATATTCTAC TAGTCTAAAC TTAGCTATTT TTAACTTAGA   
  
  
- ACCTTTAACG TTCTTCTTCT GACACCTTGT CGAGTACTTG AACCAACGTA ATCTCCTTAA CCCTTTACCC   
  
  
- ATCTATACAA AGAAACAAAA CATTCAACAA TTAGAACGTT AGTTCCCGTA ATACGTGAAA ATAGATGGAT   
  
  
- TACTCCGTAT GGGTTGACGG AGGTTTCTTA AATTTTGTCT TCCTAAGGTA ACTTTGTTTC TCGATGTTCT   
  
  
- CATTGTCCTG TTACCAAAAA AGTTGAAATT AAAGATATTA AGAAATCATC AACTGAAAAT TCGAAACCAA   
  
  
- CTTACTTCGG TAGCCACAAG ATCGAGGTAG ACCAGGTGGA GGCTACGTAG ATAGGGTAGT TTTGTTACAT   
  
  
- TGTCCTGTTT TGTCTGAGAG TTGGTTTCTA TGGTTGCTAC TCATTAATTC TGTGTGATCA TGACATTCTT   
  
  
- TTAGCTAGAT AGAGATTCTA ATTAGATGTC TTAGGCTTTG GGACTTTGAC ATTGAAAGAA CCATGACTCA   
  
  
- GGAAGACTTT AATTTCATAT ATGGGTGTAC GTTATAAACG AGTTTTTGTG CTTTACACCT ACTCTCTTCC   
  
  
- GAGGTAGTCT TCATTACTCC ACCACAGGGT CACAATGTCA AATGGTTTGG ACATTGGGAA CTGCTCACAC   
  
  
- CTTAAGTCTT GATTTCAGCT TCACTTTTAT AGCATCTTAA CATTCTTGGT GTTGAACAGG GGGACTTATT   
  
  
- CTATAGAATT AATCAAATTT CTCAGTTCTC TCGTGCGTGC ATTTGTGTGT CGACGAAGGA AAAGTAGACG   
  
  
- AGGTGAGAGA GAGGAGAGAA AAAGTATCGG AAGAGCGAGA GAGAAAGAGA GAAAGAGGAA TAGCGAATTA   
  
  
- GAGGAAGAGG AGAAGAAGGG AGAGCTCAAG CAAAGGAGAA GTCATTACGT TTCGATTCCG ACACATGCAG   
  
  
- CGAATAGAAT ACTTTCCGAT CCAATATAA

+     GTGGC-motif

| Site Name | Organism | Position | Strand | Matrix score. | sequence | function |
| --- | --- | --- | --- | --- | --- | --- |
| GTGGC-motif | Arabidopsis thaliana | 112 | - | 10 | CATCGTGTGGC | part of a light responsive element |

> 2018/04/13 10:10:12  
+ CGCGGGGATC CAGGGCCAGT AGGGGGTCCG ACAAGAAGGG GACAGCTCGA ACCGCTCCAT TTTCCGACAC   
  
  
+ GTGATCATCT ACACGAGTGG GGGCCTCGAG CATTTACACA TGTCACACGA TGACAGGCAG ATCGGGCCCA   
  
  
+ CTTTATTAAA GGGTTGACTT TTTAGGGGAG AAAACGTCCA GGAAGATTTG GTGCTGGGCT GCAACTGAAC   
  
  
+ CTAAGCCCAC TCTGACAGTC AACCCCACTT TTTGTCTTAA CGTGTGAAGG GTCACGTTGG GTTCTCAACG   
  
  
+ GGTCACGAAG TCAAAGCCGC ATATGTACAA AACTTCCCTC ACTTTGAAGC AAACCACTCA TGGGGATCAT   
  
  
+ ATCATCTTGT TGTGTTGCGT TGAAGATAAA GTTGTATATT AGCCTTGTGG TGCTTGTATC ATGAATTTAA   
  
  
+ TAGATAGGCC CAATTTAAGA GAACCGAGAC ATTGAAACTT TTCTTGTCTC CTTCTCTAGA GTACATAATT   
  
  
+ TCCCCAAATT TGACCACTTT TGCCTCATGG GTATAAGATG ATCAGATTTG AATCGATAAA AATTGAATCT   
  
  
+ TGGAAATTGC AAGAAGAAGA CTGTGGAACA GCTCATGAAC TTGGTTGCAT TAGAGGAATT GGGAAATGGG   
  
  
+ TAGATATGTT TCTTTGTTTT GTAAGTTGTT AATCTTGCAA TCAAGGGCAT TATGCACTTT TATCTACCTA   
  
  
+ ATGAGGCATA CCCAACTGCC TCCAAAGAAT TTAAAACAGA AGGATTCCAT TGAAACAAAG AGCTACAAGA   
  
  
+ GTAACAGGAC AATGGTTTTT TCAACTTTAA TTTCTATAAT TCTTTAGTAG TTGACTTTTA AGCTTTGGTT   
  
  
+ GAATGAAGCC ATCGGTGTTC TAGCTCCATC TGGTCCACCT CCGATGCATC TATCCCATCA AAACAATGTA   
  
  
+ ACAGGACAAA ACAGACTCTC AACCAAAGAT ACCAACGATG AGTAATTAAG ACACACTAGT ACTGTAAGAA   
  
  
+ AATCGATCTA TCTCTAAGAT TAATCTACAG AATCCGAAAC CCTGAAACTG TAACTTTCTT GGTACTGAGT   
  
  
+ CCTTCTGAAA TTAAAGTATA TACCCACATG CAATATTTGC TCAAAAACAC GAAATGTGGA TGAGAGAAGG   
  
  
+ CTCCATCAGA AGTAATGAGG TGGTGTCCCA GTGTTACAGT TTACCAAACC TGTAACCCTT GACGAGTGTG   
  
  
+ GAATTCAGAA CTAAAGTCGA AGTGAAAATA TCGTAGAATT GTAAGAACCA CAACTTGTCC CCCTGAATAA   
  
  
+ GATATCTTAA TTAGTTTAAA GAGTCAAGAG AGCACGCACG TAAACACACA GCTGCTTCCT TTTCATCTGC   
  
  
+ TCCACTCTCT CTCCTCTCTT TTTCATAGCC TTCTCGCTCT CTCTTTCTCT CTTTCTCCTT ATCGCTTAAT   
  
  
+ CTCCTTCTCC TCTTCTTCCC TCTCGAGTTC GTTTCCTCTT CAGTAATGCA AAGCTAAGGC TGTGTACGTC   
  
  
+ GCTTATCTTA TGAAAGGCTA GGTTATATT  

- GCGCCCCTAG GTCCCGGTCA TCCCCCAGGC TGTTCTTCCC CTGTCGAGCT TGGCGAGGTA AAAGGCTGTG   
  
  
- CACTAGTAGA TGTGCTCACC CCCGGAGCTC GTAAATGTGT ACAGTGTGCT ACTGTCCGTC TAGCCCGGGT   
  
  
- GAAATAATTT CCCAACTGAA AAATCCCCTC TTTTGCAGGT CCTTCTAAAC CACGACCCGA CGTTGACTTG   
  
  
- GATTCGGGTG AGACTGTCAG TTGGGGTGAA AAACAGAATT GCACACTTCC CAGTGCAACC CAAGAGTTGC   
  
  
- CCAGTGCTTC AGTTTCGGCG TATACATGTT TTGAAGGGAG TGAAACTTCG TTTGGTGAGT ACCCCTAGTA   
  
  
- TAGTAGAACA ACACAACGCA ACTTCTATTT CAACATATAA TCGGAACACC ACGAACATAG TACTTAAATT   
  
  
- ATCTATCCGG GTTAAATTCT CTTGGCTCTG TAACTTTGAA AAGAACAGAG GAAGAGATCT CATGTATTAA   
  
  
- AGGGGTTTAA ACTGGTGAAA ACGGAGTACC CATATTCTAC TAGTCTAAAC TTAGCTATTT TTAACTTAGA   
  
  
- ACCTTTAACG TTCTTCTTCT GACACCTTGT CGAGTACTTG AACCAACGTA ATCTCCTTAA CCCTTTACCC   
  
  
- ATCTATACAA AGAAACAAAA CATTCAACAA TTAGAACGTT AGTTCCCGTA ATACGTGAAA ATAGATGGAT   
  
  
- TACTCCGTAT GGGTTGACGG AGGTTTCTTA AATTTTGTCT TCCTAAGGTA ACTTTGTTTC TCGATGTTCT   
  
  
- CATTGTCCTG TTACCAAAAA AGTTGAAATT AAAGATATTA AGAAATCATC AACTGAAAAT TCGAAACCAA   
  
  
- CTTACTTCGG TAGCCACAAG ATCGAGGTAG ACCAGGTGGA GGCTACGTAG ATAGGGTAGT TTTGTTACAT   
  
  
- TGTCCTGTTT TGTCTGAGAG TTGGTTTCTA TGGTTGCTAC TCATTAATTC TGTGTGATCA TGACATTCTT   
  
  
- TTAGCTAGAT AGAGATTCTA ATTAGATGTC TTAGGCTTTG GGACTTTGAC ATTGAAAGAA CCATGACTCA   
  
  
- GGAAGACTTT AATTTCATAT ATGGGTGTAC GTTATAAACG AGTTTTTGTG CTTTACACCT ACTCTCTTCC   
  
  
- GAGGTAGTCT TCATTACTCC ACCACAGGGT CACAATGTCA AATGGTTTGG ACATTGGGAA CTGCTCACAC   
  
  
- CTTAAGTCTT GATTTCAGCT TCACTTTTAT AGCATCTTAA CATTCTTGGT GTTGAACAGG GGGACTTATT   
  
  
- CTATAGAATT AATCAAATTT CTCAGTTCTC TCGTGCGTGC ATTTGTGTGT CGACGAAGGA AAAGTAGACG   
  
  
- AGGTGAGAGA GAGGAGAGAA AAAGTATCGG AAGAGCGAGA GAGAAAGAGA GAAAGAGGAA TAGCGAATTA   
  
  
- GAGGAAGAGG AGAAGAAGGG AGAGCTCAAG CAAAGGAGAA GTCATTACGT TTCGATTCCG ACACATGCAG   
  
  
- CGAATAGAAT ACTTTCCGAT CCAATATAA

+     I-box

| Site Name | Organism | Position | Strand | Matrix score. | sequence | function |
| --- | --- | --- | --- | --- | --- | --- |
| I-box | Gossypium hirsutum | 1451 | + | 10 | AAGATAAGGCT | part of a light responsive element |
| I-box | Pisum sativum | 347 | - | 9 | ATGATATGA | part of a light responsive element |
| I-box | Zea mays | 1385 | - | 9 | cGATAAGGCG | part of a light responsive element |

> 2018/04/13 10:10:12  
+ CGCGGGGATC CAGGGCCAGT AGGGGGTCCG ACAAGAAGGG GACAGCTCGA ACCGCTCCAT TTTCCGACAC   
  
  
+ GTGATCATCT ACACGAGTGG GGGCCTCGAG CATTTACACA TGTCACACGA TGACAGGCAG ATCGGGCCCA   
  
  
+ CTTTATTAAA GGGTTGACTT TTTAGGGGAG AAAACGTCCA GGAAGATTTG GTGCTGGGCT GCAACTGAAC   
  
  
+ CTAAGCCCAC TCTGACAGTC AACCCCACTT TTTGTCTTAA CGTGTGAAGG GTCACGTTGG GTTCTCAACG   
  
  
+ GGTCACGAAG TCAAAGCCGC ATATGTACAA AACTTCCCTC ACTTTGAAGC AAACCACTCA TGGGGATCAT   
  
  
+ ATCATCTTGT TGTGTTGCGT TGAAGATAAA GTTGTATATT AGCCTTGTGG TGCTTGTATC ATGAATTTAA   
  
  
+ TAGATAGGCC CAATTTAAGA GAACCGAGAC ATTGAAACTT TTCTTGTCTC CTTCTCTAGA GTACATAATT   
  
  
+ TCCCCAAATT TGACCACTTT TGCCTCATGG GTATAAGATG ATCAGATTTG AATCGATAAA AATTGAATCT   
  
  
+ TGGAAATTGC AAGAAGAAGA CTGTGGAACA GCTCATGAAC TTGGTTGCAT TAGAGGAATT GGGAAATGGG   
  
  
+ TAGATATGTT TCTTTGTTTT GTAAGTTGTT AATCTTGCAA TCAAGGGCAT TATGCACTTT TATCTACCTA   
  
  
+ ATGAGGCATA CCCAACTGCC TCCAAAGAAT TTAAAACAGA AGGATTCCAT TGAAACAAAG AGCTACAAGA   
  
  
+ GTAACAGGAC AATGGTTTTT TCAACTTTAA TTTCTATAAT TCTTTAGTAG TTGACTTTTA AGCTTTGGTT   
  
  
+ GAATGAAGCC ATCGGTGTTC TAGCTCCATC TGGTCCACCT CCGATGCATC TATCCCATCA AAACAATGTA   
  
  
+ ACAGGACAAA ACAGACTCTC AACCAAAGAT ACCAACGATG AGTAATTAAG ACACACTAGT ACTGTAAGAA   
  
  
+ AATCGATCTA TCTCTAAGAT TAATCTACAG AATCCGAAAC CCTGAAACTG TAACTTTCTT GGTACTGAGT   
  
  
+ CCTTCTGAAA TTAAAGTATA TACCCACATG CAATATTTGC TCAAAAACAC GAAATGTGGA TGAGAGAAGG   
  
  
+ CTCCATCAGA AGTAATGAGG TGGTGTCCCA GTGTTACAGT TTACCAAACC TGTAACCCTT GACGAGTGTG   
  
  
+ GAATTCAGAA CTAAAGTCGA AGTGAAAATA TCGTAGAATT GTAAGAACCA CAACTTGTCC CCCTGAATAA   
  
  
+ GATATCTTAA TTAGTTTAAA GAGTCAAGAG AGCACGCACG TAAACACACA GCTGCTTCCT TTTCATCTGC   
  
  
+ TCCACTCTCT CTCCTCTCTT TTTCATAGCC TTCTCGCTCT CTCTTTCTCT CTTTCTCCTT ATCGCTTAAT   
  
  
+ CTCCTTCTCC TCTTCTTCCC TCTCGAGTTC GTTTCCTCTT CAGTAATGCA AAGCTAAGGC TGTGTACGTC   
  
  
+ GCTTATCTTA TGAAAGGCTA GGTTATATT  

- GCGCCCCTAG GTCCCGGTCA TCCCCCAGGC TGTTCTTCCC CTGTCGAGCT TGGCGAGGTA AAAGGCTGTG   
  
  
- CACTAGTAGA TGTGCTCACC CCCGGAGCTC GTAAATGTGT ACAGTGTGCT ACTGTCCGTC TAGCCCGGGT   
  
  
- GAAATAATTT CCCAACTGAA AAATCCCCTC TTTTGCAGGT CCTTCTAAAC CACGACCCGA CGTTGACTTG   
  
  
- GATTCGGGTG AGACTGTCAG TTGGGGTGAA AAACAGAATT GCACACTTCC CAGTGCAACC CAAGAGTTGC   
  
  
- CCAGTGCTTC AGTTTCGGCG TATACATGTT TTGAAGGGAG TGAAACTTCG TTTGGTGAGT ACCCCTAGTA   
  
  
- TAGTAGAACA ACACAACGCA ACTTCTATTT CAACATATAA TCGGAACACC ACGAACATAG TACTTAAATT   
  
  
- ATCTATCCGG GTTAAATTCT CTTGGCTCTG TAACTTTGAA AAGAACAGAG GAAGAGATCT CATGTATTAA   
  
  
- AGGGGTTTAA ACTGGTGAAA ACGGAGTACC CATATTCTAC TAGTCTAAAC TTAGCTATTT TTAACTTAGA   
  
  
- ACCTTTAACG TTCTTCTTCT GACACCTTGT CGAGTACTTG AACCAACGTA ATCTCCTTAA CCCTTTACCC   
  
  
- ATCTATACAA AGAAACAAAA CATTCAACAA TTAGAACGTT AGTTCCCGTA ATACGTGAAA ATAGATGGAT   
  
  
- TACTCCGTAT GGGTTGACGG AGGTTTCTTA AATTTTGTCT TCCTAAGGTA ACTTTGTTTC TCGATGTTCT   
  
  
- CATTGTCCTG TTACCAAAAA AGTTGAAATT AAAGATATTA AGAAATCATC AACTGAAAAT TCGAAACCAA   
  
  
- CTTACTTCGG TAGCCACAAG ATCGAGGTAG ACCAGGTGGA GGCTACGTAG ATAGGGTAGT TTTGTTACAT   
  
  
- TGTCCTGTTT TGTCTGAGAG TTGGTTTCTA TGGTTGCTAC TCATTAATTC TGTGTGATCA TGACATTCTT   
  
  
- TTAGCTAGAT AGAGATTCTA ATTAGATGTC TTAGGCTTTG GGACTTTGAC ATTGAAAGAA CCATGACTCA   
  
  
- GGAAGACTTT AATTTCATAT ATGGGTGTAC GTTATAAACG AGTTTTTGTG CTTTACACCT ACTCTCTTCC   
  
  
- GAGGTAGTCT TCATTACTCC ACCACAGGGT CACAATGTCA AATGGTTTGG ACATTGGGAA CTGCTCACAC   
  
  
- CTTAAGTCTT GATTTCAGCT TCACTTTTAT AGCATCTTAA CATTCTTGGT GTTGAACAGG GGGACTTATT   
  
  
- CTATAGAATT AATCAAATTT CTCAGTTCTC TCGTGCGTGC ATTTGTGTGT CGACGAAGGA AAAGTAGACG   
  
  
- AGGTGAGAGA GAGGAGAGAA AAAGTATCGG AAGAGCGAGA GAGAAAGAGA GAAAGAGGAA TAGCGAATTA   
  
  
- GAGGAAGAGG AGAAGAAGGG AGAGCTCAAG CAAAGGAGAA GTCATTACGT TTCGATTCCG ACACATGCAG   
  
  
- CGAATAGAAT ACTTTCCGAT CCAATATAA

+     LTR

| Site Name | Organism | Position | Strand | Matrix score. | sequence | function |
| --- | --- | --- | --- | --- | --- | --- |
| LTR | Hordeum vulgare | 1014 | + | 6 | CCGAAA | cis-acting element involved in low-temperature responsiveness |

> 2018/04/13 10:10:12  
+ CGCGGGGATC CAGGGCCAGT AGGGGGTCCG ACAAGAAGGG GACAGCTCGA ACCGCTCCAT TTTCCGACAC   
  
  
+ GTGATCATCT ACACGAGTGG GGGCCTCGAG CATTTACACA TGTCACACGA TGACAGGCAG ATCGGGCCCA   
  
  
+ CTTTATTAAA GGGTTGACTT TTTAGGGGAG AAAACGTCCA GGAAGATTTG GTGCTGGGCT GCAACTGAAC   
  
  
+ CTAAGCCCAC TCTGACAGTC AACCCCACTT TTTGTCTTAA CGTGTGAAGG GTCACGTTGG GTTCTCAACG   
  
  
+ GGTCACGAAG TCAAAGCCGC ATATGTACAA AACTTCCCTC ACTTTGAAGC AAACCACTCA TGGGGATCAT   
  
  
+ ATCATCTTGT TGTGTTGCGT TGAAGATAAA GTTGTATATT AGCCTTGTGG TGCTTGTATC ATGAATTTAA   
  
  
+ TAGATAGGCC CAATTTAAGA GAACCGAGAC ATTGAAACTT TTCTTGTCTC CTTCTCTAGA GTACATAATT   
  
  
+ TCCCCAAATT TGACCACTTT TGCCTCATGG GTATAAGATG ATCAGATTTG AATCGATAAA AATTGAATCT   
  
  
+ TGGAAATTGC AAGAAGAAGA CTGTGGAACA GCTCATGAAC TTGGTTGCAT TAGAGGAATT GGGAAATGGG   
  
  
+ TAGATATGTT TCTTTGTTTT GTAAGTTGTT AATCTTGCAA TCAAGGGCAT TATGCACTTT TATCTACCTA   
  
  
+ ATGAGGCATA CCCAACTGCC TCCAAAGAAT TTAAAACAGA AGGATTCCAT TGAAACAAAG AGCTACAAGA   
  
  
+ GTAACAGGAC AATGGTTTTT TCAACTTTAA TTTCTATAAT TCTTTAGTAG TTGACTTTTA AGCTTTGGTT   
  
  
+ GAATGAAGCC ATCGGTGTTC TAGCTCCATC TGGTCCACCT CCGATGCATC TATCCCATCA AAACAATGTA   
  
  
+ ACAGGACAAA ACAGACTCTC AACCAAAGAT ACCAACGATG AGTAATTAAG ACACACTAGT ACTGTAAGAA   
  
  
+ AATCGATCTA TCTCTAAGAT TAATCTACAG AATCCGAAAC CCTGAAACTG TAACTTTCTT GGTACTGAGT   
  
  
+ CCTTCTGAAA TTAAAGTATA TACCCACATG CAATATTTGC TCAAAAACAC GAAATGTGGA TGAGAGAAGG   
  
  
+ CTCCATCAGA AGTAATGAGG TGGTGTCCCA GTGTTACAGT TTACCAAACC TGTAACCCTT GACGAGTGTG   
  
  
+ GAATTCAGAA CTAAAGTCGA AGTGAAAATA TCGTAGAATT GTAAGAACCA CAACTTGTCC CCCTGAATAA   
  
  
+ GATATCTTAA TTAGTTTAAA GAGTCAAGAG AGCACGCACG TAAACACACA GCTGCTTCCT TTTCATCTGC   
  
  
+ TCCACTCTCT CTCCTCTCTT TTTCATAGCC TTCTCGCTCT CTCTTTCTCT CTTTCTCCTT ATCGCTTAAT   
  
  
+ CTCCTTCTCC TCTTCTTCCC TCTCGAGTTC GTTTCCTCTT CAGTAATGCA AAGCTAAGGC TGTGTACGTC   
  
  
+ GCTTATCTTA TGAAAGGCTA GGTTATATT  

- GCGCCCCTAG GTCCCGGTCA TCCCCCAGGC TGTTCTTCCC CTGTCGAGCT TGGCGAGGTA AAAGGCTGTG   
  
  
- CACTAGTAGA TGTGCTCACC CCCGGAGCTC GTAAATGTGT ACAGTGTGCT ACTGTCCGTC TAGCCCGGGT   
  
  
- GAAATAATTT CCCAACTGAA AAATCCCCTC TTTTGCAGGT CCTTCTAAAC CACGACCCGA CGTTGACTTG   
  
  
- GATTCGGGTG AGACTGTCAG TTGGGGTGAA AAACAGAATT GCACACTTCC CAGTGCAACC CAAGAGTTGC   
  
  
- CCAGTGCTTC AGTTTCGGCG TATACATGTT TTGAAGGGAG TGAAACTTCG TTTGGTGAGT ACCCCTAGTA   
  
  
- TAGTAGAACA ACACAACGCA ACTTCTATTT CAACATATAA TCGGAACACC ACGAACATAG TACTTAAATT   
  
  
- ATCTATCCGG GTTAAATTCT CTTGGCTCTG TAACTTTGAA AAGAACAGAG GAAGAGATCT CATGTATTAA   
  
  
- AGGGGTTTAA ACTGGTGAAA ACGGAGTACC CATATTCTAC TAGTCTAAAC TTAGCTATTT TTAACTTAGA   
  
  
- ACCTTTAACG TTCTTCTTCT GACACCTTGT CGAGTACTTG AACCAACGTA ATCTCCTTAA CCCTTTACCC   
  
  
- ATCTATACAA AGAAACAAAA CATTCAACAA TTAGAACGTT AGTTCCCGTA ATACGTGAAA ATAGATGGAT   
  
  
- TACTCCGTAT GGGTTGACGG AGGTTTCTTA AATTTTGTCT TCCTAAGGTA ACTTTGTTTC TCGATGTTCT   
  
  
- CATTGTCCTG TTACCAAAAA AGTTGAAATT AAAGATATTA AGAAATCATC AACTGAAAAT TCGAAACCAA   
  
  
- CTTACTTCGG TAGCCACAAG ATCGAGGTAG ACCAGGTGGA GGCTACGTAG ATAGGGTAGT TTTGTTACAT   
  
  
- TGTCCTGTTT TGTCTGAGAG TTGGTTTCTA TGGTTGCTAC TCATTAATTC TGTGTGATCA TGACATTCTT   
  
  
- TTAGCTAGAT AGAGATTCTA ATTAGATGTC TTAGGCTTTG GGACTTTGAC ATTGAAAGAA CCATGACTCA   
  
  
- GGAAGACTTT AATTTCATAT ATGGGTGTAC GTTATAAACG AGTTTTTGTG CTTTACACCT ACTCTCTTCC   
  
  
- GAGGTAGTCT TCATTACTCC ACCACAGGGT CACAATGTCA AATGGTTTGG ACATTGGGAA CTGCTCACAC   
  
  
- CTTAAGTCTT GATTTCAGCT TCACTTTTAT AGCATCTTAA CATTCTTGGT GTTGAACAGG GGGACTTATT   
  
  
- CTATAGAATT AATCAAATTT CTCAGTTCTC TCGTGCGTGC ATTTGTGTGT CGACGAAGGA AAAGTAGACG   
  
  
- AGGTGAGAGA GAGGAGAGAA AAAGTATCGG AAGAGCGAGA GAGAAAGAGA GAAAGAGGAA TAGCGAATTA   
  
  
- GAGGAAGAGG AGAAGAAGGG AGAGCTCAAG CAAAGGAGAA GTCATTACGT TTCGATTCCG ACACATGCAG   
  
  
- CGAATAGAAT ACTTTCCGAT CCAATATAA

+     MBS

| Site Name | Organism | Position | Strand | Matrix score. | sequence | function |
| --- | --- | --- | --- | --- | --- | --- |
| MBS | Arabidopsis thaliana | 202 | + | 6 | CAACTG | MYB binding site involved in drought-inducibility |
| MBS | Arabidopsis thaliana | 713 | + | 6 | CAACTG | MYB binding site involved in drought-inducibility |

> 2018/04/13 10:10:12  
+ CGCGGGGATC CAGGGCCAGT AGGGGGTCCG ACAAGAAGGG GACAGCTCGA ACCGCTCCAT TTTCCGACAC   
  
  
+ GTGATCATCT ACACGAGTGG GGGCCTCGAG CATTTACACA TGTCACACGA TGACAGGCAG ATCGGGCCCA   
  
  
+ CTTTATTAAA GGGTTGACTT TTTAGGGGAG AAAACGTCCA GGAAGATTTG GTGCTGGGCT GCAACTGAAC   
  
  
+ CTAAGCCCAC TCTGACAGTC AACCCCACTT TTTGTCTTAA CGTGTGAAGG GTCACGTTGG GTTCTCAACG   
  
  
+ GGTCACGAAG TCAAAGCCGC ATATGTACAA AACTTCCCTC ACTTTGAAGC AAACCACTCA TGGGGATCAT   
  
  
+ ATCATCTTGT TGTGTTGCGT TGAAGATAAA GTTGTATATT AGCCTTGTGG TGCTTGTATC ATGAATTTAA   
  
  
+ TAGATAGGCC CAATTTAAGA GAACCGAGAC ATTGAAACTT TTCTTGTCTC CTTCTCTAGA GTACATAATT   
  
  
+ TCCCCAAATT TGACCACTTT TGCCTCATGG GTATAAGATG ATCAGATTTG AATCGATAAA AATTGAATCT   
  
  
+ TGGAAATTGC AAGAAGAAGA CTGTGGAACA GCTCATGAAC TTGGTTGCAT TAGAGGAATT GGGAAATGGG   
  
  
+ TAGATATGTT TCTTTGTTTT GTAAGTTGTT AATCTTGCAA TCAAGGGCAT TATGCACTTT TATCTACCTA   
  
  
+ ATGAGGCATA CCCAACTGCC TCCAAAGAAT TTAAAACAGA AGGATTCCAT TGAAACAAAG AGCTACAAGA   
  
  
+ GTAACAGGAC AATGGTTTTT TCAACTTTAA TTTCTATAAT TCTTTAGTAG TTGACTTTTA AGCTTTGGTT   
  
  
+ GAATGAAGCC ATCGGTGTTC TAGCTCCATC TGGTCCACCT CCGATGCATC TATCCCATCA AAACAATGTA   
  
  
+ ACAGGACAAA ACAGACTCTC AACCAAAGAT ACCAACGATG AGTAATTAAG ACACACTAGT ACTGTAAGAA   
  
  
+ AATCGATCTA TCTCTAAGAT TAATCTACAG AATCCGAAAC CCTGAAACTG TAACTTTCTT GGTACTGAGT   
  
  
+ CCTTCTGAAA TTAAAGTATA TACCCACATG CAATATTTGC TCAAAAACAC GAAATGTGGA TGAGAGAAGG   
  
  
+ CTCCATCAGA AGTAATGAGG TGGTGTCCCA GTGTTACAGT TTACCAAACC TGTAACCCTT GACGAGTGTG   
  
  
+ GAATTCAGAA CTAAAGTCGA AGTGAAAATA TCGTAGAATT GTAAGAACCA CAACTTGTCC CCCTGAATAA   
  
  
+ GATATCTTAA TTAGTTTAAA GAGTCAAGAG AGCACGCACG TAAACACACA GCTGCTTCCT TTTCATCTGC   
  
  
+ TCCACTCTCT CTCCTCTCTT TTTCATAGCC TTCTCGCTCT CTCTTTCTCT CTTTCTCCTT ATCGCTTAAT   
  
  
+ CTCCTTCTCC TCTTCTTCCC TCTCGAGTTC GTTTCCTCTT CAGTAATGCA AAGCTAAGGC TGTGTACGTC   
  
  
+ GCTTATCTTA TGAAAGGCTA GGTTATATT  

- GCGCCCCTAG GTCCCGGTCA TCCCCCAGGC TGTTCTTCCC CTGTCGAGCT TGGCGAGGTA AAAGGCTGTG   
  
  
- CACTAGTAGA TGTGCTCACC CCCGGAGCTC GTAAATGTGT ACAGTGTGCT ACTGTCCGTC TAGCCCGGGT   
  
  
- GAAATAATTT CCCAACTGAA AAATCCCCTC TTTTGCAGGT CCTTCTAAAC CACGACCCGA CGTTGACTTG   
  
  
- GATTCGGGTG AGACTGTCAG TTGGGGTGAA AAACAGAATT GCACACTTCC CAGTGCAACC CAAGAGTTGC   
  
  
- CCAGTGCTTC AGTTTCGGCG TATACATGTT TTGAAGGGAG TGAAACTTCG TTTGGTGAGT ACCCCTAGTA   
  
  
- TAGTAGAACA ACACAACGCA ACTTCTATTT CAACATATAA TCGGAACACC ACGAACATAG TACTTAAATT   
  
  
- ATCTATCCGG GTTAAATTCT CTTGGCTCTG TAACTTTGAA AAGAACAGAG GAAGAGATCT CATGTATTAA   
  
  
- AGGGGTTTAA ACTGGTGAAA ACGGAGTACC CATATTCTAC TAGTCTAAAC TTAGCTATTT TTAACTTAGA   
  
  
- ACCTTTAACG TTCTTCTTCT GACACCTTGT CGAGTACTTG AACCAACGTA ATCTCCTTAA CCCTTTACCC   
  
  
- ATCTATACAA AGAAACAAAA CATTCAACAA TTAGAACGTT AGTTCCCGTA ATACGTGAAA ATAGATGGAT   
  
  
- TACTCCGTAT GGGTTGACGG AGGTTTCTTA AATTTTGTCT TCCTAAGGTA ACTTTGTTTC TCGATGTTCT   
  
  
- CATTGTCCTG TTACCAAAAA AGTTGAAATT AAAGATATTA AGAAATCATC AACTGAAAAT TCGAAACCAA   
  
  
- CTTACTTCGG TAGCCACAAG ATCGAGGTAG ACCAGGTGGA GGCTACGTAG ATAGGGTAGT TTTGTTACAT   
  
  
- TGTCCTGTTT TGTCTGAGAG TTGGTTTCTA TGGTTGCTAC TCATTAATTC TGTGTGATCA TGACATTCTT   
  
  
- TTAGCTAGAT AGAGATTCTA ATTAGATGTC TTAGGCTTTG GGACTTTGAC ATTGAAAGAA CCATGACTCA   
  
  
- GGAAGACTTT AATTTCATAT ATGGGTGTAC GTTATAAACG AGTTTTTGTG CTTTACACCT ACTCTCTTCC   
  
  
- GAGGTAGTCT TCATTACTCC ACCACAGGGT CACAATGTCA AATGGTTTGG ACATTGGGAA CTGCTCACAC   
  
  
- CTTAAGTCTT GATTTCAGCT TCACTTTTAT AGCATCTTAA CATTCTTGGT GTTGAACAGG GGGACTTATT   
  
  
- CTATAGAATT AATCAAATTT CTCAGTTCTC TCGTGCGTGC ATTTGTGTGT CGACGAAGGA AAAGTAGACG   
  
  
- AGGTGAGAGA GAGGAGAGAA AAAGTATCGG AAGAGCGAGA GAGAAAGAGA GAAAGAGGAA TAGCGAATTA   
  
  
- GAGGAAGAGG AGAAGAAGGG AGAGCTCAAG CAAAGGAGAA GTCATTACGT TTCGATTCCG ACACATGCAG   
  
  
- CGAATAGAAT ACTTTCCGAT CCAATATAA

+     MRE

| Site Name | Organism | Position | Strand | Matrix score. | sequence | function |
| --- | --- | --- | --- | --- | --- | --- |
| MRE | Petroselinum crispum | 208 | + | 7 | AACCTAA | MYB binding site involved in light responsiveness |

> 2018/04/13 10:10:12  
+ CGCGGGGATC CAGGGCCAGT AGGGGGTCCG ACAAGAAGGG GACAGCTCGA ACCGCTCCAT TTTCCGACAC   
  
  
+ GTGATCATCT ACACGAGTGG GGGCCTCGAG CATTTACACA TGTCACACGA TGACAGGCAG ATCGGGCCCA   
  
  
+ CTTTATTAAA GGGTTGACTT TTTAGGGGAG AAAACGTCCA GGAAGATTTG GTGCTGGGCT GCAACTGAAC   
  
  
+ CTAAGCCCAC TCTGACAGTC AACCCCACTT TTTGTCTTAA CGTGTGAAGG GTCACGTTGG GTTCTCAACG   
  
  
+ GGTCACGAAG TCAAAGCCGC ATATGTACAA AACTTCCCTC ACTTTGAAGC AAACCACTCA TGGGGATCAT   
  
  
+ ATCATCTTGT TGTGTTGCGT TGAAGATAAA GTTGTATATT AGCCTTGTGG TGCTTGTATC ATGAATTTAA   
  
  
+ TAGATAGGCC CAATTTAAGA GAACCGAGAC ATTGAAACTT TTCTTGTCTC CTTCTCTAGA GTACATAATT   
  
  
+ TCCCCAAATT TGACCACTTT TGCCTCATGG GTATAAGATG ATCAGATTTG AATCGATAAA AATTGAATCT   
  
  
+ TGGAAATTGC AAGAAGAAGA CTGTGGAACA GCTCATGAAC TTGGTTGCAT TAGAGGAATT GGGAAATGGG   
  
  
+ TAGATATGTT TCTTTGTTTT GTAAGTTGTT AATCTTGCAA TCAAGGGCAT TATGCACTTT TATCTACCTA   
  
  
+ ATGAGGCATA CCCAACTGCC TCCAAAGAAT TTAAAACAGA AGGATTCCAT TGAAACAAAG AGCTACAAGA   
  
  
+ GTAACAGGAC AATGGTTTTT TCAACTTTAA TTTCTATAAT TCTTTAGTAG TTGACTTTTA AGCTTTGGTT   
  
  
+ GAATGAAGCC ATCGGTGTTC TAGCTCCATC TGGTCCACCT CCGATGCATC TATCCCATCA AAACAATGTA   
  
  
+ ACAGGACAAA ACAGACTCTC AACCAAAGAT ACCAACGATG AGTAATTAAG ACACACTAGT ACTGTAAGAA   
  
  
+ AATCGATCTA TCTCTAAGAT TAATCTACAG AATCCGAAAC CCTGAAACTG TAACTTTCTT GGTACTGAGT   
  
  
+ CCTTCTGAAA TTAAAGTATA TACCCACATG CAATATTTGC TCAAAAACAC GAAATGTGGA TGAGAGAAGG   
  
  
+ CTCCATCAGA AGTAATGAGG TGGTGTCCCA GTGTTACAGT TTACCAAACC TGTAACCCTT GACGAGTGTG   
  
  
+ GAATTCAGAA CTAAAGTCGA AGTGAAAATA TCGTAGAATT GTAAGAACCA CAACTTGTCC CCCTGAATAA   
  
  
+ GATATCTTAA TTAGTTTAAA GAGTCAAGAG AGCACGCACG TAAACACACA GCTGCTTCCT TTTCATCTGC   
  
  
+ TCCACTCTCT CTCCTCTCTT TTTCATAGCC TTCTCGCTCT CTCTTTCTCT CTTTCTCCTT ATCGCTTAAT   
  
  
+ CTCCTTCTCC TCTTCTTCCC TCTCGAGTTC GTTTCCTCTT CAGTAATGCA AAGCTAAGGC TGTGTACGTC   
  
  
+ GCTTATCTTA TGAAAGGCTA GGTTATATT  

- GCGCCCCTAG GTCCCGGTCA TCCCCCAGGC TGTTCTTCCC CTGTCGAGCT TGGCGAGGTA AAAGGCTGTG   
  
  
- CACTAGTAGA TGTGCTCACC CCCGGAGCTC GTAAATGTGT ACAGTGTGCT ACTGTCCGTC TAGCCCGGGT   
  
  
- GAAATAATTT CCCAACTGAA AAATCCCCTC TTTTGCAGGT CCTTCTAAAC CACGACCCGA CGTTGACTTG   
  
  
- GATTCGGGTG AGACTGTCAG TTGGGGTGAA AAACAGAATT GCACACTTCC CAGTGCAACC CAAGAGTTGC   
  
  
- CCAGTGCTTC AGTTTCGGCG TATACATGTT TTGAAGGGAG TGAAACTTCG TTTGGTGAGT ACCCCTAGTA   
  
  
- TAGTAGAACA ACACAACGCA ACTTCTATTT CAACATATAA TCGGAACACC ACGAACATAG TACTTAAATT   
  
  
- ATCTATCCGG GTTAAATTCT CTTGGCTCTG TAACTTTGAA AAGAACAGAG GAAGAGATCT CATGTATTAA   
  
  
- AGGGGTTTAA ACTGGTGAAA ACGGAGTACC CATATTCTAC TAGTCTAAAC TTAGCTATTT TTAACTTAGA   
  
  
- ACCTTTAACG TTCTTCTTCT GACACCTTGT CGAGTACTTG AACCAACGTA ATCTCCTTAA CCCTTTACCC   
  
  
- ATCTATACAA AGAAACAAAA CATTCAACAA TTAGAACGTT AGTTCCCGTA ATACGTGAAA ATAGATGGAT   
  
  
- TACTCCGTAT GGGTTGACGG AGGTTTCTTA AATTTTGTCT TCCTAAGGTA ACTTTGTTTC TCGATGTTCT   
  
  
- CATTGTCCTG TTACCAAAAA AGTTGAAATT AAAGATATTA AGAAATCATC AACTGAAAAT TCGAAACCAA   
  
  
- CTTACTTCGG TAGCCACAAG ATCGAGGTAG ACCAGGTGGA GGCTACGTAG ATAGGGTAGT TTTGTTACAT   
  
  
- TGTCCTGTTT TGTCTGAGAG TTGGTTTCTA TGGTTGCTAC TCATTAATTC TGTGTGATCA TGACATTCTT   
  
  
- TTAGCTAGAT AGAGATTCTA ATTAGATGTC TTAGGCTTTG GGACTTTGAC ATTGAAAGAA CCATGACTCA   
  
  
- GGAAGACTTT AATTTCATAT ATGGGTGTAC GTTATAAACG AGTTTTTGTG CTTTACACCT ACTCTCTTCC   
  
  
- GAGGTAGTCT TCATTACTCC ACCACAGGGT CACAATGTCA AATGGTTTGG ACATTGGGAA CTGCTCACAC   
  
  
- CTTAAGTCTT GATTTCAGCT TCACTTTTAT AGCATCTTAA CATTCTTGGT GTTGAACAGG GGGACTTATT   
  
  
- CTATAGAATT AATCAAATTT CTCAGTTCTC TCGTGCGTGC ATTTGTGTGT CGACGAAGGA AAAGTAGACG   
  
  
- AGGTGAGAGA GAGGAGAGAA AAAGTATCGG AAGAGCGAGA GAGAAAGAGA GAAAGAGGAA TAGCGAATTA   
  
  
- GAGGAAGAGG AGAAGAAGGG AGAGCTCAAG CAAAGGAGAA GTCATTACGT TTCGATTCCG ACACATGCAG   
  
  
- CGAATAGAAT ACTTTCCGAT CCAATATAA

+     O2-site

| Site Name | Organism | Position | Strand | Matrix score. | sequence | function |
| --- | --- | --- | --- | --- | --- | --- |
| O2-site | Zea mays | 347 | - | 9 | GATGACATGA | cis-acting regulatory element involved in zein metabolism regulation |

> 2018/04/13 10:10:12  
+ CGCGGGGATC CAGGGCCAGT AGGGGGTCCG ACAAGAAGGG GACAGCTCGA ACCGCTCCAT TTTCCGACAC   
  
  
+ GTGATCATCT ACACGAGTGG GGGCCTCGAG CATTTACACA TGTCACACGA TGACAGGCAG ATCGGGCCCA   
  
  
+ CTTTATTAAA GGGTTGACTT TTTAGGGGAG AAAACGTCCA GGAAGATTTG GTGCTGGGCT GCAACTGAAC   
  
  
+ CTAAGCCCAC TCTGACAGTC AACCCCACTT TTTGTCTTAA CGTGTGAAGG GTCACGTTGG GTTCTCAACG   
  
  
+ GGTCACGAAG TCAAAGCCGC ATATGTACAA AACTTCCCTC ACTTTGAAGC AAACCACTCA TGGGGATCAT   
  
  
+ ATCATCTTGT TGTGTTGCGT TGAAGATAAA GTTGTATATT AGCCTTGTGG TGCTTGTATC ATGAATTTAA   
  
  
+ TAGATAGGCC CAATTTAAGA GAACCGAGAC ATTGAAACTT TTCTTGTCTC CTTCTCTAGA GTACATAATT   
  
  
+ TCCCCAAATT TGACCACTTT TGCCTCATGG GTATAAGATG ATCAGATTTG AATCGATAAA AATTGAATCT   
  
  
+ TGGAAATTGC AAGAAGAAGA CTGTGGAACA GCTCATGAAC TTGGTTGCAT TAGAGGAATT GGGAAATGGG   
  
  
+ TAGATATGTT TCTTTGTTTT GTAAGTTGTT AATCTTGCAA TCAAGGGCAT TATGCACTTT TATCTACCTA   
  
  
+ ATGAGGCATA CCCAACTGCC TCCAAAGAAT TTAAAACAGA AGGATTCCAT TGAAACAAAG AGCTACAAGA   
  
  
+ GTAACAGGAC AATGGTTTTT TCAACTTTAA TTTCTATAAT TCTTTAGTAG TTGACTTTTA AGCTTTGGTT   
  
  
+ GAATGAAGCC ATCGGTGTTC TAGCTCCATC TGGTCCACCT CCGATGCATC TATCCCATCA AAACAATGTA   
  
  
+ ACAGGACAAA ACAGACTCTC AACCAAAGAT ACCAACGATG AGTAATTAAG ACACACTAGT ACTGTAAGAA   
  
  
+ AATCGATCTA TCTCTAAGAT TAATCTACAG AATCCGAAAC CCTGAAACTG TAACTTTCTT GGTACTGAGT   
  
  
+ CCTTCTGAAA TTAAAGTATA TACCCACATG CAATATTTGC TCAAAAACAC GAAATGTGGA TGAGAGAAGG   
  
  
+ CTCCATCAGA AGTAATGAGG TGGTGTCCCA GTGTTACAGT TTACCAAACC TGTAACCCTT GACGAGTGTG   
  
  
+ GAATTCAGAA CTAAAGTCGA AGTGAAAATA TCGTAGAATT GTAAGAACCA CAACTTGTCC CCCTGAATAA   
  
  
+ GATATCTTAA TTAGTTTAAA GAGTCAAGAG AGCACGCACG TAAACACACA GCTGCTTCCT TTTCATCTGC   
  
  
+ TCCACTCTCT CTCCTCTCTT TTTCATAGCC TTCTCGCTCT CTCTTTCTCT CTTTCTCCTT ATCGCTTAAT   
  
  
+ CTCCTTCTCC TCTTCTTCCC TCTCGAGTTC GTTTCCTCTT CAGTAATGCA AAGCTAAGGC TGTGTACGTC   
  
  
+ GCTTATCTTA TGAAAGGCTA GGTTATATT  

- GCGCCCCTAG GTCCCGGTCA TCCCCCAGGC TGTTCTTCCC CTGTCGAGCT TGGCGAGGTA AAAGGCTGTG   
  
  
- CACTAGTAGA TGTGCTCACC CCCGGAGCTC GTAAATGTGT ACAGTGTGCT ACTGTCCGTC TAGCCCGGGT   
  
  
- GAAATAATTT CCCAACTGAA AAATCCCCTC TTTTGCAGGT CCTTCTAAAC CACGACCCGA CGTTGACTTG   
  
  
- GATTCGGGTG AGACTGTCAG TTGGGGTGAA AAACAGAATT GCACACTTCC CAGTGCAACC CAAGAGTTGC   
  
  
- CCAGTGCTTC AGTTTCGGCG TATACATGTT TTGAAGGGAG TGAAACTTCG TTTGGTGAGT ACCCCTAGTA   
  
  
- TAGTAGAACA ACACAACGCA ACTTCTATTT CAACATATAA TCGGAACACC ACGAACATAG TACTTAAATT   
  
  
- ATCTATCCGG GTTAAATTCT CTTGGCTCTG TAACTTTGAA AAGAACAGAG GAAGAGATCT CATGTATTAA   
  
  
- AGGGGTTTAA ACTGGTGAAA ACGGAGTACC CATATTCTAC TAGTCTAAAC TTAGCTATTT TTAACTTAGA   
  
  
- ACCTTTAACG TTCTTCTTCT GACACCTTGT CGAGTACTTG AACCAACGTA ATCTCCTTAA CCCTTTACCC   
  
  
- ATCTATACAA AGAAACAAAA CATTCAACAA TTAGAACGTT AGTTCCCGTA ATACGTGAAA ATAGATGGAT   
  
  
- TACTCCGTAT GGGTTGACGG AGGTTTCTTA AATTTTGTCT TCCTAAGGTA ACTTTGTTTC TCGATGTTCT   
  
  
- CATTGTCCTG TTACCAAAAA AGTTGAAATT AAAGATATTA AGAAATCATC AACTGAAAAT TCGAAACCAA   
  
  
- CTTACTTCGG TAGCCACAAG ATCGAGGTAG ACCAGGTGGA GGCTACGTAG ATAGGGTAGT TTTGTTACAT   
  
  
- TGTCCTGTTT TGTCTGAGAG TTGGTTTCTA TGGTTGCTAC TCATTAATTC TGTGTGATCA TGACATTCTT   
  
  
- TTAGCTAGAT AGAGATTCTA ATTAGATGTC TTAGGCTTTG GGACTTTGAC ATTGAAAGAA CCATGACTCA   
  
  
- GGAAGACTTT AATTTCATAT ATGGGTGTAC GTTATAAACG AGTTTTTGTG CTTTACACCT ACTCTCTTCC   
  
  
- GAGGTAGTCT TCATTACTCC ACCACAGGGT CACAATGTCA AATGGTTTGG ACATTGGGAA CTGCTCACAC   
  
  
- CTTAAGTCTT GATTTCAGCT TCACTTTTAT AGCATCTTAA CATTCTTGGT GTTGAACAGG GGGACTTATT   
  
  
- CTATAGAATT AATCAAATTT CTCAGTTCTC TCGTGCGTGC ATTTGTGTGT CGACGAAGGA AAAGTAGACG   
  
  
- AGGTGAGAGA GAGGAGAGAA AAAGTATCGG AAGAGCGAGA GAGAAAGAGA GAAAGAGGAA TAGCGAATTA   
  
  
- GAGGAAGAGG AGAAGAAGGG AGAGCTCAAG CAAAGGAGAA GTCATTACGT TTCGATTCCG ACACATGCAG   
  
  
- CGAATAGAAT ACTTTCCGAT CCAATATAA

+     Skn-1\_motif

| Site Name | Organism | Position | Strand | Matrix score. | sequence | function |
| --- | --- | --- | --- | --- | --- | --- |
| Skn-1\_motif | Oryza sativa | 120 | - | 5 | GTCAT | cis-acting regulatory element required for endosperm expression |

> 2018/04/13 10:10:12  
+ CGCGGGGATC CAGGGCCAGT AGGGGGTCCG ACAAGAAGGG GACAGCTCGA ACCGCTCCAT TTTCCGACAC   
  
  
+ GTGATCATCT ACACGAGTGG GGGCCTCGAG CATTTACACA TGTCACACGA TGACAGGCAG ATCGGGCCCA   
  
  
+ CTTTATTAAA GGGTTGACTT TTTAGGGGAG AAAACGTCCA GGAAGATTTG GTGCTGGGCT GCAACTGAAC   
  
  
+ CTAAGCCCAC TCTGACAGTC AACCCCACTT TTTGTCTTAA CGTGTGAAGG GTCACGTTGG GTTCTCAACG   
  
  
+ GGTCACGAAG TCAAAGCCGC ATATGTACAA AACTTCCCTC ACTTTGAAGC AAACCACTCA TGGGGATCAT   
  
  
+ ATCATCTTGT TGTGTTGCGT TGAAGATAAA GTTGTATATT AGCCTTGTGG TGCTTGTATC ATGAATTTAA   
  
  
+ TAGATAGGCC CAATTTAAGA GAACCGAGAC ATTGAAACTT TTCTTGTCTC CTTCTCTAGA GTACATAATT   
  
  
+ TCCCCAAATT TGACCACTTT TGCCTCATGG GTATAAGATG ATCAGATTTG AATCGATAAA AATTGAATCT   
  
  
+ TGGAAATTGC AAGAAGAAGA CTGTGGAACA GCTCATGAAC TTGGTTGCAT TAGAGGAATT GGGAAATGGG   
  
  
+ TAGATATGTT TCTTTGTTTT GTAAGTTGTT AATCTTGCAA TCAAGGGCAT TATGCACTTT TATCTACCTA   
  
  
+ ATGAGGCATA CCCAACTGCC TCCAAAGAAT TTAAAACAGA AGGATTCCAT TGAAACAAAG AGCTACAAGA   
  
  
+ GTAACAGGAC AATGGTTTTT TCAACTTTAA TTTCTATAAT TCTTTAGTAG TTGACTTTTA AGCTTTGGTT   
  
  
+ GAATGAAGCC ATCGGTGTTC TAGCTCCATC TGGTCCACCT CCGATGCATC TATCCCATCA AAACAATGTA   
  
  
+ ACAGGACAAA ACAGACTCTC AACCAAAGAT ACCAACGATG AGTAATTAAG ACACACTAGT ACTGTAAGAA   
  
  
+ AATCGATCTA TCTCTAAGAT TAATCTACAG AATCCGAAAC CCTGAAACTG TAACTTTCTT GGTACTGAGT   
  
  
+ CCTTCTGAAA TTAAAGTATA TACCCACATG CAATATTTGC TCAAAAACAC GAAATGTGGA TGAGAGAAGG   
  
  
+ CTCCATCAGA AGTAATGAGG TGGTGTCCCA GTGTTACAGT TTACCAAACC TGTAACCCTT GACGAGTGTG   
  
  
+ GAATTCAGAA CTAAAGTCGA AGTGAAAATA TCGTAGAATT GTAAGAACCA CAACTTGTCC CCCTGAATAA   
  
  
+ GATATCTTAA TTAGTTTAAA GAGTCAAGAG AGCACGCACG TAAACACACA GCTGCTTCCT TTTCATCTGC   
  
  
+ TCCACTCTCT CTCCTCTCTT TTTCATAGCC TTCTCGCTCT CTCTTTCTCT CTTTCTCCTT ATCGCTTAAT   
  
  
+ CTCCTTCTCC TCTTCTTCCC TCTCGAGTTC GTTTCCTCTT CAGTAATGCA AAGCTAAGGC TGTGTACGTC   
  
  
+ GCTTATCTTA TGAAAGGCTA GGTTATATT  

- GCGCCCCTAG GTCCCGGTCA TCCCCCAGGC TGTTCTTCCC CTGTCGAGCT TGGCGAGGTA AAAGGCTGTG   
  
  
- CACTAGTAGA TGTGCTCACC CCCGGAGCTC GTAAATGTGT ACAGTGTGCT ACTGTCCGTC TAGCCCGGGT   
  
  
- GAAATAATTT CCCAACTGAA AAATCCCCTC TTTTGCAGGT CCTTCTAAAC CACGACCCGA CGTTGACTTG   
  
  
- GATTCGGGTG AGACTGTCAG TTGGGGTGAA AAACAGAATT GCACACTTCC CAGTGCAACC CAAGAGTTGC   
  
  
- CCAGTGCTTC AGTTTCGGCG TATACATGTT TTGAAGGGAG TGAAACTTCG TTTGGTGAGT ACCCCTAGTA   
  
  
- TAGTAGAACA ACACAACGCA ACTTCTATTT CAACATATAA TCGGAACACC ACGAACATAG TACTTAAATT   
  
  
- ATCTATCCGG GTTAAATTCT CTTGGCTCTG TAACTTTGAA AAGAACAGAG GAAGAGATCT CATGTATTAA   
  
  
- AGGGGTTTAA ACTGGTGAAA ACGGAGTACC CATATTCTAC TAGTCTAAAC TTAGCTATTT TTAACTTAGA   
  
  
- ACCTTTAACG TTCTTCTTCT GACACCTTGT CGAGTACTTG AACCAACGTA ATCTCCTTAA CCCTTTACCC   
  
  
- ATCTATACAA AGAAACAAAA CATTCAACAA TTAGAACGTT AGTTCCCGTA ATACGTGAAA ATAGATGGAT   
  
  
- TACTCCGTAT GGGTTGACGG AGGTTTCTTA AATTTTGTCT TCCTAAGGTA ACTTTGTTTC TCGATGTTCT   
  
  
- CATTGTCCTG TTACCAAAAA AGTTGAAATT AAAGATATTA AGAAATCATC AACTGAAAAT TCGAAACCAA   
  
  
- CTTACTTCGG TAGCCACAAG ATCGAGGTAG ACCAGGTGGA GGCTACGTAG ATAGGGTAGT TTTGTTACAT   
  
  
- TGTCCTGTTT TGTCTGAGAG TTGGTTTCTA TGGTTGCTAC TCATTAATTC TGTGTGATCA TGACATTCTT   
  
  
- TTAGCTAGAT AGAGATTCTA ATTAGATGTC TTAGGCTTTG GGACTTTGAC ATTGAAAGAA CCATGACTCA   
  
  
- GGAAGACTTT AATTTCATAT ATGGGTGTAC GTTATAAACG AGTTTTTGTG CTTTACACCT ACTCTCTTCC   
  
  
- GAGGTAGTCT TCATTACTCC ACCACAGGGT CACAATGTCA AATGGTTTGG ACATTGGGAA CTGCTCACAC   
  
  
- CTTAAGTCTT GATTTCAGCT TCACTTTTAT AGCATCTTAA CATTCTTGGT GTTGAACAGG GGGACTTATT   
  
  
- CTATAGAATT AATCAAATTT CTCAGTTCTC TCGTGCGTGC ATTTGTGTGT CGACGAAGGA AAAGTAGACG   
  
  
- AGGTGAGAGA GAGGAGAGAA AAAGTATCGG AAGAGCGAGA GAGAAAGAGA GAAAGAGGAA TAGCGAATTA   
  
  
- GAGGAAGAGG AGAAGAAGGG AGAGCTCAAG CAAAGGAGAA GTCATTACGT TTCGATTCCG ACACATGCAG   
  
  
- CGAATAGAAT ACTTTCCGAT CCAATATAA

+     TATA-box

| Site Name | Organism | Position | Strand | Matrix score. | sequence | function |
| --- | --- | --- | --- | --- | --- | --- |
| TATA-box | Oryza sativa | 647 | - | 7 | TACAAAA | core promoter element around -30 of transcription start |
| TATA-box | Glycine max | 418 | + | 5 | TAATA | core promoter element around -30 of transcription start |
| TATA-box | Glycine max | 1496 | - | 5 | TAATA | core promoter element around -30 of transcription start |
| TATA-box | Oryza sativa | 306 | + | 7 | TACAAAA | core promoter element around -30 of transcription start |
| TATA-box | Glycine max | 387 | - | 5 | TAATA | core promoter element around -30 of transcription start |
| TATA-box | Lycopersicon esculentum | 826 | + | 5 | TTTTA | core promoter element around -30 of transcription start |
| TATA-box | Lycopersicon esculentum | 547 | - | 5 | TTTTA | core promoter element around -30 of transcription start |
| TATA-box | Arabidopsis thaliana | 1069 | - | 4 | TATA | core promoter element around -30 of transcription start |
| TATA-box | Arabidopsis thaliana | 805 | - | 4 | TATA | core promoter element around -30 of transcription start |
| TATA-box | Helianthus annuus | 383 | - | 6 | TATACA | core promoter element around -30 of transcription start |
| TATA-box | Arabidopsis thaliana | 1067 | - | 4 | TATA | core promoter element around -30 of transcription start |
| TATA-box | Lycopersicon esculentum | 160 | + | 5 | TTTTA | core promoter element around -30 of transcription start |
| TATA-box | Arabidopsis thaliana | 385 | + | 4 | TATA | core promoter element around -30 of transcription start |
| TATA-box | Arabidopsis thaliana | 1493 | - | 5 | TATAA | core promoter element around -30 of transcription start |
| TATA-box | Glycine max | 144 | - | 5 | TAATA | core promoter element around -30 of transcription start |
| TATA-box | Arabidopsis thaliana | 1494 | - | 4 | TATA | core promoter element around -30 of transcription start |
| TATA-box | Arabidopsis thaliana | 522 | + | 4 | TATA | core promoter element around -30 of transcription start |
| TATA-box | Lycopersicon esculentum | 688 | + | 5 | TTTTA | core promoter element around -30 of transcription start |
| TATA-box | Lycopersicon esculentum | 732 | - | 5 | TTTTA | core promoter element around -30 of transcription start |

> 2018/04/13 10:10:12  
+ CGCGGGGATC CAGGGCCAGT AGGGGGTCCG ACAAGAAGGG GACAGCTCGA ACCGCTCCAT TTTCCGACAC   
  
  
+ GTGATCATCT ACACGAGTGG GGGCCTCGAG CATTTACACA TGTCACACGA TGACAGGCAG ATCGGGCCCA   
  
  
+ CTTTATTAAA GGGTTGACTT TTTAGGGGAG AAAACGTCCA GGAAGATTTG GTGCTGGGCT GCAACTGAAC   
  
  
+ CTAAGCCCAC TCTGACAGTC AACCCCACTT TTTGTCTTAA CGTGTGAAGG GTCACGTTGG GTTCTCAACG   
  
  
+ GGTCACGAAG TCAAAGCCGC ATATGTACAA AACTTCCCTC ACTTTGAAGC AAACCACTCA TGGGGATCAT   
  
  
+ ATCATCTTGT TGTGTTGCGT TGAAGATAAA GTTGTATATT AGCCTTGTGG TGCTTGTATC ATGAATTTAA   
  
  
+ TAGATAGGCC CAATTTAAGA GAACCGAGAC ATTGAAACTT TTCTTGTCTC CTTCTCTAGA GTACATAATT   
  
  
+ TCCCCAAATT TGACCACTTT TGCCTCATGG GTATAAGATG ATCAGATTTG AATCGATAAA AATTGAATCT   
  
  
+ TGGAAATTGC AAGAAGAAGA CTGTGGAACA GCTCATGAAC TTGGTTGCAT TAGAGGAATT GGGAAATGGG   
  
  
+ TAGATATGTT TCTTTGTTTT GTAAGTTGTT AATCTTGCAA TCAAGGGCAT TATGCACTTT TATCTACCTA   
  
  
+ ATGAGGCATA CCCAACTGCC TCCAAAGAAT TTAAAACAGA AGGATTCCAT TGAAACAAAG AGCTACAAGA   
  
  
+ GTAACAGGAC AATGGTTTTT TCAACTTTAA TTTCTATAAT TCTTTAGTAG TTGACTTTTA AGCTTTGGTT   
  
  
+ GAATGAAGCC ATCGGTGTTC TAGCTCCATC TGGTCCACCT CCGATGCATC TATCCCATCA AAACAATGTA   
  
  
+ ACAGGACAAA ACAGACTCTC AACCAAAGAT ACCAACGATG AGTAATTAAG ACACACTAGT ACTGTAAGAA   
  
  
+ AATCGATCTA TCTCTAAGAT TAATCTACAG AATCCGAAAC CCTGAAACTG TAACTTTCTT GGTACTGAGT   
  
  
+ CCTTCTGAAA TTAAAGTATA TACCCACATG CAATATTTGC TCAAAAACAC GAAATGTGGA TGAGAGAAGG   
  
  
+ CTCCATCAGA AGTAATGAGG TGGTGTCCCA GTGTTACAGT TTACCAAACC TGTAACCCTT GACGAGTGTG   
  
  
+ GAATTCAGAA CTAAAGTCGA AGTGAAAATA TCGTAGAATT GTAAGAACCA CAACTTGTCC CCCTGAATAA   
  
  
+ GATATCTTAA TTAGTTTAAA GAGTCAAGAG AGCACGCACG TAAACACACA GCTGCTTCCT TTTCATCTGC   
  
  
+ TCCACTCTCT CTCCTCTCTT TTTCATAGCC TTCTCGCTCT CTCTTTCTCT CTTTCTCCTT ATCGCTTAAT   
  
  
+ CTCCTTCTCC TCTTCTTCCC TCTCGAGTTC GTTTCCTCTT CAGTAATGCA AAGCTAAGGC TGTGTACGTC   
  
  
+ GCTTATCTTA TGAAAGGCTA GGTTATATT  

- GCGCCCCTAG GTCCCGGTCA TCCCCCAGGC TGTTCTTCCC CTGTCGAGCT TGGCGAGGTA AAAGGCTGTG   
  
  
- CACTAGTAGA TGTGCTCACC CCCGGAGCTC GTAAATGTGT ACAGTGTGCT ACTGTCCGTC TAGCCCGGGT   
  
  
- GAAATAATTT CCCAACTGAA AAATCCCCTC TTTTGCAGGT CCTTCTAAAC CACGACCCGA CGTTGACTTG   
  
  
- GATTCGGGTG AGACTGTCAG TTGGGGTGAA AAACAGAATT GCACACTTCC CAGTGCAACC CAAGAGTTGC   
  
  
- CCAGTGCTTC AGTTTCGGCG TATACATGTT TTGAAGGGAG TGAAACTTCG TTTGGTGAGT ACCCCTAGTA   
  
  
- TAGTAGAACA ACACAACGCA ACTTCTATTT CAACATATAA TCGGAACACC ACGAACATAG TACTTAAATT   
  
  
- ATCTATCCGG GTTAAATTCT CTTGGCTCTG TAACTTTGAA AAGAACAGAG GAAGAGATCT CATGTATTAA   
  
  
- AGGGGTTTAA ACTGGTGAAA ACGGAGTACC CATATTCTAC TAGTCTAAAC TTAGCTATTT TTAACTTAGA   
  
  
- ACCTTTAACG TTCTTCTTCT GACACCTTGT CGAGTACTTG AACCAACGTA ATCTCCTTAA CCCTTTACCC   
  
  
- ATCTATACAA AGAAACAAAA CATTCAACAA TTAGAACGTT AGTTCCCGTA ATACGTGAAA ATAGATGGAT   
  
  
- TACTCCGTAT GGGTTGACGG AGGTTTCTTA AATTTTGTCT TCCTAAGGTA ACTTTGTTTC TCGATGTTCT   
  
  
- CATTGTCCTG TTACCAAAAA AGTTGAAATT AAAGATATTA AGAAATCATC AACTGAAAAT TCGAAACCAA   
  
  
- CTTACTTCGG TAGCCACAAG ATCGAGGTAG ACCAGGTGGA GGCTACGTAG ATAGGGTAGT TTTGTTACAT   
  
  
- TGTCCTGTTT TGTCTGAGAG TTGGTTTCTA TGGTTGCTAC TCATTAATTC TGTGTGATCA TGACATTCTT   
  
  
- TTAGCTAGAT AGAGATTCTA ATTAGATGTC TTAGGCTTTG GGACTTTGAC ATTGAAAGAA CCATGACTCA   
  
  
- GGAAGACTTT AATTTCATAT ATGGGTGTAC GTTATAAACG AGTTTTTGTG CTTTACACCT ACTCTCTTCC   
  
  
- GAGGTAGTCT TCATTACTCC ACCACAGGGT CACAATGTCA AATGGTTTGG ACATTGGGAA CTGCTCACAC   
  
  
- CTTAAGTCTT GATTTCAGCT TCACTTTTAT AGCATCTTAA CATTCTTGGT GTTGAACAGG GGGACTTATT   
  
  
- CTATAGAATT AATCAAATTT CTCAGTTCTC TCGTGCGTGC ATTTGTGTGT CGACGAAGGA AAAGTAGACG   
  
  
- AGGTGAGAGA GAGGAGAGAA AAAGTATCGG AAGAGCGAGA GAGAAAGAGA GAAAGAGGAA TAGCGAATTA   
  
  
- GAGGAAGAGG AGAAGAAGGG AGAGCTCAAG CAAAGGAGAA GTCATTACGT TTCGATTCCG ACACATGCAG   
  
  
- CGAATAGAAT ACTTTCCGAT CCAATATAAT

+     TATC-box

| Site Name | Organism | Position | Strand | Matrix score. | sequence | function |
| --- | --- | --- | --- | --- | --- | --- |
| TATC-box | Oryza sativa | 891 | + | 7 | TATCCCA | cis-acting element involved in gibberellin-responsiveness |

> 2018/04/13 10:10:12  
+ CGCGGGGATC CAGGGCCAGT AGGGGGTCCG ACAAGAAGGG GACAGCTCGA ACCGCTCCAT TTTCCGACAC   
  
  
+ GTGATCATCT ACACGAGTGG GGGCCTCGAG CATTTACACA TGTCACACGA TGACAGGCAG ATCGGGCCCA   
  
  
+ CTTTATTAAA GGGTTGACTT TTTAGGGGAG AAAACGTCCA GGAAGATTTG GTGCTGGGCT GCAACTGAAC   
  
  
+ CTAAGCCCAC TCTGACAGTC AACCCCACTT TTTGTCTTAA CGTGTGAAGG GTCACGTTGG GTTCTCAACG   
  
  
+ GGTCACGAAG TCAAAGCCGC ATATGTACAA AACTTCCCTC ACTTTGAAGC AAACCACTCA TGGGGATCAT   
  
  
+ ATCATCTTGT TGTGTTGCGT TGAAGATAAA GTTGTATATT AGCCTTGTGG TGCTTGTATC ATGAATTTAA   
  
  
+ TAGATAGGCC CAATTTAAGA GAACCGAGAC ATTGAAACTT TTCTTGTCTC CTTCTCTAGA GTACATAATT   
  
  
+ TCCCCAAATT TGACCACTTT TGCCTCATGG GTATAAGATG ATCAGATTTG AATCGATAAA AATTGAATCT   
  
  
+ TGGAAATTGC AAGAAGAAGA CTGTGGAACA GCTCATGAAC TTGGTTGCAT TAGAGGAATT GGGAAATGGG   
  
  
+ TAGATATGTT TCTTTGTTTT GTAAGTTGTT AATCTTGCAA TCAAGGGCAT TATGCACTTT TATCTACCTA   
  
  
+ ATGAGGCATA CCCAACTGCC TCCAAAGAAT TTAAAACAGA AGGATTCCAT TGAAACAAAG AGCTACAAGA   
  
  
+ GTAACAGGAC AATGGTTTTT TCAACTTTAA TTTCTATAAT TCTTTAGTAG TTGACTTTTA AGCTTTGGTT   
  
  
+ GAATGAAGCC ATCGGTGTTC TAGCTCCATC TGGTCCACCT CCGATGCATC TATCCCATCA AAACAATGTA   
  
  
+ ACAGGACAAA ACAGACTCTC AACCAAAGAT ACCAACGATG AGTAATTAAG ACACACTAGT ACTGTAAGAA   
  
  
+ AATCGATCTA TCTCTAAGAT TAATCTACAG AATCCGAAAC CCTGAAACTG TAACTTTCTT GGTACTGAGT   
  
  
+ CCTTCTGAAA TTAAAGTATA TACCCACATG CAATATTTGC TCAAAAACAC GAAATGTGGA TGAGAGAAGG   
  
  
+ CTCCATCAGA AGTAATGAGG TGGTGTCCCA GTGTTACAGT TTACCAAACC TGTAACCCTT GACGAGTGTG   
  
  
+ GAATTCAGAA CTAAAGTCGA AGTGAAAATA TCGTAGAATT GTAAGAACCA CAACTTGTCC CCCTGAATAA   
  
  
+ GATATCTTAA TTAGTTTAAA GAGTCAAGAG AGCACGCACG TAAACACACA GCTGCTTCCT TTTCATCTGC   
  
  
+ TCCACTCTCT CTCCTCTCTT TTTCATAGCC TTCTCGCTCT CTCTTTCTCT CTTTCTCCTT ATCGCTTAAT   
  
  
+ CTCCTTCTCC TCTTCTTCCC TCTCGAGTTC GTTTCCTCTT CAGTAATGCA AAGCTAAGGC TGTGTACGTC   
  
  
+ GCTTATCTTA TGAAAGGCTA GGTTATATT  

- GCGCCCCTAG GTCCCGGTCA TCCCCCAGGC TGTTCTTCCC CTGTCGAGCT TGGCGAGGTA AAAGGCTGTG   
  
  
- CACTAGTAGA TGTGCTCACC CCCGGAGCTC GTAAATGTGT ACAGTGTGCT ACTGTCCGTC TAGCCCGGGT   
  
  
- GAAATAATTT CCCAACTGAA AAATCCCCTC TTTTGCAGGT CCTTCTAAAC CACGACCCGA CGTTGACTTG   
  
  
- GATTCGGGTG AGACTGTCAG TTGGGGTGAA AAACAGAATT GCACACTTCC CAGTGCAACC CAAGAGTTGC   
  
  
- CCAGTGCTTC AGTTTCGGCG TATACATGTT TTGAAGGGAG TGAAACTTCG TTTGGTGAGT ACCCCTAGTA   
  
  
- TAGTAGAACA ACACAACGCA ACTTCTATTT CAACATATAA TCGGAACACC ACGAACATAG TACTTAAATT   
  
  
- ATCTATCCGG GTTAAATTCT CTTGGCTCTG TAACTTTGAA AAGAACAGAG GAAGAGATCT CATGTATTAA   
  
  
- AGGGGTTTAA ACTGGTGAAA ACGGAGTACC CATATTCTAC TAGTCTAAAC TTAGCTATTT TTAACTTAGA   
  
  
- ACCTTTAACG TTCTTCTTCT GACACCTTGT CGAGTACTTG AACCAACGTA ATCTCCTTAA CCCTTTACCC   
  
  
- ATCTATACAA AGAAACAAAA CATTCAACAA TTAGAACGTT AGTTCCCGTA ATACGTGAAA ATAGATGGAT   
  
  
- TACTCCGTAT GGGTTGACGG AGGTTTCTTA AATTTTGTCT TCCTAAGGTA ACTTTGTTTC TCGATGTTCT   
  
  
- CATTGTCCTG TTACCAAAAA AGTTGAAATT AAAGATATTA AGAAATCATC AACTGAAAAT TCGAAACCAA   
  
  
- CTTACTTCGG TAGCCACAAG ATCGAGGTAG ACCAGGTGGA GGCTACGTAG ATAGGGTAGT TTTGTTACAT   
  
  
- TGTCCTGTTT TGTCTGAGAG TTGGTTTCTA TGGTTGCTAC TCATTAATTC TGTGTGATCA TGACATTCTT   
  
  
- TTAGCTAGAT AGAGATTCTA ATTAGATGTC TTAGGCTTTG GGACTTTGAC ATTGAAAGAA CCATGACTCA   
  
  
- GGAAGACTTT AATTTCATAT ATGGGTGTAC GTTATAAACG AGTTTTTGTG CTTTACACCT ACTCTCTTCC   
  
  
- GAGGTAGTCT TCATTACTCC ACCACAGGGT CACAATGTCA AATGGTTTGG ACATTGGGAA CTGCTCACAC   
  
  
- CTTAAGTCTT GATTTCAGCT TCACTTTTAT AGCATCTTAA CATTCTTGGT GTTGAACAGG GGGACTTATT   
  
  
- CTATAGAATT AATCAAATTT CTCAGTTCTC TCGTGCGTGC ATTTGTGTGT CGACGAAGGA AAAGTAGACG   
  
  
- AGGTGAGAGA GAGGAGAGAA AAAGTATCGG AAGAGCGAGA GAGAAAGAGA GAAAGAGGAA TAGCGAATTA   
  
  
- GAGGAAGAGG AGAAGAAGGG AGAGCTCAAG CAAAGGAGAA GTCATTACGT TTCGATTCCG ACACATGCAG   
  
  
- CGAATAGAAT ACTTTCCGAT CCAATATAA

+     TC-rich repeats

| Site Name | Organism | Position | Strand | Matrix score. | sequence | function |
| --- | --- | --- | --- | --- | --- | --- |
| TC-rich repeats | Nicotiana tabacum | 974 | - | 9 | GTTTTCTTAC | cis-acting element involved in defense and stress responsiveness |

> 2018/04/13 10:10:12  
+ CGCGGGGATC CAGGGCCAGT AGGGGGTCCG ACAAGAAGGG GACAGCTCGA ACCGCTCCAT TTTCCGACAC   
  
  
+ GTGATCATCT ACACGAGTGG GGGCCTCGAG CATTTACACA TGTCACACGA TGACAGGCAG ATCGGGCCCA   
  
  
+ CTTTATTAAA GGGTTGACTT TTTAGGGGAG AAAACGTCCA GGAAGATTTG GTGCTGGGCT GCAACTGAAC   
  
  
+ CTAAGCCCAC TCTGACAGTC AACCCCACTT TTTGTCTTAA CGTGTGAAGG GTCACGTTGG GTTCTCAACG   
  
  
+ GGTCACGAAG TCAAAGCCGC ATATGTACAA AACTTCCCTC ACTTTGAAGC AAACCACTCA TGGGGATCAT   
  
  
+ ATCATCTTGT TGTGTTGCGT TGAAGATAAA GTTGTATATT AGCCTTGTGG TGCTTGTATC ATGAATTTAA   
  
  
+ TAGATAGGCC CAATTTAAGA GAACCGAGAC ATTGAAACTT TTCTTGTCTC CTTCTCTAGA GTACATAATT   
  
  
+ TCCCCAAATT TGACCACTTT TGCCTCATGG GTATAAGATG ATCAGATTTG AATCGATAAA AATTGAATCT   
  
  
+ TGGAAATTGC AAGAAGAAGA CTGTGGAACA GCTCATGAAC TTGGTTGCAT TAGAGGAATT GGGAAATGGG   
  
  
+ TAGATATGTT TCTTTGTTTT GTAAGTTGTT AATCTTGCAA TCAAGGGCAT TATGCACTTT TATCTACCTA   
  
  
+ ATGAGGCATA CCCAACTGCC TCCAAAGAAT TTAAAACAGA AGGATTCCAT TGAAACAAAG AGCTACAAGA   
  
  
+ GTAACAGGAC AATGGTTTTT TCAACTTTAA TTTCTATAAT TCTTTAGTAG TTGACTTTTA AGCTTTGGTT   
  
  
+ GAATGAAGCC ATCGGTGTTC TAGCTCCATC TGGTCCACCT CCGATGCATC TATCCCATCA AAACAATGTA   
  
  
+ ACAGGACAAA ACAGACTCTC AACCAAAGAT ACCAACGATG AGTAATTAAG ACACACTAGT ACTGTAAGAA   
  
  
+ AATCGATCTA TCTCTAAGAT TAATCTACAG AATCCGAAAC CCTGAAACTG TAACTTTCTT GGTACTGAGT   
  
  
+ CCTTCTGAAA TTAAAGTATA TACCCACATG CAATATTTGC TCAAAAACAC GAAATGTGGA TGAGAGAAGG   
  
  
+ CTCCATCAGA AGTAATGAGG TGGTGTCCCA GTGTTACAGT TTACCAAACC TGTAACCCTT GACGAGTGTG   
  
  
+ GAATTCAGAA CTAAAGTCGA AGTGAAAATA TCGTAGAATT GTAAGAACCA CAACTTGTCC CCCTGAATAA   
  
  
+ GATATCTTAA TTAGTTTAAA GAGTCAAGAG AGCACGCACG TAAACACACA GCTGCTTCCT TTTCATCTGC   
  
  
+ TCCACTCTCT CTCCTCTCTT TTTCATAGCC TTCTCGCTCT CTCTTTCTCT CTTTCTCCTT ATCGCTTAAT   
  
  
+ CTCCTTCTCC TCTTCTTCCC TCTCGAGTTC GTTTCCTCTT CAGTAATGCA AAGCTAAGGC TGTGTACGTC   
  
  
+ GCTTATCTTA TGAAAGGCTA GGTTATATT  

- GCGCCCCTAG GTCCCGGTCA TCCCCCAGGC TGTTCTTCCC CTGTCGAGCT TGGCGAGGTA AAAGGCTGTG   
  
  
- CACTAGTAGA TGTGCTCACC CCCGGAGCTC GTAAATGTGT ACAGTGTGCT ACTGTCCGTC TAGCCCGGGT   
  
  
- GAAATAATTT CCCAACTGAA AAATCCCCTC TTTTGCAGGT CCTTCTAAAC CACGACCCGA CGTTGACTTG   
  
  
- GATTCGGGTG AGACTGTCAG TTGGGGTGAA AAACAGAATT GCACACTTCC CAGTGCAACC CAAGAGTTGC   
  
  
- CCAGTGCTTC AGTTTCGGCG TATACATGTT TTGAAGGGAG TGAAACTTCG TTTGGTGAGT ACCCCTAGTA   
  
  
- TAGTAGAACA ACACAACGCA ACTTCTATTT CAACATATAA TCGGAACACC ACGAACATAG TACTTAAATT   
  
  
- ATCTATCCGG GTTAAATTCT CTTGGCTCTG TAACTTTGAA AAGAACAGAG GAAGAGATCT CATGTATTAA   
  
  
- AGGGGTTTAA ACTGGTGAAA ACGGAGTACC CATATTCTAC TAGTCTAAAC TTAGCTATTT TTAACTTAGA   
  
  
- ACCTTTAACG TTCTTCTTCT GACACCTTGT CGAGTACTTG AACCAACGTA ATCTCCTTAA CCCTTTACCC   
  
  
- ATCTATACAA AGAAACAAAA CATTCAACAA TTAGAACGTT AGTTCCCGTA ATACGTGAAA ATAGATGGAT   
  
  
- TACTCCGTAT GGGTTGACGG AGGTTTCTTA AATTTTGTCT TCCTAAGGTA ACTTTGTTTC TCGATGTTCT   
  
  
- CATTGTCCTG TTACCAAAAA AGTTGAAATT AAAGATATTA AGAAATCATC AACTGAAAAT TCGAAACCAA   
  
  
- CTTACTTCGG TAGCCACAAG ATCGAGGTAG ACCAGGTGGA GGCTACGTAG ATAGGGTAGT TTTGTTACAT   
  
  
- TGTCCTGTTT TGTCTGAGAG TTGGTTTCTA TGGTTGCTAC TCATTAATTC TGTGTGATCA TGACATTCTT   
  
  
- TTAGCTAGAT AGAGATTCTA ATTAGATGTC TTAGGCTTTG GGACTTTGAC ATTGAAAGAA CCATGACTCA   
  
  
- GGAAGACTTT AATTTCATAT ATGGGTGTAC GTTATAAACG AGTTTTTGTG CTTTACACCT ACTCTCTTCC   
  
  
- GAGGTAGTCT TCATTACTCC ACCACAGGGT CACAATGTCA AATGGTTTGG ACATTGGGAA CTGCTCACAC   
  
  
- CTTAAGTCTT GATTTCAGCT TCACTTTTAT AGCATCTTAA CATTCTTGGT GTTGAACAGG GGGACTTATT   
  
  
- CTATAGAATT AATCAAATTT CTCAGTTCTC TCGTGCGTGC ATTTGTGTGT CGACGAAGGA AAAGTAGACG   
  
  
- AGGTGAGAGA GAGGAGAGAA AAAGTATCGG AAGAGCGAGA GAGAAAGAGA GAAAGAGGAA TAGCGAATTA   
  
  
- GAGGAAGAGG AGAAGAAGGG AGAGCTCAAG CAAAGGAGAA GTCATTACGT TTCGATTCCG ACACATGCAG   
  
  
- CGAATAGAAT ACTTTCCGAT CCAATATAA

+     TCCACCT-motif

| Site Name | Organism | Position | Strand | Matrix score. | sequence | function |
| --- | --- | --- | --- | --- | --- | --- |
| TCCACCT-motif | Petroselinum hortense | 874 | + | 7 | TCCACCT |  |

> 2018/04/13 10:10:12  
+ CGCGGGGATC CAGGGCCAGT AGGGGGTCCG ACAAGAAGGG GACAGCTCGA ACCGCTCCAT TTTCCGACAC   
  
  
+ GTGATCATCT ACACGAGTGG GGGCCTCGAG CATTTACACA TGTCACACGA TGACAGGCAG ATCGGGCCCA   
  
  
+ CTTTATTAAA GGGTTGACTT TTTAGGGGAG AAAACGTCCA GGAAGATTTG GTGCTGGGCT GCAACTGAAC   
  
  
+ CTAAGCCCAC TCTGACAGTC AACCCCACTT TTTGTCTTAA CGTGTGAAGG GTCACGTTGG GTTCTCAACG   
  
  
+ GGTCACGAAG TCAAAGCCGC ATATGTACAA AACTTCCCTC ACTTTGAAGC AAACCACTCA TGGGGATCAT   
  
  
+ ATCATCTTGT TGTGTTGCGT TGAAGATAAA GTTGTATATT AGCCTTGTGG TGCTTGTATC ATGAATTTAA   
  
  
+ TAGATAGGCC CAATTTAAGA GAACCGAGAC ATTGAAACTT TTCTTGTCTC CTTCTCTAGA GTACATAATT   
  
  
+ TCCCCAAATT TGACCACTTT TGCCTCATGG GTATAAGATG ATCAGATTTG AATCGATAAA AATTGAATCT   
  
  
+ TGGAAATTGC AAGAAGAAGA CTGTGGAACA GCTCATGAAC TTGGTTGCAT TAGAGGAATT GGGAAATGGG   
  
  
+ TAGATATGTT TCTTTGTTTT GTAAGTTGTT AATCTTGCAA TCAAGGGCAT TATGCACTTT TATCTACCTA   
  
  
+ ATGAGGCATA CCCAACTGCC TCCAAAGAAT TTAAAACAGA AGGATTCCAT TGAAACAAAG AGCTACAAGA   
  
  
+ GTAACAGGAC AATGGTTTTT TCAACTTTAA TTTCTATAAT TCTTTAGTAG TTGACTTTTA AGCTTTGGTT   
  
  
+ GAATGAAGCC ATCGGTGTTC TAGCTCCATC TGGTCCACCT CCGATGCATC TATCCCATCA AAACAATGTA   
  
  
+ ACAGGACAAA ACAGACTCTC AACCAAAGAT ACCAACGATG AGTAATTAAG ACACACTAGT ACTGTAAGAA   
  
  
+ AATCGATCTA TCTCTAAGAT TAATCTACAG AATCCGAAAC CCTGAAACTG TAACTTTCTT GGTACTGAGT   
  
  
+ CCTTCTGAAA TTAAAGTATA TACCCACATG CAATATTTGC TCAAAAACAC GAAATGTGGA TGAGAGAAGG   
  
  
+ CTCCATCAGA AGTAATGAGG TGGTGTCCCA GTGTTACAGT TTACCAAACC TGTAACCCTT GACGAGTGTG   
  
  
+ GAATTCAGAA CTAAAGTCGA AGTGAAAATA TCGTAGAATT GTAAGAACCA CAACTTGTCC CCCTGAATAA   
  
  
+ GATATCTTAA TTAGTTTAAA GAGTCAAGAG AGCACGCACG TAAACACACA GCTGCTTCCT TTTCATCTGC   
  
  
+ TCCACTCTCT CTCCTCTCTT TTTCATAGCC TTCTCGCTCT CTCTTTCTCT CTTTCTCCTT ATCGCTTAAT   
  
  
+ CTCCTTCTCC TCTTCTTCCC TCTCGAGTTC GTTTCCTCTT CAGTAATGCA AAGCTAAGGC TGTGTACGTC   
  
  
+ GCTTATCTTA TGAAAGGCTA GGTTATATT  

- GCGCCCCTAG GTCCCGGTCA TCCCCCAGGC TGTTCTTCCC CTGTCGAGCT TGGCGAGGTA AAAGGCTGTG   
  
  
- CACTAGTAGA TGTGCTCACC CCCGGAGCTC GTAAATGTGT ACAGTGTGCT ACTGTCCGTC TAGCCCGGGT   
  
  
- GAAATAATTT CCCAACTGAA AAATCCCCTC TTTTGCAGGT CCTTCTAAAC CACGACCCGA CGTTGACTTG   
  
  
- GATTCGGGTG AGACTGTCAG TTGGGGTGAA AAACAGAATT GCACACTTCC CAGTGCAACC CAAGAGTTGC   
  
  
- CCAGTGCTTC AGTTTCGGCG TATACATGTT TTGAAGGGAG TGAAACTTCG TTTGGTGAGT ACCCCTAGTA   
  
  
- TAGTAGAACA ACACAACGCA ACTTCTATTT CAACATATAA TCGGAACACC ACGAACATAG TACTTAAATT   
  
  
- ATCTATCCGG GTTAAATTCT CTTGGCTCTG TAACTTTGAA AAGAACAGAG GAAGAGATCT CATGTATTAA   
  
  
- AGGGGTTTAA ACTGGTGAAA ACGGAGTACC CATATTCTAC TAGTCTAAAC TTAGCTATTT TTAACTTAGA   
  
  
- ACCTTTAACG TTCTTCTTCT GACACCTTGT CGAGTACTTG AACCAACGTA ATCTCCTTAA CCCTTTACCC   
  
  
- ATCTATACAA AGAAACAAAA CATTCAACAA TTAGAACGTT AGTTCCCGTA ATACGTGAAA ATAGATGGAT   
  
  
- TACTCCGTAT GGGTTGACGG AGGTTTCTTA AATTTTGTCT TCCTAAGGTA ACTTTGTTTC TCGATGTTCT   
  
  
- CATTGTCCTG TTACCAAAAA AGTTGAAATT AAAGATATTA AGAAATCATC AACTGAAAAT TCGAAACCAA   
  
  
- CTTACTTCGG TAGCCACAAG ATCGAGGTAG ACCAGGTGGA GGCTACGTAG ATAGGGTAGT TTTGTTACAT   
  
  
- TGTCCTGTTT TGTCTGAGAG TTGGTTTCTA TGGTTGCTAC TCATTAATTC TGTGTGATCA TGACATTCTT   
  
  
- TTAGCTAGAT AGAGATTCTA ATTAGATGTC TTAGGCTTTG GGACTTTGAC ATTGAAAGAA CCATGACTCA   
  
  
- GGAAGACTTT AATTTCATAT ATGGGTGTAC GTTATAAACG AGTTTTTGTG CTTTACACCT ACTCTCTTCC   
  
  
- GAGGTAGTCT TCATTACTCC ACCACAGGGT CACAATGTCA AATGGTTTGG ACATTGGGAA CTGCTCACAC   
  
  
- CTTAAGTCTT GATTTCAGCT TCACTTTTAT AGCATCTTAA CATTCTTGGT GTTGAACAGG GGGACTTATT   
  
  
- CTATAGAATT AATCAAATTT CTCAGTTCTC TCGTGCGTGC ATTTGTGTGT CGACGAAGGA AAAGTAGACG   
  
  
- AGGTGAGAGA GAGGAGAGAA AAAGTATCGG AAGAGCGAGA GAGAAAGAGA GAAAGAGGAA TAGCGAATTA   
  
  
- GAGGAAGAGG AGAAGAAGGG AGAGCTCAAG CAAAGGAGAA GTCATTACGT TTCGATTCCG ACACATGCAG   
  
  
- CGAATAGAAT ACTTTCCGAT CCAATATAA

+     TCT-motif

| Site Name | Organism | Position | Strand | Matrix score. | sequence | function |
| --- | --- | --- | --- | --- | --- | --- |
| TCT-motif | Arabidopsis thaliana | 974 | - | 6 | TCTTAC | part of a light responsive element |
| TCT-motif | Arabidopsis thaliana | 1231 | - | 6 | TCTTAC | part of a light responsive element |

> 2018/04/13 10:10:12  
+ CGCGGGGATC CAGGGCCAGT AGGGGGTCCG ACAAGAAGGG GACAGCTCGA ACCGCTCCAT TTTCCGACAC   
  
  
+ GTGATCATCT ACACGAGTGG GGGCCTCGAG CATTTACACA TGTCACACGA TGACAGGCAG ATCGGGCCCA   
  
  
+ CTTTATTAAA GGGTTGACTT TTTAGGGGAG AAAACGTCCA GGAAGATTTG GTGCTGGGCT GCAACTGAAC   
  
  
+ CTAAGCCCAC TCTGACAGTC AACCCCACTT TTTGTCTTAA CGTGTGAAGG GTCACGTTGG GTTCTCAACG   
  
  
+ GGTCACGAAG TCAAAGCCGC ATATGTACAA AACTTCCCTC ACTTTGAAGC AAACCACTCA TGGGGATCAT   
  
  
+ ATCATCTTGT TGTGTTGCGT TGAAGATAAA GTTGTATATT AGCCTTGTGG TGCTTGTATC ATGAATTTAA   
  
  
+ TAGATAGGCC CAATTTAAGA GAACCGAGAC ATTGAAACTT TTCTTGTCTC CTTCTCTAGA GTACATAATT   
  
  
+ TCCCCAAATT TGACCACTTT TGCCTCATGG GTATAAGATG ATCAGATTTG AATCGATAAA AATTGAATCT   
  
  
+ TGGAAATTGC AAGAAGAAGA CTGTGGAACA GCTCATGAAC TTGGTTGCAT TAGAGGAATT GGGAAATGGG   
  
  
+ TAGATATGTT TCTTTGTTTT GTAAGTTGTT AATCTTGCAA TCAAGGGCAT TATGCACTTT TATCTACCTA   
  
  
+ ATGAGGCATA CCCAACTGCC TCCAAAGAAT TTAAAACAGA AGGATTCCAT TGAAACAAAG AGCTACAAGA   
  
  
+ GTAACAGGAC AATGGTTTTT TCAACTTTAA TTTCTATAAT TCTTTAGTAG TTGACTTTTA AGCTTTGGTT   
  
  
+ GAATGAAGCC ATCGGTGTTC TAGCTCCATC TGGTCCACCT CCGATGCATC TATCCCATCA AAACAATGTA   
  
  
+ ACAGGACAAA ACAGACTCTC AACCAAAGAT ACCAACGATG AGTAATTAAG ACACACTAGT ACTGTAAGAA   
  
  
+ AATCGATCTA TCTCTAAGAT TAATCTACAG AATCCGAAAC CCTGAAACTG TAACTTTCTT GGTACTGAGT   
  
  
+ CCTTCTGAAA TTAAAGTATA TACCCACATG CAATATTTGC TCAAAAACAC GAAATGTGGA TGAGAGAAGG   
  
  
+ CTCCATCAGA AGTAATGAGG TGGTGTCCCA GTGTTACAGT TTACCAAACC TGTAACCCTT GACGAGTGTG   
  
  
+ GAATTCAGAA CTAAAGTCGA AGTGAAAATA TCGTAGAATT GTAAGAACCA CAACTTGTCC CCCTGAATAA   
  
  
+ GATATCTTAA TTAGTTTAAA GAGTCAAGAG AGCACGCACG TAAACACACA GCTGCTTCCT TTTCATCTGC   
  
  
+ TCCACTCTCT CTCCTCTCTT TTTCATAGCC TTCTCGCTCT CTCTTTCTCT CTTTCTCCTT ATCGCTTAAT   
  
  
+ CTCCTTCTCC TCTTCTTCCC TCTCGAGTTC GTTTCCTCTT CAGTAATGCA AAGCTAAGGC TGTGTACGTC   
  
  
+ GCTTATCTTA TGAAAGGCTA GGTTATATT  

- GCGCCCCTAG GTCCCGGTCA TCCCCCAGGC TGTTCTTCCC CTGTCGAGCT TGGCGAGGTA AAAGGCTGTG   
  
  
- CACTAGTAGA TGTGCTCACC CCCGGAGCTC GTAAATGTGT ACAGTGTGCT ACTGTCCGTC TAGCCCGGGT   
  
  
- GAAATAATTT CCCAACTGAA AAATCCCCTC TTTTGCAGGT CCTTCTAAAC CACGACCCGA CGTTGACTTG   
  
  
- GATTCGGGTG AGACTGTCAG TTGGGGTGAA AAACAGAATT GCACACTTCC CAGTGCAACC CAAGAGTTGC   
  
  
- CCAGTGCTTC AGTTTCGGCG TATACATGTT TTGAAGGGAG TGAAACTTCG TTTGGTGAGT ACCCCTAGTA   
  
  
- TAGTAGAACA ACACAACGCA ACTTCTATTT CAACATATAA TCGGAACACC ACGAACATAG TACTTAAATT   
  
  
- ATCTATCCGG GTTAAATTCT CTTGGCTCTG TAACTTTGAA AAGAACAGAG GAAGAGATCT CATGTATTAA   
  
  
- AGGGGTTTAA ACTGGTGAAA ACGGAGTACC CATATTCTAC TAGTCTAAAC TTAGCTATTT TTAACTTAGA   
  
  
- ACCTTTAACG TTCTTCTTCT GACACCTTGT CGAGTACTTG AACCAACGTA ATCTCCTTAA CCCTTTACCC   
  
  
- ATCTATACAA AGAAACAAAA CATTCAACAA TTAGAACGTT AGTTCCCGTA ATACGTGAAA ATAGATGGAT   
  
  
- TACTCCGTAT GGGTTGACGG AGGTTTCTTA AATTTTGTCT TCCTAAGGTA ACTTTGTTTC TCGATGTTCT   
  
  
- CATTGTCCTG TTACCAAAAA AGTTGAAATT AAAGATATTA AGAAATCATC AACTGAAAAT TCGAAACCAA   
  
  
- CTTACTTCGG TAGCCACAAG ATCGAGGTAG ACCAGGTGGA GGCTACGTAG ATAGGGTAGT TTTGTTACAT   
  
  
- TGTCCTGTTT TGTCTGAGAG TTGGTTTCTA TGGTTGCTAC TCATTAATTC TGTGTGATCA TGACATTCTT   
  
  
- TTAGCTAGAT AGAGATTCTA ATTAGATGTC TTAGGCTTTG GGACTTTGAC ATTGAAAGAA CCATGACTCA   
  
  
- GGAAGACTTT AATTTCATAT ATGGGTGTAC GTTATAAACG AGTTTTTGTG CTTTACACCT ACTCTCTTCC   
  
  
- GAGGTAGTCT TCATTACTCC ACCACAGGGT CACAATGTCA AATGGTTTGG ACATTGGGAA CTGCTCACAC   
  
  
- CTTAAGTCTT GATTTCAGCT TCACTTTTAT AGCATCTTAA CATTCTTGGT GTTGAACAGG GGGACTTATT   
  
  
- CTATAGAATT AATCAAATTT CTCAGTTCTC TCGTGCGTGC ATTTGTGTGT CGACGAAGGA AAAGTAGACG   
  
  
- AGGTGAGAGA GAGGAGAGAA AAAGTATCGG AAGAGCGAGA GAGAAAGAGA GAAAGAGGAA TAGCGAATTA   
  
  
- GAGGAAGAGG AGAAGAAGGG AGAGCTCAAG CAAAGGAGAA GTCATTACGT TTCGATTCCG ACACATGCAG   
  
  
- CGAATAGAAT ACTTTCCGAT CCAATATAA

+     TGACG-motif

| Site Name | Organism | Position | Strand | Matrix score. | sequence | function |
| --- | --- | --- | --- | --- | --- | --- |
| TGACG-motif | Hordeum vulgare | 1180 | + | 5 | TGACG | cis-acting regulatory element involved in the MeJA-responsiveness |

> 2018/04/13 10:10:12  
+ CGCGGGGATC CAGGGCCAGT AGGGGGTCCG ACAAGAAGGG GACAGCTCGA ACCGCTCCAT TTTCCGACAC   
  
  
+ GTGATCATCT ACACGAGTGG GGGCCTCGAG CATTTACACA TGTCACACGA TGACAGGCAG ATCGGGCCCA   
  
  
+ CTTTATTAAA GGGTTGACTT TTTAGGGGAG AAAACGTCCA GGAAGATTTG GTGCTGGGCT GCAACTGAAC   
  
  
+ CTAAGCCCAC TCTGACAGTC AACCCCACTT TTTGTCTTAA CGTGTGAAGG GTCACGTTGG GTTCTCAACG   
  
  
+ GGTCACGAAG TCAAAGCCGC ATATGTACAA AACTTCCCTC ACTTTGAAGC AAACCACTCA TGGGGATCAT   
  
  
+ ATCATCTTGT TGTGTTGCGT TGAAGATAAA GTTGTATATT AGCCTTGTGG TGCTTGTATC ATGAATTTAA   
  
  
+ TAGATAGGCC CAATTTAAGA GAACCGAGAC ATTGAAACTT TTCTTGTCTC CTTCTCTAGA GTACATAATT   
  
  
+ TCCCCAAATT TGACCACTTT TGCCTCATGG GTATAAGATG ATCAGATTTG AATCGATAAA AATTGAATCT   
  
  
+ TGGAAATTGC AAGAAGAAGA CTGTGGAACA GCTCATGAAC TTGGTTGCAT TAGAGGAATT GGGAAATGGG   
  
  
+ TAGATATGTT TCTTTGTTTT GTAAGTTGTT AATCTTGCAA TCAAGGGCAT TATGCACTTT TATCTACCTA   
  
  
+ ATGAGGCATA CCCAACTGCC TCCAAAGAAT TTAAAACAGA AGGATTCCAT TGAAACAAAG AGCTACAAGA   
  
  
+ GTAACAGGAC AATGGTTTTT TCAACTTTAA TTTCTATAAT TCTTTAGTAG TTGACTTTTA AGCTTTGGTT   
  
  
+ GAATGAAGCC ATCGGTGTTC TAGCTCCATC TGGTCCACCT CCGATGCATC TATCCCATCA AAACAATGTA   
  
  
+ ACAGGACAAA ACAGACTCTC AACCAAAGAT ACCAACGATG AGTAATTAAG ACACACTAGT ACTGTAAGAA   
  
  
+ AATCGATCTA TCTCTAAGAT TAATCTACAG AATCCGAAAC CCTGAAACTG TAACTTTCTT GGTACTGAGT   
  
  
+ CCTTCTGAAA TTAAAGTATA TACCCACATG CAATATTTGC TCAAAAACAC GAAATGTGGA TGAGAGAAGG   
  
  
+ CTCCATCAGA AGTAATGAGG TGGTGTCCCA GTGTTACAGT TTACCAAACC TGTAACCCTT GACGAGTGTG   
  
  
+ GAATTCAGAA CTAAAGTCGA AGTGAAAATA TCGTAGAATT GTAAGAACCA CAACTTGTCC CCCTGAATAA   
  
  
+ GATATCTTAA TTAGTTTAAA GAGTCAAGAG AGCACGCACG TAAACACACA GCTGCTTCCT TTTCATCTGC   
  
  
+ TCCACTCTCT CTCCTCTCTT TTTCATAGCC TTCTCGCTCT CTCTTTCTCT CTTTCTCCTT ATCGCTTAAT   
  
  
+ CTCCTTCTCC TCTTCTTCCC TCTCGAGTTC GTTTCCTCTT CAGTAATGCA AAGCTAAGGC TGTGTACGTC   
  
  
+ GCTTATCTTA TGAAAGGCTA GGTTATATT  

- GCGCCCCTAG GTCCCGGTCA TCCCCCAGGC TGTTCTTCCC CTGTCGAGCT TGGCGAGGTA AAAGGCTGTG   
  
  
- CACTAGTAGA TGTGCTCACC CCCGGAGCTC GTAAATGTGT ACAGTGTGCT ACTGTCCGTC TAGCCCGGGT   
  
  
- GAAATAATTT CCCAACTGAA AAATCCCCTC TTTTGCAGGT CCTTCTAAAC CACGACCCGA CGTTGACTTG   
  
  
- GATTCGGGTG AGACTGTCAG TTGGGGTGAA AAACAGAATT GCACACTTCC CAGTGCAACC CAAGAGTTGC   
  
  
- CCAGTGCTTC AGTTTCGGCG TATACATGTT TTGAAGGGAG TGAAACTTCG TTTGGTGAGT ACCCCTAGTA   
  
  
- TAGTAGAACA ACACAACGCA ACTTCTATTT CAACATATAA TCGGAACACC ACGAACATAG TACTTAAATT   
  
  
- ATCTATCCGG GTTAAATTCT CTTGGCTCTG TAACTTTGAA AAGAACAGAG GAAGAGATCT CATGTATTAA   
  
  
- AGGGGTTTAA ACTGGTGAAA ACGGAGTACC CATATTCTAC TAGTCTAAAC TTAGCTATTT TTAACTTAGA   
  
  
- ACCTTTAACG TTCTTCTTCT GACACCTTGT CGAGTACTTG AACCAACGTA ATCTCCTTAA CCCTTTACCC   
  
  
- ATCTATACAA AGAAACAAAA CATTCAACAA TTAGAACGTT AGTTCCCGTA ATACGTGAAA ATAGATGGAT   
  
  
- TACTCCGTAT GGGTTGACGG AGGTTTCTTA AATTTTGTCT TCCTAAGGTA ACTTTGTTTC TCGATGTTCT   
  
  
- CATTGTCCTG TTACCAAAAA AGTTGAAATT AAAGATATTA AGAAATCATC AACTGAAAAT TCGAAACCAA   
  
  
- CTTACTTCGG TAGCCACAAG ATCGAGGTAG ACCAGGTGGA GGCTACGTAG ATAGGGTAGT TTTGTTACAT   
  
  
- TGTCCTGTTT TGTCTGAGAG TTGGTTTCTA TGGTTGCTAC TCATTAATTC TGTGTGATCA TGACATTCTT   
  
  
- TTAGCTAGAT AGAGATTCTA ATTAGATGTC TTAGGCTTTG GGACTTTGAC ATTGAAAGAA CCATGACTCA   
  
  
- GGAAGACTTT AATTTCATAT ATGGGTGTAC GTTATAAACG AGTTTTTGTG CTTTACACCT ACTCTCTTCC   
  
  
- GAGGTAGTCT TCATTACTCC ACCACAGGGT CACAATGTCA AATGGTTTGG ACATTGGGAA CTGCTCACAC   
  
  
- CTTAAGTCTT GATTTCAGCT TCACTTTTAT AGCATCTTAA CATTCTTGGT GTTGAACAGG GGGACTTATT   
  
  
- CTATAGAATT AATCAAATTT CTCAGTTCTC TCGTGCGTGC ATTTGTGTGT CGACGAAGGA AAAGTAGACG   
  
  
- AGGTGAGAGA GAGGAGAGAA AAAGTATCGG AAGAGCGAGA GAGAAAGAGA GAAAGAGGAA TAGCGAATTA   
  
  
- GAGGAAGAGG AGAAGAAGGG AGAGCTCAAG CAAAGGAGAA GTCATTACGT TTCGATTCCG ACACATGCAG   
  
  
- CGAATAGAAT ACTTTCCGAT CCAATATAA

+     Unnamed\_\_2

| Site Name | Organism | Position | Strand | Matrix score. | sequence | function |
| --- | --- | --- | --- | --- | --- | --- |
| Unnamed\_\_2 | Petroselinum hortense | 1485 | - | 9 | AACCTAACCT |  |

> 2018/04/13 10:10:12  
+ CGCGGGGATC CAGGGCCAGT AGGGGGTCCG ACAAGAAGGG GACAGCTCGA ACCGCTCCAT TTTCCGACAC   
  
  
+ GTGATCATCT ACACGAGTGG GGGCCTCGAG CATTTACACA TGTCACACGA TGACAGGCAG ATCGGGCCCA   
  
  
+ CTTTATTAAA GGGTTGACTT TTTAGGGGAG AAAACGTCCA GGAAGATTTG GTGCTGGGCT GCAACTGAAC   
  
  
+ CTAAGCCCAC TCTGACAGTC AACCCCACTT TTTGTCTTAA CGTGTGAAGG GTCACGTTGG GTTCTCAACG   
  
  
+ GGTCACGAAG TCAAAGCCGC ATATGTACAA AACTTCCCTC ACTTTGAAGC AAACCACTCA TGGGGATCAT   
  
  
+ ATCATCTTGT TGTGTTGCGT TGAAGATAAA GTTGTATATT AGCCTTGTGG TGCTTGTATC ATGAATTTAA   
  
  
+ TAGATAGGCC CAATTTAAGA GAACCGAGAC ATTGAAACTT TTCTTGTCTC CTTCTCTAGA GTACATAATT   
  
  
+ TCCCCAAATT TGACCACTTT TGCCTCATGG GTATAAGATG ATCAGATTTG AATCGATAAA AATTGAATCT   
  
  
+ TGGAAATTGC AAGAAGAAGA CTGTGGAACA GCTCATGAAC TTGGTTGCAT TAGAGGAATT GGGAAATGGG   
  
  
+ TAGATATGTT TCTTTGTTTT GTAAGTTGTT AATCTTGCAA TCAAGGGCAT TATGCACTTT TATCTACCTA   
  
  
+ ATGAGGCATA CCCAACTGCC TCCAAAGAAT TTAAAACAGA AGGATTCCAT TGAAACAAAG AGCTACAAGA   
  
  
+ GTAACAGGAC AATGGTTTTT TCAACTTTAA TTTCTATAAT TCTTTAGTAG TTGACTTTTA AGCTTTGGTT   
  
  
+ GAATGAAGCC ATCGGTGTTC TAGCTCCATC TGGTCCACCT CCGATGCATC TATCCCATCA AAACAATGTA   
  
  
+ ACAGGACAAA ACAGACTCTC AACCAAAGAT ACCAACGATG AGTAATTAAG ACACACTAGT ACTGTAAGAA   
  
  
+ AATCGATCTA TCTCTAAGAT TAATCTACAG AATCCGAAAC CCTGAAACTG TAACTTTCTT GGTACTGAGT   
  
  
+ CCTTCTGAAA TTAAAGTATA TACCCACATG CAATATTTGC TCAAAAACAC GAAATGTGGA TGAGAGAAGG   
  
  
+ CTCCATCAGA AGTAATGAGG TGGTGTCCCA GTGTTACAGT TTACCAAACC TGTAACCCTT GACGAGTGTG   
  
  
+ GAATTCAGAA CTAAAGTCGA AGTGAAAATA TCGTAGAATT GTAAGAACCA CAACTTGTCC CCCTGAATAA   
  
  
+ GATATCTTAA TTAGTTTAAA GAGTCAAGAG AGCACGCACG TAAACACACA GCTGCTTCCT TTTCATCTGC   
  
  
+ TCCACTCTCT CTCCTCTCTT TTTCATAGCC TTCTCGCTCT CTCTTTCTCT CTTTCTCCTT ATCGCTTAAT   
  
  
+ CTCCTTCTCC TCTTCTTCCC TCTCGAGTTC GTTTCCTCTT CAGTAATGCA AAGCTAAGGC TGTGTACGTC   
  
  
+ GCTTATCTTA TGAAAGGCTA GGTTATATT  

- GCGCCCCTAG GTCCCGGTCA TCCCCCAGGC TGTTCTTCCC CTGTCGAGCT TGGCGAGGTA AAAGGCTGTG   
  
  
- CACTAGTAGA TGTGCTCACC CCCGGAGCTC GTAAATGTGT ACAGTGTGCT ACTGTCCGTC TAGCCCGGGT   
  
  
- GAAATAATTT CCCAACTGAA AAATCCCCTC TTTTGCAGGT CCTTCTAAAC CACGACCCGA CGTTGACTTG   
  
  
- GATTCGGGTG AGACTGTCAG TTGGGGTGAA AAACAGAATT GCACACTTCC CAGTGCAACC CAAGAGTTGC   
  
  
- CCAGTGCTTC AGTTTCGGCG TATACATGTT TTGAAGGGAG TGAAACTTCG TTTGGTGAGT ACCCCTAGTA   
  
  
- TAGTAGAACA ACACAACGCA ACTTCTATTT CAACATATAA TCGGAACACC ACGAACATAG TACTTAAATT   
  
  
- ATCTATCCGG GTTAAATTCT CTTGGCTCTG TAACTTTGAA AAGAACAGAG GAAGAGATCT CATGTATTAA   
  
  
- AGGGGTTTAA ACTGGTGAAA ACGGAGTACC CATATTCTAC TAGTCTAAAC TTAGCTATTT TTAACTTAGA   
  
  
- ACCTTTAACG TTCTTCTTCT GACACCTTGT CGAGTACTTG AACCAACGTA ATCTCCTTAA CCCTTTACCC   
  
  
- ATCTATACAA AGAAACAAAA CATTCAACAA TTAGAACGTT AGTTCCCGTA ATACGTGAAA ATAGATGGAT   
  
  
- TACTCCGTAT GGGTTGACGG AGGTTTCTTA AATTTTGTCT TCCTAAGGTA ACTTTGTTTC TCGATGTTCT   
  
  
- CATTGTCCTG TTACCAAAAA AGTTGAAATT AAAGATATTA AGAAATCATC AACTGAAAAT TCGAAACCAA   
  
  
- CTTACTTCGG TAGCCACAAG ATCGAGGTAG ACCAGGTGGA GGCTACGTAG ATAGGGTAGT TTTGTTACAT   
  
  
- TGTCCTGTTT TGTCTGAGAG TTGGTTTCTA TGGTTGCTAC TCATTAATTC TGTGTGATCA TGACATTCTT   
  
  
- TTAGCTAGAT AGAGATTCTA ATTAGATGTC TTAGGCTTTG GGACTTTGAC ATTGAAAGAA CCATGACTCA   
  
  
- GGAAGACTTT AATTTCATAT ATGGGTGTAC GTTATAAACG AGTTTTTGTG CTTTACACCT ACTCTCTTCC   
  
  
- GAGGTAGTCT TCATTACTCC ACCACAGGGT CACAATGTCA AATGGTTTGG ACATTGGGAA CTGCTCACAC   
  
  
- CTTAAGTCTT GATTTCAGCT TCACTTTTAT AGCATCTTAA CATTCTTGGT GTTGAACAGG GGGACTTATT   
  
  
- CTATAGAATT AATCAAATTT CTCAGTTCTC TCGTGCGTGC ATTTGTGTGT CGACGAAGGA AAAGTAGACG   
  
  
- AGGTGAGAGA GAGGAGAGAA AAAGTATCGG AAGAGCGAGA GAGAAAGAGA GAAAGAGGAA TAGCGAATTA   
  
  
- GAGGAAGAGG AGAAGAAGGG AGAGCTCAAG CAAAGGAGAA GTCATTACGT TTCGATTCCG ACACATGCAG   
  
  
- CGAATAGAAT ACTTTCCGAT CCAATATAA

+     Unnamed\_\_4

| Site Name | Organism | Position | Strand | Matrix score. | sequence | function |
| --- | --- | --- | --- | --- | --- | --- |
| Unnamed\_\_4 | Petroselinum hortense | 1401 | + | 4 | CTCC |  |
| Unnamed\_\_4 | Petroselinum hortense | 720 | + | 4 | CTCC |  |
| Unnamed\_\_4 | Petroselinum hortense | 55 | + | 4 | CTCC |  |
| Unnamed\_\_4 | Petroselinum hortense | 468 | + | 4 | CTCC |  |
| Unnamed\_\_4 | Petroselinum hortense | 864 | + | 4 | CTCC |  |
| Unnamed\_\_4 | Petroselinum hortense | 879 | + | 4 | CTCC |  |
| Unnamed\_\_4 | Petroselinum hortense | 1407 | + | 4 | CTCC |  |
| Unnamed\_\_4 | Petroselinum hortense | 1341 | + | 4 | CTCC |  |
| Unnamed\_\_4 | Petroselinum hortense | 1121 | + | 4 | CTCC |  |
| Unnamed\_\_4 | Petroselinum hortense | 167 | - | 4 | CTCC |  |
| Unnamed\_\_4 | Petroselinum hortense | 1330 | + | 4 | CTCC |  |
| Unnamed\_\_4 | Petroselinum hortense | 1385 | + | 4 | CTCC |  |

> 2018/04/13 10:10:12  
+ CGCGGGGATC CAGGGCCAGT AGGGGGTCCG ACAAGAAGGG GACAGCTCGA ACCGCTCCAT TTTCCGACAC   
  
  
+ GTGATCATCT ACACGAGTGG GGGCCTCGAG CATTTACACA TGTCACACGA TGACAGGCAG ATCGGGCCCA   
  
  
+ CTTTATTAAA GGGTTGACTT TTTAGGGGAG AAAACGTCCA GGAAGATTTG GTGCTGGGCT GCAACTGAAC   
  
  
+ CTAAGCCCAC TCTGACAGTC AACCCCACTT TTTGTCTTAA CGTGTGAAGG GTCACGTTGG GTTCTCAACG   
  
  
+ GGTCACGAAG TCAAAGCCGC ATATGTACAA AACTTCCCTC ACTTTGAAGC AAACCACTCA TGGGGATCAT   
  
  
+ ATCATCTTGT TGTGTTGCGT TGAAGATAAA GTTGTATATT AGCCTTGTGG TGCTTGTATC ATGAATTTAA   
  
  
+ TAGATAGGCC CAATTTAAGA GAACCGAGAC ATTGAAACTT TTCTTGTCTC CTTCTCTAGA GTACATAATT   
  
  
+ TCCCCAAATT TGACCACTTT TGCCTCATGG GTATAAGATG ATCAGATTTG AATCGATAAA AATTGAATCT   
  
  
+ TGGAAATTGC AAGAAGAAGA CTGTGGAACA GCTCATGAAC TTGGTTGCAT TAGAGGAATT GGGAAATGGG   
  
  
+ TAGATATGTT TCTTTGTTTT GTAAGTTGTT AATCTTGCAA TCAAGGGCAT TATGCACTTT TATCTACCTA   
  
  
+ ATGAGGCATA CCCAACTGCC TCCAAAGAAT TTAAAACAGA AGGATTCCAT TGAAACAAAG AGCTACAAGA   
  
  
+ GTAACAGGAC AATGGTTTTT TCAACTTTAA TTTCTATAAT TCTTTAGTAG TTGACTTTTA AGCTTTGGTT   
  
  
+ GAATGAAGCC ATCGGTGTTC TAGCTCCATC TGGTCCACCT CCGATGCATC TATCCCATCA AAACAATGTA   
  
  
+ ACAGGACAAA ACAGACTCTC AACCAAAGAT ACCAACGATG AGTAATTAAG ACACACTAGT ACTGTAAGAA   
  
  
+ AATCGATCTA TCTCTAAGAT TAATCTACAG AATCCGAAAC CCTGAAACTG TAACTTTCTT GGTACTGAGT   
  
  
+ CCTTCTGAAA TTAAAGTATA TACCCACATG CAATATTTGC TCAAAAACAC GAAATGTGGA TGAGAGAAGG   
  
  
+ CTCCATCAGA AGTAATGAGG TGGTGTCCCA GTGTTACAGT TTACCAAACC TGTAACCCTT GACGAGTGTG   
  
  
+ GAATTCAGAA CTAAAGTCGA AGTGAAAATA TCGTAGAATT GTAAGAACCA CAACTTGTCC CCCTGAATAA   
  
  
+ GATATCTTAA TTAGTTTAAA GAGTCAAGAG AGCACGCACG TAAACACACA GCTGCTTCCT TTTCATCTGC   
  
  
+ TCCACTCTCT CTCCTCTCTT TTTCATAGCC TTCTCGCTCT CTCTTTCTCT CTTTCTCCTT ATCGCTTAAT   
  
  
+ CTCCTTCTCC TCTTCTTCCC TCTCGAGTTC GTTTCCTCTT CAGTAATGCA AAGCTAAGGC TGTGTACGTC   
  
  
+ GCTTATCTTA TGAAAGGCTA GGTTATATT  

- GCGCCCCTAG GTCCCGGTCA TCCCCCAGGC TGTTCTTCCC CTGTCGAGCT TGGCGAGGTA AAAGGCTGTG   
  
  
- CACTAGTAGA TGTGCTCACC CCCGGAGCTC GTAAATGTGT ACAGTGTGCT ACTGTCCGTC TAGCCCGGGT   
  
  
- GAAATAATTT CCCAACTGAA AAATCCCCTC TTTTGCAGGT CCTTCTAAAC CACGACCCGA CGTTGACTTG   
  
  
- GATTCGGGTG AGACTGTCAG TTGGGGTGAA AAACAGAATT GCACACTTCC CAGTGCAACC CAAGAGTTGC   
  
  
- CCAGTGCTTC AGTTTCGGCG TATACATGTT TTGAAGGGAG TGAAACTTCG TTTGGTGAGT ACCCCTAGTA   
  
  
- TAGTAGAACA ACACAACGCA ACTTCTATTT CAACATATAA TCGGAACACC ACGAACATAG TACTTAAATT   
  
  
- ATCTATCCGG GTTAAATTCT CTTGGCTCTG TAACTTTGAA AAGAACAGAG GAAGAGATCT CATGTATTAA   
  
  
- AGGGGTTTAA ACTGGTGAAA ACGGAGTACC CATATTCTAC TAGTCTAAAC TTAGCTATTT TTAACTTAGA   
  
  
- ACCTTTAACG TTCTTCTTCT GACACCTTGT CGAGTACTTG AACCAACGTA ATCTCCTTAA CCCTTTACCC   
  
  
- ATCTATACAA AGAAACAAAA CATTCAACAA TTAGAACGTT AGTTCCCGTA ATACGTGAAA ATAGATGGAT   
  
  
- TACTCCGTAT GGGTTGACGG AGGTTTCTTA AATTTTGTCT TCCTAAGGTA ACTTTGTTTC TCGATGTTCT   
  
  
- CATTGTCCTG TTACCAAAAA AGTTGAAATT AAAGATATTA AGAAATCATC AACTGAAAAT TCGAAACCAA   
  
  
- CTTACTTCGG TAGCCACAAG ATCGAGGTAG ACCAGGTGGA GGCTACGTAG ATAGGGTAGT TTTGTTACAT   
  
  
- TGTCCTGTTT TGTCTGAGAG TTGGTTTCTA TGGTTGCTAC TCATTAATTC TGTGTGATCA TGACATTCTT   
  
  
- TTAGCTAGAT AGAGATTCTA ATTAGATGTC TTAGGCTTTG GGACTTTGAC ATTGAAAGAA CCATGACTCA   
  
  
- GGAAGACTTT AATTTCATAT ATGGGTGTAC GTTATAAACG AGTTTTTGTG CTTTACACCT ACTCTCTTCC   
  
  
- GAGGTAGTCT TCATTACTCC ACCACAGGGT CACAATGTCA AATGGTTTGG ACATTGGGAA CTGCTCACAC   
  
  
- CTTAAGTCTT GATTTCAGCT TCACTTTTAT AGCATCTTAA CATTCTTGGT GTTGAACAGG GGGACTTATT   
  
  
- CTATAGAATT AATCAAATTT CTCAGTTCTC TCGTGCGTGC ATTTGTGTGT CGACGAAGGA AAAGTAGACG   
  
  
- AGGTGAGAGA GAGGAGAGAA AAAGTATCGG AAGAGCGAGA GAGAAAGAGA GAAAGAGGAA TAGCGAATTA   
  
  
- GAGGAAGAGG AGAAGAAGGG AGAGCTCAAG CAAAGGAGAA GTCATTACGT TTCGATTCCG ACACATGCAG   
  
  
- CGAATAGAAT ACTTTCCGAT CCAATATAA

+     W box

| Site Name | Organism | Position | Strand | Matrix score. | sequence | function |
| --- | --- | --- | --- | --- | --- | --- |
| W box | Arabidopsis thaliana | 500 | + | 6 | TTGACC |  |

> 2018/04/13 10:10:12  
+ CGCGGGGATC CAGGGCCAGT AGGGGGTCCG ACAAGAAGGG GACAGCTCGA ACCGCTCCAT TTTCCGACAC   
  
  
+ GTGATCATCT ACACGAGTGG GGGCCTCGAG CATTTACACA TGTCACACGA TGACAGGCAG ATCGGGCCCA   
  
  
+ CTTTATTAAA GGGTTGACTT TTTAGGGGAG AAAACGTCCA GGAAGATTTG GTGCTGGGCT GCAACTGAAC   
  
  
+ CTAAGCCCAC TCTGACAGTC AACCCCACTT TTTGTCTTAA CGTGTGAAGG GTCACGTTGG GTTCTCAACG   
  
  
+ GGTCACGAAG TCAAAGCCGC ATATGTACAA AACTTCCCTC ACTTTGAAGC AAACCACTCA TGGGGATCAT   
  
  
+ ATCATCTTGT TGTGTTGCGT TGAAGATAAA GTTGTATATT AGCCTTGTGG TGCTTGTATC ATGAATTTAA   
  
  
+ TAGATAGGCC CAATTTAAGA GAACCGAGAC ATTGAAACTT TTCTTGTCTC CTTCTCTAGA GTACATAATT   
  
  
+ TCCCCAAATT TGACCACTTT TGCCTCATGG GTATAAGATG ATCAGATTTG AATCGATAAA AATTGAATCT   
  
  
+ TGGAAATTGC AAGAAGAAGA CTGTGGAACA GCTCATGAAC TTGGTTGCAT TAGAGGAATT GGGAAATGGG   
  
  
+ TAGATATGTT TCTTTGTTTT GTAAGTTGTT AATCTTGCAA TCAAGGGCAT TATGCACTTT TATCTACCTA   
  
  
+ ATGAGGCATA CCCAACTGCC TCCAAAGAAT TTAAAACAGA AGGATTCCAT TGAAACAAAG AGCTACAAGA   
  
  
+ GTAACAGGAC AATGGTTTTT TCAACTTTAA TTTCTATAAT TCTTTAGTAG TTGACTTTTA AGCTTTGGTT   
  
  
+ GAATGAAGCC ATCGGTGTTC TAGCTCCATC TGGTCCACCT CCGATGCATC TATCCCATCA AAACAATGTA   
  
  
+ ACAGGACAAA ACAGACTCTC AACCAAAGAT ACCAACGATG AGTAATTAAG ACACACTAGT ACTGTAAGAA   
  
  
+ AATCGATCTA TCTCTAAGAT TAATCTACAG AATCCGAAAC CCTGAAACTG TAACTTTCTT GGTACTGAGT   
  
  
+ CCTTCTGAAA TTAAAGTATA TACCCACATG CAATATTTGC TCAAAAACAC GAAATGTGGA TGAGAGAAGG   
  
  
+ CTCCATCAGA AGTAATGAGG TGGTGTCCCA GTGTTACAGT TTACCAAACC TGTAACCCTT GACGAGTGTG   
  
  
+ GAATTCAGAA CTAAAGTCGA AGTGAAAATA TCGTAGAATT GTAAGAACCA CAACTTGTCC CCCTGAATAA   
  
  
+ GATATCTTAA TTAGTTTAAA GAGTCAAGAG AGCACGCACG TAAACACACA GCTGCTTCCT TTTCATCTGC   
  
  
+ TCCACTCTCT CTCCTCTCTT TTTCATAGCC TTCTCGCTCT CTCTTTCTCT CTTTCTCCTT ATCGCTTAAT   
  
  
+ CTCCTTCTCC TCTTCTTCCC TCTCGAGTTC GTTTCCTCTT CAGTAATGCA AAGCTAAGGC TGTGTACGTC   
  
  
+ GCTTATCTTA TGAAAGGCTA GGTTATATT  

- GCGCCCCTAG GTCCCGGTCA TCCCCCAGGC TGTTCTTCCC CTGTCGAGCT TGGCGAGGTA AAAGGCTGTG   
  
  
- CACTAGTAGA TGTGCTCACC CCCGGAGCTC GTAAATGTGT ACAGTGTGCT ACTGTCCGTC TAGCCCGGGT   
  
  
- GAAATAATTT CCCAACTGAA AAATCCCCTC TTTTGCAGGT CCTTCTAAAC CACGACCCGA CGTTGACTTG   
  
  
- GATTCGGGTG AGACTGTCAG TTGGGGTGAA AAACAGAATT GCACACTTCC CAGTGCAACC CAAGAGTTGC   
  
  
- CCAGTGCTTC AGTTTCGGCG TATACATGTT TTGAAGGGAG TGAAACTTCG TTTGGTGAGT ACCCCTAGTA   
  
  
- TAGTAGAACA ACACAACGCA ACTTCTATTT CAACATATAA TCGGAACACC ACGAACATAG TACTTAAATT   
  
  
- ATCTATCCGG GTTAAATTCT CTTGGCTCTG TAACTTTGAA AAGAACAGAG GAAGAGATCT CATGTATTAA   
  
  
- AGGGGTTTAA ACTGGTGAAA ACGGAGTACC CATATTCTAC TAGTCTAAAC TTAGCTATTT TTAACTTAGA   
  
  
- ACCTTTAACG TTCTTCTTCT GACACCTTGT CGAGTACTTG AACCAACGTA ATCTCCTTAA CCCTTTACCC   
  
  
- ATCTATACAA AGAAACAAAA CATTCAACAA TTAGAACGTT AGTTCCCGTA ATACGTGAAA ATAGATGGAT   
  
  
- TACTCCGTAT GGGTTGACGG AGGTTTCTTA AATTTTGTCT TCCTAAGGTA ACTTTGTTTC TCGATGTTCT   
  
  
- CATTGTCCTG TTACCAAAAA AGTTGAAATT AAAGATATTA AGAAATCATC AACTGAAAAT TCGAAACCAA   
  
  
- CTTACTTCGG TAGCCACAAG ATCGAGGTAG ACCAGGTGGA GGCTACGTAG ATAGGGTAGT TTTGTTACAT   
  
  
- TGTCCTGTTT TGTCTGAGAG TTGGTTTCTA TGGTTGCTAC TCATTAATTC TGTGTGATCA TGACATTCTT   
  
  
- TTAGCTAGAT AGAGATTCTA ATTAGATGTC TTAGGCTTTG GGACTTTGAC ATTGAAAGAA CCATGACTCA   
  
  
- GGAAGACTTT AATTTCATAT ATGGGTGTAC GTTATAAACG AGTTTTTGTG CTTTACACCT ACTCTCTTCC   
  
  
- GAGGTAGTCT TCATTACTCC ACCACAGGGT CACAATGTCA AATGGTTTGG ACATTGGGAA CTGCTCACAC   
  
  
- CTTAAGTCTT GATTTCAGCT TCACTTTTAT AGCATCTTAA CATTCTTGGT GTTGAACAGG GGGACTTATT   
  
  
- CTATAGAATT AATCAAATTT CTCAGTTCTC TCGTGCGTGC ATTTGTGTGT CGACGAAGGA AAAGTAGACG   
  
  
- AGGTGAGAGA GAGGAGAGAA AAAGTATCGG AAGAGCGAGA GAGAAAGAGA GAAAGAGGAA TAGCGAATTA   
  
  
- GAGGAAGAGG AGAAGAAGGG AGAGCTCAAG CAAAGGAGAA GTCATTACGT TTCGATTCCG ACACATGCAG   
  
  
- CGAATAGAAT ACTTTCCGAT CCAATATAA

+     circadian

| Site Name | Organism | Position | Strand | Matrix score. | sequence | function |
| --- | --- | --- | --- | --- | --- | --- |
| circadian | Lycopersicon esculentum | 375 | - | 6 | CAANNNNATC | cis-acting regulatory element involved in circadian control |
| circadian | Lycopersicon esculentum | 743 | - | 6 | CAANNNNATC | cis-acting regulatory element involved in circadian control |
| circadian | Lycopersicon esculentum | 934 | + | 9 | CAAAGATATC | cis-acting regulatory element involved in circadian control |

> 2018/04/13 10:10:12  
+ CGCGGGGATC CAGGGCCAGT AGGGGGTCCG ACAAGAAGGG GACAGCTCGA ACCGCTCCAT TTTCCGACAC   
  
  
+ GTGATCATCT ACACGAGTGG GGGCCTCGAG CATTTACACA TGTCACACGA TGACAGGCAG ATCGGGCCCA   
  
  
+ CTTTATTAAA GGGTTGACTT TTTAGGGGAG AAAACGTCCA GGAAGATTTG GTGCTGGGCT GCAACTGAAC   
  
  
+ CTAAGCCCAC TCTGACAGTC AACCCCACTT TTTGTCTTAA CGTGTGAAGG GTCACGTTGG GTTCTCAACG   
  
  
+ GGTCACGAAG TCAAAGCCGC ATATGTACAA AACTTCCCTC ACTTTGAAGC AAACCACTCA TGGGGATCAT   
  
  
+ ATCATCTTGT TGTGTTGCGT TGAAGATAAA GTTGTATATT AGCCTTGTGG TGCTTGTATC ATGAATTTAA   
  
  
+ TAGATAGGCC CAATTTAAGA GAACCGAGAC ATTGAAACTT TTCTTGTCTC CTTCTCTAGA GTACATAATT   
  
  
+ TCCCCAAATT TGACCACTTT TGCCTCATGG GTATAAGATG ATCAGATTTG AATCGATAAA AATTGAATCT   
  
  
+ TGGAAATTGC AAGAAGAAGA CTGTGGAACA GCTCATGAAC TTGGTTGCAT TAGAGGAATT GGGAAATGGG   
  
  
+ TAGATATGTT TCTTTGTTTT GTAAGTTGTT AATCTTGCAA TCAAGGGCAT TATGCACTTT TATCTACCTA   
  
  
+ ATGAGGCATA CCCAACTGCC TCCAAAGAAT TTAAAACAGA AGGATTCCAT TGAAACAAAG AGCTACAAGA   
  
  
+ GTAACAGGAC AATGGTTTTT TCAACTTTAA TTTCTATAAT TCTTTAGTAG TTGACTTTTA AGCTTTGGTT   
  
  
+ GAATGAAGCC ATCGGTGTTC TAGCTCCATC TGGTCCACCT CCGATGCATC TATCCCATCA AAACAATGTA   
  
  
+ ACAGGACAAA ACAGACTCTC AACCAAAGAT ACCAACGATG AGTAATTAAG ACACACTAGT ACTGTAAGAA   
  
  
+ AATCGATCTA TCTCTAAGAT TAATCTACAG AATCCGAAAC CCTGAAACTG TAACTTTCTT GGTACTGAGT   
  
  
+ CCTTCTGAAA TTAAAGTATA TACCCACATG CAATATTTGC TCAAAAACAC GAAATGTGGA TGAGAGAAGG   
  
  
+ CTCCATCAGA AGTAATGAGG TGGTGTCCCA GTGTTACAGT TTACCAAACC TGTAACCCTT GACGAGTGTG   
  
  
+ GAATTCAGAA CTAAAGTCGA AGTGAAAATA TCGTAGAATT GTAAGAACCA CAACTTGTCC CCCTGAATAA   
  
  
+ GATATCTTAA TTAGTTTAAA GAGTCAAGAG AGCACGCACG TAAACACACA GCTGCTTCCT TTTCATCTGC   
  
  
+ TCCACTCTCT CTCCTCTCTT TTTCATAGCC TTCTCGCTCT CTCTTTCTCT CTTTCTCCTT ATCGCTTAAT   
  
  
+ CTCCTTCTCC TCTTCTTCCC TCTCGAGTTC GTTTCCTCTT CAGTAATGCA AAGCTAAGGC TGTGTACGTC   
  
  
+ GCTTATCTTA TGAAAGGCTA GGTTATATT  

- GCGCCCCTAG GTCCCGGTCA TCCCCCAGGC TGTTCTTCCC CTGTCGAGCT TGGCGAGGTA AAAGGCTGTG   
  
  
- CACTAGTAGA TGTGCTCACC CCCGGAGCTC GTAAATGTGT ACAGTGTGCT ACTGTCCGTC TAGCCCGGGT   
  
  
- GAAATAATTT CCCAACTGAA AAATCCCCTC TTTTGCAGGT CCTTCTAAAC CACGACCCGA CGTTGACTTG   
  
  
- GATTCGGGTG AGACTGTCAG TTGGGGTGAA AAACAGAATT GCACACTTCC CAGTGCAACC CAAGAGTTGC   
  
  
- CCAGTGCTTC AGTTTCGGCG TATACATGTT TTGAAGGGAG TGAAACTTCG TTTGGTGAGT ACCCCTAGTA   
  
  
- TAGTAGAACA ACACAACGCA ACTTCTATTT CAACATATAA TCGGAACACC ACGAACATAG TACTTAAATT   
  
  
- ATCTATCCGG GTTAAATTCT CTTGGCTCTG TAACTTTGAA AAGAACAGAG GAAGAGATCT CATGTATTAA   
  
  
- AGGGGTTTAA ACTGGTGAAA ACGGAGTACC CATATTCTAC TAGTCTAAAC TTAGCTATTT TTAACTTAGA   
  
  
- ACCTTTAACG TTCTTCTTCT GACACCTTGT CGAGTACTTG AACCAACGTA ATCTCCTTAA CCCTTTACCC   
  
  
- ATCTATACAA AGAAACAAAA CATTCAACAA TTAGAACGTT AGTTCCCGTA ATACGTGAAA ATAGATGGAT   
  
  
- TACTCCGTAT GGGTTGACGG AGGTTTCTTA AATTTTGTCT TCCTAAGGTA ACTTTGTTTC TCGATGTTCT   
  
  
- CATTGTCCTG TTACCAAAAA AGTTGAAATT AAAGATATTA AGAAATCATC AACTGAAAAT TCGAAACCAA   
  
  
- CTTACTTCGG TAGCCACAAG ATCGAGGTAG ACCAGGTGGA GGCTACGTAG ATAGGGTAGT TTTGTTACAT   
  
  
- TGTCCTGTTT TGTCTGAGAG TTGGTTTCTA TGGTTGCTAC TCATTAATTC TGTGTGATCA TGACATTCTT   
  
  
- TTAGCTAGAT AGAGATTCTA ATTAGATGTC TTAGGCTTTG GGACTTTGAC ATTGAAAGAA CCATGACTCA   
  
  
- GGAAGACTTT AATTTCATAT ATGGGTGTAC GTTATAAACG AGTTTTTGTG CTTTACACCT ACTCTCTTCC   
  
  
- GAGGTAGTCT TCATTACTCC ACCACAGGGT CACAATGTCA AATGGTTTGG ACATTGGGAA CTGCTCACAC   
  
  
- CTTAAGTCTT GATTTCAGCT TCACTTTTAT AGCATCTTAA CATTCTTGGT GTTGAACAGG GGGACTTATT   
  
  
- CTATAGAATT AATCAAATTT CTCAGTTCTC TCGTGCGTGC ATTTGTGTGT CGACGAAGGA AAAGTAGACG   
  
  
- AGGTGAGAGA GAGGAGAGAA AAAGTATCGG AAGAGCGAGA GAGAAAGAGA GAAAGAGGAA TAGCGAATTA   
  
  
- GAGGAAGAGG AGAAGAAGGG AGAGCTCAAG CAAAGGAGAA GTCATTACGT TTCGATTCCG ACACATGCAG   
  
  
- CGAATAGAAT ACTTTCCGAT CCAATATAA

+     rbcS-CMA7a

| Site Name | Organism | Position | Strand | Matrix score. | sequence | function |
| --- | --- | --- | --- | --- | --- | --- |
| rbcS-CMA7a | Zea mays | 1387 | - | 9 | GGCGATAAGG | part of a light responsive element |

> 2018/04/13 10:10:12  
+ CGCGGGGATC CAGGGCCAGT AGGGGGTCCG ACAAGAAGGG GACAGCTCGA ACCGCTCCAT TTTCCGACAC   
  
  
+ GTGATCATCT ACACGAGTGG GGGCCTCGAG CATTTACACA TGTCACACGA TGACAGGCAG ATCGGGCCCA   
  
  
+ CTTTATTAAA GGGTTGACTT TTTAGGGGAG AAAACGTCCA GGAAGATTTG GTGCTGGGCT GCAACTGAAC   
  
  
+ CTAAGCCCAC TCTGACAGTC AACCCCACTT TTTGTCTTAA CGTGTGAAGG GTCACGTTGG GTTCTCAACG   
  
  
+ GGTCACGAAG TCAAAGCCGC ATATGTACAA AACTTCCCTC ACTTTGAAGC AAACCACTCA TGGGGATCAT   
  
  
+ ATCATCTTGT TGTGTTGCGT TGAAGATAAA GTTGTATATT AGCCTTGTGG TGCTTGTATC ATGAATTTAA   
  
  
+ TAGATAGGCC CAATTTAAGA GAACCGAGAC ATTGAAACTT TTCTTGTCTC CTTCTCTAGA GTACATAATT   
  
  
+ TCCCCAAATT TGACCACTTT TGCCTCATGG GTATAAGATG ATCAGATTTG AATCGATAAA AATTGAATCT   
  
  
+ TGGAAATTGC AAGAAGAAGA CTGTGGAACA GCTCATGAAC TTGGTTGCAT TAGAGGAATT GGGAAATGGG   
  
  
+ TAGATATGTT TCTTTGTTTT GTAAGTTGTT AATCTTGCAA TCAAGGGCAT TATGCACTTT TATCTACCTA   
  
  
+ ATGAGGCATA CCCAACTGCC TCCAAAGAAT TTAAAACAGA AGGATTCCAT TGAAACAAAG AGCTACAAGA   
  
  
+ GTAACAGGAC AATGGTTTTT TCAACTTTAA TTTCTATAAT TCTTTAGTAG TTGACTTTTA AGCTTTGGTT   
  
  
+ GAATGAAGCC ATCGGTGTTC TAGCTCCATC TGGTCCACCT CCGATGCATC TATCCCATCA AAACAATGTA   
  
  
+ ACAGGACAAA ACAGACTCTC AACCAAAGAT ACCAACGATG AGTAATTAAG ACACACTAGT ACTGTAAGAA   
  
  
+ AATCGATCTA TCTCTAAGAT TAATCTACAG AATCCGAAAC CCTGAAACTG TAACTTTCTT GGTACTGAGT   
  
  
+ CCTTCTGAAA TTAAAGTATA TACCCACATG CAATATTTGC TCAAAAACAC GAAATGTGGA TGAGAGAAGG   
  
  
+ CTCCATCAGA AGTAATGAGG TGGTGTCCCA GTGTTACAGT TTACCAAACC TGTAACCCTT GACGAGTGTG   
  
  
+ GAATTCAGAA CTAAAGTCGA AGTGAAAATA TCGTAGAATT GTAAGAACCA CAACTTGTCC CCCTGAATAA   
  
  
+ GATATCTTAA TTAGTTTAAA GAGTCAAGAG AGCACGCACG TAAACACACA GCTGCTTCCT TTTCATCTGC   
  
  
+ TCCACTCTCT CTCCTCTCTT TTTCATAGCC TTCTCGCTCT CTCTTTCTCT CTTTCTCCTT ATCGCTTAAT   
  
  
+ CTCCTTCTCC TCTTCTTCCC TCTCGAGTTC GTTTCCTCTT CAGTAATGCA AAGCTAAGGC TGTGTACGTC   
  
  
+ GCTTATCTTA TGAAAGGCTA GGTTATATT  

- GCGCCCCTAG GTCCCGGTCA TCCCCCAGGC TGTTCTTCCC CTGTCGAGCT TGGCGAGGTA AAAGGCTGTG   
  
  
- CACTAGTAGA TGTGCTCACC CCCGGAGCTC GTAAATGTGT ACAGTGTGCT ACTGTCCGTC TAGCCCGGGT   
  
  
- GAAATAATTT CCCAACTGAA AAATCCCCTC TTTTGCAGGT CCTTCTAAAC CACGACCCGA CGTTGACTTG   
  
  
- GATTCGGGTG AGACTGTCAG TTGGGGTGAA AAACAGAATT GCACACTTCC CAGTGCAACC CAAGAGTTGC   
  
  
- CCAGTGCTTC AGTTTCGGCG TATACATGTT TTGAAGGGAG TGAAACTTCG TTTGGTGAGT ACCCCTAGTA   
  
  
- TAGTAGAACA ACACAACGCA ACTTCTATTT CAACATATAA TCGGAACACC ACGAACATAG TACTTAAATT   
  
  
- ATCTATCCGG GTTAAATTCT CTTGGCTCTG TAACTTTGAA AAGAACAGAG GAAGAGATCT CATGTATTAA   
  
  
- AGGGGTTTAA ACTGGTGAAA ACGGAGTACC CATATTCTAC TAGTCTAAAC TTAGCTATTT TTAACTTAGA   
  
  
- ACCTTTAACG TTCTTCTTCT GACACCTTGT CGAGTACTTG AACCAACGTA ATCTCCTTAA CCCTTTACCC   
  
  
- ATCTATACAA AGAAACAAAA CATTCAACAA TTAGAACGTT AGTTCCCGTA ATACGTGAAA ATAGATGGAT   
  
  
- TACTCCGTAT GGGTTGACGG AGGTTTCTTA AATTTTGTCT TCCTAAGGTA ACTTTGTTTC TCGATGTTCT   
  
  
- CATTGTCCTG TTACCAAAAA AGTTGAAATT AAAGATATTA AGAAATCATC AACTGAAAAT TCGAAACCAA   
  
  
- CTTACTTCGG TAGCCACAAG ATCGAGGTAG ACCAGGTGGA GGCTACGTAG ATAGGGTAGT TTTGTTACAT   
  
  
- TGTCCTGTTT TGTCTGAGAG TTGGTTTCTA TGGTTGCTAC TCATTAATTC TGTGTGATCA TGACATTCTT   
  
  
- TTAGCTAGAT AGAGATTCTA ATTAGATGTC TTAGGCTTTG GGACTTTGAC ATTGAAAGAA CCATGACTCA   
  
  
- GGAAGACTTT AATTTCATAT ATGGGTGTAC GTTATAAACG AGTTTTTGTG CTTTACACCT ACTCTCTTCC   
  
  
- GAGGTAGTCT TCATTACTCC ACCACAGGGT CACAATGTCA AATGGTTTGG ACATTGGGAA CTGCTCACAC   
  
  
- CTTAAGTCTT GATTTCAGCT TCACTTTTAT AGCATCTTAA CATTCTTGGT GTTGAACAGG GGGACTTATT   
  
  
- CTATAGAATT AATCAAATTT CTCAGTTCTC TCGTGCGTGC ATTTGTGTGT CGACGAAGGA AAAGTAGACG   
  
  
- AGGTGAGAGA GAGGAGAGAA AAAGTATCGG AAGAGCGAGA GAGAAAGAGA GAAAGAGGAA TAGCGAATTA   
  
  
- GAGGAAGAGG AGAAGAAGGG AGAGCTCAAG CAAAGGAGAA GTCATTACGT TTCGATTCCG ACACATGCAG   
  
  
- CGAATAGAAT ACTTTCCGAT CCAATATAA
