# Supplementary material for: Genome-wide analysis of the WRKY gene family in drumstick (Moringa oleifera Lam.)
Source: PeerJ. 2019 Jun 10;7:e7063. doi: 10.7717/peerj.7063 (PMC6563795; doi:10.7717/peerj.7063)
Supplement: Supplemental Information 1 [file peerj-07-7063-s003.gz › MoWRKY18_plantcare.html]

Content-Type: text/html; charset=ISO-8859-1


CallMat\_Firefox


Webmaster Firefox specific output  
To save the result:
click on the frame with the right mouse button and save the source code as a text file with extension .html  
REFERENCE:PlantCARE: a database of plant cis-acting regulatory elements and a portal to tools for in silico analysis of promoter sequences.  
Lescot, M., Déhais, P., Moreau, Y., De Moor, B., Rouzé ,P.,and Rombauts, S.  
Nucleic Acids Res., Database issue(2002), 30(1):325-327.   


---

> 2018/04/13 10:10:12  
+ GTTGCTTGCT TTAATTAAAG AAGGAAGCAA AACAGATGGT TGGATTGAAA AGGACAACCC ATAAGGCATA   
  
  
+ TAATAATCAG CAGTAATTAA GTTTCACTTG TTTTTGATAG ACTTAATTAT ATATGTGCAG TGCATGTTTG   
  
  
+ CAGACCAGCA TGGCAAGCAT AAAACTTCAC AAGATCTCTT CCAAAGATTG TATTTGTAGA TGGCTTCAGA   
  
  
+ CATGACACAA AGTGCTACCC ACAATCAACT TACTGCTGTC TGGTAAACGT TGAAACTGGT GTCCCATAAC   
  
  
+ ACACTGTAGT ACTCTCTCCC ACACCTTGTA ACAAGTTTAC TTCATTTGGT TCTCTGGTAT TTAATTTAGT   
  
  
+ GAAGAAGATC ATTAAGCTAC TCAACAGGAA GCTTTTCAAG TAAATGCAAC TTGTATGACA ACATTCTTCT   
  
  
+ TCTGTCTGTC TCACACCACA GACTTCACTA GTTACATCAT TAATTAGGCA TGTAGGCCAA TGGTTTATCA   
  
  
+ GCTGTAATAA GGTGATATGA TCAATATGAA AAGACCAGCT TCAAATCAGA TAGGCTGTGG ATTTATTCCC   
  
  
+ CAACCACGGA CTTTAATTAG CCTCCAAGTG TACAATATAT ATGAAATGTG CATTGCTCAA TGGTATAATT   
  
  
+ AATTAGCTAG AACATACATG CGGATAGTGT CTGTGTGCAG GGAGAGATAA AGAAATCAGA GGGAGGGAGA   
  
  
+ GTTGTTGTAT ATATGTATGG ATCGATCCTG GATGTGCTGA ATCAGATTAG AAATCAATTC AACAAGCAAA   
  
  
+ TATCATTCTC ATCTTTCACA TCCACATCAT TCCAACATGA AAACTACATC TTTGTCCAGG AACGGCCACA   
  
  
+ AGTAGTGTGA AAGTATATCA GCACTCAAAT TGCTTTTCAA TAATGATGCT ATCTATTGTG ATAAGATGTT   
  
  
+ ATTTATTAGG AAGAAATTGC ATATAACAAT CTTGTTTAAG ATAAACTGGA CTGTGAACCA AGAAGGACCA   
  
  
+ AAGATGGACA AGCTATGAAG TGAAATGTCT ATGATTTTCA CTCCGAATAT GTCAGAATAG ATTGGTTTTG   
  
  
+ ACGCACCCGC ATACAGTAAT AGCCAGTCAA GGATATAAAC GTTAGTGTTG CCTTGTGCTT CTTCCATGTA   
  
  
+ TTACAGTGCT TAAGAAATCA GACTATATAT TAATCAGTAA CTCTTTTCTT GCGCACAGCC ACACATCCCC   
  
  
+ TTAATAATTC CTCAGCACTA TCAATGTTTG AGAGGGCACA GAGAGAGAGA GAGAGAGAGA GAGAGAGAGA   
  
  
+ AAGAGAGGTG AGTCTGCAAG TCTTTCCTCT GCGGTACCTG TGAGAAGATA AGAAGGAATA ACTTGCAGAA   
  
  
+ AACATTCCGT TTAGCGTTTA CTGGTTGACC AGGCTTACAT CTCAAAACAA AGGGCTATTT CTGCAGACGA   
  
  
+ TCGAATAGAG CATCAACTTG GTCATACCTG CTATTGAACC AGACCCTTTT GACAGGAAGA ATATATTAAG   
  
  
+ AAGTAGGGCT TGCTGGGTTA TCGAAAGAA  

- CAACGAACGA AATTAATTTC TTCCTTCGTT TTGTCTACCA ACCTAACTTT TCCTGTTGGG TATTCCGTAT   
  
  
- ATTATTAGTC GTCATTAATT CAAAGTGAAC AAAAACTATC TGAATTAATA TATACACGTC ACGTACAAAC   
  
  
- GTCTGGTCGT ACCGTTCGTA TTTTGAAGTG TTCTAGAGAA GGTTTCTAAC ATAAACATCT ACCGAAGTCT   
  
  
- GTACTGTGTT TCACGATGGG TGTTAGTTGA ATGACGACAG ACCATTTGCA ACTTTGACCA CAGGGTATTG   
  
  
- TGTGACATCA TGAGAGAGGG TGTGGAACAT TGTTCAAATG AAGTAAACCA AGAGACCATA AATTAAATCA   
  
  
- CTTCTTCTAG TAATTCGATG AGTTGTCCTT CGAAAAGTTC ATTTACGTTG AACATACTGT TGTAAGAAGA   
  
  
- AGACAGACAG AGTGTGGTGT CTGAAGTGAT CAATGTAGTA ATTAATCCGT ACATCCGGTT ACCAAATAGT   
  
  
- CGACATTATT CCACTATACT AGTTATACTT TTCTGGTCGA AGTTTAGTCT ATCCGACACC TAAATAAGGG   
  
  
- GTTGGTGCCT GAAATTAATC GGAGGTTCAC ATGTTATATA TACTTTACAC GTAACGAGTT ACCATATTAA   
  
  
- TTAATCGATC TTGTATGTAC GCCTATCACA GACACACGTC CCTCTCTATT TCTTTAGTCT CCCTCCCTCT   
  
  
- CAACAACATA TATACATACC TAGCTAGGAC CTACACGACT TAGTCTAATC TTTAGTTAAG TTGTTCGTTT   
  
  
- ATAGTAAGAG TAGAAAGTGT AGGTGTAGTA AGGTTGTACT TTTGATGTAG AAACAGGTCC TTGCCGGTGT   
  
  
- TCATCACACT TTCATATAGT CGTGAGTTTA ACGAAAAGTT ATTACTACGA TAGATAACAC TATTCTACAA   
  
  
- TAAATAATCC TTCTTTAACG TATATTGTTA GAACAAATTC TATTTGACCT GACACTTGGT TCTTCCTGGT   
  
  
- TTCTACCTGT TCGATACTTC ACTTTACAGA TACTAAAAGT GAGGCTTATA CAGTCTTATC TAACCAAAAC   
  
  
- TGCGTGGGCG TATGTCATTA TCGGTCAGTT CCTATATTTG CAATCACAAC GGAACACGAA GAAGGTACAT   
  
  
- AATGTCACGA ATTCTTTAGT CTGATATATA ATTAGTCATT GAGAAAAGAA CGCGTGTCGG TGTGTAGGGG   
  
  
- AATTATTAAG GAGTCGTGAT AGTTACAAAC TCTCCCGTGT CTCTCTCTCT CTCTCTCTCT CTCTCTCTCT   
  
  
- TTCTCTCCAC TCAGACGTTC AGAAAGGAGA CGCCATGGAC ACTCTTCTAT TCTTCCTTAT TGAACGTCTT   
  
  
- TTGTAAGGCA AATCGCAAAT GACCAACTGG TCCGAATGTA GAGTTTTGTT TCCCGATAAA GACGTCTGCT   
  
  
- AGCTTATCTC GTAGTTGAAC CAGTATGGAC GATAACTTGG TCTGGGAAAA CTGTCCTTCT TATATAATTC   
  
  
- TTCATCCCGA ACGACCCAAT AGCTTTCTT

  
  
Motifs Found  

+     5UTR Py-rich stretch

| Site Name | Organism | Position | Strand | Matrix score. | sequence | function |
| --- | --- | --- | --- | --- | --- | --- |
| 5UTR Py-rich stretch | Lycopersicon esculentum | 1245 | - | 13 | TTTCTCTCTCTCTC | cis-acting element conferring high transcription levels |
| 5UTR Py-rich stretch | Lycopersicon esculentum | 1243 | - | 13 | TTTCTCTCTCTCTC | cis-acting element conferring high transcription levels |
| 5UTR Py-rich stretch | Lycopersicon esculentum | 1249 | - | 14 | TTTCTCTCTCTCTC | cis-acting element conferring high transcription levels |
| 5UTR Py-rich stretch | Lycopersicon esculentum | 1247 | - | 13 | TTTCTCTCTCTCTC | cis-acting element conferring high transcription levels |
| 5UTR Py-rich stretch | Lycopersicon esculentum | 1239 | - | 13 | TTTCTCTCTCTCTC | cis-acting element conferring high transcription levels |
| 5UTR Py-rich stretch | Lycopersicon esculentum | 1237 | - | 13 | TTTCTCTCTCTCTC | cis-acting element conferring high transcription levels |
| 5UTR Py-rich stretch | Lycopersicon esculentum | 1233 | - | 13 | TTTCTCTCTCTCTC | cis-acting element conferring high transcription levels |
| 5UTR Py-rich stretch | Lycopersicon esculentum | 1241 | - | 13 | TTTCTCTCTCTCTC | cis-acting element conferring high transcription levels |
| 5UTR Py-rich stretch | Lycopersicon esculentum | 1231 | - | 13 | TTTCTCTCTCTCTC | cis-acting element conferring high transcription levels |
| 5UTR Py-rich stretch | Lycopersicon esculentum | 1235 | - | 13 | TTTCTCTCTCTCTC | cis-acting element conferring high transcription levels |

> 2018/04/13 10:10:12  
+ GTTGCTTGCT TTAATTAAAG AAGGAAGCAA AACAGATGGT TGGATTGAAA AGGACAACCC ATAAGGCATA   
  
  
+ TAATAATCAG CAGTAATTAA GTTTCACTTG TTTTTGATAG ACTTAATTAT ATATGTGCAG TGCATGTTTG   
  
  
+ CAGACCAGCA TGGCAAGCAT AAAACTTCAC AAGATCTCTT CCAAAGATTG TATTTGTAGA TGGCTTCAGA   
  
  
+ CATGACACAA AGTGCTACCC ACAATCAACT TACTGCTGTC TGGTAAACGT TGAAACTGGT GTCCCATAAC   
  
  
+ ACACTGTAGT ACTCTCTCCC ACACCTTGTA ACAAGTTTAC TTCATTTGGT TCTCTGGTAT TTAATTTAGT   
  
  
+ GAAGAAGATC ATTAAGCTAC TCAACAGGAA GCTTTTCAAG TAAATGCAAC TTGTATGACA ACATTCTTCT   
  
  
+ TCTGTCTGTC TCACACCACA GACTTCACTA GTTACATCAT TAATTAGGCA TGTAGGCCAA TGGTTTATCA   
  
  
+ GCTGTAATAA GGTGATATGA TCAATATGAA AAGACCAGCT TCAAATCAGA TAGGCTGTGG ATTTATTCCC   
  
  
+ CAACCACGGA CTTTAATTAG CCTCCAAGTG TACAATATAT ATGAAATGTG CATTGCTCAA TGGTATAATT   
  
  
+ AATTAGCTAG AACATACATG CGGATAGTGT CTGTGTGCAG GGAGAGATAA AGAAATCAGA GGGAGGGAGA   
  
  
+ GTTGTTGTAT ATATGTATGG ATCGATCCTG GATGTGCTGA ATCAGATTAG AAATCAATTC AACAAGCAAA   
  
  
+ TATCATTCTC ATCTTTCACA TCCACATCAT TCCAACATGA AAACTACATC TTTGTCCAGG AACGGCCACA   
  
  
+ AGTAGTGTGA AAGTATATCA GCACTCAAAT TGCTTTTCAA TAATGATGCT ATCTATTGTG ATAAGATGTT   
  
  
+ ATTTATTAGG AAGAAATTGC ATATAACAAT CTTGTTTAAG ATAAACTGGA CTGTGAACCA AGAAGGACCA   
  
  
+ AAGATGGACA AGCTATGAAG TGAAATGTCT ATGATTTTCA CTCCGAATAT GTCAGAATAG ATTGGTTTTG   
  
  
+ ACGCACCCGC ATACAGTAAT AGCCAGTCAA GGATATAAAC GTTAGTGTTG CCTTGTGCTT CTTCCATGTA   
  
  
+ TTACAGTGCT TAAGAAATCA GACTATATAT TAATCAGTAA CTCTTTTCTT GCGCACAGCC ACACATCCCC   
  
  
+ TTAATAATTC CTCAGCACTA TCAATGTTTG AGAGGGCACA GAGAGAGAGA GAGAGAGAGA GAGAGAGAGA   
  
  
+ AAGAGAGGTG AGTCTGCAAG TCTTTCCTCT GCGGTACCTG TGAGAAGATA AGAAGGAATA ACTTGCAGAA   
  
  
+ AACATTCCGT TTAGCGTTTA CTGGTTGACC AGGCTTACAT CTCAAAACAA AGGGCTATTT CTGCAGACGA   
  
  
+ TCGAATAGAG CATCAACTTG GTCATACCTG CTATTGAACC AGACCCTTTT GACAGGAAGA ATATATTAAG   
  
  
+ AAGTAGGGCT TGCTGGGTTA TCGAAAGAA  

- CAACGAACGA AATTAATTTC TTCCTTCGTT TTGTCTACCA ACCTAACTTT TCCTGTTGGG TATTCCGTAT   
  
  
- ATTATTAGTC GTCATTAATT CAAAGTGAAC AAAAACTATC TGAATTAATA TATACACGTC ACGTACAAAC   
  
  
- GTCTGGTCGT ACCGTTCGTA TTTTGAAGTG TTCTAGAGAA GGTTTCTAAC ATAAACATCT ACCGAAGTCT   
  
  
- GTACTGTGTT TCACGATGGG TGTTAGTTGA ATGACGACAG ACCATTTGCA ACTTTGACCA CAGGGTATTG   
  
  
- TGTGACATCA TGAGAGAGGG TGTGGAACAT TGTTCAAATG AAGTAAACCA AGAGACCATA AATTAAATCA   
  
  
- CTTCTTCTAG TAATTCGATG AGTTGTCCTT CGAAAAGTTC ATTTACGTTG AACATACTGT TGTAAGAAGA   
  
  
- AGACAGACAG AGTGTGGTGT CTGAAGTGAT CAATGTAGTA ATTAATCCGT ACATCCGGTT ACCAAATAGT   
  
  
- CGACATTATT CCACTATACT AGTTATACTT TTCTGGTCGA AGTTTAGTCT ATCCGACACC TAAATAAGGG   
  
  
- GTTGGTGCCT GAAATTAATC GGAGGTTCAC ATGTTATATA TACTTTACAC GTAACGAGTT ACCATATTAA   
  
  
- TTAATCGATC TTGTATGTAC GCCTATCACA GACACACGTC CCTCTCTATT TCTTTAGTCT CCCTCCCTCT   
  
  
- CAACAACATA TATACATACC TAGCTAGGAC CTACACGACT TAGTCTAATC TTTAGTTAAG TTGTTCGTTT   
  
  
- ATAGTAAGAG TAGAAAGTGT AGGTGTAGTA AGGTTGTACT TTTGATGTAG AAACAGGTCC TTGCCGGTGT   
  
  
- TCATCACACT TTCATATAGT CGTGAGTTTA ACGAAAAGTT ATTACTACGA TAGATAACAC TATTCTACAA   
  
  
- TAAATAATCC TTCTTTAACG TATATTGTTA GAACAAATTC TATTTGACCT GACACTTGGT TCTTCCTGGT   
  
  
- TTCTACCTGT TCGATACTTC ACTTTACAGA TACTAAAAGT GAGGCTTATA CAGTCTTATC TAACCAAAAC   
  
  
- TGCGTGGGCG TATGTCATTA TCGGTCAGTT CCTATATTTG CAATCACAAC GGAACACGAA GAAGGTACAT   
  
  
- AATGTCACGA ATTCTTTAGT CTGATATATA ATTAGTCATT GAGAAAAGAA CGCGTGTCGG TGTGTAGGGG   
  
  
- AATTATTAAG GAGTCGTGAT AGTTACAAAC TCTCCCGTGT CTCTCTCTCT CTCTCTCTCT CTCTCTCTCT   
  
  
- TTCTCTCCAC TCAGACGTTC AGAAAGGAGA CGCCATGGAC ACTCTTCTAT TCTTCCTTAT TGAACGTCTT   
  
  
- TTGTAAGGCA AATCGCAAAT GACCAACTGG TCCGAATGTA GAGTTTTGTT TCCCGATAAA GACGTCTGCT   
  
  
- AGCTTATCTC GTAGTTGAAC CAGTATGGAC GATAACTTGG TCTGGGAAAA CTGTCCTTCT TATATAATTC   
  
  
- TTCATCCCGA ACGACCCAAT AGCTTTCTT

+     AAGAA-motif

| Site Name | Organism | Position | Strand | Matrix score. | sequence | function |
| --- | --- | --- | --- | --- | --- | --- |
| AAGAA-motif | Avena sativa | 1493 | + | 7 | GAAAGAA |  |
| AAGAA-motif | Avena sativa | 676 | + | 9 | gGTAAAGAAA |  |

> 2018/04/13 10:10:12  
+ GTTGCTTGCT TTAATTAAAG AAGGAAGCAA AACAGATGGT TGGATTGAAA AGGACAACCC ATAAGGCATA   
  
  
+ TAATAATCAG CAGTAATTAA GTTTCACTTG TTTTTGATAG ACTTAATTAT ATATGTGCAG TGCATGTTTG   
  
  
+ CAGACCAGCA TGGCAAGCAT AAAACTTCAC AAGATCTCTT CCAAAGATTG TATTTGTAGA TGGCTTCAGA   
  
  
+ CATGACACAA AGTGCTACCC ACAATCAACT TACTGCTGTC TGGTAAACGT TGAAACTGGT GTCCCATAAC   
  
  
+ ACACTGTAGT ACTCTCTCCC ACACCTTGTA ACAAGTTTAC TTCATTTGGT TCTCTGGTAT TTAATTTAGT   
  
  
+ GAAGAAGATC ATTAAGCTAC TCAACAGGAA GCTTTTCAAG TAAATGCAAC TTGTATGACA ACATTCTTCT   
  
  
+ TCTGTCTGTC TCACACCACA GACTTCACTA GTTACATCAT TAATTAGGCA TGTAGGCCAA TGGTTTATCA   
  
  
+ GCTGTAATAA GGTGATATGA TCAATATGAA AAGACCAGCT TCAAATCAGA TAGGCTGTGG ATTTATTCCC   
  
  
+ CAACCACGGA CTTTAATTAG CCTCCAAGTG TACAATATAT ATGAAATGTG CATTGCTCAA TGGTATAATT   
  
  
+ AATTAGCTAG AACATACATG CGGATAGTGT CTGTGTGCAG GGAGAGATAA AGAAATCAGA GGGAGGGAGA   
  
  
+ GTTGTTGTAT ATATGTATGG ATCGATCCTG GATGTGCTGA ATCAGATTAG AAATCAATTC AACAAGCAAA   
  
  
+ TATCATTCTC ATCTTTCACA TCCACATCAT TCCAACATGA AAACTACATC TTTGTCCAGG AACGGCCACA   
  
  
+ AGTAGTGTGA AAGTATATCA GCACTCAAAT TGCTTTTCAA TAATGATGCT ATCTATTGTG ATAAGATGTT   
  
  
+ ATTTATTAGG AAGAAATTGC ATATAACAAT CTTGTTTAAG ATAAACTGGA CTGTGAACCA AGAAGGACCA   
  
  
+ AAGATGGACA AGCTATGAAG TGAAATGTCT ATGATTTTCA CTCCGAATAT GTCAGAATAG ATTGGTTTTG   
  
  
+ ACGCACCCGC ATACAGTAAT AGCCAGTCAA GGATATAAAC GTTAGTGTTG CCTTGTGCTT CTTCCATGTA   
  
  
+ TTACAGTGCT TAAGAAATCA GACTATATAT TAATCAGTAA CTCTTTTCTT GCGCACAGCC ACACATCCCC   
  
  
+ TTAATAATTC CTCAGCACTA TCAATGTTTG AGAGGGCACA GAGAGAGAGA GAGAGAGAGA GAGAGAGAGA   
  
  
+ AAGAGAGGTG AGTCTGCAAG TCTTTCCTCT GCGGTACCTG TGAGAAGATA AGAAGGAATA ACTTGCAGAA   
  
  
+ AACATTCCGT TTAGCGTTTA CTGGTTGACC AGGCTTACAT CTCAAAACAA AGGGCTATTT CTGCAGACGA   
  
  
+ TCGAATAGAG CATCAACTTG GTCATACCTG CTATTGAACC AGACCCTTTT GACAGGAAGA ATATATTAAG   
  
  
+ AAGTAGGGCT TGCTGGGTTA TCGAAAGAA  

- CAACGAACGA AATTAATTTC TTCCTTCGTT TTGTCTACCA ACCTAACTTT TCCTGTTGGG TATTCCGTAT   
  
  
- ATTATTAGTC GTCATTAATT CAAAGTGAAC AAAAACTATC TGAATTAATA TATACACGTC ACGTACAAAC   
  
  
- GTCTGGTCGT ACCGTTCGTA TTTTGAAGTG TTCTAGAGAA GGTTTCTAAC ATAAACATCT ACCGAAGTCT   
  
  
- GTACTGTGTT TCACGATGGG TGTTAGTTGA ATGACGACAG ACCATTTGCA ACTTTGACCA CAGGGTATTG   
  
  
- TGTGACATCA TGAGAGAGGG TGTGGAACAT TGTTCAAATG AAGTAAACCA AGAGACCATA AATTAAATCA   
  
  
- CTTCTTCTAG TAATTCGATG AGTTGTCCTT CGAAAAGTTC ATTTACGTTG AACATACTGT TGTAAGAAGA   
  
  
- AGACAGACAG AGTGTGGTGT CTGAAGTGAT CAATGTAGTA ATTAATCCGT ACATCCGGTT ACCAAATAGT   
  
  
- CGACATTATT CCACTATACT AGTTATACTT TTCTGGTCGA AGTTTAGTCT ATCCGACACC TAAATAAGGG   
  
  
- GTTGGTGCCT GAAATTAATC GGAGGTTCAC ATGTTATATA TACTTTACAC GTAACGAGTT ACCATATTAA   
  
  
- TTAATCGATC TTGTATGTAC GCCTATCACA GACACACGTC CCTCTCTATT TCTTTAGTCT CCCTCCCTCT   
  
  
- CAACAACATA TATACATACC TAGCTAGGAC CTACACGACT TAGTCTAATC TTTAGTTAAG TTGTTCGTTT   
  
  
- ATAGTAAGAG TAGAAAGTGT AGGTGTAGTA AGGTTGTACT TTTGATGTAG AAACAGGTCC TTGCCGGTGT   
  
  
- TCATCACACT TTCATATAGT CGTGAGTTTA ACGAAAAGTT ATTACTACGA TAGATAACAC TATTCTACAA   
  
  
- TAAATAATCC TTCTTTAACG TATATTGTTA GAACAAATTC TATTTGACCT GACACTTGGT TCTTCCTGGT   
  
  
- TTCTACCTGT TCGATACTTC ACTTTACAGA TACTAAAAGT GAGGCTTATA CAGTCTTATC TAACCAAAAC   
  
  
- TGCGTGGGCG TATGTCATTA TCGGTCAGTT CCTATATTTG CAATCACAAC GGAACACGAA GAAGGTACAT   
  
  
- AATGTCACGA ATTCTTTAGT CTGATATATA ATTAGTCATT GAGAAAAGAA CGCGTGTCGG TGTGTAGGGG   
  
  
- AATTATTAAG GAGTCGTGAT AGTTACAAAC TCTCCCGTGT CTCTCTCTCT CTCTCTCTCT CTCTCTCTCT   
  
  
- TTCTCTCCAC TCAGACGTTC AGAAAGGAGA CGCCATGGAC ACTCTTCTAT TCTTCCTTAT TGAACGTCTT   
  
  
- TTGTAAGGCA AATCGCAAAT GACCAACTGG TCCGAATGTA GAGTTTTGTT TCCCGATAAA GACGTCTGCT   
  
  
- AGCTTATCTC GTAGTTGAAC CAGTATGGAC GATAACTTGG TCTGGGAAAA CTGTCCTTCT TATATAATTC   
  
  
- TTCATCCCGA ACGACCCAAT AGCTTTCTT

+     ARE

| Site Name | Organism | Position | Strand | Matrix score. | sequence | function |
| --- | --- | --- | --- | --- | --- | --- |
| ARE | Zea mays | 481 | + | 6 | TGGTTT | cis-acting regulatory element essential for the anaerobic induction |
| ARE | Zea mays | 1043 | + | 6 | TGGTTT | cis-acting regulatory element essential for the anaerobic induction |

> 2018/04/13 10:10:12  
+ GTTGCTTGCT TTAATTAAAG AAGGAAGCAA AACAGATGGT TGGATTGAAA AGGACAACCC ATAAGGCATA   
  
  
+ TAATAATCAG CAGTAATTAA GTTTCACTTG TTTTTGATAG ACTTAATTAT ATATGTGCAG TGCATGTTTG   
  
  
+ CAGACCAGCA TGGCAAGCAT AAAACTTCAC AAGATCTCTT CCAAAGATTG TATTTGTAGA TGGCTTCAGA   
  
  
+ CATGACACAA AGTGCTACCC ACAATCAACT TACTGCTGTC TGGTAAACGT TGAAACTGGT GTCCCATAAC   
  
  
+ ACACTGTAGT ACTCTCTCCC ACACCTTGTA ACAAGTTTAC TTCATTTGGT TCTCTGGTAT TTAATTTAGT   
  
  
+ GAAGAAGATC ATTAAGCTAC TCAACAGGAA GCTTTTCAAG TAAATGCAAC TTGTATGACA ACATTCTTCT   
  
  
+ TCTGTCTGTC TCACACCACA GACTTCACTA GTTACATCAT TAATTAGGCA TGTAGGCCAA TGGTTTATCA   
  
  
+ GCTGTAATAA GGTGATATGA TCAATATGAA AAGACCAGCT TCAAATCAGA TAGGCTGTGG ATTTATTCCC   
  
  
+ CAACCACGGA CTTTAATTAG CCTCCAAGTG TACAATATAT ATGAAATGTG CATTGCTCAA TGGTATAATT   
  
  
+ AATTAGCTAG AACATACATG CGGATAGTGT CTGTGTGCAG GGAGAGATAA AGAAATCAGA GGGAGGGAGA   
  
  
+ GTTGTTGTAT ATATGTATGG ATCGATCCTG GATGTGCTGA ATCAGATTAG AAATCAATTC AACAAGCAAA   
  
  
+ TATCATTCTC ATCTTTCACA TCCACATCAT TCCAACATGA AAACTACATC TTTGTCCAGG AACGGCCACA   
  
  
+ AGTAGTGTGA AAGTATATCA GCACTCAAAT TGCTTTTCAA TAATGATGCT ATCTATTGTG ATAAGATGTT   
  
  
+ ATTTATTAGG AAGAAATTGC ATATAACAAT CTTGTTTAAG ATAAACTGGA CTGTGAACCA AGAAGGACCA   
  
  
+ AAGATGGACA AGCTATGAAG TGAAATGTCT ATGATTTTCA CTCCGAATAT GTCAGAATAG ATTGGTTTTG   
  
  
+ ACGCACCCGC ATACAGTAAT AGCCAGTCAA GGATATAAAC GTTAGTGTTG CCTTGTGCTT CTTCCATGTA   
  
  
+ TTACAGTGCT TAAGAAATCA GACTATATAT TAATCAGTAA CTCTTTTCTT GCGCACAGCC ACACATCCCC   
  
  
+ TTAATAATTC CTCAGCACTA TCAATGTTTG AGAGGGCACA GAGAGAGAGA GAGAGAGAGA GAGAGAGAGA   
  
  
+ AAGAGAGGTG AGTCTGCAAG TCTTTCCTCT GCGGTACCTG TGAGAAGATA AGAAGGAATA ACTTGCAGAA   
  
  
+ AACATTCCGT TTAGCGTTTA CTGGTTGACC AGGCTTACAT CTCAAAACAA AGGGCTATTT CTGCAGACGA   
  
  
+ TCGAATAGAG CATCAACTTG GTCATACCTG CTATTGAACC AGACCCTTTT GACAGGAAGA ATATATTAAG   
  
  
+ AAGTAGGGCT TGCTGGGTTA TCGAAAGAA  

- CAACGAACGA AATTAATTTC TTCCTTCGTT TTGTCTACCA ACCTAACTTT TCCTGTTGGG TATTCCGTAT   
  
  
- ATTATTAGTC GTCATTAATT CAAAGTGAAC AAAAACTATC TGAATTAATA TATACACGTC ACGTACAAAC   
  
  
- GTCTGGTCGT ACCGTTCGTA TTTTGAAGTG TTCTAGAGAA GGTTTCTAAC ATAAACATCT ACCGAAGTCT   
  
  
- GTACTGTGTT TCACGATGGG TGTTAGTTGA ATGACGACAG ACCATTTGCA ACTTTGACCA CAGGGTATTG   
  
  
- TGTGACATCA TGAGAGAGGG TGTGGAACAT TGTTCAAATG AAGTAAACCA AGAGACCATA AATTAAATCA   
  
  
- CTTCTTCTAG TAATTCGATG AGTTGTCCTT CGAAAAGTTC ATTTACGTTG AACATACTGT TGTAAGAAGA   
  
  
- AGACAGACAG AGTGTGGTGT CTGAAGTGAT CAATGTAGTA ATTAATCCGT ACATCCGGTT ACCAAATAGT   
  
  
- CGACATTATT CCACTATACT AGTTATACTT TTCTGGTCGA AGTTTAGTCT ATCCGACACC TAAATAAGGG   
  
  
- GTTGGTGCCT GAAATTAATC GGAGGTTCAC ATGTTATATA TACTTTACAC GTAACGAGTT ACCATATTAA   
  
  
- TTAATCGATC TTGTATGTAC GCCTATCACA GACACACGTC CCTCTCTATT TCTTTAGTCT CCCTCCCTCT   
  
  
- CAACAACATA TATACATACC TAGCTAGGAC CTACACGACT TAGTCTAATC TTTAGTTAAG TTGTTCGTTT   
  
  
- ATAGTAAGAG TAGAAAGTGT AGGTGTAGTA AGGTTGTACT TTTGATGTAG AAACAGGTCC TTGCCGGTGT   
  
  
- TCATCACACT TTCATATAGT CGTGAGTTTA ACGAAAAGTT ATTACTACGA TAGATAACAC TATTCTACAA   
  
  
- TAAATAATCC TTCTTTAACG TATATTGTTA GAACAAATTC TATTTGACCT GACACTTGGT TCTTCCTGGT   
  
  
- TTCTACCTGT TCGATACTTC ACTTTACAGA TACTAAAAGT GAGGCTTATA CAGTCTTATC TAACCAAAAC   
  
  
- TGCGTGGGCG TATGTCATTA TCGGTCAGTT CCTATATTTG CAATCACAAC GGAACACGAA GAAGGTACAT   
  
  
- AATGTCACGA ATTCTTTAGT CTGATATATA ATTAGTCATT GAGAAAAGAA CGCGTGTCGG TGTGTAGGGG   
  
  
- AATTATTAAG GAGTCGTGAT AGTTACAAAC TCTCCCGTGT CTCTCTCTCT CTCTCTCTCT CTCTCTCTCT   
  
  
- TTCTCTCCAC TCAGACGTTC AGAAAGGAGA CGCCATGGAC ACTCTTCTAT TCTTCCTTAT TGAACGTCTT   
  
  
- TTGTAAGGCA AATCGCAAAT GACCAACTGG TCCGAATGTA GAGTTTTGTT TCCCGATAAA GACGTCTGCT   
  
  
- AGCTTATCTC GTAGTTGAAC CAGTATGGAC GATAACTTGG TCTGGGAAAA CTGTCCTTCT TATATAATTC   
  
  
- TTCATCCCGA ACGACCCAAT AGCTTTCTT

+     AT-rich element

| Site Name | Organism | Position | Strand | Matrix score. | sequence | function |
| --- | --- | --- | --- | --- | --- | --- |
| AT-rich element | Glycine max | 747 | + | 10 | ATAGAAATCAA | binding site of AT-rich DNA binding protein (ATBP-1) |

> 2018/04/13 10:10:12  
+ GTTGCTTGCT TTAATTAAAG AAGGAAGCAA AACAGATGGT TGGATTGAAA AGGACAACCC ATAAGGCATA   
  
  
+ TAATAATCAG CAGTAATTAA GTTTCACTTG TTTTTGATAG ACTTAATTAT ATATGTGCAG TGCATGTTTG   
  
  
+ CAGACCAGCA TGGCAAGCAT AAAACTTCAC AAGATCTCTT CCAAAGATTG TATTTGTAGA TGGCTTCAGA   
  
  
+ CATGACACAA AGTGCTACCC ACAATCAACT TACTGCTGTC TGGTAAACGT TGAAACTGGT GTCCCATAAC   
  
  
+ ACACTGTAGT ACTCTCTCCC ACACCTTGTA ACAAGTTTAC TTCATTTGGT TCTCTGGTAT TTAATTTAGT   
  
  
+ GAAGAAGATC ATTAAGCTAC TCAACAGGAA GCTTTTCAAG TAAATGCAAC TTGTATGACA ACATTCTTCT   
  
  
+ TCTGTCTGTC TCACACCACA GACTTCACTA GTTACATCAT TAATTAGGCA TGTAGGCCAA TGGTTTATCA   
  
  
+ GCTGTAATAA GGTGATATGA TCAATATGAA AAGACCAGCT TCAAATCAGA TAGGCTGTGG ATTTATTCCC   
  
  
+ CAACCACGGA CTTTAATTAG CCTCCAAGTG TACAATATAT ATGAAATGTG CATTGCTCAA TGGTATAATT   
  
  
+ AATTAGCTAG AACATACATG CGGATAGTGT CTGTGTGCAG GGAGAGATAA AGAAATCAGA GGGAGGGAGA   
  
  
+ GTTGTTGTAT ATATGTATGG ATCGATCCTG GATGTGCTGA ATCAGATTAG AAATCAATTC AACAAGCAAA   
  
  
+ TATCATTCTC ATCTTTCACA TCCACATCAT TCCAACATGA AAACTACATC TTTGTCCAGG AACGGCCACA   
  
  
+ AGTAGTGTGA AAGTATATCA GCACTCAAAT TGCTTTTCAA TAATGATGCT ATCTATTGTG ATAAGATGTT   
  
  
+ ATTTATTAGG AAGAAATTGC ATATAACAAT CTTGTTTAAG ATAAACTGGA CTGTGAACCA AGAAGGACCA   
  
  
+ AAGATGGACA AGCTATGAAG TGAAATGTCT ATGATTTTCA CTCCGAATAT GTCAGAATAG ATTGGTTTTG   
  
  
+ ACGCACCCGC ATACAGTAAT AGCCAGTCAA GGATATAAAC GTTAGTGTTG CCTTGTGCTT CTTCCATGTA   
  
  
+ TTACAGTGCT TAAGAAATCA GACTATATAT TAATCAGTAA CTCTTTTCTT GCGCACAGCC ACACATCCCC   
  
  
+ TTAATAATTC CTCAGCACTA TCAATGTTTG AGAGGGCACA GAGAGAGAGA GAGAGAGAGA GAGAGAGAGA   
  
  
+ AAGAGAGGTG AGTCTGCAAG TCTTTCCTCT GCGGTACCTG TGAGAAGATA AGAAGGAATA ACTTGCAGAA   
  
  
+ AACATTCCGT TTAGCGTTTA CTGGTTGACC AGGCTTACAT CTCAAAACAA AGGGCTATTT CTGCAGACGA   
  
  
+ TCGAATAGAG CATCAACTTG GTCATACCTG CTATTGAACC AGACCCTTTT GACAGGAAGA ATATATTAAG   
  
  
+ AAGTAGGGCT TGCTGGGTTA TCGAAAGAA  

- CAACGAACGA AATTAATTTC TTCCTTCGTT TTGTCTACCA ACCTAACTTT TCCTGTTGGG TATTCCGTAT   
  
  
- ATTATTAGTC GTCATTAATT CAAAGTGAAC AAAAACTATC TGAATTAATA TATACACGTC ACGTACAAAC   
  
  
- GTCTGGTCGT ACCGTTCGTA TTTTGAAGTG TTCTAGAGAA GGTTTCTAAC ATAAACATCT ACCGAAGTCT   
  
  
- GTACTGTGTT TCACGATGGG TGTTAGTTGA ATGACGACAG ACCATTTGCA ACTTTGACCA CAGGGTATTG   
  
  
- TGTGACATCA TGAGAGAGGG TGTGGAACAT TGTTCAAATG AAGTAAACCA AGAGACCATA AATTAAATCA   
  
  
- CTTCTTCTAG TAATTCGATG AGTTGTCCTT CGAAAAGTTC ATTTACGTTG AACATACTGT TGTAAGAAGA   
  
  
- AGACAGACAG AGTGTGGTGT CTGAAGTGAT CAATGTAGTA ATTAATCCGT ACATCCGGTT ACCAAATAGT   
  
  
- CGACATTATT CCACTATACT AGTTATACTT TTCTGGTCGA AGTTTAGTCT ATCCGACACC TAAATAAGGG   
  
  
- GTTGGTGCCT GAAATTAATC GGAGGTTCAC ATGTTATATA TACTTTACAC GTAACGAGTT ACCATATTAA   
  
  
- TTAATCGATC TTGTATGTAC GCCTATCACA GACACACGTC CCTCTCTATT TCTTTAGTCT CCCTCCCTCT   
  
  
- CAACAACATA TATACATACC TAGCTAGGAC CTACACGACT TAGTCTAATC TTTAGTTAAG TTGTTCGTTT   
  
  
- ATAGTAAGAG TAGAAAGTGT AGGTGTAGTA AGGTTGTACT TTTGATGTAG AAACAGGTCC TTGCCGGTGT   
  
  
- TCATCACACT TTCATATAGT CGTGAGTTTA ACGAAAAGTT ATTACTACGA TAGATAACAC TATTCTACAA   
  
  
- TAAATAATCC TTCTTTAACG TATATTGTTA GAACAAATTC TATTTGACCT GACACTTGGT TCTTCCTGGT   
  
  
- TTCTACCTGT TCGATACTTC ACTTTACAGA TACTAAAAGT GAGGCTTATA CAGTCTTATC TAACCAAAAC   
  
  
- TGCGTGGGCG TATGTCATTA TCGGTCAGTT CCTATATTTG CAATCACAAC GGAACACGAA GAAGGTACAT   
  
  
- AATGTCACGA ATTCTTTAGT CTGATATATA ATTAGTCATT GAGAAAAGAA CGCGTGTCGG TGTGTAGGGG   
  
  
- AATTATTAAG GAGTCGTGAT AGTTACAAAC TCTCCCGTGT CTCTCTCTCT CTCTCTCTCT CTCTCTCTCT   
  
  
- TTCTCTCCAC TCAGACGTTC AGAAAGGAGA CGCCATGGAC ACTCTTCTAT TCTTCCTTAT TGAACGTCTT   
  
  
- TTGTAAGGCA AATCGCAAAT GACCAACTGG TCCGAATGTA GAGTTTTGTT TCCCGATAAA GACGTCTGCT   
  
  
- AGCTTATCTC GTAGTTGAAC CAGTATGGAC GATAACTTGG TCTGGGAAAA CTGTCCTTCT TATATAATTC   
  
  
- TTCATCCCGA ACGACCCAAT AGCTTTCTT

+     ATCT-motif

| Site Name | Organism | Position | Strand | Matrix score. | sequence | function |
| --- | --- | --- | --- | --- | --- | --- |
| ATCT-motif | Arabidopsis thaliana | 1034 | - | 9 | AATCTAATCT | part of a conserved DNA module involved in light responsiveness |

> 2018/04/13 10:10:12  
+ GTTGCTTGCT TTAATTAAAG AAGGAAGCAA AACAGATGGT TGGATTGAAA AGGACAACCC ATAAGGCATA   
  
  
+ TAATAATCAG CAGTAATTAA GTTTCACTTG TTTTTGATAG ACTTAATTAT ATATGTGCAG TGCATGTTTG   
  
  
+ CAGACCAGCA TGGCAAGCAT AAAACTTCAC AAGATCTCTT CCAAAGATTG TATTTGTAGA TGGCTTCAGA   
  
  
+ CATGACACAA AGTGCTACCC ACAATCAACT TACTGCTGTC TGGTAAACGT TGAAACTGGT GTCCCATAAC   
  
  
+ ACACTGTAGT ACTCTCTCCC ACACCTTGTA ACAAGTTTAC TTCATTTGGT TCTCTGGTAT TTAATTTAGT   
  
  
+ GAAGAAGATC ATTAAGCTAC TCAACAGGAA GCTTTTCAAG TAAATGCAAC TTGTATGACA ACATTCTTCT   
  
  
+ TCTGTCTGTC TCACACCACA GACTTCACTA GTTACATCAT TAATTAGGCA TGTAGGCCAA TGGTTTATCA   
  
  
+ GCTGTAATAA GGTGATATGA TCAATATGAA AAGACCAGCT TCAAATCAGA TAGGCTGTGG ATTTATTCCC   
  
  
+ CAACCACGGA CTTTAATTAG CCTCCAAGTG TACAATATAT ATGAAATGTG CATTGCTCAA TGGTATAATT   
  
  
+ AATTAGCTAG AACATACATG CGGATAGTGT CTGTGTGCAG GGAGAGATAA AGAAATCAGA GGGAGGGAGA   
  
  
+ GTTGTTGTAT ATATGTATGG ATCGATCCTG GATGTGCTGA ATCAGATTAG AAATCAATTC AACAAGCAAA   
  
  
+ TATCATTCTC ATCTTTCACA TCCACATCAT TCCAACATGA AAACTACATC TTTGTCCAGG AACGGCCACA   
  
  
+ AGTAGTGTGA AAGTATATCA GCACTCAAAT TGCTTTTCAA TAATGATGCT ATCTATTGTG ATAAGATGTT   
  
  
+ ATTTATTAGG AAGAAATTGC ATATAACAAT CTTGTTTAAG ATAAACTGGA CTGTGAACCA AGAAGGACCA   
  
  
+ AAGATGGACA AGCTATGAAG TGAAATGTCT ATGATTTTCA CTCCGAATAT GTCAGAATAG ATTGGTTTTG   
  
  
+ ACGCACCCGC ATACAGTAAT AGCCAGTCAA GGATATAAAC GTTAGTGTTG CCTTGTGCTT CTTCCATGTA   
  
  
+ TTACAGTGCT TAAGAAATCA GACTATATAT TAATCAGTAA CTCTTTTCTT GCGCACAGCC ACACATCCCC   
  
  
+ TTAATAATTC CTCAGCACTA TCAATGTTTG AGAGGGCACA GAGAGAGAGA GAGAGAGAGA GAGAGAGAGA   
  
  
+ AAGAGAGGTG AGTCTGCAAG TCTTTCCTCT GCGGTACCTG TGAGAAGATA AGAAGGAATA ACTTGCAGAA   
  
  
+ AACATTCCGT TTAGCGTTTA CTGGTTGACC AGGCTTACAT CTCAAAACAA AGGGCTATTT CTGCAGACGA   
  
  
+ TCGAATAGAG CATCAACTTG GTCATACCTG CTATTGAACC AGACCCTTTT GACAGGAAGA ATATATTAAG   
  
  
+ AAGTAGGGCT TGCTGGGTTA TCGAAAGAA  

- CAACGAACGA AATTAATTTC TTCCTTCGTT TTGTCTACCA ACCTAACTTT TCCTGTTGGG TATTCCGTAT   
  
  
- ATTATTAGTC GTCATTAATT CAAAGTGAAC AAAAACTATC TGAATTAATA TATACACGTC ACGTACAAAC   
  
  
- GTCTGGTCGT ACCGTTCGTA TTTTGAAGTG TTCTAGAGAA GGTTTCTAAC ATAAACATCT ACCGAAGTCT   
  
  
- GTACTGTGTT TCACGATGGG TGTTAGTTGA ATGACGACAG ACCATTTGCA ACTTTGACCA CAGGGTATTG   
  
  
- TGTGACATCA TGAGAGAGGG TGTGGAACAT TGTTCAAATG AAGTAAACCA AGAGACCATA AATTAAATCA   
  
  
- CTTCTTCTAG TAATTCGATG AGTTGTCCTT CGAAAAGTTC ATTTACGTTG AACATACTGT TGTAAGAAGA   
  
  
- AGACAGACAG AGTGTGGTGT CTGAAGTGAT CAATGTAGTA ATTAATCCGT ACATCCGGTT ACCAAATAGT   
  
  
- CGACATTATT CCACTATACT AGTTATACTT TTCTGGTCGA AGTTTAGTCT ATCCGACACC TAAATAAGGG   
  
  
- GTTGGTGCCT GAAATTAATC GGAGGTTCAC ATGTTATATA TACTTTACAC GTAACGAGTT ACCATATTAA   
  
  
- TTAATCGATC TTGTATGTAC GCCTATCACA GACACACGTC CCTCTCTATT TCTTTAGTCT CCCTCCCTCT   
  
  
- CAACAACATA TATACATACC TAGCTAGGAC CTACACGACT TAGTCTAATC TTTAGTTAAG TTGTTCGTTT   
  
  
- ATAGTAAGAG TAGAAAGTGT AGGTGTAGTA AGGTTGTACT TTTGATGTAG AAACAGGTCC TTGCCGGTGT   
  
  
- TCATCACACT TTCATATAGT CGTGAGTTTA ACGAAAAGTT ATTACTACGA TAGATAACAC TATTCTACAA   
  
  
- TAAATAATCC TTCTTTAACG TATATTGTTA GAACAAATTC TATTTGACCT GACACTTGGT TCTTCCTGGT   
  
  
- TTCTACCTGT TCGATACTTC ACTTTACAGA TACTAAAAGT GAGGCTTATA CAGTCTTATC TAACCAAAAC   
  
  
- TGCGTGGGCG TATGTCATTA TCGGTCAGTT CCTATATTTG CAATCACAAC GGAACACGAA GAAGGTACAT   
  
  
- AATGTCACGA ATTCTTTAGT CTGATATATA ATTAGTCATT GAGAAAAGAA CGCGTGTCGG TGTGTAGGGG   
  
  
- AATTATTAAG GAGTCGTGAT AGTTACAAAC TCTCCCGTGT CTCTCTCTCT CTCTCTCTCT CTCTCTCTCT   
  
  
- TTCTCTCCAC TCAGACGTTC AGAAAGGAGA CGCCATGGAC ACTCTTCTAT TCTTCCTTAT TGAACGTCTT   
  
  
- TTGTAAGGCA AATCGCAAAT GACCAACTGG TCCGAATGTA GAGTTTTGTT TCCCGATAAA GACGTCTGCT   
  
  
- AGCTTATCTC GTAGTTGAAC CAGTATGGAC GATAACTTGG TCTGGGAAAA CTGTCCTTCT TATATAATTC   
  
  
- TTCATCCCGA ACGACCCAAT AGCTTTCTT

+     Box 4

| Site Name | Organism | Position | Strand | Matrix score. | sequence | function |
| --- | --- | --- | --- | --- | --- | --- |
| Box 4 | Petroselinum crispum | 1149 | - | 6 | ATTAAT | part of a conserved DNA module involved in light responsiveness |
| Box 4 | Petroselinum crispum | 459 | + | 6 | ATTAAT | part of a conserved DNA module involved in light responsiveness |
| Box 4 | Petroselinum crispum | 628 | + | 6 | ATTAAT | part of a conserved DNA module involved in light responsiveness |

> 2018/04/13 10:10:12  
+ GTTGCTTGCT TTAATTAAAG AAGGAAGCAA AACAGATGGT TGGATTGAAA AGGACAACCC ATAAGGCATA   
  
  
+ TAATAATCAG CAGTAATTAA GTTTCACTTG TTTTTGATAG ACTTAATTAT ATATGTGCAG TGCATGTTTG   
  
  
+ CAGACCAGCA TGGCAAGCAT AAAACTTCAC AAGATCTCTT CCAAAGATTG TATTTGTAGA TGGCTTCAGA   
  
  
+ CATGACACAA AGTGCTACCC ACAATCAACT TACTGCTGTC TGGTAAACGT TGAAACTGGT GTCCCATAAC   
  
  
+ ACACTGTAGT ACTCTCTCCC ACACCTTGTA ACAAGTTTAC TTCATTTGGT TCTCTGGTAT TTAATTTAGT   
  
  
+ GAAGAAGATC ATTAAGCTAC TCAACAGGAA GCTTTTCAAG TAAATGCAAC TTGTATGACA ACATTCTTCT   
  
  
+ TCTGTCTGTC TCACACCACA GACTTCACTA GTTACATCAT TAATTAGGCA TGTAGGCCAA TGGTTTATCA   
  
  
+ GCTGTAATAA GGTGATATGA TCAATATGAA AAGACCAGCT TCAAATCAGA TAGGCTGTGG ATTTATTCCC   
  
  
+ CAACCACGGA CTTTAATTAG CCTCCAAGTG TACAATATAT ATGAAATGTG CATTGCTCAA TGGTATAATT   
  
  
+ AATTAGCTAG AACATACATG CGGATAGTGT CTGTGTGCAG GGAGAGATAA AGAAATCAGA GGGAGGGAGA   
  
  
+ GTTGTTGTAT ATATGTATGG ATCGATCCTG GATGTGCTGA ATCAGATTAG AAATCAATTC AACAAGCAAA   
  
  
+ TATCATTCTC ATCTTTCACA TCCACATCAT TCCAACATGA AAACTACATC TTTGTCCAGG AACGGCCACA   
  
  
+ AGTAGTGTGA AAGTATATCA GCACTCAAAT TGCTTTTCAA TAATGATGCT ATCTATTGTG ATAAGATGTT   
  
  
+ ATTTATTAGG AAGAAATTGC ATATAACAAT CTTGTTTAAG ATAAACTGGA CTGTGAACCA AGAAGGACCA   
  
  
+ AAGATGGACA AGCTATGAAG TGAAATGTCT ATGATTTTCA CTCCGAATAT GTCAGAATAG ATTGGTTTTG   
  
  
+ ACGCACCCGC ATACAGTAAT AGCCAGTCAA GGATATAAAC GTTAGTGTTG CCTTGTGCTT CTTCCATGTA   
  
  
+ TTACAGTGCT TAAGAAATCA GACTATATAT TAATCAGTAA CTCTTTTCTT GCGCACAGCC ACACATCCCC   
  
  
+ TTAATAATTC CTCAGCACTA TCAATGTTTG AGAGGGCACA GAGAGAGAGA GAGAGAGAGA GAGAGAGAGA   
  
  
+ AAGAGAGGTG AGTCTGCAAG TCTTTCCTCT GCGGTACCTG TGAGAAGATA AGAAGGAATA ACTTGCAGAA   
  
  
+ AACATTCCGT TTAGCGTTTA CTGGTTGACC AGGCTTACAT CTCAAAACAA AGGGCTATTT CTGCAGACGA   
  
  
+ TCGAATAGAG CATCAACTTG GTCATACCTG CTATTGAACC AGACCCTTTT GACAGGAAGA ATATATTAAG   
  
  
+ AAGTAGGGCT TGCTGGGTTA TCGAAAGAA  

- CAACGAACGA AATTAATTTC TTCCTTCGTT TTGTCTACCA ACCTAACTTT TCCTGTTGGG TATTCCGTAT   
  
  
- ATTATTAGTC GTCATTAATT CAAAGTGAAC AAAAACTATC TGAATTAATA TATACACGTC ACGTACAAAC   
  
  
- GTCTGGTCGT ACCGTTCGTA TTTTGAAGTG TTCTAGAGAA GGTTTCTAAC ATAAACATCT ACCGAAGTCT   
  
  
- GTACTGTGTT TCACGATGGG TGTTAGTTGA ATGACGACAG ACCATTTGCA ACTTTGACCA CAGGGTATTG   
  
  
- TGTGACATCA TGAGAGAGGG TGTGGAACAT TGTTCAAATG AAGTAAACCA AGAGACCATA AATTAAATCA   
  
  
- CTTCTTCTAG TAATTCGATG AGTTGTCCTT CGAAAAGTTC ATTTACGTTG AACATACTGT TGTAAGAAGA   
  
  
- AGACAGACAG AGTGTGGTGT CTGAAGTGAT CAATGTAGTA ATTAATCCGT ACATCCGGTT ACCAAATAGT   
  
  
- CGACATTATT CCACTATACT AGTTATACTT TTCTGGTCGA AGTTTAGTCT ATCCGACACC TAAATAAGGG   
  
  
- GTTGGTGCCT GAAATTAATC GGAGGTTCAC ATGTTATATA TACTTTACAC GTAACGAGTT ACCATATTAA   
  
  
- TTAATCGATC TTGTATGTAC GCCTATCACA GACACACGTC CCTCTCTATT TCTTTAGTCT CCCTCCCTCT   
  
  
- CAACAACATA TATACATACC TAGCTAGGAC CTACACGACT TAGTCTAATC TTTAGTTAAG TTGTTCGTTT   
  
  
- ATAGTAAGAG TAGAAAGTGT AGGTGTAGTA AGGTTGTACT TTTGATGTAG AAACAGGTCC TTGCCGGTGT   
  
  
- TCATCACACT TTCATATAGT CGTGAGTTTA ACGAAAAGTT ATTACTACGA TAGATAACAC TATTCTACAA   
  
  
- TAAATAATCC TTCTTTAACG TATATTGTTA GAACAAATTC TATTTGACCT GACACTTGGT TCTTCCTGGT   
  
  
- TTCTACCTGT TCGATACTTC ACTTTACAGA TACTAAAAGT GAGGCTTATA CAGTCTTATC TAACCAAAAC   
  
  
- TGCGTGGGCG TATGTCATTA TCGGTCAGTT CCTATATTTG CAATCACAAC GGAACACGAA GAAGGTACAT   
  
  
- AATGTCACGA ATTCTTTAGT CTGATATATA ATTAGTCATT GAGAAAAGAA CGCGTGTCGG TGTGTAGGGG   
  
  
- AATTATTAAG GAGTCGTGAT AGTTACAAAC TCTCCCGTGT CTCTCTCTCT CTCTCTCTCT CTCTCTCTCT   
  
  
- TTCTCTCCAC TCAGACGTTC AGAAAGGAGA CGCCATGGAC ACTCTTCTAT TCTTCCTTAT TGAACGTCTT   
  
  
- TTGTAAGGCA AATCGCAAAT GACCAACTGG TCCGAATGTA GAGTTTTGTT TCCCGATAAA GACGTCTGCT   
  
  
- AGCTTATCTC GTAGTTGAAC CAGTATGGAC GATAACTTGG TCTGGGAAAA CTGTCCTTCT TATATAATTC   
  
  
- TTCATCCCGA ACGACCCAAT AGCTTTCTT

+     Box III

| Site Name | Organism | Position | Strand | Matrix score. | sequence | function |
| --- | --- | --- | --- | --- | --- | --- |
| Box III | Pisum sativum | 1011 | + | 11 | atCATTTTCACt | protein binding site |

> 2018/04/13 10:10:12  
+ GTTGCTTGCT TTAATTAAAG AAGGAAGCAA AACAGATGGT TGGATTGAAA AGGACAACCC ATAAGGCATA   
  
  
+ TAATAATCAG CAGTAATTAA GTTTCACTTG TTTTTGATAG ACTTAATTAT ATATGTGCAG TGCATGTTTG   
  
  
+ CAGACCAGCA TGGCAAGCAT AAAACTTCAC AAGATCTCTT CCAAAGATTG TATTTGTAGA TGGCTTCAGA   
  
  
+ CATGACACAA AGTGCTACCC ACAATCAACT TACTGCTGTC TGGTAAACGT TGAAACTGGT GTCCCATAAC   
  
  
+ ACACTGTAGT ACTCTCTCCC ACACCTTGTA ACAAGTTTAC TTCATTTGGT TCTCTGGTAT TTAATTTAGT   
  
  
+ GAAGAAGATC ATTAAGCTAC TCAACAGGAA GCTTTTCAAG TAAATGCAAC TTGTATGACA ACATTCTTCT   
  
  
+ TCTGTCTGTC TCACACCACA GACTTCACTA GTTACATCAT TAATTAGGCA TGTAGGCCAA TGGTTTATCA   
  
  
+ GCTGTAATAA GGTGATATGA TCAATATGAA AAGACCAGCT TCAAATCAGA TAGGCTGTGG ATTTATTCCC   
  
  
+ CAACCACGGA CTTTAATTAG CCTCCAAGTG TACAATATAT ATGAAATGTG CATTGCTCAA TGGTATAATT   
  
  
+ AATTAGCTAG AACATACATG CGGATAGTGT CTGTGTGCAG GGAGAGATAA AGAAATCAGA GGGAGGGAGA   
  
  
+ GTTGTTGTAT ATATGTATGG ATCGATCCTG GATGTGCTGA ATCAGATTAG AAATCAATTC AACAAGCAAA   
  
  
+ TATCATTCTC ATCTTTCACA TCCACATCAT TCCAACATGA AAACTACATC TTTGTCCAGG AACGGCCACA   
  
  
+ AGTAGTGTGA AAGTATATCA GCACTCAAAT TGCTTTTCAA TAATGATGCT ATCTATTGTG ATAAGATGTT   
  
  
+ ATTTATTAGG AAGAAATTGC ATATAACAAT CTTGTTTAAG ATAAACTGGA CTGTGAACCA AGAAGGACCA   
  
  
+ AAGATGGACA AGCTATGAAG TGAAATGTCT ATGATTTTCA CTCCGAATAT GTCAGAATAG ATTGGTTTTG   
  
  
+ ACGCACCCGC ATACAGTAAT AGCCAGTCAA GGATATAAAC GTTAGTGTTG CCTTGTGCTT CTTCCATGTA   
  
  
+ TTACAGTGCT TAAGAAATCA GACTATATAT TAATCAGTAA CTCTTTTCTT GCGCACAGCC ACACATCCCC   
  
  
+ TTAATAATTC CTCAGCACTA TCAATGTTTG AGAGGGCACA GAGAGAGAGA GAGAGAGAGA GAGAGAGAGA   
  
  
+ AAGAGAGGTG AGTCTGCAAG TCTTTCCTCT GCGGTACCTG TGAGAAGATA AGAAGGAATA ACTTGCAGAA   
  
  
+ AACATTCCGT TTAGCGTTTA CTGGTTGACC AGGCTTACAT CTCAAAACAA AGGGCTATTT CTGCAGACGA   
  
  
+ TCGAATAGAG CATCAACTTG GTCATACCTG CTATTGAACC AGACCCTTTT GACAGGAAGA ATATATTAAG   
  
  
+ AAGTAGGGCT TGCTGGGTTA TCGAAAGAA  

- CAACGAACGA AATTAATTTC TTCCTTCGTT TTGTCTACCA ACCTAACTTT TCCTGTTGGG TATTCCGTAT   
  
  
- ATTATTAGTC GTCATTAATT CAAAGTGAAC AAAAACTATC TGAATTAATA TATACACGTC ACGTACAAAC   
  
  
- GTCTGGTCGT ACCGTTCGTA TTTTGAAGTG TTCTAGAGAA GGTTTCTAAC ATAAACATCT ACCGAAGTCT   
  
  
- GTACTGTGTT TCACGATGGG TGTTAGTTGA ATGACGACAG ACCATTTGCA ACTTTGACCA CAGGGTATTG   
  
  
- TGTGACATCA TGAGAGAGGG TGTGGAACAT TGTTCAAATG AAGTAAACCA AGAGACCATA AATTAAATCA   
  
  
- CTTCTTCTAG TAATTCGATG AGTTGTCCTT CGAAAAGTTC ATTTACGTTG AACATACTGT TGTAAGAAGA   
  
  
- AGACAGACAG AGTGTGGTGT CTGAAGTGAT CAATGTAGTA ATTAATCCGT ACATCCGGTT ACCAAATAGT   
  
  
- CGACATTATT CCACTATACT AGTTATACTT TTCTGGTCGA AGTTTAGTCT ATCCGACACC TAAATAAGGG   
  
  
- GTTGGTGCCT GAAATTAATC GGAGGTTCAC ATGTTATATA TACTTTACAC GTAACGAGTT ACCATATTAA   
  
  
- TTAATCGATC TTGTATGTAC GCCTATCACA GACACACGTC CCTCTCTATT TCTTTAGTCT CCCTCCCTCT   
  
  
- CAACAACATA TATACATACC TAGCTAGGAC CTACACGACT TAGTCTAATC TTTAGTTAAG TTGTTCGTTT   
  
  
- ATAGTAAGAG TAGAAAGTGT AGGTGTAGTA AGGTTGTACT TTTGATGTAG AAACAGGTCC TTGCCGGTGT   
  
  
- TCATCACACT TTCATATAGT CGTGAGTTTA ACGAAAAGTT ATTACTACGA TAGATAACAC TATTCTACAA   
  
  
- TAAATAATCC TTCTTTAACG TATATTGTTA GAACAAATTC TATTTGACCT GACACTTGGT TCTTCCTGGT   
  
  
- TTCTACCTGT TCGATACTTC ACTTTACAGA TACTAAAAGT GAGGCTTATA CAGTCTTATC TAACCAAAAC   
  
  
- TGCGTGGGCG TATGTCATTA TCGGTCAGTT CCTATATTTG CAATCACAAC GGAACACGAA GAAGGTACAT   
  
  
- AATGTCACGA ATTCTTTAGT CTGATATATA ATTAGTCATT GAGAAAAGAA CGCGTGTCGG TGTGTAGGGG   
  
  
- AATTATTAAG GAGTCGTGAT AGTTACAAAC TCTCCCGTGT CTCTCTCTCT CTCTCTCTCT CTCTCTCTCT   
  
  
- TTCTCTCCAC TCAGACGTTC AGAAAGGAGA CGCCATGGAC ACTCTTCTAT TCTTCCTTAT TGAACGTCTT   
  
  
- TTGTAAGGCA AATCGCAAAT GACCAACTGG TCCGAATGTA GAGTTTTGTT TCCCGATAAA GACGTCTGCT   
  
  
- AGCTTATCTC GTAGTTGAAC CAGTATGGAC GATAACTTGG TCTGGGAAAA CTGTCCTTCT TATATAATTC   
  
  
- TTCATCCCGA ACGACCCAAT AGCTTTCTT

+     Box-W1

| Site Name | Organism | Position | Strand | Matrix score. | sequence | function |
| --- | --- | --- | --- | --- | --- | --- |
| Box-W1 | Petroselinum crispum | 1355 | + | 6 | TTGACC | fungal elicitor responsive element |

> 2018/04/13 10:10:12  
+ GTTGCTTGCT TTAATTAAAG AAGGAAGCAA AACAGATGGT TGGATTGAAA AGGACAACCC ATAAGGCATA   
  
  
+ TAATAATCAG CAGTAATTAA GTTTCACTTG TTTTTGATAG ACTTAATTAT ATATGTGCAG TGCATGTTTG   
  
  
+ CAGACCAGCA TGGCAAGCAT AAAACTTCAC AAGATCTCTT CCAAAGATTG TATTTGTAGA TGGCTTCAGA   
  
  
+ CATGACACAA AGTGCTACCC ACAATCAACT TACTGCTGTC TGGTAAACGT TGAAACTGGT GTCCCATAAC   
  
  
+ ACACTGTAGT ACTCTCTCCC ACACCTTGTA ACAAGTTTAC TTCATTTGGT TCTCTGGTAT TTAATTTAGT   
  
  
+ GAAGAAGATC ATTAAGCTAC TCAACAGGAA GCTTTTCAAG TAAATGCAAC TTGTATGACA ACATTCTTCT   
  
  
+ TCTGTCTGTC TCACACCACA GACTTCACTA GTTACATCAT TAATTAGGCA TGTAGGCCAA TGGTTTATCA   
  
  
+ GCTGTAATAA GGTGATATGA TCAATATGAA AAGACCAGCT TCAAATCAGA TAGGCTGTGG ATTTATTCCC   
  
  
+ CAACCACGGA CTTTAATTAG CCTCCAAGTG TACAATATAT ATGAAATGTG CATTGCTCAA TGGTATAATT   
  
  
+ AATTAGCTAG AACATACATG CGGATAGTGT CTGTGTGCAG GGAGAGATAA AGAAATCAGA GGGAGGGAGA   
  
  
+ GTTGTTGTAT ATATGTATGG ATCGATCCTG GATGTGCTGA ATCAGATTAG AAATCAATTC AACAAGCAAA   
  
  
+ TATCATTCTC ATCTTTCACA TCCACATCAT TCCAACATGA AAACTACATC TTTGTCCAGG AACGGCCACA   
  
  
+ AGTAGTGTGA AAGTATATCA GCACTCAAAT TGCTTTTCAA TAATGATGCT ATCTATTGTG ATAAGATGTT   
  
  
+ ATTTATTAGG AAGAAATTGC ATATAACAAT CTTGTTTAAG ATAAACTGGA CTGTGAACCA AGAAGGACCA   
  
  
+ AAGATGGACA AGCTATGAAG TGAAATGTCT ATGATTTTCA CTCCGAATAT GTCAGAATAG ATTGGTTTTG   
  
  
+ ACGCACCCGC ATACAGTAAT AGCCAGTCAA GGATATAAAC GTTAGTGTTG CCTTGTGCTT CTTCCATGTA   
  
  
+ TTACAGTGCT TAAGAAATCA GACTATATAT TAATCAGTAA CTCTTTTCTT GCGCACAGCC ACACATCCCC   
  
  
+ TTAATAATTC CTCAGCACTA TCAATGTTTG AGAGGGCACA GAGAGAGAGA GAGAGAGAGA GAGAGAGAGA   
  
  
+ AAGAGAGGTG AGTCTGCAAG TCTTTCCTCT GCGGTACCTG TGAGAAGATA AGAAGGAATA ACTTGCAGAA   
  
  
+ AACATTCCGT TTAGCGTTTA CTGGTTGACC AGGCTTACAT CTCAAAACAA AGGGCTATTT CTGCAGACGA   
  
  
+ TCGAATAGAG CATCAACTTG GTCATACCTG CTATTGAACC AGACCCTTTT GACAGGAAGA ATATATTAAG   
  
  
+ AAGTAGGGCT TGCTGGGTTA TCGAAAGAA  

- CAACGAACGA AATTAATTTC TTCCTTCGTT TTGTCTACCA ACCTAACTTT TCCTGTTGGG TATTCCGTAT   
  
  
- ATTATTAGTC GTCATTAATT CAAAGTGAAC AAAAACTATC TGAATTAATA TATACACGTC ACGTACAAAC   
  
  
- GTCTGGTCGT ACCGTTCGTA TTTTGAAGTG TTCTAGAGAA GGTTTCTAAC ATAAACATCT ACCGAAGTCT   
  
  
- GTACTGTGTT TCACGATGGG TGTTAGTTGA ATGACGACAG ACCATTTGCA ACTTTGACCA CAGGGTATTG   
  
  
- TGTGACATCA TGAGAGAGGG TGTGGAACAT TGTTCAAATG AAGTAAACCA AGAGACCATA AATTAAATCA   
  
  
- CTTCTTCTAG TAATTCGATG AGTTGTCCTT CGAAAAGTTC ATTTACGTTG AACATACTGT TGTAAGAAGA   
  
  
- AGACAGACAG AGTGTGGTGT CTGAAGTGAT CAATGTAGTA ATTAATCCGT ACATCCGGTT ACCAAATAGT   
  
  
- CGACATTATT CCACTATACT AGTTATACTT TTCTGGTCGA AGTTTAGTCT ATCCGACACC TAAATAAGGG   
  
  
- GTTGGTGCCT GAAATTAATC GGAGGTTCAC ATGTTATATA TACTTTACAC GTAACGAGTT ACCATATTAA   
  
  
- TTAATCGATC TTGTATGTAC GCCTATCACA GACACACGTC CCTCTCTATT TCTTTAGTCT CCCTCCCTCT   
  
  
- CAACAACATA TATACATACC TAGCTAGGAC CTACACGACT TAGTCTAATC TTTAGTTAAG TTGTTCGTTT   
  
  
- ATAGTAAGAG TAGAAAGTGT AGGTGTAGTA AGGTTGTACT TTTGATGTAG AAACAGGTCC TTGCCGGTGT   
  
  
- TCATCACACT TTCATATAGT CGTGAGTTTA ACGAAAAGTT ATTACTACGA TAGATAACAC TATTCTACAA   
  
  
- TAAATAATCC TTCTTTAACG TATATTGTTA GAACAAATTC TATTTGACCT GACACTTGGT TCTTCCTGGT   
  
  
- TTCTACCTGT TCGATACTTC ACTTTACAGA TACTAAAAGT GAGGCTTATA CAGTCTTATC TAACCAAAAC   
  
  
- TGCGTGGGCG TATGTCATTA TCGGTCAGTT CCTATATTTG CAATCACAAC GGAACACGAA GAAGGTACAT   
  
  
- AATGTCACGA ATTCTTTAGT CTGATATATA ATTAGTCATT GAGAAAAGAA CGCGTGTCGG TGTGTAGGGG   
  
  
- AATTATTAAG GAGTCGTGAT AGTTACAAAC TCTCCCGTGT CTCTCTCTCT CTCTCTCTCT CTCTCTCTCT   
  
  
- TTCTCTCCAC TCAGACGTTC AGAAAGGAGA CGCCATGGAC ACTCTTCTAT TCTTCCTTAT TGAACGTCTT   
  
  
- TTGTAAGGCA AATCGCAAAT GACCAACTGG TCCGAATGTA GAGTTTTGTT TCCCGATAAA GACGTCTGCT   
  
  
- AGCTTATCTC GTAGTTGAAC CAGTATGGAC GATAACTTGG TCTGGGAAAA CTGTCCTTCT TATATAATTC   
  
  
- TTCATCCCGA ACGACCCAAT AGCTTTCTT

+     CAAT-box

| Site Name | Organism | Position | Strand | Matrix score. | sequence | function |
| --- | --- | --- | --- | --- | --- | --- |
| CAAT-box | Hordeum vulgare | 1433 | - | 4 | CAAT | common cis-acting element in promoter and enhancer regions |
| CAAT-box | Hordeum vulgare | 232 | + | 4 | CAAT | common cis-acting element in promoter and enhancer regions |
| CAAT-box | Brassica rapa | 767 | + | 5 | CAAAT | common cis-acting element in promoter and enhancer regions |
| CAAT-box | Hordeum vulgare | 895 | - | 4 | CAAT | common cis-acting element in promoter and enhancer regions |
| CAAT-box | Arabidopsis thaliana | 477 | + | 5 | CCAAT | common cis-acting element in promoter and enhancer regions |
| CAAT-box | Brassica rapa | 532 | + | 5 | CAAAT | common cis-acting element in promoter and enhancer regions |
| CAAT-box | Hordeum vulgare | 593 | + | 4 | CAAT | common cis-acting element in promoter and enhancer regions |
| CAAT-box | Hordeum vulgare | 512 | + | 4 | CAAT | common cis-acting element in promoter and enhancer regions |
| CAAT-box | Arabidopsis thaliana | 1041 | - | 5 | CCAAT | common cis-acting element in promoter and enhancer regions |
| CAAT-box | Hordeum vulgare | 926 | - | 4 | CAAT | common cis-acting element in promoter and enhancer regions |
| CAAT-box | Hordeum vulgare | 878 | + | 4 | CAAT | common cis-acting element in promoter and enhancer regions |
| CAAT-box | Glycine max | 755 | + | 5 | CAATT | common cis-acting element in promoter and enhancer regions |
| CAAT-box | Brassica rapa | 192 | - | 5 | CAAAT | common cis-acting element in promoter and enhancer regions |
| CAAT-box | Brassica rapa | 324 | - | 5 | CAAAT | common cis-acting element in promoter and enhancer regions |
| CAAT-box | Hordeum vulgare | 937 | + | 4 | CAAT | common cis-acting element in promoter and enhancer regions |
| CAAT-box | Glycine max | 925 | - | 5 | CAATT | common cis-acting element in promoter and enhancer regions |
| CAAT-box | Glycine max | 868 | - | 5 | CAATT | common cis-acting element in promoter and enhancer regions |
| CAAT-box | Hordeum vulgare | 612 | - | 4 | CAAT | common cis-acting element in promoter and enhancer regions |
| CAAT-box | Hordeum vulgare | 618 | + | 4 | CAAT | common cis-acting element in promoter and enhancer regions |
| CAAT-box | Hordeum vulgare | 478 | + | 4 | CAAT | common cis-acting element in promoter and enhancer regions |
| CAAT-box | Brassica rapa | 866 | + | 5 | CAAAT | common cis-acting element in promoter and enhancer regions |
| CAAT-box | Hordeum vulgare | 44 | - | 4 | CAAT | common cis-acting element in promoter and enhancer regions |
| CAAT-box | Hordeum vulgare | 869 | - | 4 | CAAT | common cis-acting element in promoter and enhancer regions |
| CAAT-box | Hordeum vulgare | 187 | - | 4 | CAAT | common cis-acting element in promoter and enhancer regions |
| CAAT-box | Hordeum vulgare | 1212 | + | 4 | CAAT | common cis-acting element in promoter and enhancer regions |

> 2018/04/13 10:10:12  
+ GTTGCTTGCT TTAATTAAAG AAGGAAGCAA AACAGATGGT TGGATTGAAA AGGACAACCC ATAAGGCATA   
  
  
+ TAATAATCAG CAGTAATTAA GTTTCACTTG TTTTTGATAG ACTTAATTAT ATATGTGCAG TGCATGTTTG   
  
  
+ CAGACCAGCA TGGCAAGCAT AAAACTTCAC AAGATCTCTT CCAAAGATTG TATTTGTAGA TGGCTTCAGA   
  
  
+ CATGACACAA AGTGCTACCC ACAATCAACT TACTGCTGTC TGGTAAACGT TGAAACTGGT GTCCCATAAC   
  
  
+ ACACTGTAGT ACTCTCTCCC ACACCTTGTA ACAAGTTTAC TTCATTTGGT TCTCTGGTAT TTAATTTAGT   
  
  
+ GAAGAAGATC ATTAAGCTAC TCAACAGGAA GCTTTTCAAG TAAATGCAAC TTGTATGACA ACATTCTTCT   
  
  
+ TCTGTCTGTC TCACACCACA GACTTCACTA GTTACATCAT TAATTAGGCA TGTAGGCCAA TGGTTTATCA   
  
  
+ GCTGTAATAA GGTGATATGA TCAATATGAA AAGACCAGCT TCAAATCAGA TAGGCTGTGG ATTTATTCCC   
  
  
+ CAACCACGGA CTTTAATTAG CCTCCAAGTG TACAATATAT ATGAAATGTG CATTGCTCAA TGGTATAATT   
  
  
+ AATTAGCTAG AACATACATG CGGATAGTGT CTGTGTGCAG GGAGAGATAA AGAAATCAGA GGGAGGGAGA   
  
  
+ GTTGTTGTAT ATATGTATGG ATCGATCCTG GATGTGCTGA ATCAGATTAG AAATCAATTC AACAAGCAAA   
  
  
+ TATCATTCTC ATCTTTCACA TCCACATCAT TCCAACATGA AAACTACATC TTTGTCCAGG AACGGCCACA   
  
  
+ AGTAGTGTGA AAGTATATCA GCACTCAAAT TGCTTTTCAA TAATGATGCT ATCTATTGTG ATAAGATGTT   
  
  
+ ATTTATTAGG AAGAAATTGC ATATAACAAT CTTGTTTAAG ATAAACTGGA CTGTGAACCA AGAAGGACCA   
  
  
+ AAGATGGACA AGCTATGAAG TGAAATGTCT ATGATTTTCA CTCCGAATAT GTCAGAATAG ATTGGTTTTG   
  
  
+ ACGCACCCGC ATACAGTAAT AGCCAGTCAA GGATATAAAC GTTAGTGTTG CCTTGTGCTT CTTCCATGTA   
  
  
+ TTACAGTGCT TAAGAAATCA GACTATATAT TAATCAGTAA CTCTTTTCTT GCGCACAGCC ACACATCCCC   
  
  
+ TTAATAATTC CTCAGCACTA TCAATGTTTG AGAGGGCACA GAGAGAGAGA GAGAGAGAGA GAGAGAGAGA   
  
  
+ AAGAGAGGTG AGTCTGCAAG TCTTTCCTCT GCGGTACCTG TGAGAAGATA AGAAGGAATA ACTTGCAGAA   
  
  
+ AACATTCCGT TTAGCGTTTA CTGGTTGACC AGGCTTACAT CTCAAAACAA AGGGCTATTT CTGCAGACGA   
  
  
+ TCGAATAGAG CATCAACTTG GTCATACCTG CTATTGAACC AGACCCTTTT GACAGGAAGA ATATATTAAG   
  
  
+ AAGTAGGGCT TGCTGGGTTA TCGAAAGAA  

- CAACGAACGA AATTAATTTC TTCCTTCGTT TTGTCTACCA ACCTAACTTT TCCTGTTGGG TATTCCGTAT   
  
  
- ATTATTAGTC GTCATTAATT CAAAGTGAAC AAAAACTATC TGAATTAATA TATACACGTC ACGTACAAAC   
  
  
- GTCTGGTCGT ACCGTTCGTA TTTTGAAGTG TTCTAGAGAA GGTTTCTAAC ATAAACATCT ACCGAAGTCT   
  
  
- GTACTGTGTT TCACGATGGG TGTTAGTTGA ATGACGACAG ACCATTTGCA ACTTTGACCA CAGGGTATTG   
  
  
- TGTGACATCA TGAGAGAGGG TGTGGAACAT TGTTCAAATG AAGTAAACCA AGAGACCATA AATTAAATCA   
  
  
- CTTCTTCTAG TAATTCGATG AGTTGTCCTT CGAAAAGTTC ATTTACGTTG AACATACTGT TGTAAGAAGA   
  
  
- AGACAGACAG AGTGTGGTGT CTGAAGTGAT CAATGTAGTA ATTAATCCGT ACATCCGGTT ACCAAATAGT   
  
  
- CGACATTATT CCACTATACT AGTTATACTT TTCTGGTCGA AGTTTAGTCT ATCCGACACC TAAATAAGGG   
  
  
- GTTGGTGCCT GAAATTAATC GGAGGTTCAC ATGTTATATA TACTTTACAC GTAACGAGTT ACCATATTAA   
  
  
- TTAATCGATC TTGTATGTAC GCCTATCACA GACACACGTC CCTCTCTATT TCTTTAGTCT CCCTCCCTCT   
  
  
- CAACAACATA TATACATACC TAGCTAGGAC CTACACGACT TAGTCTAATC TTTAGTTAAG TTGTTCGTTT   
  
  
- ATAGTAAGAG TAGAAAGTGT AGGTGTAGTA AGGTTGTACT TTTGATGTAG AAACAGGTCC TTGCCGGTGT   
  
  
- TCATCACACT TTCATATAGT CGTGAGTTTA ACGAAAAGTT ATTACTACGA TAGATAACAC TATTCTACAA   
  
  
- TAAATAATCC TTCTTTAACG TATATTGTTA GAACAAATTC TATTTGACCT GACACTTGGT TCTTCCTGGT   
  
  
- TTCTACCTGT TCGATACTTC ACTTTACAGA TACTAAAAGT GAGGCTTATA CAGTCTTATC TAACCAAAAC   
  
  
- TGCGTGGGCG TATGTCATTA TCGGTCAGTT CCTATATTTG CAATCACAAC GGAACACGAA GAAGGTACAT   
  
  
- AATGTCACGA ATTCTTTAGT CTGATATATA ATTAGTCATT GAGAAAAGAA CGCGTGTCGG TGTGTAGGGG   
  
  
- AATTATTAAG GAGTCGTGAT AGTTACAAAC TCTCCCGTGT CTCTCTCTCT CTCTCTCTCT CTCTCTCTCT   
  
  
- TTCTCTCCAC TCAGACGTTC AGAAAGGAGA CGCCATGGAC ACTCTTCTAT TCTTCCTTAT TGAACGTCTT   
  
  
- TTGTAAGGCA AATCGCAAAT GACCAACTGG TCCGAATGTA GAGTTTTGTT TCCCGATAAA GACGTCTGCT   
  
  
- AGCTTATCTC GTAGTTGAAC CAGTATGGAC GATAACTTGG TCTGGGAAAA CTGTCCTTCT TATATAATTC   
  
  
- TTCATCCCGA ACGACCCAAT AGCTTTCTT

+     CGTCA-motif

| Site Name | Organism | Position | Strand | Matrix score. | sequence | function |
| --- | --- | --- | --- | --- | --- | --- |
| CGTCA-motif | Hordeum vulgare | 1049 | - | 5 | CGTCA | cis-acting regulatory element involved in the MeJA-responsiveness |

> 2018/04/13 10:10:12  
+ GTTGCTTGCT TTAATTAAAG AAGGAAGCAA AACAGATGGT TGGATTGAAA AGGACAACCC ATAAGGCATA   
  
  
+ TAATAATCAG CAGTAATTAA GTTTCACTTG TTTTTGATAG ACTTAATTAT ATATGTGCAG TGCATGTTTG   
  
  
+ CAGACCAGCA TGGCAAGCAT AAAACTTCAC AAGATCTCTT CCAAAGATTG TATTTGTAGA TGGCTTCAGA   
  
  
+ CATGACACAA AGTGCTACCC ACAATCAACT TACTGCTGTC TGGTAAACGT TGAAACTGGT GTCCCATAAC   
  
  
+ ACACTGTAGT ACTCTCTCCC ACACCTTGTA ACAAGTTTAC TTCATTTGGT TCTCTGGTAT TTAATTTAGT   
  
  
+ GAAGAAGATC ATTAAGCTAC TCAACAGGAA GCTTTTCAAG TAAATGCAAC TTGTATGACA ACATTCTTCT   
  
  
+ TCTGTCTGTC TCACACCACA GACTTCACTA GTTACATCAT TAATTAGGCA TGTAGGCCAA TGGTTTATCA   
  
  
+ GCTGTAATAA GGTGATATGA TCAATATGAA AAGACCAGCT TCAAATCAGA TAGGCTGTGG ATTTATTCCC   
  
  
+ CAACCACGGA CTTTAATTAG CCTCCAAGTG TACAATATAT ATGAAATGTG CATTGCTCAA TGGTATAATT   
  
  
+ AATTAGCTAG AACATACATG CGGATAGTGT CTGTGTGCAG GGAGAGATAA AGAAATCAGA GGGAGGGAGA   
  
  
+ GTTGTTGTAT ATATGTATGG ATCGATCCTG GATGTGCTGA ATCAGATTAG AAATCAATTC AACAAGCAAA   
  
  
+ TATCATTCTC ATCTTTCACA TCCACATCAT TCCAACATGA AAACTACATC TTTGTCCAGG AACGGCCACA   
  
  
+ AGTAGTGTGA AAGTATATCA GCACTCAAAT TGCTTTTCAA TAATGATGCT ATCTATTGTG ATAAGATGTT   
  
  
+ ATTTATTAGG AAGAAATTGC ATATAACAAT CTTGTTTAAG ATAAACTGGA CTGTGAACCA AGAAGGACCA   
  
  
+ AAGATGGACA AGCTATGAAG TGAAATGTCT ATGATTTTCA CTCCGAATAT GTCAGAATAG ATTGGTTTTG   
  
  
+ ACGCACCCGC ATACAGTAAT AGCCAGTCAA GGATATAAAC GTTAGTGTTG CCTTGTGCTT CTTCCATGTA   
  
  
+ TTACAGTGCT TAAGAAATCA GACTATATAT TAATCAGTAA CTCTTTTCTT GCGCACAGCC ACACATCCCC   
  
  
+ TTAATAATTC CTCAGCACTA TCAATGTTTG AGAGGGCACA GAGAGAGAGA GAGAGAGAGA GAGAGAGAGA   
  
  
+ AAGAGAGGTG AGTCTGCAAG TCTTTCCTCT GCGGTACCTG TGAGAAGATA AGAAGGAATA ACTTGCAGAA   
  
  
+ AACATTCCGT TTAGCGTTTA CTGGTTGACC AGGCTTACAT CTCAAAACAA AGGGCTATTT CTGCAGACGA   
  
  
+ TCGAATAGAG CATCAACTTG GTCATACCTG CTATTGAACC AGACCCTTTT GACAGGAAGA ATATATTAAG   
  
  
+ AAGTAGGGCT TGCTGGGTTA TCGAAAGAA  

- CAACGAACGA AATTAATTTC TTCCTTCGTT TTGTCTACCA ACCTAACTTT TCCTGTTGGG TATTCCGTAT   
  
  
- ATTATTAGTC GTCATTAATT CAAAGTGAAC AAAAACTATC TGAATTAATA TATACACGTC ACGTACAAAC   
  
  
- GTCTGGTCGT ACCGTTCGTA TTTTGAAGTG TTCTAGAGAA GGTTTCTAAC ATAAACATCT ACCGAAGTCT   
  
  
- GTACTGTGTT TCACGATGGG TGTTAGTTGA ATGACGACAG ACCATTTGCA ACTTTGACCA CAGGGTATTG   
  
  
- TGTGACATCA TGAGAGAGGG TGTGGAACAT TGTTCAAATG AAGTAAACCA AGAGACCATA AATTAAATCA   
  
  
- CTTCTTCTAG TAATTCGATG AGTTGTCCTT CGAAAAGTTC ATTTACGTTG AACATACTGT TGTAAGAAGA   
  
  
- AGACAGACAG AGTGTGGTGT CTGAAGTGAT CAATGTAGTA ATTAATCCGT ACATCCGGTT ACCAAATAGT   
  
  
- CGACATTATT CCACTATACT AGTTATACTT TTCTGGTCGA AGTTTAGTCT ATCCGACACC TAAATAAGGG   
  
  
- GTTGGTGCCT GAAATTAATC GGAGGTTCAC ATGTTATATA TACTTTACAC GTAACGAGTT ACCATATTAA   
  
  
- TTAATCGATC TTGTATGTAC GCCTATCACA GACACACGTC CCTCTCTATT TCTTTAGTCT CCCTCCCTCT   
  
  
- CAACAACATA TATACATACC TAGCTAGGAC CTACACGACT TAGTCTAATC TTTAGTTAAG TTGTTCGTTT   
  
  
- ATAGTAAGAG TAGAAAGTGT AGGTGTAGTA AGGTTGTACT TTTGATGTAG AAACAGGTCC TTGCCGGTGT   
  
  
- TCATCACACT TTCATATAGT CGTGAGTTTA ACGAAAAGTT ATTACTACGA TAGATAACAC TATTCTACAA   
  
  
- TAAATAATCC TTCTTTAACG TATATTGTTA GAACAAATTC TATTTGACCT GACACTTGGT TCTTCCTGGT   
  
  
- TTCTACCTGT TCGATACTTC ACTTTACAGA TACTAAAAGT GAGGCTTATA CAGTCTTATC TAACCAAAAC   
  
  
- TGCGTGGGCG TATGTCATTA TCGGTCAGTT CCTATATTTG CAATCACAAC GGAACACGAA GAAGGTACAT   
  
  
- AATGTCACGA ATTCTTTAGT CTGATATATA ATTAGTCATT GAGAAAAGAA CGCGTGTCGG TGTGTAGGGG   
  
  
- AATTATTAAG GAGTCGTGAT AGTTACAAAC TCTCCCGTGT CTCTCTCTCT CTCTCTCTCT CTCTCTCTCT   
  
  
- TTCTCTCCAC TCAGACGTTC AGAAAGGAGA CGCCATGGAC ACTCTTCTAT TCTTCCTTAT TGAACGTCTT   
  
  
- TTGTAAGGCA AATCGCAAAT GACCAACTGG TCCGAATGTA GAGTTTTGTT TCCCGATAAA GACGTCTGCT   
  
  
- AGCTTATCTC GTAGTTGAAC CAGTATGGAC GATAACTTGG TCTGGGAAAA CTGTCCTTCT TATATAATTC   
  
  
- TTCATCCCGA ACGACCCAAT AGCTTTCTT

+     CTAG-motif

| Site Name | Organism | Position | Strand | Matrix score. | sequence | function |
| --- | --- | --- | --- | --- | --- | --- |
| CTAG-motif | Avena sativa | 1321 | + | 9 | ACTAGCAGAA |  |

> 2018/04/13 10:10:12  
+ GTTGCTTGCT TTAATTAAAG AAGGAAGCAA AACAGATGGT TGGATTGAAA AGGACAACCC ATAAGGCATA   
  
  
+ TAATAATCAG CAGTAATTAA GTTTCACTTG TTTTTGATAG ACTTAATTAT ATATGTGCAG TGCATGTTTG   
  
  
+ CAGACCAGCA TGGCAAGCAT AAAACTTCAC AAGATCTCTT CCAAAGATTG TATTTGTAGA TGGCTTCAGA   
  
  
+ CATGACACAA AGTGCTACCC ACAATCAACT TACTGCTGTC TGGTAAACGT TGAAACTGGT GTCCCATAAC   
  
  
+ ACACTGTAGT ACTCTCTCCC ACACCTTGTA ACAAGTTTAC TTCATTTGGT TCTCTGGTAT TTAATTTAGT   
  
  
+ GAAGAAGATC ATTAAGCTAC TCAACAGGAA GCTTTTCAAG TAAATGCAAC TTGTATGACA ACATTCTTCT   
  
  
+ TCTGTCTGTC TCACACCACA GACTTCACTA GTTACATCAT TAATTAGGCA TGTAGGCCAA TGGTTTATCA   
  
  
+ GCTGTAATAA GGTGATATGA TCAATATGAA AAGACCAGCT TCAAATCAGA TAGGCTGTGG ATTTATTCCC   
  
  
+ CAACCACGGA CTTTAATTAG CCTCCAAGTG TACAATATAT ATGAAATGTG CATTGCTCAA TGGTATAATT   
  
  
+ AATTAGCTAG AACATACATG CGGATAGTGT CTGTGTGCAG GGAGAGATAA AGAAATCAGA GGGAGGGAGA   
  
  
+ GTTGTTGTAT ATATGTATGG ATCGATCCTG GATGTGCTGA ATCAGATTAG AAATCAATTC AACAAGCAAA   
  
  
+ TATCATTCTC ATCTTTCACA TCCACATCAT TCCAACATGA AAACTACATC TTTGTCCAGG AACGGCCACA   
  
  
+ AGTAGTGTGA AAGTATATCA GCACTCAAAT TGCTTTTCAA TAATGATGCT ATCTATTGTG ATAAGATGTT   
  
  
+ ATTTATTAGG AAGAAATTGC ATATAACAAT CTTGTTTAAG ATAAACTGGA CTGTGAACCA AGAAGGACCA   
  
  
+ AAGATGGACA AGCTATGAAG TGAAATGTCT ATGATTTTCA CTCCGAATAT GTCAGAATAG ATTGGTTTTG   
  
  
+ ACGCACCCGC ATACAGTAAT AGCCAGTCAA GGATATAAAC GTTAGTGTTG CCTTGTGCTT CTTCCATGTA   
  
  
+ TTACAGTGCT TAAGAAATCA GACTATATAT TAATCAGTAA CTCTTTTCTT GCGCACAGCC ACACATCCCC   
  
  
+ TTAATAATTC CTCAGCACTA TCAATGTTTG AGAGGGCACA GAGAGAGAGA GAGAGAGAGA GAGAGAGAGA   
  
  
+ AAGAGAGGTG AGTCTGCAAG TCTTTCCTCT GCGGTACCTG TGAGAAGATA AGAAGGAATA ACTTGCAGAA   
  
  
+ AACATTCCGT TTAGCGTTTA CTGGTTGACC AGGCTTACAT CTCAAAACAA AGGGCTATTT CTGCAGACGA   
  
  
+ TCGAATAGAG CATCAACTTG GTCATACCTG CTATTGAACC AGACCCTTTT GACAGGAAGA ATATATTAAG   
  
  
+ AAGTAGGGCT TGCTGGGTTA TCGAAAGAA  

- CAACGAACGA AATTAATTTC TTCCTTCGTT TTGTCTACCA ACCTAACTTT TCCTGTTGGG TATTCCGTAT   
  
  
- ATTATTAGTC GTCATTAATT CAAAGTGAAC AAAAACTATC TGAATTAATA TATACACGTC ACGTACAAAC   
  
  
- GTCTGGTCGT ACCGTTCGTA TTTTGAAGTG TTCTAGAGAA GGTTTCTAAC ATAAACATCT ACCGAAGTCT   
  
  
- GTACTGTGTT TCACGATGGG TGTTAGTTGA ATGACGACAG ACCATTTGCA ACTTTGACCA CAGGGTATTG   
  
  
- TGTGACATCA TGAGAGAGGG TGTGGAACAT TGTTCAAATG AAGTAAACCA AGAGACCATA AATTAAATCA   
  
  
- CTTCTTCTAG TAATTCGATG AGTTGTCCTT CGAAAAGTTC ATTTACGTTG AACATACTGT TGTAAGAAGA   
  
  
- AGACAGACAG AGTGTGGTGT CTGAAGTGAT CAATGTAGTA ATTAATCCGT ACATCCGGTT ACCAAATAGT   
  
  
- CGACATTATT CCACTATACT AGTTATACTT TTCTGGTCGA AGTTTAGTCT ATCCGACACC TAAATAAGGG   
  
  
- GTTGGTGCCT GAAATTAATC GGAGGTTCAC ATGTTATATA TACTTTACAC GTAACGAGTT ACCATATTAA   
  
  
- TTAATCGATC TTGTATGTAC GCCTATCACA GACACACGTC CCTCTCTATT TCTTTAGTCT CCCTCCCTCT   
  
  
- CAACAACATA TATACATACC TAGCTAGGAC CTACACGACT TAGTCTAATC TTTAGTTAAG TTGTTCGTTT   
  
  
- ATAGTAAGAG TAGAAAGTGT AGGTGTAGTA AGGTTGTACT TTTGATGTAG AAACAGGTCC TTGCCGGTGT   
  
  
- TCATCACACT TTCATATAGT CGTGAGTTTA ACGAAAAGTT ATTACTACGA TAGATAACAC TATTCTACAA   
  
  
- TAAATAATCC TTCTTTAACG TATATTGTTA GAACAAATTC TATTTGACCT GACACTTGGT TCTTCCTGGT   
  
  
- TTCTACCTGT TCGATACTTC ACTTTACAGA TACTAAAAGT GAGGCTTATA CAGTCTTATC TAACCAAAAC   
  
  
- TGCGTGGGCG TATGTCATTA TCGGTCAGTT CCTATATTTG CAATCACAAC GGAACACGAA GAAGGTACAT   
  
  
- AATGTCACGA ATTCTTTAGT CTGATATATA ATTAGTCATT GAGAAAAGAA CGCGTGTCGG TGTGTAGGGG   
  
  
- AATTATTAAG GAGTCGTGAT AGTTACAAAC TCTCCCGTGT CTCTCTCTCT CTCTCTCTCT CTCTCTCTCT   
  
  
- TTCTCTCCAC TCAGACGTTC AGAAAGGAGA CGCCATGGAC ACTCTTCTAT TCTTCCTTAT TGAACGTCTT   
  
  
- TTGTAAGGCA AATCGCAAAT GACCAACTGG TCCGAATGTA GAGTTTTGTT TCCCGATAAA GACGTCTGCT   
  
  
- AGCTTATCTC GTAGTTGAAC CAGTATGGAC GATAACTTGG TCTGGGAAAA CTGTCCTTCT TATATAATTC   
  
  
- TTCATCCCGA ACGACCCAAT AGCTTTCTT

+     GA-motif

| Site Name | Organism | Position | Strand | Matrix score. | sequence | function |
| --- | --- | --- | --- | --- | --- | --- |
| GA-motif | Helianthus annuus | 779 | - | 8 | AAAGATGA | part of a light responsive element |

> 2018/04/13 10:10:12  
+ GTTGCTTGCT TTAATTAAAG AAGGAAGCAA AACAGATGGT TGGATTGAAA AGGACAACCC ATAAGGCATA   
  
  
+ TAATAATCAG CAGTAATTAA GTTTCACTTG TTTTTGATAG ACTTAATTAT ATATGTGCAG TGCATGTTTG   
  
  
+ CAGACCAGCA TGGCAAGCAT AAAACTTCAC AAGATCTCTT CCAAAGATTG TATTTGTAGA TGGCTTCAGA   
  
  
+ CATGACACAA AGTGCTACCC ACAATCAACT TACTGCTGTC TGGTAAACGT TGAAACTGGT GTCCCATAAC   
  
  
+ ACACTGTAGT ACTCTCTCCC ACACCTTGTA ACAAGTTTAC TTCATTTGGT TCTCTGGTAT TTAATTTAGT   
  
  
+ GAAGAAGATC ATTAAGCTAC TCAACAGGAA GCTTTTCAAG TAAATGCAAC TTGTATGACA ACATTCTTCT   
  
  
+ TCTGTCTGTC TCACACCACA GACTTCACTA GTTACATCAT TAATTAGGCA TGTAGGCCAA TGGTTTATCA   
  
  
+ GCTGTAATAA GGTGATATGA TCAATATGAA AAGACCAGCT TCAAATCAGA TAGGCTGTGG ATTTATTCCC   
  
  
+ CAACCACGGA CTTTAATTAG CCTCCAAGTG TACAATATAT ATGAAATGTG CATTGCTCAA TGGTATAATT   
  
  
+ AATTAGCTAG AACATACATG CGGATAGTGT CTGTGTGCAG GGAGAGATAA AGAAATCAGA GGGAGGGAGA   
  
  
+ GTTGTTGTAT ATATGTATGG ATCGATCCTG GATGTGCTGA ATCAGATTAG AAATCAATTC AACAAGCAAA   
  
  
+ TATCATTCTC ATCTTTCACA TCCACATCAT TCCAACATGA AAACTACATC TTTGTCCAGG AACGGCCACA   
  
  
+ AGTAGTGTGA AAGTATATCA GCACTCAAAT TGCTTTTCAA TAATGATGCT ATCTATTGTG ATAAGATGTT   
  
  
+ ATTTATTAGG AAGAAATTGC ATATAACAAT CTTGTTTAAG ATAAACTGGA CTGTGAACCA AGAAGGACCA   
  
  
+ AAGATGGACA AGCTATGAAG TGAAATGTCT ATGATTTTCA CTCCGAATAT GTCAGAATAG ATTGGTTTTG   
  
  
+ ACGCACCCGC ATACAGTAAT AGCCAGTCAA GGATATAAAC GTTAGTGTTG CCTTGTGCTT CTTCCATGTA   
  
  
+ TTACAGTGCT TAAGAAATCA GACTATATAT TAATCAGTAA CTCTTTTCTT GCGCACAGCC ACACATCCCC   
  
  
+ TTAATAATTC CTCAGCACTA TCAATGTTTG AGAGGGCACA GAGAGAGAGA GAGAGAGAGA GAGAGAGAGA   
  
  
+ AAGAGAGGTG AGTCTGCAAG TCTTTCCTCT GCGGTACCTG TGAGAAGATA AGAAGGAATA ACTTGCAGAA   
  
  
+ AACATTCCGT TTAGCGTTTA CTGGTTGACC AGGCTTACAT CTCAAAACAA AGGGCTATTT CTGCAGACGA   
  
  
+ TCGAATAGAG CATCAACTTG GTCATACCTG CTATTGAACC AGACCCTTTT GACAGGAAGA ATATATTAAG   
  
  
+ AAGTAGGGCT TGCTGGGTTA TCGAAAGAA  

- CAACGAACGA AATTAATTTC TTCCTTCGTT TTGTCTACCA ACCTAACTTT TCCTGTTGGG TATTCCGTAT   
  
  
- ATTATTAGTC GTCATTAATT CAAAGTGAAC AAAAACTATC TGAATTAATA TATACACGTC ACGTACAAAC   
  
  
- GTCTGGTCGT ACCGTTCGTA TTTTGAAGTG TTCTAGAGAA GGTTTCTAAC ATAAACATCT ACCGAAGTCT   
  
  
- GTACTGTGTT TCACGATGGG TGTTAGTTGA ATGACGACAG ACCATTTGCA ACTTTGACCA CAGGGTATTG   
  
  
- TGTGACATCA TGAGAGAGGG TGTGGAACAT TGTTCAAATG AAGTAAACCA AGAGACCATA AATTAAATCA   
  
  
- CTTCTTCTAG TAATTCGATG AGTTGTCCTT CGAAAAGTTC ATTTACGTTG AACATACTGT TGTAAGAAGA   
  
  
- AGACAGACAG AGTGTGGTGT CTGAAGTGAT CAATGTAGTA ATTAATCCGT ACATCCGGTT ACCAAATAGT   
  
  
- CGACATTATT CCACTATACT AGTTATACTT TTCTGGTCGA AGTTTAGTCT ATCCGACACC TAAATAAGGG   
  
  
- GTTGGTGCCT GAAATTAATC GGAGGTTCAC ATGTTATATA TACTTTACAC GTAACGAGTT ACCATATTAA   
  
  
- TTAATCGATC TTGTATGTAC GCCTATCACA GACACACGTC CCTCTCTATT TCTTTAGTCT CCCTCCCTCT   
  
  
- CAACAACATA TATACATACC TAGCTAGGAC CTACACGACT TAGTCTAATC TTTAGTTAAG TTGTTCGTTT   
  
  
- ATAGTAAGAG TAGAAAGTGT AGGTGTAGTA AGGTTGTACT TTTGATGTAG AAACAGGTCC TTGCCGGTGT   
  
  
- TCATCACACT TTCATATAGT CGTGAGTTTA ACGAAAAGTT ATTACTACGA TAGATAACAC TATTCTACAA   
  
  
- TAAATAATCC TTCTTTAACG TATATTGTTA GAACAAATTC TATTTGACCT GACACTTGGT TCTTCCTGGT   
  
  
- TTCTACCTGT TCGATACTTC ACTTTACAGA TACTAAAAGT GAGGCTTATA CAGTCTTATC TAACCAAAAC   
  
  
- TGCGTGGGCG TATGTCATTA TCGGTCAGTT CCTATATTTG CAATCACAAC GGAACACGAA GAAGGTACAT   
  
  
- AATGTCACGA ATTCTTTAGT CTGATATATA ATTAGTCATT GAGAAAAGAA CGCGTGTCGG TGTGTAGGGG   
  
  
- AATTATTAAG GAGTCGTGAT AGTTACAAAC TCTCCCGTGT CTCTCTCTCT CTCTCTCTCT CTCTCTCTCT   
  
  
- TTCTCTCCAC TCAGACGTTC AGAAAGGAGA CGCCATGGAC ACTCTTCTAT TCTTCCTTAT TGAACGTCTT   
  
  
- TTGTAAGGCA AATCGCAAAT GACCAACTGG TCCGAATGTA GAGTTTTGTT TCCCGATAAA GACGTCTGCT   
  
  
- AGCTTATCTC GTAGTTGAAC CAGTATGGAC GATAACTTGG TCTGGGAAAA CTGTCCTTCT TATATAATTC   
  
  
- TTCATCCCGA ACGACCCAAT AGCTTTCTT

+     GAG-motif

| Site Name | Organism | Position | Strand | Matrix score. | sequence | function |
| --- | --- | --- | --- | --- | --- | --- |
| GAG-motif | Arabidopsis thaliana | 291 | - | 7 | AGAGAGT | part of a light responsive element |

> 2018/04/13 10:10:12  
+ GTTGCTTGCT TTAATTAAAG AAGGAAGCAA AACAGATGGT TGGATTGAAA AGGACAACCC ATAAGGCATA   
  
  
+ TAATAATCAG CAGTAATTAA GTTTCACTTG TTTTTGATAG ACTTAATTAT ATATGTGCAG TGCATGTTTG   
  
  
+ CAGACCAGCA TGGCAAGCAT AAAACTTCAC AAGATCTCTT CCAAAGATTG TATTTGTAGA TGGCTTCAGA   
  
  
+ CATGACACAA AGTGCTACCC ACAATCAACT TACTGCTGTC TGGTAAACGT TGAAACTGGT GTCCCATAAC   
  
  
+ ACACTGTAGT ACTCTCTCCC ACACCTTGTA ACAAGTTTAC TTCATTTGGT TCTCTGGTAT TTAATTTAGT   
  
  
+ GAAGAAGATC ATTAAGCTAC TCAACAGGAA GCTTTTCAAG TAAATGCAAC TTGTATGACA ACATTCTTCT   
  
  
+ TCTGTCTGTC TCACACCACA GACTTCACTA GTTACATCAT TAATTAGGCA TGTAGGCCAA TGGTTTATCA   
  
  
+ GCTGTAATAA GGTGATATGA TCAATATGAA AAGACCAGCT TCAAATCAGA TAGGCTGTGG ATTTATTCCC   
  
  
+ CAACCACGGA CTTTAATTAG CCTCCAAGTG TACAATATAT ATGAAATGTG CATTGCTCAA TGGTATAATT   
  
  
+ AATTAGCTAG AACATACATG CGGATAGTGT CTGTGTGCAG GGAGAGATAA AGAAATCAGA GGGAGGGAGA   
  
  
+ GTTGTTGTAT ATATGTATGG ATCGATCCTG GATGTGCTGA ATCAGATTAG AAATCAATTC AACAAGCAAA   
  
  
+ TATCATTCTC ATCTTTCACA TCCACATCAT TCCAACATGA AAACTACATC TTTGTCCAGG AACGGCCACA   
  
  
+ AGTAGTGTGA AAGTATATCA GCACTCAAAT TGCTTTTCAA TAATGATGCT ATCTATTGTG ATAAGATGTT   
  
  
+ ATTTATTAGG AAGAAATTGC ATATAACAAT CTTGTTTAAG ATAAACTGGA CTGTGAACCA AGAAGGACCA   
  
  
+ AAGATGGACA AGCTATGAAG TGAAATGTCT ATGATTTTCA CTCCGAATAT GTCAGAATAG ATTGGTTTTG   
  
  
+ ACGCACCCGC ATACAGTAAT AGCCAGTCAA GGATATAAAC GTTAGTGTTG CCTTGTGCTT CTTCCATGTA   
  
  
+ TTACAGTGCT TAAGAAATCA GACTATATAT TAATCAGTAA CTCTTTTCTT GCGCACAGCC ACACATCCCC   
  
  
+ TTAATAATTC CTCAGCACTA TCAATGTTTG AGAGGGCACA GAGAGAGAGA GAGAGAGAGA GAGAGAGAGA   
  
  
+ AAGAGAGGTG AGTCTGCAAG TCTTTCCTCT GCGGTACCTG TGAGAAGATA AGAAGGAATA ACTTGCAGAA   
  
  
+ AACATTCCGT TTAGCGTTTA CTGGTTGACC AGGCTTACAT CTCAAAACAA AGGGCTATTT CTGCAGACGA   
  
  
+ TCGAATAGAG CATCAACTTG GTCATACCTG CTATTGAACC AGACCCTTTT GACAGGAAGA ATATATTAAG   
  
  
+ AAGTAGGGCT TGCTGGGTTA TCGAAAGAA  

- CAACGAACGA AATTAATTTC TTCCTTCGTT TTGTCTACCA ACCTAACTTT TCCTGTTGGG TATTCCGTAT   
  
  
- ATTATTAGTC GTCATTAATT CAAAGTGAAC AAAAACTATC TGAATTAATA TATACACGTC ACGTACAAAC   
  
  
- GTCTGGTCGT ACCGTTCGTA TTTTGAAGTG TTCTAGAGAA GGTTTCTAAC ATAAACATCT ACCGAAGTCT   
  
  
- GTACTGTGTT TCACGATGGG TGTTAGTTGA ATGACGACAG ACCATTTGCA ACTTTGACCA CAGGGTATTG   
  
  
- TGTGACATCA TGAGAGAGGG TGTGGAACAT TGTTCAAATG AAGTAAACCA AGAGACCATA AATTAAATCA   
  
  
- CTTCTTCTAG TAATTCGATG AGTTGTCCTT CGAAAAGTTC ATTTACGTTG AACATACTGT TGTAAGAAGA   
  
  
- AGACAGACAG AGTGTGGTGT CTGAAGTGAT CAATGTAGTA ATTAATCCGT ACATCCGGTT ACCAAATAGT   
  
  
- CGACATTATT CCACTATACT AGTTATACTT TTCTGGTCGA AGTTTAGTCT ATCCGACACC TAAATAAGGG   
  
  
- GTTGGTGCCT GAAATTAATC GGAGGTTCAC ATGTTATATA TACTTTACAC GTAACGAGTT ACCATATTAA   
  
  
- TTAATCGATC TTGTATGTAC GCCTATCACA GACACACGTC CCTCTCTATT TCTTTAGTCT CCCTCCCTCT   
  
  
- CAACAACATA TATACATACC TAGCTAGGAC CTACACGACT TAGTCTAATC TTTAGTTAAG TTGTTCGTTT   
  
  
- ATAGTAAGAG TAGAAAGTGT AGGTGTAGTA AGGTTGTACT TTTGATGTAG AAACAGGTCC TTGCCGGTGT   
  
  
- TCATCACACT TTCATATAGT CGTGAGTTTA ACGAAAAGTT ATTACTACGA TAGATAACAC TATTCTACAA   
  
  
- TAAATAATCC TTCTTTAACG TATATTGTTA GAACAAATTC TATTTGACCT GACACTTGGT TCTTCCTGGT   
  
  
- TTCTACCTGT TCGATACTTC ACTTTACAGA TACTAAAAGT GAGGCTTATA CAGTCTTATC TAACCAAAAC   
  
  
- TGCGTGGGCG TATGTCATTA TCGGTCAGTT CCTATATTTG CAATCACAAC GGAACACGAA GAAGGTACAT   
  
  
- AATGTCACGA ATTCTTTAGT CTGATATATA ATTAGTCATT GAGAAAAGAA CGCGTGTCGG TGTGTAGGGG   
  
  
- AATTATTAAG GAGTCGTGAT AGTTACAAAC TCTCCCGTGT CTCTCTCTCT CTCTCTCTCT CTCTCTCTCT   
  
  
- TTCTCTCCAC TCAGACGTTC AGAAAGGAGA CGCCATGGAC ACTCTTCTAT TCTTCCTTAT TGAACGTCTT   
  
  
- TTGTAAGGCA AATCGCAAAT GACCAACTGG TCCGAATGTA GAGTTTTGTT TCCCGATAAA GACGTCTGCT   
  
  
- AGCTTATCTC GTAGTTGAAC CAGTATGGAC GATAACTTGG TCTGGGAAAA CTGTCCTTCT TATATAATTC   
  
  
- TTCATCCCGA ACGACCCAAT AGCTTTCTT

+     GARE-motif

| Site Name | Organism | Position | Strand | Matrix score. | sequence | function |
| --- | --- | --- | --- | --- | --- | --- |
| GARE-motif | Brassica oleracea | 30 | + | 7 | AAACAGA | gibberellin-responsive element |

> 2018/04/13 10:10:12  
+ GTTGCTTGCT TTAATTAAAG AAGGAAGCAA AACAGATGGT TGGATTGAAA AGGACAACCC ATAAGGCATA   
  
  
+ TAATAATCAG CAGTAATTAA GTTTCACTTG TTTTTGATAG ACTTAATTAT ATATGTGCAG TGCATGTTTG   
  
  
+ CAGACCAGCA TGGCAAGCAT AAAACTTCAC AAGATCTCTT CCAAAGATTG TATTTGTAGA TGGCTTCAGA   
  
  
+ CATGACACAA AGTGCTACCC ACAATCAACT TACTGCTGTC TGGTAAACGT TGAAACTGGT GTCCCATAAC   
  
  
+ ACACTGTAGT ACTCTCTCCC ACACCTTGTA ACAAGTTTAC TTCATTTGGT TCTCTGGTAT TTAATTTAGT   
  
  
+ GAAGAAGATC ATTAAGCTAC TCAACAGGAA GCTTTTCAAG TAAATGCAAC TTGTATGACA ACATTCTTCT   
  
  
+ TCTGTCTGTC TCACACCACA GACTTCACTA GTTACATCAT TAATTAGGCA TGTAGGCCAA TGGTTTATCA   
  
  
+ GCTGTAATAA GGTGATATGA TCAATATGAA AAGACCAGCT TCAAATCAGA TAGGCTGTGG ATTTATTCCC   
  
  
+ CAACCACGGA CTTTAATTAG CCTCCAAGTG TACAATATAT ATGAAATGTG CATTGCTCAA TGGTATAATT   
  
  
+ AATTAGCTAG AACATACATG CGGATAGTGT CTGTGTGCAG GGAGAGATAA AGAAATCAGA GGGAGGGAGA   
  
  
+ GTTGTTGTAT ATATGTATGG ATCGATCCTG GATGTGCTGA ATCAGATTAG AAATCAATTC AACAAGCAAA   
  
  
+ TATCATTCTC ATCTTTCACA TCCACATCAT TCCAACATGA AAACTACATC TTTGTCCAGG AACGGCCACA   
  
  
+ AGTAGTGTGA AAGTATATCA GCACTCAAAT TGCTTTTCAA TAATGATGCT ATCTATTGTG ATAAGATGTT   
  
  
+ ATTTATTAGG AAGAAATTGC ATATAACAAT CTTGTTTAAG ATAAACTGGA CTGTGAACCA AGAAGGACCA   
  
  
+ AAGATGGACA AGCTATGAAG TGAAATGTCT ATGATTTTCA CTCCGAATAT GTCAGAATAG ATTGGTTTTG   
  
  
+ ACGCACCCGC ATACAGTAAT AGCCAGTCAA GGATATAAAC GTTAGTGTTG CCTTGTGCTT CTTCCATGTA   
  
  
+ TTACAGTGCT TAAGAAATCA GACTATATAT TAATCAGTAA CTCTTTTCTT GCGCACAGCC ACACATCCCC   
  
  
+ TTAATAATTC CTCAGCACTA TCAATGTTTG AGAGGGCACA GAGAGAGAGA GAGAGAGAGA GAGAGAGAGA   
  
  
+ AAGAGAGGTG AGTCTGCAAG TCTTTCCTCT GCGGTACCTG TGAGAAGATA AGAAGGAATA ACTTGCAGAA   
  
  
+ AACATTCCGT TTAGCGTTTA CTGGTTGACC AGGCTTACAT CTCAAAACAA AGGGCTATTT CTGCAGACGA   
  
  
+ TCGAATAGAG CATCAACTTG GTCATACCTG CTATTGAACC AGACCCTTTT GACAGGAAGA ATATATTAAG   
  
  
+ AAGTAGGGCT TGCTGGGTTA TCGAAAGAA  

- CAACGAACGA AATTAATTTC TTCCTTCGTT TTGTCTACCA ACCTAACTTT TCCTGTTGGG TATTCCGTAT   
  
  
- ATTATTAGTC GTCATTAATT CAAAGTGAAC AAAAACTATC TGAATTAATA TATACACGTC ACGTACAAAC   
  
  
- GTCTGGTCGT ACCGTTCGTA TTTTGAAGTG TTCTAGAGAA GGTTTCTAAC ATAAACATCT ACCGAAGTCT   
  
  
- GTACTGTGTT TCACGATGGG TGTTAGTTGA ATGACGACAG ACCATTTGCA ACTTTGACCA CAGGGTATTG   
  
  
- TGTGACATCA TGAGAGAGGG TGTGGAACAT TGTTCAAATG AAGTAAACCA AGAGACCATA AATTAAATCA   
  
  
- CTTCTTCTAG TAATTCGATG AGTTGTCCTT CGAAAAGTTC ATTTACGTTG AACATACTGT TGTAAGAAGA   
  
  
- AGACAGACAG AGTGTGGTGT CTGAAGTGAT CAATGTAGTA ATTAATCCGT ACATCCGGTT ACCAAATAGT   
  
  
- CGACATTATT CCACTATACT AGTTATACTT TTCTGGTCGA AGTTTAGTCT ATCCGACACC TAAATAAGGG   
  
  
- GTTGGTGCCT GAAATTAATC GGAGGTTCAC ATGTTATATA TACTTTACAC GTAACGAGTT ACCATATTAA   
  
  
- TTAATCGATC TTGTATGTAC GCCTATCACA GACACACGTC CCTCTCTATT TCTTTAGTCT CCCTCCCTCT   
  
  
- CAACAACATA TATACATACC TAGCTAGGAC CTACACGACT TAGTCTAATC TTTAGTTAAG TTGTTCGTTT   
  
  
- ATAGTAAGAG TAGAAAGTGT AGGTGTAGTA AGGTTGTACT TTTGATGTAG AAACAGGTCC TTGCCGGTGT   
  
  
- TCATCACACT TTCATATAGT CGTGAGTTTA ACGAAAAGTT ATTACTACGA TAGATAACAC TATTCTACAA   
  
  
- TAAATAATCC TTCTTTAACG TATATTGTTA GAACAAATTC TATTTGACCT GACACTTGGT TCTTCCTGGT   
  
  
- TTCTACCTGT TCGATACTTC ACTTTACAGA TACTAAAAGT GAGGCTTATA CAGTCTTATC TAACCAAAAC   
  
  
- TGCGTGGGCG TATGTCATTA TCGGTCAGTT CCTATATTTG CAATCACAAC GGAACACGAA GAAGGTACAT   
  
  
- AATGTCACGA ATTCTTTAGT CTGATATATA ATTAGTCATT GAGAAAAGAA CGCGTGTCGG TGTGTAGGGG   
  
  
- AATTATTAAG GAGTCGTGAT AGTTACAAAC TCTCCCGTGT CTCTCTCTCT CTCTCTCTCT CTCTCTCTCT   
  
  
- TTCTCTCCAC TCAGACGTTC AGAAAGGAGA CGCCATGGAC ACTCTTCTAT TCTTCCTTAT TGAACGTCTT   
  
  
- TTGTAAGGCA AATCGCAAAT GACCAACTGG TCCGAATGTA GAGTTTTGTT TCCCGATAAA GACGTCTGCT   
  
  
- AGCTTATCTC GTAGTTGAAC CAGTATGGAC GATAACTTGG TCTGGGAAAA CTGTCCTTCT TATATAATTC   
  
  
- TTCATCCCGA ACGACCCAAT AGCTTTCTT

+     GCN4\_motif

| Site Name | Organism | Position | Strand | Matrix score. | sequence | function |
| --- | --- | --- | --- | --- | --- | --- |
| GCN4\_motif | Oryza sativa | 213 | - | 7 | TGTGTCA | cis-regulatory element involved in endosperm expression |

> 2018/04/13 10:10:12  
+ GTTGCTTGCT TTAATTAAAG AAGGAAGCAA AACAGATGGT TGGATTGAAA AGGACAACCC ATAAGGCATA   
  
  
+ TAATAATCAG CAGTAATTAA GTTTCACTTG TTTTTGATAG ACTTAATTAT ATATGTGCAG TGCATGTTTG   
  
  
+ CAGACCAGCA TGGCAAGCAT AAAACTTCAC AAGATCTCTT CCAAAGATTG TATTTGTAGA TGGCTTCAGA   
  
  
+ CATGACACAA AGTGCTACCC ACAATCAACT TACTGCTGTC TGGTAAACGT TGAAACTGGT GTCCCATAAC   
  
  
+ ACACTGTAGT ACTCTCTCCC ACACCTTGTA ACAAGTTTAC TTCATTTGGT TCTCTGGTAT TTAATTTAGT   
  
  
+ GAAGAAGATC ATTAAGCTAC TCAACAGGAA GCTTTTCAAG TAAATGCAAC TTGTATGACA ACATTCTTCT   
  
  
+ TCTGTCTGTC TCACACCACA GACTTCACTA GTTACATCAT TAATTAGGCA TGTAGGCCAA TGGTTTATCA   
  
  
+ GCTGTAATAA GGTGATATGA TCAATATGAA AAGACCAGCT TCAAATCAGA TAGGCTGTGG ATTTATTCCC   
  
  
+ CAACCACGGA CTTTAATTAG CCTCCAAGTG TACAATATAT ATGAAATGTG CATTGCTCAA TGGTATAATT   
  
  
+ AATTAGCTAG AACATACATG CGGATAGTGT CTGTGTGCAG GGAGAGATAA AGAAATCAGA GGGAGGGAGA   
  
  
+ GTTGTTGTAT ATATGTATGG ATCGATCCTG GATGTGCTGA ATCAGATTAG AAATCAATTC AACAAGCAAA   
  
  
+ TATCATTCTC ATCTTTCACA TCCACATCAT TCCAACATGA AAACTACATC TTTGTCCAGG AACGGCCACA   
  
  
+ AGTAGTGTGA AAGTATATCA GCACTCAAAT TGCTTTTCAA TAATGATGCT ATCTATTGTG ATAAGATGTT   
  
  
+ ATTTATTAGG AAGAAATTGC ATATAACAAT CTTGTTTAAG ATAAACTGGA CTGTGAACCA AGAAGGACCA   
  
  
+ AAGATGGACA AGCTATGAAG TGAAATGTCT ATGATTTTCA CTCCGAATAT GTCAGAATAG ATTGGTTTTG   
  
  
+ ACGCACCCGC ATACAGTAAT AGCCAGTCAA GGATATAAAC GTTAGTGTTG CCTTGTGCTT CTTCCATGTA   
  
  
+ TTACAGTGCT TAAGAAATCA GACTATATAT TAATCAGTAA CTCTTTTCTT GCGCACAGCC ACACATCCCC   
  
  
+ TTAATAATTC CTCAGCACTA TCAATGTTTG AGAGGGCACA GAGAGAGAGA GAGAGAGAGA GAGAGAGAGA   
  
  
+ AAGAGAGGTG AGTCTGCAAG TCTTTCCTCT GCGGTACCTG TGAGAAGATA AGAAGGAATA ACTTGCAGAA   
  
  
+ AACATTCCGT TTAGCGTTTA CTGGTTGACC AGGCTTACAT CTCAAAACAA AGGGCTATTT CTGCAGACGA   
  
  
+ TCGAATAGAG CATCAACTTG GTCATACCTG CTATTGAACC AGACCCTTTT GACAGGAAGA ATATATTAAG   
  
  
+ AAGTAGGGCT TGCTGGGTTA TCGAAAGAA  

- CAACGAACGA AATTAATTTC TTCCTTCGTT TTGTCTACCA ACCTAACTTT TCCTGTTGGG TATTCCGTAT   
  
  
- ATTATTAGTC GTCATTAATT CAAAGTGAAC AAAAACTATC TGAATTAATA TATACACGTC ACGTACAAAC   
  
  
- GTCTGGTCGT ACCGTTCGTA TTTTGAAGTG TTCTAGAGAA GGTTTCTAAC ATAAACATCT ACCGAAGTCT   
  
  
- GTACTGTGTT TCACGATGGG TGTTAGTTGA ATGACGACAG ACCATTTGCA ACTTTGACCA CAGGGTATTG   
  
  
- TGTGACATCA TGAGAGAGGG TGTGGAACAT TGTTCAAATG AAGTAAACCA AGAGACCATA AATTAAATCA   
  
  
- CTTCTTCTAG TAATTCGATG AGTTGTCCTT CGAAAAGTTC ATTTACGTTG AACATACTGT TGTAAGAAGA   
  
  
- AGACAGACAG AGTGTGGTGT CTGAAGTGAT CAATGTAGTA ATTAATCCGT ACATCCGGTT ACCAAATAGT   
  
  
- CGACATTATT CCACTATACT AGTTATACTT TTCTGGTCGA AGTTTAGTCT ATCCGACACC TAAATAAGGG   
  
  
- GTTGGTGCCT GAAATTAATC GGAGGTTCAC ATGTTATATA TACTTTACAC GTAACGAGTT ACCATATTAA   
  
  
- TTAATCGATC TTGTATGTAC GCCTATCACA GACACACGTC CCTCTCTATT TCTTTAGTCT CCCTCCCTCT   
  
  
- CAACAACATA TATACATACC TAGCTAGGAC CTACACGACT TAGTCTAATC TTTAGTTAAG TTGTTCGTTT   
  
  
- ATAGTAAGAG TAGAAAGTGT AGGTGTAGTA AGGTTGTACT TTTGATGTAG AAACAGGTCC TTGCCGGTGT   
  
  
- TCATCACACT TTCATATAGT CGTGAGTTTA ACGAAAAGTT ATTACTACGA TAGATAACAC TATTCTACAA   
  
  
- TAAATAATCC TTCTTTAACG TATATTGTTA GAACAAATTC TATTTGACCT GACACTTGGT TCTTCCTGGT   
  
  
- TTCTACCTGT TCGATACTTC ACTTTACAGA TACTAAAAGT GAGGCTTATA CAGTCTTATC TAACCAAAAC   
  
  
- TGCGTGGGCG TATGTCATTA TCGGTCAGTT CCTATATTTG CAATCACAAC GGAACACGAA GAAGGTACAT   
  
  
- AATGTCACGA ATTCTTTAGT CTGATATATA ATTAGTCATT GAGAAAAGAA CGCGTGTCGG TGTGTAGGGG   
  
  
- AATTATTAAG GAGTCGTGAT AGTTACAAAC TCTCCCGTGT CTCTCTCTCT CTCTCTCTCT CTCTCTCTCT   
  
  
- TTCTCTCCAC TCAGACGTTC AGAAAGGAGA CGCCATGGAC ACTCTTCTAT TCTTCCTTAT TGAACGTCTT   
  
  
- TTGTAAGGCA AATCGCAAAT GACCAACTGG TCCGAATGTA GAGTTTTGTT TCCCGATAAA GACGTCTGCT   
  
  
- AGCTTATCTC GTAGTTGAAC CAGTATGGAC GATAACTTGG TCTGGGAAAA CTGTCCTTCT TATATAATTC   
  
  
- TTCATCCCGA ACGACCCAAT AGCTTTCTT

+     GT1-motif

| Site Name | Organism | Position | Strand | Matrix score. | sequence | function |
| --- | --- | --- | --- | --- | --- | --- |
| GT1-motif | Solanum tuberosum | 546 | - | 8 | AATCCACA | light responsive element |

> 2018/04/13 10:10:12  
+ GTTGCTTGCT TTAATTAAAG AAGGAAGCAA AACAGATGGT TGGATTGAAA AGGACAACCC ATAAGGCATA   
  
  
+ TAATAATCAG CAGTAATTAA GTTTCACTTG TTTTTGATAG ACTTAATTAT ATATGTGCAG TGCATGTTTG   
  
  
+ CAGACCAGCA TGGCAAGCAT AAAACTTCAC AAGATCTCTT CCAAAGATTG TATTTGTAGA TGGCTTCAGA   
  
  
+ CATGACACAA AGTGCTACCC ACAATCAACT TACTGCTGTC TGGTAAACGT TGAAACTGGT GTCCCATAAC   
  
  
+ ACACTGTAGT ACTCTCTCCC ACACCTTGTA ACAAGTTTAC TTCATTTGGT TCTCTGGTAT TTAATTTAGT   
  
  
+ GAAGAAGATC ATTAAGCTAC TCAACAGGAA GCTTTTCAAG TAAATGCAAC TTGTATGACA ACATTCTTCT   
  
  
+ TCTGTCTGTC TCACACCACA GACTTCACTA GTTACATCAT TAATTAGGCA TGTAGGCCAA TGGTTTATCA   
  
  
+ GCTGTAATAA GGTGATATGA TCAATATGAA AAGACCAGCT TCAAATCAGA TAGGCTGTGG ATTTATTCCC   
  
  
+ CAACCACGGA CTTTAATTAG CCTCCAAGTG TACAATATAT ATGAAATGTG CATTGCTCAA TGGTATAATT   
  
  
+ AATTAGCTAG AACATACATG CGGATAGTGT CTGTGTGCAG GGAGAGATAA AGAAATCAGA GGGAGGGAGA   
  
  
+ GTTGTTGTAT ATATGTATGG ATCGATCCTG GATGTGCTGA ATCAGATTAG AAATCAATTC AACAAGCAAA   
  
  
+ TATCATTCTC ATCTTTCACA TCCACATCAT TCCAACATGA AAACTACATC TTTGTCCAGG AACGGCCACA   
  
  
+ AGTAGTGTGA AAGTATATCA GCACTCAAAT TGCTTTTCAA TAATGATGCT ATCTATTGTG ATAAGATGTT   
  
  
+ ATTTATTAGG AAGAAATTGC ATATAACAAT CTTGTTTAAG ATAAACTGGA CTGTGAACCA AGAAGGACCA   
  
  
+ AAGATGGACA AGCTATGAAG TGAAATGTCT ATGATTTTCA CTCCGAATAT GTCAGAATAG ATTGGTTTTG   
  
  
+ ACGCACCCGC ATACAGTAAT AGCCAGTCAA GGATATAAAC GTTAGTGTTG CCTTGTGCTT CTTCCATGTA   
  
  
+ TTACAGTGCT TAAGAAATCA GACTATATAT TAATCAGTAA CTCTTTTCTT GCGCACAGCC ACACATCCCC   
  
  
+ TTAATAATTC CTCAGCACTA TCAATGTTTG AGAGGGCACA GAGAGAGAGA GAGAGAGAGA GAGAGAGAGA   
  
  
+ AAGAGAGGTG AGTCTGCAAG TCTTTCCTCT GCGGTACCTG TGAGAAGATA AGAAGGAATA ACTTGCAGAA   
  
  
+ AACATTCCGT TTAGCGTTTA CTGGTTGACC AGGCTTACAT CTCAAAACAA AGGGCTATTT CTGCAGACGA   
  
  
+ TCGAATAGAG CATCAACTTG GTCATACCTG CTATTGAACC AGACCCTTTT GACAGGAAGA ATATATTAAG   
  
  
+ AAGTAGGGCT TGCTGGGTTA TCGAAAGAA  

- CAACGAACGA AATTAATTTC TTCCTTCGTT TTGTCTACCA ACCTAACTTT TCCTGTTGGG TATTCCGTAT   
  
  
- ATTATTAGTC GTCATTAATT CAAAGTGAAC AAAAACTATC TGAATTAATA TATACACGTC ACGTACAAAC   
  
  
- GTCTGGTCGT ACCGTTCGTA TTTTGAAGTG TTCTAGAGAA GGTTTCTAAC ATAAACATCT ACCGAAGTCT   
  
  
- GTACTGTGTT TCACGATGGG TGTTAGTTGA ATGACGACAG ACCATTTGCA ACTTTGACCA CAGGGTATTG   
  
  
- TGTGACATCA TGAGAGAGGG TGTGGAACAT TGTTCAAATG AAGTAAACCA AGAGACCATA AATTAAATCA   
  
  
- CTTCTTCTAG TAATTCGATG AGTTGTCCTT CGAAAAGTTC ATTTACGTTG AACATACTGT TGTAAGAAGA   
  
  
- AGACAGACAG AGTGTGGTGT CTGAAGTGAT CAATGTAGTA ATTAATCCGT ACATCCGGTT ACCAAATAGT   
  
  
- CGACATTATT CCACTATACT AGTTATACTT TTCTGGTCGA AGTTTAGTCT ATCCGACACC TAAATAAGGG   
  
  
- GTTGGTGCCT GAAATTAATC GGAGGTTCAC ATGTTATATA TACTTTACAC GTAACGAGTT ACCATATTAA   
  
  
- TTAATCGATC TTGTATGTAC GCCTATCACA GACACACGTC CCTCTCTATT TCTTTAGTCT CCCTCCCTCT   
  
  
- CAACAACATA TATACATACC TAGCTAGGAC CTACACGACT TAGTCTAATC TTTAGTTAAG TTGTTCGTTT   
  
  
- ATAGTAAGAG TAGAAAGTGT AGGTGTAGTA AGGTTGTACT TTTGATGTAG AAACAGGTCC TTGCCGGTGT   
  
  
- TCATCACACT TTCATATAGT CGTGAGTTTA ACGAAAAGTT ATTACTACGA TAGATAACAC TATTCTACAA   
  
  
- TAAATAATCC TTCTTTAACG TATATTGTTA GAACAAATTC TATTTGACCT GACACTTGGT TCTTCCTGGT   
  
  
- TTCTACCTGT TCGATACTTC ACTTTACAGA TACTAAAAGT GAGGCTTATA CAGTCTTATC TAACCAAAAC   
  
  
- TGCGTGGGCG TATGTCATTA TCGGTCAGTT CCTATATTTG CAATCACAAC GGAACACGAA GAAGGTACAT   
  
  
- AATGTCACGA ATTCTTTAGT CTGATATATA ATTAGTCATT GAGAAAAGAA CGCGTGTCGG TGTGTAGGGG   
  
  
- AATTATTAAG GAGTCGTGAT AGTTACAAAC TCTCCCGTGT CTCTCTCTCT CTCTCTCTCT CTCTCTCTCT   
  
  
- TTCTCTCCAC TCAGACGTTC AGAAAGGAGA CGCCATGGAC ACTCTTCTAT TCTTCCTTAT TGAACGTCTT   
  
  
- TTGTAAGGCA AATCGCAAAT GACCAACTGG TCCGAATGTA GAGTTTTGTT TCCCGATAAA GACGTCTGCT   
  
  
- AGCTTATCTC GTAGTTGAAC CAGTATGGAC GATAACTTGG TCTGGGAAAA CTGTCCTTCT TATATAATTC   
  
  
- TTCATCCCGA ACGACCCAAT AGCTTTCTT

+     I-box

| Site Name | Organism | Position | Strand | Matrix score. | sequence | function |
| --- | --- | --- | --- | --- | --- | --- |
| I-box | Triticum aestivum | 1305 | + | 9 | aAGATAAGA | part of a light responsive element |

> 2018/04/13 10:10:12  
+ GTTGCTTGCT TTAATTAAAG AAGGAAGCAA AACAGATGGT TGGATTGAAA AGGACAACCC ATAAGGCATA   
  
  
+ TAATAATCAG CAGTAATTAA GTTTCACTTG TTTTTGATAG ACTTAATTAT ATATGTGCAG TGCATGTTTG   
  
  
+ CAGACCAGCA TGGCAAGCAT AAAACTTCAC AAGATCTCTT CCAAAGATTG TATTTGTAGA TGGCTTCAGA   
  
  
+ CATGACACAA AGTGCTACCC ACAATCAACT TACTGCTGTC TGGTAAACGT TGAAACTGGT GTCCCATAAC   
  
  
+ ACACTGTAGT ACTCTCTCCC ACACCTTGTA ACAAGTTTAC TTCATTTGGT TCTCTGGTAT TTAATTTAGT   
  
  
+ GAAGAAGATC ATTAAGCTAC TCAACAGGAA GCTTTTCAAG TAAATGCAAC TTGTATGACA ACATTCTTCT   
  
  
+ TCTGTCTGTC TCACACCACA GACTTCACTA GTTACATCAT TAATTAGGCA TGTAGGCCAA TGGTTTATCA   
  
  
+ GCTGTAATAA GGTGATATGA TCAATATGAA AAGACCAGCT TCAAATCAGA TAGGCTGTGG ATTTATTCCC   
  
  
+ CAACCACGGA CTTTAATTAG CCTCCAAGTG TACAATATAT ATGAAATGTG CATTGCTCAA TGGTATAATT   
  
  
+ AATTAGCTAG AACATACATG CGGATAGTGT CTGTGTGCAG GGAGAGATAA AGAAATCAGA GGGAGGGAGA   
  
  
+ GTTGTTGTAT ATATGTATGG ATCGATCCTG GATGTGCTGA ATCAGATTAG AAATCAATTC AACAAGCAAA   
  
  
+ TATCATTCTC ATCTTTCACA TCCACATCAT TCCAACATGA AAACTACATC TTTGTCCAGG AACGGCCACA   
  
  
+ AGTAGTGTGA AAGTATATCA GCACTCAAAT TGCTTTTCAA TAATGATGCT ATCTATTGTG ATAAGATGTT   
  
  
+ ATTTATTAGG AAGAAATTGC ATATAACAAT CTTGTTTAAG ATAAACTGGA CTGTGAACCA AGAAGGACCA   
  
  
+ AAGATGGACA AGCTATGAAG TGAAATGTCT ATGATTTTCA CTCCGAATAT GTCAGAATAG ATTGGTTTTG   
  
  
+ ACGCACCCGC ATACAGTAAT AGCCAGTCAA GGATATAAAC GTTAGTGTTG CCTTGTGCTT CTTCCATGTA   
  
  
+ TTACAGTGCT TAAGAAATCA GACTATATAT TAATCAGTAA CTCTTTTCTT GCGCACAGCC ACACATCCCC   
  
  
+ TTAATAATTC CTCAGCACTA TCAATGTTTG AGAGGGCACA GAGAGAGAGA GAGAGAGAGA GAGAGAGAGA   
  
  
+ AAGAGAGGTG AGTCTGCAAG TCTTTCCTCT GCGGTACCTG TGAGAAGATA AGAAGGAATA ACTTGCAGAA   
  
  
+ AACATTCCGT TTAGCGTTTA CTGGTTGACC AGGCTTACAT CTCAAAACAA AGGGCTATTT CTGCAGACGA   
  
  
+ TCGAATAGAG CATCAACTTG GTCATACCTG CTATTGAACC AGACCCTTTT GACAGGAAGA ATATATTAAG   
  
  
+ AAGTAGGGCT TGCTGGGTTA TCGAAAGAA  

- CAACGAACGA AATTAATTTC TTCCTTCGTT TTGTCTACCA ACCTAACTTT TCCTGTTGGG TATTCCGTAT   
  
  
- ATTATTAGTC GTCATTAATT CAAAGTGAAC AAAAACTATC TGAATTAATA TATACACGTC ACGTACAAAC   
  
  
- GTCTGGTCGT ACCGTTCGTA TTTTGAAGTG TTCTAGAGAA GGTTTCTAAC ATAAACATCT ACCGAAGTCT   
  
  
- GTACTGTGTT TCACGATGGG TGTTAGTTGA ATGACGACAG ACCATTTGCA ACTTTGACCA CAGGGTATTG   
  
  
- TGTGACATCA TGAGAGAGGG TGTGGAACAT TGTTCAAATG AAGTAAACCA AGAGACCATA AATTAAATCA   
  
  
- CTTCTTCTAG TAATTCGATG AGTTGTCCTT CGAAAAGTTC ATTTACGTTG AACATACTGT TGTAAGAAGA   
  
  
- AGACAGACAG AGTGTGGTGT CTGAAGTGAT CAATGTAGTA ATTAATCCGT ACATCCGGTT ACCAAATAGT   
  
  
- CGACATTATT CCACTATACT AGTTATACTT TTCTGGTCGA AGTTTAGTCT ATCCGACACC TAAATAAGGG   
  
  
- GTTGGTGCCT GAAATTAATC GGAGGTTCAC ATGTTATATA TACTTTACAC GTAACGAGTT ACCATATTAA   
  
  
- TTAATCGATC TTGTATGTAC GCCTATCACA GACACACGTC CCTCTCTATT TCTTTAGTCT CCCTCCCTCT   
  
  
- CAACAACATA TATACATACC TAGCTAGGAC CTACACGACT TAGTCTAATC TTTAGTTAAG TTGTTCGTTT   
  
  
- ATAGTAAGAG TAGAAAGTGT AGGTGTAGTA AGGTTGTACT TTTGATGTAG AAACAGGTCC TTGCCGGTGT   
  
  
- TCATCACACT TTCATATAGT CGTGAGTTTA ACGAAAAGTT ATTACTACGA TAGATAACAC TATTCTACAA   
  
  
- TAAATAATCC TTCTTTAACG TATATTGTTA GAACAAATTC TATTTGACCT GACACTTGGT TCTTCCTGGT   
  
  
- TTCTACCTGT TCGATACTTC ACTTTACAGA TACTAAAAGT GAGGCTTATA CAGTCTTATC TAACCAAAAC   
  
  
- TGCGTGGGCG TATGTCATTA TCGGTCAGTT CCTATATTTG CAATCACAAC GGAACACGAA GAAGGTACAT   
  
  
- AATGTCACGA ATTCTTTAGT CTGATATATA ATTAGTCATT GAGAAAAGAA CGCGTGTCGG TGTGTAGGGG   
  
  
- AATTATTAAG GAGTCGTGAT AGTTACAAAC TCTCCCGTGT CTCTCTCTCT CTCTCTCTCT CTCTCTCTCT   
  
  
- TTCTCTCCAC TCAGACGTTC AGAAAGGAGA CGCCATGGAC ACTCTTCTAT TCTTCCTTAT TGAACGTCTT   
  
  
- TTGTAAGGCA AATCGCAAAT GACCAACTGG TCCGAATGTA GAGTTTTGTT TCCCGATAAA GACGTCTGCT   
  
  
- AGCTTATCTC GTAGTTGAAC CAGTATGGAC GATAACTTGG TCTGGGAAAA CTGTCCTTCT TATATAATTC   
  
  
- TTCATCCCGA ACGACCCAAT AGCTTTCTT

+     MNF1

| Site Name | Organism | Position | Strand | Matrix score. | sequence | function |
| --- | --- | --- | --- | --- | --- | --- |
| MNF1 | Zea mays | 1222 | - | 6.5 | GTGCCC(A/T)(A/T) | light responsive element |

> 2018/04/13 10:10:12  
+ GTTGCTTGCT TTAATTAAAG AAGGAAGCAA AACAGATGGT TGGATTGAAA AGGACAACCC ATAAGGCATA   
  
  
+ TAATAATCAG CAGTAATTAA GTTTCACTTG TTTTTGATAG ACTTAATTAT ATATGTGCAG TGCATGTTTG   
  
  
+ CAGACCAGCA TGGCAAGCAT AAAACTTCAC AAGATCTCTT CCAAAGATTG TATTTGTAGA TGGCTTCAGA   
  
  
+ CATGACACAA AGTGCTACCC ACAATCAACT TACTGCTGTC TGGTAAACGT TGAAACTGGT GTCCCATAAC   
  
  
+ ACACTGTAGT ACTCTCTCCC ACACCTTGTA ACAAGTTTAC TTCATTTGGT TCTCTGGTAT TTAATTTAGT   
  
  
+ GAAGAAGATC ATTAAGCTAC TCAACAGGAA GCTTTTCAAG TAAATGCAAC TTGTATGACA ACATTCTTCT   
  
  
+ TCTGTCTGTC TCACACCACA GACTTCACTA GTTACATCAT TAATTAGGCA TGTAGGCCAA TGGTTTATCA   
  
  
+ GCTGTAATAA GGTGATATGA TCAATATGAA AAGACCAGCT TCAAATCAGA TAGGCTGTGG ATTTATTCCC   
  
  
+ CAACCACGGA CTTTAATTAG CCTCCAAGTG TACAATATAT ATGAAATGTG CATTGCTCAA TGGTATAATT   
  
  
+ AATTAGCTAG AACATACATG CGGATAGTGT CTGTGTGCAG GGAGAGATAA AGAAATCAGA GGGAGGGAGA   
  
  
+ GTTGTTGTAT ATATGTATGG ATCGATCCTG GATGTGCTGA ATCAGATTAG AAATCAATTC AACAAGCAAA   
  
  
+ TATCATTCTC ATCTTTCACA TCCACATCAT TCCAACATGA AAACTACATC TTTGTCCAGG AACGGCCACA   
  
  
+ AGTAGTGTGA AAGTATATCA GCACTCAAAT TGCTTTTCAA TAATGATGCT ATCTATTGTG ATAAGATGTT   
  
  
+ ATTTATTAGG AAGAAATTGC ATATAACAAT CTTGTTTAAG ATAAACTGGA CTGTGAACCA AGAAGGACCA   
  
  
+ AAGATGGACA AGCTATGAAG TGAAATGTCT ATGATTTTCA CTCCGAATAT GTCAGAATAG ATTGGTTTTG   
  
  
+ ACGCACCCGC ATACAGTAAT AGCCAGTCAA GGATATAAAC GTTAGTGTTG CCTTGTGCTT CTTCCATGTA   
  
  
+ TTACAGTGCT TAAGAAATCA GACTATATAT TAATCAGTAA CTCTTTTCTT GCGCACAGCC ACACATCCCC   
  
  
+ TTAATAATTC CTCAGCACTA TCAATGTTTG AGAGGGCACA GAGAGAGAGA GAGAGAGAGA GAGAGAGAGA   
  
  
+ AAGAGAGGTG AGTCTGCAAG TCTTTCCTCT GCGGTACCTG TGAGAAGATA AGAAGGAATA ACTTGCAGAA   
  
  
+ AACATTCCGT TTAGCGTTTA CTGGTTGACC AGGCTTACAT CTCAAAACAA AGGGCTATTT CTGCAGACGA   
  
  
+ TCGAATAGAG CATCAACTTG GTCATACCTG CTATTGAACC AGACCCTTTT GACAGGAAGA ATATATTAAG   
  
  
+ AAGTAGGGCT TGCTGGGTTA TCGAAAGAA  

- CAACGAACGA AATTAATTTC TTCCTTCGTT TTGTCTACCA ACCTAACTTT TCCTGTTGGG TATTCCGTAT   
  
  
- ATTATTAGTC GTCATTAATT CAAAGTGAAC AAAAACTATC TGAATTAATA TATACACGTC ACGTACAAAC   
  
  
- GTCTGGTCGT ACCGTTCGTA TTTTGAAGTG TTCTAGAGAA GGTTTCTAAC ATAAACATCT ACCGAAGTCT   
  
  
- GTACTGTGTT TCACGATGGG TGTTAGTTGA ATGACGACAG ACCATTTGCA ACTTTGACCA CAGGGTATTG   
  
  
- TGTGACATCA TGAGAGAGGG TGTGGAACAT TGTTCAAATG AAGTAAACCA AGAGACCATA AATTAAATCA   
  
  
- CTTCTTCTAG TAATTCGATG AGTTGTCCTT CGAAAAGTTC ATTTACGTTG AACATACTGT TGTAAGAAGA   
  
  
- AGACAGACAG AGTGTGGTGT CTGAAGTGAT CAATGTAGTA ATTAATCCGT ACATCCGGTT ACCAAATAGT   
  
  
- CGACATTATT CCACTATACT AGTTATACTT TTCTGGTCGA AGTTTAGTCT ATCCGACACC TAAATAAGGG   
  
  
- GTTGGTGCCT GAAATTAATC GGAGGTTCAC ATGTTATATA TACTTTACAC GTAACGAGTT ACCATATTAA   
  
  
- TTAATCGATC TTGTATGTAC GCCTATCACA GACACACGTC CCTCTCTATT TCTTTAGTCT CCCTCCCTCT   
  
  
- CAACAACATA TATACATACC TAGCTAGGAC CTACACGACT TAGTCTAATC TTTAGTTAAG TTGTTCGTTT   
  
  
- ATAGTAAGAG TAGAAAGTGT AGGTGTAGTA AGGTTGTACT TTTGATGTAG AAACAGGTCC TTGCCGGTGT   
  
  
- TCATCACACT TTCATATAGT CGTGAGTTTA ACGAAAAGTT ATTACTACGA TAGATAACAC TATTCTACAA   
  
  
- TAAATAATCC TTCTTTAACG TATATTGTTA GAACAAATTC TATTTGACCT GACACTTGGT TCTTCCTGGT   
  
  
- TTCTACCTGT TCGATACTTC ACTTTACAGA TACTAAAAGT GAGGCTTATA CAGTCTTATC TAACCAAAAC   
  
  
- TGCGTGGGCG TATGTCATTA TCGGTCAGTT CCTATATTTG CAATCACAAC GGAACACGAA GAAGGTACAT   
  
  
- AATGTCACGA ATTCTTTAGT CTGATATATA ATTAGTCATT GAGAAAAGAA CGCGTGTCGG TGTGTAGGGG   
  
  
- AATTATTAAG GAGTCGTGAT AGTTACAAAC TCTCCCGTGT CTCTCTCTCT CTCTCTCTCT CTCTCTCTCT   
  
  
- TTCTCTCCAC TCAGACGTTC AGAAAGGAGA CGCCATGGAC ACTCTTCTAT TCTTCCTTAT TGAACGTCTT   
  
  
- TTGTAAGGCA AATCGCAAAT GACCAACTGG TCCGAATGTA GAGTTTTGTT TCCCGATAAA GACGTCTGCT   
  
  
- AGCTTATCTC GTAGTTGAAC CAGTATGGAC GATAACTTGG TCTGGGAAAA CTGTCCTTCT TATATAATTC   
  
  
- TTCATCCCGA ACGACCCAAT AGCTTTCTT

+     O2-site

| Site Name | Organism | Position | Strand | Matrix score. | sequence | function |
| --- | --- | --- | --- | --- | --- | --- |
| O2-site | Zea mays | 792 | - | 9 | GATGATGTGG | cis-acting regulatory element involved in zein metabolism regulation |

> 2018/04/13 10:10:12  
+ GTTGCTTGCT TTAATTAAAG AAGGAAGCAA AACAGATGGT TGGATTGAAA AGGACAACCC ATAAGGCATA   
  
  
+ TAATAATCAG CAGTAATTAA GTTTCACTTG TTTTTGATAG ACTTAATTAT ATATGTGCAG TGCATGTTTG   
  
  
+ CAGACCAGCA TGGCAAGCAT AAAACTTCAC AAGATCTCTT CCAAAGATTG TATTTGTAGA TGGCTTCAGA   
  
  
+ CATGACACAA AGTGCTACCC ACAATCAACT TACTGCTGTC TGGTAAACGT TGAAACTGGT GTCCCATAAC   
  
  
+ ACACTGTAGT ACTCTCTCCC ACACCTTGTA ACAAGTTTAC TTCATTTGGT TCTCTGGTAT TTAATTTAGT   
  
  
+ GAAGAAGATC ATTAAGCTAC TCAACAGGAA GCTTTTCAAG TAAATGCAAC TTGTATGACA ACATTCTTCT   
  
  
+ TCTGTCTGTC TCACACCACA GACTTCACTA GTTACATCAT TAATTAGGCA TGTAGGCCAA TGGTTTATCA   
  
  
+ GCTGTAATAA GGTGATATGA TCAATATGAA AAGACCAGCT TCAAATCAGA TAGGCTGTGG ATTTATTCCC   
  
  
+ CAACCACGGA CTTTAATTAG CCTCCAAGTG TACAATATAT ATGAAATGTG CATTGCTCAA TGGTATAATT   
  
  
+ AATTAGCTAG AACATACATG CGGATAGTGT CTGTGTGCAG GGAGAGATAA AGAAATCAGA GGGAGGGAGA   
  
  
+ GTTGTTGTAT ATATGTATGG ATCGATCCTG GATGTGCTGA ATCAGATTAG AAATCAATTC AACAAGCAAA   
  
  
+ TATCATTCTC ATCTTTCACA TCCACATCAT TCCAACATGA AAACTACATC TTTGTCCAGG AACGGCCACA   
  
  
+ AGTAGTGTGA AAGTATATCA GCACTCAAAT TGCTTTTCAA TAATGATGCT ATCTATTGTG ATAAGATGTT   
  
  
+ ATTTATTAGG AAGAAATTGC ATATAACAAT CTTGTTTAAG ATAAACTGGA CTGTGAACCA AGAAGGACCA   
  
  
+ AAGATGGACA AGCTATGAAG TGAAATGTCT ATGATTTTCA CTCCGAATAT GTCAGAATAG ATTGGTTTTG   
  
  
+ ACGCACCCGC ATACAGTAAT AGCCAGTCAA GGATATAAAC GTTAGTGTTG CCTTGTGCTT CTTCCATGTA   
  
  
+ TTACAGTGCT TAAGAAATCA GACTATATAT TAATCAGTAA CTCTTTTCTT GCGCACAGCC ACACATCCCC   
  
  
+ TTAATAATTC CTCAGCACTA TCAATGTTTG AGAGGGCACA GAGAGAGAGA GAGAGAGAGA GAGAGAGAGA   
  
  
+ AAGAGAGGTG AGTCTGCAAG TCTTTCCTCT GCGGTACCTG TGAGAAGATA AGAAGGAATA ACTTGCAGAA   
  
  
+ AACATTCCGT TTAGCGTTTA CTGGTTGACC AGGCTTACAT CTCAAAACAA AGGGCTATTT CTGCAGACGA   
  
  
+ TCGAATAGAG CATCAACTTG GTCATACCTG CTATTGAACC AGACCCTTTT GACAGGAAGA ATATATTAAG   
  
  
+ AAGTAGGGCT TGCTGGGTTA TCGAAAGAA  

- CAACGAACGA AATTAATTTC TTCCTTCGTT TTGTCTACCA ACCTAACTTT TCCTGTTGGG TATTCCGTAT   
  
  
- ATTATTAGTC GTCATTAATT CAAAGTGAAC AAAAACTATC TGAATTAATA TATACACGTC ACGTACAAAC   
  
  
- GTCTGGTCGT ACCGTTCGTA TTTTGAAGTG TTCTAGAGAA GGTTTCTAAC ATAAACATCT ACCGAAGTCT   
  
  
- GTACTGTGTT TCACGATGGG TGTTAGTTGA ATGACGACAG ACCATTTGCA ACTTTGACCA CAGGGTATTG   
  
  
- TGTGACATCA TGAGAGAGGG TGTGGAACAT TGTTCAAATG AAGTAAACCA AGAGACCATA AATTAAATCA   
  
  
- CTTCTTCTAG TAATTCGATG AGTTGTCCTT CGAAAAGTTC ATTTACGTTG AACATACTGT TGTAAGAAGA   
  
  
- AGACAGACAG AGTGTGGTGT CTGAAGTGAT CAATGTAGTA ATTAATCCGT ACATCCGGTT ACCAAATAGT   
  
  
- CGACATTATT CCACTATACT AGTTATACTT TTCTGGTCGA AGTTTAGTCT ATCCGACACC TAAATAAGGG   
  
  
- GTTGGTGCCT GAAATTAATC GGAGGTTCAC ATGTTATATA TACTTTACAC GTAACGAGTT ACCATATTAA   
  
  
- TTAATCGATC TTGTATGTAC GCCTATCACA GACACACGTC CCTCTCTATT TCTTTAGTCT CCCTCCCTCT   
  
  
- CAACAACATA TATACATACC TAGCTAGGAC CTACACGACT TAGTCTAATC TTTAGTTAAG TTGTTCGTTT   
  
  
- ATAGTAAGAG TAGAAAGTGT AGGTGTAGTA AGGTTGTACT TTTGATGTAG AAACAGGTCC TTGCCGGTGT   
  
  
- TCATCACACT TTCATATAGT CGTGAGTTTA ACGAAAAGTT ATTACTACGA TAGATAACAC TATTCTACAA   
  
  
- TAAATAATCC TTCTTTAACG TATATTGTTA GAACAAATTC TATTTGACCT GACACTTGGT TCTTCCTGGT   
  
  
- TTCTACCTGT TCGATACTTC ACTTTACAGA TACTAAAAGT GAGGCTTATA CAGTCTTATC TAACCAAAAC   
  
  
- TGCGTGGGCG TATGTCATTA TCGGTCAGTT CCTATATTTG CAATCACAAC GGAACACGAA GAAGGTACAT   
  
  
- AATGTCACGA ATTCTTTAGT CTGATATATA ATTAGTCATT GAGAAAAGAA CGCGTGTCGG TGTGTAGGGG   
  
  
- AATTATTAAG GAGTCGTGAT AGTTACAAAC TCTCCCGTGT CTCTCTCTCT CTCTCTCTCT CTCTCTCTCT   
  
  
- TTCTCTCCAC TCAGACGTTC AGAAAGGAGA CGCCATGGAC ACTCTTCTAT TCTTCCTTAT TGAACGTCTT   
  
  
- TTGTAAGGCA AATCGCAAAT GACCAACTGG TCCGAATGTA GAGTTTTGTT TCCCGATAAA GACGTCTGCT   
  
  
- AGCTTATCTC GTAGTTGAAC CAGTATGGAC GATAACTTGG TCTGGGAAAA CTGTCCTTCT TATATAATTC   
  
  
- TTCATCCCGA ACGACCCAAT AGCTTTCTT

+     P-box

| Site Name | Organism | Position | Strand | Matrix score. | sequence | function |
| --- | --- | --- | --- | --- | --- | --- |
| P-box | Oryza sativa | 1445 | + | 7 | CCTTTTG | gibberellin-responsive element |

> 2018/04/13 10:10:12  
+ GTTGCTTGCT TTAATTAAAG AAGGAAGCAA AACAGATGGT TGGATTGAAA AGGACAACCC ATAAGGCATA   
  
  
+ TAATAATCAG CAGTAATTAA GTTTCACTTG TTTTTGATAG ACTTAATTAT ATATGTGCAG TGCATGTTTG   
  
  
+ CAGACCAGCA TGGCAAGCAT AAAACTTCAC AAGATCTCTT CCAAAGATTG TATTTGTAGA TGGCTTCAGA   
  
  
+ CATGACACAA AGTGCTACCC ACAATCAACT TACTGCTGTC TGGTAAACGT TGAAACTGGT GTCCCATAAC   
  
  
+ ACACTGTAGT ACTCTCTCCC ACACCTTGTA ACAAGTTTAC TTCATTTGGT TCTCTGGTAT TTAATTTAGT   
  
  
+ GAAGAAGATC ATTAAGCTAC TCAACAGGAA GCTTTTCAAG TAAATGCAAC TTGTATGACA ACATTCTTCT   
  
  
+ TCTGTCTGTC TCACACCACA GACTTCACTA GTTACATCAT TAATTAGGCA TGTAGGCCAA TGGTTTATCA   
  
  
+ GCTGTAATAA GGTGATATGA TCAATATGAA AAGACCAGCT TCAAATCAGA TAGGCTGTGG ATTTATTCCC   
  
  
+ CAACCACGGA CTTTAATTAG CCTCCAAGTG TACAATATAT ATGAAATGTG CATTGCTCAA TGGTATAATT   
  
  
+ AATTAGCTAG AACATACATG CGGATAGTGT CTGTGTGCAG GGAGAGATAA AGAAATCAGA GGGAGGGAGA   
  
  
+ GTTGTTGTAT ATATGTATGG ATCGATCCTG GATGTGCTGA ATCAGATTAG AAATCAATTC AACAAGCAAA   
  
  
+ TATCATTCTC ATCTTTCACA TCCACATCAT TCCAACATGA AAACTACATC TTTGTCCAGG AACGGCCACA   
  
  
+ AGTAGTGTGA AAGTATATCA GCACTCAAAT TGCTTTTCAA TAATGATGCT ATCTATTGTG ATAAGATGTT   
  
  
+ ATTTATTAGG AAGAAATTGC ATATAACAAT CTTGTTTAAG ATAAACTGGA CTGTGAACCA AGAAGGACCA   
  
  
+ AAGATGGACA AGCTATGAAG TGAAATGTCT ATGATTTTCA CTCCGAATAT GTCAGAATAG ATTGGTTTTG   
  
  
+ ACGCACCCGC ATACAGTAAT AGCCAGTCAA GGATATAAAC GTTAGTGTTG CCTTGTGCTT CTTCCATGTA   
  
  
+ TTACAGTGCT TAAGAAATCA GACTATATAT TAATCAGTAA CTCTTTTCTT GCGCACAGCC ACACATCCCC   
  
  
+ TTAATAATTC CTCAGCACTA TCAATGTTTG AGAGGGCACA GAGAGAGAGA GAGAGAGAGA GAGAGAGAGA   
  
  
+ AAGAGAGGTG AGTCTGCAAG TCTTTCCTCT GCGGTACCTG TGAGAAGATA AGAAGGAATA ACTTGCAGAA   
  
  
+ AACATTCCGT TTAGCGTTTA CTGGTTGACC AGGCTTACAT CTCAAAACAA AGGGCTATTT CTGCAGACGA   
  
  
+ TCGAATAGAG CATCAACTTG GTCATACCTG CTATTGAACC AGACCCTTTT GACAGGAAGA ATATATTAAG   
  
  
+ AAGTAGGGCT TGCTGGGTTA TCGAAAGAA  

- CAACGAACGA AATTAATTTC TTCCTTCGTT TTGTCTACCA ACCTAACTTT TCCTGTTGGG TATTCCGTAT   
  
  
- ATTATTAGTC GTCATTAATT CAAAGTGAAC AAAAACTATC TGAATTAATA TATACACGTC ACGTACAAAC   
  
  
- GTCTGGTCGT ACCGTTCGTA TTTTGAAGTG TTCTAGAGAA GGTTTCTAAC ATAAACATCT ACCGAAGTCT   
  
  
- GTACTGTGTT TCACGATGGG TGTTAGTTGA ATGACGACAG ACCATTTGCA ACTTTGACCA CAGGGTATTG   
  
  
- TGTGACATCA TGAGAGAGGG TGTGGAACAT TGTTCAAATG AAGTAAACCA AGAGACCATA AATTAAATCA   
  
  
- CTTCTTCTAG TAATTCGATG AGTTGTCCTT CGAAAAGTTC ATTTACGTTG AACATACTGT TGTAAGAAGA   
  
  
- AGACAGACAG AGTGTGGTGT CTGAAGTGAT CAATGTAGTA ATTAATCCGT ACATCCGGTT ACCAAATAGT   
  
  
- CGACATTATT CCACTATACT AGTTATACTT TTCTGGTCGA AGTTTAGTCT ATCCGACACC TAAATAAGGG   
  
  
- GTTGGTGCCT GAAATTAATC GGAGGTTCAC ATGTTATATA TACTTTACAC GTAACGAGTT ACCATATTAA   
  
  
- TTAATCGATC TTGTATGTAC GCCTATCACA GACACACGTC CCTCTCTATT TCTTTAGTCT CCCTCCCTCT   
  
  
- CAACAACATA TATACATACC TAGCTAGGAC CTACACGACT TAGTCTAATC TTTAGTTAAG TTGTTCGTTT   
  
  
- ATAGTAAGAG TAGAAAGTGT AGGTGTAGTA AGGTTGTACT TTTGATGTAG AAACAGGTCC TTGCCGGTGT   
  
  
- TCATCACACT TTCATATAGT CGTGAGTTTA ACGAAAAGTT ATTACTACGA TAGATAACAC TATTCTACAA   
  
  
- TAAATAATCC TTCTTTAACG TATATTGTTA GAACAAATTC TATTTGACCT GACACTTGGT TCTTCCTGGT   
  
  
- TTCTACCTGT TCGATACTTC ACTTTACAGA TACTAAAAGT GAGGCTTATA CAGTCTTATC TAACCAAAAC   
  
  
- TGCGTGGGCG TATGTCATTA TCGGTCAGTT CCTATATTTG CAATCACAAC GGAACACGAA GAAGGTACAT   
  
  
- AATGTCACGA ATTCTTTAGT CTGATATATA ATTAGTCATT GAGAAAAGAA CGCGTGTCGG TGTGTAGGGG   
  
  
- AATTATTAAG GAGTCGTGAT AGTTACAAAC TCTCCCGTGT CTCTCTCTCT CTCTCTCTCT CTCTCTCTCT   
  
  
- TTCTCTCCAC TCAGACGTTC AGAAAGGAGA CGCCATGGAC ACTCTTCTAT TCTTCCTTAT TGAACGTCTT   
  
  
- TTGTAAGGCA AATCGCAAAT GACCAACTGG TCCGAATGTA GAGTTTTGTT TCCCGATAAA GACGTCTGCT   
  
  
- AGCTTATCTC GTAGTTGAAC CAGTATGGAC GATAACTTGG TCTGGGAAAA CTGTCCTTCT TATATAATTC   
  
  
- TTCATCCCGA ACGACCCAAT AGCTTTCTT

+     Skn-1\_motif

| Site Name | Organism | Position | Strand | Matrix score. | sequence | function |
| --- | --- | --- | --- | --- | --- | --- |
| Skn-1\_motif | Oryza sativa | 212 | - | 5 | GTCAT | cis-acting regulatory element required for endosperm expression |
| Skn-1\_motif | Oryza sativa | 1421 | + | 5 | GTCAT | cis-acting regulatory element required for endosperm expression |
| Skn-1\_motif | Oryza sativa | 405 | - | 5 | GTCAT | cis-acting regulatory element required for endosperm expression |

> 2018/04/13 10:10:12  
+ GTTGCTTGCT TTAATTAAAG AAGGAAGCAA AACAGATGGT TGGATTGAAA AGGACAACCC ATAAGGCATA   
  
  
+ TAATAATCAG CAGTAATTAA GTTTCACTTG TTTTTGATAG ACTTAATTAT ATATGTGCAG TGCATGTTTG   
  
  
+ CAGACCAGCA TGGCAAGCAT AAAACTTCAC AAGATCTCTT CCAAAGATTG TATTTGTAGA TGGCTTCAGA   
  
  
+ CATGACACAA AGTGCTACCC ACAATCAACT TACTGCTGTC TGGTAAACGT TGAAACTGGT GTCCCATAAC   
  
  
+ ACACTGTAGT ACTCTCTCCC ACACCTTGTA ACAAGTTTAC TTCATTTGGT TCTCTGGTAT TTAATTTAGT   
  
  
+ GAAGAAGATC ATTAAGCTAC TCAACAGGAA GCTTTTCAAG TAAATGCAAC TTGTATGACA ACATTCTTCT   
  
  
+ TCTGTCTGTC TCACACCACA GACTTCACTA GTTACATCAT TAATTAGGCA TGTAGGCCAA TGGTTTATCA   
  
  
+ GCTGTAATAA GGTGATATGA TCAATATGAA AAGACCAGCT TCAAATCAGA TAGGCTGTGG ATTTATTCCC   
  
  
+ CAACCACGGA CTTTAATTAG CCTCCAAGTG TACAATATAT ATGAAATGTG CATTGCTCAA TGGTATAATT   
  
  
+ AATTAGCTAG AACATACATG CGGATAGTGT CTGTGTGCAG GGAGAGATAA AGAAATCAGA GGGAGGGAGA   
  
  
+ GTTGTTGTAT ATATGTATGG ATCGATCCTG GATGTGCTGA ATCAGATTAG AAATCAATTC AACAAGCAAA   
  
  
+ TATCATTCTC ATCTTTCACA TCCACATCAT TCCAACATGA AAACTACATC TTTGTCCAGG AACGGCCACA   
  
  
+ AGTAGTGTGA AAGTATATCA GCACTCAAAT TGCTTTTCAA TAATGATGCT ATCTATTGTG ATAAGATGTT   
  
  
+ ATTTATTAGG AAGAAATTGC ATATAACAAT CTTGTTTAAG ATAAACTGGA CTGTGAACCA AGAAGGACCA   
  
  
+ AAGATGGACA AGCTATGAAG TGAAATGTCT ATGATTTTCA CTCCGAATAT GTCAGAATAG ATTGGTTTTG   
  
  
+ ACGCACCCGC ATACAGTAAT AGCCAGTCAA GGATATAAAC GTTAGTGTTG CCTTGTGCTT CTTCCATGTA   
  
  
+ TTACAGTGCT TAAGAAATCA GACTATATAT TAATCAGTAA CTCTTTTCTT GCGCACAGCC ACACATCCCC   
  
  
+ TTAATAATTC CTCAGCACTA TCAATGTTTG AGAGGGCACA GAGAGAGAGA GAGAGAGAGA GAGAGAGAGA   
  
  
+ AAGAGAGGTG AGTCTGCAAG TCTTTCCTCT GCGGTACCTG TGAGAAGATA AGAAGGAATA ACTTGCAGAA   
  
  
+ AACATTCCGT TTAGCGTTTA CTGGTTGACC AGGCTTACAT CTCAAAACAA AGGGCTATTT CTGCAGACGA   
  
  
+ TCGAATAGAG CATCAACTTG GTCATACCTG CTATTGAACC AGACCCTTTT GACAGGAAGA ATATATTAAG   
  
  
+ AAGTAGGGCT TGCTGGGTTA TCGAAAGAA  

- CAACGAACGA AATTAATTTC TTCCTTCGTT TTGTCTACCA ACCTAACTTT TCCTGTTGGG TATTCCGTAT   
  
  
- ATTATTAGTC GTCATTAATT CAAAGTGAAC AAAAACTATC TGAATTAATA TATACACGTC ACGTACAAAC   
  
  
- GTCTGGTCGT ACCGTTCGTA TTTTGAAGTG TTCTAGAGAA GGTTTCTAAC ATAAACATCT ACCGAAGTCT   
  
  
- GTACTGTGTT TCACGATGGG TGTTAGTTGA ATGACGACAG ACCATTTGCA ACTTTGACCA CAGGGTATTG   
  
  
- TGTGACATCA TGAGAGAGGG TGTGGAACAT TGTTCAAATG AAGTAAACCA AGAGACCATA AATTAAATCA   
  
  
- CTTCTTCTAG TAATTCGATG AGTTGTCCTT CGAAAAGTTC ATTTACGTTG AACATACTGT TGTAAGAAGA   
  
  
- AGACAGACAG AGTGTGGTGT CTGAAGTGAT CAATGTAGTA ATTAATCCGT ACATCCGGTT ACCAAATAGT   
  
  
- CGACATTATT CCACTATACT AGTTATACTT TTCTGGTCGA AGTTTAGTCT ATCCGACACC TAAATAAGGG   
  
  
- GTTGGTGCCT GAAATTAATC GGAGGTTCAC ATGTTATATA TACTTTACAC GTAACGAGTT ACCATATTAA   
  
  
- TTAATCGATC TTGTATGTAC GCCTATCACA GACACACGTC CCTCTCTATT TCTTTAGTCT CCCTCCCTCT   
  
  
- CAACAACATA TATACATACC TAGCTAGGAC CTACACGACT TAGTCTAATC TTTAGTTAAG TTGTTCGTTT   
  
  
- ATAGTAAGAG TAGAAAGTGT AGGTGTAGTA AGGTTGTACT TTTGATGTAG AAACAGGTCC TTGCCGGTGT   
  
  
- TCATCACACT TTCATATAGT CGTGAGTTTA ACGAAAAGTT ATTACTACGA TAGATAACAC TATTCTACAA   
  
  
- TAAATAATCC TTCTTTAACG TATATTGTTA GAACAAATTC TATTTGACCT GACACTTGGT TCTTCCTGGT   
  
  
- TTCTACCTGT TCGATACTTC ACTTTACAGA TACTAAAAGT GAGGCTTATA CAGTCTTATC TAACCAAAAC   
  
  
- TGCGTGGGCG TATGTCATTA TCGGTCAGTT CCTATATTTG CAATCACAAC GGAACACGAA GAAGGTACAT   
  
  
- AATGTCACGA ATTCTTTAGT CTGATATATA ATTAGTCATT GAGAAAAGAA CGCGTGTCGG TGTGTAGGGG   
  
  
- AATTATTAAG GAGTCGTGAT AGTTACAAAC TCTCCCGTGT CTCTCTCTCT CTCTCTCTCT CTCTCTCTCT   
  
  
- TTCTCTCCAC TCAGACGTTC AGAAAGGAGA CGCCATGGAC ACTCTTCTAT TCTTCCTTAT TGAACGTCTT   
  
  
- TTGTAAGGCA AATCGCAAAT GACCAACTGG TCCGAATGTA GAGTTTTGTT TCCCGATAAA GACGTCTGCT   
  
  
- AGCTTATCTC GTAGTTGAAC CAGTATGGAC GATAACTTGG TCTGGGAAAA CTGTCCTTCT TATATAATTC   
  
  
- TTCATCCCGA ACGACCCAAT AGCTTTCTT

+     Sp1

| Site Name | Organism | Position | Strand | Matrix score. | sequence | function |
| --- | --- | --- | --- | --- | --- | --- |
| Sp1 | Zea mays | 691 | - | 5 | CC(G/A)CCC | light responsive element |

> 2018/04/13 10:10:12  
+ GTTGCTTGCT TTAATTAAAG AAGGAAGCAA AACAGATGGT TGGATTGAAA AGGACAACCC ATAAGGCATA   
  
  
+ TAATAATCAG CAGTAATTAA GTTTCACTTG TTTTTGATAG ACTTAATTAT ATATGTGCAG TGCATGTTTG   
  
  
+ CAGACCAGCA TGGCAAGCAT AAAACTTCAC AAGATCTCTT CCAAAGATTG TATTTGTAGA TGGCTTCAGA   
  
  
+ CATGACACAA AGTGCTACCC ACAATCAACT TACTGCTGTC TGGTAAACGT TGAAACTGGT GTCCCATAAC   
  
  
+ ACACTGTAGT ACTCTCTCCC ACACCTTGTA ACAAGTTTAC TTCATTTGGT TCTCTGGTAT TTAATTTAGT   
  
  
+ GAAGAAGATC ATTAAGCTAC TCAACAGGAA GCTTTTCAAG TAAATGCAAC TTGTATGACA ACATTCTTCT   
  
  
+ TCTGTCTGTC TCACACCACA GACTTCACTA GTTACATCAT TAATTAGGCA TGTAGGCCAA TGGTTTATCA   
  
  
+ GCTGTAATAA GGTGATATGA TCAATATGAA AAGACCAGCT TCAAATCAGA TAGGCTGTGG ATTTATTCCC   
  
  
+ CAACCACGGA CTTTAATTAG CCTCCAAGTG TACAATATAT ATGAAATGTG CATTGCTCAA TGGTATAATT   
  
  
+ AATTAGCTAG AACATACATG CGGATAGTGT CTGTGTGCAG GGAGAGATAA AGAAATCAGA GGGAGGGAGA   
  
  
+ GTTGTTGTAT ATATGTATGG ATCGATCCTG GATGTGCTGA ATCAGATTAG AAATCAATTC AACAAGCAAA   
  
  
+ TATCATTCTC ATCTTTCACA TCCACATCAT TCCAACATGA AAACTACATC TTTGTCCAGG AACGGCCACA   
  
  
+ AGTAGTGTGA AAGTATATCA GCACTCAAAT TGCTTTTCAA TAATGATGCT ATCTATTGTG ATAAGATGTT   
  
  
+ ATTTATTAGG AAGAAATTGC ATATAACAAT CTTGTTTAAG ATAAACTGGA CTGTGAACCA AGAAGGACCA   
  
  
+ AAGATGGACA AGCTATGAAG TGAAATGTCT ATGATTTTCA CTCCGAATAT GTCAGAATAG ATTGGTTTTG   
  
  
+ ACGCACCCGC ATACAGTAAT AGCCAGTCAA GGATATAAAC GTTAGTGTTG CCTTGTGCTT CTTCCATGTA   
  
  
+ TTACAGTGCT TAAGAAATCA GACTATATAT TAATCAGTAA CTCTTTTCTT GCGCACAGCC ACACATCCCC   
  
  
+ TTAATAATTC CTCAGCACTA TCAATGTTTG AGAGGGCACA GAGAGAGAGA GAGAGAGAGA GAGAGAGAGA   
  
  
+ AAGAGAGGTG AGTCTGCAAG TCTTTCCTCT GCGGTACCTG TGAGAAGATA AGAAGGAATA ACTTGCAGAA   
  
  
+ AACATTCCGT TTAGCGTTTA CTGGTTGACC AGGCTTACAT CTCAAAACAA AGGGCTATTT CTGCAGACGA   
  
  
+ TCGAATAGAG CATCAACTTG GTCATACCTG CTATTGAACC AGACCCTTTT GACAGGAAGA ATATATTAAG   
  
  
+ AAGTAGGGCT TGCTGGGTTA TCGAAAGAA  

- CAACGAACGA AATTAATTTC TTCCTTCGTT TTGTCTACCA ACCTAACTTT TCCTGTTGGG TATTCCGTAT   
  
  
- ATTATTAGTC GTCATTAATT CAAAGTGAAC AAAAACTATC TGAATTAATA TATACACGTC ACGTACAAAC   
  
  
- GTCTGGTCGT ACCGTTCGTA TTTTGAAGTG TTCTAGAGAA GGTTTCTAAC ATAAACATCT ACCGAAGTCT   
  
  
- GTACTGTGTT TCACGATGGG TGTTAGTTGA ATGACGACAG ACCATTTGCA ACTTTGACCA CAGGGTATTG   
  
  
- TGTGACATCA TGAGAGAGGG TGTGGAACAT TGTTCAAATG AAGTAAACCA AGAGACCATA AATTAAATCA   
  
  
- CTTCTTCTAG TAATTCGATG AGTTGTCCTT CGAAAAGTTC ATTTACGTTG AACATACTGT TGTAAGAAGA   
  
  
- AGACAGACAG AGTGTGGTGT CTGAAGTGAT CAATGTAGTA ATTAATCCGT ACATCCGGTT ACCAAATAGT   
  
  
- CGACATTATT CCACTATACT AGTTATACTT TTCTGGTCGA AGTTTAGTCT ATCCGACACC TAAATAAGGG   
  
  
- GTTGGTGCCT GAAATTAATC GGAGGTTCAC ATGTTATATA TACTTTACAC GTAACGAGTT ACCATATTAA   
  
  
- TTAATCGATC TTGTATGTAC GCCTATCACA GACACACGTC CCTCTCTATT TCTTTAGTCT CCCTCCCTCT   
  
  
- CAACAACATA TATACATACC TAGCTAGGAC CTACACGACT TAGTCTAATC TTTAGTTAAG TTGTTCGTTT   
  
  
- ATAGTAAGAG TAGAAAGTGT AGGTGTAGTA AGGTTGTACT TTTGATGTAG AAACAGGTCC TTGCCGGTGT   
  
  
- TCATCACACT TTCATATAGT CGTGAGTTTA ACGAAAAGTT ATTACTACGA TAGATAACAC TATTCTACAA   
  
  
- TAAATAATCC TTCTTTAACG TATATTGTTA GAACAAATTC TATTTGACCT GACACTTGGT TCTTCCTGGT   
  
  
- TTCTACCTGT TCGATACTTC ACTTTACAGA TACTAAAAGT GAGGCTTATA CAGTCTTATC TAACCAAAAC   
  
  
- TGCGTGGGCG TATGTCATTA TCGGTCAGTT CCTATATTTG CAATCACAAC GGAACACGAA GAAGGTACAT   
  
  
- AATGTCACGA ATTCTTTAGT CTGATATATA ATTAGTCATT GAGAAAAGAA CGCGTGTCGG TGTGTAGGGG   
  
  
- AATTATTAAG GAGTCGTGAT AGTTACAAAC TCTCCCGTGT CTCTCTCTCT CTCTCTCTCT CTCTCTCTCT   
  
  
- TTCTCTCCAC TCAGACGTTC AGAAAGGAGA CGCCATGGAC ACTCTTCTAT TCTTCCTTAT TGAACGTCTT   
  
  
- TTGTAAGGCA AATCGCAAAT GACCAACTGG TCCGAATGTA GAGTTTTGTT TCCCGATAAA GACGTCTGCT   
  
  
- AGCTTATCTC GTAGTTGAAC CAGTATGGAC GATAACTTGG TCTGGGAAAA CTGTCCTTCT TATATAATTC   
  
  
- TTCATCCCGA ACGACCCAAT AGCTTTCTT

+     TATA-box

| Site Name | Organism | Position | Strand | Matrix score. | sequence | function |
| --- | --- | --- | --- | --- | --- | --- |
| TATA-box | Glycine max | 1119 | - | 5 | TAATA | core promoter element around -30 of transcription start |
| TATA-box | Glycine max | 71 | + | 5 | TAATA | core promoter element around -30 of transcription start |
| TATA-box | Arabidopsis thaliana | 69 | + | 4 | TATA | core promoter element around -30 of transcription start |
| TATA-box | Brassica oleracea | 68 | + | 7 | ATATAAT | core promoter element around -30 of transcription start |
| TATA-box | Brassica napus | 116 | + | 6 | ATTATA | core promoter element around -30 of transcription start |
| TATA-box | Arabidopsis thaliana | 596 | + | 4 | TATA | core promoter element around -30 of transcription start |
| TATA-box | Glycine max | 495 | + | 5 | TAATA | core promoter element around -30 of transcription start |
| TATA-box | Arabidopsis thaliana | 120 | + | 4 | TATA | core promoter element around -30 of transcription start |
| TATA-box | Arabidopsis thaliana | 117 | - | 7 | TATATAA | core promoter element around -30 of transcription start |
| TATA-box | Glycine max | 1464 | - | 5 | TAATA | core promoter element around -30 of transcription start |
| TATA-box | Brassica napus | 119 | + | 6 | ATATAT | core promoter element around -30 of transcription start |
| TATA-box | Arabidopsis thaliana | 708 | + | 4 | TATA | core promoter element around -30 of transcription start |
| TATA-box | Brassica napus | 1145 | - | 6 | ATATAT | core promoter element around -30 of transcription start |
| TATA-box | Arabidopsis thaliana | 1142 | + | 9 | tcTATATAtt | core promoter element around -30 of transcription start |
| TATA-box | Glycine max | 1192 | + | 5 | TAATA | core promoter element around -30 of transcription start |
| TATA-box | Brassica oleracea | 931 | + | 6 | ATATAA | core promoter element around -30 of transcription start |
| TATA-box | Arabidopsis thaliana | 932 | - | 4 | TATA | core promoter element around -30 of transcription start |
| TATA-box | Glycine max | 914 | - | 5 | TAATA | core promoter element around -30 of transcription start |
| TATA-box | Arabidopsis thaliana | 854 | - | 4 | TATA | core promoter element around -30 of transcription start |
| TATA-box | Arabidopsis thaliana | 598 | + | 4 | TATA | core promoter element around -30 of transcription start |
| TATA-box | Lycopersicon esculentum | 160 | - | 5 | TTTTA | core promoter element around -30 of transcription start |
| TATA-box | Brassica napus | 1461 | - | 6 | ATATAT | core promoter element around -30 of transcription start |
| TATA-box | Arabidopsis thaliana | 1146 | + | 9 | taTATAAAtc | core promoter element around -30 of transcription start |
| TATA-box | Brassica napus | 709 | + | 6 | ATATAT | core promoter element around -30 of transcription start |
| TATA-box | Arabidopsis thaliana | 1084 | + | 6 | TATAAA | core promoter element around -30 of transcription start |
| TATA-box | Helianthus annuus | 706 | - | 6 | TATACA | core promoter element around -30 of transcription start |
| TATA-box | Arabidopsis thaliana | 118 | + | 4 | TATA | core promoter element around -30 of transcription start |
| TATA-box | Arabidopsis thaliana | 1462 | - | 4 | TATA | core promoter element around -30 of transcription start |
| TATA-box | Brassica napus | 595 | + | 6 | ATATAT | core promoter element around -30 of transcription start |
| TATA-box | Glycine max | 1067 | + | 5 | TAATA | core promoter element around -30 of transcription start |
| TATA-box | Glycine max | 1148 | - | 5 | TAATA | core promoter element around -30 of transcription start |
| TATA-box | Brassica oleracea | 1083 | + | 6 | ATATAA | core promoter element around -30 of transcription start |
| TATA-box | Nicotiana tabacum | 191 | - | 9 | tcTATAAAta | core promoter element around -30 of transcription start |
| TATA-box | Arabidopsis thaliana | 624 | + | 4 | TATA | core promoter element around -30 of transcription start |
| TATA-box | Arabidopsis thaliana | 1144 | - | 4 | TATA | core promoter element around -30 of transcription start |
| TATA-box | Arabidopsis thaliana | 710 | + | 4 | TATA | core promoter element around -30 of transcription start |
| TATA-box | Brassica napus | 597 | + | 6 | ATATAT | core promoter element around -30 of transcription start |

> 2018/04/13 10:10:12  
+ GTTGCTTGCT TTAATTAAAG AAGGAAGCAA AACAGATGGT TGGATTGAAA AGGACAACCC ATAAGGCATA   
  
  
+ TAATAATCAG CAGTAATTAA GTTTCACTTG TTTTTGATAG ACTTAATTAT ATATGTGCAG TGCATGTTTG   
  
  
+ CAGACCAGCA TGGCAAGCAT AAAACTTCAC AAGATCTCTT CCAAAGATTG TATTTGTAGA TGGCTTCAGA   
  
  
+ CATGACACAA AGTGCTACCC ACAATCAACT TACTGCTGTC TGGTAAACGT TGAAACTGGT GTCCCATAAC   
  
  
+ ACACTGTAGT ACTCTCTCCC ACACCTTGTA ACAAGTTTAC TTCATTTGGT TCTCTGGTAT TTAATTTAGT   
  
  
+ GAAGAAGATC ATTAAGCTAC TCAACAGGAA GCTTTTCAAG TAAATGCAAC TTGTATGACA ACATTCTTCT   
  
  
+ TCTGTCTGTC TCACACCACA GACTTCACTA GTTACATCAT TAATTAGGCA TGTAGGCCAA TGGTTTATCA   
  
  
+ GCTGTAATAA GGTGATATGA TCAATATGAA AAGACCAGCT TCAAATCAGA TAGGCTGTGG ATTTATTCCC   
  
  
+ CAACCACGGA CTTTAATTAG CCTCCAAGTG TACAATATAT ATGAAATGTG CATTGCTCAA TGGTATAATT   
  
  
+ AATTAGCTAG AACATACATG CGGATAGTGT CTGTGTGCAG GGAGAGATAA AGAAATCAGA GGGAGGGAGA   
  
  
+ GTTGTTGTAT ATATGTATGG ATCGATCCTG GATGTGCTGA ATCAGATTAG AAATCAATTC AACAAGCAAA   
  
  
+ TATCATTCTC ATCTTTCACA TCCACATCAT TCCAACATGA AAACTACATC TTTGTCCAGG AACGGCCACA   
  
  
+ AGTAGTGTGA AAGTATATCA GCACTCAAAT TGCTTTTCAA TAATGATGCT ATCTATTGTG ATAAGATGTT   
  
  
+ ATTTATTAGG AAGAAATTGC ATATAACAAT CTTGTTTAAG ATAAACTGGA CTGTGAACCA AGAAGGACCA   
  
  
+ AAGATGGACA AGCTATGAAG TGAAATGTCT ATGATTTTCA CTCCGAATAT GTCAGAATAG ATTGGTTTTG   
  
  
+ ACGCACCCGC ATACAGTAAT AGCCAGTCAA GGATATAAAC GTTAGTGTTG CCTTGTGCTT CTTCCATGTA   
  
  
+ TTACAGTGCT TAAGAAATCA GACTATATAT TAATCAGTAA CTCTTTTCTT GCGCACAGCC ACACATCCCC   
  
  
+ TTAATAATTC CTCAGCACTA TCAATGTTTG AGAGGGCACA GAGAGAGAGA GAGAGAGAGA GAGAGAGAGA   
  
  
+ AAGAGAGGTG AGTCTGCAAG TCTTTCCTCT GCGGTACCTG TGAGAAGATA AGAAGGAATA ACTTGCAGAA   
  
  
+ AACATTCCGT TTAGCGTTTA CTGGTTGACC AGGCTTACAT CTCAAAACAA AGGGCTATTT CTGCAGACGA   
  
  
+ TCGAATAGAG CATCAACTTG GTCATACCTG CTATTGAACC AGACCCTTTT GACAGGAAGA ATATATTAAG   
  
  
+ AAGTAGGGCT TGCTGGGTTA TCGAAAGAA  

- CAACGAACGA AATTAATTTC TTCCTTCGTT TTGTCTACCA ACCTAACTTT TCCTGTTGGG TATTCCGTAT   
  
  
- ATTATTAGTC GTCATTAATT CAAAGTGAAC AAAAACTATC TGAATTAATA TATACACGTC ACGTACAAAC   
  
  
- GTCTGGTCGT ACCGTTCGTA TTTTGAAGTG TTCTAGAGAA GGTTTCTAAC ATAAACATCT ACCGAAGTCT   
  
  
- GTACTGTGTT TCACGATGGG TGTTAGTTGA ATGACGACAG ACCATTTGCA ACTTTGACCA CAGGGTATTG   
  
  
- TGTGACATCA TGAGAGAGGG TGTGGAACAT TGTTCAAATG AAGTAAACCA AGAGACCATA AATTAAATCA   
  
  
- CTTCTTCTAG TAATTCGATG AGTTGTCCTT CGAAAAGTTC ATTTACGTTG AACATACTGT TGTAAGAAGA   
  
  
- AGACAGACAG AGTGTGGTGT CTGAAGTGAT CAATGTAGTA ATTAATCCGT ACATCCGGTT ACCAAATAGT   
  
  
- CGACATTATT CCACTATACT AGTTATACTT TTCTGGTCGA AGTTTAGTCT ATCCGACACC TAAATAAGGG   
  
  
- GTTGGTGCCT GAAATTAATC GGAGGTTCAC ATGTTATATA TACTTTACAC GTAACGAGTT ACCATATTAA   
  
  
- TTAATCGATC TTGTATGTAC GCCTATCACA GACACACGTC CCTCTCTATT TCTTTAGTCT CCCTCCCTCT   
  
  
- CAACAACATA TATACATACC TAGCTAGGAC CTACACGACT TAGTCTAATC TTTAGTTAAG TTGTTCGTTT   
  
  
- ATAGTAAGAG TAGAAAGTGT AGGTGTAGTA AGGTTGTACT TTTGATGTAG AAACAGGTCC TTGCCGGTGT   
  
  
- TCATCACACT TTCATATAGT CGTGAGTTTA ACGAAAAGTT ATTACTACGA TAGATAACAC TATTCTACAA   
  
  
- TAAATAATCC TTCTTTAACG TATATTGTTA GAACAAATTC TATTTGACCT GACACTTGGT TCTTCCTGGT   
  
  
- TTCTACCTGT TCGATACTTC ACTTTACAGA TACTAAAAGT GAGGCTTATA CAGTCTTATC TAACCAAAAC   
  
  
- TGCGTGGGCG TATGTCATTA TCGGTCAGTT CCTATATTTG CAATCACAAC GGAACACGAA GAAGGTACAT   
  
  
- AATGTCACGA ATTCTTTAGT CTGATATATA ATTAGTCATT GAGAAAAGAA CGCGTGTCGG TGTGTAGGGG   
  
  
- AATTATTAAG GAGTCGTGAT AGTTACAAAC TCTCCCGTGT CTCTCTCTCT CTCTCTCTCT CTCTCTCTCT   
  
  
- TTCTCTCCAC TCAGACGTTC AGAAAGGAGA CGCCATGGAC ACTCTTCTAT TCTTCCTTAT TGAACGTCTT   
  
  
- TTGTAAGGCA AATCGCAAAT GACCAACTGG TCCGAATGTA GAGTTTTGTT TCCCGATAAA GACGTCTGCT   
  
  
- AGCTTATCTC GTAGTTGAAC CAGTATGGAC GATAACTTGG TCTGGGAAAA CTGTCCTTCT TATATAATTC   
  
  
- TTCATCCCGA ACGACCCAAT AGCTTTCTT

+     TC-rich repeats

| Site Name | Organism | Position | Strand | Matrix score. | sequence | function |
| --- | --- | --- | --- | --- | --- | --- |
| TC-rich repeats | Nicotiana tabacum | 350 | - | 9 | ATTTTCTTCA | cis-acting element involved in defense and stress responsiveness |

> 2018/04/13 10:10:12  
+ GTTGCTTGCT TTAATTAAAG AAGGAAGCAA AACAGATGGT TGGATTGAAA AGGACAACCC ATAAGGCATA   
  
  
+ TAATAATCAG CAGTAATTAA GTTTCACTTG TTTTTGATAG ACTTAATTAT ATATGTGCAG TGCATGTTTG   
  
  
+ CAGACCAGCA TGGCAAGCAT AAAACTTCAC AAGATCTCTT CCAAAGATTG TATTTGTAGA TGGCTTCAGA   
  
  
+ CATGACACAA AGTGCTACCC ACAATCAACT TACTGCTGTC TGGTAAACGT TGAAACTGGT GTCCCATAAC   
  
  
+ ACACTGTAGT ACTCTCTCCC ACACCTTGTA ACAAGTTTAC TTCATTTGGT TCTCTGGTAT TTAATTTAGT   
  
  
+ GAAGAAGATC ATTAAGCTAC TCAACAGGAA GCTTTTCAAG TAAATGCAAC TTGTATGACA ACATTCTTCT   
  
  
+ TCTGTCTGTC TCACACCACA GACTTCACTA GTTACATCAT TAATTAGGCA TGTAGGCCAA TGGTTTATCA   
  
  
+ GCTGTAATAA GGTGATATGA TCAATATGAA AAGACCAGCT TCAAATCAGA TAGGCTGTGG ATTTATTCCC   
  
  
+ CAACCACGGA CTTTAATTAG CCTCCAAGTG TACAATATAT ATGAAATGTG CATTGCTCAA TGGTATAATT   
  
  
+ AATTAGCTAG AACATACATG CGGATAGTGT CTGTGTGCAG GGAGAGATAA AGAAATCAGA GGGAGGGAGA   
  
  
+ GTTGTTGTAT ATATGTATGG ATCGATCCTG GATGTGCTGA ATCAGATTAG AAATCAATTC AACAAGCAAA   
  
  
+ TATCATTCTC ATCTTTCACA TCCACATCAT TCCAACATGA AAACTACATC TTTGTCCAGG AACGGCCACA   
  
  
+ AGTAGTGTGA AAGTATATCA GCACTCAAAT TGCTTTTCAA TAATGATGCT ATCTATTGTG ATAAGATGTT   
  
  
+ ATTTATTAGG AAGAAATTGC ATATAACAAT CTTGTTTAAG ATAAACTGGA CTGTGAACCA AGAAGGACCA   
  
  
+ AAGATGGACA AGCTATGAAG TGAAATGTCT ATGATTTTCA CTCCGAATAT GTCAGAATAG ATTGGTTTTG   
  
  
+ ACGCACCCGC ATACAGTAAT AGCCAGTCAA GGATATAAAC GTTAGTGTTG CCTTGTGCTT CTTCCATGTA   
  
  
+ TTACAGTGCT TAAGAAATCA GACTATATAT TAATCAGTAA CTCTTTTCTT GCGCACAGCC ACACATCCCC   
  
  
+ TTAATAATTC CTCAGCACTA TCAATGTTTG AGAGGGCACA GAGAGAGAGA GAGAGAGAGA GAGAGAGAGA   
  
  
+ AAGAGAGGTG AGTCTGCAAG TCTTTCCTCT GCGGTACCTG TGAGAAGATA AGAAGGAATA ACTTGCAGAA   
  
  
+ AACATTCCGT TTAGCGTTTA CTGGTTGACC AGGCTTACAT CTCAAAACAA AGGGCTATTT CTGCAGACGA   
  
  
+ TCGAATAGAG CATCAACTTG GTCATACCTG CTATTGAACC AGACCCTTTT GACAGGAAGA ATATATTAAG   
  
  
+ AAGTAGGGCT TGCTGGGTTA TCGAAAGAA  

- CAACGAACGA AATTAATTTC TTCCTTCGTT TTGTCTACCA ACCTAACTTT TCCTGTTGGG TATTCCGTAT   
  
  
- ATTATTAGTC GTCATTAATT CAAAGTGAAC AAAAACTATC TGAATTAATA TATACACGTC ACGTACAAAC   
  
  
- GTCTGGTCGT ACCGTTCGTA TTTTGAAGTG TTCTAGAGAA GGTTTCTAAC ATAAACATCT ACCGAAGTCT   
  
  
- GTACTGTGTT TCACGATGGG TGTTAGTTGA ATGACGACAG ACCATTTGCA ACTTTGACCA CAGGGTATTG   
  
  
- TGTGACATCA TGAGAGAGGG TGTGGAACAT TGTTCAAATG AAGTAAACCA AGAGACCATA AATTAAATCA   
  
  
- CTTCTTCTAG TAATTCGATG AGTTGTCCTT CGAAAAGTTC ATTTACGTTG AACATACTGT TGTAAGAAGA   
  
  
- AGACAGACAG AGTGTGGTGT CTGAAGTGAT CAATGTAGTA ATTAATCCGT ACATCCGGTT ACCAAATAGT   
  
  
- CGACATTATT CCACTATACT AGTTATACTT TTCTGGTCGA AGTTTAGTCT ATCCGACACC TAAATAAGGG   
  
  
- GTTGGTGCCT GAAATTAATC GGAGGTTCAC ATGTTATATA TACTTTACAC GTAACGAGTT ACCATATTAA   
  
  
- TTAATCGATC TTGTATGTAC GCCTATCACA GACACACGTC CCTCTCTATT TCTTTAGTCT CCCTCCCTCT   
  
  
- CAACAACATA TATACATACC TAGCTAGGAC CTACACGACT TAGTCTAATC TTTAGTTAAG TTGTTCGTTT   
  
  
- ATAGTAAGAG TAGAAAGTGT AGGTGTAGTA AGGTTGTACT TTTGATGTAG AAACAGGTCC TTGCCGGTGT   
  
  
- TCATCACACT TTCATATAGT CGTGAGTTTA ACGAAAAGTT ATTACTACGA TAGATAACAC TATTCTACAA   
  
  
- TAAATAATCC TTCTTTAACG TATATTGTTA GAACAAATTC TATTTGACCT GACACTTGGT TCTTCCTGGT   
  
  
- TTCTACCTGT TCGATACTTC ACTTTACAGA TACTAAAAGT GAGGCTTATA CAGTCTTATC TAACCAAAAC   
  
  
- TGCGTGGGCG TATGTCATTA TCGGTCAGTT CCTATATTTG CAATCACAAC GGAACACGAA GAAGGTACAT   
  
  
- AATGTCACGA ATTCTTTAGT CTGATATATA ATTAGTCATT GAGAAAAGAA CGCGTGTCGG TGTGTAGGGG   
  
  
- AATTATTAAG GAGTCGTGAT AGTTACAAAC TCTCCCGTGT CTCTCTCTCT CTCTCTCTCT CTCTCTCTCT   
  
  
- TTCTCTCCAC TCAGACGTTC AGAAAGGAGA CGCCATGGAC ACTCTTCTAT TCTTCCTTAT TGAACGTCTT   
  
  
- TTGTAAGGCA AATCGCAAAT GACCAACTGG TCCGAATGTA GAGTTTTGTT TCCCGATAAA GACGTCTGCT   
  
  
- AGCTTATCTC GTAGTTGAAC CAGTATGGAC GATAACTTGG TCTGGGAAAA CTGTCCTTCT TATATAATTC   
  
  
- TTCATCCCGA ACGACCCAAT AGCTTTCTT

+     TCA-element

| Site Name | Organism | Position | Strand | Matrix score. | sequence | function |
| --- | --- | --- | --- | --- | --- | --- |
| TCA-element | Nicotiana tabacum | 30 | - | 9 | CCATCTTTTT | cis-acting element involved in salicylic acid responsiveness |

> 2018/04/13 10:10:12  
+ GTTGCTTGCT TTAATTAAAG AAGGAAGCAA AACAGATGGT TGGATTGAAA AGGACAACCC ATAAGGCATA   
  
  
+ TAATAATCAG CAGTAATTAA GTTTCACTTG TTTTTGATAG ACTTAATTAT ATATGTGCAG TGCATGTTTG   
  
  
+ CAGACCAGCA TGGCAAGCAT AAAACTTCAC AAGATCTCTT CCAAAGATTG TATTTGTAGA TGGCTTCAGA   
  
  
+ CATGACACAA AGTGCTACCC ACAATCAACT TACTGCTGTC TGGTAAACGT TGAAACTGGT GTCCCATAAC   
  
  
+ ACACTGTAGT ACTCTCTCCC ACACCTTGTA ACAAGTTTAC TTCATTTGGT TCTCTGGTAT TTAATTTAGT   
  
  
+ GAAGAAGATC ATTAAGCTAC TCAACAGGAA GCTTTTCAAG TAAATGCAAC TTGTATGACA ACATTCTTCT   
  
  
+ TCTGTCTGTC TCACACCACA GACTTCACTA GTTACATCAT TAATTAGGCA TGTAGGCCAA TGGTTTATCA   
  
  
+ GCTGTAATAA GGTGATATGA TCAATATGAA AAGACCAGCT TCAAATCAGA TAGGCTGTGG ATTTATTCCC   
  
  
+ CAACCACGGA CTTTAATTAG CCTCCAAGTG TACAATATAT ATGAAATGTG CATTGCTCAA TGGTATAATT   
  
  
+ AATTAGCTAG AACATACATG CGGATAGTGT CTGTGTGCAG GGAGAGATAA AGAAATCAGA GGGAGGGAGA   
  
  
+ GTTGTTGTAT ATATGTATGG ATCGATCCTG GATGTGCTGA ATCAGATTAG AAATCAATTC AACAAGCAAA   
  
  
+ TATCATTCTC ATCTTTCACA TCCACATCAT TCCAACATGA AAACTACATC TTTGTCCAGG AACGGCCACA   
  
  
+ AGTAGTGTGA AAGTATATCA GCACTCAAAT TGCTTTTCAA TAATGATGCT ATCTATTGTG ATAAGATGTT   
  
  
+ ATTTATTAGG AAGAAATTGC ATATAACAAT CTTGTTTAAG ATAAACTGGA CTGTGAACCA AGAAGGACCA   
  
  
+ AAGATGGACA AGCTATGAAG TGAAATGTCT ATGATTTTCA CTCCGAATAT GTCAGAATAG ATTGGTTTTG   
  
  
+ ACGCACCCGC ATACAGTAAT AGCCAGTCAA GGATATAAAC GTTAGTGTTG CCTTGTGCTT CTTCCATGTA   
  
  
+ TTACAGTGCT TAAGAAATCA GACTATATAT TAATCAGTAA CTCTTTTCTT GCGCACAGCC ACACATCCCC   
  
  
+ TTAATAATTC CTCAGCACTA TCAATGTTTG AGAGGGCACA GAGAGAGAGA GAGAGAGAGA GAGAGAGAGA   
  
  
+ AAGAGAGGTG AGTCTGCAAG TCTTTCCTCT GCGGTACCTG TGAGAAGATA AGAAGGAATA ACTTGCAGAA   
  
  
+ AACATTCCGT TTAGCGTTTA CTGGTTGACC AGGCTTACAT CTCAAAACAA AGGGCTATTT CTGCAGACGA   
  
  
+ TCGAATAGAG CATCAACTTG GTCATACCTG CTATTGAACC AGACCCTTTT GACAGGAAGA ATATATTAAG   
  
  
+ AAGTAGGGCT TGCTGGGTTA TCGAAAGAA  

- CAACGAACGA AATTAATTTC TTCCTTCGTT TTGTCTACCA ACCTAACTTT TCCTGTTGGG TATTCCGTAT   
  
  
- ATTATTAGTC GTCATTAATT CAAAGTGAAC AAAAACTATC TGAATTAATA TATACACGTC ACGTACAAAC   
  
  
- GTCTGGTCGT ACCGTTCGTA TTTTGAAGTG TTCTAGAGAA GGTTTCTAAC ATAAACATCT ACCGAAGTCT   
  
  
- GTACTGTGTT TCACGATGGG TGTTAGTTGA ATGACGACAG ACCATTTGCA ACTTTGACCA CAGGGTATTG   
  
  
- TGTGACATCA TGAGAGAGGG TGTGGAACAT TGTTCAAATG AAGTAAACCA AGAGACCATA AATTAAATCA   
  
  
- CTTCTTCTAG TAATTCGATG AGTTGTCCTT CGAAAAGTTC ATTTACGTTG AACATACTGT TGTAAGAAGA   
  
  
- AGACAGACAG AGTGTGGTGT CTGAAGTGAT CAATGTAGTA ATTAATCCGT ACATCCGGTT ACCAAATAGT   
  
  
- CGACATTATT CCACTATACT AGTTATACTT TTCTGGTCGA AGTTTAGTCT ATCCGACACC TAAATAAGGG   
  
  
- GTTGGTGCCT GAAATTAATC GGAGGTTCAC ATGTTATATA TACTTTACAC GTAACGAGTT ACCATATTAA   
  
  
- TTAATCGATC TTGTATGTAC GCCTATCACA GACACACGTC CCTCTCTATT TCTTTAGTCT CCCTCCCTCT   
  
  
- CAACAACATA TATACATACC TAGCTAGGAC CTACACGACT TAGTCTAATC TTTAGTTAAG TTGTTCGTTT   
  
  
- ATAGTAAGAG TAGAAAGTGT AGGTGTAGTA AGGTTGTACT TTTGATGTAG AAACAGGTCC TTGCCGGTGT   
  
  
- TCATCACACT TTCATATAGT CGTGAGTTTA ACGAAAAGTT ATTACTACGA TAGATAACAC TATTCTACAA   
  
  
- TAAATAATCC TTCTTTAACG TATATTGTTA GAACAAATTC TATTTGACCT GACACTTGGT TCTTCCTGGT   
  
  
- TTCTACCTGT TCGATACTTC ACTTTACAGA TACTAAAAGT GAGGCTTATA CAGTCTTATC TAACCAAAAC   
  
  
- TGCGTGGGCG TATGTCATTA TCGGTCAGTT CCTATATTTG CAATCACAAC GGAACACGAA GAAGGTACAT   
  
  
- AATGTCACGA ATTCTTTAGT CTGATATATA ATTAGTCATT GAGAAAAGAA CGCGTGTCGG TGTGTAGGGG   
  
  
- AATTATTAAG GAGTCGTGAT AGTTACAAAC TCTCCCGTGT CTCTCTCTCT CTCTCTCTCT CTCTCTCTCT   
  
  
- TTCTCTCCAC TCAGACGTTC AGAAAGGAGA CGCCATGGAC ACTCTTCTAT TCTTCCTTAT TGAACGTCTT   
  
  
- TTGTAAGGCA AATCGCAAAT GACCAACTGG TCCGAATGTA GAGTTTTGTT TCCCGATAAA GACGTCTGCT   
  
  
- AGCTTATCTC GTAGTTGAAC CAGTATGGAC GATAACTTGG TCTGGGAAAA CTGTCCTTCT TATATAATTC   
  
  
- TTCATCCCGA ACGACCCAAT AGCTTTCTT

+     TCCC-motif

| Site Name | Organism | Position | Strand | Matrix score. | sequence | function |
| --- | --- | --- | --- | --- | --- | --- |
| TCCC-motif | Spinacia oleracea | 669 | - | 7 | TCTCCCT | part of a light responsive element |
| TCCC-motif | Spinacia oleracea | 694 | - | 7 | TCTCCCT | part of a light responsive element |

> 2018/04/13 10:10:12  
+ GTTGCTTGCT TTAATTAAAG AAGGAAGCAA AACAGATGGT TGGATTGAAA AGGACAACCC ATAAGGCATA   
  
  
+ TAATAATCAG CAGTAATTAA GTTTCACTTG TTTTTGATAG ACTTAATTAT ATATGTGCAG TGCATGTTTG   
  
  
+ CAGACCAGCA TGGCAAGCAT AAAACTTCAC AAGATCTCTT CCAAAGATTG TATTTGTAGA TGGCTTCAGA   
  
  
+ CATGACACAA AGTGCTACCC ACAATCAACT TACTGCTGTC TGGTAAACGT TGAAACTGGT GTCCCATAAC   
  
  
+ ACACTGTAGT ACTCTCTCCC ACACCTTGTA ACAAGTTTAC TTCATTTGGT TCTCTGGTAT TTAATTTAGT   
  
  
+ GAAGAAGATC ATTAAGCTAC TCAACAGGAA GCTTTTCAAG TAAATGCAAC TTGTATGACA ACATTCTTCT   
  
  
+ TCTGTCTGTC TCACACCACA GACTTCACTA GTTACATCAT TAATTAGGCA TGTAGGCCAA TGGTTTATCA   
  
  
+ GCTGTAATAA GGTGATATGA TCAATATGAA AAGACCAGCT TCAAATCAGA TAGGCTGTGG ATTTATTCCC   
  
  
+ CAACCACGGA CTTTAATTAG CCTCCAAGTG TACAATATAT ATGAAATGTG CATTGCTCAA TGGTATAATT   
  
  
+ AATTAGCTAG AACATACATG CGGATAGTGT CTGTGTGCAG GGAGAGATAA AGAAATCAGA GGGAGGGAGA   
  
  
+ GTTGTTGTAT ATATGTATGG ATCGATCCTG GATGTGCTGA ATCAGATTAG AAATCAATTC AACAAGCAAA   
  
  
+ TATCATTCTC ATCTTTCACA TCCACATCAT TCCAACATGA AAACTACATC TTTGTCCAGG AACGGCCACA   
  
  
+ AGTAGTGTGA AAGTATATCA GCACTCAAAT TGCTTTTCAA TAATGATGCT ATCTATTGTG ATAAGATGTT   
  
  
+ ATTTATTAGG AAGAAATTGC ATATAACAAT CTTGTTTAAG ATAAACTGGA CTGTGAACCA AGAAGGACCA   
  
  
+ AAGATGGACA AGCTATGAAG TGAAATGTCT ATGATTTTCA CTCCGAATAT GTCAGAATAG ATTGGTTTTG   
  
  
+ ACGCACCCGC ATACAGTAAT AGCCAGTCAA GGATATAAAC GTTAGTGTTG CCTTGTGCTT CTTCCATGTA   
  
  
+ TTACAGTGCT TAAGAAATCA GACTATATAT TAATCAGTAA CTCTTTTCTT GCGCACAGCC ACACATCCCC   
  
  
+ TTAATAATTC CTCAGCACTA TCAATGTTTG AGAGGGCACA GAGAGAGAGA GAGAGAGAGA GAGAGAGAGA   
  
  
+ AAGAGAGGTG AGTCTGCAAG TCTTTCCTCT GCGGTACCTG TGAGAAGATA AGAAGGAATA ACTTGCAGAA   
  
  
+ AACATTCCGT TTAGCGTTTA CTGGTTGACC AGGCTTACAT CTCAAAACAA AGGGCTATTT CTGCAGACGA   
  
  
+ TCGAATAGAG CATCAACTTG GTCATACCTG CTATTGAACC AGACCCTTTT GACAGGAAGA ATATATTAAG   
  
  
+ AAGTAGGGCT TGCTGGGTTA TCGAAAGAA  

- CAACGAACGA AATTAATTTC TTCCTTCGTT TTGTCTACCA ACCTAACTTT TCCTGTTGGG TATTCCGTAT   
  
  
- ATTATTAGTC GTCATTAATT CAAAGTGAAC AAAAACTATC TGAATTAATA TATACACGTC ACGTACAAAC   
  
  
- GTCTGGTCGT ACCGTTCGTA TTTTGAAGTG TTCTAGAGAA GGTTTCTAAC ATAAACATCT ACCGAAGTCT   
  
  
- GTACTGTGTT TCACGATGGG TGTTAGTTGA ATGACGACAG ACCATTTGCA ACTTTGACCA CAGGGTATTG   
  
  
- TGTGACATCA TGAGAGAGGG TGTGGAACAT TGTTCAAATG AAGTAAACCA AGAGACCATA AATTAAATCA   
  
  
- CTTCTTCTAG TAATTCGATG AGTTGTCCTT CGAAAAGTTC ATTTACGTTG AACATACTGT TGTAAGAAGA   
  
  
- AGACAGACAG AGTGTGGTGT CTGAAGTGAT CAATGTAGTA ATTAATCCGT ACATCCGGTT ACCAAATAGT   
  
  
- CGACATTATT CCACTATACT AGTTATACTT TTCTGGTCGA AGTTTAGTCT ATCCGACACC TAAATAAGGG   
  
  
- GTTGGTGCCT GAAATTAATC GGAGGTTCAC ATGTTATATA TACTTTACAC GTAACGAGTT ACCATATTAA   
  
  
- TTAATCGATC TTGTATGTAC GCCTATCACA GACACACGTC CCTCTCTATT TCTTTAGTCT CCCTCCCTCT   
  
  
- CAACAACATA TATACATACC TAGCTAGGAC CTACACGACT TAGTCTAATC TTTAGTTAAG TTGTTCGTTT   
  
  
- ATAGTAAGAG TAGAAAGTGT AGGTGTAGTA AGGTTGTACT TTTGATGTAG AAACAGGTCC TTGCCGGTGT   
  
  
- TCATCACACT TTCATATAGT CGTGAGTTTA ACGAAAAGTT ATTACTACGA TAGATAACAC TATTCTACAA   
  
  
- TAAATAATCC TTCTTTAACG TATATTGTTA GAACAAATTC TATTTGACCT GACACTTGGT TCTTCCTGGT   
  
  
- TTCTACCTGT TCGATACTTC ACTTTACAGA TACTAAAAGT GAGGCTTATA CAGTCTTATC TAACCAAAAC   
  
  
- TGCGTGGGCG TATGTCATTA TCGGTCAGTT CCTATATTTG CAATCACAAC GGAACACGAA GAAGGTACAT   
  
  
- AATGTCACGA ATTCTTTAGT CTGATATATA ATTAGTCATT GAGAAAAGAA CGCGTGTCGG TGTGTAGGGG   
  
  
- AATTATTAAG GAGTCGTGAT AGTTACAAAC TCTCCCGTGT CTCTCTCTCT CTCTCTCTCT CTCTCTCTCT   
  
  
- TTCTCTCCAC TCAGACGTTC AGAAAGGAGA CGCCATGGAC ACTCTTCTAT TCTTCCTTAT TGAACGTCTT   
  
  
- TTGTAAGGCA AATCGCAAAT GACCAACTGG TCCGAATGTA GAGTTTTGTT TCCCGATAAA GACGTCTGCT   
  
  
- AGCTTATCTC GTAGTTGAAC CAGTATGGAC GATAACTTGG TCTGGGAAAA CTGTCCTTCT TATATAATTC   
  
  
- TTCATCCCGA ACGACCCAAT AGCTTTCTT

+     TGACG-motif

| Site Name | Organism | Position | Strand | Matrix score. | sequence | function |
| --- | --- | --- | --- | --- | --- | --- |
| TGACG-motif | Hordeum vulgare | 1049 | + | 5 | TGACG | cis-acting regulatory element involved in the MeJA-responsiveness |

> 2018/04/13 10:10:12  
+ GTTGCTTGCT TTAATTAAAG AAGGAAGCAA AACAGATGGT TGGATTGAAA AGGACAACCC ATAAGGCATA   
  
  
+ TAATAATCAG CAGTAATTAA GTTTCACTTG TTTTTGATAG ACTTAATTAT ATATGTGCAG TGCATGTTTG   
  
  
+ CAGACCAGCA TGGCAAGCAT AAAACTTCAC AAGATCTCTT CCAAAGATTG TATTTGTAGA TGGCTTCAGA   
  
  
+ CATGACACAA AGTGCTACCC ACAATCAACT TACTGCTGTC TGGTAAACGT TGAAACTGGT GTCCCATAAC   
  
  
+ ACACTGTAGT ACTCTCTCCC ACACCTTGTA ACAAGTTTAC TTCATTTGGT TCTCTGGTAT TTAATTTAGT   
  
  
+ GAAGAAGATC ATTAAGCTAC TCAACAGGAA GCTTTTCAAG TAAATGCAAC TTGTATGACA ACATTCTTCT   
  
  
+ TCTGTCTGTC TCACACCACA GACTTCACTA GTTACATCAT TAATTAGGCA TGTAGGCCAA TGGTTTATCA   
  
  
+ GCTGTAATAA GGTGATATGA TCAATATGAA AAGACCAGCT TCAAATCAGA TAGGCTGTGG ATTTATTCCC   
  
  
+ CAACCACGGA CTTTAATTAG CCTCCAAGTG TACAATATAT ATGAAATGTG CATTGCTCAA TGGTATAATT   
  
  
+ AATTAGCTAG AACATACATG CGGATAGTGT CTGTGTGCAG GGAGAGATAA AGAAATCAGA GGGAGGGAGA   
  
  
+ GTTGTTGTAT ATATGTATGG ATCGATCCTG GATGTGCTGA ATCAGATTAG AAATCAATTC AACAAGCAAA   
  
  
+ TATCATTCTC ATCTTTCACA TCCACATCAT TCCAACATGA AAACTACATC TTTGTCCAGG AACGGCCACA   
  
  
+ AGTAGTGTGA AAGTATATCA GCACTCAAAT TGCTTTTCAA TAATGATGCT ATCTATTGTG ATAAGATGTT   
  
  
+ ATTTATTAGG AAGAAATTGC ATATAACAAT CTTGTTTAAG ATAAACTGGA CTGTGAACCA AGAAGGACCA   
  
  
+ AAGATGGACA AGCTATGAAG TGAAATGTCT ATGATTTTCA CTCCGAATAT GTCAGAATAG ATTGGTTTTG   
  
  
+ ACGCACCCGC ATACAGTAAT AGCCAGTCAA GGATATAAAC GTTAGTGTTG CCTTGTGCTT CTTCCATGTA   
  
  
+ TTACAGTGCT TAAGAAATCA GACTATATAT TAATCAGTAA CTCTTTTCTT GCGCACAGCC ACACATCCCC   
  
  
+ TTAATAATTC CTCAGCACTA TCAATGTTTG AGAGGGCACA GAGAGAGAGA GAGAGAGAGA GAGAGAGAGA   
  
  
+ AAGAGAGGTG AGTCTGCAAG TCTTTCCTCT GCGGTACCTG TGAGAAGATA AGAAGGAATA ACTTGCAGAA   
  
  
+ AACATTCCGT TTAGCGTTTA CTGGTTGACC AGGCTTACAT CTCAAAACAA AGGGCTATTT CTGCAGACGA   
  
  
+ TCGAATAGAG CATCAACTTG GTCATACCTG CTATTGAACC AGACCCTTTT GACAGGAAGA ATATATTAAG   
  
  
+ AAGTAGGGCT TGCTGGGTTA TCGAAAGAA  

- CAACGAACGA AATTAATTTC TTCCTTCGTT TTGTCTACCA ACCTAACTTT TCCTGTTGGG TATTCCGTAT   
  
  
- ATTATTAGTC GTCATTAATT CAAAGTGAAC AAAAACTATC TGAATTAATA TATACACGTC ACGTACAAAC   
  
  
- GTCTGGTCGT ACCGTTCGTA TTTTGAAGTG TTCTAGAGAA GGTTTCTAAC ATAAACATCT ACCGAAGTCT   
  
  
- GTACTGTGTT TCACGATGGG TGTTAGTTGA ATGACGACAG ACCATTTGCA ACTTTGACCA CAGGGTATTG   
  
  
- TGTGACATCA TGAGAGAGGG TGTGGAACAT TGTTCAAATG AAGTAAACCA AGAGACCATA AATTAAATCA   
  
  
- CTTCTTCTAG TAATTCGATG AGTTGTCCTT CGAAAAGTTC ATTTACGTTG AACATACTGT TGTAAGAAGA   
  
  
- AGACAGACAG AGTGTGGTGT CTGAAGTGAT CAATGTAGTA ATTAATCCGT ACATCCGGTT ACCAAATAGT   
  
  
- CGACATTATT CCACTATACT AGTTATACTT TTCTGGTCGA AGTTTAGTCT ATCCGACACC TAAATAAGGG   
  
  
- GTTGGTGCCT GAAATTAATC GGAGGTTCAC ATGTTATATA TACTTTACAC GTAACGAGTT ACCATATTAA   
  
  
- TTAATCGATC TTGTATGTAC GCCTATCACA GACACACGTC CCTCTCTATT TCTTTAGTCT CCCTCCCTCT   
  
  
- CAACAACATA TATACATACC TAGCTAGGAC CTACACGACT TAGTCTAATC TTTAGTTAAG TTGTTCGTTT   
  
  
- ATAGTAAGAG TAGAAAGTGT AGGTGTAGTA AGGTTGTACT TTTGATGTAG AAACAGGTCC TTGCCGGTGT   
  
  
- TCATCACACT TTCATATAGT CGTGAGTTTA ACGAAAAGTT ATTACTACGA TAGATAACAC TATTCTACAA   
  
  
- TAAATAATCC TTCTTTAACG TATATTGTTA GAACAAATTC TATTTGACCT GACACTTGGT TCTTCCTGGT   
  
  
- TTCTACCTGT TCGATACTTC ACTTTACAGA TACTAAAAGT GAGGCTTATA CAGTCTTATC TAACCAAAAC   
  
  
- TGCGTGGGCG TATGTCATTA TCGGTCAGTT CCTATATTTG CAATCACAAC GGAACACGAA GAAGGTACAT   
  
  
- AATGTCACGA ATTCTTTAGT CTGATATATA ATTAGTCATT GAGAAAAGAA CGCGTGTCGG TGTGTAGGGG   
  
  
- AATTATTAAG GAGTCGTGAT AGTTACAAAC TCTCCCGTGT CTCTCTCTCT CTCTCTCTCT CTCTCTCTCT   
  
  
- TTCTCTCCAC TCAGACGTTC AGAAAGGAGA CGCCATGGAC ACTCTTCTAT TCTTCCTTAT TGAACGTCTT   
  
  
- TTGTAAGGCA AATCGCAAAT GACCAACTGG TCCGAATGTA GAGTTTTGTT TCCCGATAAA GACGTCTGCT   
  
  
- AGCTTATCTC GTAGTTGAAC CAGTATGGAC GATAACTTGG TCTGGGAAAA CTGTCCTTCT TATATAATTC   
  
  
- TTCATCCCGA ACGACCCAAT AGCTTTCTT

+     Unnamed\_\_1

| Site Name | Organism | Position | Strand | Matrix score. | sequence | function |
| --- | --- | --- | --- | --- | --- | --- |
| Unnamed\_\_1 | Zea mays | 564 | - | 5 | CGTGG |  |

> 2018/04/13 10:10:12  
+ GTTGCTTGCT TTAATTAAAG AAGGAAGCAA AACAGATGGT TGGATTGAAA AGGACAACCC ATAAGGCATA   
  
  
+ TAATAATCAG CAGTAATTAA GTTTCACTTG TTTTTGATAG ACTTAATTAT ATATGTGCAG TGCATGTTTG   
  
  
+ CAGACCAGCA TGGCAAGCAT AAAACTTCAC AAGATCTCTT CCAAAGATTG TATTTGTAGA TGGCTTCAGA   
  
  
+ CATGACACAA AGTGCTACCC ACAATCAACT TACTGCTGTC TGGTAAACGT TGAAACTGGT GTCCCATAAC   
  
  
+ ACACTGTAGT ACTCTCTCCC ACACCTTGTA ACAAGTTTAC TTCATTTGGT TCTCTGGTAT TTAATTTAGT   
  
  
+ GAAGAAGATC ATTAAGCTAC TCAACAGGAA GCTTTTCAAG TAAATGCAAC TTGTATGACA ACATTCTTCT   
  
  
+ TCTGTCTGTC TCACACCACA GACTTCACTA GTTACATCAT TAATTAGGCA TGTAGGCCAA TGGTTTATCA   
  
  
+ GCTGTAATAA GGTGATATGA TCAATATGAA AAGACCAGCT TCAAATCAGA TAGGCTGTGG ATTTATTCCC   
  
  
+ CAACCACGGA CTTTAATTAG CCTCCAAGTG TACAATATAT ATGAAATGTG CATTGCTCAA TGGTATAATT   
  
  
+ AATTAGCTAG AACATACATG CGGATAGTGT CTGTGTGCAG GGAGAGATAA AGAAATCAGA GGGAGGGAGA   
  
  
+ GTTGTTGTAT ATATGTATGG ATCGATCCTG GATGTGCTGA ATCAGATTAG AAATCAATTC AACAAGCAAA   
  
  
+ TATCATTCTC ATCTTTCACA TCCACATCAT TCCAACATGA AAACTACATC TTTGTCCAGG AACGGCCACA   
  
  
+ AGTAGTGTGA AAGTATATCA GCACTCAAAT TGCTTTTCAA TAATGATGCT ATCTATTGTG ATAAGATGTT   
  
  
+ ATTTATTAGG AAGAAATTGC ATATAACAAT CTTGTTTAAG ATAAACTGGA CTGTGAACCA AGAAGGACCA   
  
  
+ AAGATGGACA AGCTATGAAG TGAAATGTCT ATGATTTTCA CTCCGAATAT GTCAGAATAG ATTGGTTTTG   
  
  
+ ACGCACCCGC ATACAGTAAT AGCCAGTCAA GGATATAAAC GTTAGTGTTG CCTTGTGCTT CTTCCATGTA   
  
  
+ TTACAGTGCT TAAGAAATCA GACTATATAT TAATCAGTAA CTCTTTTCTT GCGCACAGCC ACACATCCCC   
  
  
+ TTAATAATTC CTCAGCACTA TCAATGTTTG AGAGGGCACA GAGAGAGAGA GAGAGAGAGA GAGAGAGAGA   
  
  
+ AAGAGAGGTG AGTCTGCAAG TCTTTCCTCT GCGGTACCTG TGAGAAGATA AGAAGGAATA ACTTGCAGAA   
  
  
+ AACATTCCGT TTAGCGTTTA CTGGTTGACC AGGCTTACAT CTCAAAACAA AGGGCTATTT CTGCAGACGA   
  
  
+ TCGAATAGAG CATCAACTTG GTCATACCTG CTATTGAACC AGACCCTTTT GACAGGAAGA ATATATTAAG   
  
  
+ AAGTAGGGCT TGCTGGGTTA TCGAAAGAA  

- CAACGAACGA AATTAATTTC TTCCTTCGTT TTGTCTACCA ACCTAACTTT TCCTGTTGGG TATTCCGTAT   
  
  
- ATTATTAGTC GTCATTAATT CAAAGTGAAC AAAAACTATC TGAATTAATA TATACACGTC ACGTACAAAC   
  
  
- GTCTGGTCGT ACCGTTCGTA TTTTGAAGTG TTCTAGAGAA GGTTTCTAAC ATAAACATCT ACCGAAGTCT   
  
  
- GTACTGTGTT TCACGATGGG TGTTAGTTGA ATGACGACAG ACCATTTGCA ACTTTGACCA CAGGGTATTG   
  
  
- TGTGACATCA TGAGAGAGGG TGTGGAACAT TGTTCAAATG AAGTAAACCA AGAGACCATA AATTAAATCA   
  
  
- CTTCTTCTAG TAATTCGATG AGTTGTCCTT CGAAAAGTTC ATTTACGTTG AACATACTGT TGTAAGAAGA   
  
  
- AGACAGACAG AGTGTGGTGT CTGAAGTGAT CAATGTAGTA ATTAATCCGT ACATCCGGTT ACCAAATAGT   
  
  
- CGACATTATT CCACTATACT AGTTATACTT TTCTGGTCGA AGTTTAGTCT ATCCGACACC TAAATAAGGG   
  
  
- GTTGGTGCCT GAAATTAATC GGAGGTTCAC ATGTTATATA TACTTTACAC GTAACGAGTT ACCATATTAA   
  
  
- TTAATCGATC TTGTATGTAC GCCTATCACA GACACACGTC CCTCTCTATT TCTTTAGTCT CCCTCCCTCT   
  
  
- CAACAACATA TATACATACC TAGCTAGGAC CTACACGACT TAGTCTAATC TTTAGTTAAG TTGTTCGTTT   
  
  
- ATAGTAAGAG TAGAAAGTGT AGGTGTAGTA AGGTTGTACT TTTGATGTAG AAACAGGTCC TTGCCGGTGT   
  
  
- TCATCACACT TTCATATAGT CGTGAGTTTA ACGAAAAGTT ATTACTACGA TAGATAACAC TATTCTACAA   
  
  
- TAAATAATCC TTCTTTAACG TATATTGTTA GAACAAATTC TATTTGACCT GACACTTGGT TCTTCCTGGT   
  
  
- TTCTACCTGT TCGATACTTC ACTTTACAGA TACTAAAAGT GAGGCTTATA CAGTCTTATC TAACCAAAAC   
  
  
- TGCGTGGGCG TATGTCATTA TCGGTCAGTT CCTATATTTG CAATCACAAC GGAACACGAA GAAGGTACAT   
  
  
- AATGTCACGA ATTCTTTAGT CTGATATATA ATTAGTCATT GAGAAAAGAA CGCGTGTCGG TGTGTAGGGG   
  
  
- AATTATTAAG GAGTCGTGAT AGTTACAAAC TCTCCCGTGT CTCTCTCTCT CTCTCTCTCT CTCTCTCTCT   
  
  
- TTCTCTCCAC TCAGACGTTC AGAAAGGAGA CGCCATGGAC ACTCTTCTAT TCTTCCTTAT TGAACGTCTT   
  
  
- TTGTAAGGCA AATCGCAAAT GACCAACTGG TCCGAATGTA GAGTTTTGTT TCCCGATAAA GACGTCTGCT   
  
  
- AGCTTATCTC GTAGTTGAAC CAGTATGGAC GATAACTTGG TCTGGGAAAA CTGTCCTTCT TATATAATTC   
  
  
- TTCATCCCGA ACGACCCAAT AGCTTTCTT

+     Unnamed\_\_13

| Site Name | Organism | Position | Strand | Matrix score. | sequence | function |
| --- | --- | --- | --- | --- | --- | --- |
| Unnamed\_\_13 | Zea mays | 583 | + | 9 | TCCAAGTATA |  |

> 2018/04/13 10:10:12  
+ GTTGCTTGCT TTAATTAAAG AAGGAAGCAA AACAGATGGT TGGATTGAAA AGGACAACCC ATAAGGCATA   
  
  
+ TAATAATCAG CAGTAATTAA GTTTCACTTG TTTTTGATAG ACTTAATTAT ATATGTGCAG TGCATGTTTG   
  
  
+ CAGACCAGCA TGGCAAGCAT AAAACTTCAC AAGATCTCTT CCAAAGATTG TATTTGTAGA TGGCTTCAGA   
  
  
+ CATGACACAA AGTGCTACCC ACAATCAACT TACTGCTGTC TGGTAAACGT TGAAACTGGT GTCCCATAAC   
  
  
+ ACACTGTAGT ACTCTCTCCC ACACCTTGTA ACAAGTTTAC TTCATTTGGT TCTCTGGTAT TTAATTTAGT   
  
  
+ GAAGAAGATC ATTAAGCTAC TCAACAGGAA GCTTTTCAAG TAAATGCAAC TTGTATGACA ACATTCTTCT   
  
  
+ TCTGTCTGTC TCACACCACA GACTTCACTA GTTACATCAT TAATTAGGCA TGTAGGCCAA TGGTTTATCA   
  
  
+ GCTGTAATAA GGTGATATGA TCAATATGAA AAGACCAGCT TCAAATCAGA TAGGCTGTGG ATTTATTCCC   
  
  
+ CAACCACGGA CTTTAATTAG CCTCCAAGTG TACAATATAT ATGAAATGTG CATTGCTCAA TGGTATAATT   
  
  
+ AATTAGCTAG AACATACATG CGGATAGTGT CTGTGTGCAG GGAGAGATAA AGAAATCAGA GGGAGGGAGA   
  
  
+ GTTGTTGTAT ATATGTATGG ATCGATCCTG GATGTGCTGA ATCAGATTAG AAATCAATTC AACAAGCAAA   
  
  
+ TATCATTCTC ATCTTTCACA TCCACATCAT TCCAACATGA AAACTACATC TTTGTCCAGG AACGGCCACA   
  
  
+ AGTAGTGTGA AAGTATATCA GCACTCAAAT TGCTTTTCAA TAATGATGCT ATCTATTGTG ATAAGATGTT   
  
  
+ ATTTATTAGG AAGAAATTGC ATATAACAAT CTTGTTTAAG ATAAACTGGA CTGTGAACCA AGAAGGACCA   
  
  
+ AAGATGGACA AGCTATGAAG TGAAATGTCT ATGATTTTCA CTCCGAATAT GTCAGAATAG ATTGGTTTTG   
  
  
+ ACGCACCCGC ATACAGTAAT AGCCAGTCAA GGATATAAAC GTTAGTGTTG CCTTGTGCTT CTTCCATGTA   
  
  
+ TTACAGTGCT TAAGAAATCA GACTATATAT TAATCAGTAA CTCTTTTCTT GCGCACAGCC ACACATCCCC   
  
  
+ TTAATAATTC CTCAGCACTA TCAATGTTTG AGAGGGCACA GAGAGAGAGA GAGAGAGAGA GAGAGAGAGA   
  
  
+ AAGAGAGGTG AGTCTGCAAG TCTTTCCTCT GCGGTACCTG TGAGAAGATA AGAAGGAATA ACTTGCAGAA   
  
  
+ AACATTCCGT TTAGCGTTTA CTGGTTGACC AGGCTTACAT CTCAAAACAA AGGGCTATTT CTGCAGACGA   
  
  
+ TCGAATAGAG CATCAACTTG GTCATACCTG CTATTGAACC AGACCCTTTT GACAGGAAGA ATATATTAAG   
  
  
+ AAGTAGGGCT TGCTGGGTTA TCGAAAGAA  

- CAACGAACGA AATTAATTTC TTCCTTCGTT TTGTCTACCA ACCTAACTTT TCCTGTTGGG TATTCCGTAT   
  
  
- ATTATTAGTC GTCATTAATT CAAAGTGAAC AAAAACTATC TGAATTAATA TATACACGTC ACGTACAAAC   
  
  
- GTCTGGTCGT ACCGTTCGTA TTTTGAAGTG TTCTAGAGAA GGTTTCTAAC ATAAACATCT ACCGAAGTCT   
  
  
- GTACTGTGTT TCACGATGGG TGTTAGTTGA ATGACGACAG ACCATTTGCA ACTTTGACCA CAGGGTATTG   
  
  
- TGTGACATCA TGAGAGAGGG TGTGGAACAT TGTTCAAATG AAGTAAACCA AGAGACCATA AATTAAATCA   
  
  
- CTTCTTCTAG TAATTCGATG AGTTGTCCTT CGAAAAGTTC ATTTACGTTG AACATACTGT TGTAAGAAGA   
  
  
- AGACAGACAG AGTGTGGTGT CTGAAGTGAT CAATGTAGTA ATTAATCCGT ACATCCGGTT ACCAAATAGT   
  
  
- CGACATTATT CCACTATACT AGTTATACTT TTCTGGTCGA AGTTTAGTCT ATCCGACACC TAAATAAGGG   
  
  
- GTTGGTGCCT GAAATTAATC GGAGGTTCAC ATGTTATATA TACTTTACAC GTAACGAGTT ACCATATTAA   
  
  
- TTAATCGATC TTGTATGTAC GCCTATCACA GACACACGTC CCTCTCTATT TCTTTAGTCT CCCTCCCTCT   
  
  
- CAACAACATA TATACATACC TAGCTAGGAC CTACACGACT TAGTCTAATC TTTAGTTAAG TTGTTCGTTT   
  
  
- ATAGTAAGAG TAGAAAGTGT AGGTGTAGTA AGGTTGTACT TTTGATGTAG AAACAGGTCC TTGCCGGTGT   
  
  
- TCATCACACT TTCATATAGT CGTGAGTTTA ACGAAAAGTT ATTACTACGA TAGATAACAC TATTCTACAA   
  
  
- TAAATAATCC TTCTTTAACG TATATTGTTA GAACAAATTC TATTTGACCT GACACTTGGT TCTTCCTGGT   
  
  
- TTCTACCTGT TCGATACTTC ACTTTACAGA TACTAAAAGT GAGGCTTATA CAGTCTTATC TAACCAAAAC   
  
  
- TGCGTGGGCG TATGTCATTA TCGGTCAGTT CCTATATTTG CAATCACAAC GGAACACGAA GAAGGTACAT   
  
  
- AATGTCACGA ATTCTTTAGT CTGATATATA ATTAGTCATT GAGAAAAGAA CGCGTGTCGG TGTGTAGGGG   
  
  
- AATTATTAAG GAGTCGTGAT AGTTACAAAC TCTCCCGTGT CTCTCTCTCT CTCTCTCTCT CTCTCTCTCT   
  
  
- TTCTCTCCAC TCAGACGTTC AGAAAGGAGA CGCCATGGAC ACTCTTCTAT TCTTCCTTAT TGAACGTCTT   
  
  
- TTGTAAGGCA AATCGCAAAT GACCAACTGG TCCGAATGTA GAGTTTTGTT TCCCGATAAA GACGTCTGCT   
  
  
- AGCTTATCTC GTAGTTGAAC CAGTATGGAC GATAACTTGG TCTGGGAAAA CTGTCCTTCT TATATAATTC   
  
  
- TTCATCCCGA ACGACCCAAT AGCTTTCTT

+     Unnamed\_\_3

| Site Name | Organism | Position | Strand | Matrix score. | sequence | function |
| --- | --- | --- | --- | --- | --- | --- |
| Unnamed\_\_3 | Zea mays | 564 | - | 5 | CGTGG |  |

> 2018/04/13 10:10:12  
+ GTTGCTTGCT TTAATTAAAG AAGGAAGCAA AACAGATGGT TGGATTGAAA AGGACAACCC ATAAGGCATA   
  
  
+ TAATAATCAG CAGTAATTAA GTTTCACTTG TTTTTGATAG ACTTAATTAT ATATGTGCAG TGCATGTTTG   
  
  
+ CAGACCAGCA TGGCAAGCAT AAAACTTCAC AAGATCTCTT CCAAAGATTG TATTTGTAGA TGGCTTCAGA   
  
  
+ CATGACACAA AGTGCTACCC ACAATCAACT TACTGCTGTC TGGTAAACGT TGAAACTGGT GTCCCATAAC   
  
  
+ ACACTGTAGT ACTCTCTCCC ACACCTTGTA ACAAGTTTAC TTCATTTGGT TCTCTGGTAT TTAATTTAGT   
  
  
+ GAAGAAGATC ATTAAGCTAC TCAACAGGAA GCTTTTCAAG TAAATGCAAC TTGTATGACA ACATTCTTCT   
  
  
+ TCTGTCTGTC TCACACCACA GACTTCACTA GTTACATCAT TAATTAGGCA TGTAGGCCAA TGGTTTATCA   
  
  
+ GCTGTAATAA GGTGATATGA TCAATATGAA AAGACCAGCT TCAAATCAGA TAGGCTGTGG ATTTATTCCC   
  
  
+ CAACCACGGA CTTTAATTAG CCTCCAAGTG TACAATATAT ATGAAATGTG CATTGCTCAA TGGTATAATT   
  
  
+ AATTAGCTAG AACATACATG CGGATAGTGT CTGTGTGCAG GGAGAGATAA AGAAATCAGA GGGAGGGAGA   
  
  
+ GTTGTTGTAT ATATGTATGG ATCGATCCTG GATGTGCTGA ATCAGATTAG AAATCAATTC AACAAGCAAA   
  
  
+ TATCATTCTC ATCTTTCACA TCCACATCAT TCCAACATGA AAACTACATC TTTGTCCAGG AACGGCCACA   
  
  
+ AGTAGTGTGA AAGTATATCA GCACTCAAAT TGCTTTTCAA TAATGATGCT ATCTATTGTG ATAAGATGTT   
  
  
+ ATTTATTAGG AAGAAATTGC ATATAACAAT CTTGTTTAAG ATAAACTGGA CTGTGAACCA AGAAGGACCA   
  
  
+ AAGATGGACA AGCTATGAAG TGAAATGTCT ATGATTTTCA CTCCGAATAT GTCAGAATAG ATTGGTTTTG   
  
  
+ ACGCACCCGC ATACAGTAAT AGCCAGTCAA GGATATAAAC GTTAGTGTTG CCTTGTGCTT CTTCCATGTA   
  
  
+ TTACAGTGCT TAAGAAATCA GACTATATAT TAATCAGTAA CTCTTTTCTT GCGCACAGCC ACACATCCCC   
  
  
+ TTAATAATTC CTCAGCACTA TCAATGTTTG AGAGGGCACA GAGAGAGAGA GAGAGAGAGA GAGAGAGAGA   
  
  
+ AAGAGAGGTG AGTCTGCAAG TCTTTCCTCT GCGGTACCTG TGAGAAGATA AGAAGGAATA ACTTGCAGAA   
  
  
+ AACATTCCGT TTAGCGTTTA CTGGTTGACC AGGCTTACAT CTCAAAACAA AGGGCTATTT CTGCAGACGA   
  
  
+ TCGAATAGAG CATCAACTTG GTCATACCTG CTATTGAACC AGACCCTTTT GACAGGAAGA ATATATTAAG   
  
  
+ AAGTAGGGCT TGCTGGGTTA TCGAAAGAA  

- CAACGAACGA AATTAATTTC TTCCTTCGTT TTGTCTACCA ACCTAACTTT TCCTGTTGGG TATTCCGTAT   
  
  
- ATTATTAGTC GTCATTAATT CAAAGTGAAC AAAAACTATC TGAATTAATA TATACACGTC ACGTACAAAC   
  
  
- GTCTGGTCGT ACCGTTCGTA TTTTGAAGTG TTCTAGAGAA GGTTTCTAAC ATAAACATCT ACCGAAGTCT   
  
  
- GTACTGTGTT TCACGATGGG TGTTAGTTGA ATGACGACAG ACCATTTGCA ACTTTGACCA CAGGGTATTG   
  
  
- TGTGACATCA TGAGAGAGGG TGTGGAACAT TGTTCAAATG AAGTAAACCA AGAGACCATA AATTAAATCA   
  
  
- CTTCTTCTAG TAATTCGATG AGTTGTCCTT CGAAAAGTTC ATTTACGTTG AACATACTGT TGTAAGAAGA   
  
  
- AGACAGACAG AGTGTGGTGT CTGAAGTGAT CAATGTAGTA ATTAATCCGT ACATCCGGTT ACCAAATAGT   
  
  
- CGACATTATT CCACTATACT AGTTATACTT TTCTGGTCGA AGTTTAGTCT ATCCGACACC TAAATAAGGG   
  
  
- GTTGGTGCCT GAAATTAATC GGAGGTTCAC ATGTTATATA TACTTTACAC GTAACGAGTT ACCATATTAA   
  
  
- TTAATCGATC TTGTATGTAC GCCTATCACA GACACACGTC CCTCTCTATT TCTTTAGTCT CCCTCCCTCT   
  
  
- CAACAACATA TATACATACC TAGCTAGGAC CTACACGACT TAGTCTAATC TTTAGTTAAG TTGTTCGTTT   
  
  
- ATAGTAAGAG TAGAAAGTGT AGGTGTAGTA AGGTTGTACT TTTGATGTAG AAACAGGTCC TTGCCGGTGT   
  
  
- TCATCACACT TTCATATAGT CGTGAGTTTA ACGAAAAGTT ATTACTACGA TAGATAACAC TATTCTACAA   
  
  
- TAAATAATCC TTCTTTAACG TATATTGTTA GAACAAATTC TATTTGACCT GACACTTGGT TCTTCCTGGT   
  
  
- TTCTACCTGT TCGATACTTC ACTTTACAGA TACTAAAAGT GAGGCTTATA CAGTCTTATC TAACCAAAAC   
  
  
- TGCGTGGGCG TATGTCATTA TCGGTCAGTT CCTATATTTG CAATCACAAC GGAACACGAA GAAGGTACAT   
  
  
- AATGTCACGA ATTCTTTAGT CTGATATATA ATTAGTCATT GAGAAAAGAA CGCGTGTCGG TGTGTAGGGG   
  
  
- AATTATTAAG GAGTCGTGAT AGTTACAAAC TCTCCCGTGT CTCTCTCTCT CTCTCTCTCT CTCTCTCTCT   
  
  
- TTCTCTCCAC TCAGACGTTC AGAAAGGAGA CGCCATGGAC ACTCTTCTAT TCTTCCTTAT TGAACGTCTT   
  
  
- TTGTAAGGCA AATCGCAAAT GACCAACTGG TCCGAATGTA GAGTTTTGTT TCCCGATAAA GACGTCTGCT   
  
  
- AGCTTATCTC GTAGTTGAAC CAGTATGGAC GATAACTTGG TCTGGGAAAA CTGTCCTTCT TATATAATTC   
  
  
- TTCATCCCGA ACGACCCAAT AGCTTTCTT

+     Unnamed\_\_4

| Site Name | Organism | Position | Strand | Matrix score. | sequence | function |
| --- | --- | --- | --- | --- | --- | --- |
| Unnamed\_\_4 | Petroselinum hortense | 692 | - | 4 | CTCC |  |
| Unnamed\_\_4 | Petroselinum hortense | 671 | - | 4 | CTCC |  |
| Unnamed\_\_4 | Petroselinum hortense | 696 | - | 4 | CTCC |  |
| Unnamed\_\_4 | Petroselinum hortense | 296 | + | 4 | CTCC |  |
| Unnamed\_\_4 | Petroselinum hortense | 1021 | + | 4 | CTCC |  |
| Unnamed\_\_4 | Petroselinum hortense | 582 | + | 4 | CTCC |  |

> 2018/04/13 10:10:12  
+ GTTGCTTGCT TTAATTAAAG AAGGAAGCAA AACAGATGGT TGGATTGAAA AGGACAACCC ATAAGGCATA   
  
  
+ TAATAATCAG CAGTAATTAA GTTTCACTTG TTTTTGATAG ACTTAATTAT ATATGTGCAG TGCATGTTTG   
  
  
+ CAGACCAGCA TGGCAAGCAT AAAACTTCAC AAGATCTCTT CCAAAGATTG TATTTGTAGA TGGCTTCAGA   
  
  
+ CATGACACAA AGTGCTACCC ACAATCAACT TACTGCTGTC TGGTAAACGT TGAAACTGGT GTCCCATAAC   
  
  
+ ACACTGTAGT ACTCTCTCCC ACACCTTGTA ACAAGTTTAC TTCATTTGGT TCTCTGGTAT TTAATTTAGT   
  
  
+ GAAGAAGATC ATTAAGCTAC TCAACAGGAA GCTTTTCAAG TAAATGCAAC TTGTATGACA ACATTCTTCT   
  
  
+ TCTGTCTGTC TCACACCACA GACTTCACTA GTTACATCAT TAATTAGGCA TGTAGGCCAA TGGTTTATCA   
  
  
+ GCTGTAATAA GGTGATATGA TCAATATGAA AAGACCAGCT TCAAATCAGA TAGGCTGTGG ATTTATTCCC   
  
  
+ CAACCACGGA CTTTAATTAG CCTCCAAGTG TACAATATAT ATGAAATGTG CATTGCTCAA TGGTATAATT   
  
  
+ AATTAGCTAG AACATACATG CGGATAGTGT CTGTGTGCAG GGAGAGATAA AGAAATCAGA GGGAGGGAGA   
  
  
+ GTTGTTGTAT ATATGTATGG ATCGATCCTG GATGTGCTGA ATCAGATTAG AAATCAATTC AACAAGCAAA   
  
  
+ TATCATTCTC ATCTTTCACA TCCACATCAT TCCAACATGA AAACTACATC TTTGTCCAGG AACGGCCACA   
  
  
+ AGTAGTGTGA AAGTATATCA GCACTCAAAT TGCTTTTCAA TAATGATGCT ATCTATTGTG ATAAGATGTT   
  
  
+ ATTTATTAGG AAGAAATTGC ATATAACAAT CTTGTTTAAG ATAAACTGGA CTGTGAACCA AGAAGGACCA   
  
  
+ AAGATGGACA AGCTATGAAG TGAAATGTCT ATGATTTTCA CTCCGAATAT GTCAGAATAG ATTGGTTTTG   
  
  
+ ACGCACCCGC ATACAGTAAT AGCCAGTCAA GGATATAAAC GTTAGTGTTG CCTTGTGCTT CTTCCATGTA   
  
  
+ TTACAGTGCT TAAGAAATCA GACTATATAT TAATCAGTAA CTCTTTTCTT GCGCACAGCC ACACATCCCC   
  
  
+ TTAATAATTC CTCAGCACTA TCAATGTTTG AGAGGGCACA GAGAGAGAGA GAGAGAGAGA GAGAGAGAGA   
  
  
+ AAGAGAGGTG AGTCTGCAAG TCTTTCCTCT GCGGTACCTG TGAGAAGATA AGAAGGAATA ACTTGCAGAA   
  
  
+ AACATTCCGT TTAGCGTTTA CTGGTTGACC AGGCTTACAT CTCAAAACAA AGGGCTATTT CTGCAGACGA   
  
  
+ TCGAATAGAG CATCAACTTG GTCATACCTG CTATTGAACC AGACCCTTTT GACAGGAAGA ATATATTAAG   
  
  
+ AAGTAGGGCT TGCTGGGTTA TCGAAAGAA  

- CAACGAACGA AATTAATTTC TTCCTTCGTT TTGTCTACCA ACCTAACTTT TCCTGTTGGG TATTCCGTAT   
  
  
- ATTATTAGTC GTCATTAATT CAAAGTGAAC AAAAACTATC TGAATTAATA TATACACGTC ACGTACAAAC   
  
  
- GTCTGGTCGT ACCGTTCGTA TTTTGAAGTG TTCTAGAGAA GGTTTCTAAC ATAAACATCT ACCGAAGTCT   
  
  
- GTACTGTGTT TCACGATGGG TGTTAGTTGA ATGACGACAG ACCATTTGCA ACTTTGACCA CAGGGTATTG   
  
  
- TGTGACATCA TGAGAGAGGG TGTGGAACAT TGTTCAAATG AAGTAAACCA AGAGACCATA AATTAAATCA   
  
  
- CTTCTTCTAG TAATTCGATG AGTTGTCCTT CGAAAAGTTC ATTTACGTTG AACATACTGT TGTAAGAAGA   
  
  
- AGACAGACAG AGTGTGGTGT CTGAAGTGAT CAATGTAGTA ATTAATCCGT ACATCCGGTT ACCAAATAGT   
  
  
- CGACATTATT CCACTATACT AGTTATACTT TTCTGGTCGA AGTTTAGTCT ATCCGACACC TAAATAAGGG   
  
  
- GTTGGTGCCT GAAATTAATC GGAGGTTCAC ATGTTATATA TACTTTACAC GTAACGAGTT ACCATATTAA   
  
  
- TTAATCGATC TTGTATGTAC GCCTATCACA GACACACGTC CCTCTCTATT TCTTTAGTCT CCCTCCCTCT   
  
  
- CAACAACATA TATACATACC TAGCTAGGAC CTACACGACT TAGTCTAATC TTTAGTTAAG TTGTTCGTTT   
  
  
- ATAGTAAGAG TAGAAAGTGT AGGTGTAGTA AGGTTGTACT TTTGATGTAG AAACAGGTCC TTGCCGGTGT   
  
  
- TCATCACACT TTCATATAGT CGTGAGTTTA ACGAAAAGTT ATTACTACGA TAGATAACAC TATTCTACAA   
  
  
- TAAATAATCC TTCTTTAACG TATATTGTTA GAACAAATTC TATTTGACCT GACACTTGGT TCTTCCTGGT   
  
  
- TTCTACCTGT TCGATACTTC ACTTTACAGA TACTAAAAGT GAGGCTTATA CAGTCTTATC TAACCAAAAC   
  
  
- TGCGTGGGCG TATGTCATTA TCGGTCAGTT CCTATATTTG CAATCACAAC GGAACACGAA GAAGGTACAT   
  
  
- AATGTCACGA ATTCTTTAGT CTGATATATA ATTAGTCATT GAGAAAAGAA CGCGTGTCGG TGTGTAGGGG   
  
  
- AATTATTAAG GAGTCGTGAT AGTTACAAAC TCTCCCGTGT CTCTCTCTCT CTCTCTCTCT CTCTCTCTCT   
  
  
- TTCTCTCCAC TCAGACGTTC AGAAAGGAGA CGCCATGGAC ACTCTTCTAT TCTTCCTTAT TGAACGTCTT   
  
  
- TTGTAAGGCA AATCGCAAAT GACCAACTGG TCCGAATGTA GAGTTTTGTT TCCCGATAAA GACGTCTGCT   
  
  
- AGCTTATCTC GTAGTTGAAC CAGTATGGAC GATAACTTGG TCTGGGAAAA CTGTCCTTCT TATATAATTC   
  
  
- TTCATCCCGA ACGACCCAAT AGCTTTCTT

+     W box

| Site Name | Organism | Position | Strand | Matrix score. | sequence | function |
| --- | --- | --- | --- | --- | --- | --- |
| W box | Arabidopsis thaliana | 1355 | + | 6 | TTGACC |  |

> 2018/04/13 10:10:12  
+ GTTGCTTGCT TTAATTAAAG AAGGAAGCAA AACAGATGGT TGGATTGAAA AGGACAACCC ATAAGGCATA   
  
  
+ TAATAATCAG CAGTAATTAA GTTTCACTTG TTTTTGATAG ACTTAATTAT ATATGTGCAG TGCATGTTTG   
  
  
+ CAGACCAGCA TGGCAAGCAT AAAACTTCAC AAGATCTCTT CCAAAGATTG TATTTGTAGA TGGCTTCAGA   
  
  
+ CATGACACAA AGTGCTACCC ACAATCAACT TACTGCTGTC TGGTAAACGT TGAAACTGGT GTCCCATAAC   
  
  
+ ACACTGTAGT ACTCTCTCCC ACACCTTGTA ACAAGTTTAC TTCATTTGGT TCTCTGGTAT TTAATTTAGT   
  
  
+ GAAGAAGATC ATTAAGCTAC TCAACAGGAA GCTTTTCAAG TAAATGCAAC TTGTATGACA ACATTCTTCT   
  
  
+ TCTGTCTGTC TCACACCACA GACTTCACTA GTTACATCAT TAATTAGGCA TGTAGGCCAA TGGTTTATCA   
  
  
+ GCTGTAATAA GGTGATATGA TCAATATGAA AAGACCAGCT TCAAATCAGA TAGGCTGTGG ATTTATTCCC   
  
  
+ CAACCACGGA CTTTAATTAG CCTCCAAGTG TACAATATAT ATGAAATGTG CATTGCTCAA TGGTATAATT   
  
  
+ AATTAGCTAG AACATACATG CGGATAGTGT CTGTGTGCAG GGAGAGATAA AGAAATCAGA GGGAGGGAGA   
  
  
+ GTTGTTGTAT ATATGTATGG ATCGATCCTG GATGTGCTGA ATCAGATTAG AAATCAATTC AACAAGCAAA   
  
  
+ TATCATTCTC ATCTTTCACA TCCACATCAT TCCAACATGA AAACTACATC TTTGTCCAGG AACGGCCACA   
  
  
+ AGTAGTGTGA AAGTATATCA GCACTCAAAT TGCTTTTCAA TAATGATGCT ATCTATTGTG ATAAGATGTT   
  
  
+ ATTTATTAGG AAGAAATTGC ATATAACAAT CTTGTTTAAG ATAAACTGGA CTGTGAACCA AGAAGGACCA   
  
  
+ AAGATGGACA AGCTATGAAG TGAAATGTCT ATGATTTTCA CTCCGAATAT GTCAGAATAG ATTGGTTTTG   
  
  
+ ACGCACCCGC ATACAGTAAT AGCCAGTCAA GGATATAAAC GTTAGTGTTG CCTTGTGCTT CTTCCATGTA   
  
  
+ TTACAGTGCT TAAGAAATCA GACTATATAT TAATCAGTAA CTCTTTTCTT GCGCACAGCC ACACATCCCC   
  
  
+ TTAATAATTC CTCAGCACTA TCAATGTTTG AGAGGGCACA GAGAGAGAGA GAGAGAGAGA GAGAGAGAGA   
  
  
+ AAGAGAGGTG AGTCTGCAAG TCTTTCCTCT GCGGTACCTG TGAGAAGATA AGAAGGAATA ACTTGCAGAA   
  
  
+ AACATTCCGT TTAGCGTTTA CTGGTTGACC AGGCTTACAT CTCAAAACAA AGGGCTATTT CTGCAGACGA   
  
  
+ TCGAATAGAG CATCAACTTG GTCATACCTG CTATTGAACC AGACCCTTTT GACAGGAAGA ATATATTAAG   
  
  
+ AAGTAGGGCT TGCTGGGTTA TCGAAAGAA  

- CAACGAACGA AATTAATTTC TTCCTTCGTT TTGTCTACCA ACCTAACTTT TCCTGTTGGG TATTCCGTAT   
  
  
- ATTATTAGTC GTCATTAATT CAAAGTGAAC AAAAACTATC TGAATTAATA TATACACGTC ACGTACAAAC   
  
  
- GTCTGGTCGT ACCGTTCGTA TTTTGAAGTG TTCTAGAGAA GGTTTCTAAC ATAAACATCT ACCGAAGTCT   
  
  
- GTACTGTGTT TCACGATGGG TGTTAGTTGA ATGACGACAG ACCATTTGCA ACTTTGACCA CAGGGTATTG   
  
  
- TGTGACATCA TGAGAGAGGG TGTGGAACAT TGTTCAAATG AAGTAAACCA AGAGACCATA AATTAAATCA   
  
  
- CTTCTTCTAG TAATTCGATG AGTTGTCCTT CGAAAAGTTC ATTTACGTTG AACATACTGT TGTAAGAAGA   
  
  
- AGACAGACAG AGTGTGGTGT CTGAAGTGAT CAATGTAGTA ATTAATCCGT ACATCCGGTT ACCAAATAGT   
  
  
- CGACATTATT CCACTATACT AGTTATACTT TTCTGGTCGA AGTTTAGTCT ATCCGACACC TAAATAAGGG   
  
  
- GTTGGTGCCT GAAATTAATC GGAGGTTCAC ATGTTATATA TACTTTACAC GTAACGAGTT ACCATATTAA   
  
  
- TTAATCGATC TTGTATGTAC GCCTATCACA GACACACGTC CCTCTCTATT TCTTTAGTCT CCCTCCCTCT   
  
  
- CAACAACATA TATACATACC TAGCTAGGAC CTACACGACT TAGTCTAATC TTTAGTTAAG TTGTTCGTTT   
  
  
- ATAGTAAGAG TAGAAAGTGT AGGTGTAGTA AGGTTGTACT TTTGATGTAG AAACAGGTCC TTGCCGGTGT   
  
  
- TCATCACACT TTCATATAGT CGTGAGTTTA ACGAAAAGTT ATTACTACGA TAGATAACAC TATTCTACAA   
  
  
- TAAATAATCC TTCTTTAACG TATATTGTTA GAACAAATTC TATTTGACCT GACACTTGGT TCTTCCTGGT   
  
  
- TTCTACCTGT TCGATACTTC ACTTTACAGA TACTAAAAGT GAGGCTTATA CAGTCTTATC TAACCAAAAC   
  
  
- TGCGTGGGCG TATGTCATTA TCGGTCAGTT CCTATATTTG CAATCACAAC GGAACACGAA GAAGGTACAT   
  
  
- AATGTCACGA ATTCTTTAGT CTGATATATA ATTAGTCATT GAGAAAAGAA CGCGTGTCGG TGTGTAGGGG   
  
  
- AATTATTAAG GAGTCGTGAT AGTTACAAAC TCTCCCGTGT CTCTCTCTCT CTCTCTCTCT CTCTCTCTCT   
  
  
- TTCTCTCCAC TCAGACGTTC AGAAAGGAGA CGCCATGGAC ACTCTTCTAT TCTTCCTTAT TGAACGTCTT   
  
  
- TTGTAAGGCA AATCGCAAAT GACCAACTGG TCCGAATGTA GAGTTTTGTT TCCCGATAAA GACGTCTGCT   
  
  
- AGCTTATCTC GTAGTTGAAC CAGTATGGAC GATAACTTGG TCTGGGAAAA CTGTCCTTCT TATATAATTC   
  
  
- TTCATCCCGA ACGACCCAAT AGCTTTCTT

+     as-2-box

| Site Name | Organism | Position | Strand | Matrix score. | sequence | function |
| --- | --- | --- | --- | --- | --- | --- |
| as-2-box | Nicotiana tabacum | 879 | + | 9 | GATAatGATG | involved in shoot-specific expression and light responsiveness |

> 2018/04/13 10:10:12  
+ GTTGCTTGCT TTAATTAAAG AAGGAAGCAA AACAGATGGT TGGATTGAAA AGGACAACCC ATAAGGCATA   
  
  
+ TAATAATCAG CAGTAATTAA GTTTCACTTG TTTTTGATAG ACTTAATTAT ATATGTGCAG TGCATGTTTG   
  
  
+ CAGACCAGCA TGGCAAGCAT AAAACTTCAC AAGATCTCTT CCAAAGATTG TATTTGTAGA TGGCTTCAGA   
  
  
+ CATGACACAA AGTGCTACCC ACAATCAACT TACTGCTGTC TGGTAAACGT TGAAACTGGT GTCCCATAAC   
  
  
+ ACACTGTAGT ACTCTCTCCC ACACCTTGTA ACAAGTTTAC TTCATTTGGT TCTCTGGTAT TTAATTTAGT   
  
  
+ GAAGAAGATC ATTAAGCTAC TCAACAGGAA GCTTTTCAAG TAAATGCAAC TTGTATGACA ACATTCTTCT   
  
  
+ TCTGTCTGTC TCACACCACA GACTTCACTA GTTACATCAT TAATTAGGCA TGTAGGCCAA TGGTTTATCA   
  
  
+ GCTGTAATAA GGTGATATGA TCAATATGAA AAGACCAGCT TCAAATCAGA TAGGCTGTGG ATTTATTCCC   
  
  
+ CAACCACGGA CTTTAATTAG CCTCCAAGTG TACAATATAT ATGAAATGTG CATTGCTCAA TGGTATAATT   
  
  
+ AATTAGCTAG AACATACATG CGGATAGTGT CTGTGTGCAG GGAGAGATAA AGAAATCAGA GGGAGGGAGA   
  
  
+ GTTGTTGTAT ATATGTATGG ATCGATCCTG GATGTGCTGA ATCAGATTAG AAATCAATTC AACAAGCAAA   
  
  
+ TATCATTCTC ATCTTTCACA TCCACATCAT TCCAACATGA AAACTACATC TTTGTCCAGG AACGGCCACA   
  
  
+ AGTAGTGTGA AAGTATATCA GCACTCAAAT TGCTTTTCAA TAATGATGCT ATCTATTGTG ATAAGATGTT   
  
  
+ ATTTATTAGG AAGAAATTGC ATATAACAAT CTTGTTTAAG ATAAACTGGA CTGTGAACCA AGAAGGACCA   
  
  
+ AAGATGGACA AGCTATGAAG TGAAATGTCT ATGATTTTCA CTCCGAATAT GTCAGAATAG ATTGGTTTTG   
  
  
+ ACGCACCCGC ATACAGTAAT AGCCAGTCAA GGATATAAAC GTTAGTGTTG CCTTGTGCTT CTTCCATGTA   
  
  
+ TTACAGTGCT TAAGAAATCA GACTATATAT TAATCAGTAA CTCTTTTCTT GCGCACAGCC ACACATCCCC   
  
  
+ TTAATAATTC CTCAGCACTA TCAATGTTTG AGAGGGCACA GAGAGAGAGA GAGAGAGAGA GAGAGAGAGA   
  
  
+ AAGAGAGGTG AGTCTGCAAG TCTTTCCTCT GCGGTACCTG TGAGAAGATA AGAAGGAATA ACTTGCAGAA   
  
  
+ AACATTCCGT TTAGCGTTTA CTGGTTGACC AGGCTTACAT CTCAAAACAA AGGGCTATTT CTGCAGACGA   
  
  
+ TCGAATAGAG CATCAACTTG GTCATACCTG CTATTGAACC AGACCCTTTT GACAGGAAGA ATATATTAAG   
  
  
+ AAGTAGGGCT TGCTGGGTTA TCGAAAGAA  

- CAACGAACGA AATTAATTTC TTCCTTCGTT TTGTCTACCA ACCTAACTTT TCCTGTTGGG TATTCCGTAT   
  
  
- ATTATTAGTC GTCATTAATT CAAAGTGAAC AAAAACTATC TGAATTAATA TATACACGTC ACGTACAAAC   
  
  
- GTCTGGTCGT ACCGTTCGTA TTTTGAAGTG TTCTAGAGAA GGTTTCTAAC ATAAACATCT ACCGAAGTCT   
  
  
- GTACTGTGTT TCACGATGGG TGTTAGTTGA ATGACGACAG ACCATTTGCA ACTTTGACCA CAGGGTATTG   
  
  
- TGTGACATCA TGAGAGAGGG TGTGGAACAT TGTTCAAATG AAGTAAACCA AGAGACCATA AATTAAATCA   
  
  
- CTTCTTCTAG TAATTCGATG AGTTGTCCTT CGAAAAGTTC ATTTACGTTG AACATACTGT TGTAAGAAGA   
  
  
- AGACAGACAG AGTGTGGTGT CTGAAGTGAT CAATGTAGTA ATTAATCCGT ACATCCGGTT ACCAAATAGT   
  
  
- CGACATTATT CCACTATACT AGTTATACTT TTCTGGTCGA AGTTTAGTCT ATCCGACACC TAAATAAGGG   
  
  
- GTTGGTGCCT GAAATTAATC GGAGGTTCAC ATGTTATATA TACTTTACAC GTAACGAGTT ACCATATTAA   
  
  
- TTAATCGATC TTGTATGTAC GCCTATCACA GACACACGTC CCTCTCTATT TCTTTAGTCT CCCTCCCTCT   
  
  
- CAACAACATA TATACATACC TAGCTAGGAC CTACACGACT TAGTCTAATC TTTAGTTAAG TTGTTCGTTT   
  
  
- ATAGTAAGAG TAGAAAGTGT AGGTGTAGTA AGGTTGTACT TTTGATGTAG AAACAGGTCC TTGCCGGTGT   
  
  
- TCATCACACT TTCATATAGT CGTGAGTTTA ACGAAAAGTT ATTACTACGA TAGATAACAC TATTCTACAA   
  
  
- TAAATAATCC TTCTTTAACG TATATTGTTA GAACAAATTC TATTTGACCT GACACTTGGT TCTTCCTGGT   
  
  
- TTCTACCTGT TCGATACTTC ACTTTACAGA TACTAAAAGT GAGGCTTATA CAGTCTTATC TAACCAAAAC   
  
  
- TGCGTGGGCG TATGTCATTA TCGGTCAGTT CCTATATTTG CAATCACAAC GGAACACGAA GAAGGTACAT   
  
  
- AATGTCACGA ATTCTTTAGT CTGATATATA ATTAGTCATT GAGAAAAGAA CGCGTGTCGG TGTGTAGGGG   
  
  
- AATTATTAAG GAGTCGTGAT AGTTACAAAC TCTCCCGTGT CTCTCTCTCT CTCTCTCTCT CTCTCTCTCT   
  
  
- TTCTCTCCAC TCAGACGTTC AGAAAGGAGA CGCCATGGAC ACTCTTCTAT TCTTCCTTAT TGAACGTCTT   
  
  
- TTGTAAGGCA AATCGCAAAT GACCAACTGG TCCGAATGTA GAGTTTTGTT TCCCGATAAA GACGTCTGCT   
  
  
- AGCTTATCTC GTAGTTGAAC CAGTATGGAC GATAACTTGG TCTGGGAAAA CTGTCCTTCT TATATAATTC   
  
  
- TTCATCCCGA ACGACCCAAT AGCTTTCTT

+     box E

| Site Name | Organism | Position | Strand | Matrix score. | sequence | function |
| --- | --- | --- | --- | --- | --- | --- |
| box E | Petroselinum crispum | 1479 | - | 9 | ACCCATCAAG |  |

> 2018/04/13 10:10:12  
+ GTTGCTTGCT TTAATTAAAG AAGGAAGCAA AACAGATGGT TGGATTGAAA AGGACAACCC ATAAGGCATA   
  
  
+ TAATAATCAG CAGTAATTAA GTTTCACTTG TTTTTGATAG ACTTAATTAT ATATGTGCAG TGCATGTTTG   
  
  
+ CAGACCAGCA TGGCAAGCAT AAAACTTCAC AAGATCTCTT CCAAAGATTG TATTTGTAGA TGGCTTCAGA   
  
  
+ CATGACACAA AGTGCTACCC ACAATCAACT TACTGCTGTC TGGTAAACGT TGAAACTGGT GTCCCATAAC   
  
  
+ ACACTGTAGT ACTCTCTCCC ACACCTTGTA ACAAGTTTAC TTCATTTGGT TCTCTGGTAT TTAATTTAGT   
  
  
+ GAAGAAGATC ATTAAGCTAC TCAACAGGAA GCTTTTCAAG TAAATGCAAC TTGTATGACA ACATTCTTCT   
  
  
+ TCTGTCTGTC TCACACCACA GACTTCACTA GTTACATCAT TAATTAGGCA TGTAGGCCAA TGGTTTATCA   
  
  
+ GCTGTAATAA GGTGATATGA TCAATATGAA AAGACCAGCT TCAAATCAGA TAGGCTGTGG ATTTATTCCC   
  
  
+ CAACCACGGA CTTTAATTAG CCTCCAAGTG TACAATATAT ATGAAATGTG CATTGCTCAA TGGTATAATT   
  
  
+ AATTAGCTAG AACATACATG CGGATAGTGT CTGTGTGCAG GGAGAGATAA AGAAATCAGA GGGAGGGAGA   
  
  
+ GTTGTTGTAT ATATGTATGG ATCGATCCTG GATGTGCTGA ATCAGATTAG AAATCAATTC AACAAGCAAA   
  
  
+ TATCATTCTC ATCTTTCACA TCCACATCAT TCCAACATGA AAACTACATC TTTGTCCAGG AACGGCCACA   
  
  
+ AGTAGTGTGA AAGTATATCA GCACTCAAAT TGCTTTTCAA TAATGATGCT ATCTATTGTG ATAAGATGTT   
  
  
+ ATTTATTAGG AAGAAATTGC ATATAACAAT CTTGTTTAAG ATAAACTGGA CTGTGAACCA AGAAGGACCA   
  
  
+ AAGATGGACA AGCTATGAAG TGAAATGTCT ATGATTTTCA CTCCGAATAT GTCAGAATAG ATTGGTTTTG   
  
  
+ ACGCACCCGC ATACAGTAAT AGCCAGTCAA GGATATAAAC GTTAGTGTTG CCTTGTGCTT CTTCCATGTA   
  
  
+ TTACAGTGCT TAAGAAATCA GACTATATAT TAATCAGTAA CTCTTTTCTT GCGCACAGCC ACACATCCCC   
  
  
+ TTAATAATTC CTCAGCACTA TCAATGTTTG AGAGGGCACA GAGAGAGAGA GAGAGAGAGA GAGAGAGAGA   
  
  
+ AAGAGAGGTG AGTCTGCAAG TCTTTCCTCT GCGGTACCTG TGAGAAGATA AGAAGGAATA ACTTGCAGAA   
  
  
+ AACATTCCGT TTAGCGTTTA CTGGTTGACC AGGCTTACAT CTCAAAACAA AGGGCTATTT CTGCAGACGA   
  
  
+ TCGAATAGAG CATCAACTTG GTCATACCTG CTATTGAACC AGACCCTTTT GACAGGAAGA ATATATTAAG   
  
  
+ AAGTAGGGCT TGCTGGGTTA TCGAAAGAA  

- CAACGAACGA AATTAATTTC TTCCTTCGTT TTGTCTACCA ACCTAACTTT TCCTGTTGGG TATTCCGTAT   
  
  
- ATTATTAGTC GTCATTAATT CAAAGTGAAC AAAAACTATC TGAATTAATA TATACACGTC ACGTACAAAC   
  
  
- GTCTGGTCGT ACCGTTCGTA TTTTGAAGTG TTCTAGAGAA GGTTTCTAAC ATAAACATCT ACCGAAGTCT   
  
  
- GTACTGTGTT TCACGATGGG TGTTAGTTGA ATGACGACAG ACCATTTGCA ACTTTGACCA CAGGGTATTG   
  
  
- TGTGACATCA TGAGAGAGGG TGTGGAACAT TGTTCAAATG AAGTAAACCA AGAGACCATA AATTAAATCA   
  
  
- CTTCTTCTAG TAATTCGATG AGTTGTCCTT CGAAAAGTTC ATTTACGTTG AACATACTGT TGTAAGAAGA   
  
  
- AGACAGACAG AGTGTGGTGT CTGAAGTGAT CAATGTAGTA ATTAATCCGT ACATCCGGTT ACCAAATAGT   
  
  
- CGACATTATT CCACTATACT AGTTATACTT TTCTGGTCGA AGTTTAGTCT ATCCGACACC TAAATAAGGG   
  
  
- GTTGGTGCCT GAAATTAATC GGAGGTTCAC ATGTTATATA TACTTTACAC GTAACGAGTT ACCATATTAA   
  
  
- TTAATCGATC TTGTATGTAC GCCTATCACA GACACACGTC CCTCTCTATT TCTTTAGTCT CCCTCCCTCT   
  
  
- CAACAACATA TATACATACC TAGCTAGGAC CTACACGACT TAGTCTAATC TTTAGTTAAG TTGTTCGTTT   
  
  
- ATAGTAAGAG TAGAAAGTGT AGGTGTAGTA AGGTTGTACT TTTGATGTAG AAACAGGTCC TTGCCGGTGT   
  
  
- TCATCACACT TTCATATAGT CGTGAGTTTA ACGAAAAGTT ATTACTACGA TAGATAACAC TATTCTACAA   
  
  
- TAAATAATCC TTCTTTAACG TATATTGTTA GAACAAATTC TATTTGACCT GACACTTGGT TCTTCCTGGT   
  
  
- TTCTACCTGT TCGATACTTC ACTTTACAGA TACTAAAAGT GAGGCTTATA CAGTCTTATC TAACCAAAAC   
  
  
- TGCGTGGGCG TATGTCATTA TCGGTCAGTT CCTATATTTG CAATCACAAC GGAACACGAA GAAGGTACAT   
  
  
- AATGTCACGA ATTCTTTAGT CTGATATATA ATTAGTCATT GAGAAAAGAA CGCGTGTCGG TGTGTAGGGG   
  
  
- AATTATTAAG GAGTCGTGAT AGTTACAAAC TCTCCCGTGT CTCTCTCTCT CTCTCTCTCT CTCTCTCTCT   
  
  
- TTCTCTCCAC TCAGACGTTC AGAAAGGAGA CGCCATGGAC ACTCTTCTAT TCTTCCTTAT TGAACGTCTT   
  
  
- TTGTAAGGCA AATCGCAAAT GACCAACTGG TCCGAATGTA GAGTTTTGTT TCCCGATAAA GACGTCTGCT   
  
  
- AGCTTATCTC GTAGTTGAAC CAGTATGGAC GATAACTTGG TCTGGGAAAA CTGTCCTTCT TATATAATTC   
  
  
- TTCATCCCGA ACGACCCAAT AGCTTTCTT
