# Supplementary material for: Genome-wide analysis of the WRKY gene family in drumstick (Moringa oleifera Lam.)
Source: PeerJ. 2019 Jun 10;7:e7063. doi: 10.7717/peerj.7063 (PMC6563795; doi:10.7717/peerj.7063)
Supplement: Supplemental Information 1 [file peerj-07-7063-s003.gz › MoWRKY19_plantcare.html]

Content-Type: text/html; charset=ISO-8859-1


CallMat\_Firefox


Webmaster Firefox specific output  
To save the result:
click on the frame with the right mouse button and save the source code as a text file with extension .html  
REFERENCE:PlantCARE: a database of plant cis-acting regulatory elements and a portal to tools for in silico analysis of promoter sequences.  
Lescot, M., Déhais, P., Moreau, Y., De Moor, B., Rouzé ,P.,and Rombauts, S.  
Nucleic Acids Res., Database issue(2002), 30(1):325-327.   


---

> 2018/04/13 10:10:12  
+ AAAAGGCAAC CTTTTCCTTT CTTTCTTCTT TCCTTAGCTT TTTAGATTTA TGAAGAAGGG CTGCTACGAC   
  
  
+ TTTCCTGCTT TTCCACATTT CAAACGCTCG TGTTATCGTC CTTTCATTCT CCCACTTCGT GTCCATCAGC   
  
  
+ TCCTTTTTAA AAAAATATAT TTATTTATTT TATTAAATTA TCATTTATTA TTATTTTTTA AAAAAAATTT   
  
  
+ AATACAGTTT TTTGGGGGAA AAAAAGAAAA AAAACTCATC CATCTGATTT GCTCTCTCTC TGATTTCCAA   
  
  
+ CCGAATGCAT GAGGAGAGCA TTCAGCGGCA ATCAAGCAGA GAGGACTGAC TGTCTTATGG GCCCTATCTC   
  
  
+ TTCTTGGATT CCAGACTATT CTTGTTTTTA GTAGCTCTCA AATCAATAAC CAGATCTAAT CCTAATACTC   
  
  
+ TCTTCATAAT CATAACTTAA AGAGGGAATT AATTAACCAA ATCAGGTAAA CAAACATCAT TAATTACAGC   
  
  
+ CACCTCGTTT GATCGATCCC GATCACCCAT GATCGTCACT TCCAGATCTT CACGTGTGCA ACCAATGAGC   
  
  
+ CATTAGTTGT AAATCAATTA ACATGTTTAA TCATAAAGAC TTCAAATTTT TGAAGTCAAC ATTCAAATGA   
  
  
+ TGCAGTTTGC TGAGATGGTA TTAGTGGCAC CACACAGCCC ACCCAAATGA ATTAACCATG AGACCTATTT   
  
  
+ ATGATCCTTC ATGGAAGTTC CTTAAGCCGC GCAGACATTC ACTATATAAT AGTAATTATA ACTTTCCACC   
  
  
+ TACCAACCAA GCATATGTAT ACTTTCAATA AATACATGTG CCCAAATTCC ATAAAATTAT AGAACAATTA   
  
  
+ TTGAATGAAT TTAATTATCA GATCAGTCCA AGACAGGCGC TTAGCTAGGT TAGCATAAAA TATCGTGCAC   
  
  
+ CTGTTATATC CTTATCAGCT CAGAGGCCTA ATACTGTTGC TAGTCTGGTA GCTCTACGAA TTTATTTCCA   
  
  
+ TGTAAATCAG AATTAAAAAT TATTATCATC TGTATAATCG TATGTGTTAA CGTCGCTTTC CTAATGATGA   
  
  
+ AGCCGCCAGC TCCATTTTCC ACCCTACATT TTTAAATTAT TATCATCTGT ATAATCGTAT GTGTTAACGT   
  
  
+ CGCTTTCCTA ATGATGAAGC CGCCAGCTCC ATTTTCCACC CTACATTTTT TCCAATAGAT CAATCCGCTG   
  
  
+ ATGAAGCTAT ATGTACATTC AATTTTTTTC TTGAGCTAAC TGGAAAATAT TTTCGTGCCG TCTACGACAC   
  
  
+ CTGTTAACCC TATATATATC TCTTTGGATT TTTCTGTCTA TTTTTGTATA CTTCTTCAGG AAAATATTTT   
  
  
+ CGTGCCGTCT ACGACACCTG TTAACCCTAT ATATATCTCT TTGGATTTTT CTGTCTATTT TTGTATACTT   
  
  
+ CTTCTGGATC AATCTCCATC TTTATCTTCT TCCTCTTCTT CTGCTTGAAG CTTGATCTTC TTCTGTAACT   
  
  
+ ATACAGTGCG TTGTTTCTGT TTCCTTTTC  

- TTTTCCGTTG GAAAAGGAAA GAAAGAAGAA AGGAATCGAA AAATCTAAAT ACTTCTTCCC GACGATGCTG   
  
  
- AAAGGACGAA AAGGTGTAAA GTTTGCGAGC ACAATAGCAG GAAAGTAAGA GGGTGAAGCA CAGGTAGTCG   
  
  
- AGGAAAAATT TTTTTATATA AATAAATAAA ATAATTTAAT AGTAAATAAT AATAAAAAAT TTTTTTTAAA   
  
  
- TTATGTCAAA AAACCCCCTT TTTTTCTTTT TTTTGAGTAG GTAGACTAAA CGAGAGAGAG ACTAAAGGTT   
  
  
- GGCTTACGTA CTCCTCTCGT AAGTCGCCGT TAGTTCGTCT CTCCTGACTG ACAGAATACC CGGGATAGAG   
  
  
- AAGAACCTAA GGTCTGATAA GAACAAAAAT CATCGAGAGT TTAGTTATTG GTCTAGATTA GGATTATGAG   
  
  
- AGAAGTATTA GTATTGAATT TCTCCCTTAA TTAATTGGTT TAGTCCATTT GTTTGTAGTA ATTAATGTCG   
  
  
- GTGGAGCAAA CTAGCTAGGG CTAGTGGGTA CTAGCAGTGA AGGTCTAGAA GTGCACACGT TGGTTACTCG   
  
  
- GTAATCAACA TTTAGTTAAT TGTACAAATT AGTATTTCTG AAGTTTAAAA ACTTCAGTTG TAAGTTTACT   
  
  
- ACGTCAAACG ACTCTACCAT AATCACCGTG GTGTGTCGGG TGGGTTTACT TAATTGGTAC TCTGGATAAA   
  
  
- TACTAGGAAG TACCTTCAAG GAATTCGGCG CGTCTGTAAG TGATATATTA TCATTAATAT TGAAAGGTGG   
  
  
- ATGGTTGGTT CGTATACATA TGAAAGTTAT TTATGTACAC GGGTTTAAGG TATTTTAATA TCTTGTTAAT   
  
  
- AACTTACTTA AATTAATAGT CTAGTCAGGT TCTGTCCGCG AATCGATCCA ATCGTATTTT ATAGCACGTG   
  
  
- GACAATATAG GAATAGTCGA GTCTCCGGAT TATGACAACG ATCAGACCAT CGAGATGCTT AAATAAAGGT   
  
  
- ACATTTAGTC TTAATTTTTA ATAATAGTAG ACATATTAGC ATACACAATT GCAGCGAAAG GATTACTACT   
  
  
- TCGGCGGTCG AGGTAAAAGG TGGGATGTAA AAATTTAATA ATAGTAGACA TATTAGCATA CACAATTGCA   
  
  
- GCGAAAGGAT TACTACTTCG GCGGTCGAGG TAAAAGGTGG GATGTAAAAA AGGTTATCTA GTTAGGCGAC   
  
  
- TACTTCGATA TACATGTAAG TTAAAAAAAG AACTCGATTG ACCTTTTATA AAAGCACGGC AGATGCTGTG   
  
  
- GACAATTGGG ATATATATAG AGAAACCTAA AAAGACAGAT AAAAACATAT GAAGAAGTCC TTTTATAAAA   
  
  
- GCACGGCAGA TGCTGTGGAC AATTGGGATA TATATAGAGA AACCTAAAAA GACAGATAAA AACATATGAA   
  
  
- GAAGACCTAG TTAGAGGTAG AAATAGAAGA AGGAGAAGAA GACGAACTTC GAACTAGAAG AAGACATTGA   
  
  
- TATGTCACGC AACAAAGACA AAGGAAAAG

  
  
Motifs Found  

+     3-AF1 binding site

| Site Name | Organism | Position | Strand | Matrix score. | sequence | function |
| --- | --- | --- | --- | --- | --- | --- |
| 3-AF1 binding site | Solanum tuberosum | 1361 | - | 10 | AAGAGATATTT | light responsive element |
| 3-AF1 binding site | Solanum tuberosum | 1274 | - | 10 | AAGAGATATTT | light responsive element |

> 2018/04/13 10:10:12  
+ AAAAGGCAAC CTTTTCCTTT CTTTCTTCTT TCCTTAGCTT TTTAGATTTA TGAAGAAGGG CTGCTACGAC   
  
  
+ TTTCCTGCTT TTCCACATTT CAAACGCTCG TGTTATCGTC CTTTCATTCT CCCACTTCGT GTCCATCAGC   
  
  
+ TCCTTTTTAA AAAAATATAT TTATTTATTT TATTAAATTA TCATTTATTA TTATTTTTTA AAAAAAATTT   
  
  
+ AATACAGTTT TTTGGGGGAA AAAAAGAAAA AAAACTCATC CATCTGATTT GCTCTCTCTC TGATTTCCAA   
  
  
+ CCGAATGCAT GAGGAGAGCA TTCAGCGGCA ATCAAGCAGA GAGGACTGAC TGTCTTATGG GCCCTATCTC   
  
  
+ TTCTTGGATT CCAGACTATT CTTGTTTTTA GTAGCTCTCA AATCAATAAC CAGATCTAAT CCTAATACTC   
  
  
+ TCTTCATAAT CATAACTTAA AGAGGGAATT AATTAACCAA ATCAGGTAAA CAAACATCAT TAATTACAGC   
  
  
+ CACCTCGTTT GATCGATCCC GATCACCCAT GATCGTCACT TCCAGATCTT CACGTGTGCA ACCAATGAGC   
  
  
+ CATTAGTTGT AAATCAATTA ACATGTTTAA TCATAAAGAC TTCAAATTTT TGAAGTCAAC ATTCAAATGA   
  
  
+ TGCAGTTTGC TGAGATGGTA TTAGTGGCAC CACACAGCCC ACCCAAATGA ATTAACCATG AGACCTATTT   
  
  
+ ATGATCCTTC ATGGAAGTTC CTTAAGCCGC GCAGACATTC ACTATATAAT AGTAATTATA ACTTTCCACC   
  
  
+ TACCAACCAA GCATATGTAT ACTTTCAATA AATACATGTG CCCAAATTCC ATAAAATTAT AGAACAATTA   
  
  
+ TTGAATGAAT TTAATTATCA GATCAGTCCA AGACAGGCGC TTAGCTAGGT TAGCATAAAA TATCGTGCAC   
  
  
+ CTGTTATATC CTTATCAGCT CAGAGGCCTA ATACTGTTGC TAGTCTGGTA GCTCTACGAA TTTATTTCCA   
  
  
+ TGTAAATCAG AATTAAAAAT TATTATCATC TGTATAATCG TATGTGTTAA CGTCGCTTTC CTAATGATGA   
  
  
+ AGCCGCCAGC TCCATTTTCC ACCCTACATT TTTAAATTAT TATCATCTGT ATAATCGTAT GTGTTAACGT   
  
  
+ CGCTTTCCTA ATGATGAAGC CGCCAGCTCC ATTTTCCACC CTACATTTTT TCCAATAGAT CAATCCGCTG   
  
  
+ ATGAAGCTAT ATGTACATTC AATTTTTTTC TTGAGCTAAC TGGAAAATAT TTTCGTGCCG TCTACGACAC   
  
  
+ CTGTTAACCC TATATATATC TCTTTGGATT TTTCTGTCTA TTTTTGTATA CTTCTTCAGG AAAATATTTT   
  
  
+ CGTGCCGTCT ACGACACCTG TTAACCCTAT ATATATCTCT TTGGATTTTT CTGTCTATTT TTGTATACTT   
  
  
+ CTTCTGGATC AATCTCCATC TTTATCTTCT TCCTCTTCTT CTGCTTGAAG CTTGATCTTC TTCTGTAACT   
  
  
+ ATACAGTGCG TTGTTTCTGT TTCCTTTTC  

- TTTTCCGTTG GAAAAGGAAA GAAAGAAGAA AGGAATCGAA AAATCTAAAT ACTTCTTCCC GACGATGCTG   
  
  
- AAAGGACGAA AAGGTGTAAA GTTTGCGAGC ACAATAGCAG GAAAGTAAGA GGGTGAAGCA CAGGTAGTCG   
  
  
- AGGAAAAATT TTTTTATATA AATAAATAAA ATAATTTAAT AGTAAATAAT AATAAAAAAT TTTTTTTAAA   
  
  
- TTATGTCAAA AAACCCCCTT TTTTTCTTTT TTTTGAGTAG GTAGACTAAA CGAGAGAGAG ACTAAAGGTT   
  
  
- GGCTTACGTA CTCCTCTCGT AAGTCGCCGT TAGTTCGTCT CTCCTGACTG ACAGAATACC CGGGATAGAG   
  
  
- AAGAACCTAA GGTCTGATAA GAACAAAAAT CATCGAGAGT TTAGTTATTG GTCTAGATTA GGATTATGAG   
  
  
- AGAAGTATTA GTATTGAATT TCTCCCTTAA TTAATTGGTT TAGTCCATTT GTTTGTAGTA ATTAATGTCG   
  
  
- GTGGAGCAAA CTAGCTAGGG CTAGTGGGTA CTAGCAGTGA AGGTCTAGAA GTGCACACGT TGGTTACTCG   
  
  
- GTAATCAACA TTTAGTTAAT TGTACAAATT AGTATTTCTG AAGTTTAAAA ACTTCAGTTG TAAGTTTACT   
  
  
- ACGTCAAACG ACTCTACCAT AATCACCGTG GTGTGTCGGG TGGGTTTACT TAATTGGTAC TCTGGATAAA   
  
  
- TACTAGGAAG TACCTTCAAG GAATTCGGCG CGTCTGTAAG TGATATATTA TCATTAATAT TGAAAGGTGG   
  
  
- ATGGTTGGTT CGTATACATA TGAAAGTTAT TTATGTACAC GGGTTTAAGG TATTTTAATA TCTTGTTAAT   
  
  
- AACTTACTTA AATTAATAGT CTAGTCAGGT TCTGTCCGCG AATCGATCCA ATCGTATTTT ATAGCACGTG   
  
  
- GACAATATAG GAATAGTCGA GTCTCCGGAT TATGACAACG ATCAGACCAT CGAGATGCTT AAATAAAGGT   
  
  
- ACATTTAGTC TTAATTTTTA ATAATAGTAG ACATATTAGC ATACACAATT GCAGCGAAAG GATTACTACT   
  
  
- TCGGCGGTCG AGGTAAAAGG TGGGATGTAA AAATTTAATA ATAGTAGACA TATTAGCATA CACAATTGCA   
  
  
- GCGAAAGGAT TACTACTTCG GCGGTCGAGG TAAAAGGTGG GATGTAAAAA AGGTTATCTA GTTAGGCGAC   
  
  
- TACTTCGATA TACATGTAAG TTAAAAAAAG AACTCGATTG ACCTTTTATA AAAGCACGGC AGATGCTGTG   
  
  
- GACAATTGGG ATATATATAG AGAAACCTAA AAAGACAGAT AAAAACATAT GAAGAAGTCC TTTTATAAAA   
  
  
- GCACGGCAGA TGCTGTGGAC AATTGGGATA TATATAGAGA AACCTAAAAA GACAGATAAA AACATATGAA   
  
  
- GAAGACCTAG TTAGAGGTAG AAATAGAAGA AGGAGAAGAA GACGAACTTC GAACTAGAAG AAGACATTGA   
  
  
- TATGTCACGC AACAAAGACA AAGGAAAAG

+     5UTR Py-rich stretch

| Site Name | Organism | Position | Strand | Matrix score. | sequence | function |
| --- | --- | --- | --- | --- | --- | --- |
| 5UTR Py-rich stretch | Lycopersicon esculentum | 22 | + | 9 | TTTCTTCTCT | cis-acting element conferring high transcription levels |

> 2018/04/13 10:10:12  
+ AAAAGGCAAC CTTTTCCTTT CTTTCTTCTT TCCTTAGCTT TTTAGATTTA TGAAGAAGGG CTGCTACGAC   
  
  
+ TTTCCTGCTT TTCCACATTT CAAACGCTCG TGTTATCGTC CTTTCATTCT CCCACTTCGT GTCCATCAGC   
  
  
+ TCCTTTTTAA AAAAATATAT TTATTTATTT TATTAAATTA TCATTTATTA TTATTTTTTA AAAAAAATTT   
  
  
+ AATACAGTTT TTTGGGGGAA AAAAAGAAAA AAAACTCATC CATCTGATTT GCTCTCTCTC TGATTTCCAA   
  
  
+ CCGAATGCAT GAGGAGAGCA TTCAGCGGCA ATCAAGCAGA GAGGACTGAC TGTCTTATGG GCCCTATCTC   
  
  
+ TTCTTGGATT CCAGACTATT CTTGTTTTTA GTAGCTCTCA AATCAATAAC CAGATCTAAT CCTAATACTC   
  
  
+ TCTTCATAAT CATAACTTAA AGAGGGAATT AATTAACCAA ATCAGGTAAA CAAACATCAT TAATTACAGC   
  
  
+ CACCTCGTTT GATCGATCCC GATCACCCAT GATCGTCACT TCCAGATCTT CACGTGTGCA ACCAATGAGC   
  
  
+ CATTAGTTGT AAATCAATTA ACATGTTTAA TCATAAAGAC TTCAAATTTT TGAAGTCAAC ATTCAAATGA   
  
  
+ TGCAGTTTGC TGAGATGGTA TTAGTGGCAC CACACAGCCC ACCCAAATGA ATTAACCATG AGACCTATTT   
  
  
+ ATGATCCTTC ATGGAAGTTC CTTAAGCCGC GCAGACATTC ACTATATAAT AGTAATTATA ACTTTCCACC   
  
  
+ TACCAACCAA GCATATGTAT ACTTTCAATA AATACATGTG CCCAAATTCC ATAAAATTAT AGAACAATTA   
  
  
+ TTGAATGAAT TTAATTATCA GATCAGTCCA AGACAGGCGC TTAGCTAGGT TAGCATAAAA TATCGTGCAC   
  
  
+ CTGTTATATC CTTATCAGCT CAGAGGCCTA ATACTGTTGC TAGTCTGGTA GCTCTACGAA TTTATTTCCA   
  
  
+ TGTAAATCAG AATTAAAAAT TATTATCATC TGTATAATCG TATGTGTTAA CGTCGCTTTC CTAATGATGA   
  
  
+ AGCCGCCAGC TCCATTTTCC ACCCTACATT TTTAAATTAT TATCATCTGT ATAATCGTAT GTGTTAACGT   
  
  
+ CGCTTTCCTA ATGATGAAGC CGCCAGCTCC ATTTTCCACC CTACATTTTT TCCAATAGAT CAATCCGCTG   
  
  
+ ATGAAGCTAT ATGTACATTC AATTTTTTTC TTGAGCTAAC TGGAAAATAT TTTCGTGCCG TCTACGACAC   
  
  
+ CTGTTAACCC TATATATATC TCTTTGGATT TTTCTGTCTA TTTTTGTATA CTTCTTCAGG AAAATATTTT   
  
  
+ CGTGCCGTCT ACGACACCTG TTAACCCTAT ATATATCTCT TTGGATTTTT CTGTCTATTT TTGTATACTT   
  
  
+ CTTCTGGATC AATCTCCATC TTTATCTTCT TCCTCTTCTT CTGCTTGAAG CTTGATCTTC TTCTGTAACT   
  
  
+ ATACAGTGCG TTGTTTCTGT TTCCTTTTC  

- TTTTCCGTTG GAAAAGGAAA GAAAGAAGAA AGGAATCGAA AAATCTAAAT ACTTCTTCCC GACGATGCTG   
  
  
- AAAGGACGAA AAGGTGTAAA GTTTGCGAGC ACAATAGCAG GAAAGTAAGA GGGTGAAGCA CAGGTAGTCG   
  
  
- AGGAAAAATT TTTTTATATA AATAAATAAA ATAATTTAAT AGTAAATAAT AATAAAAAAT TTTTTTTAAA   
  
  
- TTATGTCAAA AAACCCCCTT TTTTTCTTTT TTTTGAGTAG GTAGACTAAA CGAGAGAGAG ACTAAAGGTT   
  
  
- GGCTTACGTA CTCCTCTCGT AAGTCGCCGT TAGTTCGTCT CTCCTGACTG ACAGAATACC CGGGATAGAG   
  
  
- AAGAACCTAA GGTCTGATAA GAACAAAAAT CATCGAGAGT TTAGTTATTG GTCTAGATTA GGATTATGAG   
  
  
- AGAAGTATTA GTATTGAATT TCTCCCTTAA TTAATTGGTT TAGTCCATTT GTTTGTAGTA ATTAATGTCG   
  
  
- GTGGAGCAAA CTAGCTAGGG CTAGTGGGTA CTAGCAGTGA AGGTCTAGAA GTGCACACGT TGGTTACTCG   
  
  
- GTAATCAACA TTTAGTTAAT TGTACAAATT AGTATTTCTG AAGTTTAAAA ACTTCAGTTG TAAGTTTACT   
  
  
- ACGTCAAACG ACTCTACCAT AATCACCGTG GTGTGTCGGG TGGGTTTACT TAATTGGTAC TCTGGATAAA   
  
  
- TACTAGGAAG TACCTTCAAG GAATTCGGCG CGTCTGTAAG TGATATATTA TCATTAATAT TGAAAGGTGG   
  
  
- ATGGTTGGTT CGTATACATA TGAAAGTTAT TTATGTACAC GGGTTTAAGG TATTTTAATA TCTTGTTAAT   
  
  
- AACTTACTTA AATTAATAGT CTAGTCAGGT TCTGTCCGCG AATCGATCCA ATCGTATTTT ATAGCACGTG   
  
  
- GACAATATAG GAATAGTCGA GTCTCCGGAT TATGACAACG ATCAGACCAT CGAGATGCTT AAATAAAGGT   
  
  
- ACATTTAGTC TTAATTTTTA ATAATAGTAG ACATATTAGC ATACACAATT GCAGCGAAAG GATTACTACT   
  
  
- TCGGCGGTCG AGGTAAAAGG TGGGATGTAA AAATTTAATA ATAGTAGACA TATTAGCATA CACAATTGCA   
  
  
- GCGAAAGGAT TACTACTTCG GCGGTCGAGG TAAAAGGTGG GATGTAAAAA AGGTTATCTA GTTAGGCGAC   
  
  
- TACTTCGATA TACATGTAAG TTAAAAAAAG AACTCGATTG ACCTTTTATA AAAGCACGGC AGATGCTGTG   
  
  
- GACAATTGGG ATATATATAG AGAAACCTAA AAAGACAGAT AAAAACATAT GAAGAAGTCC TTTTATAAAA   
  
  
- GCACGGCAGA TGCTGTGGAC AATTGGGATA TATATAGAGA AACCTAAAAA GACAGATAAA AACATATGAA   
  
  
- GAAGACCTAG TTAGAGGTAG AAATAGAAGA AGGAGAAGAA GACGAACTTC GAACTAGAAG AAGACATTGA   
  
  
- TATGTCACGC AACAAAGACA AAGGAAAAG

+     AAGAA-motif

| Site Name | Organism | Position | Strand | Matrix score. | sequence | function |
| --- | --- | --- | --- | --- | --- | --- |
| AAGAA-motif | Avena sativa | 465 | + | 9 | gGTAAAGAAA |  |
| AAGAA-motif | Avena sativa | 19 | - | 7 | GAAAGAA |  |
| AAGAA-motif | Avena sativa | 26 | - | 7 | GAAAGAA |  |

> 2018/04/13 10:10:12  
+ AAAAGGCAAC CTTTTCCTTT CTTTCTTCTT TCCTTAGCTT TTTAGATTTA TGAAGAAGGG CTGCTACGAC   
  
  
+ TTTCCTGCTT TTCCACATTT CAAACGCTCG TGTTATCGTC CTTTCATTCT CCCACTTCGT GTCCATCAGC   
  
  
+ TCCTTTTTAA AAAAATATAT TTATTTATTT TATTAAATTA TCATTTATTA TTATTTTTTA AAAAAAATTT   
  
  
+ AATACAGTTT TTTGGGGGAA AAAAAGAAAA AAAACTCATC CATCTGATTT GCTCTCTCTC TGATTTCCAA   
  
  
+ CCGAATGCAT GAGGAGAGCA TTCAGCGGCA ATCAAGCAGA GAGGACTGAC TGTCTTATGG GCCCTATCTC   
  
  
+ TTCTTGGATT CCAGACTATT CTTGTTTTTA GTAGCTCTCA AATCAATAAC CAGATCTAAT CCTAATACTC   
  
  
+ TCTTCATAAT CATAACTTAA AGAGGGAATT AATTAACCAA ATCAGGTAAA CAAACATCAT TAATTACAGC   
  
  
+ CACCTCGTTT GATCGATCCC GATCACCCAT GATCGTCACT TCCAGATCTT CACGTGTGCA ACCAATGAGC   
  
  
+ CATTAGTTGT AAATCAATTA ACATGTTTAA TCATAAAGAC TTCAAATTTT TGAAGTCAAC ATTCAAATGA   
  
  
+ TGCAGTTTGC TGAGATGGTA TTAGTGGCAC CACACAGCCC ACCCAAATGA ATTAACCATG AGACCTATTT   
  
  
+ ATGATCCTTC ATGGAAGTTC CTTAAGCCGC GCAGACATTC ACTATATAAT AGTAATTATA ACTTTCCACC   
  
  
+ TACCAACCAA GCATATGTAT ACTTTCAATA AATACATGTG CCCAAATTCC ATAAAATTAT AGAACAATTA   
  
  
+ TTGAATGAAT TTAATTATCA GATCAGTCCA AGACAGGCGC TTAGCTAGGT TAGCATAAAA TATCGTGCAC   
  
  
+ CTGTTATATC CTTATCAGCT CAGAGGCCTA ATACTGTTGC TAGTCTGGTA GCTCTACGAA TTTATTTCCA   
  
  
+ TGTAAATCAG AATTAAAAAT TATTATCATC TGTATAATCG TATGTGTTAA CGTCGCTTTC CTAATGATGA   
  
  
+ AGCCGCCAGC TCCATTTTCC ACCCTACATT TTTAAATTAT TATCATCTGT ATAATCGTAT GTGTTAACGT   
  
  
+ CGCTTTCCTA ATGATGAAGC CGCCAGCTCC ATTTTCCACC CTACATTTTT TCCAATAGAT CAATCCGCTG   
  
  
+ ATGAAGCTAT ATGTACATTC AATTTTTTTC TTGAGCTAAC TGGAAAATAT TTTCGTGCCG TCTACGACAC   
  
  
+ CTGTTAACCC TATATATATC TCTTTGGATT TTTCTGTCTA TTTTTGTATA CTTCTTCAGG AAAATATTTT   
  
  
+ CGTGCCGTCT ACGACACCTG TTAACCCTAT ATATATCTCT TTGGATTTTT CTGTCTATTT TTGTATACTT   
  
  
+ CTTCTGGATC AATCTCCATC TTTATCTTCT TCCTCTTCTT CTGCTTGAAG CTTGATCTTC TTCTGTAACT   
  
  
+ ATACAGTGCG TTGTTTCTGT TTCCTTTTC  

- TTTTCCGTTG GAAAAGGAAA GAAAGAAGAA AGGAATCGAA AAATCTAAAT ACTTCTTCCC GACGATGCTG   
  
  
- AAAGGACGAA AAGGTGTAAA GTTTGCGAGC ACAATAGCAG GAAAGTAAGA GGGTGAAGCA CAGGTAGTCG   
  
  
- AGGAAAAATT TTTTTATATA AATAAATAAA ATAATTTAAT AGTAAATAAT AATAAAAAAT TTTTTTTAAA   
  
  
- TTATGTCAAA AAACCCCCTT TTTTTCTTTT TTTTGAGTAG GTAGACTAAA CGAGAGAGAG ACTAAAGGTT   
  
  
- GGCTTACGTA CTCCTCTCGT AAGTCGCCGT TAGTTCGTCT CTCCTGACTG ACAGAATACC CGGGATAGAG   
  
  
- AAGAACCTAA GGTCTGATAA GAACAAAAAT CATCGAGAGT TTAGTTATTG GTCTAGATTA GGATTATGAG   
  
  
- AGAAGTATTA GTATTGAATT TCTCCCTTAA TTAATTGGTT TAGTCCATTT GTTTGTAGTA ATTAATGTCG   
  
  
- GTGGAGCAAA CTAGCTAGGG CTAGTGGGTA CTAGCAGTGA AGGTCTAGAA GTGCACACGT TGGTTACTCG   
  
  
- GTAATCAACA TTTAGTTAAT TGTACAAATT AGTATTTCTG AAGTTTAAAA ACTTCAGTTG TAAGTTTACT   
  
  
- ACGTCAAACG ACTCTACCAT AATCACCGTG GTGTGTCGGG TGGGTTTACT TAATTGGTAC TCTGGATAAA   
  
  
- TACTAGGAAG TACCTTCAAG GAATTCGGCG CGTCTGTAAG TGATATATTA TCATTAATAT TGAAAGGTGG   
  
  
- ATGGTTGGTT CGTATACATA TGAAAGTTAT TTATGTACAC GGGTTTAAGG TATTTTAATA TCTTGTTAAT   
  
  
- AACTTACTTA AATTAATAGT CTAGTCAGGT TCTGTCCGCG AATCGATCCA ATCGTATTTT ATAGCACGTG   
  
  
- GACAATATAG GAATAGTCGA GTCTCCGGAT TATGACAACG ATCAGACCAT CGAGATGCTT AAATAAAGGT   
  
  
- ACATTTAGTC TTAATTTTTA ATAATAGTAG ACATATTAGC ATACACAATT GCAGCGAAAG GATTACTACT   
  
  
- TCGGCGGTCG AGGTAAAAGG TGGGATGTAA AAATTTAATA ATAGTAGACA TATTAGCATA CACAATTGCA   
  
  
- GCGAAAGGAT TACTACTTCG GCGGTCGAGG TAAAAGGTGG GATGTAAAAA AGGTTATCTA GTTAGGCGAC   
  
  
- TACTTCGATA TACATGTAAG TTAAAAAAAG AACTCGATTG ACCTTTTATA AAAGCACGGC AGATGCTGTG   
  
  
- GACAATTGGG ATATATATAG AGAAACCTAA AAAGACAGAT AAAAACATAT GAAGAAGTCC TTTTATAAAA   
  
  
- GCACGGCAGA TGCTGTGGAC AATTGGGATA TATATAGAGA AACCTAAAAA GACAGATAAA AACATATGAA   
  
  
- GAAGACCTAG TTAGAGGTAG AAATAGAAGA AGGAGAAGAA GACGAACTTC GAACTAGAAG AAGACATTGA   
  
  
- TATGTCACGC AACAAAGACA AAGGAAAAG

+     ABRE

| Site Name | Organism | Position | Strand | Matrix score. | sequence | function |
| --- | --- | --- | --- | --- | --- | --- |
| ABRE | Arabidopsis thaliana | 541 | + | 6 | CACGTG | cis-acting element involved in the abscisic acid responsiveness |

> 2018/04/13 10:10:12  
+ AAAAGGCAAC CTTTTCCTTT CTTTCTTCTT TCCTTAGCTT TTTAGATTTA TGAAGAAGGG CTGCTACGAC   
  
  
+ TTTCCTGCTT TTCCACATTT CAAACGCTCG TGTTATCGTC CTTTCATTCT CCCACTTCGT GTCCATCAGC   
  
  
+ TCCTTTTTAA AAAAATATAT TTATTTATTT TATTAAATTA TCATTTATTA TTATTTTTTA AAAAAAATTT   
  
  
+ AATACAGTTT TTTGGGGGAA AAAAAGAAAA AAAACTCATC CATCTGATTT GCTCTCTCTC TGATTTCCAA   
  
  
+ CCGAATGCAT GAGGAGAGCA TTCAGCGGCA ATCAAGCAGA GAGGACTGAC TGTCTTATGG GCCCTATCTC   
  
  
+ TTCTTGGATT CCAGACTATT CTTGTTTTTA GTAGCTCTCA AATCAATAAC CAGATCTAAT CCTAATACTC   
  
  
+ TCTTCATAAT CATAACTTAA AGAGGGAATT AATTAACCAA ATCAGGTAAA CAAACATCAT TAATTACAGC   
  
  
+ CACCTCGTTT GATCGATCCC GATCACCCAT GATCGTCACT TCCAGATCTT CACGTGTGCA ACCAATGAGC   
  
  
+ CATTAGTTGT AAATCAATTA ACATGTTTAA TCATAAAGAC TTCAAATTTT TGAAGTCAAC ATTCAAATGA   
  
  
+ TGCAGTTTGC TGAGATGGTA TTAGTGGCAC CACACAGCCC ACCCAAATGA ATTAACCATG AGACCTATTT   
  
  
+ ATGATCCTTC ATGGAAGTTC CTTAAGCCGC GCAGACATTC ACTATATAAT AGTAATTATA ACTTTCCACC   
  
  
+ TACCAACCAA GCATATGTAT ACTTTCAATA AATACATGTG CCCAAATTCC ATAAAATTAT AGAACAATTA   
  
  
+ TTGAATGAAT TTAATTATCA GATCAGTCCA AGACAGGCGC TTAGCTAGGT TAGCATAAAA TATCGTGCAC   
  
  
+ CTGTTATATC CTTATCAGCT CAGAGGCCTA ATACTGTTGC TAGTCTGGTA GCTCTACGAA TTTATTTCCA   
  
  
+ TGTAAATCAG AATTAAAAAT TATTATCATC TGTATAATCG TATGTGTTAA CGTCGCTTTC CTAATGATGA   
  
  
+ AGCCGCCAGC TCCATTTTCC ACCCTACATT TTTAAATTAT TATCATCTGT ATAATCGTAT GTGTTAACGT   
  
  
+ CGCTTTCCTA ATGATGAAGC CGCCAGCTCC ATTTTCCACC CTACATTTTT TCCAATAGAT CAATCCGCTG   
  
  
+ ATGAAGCTAT ATGTACATTC AATTTTTTTC TTGAGCTAAC TGGAAAATAT TTTCGTGCCG TCTACGACAC   
  
  
+ CTGTTAACCC TATATATATC TCTTTGGATT TTTCTGTCTA TTTTTGTATA CTTCTTCAGG AAAATATTTT   
  
  
+ CGTGCCGTCT ACGACACCTG TTAACCCTAT ATATATCTCT TTGGATTTTT CTGTCTATTT TTGTATACTT   
  
  
+ CTTCTGGATC AATCTCCATC TTTATCTTCT TCCTCTTCTT CTGCTTGAAG CTTGATCTTC TTCTGTAACT   
  
  
+ ATACAGTGCG TTGTTTCTGT TTCCTTTTC  

- TTTTCCGTTG GAAAAGGAAA GAAAGAAGAA AGGAATCGAA AAATCTAAAT ACTTCTTCCC GACGATGCTG   
  
  
- AAAGGACGAA AAGGTGTAAA GTTTGCGAGC ACAATAGCAG GAAAGTAAGA GGGTGAAGCA CAGGTAGTCG   
  
  
- AGGAAAAATT TTTTTATATA AATAAATAAA ATAATTTAAT AGTAAATAAT AATAAAAAAT TTTTTTTAAA   
  
  
- TTATGTCAAA AAACCCCCTT TTTTTCTTTT TTTTGAGTAG GTAGACTAAA CGAGAGAGAG ACTAAAGGTT   
  
  
- GGCTTACGTA CTCCTCTCGT AAGTCGCCGT TAGTTCGTCT CTCCTGACTG ACAGAATACC CGGGATAGAG   
  
  
- AAGAACCTAA GGTCTGATAA GAACAAAAAT CATCGAGAGT TTAGTTATTG GTCTAGATTA GGATTATGAG   
  
  
- AGAAGTATTA GTATTGAATT TCTCCCTTAA TTAATTGGTT TAGTCCATTT GTTTGTAGTA ATTAATGTCG   
  
  
- GTGGAGCAAA CTAGCTAGGG CTAGTGGGTA CTAGCAGTGA AGGTCTAGAA GTGCACACGT TGGTTACTCG   
  
  
- GTAATCAACA TTTAGTTAAT TGTACAAATT AGTATTTCTG AAGTTTAAAA ACTTCAGTTG TAAGTTTACT   
  
  
- ACGTCAAACG ACTCTACCAT AATCACCGTG GTGTGTCGGG TGGGTTTACT TAATTGGTAC TCTGGATAAA   
  
  
- TACTAGGAAG TACCTTCAAG GAATTCGGCG CGTCTGTAAG TGATATATTA TCATTAATAT TGAAAGGTGG   
  
  
- ATGGTTGGTT CGTATACATA TGAAAGTTAT TTATGTACAC GGGTTTAAGG TATTTTAATA TCTTGTTAAT   
  
  
- AACTTACTTA AATTAATAGT CTAGTCAGGT TCTGTCCGCG AATCGATCCA ATCGTATTTT ATAGCACGTG   
  
  
- GACAATATAG GAATAGTCGA GTCTCCGGAT TATGACAACG ATCAGACCAT CGAGATGCTT AAATAAAGGT   
  
  
- ACATTTAGTC TTAATTTTTA ATAATAGTAG ACATATTAGC ATACACAATT GCAGCGAAAG GATTACTACT   
  
  
- TCGGCGGTCG AGGTAAAAGG TGGGATGTAA AAATTTAATA ATAGTAGACA TATTAGCATA CACAATTGCA   
  
  
- GCGAAAGGAT TACTACTTCG GCGGTCGAGG TAAAAGGTGG GATGTAAAAA AGGTTATCTA GTTAGGCGAC   
  
  
- TACTTCGATA TACATGTAAG TTAAAAAAAG AACTCGATTG ACCTTTTATA AAAGCACGGC AGATGCTGTG   
  
  
- GACAATTGGG ATATATATAG AGAAACCTAA AAAGACAGAT AAAAACATAT GAAGAAGTCC TTTTATAAAA   
  
  
- GCACGGCAGA TGCTGTGGAC AATTGGGATA TATATAGAGA AACCTAAAAA GACAGATAAA AACATATGAA   
  
  
- GAAGACCTAG TTAGAGGTAG AAATAGAAGA AGGAGAAGAA GACGAACTTC GAACTAGAAG AAGACATTGA   
  
  
- TATGTCACGC AACAAAGACA AAGGAAAAG

+     AC-I

| Site Name | Organism | Position | Strand | Matrix score. | sequence | function |
| --- | --- | --- | --- | --- | --- | --- |
| AC-I | Phaseolus vulgaris | 765 | + | 9 | CCCACCTACC |  |

> 2018/04/13 10:10:12  
+ AAAAGGCAAC CTTTTCCTTT CTTTCTTCTT TCCTTAGCTT TTTAGATTTA TGAAGAAGGG CTGCTACGAC   
  
  
+ TTTCCTGCTT TTCCACATTT CAAACGCTCG TGTTATCGTC CTTTCATTCT CCCACTTCGT GTCCATCAGC   
  
  
+ TCCTTTTTAA AAAAATATAT TTATTTATTT TATTAAATTA TCATTTATTA TTATTTTTTA AAAAAAATTT   
  
  
+ AATACAGTTT TTTGGGGGAA AAAAAGAAAA AAAACTCATC CATCTGATTT GCTCTCTCTC TGATTTCCAA   
  
  
+ CCGAATGCAT GAGGAGAGCA TTCAGCGGCA ATCAAGCAGA GAGGACTGAC TGTCTTATGG GCCCTATCTC   
  
  
+ TTCTTGGATT CCAGACTATT CTTGTTTTTA GTAGCTCTCA AATCAATAAC CAGATCTAAT CCTAATACTC   
  
  
+ TCTTCATAAT CATAACTTAA AGAGGGAATT AATTAACCAA ATCAGGTAAA CAAACATCAT TAATTACAGC   
  
  
+ CACCTCGTTT GATCGATCCC GATCACCCAT GATCGTCACT TCCAGATCTT CACGTGTGCA ACCAATGAGC   
  
  
+ CATTAGTTGT AAATCAATTA ACATGTTTAA TCATAAAGAC TTCAAATTTT TGAAGTCAAC ATTCAAATGA   
  
  
+ TGCAGTTTGC TGAGATGGTA TTAGTGGCAC CACACAGCCC ACCCAAATGA ATTAACCATG AGACCTATTT   
  
  
+ ATGATCCTTC ATGGAAGTTC CTTAAGCCGC GCAGACATTC ACTATATAAT AGTAATTATA ACTTTCCACC   
  
  
+ TACCAACCAA GCATATGTAT ACTTTCAATA AATACATGTG CCCAAATTCC ATAAAATTAT AGAACAATTA   
  
  
+ TTGAATGAAT TTAATTATCA GATCAGTCCA AGACAGGCGC TTAGCTAGGT TAGCATAAAA TATCGTGCAC   
  
  
+ CTGTTATATC CTTATCAGCT CAGAGGCCTA ATACTGTTGC TAGTCTGGTA GCTCTACGAA TTTATTTCCA   
  
  
+ TGTAAATCAG AATTAAAAAT TATTATCATC TGTATAATCG TATGTGTTAA CGTCGCTTTC CTAATGATGA   
  
  
+ AGCCGCCAGC TCCATTTTCC ACCCTACATT TTTAAATTAT TATCATCTGT ATAATCGTAT GTGTTAACGT   
  
  
+ CGCTTTCCTA ATGATGAAGC CGCCAGCTCC ATTTTCCACC CTACATTTTT TCCAATAGAT CAATCCGCTG   
  
  
+ ATGAAGCTAT ATGTACATTC AATTTTTTTC TTGAGCTAAC TGGAAAATAT TTTCGTGCCG TCTACGACAC   
  
  
+ CTGTTAACCC TATATATATC TCTTTGGATT TTTCTGTCTA TTTTTGTATA CTTCTTCAGG AAAATATTTT   
  
  
+ CGTGCCGTCT ACGACACCTG TTAACCCTAT ATATATCTCT TTGGATTTTT CTGTCTATTT TTGTATACTT   
  
  
+ CTTCTGGATC AATCTCCATC TTTATCTTCT TCCTCTTCTT CTGCTTGAAG CTTGATCTTC TTCTGTAACT   
  
  
+ ATACAGTGCG TTGTTTCTGT TTCCTTTTC  

- TTTTCCGTTG GAAAAGGAAA GAAAGAAGAA AGGAATCGAA AAATCTAAAT ACTTCTTCCC GACGATGCTG   
  
  
- AAAGGACGAA AAGGTGTAAA GTTTGCGAGC ACAATAGCAG GAAAGTAAGA GGGTGAAGCA CAGGTAGTCG   
  
  
- AGGAAAAATT TTTTTATATA AATAAATAAA ATAATTTAAT AGTAAATAAT AATAAAAAAT TTTTTTTAAA   
  
  
- TTATGTCAAA AAACCCCCTT TTTTTCTTTT TTTTGAGTAG GTAGACTAAA CGAGAGAGAG ACTAAAGGTT   
  
  
- GGCTTACGTA CTCCTCTCGT AAGTCGCCGT TAGTTCGTCT CTCCTGACTG ACAGAATACC CGGGATAGAG   
  
  
- AAGAACCTAA GGTCTGATAA GAACAAAAAT CATCGAGAGT TTAGTTATTG GTCTAGATTA GGATTATGAG   
  
  
- AGAAGTATTA GTATTGAATT TCTCCCTTAA TTAATTGGTT TAGTCCATTT GTTTGTAGTA ATTAATGTCG   
  
  
- GTGGAGCAAA CTAGCTAGGG CTAGTGGGTA CTAGCAGTGA AGGTCTAGAA GTGCACACGT TGGTTACTCG   
  
  
- GTAATCAACA TTTAGTTAAT TGTACAAATT AGTATTTCTG AAGTTTAAAA ACTTCAGTTG TAAGTTTACT   
  
  
- ACGTCAAACG ACTCTACCAT AATCACCGTG GTGTGTCGGG TGGGTTTACT TAATTGGTAC TCTGGATAAA   
  
  
- TACTAGGAAG TACCTTCAAG GAATTCGGCG CGTCTGTAAG TGATATATTA TCATTAATAT TGAAAGGTGG   
  
  
- ATGGTTGGTT CGTATACATA TGAAAGTTAT TTATGTACAC GGGTTTAAGG TATTTTAATA TCTTGTTAAT   
  
  
- AACTTACTTA AATTAATAGT CTAGTCAGGT TCTGTCCGCG AATCGATCCA ATCGTATTTT ATAGCACGTG   
  
  
- GACAATATAG GAATAGTCGA GTCTCCGGAT TATGACAACG ATCAGACCAT CGAGATGCTT AAATAAAGGT   
  
  
- ACATTTAGTC TTAATTTTTA ATAATAGTAG ACATATTAGC ATACACAATT GCAGCGAAAG GATTACTACT   
  
  
- TCGGCGGTCG AGGTAAAAGG TGGGATGTAA AAATTTAATA ATAGTAGACA TATTAGCATA CACAATTGCA   
  
  
- GCGAAAGGAT TACTACTTCG GCGGTCGAGG TAAAAGGTGG GATGTAAAAA AGGTTATCTA GTTAGGCGAC   
  
  
- TACTTCGATA TACATGTAAG TTAAAAAAAG AACTCGATTG ACCTTTTATA AAAGCACGGC AGATGCTGTG   
  
  
- GACAATTGGG ATATATATAG AGAAACCTAA AAAGACAGAT AAAAACATAT GAAGAAGTCC TTTTATAAAA   
  
  
- GCACGGCAGA TGCTGTGGAC AATTGGGATA TATATAGAGA AACCTAAAAA GACAGATAAA AACATATGAA   
  
  
- GAAGACCTAG TTAGAGGTAG AAATAGAAGA AGGAGAAGAA GACGAACTTC GAACTAGAAG AAGACATTGA   
  
  
- TATGTCACGC AACAAAGACA AAGGAAAAG

+     AC-II

| Site Name | Organism | Position | Strand | Matrix score. | sequence | function |
| --- | --- | --- | --- | --- | --- | --- |
| AC-II | Phaseolus vulgaris | 764 | + | 9 | (C/T)T(T/C)(C/T)(A/C)(A/C)C(A/C)A(A/C)C(C/A)(C/A)C |  |

> 2018/04/13 10:10:12  
+ AAAAGGCAAC CTTTTCCTTT CTTTCTTCTT TCCTTAGCTT TTTAGATTTA TGAAGAAGGG CTGCTACGAC   
  
  
+ TTTCCTGCTT TTCCACATTT CAAACGCTCG TGTTATCGTC CTTTCATTCT CCCACTTCGT GTCCATCAGC   
  
  
+ TCCTTTTTAA AAAAATATAT TTATTTATTT TATTAAATTA TCATTTATTA TTATTTTTTA AAAAAAATTT   
  
  
+ AATACAGTTT TTTGGGGGAA AAAAAGAAAA AAAACTCATC CATCTGATTT GCTCTCTCTC TGATTTCCAA   
  
  
+ CCGAATGCAT GAGGAGAGCA TTCAGCGGCA ATCAAGCAGA GAGGACTGAC TGTCTTATGG GCCCTATCTC   
  
  
+ TTCTTGGATT CCAGACTATT CTTGTTTTTA GTAGCTCTCA AATCAATAAC CAGATCTAAT CCTAATACTC   
  
  
+ TCTTCATAAT CATAACTTAA AGAGGGAATT AATTAACCAA ATCAGGTAAA CAAACATCAT TAATTACAGC   
  
  
+ CACCTCGTTT GATCGATCCC GATCACCCAT GATCGTCACT TCCAGATCTT CACGTGTGCA ACCAATGAGC   
  
  
+ CATTAGTTGT AAATCAATTA ACATGTTTAA TCATAAAGAC TTCAAATTTT TGAAGTCAAC ATTCAAATGA   
  
  
+ TGCAGTTTGC TGAGATGGTA TTAGTGGCAC CACACAGCCC ACCCAAATGA ATTAACCATG AGACCTATTT   
  
  
+ ATGATCCTTC ATGGAAGTTC CTTAAGCCGC GCAGACATTC ACTATATAAT AGTAATTATA ACTTTCCACC   
  
  
+ TACCAACCAA GCATATGTAT ACTTTCAATA AATACATGTG CCCAAATTCC ATAAAATTAT AGAACAATTA   
  
  
+ TTGAATGAAT TTAATTATCA GATCAGTCCA AGACAGGCGC TTAGCTAGGT TAGCATAAAA TATCGTGCAC   
  
  
+ CTGTTATATC CTTATCAGCT CAGAGGCCTA ATACTGTTGC TAGTCTGGTA GCTCTACGAA TTTATTTCCA   
  
  
+ TGTAAATCAG AATTAAAAAT TATTATCATC TGTATAATCG TATGTGTTAA CGTCGCTTTC CTAATGATGA   
  
  
+ AGCCGCCAGC TCCATTTTCC ACCCTACATT TTTAAATTAT TATCATCTGT ATAATCGTAT GTGTTAACGT   
  
  
+ CGCTTTCCTA ATGATGAAGC CGCCAGCTCC ATTTTCCACC CTACATTTTT TCCAATAGAT CAATCCGCTG   
  
  
+ ATGAAGCTAT ATGTACATTC AATTTTTTTC TTGAGCTAAC TGGAAAATAT TTTCGTGCCG TCTACGACAC   
  
  
+ CTGTTAACCC TATATATATC TCTTTGGATT TTTCTGTCTA TTTTTGTATA CTTCTTCAGG AAAATATTTT   
  
  
+ CGTGCCGTCT ACGACACCTG TTAACCCTAT ATATATCTCT TTGGATTTTT CTGTCTATTT TTGTATACTT   
  
  
+ CTTCTGGATC AATCTCCATC TTTATCTTCT TCCTCTTCTT CTGCTTGAAG CTTGATCTTC TTCTGTAACT   
  
  
+ ATACAGTGCG TTGTTTCTGT TTCCTTTTC  

- TTTTCCGTTG GAAAAGGAAA GAAAGAAGAA AGGAATCGAA AAATCTAAAT ACTTCTTCCC GACGATGCTG   
  
  
- AAAGGACGAA AAGGTGTAAA GTTTGCGAGC ACAATAGCAG GAAAGTAAGA GGGTGAAGCA CAGGTAGTCG   
  
  
- AGGAAAAATT TTTTTATATA AATAAATAAA ATAATTTAAT AGTAAATAAT AATAAAAAAT TTTTTTTAAA   
  
  
- TTATGTCAAA AAACCCCCTT TTTTTCTTTT TTTTGAGTAG GTAGACTAAA CGAGAGAGAG ACTAAAGGTT   
  
  
- GGCTTACGTA CTCCTCTCGT AAGTCGCCGT TAGTTCGTCT CTCCTGACTG ACAGAATACC CGGGATAGAG   
  
  
- AAGAACCTAA GGTCTGATAA GAACAAAAAT CATCGAGAGT TTAGTTATTG GTCTAGATTA GGATTATGAG   
  
  
- AGAAGTATTA GTATTGAATT TCTCCCTTAA TTAATTGGTT TAGTCCATTT GTTTGTAGTA ATTAATGTCG   
  
  
- GTGGAGCAAA CTAGCTAGGG CTAGTGGGTA CTAGCAGTGA AGGTCTAGAA GTGCACACGT TGGTTACTCG   
  
  
- GTAATCAACA TTTAGTTAAT TGTACAAATT AGTATTTCTG AAGTTTAAAA ACTTCAGTTG TAAGTTTACT   
  
  
- ACGTCAAACG ACTCTACCAT AATCACCGTG GTGTGTCGGG TGGGTTTACT TAATTGGTAC TCTGGATAAA   
  
  
- TACTAGGAAG TACCTTCAAG GAATTCGGCG CGTCTGTAAG TGATATATTA TCATTAATAT TGAAAGGTGG   
  
  
- ATGGTTGGTT CGTATACATA TGAAAGTTAT TTATGTACAC GGGTTTAAGG TATTTTAATA TCTTGTTAAT   
  
  
- AACTTACTTA AATTAATAGT CTAGTCAGGT TCTGTCCGCG AATCGATCCA ATCGTATTTT ATAGCACGTG   
  
  
- GACAATATAG GAATAGTCGA GTCTCCGGAT TATGACAACG ATCAGACCAT CGAGATGCTT AAATAAAGGT   
  
  
- ACATTTAGTC TTAATTTTTA ATAATAGTAG ACATATTAGC ATACACAATT GCAGCGAAAG GATTACTACT   
  
  
- TCGGCGGTCG AGGTAAAAGG TGGGATGTAA AAATTTAATA ATAGTAGACA TATTAGCATA CACAATTGCA   
  
  
- GCGAAAGGAT TACTACTTCG GCGGTCGAGG TAAAAGGTGG GATGTAAAAA AGGTTATCTA GTTAGGCGAC   
  
  
- TACTTCGATA TACATGTAAG TTAAAAAAAG AACTCGATTG ACCTTTTATA AAAGCACGGC AGATGCTGTG   
  
  
- GACAATTGGG ATATATATAG AGAAACCTAA AAAGACAGAT AAAAACATAT GAAGAAGTCC TTTTATAAAA   
  
  
- GCACGGCAGA TGCTGTGGAC AATTGGGATA TATATAGAGA AACCTAAAAA GACAGATAAA AACATATGAA   
  
  
- GAAGACCTAG TTAGAGGTAG AAATAGAAGA AGGAGAAGAA GACGAACTTC GAACTAGAAG AAGACATTGA   
  
  
- TATGTCACGC AACAAAGACA AAGGAAAAG

+     AE-box

| Site Name | Organism | Position | Strand | Matrix score. | sequence | function |
| --- | --- | --- | --- | --- | --- | --- |
| AE-box | Arabidopsis thaliana | 1481 | - | 8 | AGAAACAA | part of a module for light response |

> 2018/04/13 10:10:12  
+ AAAAGGCAAC CTTTTCCTTT CTTTCTTCTT TCCTTAGCTT TTTAGATTTA TGAAGAAGGG CTGCTACGAC   
  
  
+ TTTCCTGCTT TTCCACATTT CAAACGCTCG TGTTATCGTC CTTTCATTCT CCCACTTCGT GTCCATCAGC   
  
  
+ TCCTTTTTAA AAAAATATAT TTATTTATTT TATTAAATTA TCATTTATTA TTATTTTTTA AAAAAAATTT   
  
  
+ AATACAGTTT TTTGGGGGAA AAAAAGAAAA AAAACTCATC CATCTGATTT GCTCTCTCTC TGATTTCCAA   
  
  
+ CCGAATGCAT GAGGAGAGCA TTCAGCGGCA ATCAAGCAGA GAGGACTGAC TGTCTTATGG GCCCTATCTC   
  
  
+ TTCTTGGATT CCAGACTATT CTTGTTTTTA GTAGCTCTCA AATCAATAAC CAGATCTAAT CCTAATACTC   
  
  
+ TCTTCATAAT CATAACTTAA AGAGGGAATT AATTAACCAA ATCAGGTAAA CAAACATCAT TAATTACAGC   
  
  
+ CACCTCGTTT GATCGATCCC GATCACCCAT GATCGTCACT TCCAGATCTT CACGTGTGCA ACCAATGAGC   
  
  
+ CATTAGTTGT AAATCAATTA ACATGTTTAA TCATAAAGAC TTCAAATTTT TGAAGTCAAC ATTCAAATGA   
  
  
+ TGCAGTTTGC TGAGATGGTA TTAGTGGCAC CACACAGCCC ACCCAAATGA ATTAACCATG AGACCTATTT   
  
  
+ ATGATCCTTC ATGGAAGTTC CTTAAGCCGC GCAGACATTC ACTATATAAT AGTAATTATA ACTTTCCACC   
  
  
+ TACCAACCAA GCATATGTAT ACTTTCAATA AATACATGTG CCCAAATTCC ATAAAATTAT AGAACAATTA   
  
  
+ TTGAATGAAT TTAATTATCA GATCAGTCCA AGACAGGCGC TTAGCTAGGT TAGCATAAAA TATCGTGCAC   
  
  
+ CTGTTATATC CTTATCAGCT CAGAGGCCTA ATACTGTTGC TAGTCTGGTA GCTCTACGAA TTTATTTCCA   
  
  
+ TGTAAATCAG AATTAAAAAT TATTATCATC TGTATAATCG TATGTGTTAA CGTCGCTTTC CTAATGATGA   
  
  
+ AGCCGCCAGC TCCATTTTCC ACCCTACATT TTTAAATTAT TATCATCTGT ATAATCGTAT GTGTTAACGT   
  
  
+ CGCTTTCCTA ATGATGAAGC CGCCAGCTCC ATTTTCCACC CTACATTTTT TCCAATAGAT CAATCCGCTG   
  
  
+ ATGAAGCTAT ATGTACATTC AATTTTTTTC TTGAGCTAAC TGGAAAATAT TTTCGTGCCG TCTACGACAC   
  
  
+ CTGTTAACCC TATATATATC TCTTTGGATT TTTCTGTCTA TTTTTGTATA CTTCTTCAGG AAAATATTTT   
  
  
+ CGTGCCGTCT ACGACACCTG TTAACCCTAT ATATATCTCT TTGGATTTTT CTGTCTATTT TTGTATACTT   
  
  
+ CTTCTGGATC AATCTCCATC TTTATCTTCT TCCTCTTCTT CTGCTTGAAG CTTGATCTTC TTCTGTAACT   
  
  
+ ATACAGTGCG TTGTTTCTGT TTCCTTTTC  

- TTTTCCGTTG GAAAAGGAAA GAAAGAAGAA AGGAATCGAA AAATCTAAAT ACTTCTTCCC GACGATGCTG   
  
  
- AAAGGACGAA AAGGTGTAAA GTTTGCGAGC ACAATAGCAG GAAAGTAAGA GGGTGAAGCA CAGGTAGTCG   
  
  
- AGGAAAAATT TTTTTATATA AATAAATAAA ATAATTTAAT AGTAAATAAT AATAAAAAAT TTTTTTTAAA   
  
  
- TTATGTCAAA AAACCCCCTT TTTTTCTTTT TTTTGAGTAG GTAGACTAAA CGAGAGAGAG ACTAAAGGTT   
  
  
- GGCTTACGTA CTCCTCTCGT AAGTCGCCGT TAGTTCGTCT CTCCTGACTG ACAGAATACC CGGGATAGAG   
  
  
- AAGAACCTAA GGTCTGATAA GAACAAAAAT CATCGAGAGT TTAGTTATTG GTCTAGATTA GGATTATGAG   
  
  
- AGAAGTATTA GTATTGAATT TCTCCCTTAA TTAATTGGTT TAGTCCATTT GTTTGTAGTA ATTAATGTCG   
  
  
- GTGGAGCAAA CTAGCTAGGG CTAGTGGGTA CTAGCAGTGA AGGTCTAGAA GTGCACACGT TGGTTACTCG   
  
  
- GTAATCAACA TTTAGTTAAT TGTACAAATT AGTATTTCTG AAGTTTAAAA ACTTCAGTTG TAAGTTTACT   
  
  
- ACGTCAAACG ACTCTACCAT AATCACCGTG GTGTGTCGGG TGGGTTTACT TAATTGGTAC TCTGGATAAA   
  
  
- TACTAGGAAG TACCTTCAAG GAATTCGGCG CGTCTGTAAG TGATATATTA TCATTAATAT TGAAAGGTGG   
  
  
- ATGGTTGGTT CGTATACATA TGAAAGTTAT TTATGTACAC GGGTTTAAGG TATTTTAATA TCTTGTTAAT   
  
  
- AACTTACTTA AATTAATAGT CTAGTCAGGT TCTGTCCGCG AATCGATCCA ATCGTATTTT ATAGCACGTG   
  
  
- GACAATATAG GAATAGTCGA GTCTCCGGAT TATGACAACG ATCAGACCAT CGAGATGCTT AAATAAAGGT   
  
  
- ACATTTAGTC TTAATTTTTA ATAATAGTAG ACATATTAGC ATACACAATT GCAGCGAAAG GATTACTACT   
  
  
- TCGGCGGTCG AGGTAAAAGG TGGGATGTAA AAATTTAATA ATAGTAGACA TATTAGCATA CACAATTGCA   
  
  
- GCGAAAGGAT TACTACTTCG GCGGTCGAGG TAAAAGGTGG GATGTAAAAA AGGTTATCTA GTTAGGCGAC   
  
  
- TACTTCGATA TACATGTAAG TTAAAAAAAG AACTCGATTG ACCTTTTATA AAAGCACGGC AGATGCTGTG   
  
  
- GACAATTGGG ATATATATAG AGAAACCTAA AAAGACAGAT AAAAACATAT GAAGAAGTCC TTTTATAAAA   
  
  
- GCACGGCAGA TGCTGTGGAC AATTGGGATA TATATAGAGA AACCTAAAAA GACAGATAAA AACATATGAA   
  
  
- GAAGACCTAG TTAGAGGTAG AAATAGAAGA AGGAGAAGAA GACGAACTTC GAACTAGAAG AAGACATTGA   
  
  
- TATGTCACGC AACAAAGACA AAGGAAAAG

+     ATCT-motif

| Site Name | Organism | Position | Strand | Matrix score. | sequence | function |
| --- | --- | --- | --- | --- | --- | --- |
| ATCT-motif | Pisum sativum | 403 | + | 9 | AATCTAATCC | part of a conserved DNA module involved in light responsiveness |

> 2018/04/13 10:10:12  
+ AAAAGGCAAC CTTTTCCTTT CTTTCTTCTT TCCTTAGCTT TTTAGATTTA TGAAGAAGGG CTGCTACGAC   
  
  
+ TTTCCTGCTT TTCCACATTT CAAACGCTCG TGTTATCGTC CTTTCATTCT CCCACTTCGT GTCCATCAGC   
  
  
+ TCCTTTTTAA AAAAATATAT TTATTTATTT TATTAAATTA TCATTTATTA TTATTTTTTA AAAAAAATTT   
  
  
+ AATACAGTTT TTTGGGGGAA AAAAAGAAAA AAAACTCATC CATCTGATTT GCTCTCTCTC TGATTTCCAA   
  
  
+ CCGAATGCAT GAGGAGAGCA TTCAGCGGCA ATCAAGCAGA GAGGACTGAC TGTCTTATGG GCCCTATCTC   
  
  
+ TTCTTGGATT CCAGACTATT CTTGTTTTTA GTAGCTCTCA AATCAATAAC CAGATCTAAT CCTAATACTC   
  
  
+ TCTTCATAAT CATAACTTAA AGAGGGAATT AATTAACCAA ATCAGGTAAA CAAACATCAT TAATTACAGC   
  
  
+ CACCTCGTTT GATCGATCCC GATCACCCAT GATCGTCACT TCCAGATCTT CACGTGTGCA ACCAATGAGC   
  
  
+ CATTAGTTGT AAATCAATTA ACATGTTTAA TCATAAAGAC TTCAAATTTT TGAAGTCAAC ATTCAAATGA   
  
  
+ TGCAGTTTGC TGAGATGGTA TTAGTGGCAC CACACAGCCC ACCCAAATGA ATTAACCATG AGACCTATTT   
  
  
+ ATGATCCTTC ATGGAAGTTC CTTAAGCCGC GCAGACATTC ACTATATAAT AGTAATTATA ACTTTCCACC   
  
  
+ TACCAACCAA GCATATGTAT ACTTTCAATA AATACATGTG CCCAAATTCC ATAAAATTAT AGAACAATTA   
  
  
+ TTGAATGAAT TTAATTATCA GATCAGTCCA AGACAGGCGC TTAGCTAGGT TAGCATAAAA TATCGTGCAC   
  
  
+ CTGTTATATC CTTATCAGCT CAGAGGCCTA ATACTGTTGC TAGTCTGGTA GCTCTACGAA TTTATTTCCA   
  
  
+ TGTAAATCAG AATTAAAAAT TATTATCATC TGTATAATCG TATGTGTTAA CGTCGCTTTC CTAATGATGA   
  
  
+ AGCCGCCAGC TCCATTTTCC ACCCTACATT TTTAAATTAT TATCATCTGT ATAATCGTAT GTGTTAACGT   
  
  
+ CGCTTTCCTA ATGATGAAGC CGCCAGCTCC ATTTTCCACC CTACATTTTT TCCAATAGAT CAATCCGCTG   
  
  
+ ATGAAGCTAT ATGTACATTC AATTTTTTTC TTGAGCTAAC TGGAAAATAT TTTCGTGCCG TCTACGACAC   
  
  
+ CTGTTAACCC TATATATATC TCTTTGGATT TTTCTGTCTA TTTTTGTATA CTTCTTCAGG AAAATATTTT   
  
  
+ CGTGCCGTCT ACGACACCTG TTAACCCTAT ATATATCTCT TTGGATTTTT CTGTCTATTT TTGTATACTT   
  
  
+ CTTCTGGATC AATCTCCATC TTTATCTTCT TCCTCTTCTT CTGCTTGAAG CTTGATCTTC TTCTGTAACT   
  
  
+ ATACAGTGCG TTGTTTCTGT TTCCTTTTC  

- TTTTCCGTTG GAAAAGGAAA GAAAGAAGAA AGGAATCGAA AAATCTAAAT ACTTCTTCCC GACGATGCTG   
  
  
- AAAGGACGAA AAGGTGTAAA GTTTGCGAGC ACAATAGCAG GAAAGTAAGA GGGTGAAGCA CAGGTAGTCG   
  
  
- AGGAAAAATT TTTTTATATA AATAAATAAA ATAATTTAAT AGTAAATAAT AATAAAAAAT TTTTTTTAAA   
  
  
- TTATGTCAAA AAACCCCCTT TTTTTCTTTT TTTTGAGTAG GTAGACTAAA CGAGAGAGAG ACTAAAGGTT   
  
  
- GGCTTACGTA CTCCTCTCGT AAGTCGCCGT TAGTTCGTCT CTCCTGACTG ACAGAATACC CGGGATAGAG   
  
  
- AAGAACCTAA GGTCTGATAA GAACAAAAAT CATCGAGAGT TTAGTTATTG GTCTAGATTA GGATTATGAG   
  
  
- AGAAGTATTA GTATTGAATT TCTCCCTTAA TTAATTGGTT TAGTCCATTT GTTTGTAGTA ATTAATGTCG   
  
  
- GTGGAGCAAA CTAGCTAGGG CTAGTGGGTA CTAGCAGTGA AGGTCTAGAA GTGCACACGT TGGTTACTCG   
  
  
- GTAATCAACA TTTAGTTAAT TGTACAAATT AGTATTTCTG AAGTTTAAAA ACTTCAGTTG TAAGTTTACT   
  
  
- ACGTCAAACG ACTCTACCAT AATCACCGTG GTGTGTCGGG TGGGTTTACT TAATTGGTAC TCTGGATAAA   
  
  
- TACTAGGAAG TACCTTCAAG GAATTCGGCG CGTCTGTAAG TGATATATTA TCATTAATAT TGAAAGGTGG   
  
  
- ATGGTTGGTT CGTATACATA TGAAAGTTAT TTATGTACAC GGGTTTAAGG TATTTTAATA TCTTGTTAAT   
  
  
- AACTTACTTA AATTAATAGT CTAGTCAGGT TCTGTCCGCG AATCGATCCA ATCGTATTTT ATAGCACGTG   
  
  
- GACAATATAG GAATAGTCGA GTCTCCGGAT TATGACAACG ATCAGACCAT CGAGATGCTT AAATAAAGGT   
  
  
- ACATTTAGTC TTAATTTTTA ATAATAGTAG ACATATTAGC ATACACAATT GCAGCGAAAG GATTACTACT   
  
  
- TCGGCGGTCG AGGTAAAAGG TGGGATGTAA AAATTTAATA ATAGTAGACA TATTAGCATA CACAATTGCA   
  
  
- GCGAAAGGAT TACTACTTCG GCGGTCGAGG TAAAAGGTGG GATGTAAAAA AGGTTATCTA GTTAGGCGAC   
  
  
- TACTTCGATA TACATGTAAG TTAAAAAAAG AACTCGATTG ACCTTTTATA AAAGCACGGC AGATGCTGTG   
  
  
- GACAATTGGG ATATATATAG AGAAACCTAA AAAGACAGAT AAAAACATAT GAAGAAGTCC TTTTATAAAA   
  
  
- GCACGGCAGA TGCTGTGGAC AATTGGGATA TATATAGAGA AACCTAAAAA GACAGATAAA AACATATGAA   
  
  
- GAAGACCTAG TTAGAGGTAG AAATAGAAGA AGGAGAAGAA GACGAACTTC GAACTAGAAG AAGACATTGA   
  
  
- TATGTCACGC AACAAAGACA AAGGAAAAG

+     Box 4

| Site Name | Organism | Position | Strand | Matrix score. | sequence | function |
| --- | --- | --- | --- | --- | --- | --- |
| Box 4 | Petroselinum crispum | 448 | + | 6 | ATTAAT | part of a conserved DNA module involved in light responsiveness |
| Box 4 | Petroselinum crispum | 479 | + | 6 | ATTAAT | part of a conserved DNA module involved in light responsiveness |

> 2018/04/13 10:10:12  
+ AAAAGGCAAC CTTTTCCTTT CTTTCTTCTT TCCTTAGCTT TTTAGATTTA TGAAGAAGGG CTGCTACGAC   
  
  
+ TTTCCTGCTT TTCCACATTT CAAACGCTCG TGTTATCGTC CTTTCATTCT CCCACTTCGT GTCCATCAGC   
  
  
+ TCCTTTTTAA AAAAATATAT TTATTTATTT TATTAAATTA TCATTTATTA TTATTTTTTA AAAAAAATTT   
  
  
+ AATACAGTTT TTTGGGGGAA AAAAAGAAAA AAAACTCATC CATCTGATTT GCTCTCTCTC TGATTTCCAA   
  
  
+ CCGAATGCAT GAGGAGAGCA TTCAGCGGCA ATCAAGCAGA GAGGACTGAC TGTCTTATGG GCCCTATCTC   
  
  
+ TTCTTGGATT CCAGACTATT CTTGTTTTTA GTAGCTCTCA AATCAATAAC CAGATCTAAT CCTAATACTC   
  
  
+ TCTTCATAAT CATAACTTAA AGAGGGAATT AATTAACCAA ATCAGGTAAA CAAACATCAT TAATTACAGC   
  
  
+ CACCTCGTTT GATCGATCCC GATCACCCAT GATCGTCACT TCCAGATCTT CACGTGTGCA ACCAATGAGC   
  
  
+ CATTAGTTGT AAATCAATTA ACATGTTTAA TCATAAAGAC TTCAAATTTT TGAAGTCAAC ATTCAAATGA   
  
  
+ TGCAGTTTGC TGAGATGGTA TTAGTGGCAC CACACAGCCC ACCCAAATGA ATTAACCATG AGACCTATTT   
  
  
+ ATGATCCTTC ATGGAAGTTC CTTAAGCCGC GCAGACATTC ACTATATAAT AGTAATTATA ACTTTCCACC   
  
  
+ TACCAACCAA GCATATGTAT ACTTTCAATA AATACATGTG CCCAAATTCC ATAAAATTAT AGAACAATTA   
  
  
+ TTGAATGAAT TTAATTATCA GATCAGTCCA AGACAGGCGC TTAGCTAGGT TAGCATAAAA TATCGTGCAC   
  
  
+ CTGTTATATC CTTATCAGCT CAGAGGCCTA ATACTGTTGC TAGTCTGGTA GCTCTACGAA TTTATTTCCA   
  
  
+ TGTAAATCAG AATTAAAAAT TATTATCATC TGTATAATCG TATGTGTTAA CGTCGCTTTC CTAATGATGA   
  
  
+ AGCCGCCAGC TCCATTTTCC ACCCTACATT TTTAAATTAT TATCATCTGT ATAATCGTAT GTGTTAACGT   
  
  
+ CGCTTTCCTA ATGATGAAGC CGCCAGCTCC ATTTTCCACC CTACATTTTT TCCAATAGAT CAATCCGCTG   
  
  
+ ATGAAGCTAT ATGTACATTC AATTTTTTTC TTGAGCTAAC TGGAAAATAT TTTCGTGCCG TCTACGACAC   
  
  
+ CTGTTAACCC TATATATATC TCTTTGGATT TTTCTGTCTA TTTTTGTATA CTTCTTCAGG AAAATATTTT   
  
  
+ CGTGCCGTCT ACGACACCTG TTAACCCTAT ATATATCTCT TTGGATTTTT CTGTCTATTT TTGTATACTT   
  
  
+ CTTCTGGATC AATCTCCATC TTTATCTTCT TCCTCTTCTT CTGCTTGAAG CTTGATCTTC TTCTGTAACT   
  
  
+ ATACAGTGCG TTGTTTCTGT TTCCTTTTC  

- TTTTCCGTTG GAAAAGGAAA GAAAGAAGAA AGGAATCGAA AAATCTAAAT ACTTCTTCCC GACGATGCTG   
  
  
- AAAGGACGAA AAGGTGTAAA GTTTGCGAGC ACAATAGCAG GAAAGTAAGA GGGTGAAGCA CAGGTAGTCG   
  
  
- AGGAAAAATT TTTTTATATA AATAAATAAA ATAATTTAAT AGTAAATAAT AATAAAAAAT TTTTTTTAAA   
  
  
- TTATGTCAAA AAACCCCCTT TTTTTCTTTT TTTTGAGTAG GTAGACTAAA CGAGAGAGAG ACTAAAGGTT   
  
  
- GGCTTACGTA CTCCTCTCGT AAGTCGCCGT TAGTTCGTCT CTCCTGACTG ACAGAATACC CGGGATAGAG   
  
  
- AAGAACCTAA GGTCTGATAA GAACAAAAAT CATCGAGAGT TTAGTTATTG GTCTAGATTA GGATTATGAG   
  
  
- AGAAGTATTA GTATTGAATT TCTCCCTTAA TTAATTGGTT TAGTCCATTT GTTTGTAGTA ATTAATGTCG   
  
  
- GTGGAGCAAA CTAGCTAGGG CTAGTGGGTA CTAGCAGTGA AGGTCTAGAA GTGCACACGT TGGTTACTCG   
  
  
- GTAATCAACA TTTAGTTAAT TGTACAAATT AGTATTTCTG AAGTTTAAAA ACTTCAGTTG TAAGTTTACT   
  
  
- ACGTCAAACG ACTCTACCAT AATCACCGTG GTGTGTCGGG TGGGTTTACT TAATTGGTAC TCTGGATAAA   
  
  
- TACTAGGAAG TACCTTCAAG GAATTCGGCG CGTCTGTAAG TGATATATTA TCATTAATAT TGAAAGGTGG   
  
  
- ATGGTTGGTT CGTATACATA TGAAAGTTAT TTATGTACAC GGGTTTAAGG TATTTTAATA TCTTGTTAAT   
  
  
- AACTTACTTA AATTAATAGT CTAGTCAGGT TCTGTCCGCG AATCGATCCA ATCGTATTTT ATAGCACGTG   
  
  
- GACAATATAG GAATAGTCGA GTCTCCGGAT TATGACAACG ATCAGACCAT CGAGATGCTT AAATAAAGGT   
  
  
- ACATTTAGTC TTAATTTTTA ATAATAGTAG ACATATTAGC ATACACAATT GCAGCGAAAG GATTACTACT   
  
  
- TCGGCGGTCG AGGTAAAAGG TGGGATGTAA AAATTTAATA ATAGTAGACA TATTAGCATA CACAATTGCA   
  
  
- GCGAAAGGAT TACTACTTCG GCGGTCGAGG TAAAAGGTGG GATGTAAAAA AGGTTATCTA GTTAGGCGAC   
  
  
- TACTTCGATA TACATGTAAG TTAAAAAAAG AACTCGATTG ACCTTTTATA AAAGCACGGC AGATGCTGTG   
  
  
- GACAATTGGG ATATATATAG AGAAACCTAA AAAGACAGAT AAAAACATAT GAAGAAGTCC TTTTATAAAA   
  
  
- GCACGGCAGA TGCTGTGGAC AATTGGGATA TATATAGAGA AACCTAAAAA GACAGATAAA AACATATGAA   
  
  
- GAAGACCTAG TTAGAGGTAG AAATAGAAGA AGGAGAAGAA GACGAACTTC GAACTAGAAG AAGACATTGA   
  
  
- TATGTCACGC AACAAAGACA AAGGAAAAG

+     Box I

| Site Name | Organism | Position | Strand | Matrix score. | sequence | function |
| --- | --- | --- | --- | --- | --- | --- |
| Box I | Pisum sativum | 88 | + | 7 | TTTCAAA | light responsive element |

> 2018/04/13 10:10:12  
+ AAAAGGCAAC CTTTTCCTTT CTTTCTTCTT TCCTTAGCTT TTTAGATTTA TGAAGAAGGG CTGCTACGAC   
  
  
+ TTTCCTGCTT TTCCACATTT CAAACGCTCG TGTTATCGTC CTTTCATTCT CCCACTTCGT GTCCATCAGC   
  
  
+ TCCTTTTTAA AAAAATATAT TTATTTATTT TATTAAATTA TCATTTATTA TTATTTTTTA AAAAAAATTT   
  
  
+ AATACAGTTT TTTGGGGGAA AAAAAGAAAA AAAACTCATC CATCTGATTT GCTCTCTCTC TGATTTCCAA   
  
  
+ CCGAATGCAT GAGGAGAGCA TTCAGCGGCA ATCAAGCAGA GAGGACTGAC TGTCTTATGG GCCCTATCTC   
  
  
+ TTCTTGGATT CCAGACTATT CTTGTTTTTA GTAGCTCTCA AATCAATAAC CAGATCTAAT CCTAATACTC   
  
  
+ TCTTCATAAT CATAACTTAA AGAGGGAATT AATTAACCAA ATCAGGTAAA CAAACATCAT TAATTACAGC   
  
  
+ CACCTCGTTT GATCGATCCC GATCACCCAT GATCGTCACT TCCAGATCTT CACGTGTGCA ACCAATGAGC   
  
  
+ CATTAGTTGT AAATCAATTA ACATGTTTAA TCATAAAGAC TTCAAATTTT TGAAGTCAAC ATTCAAATGA   
  
  
+ TGCAGTTTGC TGAGATGGTA TTAGTGGCAC CACACAGCCC ACCCAAATGA ATTAACCATG AGACCTATTT   
  
  
+ ATGATCCTTC ATGGAAGTTC CTTAAGCCGC GCAGACATTC ACTATATAAT AGTAATTATA ACTTTCCACC   
  
  
+ TACCAACCAA GCATATGTAT ACTTTCAATA AATACATGTG CCCAAATTCC ATAAAATTAT AGAACAATTA   
  
  
+ TTGAATGAAT TTAATTATCA GATCAGTCCA AGACAGGCGC TTAGCTAGGT TAGCATAAAA TATCGTGCAC   
  
  
+ CTGTTATATC CTTATCAGCT CAGAGGCCTA ATACTGTTGC TAGTCTGGTA GCTCTACGAA TTTATTTCCA   
  
  
+ TGTAAATCAG AATTAAAAAT TATTATCATC TGTATAATCG TATGTGTTAA CGTCGCTTTC CTAATGATGA   
  
  
+ AGCCGCCAGC TCCATTTTCC ACCCTACATT TTTAAATTAT TATCATCTGT ATAATCGTAT GTGTTAACGT   
  
  
+ CGCTTTCCTA ATGATGAAGC CGCCAGCTCC ATTTTCCACC CTACATTTTT TCCAATAGAT CAATCCGCTG   
  
  
+ ATGAAGCTAT ATGTACATTC AATTTTTTTC TTGAGCTAAC TGGAAAATAT TTTCGTGCCG TCTACGACAC   
  
  
+ CTGTTAACCC TATATATATC TCTTTGGATT TTTCTGTCTA TTTTTGTATA CTTCTTCAGG AAAATATTTT   
  
  
+ CGTGCCGTCT ACGACACCTG TTAACCCTAT ATATATCTCT TTGGATTTTT CTGTCTATTT TTGTATACTT   
  
  
+ CTTCTGGATC AATCTCCATC TTTATCTTCT TCCTCTTCTT CTGCTTGAAG CTTGATCTTC TTCTGTAACT   
  
  
+ ATACAGTGCG TTGTTTCTGT TTCCTTTTC  

- TTTTCCGTTG GAAAAGGAAA GAAAGAAGAA AGGAATCGAA AAATCTAAAT ACTTCTTCCC GACGATGCTG   
  
  
- AAAGGACGAA AAGGTGTAAA GTTTGCGAGC ACAATAGCAG GAAAGTAAGA GGGTGAAGCA CAGGTAGTCG   
  
  
- AGGAAAAATT TTTTTATATA AATAAATAAA ATAATTTAAT AGTAAATAAT AATAAAAAAT TTTTTTTAAA   
  
  
- TTATGTCAAA AAACCCCCTT TTTTTCTTTT TTTTGAGTAG GTAGACTAAA CGAGAGAGAG ACTAAAGGTT   
  
  
- GGCTTACGTA CTCCTCTCGT AAGTCGCCGT TAGTTCGTCT CTCCTGACTG ACAGAATACC CGGGATAGAG   
  
  
- AAGAACCTAA GGTCTGATAA GAACAAAAAT CATCGAGAGT TTAGTTATTG GTCTAGATTA GGATTATGAG   
  
  
- AGAAGTATTA GTATTGAATT TCTCCCTTAA TTAATTGGTT TAGTCCATTT GTTTGTAGTA ATTAATGTCG   
  
  
- GTGGAGCAAA CTAGCTAGGG CTAGTGGGTA CTAGCAGTGA AGGTCTAGAA GTGCACACGT TGGTTACTCG   
  
  
- GTAATCAACA TTTAGTTAAT TGTACAAATT AGTATTTCTG AAGTTTAAAA ACTTCAGTTG TAAGTTTACT   
  
  
- ACGTCAAACG ACTCTACCAT AATCACCGTG GTGTGTCGGG TGGGTTTACT TAATTGGTAC TCTGGATAAA   
  
  
- TACTAGGAAG TACCTTCAAG GAATTCGGCG CGTCTGTAAG TGATATATTA TCATTAATAT TGAAAGGTGG   
  
  
- ATGGTTGGTT CGTATACATA TGAAAGTTAT TTATGTACAC GGGTTTAAGG TATTTTAATA TCTTGTTAAT   
  
  
- AACTTACTTA AATTAATAGT CTAGTCAGGT TCTGTCCGCG AATCGATCCA ATCGTATTTT ATAGCACGTG   
  
  
- GACAATATAG GAATAGTCGA GTCTCCGGAT TATGACAACG ATCAGACCAT CGAGATGCTT AAATAAAGGT   
  
  
- ACATTTAGTC TTAATTTTTA ATAATAGTAG ACATATTAGC ATACACAATT GCAGCGAAAG GATTACTACT   
  
  
- TCGGCGGTCG AGGTAAAAGG TGGGATGTAA AAATTTAATA ATAGTAGACA TATTAGCATA CACAATTGCA   
  
  
- GCGAAAGGAT TACTACTTCG GCGGTCGAGG TAAAAGGTGG GATGTAAAAA AGGTTATCTA GTTAGGCGAC   
  
  
- TACTTCGATA TACATGTAAG TTAAAAAAAG AACTCGATTG ACCTTTTATA AAAGCACGGC AGATGCTGTG   
  
  
- GACAATTGGG ATATATATAG AGAAACCTAA AAAGACAGAT AAAAACATAT GAAGAAGTCC TTTTATAAAA   
  
  
- GCACGGCAGA TGCTGTGGAC AATTGGGATA TATATAGAGA AACCTAAAAA GACAGATAAA AACATATGAA   
  
  
- GAAGACCTAG TTAGAGGTAG AAATAGAAGA AGGAGAAGAA GACGAACTTC GAACTAGAAG AAGACATTGA   
  
  
- TATGTCACGC AACAAAGACA AAGGAAAAG

+     CAAT-box

| Site Name | Organism | Position | Strand | Matrix score. | sequence | function |
| --- | --- | --- | --- | --- | --- | --- |
| CAAT-box | Hordeum vulgare | 1410 | + | 4 | CAAT | common cis-acting element in promoter and enhancer regions |
| CAAT-box | Hordeum vulgare | 1181 | + | 4 | CAAT | common cis-acting element in promoter and enhancer regions |
| CAAT-box | Glycine max | 1210 | + | 5 | CAATT | common cis-acting element in promoter and enhancer regions |
| CAAT-box | Brassica rapa | 674 | + | 5 | CAAAT | common cis-acting element in promoter and enhancer regions |
| CAAT-box | Glycine max | 835 | + | 5 | CAATT | common cis-acting element in promoter and enhancer regions |
| CAAT-box | Brassica rapa | 813 | + | 5 | CAAAT | common cis-acting element in promoter and enhancer regions |
| CAAT-box | Hordeum vulgare | 1173 | + | 4 | CAAT | common cis-acting element in promoter and enhancer regions |
| CAAT-box | Hordeum vulgare | 796 | + | 4 | CAAT | common cis-acting element in promoter and enhancer regions |
| CAAT-box | Brassica rapa | 624 | + | 5 | CAAAT | common cis-acting element in promoter and enhancer regions |
| CAAT-box | Brassica rapa | 603 | + | 5 | CAAAT | common cis-acting element in promoter and enhancer regions |
| CAAT-box | Arabidopsis thaliana | 1172 | + | 5 | CCAAT | common cis-acting element in promoter and enhancer regions |
| CAAT-box | Hordeum vulgare | 840 | - | 4 | CAAT | common cis-acting element in promoter and enhancer regions |
| CAAT-box | Brassica rapa | 458 | + | 5 | CAAAT | common cis-acting element in promoter and enhancer regions |
| CAAT-box | Hordeum vulgare | 394 | + | 4 | CAAT | common cis-acting element in promoter and enhancer regions |
| CAAT-box | Brassica rapa | 389 | + | 5 | CAAAT | common cis-acting element in promoter and enhancer regions |
| CAAT-box | Arabidopsis thaliana | 307 | + | 6 | gGCAAT | common cis-acting element in promoter and enhancer regions |
| CAAT-box | Brassica rapa | 257 | - | 5 | CAAAT | common cis-acting element in promoter and enhancer regions |
| CAAT-box | Glycine max | 575 | + | 5 | CAATT | common cis-acting element in promoter and enhancer regions |
| CAAT-box | Arabidopsis thaliana | 552 | + | 5 | CCAAT | common cis-acting element in promoter and enhancer regions |
| CAAT-box | Hordeum vulgare | 309 | + | 4 | CAAT | common cis-acting element in promoter and enhancer regions |
| CAAT-box | Hordeum vulgare | 553 | + | 4 | CAAT | common cis-acting element in promoter and enhancer regions |

> 2018/04/13 10:10:12  
+ AAAAGGCAAC CTTTTCCTTT CTTTCTTCTT TCCTTAGCTT TTTAGATTTA TGAAGAAGGG CTGCTACGAC   
  
  
+ TTTCCTGCTT TTCCACATTT CAAACGCTCG TGTTATCGTC CTTTCATTCT CCCACTTCGT GTCCATCAGC   
  
  
+ TCCTTTTTAA AAAAATATAT TTATTTATTT TATTAAATTA TCATTTATTA TTATTTTTTA AAAAAAATTT   
  
  
+ AATACAGTTT TTTGGGGGAA AAAAAGAAAA AAAACTCATC CATCTGATTT GCTCTCTCTC TGATTTCCAA   
  
  
+ CCGAATGCAT GAGGAGAGCA TTCAGCGGCA ATCAAGCAGA GAGGACTGAC TGTCTTATGG GCCCTATCTC   
  
  
+ TTCTTGGATT CCAGACTATT CTTGTTTTTA GTAGCTCTCA AATCAATAAC CAGATCTAAT CCTAATACTC   
  
  
+ TCTTCATAAT CATAACTTAA AGAGGGAATT AATTAACCAA ATCAGGTAAA CAAACATCAT TAATTACAGC   
  
  
+ CACCTCGTTT GATCGATCCC GATCACCCAT GATCGTCACT TCCAGATCTT CACGTGTGCA ACCAATGAGC   
  
  
+ CATTAGTTGT AAATCAATTA ACATGTTTAA TCATAAAGAC TTCAAATTTT TGAAGTCAAC ATTCAAATGA   
  
  
+ TGCAGTTTGC TGAGATGGTA TTAGTGGCAC CACACAGCCC ACCCAAATGA ATTAACCATG AGACCTATTT   
  
  
+ ATGATCCTTC ATGGAAGTTC CTTAAGCCGC GCAGACATTC ACTATATAAT AGTAATTATA ACTTTCCACC   
  
  
+ TACCAACCAA GCATATGTAT ACTTTCAATA AATACATGTG CCCAAATTCC ATAAAATTAT AGAACAATTA   
  
  
+ TTGAATGAAT TTAATTATCA GATCAGTCCA AGACAGGCGC TTAGCTAGGT TAGCATAAAA TATCGTGCAC   
  
  
+ CTGTTATATC CTTATCAGCT CAGAGGCCTA ATACTGTTGC TAGTCTGGTA GCTCTACGAA TTTATTTCCA   
  
  
+ TGTAAATCAG AATTAAAAAT TATTATCATC TGTATAATCG TATGTGTTAA CGTCGCTTTC CTAATGATGA   
  
  
+ AGCCGCCAGC TCCATTTTCC ACCCTACATT TTTAAATTAT TATCATCTGT ATAATCGTAT GTGTTAACGT   
  
  
+ CGCTTTCCTA ATGATGAAGC CGCCAGCTCC ATTTTCCACC CTACATTTTT TCCAATAGAT CAATCCGCTG   
  
  
+ ATGAAGCTAT ATGTACATTC AATTTTTTTC TTGAGCTAAC TGGAAAATAT TTTCGTGCCG TCTACGACAC   
  
  
+ CTGTTAACCC TATATATATC TCTTTGGATT TTTCTGTCTA TTTTTGTATA CTTCTTCAGG AAAATATTTT   
  
  
+ CGTGCCGTCT ACGACACCTG TTAACCCTAT ATATATCTCT TTGGATTTTT CTGTCTATTT TTGTATACTT   
  
  
+ CTTCTGGATC AATCTCCATC TTTATCTTCT TCCTCTTCTT CTGCTTGAAG CTTGATCTTC TTCTGTAACT   
  
  
+ ATACAGTGCG TTGTTTCTGT TTCCTTTTC  

- TTTTCCGTTG GAAAAGGAAA GAAAGAAGAA AGGAATCGAA AAATCTAAAT ACTTCTTCCC GACGATGCTG   
  
  
- AAAGGACGAA AAGGTGTAAA GTTTGCGAGC ACAATAGCAG GAAAGTAAGA GGGTGAAGCA CAGGTAGTCG   
  
  
- AGGAAAAATT TTTTTATATA AATAAATAAA ATAATTTAAT AGTAAATAAT AATAAAAAAT TTTTTTTAAA   
  
  
- TTATGTCAAA AAACCCCCTT TTTTTCTTTT TTTTGAGTAG GTAGACTAAA CGAGAGAGAG ACTAAAGGTT   
  
  
- GGCTTACGTA CTCCTCTCGT AAGTCGCCGT TAGTTCGTCT CTCCTGACTG ACAGAATACC CGGGATAGAG   
  
  
- AAGAACCTAA GGTCTGATAA GAACAAAAAT CATCGAGAGT TTAGTTATTG GTCTAGATTA GGATTATGAG   
  
  
- AGAAGTATTA GTATTGAATT TCTCCCTTAA TTAATTGGTT TAGTCCATTT GTTTGTAGTA ATTAATGTCG   
  
  
- GTGGAGCAAA CTAGCTAGGG CTAGTGGGTA CTAGCAGTGA AGGTCTAGAA GTGCACACGT TGGTTACTCG   
  
  
- GTAATCAACA TTTAGTTAAT TGTACAAATT AGTATTTCTG AAGTTTAAAA ACTTCAGTTG TAAGTTTACT   
  
  
- ACGTCAAACG ACTCTACCAT AATCACCGTG GTGTGTCGGG TGGGTTTACT TAATTGGTAC TCTGGATAAA   
  
  
- TACTAGGAAG TACCTTCAAG GAATTCGGCG CGTCTGTAAG TGATATATTA TCATTAATAT TGAAAGGTGG   
  
  
- ATGGTTGGTT CGTATACATA TGAAAGTTAT TTATGTACAC GGGTTTAAGG TATTTTAATA TCTTGTTAAT   
  
  
- AACTTACTTA AATTAATAGT CTAGTCAGGT TCTGTCCGCG AATCGATCCA ATCGTATTTT ATAGCACGTG   
  
  
- GACAATATAG GAATAGTCGA GTCTCCGGAT TATGACAACG ATCAGACCAT CGAGATGCTT AAATAAAGGT   
  
  
- ACATTTAGTC TTAATTTTTA ATAATAGTAG ACATATTAGC ATACACAATT GCAGCGAAAG GATTACTACT   
  
  
- TCGGCGGTCG AGGTAAAAGG TGGGATGTAA AAATTTAATA ATAGTAGACA TATTAGCATA CACAATTGCA   
  
  
- GCGAAAGGAT TACTACTTCG GCGGTCGAGG TAAAAGGTGG GATGTAAAAA AGGTTATCTA GTTAGGCGAC   
  
  
- TACTTCGATA TACATGTAAG TTAAAAAAAG AACTCGATTG ACCTTTTATA AAAGCACGGC AGATGCTGTG   
  
  
- GACAATTGGG ATATATATAG AGAAACCTAA AAAGACAGAT AAAAACATAT GAAGAAGTCC TTTTATAAAA   
  
  
- GCACGGCAGA TGCTGTGGAC AATTGGGATA TATATAGAGA AACCTAAAAA GACAGATAAA AACATATGAA   
  
  
- GAAGACCTAG TTAGAGGTAG AAATAGAAGA AGGAGAAGAA GACGAACTTC GAACTAGAAG AAGACATTGA   
  
  
- TATGTCACGC AACAAAGACA AAGGAAAAG

+     CAT-box

| Site Name | Organism | Position | Strand | Matrix score. | sequence | function |
| --- | --- | --- | --- | --- | --- | --- |
| CAT-box | Arabidopsis thaliana | 653 | - | 6 | GCCACT | cis-acting regulatory element related to meristem expression |

> 2018/04/13 10:10:12  
+ AAAAGGCAAC CTTTTCCTTT CTTTCTTCTT TCCTTAGCTT TTTAGATTTA TGAAGAAGGG CTGCTACGAC   
  
  
+ TTTCCTGCTT TTCCACATTT CAAACGCTCG TGTTATCGTC CTTTCATTCT CCCACTTCGT GTCCATCAGC   
  
  
+ TCCTTTTTAA AAAAATATAT TTATTTATTT TATTAAATTA TCATTTATTA TTATTTTTTA AAAAAAATTT   
  
  
+ AATACAGTTT TTTGGGGGAA AAAAAGAAAA AAAACTCATC CATCTGATTT GCTCTCTCTC TGATTTCCAA   
  
  
+ CCGAATGCAT GAGGAGAGCA TTCAGCGGCA ATCAAGCAGA GAGGACTGAC TGTCTTATGG GCCCTATCTC   
  
  
+ TTCTTGGATT CCAGACTATT CTTGTTTTTA GTAGCTCTCA AATCAATAAC CAGATCTAAT CCTAATACTC   
  
  
+ TCTTCATAAT CATAACTTAA AGAGGGAATT AATTAACCAA ATCAGGTAAA CAAACATCAT TAATTACAGC   
  
  
+ CACCTCGTTT GATCGATCCC GATCACCCAT GATCGTCACT TCCAGATCTT CACGTGTGCA ACCAATGAGC   
  
  
+ CATTAGTTGT AAATCAATTA ACATGTTTAA TCATAAAGAC TTCAAATTTT TGAAGTCAAC ATTCAAATGA   
  
  
+ TGCAGTTTGC TGAGATGGTA TTAGTGGCAC CACACAGCCC ACCCAAATGA ATTAACCATG AGACCTATTT   
  
  
+ ATGATCCTTC ATGGAAGTTC CTTAAGCCGC GCAGACATTC ACTATATAAT AGTAATTATA ACTTTCCACC   
  
  
+ TACCAACCAA GCATATGTAT ACTTTCAATA AATACATGTG CCCAAATTCC ATAAAATTAT AGAACAATTA   
  
  
+ TTGAATGAAT TTAATTATCA GATCAGTCCA AGACAGGCGC TTAGCTAGGT TAGCATAAAA TATCGTGCAC   
  
  
+ CTGTTATATC CTTATCAGCT CAGAGGCCTA ATACTGTTGC TAGTCTGGTA GCTCTACGAA TTTATTTCCA   
  
  
+ TGTAAATCAG AATTAAAAAT TATTATCATC TGTATAATCG TATGTGTTAA CGTCGCTTTC CTAATGATGA   
  
  
+ AGCCGCCAGC TCCATTTTCC ACCCTACATT TTTAAATTAT TATCATCTGT ATAATCGTAT GTGTTAACGT   
  
  
+ CGCTTTCCTA ATGATGAAGC CGCCAGCTCC ATTTTCCACC CTACATTTTT TCCAATAGAT CAATCCGCTG   
  
  
+ ATGAAGCTAT ATGTACATTC AATTTTTTTC TTGAGCTAAC TGGAAAATAT TTTCGTGCCG TCTACGACAC   
  
  
+ CTGTTAACCC TATATATATC TCTTTGGATT TTTCTGTCTA TTTTTGTATA CTTCTTCAGG AAAATATTTT   
  
  
+ CGTGCCGTCT ACGACACCTG TTAACCCTAT ATATATCTCT TTGGATTTTT CTGTCTATTT TTGTATACTT   
  
  
+ CTTCTGGATC AATCTCCATC TTTATCTTCT TCCTCTTCTT CTGCTTGAAG CTTGATCTTC TTCTGTAACT   
  
  
+ ATACAGTGCG TTGTTTCTGT TTCCTTTTC  

- TTTTCCGTTG GAAAAGGAAA GAAAGAAGAA AGGAATCGAA AAATCTAAAT ACTTCTTCCC GACGATGCTG   
  
  
- AAAGGACGAA AAGGTGTAAA GTTTGCGAGC ACAATAGCAG GAAAGTAAGA GGGTGAAGCA CAGGTAGTCG   
  
  
- AGGAAAAATT TTTTTATATA AATAAATAAA ATAATTTAAT AGTAAATAAT AATAAAAAAT TTTTTTTAAA   
  
  
- TTATGTCAAA AAACCCCCTT TTTTTCTTTT TTTTGAGTAG GTAGACTAAA CGAGAGAGAG ACTAAAGGTT   
  
  
- GGCTTACGTA CTCCTCTCGT AAGTCGCCGT TAGTTCGTCT CTCCTGACTG ACAGAATACC CGGGATAGAG   
  
  
- AAGAACCTAA GGTCTGATAA GAACAAAAAT CATCGAGAGT TTAGTTATTG GTCTAGATTA GGATTATGAG   
  
  
- AGAAGTATTA GTATTGAATT TCTCCCTTAA TTAATTGGTT TAGTCCATTT GTTTGTAGTA ATTAATGTCG   
  
  
- GTGGAGCAAA CTAGCTAGGG CTAGTGGGTA CTAGCAGTGA AGGTCTAGAA GTGCACACGT TGGTTACTCG   
  
  
- GTAATCAACA TTTAGTTAAT TGTACAAATT AGTATTTCTG AAGTTTAAAA ACTTCAGTTG TAAGTTTACT   
  
  
- ACGTCAAACG ACTCTACCAT AATCACCGTG GTGTGTCGGG TGGGTTTACT TAATTGGTAC TCTGGATAAA   
  
  
- TACTAGGAAG TACCTTCAAG GAATTCGGCG CGTCTGTAAG TGATATATTA TCATTAATAT TGAAAGGTGG   
  
  
- ATGGTTGGTT CGTATACATA TGAAAGTTAT TTATGTACAC GGGTTTAAGG TATTTTAATA TCTTGTTAAT   
  
  
- AACTTACTTA AATTAATAGT CTAGTCAGGT TCTGTCCGCG AATCGATCCA ATCGTATTTT ATAGCACGTG   
  
  
- GACAATATAG GAATAGTCGA GTCTCCGGAT TATGACAACG ATCAGACCAT CGAGATGCTT AAATAAAGGT   
  
  
- ACATTTAGTC TTAATTTTTA ATAATAGTAG ACATATTAGC ATACACAATT GCAGCGAAAG GATTACTACT   
  
  
- TCGGCGGTCG AGGTAAAAGG TGGGATGTAA AAATTTAATA ATAGTAGACA TATTAGCATA CACAATTGCA   
  
  
- GCGAAAGGAT TACTACTTCG GCGGTCGAGG TAAAAGGTGG GATGTAAAAA AGGTTATCTA GTTAGGCGAC   
  
  
- TACTTCGATA TACATGTAAG TTAAAAAAAG AACTCGATTG ACCTTTTATA AAAGCACGGC AGATGCTGTG   
  
  
- GACAATTGGG ATATATATAG AGAAACCTAA AAAGACAGAT AAAAACATAT GAAGAAGTCC TTTTATAAAA   
  
  
- GCACGGCAGA TGCTGTGGAC AATTGGGATA TATATAGAGA AACCTAAAAA GACAGATAAA AACATATGAA   
  
  
- GAAGACCTAG TTAGAGGTAG AAATAGAAGA AGGAGAAGAA GACGAACTTC GAACTAGAAG AAGACATTGA   
  
  
- TATGTCACGC AACAAAGACA AAGGAAAAG

+     CATT-motif

| Site Name | Organism | Position | Strand | Matrix score. | sequence | function |
| --- | --- | --- | --- | --- | --- | --- |
| CATT-motif | Zea mays | 283 | - | 6 | GCATTC | part of a light responsive element |
| CATT-motif | Zea mays | 298 | + | 6 | GCATTC | part of a light responsive element |

> 2018/04/13 10:10:12  
+ AAAAGGCAAC CTTTTCCTTT CTTTCTTCTT TCCTTAGCTT TTTAGATTTA TGAAGAAGGG CTGCTACGAC   
  
  
+ TTTCCTGCTT TTCCACATTT CAAACGCTCG TGTTATCGTC CTTTCATTCT CCCACTTCGT GTCCATCAGC   
  
  
+ TCCTTTTTAA AAAAATATAT TTATTTATTT TATTAAATTA TCATTTATTA TTATTTTTTA AAAAAAATTT   
  
  
+ AATACAGTTT TTTGGGGGAA AAAAAGAAAA AAAACTCATC CATCTGATTT GCTCTCTCTC TGATTTCCAA   
  
  
+ CCGAATGCAT GAGGAGAGCA TTCAGCGGCA ATCAAGCAGA GAGGACTGAC TGTCTTATGG GCCCTATCTC   
  
  
+ TTCTTGGATT CCAGACTATT CTTGTTTTTA GTAGCTCTCA AATCAATAAC CAGATCTAAT CCTAATACTC   
  
  
+ TCTTCATAAT CATAACTTAA AGAGGGAATT AATTAACCAA ATCAGGTAAA CAAACATCAT TAATTACAGC   
  
  
+ CACCTCGTTT GATCGATCCC GATCACCCAT GATCGTCACT TCCAGATCTT CACGTGTGCA ACCAATGAGC   
  
  
+ CATTAGTTGT AAATCAATTA ACATGTTTAA TCATAAAGAC TTCAAATTTT TGAAGTCAAC ATTCAAATGA   
  
  
+ TGCAGTTTGC TGAGATGGTA TTAGTGGCAC CACACAGCCC ACCCAAATGA ATTAACCATG AGACCTATTT   
  
  
+ ATGATCCTTC ATGGAAGTTC CTTAAGCCGC GCAGACATTC ACTATATAAT AGTAATTATA ACTTTCCACC   
  
  
+ TACCAACCAA GCATATGTAT ACTTTCAATA AATACATGTG CCCAAATTCC ATAAAATTAT AGAACAATTA   
  
  
+ TTGAATGAAT TTAATTATCA GATCAGTCCA AGACAGGCGC TTAGCTAGGT TAGCATAAAA TATCGTGCAC   
  
  
+ CTGTTATATC CTTATCAGCT CAGAGGCCTA ATACTGTTGC TAGTCTGGTA GCTCTACGAA TTTATTTCCA   
  
  
+ TGTAAATCAG AATTAAAAAT TATTATCATC TGTATAATCG TATGTGTTAA CGTCGCTTTC CTAATGATGA   
  
  
+ AGCCGCCAGC TCCATTTTCC ACCCTACATT TTTAAATTAT TATCATCTGT ATAATCGTAT GTGTTAACGT   
  
  
+ CGCTTTCCTA ATGATGAAGC CGCCAGCTCC ATTTTCCACC CTACATTTTT TCCAATAGAT CAATCCGCTG   
  
  
+ ATGAAGCTAT ATGTACATTC AATTTTTTTC TTGAGCTAAC TGGAAAATAT TTTCGTGCCG TCTACGACAC   
  
  
+ CTGTTAACCC TATATATATC TCTTTGGATT TTTCTGTCTA TTTTTGTATA CTTCTTCAGG AAAATATTTT   
  
  
+ CGTGCCGTCT ACGACACCTG TTAACCCTAT ATATATCTCT TTGGATTTTT CTGTCTATTT TTGTATACTT   
  
  
+ CTTCTGGATC AATCTCCATC TTTATCTTCT TCCTCTTCTT CTGCTTGAAG CTTGATCTTC TTCTGTAACT   
  
  
+ ATACAGTGCG TTGTTTCTGT TTCCTTTTC  

- TTTTCCGTTG GAAAAGGAAA GAAAGAAGAA AGGAATCGAA AAATCTAAAT ACTTCTTCCC GACGATGCTG   
  
  
- AAAGGACGAA AAGGTGTAAA GTTTGCGAGC ACAATAGCAG GAAAGTAAGA GGGTGAAGCA CAGGTAGTCG   
  
  
- AGGAAAAATT TTTTTATATA AATAAATAAA ATAATTTAAT AGTAAATAAT AATAAAAAAT TTTTTTTAAA   
  
  
- TTATGTCAAA AAACCCCCTT TTTTTCTTTT TTTTGAGTAG GTAGACTAAA CGAGAGAGAG ACTAAAGGTT   
  
  
- GGCTTACGTA CTCCTCTCGT AAGTCGCCGT TAGTTCGTCT CTCCTGACTG ACAGAATACC CGGGATAGAG   
  
  
- AAGAACCTAA GGTCTGATAA GAACAAAAAT CATCGAGAGT TTAGTTATTG GTCTAGATTA GGATTATGAG   
  
  
- AGAAGTATTA GTATTGAATT TCTCCCTTAA TTAATTGGTT TAGTCCATTT GTTTGTAGTA ATTAATGTCG   
  
  
- GTGGAGCAAA CTAGCTAGGG CTAGTGGGTA CTAGCAGTGA AGGTCTAGAA GTGCACACGT TGGTTACTCG   
  
  
- GTAATCAACA TTTAGTTAAT TGTACAAATT AGTATTTCTG AAGTTTAAAA ACTTCAGTTG TAAGTTTACT   
  
  
- ACGTCAAACG ACTCTACCAT AATCACCGTG GTGTGTCGGG TGGGTTTACT TAATTGGTAC TCTGGATAAA   
  
  
- TACTAGGAAG TACCTTCAAG GAATTCGGCG CGTCTGTAAG TGATATATTA TCATTAATAT TGAAAGGTGG   
  
  
- ATGGTTGGTT CGTATACATA TGAAAGTTAT TTATGTACAC GGGTTTAAGG TATTTTAATA TCTTGTTAAT   
  
  
- AACTTACTTA AATTAATAGT CTAGTCAGGT TCTGTCCGCG AATCGATCCA ATCGTATTTT ATAGCACGTG   
  
  
- GACAATATAG GAATAGTCGA GTCTCCGGAT TATGACAACG ATCAGACCAT CGAGATGCTT AAATAAAGGT   
  
  
- ACATTTAGTC TTAATTTTTA ATAATAGTAG ACATATTAGC ATACACAATT GCAGCGAAAG GATTACTACT   
  
  
- TCGGCGGTCG AGGTAAAAGG TGGGATGTAA AAATTTAATA ATAGTAGACA TATTAGCATA CACAATTGCA   
  
  
- GCGAAAGGAT TACTACTTCG GCGGTCGAGG TAAAAGGTGG GATGTAAAAA AGGTTATCTA GTTAGGCGAC   
  
  
- TACTTCGATA TACATGTAAG TTAAAAAAAG AACTCGATTG ACCTTTTATA AAAGCACGGC AGATGCTGTG   
  
  
- GACAATTGGG ATATATATAG AGAAACCTAA AAAGACAGAT AAAAACATAT GAAGAAGTCC TTTTATAAAA   
  
  
- GCACGGCAGA TGCTGTGGAC AATTGGGATA TATATAGAGA AACCTAAAAA GACAGATAAA AACATATGAA   
  
  
- GAAGACCTAG TTAGAGGTAG AAATAGAAGA AGGAGAAGAA GACGAACTTC GAACTAGAAG AAGACATTGA   
  
  
- TATGTCACGC AACAAAGACA AAGGAAAAG

+     CGTCA-motif

| Site Name | Organism | Position | Strand | Matrix score. | sequence | function |
| --- | --- | --- | --- | --- | --- | --- |
| CGTCA-motif | Hordeum vulgare | 524 | + | 5 | CGTCA | cis-acting regulatory element involved in the MeJA-responsiveness |

> 2018/04/13 10:10:12  
+ AAAAGGCAAC CTTTTCCTTT CTTTCTTCTT TCCTTAGCTT TTTAGATTTA TGAAGAAGGG CTGCTACGAC   
  
  
+ TTTCCTGCTT TTCCACATTT CAAACGCTCG TGTTATCGTC CTTTCATTCT CCCACTTCGT GTCCATCAGC   
  
  
+ TCCTTTTTAA AAAAATATAT TTATTTATTT TATTAAATTA TCATTTATTA TTATTTTTTA AAAAAAATTT   
  
  
+ AATACAGTTT TTTGGGGGAA AAAAAGAAAA AAAACTCATC CATCTGATTT GCTCTCTCTC TGATTTCCAA   
  
  
+ CCGAATGCAT GAGGAGAGCA TTCAGCGGCA ATCAAGCAGA GAGGACTGAC TGTCTTATGG GCCCTATCTC   
  
  
+ TTCTTGGATT CCAGACTATT CTTGTTTTTA GTAGCTCTCA AATCAATAAC CAGATCTAAT CCTAATACTC   
  
  
+ TCTTCATAAT CATAACTTAA AGAGGGAATT AATTAACCAA ATCAGGTAAA CAAACATCAT TAATTACAGC   
  
  
+ CACCTCGTTT GATCGATCCC GATCACCCAT GATCGTCACT TCCAGATCTT CACGTGTGCA ACCAATGAGC   
  
  
+ CATTAGTTGT AAATCAATTA ACATGTTTAA TCATAAAGAC TTCAAATTTT TGAAGTCAAC ATTCAAATGA   
  
  
+ TGCAGTTTGC TGAGATGGTA TTAGTGGCAC CACACAGCCC ACCCAAATGA ATTAACCATG AGACCTATTT   
  
  
+ ATGATCCTTC ATGGAAGTTC CTTAAGCCGC GCAGACATTC ACTATATAAT AGTAATTATA ACTTTCCACC   
  
  
+ TACCAACCAA GCATATGTAT ACTTTCAATA AATACATGTG CCCAAATTCC ATAAAATTAT AGAACAATTA   
  
  
+ TTGAATGAAT TTAATTATCA GATCAGTCCA AGACAGGCGC TTAGCTAGGT TAGCATAAAA TATCGTGCAC   
  
  
+ CTGTTATATC CTTATCAGCT CAGAGGCCTA ATACTGTTGC TAGTCTGGTA GCTCTACGAA TTTATTTCCA   
  
  
+ TGTAAATCAG AATTAAAAAT TATTATCATC TGTATAATCG TATGTGTTAA CGTCGCTTTC CTAATGATGA   
  
  
+ AGCCGCCAGC TCCATTTTCC ACCCTACATT TTTAAATTAT TATCATCTGT ATAATCGTAT GTGTTAACGT   
  
  
+ CGCTTTCCTA ATGATGAAGC CGCCAGCTCC ATTTTCCACC CTACATTTTT TCCAATAGAT CAATCCGCTG   
  
  
+ ATGAAGCTAT ATGTACATTC AATTTTTTTC TTGAGCTAAC TGGAAAATAT TTTCGTGCCG TCTACGACAC   
  
  
+ CTGTTAACCC TATATATATC TCTTTGGATT TTTCTGTCTA TTTTTGTATA CTTCTTCAGG AAAATATTTT   
  
  
+ CGTGCCGTCT ACGACACCTG TTAACCCTAT ATATATCTCT TTGGATTTTT CTGTCTATTT TTGTATACTT   
  
  
+ CTTCTGGATC AATCTCCATC TTTATCTTCT TCCTCTTCTT CTGCTTGAAG CTTGATCTTC TTCTGTAACT   
  
  
+ ATACAGTGCG TTGTTTCTGT TTCCTTTTC  

- TTTTCCGTTG GAAAAGGAAA GAAAGAAGAA AGGAATCGAA AAATCTAAAT ACTTCTTCCC GACGATGCTG   
  
  
- AAAGGACGAA AAGGTGTAAA GTTTGCGAGC ACAATAGCAG GAAAGTAAGA GGGTGAAGCA CAGGTAGTCG   
  
  
- AGGAAAAATT TTTTTATATA AATAAATAAA ATAATTTAAT AGTAAATAAT AATAAAAAAT TTTTTTTAAA   
  
  
- TTATGTCAAA AAACCCCCTT TTTTTCTTTT TTTTGAGTAG GTAGACTAAA CGAGAGAGAG ACTAAAGGTT   
  
  
- GGCTTACGTA CTCCTCTCGT AAGTCGCCGT TAGTTCGTCT CTCCTGACTG ACAGAATACC CGGGATAGAG   
  
  
- AAGAACCTAA GGTCTGATAA GAACAAAAAT CATCGAGAGT TTAGTTATTG GTCTAGATTA GGATTATGAG   
  
  
- AGAAGTATTA GTATTGAATT TCTCCCTTAA TTAATTGGTT TAGTCCATTT GTTTGTAGTA ATTAATGTCG   
  
  
- GTGGAGCAAA CTAGCTAGGG CTAGTGGGTA CTAGCAGTGA AGGTCTAGAA GTGCACACGT TGGTTACTCG   
  
  
- GTAATCAACA TTTAGTTAAT TGTACAAATT AGTATTTCTG AAGTTTAAAA ACTTCAGTTG TAAGTTTACT   
  
  
- ACGTCAAACG ACTCTACCAT AATCACCGTG GTGTGTCGGG TGGGTTTACT TAATTGGTAC TCTGGATAAA   
  
  
- TACTAGGAAG TACCTTCAAG GAATTCGGCG CGTCTGTAAG TGATATATTA TCATTAATAT TGAAAGGTGG   
  
  
- ATGGTTGGTT CGTATACATA TGAAAGTTAT TTATGTACAC GGGTTTAAGG TATTTTAATA TCTTGTTAAT   
  
  
- AACTTACTTA AATTAATAGT CTAGTCAGGT TCTGTCCGCG AATCGATCCA ATCGTATTTT ATAGCACGTG   
  
  
- GACAATATAG GAATAGTCGA GTCTCCGGAT TATGACAACG ATCAGACCAT CGAGATGCTT AAATAAAGGT   
  
  
- ACATTTAGTC TTAATTTTTA ATAATAGTAG ACATATTAGC ATACACAATT GCAGCGAAAG GATTACTACT   
  
  
- TCGGCGGTCG AGGTAAAAGG TGGGATGTAA AAATTTAATA ATAGTAGACA TATTAGCATA CACAATTGCA   
  
  
- GCGAAAGGAT TACTACTTCG GCGGTCGAGG TAAAAGGTGG GATGTAAAAA AGGTTATCTA GTTAGGCGAC   
  
  
- TACTTCGATA TACATGTAAG TTAAAAAAAG AACTCGATTG ACCTTTTATA AAAGCACGGC AGATGCTGTG   
  
  
- GACAATTGGG ATATATATAG AGAAACCTAA AAAGACAGAT AAAAACATAT GAAGAAGTCC TTTTATAAAA   
  
  
- GCACGGCAGA TGCTGTGGAC AATTGGGATA TATATAGAGA AACCTAAAAA GACAGATAAA AACATATGAA   
  
  
- GAAGACCTAG TTAGAGGTAG AAATAGAAGA AGGAGAAGAA GACGAACTTC GAACTAGAAG AAGACATTGA   
  
  
- TATGTCACGC AACAAAGACA AAGGAAAAG

+     ERE

| Site Name | Organism | Position | Strand | Matrix score. | sequence | function |
| --- | --- | --- | --- | --- | --- | --- |
| ERE | Dianthus caryophyllus | 87 | + | 8 | ATTTCAAA | ethylene-responsive element |

> 2018/04/13 10:10:12  
+ AAAAGGCAAC CTTTTCCTTT CTTTCTTCTT TCCTTAGCTT TTTAGATTTA TGAAGAAGGG CTGCTACGAC   
  
  
+ TTTCCTGCTT TTCCACATTT CAAACGCTCG TGTTATCGTC CTTTCATTCT CCCACTTCGT GTCCATCAGC   
  
  
+ TCCTTTTTAA AAAAATATAT TTATTTATTT TATTAAATTA TCATTTATTA TTATTTTTTA AAAAAAATTT   
  
  
+ AATACAGTTT TTTGGGGGAA AAAAAGAAAA AAAACTCATC CATCTGATTT GCTCTCTCTC TGATTTCCAA   
  
  
+ CCGAATGCAT GAGGAGAGCA TTCAGCGGCA ATCAAGCAGA GAGGACTGAC TGTCTTATGG GCCCTATCTC   
  
  
+ TTCTTGGATT CCAGACTATT CTTGTTTTTA GTAGCTCTCA AATCAATAAC CAGATCTAAT CCTAATACTC   
  
  
+ TCTTCATAAT CATAACTTAA AGAGGGAATT AATTAACCAA ATCAGGTAAA CAAACATCAT TAATTACAGC   
  
  
+ CACCTCGTTT GATCGATCCC GATCACCCAT GATCGTCACT TCCAGATCTT CACGTGTGCA ACCAATGAGC   
  
  
+ CATTAGTTGT AAATCAATTA ACATGTTTAA TCATAAAGAC TTCAAATTTT TGAAGTCAAC ATTCAAATGA   
  
  
+ TGCAGTTTGC TGAGATGGTA TTAGTGGCAC CACACAGCCC ACCCAAATGA ATTAACCATG AGACCTATTT   
  
  
+ ATGATCCTTC ATGGAAGTTC CTTAAGCCGC GCAGACATTC ACTATATAAT AGTAATTATA ACTTTCCACC   
  
  
+ TACCAACCAA GCATATGTAT ACTTTCAATA AATACATGTG CCCAAATTCC ATAAAATTAT AGAACAATTA   
  
  
+ TTGAATGAAT TTAATTATCA GATCAGTCCA AGACAGGCGC TTAGCTAGGT TAGCATAAAA TATCGTGCAC   
  
  
+ CTGTTATATC CTTATCAGCT CAGAGGCCTA ATACTGTTGC TAGTCTGGTA GCTCTACGAA TTTATTTCCA   
  
  
+ TGTAAATCAG AATTAAAAAT TATTATCATC TGTATAATCG TATGTGTTAA CGTCGCTTTC CTAATGATGA   
  
  
+ AGCCGCCAGC TCCATTTTCC ACCCTACATT TTTAAATTAT TATCATCTGT ATAATCGTAT GTGTTAACGT   
  
  
+ CGCTTTCCTA ATGATGAAGC CGCCAGCTCC ATTTTCCACC CTACATTTTT TCCAATAGAT CAATCCGCTG   
  
  
+ ATGAAGCTAT ATGTACATTC AATTTTTTTC TTGAGCTAAC TGGAAAATAT TTTCGTGCCG TCTACGACAC   
  
  
+ CTGTTAACCC TATATATATC TCTTTGGATT TTTCTGTCTA TTTTTGTATA CTTCTTCAGG AAAATATTTT   
  
  
+ CGTGCCGTCT ACGACACCTG TTAACCCTAT ATATATCTCT TTGGATTTTT CTGTCTATTT TTGTATACTT   
  
  
+ CTTCTGGATC AATCTCCATC TTTATCTTCT TCCTCTTCTT CTGCTTGAAG CTTGATCTTC TTCTGTAACT   
  
  
+ ATACAGTGCG TTGTTTCTGT TTCCTTTTC  

- TTTTCCGTTG GAAAAGGAAA GAAAGAAGAA AGGAATCGAA AAATCTAAAT ACTTCTTCCC GACGATGCTG   
  
  
- AAAGGACGAA AAGGTGTAAA GTTTGCGAGC ACAATAGCAG GAAAGTAAGA GGGTGAAGCA CAGGTAGTCG   
  
  
- AGGAAAAATT TTTTTATATA AATAAATAAA ATAATTTAAT AGTAAATAAT AATAAAAAAT TTTTTTTAAA   
  
  
- TTATGTCAAA AAACCCCCTT TTTTTCTTTT TTTTGAGTAG GTAGACTAAA CGAGAGAGAG ACTAAAGGTT   
  
  
- GGCTTACGTA CTCCTCTCGT AAGTCGCCGT TAGTTCGTCT CTCCTGACTG ACAGAATACC CGGGATAGAG   
  
  
- AAGAACCTAA GGTCTGATAA GAACAAAAAT CATCGAGAGT TTAGTTATTG GTCTAGATTA GGATTATGAG   
  
  
- AGAAGTATTA GTATTGAATT TCTCCCTTAA TTAATTGGTT TAGTCCATTT GTTTGTAGTA ATTAATGTCG   
  
  
- GTGGAGCAAA CTAGCTAGGG CTAGTGGGTA CTAGCAGTGA AGGTCTAGAA GTGCACACGT TGGTTACTCG   
  
  
- GTAATCAACA TTTAGTTAAT TGTACAAATT AGTATTTCTG AAGTTTAAAA ACTTCAGTTG TAAGTTTACT   
  
  
- ACGTCAAACG ACTCTACCAT AATCACCGTG GTGTGTCGGG TGGGTTTACT TAATTGGTAC TCTGGATAAA   
  
  
- TACTAGGAAG TACCTTCAAG GAATTCGGCG CGTCTGTAAG TGATATATTA TCATTAATAT TGAAAGGTGG   
  
  
- ATGGTTGGTT CGTATACATA TGAAAGTTAT TTATGTACAC GGGTTTAAGG TATTTTAATA TCTTGTTAAT   
  
  
- AACTTACTTA AATTAATAGT CTAGTCAGGT TCTGTCCGCG AATCGATCCA ATCGTATTTT ATAGCACGTG   
  
  
- GACAATATAG GAATAGTCGA GTCTCCGGAT TATGACAACG ATCAGACCAT CGAGATGCTT AAATAAAGGT   
  
  
- ACATTTAGTC TTAATTTTTA ATAATAGTAG ACATATTAGC ATACACAATT GCAGCGAAAG GATTACTACT   
  
  
- TCGGCGGTCG AGGTAAAAGG TGGGATGTAA AAATTTAATA ATAGTAGACA TATTAGCATA CACAATTGCA   
  
  
- GCGAAAGGAT TACTACTTCG GCGGTCGAGG TAAAAGGTGG GATGTAAAAA AGGTTATCTA GTTAGGCGAC   
  
  
- TACTTCGATA TACATGTAAG TTAAAAAAAG AACTCGATTG ACCTTTTATA AAAGCACGGC AGATGCTGTG   
  
  
- GACAATTGGG ATATATATAG AGAAACCTAA AAAGACAGAT AAAAACATAT GAAGAAGTCC TTTTATAAAA   
  
  
- GCACGGCAGA TGCTGTGGAC AATTGGGATA TATATAGAGA AACCTAAAAA GACAGATAAA AACATATGAA   
  
  
- GAAGACCTAG TTAGAGGTAG AAATAGAAGA AGGAGAAGAA GACGAACTTC GAACTAGAAG AAGACATTGA   
  
  
- TATGTCACGC AACAAAGACA AAGGAAAAG

+     G-Box

| Site Name | Organism | Position | Strand | Matrix score. | sequence | function |
| --- | --- | --- | --- | --- | --- | --- |
| G-Box | Pisum sativum | 541 | + | 6 | CACGTG | cis-acting regulatory element involved in light responsiveness |

> 2018/04/13 10:10:12  
+ AAAAGGCAAC CTTTTCCTTT CTTTCTTCTT TCCTTAGCTT TTTAGATTTA TGAAGAAGGG CTGCTACGAC   
  
  
+ TTTCCTGCTT TTCCACATTT CAAACGCTCG TGTTATCGTC CTTTCATTCT CCCACTTCGT GTCCATCAGC   
  
  
+ TCCTTTTTAA AAAAATATAT TTATTTATTT TATTAAATTA TCATTTATTA TTATTTTTTA AAAAAAATTT   
  
  
+ AATACAGTTT TTTGGGGGAA AAAAAGAAAA AAAACTCATC CATCTGATTT GCTCTCTCTC TGATTTCCAA   
  
  
+ CCGAATGCAT GAGGAGAGCA TTCAGCGGCA ATCAAGCAGA GAGGACTGAC TGTCTTATGG GCCCTATCTC   
  
  
+ TTCTTGGATT CCAGACTATT CTTGTTTTTA GTAGCTCTCA AATCAATAAC CAGATCTAAT CCTAATACTC   
  
  
+ TCTTCATAAT CATAACTTAA AGAGGGAATT AATTAACCAA ATCAGGTAAA CAAACATCAT TAATTACAGC   
  
  
+ CACCTCGTTT GATCGATCCC GATCACCCAT GATCGTCACT TCCAGATCTT CACGTGTGCA ACCAATGAGC   
  
  
+ CATTAGTTGT AAATCAATTA ACATGTTTAA TCATAAAGAC TTCAAATTTT TGAAGTCAAC ATTCAAATGA   
  
  
+ TGCAGTTTGC TGAGATGGTA TTAGTGGCAC CACACAGCCC ACCCAAATGA ATTAACCATG AGACCTATTT   
  
  
+ ATGATCCTTC ATGGAAGTTC CTTAAGCCGC GCAGACATTC ACTATATAAT AGTAATTATA ACTTTCCACC   
  
  
+ TACCAACCAA GCATATGTAT ACTTTCAATA AATACATGTG CCCAAATTCC ATAAAATTAT AGAACAATTA   
  
  
+ TTGAATGAAT TTAATTATCA GATCAGTCCA AGACAGGCGC TTAGCTAGGT TAGCATAAAA TATCGTGCAC   
  
  
+ CTGTTATATC CTTATCAGCT CAGAGGCCTA ATACTGTTGC TAGTCTGGTA GCTCTACGAA TTTATTTCCA   
  
  
+ TGTAAATCAG AATTAAAAAT TATTATCATC TGTATAATCG TATGTGTTAA CGTCGCTTTC CTAATGATGA   
  
  
+ AGCCGCCAGC TCCATTTTCC ACCCTACATT TTTAAATTAT TATCATCTGT ATAATCGTAT GTGTTAACGT   
  
  
+ CGCTTTCCTA ATGATGAAGC CGCCAGCTCC ATTTTCCACC CTACATTTTT TCCAATAGAT CAATCCGCTG   
  
  
+ ATGAAGCTAT ATGTACATTC AATTTTTTTC TTGAGCTAAC TGGAAAATAT TTTCGTGCCG TCTACGACAC   
  
  
+ CTGTTAACCC TATATATATC TCTTTGGATT TTTCTGTCTA TTTTTGTATA CTTCTTCAGG AAAATATTTT   
  
  
+ CGTGCCGTCT ACGACACCTG TTAACCCTAT ATATATCTCT TTGGATTTTT CTGTCTATTT TTGTATACTT   
  
  
+ CTTCTGGATC AATCTCCATC TTTATCTTCT TCCTCTTCTT CTGCTTGAAG CTTGATCTTC TTCTGTAACT   
  
  
+ ATACAGTGCG TTGTTTCTGT TTCCTTTTC  

- TTTTCCGTTG GAAAAGGAAA GAAAGAAGAA AGGAATCGAA AAATCTAAAT ACTTCTTCCC GACGATGCTG   
  
  
- AAAGGACGAA AAGGTGTAAA GTTTGCGAGC ACAATAGCAG GAAAGTAAGA GGGTGAAGCA CAGGTAGTCG   
  
  
- AGGAAAAATT TTTTTATATA AATAAATAAA ATAATTTAAT AGTAAATAAT AATAAAAAAT TTTTTTTAAA   
  
  
- TTATGTCAAA AAACCCCCTT TTTTTCTTTT TTTTGAGTAG GTAGACTAAA CGAGAGAGAG ACTAAAGGTT   
  
  
- GGCTTACGTA CTCCTCTCGT AAGTCGCCGT TAGTTCGTCT CTCCTGACTG ACAGAATACC CGGGATAGAG   
  
  
- AAGAACCTAA GGTCTGATAA GAACAAAAAT CATCGAGAGT TTAGTTATTG GTCTAGATTA GGATTATGAG   
  
  
- AGAAGTATTA GTATTGAATT TCTCCCTTAA TTAATTGGTT TAGTCCATTT GTTTGTAGTA ATTAATGTCG   
  
  
- GTGGAGCAAA CTAGCTAGGG CTAGTGGGTA CTAGCAGTGA AGGTCTAGAA GTGCACACGT TGGTTACTCG   
  
  
- GTAATCAACA TTTAGTTAAT TGTACAAATT AGTATTTCTG AAGTTTAAAA ACTTCAGTTG TAAGTTTACT   
  
  
- ACGTCAAACG ACTCTACCAT AATCACCGTG GTGTGTCGGG TGGGTTTACT TAATTGGTAC TCTGGATAAA   
  
  
- TACTAGGAAG TACCTTCAAG GAATTCGGCG CGTCTGTAAG TGATATATTA TCATTAATAT TGAAAGGTGG   
  
  
- ATGGTTGGTT CGTATACATA TGAAAGTTAT TTATGTACAC GGGTTTAAGG TATTTTAATA TCTTGTTAAT   
  
  
- AACTTACTTA AATTAATAGT CTAGTCAGGT TCTGTCCGCG AATCGATCCA ATCGTATTTT ATAGCACGTG   
  
  
- GACAATATAG GAATAGTCGA GTCTCCGGAT TATGACAACG ATCAGACCAT CGAGATGCTT AAATAAAGGT   
  
  
- ACATTTAGTC TTAATTTTTA ATAATAGTAG ACATATTAGC ATACACAATT GCAGCGAAAG GATTACTACT   
  
  
- TCGGCGGTCG AGGTAAAAGG TGGGATGTAA AAATTTAATA ATAGTAGACA TATTAGCATA CACAATTGCA   
  
  
- GCGAAAGGAT TACTACTTCG GCGGTCGAGG TAAAAGGTGG GATGTAAAAA AGGTTATCTA GTTAGGCGAC   
  
  
- TACTTCGATA TACATGTAAG TTAAAAAAAG AACTCGATTG ACCTTTTATA AAAGCACGGC AGATGCTGTG   
  
  
- GACAATTGGG ATATATATAG AGAAACCTAA AAAGACAGAT AAAAACATAT GAAGAAGTCC TTTTATAAAA   
  
  
- GCACGGCAGA TGCTGTGGAC AATTGGGATA TATATAGAGA AACCTAAAAA GACAGATAAA AACATATGAA   
  
  
- GAAGACCTAG TTAGAGGTAG AAATAGAAGA AGGAGAAGAA GACGAACTTC GAACTAGAAG AAGACATTGA   
  
  
- TATGTCACGC AACAAAGACA AAGGAAAAG

+     G-box

| Site Name | Organism | Position | Strand | Matrix score. | sequence | function |
| --- | --- | --- | --- | --- | --- | --- |
| G-box | Arabidopsis thaliana | 541 | + | 6 | CACGTG | cis-acting regulatory element involved in light responsiveness |
| G-box | Larix laricina | 124 | - | 9 | GACACGTAGT | cis-acting regulatory element involved in light responsiveness |

> 2018/04/13 10:10:12  
+ AAAAGGCAAC CTTTTCCTTT CTTTCTTCTT TCCTTAGCTT TTTAGATTTA TGAAGAAGGG CTGCTACGAC   
  
  
+ TTTCCTGCTT TTCCACATTT CAAACGCTCG TGTTATCGTC CTTTCATTCT CCCACTTCGT GTCCATCAGC   
  
  
+ TCCTTTTTAA AAAAATATAT TTATTTATTT TATTAAATTA TCATTTATTA TTATTTTTTA AAAAAAATTT   
  
  
+ AATACAGTTT TTTGGGGGAA AAAAAGAAAA AAAACTCATC CATCTGATTT GCTCTCTCTC TGATTTCCAA   
  
  
+ CCGAATGCAT GAGGAGAGCA TTCAGCGGCA ATCAAGCAGA GAGGACTGAC TGTCTTATGG GCCCTATCTC   
  
  
+ TTCTTGGATT CCAGACTATT CTTGTTTTTA GTAGCTCTCA AATCAATAAC CAGATCTAAT CCTAATACTC   
  
  
+ TCTTCATAAT CATAACTTAA AGAGGGAATT AATTAACCAA ATCAGGTAAA CAAACATCAT TAATTACAGC   
  
  
+ CACCTCGTTT GATCGATCCC GATCACCCAT GATCGTCACT TCCAGATCTT CACGTGTGCA ACCAATGAGC   
  
  
+ CATTAGTTGT AAATCAATTA ACATGTTTAA TCATAAAGAC TTCAAATTTT TGAAGTCAAC ATTCAAATGA   
  
  
+ TGCAGTTTGC TGAGATGGTA TTAGTGGCAC CACACAGCCC ACCCAAATGA ATTAACCATG AGACCTATTT   
  
  
+ ATGATCCTTC ATGGAAGTTC CTTAAGCCGC GCAGACATTC ACTATATAAT AGTAATTATA ACTTTCCACC   
  
  
+ TACCAACCAA GCATATGTAT ACTTTCAATA AATACATGTG CCCAAATTCC ATAAAATTAT AGAACAATTA   
  
  
+ TTGAATGAAT TTAATTATCA GATCAGTCCA AGACAGGCGC TTAGCTAGGT TAGCATAAAA TATCGTGCAC   
  
  
+ CTGTTATATC CTTATCAGCT CAGAGGCCTA ATACTGTTGC TAGTCTGGTA GCTCTACGAA TTTATTTCCA   
  
  
+ TGTAAATCAG AATTAAAAAT TATTATCATC TGTATAATCG TATGTGTTAA CGTCGCTTTC CTAATGATGA   
  
  
+ AGCCGCCAGC TCCATTTTCC ACCCTACATT TTTAAATTAT TATCATCTGT ATAATCGTAT GTGTTAACGT   
  
  
+ CGCTTTCCTA ATGATGAAGC CGCCAGCTCC ATTTTCCACC CTACATTTTT TCCAATAGAT CAATCCGCTG   
  
  
+ ATGAAGCTAT ATGTACATTC AATTTTTTTC TTGAGCTAAC TGGAAAATAT TTTCGTGCCG TCTACGACAC   
  
  
+ CTGTTAACCC TATATATATC TCTTTGGATT TTTCTGTCTA TTTTTGTATA CTTCTTCAGG AAAATATTTT   
  
  
+ CGTGCCGTCT ACGACACCTG TTAACCCTAT ATATATCTCT TTGGATTTTT CTGTCTATTT TTGTATACTT   
  
  
+ CTTCTGGATC AATCTCCATC TTTATCTTCT TCCTCTTCTT CTGCTTGAAG CTTGATCTTC TTCTGTAACT   
  
  
+ ATACAGTGCG TTGTTTCTGT TTCCTTTTC  

- TTTTCCGTTG GAAAAGGAAA GAAAGAAGAA AGGAATCGAA AAATCTAAAT ACTTCTTCCC GACGATGCTG   
  
  
- AAAGGACGAA AAGGTGTAAA GTTTGCGAGC ACAATAGCAG GAAAGTAAGA GGGTGAAGCA CAGGTAGTCG   
  
  
- AGGAAAAATT TTTTTATATA AATAAATAAA ATAATTTAAT AGTAAATAAT AATAAAAAAT TTTTTTTAAA   
  
  
- TTATGTCAAA AAACCCCCTT TTTTTCTTTT TTTTGAGTAG GTAGACTAAA CGAGAGAGAG ACTAAAGGTT   
  
  
- GGCTTACGTA CTCCTCTCGT AAGTCGCCGT TAGTTCGTCT CTCCTGACTG ACAGAATACC CGGGATAGAG   
  
  
- AAGAACCTAA GGTCTGATAA GAACAAAAAT CATCGAGAGT TTAGTTATTG GTCTAGATTA GGATTATGAG   
  
  
- AGAAGTATTA GTATTGAATT TCTCCCTTAA TTAATTGGTT TAGTCCATTT GTTTGTAGTA ATTAATGTCG   
  
  
- GTGGAGCAAA CTAGCTAGGG CTAGTGGGTA CTAGCAGTGA AGGTCTAGAA GTGCACACGT TGGTTACTCG   
  
  
- GTAATCAACA TTTAGTTAAT TGTACAAATT AGTATTTCTG AAGTTTAAAA ACTTCAGTTG TAAGTTTACT   
  
  
- ACGTCAAACG ACTCTACCAT AATCACCGTG GTGTGTCGGG TGGGTTTACT TAATTGGTAC TCTGGATAAA   
  
  
- TACTAGGAAG TACCTTCAAG GAATTCGGCG CGTCTGTAAG TGATATATTA TCATTAATAT TGAAAGGTGG   
  
  
- ATGGTTGGTT CGTATACATA TGAAAGTTAT TTATGTACAC GGGTTTAAGG TATTTTAATA TCTTGTTAAT   
  
  
- AACTTACTTA AATTAATAGT CTAGTCAGGT TCTGTCCGCG AATCGATCCA ATCGTATTTT ATAGCACGTG   
  
  
- GACAATATAG GAATAGTCGA GTCTCCGGAT TATGACAACG ATCAGACCAT CGAGATGCTT AAATAAAGGT   
  
  
- ACATTTAGTC TTAATTTTTA ATAATAGTAG ACATATTAGC ATACACAATT GCAGCGAAAG GATTACTACT   
  
  
- TCGGCGGTCG AGGTAAAAGG TGGGATGTAA AAATTTAATA ATAGTAGACA TATTAGCATA CACAATTGCA   
  
  
- GCGAAAGGAT TACTACTTCG GCGGTCGAGG TAAAAGGTGG GATGTAAAAA AGGTTATCTA GTTAGGCGAC   
  
  
- TACTTCGATA TACATGTAAG TTAAAAAAAG AACTCGATTG ACCTTTTATA AAAGCACGGC AGATGCTGTG   
  
  
- GACAATTGGG ATATATATAG AGAAACCTAA AAAGACAGAT AAAAACATAT GAAGAAGTCC TTTTATAAAA   
  
  
- GCACGGCAGA TGCTGTGGAC AATTGGGATA TATATAGAGA AACCTAAAAA GACAGATAAA AACATATGAA   
  
  
- GAAGACCTAG TTAGAGGTAG AAATAGAAGA AGGAGAAGAA GACGAACTTC GAACTAGAAG AAGACATTGA   
  
  
- TATGTCACGC AACAAAGACA AAGGAAAAG

+     GAG-motif

| Site Name | Organism | Position | Strand | Matrix score. | sequence | function |
| --- | --- | --- | --- | --- | --- | --- |
| GAG-motif | Arabidopsis thaliana | 417 | - | 7 | AGAGAGT | part of a light responsive element |

> 2018/04/13 10:10:12  
+ AAAAGGCAAC CTTTTCCTTT CTTTCTTCTT TCCTTAGCTT TTTAGATTTA TGAAGAAGGG CTGCTACGAC   
  
  
+ TTTCCTGCTT TTCCACATTT CAAACGCTCG TGTTATCGTC CTTTCATTCT CCCACTTCGT GTCCATCAGC   
  
  
+ TCCTTTTTAA AAAAATATAT TTATTTATTT TATTAAATTA TCATTTATTA TTATTTTTTA AAAAAAATTT   
  
  
+ AATACAGTTT TTTGGGGGAA AAAAAGAAAA AAAACTCATC CATCTGATTT GCTCTCTCTC TGATTTCCAA   
  
  
+ CCGAATGCAT GAGGAGAGCA TTCAGCGGCA ATCAAGCAGA GAGGACTGAC TGTCTTATGG GCCCTATCTC   
  
  
+ TTCTTGGATT CCAGACTATT CTTGTTTTTA GTAGCTCTCA AATCAATAAC CAGATCTAAT CCTAATACTC   
  
  
+ TCTTCATAAT CATAACTTAA AGAGGGAATT AATTAACCAA ATCAGGTAAA CAAACATCAT TAATTACAGC   
  
  
+ CACCTCGTTT GATCGATCCC GATCACCCAT GATCGTCACT TCCAGATCTT CACGTGTGCA ACCAATGAGC   
  
  
+ CATTAGTTGT AAATCAATTA ACATGTTTAA TCATAAAGAC TTCAAATTTT TGAAGTCAAC ATTCAAATGA   
  
  
+ TGCAGTTTGC TGAGATGGTA TTAGTGGCAC CACACAGCCC ACCCAAATGA ATTAACCATG AGACCTATTT   
  
  
+ ATGATCCTTC ATGGAAGTTC CTTAAGCCGC GCAGACATTC ACTATATAAT AGTAATTATA ACTTTCCACC   
  
  
+ TACCAACCAA GCATATGTAT ACTTTCAATA AATACATGTG CCCAAATTCC ATAAAATTAT AGAACAATTA   
  
  
+ TTGAATGAAT TTAATTATCA GATCAGTCCA AGACAGGCGC TTAGCTAGGT TAGCATAAAA TATCGTGCAC   
  
  
+ CTGTTATATC CTTATCAGCT CAGAGGCCTA ATACTGTTGC TAGTCTGGTA GCTCTACGAA TTTATTTCCA   
  
  
+ TGTAAATCAG AATTAAAAAT TATTATCATC TGTATAATCG TATGTGTTAA CGTCGCTTTC CTAATGATGA   
  
  
+ AGCCGCCAGC TCCATTTTCC ACCCTACATT TTTAAATTAT TATCATCTGT ATAATCGTAT GTGTTAACGT   
  
  
+ CGCTTTCCTA ATGATGAAGC CGCCAGCTCC ATTTTCCACC CTACATTTTT TCCAATAGAT CAATCCGCTG   
  
  
+ ATGAAGCTAT ATGTACATTC AATTTTTTTC TTGAGCTAAC TGGAAAATAT TTTCGTGCCG TCTACGACAC   
  
  
+ CTGTTAACCC TATATATATC TCTTTGGATT TTTCTGTCTA TTTTTGTATA CTTCTTCAGG AAAATATTTT   
  
  
+ CGTGCCGTCT ACGACACCTG TTAACCCTAT ATATATCTCT TTGGATTTTT CTGTCTATTT TTGTATACTT   
  
  
+ CTTCTGGATC AATCTCCATC TTTATCTTCT TCCTCTTCTT CTGCTTGAAG CTTGATCTTC TTCTGTAACT   
  
  
+ ATACAGTGCG TTGTTTCTGT TTCCTTTTC  

- TTTTCCGTTG GAAAAGGAAA GAAAGAAGAA AGGAATCGAA AAATCTAAAT ACTTCTTCCC GACGATGCTG   
  
  
- AAAGGACGAA AAGGTGTAAA GTTTGCGAGC ACAATAGCAG GAAAGTAAGA GGGTGAAGCA CAGGTAGTCG   
  
  
- AGGAAAAATT TTTTTATATA AATAAATAAA ATAATTTAAT AGTAAATAAT AATAAAAAAT TTTTTTTAAA   
  
  
- TTATGTCAAA AAACCCCCTT TTTTTCTTTT TTTTGAGTAG GTAGACTAAA CGAGAGAGAG ACTAAAGGTT   
  
  
- GGCTTACGTA CTCCTCTCGT AAGTCGCCGT TAGTTCGTCT CTCCTGACTG ACAGAATACC CGGGATAGAG   
  
  
- AAGAACCTAA GGTCTGATAA GAACAAAAAT CATCGAGAGT TTAGTTATTG GTCTAGATTA GGATTATGAG   
  
  
- AGAAGTATTA GTATTGAATT TCTCCCTTAA TTAATTGGTT TAGTCCATTT GTTTGTAGTA ATTAATGTCG   
  
  
- GTGGAGCAAA CTAGCTAGGG CTAGTGGGTA CTAGCAGTGA AGGTCTAGAA GTGCACACGT TGGTTACTCG   
  
  
- GTAATCAACA TTTAGTTAAT TGTACAAATT AGTATTTCTG AAGTTTAAAA ACTTCAGTTG TAAGTTTACT   
  
  
- ACGTCAAACG ACTCTACCAT AATCACCGTG GTGTGTCGGG TGGGTTTACT TAATTGGTAC TCTGGATAAA   
  
  
- TACTAGGAAG TACCTTCAAG GAATTCGGCG CGTCTGTAAG TGATATATTA TCATTAATAT TGAAAGGTGG   
  
  
- ATGGTTGGTT CGTATACATA TGAAAGTTAT TTATGTACAC GGGTTTAAGG TATTTTAATA TCTTGTTAAT   
  
  
- AACTTACTTA AATTAATAGT CTAGTCAGGT TCTGTCCGCG AATCGATCCA ATCGTATTTT ATAGCACGTG   
  
  
- GACAATATAG GAATAGTCGA GTCTCCGGAT TATGACAACG ATCAGACCAT CGAGATGCTT AAATAAAGGT   
  
  
- ACATTTAGTC TTAATTTTTA ATAATAGTAG ACATATTAGC ATACACAATT GCAGCGAAAG GATTACTACT   
  
  
- TCGGCGGTCG AGGTAAAAGG TGGGATGTAA AAATTTAATA ATAGTAGACA TATTAGCATA CACAATTGCA   
  
  
- GCGAAAGGAT TACTACTTCG GCGGTCGAGG TAAAAGGTGG GATGTAAAAA AGGTTATCTA GTTAGGCGAC   
  
  
- TACTTCGATA TACATGTAAG TTAAAAAAAG AACTCGATTG ACCTTTTATA AAAGCACGGC AGATGCTGTG   
  
  
- GACAATTGGG ATATATATAG AGAAACCTAA AAAGACAGAT AAAAACATAT GAAGAAGTCC TTTTATAAAA   
  
  
- GCACGGCAGA TGCTGTGGAC AATTGGGATA TATATAGAGA AACCTAAAAA GACAGATAAA AACATATGAA   
  
  
- GAAGACCTAG TTAGAGGTAG AAATAGAAGA AGGAGAAGAA GACGAACTTC GAACTAGAAG AAGACATTGA   
  
  
- TATGTCACGC AACAAAGACA AAGGAAAAG

+     GARE-motif

| Site Name | Organism | Position | Strand | Matrix score. | sequence | function |
| --- | --- | --- | --- | --- | --- | --- |
| GARE-motif | Brassica oleracea | 1486 | - | 7 | AAACAGA | gibberellin-responsive element |

> 2018/04/13 10:10:12  
+ AAAAGGCAAC CTTTTCCTTT CTTTCTTCTT TCCTTAGCTT TTTAGATTTA TGAAGAAGGG CTGCTACGAC   
  
  
+ TTTCCTGCTT TTCCACATTT CAAACGCTCG TGTTATCGTC CTTTCATTCT CCCACTTCGT GTCCATCAGC   
  
  
+ TCCTTTTTAA AAAAATATAT TTATTTATTT TATTAAATTA TCATTTATTA TTATTTTTTA AAAAAAATTT   
  
  
+ AATACAGTTT TTTGGGGGAA AAAAAGAAAA AAAACTCATC CATCTGATTT GCTCTCTCTC TGATTTCCAA   
  
  
+ CCGAATGCAT GAGGAGAGCA TTCAGCGGCA ATCAAGCAGA GAGGACTGAC TGTCTTATGG GCCCTATCTC   
  
  
+ TTCTTGGATT CCAGACTATT CTTGTTTTTA GTAGCTCTCA AATCAATAAC CAGATCTAAT CCTAATACTC   
  
  
+ TCTTCATAAT CATAACTTAA AGAGGGAATT AATTAACCAA ATCAGGTAAA CAAACATCAT TAATTACAGC   
  
  
+ CACCTCGTTT GATCGATCCC GATCACCCAT GATCGTCACT TCCAGATCTT CACGTGTGCA ACCAATGAGC   
  
  
+ CATTAGTTGT AAATCAATTA ACATGTTTAA TCATAAAGAC TTCAAATTTT TGAAGTCAAC ATTCAAATGA   
  
  
+ TGCAGTTTGC TGAGATGGTA TTAGTGGCAC CACACAGCCC ACCCAAATGA ATTAACCATG AGACCTATTT   
  
  
+ ATGATCCTTC ATGGAAGTTC CTTAAGCCGC GCAGACATTC ACTATATAAT AGTAATTATA ACTTTCCACC   
  
  
+ TACCAACCAA GCATATGTAT ACTTTCAATA AATACATGTG CCCAAATTCC ATAAAATTAT AGAACAATTA   
  
  
+ TTGAATGAAT TTAATTATCA GATCAGTCCA AGACAGGCGC TTAGCTAGGT TAGCATAAAA TATCGTGCAC   
  
  
+ CTGTTATATC CTTATCAGCT CAGAGGCCTA ATACTGTTGC TAGTCTGGTA GCTCTACGAA TTTATTTCCA   
  
  
+ TGTAAATCAG AATTAAAAAT TATTATCATC TGTATAATCG TATGTGTTAA CGTCGCTTTC CTAATGATGA   
  
  
+ AGCCGCCAGC TCCATTTTCC ACCCTACATT TTTAAATTAT TATCATCTGT ATAATCGTAT GTGTTAACGT   
  
  
+ CGCTTTCCTA ATGATGAAGC CGCCAGCTCC ATTTTCCACC CTACATTTTT TCCAATAGAT CAATCCGCTG   
  
  
+ ATGAAGCTAT ATGTACATTC AATTTTTTTC TTGAGCTAAC TGGAAAATAT TTTCGTGCCG TCTACGACAC   
  
  
+ CTGTTAACCC TATATATATC TCTTTGGATT TTTCTGTCTA TTTTTGTATA CTTCTTCAGG AAAATATTTT   
  
  
+ CGTGCCGTCT ACGACACCTG TTAACCCTAT ATATATCTCT TTGGATTTTT CTGTCTATTT TTGTATACTT   
  
  
+ CTTCTGGATC AATCTCCATC TTTATCTTCT TCCTCTTCTT CTGCTTGAAG CTTGATCTTC TTCTGTAACT   
  
  
+ ATACAGTGCG TTGTTTCTGT TTCCTTTTC  

- TTTTCCGTTG GAAAAGGAAA GAAAGAAGAA AGGAATCGAA AAATCTAAAT ACTTCTTCCC GACGATGCTG   
  
  
- AAAGGACGAA AAGGTGTAAA GTTTGCGAGC ACAATAGCAG GAAAGTAAGA GGGTGAAGCA CAGGTAGTCG   
  
  
- AGGAAAAATT TTTTTATATA AATAAATAAA ATAATTTAAT AGTAAATAAT AATAAAAAAT TTTTTTTAAA   
  
  
- TTATGTCAAA AAACCCCCTT TTTTTCTTTT TTTTGAGTAG GTAGACTAAA CGAGAGAGAG ACTAAAGGTT   
  
  
- GGCTTACGTA CTCCTCTCGT AAGTCGCCGT TAGTTCGTCT CTCCTGACTG ACAGAATACC CGGGATAGAG   
  
  
- AAGAACCTAA GGTCTGATAA GAACAAAAAT CATCGAGAGT TTAGTTATTG GTCTAGATTA GGATTATGAG   
  
  
- AGAAGTATTA GTATTGAATT TCTCCCTTAA TTAATTGGTT TAGTCCATTT GTTTGTAGTA ATTAATGTCG   
  
  
- GTGGAGCAAA CTAGCTAGGG CTAGTGGGTA CTAGCAGTGA AGGTCTAGAA GTGCACACGT TGGTTACTCG   
  
  
- GTAATCAACA TTTAGTTAAT TGTACAAATT AGTATTTCTG AAGTTTAAAA ACTTCAGTTG TAAGTTTACT   
  
  
- ACGTCAAACG ACTCTACCAT AATCACCGTG GTGTGTCGGG TGGGTTTACT TAATTGGTAC TCTGGATAAA   
  
  
- TACTAGGAAG TACCTTCAAG GAATTCGGCG CGTCTGTAAG TGATATATTA TCATTAATAT TGAAAGGTGG   
  
  
- ATGGTTGGTT CGTATACATA TGAAAGTTAT TTATGTACAC GGGTTTAAGG TATTTTAATA TCTTGTTAAT   
  
  
- AACTTACTTA AATTAATAGT CTAGTCAGGT TCTGTCCGCG AATCGATCCA ATCGTATTTT ATAGCACGTG   
  
  
- GACAATATAG GAATAGTCGA GTCTCCGGAT TATGACAACG ATCAGACCAT CGAGATGCTT AAATAAAGGT   
  
  
- ACATTTAGTC TTAATTTTTA ATAATAGTAG ACATATTAGC ATACACAATT GCAGCGAAAG GATTACTACT   
  
  
- TCGGCGGTCG AGGTAAAAGG TGGGATGTAA AAATTTAATA ATAGTAGACA TATTAGCATA CACAATTGCA   
  
  
- GCGAAAGGAT TACTACTTCG GCGGTCGAGG TAAAAGGTGG GATGTAAAAA AGGTTATCTA GTTAGGCGAC   
  
  
- TACTTCGATA TACATGTAAG TTAAAAAAAG AACTCGATTG ACCTTTTATA AAAGCACGGC AGATGCTGTG   
  
  
- GACAATTGGG ATATATATAG AGAAACCTAA AAAGACAGAT AAAAACATAT GAAGAAGTCC TTTTATAAAA   
  
  
- GCACGGCAGA TGCTGTGGAC AATTGGGATA TATATAGAGA AACCTAAAAA GACAGATAAA AACATATGAA   
  
  
- GAAGACCTAG TTAGAGGTAG AAATAGAAGA AGGAGAAGAA GACGAACTTC GAACTAGAAG AAGACATTGA   
  
  
- TATGTCACGC AACAAAGACA AAGGAAAAG

+     GATA-motif

| Site Name | Organism | Position | Strand | Matrix score. | sequence | function |
| --- | --- | --- | --- | --- | --- | --- |
| GATA-motif | Solanum tuberosum | 10 | - | 9 | AAGGATAAGG | part of a light responsive element |
| GATA-motif | Pisum sativum | 342 | - | 7 | GATAGGG | part of a light responsive element |

> 2018/04/13 10:10:12  
+ AAAAGGCAAC CTTTTCCTTT CTTTCTTCTT TCCTTAGCTT TTTAGATTTA TGAAGAAGGG CTGCTACGAC   
  
  
+ TTTCCTGCTT TTCCACATTT CAAACGCTCG TGTTATCGTC CTTTCATTCT CCCACTTCGT GTCCATCAGC   
  
  
+ TCCTTTTTAA AAAAATATAT TTATTTATTT TATTAAATTA TCATTTATTA TTATTTTTTA AAAAAAATTT   
  
  
+ AATACAGTTT TTTGGGGGAA AAAAAGAAAA AAAACTCATC CATCTGATTT GCTCTCTCTC TGATTTCCAA   
  
  
+ CCGAATGCAT GAGGAGAGCA TTCAGCGGCA ATCAAGCAGA GAGGACTGAC TGTCTTATGG GCCCTATCTC   
  
  
+ TTCTTGGATT CCAGACTATT CTTGTTTTTA GTAGCTCTCA AATCAATAAC CAGATCTAAT CCTAATACTC   
  
  
+ TCTTCATAAT CATAACTTAA AGAGGGAATT AATTAACCAA ATCAGGTAAA CAAACATCAT TAATTACAGC   
  
  
+ CACCTCGTTT GATCGATCCC GATCACCCAT GATCGTCACT TCCAGATCTT CACGTGTGCA ACCAATGAGC   
  
  
+ CATTAGTTGT AAATCAATTA ACATGTTTAA TCATAAAGAC TTCAAATTTT TGAAGTCAAC ATTCAAATGA   
  
  
+ TGCAGTTTGC TGAGATGGTA TTAGTGGCAC CACACAGCCC ACCCAAATGA ATTAACCATG AGACCTATTT   
  
  
+ ATGATCCTTC ATGGAAGTTC CTTAAGCCGC GCAGACATTC ACTATATAAT AGTAATTATA ACTTTCCACC   
  
  
+ TACCAACCAA GCATATGTAT ACTTTCAATA AATACATGTG CCCAAATTCC ATAAAATTAT AGAACAATTA   
  
  
+ TTGAATGAAT TTAATTATCA GATCAGTCCA AGACAGGCGC TTAGCTAGGT TAGCATAAAA TATCGTGCAC   
  
  
+ CTGTTATATC CTTATCAGCT CAGAGGCCTA ATACTGTTGC TAGTCTGGTA GCTCTACGAA TTTATTTCCA   
  
  
+ TGTAAATCAG AATTAAAAAT TATTATCATC TGTATAATCG TATGTGTTAA CGTCGCTTTC CTAATGATGA   
  
  
+ AGCCGCCAGC TCCATTTTCC ACCCTACATT TTTAAATTAT TATCATCTGT ATAATCGTAT GTGTTAACGT   
  
  
+ CGCTTTCCTA ATGATGAAGC CGCCAGCTCC ATTTTCCACC CTACATTTTT TCCAATAGAT CAATCCGCTG   
  
  
+ ATGAAGCTAT ATGTACATTC AATTTTTTTC TTGAGCTAAC TGGAAAATAT TTTCGTGCCG TCTACGACAC   
  
  
+ CTGTTAACCC TATATATATC TCTTTGGATT TTTCTGTCTA TTTTTGTATA CTTCTTCAGG AAAATATTTT   
  
  
+ CGTGCCGTCT ACGACACCTG TTAACCCTAT ATATATCTCT TTGGATTTTT CTGTCTATTT TTGTATACTT   
  
  
+ CTTCTGGATC AATCTCCATC TTTATCTTCT TCCTCTTCTT CTGCTTGAAG CTTGATCTTC TTCTGTAACT   
  
  
+ ATACAGTGCG TTGTTTCTGT TTCCTTTTC  

- TTTTCCGTTG GAAAAGGAAA GAAAGAAGAA AGGAATCGAA AAATCTAAAT ACTTCTTCCC GACGATGCTG   
  
  
- AAAGGACGAA AAGGTGTAAA GTTTGCGAGC ACAATAGCAG GAAAGTAAGA GGGTGAAGCA CAGGTAGTCG   
  
  
- AGGAAAAATT TTTTTATATA AATAAATAAA ATAATTTAAT AGTAAATAAT AATAAAAAAT TTTTTTTAAA   
  
  
- TTATGTCAAA AAACCCCCTT TTTTTCTTTT TTTTGAGTAG GTAGACTAAA CGAGAGAGAG ACTAAAGGTT   
  
  
- GGCTTACGTA CTCCTCTCGT AAGTCGCCGT TAGTTCGTCT CTCCTGACTG ACAGAATACC CGGGATAGAG   
  
  
- AAGAACCTAA GGTCTGATAA GAACAAAAAT CATCGAGAGT TTAGTTATTG GTCTAGATTA GGATTATGAG   
  
  
- AGAAGTATTA GTATTGAATT TCTCCCTTAA TTAATTGGTT TAGTCCATTT GTTTGTAGTA ATTAATGTCG   
  
  
- GTGGAGCAAA CTAGCTAGGG CTAGTGGGTA CTAGCAGTGA AGGTCTAGAA GTGCACACGT TGGTTACTCG   
  
  
- GTAATCAACA TTTAGTTAAT TGTACAAATT AGTATTTCTG AAGTTTAAAA ACTTCAGTTG TAAGTTTACT   
  
  
- ACGTCAAACG ACTCTACCAT AATCACCGTG GTGTGTCGGG TGGGTTTACT TAATTGGTAC TCTGGATAAA   
  
  
- TACTAGGAAG TACCTTCAAG GAATTCGGCG CGTCTGTAAG TGATATATTA TCATTAATAT TGAAAGGTGG   
  
  
- ATGGTTGGTT CGTATACATA TGAAAGTTAT TTATGTACAC GGGTTTAAGG TATTTTAATA TCTTGTTAAT   
  
  
- AACTTACTTA AATTAATAGT CTAGTCAGGT TCTGTCCGCG AATCGATCCA ATCGTATTTT ATAGCACGTG   
  
  
- GACAATATAG GAATAGTCGA GTCTCCGGAT TATGACAACG ATCAGACCAT CGAGATGCTT AAATAAAGGT   
  
  
- ACATTTAGTC TTAATTTTTA ATAATAGTAG ACATATTAGC ATACACAATT GCAGCGAAAG GATTACTACT   
  
  
- TCGGCGGTCG AGGTAAAAGG TGGGATGTAA AAATTTAATA ATAGTAGACA TATTAGCATA CACAATTGCA   
  
  
- GCGAAAGGAT TACTACTTCG GCGGTCGAGG TAAAAGGTGG GATGTAAAAA AGGTTATCTA GTTAGGCGAC   
  
  
- TACTTCGATA TACATGTAAG TTAAAAAAAG AACTCGATTG ACCTTTTATA AAAGCACGGC AGATGCTGTG   
  
  
- GACAATTGGG ATATATATAG AGAAACCTAA AAAGACAGAT AAAAACATAT GAAGAAGTCC TTTTATAAAA   
  
  
- GCACGGCAGA TGCTGTGGAC AATTGGGATA TATATAGAGA AACCTAAAAA GACAGATAAA AACATATGAA   
  
  
- GAAGACCTAG TTAGAGGTAG AAATAGAAGA AGGAGAAGAA GACGAACTTC GAACTAGAAG AAGACATTGA   
  
  
- TATGTCACGC AACAAAGACA AAGGAAAAG

+     GCC box

| Site Name | Organism | Position | Strand | Matrix score. | sequence | function |
| --- | --- | --- | --- | --- | --- | --- |
| GCC box | Arabidopsis thaliana | 1138 | + | 7 | AGCCGCC |  |
| GCC box | Arabidopsis thaliana | 1051 | + | 7 | AGCCGCC |  |

> 2018/04/13 10:10:12  
+ AAAAGGCAAC CTTTTCCTTT CTTTCTTCTT TCCTTAGCTT TTTAGATTTA TGAAGAAGGG CTGCTACGAC   
  
  
+ TTTCCTGCTT TTCCACATTT CAAACGCTCG TGTTATCGTC CTTTCATTCT CCCACTTCGT GTCCATCAGC   
  
  
+ TCCTTTTTAA AAAAATATAT TTATTTATTT TATTAAATTA TCATTTATTA TTATTTTTTA AAAAAAATTT   
  
  
+ AATACAGTTT TTTGGGGGAA AAAAAGAAAA AAAACTCATC CATCTGATTT GCTCTCTCTC TGATTTCCAA   
  
  
+ CCGAATGCAT GAGGAGAGCA TTCAGCGGCA ATCAAGCAGA GAGGACTGAC TGTCTTATGG GCCCTATCTC   
  
  
+ TTCTTGGATT CCAGACTATT CTTGTTTTTA GTAGCTCTCA AATCAATAAC CAGATCTAAT CCTAATACTC   
  
  
+ TCTTCATAAT CATAACTTAA AGAGGGAATT AATTAACCAA ATCAGGTAAA CAAACATCAT TAATTACAGC   
  
  
+ CACCTCGTTT GATCGATCCC GATCACCCAT GATCGTCACT TCCAGATCTT CACGTGTGCA ACCAATGAGC   
  
  
+ CATTAGTTGT AAATCAATTA ACATGTTTAA TCATAAAGAC TTCAAATTTT TGAAGTCAAC ATTCAAATGA   
  
  
+ TGCAGTTTGC TGAGATGGTA TTAGTGGCAC CACACAGCCC ACCCAAATGA ATTAACCATG AGACCTATTT   
  
  
+ ATGATCCTTC ATGGAAGTTC CTTAAGCCGC GCAGACATTC ACTATATAAT AGTAATTATA ACTTTCCACC   
  
  
+ TACCAACCAA GCATATGTAT ACTTTCAATA AATACATGTG CCCAAATTCC ATAAAATTAT AGAACAATTA   
  
  
+ TTGAATGAAT TTAATTATCA GATCAGTCCA AGACAGGCGC TTAGCTAGGT TAGCATAAAA TATCGTGCAC   
  
  
+ CTGTTATATC CTTATCAGCT CAGAGGCCTA ATACTGTTGC TAGTCTGGTA GCTCTACGAA TTTATTTCCA   
  
  
+ TGTAAATCAG AATTAAAAAT TATTATCATC TGTATAATCG TATGTGTTAA CGTCGCTTTC CTAATGATGA   
  
  
+ AGCCGCCAGC TCCATTTTCC ACCCTACATT TTTAAATTAT TATCATCTGT ATAATCGTAT GTGTTAACGT   
  
  
+ CGCTTTCCTA ATGATGAAGC CGCCAGCTCC ATTTTCCACC CTACATTTTT TCCAATAGAT CAATCCGCTG   
  
  
+ ATGAAGCTAT ATGTACATTC AATTTTTTTC TTGAGCTAAC TGGAAAATAT TTTCGTGCCG TCTACGACAC   
  
  
+ CTGTTAACCC TATATATATC TCTTTGGATT TTTCTGTCTA TTTTTGTATA CTTCTTCAGG AAAATATTTT   
  
  
+ CGTGCCGTCT ACGACACCTG TTAACCCTAT ATATATCTCT TTGGATTTTT CTGTCTATTT TTGTATACTT   
  
  
+ CTTCTGGATC AATCTCCATC TTTATCTTCT TCCTCTTCTT CTGCTTGAAG CTTGATCTTC TTCTGTAACT   
  
  
+ ATACAGTGCG TTGTTTCTGT TTCCTTTTC  

- TTTTCCGTTG GAAAAGGAAA GAAAGAAGAA AGGAATCGAA AAATCTAAAT ACTTCTTCCC GACGATGCTG   
  
  
- AAAGGACGAA AAGGTGTAAA GTTTGCGAGC ACAATAGCAG GAAAGTAAGA GGGTGAAGCA CAGGTAGTCG   
  
  
- AGGAAAAATT TTTTTATATA AATAAATAAA ATAATTTAAT AGTAAATAAT AATAAAAAAT TTTTTTTAAA   
  
  
- TTATGTCAAA AAACCCCCTT TTTTTCTTTT TTTTGAGTAG GTAGACTAAA CGAGAGAGAG ACTAAAGGTT   
  
  
- GGCTTACGTA CTCCTCTCGT AAGTCGCCGT TAGTTCGTCT CTCCTGACTG ACAGAATACC CGGGATAGAG   
  
  
- AAGAACCTAA GGTCTGATAA GAACAAAAAT CATCGAGAGT TTAGTTATTG GTCTAGATTA GGATTATGAG   
  
  
- AGAAGTATTA GTATTGAATT TCTCCCTTAA TTAATTGGTT TAGTCCATTT GTTTGTAGTA ATTAATGTCG   
  
  
- GTGGAGCAAA CTAGCTAGGG CTAGTGGGTA CTAGCAGTGA AGGTCTAGAA GTGCACACGT TGGTTACTCG   
  
  
- GTAATCAACA TTTAGTTAAT TGTACAAATT AGTATTTCTG AAGTTTAAAA ACTTCAGTTG TAAGTTTACT   
  
  
- ACGTCAAACG ACTCTACCAT AATCACCGTG GTGTGTCGGG TGGGTTTACT TAATTGGTAC TCTGGATAAA   
  
  
- TACTAGGAAG TACCTTCAAG GAATTCGGCG CGTCTGTAAG TGATATATTA TCATTAATAT TGAAAGGTGG   
  
  
- ATGGTTGGTT CGTATACATA TGAAAGTTAT TTATGTACAC GGGTTTAAGG TATTTTAATA TCTTGTTAAT   
  
  
- AACTTACTTA AATTAATAGT CTAGTCAGGT TCTGTCCGCG AATCGATCCA ATCGTATTTT ATAGCACGTG   
  
  
- GACAATATAG GAATAGTCGA GTCTCCGGAT TATGACAACG ATCAGACCAT CGAGATGCTT AAATAAAGGT   
  
  
- ACATTTAGTC TTAATTTTTA ATAATAGTAG ACATATTAGC ATACACAATT GCAGCGAAAG GATTACTACT   
  
  
- TCGGCGGTCG AGGTAAAAGG TGGGATGTAA AAATTTAATA ATAGTAGACA TATTAGCATA CACAATTGCA   
  
  
- GCGAAAGGAT TACTACTTCG GCGGTCGAGG TAAAAGGTGG GATGTAAAAA AGGTTATCTA GTTAGGCGAC   
  
  
- TACTTCGATA TACATGTAAG TTAAAAAAAG AACTCGATTG ACCTTTTATA AAAGCACGGC AGATGCTGTG   
  
  
- GACAATTGGG ATATATATAG AGAAACCTAA AAAGACAGAT AAAAACATAT GAAGAAGTCC TTTTATAAAA   
  
  
- GCACGGCAGA TGCTGTGGAC AATTGGGATA TATATAGAGA AACCTAAAAA GACAGATAAA AACATATGAA   
  
  
- GAAGACCTAG TTAGAGGTAG AAATAGAAGA AGGAGAAGAA GACGAACTTC GAACTAGAAG AAGACATTGA   
  
  
- TATGTCACGC AACAAAGACA AAGGAAAAG

+     GT1-motif

| Site Name | Organism | Position | Strand | Matrix score. | sequence | function |
| --- | --- | --- | --- | --- | --- | --- |
| GT1-motif | Arabidopsis thaliana | 1264 | - | 6 | GGTTAA | light responsive element |
| GT1-motif | Arabidopsis thaliana | 1351 | - | 6 | GGTTAA | light responsive element |
| GT1-motif | Arabidopsis thaliana | 682 | - | 6 | GGTTAA | light responsive element |
| GT1-motif | Avena sativa | 681 | - | 7 | GGTTAAT | light responsive element |
| GT1-motif | Arabidopsis thaliana | 453 | - | 6 | GGTTAA | light responsive element |
| GT1-motif | Avena sativa | 452 | - | 7 | GGTTAAT | light responsive element |

> 2018/04/13 10:10:12  
+ AAAAGGCAAC CTTTTCCTTT CTTTCTTCTT TCCTTAGCTT TTTAGATTTA TGAAGAAGGG CTGCTACGAC   
  
  
+ TTTCCTGCTT TTCCACATTT CAAACGCTCG TGTTATCGTC CTTTCATTCT CCCACTTCGT GTCCATCAGC   
  
  
+ TCCTTTTTAA AAAAATATAT TTATTTATTT TATTAAATTA TCATTTATTA TTATTTTTTA AAAAAAATTT   
  
  
+ AATACAGTTT TTTGGGGGAA AAAAAGAAAA AAAACTCATC CATCTGATTT GCTCTCTCTC TGATTTCCAA   
  
  
+ CCGAATGCAT GAGGAGAGCA TTCAGCGGCA ATCAAGCAGA GAGGACTGAC TGTCTTATGG GCCCTATCTC   
  
  
+ TTCTTGGATT CCAGACTATT CTTGTTTTTA GTAGCTCTCA AATCAATAAC CAGATCTAAT CCTAATACTC   
  
  
+ TCTTCATAAT CATAACTTAA AGAGGGAATT AATTAACCAA ATCAGGTAAA CAAACATCAT TAATTACAGC   
  
  
+ CACCTCGTTT GATCGATCCC GATCACCCAT GATCGTCACT TCCAGATCTT CACGTGTGCA ACCAATGAGC   
  
  
+ CATTAGTTGT AAATCAATTA ACATGTTTAA TCATAAAGAC TTCAAATTTT TGAAGTCAAC ATTCAAATGA   
  
  
+ TGCAGTTTGC TGAGATGGTA TTAGTGGCAC CACACAGCCC ACCCAAATGA ATTAACCATG AGACCTATTT   
  
  
+ ATGATCCTTC ATGGAAGTTC CTTAAGCCGC GCAGACATTC ACTATATAAT AGTAATTATA ACTTTCCACC   
  
  
+ TACCAACCAA GCATATGTAT ACTTTCAATA AATACATGTG CCCAAATTCC ATAAAATTAT AGAACAATTA   
  
  
+ TTGAATGAAT TTAATTATCA GATCAGTCCA AGACAGGCGC TTAGCTAGGT TAGCATAAAA TATCGTGCAC   
  
  
+ CTGTTATATC CTTATCAGCT CAGAGGCCTA ATACTGTTGC TAGTCTGGTA GCTCTACGAA TTTATTTCCA   
  
  
+ TGTAAATCAG AATTAAAAAT TATTATCATC TGTATAATCG TATGTGTTAA CGTCGCTTTC CTAATGATGA   
  
  
+ AGCCGCCAGC TCCATTTTCC ACCCTACATT TTTAAATTAT TATCATCTGT ATAATCGTAT GTGTTAACGT   
  
  
+ CGCTTTCCTA ATGATGAAGC CGCCAGCTCC ATTTTCCACC CTACATTTTT TCCAATAGAT CAATCCGCTG   
  
  
+ ATGAAGCTAT ATGTACATTC AATTTTTTTC TTGAGCTAAC TGGAAAATAT TTTCGTGCCG TCTACGACAC   
  
  
+ CTGTTAACCC TATATATATC TCTTTGGATT TTTCTGTCTA TTTTTGTATA CTTCTTCAGG AAAATATTTT   
  
  
+ CGTGCCGTCT ACGACACCTG TTAACCCTAT ATATATCTCT TTGGATTTTT CTGTCTATTT TTGTATACTT   
  
  
+ CTTCTGGATC AATCTCCATC TTTATCTTCT TCCTCTTCTT CTGCTTGAAG CTTGATCTTC TTCTGTAACT   
  
  
+ ATACAGTGCG TTGTTTCTGT TTCCTTTTC  

- TTTTCCGTTG GAAAAGGAAA GAAAGAAGAA AGGAATCGAA AAATCTAAAT ACTTCTTCCC GACGATGCTG   
  
  
- AAAGGACGAA AAGGTGTAAA GTTTGCGAGC ACAATAGCAG GAAAGTAAGA GGGTGAAGCA CAGGTAGTCG   
  
  
- AGGAAAAATT TTTTTATATA AATAAATAAA ATAATTTAAT AGTAAATAAT AATAAAAAAT TTTTTTTAAA   
  
  
- TTATGTCAAA AAACCCCCTT TTTTTCTTTT TTTTGAGTAG GTAGACTAAA CGAGAGAGAG ACTAAAGGTT   
  
  
- GGCTTACGTA CTCCTCTCGT AAGTCGCCGT TAGTTCGTCT CTCCTGACTG ACAGAATACC CGGGATAGAG   
  
  
- AAGAACCTAA GGTCTGATAA GAACAAAAAT CATCGAGAGT TTAGTTATTG GTCTAGATTA GGATTATGAG   
  
  
- AGAAGTATTA GTATTGAATT TCTCCCTTAA TTAATTGGTT TAGTCCATTT GTTTGTAGTA ATTAATGTCG   
  
  
- GTGGAGCAAA CTAGCTAGGG CTAGTGGGTA CTAGCAGTGA AGGTCTAGAA GTGCACACGT TGGTTACTCG   
  
  
- GTAATCAACA TTTAGTTAAT TGTACAAATT AGTATTTCTG AAGTTTAAAA ACTTCAGTTG TAAGTTTACT   
  
  
- ACGTCAAACG ACTCTACCAT AATCACCGTG GTGTGTCGGG TGGGTTTACT TAATTGGTAC TCTGGATAAA   
  
  
- TACTAGGAAG TACCTTCAAG GAATTCGGCG CGTCTGTAAG TGATATATTA TCATTAATAT TGAAAGGTGG   
  
  
- ATGGTTGGTT CGTATACATA TGAAAGTTAT TTATGTACAC GGGTTTAAGG TATTTTAATA TCTTGTTAAT   
  
  
- AACTTACTTA AATTAATAGT CTAGTCAGGT TCTGTCCGCG AATCGATCCA ATCGTATTTT ATAGCACGTG   
  
  
- GACAATATAG GAATAGTCGA GTCTCCGGAT TATGACAACG ATCAGACCAT CGAGATGCTT AAATAAAGGT   
  
  
- ACATTTAGTC TTAATTTTTA ATAATAGTAG ACATATTAGC ATACACAATT GCAGCGAAAG GATTACTACT   
  
  
- TCGGCGGTCG AGGTAAAAGG TGGGATGTAA AAATTTAATA ATAGTAGACA TATTAGCATA CACAATTGCA   
  
  
- GCGAAAGGAT TACTACTTCG GCGGTCGAGG TAAAAGGTGG GATGTAAAAA AGGTTATCTA GTTAGGCGAC   
  
  
- TACTTCGATA TACATGTAAG TTAAAAAAAG AACTCGATTG ACCTTTTATA AAAGCACGGC AGATGCTGTG   
  
  
- GACAATTGGG ATATATATAG AGAAACCTAA AAAGACAGAT AAAAACATAT GAAGAAGTCC TTTTATAAAA   
  
  
- GCACGGCAGA TGCTGTGGAC AATTGGGATA TATATAGAGA AACCTAAAAA GACAGATAAA AACATATGAA   
  
  
- GAAGACCTAG TTAGAGGTAG AAATAGAAGA AGGAGAAGAA GACGAACTTC GAACTAGAAG AAGACATTGA   
  
  
- TATGTCACGC AACAAAGACA AAGGAAAAG

+     HD-Zip 1

| Site Name | Organism | Position | Strand | Matrix score. | sequence | function |
| --- | --- | --- | --- | --- | --- | --- |
| HD-Zip 1 | Arabidopsis thaliana | 835 | - | 8.5 | CAAT(A/T)ATTG | element involved in differentiation of the palisade mesophyll cells |

> 2018/04/13 10:10:12  
+ AAAAGGCAAC CTTTTCCTTT CTTTCTTCTT TCCTTAGCTT TTTAGATTTA TGAAGAAGGG CTGCTACGAC   
  
  
+ TTTCCTGCTT TTCCACATTT CAAACGCTCG TGTTATCGTC CTTTCATTCT CCCACTTCGT GTCCATCAGC   
  
  
+ TCCTTTTTAA AAAAATATAT TTATTTATTT TATTAAATTA TCATTTATTA TTATTTTTTA AAAAAAATTT   
  
  
+ AATACAGTTT TTTGGGGGAA AAAAAGAAAA AAAACTCATC CATCTGATTT GCTCTCTCTC TGATTTCCAA   
  
  
+ CCGAATGCAT GAGGAGAGCA TTCAGCGGCA ATCAAGCAGA GAGGACTGAC TGTCTTATGG GCCCTATCTC   
  
  
+ TTCTTGGATT CCAGACTATT CTTGTTTTTA GTAGCTCTCA AATCAATAAC CAGATCTAAT CCTAATACTC   
  
  
+ TCTTCATAAT CATAACTTAA AGAGGGAATT AATTAACCAA ATCAGGTAAA CAAACATCAT TAATTACAGC   
  
  
+ CACCTCGTTT GATCGATCCC GATCACCCAT GATCGTCACT TCCAGATCTT CACGTGTGCA ACCAATGAGC   
  
  
+ CATTAGTTGT AAATCAATTA ACATGTTTAA TCATAAAGAC TTCAAATTTT TGAAGTCAAC ATTCAAATGA   
  
  
+ TGCAGTTTGC TGAGATGGTA TTAGTGGCAC CACACAGCCC ACCCAAATGA ATTAACCATG AGACCTATTT   
  
  
+ ATGATCCTTC ATGGAAGTTC CTTAAGCCGC GCAGACATTC ACTATATAAT AGTAATTATA ACTTTCCACC   
  
  
+ TACCAACCAA GCATATGTAT ACTTTCAATA AATACATGTG CCCAAATTCC ATAAAATTAT AGAACAATTA   
  
  
+ TTGAATGAAT TTAATTATCA GATCAGTCCA AGACAGGCGC TTAGCTAGGT TAGCATAAAA TATCGTGCAC   
  
  
+ CTGTTATATC CTTATCAGCT CAGAGGCCTA ATACTGTTGC TAGTCTGGTA GCTCTACGAA TTTATTTCCA   
  
  
+ TGTAAATCAG AATTAAAAAT TATTATCATC TGTATAATCG TATGTGTTAA CGTCGCTTTC CTAATGATGA   
  
  
+ AGCCGCCAGC TCCATTTTCC ACCCTACATT TTTAAATTAT TATCATCTGT ATAATCGTAT GTGTTAACGT   
  
  
+ CGCTTTCCTA ATGATGAAGC CGCCAGCTCC ATTTTCCACC CTACATTTTT TCCAATAGAT CAATCCGCTG   
  
  
+ ATGAAGCTAT ATGTACATTC AATTTTTTTC TTGAGCTAAC TGGAAAATAT TTTCGTGCCG TCTACGACAC   
  
  
+ CTGTTAACCC TATATATATC TCTTTGGATT TTTCTGTCTA TTTTTGTATA CTTCTTCAGG AAAATATTTT   
  
  
+ CGTGCCGTCT ACGACACCTG TTAACCCTAT ATATATCTCT TTGGATTTTT CTGTCTATTT TTGTATACTT   
  
  
+ CTTCTGGATC AATCTCCATC TTTATCTTCT TCCTCTTCTT CTGCTTGAAG CTTGATCTTC TTCTGTAACT   
  
  
+ ATACAGTGCG TTGTTTCTGT TTCCTTTTC  

- TTTTCCGTTG GAAAAGGAAA GAAAGAAGAA AGGAATCGAA AAATCTAAAT ACTTCTTCCC GACGATGCTG   
  
  
- AAAGGACGAA AAGGTGTAAA GTTTGCGAGC ACAATAGCAG GAAAGTAAGA GGGTGAAGCA CAGGTAGTCG   
  
  
- AGGAAAAATT TTTTTATATA AATAAATAAA ATAATTTAAT AGTAAATAAT AATAAAAAAT TTTTTTTAAA   
  
  
- TTATGTCAAA AAACCCCCTT TTTTTCTTTT TTTTGAGTAG GTAGACTAAA CGAGAGAGAG ACTAAAGGTT   
  
  
- GGCTTACGTA CTCCTCTCGT AAGTCGCCGT TAGTTCGTCT CTCCTGACTG ACAGAATACC CGGGATAGAG   
  
  
- AAGAACCTAA GGTCTGATAA GAACAAAAAT CATCGAGAGT TTAGTTATTG GTCTAGATTA GGATTATGAG   
  
  
- AGAAGTATTA GTATTGAATT TCTCCCTTAA TTAATTGGTT TAGTCCATTT GTTTGTAGTA ATTAATGTCG   
  
  
- GTGGAGCAAA CTAGCTAGGG CTAGTGGGTA CTAGCAGTGA AGGTCTAGAA GTGCACACGT TGGTTACTCG   
  
  
- GTAATCAACA TTTAGTTAAT TGTACAAATT AGTATTTCTG AAGTTTAAAA ACTTCAGTTG TAAGTTTACT   
  
  
- ACGTCAAACG ACTCTACCAT AATCACCGTG GTGTGTCGGG TGGGTTTACT TAATTGGTAC TCTGGATAAA   
  
  
- TACTAGGAAG TACCTTCAAG GAATTCGGCG CGTCTGTAAG TGATATATTA TCATTAATAT TGAAAGGTGG   
  
  
- ATGGTTGGTT CGTATACATA TGAAAGTTAT TTATGTACAC GGGTTTAAGG TATTTTAATA TCTTGTTAAT   
  
  
- AACTTACTTA AATTAATAGT CTAGTCAGGT TCTGTCCGCG AATCGATCCA ATCGTATTTT ATAGCACGTG   
  
  
- GACAATATAG GAATAGTCGA GTCTCCGGAT TATGACAACG ATCAGACCAT CGAGATGCTT AAATAAAGGT   
  
  
- ACATTTAGTC TTAATTTTTA ATAATAGTAG ACATATTAGC ATACACAATT GCAGCGAAAG GATTACTACT   
  
  
- TCGGCGGTCG AGGTAAAAGG TGGGATGTAA AAATTTAATA ATAGTAGACA TATTAGCATA CACAATTGCA   
  
  
- GCGAAAGGAT TACTACTTCG GCGGTCGAGG TAAAAGGTGG GATGTAAAAA AGGTTATCTA GTTAGGCGAC   
  
  
- TACTTCGATA TACATGTAAG TTAAAAAAAG AACTCGATTG ACCTTTTATA AAAGCACGGC AGATGCTGTG   
  
  
- GACAATTGGG ATATATATAG AGAAACCTAA AAAGACAGAT AAAAACATAT GAAGAAGTCC TTTTATAAAA   
  
  
- GCACGGCAGA TGCTGTGGAC AATTGGGATA TATATAGAGA AACCTAAAAA GACAGATAAA AACATATGAA   
  
  
- GAAGACCTAG TTAGAGGTAG AAATAGAAGA AGGAGAAGAA GACGAACTTC GAACTAGAAG AAGACATTGA   
  
  
- TATGTCACGC AACAAAGACA AAGGAAAAG

+     HD-Zip 2

| Site Name | Organism | Position | Strand | Matrix score. | sequence | function |
| --- | --- | --- | --- | --- | --- | --- |
| HD-Zip 2 | Arabidopsis thaliana | 835 | - | 8 | CAAT(G/C)ATTG | element involved in the control of leaf morphology development |

> 2018/04/13 10:10:12  
+ AAAAGGCAAC CTTTTCCTTT CTTTCTTCTT TCCTTAGCTT TTTAGATTTA TGAAGAAGGG CTGCTACGAC   
  
  
+ TTTCCTGCTT TTCCACATTT CAAACGCTCG TGTTATCGTC CTTTCATTCT CCCACTTCGT GTCCATCAGC   
  
  
+ TCCTTTTTAA AAAAATATAT TTATTTATTT TATTAAATTA TCATTTATTA TTATTTTTTA AAAAAAATTT   
  
  
+ AATACAGTTT TTTGGGGGAA AAAAAGAAAA AAAACTCATC CATCTGATTT GCTCTCTCTC TGATTTCCAA   
  
  
+ CCGAATGCAT GAGGAGAGCA TTCAGCGGCA ATCAAGCAGA GAGGACTGAC TGTCTTATGG GCCCTATCTC   
  
  
+ TTCTTGGATT CCAGACTATT CTTGTTTTTA GTAGCTCTCA AATCAATAAC CAGATCTAAT CCTAATACTC   
  
  
+ TCTTCATAAT CATAACTTAA AGAGGGAATT AATTAACCAA ATCAGGTAAA CAAACATCAT TAATTACAGC   
  
  
+ CACCTCGTTT GATCGATCCC GATCACCCAT GATCGTCACT TCCAGATCTT CACGTGTGCA ACCAATGAGC   
  
  
+ CATTAGTTGT AAATCAATTA ACATGTTTAA TCATAAAGAC TTCAAATTTT TGAAGTCAAC ATTCAAATGA   
  
  
+ TGCAGTTTGC TGAGATGGTA TTAGTGGCAC CACACAGCCC ACCCAAATGA ATTAACCATG AGACCTATTT   
  
  
+ ATGATCCTTC ATGGAAGTTC CTTAAGCCGC GCAGACATTC ACTATATAAT AGTAATTATA ACTTTCCACC   
  
  
+ TACCAACCAA GCATATGTAT ACTTTCAATA AATACATGTG CCCAAATTCC ATAAAATTAT AGAACAATTA   
  
  
+ TTGAATGAAT TTAATTATCA GATCAGTCCA AGACAGGCGC TTAGCTAGGT TAGCATAAAA TATCGTGCAC   
  
  
+ CTGTTATATC CTTATCAGCT CAGAGGCCTA ATACTGTTGC TAGTCTGGTA GCTCTACGAA TTTATTTCCA   
  
  
+ TGTAAATCAG AATTAAAAAT TATTATCATC TGTATAATCG TATGTGTTAA CGTCGCTTTC CTAATGATGA   
  
  
+ AGCCGCCAGC TCCATTTTCC ACCCTACATT TTTAAATTAT TATCATCTGT ATAATCGTAT GTGTTAACGT   
  
  
+ CGCTTTCCTA ATGATGAAGC CGCCAGCTCC ATTTTCCACC CTACATTTTT TCCAATAGAT CAATCCGCTG   
  
  
+ ATGAAGCTAT ATGTACATTC AATTTTTTTC TTGAGCTAAC TGGAAAATAT TTTCGTGCCG TCTACGACAC   
  
  
+ CTGTTAACCC TATATATATC TCTTTGGATT TTTCTGTCTA TTTTTGTATA CTTCTTCAGG AAAATATTTT   
  
  
+ CGTGCCGTCT ACGACACCTG TTAACCCTAT ATATATCTCT TTGGATTTTT CTGTCTATTT TTGTATACTT   
  
  
+ CTTCTGGATC AATCTCCATC TTTATCTTCT TCCTCTTCTT CTGCTTGAAG CTTGATCTTC TTCTGTAACT   
  
  
+ ATACAGTGCG TTGTTTCTGT TTCCTTTTC  

- TTTTCCGTTG GAAAAGGAAA GAAAGAAGAA AGGAATCGAA AAATCTAAAT ACTTCTTCCC GACGATGCTG   
  
  
- AAAGGACGAA AAGGTGTAAA GTTTGCGAGC ACAATAGCAG GAAAGTAAGA GGGTGAAGCA CAGGTAGTCG   
  
  
- AGGAAAAATT TTTTTATATA AATAAATAAA ATAATTTAAT AGTAAATAAT AATAAAAAAT TTTTTTTAAA   
  
  
- TTATGTCAAA AAACCCCCTT TTTTTCTTTT TTTTGAGTAG GTAGACTAAA CGAGAGAGAG ACTAAAGGTT   
  
  
- GGCTTACGTA CTCCTCTCGT AAGTCGCCGT TAGTTCGTCT CTCCTGACTG ACAGAATACC CGGGATAGAG   
  
  
- AAGAACCTAA GGTCTGATAA GAACAAAAAT CATCGAGAGT TTAGTTATTG GTCTAGATTA GGATTATGAG   
  
  
- AGAAGTATTA GTATTGAATT TCTCCCTTAA TTAATTGGTT TAGTCCATTT GTTTGTAGTA ATTAATGTCG   
  
  
- GTGGAGCAAA CTAGCTAGGG CTAGTGGGTA CTAGCAGTGA AGGTCTAGAA GTGCACACGT TGGTTACTCG   
  
  
- GTAATCAACA TTTAGTTAAT TGTACAAATT AGTATTTCTG AAGTTTAAAA ACTTCAGTTG TAAGTTTACT   
  
  
- ACGTCAAACG ACTCTACCAT AATCACCGTG GTGTGTCGGG TGGGTTTACT TAATTGGTAC TCTGGATAAA   
  
  
- TACTAGGAAG TACCTTCAAG GAATTCGGCG CGTCTGTAAG TGATATATTA TCATTAATAT TGAAAGGTGG   
  
  
- ATGGTTGGTT CGTATACATA TGAAAGTTAT TTATGTACAC GGGTTTAAGG TATTTTAATA TCTTGTTAAT   
  
  
- AACTTACTTA AATTAATAGT CTAGTCAGGT TCTGTCCGCG AATCGATCCA ATCGTATTTT ATAGCACGTG   
  
  
- GACAATATAG GAATAGTCGA GTCTCCGGAT TATGACAACG ATCAGACCAT CGAGATGCTT AAATAAAGGT   
  
  
- ACATTTAGTC TTAATTTTTA ATAATAGTAG ACATATTAGC ATACACAATT GCAGCGAAAG GATTACTACT   
  
  
- TCGGCGGTCG AGGTAAAAGG TGGGATGTAA AAATTTAATA ATAGTAGACA TATTAGCATA CACAATTGCA   
  
  
- GCGAAAGGAT TACTACTTCG GCGGTCGAGG TAAAAGGTGG GATGTAAAAA AGGTTATCTA GTTAGGCGAC   
  
  
- TACTTCGATA TACATGTAAG TTAAAAAAAG AACTCGATTG ACCTTTTATA AAAGCACGGC AGATGCTGTG   
  
  
- GACAATTGGG ATATATATAG AGAAACCTAA AAAGACAGAT AAAAACATAT GAAGAAGTCC TTTTATAAAA   
  
  
- GCACGGCAGA TGCTGTGGAC AATTGGGATA TATATAGAGA AACCTAAAAA GACAGATAAA AACATATGAA   
  
  
- GAAGACCTAG TTAGAGGTAG AAATAGAAGA AGGAGAAGAA GACGAACTTC GAACTAGAAG AAGACATTGA   
  
  
- TATGTCACGC AACAAAGACA AAGGAAAAG

+     HSE

| Site Name | Organism | Position | Strand | Matrix score. | sequence | function |
| --- | --- | --- | --- | --- | --- | --- |
| HSE | Brassica oleracea | 202 | + | 9 | AAAAAATTTC | cis-acting element involved in heat stress responsiveness |

> 2018/04/13 10:10:12  
+ AAAAGGCAAC CTTTTCCTTT CTTTCTTCTT TCCTTAGCTT TTTAGATTTA TGAAGAAGGG CTGCTACGAC   
  
  
+ TTTCCTGCTT TTCCACATTT CAAACGCTCG TGTTATCGTC CTTTCATTCT CCCACTTCGT GTCCATCAGC   
  
  
+ TCCTTTTTAA AAAAATATAT TTATTTATTT TATTAAATTA TCATTTATTA TTATTTTTTA AAAAAAATTT   
  
  
+ AATACAGTTT TTTGGGGGAA AAAAAGAAAA AAAACTCATC CATCTGATTT GCTCTCTCTC TGATTTCCAA   
  
  
+ CCGAATGCAT GAGGAGAGCA TTCAGCGGCA ATCAAGCAGA GAGGACTGAC TGTCTTATGG GCCCTATCTC   
  
  
+ TTCTTGGATT CCAGACTATT CTTGTTTTTA GTAGCTCTCA AATCAATAAC CAGATCTAAT CCTAATACTC   
  
  
+ TCTTCATAAT CATAACTTAA AGAGGGAATT AATTAACCAA ATCAGGTAAA CAAACATCAT TAATTACAGC   
  
  
+ CACCTCGTTT GATCGATCCC GATCACCCAT GATCGTCACT TCCAGATCTT CACGTGTGCA ACCAATGAGC   
  
  
+ CATTAGTTGT AAATCAATTA ACATGTTTAA TCATAAAGAC TTCAAATTTT TGAAGTCAAC ATTCAAATGA   
  
  
+ TGCAGTTTGC TGAGATGGTA TTAGTGGCAC CACACAGCCC ACCCAAATGA ATTAACCATG AGACCTATTT   
  
  
+ ATGATCCTTC ATGGAAGTTC CTTAAGCCGC GCAGACATTC ACTATATAAT AGTAATTATA ACTTTCCACC   
  
  
+ TACCAACCAA GCATATGTAT ACTTTCAATA AATACATGTG CCCAAATTCC ATAAAATTAT AGAACAATTA   
  
  
+ TTGAATGAAT TTAATTATCA GATCAGTCCA AGACAGGCGC TTAGCTAGGT TAGCATAAAA TATCGTGCAC   
  
  
+ CTGTTATATC CTTATCAGCT CAGAGGCCTA ATACTGTTGC TAGTCTGGTA GCTCTACGAA TTTATTTCCA   
  
  
+ TGTAAATCAG AATTAAAAAT TATTATCATC TGTATAATCG TATGTGTTAA CGTCGCTTTC CTAATGATGA   
  
  
+ AGCCGCCAGC TCCATTTTCC ACCCTACATT TTTAAATTAT TATCATCTGT ATAATCGTAT GTGTTAACGT   
  
  
+ CGCTTTCCTA ATGATGAAGC CGCCAGCTCC ATTTTCCACC CTACATTTTT TCCAATAGAT CAATCCGCTG   
  
  
+ ATGAAGCTAT ATGTACATTC AATTTTTTTC TTGAGCTAAC TGGAAAATAT TTTCGTGCCG TCTACGACAC   
  
  
+ CTGTTAACCC TATATATATC TCTTTGGATT TTTCTGTCTA TTTTTGTATA CTTCTTCAGG AAAATATTTT   
  
  
+ CGTGCCGTCT ACGACACCTG TTAACCCTAT ATATATCTCT TTGGATTTTT CTGTCTATTT TTGTATACTT   
  
  
+ CTTCTGGATC AATCTCCATC TTTATCTTCT TCCTCTTCTT CTGCTTGAAG CTTGATCTTC TTCTGTAACT   
  
  
+ ATACAGTGCG TTGTTTCTGT TTCCTTTTC  

- TTTTCCGTTG GAAAAGGAAA GAAAGAAGAA AGGAATCGAA AAATCTAAAT ACTTCTTCCC GACGATGCTG   
  
  
- AAAGGACGAA AAGGTGTAAA GTTTGCGAGC ACAATAGCAG GAAAGTAAGA GGGTGAAGCA CAGGTAGTCG   
  
  
- AGGAAAAATT TTTTTATATA AATAAATAAA ATAATTTAAT AGTAAATAAT AATAAAAAAT TTTTTTTAAA   
  
  
- TTATGTCAAA AAACCCCCTT TTTTTCTTTT TTTTGAGTAG GTAGACTAAA CGAGAGAGAG ACTAAAGGTT   
  
  
- GGCTTACGTA CTCCTCTCGT AAGTCGCCGT TAGTTCGTCT CTCCTGACTG ACAGAATACC CGGGATAGAG   
  
  
- AAGAACCTAA GGTCTGATAA GAACAAAAAT CATCGAGAGT TTAGTTATTG GTCTAGATTA GGATTATGAG   
  
  
- AGAAGTATTA GTATTGAATT TCTCCCTTAA TTAATTGGTT TAGTCCATTT GTTTGTAGTA ATTAATGTCG   
  
  
- GTGGAGCAAA CTAGCTAGGG CTAGTGGGTA CTAGCAGTGA AGGTCTAGAA GTGCACACGT TGGTTACTCG   
  
  
- GTAATCAACA TTTAGTTAAT TGTACAAATT AGTATTTCTG AAGTTTAAAA ACTTCAGTTG TAAGTTTACT   
  
  
- ACGTCAAACG ACTCTACCAT AATCACCGTG GTGTGTCGGG TGGGTTTACT TAATTGGTAC TCTGGATAAA   
  
  
- TACTAGGAAG TACCTTCAAG GAATTCGGCG CGTCTGTAAG TGATATATTA TCATTAATAT TGAAAGGTGG   
  
  
- ATGGTTGGTT CGTATACATA TGAAAGTTAT TTATGTACAC GGGTTTAAGG TATTTTAATA TCTTGTTAAT   
  
  
- AACTTACTTA AATTAATAGT CTAGTCAGGT TCTGTCCGCG AATCGATCCA ATCGTATTTT ATAGCACGTG   
  
  
- GACAATATAG GAATAGTCGA GTCTCCGGAT TATGACAACG ATCAGACCAT CGAGATGCTT AAATAAAGGT   
  
  
- ACATTTAGTC TTAATTTTTA ATAATAGTAG ACATATTAGC ATACACAATT GCAGCGAAAG GATTACTACT   
  
  
- TCGGCGGTCG AGGTAAAAGG TGGGATGTAA AAATTTAATA ATAGTAGACA TATTAGCATA CACAATTGCA   
  
  
- GCGAAAGGAT TACTACTTCG GCGGTCGAGG TAAAAGGTGG GATGTAAAAA AGGTTATCTA GTTAGGCGAC   
  
  
- TACTTCGATA TACATGTAAG TTAAAAAAAG AACTCGATTG ACCTTTTATA AAAGCACGGC AGATGCTGTG   
  
  
- GACAATTGGG ATATATATAG AGAAACCTAA AAAGACAGAT AAAAACATAT GAAGAAGTCC TTTTATAAAA   
  
  
- GCACGGCAGA TGCTGTGGAC AATTGGGATA TATATAGAGA AACCTAAAAA GACAGATAAA AACATATGAA   
  
  
- GAAGACCTAG TTAGAGGTAG AAATAGAAGA AGGAGAAGAA GACGAACTTC GAACTAGAAG AAGACATTGA   
  
  
- TATGTCACGC AACAAAGACA AAGGAAAAG

+     I-box

| Site Name | Organism | Position | Strand | Matrix score. | sequence | function |
| --- | --- | --- | --- | --- | --- | --- |
| I-box | Larix laricina | 935 | - | 9 | GTATAAGGCC | part of a light responsive element |
| I-box | Zea mays | 342 | - | 7 | GATAGGG | part of a light responsive element |

> 2018/04/13 10:10:12  
+ AAAAGGCAAC CTTTTCCTTT CTTTCTTCTT TCCTTAGCTT TTTAGATTTA TGAAGAAGGG CTGCTACGAC   
  
  
+ TTTCCTGCTT TTCCACATTT CAAACGCTCG TGTTATCGTC CTTTCATTCT CCCACTTCGT GTCCATCAGC   
  
  
+ TCCTTTTTAA AAAAATATAT TTATTTATTT TATTAAATTA TCATTTATTA TTATTTTTTA AAAAAAATTT   
  
  
+ AATACAGTTT TTTGGGGGAA AAAAAGAAAA AAAACTCATC CATCTGATTT GCTCTCTCTC TGATTTCCAA   
  
  
+ CCGAATGCAT GAGGAGAGCA TTCAGCGGCA ATCAAGCAGA GAGGACTGAC TGTCTTATGG GCCCTATCTC   
  
  
+ TTCTTGGATT CCAGACTATT CTTGTTTTTA GTAGCTCTCA AATCAATAAC CAGATCTAAT CCTAATACTC   
  
  
+ TCTTCATAAT CATAACTTAA AGAGGGAATT AATTAACCAA ATCAGGTAAA CAAACATCAT TAATTACAGC   
  
  
+ CACCTCGTTT GATCGATCCC GATCACCCAT GATCGTCACT TCCAGATCTT CACGTGTGCA ACCAATGAGC   
  
  
+ CATTAGTTGT AAATCAATTA ACATGTTTAA TCATAAAGAC TTCAAATTTT TGAAGTCAAC ATTCAAATGA   
  
  
+ TGCAGTTTGC TGAGATGGTA TTAGTGGCAC CACACAGCCC ACCCAAATGA ATTAACCATG AGACCTATTT   
  
  
+ ATGATCCTTC ATGGAAGTTC CTTAAGCCGC GCAGACATTC ACTATATAAT AGTAATTATA ACTTTCCACC   
  
  
+ TACCAACCAA GCATATGTAT ACTTTCAATA AATACATGTG CCCAAATTCC ATAAAATTAT AGAACAATTA   
  
  
+ TTGAATGAAT TTAATTATCA GATCAGTCCA AGACAGGCGC TTAGCTAGGT TAGCATAAAA TATCGTGCAC   
  
  
+ CTGTTATATC CTTATCAGCT CAGAGGCCTA ATACTGTTGC TAGTCTGGTA GCTCTACGAA TTTATTTCCA   
  
  
+ TGTAAATCAG AATTAAAAAT TATTATCATC TGTATAATCG TATGTGTTAA CGTCGCTTTC CTAATGATGA   
  
  
+ AGCCGCCAGC TCCATTTTCC ACCCTACATT TTTAAATTAT TATCATCTGT ATAATCGTAT GTGTTAACGT   
  
  
+ CGCTTTCCTA ATGATGAAGC CGCCAGCTCC ATTTTCCACC CTACATTTTT TCCAATAGAT CAATCCGCTG   
  
  
+ ATGAAGCTAT ATGTACATTC AATTTTTTTC TTGAGCTAAC TGGAAAATAT TTTCGTGCCG TCTACGACAC   
  
  
+ CTGTTAACCC TATATATATC TCTTTGGATT TTTCTGTCTA TTTTTGTATA CTTCTTCAGG AAAATATTTT   
  
  
+ CGTGCCGTCT ACGACACCTG TTAACCCTAT ATATATCTCT TTGGATTTTT CTGTCTATTT TTGTATACTT   
  
  
+ CTTCTGGATC AATCTCCATC TTTATCTTCT TCCTCTTCTT CTGCTTGAAG CTTGATCTTC TTCTGTAACT   
  
  
+ ATACAGTGCG TTGTTTCTGT TTCCTTTTC  

- TTTTCCGTTG GAAAAGGAAA GAAAGAAGAA AGGAATCGAA AAATCTAAAT ACTTCTTCCC GACGATGCTG   
  
  
- AAAGGACGAA AAGGTGTAAA GTTTGCGAGC ACAATAGCAG GAAAGTAAGA GGGTGAAGCA CAGGTAGTCG   
  
  
- AGGAAAAATT TTTTTATATA AATAAATAAA ATAATTTAAT AGTAAATAAT AATAAAAAAT TTTTTTTAAA   
  
  
- TTATGTCAAA AAACCCCCTT TTTTTCTTTT TTTTGAGTAG GTAGACTAAA CGAGAGAGAG ACTAAAGGTT   
  
  
- GGCTTACGTA CTCCTCTCGT AAGTCGCCGT TAGTTCGTCT CTCCTGACTG ACAGAATACC CGGGATAGAG   
  
  
- AAGAACCTAA GGTCTGATAA GAACAAAAAT CATCGAGAGT TTAGTTATTG GTCTAGATTA GGATTATGAG   
  
  
- AGAAGTATTA GTATTGAATT TCTCCCTTAA TTAATTGGTT TAGTCCATTT GTTTGTAGTA ATTAATGTCG   
  
  
- GTGGAGCAAA CTAGCTAGGG CTAGTGGGTA CTAGCAGTGA AGGTCTAGAA GTGCACACGT TGGTTACTCG   
  
  
- GTAATCAACA TTTAGTTAAT TGTACAAATT AGTATTTCTG AAGTTTAAAA ACTTCAGTTG TAAGTTTACT   
  
  
- ACGTCAAACG ACTCTACCAT AATCACCGTG GTGTGTCGGG TGGGTTTACT TAATTGGTAC TCTGGATAAA   
  
  
- TACTAGGAAG TACCTTCAAG GAATTCGGCG CGTCTGTAAG TGATATATTA TCATTAATAT TGAAAGGTGG   
  
  
- ATGGTTGGTT CGTATACATA TGAAAGTTAT TTATGTACAC GGGTTTAAGG TATTTTAATA TCTTGTTAAT   
  
  
- AACTTACTTA AATTAATAGT CTAGTCAGGT TCTGTCCGCG AATCGATCCA ATCGTATTTT ATAGCACGTG   
  
  
- GACAATATAG GAATAGTCGA GTCTCCGGAT TATGACAACG ATCAGACCAT CGAGATGCTT AAATAAAGGT   
  
  
- ACATTTAGTC TTAATTTTTA ATAATAGTAG ACATATTAGC ATACACAATT GCAGCGAAAG GATTACTACT   
  
  
- TCGGCGGTCG AGGTAAAAGG TGGGATGTAA AAATTTAATA ATAGTAGACA TATTAGCATA CACAATTGCA   
  
  
- GCGAAAGGAT TACTACTTCG GCGGTCGAGG TAAAAGGTGG GATGTAAAAA AGGTTATCTA GTTAGGCGAC   
  
  
- TACTTCGATA TACATGTAAG TTAAAAAAAG AACTCGATTG ACCTTTTATA AAAGCACGGC AGATGCTGTG   
  
  
- GACAATTGGG ATATATATAG AGAAACCTAA AAAGACAGAT AAAAACATAT GAAGAAGTCC TTTTATAAAA   
  
  
- GCACGGCAGA TGCTGTGGAC AATTGGGATA TATATAGAGA AACCTAAAAA GACAGATAAA AACATATGAA   
  
  
- GAAGACCTAG TTAGAGGTAG AAATAGAAGA AGGAGAAGAA GACGAACTTC GAACTAGAAG AAGACATTGA   
  
  
- TATGTCACGC AACAAAGACA AAGGAAAAG

+     MBS

| Site Name | Organism | Position | Strand | Matrix score. | sequence | function |
| --- | --- | --- | --- | --- | --- | --- |
| MBS | Arabidopsis thaliana | 1227 | + | 6 | TAACTG | MYB binding site involved in drought-inducibility |

> 2018/04/13 10:10:12  
+ AAAAGGCAAC CTTTTCCTTT CTTTCTTCTT TCCTTAGCTT TTTAGATTTA TGAAGAAGGG CTGCTACGAC   
  
  
+ TTTCCTGCTT TTCCACATTT CAAACGCTCG TGTTATCGTC CTTTCATTCT CCCACTTCGT GTCCATCAGC   
  
  
+ TCCTTTTTAA AAAAATATAT TTATTTATTT TATTAAATTA TCATTTATTA TTATTTTTTA AAAAAAATTT   
  
  
+ AATACAGTTT TTTGGGGGAA AAAAAGAAAA AAAACTCATC CATCTGATTT GCTCTCTCTC TGATTTCCAA   
  
  
+ CCGAATGCAT GAGGAGAGCA TTCAGCGGCA ATCAAGCAGA GAGGACTGAC TGTCTTATGG GCCCTATCTC   
  
  
+ TTCTTGGATT CCAGACTATT CTTGTTTTTA GTAGCTCTCA AATCAATAAC CAGATCTAAT CCTAATACTC   
  
  
+ TCTTCATAAT CATAACTTAA AGAGGGAATT AATTAACCAA ATCAGGTAAA CAAACATCAT TAATTACAGC   
  
  
+ CACCTCGTTT GATCGATCCC GATCACCCAT GATCGTCACT TCCAGATCTT CACGTGTGCA ACCAATGAGC   
  
  
+ CATTAGTTGT AAATCAATTA ACATGTTTAA TCATAAAGAC TTCAAATTTT TGAAGTCAAC ATTCAAATGA   
  
  
+ TGCAGTTTGC TGAGATGGTA TTAGTGGCAC CACACAGCCC ACCCAAATGA ATTAACCATG AGACCTATTT   
  
  
+ ATGATCCTTC ATGGAAGTTC CTTAAGCCGC GCAGACATTC ACTATATAAT AGTAATTATA ACTTTCCACC   
  
  
+ TACCAACCAA GCATATGTAT ACTTTCAATA AATACATGTG CCCAAATTCC ATAAAATTAT AGAACAATTA   
  
  
+ TTGAATGAAT TTAATTATCA GATCAGTCCA AGACAGGCGC TTAGCTAGGT TAGCATAAAA TATCGTGCAC   
  
  
+ CTGTTATATC CTTATCAGCT CAGAGGCCTA ATACTGTTGC TAGTCTGGTA GCTCTACGAA TTTATTTCCA   
  
  
+ TGTAAATCAG AATTAAAAAT TATTATCATC TGTATAATCG TATGTGTTAA CGTCGCTTTC CTAATGATGA   
  
  
+ AGCCGCCAGC TCCATTTTCC ACCCTACATT TTTAAATTAT TATCATCTGT ATAATCGTAT GTGTTAACGT   
  
  
+ CGCTTTCCTA ATGATGAAGC CGCCAGCTCC ATTTTCCACC CTACATTTTT TCCAATAGAT CAATCCGCTG   
  
  
+ ATGAAGCTAT ATGTACATTC AATTTTTTTC TTGAGCTAAC TGGAAAATAT TTTCGTGCCG TCTACGACAC   
  
  
+ CTGTTAACCC TATATATATC TCTTTGGATT TTTCTGTCTA TTTTTGTATA CTTCTTCAGG AAAATATTTT   
  
  
+ CGTGCCGTCT ACGACACCTG TTAACCCTAT ATATATCTCT TTGGATTTTT CTGTCTATTT TTGTATACTT   
  
  
+ CTTCTGGATC AATCTCCATC TTTATCTTCT TCCTCTTCTT CTGCTTGAAG CTTGATCTTC TTCTGTAACT   
  
  
+ ATACAGTGCG TTGTTTCTGT TTCCTTTTC  

- TTTTCCGTTG GAAAAGGAAA GAAAGAAGAA AGGAATCGAA AAATCTAAAT ACTTCTTCCC GACGATGCTG   
  
  
- AAAGGACGAA AAGGTGTAAA GTTTGCGAGC ACAATAGCAG GAAAGTAAGA GGGTGAAGCA CAGGTAGTCG   
  
  
- AGGAAAAATT TTTTTATATA AATAAATAAA ATAATTTAAT AGTAAATAAT AATAAAAAAT TTTTTTTAAA   
  
  
- TTATGTCAAA AAACCCCCTT TTTTTCTTTT TTTTGAGTAG GTAGACTAAA CGAGAGAGAG ACTAAAGGTT   
  
  
- GGCTTACGTA CTCCTCTCGT AAGTCGCCGT TAGTTCGTCT CTCCTGACTG ACAGAATACC CGGGATAGAG   
  
  
- AAGAACCTAA GGTCTGATAA GAACAAAAAT CATCGAGAGT TTAGTTATTG GTCTAGATTA GGATTATGAG   
  
  
- AGAAGTATTA GTATTGAATT TCTCCCTTAA TTAATTGGTT TAGTCCATTT GTTTGTAGTA ATTAATGTCG   
  
  
- GTGGAGCAAA CTAGCTAGGG CTAGTGGGTA CTAGCAGTGA AGGTCTAGAA GTGCACACGT TGGTTACTCG   
  
  
- GTAATCAACA TTTAGTTAAT TGTACAAATT AGTATTTCTG AAGTTTAAAA ACTTCAGTTG TAAGTTTACT   
  
  
- ACGTCAAACG ACTCTACCAT AATCACCGTG GTGTGTCGGG TGGGTTTACT TAATTGGTAC TCTGGATAAA   
  
  
- TACTAGGAAG TACCTTCAAG GAATTCGGCG CGTCTGTAAG TGATATATTA TCATTAATAT TGAAAGGTGG   
  
  
- ATGGTTGGTT CGTATACATA TGAAAGTTAT TTATGTACAC GGGTTTAAGG TATTTTAATA TCTTGTTAAT   
  
  
- AACTTACTTA AATTAATAGT CTAGTCAGGT TCTGTCCGCG AATCGATCCA ATCGTATTTT ATAGCACGTG   
  
  
- GACAATATAG GAATAGTCGA GTCTCCGGAT TATGACAACG ATCAGACCAT CGAGATGCTT AAATAAAGGT   
  
  
- ACATTTAGTC TTAATTTTTA ATAATAGTAG ACATATTAGC ATACACAATT GCAGCGAAAG GATTACTACT   
  
  
- TCGGCGGTCG AGGTAAAAGG TGGGATGTAA AAATTTAATA ATAGTAGACA TATTAGCATA CACAATTGCA   
  
  
- GCGAAAGGAT TACTACTTCG GCGGTCGAGG TAAAAGGTGG GATGTAAAAA AGGTTATCTA GTTAGGCGAC   
  
  
- TACTTCGATA TACATGTAAG TTAAAAAAAG AACTCGATTG ACCTTTTATA AAAGCACGGC AGATGCTGTG   
  
  
- GACAATTGGG ATATATATAG AGAAACCTAA AAAGACAGAT AAAAACATAT GAAGAAGTCC TTTTATAAAA   
  
  
- GCACGGCAGA TGCTGTGGAC AATTGGGATA TATATAGAGA AACCTAAAAA GACAGATAAA AACATATGAA   
  
  
- GAAGACCTAG TTAGAGGTAG AAATAGAAGA AGGAGAAGAA GACGAACTTC GAACTAGAAG AAGACATTGA   
  
  
- TATGTCACGC AACAAAGACA AAGGAAAAG

+     MNF1

| Site Name | Organism | Position | Strand | Matrix score. | sequence | function |
| --- | --- | --- | --- | --- | --- | --- |
| MNF1 | Zea mays | 808 | + | 7 | GTGCCC(A/T)(A/T) | light responsive element |

> 2018/04/13 10:10:12  
+ AAAAGGCAAC CTTTTCCTTT CTTTCTTCTT TCCTTAGCTT TTTAGATTTA TGAAGAAGGG CTGCTACGAC   
  
  
+ TTTCCTGCTT TTCCACATTT CAAACGCTCG TGTTATCGTC CTTTCATTCT CCCACTTCGT GTCCATCAGC   
  
  
+ TCCTTTTTAA AAAAATATAT TTATTTATTT TATTAAATTA TCATTTATTA TTATTTTTTA AAAAAAATTT   
  
  
+ AATACAGTTT TTTGGGGGAA AAAAAGAAAA AAAACTCATC CATCTGATTT GCTCTCTCTC TGATTTCCAA   
  
  
+ CCGAATGCAT GAGGAGAGCA TTCAGCGGCA ATCAAGCAGA GAGGACTGAC TGTCTTATGG GCCCTATCTC   
  
  
+ TTCTTGGATT CCAGACTATT CTTGTTTTTA GTAGCTCTCA AATCAATAAC CAGATCTAAT CCTAATACTC   
  
  
+ TCTTCATAAT CATAACTTAA AGAGGGAATT AATTAACCAA ATCAGGTAAA CAAACATCAT TAATTACAGC   
  
  
+ CACCTCGTTT GATCGATCCC GATCACCCAT GATCGTCACT TCCAGATCTT CACGTGTGCA ACCAATGAGC   
  
  
+ CATTAGTTGT AAATCAATTA ACATGTTTAA TCATAAAGAC TTCAAATTTT TGAAGTCAAC ATTCAAATGA   
  
  
+ TGCAGTTTGC TGAGATGGTA TTAGTGGCAC CACACAGCCC ACCCAAATGA ATTAACCATG AGACCTATTT   
  
  
+ ATGATCCTTC ATGGAAGTTC CTTAAGCCGC GCAGACATTC ACTATATAAT AGTAATTATA ACTTTCCACC   
  
  
+ TACCAACCAA GCATATGTAT ACTTTCAATA AATACATGTG CCCAAATTCC ATAAAATTAT AGAACAATTA   
  
  
+ TTGAATGAAT TTAATTATCA GATCAGTCCA AGACAGGCGC TTAGCTAGGT TAGCATAAAA TATCGTGCAC   
  
  
+ CTGTTATATC CTTATCAGCT CAGAGGCCTA ATACTGTTGC TAGTCTGGTA GCTCTACGAA TTTATTTCCA   
  
  
+ TGTAAATCAG AATTAAAAAT TATTATCATC TGTATAATCG TATGTGTTAA CGTCGCTTTC CTAATGATGA   
  
  
+ AGCCGCCAGC TCCATTTTCC ACCCTACATT TTTAAATTAT TATCATCTGT ATAATCGTAT GTGTTAACGT   
  
  
+ CGCTTTCCTA ATGATGAAGC CGCCAGCTCC ATTTTCCACC CTACATTTTT TCCAATAGAT CAATCCGCTG   
  
  
+ ATGAAGCTAT ATGTACATTC AATTTTTTTC TTGAGCTAAC TGGAAAATAT TTTCGTGCCG TCTACGACAC   
  
  
+ CTGTTAACCC TATATATATC TCTTTGGATT TTTCTGTCTA TTTTTGTATA CTTCTTCAGG AAAATATTTT   
  
  
+ CGTGCCGTCT ACGACACCTG TTAACCCTAT ATATATCTCT TTGGATTTTT CTGTCTATTT TTGTATACTT   
  
  
+ CTTCTGGATC AATCTCCATC TTTATCTTCT TCCTCTTCTT CTGCTTGAAG CTTGATCTTC TTCTGTAACT   
  
  
+ ATACAGTGCG TTGTTTCTGT TTCCTTTTC  

- TTTTCCGTTG GAAAAGGAAA GAAAGAAGAA AGGAATCGAA AAATCTAAAT ACTTCTTCCC GACGATGCTG   
  
  
- AAAGGACGAA AAGGTGTAAA GTTTGCGAGC ACAATAGCAG GAAAGTAAGA GGGTGAAGCA CAGGTAGTCG   
  
  
- AGGAAAAATT TTTTTATATA AATAAATAAA ATAATTTAAT AGTAAATAAT AATAAAAAAT TTTTTTTAAA   
  
  
- TTATGTCAAA AAACCCCCTT TTTTTCTTTT TTTTGAGTAG GTAGACTAAA CGAGAGAGAG ACTAAAGGTT   
  
  
- GGCTTACGTA CTCCTCTCGT AAGTCGCCGT TAGTTCGTCT CTCCTGACTG ACAGAATACC CGGGATAGAG   
  
  
- AAGAACCTAA GGTCTGATAA GAACAAAAAT CATCGAGAGT TTAGTTATTG GTCTAGATTA GGATTATGAG   
  
  
- AGAAGTATTA GTATTGAATT TCTCCCTTAA TTAATTGGTT TAGTCCATTT GTTTGTAGTA ATTAATGTCG   
  
  
- GTGGAGCAAA CTAGCTAGGG CTAGTGGGTA CTAGCAGTGA AGGTCTAGAA GTGCACACGT TGGTTACTCG   
  
  
- GTAATCAACA TTTAGTTAAT TGTACAAATT AGTATTTCTG AAGTTTAAAA ACTTCAGTTG TAAGTTTACT   
  
  
- ACGTCAAACG ACTCTACCAT AATCACCGTG GTGTGTCGGG TGGGTTTACT TAATTGGTAC TCTGGATAAA   
  
  
- TACTAGGAAG TACCTTCAAG GAATTCGGCG CGTCTGTAAG TGATATATTA TCATTAATAT TGAAAGGTGG   
  
  
- ATGGTTGGTT CGTATACATA TGAAAGTTAT TTATGTACAC GGGTTTAAGG TATTTTAATA TCTTGTTAAT   
  
  
- AACTTACTTA AATTAATAGT CTAGTCAGGT TCTGTCCGCG AATCGATCCA ATCGTATTTT ATAGCACGTG   
  
  
- GACAATATAG GAATAGTCGA GTCTCCGGAT TATGACAACG ATCAGACCAT CGAGATGCTT AAATAAAGGT   
  
  
- ACATTTAGTC TTAATTTTTA ATAATAGTAG ACATATTAGC ATACACAATT GCAGCGAAAG GATTACTACT   
  
  
- TCGGCGGTCG AGGTAAAAGG TGGGATGTAA AAATTTAATA ATAGTAGACA TATTAGCATA CACAATTGCA   
  
  
- GCGAAAGGAT TACTACTTCG GCGGTCGAGG TAAAAGGTGG GATGTAAAAA AGGTTATCTA GTTAGGCGAC   
  
  
- TACTTCGATA TACATGTAAG TTAAAAAAAG AACTCGATTG ACCTTTTATA AAAGCACGGC AGATGCTGTG   
  
  
- GACAATTGGG ATATATATAG AGAAACCTAA AAAGACAGAT AAAAACATAT GAAGAAGTCC TTTTATAAAA   
  
  
- GCACGGCAGA TGCTGTGGAC AATTGGGATA TATATAGAGA AACCTAAAAA GACAGATAAA AACATATGAA   
  
  
- GAAGACCTAG TTAGAGGTAG AAATAGAAGA AGGAGAAGAA GACGAACTTC GAACTAGAAG AAGACATTGA   
  
  
- TATGTCACGC AACAAAGACA AAGGAAAAG

+     Sp1

| Site Name | Organism | Position | Strand | Matrix score. | sequence | function |
| --- | --- | --- | --- | --- | --- | --- |
| Sp1 | Zea mays | 1156 | + | 5.5 | CC(G/A)CCC | light responsive element |
| Sp1 | Zea mays | 669 | + | 5.5 | CC(G/A)CCC | light responsive element |
| Sp1 | Zea mays | 1069 | + | 5.5 | CC(G/A)CCC | light responsive element |

> 2018/04/13 10:10:12  
+ AAAAGGCAAC CTTTTCCTTT CTTTCTTCTT TCCTTAGCTT TTTAGATTTA TGAAGAAGGG CTGCTACGAC   
  
  
+ TTTCCTGCTT TTCCACATTT CAAACGCTCG TGTTATCGTC CTTTCATTCT CCCACTTCGT GTCCATCAGC   
  
  
+ TCCTTTTTAA AAAAATATAT TTATTTATTT TATTAAATTA TCATTTATTA TTATTTTTTA AAAAAAATTT   
  
  
+ AATACAGTTT TTTGGGGGAA AAAAAGAAAA AAAACTCATC CATCTGATTT GCTCTCTCTC TGATTTCCAA   
  
  
+ CCGAATGCAT GAGGAGAGCA TTCAGCGGCA ATCAAGCAGA GAGGACTGAC TGTCTTATGG GCCCTATCTC   
  
  
+ TTCTTGGATT CCAGACTATT CTTGTTTTTA GTAGCTCTCA AATCAATAAC CAGATCTAAT CCTAATACTC   
  
  
+ TCTTCATAAT CATAACTTAA AGAGGGAATT AATTAACCAA ATCAGGTAAA CAAACATCAT TAATTACAGC   
  
  
+ CACCTCGTTT GATCGATCCC GATCACCCAT GATCGTCACT TCCAGATCTT CACGTGTGCA ACCAATGAGC   
  
  
+ CATTAGTTGT AAATCAATTA ACATGTTTAA TCATAAAGAC TTCAAATTTT TGAAGTCAAC ATTCAAATGA   
  
  
+ TGCAGTTTGC TGAGATGGTA TTAGTGGCAC CACACAGCCC ACCCAAATGA ATTAACCATG AGACCTATTT   
  
  
+ ATGATCCTTC ATGGAAGTTC CTTAAGCCGC GCAGACATTC ACTATATAAT AGTAATTATA ACTTTCCACC   
  
  
+ TACCAACCAA GCATATGTAT ACTTTCAATA AATACATGTG CCCAAATTCC ATAAAATTAT AGAACAATTA   
  
  
+ TTGAATGAAT TTAATTATCA GATCAGTCCA AGACAGGCGC TTAGCTAGGT TAGCATAAAA TATCGTGCAC   
  
  
+ CTGTTATATC CTTATCAGCT CAGAGGCCTA ATACTGTTGC TAGTCTGGTA GCTCTACGAA TTTATTTCCA   
  
  
+ TGTAAATCAG AATTAAAAAT TATTATCATC TGTATAATCG TATGTGTTAA CGTCGCTTTC CTAATGATGA   
  
  
+ AGCCGCCAGC TCCATTTTCC ACCCTACATT TTTAAATTAT TATCATCTGT ATAATCGTAT GTGTTAACGT   
  
  
+ CGCTTTCCTA ATGATGAAGC CGCCAGCTCC ATTTTCCACC CTACATTTTT TCCAATAGAT CAATCCGCTG   
  
  
+ ATGAAGCTAT ATGTACATTC AATTTTTTTC TTGAGCTAAC TGGAAAATAT TTTCGTGCCG TCTACGACAC   
  
  
+ CTGTTAACCC TATATATATC TCTTTGGATT TTTCTGTCTA TTTTTGTATA CTTCTTCAGG AAAATATTTT   
  
  
+ CGTGCCGTCT ACGACACCTG TTAACCCTAT ATATATCTCT TTGGATTTTT CTGTCTATTT TTGTATACTT   
  
  
+ CTTCTGGATC AATCTCCATC TTTATCTTCT TCCTCTTCTT CTGCTTGAAG CTTGATCTTC TTCTGTAACT   
  
  
+ ATACAGTGCG TTGTTTCTGT TTCCTTTTC  

- TTTTCCGTTG GAAAAGGAAA GAAAGAAGAA AGGAATCGAA AAATCTAAAT ACTTCTTCCC GACGATGCTG   
  
  
- AAAGGACGAA AAGGTGTAAA GTTTGCGAGC ACAATAGCAG GAAAGTAAGA GGGTGAAGCA CAGGTAGTCG   
  
  
- AGGAAAAATT TTTTTATATA AATAAATAAA ATAATTTAAT AGTAAATAAT AATAAAAAAT TTTTTTTAAA   
  
  
- TTATGTCAAA AAACCCCCTT TTTTTCTTTT TTTTGAGTAG GTAGACTAAA CGAGAGAGAG ACTAAAGGTT   
  
  
- GGCTTACGTA CTCCTCTCGT AAGTCGCCGT TAGTTCGTCT CTCCTGACTG ACAGAATACC CGGGATAGAG   
  
  
- AAGAACCTAA GGTCTGATAA GAACAAAAAT CATCGAGAGT TTAGTTATTG GTCTAGATTA GGATTATGAG   
  
  
- AGAAGTATTA GTATTGAATT TCTCCCTTAA TTAATTGGTT TAGTCCATTT GTTTGTAGTA ATTAATGTCG   
  
  
- GTGGAGCAAA CTAGCTAGGG CTAGTGGGTA CTAGCAGTGA AGGTCTAGAA GTGCACACGT TGGTTACTCG   
  
  
- GTAATCAACA TTTAGTTAAT TGTACAAATT AGTATTTCTG AAGTTTAAAA ACTTCAGTTG TAAGTTTACT   
  
  
- ACGTCAAACG ACTCTACCAT AATCACCGTG GTGTGTCGGG TGGGTTTACT TAATTGGTAC TCTGGATAAA   
  
  
- TACTAGGAAG TACCTTCAAG GAATTCGGCG CGTCTGTAAG TGATATATTA TCATTAATAT TGAAAGGTGG   
  
  
- ATGGTTGGTT CGTATACATA TGAAAGTTAT TTATGTACAC GGGTTTAAGG TATTTTAATA TCTTGTTAAT   
  
  
- AACTTACTTA AATTAATAGT CTAGTCAGGT TCTGTCCGCG AATCGATCCA ATCGTATTTT ATAGCACGTG   
  
  
- GACAATATAG GAATAGTCGA GTCTCCGGAT TATGACAACG ATCAGACCAT CGAGATGCTT AAATAAAGGT   
  
  
- ACATTTAGTC TTAATTTTTA ATAATAGTAG ACATATTAGC ATACACAATT GCAGCGAAAG GATTACTACT   
  
  
- TCGGCGGTCG AGGTAAAAGG TGGGATGTAA AAATTTAATA ATAGTAGACA TATTAGCATA CACAATTGCA   
  
  
- GCGAAAGGAT TACTACTTCG GCGGTCGAGG TAAAAGGTGG GATGTAAAAA AGGTTATCTA GTTAGGCGAC   
  
  
- TACTTCGATA TACATGTAAG TTAAAAAAAG AACTCGATTG ACCTTTTATA AAAGCACGGC AGATGCTGTG   
  
  
- GACAATTGGG ATATATATAG AGAAACCTAA AAAGACAGAT AAAAACATAT GAAGAAGTCC TTTTATAAAA   
  
  
- GCACGGCAGA TGCTGTGGAC AATTGGGATA TATATAGAGA AACCTAAAAA GACAGATAAA AACATATGAA   
  
  
- GAAGACCTAG TTAGAGGTAG AAATAGAAGA AGGAGAAGAA GACGAACTTC GAACTAGAAG AAGACATTGA   
  
  
- TATGTCACGC AACAAAGACA AAGGAAAAG

+     TATA-box

| Site Name | Organism | Position | Strand | Matrix score. | sequence | function |
| --- | --- | --- | --- | --- | --- | --- |
| TATA-box | Arabidopsis thaliana | 1394 | - | 4 | TATA | core promoter element around -30 of transcription start |
| TATA-box | Oryza sativa | 1389 | - | 7 | TACAAAA | core promoter element around -30 of transcription start |
| TATA-box | Helianthus annuus | 1392 | - | 6 | TATACA | core promoter element around -30 of transcription start |
| TATA-box | Arabidopsis thaliana | 1362 | - | 4 | TATA | core promoter element around -30 of transcription start |
| TATA-box | Arabidopsis thaliana | 1470 | - | 4 | TATA | core promoter element around -30 of transcription start |
| TATA-box | Brassica oleracea | 744 | + | 7 | ATATAAT | core promoter element around -30 of transcription start |
| TATA-box | Brassica napus | 1361 | - | 6 | ATATAT | core promoter element around -30 of transcription start |
| TATA-box | Arabidopsis thaliana | 1356 | - | 9 | taTATAAAgg | core promoter element around -30 of transcription start |
| TATA-box | Arabidopsis thaliana | 1360 | - | 4 | TATA | core promoter element around -30 of transcription start |
| TATA-box | Lycopersicon esculentum | 994 | - | 5 | TTTTA | core promoter element around -30 of transcription start |
| TATA-box | Glycine max | 649 | - | 5 | TAATA | core promoter element around -30 of transcription start |
| TATA-box | Brassica napus | 1359 | - | 6 | ATATAT | core promoter element around -30 of transcription start |
| TATA-box | Arabidopsis thaliana | 156 | + | 4 | TATA | core promoter element around -30 of transcription start |
| TATA-box | Helianthus annuus | 1011 | - | 6 | TATACA | core promoter element around -30 of transcription start |
| TATA-box | Lycopersicon esculentum | 822 | - | 5 | TTTTA | core promoter element around -30 of transcription start |
| TATA-box | Nicotiana tabacum | 795 | + | 9 | tcTATAAAta | core promoter element around -30 of transcription start |
| TATA-box | Helianthus annuus | 786 | - | 6 | TATACA | core promoter element around -30 of transcription start |
| TATA-box | Lycopersicon esculentum | 145 | + | 5 | TTTTA | core promoter element around -30 of transcription start |
| TATA-box | Zea mays | 195 | - | 8 | TTTAAAAA | core promoter element around -30 of transcription start |
| TATA-box | Glycine max | 189 | - | 5 | TAATA | core promoter element around -30 of transcription start |
| TATA-box | Brassica napus | 1274 | - | 6 | ATATAT | core promoter element around -30 of transcription start |
| TATA-box | Arabidopsis thaliana | 1269 | - | 9 | taTATAAAgg | core promoter element around -30 of transcription start |
| TATA-box | Glycine max | 186 | - | 5 | TAATA | core promoter element around -30 of transcription start |
| TATA-box | Glycine max | 171 | - | 5 | TAATA | core promoter element around -30 of transcription start |
| TATA-box | Glycine max | 1088 | - | 5 | TAATA | core promoter element around -30 of transcription start |
| TATA-box | Arabidopsis thaliana | 743 | + | 4 | TATA | core promoter element around -30 of transcription start |
| TATA-box | Lycopersicon esculentum | 1080 | + | 5 | TTTTA | core promoter element around -30 of transcription start |
| TATA-box | Helianthus annuus | 1098 | - | 6 | TATACA | core promoter element around -30 of transcription start |
| TATA-box | Lycopersicon esculentum | 199 | - | 5 | TTTTA | core promoter element around -30 of transcription start |
| TATA-box | Helianthus annuus | 1305 | - | 6 | TATACA | core promoter element around -30 of transcription start |
| TATA-box | Arabidopsis thaliana | 757 | - | 4 | TATA | core promoter element around -30 of transcription start |
| TATA-box | Arabidopsis thaliana | 915 | - | 4 | TATA | core promoter element around -30 of transcription start |
| TATA-box | Brassica napus | 1272 | - | 6 | ATATAT | core promoter element around -30 of transcription start |
| TATA-box | Arabidopsis thaliana | 1271 | - | 8 | TATATATA | core promoter element around -30 of transcription start |
| TATA-box | Arabidopsis thaliana | 756 | - | 5 | TATAA | core promoter element around -30 of transcription start |
| TATA-box | Glycine max | 747 | + | 5 | TAATA | core promoter element around -30 of transcription start |
| TATA-box | Arabidopsis thaliana | 1358 | - | 8 | TATATATA | core promoter element around -30 of transcription start |
| TATA-box | Arabidopsis thaliana | 914 | - | 5 | TATAA | core promoter element around -30 of transcription start |
| TATA-box | Zea mays | 1079 | - | 8 | TTTAAAAA | core promoter element around -30 of transcription start |
| TATA-box | Lycopersicon esculentum | 148 | - | 5 | TTTTA | core promoter element around -30 of transcription start |
| TATA-box | Brassica napus | 155 | + | 6 | ATATAT | core promoter element around -30 of transcription start |
| TATA-box | Lycopersicon esculentum | 40 | + | 5 | TTTTA | core promoter element around -30 of transcription start |
| TATA-box | Arabidopsis thaliana | 1275 | - | 4 | TATA | core promoter element around -30 of transcription start |
| TATA-box | Oryza sativa | 1302 | - | 7 | TACAAAA | core promoter element around -30 of transcription start |
| TATA-box | Lycopersicon esculentum | 168 | + | 5 | TTTTA | core promoter element around -30 of transcription start |
| TATA-box | Arabidopsis thaliana | 745 | + | 4 | TATA | core promoter element around -30 of transcription start |
| TATA-box | Arabidopsis thaliana | 1100 | - | 4 | TATA | core promoter element around -30 of transcription start |
| TATA-box | Zea mays | 144 | - | 8 | TTTAAAAA | core promoter element around -30 of transcription start |
| TATA-box | Arabidopsis thaliana | 1198 | - | 4 | TATA | core promoter element around -30 of transcription start |
| TATA-box | Lycopersicon esculentum | 196 | + | 5 | TTTTA | core promoter element around -30 of transcription start |
| TATA-box | Arabidopsis thaliana | 1273 | - | 4 | TATA | core promoter element around -30 of transcription start |
| TATA-box | Glycine max | 413 | + | 5 | TAATA | core promoter element around -30 of transcription start |
| TATA-box | Glycine max | 939 | + | 5 | TAATA | core promoter element around -30 of transcription start |
| TATA-box | Zea mays | 146 | + | 8 | TTTAAAAA | core promoter element around -30 of transcription start |
| TATA-box | Arabidopsis thaliana | 1307 | - | 4 | TATA | core promoter element around -30 of transcription start |
| TATA-box | Arabidopsis thaliana | 788 | - | 4 | TATA | core promoter element around -30 of transcription start |
| TATA-box | Lycopersicon esculentum | 896 | - | 5 | TTTTA | core promoter element around -30 of transcription start |
| TATA-box | Glycine max | 1001 | - | 5 | TAATA | core promoter element around -30 of transcription start |
| TATA-box | Glycine max | 210 | + | 5 | TAATA | core promoter element around -30 of transcription start |
| TATA-box | Lycopersicon esculentum | 376 | + | 5 | TTTTA | core promoter element around -30 of transcription start |
| TATA-box | Arabidopsis thaliana | 828 | - | 4 | TATA | core promoter element around -30 of transcription start |
| TATA-box | Brassica napus | 826 | + | 6 | ATTATA | core promoter element around -30 of transcription start |
| TATA-box | Arabidopsis thaliana | 1013 | - | 4 | TATA | core promoter element around -30 of transcription start |
| TATA-box | Zea mays | 197 | + | 8 | TTTAAAAA | core promoter element around -30 of transcription start |
| TATA-box | Arabidopsis thaliana | 827 | - | 5 | TATAA | core promoter element around -30 of transcription start |
| TATA-box | Brassica napus | 755 | + | 6 | ATTATA | core promoter element around -30 of transcription start |

> 2018/04/13 10:10:12  
+ AAAAGGCAAC CTTTTCCTTT CTTTCTTCTT TCCTTAGCTT TTTAGATTTA TGAAGAAGGG CTGCTACGAC   
  
  
+ TTTCCTGCTT TTCCACATTT CAAACGCTCG TGTTATCGTC CTTTCATTCT CCCACTTCGT GTCCATCAGC   
  
  
+ TCCTTTTTAA AAAAATATAT TTATTTATTT TATTAAATTA TCATTTATTA TTATTTTTTA AAAAAAATTT   
  
  
+ AATACAGTTT TTTGGGGGAA AAAAAGAAAA AAAACTCATC CATCTGATTT GCTCTCTCTC TGATTTCCAA   
  
  
+ CCGAATGCAT GAGGAGAGCA TTCAGCGGCA ATCAAGCAGA GAGGACTGAC TGTCTTATGG GCCCTATCTC   
  
  
+ TTCTTGGATT CCAGACTATT CTTGTTTTTA GTAGCTCTCA AATCAATAAC CAGATCTAAT CCTAATACTC   
  
  
+ TCTTCATAAT CATAACTTAA AGAGGGAATT AATTAACCAA ATCAGGTAAA CAAACATCAT TAATTACAGC   
  
  
+ CACCTCGTTT GATCGATCCC GATCACCCAT GATCGTCACT TCCAGATCTT CACGTGTGCA ACCAATGAGC   
  
  
+ CATTAGTTGT AAATCAATTA ACATGTTTAA TCATAAAGAC TTCAAATTTT TGAAGTCAAC ATTCAAATGA   
  
  
+ TGCAGTTTGC TGAGATGGTA TTAGTGGCAC CACACAGCCC ACCCAAATGA ATTAACCATG AGACCTATTT   
  
  
+ ATGATCCTTC ATGGAAGTTC CTTAAGCCGC GCAGACATTC ACTATATAAT AGTAATTATA ACTTTCCACC   
  
  
+ TACCAACCAA GCATATGTAT ACTTTCAATA AATACATGTG CCCAAATTCC ATAAAATTAT AGAACAATTA   
  
  
+ TTGAATGAAT TTAATTATCA GATCAGTCCA AGACAGGCGC TTAGCTAGGT TAGCATAAAA TATCGTGCAC   
  
  
+ CTGTTATATC CTTATCAGCT CAGAGGCCTA ATACTGTTGC TAGTCTGGTA GCTCTACGAA TTTATTTCCA   
  
  
+ TGTAAATCAG AATTAAAAAT TATTATCATC TGTATAATCG TATGTGTTAA CGTCGCTTTC CTAATGATGA   
  
  
+ AGCCGCCAGC TCCATTTTCC ACCCTACATT TTTAAATTAT TATCATCTGT ATAATCGTAT GTGTTAACGT   
  
  
+ CGCTTTCCTA ATGATGAAGC CGCCAGCTCC ATTTTCCACC CTACATTTTT TCCAATAGAT CAATCCGCTG   
  
  
+ ATGAAGCTAT ATGTACATTC AATTTTTTTC TTGAGCTAAC TGGAAAATAT TTTCGTGCCG TCTACGACAC   
  
  
+ CTGTTAACCC TATATATATC TCTTTGGATT TTTCTGTCTA TTTTTGTATA CTTCTTCAGG AAAATATTTT   
  
  
+ CGTGCCGTCT ACGACACCTG TTAACCCTAT ATATATCTCT TTGGATTTTT CTGTCTATTT TTGTATACTT   
  
  
+ CTTCTGGATC AATCTCCATC TTTATCTTCT TCCTCTTCTT CTGCTTGAAG CTTGATCTTC TTCTGTAACT   
  
  
+ ATACAGTGCG TTGTTTCTGT TTCCTTTTC  

- TTTTCCGTTG GAAAAGGAAA GAAAGAAGAA AGGAATCGAA AAATCTAAAT ACTTCTTCCC GACGATGCTG   
  
  
- AAAGGACGAA AAGGTGTAAA GTTTGCGAGC ACAATAGCAG GAAAGTAAGA GGGTGAAGCA CAGGTAGTCG   
  
  
- AGGAAAAATT TTTTTATATA AATAAATAAA ATAATTTAAT AGTAAATAAT AATAAAAAAT TTTTTTTAAA   
  
  
- TTATGTCAAA AAACCCCCTT TTTTTCTTTT TTTTGAGTAG GTAGACTAAA CGAGAGAGAG ACTAAAGGTT   
  
  
- GGCTTACGTA CTCCTCTCGT AAGTCGCCGT TAGTTCGTCT CTCCTGACTG ACAGAATACC CGGGATAGAG   
  
  
- AAGAACCTAA GGTCTGATAA GAACAAAAAT CATCGAGAGT TTAGTTATTG GTCTAGATTA GGATTATGAG   
  
  
- AGAAGTATTA GTATTGAATT TCTCCCTTAA TTAATTGGTT TAGTCCATTT GTTTGTAGTA ATTAATGTCG   
  
  
- GTGGAGCAAA CTAGCTAGGG CTAGTGGGTA CTAGCAGTGA AGGTCTAGAA GTGCACACGT TGGTTACTCG   
  
  
- GTAATCAACA TTTAGTTAAT TGTACAAATT AGTATTTCTG AAGTTTAAAA ACTTCAGTTG TAAGTTTACT   
  
  
- ACGTCAAACG ACTCTACCAT AATCACCGTG GTGTGTCGGG TGGGTTTACT TAATTGGTAC TCTGGATAAA   
  
  
- TACTAGGAAG TACCTTCAAG GAATTCGGCG CGTCTGTAAG TGATATATTA TCATTAATAT TGAAAGGTGG   
  
  
- ATGGTTGGTT CGTATACATA TGAAAGTTAT TTATGTACAC GGGTTTAAGG TATTTTAATA TCTTGTTAAT   
  
  
- AACTTACTTA AATTAATAGT CTAGTCAGGT TCTGTCCGCG AATCGATCCA ATCGTATTTT ATAGCACGTG   
  
  
- GACAATATAG GAATAGTCGA GTCTCCGGAT TATGACAACG ATCAGACCAT CGAGATGCTT AAATAAAGGT   
  
  
- ACATTTAGTC TTAATTTTTA ATAATAGTAG ACATATTAGC ATACACAATT GCAGCGAAAG GATTACTACT   
  
  
- TCGGCGGTCG AGGTAAAAGG TGGGATGTAA AAATTTAATA ATAGTAGACA TATTAGCATA CACAATTGCA   
  
  
- GCGAAAGGAT TACTACTTCG GCGGTCGAGG TAAAAGGTGG GATGTAAAAA AGGTTATCTA GTTAGGCGAC   
  
  
- TACTTCGATA TACATGTAAG TTAAAAAAAG AACTCGATTG ACCTTTTATA AAAGCACGGC AGATGCTGTG   
  
  
- GACAATTGGG ATATATATAG AGAAACCTAA AAAGACAGAT AAAAACATAT GAAGAAGTCC TTTTATAAAA   
  
  
- GCACGGCAGA TGCTGTGGAC AATTGGGATA TATATAGAGA AACCTAAAAA GACAGATAAA AACATATGAA   
  
  
- GAAGACCTAG TTAGAGGTAG AAATAGAAGA AGGAGAAGAA GACGAACTTC GAACTAGAAG AAGACATTGA   
  
  
- TATGTCACGC AACAAAGACA AAGGAAAAG

+     TC-rich repeats

| Site Name | Organism | Position | Strand | Matrix score. | sequence | function |
| --- | --- | --- | --- | --- | --- | --- |
| TC-rich repeats | Nicotiana tabacum | 1165 | + | 9 | ATTTTCTCCA | cis-acting element involved in defense and stress responsiveness |

> 2018/04/13 10:10:12  
+ AAAAGGCAAC CTTTTCCTTT CTTTCTTCTT TCCTTAGCTT TTTAGATTTA TGAAGAAGGG CTGCTACGAC   
  
  
+ TTTCCTGCTT TTCCACATTT CAAACGCTCG TGTTATCGTC CTTTCATTCT CCCACTTCGT GTCCATCAGC   
  
  
+ TCCTTTTTAA AAAAATATAT TTATTTATTT TATTAAATTA TCATTTATTA TTATTTTTTA AAAAAAATTT   
  
  
+ AATACAGTTT TTTGGGGGAA AAAAAGAAAA AAAACTCATC CATCTGATTT GCTCTCTCTC TGATTTCCAA   
  
  
+ CCGAATGCAT GAGGAGAGCA TTCAGCGGCA ATCAAGCAGA GAGGACTGAC TGTCTTATGG GCCCTATCTC   
  
  
+ TTCTTGGATT CCAGACTATT CTTGTTTTTA GTAGCTCTCA AATCAATAAC CAGATCTAAT CCTAATACTC   
  
  
+ TCTTCATAAT CATAACTTAA AGAGGGAATT AATTAACCAA ATCAGGTAAA CAAACATCAT TAATTACAGC   
  
  
+ CACCTCGTTT GATCGATCCC GATCACCCAT GATCGTCACT TCCAGATCTT CACGTGTGCA ACCAATGAGC   
  
  
+ CATTAGTTGT AAATCAATTA ACATGTTTAA TCATAAAGAC TTCAAATTTT TGAAGTCAAC ATTCAAATGA   
  
  
+ TGCAGTTTGC TGAGATGGTA TTAGTGGCAC CACACAGCCC ACCCAAATGA ATTAACCATG AGACCTATTT   
  
  
+ ATGATCCTTC ATGGAAGTTC CTTAAGCCGC GCAGACATTC ACTATATAAT AGTAATTATA ACTTTCCACC   
  
  
+ TACCAACCAA GCATATGTAT ACTTTCAATA AATACATGTG CCCAAATTCC ATAAAATTAT AGAACAATTA   
  
  
+ TTGAATGAAT TTAATTATCA GATCAGTCCA AGACAGGCGC TTAGCTAGGT TAGCATAAAA TATCGTGCAC   
  
  
+ CTGTTATATC CTTATCAGCT CAGAGGCCTA ATACTGTTGC TAGTCTGGTA GCTCTACGAA TTTATTTCCA   
  
  
+ TGTAAATCAG AATTAAAAAT TATTATCATC TGTATAATCG TATGTGTTAA CGTCGCTTTC CTAATGATGA   
  
  
+ AGCCGCCAGC TCCATTTTCC ACCCTACATT TTTAAATTAT TATCATCTGT ATAATCGTAT GTGTTAACGT   
  
  
+ CGCTTTCCTA ATGATGAAGC CGCCAGCTCC ATTTTCCACC CTACATTTTT TCCAATAGAT CAATCCGCTG   
  
  
+ ATGAAGCTAT ATGTACATTC AATTTTTTTC TTGAGCTAAC TGGAAAATAT TTTCGTGCCG TCTACGACAC   
  
  
+ CTGTTAACCC TATATATATC TCTTTGGATT TTTCTGTCTA TTTTTGTATA CTTCTTCAGG AAAATATTTT   
  
  
+ CGTGCCGTCT ACGACACCTG TTAACCCTAT ATATATCTCT TTGGATTTTT CTGTCTATTT TTGTATACTT   
  
  
+ CTTCTGGATC AATCTCCATC TTTATCTTCT TCCTCTTCTT CTGCTTGAAG CTTGATCTTC TTCTGTAACT   
  
  
+ ATACAGTGCG TTGTTTCTGT TTCCTTTTC  

- TTTTCCGTTG GAAAAGGAAA GAAAGAAGAA AGGAATCGAA AAATCTAAAT ACTTCTTCCC GACGATGCTG   
  
  
- AAAGGACGAA AAGGTGTAAA GTTTGCGAGC ACAATAGCAG GAAAGTAAGA GGGTGAAGCA CAGGTAGTCG   
  
  
- AGGAAAAATT TTTTTATATA AATAAATAAA ATAATTTAAT AGTAAATAAT AATAAAAAAT TTTTTTTAAA   
  
  
- TTATGTCAAA AAACCCCCTT TTTTTCTTTT TTTTGAGTAG GTAGACTAAA CGAGAGAGAG ACTAAAGGTT   
  
  
- GGCTTACGTA CTCCTCTCGT AAGTCGCCGT TAGTTCGTCT CTCCTGACTG ACAGAATACC CGGGATAGAG   
  
  
- AAGAACCTAA GGTCTGATAA GAACAAAAAT CATCGAGAGT TTAGTTATTG GTCTAGATTA GGATTATGAG   
  
  
- AGAAGTATTA GTATTGAATT TCTCCCTTAA TTAATTGGTT TAGTCCATTT GTTTGTAGTA ATTAATGTCG   
  
  
- GTGGAGCAAA CTAGCTAGGG CTAGTGGGTA CTAGCAGTGA AGGTCTAGAA GTGCACACGT TGGTTACTCG   
  
  
- GTAATCAACA TTTAGTTAAT TGTACAAATT AGTATTTCTG AAGTTTAAAA ACTTCAGTTG TAAGTTTACT   
  
  
- ACGTCAAACG ACTCTACCAT AATCACCGTG GTGTGTCGGG TGGGTTTACT TAATTGGTAC TCTGGATAAA   
  
  
- TACTAGGAAG TACCTTCAAG GAATTCGGCG CGTCTGTAAG TGATATATTA TCATTAATAT TGAAAGGTGG   
  
  
- ATGGTTGGTT CGTATACATA TGAAAGTTAT TTATGTACAC GGGTTTAAGG TATTTTAATA TCTTGTTAAT   
  
  
- AACTTACTTA AATTAATAGT CTAGTCAGGT TCTGTCCGCG AATCGATCCA ATCGTATTTT ATAGCACGTG   
  
  
- GACAATATAG GAATAGTCGA GTCTCCGGAT TATGACAACG ATCAGACCAT CGAGATGCTT AAATAAAGGT   
  
  
- ACATTTAGTC TTAATTTTTA ATAATAGTAG ACATATTAGC ATACACAATT GCAGCGAAAG GATTACTACT   
  
  
- TCGGCGGTCG AGGTAAAAGG TGGGATGTAA AAATTTAATA ATAGTAGACA TATTAGCATA CACAATTGCA   
  
  
- GCGAAAGGAT TACTACTTCG GCGGTCGAGG TAAAAGGTGG GATGTAAAAA AGGTTATCTA GTTAGGCGAC   
  
  
- TACTTCGATA TACATGTAAG TTAAAAAAAG AACTCGATTG ACCTTTTATA AAAGCACGGC AGATGCTGTG   
  
  
- GACAATTGGG ATATATATAG AGAAACCTAA AAAGACAGAT AAAAACATAT GAAGAAGTCC TTTTATAAAA   
  
  
- GCACGGCAGA TGCTGTGGAC AATTGGGATA TATATAGAGA AACCTAAAAA GACAGATAAA AACATATGAA   
  
  
- GAAGACCTAG TTAGAGGTAG AAATAGAAGA AGGAGAAGAA GACGAACTTC GAACTAGAAG AAGACATTGA   
  
  
- TATGTCACGC AACAAAGACA AAGGAAAAG

+     TCA-element

| Site Name | Organism | Position | Strand | Matrix score. | sequence | function |
| --- | --- | --- | --- | --- | --- | --- |
| TCA-element | Nicotiana tabacum | 1416 | + | 9 | CCATCTTTTT | cis-acting element involved in salicylic acid responsiveness |

> 2018/04/13 10:10:12  
+ AAAAGGCAAC CTTTTCCTTT CTTTCTTCTT TCCTTAGCTT TTTAGATTTA TGAAGAAGGG CTGCTACGAC   
  
  
+ TTTCCTGCTT TTCCACATTT CAAACGCTCG TGTTATCGTC CTTTCATTCT CCCACTTCGT GTCCATCAGC   
  
  
+ TCCTTTTTAA AAAAATATAT TTATTTATTT TATTAAATTA TCATTTATTA TTATTTTTTA AAAAAAATTT   
  
  
+ AATACAGTTT TTTGGGGGAA AAAAAGAAAA AAAACTCATC CATCTGATTT GCTCTCTCTC TGATTTCCAA   
  
  
+ CCGAATGCAT GAGGAGAGCA TTCAGCGGCA ATCAAGCAGA GAGGACTGAC TGTCTTATGG GCCCTATCTC   
  
  
+ TTCTTGGATT CCAGACTATT CTTGTTTTTA GTAGCTCTCA AATCAATAAC CAGATCTAAT CCTAATACTC   
  
  
+ TCTTCATAAT CATAACTTAA AGAGGGAATT AATTAACCAA ATCAGGTAAA CAAACATCAT TAATTACAGC   
  
  
+ CACCTCGTTT GATCGATCCC GATCACCCAT GATCGTCACT TCCAGATCTT CACGTGTGCA ACCAATGAGC   
  
  
+ CATTAGTTGT AAATCAATTA ACATGTTTAA TCATAAAGAC TTCAAATTTT TGAAGTCAAC ATTCAAATGA   
  
  
+ TGCAGTTTGC TGAGATGGTA TTAGTGGCAC CACACAGCCC ACCCAAATGA ATTAACCATG AGACCTATTT   
  
  
+ ATGATCCTTC ATGGAAGTTC CTTAAGCCGC GCAGACATTC ACTATATAAT AGTAATTATA ACTTTCCACC   
  
  
+ TACCAACCAA GCATATGTAT ACTTTCAATA AATACATGTG CCCAAATTCC ATAAAATTAT AGAACAATTA   
  
  
+ TTGAATGAAT TTAATTATCA GATCAGTCCA AGACAGGCGC TTAGCTAGGT TAGCATAAAA TATCGTGCAC   
  
  
+ CTGTTATATC CTTATCAGCT CAGAGGCCTA ATACTGTTGC TAGTCTGGTA GCTCTACGAA TTTATTTCCA   
  
  
+ TGTAAATCAG AATTAAAAAT TATTATCATC TGTATAATCG TATGTGTTAA CGTCGCTTTC CTAATGATGA   
  
  
+ AGCCGCCAGC TCCATTTTCC ACCCTACATT TTTAAATTAT TATCATCTGT ATAATCGTAT GTGTTAACGT   
  
  
+ CGCTTTCCTA ATGATGAAGC CGCCAGCTCC ATTTTCCACC CTACATTTTT TCCAATAGAT CAATCCGCTG   
  
  
+ ATGAAGCTAT ATGTACATTC AATTTTTTTC TTGAGCTAAC TGGAAAATAT TTTCGTGCCG TCTACGACAC   
  
  
+ CTGTTAACCC TATATATATC TCTTTGGATT TTTCTGTCTA TTTTTGTATA CTTCTTCAGG AAAATATTTT   
  
  
+ CGTGCCGTCT ACGACACCTG TTAACCCTAT ATATATCTCT TTGGATTTTT CTGTCTATTT TTGTATACTT   
  
  
+ CTTCTGGATC AATCTCCATC TTTATCTTCT TCCTCTTCTT CTGCTTGAAG CTTGATCTTC TTCTGTAACT   
  
  
+ ATACAGTGCG TTGTTTCTGT TTCCTTTTC  

- TTTTCCGTTG GAAAAGGAAA GAAAGAAGAA AGGAATCGAA AAATCTAAAT ACTTCTTCCC GACGATGCTG   
  
  
- AAAGGACGAA AAGGTGTAAA GTTTGCGAGC ACAATAGCAG GAAAGTAAGA GGGTGAAGCA CAGGTAGTCG   
  
  
- AGGAAAAATT TTTTTATATA AATAAATAAA ATAATTTAAT AGTAAATAAT AATAAAAAAT TTTTTTTAAA   
  
  
- TTATGTCAAA AAACCCCCTT TTTTTCTTTT TTTTGAGTAG GTAGACTAAA CGAGAGAGAG ACTAAAGGTT   
  
  
- GGCTTACGTA CTCCTCTCGT AAGTCGCCGT TAGTTCGTCT CTCCTGACTG ACAGAATACC CGGGATAGAG   
  
  
- AAGAACCTAA GGTCTGATAA GAACAAAAAT CATCGAGAGT TTAGTTATTG GTCTAGATTA GGATTATGAG   
  
  
- AGAAGTATTA GTATTGAATT TCTCCCTTAA TTAATTGGTT TAGTCCATTT GTTTGTAGTA ATTAATGTCG   
  
  
- GTGGAGCAAA CTAGCTAGGG CTAGTGGGTA CTAGCAGTGA AGGTCTAGAA GTGCACACGT TGGTTACTCG   
  
  
- GTAATCAACA TTTAGTTAAT TGTACAAATT AGTATTTCTG AAGTTTAAAA ACTTCAGTTG TAAGTTTACT   
  
  
- ACGTCAAACG ACTCTACCAT AATCACCGTG GTGTGTCGGG TGGGTTTACT TAATTGGTAC TCTGGATAAA   
  
  
- TACTAGGAAG TACCTTCAAG GAATTCGGCG CGTCTGTAAG TGATATATTA TCATTAATAT TGAAAGGTGG   
  
  
- ATGGTTGGTT CGTATACATA TGAAAGTTAT TTATGTACAC GGGTTTAAGG TATTTTAATA TCTTGTTAAT   
  
  
- AACTTACTTA AATTAATAGT CTAGTCAGGT TCTGTCCGCG AATCGATCCA ATCGTATTTT ATAGCACGTG   
  
  
- GACAATATAG GAATAGTCGA GTCTCCGGAT TATGACAACG ATCAGACCAT CGAGATGCTT AAATAAAGGT   
  
  
- ACATTTAGTC TTAATTTTTA ATAATAGTAG ACATATTAGC ATACACAATT GCAGCGAAAG GATTACTACT   
  
  
- TCGGCGGTCG AGGTAAAAGG TGGGATGTAA AAATTTAATA ATAGTAGACA TATTAGCATA CACAATTGCA   
  
  
- GCGAAAGGAT TACTACTTCG GCGGTCGAGG TAAAAGGTGG GATGTAAAAA AGGTTATCTA GTTAGGCGAC   
  
  
- TACTTCGATA TACATGTAAG TTAAAAAAAG AACTCGATTG ACCTTTTATA AAAGCACGGC AGATGCTGTG   
  
  
- GACAATTGGG ATATATATAG AGAAACCTAA AAAGACAGAT AAAAACATAT GAAGAAGTCC TTTTATAAAA   
  
  
- GCACGGCAGA TGCTGTGGAC AATTGGGATA TATATAGAGA AACCTAAAAA GACAGATAAA AACATATGAA   
  
  
- GAAGACCTAG TTAGAGGTAG AAATAGAAGA AGGAGAAGAA GACGAACTTC GAACTAGAAG AAGACATTGA   
  
  
- TATGTCACGC AACAAAGACA AAGGAAAAG

+     TCCACCT-motif

| Site Name | Organism | Position | Strand | Matrix score. | sequence | function |
| --- | --- | --- | --- | --- | --- | --- |
| TCCACCT-motif | Petroselinum hortense | 765 | + | 7 | TCCACCT |  |

> 2018/04/13 10:10:12  
+ AAAAGGCAAC CTTTTCCTTT CTTTCTTCTT TCCTTAGCTT TTTAGATTTA TGAAGAAGGG CTGCTACGAC   
  
  
+ TTTCCTGCTT TTCCACATTT CAAACGCTCG TGTTATCGTC CTTTCATTCT CCCACTTCGT GTCCATCAGC   
  
  
+ TCCTTTTTAA AAAAATATAT TTATTTATTT TATTAAATTA TCATTTATTA TTATTTTTTA AAAAAAATTT   
  
  
+ AATACAGTTT TTTGGGGGAA AAAAAGAAAA AAAACTCATC CATCTGATTT GCTCTCTCTC TGATTTCCAA   
  
  
+ CCGAATGCAT GAGGAGAGCA TTCAGCGGCA ATCAAGCAGA GAGGACTGAC TGTCTTATGG GCCCTATCTC   
  
  
+ TTCTTGGATT CCAGACTATT CTTGTTTTTA GTAGCTCTCA AATCAATAAC CAGATCTAAT CCTAATACTC   
  
  
+ TCTTCATAAT CATAACTTAA AGAGGGAATT AATTAACCAA ATCAGGTAAA CAAACATCAT TAATTACAGC   
  
  
+ CACCTCGTTT GATCGATCCC GATCACCCAT GATCGTCACT TCCAGATCTT CACGTGTGCA ACCAATGAGC   
  
  
+ CATTAGTTGT AAATCAATTA ACATGTTTAA TCATAAAGAC TTCAAATTTT TGAAGTCAAC ATTCAAATGA   
  
  
+ TGCAGTTTGC TGAGATGGTA TTAGTGGCAC CACACAGCCC ACCCAAATGA ATTAACCATG AGACCTATTT   
  
  
+ ATGATCCTTC ATGGAAGTTC CTTAAGCCGC GCAGACATTC ACTATATAAT AGTAATTATA ACTTTCCACC   
  
  
+ TACCAACCAA GCATATGTAT ACTTTCAATA AATACATGTG CCCAAATTCC ATAAAATTAT AGAACAATTA   
  
  
+ TTGAATGAAT TTAATTATCA GATCAGTCCA AGACAGGCGC TTAGCTAGGT TAGCATAAAA TATCGTGCAC   
  
  
+ CTGTTATATC CTTATCAGCT CAGAGGCCTA ATACTGTTGC TAGTCTGGTA GCTCTACGAA TTTATTTCCA   
  
  
+ TGTAAATCAG AATTAAAAAT TATTATCATC TGTATAATCG TATGTGTTAA CGTCGCTTTC CTAATGATGA   
  
  
+ AGCCGCCAGC TCCATTTTCC ACCCTACATT TTTAAATTAT TATCATCTGT ATAATCGTAT GTGTTAACGT   
  
  
+ CGCTTTCCTA ATGATGAAGC CGCCAGCTCC ATTTTCCACC CTACATTTTT TCCAATAGAT CAATCCGCTG   
  
  
+ ATGAAGCTAT ATGTACATTC AATTTTTTTC TTGAGCTAAC TGGAAAATAT TTTCGTGCCG TCTACGACAC   
  
  
+ CTGTTAACCC TATATATATC TCTTTGGATT TTTCTGTCTA TTTTTGTATA CTTCTTCAGG AAAATATTTT   
  
  
+ CGTGCCGTCT ACGACACCTG TTAACCCTAT ATATATCTCT TTGGATTTTT CTGTCTATTT TTGTATACTT   
  
  
+ CTTCTGGATC AATCTCCATC TTTATCTTCT TCCTCTTCTT CTGCTTGAAG CTTGATCTTC TTCTGTAACT   
  
  
+ ATACAGTGCG TTGTTTCTGT TTCCTTTTC  

- TTTTCCGTTG GAAAAGGAAA GAAAGAAGAA AGGAATCGAA AAATCTAAAT ACTTCTTCCC GACGATGCTG   
  
  
- AAAGGACGAA AAGGTGTAAA GTTTGCGAGC ACAATAGCAG GAAAGTAAGA GGGTGAAGCA CAGGTAGTCG   
  
  
- AGGAAAAATT TTTTTATATA AATAAATAAA ATAATTTAAT AGTAAATAAT AATAAAAAAT TTTTTTTAAA   
  
  
- TTATGTCAAA AAACCCCCTT TTTTTCTTTT TTTTGAGTAG GTAGACTAAA CGAGAGAGAG ACTAAAGGTT   
  
  
- GGCTTACGTA CTCCTCTCGT AAGTCGCCGT TAGTTCGTCT CTCCTGACTG ACAGAATACC CGGGATAGAG   
  
  
- AAGAACCTAA GGTCTGATAA GAACAAAAAT CATCGAGAGT TTAGTTATTG GTCTAGATTA GGATTATGAG   
  
  
- AGAAGTATTA GTATTGAATT TCTCCCTTAA TTAATTGGTT TAGTCCATTT GTTTGTAGTA ATTAATGTCG   
  
  
- GTGGAGCAAA CTAGCTAGGG CTAGTGGGTA CTAGCAGTGA AGGTCTAGAA GTGCACACGT TGGTTACTCG   
  
  
- GTAATCAACA TTTAGTTAAT TGTACAAATT AGTATTTCTG AAGTTTAAAA ACTTCAGTTG TAAGTTTACT   
  
  
- ACGTCAAACG ACTCTACCAT AATCACCGTG GTGTGTCGGG TGGGTTTACT TAATTGGTAC TCTGGATAAA   
  
  
- TACTAGGAAG TACCTTCAAG GAATTCGGCG CGTCTGTAAG TGATATATTA TCATTAATAT TGAAAGGTGG   
  
  
- ATGGTTGGTT CGTATACATA TGAAAGTTAT TTATGTACAC GGGTTTAAGG TATTTTAATA TCTTGTTAAT   
  
  
- AACTTACTTA AATTAATAGT CTAGTCAGGT TCTGTCCGCG AATCGATCCA ATCGTATTTT ATAGCACGTG   
  
  
- GACAATATAG GAATAGTCGA GTCTCCGGAT TATGACAACG ATCAGACCAT CGAGATGCTT AAATAAAGGT   
  
  
- ACATTTAGTC TTAATTTTTA ATAATAGTAG ACATATTAGC ATACACAATT GCAGCGAAAG GATTACTACT   
  
  
- TCGGCGGTCG AGGTAAAAGG TGGGATGTAA AAATTTAATA ATAGTAGACA TATTAGCATA CACAATTGCA   
  
  
- GCGAAAGGAT TACTACTTCG GCGGTCGAGG TAAAAGGTGG GATGTAAAAA AGGTTATCTA GTTAGGCGAC   
  
  
- TACTTCGATA TACATGTAAG TTAAAAAAAG AACTCGATTG ACCTTTTATA AAAGCACGGC AGATGCTGTG   
  
  
- GACAATTGGG ATATATATAG AGAAACCTAA AAAGACAGAT AAAAACATAT GAAGAAGTCC TTTTATAAAA   
  
  
- GCACGGCAGA TGCTGTGGAC AATTGGGATA TATATAGAGA AACCTAAAAA GACAGATAAA AACATATGAA   
  
  
- GAAGACCTAG TTAGAGGTAG AAATAGAAGA AGGAGAAGAA GACGAACTTC GAACTAGAAG AAGACATTGA   
  
  
- TATGTCACGC AACAAAGACA AAGGAAAAG

+     TGACG-motif

| Site Name | Organism | Position | Strand | Matrix score. | sequence | function |
| --- | --- | --- | --- | --- | --- | --- |
| TGACG-motif | Hordeum vulgare | 524 | - | 5 | TGACG | cis-acting regulatory element involved in the MeJA-responsiveness |

> 2018/04/13 10:10:12  
+ AAAAGGCAAC CTTTTCCTTT CTTTCTTCTT TCCTTAGCTT TTTAGATTTA TGAAGAAGGG CTGCTACGAC   
  
  
+ TTTCCTGCTT TTCCACATTT CAAACGCTCG TGTTATCGTC CTTTCATTCT CCCACTTCGT GTCCATCAGC   
  
  
+ TCCTTTTTAA AAAAATATAT TTATTTATTT TATTAAATTA TCATTTATTA TTATTTTTTA AAAAAAATTT   
  
  
+ AATACAGTTT TTTGGGGGAA AAAAAGAAAA AAAACTCATC CATCTGATTT GCTCTCTCTC TGATTTCCAA   
  
  
+ CCGAATGCAT GAGGAGAGCA TTCAGCGGCA ATCAAGCAGA GAGGACTGAC TGTCTTATGG GCCCTATCTC   
  
  
+ TTCTTGGATT CCAGACTATT CTTGTTTTTA GTAGCTCTCA AATCAATAAC CAGATCTAAT CCTAATACTC   
  
  
+ TCTTCATAAT CATAACTTAA AGAGGGAATT AATTAACCAA ATCAGGTAAA CAAACATCAT TAATTACAGC   
  
  
+ CACCTCGTTT GATCGATCCC GATCACCCAT GATCGTCACT TCCAGATCTT CACGTGTGCA ACCAATGAGC   
  
  
+ CATTAGTTGT AAATCAATTA ACATGTTTAA TCATAAAGAC TTCAAATTTT TGAAGTCAAC ATTCAAATGA   
  
  
+ TGCAGTTTGC TGAGATGGTA TTAGTGGCAC CACACAGCCC ACCCAAATGA ATTAACCATG AGACCTATTT   
  
  
+ ATGATCCTTC ATGGAAGTTC CTTAAGCCGC GCAGACATTC ACTATATAAT AGTAATTATA ACTTTCCACC   
  
  
+ TACCAACCAA GCATATGTAT ACTTTCAATA AATACATGTG CCCAAATTCC ATAAAATTAT AGAACAATTA   
  
  
+ TTGAATGAAT TTAATTATCA GATCAGTCCA AGACAGGCGC TTAGCTAGGT TAGCATAAAA TATCGTGCAC   
  
  
+ CTGTTATATC CTTATCAGCT CAGAGGCCTA ATACTGTTGC TAGTCTGGTA GCTCTACGAA TTTATTTCCA   
  
  
+ TGTAAATCAG AATTAAAAAT TATTATCATC TGTATAATCG TATGTGTTAA CGTCGCTTTC CTAATGATGA   
  
  
+ AGCCGCCAGC TCCATTTTCC ACCCTACATT TTTAAATTAT TATCATCTGT ATAATCGTAT GTGTTAACGT   
  
  
+ CGCTTTCCTA ATGATGAAGC CGCCAGCTCC ATTTTCCACC CTACATTTTT TCCAATAGAT CAATCCGCTG   
  
  
+ ATGAAGCTAT ATGTACATTC AATTTTTTTC TTGAGCTAAC TGGAAAATAT TTTCGTGCCG TCTACGACAC   
  
  
+ CTGTTAACCC TATATATATC TCTTTGGATT TTTCTGTCTA TTTTTGTATA CTTCTTCAGG AAAATATTTT   
  
  
+ CGTGCCGTCT ACGACACCTG TTAACCCTAT ATATATCTCT TTGGATTTTT CTGTCTATTT TTGTATACTT   
  
  
+ CTTCTGGATC AATCTCCATC TTTATCTTCT TCCTCTTCTT CTGCTTGAAG CTTGATCTTC TTCTGTAACT   
  
  
+ ATACAGTGCG TTGTTTCTGT TTCCTTTTC  

- TTTTCCGTTG GAAAAGGAAA GAAAGAAGAA AGGAATCGAA AAATCTAAAT ACTTCTTCCC GACGATGCTG   
  
  
- AAAGGACGAA AAGGTGTAAA GTTTGCGAGC ACAATAGCAG GAAAGTAAGA GGGTGAAGCA CAGGTAGTCG   
  
  
- AGGAAAAATT TTTTTATATA AATAAATAAA ATAATTTAAT AGTAAATAAT AATAAAAAAT TTTTTTTAAA   
  
  
- TTATGTCAAA AAACCCCCTT TTTTTCTTTT TTTTGAGTAG GTAGACTAAA CGAGAGAGAG ACTAAAGGTT   
  
  
- GGCTTACGTA CTCCTCTCGT AAGTCGCCGT TAGTTCGTCT CTCCTGACTG ACAGAATACC CGGGATAGAG   
  
  
- AAGAACCTAA GGTCTGATAA GAACAAAAAT CATCGAGAGT TTAGTTATTG GTCTAGATTA GGATTATGAG   
  
  
- AGAAGTATTA GTATTGAATT TCTCCCTTAA TTAATTGGTT TAGTCCATTT GTTTGTAGTA ATTAATGTCG   
  
  
- GTGGAGCAAA CTAGCTAGGG CTAGTGGGTA CTAGCAGTGA AGGTCTAGAA GTGCACACGT TGGTTACTCG   
  
  
- GTAATCAACA TTTAGTTAAT TGTACAAATT AGTATTTCTG AAGTTTAAAA ACTTCAGTTG TAAGTTTACT   
  
  
- ACGTCAAACG ACTCTACCAT AATCACCGTG GTGTGTCGGG TGGGTTTACT TAATTGGTAC TCTGGATAAA   
  
  
- TACTAGGAAG TACCTTCAAG GAATTCGGCG CGTCTGTAAG TGATATATTA TCATTAATAT TGAAAGGTGG   
  
  
- ATGGTTGGTT CGTATACATA TGAAAGTTAT TTATGTACAC GGGTTTAAGG TATTTTAATA TCTTGTTAAT   
  
  
- AACTTACTTA AATTAATAGT CTAGTCAGGT TCTGTCCGCG AATCGATCCA ATCGTATTTT ATAGCACGTG   
  
  
- GACAATATAG GAATAGTCGA GTCTCCGGAT TATGACAACG ATCAGACCAT CGAGATGCTT AAATAAAGGT   
  
  
- ACATTTAGTC TTAATTTTTA ATAATAGTAG ACATATTAGC ATACACAATT GCAGCGAAAG GATTACTACT   
  
  
- TCGGCGGTCG AGGTAAAAGG TGGGATGTAA AAATTTAATA ATAGTAGACA TATTAGCATA CACAATTGCA   
  
  
- GCGAAAGGAT TACTACTTCG GCGGTCGAGG TAAAAGGTGG GATGTAAAAA AGGTTATCTA GTTAGGCGAC   
  
  
- TACTTCGATA TACATGTAAG TTAAAAAAAG AACTCGATTG ACCTTTTATA AAAGCACGGC AGATGCTGTG   
  
  
- GACAATTGGG ATATATATAG AGAAACCTAA AAAGACAGAT AAAAACATAT GAAGAAGTCC TTTTATAAAA   
  
  
- GCACGGCAGA TGCTGTGGAC AATTGGGATA TATATAGAGA AACCTAAAAA GACAGATAAA AACATATGAA   
  
  
- GAAGACCTAG TTAGAGGTAG AAATAGAAGA AGGAGAAGAA GACGAACTTC GAACTAGAAG AAGACATTGA   
  
  
- TATGTCACGC AACAAAGACA AAGGAAAAG

+     Unnamed\_\_1

| Site Name | Organism | Position | Strand | Matrix score. | sequence | function |
| --- | --- | --- | --- | --- | --- | --- |
| Unnamed\_\_1 | Glycine max | 847 | + | 11 | GAATTTAATTAA | 60K protein binding site |

> 2018/04/13 10:10:12  
+ AAAAGGCAAC CTTTTCCTTT CTTTCTTCTT TCCTTAGCTT TTTAGATTTA TGAAGAAGGG CTGCTACGAC   
  
  
+ TTTCCTGCTT TTCCACATTT CAAACGCTCG TGTTATCGTC CTTTCATTCT CCCACTTCGT GTCCATCAGC   
  
  
+ TCCTTTTTAA AAAAATATAT TTATTTATTT TATTAAATTA TCATTTATTA TTATTTTTTA AAAAAAATTT   
  
  
+ AATACAGTTT TTTGGGGGAA AAAAAGAAAA AAAACTCATC CATCTGATTT GCTCTCTCTC TGATTTCCAA   
  
  
+ CCGAATGCAT GAGGAGAGCA TTCAGCGGCA ATCAAGCAGA GAGGACTGAC TGTCTTATGG GCCCTATCTC   
  
  
+ TTCTTGGATT CCAGACTATT CTTGTTTTTA GTAGCTCTCA AATCAATAAC CAGATCTAAT CCTAATACTC   
  
  
+ TCTTCATAAT CATAACTTAA AGAGGGAATT AATTAACCAA ATCAGGTAAA CAAACATCAT TAATTACAGC   
  
  
+ CACCTCGTTT GATCGATCCC GATCACCCAT GATCGTCACT TCCAGATCTT CACGTGTGCA ACCAATGAGC   
  
  
+ CATTAGTTGT AAATCAATTA ACATGTTTAA TCATAAAGAC TTCAAATTTT TGAAGTCAAC ATTCAAATGA   
  
  
+ TGCAGTTTGC TGAGATGGTA TTAGTGGCAC CACACAGCCC ACCCAAATGA ATTAACCATG AGACCTATTT   
  
  
+ ATGATCCTTC ATGGAAGTTC CTTAAGCCGC GCAGACATTC ACTATATAAT AGTAATTATA ACTTTCCACC   
  
  
+ TACCAACCAA GCATATGTAT ACTTTCAATA AATACATGTG CCCAAATTCC ATAAAATTAT AGAACAATTA   
  
  
+ TTGAATGAAT TTAATTATCA GATCAGTCCA AGACAGGCGC TTAGCTAGGT TAGCATAAAA TATCGTGCAC   
  
  
+ CTGTTATATC CTTATCAGCT CAGAGGCCTA ATACTGTTGC TAGTCTGGTA GCTCTACGAA TTTATTTCCA   
  
  
+ TGTAAATCAG AATTAAAAAT TATTATCATC TGTATAATCG TATGTGTTAA CGTCGCTTTC CTAATGATGA   
  
  
+ AGCCGCCAGC TCCATTTTCC ACCCTACATT TTTAAATTAT TATCATCTGT ATAATCGTAT GTGTTAACGT   
  
  
+ CGCTTTCCTA ATGATGAAGC CGCCAGCTCC ATTTTCCACC CTACATTTTT TCCAATAGAT CAATCCGCTG   
  
  
+ ATGAAGCTAT ATGTACATTC AATTTTTTTC TTGAGCTAAC TGGAAAATAT TTTCGTGCCG TCTACGACAC   
  
  
+ CTGTTAACCC TATATATATC TCTTTGGATT TTTCTGTCTA TTTTTGTATA CTTCTTCAGG AAAATATTTT   
  
  
+ CGTGCCGTCT ACGACACCTG TTAACCCTAT ATATATCTCT TTGGATTTTT CTGTCTATTT TTGTATACTT   
  
  
+ CTTCTGGATC AATCTCCATC TTTATCTTCT TCCTCTTCTT CTGCTTGAAG CTTGATCTTC TTCTGTAACT   
  
  
+ ATACAGTGCG TTGTTTCTGT TTCCTTTTC  

- TTTTCCGTTG GAAAAGGAAA GAAAGAAGAA AGGAATCGAA AAATCTAAAT ACTTCTTCCC GACGATGCTG   
  
  
- AAAGGACGAA AAGGTGTAAA GTTTGCGAGC ACAATAGCAG GAAAGTAAGA GGGTGAAGCA CAGGTAGTCG   
  
  
- AGGAAAAATT TTTTTATATA AATAAATAAA ATAATTTAAT AGTAAATAAT AATAAAAAAT TTTTTTTAAA   
  
  
- TTATGTCAAA AAACCCCCTT TTTTTCTTTT TTTTGAGTAG GTAGACTAAA CGAGAGAGAG ACTAAAGGTT   
  
  
- GGCTTACGTA CTCCTCTCGT AAGTCGCCGT TAGTTCGTCT CTCCTGACTG ACAGAATACC CGGGATAGAG   
  
  
- AAGAACCTAA GGTCTGATAA GAACAAAAAT CATCGAGAGT TTAGTTATTG GTCTAGATTA GGATTATGAG   
  
  
- AGAAGTATTA GTATTGAATT TCTCCCTTAA TTAATTGGTT TAGTCCATTT GTTTGTAGTA ATTAATGTCG   
  
  
- GTGGAGCAAA CTAGCTAGGG CTAGTGGGTA CTAGCAGTGA AGGTCTAGAA GTGCACACGT TGGTTACTCG   
  
  
- GTAATCAACA TTTAGTTAAT TGTACAAATT AGTATTTCTG AAGTTTAAAA ACTTCAGTTG TAAGTTTACT   
  
  
- ACGTCAAACG ACTCTACCAT AATCACCGTG GTGTGTCGGG TGGGTTTACT TAATTGGTAC TCTGGATAAA   
  
  
- TACTAGGAAG TACCTTCAAG GAATTCGGCG CGTCTGTAAG TGATATATTA TCATTAATAT TGAAAGGTGG   
  
  
- ATGGTTGGTT CGTATACATA TGAAAGTTAT TTATGTACAC GGGTTTAAGG TATTTTAATA TCTTGTTAAT   
  
  
- AACTTACTTA AATTAATAGT CTAGTCAGGT TCTGTCCGCG AATCGATCCA ATCGTATTTT ATAGCACGTG   
  
  
- GACAATATAG GAATAGTCGA GTCTCCGGAT TATGACAACG ATCAGACCAT CGAGATGCTT AAATAAAGGT   
  
  
- ACATTTAGTC TTAATTTTTA ATAATAGTAG ACATATTAGC ATACACAATT GCAGCGAAAG GATTACTACT   
  
  
- TCGGCGGTCG AGGTAAAAGG TGGGATGTAA AAATTTAATA ATAGTAGACA TATTAGCATA CACAATTGCA   
  
  
- GCGAAAGGAT TACTACTTCG GCGGTCGAGG TAAAAGGTGG GATGTAAAAA AGGTTATCTA GTTAGGCGAC   
  
  
- TACTTCGATA TACATGTAAG TTAAAAAAAG AACTCGATTG ACCTTTTATA AAAGCACGGC AGATGCTGTG   
  
  
- GACAATTGGG ATATATATAG AGAAACCTAA AAAGACAGAT AAAAACATAT GAAGAAGTCC TTTTATAAAA   
  
  
- GCACGGCAGA TGCTGTGGAC AATTGGGATA TATATAGAGA AACCTAAAAA GACAGATAAA AACATATGAA   
  
  
- GAAGACCTAG TTAGAGGTAG AAATAGAAGA AGGAGAAGAA GACGAACTTC GAACTAGAAG AAGACATTGA   
  
  
- TATGTCACGC AACAAAGACA AAGGAAAAG

+     Unnamed\_\_4

| Site Name | Organism | Position | Strand | Matrix score. | sequence | function |
| --- | --- | --- | --- | --- | --- | --- |
| Unnamed\_\_4 | Petroselinum hortense | 1414 | + | 4 | CTCC |  |
| Unnamed\_\_4 | Petroselinum hortense | 1147 | + | 4 | CTCC |  |
| Unnamed\_\_4 | Petroselinum hortense | 293 | - | 4 | CTCC |  |
| Unnamed\_\_4 | Petroselinum hortense | 140 | + | 4 | CTCC |  |
| Unnamed\_\_4 | Petroselinum hortense | 119 | + | 4 | CTCC |  |
| Unnamed\_\_4 | Petroselinum hortense | 1060 | + | 4 | CTCC |  |

> 2018/04/13 10:10:12  
+ AAAAGGCAAC CTTTTCCTTT CTTTCTTCTT TCCTTAGCTT TTTAGATTTA TGAAGAAGGG CTGCTACGAC   
  
  
+ TTTCCTGCTT TTCCACATTT CAAACGCTCG TGTTATCGTC CTTTCATTCT CCCACTTCGT GTCCATCAGC   
  
  
+ TCCTTTTTAA AAAAATATAT TTATTTATTT TATTAAATTA TCATTTATTA TTATTTTTTA AAAAAAATTT   
  
  
+ AATACAGTTT TTTGGGGGAA AAAAAGAAAA AAAACTCATC CATCTGATTT GCTCTCTCTC TGATTTCCAA   
  
  
+ CCGAATGCAT GAGGAGAGCA TTCAGCGGCA ATCAAGCAGA GAGGACTGAC TGTCTTATGG GCCCTATCTC   
  
  
+ TTCTTGGATT CCAGACTATT CTTGTTTTTA GTAGCTCTCA AATCAATAAC CAGATCTAAT CCTAATACTC   
  
  
+ TCTTCATAAT CATAACTTAA AGAGGGAATT AATTAACCAA ATCAGGTAAA CAAACATCAT TAATTACAGC   
  
  
+ CACCTCGTTT GATCGATCCC GATCACCCAT GATCGTCACT TCCAGATCTT CACGTGTGCA ACCAATGAGC   
  
  
+ CATTAGTTGT AAATCAATTA ACATGTTTAA TCATAAAGAC TTCAAATTTT TGAAGTCAAC ATTCAAATGA   
  
  
+ TGCAGTTTGC TGAGATGGTA TTAGTGGCAC CACACAGCCC ACCCAAATGA ATTAACCATG AGACCTATTT   
  
  
+ ATGATCCTTC ATGGAAGTTC CTTAAGCCGC GCAGACATTC ACTATATAAT AGTAATTATA ACTTTCCACC   
  
  
+ TACCAACCAA GCATATGTAT ACTTTCAATA AATACATGTG CCCAAATTCC ATAAAATTAT AGAACAATTA   
  
  
+ TTGAATGAAT TTAATTATCA GATCAGTCCA AGACAGGCGC TTAGCTAGGT TAGCATAAAA TATCGTGCAC   
  
  
+ CTGTTATATC CTTATCAGCT CAGAGGCCTA ATACTGTTGC TAGTCTGGTA GCTCTACGAA TTTATTTCCA   
  
  
+ TGTAAATCAG AATTAAAAAT TATTATCATC TGTATAATCG TATGTGTTAA CGTCGCTTTC CTAATGATGA   
  
  
+ AGCCGCCAGC TCCATTTTCC ACCCTACATT TTTAAATTAT TATCATCTGT ATAATCGTAT GTGTTAACGT   
  
  
+ CGCTTTCCTA ATGATGAAGC CGCCAGCTCC ATTTTCCACC CTACATTTTT TCCAATAGAT CAATCCGCTG   
  
  
+ ATGAAGCTAT ATGTACATTC AATTTTTTTC TTGAGCTAAC TGGAAAATAT TTTCGTGCCG TCTACGACAC   
  
  
+ CTGTTAACCC TATATATATC TCTTTGGATT TTTCTGTCTA TTTTTGTATA CTTCTTCAGG AAAATATTTT   
  
  
+ CGTGCCGTCT ACGACACCTG TTAACCCTAT ATATATCTCT TTGGATTTTT CTGTCTATTT TTGTATACTT   
  
  
+ CTTCTGGATC AATCTCCATC TTTATCTTCT TCCTCTTCTT CTGCTTGAAG CTTGATCTTC TTCTGTAACT   
  
  
+ ATACAGTGCG TTGTTTCTGT TTCCTTTTC  

- TTTTCCGTTG GAAAAGGAAA GAAAGAAGAA AGGAATCGAA AAATCTAAAT ACTTCTTCCC GACGATGCTG   
  
  
- AAAGGACGAA AAGGTGTAAA GTTTGCGAGC ACAATAGCAG GAAAGTAAGA GGGTGAAGCA CAGGTAGTCG   
  
  
- AGGAAAAATT TTTTTATATA AATAAATAAA ATAATTTAAT AGTAAATAAT AATAAAAAAT TTTTTTTAAA   
  
  
- TTATGTCAAA AAACCCCCTT TTTTTCTTTT TTTTGAGTAG GTAGACTAAA CGAGAGAGAG ACTAAAGGTT   
  
  
- GGCTTACGTA CTCCTCTCGT AAGTCGCCGT TAGTTCGTCT CTCCTGACTG ACAGAATACC CGGGATAGAG   
  
  
- AAGAACCTAA GGTCTGATAA GAACAAAAAT CATCGAGAGT TTAGTTATTG GTCTAGATTA GGATTATGAG   
  
  
- AGAAGTATTA GTATTGAATT TCTCCCTTAA TTAATTGGTT TAGTCCATTT GTTTGTAGTA ATTAATGTCG   
  
  
- GTGGAGCAAA CTAGCTAGGG CTAGTGGGTA CTAGCAGTGA AGGTCTAGAA GTGCACACGT TGGTTACTCG   
  
  
- GTAATCAACA TTTAGTTAAT TGTACAAATT AGTATTTCTG AAGTTTAAAA ACTTCAGTTG TAAGTTTACT   
  
  
- ACGTCAAACG ACTCTACCAT AATCACCGTG GTGTGTCGGG TGGGTTTACT TAATTGGTAC TCTGGATAAA   
  
  
- TACTAGGAAG TACCTTCAAG GAATTCGGCG CGTCTGTAAG TGATATATTA TCATTAATAT TGAAAGGTGG   
  
  
- ATGGTTGGTT CGTATACATA TGAAAGTTAT TTATGTACAC GGGTTTAAGG TATTTTAATA TCTTGTTAAT   
  
  
- AACTTACTTA AATTAATAGT CTAGTCAGGT TCTGTCCGCG AATCGATCCA ATCGTATTTT ATAGCACGTG   
  
  
- GACAATATAG GAATAGTCGA GTCTCCGGAT TATGACAACG ATCAGACCAT CGAGATGCTT AAATAAAGGT   
  
  
- ACATTTAGTC TTAATTTTTA ATAATAGTAG ACATATTAGC ATACACAATT GCAGCGAAAG GATTACTACT   
  
  
- TCGGCGGTCG AGGTAAAAGG TGGGATGTAA AAATTTAATA ATAGTAGACA TATTAGCATA CACAATTGCA   
  
  
- GCGAAAGGAT TACTACTTCG GCGGTCGAGG TAAAAGGTGG GATGTAAAAA AGGTTATCTA GTTAGGCGAC   
  
  
- TACTTCGATA TACATGTAAG TTAAAAAAAG AACTCGATTG ACCTTTTATA AAAGCACGGC AGATGCTGTG   
  
  
- GACAATTGGG ATATATATAG AGAAACCTAA AAAGACAGAT AAAAACATAT GAAGAAGTCC TTTTATAAAA   
  
  
- GCACGGCAGA TGCTGTGGAC AATTGGGATA TATATAGAGA AACCTAAAAA GACAGATAAA AACATATGAA   
  
  
- GAAGACCTAG TTAGAGGTAG AAATAGAAGA AGGAGAAGAA GACGAACTTC GAACTAGAAG AAGACATTGA   
  
  
- TATGTCACGC AACAAAGACA AAGGAAAAG

+     Unnamed\_\_6

| Site Name | Organism | Position | Strand | Matrix score. | sequence | function |
| --- | --- | --- | --- | --- | --- | --- |
| Unnamed\_\_6 | Zea mays | 1271 | + | 10 | taTAAATATct |  |
| Unnamed\_\_6 | Zea mays | 1358 | + | 10 | taTAAATATct |  |

> 2018/04/13 10:10:12  
+ AAAAGGCAAC CTTTTCCTTT CTTTCTTCTT TCCTTAGCTT TTTAGATTTA TGAAGAAGGG CTGCTACGAC   
  
  
+ TTTCCTGCTT TTCCACATTT CAAACGCTCG TGTTATCGTC CTTTCATTCT CCCACTTCGT GTCCATCAGC   
  
  
+ TCCTTTTTAA AAAAATATAT TTATTTATTT TATTAAATTA TCATTTATTA TTATTTTTTA AAAAAAATTT   
  
  
+ AATACAGTTT TTTGGGGGAA AAAAAGAAAA AAAACTCATC CATCTGATTT GCTCTCTCTC TGATTTCCAA   
  
  
+ CCGAATGCAT GAGGAGAGCA TTCAGCGGCA ATCAAGCAGA GAGGACTGAC TGTCTTATGG GCCCTATCTC   
  
  
+ TTCTTGGATT CCAGACTATT CTTGTTTTTA GTAGCTCTCA AATCAATAAC CAGATCTAAT CCTAATACTC   
  
  
+ TCTTCATAAT CATAACTTAA AGAGGGAATT AATTAACCAA ATCAGGTAAA CAAACATCAT TAATTACAGC   
  
  
+ CACCTCGTTT GATCGATCCC GATCACCCAT GATCGTCACT TCCAGATCTT CACGTGTGCA ACCAATGAGC   
  
  
+ CATTAGTTGT AAATCAATTA ACATGTTTAA TCATAAAGAC TTCAAATTTT TGAAGTCAAC ATTCAAATGA   
  
  
+ TGCAGTTTGC TGAGATGGTA TTAGTGGCAC CACACAGCCC ACCCAAATGA ATTAACCATG AGACCTATTT   
  
  
+ ATGATCCTTC ATGGAAGTTC CTTAAGCCGC GCAGACATTC ACTATATAAT AGTAATTATA ACTTTCCACC   
  
  
+ TACCAACCAA GCATATGTAT ACTTTCAATA AATACATGTG CCCAAATTCC ATAAAATTAT AGAACAATTA   
  
  
+ TTGAATGAAT TTAATTATCA GATCAGTCCA AGACAGGCGC TTAGCTAGGT TAGCATAAAA TATCGTGCAC   
  
  
+ CTGTTATATC CTTATCAGCT CAGAGGCCTA ATACTGTTGC TAGTCTGGTA GCTCTACGAA TTTATTTCCA   
  
  
+ TGTAAATCAG AATTAAAAAT TATTATCATC TGTATAATCG TATGTGTTAA CGTCGCTTTC CTAATGATGA   
  
  
+ AGCCGCCAGC TCCATTTTCC ACCCTACATT TTTAAATTAT TATCATCTGT ATAATCGTAT GTGTTAACGT   
  
  
+ CGCTTTCCTA ATGATGAAGC CGCCAGCTCC ATTTTCCACC CTACATTTTT TCCAATAGAT CAATCCGCTG   
  
  
+ ATGAAGCTAT ATGTACATTC AATTTTTTTC TTGAGCTAAC TGGAAAATAT TTTCGTGCCG TCTACGACAC   
  
  
+ CTGTTAACCC TATATATATC TCTTTGGATT TTTCTGTCTA TTTTTGTATA CTTCTTCAGG AAAATATTTT   
  
  
+ CGTGCCGTCT ACGACACCTG TTAACCCTAT ATATATCTCT TTGGATTTTT CTGTCTATTT TTGTATACTT   
  
  
+ CTTCTGGATC AATCTCCATC TTTATCTTCT TCCTCTTCTT CTGCTTGAAG CTTGATCTTC TTCTGTAACT   
  
  
+ ATACAGTGCG TTGTTTCTGT TTCCTTTTC  

- TTTTCCGTTG GAAAAGGAAA GAAAGAAGAA AGGAATCGAA AAATCTAAAT ACTTCTTCCC GACGATGCTG   
  
  
- AAAGGACGAA AAGGTGTAAA GTTTGCGAGC ACAATAGCAG GAAAGTAAGA GGGTGAAGCA CAGGTAGTCG   
  
  
- AGGAAAAATT TTTTTATATA AATAAATAAA ATAATTTAAT AGTAAATAAT AATAAAAAAT TTTTTTTAAA   
  
  
- TTATGTCAAA AAACCCCCTT TTTTTCTTTT TTTTGAGTAG GTAGACTAAA CGAGAGAGAG ACTAAAGGTT   
  
  
- GGCTTACGTA CTCCTCTCGT AAGTCGCCGT TAGTTCGTCT CTCCTGACTG ACAGAATACC CGGGATAGAG   
  
  
- AAGAACCTAA GGTCTGATAA GAACAAAAAT CATCGAGAGT TTAGTTATTG GTCTAGATTA GGATTATGAG   
  
  
- AGAAGTATTA GTATTGAATT TCTCCCTTAA TTAATTGGTT TAGTCCATTT GTTTGTAGTA ATTAATGTCG   
  
  
- GTGGAGCAAA CTAGCTAGGG CTAGTGGGTA CTAGCAGTGA AGGTCTAGAA GTGCACACGT TGGTTACTCG   
  
  
- GTAATCAACA TTTAGTTAAT TGTACAAATT AGTATTTCTG AAGTTTAAAA ACTTCAGTTG TAAGTTTACT   
  
  
- ACGTCAAACG ACTCTACCAT AATCACCGTG GTGTGTCGGG TGGGTTTACT TAATTGGTAC TCTGGATAAA   
  
  
- TACTAGGAAG TACCTTCAAG GAATTCGGCG CGTCTGTAAG TGATATATTA TCATTAATAT TGAAAGGTGG   
  
  
- ATGGTTGGTT CGTATACATA TGAAAGTTAT TTATGTACAC GGGTTTAAGG TATTTTAATA TCTTGTTAAT   
  
  
- AACTTACTTA AATTAATAGT CTAGTCAGGT TCTGTCCGCG AATCGATCCA ATCGTATTTT ATAGCACGTG   
  
  
- GACAATATAG GAATAGTCGA GTCTCCGGAT TATGACAACG ATCAGACCAT CGAGATGCTT AAATAAAGGT   
  
  
- ACATTTAGTC TTAATTTTTA ATAATAGTAG ACATATTAGC ATACACAATT GCAGCGAAAG GATTACTACT   
  
  
- TCGGCGGTCG AGGTAAAAGG TGGGATGTAA AAATTTAATA ATAGTAGACA TATTAGCATA CACAATTGCA   
  
  
- GCGAAAGGAT TACTACTTCG GCGGTCGAGG TAAAAGGTGG GATGTAAAAA AGGTTATCTA GTTAGGCGAC   
  
  
- TACTTCGATA TACATGTAAG TTAAAAAAAG AACTCGATTG ACCTTTTATA AAAGCACGGC AGATGCTGTG   
  
  
- GACAATTGGG ATATATATAG AGAAACCTAA AAAGACAGAT AAAAACATAT GAAGAAGTCC TTTTATAAAA   
  
  
- GCACGGCAGA TGCTGTGGAC AATTGGGATA TATATAGAGA AACCTAAAAA GACAGATAAA AACATATGAA   
  
  
- GAAGACCTAG TTAGAGGTAG AAATAGAAGA AGGAGAAGAA GACGAACTTC GAACTAGAAG AAGACATTGA   
  
  
- TATGTCACGC AACAAAGACA AAGGAAAAG

+     WUN-motif

| Site Name | Organism | Position | Strand | Matrix score. | sequence | function |
| --- | --- | --- | --- | --- | --- | --- |
| WUN-motif | Brassica oleracea | 1038 | - | 9 | TCATTACGAA | wound-responsive element |
| WUN-motif | Brassica oleracea | 1125 | - | 9 | TCATTACGAA | wound-responsive element |

> 2018/04/13 10:10:12  
+ AAAAGGCAAC CTTTTCCTTT CTTTCTTCTT TCCTTAGCTT TTTAGATTTA TGAAGAAGGG CTGCTACGAC   
  
  
+ TTTCCTGCTT TTCCACATTT CAAACGCTCG TGTTATCGTC CTTTCATTCT CCCACTTCGT GTCCATCAGC   
  
  
+ TCCTTTTTAA AAAAATATAT TTATTTATTT TATTAAATTA TCATTTATTA TTATTTTTTA AAAAAAATTT   
  
  
+ AATACAGTTT TTTGGGGGAA AAAAAGAAAA AAAACTCATC CATCTGATTT GCTCTCTCTC TGATTTCCAA   
  
  
+ CCGAATGCAT GAGGAGAGCA TTCAGCGGCA ATCAAGCAGA GAGGACTGAC TGTCTTATGG GCCCTATCTC   
  
  
+ TTCTTGGATT CCAGACTATT CTTGTTTTTA GTAGCTCTCA AATCAATAAC CAGATCTAAT CCTAATACTC   
  
  
+ TCTTCATAAT CATAACTTAA AGAGGGAATT AATTAACCAA ATCAGGTAAA CAAACATCAT TAATTACAGC   
  
  
+ CACCTCGTTT GATCGATCCC GATCACCCAT GATCGTCACT TCCAGATCTT CACGTGTGCA ACCAATGAGC   
  
  
+ CATTAGTTGT AAATCAATTA ACATGTTTAA TCATAAAGAC TTCAAATTTT TGAAGTCAAC ATTCAAATGA   
  
  
+ TGCAGTTTGC TGAGATGGTA TTAGTGGCAC CACACAGCCC ACCCAAATGA ATTAACCATG AGACCTATTT   
  
  
+ ATGATCCTTC ATGGAAGTTC CTTAAGCCGC GCAGACATTC ACTATATAAT AGTAATTATA ACTTTCCACC   
  
  
+ TACCAACCAA GCATATGTAT ACTTTCAATA AATACATGTG CCCAAATTCC ATAAAATTAT AGAACAATTA   
  
  
+ TTGAATGAAT TTAATTATCA GATCAGTCCA AGACAGGCGC TTAGCTAGGT TAGCATAAAA TATCGTGCAC   
  
  
+ CTGTTATATC CTTATCAGCT CAGAGGCCTA ATACTGTTGC TAGTCTGGTA GCTCTACGAA TTTATTTCCA   
  
  
+ TGTAAATCAG AATTAAAAAT TATTATCATC TGTATAATCG TATGTGTTAA CGTCGCTTTC CTAATGATGA   
  
  
+ AGCCGCCAGC TCCATTTTCC ACCCTACATT TTTAAATTAT TATCATCTGT ATAATCGTAT GTGTTAACGT   
  
  
+ CGCTTTCCTA ATGATGAAGC CGCCAGCTCC ATTTTCCACC CTACATTTTT TCCAATAGAT CAATCCGCTG   
  
  
+ ATGAAGCTAT ATGTACATTC AATTTTTTTC TTGAGCTAAC TGGAAAATAT TTTCGTGCCG TCTACGACAC   
  
  
+ CTGTTAACCC TATATATATC TCTTTGGATT TTTCTGTCTA TTTTTGTATA CTTCTTCAGG AAAATATTTT   
  
  
+ CGTGCCGTCT ACGACACCTG TTAACCCTAT ATATATCTCT TTGGATTTTT CTGTCTATTT TTGTATACTT   
  
  
+ CTTCTGGATC AATCTCCATC TTTATCTTCT TCCTCTTCTT CTGCTTGAAG CTTGATCTTC TTCTGTAACT   
  
  
+ ATACAGTGCG TTGTTTCTGT TTCCTTTTC  

- TTTTCCGTTG GAAAAGGAAA GAAAGAAGAA AGGAATCGAA AAATCTAAAT ACTTCTTCCC GACGATGCTG   
  
  
- AAAGGACGAA AAGGTGTAAA GTTTGCGAGC ACAATAGCAG GAAAGTAAGA GGGTGAAGCA CAGGTAGTCG   
  
  
- AGGAAAAATT TTTTTATATA AATAAATAAA ATAATTTAAT AGTAAATAAT AATAAAAAAT TTTTTTTAAA   
  
  
- TTATGTCAAA AAACCCCCTT TTTTTCTTTT TTTTGAGTAG GTAGACTAAA CGAGAGAGAG ACTAAAGGTT   
  
  
- GGCTTACGTA CTCCTCTCGT AAGTCGCCGT TAGTTCGTCT CTCCTGACTG ACAGAATACC CGGGATAGAG   
  
  
- AAGAACCTAA GGTCTGATAA GAACAAAAAT CATCGAGAGT TTAGTTATTG GTCTAGATTA GGATTATGAG   
  
  
- AGAAGTATTA GTATTGAATT TCTCCCTTAA TTAATTGGTT TAGTCCATTT GTTTGTAGTA ATTAATGTCG   
  
  
- GTGGAGCAAA CTAGCTAGGG CTAGTGGGTA CTAGCAGTGA AGGTCTAGAA GTGCACACGT TGGTTACTCG   
  
  
- GTAATCAACA TTTAGTTAAT TGTACAAATT AGTATTTCTG AAGTTTAAAA ACTTCAGTTG TAAGTTTACT   
  
  
- ACGTCAAACG ACTCTACCAT AATCACCGTG GTGTGTCGGG TGGGTTTACT TAATTGGTAC TCTGGATAAA   
  
  
- TACTAGGAAG TACCTTCAAG GAATTCGGCG CGTCTGTAAG TGATATATTA TCATTAATAT TGAAAGGTGG   
  
  
- ATGGTTGGTT CGTATACATA TGAAAGTTAT TTATGTACAC GGGTTTAAGG TATTTTAATA TCTTGTTAAT   
  
  
- AACTTACTTA AATTAATAGT CTAGTCAGGT TCTGTCCGCG AATCGATCCA ATCGTATTTT ATAGCACGTG   
  
  
- GACAATATAG GAATAGTCGA GTCTCCGGAT TATGACAACG ATCAGACCAT CGAGATGCTT AAATAAAGGT   
  
  
- ACATTTAGTC TTAATTTTTA ATAATAGTAG ACATATTAGC ATACACAATT GCAGCGAAAG GATTACTACT   
  
  
- TCGGCGGTCG AGGTAAAAGG TGGGATGTAA AAATTTAATA ATAGTAGACA TATTAGCATA CACAATTGCA   
  
  
- GCGAAAGGAT TACTACTTCG GCGGTCGAGG TAAAAGGTGG GATGTAAAAA AGGTTATCTA GTTAGGCGAC   
  
  
- TACTTCGATA TACATGTAAG TTAAAAAAAG AACTCGATTG ACCTTTTATA AAAGCACGGC AGATGCTGTG   
  
  
- GACAATTGGG ATATATATAG AGAAACCTAA AAAGACAGAT AAAAACATAT GAAGAAGTCC TTTTATAAAA   
  
  
- GCACGGCAGA TGCTGTGGAC AATTGGGATA TATATAGAGA AACCTAAAAA GACAGATAAA AACATATGAA   
  
  
- GAAGACCTAG TTAGAGGTAG AAATAGAAGA AGGAGAAGAA GACGAACTTC GAACTAGAAG AAGACATTGA   
  
  
- TATGTCACGC AACAAAGACA AAGGAAAAG

+     as-2-box

| Site Name | Organism | Position | Strand | Matrix score. | sequence | function |
| --- | --- | --- | --- | --- | --- | --- |
| as-2-box | Nicotiana tabacum | 1417 | - | 9 | GATAatGATG | involved in shoot-specific expression and light responsiveness |

> 2018/04/13 10:10:12  
+ AAAAGGCAAC CTTTTCCTTT CTTTCTTCTT TCCTTAGCTT TTTAGATTTA TGAAGAAGGG CTGCTACGAC   
  
  
+ TTTCCTGCTT TTCCACATTT CAAACGCTCG TGTTATCGTC CTTTCATTCT CCCACTTCGT GTCCATCAGC   
  
  
+ TCCTTTTTAA AAAAATATAT TTATTTATTT TATTAAATTA TCATTTATTA TTATTTTTTA AAAAAAATTT   
  
  
+ AATACAGTTT TTTGGGGGAA AAAAAGAAAA AAAACTCATC CATCTGATTT GCTCTCTCTC TGATTTCCAA   
  
  
+ CCGAATGCAT GAGGAGAGCA TTCAGCGGCA ATCAAGCAGA GAGGACTGAC TGTCTTATGG GCCCTATCTC   
  
  
+ TTCTTGGATT CCAGACTATT CTTGTTTTTA GTAGCTCTCA AATCAATAAC CAGATCTAAT CCTAATACTC   
  
  
+ TCTTCATAAT CATAACTTAA AGAGGGAATT AATTAACCAA ATCAGGTAAA CAAACATCAT TAATTACAGC   
  
  
+ CACCTCGTTT GATCGATCCC GATCACCCAT GATCGTCACT TCCAGATCTT CACGTGTGCA ACCAATGAGC   
  
  
+ CATTAGTTGT AAATCAATTA ACATGTTTAA TCATAAAGAC TTCAAATTTT TGAAGTCAAC ATTCAAATGA   
  
  
+ TGCAGTTTGC TGAGATGGTA TTAGTGGCAC CACACAGCCC ACCCAAATGA ATTAACCATG AGACCTATTT   
  
  
+ ATGATCCTTC ATGGAAGTTC CTTAAGCCGC GCAGACATTC ACTATATAAT AGTAATTATA ACTTTCCACC   
  
  
+ TACCAACCAA GCATATGTAT ACTTTCAATA AATACATGTG CCCAAATTCC ATAAAATTAT AGAACAATTA   
  
  
+ TTGAATGAAT TTAATTATCA GATCAGTCCA AGACAGGCGC TTAGCTAGGT TAGCATAAAA TATCGTGCAC   
  
  
+ CTGTTATATC CTTATCAGCT CAGAGGCCTA ATACTGTTGC TAGTCTGGTA GCTCTACGAA TTTATTTCCA   
  
  
+ TGTAAATCAG AATTAAAAAT TATTATCATC TGTATAATCG TATGTGTTAA CGTCGCTTTC CTAATGATGA   
  
  
+ AGCCGCCAGC TCCATTTTCC ACCCTACATT TTTAAATTAT TATCATCTGT ATAATCGTAT GTGTTAACGT   
  
  
+ CGCTTTCCTA ATGATGAAGC CGCCAGCTCC ATTTTCCACC CTACATTTTT TCCAATAGAT CAATCCGCTG   
  
  
+ ATGAAGCTAT ATGTACATTC AATTTTTTTC TTGAGCTAAC TGGAAAATAT TTTCGTGCCG TCTACGACAC   
  
  
+ CTGTTAACCC TATATATATC TCTTTGGATT TTTCTGTCTA TTTTTGTATA CTTCTTCAGG AAAATATTTT   
  
  
+ CGTGCCGTCT ACGACACCTG TTAACCCTAT ATATATCTCT TTGGATTTTT CTGTCTATTT TTGTATACTT   
  
  
+ CTTCTGGATC AATCTCCATC TTTATCTTCT TCCTCTTCTT CTGCTTGAAG CTTGATCTTC TTCTGTAACT   
  
  
+ ATACAGTGCG TTGTTTCTGT TTCCTTTTC  

- TTTTCCGTTG GAAAAGGAAA GAAAGAAGAA AGGAATCGAA AAATCTAAAT ACTTCTTCCC GACGATGCTG   
  
  
- AAAGGACGAA AAGGTGTAAA GTTTGCGAGC ACAATAGCAG GAAAGTAAGA GGGTGAAGCA CAGGTAGTCG   
  
  
- AGGAAAAATT TTTTTATATA AATAAATAAA ATAATTTAAT AGTAAATAAT AATAAAAAAT TTTTTTTAAA   
  
  
- TTATGTCAAA AAACCCCCTT TTTTTCTTTT TTTTGAGTAG GTAGACTAAA CGAGAGAGAG ACTAAAGGTT   
  
  
- GGCTTACGTA CTCCTCTCGT AAGTCGCCGT TAGTTCGTCT CTCCTGACTG ACAGAATACC CGGGATAGAG   
  
  
- AAGAACCTAA GGTCTGATAA GAACAAAAAT CATCGAGAGT TTAGTTATTG GTCTAGATTA GGATTATGAG   
  
  
- AGAAGTATTA GTATTGAATT TCTCCCTTAA TTAATTGGTT TAGTCCATTT GTTTGTAGTA ATTAATGTCG   
  
  
- GTGGAGCAAA CTAGCTAGGG CTAGTGGGTA CTAGCAGTGA AGGTCTAGAA GTGCACACGT TGGTTACTCG   
  
  
- GTAATCAACA TTTAGTTAAT TGTACAAATT AGTATTTCTG AAGTTTAAAA ACTTCAGTTG TAAGTTTACT   
  
  
- ACGTCAAACG ACTCTACCAT AATCACCGTG GTGTGTCGGG TGGGTTTACT TAATTGGTAC TCTGGATAAA   
  
  
- TACTAGGAAG TACCTTCAAG GAATTCGGCG CGTCTGTAAG TGATATATTA TCATTAATAT TGAAAGGTGG   
  
  
- ATGGTTGGTT CGTATACATA TGAAAGTTAT TTATGTACAC GGGTTTAAGG TATTTTAATA TCTTGTTAAT   
  
  
- AACTTACTTA AATTAATAGT CTAGTCAGGT TCTGTCCGCG AATCGATCCA ATCGTATTTT ATAGCACGTG   
  
  
- GACAATATAG GAATAGTCGA GTCTCCGGAT TATGACAACG ATCAGACCAT CGAGATGCTT AAATAAAGGT   
  
  
- ACATTTAGTC TTAATTTTTA ATAATAGTAG ACATATTAGC ATACACAATT GCAGCGAAAG GATTACTACT   
  
  
- TCGGCGGTCG AGGTAAAAGG TGGGATGTAA AAATTTAATA ATAGTAGACA TATTAGCATA CACAATTGCA   
  
  
- GCGAAAGGAT TACTACTTCG GCGGTCGAGG TAAAAGGTGG GATGTAAAAA AGGTTATCTA GTTAGGCGAC   
  
  
- TACTTCGATA TACATGTAAG TTAAAAAAAG AACTCGATTG ACCTTTTATA AAAGCACGGC AGATGCTGTG   
  
  
- GACAATTGGG ATATATATAG AGAAACCTAA AAAGACAGAT AAAAACATAT GAAGAAGTCC TTTTATAAAA   
  
  
- GCACGGCAGA TGCTGTGGAC AATTGGGATA TATATAGAGA AACCTAAAAA GACAGATAAA AACATATGAA   
  
  
- GAAGACCTAG TTAGAGGTAG AAATAGAAGA AGGAGAAGAA GACGAACTTC GAACTAGAAG AAGACATTGA   
  
  
- TATGTCACGC AACAAAGACA AAGGAAAAG

+     box S

| Site Name | Organism | Position | Strand | Matrix score. | sequence | function |
| --- | --- | --- | --- | --- | --- | --- |
| box S | Arabidopsis thaliana | 488 | + | 7 | AGCCACC |  |

> 2018/04/13 10:10:12  
+ AAAAGGCAAC CTTTTCCTTT CTTTCTTCTT TCCTTAGCTT TTTAGATTTA TGAAGAAGGG CTGCTACGAC   
  
  
+ TTTCCTGCTT TTCCACATTT CAAACGCTCG TGTTATCGTC CTTTCATTCT CCCACTTCGT GTCCATCAGC   
  
  
+ TCCTTTTTAA AAAAATATAT TTATTTATTT TATTAAATTA TCATTTATTA TTATTTTTTA AAAAAAATTT   
  
  
+ AATACAGTTT TTTGGGGGAA AAAAAGAAAA AAAACTCATC CATCTGATTT GCTCTCTCTC TGATTTCCAA   
  
  
+ CCGAATGCAT GAGGAGAGCA TTCAGCGGCA ATCAAGCAGA GAGGACTGAC TGTCTTATGG GCCCTATCTC   
  
  
+ TTCTTGGATT CCAGACTATT CTTGTTTTTA GTAGCTCTCA AATCAATAAC CAGATCTAAT CCTAATACTC   
  
  
+ TCTTCATAAT CATAACTTAA AGAGGGAATT AATTAACCAA ATCAGGTAAA CAAACATCAT TAATTACAGC   
  
  
+ CACCTCGTTT GATCGATCCC GATCACCCAT GATCGTCACT TCCAGATCTT CACGTGTGCA ACCAATGAGC   
  
  
+ CATTAGTTGT AAATCAATTA ACATGTTTAA TCATAAAGAC TTCAAATTTT TGAAGTCAAC ATTCAAATGA   
  
  
+ TGCAGTTTGC TGAGATGGTA TTAGTGGCAC CACACAGCCC ACCCAAATGA ATTAACCATG AGACCTATTT   
  
  
+ ATGATCCTTC ATGGAAGTTC CTTAAGCCGC GCAGACATTC ACTATATAAT AGTAATTATA ACTTTCCACC   
  
  
+ TACCAACCAA GCATATGTAT ACTTTCAATA AATACATGTG CCCAAATTCC ATAAAATTAT AGAACAATTA   
  
  
+ TTGAATGAAT TTAATTATCA GATCAGTCCA AGACAGGCGC TTAGCTAGGT TAGCATAAAA TATCGTGCAC   
  
  
+ CTGTTATATC CTTATCAGCT CAGAGGCCTA ATACTGTTGC TAGTCTGGTA GCTCTACGAA TTTATTTCCA   
  
  
+ TGTAAATCAG AATTAAAAAT TATTATCATC TGTATAATCG TATGTGTTAA CGTCGCTTTC CTAATGATGA   
  
  
+ AGCCGCCAGC TCCATTTTCC ACCCTACATT TTTAAATTAT TATCATCTGT ATAATCGTAT GTGTTAACGT   
  
  
+ CGCTTTCCTA ATGATGAAGC CGCCAGCTCC ATTTTCCACC CTACATTTTT TCCAATAGAT CAATCCGCTG   
  
  
+ ATGAAGCTAT ATGTACATTC AATTTTTTTC TTGAGCTAAC TGGAAAATAT TTTCGTGCCG TCTACGACAC   
  
  
+ CTGTTAACCC TATATATATC TCTTTGGATT TTTCTGTCTA TTTTTGTATA CTTCTTCAGG AAAATATTTT   
  
  
+ CGTGCCGTCT ACGACACCTG TTAACCCTAT ATATATCTCT TTGGATTTTT CTGTCTATTT TTGTATACTT   
  
  
+ CTTCTGGATC AATCTCCATC TTTATCTTCT TCCTCTTCTT CTGCTTGAAG CTTGATCTTC TTCTGTAACT   
  
  
+ ATACAGTGCG TTGTTTCTGT TTCCTTTTC  

- TTTTCCGTTG GAAAAGGAAA GAAAGAAGAA AGGAATCGAA AAATCTAAAT ACTTCTTCCC GACGATGCTG   
  
  
- AAAGGACGAA AAGGTGTAAA GTTTGCGAGC ACAATAGCAG GAAAGTAAGA GGGTGAAGCA CAGGTAGTCG   
  
  
- AGGAAAAATT TTTTTATATA AATAAATAAA ATAATTTAAT AGTAAATAAT AATAAAAAAT TTTTTTTAAA   
  
  
- TTATGTCAAA AAACCCCCTT TTTTTCTTTT TTTTGAGTAG GTAGACTAAA CGAGAGAGAG ACTAAAGGTT   
  
  
- GGCTTACGTA CTCCTCTCGT AAGTCGCCGT TAGTTCGTCT CTCCTGACTG ACAGAATACC CGGGATAGAG   
  
  
- AAGAACCTAA GGTCTGATAA GAACAAAAAT CATCGAGAGT TTAGTTATTG GTCTAGATTA GGATTATGAG   
  
  
- AGAAGTATTA GTATTGAATT TCTCCCTTAA TTAATTGGTT TAGTCCATTT GTTTGTAGTA ATTAATGTCG   
  
  
- GTGGAGCAAA CTAGCTAGGG CTAGTGGGTA CTAGCAGTGA AGGTCTAGAA GTGCACACGT TGGTTACTCG   
  
  
- GTAATCAACA TTTAGTTAAT TGTACAAATT AGTATTTCTG AAGTTTAAAA ACTTCAGTTG TAAGTTTACT   
  
  
- ACGTCAAACG ACTCTACCAT AATCACCGTG GTGTGTCGGG TGGGTTTACT TAATTGGTAC TCTGGATAAA   
  
  
- TACTAGGAAG TACCTTCAAG GAATTCGGCG CGTCTGTAAG TGATATATTA TCATTAATAT TGAAAGGTGG   
  
  
- ATGGTTGGTT CGTATACATA TGAAAGTTAT TTATGTACAC GGGTTTAAGG TATTTTAATA TCTTGTTAAT   
  
  
- AACTTACTTA AATTAATAGT CTAGTCAGGT TCTGTCCGCG AATCGATCCA ATCGTATTTT ATAGCACGTG   
  
  
- GACAATATAG GAATAGTCGA GTCTCCGGAT TATGACAACG ATCAGACCAT CGAGATGCTT AAATAAAGGT   
  
  
- ACATTTAGTC TTAATTTTTA ATAATAGTAG ACATATTAGC ATACACAATT GCAGCGAAAG GATTACTACT   
  
  
- TCGGCGGTCG AGGTAAAAGG TGGGATGTAA AAATTTAATA ATAGTAGACA TATTAGCATA CACAATTGCA   
  
  
- GCGAAAGGAT TACTACTTCG GCGGTCGAGG TAAAAGGTGG GATGTAAAAA AGGTTATCTA GTTAGGCGAC   
  
  
- TACTTCGATA TACATGTAAG TTAAAAAAAG AACTCGATTG ACCTTTTATA AAAGCACGGC AGATGCTGTG   
  
  
- GACAATTGGG ATATATATAG AGAAACCTAA AAAGACAGAT AAAAACATAT GAAGAAGTCC TTTTATAAAA   
  
  
- GCACGGCAGA TGCTGTGGAC AATTGGGATA TATATAGAGA AACCTAAAAA GACAGATAAA AACATATGAA   
  
  
- GAAGACCTAG TTAGAGGTAG AAATAGAAGA AGGAGAAGAA GACGAACTTC GAACTAGAAG AAGACATTGA   
  
  
- TATGTCACGC AACAAAGACA AAGGAAAAG
