# Supplementary material for: Genome-wide analysis of the WRKY gene family in drumstick (Moringa oleifera Lam.)
Source: PeerJ. 2019 Jun 10;7:e7063. doi: 10.7717/peerj.7063 (PMC6563795; doi:10.7717/peerj.7063)
Supplement: Supplemental Information 1 [file peerj-07-7063-s003.gz › MoWRKY1_plantcare.html]

Content-Type: text/html; charset=ISO-8859-1


CallMat\_Firefox


Webmaster Firefox specific output  
To save the result:
click on the frame with the right mouse button and save the source code as a text file with extension .html  
REFERENCE:PlantCARE: a database of plant cis-acting regulatory elements and a portal to tools for in silico analysis of promoter sequences.  
Lescot, M., Déhais, P., Moreau, Y., De Moor, B., Rouzé ,P.,and Rombauts, S.  
Nucleic Acids Res., Database issue(2002), 30(1):325-327.   


---

> 2018/04/13 10:10:12  
+ ACTAATTTTA GTGGGTTTAG GTTGTTCGTT TTTTTCTCCT TGAGAAATAC AGGATAGACA AAGCAAGAGC   
  
  
+ ACCAACAACA GCACAACGAA ACGGAGGAAT TGAAAAGCTA CAGACCCAAC ACGAAGCGCC TAGACCTTTT   
  
  
+ AGAAGTCATT TTTATTTTTC AGGTGCGGCG TCTATGCTTT TTCGAGAGAA GAAACTTAAA GACGCAAACG   
  
  
+ GGAAAAAGAA AAGAGACAAA ACAGAGAAAA TCCTCTCTCT ACTCACTACC ACTACTACTA CGACCTATGA   
  
  
+ CGTTGCACTA ACCNCCGACT ACCGCCGGAC CAGACATGGA AAATACACTA AACTGAGAAG ACAGGGGCTG   
  
  
+ CTGAGAGTGT TACTGGCGTC GTGGCGTCAG TCACCGTCGA CCGGATTCGT CGTCAGTGTG AACGAAAAAG   
  
  
+ AAAAAAGAGA AATTAGAAAA AAAAAAAAGG GACGACAATG GACGTCTATT GCCCTTTCTC TTTCCCCCAC   
  
  
+ CGTGATCGTA AAGGGTAAAA CGGTGCACCG CGCTGTCATT CATCAACTCT AACCGTCCTA TACTAACCTC   
  
  
+ CTTTTTGTTG GTTAAAAAAA AAAAAGGGGA AGACAATGGA CGTCTATTGC CCTTTCTCTT TCTCCCACCG   
  
  
+ TGATCGTAAA GGGTAAAACG GTGCACCGCG CTGTCATTCA TCAACTCTAA CCGTCCTATA CTAACCTCCT   
  
  
+ TTTTGTTGGT GTGCGAGGAC GCTCAGCATG GGTATTAGTT AAAAGGGGTG ATATGGGGAT ATAATAAAAA   
  
  
+ ATTATTAATA AAATTAAAAA CTCTATTTTA TTTACGAATG TAATTATACG GCACACGCAC GTAGACACAC   
  
  
+ CTTCCGTTAC CACGTCGCGT GCATCCTGGT ACTGGGTAAT TTAGAAACTT TCTCTGTTCA TTTACATAAA   
  
  
+ TAAATATATA CAACCGTTTC AGTTCCCTTG TGTAATTAAA ACCATTCAGG AAAGTTAATT TTGGTTTG  

- TGATTAAAAT CACCCAAATC CAACAAGCAA AAAAAGAGGA ACTCTTTATG TCCTATCTGT TTCGTTCTCG   
  
  
- TGGTTGTTGT CGTGTTGCTT TGCCTCCTTA ACTTTTCGAT GTCTGGGTTG TGCTTCGCGG ATCTGGAAAA   
  
  
- TCTTCAGTAA AAATAAAAAG TCCACGCCGC AGATACGAAA AAGCTCTCTT CTTTGAATTT CTGCGTTTGC   
  
  
- CCTTTTTCTT TTCTCTGTTT TGTCTCTTTT AGGAGAGAGA TGAGTGATGG TGATGATGAT GCTGGATACT   
  
  
- GCAACGTGAT TGGNGGCTGA TGGCGGCCTG GTCTGTACCT TTTATGTGAT TTGACTCTTC TGTCCCCGAC   
  
  
- GACTCTCACA ATGACCGCAG CACCGCAGTC AGTGGCAGCT GGCCTAAGCA GCAGTCACAC TTGCTTTTTC   
  
  
- TTTTTTCTCT TTAATCTTTT TTTTTTTTCC CTGCTGTTAC CTGCAGATAA CGGGAAAGAG AAAGGGGGTG   
  
  
- GCACTAGCAT TTCCCATTTT GCCACGTGGC GCGACAGTAA GTAGTTGAGA TTGGCAGGAT ATGATTGGAG   
  
  
- GAAAAACAAC CAATTTTTTT TTTTTCCCCT TCTGTTACCT GCAGATAACG GGAAAGAGAA AGAGGGTGGC   
  
  
- ACTAGCATTT CCCATTTTGC CACGTGGCGC GACAGTAAGT AGTTGAGATT GGCAGGATAT GATTGGAGGA   
  
  
- AAAACAACCA CACGCTCCTG CGAGTCGTAC CCATAATCAA TTTTCCCCAC TATACCCCTA TATTATTTTT   
  
  
- TAATAATTAT TTTAATTTTT GAGATAAAAT AAATGCTTAC ATTAATATGC CGTGTGCGTG CATCTGTGTG   
  
  
- GAAGGCAATG GTGCAGCGCA CGTAGGACCA TGACCCATTA AATCTTTGAA AGAGACAAGT AAATGTATTT   
  
  
- ATTTATATAT GTTGGCAAAG TCAAGGGAAC ACATTAATTT TGGTAAGTCC TTTCAATTAA AACCAAAC

  
  
Motifs Found  

+     5UTR Py-rich stretch

| Site Name | Organism | Position | Strand | Matrix score. | sequence | function |
| --- | --- | --- | --- | --- | --- | --- |
| 5UTR Py-rich stretch | Lycopersicon esculentum | 185 | - | 10 | TTTCTTCTCT | cis-acting element conferring high transcription levels |

> 2018/04/13 10:10:12  
+ ACTAATTTTA GTGGGTTTAG GTTGTTCGTT TTTTTCTCCT TGAGAAATAC AGGATAGACA AAGCAAGAGC   
  
  
+ ACCAACAACA GCACAACGAA ACGGAGGAAT TGAAAAGCTA CAGACCCAAC ACGAAGCGCC TAGACCTTTT   
  
  
+ AGAAGTCATT TTTATTTTTC AGGTGCGGCG TCTATGCTTT TTCGAGAGAA GAAACTTAAA GACGCAAACG   
  
  
+ GGAAAAAGAA AAGAGACAAA ACAGAGAAAA TCCTCTCTCT ACTCACTACC ACTACTACTA CGACCTATGA   
  
  
+ CGTTGCACTA ACCNCCGACT ACCGCCGGAC CAGACATGGA AAATACACTA AACTGAGAAG ACAGGGGCTG   
  
  
+ CTGAGAGTGT TACTGGCGTC GTGGCGTCAG TCACCGTCGA CCGGATTCGT CGTCAGTGTG AACGAAAAAG   
  
  
+ AAAAAAGAGA AATTAGAAAA AAAAAAAAGG GACGACAATG GACGTCTATT GCCCTTTCTC TTTCCCCCAC   
  
  
+ CGTGATCGTA AAGGGTAAAA CGGTGCACCG CGCTGTCATT CATCAACTCT AACCGTCCTA TACTAACCTC   
  
  
+ CTTTTTGTTG GTTAAAAAAA AAAAAGGGGA AGACAATGGA CGTCTATTGC CCTTTCTCTT TCTCCCACCG   
  
  
+ TGATCGTAAA GGGTAAAACG GTGCACCGCG CTGTCATTCA TCAACTCTAA CCGTCCTATA CTAACCTCCT   
  
  
+ TTTTGTTGGT GTGCGAGGAC GCTCAGCATG GGTATTAGTT AAAAGGGGTG ATATGGGGAT ATAATAAAAA   
  
  
+ ATTATTAATA AAATTAAAAA CTCTATTTTA TTTACGAATG TAATTATACG GCACACGCAC GTAGACACAC   
  
  
+ CTTCCGTTAC CACGTCGCGT GCATCCTGGT ACTGGGTAAT TTAGAAACTT TCTCTGTTCA TTTACATAAA   
  
  
+ TAAATATATA CAACCGTTTC AGTTCCCTTG TGTAATTAAA ACCATTCAGG AAAGTTAATT TTGGTTTG  

- TGATTAAAAT CACCCAAATC CAACAAGCAA AAAAAGAGGA ACTCTTTATG TCCTATCTGT TTCGTTCTCG   
  
  
- TGGTTGTTGT CGTGTTGCTT TGCCTCCTTA ACTTTTCGAT GTCTGGGTTG TGCTTCGCGG ATCTGGAAAA   
  
  
- TCTTCAGTAA AAATAAAAAG TCCACGCCGC AGATACGAAA AAGCTCTCTT CTTTGAATTT CTGCGTTTGC   
  
  
- CCTTTTTCTT TTCTCTGTTT TGTCTCTTTT AGGAGAGAGA TGAGTGATGG TGATGATGAT GCTGGATACT   
  
  
- GCAACGTGAT TGGNGGCTGA TGGCGGCCTG GTCTGTACCT TTTATGTGAT TTGACTCTTC TGTCCCCGAC   
  
  
- GACTCTCACA ATGACCGCAG CACCGCAGTC AGTGGCAGCT GGCCTAAGCA GCAGTCACAC TTGCTTTTTC   
  
  
- TTTTTTCTCT TTAATCTTTT TTTTTTTTCC CTGCTGTTAC CTGCAGATAA CGGGAAAGAG AAAGGGGGTG   
  
  
- GCACTAGCAT TTCCCATTTT GCCACGTGGC GCGACAGTAA GTAGTTGAGA TTGGCAGGAT ATGATTGGAG   
  
  
- GAAAAACAAC CAATTTTTTT TTTTTCCCCT TCTGTTACCT GCAGATAACG GGAAAGAGAA AGAGGGTGGC   
  
  
- ACTAGCATTT CCCATTTTGC CACGTGGCGC GACAGTAAGT AGTTGAGATT GGCAGGATAT GATTGGAGGA   
  
  
- AAAACAACCA CACGCTCCTG CGAGTCGTAC CCATAATCAA TTTTCCCCAC TATACCCCTA TATTATTTTT   
  
  
- TAATAATTAT TTTAATTTTT GAGATAAAAT AAATGCTTAC ATTAATATGC CGTGTGCGTG CATCTGTGTG   
  
  
- GAAGGCAATG GTGCAGCGCA CGTAGGACCA TGACCCATTA AATCTTTGAA AGAGACAAGT AAATGTATTT   
  
  
- ATTTATATAT GTTGGCAAAG TCAAGGGAAC ACATTAATTT TGGTAAGTCC TTTCAATTAA AACCAAAC

+     A-box

| Site Name | Organism | Position | Strand | Matrix score. | sequence | function |
| --- | --- | --- | --- | --- | --- | --- |
| A-box | Petroselinum crispum | 543 | + | 6 | CCGTCC | cis-acting regulatory element |
| A-box | Petroselinum crispum | 681 | + | 6 | CCGTCC | cis-acting regulatory element |

> 2018/04/13 10:10:12  
+ ACTAATTTTA GTGGGTTTAG GTTGTTCGTT TTTTTCTCCT TGAGAAATAC AGGATAGACA AAGCAAGAGC   
  
  
+ ACCAACAACA GCACAACGAA ACGGAGGAAT TGAAAAGCTA CAGACCCAAC ACGAAGCGCC TAGACCTTTT   
  
  
+ AGAAGTCATT TTTATTTTTC AGGTGCGGCG TCTATGCTTT TTCGAGAGAA GAAACTTAAA GACGCAAACG   
  
  
+ GGAAAAAGAA AAGAGACAAA ACAGAGAAAA TCCTCTCTCT ACTCACTACC ACTACTACTA CGACCTATGA   
  
  
+ CGTTGCACTA ACCNCCGACT ACCGCCGGAC CAGACATGGA AAATACACTA AACTGAGAAG ACAGGGGCTG   
  
  
+ CTGAGAGTGT TACTGGCGTC GTGGCGTCAG TCACCGTCGA CCGGATTCGT CGTCAGTGTG AACGAAAAAG   
  
  
+ AAAAAAGAGA AATTAGAAAA AAAAAAAAGG GACGACAATG GACGTCTATT GCCCTTTCTC TTTCCCCCAC   
  
  
+ CGTGATCGTA AAGGGTAAAA CGGTGCACCG CGCTGTCATT CATCAACTCT AACCGTCCTA TACTAACCTC   
  
  
+ CTTTTTGTTG GTTAAAAAAA AAAAAGGGGA AGACAATGGA CGTCTATTGC CCTTTCTCTT TCTCCCACCG   
  
  
+ TGATCGTAAA GGGTAAAACG GTGCACCGCG CTGTCATTCA TCAACTCTAA CCGTCCTATA CTAACCTCCT   
  
  
+ TTTTGTTGGT GTGCGAGGAC GCTCAGCATG GGTATTAGTT AAAAGGGGTG ATATGGGGAT ATAATAAAAA   
  
  
+ ATTATTAATA AAATTAAAAA CTCTATTTTA TTTACGAATG TAATTATACG GCACACGCAC GTAGACACAC   
  
  
+ CTTCCGTTAC CACGTCGCGT GCATCCTGGT ACTGGGTAAT TTAGAAACTT TCTCTGTTCA TTTACATAAA   
  
  
+ TAAATATATA CAACCGTTTC AGTTCCCTTG TGTAATTAAA ACCATTCAGG AAAGTTAATT TTGGTTTG  

- TGATTAAAAT CACCCAAATC CAACAAGCAA AAAAAGAGGA ACTCTTTATG TCCTATCTGT TTCGTTCTCG   
  
  
- TGGTTGTTGT CGTGTTGCTT TGCCTCCTTA ACTTTTCGAT GTCTGGGTTG TGCTTCGCGG ATCTGGAAAA   
  
  
- TCTTCAGTAA AAATAAAAAG TCCACGCCGC AGATACGAAA AAGCTCTCTT CTTTGAATTT CTGCGTTTGC   
  
  
- CCTTTTTCTT TTCTCTGTTT TGTCTCTTTT AGGAGAGAGA TGAGTGATGG TGATGATGAT GCTGGATACT   
  
  
- GCAACGTGAT TGGNGGCTGA TGGCGGCCTG GTCTGTACCT TTTATGTGAT TTGACTCTTC TGTCCCCGAC   
  
  
- GACTCTCACA ATGACCGCAG CACCGCAGTC AGTGGCAGCT GGCCTAAGCA GCAGTCACAC TTGCTTTTTC   
  
  
- TTTTTTCTCT TTAATCTTTT TTTTTTTTCC CTGCTGTTAC CTGCAGATAA CGGGAAAGAG AAAGGGGGTG   
  
  
- GCACTAGCAT TTCCCATTTT GCCACGTGGC GCGACAGTAA GTAGTTGAGA TTGGCAGGAT ATGATTGGAG   
  
  
- GAAAAACAAC CAATTTTTTT TTTTTCCCCT TCTGTTACCT GCAGATAACG GGAAAGAGAA AGAGGGTGGC   
  
  
- ACTAGCATTT CCCATTTTGC CACGTGGCGC GACAGTAAGT AGTTGAGATT GGCAGGATAT GATTGGAGGA   
  
  
- AAAACAACCA CACGCTCCTG CGAGTCGTAC CCATAATCAA TTTTCCCCAC TATACCCCTA TATTATTTTT   
  
  
- TAATAATTAT TTTAATTTTT GAGATAAAAT AAATGCTTAC ATTAATATGC CGTGTGCGTG CATCTGTGTG   
  
  
- GAAGGCAATG GTGCAGCGCA CGTAGGACCA TGACCCATTA AATCTTTGAA AGAGACAAGT AAATGTATTT   
  
  
- ATTTATATAT GTTGGCAAAG TCAAGGGAAC ACATTAATTT TGGTAAGTCC TTTCAATTAA AACCAAAC

+     ABRE

| Site Name | Organism | Position | Strand | Matrix score. | sequence | function |
| --- | --- | --- | --- | --- | --- | --- |
| ABRE | Arabidopsis thaliana | 828 | - | 6 | TACGTG | cis-acting element involved in the abscisic acid responsiveness |

> 2018/04/13 10:10:12  
+ ACTAATTTTA GTGGGTTTAG GTTGTTCGTT TTTTTCTCCT TGAGAAATAC AGGATAGACA AAGCAAGAGC   
  
  
+ ACCAACAACA GCACAACGAA ACGGAGGAAT TGAAAAGCTA CAGACCCAAC ACGAAGCGCC TAGACCTTTT   
  
  
+ AGAAGTCATT TTTATTTTTC AGGTGCGGCG TCTATGCTTT TTCGAGAGAA GAAACTTAAA GACGCAAACG   
  
  
+ GGAAAAAGAA AAGAGACAAA ACAGAGAAAA TCCTCTCTCT ACTCACTACC ACTACTACTA CGACCTATGA   
  
  
+ CGTTGCACTA ACCNCCGACT ACCGCCGGAC CAGACATGGA AAATACACTA AACTGAGAAG ACAGGGGCTG   
  
  
+ CTGAGAGTGT TACTGGCGTC GTGGCGTCAG TCACCGTCGA CCGGATTCGT CGTCAGTGTG AACGAAAAAG   
  
  
+ AAAAAAGAGA AATTAGAAAA AAAAAAAAGG GACGACAATG GACGTCTATT GCCCTTTCTC TTTCCCCCAC   
  
  
+ CGTGATCGTA AAGGGTAAAA CGGTGCACCG CGCTGTCATT CATCAACTCT AACCGTCCTA TACTAACCTC   
  
  
+ CTTTTTGTTG GTTAAAAAAA AAAAAGGGGA AGACAATGGA CGTCTATTGC CCTTTCTCTT TCTCCCACCG   
  
  
+ TGATCGTAAA GGGTAAAACG GTGCACCGCG CTGTCATTCA TCAACTCTAA CCGTCCTATA CTAACCTCCT   
  
  
+ TTTTGTTGGT GTGCGAGGAC GCTCAGCATG GGTATTAGTT AAAAGGGGTG ATATGGGGAT ATAATAAAAA   
  
  
+ ATTATTAATA AAATTAAAAA CTCTATTTTA TTTACGAATG TAATTATACG GCACACGCAC GTAGACACAC   
  
  
+ CTTCCGTTAC CACGTCGCGT GCATCCTGGT ACTGGGTAAT TTAGAAACTT TCTCTGTTCA TTTACATAAA   
  
  
+ TAAATATATA CAACCGTTTC AGTTCCCTTG TGTAATTAAA ACCATTCAGG AAAGTTAATT TTGGTTTG  

- TGATTAAAAT CACCCAAATC CAACAAGCAA AAAAAGAGGA ACTCTTTATG TCCTATCTGT TTCGTTCTCG   
  
  
- TGGTTGTTGT CGTGTTGCTT TGCCTCCTTA ACTTTTCGAT GTCTGGGTTG TGCTTCGCGG ATCTGGAAAA   
  
  
- TCTTCAGTAA AAATAAAAAG TCCACGCCGC AGATACGAAA AAGCTCTCTT CTTTGAATTT CTGCGTTTGC   
  
  
- CCTTTTTCTT TTCTCTGTTT TGTCTCTTTT AGGAGAGAGA TGAGTGATGG TGATGATGAT GCTGGATACT   
  
  
- GCAACGTGAT TGGNGGCTGA TGGCGGCCTG GTCTGTACCT TTTATGTGAT TTGACTCTTC TGTCCCCGAC   
  
  
- GACTCTCACA ATGACCGCAG CACCGCAGTC AGTGGCAGCT GGCCTAAGCA GCAGTCACAC TTGCTTTTTC   
  
  
- TTTTTTCTCT TTAATCTTTT TTTTTTTTCC CTGCTGTTAC CTGCAGATAA CGGGAAAGAG AAAGGGGGTG   
  
  
- GCACTAGCAT TTCCCATTTT GCCACGTGGC GCGACAGTAA GTAGTTGAGA TTGGCAGGAT ATGATTGGAG   
  
  
- GAAAAACAAC CAATTTTTTT TTTTTCCCCT TCTGTTACCT GCAGATAACG GGAAAGAGAA AGAGGGTGGC   
  
  
- ACTAGCATTT CCCATTTTGC CACGTGGCGC GACAGTAAGT AGTTGAGATT GGCAGGATAT GATTGGAGGA   
  
  
- AAAACAACCA CACGCTCCTG CGAGTCGTAC CCATAATCAA TTTTCCCCAC TATACCCCTA TATTATTTTT   
  
  
- TAATAATTAT TTTAATTTTT GAGATAAAAT AAATGCTTAC ATTAATATGC CGTGTGCGTG CATCTGTGTG   
  
  
- GAAGGCAATG GTGCAGCGCA CGTAGGACCA TGACCCATTA AATCTTTGAA AGAGACAAGT AAATGTATTT   
  
  
- ATTTATATAT GTTGGCAAAG TCAAGGGAAC ACATTAATTT TGGTAAGTCC TTTCAATTAA AACCAAAC

+     AE-box

| Site Name | Organism | Position | Strand | Matrix score. | sequence | function |
| --- | --- | --- | --- | --- | --- | --- |
| AE-box | Arabidopsis thaliana | 883 | + | 8 | AGAAACTT | part of a module for light response |
| AE-box | Arabidopsis thaliana | 190 | + | 8 | AGAAACTT | part of a module for light response |

> 2018/04/13 10:10:12  
+ ACTAATTTTA GTGGGTTTAG GTTGTTCGTT TTTTTCTCCT TGAGAAATAC AGGATAGACA AAGCAAGAGC   
  
  
+ ACCAACAACA GCACAACGAA ACGGAGGAAT TGAAAAGCTA CAGACCCAAC ACGAAGCGCC TAGACCTTTT   
  
  
+ AGAAGTCATT TTTATTTTTC AGGTGCGGCG TCTATGCTTT TTCGAGAGAA GAAACTTAAA GACGCAAACG   
  
  
+ GGAAAAAGAA AAGAGACAAA ACAGAGAAAA TCCTCTCTCT ACTCACTACC ACTACTACTA CGACCTATGA   
  
  
+ CGTTGCACTA ACCNCCGACT ACCGCCGGAC CAGACATGGA AAATACACTA AACTGAGAAG ACAGGGGCTG   
  
  
+ CTGAGAGTGT TACTGGCGTC GTGGCGTCAG TCACCGTCGA CCGGATTCGT CGTCAGTGTG AACGAAAAAG   
  
  
+ AAAAAAGAGA AATTAGAAAA AAAAAAAAGG GACGACAATG GACGTCTATT GCCCTTTCTC TTTCCCCCAC   
  
  
+ CGTGATCGTA AAGGGTAAAA CGGTGCACCG CGCTGTCATT CATCAACTCT AACCGTCCTA TACTAACCTC   
  
  
+ CTTTTTGTTG GTTAAAAAAA AAAAAGGGGA AGACAATGGA CGTCTATTGC CCTTTCTCTT TCTCCCACCG   
  
  
+ TGATCGTAAA GGGTAAAACG GTGCACCGCG CTGTCATTCA TCAACTCTAA CCGTCCTATA CTAACCTCCT   
  
  
+ TTTTGTTGGT GTGCGAGGAC GCTCAGCATG GGTATTAGTT AAAAGGGGTG ATATGGGGAT ATAATAAAAA   
  
  
+ ATTATTAATA AAATTAAAAA CTCTATTTTA TTTACGAATG TAATTATACG GCACACGCAC GTAGACACAC   
  
  
+ CTTCCGTTAC CACGTCGCGT GCATCCTGGT ACTGGGTAAT TTAGAAACTT TCTCTGTTCA TTTACATAAA   
  
  
+ TAAATATATA CAACCGTTTC AGTTCCCTTG TGTAATTAAA ACCATTCAGG AAAGTTAATT TTGGTTTG  

- TGATTAAAAT CACCCAAATC CAACAAGCAA AAAAAGAGGA ACTCTTTATG TCCTATCTGT TTCGTTCTCG   
  
  
- TGGTTGTTGT CGTGTTGCTT TGCCTCCTTA ACTTTTCGAT GTCTGGGTTG TGCTTCGCGG ATCTGGAAAA   
  
  
- TCTTCAGTAA AAATAAAAAG TCCACGCCGC AGATACGAAA AAGCTCTCTT CTTTGAATTT CTGCGTTTGC   
  
  
- CCTTTTTCTT TTCTCTGTTT TGTCTCTTTT AGGAGAGAGA TGAGTGATGG TGATGATGAT GCTGGATACT   
  
  
- GCAACGTGAT TGGNGGCTGA TGGCGGCCTG GTCTGTACCT TTTATGTGAT TTGACTCTTC TGTCCCCGAC   
  
  
- GACTCTCACA ATGACCGCAG CACCGCAGTC AGTGGCAGCT GGCCTAAGCA GCAGTCACAC TTGCTTTTTC   
  
  
- TTTTTTCTCT TTAATCTTTT TTTTTTTTCC CTGCTGTTAC CTGCAGATAA CGGGAAAGAG AAAGGGGGTG   
  
  
- GCACTAGCAT TTCCCATTTT GCCACGTGGC GCGACAGTAA GTAGTTGAGA TTGGCAGGAT ATGATTGGAG   
  
  
- GAAAAACAAC CAATTTTTTT TTTTTCCCCT TCTGTTACCT GCAGATAACG GGAAAGAGAA AGAGGGTGGC   
  
  
- ACTAGCATTT CCCATTTTGC CACGTGGCGC GACAGTAAGT AGTTGAGATT GGCAGGATAT GATTGGAGGA   
  
  
- AAAACAACCA CACGCTCCTG CGAGTCGTAC CCATAATCAA TTTTCCCCAC TATACCCCTA TATTATTTTT   
  
  
- TAATAATTAT TTTAATTTTT GAGATAAAAT AAATGCTTAC ATTAATATGC CGTGTGCGTG CATCTGTGTG   
  
  
- GAAGGCAATG GTGCAGCGCA CGTAGGACCA TGACCCATTA AATCTTTGAA AGAGACAAGT AAATGTATTT   
  
  
- ATTTATATAT GTTGGCAAAG TCAAGGGAAC ACATTAATTT TGGTAAGTCC TTTCAATTAA AACCAAAC

+     ARE

| Site Name | Organism | Position | Strand | Matrix score. | sequence | function |
| --- | --- | --- | --- | --- | --- | --- |
| ARE | Zea mays | 949 | - | 6 | TGGTTT | cis-acting regulatory element essential for the anaerobic induction |
| ARE | Zea mays | 972 | + | 6 | TGGTTT | cis-acting regulatory element essential for the anaerobic induction |

> 2018/04/13 10:10:12  
+ ACTAATTTTA GTGGGTTTAG GTTGTTCGTT TTTTTCTCCT TGAGAAATAC AGGATAGACA AAGCAAGAGC   
  
  
+ ACCAACAACA GCACAACGAA ACGGAGGAAT TGAAAAGCTA CAGACCCAAC ACGAAGCGCC TAGACCTTTT   
  
  
+ AGAAGTCATT TTTATTTTTC AGGTGCGGCG TCTATGCTTT TTCGAGAGAA GAAACTTAAA GACGCAAACG   
  
  
+ GGAAAAAGAA AAGAGACAAA ACAGAGAAAA TCCTCTCTCT ACTCACTACC ACTACTACTA CGACCTATGA   
  
  
+ CGTTGCACTA ACCNCCGACT ACCGCCGGAC CAGACATGGA AAATACACTA AACTGAGAAG ACAGGGGCTG   
  
  
+ CTGAGAGTGT TACTGGCGTC GTGGCGTCAG TCACCGTCGA CCGGATTCGT CGTCAGTGTG AACGAAAAAG   
  
  
+ AAAAAAGAGA AATTAGAAAA AAAAAAAAGG GACGACAATG GACGTCTATT GCCCTTTCTC TTTCCCCCAC   
  
  
+ CGTGATCGTA AAGGGTAAAA CGGTGCACCG CGCTGTCATT CATCAACTCT AACCGTCCTA TACTAACCTC   
  
  
+ CTTTTTGTTG GTTAAAAAAA AAAAAGGGGA AGACAATGGA CGTCTATTGC CCTTTCTCTT TCTCCCACCG   
  
  
+ TGATCGTAAA GGGTAAAACG GTGCACCGCG CTGTCATTCA TCAACTCTAA CCGTCCTATA CTAACCTCCT   
  
  
+ TTTTGTTGGT GTGCGAGGAC GCTCAGCATG GGTATTAGTT AAAAGGGGTG ATATGGGGAT ATAATAAAAA   
  
  
+ ATTATTAATA AAATTAAAAA CTCTATTTTA TTTACGAATG TAATTATACG GCACACGCAC GTAGACACAC   
  
  
+ CTTCCGTTAC CACGTCGCGT GCATCCTGGT ACTGGGTAAT TTAGAAACTT TCTCTGTTCA TTTACATAAA   
  
  
+ TAAATATATA CAACCGTTTC AGTTCCCTTG TGTAATTAAA ACCATTCAGG AAAGTTAATT TTGGTTTG  

- TGATTAAAAT CACCCAAATC CAACAAGCAA AAAAAGAGGA ACTCTTTATG TCCTATCTGT TTCGTTCTCG   
  
  
- TGGTTGTTGT CGTGTTGCTT TGCCTCCTTA ACTTTTCGAT GTCTGGGTTG TGCTTCGCGG ATCTGGAAAA   
  
  
- TCTTCAGTAA AAATAAAAAG TCCACGCCGC AGATACGAAA AAGCTCTCTT CTTTGAATTT CTGCGTTTGC   
  
  
- CCTTTTTCTT TTCTCTGTTT TGTCTCTTTT AGGAGAGAGA TGAGTGATGG TGATGATGAT GCTGGATACT   
  
  
- GCAACGTGAT TGGNGGCTGA TGGCGGCCTG GTCTGTACCT TTTATGTGAT TTGACTCTTC TGTCCCCGAC   
  
  
- GACTCTCACA ATGACCGCAG CACCGCAGTC AGTGGCAGCT GGCCTAAGCA GCAGTCACAC TTGCTTTTTC   
  
  
- TTTTTTCTCT TTAATCTTTT TTTTTTTTCC CTGCTGTTAC CTGCAGATAA CGGGAAAGAG AAAGGGGGTG   
  
  
- GCACTAGCAT TTCCCATTTT GCCACGTGGC GCGACAGTAA GTAGTTGAGA TTGGCAGGAT ATGATTGGAG   
  
  
- GAAAAACAAC CAATTTTTTT TTTTTCCCCT TCTGTTACCT GCAGATAACG GGAAAGAGAA AGAGGGTGGC   
  
  
- ACTAGCATTT CCCATTTTGC CACGTGGCGC GACAGTAAGT AGTTGAGATT GGCAGGATAT GATTGGAGGA   
  
  
- AAAACAACCA CACGCTCCTG CGAGTCGTAC CCATAATCAA TTTTCCCCAC TATACCCCTA TATTATTTTT   
  
  
- TAATAATTAT TTTAATTTTT GAGATAAAAT AAATGCTTAC ATTAATATGC CGTGTGCGTG CATCTGTGTG   
  
  
- GAAGGCAATG GTGCAGCGCA CGTAGGACCA TGACCCATTA AATCTTTGAA AGAGACAAGT AAATGTATTT   
  
  
- ATTTATATAT GTTGGCAAAG TCAAGGGAAC ACATTAATTT TGGTAAGTCC TTTCAATTAA AACCAAAC

+     AT1-motif

| Site Name | Organism | Position | Strand | Matrix score. | sequence | function |
| --- | --- | --- | --- | --- | --- | --- |
| AT1-motif | Solanum tuberosum | 763 | - | 13 | AATTATTTTTTATT | part of a light responsive module |

> 2018/04/13 10:10:12  
+ ACTAATTTTA GTGGGTTTAG GTTGTTCGTT TTTTTCTCCT TGAGAAATAC AGGATAGACA AAGCAAGAGC   
  
  
+ ACCAACAACA GCACAACGAA ACGGAGGAAT TGAAAAGCTA CAGACCCAAC ACGAAGCGCC TAGACCTTTT   
  
  
+ AGAAGTCATT TTTATTTTTC AGGTGCGGCG TCTATGCTTT TTCGAGAGAA GAAACTTAAA GACGCAAACG   
  
  
+ GGAAAAAGAA AAGAGACAAA ACAGAGAAAA TCCTCTCTCT ACTCACTACC ACTACTACTA CGACCTATGA   
  
  
+ CGTTGCACTA ACCNCCGACT ACCGCCGGAC CAGACATGGA AAATACACTA AACTGAGAAG ACAGGGGCTG   
  
  
+ CTGAGAGTGT TACTGGCGTC GTGGCGTCAG TCACCGTCGA CCGGATTCGT CGTCAGTGTG AACGAAAAAG   
  
  
+ AAAAAAGAGA AATTAGAAAA AAAAAAAAGG GACGACAATG GACGTCTATT GCCCTTTCTC TTTCCCCCAC   
  
  
+ CGTGATCGTA AAGGGTAAAA CGGTGCACCG CGCTGTCATT CATCAACTCT AACCGTCCTA TACTAACCTC   
  
  
+ CTTTTTGTTG GTTAAAAAAA AAAAAGGGGA AGACAATGGA CGTCTATTGC CCTTTCTCTT TCTCCCACCG   
  
  
+ TGATCGTAAA GGGTAAAACG GTGCACCGCG CTGTCATTCA TCAACTCTAA CCGTCCTATA CTAACCTCCT   
  
  
+ TTTTGTTGGT GTGCGAGGAC GCTCAGCATG GGTATTAGTT AAAAGGGGTG ATATGGGGAT ATAATAAAAA   
  
  
+ ATTATTAATA AAATTAAAAA CTCTATTTTA TTTACGAATG TAATTATACG GCACACGCAC GTAGACACAC   
  
  
+ CTTCCGTTAC CACGTCGCGT GCATCCTGGT ACTGGGTAAT TTAGAAACTT TCTCTGTTCA TTTACATAAA   
  
  
+ TAAATATATA CAACCGTTTC AGTTCCCTTG TGTAATTAAA ACCATTCAGG AAAGTTAATT TTGGTTTG  

- TGATTAAAAT CACCCAAATC CAACAAGCAA AAAAAGAGGA ACTCTTTATG TCCTATCTGT TTCGTTCTCG   
  
  
- TGGTTGTTGT CGTGTTGCTT TGCCTCCTTA ACTTTTCGAT GTCTGGGTTG TGCTTCGCGG ATCTGGAAAA   
  
  
- TCTTCAGTAA AAATAAAAAG TCCACGCCGC AGATACGAAA AAGCTCTCTT CTTTGAATTT CTGCGTTTGC   
  
  
- CCTTTTTCTT TTCTCTGTTT TGTCTCTTTT AGGAGAGAGA TGAGTGATGG TGATGATGAT GCTGGATACT   
  
  
- GCAACGTGAT TGGNGGCTGA TGGCGGCCTG GTCTGTACCT TTTATGTGAT TTGACTCTTC TGTCCCCGAC   
  
  
- GACTCTCACA ATGACCGCAG CACCGCAGTC AGTGGCAGCT GGCCTAAGCA GCAGTCACAC TTGCTTTTTC   
  
  
- TTTTTTCTCT TTAATCTTTT TTTTTTTTCC CTGCTGTTAC CTGCAGATAA CGGGAAAGAG AAAGGGGGTG   
  
  
- GCACTAGCAT TTCCCATTTT GCCACGTGGC GCGACAGTAA GTAGTTGAGA TTGGCAGGAT ATGATTGGAG   
  
  
- GAAAAACAAC CAATTTTTTT TTTTTCCCCT TCTGTTACCT GCAGATAACG GGAAAGAGAA AGAGGGTGGC   
  
  
- ACTAGCATTT CCCATTTTGC CACGTGGCGC GACAGTAAGT AGTTGAGATT GGCAGGATAT GATTGGAGGA   
  
  
- AAAACAACCA CACGCTCCTG CGAGTCGTAC CCATAATCAA TTTTCCCCAC TATACCCCTA TATTATTTTT   
  
  
- TAATAATTAT TTTAATTTTT GAGATAAAAT AAATGCTTAC ATTAATATGC CGTGTGCGTG CATCTGTGTG   
  
  
- GAAGGCAATG GTGCAGCGCA CGTAGGACCA TGACCCATTA AATCTTTGAA AGAGACAAGT AAATGTATTT   
  
  
- ATTTATATAT GTTGGCAAAG TCAAGGGAAC ACATTAATTT TGGTAAGTCC TTTCAATTAA AACCAAAC

+     Box 4

| Site Name | Organism | Position | Strand | Matrix score. | sequence | function |
| --- | --- | --- | --- | --- | --- | --- |
| Box 4 | Petroselinum crispum | 774 | - | 6 | ATTAAT | part of a conserved DNA module involved in light responsiveness |

> 2018/04/13 10:10:12  
+ ACTAATTTTA GTGGGTTTAG GTTGTTCGTT TTTTTCTCCT TGAGAAATAC AGGATAGACA AAGCAAGAGC   
  
  
+ ACCAACAACA GCACAACGAA ACGGAGGAAT TGAAAAGCTA CAGACCCAAC ACGAAGCGCC TAGACCTTTT   
  
  
+ AGAAGTCATT TTTATTTTTC AGGTGCGGCG TCTATGCTTT TTCGAGAGAA GAAACTTAAA GACGCAAACG   
  
  
+ GGAAAAAGAA AAGAGACAAA ACAGAGAAAA TCCTCTCTCT ACTCACTACC ACTACTACTA CGACCTATGA   
  
  
+ CGTTGCACTA ACCNCCGACT ACCGCCGGAC CAGACATGGA AAATACACTA AACTGAGAAG ACAGGGGCTG   
  
  
+ CTGAGAGTGT TACTGGCGTC GTGGCGTCAG TCACCGTCGA CCGGATTCGT CGTCAGTGTG AACGAAAAAG   
  
  
+ AAAAAAGAGA AATTAGAAAA AAAAAAAAGG GACGACAATG GACGTCTATT GCCCTTTCTC TTTCCCCCAC   
  
  
+ CGTGATCGTA AAGGGTAAAA CGGTGCACCG CGCTGTCATT CATCAACTCT AACCGTCCTA TACTAACCTC   
  
  
+ CTTTTTGTTG GTTAAAAAAA AAAAAGGGGA AGACAATGGA CGTCTATTGC CCTTTCTCTT TCTCCCACCG   
  
  
+ TGATCGTAAA GGGTAAAACG GTGCACCGCG CTGTCATTCA TCAACTCTAA CCGTCCTATA CTAACCTCCT   
  
  
+ TTTTGTTGGT GTGCGAGGAC GCTCAGCATG GGTATTAGTT AAAAGGGGTG ATATGGGGAT ATAATAAAAA   
  
  
+ ATTATTAATA AAATTAAAAA CTCTATTTTA TTTACGAATG TAATTATACG GCACACGCAC GTAGACACAC   
  
  
+ CTTCCGTTAC CACGTCGCGT GCATCCTGGT ACTGGGTAAT TTAGAAACTT TCTCTGTTCA TTTACATAAA   
  
  
+ TAAATATATA CAACCGTTTC AGTTCCCTTG TGTAATTAAA ACCATTCAGG AAAGTTAATT TTGGTTTG  

- TGATTAAAAT CACCCAAATC CAACAAGCAA AAAAAGAGGA ACTCTTTATG TCCTATCTGT TTCGTTCTCG   
  
  
- TGGTTGTTGT CGTGTTGCTT TGCCTCCTTA ACTTTTCGAT GTCTGGGTTG TGCTTCGCGG ATCTGGAAAA   
  
  
- TCTTCAGTAA AAATAAAAAG TCCACGCCGC AGATACGAAA AAGCTCTCTT CTTTGAATTT CTGCGTTTGC   
  
  
- CCTTTTTCTT TTCTCTGTTT TGTCTCTTTT AGGAGAGAGA TGAGTGATGG TGATGATGAT GCTGGATACT   
  
  
- GCAACGTGAT TGGNGGCTGA TGGCGGCCTG GTCTGTACCT TTTATGTGAT TTGACTCTTC TGTCCCCGAC   
  
  
- GACTCTCACA ATGACCGCAG CACCGCAGTC AGTGGCAGCT GGCCTAAGCA GCAGTCACAC TTGCTTTTTC   
  
  
- TTTTTTCTCT TTAATCTTTT TTTTTTTTCC CTGCTGTTAC CTGCAGATAA CGGGAAAGAG AAAGGGGGTG   
  
  
- GCACTAGCAT TTCCCATTTT GCCACGTGGC GCGACAGTAA GTAGTTGAGA TTGGCAGGAT ATGATTGGAG   
  
  
- GAAAAACAAC CAATTTTTTT TTTTTCCCCT TCTGTTACCT GCAGATAACG GGAAAGAGAA AGAGGGTGGC   
  
  
- ACTAGCATTT CCCATTTTGC CACGTGGCGC GACAGTAAGT AGTTGAGATT GGCAGGATAT GATTGGAGGA   
  
  
- AAAACAACCA CACGCTCCTG CGAGTCGTAC CCATAATCAA TTTTCCCCAC TATACCCCTA TATTATTTTT   
  
  
- TAATAATTAT TTTAATTTTT GAGATAAAAT AAATGCTTAC ATTAATATGC CGTGTGCGTG CATCTGTGTG   
  
  
- GAAGGCAATG GTGCAGCGCA CGTAGGACCA TGACCCATTA AATCTTTGAA AGAGACAAGT AAATGTATTT   
  
  
- ATTTATATAT GTTGGCAAAG TCAAGGGAAC ACATTAATTT TGGTAAGTCC TTTCAATTAA AACCAAAC

+     CAAT-box

| Site Name | Organism | Position | Strand | Matrix score. | sequence | function |
| --- | --- | --- | --- | --- | --- | --- |
| CAAT-box | Hordeum vulgare | 456 | + | 4 | CAAT | common cis-acting element in promoter and enhancer regions |
| CAAT-box | Hordeum vulgare | 594 | + | 4 | CAAT | common cis-acting element in promoter and enhancer regions |
| CAAT-box | Arabidopsis thaliana | 468 | - | 6 | gGCAAT | common cis-acting element in promoter and enhancer regions |
| CAAT-box | Arabidopsis thaliana | 606 | - | 6 | gGCAAT | common cis-acting element in promoter and enhancer regions |
| CAAT-box | Hordeum vulgare | 99 | - | 4 | CAAT | common cis-acting element in promoter and enhancer regions |
| CAAT-box | Glycine max | 98 | - | 5 | CAATT | common cis-acting element in promoter and enhancer regions |

> 2018/04/13 10:10:12  
+ ACTAATTTTA GTGGGTTTAG GTTGTTCGTT TTTTTCTCCT TGAGAAATAC AGGATAGACA AAGCAAGAGC   
  
  
+ ACCAACAACA GCACAACGAA ACGGAGGAAT TGAAAAGCTA CAGACCCAAC ACGAAGCGCC TAGACCTTTT   
  
  
+ AGAAGTCATT TTTATTTTTC AGGTGCGGCG TCTATGCTTT TTCGAGAGAA GAAACTTAAA GACGCAAACG   
  
  
+ GGAAAAAGAA AAGAGACAAA ACAGAGAAAA TCCTCTCTCT ACTCACTACC ACTACTACTA CGACCTATGA   
  
  
+ CGTTGCACTA ACCNCCGACT ACCGCCGGAC CAGACATGGA AAATACACTA AACTGAGAAG ACAGGGGCTG   
  
  
+ CTGAGAGTGT TACTGGCGTC GTGGCGTCAG TCACCGTCGA CCGGATTCGT CGTCAGTGTG AACGAAAAAG   
  
  
+ AAAAAAGAGA AATTAGAAAA AAAAAAAAGG GACGACAATG GACGTCTATT GCCCTTTCTC TTTCCCCCAC   
  
  
+ CGTGATCGTA AAGGGTAAAA CGGTGCACCG CGCTGTCATT CATCAACTCT AACCGTCCTA TACTAACCTC   
  
  
+ CTTTTTGTTG GTTAAAAAAA AAAAAGGGGA AGACAATGGA CGTCTATTGC CCTTTCTCTT TCTCCCACCG   
  
  
+ TGATCGTAAA GGGTAAAACG GTGCACCGCG CTGTCATTCA TCAACTCTAA CCGTCCTATA CTAACCTCCT   
  
  
+ TTTTGTTGGT GTGCGAGGAC GCTCAGCATG GGTATTAGTT AAAAGGGGTG ATATGGGGAT ATAATAAAAA   
  
  
+ ATTATTAATA AAATTAAAAA CTCTATTTTA TTTACGAATG TAATTATACG GCACACGCAC GTAGACACAC   
  
  
+ CTTCCGTTAC CACGTCGCGT GCATCCTGGT ACTGGGTAAT TTAGAAACTT TCTCTGTTCA TTTACATAAA   
  
  
+ TAAATATATA CAACCGTTTC AGTTCCCTTG TGTAATTAAA ACCATTCAGG AAAGTTAATT TTGGTTTG  

- TGATTAAAAT CACCCAAATC CAACAAGCAA AAAAAGAGGA ACTCTTTATG TCCTATCTGT TTCGTTCTCG   
  
  
- TGGTTGTTGT CGTGTTGCTT TGCCTCCTTA ACTTTTCGAT GTCTGGGTTG TGCTTCGCGG ATCTGGAAAA   
  
  
- TCTTCAGTAA AAATAAAAAG TCCACGCCGC AGATACGAAA AAGCTCTCTT CTTTGAATTT CTGCGTTTGC   
  
  
- CCTTTTTCTT TTCTCTGTTT TGTCTCTTTT AGGAGAGAGA TGAGTGATGG TGATGATGAT GCTGGATACT   
  
  
- GCAACGTGAT TGGNGGCTGA TGGCGGCCTG GTCTGTACCT TTTATGTGAT TTGACTCTTC TGTCCCCGAC   
  
  
- GACTCTCACA ATGACCGCAG CACCGCAGTC AGTGGCAGCT GGCCTAAGCA GCAGTCACAC TTGCTTTTTC   
  
  
- TTTTTTCTCT TTAATCTTTT TTTTTTTTCC CTGCTGTTAC CTGCAGATAA CGGGAAAGAG AAAGGGGGTG   
  
  
- GCACTAGCAT TTCCCATTTT GCCACGTGGC GCGACAGTAA GTAGTTGAGA TTGGCAGGAT ATGATTGGAG   
  
  
- GAAAAACAAC CAATTTTTTT TTTTTCCCCT TCTGTTACCT GCAGATAACG GGAAAGAGAA AGAGGGTGGC   
  
  
- ACTAGCATTT CCCATTTTGC CACGTGGCGC GACAGTAAGT AGTTGAGATT GGCAGGATAT GATTGGAGGA   
  
  
- AAAACAACCA CACGCTCCTG CGAGTCGTAC CCATAATCAA TTTTCCCCAC TATACCCCTA TATTATTTTT   
  
  
- TAATAATTAT TTTAATTTTT GAGATAAAAT AAATGCTTAC ATTAATATGC CGTGTGCGTG CATCTGTGTG   
  
  
- GAAGGCAATG GTGCAGCGCA CGTAGGACCA TGACCCATTA AATCTTTGAA AGAGACAAGT AAATGTATTT   
  
  
- ATTTATATAT GTTGGCAAAG TCAAGGGAAC ACATTAATTT TGGTAAGTCC TTTCAATTAA AACCAAAC

+     CCGTCC-box

| Site Name | Organism | Position | Strand | Matrix score. | sequence | function |
| --- | --- | --- | --- | --- | --- | --- |
| CCGTCC-box | Arabidopsis thaliana | 681 | + | 6 | CCGTCC | cis-acting regulatory element related to meristem specific activation |
| CCGTCC-box | Arabidopsis thaliana | 543 | + | 6 | CCGTCC | cis-acting regulatory element related to meristem specific activation |

> 2018/04/13 10:10:12  
+ ACTAATTTTA GTGGGTTTAG GTTGTTCGTT TTTTTCTCCT TGAGAAATAC AGGATAGACA AAGCAAGAGC   
  
  
+ ACCAACAACA GCACAACGAA ACGGAGGAAT TGAAAAGCTA CAGACCCAAC ACGAAGCGCC TAGACCTTTT   
  
  
+ AGAAGTCATT TTTATTTTTC AGGTGCGGCG TCTATGCTTT TTCGAGAGAA GAAACTTAAA GACGCAAACG   
  
  
+ GGAAAAAGAA AAGAGACAAA ACAGAGAAAA TCCTCTCTCT ACTCACTACC ACTACTACTA CGACCTATGA   
  
  
+ CGTTGCACTA ACCNCCGACT ACCGCCGGAC CAGACATGGA AAATACACTA AACTGAGAAG ACAGGGGCTG   
  
  
+ CTGAGAGTGT TACTGGCGTC GTGGCGTCAG TCACCGTCGA CCGGATTCGT CGTCAGTGTG AACGAAAAAG   
  
  
+ AAAAAAGAGA AATTAGAAAA AAAAAAAAGG GACGACAATG GACGTCTATT GCCCTTTCTC TTTCCCCCAC   
  
  
+ CGTGATCGTA AAGGGTAAAA CGGTGCACCG CGCTGTCATT CATCAACTCT AACCGTCCTA TACTAACCTC   
  
  
+ CTTTTTGTTG GTTAAAAAAA AAAAAGGGGA AGACAATGGA CGTCTATTGC CCTTTCTCTT TCTCCCACCG   
  
  
+ TGATCGTAAA GGGTAAAACG GTGCACCGCG CTGTCATTCA TCAACTCTAA CCGTCCTATA CTAACCTCCT   
  
  
+ TTTTGTTGGT GTGCGAGGAC GCTCAGCATG GGTATTAGTT AAAAGGGGTG ATATGGGGAT ATAATAAAAA   
  
  
+ ATTATTAATA AAATTAAAAA CTCTATTTTA TTTACGAATG TAATTATACG GCACACGCAC GTAGACACAC   
  
  
+ CTTCCGTTAC CACGTCGCGT GCATCCTGGT ACTGGGTAAT TTAGAAACTT TCTCTGTTCA TTTACATAAA   
  
  
+ TAAATATATA CAACCGTTTC AGTTCCCTTG TGTAATTAAA ACCATTCAGG AAAGTTAATT TTGGTTTG  

- TGATTAAAAT CACCCAAATC CAACAAGCAA AAAAAGAGGA ACTCTTTATG TCCTATCTGT TTCGTTCTCG   
  
  
- TGGTTGTTGT CGTGTTGCTT TGCCTCCTTA ACTTTTCGAT GTCTGGGTTG TGCTTCGCGG ATCTGGAAAA   
  
  
- TCTTCAGTAA AAATAAAAAG TCCACGCCGC AGATACGAAA AAGCTCTCTT CTTTGAATTT CTGCGTTTGC   
  
  
- CCTTTTTCTT TTCTCTGTTT TGTCTCTTTT AGGAGAGAGA TGAGTGATGG TGATGATGAT GCTGGATACT   
  
  
- GCAACGTGAT TGGNGGCTGA TGGCGGCCTG GTCTGTACCT TTTATGTGAT TTGACTCTTC TGTCCCCGAC   
  
  
- GACTCTCACA ATGACCGCAG CACCGCAGTC AGTGGCAGCT GGCCTAAGCA GCAGTCACAC TTGCTTTTTC   
  
  
- TTTTTTCTCT TTAATCTTTT TTTTTTTTCC CTGCTGTTAC CTGCAGATAA CGGGAAAGAG AAAGGGGGTG   
  
  
- GCACTAGCAT TTCCCATTTT GCCACGTGGC GCGACAGTAA GTAGTTGAGA TTGGCAGGAT ATGATTGGAG   
  
  
- GAAAAACAAC CAATTTTTTT TTTTTCCCCT TCTGTTACCT GCAGATAACG GGAAAGAGAA AGAGGGTGGC   
  
  
- ACTAGCATTT CCCATTTTGC CACGTGGCGC GACAGTAAGT AGTTGAGATT GGCAGGATAT GATTGGAGGA   
  
  
- AAAACAACCA CACGCTCCTG CGAGTCGTAC CCATAATCAA TTTTCCCCAC TATACCCCTA TATTATTTTT   
  
  
- TAATAATTAT TTTAATTTTT GAGATAAAAT AAATGCTTAC ATTAATATGC CGTGTGCGTG CATCTGTGTG   
  
  
- GAAGGCAATG GTGCAGCGCA CGTAGGACCA TGACCCATTA AATCTTTGAA AGAGACAAGT AAATGTATTT   
  
  
- ATTTATATAT GTTGGCAAAG TCAAGGGAAC ACATTAATTT TGGTAAGTCC TTTCAATTAA AACCAAAC

+     CGTCA-motif

| Site Name | Organism | Position | Strand | Matrix score. | sequence | function |
| --- | --- | --- | --- | --- | --- | --- |
| CGTCA-motif | Hordeum vulgare | 401 | + | 5 | CGTCA | cis-acting regulatory element involved in the MeJA-responsiveness |
| CGTCA-motif | Hordeum vulgare | 375 | + | 5 | CGTCA | cis-acting regulatory element involved in the MeJA-responsiveness |
| CGTCA-motif | Hordeum vulgare | 278 | - | 5 | CGTCA | cis-acting regulatory element involved in the MeJA-responsiveness |

> 2018/04/13 10:10:12  
+ ACTAATTTTA GTGGGTTTAG GTTGTTCGTT TTTTTCTCCT TGAGAAATAC AGGATAGACA AAGCAAGAGC   
  
  
+ ACCAACAACA GCACAACGAA ACGGAGGAAT TGAAAAGCTA CAGACCCAAC ACGAAGCGCC TAGACCTTTT   
  
  
+ AGAAGTCATT TTTATTTTTC AGGTGCGGCG TCTATGCTTT TTCGAGAGAA GAAACTTAAA GACGCAAACG   
  
  
+ GGAAAAAGAA AAGAGACAAA ACAGAGAAAA TCCTCTCTCT ACTCACTACC ACTACTACTA CGACCTATGA   
  
  
+ CGTTGCACTA ACCNCCGACT ACCGCCGGAC CAGACATGGA AAATACACTA AACTGAGAAG ACAGGGGCTG   
  
  
+ CTGAGAGTGT TACTGGCGTC GTGGCGTCAG TCACCGTCGA CCGGATTCGT CGTCAGTGTG AACGAAAAAG   
  
  
+ AAAAAAGAGA AATTAGAAAA AAAAAAAAGG GACGACAATG GACGTCTATT GCCCTTTCTC TTTCCCCCAC   
  
  
+ CGTGATCGTA AAGGGTAAAA CGGTGCACCG CGCTGTCATT CATCAACTCT AACCGTCCTA TACTAACCTC   
  
  
+ CTTTTTGTTG GTTAAAAAAA AAAAAGGGGA AGACAATGGA CGTCTATTGC CCTTTCTCTT TCTCCCACCG   
  
  
+ TGATCGTAAA GGGTAAAACG GTGCACCGCG CTGTCATTCA TCAACTCTAA CCGTCCTATA CTAACCTCCT   
  
  
+ TTTTGTTGGT GTGCGAGGAC GCTCAGCATG GGTATTAGTT AAAAGGGGTG ATATGGGGAT ATAATAAAAA   
  
  
+ ATTATTAATA AAATTAAAAA CTCTATTTTA TTTACGAATG TAATTATACG GCACACGCAC GTAGACACAC   
  
  
+ CTTCCGTTAC CACGTCGCGT GCATCCTGGT ACTGGGTAAT TTAGAAACTT TCTCTGTTCA TTTACATAAA   
  
  
+ TAAATATATA CAACCGTTTC AGTTCCCTTG TGTAATTAAA ACCATTCAGG AAAGTTAATT TTGGTTTG  

- TGATTAAAAT CACCCAAATC CAACAAGCAA AAAAAGAGGA ACTCTTTATG TCCTATCTGT TTCGTTCTCG   
  
  
- TGGTTGTTGT CGTGTTGCTT TGCCTCCTTA ACTTTTCGAT GTCTGGGTTG TGCTTCGCGG ATCTGGAAAA   
  
  
- TCTTCAGTAA AAATAAAAAG TCCACGCCGC AGATACGAAA AAGCTCTCTT CTTTGAATTT CTGCGTTTGC   
  
  
- CCTTTTTCTT TTCTCTGTTT TGTCTCTTTT AGGAGAGAGA TGAGTGATGG TGATGATGAT GCTGGATACT   
  
  
- GCAACGTGAT TGGNGGCTGA TGGCGGCCTG GTCTGTACCT TTTATGTGAT TTGACTCTTC TGTCCCCGAC   
  
  
- GACTCTCACA ATGACCGCAG CACCGCAGTC AGTGGCAGCT GGCCTAAGCA GCAGTCACAC TTGCTTTTTC   
  
  
- TTTTTTCTCT TTAATCTTTT TTTTTTTTCC CTGCTGTTAC CTGCAGATAA CGGGAAAGAG AAAGGGGGTG   
  
  
- GCACTAGCAT TTCCCATTTT GCCACGTGGC GCGACAGTAA GTAGTTGAGA TTGGCAGGAT ATGATTGGAG   
  
  
- GAAAAACAAC CAATTTTTTT TTTTTCCCCT TCTGTTACCT GCAGATAACG GGAAAGAGAA AGAGGGTGGC   
  
  
- ACTAGCATTT CCCATTTTGC CACGTGGCGC GACAGTAAGT AGTTGAGATT GGCAGGATAT GATTGGAGGA   
  
  
- AAAACAACCA CACGCTCCTG CGAGTCGTAC CCATAATCAA TTTTCCCCAC TATACCCCTA TATTATTTTT   
  
  
- TAATAATTAT TTTAATTTTT GAGATAAAAT AAATGCTTAC ATTAATATGC CGTGTGCGTG CATCTGTGTG   
  
  
- GAAGGCAATG GTGCAGCGCA CGTAGGACCA TGACCCATTA AATCTTTGAA AGAGACAAGT AAATGTATTT   
  
  
- ATTTATATAT GTTGGCAAAG TCAAGGGAAC ACATTAATTT TGGTAAGTCC TTTCAATTAA AACCAAAC

+     G-Box

| Site Name | Organism | Position | Strand | Matrix score. | sequence | function |
| --- | --- | --- | --- | --- | --- | --- |
| G-Box | Pisum sativum | 311 | + | 10 | CACACATGGAA | cis-acting regulatory element involved in light responsiveness |
| G-Box | Antirrhinum majus | 828 | + | 6 | CACGTA | cis-acting regulatory element involved in light responsiveness |

> 2018/04/13 10:10:12  
+ ACTAATTTTA GTGGGTTTAG GTTGTTCGTT TTTTTCTCCT TGAGAAATAC AGGATAGACA AAGCAAGAGC   
  
  
+ ACCAACAACA GCACAACGAA ACGGAGGAAT TGAAAAGCTA CAGACCCAAC ACGAAGCGCC TAGACCTTTT   
  
  
+ AGAAGTCATT TTTATTTTTC AGGTGCGGCG TCTATGCTTT TTCGAGAGAA GAAACTTAAA GACGCAAACG   
  
  
+ GGAAAAAGAA AAGAGACAAA ACAGAGAAAA TCCTCTCTCT ACTCACTACC ACTACTACTA CGACCTATGA   
  
  
+ CGTTGCACTA ACCNCCGACT ACCGCCGGAC CAGACATGGA AAATACACTA AACTGAGAAG ACAGGGGCTG   
  
  
+ CTGAGAGTGT TACTGGCGTC GTGGCGTCAG TCACCGTCGA CCGGATTCGT CGTCAGTGTG AACGAAAAAG   
  
  
+ AAAAAAGAGA AATTAGAAAA AAAAAAAAGG GACGACAATG GACGTCTATT GCCCTTTCTC TTTCCCCCAC   
  
  
+ CGTGATCGTA AAGGGTAAAA CGGTGCACCG CGCTGTCATT CATCAACTCT AACCGTCCTA TACTAACCTC   
  
  
+ CTTTTTGTTG GTTAAAAAAA AAAAAGGGGA AGACAATGGA CGTCTATTGC CCTTTCTCTT TCTCCCACCG   
  
  
+ TGATCGTAAA GGGTAAAACG GTGCACCGCG CTGTCATTCA TCAACTCTAA CCGTCCTATA CTAACCTCCT   
  
  
+ TTTTGTTGGT GTGCGAGGAC GCTCAGCATG GGTATTAGTT AAAAGGGGTG ATATGGGGAT ATAATAAAAA   
  
  
+ ATTATTAATA AAATTAAAAA CTCTATTTTA TTTACGAATG TAATTATACG GCACACGCAC GTAGACACAC   
  
  
+ CTTCCGTTAC CACGTCGCGT GCATCCTGGT ACTGGGTAAT TTAGAAACTT TCTCTGTTCA TTTACATAAA   
  
  
+ TAAATATATA CAACCGTTTC AGTTCCCTTG TGTAATTAAA ACCATTCAGG AAAGTTAATT TTGGTTTG  

- TGATTAAAAT CACCCAAATC CAACAAGCAA AAAAAGAGGA ACTCTTTATG TCCTATCTGT TTCGTTCTCG   
  
  
- TGGTTGTTGT CGTGTTGCTT TGCCTCCTTA ACTTTTCGAT GTCTGGGTTG TGCTTCGCGG ATCTGGAAAA   
  
  
- TCTTCAGTAA AAATAAAAAG TCCACGCCGC AGATACGAAA AAGCTCTCTT CTTTGAATTT CTGCGTTTGC   
  
  
- CCTTTTTCTT TTCTCTGTTT TGTCTCTTTT AGGAGAGAGA TGAGTGATGG TGATGATGAT GCTGGATACT   
  
  
- GCAACGTGAT TGGNGGCTGA TGGCGGCCTG GTCTGTACCT TTTATGTGAT TTGACTCTTC TGTCCCCGAC   
  
  
- GACTCTCACA ATGACCGCAG CACCGCAGTC AGTGGCAGCT GGCCTAAGCA GCAGTCACAC TTGCTTTTTC   
  
  
- TTTTTTCTCT TTAATCTTTT TTTTTTTTCC CTGCTGTTAC CTGCAGATAA CGGGAAAGAG AAAGGGGGTG   
  
  
- GCACTAGCAT TTCCCATTTT GCCACGTGGC GCGACAGTAA GTAGTTGAGA TTGGCAGGAT ATGATTGGAG   
  
  
- GAAAAACAAC CAATTTTTTT TTTTTCCCCT TCTGTTACCT GCAGATAACG GGAAAGAGAA AGAGGGTGGC   
  
  
- ACTAGCATTT CCCATTTTGC CACGTGGCGC GACAGTAAGT AGTTGAGATT GGCAGGATAT GATTGGAGGA   
  
  
- AAAACAACCA CACGCTCCTG CGAGTCGTAC CCATAATCAA TTTTCCCCAC TATACCCCTA TATTATTTTT   
  
  
- TAATAATTAT TTTAATTTTT GAGATAAAAT AAATGCTTAC ATTAATATGC CGTGTGCGTG CATCTGTGTG   
  
  
- GAAGGCAATG GTGCAGCGCA CGTAGGACCA TGACCCATTA AATCTTTGAA AGAGACAAGT AAATGTATTT   
  
  
- ATTTATATAT GTTGGCAAAG TCAAGGGAAC ACATTAATTT TGGTAAGTCC TTTCAATTAA AACCAAAC

+     G-box

| Site Name | Organism | Position | Strand | Matrix score. | sequence | function |
| --- | --- | --- | --- | --- | --- | --- |
| G-box | Zea mays | 368 | - | 6 | CACGAC | cis-acting regulatory element involved in light responsiveness |
| G-box | Arabidopsis thaliana | 848 | - | 10 | GCCACGTGGTA | cis-acting regulatory element involved in light responsiveness |
| G-box | Daucus carota | 828 | - | 6 | TACGTG | cis-acting regulatory element involved in light responsiveness |
| G-box | Zea mays | 851 | + | 6 | CACGTC | cis-acting regulatory element involved in light responsiveness |

> 2018/04/13 10:10:12  
+ ACTAATTTTA GTGGGTTTAG GTTGTTCGTT TTTTTCTCCT TGAGAAATAC AGGATAGACA AAGCAAGAGC   
  
  
+ ACCAACAACA GCACAACGAA ACGGAGGAAT TGAAAAGCTA CAGACCCAAC ACGAAGCGCC TAGACCTTTT   
  
  
+ AGAAGTCATT TTTATTTTTC AGGTGCGGCG TCTATGCTTT TTCGAGAGAA GAAACTTAAA GACGCAAACG   
  
  
+ GGAAAAAGAA AAGAGACAAA ACAGAGAAAA TCCTCTCTCT ACTCACTACC ACTACTACTA CGACCTATGA   
  
  
+ CGTTGCACTA ACCNCCGACT ACCGCCGGAC CAGACATGGA AAATACACTA AACTGAGAAG ACAGGGGCTG   
  
  
+ CTGAGAGTGT TACTGGCGTC GTGGCGTCAG TCACCGTCGA CCGGATTCGT CGTCAGTGTG AACGAAAAAG   
  
  
+ AAAAAAGAGA AATTAGAAAA AAAAAAAAGG GACGACAATG GACGTCTATT GCCCTTTCTC TTTCCCCCAC   
  
  
+ CGTGATCGTA AAGGGTAAAA CGGTGCACCG CGCTGTCATT CATCAACTCT AACCGTCCTA TACTAACCTC   
  
  
+ CTTTTTGTTG GTTAAAAAAA AAAAAGGGGA AGACAATGGA CGTCTATTGC CCTTTCTCTT TCTCCCACCG   
  
  
+ TGATCGTAAA GGGTAAAACG GTGCACCGCG CTGTCATTCA TCAACTCTAA CCGTCCTATA CTAACCTCCT   
  
  
+ TTTTGTTGGT GTGCGAGGAC GCTCAGCATG GGTATTAGTT AAAAGGGGTG ATATGGGGAT ATAATAAAAA   
  
  
+ ATTATTAATA AAATTAAAAA CTCTATTTTA TTTACGAATG TAATTATACG GCACACGCAC GTAGACACAC   
  
  
+ CTTCCGTTAC CACGTCGCGT GCATCCTGGT ACTGGGTAAT TTAGAAACTT TCTCTGTTCA TTTACATAAA   
  
  
+ TAAATATATA CAACCGTTTC AGTTCCCTTG TGTAATTAAA ACCATTCAGG AAAGTTAATT TTGGTTTG  

- TGATTAAAAT CACCCAAATC CAACAAGCAA AAAAAGAGGA ACTCTTTATG TCCTATCTGT TTCGTTCTCG   
  
  
- TGGTTGTTGT CGTGTTGCTT TGCCTCCTTA ACTTTTCGAT GTCTGGGTTG TGCTTCGCGG ATCTGGAAAA   
  
  
- TCTTCAGTAA AAATAAAAAG TCCACGCCGC AGATACGAAA AAGCTCTCTT CTTTGAATTT CTGCGTTTGC   
  
  
- CCTTTTTCTT TTCTCTGTTT TGTCTCTTTT AGGAGAGAGA TGAGTGATGG TGATGATGAT GCTGGATACT   
  
  
- GCAACGTGAT TGGNGGCTGA TGGCGGCCTG GTCTGTACCT TTTATGTGAT TTGACTCTTC TGTCCCCGAC   
  
  
- GACTCTCACA ATGACCGCAG CACCGCAGTC AGTGGCAGCT GGCCTAAGCA GCAGTCACAC TTGCTTTTTC   
  
  
- TTTTTTCTCT TTAATCTTTT TTTTTTTTCC CTGCTGTTAC CTGCAGATAA CGGGAAAGAG AAAGGGGGTG   
  
  
- GCACTAGCAT TTCCCATTTT GCCACGTGGC GCGACAGTAA GTAGTTGAGA TTGGCAGGAT ATGATTGGAG   
  
  
- GAAAAACAAC CAATTTTTTT TTTTTCCCCT TCTGTTACCT GCAGATAACG GGAAAGAGAA AGAGGGTGGC   
  
  
- ACTAGCATTT CCCATTTTGC CACGTGGCGC GACAGTAAGT AGTTGAGATT GGCAGGATAT GATTGGAGGA   
  
  
- AAAACAACCA CACGCTCCTG CGAGTCGTAC CCATAATCAA TTTTCCCCAC TATACCCCTA TATTATTTTT   
  
  
- TAATAATTAT TTTAATTTTT GAGATAAAAT AAATGCTTAC ATTAATATGC CGTGTGCGTG CATCTGTGTG   
  
  
- GAAGGCAATG GTGCAGCGCA CGTAGGACCA TGACCCATTA AATCTTTGAA AGAGACAAGT AAATGTATTT   
  
  
- ATTTATATAT GTTGGCAAAG TCAAGGGAAC ACATTAATTT TGGTAAGTCC TTTCAATTAA AACCAAAC

+     GARE-motif

| Site Name | Organism | Position | Strand | Matrix score. | sequence | function |
| --- | --- | --- | --- | --- | --- | --- |
| GARE-motif | Brassica oleracea | 229 | + | 7 | AAACAGA | gibberellin-responsive element |

> 2018/04/13 10:10:12  
+ ACTAATTTTA GTGGGTTTAG GTTGTTCGTT TTTTTCTCCT TGAGAAATAC AGGATAGACA AAGCAAGAGC   
  
  
+ ACCAACAACA GCACAACGAA ACGGAGGAAT TGAAAAGCTA CAGACCCAAC ACGAAGCGCC TAGACCTTTT   
  
  
+ AGAAGTCATT TTTATTTTTC AGGTGCGGCG TCTATGCTTT TTCGAGAGAA GAAACTTAAA GACGCAAACG   
  
  
+ GGAAAAAGAA AAGAGACAAA ACAGAGAAAA TCCTCTCTCT ACTCACTACC ACTACTACTA CGACCTATGA   
  
  
+ CGTTGCACTA ACCNCCGACT ACCGCCGGAC CAGACATGGA AAATACACTA AACTGAGAAG ACAGGGGCTG   
  
  
+ CTGAGAGTGT TACTGGCGTC GTGGCGTCAG TCACCGTCGA CCGGATTCGT CGTCAGTGTG AACGAAAAAG   
  
  
+ AAAAAAGAGA AATTAGAAAA AAAAAAAAGG GACGACAATG GACGTCTATT GCCCTTTCTC TTTCCCCCAC   
  
  
+ CGTGATCGTA AAGGGTAAAA CGGTGCACCG CGCTGTCATT CATCAACTCT AACCGTCCTA TACTAACCTC   
  
  
+ CTTTTTGTTG GTTAAAAAAA AAAAAGGGGA AGACAATGGA CGTCTATTGC CCTTTCTCTT TCTCCCACCG   
  
  
+ TGATCGTAAA GGGTAAAACG GTGCACCGCG CTGTCATTCA TCAACTCTAA CCGTCCTATA CTAACCTCCT   
  
  
+ TTTTGTTGGT GTGCGAGGAC GCTCAGCATG GGTATTAGTT AAAAGGGGTG ATATGGGGAT ATAATAAAAA   
  
  
+ ATTATTAATA AAATTAAAAA CTCTATTTTA TTTACGAATG TAATTATACG GCACACGCAC GTAGACACAC   
  
  
+ CTTCCGTTAC CACGTCGCGT GCATCCTGGT ACTGGGTAAT TTAGAAACTT TCTCTGTTCA TTTACATAAA   
  
  
+ TAAATATATA CAACCGTTTC AGTTCCCTTG TGTAATTAAA ACCATTCAGG AAAGTTAATT TTGGTTTG  

- TGATTAAAAT CACCCAAATC CAACAAGCAA AAAAAGAGGA ACTCTTTATG TCCTATCTGT TTCGTTCTCG   
  
  
- TGGTTGTTGT CGTGTTGCTT TGCCTCCTTA ACTTTTCGAT GTCTGGGTTG TGCTTCGCGG ATCTGGAAAA   
  
  
- TCTTCAGTAA AAATAAAAAG TCCACGCCGC AGATACGAAA AAGCTCTCTT CTTTGAATTT CTGCGTTTGC   
  
  
- CCTTTTTCTT TTCTCTGTTT TGTCTCTTTT AGGAGAGAGA TGAGTGATGG TGATGATGAT GCTGGATACT   
  
  
- GCAACGTGAT TGGNGGCTGA TGGCGGCCTG GTCTGTACCT TTTATGTGAT TTGACTCTTC TGTCCCCGAC   
  
  
- GACTCTCACA ATGACCGCAG CACCGCAGTC AGTGGCAGCT GGCCTAAGCA GCAGTCACAC TTGCTTTTTC   
  
  
- TTTTTTCTCT TTAATCTTTT TTTTTTTTCC CTGCTGTTAC CTGCAGATAA CGGGAAAGAG AAAGGGGGTG   
  
  
- GCACTAGCAT TTCCCATTTT GCCACGTGGC GCGACAGTAA GTAGTTGAGA TTGGCAGGAT ATGATTGGAG   
  
  
- GAAAAACAAC CAATTTTTTT TTTTTCCCCT TCTGTTACCT GCAGATAACG GGAAAGAGAA AGAGGGTGGC   
  
  
- ACTAGCATTT CCCATTTTGC CACGTGGCGC GACAGTAAGT AGTTGAGATT GGCAGGATAT GATTGGAGGA   
  
  
- AAAACAACCA CACGCTCCTG CGAGTCGTAC CCATAATCAA TTTTCCCCAC TATACCCCTA TATTATTTTT   
  
  
- TAATAATTAT TTTAATTTTT GAGATAAAAT AAATGCTTAC ATTAATATGC CGTGTGCGTG CATCTGTGTG   
  
  
- GAAGGCAATG GTGCAGCGCA CGTAGGACCA TGACCCATTA AATCTTTGAA AGAGACAAGT AAATGTATTT   
  
  
- ATTTATATAT GTTGGCAAAG TCAAGGGAAC ACATTAATTT TGGTAAGTCC TTTCAATTAA AACCAAAC

+     GT1-motif

| Site Name | Organism | Position | Strand | Matrix score. | sequence | function |
| --- | --- | --- | --- | --- | --- | --- |
| GT1-motif | Arabidopsis thaliana | 570 | + | 6 | GGTTAA | light responsive element |

> 2018/04/13 10:10:12  
+ ACTAATTTTA GTGGGTTTAG GTTGTTCGTT TTTTTCTCCT TGAGAAATAC AGGATAGACA AAGCAAGAGC   
  
  
+ ACCAACAACA GCACAACGAA ACGGAGGAAT TGAAAAGCTA CAGACCCAAC ACGAAGCGCC TAGACCTTTT   
  
  
+ AGAAGTCATT TTTATTTTTC AGGTGCGGCG TCTATGCTTT TTCGAGAGAA GAAACTTAAA GACGCAAACG   
  
  
+ GGAAAAAGAA AAGAGACAAA ACAGAGAAAA TCCTCTCTCT ACTCACTACC ACTACTACTA CGACCTATGA   
  
  
+ CGTTGCACTA ACCNCCGACT ACCGCCGGAC CAGACATGGA AAATACACTA AACTGAGAAG ACAGGGGCTG   
  
  
+ CTGAGAGTGT TACTGGCGTC GTGGCGTCAG TCACCGTCGA CCGGATTCGT CGTCAGTGTG AACGAAAAAG   
  
  
+ AAAAAAGAGA AATTAGAAAA AAAAAAAAGG GACGACAATG GACGTCTATT GCCCTTTCTC TTTCCCCCAC   
  
  
+ CGTGATCGTA AAGGGTAAAA CGGTGCACCG CGCTGTCATT CATCAACTCT AACCGTCCTA TACTAACCTC   
  
  
+ CTTTTTGTTG GTTAAAAAAA AAAAAGGGGA AGACAATGGA CGTCTATTGC CCTTTCTCTT TCTCCCACCG   
  
  
+ TGATCGTAAA GGGTAAAACG GTGCACCGCG CTGTCATTCA TCAACTCTAA CCGTCCTATA CTAACCTCCT   
  
  
+ TTTTGTTGGT GTGCGAGGAC GCTCAGCATG GGTATTAGTT AAAAGGGGTG ATATGGGGAT ATAATAAAAA   
  
  
+ ATTATTAATA AAATTAAAAA CTCTATTTTA TTTACGAATG TAATTATACG GCACACGCAC GTAGACACAC   
  
  
+ CTTCCGTTAC CACGTCGCGT GCATCCTGGT ACTGGGTAAT TTAGAAACTT TCTCTGTTCA TTTACATAAA   
  
  
+ TAAATATATA CAACCGTTTC AGTTCCCTTG TGTAATTAAA ACCATTCAGG AAAGTTAATT TTGGTTTG  

- TGATTAAAAT CACCCAAATC CAACAAGCAA AAAAAGAGGA ACTCTTTATG TCCTATCTGT TTCGTTCTCG   
  
  
- TGGTTGTTGT CGTGTTGCTT TGCCTCCTTA ACTTTTCGAT GTCTGGGTTG TGCTTCGCGG ATCTGGAAAA   
  
  
- TCTTCAGTAA AAATAAAAAG TCCACGCCGC AGATACGAAA AAGCTCTCTT CTTTGAATTT CTGCGTTTGC   
  
  
- CCTTTTTCTT TTCTCTGTTT TGTCTCTTTT AGGAGAGAGA TGAGTGATGG TGATGATGAT GCTGGATACT   
  
  
- GCAACGTGAT TGGNGGCTGA TGGCGGCCTG GTCTGTACCT TTTATGTGAT TTGACTCTTC TGTCCCCGAC   
  
  
- GACTCTCACA ATGACCGCAG CACCGCAGTC AGTGGCAGCT GGCCTAAGCA GCAGTCACAC TTGCTTTTTC   
  
  
- TTTTTTCTCT TTAATCTTTT TTTTTTTTCC CTGCTGTTAC CTGCAGATAA CGGGAAAGAG AAAGGGGGTG   
  
  
- GCACTAGCAT TTCCCATTTT GCCACGTGGC GCGACAGTAA GTAGTTGAGA TTGGCAGGAT ATGATTGGAG   
  
  
- GAAAAACAAC CAATTTTTTT TTTTTCCCCT TCTGTTACCT GCAGATAACG GGAAAGAGAA AGAGGGTGGC   
  
  
- ACTAGCATTT CCCATTTTGC CACGTGGCGC GACAGTAAGT AGTTGAGATT GGCAGGATAT GATTGGAGGA   
  
  
- AAAACAACCA CACGCTCCTG CGAGTCGTAC CCATAATCAA TTTTCCCCAC TATACCCCTA TATTATTTTT   
  
  
- TAATAATTAT TTTAATTTTT GAGATAAAAT AAATGCTTAC ATTAATATGC CGTGTGCGTG CATCTGTGTG   
  
  
- GAAGGCAATG GTGCAGCGCA CGTAGGACCA TGACCCATTA AATCTTTGAA AGAGACAAGT AAATGTATTT   
  
  
- ATTTATATAT GTTGGCAAAG TCAAGGGAAC ACATTAATTT TGGTAAGTCC TTTCAATTAA AACCAAAC

+     I-box

| Site Name | Organism | Position | Strand | Matrix score. | sequence | function |
| --- | --- | --- | --- | --- | --- | --- |
| I-box | Flaveria trinervia | 750 | + | 7 | GATATGG | part of a light responsive element |

> 2018/04/13 10:10:12  
+ ACTAATTTTA GTGGGTTTAG GTTGTTCGTT TTTTTCTCCT TGAGAAATAC AGGATAGACA AAGCAAGAGC   
  
  
+ ACCAACAACA GCACAACGAA ACGGAGGAAT TGAAAAGCTA CAGACCCAAC ACGAAGCGCC TAGACCTTTT   
  
  
+ AGAAGTCATT TTTATTTTTC AGGTGCGGCG TCTATGCTTT TTCGAGAGAA GAAACTTAAA GACGCAAACG   
  
  
+ GGAAAAAGAA AAGAGACAAA ACAGAGAAAA TCCTCTCTCT ACTCACTACC ACTACTACTA CGACCTATGA   
  
  
+ CGTTGCACTA ACCNCCGACT ACCGCCGGAC CAGACATGGA AAATACACTA AACTGAGAAG ACAGGGGCTG   
  
  
+ CTGAGAGTGT TACTGGCGTC GTGGCGTCAG TCACCGTCGA CCGGATTCGT CGTCAGTGTG AACGAAAAAG   
  
  
+ AAAAAAGAGA AATTAGAAAA AAAAAAAAGG GACGACAATG GACGTCTATT GCCCTTTCTC TTTCCCCCAC   
  
  
+ CGTGATCGTA AAGGGTAAAA CGGTGCACCG CGCTGTCATT CATCAACTCT AACCGTCCTA TACTAACCTC   
  
  
+ CTTTTTGTTG GTTAAAAAAA AAAAAGGGGA AGACAATGGA CGTCTATTGC CCTTTCTCTT TCTCCCACCG   
  
  
+ TGATCGTAAA GGGTAAAACG GTGCACCGCG CTGTCATTCA TCAACTCTAA CCGTCCTATA CTAACCTCCT   
  
  
+ TTTTGTTGGT GTGCGAGGAC GCTCAGCATG GGTATTAGTT AAAAGGGGTG ATATGGGGAT ATAATAAAAA   
  
  
+ ATTATTAATA AAATTAAAAA CTCTATTTTA TTTACGAATG TAATTATACG GCACACGCAC GTAGACACAC   
  
  
+ CTTCCGTTAC CACGTCGCGT GCATCCTGGT ACTGGGTAAT TTAGAAACTT TCTCTGTTCA TTTACATAAA   
  
  
+ TAAATATATA CAACCGTTTC AGTTCCCTTG TGTAATTAAA ACCATTCAGG AAAGTTAATT TTGGTTTG  

- TGATTAAAAT CACCCAAATC CAACAAGCAA AAAAAGAGGA ACTCTTTATG TCCTATCTGT TTCGTTCTCG   
  
  
- TGGTTGTTGT CGTGTTGCTT TGCCTCCTTA ACTTTTCGAT GTCTGGGTTG TGCTTCGCGG ATCTGGAAAA   
  
  
- TCTTCAGTAA AAATAAAAAG TCCACGCCGC AGATACGAAA AAGCTCTCTT CTTTGAATTT CTGCGTTTGC   
  
  
- CCTTTTTCTT TTCTCTGTTT TGTCTCTTTT AGGAGAGAGA TGAGTGATGG TGATGATGAT GCTGGATACT   
  
  
- GCAACGTGAT TGGNGGCTGA TGGCGGCCTG GTCTGTACCT TTTATGTGAT TTGACTCTTC TGTCCCCGAC   
  
  
- GACTCTCACA ATGACCGCAG CACCGCAGTC AGTGGCAGCT GGCCTAAGCA GCAGTCACAC TTGCTTTTTC   
  
  
- TTTTTTCTCT TTAATCTTTT TTTTTTTTCC CTGCTGTTAC CTGCAGATAA CGGGAAAGAG AAAGGGGGTG   
  
  
- GCACTAGCAT TTCCCATTTT GCCACGTGGC GCGACAGTAA GTAGTTGAGA TTGGCAGGAT ATGATTGGAG   
  
  
- GAAAAACAAC CAATTTTTTT TTTTTCCCCT TCTGTTACCT GCAGATAACG GGAAAGAGAA AGAGGGTGGC   
  
  
- ACTAGCATTT CCCATTTTGC CACGTGGCGC GACAGTAAGT AGTTGAGATT GGCAGGATAT GATTGGAGGA   
  
  
- AAAACAACCA CACGCTCCTG CGAGTCGTAC CCATAATCAA TTTTCCCCAC TATACCCCTA TATTATTTTT   
  
  
- TAATAATTAT TTTAATTTTT GAGATAAAAT AAATGCTTAC ATTAATATGC CGTGTGCGTG CATCTGTGTG   
  
  
- GAAGGCAATG GTGCAGCGCA CGTAGGACCA TGACCCATTA AATCTTTGAA AGAGACAAGT AAATGTATTT   
  
  
- ATTTATATAT GTTGGCAAAG TCAAGGGAAC ACATTAATTT TGGTAAGTCC TTTCAATTAA AACCAAAC

+     MRE

| Site Name | Organism | Position | Strand | Matrix score. | sequence | function |
| --- | --- | --- | --- | --- | --- | --- |
| MRE | Petroselinum crispum | 17 | - | 7 | AACCTAA | MYB binding site involved in light responsiveness |

> 2018/04/13 10:10:12  
+ ACTAATTTTA GTGGGTTTAG GTTGTTCGTT TTTTTCTCCT TGAGAAATAC AGGATAGACA AAGCAAGAGC   
  
  
+ ACCAACAACA GCACAACGAA ACGGAGGAAT TGAAAAGCTA CAGACCCAAC ACGAAGCGCC TAGACCTTTT   
  
  
+ AGAAGTCATT TTTATTTTTC AGGTGCGGCG TCTATGCTTT TTCGAGAGAA GAAACTTAAA GACGCAAACG   
  
  
+ GGAAAAAGAA AAGAGACAAA ACAGAGAAAA TCCTCTCTCT ACTCACTACC ACTACTACTA CGACCTATGA   
  
  
+ CGTTGCACTA ACCNCCGACT ACCGCCGGAC CAGACATGGA AAATACACTA AACTGAGAAG ACAGGGGCTG   
  
  
+ CTGAGAGTGT TACTGGCGTC GTGGCGTCAG TCACCGTCGA CCGGATTCGT CGTCAGTGTG AACGAAAAAG   
  
  
+ AAAAAAGAGA AATTAGAAAA AAAAAAAAGG GACGACAATG GACGTCTATT GCCCTTTCTC TTTCCCCCAC   
  
  
+ CGTGATCGTA AAGGGTAAAA CGGTGCACCG CGCTGTCATT CATCAACTCT AACCGTCCTA TACTAACCTC   
  
  
+ CTTTTTGTTG GTTAAAAAAA AAAAAGGGGA AGACAATGGA CGTCTATTGC CCTTTCTCTT TCTCCCACCG   
  
  
+ TGATCGTAAA GGGTAAAACG GTGCACCGCG CTGTCATTCA TCAACTCTAA CCGTCCTATA CTAACCTCCT   
  
  
+ TTTTGTTGGT GTGCGAGGAC GCTCAGCATG GGTATTAGTT AAAAGGGGTG ATATGGGGAT ATAATAAAAA   
  
  
+ ATTATTAATA AAATTAAAAA CTCTATTTTA TTTACGAATG TAATTATACG GCACACGCAC GTAGACACAC   
  
  
+ CTTCCGTTAC CACGTCGCGT GCATCCTGGT ACTGGGTAAT TTAGAAACTT TCTCTGTTCA TTTACATAAA   
  
  
+ TAAATATATA CAACCGTTTC AGTTCCCTTG TGTAATTAAA ACCATTCAGG AAAGTTAATT TTGGTTTG  

- TGATTAAAAT CACCCAAATC CAACAAGCAA AAAAAGAGGA ACTCTTTATG TCCTATCTGT TTCGTTCTCG   
  
  
- TGGTTGTTGT CGTGTTGCTT TGCCTCCTTA ACTTTTCGAT GTCTGGGTTG TGCTTCGCGG ATCTGGAAAA   
  
  
- TCTTCAGTAA AAATAAAAAG TCCACGCCGC AGATACGAAA AAGCTCTCTT CTTTGAATTT CTGCGTTTGC   
  
  
- CCTTTTTCTT TTCTCTGTTT TGTCTCTTTT AGGAGAGAGA TGAGTGATGG TGATGATGAT GCTGGATACT   
  
  
- GCAACGTGAT TGGNGGCTGA TGGCGGCCTG GTCTGTACCT TTTATGTGAT TTGACTCTTC TGTCCCCGAC   
  
  
- GACTCTCACA ATGACCGCAG CACCGCAGTC AGTGGCAGCT GGCCTAAGCA GCAGTCACAC TTGCTTTTTC   
  
  
- TTTTTTCTCT TTAATCTTTT TTTTTTTTCC CTGCTGTTAC CTGCAGATAA CGGGAAAGAG AAAGGGGGTG   
  
  
- GCACTAGCAT TTCCCATTTT GCCACGTGGC GCGACAGTAA GTAGTTGAGA TTGGCAGGAT ATGATTGGAG   
  
  
- GAAAAACAAC CAATTTTTTT TTTTTCCCCT TCTGTTACCT GCAGATAACG GGAAAGAGAA AGAGGGTGGC   
  
  
- ACTAGCATTT CCCATTTTGC CACGTGGCGC GACAGTAAGT AGTTGAGATT GGCAGGATAT GATTGGAGGA   
  
  
- AAAACAACCA CACGCTCCTG CGAGTCGTAC CCATAATCAA TTTTCCCCAC TATACCCCTA TATTATTTTT   
  
  
- TAATAATTAT TTTAATTTTT GAGATAAAAT AAATGCTTAC ATTAATATGC CGTGTGCGTG CATCTGTGTG   
  
  
- GAAGGCAATG GTGCAGCGCA CGTAGGACCA TGACCCATTA AATCTTTGAA AGAGACAAGT AAATGTATTT   
  
  
- ATTTATATAT GTTGGCAAAG TCAAGGGAAC ACATTAATTT TGGTAAGTCC TTTCAATTAA AACCAAAC

+     O2-site

| Site Name | Organism | Position | Strand | Matrix score. | sequence | function |
| --- | --- | --- | --- | --- | --- | --- |
| O2-site | Zea mays | 747 | + | 9 | GATGATATGG | cis-acting regulatory element involved in zein metabolism regulation |

> 2018/04/13 10:10:12  
+ ACTAATTTTA GTGGGTTTAG GTTGTTCGTT TTTTTCTCCT TGAGAAATAC AGGATAGACA AAGCAAGAGC   
  
  
+ ACCAACAACA GCACAACGAA ACGGAGGAAT TGAAAAGCTA CAGACCCAAC ACGAAGCGCC TAGACCTTTT   
  
  
+ AGAAGTCATT TTTATTTTTC AGGTGCGGCG TCTATGCTTT TTCGAGAGAA GAAACTTAAA GACGCAAACG   
  
  
+ GGAAAAAGAA AAGAGACAAA ACAGAGAAAA TCCTCTCTCT ACTCACTACC ACTACTACTA CGACCTATGA   
  
  
+ CGTTGCACTA ACCNCCGACT ACCGCCGGAC CAGACATGGA AAATACACTA AACTGAGAAG ACAGGGGCTG   
  
  
+ CTGAGAGTGT TACTGGCGTC GTGGCGTCAG TCACCGTCGA CCGGATTCGT CGTCAGTGTG AACGAAAAAG   
  
  
+ AAAAAAGAGA AATTAGAAAA AAAAAAAAGG GACGACAATG GACGTCTATT GCCCTTTCTC TTTCCCCCAC   
  
  
+ CGTGATCGTA AAGGGTAAAA CGGTGCACCG CGCTGTCATT CATCAACTCT AACCGTCCTA TACTAACCTC   
  
  
+ CTTTTTGTTG GTTAAAAAAA AAAAAGGGGA AGACAATGGA CGTCTATTGC CCTTTCTCTT TCTCCCACCG   
  
  
+ TGATCGTAAA GGGTAAAACG GTGCACCGCG CTGTCATTCA TCAACTCTAA CCGTCCTATA CTAACCTCCT   
  
  
+ TTTTGTTGGT GTGCGAGGAC GCTCAGCATG GGTATTAGTT AAAAGGGGTG ATATGGGGAT ATAATAAAAA   
  
  
+ ATTATTAATA AAATTAAAAA CTCTATTTTA TTTACGAATG TAATTATACG GCACACGCAC GTAGACACAC   
  
  
+ CTTCCGTTAC CACGTCGCGT GCATCCTGGT ACTGGGTAAT TTAGAAACTT TCTCTGTTCA TTTACATAAA   
  
  
+ TAAATATATA CAACCGTTTC AGTTCCCTTG TGTAATTAAA ACCATTCAGG AAAGTTAATT TTGGTTTG  

- TGATTAAAAT CACCCAAATC CAACAAGCAA AAAAAGAGGA ACTCTTTATG TCCTATCTGT TTCGTTCTCG   
  
  
- TGGTTGTTGT CGTGTTGCTT TGCCTCCTTA ACTTTTCGAT GTCTGGGTTG TGCTTCGCGG ATCTGGAAAA   
  
  
- TCTTCAGTAA AAATAAAAAG TCCACGCCGC AGATACGAAA AAGCTCTCTT CTTTGAATTT CTGCGTTTGC   
  
  
- CCTTTTTCTT TTCTCTGTTT TGTCTCTTTT AGGAGAGAGA TGAGTGATGG TGATGATGAT GCTGGATACT   
  
  
- GCAACGTGAT TGGNGGCTGA TGGCGGCCTG GTCTGTACCT TTTATGTGAT TTGACTCTTC TGTCCCCGAC   
  
  
- GACTCTCACA ATGACCGCAG CACCGCAGTC AGTGGCAGCT GGCCTAAGCA GCAGTCACAC TTGCTTTTTC   
  
  
- TTTTTTCTCT TTAATCTTTT TTTTTTTTCC CTGCTGTTAC CTGCAGATAA CGGGAAAGAG AAAGGGGGTG   
  
  
- GCACTAGCAT TTCCCATTTT GCCACGTGGC GCGACAGTAA GTAGTTGAGA TTGGCAGGAT ATGATTGGAG   
  
  
- GAAAAACAAC CAATTTTTTT TTTTTCCCCT TCTGTTACCT GCAGATAACG GGAAAGAGAA AGAGGGTGGC   
  
  
- ACTAGCATTT CCCATTTTGC CACGTGGCGC GACAGTAAGT AGTTGAGATT GGCAGGATAT GATTGGAGGA   
  
  
- AAAACAACCA CACGCTCCTG CGAGTCGTAC CCATAATCAA TTTTCCCCAC TATACCCCTA TATTATTTTT   
  
  
- TAATAATTAT TTTAATTTTT GAGATAAAAT AAATGCTTAC ATTAATATGC CGTGTGCGTG CATCTGTGTG   
  
  
- GAAGGCAATG GTGCAGCGCA CGTAGGACCA TGACCCATTA AATCTTTGAA AGAGACAAGT AAATGTATTT   
  
  
- ATTTATATAT GTTGGCAAAG TCAAGGGAAC ACATTAATTT TGGTAAGTCC TTTCAATTAA AACCAAAC

+     Skn-1\_motif

| Site Name | Organism | Position | Strand | Matrix score. | sequence | function |
| --- | --- | --- | --- | --- | --- | --- |
| Skn-1\_motif | Oryza sativa | 277 | - | 5 | GTCAT | cis-acting regulatory element required for endosperm expression |
| Skn-1\_motif | Oryza sativa | 663 | + | 5 | GTCAT | cis-acting regulatory element required for endosperm expression |
| Skn-1\_motif | Oryza sativa | 525 | + | 5 | GTCAT | cis-acting regulatory element required for endosperm expression |
| Skn-1\_motif | Oryza sativa | 145 | + | 5 | GTCAT | cis-acting regulatory element required for endosperm expression |

> 2018/04/13 10:10:12  
+ ACTAATTTTA GTGGGTTTAG GTTGTTCGTT TTTTTCTCCT TGAGAAATAC AGGATAGACA AAGCAAGAGC   
  
  
+ ACCAACAACA GCACAACGAA ACGGAGGAAT TGAAAAGCTA CAGACCCAAC ACGAAGCGCC TAGACCTTTT   
  
  
+ AGAAGTCATT TTTATTTTTC AGGTGCGGCG TCTATGCTTT TTCGAGAGAA GAAACTTAAA GACGCAAACG   
  
  
+ GGAAAAAGAA AAGAGACAAA ACAGAGAAAA TCCTCTCTCT ACTCACTACC ACTACTACTA CGACCTATGA   
  
  
+ CGTTGCACTA ACCNCCGACT ACCGCCGGAC CAGACATGGA AAATACACTA AACTGAGAAG ACAGGGGCTG   
  
  
+ CTGAGAGTGT TACTGGCGTC GTGGCGTCAG TCACCGTCGA CCGGATTCGT CGTCAGTGTG AACGAAAAAG   
  
  
+ AAAAAAGAGA AATTAGAAAA AAAAAAAAGG GACGACAATG GACGTCTATT GCCCTTTCTC TTTCCCCCAC   
  
  
+ CGTGATCGTA AAGGGTAAAA CGGTGCACCG CGCTGTCATT CATCAACTCT AACCGTCCTA TACTAACCTC   
  
  
+ CTTTTTGTTG GTTAAAAAAA AAAAAGGGGA AGACAATGGA CGTCTATTGC CCTTTCTCTT TCTCCCACCG   
  
  
+ TGATCGTAAA GGGTAAAACG GTGCACCGCG CTGTCATTCA TCAACTCTAA CCGTCCTATA CTAACCTCCT   
  
  
+ TTTTGTTGGT GTGCGAGGAC GCTCAGCATG GGTATTAGTT AAAAGGGGTG ATATGGGGAT ATAATAAAAA   
  
  
+ ATTATTAATA AAATTAAAAA CTCTATTTTA TTTACGAATG TAATTATACG GCACACGCAC GTAGACACAC   
  
  
+ CTTCCGTTAC CACGTCGCGT GCATCCTGGT ACTGGGTAAT TTAGAAACTT TCTCTGTTCA TTTACATAAA   
  
  
+ TAAATATATA CAACCGTTTC AGTTCCCTTG TGTAATTAAA ACCATTCAGG AAAGTTAATT TTGGTTTG  

- TGATTAAAAT CACCCAAATC CAACAAGCAA AAAAAGAGGA ACTCTTTATG TCCTATCTGT TTCGTTCTCG   
  
  
- TGGTTGTTGT CGTGTTGCTT TGCCTCCTTA ACTTTTCGAT GTCTGGGTTG TGCTTCGCGG ATCTGGAAAA   
  
  
- TCTTCAGTAA AAATAAAAAG TCCACGCCGC AGATACGAAA AAGCTCTCTT CTTTGAATTT CTGCGTTTGC   
  
  
- CCTTTTTCTT TTCTCTGTTT TGTCTCTTTT AGGAGAGAGA TGAGTGATGG TGATGATGAT GCTGGATACT   
  
  
- GCAACGTGAT TGGNGGCTGA TGGCGGCCTG GTCTGTACCT TTTATGTGAT TTGACTCTTC TGTCCCCGAC   
  
  
- GACTCTCACA ATGACCGCAG CACCGCAGTC AGTGGCAGCT GGCCTAAGCA GCAGTCACAC TTGCTTTTTC   
  
  
- TTTTTTCTCT TTAATCTTTT TTTTTTTTCC CTGCTGTTAC CTGCAGATAA CGGGAAAGAG AAAGGGGGTG   
  
  
- GCACTAGCAT TTCCCATTTT GCCACGTGGC GCGACAGTAA GTAGTTGAGA TTGGCAGGAT ATGATTGGAG   
  
  
- GAAAAACAAC CAATTTTTTT TTTTTCCCCT TCTGTTACCT GCAGATAACG GGAAAGAGAA AGAGGGTGGC   
  
  
- ACTAGCATTT CCCATTTTGC CACGTGGCGC GACAGTAAGT AGTTGAGATT GGCAGGATAT GATTGGAGGA   
  
  
- AAAACAACCA CACGCTCCTG CGAGTCGTAC CCATAATCAA TTTTCCCCAC TATACCCCTA TATTATTTTT   
  
  
- TAATAATTAT TTTAATTTTT GAGATAAAAT AAATGCTTAC ATTAATATGC CGTGTGCGTG CATCTGTGTG   
  
  
- GAAGGCAATG GTGCAGCGCA CGTAGGACCA TGACCCATTA AATCTTTGAA AGAGACAAGT AAATGTATTT   
  
  
- ATTTATATAT GTTGGCAAAG TCAAGGGAAC ACATTAATTT TGGTAAGTCC TTTCAATTAA AACCAAAC

+     TATA-box

| Site Name | Organism | Position | Strand | Matrix score. | sequence | function |
| --- | --- | --- | --- | --- | --- | --- |
| TATA-box | Lycopersicon esculentum | 796 | + | 5 | TTTTA | core promoter element around -30 of transcription start |
| TATA-box | Glycine max | 762 | + | 5 | TAATA | core promoter element around -30 of transcription start |
| TATA-box | Brassica oleracea | 759 | + | 7 | ATATAAT | core promoter element around -30 of transcription start |
| TATA-box | Arabidopsis thaliana | 687 | - | 4 | TATA | core promoter element around -30 of transcription start |
| TATA-box | Lycopersicon esculentum | 740 | - | 5 | TTTTA | core promoter element around -30 of transcription start |
| TATA-box | Lycopersicon esculentum | 785 | - | 5 | TTTTA | core promoter element around -30 of transcription start |
| TATA-box | Glycine max | 776 | + | 5 | TAATA | core promoter element around -30 of transcription start |
| TATA-box | Glycine max | 733 | - | 5 | TAATA | core promoter element around -30 of transcription start |
| TATA-box | Lycopersicon esculentum | 765 | - | 5 | TTTTA | core promoter element around -30 of transcription start |
| TATA-box | Lycopersicon esculentum | 573 | - | 5 | TTTTA | core promoter element around -30 of transcription start |
| TATA-box | Lycopersicon esculentum | 150 | + | 5 | TTTTA | core promoter element around -30 of transcription start |
| TATA-box | Arabidopsis thaliana | 549 | - | 4 | TATA | core promoter element around -30 of transcription start |
| TATA-box | Lycopersicon esculentum | 947 | - | 5 | TTTTA | core promoter element around -30 of transcription start |
| TATA-box | Oryza sativa | 903 | + | 8 | TACATAAA | core promoter element around -30 of transcription start |
| TATA-box | Brassica napus | 914 | - | 6 | ATATAT | core promoter element around -30 of transcription start |
| TATA-box | Arabidopsis thaliana | 913 | - | 9 | tcTATATAtt | core promoter element around -30 of transcription start |
| TATA-box | Lycopersicon esculentum | 779 | - | 5 | TTTTA | core promoter element around -30 of transcription start |
| TATA-box | Arabidopsis thaliana | 760 | - | 4 | TATA | core promoter element around -30 of transcription start |
| TATA-box | Lycopersicon esculentum | 644 | - | 5 | TTTTA | core promoter element around -30 of transcription start |
| TATA-box | Lycopersicon esculentum | 506 | - | 5 | TTTTA | core promoter element around -30 of transcription start |
| TATA-box | Lycopersicon esculentum | 6 | + | 5 | TTTTA | core promoter element around -30 of transcription start |
| TATA-box | Arabidopsis thaliana | 815 | - | 4 | TATA | core promoter element around -30 of transcription start |
| TATA-box | Lycopersicon esculentum | 137 | + | 5 | TTTTA | core promoter element around -30 of transcription start |
| TATA-box | Glycine max | 773 | - | 5 | TAATA | core promoter element around -30 of transcription start |
| TATA-box | Arabidopsis thaliana | 814 | - | 5 | TATAA | core promoter element around -30 of transcription start |
| TATA-box | Arabidopsis thaliana | 917 | - | 4 | TATA | core promoter element around -30 of transcription start |
| TATA-box | Arabidopsis thaliana | 915 | - | 4 | TATA | core promoter element around -30 of transcription start |
| TATA-box | Brassica napus | 813 | + | 6 | ATTATA | core promoter element around -30 of transcription start |

> 2018/04/13 10:10:12  
+ ACTAATTTTA GTGGGTTTAG GTTGTTCGTT TTTTTCTCCT TGAGAAATAC AGGATAGACA AAGCAAGAGC   
  
  
+ ACCAACAACA GCACAACGAA ACGGAGGAAT TGAAAAGCTA CAGACCCAAC ACGAAGCGCC TAGACCTTTT   
  
  
+ AGAAGTCATT TTTATTTTTC AGGTGCGGCG TCTATGCTTT TTCGAGAGAA GAAACTTAAA GACGCAAACG   
  
  
+ GGAAAAAGAA AAGAGACAAA ACAGAGAAAA TCCTCTCTCT ACTCACTACC ACTACTACTA CGACCTATGA   
  
  
+ CGTTGCACTA ACCNCCGACT ACCGCCGGAC CAGACATGGA AAATACACTA AACTGAGAAG ACAGGGGCTG   
  
  
+ CTGAGAGTGT TACTGGCGTC GTGGCGTCAG TCACCGTCGA CCGGATTCGT CGTCAGTGTG AACGAAAAAG   
  
  
+ AAAAAAGAGA AATTAGAAAA AAAAAAAAGG GACGACAATG GACGTCTATT GCCCTTTCTC TTTCCCCCAC   
  
  
+ CGTGATCGTA AAGGGTAAAA CGGTGCACCG CGCTGTCATT CATCAACTCT AACCGTCCTA TACTAACCTC   
  
  
+ CTTTTTGTTG GTTAAAAAAA AAAAAGGGGA AGACAATGGA CGTCTATTGC CCTTTCTCTT TCTCCCACCG   
  
  
+ TGATCGTAAA GGGTAAAACG GTGCACCGCG CTGTCATTCA TCAACTCTAA CCGTCCTATA CTAACCTCCT   
  
  
+ TTTTGTTGGT GTGCGAGGAC GCTCAGCATG GGTATTAGTT AAAAGGGGTG ATATGGGGAT ATAATAAAAA   
  
  
+ ATTATTAATA AAATTAAAAA CTCTATTTTA TTTACGAATG TAATTATACG GCACACGCAC GTAGACACAC   
  
  
+ CTTCCGTTAC CACGTCGCGT GCATCCTGGT ACTGGGTAAT TTAGAAACTT TCTCTGTTCA TTTACATAAA   
  
  
+ TAAATATATA CAACCGTTTC AGTTCCCTTG TGTAATTAAA ACCATTCAGG AAAGTTAATT TTGGTTTG  

- TGATTAAAAT CACCCAAATC CAACAAGCAA AAAAAGAGGA ACTCTTTATG TCCTATCTGT TTCGTTCTCG   
  
  
- TGGTTGTTGT CGTGTTGCTT TGCCTCCTTA ACTTTTCGAT GTCTGGGTTG TGCTTCGCGG ATCTGGAAAA   
  
  
- TCTTCAGTAA AAATAAAAAG TCCACGCCGC AGATACGAAA AAGCTCTCTT CTTTGAATTT CTGCGTTTGC   
  
  
- CCTTTTTCTT TTCTCTGTTT TGTCTCTTTT AGGAGAGAGA TGAGTGATGG TGATGATGAT GCTGGATACT   
  
  
- GCAACGTGAT TGGNGGCTGA TGGCGGCCTG GTCTGTACCT TTTATGTGAT TTGACTCTTC TGTCCCCGAC   
  
  
- GACTCTCACA ATGACCGCAG CACCGCAGTC AGTGGCAGCT GGCCTAAGCA GCAGTCACAC TTGCTTTTTC   
  
  
- TTTTTTCTCT TTAATCTTTT TTTTTTTTCC CTGCTGTTAC CTGCAGATAA CGGGAAAGAG AAAGGGGGTG   
  
  
- GCACTAGCAT TTCCCATTTT GCCACGTGGC GCGACAGTAA GTAGTTGAGA TTGGCAGGAT ATGATTGGAG   
  
  
- GAAAAACAAC CAATTTTTTT TTTTTCCCCT TCTGTTACCT GCAGATAACG GGAAAGAGAA AGAGGGTGGC   
  
  
- ACTAGCATTT CCCATTTTGC CACGTGGCGC GACAGTAAGT AGTTGAGATT GGCAGGATAT GATTGGAGGA   
  
  
- AAAACAACCA CACGCTCCTG CGAGTCGTAC CCATAATCAA TTTTCCCCAC TATACCCCTA TATTATTTTT   
  
  
- TAATAATTAT TTTAATTTTT GAGATAAAAT AAATGCTTAC ATTAATATGC CGTGTGCGTG CATCTGTGTG   
  
  
- GAAGGCAATG GTGCAGCGCA CGTAGGACCA TGACCCATTA AATCTTTGAA AGAGACAAGT AAATGTATTT   
  
  
- ATTTATATAT GTTGGCAAAG TCAAGGGAAC ACATTAATTT TGGTAAGTCC TTTCAATTAA AACCAAAC

+     TGACG-motif

| Site Name | Organism | Position | Strand | Matrix score. | sequence | function |
| --- | --- | --- | --- | --- | --- | --- |
| TGACG-motif | Hordeum vulgare | 278 | + | 5 | TGACG | cis-acting regulatory element involved in the MeJA-responsiveness |
| TGACG-motif | Hordeum vulgare | 375 | - | 5 | TGACG | cis-acting regulatory element involved in the MeJA-responsiveness |
| TGACG-motif | Hordeum vulgare | 401 | - | 5 | TGACG | cis-acting regulatory element involved in the MeJA-responsiveness |

> 2018/04/13 10:10:12  
+ ACTAATTTTA GTGGGTTTAG GTTGTTCGTT TTTTTCTCCT TGAGAAATAC AGGATAGACA AAGCAAGAGC   
  
  
+ ACCAACAACA GCACAACGAA ACGGAGGAAT TGAAAAGCTA CAGACCCAAC ACGAAGCGCC TAGACCTTTT   
  
  
+ AGAAGTCATT TTTATTTTTC AGGTGCGGCG TCTATGCTTT TTCGAGAGAA GAAACTTAAA GACGCAAACG   
  
  
+ GGAAAAAGAA AAGAGACAAA ACAGAGAAAA TCCTCTCTCT ACTCACTACC ACTACTACTA CGACCTATGA   
  
  
+ CGTTGCACTA ACCNCCGACT ACCGCCGGAC CAGACATGGA AAATACACTA AACTGAGAAG ACAGGGGCTG   
  
  
+ CTGAGAGTGT TACTGGCGTC GTGGCGTCAG TCACCGTCGA CCGGATTCGT CGTCAGTGTG AACGAAAAAG   
  
  
+ AAAAAAGAGA AATTAGAAAA AAAAAAAAGG GACGACAATG GACGTCTATT GCCCTTTCTC TTTCCCCCAC   
  
  
+ CGTGATCGTA AAGGGTAAAA CGGTGCACCG CGCTGTCATT CATCAACTCT AACCGTCCTA TACTAACCTC   
  
  
+ CTTTTTGTTG GTTAAAAAAA AAAAAGGGGA AGACAATGGA CGTCTATTGC CCTTTCTCTT TCTCCCACCG   
  
  
+ TGATCGTAAA GGGTAAAACG GTGCACCGCG CTGTCATTCA TCAACTCTAA CCGTCCTATA CTAACCTCCT   
  
  
+ TTTTGTTGGT GTGCGAGGAC GCTCAGCATG GGTATTAGTT AAAAGGGGTG ATATGGGGAT ATAATAAAAA   
  
  
+ ATTATTAATA AAATTAAAAA CTCTATTTTA TTTACGAATG TAATTATACG GCACACGCAC GTAGACACAC   
  
  
+ CTTCCGTTAC CACGTCGCGT GCATCCTGGT ACTGGGTAAT TTAGAAACTT TCTCTGTTCA TTTACATAAA   
  
  
+ TAAATATATA CAACCGTTTC AGTTCCCTTG TGTAATTAAA ACCATTCAGG AAAGTTAATT TTGGTTTG  

- TGATTAAAAT CACCCAAATC CAACAAGCAA AAAAAGAGGA ACTCTTTATG TCCTATCTGT TTCGTTCTCG   
  
  
- TGGTTGTTGT CGTGTTGCTT TGCCTCCTTA ACTTTTCGAT GTCTGGGTTG TGCTTCGCGG ATCTGGAAAA   
  
  
- TCTTCAGTAA AAATAAAAAG TCCACGCCGC AGATACGAAA AAGCTCTCTT CTTTGAATTT CTGCGTTTGC   
  
  
- CCTTTTTCTT TTCTCTGTTT TGTCTCTTTT AGGAGAGAGA TGAGTGATGG TGATGATGAT GCTGGATACT   
  
  
- GCAACGTGAT TGGNGGCTGA TGGCGGCCTG GTCTGTACCT TTTATGTGAT TTGACTCTTC TGTCCCCGAC   
  
  
- GACTCTCACA ATGACCGCAG CACCGCAGTC AGTGGCAGCT GGCCTAAGCA GCAGTCACAC TTGCTTTTTC   
  
  
- TTTTTTCTCT TTAATCTTTT TTTTTTTTCC CTGCTGTTAC CTGCAGATAA CGGGAAAGAG AAAGGGGGTG   
  
  
- GCACTAGCAT TTCCCATTTT GCCACGTGGC GCGACAGTAA GTAGTTGAGA TTGGCAGGAT ATGATTGGAG   
  
  
- GAAAAACAAC CAATTTTTTT TTTTTCCCCT TCTGTTACCT GCAGATAACG GGAAAGAGAA AGAGGGTGGC   
  
  
- ACTAGCATTT CCCATTTTGC CACGTGGCGC GACAGTAAGT AGTTGAGATT GGCAGGATAT GATTGGAGGA   
  
  
- AAAACAACCA CACGCTCCTG CGAGTCGTAC CCATAATCAA TTTTCCCCAC TATACCCCTA TATTATTTTT   
  
  
- TAATAATTAT TTTAATTTTT GAGATAAAAT AAATGCTTAC ATTAATATGC CGTGTGCGTG CATCTGTGTG   
  
  
- GAAGGCAATG GTGCAGCGCA CGTAGGACCA TGACCCATTA AATCTTTGAA AGAGACAAGT AAATGTATTT   
  
  
- ATTTATATAT GTTGGCAAAG TCAAGGGAAC ACATTAATTT TGGTAAGTCC TTTCAATTAA AACCAAAC

+     Unnamed\_\_1

| Site Name | Organism | Position | Strand | Matrix score. | sequence | function |
| --- | --- | --- | --- | --- | --- | --- |
| Unnamed\_\_1 | Zea mays | 370 | + | 5 | CGTGG |  |
| Unnamed\_\_1 | Zea mays | 850 | - | 5 | CGTGG |  |

> 2018/04/13 10:10:12  
+ ACTAATTTTA GTGGGTTTAG GTTGTTCGTT TTTTTCTCCT TGAGAAATAC AGGATAGACA AAGCAAGAGC   
  
  
+ ACCAACAACA GCACAACGAA ACGGAGGAAT TGAAAAGCTA CAGACCCAAC ACGAAGCGCC TAGACCTTTT   
  
  
+ AGAAGTCATT TTTATTTTTC AGGTGCGGCG TCTATGCTTT TTCGAGAGAA GAAACTTAAA GACGCAAACG   
  
  
+ GGAAAAAGAA AAGAGACAAA ACAGAGAAAA TCCTCTCTCT ACTCACTACC ACTACTACTA CGACCTATGA   
  
  
+ CGTTGCACTA ACCNCCGACT ACCGCCGGAC CAGACATGGA AAATACACTA AACTGAGAAG ACAGGGGCTG   
  
  
+ CTGAGAGTGT TACTGGCGTC GTGGCGTCAG TCACCGTCGA CCGGATTCGT CGTCAGTGTG AACGAAAAAG   
  
  
+ AAAAAAGAGA AATTAGAAAA AAAAAAAAGG GACGACAATG GACGTCTATT GCCCTTTCTC TTTCCCCCAC   
  
  
+ CGTGATCGTA AAGGGTAAAA CGGTGCACCG CGCTGTCATT CATCAACTCT AACCGTCCTA TACTAACCTC   
  
  
+ CTTTTTGTTG GTTAAAAAAA AAAAAGGGGA AGACAATGGA CGTCTATTGC CCTTTCTCTT TCTCCCACCG   
  
  
+ TGATCGTAAA GGGTAAAACG GTGCACCGCG CTGTCATTCA TCAACTCTAA CCGTCCTATA CTAACCTCCT   
  
  
+ TTTTGTTGGT GTGCGAGGAC GCTCAGCATG GGTATTAGTT AAAAGGGGTG ATATGGGGAT ATAATAAAAA   
  
  
+ ATTATTAATA AAATTAAAAA CTCTATTTTA TTTACGAATG TAATTATACG GCACACGCAC GTAGACACAC   
  
  
+ CTTCCGTTAC CACGTCGCGT GCATCCTGGT ACTGGGTAAT TTAGAAACTT TCTCTGTTCA TTTACATAAA   
  
  
+ TAAATATATA CAACCGTTTC AGTTCCCTTG TGTAATTAAA ACCATTCAGG AAAGTTAATT TTGGTTTG  

- TGATTAAAAT CACCCAAATC CAACAAGCAA AAAAAGAGGA ACTCTTTATG TCCTATCTGT TTCGTTCTCG   
  
  
- TGGTTGTTGT CGTGTTGCTT TGCCTCCTTA ACTTTTCGAT GTCTGGGTTG TGCTTCGCGG ATCTGGAAAA   
  
  
- TCTTCAGTAA AAATAAAAAG TCCACGCCGC AGATACGAAA AAGCTCTCTT CTTTGAATTT CTGCGTTTGC   
  
  
- CCTTTTTCTT TTCTCTGTTT TGTCTCTTTT AGGAGAGAGA TGAGTGATGG TGATGATGAT GCTGGATACT   
  
  
- GCAACGTGAT TGGNGGCTGA TGGCGGCCTG GTCTGTACCT TTTATGTGAT TTGACTCTTC TGTCCCCGAC   
  
  
- GACTCTCACA ATGACCGCAG CACCGCAGTC AGTGGCAGCT GGCCTAAGCA GCAGTCACAC TTGCTTTTTC   
  
  
- TTTTTTCTCT TTAATCTTTT TTTTTTTTCC CTGCTGTTAC CTGCAGATAA CGGGAAAGAG AAAGGGGGTG   
  
  
- GCACTAGCAT TTCCCATTTT GCCACGTGGC GCGACAGTAA GTAGTTGAGA TTGGCAGGAT ATGATTGGAG   
  
  
- GAAAAACAAC CAATTTTTTT TTTTTCCCCT TCTGTTACCT GCAGATAACG GGAAAGAGAA AGAGGGTGGC   
  
  
- ACTAGCATTT CCCATTTTGC CACGTGGCGC GACAGTAAGT AGTTGAGATT GGCAGGATAT GATTGGAGGA   
  
  
- AAAACAACCA CACGCTCCTG CGAGTCGTAC CCATAATCAA TTTTCCCCAC TATACCCCTA TATTATTTTT   
  
  
- TAATAATTAT TTTAATTTTT GAGATAAAAT AAATGCTTAC ATTAATATGC CGTGTGCGTG CATCTGTGTG   
  
  
- GAAGGCAATG GTGCAGCGCA CGTAGGACCA TGACCCATTA AATCTTTGAA AGAGACAAGT AAATGTATTT   
  
  
- ATTTATATAT GTTGGCAAAG TCAAGGGAAC ACATTAATTT TGGTAAGTCC TTTCAATTAA AACCAAAC

+     Unnamed\_\_3

| Site Name | Organism | Position | Strand | Matrix score. | sequence | function |
| --- | --- | --- | --- | --- | --- | --- |
| Unnamed\_\_3 | Zea mays | 850 | - | 5 | CGTGG |  |
| Unnamed\_\_3 | Zea mays | 370 | + | 5 | CGTGG |  |

> 2018/04/13 10:10:12  
+ ACTAATTTTA GTGGGTTTAG GTTGTTCGTT TTTTTCTCCT TGAGAAATAC AGGATAGACA AAGCAAGAGC   
  
  
+ ACCAACAACA GCACAACGAA ACGGAGGAAT TGAAAAGCTA CAGACCCAAC ACGAAGCGCC TAGACCTTTT   
  
  
+ AGAAGTCATT TTTATTTTTC AGGTGCGGCG TCTATGCTTT TTCGAGAGAA GAAACTTAAA GACGCAAACG   
  
  
+ GGAAAAAGAA AAGAGACAAA ACAGAGAAAA TCCTCTCTCT ACTCACTACC ACTACTACTA CGACCTATGA   
  
  
+ CGTTGCACTA ACCNCCGACT ACCGCCGGAC CAGACATGGA AAATACACTA AACTGAGAAG ACAGGGGCTG   
  
  
+ CTGAGAGTGT TACTGGCGTC GTGGCGTCAG TCACCGTCGA CCGGATTCGT CGTCAGTGTG AACGAAAAAG   
  
  
+ AAAAAAGAGA AATTAGAAAA AAAAAAAAGG GACGACAATG GACGTCTATT GCCCTTTCTC TTTCCCCCAC   
  
  
+ CGTGATCGTA AAGGGTAAAA CGGTGCACCG CGCTGTCATT CATCAACTCT AACCGTCCTA TACTAACCTC   
  
  
+ CTTTTTGTTG GTTAAAAAAA AAAAAGGGGA AGACAATGGA CGTCTATTGC CCTTTCTCTT TCTCCCACCG   
  
  
+ TGATCGTAAA GGGTAAAACG GTGCACCGCG CTGTCATTCA TCAACTCTAA CCGTCCTATA CTAACCTCCT   
  
  
+ TTTTGTTGGT GTGCGAGGAC GCTCAGCATG GGTATTAGTT AAAAGGGGTG ATATGGGGAT ATAATAAAAA   
  
  
+ ATTATTAATA AAATTAAAAA CTCTATTTTA TTTACGAATG TAATTATACG GCACACGCAC GTAGACACAC   
  
  
+ CTTCCGTTAC CACGTCGCGT GCATCCTGGT ACTGGGTAAT TTAGAAACTT TCTCTGTTCA TTTACATAAA   
  
  
+ TAAATATATA CAACCGTTTC AGTTCCCTTG TGTAATTAAA ACCATTCAGG AAAGTTAATT TTGGTTTG  

- TGATTAAAAT CACCCAAATC CAACAAGCAA AAAAAGAGGA ACTCTTTATG TCCTATCTGT TTCGTTCTCG   
  
  
- TGGTTGTTGT CGTGTTGCTT TGCCTCCTTA ACTTTTCGAT GTCTGGGTTG TGCTTCGCGG ATCTGGAAAA   
  
  
- TCTTCAGTAA AAATAAAAAG TCCACGCCGC AGATACGAAA AAGCTCTCTT CTTTGAATTT CTGCGTTTGC   
  
  
- CCTTTTTCTT TTCTCTGTTT TGTCTCTTTT AGGAGAGAGA TGAGTGATGG TGATGATGAT GCTGGATACT   
  
  
- GCAACGTGAT TGGNGGCTGA TGGCGGCCTG GTCTGTACCT TTTATGTGAT TTGACTCTTC TGTCCCCGAC   
  
  
- GACTCTCACA ATGACCGCAG CACCGCAGTC AGTGGCAGCT GGCCTAAGCA GCAGTCACAC TTGCTTTTTC   
  
  
- TTTTTTCTCT TTAATCTTTT TTTTTTTTCC CTGCTGTTAC CTGCAGATAA CGGGAAAGAG AAAGGGGGTG   
  
  
- GCACTAGCAT TTCCCATTTT GCCACGTGGC GCGACAGTAA GTAGTTGAGA TTGGCAGGAT ATGATTGGAG   
  
  
- GAAAAACAAC CAATTTTTTT TTTTTCCCCT TCTGTTACCT GCAGATAACG GGAAAGAGAA AGAGGGTGGC   
  
  
- ACTAGCATTT CCCATTTTGC CACGTGGCGC GACAGTAAGT AGTTGAGATT GGCAGGATAT GATTGGAGGA   
  
  
- AAAACAACCA CACGCTCCTG CGAGTCGTAC CCATAATCAA TTTTCCCCAC TATACCCCTA TATTATTTTT   
  
  
- TAATAATTAT TTTAATTTTT GAGATAAAAT AAATGCTTAC ATTAATATGC CGTGTGCGTG CATCTGTGTG   
  
  
- GAAGGCAATG GTGCAGCGCA CGTAGGACCA TGACCCATTA AATCTTTGAA AGAGACAAGT AAATGTATTT   
  
  
- ATTTATATAT GTTGGCAAAG TCAAGGGAAC ACATTAATTT TGGTAAGTCC TTTCAATTAA AACCAAAC

+     Unnamed\_\_4

| Site Name | Organism | Position | Strand | Matrix score. | sequence | function |
| --- | --- | --- | --- | --- | --- | --- |
| Unnamed\_\_4 | Petroselinum hortense | 93 | - | 4 | CTCC |  |
| Unnamed\_\_4 | Petroselinum hortense | 696 | + | 4 | CTCC |  |
| Unnamed\_\_4 | Petroselinum hortense | 36 | + | 4 | CTCC |  |
| Unnamed\_\_4 | Petroselinum hortense | 558 | + | 4 | CTCC |  |
| Unnamed\_\_4 | Petroselinum hortense | 622 | + | 4 | CTCC |  |

> 2018/04/13 10:10:12  
+ ACTAATTTTA GTGGGTTTAG GTTGTTCGTT TTTTTCTCCT TGAGAAATAC AGGATAGACA AAGCAAGAGC   
  
  
+ ACCAACAACA GCACAACGAA ACGGAGGAAT TGAAAAGCTA CAGACCCAAC ACGAAGCGCC TAGACCTTTT   
  
  
+ AGAAGTCATT TTTATTTTTC AGGTGCGGCG TCTATGCTTT TTCGAGAGAA GAAACTTAAA GACGCAAACG   
  
  
+ GGAAAAAGAA AAGAGACAAA ACAGAGAAAA TCCTCTCTCT ACTCACTACC ACTACTACTA CGACCTATGA   
  
  
+ CGTTGCACTA ACCNCCGACT ACCGCCGGAC CAGACATGGA AAATACACTA AACTGAGAAG ACAGGGGCTG   
  
  
+ CTGAGAGTGT TACTGGCGTC GTGGCGTCAG TCACCGTCGA CCGGATTCGT CGTCAGTGTG AACGAAAAAG   
  
  
+ AAAAAAGAGA AATTAGAAAA AAAAAAAAGG GACGACAATG GACGTCTATT GCCCTTTCTC TTTCCCCCAC   
  
  
+ CGTGATCGTA AAGGGTAAAA CGGTGCACCG CGCTGTCATT CATCAACTCT AACCGTCCTA TACTAACCTC   
  
  
+ CTTTTTGTTG GTTAAAAAAA AAAAAGGGGA AGACAATGGA CGTCTATTGC CCTTTCTCTT TCTCCCACCG   
  
  
+ TGATCGTAAA GGGTAAAACG GTGCACCGCG CTGTCATTCA TCAACTCTAA CCGTCCTATA CTAACCTCCT   
  
  
+ TTTTGTTGGT GTGCGAGGAC GCTCAGCATG GGTATTAGTT AAAAGGGGTG ATATGGGGAT ATAATAAAAA   
  
  
+ ATTATTAATA AAATTAAAAA CTCTATTTTA TTTACGAATG TAATTATACG GCACACGCAC GTAGACACAC   
  
  
+ CTTCCGTTAC CACGTCGCGT GCATCCTGGT ACTGGGTAAT TTAGAAACTT TCTCTGTTCA TTTACATAAA   
  
  
+ TAAATATATA CAACCGTTTC AGTTCCCTTG TGTAATTAAA ACCATTCAGG AAAGTTAATT TTGGTTTG  

- TGATTAAAAT CACCCAAATC CAACAAGCAA AAAAAGAGGA ACTCTTTATG TCCTATCTGT TTCGTTCTCG   
  
  
- TGGTTGTTGT CGTGTTGCTT TGCCTCCTTA ACTTTTCGAT GTCTGGGTTG TGCTTCGCGG ATCTGGAAAA   
  
  
- TCTTCAGTAA AAATAAAAAG TCCACGCCGC AGATACGAAA AAGCTCTCTT CTTTGAATTT CTGCGTTTGC   
  
  
- CCTTTTTCTT TTCTCTGTTT TGTCTCTTTT AGGAGAGAGA TGAGTGATGG TGATGATGAT GCTGGATACT   
  
  
- GCAACGTGAT TGGNGGCTGA TGGCGGCCTG GTCTGTACCT TTTATGTGAT TTGACTCTTC TGTCCCCGAC   
  
  
- GACTCTCACA ATGACCGCAG CACCGCAGTC AGTGGCAGCT GGCCTAAGCA GCAGTCACAC TTGCTTTTTC   
  
  
- TTTTTTCTCT TTAATCTTTT TTTTTTTTCC CTGCTGTTAC CTGCAGATAA CGGGAAAGAG AAAGGGGGTG   
  
  
- GCACTAGCAT TTCCCATTTT GCCACGTGGC GCGACAGTAA GTAGTTGAGA TTGGCAGGAT ATGATTGGAG   
  
  
- GAAAAACAAC CAATTTTTTT TTTTTCCCCT TCTGTTACCT GCAGATAACG GGAAAGAGAA AGAGGGTGGC   
  
  
- ACTAGCATTT CCCATTTTGC CACGTGGCGC GACAGTAAGT AGTTGAGATT GGCAGGATAT GATTGGAGGA   
  
  
- AAAACAACCA CACGCTCCTG CGAGTCGTAC CCATAATCAA TTTTCCCCAC TATACCCCTA TATTATTTTT   
  
  
- TAATAATTAT TTTAATTTTT GAGATAAAAT AAATGCTTAC ATTAATATGC CGTGTGCGTG CATCTGTGTG   
  
  
- GAAGGCAATG GTGCAGCGCA CGTAGGACCA TGACCCATTA AATCTTTGAA AGAGACAAGT AAATGTATTT   
  
  
- ATTTATATAT GTTGGCAAAG TCAAGGGAAC ACATTAATTT TGGTAAGTCC TTTCAATTAA AACCAAAC
