# Supplementary material for: Genome-wide analysis of the WRKY gene family in drumstick (Moringa oleifera Lam.)
Source: PeerJ. 2019 Jun 10;7:e7063. doi: 10.7717/peerj.7063 (PMC6563795; doi:10.7717/peerj.7063)
Supplement: Supplemental Information 1 [file peerj-07-7063-s003.gz › MoWRKY20_plantcare.html]

Content-Type: text/html; charset=ISO-8859-1


CallMat\_Firefox


Webmaster Firefox specific output  
To save the result:
click on the frame with the right mouse button and save the source code as a text file with extension .html  
REFERENCE:PlantCARE: a database of plant cis-acting regulatory elements and a portal to tools for in silico analysis of promoter sequences.  
Lescot, M., Déhais, P., Moreau, Y., De Moor, B., Rouzé ,P.,and Rombauts, S.  
Nucleic Acids Res., Database issue(2002), 30(1):325-327.   


---

> 2018/04/13 10:10:12  
+ GAGAGGTTTT GGTAGGTTCT GTTGTGGGAA TCTATACCTA CCCATATTTA TATGTTTCTG GTTCTTCTCT   
  
  
+ CTTTTAGAAA TTTTTCCTTT CTACTACTTC GCTCGTGTTT GGCTCTTTTA TTTATGTTTT TCCTTTTCGT   
  
  
+ CTCTACTACT CTTTTCCCCC TTCCTCTTCC CCCTCCCCTT CCCCTTCCCC TTCCCAGCTC TCAGTTTGAC   
  
  
+ TACGGGAGGG TTACGTTCCG TAGCACAGCA AATACTCCTA TAACGGCAGT TTGGGGGCTA GGTAAGCATA   
  
  
+ TTGCGGTGCA CAGTCCCGAA AACAGTCCTG TAACTGGCGG AGAAATTCAC GCCCAGGTAC CGTTGCCCTA   
  
  
+ TTTTGGTCAA CCTCTGGCAC GTCTCTACTA CGGTTACTTC CTATTTCAGT TTTCTAACTC TACTTGGCAA   
  
  
+ CCTAACCATC GTCTTCTCTC TCTCTCTCTG TCTCTCTCTC TCTCTCCCAA AATCAACCTC TGTCACTCCA   
  
  
+ TCCCAACTGG AACTTGTCGA AAATTACGTT TTCAGTAACT CACGCCTCTA CCCCACATAC CCAGGCGGTC   
  
  
+ CGAGCAGAAA ATGGCCTGTG GCCTTCGACG CAGTTTCGCA GTAGGGTGTT CGCTGTACCC TAAGAAAGAG   
  
  
+ AGAGAAATCA AAAAGAAGAT GCCAAAAGAT GAGCACCGGG GGTGGTTGTA AAAGGTACTA GTGTAGACGT   
  
  
+ GGCAAGTATG AACAGCTCTA GCTAAGACCA TCCTCTCCAG TGTGGTACGT ACCTGAACAC TCCACCACCG   
  
  
+ GCAACTTTTT CAAAGAGCGG TCAATGAAGT ATATGTCATT ACTCTTCACT TCCTCAAACC AGTTTTGATT   
  
  
+ TGAACTAAAG TTTGTAAAGC GGCCTTAAAC ACGCTTAGTC TATTAATTCT AAAATAATTA TTTTTGGGTG   
  
  
+ AATAGTAAAG CCGATGTAAT TACCTTATTT ACCAACCTTG CTTCTCTAGG TGTGCGGTTT CGTTTAGCTA   
  
  
+ GTCTTTATTT CCAGTTGAAG TGTGCGAAAG AGTTCGAAGA GACTAGTTTA AAGTTAATTA CTGGTTTGGT   
  
  
+ TTTTGACAAG TGTCTAAACC GTTATCGATA CAAAGAAGAC AGAAGGAAAT AGGGTTAAAT TTTTGTCAAA   
  
  
+ TATATATCAT AAAAAATTAA ATATTATTAT GATATTTTTA TTTTTTTGAA TAAATAAATA AAATATTTTA   
  
  
+ ATAGTTCATA ATATAAAAAA TTTTTTTAAT TTGGGTGAAA TTTAATTAAC TTCTATATTG GCTTTCACAA   
  
  
+ ATATAACTTT TTTTTTTCAC ATTAACTATG AATTGTGCCA ACCTCTTTTC CTCCCTTCGA ATGTTCGTTT   
  
  
+ ATCGAACTGA ACCGTAAACT ACTCGCTGAA GTAAAACTTG AATTTCCAAC TACCCGGTGT AAGTGTAATA   
  
  
+ ATCAGTATGG TATAAAAACA ATGAAAATTA CCTTCTTTTG GGTTTTTTTT TTTTTTTTTC GTTTTTCTTC   
  
  
+ GTTTGGGAGA AATTGTTTAA AATAATTTA  

- CTCTCCAAAA CCATCCAAGA CAACACCCTT AGATATGGAT GGGTATAAAT ATACAAAGAC CAAGAAGAGA   
  
  
- GAAAATCTTT AAAAAGGAAA GATGATGAAG CGAGCACAAA CCGAGAAAAT AAATACAAAA AGGAAAAGCA   
  
  
- GAGATGATGA GAAAAGGGGG AAGGAGAAGG GGGAGGGGAA GGGGAAGGGG AAGGGTCGAG AGTCAAACTG   
  
  
- ATGCCCTCCC AATGCAAGGC ATCGTGTCGT TTATGAGGAT ATTGCCGTCA AACCCCCGAT CCATTCGTAT   
  
  
- AACGCCACGT GTCAGGGCTT TTGTCAGGAC ATTGACCGCC TCTTTAAGTG CGGGTCCATG GCAACGGGAT   
  
  
- AAAACCAGTT GGAGACCGTG CAGAGATGAT GCCAATGAAG GATAAAGTCA AAAGATTGAG ATGAACCGTT   
  
  
- GGATTGGTAG CAGAAGAGAG AGAGAGAGAC AGAGAGAGAG AGAGAGGGTT TTAGTTGGAG ACAGTGAGGT   
  
  
- AGGGTTGACC TTGAACAGCT TTTAATGCAA AAGTCATTGA GTGCGGAGAT GGGGTGTATG GGTCCGCCAG   
  
  
- GCTCGTCTTT TACCGGACAC CGGAAGCTGC GTCAAAGCGT CATCCCACAA GCGACATGGG ATTCTTTCTC   
  
  
- TCTCTTTAGT TTTTCTTCTA CGGTTTTCTA CTCGTGGCCC CCACCAACAT TTTCCATGAT CACATCTGCA   
  
  
- CCGTTCATAC TTGTCGAGAT CGATTCTGGT AGGAGAGGTC ACACCATGCA TGGACTTGTG AGGTGGTGGC   
  
  
- CGTTGAAAAA GTTTCTCGCC AGTTACTTCA TATACAGTAA TGAGAAGTGA AGGAGTTTGG TCAAAACTAA   
  
  
- ACTTGATTTC AAACATTTCG CCGGAATTTG TGCGAATCAG ATAATTAAGA TTTTATTAAT AAAAACCCAC   
  
  
- TTATCATTTC GGCTACATTA ATGGAATAAA TGGTTGGAAC GAAGAGATCC ACACGCCAAA GCAAATCGAT   
  
  
- CAGAAATAAA GGTCAACTTC ACACGCTTTC TCAAGCTTCT CTGATCAAAT TTCAATTAAT GACCAAACCA   
  
  
- AAAACTGTTC ACAGATTTGG CAATAGCTAT GTTTCTTCTG TCTTCCTTTA TCCCAATTTA AAAACAGTTT   
  
  
- ATATATAGTA TTTTTTAATT TATAATAATA CTATAAAAAT AAAAAAACTT ATTTATTTAT TTTATAAAAT   
  
  
- TATCAAGTAT TATATTTTTT AAAAAAATTA AACCCACTTT AAATTAATTG AAGATATAAC CGAAAGTGTT   
  
  
- TATATTGAAA AAAAAAAGTG TAATTGATAC TTAACACGGT TGGAGAAAAG GAGGGAAGCT TACAAGCAAA   
  
  
- TAGCTTGACT TGGCATTTGA TGAGCGACTT CATTTTGAAC TTAAAGGTTG ATGGGCCACA TTCACATTAT   
  
  
- TAGTCATACC ATATTTTTGT TACTTTTAAT GGAAGAAAAC CCAAAAAAAA AAAAAAAAAG CAAAAAGAAG   
  
  
- CAAACCCTCT TTAACAAATT TTATTAAAT

  
  
Motifs Found  

+     5UTR Py-rich stretch

| Site Name | Organism | Position | Strand | Matrix score. | sequence | function |
| --- | --- | --- | --- | --- | --- | --- |
| 5UTR Py-rich stretch | Lycopersicon esculentum | 948 | + | 9 | TTTCTTCTCT | cis-acting element conferring high transcription levels |
| 5UTR Py-rich stretch | Lycopersicon esculentum | 451 | + | 13 | TTTCTCTCTCTCTC | cis-acting element conferring high transcription levels |
| 5UTR Py-rich stretch | Lycopersicon esculentum | 453 | + | 13 | TTTCTCTCTCTCTC | cis-acting element conferring high transcription levels |
| 5UTR Py-rich stretch | Lycopersicon esculentum | 624 | - | 13 | TTTCTCTCTCTCTC | cis-acting element conferring high transcription levels |
| 5UTR Py-rich stretch | Lycopersicon esculentum | 449 | + | 13 | TTTCTCTCTCTCTC | cis-acting element conferring high transcription levels |
| 5UTR Py-rich stretch | Lycopersicon esculentum | 435 | + | 13 | TTTCTCTCTCTCTC | cis-acting element conferring high transcription levels |
| 5UTR Py-rich stretch | Lycopersicon esculentum | 433 | + | 13 | TTTCTCTCTCTCTC | cis-acting element conferring high transcription levels |
| 5UTR Py-rich stretch | Lycopersicon esculentum | 135 | + | 9 | TTTCTTCTCT | cis-acting element conferring high transcription levels |
| 5UTR Py-rich stretch | Lycopersicon esculentum | 61 | + | 9 | TTTCTTCTCT | cis-acting element conferring high transcription levels |

> 2018/04/13 10:10:12  
+ GAGAGGTTTT GGTAGGTTCT GTTGTGGGAA TCTATACCTA CCCATATTTA TATGTTTCTG GTTCTTCTCT   
  
  
+ CTTTTAGAAA TTTTTCCTTT CTACTACTTC GCTCGTGTTT GGCTCTTTTA TTTATGTTTT TCCTTTTCGT   
  
  
+ CTCTACTACT CTTTTCCCCC TTCCTCTTCC CCCTCCCCTT CCCCTTCCCC TTCCCAGCTC TCAGTTTGAC   
  
  
+ TACGGGAGGG TTACGTTCCG TAGCACAGCA AATACTCCTA TAACGGCAGT TTGGGGGCTA GGTAAGCATA   
  
  
+ TTGCGGTGCA CAGTCCCGAA AACAGTCCTG TAACTGGCGG AGAAATTCAC GCCCAGGTAC CGTTGCCCTA   
  
  
+ TTTTGGTCAA CCTCTGGCAC GTCTCTACTA CGGTTACTTC CTATTTCAGT TTTCTAACTC TACTTGGCAA   
  
  
+ CCTAACCATC GTCTTCTCTC TCTCTCTCTG TCTCTCTCTC TCTCTCCCAA AATCAACCTC TGTCACTCCA   
  
  
+ TCCCAACTGG AACTTGTCGA AAATTACGTT TTCAGTAACT CACGCCTCTA CCCCACATAC CCAGGCGGTC   
  
  
+ CGAGCAGAAA ATGGCCTGTG GCCTTCGACG CAGTTTCGCA GTAGGGTGTT CGCTGTACCC TAAGAAAGAG   
  
  
+ AGAGAAATCA AAAAGAAGAT GCCAAAAGAT GAGCACCGGG GGTGGTTGTA AAAGGTACTA GTGTAGACGT   
  
  
+ GGCAAGTATG AACAGCTCTA GCTAAGACCA TCCTCTCCAG TGTGGTACGT ACCTGAACAC TCCACCACCG   
  
  
+ GCAACTTTTT CAAAGAGCGG TCAATGAAGT ATATGTCATT ACTCTTCACT TCCTCAAACC AGTTTTGATT   
  
  
+ TGAACTAAAG TTTGTAAAGC GGCCTTAAAC ACGCTTAGTC TATTAATTCT AAAATAATTA TTTTTGGGTG   
  
  
+ AATAGTAAAG CCGATGTAAT TACCTTATTT ACCAACCTTG CTTCTCTAGG TGTGCGGTTT CGTTTAGCTA   
  
  
+ GTCTTTATTT CCAGTTGAAG TGTGCGAAAG AGTTCGAAGA GACTAGTTTA AAGTTAATTA CTGGTTTGGT   
  
  
+ TTTTGACAAG TGTCTAAACC GTTATCGATA CAAAGAAGAC AGAAGGAAAT AGGGTTAAAT TTTTGTCAAA   
  
  
+ TATATATCAT AAAAAATTAA ATATTATTAT GATATTTTTA TTTTTTTGAA TAAATAAATA AAATATTTTA   
  
  
+ ATAGTTCATA ATATAAAAAA TTTTTTTAAT TTGGGTGAAA TTTAATTAAC TTCTATATTG GCTTTCACAA   
  
  
+ ATATAACTTT TTTTTTTCAC ATTAACTATG AATTGTGCCA ACCTCTTTTC CTCCCTTCGA ATGTTCGTTT   
  
  
+ ATCGAACTGA ACCGTAAACT ACTCGCTGAA GTAAAACTTG AATTTCCAAC TACCCGGTGT AAGTGTAATA   
  
  
+ ATCAGTATGG TATAAAAACA ATGAAAATTA CCTTCTTTTG GGTTTTTTTT TTTTTTTTTC GTTTTTCTTC   
  
  
+ GTTTGGGAGA AATTGTTTAA AATAATTTA  

- CTCTCCAAAA CCATCCAAGA CAACACCCTT AGATATGGAT GGGTATAAAT ATACAAAGAC CAAGAAGAGA   
  
  
- GAAAATCTTT AAAAAGGAAA GATGATGAAG CGAGCACAAA CCGAGAAAAT AAATACAAAA AGGAAAAGCA   
  
  
- GAGATGATGA GAAAAGGGGG AAGGAGAAGG GGGAGGGGAA GGGGAAGGGG AAGGGTCGAG AGTCAAACTG   
  
  
- ATGCCCTCCC AATGCAAGGC ATCGTGTCGT TTATGAGGAT ATTGCCGTCA AACCCCCGAT CCATTCGTAT   
  
  
- AACGCCACGT GTCAGGGCTT TTGTCAGGAC ATTGACCGCC TCTTTAAGTG CGGGTCCATG GCAACGGGAT   
  
  
- AAAACCAGTT GGAGACCGTG CAGAGATGAT GCCAATGAAG GATAAAGTCA AAAGATTGAG ATGAACCGTT   
  
  
- GGATTGGTAG CAGAAGAGAG AGAGAGAGAC AGAGAGAGAG AGAGAGGGTT TTAGTTGGAG ACAGTGAGGT   
  
  
- AGGGTTGACC TTGAACAGCT TTTAATGCAA AAGTCATTGA GTGCGGAGAT GGGGTGTATG GGTCCGCCAG   
  
  
- GCTCGTCTTT TACCGGACAC CGGAAGCTGC GTCAAAGCGT CATCCCACAA GCGACATGGG ATTCTTTCTC   
  
  
- TCTCTTTAGT TTTTCTTCTA CGGTTTTCTA CTCGTGGCCC CCACCAACAT TTTCCATGAT CACATCTGCA   
  
  
- CCGTTCATAC TTGTCGAGAT CGATTCTGGT AGGAGAGGTC ACACCATGCA TGGACTTGTG AGGTGGTGGC   
  
  
- CGTTGAAAAA GTTTCTCGCC AGTTACTTCA TATACAGTAA TGAGAAGTGA AGGAGTTTGG TCAAAACTAA   
  
  
- ACTTGATTTC AAACATTTCG CCGGAATTTG TGCGAATCAG ATAATTAAGA TTTTATTAAT AAAAACCCAC   
  
  
- TTATCATTTC GGCTACATTA ATGGAATAAA TGGTTGGAAC GAAGAGATCC ACACGCCAAA GCAAATCGAT   
  
  
- CAGAAATAAA GGTCAACTTC ACACGCTTTC TCAAGCTTCT CTGATCAAAT TTCAATTAAT GACCAAACCA   
  
  
- AAAACTGTTC ACAGATTTGG CAATAGCTAT GTTTCTTCTG TCTTCCTTTA TCCCAATTTA AAAACAGTTT   
  
  
- ATATATAGTA TTTTTTAATT TATAATAATA CTATAAAAAT AAAAAAACTT ATTTATTTAT TTTATAAAAT   
  
  
- TATCAAGTAT TATATTTTTT AAAAAAATTA AACCCACTTT AAATTAATTG AAGATATAAC CGAAAGTGTT   
  
  
- TATATTGAAA AAAAAAAGTG TAATTGATAC TTAACACGGT TGGAGAAAAG GAGGGAAGCT TACAAGCAAA   
  
  
- TAGCTTGACT TGGCATTTGA TGAGCGACTT CATTTTGAAC TTAAAGGTTG ATGGGCCACA TTCACATTAT   
  
  
- TAGTCATACC ATATTTTTGT TACTTTTAAT GGAAGAAAAC CCAAAAAAAA AAAAAAAAAG CAAAAAGAAG   
  
  
- CAAACCCTCT TTAACAAATT TTATTAAAT

+     ABRE

| Site Name | Organism | Position | Strand | Matrix score. | sequence | function |
| --- | --- | --- | --- | --- | --- | --- |
| ABRE | Arabidopsis thaliana | 697 | + | 7 | ACGTGGC | cis-acting element involved in the abscisic acid responsiveness |

> 2018/04/13 10:10:12  
+ GAGAGGTTTT GGTAGGTTCT GTTGTGGGAA TCTATACCTA CCCATATTTA TATGTTTCTG GTTCTTCTCT   
  
  
+ CTTTTAGAAA TTTTTCCTTT CTACTACTTC GCTCGTGTTT GGCTCTTTTA TTTATGTTTT TCCTTTTCGT   
  
  
+ CTCTACTACT CTTTTCCCCC TTCCTCTTCC CCCTCCCCTT CCCCTTCCCC TTCCCAGCTC TCAGTTTGAC   
  
  
+ TACGGGAGGG TTACGTTCCG TAGCACAGCA AATACTCCTA TAACGGCAGT TTGGGGGCTA GGTAAGCATA   
  
  
+ TTGCGGTGCA CAGTCCCGAA AACAGTCCTG TAACTGGCGG AGAAATTCAC GCCCAGGTAC CGTTGCCCTA   
  
  
+ TTTTGGTCAA CCTCTGGCAC GTCTCTACTA CGGTTACTTC CTATTTCAGT TTTCTAACTC TACTTGGCAA   
  
  
+ CCTAACCATC GTCTTCTCTC TCTCTCTCTG TCTCTCTCTC TCTCTCCCAA AATCAACCTC TGTCACTCCA   
  
  
+ TCCCAACTGG AACTTGTCGA AAATTACGTT TTCAGTAACT CACGCCTCTA CCCCACATAC CCAGGCGGTC   
  
  
+ CGAGCAGAAA ATGGCCTGTG GCCTTCGACG CAGTTTCGCA GTAGGGTGTT CGCTGTACCC TAAGAAAGAG   
  
  
+ AGAGAAATCA AAAAGAAGAT GCCAAAAGAT GAGCACCGGG GGTGGTTGTA AAAGGTACTA GTGTAGACGT   
  
  
+ GGCAAGTATG AACAGCTCTA GCTAAGACCA TCCTCTCCAG TGTGGTACGT ACCTGAACAC TCCACCACCG   
  
  
+ GCAACTTTTT CAAAGAGCGG TCAATGAAGT ATATGTCATT ACTCTTCACT TCCTCAAACC AGTTTTGATT   
  
  
+ TGAACTAAAG TTTGTAAAGC GGCCTTAAAC ACGCTTAGTC TATTAATTCT AAAATAATTA TTTTTGGGTG   
  
  
+ AATAGTAAAG CCGATGTAAT TACCTTATTT ACCAACCTTG CTTCTCTAGG TGTGCGGTTT CGTTTAGCTA   
  
  
+ GTCTTTATTT CCAGTTGAAG TGTGCGAAAG AGTTCGAAGA GACTAGTTTA AAGTTAATTA CTGGTTTGGT   
  
  
+ TTTTGACAAG TGTCTAAACC GTTATCGATA CAAAGAAGAC AGAAGGAAAT AGGGTTAAAT TTTTGTCAAA   
  
  
+ TATATATCAT AAAAAATTAA ATATTATTAT GATATTTTTA TTTTTTTGAA TAAATAAATA AAATATTTTA   
  
  
+ ATAGTTCATA ATATAAAAAA TTTTTTTAAT TTGGGTGAAA TTTAATTAAC TTCTATATTG GCTTTCACAA   
  
  
+ ATATAACTTT TTTTTTTCAC ATTAACTATG AATTGTGCCA ACCTCTTTTC CTCCCTTCGA ATGTTCGTTT   
  
  
+ ATCGAACTGA ACCGTAAACT ACTCGCTGAA GTAAAACTTG AATTTCCAAC TACCCGGTGT AAGTGTAATA   
  
  
+ ATCAGTATGG TATAAAAACA ATGAAAATTA CCTTCTTTTG GGTTTTTTTT TTTTTTTTTC GTTTTTCTTC   
  
  
+ GTTTGGGAGA AATTGTTTAA AATAATTTA  

- CTCTCCAAAA CCATCCAAGA CAACACCCTT AGATATGGAT GGGTATAAAT ATACAAAGAC CAAGAAGAGA   
  
  
- GAAAATCTTT AAAAAGGAAA GATGATGAAG CGAGCACAAA CCGAGAAAAT AAATACAAAA AGGAAAAGCA   
  
  
- GAGATGATGA GAAAAGGGGG AAGGAGAAGG GGGAGGGGAA GGGGAAGGGG AAGGGTCGAG AGTCAAACTG   
  
  
- ATGCCCTCCC AATGCAAGGC ATCGTGTCGT TTATGAGGAT ATTGCCGTCA AACCCCCGAT CCATTCGTAT   
  
  
- AACGCCACGT GTCAGGGCTT TTGTCAGGAC ATTGACCGCC TCTTTAAGTG CGGGTCCATG GCAACGGGAT   
  
  
- AAAACCAGTT GGAGACCGTG CAGAGATGAT GCCAATGAAG GATAAAGTCA AAAGATTGAG ATGAACCGTT   
  
  
- GGATTGGTAG CAGAAGAGAG AGAGAGAGAC AGAGAGAGAG AGAGAGGGTT TTAGTTGGAG ACAGTGAGGT   
  
  
- AGGGTTGACC TTGAACAGCT TTTAATGCAA AAGTCATTGA GTGCGGAGAT GGGGTGTATG GGTCCGCCAG   
  
  
- GCTCGTCTTT TACCGGACAC CGGAAGCTGC GTCAAAGCGT CATCCCACAA GCGACATGGG ATTCTTTCTC   
  
  
- TCTCTTTAGT TTTTCTTCTA CGGTTTTCTA CTCGTGGCCC CCACCAACAT TTTCCATGAT CACATCTGCA   
  
  
- CCGTTCATAC TTGTCGAGAT CGATTCTGGT AGGAGAGGTC ACACCATGCA TGGACTTGTG AGGTGGTGGC   
  
  
- CGTTGAAAAA GTTTCTCGCC AGTTACTTCA TATACAGTAA TGAGAAGTGA AGGAGTTTGG TCAAAACTAA   
  
  
- ACTTGATTTC AAACATTTCG CCGGAATTTG TGCGAATCAG ATAATTAAGA TTTTATTAAT AAAAACCCAC   
  
  
- TTATCATTTC GGCTACATTA ATGGAATAAA TGGTTGGAAC GAAGAGATCC ACACGCCAAA GCAAATCGAT   
  
  
- CAGAAATAAA GGTCAACTTC ACACGCTTTC TCAAGCTTCT CTGATCAAAT TTCAATTAAT GACCAAACCA   
  
  
- AAAACTGTTC ACAGATTTGG CAATAGCTAT GTTTCTTCTG TCTTCCTTTA TCCCAATTTA AAAACAGTTT   
  
  
- ATATATAGTA TTTTTTAATT TATAATAATA CTATAAAAAT AAAAAAACTT ATTTATTTAT TTTATAAAAT   
  
  
- TATCAAGTAT TATATTTTTT AAAAAAATTA AACCCACTTT AAATTAATTG AAGATATAAC CGAAAGTGTT   
  
  
- TATATTGAAA AAAAAAAGTG TAATTGATAC TTAACACGGT TGGAGAAAAG GAGGGAAGCT TACAAGCAAA   
  
  
- TAGCTTGACT TGGCATTTGA TGAGCGACTT CATTTTGAAC TTAAAGGTTG ATGGGCCACA TTCACATTAT   
  
  
- TAGTCATACC ATATTTTTGT TACTTTTAAT GGAAGAAAAC CCAAAAAAAA AAAAAAAAAG CAAAAAGAAG   
  
  
- CAAACCCTCT TTAACAAATT TTATTAAAT

+     AC-I

| Site Name | Organism | Position | Strand | Matrix score. | sequence | function |
| --- | --- | --- | --- | --- | --- | --- |
| AC-I | Phaseolus vulgaris | 542 | + | 9 | CCCACCTACC |  |

> 2018/04/13 10:10:12  
+ GAGAGGTTTT GGTAGGTTCT GTTGTGGGAA TCTATACCTA CCCATATTTA TATGTTTCTG GTTCTTCTCT   
  
  
+ CTTTTAGAAA TTTTTCCTTT CTACTACTTC GCTCGTGTTT GGCTCTTTTA TTTATGTTTT TCCTTTTCGT   
  
  
+ CTCTACTACT CTTTTCCCCC TTCCTCTTCC CCCTCCCCTT CCCCTTCCCC TTCCCAGCTC TCAGTTTGAC   
  
  
+ TACGGGAGGG TTACGTTCCG TAGCACAGCA AATACTCCTA TAACGGCAGT TTGGGGGCTA GGTAAGCATA   
  
  
+ TTGCGGTGCA CAGTCCCGAA AACAGTCCTG TAACTGGCGG AGAAATTCAC GCCCAGGTAC CGTTGCCCTA   
  
  
+ TTTTGGTCAA CCTCTGGCAC GTCTCTACTA CGGTTACTTC CTATTTCAGT TTTCTAACTC TACTTGGCAA   
  
  
+ CCTAACCATC GTCTTCTCTC TCTCTCTCTG TCTCTCTCTC TCTCTCCCAA AATCAACCTC TGTCACTCCA   
  
  
+ TCCCAACTGG AACTTGTCGA AAATTACGTT TTCAGTAACT CACGCCTCTA CCCCACATAC CCAGGCGGTC   
  
  
+ CGAGCAGAAA ATGGCCTGTG GCCTTCGACG CAGTTTCGCA GTAGGGTGTT CGCTGTACCC TAAGAAAGAG   
  
  
+ AGAGAAATCA AAAAGAAGAT GCCAAAAGAT GAGCACCGGG GGTGGTTGTA AAAGGTACTA GTGTAGACGT   
  
  
+ GGCAAGTATG AACAGCTCTA GCTAAGACCA TCCTCTCCAG TGTGGTACGT ACCTGAACAC TCCACCACCG   
  
  
+ GCAACTTTTT CAAAGAGCGG TCAATGAAGT ATATGTCATT ACTCTTCACT TCCTCAAACC AGTTTTGATT   
  
  
+ TGAACTAAAG TTTGTAAAGC GGCCTTAAAC ACGCTTAGTC TATTAATTCT AAAATAATTA TTTTTGGGTG   
  
  
+ AATAGTAAAG CCGATGTAAT TACCTTATTT ACCAACCTTG CTTCTCTAGG TGTGCGGTTT CGTTTAGCTA   
  
  
+ GTCTTTATTT CCAGTTGAAG TGTGCGAAAG AGTTCGAAGA GACTAGTTTA AAGTTAATTA CTGGTTTGGT   
  
  
+ TTTTGACAAG TGTCTAAACC GTTATCGATA CAAAGAAGAC AGAAGGAAAT AGGGTTAAAT TTTTGTCAAA   
  
  
+ TATATATCAT AAAAAATTAA ATATTATTAT GATATTTTTA TTTTTTTGAA TAAATAAATA AAATATTTTA   
  
  
+ ATAGTTCATA ATATAAAAAA TTTTTTTAAT TTGGGTGAAA TTTAATTAAC TTCTATATTG GCTTTCACAA   
  
  
+ ATATAACTTT TTTTTTTCAC ATTAACTATG AATTGTGCCA ACCTCTTTTC CTCCCTTCGA ATGTTCGTTT   
  
  
+ ATCGAACTGA ACCGTAAACT ACTCGCTGAA GTAAAACTTG AATTTCCAAC TACCCGGTGT AAGTGTAATA   
  
  
+ ATCAGTATGG TATAAAAACA ATGAAAATTA CCTTCTTTTG GGTTTTTTTT TTTTTTTTTC GTTTTTCTTC   
  
  
+ GTTTGGGAGA AATTGTTTAA AATAATTTA  

- CTCTCCAAAA CCATCCAAGA CAACACCCTT AGATATGGAT GGGTATAAAT ATACAAAGAC CAAGAAGAGA   
  
  
- GAAAATCTTT AAAAAGGAAA GATGATGAAG CGAGCACAAA CCGAGAAAAT AAATACAAAA AGGAAAAGCA   
  
  
- GAGATGATGA GAAAAGGGGG AAGGAGAAGG GGGAGGGGAA GGGGAAGGGG AAGGGTCGAG AGTCAAACTG   
  
  
- ATGCCCTCCC AATGCAAGGC ATCGTGTCGT TTATGAGGAT ATTGCCGTCA AACCCCCGAT CCATTCGTAT   
  
  
- AACGCCACGT GTCAGGGCTT TTGTCAGGAC ATTGACCGCC TCTTTAAGTG CGGGTCCATG GCAACGGGAT   
  
  
- AAAACCAGTT GGAGACCGTG CAGAGATGAT GCCAATGAAG GATAAAGTCA AAAGATTGAG ATGAACCGTT   
  
  
- GGATTGGTAG CAGAAGAGAG AGAGAGAGAC AGAGAGAGAG AGAGAGGGTT TTAGTTGGAG ACAGTGAGGT   
  
  
- AGGGTTGACC TTGAACAGCT TTTAATGCAA AAGTCATTGA GTGCGGAGAT GGGGTGTATG GGTCCGCCAG   
  
  
- GCTCGTCTTT TACCGGACAC CGGAAGCTGC GTCAAAGCGT CATCCCACAA GCGACATGGG ATTCTTTCTC   
  
  
- TCTCTTTAGT TTTTCTTCTA CGGTTTTCTA CTCGTGGCCC CCACCAACAT TTTCCATGAT CACATCTGCA   
  
  
- CCGTTCATAC TTGTCGAGAT CGATTCTGGT AGGAGAGGTC ACACCATGCA TGGACTTGTG AGGTGGTGGC   
  
  
- CGTTGAAAAA GTTTCTCGCC AGTTACTTCA TATACAGTAA TGAGAAGTGA AGGAGTTTGG TCAAAACTAA   
  
  
- ACTTGATTTC AAACATTTCG CCGGAATTTG TGCGAATCAG ATAATTAAGA TTTTATTAAT AAAAACCCAC   
  
  
- TTATCATTTC GGCTACATTA ATGGAATAAA TGGTTGGAAC GAAGAGATCC ACACGCCAAA GCAAATCGAT   
  
  
- CAGAAATAAA GGTCAACTTC ACACGCTTTC TCAAGCTTCT CTGATCAAAT TTCAATTAAT GACCAAACCA   
  
  
- AAAACTGTTC ACAGATTTGG CAATAGCTAT GTTTCTTCTG TCTTCCTTTA TCCCAATTTA AAAACAGTTT   
  
  
- ATATATAGTA TTTTTTAATT TATAATAATA CTATAAAAAT AAAAAAACTT ATTTATTTAT TTTATAAAAT   
  
  
- TATCAAGTAT TATATTTTTT AAAAAAATTA AACCCACTTT AAATTAATTG AAGATATAAC CGAAAGTGTT   
  
  
- TATATTGAAA AAAAAAAGTG TAATTGATAC TTAACACGGT TGGAGAAAAG GAGGGAAGCT TACAAGCAAA   
  
  
- TAGCTTGACT TGGCATTTGA TGAGCGACTT CATTTTGAAC TTAAAGGTTG ATGGGCCACA TTCACATTAT   
  
  
- TAGTCATACC ATATTTTTGT TACTTTTAAT GGAAGAAAAC CCAAAAAAAA AAAAAAAAAG CAAAAAGAAG   
  
  
- CAAACCCTCT TTAACAAATT TTATTAAAT

+     AC-II

| Site Name | Organism | Position | Strand | Matrix score. | sequence | function |
| --- | --- | --- | --- | --- | --- | --- |
| AC-II | Phaseolus vulgaris | 671 | - | 9 | (C/T)T(T/C)(C/T)(A/C)(A/C)C(A/C)A(A/C)C(C/A)(C/A)C |  |
| AC-II | Phaseolus vulgaris | 668 | - | 9 | (C/T)T(T/C)(C/T)(A/C)(A/C)C(A/C)A(A/C)C(C/A)(C/A)C |  |

> 2018/04/13 10:10:12  
+ GAGAGGTTTT GGTAGGTTCT GTTGTGGGAA TCTATACCTA CCCATATTTA TATGTTTCTG GTTCTTCTCT   
  
  
+ CTTTTAGAAA TTTTTCCTTT CTACTACTTC GCTCGTGTTT GGCTCTTTTA TTTATGTTTT TCCTTTTCGT   
  
  
+ CTCTACTACT CTTTTCCCCC TTCCTCTTCC CCCTCCCCTT CCCCTTCCCC TTCCCAGCTC TCAGTTTGAC   
  
  
+ TACGGGAGGG TTACGTTCCG TAGCACAGCA AATACTCCTA TAACGGCAGT TTGGGGGCTA GGTAAGCATA   
  
  
+ TTGCGGTGCA CAGTCCCGAA AACAGTCCTG TAACTGGCGG AGAAATTCAC GCCCAGGTAC CGTTGCCCTA   
  
  
+ TTTTGGTCAA CCTCTGGCAC GTCTCTACTA CGGTTACTTC CTATTTCAGT TTTCTAACTC TACTTGGCAA   
  
  
+ CCTAACCATC GTCTTCTCTC TCTCTCTCTG TCTCTCTCTC TCTCTCCCAA AATCAACCTC TGTCACTCCA   
  
  
+ TCCCAACTGG AACTTGTCGA AAATTACGTT TTCAGTAACT CACGCCTCTA CCCCACATAC CCAGGCGGTC   
  
  
+ CGAGCAGAAA ATGGCCTGTG GCCTTCGACG CAGTTTCGCA GTAGGGTGTT CGCTGTACCC TAAGAAAGAG   
  
  
+ AGAGAAATCA AAAAGAAGAT GCCAAAAGAT GAGCACCGGG GGTGGTTGTA AAAGGTACTA GTGTAGACGT   
  
  
+ GGCAAGTATG AACAGCTCTA GCTAAGACCA TCCTCTCCAG TGTGGTACGT ACCTGAACAC TCCACCACCG   
  
  
+ GCAACTTTTT CAAAGAGCGG TCAATGAAGT ATATGTCATT ACTCTTCACT TCCTCAAACC AGTTTTGATT   
  
  
+ TGAACTAAAG TTTGTAAAGC GGCCTTAAAC ACGCTTAGTC TATTAATTCT AAAATAATTA TTTTTGGGTG   
  
  
+ AATAGTAAAG CCGATGTAAT TACCTTATTT ACCAACCTTG CTTCTCTAGG TGTGCGGTTT CGTTTAGCTA   
  
  
+ GTCTTTATTT CCAGTTGAAG TGTGCGAAAG AGTTCGAAGA GACTAGTTTA AAGTTAATTA CTGGTTTGGT   
  
  
+ TTTTGACAAG TGTCTAAACC GTTATCGATA CAAAGAAGAC AGAAGGAAAT AGGGTTAAAT TTTTGTCAAA   
  
  
+ TATATATCAT AAAAAATTAA ATATTATTAT GATATTTTTA TTTTTTTGAA TAAATAAATA AAATATTTTA   
  
  
+ ATAGTTCATA ATATAAAAAA TTTTTTTAAT TTGGGTGAAA TTTAATTAAC TTCTATATTG GCTTTCACAA   
  
  
+ ATATAACTTT TTTTTTTCAC ATTAACTATG AATTGTGCCA ACCTCTTTTC CTCCCTTCGA ATGTTCGTTT   
  
  
+ ATCGAACTGA ACCGTAAACT ACTCGCTGAA GTAAAACTTG AATTTCCAAC TACCCGGTGT AAGTGTAATA   
  
  
+ ATCAGTATGG TATAAAAACA ATGAAAATTA CCTTCTTTTG GGTTTTTTTT TTTTTTTTTC GTTTTTCTTC   
  
  
+ GTTTGGGAGA AATTGTTTAA AATAATTTA  

- CTCTCCAAAA CCATCCAAGA CAACACCCTT AGATATGGAT GGGTATAAAT ATACAAAGAC CAAGAAGAGA   
  
  
- GAAAATCTTT AAAAAGGAAA GATGATGAAG CGAGCACAAA CCGAGAAAAT AAATACAAAA AGGAAAAGCA   
  
  
- GAGATGATGA GAAAAGGGGG AAGGAGAAGG GGGAGGGGAA GGGGAAGGGG AAGGGTCGAG AGTCAAACTG   
  
  
- ATGCCCTCCC AATGCAAGGC ATCGTGTCGT TTATGAGGAT ATTGCCGTCA AACCCCCGAT CCATTCGTAT   
  
  
- AACGCCACGT GTCAGGGCTT TTGTCAGGAC ATTGACCGCC TCTTTAAGTG CGGGTCCATG GCAACGGGAT   
  
  
- AAAACCAGTT GGAGACCGTG CAGAGATGAT GCCAATGAAG GATAAAGTCA AAAGATTGAG ATGAACCGTT   
  
  
- GGATTGGTAG CAGAAGAGAG AGAGAGAGAC AGAGAGAGAG AGAGAGGGTT TTAGTTGGAG ACAGTGAGGT   
  
  
- AGGGTTGACC TTGAACAGCT TTTAATGCAA AAGTCATTGA GTGCGGAGAT GGGGTGTATG GGTCCGCCAG   
  
  
- GCTCGTCTTT TACCGGACAC CGGAAGCTGC GTCAAAGCGT CATCCCACAA GCGACATGGG ATTCTTTCTC   
  
  
- TCTCTTTAGT TTTTCTTCTA CGGTTTTCTA CTCGTGGCCC CCACCAACAT TTTCCATGAT CACATCTGCA   
  
  
- CCGTTCATAC TTGTCGAGAT CGATTCTGGT AGGAGAGGTC ACACCATGCA TGGACTTGTG AGGTGGTGGC   
  
  
- CGTTGAAAAA GTTTCTCGCC AGTTACTTCA TATACAGTAA TGAGAAGTGA AGGAGTTTGG TCAAAACTAA   
  
  
- ACTTGATTTC AAACATTTCG CCGGAATTTG TGCGAATCAG ATAATTAAGA TTTTATTAAT AAAAACCCAC   
  
  
- TTATCATTTC GGCTACATTA ATGGAATAAA TGGTTGGAAC GAAGAGATCC ACACGCCAAA GCAAATCGAT   
  
  
- CAGAAATAAA GGTCAACTTC ACACGCTTTC TCAAGCTTCT CTGATCAAAT TTCAATTAAT GACCAAACCA   
  
  
- AAAACTGTTC ACAGATTTGG CAATAGCTAT GTTTCTTCTG TCTTCCTTTA TCCCAATTTA AAAACAGTTT   
  
  
- ATATATAGTA TTTTTTAATT TATAATAATA CTATAAAAAT AAAAAAACTT ATTTATTTAT TTTATAAAAT   
  
  
- TATCAAGTAT TATATTTTTT AAAAAAATTA AACCCACTTT AAATTAATTG AAGATATAAC CGAAAGTGTT   
  
  
- TATATTGAAA AAAAAAAGTG TAATTGATAC TTAACACGGT TGGAGAAAAG GAGGGAAGCT TACAAGCAAA   
  
  
- TAGCTTGACT TGGCATTTGA TGAGCGACTT CATTTTGAAC TTAAAGGTTG ATGGGCCACA TTCACATTAT   
  
  
- TAGTCATACC ATATTTTTGT TACTTTTAAT GGAAGAAAAC CCAAAAAAAA AAAAAAAAAG CAAAAAGAAG   
  
  
- CAAACCCTCT TTAACAAATT TTATTAAAT

+     AE-box

| Site Name | Organism | Position | Strand | Matrix score. | sequence | function |
| --- | --- | --- | --- | --- | --- | --- |
| AE-box | Arabidopsis thaliana | 52 | - | 8 | AGAAACAT | part of a module for light response |

> 2018/04/13 10:10:12  
+ GAGAGGTTTT GGTAGGTTCT GTTGTGGGAA TCTATACCTA CCCATATTTA TATGTTTCTG GTTCTTCTCT   
  
  
+ CTTTTAGAAA TTTTTCCTTT CTACTACTTC GCTCGTGTTT GGCTCTTTTA TTTATGTTTT TCCTTTTCGT   
  
  
+ CTCTACTACT CTTTTCCCCC TTCCTCTTCC CCCTCCCCTT CCCCTTCCCC TTCCCAGCTC TCAGTTTGAC   
  
  
+ TACGGGAGGG TTACGTTCCG TAGCACAGCA AATACTCCTA TAACGGCAGT TTGGGGGCTA GGTAAGCATA   
  
  
+ TTGCGGTGCA CAGTCCCGAA AACAGTCCTG TAACTGGCGG AGAAATTCAC GCCCAGGTAC CGTTGCCCTA   
  
  
+ TTTTGGTCAA CCTCTGGCAC GTCTCTACTA CGGTTACTTC CTATTTCAGT TTTCTAACTC TACTTGGCAA   
  
  
+ CCTAACCATC GTCTTCTCTC TCTCTCTCTG TCTCTCTCTC TCTCTCCCAA AATCAACCTC TGTCACTCCA   
  
  
+ TCCCAACTGG AACTTGTCGA AAATTACGTT TTCAGTAACT CACGCCTCTA CCCCACATAC CCAGGCGGTC   
  
  
+ CGAGCAGAAA ATGGCCTGTG GCCTTCGACG CAGTTTCGCA GTAGGGTGTT CGCTGTACCC TAAGAAAGAG   
  
  
+ AGAGAAATCA AAAAGAAGAT GCCAAAAGAT GAGCACCGGG GGTGGTTGTA AAAGGTACTA GTGTAGACGT   
  
  
+ GGCAAGTATG AACAGCTCTA GCTAAGACCA TCCTCTCCAG TGTGGTACGT ACCTGAACAC TCCACCACCG   
  
  
+ GCAACTTTTT CAAAGAGCGG TCAATGAAGT ATATGTCATT ACTCTTCACT TCCTCAAACC AGTTTTGATT   
  
  
+ TGAACTAAAG TTTGTAAAGC GGCCTTAAAC ACGCTTAGTC TATTAATTCT AAAATAATTA TTTTTGGGTG   
  
  
+ AATAGTAAAG CCGATGTAAT TACCTTATTT ACCAACCTTG CTTCTCTAGG TGTGCGGTTT CGTTTAGCTA   
  
  
+ GTCTTTATTT CCAGTTGAAG TGTGCGAAAG AGTTCGAAGA GACTAGTTTA AAGTTAATTA CTGGTTTGGT   
  
  
+ TTTTGACAAG TGTCTAAACC GTTATCGATA CAAAGAAGAC AGAAGGAAAT AGGGTTAAAT TTTTGTCAAA   
  
  
+ TATATATCAT AAAAAATTAA ATATTATTAT GATATTTTTA TTTTTTTGAA TAAATAAATA AAATATTTTA   
  
  
+ ATAGTTCATA ATATAAAAAA TTTTTTTAAT TTGGGTGAAA TTTAATTAAC TTCTATATTG GCTTTCACAA   
  
  
+ ATATAACTTT TTTTTTTCAC ATTAACTATG AATTGTGCCA ACCTCTTTTC CTCCCTTCGA ATGTTCGTTT   
  
  
+ ATCGAACTGA ACCGTAAACT ACTCGCTGAA GTAAAACTTG AATTTCCAAC TACCCGGTGT AAGTGTAATA   
  
  
+ ATCAGTATGG TATAAAAACA ATGAAAATTA CCTTCTTTTG GGTTTTTTTT TTTTTTTTTC GTTTTTCTTC   
  
  
+ GTTTGGGAGA AATTGTTTAA AATAATTTA  

- CTCTCCAAAA CCATCCAAGA CAACACCCTT AGATATGGAT GGGTATAAAT ATACAAAGAC CAAGAAGAGA   
  
  
- GAAAATCTTT AAAAAGGAAA GATGATGAAG CGAGCACAAA CCGAGAAAAT AAATACAAAA AGGAAAAGCA   
  
  
- GAGATGATGA GAAAAGGGGG AAGGAGAAGG GGGAGGGGAA GGGGAAGGGG AAGGGTCGAG AGTCAAACTG   
  
  
- ATGCCCTCCC AATGCAAGGC ATCGTGTCGT TTATGAGGAT ATTGCCGTCA AACCCCCGAT CCATTCGTAT   
  
  
- AACGCCACGT GTCAGGGCTT TTGTCAGGAC ATTGACCGCC TCTTTAAGTG CGGGTCCATG GCAACGGGAT   
  
  
- AAAACCAGTT GGAGACCGTG CAGAGATGAT GCCAATGAAG GATAAAGTCA AAAGATTGAG ATGAACCGTT   
  
  
- GGATTGGTAG CAGAAGAGAG AGAGAGAGAC AGAGAGAGAG AGAGAGGGTT TTAGTTGGAG ACAGTGAGGT   
  
  
- AGGGTTGACC TTGAACAGCT TTTAATGCAA AAGTCATTGA GTGCGGAGAT GGGGTGTATG GGTCCGCCAG   
  
  
- GCTCGTCTTT TACCGGACAC CGGAAGCTGC GTCAAAGCGT CATCCCACAA GCGACATGGG ATTCTTTCTC   
  
  
- TCTCTTTAGT TTTTCTTCTA CGGTTTTCTA CTCGTGGCCC CCACCAACAT TTTCCATGAT CACATCTGCA   
  
  
- CCGTTCATAC TTGTCGAGAT CGATTCTGGT AGGAGAGGTC ACACCATGCA TGGACTTGTG AGGTGGTGGC   
  
  
- CGTTGAAAAA GTTTCTCGCC AGTTACTTCA TATACAGTAA TGAGAAGTGA AGGAGTTTGG TCAAAACTAA   
  
  
- ACTTGATTTC AAACATTTCG CCGGAATTTG TGCGAATCAG ATAATTAAGA TTTTATTAAT AAAAACCCAC   
  
  
- TTATCATTTC GGCTACATTA ATGGAATAAA TGGTTGGAAC GAAGAGATCC ACACGCCAAA GCAAATCGAT   
  
  
- CAGAAATAAA GGTCAACTTC ACACGCTTTC TCAAGCTTCT CTGATCAAAT TTCAATTAAT GACCAAACCA   
  
  
- AAAACTGTTC ACAGATTTGG CAATAGCTAT GTTTCTTCTG TCTTCCTTTA TCCCAATTTA AAAACAGTTT   
  
  
- ATATATAGTA TTTTTTAATT TATAATAATA CTATAAAAAT AAAAAAACTT ATTTATTTAT TTTATAAAAT   
  
  
- TATCAAGTAT TATATTTTTT AAAAAAATTA AACCCACTTT AAATTAATTG AAGATATAAC CGAAAGTGTT   
  
  
- TATATTGAAA AAAAAAAGTG TAATTGATAC TTAACACGGT TGGAGAAAAG GAGGGAAGCT TACAAGCAAA   
  
  
- TAGCTTGACT TGGCATTTGA TGAGCGACTT CATTTTGAAC TTAAAGGTTG ATGGGCCACA TTCACATTAT   
  
  
- TAGTCATACC ATATTTTTGT TACTTTTAAT GGAAGAAAAC CCAAAAAAAA AAAAAAAAAG CAAAAAGAAG   
  
  
- CAAACCCTCT TTAACAAATT TTATTAAAT

+     ARE

| Site Name | Organism | Position | Strand | Matrix score. | sequence | function |
| --- | --- | --- | --- | --- | --- | --- |
| ARE | Zea mays | 1042 | + | 6 | TGGTTT | cis-acting regulatory element essential for the anaerobic induction |
| ARE | Zea mays | 1047 | + | 6 | TGGTTT | cis-acting regulatory element essential for the anaerobic induction |
| ARE | Zea mays | 826 | - | 6 | TGGTTT | cis-acting regulatory element essential for the anaerobic induction |

> 2018/04/13 10:10:12  
+ GAGAGGTTTT GGTAGGTTCT GTTGTGGGAA TCTATACCTA CCCATATTTA TATGTTTCTG GTTCTTCTCT   
  
  
+ CTTTTAGAAA TTTTTCCTTT CTACTACTTC GCTCGTGTTT GGCTCTTTTA TTTATGTTTT TCCTTTTCGT   
  
  
+ CTCTACTACT CTTTTCCCCC TTCCTCTTCC CCCTCCCCTT CCCCTTCCCC TTCCCAGCTC TCAGTTTGAC   
  
  
+ TACGGGAGGG TTACGTTCCG TAGCACAGCA AATACTCCTA TAACGGCAGT TTGGGGGCTA GGTAAGCATA   
  
  
+ TTGCGGTGCA CAGTCCCGAA AACAGTCCTG TAACTGGCGG AGAAATTCAC GCCCAGGTAC CGTTGCCCTA   
  
  
+ TTTTGGTCAA CCTCTGGCAC GTCTCTACTA CGGTTACTTC CTATTTCAGT TTTCTAACTC TACTTGGCAA   
  
  
+ CCTAACCATC GTCTTCTCTC TCTCTCTCTG TCTCTCTCTC TCTCTCCCAA AATCAACCTC TGTCACTCCA   
  
  
+ TCCCAACTGG AACTTGTCGA AAATTACGTT TTCAGTAACT CACGCCTCTA CCCCACATAC CCAGGCGGTC   
  
  
+ CGAGCAGAAA ATGGCCTGTG GCCTTCGACG CAGTTTCGCA GTAGGGTGTT CGCTGTACCC TAAGAAAGAG   
  
  
+ AGAGAAATCA AAAAGAAGAT GCCAAAAGAT GAGCACCGGG GGTGGTTGTA AAAGGTACTA GTGTAGACGT   
  
  
+ GGCAAGTATG AACAGCTCTA GCTAAGACCA TCCTCTCCAG TGTGGTACGT ACCTGAACAC TCCACCACCG   
  
  
+ GCAACTTTTT CAAAGAGCGG TCAATGAAGT ATATGTCATT ACTCTTCACT TCCTCAAACC AGTTTTGATT   
  
  
+ TGAACTAAAG TTTGTAAAGC GGCCTTAAAC ACGCTTAGTC TATTAATTCT AAAATAATTA TTTTTGGGTG   
  
  
+ AATAGTAAAG CCGATGTAAT TACCTTATTT ACCAACCTTG CTTCTCTAGG TGTGCGGTTT CGTTTAGCTA   
  
  
+ GTCTTTATTT CCAGTTGAAG TGTGCGAAAG AGTTCGAAGA GACTAGTTTA AAGTTAATTA CTGGTTTGGT   
  
  
+ TTTTGACAAG TGTCTAAACC GTTATCGATA CAAAGAAGAC AGAAGGAAAT AGGGTTAAAT TTTTGTCAAA   
  
  
+ TATATATCAT AAAAAATTAA ATATTATTAT GATATTTTTA TTTTTTTGAA TAAATAAATA AAATATTTTA   
  
  
+ ATAGTTCATA ATATAAAAAA TTTTTTTAAT TTGGGTGAAA TTTAATTAAC TTCTATATTG GCTTTCACAA   
  
  
+ ATATAACTTT TTTTTTTCAC ATTAACTATG AATTGTGCCA ACCTCTTTTC CTCCCTTCGA ATGTTCGTTT   
  
  
+ ATCGAACTGA ACCGTAAACT ACTCGCTGAA GTAAAACTTG AATTTCCAAC TACCCGGTGT AAGTGTAATA   
  
  
+ ATCAGTATGG TATAAAAACA ATGAAAATTA CCTTCTTTTG GGTTTTTTTT TTTTTTTTTC GTTTTTCTTC   
  
  
+ GTTTGGGAGA AATTGTTTAA AATAATTTA  

- CTCTCCAAAA CCATCCAAGA CAACACCCTT AGATATGGAT GGGTATAAAT ATACAAAGAC CAAGAAGAGA   
  
  
- GAAAATCTTT AAAAAGGAAA GATGATGAAG CGAGCACAAA CCGAGAAAAT AAATACAAAA AGGAAAAGCA   
  
  
- GAGATGATGA GAAAAGGGGG AAGGAGAAGG GGGAGGGGAA GGGGAAGGGG AAGGGTCGAG AGTCAAACTG   
  
  
- ATGCCCTCCC AATGCAAGGC ATCGTGTCGT TTATGAGGAT ATTGCCGTCA AACCCCCGAT CCATTCGTAT   
  
  
- AACGCCACGT GTCAGGGCTT TTGTCAGGAC ATTGACCGCC TCTTTAAGTG CGGGTCCATG GCAACGGGAT   
  
  
- AAAACCAGTT GGAGACCGTG CAGAGATGAT GCCAATGAAG GATAAAGTCA AAAGATTGAG ATGAACCGTT   
  
  
- GGATTGGTAG CAGAAGAGAG AGAGAGAGAC AGAGAGAGAG AGAGAGGGTT TTAGTTGGAG ACAGTGAGGT   
  
  
- AGGGTTGACC TTGAACAGCT TTTAATGCAA AAGTCATTGA GTGCGGAGAT GGGGTGTATG GGTCCGCCAG   
  
  
- GCTCGTCTTT TACCGGACAC CGGAAGCTGC GTCAAAGCGT CATCCCACAA GCGACATGGG ATTCTTTCTC   
  
  
- TCTCTTTAGT TTTTCTTCTA CGGTTTTCTA CTCGTGGCCC CCACCAACAT TTTCCATGAT CACATCTGCA   
  
  
- CCGTTCATAC TTGTCGAGAT CGATTCTGGT AGGAGAGGTC ACACCATGCA TGGACTTGTG AGGTGGTGGC   
  
  
- CGTTGAAAAA GTTTCTCGCC AGTTACTTCA TATACAGTAA TGAGAAGTGA AGGAGTTTGG TCAAAACTAA   
  
  
- ACTTGATTTC AAACATTTCG CCGGAATTTG TGCGAATCAG ATAATTAAGA TTTTATTAAT AAAAACCCAC   
  
  
- TTATCATTTC GGCTACATTA ATGGAATAAA TGGTTGGAAC GAAGAGATCC ACACGCCAAA GCAAATCGAT   
  
  
- CAGAAATAAA GGTCAACTTC ACACGCTTTC TCAAGCTTCT CTGATCAAAT TTCAATTAAT GACCAAACCA   
  
  
- AAAACTGTTC ACAGATTTGG CAATAGCTAT GTTTCTTCTG TCTTCCTTTA TCCCAATTTA AAAACAGTTT   
  
  
- ATATATAGTA TTTTTTAATT TATAATAATA CTATAAAAAT AAAAAAACTT ATTTATTTAT TTTATAAAAT   
  
  
- TATCAAGTAT TATATTTTTT AAAAAAATTA AACCCACTTT AAATTAATTG AAGATATAAC CGAAAGTGTT   
  
  
- TATATTGAAA AAAAAAAGTG TAATTGATAC TTAACACGGT TGGAGAAAAG GAGGGAAGCT TACAAGCAAA   
  
  
- TAGCTTGACT TGGCATTTGA TGAGCGACTT CATTTTGAAC TTAAAGGTTG ATGGGCCACA TTCACATTAT   
  
  
- TAGTCATACC ATATTTTTGT TACTTTTAAT GGAAGAAAAC CCAAAAAAAA AAAAAAAAAG CAAAAAGAAG   
  
  
- CAAACCCTCT TTAACAAATT TTATTAAAT

+     AT-rich element

| Site Name | Organism | Position | Strand | Matrix score. | sequence | function |
| --- | --- | --- | --- | --- | --- | --- |
| AT-rich element | Glycine max | 631 | + | 10 | ATAGAAATCAA | binding site of AT-rich DNA binding protein (ATBP-1) |

> 2018/04/13 10:10:12  
+ GAGAGGTTTT GGTAGGTTCT GTTGTGGGAA TCTATACCTA CCCATATTTA TATGTTTCTG GTTCTTCTCT   
  
  
+ CTTTTAGAAA TTTTTCCTTT CTACTACTTC GCTCGTGTTT GGCTCTTTTA TTTATGTTTT TCCTTTTCGT   
  
  
+ CTCTACTACT CTTTTCCCCC TTCCTCTTCC CCCTCCCCTT CCCCTTCCCC TTCCCAGCTC TCAGTTTGAC   
  
  
+ TACGGGAGGG TTACGTTCCG TAGCACAGCA AATACTCCTA TAACGGCAGT TTGGGGGCTA GGTAAGCATA   
  
  
+ TTGCGGTGCA CAGTCCCGAA AACAGTCCTG TAACTGGCGG AGAAATTCAC GCCCAGGTAC CGTTGCCCTA   
  
  
+ TTTTGGTCAA CCTCTGGCAC GTCTCTACTA CGGTTACTTC CTATTTCAGT TTTCTAACTC TACTTGGCAA   
  
  
+ CCTAACCATC GTCTTCTCTC TCTCTCTCTG TCTCTCTCTC TCTCTCCCAA AATCAACCTC TGTCACTCCA   
  
  
+ TCCCAACTGG AACTTGTCGA AAATTACGTT TTCAGTAACT CACGCCTCTA CCCCACATAC CCAGGCGGTC   
  
  
+ CGAGCAGAAA ATGGCCTGTG GCCTTCGACG CAGTTTCGCA GTAGGGTGTT CGCTGTACCC TAAGAAAGAG   
  
  
+ AGAGAAATCA AAAAGAAGAT GCCAAAAGAT GAGCACCGGG GGTGGTTGTA AAAGGTACTA GTGTAGACGT   
  
  
+ GGCAAGTATG AACAGCTCTA GCTAAGACCA TCCTCTCCAG TGTGGTACGT ACCTGAACAC TCCACCACCG   
  
  
+ GCAACTTTTT CAAAGAGCGG TCAATGAAGT ATATGTCATT ACTCTTCACT TCCTCAAACC AGTTTTGATT   
  
  
+ TGAACTAAAG TTTGTAAAGC GGCCTTAAAC ACGCTTAGTC TATTAATTCT AAAATAATTA TTTTTGGGTG   
  
  
+ AATAGTAAAG CCGATGTAAT TACCTTATTT ACCAACCTTG CTTCTCTAGG TGTGCGGTTT CGTTTAGCTA   
  
  
+ GTCTTTATTT CCAGTTGAAG TGTGCGAAAG AGTTCGAAGA GACTAGTTTA AAGTTAATTA CTGGTTTGGT   
  
  
+ TTTTGACAAG TGTCTAAACC GTTATCGATA CAAAGAAGAC AGAAGGAAAT AGGGTTAAAT TTTTGTCAAA   
  
  
+ TATATATCAT AAAAAATTAA ATATTATTAT GATATTTTTA TTTTTTTGAA TAAATAAATA AAATATTTTA   
  
  
+ ATAGTTCATA ATATAAAAAA TTTTTTTAAT TTGGGTGAAA TTTAATTAAC TTCTATATTG GCTTTCACAA   
  
  
+ ATATAACTTT TTTTTTTCAC ATTAACTATG AATTGTGCCA ACCTCTTTTC CTCCCTTCGA ATGTTCGTTT   
  
  
+ ATCGAACTGA ACCGTAAACT ACTCGCTGAA GTAAAACTTG AATTTCCAAC TACCCGGTGT AAGTGTAATA   
  
  
+ ATCAGTATGG TATAAAAACA ATGAAAATTA CCTTCTTTTG GGTTTTTTTT TTTTTTTTTC GTTTTTCTTC   
  
  
+ GTTTGGGAGA AATTGTTTAA AATAATTTA  

- CTCTCCAAAA CCATCCAAGA CAACACCCTT AGATATGGAT GGGTATAAAT ATACAAAGAC CAAGAAGAGA   
  
  
- GAAAATCTTT AAAAAGGAAA GATGATGAAG CGAGCACAAA CCGAGAAAAT AAATACAAAA AGGAAAAGCA   
  
  
- GAGATGATGA GAAAAGGGGG AAGGAGAAGG GGGAGGGGAA GGGGAAGGGG AAGGGTCGAG AGTCAAACTG   
  
  
- ATGCCCTCCC AATGCAAGGC ATCGTGTCGT TTATGAGGAT ATTGCCGTCA AACCCCCGAT CCATTCGTAT   
  
  
- AACGCCACGT GTCAGGGCTT TTGTCAGGAC ATTGACCGCC TCTTTAAGTG CGGGTCCATG GCAACGGGAT   
  
  
- AAAACCAGTT GGAGACCGTG CAGAGATGAT GCCAATGAAG GATAAAGTCA AAAGATTGAG ATGAACCGTT   
  
  
- GGATTGGTAG CAGAAGAGAG AGAGAGAGAC AGAGAGAGAG AGAGAGGGTT TTAGTTGGAG ACAGTGAGGT   
  
  
- AGGGTTGACC TTGAACAGCT TTTAATGCAA AAGTCATTGA GTGCGGAGAT GGGGTGTATG GGTCCGCCAG   
  
  
- GCTCGTCTTT TACCGGACAC CGGAAGCTGC GTCAAAGCGT CATCCCACAA GCGACATGGG ATTCTTTCTC   
  
  
- TCTCTTTAGT TTTTCTTCTA CGGTTTTCTA CTCGTGGCCC CCACCAACAT TTTCCATGAT CACATCTGCA   
  
  
- CCGTTCATAC TTGTCGAGAT CGATTCTGGT AGGAGAGGTC ACACCATGCA TGGACTTGTG AGGTGGTGGC   
  
  
- CGTTGAAAAA GTTTCTCGCC AGTTACTTCA TATACAGTAA TGAGAAGTGA AGGAGTTTGG TCAAAACTAA   
  
  
- ACTTGATTTC AAACATTTCG CCGGAATTTG TGCGAATCAG ATAATTAAGA TTTTATTAAT AAAAACCCAC   
  
  
- TTATCATTTC GGCTACATTA ATGGAATAAA TGGTTGGAAC GAAGAGATCC ACACGCCAAA GCAAATCGAT   
  
  
- CAGAAATAAA GGTCAACTTC ACACGCTTTC TCAAGCTTCT CTGATCAAAT TTCAATTAAT GACCAAACCA   
  
  
- AAAACTGTTC ACAGATTTGG CAATAGCTAT GTTTCTTCTG TCTTCCTTTA TCCCAATTTA AAAACAGTTT   
  
  
- ATATATAGTA TTTTTTAATT TATAATAATA CTATAAAAAT AAAAAAACTT ATTTATTTAT TTTATAAAAT   
  
  
- TATCAAGTAT TATATTTTTT AAAAAAATTA AACCCACTTT AAATTAATTG AAGATATAAC CGAAAGTGTT   
  
  
- TATATTGAAA AAAAAAAGTG TAATTGATAC TTAACACGGT TGGAGAAAAG GAGGGAAGCT TACAAGCAAA   
  
  
- TAGCTTGACT TGGCATTTGA TGAGCGACTT CATTTTGAAC TTAAAGGTTG ATGGGCCACA TTCACATTAT   
  
  
- TAGTCATACC ATATTTTTGT TACTTTTAAT GGAAGAAAAC CCAAAAAAAA AAAAAAAAAG CAAAAAGAAG   
  
  
- CAAACCCTCT TTAACAAATT TTATTAAAT

+     Box 4

| Site Name | Organism | Position | Strand | Matrix score. | sequence | function |
| --- | --- | --- | --- | --- | --- | --- |
| Box 4 | Petroselinum crispum | 882 | - | 6 | ATTAAT | part of a conserved DNA module involved in light responsiveness |

> 2018/04/13 10:10:12  
+ GAGAGGTTTT GGTAGGTTCT GTTGTGGGAA TCTATACCTA CCCATATTTA TATGTTTCTG GTTCTTCTCT   
  
  
+ CTTTTAGAAA TTTTTCCTTT CTACTACTTC GCTCGTGTTT GGCTCTTTTA TTTATGTTTT TCCTTTTCGT   
  
  
+ CTCTACTACT CTTTTCCCCC TTCCTCTTCC CCCTCCCCTT CCCCTTCCCC TTCCCAGCTC TCAGTTTGAC   
  
  
+ TACGGGAGGG TTACGTTCCG TAGCACAGCA AATACTCCTA TAACGGCAGT TTGGGGGCTA GGTAAGCATA   
  
  
+ TTGCGGTGCA CAGTCCCGAA AACAGTCCTG TAACTGGCGG AGAAATTCAC GCCCAGGTAC CGTTGCCCTA   
  
  
+ TTTTGGTCAA CCTCTGGCAC GTCTCTACTA CGGTTACTTC CTATTTCAGT TTTCTAACTC TACTTGGCAA   
  
  
+ CCTAACCATC GTCTTCTCTC TCTCTCTCTG TCTCTCTCTC TCTCTCCCAA AATCAACCTC TGTCACTCCA   
  
  
+ TCCCAACTGG AACTTGTCGA AAATTACGTT TTCAGTAACT CACGCCTCTA CCCCACATAC CCAGGCGGTC   
  
  
+ CGAGCAGAAA ATGGCCTGTG GCCTTCGACG CAGTTTCGCA GTAGGGTGTT CGCTGTACCC TAAGAAAGAG   
  
  
+ AGAGAAATCA AAAAGAAGAT GCCAAAAGAT GAGCACCGGG GGTGGTTGTA AAAGGTACTA GTGTAGACGT   
  
  
+ GGCAAGTATG AACAGCTCTA GCTAAGACCA TCCTCTCCAG TGTGGTACGT ACCTGAACAC TCCACCACCG   
  
  
+ GCAACTTTTT CAAAGAGCGG TCAATGAAGT ATATGTCATT ACTCTTCACT TCCTCAAACC AGTTTTGATT   
  
  
+ TGAACTAAAG TTTGTAAAGC GGCCTTAAAC ACGCTTAGTC TATTAATTCT AAAATAATTA TTTTTGGGTG   
  
  
+ AATAGTAAAG CCGATGTAAT TACCTTATTT ACCAACCTTG CTTCTCTAGG TGTGCGGTTT CGTTTAGCTA   
  
  
+ GTCTTTATTT CCAGTTGAAG TGTGCGAAAG AGTTCGAAGA GACTAGTTTA AAGTTAATTA CTGGTTTGGT   
  
  
+ TTTTGACAAG TGTCTAAACC GTTATCGATA CAAAGAAGAC AGAAGGAAAT AGGGTTAAAT TTTTGTCAAA   
  
  
+ TATATATCAT AAAAAATTAA ATATTATTAT GATATTTTTA TTTTTTTGAA TAAATAAATA AAATATTTTA   
  
  
+ ATAGTTCATA ATATAAAAAA TTTTTTTAAT TTGGGTGAAA TTTAATTAAC TTCTATATTG GCTTTCACAA   
  
  
+ ATATAACTTT TTTTTTTCAC ATTAACTATG AATTGTGCCA ACCTCTTTTC CTCCCTTCGA ATGTTCGTTT   
  
  
+ ATCGAACTGA ACCGTAAACT ACTCGCTGAA GTAAAACTTG AATTTCCAAC TACCCGGTGT AAGTGTAATA   
  
  
+ ATCAGTATGG TATAAAAACA ATGAAAATTA CCTTCTTTTG GGTTTTTTTT TTTTTTTTTC GTTTTTCTTC   
  
  
+ GTTTGGGAGA AATTGTTTAA AATAATTTA  

- CTCTCCAAAA CCATCCAAGA CAACACCCTT AGATATGGAT GGGTATAAAT ATACAAAGAC CAAGAAGAGA   
  
  
- GAAAATCTTT AAAAAGGAAA GATGATGAAG CGAGCACAAA CCGAGAAAAT AAATACAAAA AGGAAAAGCA   
  
  
- GAGATGATGA GAAAAGGGGG AAGGAGAAGG GGGAGGGGAA GGGGAAGGGG AAGGGTCGAG AGTCAAACTG   
  
  
- ATGCCCTCCC AATGCAAGGC ATCGTGTCGT TTATGAGGAT ATTGCCGTCA AACCCCCGAT CCATTCGTAT   
  
  
- AACGCCACGT GTCAGGGCTT TTGTCAGGAC ATTGACCGCC TCTTTAAGTG CGGGTCCATG GCAACGGGAT   
  
  
- AAAACCAGTT GGAGACCGTG CAGAGATGAT GCCAATGAAG GATAAAGTCA AAAGATTGAG ATGAACCGTT   
  
  
- GGATTGGTAG CAGAAGAGAG AGAGAGAGAC AGAGAGAGAG AGAGAGGGTT TTAGTTGGAG ACAGTGAGGT   
  
  
- AGGGTTGACC TTGAACAGCT TTTAATGCAA AAGTCATTGA GTGCGGAGAT GGGGTGTATG GGTCCGCCAG   
  
  
- GCTCGTCTTT TACCGGACAC CGGAAGCTGC GTCAAAGCGT CATCCCACAA GCGACATGGG ATTCTTTCTC   
  
  
- TCTCTTTAGT TTTTCTTCTA CGGTTTTCTA CTCGTGGCCC CCACCAACAT TTTCCATGAT CACATCTGCA   
  
  
- CCGTTCATAC TTGTCGAGAT CGATTCTGGT AGGAGAGGTC ACACCATGCA TGGACTTGTG AGGTGGTGGC   
  
  
- CGTTGAAAAA GTTTCTCGCC AGTTACTTCA TATACAGTAA TGAGAAGTGA AGGAGTTTGG TCAAAACTAA   
  
  
- ACTTGATTTC AAACATTTCG CCGGAATTTG TGCGAATCAG ATAATTAAGA TTTTATTAAT AAAAACCCAC   
  
  
- TTATCATTTC GGCTACATTA ATGGAATAAA TGGTTGGAAC GAAGAGATCC ACACGCCAAA GCAAATCGAT   
  
  
- CAGAAATAAA GGTCAACTTC ACACGCTTTC TCAAGCTTCT CTGATCAAAT TTCAATTAAT GACCAAACCA   
  
  
- AAAACTGTTC ACAGATTTGG CAATAGCTAT GTTTCTTCTG TCTTCCTTTA TCCCAATTTA AAAACAGTTT   
  
  
- ATATATAGTA TTTTTTAATT TATAATAATA CTATAAAAAT AAAAAAACTT ATTTATTTAT TTTATAAAAT   
  
  
- TATCAAGTAT TATATTTTTT AAAAAAATTA AACCCACTTT AAATTAATTG AAGATATAAC CGAAAGTGTT   
  
  
- TATATTGAAA AAAAAAAGTG TAATTGATAC TTAACACGGT TGGAGAAAAG GAGGGAAGCT TACAAGCAAA   
  
  
- TAGCTTGACT TGGCATTTGA TGAGCGACTT CATTTTGAAC TTAAAGGTTG ATGGGCCACA TTCACATTAT   
  
  
- TAGTCATACC ATATTTTTGT TACTTTTAAT GGAAGAAAAC CCAAAAAAAA AAAAAAAAAG CAAAAAGAAG   
  
  
- CAAACCCTCT TTAACAAATT TTATTAAAT

+     Box I

| Site Name | Organism | Position | Strand | Matrix score. | sequence | function |
| --- | --- | --- | --- | --- | --- | --- |
| Box I | Pisum sativum | 778 | + | 7 | TTTCAAA | light responsive element |

> 2018/04/13 10:10:12  
+ GAGAGGTTTT GGTAGGTTCT GTTGTGGGAA TCTATACCTA CCCATATTTA TATGTTTCTG GTTCTTCTCT   
  
  
+ CTTTTAGAAA TTTTTCCTTT CTACTACTTC GCTCGTGTTT GGCTCTTTTA TTTATGTTTT TCCTTTTCGT   
  
  
+ CTCTACTACT CTTTTCCCCC TTCCTCTTCC CCCTCCCCTT CCCCTTCCCC TTCCCAGCTC TCAGTTTGAC   
  
  
+ TACGGGAGGG TTACGTTCCG TAGCACAGCA AATACTCCTA TAACGGCAGT TTGGGGGCTA GGTAAGCATA   
  
  
+ TTGCGGTGCA CAGTCCCGAA AACAGTCCTG TAACTGGCGG AGAAATTCAC GCCCAGGTAC CGTTGCCCTA   
  
  
+ TTTTGGTCAA CCTCTGGCAC GTCTCTACTA CGGTTACTTC CTATTTCAGT TTTCTAACTC TACTTGGCAA   
  
  
+ CCTAACCATC GTCTTCTCTC TCTCTCTCTG TCTCTCTCTC TCTCTCCCAA AATCAACCTC TGTCACTCCA   
  
  
+ TCCCAACTGG AACTTGTCGA AAATTACGTT TTCAGTAACT CACGCCTCTA CCCCACATAC CCAGGCGGTC   
  
  
+ CGAGCAGAAA ATGGCCTGTG GCCTTCGACG CAGTTTCGCA GTAGGGTGTT CGCTGTACCC TAAGAAAGAG   
  
  
+ AGAGAAATCA AAAAGAAGAT GCCAAAAGAT GAGCACCGGG GGTGGTTGTA AAAGGTACTA GTGTAGACGT   
  
  
+ GGCAAGTATG AACAGCTCTA GCTAAGACCA TCCTCTCCAG TGTGGTACGT ACCTGAACAC TCCACCACCG   
  
  
+ GCAACTTTTT CAAAGAGCGG TCAATGAAGT ATATGTCATT ACTCTTCACT TCCTCAAACC AGTTTTGATT   
  
  
+ TGAACTAAAG TTTGTAAAGC GGCCTTAAAC ACGCTTAGTC TATTAATTCT AAAATAATTA TTTTTGGGTG   
  
  
+ AATAGTAAAG CCGATGTAAT TACCTTATTT ACCAACCTTG CTTCTCTAGG TGTGCGGTTT CGTTTAGCTA   
  
  
+ GTCTTTATTT CCAGTTGAAG TGTGCGAAAG AGTTCGAAGA GACTAGTTTA AAGTTAATTA CTGGTTTGGT   
  
  
+ TTTTGACAAG TGTCTAAACC GTTATCGATA CAAAGAAGAC AGAAGGAAAT AGGGTTAAAT TTTTGTCAAA   
  
  
+ TATATATCAT AAAAAATTAA ATATTATTAT GATATTTTTA TTTTTTTGAA TAAATAAATA AAATATTTTA   
  
  
+ ATAGTTCATA ATATAAAAAA TTTTTTTAAT TTGGGTGAAA TTTAATTAAC TTCTATATTG GCTTTCACAA   
  
  
+ ATATAACTTT TTTTTTTCAC ATTAACTATG AATTGTGCCA ACCTCTTTTC CTCCCTTCGA ATGTTCGTTT   
  
  
+ ATCGAACTGA ACCGTAAACT ACTCGCTGAA GTAAAACTTG AATTTCCAAC TACCCGGTGT AAGTGTAATA   
  
  
+ ATCAGTATGG TATAAAAACA ATGAAAATTA CCTTCTTTTG GGTTTTTTTT TTTTTTTTTC GTTTTTCTTC   
  
  
+ GTTTGGGAGA AATTGTTTAA AATAATTTA  

- CTCTCCAAAA CCATCCAAGA CAACACCCTT AGATATGGAT GGGTATAAAT ATACAAAGAC CAAGAAGAGA   
  
  
- GAAAATCTTT AAAAAGGAAA GATGATGAAG CGAGCACAAA CCGAGAAAAT AAATACAAAA AGGAAAAGCA   
  
  
- GAGATGATGA GAAAAGGGGG AAGGAGAAGG GGGAGGGGAA GGGGAAGGGG AAGGGTCGAG AGTCAAACTG   
  
  
- ATGCCCTCCC AATGCAAGGC ATCGTGTCGT TTATGAGGAT ATTGCCGTCA AACCCCCGAT CCATTCGTAT   
  
  
- AACGCCACGT GTCAGGGCTT TTGTCAGGAC ATTGACCGCC TCTTTAAGTG CGGGTCCATG GCAACGGGAT   
  
  
- AAAACCAGTT GGAGACCGTG CAGAGATGAT GCCAATGAAG GATAAAGTCA AAAGATTGAG ATGAACCGTT   
  
  
- GGATTGGTAG CAGAAGAGAG AGAGAGAGAC AGAGAGAGAG AGAGAGGGTT TTAGTTGGAG ACAGTGAGGT   
  
  
- AGGGTTGACC TTGAACAGCT TTTAATGCAA AAGTCATTGA GTGCGGAGAT GGGGTGTATG GGTCCGCCAG   
  
  
- GCTCGTCTTT TACCGGACAC CGGAAGCTGC GTCAAAGCGT CATCCCACAA GCGACATGGG ATTCTTTCTC   
  
  
- TCTCTTTAGT TTTTCTTCTA CGGTTTTCTA CTCGTGGCCC CCACCAACAT TTTCCATGAT CACATCTGCA   
  
  
- CCGTTCATAC TTGTCGAGAT CGATTCTGGT AGGAGAGGTC ACACCATGCA TGGACTTGTG AGGTGGTGGC   
  
  
- CGTTGAAAAA GTTTCTCGCC AGTTACTTCA TATACAGTAA TGAGAAGTGA AGGAGTTTGG TCAAAACTAA   
  
  
- ACTTGATTTC AAACATTTCG CCGGAATTTG TGCGAATCAG ATAATTAAGA TTTTATTAAT AAAAACCCAC   
  
  
- TTATCATTTC GGCTACATTA ATGGAATAAA TGGTTGGAAC GAAGAGATCC ACACGCCAAA GCAAATCGAT   
  
  
- CAGAAATAAA GGTCAACTTC ACACGCTTTC TCAAGCTTCT CTGATCAAAT TTCAATTAAT GACCAAACCA   
  
  
- AAAACTGTTC ACAGATTTGG CAATAGCTAT GTTTCTTCTG TCTTCCTTTA TCCCAATTTA AAAACAGTTT   
  
  
- ATATATAGTA TTTTTTAATT TATAATAATA CTATAAAAAT AAAAAAACTT ATTTATTTAT TTTATAAAAT   
  
  
- TATCAAGTAT TATATTTTTT AAAAAAATTA AACCCACTTT AAATTAATTG AAGATATAAC CGAAAGTGTT   
  
  
- TATATTGAAA AAAAAAAGTG TAATTGATAC TTAACACGGT TGGAGAAAAG GAGGGAAGCT TACAAGCAAA   
  
  
- TAGCTTGACT TGGCATTTGA TGAGCGACTT CATTTTGAAC TTAAAGGTTG ATGGGCCACA TTCACATTAT   
  
  
- TAGTCATACC ATATTTTTGT TACTTTTAAT GGAAGAAAAC CCAAAAAAAA AAAAAAAAAG CAAAAAGAAG   
  
  
- CAAACCCTCT TTAACAAATT TTATTAAAT

+     Box-W1

| Site Name | Organism | Position | Strand | Matrix score. | sequence | function |
| --- | --- | --- | --- | --- | --- | --- |
| Box-W1 | Petroselinum crispum | 789 | - | 6 | TTGACC | fungal elicitor responsive element |
| Box-W1 | Petroselinum crispum | 355 | - | 6 | TTGACC | fungal elicitor responsive element |

> 2018/04/13 10:10:12  
+ GAGAGGTTTT GGTAGGTTCT GTTGTGGGAA TCTATACCTA CCCATATTTA TATGTTTCTG GTTCTTCTCT   
  
  
+ CTTTTAGAAA TTTTTCCTTT CTACTACTTC GCTCGTGTTT GGCTCTTTTA TTTATGTTTT TCCTTTTCGT   
  
  
+ CTCTACTACT CTTTTCCCCC TTCCTCTTCC CCCTCCCCTT CCCCTTCCCC TTCCCAGCTC TCAGTTTGAC   
  
  
+ TACGGGAGGG TTACGTTCCG TAGCACAGCA AATACTCCTA TAACGGCAGT TTGGGGGCTA GGTAAGCATA   
  
  
+ TTGCGGTGCA CAGTCCCGAA AACAGTCCTG TAACTGGCGG AGAAATTCAC GCCCAGGTAC CGTTGCCCTA   
  
  
+ TTTTGGTCAA CCTCTGGCAC GTCTCTACTA CGGTTACTTC CTATTTCAGT TTTCTAACTC TACTTGGCAA   
  
  
+ CCTAACCATC GTCTTCTCTC TCTCTCTCTG TCTCTCTCTC TCTCTCCCAA AATCAACCTC TGTCACTCCA   
  
  
+ TCCCAACTGG AACTTGTCGA AAATTACGTT TTCAGTAACT CACGCCTCTA CCCCACATAC CCAGGCGGTC   
  
  
+ CGAGCAGAAA ATGGCCTGTG GCCTTCGACG CAGTTTCGCA GTAGGGTGTT CGCTGTACCC TAAGAAAGAG   
  
  
+ AGAGAAATCA AAAAGAAGAT GCCAAAAGAT GAGCACCGGG GGTGGTTGTA AAAGGTACTA GTGTAGACGT   
  
  
+ GGCAAGTATG AACAGCTCTA GCTAAGACCA TCCTCTCCAG TGTGGTACGT ACCTGAACAC TCCACCACCG   
  
  
+ GCAACTTTTT CAAAGAGCGG TCAATGAAGT ATATGTCATT ACTCTTCACT TCCTCAAACC AGTTTTGATT   
  
  
+ TGAACTAAAG TTTGTAAAGC GGCCTTAAAC ACGCTTAGTC TATTAATTCT AAAATAATTA TTTTTGGGTG   
  
  
+ AATAGTAAAG CCGATGTAAT TACCTTATTT ACCAACCTTG CTTCTCTAGG TGTGCGGTTT CGTTTAGCTA   
  
  
+ GTCTTTATTT CCAGTTGAAG TGTGCGAAAG AGTTCGAAGA GACTAGTTTA AAGTTAATTA CTGGTTTGGT   
  
  
+ TTTTGACAAG TGTCTAAACC GTTATCGATA CAAAGAAGAC AGAAGGAAAT AGGGTTAAAT TTTTGTCAAA   
  
  
+ TATATATCAT AAAAAATTAA ATATTATTAT GATATTTTTA TTTTTTTGAA TAAATAAATA AAATATTTTA   
  
  
+ ATAGTTCATA ATATAAAAAA TTTTTTTAAT TTGGGTGAAA TTTAATTAAC TTCTATATTG GCTTTCACAA   
  
  
+ ATATAACTTT TTTTTTTCAC ATTAACTATG AATTGTGCCA ACCTCTTTTC CTCCCTTCGA ATGTTCGTTT   
  
  
+ ATCGAACTGA ACCGTAAACT ACTCGCTGAA GTAAAACTTG AATTTCCAAC TACCCGGTGT AAGTGTAATA   
  
  
+ ATCAGTATGG TATAAAAACA ATGAAAATTA CCTTCTTTTG GGTTTTTTTT TTTTTTTTTC GTTTTTCTTC   
  
  
+ GTTTGGGAGA AATTGTTTAA AATAATTTA  

- CTCTCCAAAA CCATCCAAGA CAACACCCTT AGATATGGAT GGGTATAAAT ATACAAAGAC CAAGAAGAGA   
  
  
- GAAAATCTTT AAAAAGGAAA GATGATGAAG CGAGCACAAA CCGAGAAAAT AAATACAAAA AGGAAAAGCA   
  
  
- GAGATGATGA GAAAAGGGGG AAGGAGAAGG GGGAGGGGAA GGGGAAGGGG AAGGGTCGAG AGTCAAACTG   
  
  
- ATGCCCTCCC AATGCAAGGC ATCGTGTCGT TTATGAGGAT ATTGCCGTCA AACCCCCGAT CCATTCGTAT   
  
  
- AACGCCACGT GTCAGGGCTT TTGTCAGGAC ATTGACCGCC TCTTTAAGTG CGGGTCCATG GCAACGGGAT   
  
  
- AAAACCAGTT GGAGACCGTG CAGAGATGAT GCCAATGAAG GATAAAGTCA AAAGATTGAG ATGAACCGTT   
  
  
- GGATTGGTAG CAGAAGAGAG AGAGAGAGAC AGAGAGAGAG AGAGAGGGTT TTAGTTGGAG ACAGTGAGGT   
  
  
- AGGGTTGACC TTGAACAGCT TTTAATGCAA AAGTCATTGA GTGCGGAGAT GGGGTGTATG GGTCCGCCAG   
  
  
- GCTCGTCTTT TACCGGACAC CGGAAGCTGC GTCAAAGCGT CATCCCACAA GCGACATGGG ATTCTTTCTC   
  
  
- TCTCTTTAGT TTTTCTTCTA CGGTTTTCTA CTCGTGGCCC CCACCAACAT TTTCCATGAT CACATCTGCA   
  
  
- CCGTTCATAC TTGTCGAGAT CGATTCTGGT AGGAGAGGTC ACACCATGCA TGGACTTGTG AGGTGGTGGC   
  
  
- CGTTGAAAAA GTTTCTCGCC AGTTACTTCA TATACAGTAA TGAGAAGTGA AGGAGTTTGG TCAAAACTAA   
  
  
- ACTTGATTTC AAACATTTCG CCGGAATTTG TGCGAATCAG ATAATTAAGA TTTTATTAAT AAAAACCCAC   
  
  
- TTATCATTTC GGCTACATTA ATGGAATAAA TGGTTGGAAC GAAGAGATCC ACACGCCAAA GCAAATCGAT   
  
  
- CAGAAATAAA GGTCAACTTC ACACGCTTTC TCAAGCTTCT CTGATCAAAT TTCAATTAAT GACCAAACCA   
  
  
- AAAACTGTTC ACAGATTTGG CAATAGCTAT GTTTCTTCTG TCTTCCTTTA TCCCAATTTA AAAACAGTTT   
  
  
- ATATATAGTA TTTTTTAATT TATAATAATA CTATAAAAAT AAAAAAACTT ATTTATTTAT TTTATAAAAT   
  
  
- TATCAAGTAT TATATTTTTT AAAAAAATTA AACCCACTTT AAATTAATTG AAGATATAAC CGAAAGTGTT   
  
  
- TATATTGAAA AAAAAAAGTG TAATTGATAC TTAACACGGT TGGAGAAAAG GAGGGAAGCT TACAAGCAAA   
  
  
- TAGCTTGACT TGGCATTTGA TGAGCGACTT CATTTTGAAC TTAAAGGTTG ATGGGCCACA TTCACATTAT   
  
  
- TAGTCATACC ATATTTTTGT TACTTTTAAT GGAAGAAAAC CCAAAAAAAA AAAAAAAAAG CAAAAAGAAG   
  
  
- CAAACCCTCT TTAACAAATT TTATTAAAT

+     CAAT-box

| Site Name | Organism | Position | Strand | Matrix score. | sequence | function |
| --- | --- | --- | --- | --- | --- | --- |
| CAAT-box | Hordeum vulgare | 1292 | - | 4 | CAAT | common cis-acting element in promoter and enhancer regions |
| CAAT-box | Glycine max | 1291 | - | 5 | CAATT | common cis-acting element in promoter and enhancer regions |
| CAAT-box | Brassica rapa | 1258 | + | 5 | CAAAT | common cis-acting element in promoter and enhancer regions |
| CAAT-box | Hordeum vulgare | 1419 | + | 4 | CAAT | common cis-acting element in promoter and enhancer regions |
| CAAT-box | Glycine max | 1481 | - | 5 | CAATT | common cis-acting element in promoter and enhancer regions |
| CAAT-box | Arabidopsis thaliana | 1247 | - | 5 | CCAAT | common cis-acting element in promoter and enhancer regions |
| CAAT-box | Hordeum vulgare | 1482 | - | 4 | CAAT | common cis-acting element in promoter and enhancer regions |
| CAAT-box | Petunia hybrida | 1296 | + | 7 | TGCCAAC | common cis-acting element in promoter and enhancer regions |
| CAAT-box | Brassica rapa | 1117 | + | 5 | CAAAT | common cis-acting element in promoter and enhancer regions |
| CAAT-box | Hordeum vulgare | 792 | + | 4 | CAAT | common cis-acting element in promoter and enhancer regions |
| CAAT-box | Brassica rapa | 1219 | - | 5 | CAAAT | common cis-acting element in promoter and enhancer regions |
| CAAT-box | Brassica rapa | 838 | - | 5 | CAAAT | common cis-acting element in promoter and enhancer regions |
| CAAT-box | Hordeum vulgare | 280 | - | 4 | CAAT | common cis-acting element in promoter and enhancer regions |
| CAAT-box | Brassica rapa | 239 | + | 5 | CAAAT | common cis-acting element in promoter and enhancer regions |

> 2018/04/13 10:10:12  
+ GAGAGGTTTT GGTAGGTTCT GTTGTGGGAA TCTATACCTA CCCATATTTA TATGTTTCTG GTTCTTCTCT   
  
  
+ CTTTTAGAAA TTTTTCCTTT CTACTACTTC GCTCGTGTTT GGCTCTTTTA TTTATGTTTT TCCTTTTCGT   
  
  
+ CTCTACTACT CTTTTCCCCC TTCCTCTTCC CCCTCCCCTT CCCCTTCCCC TTCCCAGCTC TCAGTTTGAC   
  
  
+ TACGGGAGGG TTACGTTCCG TAGCACAGCA AATACTCCTA TAACGGCAGT TTGGGGGCTA GGTAAGCATA   
  
  
+ TTGCGGTGCA CAGTCCCGAA AACAGTCCTG TAACTGGCGG AGAAATTCAC GCCCAGGTAC CGTTGCCCTA   
  
  
+ TTTTGGTCAA CCTCTGGCAC GTCTCTACTA CGGTTACTTC CTATTTCAGT TTTCTAACTC TACTTGGCAA   
  
  
+ CCTAACCATC GTCTTCTCTC TCTCTCTCTG TCTCTCTCTC TCTCTCCCAA AATCAACCTC TGTCACTCCA   
  
  
+ TCCCAACTGG AACTTGTCGA AAATTACGTT TTCAGTAACT CACGCCTCTA CCCCACATAC CCAGGCGGTC   
  
  
+ CGAGCAGAAA ATGGCCTGTG GCCTTCGACG CAGTTTCGCA GTAGGGTGTT CGCTGTACCC TAAGAAAGAG   
  
  
+ AGAGAAATCA AAAAGAAGAT GCCAAAAGAT GAGCACCGGG GGTGGTTGTA AAAGGTACTA GTGTAGACGT   
  
  
+ GGCAAGTATG AACAGCTCTA GCTAAGACCA TCCTCTCCAG TGTGGTACGT ACCTGAACAC TCCACCACCG   
  
  
+ GCAACTTTTT CAAAGAGCGG TCAATGAAGT ATATGTCATT ACTCTTCACT TCCTCAAACC AGTTTTGATT   
  
  
+ TGAACTAAAG TTTGTAAAGC GGCCTTAAAC ACGCTTAGTC TATTAATTCT AAAATAATTA TTTTTGGGTG   
  
  
+ AATAGTAAAG CCGATGTAAT TACCTTATTT ACCAACCTTG CTTCTCTAGG TGTGCGGTTT CGTTTAGCTA   
  
  
+ GTCTTTATTT CCAGTTGAAG TGTGCGAAAG AGTTCGAAGA GACTAGTTTA AAGTTAATTA CTGGTTTGGT   
  
  
+ TTTTGACAAG TGTCTAAACC GTTATCGATA CAAAGAAGAC AGAAGGAAAT AGGGTTAAAT TTTTGTCAAA   
  
  
+ TATATATCAT AAAAAATTAA ATATTATTAT GATATTTTTA TTTTTTTGAA TAAATAAATA AAATATTTTA   
  
  
+ ATAGTTCATA ATATAAAAAA TTTTTTTAAT TTGGGTGAAA TTTAATTAAC TTCTATATTG GCTTTCACAA   
  
  
+ ATATAACTTT TTTTTTTCAC ATTAACTATG AATTGTGCCA ACCTCTTTTC CTCCCTTCGA ATGTTCGTTT   
  
  
+ ATCGAACTGA ACCGTAAACT ACTCGCTGAA GTAAAACTTG AATTTCCAAC TACCCGGTGT AAGTGTAATA   
  
  
+ ATCAGTATGG TATAAAAACA ATGAAAATTA CCTTCTTTTG GGTTTTTTTT TTTTTTTTTC GTTTTTCTTC   
  
  
+ GTTTGGGAGA AATTGTTTAA AATAATTTA  

- CTCTCCAAAA CCATCCAAGA CAACACCCTT AGATATGGAT GGGTATAAAT ATACAAAGAC CAAGAAGAGA   
  
  
- GAAAATCTTT AAAAAGGAAA GATGATGAAG CGAGCACAAA CCGAGAAAAT AAATACAAAA AGGAAAAGCA   
  
  
- GAGATGATGA GAAAAGGGGG AAGGAGAAGG GGGAGGGGAA GGGGAAGGGG AAGGGTCGAG AGTCAAACTG   
  
  
- ATGCCCTCCC AATGCAAGGC ATCGTGTCGT TTATGAGGAT ATTGCCGTCA AACCCCCGAT CCATTCGTAT   
  
  
- AACGCCACGT GTCAGGGCTT TTGTCAGGAC ATTGACCGCC TCTTTAAGTG CGGGTCCATG GCAACGGGAT   
  
  
- AAAACCAGTT GGAGACCGTG CAGAGATGAT GCCAATGAAG GATAAAGTCA AAAGATTGAG ATGAACCGTT   
  
  
- GGATTGGTAG CAGAAGAGAG AGAGAGAGAC AGAGAGAGAG AGAGAGGGTT TTAGTTGGAG ACAGTGAGGT   
  
  
- AGGGTTGACC TTGAACAGCT TTTAATGCAA AAGTCATTGA GTGCGGAGAT GGGGTGTATG GGTCCGCCAG   
  
  
- GCTCGTCTTT TACCGGACAC CGGAAGCTGC GTCAAAGCGT CATCCCACAA GCGACATGGG ATTCTTTCTC   
  
  
- TCTCTTTAGT TTTTCTTCTA CGGTTTTCTA CTCGTGGCCC CCACCAACAT TTTCCATGAT CACATCTGCA   
  
  
- CCGTTCATAC TTGTCGAGAT CGATTCTGGT AGGAGAGGTC ACACCATGCA TGGACTTGTG AGGTGGTGGC   
  
  
- CGTTGAAAAA GTTTCTCGCC AGTTACTTCA TATACAGTAA TGAGAAGTGA AGGAGTTTGG TCAAAACTAA   
  
  
- ACTTGATTTC AAACATTTCG CCGGAATTTG TGCGAATCAG ATAATTAAGA TTTTATTAAT AAAAACCCAC   
  
  
- TTATCATTTC GGCTACATTA ATGGAATAAA TGGTTGGAAC GAAGAGATCC ACACGCCAAA GCAAATCGAT   
  
  
- CAGAAATAAA GGTCAACTTC ACACGCTTTC TCAAGCTTCT CTGATCAAAT TTCAATTAAT GACCAAACCA   
  
  
- AAAACTGTTC ACAGATTTGG CAATAGCTAT GTTTCTTCTG TCTTCCTTTA TCCCAATTTA AAAACAGTTT   
  
  
- ATATATAGTA TTTTTTAATT TATAATAATA CTATAAAAAT AAAAAAACTT ATTTATTTAT TTTATAAAAT   
  
  
- TATCAAGTAT TATATTTTTT AAAAAAATTA AACCCACTTT AAATTAATTG AAGATATAAC CGAAAGTGTT   
  
  
- TATATTGAAA AAAAAAAGTG TAATTGATAC TTAACACGGT TGGAGAAAAG GAGGGAAGCT TACAAGCAAA   
  
  
- TAGCTTGACT TGGCATTTGA TGAGCGACTT CATTTTGAAC TTAAAGGTTG ATGGGCCACA TTCACATTAT   
  
  
- TAGTCATACC ATATTTTTGT TACTTTTAAT GGAAGAAAAC CCAAAAAAAA AAAAAAAAAG CAAAAAGAAG   
  
  
- CAAACCCTCT TTAACAAATT TTATTAAAT

+     CCAAT-box

| Site Name | Organism | Position | Strand | Matrix score. | sequence | function |
| --- | --- | --- | --- | --- | --- | --- |
| CCAAT-box | Hordeum vulgare | 340 | - | 6 | CAACGG | MYBHv1 binding site |

> 2018/04/13 10:10:12  
+ GAGAGGTTTT GGTAGGTTCT GTTGTGGGAA TCTATACCTA CCCATATTTA TATGTTTCTG GTTCTTCTCT   
  
  
+ CTTTTAGAAA TTTTTCCTTT CTACTACTTC GCTCGTGTTT GGCTCTTTTA TTTATGTTTT TCCTTTTCGT   
  
  
+ CTCTACTACT CTTTTCCCCC TTCCTCTTCC CCCTCCCCTT CCCCTTCCCC TTCCCAGCTC TCAGTTTGAC   
  
  
+ TACGGGAGGG TTACGTTCCG TAGCACAGCA AATACTCCTA TAACGGCAGT TTGGGGGCTA GGTAAGCATA   
  
  
+ TTGCGGTGCA CAGTCCCGAA AACAGTCCTG TAACTGGCGG AGAAATTCAC GCCCAGGTAC CGTTGCCCTA   
  
  
+ TTTTGGTCAA CCTCTGGCAC GTCTCTACTA CGGTTACTTC CTATTTCAGT TTTCTAACTC TACTTGGCAA   
  
  
+ CCTAACCATC GTCTTCTCTC TCTCTCTCTG TCTCTCTCTC TCTCTCCCAA AATCAACCTC TGTCACTCCA   
  
  
+ TCCCAACTGG AACTTGTCGA AAATTACGTT TTCAGTAACT CACGCCTCTA CCCCACATAC CCAGGCGGTC   
  
  
+ CGAGCAGAAA ATGGCCTGTG GCCTTCGACG CAGTTTCGCA GTAGGGTGTT CGCTGTACCC TAAGAAAGAG   
  
  
+ AGAGAAATCA AAAAGAAGAT GCCAAAAGAT GAGCACCGGG GGTGGTTGTA AAAGGTACTA GTGTAGACGT   
  
  
+ GGCAAGTATG AACAGCTCTA GCTAAGACCA TCCTCTCCAG TGTGGTACGT ACCTGAACAC TCCACCACCG   
  
  
+ GCAACTTTTT CAAAGAGCGG TCAATGAAGT ATATGTCATT ACTCTTCACT TCCTCAAACC AGTTTTGATT   
  
  
+ TGAACTAAAG TTTGTAAAGC GGCCTTAAAC ACGCTTAGTC TATTAATTCT AAAATAATTA TTTTTGGGTG   
  
  
+ AATAGTAAAG CCGATGTAAT TACCTTATTT ACCAACCTTG CTTCTCTAGG TGTGCGGTTT CGTTTAGCTA   
  
  
+ GTCTTTATTT CCAGTTGAAG TGTGCGAAAG AGTTCGAAGA GACTAGTTTA AAGTTAATTA CTGGTTTGGT   
  
  
+ TTTTGACAAG TGTCTAAACC GTTATCGATA CAAAGAAGAC AGAAGGAAAT AGGGTTAAAT TTTTGTCAAA   
  
  
+ TATATATCAT AAAAAATTAA ATATTATTAT GATATTTTTA TTTTTTTGAA TAAATAAATA AAATATTTTA   
  
  
+ ATAGTTCATA ATATAAAAAA TTTTTTTAAT TTGGGTGAAA TTTAATTAAC TTCTATATTG GCTTTCACAA   
  
  
+ ATATAACTTT TTTTTTTCAC ATTAACTATG AATTGTGCCA ACCTCTTTTC CTCCCTTCGA ATGTTCGTTT   
  
  
+ ATCGAACTGA ACCGTAAACT ACTCGCTGAA GTAAAACTTG AATTTCCAAC TACCCGGTGT AAGTGTAATA   
  
  
+ ATCAGTATGG TATAAAAACA ATGAAAATTA CCTTCTTTTG GGTTTTTTTT TTTTTTTTTC GTTTTTCTTC   
  
  
+ GTTTGGGAGA AATTGTTTAA AATAATTTA  

- CTCTCCAAAA CCATCCAAGA CAACACCCTT AGATATGGAT GGGTATAAAT ATACAAAGAC CAAGAAGAGA   
  
  
- GAAAATCTTT AAAAAGGAAA GATGATGAAG CGAGCACAAA CCGAGAAAAT AAATACAAAA AGGAAAAGCA   
  
  
- GAGATGATGA GAAAAGGGGG AAGGAGAAGG GGGAGGGGAA GGGGAAGGGG AAGGGTCGAG AGTCAAACTG   
  
  
- ATGCCCTCCC AATGCAAGGC ATCGTGTCGT TTATGAGGAT ATTGCCGTCA AACCCCCGAT CCATTCGTAT   
  
  
- AACGCCACGT GTCAGGGCTT TTGTCAGGAC ATTGACCGCC TCTTTAAGTG CGGGTCCATG GCAACGGGAT   
  
  
- AAAACCAGTT GGAGACCGTG CAGAGATGAT GCCAATGAAG GATAAAGTCA AAAGATTGAG ATGAACCGTT   
  
  
- GGATTGGTAG CAGAAGAGAG AGAGAGAGAC AGAGAGAGAG AGAGAGGGTT TTAGTTGGAG ACAGTGAGGT   
  
  
- AGGGTTGACC TTGAACAGCT TTTAATGCAA AAGTCATTGA GTGCGGAGAT GGGGTGTATG GGTCCGCCAG   
  
  
- GCTCGTCTTT TACCGGACAC CGGAAGCTGC GTCAAAGCGT CATCCCACAA GCGACATGGG ATTCTTTCTC   
  
  
- TCTCTTTAGT TTTTCTTCTA CGGTTTTCTA CTCGTGGCCC CCACCAACAT TTTCCATGAT CACATCTGCA   
  
  
- CCGTTCATAC TTGTCGAGAT CGATTCTGGT AGGAGAGGTC ACACCATGCA TGGACTTGTG AGGTGGTGGC   
  
  
- CGTTGAAAAA GTTTCTCGCC AGTTACTTCA TATACAGTAA TGAGAAGTGA AGGAGTTTGG TCAAAACTAA   
  
  
- ACTTGATTTC AAACATTTCG CCGGAATTTG TGCGAATCAG ATAATTAAGA TTTTATTAAT AAAAACCCAC   
  
  
- TTATCATTTC GGCTACATTA ATGGAATAAA TGGTTGGAAC GAAGAGATCC ACACGCCAAA GCAAATCGAT   
  
  
- CAGAAATAAA GGTCAACTTC ACACGCTTTC TCAAGCTTCT CTGATCAAAT TTCAATTAAT GACCAAACCA   
  
  
- AAAACTGTTC ACAGATTTGG CAATAGCTAT GTTTCTTCTG TCTTCCTTTA TCCCAATTTA AAAACAGTTT   
  
  
- ATATATAGTA TTTTTTAATT TATAATAATA CTATAAAAAT AAAAAAACTT ATTTATTTAT TTTATAAAAT   
  
  
- TATCAAGTAT TATATTTTTT AAAAAAATTA AACCCACTTT AAATTAATTG AAGATATAAC CGAAAGTGTT   
  
  
- TATATTGAAA AAAAAAAGTG TAATTGATAC TTAACACGGT TGGAGAAAAG GAGGGAAGCT TACAAGCAAA   
  
  
- TAGCTTGACT TGGCATTTGA TGAGCGACTT CATTTTGAAC TTAAAGGTTG ATGGGCCACA TTCACATTAT   
  
  
- TAGTCATACC ATATTTTTGT TACTTTTAAT GGAAGAAAAC CCAAAAAAAA AAAAAAAAAG CAAAAAGAAG   
  
  
- CAAACCCTCT TTAACAAATT TTATTAAAT

+     G-box

| Site Name | Organism | Position | Strand | Matrix score. | sequence | function |
| --- | --- | --- | --- | --- | --- | --- |
| G-box | Zea mays | 696 | - | 6 | CACGTC | cis-acting regulatory element involved in light responsiveness |
| G-box | Zea mays | 368 | + | 6 | CACGTC | cis-acting regulatory element involved in light responsiveness |
| G-box | Nicotiana plumbaginifolia | 694 | + | 10 | CAGACGTGGCA | cis-acting regulatory element involved in light responsiveness |

> 2018/04/13 10:10:12  
+ GAGAGGTTTT GGTAGGTTCT GTTGTGGGAA TCTATACCTA CCCATATTTA TATGTTTCTG GTTCTTCTCT   
  
  
+ CTTTTAGAAA TTTTTCCTTT CTACTACTTC GCTCGTGTTT GGCTCTTTTA TTTATGTTTT TCCTTTTCGT   
  
  
+ CTCTACTACT CTTTTCCCCC TTCCTCTTCC CCCTCCCCTT CCCCTTCCCC TTCCCAGCTC TCAGTTTGAC   
  
  
+ TACGGGAGGG TTACGTTCCG TAGCACAGCA AATACTCCTA TAACGGCAGT TTGGGGGCTA GGTAAGCATA   
  
  
+ TTGCGGTGCA CAGTCCCGAA AACAGTCCTG TAACTGGCGG AGAAATTCAC GCCCAGGTAC CGTTGCCCTA   
  
  
+ TTTTGGTCAA CCTCTGGCAC GTCTCTACTA CGGTTACTTC CTATTTCAGT TTTCTAACTC TACTTGGCAA   
  
  
+ CCTAACCATC GTCTTCTCTC TCTCTCTCTG TCTCTCTCTC TCTCTCCCAA AATCAACCTC TGTCACTCCA   
  
  
+ TCCCAACTGG AACTTGTCGA AAATTACGTT TTCAGTAACT CACGCCTCTA CCCCACATAC CCAGGCGGTC   
  
  
+ CGAGCAGAAA ATGGCCTGTG GCCTTCGACG CAGTTTCGCA GTAGGGTGTT CGCTGTACCC TAAGAAAGAG   
  
  
+ AGAGAAATCA AAAAGAAGAT GCCAAAAGAT GAGCACCGGG GGTGGTTGTA AAAGGTACTA GTGTAGACGT   
  
  
+ GGCAAGTATG AACAGCTCTA GCTAAGACCA TCCTCTCCAG TGTGGTACGT ACCTGAACAC TCCACCACCG   
  
  
+ GCAACTTTTT CAAAGAGCGG TCAATGAAGT ATATGTCATT ACTCTTCACT TCCTCAAACC AGTTTTGATT   
  
  
+ TGAACTAAAG TTTGTAAAGC GGCCTTAAAC ACGCTTAGTC TATTAATTCT AAAATAATTA TTTTTGGGTG   
  
  
+ AATAGTAAAG CCGATGTAAT TACCTTATTT ACCAACCTTG CTTCTCTAGG TGTGCGGTTT CGTTTAGCTA   
  
  
+ GTCTTTATTT CCAGTTGAAG TGTGCGAAAG AGTTCGAAGA GACTAGTTTA AAGTTAATTA CTGGTTTGGT   
  
  
+ TTTTGACAAG TGTCTAAACC GTTATCGATA CAAAGAAGAC AGAAGGAAAT AGGGTTAAAT TTTTGTCAAA   
  
  
+ TATATATCAT AAAAAATTAA ATATTATTAT GATATTTTTA TTTTTTTGAA TAAATAAATA AAATATTTTA   
  
  
+ ATAGTTCATA ATATAAAAAA TTTTTTTAAT TTGGGTGAAA TTTAATTAAC TTCTATATTG GCTTTCACAA   
  
  
+ ATATAACTTT TTTTTTTCAC ATTAACTATG AATTGTGCCA ACCTCTTTTC CTCCCTTCGA ATGTTCGTTT   
  
  
+ ATCGAACTGA ACCGTAAACT ACTCGCTGAA GTAAAACTTG AATTTCCAAC TACCCGGTGT AAGTGTAATA   
  
  
+ ATCAGTATGG TATAAAAACA ATGAAAATTA CCTTCTTTTG GGTTTTTTTT TTTTTTTTTC GTTTTTCTTC   
  
  
+ GTTTGGGAGA AATTGTTTAA AATAATTTA  

- CTCTCCAAAA CCATCCAAGA CAACACCCTT AGATATGGAT GGGTATAAAT ATACAAAGAC CAAGAAGAGA   
  
  
- GAAAATCTTT AAAAAGGAAA GATGATGAAG CGAGCACAAA CCGAGAAAAT AAATACAAAA AGGAAAAGCA   
  
  
- GAGATGATGA GAAAAGGGGG AAGGAGAAGG GGGAGGGGAA GGGGAAGGGG AAGGGTCGAG AGTCAAACTG   
  
  
- ATGCCCTCCC AATGCAAGGC ATCGTGTCGT TTATGAGGAT ATTGCCGTCA AACCCCCGAT CCATTCGTAT   
  
  
- AACGCCACGT GTCAGGGCTT TTGTCAGGAC ATTGACCGCC TCTTTAAGTG CGGGTCCATG GCAACGGGAT   
  
  
- AAAACCAGTT GGAGACCGTG CAGAGATGAT GCCAATGAAG GATAAAGTCA AAAGATTGAG ATGAACCGTT   
  
  
- GGATTGGTAG CAGAAGAGAG AGAGAGAGAC AGAGAGAGAG AGAGAGGGTT TTAGTTGGAG ACAGTGAGGT   
  
  
- AGGGTTGACC TTGAACAGCT TTTAATGCAA AAGTCATTGA GTGCGGAGAT GGGGTGTATG GGTCCGCCAG   
  
  
- GCTCGTCTTT TACCGGACAC CGGAAGCTGC GTCAAAGCGT CATCCCACAA GCGACATGGG ATTCTTTCTC   
  
  
- TCTCTTTAGT TTTTCTTCTA CGGTTTTCTA CTCGTGGCCC CCACCAACAT TTTCCATGAT CACATCTGCA   
  
  
- CCGTTCATAC TTGTCGAGAT CGATTCTGGT AGGAGAGGTC ACACCATGCA TGGACTTGTG AGGTGGTGGC   
  
  
- CGTTGAAAAA GTTTCTCGCC AGTTACTTCA TATACAGTAA TGAGAAGTGA AGGAGTTTGG TCAAAACTAA   
  
  
- ACTTGATTTC AAACATTTCG CCGGAATTTG TGCGAATCAG ATAATTAAGA TTTTATTAAT AAAAACCCAC   
  
  
- TTATCATTTC GGCTACATTA ATGGAATAAA TGGTTGGAAC GAAGAGATCC ACACGCCAAA GCAAATCGAT   
  
  
- CAGAAATAAA GGTCAACTTC ACACGCTTTC TCAAGCTTCT CTGATCAAAT TTCAATTAAT GACCAAACCA   
  
  
- AAAACTGTTC ACAGATTTGG CAATAGCTAT GTTTCTTCTG TCTTCCTTTA TCCCAATTTA AAAACAGTTT   
  
  
- ATATATAGTA TTTTTTAATT TATAATAATA CTATAAAAAT AAAAAAACTT ATTTATTTAT TTTATAAAAT   
  
  
- TATCAAGTAT TATATTTTTT AAAAAAATTA AACCCACTTT AAATTAATTG AAGATATAAC CGAAAGTGTT   
  
  
- TATATTGAAA AAAAAAAGTG TAATTGATAC TTAACACGGT TGGAGAAAAG GAGGGAAGCT TACAAGCAAA   
  
  
- TAGCTTGACT TGGCATTTGA TGAGCGACTT CATTTTGAAC TTAAAGGTTG ATGGGCCACA TTCACATTAT   
  
  
- TAGTCATACC ATATTTTTGT TACTTTTAAT GGAAGAAAAC CCAAAAAAAA AAAAAAAAAG CAAAAAGAAG   
  
  
- CAAACCCTCT TTAACAAATT TTATTAAAT

+     GA-motif

| Site Name | Organism | Position | Strand | Matrix score. | sequence | function |
| --- | --- | --- | --- | --- | --- | --- |
| GA-motif | Helianthus annuus | 655 | + | 8 | AAAGATGA | part of a light responsive element |

> 2018/04/13 10:10:12  
+ GAGAGGTTTT GGTAGGTTCT GTTGTGGGAA TCTATACCTA CCCATATTTA TATGTTTCTG GTTCTTCTCT   
  
  
+ CTTTTAGAAA TTTTTCCTTT CTACTACTTC GCTCGTGTTT GGCTCTTTTA TTTATGTTTT TCCTTTTCGT   
  
  
+ CTCTACTACT CTTTTCCCCC TTCCTCTTCC CCCTCCCCTT CCCCTTCCCC TTCCCAGCTC TCAGTTTGAC   
  
  
+ TACGGGAGGG TTACGTTCCG TAGCACAGCA AATACTCCTA TAACGGCAGT TTGGGGGCTA GGTAAGCATA   
  
  
+ TTGCGGTGCA CAGTCCCGAA AACAGTCCTG TAACTGGCGG AGAAATTCAC GCCCAGGTAC CGTTGCCCTA   
  
  
+ TTTTGGTCAA CCTCTGGCAC GTCTCTACTA CGGTTACTTC CTATTTCAGT TTTCTAACTC TACTTGGCAA   
  
  
+ CCTAACCATC GTCTTCTCTC TCTCTCTCTG TCTCTCTCTC TCTCTCCCAA AATCAACCTC TGTCACTCCA   
  
  
+ TCCCAACTGG AACTTGTCGA AAATTACGTT TTCAGTAACT CACGCCTCTA CCCCACATAC CCAGGCGGTC   
  
  
+ CGAGCAGAAA ATGGCCTGTG GCCTTCGACG CAGTTTCGCA GTAGGGTGTT CGCTGTACCC TAAGAAAGAG   
  
  
+ AGAGAAATCA AAAAGAAGAT GCCAAAAGAT GAGCACCGGG GGTGGTTGTA AAAGGTACTA GTGTAGACGT   
  
  
+ GGCAAGTATG AACAGCTCTA GCTAAGACCA TCCTCTCCAG TGTGGTACGT ACCTGAACAC TCCACCACCG   
  
  
+ GCAACTTTTT CAAAGAGCGG TCAATGAAGT ATATGTCATT ACTCTTCACT TCCTCAAACC AGTTTTGATT   
  
  
+ TGAACTAAAG TTTGTAAAGC GGCCTTAAAC ACGCTTAGTC TATTAATTCT AAAATAATTA TTTTTGGGTG   
  
  
+ AATAGTAAAG CCGATGTAAT TACCTTATTT ACCAACCTTG CTTCTCTAGG TGTGCGGTTT CGTTTAGCTA   
  
  
+ GTCTTTATTT CCAGTTGAAG TGTGCGAAAG AGTTCGAAGA GACTAGTTTA AAGTTAATTA CTGGTTTGGT   
  
  
+ TTTTGACAAG TGTCTAAACC GTTATCGATA CAAAGAAGAC AGAAGGAAAT AGGGTTAAAT TTTTGTCAAA   
  
  
+ TATATATCAT AAAAAATTAA ATATTATTAT GATATTTTTA TTTTTTTGAA TAAATAAATA AAATATTTTA   
  
  
+ ATAGTTCATA ATATAAAAAA TTTTTTTAAT TTGGGTGAAA TTTAATTAAC TTCTATATTG GCTTTCACAA   
  
  
+ ATATAACTTT TTTTTTTCAC ATTAACTATG AATTGTGCCA ACCTCTTTTC CTCCCTTCGA ATGTTCGTTT   
  
  
+ ATCGAACTGA ACCGTAAACT ACTCGCTGAA GTAAAACTTG AATTTCCAAC TACCCGGTGT AAGTGTAATA   
  
  
+ ATCAGTATGG TATAAAAACA ATGAAAATTA CCTTCTTTTG GGTTTTTTTT TTTTTTTTTC GTTTTTCTTC   
  
  
+ GTTTGGGAGA AATTGTTTAA AATAATTTA  

- CTCTCCAAAA CCATCCAAGA CAACACCCTT AGATATGGAT GGGTATAAAT ATACAAAGAC CAAGAAGAGA   
  
  
- GAAAATCTTT AAAAAGGAAA GATGATGAAG CGAGCACAAA CCGAGAAAAT AAATACAAAA AGGAAAAGCA   
  
  
- GAGATGATGA GAAAAGGGGG AAGGAGAAGG GGGAGGGGAA GGGGAAGGGG AAGGGTCGAG AGTCAAACTG   
  
  
- ATGCCCTCCC AATGCAAGGC ATCGTGTCGT TTATGAGGAT ATTGCCGTCA AACCCCCGAT CCATTCGTAT   
  
  
- AACGCCACGT GTCAGGGCTT TTGTCAGGAC ATTGACCGCC TCTTTAAGTG CGGGTCCATG GCAACGGGAT   
  
  
- AAAACCAGTT GGAGACCGTG CAGAGATGAT GCCAATGAAG GATAAAGTCA AAAGATTGAG ATGAACCGTT   
  
  
- GGATTGGTAG CAGAAGAGAG AGAGAGAGAC AGAGAGAGAG AGAGAGGGTT TTAGTTGGAG ACAGTGAGGT   
  
  
- AGGGTTGACC TTGAACAGCT TTTAATGCAA AAGTCATTGA GTGCGGAGAT GGGGTGTATG GGTCCGCCAG   
  
  
- GCTCGTCTTT TACCGGACAC CGGAAGCTGC GTCAAAGCGT CATCCCACAA GCGACATGGG ATTCTTTCTC   
  
  
- TCTCTTTAGT TTTTCTTCTA CGGTTTTCTA CTCGTGGCCC CCACCAACAT TTTCCATGAT CACATCTGCA   
  
  
- CCGTTCATAC TTGTCGAGAT CGATTCTGGT AGGAGAGGTC ACACCATGCA TGGACTTGTG AGGTGGTGGC   
  
  
- CGTTGAAAAA GTTTCTCGCC AGTTACTTCA TATACAGTAA TGAGAAGTGA AGGAGTTTGG TCAAAACTAA   
  
  
- ACTTGATTTC AAACATTTCG CCGGAATTTG TGCGAATCAG ATAATTAAGA TTTTATTAAT AAAAACCCAC   
  
  
- TTATCATTTC GGCTACATTA ATGGAATAAA TGGTTGGAAC GAAGAGATCC ACACGCCAAA GCAAATCGAT   
  
  
- CAGAAATAAA GGTCAACTTC ACACGCTTTC TCAAGCTTCT CTGATCAAAT TTCAATTAAT GACCAAACCA   
  
  
- AAAACTGTTC ACAGATTTGG CAATAGCTAT GTTTCTTCTG TCTTCCTTTA TCCCAATTTA AAAACAGTTT   
  
  
- ATATATAGTA TTTTTTAATT TATAATAATA CTATAAAAAT AAAAAAACTT ATTTATTTAT TTTATAAAAT   
  
  
- TATCAAGTAT TATATTTTTT AAAAAAATTA AACCCACTTT AAATTAATTG AAGATATAAC CGAAAGTGTT   
  
  
- TATATTGAAA AAAAAAAGTG TAATTGATAC TTAACACGGT TGGAGAAAAG GAGGGAAGCT TACAAGCAAA   
  
  
- TAGCTTGACT TGGCATTTGA TGAGCGACTT CATTTTGAAC TTAAAGGTTG ATGGGCCACA TTCACATTAT   
  
  
- TAGTCATACC ATATTTTTGT TACTTTTAAT GGAAGAAAAC CCAAAAAAAA AAAAAAAAAG CAAAAAGAAG   
  
  
- CAAACCCTCT TTAACAAATT TTATTAAAT

+     GARE-motif

| Site Name | Organism | Position | Strand | Matrix score. | sequence | function |
| --- | --- | --- | --- | --- | --- | --- |
| GARE-motif | Brassica oleracea | 18 | + | 7 | TCTGTTG | gibberellin-responsive element |

> 2018/04/13 10:10:12  
+ GAGAGGTTTT GGTAGGTTCT GTTGTGGGAA TCTATACCTA CCCATATTTA TATGTTTCTG GTTCTTCTCT   
  
  
+ CTTTTAGAAA TTTTTCCTTT CTACTACTTC GCTCGTGTTT GGCTCTTTTA TTTATGTTTT TCCTTTTCGT   
  
  
+ CTCTACTACT CTTTTCCCCC TTCCTCTTCC CCCTCCCCTT CCCCTTCCCC TTCCCAGCTC TCAGTTTGAC   
  
  
+ TACGGGAGGG TTACGTTCCG TAGCACAGCA AATACTCCTA TAACGGCAGT TTGGGGGCTA GGTAAGCATA   
  
  
+ TTGCGGTGCA CAGTCCCGAA AACAGTCCTG TAACTGGCGG AGAAATTCAC GCCCAGGTAC CGTTGCCCTA   
  
  
+ TTTTGGTCAA CCTCTGGCAC GTCTCTACTA CGGTTACTTC CTATTTCAGT TTTCTAACTC TACTTGGCAA   
  
  
+ CCTAACCATC GTCTTCTCTC TCTCTCTCTG TCTCTCTCTC TCTCTCCCAA AATCAACCTC TGTCACTCCA   
  
  
+ TCCCAACTGG AACTTGTCGA AAATTACGTT TTCAGTAACT CACGCCTCTA CCCCACATAC CCAGGCGGTC   
  
  
+ CGAGCAGAAA ATGGCCTGTG GCCTTCGACG CAGTTTCGCA GTAGGGTGTT CGCTGTACCC TAAGAAAGAG   
  
  
+ AGAGAAATCA AAAAGAAGAT GCCAAAAGAT GAGCACCGGG GGTGGTTGTA AAAGGTACTA GTGTAGACGT   
  
  
+ GGCAAGTATG AACAGCTCTA GCTAAGACCA TCCTCTCCAG TGTGGTACGT ACCTGAACAC TCCACCACCG   
  
  
+ GCAACTTTTT CAAAGAGCGG TCAATGAAGT ATATGTCATT ACTCTTCACT TCCTCAAACC AGTTTTGATT   
  
  
+ TGAACTAAAG TTTGTAAAGC GGCCTTAAAC ACGCTTAGTC TATTAATTCT AAAATAATTA TTTTTGGGTG   
  
  
+ AATAGTAAAG CCGATGTAAT TACCTTATTT ACCAACCTTG CTTCTCTAGG TGTGCGGTTT CGTTTAGCTA   
  
  
+ GTCTTTATTT CCAGTTGAAG TGTGCGAAAG AGTTCGAAGA GACTAGTTTA AAGTTAATTA CTGGTTTGGT   
  
  
+ TTTTGACAAG TGTCTAAACC GTTATCGATA CAAAGAAGAC AGAAGGAAAT AGGGTTAAAT TTTTGTCAAA   
  
  
+ TATATATCAT AAAAAATTAA ATATTATTAT GATATTTTTA TTTTTTTGAA TAAATAAATA AAATATTTTA   
  
  
+ ATAGTTCATA ATATAAAAAA TTTTTTTAAT TTGGGTGAAA TTTAATTAAC TTCTATATTG GCTTTCACAA   
  
  
+ ATATAACTTT TTTTTTTCAC ATTAACTATG AATTGTGCCA ACCTCTTTTC CTCCCTTCGA ATGTTCGTTT   
  
  
+ ATCGAACTGA ACCGTAAACT ACTCGCTGAA GTAAAACTTG AATTTCCAAC TACCCGGTGT AAGTGTAATA   
  
  
+ ATCAGTATGG TATAAAAACA ATGAAAATTA CCTTCTTTTG GGTTTTTTTT TTTTTTTTTC GTTTTTCTTC   
  
  
+ GTTTGGGAGA AATTGTTTAA AATAATTTA  

- CTCTCCAAAA CCATCCAAGA CAACACCCTT AGATATGGAT GGGTATAAAT ATACAAAGAC CAAGAAGAGA   
  
  
- GAAAATCTTT AAAAAGGAAA GATGATGAAG CGAGCACAAA CCGAGAAAAT AAATACAAAA AGGAAAAGCA   
  
  
- GAGATGATGA GAAAAGGGGG AAGGAGAAGG GGGAGGGGAA GGGGAAGGGG AAGGGTCGAG AGTCAAACTG   
  
  
- ATGCCCTCCC AATGCAAGGC ATCGTGTCGT TTATGAGGAT ATTGCCGTCA AACCCCCGAT CCATTCGTAT   
  
  
- AACGCCACGT GTCAGGGCTT TTGTCAGGAC ATTGACCGCC TCTTTAAGTG CGGGTCCATG GCAACGGGAT   
  
  
- AAAACCAGTT GGAGACCGTG CAGAGATGAT GCCAATGAAG GATAAAGTCA AAAGATTGAG ATGAACCGTT   
  
  
- GGATTGGTAG CAGAAGAGAG AGAGAGAGAC AGAGAGAGAG AGAGAGGGTT TTAGTTGGAG ACAGTGAGGT   
  
  
- AGGGTTGACC TTGAACAGCT TTTAATGCAA AAGTCATTGA GTGCGGAGAT GGGGTGTATG GGTCCGCCAG   
  
  
- GCTCGTCTTT TACCGGACAC CGGAAGCTGC GTCAAAGCGT CATCCCACAA GCGACATGGG ATTCTTTCTC   
  
  
- TCTCTTTAGT TTTTCTTCTA CGGTTTTCTA CTCGTGGCCC CCACCAACAT TTTCCATGAT CACATCTGCA   
  
  
- CCGTTCATAC TTGTCGAGAT CGATTCTGGT AGGAGAGGTC ACACCATGCA TGGACTTGTG AGGTGGTGGC   
  
  
- CGTTGAAAAA GTTTCTCGCC AGTTACTTCA TATACAGTAA TGAGAAGTGA AGGAGTTTGG TCAAAACTAA   
  
  
- ACTTGATTTC AAACATTTCG CCGGAATTTG TGCGAATCAG ATAATTAAGA TTTTATTAAT AAAAACCCAC   
  
  
- TTATCATTTC GGCTACATTA ATGGAATAAA TGGTTGGAAC GAAGAGATCC ACACGCCAAA GCAAATCGAT   
  
  
- CAGAAATAAA GGTCAACTTC ACACGCTTTC TCAAGCTTCT CTGATCAAAT TTCAATTAAT GACCAAACCA   
  
  
- AAAACTGTTC ACAGATTTGG CAATAGCTAT GTTTCTTCTG TCTTCCTTTA TCCCAATTTA AAAACAGTTT   
  
  
- ATATATAGTA TTTTTTAATT TATAATAATA CTATAAAAAT AAAAAAACTT ATTTATTTAT TTTATAAAAT   
  
  
- TATCAAGTAT TATATTTTTT AAAAAAATTA AACCCACTTT AAATTAATTG AAGATATAAC CGAAAGTGTT   
  
  
- TATATTGAAA AAAAAAAGTG TAATTGATAC TTAACACGGT TGGAGAAAAG GAGGGAAGCT TACAAGCAAA   
  
  
- TAGCTTGACT TGGCATTTGA TGAGCGACTT CATTTTGAAC TTAAAGGTTG ATGGGCCACA TTCACATTAT   
  
  
- TAGTCATACC ATATTTTTGT TACTTTTAAT GGAAGAAAAC CCAAAAAAAA AAAAAAAAAG CAAAAAGAAG   
  
  
- CAAACCCTCT TTAACAAATT TTATTAAAT

+     GC-motif

| Site Name | Organism | Position | Strand | Matrix score. | sequence | function |
| --- | --- | --- | --- | --- | --- | --- |
| GC-motif | Zea mays | 667 | - | 6 | CCCCCG | enhancer-like element involved in anoxic specific inducibility |

> 2018/04/13 10:10:12  
+ GAGAGGTTTT GGTAGGTTCT GTTGTGGGAA TCTATACCTA CCCATATTTA TATGTTTCTG GTTCTTCTCT   
  
  
+ CTTTTAGAAA TTTTTCCTTT CTACTACTTC GCTCGTGTTT GGCTCTTTTA TTTATGTTTT TCCTTTTCGT   
  
  
+ CTCTACTACT CTTTTCCCCC TTCCTCTTCC CCCTCCCCTT CCCCTTCCCC TTCCCAGCTC TCAGTTTGAC   
  
  
+ TACGGGAGGG TTACGTTCCG TAGCACAGCA AATACTCCTA TAACGGCAGT TTGGGGGCTA GGTAAGCATA   
  
  
+ TTGCGGTGCA CAGTCCCGAA AACAGTCCTG TAACTGGCGG AGAAATTCAC GCCCAGGTAC CGTTGCCCTA   
  
  
+ TTTTGGTCAA CCTCTGGCAC GTCTCTACTA CGGTTACTTC CTATTTCAGT TTTCTAACTC TACTTGGCAA   
  
  
+ CCTAACCATC GTCTTCTCTC TCTCTCTCTG TCTCTCTCTC TCTCTCCCAA AATCAACCTC TGTCACTCCA   
  
  
+ TCCCAACTGG AACTTGTCGA AAATTACGTT TTCAGTAACT CACGCCTCTA CCCCACATAC CCAGGCGGTC   
  
  
+ CGAGCAGAAA ATGGCCTGTG GCCTTCGACG CAGTTTCGCA GTAGGGTGTT CGCTGTACCC TAAGAAAGAG   
  
  
+ AGAGAAATCA AAAAGAAGAT GCCAAAAGAT GAGCACCGGG GGTGGTTGTA AAAGGTACTA GTGTAGACGT   
  
  
+ GGCAAGTATG AACAGCTCTA GCTAAGACCA TCCTCTCCAG TGTGGTACGT ACCTGAACAC TCCACCACCG   
  
  
+ GCAACTTTTT CAAAGAGCGG TCAATGAAGT ATATGTCATT ACTCTTCACT TCCTCAAACC AGTTTTGATT   
  
  
+ TGAACTAAAG TTTGTAAAGC GGCCTTAAAC ACGCTTAGTC TATTAATTCT AAAATAATTA TTTTTGGGTG   
  
  
+ AATAGTAAAG CCGATGTAAT TACCTTATTT ACCAACCTTG CTTCTCTAGG TGTGCGGTTT CGTTTAGCTA   
  
  
+ GTCTTTATTT CCAGTTGAAG TGTGCGAAAG AGTTCGAAGA GACTAGTTTA AAGTTAATTA CTGGTTTGGT   
  
  
+ TTTTGACAAG TGTCTAAACC GTTATCGATA CAAAGAAGAC AGAAGGAAAT AGGGTTAAAT TTTTGTCAAA   
  
  
+ TATATATCAT AAAAAATTAA ATATTATTAT GATATTTTTA TTTTTTTGAA TAAATAAATA AAATATTTTA   
  
  
+ ATAGTTCATA ATATAAAAAA TTTTTTTAAT TTGGGTGAAA TTTAATTAAC TTCTATATTG GCTTTCACAA   
  
  
+ ATATAACTTT TTTTTTTCAC ATTAACTATG AATTGTGCCA ACCTCTTTTC CTCCCTTCGA ATGTTCGTTT   
  
  
+ ATCGAACTGA ACCGTAAACT ACTCGCTGAA GTAAAACTTG AATTTCCAAC TACCCGGTGT AAGTGTAATA   
  
  
+ ATCAGTATGG TATAAAAACA ATGAAAATTA CCTTCTTTTG GGTTTTTTTT TTTTTTTTTC GTTTTTCTTC   
  
  
+ GTTTGGGAGA AATTGTTTAA AATAATTTA  

- CTCTCCAAAA CCATCCAAGA CAACACCCTT AGATATGGAT GGGTATAAAT ATACAAAGAC CAAGAAGAGA   
  
  
- GAAAATCTTT AAAAAGGAAA GATGATGAAG CGAGCACAAA CCGAGAAAAT AAATACAAAA AGGAAAAGCA   
  
  
- GAGATGATGA GAAAAGGGGG AAGGAGAAGG GGGAGGGGAA GGGGAAGGGG AAGGGTCGAG AGTCAAACTG   
  
  
- ATGCCCTCCC AATGCAAGGC ATCGTGTCGT TTATGAGGAT ATTGCCGTCA AACCCCCGAT CCATTCGTAT   
  
  
- AACGCCACGT GTCAGGGCTT TTGTCAGGAC ATTGACCGCC TCTTTAAGTG CGGGTCCATG GCAACGGGAT   
  
  
- AAAACCAGTT GGAGACCGTG CAGAGATGAT GCCAATGAAG GATAAAGTCA AAAGATTGAG ATGAACCGTT   
  
  
- GGATTGGTAG CAGAAGAGAG AGAGAGAGAC AGAGAGAGAG AGAGAGGGTT TTAGTTGGAG ACAGTGAGGT   
  
  
- AGGGTTGACC TTGAACAGCT TTTAATGCAA AAGTCATTGA GTGCGGAGAT GGGGTGTATG GGTCCGCCAG   
  
  
- GCTCGTCTTT TACCGGACAC CGGAAGCTGC GTCAAAGCGT CATCCCACAA GCGACATGGG ATTCTTTCTC   
  
  
- TCTCTTTAGT TTTTCTTCTA CGGTTTTCTA CTCGTGGCCC CCACCAACAT TTTCCATGAT CACATCTGCA   
  
  
- CCGTTCATAC TTGTCGAGAT CGATTCTGGT AGGAGAGGTC ACACCATGCA TGGACTTGTG AGGTGGTGGC   
  
  
- CGTTGAAAAA GTTTCTCGCC AGTTACTTCA TATACAGTAA TGAGAAGTGA AGGAGTTTGG TCAAAACTAA   
  
  
- ACTTGATTTC AAACATTTCG CCGGAATTTG TGCGAATCAG ATAATTAAGA TTTTATTAAT AAAAACCCAC   
  
  
- TTATCATTTC GGCTACATTA ATGGAATAAA TGGTTGGAAC GAAGAGATCC ACACGCCAAA GCAAATCGAT   
  
  
- CAGAAATAAA GGTCAACTTC ACACGCTTTC TCAAGCTTCT CTGATCAAAT TTCAATTAAT GACCAAACCA   
  
  
- AAAACTGTTC ACAGATTTGG CAATAGCTAT GTTTCTTCTG TCTTCCTTTA TCCCAATTTA AAAACAGTTT   
  
  
- ATATATAGTA TTTTTTAATT TATAATAATA CTATAAAAAT AAAAAAACTT ATTTATTTAT TTTATAAAAT   
  
  
- TATCAAGTAT TATATTTTTT AAAAAAATTA AACCCACTTT AAATTAATTG AAGATATAAC CGAAAGTGTT   
  
  
- TATATTGAAA AAAAAAAGTG TAATTGATAC TTAACACGGT TGGAGAAAAG GAGGGAAGCT TACAAGCAAA   
  
  
- TAGCTTGACT TGGCATTTGA TGAGCGACTT CATTTTGAAC TTAAAGGTTG ATGGGCCACA TTCACATTAT   
  
  
- TAGTCATACC ATATTTTTGT TACTTTTAAT GGAAGAAAAC CCAAAAAAAA AAAAAAAAAG CAAAAAGAAG   
  
  
- CAAACCCTCT TTAACAAATT TTATTAAAT

+     GT1-motif

| Site Name | Organism | Position | Strand | Matrix score. | sequence | function |
| --- | --- | --- | --- | --- | --- | --- |
| GT1-motif | Arabidopsis thaliana | 1103 | + | 6 | GGTTAA | light responsive element |

> 2018/04/13 10:10:12  
+ GAGAGGTTTT GGTAGGTTCT GTTGTGGGAA TCTATACCTA CCCATATTTA TATGTTTCTG GTTCTTCTCT   
  
  
+ CTTTTAGAAA TTTTTCCTTT CTACTACTTC GCTCGTGTTT GGCTCTTTTA TTTATGTTTT TCCTTTTCGT   
  
  
+ CTCTACTACT CTTTTCCCCC TTCCTCTTCC CCCTCCCCTT CCCCTTCCCC TTCCCAGCTC TCAGTTTGAC   
  
  
+ TACGGGAGGG TTACGTTCCG TAGCACAGCA AATACTCCTA TAACGGCAGT TTGGGGGCTA GGTAAGCATA   
  
  
+ TTGCGGTGCA CAGTCCCGAA AACAGTCCTG TAACTGGCGG AGAAATTCAC GCCCAGGTAC CGTTGCCCTA   
  
  
+ TTTTGGTCAA CCTCTGGCAC GTCTCTACTA CGGTTACTTC CTATTTCAGT TTTCTAACTC TACTTGGCAA   
  
  
+ CCTAACCATC GTCTTCTCTC TCTCTCTCTG TCTCTCTCTC TCTCTCCCAA AATCAACCTC TGTCACTCCA   
  
  
+ TCCCAACTGG AACTTGTCGA AAATTACGTT TTCAGTAACT CACGCCTCTA CCCCACATAC CCAGGCGGTC   
  
  
+ CGAGCAGAAA ATGGCCTGTG GCCTTCGACG CAGTTTCGCA GTAGGGTGTT CGCTGTACCC TAAGAAAGAG   
  
  
+ AGAGAAATCA AAAAGAAGAT GCCAAAAGAT GAGCACCGGG GGTGGTTGTA AAAGGTACTA GTGTAGACGT   
  
  
+ GGCAAGTATG AACAGCTCTA GCTAAGACCA TCCTCTCCAG TGTGGTACGT ACCTGAACAC TCCACCACCG   
  
  
+ GCAACTTTTT CAAAGAGCGG TCAATGAAGT ATATGTCATT ACTCTTCACT TCCTCAAACC AGTTTTGATT   
  
  
+ TGAACTAAAG TTTGTAAAGC GGCCTTAAAC ACGCTTAGTC TATTAATTCT AAAATAATTA TTTTTGGGTG   
  
  
+ AATAGTAAAG CCGATGTAAT TACCTTATTT ACCAACCTTG CTTCTCTAGG TGTGCGGTTT CGTTTAGCTA   
  
  
+ GTCTTTATTT CCAGTTGAAG TGTGCGAAAG AGTTCGAAGA GACTAGTTTA AAGTTAATTA CTGGTTTGGT   
  
  
+ TTTTGACAAG TGTCTAAACC GTTATCGATA CAAAGAAGAC AGAAGGAAAT AGGGTTAAAT TTTTGTCAAA   
  
  
+ TATATATCAT AAAAAATTAA ATATTATTAT GATATTTTTA TTTTTTTGAA TAAATAAATA AAATATTTTA   
  
  
+ ATAGTTCATA ATATAAAAAA TTTTTTTAAT TTGGGTGAAA TTTAATTAAC TTCTATATTG GCTTTCACAA   
  
  
+ ATATAACTTT TTTTTTTCAC ATTAACTATG AATTGTGCCA ACCTCTTTTC CTCCCTTCGA ATGTTCGTTT   
  
  
+ ATCGAACTGA ACCGTAAACT ACTCGCTGAA GTAAAACTTG AATTTCCAAC TACCCGGTGT AAGTGTAATA   
  
  
+ ATCAGTATGG TATAAAAACA ATGAAAATTA CCTTCTTTTG GGTTTTTTTT TTTTTTTTTC GTTTTTCTTC   
  
  
+ GTTTGGGAGA AATTGTTTAA AATAATTTA  

- CTCTCCAAAA CCATCCAAGA CAACACCCTT AGATATGGAT GGGTATAAAT ATACAAAGAC CAAGAAGAGA   
  
  
- GAAAATCTTT AAAAAGGAAA GATGATGAAG CGAGCACAAA CCGAGAAAAT AAATACAAAA AGGAAAAGCA   
  
  
- GAGATGATGA GAAAAGGGGG AAGGAGAAGG GGGAGGGGAA GGGGAAGGGG AAGGGTCGAG AGTCAAACTG   
  
  
- ATGCCCTCCC AATGCAAGGC ATCGTGTCGT TTATGAGGAT ATTGCCGTCA AACCCCCGAT CCATTCGTAT   
  
  
- AACGCCACGT GTCAGGGCTT TTGTCAGGAC ATTGACCGCC TCTTTAAGTG CGGGTCCATG GCAACGGGAT   
  
  
- AAAACCAGTT GGAGACCGTG CAGAGATGAT GCCAATGAAG GATAAAGTCA AAAGATTGAG ATGAACCGTT   
  
  
- GGATTGGTAG CAGAAGAGAG AGAGAGAGAC AGAGAGAGAG AGAGAGGGTT TTAGTTGGAG ACAGTGAGGT   
  
  
- AGGGTTGACC TTGAACAGCT TTTAATGCAA AAGTCATTGA GTGCGGAGAT GGGGTGTATG GGTCCGCCAG   
  
  
- GCTCGTCTTT TACCGGACAC CGGAAGCTGC GTCAAAGCGT CATCCCACAA GCGACATGGG ATTCTTTCTC   
  
  
- TCTCTTTAGT TTTTCTTCTA CGGTTTTCTA CTCGTGGCCC CCACCAACAT TTTCCATGAT CACATCTGCA   
  
  
- CCGTTCATAC TTGTCGAGAT CGATTCTGGT AGGAGAGGTC ACACCATGCA TGGACTTGTG AGGTGGTGGC   
  
  
- CGTTGAAAAA GTTTCTCGCC AGTTACTTCA TATACAGTAA TGAGAAGTGA AGGAGTTTGG TCAAAACTAA   
  
  
- ACTTGATTTC AAACATTTCG CCGGAATTTG TGCGAATCAG ATAATTAAGA TTTTATTAAT AAAAACCCAC   
  
  
- TTATCATTTC GGCTACATTA ATGGAATAAA TGGTTGGAAC GAAGAGATCC ACACGCCAAA GCAAATCGAT   
  
  
- CAGAAATAAA GGTCAACTTC ACACGCTTTC TCAAGCTTCT CTGATCAAAT TTCAATTAAT GACCAAACCA   
  
  
- AAAACTGTTC ACAGATTTGG CAATAGCTAT GTTTCTTCTG TCTTCCTTTA TCCCAATTTA AAAACAGTTT   
  
  
- ATATATAGTA TTTTTTAATT TATAATAATA CTATAAAAAT AAAAAAACTT ATTTATTTAT TTTATAAAAT   
  
  
- TATCAAGTAT TATATTTTTT AAAAAAATTA AACCCACTTT AAATTAATTG AAGATATAAC CGAAAGTGTT   
  
  
- TATATTGAAA AAAAAAAGTG TAATTGATAC TTAACACGGT TGGAGAAAAG GAGGGAAGCT TACAAGCAAA   
  
  
- TAGCTTGACT TGGCATTTGA TGAGCGACTT CATTTTGAAC TTAAAGGTTG ATGGGCCACA TTCACATTAT   
  
  
- TAGTCATACC ATATTTTTGT TACTTTTAAT GGAAGAAAAC CCAAAAAAAA AAAAAAAAAG CAAAAAGAAG   
  
  
- CAAACCCTCT TTAACAAATT TTATTAAAT

+     HSE

| Site Name | Organism | Position | Strand | Matrix score. | sequence | function |
| --- | --- | --- | --- | --- | --- | --- |
| HSE | Brassica oleracea | 1479 | - | 9 | AAAAAATTTC | cis-acting element involved in heat stress responsiveness |
| HSE | Brassica oleracea | 1207 | - | 9 | AAAAAATTTC | cis-acting element involved in heat stress responsiveness |
| HSE | Brassica oleracea | 1205 | + | 9 | AAAAAATTTC | cis-acting element involved in heat stress responsiveness |
| HSE | Brassica oleracea | 77 | - | 9 | AAAAAATTTC | cis-acting element involved in heat stress responsiveness |

> 2018/04/13 10:10:12  
+ GAGAGGTTTT GGTAGGTTCT GTTGTGGGAA TCTATACCTA CCCATATTTA TATGTTTCTG GTTCTTCTCT   
  
  
+ CTTTTAGAAA TTTTTCCTTT CTACTACTTC GCTCGTGTTT GGCTCTTTTA TTTATGTTTT TCCTTTTCGT   
  
  
+ CTCTACTACT CTTTTCCCCC TTCCTCTTCC CCCTCCCCTT CCCCTTCCCC TTCCCAGCTC TCAGTTTGAC   
  
  
+ TACGGGAGGG TTACGTTCCG TAGCACAGCA AATACTCCTA TAACGGCAGT TTGGGGGCTA GGTAAGCATA   
  
  
+ TTGCGGTGCA CAGTCCCGAA AACAGTCCTG TAACTGGCGG AGAAATTCAC GCCCAGGTAC CGTTGCCCTA   
  
  
+ TTTTGGTCAA CCTCTGGCAC GTCTCTACTA CGGTTACTTC CTATTTCAGT TTTCTAACTC TACTTGGCAA   
  
  
+ CCTAACCATC GTCTTCTCTC TCTCTCTCTG TCTCTCTCTC TCTCTCCCAA AATCAACCTC TGTCACTCCA   
  
  
+ TCCCAACTGG AACTTGTCGA AAATTACGTT TTCAGTAACT CACGCCTCTA CCCCACATAC CCAGGCGGTC   
  
  
+ CGAGCAGAAA ATGGCCTGTG GCCTTCGACG CAGTTTCGCA GTAGGGTGTT CGCTGTACCC TAAGAAAGAG   
  
  
+ AGAGAAATCA AAAAGAAGAT GCCAAAAGAT GAGCACCGGG GGTGGTTGTA AAAGGTACTA GTGTAGACGT   
  
  
+ GGCAAGTATG AACAGCTCTA GCTAAGACCA TCCTCTCCAG TGTGGTACGT ACCTGAACAC TCCACCACCG   
  
  
+ GCAACTTTTT CAAAGAGCGG TCAATGAAGT ATATGTCATT ACTCTTCACT TCCTCAAACC AGTTTTGATT   
  
  
+ TGAACTAAAG TTTGTAAAGC GGCCTTAAAC ACGCTTAGTC TATTAATTCT AAAATAATTA TTTTTGGGTG   
  
  
+ AATAGTAAAG CCGATGTAAT TACCTTATTT ACCAACCTTG CTTCTCTAGG TGTGCGGTTT CGTTTAGCTA   
  
  
+ GTCTTTATTT CCAGTTGAAG TGTGCGAAAG AGTTCGAAGA GACTAGTTTA AAGTTAATTA CTGGTTTGGT   
  
  
+ TTTTGACAAG TGTCTAAACC GTTATCGATA CAAAGAAGAC AGAAGGAAAT AGGGTTAAAT TTTTGTCAAA   
  
  
+ TATATATCAT AAAAAATTAA ATATTATTAT GATATTTTTA TTTTTTTGAA TAAATAAATA AAATATTTTA   
  
  
+ ATAGTTCATA ATATAAAAAA TTTTTTTAAT TTGGGTGAAA TTTAATTAAC TTCTATATTG GCTTTCACAA   
  
  
+ ATATAACTTT TTTTTTTCAC ATTAACTATG AATTGTGCCA ACCTCTTTTC CTCCCTTCGA ATGTTCGTTT   
  
  
+ ATCGAACTGA ACCGTAAACT ACTCGCTGAA GTAAAACTTG AATTTCCAAC TACCCGGTGT AAGTGTAATA   
  
  
+ ATCAGTATGG TATAAAAACA ATGAAAATTA CCTTCTTTTG GGTTTTTTTT TTTTTTTTTC GTTTTTCTTC   
  
  
+ GTTTGGGAGA AATTGTTTAA AATAATTTA  

- CTCTCCAAAA CCATCCAAGA CAACACCCTT AGATATGGAT GGGTATAAAT ATACAAAGAC CAAGAAGAGA   
  
  
- GAAAATCTTT AAAAAGGAAA GATGATGAAG CGAGCACAAA CCGAGAAAAT AAATACAAAA AGGAAAAGCA   
  
  
- GAGATGATGA GAAAAGGGGG AAGGAGAAGG GGGAGGGGAA GGGGAAGGGG AAGGGTCGAG AGTCAAACTG   
  
  
- ATGCCCTCCC AATGCAAGGC ATCGTGTCGT TTATGAGGAT ATTGCCGTCA AACCCCCGAT CCATTCGTAT   
  
  
- AACGCCACGT GTCAGGGCTT TTGTCAGGAC ATTGACCGCC TCTTTAAGTG CGGGTCCATG GCAACGGGAT   
  
  
- AAAACCAGTT GGAGACCGTG CAGAGATGAT GCCAATGAAG GATAAAGTCA AAAGATTGAG ATGAACCGTT   
  
  
- GGATTGGTAG CAGAAGAGAG AGAGAGAGAC AGAGAGAGAG AGAGAGGGTT TTAGTTGGAG ACAGTGAGGT   
  
  
- AGGGTTGACC TTGAACAGCT TTTAATGCAA AAGTCATTGA GTGCGGAGAT GGGGTGTATG GGTCCGCCAG   
  
  
- GCTCGTCTTT TACCGGACAC CGGAAGCTGC GTCAAAGCGT CATCCCACAA GCGACATGGG ATTCTTTCTC   
  
  
- TCTCTTTAGT TTTTCTTCTA CGGTTTTCTA CTCGTGGCCC CCACCAACAT TTTCCATGAT CACATCTGCA   
  
  
- CCGTTCATAC TTGTCGAGAT CGATTCTGGT AGGAGAGGTC ACACCATGCA TGGACTTGTG AGGTGGTGGC   
  
  
- CGTTGAAAAA GTTTCTCGCC AGTTACTTCA TATACAGTAA TGAGAAGTGA AGGAGTTTGG TCAAAACTAA   
  
  
- ACTTGATTTC AAACATTTCG CCGGAATTTG TGCGAATCAG ATAATTAAGA TTTTATTAAT AAAAACCCAC   
  
  
- TTATCATTTC GGCTACATTA ATGGAATAAA TGGTTGGAAC GAAGAGATCC ACACGCCAAA GCAAATCGAT   
  
  
- CAGAAATAAA GGTCAACTTC ACACGCTTTC TCAAGCTTCT CTGATCAAAT TTCAATTAAT GACCAAACCA   
  
  
- AAAACTGTTC ACAGATTTGG CAATAGCTAT GTTTCTTCTG TCTTCCTTTA TCCCAATTTA AAAACAGTTT   
  
  
- ATATATAGTA TTTTTTAATT TATAATAATA CTATAAAAAT AAAAAAACTT ATTTATTTAT TTTATAAAAT   
  
  
- TATCAAGTAT TATATTTTTT AAAAAAATTA AACCCACTTT AAATTAATTG AAGATATAAC CGAAAGTGTT   
  
  
- TATATTGAAA AAAAAAAGTG TAATTGATAC TTAACACGGT TGGAGAAAAG GAGGGAAGCT TACAAGCAAA   
  
  
- TAGCTTGACT TGGCATTTGA TGAGCGACTT CATTTTGAAC TTAAAGGTTG ATGGGCCACA TTCACATTAT   
  
  
- TAGTCATACC ATATTTTTGT TACTTTTAAT GGAAGAAAAC CCAAAAAAAA AAAAAAAAAG CAAAAAGAAG   
  
  
- CAAACCCTCT TTAACAAATT TTATTAAAT

+     I-box

| Site Name | Organism | Position | Strand | Matrix score. | sequence | function |
| --- | --- | --- | --- | --- | --- | --- |
| I-box | Larix laricina | 861 | - | 9 | GTATAAGGCC | part of a light responsive element |

> 2018/04/13 10:10:12  
+ GAGAGGTTTT GGTAGGTTCT GTTGTGGGAA TCTATACCTA CCCATATTTA TATGTTTCTG GTTCTTCTCT   
  
  
+ CTTTTAGAAA TTTTTCCTTT CTACTACTTC GCTCGTGTTT GGCTCTTTTA TTTATGTTTT TCCTTTTCGT   
  
  
+ CTCTACTACT CTTTTCCCCC TTCCTCTTCC CCCTCCCCTT CCCCTTCCCC TTCCCAGCTC TCAGTTTGAC   
  
  
+ TACGGGAGGG TTACGTTCCG TAGCACAGCA AATACTCCTA TAACGGCAGT TTGGGGGCTA GGTAAGCATA   
  
  
+ TTGCGGTGCA CAGTCCCGAA AACAGTCCTG TAACTGGCGG AGAAATTCAC GCCCAGGTAC CGTTGCCCTA   
  
  
+ TTTTGGTCAA CCTCTGGCAC GTCTCTACTA CGGTTACTTC CTATTTCAGT TTTCTAACTC TACTTGGCAA   
  
  
+ CCTAACCATC GTCTTCTCTC TCTCTCTCTG TCTCTCTCTC TCTCTCCCAA AATCAACCTC TGTCACTCCA   
  
  
+ TCCCAACTGG AACTTGTCGA AAATTACGTT TTCAGTAACT CACGCCTCTA CCCCACATAC CCAGGCGGTC   
  
  
+ CGAGCAGAAA ATGGCCTGTG GCCTTCGACG CAGTTTCGCA GTAGGGTGTT CGCTGTACCC TAAGAAAGAG   
  
  
+ AGAGAAATCA AAAAGAAGAT GCCAAAAGAT GAGCACCGGG GGTGGTTGTA AAAGGTACTA GTGTAGACGT   
  
  
+ GGCAAGTATG AACAGCTCTA GCTAAGACCA TCCTCTCCAG TGTGGTACGT ACCTGAACAC TCCACCACCG   
  
  
+ GCAACTTTTT CAAAGAGCGG TCAATGAAGT ATATGTCATT ACTCTTCACT TCCTCAAACC AGTTTTGATT   
  
  
+ TGAACTAAAG TTTGTAAAGC GGCCTTAAAC ACGCTTAGTC TATTAATTCT AAAATAATTA TTTTTGGGTG   
  
  
+ AATAGTAAAG CCGATGTAAT TACCTTATTT ACCAACCTTG CTTCTCTAGG TGTGCGGTTT CGTTTAGCTA   
  
  
+ GTCTTTATTT CCAGTTGAAG TGTGCGAAAG AGTTCGAAGA GACTAGTTTA AAGTTAATTA CTGGTTTGGT   
  
  
+ TTTTGACAAG TGTCTAAACC GTTATCGATA CAAAGAAGAC AGAAGGAAAT AGGGTTAAAT TTTTGTCAAA   
  
  
+ TATATATCAT AAAAAATTAA ATATTATTAT GATATTTTTA TTTTTTTGAA TAAATAAATA AAATATTTTA   
  
  
+ ATAGTTCATA ATATAAAAAA TTTTTTTAAT TTGGGTGAAA TTTAATTAAC TTCTATATTG GCTTTCACAA   
  
  
+ ATATAACTTT TTTTTTTCAC ATTAACTATG AATTGTGCCA ACCTCTTTTC CTCCCTTCGA ATGTTCGTTT   
  
  
+ ATCGAACTGA ACCGTAAACT ACTCGCTGAA GTAAAACTTG AATTTCCAAC TACCCGGTGT AAGTGTAATA   
  
  
+ ATCAGTATGG TATAAAAACA ATGAAAATTA CCTTCTTTTG GGTTTTTTTT TTTTTTTTTC GTTTTTCTTC   
  
  
+ GTTTGGGAGA AATTGTTTAA AATAATTTA  

- CTCTCCAAAA CCATCCAAGA CAACACCCTT AGATATGGAT GGGTATAAAT ATACAAAGAC CAAGAAGAGA   
  
  
- GAAAATCTTT AAAAAGGAAA GATGATGAAG CGAGCACAAA CCGAGAAAAT AAATACAAAA AGGAAAAGCA   
  
  
- GAGATGATGA GAAAAGGGGG AAGGAGAAGG GGGAGGGGAA GGGGAAGGGG AAGGGTCGAG AGTCAAACTG   
  
  
- ATGCCCTCCC AATGCAAGGC ATCGTGTCGT TTATGAGGAT ATTGCCGTCA AACCCCCGAT CCATTCGTAT   
  
  
- AACGCCACGT GTCAGGGCTT TTGTCAGGAC ATTGACCGCC TCTTTAAGTG CGGGTCCATG GCAACGGGAT   
  
  
- AAAACCAGTT GGAGACCGTG CAGAGATGAT GCCAATGAAG GATAAAGTCA AAAGATTGAG ATGAACCGTT   
  
  
- GGATTGGTAG CAGAAGAGAG AGAGAGAGAC AGAGAGAGAG AGAGAGGGTT TTAGTTGGAG ACAGTGAGGT   
  
  
- AGGGTTGACC TTGAACAGCT TTTAATGCAA AAGTCATTGA GTGCGGAGAT GGGGTGTATG GGTCCGCCAG   
  
  
- GCTCGTCTTT TACCGGACAC CGGAAGCTGC GTCAAAGCGT CATCCCACAA GCGACATGGG ATTCTTTCTC   
  
  
- TCTCTTTAGT TTTTCTTCTA CGGTTTTCTA CTCGTGGCCC CCACCAACAT TTTCCATGAT CACATCTGCA   
  
  
- CCGTTCATAC TTGTCGAGAT CGATTCTGGT AGGAGAGGTC ACACCATGCA TGGACTTGTG AGGTGGTGGC   
  
  
- CGTTGAAAAA GTTTCTCGCC AGTTACTTCA TATACAGTAA TGAGAAGTGA AGGAGTTTGG TCAAAACTAA   
  
  
- ACTTGATTTC AAACATTTCG CCGGAATTTG TGCGAATCAG ATAATTAAGA TTTTATTAAT AAAAACCCAC   
  
  
- TTATCATTTC GGCTACATTA ATGGAATAAA TGGTTGGAAC GAAGAGATCC ACACGCCAAA GCAAATCGAT   
  
  
- CAGAAATAAA GGTCAACTTC ACACGCTTTC TCAAGCTTCT CTGATCAAAT TTCAATTAAT GACCAAACCA   
  
  
- AAAACTGTTC ACAGATTTGG CAATAGCTAT GTTTCTTCTG TCTTCCTTTA TCCCAATTTA AAAACAGTTT   
  
  
- ATATATAGTA TTTTTTAATT TATAATAATA CTATAAAAAT AAAAAAACTT ATTTATTTAT TTTATAAAAT   
  
  
- TATCAAGTAT TATATTTTTT AAAAAAATTA AACCCACTTT AAATTAATTG AAGATATAAC CGAAAGTGTT   
  
  
- TATATTGAAA AAAAAAAGTG TAATTGATAC TTAACACGGT TGGAGAAAAG GAGGGAAGCT TACAAGCAAA   
  
  
- TAGCTTGACT TGGCATTTGA TGAGCGACTT CATTTTGAAC TTAAAGGTTG ATGGGCCACA TTCACATTAT   
  
  
- TAGTCATACC ATATTTTTGT TACTTTTAAT GGAAGAAAAC CCAAAAAAAA AAAAAAAAAG CAAAAAGAAG   
  
  
- CAAACCCTCT TTAACAAATT TTATTAAAT

+     LTR

| Site Name | Organism | Position | Strand | Matrix score. | sequence | function |
| --- | --- | --- | --- | --- | --- | --- |
| LTR | Hordeum vulgare | 296 | + | 6 | CCGAAA | cis-acting element involved in low-temperature responsiveness |

> 2018/04/13 10:10:12  
+ GAGAGGTTTT GGTAGGTTCT GTTGTGGGAA TCTATACCTA CCCATATTTA TATGTTTCTG GTTCTTCTCT   
  
  
+ CTTTTAGAAA TTTTTCCTTT CTACTACTTC GCTCGTGTTT GGCTCTTTTA TTTATGTTTT TCCTTTTCGT   
  
  
+ CTCTACTACT CTTTTCCCCC TTCCTCTTCC CCCTCCCCTT CCCCTTCCCC TTCCCAGCTC TCAGTTTGAC   
  
  
+ TACGGGAGGG TTACGTTCCG TAGCACAGCA AATACTCCTA TAACGGCAGT TTGGGGGCTA GGTAAGCATA   
  
  
+ TTGCGGTGCA CAGTCCCGAA AACAGTCCTG TAACTGGCGG AGAAATTCAC GCCCAGGTAC CGTTGCCCTA   
  
  
+ TTTTGGTCAA CCTCTGGCAC GTCTCTACTA CGGTTACTTC CTATTTCAGT TTTCTAACTC TACTTGGCAA   
  
  
+ CCTAACCATC GTCTTCTCTC TCTCTCTCTG TCTCTCTCTC TCTCTCCCAA AATCAACCTC TGTCACTCCA   
  
  
+ TCCCAACTGG AACTTGTCGA AAATTACGTT TTCAGTAACT CACGCCTCTA CCCCACATAC CCAGGCGGTC   
  
  
+ CGAGCAGAAA ATGGCCTGTG GCCTTCGACG CAGTTTCGCA GTAGGGTGTT CGCTGTACCC TAAGAAAGAG   
  
  
+ AGAGAAATCA AAAAGAAGAT GCCAAAAGAT GAGCACCGGG GGTGGTTGTA AAAGGTACTA GTGTAGACGT   
  
  
+ GGCAAGTATG AACAGCTCTA GCTAAGACCA TCCTCTCCAG TGTGGTACGT ACCTGAACAC TCCACCACCG   
  
  
+ GCAACTTTTT CAAAGAGCGG TCAATGAAGT ATATGTCATT ACTCTTCACT TCCTCAAACC AGTTTTGATT   
  
  
+ TGAACTAAAG TTTGTAAAGC GGCCTTAAAC ACGCTTAGTC TATTAATTCT AAAATAATTA TTTTTGGGTG   
  
  
+ AATAGTAAAG CCGATGTAAT TACCTTATTT ACCAACCTTG CTTCTCTAGG TGTGCGGTTT CGTTTAGCTA   
  
  
+ GTCTTTATTT CCAGTTGAAG TGTGCGAAAG AGTTCGAAGA GACTAGTTTA AAGTTAATTA CTGGTTTGGT   
  
  
+ TTTTGACAAG TGTCTAAACC GTTATCGATA CAAAGAAGAC AGAAGGAAAT AGGGTTAAAT TTTTGTCAAA   
  
  
+ TATATATCAT AAAAAATTAA ATATTATTAT GATATTTTTA TTTTTTTGAA TAAATAAATA AAATATTTTA   
  
  
+ ATAGTTCATA ATATAAAAAA TTTTTTTAAT TTGGGTGAAA TTTAATTAAC TTCTATATTG GCTTTCACAA   
  
  
+ ATATAACTTT TTTTTTTCAC ATTAACTATG AATTGTGCCA ACCTCTTTTC CTCCCTTCGA ATGTTCGTTT   
  
  
+ ATCGAACTGA ACCGTAAACT ACTCGCTGAA GTAAAACTTG AATTTCCAAC TACCCGGTGT AAGTGTAATA   
  
  
+ ATCAGTATGG TATAAAAACA ATGAAAATTA CCTTCTTTTG GGTTTTTTTT TTTTTTTTTC GTTTTTCTTC   
  
  
+ GTTTGGGAGA AATTGTTTAA AATAATTTA  

- CTCTCCAAAA CCATCCAAGA CAACACCCTT AGATATGGAT GGGTATAAAT ATACAAAGAC CAAGAAGAGA   
  
  
- GAAAATCTTT AAAAAGGAAA GATGATGAAG CGAGCACAAA CCGAGAAAAT AAATACAAAA AGGAAAAGCA   
  
  
- GAGATGATGA GAAAAGGGGG AAGGAGAAGG GGGAGGGGAA GGGGAAGGGG AAGGGTCGAG AGTCAAACTG   
  
  
- ATGCCCTCCC AATGCAAGGC ATCGTGTCGT TTATGAGGAT ATTGCCGTCA AACCCCCGAT CCATTCGTAT   
  
  
- AACGCCACGT GTCAGGGCTT TTGTCAGGAC ATTGACCGCC TCTTTAAGTG CGGGTCCATG GCAACGGGAT   
  
  
- AAAACCAGTT GGAGACCGTG CAGAGATGAT GCCAATGAAG GATAAAGTCA AAAGATTGAG ATGAACCGTT   
  
  
- GGATTGGTAG CAGAAGAGAG AGAGAGAGAC AGAGAGAGAG AGAGAGGGTT TTAGTTGGAG ACAGTGAGGT   
  
  
- AGGGTTGACC TTGAACAGCT TTTAATGCAA AAGTCATTGA GTGCGGAGAT GGGGTGTATG GGTCCGCCAG   
  
  
- GCTCGTCTTT TACCGGACAC CGGAAGCTGC GTCAAAGCGT CATCCCACAA GCGACATGGG ATTCTTTCTC   
  
  
- TCTCTTTAGT TTTTCTTCTA CGGTTTTCTA CTCGTGGCCC CCACCAACAT TTTCCATGAT CACATCTGCA   
  
  
- CCGTTCATAC TTGTCGAGAT CGATTCTGGT AGGAGAGGTC ACACCATGCA TGGACTTGTG AGGTGGTGGC   
  
  
- CGTTGAAAAA GTTTCTCGCC AGTTACTTCA TATACAGTAA TGAGAAGTGA AGGAGTTTGG TCAAAACTAA   
  
  
- ACTTGATTTC AAACATTTCG CCGGAATTTG TGCGAATCAG ATAATTAAGA TTTTATTAAT AAAAACCCAC   
  
  
- TTATCATTTC GGCTACATTA ATGGAATAAA TGGTTGGAAC GAAGAGATCC ACACGCCAAA GCAAATCGAT   
  
  
- CAGAAATAAA GGTCAACTTC ACACGCTTTC TCAAGCTTCT CTGATCAAAT TTCAATTAAT GACCAAACCA   
  
  
- AAAACTGTTC ACAGATTTGG CAATAGCTAT GTTTCTTCTG TCTTCCTTTA TCCCAATTTA AAAACAGTTT   
  
  
- ATATATAGTA TTTTTTAATT TATAATAATA CTATAAAAAT AAAAAAACTT ATTTATTTAT TTTATAAAAT   
  
  
- TATCAAGTAT TATATTTTTT AAAAAAATTA AACCCACTTT AAATTAATTG AAGATATAAC CGAAAGTGTT   
  
  
- TATATTGAAA AAAAAAAGTG TAATTGATAC TTAACACGGT TGGAGAAAAG GAGGGAAGCT TACAAGCAAA   
  
  
- TAGCTTGACT TGGCATTTGA TGAGCGACTT CATTTTGAAC TTAAAGGTTG ATGGGCCACA TTCACATTAT   
  
  
- TAGTCATACC ATATTTTTGT TACTTTTAAT GGAAGAAAAC CCAAAAAAAA AAAAAAAAAG CAAAAAGAAG   
  
  
- CAAACCCTCT TTAACAAATT TTATTAAAT

+     MBS

| Site Name | Organism | Position | Strand | Matrix score. | sequence | function |
| --- | --- | --- | --- | --- | --- | --- |
| MBS | Arabidopsis thaliana | 311 | + | 6 | TAACTG | MYB binding site involved in drought-inducibility |
| MBS | Arabidopsis thaliana | 494 | + | 6 | CAACTG | MYB binding site involved in drought-inducibility |
| MBS | Arabidopsis thaliana | 992 | - | 6 | CAACTG | MYB binding site involved in drought-inducibility |
| MBS | Zea mays | 788 | + | 6 | CGGTCA | MYB Binding Site |

> 2018/04/13 10:10:12  
+ GAGAGGTTTT GGTAGGTTCT GTTGTGGGAA TCTATACCTA CCCATATTTA TATGTTTCTG GTTCTTCTCT   
  
  
+ CTTTTAGAAA TTTTTCCTTT CTACTACTTC GCTCGTGTTT GGCTCTTTTA TTTATGTTTT TCCTTTTCGT   
  
  
+ CTCTACTACT CTTTTCCCCC TTCCTCTTCC CCCTCCCCTT CCCCTTCCCC TTCCCAGCTC TCAGTTTGAC   
  
  
+ TACGGGAGGG TTACGTTCCG TAGCACAGCA AATACTCCTA TAACGGCAGT TTGGGGGCTA GGTAAGCATA   
  
  
+ TTGCGGTGCA CAGTCCCGAA AACAGTCCTG TAACTGGCGG AGAAATTCAC GCCCAGGTAC CGTTGCCCTA   
  
  
+ TTTTGGTCAA CCTCTGGCAC GTCTCTACTA CGGTTACTTC CTATTTCAGT TTTCTAACTC TACTTGGCAA   
  
  
+ CCTAACCATC GTCTTCTCTC TCTCTCTCTG TCTCTCTCTC TCTCTCCCAA AATCAACCTC TGTCACTCCA   
  
  
+ TCCCAACTGG AACTTGTCGA AAATTACGTT TTCAGTAACT CACGCCTCTA CCCCACATAC CCAGGCGGTC   
  
  
+ CGAGCAGAAA ATGGCCTGTG GCCTTCGACG CAGTTTCGCA GTAGGGTGTT CGCTGTACCC TAAGAAAGAG   
  
  
+ AGAGAAATCA AAAAGAAGAT GCCAAAAGAT GAGCACCGGG GGTGGTTGTA AAAGGTACTA GTGTAGACGT   
  
  
+ GGCAAGTATG AACAGCTCTA GCTAAGACCA TCCTCTCCAG TGTGGTACGT ACCTGAACAC TCCACCACCG   
  
  
+ GCAACTTTTT CAAAGAGCGG TCAATGAAGT ATATGTCATT ACTCTTCACT TCCTCAAACC AGTTTTGATT   
  
  
+ TGAACTAAAG TTTGTAAAGC GGCCTTAAAC ACGCTTAGTC TATTAATTCT AAAATAATTA TTTTTGGGTG   
  
  
+ AATAGTAAAG CCGATGTAAT TACCTTATTT ACCAACCTTG CTTCTCTAGG TGTGCGGTTT CGTTTAGCTA   
  
  
+ GTCTTTATTT CCAGTTGAAG TGTGCGAAAG AGTTCGAAGA GACTAGTTTA AAGTTAATTA CTGGTTTGGT   
  
  
+ TTTTGACAAG TGTCTAAACC GTTATCGATA CAAAGAAGAC AGAAGGAAAT AGGGTTAAAT TTTTGTCAAA   
  
  
+ TATATATCAT AAAAAATTAA ATATTATTAT GATATTTTTA TTTTTTTGAA TAAATAAATA AAATATTTTA   
  
  
+ ATAGTTCATA ATATAAAAAA TTTTTTTAAT TTGGGTGAAA TTTAATTAAC TTCTATATTG GCTTTCACAA   
  
  
+ ATATAACTTT TTTTTTTCAC ATTAACTATG AATTGTGCCA ACCTCTTTTC CTCCCTTCGA ATGTTCGTTT   
  
  
+ ATCGAACTGA ACCGTAAACT ACTCGCTGAA GTAAAACTTG AATTTCCAAC TACCCGGTGT AAGTGTAATA   
  
  
+ ATCAGTATGG TATAAAAACA ATGAAAATTA CCTTCTTTTG GGTTTTTTTT TTTTTTTTTC GTTTTTCTTC   
  
  
+ GTTTGGGAGA AATTGTTTAA AATAATTTA  

- CTCTCCAAAA CCATCCAAGA CAACACCCTT AGATATGGAT GGGTATAAAT ATACAAAGAC CAAGAAGAGA   
  
  
- GAAAATCTTT AAAAAGGAAA GATGATGAAG CGAGCACAAA CCGAGAAAAT AAATACAAAA AGGAAAAGCA   
  
  
- GAGATGATGA GAAAAGGGGG AAGGAGAAGG GGGAGGGGAA GGGGAAGGGG AAGGGTCGAG AGTCAAACTG   
  
  
- ATGCCCTCCC AATGCAAGGC ATCGTGTCGT TTATGAGGAT ATTGCCGTCA AACCCCCGAT CCATTCGTAT   
  
  
- AACGCCACGT GTCAGGGCTT TTGTCAGGAC ATTGACCGCC TCTTTAAGTG CGGGTCCATG GCAACGGGAT   
  
  
- AAAACCAGTT GGAGACCGTG CAGAGATGAT GCCAATGAAG GATAAAGTCA AAAGATTGAG ATGAACCGTT   
  
  
- GGATTGGTAG CAGAAGAGAG AGAGAGAGAC AGAGAGAGAG AGAGAGGGTT TTAGTTGGAG ACAGTGAGGT   
  
  
- AGGGTTGACC TTGAACAGCT TTTAATGCAA AAGTCATTGA GTGCGGAGAT GGGGTGTATG GGTCCGCCAG   
  
  
- GCTCGTCTTT TACCGGACAC CGGAAGCTGC GTCAAAGCGT CATCCCACAA GCGACATGGG ATTCTTTCTC   
  
  
- TCTCTTTAGT TTTTCTTCTA CGGTTTTCTA CTCGTGGCCC CCACCAACAT TTTCCATGAT CACATCTGCA   
  
  
- CCGTTCATAC TTGTCGAGAT CGATTCTGGT AGGAGAGGTC ACACCATGCA TGGACTTGTG AGGTGGTGGC   
  
  
- CGTTGAAAAA GTTTCTCGCC AGTTACTTCA TATACAGTAA TGAGAAGTGA AGGAGTTTGG TCAAAACTAA   
  
  
- ACTTGATTTC AAACATTTCG CCGGAATTTG TGCGAATCAG ATAATTAAGA TTTTATTAAT AAAAACCCAC   
  
  
- TTATCATTTC GGCTACATTA ATGGAATAAA TGGTTGGAAC GAAGAGATCC ACACGCCAAA GCAAATCGAT   
  
  
- CAGAAATAAA GGTCAACTTC ACACGCTTTC TCAAGCTTCT CTGATCAAAT TTCAATTAAT GACCAAACCA   
  
  
- AAAACTGTTC ACAGATTTGG CAATAGCTAT GTTTCTTCTG TCTTCCTTTA TCCCAATTTA AAAACAGTTT   
  
  
- ATATATAGTA TTTTTTAATT TATAATAATA CTATAAAAAT AAAAAAACTT ATTTATTTAT TTTATAAAAT   
  
  
- TATCAAGTAT TATATTTTTT AAAAAAATTA AACCCACTTT AAATTAATTG AAGATATAAC CGAAAGTGTT   
  
  
- TATATTGAAA AAAAAAAGTG TAATTGATAC TTAACACGGT TGGAGAAAAG GAGGGAAGCT TACAAGCAAA   
  
  
- TAGCTTGACT TGGCATTTGA TGAGCGACTT CATTTTGAAC TTAAAGGTTG ATGGGCCACA TTCACATTAT   
  
  
- TAGTCATACC ATATTTTTGT TACTTTTAAT GGAAGAAAAC CCAAAAAAAA AAAAAAAAAG CAAAAAGAAG   
  
  
- CAAACCCTCT TTAACAAATT TTATTAAAT

+     MRE

| Site Name | Organism | Position | Strand | Matrix score. | sequence | function |
| --- | --- | --- | --- | --- | --- | --- |
| MRE | Petroselinum crispum | 419 | + | 7 | AACCTAA | MYB binding site involved in light responsiveness |

> 2018/04/13 10:10:12  
+ GAGAGGTTTT GGTAGGTTCT GTTGTGGGAA TCTATACCTA CCCATATTTA TATGTTTCTG GTTCTTCTCT   
  
  
+ CTTTTAGAAA TTTTTCCTTT CTACTACTTC GCTCGTGTTT GGCTCTTTTA TTTATGTTTT TCCTTTTCGT   
  
  
+ CTCTACTACT CTTTTCCCCC TTCCTCTTCC CCCTCCCCTT CCCCTTCCCC TTCCCAGCTC TCAGTTTGAC   
  
  
+ TACGGGAGGG TTACGTTCCG TAGCACAGCA AATACTCCTA TAACGGCAGT TTGGGGGCTA GGTAAGCATA   
  
  
+ TTGCGGTGCA CAGTCCCGAA AACAGTCCTG TAACTGGCGG AGAAATTCAC GCCCAGGTAC CGTTGCCCTA   
  
  
+ TTTTGGTCAA CCTCTGGCAC GTCTCTACTA CGGTTACTTC CTATTTCAGT TTTCTAACTC TACTTGGCAA   
  
  
+ CCTAACCATC GTCTTCTCTC TCTCTCTCTG TCTCTCTCTC TCTCTCCCAA AATCAACCTC TGTCACTCCA   
  
  
+ TCCCAACTGG AACTTGTCGA AAATTACGTT TTCAGTAACT CACGCCTCTA CCCCACATAC CCAGGCGGTC   
  
  
+ CGAGCAGAAA ATGGCCTGTG GCCTTCGACG CAGTTTCGCA GTAGGGTGTT CGCTGTACCC TAAGAAAGAG   
  
  
+ AGAGAAATCA AAAAGAAGAT GCCAAAAGAT GAGCACCGGG GGTGGTTGTA AAAGGTACTA GTGTAGACGT   
  
  
+ GGCAAGTATG AACAGCTCTA GCTAAGACCA TCCTCTCCAG TGTGGTACGT ACCTGAACAC TCCACCACCG   
  
  
+ GCAACTTTTT CAAAGAGCGG TCAATGAAGT ATATGTCATT ACTCTTCACT TCCTCAAACC AGTTTTGATT   
  
  
+ TGAACTAAAG TTTGTAAAGC GGCCTTAAAC ACGCTTAGTC TATTAATTCT AAAATAATTA TTTTTGGGTG   
  
  
+ AATAGTAAAG CCGATGTAAT TACCTTATTT ACCAACCTTG CTTCTCTAGG TGTGCGGTTT CGTTTAGCTA   
  
  
+ GTCTTTATTT CCAGTTGAAG TGTGCGAAAG AGTTCGAAGA GACTAGTTTA AAGTTAATTA CTGGTTTGGT   
  
  
+ TTTTGACAAG TGTCTAAACC GTTATCGATA CAAAGAAGAC AGAAGGAAAT AGGGTTAAAT TTTTGTCAAA   
  
  
+ TATATATCAT AAAAAATTAA ATATTATTAT GATATTTTTA TTTTTTTGAA TAAATAAATA AAATATTTTA   
  
  
+ ATAGTTCATA ATATAAAAAA TTTTTTTAAT TTGGGTGAAA TTTAATTAAC TTCTATATTG GCTTTCACAA   
  
  
+ ATATAACTTT TTTTTTTCAC ATTAACTATG AATTGTGCCA ACCTCTTTTC CTCCCTTCGA ATGTTCGTTT   
  
  
+ ATCGAACTGA ACCGTAAACT ACTCGCTGAA GTAAAACTTG AATTTCCAAC TACCCGGTGT AAGTGTAATA   
  
  
+ ATCAGTATGG TATAAAAACA ATGAAAATTA CCTTCTTTTG GGTTTTTTTT TTTTTTTTTC GTTTTTCTTC   
  
  
+ GTTTGGGAGA AATTGTTTAA AATAATTTA  

- CTCTCCAAAA CCATCCAAGA CAACACCCTT AGATATGGAT GGGTATAAAT ATACAAAGAC CAAGAAGAGA   
  
  
- GAAAATCTTT AAAAAGGAAA GATGATGAAG CGAGCACAAA CCGAGAAAAT AAATACAAAA AGGAAAAGCA   
  
  
- GAGATGATGA GAAAAGGGGG AAGGAGAAGG GGGAGGGGAA GGGGAAGGGG AAGGGTCGAG AGTCAAACTG   
  
  
- ATGCCCTCCC AATGCAAGGC ATCGTGTCGT TTATGAGGAT ATTGCCGTCA AACCCCCGAT CCATTCGTAT   
  
  
- AACGCCACGT GTCAGGGCTT TTGTCAGGAC ATTGACCGCC TCTTTAAGTG CGGGTCCATG GCAACGGGAT   
  
  
- AAAACCAGTT GGAGACCGTG CAGAGATGAT GCCAATGAAG GATAAAGTCA AAAGATTGAG ATGAACCGTT   
  
  
- GGATTGGTAG CAGAAGAGAG AGAGAGAGAC AGAGAGAGAG AGAGAGGGTT TTAGTTGGAG ACAGTGAGGT   
  
  
- AGGGTTGACC TTGAACAGCT TTTAATGCAA AAGTCATTGA GTGCGGAGAT GGGGTGTATG GGTCCGCCAG   
  
  
- GCTCGTCTTT TACCGGACAC CGGAAGCTGC GTCAAAGCGT CATCCCACAA GCGACATGGG ATTCTTTCTC   
  
  
- TCTCTTTAGT TTTTCTTCTA CGGTTTTCTA CTCGTGGCCC CCACCAACAT TTTCCATGAT CACATCTGCA   
  
  
- CCGTTCATAC TTGTCGAGAT CGATTCTGGT AGGAGAGGTC ACACCATGCA TGGACTTGTG AGGTGGTGGC   
  
  
- CGTTGAAAAA GTTTCTCGCC AGTTACTTCA TATACAGTAA TGAGAAGTGA AGGAGTTTGG TCAAAACTAA   
  
  
- ACTTGATTTC AAACATTTCG CCGGAATTTG TGCGAATCAG ATAATTAAGA TTTTATTAAT AAAAACCCAC   
  
  
- TTATCATTTC GGCTACATTA ATGGAATAAA TGGTTGGAAC GAAGAGATCC ACACGCCAAA GCAAATCGAT   
  
  
- CAGAAATAAA GGTCAACTTC ACACGCTTTC TCAAGCTTCT CTGATCAAAT TTCAATTAAT GACCAAACCA   
  
  
- AAAACTGTTC ACAGATTTGG CAATAGCTAT GTTTCTTCTG TCTTCCTTTA TCCCAATTTA AAAACAGTTT   
  
  
- ATATATAGTA TTTTTTAATT TATAATAATA CTATAAAAAT AAAAAAACTT ATTTATTTAT TTTATAAAAT   
  
  
- TATCAAGTAT TATATTTTTT AAAAAAATTA AACCCACTTT AAATTAATTG AAGATATAAC CGAAAGTGTT   
  
  
- TATATTGAAA AAAAAAAGTG TAATTGATAC TTAACACGGT TGGAGAAAAG GAGGGAAGCT TACAAGCAAA   
  
  
- TAGCTTGACT TGGCATTTGA TGAGCGACTT CATTTTGAAC TTAAAGGTTG ATGGGCCACA TTCACATTAT   
  
  
- TAGTCATACC ATATTTTTGT TACTTTTAAT GGAAGAAAAC CCAAAAAAAA AAAAAAAAAG CAAAAAGAAG   
  
  
- CAAACCCTCT TTAACAAATT TTATTAAAT

+     Skn-1\_motif

| Site Name | Organism | Position | Strand | Matrix score. | sequence | function |
| --- | --- | --- | --- | --- | --- | --- |
| Skn-1\_motif | Oryza sativa | 805 | + | 5 | GTCAT | cis-acting regulatory element required for endosperm expression |

> 2018/04/13 10:10:12  
+ GAGAGGTTTT GGTAGGTTCT GTTGTGGGAA TCTATACCTA CCCATATTTA TATGTTTCTG GTTCTTCTCT   
  
  
+ CTTTTAGAAA TTTTTCCTTT CTACTACTTC GCTCGTGTTT GGCTCTTTTA TTTATGTTTT TCCTTTTCGT   
  
  
+ CTCTACTACT CTTTTCCCCC TTCCTCTTCC CCCTCCCCTT CCCCTTCCCC TTCCCAGCTC TCAGTTTGAC   
  
  
+ TACGGGAGGG TTACGTTCCG TAGCACAGCA AATACTCCTA TAACGGCAGT TTGGGGGCTA GGTAAGCATA   
  
  
+ TTGCGGTGCA CAGTCCCGAA AACAGTCCTG TAACTGGCGG AGAAATTCAC GCCCAGGTAC CGTTGCCCTA   
  
  
+ TTTTGGTCAA CCTCTGGCAC GTCTCTACTA CGGTTACTTC CTATTTCAGT TTTCTAACTC TACTTGGCAA   
  
  
+ CCTAACCATC GTCTTCTCTC TCTCTCTCTG TCTCTCTCTC TCTCTCCCAA AATCAACCTC TGTCACTCCA   
  
  
+ TCCCAACTGG AACTTGTCGA AAATTACGTT TTCAGTAACT CACGCCTCTA CCCCACATAC CCAGGCGGTC   
  
  
+ CGAGCAGAAA ATGGCCTGTG GCCTTCGACG CAGTTTCGCA GTAGGGTGTT CGCTGTACCC TAAGAAAGAG   
  
  
+ AGAGAAATCA AAAAGAAGAT GCCAAAAGAT GAGCACCGGG GGTGGTTGTA AAAGGTACTA GTGTAGACGT   
  
  
+ GGCAAGTATG AACAGCTCTA GCTAAGACCA TCCTCTCCAG TGTGGTACGT ACCTGAACAC TCCACCACCG   
  
  
+ GCAACTTTTT CAAAGAGCGG TCAATGAAGT ATATGTCATT ACTCTTCACT TCCTCAAACC AGTTTTGATT   
  
  
+ TGAACTAAAG TTTGTAAAGC GGCCTTAAAC ACGCTTAGTC TATTAATTCT AAAATAATTA TTTTTGGGTG   
  
  
+ AATAGTAAAG CCGATGTAAT TACCTTATTT ACCAACCTTG CTTCTCTAGG TGTGCGGTTT CGTTTAGCTA   
  
  
+ GTCTTTATTT CCAGTTGAAG TGTGCGAAAG AGTTCGAAGA GACTAGTTTA AAGTTAATTA CTGGTTTGGT   
  
  
+ TTTTGACAAG TGTCTAAACC GTTATCGATA CAAAGAAGAC AGAAGGAAAT AGGGTTAAAT TTTTGTCAAA   
  
  
+ TATATATCAT AAAAAATTAA ATATTATTAT GATATTTTTA TTTTTTTGAA TAAATAAATA AAATATTTTA   
  
  
+ ATAGTTCATA ATATAAAAAA TTTTTTTAAT TTGGGTGAAA TTTAATTAAC TTCTATATTG GCTTTCACAA   
  
  
+ ATATAACTTT TTTTTTTCAC ATTAACTATG AATTGTGCCA ACCTCTTTTC CTCCCTTCGA ATGTTCGTTT   
  
  
+ ATCGAACTGA ACCGTAAACT ACTCGCTGAA GTAAAACTTG AATTTCCAAC TACCCGGTGT AAGTGTAATA   
  
  
+ ATCAGTATGG TATAAAAACA ATGAAAATTA CCTTCTTTTG GGTTTTTTTT TTTTTTTTTC GTTTTTCTTC   
  
  
+ GTTTGGGAGA AATTGTTTAA AATAATTTA  

- CTCTCCAAAA CCATCCAAGA CAACACCCTT AGATATGGAT GGGTATAAAT ATACAAAGAC CAAGAAGAGA   
  
  
- GAAAATCTTT AAAAAGGAAA GATGATGAAG CGAGCACAAA CCGAGAAAAT AAATACAAAA AGGAAAAGCA   
  
  
- GAGATGATGA GAAAAGGGGG AAGGAGAAGG GGGAGGGGAA GGGGAAGGGG AAGGGTCGAG AGTCAAACTG   
  
  
- ATGCCCTCCC AATGCAAGGC ATCGTGTCGT TTATGAGGAT ATTGCCGTCA AACCCCCGAT CCATTCGTAT   
  
  
- AACGCCACGT GTCAGGGCTT TTGTCAGGAC ATTGACCGCC TCTTTAAGTG CGGGTCCATG GCAACGGGAT   
  
  
- AAAACCAGTT GGAGACCGTG CAGAGATGAT GCCAATGAAG GATAAAGTCA AAAGATTGAG ATGAACCGTT   
  
  
- GGATTGGTAG CAGAAGAGAG AGAGAGAGAC AGAGAGAGAG AGAGAGGGTT TTAGTTGGAG ACAGTGAGGT   
  
  
- AGGGTTGACC TTGAACAGCT TTTAATGCAA AAGTCATTGA GTGCGGAGAT GGGGTGTATG GGTCCGCCAG   
  
  
- GCTCGTCTTT TACCGGACAC CGGAAGCTGC GTCAAAGCGT CATCCCACAA GCGACATGGG ATTCTTTCTC   
  
  
- TCTCTTTAGT TTTTCTTCTA CGGTTTTCTA CTCGTGGCCC CCACCAACAT TTTCCATGAT CACATCTGCA   
  
  
- CCGTTCATAC TTGTCGAGAT CGATTCTGGT AGGAGAGGTC ACACCATGCA TGGACTTGTG AGGTGGTGGC   
  
  
- CGTTGAAAAA GTTTCTCGCC AGTTACTTCA TATACAGTAA TGAGAAGTGA AGGAGTTTGG TCAAAACTAA   
  
  
- ACTTGATTTC AAACATTTCG CCGGAATTTG TGCGAATCAG ATAATTAAGA TTTTATTAAT AAAAACCCAC   
  
  
- TTATCATTTC GGCTACATTA ATGGAATAAA TGGTTGGAAC GAAGAGATCC ACACGCCAAA GCAAATCGAT   
  
  
- CAGAAATAAA GGTCAACTTC ACACGCTTTC TCAAGCTTCT CTGATCAAAT TTCAATTAAT GACCAAACCA   
  
  
- AAAACTGTTC ACAGATTTGG CAATAGCTAT GTTTCTTCTG TCTTCCTTTA TCCCAATTTA AAAACAGTTT   
  
  
- ATATATAGTA TTTTTTAATT TATAATAATA CTATAAAAAT AAAAAAACTT ATTTATTTAT TTTATAAAAT   
  
  
- TATCAAGTAT TATATTTTTT AAAAAAATTA AACCCACTTT AAATTAATTG AAGATATAAC CGAAAGTGTT   
  
  
- TATATTGAAA AAAAAAAGTG TAATTGATAC TTAACACGGT TGGAGAAAAG GAGGGAAGCT TACAAGCAAA   
  
  
- TAGCTTGACT TGGCATTTGA TGAGCGACTT CATTTTGAAC TTAAAGGTTG ATGGGCCACA TTCACATTAT   
  
  
- TAGTCATACC ATATTTTTGT TACTTTTAAT GGAAGAAAAC CCAAAAAAAA AAAAAAAAAG CAAAAAGAAG   
  
  
- CAAACCCTCT TTAACAAATT TTATTAAAT

+     Sp1

| Site Name | Organism | Position | Strand | Matrix score. | sequence | function |
| --- | --- | --- | --- | --- | --- | --- |
| Sp1 | Zea mays | 1310 | + | 5 | CC(G/A)CCC | light responsive element |
| Sp1 | Zea mays | 172 | + | 5 | CC(G/A)CCC | light responsive element |
| Sp1 | Zea mays | 214 | - | 5 | CC(G/A)CCC | light responsive element |
| Sp1 | Zea mays | 670 | - | 5.5 | CC(G/A)CCC | light responsive element |

> 2018/04/13 10:10:12  
+ GAGAGGTTTT GGTAGGTTCT GTTGTGGGAA TCTATACCTA CCCATATTTA TATGTTTCTG GTTCTTCTCT   
  
  
+ CTTTTAGAAA TTTTTCCTTT CTACTACTTC GCTCGTGTTT GGCTCTTTTA TTTATGTTTT TCCTTTTCGT   
  
  
+ CTCTACTACT CTTTTCCCCC TTCCTCTTCC CCCTCCCCTT CCCCTTCCCC TTCCCAGCTC TCAGTTTGAC   
  
  
+ TACGGGAGGG TTACGTTCCG TAGCACAGCA AATACTCCTA TAACGGCAGT TTGGGGGCTA GGTAAGCATA   
  
  
+ TTGCGGTGCA CAGTCCCGAA AACAGTCCTG TAACTGGCGG AGAAATTCAC GCCCAGGTAC CGTTGCCCTA   
  
  
+ TTTTGGTCAA CCTCTGGCAC GTCTCTACTA CGGTTACTTC CTATTTCAGT TTTCTAACTC TACTTGGCAA   
  
  
+ CCTAACCATC GTCTTCTCTC TCTCTCTCTG TCTCTCTCTC TCTCTCCCAA AATCAACCTC TGTCACTCCA   
  
  
+ TCCCAACTGG AACTTGTCGA AAATTACGTT TTCAGTAACT CACGCCTCTA CCCCACATAC CCAGGCGGTC   
  
  
+ CGAGCAGAAA ATGGCCTGTG GCCTTCGACG CAGTTTCGCA GTAGGGTGTT CGCTGTACCC TAAGAAAGAG   
  
  
+ AGAGAAATCA AAAAGAAGAT GCCAAAAGAT GAGCACCGGG GGTGGTTGTA AAAGGTACTA GTGTAGACGT   
  
  
+ GGCAAGTATG AACAGCTCTA GCTAAGACCA TCCTCTCCAG TGTGGTACGT ACCTGAACAC TCCACCACCG   
  
  
+ GCAACTTTTT CAAAGAGCGG TCAATGAAGT ATATGTCATT ACTCTTCACT TCCTCAAACC AGTTTTGATT   
  
  
+ TGAACTAAAG TTTGTAAAGC GGCCTTAAAC ACGCTTAGTC TATTAATTCT AAAATAATTA TTTTTGGGTG   
  
  
+ AATAGTAAAG CCGATGTAAT TACCTTATTT ACCAACCTTG CTTCTCTAGG TGTGCGGTTT CGTTTAGCTA   
  
  
+ GTCTTTATTT CCAGTTGAAG TGTGCGAAAG AGTTCGAAGA GACTAGTTTA AAGTTAATTA CTGGTTTGGT   
  
  
+ TTTTGACAAG TGTCTAAACC GTTATCGATA CAAAGAAGAC AGAAGGAAAT AGGGTTAAAT TTTTGTCAAA   
  
  
+ TATATATCAT AAAAAATTAA ATATTATTAT GATATTTTTA TTTTTTTGAA TAAATAAATA AAATATTTTA   
  
  
+ ATAGTTCATA ATATAAAAAA TTTTTTTAAT TTGGGTGAAA TTTAATTAAC TTCTATATTG GCTTTCACAA   
  
  
+ ATATAACTTT TTTTTTTCAC ATTAACTATG AATTGTGCCA ACCTCTTTTC CTCCCTTCGA ATGTTCGTTT   
  
  
+ ATCGAACTGA ACCGTAAACT ACTCGCTGAA GTAAAACTTG AATTTCCAAC TACCCGGTGT AAGTGTAATA   
  
  
+ ATCAGTATGG TATAAAAACA ATGAAAATTA CCTTCTTTTG GGTTTTTTTT TTTTTTTTTC GTTTTTCTTC   
  
  
+ GTTTGGGAGA AATTGTTTAA AATAATTTA  

- CTCTCCAAAA CCATCCAAGA CAACACCCTT AGATATGGAT GGGTATAAAT ATACAAAGAC CAAGAAGAGA   
  
  
- GAAAATCTTT AAAAAGGAAA GATGATGAAG CGAGCACAAA CCGAGAAAAT AAATACAAAA AGGAAAAGCA   
  
  
- GAGATGATGA GAAAAGGGGG AAGGAGAAGG GGGAGGGGAA GGGGAAGGGG AAGGGTCGAG AGTCAAACTG   
  
  
- ATGCCCTCCC AATGCAAGGC ATCGTGTCGT TTATGAGGAT ATTGCCGTCA AACCCCCGAT CCATTCGTAT   
  
  
- AACGCCACGT GTCAGGGCTT TTGTCAGGAC ATTGACCGCC TCTTTAAGTG CGGGTCCATG GCAACGGGAT   
  
  
- AAAACCAGTT GGAGACCGTG CAGAGATGAT GCCAATGAAG GATAAAGTCA AAAGATTGAG ATGAACCGTT   
  
  
- GGATTGGTAG CAGAAGAGAG AGAGAGAGAC AGAGAGAGAG AGAGAGGGTT TTAGTTGGAG ACAGTGAGGT   
  
  
- AGGGTTGACC TTGAACAGCT TTTAATGCAA AAGTCATTGA GTGCGGAGAT GGGGTGTATG GGTCCGCCAG   
  
  
- GCTCGTCTTT TACCGGACAC CGGAAGCTGC GTCAAAGCGT CATCCCACAA GCGACATGGG ATTCTTTCTC   
  
  
- TCTCTTTAGT TTTTCTTCTA CGGTTTTCTA CTCGTGGCCC CCACCAACAT TTTCCATGAT CACATCTGCA   
  
  
- CCGTTCATAC TTGTCGAGAT CGATTCTGGT AGGAGAGGTC ACACCATGCA TGGACTTGTG AGGTGGTGGC   
  
  
- CGTTGAAAAA GTTTCTCGCC AGTTACTTCA TATACAGTAA TGAGAAGTGA AGGAGTTTGG TCAAAACTAA   
  
  
- ACTTGATTTC AAACATTTCG CCGGAATTTG TGCGAATCAG ATAATTAAGA TTTTATTAAT AAAAACCCAC   
  
  
- TTATCATTTC GGCTACATTA ATGGAATAAA TGGTTGGAAC GAAGAGATCC ACACGCCAAA GCAAATCGAT   
  
  
- CAGAAATAAA GGTCAACTTC ACACGCTTTC TCAAGCTTCT CTGATCAAAT TTCAATTAAT GACCAAACCA   
  
  
- AAAACTGTTC ACAGATTTGG CAATAGCTAT GTTTCTTCTG TCTTCCTTTA TCCCAATTTA AAAACAGTTT   
  
  
- ATATATAGTA TTTTTTAATT TATAATAATA CTATAAAAAT AAAAAAACTT ATTTATTTAT TTTATAAAAT   
  
  
- TATCAAGTAT TATATTTTTT AAAAAAATTA AACCCACTTT AAATTAATTG AAGATATAAC CGAAAGTGTT   
  
  
- TATATTGAAA AAAAAAAGTG TAATTGATAC TTAACACGGT TGGAGAAAAG GAGGGAAGCT TACAAGCAAA   
  
  
- TAGCTTGACT TGGCATTTGA TGAGCGACTT CATTTTGAAC TTAAAGGTTG ATGGGCCACA TTCACATTAT   
  
  
- TAGTCATACC ATATTTTTGT TACTTTTAAT GGAAGAAAAC CCAAAAAAAA AAAAAAAAAG CAAAAAGAAG   
  
  
- CAAACCCTCT TTAACAAATT TTATTAAAT

+     TATA-box

| Site Name | Organism | Position | Strand | Matrix score. | sequence | function |
| --- | --- | --- | --- | --- | --- | --- |
| TATA-box | Lycopersicon esculentum | 1488 | - | 5 | TTTTA | core promoter element around -30 of transcription start |
| TATA-box | Glycine max | 1396 | + | 5 | TAATA | core promoter element around -30 of transcription start |
| TATA-box | Lycopersicon esculentum | 1413 | - | 5 | TTTTA | core promoter element around -30 of transcription start |
| TATA-box | Arabidopsis thaliana | 1244 | - | 4 | TATA | core promoter element around -30 of transcription start |
| TATA-box | Glycine max | 1142 | - | 5 | TAATA | core promoter element around -30 of transcription start |
| TATA-box | Arabidopsis thaliana | 1123 | - | 4 | TATA | core promoter element around -30 of transcription start |
| TATA-box | Lycopersicon esculentum | 679 | - | 5 | TTTTA | core promoter element around -30 of transcription start |
| TATA-box | Arabidopsis thaliana | 249 | + | 4 | TATA | core promoter element around -30 of transcription start |
| TATA-box | Lycopersicon esculentum | 72 | + | 5 | TTTTA | core promoter element around -30 of transcription start |
| TATA-box | Arabidopsis thaliana | 1411 | + | 6 | TATAAA | core promoter element around -30 of transcription start |
| TATA-box | Arabidopsis thaliana | 48 | - | 5 | TATAA | core promoter element around -30 of transcription start |
| TATA-box | Glycine max | 1199 | + | 5 | TAATA | core promoter element around -30 of transcription start |
| TATA-box | Lycopersicon esculentum | 1130 | - | 5 | TTTTA | core promoter element around -30 of transcription start |
| TATA-box | Lycopersicon esculentum | 116 | + | 5 | TTTTA | core promoter element around -30 of transcription start |
| TATA-box | Arabidopsis thaliana | 47 | - | 6 | TATAAA | core promoter element around -30 of transcription start |
| TATA-box | Brassica oleracea | 1201 | + | 6 | ATATAA | core promoter element around -30 of transcription start |
| TATA-box | Arabidopsis thaliana | 49 | + | 4 | TATA | core promoter element around -30 of transcription start |
| TATA-box | Glycine max | 1145 | - | 5 | TAATA | core promoter element around -30 of transcription start |
| TATA-box | Brassica oleracea | 1261 | + | 6 | ATATAA | core promoter element around -30 of transcription start |
| TATA-box | Lycopersicon esculentum | 1204 | - | 5 | TTTTA | core promoter element around -30 of transcription start |
| TATA-box | Lycopersicon esculentum | 1362 | - | 5 | TTTTA | core promoter element around -30 of transcription start |
| TATA-box | Brassica napus | 1122 | - | 6 | ATATAT | core promoter element around -30 of transcription start |
| TATA-box | Lycopersicon esculentum | 890 | - | 5 | TTTTA | core promoter element around -30 of transcription start |
| TATA-box | Arabidopsis thaliana | 1262 | - | 4 | TATA | core promoter element around -30 of transcription start |
| TATA-box | Arabidopsis thaliana | 1121 | - | 4 | TATA | core promoter element around -30 of transcription start |
| TATA-box | Lycopersicon esculentum | 1179 | - | 5 | TTTTA | core promoter element around -30 of transcription start |
| TATA-box | Brassica napus | 1120 | - | 6 | ATATAT | core promoter element around -30 of transcription start |
| TATA-box | Arabidopsis thaliana | 1202 | + | 6 | TATAAA | core promoter element around -30 of transcription start |
| TATA-box | Arabidopsis thaliana | 800 | - | 4 | TATA | core promoter element around -30 of transcription start |
| TATA-box | Arabidopsis thaliana | 33 | + | 4 | TATA | core promoter element around -30 of transcription start |
| TATA-box | Daucus carota | 45 | - | 8 | TATAAATA | core promoter element around -30 of transcription start |
| TATA-box | Ac | 46 | - | 7 | TATAAAT | core promoter element around -30 of transcription start |
| TATA-box | Glycine max | 1189 | + | 5 | TAATA | core promoter element around -30 of transcription start |
| TATA-box | Glycine max | 881 | - | 5 | TAATA | core promoter element around -30 of transcription start |
| TATA-box | Lycopersicon esculentum | 1214 | + | 5 | TTTTA | core promoter element around -30 of transcription start |
| TATA-box | Lycopersicon esculentum | 1156 | + | 5 | TTTTA | core promoter element around -30 of transcription start |
| TATA-box | Lycopersicon esculentum | 1186 | + | 5 | TTTTA | core promoter element around -30 of transcription start |

> 2018/04/13 10:10:12  
+ GAGAGGTTTT GGTAGGTTCT GTTGTGGGAA TCTATACCTA CCCATATTTA TATGTTTCTG GTTCTTCTCT   
  
  
+ CTTTTAGAAA TTTTTCCTTT CTACTACTTC GCTCGTGTTT GGCTCTTTTA TTTATGTTTT TCCTTTTCGT   
  
  
+ CTCTACTACT CTTTTCCCCC TTCCTCTTCC CCCTCCCCTT CCCCTTCCCC TTCCCAGCTC TCAGTTTGAC   
  
  
+ TACGGGAGGG TTACGTTCCG TAGCACAGCA AATACTCCTA TAACGGCAGT TTGGGGGCTA GGTAAGCATA   
  
  
+ TTGCGGTGCA CAGTCCCGAA AACAGTCCTG TAACTGGCGG AGAAATTCAC GCCCAGGTAC CGTTGCCCTA   
  
  
+ TTTTGGTCAA CCTCTGGCAC GTCTCTACTA CGGTTACTTC CTATTTCAGT TTTCTAACTC TACTTGGCAA   
  
  
+ CCTAACCATC GTCTTCTCTC TCTCTCTCTG TCTCTCTCTC TCTCTCCCAA AATCAACCTC TGTCACTCCA   
  
  
+ TCCCAACTGG AACTTGTCGA AAATTACGTT TTCAGTAACT CACGCCTCTA CCCCACATAC CCAGGCGGTC   
  
  
+ CGAGCAGAAA ATGGCCTGTG GCCTTCGACG CAGTTTCGCA GTAGGGTGTT CGCTGTACCC TAAGAAAGAG   
  
  
+ AGAGAAATCA AAAAGAAGAT GCCAAAAGAT GAGCACCGGG GGTGGTTGTA AAAGGTACTA GTGTAGACGT   
  
  
+ GGCAAGTATG AACAGCTCTA GCTAAGACCA TCCTCTCCAG TGTGGTACGT ACCTGAACAC TCCACCACCG   
  
  
+ GCAACTTTTT CAAAGAGCGG TCAATGAAGT ATATGTCATT ACTCTTCACT TCCTCAAACC AGTTTTGATT   
  
  
+ TGAACTAAAG TTTGTAAAGC GGCCTTAAAC ACGCTTAGTC TATTAATTCT AAAATAATTA TTTTTGGGTG   
  
  
+ AATAGTAAAG CCGATGTAAT TACCTTATTT ACCAACCTTG CTTCTCTAGG TGTGCGGTTT CGTTTAGCTA   
  
  
+ GTCTTTATTT CCAGTTGAAG TGTGCGAAAG AGTTCGAAGA GACTAGTTTA AAGTTAATTA CTGGTTTGGT   
  
  
+ TTTTGACAAG TGTCTAAACC GTTATCGATA CAAAGAAGAC AGAAGGAAAT AGGGTTAAAT TTTTGTCAAA   
  
  
+ TATATATCAT AAAAAATTAA ATATTATTAT GATATTTTTA TTTTTTTGAA TAAATAAATA AAATATTTTA   
  
  
+ ATAGTTCATA ATATAAAAAA TTTTTTTAAT TTGGGTGAAA TTTAATTAAC TTCTATATTG GCTTTCACAA   
  
  
+ ATATAACTTT TTTTTTTCAC ATTAACTATG AATTGTGCCA ACCTCTTTTC CTCCCTTCGA ATGTTCGTTT   
  
  
+ ATCGAACTGA ACCGTAAACT ACTCGCTGAA GTAAAACTTG AATTTCCAAC TACCCGGTGT AAGTGTAATA   
  
  
+ ATCAGTATGG TATAAAAACA ATGAAAATTA CCTTCTTTTG GGTTTTTTTT TTTTTTTTTC GTTTTTCTTC   
  
  
+ GTTTGGGAGA AATTGTTTAA AATAATTTA  

- CTCTCCAAAA CCATCCAAGA CAACACCCTT AGATATGGAT GGGTATAAAT ATACAAAGAC CAAGAAGAGA   
  
  
- GAAAATCTTT AAAAAGGAAA GATGATGAAG CGAGCACAAA CCGAGAAAAT AAATACAAAA AGGAAAAGCA   
  
  
- GAGATGATGA GAAAAGGGGG AAGGAGAAGG GGGAGGGGAA GGGGAAGGGG AAGGGTCGAG AGTCAAACTG   
  
  
- ATGCCCTCCC AATGCAAGGC ATCGTGTCGT TTATGAGGAT ATTGCCGTCA AACCCCCGAT CCATTCGTAT   
  
  
- AACGCCACGT GTCAGGGCTT TTGTCAGGAC ATTGACCGCC TCTTTAAGTG CGGGTCCATG GCAACGGGAT   
  
  
- AAAACCAGTT GGAGACCGTG CAGAGATGAT GCCAATGAAG GATAAAGTCA AAAGATTGAG ATGAACCGTT   
  
  
- GGATTGGTAG CAGAAGAGAG AGAGAGAGAC AGAGAGAGAG AGAGAGGGTT TTAGTTGGAG ACAGTGAGGT   
  
  
- AGGGTTGACC TTGAACAGCT TTTAATGCAA AAGTCATTGA GTGCGGAGAT GGGGTGTATG GGTCCGCCAG   
  
  
- GCTCGTCTTT TACCGGACAC CGGAAGCTGC GTCAAAGCGT CATCCCACAA GCGACATGGG ATTCTTTCTC   
  
  
- TCTCTTTAGT TTTTCTTCTA CGGTTTTCTA CTCGTGGCCC CCACCAACAT TTTCCATGAT CACATCTGCA   
  
  
- CCGTTCATAC TTGTCGAGAT CGATTCTGGT AGGAGAGGTC ACACCATGCA TGGACTTGTG AGGTGGTGGC   
  
  
- CGTTGAAAAA GTTTCTCGCC AGTTACTTCA TATACAGTAA TGAGAAGTGA AGGAGTTTGG TCAAAACTAA   
  
  
- ACTTGATTTC AAACATTTCG CCGGAATTTG TGCGAATCAG ATAATTAAGA TTTTATTAAT AAAAACCCAC   
  
  
- TTATCATTTC GGCTACATTA ATGGAATAAA TGGTTGGAAC GAAGAGATCC ACACGCCAAA GCAAATCGAT   
  
  
- CAGAAATAAA GGTCAACTTC ACACGCTTTC TCAAGCTTCT CTGATCAAAT TTCAATTAAT GACCAAACCA   
  
  
- AAAACTGTTC ACAGATTTGG CAATAGCTAT GTTTCTTCTG TCTTCCTTTA TCCCAATTTA AAAACAGTTT   
  
  
- ATATATAGTA TTTTTTAATT TATAATAATA CTATAAAAAT AAAAAAACTT ATTTATTTAT TTTATAAAAT   
  
  
- TATCAAGTAT TATATTTTTT AAAAAAATTA AACCCACTTT AAATTAATTG AAGATATAAC CGAAAGTGTT   
  
  
- TATATTGAAA AAAAAAAGTG TAATTGATAC TTAACACGGT TGGAGAAAAG GAGGGAAGCT TACAAGCAAA   
  
  
- TAGCTTGACT TGGCATTTGA TGAGCGACTT CATTTTGAAC TTAAAGGTTG ATGGGCCACA TTCACATTAT   
  
  
- TAGTCATACC ATATTTTTGT TACTTTTAAT GGAAGAAAAC CCAAAAAAAA AAAAAAAAAG CAAAAAGAAG   
  
  
- CAAACCCTCT TTAACAAATT TTATTAAAT

+     TC-rich repeats

| Site Name | Organism | Position | Strand | Matrix score. | sequence | function |
| --- | --- | --- | --- | --- | --- | --- |
| TC-rich repeats | Nicotiana tabacum | 399 | + | 9 | GTTTTCTTAC | cis-acting element involved in defense and stress responsiveness |

> 2018/04/13 10:10:12  
+ GAGAGGTTTT GGTAGGTTCT GTTGTGGGAA TCTATACCTA CCCATATTTA TATGTTTCTG GTTCTTCTCT   
  
  
+ CTTTTAGAAA TTTTTCCTTT CTACTACTTC GCTCGTGTTT GGCTCTTTTA TTTATGTTTT TCCTTTTCGT   
  
  
+ CTCTACTACT CTTTTCCCCC TTCCTCTTCC CCCTCCCCTT CCCCTTCCCC TTCCCAGCTC TCAGTTTGAC   
  
  
+ TACGGGAGGG TTACGTTCCG TAGCACAGCA AATACTCCTA TAACGGCAGT TTGGGGGCTA GGTAAGCATA   
  
  
+ TTGCGGTGCA CAGTCCCGAA AACAGTCCTG TAACTGGCGG AGAAATTCAC GCCCAGGTAC CGTTGCCCTA   
  
  
+ TTTTGGTCAA CCTCTGGCAC GTCTCTACTA CGGTTACTTC CTATTTCAGT TTTCTAACTC TACTTGGCAA   
  
  
+ CCTAACCATC GTCTTCTCTC TCTCTCTCTG TCTCTCTCTC TCTCTCCCAA AATCAACCTC TGTCACTCCA   
  
  
+ TCCCAACTGG AACTTGTCGA AAATTACGTT TTCAGTAACT CACGCCTCTA CCCCACATAC CCAGGCGGTC   
  
  
+ CGAGCAGAAA ATGGCCTGTG GCCTTCGACG CAGTTTCGCA GTAGGGTGTT CGCTGTACCC TAAGAAAGAG   
  
  
+ AGAGAAATCA AAAAGAAGAT GCCAAAAGAT GAGCACCGGG GGTGGTTGTA AAAGGTACTA GTGTAGACGT   
  
  
+ GGCAAGTATG AACAGCTCTA GCTAAGACCA TCCTCTCCAG TGTGGTACGT ACCTGAACAC TCCACCACCG   
  
  
+ GCAACTTTTT CAAAGAGCGG TCAATGAAGT ATATGTCATT ACTCTTCACT TCCTCAAACC AGTTTTGATT   
  
  
+ TGAACTAAAG TTTGTAAAGC GGCCTTAAAC ACGCTTAGTC TATTAATTCT AAAATAATTA TTTTTGGGTG   
  
  
+ AATAGTAAAG CCGATGTAAT TACCTTATTT ACCAACCTTG CTTCTCTAGG TGTGCGGTTT CGTTTAGCTA   
  
  
+ GTCTTTATTT CCAGTTGAAG TGTGCGAAAG AGTTCGAAGA GACTAGTTTA AAGTTAATTA CTGGTTTGGT   
  
  
+ TTTTGACAAG TGTCTAAACC GTTATCGATA CAAAGAAGAC AGAAGGAAAT AGGGTTAAAT TTTTGTCAAA   
  
  
+ TATATATCAT AAAAAATTAA ATATTATTAT GATATTTTTA TTTTTTTGAA TAAATAAATA AAATATTTTA   
  
  
+ ATAGTTCATA ATATAAAAAA TTTTTTTAAT TTGGGTGAAA TTTAATTAAC TTCTATATTG GCTTTCACAA   
  
  
+ ATATAACTTT TTTTTTTCAC ATTAACTATG AATTGTGCCA ACCTCTTTTC CTCCCTTCGA ATGTTCGTTT   
  
  
+ ATCGAACTGA ACCGTAAACT ACTCGCTGAA GTAAAACTTG AATTTCCAAC TACCCGGTGT AAGTGTAATA   
  
  
+ ATCAGTATGG TATAAAAACA ATGAAAATTA CCTTCTTTTG GGTTTTTTTT TTTTTTTTTC GTTTTTCTTC   
  
  
+ GTTTGGGAGA AATTGTTTAA AATAATTTA  

- CTCTCCAAAA CCATCCAAGA CAACACCCTT AGATATGGAT GGGTATAAAT ATACAAAGAC CAAGAAGAGA   
  
  
- GAAAATCTTT AAAAAGGAAA GATGATGAAG CGAGCACAAA CCGAGAAAAT AAATACAAAA AGGAAAAGCA   
  
  
- GAGATGATGA GAAAAGGGGG AAGGAGAAGG GGGAGGGGAA GGGGAAGGGG AAGGGTCGAG AGTCAAACTG   
  
  
- ATGCCCTCCC AATGCAAGGC ATCGTGTCGT TTATGAGGAT ATTGCCGTCA AACCCCCGAT CCATTCGTAT   
  
  
- AACGCCACGT GTCAGGGCTT TTGTCAGGAC ATTGACCGCC TCTTTAAGTG CGGGTCCATG GCAACGGGAT   
  
  
- AAAACCAGTT GGAGACCGTG CAGAGATGAT GCCAATGAAG GATAAAGTCA AAAGATTGAG ATGAACCGTT   
  
  
- GGATTGGTAG CAGAAGAGAG AGAGAGAGAC AGAGAGAGAG AGAGAGGGTT TTAGTTGGAG ACAGTGAGGT   
  
  
- AGGGTTGACC TTGAACAGCT TTTAATGCAA AAGTCATTGA GTGCGGAGAT GGGGTGTATG GGTCCGCCAG   
  
  
- GCTCGTCTTT TACCGGACAC CGGAAGCTGC GTCAAAGCGT CATCCCACAA GCGACATGGG ATTCTTTCTC   
  
  
- TCTCTTTAGT TTTTCTTCTA CGGTTTTCTA CTCGTGGCCC CCACCAACAT TTTCCATGAT CACATCTGCA   
  
  
- CCGTTCATAC TTGTCGAGAT CGATTCTGGT AGGAGAGGTC ACACCATGCA TGGACTTGTG AGGTGGTGGC   
  
  
- CGTTGAAAAA GTTTCTCGCC AGTTACTTCA TATACAGTAA TGAGAAGTGA AGGAGTTTGG TCAAAACTAA   
  
  
- ACTTGATTTC AAACATTTCG CCGGAATTTG TGCGAATCAG ATAATTAAGA TTTTATTAAT AAAAACCCAC   
  
  
- TTATCATTTC GGCTACATTA ATGGAATAAA TGGTTGGAAC GAAGAGATCC ACACGCCAAA GCAAATCGAT   
  
  
- CAGAAATAAA GGTCAACTTC ACACGCTTTC TCAAGCTTCT CTGATCAAAT TTCAATTAAT GACCAAACCA   
  
  
- AAAACTGTTC ACAGATTTGG CAATAGCTAT GTTTCTTCTG TCTTCCTTTA TCCCAATTTA AAAACAGTTT   
  
  
- ATATATAGTA TTTTTTAATT TATAATAATA CTATAAAAAT AAAAAAACTT ATTTATTTAT TTTATAAAAT   
  
  
- TATCAAGTAT TATATTTTTT AAAAAAATTA AACCCACTTT AAATTAATTG AAGATATAAC CGAAAGTGTT   
  
  
- TATATTGAAA AAAAAAAGTG TAATTGATAC TTAACACGGT TGGAGAAAAG GAGGGAAGCT TACAAGCAAA   
  
  
- TAGCTTGACT TGGCATTTGA TGAGCGACTT CATTTTGAAC TTAAAGGTTG ATGGGCCACA TTCACATTAT   
  
  
- TAGTCATACC ATATTTTTGT TACTTTTAAT GGAAGAAAAC CCAAAAAAAA AAAAAAAAAG CAAAAAGAAG   
  
  
- CAAACCCTCT TTAACAAATT TTATTAAAT

+     TGG-motif

| Site Name | Organism | Position | Strand | Matrix score. | sequence | function |
| --- | --- | --- | --- | --- | --- | --- |
| TGG-motif | Gossypium hirsutum | 415 | - | 8 | GGTTGCCA | part of a light responsive element |

> 2018/04/13 10:10:12  
+ GAGAGGTTTT GGTAGGTTCT GTTGTGGGAA TCTATACCTA CCCATATTTA TATGTTTCTG GTTCTTCTCT   
  
  
+ CTTTTAGAAA TTTTTCCTTT CTACTACTTC GCTCGTGTTT GGCTCTTTTA TTTATGTTTT TCCTTTTCGT   
  
  
+ CTCTACTACT CTTTTCCCCC TTCCTCTTCC CCCTCCCCTT CCCCTTCCCC TTCCCAGCTC TCAGTTTGAC   
  
  
+ TACGGGAGGG TTACGTTCCG TAGCACAGCA AATACTCCTA TAACGGCAGT TTGGGGGCTA GGTAAGCATA   
  
  
+ TTGCGGTGCA CAGTCCCGAA AACAGTCCTG TAACTGGCGG AGAAATTCAC GCCCAGGTAC CGTTGCCCTA   
  
  
+ TTTTGGTCAA CCTCTGGCAC GTCTCTACTA CGGTTACTTC CTATTTCAGT TTTCTAACTC TACTTGGCAA   
  
  
+ CCTAACCATC GTCTTCTCTC TCTCTCTCTG TCTCTCTCTC TCTCTCCCAA AATCAACCTC TGTCACTCCA   
  
  
+ TCCCAACTGG AACTTGTCGA AAATTACGTT TTCAGTAACT CACGCCTCTA CCCCACATAC CCAGGCGGTC   
  
  
+ CGAGCAGAAA ATGGCCTGTG GCCTTCGACG CAGTTTCGCA GTAGGGTGTT CGCTGTACCC TAAGAAAGAG   
  
  
+ AGAGAAATCA AAAAGAAGAT GCCAAAAGAT GAGCACCGGG GGTGGTTGTA AAAGGTACTA GTGTAGACGT   
  
  
+ GGCAAGTATG AACAGCTCTA GCTAAGACCA TCCTCTCCAG TGTGGTACGT ACCTGAACAC TCCACCACCG   
  
  
+ GCAACTTTTT CAAAGAGCGG TCAATGAAGT ATATGTCATT ACTCTTCACT TCCTCAAACC AGTTTTGATT   
  
  
+ TGAACTAAAG TTTGTAAAGC GGCCTTAAAC ACGCTTAGTC TATTAATTCT AAAATAATTA TTTTTGGGTG   
  
  
+ AATAGTAAAG CCGATGTAAT TACCTTATTT ACCAACCTTG CTTCTCTAGG TGTGCGGTTT CGTTTAGCTA   
  
  
+ GTCTTTATTT CCAGTTGAAG TGTGCGAAAG AGTTCGAAGA GACTAGTTTA AAGTTAATTA CTGGTTTGGT   
  
  
+ TTTTGACAAG TGTCTAAACC GTTATCGATA CAAAGAAGAC AGAAGGAAAT AGGGTTAAAT TTTTGTCAAA   
  
  
+ TATATATCAT AAAAAATTAA ATATTATTAT GATATTTTTA TTTTTTTGAA TAAATAAATA AAATATTTTA   
  
  
+ ATAGTTCATA ATATAAAAAA TTTTTTTAAT TTGGGTGAAA TTTAATTAAC TTCTATATTG GCTTTCACAA   
  
  
+ ATATAACTTT TTTTTTTCAC ATTAACTATG AATTGTGCCA ACCTCTTTTC CTCCCTTCGA ATGTTCGTTT   
  
  
+ ATCGAACTGA ACCGTAAACT ACTCGCTGAA GTAAAACTTG AATTTCCAAC TACCCGGTGT AAGTGTAATA   
  
  
+ ATCAGTATGG TATAAAAACA ATGAAAATTA CCTTCTTTTG GGTTTTTTTT TTTTTTTTTC GTTTTTCTTC   
  
  
+ GTTTGGGAGA AATTGTTTAA AATAATTTA  

- CTCTCCAAAA CCATCCAAGA CAACACCCTT AGATATGGAT GGGTATAAAT ATACAAAGAC CAAGAAGAGA   
  
  
- GAAAATCTTT AAAAAGGAAA GATGATGAAG CGAGCACAAA CCGAGAAAAT AAATACAAAA AGGAAAAGCA   
  
  
- GAGATGATGA GAAAAGGGGG AAGGAGAAGG GGGAGGGGAA GGGGAAGGGG AAGGGTCGAG AGTCAAACTG   
  
  
- ATGCCCTCCC AATGCAAGGC ATCGTGTCGT TTATGAGGAT ATTGCCGTCA AACCCCCGAT CCATTCGTAT   
  
  
- AACGCCACGT GTCAGGGCTT TTGTCAGGAC ATTGACCGCC TCTTTAAGTG CGGGTCCATG GCAACGGGAT   
  
  
- AAAACCAGTT GGAGACCGTG CAGAGATGAT GCCAATGAAG GATAAAGTCA AAAGATTGAG ATGAACCGTT   
  
  
- GGATTGGTAG CAGAAGAGAG AGAGAGAGAC AGAGAGAGAG AGAGAGGGTT TTAGTTGGAG ACAGTGAGGT   
  
  
- AGGGTTGACC TTGAACAGCT TTTAATGCAA AAGTCATTGA GTGCGGAGAT GGGGTGTATG GGTCCGCCAG   
  
  
- GCTCGTCTTT TACCGGACAC CGGAAGCTGC GTCAAAGCGT CATCCCACAA GCGACATGGG ATTCTTTCTC   
  
  
- TCTCTTTAGT TTTTCTTCTA CGGTTTTCTA CTCGTGGCCC CCACCAACAT TTTCCATGAT CACATCTGCA   
  
  
- CCGTTCATAC TTGTCGAGAT CGATTCTGGT AGGAGAGGTC ACACCATGCA TGGACTTGTG AGGTGGTGGC   
  
  
- CGTTGAAAAA GTTTCTCGCC AGTTACTTCA TATACAGTAA TGAGAAGTGA AGGAGTTTGG TCAAAACTAA   
  
  
- ACTTGATTTC AAACATTTCG CCGGAATTTG TGCGAATCAG ATAATTAAGA TTTTATTAAT AAAAACCCAC   
  
  
- TTATCATTTC GGCTACATTA ATGGAATAAA TGGTTGGAAC GAAGAGATCC ACACGCCAAA GCAAATCGAT   
  
  
- CAGAAATAAA GGTCAACTTC ACACGCTTTC TCAAGCTTCT CTGATCAAAT TTCAATTAAT GACCAAACCA   
  
  
- AAAACTGTTC ACAGATTTGG CAATAGCTAT GTTTCTTCTG TCTTCCTTTA TCCCAATTTA AAAACAGTTT   
  
  
- ATATATAGTA TTTTTTAATT TATAATAATA CTATAAAAAT AAAAAAACTT ATTTATTTAT TTTATAAAAT   
  
  
- TATCAAGTAT TATATTTTTT AAAAAAATTA AACCCACTTT AAATTAATTG AAGATATAAC CGAAAGTGTT   
  
  
- TATATTGAAA AAAAAAAGTG TAATTGATAC TTAACACGGT TGGAGAAAAG GAGGGAAGCT TACAAGCAAA   
  
  
- TAGCTTGACT TGGCATTTGA TGAGCGACTT CATTTTGAAC TTAAAGGTTG ATGGGCCACA TTCACATTAT   
  
  
- TAGTCATACC ATATTTTTGT TACTTTTAAT GGAAGAAAAC CCAAAAAAAA AAAAAAAAAG CAAAAAGAAG   
  
  
- CAAACCCTCT TTAACAAATT TTATTAAAT

+     Unnamed\_\_1

| Site Name | Organism | Position | Strand | Matrix score. | sequence | function |
| --- | --- | --- | --- | --- | --- | --- |
| Unnamed\_\_1 | Glycine max | 1232 | - | 11 | GAATTTAATTAA | 60K protein binding site |
| Unnamed\_\_1 | Zea mays | 698 | + | 5 | CGTGG |  |
| Unnamed\_\_1 | Glycine max | 1228 | + | 11 | GAATTTAATTAA | 60K protein binding site |

> 2018/04/13 10:10:12  
+ GAGAGGTTTT GGTAGGTTCT GTTGTGGGAA TCTATACCTA CCCATATTTA TATGTTTCTG GTTCTTCTCT   
  
  
+ CTTTTAGAAA TTTTTCCTTT CTACTACTTC GCTCGTGTTT GGCTCTTTTA TTTATGTTTT TCCTTTTCGT   
  
  
+ CTCTACTACT CTTTTCCCCC TTCCTCTTCC CCCTCCCCTT CCCCTTCCCC TTCCCAGCTC TCAGTTTGAC   
  
  
+ TACGGGAGGG TTACGTTCCG TAGCACAGCA AATACTCCTA TAACGGCAGT TTGGGGGCTA GGTAAGCATA   
  
  
+ TTGCGGTGCA CAGTCCCGAA AACAGTCCTG TAACTGGCGG AGAAATTCAC GCCCAGGTAC CGTTGCCCTA   
  
  
+ TTTTGGTCAA CCTCTGGCAC GTCTCTACTA CGGTTACTTC CTATTTCAGT TTTCTAACTC TACTTGGCAA   
  
  
+ CCTAACCATC GTCTTCTCTC TCTCTCTCTG TCTCTCTCTC TCTCTCCCAA AATCAACCTC TGTCACTCCA   
  
  
+ TCCCAACTGG AACTTGTCGA AAATTACGTT TTCAGTAACT CACGCCTCTA CCCCACATAC CCAGGCGGTC   
  
  
+ CGAGCAGAAA ATGGCCTGTG GCCTTCGACG CAGTTTCGCA GTAGGGTGTT CGCTGTACCC TAAGAAAGAG   
  
  
+ AGAGAAATCA AAAAGAAGAT GCCAAAAGAT GAGCACCGGG GGTGGTTGTA AAAGGTACTA GTGTAGACGT   
  
  
+ GGCAAGTATG AACAGCTCTA GCTAAGACCA TCCTCTCCAG TGTGGTACGT ACCTGAACAC TCCACCACCG   
  
  
+ GCAACTTTTT CAAAGAGCGG TCAATGAAGT ATATGTCATT ACTCTTCACT TCCTCAAACC AGTTTTGATT   
  
  
+ TGAACTAAAG TTTGTAAAGC GGCCTTAAAC ACGCTTAGTC TATTAATTCT AAAATAATTA TTTTTGGGTG   
  
  
+ AATAGTAAAG CCGATGTAAT TACCTTATTT ACCAACCTTG CTTCTCTAGG TGTGCGGTTT CGTTTAGCTA   
  
  
+ GTCTTTATTT CCAGTTGAAG TGTGCGAAAG AGTTCGAAGA GACTAGTTTA AAGTTAATTA CTGGTTTGGT   
  
  
+ TTTTGACAAG TGTCTAAACC GTTATCGATA CAAAGAAGAC AGAAGGAAAT AGGGTTAAAT TTTTGTCAAA   
  
  
+ TATATATCAT AAAAAATTAA ATATTATTAT GATATTTTTA TTTTTTTGAA TAAATAAATA AAATATTTTA   
  
  
+ ATAGTTCATA ATATAAAAAA TTTTTTTAAT TTGGGTGAAA TTTAATTAAC TTCTATATTG GCTTTCACAA   
  
  
+ ATATAACTTT TTTTTTTCAC ATTAACTATG AATTGTGCCA ACCTCTTTTC CTCCCTTCGA ATGTTCGTTT   
  
  
+ ATCGAACTGA ACCGTAAACT ACTCGCTGAA GTAAAACTTG AATTTCCAAC TACCCGGTGT AAGTGTAATA   
  
  
+ ATCAGTATGG TATAAAAACA ATGAAAATTA CCTTCTTTTG GGTTTTTTTT TTTTTTTTTC GTTTTTCTTC   
  
  
+ GTTTGGGAGA AATTGTTTAA AATAATTTA  

- CTCTCCAAAA CCATCCAAGA CAACACCCTT AGATATGGAT GGGTATAAAT ATACAAAGAC CAAGAAGAGA   
  
  
- GAAAATCTTT AAAAAGGAAA GATGATGAAG CGAGCACAAA CCGAGAAAAT AAATACAAAA AGGAAAAGCA   
  
  
- GAGATGATGA GAAAAGGGGG AAGGAGAAGG GGGAGGGGAA GGGGAAGGGG AAGGGTCGAG AGTCAAACTG   
  
  
- ATGCCCTCCC AATGCAAGGC ATCGTGTCGT TTATGAGGAT ATTGCCGTCA AACCCCCGAT CCATTCGTAT   
  
  
- AACGCCACGT GTCAGGGCTT TTGTCAGGAC ATTGACCGCC TCTTTAAGTG CGGGTCCATG GCAACGGGAT   
  
  
- AAAACCAGTT GGAGACCGTG CAGAGATGAT GCCAATGAAG GATAAAGTCA AAAGATTGAG ATGAACCGTT   
  
  
- GGATTGGTAG CAGAAGAGAG AGAGAGAGAC AGAGAGAGAG AGAGAGGGTT TTAGTTGGAG ACAGTGAGGT   
  
  
- AGGGTTGACC TTGAACAGCT TTTAATGCAA AAGTCATTGA GTGCGGAGAT GGGGTGTATG GGTCCGCCAG   
  
  
- GCTCGTCTTT TACCGGACAC CGGAAGCTGC GTCAAAGCGT CATCCCACAA GCGACATGGG ATTCTTTCTC   
  
  
- TCTCTTTAGT TTTTCTTCTA CGGTTTTCTA CTCGTGGCCC CCACCAACAT TTTCCATGAT CACATCTGCA   
  
  
- CCGTTCATAC TTGTCGAGAT CGATTCTGGT AGGAGAGGTC ACACCATGCA TGGACTTGTG AGGTGGTGGC   
  
  
- CGTTGAAAAA GTTTCTCGCC AGTTACTTCA TATACAGTAA TGAGAAGTGA AGGAGTTTGG TCAAAACTAA   
  
  
- ACTTGATTTC AAACATTTCG CCGGAATTTG TGCGAATCAG ATAATTAAGA TTTTATTAAT AAAAACCCAC   
  
  
- TTATCATTTC GGCTACATTA ATGGAATAAA TGGTTGGAAC GAAGAGATCC ACACGCCAAA GCAAATCGAT   
  
  
- CAGAAATAAA GGTCAACTTC ACACGCTTTC TCAAGCTTCT CTGATCAAAT TTCAATTAAT GACCAAACCA   
  
  
- AAAACTGTTC ACAGATTTGG CAATAGCTAT GTTTCTTCTG TCTTCCTTTA TCCCAATTTA AAAACAGTTT   
  
  
- ATATATAGTA TTTTTTAATT TATAATAATA CTATAAAAAT AAAAAAACTT ATTTATTTAT TTTATAAAAT   
  
  
- TATCAAGTAT TATATTTTTT AAAAAAATTA AACCCACTTT AAATTAATTG AAGATATAAC CGAAAGTGTT   
  
  
- TATATTGAAA AAAAAAAGTG TAATTGATAC TTAACACGGT TGGAGAAAAG GAGGGAAGCT TACAAGCAAA   
  
  
- TAGCTTGACT TGGCATTTGA TGAGCGACTT CATTTTGAAC TTAAAGGTTG ATGGGCCACA TTCACATTAT   
  
  
- TAGTCATACC ATATTTTTGT TACTTTTAAT GGAAGAAAAC CCAAAAAAAA AAAAAAAAAG CAAAAAGAAG   
  
  
- CAAACCCTCT TTAACAAATT TTATTAAAT

+     Unnamed\_\_2

| Site Name | Organism | Position | Strand | Matrix score. | sequence | function |
| --- | --- | --- | --- | --- | --- | --- |
| Unnamed\_\_2 | Petroselinum hortense | 419 | + | 9 | AACCTAACCT |  |
| Unnamed\_\_2 | Zea mays | 666 | - | 6 | CCCCGG |  |

> 2018/04/13 10:10:12  
+ GAGAGGTTTT GGTAGGTTCT GTTGTGGGAA TCTATACCTA CCCATATTTA TATGTTTCTG GTTCTTCTCT   
  
  
+ CTTTTAGAAA TTTTTCCTTT CTACTACTTC GCTCGTGTTT GGCTCTTTTA TTTATGTTTT TCCTTTTCGT   
  
  
+ CTCTACTACT CTTTTCCCCC TTCCTCTTCC CCCTCCCCTT CCCCTTCCCC TTCCCAGCTC TCAGTTTGAC   
  
  
+ TACGGGAGGG TTACGTTCCG TAGCACAGCA AATACTCCTA TAACGGCAGT TTGGGGGCTA GGTAAGCATA   
  
  
+ TTGCGGTGCA CAGTCCCGAA AACAGTCCTG TAACTGGCGG AGAAATTCAC GCCCAGGTAC CGTTGCCCTA   
  
  
+ TTTTGGTCAA CCTCTGGCAC GTCTCTACTA CGGTTACTTC CTATTTCAGT TTTCTAACTC TACTTGGCAA   
  
  
+ CCTAACCATC GTCTTCTCTC TCTCTCTCTG TCTCTCTCTC TCTCTCCCAA AATCAACCTC TGTCACTCCA   
  
  
+ TCCCAACTGG AACTTGTCGA AAATTACGTT TTCAGTAACT CACGCCTCTA CCCCACATAC CCAGGCGGTC   
  
  
+ CGAGCAGAAA ATGGCCTGTG GCCTTCGACG CAGTTTCGCA GTAGGGTGTT CGCTGTACCC TAAGAAAGAG   
  
  
+ AGAGAAATCA AAAAGAAGAT GCCAAAAGAT GAGCACCGGG GGTGGTTGTA AAAGGTACTA GTGTAGACGT   
  
  
+ GGCAAGTATG AACAGCTCTA GCTAAGACCA TCCTCTCCAG TGTGGTACGT ACCTGAACAC TCCACCACCG   
  
  
+ GCAACTTTTT CAAAGAGCGG TCAATGAAGT ATATGTCATT ACTCTTCACT TCCTCAAACC AGTTTTGATT   
  
  
+ TGAACTAAAG TTTGTAAAGC GGCCTTAAAC ACGCTTAGTC TATTAATTCT AAAATAATTA TTTTTGGGTG   
  
  
+ AATAGTAAAG CCGATGTAAT TACCTTATTT ACCAACCTTG CTTCTCTAGG TGTGCGGTTT CGTTTAGCTA   
  
  
+ GTCTTTATTT CCAGTTGAAG TGTGCGAAAG AGTTCGAAGA GACTAGTTTA AAGTTAATTA CTGGTTTGGT   
  
  
+ TTTTGACAAG TGTCTAAACC GTTATCGATA CAAAGAAGAC AGAAGGAAAT AGGGTTAAAT TTTTGTCAAA   
  
  
+ TATATATCAT AAAAAATTAA ATATTATTAT GATATTTTTA TTTTTTTGAA TAAATAAATA AAATATTTTA   
  
  
+ ATAGTTCATA ATATAAAAAA TTTTTTTAAT TTGGGTGAAA TTTAATTAAC TTCTATATTG GCTTTCACAA   
  
  
+ ATATAACTTT TTTTTTTCAC ATTAACTATG AATTGTGCCA ACCTCTTTTC CTCCCTTCGA ATGTTCGTTT   
  
  
+ ATCGAACTGA ACCGTAAACT ACTCGCTGAA GTAAAACTTG AATTTCCAAC TACCCGGTGT AAGTGTAATA   
  
  
+ ATCAGTATGG TATAAAAACA ATGAAAATTA CCTTCTTTTG GGTTTTTTTT TTTTTTTTTC GTTTTTCTTC   
  
  
+ GTTTGGGAGA AATTGTTTAA AATAATTTA  

- CTCTCCAAAA CCATCCAAGA CAACACCCTT AGATATGGAT GGGTATAAAT ATACAAAGAC CAAGAAGAGA   
  
  
- GAAAATCTTT AAAAAGGAAA GATGATGAAG CGAGCACAAA CCGAGAAAAT AAATACAAAA AGGAAAAGCA   
  
  
- GAGATGATGA GAAAAGGGGG AAGGAGAAGG GGGAGGGGAA GGGGAAGGGG AAGGGTCGAG AGTCAAACTG   
  
  
- ATGCCCTCCC AATGCAAGGC ATCGTGTCGT TTATGAGGAT ATTGCCGTCA AACCCCCGAT CCATTCGTAT   
  
  
- AACGCCACGT GTCAGGGCTT TTGTCAGGAC ATTGACCGCC TCTTTAAGTG CGGGTCCATG GCAACGGGAT   
  
  
- AAAACCAGTT GGAGACCGTG CAGAGATGAT GCCAATGAAG GATAAAGTCA AAAGATTGAG ATGAACCGTT   
  
  
- GGATTGGTAG CAGAAGAGAG AGAGAGAGAC AGAGAGAGAG AGAGAGGGTT TTAGTTGGAG ACAGTGAGGT   
  
  
- AGGGTTGACC TTGAACAGCT TTTAATGCAA AAGTCATTGA GTGCGGAGAT GGGGTGTATG GGTCCGCCAG   
  
  
- GCTCGTCTTT TACCGGACAC CGGAAGCTGC GTCAAAGCGT CATCCCACAA GCGACATGGG ATTCTTTCTC   
  
  
- TCTCTTTAGT TTTTCTTCTA CGGTTTTCTA CTCGTGGCCC CCACCAACAT TTTCCATGAT CACATCTGCA   
  
  
- CCGTTCATAC TTGTCGAGAT CGATTCTGGT AGGAGAGGTC ACACCATGCA TGGACTTGTG AGGTGGTGGC   
  
  
- CGTTGAAAAA GTTTCTCGCC AGTTACTTCA TATACAGTAA TGAGAAGTGA AGGAGTTTGG TCAAAACTAA   
  
  
- ACTTGATTTC AAACATTTCG CCGGAATTTG TGCGAATCAG ATAATTAAGA TTTTATTAAT AAAAACCCAC   
  
  
- TTATCATTTC GGCTACATTA ATGGAATAAA TGGTTGGAAC GAAGAGATCC ACACGCCAAA GCAAATCGAT   
  
  
- CAGAAATAAA GGTCAACTTC ACACGCTTTC TCAAGCTTCT CTGATCAAAT TTCAATTAAT GACCAAACCA   
  
  
- AAAACTGTTC ACAGATTTGG CAATAGCTAT GTTTCTTCTG TCTTCCTTTA TCCCAATTTA AAAACAGTTT   
  
  
- ATATATAGTA TTTTTTAATT TATAATAATA CTATAAAAAT AAAAAAACTT ATTTATTTAT TTTATAAAAT   
  
  
- TATCAAGTAT TATATTTTTT AAAAAAATTA AACCCACTTT AAATTAATTG AAGATATAAC CGAAAGTGTT   
  
  
- TATATTGAAA AAAAAAAGTG TAATTGATAC TTAACACGGT TGGAGAAAAG GAGGGAAGCT TACAAGCAAA   
  
  
- TAGCTTGACT TGGCATTTGA TGAGCGACTT CATTTTGAAC TTAAAGGTTG ATGGGCCACA TTCACATTAT   
  
  
- TAGTCATACC ATATTTTTGT TACTTTTAAT GGAAGAAAAC CCAAAAAAAA AAAAAAAAAG CAAAAAGAAG   
  
  
- CAAACCCTCT TTAACAAATT TTATTAAAT

+     Unnamed\_\_3

| Site Name | Organism | Position | Strand | Matrix score. | sequence | function |
| --- | --- | --- | --- | --- | --- | --- |
| Unnamed\_\_3 | Zea mays | 698 | + | 5 | CGTGG |  |

> 2018/04/13 10:10:12  
+ GAGAGGTTTT GGTAGGTTCT GTTGTGGGAA TCTATACCTA CCCATATTTA TATGTTTCTG GTTCTTCTCT   
  
  
+ CTTTTAGAAA TTTTTCCTTT CTACTACTTC GCTCGTGTTT GGCTCTTTTA TTTATGTTTT TCCTTTTCGT   
  
  
+ CTCTACTACT CTTTTCCCCC TTCCTCTTCC CCCTCCCCTT CCCCTTCCCC TTCCCAGCTC TCAGTTTGAC   
  
  
+ TACGGGAGGG TTACGTTCCG TAGCACAGCA AATACTCCTA TAACGGCAGT TTGGGGGCTA GGTAAGCATA   
  
  
+ TTGCGGTGCA CAGTCCCGAA AACAGTCCTG TAACTGGCGG AGAAATTCAC GCCCAGGTAC CGTTGCCCTA   
  
  
+ TTTTGGTCAA CCTCTGGCAC GTCTCTACTA CGGTTACTTC CTATTTCAGT TTTCTAACTC TACTTGGCAA   
  
  
+ CCTAACCATC GTCTTCTCTC TCTCTCTCTG TCTCTCTCTC TCTCTCCCAA AATCAACCTC TGTCACTCCA   
  
  
+ TCCCAACTGG AACTTGTCGA AAATTACGTT TTCAGTAACT CACGCCTCTA CCCCACATAC CCAGGCGGTC   
  
  
+ CGAGCAGAAA ATGGCCTGTG GCCTTCGACG CAGTTTCGCA GTAGGGTGTT CGCTGTACCC TAAGAAAGAG   
  
  
+ AGAGAAATCA AAAAGAAGAT GCCAAAAGAT GAGCACCGGG GGTGGTTGTA AAAGGTACTA GTGTAGACGT   
  
  
+ GGCAAGTATG AACAGCTCTA GCTAAGACCA TCCTCTCCAG TGTGGTACGT ACCTGAACAC TCCACCACCG   
  
  
+ GCAACTTTTT CAAAGAGCGG TCAATGAAGT ATATGTCATT ACTCTTCACT TCCTCAAACC AGTTTTGATT   
  
  
+ TGAACTAAAG TTTGTAAAGC GGCCTTAAAC ACGCTTAGTC TATTAATTCT AAAATAATTA TTTTTGGGTG   
  
  
+ AATAGTAAAG CCGATGTAAT TACCTTATTT ACCAACCTTG CTTCTCTAGG TGTGCGGTTT CGTTTAGCTA   
  
  
+ GTCTTTATTT CCAGTTGAAG TGTGCGAAAG AGTTCGAAGA GACTAGTTTA AAGTTAATTA CTGGTTTGGT   
  
  
+ TTTTGACAAG TGTCTAAACC GTTATCGATA CAAAGAAGAC AGAAGGAAAT AGGGTTAAAT TTTTGTCAAA   
  
  
+ TATATATCAT AAAAAATTAA ATATTATTAT GATATTTTTA TTTTTTTGAA TAAATAAATA AAATATTTTA   
  
  
+ ATAGTTCATA ATATAAAAAA TTTTTTTAAT TTGGGTGAAA TTTAATTAAC TTCTATATTG GCTTTCACAA   
  
  
+ ATATAACTTT TTTTTTTCAC ATTAACTATG AATTGTGCCA ACCTCTTTTC CTCCCTTCGA ATGTTCGTTT   
  
  
+ ATCGAACTGA ACCGTAAACT ACTCGCTGAA GTAAAACTTG AATTTCCAAC TACCCGGTGT AAGTGTAATA   
  
  
+ ATCAGTATGG TATAAAAACA ATGAAAATTA CCTTCTTTTG GGTTTTTTTT TTTTTTTTTC GTTTTTCTTC   
  
  
+ GTTTGGGAGA AATTGTTTAA AATAATTTA  

- CTCTCCAAAA CCATCCAAGA CAACACCCTT AGATATGGAT GGGTATAAAT ATACAAAGAC CAAGAAGAGA   
  
  
- GAAAATCTTT AAAAAGGAAA GATGATGAAG CGAGCACAAA CCGAGAAAAT AAATACAAAA AGGAAAAGCA   
  
  
- GAGATGATGA GAAAAGGGGG AAGGAGAAGG GGGAGGGGAA GGGGAAGGGG AAGGGTCGAG AGTCAAACTG   
  
  
- ATGCCCTCCC AATGCAAGGC ATCGTGTCGT TTATGAGGAT ATTGCCGTCA AACCCCCGAT CCATTCGTAT   
  
  
- AACGCCACGT GTCAGGGCTT TTGTCAGGAC ATTGACCGCC TCTTTAAGTG CGGGTCCATG GCAACGGGAT   
  
  
- AAAACCAGTT GGAGACCGTG CAGAGATGAT GCCAATGAAG GATAAAGTCA AAAGATTGAG ATGAACCGTT   
  
  
- GGATTGGTAG CAGAAGAGAG AGAGAGAGAC AGAGAGAGAG AGAGAGGGTT TTAGTTGGAG ACAGTGAGGT   
  
  
- AGGGTTGACC TTGAACAGCT TTTAATGCAA AAGTCATTGA GTGCGGAGAT GGGGTGTATG GGTCCGCCAG   
  
  
- GCTCGTCTTT TACCGGACAC CGGAAGCTGC GTCAAAGCGT CATCCCACAA GCGACATGGG ATTCTTTCTC   
  
  
- TCTCTTTAGT TTTTCTTCTA CGGTTTTCTA CTCGTGGCCC CCACCAACAT TTTCCATGAT CACATCTGCA   
  
  
- CCGTTCATAC TTGTCGAGAT CGATTCTGGT AGGAGAGGTC ACACCATGCA TGGACTTGTG AGGTGGTGGC   
  
  
- CGTTGAAAAA GTTTCTCGCC AGTTACTTCA TATACAGTAA TGAGAAGTGA AGGAGTTTGG TCAAAACTAA   
  
  
- ACTTGATTTC AAACATTTCG CCGGAATTTG TGCGAATCAG ATAATTAAGA TTTTATTAAT AAAAACCCAC   
  
  
- TTATCATTTC GGCTACATTA ATGGAATAAA TGGTTGGAAC GAAGAGATCC ACACGCCAAA GCAAATCGAT   
  
  
- CAGAAATAAA GGTCAACTTC ACACGCTTTC TCAAGCTTCT CTGATCAAAT TTCAATTAAT GACCAAACCA   
  
  
- AAAACTGTTC ACAGATTTGG CAATAGCTAT GTTTCTTCTG TCTTCCTTTA TCCCAATTTA AAAACAGTTT   
  
  
- ATATATAGTA TTTTTTAATT TATAATAATA CTATAAAAAT AAAAAAACTT ATTTATTTAT TTTATAAAAT   
  
  
- TATCAAGTAT TATATTTTTT AAAAAAATTA AACCCACTTT AAATTAATTG AAGATATAAC CGAAAGTGTT   
  
  
- TATATTGAAA AAAAAAAGTG TAATTGATAC TTAACACGGT TGGAGAAAAG GAGGGAAGCT TACAAGCAAA   
  
  
- TAGCTTGACT TGGCATTTGA TGAGCGACTT CATTTTGAAC TTAAAGGTTG ATGGGCCACA TTCACATTAT   
  
  
- TAGTCATACC ATATTTTTGT TACTTTTAAT GGAAGAAAAC CCAAAAAAAA AAAAAAAAAG CAAAAAGAAG   
  
  
- CAAACCCTCT TTAACAAATT TTATTAAAT

+     Unnamed\_\_4

| Site Name | Organism | Position | Strand | Matrix score. | sequence | function |
| --- | --- | --- | --- | --- | --- | --- |
| Unnamed\_\_4 | Petroselinum hortense | 1311 | + | 4 | CTCC |  |
| Unnamed\_\_4 | Petroselinum hortense | 735 | + | 4 | CTCC |  |
| Unnamed\_\_4 | Petroselinum hortense | 486 | + | 4 | CTCC |  |
| Unnamed\_\_4 | Petroselinum hortense | 760 | + | 4 | CTCC |  |
| Unnamed\_\_4 | Petroselinum hortense | 245 | + | 4 | CTCC |  |
| Unnamed\_\_4 | Petroselinum hortense | 1476 | - | 4 | CTCC |  |
| Unnamed\_\_4 | Petroselinum hortense | 319 | - | 4 | CTCC |  |
| Unnamed\_\_4 | Petroselinum hortense | 464 | + | 4 | CTCC |  |
| Unnamed\_\_4 | Petroselinum hortense | 215 | - | 4 | CTCC |  |
| Unnamed\_\_4 | Petroselinum hortense | 173 | + | 4 | CTCC |  |

> 2018/04/13 10:10:12  
+ GAGAGGTTTT GGTAGGTTCT GTTGTGGGAA TCTATACCTA CCCATATTTA TATGTTTCTG GTTCTTCTCT   
  
  
+ CTTTTAGAAA TTTTTCCTTT CTACTACTTC GCTCGTGTTT GGCTCTTTTA TTTATGTTTT TCCTTTTCGT   
  
  
+ CTCTACTACT CTTTTCCCCC TTCCTCTTCC CCCTCCCCTT CCCCTTCCCC TTCCCAGCTC TCAGTTTGAC   
  
  
+ TACGGGAGGG TTACGTTCCG TAGCACAGCA AATACTCCTA TAACGGCAGT TTGGGGGCTA GGTAAGCATA   
  
  
+ TTGCGGTGCA CAGTCCCGAA AACAGTCCTG TAACTGGCGG AGAAATTCAC GCCCAGGTAC CGTTGCCCTA   
  
  
+ TTTTGGTCAA CCTCTGGCAC GTCTCTACTA CGGTTACTTC CTATTTCAGT TTTCTAACTC TACTTGGCAA   
  
  
+ CCTAACCATC GTCTTCTCTC TCTCTCTCTG TCTCTCTCTC TCTCTCCCAA AATCAACCTC TGTCACTCCA   
  
  
+ TCCCAACTGG AACTTGTCGA AAATTACGTT TTCAGTAACT CACGCCTCTA CCCCACATAC CCAGGCGGTC   
  
  
+ CGAGCAGAAA ATGGCCTGTG GCCTTCGACG CAGTTTCGCA GTAGGGTGTT CGCTGTACCC TAAGAAAGAG   
  
  
+ AGAGAAATCA AAAAGAAGAT GCCAAAAGAT GAGCACCGGG GGTGGTTGTA AAAGGTACTA GTGTAGACGT   
  
  
+ GGCAAGTATG AACAGCTCTA GCTAAGACCA TCCTCTCCAG TGTGGTACGT ACCTGAACAC TCCACCACCG   
  
  
+ GCAACTTTTT CAAAGAGCGG TCAATGAAGT ATATGTCATT ACTCTTCACT TCCTCAAACC AGTTTTGATT   
  
  
+ TGAACTAAAG TTTGTAAAGC GGCCTTAAAC ACGCTTAGTC TATTAATTCT AAAATAATTA TTTTTGGGTG   
  
  
+ AATAGTAAAG CCGATGTAAT TACCTTATTT ACCAACCTTG CTTCTCTAGG TGTGCGGTTT CGTTTAGCTA   
  
  
+ GTCTTTATTT CCAGTTGAAG TGTGCGAAAG AGTTCGAAGA GACTAGTTTA AAGTTAATTA CTGGTTTGGT   
  
  
+ TTTTGACAAG TGTCTAAACC GTTATCGATA CAAAGAAGAC AGAAGGAAAT AGGGTTAAAT TTTTGTCAAA   
  
  
+ TATATATCAT AAAAAATTAA ATATTATTAT GATATTTTTA TTTTTTTGAA TAAATAAATA AAATATTTTA   
  
  
+ ATAGTTCATA ATATAAAAAA TTTTTTTAAT TTGGGTGAAA TTTAATTAAC TTCTATATTG GCTTTCACAA   
  
  
+ ATATAACTTT TTTTTTTCAC ATTAACTATG AATTGTGCCA ACCTCTTTTC CTCCCTTCGA ATGTTCGTTT   
  
  
+ ATCGAACTGA ACCGTAAACT ACTCGCTGAA GTAAAACTTG AATTTCCAAC TACCCGGTGT AAGTGTAATA   
  
  
+ ATCAGTATGG TATAAAAACA ATGAAAATTA CCTTCTTTTG GGTTTTTTTT TTTTTTTTTC GTTTTTCTTC   
  
  
+ GTTTGGGAGA AATTGTTTAA AATAATTTA  

- CTCTCCAAAA CCATCCAAGA CAACACCCTT AGATATGGAT GGGTATAAAT ATACAAAGAC CAAGAAGAGA   
  
  
- GAAAATCTTT AAAAAGGAAA GATGATGAAG CGAGCACAAA CCGAGAAAAT AAATACAAAA AGGAAAAGCA   
  
  
- GAGATGATGA GAAAAGGGGG AAGGAGAAGG GGGAGGGGAA GGGGAAGGGG AAGGGTCGAG AGTCAAACTG   
  
  
- ATGCCCTCCC AATGCAAGGC ATCGTGTCGT TTATGAGGAT ATTGCCGTCA AACCCCCGAT CCATTCGTAT   
  
  
- AACGCCACGT GTCAGGGCTT TTGTCAGGAC ATTGACCGCC TCTTTAAGTG CGGGTCCATG GCAACGGGAT   
  
  
- AAAACCAGTT GGAGACCGTG CAGAGATGAT GCCAATGAAG GATAAAGTCA AAAGATTGAG ATGAACCGTT   
  
  
- GGATTGGTAG CAGAAGAGAG AGAGAGAGAC AGAGAGAGAG AGAGAGGGTT TTAGTTGGAG ACAGTGAGGT   
  
  
- AGGGTTGACC TTGAACAGCT TTTAATGCAA AAGTCATTGA GTGCGGAGAT GGGGTGTATG GGTCCGCCAG   
  
  
- GCTCGTCTTT TACCGGACAC CGGAAGCTGC GTCAAAGCGT CATCCCACAA GCGACATGGG ATTCTTTCTC   
  
  
- TCTCTTTAGT TTTTCTTCTA CGGTTTTCTA CTCGTGGCCC CCACCAACAT TTTCCATGAT CACATCTGCA   
  
  
- CCGTTCATAC TTGTCGAGAT CGATTCTGGT AGGAGAGGTC ACACCATGCA TGGACTTGTG AGGTGGTGGC   
  
  
- CGTTGAAAAA GTTTCTCGCC AGTTACTTCA TATACAGTAA TGAGAAGTGA AGGAGTTTGG TCAAAACTAA   
  
  
- ACTTGATTTC AAACATTTCG CCGGAATTTG TGCGAATCAG ATAATTAAGA TTTTATTAAT AAAAACCCAC   
  
  
- TTATCATTTC GGCTACATTA ATGGAATAAA TGGTTGGAAC GAAGAGATCC ACACGCCAAA GCAAATCGAT   
  
  
- CAGAAATAAA GGTCAACTTC ACACGCTTTC TCAAGCTTCT CTGATCAAAT TTCAATTAAT GACCAAACCA   
  
  
- AAAACTGTTC ACAGATTTGG CAATAGCTAT GTTTCTTCTG TCTTCCTTTA TCCCAATTTA AAAACAGTTT   
  
  
- ATATATAGTA TTTTTTAATT TATAATAATA CTATAAAAAT AAAAAAACTT ATTTATTTAT TTTATAAAAT   
  
  
- TATCAAGTAT TATATTTTTT AAAAAAATTA AACCCACTTT AAATTAATTG AAGATATAAC CGAAAGTGTT   
  
  
- TATATTGAAA AAAAAAAGTG TAATTGATAC TTAACACGGT TGGAGAAAAG GAGGGAAGCT TACAAGCAAA   
  
  
- TAGCTTGACT TGGCATTTGA TGAGCGACTT CATTTTGAAC TTAAAGGTTG ATGGGCCACA TTCACATTAT   
  
  
- TAGTCATACC ATATTTTTGT TACTTTTAAT GGAAGAAAAC CCAAAAAAAA AAAAAAAAAG CAAAAAGAAG   
  
  
- CAAACCCTCT TTAACAAATT TTATTAAAT

+     W box

| Site Name | Organism | Position | Strand | Matrix score. | sequence | function |
| --- | --- | --- | --- | --- | --- | --- |
| W box | Arabidopsis thaliana | 355 | - | 6 | TTGACC |  |
| W box | Arabidopsis thaliana | 789 | - | 6 | TTGACC |  |

> 2018/04/13 10:10:12  
+ GAGAGGTTTT GGTAGGTTCT GTTGTGGGAA TCTATACCTA CCCATATTTA TATGTTTCTG GTTCTTCTCT   
  
  
+ CTTTTAGAAA TTTTTCCTTT CTACTACTTC GCTCGTGTTT GGCTCTTTTA TTTATGTTTT TCCTTTTCGT   
  
  
+ CTCTACTACT CTTTTCCCCC TTCCTCTTCC CCCTCCCCTT CCCCTTCCCC TTCCCAGCTC TCAGTTTGAC   
  
  
+ TACGGGAGGG TTACGTTCCG TAGCACAGCA AATACTCCTA TAACGGCAGT TTGGGGGCTA GGTAAGCATA   
  
  
+ TTGCGGTGCA CAGTCCCGAA AACAGTCCTG TAACTGGCGG AGAAATTCAC GCCCAGGTAC CGTTGCCCTA   
  
  
+ TTTTGGTCAA CCTCTGGCAC GTCTCTACTA CGGTTACTTC CTATTTCAGT TTTCTAACTC TACTTGGCAA   
  
  
+ CCTAACCATC GTCTTCTCTC TCTCTCTCTG TCTCTCTCTC TCTCTCCCAA AATCAACCTC TGTCACTCCA   
  
  
+ TCCCAACTGG AACTTGTCGA AAATTACGTT TTCAGTAACT CACGCCTCTA CCCCACATAC CCAGGCGGTC   
  
  
+ CGAGCAGAAA ATGGCCTGTG GCCTTCGACG CAGTTTCGCA GTAGGGTGTT CGCTGTACCC TAAGAAAGAG   
  
  
+ AGAGAAATCA AAAAGAAGAT GCCAAAAGAT GAGCACCGGG GGTGGTTGTA AAAGGTACTA GTGTAGACGT   
  
  
+ GGCAAGTATG AACAGCTCTA GCTAAGACCA TCCTCTCCAG TGTGGTACGT ACCTGAACAC TCCACCACCG   
  
  
+ GCAACTTTTT CAAAGAGCGG TCAATGAAGT ATATGTCATT ACTCTTCACT TCCTCAAACC AGTTTTGATT   
  
  
+ TGAACTAAAG TTTGTAAAGC GGCCTTAAAC ACGCTTAGTC TATTAATTCT AAAATAATTA TTTTTGGGTG   
  
  
+ AATAGTAAAG CCGATGTAAT TACCTTATTT ACCAACCTTG CTTCTCTAGG TGTGCGGTTT CGTTTAGCTA   
  
  
+ GTCTTTATTT CCAGTTGAAG TGTGCGAAAG AGTTCGAAGA GACTAGTTTA AAGTTAATTA CTGGTTTGGT   
  
  
+ TTTTGACAAG TGTCTAAACC GTTATCGATA CAAAGAAGAC AGAAGGAAAT AGGGTTAAAT TTTTGTCAAA   
  
  
+ TATATATCAT AAAAAATTAA ATATTATTAT GATATTTTTA TTTTTTTGAA TAAATAAATA AAATATTTTA   
  
  
+ ATAGTTCATA ATATAAAAAA TTTTTTTAAT TTGGGTGAAA TTTAATTAAC TTCTATATTG GCTTTCACAA   
  
  
+ ATATAACTTT TTTTTTTCAC ATTAACTATG AATTGTGCCA ACCTCTTTTC CTCCCTTCGA ATGTTCGTTT   
  
  
+ ATCGAACTGA ACCGTAAACT ACTCGCTGAA GTAAAACTTG AATTTCCAAC TACCCGGTGT AAGTGTAATA   
  
  
+ ATCAGTATGG TATAAAAACA ATGAAAATTA CCTTCTTTTG GGTTTTTTTT TTTTTTTTTC GTTTTTCTTC   
  
  
+ GTTTGGGAGA AATTGTTTAA AATAATTTA  

- CTCTCCAAAA CCATCCAAGA CAACACCCTT AGATATGGAT GGGTATAAAT ATACAAAGAC CAAGAAGAGA   
  
  
- GAAAATCTTT AAAAAGGAAA GATGATGAAG CGAGCACAAA CCGAGAAAAT AAATACAAAA AGGAAAAGCA   
  
  
- GAGATGATGA GAAAAGGGGG AAGGAGAAGG GGGAGGGGAA GGGGAAGGGG AAGGGTCGAG AGTCAAACTG   
  
  
- ATGCCCTCCC AATGCAAGGC ATCGTGTCGT TTATGAGGAT ATTGCCGTCA AACCCCCGAT CCATTCGTAT   
  
  
- AACGCCACGT GTCAGGGCTT TTGTCAGGAC ATTGACCGCC TCTTTAAGTG CGGGTCCATG GCAACGGGAT   
  
  
- AAAACCAGTT GGAGACCGTG CAGAGATGAT GCCAATGAAG GATAAAGTCA AAAGATTGAG ATGAACCGTT   
  
  
- GGATTGGTAG CAGAAGAGAG AGAGAGAGAC AGAGAGAGAG AGAGAGGGTT TTAGTTGGAG ACAGTGAGGT   
  
  
- AGGGTTGACC TTGAACAGCT TTTAATGCAA AAGTCATTGA GTGCGGAGAT GGGGTGTATG GGTCCGCCAG   
  
  
- GCTCGTCTTT TACCGGACAC CGGAAGCTGC GTCAAAGCGT CATCCCACAA GCGACATGGG ATTCTTTCTC   
  
  
- TCTCTTTAGT TTTTCTTCTA CGGTTTTCTA CTCGTGGCCC CCACCAACAT TTTCCATGAT CACATCTGCA   
  
  
- CCGTTCATAC TTGTCGAGAT CGATTCTGGT AGGAGAGGTC ACACCATGCA TGGACTTGTG AGGTGGTGGC   
  
  
- CGTTGAAAAA GTTTCTCGCC AGTTACTTCA TATACAGTAA TGAGAAGTGA AGGAGTTTGG TCAAAACTAA   
  
  
- ACTTGATTTC AAACATTTCG CCGGAATTTG TGCGAATCAG ATAATTAAGA TTTTATTAAT AAAAACCCAC   
  
  
- TTATCATTTC GGCTACATTA ATGGAATAAA TGGTTGGAAC GAAGAGATCC ACACGCCAAA GCAAATCGAT   
  
  
- CAGAAATAAA GGTCAACTTC ACACGCTTTC TCAAGCTTCT CTGATCAAAT TTCAATTAAT GACCAAACCA   
  
  
- AAAACTGTTC ACAGATTTGG CAATAGCTAT GTTTCTTCTG TCTTCCTTTA TCCCAATTTA AAAACAGTTT   
  
  
- ATATATAGTA TTTTTTAATT TATAATAATA CTATAAAAAT AAAAAAACTT ATTTATTTAT TTTATAAAAT   
  
  
- TATCAAGTAT TATATTTTTT AAAAAAATTA AACCCACTTT AAATTAATTG AAGATATAAC CGAAAGTGTT   
  
  
- TATATTGAAA AAAAAAAGTG TAATTGATAC TTAACACGGT TGGAGAAAAG GAGGGAAGCT TACAAGCAAA   
  
  
- TAGCTTGACT TGGCATTTGA TGAGCGACTT CATTTTGAAC TTAAAGGTTG ATGGGCCACA TTCACATTAT   
  
  
- TAGTCATACC ATATTTTTGT TACTTTTAAT GGAAGAAAAC CCAAAAAAAA AAAAAAAAAG CAAAAAGAAG   
  
  
- CAAACCCTCT TTAACAAATT TTATTAAAT

+     chs-Unit 1 m1

| Site Name | Organism | Position | Strand | Matrix score. | sequence | function |
| --- | --- | --- | --- | --- | --- | --- |
| chs-Unit 1 m1 | Arabidopsis thaliana | 740 | - | 10 | ACCTACCACAC | part of a light responsive element |

> 2018/04/13 10:10:12  
+ GAGAGGTTTT GGTAGGTTCT GTTGTGGGAA TCTATACCTA CCCATATTTA TATGTTTCTG GTTCTTCTCT   
  
  
+ CTTTTAGAAA TTTTTCCTTT CTACTACTTC GCTCGTGTTT GGCTCTTTTA TTTATGTTTT TCCTTTTCGT   
  
  
+ CTCTACTACT CTTTTCCCCC TTCCTCTTCC CCCTCCCCTT CCCCTTCCCC TTCCCAGCTC TCAGTTTGAC   
  
  
+ TACGGGAGGG TTACGTTCCG TAGCACAGCA AATACTCCTA TAACGGCAGT TTGGGGGCTA GGTAAGCATA   
  
  
+ TTGCGGTGCA CAGTCCCGAA AACAGTCCTG TAACTGGCGG AGAAATTCAC GCCCAGGTAC CGTTGCCCTA   
  
  
+ TTTTGGTCAA CCTCTGGCAC GTCTCTACTA CGGTTACTTC CTATTTCAGT TTTCTAACTC TACTTGGCAA   
  
  
+ CCTAACCATC GTCTTCTCTC TCTCTCTCTG TCTCTCTCTC TCTCTCCCAA AATCAACCTC TGTCACTCCA   
  
  
+ TCCCAACTGG AACTTGTCGA AAATTACGTT TTCAGTAACT CACGCCTCTA CCCCACATAC CCAGGCGGTC   
  
  
+ CGAGCAGAAA ATGGCCTGTG GCCTTCGACG CAGTTTCGCA GTAGGGTGTT CGCTGTACCC TAAGAAAGAG   
  
  
+ AGAGAAATCA AAAAGAAGAT GCCAAAAGAT GAGCACCGGG GGTGGTTGTA AAAGGTACTA GTGTAGACGT   
  
  
+ GGCAAGTATG AACAGCTCTA GCTAAGACCA TCCTCTCCAG TGTGGTACGT ACCTGAACAC TCCACCACCG   
  
  
+ GCAACTTTTT CAAAGAGCGG TCAATGAAGT ATATGTCATT ACTCTTCACT TCCTCAAACC AGTTTTGATT   
  
  
+ TGAACTAAAG TTTGTAAAGC GGCCTTAAAC ACGCTTAGTC TATTAATTCT AAAATAATTA TTTTTGGGTG   
  
  
+ AATAGTAAAG CCGATGTAAT TACCTTATTT ACCAACCTTG CTTCTCTAGG TGTGCGGTTT CGTTTAGCTA   
  
  
+ GTCTTTATTT CCAGTTGAAG TGTGCGAAAG AGTTCGAAGA GACTAGTTTA AAGTTAATTA CTGGTTTGGT   
  
  
+ TTTTGACAAG TGTCTAAACC GTTATCGATA CAAAGAAGAC AGAAGGAAAT AGGGTTAAAT TTTTGTCAAA   
  
  
+ TATATATCAT AAAAAATTAA ATATTATTAT GATATTTTTA TTTTTTTGAA TAAATAAATA AAATATTTTA   
  
  
+ ATAGTTCATA ATATAAAAAA TTTTTTTAAT TTGGGTGAAA TTTAATTAAC TTCTATATTG GCTTTCACAA   
  
  
+ ATATAACTTT TTTTTTTCAC ATTAACTATG AATTGTGCCA ACCTCTTTTC CTCCCTTCGA ATGTTCGTTT   
  
  
+ ATCGAACTGA ACCGTAAACT ACTCGCTGAA GTAAAACTTG AATTTCCAAC TACCCGGTGT AAGTGTAATA   
  
  
+ ATCAGTATGG TATAAAAACA ATGAAAATTA CCTTCTTTTG GGTTTTTTTT TTTTTTTTTC GTTTTTCTTC   
  
  
+ GTTTGGGAGA AATTGTTTAA AATAATTTA  

- CTCTCCAAAA CCATCCAAGA CAACACCCTT AGATATGGAT GGGTATAAAT ATACAAAGAC CAAGAAGAGA   
  
  
- GAAAATCTTT AAAAAGGAAA GATGATGAAG CGAGCACAAA CCGAGAAAAT AAATACAAAA AGGAAAAGCA   
  
  
- GAGATGATGA GAAAAGGGGG AAGGAGAAGG GGGAGGGGAA GGGGAAGGGG AAGGGTCGAG AGTCAAACTG   
  
  
- ATGCCCTCCC AATGCAAGGC ATCGTGTCGT TTATGAGGAT ATTGCCGTCA AACCCCCGAT CCATTCGTAT   
  
  
- AACGCCACGT GTCAGGGCTT TTGTCAGGAC ATTGACCGCC TCTTTAAGTG CGGGTCCATG GCAACGGGAT   
  
  
- AAAACCAGTT GGAGACCGTG CAGAGATGAT GCCAATGAAG GATAAAGTCA AAAGATTGAG ATGAACCGTT   
  
  
- GGATTGGTAG CAGAAGAGAG AGAGAGAGAC AGAGAGAGAG AGAGAGGGTT TTAGTTGGAG ACAGTGAGGT   
  
  
- AGGGTTGACC TTGAACAGCT TTTAATGCAA AAGTCATTGA GTGCGGAGAT GGGGTGTATG GGTCCGCCAG   
  
  
- GCTCGTCTTT TACCGGACAC CGGAAGCTGC GTCAAAGCGT CATCCCACAA GCGACATGGG ATTCTTTCTC   
  
  
- TCTCTTTAGT TTTTCTTCTA CGGTTTTCTA CTCGTGGCCC CCACCAACAT TTTCCATGAT CACATCTGCA   
  
  
- CCGTTCATAC TTGTCGAGAT CGATTCTGGT AGGAGAGGTC ACACCATGCA TGGACTTGTG AGGTGGTGGC   
  
  
- CGTTGAAAAA GTTTCTCGCC AGTTACTTCA TATACAGTAA TGAGAAGTGA AGGAGTTTGG TCAAAACTAA   
  
  
- ACTTGATTTC AAACATTTCG CCGGAATTTG TGCGAATCAG ATAATTAAGA TTTTATTAAT AAAAACCCAC   
  
  
- TTATCATTTC GGCTACATTA ATGGAATAAA TGGTTGGAAC GAAGAGATCC ACACGCCAAA GCAAATCGAT   
  
  
- CAGAAATAAA GGTCAACTTC ACACGCTTTC TCAAGCTTCT CTGATCAAAT TTCAATTAAT GACCAAACCA   
  
  
- AAAACTGTTC ACAGATTTGG CAATAGCTAT GTTTCTTCTG TCTTCCTTTA TCCCAATTTA AAAACAGTTT   
  
  
- ATATATAGTA TTTTTTAATT TATAATAATA CTATAAAAAT AAAAAAACTT ATTTATTTAT TTTATAAAAT   
  
  
- TATCAAGTAT TATATTTTTT AAAAAAATTA AACCCACTTT AAATTAATTG AAGATATAAC CGAAAGTGTT   
  
  
- TATATTGAAA AAAAAAAGTG TAATTGATAC TTAACACGGT TGGAGAAAAG GAGGGAAGCT TACAAGCAAA   
  
  
- TAGCTTGACT TGGCATTTGA TGAGCGACTT CATTTTGAAC TTAAAGGTTG ATGGGCCACA TTCACATTAT   
  
  
- TAGTCATACC ATATTTTTGT TACTTTTAAT GGAAGAAAAC CCAAAAAAAA AAAAAAAAAG CAAAAAGAAG   
  
  
- CAAACCCTCT TTAACAAATT TTATTAAAT
