# Supplementary material for: Genome-wide analysis of the WRKY gene family in drumstick (Moringa oleifera Lam.)
Source: PeerJ. 2019 Jun 10;7:e7063. doi: 10.7717/peerj.7063 (PMC6563795; doi:10.7717/peerj.7063)
Supplement: Supplemental Information 1 [file peerj-07-7063-s003.gz › MoWRKY21_plantcare.html]

Content-Type: text/html; charset=ISO-8859-1


CallMat\_Firefox


Webmaster Firefox specific output  
To save the result:
click on the frame with the right mouse button and save the source code as a text file with extension .html  
REFERENCE:PlantCARE: a database of plant cis-acting regulatory elements and a portal to tools for in silico analysis of promoter sequences.  
Lescot, M., Déhais, P., Moreau, Y., De Moor, B., Rouzé ,P.,and Rombauts, S.  
Nucleic Acids Res., Database issue(2002), 30(1):325-327.   


---

> 2018/04/13 10:10:12  
+ GACACAGAGG AAGGGGAGAG AGAGAGAGAG AGATAGCTAC CTTTTCAACG TCTTCTAGTT TAAGTTTGTC   
  
  
+ AAATTATCCC TTTCTTTATC CCTTTCTTAG ACGACTTCTA TATATATGCT AGAGATCTTC TACTTCTGGA   
  
  
+ ATTTGGTATA TGTCATTTAA TTCTCTAGGA GGGAGAGAAA GAGAAGTTGA AGATCCTATC TACCTGTCTT   
  
  
+ GAATCTATCT ATACTTTCAT TGGTTTATGG ATATATTTAT ACTGGGTTGG TGAAAGGGAA AGTTGTCGTA   
  
  
+ ATTAACTCTT AAAGATCAGT AAATATAACT CCTTCTCACT CGTATTTAAT TTCTTTTTCT TTCGTCTTCG   
  
  
+ GTTTTTATCT TTAATTCTAT GTCTTTGAGG TAGCCTATCA TCAACCCATA ACACTTTTAA TTGTACAAGA   
  
  
+ TCATTAGTTT TCGTATCTTA AAGTACACAT TCCGAATATC ACCCTTCAAT GACATCACTC CTTCTCCAGT   
  
  
+ TGTTATCTGC TGTTTTCCTA ACTCGTCATT CCCAAAACAA AACGATTTAA CTTACTTTTT CGTCACGTGA   
  
  
+ AAAGAGAGTA ATTTCCTATT TTCCCTTTTG GCCTACTCAT CTGTTATTAT CTCGTTTTAG AGAGTACATT   
  
  
+ CACGGGTTCA CTTTTAATAG TAAGTGTGTA TAGACGCAGA GTTTTCCTTT TTCTTCTCTC TGTTTACCAC   
  
  
+ TATTTATATA GATAGTGAAA GATCCCAAAG AAATCAAAAA GAAAAAAGAT CCCAAAGAAA AACTCATGTA   
  
  
+ TGGGATTACT TTTCTTCATA CATAGTTACC GTTACTACTA CTTAGAACCC GCGTTTAGAG AAGTGACACG   
  
  
+ TAGTAGCCGC CACTTCTATA GTAATACTAA CTACTTTCTC CGGGATAAAC TGAAAACCGT CTCTTTTTCG   
  
  
+ AAGAGTGGAA AGAGGTAGAG GAAAACGATA AAGTGGAAAA GAAATTATGA AGGAGGTTTG GGGTGTAGAT   
  
  
+ GGGTGGTGAG GAGTTTAAGG TTTTGGGTCA TGGAATAATA TAAGAATAGT AGGGGAAGTT AATTCGTGGT   
  
  
+ ACGATTATTA ATTAGTAGGT GGGAAACGAA TAGTATTAAT CAATTTAAAT ATATTATGTG AGAGACACCT   
  
  
+ GGCTGGACTG CGGAACGATA CATACAACTA ACGAGTACTT GGGGTTGACA AACCTATAAC CTGGTTGACC   
  
  
+ CAATTAAATG AATGGTAAAC TAATAGTACA AGAACAACAT GAACTATCAC TAGAATTAAT CTACTTGTAC   
  
  
+ TAATTCAAGT AAACTAGCCT TTATTACTGA CTTCGTTTAG ATCCCGTGGT ATACTACCAC TTTTTAGTAT   
  
  
+ TCCGGTCGTC AATTTATAAA TTCTACAGTA CCTAGTCGTC TAATTATTTC TATAGACTTA AAACTAATTG   
  
  
+ GGTAAAGTTG TATCCTCTAG CGTATTTTAC TAAATCGATA GAATCGATCG TACGTTTTCG TTTGTTATTA   
  
  
+ ATCTACTAAA AAATTTTTAA TTTAAAGGT  

- CTGTGTCTCC TTCCCCTCTC TCTCTCTCTC TCTATCGATG GAAAAGTTGC AGAAGATCAA ATTCAAACAG   
  
  
- TTTAATAGGG AAAGAAATAG GGAAAGAATC TGCTGAAGAT ATATATACGA TCTCTAGAAG ATGAAGACCT   
  
  
- TAAACCATAT ACAGTAAATT AAGAGATCCT CCCTCTCTTT CTCTTCAACT TCTAGGATAG ATGGACAGAA   
  
  
- CTTAGATAGA TATGAAAGTA ACCAAATACC TATATAAATA TGACCCAACC ACTTTCCCTT TCAACAGCAT   
  
  
- TAATTGAGAA TTTCTAGTCA TTTATATTGA GGAAGAGTGA GCATAAATTA AAGAAAAAGA AAGCAGAAGC   
  
  
- CAAAAATAGA AATTAAGATA CAGAAACTCC ATCGGATAGT AGTTGGGTAT TGTGAAAATT AACATGTTCT   
  
  
- AGTAATCAAA AGCATAGAAT TTCATGTGTA AGGCTTATAG TGGGAAGTTA CTGTAGTGAG GAAGAGGTCA   
  
  
- ACAATAGACG ACAAAAGGAT TGAGCAGTAA GGGTTTTGTT TTGCTAAATT GAATGAAAAA GCAGTGCACT   
  
  
- TTTCTCTCAT TAAAGGATAA AAGGGAAAAC CGGATGAGTA GACAATAATA GAGCAAAATC TCTCATGTAA   
  
  
- GTGCCCAAGT GAAAATTATC ATTCACACAT ATCTGCGTCT CAAAAGGAAA AAGAAGAGAG ACAAATGGTG   
  
  
- ATAAATATAT CTATCACTTT CTAGGGTTTC TTTAGTTTTT CTTTTTTCTA GGGTTTCTTT TTGAGTACAT   
  
  
- ACCCTAATGA AAAGAAGTAT GTATCAATGG CAATGATGAT GAATCTTGGG CGCAAATCTC TTCACTGTGC   
  
  
- ATCATCGGCG GTGAAGATAT CATTATGATT GATGAAAGAG GCCCTATTTG ACTTTTGGCA GAGAAAAAGC   
  
  
- TTCTCACCTT TCTCCATCTC CTTTTGCTAT TTCACCTTTT CTTTAATACT TCCTCCAAAC CCCACATCTA   
  
  
- CCCACCACTC CTCAAATTCC AAAACCCAGT ACCTTATTAT ATTCTTATCA TCCCCTTCAA TTAAGCACCA   
  
  
- TGCTAATAAT TAATCATCCA CCCTTTGCTT ATCATAATTA GTTAAATTTA TATAATACAC TCTCTGTGGA   
  
  
- CCGACCTGAC GCCTTGCTAT GTATGTTGAT TGCTCATGAA CCCCAACTGT TTGGATATTG GACCAACTGG   
  
  
- GTTAATTTAC TTACCATTTG ATTATCATGT TCTTGTTGTA CTTGATAGTG ATCTTAATTA GATGAACATG   
  
  
- ATTAAGTTCA TTTGATCGGA AATAATGACT GAAGCAAATC TAGGGCACCA TATGATGGTG AAAAATCATA   
  
  
- AGGCCAGCAG TTAAATATTT AAGATGTCAT GGATCAGCAG ATTAATAAAG ATATCTGAAT TTTGATTAAC   
  
  
- CCATTTCAAC ATAGGAGATC GCATAAAATG ATTTAGCTAT CTTAGCTAGC ATGCAAAAGC AAACAATAAT   
  
  
- TAGATGATTT TTTAAAAATT AAATTTCCA

  
  
Motifs Found  

+     4cl-CMA2b

| Site Name | Organism | Position | Strand | Matrix score. | sequence | function |
| --- | --- | --- | --- | --- | --- | --- |
| 4cl-CMA2b | Petroselinum crispum | 255 | - | 10 | TCTCACCAACC | light responsive element |

> 2018/04/13 10:10:12  
+ GACACAGAGG AAGGGGAGAG AGAGAGAGAG AGATAGCTAC CTTTTCAACG TCTTCTAGTT TAAGTTTGTC   
  
  
+ AAATTATCCC TTTCTTTATC CCTTTCTTAG ACGACTTCTA TATATATGCT AGAGATCTTC TACTTCTGGA   
  
  
+ ATTTGGTATA TGTCATTTAA TTCTCTAGGA GGGAGAGAAA GAGAAGTTGA AGATCCTATC TACCTGTCTT   
  
  
+ GAATCTATCT ATACTTTCAT TGGTTTATGG ATATATTTAT ACTGGGTTGG TGAAAGGGAA AGTTGTCGTA   
  
  
+ ATTAACTCTT AAAGATCAGT AAATATAACT CCTTCTCACT CGTATTTAAT TTCTTTTTCT TTCGTCTTCG   
  
  
+ GTTTTTATCT TTAATTCTAT GTCTTTGAGG TAGCCTATCA TCAACCCATA ACACTTTTAA TTGTACAAGA   
  
  
+ TCATTAGTTT TCGTATCTTA AAGTACACAT TCCGAATATC ACCCTTCAAT GACATCACTC CTTCTCCAGT   
  
  
+ TGTTATCTGC TGTTTTCCTA ACTCGTCATT CCCAAAACAA AACGATTTAA CTTACTTTTT CGTCACGTGA   
  
  
+ AAAGAGAGTA ATTTCCTATT TTCCCTTTTG GCCTACTCAT CTGTTATTAT CTCGTTTTAG AGAGTACATT   
  
  
+ CACGGGTTCA CTTTTAATAG TAAGTGTGTA TAGACGCAGA GTTTTCCTTT TTCTTCTCTC TGTTTACCAC   
  
  
+ TATTTATATA GATAGTGAAA GATCCCAAAG AAATCAAAAA GAAAAAAGAT CCCAAAGAAA AACTCATGTA   
  
  
+ TGGGATTACT TTTCTTCATA CATAGTTACC GTTACTACTA CTTAGAACCC GCGTTTAGAG AAGTGACACG   
  
  
+ TAGTAGCCGC CACTTCTATA GTAATACTAA CTACTTTCTC CGGGATAAAC TGAAAACCGT CTCTTTTTCG   
  
  
+ AAGAGTGGAA AGAGGTAGAG GAAAACGATA AAGTGGAAAA GAAATTATGA AGGAGGTTTG GGGTGTAGAT   
  
  
+ GGGTGGTGAG GAGTTTAAGG TTTTGGGTCA TGGAATAATA TAAGAATAGT AGGGGAAGTT AATTCGTGGT   
  
  
+ ACGATTATTA ATTAGTAGGT GGGAAACGAA TAGTATTAAT CAATTTAAAT ATATTATGTG AGAGACACCT   
  
  
+ GGCTGGACTG CGGAACGATA CATACAACTA ACGAGTACTT GGGGTTGACA AACCTATAAC CTGGTTGACC   
  
  
+ CAATTAAATG AATGGTAAAC TAATAGTACA AGAACAACAT GAACTATCAC TAGAATTAAT CTACTTGTAC   
  
  
+ TAATTCAAGT AAACTAGCCT TTATTACTGA CTTCGTTTAG ATCCCGTGGT ATACTACCAC TTTTTAGTAT   
  
  
+ TCCGGTCGTC AATTTATAAA TTCTACAGTA CCTAGTCGTC TAATTATTTC TATAGACTTA AAACTAATTG   
  
  
+ GGTAAAGTTG TATCCTCTAG CGTATTTTAC TAAATCGATA GAATCGATCG TACGTTTTCG TTTGTTATTA   
  
  
+ ATCTACTAAA AAATTTTTAA TTTAAAGGT  

- CTGTGTCTCC TTCCCCTCTC TCTCTCTCTC TCTATCGATG GAAAAGTTGC AGAAGATCAA ATTCAAACAG   
  
  
- TTTAATAGGG AAAGAAATAG GGAAAGAATC TGCTGAAGAT ATATATACGA TCTCTAGAAG ATGAAGACCT   
  
  
- TAAACCATAT ACAGTAAATT AAGAGATCCT CCCTCTCTTT CTCTTCAACT TCTAGGATAG ATGGACAGAA   
  
  
- CTTAGATAGA TATGAAAGTA ACCAAATACC TATATAAATA TGACCCAACC ACTTTCCCTT TCAACAGCAT   
  
  
- TAATTGAGAA TTTCTAGTCA TTTATATTGA GGAAGAGTGA GCATAAATTA AAGAAAAAGA AAGCAGAAGC   
  
  
- CAAAAATAGA AATTAAGATA CAGAAACTCC ATCGGATAGT AGTTGGGTAT TGTGAAAATT AACATGTTCT   
  
  
- AGTAATCAAA AGCATAGAAT TTCATGTGTA AGGCTTATAG TGGGAAGTTA CTGTAGTGAG GAAGAGGTCA   
  
  
- ACAATAGACG ACAAAAGGAT TGAGCAGTAA GGGTTTTGTT TTGCTAAATT GAATGAAAAA GCAGTGCACT   
  
  
- TTTCTCTCAT TAAAGGATAA AAGGGAAAAC CGGATGAGTA GACAATAATA GAGCAAAATC TCTCATGTAA   
  
  
- GTGCCCAAGT GAAAATTATC ATTCACACAT ATCTGCGTCT CAAAAGGAAA AAGAAGAGAG ACAAATGGTG   
  
  
- ATAAATATAT CTATCACTTT CTAGGGTTTC TTTAGTTTTT CTTTTTTCTA GGGTTTCTTT TTGAGTACAT   
  
  
- ACCCTAATGA AAAGAAGTAT GTATCAATGG CAATGATGAT GAATCTTGGG CGCAAATCTC TTCACTGTGC   
  
  
- ATCATCGGCG GTGAAGATAT CATTATGATT GATGAAAGAG GCCCTATTTG ACTTTTGGCA GAGAAAAAGC   
  
  
- TTCTCACCTT TCTCCATCTC CTTTTGCTAT TTCACCTTTT CTTTAATACT TCCTCCAAAC CCCACATCTA   
  
  
- CCCACCACTC CTCAAATTCC AAAACCCAGT ACCTTATTAT ATTCTTATCA TCCCCTTCAA TTAAGCACCA   
  
  
- TGCTAATAAT TAATCATCCA CCCTTTGCTT ATCATAATTA GTTAAATTTA TATAATACAC TCTCTGTGGA   
  
  
- CCGACCTGAC GCCTTGCTAT GTATGTTGAT TGCTCATGAA CCCCAACTGT TTGGATATTG GACCAACTGG   
  
  
- GTTAATTTAC TTACCATTTG ATTATCATGT TCTTGTTGTA CTTGATAGTG ATCTTAATTA GATGAACATG   
  
  
- ATTAAGTTCA TTTGATCGGA AATAATGACT GAAGCAAATC TAGGGCACCA TATGATGGTG AAAAATCATA   
  
  
- AGGCCAGCAG TTAAATATTT AAGATGTCAT GGATCAGCAG ATTAATAAAG ATATCTGAAT TTTGATTAAC   
  
  
- CCATTTCAAC ATAGGAGATC GCATAAAATG ATTTAGCTAT CTTAGCTAGC ATGCAAAAGC AAACAATAAT   
  
  
- TAGATGATTT TTTAAAAATT AAATTTCCA

+     5UTR Py-rich stretch

| Site Name | Organism | Position | Strand | Matrix score. | sequence | function |
| --- | --- | --- | --- | --- | --- | --- |
| 5UTR Py-rich stretch | Lycopersicon esculentum | 20 | - | 13 | TTTCTCTCTCTCTC | cis-acting element conferring high transcription levels |
| 5UTR Py-rich stretch | Lycopersicon esculentum | 680 | + | 10 | TTTCTTCTCT | cis-acting element conferring high transcription levels |
| 5UTR Py-rich stretch | Lycopersicon esculentum | 16 | - | 13 | TTTCTCTCTCTCTC | cis-acting element conferring high transcription levels |
| 5UTR Py-rich stretch | Lycopersicon esculentum | 18 | - | 13 | TTTCTCTCTCTCTC | cis-acting element conferring high transcription levels |
| 5UTR Py-rich stretch | Lycopersicon esculentum | 22 | - | 13 | TTTCTCTCTCTCTC | cis-acting element conferring high transcription levels |

> 2018/04/13 10:10:12  
+ GACACAGAGG AAGGGGAGAG AGAGAGAGAG AGATAGCTAC CTTTTCAACG TCTTCTAGTT TAAGTTTGTC   
  
  
+ AAATTATCCC TTTCTTTATC CCTTTCTTAG ACGACTTCTA TATATATGCT AGAGATCTTC TACTTCTGGA   
  
  
+ ATTTGGTATA TGTCATTTAA TTCTCTAGGA GGGAGAGAAA GAGAAGTTGA AGATCCTATC TACCTGTCTT   
  
  
+ GAATCTATCT ATACTTTCAT TGGTTTATGG ATATATTTAT ACTGGGTTGG TGAAAGGGAA AGTTGTCGTA   
  
  
+ ATTAACTCTT AAAGATCAGT AAATATAACT CCTTCTCACT CGTATTTAAT TTCTTTTTCT TTCGTCTTCG   
  
  
+ GTTTTTATCT TTAATTCTAT GTCTTTGAGG TAGCCTATCA TCAACCCATA ACACTTTTAA TTGTACAAGA   
  
  
+ TCATTAGTTT TCGTATCTTA AAGTACACAT TCCGAATATC ACCCTTCAAT GACATCACTC CTTCTCCAGT   
  
  
+ TGTTATCTGC TGTTTTCCTA ACTCGTCATT CCCAAAACAA AACGATTTAA CTTACTTTTT CGTCACGTGA   
  
  
+ AAAGAGAGTA ATTTCCTATT TTCCCTTTTG GCCTACTCAT CTGTTATTAT CTCGTTTTAG AGAGTACATT   
  
  
+ CACGGGTTCA CTTTTAATAG TAAGTGTGTA TAGACGCAGA GTTTTCCTTT TTCTTCTCTC TGTTTACCAC   
  
  
+ TATTTATATA GATAGTGAAA GATCCCAAAG AAATCAAAAA GAAAAAAGAT CCCAAAGAAA AACTCATGTA   
  
  
+ TGGGATTACT TTTCTTCATA CATAGTTACC GTTACTACTA CTTAGAACCC GCGTTTAGAG AAGTGACACG   
  
  
+ TAGTAGCCGC CACTTCTATA GTAATACTAA CTACTTTCTC CGGGATAAAC TGAAAACCGT CTCTTTTTCG   
  
  
+ AAGAGTGGAA AGAGGTAGAG GAAAACGATA AAGTGGAAAA GAAATTATGA AGGAGGTTTG GGGTGTAGAT   
  
  
+ GGGTGGTGAG GAGTTTAAGG TTTTGGGTCA TGGAATAATA TAAGAATAGT AGGGGAAGTT AATTCGTGGT   
  
  
+ ACGATTATTA ATTAGTAGGT GGGAAACGAA TAGTATTAAT CAATTTAAAT ATATTATGTG AGAGACACCT   
  
  
+ GGCTGGACTG CGGAACGATA CATACAACTA ACGAGTACTT GGGGTTGACA AACCTATAAC CTGGTTGACC   
  
  
+ CAATTAAATG AATGGTAAAC TAATAGTACA AGAACAACAT GAACTATCAC TAGAATTAAT CTACTTGTAC   
  
  
+ TAATTCAAGT AAACTAGCCT TTATTACTGA CTTCGTTTAG ATCCCGTGGT ATACTACCAC TTTTTAGTAT   
  
  
+ TCCGGTCGTC AATTTATAAA TTCTACAGTA CCTAGTCGTC TAATTATTTC TATAGACTTA AAACTAATTG   
  
  
+ GGTAAAGTTG TATCCTCTAG CGTATTTTAC TAAATCGATA GAATCGATCG TACGTTTTCG TTTGTTATTA   
  
  
+ ATCTACTAAA AAATTTTTAA TTTAAAGGT  

- CTGTGTCTCC TTCCCCTCTC TCTCTCTCTC TCTATCGATG GAAAAGTTGC AGAAGATCAA ATTCAAACAG   
  
  
- TTTAATAGGG AAAGAAATAG GGAAAGAATC TGCTGAAGAT ATATATACGA TCTCTAGAAG ATGAAGACCT   
  
  
- TAAACCATAT ACAGTAAATT AAGAGATCCT CCCTCTCTTT CTCTTCAACT TCTAGGATAG ATGGACAGAA   
  
  
- CTTAGATAGA TATGAAAGTA ACCAAATACC TATATAAATA TGACCCAACC ACTTTCCCTT TCAACAGCAT   
  
  
- TAATTGAGAA TTTCTAGTCA TTTATATTGA GGAAGAGTGA GCATAAATTA AAGAAAAAGA AAGCAGAAGC   
  
  
- CAAAAATAGA AATTAAGATA CAGAAACTCC ATCGGATAGT AGTTGGGTAT TGTGAAAATT AACATGTTCT   
  
  
- AGTAATCAAA AGCATAGAAT TTCATGTGTA AGGCTTATAG TGGGAAGTTA CTGTAGTGAG GAAGAGGTCA   
  
  
- ACAATAGACG ACAAAAGGAT TGAGCAGTAA GGGTTTTGTT TTGCTAAATT GAATGAAAAA GCAGTGCACT   
  
  
- TTTCTCTCAT TAAAGGATAA AAGGGAAAAC CGGATGAGTA GACAATAATA GAGCAAAATC TCTCATGTAA   
  
  
- GTGCCCAAGT GAAAATTATC ATTCACACAT ATCTGCGTCT CAAAAGGAAA AAGAAGAGAG ACAAATGGTG   
  
  
- ATAAATATAT CTATCACTTT CTAGGGTTTC TTTAGTTTTT CTTTTTTCTA GGGTTTCTTT TTGAGTACAT   
  
  
- ACCCTAATGA AAAGAAGTAT GTATCAATGG CAATGATGAT GAATCTTGGG CGCAAATCTC TTCACTGTGC   
  
  
- ATCATCGGCG GTGAAGATAT CATTATGATT GATGAAAGAG GCCCTATTTG ACTTTTGGCA GAGAAAAAGC   
  
  
- TTCTCACCTT TCTCCATCTC CTTTTGCTAT TTCACCTTTT CTTTAATACT TCCTCCAAAC CCCACATCTA   
  
  
- CCCACCACTC CTCAAATTCC AAAACCCAGT ACCTTATTAT ATTCTTATCA TCCCCTTCAA TTAAGCACCA   
  
  
- TGCTAATAAT TAATCATCCA CCCTTTGCTT ATCATAATTA GTTAAATTTA TATAATACAC TCTCTGTGGA   
  
  
- CCGACCTGAC GCCTTGCTAT GTATGTTGAT TGCTCATGAA CCCCAACTGT TTGGATATTG GACCAACTGG   
  
  
- GTTAATTTAC TTACCATTTG ATTATCATGT TCTTGTTGTA CTTGATAGTG ATCTTAATTA GATGAACATG   
  
  
- ATTAAGTTCA TTTGATCGGA AATAATGACT GAAGCAAATC TAGGGCACCA TATGATGGTG AAAAATCATA   
  
  
- AGGCCAGCAG TTAAATATTT AAGATGTCAT GGATCAGCAG ATTAATAAAG ATATCTGAAT TTTGATTAAC   
  
  
- CCATTTCAAC ATAGGAGATC GCATAAAATG ATTTAGCTAT CTTAGCTAGC ATGCAAAAGC AAACAATAAT   
  
  
- TAGATGATTT TTTAAAAATT AAATTTCCA

+     AAGAA-motif

| Site Name | Organism | Position | Strand | Matrix score. | sequence | function |
| --- | --- | --- | --- | --- | --- | --- |
| AAGAA-motif | Avena sativa | 945 | + | 9 | gGTAAAGAAA |  |
| AAGAA-motif | Avena sativa | 81 | - | 9 | gGTAAAGAAA |  |
| AAGAA-motif | Avena sativa | 337 | - | 7 | GAAAGAA |  |

> 2018/04/13 10:10:12  
+ GACACAGAGG AAGGGGAGAG AGAGAGAGAG AGATAGCTAC CTTTTCAACG TCTTCTAGTT TAAGTTTGTC   
  
  
+ AAATTATCCC TTTCTTTATC CCTTTCTTAG ACGACTTCTA TATATATGCT AGAGATCTTC TACTTCTGGA   
  
  
+ ATTTGGTATA TGTCATTTAA TTCTCTAGGA GGGAGAGAAA GAGAAGTTGA AGATCCTATC TACCTGTCTT   
  
  
+ GAATCTATCT ATACTTTCAT TGGTTTATGG ATATATTTAT ACTGGGTTGG TGAAAGGGAA AGTTGTCGTA   
  
  
+ ATTAACTCTT AAAGATCAGT AAATATAACT CCTTCTCACT CGTATTTAAT TTCTTTTTCT TTCGTCTTCG   
  
  
+ GTTTTTATCT TTAATTCTAT GTCTTTGAGG TAGCCTATCA TCAACCCATA ACACTTTTAA TTGTACAAGA   
  
  
+ TCATTAGTTT TCGTATCTTA AAGTACACAT TCCGAATATC ACCCTTCAAT GACATCACTC CTTCTCCAGT   
  
  
+ TGTTATCTGC TGTTTTCCTA ACTCGTCATT CCCAAAACAA AACGATTTAA CTTACTTTTT CGTCACGTGA   
  
  
+ AAAGAGAGTA ATTTCCTATT TTCCCTTTTG GCCTACTCAT CTGTTATTAT CTCGTTTTAG AGAGTACATT   
  
  
+ CACGGGTTCA CTTTTAATAG TAAGTGTGTA TAGACGCAGA GTTTTCCTTT TTCTTCTCTC TGTTTACCAC   
  
  
+ TATTTATATA GATAGTGAAA GATCCCAAAG AAATCAAAAA GAAAAAAGAT CCCAAAGAAA AACTCATGTA   
  
  
+ TGGGATTACT TTTCTTCATA CATAGTTACC GTTACTACTA CTTAGAACCC GCGTTTAGAG AAGTGACACG   
  
  
+ TAGTAGCCGC CACTTCTATA GTAATACTAA CTACTTTCTC CGGGATAAAC TGAAAACCGT CTCTTTTTCG   
  
  
+ AAGAGTGGAA AGAGGTAGAG GAAAACGATA AAGTGGAAAA GAAATTATGA AGGAGGTTTG GGGTGTAGAT   
  
  
+ GGGTGGTGAG GAGTTTAAGG TTTTGGGTCA TGGAATAATA TAAGAATAGT AGGGGAAGTT AATTCGTGGT   
  
  
+ ACGATTATTA ATTAGTAGGT GGGAAACGAA TAGTATTAAT CAATTTAAAT ATATTATGTG AGAGACACCT   
  
  
+ GGCTGGACTG CGGAACGATA CATACAACTA ACGAGTACTT GGGGTTGACA AACCTATAAC CTGGTTGACC   
  
  
+ CAATTAAATG AATGGTAAAC TAATAGTACA AGAACAACAT GAACTATCAC TAGAATTAAT CTACTTGTAC   
  
  
+ TAATTCAAGT AAACTAGCCT TTATTACTGA CTTCGTTTAG ATCCCGTGGT ATACTACCAC TTTTTAGTAT   
  
  
+ TCCGGTCGTC AATTTATAAA TTCTACAGTA CCTAGTCGTC TAATTATTTC TATAGACTTA AAACTAATTG   
  
  
+ GGTAAAGTTG TATCCTCTAG CGTATTTTAC TAAATCGATA GAATCGATCG TACGTTTTCG TTTGTTATTA   
  
  
+ ATCTACTAAA AAATTTTTAA TTTAAAGGT  

- CTGTGTCTCC TTCCCCTCTC TCTCTCTCTC TCTATCGATG GAAAAGTTGC AGAAGATCAA ATTCAAACAG   
  
  
- TTTAATAGGG AAAGAAATAG GGAAAGAATC TGCTGAAGAT ATATATACGA TCTCTAGAAG ATGAAGACCT   
  
  
- TAAACCATAT ACAGTAAATT AAGAGATCCT CCCTCTCTTT CTCTTCAACT TCTAGGATAG ATGGACAGAA   
  
  
- CTTAGATAGA TATGAAAGTA ACCAAATACC TATATAAATA TGACCCAACC ACTTTCCCTT TCAACAGCAT   
  
  
- TAATTGAGAA TTTCTAGTCA TTTATATTGA GGAAGAGTGA GCATAAATTA AAGAAAAAGA AAGCAGAAGC   
  
  
- CAAAAATAGA AATTAAGATA CAGAAACTCC ATCGGATAGT AGTTGGGTAT TGTGAAAATT AACATGTTCT   
  
  
- AGTAATCAAA AGCATAGAAT TTCATGTGTA AGGCTTATAG TGGGAAGTTA CTGTAGTGAG GAAGAGGTCA   
  
  
- ACAATAGACG ACAAAAGGAT TGAGCAGTAA GGGTTTTGTT TTGCTAAATT GAATGAAAAA GCAGTGCACT   
  
  
- TTTCTCTCAT TAAAGGATAA AAGGGAAAAC CGGATGAGTA GACAATAATA GAGCAAAATC TCTCATGTAA   
  
  
- GTGCCCAAGT GAAAATTATC ATTCACACAT ATCTGCGTCT CAAAAGGAAA AAGAAGAGAG ACAAATGGTG   
  
  
- ATAAATATAT CTATCACTTT CTAGGGTTTC TTTAGTTTTT CTTTTTTCTA GGGTTTCTTT TTGAGTACAT   
  
  
- ACCCTAATGA AAAGAAGTAT GTATCAATGG CAATGATGAT GAATCTTGGG CGCAAATCTC TTCACTGTGC   
  
  
- ATCATCGGCG GTGAAGATAT CATTATGATT GATGAAAGAG GCCCTATTTG ACTTTTGGCA GAGAAAAAGC   
  
  
- TTCTCACCTT TCTCCATCTC CTTTTGCTAT TTCACCTTTT CTTTAATACT TCCTCCAAAC CCCACATCTA   
  
  
- CCCACCACTC CTCAAATTCC AAAACCCAGT ACCTTATTAT ATTCTTATCA TCCCCTTCAA TTAAGCACCA   
  
  
- TGCTAATAAT TAATCATCCA CCCTTTGCTT ATCATAATTA GTTAAATTTA TATAATACAC TCTCTGTGGA   
  
  
- CCGACCTGAC GCCTTGCTAT GTATGTTGAT TGCTCATGAA CCCCAACTGT TTGGATATTG GACCAACTGG   
  
  
- GTTAATTTAC TTACCATTTG ATTATCATGT TCTTGTTGTA CTTGATAGTG ATCTTAATTA GATGAACATG   
  
  
- ATTAAGTTCA TTTGATCGGA AATAATGACT GAAGCAAATC TAGGGCACCA TATGATGGTG AAAAATCATA   
  
  
- AGGCCAGCAG TTAAATATTT AAGATGTCAT GGATCAGCAG ATTAATAAAG ATATCTGAAT TTTGATTAAC   
  
  
- CCATTTCAAC ATAGGAGATC GCATAAAATG ATTTAGCTAT CTTAGCTAGC ATGCAAAAGC AAACAATAAT   
  
  
- TAGATGATTT TTTAAAAATT AAATTTCCA

+     ABRE

| Site Name | Organism | Position | Strand | Matrix score. | sequence | function |
| --- | --- | --- | --- | --- | --- | --- |
| ABRE | Arabidopsis thaliana | 835 | - | 8 | TACGTGTC | cis-acting element involved in the abscisic acid responsiveness |
| ABRE | Arabidopsis thaliana | 837 | - | 6 | TACGTG | cis-acting element involved in the abscisic acid responsiveness |
| ABRE | Arabidopsis thaliana | 554 | + | 6 | CACGTG | cis-acting element involved in the abscisic acid responsiveness |
| ABRE | Triticum aestivum | 1114 | + | 9 | GACACGTGGC | cis-acting element involved in the abscisic acid responsiveness |

> 2018/04/13 10:10:12  
+ GACACAGAGG AAGGGGAGAG AGAGAGAGAG AGATAGCTAC CTTTTCAACG TCTTCTAGTT TAAGTTTGTC   
  
  
+ AAATTATCCC TTTCTTTATC CCTTTCTTAG ACGACTTCTA TATATATGCT AGAGATCTTC TACTTCTGGA   
  
  
+ ATTTGGTATA TGTCATTTAA TTCTCTAGGA GGGAGAGAAA GAGAAGTTGA AGATCCTATC TACCTGTCTT   
  
  
+ GAATCTATCT ATACTTTCAT TGGTTTATGG ATATATTTAT ACTGGGTTGG TGAAAGGGAA AGTTGTCGTA   
  
  
+ ATTAACTCTT AAAGATCAGT AAATATAACT CCTTCTCACT CGTATTTAAT TTCTTTTTCT TTCGTCTTCG   
  
  
+ GTTTTTATCT TTAATTCTAT GTCTTTGAGG TAGCCTATCA TCAACCCATA ACACTTTTAA TTGTACAAGA   
  
  
+ TCATTAGTTT TCGTATCTTA AAGTACACAT TCCGAATATC ACCCTTCAAT GACATCACTC CTTCTCCAGT   
  
  
+ TGTTATCTGC TGTTTTCCTA ACTCGTCATT CCCAAAACAA AACGATTTAA CTTACTTTTT CGTCACGTGA   
  
  
+ AAAGAGAGTA ATTTCCTATT TTCCCTTTTG GCCTACTCAT CTGTTATTAT CTCGTTTTAG AGAGTACATT   
  
  
+ CACGGGTTCA CTTTTAATAG TAAGTGTGTA TAGACGCAGA GTTTTCCTTT TTCTTCTCTC TGTTTACCAC   
  
  
+ TATTTATATA GATAGTGAAA GATCCCAAAG AAATCAAAAA GAAAAAAGAT CCCAAAGAAA AACTCATGTA   
  
  
+ TGGGATTACT TTTCTTCATA CATAGTTACC GTTACTACTA CTTAGAACCC GCGTTTAGAG AAGTGACACG   
  
  
+ TAGTAGCCGC CACTTCTATA GTAATACTAA CTACTTTCTC CGGGATAAAC TGAAAACCGT CTCTTTTTCG   
  
  
+ AAGAGTGGAA AGAGGTAGAG GAAAACGATA AAGTGGAAAA GAAATTATGA AGGAGGTTTG GGGTGTAGAT   
  
  
+ GGGTGGTGAG GAGTTTAAGG TTTTGGGTCA TGGAATAATA TAAGAATAGT AGGGGAAGTT AATTCGTGGT   
  
  
+ ACGATTATTA ATTAGTAGGT GGGAAACGAA TAGTATTAAT CAATTTAAAT ATATTATGTG AGAGACACCT   
  
  
+ GGCTGGACTG CGGAACGATA CATACAACTA ACGAGTACTT GGGGTTGACA AACCTATAAC CTGGTTGACC   
  
  
+ CAATTAAATG AATGGTAAAC TAATAGTACA AGAACAACAT GAACTATCAC TAGAATTAAT CTACTTGTAC   
  
  
+ TAATTCAAGT AAACTAGCCT TTATTACTGA CTTCGTTTAG ATCCCGTGGT ATACTACCAC TTTTTAGTAT   
  
  
+ TCCGGTCGTC AATTTATAAA TTCTACAGTA CCTAGTCGTC TAATTATTTC TATAGACTTA AAACTAATTG   
  
  
+ GGTAAAGTTG TATCCTCTAG CGTATTTTAC TAAATCGATA GAATCGATCG TACGTTTTCG TTTGTTATTA   
  
  
+ ATCTACTAAA AAATTTTTAA TTTAAAGGT  

- CTGTGTCTCC TTCCCCTCTC TCTCTCTCTC TCTATCGATG GAAAAGTTGC AGAAGATCAA ATTCAAACAG   
  
  
- TTTAATAGGG AAAGAAATAG GGAAAGAATC TGCTGAAGAT ATATATACGA TCTCTAGAAG ATGAAGACCT   
  
  
- TAAACCATAT ACAGTAAATT AAGAGATCCT CCCTCTCTTT CTCTTCAACT TCTAGGATAG ATGGACAGAA   
  
  
- CTTAGATAGA TATGAAAGTA ACCAAATACC TATATAAATA TGACCCAACC ACTTTCCCTT TCAACAGCAT   
  
  
- TAATTGAGAA TTTCTAGTCA TTTATATTGA GGAAGAGTGA GCATAAATTA AAGAAAAAGA AAGCAGAAGC   
  
  
- CAAAAATAGA AATTAAGATA CAGAAACTCC ATCGGATAGT AGTTGGGTAT TGTGAAAATT AACATGTTCT   
  
  
- AGTAATCAAA AGCATAGAAT TTCATGTGTA AGGCTTATAG TGGGAAGTTA CTGTAGTGAG GAAGAGGTCA   
  
  
- ACAATAGACG ACAAAAGGAT TGAGCAGTAA GGGTTTTGTT TTGCTAAATT GAATGAAAAA GCAGTGCACT   
  
  
- TTTCTCTCAT TAAAGGATAA AAGGGAAAAC CGGATGAGTA GACAATAATA GAGCAAAATC TCTCATGTAA   
  
  
- GTGCCCAAGT GAAAATTATC ATTCACACAT ATCTGCGTCT CAAAAGGAAA AAGAAGAGAG ACAAATGGTG   
  
  
- ATAAATATAT CTATCACTTT CTAGGGTTTC TTTAGTTTTT CTTTTTTCTA GGGTTTCTTT TTGAGTACAT   
  
  
- ACCCTAATGA AAAGAAGTAT GTATCAATGG CAATGATGAT GAATCTTGGG CGCAAATCTC TTCACTGTGC   
  
  
- ATCATCGGCG GTGAAGATAT CATTATGATT GATGAAAGAG GCCCTATTTG ACTTTTGGCA GAGAAAAAGC   
  
  
- TTCTCACCTT TCTCCATCTC CTTTTGCTAT TTCACCTTTT CTTTAATACT TCCTCCAAAC CCCACATCTA   
  
  
- CCCACCACTC CTCAAATTCC AAAACCCAGT ACCTTATTAT ATTCTTATCA TCCCCTTCAA TTAAGCACCA   
  
  
- TGCTAATAAT TAATCATCCA CCCTTTGCTT ATCATAATTA GTTAAATTTA TATAATACAC TCTCTGTGGA   
  
  
- CCGACCTGAC GCCTTGCTAT GTATGTTGAT TGCTCATGAA CCCCAACTGT TTGGATATTG GACCAACTGG   
  
  
- GTTAATTTAC TTACCATTTG ATTATCATGT TCTTGTTGTA CTTGATAGTG ATCTTAATTA GATGAACATG   
  
  
- ATTAAGTTCA TTTGATCGGA AATAATGACT GAAGCAAATC TAGGGCACCA TATGATGGTG AAAAATCATA   
  
  
- AGGCCAGCAG TTAAATATTT AAGATGTCAT GGATCAGCAG ATTAATAAAG ATATCTGAAT TTTGATTAAC   
  
  
- CCATTTCAAC ATAGGAGATC GCATAAAATG ATTTAGCTAT CTTAGCTAGC ATGCAAAAGC AAACAATAAT   
  
  
- TAGATGATTT TTTAAAAATT AAATTTCCA

+     AC-I

| Site Name | Organism | Position | Strand | Matrix score. | sequence | function |
| --- | --- | --- | --- | --- | --- | --- |
| AC-I | Phaseolus vulgaris | 1064 | - | 9 | CCCACCTACC |  |
| AC-I | Populus tremuloides | 255 | - | 10 | TCTCACCAACC |  |

> 2018/04/13 10:10:12  
+ GACACAGAGG AAGGGGAGAG AGAGAGAGAG AGATAGCTAC CTTTTCAACG TCTTCTAGTT TAAGTTTGTC   
  
  
+ AAATTATCCC TTTCTTTATC CCTTTCTTAG ACGACTTCTA TATATATGCT AGAGATCTTC TACTTCTGGA   
  
  
+ ATTTGGTATA TGTCATTTAA TTCTCTAGGA GGGAGAGAAA GAGAAGTTGA AGATCCTATC TACCTGTCTT   
  
  
+ GAATCTATCT ATACTTTCAT TGGTTTATGG ATATATTTAT ACTGGGTTGG TGAAAGGGAA AGTTGTCGTA   
  
  
+ ATTAACTCTT AAAGATCAGT AAATATAACT CCTTCTCACT CGTATTTAAT TTCTTTTTCT TTCGTCTTCG   
  
  
+ GTTTTTATCT TTAATTCTAT GTCTTTGAGG TAGCCTATCA TCAACCCATA ACACTTTTAA TTGTACAAGA   
  
  
+ TCATTAGTTT TCGTATCTTA AAGTACACAT TCCGAATATC ACCCTTCAAT GACATCACTC CTTCTCCAGT   
  
  
+ TGTTATCTGC TGTTTTCCTA ACTCGTCATT CCCAAAACAA AACGATTTAA CTTACTTTTT CGTCACGTGA   
  
  
+ AAAGAGAGTA ATTTCCTATT TTCCCTTTTG GCCTACTCAT CTGTTATTAT CTCGTTTTAG AGAGTACATT   
  
  
+ CACGGGTTCA CTTTTAATAG TAAGTGTGTA TAGACGCAGA GTTTTCCTTT TTCTTCTCTC TGTTTACCAC   
  
  
+ TATTTATATA GATAGTGAAA GATCCCAAAG AAATCAAAAA GAAAAAAGAT CCCAAAGAAA AACTCATGTA   
  
  
+ TGGGATTACT TTTCTTCATA CATAGTTACC GTTACTACTA CTTAGAACCC GCGTTTAGAG AAGTGACACG   
  
  
+ TAGTAGCCGC CACTTCTATA GTAATACTAA CTACTTTCTC CGGGATAAAC TGAAAACCGT CTCTTTTTCG   
  
  
+ AAGAGTGGAA AGAGGTAGAG GAAAACGATA AAGTGGAAAA GAAATTATGA AGGAGGTTTG GGGTGTAGAT   
  
  
+ GGGTGGTGAG GAGTTTAAGG TTTTGGGTCA TGGAATAATA TAAGAATAGT AGGGGAAGTT AATTCGTGGT   
  
  
+ ACGATTATTA ATTAGTAGGT GGGAAACGAA TAGTATTAAT CAATTTAAAT ATATTATGTG AGAGACACCT   
  
  
+ GGCTGGACTG CGGAACGATA CATACAACTA ACGAGTACTT GGGGTTGACA AACCTATAAC CTGGTTGACC   
  
  
+ CAATTAAATG AATGGTAAAC TAATAGTACA AGAACAACAT GAACTATCAC TAGAATTAAT CTACTTGTAC   
  
  
+ TAATTCAAGT AAACTAGCCT TTATTACTGA CTTCGTTTAG ATCCCGTGGT ATACTACCAC TTTTTAGTAT   
  
  
+ TCCGGTCGTC AATTTATAAA TTCTACAGTA CCTAGTCGTC TAATTATTTC TATAGACTTA AAACTAATTG   
  
  
+ GGTAAAGTTG TATCCTCTAG CGTATTTTAC TAAATCGATA GAATCGATCG TACGTTTTCG TTTGTTATTA   
  
  
+ ATCTACTAAA AAATTTTTAA TTTAAAGGT  

- CTGTGTCTCC TTCCCCTCTC TCTCTCTCTC TCTATCGATG GAAAAGTTGC AGAAGATCAA ATTCAAACAG   
  
  
- TTTAATAGGG AAAGAAATAG GGAAAGAATC TGCTGAAGAT ATATATACGA TCTCTAGAAG ATGAAGACCT   
  
  
- TAAACCATAT ACAGTAAATT AAGAGATCCT CCCTCTCTTT CTCTTCAACT TCTAGGATAG ATGGACAGAA   
  
  
- CTTAGATAGA TATGAAAGTA ACCAAATACC TATATAAATA TGACCCAACC ACTTTCCCTT TCAACAGCAT   
  
  
- TAATTGAGAA TTTCTAGTCA TTTATATTGA GGAAGAGTGA GCATAAATTA AAGAAAAAGA AAGCAGAAGC   
  
  
- CAAAAATAGA AATTAAGATA CAGAAACTCC ATCGGATAGT AGTTGGGTAT TGTGAAAATT AACATGTTCT   
  
  
- AGTAATCAAA AGCATAGAAT TTCATGTGTA AGGCTTATAG TGGGAAGTTA CTGTAGTGAG GAAGAGGTCA   
  
  
- ACAATAGACG ACAAAAGGAT TGAGCAGTAA GGGTTTTGTT TTGCTAAATT GAATGAAAAA GCAGTGCACT   
  
  
- TTTCTCTCAT TAAAGGATAA AAGGGAAAAC CGGATGAGTA GACAATAATA GAGCAAAATC TCTCATGTAA   
  
  
- GTGCCCAAGT GAAAATTATC ATTCACACAT ATCTGCGTCT CAAAAGGAAA AAGAAGAGAG ACAAATGGTG   
  
  
- ATAAATATAT CTATCACTTT CTAGGGTTTC TTTAGTTTTT CTTTTTTCTA GGGTTTCTTT TTGAGTACAT   
  
  
- ACCCTAATGA AAAGAAGTAT GTATCAATGG CAATGATGAT GAATCTTGGG CGCAAATCTC TTCACTGTGC   
  
  
- ATCATCGGCG GTGAAGATAT CATTATGATT GATGAAAGAG GCCCTATTTG ACTTTTGGCA GAGAAAAAGC   
  
  
- TTCTCACCTT TCTCCATCTC CTTTTGCTAT TTCACCTTTT CTTTAATACT TCCTCCAAAC CCCACATCTA   
  
  
- CCCACCACTC CTCAAATTCC AAAACCCAGT ACCTTATTAT ATTCTTATCA TCCCCTTCAA TTAAGCACCA   
  
  
- TGCTAATAAT TAATCATCCA CCCTTTGCTT ATCATAATTA GTTAAATTTA TATAATACAC TCTCTGTGGA   
  
  
- CCGACCTGAC GCCTTGCTAT GTATGTTGAT TGCTCATGAA CCCCAACTGT TTGGATATTG GACCAACTGG   
  
  
- GTTAATTTAC TTACCATTTG ATTATCATGT TCTTGTTGTA CTTGATAGTG ATCTTAATTA GATGAACATG   
  
  
- ATTAAGTTCA TTTGATCGGA AATAATGACT GAAGCAAATC TAGGGCACCA TATGATGGTG AAAAATCATA   
  
  
- AGGCCAGCAG TTAAATATTT AAGATGTCAT GGATCAGCAG ATTAATAAAG ATATCTGAAT TTTGATTAAC   
  
  
- CCATTTCAAC ATAGGAGATC GCATAAAATG ATTTAGCTAT CTTAGCTAGC ATGCAAAAGC AAACAATAAT   
  
  
- TAGATGATTT TTTAAAAATT AAATTTCCA

+     ACE

| Site Name | Organism | Position | Strand | Matrix score. | sequence | function |
| --- | --- | --- | --- | --- | --- | --- |
| ACE | Petroselinum crispum | 1417 | + | 9 | CTAACGTATT | cis-acting element involved in light responsiveness |

> 2018/04/13 10:10:12  
+ GACACAGAGG AAGGGGAGAG AGAGAGAGAG AGATAGCTAC CTTTTCAACG TCTTCTAGTT TAAGTTTGTC   
  
  
+ AAATTATCCC TTTCTTTATC CCTTTCTTAG ACGACTTCTA TATATATGCT AGAGATCTTC TACTTCTGGA   
  
  
+ ATTTGGTATA TGTCATTTAA TTCTCTAGGA GGGAGAGAAA GAGAAGTTGA AGATCCTATC TACCTGTCTT   
  
  
+ GAATCTATCT ATACTTTCAT TGGTTTATGG ATATATTTAT ACTGGGTTGG TGAAAGGGAA AGTTGTCGTA   
  
  
+ ATTAACTCTT AAAGATCAGT AAATATAACT CCTTCTCACT CGTATTTAAT TTCTTTTTCT TTCGTCTTCG   
  
  
+ GTTTTTATCT TTAATTCTAT GTCTTTGAGG TAGCCTATCA TCAACCCATA ACACTTTTAA TTGTACAAGA   
  
  
+ TCATTAGTTT TCGTATCTTA AAGTACACAT TCCGAATATC ACCCTTCAAT GACATCACTC CTTCTCCAGT   
  
  
+ TGTTATCTGC TGTTTTCCTA ACTCGTCATT CCCAAAACAA AACGATTTAA CTTACTTTTT CGTCACGTGA   
  
  
+ AAAGAGAGTA ATTTCCTATT TTCCCTTTTG GCCTACTCAT CTGTTATTAT CTCGTTTTAG AGAGTACATT   
  
  
+ CACGGGTTCA CTTTTAATAG TAAGTGTGTA TAGACGCAGA GTTTTCCTTT TTCTTCTCTC TGTTTACCAC   
  
  
+ TATTTATATA GATAGTGAAA GATCCCAAAG AAATCAAAAA GAAAAAAGAT CCCAAAGAAA AACTCATGTA   
  
  
+ TGGGATTACT TTTCTTCATA CATAGTTACC GTTACTACTA CTTAGAACCC GCGTTTAGAG AAGTGACACG   
  
  
+ TAGTAGCCGC CACTTCTATA GTAATACTAA CTACTTTCTC CGGGATAAAC TGAAAACCGT CTCTTTTTCG   
  
  
+ AAGAGTGGAA AGAGGTAGAG GAAAACGATA AAGTGGAAAA GAAATTATGA AGGAGGTTTG GGGTGTAGAT   
  
  
+ GGGTGGTGAG GAGTTTAAGG TTTTGGGTCA TGGAATAATA TAAGAATAGT AGGGGAAGTT AATTCGTGGT   
  
  
+ ACGATTATTA ATTAGTAGGT GGGAAACGAA TAGTATTAAT CAATTTAAAT ATATTATGTG AGAGACACCT   
  
  
+ GGCTGGACTG CGGAACGATA CATACAACTA ACGAGTACTT GGGGTTGACA AACCTATAAC CTGGTTGACC   
  
  
+ CAATTAAATG AATGGTAAAC TAATAGTACA AGAACAACAT GAACTATCAC TAGAATTAAT CTACTTGTAC   
  
  
+ TAATTCAAGT AAACTAGCCT TTATTACTGA CTTCGTTTAG ATCCCGTGGT ATACTACCAC TTTTTAGTAT   
  
  
+ TCCGGTCGTC AATTTATAAA TTCTACAGTA CCTAGTCGTC TAATTATTTC TATAGACTTA AAACTAATTG   
  
  
+ GGTAAAGTTG TATCCTCTAG CGTATTTTAC TAAATCGATA GAATCGATCG TACGTTTTCG TTTGTTATTA   
  
  
+ ATCTACTAAA AAATTTTTAA TTTAAAGGT  

- CTGTGTCTCC TTCCCCTCTC TCTCTCTCTC TCTATCGATG GAAAAGTTGC AGAAGATCAA ATTCAAACAG   
  
  
- TTTAATAGGG AAAGAAATAG GGAAAGAATC TGCTGAAGAT ATATATACGA TCTCTAGAAG ATGAAGACCT   
  
  
- TAAACCATAT ACAGTAAATT AAGAGATCCT CCCTCTCTTT CTCTTCAACT TCTAGGATAG ATGGACAGAA   
  
  
- CTTAGATAGA TATGAAAGTA ACCAAATACC TATATAAATA TGACCCAACC ACTTTCCCTT TCAACAGCAT   
  
  
- TAATTGAGAA TTTCTAGTCA TTTATATTGA GGAAGAGTGA GCATAAATTA AAGAAAAAGA AAGCAGAAGC   
  
  
- CAAAAATAGA AATTAAGATA CAGAAACTCC ATCGGATAGT AGTTGGGTAT TGTGAAAATT AACATGTTCT   
  
  
- AGTAATCAAA AGCATAGAAT TTCATGTGTA AGGCTTATAG TGGGAAGTTA CTGTAGTGAG GAAGAGGTCA   
  
  
- ACAATAGACG ACAAAAGGAT TGAGCAGTAA GGGTTTTGTT TTGCTAAATT GAATGAAAAA GCAGTGCACT   
  
  
- TTTCTCTCAT TAAAGGATAA AAGGGAAAAC CGGATGAGTA GACAATAATA GAGCAAAATC TCTCATGTAA   
  
  
- GTGCCCAAGT GAAAATTATC ATTCACACAT ATCTGCGTCT CAAAAGGAAA AAGAAGAGAG ACAAATGGTG   
  
  
- ATAAATATAT CTATCACTTT CTAGGGTTTC TTTAGTTTTT CTTTTTTCTA GGGTTTCTTT TTGAGTACAT   
  
  
- ACCCTAATGA AAAGAAGTAT GTATCAATGG CAATGATGAT GAATCTTGGG CGCAAATCTC TTCACTGTGC   
  
  
- ATCATCGGCG GTGAAGATAT CATTATGATT GATGAAAGAG GCCCTATTTG ACTTTTGGCA GAGAAAAAGC   
  
  
- TTCTCACCTT TCTCCATCTC CTTTTGCTAT TTCACCTTTT CTTTAATACT TCCTCCAAAC CCCACATCTA   
  
  
- CCCACCACTC CTCAAATTCC AAAACCCAGT ACCTTATTAT ATTCTTATCA TCCCCTTCAA TTAAGCACCA   
  
  
- TGCTAATAAT TAATCATCCA CCCTTTGCTT ATCATAATTA GTTAAATTTA TATAATACAC TCTCTGTGGA   
  
  
- CCGACCTGAC GCCTTGCTAT GTATGTTGAT TGCTCATGAA CCCCAACTGT TTGGATATTG GACCAACTGG   
  
  
- GTTAATTTAC TTACCATTTG ATTATCATGT TCTTGTTGTA CTTGATAGTG ATCTTAATTA GATGAACATG   
  
  
- ATTAAGTTCA TTTGATCGGA AATAATGACT GAAGCAAATC TAGGGCACCA TATGATGGTG AAAAATCATA   
  
  
- AGGCCAGCAG TTAAATATTT AAGATGTCAT GGATCAGCAG ATTAATAAAG ATATCTGAAT TTTGATTAAC   
  
  
- CCATTTCAAC ATAGGAGATC GCATAAAATG ATTTAGCTAT CTTAGCTAGC ATGCAAAAGC AAACAATAAT   
  
  
- TAGATGATTT TTTAAAAATT AAATTTCCA

+     ARE

| Site Name | Organism | Position | Strand | Matrix score. | sequence | function |
| --- | --- | --- | --- | --- | --- | --- |
| ARE | Zea mays | 231 | + | 6 | TGGTTT | cis-acting regulatory element essential for the anaerobic induction |

> 2018/04/13 10:10:12  
+ GACACAGAGG AAGGGGAGAG AGAGAGAGAG AGATAGCTAC CTTTTCAACG TCTTCTAGTT TAAGTTTGTC   
  
  
+ AAATTATCCC TTTCTTTATC CCTTTCTTAG ACGACTTCTA TATATATGCT AGAGATCTTC TACTTCTGGA   
  
  
+ ATTTGGTATA TGTCATTTAA TTCTCTAGGA GGGAGAGAAA GAGAAGTTGA AGATCCTATC TACCTGTCTT   
  
  
+ GAATCTATCT ATACTTTCAT TGGTTTATGG ATATATTTAT ACTGGGTTGG TGAAAGGGAA AGTTGTCGTA   
  
  
+ ATTAACTCTT AAAGATCAGT AAATATAACT CCTTCTCACT CGTATTTAAT TTCTTTTTCT TTCGTCTTCG   
  
  
+ GTTTTTATCT TTAATTCTAT GTCTTTGAGG TAGCCTATCA TCAACCCATA ACACTTTTAA TTGTACAAGA   
  
  
+ TCATTAGTTT TCGTATCTTA AAGTACACAT TCCGAATATC ACCCTTCAAT GACATCACTC CTTCTCCAGT   
  
  
+ TGTTATCTGC TGTTTTCCTA ACTCGTCATT CCCAAAACAA AACGATTTAA CTTACTTTTT CGTCACGTGA   
  
  
+ AAAGAGAGTA ATTTCCTATT TTCCCTTTTG GCCTACTCAT CTGTTATTAT CTCGTTTTAG AGAGTACATT   
  
  
+ CACGGGTTCA CTTTTAATAG TAAGTGTGTA TAGACGCAGA GTTTTCCTTT TTCTTCTCTC TGTTTACCAC   
  
  
+ TATTTATATA GATAGTGAAA GATCCCAAAG AAATCAAAAA GAAAAAAGAT CCCAAAGAAA AACTCATGTA   
  
  
+ TGGGATTACT TTTCTTCATA CATAGTTACC GTTACTACTA CTTAGAACCC GCGTTTAGAG AAGTGACACG   
  
  
+ TAGTAGCCGC CACTTCTATA GTAATACTAA CTACTTTCTC CGGGATAAAC TGAAAACCGT CTCTTTTTCG   
  
  
+ AAGAGTGGAA AGAGGTAGAG GAAAACGATA AAGTGGAAAA GAAATTATGA AGGAGGTTTG GGGTGTAGAT   
  
  
+ GGGTGGTGAG GAGTTTAAGG TTTTGGGTCA TGGAATAATA TAAGAATAGT AGGGGAAGTT AATTCGTGGT   
  
  
+ ACGATTATTA ATTAGTAGGT GGGAAACGAA TAGTATTAAT CAATTTAAAT ATATTATGTG AGAGACACCT   
  
  
+ GGCTGGACTG CGGAACGATA CATACAACTA ACGAGTACTT GGGGTTGACA AACCTATAAC CTGGTTGACC   
  
  
+ CAATTAAATG AATGGTAAAC TAATAGTACA AGAACAACAT GAACTATCAC TAGAATTAAT CTACTTGTAC   
  
  
+ TAATTCAAGT AAACTAGCCT TTATTACTGA CTTCGTTTAG ATCCCGTGGT ATACTACCAC TTTTTAGTAT   
  
  
+ TCCGGTCGTC AATTTATAAA TTCTACAGTA CCTAGTCGTC TAATTATTTC TATAGACTTA AAACTAATTG   
  
  
+ GGTAAAGTTG TATCCTCTAG CGTATTTTAC TAAATCGATA GAATCGATCG TACGTTTTCG TTTGTTATTA   
  
  
+ ATCTACTAAA AAATTTTTAA TTTAAAGGT  

- CTGTGTCTCC TTCCCCTCTC TCTCTCTCTC TCTATCGATG GAAAAGTTGC AGAAGATCAA ATTCAAACAG   
  
  
- TTTAATAGGG AAAGAAATAG GGAAAGAATC TGCTGAAGAT ATATATACGA TCTCTAGAAG ATGAAGACCT   
  
  
- TAAACCATAT ACAGTAAATT AAGAGATCCT CCCTCTCTTT CTCTTCAACT TCTAGGATAG ATGGACAGAA   
  
  
- CTTAGATAGA TATGAAAGTA ACCAAATACC TATATAAATA TGACCCAACC ACTTTCCCTT TCAACAGCAT   
  
  
- TAATTGAGAA TTTCTAGTCA TTTATATTGA GGAAGAGTGA GCATAAATTA AAGAAAAAGA AAGCAGAAGC   
  
  
- CAAAAATAGA AATTAAGATA CAGAAACTCC ATCGGATAGT AGTTGGGTAT TGTGAAAATT AACATGTTCT   
  
  
- AGTAATCAAA AGCATAGAAT TTCATGTGTA AGGCTTATAG TGGGAAGTTA CTGTAGTGAG GAAGAGGTCA   
  
  
- ACAATAGACG ACAAAAGGAT TGAGCAGTAA GGGTTTTGTT TTGCTAAATT GAATGAAAAA GCAGTGCACT   
  
  
- TTTCTCTCAT TAAAGGATAA AAGGGAAAAC CGGATGAGTA GACAATAATA GAGCAAAATC TCTCATGTAA   
  
  
- GTGCCCAAGT GAAAATTATC ATTCACACAT ATCTGCGTCT CAAAAGGAAA AAGAAGAGAG ACAAATGGTG   
  
  
- ATAAATATAT CTATCACTTT CTAGGGTTTC TTTAGTTTTT CTTTTTTCTA GGGTTTCTTT TTGAGTACAT   
  
  
- ACCCTAATGA AAAGAAGTAT GTATCAATGG CAATGATGAT GAATCTTGGG CGCAAATCTC TTCACTGTGC   
  
  
- ATCATCGGCG GTGAAGATAT CATTATGATT GATGAAAGAG GCCCTATTTG ACTTTTGGCA GAGAAAAAGC   
  
  
- TTCTCACCTT TCTCCATCTC CTTTTGCTAT TTCACCTTTT CTTTAATACT TCCTCCAAAC CCCACATCTA   
  
  
- CCCACCACTC CTCAAATTCC AAAACCCAGT ACCTTATTAT ATTCTTATCA TCCCCTTCAA TTAAGCACCA   
  
  
- TGCTAATAAT TAATCATCCA CCCTTTGCTT ATCATAATTA GTTAAATTTA TATAATACAC TCTCTGTGGA   
  
  
- CCGACCTGAC GCCTTGCTAT GTATGTTGAT TGCTCATGAA CCCCAACTGT TTGGATATTG GACCAACTGG   
  
  
- GTTAATTTAC TTACCATTTG ATTATCATGT TCTTGTTGTA CTTGATAGTG ATCTTAATTA GATGAACATG   
  
  
- ATTAAGTTCA TTTGATCGGA AATAATGACT GAAGCAAATC TAGGGCACCA TATGATGGTG AAAAATCATA   
  
  
- AGGCCAGCAG TTAAATATTT AAGATGTCAT GGATCAGCAG ATTAATAAAG ATATCTGAAT TTTGATTAAC   
  
  
- CCATTTCAAC ATAGGAGATC GCATAAAATG ATTTAGCTAT CTTAGCTAGC ATGCAAAAGC AAACAATAAT   
  
  
- TAGATGATTT TTTAAAAATT AAATTTCCA

+     AT-rich element

| Site Name | Organism | Position | Strand | Matrix score. | sequence | function |
| --- | --- | --- | --- | --- | --- | --- |
| AT-rich element | Glycine max | 727 | + | 10 | ATAGAAATCAA | binding site of AT-rich DNA binding protein (ATBP-1) |

> 2018/04/13 10:10:12  
+ GACACAGAGG AAGGGGAGAG AGAGAGAGAG AGATAGCTAC CTTTTCAACG TCTTCTAGTT TAAGTTTGTC   
  
  
+ AAATTATCCC TTTCTTTATC CCTTTCTTAG ACGACTTCTA TATATATGCT AGAGATCTTC TACTTCTGGA   
  
  
+ ATTTGGTATA TGTCATTTAA TTCTCTAGGA GGGAGAGAAA GAGAAGTTGA AGATCCTATC TACCTGTCTT   
  
  
+ GAATCTATCT ATACTTTCAT TGGTTTATGG ATATATTTAT ACTGGGTTGG TGAAAGGGAA AGTTGTCGTA   
  
  
+ ATTAACTCTT AAAGATCAGT AAATATAACT CCTTCTCACT CGTATTTAAT TTCTTTTTCT TTCGTCTTCG   
  
  
+ GTTTTTATCT TTAATTCTAT GTCTTTGAGG TAGCCTATCA TCAACCCATA ACACTTTTAA TTGTACAAGA   
  
  
+ TCATTAGTTT TCGTATCTTA AAGTACACAT TCCGAATATC ACCCTTCAAT GACATCACTC CTTCTCCAGT   
  
  
+ TGTTATCTGC TGTTTTCCTA ACTCGTCATT CCCAAAACAA AACGATTTAA CTTACTTTTT CGTCACGTGA   
  
  
+ AAAGAGAGTA ATTTCCTATT TTCCCTTTTG GCCTACTCAT CTGTTATTAT CTCGTTTTAG AGAGTACATT   
  
  
+ CACGGGTTCA CTTTTAATAG TAAGTGTGTA TAGACGCAGA GTTTTCCTTT TTCTTCTCTC TGTTTACCAC   
  
  
+ TATTTATATA GATAGTGAAA GATCCCAAAG AAATCAAAAA GAAAAAAGAT CCCAAAGAAA AACTCATGTA   
  
  
+ TGGGATTACT TTTCTTCATA CATAGTTACC GTTACTACTA CTTAGAACCC GCGTTTAGAG AAGTGACACG   
  
  
+ TAGTAGCCGC CACTTCTATA GTAATACTAA CTACTTTCTC CGGGATAAAC TGAAAACCGT CTCTTTTTCG   
  
  
+ AAGAGTGGAA AGAGGTAGAG GAAAACGATA AAGTGGAAAA GAAATTATGA AGGAGGTTTG GGGTGTAGAT   
  
  
+ GGGTGGTGAG GAGTTTAAGG TTTTGGGTCA TGGAATAATA TAAGAATAGT AGGGGAAGTT AATTCGTGGT   
  
  
+ ACGATTATTA ATTAGTAGGT GGGAAACGAA TAGTATTAAT CAATTTAAAT ATATTATGTG AGAGACACCT   
  
  
+ GGCTGGACTG CGGAACGATA CATACAACTA ACGAGTACTT GGGGTTGACA AACCTATAAC CTGGTTGACC   
  
  
+ CAATTAAATG AATGGTAAAC TAATAGTACA AGAACAACAT GAACTATCAC TAGAATTAAT CTACTTGTAC   
  
  
+ TAATTCAAGT AAACTAGCCT TTATTACTGA CTTCGTTTAG ATCCCGTGGT ATACTACCAC TTTTTAGTAT   
  
  
+ TCCGGTCGTC AATTTATAAA TTCTACAGTA CCTAGTCGTC TAATTATTTC TATAGACTTA AAACTAATTG   
  
  
+ GGTAAAGTTG TATCCTCTAG CGTATTTTAC TAAATCGATA GAATCGATCG TACGTTTTCG TTTGTTATTA   
  
  
+ ATCTACTAAA AAATTTTTAA TTTAAAGGT  

- CTGTGTCTCC TTCCCCTCTC TCTCTCTCTC TCTATCGATG GAAAAGTTGC AGAAGATCAA ATTCAAACAG   
  
  
- TTTAATAGGG AAAGAAATAG GGAAAGAATC TGCTGAAGAT ATATATACGA TCTCTAGAAG ATGAAGACCT   
  
  
- TAAACCATAT ACAGTAAATT AAGAGATCCT CCCTCTCTTT CTCTTCAACT TCTAGGATAG ATGGACAGAA   
  
  
- CTTAGATAGA TATGAAAGTA ACCAAATACC TATATAAATA TGACCCAACC ACTTTCCCTT TCAACAGCAT   
  
  
- TAATTGAGAA TTTCTAGTCA TTTATATTGA GGAAGAGTGA GCATAAATTA AAGAAAAAGA AAGCAGAAGC   
  
  
- CAAAAATAGA AATTAAGATA CAGAAACTCC ATCGGATAGT AGTTGGGTAT TGTGAAAATT AACATGTTCT   
  
  
- AGTAATCAAA AGCATAGAAT TTCATGTGTA AGGCTTATAG TGGGAAGTTA CTGTAGTGAG GAAGAGGTCA   
  
  
- ACAATAGACG ACAAAAGGAT TGAGCAGTAA GGGTTTTGTT TTGCTAAATT GAATGAAAAA GCAGTGCACT   
  
  
- TTTCTCTCAT TAAAGGATAA AAGGGAAAAC CGGATGAGTA GACAATAATA GAGCAAAATC TCTCATGTAA   
  
  
- GTGCCCAAGT GAAAATTATC ATTCACACAT ATCTGCGTCT CAAAAGGAAA AAGAAGAGAG ACAAATGGTG   
  
  
- ATAAATATAT CTATCACTTT CTAGGGTTTC TTTAGTTTTT CTTTTTTCTA GGGTTTCTTT TTGAGTACAT   
  
  
- ACCCTAATGA AAAGAAGTAT GTATCAATGG CAATGATGAT GAATCTTGGG CGCAAATCTC TTCACTGTGC   
  
  
- ATCATCGGCG GTGAAGATAT CATTATGATT GATGAAAGAG GCCCTATTTG ACTTTTGGCA GAGAAAAAGC   
  
  
- TTCTCACCTT TCTCCATCTC CTTTTGCTAT TTCACCTTTT CTTTAATACT TCCTCCAAAC CCCACATCTA   
  
  
- CCCACCACTC CTCAAATTCC AAAACCCAGT ACCTTATTAT ATTCTTATCA TCCCCTTCAA TTAAGCACCA   
  
  
- TGCTAATAAT TAATCATCCA CCCTTTGCTT ATCATAATTA GTTAAATTTA TATAATACAC TCTCTGTGGA   
  
  
- CCGACCTGAC GCCTTGCTAT GTATGTTGAT TGCTCATGAA CCCCAACTGT TTGGATATTG GACCAACTGG   
  
  
- GTTAATTTAC TTACCATTTG ATTATCATGT TCTTGTTGTA CTTGATAGTG ATCTTAATTA GATGAACATG   
  
  
- ATTAAGTTCA TTTGATCGGA AATAATGACT GAAGCAAATC TAGGGCACCA TATGATGGTG AAAAATCATA   
  
  
- AGGCCAGCAG TTAAATATTT AAGATGTCAT GGATCAGCAG ATTAATAAAG ATATCTGAAT TTTGATTAAC   
  
  
- CCATTTCAAC ATAGGAGATC GCATAAAATG ATTTAGCTAT CTTAGCTAGC ATGCAAAAGC AAACAATAAT   
  
  
- TAGATGATTT TTTAAAAATT AAATTTCCA

+     Box 4

| Site Name | Organism | Position | Strand | Matrix score. | sequence | function |
| --- | --- | --- | --- | --- | --- | --- |
| Box 4 | Petroselinum crispum | 1085 | - | 6 | ATTAAT | part of a conserved DNA module involved in light responsiveness |
| Box 4 | Petroselinum crispum | 1467 | - | 6 | ATTAAT | part of a conserved DNA module involved in light responsiveness |
| Box 4 | Petroselinum crispum | 1057 | - | 6 | ATTAAT | part of a conserved DNA module involved in light responsiveness |
| Box 4 | Petroselinum crispum | 1245 | - | 6 | ATTAAT | part of a conserved DNA module involved in light responsiveness |

> 2018/04/13 10:10:12  
+ GACACAGAGG AAGGGGAGAG AGAGAGAGAG AGATAGCTAC CTTTTCAACG TCTTCTAGTT TAAGTTTGTC   
  
  
+ AAATTATCCC TTTCTTTATC CCTTTCTTAG ACGACTTCTA TATATATGCT AGAGATCTTC TACTTCTGGA   
  
  
+ ATTTGGTATA TGTCATTTAA TTCTCTAGGA GGGAGAGAAA GAGAAGTTGA AGATCCTATC TACCTGTCTT   
  
  
+ GAATCTATCT ATACTTTCAT TGGTTTATGG ATATATTTAT ACTGGGTTGG TGAAAGGGAA AGTTGTCGTA   
  
  
+ ATTAACTCTT AAAGATCAGT AAATATAACT CCTTCTCACT CGTATTTAAT TTCTTTTTCT TTCGTCTTCG   
  
  
+ GTTTTTATCT TTAATTCTAT GTCTTTGAGG TAGCCTATCA TCAACCCATA ACACTTTTAA TTGTACAAGA   
  
  
+ TCATTAGTTT TCGTATCTTA AAGTACACAT TCCGAATATC ACCCTTCAAT GACATCACTC CTTCTCCAGT   
  
  
+ TGTTATCTGC TGTTTTCCTA ACTCGTCATT CCCAAAACAA AACGATTTAA CTTACTTTTT CGTCACGTGA   
  
  
+ AAAGAGAGTA ATTTCCTATT TTCCCTTTTG GCCTACTCAT CTGTTATTAT CTCGTTTTAG AGAGTACATT   
  
  
+ CACGGGTTCA CTTTTAATAG TAAGTGTGTA TAGACGCAGA GTTTTCCTTT TTCTTCTCTC TGTTTACCAC   
  
  
+ TATTTATATA GATAGTGAAA GATCCCAAAG AAATCAAAAA GAAAAAAGAT CCCAAAGAAA AACTCATGTA   
  
  
+ TGGGATTACT TTTCTTCATA CATAGTTACC GTTACTACTA CTTAGAACCC GCGTTTAGAG AAGTGACACG   
  
  
+ TAGTAGCCGC CACTTCTATA GTAATACTAA CTACTTTCTC CGGGATAAAC TGAAAACCGT CTCTTTTTCG   
  
  
+ AAGAGTGGAA AGAGGTAGAG GAAAACGATA AAGTGGAAAA GAAATTATGA AGGAGGTTTG GGGTGTAGAT   
  
  
+ GGGTGGTGAG GAGTTTAAGG TTTTGGGTCA TGGAATAATA TAAGAATAGT AGGGGAAGTT AATTCGTGGT   
  
  
+ ACGATTATTA ATTAGTAGGT GGGAAACGAA TAGTATTAAT CAATTTAAAT ATATTATGTG AGAGACACCT   
  
  
+ GGCTGGACTG CGGAACGATA CATACAACTA ACGAGTACTT GGGGTTGACA AACCTATAAC CTGGTTGACC   
  
  
+ CAATTAAATG AATGGTAAAC TAATAGTACA AGAACAACAT GAACTATCAC TAGAATTAAT CTACTTGTAC   
  
  
+ TAATTCAAGT AAACTAGCCT TTATTACTGA CTTCGTTTAG ATCCCGTGGT ATACTACCAC TTTTTAGTAT   
  
  
+ TCCGGTCGTC AATTTATAAA TTCTACAGTA CCTAGTCGTC TAATTATTTC TATAGACTTA AAACTAATTG   
  
  
+ GGTAAAGTTG TATCCTCTAG CGTATTTTAC TAAATCGATA GAATCGATCG TACGTTTTCG TTTGTTATTA   
  
  
+ ATCTACTAAA AAATTTTTAA TTTAAAGGT  

- CTGTGTCTCC TTCCCCTCTC TCTCTCTCTC TCTATCGATG GAAAAGTTGC AGAAGATCAA ATTCAAACAG   
  
  
- TTTAATAGGG AAAGAAATAG GGAAAGAATC TGCTGAAGAT ATATATACGA TCTCTAGAAG ATGAAGACCT   
  
  
- TAAACCATAT ACAGTAAATT AAGAGATCCT CCCTCTCTTT CTCTTCAACT TCTAGGATAG ATGGACAGAA   
  
  
- CTTAGATAGA TATGAAAGTA ACCAAATACC TATATAAATA TGACCCAACC ACTTTCCCTT TCAACAGCAT   
  
  
- TAATTGAGAA TTTCTAGTCA TTTATATTGA GGAAGAGTGA GCATAAATTA AAGAAAAAGA AAGCAGAAGC   
  
  
- CAAAAATAGA AATTAAGATA CAGAAACTCC ATCGGATAGT AGTTGGGTAT TGTGAAAATT AACATGTTCT   
  
  
- AGTAATCAAA AGCATAGAAT TTCATGTGTA AGGCTTATAG TGGGAAGTTA CTGTAGTGAG GAAGAGGTCA   
  
  
- ACAATAGACG ACAAAAGGAT TGAGCAGTAA GGGTTTTGTT TTGCTAAATT GAATGAAAAA GCAGTGCACT   
  
  
- TTTCTCTCAT TAAAGGATAA AAGGGAAAAC CGGATGAGTA GACAATAATA GAGCAAAATC TCTCATGTAA   
  
  
- GTGCCCAAGT GAAAATTATC ATTCACACAT ATCTGCGTCT CAAAAGGAAA AAGAAGAGAG ACAAATGGTG   
  
  
- ATAAATATAT CTATCACTTT CTAGGGTTTC TTTAGTTTTT CTTTTTTCTA GGGTTTCTTT TTGAGTACAT   
  
  
- ACCCTAATGA AAAGAAGTAT GTATCAATGG CAATGATGAT GAATCTTGGG CGCAAATCTC TTCACTGTGC   
  
  
- ATCATCGGCG GTGAAGATAT CATTATGATT GATGAAAGAG GCCCTATTTG ACTTTTGGCA GAGAAAAAGC   
  
  
- TTCTCACCTT TCTCCATCTC CTTTTGCTAT TTCACCTTTT CTTTAATACT TCCTCCAAAC CCCACATCTA   
  
  
- CCCACCACTC CTCAAATTCC AAAACCCAGT ACCTTATTAT ATTCTTATCA TCCCCTTCAA TTAAGCACCA   
  
  
- TGCTAATAAT TAATCATCCA CCCTTTGCTT ATCATAATTA GTTAAATTTA TATAATACAC TCTCTGTGGA   
  
  
- CCGACCTGAC GCCTTGCTAT GTATGTTGAT TGCTCATGAA CCCCAACTGT TTGGATATTG GACCAACTGG   
  
  
- GTTAATTTAC TTACCATTTG ATTATCATGT TCTTGTTGTA CTTGATAGTG ATCTTAATTA GATGAACATG   
  
  
- ATTAAGTTCA TTTGATCGGA AATAATGACT GAAGCAAATC TAGGGCACCA TATGATGGTG AAAAATCATA   
  
  
- AGGCCAGCAG TTAAATATTT AAGATGTCAT GGATCAGCAG ATTAATAAAG ATATCTGAAT TTTGATTAAC   
  
  
- CCATTTCAAC ATAGGAGATC GCATAAAATG ATTTAGCTAT CTTAGCTAGC ATGCAAAAGC AAACAATAAT   
  
  
- TAGATGATTT TTTAAAAATT AAATTTCCA

+     Box-W1

| Site Name | Organism | Position | Strand | Matrix score. | sequence | function |
| --- | --- | --- | --- | --- | --- | --- |
| Box-W1 | Petroselinum crispum | 1185 | + | 6 | TTGACC | fungal elicitor responsive element |

> 2018/04/13 10:10:12  
+ GACACAGAGG AAGGGGAGAG AGAGAGAGAG AGATAGCTAC CTTTTCAACG TCTTCTAGTT TAAGTTTGTC   
  
  
+ AAATTATCCC TTTCTTTATC CCTTTCTTAG ACGACTTCTA TATATATGCT AGAGATCTTC TACTTCTGGA   
  
  
+ ATTTGGTATA TGTCATTTAA TTCTCTAGGA GGGAGAGAAA GAGAAGTTGA AGATCCTATC TACCTGTCTT   
  
  
+ GAATCTATCT ATACTTTCAT TGGTTTATGG ATATATTTAT ACTGGGTTGG TGAAAGGGAA AGTTGTCGTA   
  
  
+ ATTAACTCTT AAAGATCAGT AAATATAACT CCTTCTCACT CGTATTTAAT TTCTTTTTCT TTCGTCTTCG   
  
  
+ GTTTTTATCT TTAATTCTAT GTCTTTGAGG TAGCCTATCA TCAACCCATA ACACTTTTAA TTGTACAAGA   
  
  
+ TCATTAGTTT TCGTATCTTA AAGTACACAT TCCGAATATC ACCCTTCAAT GACATCACTC CTTCTCCAGT   
  
  
+ TGTTATCTGC TGTTTTCCTA ACTCGTCATT CCCAAAACAA AACGATTTAA CTTACTTTTT CGTCACGTGA   
  
  
+ AAAGAGAGTA ATTTCCTATT TTCCCTTTTG GCCTACTCAT CTGTTATTAT CTCGTTTTAG AGAGTACATT   
  
  
+ CACGGGTTCA CTTTTAATAG TAAGTGTGTA TAGACGCAGA GTTTTCCTTT TTCTTCTCTC TGTTTACCAC   
  
  
+ TATTTATATA GATAGTGAAA GATCCCAAAG AAATCAAAAA GAAAAAAGAT CCCAAAGAAA AACTCATGTA   
  
  
+ TGGGATTACT TTTCTTCATA CATAGTTACC GTTACTACTA CTTAGAACCC GCGTTTAGAG AAGTGACACG   
  
  
+ TAGTAGCCGC CACTTCTATA GTAATACTAA CTACTTTCTC CGGGATAAAC TGAAAACCGT CTCTTTTTCG   
  
  
+ AAGAGTGGAA AGAGGTAGAG GAAAACGATA AAGTGGAAAA GAAATTATGA AGGAGGTTTG GGGTGTAGAT   
  
  
+ GGGTGGTGAG GAGTTTAAGG TTTTGGGTCA TGGAATAATA TAAGAATAGT AGGGGAAGTT AATTCGTGGT   
  
  
+ ACGATTATTA ATTAGTAGGT GGGAAACGAA TAGTATTAAT CAATTTAAAT ATATTATGTG AGAGACACCT   
  
  
+ GGCTGGACTG CGGAACGATA CATACAACTA ACGAGTACTT GGGGTTGACA AACCTATAAC CTGGTTGACC   
  
  
+ CAATTAAATG AATGGTAAAC TAATAGTACA AGAACAACAT GAACTATCAC TAGAATTAAT CTACTTGTAC   
  
  
+ TAATTCAAGT AAACTAGCCT TTATTACTGA CTTCGTTTAG ATCCCGTGGT ATACTACCAC TTTTTAGTAT   
  
  
+ TCCGGTCGTC AATTTATAAA TTCTACAGTA CCTAGTCGTC TAATTATTTC TATAGACTTA AAACTAATTG   
  
  
+ GGTAAAGTTG TATCCTCTAG CGTATTTTAC TAAATCGATA GAATCGATCG TACGTTTTCG TTTGTTATTA   
  
  
+ ATCTACTAAA AAATTTTTAA TTTAAAGGT  

- CTGTGTCTCC TTCCCCTCTC TCTCTCTCTC TCTATCGATG GAAAAGTTGC AGAAGATCAA ATTCAAACAG   
  
  
- TTTAATAGGG AAAGAAATAG GGAAAGAATC TGCTGAAGAT ATATATACGA TCTCTAGAAG ATGAAGACCT   
  
  
- TAAACCATAT ACAGTAAATT AAGAGATCCT CCCTCTCTTT CTCTTCAACT TCTAGGATAG ATGGACAGAA   
  
  
- CTTAGATAGA TATGAAAGTA ACCAAATACC TATATAAATA TGACCCAACC ACTTTCCCTT TCAACAGCAT   
  
  
- TAATTGAGAA TTTCTAGTCA TTTATATTGA GGAAGAGTGA GCATAAATTA AAGAAAAAGA AAGCAGAAGC   
  
  
- CAAAAATAGA AATTAAGATA CAGAAACTCC ATCGGATAGT AGTTGGGTAT TGTGAAAATT AACATGTTCT   
  
  
- AGTAATCAAA AGCATAGAAT TTCATGTGTA AGGCTTATAG TGGGAAGTTA CTGTAGTGAG GAAGAGGTCA   
  
  
- ACAATAGACG ACAAAAGGAT TGAGCAGTAA GGGTTTTGTT TTGCTAAATT GAATGAAAAA GCAGTGCACT   
  
  
- TTTCTCTCAT TAAAGGATAA AAGGGAAAAC CGGATGAGTA GACAATAATA GAGCAAAATC TCTCATGTAA   
  
  
- GTGCCCAAGT GAAAATTATC ATTCACACAT ATCTGCGTCT CAAAAGGAAA AAGAAGAGAG ACAAATGGTG   
  
  
- ATAAATATAT CTATCACTTT CTAGGGTTTC TTTAGTTTTT CTTTTTTCTA GGGTTTCTTT TTGAGTACAT   
  
  
- ACCCTAATGA AAAGAAGTAT GTATCAATGG CAATGATGAT GAATCTTGGG CGCAAATCTC TTCACTGTGC   
  
  
- ATCATCGGCG GTGAAGATAT CATTATGATT GATGAAAGAG GCCCTATTTG ACTTTTGGCA GAGAAAAAGC   
  
  
- TTCTCACCTT TCTCCATCTC CTTTTGCTAT TTCACCTTTT CTTTAATACT TCCTCCAAAC CCCACATCTA   
  
  
- CCCACCACTC CTCAAATTCC AAAACCCAGT ACCTTATTAT ATTCTTATCA TCCCCTTCAA TTAAGCACCA   
  
  
- TGCTAATAAT TAATCATCCA CCCTTTGCTT ATCATAATTA GTTAAATTTA TATAATACAC TCTCTGTGGA   
  
  
- CCGACCTGAC GCCTTGCTAT GTATGTTGAT TGCTCATGAA CCCCAACTGT TTGGATATTG GACCAACTGG   
  
  
- GTTAATTTAC TTACCATTTG ATTATCATGT TCTTGTTGTA CTTGATAGTG ATCTTAATTA GATGAACATG   
  
  
- ATTAAGTTCA TTTGATCGGA AATAATGACT GAAGCAAATC TAGGGCACCA TATGATGGTG AAAAATCATA   
  
  
- AGGCCAGCAG TTAAATATTT AAGATGTCAT GGATCAGCAG ATTAATAAAG ATATCTGAAT TTTGATTAAC   
  
  
- CCATTTCAAC ATAGGAGATC GCATAAAATG ATTTAGCTAT CTTAGCTAGC ATGCAAAAGC AAACAATAAT   
  
  
- TAGATGATTT TTTAAAAATT AAATTTCCA

+     CAAT-box

| Site Name | Organism | Position | Strand | Matrix score. | sequence | function |
| --- | --- | --- | --- | --- | --- | --- |
| CAAT-box | Glycine max | 1396 | - | 5 | CAATT | common cis-acting element in promoter and enhancer regions |
| CAAT-box | Arabidopsis thaliana | 1397 | - | 5 | CCAAT | common cis-acting element in promoter and enhancer regions |
| CAAT-box | Brassica rapa | 141 | - | 5 | CAAAT | common cis-acting element in promoter and enhancer regions |
| CAAT-box | Glycine max | 1091 | + | 5 | CAATT | common cis-acting element in promoter and enhancer regions |
| CAAT-box | Glycine max | 409 | - | 5 | CAATT | common cis-acting element in promoter and enhancer regions |
| CAAT-box | Brassica rapa | 70 | + | 5 | CAAAT | common cis-acting element in promoter and enhancer regions |
| CAAT-box | Arabidopsis thaliana | 229 | - | 5 | CCAAT | common cis-acting element in promoter and enhancer regions |
| CAAT-box | Arabidopsis thaliana | 1190 | + | 5 | CCAAT | common cis-acting element in promoter and enhancer regions |
| CAAT-box | Hordeum vulgare | 467 | + | 4 | CAAT | common cis-acting element in promoter and enhancer regions |
| CAAT-box | Glycine max | 1191 | + | 5 | CAATT | common cis-acting element in promoter and enhancer regions |
| CAAT-box | Hordeum vulgare | 410 | - | 4 | CAAT | common cis-acting element in promoter and enhancer regions |
| CAAT-box | Glycine max | 1340 | + | 5 | CAATT | common cis-acting element in promoter and enhancer regions |

> 2018/04/13 10:10:12  
+ GACACAGAGG AAGGGGAGAG AGAGAGAGAG AGATAGCTAC CTTTTCAACG TCTTCTAGTT TAAGTTTGTC   
  
  
+ AAATTATCCC TTTCTTTATC CCTTTCTTAG ACGACTTCTA TATATATGCT AGAGATCTTC TACTTCTGGA   
  
  
+ ATTTGGTATA TGTCATTTAA TTCTCTAGGA GGGAGAGAAA GAGAAGTTGA AGATCCTATC TACCTGTCTT   
  
  
+ GAATCTATCT ATACTTTCAT TGGTTTATGG ATATATTTAT ACTGGGTTGG TGAAAGGGAA AGTTGTCGTA   
  
  
+ ATTAACTCTT AAAGATCAGT AAATATAACT CCTTCTCACT CGTATTTAAT TTCTTTTTCT TTCGTCTTCG   
  
  
+ GTTTTTATCT TTAATTCTAT GTCTTTGAGG TAGCCTATCA TCAACCCATA ACACTTTTAA TTGTACAAGA   
  
  
+ TCATTAGTTT TCGTATCTTA AAGTACACAT TCCGAATATC ACCCTTCAAT GACATCACTC CTTCTCCAGT   
  
  
+ TGTTATCTGC TGTTTTCCTA ACTCGTCATT CCCAAAACAA AACGATTTAA CTTACTTTTT CGTCACGTGA   
  
  
+ AAAGAGAGTA ATTTCCTATT TTCCCTTTTG GCCTACTCAT CTGTTATTAT CTCGTTTTAG AGAGTACATT   
  
  
+ CACGGGTTCA CTTTTAATAG TAAGTGTGTA TAGACGCAGA GTTTTCCTTT TTCTTCTCTC TGTTTACCAC   
  
  
+ TATTTATATA GATAGTGAAA GATCCCAAAG AAATCAAAAA GAAAAAAGAT CCCAAAGAAA AACTCATGTA   
  
  
+ TGGGATTACT TTTCTTCATA CATAGTTACC GTTACTACTA CTTAGAACCC GCGTTTAGAG AAGTGACACG   
  
  
+ TAGTAGCCGC CACTTCTATA GTAATACTAA CTACTTTCTC CGGGATAAAC TGAAAACCGT CTCTTTTTCG   
  
  
+ AAGAGTGGAA AGAGGTAGAG GAAAACGATA AAGTGGAAAA GAAATTATGA AGGAGGTTTG GGGTGTAGAT   
  
  
+ GGGTGGTGAG GAGTTTAAGG TTTTGGGTCA TGGAATAATA TAAGAATAGT AGGGGAAGTT AATTCGTGGT   
  
  
+ ACGATTATTA ATTAGTAGGT GGGAAACGAA TAGTATTAAT CAATTTAAAT ATATTATGTG AGAGACACCT   
  
  
+ GGCTGGACTG CGGAACGATA CATACAACTA ACGAGTACTT GGGGTTGACA AACCTATAAC CTGGTTGACC   
  
  
+ CAATTAAATG AATGGTAAAC TAATAGTACA AGAACAACAT GAACTATCAC TAGAATTAAT CTACTTGTAC   
  
  
+ TAATTCAAGT AAACTAGCCT TTATTACTGA CTTCGTTTAG ATCCCGTGGT ATACTACCAC TTTTTAGTAT   
  
  
+ TCCGGTCGTC AATTTATAAA TTCTACAGTA CCTAGTCGTC TAATTATTTC TATAGACTTA AAACTAATTG   
  
  
+ GGTAAAGTTG TATCCTCTAG CGTATTTTAC TAAATCGATA GAATCGATCG TACGTTTTCG TTTGTTATTA   
  
  
+ ATCTACTAAA AAATTTTTAA TTTAAAGGT  

- CTGTGTCTCC TTCCCCTCTC TCTCTCTCTC TCTATCGATG GAAAAGTTGC AGAAGATCAA ATTCAAACAG   
  
  
- TTTAATAGGG AAAGAAATAG GGAAAGAATC TGCTGAAGAT ATATATACGA TCTCTAGAAG ATGAAGACCT   
  
  
- TAAACCATAT ACAGTAAATT AAGAGATCCT CCCTCTCTTT CTCTTCAACT TCTAGGATAG ATGGACAGAA   
  
  
- CTTAGATAGA TATGAAAGTA ACCAAATACC TATATAAATA TGACCCAACC ACTTTCCCTT TCAACAGCAT   
  
  
- TAATTGAGAA TTTCTAGTCA TTTATATTGA GGAAGAGTGA GCATAAATTA AAGAAAAAGA AAGCAGAAGC   
  
  
- CAAAAATAGA AATTAAGATA CAGAAACTCC ATCGGATAGT AGTTGGGTAT TGTGAAAATT AACATGTTCT   
  
  
- AGTAATCAAA AGCATAGAAT TTCATGTGTA AGGCTTATAG TGGGAAGTTA CTGTAGTGAG GAAGAGGTCA   
  
  
- ACAATAGACG ACAAAAGGAT TGAGCAGTAA GGGTTTTGTT TTGCTAAATT GAATGAAAAA GCAGTGCACT   
  
  
- TTTCTCTCAT TAAAGGATAA AAGGGAAAAC CGGATGAGTA GACAATAATA GAGCAAAATC TCTCATGTAA   
  
  
- GTGCCCAAGT GAAAATTATC ATTCACACAT ATCTGCGTCT CAAAAGGAAA AAGAAGAGAG ACAAATGGTG   
  
  
- ATAAATATAT CTATCACTTT CTAGGGTTTC TTTAGTTTTT CTTTTTTCTA GGGTTTCTTT TTGAGTACAT   
  
  
- ACCCTAATGA AAAGAAGTAT GTATCAATGG CAATGATGAT GAATCTTGGG CGCAAATCTC TTCACTGTGC   
  
  
- ATCATCGGCG GTGAAGATAT CATTATGATT GATGAAAGAG GCCCTATTTG ACTTTTGGCA GAGAAAAAGC   
  
  
- TTCTCACCTT TCTCCATCTC CTTTTGCTAT TTCACCTTTT CTTTAATACT TCCTCCAAAC CCCACATCTA   
  
  
- CCCACCACTC CTCAAATTCC AAAACCCAGT ACCTTATTAT ATTCTTATCA TCCCCTTCAA TTAAGCACCA   
  
  
- TGCTAATAAT TAATCATCCA CCCTTTGCTT ATCATAATTA GTTAAATTTA TATAATACAC TCTCTGTGGA   
  
  
- CCGACCTGAC GCCTTGCTAT GTATGTTGAT TGCTCATGAA CCCCAACTGT TTGGATATTG GACCAACTGG   
  
  
- GTTAATTTAC TTACCATTTG ATTATCATGT TCTTGTTGTA CTTGATAGTG ATCTTAATTA GATGAACATG   
  
  
- ATTAAGTTCA TTTGATCGGA AATAATGACT GAAGCAAATC TAGGGCACCA TATGATGGTG AAAAATCATA   
  
  
- AGGCCAGCAG TTAAATATTT AAGATGTCAT GGATCAGCAG ATTAATAAAG ATATCTGAAT TTTGATTAAC   
  
  
- CCATTTCAAC ATAGGAGATC GCATAAAATG ATTTAGCTAT CTTAGCTAGC ATGCAAAAGC AAACAATAAT   
  
  
- TAGATGATTT TTTAAAAATT AAATTTCCA

+     CAT-box

| Site Name | Organism | Position | Strand | Matrix score. | sequence | function |
| --- | --- | --- | --- | --- | --- | --- |
| CAT-box | Arabidopsis thaliana | 849 | + | 6 | GCCACT | cis-acting regulatory element related to meristem expression |

> 2018/04/13 10:10:12  
+ GACACAGAGG AAGGGGAGAG AGAGAGAGAG AGATAGCTAC CTTTTCAACG TCTTCTAGTT TAAGTTTGTC   
  
  
+ AAATTATCCC TTTCTTTATC CCTTTCTTAG ACGACTTCTA TATATATGCT AGAGATCTTC TACTTCTGGA   
  
  
+ ATTTGGTATA TGTCATTTAA TTCTCTAGGA GGGAGAGAAA GAGAAGTTGA AGATCCTATC TACCTGTCTT   
  
  
+ GAATCTATCT ATACTTTCAT TGGTTTATGG ATATATTTAT ACTGGGTTGG TGAAAGGGAA AGTTGTCGTA   
  
  
+ ATTAACTCTT AAAGATCAGT AAATATAACT CCTTCTCACT CGTATTTAAT TTCTTTTTCT TTCGTCTTCG   
  
  
+ GTTTTTATCT TTAATTCTAT GTCTTTGAGG TAGCCTATCA TCAACCCATA ACACTTTTAA TTGTACAAGA   
  
  
+ TCATTAGTTT TCGTATCTTA AAGTACACAT TCCGAATATC ACCCTTCAAT GACATCACTC CTTCTCCAGT   
  
  
+ TGTTATCTGC TGTTTTCCTA ACTCGTCATT CCCAAAACAA AACGATTTAA CTTACTTTTT CGTCACGTGA   
  
  
+ AAAGAGAGTA ATTTCCTATT TTCCCTTTTG GCCTACTCAT CTGTTATTAT CTCGTTTTAG AGAGTACATT   
  
  
+ CACGGGTTCA CTTTTAATAG TAAGTGTGTA TAGACGCAGA GTTTTCCTTT TTCTTCTCTC TGTTTACCAC   
  
  
+ TATTTATATA GATAGTGAAA GATCCCAAAG AAATCAAAAA GAAAAAAGAT CCCAAAGAAA AACTCATGTA   
  
  
+ TGGGATTACT TTTCTTCATA CATAGTTACC GTTACTACTA CTTAGAACCC GCGTTTAGAG AAGTGACACG   
  
  
+ TAGTAGCCGC CACTTCTATA GTAATACTAA CTACTTTCTC CGGGATAAAC TGAAAACCGT CTCTTTTTCG   
  
  
+ AAGAGTGGAA AGAGGTAGAG GAAAACGATA AAGTGGAAAA GAAATTATGA AGGAGGTTTG GGGTGTAGAT   
  
  
+ GGGTGGTGAG GAGTTTAAGG TTTTGGGTCA TGGAATAATA TAAGAATAGT AGGGGAAGTT AATTCGTGGT   
  
  
+ ACGATTATTA ATTAGTAGGT GGGAAACGAA TAGTATTAAT CAATTTAAAT ATATTATGTG AGAGACACCT   
  
  
+ GGCTGGACTG CGGAACGATA CATACAACTA ACGAGTACTT GGGGTTGACA AACCTATAAC CTGGTTGACC   
  
  
+ CAATTAAATG AATGGTAAAC TAATAGTACA AGAACAACAT GAACTATCAC TAGAATTAAT CTACTTGTAC   
  
  
+ TAATTCAAGT AAACTAGCCT TTATTACTGA CTTCGTTTAG ATCCCGTGGT ATACTACCAC TTTTTAGTAT   
  
  
+ TCCGGTCGTC AATTTATAAA TTCTACAGTA CCTAGTCGTC TAATTATTTC TATAGACTTA AAACTAATTG   
  
  
+ GGTAAAGTTG TATCCTCTAG CGTATTTTAC TAAATCGATA GAATCGATCG TACGTTTTCG TTTGTTATTA   
  
  
+ ATCTACTAAA AAATTTTTAA TTTAAAGGT  

- CTGTGTCTCC TTCCCCTCTC TCTCTCTCTC TCTATCGATG GAAAAGTTGC AGAAGATCAA ATTCAAACAG   
  
  
- TTTAATAGGG AAAGAAATAG GGAAAGAATC TGCTGAAGAT ATATATACGA TCTCTAGAAG ATGAAGACCT   
  
  
- TAAACCATAT ACAGTAAATT AAGAGATCCT CCCTCTCTTT CTCTTCAACT TCTAGGATAG ATGGACAGAA   
  
  
- CTTAGATAGA TATGAAAGTA ACCAAATACC TATATAAATA TGACCCAACC ACTTTCCCTT TCAACAGCAT   
  
  
- TAATTGAGAA TTTCTAGTCA TTTATATTGA GGAAGAGTGA GCATAAATTA AAGAAAAAGA AAGCAGAAGC   
  
  
- CAAAAATAGA AATTAAGATA CAGAAACTCC ATCGGATAGT AGTTGGGTAT TGTGAAAATT AACATGTTCT   
  
  
- AGTAATCAAA AGCATAGAAT TTCATGTGTA AGGCTTATAG TGGGAAGTTA CTGTAGTGAG GAAGAGGTCA   
  
  
- ACAATAGACG ACAAAAGGAT TGAGCAGTAA GGGTTTTGTT TTGCTAAATT GAATGAAAAA GCAGTGCACT   
  
  
- TTTCTCTCAT TAAAGGATAA AAGGGAAAAC CGGATGAGTA GACAATAATA GAGCAAAATC TCTCATGTAA   
  
  
- GTGCCCAAGT GAAAATTATC ATTCACACAT ATCTGCGTCT CAAAAGGAAA AAGAAGAGAG ACAAATGGTG   
  
  
- ATAAATATAT CTATCACTTT CTAGGGTTTC TTTAGTTTTT CTTTTTTCTA GGGTTTCTTT TTGAGTACAT   
  
  
- ACCCTAATGA AAAGAAGTAT GTATCAATGG CAATGATGAT GAATCTTGGG CGCAAATCTC TTCACTGTGC   
  
  
- ATCATCGGCG GTGAAGATAT CATTATGATT GATGAAAGAG GCCCTATTTG ACTTTTGGCA GAGAAAAAGC   
  
  
- TTCTCACCTT TCTCCATCTC CTTTTGCTAT TTCACCTTTT CTTTAATACT TCCTCCAAAC CCCACATCTA   
  
  
- CCCACCACTC CTCAAATTCC AAAACCCAGT ACCTTATTAT ATTCTTATCA TCCCCTTCAA TTAAGCACCA   
  
  
- TGCTAATAAT TAATCATCCA CCCTTTGCTT ATCATAATTA GTTAAATTTA TATAATACAC TCTCTGTGGA   
  
  
- CCGACCTGAC GCCTTGCTAT GTATGTTGAT TGCTCATGAA CCCCAACTGT TTGGATATTG GACCAACTGG   
  
  
- GTTAATTTAC TTACCATTTG ATTATCATGT TCTTGTTGTA CTTGATAGTG ATCTTAATTA GATGAACATG   
  
  
- ATTAAGTTCA TTTGATCGGA AATAATGACT GAAGCAAATC TAGGGCACCA TATGATGGTG AAAAATCATA   
  
  
- AGGCCAGCAG TTAAATATTT AAGATGTCAT GGATCAGCAG ATTAATAAAG ATATCTGAAT TTTGATTAAC   
  
  
- CCATTTCAAC ATAGGAGATC GCATAAAATG ATTTAGCTAT CTTAGCTAGC ATGCAAAAGC AAACAATAAT   
  
  
- TAGATGATTT TTTAAAAATT AAATTTCCA

+     CGTCA-motif

| Site Name | Organism | Position | Strand | Matrix score. | sequence | function |
| --- | --- | --- | --- | --- | --- | --- |
| CGTCA-motif | Hordeum vulgare | 1337 | + | 5 | CGTCA | cis-acting regulatory element involved in the MeJA-responsiveness |
| CGTCA-motif | Hordeum vulgare | 551 | + | 5 | CGTCA | cis-acting regulatory element involved in the MeJA-responsiveness |
| CGTCA-motif | Hordeum vulgare | 514 | + | 5 | CGTCA | cis-acting regulatory element involved in the MeJA-responsiveness |

> 2018/04/13 10:10:12  
+ GACACAGAGG AAGGGGAGAG AGAGAGAGAG AGATAGCTAC CTTTTCAACG TCTTCTAGTT TAAGTTTGTC   
  
  
+ AAATTATCCC TTTCTTTATC CCTTTCTTAG ACGACTTCTA TATATATGCT AGAGATCTTC TACTTCTGGA   
  
  
+ ATTTGGTATA TGTCATTTAA TTCTCTAGGA GGGAGAGAAA GAGAAGTTGA AGATCCTATC TACCTGTCTT   
  
  
+ GAATCTATCT ATACTTTCAT TGGTTTATGG ATATATTTAT ACTGGGTTGG TGAAAGGGAA AGTTGTCGTA   
  
  
+ ATTAACTCTT AAAGATCAGT AAATATAACT CCTTCTCACT CGTATTTAAT TTCTTTTTCT TTCGTCTTCG   
  
  
+ GTTTTTATCT TTAATTCTAT GTCTTTGAGG TAGCCTATCA TCAACCCATA ACACTTTTAA TTGTACAAGA   
  
  
+ TCATTAGTTT TCGTATCTTA AAGTACACAT TCCGAATATC ACCCTTCAAT GACATCACTC CTTCTCCAGT   
  
  
+ TGTTATCTGC TGTTTTCCTA ACTCGTCATT CCCAAAACAA AACGATTTAA CTTACTTTTT CGTCACGTGA   
  
  
+ AAAGAGAGTA ATTTCCTATT TTCCCTTTTG GCCTACTCAT CTGTTATTAT CTCGTTTTAG AGAGTACATT   
  
  
+ CACGGGTTCA CTTTTAATAG TAAGTGTGTA TAGACGCAGA GTTTTCCTTT TTCTTCTCTC TGTTTACCAC   
  
  
+ TATTTATATA GATAGTGAAA GATCCCAAAG AAATCAAAAA GAAAAAAGAT CCCAAAGAAA AACTCATGTA   
  
  
+ TGGGATTACT TTTCTTCATA CATAGTTACC GTTACTACTA CTTAGAACCC GCGTTTAGAG AAGTGACACG   
  
  
+ TAGTAGCCGC CACTTCTATA GTAATACTAA CTACTTTCTC CGGGATAAAC TGAAAACCGT CTCTTTTTCG   
  
  
+ AAGAGTGGAA AGAGGTAGAG GAAAACGATA AAGTGGAAAA GAAATTATGA AGGAGGTTTG GGGTGTAGAT   
  
  
+ GGGTGGTGAG GAGTTTAAGG TTTTGGGTCA TGGAATAATA TAAGAATAGT AGGGGAAGTT AATTCGTGGT   
  
  
+ ACGATTATTA ATTAGTAGGT GGGAAACGAA TAGTATTAAT CAATTTAAAT ATATTATGTG AGAGACACCT   
  
  
+ GGCTGGACTG CGGAACGATA CATACAACTA ACGAGTACTT GGGGTTGACA AACCTATAAC CTGGTTGACC   
  
  
+ CAATTAAATG AATGGTAAAC TAATAGTACA AGAACAACAT GAACTATCAC TAGAATTAAT CTACTTGTAC   
  
  
+ TAATTCAAGT AAACTAGCCT TTATTACTGA CTTCGTTTAG ATCCCGTGGT ATACTACCAC TTTTTAGTAT   
  
  
+ TCCGGTCGTC AATTTATAAA TTCTACAGTA CCTAGTCGTC TAATTATTTC TATAGACTTA AAACTAATTG   
  
  
+ GGTAAAGTTG TATCCTCTAG CGTATTTTAC TAAATCGATA GAATCGATCG TACGTTTTCG TTTGTTATTA   
  
  
+ ATCTACTAAA AAATTTTTAA TTTAAAGGT  

- CTGTGTCTCC TTCCCCTCTC TCTCTCTCTC TCTATCGATG GAAAAGTTGC AGAAGATCAA ATTCAAACAG   
  
  
- TTTAATAGGG AAAGAAATAG GGAAAGAATC TGCTGAAGAT ATATATACGA TCTCTAGAAG ATGAAGACCT   
  
  
- TAAACCATAT ACAGTAAATT AAGAGATCCT CCCTCTCTTT CTCTTCAACT TCTAGGATAG ATGGACAGAA   
  
  
- CTTAGATAGA TATGAAAGTA ACCAAATACC TATATAAATA TGACCCAACC ACTTTCCCTT TCAACAGCAT   
  
  
- TAATTGAGAA TTTCTAGTCA TTTATATTGA GGAAGAGTGA GCATAAATTA AAGAAAAAGA AAGCAGAAGC   
  
  
- CAAAAATAGA AATTAAGATA CAGAAACTCC ATCGGATAGT AGTTGGGTAT TGTGAAAATT AACATGTTCT   
  
  
- AGTAATCAAA AGCATAGAAT TTCATGTGTA AGGCTTATAG TGGGAAGTTA CTGTAGTGAG GAAGAGGTCA   
  
  
- ACAATAGACG ACAAAAGGAT TGAGCAGTAA GGGTTTTGTT TTGCTAAATT GAATGAAAAA GCAGTGCACT   
  
  
- TTTCTCTCAT TAAAGGATAA AAGGGAAAAC CGGATGAGTA GACAATAATA GAGCAAAATC TCTCATGTAA   
  
  
- GTGCCCAAGT GAAAATTATC ATTCACACAT ATCTGCGTCT CAAAAGGAAA AAGAAGAGAG ACAAATGGTG   
  
  
- ATAAATATAT CTATCACTTT CTAGGGTTTC TTTAGTTTTT CTTTTTTCTA GGGTTTCTTT TTGAGTACAT   
  
  
- ACCCTAATGA AAAGAAGTAT GTATCAATGG CAATGATGAT GAATCTTGGG CGCAAATCTC TTCACTGTGC   
  
  
- ATCATCGGCG GTGAAGATAT CATTATGATT GATGAAAGAG GCCCTATTTG ACTTTTGGCA GAGAAAAAGC   
  
  
- TTCTCACCTT TCTCCATCTC CTTTTGCTAT TTCACCTTTT CTTTAATACT TCCTCCAAAC CCCACATCTA   
  
  
- CCCACCACTC CTCAAATTCC AAAACCCAGT ACCTTATTAT ATTCTTATCA TCCCCTTCAA TTAAGCACCA   
  
  
- TGCTAATAAT TAATCATCCA CCCTTTGCTT ATCATAATTA GTTAAATTTA TATAATACAC TCTCTGTGGA   
  
  
- CCGACCTGAC GCCTTGCTAT GTATGTTGAT TGCTCATGAA CCCCAACTGT TTGGATATTG GACCAACTGG   
  
  
- GTTAATTTAC TTACCATTTG ATTATCATGT TCTTGTTGTA CTTGATAGTG ATCTTAATTA GATGAACATG   
  
  
- ATTAAGTTCA TTTGATCGGA AATAATGACT GAAGCAAATC TAGGGCACCA TATGATGGTG AAAAATCATA   
  
  
- AGGCCAGCAG TTAAATATTT AAGATGTCAT GGATCAGCAG ATTAATAAAG ATATCTGAAT TTTGATTAAC   
  
  
- CCATTTCAAC ATAGGAGATC GCATAAAATG ATTTAGCTAT CTTAGCTAGC ATGCAAAAGC AAACAATAAT   
  
  
- TAGATGATTT TTTAAAAATT AAATTTCCA

+     G-Box

| Site Name | Organism | Position | Strand | Matrix score. | sequence | function |
| --- | --- | --- | --- | --- | --- | --- |
| G-Box | Antirrhinum majus | 837 | + | 6 | CACGTA | cis-acting regulatory element involved in light responsiveness |
| G-Box | Pisum sativum | 554 | + | 6 | CACGTG | cis-acting regulatory element involved in light responsiveness |

> 2018/04/13 10:10:12  
+ GACACAGAGG AAGGGGAGAG AGAGAGAGAG AGATAGCTAC CTTTTCAACG TCTTCTAGTT TAAGTTTGTC   
  
  
+ AAATTATCCC TTTCTTTATC CCTTTCTTAG ACGACTTCTA TATATATGCT AGAGATCTTC TACTTCTGGA   
  
  
+ ATTTGGTATA TGTCATTTAA TTCTCTAGGA GGGAGAGAAA GAGAAGTTGA AGATCCTATC TACCTGTCTT   
  
  
+ GAATCTATCT ATACTTTCAT TGGTTTATGG ATATATTTAT ACTGGGTTGG TGAAAGGGAA AGTTGTCGTA   
  
  
+ ATTAACTCTT AAAGATCAGT AAATATAACT CCTTCTCACT CGTATTTAAT TTCTTTTTCT TTCGTCTTCG   
  
  
+ GTTTTTATCT TTAATTCTAT GTCTTTGAGG TAGCCTATCA TCAACCCATA ACACTTTTAA TTGTACAAGA   
  
  
+ TCATTAGTTT TCGTATCTTA AAGTACACAT TCCGAATATC ACCCTTCAAT GACATCACTC CTTCTCCAGT   
  
  
+ TGTTATCTGC TGTTTTCCTA ACTCGTCATT CCCAAAACAA AACGATTTAA CTTACTTTTT CGTCACGTGA   
  
  
+ AAAGAGAGTA ATTTCCTATT TTCCCTTTTG GCCTACTCAT CTGTTATTAT CTCGTTTTAG AGAGTACATT   
  
  
+ CACGGGTTCA CTTTTAATAG TAAGTGTGTA TAGACGCAGA GTTTTCCTTT TTCTTCTCTC TGTTTACCAC   
  
  
+ TATTTATATA GATAGTGAAA GATCCCAAAG AAATCAAAAA GAAAAAAGAT CCCAAAGAAA AACTCATGTA   
  
  
+ TGGGATTACT TTTCTTCATA CATAGTTACC GTTACTACTA CTTAGAACCC GCGTTTAGAG AAGTGACACG   
  
  
+ TAGTAGCCGC CACTTCTATA GTAATACTAA CTACTTTCTC CGGGATAAAC TGAAAACCGT CTCTTTTTCG   
  
  
+ AAGAGTGGAA AGAGGTAGAG GAAAACGATA AAGTGGAAAA GAAATTATGA AGGAGGTTTG GGGTGTAGAT   
  
  
+ GGGTGGTGAG GAGTTTAAGG TTTTGGGTCA TGGAATAATA TAAGAATAGT AGGGGAAGTT AATTCGTGGT   
  
  
+ ACGATTATTA ATTAGTAGGT GGGAAACGAA TAGTATTAAT CAATTTAAAT ATATTATGTG AGAGACACCT   
  
  
+ GGCTGGACTG CGGAACGATA CATACAACTA ACGAGTACTT GGGGTTGACA AACCTATAAC CTGGTTGACC   
  
  
+ CAATTAAATG AATGGTAAAC TAATAGTACA AGAACAACAT GAACTATCAC TAGAATTAAT CTACTTGTAC   
  
  
+ TAATTCAAGT AAACTAGCCT TTATTACTGA CTTCGTTTAG ATCCCGTGGT ATACTACCAC TTTTTAGTAT   
  
  
+ TCCGGTCGTC AATTTATAAA TTCTACAGTA CCTAGTCGTC TAATTATTTC TATAGACTTA AAACTAATTG   
  
  
+ GGTAAAGTTG TATCCTCTAG CGTATTTTAC TAAATCGATA GAATCGATCG TACGTTTTCG TTTGTTATTA   
  
  
+ ATCTACTAAA AAATTTTTAA TTTAAAGGT  

- CTGTGTCTCC TTCCCCTCTC TCTCTCTCTC TCTATCGATG GAAAAGTTGC AGAAGATCAA ATTCAAACAG   
  
  
- TTTAATAGGG AAAGAAATAG GGAAAGAATC TGCTGAAGAT ATATATACGA TCTCTAGAAG ATGAAGACCT   
  
  
- TAAACCATAT ACAGTAAATT AAGAGATCCT CCCTCTCTTT CTCTTCAACT TCTAGGATAG ATGGACAGAA   
  
  
- CTTAGATAGA TATGAAAGTA ACCAAATACC TATATAAATA TGACCCAACC ACTTTCCCTT TCAACAGCAT   
  
  
- TAATTGAGAA TTTCTAGTCA TTTATATTGA GGAAGAGTGA GCATAAATTA AAGAAAAAGA AAGCAGAAGC   
  
  
- CAAAAATAGA AATTAAGATA CAGAAACTCC ATCGGATAGT AGTTGGGTAT TGTGAAAATT AACATGTTCT   
  
  
- AGTAATCAAA AGCATAGAAT TTCATGTGTA AGGCTTATAG TGGGAAGTTA CTGTAGTGAG GAAGAGGTCA   
  
  
- ACAATAGACG ACAAAAGGAT TGAGCAGTAA GGGTTTTGTT TTGCTAAATT GAATGAAAAA GCAGTGCACT   
  
  
- TTTCTCTCAT TAAAGGATAA AAGGGAAAAC CGGATGAGTA GACAATAATA GAGCAAAATC TCTCATGTAA   
  
  
- GTGCCCAAGT GAAAATTATC ATTCACACAT ATCTGCGTCT CAAAAGGAAA AAGAAGAGAG ACAAATGGTG   
  
  
- ATAAATATAT CTATCACTTT CTAGGGTTTC TTTAGTTTTT CTTTTTTCTA GGGTTTCTTT TTGAGTACAT   
  
  
- ACCCTAATGA AAAGAAGTAT GTATCAATGG CAATGATGAT GAATCTTGGG CGCAAATCTC TTCACTGTGC   
  
  
- ATCATCGGCG GTGAAGATAT CATTATGATT GATGAAAGAG GCCCTATTTG ACTTTTGGCA GAGAAAAAGC   
  
  
- TTCTCACCTT TCTCCATCTC CTTTTGCTAT TTCACCTTTT CTTTAATACT TCCTCCAAAC CCCACATCTA   
  
  
- CCCACCACTC CTCAAATTCC AAAACCCAGT ACCTTATTAT ATTCTTATCA TCCCCTTCAA TTAAGCACCA   
  
  
- TGCTAATAAT TAATCATCCA CCCTTTGCTT ATCATAATTA GTTAAATTTA TATAATACAC TCTCTGTGGA   
  
  
- CCGACCTGAC GCCTTGCTAT GTATGTTGAT TGCTCATGAA CCCCAACTGT TTGGATATTG GACCAACTGG   
  
  
- GTTAATTTAC TTACCATTTG ATTATCATGT TCTTGTTGTA CTTGATAGTG ATCTTAATTA GATGAACATG   
  
  
- ATTAAGTTCA TTTGATCGGA AATAATGACT GAAGCAAATC TAGGGCACCA TATGATGGTG AAAAATCATA   
  
  
- AGGCCAGCAG TTAAATATTT AAGATGTCAT GGATCAGCAG ATTAATAAAG ATATCTGAAT TTTGATTAAC   
  
  
- CCATTTCAAC ATAGGAGATC GCATAAAATG ATTTAGCTAT CTTAGCTAGC ATGCAAAAGC AAACAATAAT   
  
  
- TAGATGATTT TTTAAAAATT AAATTTCCA

+     G-box

| Site Name | Organism | Position | Strand | Matrix score. | sequence | function |
| --- | --- | --- | --- | --- | --- | --- |
| G-box | Daucus carota | 837 | - | 6 | TACGTG | cis-acting regulatory element involved in light responsiveness |
| G-box | Arabidopsis thaliana | 554 | + | 6 | CACGTG | cis-acting regulatory element involved in light responsiveness |
| G-box | Brassica oleracea | 834 | + | 9 | TAACACGTAG | cis-acting regulatory element involved in light responsiveness |
| G-box | Larix laricina | 835 | + | 10 | GACACGTAGT | cis-acting regulatory element involved in light responsiveness |

> 2018/04/13 10:10:12  
+ GACACAGAGG AAGGGGAGAG AGAGAGAGAG AGATAGCTAC CTTTTCAACG TCTTCTAGTT TAAGTTTGTC   
  
  
+ AAATTATCCC TTTCTTTATC CCTTTCTTAG ACGACTTCTA TATATATGCT AGAGATCTTC TACTTCTGGA   
  
  
+ ATTTGGTATA TGTCATTTAA TTCTCTAGGA GGGAGAGAAA GAGAAGTTGA AGATCCTATC TACCTGTCTT   
  
  
+ GAATCTATCT ATACTTTCAT TGGTTTATGG ATATATTTAT ACTGGGTTGG TGAAAGGGAA AGTTGTCGTA   
  
  
+ ATTAACTCTT AAAGATCAGT AAATATAACT CCTTCTCACT CGTATTTAAT TTCTTTTTCT TTCGTCTTCG   
  
  
+ GTTTTTATCT TTAATTCTAT GTCTTTGAGG TAGCCTATCA TCAACCCATA ACACTTTTAA TTGTACAAGA   
  
  
+ TCATTAGTTT TCGTATCTTA AAGTACACAT TCCGAATATC ACCCTTCAAT GACATCACTC CTTCTCCAGT   
  
  
+ TGTTATCTGC TGTTTTCCTA ACTCGTCATT CCCAAAACAA AACGATTTAA CTTACTTTTT CGTCACGTGA   
  
  
+ AAAGAGAGTA ATTTCCTATT TTCCCTTTTG GCCTACTCAT CTGTTATTAT CTCGTTTTAG AGAGTACATT   
  
  
+ CACGGGTTCA CTTTTAATAG TAAGTGTGTA TAGACGCAGA GTTTTCCTTT TTCTTCTCTC TGTTTACCAC   
  
  
+ TATTTATATA GATAGTGAAA GATCCCAAAG AAATCAAAAA GAAAAAAGAT CCCAAAGAAA AACTCATGTA   
  
  
+ TGGGATTACT TTTCTTCATA CATAGTTACC GTTACTACTA CTTAGAACCC GCGTTTAGAG AAGTGACACG   
  
  
+ TAGTAGCCGC CACTTCTATA GTAATACTAA CTACTTTCTC CGGGATAAAC TGAAAACCGT CTCTTTTTCG   
  
  
+ AAGAGTGGAA AGAGGTAGAG GAAAACGATA AAGTGGAAAA GAAATTATGA AGGAGGTTTG GGGTGTAGAT   
  
  
+ GGGTGGTGAG GAGTTTAAGG TTTTGGGTCA TGGAATAATA TAAGAATAGT AGGGGAAGTT AATTCGTGGT   
  
  
+ ACGATTATTA ATTAGTAGGT GGGAAACGAA TAGTATTAAT CAATTTAAAT ATATTATGTG AGAGACACCT   
  
  
+ GGCTGGACTG CGGAACGATA CATACAACTA ACGAGTACTT GGGGTTGACA AACCTATAAC CTGGTTGACC   
  
  
+ CAATTAAATG AATGGTAAAC TAATAGTACA AGAACAACAT GAACTATCAC TAGAATTAAT CTACTTGTAC   
  
  
+ TAATTCAAGT AAACTAGCCT TTATTACTGA CTTCGTTTAG ATCCCGTGGT ATACTACCAC TTTTTAGTAT   
  
  
+ TCCGGTCGTC AATTTATAAA TTCTACAGTA CCTAGTCGTC TAATTATTTC TATAGACTTA AAACTAATTG   
  
  
+ GGTAAAGTTG TATCCTCTAG CGTATTTTAC TAAATCGATA GAATCGATCG TACGTTTTCG TTTGTTATTA   
  
  
+ ATCTACTAAA AAATTTTTAA TTTAAAGGT  

- CTGTGTCTCC TTCCCCTCTC TCTCTCTCTC TCTATCGATG GAAAAGTTGC AGAAGATCAA ATTCAAACAG   
  
  
- TTTAATAGGG AAAGAAATAG GGAAAGAATC TGCTGAAGAT ATATATACGA TCTCTAGAAG ATGAAGACCT   
  
  
- TAAACCATAT ACAGTAAATT AAGAGATCCT CCCTCTCTTT CTCTTCAACT TCTAGGATAG ATGGACAGAA   
  
  
- CTTAGATAGA TATGAAAGTA ACCAAATACC TATATAAATA TGACCCAACC ACTTTCCCTT TCAACAGCAT   
  
  
- TAATTGAGAA TTTCTAGTCA TTTATATTGA GGAAGAGTGA GCATAAATTA AAGAAAAAGA AAGCAGAAGC   
  
  
- CAAAAATAGA AATTAAGATA CAGAAACTCC ATCGGATAGT AGTTGGGTAT TGTGAAAATT AACATGTTCT   
  
  
- AGTAATCAAA AGCATAGAAT TTCATGTGTA AGGCTTATAG TGGGAAGTTA CTGTAGTGAG GAAGAGGTCA   
  
  
- ACAATAGACG ACAAAAGGAT TGAGCAGTAA GGGTTTTGTT TTGCTAAATT GAATGAAAAA GCAGTGCACT   
  
  
- TTTCTCTCAT TAAAGGATAA AAGGGAAAAC CGGATGAGTA GACAATAATA GAGCAAAATC TCTCATGTAA   
  
  
- GTGCCCAAGT GAAAATTATC ATTCACACAT ATCTGCGTCT CAAAAGGAAA AAGAAGAGAG ACAAATGGTG   
  
  
- ATAAATATAT CTATCACTTT CTAGGGTTTC TTTAGTTTTT CTTTTTTCTA GGGTTTCTTT TTGAGTACAT   
  
  
- ACCCTAATGA AAAGAAGTAT GTATCAATGG CAATGATGAT GAATCTTGGG CGCAAATCTC TTCACTGTGC   
  
  
- ATCATCGGCG GTGAAGATAT CATTATGATT GATGAAAGAG GCCCTATTTG ACTTTTGGCA GAGAAAAAGC   
  
  
- TTCTCACCTT TCTCCATCTC CTTTTGCTAT TTCACCTTTT CTTTAATACT TCCTCCAAAC CCCACATCTA   
  
  
- CCCACCACTC CTCAAATTCC AAAACCCAGT ACCTTATTAT ATTCTTATCA TCCCCTTCAA TTAAGCACCA   
  
  
- TGCTAATAAT TAATCATCCA CCCTTTGCTT ATCATAATTA GTTAAATTTA TATAATACAC TCTCTGTGGA   
  
  
- CCGACCTGAC GCCTTGCTAT GTATGTTGAT TGCTCATGAA CCCCAACTGT TTGGATATTG GACCAACTGG   
  
  
- GTTAATTTAC TTACCATTTG ATTATCATGT TCTTGTTGTA CTTGATAGTG ATCTTAATTA GATGAACATG   
  
  
- ATTAAGTTCA TTTGATCGGA AATAATGACT GAAGCAAATC TAGGGCACCA TATGATGGTG AAAAATCATA   
  
  
- AGGCCAGCAG TTAAATATTT AAGATGTCAT GGATCAGCAG ATTAATAAAG ATATCTGAAT TTTGATTAAC   
  
  
- CCATTTCAAC ATAGGAGATC GCATAAAATG ATTTAGCTAT CTTAGCTAGC ATGCAAAAGC AAACAATAAT   
  
  
- TAGATGATTT TTTAAAAATT AAATTTCCA

+     GAG-motif

| Site Name | Organism | Position | Strand | Matrix score. | sequence | function |
| --- | --- | --- | --- | --- | --- | --- |
| GAG-motif | Arabidopsis thaliana | 619 | + | 7 | AGAGAGT | part of a light responsive element |
| GAG-motif | Arabidopsis thaliana | 563 | + | 7 | AGAGAGT | part of a light responsive element |

> 2018/04/13 10:10:12  
+ GACACAGAGG AAGGGGAGAG AGAGAGAGAG AGATAGCTAC CTTTTCAACG TCTTCTAGTT TAAGTTTGTC   
  
  
+ AAATTATCCC TTTCTTTATC CCTTTCTTAG ACGACTTCTA TATATATGCT AGAGATCTTC TACTTCTGGA   
  
  
+ ATTTGGTATA TGTCATTTAA TTCTCTAGGA GGGAGAGAAA GAGAAGTTGA AGATCCTATC TACCTGTCTT   
  
  
+ GAATCTATCT ATACTTTCAT TGGTTTATGG ATATATTTAT ACTGGGTTGG TGAAAGGGAA AGTTGTCGTA   
  
  
+ ATTAACTCTT AAAGATCAGT AAATATAACT CCTTCTCACT CGTATTTAAT TTCTTTTTCT TTCGTCTTCG   
  
  
+ GTTTTTATCT TTAATTCTAT GTCTTTGAGG TAGCCTATCA TCAACCCATA ACACTTTTAA TTGTACAAGA   
  
  
+ TCATTAGTTT TCGTATCTTA AAGTACACAT TCCGAATATC ACCCTTCAAT GACATCACTC CTTCTCCAGT   
  
  
+ TGTTATCTGC TGTTTTCCTA ACTCGTCATT CCCAAAACAA AACGATTTAA CTTACTTTTT CGTCACGTGA   
  
  
+ AAAGAGAGTA ATTTCCTATT TTCCCTTTTG GCCTACTCAT CTGTTATTAT CTCGTTTTAG AGAGTACATT   
  
  
+ CACGGGTTCA CTTTTAATAG TAAGTGTGTA TAGACGCAGA GTTTTCCTTT TTCTTCTCTC TGTTTACCAC   
  
  
+ TATTTATATA GATAGTGAAA GATCCCAAAG AAATCAAAAA GAAAAAAGAT CCCAAAGAAA AACTCATGTA   
  
  
+ TGGGATTACT TTTCTTCATA CATAGTTACC GTTACTACTA CTTAGAACCC GCGTTTAGAG AAGTGACACG   
  
  
+ TAGTAGCCGC CACTTCTATA GTAATACTAA CTACTTTCTC CGGGATAAAC TGAAAACCGT CTCTTTTTCG   
  
  
+ AAGAGTGGAA AGAGGTAGAG GAAAACGATA AAGTGGAAAA GAAATTATGA AGGAGGTTTG GGGTGTAGAT   
  
  
+ GGGTGGTGAG GAGTTTAAGG TTTTGGGTCA TGGAATAATA TAAGAATAGT AGGGGAAGTT AATTCGTGGT   
  
  
+ ACGATTATTA ATTAGTAGGT GGGAAACGAA TAGTATTAAT CAATTTAAAT ATATTATGTG AGAGACACCT   
  
  
+ GGCTGGACTG CGGAACGATA CATACAACTA ACGAGTACTT GGGGTTGACA AACCTATAAC CTGGTTGACC   
  
  
+ CAATTAAATG AATGGTAAAC TAATAGTACA AGAACAACAT GAACTATCAC TAGAATTAAT CTACTTGTAC   
  
  
+ TAATTCAAGT AAACTAGCCT TTATTACTGA CTTCGTTTAG ATCCCGTGGT ATACTACCAC TTTTTAGTAT   
  
  
+ TCCGGTCGTC AATTTATAAA TTCTACAGTA CCTAGTCGTC TAATTATTTC TATAGACTTA AAACTAATTG   
  
  
+ GGTAAAGTTG TATCCTCTAG CGTATTTTAC TAAATCGATA GAATCGATCG TACGTTTTCG TTTGTTATTA   
  
  
+ ATCTACTAAA AAATTTTTAA TTTAAAGGT  

- CTGTGTCTCC TTCCCCTCTC TCTCTCTCTC TCTATCGATG GAAAAGTTGC AGAAGATCAA ATTCAAACAG   
  
  
- TTTAATAGGG AAAGAAATAG GGAAAGAATC TGCTGAAGAT ATATATACGA TCTCTAGAAG ATGAAGACCT   
  
  
- TAAACCATAT ACAGTAAATT AAGAGATCCT CCCTCTCTTT CTCTTCAACT TCTAGGATAG ATGGACAGAA   
  
  
- CTTAGATAGA TATGAAAGTA ACCAAATACC TATATAAATA TGACCCAACC ACTTTCCCTT TCAACAGCAT   
  
  
- TAATTGAGAA TTTCTAGTCA TTTATATTGA GGAAGAGTGA GCATAAATTA AAGAAAAAGA AAGCAGAAGC   
  
  
- CAAAAATAGA AATTAAGATA CAGAAACTCC ATCGGATAGT AGTTGGGTAT TGTGAAAATT AACATGTTCT   
  
  
- AGTAATCAAA AGCATAGAAT TTCATGTGTA AGGCTTATAG TGGGAAGTTA CTGTAGTGAG GAAGAGGTCA   
  
  
- ACAATAGACG ACAAAAGGAT TGAGCAGTAA GGGTTTTGTT TTGCTAAATT GAATGAAAAA GCAGTGCACT   
  
  
- TTTCTCTCAT TAAAGGATAA AAGGGAAAAC CGGATGAGTA GACAATAATA GAGCAAAATC TCTCATGTAA   
  
  
- GTGCCCAAGT GAAAATTATC ATTCACACAT ATCTGCGTCT CAAAAGGAAA AAGAAGAGAG ACAAATGGTG   
  
  
- ATAAATATAT CTATCACTTT CTAGGGTTTC TTTAGTTTTT CTTTTTTCTA GGGTTTCTTT TTGAGTACAT   
  
  
- ACCCTAATGA AAAGAAGTAT GTATCAATGG CAATGATGAT GAATCTTGGG CGCAAATCTC TTCACTGTGC   
  
  
- ATCATCGGCG GTGAAGATAT CATTATGATT GATGAAAGAG GCCCTATTTG ACTTTTGGCA GAGAAAAAGC   
  
  
- TTCTCACCTT TCTCCATCTC CTTTTGCTAT TTCACCTTTT CTTTAATACT TCCTCCAAAC CCCACATCTA   
  
  
- CCCACCACTC CTCAAATTCC AAAACCCAGT ACCTTATTAT ATTCTTATCA TCCCCTTCAA TTAAGCACCA   
  
  
- TGCTAATAAT TAATCATCCA CCCTTTGCTT ATCATAATTA GTTAAATTTA TATAATACAC TCTCTGTGGA   
  
  
- CCGACCTGAC GCCTTGCTAT GTATGTTGAT TGCTCATGAA CCCCAACTGT TTGGATATTG GACCAACTGG   
  
  
- GTTAATTTAC TTACCATTTG ATTATCATGT TCTTGTTGTA CTTGATAGTG ATCTTAATTA GATGAACATG   
  
  
- ATTAAGTTCA TTTGATCGGA AATAATGACT GAAGCAAATC TAGGGCACCA TATGATGGTG AAAAATCATA   
  
  
- AGGCCAGCAG TTAAATATTT AAGATGTCAT GGATCAGCAG ATTAATAAAG ATATCTGAAT TTTGATTAAC   
  
  
- CCATTTCAAC ATAGGAGATC GCATAAAATG ATTTAGCTAT CTTAGCTAGC ATGCAAAAGC AAACAATAAT   
  
  
- TAGATGATTT TTTAAAAATT AAATTTCCA

+     GARE-motif

| Site Name | Organism | Position | Strand | Matrix score. | sequence | function |
| --- | --- | --- | --- | --- | --- | --- |
| GARE-motif | Brassica oleracea | 689 | - | 7 | AAACAGA | gibberellin-responsive element |

> 2018/04/13 10:10:12  
+ GACACAGAGG AAGGGGAGAG AGAGAGAGAG AGATAGCTAC CTTTTCAACG TCTTCTAGTT TAAGTTTGTC   
  
  
+ AAATTATCCC TTTCTTTATC CCTTTCTTAG ACGACTTCTA TATATATGCT AGAGATCTTC TACTTCTGGA   
  
  
+ ATTTGGTATA TGTCATTTAA TTCTCTAGGA GGGAGAGAAA GAGAAGTTGA AGATCCTATC TACCTGTCTT   
  
  
+ GAATCTATCT ATACTTTCAT TGGTTTATGG ATATATTTAT ACTGGGTTGG TGAAAGGGAA AGTTGTCGTA   
  
  
+ ATTAACTCTT AAAGATCAGT AAATATAACT CCTTCTCACT CGTATTTAAT TTCTTTTTCT TTCGTCTTCG   
  
  
+ GTTTTTATCT TTAATTCTAT GTCTTTGAGG TAGCCTATCA TCAACCCATA ACACTTTTAA TTGTACAAGA   
  
  
+ TCATTAGTTT TCGTATCTTA AAGTACACAT TCCGAATATC ACCCTTCAAT GACATCACTC CTTCTCCAGT   
  
  
+ TGTTATCTGC TGTTTTCCTA ACTCGTCATT CCCAAAACAA AACGATTTAA CTTACTTTTT CGTCACGTGA   
  
  
+ AAAGAGAGTA ATTTCCTATT TTCCCTTTTG GCCTACTCAT CTGTTATTAT CTCGTTTTAG AGAGTACATT   
  
  
+ CACGGGTTCA CTTTTAATAG TAAGTGTGTA TAGACGCAGA GTTTTCCTTT TTCTTCTCTC TGTTTACCAC   
  
  
+ TATTTATATA GATAGTGAAA GATCCCAAAG AAATCAAAAA GAAAAAAGAT CCCAAAGAAA AACTCATGTA   
  
  
+ TGGGATTACT TTTCTTCATA CATAGTTACC GTTACTACTA CTTAGAACCC GCGTTTAGAG AAGTGACACG   
  
  
+ TAGTAGCCGC CACTTCTATA GTAATACTAA CTACTTTCTC CGGGATAAAC TGAAAACCGT CTCTTTTTCG   
  
  
+ AAGAGTGGAA AGAGGTAGAG GAAAACGATA AAGTGGAAAA GAAATTATGA AGGAGGTTTG GGGTGTAGAT   
  
  
+ GGGTGGTGAG GAGTTTAAGG TTTTGGGTCA TGGAATAATA TAAGAATAGT AGGGGAAGTT AATTCGTGGT   
  
  
+ ACGATTATTA ATTAGTAGGT GGGAAACGAA TAGTATTAAT CAATTTAAAT ATATTATGTG AGAGACACCT   
  
  
+ GGCTGGACTG CGGAACGATA CATACAACTA ACGAGTACTT GGGGTTGACA AACCTATAAC CTGGTTGACC   
  
  
+ CAATTAAATG AATGGTAAAC TAATAGTACA AGAACAACAT GAACTATCAC TAGAATTAAT CTACTTGTAC   
  
  
+ TAATTCAAGT AAACTAGCCT TTATTACTGA CTTCGTTTAG ATCCCGTGGT ATACTACCAC TTTTTAGTAT   
  
  
+ TCCGGTCGTC AATTTATAAA TTCTACAGTA CCTAGTCGTC TAATTATTTC TATAGACTTA AAACTAATTG   
  
  
+ GGTAAAGTTG TATCCTCTAG CGTATTTTAC TAAATCGATA GAATCGATCG TACGTTTTCG TTTGTTATTA   
  
  
+ ATCTACTAAA AAATTTTTAA TTTAAAGGT  

- CTGTGTCTCC TTCCCCTCTC TCTCTCTCTC TCTATCGATG GAAAAGTTGC AGAAGATCAA ATTCAAACAG   
  
  
- TTTAATAGGG AAAGAAATAG GGAAAGAATC TGCTGAAGAT ATATATACGA TCTCTAGAAG ATGAAGACCT   
  
  
- TAAACCATAT ACAGTAAATT AAGAGATCCT CCCTCTCTTT CTCTTCAACT TCTAGGATAG ATGGACAGAA   
  
  
- CTTAGATAGA TATGAAAGTA ACCAAATACC TATATAAATA TGACCCAACC ACTTTCCCTT TCAACAGCAT   
  
  
- TAATTGAGAA TTTCTAGTCA TTTATATTGA GGAAGAGTGA GCATAAATTA AAGAAAAAGA AAGCAGAAGC   
  
  
- CAAAAATAGA AATTAAGATA CAGAAACTCC ATCGGATAGT AGTTGGGTAT TGTGAAAATT AACATGTTCT   
  
  
- AGTAATCAAA AGCATAGAAT TTCATGTGTA AGGCTTATAG TGGGAAGTTA CTGTAGTGAG GAAGAGGTCA   
  
  
- ACAATAGACG ACAAAAGGAT TGAGCAGTAA GGGTTTTGTT TTGCTAAATT GAATGAAAAA GCAGTGCACT   
  
  
- TTTCTCTCAT TAAAGGATAA AAGGGAAAAC CGGATGAGTA GACAATAATA GAGCAAAATC TCTCATGTAA   
  
  
- GTGCCCAAGT GAAAATTATC ATTCACACAT ATCTGCGTCT CAAAAGGAAA AAGAAGAGAG ACAAATGGTG   
  
  
- ATAAATATAT CTATCACTTT CTAGGGTTTC TTTAGTTTTT CTTTTTTCTA GGGTTTCTTT TTGAGTACAT   
  
  
- ACCCTAATGA AAAGAAGTAT GTATCAATGG CAATGATGAT GAATCTTGGG CGCAAATCTC TTCACTGTGC   
  
  
- ATCATCGGCG GTGAAGATAT CATTATGATT GATGAAAGAG GCCCTATTTG ACTTTTGGCA GAGAAAAAGC   
  
  
- TTCTCACCTT TCTCCATCTC CTTTTGCTAT TTCACCTTTT CTTTAATACT TCCTCCAAAC CCCACATCTA   
  
  
- CCCACCACTC CTCAAATTCC AAAACCCAGT ACCTTATTAT ATTCTTATCA TCCCCTTCAA TTAAGCACCA   
  
  
- TGCTAATAAT TAATCATCCA CCCTTTGCTT ATCATAATTA GTTAAATTTA TATAATACAC TCTCTGTGGA   
  
  
- CCGACCTGAC GCCTTGCTAT GTATGTTGAT TGCTCATGAA CCCCAACTGT TTGGATATTG GACCAACTGG   
  
  
- GTTAATTTAC TTACCATTTG ATTATCATGT TCTTGTTGTA CTTGATAGTG ATCTTAATTA GATGAACATG   
  
  
- ATTAAGTTCA TTTGATCGGA AATAATGACT GAAGCAAATC TAGGGCACCA TATGATGGTG AAAAATCATA   
  
  
- AGGCCAGCAG TTAAATATTT AAGATGTCAT GGATCAGCAG ATTAATAAAG ATATCTGAAT TTTGATTAAC   
  
  
- CCATTTCAAC ATAGGAGATC GCATAAAATG ATTTAGCTAT CTTAGCTAGC ATGCAAAAGC AAACAATAAT   
  
  
- TAGATGATTT TTTAAAAATT AAATTTCCA

+     GATA-motif

| Site Name | Organism | Position | Strand | Matrix score. | sequence | function |
| --- | --- | --- | --- | --- | --- | --- |
| GATA-motif | Arabidopsis thaliana | 194 | - | 7 | GATAGGA | part of a light responsive element |

> 2018/04/13 10:10:12  
+ GACACAGAGG AAGGGGAGAG AGAGAGAGAG AGATAGCTAC CTTTTCAACG TCTTCTAGTT TAAGTTTGTC   
  
  
+ AAATTATCCC TTTCTTTATC CCTTTCTTAG ACGACTTCTA TATATATGCT AGAGATCTTC TACTTCTGGA   
  
  
+ ATTTGGTATA TGTCATTTAA TTCTCTAGGA GGGAGAGAAA GAGAAGTTGA AGATCCTATC TACCTGTCTT   
  
  
+ GAATCTATCT ATACTTTCAT TGGTTTATGG ATATATTTAT ACTGGGTTGG TGAAAGGGAA AGTTGTCGTA   
  
  
+ ATTAACTCTT AAAGATCAGT AAATATAACT CCTTCTCACT CGTATTTAAT TTCTTTTTCT TTCGTCTTCG   
  
  
+ GTTTTTATCT TTAATTCTAT GTCTTTGAGG TAGCCTATCA TCAACCCATA ACACTTTTAA TTGTACAAGA   
  
  
+ TCATTAGTTT TCGTATCTTA AAGTACACAT TCCGAATATC ACCCTTCAAT GACATCACTC CTTCTCCAGT   
  
  
+ TGTTATCTGC TGTTTTCCTA ACTCGTCATT CCCAAAACAA AACGATTTAA CTTACTTTTT CGTCACGTGA   
  
  
+ AAAGAGAGTA ATTTCCTATT TTCCCTTTTG GCCTACTCAT CTGTTATTAT CTCGTTTTAG AGAGTACATT   
  
  
+ CACGGGTTCA CTTTTAATAG TAAGTGTGTA TAGACGCAGA GTTTTCCTTT TTCTTCTCTC TGTTTACCAC   
  
  
+ TATTTATATA GATAGTGAAA GATCCCAAAG AAATCAAAAA GAAAAAAGAT CCCAAAGAAA AACTCATGTA   
  
  
+ TGGGATTACT TTTCTTCATA CATAGTTACC GTTACTACTA CTTAGAACCC GCGTTTAGAG AAGTGACACG   
  
  
+ TAGTAGCCGC CACTTCTATA GTAATACTAA CTACTTTCTC CGGGATAAAC TGAAAACCGT CTCTTTTTCG   
  
  
+ AAGAGTGGAA AGAGGTAGAG GAAAACGATA AAGTGGAAAA GAAATTATGA AGGAGGTTTG GGGTGTAGAT   
  
  
+ GGGTGGTGAG GAGTTTAAGG TTTTGGGTCA TGGAATAATA TAAGAATAGT AGGGGAAGTT AATTCGTGGT   
  
  
+ ACGATTATTA ATTAGTAGGT GGGAAACGAA TAGTATTAAT CAATTTAAAT ATATTATGTG AGAGACACCT   
  
  
+ GGCTGGACTG CGGAACGATA CATACAACTA ACGAGTACTT GGGGTTGACA AACCTATAAC CTGGTTGACC   
  
  
+ CAATTAAATG AATGGTAAAC TAATAGTACA AGAACAACAT GAACTATCAC TAGAATTAAT CTACTTGTAC   
  
  
+ TAATTCAAGT AAACTAGCCT TTATTACTGA CTTCGTTTAG ATCCCGTGGT ATACTACCAC TTTTTAGTAT   
  
  
+ TCCGGTCGTC AATTTATAAA TTCTACAGTA CCTAGTCGTC TAATTATTTC TATAGACTTA AAACTAATTG   
  
  
+ GGTAAAGTTG TATCCTCTAG CGTATTTTAC TAAATCGATA GAATCGATCG TACGTTTTCG TTTGTTATTA   
  
  
+ ATCTACTAAA AAATTTTTAA TTTAAAGGT  

- CTGTGTCTCC TTCCCCTCTC TCTCTCTCTC TCTATCGATG GAAAAGTTGC AGAAGATCAA ATTCAAACAG   
  
  
- TTTAATAGGG AAAGAAATAG GGAAAGAATC TGCTGAAGAT ATATATACGA TCTCTAGAAG ATGAAGACCT   
  
  
- TAAACCATAT ACAGTAAATT AAGAGATCCT CCCTCTCTTT CTCTTCAACT TCTAGGATAG ATGGACAGAA   
  
  
- CTTAGATAGA TATGAAAGTA ACCAAATACC TATATAAATA TGACCCAACC ACTTTCCCTT TCAACAGCAT   
  
  
- TAATTGAGAA TTTCTAGTCA TTTATATTGA GGAAGAGTGA GCATAAATTA AAGAAAAAGA AAGCAGAAGC   
  
  
- CAAAAATAGA AATTAAGATA CAGAAACTCC ATCGGATAGT AGTTGGGTAT TGTGAAAATT AACATGTTCT   
  
  
- AGTAATCAAA AGCATAGAAT TTCATGTGTA AGGCTTATAG TGGGAAGTTA CTGTAGTGAG GAAGAGGTCA   
  
  
- ACAATAGACG ACAAAAGGAT TGAGCAGTAA GGGTTTTGTT TTGCTAAATT GAATGAAAAA GCAGTGCACT   
  
  
- TTTCTCTCAT TAAAGGATAA AAGGGAAAAC CGGATGAGTA GACAATAATA GAGCAAAATC TCTCATGTAA   
  
  
- GTGCCCAAGT GAAAATTATC ATTCACACAT ATCTGCGTCT CAAAAGGAAA AAGAAGAGAG ACAAATGGTG   
  
  
- ATAAATATAT CTATCACTTT CTAGGGTTTC TTTAGTTTTT CTTTTTTCTA GGGTTTCTTT TTGAGTACAT   
  
  
- ACCCTAATGA AAAGAAGTAT GTATCAATGG CAATGATGAT GAATCTTGGG CGCAAATCTC TTCACTGTGC   
  
  
- ATCATCGGCG GTGAAGATAT CATTATGATT GATGAAAGAG GCCCTATTTG ACTTTTGGCA GAGAAAAAGC   
  
  
- TTCTCACCTT TCTCCATCTC CTTTTGCTAT TTCACCTTTT CTTTAATACT TCCTCCAAAC CCCACATCTA   
  
  
- CCCACCACTC CTCAAATTCC AAAACCCAGT ACCTTATTAT ATTCTTATCA TCCCCTTCAA TTAAGCACCA   
  
  
- TGCTAATAAT TAATCATCCA CCCTTTGCTT ATCATAATTA GTTAAATTTA TATAATACAC TCTCTGTGGA   
  
  
- CCGACCTGAC GCCTTGCTAT GTATGTTGAT TGCTCATGAA CCCCAACTGT TTGGATATTG GACCAACTGG   
  
  
- GTTAATTTAC TTACCATTTG ATTATCATGT TCTTGTTGTA CTTGATAGTG ATCTTAATTA GATGAACATG   
  
  
- ATTAAGTTCA TTTGATCGGA AATAATGACT GAAGCAAATC TAGGGCACCA TATGATGGTG AAAAATCATA   
  
  
- AGGCCAGCAG TTAAATATTT AAGATGTCAT GGATCAGCAG ATTAATAAAG ATATCTGAAT TTTGATTAAC   
  
  
- CCATTTCAAC ATAGGAGATC GCATAAAATG ATTTAGCTAT CTTAGCTAGC ATGCAAAAGC AAACAATAAT   
  
  
- TAGATGATTT TTTAAAAATT AAATTTCCA

+     GCC box

| Site Name | Organism | Position | Strand | Matrix score. | sequence | function |
| --- | --- | --- | --- | --- | --- | --- |
| GCC box | Arabidopsis thaliana | 845 | + | 7 | AGCCGCC |  |

> 2018/04/13 10:10:12  
+ GACACAGAGG AAGGGGAGAG AGAGAGAGAG AGATAGCTAC CTTTTCAACG TCTTCTAGTT TAAGTTTGTC   
  
  
+ AAATTATCCC TTTCTTTATC CCTTTCTTAG ACGACTTCTA TATATATGCT AGAGATCTTC TACTTCTGGA   
  
  
+ ATTTGGTATA TGTCATTTAA TTCTCTAGGA GGGAGAGAAA GAGAAGTTGA AGATCCTATC TACCTGTCTT   
  
  
+ GAATCTATCT ATACTTTCAT TGGTTTATGG ATATATTTAT ACTGGGTTGG TGAAAGGGAA AGTTGTCGTA   
  
  
+ ATTAACTCTT AAAGATCAGT AAATATAACT CCTTCTCACT CGTATTTAAT TTCTTTTTCT TTCGTCTTCG   
  
  
+ GTTTTTATCT TTAATTCTAT GTCTTTGAGG TAGCCTATCA TCAACCCATA ACACTTTTAA TTGTACAAGA   
  
  
+ TCATTAGTTT TCGTATCTTA AAGTACACAT TCCGAATATC ACCCTTCAAT GACATCACTC CTTCTCCAGT   
  
  
+ TGTTATCTGC TGTTTTCCTA ACTCGTCATT CCCAAAACAA AACGATTTAA CTTACTTTTT CGTCACGTGA   
  
  
+ AAAGAGAGTA ATTTCCTATT TTCCCTTTTG GCCTACTCAT CTGTTATTAT CTCGTTTTAG AGAGTACATT   
  
  
+ CACGGGTTCA CTTTTAATAG TAAGTGTGTA TAGACGCAGA GTTTTCCTTT TTCTTCTCTC TGTTTACCAC   
  
  
+ TATTTATATA GATAGTGAAA GATCCCAAAG AAATCAAAAA GAAAAAAGAT CCCAAAGAAA AACTCATGTA   
  
  
+ TGGGATTACT TTTCTTCATA CATAGTTACC GTTACTACTA CTTAGAACCC GCGTTTAGAG AAGTGACACG   
  
  
+ TAGTAGCCGC CACTTCTATA GTAATACTAA CTACTTTCTC CGGGATAAAC TGAAAACCGT CTCTTTTTCG   
  
  
+ AAGAGTGGAA AGAGGTAGAG GAAAACGATA AAGTGGAAAA GAAATTATGA AGGAGGTTTG GGGTGTAGAT   
  
  
+ GGGTGGTGAG GAGTTTAAGG TTTTGGGTCA TGGAATAATA TAAGAATAGT AGGGGAAGTT AATTCGTGGT   
  
  
+ ACGATTATTA ATTAGTAGGT GGGAAACGAA TAGTATTAAT CAATTTAAAT ATATTATGTG AGAGACACCT   
  
  
+ GGCTGGACTG CGGAACGATA CATACAACTA ACGAGTACTT GGGGTTGACA AACCTATAAC CTGGTTGACC   
  
  
+ CAATTAAATG AATGGTAAAC TAATAGTACA AGAACAACAT GAACTATCAC TAGAATTAAT CTACTTGTAC   
  
  
+ TAATTCAAGT AAACTAGCCT TTATTACTGA CTTCGTTTAG ATCCCGTGGT ATACTACCAC TTTTTAGTAT   
  
  
+ TCCGGTCGTC AATTTATAAA TTCTACAGTA CCTAGTCGTC TAATTATTTC TATAGACTTA AAACTAATTG   
  
  
+ GGTAAAGTTG TATCCTCTAG CGTATTTTAC TAAATCGATA GAATCGATCG TACGTTTTCG TTTGTTATTA   
  
  
+ ATCTACTAAA AAATTTTTAA TTTAAAGGT  

- CTGTGTCTCC TTCCCCTCTC TCTCTCTCTC TCTATCGATG GAAAAGTTGC AGAAGATCAA ATTCAAACAG   
  
  
- TTTAATAGGG AAAGAAATAG GGAAAGAATC TGCTGAAGAT ATATATACGA TCTCTAGAAG ATGAAGACCT   
  
  
- TAAACCATAT ACAGTAAATT AAGAGATCCT CCCTCTCTTT CTCTTCAACT TCTAGGATAG ATGGACAGAA   
  
  
- CTTAGATAGA TATGAAAGTA ACCAAATACC TATATAAATA TGACCCAACC ACTTTCCCTT TCAACAGCAT   
  
  
- TAATTGAGAA TTTCTAGTCA TTTATATTGA GGAAGAGTGA GCATAAATTA AAGAAAAAGA AAGCAGAAGC   
  
  
- CAAAAATAGA AATTAAGATA CAGAAACTCC ATCGGATAGT AGTTGGGTAT TGTGAAAATT AACATGTTCT   
  
  
- AGTAATCAAA AGCATAGAAT TTCATGTGTA AGGCTTATAG TGGGAAGTTA CTGTAGTGAG GAAGAGGTCA   
  
  
- ACAATAGACG ACAAAAGGAT TGAGCAGTAA GGGTTTTGTT TTGCTAAATT GAATGAAAAA GCAGTGCACT   
  
  
- TTTCTCTCAT TAAAGGATAA AAGGGAAAAC CGGATGAGTA GACAATAATA GAGCAAAATC TCTCATGTAA   
  
  
- GTGCCCAAGT GAAAATTATC ATTCACACAT ATCTGCGTCT CAAAAGGAAA AAGAAGAGAG ACAAATGGTG   
  
  
- ATAAATATAT CTATCACTTT CTAGGGTTTC TTTAGTTTTT CTTTTTTCTA GGGTTTCTTT TTGAGTACAT   
  
  
- ACCCTAATGA AAAGAAGTAT GTATCAATGG CAATGATGAT GAATCTTGGG CGCAAATCTC TTCACTGTGC   
  
  
- ATCATCGGCG GTGAAGATAT CATTATGATT GATGAAAGAG GCCCTATTTG ACTTTTGGCA GAGAAAAAGC   
  
  
- TTCTCACCTT TCTCCATCTC CTTTTGCTAT TTCACCTTTT CTTTAATACT TCCTCCAAAC CCCACATCTA   
  
  
- CCCACCACTC CTCAAATTCC AAAACCCAGT ACCTTATTAT ATTCTTATCA TCCCCTTCAA TTAAGCACCA   
  
  
- TGCTAATAAT TAATCATCCA CCCTTTGCTT ATCATAATTA GTTAAATTTA TATAATACAC TCTCTGTGGA   
  
  
- CCGACCTGAC GCCTTGCTAT GTATGTTGAT TGCTCATGAA CCCCAACTGT TTGGATATTG GACCAACTGG   
  
  
- GTTAATTTAC TTACCATTTG ATTATCATGT TCTTGTTGTA CTTGATAGTG ATCTTAATTA GATGAACATG   
  
  
- ATTAAGTTCA TTTGATCGGA AATAATGACT GAAGCAAATC TAGGGCACCA TATGATGGTG AAAAATCATA   
  
  
- AGGCCAGCAG TTAAATATTT AAGATGTCAT GGATCAGCAG ATTAATAAAG ATATCTGAAT TTTGATTAAC   
  
  
- CCATTTCAAC ATAGGAGATC GCATAAAATG ATTTAGCTAT CTTAGCTAGC ATGCAAAAGC AAACAATAAT   
  
  
- TAGATGATTT TTTAAAAATT AAATTTCCA

+     HSE

| Site Name | Organism | Position | Strand | Matrix score. | sequence | function |
| --- | --- | --- | --- | --- | --- | --- |
| HSE | Brassica oleracea | 1478 | + | 9 | AAAAAATTTC | cis-acting element involved in heat stress responsiveness |

> 2018/04/13 10:10:12  
+ GACACAGAGG AAGGGGAGAG AGAGAGAGAG AGATAGCTAC CTTTTCAACG TCTTCTAGTT TAAGTTTGTC   
  
  
+ AAATTATCCC TTTCTTTATC CCTTTCTTAG ACGACTTCTA TATATATGCT AGAGATCTTC TACTTCTGGA   
  
  
+ ATTTGGTATA TGTCATTTAA TTCTCTAGGA GGGAGAGAAA GAGAAGTTGA AGATCCTATC TACCTGTCTT   
  
  
+ GAATCTATCT ATACTTTCAT TGGTTTATGG ATATATTTAT ACTGGGTTGG TGAAAGGGAA AGTTGTCGTA   
  
  
+ ATTAACTCTT AAAGATCAGT AAATATAACT CCTTCTCACT CGTATTTAAT TTCTTTTTCT TTCGTCTTCG   
  
  
+ GTTTTTATCT TTAATTCTAT GTCTTTGAGG TAGCCTATCA TCAACCCATA ACACTTTTAA TTGTACAAGA   
  
  
+ TCATTAGTTT TCGTATCTTA AAGTACACAT TCCGAATATC ACCCTTCAAT GACATCACTC CTTCTCCAGT   
  
  
+ TGTTATCTGC TGTTTTCCTA ACTCGTCATT CCCAAAACAA AACGATTTAA CTTACTTTTT CGTCACGTGA   
  
  
+ AAAGAGAGTA ATTTCCTATT TTCCCTTTTG GCCTACTCAT CTGTTATTAT CTCGTTTTAG AGAGTACATT   
  
  
+ CACGGGTTCA CTTTTAATAG TAAGTGTGTA TAGACGCAGA GTTTTCCTTT TTCTTCTCTC TGTTTACCAC   
  
  
+ TATTTATATA GATAGTGAAA GATCCCAAAG AAATCAAAAA GAAAAAAGAT CCCAAAGAAA AACTCATGTA   
  
  
+ TGGGATTACT TTTCTTCATA CATAGTTACC GTTACTACTA CTTAGAACCC GCGTTTAGAG AAGTGACACG   
  
  
+ TAGTAGCCGC CACTTCTATA GTAATACTAA CTACTTTCTC CGGGATAAAC TGAAAACCGT CTCTTTTTCG   
  
  
+ AAGAGTGGAA AGAGGTAGAG GAAAACGATA AAGTGGAAAA GAAATTATGA AGGAGGTTTG GGGTGTAGAT   
  
  
+ GGGTGGTGAG GAGTTTAAGG TTTTGGGTCA TGGAATAATA TAAGAATAGT AGGGGAAGTT AATTCGTGGT   
  
  
+ ACGATTATTA ATTAGTAGGT GGGAAACGAA TAGTATTAAT CAATTTAAAT ATATTATGTG AGAGACACCT   
  
  
+ GGCTGGACTG CGGAACGATA CATACAACTA ACGAGTACTT GGGGTTGACA AACCTATAAC CTGGTTGACC   
  
  
+ CAATTAAATG AATGGTAAAC TAATAGTACA AGAACAACAT GAACTATCAC TAGAATTAAT CTACTTGTAC   
  
  
+ TAATTCAAGT AAACTAGCCT TTATTACTGA CTTCGTTTAG ATCCCGTGGT ATACTACCAC TTTTTAGTAT   
  
  
+ TCCGGTCGTC AATTTATAAA TTCTACAGTA CCTAGTCGTC TAATTATTTC TATAGACTTA AAACTAATTG   
  
  
+ GGTAAAGTTG TATCCTCTAG CGTATTTTAC TAAATCGATA GAATCGATCG TACGTTTTCG TTTGTTATTA   
  
  
+ ATCTACTAAA AAATTTTTAA TTTAAAGGT  

- CTGTGTCTCC TTCCCCTCTC TCTCTCTCTC TCTATCGATG GAAAAGTTGC AGAAGATCAA ATTCAAACAG   
  
  
- TTTAATAGGG AAAGAAATAG GGAAAGAATC TGCTGAAGAT ATATATACGA TCTCTAGAAG ATGAAGACCT   
  
  
- TAAACCATAT ACAGTAAATT AAGAGATCCT CCCTCTCTTT CTCTTCAACT TCTAGGATAG ATGGACAGAA   
  
  
- CTTAGATAGA TATGAAAGTA ACCAAATACC TATATAAATA TGACCCAACC ACTTTCCCTT TCAACAGCAT   
  
  
- TAATTGAGAA TTTCTAGTCA TTTATATTGA GGAAGAGTGA GCATAAATTA AAGAAAAAGA AAGCAGAAGC   
  
  
- CAAAAATAGA AATTAAGATA CAGAAACTCC ATCGGATAGT AGTTGGGTAT TGTGAAAATT AACATGTTCT   
  
  
- AGTAATCAAA AGCATAGAAT TTCATGTGTA AGGCTTATAG TGGGAAGTTA CTGTAGTGAG GAAGAGGTCA   
  
  
- ACAATAGACG ACAAAAGGAT TGAGCAGTAA GGGTTTTGTT TTGCTAAATT GAATGAAAAA GCAGTGCACT   
  
  
- TTTCTCTCAT TAAAGGATAA AAGGGAAAAC CGGATGAGTA GACAATAATA GAGCAAAATC TCTCATGTAA   
  
  
- GTGCCCAAGT GAAAATTATC ATTCACACAT ATCTGCGTCT CAAAAGGAAA AAGAAGAGAG ACAAATGGTG   
  
  
- ATAAATATAT CTATCACTTT CTAGGGTTTC TTTAGTTTTT CTTTTTTCTA GGGTTTCTTT TTGAGTACAT   
  
  
- ACCCTAATGA AAAGAAGTAT GTATCAATGG CAATGATGAT GAATCTTGGG CGCAAATCTC TTCACTGTGC   
  
  
- ATCATCGGCG GTGAAGATAT CATTATGATT GATGAAAGAG GCCCTATTTG ACTTTTGGCA GAGAAAAAGC   
  
  
- TTCTCACCTT TCTCCATCTC CTTTTGCTAT TTCACCTTTT CTTTAATACT TCCTCCAAAC CCCACATCTA   
  
  
- CCCACCACTC CTCAAATTCC AAAACCCAGT ACCTTATTAT ATTCTTATCA TCCCCTTCAA TTAAGCACCA   
  
  
- TGCTAATAAT TAATCATCCA CCCTTTGCTT ATCATAATTA GTTAAATTTA TATAATACAC TCTCTGTGGA   
  
  
- CCGACCTGAC GCCTTGCTAT GTATGTTGAT TGCTCATGAA CCCCAACTGT TTGGATATTG GACCAACTGG   
  
  
- GTTAATTTAC TTACCATTTG ATTATCATGT TCTTGTTGTA CTTGATAGTG ATCTTAATTA GATGAACATG   
  
  
- ATTAAGTTCA TTTGATCGGA AATAATGACT GAAGCAAATC TAGGGCACCA TATGATGGTG AAAAATCATA   
  
  
- AGGCCAGCAG TTAAATATTT AAGATGTCAT GGATCAGCAG ATTAATAAAG ATATCTGAAT TTTGATTAAC   
  
  
- CCATTTCAAC ATAGGAGATC GCATAAAATG ATTTAGCTAT CTTAGCTAGC ATGCAAAAGC AAACAATAAT   
  
  
- TAGATGATTT TTTAAAAATT AAATTTCCA

+     L-box

| Site Name | Organism | Position | Strand | Matrix score. | sequence | function |
| --- | --- | --- | --- | --- | --- | --- |
| L-box | Petroselinum crispum | 255 | - | 10 | TCTCACCAACC | part of a light responsive element |
| L-box | Petroselinum crispum | 1065 | - | 10 | ATCCCACCTAC | part of a light responsive element |

> 2018/04/13 10:10:12  
+ GACACAGAGG AAGGGGAGAG AGAGAGAGAG AGATAGCTAC CTTTTCAACG TCTTCTAGTT TAAGTTTGTC   
  
  
+ AAATTATCCC TTTCTTTATC CCTTTCTTAG ACGACTTCTA TATATATGCT AGAGATCTTC TACTTCTGGA   
  
  
+ ATTTGGTATA TGTCATTTAA TTCTCTAGGA GGGAGAGAAA GAGAAGTTGA AGATCCTATC TACCTGTCTT   
  
  
+ GAATCTATCT ATACTTTCAT TGGTTTATGG ATATATTTAT ACTGGGTTGG TGAAAGGGAA AGTTGTCGTA   
  
  
+ ATTAACTCTT AAAGATCAGT AAATATAACT CCTTCTCACT CGTATTTAAT TTCTTTTTCT TTCGTCTTCG   
  
  
+ GTTTTTATCT TTAATTCTAT GTCTTTGAGG TAGCCTATCA TCAACCCATA ACACTTTTAA TTGTACAAGA   
  
  
+ TCATTAGTTT TCGTATCTTA AAGTACACAT TCCGAATATC ACCCTTCAAT GACATCACTC CTTCTCCAGT   
  
  
+ TGTTATCTGC TGTTTTCCTA ACTCGTCATT CCCAAAACAA AACGATTTAA CTTACTTTTT CGTCACGTGA   
  
  
+ AAAGAGAGTA ATTTCCTATT TTCCCTTTTG GCCTACTCAT CTGTTATTAT CTCGTTTTAG AGAGTACATT   
  
  
+ CACGGGTTCA CTTTTAATAG TAAGTGTGTA TAGACGCAGA GTTTTCCTTT TTCTTCTCTC TGTTTACCAC   
  
  
+ TATTTATATA GATAGTGAAA GATCCCAAAG AAATCAAAAA GAAAAAAGAT CCCAAAGAAA AACTCATGTA   
  
  
+ TGGGATTACT TTTCTTCATA CATAGTTACC GTTACTACTA CTTAGAACCC GCGTTTAGAG AAGTGACACG   
  
  
+ TAGTAGCCGC CACTTCTATA GTAATACTAA CTACTTTCTC CGGGATAAAC TGAAAACCGT CTCTTTTTCG   
  
  
+ AAGAGTGGAA AGAGGTAGAG GAAAACGATA AAGTGGAAAA GAAATTATGA AGGAGGTTTG GGGTGTAGAT   
  
  
+ GGGTGGTGAG GAGTTTAAGG TTTTGGGTCA TGGAATAATA TAAGAATAGT AGGGGAAGTT AATTCGTGGT   
  
  
+ ACGATTATTA ATTAGTAGGT GGGAAACGAA TAGTATTAAT CAATTTAAAT ATATTATGTG AGAGACACCT   
  
  
+ GGCTGGACTG CGGAACGATA CATACAACTA ACGAGTACTT GGGGTTGACA AACCTATAAC CTGGTTGACC   
  
  
+ CAATTAAATG AATGGTAAAC TAATAGTACA AGAACAACAT GAACTATCAC TAGAATTAAT CTACTTGTAC   
  
  
+ TAATTCAAGT AAACTAGCCT TTATTACTGA CTTCGTTTAG ATCCCGTGGT ATACTACCAC TTTTTAGTAT   
  
  
+ TCCGGTCGTC AATTTATAAA TTCTACAGTA CCTAGTCGTC TAATTATTTC TATAGACTTA AAACTAATTG   
  
  
+ GGTAAAGTTG TATCCTCTAG CGTATTTTAC TAAATCGATA GAATCGATCG TACGTTTTCG TTTGTTATTA   
  
  
+ ATCTACTAAA AAATTTTTAA TTTAAAGGT  

- CTGTGTCTCC TTCCCCTCTC TCTCTCTCTC TCTATCGATG GAAAAGTTGC AGAAGATCAA ATTCAAACAG   
  
  
- TTTAATAGGG AAAGAAATAG GGAAAGAATC TGCTGAAGAT ATATATACGA TCTCTAGAAG ATGAAGACCT   
  
  
- TAAACCATAT ACAGTAAATT AAGAGATCCT CCCTCTCTTT CTCTTCAACT TCTAGGATAG ATGGACAGAA   
  
  
- CTTAGATAGA TATGAAAGTA ACCAAATACC TATATAAATA TGACCCAACC ACTTTCCCTT TCAACAGCAT   
  
  
- TAATTGAGAA TTTCTAGTCA TTTATATTGA GGAAGAGTGA GCATAAATTA AAGAAAAAGA AAGCAGAAGC   
  
  
- CAAAAATAGA AATTAAGATA CAGAAACTCC ATCGGATAGT AGTTGGGTAT TGTGAAAATT AACATGTTCT   
  
  
- AGTAATCAAA AGCATAGAAT TTCATGTGTA AGGCTTATAG TGGGAAGTTA CTGTAGTGAG GAAGAGGTCA   
  
  
- ACAATAGACG ACAAAAGGAT TGAGCAGTAA GGGTTTTGTT TTGCTAAATT GAATGAAAAA GCAGTGCACT   
  
  
- TTTCTCTCAT TAAAGGATAA AAGGGAAAAC CGGATGAGTA GACAATAATA GAGCAAAATC TCTCATGTAA   
  
  
- GTGCCCAAGT GAAAATTATC ATTCACACAT ATCTGCGTCT CAAAAGGAAA AAGAAGAGAG ACAAATGGTG   
  
  
- ATAAATATAT CTATCACTTT CTAGGGTTTC TTTAGTTTTT CTTTTTTCTA GGGTTTCTTT TTGAGTACAT   
  
  
- ACCCTAATGA AAAGAAGTAT GTATCAATGG CAATGATGAT GAATCTTGGG CGCAAATCTC TTCACTGTGC   
  
  
- ATCATCGGCG GTGAAGATAT CATTATGATT GATGAAAGAG GCCCTATTTG ACTTTTGGCA GAGAAAAAGC   
  
  
- TTCTCACCTT TCTCCATCTC CTTTTGCTAT TTCACCTTTT CTTTAATACT TCCTCCAAAC CCCACATCTA   
  
  
- CCCACCACTC CTCAAATTCC AAAACCCAGT ACCTTATTAT ATTCTTATCA TCCCCTTCAA TTAAGCACCA   
  
  
- TGCTAATAAT TAATCATCCA CCCTTTGCTT ATCATAATTA GTTAAATTTA TATAATACAC TCTCTGTGGA   
  
  
- CCGACCTGAC GCCTTGCTAT GTATGTTGAT TGCTCATGAA CCCCAACTGT TTGGATATTG GACCAACTGG   
  
  
- GTTAATTTAC TTACCATTTG ATTATCATGT TCTTGTTGTA CTTGATAGTG ATCTTAATTA GATGAACATG   
  
  
- ATTAAGTTCA TTTGATCGGA AATAATGACT GAAGCAAATC TAGGGCACCA TATGATGGTG AAAAATCATA   
  
  
- AGGCCAGCAG TTAAATATTT AAGATGTCAT GGATCAGCAG ATTAATAAAG ATATCTGAAT TTTGATTAAC   
  
  
- CCATTTCAAC ATAGGAGATC GCATAAAATG ATTTAGCTAT CTTAGCTAGC ATGCAAAAGC AAACAATAAT   
  
  
- TAGATGATTT TTTAAAAATT AAATTTCCA

+     MBS

| Site Name | Organism | Position | Strand | Matrix score. | sequence | function |
| --- | --- | --- | --- | --- | --- | --- |
| MBS | Arabidopsis thaliana | 487 | - | 6 | CAACTG | MYB binding site involved in drought-inducibility |

> 2018/04/13 10:10:12  
+ GACACAGAGG AAGGGGAGAG AGAGAGAGAG AGATAGCTAC CTTTTCAACG TCTTCTAGTT TAAGTTTGTC   
  
  
+ AAATTATCCC TTTCTTTATC CCTTTCTTAG ACGACTTCTA TATATATGCT AGAGATCTTC TACTTCTGGA   
  
  
+ ATTTGGTATA TGTCATTTAA TTCTCTAGGA GGGAGAGAAA GAGAAGTTGA AGATCCTATC TACCTGTCTT   
  
  
+ GAATCTATCT ATACTTTCAT TGGTTTATGG ATATATTTAT ACTGGGTTGG TGAAAGGGAA AGTTGTCGTA   
  
  
+ ATTAACTCTT AAAGATCAGT AAATATAACT CCTTCTCACT CGTATTTAAT TTCTTTTTCT TTCGTCTTCG   
  
  
+ GTTTTTATCT TTAATTCTAT GTCTTTGAGG TAGCCTATCA TCAACCCATA ACACTTTTAA TTGTACAAGA   
  
  
+ TCATTAGTTT TCGTATCTTA AAGTACACAT TCCGAATATC ACCCTTCAAT GACATCACTC CTTCTCCAGT   
  
  
+ TGTTATCTGC TGTTTTCCTA ACTCGTCATT CCCAAAACAA AACGATTTAA CTTACTTTTT CGTCACGTGA   
  
  
+ AAAGAGAGTA ATTTCCTATT TTCCCTTTTG GCCTACTCAT CTGTTATTAT CTCGTTTTAG AGAGTACATT   
  
  
+ CACGGGTTCA CTTTTAATAG TAAGTGTGTA TAGACGCAGA GTTTTCCTTT TTCTTCTCTC TGTTTACCAC   
  
  
+ TATTTATATA GATAGTGAAA GATCCCAAAG AAATCAAAAA GAAAAAAGAT CCCAAAGAAA AACTCATGTA   
  
  
+ TGGGATTACT TTTCTTCATA CATAGTTACC GTTACTACTA CTTAGAACCC GCGTTTAGAG AAGTGACACG   
  
  
+ TAGTAGCCGC CACTTCTATA GTAATACTAA CTACTTTCTC CGGGATAAAC TGAAAACCGT CTCTTTTTCG   
  
  
+ AAGAGTGGAA AGAGGTAGAG GAAAACGATA AAGTGGAAAA GAAATTATGA AGGAGGTTTG GGGTGTAGAT   
  
  
+ GGGTGGTGAG GAGTTTAAGG TTTTGGGTCA TGGAATAATA TAAGAATAGT AGGGGAAGTT AATTCGTGGT   
  
  
+ ACGATTATTA ATTAGTAGGT GGGAAACGAA TAGTATTAAT CAATTTAAAT ATATTATGTG AGAGACACCT   
  
  
+ GGCTGGACTG CGGAACGATA CATACAACTA ACGAGTACTT GGGGTTGACA AACCTATAAC CTGGTTGACC   
  
  
+ CAATTAAATG AATGGTAAAC TAATAGTACA AGAACAACAT GAACTATCAC TAGAATTAAT CTACTTGTAC   
  
  
+ TAATTCAAGT AAACTAGCCT TTATTACTGA CTTCGTTTAG ATCCCGTGGT ATACTACCAC TTTTTAGTAT   
  
  
+ TCCGGTCGTC AATTTATAAA TTCTACAGTA CCTAGTCGTC TAATTATTTC TATAGACTTA AAACTAATTG   
  
  
+ GGTAAAGTTG TATCCTCTAG CGTATTTTAC TAAATCGATA GAATCGATCG TACGTTTTCG TTTGTTATTA   
  
  
+ ATCTACTAAA AAATTTTTAA TTTAAAGGT  

- CTGTGTCTCC TTCCCCTCTC TCTCTCTCTC TCTATCGATG GAAAAGTTGC AGAAGATCAA ATTCAAACAG   
  
  
- TTTAATAGGG AAAGAAATAG GGAAAGAATC TGCTGAAGAT ATATATACGA TCTCTAGAAG ATGAAGACCT   
  
  
- TAAACCATAT ACAGTAAATT AAGAGATCCT CCCTCTCTTT CTCTTCAACT TCTAGGATAG ATGGACAGAA   
  
  
- CTTAGATAGA TATGAAAGTA ACCAAATACC TATATAAATA TGACCCAACC ACTTTCCCTT TCAACAGCAT   
  
  
- TAATTGAGAA TTTCTAGTCA TTTATATTGA GGAAGAGTGA GCATAAATTA AAGAAAAAGA AAGCAGAAGC   
  
  
- CAAAAATAGA AATTAAGATA CAGAAACTCC ATCGGATAGT AGTTGGGTAT TGTGAAAATT AACATGTTCT   
  
  
- AGTAATCAAA AGCATAGAAT TTCATGTGTA AGGCTTATAG TGGGAAGTTA CTGTAGTGAG GAAGAGGTCA   
  
  
- ACAATAGACG ACAAAAGGAT TGAGCAGTAA GGGTTTTGTT TTGCTAAATT GAATGAAAAA GCAGTGCACT   
  
  
- TTTCTCTCAT TAAAGGATAA AAGGGAAAAC CGGATGAGTA GACAATAATA GAGCAAAATC TCTCATGTAA   
  
  
- GTGCCCAAGT GAAAATTATC ATTCACACAT ATCTGCGTCT CAAAAGGAAA AAGAAGAGAG ACAAATGGTG   
  
  
- ATAAATATAT CTATCACTTT CTAGGGTTTC TTTAGTTTTT CTTTTTTCTA GGGTTTCTTT TTGAGTACAT   
  
  
- ACCCTAATGA AAAGAAGTAT GTATCAATGG CAATGATGAT GAATCTTGGG CGCAAATCTC TTCACTGTGC   
  
  
- ATCATCGGCG GTGAAGATAT CATTATGATT GATGAAAGAG GCCCTATTTG ACTTTTGGCA GAGAAAAAGC   
  
  
- TTCTCACCTT TCTCCATCTC CTTTTGCTAT TTCACCTTTT CTTTAATACT TCCTCCAAAC CCCACATCTA   
  
  
- CCCACCACTC CTCAAATTCC AAAACCCAGT ACCTTATTAT ATTCTTATCA TCCCCTTCAA TTAAGCACCA   
  
  
- TGCTAATAAT TAATCATCCA CCCTTTGCTT ATCATAATTA GTTAAATTTA TATAATACAC TCTCTGTGGA   
  
  
- CCGACCTGAC GCCTTGCTAT GTATGTTGAT TGCTCATGAA CCCCAACTGT TTGGATATTG GACCAACTGG   
  
  
- GTTAATTTAC TTACCATTTG ATTATCATGT TCTTGTTGTA CTTGATAGTG ATCTTAATTA GATGAACATG   
  
  
- ATTAAGTTCA TTTGATCGGA AATAATGACT GAAGCAAATC TAGGGCACCA TATGATGGTG AAAAATCATA   
  
  
- AGGCCAGCAG TTAAATATTT AAGATGTCAT GGATCAGCAG ATTAATAAAG ATATCTGAAT TTTGATTAAC   
  
  
- CCATTTCAAC ATAGGAGATC GCATAAAATG ATTTAGCTAT CTTAGCTAGC ATGCAAAAGC AAACAATAAT   
  
  
- TAGATGATTT TTTAAAAATT AAATTTCCA

+     MBSII

| Site Name | Organism | Position | Strand | Matrix score. | sequence | function |
| --- | --- | --- | --- | --- | --- | --- |
| MBSII | Petunia hybrida | 538 | - | 11 | AAAAGTTAGTTA | MYB binding site involved in flavonoid biosynthetic genes regulation |

> 2018/04/13 10:10:12  
+ GACACAGAGG AAGGGGAGAG AGAGAGAGAG AGATAGCTAC CTTTTCAACG TCTTCTAGTT TAAGTTTGTC   
  
  
+ AAATTATCCC TTTCTTTATC CCTTTCTTAG ACGACTTCTA TATATATGCT AGAGATCTTC TACTTCTGGA   
  
  
+ ATTTGGTATA TGTCATTTAA TTCTCTAGGA GGGAGAGAAA GAGAAGTTGA AGATCCTATC TACCTGTCTT   
  
  
+ GAATCTATCT ATACTTTCAT TGGTTTATGG ATATATTTAT ACTGGGTTGG TGAAAGGGAA AGTTGTCGTA   
  
  
+ ATTAACTCTT AAAGATCAGT AAATATAACT CCTTCTCACT CGTATTTAAT TTCTTTTTCT TTCGTCTTCG   
  
  
+ GTTTTTATCT TTAATTCTAT GTCTTTGAGG TAGCCTATCA TCAACCCATA ACACTTTTAA TTGTACAAGA   
  
  
+ TCATTAGTTT TCGTATCTTA AAGTACACAT TCCGAATATC ACCCTTCAAT GACATCACTC CTTCTCCAGT   
  
  
+ TGTTATCTGC TGTTTTCCTA ACTCGTCATT CCCAAAACAA AACGATTTAA CTTACTTTTT CGTCACGTGA   
  
  
+ AAAGAGAGTA ATTTCCTATT TTCCCTTTTG GCCTACTCAT CTGTTATTAT CTCGTTTTAG AGAGTACATT   
  
  
+ CACGGGTTCA CTTTTAATAG TAAGTGTGTA TAGACGCAGA GTTTTCCTTT TTCTTCTCTC TGTTTACCAC   
  
  
+ TATTTATATA GATAGTGAAA GATCCCAAAG AAATCAAAAA GAAAAAAGAT CCCAAAGAAA AACTCATGTA   
  
  
+ TGGGATTACT TTTCTTCATA CATAGTTACC GTTACTACTA CTTAGAACCC GCGTTTAGAG AAGTGACACG   
  
  
+ TAGTAGCCGC CACTTCTATA GTAATACTAA CTACTTTCTC CGGGATAAAC TGAAAACCGT CTCTTTTTCG   
  
  
+ AAGAGTGGAA AGAGGTAGAG GAAAACGATA AAGTGGAAAA GAAATTATGA AGGAGGTTTG GGGTGTAGAT   
  
  
+ GGGTGGTGAG GAGTTTAAGG TTTTGGGTCA TGGAATAATA TAAGAATAGT AGGGGAAGTT AATTCGTGGT   
  
  
+ ACGATTATTA ATTAGTAGGT GGGAAACGAA TAGTATTAAT CAATTTAAAT ATATTATGTG AGAGACACCT   
  
  
+ GGCTGGACTG CGGAACGATA CATACAACTA ACGAGTACTT GGGGTTGACA AACCTATAAC CTGGTTGACC   
  
  
+ CAATTAAATG AATGGTAAAC TAATAGTACA AGAACAACAT GAACTATCAC TAGAATTAAT CTACTTGTAC   
  
  
+ TAATTCAAGT AAACTAGCCT TTATTACTGA CTTCGTTTAG ATCCCGTGGT ATACTACCAC TTTTTAGTAT   
  
  
+ TCCGGTCGTC AATTTATAAA TTCTACAGTA CCTAGTCGTC TAATTATTTC TATAGACTTA AAACTAATTG   
  
  
+ GGTAAAGTTG TATCCTCTAG CGTATTTTAC TAAATCGATA GAATCGATCG TACGTTTTCG TTTGTTATTA   
  
  
+ ATCTACTAAA AAATTTTTAA TTTAAAGGT  

- CTGTGTCTCC TTCCCCTCTC TCTCTCTCTC TCTATCGATG GAAAAGTTGC AGAAGATCAA ATTCAAACAG   
  
  
- TTTAATAGGG AAAGAAATAG GGAAAGAATC TGCTGAAGAT ATATATACGA TCTCTAGAAG ATGAAGACCT   
  
  
- TAAACCATAT ACAGTAAATT AAGAGATCCT CCCTCTCTTT CTCTTCAACT TCTAGGATAG ATGGACAGAA   
  
  
- CTTAGATAGA TATGAAAGTA ACCAAATACC TATATAAATA TGACCCAACC ACTTTCCCTT TCAACAGCAT   
  
  
- TAATTGAGAA TTTCTAGTCA TTTATATTGA GGAAGAGTGA GCATAAATTA AAGAAAAAGA AAGCAGAAGC   
  
  
- CAAAAATAGA AATTAAGATA CAGAAACTCC ATCGGATAGT AGTTGGGTAT TGTGAAAATT AACATGTTCT   
  
  
- AGTAATCAAA AGCATAGAAT TTCATGTGTA AGGCTTATAG TGGGAAGTTA CTGTAGTGAG GAAGAGGTCA   
  
  
- ACAATAGACG ACAAAAGGAT TGAGCAGTAA GGGTTTTGTT TTGCTAAATT GAATGAAAAA GCAGTGCACT   
  
  
- TTTCTCTCAT TAAAGGATAA AAGGGAAAAC CGGATGAGTA GACAATAATA GAGCAAAATC TCTCATGTAA   
  
  
- GTGCCCAAGT GAAAATTATC ATTCACACAT ATCTGCGTCT CAAAAGGAAA AAGAAGAGAG ACAAATGGTG   
  
  
- ATAAATATAT CTATCACTTT CTAGGGTTTC TTTAGTTTTT CTTTTTTCTA GGGTTTCTTT TTGAGTACAT   
  
  
- ACCCTAATGA AAAGAAGTAT GTATCAATGG CAATGATGAT GAATCTTGGG CGCAAATCTC TTCACTGTGC   
  
  
- ATCATCGGCG GTGAAGATAT CATTATGATT GATGAAAGAG GCCCTATTTG ACTTTTGGCA GAGAAAAAGC   
  
  
- TTCTCACCTT TCTCCATCTC CTTTTGCTAT TTCACCTTTT CTTTAATACT TCCTCCAAAC CCCACATCTA   
  
  
- CCCACCACTC CTCAAATTCC AAAACCCAGT ACCTTATTAT ATTCTTATCA TCCCCTTCAA TTAAGCACCA   
  
  
- TGCTAATAAT TAATCATCCA CCCTTTGCTT ATCATAATTA GTTAAATTTA TATAATACAC TCTCTGTGGA   
  
  
- CCGACCTGAC GCCTTGCTAT GTATGTTGAT TGCTCATGAA CCCCAACTGT TTGGATATTG GACCAACTGG   
  
  
- GTTAATTTAC TTACCATTTG ATTATCATGT TCTTGTTGTA CTTGATAGTG ATCTTAATTA GATGAACATG   
  
  
- ATTAAGTTCA TTTGATCGGA AATAATGACT GAAGCAAATC TAGGGCACCA TATGATGGTG AAAAATCATA   
  
  
- AGGCCAGCAG TTAAATATTT AAGATGTCAT GGATCAGCAG ATTAATAAAG ATATCTGAAT TTTGATTAAC   
  
  
- CCATTTCAAC ATAGGAGATC GCATAAAATG ATTTAGCTAT CTTAGCTAGC ATGCAAAAGC AAACAATAAT   
  
  
- TAGATGATTT TTTAAAAATT AAATTTCCA

+     P-box

| Site Name | Organism | Position | Strand | Matrix score. | sequence | function |
| --- | --- | --- | --- | --- | --- | --- |
| P-box | Oryza sativa | 584 | + | 7 | CCTTTTG | gibberellin-responsive element |

> 2018/04/13 10:10:12  
+ GACACAGAGG AAGGGGAGAG AGAGAGAGAG AGATAGCTAC CTTTTCAACG TCTTCTAGTT TAAGTTTGTC   
  
  
+ AAATTATCCC TTTCTTTATC CCTTTCTTAG ACGACTTCTA TATATATGCT AGAGATCTTC TACTTCTGGA   
  
  
+ ATTTGGTATA TGTCATTTAA TTCTCTAGGA GGGAGAGAAA GAGAAGTTGA AGATCCTATC TACCTGTCTT   
  
  
+ GAATCTATCT ATACTTTCAT TGGTTTATGG ATATATTTAT ACTGGGTTGG TGAAAGGGAA AGTTGTCGTA   
  
  
+ ATTAACTCTT AAAGATCAGT AAATATAACT CCTTCTCACT CGTATTTAAT TTCTTTTTCT TTCGTCTTCG   
  
  
+ GTTTTTATCT TTAATTCTAT GTCTTTGAGG TAGCCTATCA TCAACCCATA ACACTTTTAA TTGTACAAGA   
  
  
+ TCATTAGTTT TCGTATCTTA AAGTACACAT TCCGAATATC ACCCTTCAAT GACATCACTC CTTCTCCAGT   
  
  
+ TGTTATCTGC TGTTTTCCTA ACTCGTCATT CCCAAAACAA AACGATTTAA CTTACTTTTT CGTCACGTGA   
  
  
+ AAAGAGAGTA ATTTCCTATT TTCCCTTTTG GCCTACTCAT CTGTTATTAT CTCGTTTTAG AGAGTACATT   
  
  
+ CACGGGTTCA CTTTTAATAG TAAGTGTGTA TAGACGCAGA GTTTTCCTTT TTCTTCTCTC TGTTTACCAC   
  
  
+ TATTTATATA GATAGTGAAA GATCCCAAAG AAATCAAAAA GAAAAAAGAT CCCAAAGAAA AACTCATGTA   
  
  
+ TGGGATTACT TTTCTTCATA CATAGTTACC GTTACTACTA CTTAGAACCC GCGTTTAGAG AAGTGACACG   
  
  
+ TAGTAGCCGC CACTTCTATA GTAATACTAA CTACTTTCTC CGGGATAAAC TGAAAACCGT CTCTTTTTCG   
  
  
+ AAGAGTGGAA AGAGGTAGAG GAAAACGATA AAGTGGAAAA GAAATTATGA AGGAGGTTTG GGGTGTAGAT   
  
  
+ GGGTGGTGAG GAGTTTAAGG TTTTGGGTCA TGGAATAATA TAAGAATAGT AGGGGAAGTT AATTCGTGGT   
  
  
+ ACGATTATTA ATTAGTAGGT GGGAAACGAA TAGTATTAAT CAATTTAAAT ATATTATGTG AGAGACACCT   
  
  
+ GGCTGGACTG CGGAACGATA CATACAACTA ACGAGTACTT GGGGTTGACA AACCTATAAC CTGGTTGACC   
  
  
+ CAATTAAATG AATGGTAAAC TAATAGTACA AGAACAACAT GAACTATCAC TAGAATTAAT CTACTTGTAC   
  
  
+ TAATTCAAGT AAACTAGCCT TTATTACTGA CTTCGTTTAG ATCCCGTGGT ATACTACCAC TTTTTAGTAT   
  
  
+ TCCGGTCGTC AATTTATAAA TTCTACAGTA CCTAGTCGTC TAATTATTTC TATAGACTTA AAACTAATTG   
  
  
+ GGTAAAGTTG TATCCTCTAG CGTATTTTAC TAAATCGATA GAATCGATCG TACGTTTTCG TTTGTTATTA   
  
  
+ ATCTACTAAA AAATTTTTAA TTTAAAGGT  

- CTGTGTCTCC TTCCCCTCTC TCTCTCTCTC TCTATCGATG GAAAAGTTGC AGAAGATCAA ATTCAAACAG   
  
  
- TTTAATAGGG AAAGAAATAG GGAAAGAATC TGCTGAAGAT ATATATACGA TCTCTAGAAG ATGAAGACCT   
  
  
- TAAACCATAT ACAGTAAATT AAGAGATCCT CCCTCTCTTT CTCTTCAACT TCTAGGATAG ATGGACAGAA   
  
  
- CTTAGATAGA TATGAAAGTA ACCAAATACC TATATAAATA TGACCCAACC ACTTTCCCTT TCAACAGCAT   
  
  
- TAATTGAGAA TTTCTAGTCA TTTATATTGA GGAAGAGTGA GCATAAATTA AAGAAAAAGA AAGCAGAAGC   
  
  
- CAAAAATAGA AATTAAGATA CAGAAACTCC ATCGGATAGT AGTTGGGTAT TGTGAAAATT AACATGTTCT   
  
  
- AGTAATCAAA AGCATAGAAT TTCATGTGTA AGGCTTATAG TGGGAAGTTA CTGTAGTGAG GAAGAGGTCA   
  
  
- ACAATAGACG ACAAAAGGAT TGAGCAGTAA GGGTTTTGTT TTGCTAAATT GAATGAAAAA GCAGTGCACT   
  
  
- TTTCTCTCAT TAAAGGATAA AAGGGAAAAC CGGATGAGTA GACAATAATA GAGCAAAATC TCTCATGTAA   
  
  
- GTGCCCAAGT GAAAATTATC ATTCACACAT ATCTGCGTCT CAAAAGGAAA AAGAAGAGAG ACAAATGGTG   
  
  
- ATAAATATAT CTATCACTTT CTAGGGTTTC TTTAGTTTTT CTTTTTTCTA GGGTTTCTTT TTGAGTACAT   
  
  
- ACCCTAATGA AAAGAAGTAT GTATCAATGG CAATGATGAT GAATCTTGGG CGCAAATCTC TTCACTGTGC   
  
  
- ATCATCGGCG GTGAAGATAT CATTATGATT GATGAAAGAG GCCCTATTTG ACTTTTGGCA GAGAAAAAGC   
  
  
- TTCTCACCTT TCTCCATCTC CTTTTGCTAT TTCACCTTTT CTTTAATACT TCCTCCAAAC CCCACATCTA   
  
  
- CCCACCACTC CTCAAATTCC AAAACCCAGT ACCTTATTAT ATTCTTATCA TCCCCTTCAA TTAAGCACCA   
  
  
- TGCTAATAAT TAATCATCCA CCCTTTGCTT ATCATAATTA GTTAAATTTA TATAATACAC TCTCTGTGGA   
  
  
- CCGACCTGAC GCCTTGCTAT GTATGTTGAT TGCTCATGAA CCCCAACTGT TTGGATATTG GACCAACTGG   
  
  
- GTTAATTTAC TTACCATTTG ATTATCATGT TCTTGTTGTA CTTGATAGTG ATCTTAATTA GATGAACATG   
  
  
- ATTAAGTTCA TTTGATCGGA AATAATGACT GAAGCAAATC TAGGGCACCA TATGATGGTG AAAAATCATA   
  
  
- AGGCCAGCAG TTAAATATTT AAGATGTCAT GGATCAGCAG ATTAATAAAG ATATCTGAAT TTTGATTAAC   
  
  
- CCATTTCAAC ATAGGAGATC GCATAAAATG ATTTAGCTAT CTTAGCTAGC ATGCAAAAGC AAACAATAAT   
  
  
- TAGATGATTT TTTAAAAATT AAATTTCCA

+     Skn-1\_motif

| Site Name | Organism | Position | Strand | Matrix score. | sequence | function |
| --- | --- | --- | --- | --- | --- | --- |
| Skn-1\_motif | Oryza sativa | 515 | + | 5 | GTCAT | cis-acting regulatory element required for endosperm expression |
| Skn-1\_motif | Oryza sativa | 469 | - | 5 | GTCAT | cis-acting regulatory element required for endosperm expression |
| Skn-1\_motif | Oryza sativa | 152 | + | 5 | GTCAT | cis-acting regulatory element required for endosperm expression |
| Skn-1\_motif | Oryza sativa | 1007 | + | 5 | GTCAT | cis-acting regulatory element required for endosperm expression |

> 2018/04/13 10:10:12  
+ GACACAGAGG AAGGGGAGAG AGAGAGAGAG AGATAGCTAC CTTTTCAACG TCTTCTAGTT TAAGTTTGTC   
  
  
+ AAATTATCCC TTTCTTTATC CCTTTCTTAG ACGACTTCTA TATATATGCT AGAGATCTTC TACTTCTGGA   
  
  
+ ATTTGGTATA TGTCATTTAA TTCTCTAGGA GGGAGAGAAA GAGAAGTTGA AGATCCTATC TACCTGTCTT   
  
  
+ GAATCTATCT ATACTTTCAT TGGTTTATGG ATATATTTAT ACTGGGTTGG TGAAAGGGAA AGTTGTCGTA   
  
  
+ ATTAACTCTT AAAGATCAGT AAATATAACT CCTTCTCACT CGTATTTAAT TTCTTTTTCT TTCGTCTTCG   
  
  
+ GTTTTTATCT TTAATTCTAT GTCTTTGAGG TAGCCTATCA TCAACCCATA ACACTTTTAA TTGTACAAGA   
  
  
+ TCATTAGTTT TCGTATCTTA AAGTACACAT TCCGAATATC ACCCTTCAAT GACATCACTC CTTCTCCAGT   
  
  
+ TGTTATCTGC TGTTTTCCTA ACTCGTCATT CCCAAAACAA AACGATTTAA CTTACTTTTT CGTCACGTGA   
  
  
+ AAAGAGAGTA ATTTCCTATT TTCCCTTTTG GCCTACTCAT CTGTTATTAT CTCGTTTTAG AGAGTACATT   
  
  
+ CACGGGTTCA CTTTTAATAG TAAGTGTGTA TAGACGCAGA GTTTTCCTTT TTCTTCTCTC TGTTTACCAC   
  
  
+ TATTTATATA GATAGTGAAA GATCCCAAAG AAATCAAAAA GAAAAAAGAT CCCAAAGAAA AACTCATGTA   
  
  
+ TGGGATTACT TTTCTTCATA CATAGTTACC GTTACTACTA CTTAGAACCC GCGTTTAGAG AAGTGACACG   
  
  
+ TAGTAGCCGC CACTTCTATA GTAATACTAA CTACTTTCTC CGGGATAAAC TGAAAACCGT CTCTTTTTCG   
  
  
+ AAGAGTGGAA AGAGGTAGAG GAAAACGATA AAGTGGAAAA GAAATTATGA AGGAGGTTTG GGGTGTAGAT   
  
  
+ GGGTGGTGAG GAGTTTAAGG TTTTGGGTCA TGGAATAATA TAAGAATAGT AGGGGAAGTT AATTCGTGGT   
  
  
+ ACGATTATTA ATTAGTAGGT GGGAAACGAA TAGTATTAAT CAATTTAAAT ATATTATGTG AGAGACACCT   
  
  
+ GGCTGGACTG CGGAACGATA CATACAACTA ACGAGTACTT GGGGTTGACA AACCTATAAC CTGGTTGACC   
  
  
+ CAATTAAATG AATGGTAAAC TAATAGTACA AGAACAACAT GAACTATCAC TAGAATTAAT CTACTTGTAC   
  
  
+ TAATTCAAGT AAACTAGCCT TTATTACTGA CTTCGTTTAG ATCCCGTGGT ATACTACCAC TTTTTAGTAT   
  
  
+ TCCGGTCGTC AATTTATAAA TTCTACAGTA CCTAGTCGTC TAATTATTTC TATAGACTTA AAACTAATTG   
  
  
+ GGTAAAGTTG TATCCTCTAG CGTATTTTAC TAAATCGATA GAATCGATCG TACGTTTTCG TTTGTTATTA   
  
  
+ ATCTACTAAA AAATTTTTAA TTTAAAGGT  

- CTGTGTCTCC TTCCCCTCTC TCTCTCTCTC TCTATCGATG GAAAAGTTGC AGAAGATCAA ATTCAAACAG   
  
  
- TTTAATAGGG AAAGAAATAG GGAAAGAATC TGCTGAAGAT ATATATACGA TCTCTAGAAG ATGAAGACCT   
  
  
- TAAACCATAT ACAGTAAATT AAGAGATCCT CCCTCTCTTT CTCTTCAACT TCTAGGATAG ATGGACAGAA   
  
  
- CTTAGATAGA TATGAAAGTA ACCAAATACC TATATAAATA TGACCCAACC ACTTTCCCTT TCAACAGCAT   
  
  
- TAATTGAGAA TTTCTAGTCA TTTATATTGA GGAAGAGTGA GCATAAATTA AAGAAAAAGA AAGCAGAAGC   
  
  
- CAAAAATAGA AATTAAGATA CAGAAACTCC ATCGGATAGT AGTTGGGTAT TGTGAAAATT AACATGTTCT   
  
  
- AGTAATCAAA AGCATAGAAT TTCATGTGTA AGGCTTATAG TGGGAAGTTA CTGTAGTGAG GAAGAGGTCA   
  
  
- ACAATAGACG ACAAAAGGAT TGAGCAGTAA GGGTTTTGTT TTGCTAAATT GAATGAAAAA GCAGTGCACT   
  
  
- TTTCTCTCAT TAAAGGATAA AAGGGAAAAC CGGATGAGTA GACAATAATA GAGCAAAATC TCTCATGTAA   
  
  
- GTGCCCAAGT GAAAATTATC ATTCACACAT ATCTGCGTCT CAAAAGGAAA AAGAAGAGAG ACAAATGGTG   
  
  
- ATAAATATAT CTATCACTTT CTAGGGTTTC TTTAGTTTTT CTTTTTTCTA GGGTTTCTTT TTGAGTACAT   
  
  
- ACCCTAATGA AAAGAAGTAT GTATCAATGG CAATGATGAT GAATCTTGGG CGCAAATCTC TTCACTGTGC   
  
  
- ATCATCGGCG GTGAAGATAT CATTATGATT GATGAAAGAG GCCCTATTTG ACTTTTGGCA GAGAAAAAGC   
  
  
- TTCTCACCTT TCTCCATCTC CTTTTGCTAT TTCACCTTTT CTTTAATACT TCCTCCAAAC CCCACATCTA   
  
  
- CCCACCACTC CTCAAATTCC AAAACCCAGT ACCTTATTAT ATTCTTATCA TCCCCTTCAA TTAAGCACCA   
  
  
- TGCTAATAAT TAATCATCCA CCCTTTGCTT ATCATAATTA GTTAAATTTA TATAATACAC TCTCTGTGGA   
  
  
- CCGACCTGAC GCCTTGCTAT GTATGTTGAT TGCTCATGAA CCCCAACTGT TTGGATATTG GACCAACTGG   
  
  
- GTTAATTTAC TTACCATTTG ATTATCATGT TCTTGTTGTA CTTGATAGTG ATCTTAATTA GATGAACATG   
  
  
- ATTAAGTTCA TTTGATCGGA AATAATGACT GAAGCAAATC TAGGGCACCA TATGATGGTG AAAAATCATA   
  
  
- AGGCCAGCAG TTAAATATTT AAGATGTCAT GGATCAGCAG ATTAATAAAG ATATCTGAAT TTTGATTAAC   
  
  
- CCATTTCAAC ATAGGAGATC GCATAAAATG ATTTAGCTAT CTTAGCTAGC ATGCAAAAGC AAACAATAAT   
  
  
- TAGATGATTT TTTAAAAATT AAATTTCCA

+     Sp1

| Site Name | Organism | Position | Strand | Matrix score. | sequence | function |
| --- | --- | --- | --- | --- | --- | --- |
| Sp1 | Zea mays | 981 | - | 5.5 | CC(G/A)CCC | light responsive element |

> 2018/04/13 10:10:12  
+ GACACAGAGG AAGGGGAGAG AGAGAGAGAG AGATAGCTAC CTTTTCAACG TCTTCTAGTT TAAGTTTGTC   
  
  
+ AAATTATCCC TTTCTTTATC CCTTTCTTAG ACGACTTCTA TATATATGCT AGAGATCTTC TACTTCTGGA   
  
  
+ ATTTGGTATA TGTCATTTAA TTCTCTAGGA GGGAGAGAAA GAGAAGTTGA AGATCCTATC TACCTGTCTT   
  
  
+ GAATCTATCT ATACTTTCAT TGGTTTATGG ATATATTTAT ACTGGGTTGG TGAAAGGGAA AGTTGTCGTA   
  
  
+ ATTAACTCTT AAAGATCAGT AAATATAACT CCTTCTCACT CGTATTTAAT TTCTTTTTCT TTCGTCTTCG   
  
  
+ GTTTTTATCT TTAATTCTAT GTCTTTGAGG TAGCCTATCA TCAACCCATA ACACTTTTAA TTGTACAAGA   
  
  
+ TCATTAGTTT TCGTATCTTA AAGTACACAT TCCGAATATC ACCCTTCAAT GACATCACTC CTTCTCCAGT   
  
  
+ TGTTATCTGC TGTTTTCCTA ACTCGTCATT CCCAAAACAA AACGATTTAA CTTACTTTTT CGTCACGTGA   
  
  
+ AAAGAGAGTA ATTTCCTATT TTCCCTTTTG GCCTACTCAT CTGTTATTAT CTCGTTTTAG AGAGTACATT   
  
  
+ CACGGGTTCA CTTTTAATAG TAAGTGTGTA TAGACGCAGA GTTTTCCTTT TTCTTCTCTC TGTTTACCAC   
  
  
+ TATTTATATA GATAGTGAAA GATCCCAAAG AAATCAAAAA GAAAAAAGAT CCCAAAGAAA AACTCATGTA   
  
  
+ TGGGATTACT TTTCTTCATA CATAGTTACC GTTACTACTA CTTAGAACCC GCGTTTAGAG AAGTGACACG   
  
  
+ TAGTAGCCGC CACTTCTATA GTAATACTAA CTACTTTCTC CGGGATAAAC TGAAAACCGT CTCTTTTTCG   
  
  
+ AAGAGTGGAA AGAGGTAGAG GAAAACGATA AAGTGGAAAA GAAATTATGA AGGAGGTTTG GGGTGTAGAT   
  
  
+ GGGTGGTGAG GAGTTTAAGG TTTTGGGTCA TGGAATAATA TAAGAATAGT AGGGGAAGTT AATTCGTGGT   
  
  
+ ACGATTATTA ATTAGTAGGT GGGAAACGAA TAGTATTAAT CAATTTAAAT ATATTATGTG AGAGACACCT   
  
  
+ GGCTGGACTG CGGAACGATA CATACAACTA ACGAGTACTT GGGGTTGACA AACCTATAAC CTGGTTGACC   
  
  
+ CAATTAAATG AATGGTAAAC TAATAGTACA AGAACAACAT GAACTATCAC TAGAATTAAT CTACTTGTAC   
  
  
+ TAATTCAAGT AAACTAGCCT TTATTACTGA CTTCGTTTAG ATCCCGTGGT ATACTACCAC TTTTTAGTAT   
  
  
+ TCCGGTCGTC AATTTATAAA TTCTACAGTA CCTAGTCGTC TAATTATTTC TATAGACTTA AAACTAATTG   
  
  
+ GGTAAAGTTG TATCCTCTAG CGTATTTTAC TAAATCGATA GAATCGATCG TACGTTTTCG TTTGTTATTA   
  
  
+ ATCTACTAAA AAATTTTTAA TTTAAAGGT  

- CTGTGTCTCC TTCCCCTCTC TCTCTCTCTC TCTATCGATG GAAAAGTTGC AGAAGATCAA ATTCAAACAG   
  
  
- TTTAATAGGG AAAGAAATAG GGAAAGAATC TGCTGAAGAT ATATATACGA TCTCTAGAAG ATGAAGACCT   
  
  
- TAAACCATAT ACAGTAAATT AAGAGATCCT CCCTCTCTTT CTCTTCAACT TCTAGGATAG ATGGACAGAA   
  
  
- CTTAGATAGA TATGAAAGTA ACCAAATACC TATATAAATA TGACCCAACC ACTTTCCCTT TCAACAGCAT   
  
  
- TAATTGAGAA TTTCTAGTCA TTTATATTGA GGAAGAGTGA GCATAAATTA AAGAAAAAGA AAGCAGAAGC   
  
  
- CAAAAATAGA AATTAAGATA CAGAAACTCC ATCGGATAGT AGTTGGGTAT TGTGAAAATT AACATGTTCT   
  
  
- AGTAATCAAA AGCATAGAAT TTCATGTGTA AGGCTTATAG TGGGAAGTTA CTGTAGTGAG GAAGAGGTCA   
  
  
- ACAATAGACG ACAAAAGGAT TGAGCAGTAA GGGTTTTGTT TTGCTAAATT GAATGAAAAA GCAGTGCACT   
  
  
- TTTCTCTCAT TAAAGGATAA AAGGGAAAAC CGGATGAGTA GACAATAATA GAGCAAAATC TCTCATGTAA   
  
  
- GTGCCCAAGT GAAAATTATC ATTCACACAT ATCTGCGTCT CAAAAGGAAA AAGAAGAGAG ACAAATGGTG   
  
  
- ATAAATATAT CTATCACTTT CTAGGGTTTC TTTAGTTTTT CTTTTTTCTA GGGTTTCTTT TTGAGTACAT   
  
  
- ACCCTAATGA AAAGAAGTAT GTATCAATGG CAATGATGAT GAATCTTGGG CGCAAATCTC TTCACTGTGC   
  
  
- ATCATCGGCG GTGAAGATAT CATTATGATT GATGAAAGAG GCCCTATTTG ACTTTTGGCA GAGAAAAAGC   
  
  
- TTCTCACCTT TCTCCATCTC CTTTTGCTAT TTCACCTTTT CTTTAATACT TCCTCCAAAC CCCACATCTA   
  
  
- CCCACCACTC CTCAAATTCC AAAACCCAGT ACCTTATTAT ATTCTTATCA TCCCCTTCAA TTAAGCACCA   
  
  
- TGCTAATAAT TAATCATCCA CCCTTTGCTT ATCATAATTA GTTAAATTTA TATAATACAC TCTCTGTGGA   
  
  
- CCGACCTGAC GCCTTGCTAT GTATGTTGAT TGCTCATGAA CCCCAACTGT TTGGATATTG GACCAACTGG   
  
  
- GTTAATTTAC TTACCATTTG ATTATCATGT TCTTGTTGTA CTTGATAGTG ATCTTAATTA GATGAACATG   
  
  
- ATTAAGTTCA TTTGATCGGA AATAATGACT GAAGCAAATC TAGGGCACCA TATGATGGTG AAAAATCATA   
  
  
- AGGCCAGCAG TTAAATATTT AAGATGTCAT GGATCAGCAG ATTAATAAAG ATATCTGAAT TTTGATTAAC   
  
  
- CCATTTCAAC ATAGGAGATC GCATAAAATG ATTTAGCTAT CTTAGCTAGC ATGCAAAAGC AAACAATAAT   
  
  
- TAGATGATTT TTTAAAAATT AAATTTCCA

+     TATA-box

| Site Name | Organism | Position | Strand | Matrix score. | sequence | function |
| --- | --- | --- | --- | --- | --- | --- |
| TATA-box | Glycine max | 1466 | - | 5 | TAATA | core promoter element around -30 of transcription start |
| TATA-box | Lycopersicon esculentum | 1389 | - | 5 | TTTTA | core promoter element around -30 of transcription start |
| TATA-box | Ac | 1342 | - | 7 | TATAAAT | core promoter element around -30 of transcription start |
| TATA-box | Arabidopsis thaliana | 705 | + | 4 | TATA | core promoter element around -30 of transcription start |
| TATA-box | Arabidopsis thaliana | 701 | - | 9 | taTATAAAtc | core promoter element around -30 of transcription start |
| TATA-box | Arabidopsis thaliana | 659 | + | 4 | TATA | core promoter element around -30 of transcription start |
| TATA-box | Daucus carota | 244 | - | 8 | TATAAATA | core promoter element around -30 of transcription start |
| TATA-box | Arabidopsis thaliana | 109 | + | 8 | TATATATA | core promoter element around -30 of transcription start |
| TATA-box | Arabidopsis thaliana | 220 | + | 4 | TATA | core promoter element around -30 of transcription start |
| TATA-box | Arabidopsis thaliana | 147 | + | 4 | TATA | core promoter element around -30 of transcription start |
| TATA-box | Glycine max | 1056 | - | 5 | TAATA | core promoter element around -30 of transcription start |
| TATA-box | Arabidopsis thaliana | 113 | + | 4 | TATA | core promoter element around -30 of transcription start |
| TATA-box | Brassica napus | 112 | + | 6 | ATATAT | core promoter element around -30 of transcription start |
| TATA-box | Arabidopsis thaliana | 1175 | - | 4 | TATA | core promoter element around -30 of transcription start |
| TATA-box | Arabidopsis thaliana | 704 | - | 7 | TATATAA | core promoter element around -30 of transcription start |
| TATA-box | Arabidopsis thaliana | 111 | + | 4 | TATA | core promoter element around -30 of transcription start |
| TATA-box | Daucus carota | 1489 | - | 9 | ccTATAAATT | core promoter element around -30 of transcription start |
| TATA-box | Arabidopsis thaliana | 1100 | - | 4 | TATA | core promoter element around -30 of transcription start |
| TATA-box | Brassica napus | 110 | + | 6 | ATATAT | core promoter element around -30 of transcription start |
| TATA-box | Arabidopsis thaliana | 1343 | - | 6 | TATAAA | core promoter element around -30 of transcription start |
| TATA-box | Lycopersicon esculentum | 405 | + | 5 | TTTTA | core promoter element around -30 of transcription start |
| TATA-box | Brassica napus | 241 | + | 6 | ATATAT | core promoter element around -30 of transcription start |
| TATA-box | Arabidopsis thaliana | 857 | - | 4 | TATA | core promoter element around -30 of transcription start |
| TATA-box | Lycopersicon esculentum | 642 | + | 5 | TTTTA | core promoter element around -30 of transcription start |
| TATA-box | Lycopersicon esculentum | 615 | + | 5 | TTTTA | core promoter element around -30 of transcription start |
| TATA-box | Glycine max | 1084 | - | 5 | TAATA | core promoter element around -30 of transcription start |
| TATA-box | Brassica oleracea | 1018 | + | 6 | ATATAA | core promoter element around -30 of transcription start |
| TATA-box | Arabidopsis thaliana | 707 | + | 4 | TATA | core promoter element around -30 of transcription start |
| TATA-box | Ac | 702 | - | 7 | TATAAAT | core promoter element around -30 of transcription start |
| TATA-box | Arabidopsis thaliana | 1344 | - | 5 | TATAA | core promoter element around -30 of transcription start |
| TATA-box | Arabidopsis thaliana | 107 | + | 9 | tcTATATAtt | core promoter element around -30 of transcription start |
| TATA-box | Arabidopsis thaliana | 248 | + | 4 | TATA | core promoter element around -30 of transcription start |
| TATA-box | Lycopersicon esculentum | 353 | + | 5 | TTTTA | core promoter element around -30 of transcription start |
| TATA-box | Arabidopsis thaliana | 242 | + | 4 | TATA | core promoter element around -30 of transcription start |
| TATA-box | Glycine max | 1211 | + | 5 | TAATA | core promoter element around -30 of transcription start |
| TATA-box | Lycopersicon esculentum | 1425 | + | 5 | TTTTA | core promoter element around -30 of transcription start |
| TATA-box | Arabidopsis thaliana | 703 | - | 6 | TATAAA | core promoter element around -30 of transcription start |
| TATA-box | Brassica oleracea | 303 | + | 6 | ATATAA | core promoter element around -30 of transcription start |
| TATA-box | Arabidopsis thaliana | 247 | - | 5 | TATAA | core promoter element around -30 of transcription start |
| TATA-box | Glycine max | 862 | + | 5 | TAATA | core promoter element around -30 of transcription start |
| TATA-box | Lycopersicon esculentum | 1477 | - | 5 | TTTTA | core promoter element around -30 of transcription start |
| TATA-box | Arabidopsis thaliana | 1381 | - | 4 | TATA | core promoter element around -30 of transcription start |
| TATA-box | Ac | 1345 | + | 7 | TATAAAT | core promoter element around -30 of transcription start |
| TATA-box | Lycopersicon esculentum | 1485 | + | 5 | TTTTA | core promoter element around -30 of transcription start |
| TATA-box | Arabidopsis thaliana | 304 | + | 4 | TATA | core promoter element around -30 of transcription start |
| TATA-box | Arabidopsis thaliana | 246 | - | 6 | TATAAA | core promoter element around -30 of transcription start |
| TATA-box | Lycopersicon esculentum | 1322 | + | 5 | TTTTA | core promoter element around -30 of transcription start |
| TATA-box | Glycine max | 1016 | + | 5 | TAATA | core promoter element around -30 of transcription start |
| TATA-box | Glycine max | 645 | + | 5 | TAATA | core promoter element around -30 of transcription start |
| TATA-box | Ac | 245 | - | 7 | TATAAAT | core promoter element around -30 of transcription start |
| TATA-box | Antirrhinum majus | 1341 | - | 8 | TATAAATT | core promoter element around -30 of transcription start |
| TATA-box | Helianthus annuus | 657 | - | 6 | TATACA | core promoter element around -30 of transcription start |
| TATA-box | Arabidopsis thaliana | 1019 | - | 4 | TATA | core promoter element around -30 of transcription start |
| TATA-box | Arabidopsis thaliana | 1310 | - | 4 | TATA | core promoter element around -30 of transcription start |
| TATA-box | Glycine max | 605 | - | 5 | TAATA | core promoter element around -30 of transcription start |
| TATA-box | Glycine max | 1282 | - | 5 | TAATA | core promoter element around -30 of transcription start |
| TATA-box | Brassica napus | 1099 | - | 6 | ATATAT | core promoter element around -30 of transcription start |
| TATA-box | Arabidopsis thaliana | 1094 | - | 8 | TATTTAAA | core promoter element around -30 of transcription start |
| TATA-box | Glycine max | 1102 | - | 5 | TAATA | core promoter element around -30 of transcription start |

> 2018/04/13 10:10:12  
+ GACACAGAGG AAGGGGAGAG AGAGAGAGAG AGATAGCTAC CTTTTCAACG TCTTCTAGTT TAAGTTTGTC   
  
  
+ AAATTATCCC TTTCTTTATC CCTTTCTTAG ACGACTTCTA TATATATGCT AGAGATCTTC TACTTCTGGA   
  
  
+ ATTTGGTATA TGTCATTTAA TTCTCTAGGA GGGAGAGAAA GAGAAGTTGA AGATCCTATC TACCTGTCTT   
  
  
+ GAATCTATCT ATACTTTCAT TGGTTTATGG ATATATTTAT ACTGGGTTGG TGAAAGGGAA AGTTGTCGTA   
  
  
+ ATTAACTCTT AAAGATCAGT AAATATAACT CCTTCTCACT CGTATTTAAT TTCTTTTTCT TTCGTCTTCG   
  
  
+ GTTTTTATCT TTAATTCTAT GTCTTTGAGG TAGCCTATCA TCAACCCATA ACACTTTTAA TTGTACAAGA   
  
  
+ TCATTAGTTT TCGTATCTTA AAGTACACAT TCCGAATATC ACCCTTCAAT GACATCACTC CTTCTCCAGT   
  
  
+ TGTTATCTGC TGTTTTCCTA ACTCGTCATT CCCAAAACAA AACGATTTAA CTTACTTTTT CGTCACGTGA   
  
  
+ AAAGAGAGTA ATTTCCTATT TTCCCTTTTG GCCTACTCAT CTGTTATTAT CTCGTTTTAG AGAGTACATT   
  
  
+ CACGGGTTCA CTTTTAATAG TAAGTGTGTA TAGACGCAGA GTTTTCCTTT TTCTTCTCTC TGTTTACCAC   
  
  
+ TATTTATATA GATAGTGAAA GATCCCAAAG AAATCAAAAA GAAAAAAGAT CCCAAAGAAA AACTCATGTA   
  
  
+ TGGGATTACT TTTCTTCATA CATAGTTACC GTTACTACTA CTTAGAACCC GCGTTTAGAG AAGTGACACG   
  
  
+ TAGTAGCCGC CACTTCTATA GTAATACTAA CTACTTTCTC CGGGATAAAC TGAAAACCGT CTCTTTTTCG   
  
  
+ AAGAGTGGAA AGAGGTAGAG GAAAACGATA AAGTGGAAAA GAAATTATGA AGGAGGTTTG GGGTGTAGAT   
  
  
+ GGGTGGTGAG GAGTTTAAGG TTTTGGGTCA TGGAATAATA TAAGAATAGT AGGGGAAGTT AATTCGTGGT   
  
  
+ ACGATTATTA ATTAGTAGGT GGGAAACGAA TAGTATTAAT CAATTTAAAT ATATTATGTG AGAGACACCT   
  
  
+ GGCTGGACTG CGGAACGATA CATACAACTA ACGAGTACTT GGGGTTGACA AACCTATAAC CTGGTTGACC   
  
  
+ CAATTAAATG AATGGTAAAC TAATAGTACA AGAACAACAT GAACTATCAC TAGAATTAAT CTACTTGTAC   
  
  
+ TAATTCAAGT AAACTAGCCT TTATTACTGA CTTCGTTTAG ATCCCGTGGT ATACTACCAC TTTTTAGTAT   
  
  
+ TCCGGTCGTC AATTTATAAA TTCTACAGTA CCTAGTCGTC TAATTATTTC TATAGACTTA AAACTAATTG   
  
  
+ GGTAAAGTTG TATCCTCTAG CGTATTTTAC TAAATCGATA GAATCGATCG TACGTTTTCG TTTGTTATTA   
  
  
+ ATCTACTAAA AAATTTTTAA TTTAAAGGT  

- CTGTGTCTCC TTCCCCTCTC TCTCTCTCTC TCTATCGATG GAAAAGTTGC AGAAGATCAA ATTCAAACAG   
  
  
- TTTAATAGGG AAAGAAATAG GGAAAGAATC TGCTGAAGAT ATATATACGA TCTCTAGAAG ATGAAGACCT   
  
  
- TAAACCATAT ACAGTAAATT AAGAGATCCT CCCTCTCTTT CTCTTCAACT TCTAGGATAG ATGGACAGAA   
  
  
- CTTAGATAGA TATGAAAGTA ACCAAATACC TATATAAATA TGACCCAACC ACTTTCCCTT TCAACAGCAT   
  
  
- TAATTGAGAA TTTCTAGTCA TTTATATTGA GGAAGAGTGA GCATAAATTA AAGAAAAAGA AAGCAGAAGC   
  
  
- CAAAAATAGA AATTAAGATA CAGAAACTCC ATCGGATAGT AGTTGGGTAT TGTGAAAATT AACATGTTCT   
  
  
- AGTAATCAAA AGCATAGAAT TTCATGTGTA AGGCTTATAG TGGGAAGTTA CTGTAGTGAG GAAGAGGTCA   
  
  
- ACAATAGACG ACAAAAGGAT TGAGCAGTAA GGGTTTTGTT TTGCTAAATT GAATGAAAAA GCAGTGCACT   
  
  
- TTTCTCTCAT TAAAGGATAA AAGGGAAAAC CGGATGAGTA GACAATAATA GAGCAAAATC TCTCATGTAA   
  
  
- GTGCCCAAGT GAAAATTATC ATTCACACAT ATCTGCGTCT CAAAAGGAAA AAGAAGAGAG ACAAATGGTG   
  
  
- ATAAATATAT CTATCACTTT CTAGGGTTTC TTTAGTTTTT CTTTTTTCTA GGGTTTCTTT TTGAGTACAT   
  
  
- ACCCTAATGA AAAGAAGTAT GTATCAATGG CAATGATGAT GAATCTTGGG CGCAAATCTC TTCACTGTGC   
  
  
- ATCATCGGCG GTGAAGATAT CATTATGATT GATGAAAGAG GCCCTATTTG ACTTTTGGCA GAGAAAAAGC   
  
  
- TTCTCACCTT TCTCCATCTC CTTTTGCTAT TTCACCTTTT CTTTAATACT TCCTCCAAAC CCCACATCTA   
  
  
- CCCACCACTC CTCAAATTCC AAAACCCAGT ACCTTATTAT ATTCTTATCA TCCCCTTCAA TTAAGCACCA   
  
  
- TGCTAATAAT TAATCATCCA CCCTTTGCTT ATCATAATTA GTTAAATTTA TATAATACAC TCTCTGTGGA   
  
  
- CCGACCTGAC GCCTTGCTAT GTATGTTGAT TGCTCATGAA CCCCAACTGT TTGGATATTG GACCAACTGG   
  
  
- GTTAATTTAC TTACCATTTG ATTATCATGT TCTTGTTGTA CTTGATAGTG ATCTTAATTA GATGAACATG   
  
  
- ATTAAGTTCA TTTGATCGGA AATAATGACT GAAGCAAATC TAGGGCACCA TATGATGGTG AAAAATCATA   
  
  
- AGGCCAGCAG TTAAATATTT AAGATGTCAT GGATCAGCAG ATTAATAAAG ATATCTGAAT TTTGATTAAC   
  
  
- CCATTTCAAC ATAGGAGATC GCATAAAATG ATTTAGCTAT CTTAGCTAGC ATGCAAAAGC AAACAATAAT   
  
  
- TAGATGATTT TTTAAAAATT AAATTTCCA

+     TATCCAT/C-motif

| Site Name | Organism | Position | Strand | Matrix score. | sequence | function |
| --- | --- | --- | --- | --- | --- | --- |
| TATCCAT/C-motif | Oryza sativa | 237 | - | 7 | TATCCAT |  |

> 2018/04/13 10:10:12  
+ GACACAGAGG AAGGGGAGAG AGAGAGAGAG AGATAGCTAC CTTTTCAACG TCTTCTAGTT TAAGTTTGTC   
  
  
+ AAATTATCCC TTTCTTTATC CCTTTCTTAG ACGACTTCTA TATATATGCT AGAGATCTTC TACTTCTGGA   
  
  
+ ATTTGGTATA TGTCATTTAA TTCTCTAGGA GGGAGAGAAA GAGAAGTTGA AGATCCTATC TACCTGTCTT   
  
  
+ GAATCTATCT ATACTTTCAT TGGTTTATGG ATATATTTAT ACTGGGTTGG TGAAAGGGAA AGTTGTCGTA   
  
  
+ ATTAACTCTT AAAGATCAGT AAATATAACT CCTTCTCACT CGTATTTAAT TTCTTTTTCT TTCGTCTTCG   
  
  
+ GTTTTTATCT TTAATTCTAT GTCTTTGAGG TAGCCTATCA TCAACCCATA ACACTTTTAA TTGTACAAGA   
  
  
+ TCATTAGTTT TCGTATCTTA AAGTACACAT TCCGAATATC ACCCTTCAAT GACATCACTC CTTCTCCAGT   
  
  
+ TGTTATCTGC TGTTTTCCTA ACTCGTCATT CCCAAAACAA AACGATTTAA CTTACTTTTT CGTCACGTGA   
  
  
+ AAAGAGAGTA ATTTCCTATT TTCCCTTTTG GCCTACTCAT CTGTTATTAT CTCGTTTTAG AGAGTACATT   
  
  
+ CACGGGTTCA CTTTTAATAG TAAGTGTGTA TAGACGCAGA GTTTTCCTTT TTCTTCTCTC TGTTTACCAC   
  
  
+ TATTTATATA GATAGTGAAA GATCCCAAAG AAATCAAAAA GAAAAAAGAT CCCAAAGAAA AACTCATGTA   
  
  
+ TGGGATTACT TTTCTTCATA CATAGTTACC GTTACTACTA CTTAGAACCC GCGTTTAGAG AAGTGACACG   
  
  
+ TAGTAGCCGC CACTTCTATA GTAATACTAA CTACTTTCTC CGGGATAAAC TGAAAACCGT CTCTTTTTCG   
  
  
+ AAGAGTGGAA AGAGGTAGAG GAAAACGATA AAGTGGAAAA GAAATTATGA AGGAGGTTTG GGGTGTAGAT   
  
  
+ GGGTGGTGAG GAGTTTAAGG TTTTGGGTCA TGGAATAATA TAAGAATAGT AGGGGAAGTT AATTCGTGGT   
  
  
+ ACGATTATTA ATTAGTAGGT GGGAAACGAA TAGTATTAAT CAATTTAAAT ATATTATGTG AGAGACACCT   
  
  
+ GGCTGGACTG CGGAACGATA CATACAACTA ACGAGTACTT GGGGTTGACA AACCTATAAC CTGGTTGACC   
  
  
+ CAATTAAATG AATGGTAAAC TAATAGTACA AGAACAACAT GAACTATCAC TAGAATTAAT CTACTTGTAC   
  
  
+ TAATTCAAGT AAACTAGCCT TTATTACTGA CTTCGTTTAG ATCCCGTGGT ATACTACCAC TTTTTAGTAT   
  
  
+ TCCGGTCGTC AATTTATAAA TTCTACAGTA CCTAGTCGTC TAATTATTTC TATAGACTTA AAACTAATTG   
  
  
+ GGTAAAGTTG TATCCTCTAG CGTATTTTAC TAAATCGATA GAATCGATCG TACGTTTTCG TTTGTTATTA   
  
  
+ ATCTACTAAA AAATTTTTAA TTTAAAGGT  

- CTGTGTCTCC TTCCCCTCTC TCTCTCTCTC TCTATCGATG GAAAAGTTGC AGAAGATCAA ATTCAAACAG   
  
  
- TTTAATAGGG AAAGAAATAG GGAAAGAATC TGCTGAAGAT ATATATACGA TCTCTAGAAG ATGAAGACCT   
  
  
- TAAACCATAT ACAGTAAATT AAGAGATCCT CCCTCTCTTT CTCTTCAACT TCTAGGATAG ATGGACAGAA   
  
  
- CTTAGATAGA TATGAAAGTA ACCAAATACC TATATAAATA TGACCCAACC ACTTTCCCTT TCAACAGCAT   
  
  
- TAATTGAGAA TTTCTAGTCA TTTATATTGA GGAAGAGTGA GCATAAATTA AAGAAAAAGA AAGCAGAAGC   
  
  
- CAAAAATAGA AATTAAGATA CAGAAACTCC ATCGGATAGT AGTTGGGTAT TGTGAAAATT AACATGTTCT   
  
  
- AGTAATCAAA AGCATAGAAT TTCATGTGTA AGGCTTATAG TGGGAAGTTA CTGTAGTGAG GAAGAGGTCA   
  
  
- ACAATAGACG ACAAAAGGAT TGAGCAGTAA GGGTTTTGTT TTGCTAAATT GAATGAAAAA GCAGTGCACT   
  
  
- TTTCTCTCAT TAAAGGATAA AAGGGAAAAC CGGATGAGTA GACAATAATA GAGCAAAATC TCTCATGTAA   
  
  
- GTGCCCAAGT GAAAATTATC ATTCACACAT ATCTGCGTCT CAAAAGGAAA AAGAAGAGAG ACAAATGGTG   
  
  
- ATAAATATAT CTATCACTTT CTAGGGTTTC TTTAGTTTTT CTTTTTTCTA GGGTTTCTTT TTGAGTACAT   
  
  
- ACCCTAATGA AAAGAAGTAT GTATCAATGG CAATGATGAT GAATCTTGGG CGCAAATCTC TTCACTGTGC   
  
  
- ATCATCGGCG GTGAAGATAT CATTATGATT GATGAAAGAG GCCCTATTTG ACTTTTGGCA GAGAAAAAGC   
  
  
- TTCTCACCTT TCTCCATCTC CTTTTGCTAT TTCACCTTTT CTTTAATACT TCCTCCAAAC CCCACATCTA   
  
  
- CCCACCACTC CTCAAATTCC AAAACCCAGT ACCTTATTAT ATTCTTATCA TCCCCTTCAA TTAAGCACCA   
  
  
- TGCTAATAAT TAATCATCCA CCCTTTGCTT ATCATAATTA GTTAAATTTA TATAATACAC TCTCTGTGGA   
  
  
- CCGACCTGAC GCCTTGCTAT GTATGTTGAT TGCTCATGAA CCCCAACTGT TTGGATATTG GACCAACTGG   
  
  
- GTTAATTTAC TTACCATTTG ATTATCATGT TCTTGTTGTA CTTGATAGTG ATCTTAATTA GATGAACATG   
  
  
- ATTAAGTTCA TTTGATCGGA AATAATGACT GAAGCAAATC TAGGGCACCA TATGATGGTG AAAAATCATA   
  
  
- AGGCCAGCAG TTAAATATTT AAGATGTCAT GGATCAGCAG ATTAATAAAG ATATCTGAAT TTTGATTAAC   
  
  
- CCATTTCAAC ATAGGAGATC GCATAAAATG ATTTAGCTAT CTTAGCTAGC ATGCAAAAGC AAACAATAAT   
  
  
- TAGATGATTT TTTAAAAATT AAATTTCCA

+     TC-rich repeats

| Site Name | Organism | Position | Strand | Matrix score. | sequence | function |
| --- | --- | --- | --- | --- | --- | --- |
| TC-rich repeats | Nicotiana tabacum | 779 | + | 9 | ATTTTCTTCA | cis-acting element involved in defense and stress responsiveness |

> 2018/04/13 10:10:12  
+ GACACAGAGG AAGGGGAGAG AGAGAGAGAG AGATAGCTAC CTTTTCAACG TCTTCTAGTT TAAGTTTGTC   
  
  
+ AAATTATCCC TTTCTTTATC CCTTTCTTAG ACGACTTCTA TATATATGCT AGAGATCTTC TACTTCTGGA   
  
  
+ ATTTGGTATA TGTCATTTAA TTCTCTAGGA GGGAGAGAAA GAGAAGTTGA AGATCCTATC TACCTGTCTT   
  
  
+ GAATCTATCT ATACTTTCAT TGGTTTATGG ATATATTTAT ACTGGGTTGG TGAAAGGGAA AGTTGTCGTA   
  
  
+ ATTAACTCTT AAAGATCAGT AAATATAACT CCTTCTCACT CGTATTTAAT TTCTTTTTCT TTCGTCTTCG   
  
  
+ GTTTTTATCT TTAATTCTAT GTCTTTGAGG TAGCCTATCA TCAACCCATA ACACTTTTAA TTGTACAAGA   
  
  
+ TCATTAGTTT TCGTATCTTA AAGTACACAT TCCGAATATC ACCCTTCAAT GACATCACTC CTTCTCCAGT   
  
  
+ TGTTATCTGC TGTTTTCCTA ACTCGTCATT CCCAAAACAA AACGATTTAA CTTACTTTTT CGTCACGTGA   
  
  
+ AAAGAGAGTA ATTTCCTATT TTCCCTTTTG GCCTACTCAT CTGTTATTAT CTCGTTTTAG AGAGTACATT   
  
  
+ CACGGGTTCA CTTTTAATAG TAAGTGTGTA TAGACGCAGA GTTTTCCTTT TTCTTCTCTC TGTTTACCAC   
  
  
+ TATTTATATA GATAGTGAAA GATCCCAAAG AAATCAAAAA GAAAAAAGAT CCCAAAGAAA AACTCATGTA   
  
  
+ TGGGATTACT TTTCTTCATA CATAGTTACC GTTACTACTA CTTAGAACCC GCGTTTAGAG AAGTGACACG   
  
  
+ TAGTAGCCGC CACTTCTATA GTAATACTAA CTACTTTCTC CGGGATAAAC TGAAAACCGT CTCTTTTTCG   
  
  
+ AAGAGTGGAA AGAGGTAGAG GAAAACGATA AAGTGGAAAA GAAATTATGA AGGAGGTTTG GGGTGTAGAT   
  
  
+ GGGTGGTGAG GAGTTTAAGG TTTTGGGTCA TGGAATAATA TAAGAATAGT AGGGGAAGTT AATTCGTGGT   
  
  
+ ACGATTATTA ATTAGTAGGT GGGAAACGAA TAGTATTAAT CAATTTAAAT ATATTATGTG AGAGACACCT   
  
  
+ GGCTGGACTG CGGAACGATA CATACAACTA ACGAGTACTT GGGGTTGACA AACCTATAAC CTGGTTGACC   
  
  
+ CAATTAAATG AATGGTAAAC TAATAGTACA AGAACAACAT GAACTATCAC TAGAATTAAT CTACTTGTAC   
  
  
+ TAATTCAAGT AAACTAGCCT TTATTACTGA CTTCGTTTAG ATCCCGTGGT ATACTACCAC TTTTTAGTAT   
  
  
+ TCCGGTCGTC AATTTATAAA TTCTACAGTA CCTAGTCGTC TAATTATTTC TATAGACTTA AAACTAATTG   
  
  
+ GGTAAAGTTG TATCCTCTAG CGTATTTTAC TAAATCGATA GAATCGATCG TACGTTTTCG TTTGTTATTA   
  
  
+ ATCTACTAAA AAATTTTTAA TTTAAAGGT  

- CTGTGTCTCC TTCCCCTCTC TCTCTCTCTC TCTATCGATG GAAAAGTTGC AGAAGATCAA ATTCAAACAG   
  
  
- TTTAATAGGG AAAGAAATAG GGAAAGAATC TGCTGAAGAT ATATATACGA TCTCTAGAAG ATGAAGACCT   
  
  
- TAAACCATAT ACAGTAAATT AAGAGATCCT CCCTCTCTTT CTCTTCAACT TCTAGGATAG ATGGACAGAA   
  
  
- CTTAGATAGA TATGAAAGTA ACCAAATACC TATATAAATA TGACCCAACC ACTTTCCCTT TCAACAGCAT   
  
  
- TAATTGAGAA TTTCTAGTCA TTTATATTGA GGAAGAGTGA GCATAAATTA AAGAAAAAGA AAGCAGAAGC   
  
  
- CAAAAATAGA AATTAAGATA CAGAAACTCC ATCGGATAGT AGTTGGGTAT TGTGAAAATT AACATGTTCT   
  
  
- AGTAATCAAA AGCATAGAAT TTCATGTGTA AGGCTTATAG TGGGAAGTTA CTGTAGTGAG GAAGAGGTCA   
  
  
- ACAATAGACG ACAAAAGGAT TGAGCAGTAA GGGTTTTGTT TTGCTAAATT GAATGAAAAA GCAGTGCACT   
  
  
- TTTCTCTCAT TAAAGGATAA AAGGGAAAAC CGGATGAGTA GACAATAATA GAGCAAAATC TCTCATGTAA   
  
  
- GTGCCCAAGT GAAAATTATC ATTCACACAT ATCTGCGTCT CAAAAGGAAA AAGAAGAGAG ACAAATGGTG   
  
  
- ATAAATATAT CTATCACTTT CTAGGGTTTC TTTAGTTTTT CTTTTTTCTA GGGTTTCTTT TTGAGTACAT   
  
  
- ACCCTAATGA AAAGAAGTAT GTATCAATGG CAATGATGAT GAATCTTGGG CGCAAATCTC TTCACTGTGC   
  
  
- ATCATCGGCG GTGAAGATAT CATTATGATT GATGAAAGAG GCCCTATTTG ACTTTTGGCA GAGAAAAAGC   
  
  
- TTCTCACCTT TCTCCATCTC CTTTTGCTAT TTCACCTTTT CTTTAATACT TCCTCCAAAC CCCACATCTA   
  
  
- CCCACCACTC CTCAAATTCC AAAACCCAGT ACCTTATTAT ATTCTTATCA TCCCCTTCAA TTAAGCACCA   
  
  
- TGCTAATAAT TAATCATCCA CCCTTTGCTT ATCATAATTA GTTAAATTTA TATAATACAC TCTCTGTGGA   
  
  
- CCGACCTGAC GCCTTGCTAT GTATGTTGAT TGCTCATGAA CCCCAACTGT TTGGATATTG GACCAACTGG   
  
  
- GTTAATTTAC TTACCATTTG ATTATCATGT TCTTGTTGTA CTTGATAGTG ATCTTAATTA GATGAACATG   
  
  
- ATTAAGTTCA TTTGATCGGA AATAATGACT GAAGCAAATC TAGGGCACCA TATGATGGTG AAAAATCATA   
  
  
- AGGCCAGCAG TTAAATATTT AAGATGTCAT GGATCAGCAG ATTAATAAAG ATATCTGAAT TTTGATTAAC   
  
  
- CCATTTCAAC ATAGGAGATC GCATAAAATG ATTTAGCTAT CTTAGCTAGC ATGCAAAAGC AAACAATAAT   
  
  
- TAGATGATTT TTTAAAAATT AAATTTCCA

+     TCA-element

| Site Name | Organism | Position | Strand | Matrix score. | sequence | function |
| --- | --- | --- | --- | --- | --- | --- |
| TCA-element | Brassica oleracea | 679 | - | 9 | GAGAAGAATA | cis-acting element involved in salicylic acid responsiveness |

> 2018/04/13 10:10:12  
+ GACACAGAGG AAGGGGAGAG AGAGAGAGAG AGATAGCTAC CTTTTCAACG TCTTCTAGTT TAAGTTTGTC   
  
  
+ AAATTATCCC TTTCTTTATC CCTTTCTTAG ACGACTTCTA TATATATGCT AGAGATCTTC TACTTCTGGA   
  
  
+ ATTTGGTATA TGTCATTTAA TTCTCTAGGA GGGAGAGAAA GAGAAGTTGA AGATCCTATC TACCTGTCTT   
  
  
+ GAATCTATCT ATACTTTCAT TGGTTTATGG ATATATTTAT ACTGGGTTGG TGAAAGGGAA AGTTGTCGTA   
  
  
+ ATTAACTCTT AAAGATCAGT AAATATAACT CCTTCTCACT CGTATTTAAT TTCTTTTTCT TTCGTCTTCG   
  
  
+ GTTTTTATCT TTAATTCTAT GTCTTTGAGG TAGCCTATCA TCAACCCATA ACACTTTTAA TTGTACAAGA   
  
  
+ TCATTAGTTT TCGTATCTTA AAGTACACAT TCCGAATATC ACCCTTCAAT GACATCACTC CTTCTCCAGT   
  
  
+ TGTTATCTGC TGTTTTCCTA ACTCGTCATT CCCAAAACAA AACGATTTAA CTTACTTTTT CGTCACGTGA   
  
  
+ AAAGAGAGTA ATTTCCTATT TTCCCTTTTG GCCTACTCAT CTGTTATTAT CTCGTTTTAG AGAGTACATT   
  
  
+ CACGGGTTCA CTTTTAATAG TAAGTGTGTA TAGACGCAGA GTTTTCCTTT TTCTTCTCTC TGTTTACCAC   
  
  
+ TATTTATATA GATAGTGAAA GATCCCAAAG AAATCAAAAA GAAAAAAGAT CCCAAAGAAA AACTCATGTA   
  
  
+ TGGGATTACT TTTCTTCATA CATAGTTACC GTTACTACTA CTTAGAACCC GCGTTTAGAG AAGTGACACG   
  
  
+ TAGTAGCCGC CACTTCTATA GTAATACTAA CTACTTTCTC CGGGATAAAC TGAAAACCGT CTCTTTTTCG   
  
  
+ AAGAGTGGAA AGAGGTAGAG GAAAACGATA AAGTGGAAAA GAAATTATGA AGGAGGTTTG GGGTGTAGAT   
  
  
+ GGGTGGTGAG GAGTTTAAGG TTTTGGGTCA TGGAATAATA TAAGAATAGT AGGGGAAGTT AATTCGTGGT   
  
  
+ ACGATTATTA ATTAGTAGGT GGGAAACGAA TAGTATTAAT CAATTTAAAT ATATTATGTG AGAGACACCT   
  
  
+ GGCTGGACTG CGGAACGATA CATACAACTA ACGAGTACTT GGGGTTGACA AACCTATAAC CTGGTTGACC   
  
  
+ CAATTAAATG AATGGTAAAC TAATAGTACA AGAACAACAT GAACTATCAC TAGAATTAAT CTACTTGTAC   
  
  
+ TAATTCAAGT AAACTAGCCT TTATTACTGA CTTCGTTTAG ATCCCGTGGT ATACTACCAC TTTTTAGTAT   
  
  
+ TCCGGTCGTC AATTTATAAA TTCTACAGTA CCTAGTCGTC TAATTATTTC TATAGACTTA AAACTAATTG   
  
  
+ GGTAAAGTTG TATCCTCTAG CGTATTTTAC TAAATCGATA GAATCGATCG TACGTTTTCG TTTGTTATTA   
  
  
+ ATCTACTAAA AAATTTTTAA TTTAAAGGT  

- CTGTGTCTCC TTCCCCTCTC TCTCTCTCTC TCTATCGATG GAAAAGTTGC AGAAGATCAA ATTCAAACAG   
  
  
- TTTAATAGGG AAAGAAATAG GGAAAGAATC TGCTGAAGAT ATATATACGA TCTCTAGAAG ATGAAGACCT   
  
  
- TAAACCATAT ACAGTAAATT AAGAGATCCT CCCTCTCTTT CTCTTCAACT TCTAGGATAG ATGGACAGAA   
  
  
- CTTAGATAGA TATGAAAGTA ACCAAATACC TATATAAATA TGACCCAACC ACTTTCCCTT TCAACAGCAT   
  
  
- TAATTGAGAA TTTCTAGTCA TTTATATTGA GGAAGAGTGA GCATAAATTA AAGAAAAAGA AAGCAGAAGC   
  
  
- CAAAAATAGA AATTAAGATA CAGAAACTCC ATCGGATAGT AGTTGGGTAT TGTGAAAATT AACATGTTCT   
  
  
- AGTAATCAAA AGCATAGAAT TTCATGTGTA AGGCTTATAG TGGGAAGTTA CTGTAGTGAG GAAGAGGTCA   
  
  
- ACAATAGACG ACAAAAGGAT TGAGCAGTAA GGGTTTTGTT TTGCTAAATT GAATGAAAAA GCAGTGCACT   
  
  
- TTTCTCTCAT TAAAGGATAA AAGGGAAAAC CGGATGAGTA GACAATAATA GAGCAAAATC TCTCATGTAA   
  
  
- GTGCCCAAGT GAAAATTATC ATTCACACAT ATCTGCGTCT CAAAAGGAAA AAGAAGAGAG ACAAATGGTG   
  
  
- ATAAATATAT CTATCACTTT CTAGGGTTTC TTTAGTTTTT CTTTTTTCTA GGGTTTCTTT TTGAGTACAT   
  
  
- ACCCTAATGA AAAGAAGTAT GTATCAATGG CAATGATGAT GAATCTTGGG CGCAAATCTC TTCACTGTGC   
  
  
- ATCATCGGCG GTGAAGATAT CATTATGATT GATGAAAGAG GCCCTATTTG ACTTTTGGCA GAGAAAAAGC   
  
  
- TTCTCACCTT TCTCCATCTC CTTTTGCTAT TTCACCTTTT CTTTAATACT TCCTCCAAAC CCCACATCTA   
  
  
- CCCACCACTC CTCAAATTCC AAAACCCAGT ACCTTATTAT ATTCTTATCA TCCCCTTCAA TTAAGCACCA   
  
  
- TGCTAATAAT TAATCATCCA CCCTTTGCTT ATCATAATTA GTTAAATTTA TATAATACAC TCTCTGTGGA   
  
  
- CCGACCTGAC GCCTTGCTAT GTATGTTGAT TGCTCATGAA CCCCAACTGT TTGGATATTG GACCAACTGG   
  
  
- GTTAATTTAC TTACCATTTG ATTATCATGT TCTTGTTGTA CTTGATAGTG ATCTTAATTA GATGAACATG   
  
  
- ATTAAGTTCA TTTGATCGGA AATAATGACT GAAGCAAATC TAGGGCACCA TATGATGGTG AAAAATCATA   
  
  
- AGGCCAGCAG TTAAATATTT AAGATGTCAT GGATCAGCAG ATTAATAAAG ATATCTGAAT TTTGATTAAC   
  
  
- CCATTTCAAC ATAGGAGATC GCATAAAATG ATTTAGCTAT CTTAGCTAGC ATGCAAAAGC AAACAATAAT   
  
  
- TAGATGATTT TTTAAAAATT AAATTTCCA

+     TCCC-motif

| Site Name | Organism | Position | Strand | Matrix score. | sequence | function |
| --- | --- | --- | --- | --- | --- | --- |
| TCCC-motif | Spinacia oleracea | 170 | - | 7 | TCTCCCT | part of a light responsive element |

> 2018/04/13 10:10:12  
+ GACACAGAGG AAGGGGAGAG AGAGAGAGAG AGATAGCTAC CTTTTCAACG TCTTCTAGTT TAAGTTTGTC   
  
  
+ AAATTATCCC TTTCTTTATC CCTTTCTTAG ACGACTTCTA TATATATGCT AGAGATCTTC TACTTCTGGA   
  
  
+ ATTTGGTATA TGTCATTTAA TTCTCTAGGA GGGAGAGAAA GAGAAGTTGA AGATCCTATC TACCTGTCTT   
  
  
+ GAATCTATCT ATACTTTCAT TGGTTTATGG ATATATTTAT ACTGGGTTGG TGAAAGGGAA AGTTGTCGTA   
  
  
+ ATTAACTCTT AAAGATCAGT AAATATAACT CCTTCTCACT CGTATTTAAT TTCTTTTTCT TTCGTCTTCG   
  
  
+ GTTTTTATCT TTAATTCTAT GTCTTTGAGG TAGCCTATCA TCAACCCATA ACACTTTTAA TTGTACAAGA   
  
  
+ TCATTAGTTT TCGTATCTTA AAGTACACAT TCCGAATATC ACCCTTCAAT GACATCACTC CTTCTCCAGT   
  
  
+ TGTTATCTGC TGTTTTCCTA ACTCGTCATT CCCAAAACAA AACGATTTAA CTTACTTTTT CGTCACGTGA   
  
  
+ AAAGAGAGTA ATTTCCTATT TTCCCTTTTG GCCTACTCAT CTGTTATTAT CTCGTTTTAG AGAGTACATT   
  
  
+ CACGGGTTCA CTTTTAATAG TAAGTGTGTA TAGACGCAGA GTTTTCCTTT TTCTTCTCTC TGTTTACCAC   
  
  
+ TATTTATATA GATAGTGAAA GATCCCAAAG AAATCAAAAA GAAAAAAGAT CCCAAAGAAA AACTCATGTA   
  
  
+ TGGGATTACT TTTCTTCATA CATAGTTACC GTTACTACTA CTTAGAACCC GCGTTTAGAG AAGTGACACG   
  
  
+ TAGTAGCCGC CACTTCTATA GTAATACTAA CTACTTTCTC CGGGATAAAC TGAAAACCGT CTCTTTTTCG   
  
  
+ AAGAGTGGAA AGAGGTAGAG GAAAACGATA AAGTGGAAAA GAAATTATGA AGGAGGTTTG GGGTGTAGAT   
  
  
+ GGGTGGTGAG GAGTTTAAGG TTTTGGGTCA TGGAATAATA TAAGAATAGT AGGGGAAGTT AATTCGTGGT   
  
  
+ ACGATTATTA ATTAGTAGGT GGGAAACGAA TAGTATTAAT CAATTTAAAT ATATTATGTG AGAGACACCT   
  
  
+ GGCTGGACTG CGGAACGATA CATACAACTA ACGAGTACTT GGGGTTGACA AACCTATAAC CTGGTTGACC   
  
  
+ CAATTAAATG AATGGTAAAC TAATAGTACA AGAACAACAT GAACTATCAC TAGAATTAAT CTACTTGTAC   
  
  
+ TAATTCAAGT AAACTAGCCT TTATTACTGA CTTCGTTTAG ATCCCGTGGT ATACTACCAC TTTTTAGTAT   
  
  
+ TCCGGTCGTC AATTTATAAA TTCTACAGTA CCTAGTCGTC TAATTATTTC TATAGACTTA AAACTAATTG   
  
  
+ GGTAAAGTTG TATCCTCTAG CGTATTTTAC TAAATCGATA GAATCGATCG TACGTTTTCG TTTGTTATTA   
  
  
+ ATCTACTAAA AAATTTTTAA TTTAAAGGT  

- CTGTGTCTCC TTCCCCTCTC TCTCTCTCTC TCTATCGATG GAAAAGTTGC AGAAGATCAA ATTCAAACAG   
  
  
- TTTAATAGGG AAAGAAATAG GGAAAGAATC TGCTGAAGAT ATATATACGA TCTCTAGAAG ATGAAGACCT   
  
  
- TAAACCATAT ACAGTAAATT AAGAGATCCT CCCTCTCTTT CTCTTCAACT TCTAGGATAG ATGGACAGAA   
  
  
- CTTAGATAGA TATGAAAGTA ACCAAATACC TATATAAATA TGACCCAACC ACTTTCCCTT TCAACAGCAT   
  
  
- TAATTGAGAA TTTCTAGTCA TTTATATTGA GGAAGAGTGA GCATAAATTA AAGAAAAAGA AAGCAGAAGC   
  
  
- CAAAAATAGA AATTAAGATA CAGAAACTCC ATCGGATAGT AGTTGGGTAT TGTGAAAATT AACATGTTCT   
  
  
- AGTAATCAAA AGCATAGAAT TTCATGTGTA AGGCTTATAG TGGGAAGTTA CTGTAGTGAG GAAGAGGTCA   
  
  
- ACAATAGACG ACAAAAGGAT TGAGCAGTAA GGGTTTTGTT TTGCTAAATT GAATGAAAAA GCAGTGCACT   
  
  
- TTTCTCTCAT TAAAGGATAA AAGGGAAAAC CGGATGAGTA GACAATAATA GAGCAAAATC TCTCATGTAA   
  
  
- GTGCCCAAGT GAAAATTATC ATTCACACAT ATCTGCGTCT CAAAAGGAAA AAGAAGAGAG ACAAATGGTG   
  
  
- ATAAATATAT CTATCACTTT CTAGGGTTTC TTTAGTTTTT CTTTTTTCTA GGGTTTCTTT TTGAGTACAT   
  
  
- ACCCTAATGA AAAGAAGTAT GTATCAATGG CAATGATGAT GAATCTTGGG CGCAAATCTC TTCACTGTGC   
  
  
- ATCATCGGCG GTGAAGATAT CATTATGATT GATGAAAGAG GCCCTATTTG ACTTTTGGCA GAGAAAAAGC   
  
  
- TTCTCACCTT TCTCCATCTC CTTTTGCTAT TTCACCTTTT CTTTAATACT TCCTCCAAAC CCCACATCTA   
  
  
- CCCACCACTC CTCAAATTCC AAAACCCAGT ACCTTATTAT ATTCTTATCA TCCCCTTCAA TTAAGCACCA   
  
  
- TGCTAATAAT TAATCATCCA CCCTTTGCTT ATCATAATTA GTTAAATTTA TATAATACAC TCTCTGTGGA   
  
  
- CCGACCTGAC GCCTTGCTAT GTATGTTGAT TGCTCATGAA CCCCAACTGT TTGGATATTG GACCAACTGG   
  
  
- GTTAATTTAC TTACCATTTG ATTATCATGT TCTTGTTGTA CTTGATAGTG ATCTTAATTA GATGAACATG   
  
  
- ATTAAGTTCA TTTGATCGGA AATAATGACT GAAGCAAATC TAGGGCACCA TATGATGGTG AAAAATCATA   
  
  
- AGGCCAGCAG TTAAATATTT AAGATGTCAT GGATCAGCAG ATTAATAAAG ATATCTGAAT TTTGATTAAC   
  
  
- CCATTTCAAC ATAGGAGATC GCATAAAATG ATTTAGCTAT CTTAGCTAGC ATGCAAAAGC AAACAATAAT   
  
  
- TAGATGATTT TTTAAAAATT AAATTTCCA

+     TGACG-motif

| Site Name | Organism | Position | Strand | Matrix score. | sequence | function |
| --- | --- | --- | --- | --- | --- | --- |
| TGACG-motif | Hordeum vulgare | 551 | - | 5 | TGACG | cis-acting regulatory element involved in the MeJA-responsiveness |
| TGACG-motif | Hordeum vulgare | 514 | - | 5 | TGACG | cis-acting regulatory element involved in the MeJA-responsiveness |
| TGACG-motif | Hordeum vulgare | 1337 | - | 5 | TGACG | cis-acting regulatory element involved in the MeJA-responsiveness |

> 2018/04/13 10:10:12  
+ GACACAGAGG AAGGGGAGAG AGAGAGAGAG AGATAGCTAC CTTTTCAACG TCTTCTAGTT TAAGTTTGTC   
  
  
+ AAATTATCCC TTTCTTTATC CCTTTCTTAG ACGACTTCTA TATATATGCT AGAGATCTTC TACTTCTGGA   
  
  
+ ATTTGGTATA TGTCATTTAA TTCTCTAGGA GGGAGAGAAA GAGAAGTTGA AGATCCTATC TACCTGTCTT   
  
  
+ GAATCTATCT ATACTTTCAT TGGTTTATGG ATATATTTAT ACTGGGTTGG TGAAAGGGAA AGTTGTCGTA   
  
  
+ ATTAACTCTT AAAGATCAGT AAATATAACT CCTTCTCACT CGTATTTAAT TTCTTTTTCT TTCGTCTTCG   
  
  
+ GTTTTTATCT TTAATTCTAT GTCTTTGAGG TAGCCTATCA TCAACCCATA ACACTTTTAA TTGTACAAGA   
  
  
+ TCATTAGTTT TCGTATCTTA AAGTACACAT TCCGAATATC ACCCTTCAAT GACATCACTC CTTCTCCAGT   
  
  
+ TGTTATCTGC TGTTTTCCTA ACTCGTCATT CCCAAAACAA AACGATTTAA CTTACTTTTT CGTCACGTGA   
  
  
+ AAAGAGAGTA ATTTCCTATT TTCCCTTTTG GCCTACTCAT CTGTTATTAT CTCGTTTTAG AGAGTACATT   
  
  
+ CACGGGTTCA CTTTTAATAG TAAGTGTGTA TAGACGCAGA GTTTTCCTTT TTCTTCTCTC TGTTTACCAC   
  
  
+ TATTTATATA GATAGTGAAA GATCCCAAAG AAATCAAAAA GAAAAAAGAT CCCAAAGAAA AACTCATGTA   
  
  
+ TGGGATTACT TTTCTTCATA CATAGTTACC GTTACTACTA CTTAGAACCC GCGTTTAGAG AAGTGACACG   
  
  
+ TAGTAGCCGC CACTTCTATA GTAATACTAA CTACTTTCTC CGGGATAAAC TGAAAACCGT CTCTTTTTCG   
  
  
+ AAGAGTGGAA AGAGGTAGAG GAAAACGATA AAGTGGAAAA GAAATTATGA AGGAGGTTTG GGGTGTAGAT   
  
  
+ GGGTGGTGAG GAGTTTAAGG TTTTGGGTCA TGGAATAATA TAAGAATAGT AGGGGAAGTT AATTCGTGGT   
  
  
+ ACGATTATTA ATTAGTAGGT GGGAAACGAA TAGTATTAAT CAATTTAAAT ATATTATGTG AGAGACACCT   
  
  
+ GGCTGGACTG CGGAACGATA CATACAACTA ACGAGTACTT GGGGTTGACA AACCTATAAC CTGGTTGACC   
  
  
+ CAATTAAATG AATGGTAAAC TAATAGTACA AGAACAACAT GAACTATCAC TAGAATTAAT CTACTTGTAC   
  
  
+ TAATTCAAGT AAACTAGCCT TTATTACTGA CTTCGTTTAG ATCCCGTGGT ATACTACCAC TTTTTAGTAT   
  
  
+ TCCGGTCGTC AATTTATAAA TTCTACAGTA CCTAGTCGTC TAATTATTTC TATAGACTTA AAACTAATTG   
  
  
+ GGTAAAGTTG TATCCTCTAG CGTATTTTAC TAAATCGATA GAATCGATCG TACGTTTTCG TTTGTTATTA   
  
  
+ ATCTACTAAA AAATTTTTAA TTTAAAGGT  

- CTGTGTCTCC TTCCCCTCTC TCTCTCTCTC TCTATCGATG GAAAAGTTGC AGAAGATCAA ATTCAAACAG   
  
  
- TTTAATAGGG AAAGAAATAG GGAAAGAATC TGCTGAAGAT ATATATACGA TCTCTAGAAG ATGAAGACCT   
  
  
- TAAACCATAT ACAGTAAATT AAGAGATCCT CCCTCTCTTT CTCTTCAACT TCTAGGATAG ATGGACAGAA   
  
  
- CTTAGATAGA TATGAAAGTA ACCAAATACC TATATAAATA TGACCCAACC ACTTTCCCTT TCAACAGCAT   
  
  
- TAATTGAGAA TTTCTAGTCA TTTATATTGA GGAAGAGTGA GCATAAATTA AAGAAAAAGA AAGCAGAAGC   
  
  
- CAAAAATAGA AATTAAGATA CAGAAACTCC ATCGGATAGT AGTTGGGTAT TGTGAAAATT AACATGTTCT   
  
  
- AGTAATCAAA AGCATAGAAT TTCATGTGTA AGGCTTATAG TGGGAAGTTA CTGTAGTGAG GAAGAGGTCA   
  
  
- ACAATAGACG ACAAAAGGAT TGAGCAGTAA GGGTTTTGTT TTGCTAAATT GAATGAAAAA GCAGTGCACT   
  
  
- TTTCTCTCAT TAAAGGATAA AAGGGAAAAC CGGATGAGTA GACAATAATA GAGCAAAATC TCTCATGTAA   
  
  
- GTGCCCAAGT GAAAATTATC ATTCACACAT ATCTGCGTCT CAAAAGGAAA AAGAAGAGAG ACAAATGGTG   
  
  
- ATAAATATAT CTATCACTTT CTAGGGTTTC TTTAGTTTTT CTTTTTTCTA GGGTTTCTTT TTGAGTACAT   
  
  
- ACCCTAATGA AAAGAAGTAT GTATCAATGG CAATGATGAT GAATCTTGGG CGCAAATCTC TTCACTGTGC   
  
  
- ATCATCGGCG GTGAAGATAT CATTATGATT GATGAAAGAG GCCCTATTTG ACTTTTGGCA GAGAAAAAGC   
  
  
- TTCTCACCTT TCTCCATCTC CTTTTGCTAT TTCACCTTTT CTTTAATACT TCCTCCAAAC CCCACATCTA   
  
  
- CCCACCACTC CTCAAATTCC AAAACCCAGT ACCTTATTAT ATTCTTATCA TCCCCTTCAA TTAAGCACCA   
  
  
- TGCTAATAAT TAATCATCCA CCCTTTGCTT ATCATAATTA GTTAAATTTA TATAATACAC TCTCTGTGGA   
  
  
- CCGACCTGAC GCCTTGCTAT GTATGTTGAT TGCTCATGAA CCCCAACTGT TTGGATATTG GACCAACTGG   
  
  
- GTTAATTTAC TTACCATTTG ATTATCATGT TCTTGTTGTA CTTGATAGTG ATCTTAATTA GATGAACATG   
  
  
- ATTAAGTTCA TTTGATCGGA AATAATGACT GAAGCAAATC TAGGGCACCA TATGATGGTG AAAAATCATA   
  
  
- AGGCCAGCAG TTAAATATTT AAGATGTCAT GGATCAGCAG ATTAATAAAG ATATCTGAAT TTTGATTAAC   
  
  
- CCATTTCAAC ATAGGAGATC GCATAAAATG ATTTAGCTAT CTTAGCTAGC ATGCAAAAGC AAACAATAAT   
  
  
- TAGATGATTT TTTAAAAATT AAATTTCCA

+     Unnamed\_\_1

| Site Name | Organism | Position | Strand | Matrix score. | sequence | function |
| --- | --- | --- | --- | --- | --- | --- |
| Unnamed\_\_1 | Zea mays | 1305 | + | 5 | CGTGG |  |
| Unnamed\_\_1 | Zea mays | 1045 | + | 5 | CGTGG |  |

> 2018/04/13 10:10:12  
+ GACACAGAGG AAGGGGAGAG AGAGAGAGAG AGATAGCTAC CTTTTCAACG TCTTCTAGTT TAAGTTTGTC   
  
  
+ AAATTATCCC TTTCTTTATC CCTTTCTTAG ACGACTTCTA TATATATGCT AGAGATCTTC TACTTCTGGA   
  
  
+ ATTTGGTATA TGTCATTTAA TTCTCTAGGA GGGAGAGAAA GAGAAGTTGA AGATCCTATC TACCTGTCTT   
  
  
+ GAATCTATCT ATACTTTCAT TGGTTTATGG ATATATTTAT ACTGGGTTGG TGAAAGGGAA AGTTGTCGTA   
  
  
+ ATTAACTCTT AAAGATCAGT AAATATAACT CCTTCTCACT CGTATTTAAT TTCTTTTTCT TTCGTCTTCG   
  
  
+ GTTTTTATCT TTAATTCTAT GTCTTTGAGG TAGCCTATCA TCAACCCATA ACACTTTTAA TTGTACAAGA   
  
  
+ TCATTAGTTT TCGTATCTTA AAGTACACAT TCCGAATATC ACCCTTCAAT GACATCACTC CTTCTCCAGT   
  
  
+ TGTTATCTGC TGTTTTCCTA ACTCGTCATT CCCAAAACAA AACGATTTAA CTTACTTTTT CGTCACGTGA   
  
  
+ AAAGAGAGTA ATTTCCTATT TTCCCTTTTG GCCTACTCAT CTGTTATTAT CTCGTTTTAG AGAGTACATT   
  
  
+ CACGGGTTCA CTTTTAATAG TAAGTGTGTA TAGACGCAGA GTTTTCCTTT TTCTTCTCTC TGTTTACCAC   
  
  
+ TATTTATATA GATAGTGAAA GATCCCAAAG AAATCAAAAA GAAAAAAGAT CCCAAAGAAA AACTCATGTA   
  
  
+ TGGGATTACT TTTCTTCATA CATAGTTACC GTTACTACTA CTTAGAACCC GCGTTTAGAG AAGTGACACG   
  
  
+ TAGTAGCCGC CACTTCTATA GTAATACTAA CTACTTTCTC CGGGATAAAC TGAAAACCGT CTCTTTTTCG   
  
  
+ AAGAGTGGAA AGAGGTAGAG GAAAACGATA AAGTGGAAAA GAAATTATGA AGGAGGTTTG GGGTGTAGAT   
  
  
+ GGGTGGTGAG GAGTTTAAGG TTTTGGGTCA TGGAATAATA TAAGAATAGT AGGGGAAGTT AATTCGTGGT   
  
  
+ ACGATTATTA ATTAGTAGGT GGGAAACGAA TAGTATTAAT CAATTTAAAT ATATTATGTG AGAGACACCT   
  
  
+ GGCTGGACTG CGGAACGATA CATACAACTA ACGAGTACTT GGGGTTGACA AACCTATAAC CTGGTTGACC   
  
  
+ CAATTAAATG AATGGTAAAC TAATAGTACA AGAACAACAT GAACTATCAC TAGAATTAAT CTACTTGTAC   
  
  
+ TAATTCAAGT AAACTAGCCT TTATTACTGA CTTCGTTTAG ATCCCGTGGT ATACTACCAC TTTTTAGTAT   
  
  
+ TCCGGTCGTC AATTTATAAA TTCTACAGTA CCTAGTCGTC TAATTATTTC TATAGACTTA AAACTAATTG   
  
  
+ GGTAAAGTTG TATCCTCTAG CGTATTTTAC TAAATCGATA GAATCGATCG TACGTTTTCG TTTGTTATTA   
  
  
+ ATCTACTAAA AAATTTTTAA TTTAAAGGT  

- CTGTGTCTCC TTCCCCTCTC TCTCTCTCTC TCTATCGATG GAAAAGTTGC AGAAGATCAA ATTCAAACAG   
  
  
- TTTAATAGGG AAAGAAATAG GGAAAGAATC TGCTGAAGAT ATATATACGA TCTCTAGAAG ATGAAGACCT   
  
  
- TAAACCATAT ACAGTAAATT AAGAGATCCT CCCTCTCTTT CTCTTCAACT TCTAGGATAG ATGGACAGAA   
  
  
- CTTAGATAGA TATGAAAGTA ACCAAATACC TATATAAATA TGACCCAACC ACTTTCCCTT TCAACAGCAT   
  
  
- TAATTGAGAA TTTCTAGTCA TTTATATTGA GGAAGAGTGA GCATAAATTA AAGAAAAAGA AAGCAGAAGC   
  
  
- CAAAAATAGA AATTAAGATA CAGAAACTCC ATCGGATAGT AGTTGGGTAT TGTGAAAATT AACATGTTCT   
  
  
- AGTAATCAAA AGCATAGAAT TTCATGTGTA AGGCTTATAG TGGGAAGTTA CTGTAGTGAG GAAGAGGTCA   
  
  
- ACAATAGACG ACAAAAGGAT TGAGCAGTAA GGGTTTTGTT TTGCTAAATT GAATGAAAAA GCAGTGCACT   
  
  
- TTTCTCTCAT TAAAGGATAA AAGGGAAAAC CGGATGAGTA GACAATAATA GAGCAAAATC TCTCATGTAA   
  
  
- GTGCCCAAGT GAAAATTATC ATTCACACAT ATCTGCGTCT CAAAAGGAAA AAGAAGAGAG ACAAATGGTG   
  
  
- ATAAATATAT CTATCACTTT CTAGGGTTTC TTTAGTTTTT CTTTTTTCTA GGGTTTCTTT TTGAGTACAT   
  
  
- ACCCTAATGA AAAGAAGTAT GTATCAATGG CAATGATGAT GAATCTTGGG CGCAAATCTC TTCACTGTGC   
  
  
- ATCATCGGCG GTGAAGATAT CATTATGATT GATGAAAGAG GCCCTATTTG ACTTTTGGCA GAGAAAAAGC   
  
  
- TTCTCACCTT TCTCCATCTC CTTTTGCTAT TTCACCTTTT CTTTAATACT TCCTCCAAAC CCCACATCTA   
  
  
- CCCACCACTC CTCAAATTCC AAAACCCAGT ACCTTATTAT ATTCTTATCA TCCCCTTCAA TTAAGCACCA   
  
  
- TGCTAATAAT TAATCATCCA CCCTTTGCTT ATCATAATTA GTTAAATTTA TATAATACAC TCTCTGTGGA   
  
  
- CCGACCTGAC GCCTTGCTAT GTATGTTGAT TGCTCATGAA CCCCAACTGT TTGGATATTG GACCAACTGG   
  
  
- GTTAATTTAC TTACCATTTG ATTATCATGT TCTTGTTGTA CTTGATAGTG ATCTTAATTA GATGAACATG   
  
  
- ATTAAGTTCA TTTGATCGGA AATAATGACT GAAGCAAATC TAGGGCACCA TATGATGGTG AAAAATCATA   
  
  
- AGGCCAGCAG TTAAATATTT AAGATGTCAT GGATCAGCAG ATTAATAAAG ATATCTGAAT TTTGATTAAC   
  
  
- CCATTTCAAC ATAGGAGATC GCATAAAATG ATTTAGCTAT CTTAGCTAGC ATGCAAAAGC AAACAATAAT   
  
  
- TAGATGATTT TTTAAAAATT AAATTTCCA

+     Unnamed\_\_3

| Site Name | Organism | Position | Strand | Matrix score. | sequence | function |
| --- | --- | --- | --- | --- | --- | --- |
| Unnamed\_\_3 | Zea mays | 1305 | + | 5 | CGTGG |  |
| Unnamed\_\_3 | Zea mays | 1045 | + | 5 | CGTGG |  |

> 2018/04/13 10:10:12  
+ GACACAGAGG AAGGGGAGAG AGAGAGAGAG AGATAGCTAC CTTTTCAACG TCTTCTAGTT TAAGTTTGTC   
  
  
+ AAATTATCCC TTTCTTTATC CCTTTCTTAG ACGACTTCTA TATATATGCT AGAGATCTTC TACTTCTGGA   
  
  
+ ATTTGGTATA TGTCATTTAA TTCTCTAGGA GGGAGAGAAA GAGAAGTTGA AGATCCTATC TACCTGTCTT   
  
  
+ GAATCTATCT ATACTTTCAT TGGTTTATGG ATATATTTAT ACTGGGTTGG TGAAAGGGAA AGTTGTCGTA   
  
  
+ ATTAACTCTT AAAGATCAGT AAATATAACT CCTTCTCACT CGTATTTAAT TTCTTTTTCT TTCGTCTTCG   
  
  
+ GTTTTTATCT TTAATTCTAT GTCTTTGAGG TAGCCTATCA TCAACCCATA ACACTTTTAA TTGTACAAGA   
  
  
+ TCATTAGTTT TCGTATCTTA AAGTACACAT TCCGAATATC ACCCTTCAAT GACATCACTC CTTCTCCAGT   
  
  
+ TGTTATCTGC TGTTTTCCTA ACTCGTCATT CCCAAAACAA AACGATTTAA CTTACTTTTT CGTCACGTGA   
  
  
+ AAAGAGAGTA ATTTCCTATT TTCCCTTTTG GCCTACTCAT CTGTTATTAT CTCGTTTTAG AGAGTACATT   
  
  
+ CACGGGTTCA CTTTTAATAG TAAGTGTGTA TAGACGCAGA GTTTTCCTTT TTCTTCTCTC TGTTTACCAC   
  
  
+ TATTTATATA GATAGTGAAA GATCCCAAAG AAATCAAAAA GAAAAAAGAT CCCAAAGAAA AACTCATGTA   
  
  
+ TGGGATTACT TTTCTTCATA CATAGTTACC GTTACTACTA CTTAGAACCC GCGTTTAGAG AAGTGACACG   
  
  
+ TAGTAGCCGC CACTTCTATA GTAATACTAA CTACTTTCTC CGGGATAAAC TGAAAACCGT CTCTTTTTCG   
  
  
+ AAGAGTGGAA AGAGGTAGAG GAAAACGATA AAGTGGAAAA GAAATTATGA AGGAGGTTTG GGGTGTAGAT   
  
  
+ GGGTGGTGAG GAGTTTAAGG TTTTGGGTCA TGGAATAATA TAAGAATAGT AGGGGAAGTT AATTCGTGGT   
  
  
+ ACGATTATTA ATTAGTAGGT GGGAAACGAA TAGTATTAAT CAATTTAAAT ATATTATGTG AGAGACACCT   
  
  
+ GGCTGGACTG CGGAACGATA CATACAACTA ACGAGTACTT GGGGTTGACA AACCTATAAC CTGGTTGACC   
  
  
+ CAATTAAATG AATGGTAAAC TAATAGTACA AGAACAACAT GAACTATCAC TAGAATTAAT CTACTTGTAC   
  
  
+ TAATTCAAGT AAACTAGCCT TTATTACTGA CTTCGTTTAG ATCCCGTGGT ATACTACCAC TTTTTAGTAT   
  
  
+ TCCGGTCGTC AATTTATAAA TTCTACAGTA CCTAGTCGTC TAATTATTTC TATAGACTTA AAACTAATTG   
  
  
+ GGTAAAGTTG TATCCTCTAG CGTATTTTAC TAAATCGATA GAATCGATCG TACGTTTTCG TTTGTTATTA   
  
  
+ ATCTACTAAA AAATTTTTAA TTTAAAGGT  

- CTGTGTCTCC TTCCCCTCTC TCTCTCTCTC TCTATCGATG GAAAAGTTGC AGAAGATCAA ATTCAAACAG   
  
  
- TTTAATAGGG AAAGAAATAG GGAAAGAATC TGCTGAAGAT ATATATACGA TCTCTAGAAG ATGAAGACCT   
  
  
- TAAACCATAT ACAGTAAATT AAGAGATCCT CCCTCTCTTT CTCTTCAACT TCTAGGATAG ATGGACAGAA   
  
  
- CTTAGATAGA TATGAAAGTA ACCAAATACC TATATAAATA TGACCCAACC ACTTTCCCTT TCAACAGCAT   
  
  
- TAATTGAGAA TTTCTAGTCA TTTATATTGA GGAAGAGTGA GCATAAATTA AAGAAAAAGA AAGCAGAAGC   
  
  
- CAAAAATAGA AATTAAGATA CAGAAACTCC ATCGGATAGT AGTTGGGTAT TGTGAAAATT AACATGTTCT   
  
  
- AGTAATCAAA AGCATAGAAT TTCATGTGTA AGGCTTATAG TGGGAAGTTA CTGTAGTGAG GAAGAGGTCA   
  
  
- ACAATAGACG ACAAAAGGAT TGAGCAGTAA GGGTTTTGTT TTGCTAAATT GAATGAAAAA GCAGTGCACT   
  
  
- TTTCTCTCAT TAAAGGATAA AAGGGAAAAC CGGATGAGTA GACAATAATA GAGCAAAATC TCTCATGTAA   
  
  
- GTGCCCAAGT GAAAATTATC ATTCACACAT ATCTGCGTCT CAAAAGGAAA AAGAAGAGAG ACAAATGGTG   
  
  
- ATAAATATAT CTATCACTTT CTAGGGTTTC TTTAGTTTTT CTTTTTTCTA GGGTTTCTTT TTGAGTACAT   
  
  
- ACCCTAATGA AAAGAAGTAT GTATCAATGG CAATGATGAT GAATCTTGGG CGCAAATCTC TTCACTGTGC   
  
  
- ATCATCGGCG GTGAAGATAT CATTATGATT GATGAAAGAG GCCCTATTTG ACTTTTGGCA GAGAAAAAGC   
  
  
- TTCTCACCTT TCTCCATCTC CTTTTGCTAT TTCACCTTTT CTTTAATACT TCCTCCAAAC CCCACATCTA   
  
  
- CCCACCACTC CTCAAATTCC AAAACCCAGT ACCTTATTAT ATTCTTATCA TCCCCTTCAA TTAAGCACCA   
  
  
- TGCTAATAAT TAATCATCCA CCCTTTGCTT ATCATAATTA GTTAAATTTA TATAATACAC TCTCTGTGGA   
  
  
- CCGACCTGAC GCCTTGCTAT GTATGTTGAT TGCTCATGAA CCCCAACTGT TTGGATATTG GACCAACTGG   
  
  
- GTTAATTTAC TTACCATTTG ATTATCATGT TCTTGTTGTA CTTGATAGTG ATCTTAATTA GATGAACATG   
  
  
- ATTAAGTTCA TTTGATCGGA AATAATGACT GAAGCAAATC TAGGGCACCA TATGATGGTG AAAAATCATA   
  
  
- AGGCCAGCAG TTAAATATTT AAGATGTCAT GGATCAGCAG ATTAATAAAG ATATCTGAAT TTTGATTAAC   
  
  
- CCATTTCAAC ATAGGAGATC GCATAAAATG ATTTAGCTAT CTTAGCTAGC ATGCAAAAGC AAACAATAAT   
  
  
- TAGATGATTT TTTAAAAATT AAATTTCCA

+     Unnamed\_\_4

| Site Name | Organism | Position | Strand | Matrix score. | sequence | function |
| --- | --- | --- | --- | --- | --- | --- |
| Unnamed\_\_4 | Petroselinum hortense | 990 | - | 4 | CTCC |  |
| Unnamed\_\_4 | Petroselinum hortense | 962 | - | 4 | CTCC |  |
| Unnamed\_\_4 | Petroselinum hortense | 878 | + | 4 | CTCC |  |
| Unnamed\_\_4 | Petroselinum hortense | 168 | - | 4 | CTCC |  |
| Unnamed\_\_4 | Petroselinum hortense | 15 | - | 4 | CTCC |  |
| Unnamed\_\_4 | Petroselinum hortense | 484 | + | 4 | CTCC |  |
| Unnamed\_\_4 | Petroselinum hortense | 478 | + | 4 | CTCC |  |
| Unnamed\_\_4 | Petroselinum hortense | 172 | - | 4 | CTCC |  |
| Unnamed\_\_4 | Petroselinum hortense | 309 | + | 4 | CTCC |  |

> 2018/04/13 10:10:12  
+ GACACAGAGG AAGGGGAGAG AGAGAGAGAG AGATAGCTAC CTTTTCAACG TCTTCTAGTT TAAGTTTGTC   
  
  
+ AAATTATCCC TTTCTTTATC CCTTTCTTAG ACGACTTCTA TATATATGCT AGAGATCTTC TACTTCTGGA   
  
  
+ ATTTGGTATA TGTCATTTAA TTCTCTAGGA GGGAGAGAAA GAGAAGTTGA AGATCCTATC TACCTGTCTT   
  
  
+ GAATCTATCT ATACTTTCAT TGGTTTATGG ATATATTTAT ACTGGGTTGG TGAAAGGGAA AGTTGTCGTA   
  
  
+ ATTAACTCTT AAAGATCAGT AAATATAACT CCTTCTCACT CGTATTTAAT TTCTTTTTCT TTCGTCTTCG   
  
  
+ GTTTTTATCT TTAATTCTAT GTCTTTGAGG TAGCCTATCA TCAACCCATA ACACTTTTAA TTGTACAAGA   
  
  
+ TCATTAGTTT TCGTATCTTA AAGTACACAT TCCGAATATC ACCCTTCAAT GACATCACTC CTTCTCCAGT   
  
  
+ TGTTATCTGC TGTTTTCCTA ACTCGTCATT CCCAAAACAA AACGATTTAA CTTACTTTTT CGTCACGTGA   
  
  
+ AAAGAGAGTA ATTTCCTATT TTCCCTTTTG GCCTACTCAT CTGTTATTAT CTCGTTTTAG AGAGTACATT   
  
  
+ CACGGGTTCA CTTTTAATAG TAAGTGTGTA TAGACGCAGA GTTTTCCTTT TTCTTCTCTC TGTTTACCAC   
  
  
+ TATTTATATA GATAGTGAAA GATCCCAAAG AAATCAAAAA GAAAAAAGAT CCCAAAGAAA AACTCATGTA   
  
  
+ TGGGATTACT TTTCTTCATA CATAGTTACC GTTACTACTA CTTAGAACCC GCGTTTAGAG AAGTGACACG   
  
  
+ TAGTAGCCGC CACTTCTATA GTAATACTAA CTACTTTCTC CGGGATAAAC TGAAAACCGT CTCTTTTTCG   
  
  
+ AAGAGTGGAA AGAGGTAGAG GAAAACGATA AAGTGGAAAA GAAATTATGA AGGAGGTTTG GGGTGTAGAT   
  
  
+ GGGTGGTGAG GAGTTTAAGG TTTTGGGTCA TGGAATAATA TAAGAATAGT AGGGGAAGTT AATTCGTGGT   
  
  
+ ACGATTATTA ATTAGTAGGT GGGAAACGAA TAGTATTAAT CAATTTAAAT ATATTATGTG AGAGACACCT   
  
  
+ GGCTGGACTG CGGAACGATA CATACAACTA ACGAGTACTT GGGGTTGACA AACCTATAAC CTGGTTGACC   
  
  
+ CAATTAAATG AATGGTAAAC TAATAGTACA AGAACAACAT GAACTATCAC TAGAATTAAT CTACTTGTAC   
  
  
+ TAATTCAAGT AAACTAGCCT TTATTACTGA CTTCGTTTAG ATCCCGTGGT ATACTACCAC TTTTTAGTAT   
  
  
+ TCCGGTCGTC AATTTATAAA TTCTACAGTA CCTAGTCGTC TAATTATTTC TATAGACTTA AAACTAATTG   
  
  
+ GGTAAAGTTG TATCCTCTAG CGTATTTTAC TAAATCGATA GAATCGATCG TACGTTTTCG TTTGTTATTA   
  
  
+ ATCTACTAAA AAATTTTTAA TTTAAAGGT  

- CTGTGTCTCC TTCCCCTCTC TCTCTCTCTC TCTATCGATG GAAAAGTTGC AGAAGATCAA ATTCAAACAG   
  
  
- TTTAATAGGG AAAGAAATAG GGAAAGAATC TGCTGAAGAT ATATATACGA TCTCTAGAAG ATGAAGACCT   
  
  
- TAAACCATAT ACAGTAAATT AAGAGATCCT CCCTCTCTTT CTCTTCAACT TCTAGGATAG ATGGACAGAA   
  
  
- CTTAGATAGA TATGAAAGTA ACCAAATACC TATATAAATA TGACCCAACC ACTTTCCCTT TCAACAGCAT   
  
  
- TAATTGAGAA TTTCTAGTCA TTTATATTGA GGAAGAGTGA GCATAAATTA AAGAAAAAGA AAGCAGAAGC   
  
  
- CAAAAATAGA AATTAAGATA CAGAAACTCC ATCGGATAGT AGTTGGGTAT TGTGAAAATT AACATGTTCT   
  
  
- AGTAATCAAA AGCATAGAAT TTCATGTGTA AGGCTTATAG TGGGAAGTTA CTGTAGTGAG GAAGAGGTCA   
  
  
- ACAATAGACG ACAAAAGGAT TGAGCAGTAA GGGTTTTGTT TTGCTAAATT GAATGAAAAA GCAGTGCACT   
  
  
- TTTCTCTCAT TAAAGGATAA AAGGGAAAAC CGGATGAGTA GACAATAATA GAGCAAAATC TCTCATGTAA   
  
  
- GTGCCCAAGT GAAAATTATC ATTCACACAT ATCTGCGTCT CAAAAGGAAA AAGAAGAGAG ACAAATGGTG   
  
  
- ATAAATATAT CTATCACTTT CTAGGGTTTC TTTAGTTTTT CTTTTTTCTA GGGTTTCTTT TTGAGTACAT   
  
  
- ACCCTAATGA AAAGAAGTAT GTATCAATGG CAATGATGAT GAATCTTGGG CGCAAATCTC TTCACTGTGC   
  
  
- ATCATCGGCG GTGAAGATAT CATTATGATT GATGAAAGAG GCCCTATTTG ACTTTTGGCA GAGAAAAAGC   
  
  
- TTCTCACCTT TCTCCATCTC CTTTTGCTAT TTCACCTTTT CTTTAATACT TCCTCCAAAC CCCACATCTA   
  
  
- CCCACCACTC CTCAAATTCC AAAACCCAGT ACCTTATTAT ATTCTTATCA TCCCCTTCAA TTAAGCACCA   
  
  
- TGCTAATAAT TAATCATCCA CCCTTTGCTT ATCATAATTA GTTAAATTTA TATAATACAC TCTCTGTGGA   
  
  
- CCGACCTGAC GCCTTGCTAT GTATGTTGAT TGCTCATGAA CCCCAACTGT TTGGATATTG GACCAACTGG   
  
  
- GTTAATTTAC TTACCATTTG ATTATCATGT TCTTGTTGTA CTTGATAGTG ATCTTAATTA GATGAACATG   
  
  
- ATTAAGTTCA TTTGATCGGA AATAATGACT GAAGCAAATC TAGGGCACCA TATGATGGTG AAAAATCATA   
  
  
- AGGCCAGCAG TTAAATATTT AAGATGTCAT GGATCAGCAG ATTAATAAAG ATATCTGAAT TTTGATTAAC   
  
  
- CCATTTCAAC ATAGGAGATC GCATAAAATG ATTTAGCTAT CTTAGCTAGC ATGCAAAAGC AAACAATAAT   
  
  
- TAGATGATTT TTTAAAAATT AAATTTCCA

+     Unnamed\_\_6

| Site Name | Organism | Position | Strand | Matrix score. | sequence | function |
| --- | --- | --- | --- | --- | --- | --- |
| Unnamed\_\_6 | Zea mays | 241 | - | 10 | taTAAATATct |  |

> 2018/04/13 10:10:12  
+ GACACAGAGG AAGGGGAGAG AGAGAGAGAG AGATAGCTAC CTTTTCAACG TCTTCTAGTT TAAGTTTGTC   
  
  
+ AAATTATCCC TTTCTTTATC CCTTTCTTAG ACGACTTCTA TATATATGCT AGAGATCTTC TACTTCTGGA   
  
  
+ ATTTGGTATA TGTCATTTAA TTCTCTAGGA GGGAGAGAAA GAGAAGTTGA AGATCCTATC TACCTGTCTT   
  
  
+ GAATCTATCT ATACTTTCAT TGGTTTATGG ATATATTTAT ACTGGGTTGG TGAAAGGGAA AGTTGTCGTA   
  
  
+ ATTAACTCTT AAAGATCAGT AAATATAACT CCTTCTCACT CGTATTTAAT TTCTTTTTCT TTCGTCTTCG   
  
  
+ GTTTTTATCT TTAATTCTAT GTCTTTGAGG TAGCCTATCA TCAACCCATA ACACTTTTAA TTGTACAAGA   
  
  
+ TCATTAGTTT TCGTATCTTA AAGTACACAT TCCGAATATC ACCCTTCAAT GACATCACTC CTTCTCCAGT   
  
  
+ TGTTATCTGC TGTTTTCCTA ACTCGTCATT CCCAAAACAA AACGATTTAA CTTACTTTTT CGTCACGTGA   
  
  
+ AAAGAGAGTA ATTTCCTATT TTCCCTTTTG GCCTACTCAT CTGTTATTAT CTCGTTTTAG AGAGTACATT   
  
  
+ CACGGGTTCA CTTTTAATAG TAAGTGTGTA TAGACGCAGA GTTTTCCTTT TTCTTCTCTC TGTTTACCAC   
  
  
+ TATTTATATA GATAGTGAAA GATCCCAAAG AAATCAAAAA GAAAAAAGAT CCCAAAGAAA AACTCATGTA   
  
  
+ TGGGATTACT TTTCTTCATA CATAGTTACC GTTACTACTA CTTAGAACCC GCGTTTAGAG AAGTGACACG   
  
  
+ TAGTAGCCGC CACTTCTATA GTAATACTAA CTACTTTCTC CGGGATAAAC TGAAAACCGT CTCTTTTTCG   
  
  
+ AAGAGTGGAA AGAGGTAGAG GAAAACGATA AAGTGGAAAA GAAATTATGA AGGAGGTTTG GGGTGTAGAT   
  
  
+ GGGTGGTGAG GAGTTTAAGG TTTTGGGTCA TGGAATAATA TAAGAATAGT AGGGGAAGTT AATTCGTGGT   
  
  
+ ACGATTATTA ATTAGTAGGT GGGAAACGAA TAGTATTAAT CAATTTAAAT ATATTATGTG AGAGACACCT   
  
  
+ GGCTGGACTG CGGAACGATA CATACAACTA ACGAGTACTT GGGGTTGACA AACCTATAAC CTGGTTGACC   
  
  
+ CAATTAAATG AATGGTAAAC TAATAGTACA AGAACAACAT GAACTATCAC TAGAATTAAT CTACTTGTAC   
  
  
+ TAATTCAAGT AAACTAGCCT TTATTACTGA CTTCGTTTAG ATCCCGTGGT ATACTACCAC TTTTTAGTAT   
  
  
+ TCCGGTCGTC AATTTATAAA TTCTACAGTA CCTAGTCGTC TAATTATTTC TATAGACTTA AAACTAATTG   
  
  
+ GGTAAAGTTG TATCCTCTAG CGTATTTTAC TAAATCGATA GAATCGATCG TACGTTTTCG TTTGTTATTA   
  
  
+ ATCTACTAAA AAATTTTTAA TTTAAAGGT  

- CTGTGTCTCC TTCCCCTCTC TCTCTCTCTC TCTATCGATG GAAAAGTTGC AGAAGATCAA ATTCAAACAG   
  
  
- TTTAATAGGG AAAGAAATAG GGAAAGAATC TGCTGAAGAT ATATATACGA TCTCTAGAAG ATGAAGACCT   
  
  
- TAAACCATAT ACAGTAAATT AAGAGATCCT CCCTCTCTTT CTCTTCAACT TCTAGGATAG ATGGACAGAA   
  
  
- CTTAGATAGA TATGAAAGTA ACCAAATACC TATATAAATA TGACCCAACC ACTTTCCCTT TCAACAGCAT   
  
  
- TAATTGAGAA TTTCTAGTCA TTTATATTGA GGAAGAGTGA GCATAAATTA AAGAAAAAGA AAGCAGAAGC   
  
  
- CAAAAATAGA AATTAAGATA CAGAAACTCC ATCGGATAGT AGTTGGGTAT TGTGAAAATT AACATGTTCT   
  
  
- AGTAATCAAA AGCATAGAAT TTCATGTGTA AGGCTTATAG TGGGAAGTTA CTGTAGTGAG GAAGAGGTCA   
  
  
- ACAATAGACG ACAAAAGGAT TGAGCAGTAA GGGTTTTGTT TTGCTAAATT GAATGAAAAA GCAGTGCACT   
  
  
- TTTCTCTCAT TAAAGGATAA AAGGGAAAAC CGGATGAGTA GACAATAATA GAGCAAAATC TCTCATGTAA   
  
  
- GTGCCCAAGT GAAAATTATC ATTCACACAT ATCTGCGTCT CAAAAGGAAA AAGAAGAGAG ACAAATGGTG   
  
  
- ATAAATATAT CTATCACTTT CTAGGGTTTC TTTAGTTTTT CTTTTTTCTA GGGTTTCTTT TTGAGTACAT   
  
  
- ACCCTAATGA AAAGAAGTAT GTATCAATGG CAATGATGAT GAATCTTGGG CGCAAATCTC TTCACTGTGC   
  
  
- ATCATCGGCG GTGAAGATAT CATTATGATT GATGAAAGAG GCCCTATTTG ACTTTTGGCA GAGAAAAAGC   
  
  
- TTCTCACCTT TCTCCATCTC CTTTTGCTAT TTCACCTTTT CTTTAATACT TCCTCCAAAC CCCACATCTA   
  
  
- CCCACCACTC CTCAAATTCC AAAACCCAGT ACCTTATTAT ATTCTTATCA TCCCCTTCAA TTAAGCACCA   
  
  
- TGCTAATAAT TAATCATCCA CCCTTTGCTT ATCATAATTA GTTAAATTTA TATAATACAC TCTCTGTGGA   
  
  
- CCGACCTGAC GCCTTGCTAT GTATGTTGAT TGCTCATGAA CCCCAACTGT TTGGATATTG GACCAACTGG   
  
  
- GTTAATTTAC TTACCATTTG ATTATCATGT TCTTGTTGTA CTTGATAGTG ATCTTAATTA GATGAACATG   
  
  
- ATTAAGTTCA TTTGATCGGA AATAATGACT GAAGCAAATC TAGGGCACCA TATGATGGTG AAAAATCATA   
  
  
- AGGCCAGCAG TTAAATATTT AAGATGTCAT GGATCAGCAG ATTAATAAAG ATATCTGAAT TTTGATTAAC   
  
  
- CCATTTCAAC ATAGGAGATC GCATAAAATG ATTTAGCTAT CTTAGCTAGC ATGCAAAAGC AAACAATAAT   
  
  
- TAGATGATTT TTTAAAAATT AAATTTCCA

+     W box

| Site Name | Organism | Position | Strand | Matrix score. | sequence | function |
| --- | --- | --- | --- | --- | --- | --- |
| W box | Arabidopsis thaliana | 1185 | + | 6 | TTGACC |  |

> 2018/04/13 10:10:12  
+ GACACAGAGG AAGGGGAGAG AGAGAGAGAG AGATAGCTAC CTTTTCAACG TCTTCTAGTT TAAGTTTGTC   
  
  
+ AAATTATCCC TTTCTTTATC CCTTTCTTAG ACGACTTCTA TATATATGCT AGAGATCTTC TACTTCTGGA   
  
  
+ ATTTGGTATA TGTCATTTAA TTCTCTAGGA GGGAGAGAAA GAGAAGTTGA AGATCCTATC TACCTGTCTT   
  
  
+ GAATCTATCT ATACTTTCAT TGGTTTATGG ATATATTTAT ACTGGGTTGG TGAAAGGGAA AGTTGTCGTA   
  
  
+ ATTAACTCTT AAAGATCAGT AAATATAACT CCTTCTCACT CGTATTTAAT TTCTTTTTCT TTCGTCTTCG   
  
  
+ GTTTTTATCT TTAATTCTAT GTCTTTGAGG TAGCCTATCA TCAACCCATA ACACTTTTAA TTGTACAAGA   
  
  
+ TCATTAGTTT TCGTATCTTA AAGTACACAT TCCGAATATC ACCCTTCAAT GACATCACTC CTTCTCCAGT   
  
  
+ TGTTATCTGC TGTTTTCCTA ACTCGTCATT CCCAAAACAA AACGATTTAA CTTACTTTTT CGTCACGTGA   
  
  
+ AAAGAGAGTA ATTTCCTATT TTCCCTTTTG GCCTACTCAT CTGTTATTAT CTCGTTTTAG AGAGTACATT   
  
  
+ CACGGGTTCA CTTTTAATAG TAAGTGTGTA TAGACGCAGA GTTTTCCTTT TTCTTCTCTC TGTTTACCAC   
  
  
+ TATTTATATA GATAGTGAAA GATCCCAAAG AAATCAAAAA GAAAAAAGAT CCCAAAGAAA AACTCATGTA   
  
  
+ TGGGATTACT TTTCTTCATA CATAGTTACC GTTACTACTA CTTAGAACCC GCGTTTAGAG AAGTGACACG   
  
  
+ TAGTAGCCGC CACTTCTATA GTAATACTAA CTACTTTCTC CGGGATAAAC TGAAAACCGT CTCTTTTTCG   
  
  
+ AAGAGTGGAA AGAGGTAGAG GAAAACGATA AAGTGGAAAA GAAATTATGA AGGAGGTTTG GGGTGTAGAT   
  
  
+ GGGTGGTGAG GAGTTTAAGG TTTTGGGTCA TGGAATAATA TAAGAATAGT AGGGGAAGTT AATTCGTGGT   
  
  
+ ACGATTATTA ATTAGTAGGT GGGAAACGAA TAGTATTAAT CAATTTAAAT ATATTATGTG AGAGACACCT   
  
  
+ GGCTGGACTG CGGAACGATA CATACAACTA ACGAGTACTT GGGGTTGACA AACCTATAAC CTGGTTGACC   
  
  
+ CAATTAAATG AATGGTAAAC TAATAGTACA AGAACAACAT GAACTATCAC TAGAATTAAT CTACTTGTAC   
  
  
+ TAATTCAAGT AAACTAGCCT TTATTACTGA CTTCGTTTAG ATCCCGTGGT ATACTACCAC TTTTTAGTAT   
  
  
+ TCCGGTCGTC AATTTATAAA TTCTACAGTA CCTAGTCGTC TAATTATTTC TATAGACTTA AAACTAATTG   
  
  
+ GGTAAAGTTG TATCCTCTAG CGTATTTTAC TAAATCGATA GAATCGATCG TACGTTTTCG TTTGTTATTA   
  
  
+ ATCTACTAAA AAATTTTTAA TTTAAAGGT  

- CTGTGTCTCC TTCCCCTCTC TCTCTCTCTC TCTATCGATG GAAAAGTTGC AGAAGATCAA ATTCAAACAG   
  
  
- TTTAATAGGG AAAGAAATAG GGAAAGAATC TGCTGAAGAT ATATATACGA TCTCTAGAAG ATGAAGACCT   
  
  
- TAAACCATAT ACAGTAAATT AAGAGATCCT CCCTCTCTTT CTCTTCAACT TCTAGGATAG ATGGACAGAA   
  
  
- CTTAGATAGA TATGAAAGTA ACCAAATACC TATATAAATA TGACCCAACC ACTTTCCCTT TCAACAGCAT   
  
  
- TAATTGAGAA TTTCTAGTCA TTTATATTGA GGAAGAGTGA GCATAAATTA AAGAAAAAGA AAGCAGAAGC   
  
  
- CAAAAATAGA AATTAAGATA CAGAAACTCC ATCGGATAGT AGTTGGGTAT TGTGAAAATT AACATGTTCT   
  
  
- AGTAATCAAA AGCATAGAAT TTCATGTGTA AGGCTTATAG TGGGAAGTTA CTGTAGTGAG GAAGAGGTCA   
  
  
- ACAATAGACG ACAAAAGGAT TGAGCAGTAA GGGTTTTGTT TTGCTAAATT GAATGAAAAA GCAGTGCACT   
  
  
- TTTCTCTCAT TAAAGGATAA AAGGGAAAAC CGGATGAGTA GACAATAATA GAGCAAAATC TCTCATGTAA   
  
  
- GTGCCCAAGT GAAAATTATC ATTCACACAT ATCTGCGTCT CAAAAGGAAA AAGAAGAGAG ACAAATGGTG   
  
  
- ATAAATATAT CTATCACTTT CTAGGGTTTC TTTAGTTTTT CTTTTTTCTA GGGTTTCTTT TTGAGTACAT   
  
  
- ACCCTAATGA AAAGAAGTAT GTATCAATGG CAATGATGAT GAATCTTGGG CGCAAATCTC TTCACTGTGC   
  
  
- ATCATCGGCG GTGAAGATAT CATTATGATT GATGAAAGAG GCCCTATTTG ACTTTTGGCA GAGAAAAAGC   
  
  
- TTCTCACCTT TCTCCATCTC CTTTTGCTAT TTCACCTTTT CTTTAATACT TCCTCCAAAC CCCACATCTA   
  
  
- CCCACCACTC CTCAAATTCC AAAACCCAGT ACCTTATTAT ATTCTTATCA TCCCCTTCAA TTAAGCACCA   
  
  
- TGCTAATAAT TAATCATCCA CCCTTTGCTT ATCATAATTA GTTAAATTTA TATAATACAC TCTCTGTGGA   
  
  
- CCGACCTGAC GCCTTGCTAT GTATGTTGAT TGCTCATGAA CCCCAACTGT TTGGATATTG GACCAACTGG   
  
  
- GTTAATTTAC TTACCATTTG ATTATCATGT TCTTGTTGTA CTTGATAGTG ATCTTAATTA GATGAACATG   
  
  
- ATTAAGTTCA TTTGATCGGA AATAATGACT GAAGCAAATC TAGGGCACCA TATGATGGTG AAAAATCATA   
  
  
- AGGCCAGCAG TTAAATATTT AAGATGTCAT GGATCAGCAG ATTAATAAAG ATATCTGAAT TTTGATTAAC   
  
  
- CCATTTCAAC ATAGGAGATC GCATAAAATG ATTTAGCTAT CTTAGCTAGC ATGCAAAAGC AAACAATAAT   
  
  
- TAGATGATTT TTTAAAAATT AAATTTCCA

+     circadian

| Site Name | Organism | Position | Strand | Matrix score. | sequence | function |
| --- | --- | --- | --- | --- | --- | --- |
| circadian | Lycopersicon esculentum | 726 | + | 9 | CAAAGATATC | cis-acting regulatory element involved in circadian control |
| circadian | Lycopersicon esculentum | 467 | + | 6 | CAANNNNATC | cis-acting regulatory element involved in circadian control |

> 2018/04/13 10:10:12  
+ GACACAGAGG AAGGGGAGAG AGAGAGAGAG AGATAGCTAC CTTTTCAACG TCTTCTAGTT TAAGTTTGTC   
  
  
+ AAATTATCCC TTTCTTTATC CCTTTCTTAG ACGACTTCTA TATATATGCT AGAGATCTTC TACTTCTGGA   
  
  
+ ATTTGGTATA TGTCATTTAA TTCTCTAGGA GGGAGAGAAA GAGAAGTTGA AGATCCTATC TACCTGTCTT   
  
  
+ GAATCTATCT ATACTTTCAT TGGTTTATGG ATATATTTAT ACTGGGTTGG TGAAAGGGAA AGTTGTCGTA   
  
  
+ ATTAACTCTT AAAGATCAGT AAATATAACT CCTTCTCACT CGTATTTAAT TTCTTTTTCT TTCGTCTTCG   
  
  
+ GTTTTTATCT TTAATTCTAT GTCTTTGAGG TAGCCTATCA TCAACCCATA ACACTTTTAA TTGTACAAGA   
  
  
+ TCATTAGTTT TCGTATCTTA AAGTACACAT TCCGAATATC ACCCTTCAAT GACATCACTC CTTCTCCAGT   
  
  
+ TGTTATCTGC TGTTTTCCTA ACTCGTCATT CCCAAAACAA AACGATTTAA CTTACTTTTT CGTCACGTGA   
  
  
+ AAAGAGAGTA ATTTCCTATT TTCCCTTTTG GCCTACTCAT CTGTTATTAT CTCGTTTTAG AGAGTACATT   
  
  
+ CACGGGTTCA CTTTTAATAG TAAGTGTGTA TAGACGCAGA GTTTTCCTTT TTCTTCTCTC TGTTTACCAC   
  
  
+ TATTTATATA GATAGTGAAA GATCCCAAAG AAATCAAAAA GAAAAAAGAT CCCAAAGAAA AACTCATGTA   
  
  
+ TGGGATTACT TTTCTTCATA CATAGTTACC GTTACTACTA CTTAGAACCC GCGTTTAGAG AAGTGACACG   
  
  
+ TAGTAGCCGC CACTTCTATA GTAATACTAA CTACTTTCTC CGGGATAAAC TGAAAACCGT CTCTTTTTCG   
  
  
+ AAGAGTGGAA AGAGGTAGAG GAAAACGATA AAGTGGAAAA GAAATTATGA AGGAGGTTTG GGGTGTAGAT   
  
  
+ GGGTGGTGAG GAGTTTAAGG TTTTGGGTCA TGGAATAATA TAAGAATAGT AGGGGAAGTT AATTCGTGGT   
  
  
+ ACGATTATTA ATTAGTAGGT GGGAAACGAA TAGTATTAAT CAATTTAAAT ATATTATGTG AGAGACACCT   
  
  
+ GGCTGGACTG CGGAACGATA CATACAACTA ACGAGTACTT GGGGTTGACA AACCTATAAC CTGGTTGACC   
  
  
+ CAATTAAATG AATGGTAAAC TAATAGTACA AGAACAACAT GAACTATCAC TAGAATTAAT CTACTTGTAC   
  
  
+ TAATTCAAGT AAACTAGCCT TTATTACTGA CTTCGTTTAG ATCCCGTGGT ATACTACCAC TTTTTAGTAT   
  
  
+ TCCGGTCGTC AATTTATAAA TTCTACAGTA CCTAGTCGTC TAATTATTTC TATAGACTTA AAACTAATTG   
  
  
+ GGTAAAGTTG TATCCTCTAG CGTATTTTAC TAAATCGATA GAATCGATCG TACGTTTTCG TTTGTTATTA   
  
  
+ ATCTACTAAA AAATTTTTAA TTTAAAGGT  

- CTGTGTCTCC TTCCCCTCTC TCTCTCTCTC TCTATCGATG GAAAAGTTGC AGAAGATCAA ATTCAAACAG   
  
  
- TTTAATAGGG AAAGAAATAG GGAAAGAATC TGCTGAAGAT ATATATACGA TCTCTAGAAG ATGAAGACCT   
  
  
- TAAACCATAT ACAGTAAATT AAGAGATCCT CCCTCTCTTT CTCTTCAACT TCTAGGATAG ATGGACAGAA   
  
  
- CTTAGATAGA TATGAAAGTA ACCAAATACC TATATAAATA TGACCCAACC ACTTTCCCTT TCAACAGCAT   
  
  
- TAATTGAGAA TTTCTAGTCA TTTATATTGA GGAAGAGTGA GCATAAATTA AAGAAAAAGA AAGCAGAAGC   
  
  
- CAAAAATAGA AATTAAGATA CAGAAACTCC ATCGGATAGT AGTTGGGTAT TGTGAAAATT AACATGTTCT   
  
  
- AGTAATCAAA AGCATAGAAT TTCATGTGTA AGGCTTATAG TGGGAAGTTA CTGTAGTGAG GAAGAGGTCA   
  
  
- ACAATAGACG ACAAAAGGAT TGAGCAGTAA GGGTTTTGTT TTGCTAAATT GAATGAAAAA GCAGTGCACT   
  
  
- TTTCTCTCAT TAAAGGATAA AAGGGAAAAC CGGATGAGTA GACAATAATA GAGCAAAATC TCTCATGTAA   
  
  
- GTGCCCAAGT GAAAATTATC ATTCACACAT ATCTGCGTCT CAAAAGGAAA AAGAAGAGAG ACAAATGGTG   
  
  
- ATAAATATAT CTATCACTTT CTAGGGTTTC TTTAGTTTTT CTTTTTTCTA GGGTTTCTTT TTGAGTACAT   
  
  
- ACCCTAATGA AAAGAAGTAT GTATCAATGG CAATGATGAT GAATCTTGGG CGCAAATCTC TTCACTGTGC   
  
  
- ATCATCGGCG GTGAAGATAT CATTATGATT GATGAAAGAG GCCCTATTTG ACTTTTGGCA GAGAAAAAGC   
  
  
- TTCTCACCTT TCTCCATCTC CTTTTGCTAT TTCACCTTTT CTTTAATACT TCCTCCAAAC CCCACATCTA   
  
  
- CCCACCACTC CTCAAATTCC AAAACCCAGT ACCTTATTAT ATTCTTATCA TCCCCTTCAA TTAAGCACCA   
  
  
- TGCTAATAAT TAATCATCCA CCCTTTGCTT ATCATAATTA GTTAAATTTA TATAATACAC TCTCTGTGGA   
  
  
- CCGACCTGAC GCCTTGCTAT GTATGTTGAT TGCTCATGAA CCCCAACTGT TTGGATATTG GACCAACTGG   
  
  
- GTTAATTTAC TTACCATTTG ATTATCATGT TCTTGTTGTA CTTGATAGTG ATCTTAATTA GATGAACATG   
  
  
- ATTAAGTTCA TTTGATCGGA AATAATGACT GAAGCAAATC TAGGGCACCA TATGATGGTG AAAAATCATA   
  
  
- AGGCCAGCAG TTAAATATTT AAGATGTCAT GGATCAGCAG ATTAATAAAG ATATCTGAAT TTTGATTAAC   
  
  
- CCATTTCAAC ATAGGAGATC GCATAAAATG ATTTAGCTAT CTTAGCTAGC ATGCAAAAGC AAACAATAAT   
  
  
- TAGATGATTT TTTAAAAATT AAATTTCCA
