# Supplementary material for: Genome-wide analysis of the WRKY gene family in drumstick (Moringa oleifera Lam.)
Source: PeerJ. 2019 Jun 10;7:e7063. doi: 10.7717/peerj.7063 (PMC6563795; doi:10.7717/peerj.7063)
Supplement: Supplemental Information 1 [file peerj-07-7063-s003.gz › MoWRKY22_plantcare.html]

Content-Type: text/html; charset=ISO-8859-1


CallMat\_Firefox


Webmaster Firefox specific output  
To save the result:
click on the frame with the right mouse button and save the source code as a text file with extension .html  
REFERENCE:PlantCARE: a database of plant cis-acting regulatory elements and a portal to tools for in silico analysis of promoter sequences.  
Lescot, M., Déhais, P., Moreau, Y., De Moor, B., Rouzé ,P.,and Rombauts, S.  
Nucleic Acids Res., Database issue(2002), 30(1):325-327.   


---

> 2018/04/13 10:10:12  
+ GTTTAAAAGT TGGGTAACAT CCCAGTATTG GGGGAGACTT CCGGTCCTTT AACCAAGACC TTACAATAAT   
  
  
+ AAGTAAAATG GGGGTGTCGT TAAGAGAAGT AAACGATGAT CTCAAAGATA TGTCTTTCCT AGGAAAGACA   
  
  
+ GTTGTTCTTG TTCAACTCAT AGAGGACGGT GTATGAAAAA CGATCACAGA GGACGGTGTA TAAAAAGTGA   
  
  
+ TCAGTACACC GCTTACGGCA ATCAGTAGGA ACATTGTCTA TCAAAGACGA GTAGTAGGTT CAATAGTTCT   
  
  
+ GTGTGAAGTA CATCACGCCC TTCTCGTGCG AGACTCAAAA CTCGGACGTA TTAGATCATT AGTCATTATC   
  
  
+ TTTTGTTAAG TATTAACGCT TTTCATTCGT TGACATTTAG CGTTGTAAAT TGTTTTAGTG GAGGTTGAAC   
  
  
+ AATTGAGTCT GAATTCATCC ATACTAGATA CAGATTACGT ATGACATTTG ACTATAGGTT CTTTTGTTTG   
  
  
+ TTTGTATGAT TTGTAAAAGT ATGGCGAACA AATTCTTGTC TCGTGTGATA AGCTCAGTGT CGTAAGAGAC   
  
  
+ CTAGGACATT TTACCTAGTT TCGTTTTTTA CTTCGTTAGA TGGTCTTTGT TCCCCCTTAC CATATTAGTA   
  
  
+ TGTCGAGATG GGATGGGAAT CTCTTAGTGA TAATCGAGGT GTTGCACGAT AGAGAAAGTA GTAGTCGTCT   
  
  
+ CATGCGTTGA CACATATTGA AAAGTGAGAG CATCCGAAGT GTCTCGAATC GAATCTCTTT CGGTTAGACG   
  
  
+ TCCGTAATTC AAAGCGGAGT TCCTGAGAGG AACTTTAAAG ATGTCGGTTC TTTTGTCCTT GGGTCTTATC   
  
  
+ CCTTCGAATT ATCTCCAGTT TTTTAGTTTA GTGTAATCTT TCCTTTTGTT TTAATGTATT TATTTATTTA   
  
  
+ AGTTGGGTCC CGTACCGTTG ACTTGTACAT TGTTTCTTCT TAAGTTCTTT TAAAATTAAT TAATATTTTA   
  
  
+ TATAATACAC ATGATACCAA AGGAAGTTCA ACATCTTCCT CTATGGACAG AAGAAGTCTG ACTATTGGCA   
  
  
+ ATTAGGTATC AGGTAGTTGT CTCATATTAA CTACCTCGTT TGTTTACGAA CACAATCAAT TTACTTTACT   
  
  
+ AACAAACTCA TCATATGCTA AAGCGAATGG ACCGCCACGG TACAGTCTGG CTCCAAGCTT GACGATGTGT   
  
  
+ TTCGACTTCT AAAGTTTTAA CAATACCTTT GGTGGTTCTG CTTCCGCCAT TATCTTATTT TCCGCTGGTA   
  
  
+ GGTCGCTGCA GGATGAAGTA CTCCGAGAAC GGTGCTTGTA GAGAGAAACG GATGACGGAG AGGATCTATC   
  
  
+ AATTAAGTTA TCGGTTTCTT TGTGTTCTAA GAACGAGACA AGGGCTTTGA CTGTTCATGT TGAGAAAAAA   
  
  
+ GGAAAGTTCA TAGAGTCCTT CGCTGGTACT GTCGATCATT TAGTTCCAAG TTTCGAGTGA TCGGTAAAAA   
  
  
+ TTCATCGGTG AGAAATCACC TCCTAACGT  

- CAAATTTTCA ACCCATTGTA GGGTCATAAC CCCCTCTGAA GGCCAGGAAA TTGGTTCTGG AATGTTATTA   
  
  
- TTCATTTTAC CCCCACAGCA ATTCTCTTCA TTTGCTACTA GAGTTTCTAT ACAGAAAGGA TCCTTTCTGT   
  
  
- CAACAAGAAC AAGTTGAGTA TCTCCTGCCA CATACTTTTT GCTAGTGTCT CCTGCCACAT ATTTTTCACT   
  
  
- AGTCATGTGG CGAATGCCGT TAGTCATCCT TGTAACAGAT AGTTTCTGCT CATCATCCAA GTTATCAAGA   
  
  
- CACACTTCAT GTAGTGCGGG AAGAGCACGC TCTGAGTTTT GAGCCTGCAT AATCTAGTAA TCAGTAATAG   
  
  
- AAAACAATTC ATAATTGCGA AAAGTAAGCA ACTGTAAATC GCAACATTTA ACAAAATCAC CTCCAACTTG   
  
  
- TTAACTCAGA CTTAAGTAGG TATGATCTAT GTCTAATGCA TACTGTAAAC TGATATCCAA GAAAACAAAC   
  
  
- AAACATACTA AACATTTTCA TACCGCTTGT TTAAGAACAG AGCACACTAT TCGAGTCACA GCATTCTCTG   
  
  
- GATCCTGTAA AATGGATCAA AGCAAAAAAT GAAGCAATCT ACCAGAAACA AGGGGGAATG GTATAATCAT   
  
  
- ACAGCTCTAC CCTACCCTTA GAGAATCACT ATTAGCTCCA CAACGTGCTA TCTCTTTCAT CATCAGCAGA   
  
  
- GTACGCAACT GTGTATAACT TTTCACTCTC GTAGGCTTCA CAGAGCTTAG CTTAGAGAAA GCCAATCTGC   
  
  
- AGGCATTAAG TTTCGCCTCA AGGACTCTCC TTGAAATTTC TACAGCCAAG AAAACAGGAA CCCAGAATAG   
  
  
- GGAAGCTTAA TAGAGGTCAA AAAATCAAAT CACATTAGAA AGGAAAACAA AATTACATAA ATAAATAAAT   
  
  
- TCAACCCAGG GCATGGCAAC TGAACATGTA ACAAAGAAGA ATTCAAGAAA ATTTTAATTA ATTATAAAAT   
  
  
- ATATTATGTG TACTATGGTT TCCTTCAAGT TGTAGAAGGA GATACCTGTC TTCTTCAGAC TGATAACCGT   
  
  
- TAATCCATAG TCCATCAACA GAGTATAATT GATGGAGCAA ACAAATGCTT GTGTTAGTTA AATGAAATGA   
  
  
- TTGTTTGAGT AGTATACGAT TTCGCTTACC TGGCGGTGCC ATGTCAGACC GAGGTTCGAA CTGCTACACA   
  
  
- AAGCTGAAGA TTTCAAAATT GTTATGGAAA CCACCAAGAC GAAGGCGGTA ATAGAATAAA AGGCGACCAT   
  
  
- CCAGCGACGT CCTACTTCAT GAGGCTCTTG CCACGAACAT CTCTCTTTGC CTACTGCCTC TCCTAGATAG   
  
  
- TTAATTCAAT AGCCAAAGAA ACACAAGATT CTTGCTCTGT TCCCGAAACT GACAAGTACA ACTCTTTTTT   
  
  
- CCTTTCAAGT ATCTCAGGAA GCGACCATGA CAGCTAGTAA ATCAAGGTTC AAAGCTCACT AGCCATTTTT   
  
  
- AAGTAGCCAC TCTTTAGTGG AGGATTGCA

  
  
Motifs Found  

+     5UTR Py-rich stretch

| Site Name | Organism | Position | Strand | Matrix score. | sequence | function |
| --- | --- | --- | --- | --- | --- | --- |
| 5UTR Py-rich stretch | Lycopersicon esculentum | 93 | - | 9 | TTTCTTCTCT | cis-acting element conferring high transcription levels |

> 2018/04/13 10:10:12  
+ GTTTAAAAGT TGGGTAACAT CCCAGTATTG GGGGAGACTT CCGGTCCTTT AACCAAGACC TTACAATAAT   
  
  
+ AAGTAAAATG GGGGTGTCGT TAAGAGAAGT AAACGATGAT CTCAAAGATA TGTCTTTCCT AGGAAAGACA   
  
  
+ GTTGTTCTTG TTCAACTCAT AGAGGACGGT GTATGAAAAA CGATCACAGA GGACGGTGTA TAAAAAGTGA   
  
  
+ TCAGTACACC GCTTACGGCA ATCAGTAGGA ACATTGTCTA TCAAAGACGA GTAGTAGGTT CAATAGTTCT   
  
  
+ GTGTGAAGTA CATCACGCCC TTCTCGTGCG AGACTCAAAA CTCGGACGTA TTAGATCATT AGTCATTATC   
  
  
+ TTTTGTTAAG TATTAACGCT TTTCATTCGT TGACATTTAG CGTTGTAAAT TGTTTTAGTG GAGGTTGAAC   
  
  
+ AATTGAGTCT GAATTCATCC ATACTAGATA CAGATTACGT ATGACATTTG ACTATAGGTT CTTTTGTTTG   
  
  
+ TTTGTATGAT TTGTAAAAGT ATGGCGAACA AATTCTTGTC TCGTGTGATA AGCTCAGTGT CGTAAGAGAC   
  
  
+ CTAGGACATT TTACCTAGTT TCGTTTTTTA CTTCGTTAGA TGGTCTTTGT TCCCCCTTAC CATATTAGTA   
  
  
+ TGTCGAGATG GGATGGGAAT CTCTTAGTGA TAATCGAGGT GTTGCACGAT AGAGAAAGTA GTAGTCGTCT   
  
  
+ CATGCGTTGA CACATATTGA AAAGTGAGAG CATCCGAAGT GTCTCGAATC GAATCTCTTT CGGTTAGACG   
  
  
+ TCCGTAATTC AAAGCGGAGT TCCTGAGAGG AACTTTAAAG ATGTCGGTTC TTTTGTCCTT GGGTCTTATC   
  
  
+ CCTTCGAATT ATCTCCAGTT TTTTAGTTTA GTGTAATCTT TCCTTTTGTT TTAATGTATT TATTTATTTA   
  
  
+ AGTTGGGTCC CGTACCGTTG ACTTGTACAT TGTTTCTTCT TAAGTTCTTT TAAAATTAAT TAATATTTTA   
  
  
+ TATAATACAC ATGATACCAA AGGAAGTTCA ACATCTTCCT CTATGGACAG AAGAAGTCTG ACTATTGGCA   
  
  
+ ATTAGGTATC AGGTAGTTGT CTCATATTAA CTACCTCGTT TGTTTACGAA CACAATCAAT TTACTTTACT   
  
  
+ AACAAACTCA TCATATGCTA AAGCGAATGG ACCGCCACGG TACAGTCTGG CTCCAAGCTT GACGATGTGT   
  
  
+ TTCGACTTCT AAAGTTTTAA CAATACCTTT GGTGGTTCTG CTTCCGCCAT TATCTTATTT TCCGCTGGTA   
  
  
+ GGTCGCTGCA GGATGAAGTA CTCCGAGAAC GGTGCTTGTA GAGAGAAACG GATGACGGAG AGGATCTATC   
  
  
+ AATTAAGTTA TCGGTTTCTT TGTGTTCTAA GAACGAGACA AGGGCTTTGA CTGTTCATGT TGAGAAAAAA   
  
  
+ GGAAAGTTCA TAGAGTCCTT CGCTGGTACT GTCGATCATT TAGTTCCAAG TTTCGAGTGA TCGGTAAAAA   
  
  
+ TTCATCGGTG AGAAATCACC TCCTAACGT  

- CAAATTTTCA ACCCATTGTA GGGTCATAAC CCCCTCTGAA GGCCAGGAAA TTGGTTCTGG AATGTTATTA   
  
  
- TTCATTTTAC CCCCACAGCA ATTCTCTTCA TTTGCTACTA GAGTTTCTAT ACAGAAAGGA TCCTTTCTGT   
  
  
- CAACAAGAAC AAGTTGAGTA TCTCCTGCCA CATACTTTTT GCTAGTGTCT CCTGCCACAT ATTTTTCACT   
  
  
- AGTCATGTGG CGAATGCCGT TAGTCATCCT TGTAACAGAT AGTTTCTGCT CATCATCCAA GTTATCAAGA   
  
  
- CACACTTCAT GTAGTGCGGG AAGAGCACGC TCTGAGTTTT GAGCCTGCAT AATCTAGTAA TCAGTAATAG   
  
  
- AAAACAATTC ATAATTGCGA AAAGTAAGCA ACTGTAAATC GCAACATTTA ACAAAATCAC CTCCAACTTG   
  
  
- TTAACTCAGA CTTAAGTAGG TATGATCTAT GTCTAATGCA TACTGTAAAC TGATATCCAA GAAAACAAAC   
  
  
- AAACATACTA AACATTTTCA TACCGCTTGT TTAAGAACAG AGCACACTAT TCGAGTCACA GCATTCTCTG   
  
  
- GATCCTGTAA AATGGATCAA AGCAAAAAAT GAAGCAATCT ACCAGAAACA AGGGGGAATG GTATAATCAT   
  
  
- ACAGCTCTAC CCTACCCTTA GAGAATCACT ATTAGCTCCA CAACGTGCTA TCTCTTTCAT CATCAGCAGA   
  
  
- GTACGCAACT GTGTATAACT TTTCACTCTC GTAGGCTTCA CAGAGCTTAG CTTAGAGAAA GCCAATCTGC   
  
  
- AGGCATTAAG TTTCGCCTCA AGGACTCTCC TTGAAATTTC TACAGCCAAG AAAACAGGAA CCCAGAATAG   
  
  
- GGAAGCTTAA TAGAGGTCAA AAAATCAAAT CACATTAGAA AGGAAAACAA AATTACATAA ATAAATAAAT   
  
  
- TCAACCCAGG GCATGGCAAC TGAACATGTA ACAAAGAAGA ATTCAAGAAA ATTTTAATTA ATTATAAAAT   
  
  
- ATATTATGTG TACTATGGTT TCCTTCAAGT TGTAGAAGGA GATACCTGTC TTCTTCAGAC TGATAACCGT   
  
  
- TAATCCATAG TCCATCAACA GAGTATAATT GATGGAGCAA ACAAATGCTT GTGTTAGTTA AATGAAATGA   
  
  
- TTGTTTGAGT AGTATACGAT TTCGCTTACC TGGCGGTGCC ATGTCAGACC GAGGTTCGAA CTGCTACACA   
  
  
- AAGCTGAAGA TTTCAAAATT GTTATGGAAA CCACCAAGAC GAAGGCGGTA ATAGAATAAA AGGCGACCAT   
  
  
- CCAGCGACGT CCTACTTCAT GAGGCTCTTG CCACGAACAT CTCTCTTTGC CTACTGCCTC TCCTAGATAG   
  
  
- TTAATTCAAT AGCCAAAGAA ACACAAGATT CTTGCTCTGT TCCCGAAACT GACAAGTACA ACTCTTTTTT   
  
  
- CCTTTCAAGT ATCTCAGGAA GCGACCATGA CAGCTAGTAA ATCAAGGTTC AAAGCTCACT AGCCATTTTT   
  
  
- AAGTAGCCAC TCTTTAGTGG AGGATTGCA

+     A-box

| Site Name | Organism | Position | Strand | Matrix score. | sequence | function |
| --- | --- | --- | --- | --- | --- | --- |
| A-box | Petroselinum crispum | 191 | - | 6 | CCGTCC | cis-acting regulatory element |
| A-box | Petroselinum crispum | 164 | - | 6 | CCGTCC | cis-acting regulatory element |

> 2018/04/13 10:10:12  
+ GTTTAAAAGT TGGGTAACAT CCCAGTATTG GGGGAGACTT CCGGTCCTTT AACCAAGACC TTACAATAAT   
  
  
+ AAGTAAAATG GGGGTGTCGT TAAGAGAAGT AAACGATGAT CTCAAAGATA TGTCTTTCCT AGGAAAGACA   
  
  
+ GTTGTTCTTG TTCAACTCAT AGAGGACGGT GTATGAAAAA CGATCACAGA GGACGGTGTA TAAAAAGTGA   
  
  
+ TCAGTACACC GCTTACGGCA ATCAGTAGGA ACATTGTCTA TCAAAGACGA GTAGTAGGTT CAATAGTTCT   
  
  
+ GTGTGAAGTA CATCACGCCC TTCTCGTGCG AGACTCAAAA CTCGGACGTA TTAGATCATT AGTCATTATC   
  
  
+ TTTTGTTAAG TATTAACGCT TTTCATTCGT TGACATTTAG CGTTGTAAAT TGTTTTAGTG GAGGTTGAAC   
  
  
+ AATTGAGTCT GAATTCATCC ATACTAGATA CAGATTACGT ATGACATTTG ACTATAGGTT CTTTTGTTTG   
  
  
+ TTTGTATGAT TTGTAAAAGT ATGGCGAACA AATTCTTGTC TCGTGTGATA AGCTCAGTGT CGTAAGAGAC   
  
  
+ CTAGGACATT TTACCTAGTT TCGTTTTTTA CTTCGTTAGA TGGTCTTTGT TCCCCCTTAC CATATTAGTA   
  
  
+ TGTCGAGATG GGATGGGAAT CTCTTAGTGA TAATCGAGGT GTTGCACGAT AGAGAAAGTA GTAGTCGTCT   
  
  
+ CATGCGTTGA CACATATTGA AAAGTGAGAG CATCCGAAGT GTCTCGAATC GAATCTCTTT CGGTTAGACG   
  
  
+ TCCGTAATTC AAAGCGGAGT TCCTGAGAGG AACTTTAAAG ATGTCGGTTC TTTTGTCCTT GGGTCTTATC   
  
  
+ CCTTCGAATT ATCTCCAGTT TTTTAGTTTA GTGTAATCTT TCCTTTTGTT TTAATGTATT TATTTATTTA   
  
  
+ AGTTGGGTCC CGTACCGTTG ACTTGTACAT TGTTTCTTCT TAAGTTCTTT TAAAATTAAT TAATATTTTA   
  
  
+ TATAATACAC ATGATACCAA AGGAAGTTCA ACATCTTCCT CTATGGACAG AAGAAGTCTG ACTATTGGCA   
  
  
+ ATTAGGTATC AGGTAGTTGT CTCATATTAA CTACCTCGTT TGTTTACGAA CACAATCAAT TTACTTTACT   
  
  
+ AACAAACTCA TCATATGCTA AAGCGAATGG ACCGCCACGG TACAGTCTGG CTCCAAGCTT GACGATGTGT   
  
  
+ TTCGACTTCT AAAGTTTTAA CAATACCTTT GGTGGTTCTG CTTCCGCCAT TATCTTATTT TCCGCTGGTA   
  
  
+ GGTCGCTGCA GGATGAAGTA CTCCGAGAAC GGTGCTTGTA GAGAGAAACG GATGACGGAG AGGATCTATC   
  
  
+ AATTAAGTTA TCGGTTTCTT TGTGTTCTAA GAACGAGACA AGGGCTTTGA CTGTTCATGT TGAGAAAAAA   
  
  
+ GGAAAGTTCA TAGAGTCCTT CGCTGGTACT GTCGATCATT TAGTTCCAAG TTTCGAGTGA TCGGTAAAAA   
  
  
+ TTCATCGGTG AGAAATCACC TCCTAACGT  

- CAAATTTTCA ACCCATTGTA GGGTCATAAC CCCCTCTGAA GGCCAGGAAA TTGGTTCTGG AATGTTATTA   
  
  
- TTCATTTTAC CCCCACAGCA ATTCTCTTCA TTTGCTACTA GAGTTTCTAT ACAGAAAGGA TCCTTTCTGT   
  
  
- CAACAAGAAC AAGTTGAGTA TCTCCTGCCA CATACTTTTT GCTAGTGTCT CCTGCCACAT ATTTTTCACT   
  
  
- AGTCATGTGG CGAATGCCGT TAGTCATCCT TGTAACAGAT AGTTTCTGCT CATCATCCAA GTTATCAAGA   
  
  
- CACACTTCAT GTAGTGCGGG AAGAGCACGC TCTGAGTTTT GAGCCTGCAT AATCTAGTAA TCAGTAATAG   
  
  
- AAAACAATTC ATAATTGCGA AAAGTAAGCA ACTGTAAATC GCAACATTTA ACAAAATCAC CTCCAACTTG   
  
  
- TTAACTCAGA CTTAAGTAGG TATGATCTAT GTCTAATGCA TACTGTAAAC TGATATCCAA GAAAACAAAC   
  
  
- AAACATACTA AACATTTTCA TACCGCTTGT TTAAGAACAG AGCACACTAT TCGAGTCACA GCATTCTCTG   
  
  
- GATCCTGTAA AATGGATCAA AGCAAAAAAT GAAGCAATCT ACCAGAAACA AGGGGGAATG GTATAATCAT   
  
  
- ACAGCTCTAC CCTACCCTTA GAGAATCACT ATTAGCTCCA CAACGTGCTA TCTCTTTCAT CATCAGCAGA   
  
  
- GTACGCAACT GTGTATAACT TTTCACTCTC GTAGGCTTCA CAGAGCTTAG CTTAGAGAAA GCCAATCTGC   
  
  
- AGGCATTAAG TTTCGCCTCA AGGACTCTCC TTGAAATTTC TACAGCCAAG AAAACAGGAA CCCAGAATAG   
  
  
- GGAAGCTTAA TAGAGGTCAA AAAATCAAAT CACATTAGAA AGGAAAACAA AATTACATAA ATAAATAAAT   
  
  
- TCAACCCAGG GCATGGCAAC TGAACATGTA ACAAAGAAGA ATTCAAGAAA ATTTTAATTA ATTATAAAAT   
  
  
- ATATTATGTG TACTATGGTT TCCTTCAAGT TGTAGAAGGA GATACCTGTC TTCTTCAGAC TGATAACCGT   
  
  
- TAATCCATAG TCCATCAACA GAGTATAATT GATGGAGCAA ACAAATGCTT GTGTTAGTTA AATGAAATGA   
  
  
- TTGTTTGAGT AGTATACGAT TTCGCTTACC TGGCGGTGCC ATGTCAGACC GAGGTTCGAA CTGCTACACA   
  
  
- AAGCTGAAGA TTTCAAAATT GTTATGGAAA CCACCAAGAC GAAGGCGGTA ATAGAATAAA AGGCGACCAT   
  
  
- CCAGCGACGT CCTACTTCAT GAGGCTCTTG CCACGAACAT CTCTCTTTGC CTACTGCCTC TCCTAGATAG   
  
  
- TTAATTCAAT AGCCAAAGAA ACACAAGATT CTTGCTCTGT TCCCGAAACT GACAAGTACA ACTCTTTTTT   
  
  
- CCTTTCAAGT ATCTCAGGAA GCGACCATGA CAGCTAGTAA ATCAAGGTTC AAAGCTCACT AGCCATTTTT   
  
  
- AAGTAGCCAC TCTTTAGTGG AGGATTGCA

+     ACE

| Site Name | Organism | Position | Strand | Matrix score. | sequence | function |
| --- | --- | --- | --- | --- | --- | --- |
| ACE | Petroselinum crispum | 1257 | - | 9 | GCGACGTACC | cis-acting element involved in light responsiveness |

> 2018/04/13 10:10:12  
+ GTTTAAAAGT TGGGTAACAT CCCAGTATTG GGGGAGACTT CCGGTCCTTT AACCAAGACC TTACAATAAT   
  
  
+ AAGTAAAATG GGGGTGTCGT TAAGAGAAGT AAACGATGAT CTCAAAGATA TGTCTTTCCT AGGAAAGACA   
  
  
+ GTTGTTCTTG TTCAACTCAT AGAGGACGGT GTATGAAAAA CGATCACAGA GGACGGTGTA TAAAAAGTGA   
  
  
+ TCAGTACACC GCTTACGGCA ATCAGTAGGA ACATTGTCTA TCAAAGACGA GTAGTAGGTT CAATAGTTCT   
  
  
+ GTGTGAAGTA CATCACGCCC TTCTCGTGCG AGACTCAAAA CTCGGACGTA TTAGATCATT AGTCATTATC   
  
  
+ TTTTGTTAAG TATTAACGCT TTTCATTCGT TGACATTTAG CGTTGTAAAT TGTTTTAGTG GAGGTTGAAC   
  
  
+ AATTGAGTCT GAATTCATCC ATACTAGATA CAGATTACGT ATGACATTTG ACTATAGGTT CTTTTGTTTG   
  
  
+ TTTGTATGAT TTGTAAAAGT ATGGCGAACA AATTCTTGTC TCGTGTGATA AGCTCAGTGT CGTAAGAGAC   
  
  
+ CTAGGACATT TTACCTAGTT TCGTTTTTTA CTTCGTTAGA TGGTCTTTGT TCCCCCTTAC CATATTAGTA   
  
  
+ TGTCGAGATG GGATGGGAAT CTCTTAGTGA TAATCGAGGT GTTGCACGAT AGAGAAAGTA GTAGTCGTCT   
  
  
+ CATGCGTTGA CACATATTGA AAAGTGAGAG CATCCGAAGT GTCTCGAATC GAATCTCTTT CGGTTAGACG   
  
  
+ TCCGTAATTC AAAGCGGAGT TCCTGAGAGG AACTTTAAAG ATGTCGGTTC TTTTGTCCTT GGGTCTTATC   
  
  
+ CCTTCGAATT ATCTCCAGTT TTTTAGTTTA GTGTAATCTT TCCTTTTGTT TTAATGTATT TATTTATTTA   
  
  
+ AGTTGGGTCC CGTACCGTTG ACTTGTACAT TGTTTCTTCT TAAGTTCTTT TAAAATTAAT TAATATTTTA   
  
  
+ TATAATACAC ATGATACCAA AGGAAGTTCA ACATCTTCCT CTATGGACAG AAGAAGTCTG ACTATTGGCA   
  
  
+ ATTAGGTATC AGGTAGTTGT CTCATATTAA CTACCTCGTT TGTTTACGAA CACAATCAAT TTACTTTACT   
  
  
+ AACAAACTCA TCATATGCTA AAGCGAATGG ACCGCCACGG TACAGTCTGG CTCCAAGCTT GACGATGTGT   
  
  
+ TTCGACTTCT AAAGTTTTAA CAATACCTTT GGTGGTTCTG CTTCCGCCAT TATCTTATTT TCCGCTGGTA   
  
  
+ GGTCGCTGCA GGATGAAGTA CTCCGAGAAC GGTGCTTGTA GAGAGAAACG GATGACGGAG AGGATCTATC   
  
  
+ AATTAAGTTA TCGGTTTCTT TGTGTTCTAA GAACGAGACA AGGGCTTTGA CTGTTCATGT TGAGAAAAAA   
  
  
+ GGAAAGTTCA TAGAGTCCTT CGCTGGTACT GTCGATCATT TAGTTCCAAG TTTCGAGTGA TCGGTAAAAA   
  
  
+ TTCATCGGTG AGAAATCACC TCCTAACGT  

- CAAATTTTCA ACCCATTGTA GGGTCATAAC CCCCTCTGAA GGCCAGGAAA TTGGTTCTGG AATGTTATTA   
  
  
- TTCATTTTAC CCCCACAGCA ATTCTCTTCA TTTGCTACTA GAGTTTCTAT ACAGAAAGGA TCCTTTCTGT   
  
  
- CAACAAGAAC AAGTTGAGTA TCTCCTGCCA CATACTTTTT GCTAGTGTCT CCTGCCACAT ATTTTTCACT   
  
  
- AGTCATGTGG CGAATGCCGT TAGTCATCCT TGTAACAGAT AGTTTCTGCT CATCATCCAA GTTATCAAGA   
  
  
- CACACTTCAT GTAGTGCGGG AAGAGCACGC TCTGAGTTTT GAGCCTGCAT AATCTAGTAA TCAGTAATAG   
  
  
- AAAACAATTC ATAATTGCGA AAAGTAAGCA ACTGTAAATC GCAACATTTA ACAAAATCAC CTCCAACTTG   
  
  
- TTAACTCAGA CTTAAGTAGG TATGATCTAT GTCTAATGCA TACTGTAAAC TGATATCCAA GAAAACAAAC   
  
  
- AAACATACTA AACATTTTCA TACCGCTTGT TTAAGAACAG AGCACACTAT TCGAGTCACA GCATTCTCTG   
  
  
- GATCCTGTAA AATGGATCAA AGCAAAAAAT GAAGCAATCT ACCAGAAACA AGGGGGAATG GTATAATCAT   
  
  
- ACAGCTCTAC CCTACCCTTA GAGAATCACT ATTAGCTCCA CAACGTGCTA TCTCTTTCAT CATCAGCAGA   
  
  
- GTACGCAACT GTGTATAACT TTTCACTCTC GTAGGCTTCA CAGAGCTTAG CTTAGAGAAA GCCAATCTGC   
  
  
- AGGCATTAAG TTTCGCCTCA AGGACTCTCC TTGAAATTTC TACAGCCAAG AAAACAGGAA CCCAGAATAG   
  
  
- GGAAGCTTAA TAGAGGTCAA AAAATCAAAT CACATTAGAA AGGAAAACAA AATTACATAA ATAAATAAAT   
  
  
- TCAACCCAGG GCATGGCAAC TGAACATGTA ACAAAGAAGA ATTCAAGAAA ATTTTAATTA ATTATAAAAT   
  
  
- ATATTATGTG TACTATGGTT TCCTTCAAGT TGTAGAAGGA GATACCTGTC TTCTTCAGAC TGATAACCGT   
  
  
- TAATCCATAG TCCATCAACA GAGTATAATT GATGGAGCAA ACAAATGCTT GTGTTAGTTA AATGAAATGA   
  
  
- TTGTTTGAGT AGTATACGAT TTCGCTTACC TGGCGGTGCC ATGTCAGACC GAGGTTCGAA CTGCTACACA   
  
  
- AAGCTGAAGA TTTCAAAATT GTTATGGAAA CCACCAAGAC GAAGGCGGTA ATAGAATAAA AGGCGACCAT   
  
  
- CCAGCGACGT CCTACTTCAT GAGGCTCTTG CCACGAACAT CTCTCTTTGC CTACTGCCTC TCCTAGATAG   
  
  
- TTAATTCAAT AGCCAAAGAA ACACAAGATT CTTGCTCTGT TCCCGAAACT GACAAGTACA ACTCTTTTTT   
  
  
- CCTTTCAAGT ATCTCAGGAA GCGACCATGA CAGCTAGTAA ATCAAGGTTC AAAGCTCACT AGCCATTTTT   
  
  
- AAGTAGCCAC TCTTTAGTGG AGGATTGCA

+     AE-box

| Site Name | Organism | Position | Strand | Matrix score. | sequence | function |
| --- | --- | --- | --- | --- | --- | --- |
| AE-box | Arabidopsis thaliana | 940 | - | 8 | AGAAACAA | part of a module for light response |

> 2018/04/13 10:10:12  
+ GTTTAAAAGT TGGGTAACAT CCCAGTATTG GGGGAGACTT CCGGTCCTTT AACCAAGACC TTACAATAAT   
  
  
+ AAGTAAAATG GGGGTGTCGT TAAGAGAAGT AAACGATGAT CTCAAAGATA TGTCTTTCCT AGGAAAGACA   
  
  
+ GTTGTTCTTG TTCAACTCAT AGAGGACGGT GTATGAAAAA CGATCACAGA GGACGGTGTA TAAAAAGTGA   
  
  
+ TCAGTACACC GCTTACGGCA ATCAGTAGGA ACATTGTCTA TCAAAGACGA GTAGTAGGTT CAATAGTTCT   
  
  
+ GTGTGAAGTA CATCACGCCC TTCTCGTGCG AGACTCAAAA CTCGGACGTA TTAGATCATT AGTCATTATC   
  
  
+ TTTTGTTAAG TATTAACGCT TTTCATTCGT TGACATTTAG CGTTGTAAAT TGTTTTAGTG GAGGTTGAAC   
  
  
+ AATTGAGTCT GAATTCATCC ATACTAGATA CAGATTACGT ATGACATTTG ACTATAGGTT CTTTTGTTTG   
  
  
+ TTTGTATGAT TTGTAAAAGT ATGGCGAACA AATTCTTGTC TCGTGTGATA AGCTCAGTGT CGTAAGAGAC   
  
  
+ CTAGGACATT TTACCTAGTT TCGTTTTTTA CTTCGTTAGA TGGTCTTTGT TCCCCCTTAC CATATTAGTA   
  
  
+ TGTCGAGATG GGATGGGAAT CTCTTAGTGA TAATCGAGGT GTTGCACGAT AGAGAAAGTA GTAGTCGTCT   
  
  
+ CATGCGTTGA CACATATTGA AAAGTGAGAG CATCCGAAGT GTCTCGAATC GAATCTCTTT CGGTTAGACG   
  
  
+ TCCGTAATTC AAAGCGGAGT TCCTGAGAGG AACTTTAAAG ATGTCGGTTC TTTTGTCCTT GGGTCTTATC   
  
  
+ CCTTCGAATT ATCTCCAGTT TTTTAGTTTA GTGTAATCTT TCCTTTTGTT TTAATGTATT TATTTATTTA   
  
  
+ AGTTGGGTCC CGTACCGTTG ACTTGTACAT TGTTTCTTCT TAAGTTCTTT TAAAATTAAT TAATATTTTA   
  
  
+ TATAATACAC ATGATACCAA AGGAAGTTCA ACATCTTCCT CTATGGACAG AAGAAGTCTG ACTATTGGCA   
  
  
+ ATTAGGTATC AGGTAGTTGT CTCATATTAA CTACCTCGTT TGTTTACGAA CACAATCAAT TTACTTTACT   
  
  
+ AACAAACTCA TCATATGCTA AAGCGAATGG ACCGCCACGG TACAGTCTGG CTCCAAGCTT GACGATGTGT   
  
  
+ TTCGACTTCT AAAGTTTTAA CAATACCTTT GGTGGTTCTG CTTCCGCCAT TATCTTATTT TCCGCTGGTA   
  
  
+ GGTCGCTGCA GGATGAAGTA CTCCGAGAAC GGTGCTTGTA GAGAGAAACG GATGACGGAG AGGATCTATC   
  
  
+ AATTAAGTTA TCGGTTTCTT TGTGTTCTAA GAACGAGACA AGGGCTTTGA CTGTTCATGT TGAGAAAAAA   
  
  
+ GGAAAGTTCA TAGAGTCCTT CGCTGGTACT GTCGATCATT TAGTTCCAAG TTTCGAGTGA TCGGTAAAAA   
  
  
+ TTCATCGGTG AGAAATCACC TCCTAACGT  

- CAAATTTTCA ACCCATTGTA GGGTCATAAC CCCCTCTGAA GGCCAGGAAA TTGGTTCTGG AATGTTATTA   
  
  
- TTCATTTTAC CCCCACAGCA ATTCTCTTCA TTTGCTACTA GAGTTTCTAT ACAGAAAGGA TCCTTTCTGT   
  
  
- CAACAAGAAC AAGTTGAGTA TCTCCTGCCA CATACTTTTT GCTAGTGTCT CCTGCCACAT ATTTTTCACT   
  
  
- AGTCATGTGG CGAATGCCGT TAGTCATCCT TGTAACAGAT AGTTTCTGCT CATCATCCAA GTTATCAAGA   
  
  
- CACACTTCAT GTAGTGCGGG AAGAGCACGC TCTGAGTTTT GAGCCTGCAT AATCTAGTAA TCAGTAATAG   
  
  
- AAAACAATTC ATAATTGCGA AAAGTAAGCA ACTGTAAATC GCAACATTTA ACAAAATCAC CTCCAACTTG   
  
  
- TTAACTCAGA CTTAAGTAGG TATGATCTAT GTCTAATGCA TACTGTAAAC TGATATCCAA GAAAACAAAC   
  
  
- AAACATACTA AACATTTTCA TACCGCTTGT TTAAGAACAG AGCACACTAT TCGAGTCACA GCATTCTCTG   
  
  
- GATCCTGTAA AATGGATCAA AGCAAAAAAT GAAGCAATCT ACCAGAAACA AGGGGGAATG GTATAATCAT   
  
  
- ACAGCTCTAC CCTACCCTTA GAGAATCACT ATTAGCTCCA CAACGTGCTA TCTCTTTCAT CATCAGCAGA   
  
  
- GTACGCAACT GTGTATAACT TTTCACTCTC GTAGGCTTCA CAGAGCTTAG CTTAGAGAAA GCCAATCTGC   
  
  
- AGGCATTAAG TTTCGCCTCA AGGACTCTCC TTGAAATTTC TACAGCCAAG AAAACAGGAA CCCAGAATAG   
  
  
- GGAAGCTTAA TAGAGGTCAA AAAATCAAAT CACATTAGAA AGGAAAACAA AATTACATAA ATAAATAAAT   
  
  
- TCAACCCAGG GCATGGCAAC TGAACATGTA ACAAAGAAGA ATTCAAGAAA ATTTTAATTA ATTATAAAAT   
  
  
- ATATTATGTG TACTATGGTT TCCTTCAAGT TGTAGAAGGA GATACCTGTC TTCTTCAGAC TGATAACCGT   
  
  
- TAATCCATAG TCCATCAACA GAGTATAATT GATGGAGCAA ACAAATGCTT GTGTTAGTTA AATGAAATGA   
  
  
- TTGTTTGAGT AGTATACGAT TTCGCTTACC TGGCGGTGCC ATGTCAGACC GAGGTTCGAA CTGCTACACA   
  
  
- AAGCTGAAGA TTTCAAAATT GTTATGGAAA CCACCAAGAC GAAGGCGGTA ATAGAATAAA AGGCGACCAT   
  
  
- CCAGCGACGT CCTACTTCAT GAGGCTCTTG CCACGAACAT CTCTCTTTGC CTACTGCCTC TCCTAGATAG   
  
  
- TTAATTCAAT AGCCAAAGAA ACACAAGATT CTTGCTCTGT TCCCGAAACT GACAAGTACA ACTCTTTTTT   
  
  
- CCTTTCAAGT ATCTCAGGAA GCGACCATGA CAGCTAGTAA ATCAAGGTTC AAAGCTCACT AGCCATTTTT   
  
  
- AAGTAGCCAC TCTTTAGTGG AGGATTGCA

+     AT1-motif

| Site Name | Organism | Position | Strand | Matrix score. | sequence | function |
| --- | --- | --- | --- | --- | --- | --- |
| AT1-motif | Solanum tuberosum | 959 | - | 11 | ATTAATTTTACA | part of a light responsive module |

> 2018/04/13 10:10:12  
+ GTTTAAAAGT TGGGTAACAT CCCAGTATTG GGGGAGACTT CCGGTCCTTT AACCAAGACC TTACAATAAT   
  
  
+ AAGTAAAATG GGGGTGTCGT TAAGAGAAGT AAACGATGAT CTCAAAGATA TGTCTTTCCT AGGAAAGACA   
  
  
+ GTTGTTCTTG TTCAACTCAT AGAGGACGGT GTATGAAAAA CGATCACAGA GGACGGTGTA TAAAAAGTGA   
  
  
+ TCAGTACACC GCTTACGGCA ATCAGTAGGA ACATTGTCTA TCAAAGACGA GTAGTAGGTT CAATAGTTCT   
  
  
+ GTGTGAAGTA CATCACGCCC TTCTCGTGCG AGACTCAAAA CTCGGACGTA TTAGATCATT AGTCATTATC   
  
  
+ TTTTGTTAAG TATTAACGCT TTTCATTCGT TGACATTTAG CGTTGTAAAT TGTTTTAGTG GAGGTTGAAC   
  
  
+ AATTGAGTCT GAATTCATCC ATACTAGATA CAGATTACGT ATGACATTTG ACTATAGGTT CTTTTGTTTG   
  
  
+ TTTGTATGAT TTGTAAAAGT ATGGCGAACA AATTCTTGTC TCGTGTGATA AGCTCAGTGT CGTAAGAGAC   
  
  
+ CTAGGACATT TTACCTAGTT TCGTTTTTTA CTTCGTTAGA TGGTCTTTGT TCCCCCTTAC CATATTAGTA   
  
  
+ TGTCGAGATG GGATGGGAAT CTCTTAGTGA TAATCGAGGT GTTGCACGAT AGAGAAAGTA GTAGTCGTCT   
  
  
+ CATGCGTTGA CACATATTGA AAAGTGAGAG CATCCGAAGT GTCTCGAATC GAATCTCTTT CGGTTAGACG   
  
  
+ TCCGTAATTC AAAGCGGAGT TCCTGAGAGG AACTTTAAAG ATGTCGGTTC TTTTGTCCTT GGGTCTTATC   
  
  
+ CCTTCGAATT ATCTCCAGTT TTTTAGTTTA GTGTAATCTT TCCTTTTGTT TTAATGTATT TATTTATTTA   
  
  
+ AGTTGGGTCC CGTACCGTTG ACTTGTACAT TGTTTCTTCT TAAGTTCTTT TAAAATTAAT TAATATTTTA   
  
  
+ TATAATACAC ATGATACCAA AGGAAGTTCA ACATCTTCCT CTATGGACAG AAGAAGTCTG ACTATTGGCA   
  
  
+ ATTAGGTATC AGGTAGTTGT CTCATATTAA CTACCTCGTT TGTTTACGAA CACAATCAAT TTACTTTACT   
  
  
+ AACAAACTCA TCATATGCTA AAGCGAATGG ACCGCCACGG TACAGTCTGG CTCCAAGCTT GACGATGTGT   
  
  
+ TTCGACTTCT AAAGTTTTAA CAATACCTTT GGTGGTTCTG CTTCCGCCAT TATCTTATTT TCCGCTGGTA   
  
  
+ GGTCGCTGCA GGATGAAGTA CTCCGAGAAC GGTGCTTGTA GAGAGAAACG GATGACGGAG AGGATCTATC   
  
  
+ AATTAAGTTA TCGGTTTCTT TGTGTTCTAA GAACGAGACA AGGGCTTTGA CTGTTCATGT TGAGAAAAAA   
  
  
+ GGAAAGTTCA TAGAGTCCTT CGCTGGTACT GTCGATCATT TAGTTCCAAG TTTCGAGTGA TCGGTAAAAA   
  
  
+ TTCATCGGTG AGAAATCACC TCCTAACGT  

- CAAATTTTCA ACCCATTGTA GGGTCATAAC CCCCTCTGAA GGCCAGGAAA TTGGTTCTGG AATGTTATTA   
  
  
- TTCATTTTAC CCCCACAGCA ATTCTCTTCA TTTGCTACTA GAGTTTCTAT ACAGAAAGGA TCCTTTCTGT   
  
  
- CAACAAGAAC AAGTTGAGTA TCTCCTGCCA CATACTTTTT GCTAGTGTCT CCTGCCACAT ATTTTTCACT   
  
  
- AGTCATGTGG CGAATGCCGT TAGTCATCCT TGTAACAGAT AGTTTCTGCT CATCATCCAA GTTATCAAGA   
  
  
- CACACTTCAT GTAGTGCGGG AAGAGCACGC TCTGAGTTTT GAGCCTGCAT AATCTAGTAA TCAGTAATAG   
  
  
- AAAACAATTC ATAATTGCGA AAAGTAAGCA ACTGTAAATC GCAACATTTA ACAAAATCAC CTCCAACTTG   
  
  
- TTAACTCAGA CTTAAGTAGG TATGATCTAT GTCTAATGCA TACTGTAAAC TGATATCCAA GAAAACAAAC   
  
  
- AAACATACTA AACATTTTCA TACCGCTTGT TTAAGAACAG AGCACACTAT TCGAGTCACA GCATTCTCTG   
  
  
- GATCCTGTAA AATGGATCAA AGCAAAAAAT GAAGCAATCT ACCAGAAACA AGGGGGAATG GTATAATCAT   
  
  
- ACAGCTCTAC CCTACCCTTA GAGAATCACT ATTAGCTCCA CAACGTGCTA TCTCTTTCAT CATCAGCAGA   
  
  
- GTACGCAACT GTGTATAACT TTTCACTCTC GTAGGCTTCA CAGAGCTTAG CTTAGAGAAA GCCAATCTGC   
  
  
- AGGCATTAAG TTTCGCCTCA AGGACTCTCC TTGAAATTTC TACAGCCAAG AAAACAGGAA CCCAGAATAG   
  
  
- GGAAGCTTAA TAGAGGTCAA AAAATCAAAT CACATTAGAA AGGAAAACAA AATTACATAA ATAAATAAAT   
  
  
- TCAACCCAGG GCATGGCAAC TGAACATGTA ACAAAGAAGA ATTCAAGAAA ATTTTAATTA ATTATAAAAT   
  
  
- ATATTATGTG TACTATGGTT TCCTTCAAGT TGTAGAAGGA GATACCTGTC TTCTTCAGAC TGATAACCGT   
  
  
- TAATCCATAG TCCATCAACA GAGTATAATT GATGGAGCAA ACAAATGCTT GTGTTAGTTA AATGAAATGA   
  
  
- TTGTTTGAGT AGTATACGAT TTCGCTTACC TGGCGGTGCC ATGTCAGACC GAGGTTCGAA CTGCTACACA   
  
  
- AAGCTGAAGA TTTCAAAATT GTTATGGAAA CCACCAAGAC GAAGGCGGTA ATAGAATAAA AGGCGACCAT   
  
  
- CCAGCGACGT CCTACTTCAT GAGGCTCTTG CCACGAACAT CTCTCTTTGC CTACTGCCTC TCCTAGATAG   
  
  
- TTAATTCAAT AGCCAAAGAA ACACAAGATT CTTGCTCTGT TCCCGAAACT GACAAGTACA ACTCTTTTTT   
  
  
- CCTTTCAAGT ATCTCAGGAA GCGACCATGA CAGCTAGTAA ATCAAGGTTC AAAGCTCACT AGCCATTTTT   
  
  
- AAGTAGCCAC TCTTTAGTGG AGGATTGCA

+     ATCT-motif

| Site Name | Organism | Position | Strand | Matrix score. | sequence | function |
| --- | --- | --- | --- | --- | --- | --- |
| ATCT-motif | Arabidopsis thaliana | 747 | + | 9 | AATCTAATCT | part of a conserved DNA module involved in light responsiveness |

> 2018/04/13 10:10:12  
+ GTTTAAAAGT TGGGTAACAT CCCAGTATTG GGGGAGACTT CCGGTCCTTT AACCAAGACC TTACAATAAT   
  
  
+ AAGTAAAATG GGGGTGTCGT TAAGAGAAGT AAACGATGAT CTCAAAGATA TGTCTTTCCT AGGAAAGACA   
  
  
+ GTTGTTCTTG TTCAACTCAT AGAGGACGGT GTATGAAAAA CGATCACAGA GGACGGTGTA TAAAAAGTGA   
  
  
+ TCAGTACACC GCTTACGGCA ATCAGTAGGA ACATTGTCTA TCAAAGACGA GTAGTAGGTT CAATAGTTCT   
  
  
+ GTGTGAAGTA CATCACGCCC TTCTCGTGCG AGACTCAAAA CTCGGACGTA TTAGATCATT AGTCATTATC   
  
  
+ TTTTGTTAAG TATTAACGCT TTTCATTCGT TGACATTTAG CGTTGTAAAT TGTTTTAGTG GAGGTTGAAC   
  
  
+ AATTGAGTCT GAATTCATCC ATACTAGATA CAGATTACGT ATGACATTTG ACTATAGGTT CTTTTGTTTG   
  
  
+ TTTGTATGAT TTGTAAAAGT ATGGCGAACA AATTCTTGTC TCGTGTGATA AGCTCAGTGT CGTAAGAGAC   
  
  
+ CTAGGACATT TTACCTAGTT TCGTTTTTTA CTTCGTTAGA TGGTCTTTGT TCCCCCTTAC CATATTAGTA   
  
  
+ TGTCGAGATG GGATGGGAAT CTCTTAGTGA TAATCGAGGT GTTGCACGAT AGAGAAAGTA GTAGTCGTCT   
  
  
+ CATGCGTTGA CACATATTGA AAAGTGAGAG CATCCGAAGT GTCTCGAATC GAATCTCTTT CGGTTAGACG   
  
  
+ TCCGTAATTC AAAGCGGAGT TCCTGAGAGG AACTTTAAAG ATGTCGGTTC TTTTGTCCTT GGGTCTTATC   
  
  
+ CCTTCGAATT ATCTCCAGTT TTTTAGTTTA GTGTAATCTT TCCTTTTGTT TTAATGTATT TATTTATTTA   
  
  
+ AGTTGGGTCC CGTACCGTTG ACTTGTACAT TGTTTCTTCT TAAGTTCTTT TAAAATTAAT TAATATTTTA   
  
  
+ TATAATACAC ATGATACCAA AGGAAGTTCA ACATCTTCCT CTATGGACAG AAGAAGTCTG ACTATTGGCA   
  
  
+ ATTAGGTATC AGGTAGTTGT CTCATATTAA CTACCTCGTT TGTTTACGAA CACAATCAAT TTACTTTACT   
  
  
+ AACAAACTCA TCATATGCTA AAGCGAATGG ACCGCCACGG TACAGTCTGG CTCCAAGCTT GACGATGTGT   
  
  
+ TTCGACTTCT AAAGTTTTAA CAATACCTTT GGTGGTTCTG CTTCCGCCAT TATCTTATTT TCCGCTGGTA   
  
  
+ GGTCGCTGCA GGATGAAGTA CTCCGAGAAC GGTGCTTGTA GAGAGAAACG GATGACGGAG AGGATCTATC   
  
  
+ AATTAAGTTA TCGGTTTCTT TGTGTTCTAA GAACGAGACA AGGGCTTTGA CTGTTCATGT TGAGAAAAAA   
  
  
+ GGAAAGTTCA TAGAGTCCTT CGCTGGTACT GTCGATCATT TAGTTCCAAG TTTCGAGTGA TCGGTAAAAA   
  
  
+ TTCATCGGTG AGAAATCACC TCCTAACGT  

- CAAATTTTCA ACCCATTGTA GGGTCATAAC CCCCTCTGAA GGCCAGGAAA TTGGTTCTGG AATGTTATTA   
  
  
- TTCATTTTAC CCCCACAGCA ATTCTCTTCA TTTGCTACTA GAGTTTCTAT ACAGAAAGGA TCCTTTCTGT   
  
  
- CAACAAGAAC AAGTTGAGTA TCTCCTGCCA CATACTTTTT GCTAGTGTCT CCTGCCACAT ATTTTTCACT   
  
  
- AGTCATGTGG CGAATGCCGT TAGTCATCCT TGTAACAGAT AGTTTCTGCT CATCATCCAA GTTATCAAGA   
  
  
- CACACTTCAT GTAGTGCGGG AAGAGCACGC TCTGAGTTTT GAGCCTGCAT AATCTAGTAA TCAGTAATAG   
  
  
- AAAACAATTC ATAATTGCGA AAAGTAAGCA ACTGTAAATC GCAACATTTA ACAAAATCAC CTCCAACTTG   
  
  
- TTAACTCAGA CTTAAGTAGG TATGATCTAT GTCTAATGCA TACTGTAAAC TGATATCCAA GAAAACAAAC   
  
  
- AAACATACTA AACATTTTCA TACCGCTTGT TTAAGAACAG AGCACACTAT TCGAGTCACA GCATTCTCTG   
  
  
- GATCCTGTAA AATGGATCAA AGCAAAAAAT GAAGCAATCT ACCAGAAACA AGGGGGAATG GTATAATCAT   
  
  
- ACAGCTCTAC CCTACCCTTA GAGAATCACT ATTAGCTCCA CAACGTGCTA TCTCTTTCAT CATCAGCAGA   
  
  
- GTACGCAACT GTGTATAACT TTTCACTCTC GTAGGCTTCA CAGAGCTTAG CTTAGAGAAA GCCAATCTGC   
  
  
- AGGCATTAAG TTTCGCCTCA AGGACTCTCC TTGAAATTTC TACAGCCAAG AAAACAGGAA CCCAGAATAG   
  
  
- GGAAGCTTAA TAGAGGTCAA AAAATCAAAT CACATTAGAA AGGAAAACAA AATTACATAA ATAAATAAAT   
  
  
- TCAACCCAGG GCATGGCAAC TGAACATGTA ACAAAGAAGA ATTCAAGAAA ATTTTAATTA ATTATAAAAT   
  
  
- ATATTATGTG TACTATGGTT TCCTTCAAGT TGTAGAAGGA GATACCTGTC TTCTTCAGAC TGATAACCGT   
  
  
- TAATCCATAG TCCATCAACA GAGTATAATT GATGGAGCAA ACAAATGCTT GTGTTAGTTA AATGAAATGA   
  
  
- TTGTTTGAGT AGTATACGAT TTCGCTTACC TGGCGGTGCC ATGTCAGACC GAGGTTCGAA CTGCTACACA   
  
  
- AAGCTGAAGA TTTCAAAATT GTTATGGAAA CCACCAAGAC GAAGGCGGTA ATAGAATAAA AGGCGACCAT   
  
  
- CCAGCGACGT CCTACTTCAT GAGGCTCTTG CCACGAACAT CTCTCTTTGC CTACTGCCTC TCCTAGATAG   
  
  
- TTAATTCAAT AGCCAAAGAA ACACAAGATT CTTGCTCTGT TCCCGAAACT GACAAGTACA ACTCTTTTTT   
  
  
- CCTTTCAAGT ATCTCAGGAA GCGACCATGA CAGCTAGTAA ATCAAGGTTC AAAGCTCACT AGCCATTTTT   
  
  
- AAGTAGCCAC TCTTTAGTGG AGGATTGCA

+     AuxRR-core

| Site Name | Organism | Position | Strand | Matrix score. | sequence | function |
| --- | --- | --- | --- | --- | --- | --- |
| AuxRR-core | Nicotiana tabacum | 1147 | - | 7 | GGTCCAT | cis-acting regulatory element involved in auxin responsiveness |

> 2018/04/13 10:10:12  
+ GTTTAAAAGT TGGGTAACAT CCCAGTATTG GGGGAGACTT CCGGTCCTTT AACCAAGACC TTACAATAAT   
  
  
+ AAGTAAAATG GGGGTGTCGT TAAGAGAAGT AAACGATGAT CTCAAAGATA TGTCTTTCCT AGGAAAGACA   
  
  
+ GTTGTTCTTG TTCAACTCAT AGAGGACGGT GTATGAAAAA CGATCACAGA GGACGGTGTA TAAAAAGTGA   
  
  
+ TCAGTACACC GCTTACGGCA ATCAGTAGGA ACATTGTCTA TCAAAGACGA GTAGTAGGTT CAATAGTTCT   
  
  
+ GTGTGAAGTA CATCACGCCC TTCTCGTGCG AGACTCAAAA CTCGGACGTA TTAGATCATT AGTCATTATC   
  
  
+ TTTTGTTAAG TATTAACGCT TTTCATTCGT TGACATTTAG CGTTGTAAAT TGTTTTAGTG GAGGTTGAAC   
  
  
+ AATTGAGTCT GAATTCATCC ATACTAGATA CAGATTACGT ATGACATTTG ACTATAGGTT CTTTTGTTTG   
  
  
+ TTTGTATGAT TTGTAAAAGT ATGGCGAACA AATTCTTGTC TCGTGTGATA AGCTCAGTGT CGTAAGAGAC   
  
  
+ CTAGGACATT TTACCTAGTT TCGTTTTTTA CTTCGTTAGA TGGTCTTTGT TCCCCCTTAC CATATTAGTA   
  
  
+ TGTCGAGATG GGATGGGAAT CTCTTAGTGA TAATCGAGGT GTTGCACGAT AGAGAAAGTA GTAGTCGTCT   
  
  
+ CATGCGTTGA CACATATTGA AAAGTGAGAG CATCCGAAGT GTCTCGAATC GAATCTCTTT CGGTTAGACG   
  
  
+ TCCGTAATTC AAAGCGGAGT TCCTGAGAGG AACTTTAAAG ATGTCGGTTC TTTTGTCCTT GGGTCTTATC   
  
  
+ CCTTCGAATT ATCTCCAGTT TTTTAGTTTA GTGTAATCTT TCCTTTTGTT TTAATGTATT TATTTATTTA   
  
  
+ AGTTGGGTCC CGTACCGTTG ACTTGTACAT TGTTTCTTCT TAAGTTCTTT TAAAATTAAT TAATATTTTA   
  
  
+ TATAATACAC ATGATACCAA AGGAAGTTCA ACATCTTCCT CTATGGACAG AAGAAGTCTG ACTATTGGCA   
  
  
+ ATTAGGTATC AGGTAGTTGT CTCATATTAA CTACCTCGTT TGTTTACGAA CACAATCAAT TTACTTTACT   
  
  
+ AACAAACTCA TCATATGCTA AAGCGAATGG ACCGCCACGG TACAGTCTGG CTCCAAGCTT GACGATGTGT   
  
  
+ TTCGACTTCT AAAGTTTTAA CAATACCTTT GGTGGTTCTG CTTCCGCCAT TATCTTATTT TCCGCTGGTA   
  
  
+ GGTCGCTGCA GGATGAAGTA CTCCGAGAAC GGTGCTTGTA GAGAGAAACG GATGACGGAG AGGATCTATC   
  
  
+ AATTAAGTTA TCGGTTTCTT TGTGTTCTAA GAACGAGACA AGGGCTTTGA CTGTTCATGT TGAGAAAAAA   
  
  
+ GGAAAGTTCA TAGAGTCCTT CGCTGGTACT GTCGATCATT TAGTTCCAAG TTTCGAGTGA TCGGTAAAAA   
  
  
+ TTCATCGGTG AGAAATCACC TCCTAACGT  

- CAAATTTTCA ACCCATTGTA GGGTCATAAC CCCCTCTGAA GGCCAGGAAA TTGGTTCTGG AATGTTATTA   
  
  
- TTCATTTTAC CCCCACAGCA ATTCTCTTCA TTTGCTACTA GAGTTTCTAT ACAGAAAGGA TCCTTTCTGT   
  
  
- CAACAAGAAC AAGTTGAGTA TCTCCTGCCA CATACTTTTT GCTAGTGTCT CCTGCCACAT ATTTTTCACT   
  
  
- AGTCATGTGG CGAATGCCGT TAGTCATCCT TGTAACAGAT AGTTTCTGCT CATCATCCAA GTTATCAAGA   
  
  
- CACACTTCAT GTAGTGCGGG AAGAGCACGC TCTGAGTTTT GAGCCTGCAT AATCTAGTAA TCAGTAATAG   
  
  
- AAAACAATTC ATAATTGCGA AAAGTAAGCA ACTGTAAATC GCAACATTTA ACAAAATCAC CTCCAACTTG   
  
  
- TTAACTCAGA CTTAAGTAGG TATGATCTAT GTCTAATGCA TACTGTAAAC TGATATCCAA GAAAACAAAC   
  
  
- AAACATACTA AACATTTTCA TACCGCTTGT TTAAGAACAG AGCACACTAT TCGAGTCACA GCATTCTCTG   
  
  
- GATCCTGTAA AATGGATCAA AGCAAAAAAT GAAGCAATCT ACCAGAAACA AGGGGGAATG GTATAATCAT   
  
  
- ACAGCTCTAC CCTACCCTTA GAGAATCACT ATTAGCTCCA CAACGTGCTA TCTCTTTCAT CATCAGCAGA   
  
  
- GTACGCAACT GTGTATAACT TTTCACTCTC GTAGGCTTCA CAGAGCTTAG CTTAGAGAAA GCCAATCTGC   
  
  
- AGGCATTAAG TTTCGCCTCA AGGACTCTCC TTGAAATTTC TACAGCCAAG AAAACAGGAA CCCAGAATAG   
  
  
- GGAAGCTTAA TAGAGGTCAA AAAATCAAAT CACATTAGAA AGGAAAACAA AATTACATAA ATAAATAAAT   
  
  
- TCAACCCAGG GCATGGCAAC TGAACATGTA ACAAAGAAGA ATTCAAGAAA ATTTTAATTA ATTATAAAAT   
  
  
- ATATTATGTG TACTATGGTT TCCTTCAAGT TGTAGAAGGA GATACCTGTC TTCTTCAGAC TGATAACCGT   
  
  
- TAATCCATAG TCCATCAACA GAGTATAATT GATGGAGCAA ACAAATGCTT GTGTTAGTTA AATGAAATGA   
  
  
- TTGTTTGAGT AGTATACGAT TTCGCTTACC TGGCGGTGCC ATGTCAGACC GAGGTTCGAA CTGCTACACA   
  
  
- AAGCTGAAGA TTTCAAAATT GTTATGGAAA CCACCAAGAC GAAGGCGGTA ATAGAATAAA AGGCGACCAT   
  
  
- CCAGCGACGT CCTACTTCAT GAGGCTCTTG CCACGAACAT CTCTCTTTGC CTACTGCCTC TCCTAGATAG   
  
  
- TTAATTCAAT AGCCAAAGAA ACACAAGATT CTTGCTCTGT TCCCGAAACT GACAAGTACA ACTCTTTTTT   
  
  
- CCTTTCAAGT ATCTCAGGAA GCGACCATGA CAGCTAGTAA ATCAAGGTTC AAAGCTCACT AGCCATTTTT   
  
  
- AAGTAGCCAC TCTTTAGTGG AGGATTGCA

+     Box 4

| Site Name | Organism | Position | Strand | Matrix score. | sequence | function |
| --- | --- | --- | --- | --- | --- | --- |
| Box 4 | Petroselinum crispum | 969 | - | 6 | ATTAAT | part of a conserved DNA module involved in light responsiveness |
| Box 4 | Petroselinum crispum | 965 | - | 6 | ATTAAT | part of a conserved DNA module involved in light responsiveness |

> 2018/04/13 10:10:12  
+ GTTTAAAAGT TGGGTAACAT CCCAGTATTG GGGGAGACTT CCGGTCCTTT AACCAAGACC TTACAATAAT   
  
  
+ AAGTAAAATG GGGGTGTCGT TAAGAGAAGT AAACGATGAT CTCAAAGATA TGTCTTTCCT AGGAAAGACA   
  
  
+ GTTGTTCTTG TTCAACTCAT AGAGGACGGT GTATGAAAAA CGATCACAGA GGACGGTGTA TAAAAAGTGA   
  
  
+ TCAGTACACC GCTTACGGCA ATCAGTAGGA ACATTGTCTA TCAAAGACGA GTAGTAGGTT CAATAGTTCT   
  
  
+ GTGTGAAGTA CATCACGCCC TTCTCGTGCG AGACTCAAAA CTCGGACGTA TTAGATCATT AGTCATTATC   
  
  
+ TTTTGTTAAG TATTAACGCT TTTCATTCGT TGACATTTAG CGTTGTAAAT TGTTTTAGTG GAGGTTGAAC   
  
  
+ AATTGAGTCT GAATTCATCC ATACTAGATA CAGATTACGT ATGACATTTG ACTATAGGTT CTTTTGTTTG   
  
  
+ TTTGTATGAT TTGTAAAAGT ATGGCGAACA AATTCTTGTC TCGTGTGATA AGCTCAGTGT CGTAAGAGAC   
  
  
+ CTAGGACATT TTACCTAGTT TCGTTTTTTA CTTCGTTAGA TGGTCTTTGT TCCCCCTTAC CATATTAGTA   
  
  
+ TGTCGAGATG GGATGGGAAT CTCTTAGTGA TAATCGAGGT GTTGCACGAT AGAGAAAGTA GTAGTCGTCT   
  
  
+ CATGCGTTGA CACATATTGA AAAGTGAGAG CATCCGAAGT GTCTCGAATC GAATCTCTTT CGGTTAGACG   
  
  
+ TCCGTAATTC AAAGCGGAGT TCCTGAGAGG AACTTTAAAG ATGTCGGTTC TTTTGTCCTT GGGTCTTATC   
  
  
+ CCTTCGAATT ATCTCCAGTT TTTTAGTTTA GTGTAATCTT TCCTTTTGTT TTAATGTATT TATTTATTTA   
  
  
+ AGTTGGGTCC CGTACCGTTG ACTTGTACAT TGTTTCTTCT TAAGTTCTTT TAAAATTAAT TAATATTTTA   
  
  
+ TATAATACAC ATGATACCAA AGGAAGTTCA ACATCTTCCT CTATGGACAG AAGAAGTCTG ACTATTGGCA   
  
  
+ ATTAGGTATC AGGTAGTTGT CTCATATTAA CTACCTCGTT TGTTTACGAA CACAATCAAT TTACTTTACT   
  
  
+ AACAAACTCA TCATATGCTA AAGCGAATGG ACCGCCACGG TACAGTCTGG CTCCAAGCTT GACGATGTGT   
  
  
+ TTCGACTTCT AAAGTTTTAA CAATACCTTT GGTGGTTCTG CTTCCGCCAT TATCTTATTT TCCGCTGGTA   
  
  
+ GGTCGCTGCA GGATGAAGTA CTCCGAGAAC GGTGCTTGTA GAGAGAAACG GATGACGGAG AGGATCTATC   
  
  
+ AATTAAGTTA TCGGTTTCTT TGTGTTCTAA GAACGAGACA AGGGCTTTGA CTGTTCATGT TGAGAAAAAA   
  
  
+ GGAAAGTTCA TAGAGTCCTT CGCTGGTACT GTCGATCATT TAGTTCCAAG TTTCGAGTGA TCGGTAAAAA   
  
  
+ TTCATCGGTG AGAAATCACC TCCTAACGT  

- CAAATTTTCA ACCCATTGTA GGGTCATAAC CCCCTCTGAA GGCCAGGAAA TTGGTTCTGG AATGTTATTA   
  
  
- TTCATTTTAC CCCCACAGCA ATTCTCTTCA TTTGCTACTA GAGTTTCTAT ACAGAAAGGA TCCTTTCTGT   
  
  
- CAACAAGAAC AAGTTGAGTA TCTCCTGCCA CATACTTTTT GCTAGTGTCT CCTGCCACAT ATTTTTCACT   
  
  
- AGTCATGTGG CGAATGCCGT TAGTCATCCT TGTAACAGAT AGTTTCTGCT CATCATCCAA GTTATCAAGA   
  
  
- CACACTTCAT GTAGTGCGGG AAGAGCACGC TCTGAGTTTT GAGCCTGCAT AATCTAGTAA TCAGTAATAG   
  
  
- AAAACAATTC ATAATTGCGA AAAGTAAGCA ACTGTAAATC GCAACATTTA ACAAAATCAC CTCCAACTTG   
  
  
- TTAACTCAGA CTTAAGTAGG TATGATCTAT GTCTAATGCA TACTGTAAAC TGATATCCAA GAAAACAAAC   
  
  
- AAACATACTA AACATTTTCA TACCGCTTGT TTAAGAACAG AGCACACTAT TCGAGTCACA GCATTCTCTG   
  
  
- GATCCTGTAA AATGGATCAA AGCAAAAAAT GAAGCAATCT ACCAGAAACA AGGGGGAATG GTATAATCAT   
  
  
- ACAGCTCTAC CCTACCCTTA GAGAATCACT ATTAGCTCCA CAACGTGCTA TCTCTTTCAT CATCAGCAGA   
  
  
- GTACGCAACT GTGTATAACT TTTCACTCTC GTAGGCTTCA CAGAGCTTAG CTTAGAGAAA GCCAATCTGC   
  
  
- AGGCATTAAG TTTCGCCTCA AGGACTCTCC TTGAAATTTC TACAGCCAAG AAAACAGGAA CCCAGAATAG   
  
  
- GGAAGCTTAA TAGAGGTCAA AAAATCAAAT CACATTAGAA AGGAAAACAA AATTACATAA ATAAATAAAT   
  
  
- TCAACCCAGG GCATGGCAAC TGAACATGTA ACAAAGAAGA ATTCAAGAAA ATTTTAATTA ATTATAAAAT   
  
  
- ATATTATGTG TACTATGGTT TCCTTCAAGT TGTAGAAGGA GATACCTGTC TTCTTCAGAC TGATAACCGT   
  
  
- TAATCCATAG TCCATCAACA GAGTATAATT GATGGAGCAA ACAAATGCTT GTGTTAGTTA AATGAAATGA   
  
  
- TTGTTTGAGT AGTATACGAT TTCGCTTACC TGGCGGTGCC ATGTCAGACC GAGGTTCGAA CTGCTACACA   
  
  
- AAGCTGAAGA TTTCAAAATT GTTATGGAAA CCACCAAGAC GAAGGCGGTA ATAGAATAAA AGGCGACCAT   
  
  
- CCAGCGACGT CCTACTTCAT GAGGCTCTTG CCACGAACAT CTCTCTTTGC CTACTGCCTC TCCTAGATAG   
  
  
- TTAATTCAAT AGCCAAAGAA ACACAAGATT CTTGCTCTGT TCCCGAAACT GACAAGTACA ACTCTTTTTT   
  
  
- CCTTTCAAGT ATCTCAGGAA GCGACCATGA CAGCTAGTAA ATCAAGGTTC AAAGCTCACT AGCCATTTTT   
  
  
- AAGTAGCCAC TCTTTAGTGG AGGATTGCA

+     CAAT-box

| Site Name | Organism | Position | Strand | Matrix score. | sequence | function |
| --- | --- | --- | --- | --- | --- | --- |
| CAAT-box | Hordeum vulgare | 1103 | + | 4 | CAAT | common cis-acting element in promoter and enhancer regions |
| CAAT-box | Glycine max | 420 | + | 5 | CAATT | common cis-acting element in promoter and enhancer regions |
| CAAT-box | Glycine max | 1330 | + | 5 | CAATT | common cis-acting element in promoter and enhancer regions |
| CAAT-box | Hordeum vulgare | 939 | - | 4 | CAAT | common cis-acting element in promoter and enhancer regions |
| CAAT-box | Brassica rapa | 466 | - | 5 | CAAAT | common cis-acting element in promoter and enhancer regions |
| CAAT-box | Hordeum vulgare | 716 | - | 4 | CAAT | common cis-acting element in promoter and enhancer regions |
| CAAT-box | Hordeum vulgare | 243 | - | 4 | CAAT | common cis-acting element in promoter and enhancer regions |
| CAAT-box | Hordeum vulgare | 64 | + | 4 | CAAT | common cis-acting element in promoter and enhancer regions |
| CAAT-box | Arabidopsis thaliana | 227 | + | 6 | gGCAAT | common cis-acting element in promoter and enhancer regions |
| CAAT-box | Glycine max | 1107 | + | 5 | CAATT | common cis-acting element in promoter and enhancer regions |
| CAAT-box | Glycine max | 398 | - | 5 | CAATT | common cis-acting element in promoter and enhancer regions |
| CAAT-box | Hordeum vulgare | 1211 | + | 4 | CAAT | common cis-acting element in promoter and enhancer regions |
| CAAT-box | Arabidopsis thaliana | 1044 | - | 5 | CCAAT | common cis-acting element in promoter and enhancer regions |
| CAAT-box | Arabidopsis thaliana | 27 | - | 5 | CCAAT | common cis-acting element in promoter and enhancer regions |
| CAAT-box | Hordeum vulgare | 422 | - | 4 | CAAT | common cis-acting element in promoter and enhancer regions |
| CAAT-box | Hordeum vulgare | 399 | - | 4 | CAAT | common cis-acting element in promoter and enhancer regions |
| CAAT-box | Brassica rapa | 499 | - | 5 | CAAAT | common cis-acting element in promoter and enhancer regions |
| CAAT-box | Hordeum vulgare | 271 | + | 4 | CAAT | common cis-acting element in promoter and enhancer regions |
| CAAT-box | Arabidopsis thaliana | 1047 | + | 6 | gGCAAT | common cis-acting element in promoter and enhancer regions |
| CAAT-box | Glycine max | 1049 | + | 5 | CAATT | common cis-acting element in promoter and enhancer regions |
| CAAT-box | Hordeum vulgare | 229 | + | 4 | CAAT | common cis-acting element in promoter and enhancer regions |
| CAAT-box | Brassica rapa | 519 | + | 5 | CAAAT | common cis-acting element in promoter and enhancer regions |
| CAAT-box | Glycine max | 421 | - | 5 | CAATT | common cis-acting element in promoter and enhancer regions |

> 2018/04/13 10:10:12  
+ GTTTAAAAGT TGGGTAACAT CCCAGTATTG GGGGAGACTT CCGGTCCTTT AACCAAGACC TTACAATAAT   
  
  
+ AAGTAAAATG GGGGTGTCGT TAAGAGAAGT AAACGATGAT CTCAAAGATA TGTCTTTCCT AGGAAAGACA   
  
  
+ GTTGTTCTTG TTCAACTCAT AGAGGACGGT GTATGAAAAA CGATCACAGA GGACGGTGTA TAAAAAGTGA   
  
  
+ TCAGTACACC GCTTACGGCA ATCAGTAGGA ACATTGTCTA TCAAAGACGA GTAGTAGGTT CAATAGTTCT   
  
  
+ GTGTGAAGTA CATCACGCCC TTCTCGTGCG AGACTCAAAA CTCGGACGTA TTAGATCATT AGTCATTATC   
  
  
+ TTTTGTTAAG TATTAACGCT TTTCATTCGT TGACATTTAG CGTTGTAAAT TGTTTTAGTG GAGGTTGAAC   
  
  
+ AATTGAGTCT GAATTCATCC ATACTAGATA CAGATTACGT ATGACATTTG ACTATAGGTT CTTTTGTTTG   
  
  
+ TTTGTATGAT TTGTAAAAGT ATGGCGAACA AATTCTTGTC TCGTGTGATA AGCTCAGTGT CGTAAGAGAC   
  
  
+ CTAGGACATT TTACCTAGTT TCGTTTTTTA CTTCGTTAGA TGGTCTTTGT TCCCCCTTAC CATATTAGTA   
  
  
+ TGTCGAGATG GGATGGGAAT CTCTTAGTGA TAATCGAGGT GTTGCACGAT AGAGAAAGTA GTAGTCGTCT   
  
  
+ CATGCGTTGA CACATATTGA AAAGTGAGAG CATCCGAAGT GTCTCGAATC GAATCTCTTT CGGTTAGACG   
  
  
+ TCCGTAATTC AAAGCGGAGT TCCTGAGAGG AACTTTAAAG ATGTCGGTTC TTTTGTCCTT GGGTCTTATC   
  
  
+ CCTTCGAATT ATCTCCAGTT TTTTAGTTTA GTGTAATCTT TCCTTTTGTT TTAATGTATT TATTTATTTA   
  
  
+ AGTTGGGTCC CGTACCGTTG ACTTGTACAT TGTTTCTTCT TAAGTTCTTT TAAAATTAAT TAATATTTTA   
  
  
+ TATAATACAC ATGATACCAA AGGAAGTTCA ACATCTTCCT CTATGGACAG AAGAAGTCTG ACTATTGGCA   
  
  
+ ATTAGGTATC AGGTAGTTGT CTCATATTAA CTACCTCGTT TGTTTACGAA CACAATCAAT TTACTTTACT   
  
  
+ AACAAACTCA TCATATGCTA AAGCGAATGG ACCGCCACGG TACAGTCTGG CTCCAAGCTT GACGATGTGT   
  
  
+ TTCGACTTCT AAAGTTTTAA CAATACCTTT GGTGGTTCTG CTTCCGCCAT TATCTTATTT TCCGCTGGTA   
  
  
+ GGTCGCTGCA GGATGAAGTA CTCCGAGAAC GGTGCTTGTA GAGAGAAACG GATGACGGAG AGGATCTATC   
  
  
+ AATTAAGTTA TCGGTTTCTT TGTGTTCTAA GAACGAGACA AGGGCTTTGA CTGTTCATGT TGAGAAAAAA   
  
  
+ GGAAAGTTCA TAGAGTCCTT CGCTGGTACT GTCGATCATT TAGTTCCAAG TTTCGAGTGA TCGGTAAAAA   
  
  
+ TTCATCGGTG AGAAATCACC TCCTAACGT  

- CAAATTTTCA ACCCATTGTA GGGTCATAAC CCCCTCTGAA GGCCAGGAAA TTGGTTCTGG AATGTTATTA   
  
  
- TTCATTTTAC CCCCACAGCA ATTCTCTTCA TTTGCTACTA GAGTTTCTAT ACAGAAAGGA TCCTTTCTGT   
  
  
- CAACAAGAAC AAGTTGAGTA TCTCCTGCCA CATACTTTTT GCTAGTGTCT CCTGCCACAT ATTTTTCACT   
  
  
- AGTCATGTGG CGAATGCCGT TAGTCATCCT TGTAACAGAT AGTTTCTGCT CATCATCCAA GTTATCAAGA   
  
  
- CACACTTCAT GTAGTGCGGG AAGAGCACGC TCTGAGTTTT GAGCCTGCAT AATCTAGTAA TCAGTAATAG   
  
  
- AAAACAATTC ATAATTGCGA AAAGTAAGCA ACTGTAAATC GCAACATTTA ACAAAATCAC CTCCAACTTG   
  
  
- TTAACTCAGA CTTAAGTAGG TATGATCTAT GTCTAATGCA TACTGTAAAC TGATATCCAA GAAAACAAAC   
  
  
- AAACATACTA AACATTTTCA TACCGCTTGT TTAAGAACAG AGCACACTAT TCGAGTCACA GCATTCTCTG   
  
  
- GATCCTGTAA AATGGATCAA AGCAAAAAAT GAAGCAATCT ACCAGAAACA AGGGGGAATG GTATAATCAT   
  
  
- ACAGCTCTAC CCTACCCTTA GAGAATCACT ATTAGCTCCA CAACGTGCTA TCTCTTTCAT CATCAGCAGA   
  
  
- GTACGCAACT GTGTATAACT TTTCACTCTC GTAGGCTTCA CAGAGCTTAG CTTAGAGAAA GCCAATCTGC   
  
  
- AGGCATTAAG TTTCGCCTCA AGGACTCTCC TTGAAATTTC TACAGCCAAG AAAACAGGAA CCCAGAATAG   
  
  
- GGAAGCTTAA TAGAGGTCAA AAAATCAAAT CACATTAGAA AGGAAAACAA AATTACATAA ATAAATAAAT   
  
  
- TCAACCCAGG GCATGGCAAC TGAACATGTA ACAAAGAAGA ATTCAAGAAA ATTTTAATTA ATTATAAAAT   
  
  
- ATATTATGTG TACTATGGTT TCCTTCAAGT TGTAGAAGGA GATACCTGTC TTCTTCAGAC TGATAACCGT   
  
  
- TAATCCATAG TCCATCAACA GAGTATAATT GATGGAGCAA ACAAATGCTT GTGTTAGTTA AATGAAATGA   
  
  
- TTGTTTGAGT AGTATACGAT TTCGCTTACC TGGCGGTGCC ATGTCAGACC GAGGTTCGAA CTGCTACACA   
  
  
- AAGCTGAAGA TTTCAAAATT GTTATGGAAA CCACCAAGAC GAAGGCGGTA ATAGAATAAA AGGCGACCAT   
  
  
- CCAGCGACGT CCTACTTCAT GAGGCTCTTG CCACGAACAT CTCTCTTTGC CTACTGCCTC TCCTAGATAG   
  
  
- TTAATTCAAT AGCCAAAGAA ACACAAGATT CTTGCTCTGT TCCCGAAACT GACAAGTACA ACTCTTTTTT   
  
  
- CCTTTCAAGT ATCTCAGGAA GCGACCATGA CAGCTAGTAA ATCAAGGTTC AAAGCTCACT AGCCATTTTT   
  
  
- AAGTAGCCAC TCTTTAGTGG AGGATTGCA

+     CCAAT-box

| Site Name | Organism | Position | Strand | Matrix score. | sequence | function |
| --- | --- | --- | --- | --- | --- | --- |
| CCAAT-box | Hordeum vulgare | 925 | - | 6 | CAACGG | MYBHv1 binding site |

> 2018/04/13 10:10:12  
+ GTTTAAAAGT TGGGTAACAT CCCAGTATTG GGGGAGACTT CCGGTCCTTT AACCAAGACC TTACAATAAT   
  
  
+ AAGTAAAATG GGGGTGTCGT TAAGAGAAGT AAACGATGAT CTCAAAGATA TGTCTTTCCT AGGAAAGACA   
  
  
+ GTTGTTCTTG TTCAACTCAT AGAGGACGGT GTATGAAAAA CGATCACAGA GGACGGTGTA TAAAAAGTGA   
  
  
+ TCAGTACACC GCTTACGGCA ATCAGTAGGA ACATTGTCTA TCAAAGACGA GTAGTAGGTT CAATAGTTCT   
  
  
+ GTGTGAAGTA CATCACGCCC TTCTCGTGCG AGACTCAAAA CTCGGACGTA TTAGATCATT AGTCATTATC   
  
  
+ TTTTGTTAAG TATTAACGCT TTTCATTCGT TGACATTTAG CGTTGTAAAT TGTTTTAGTG GAGGTTGAAC   
  
  
+ AATTGAGTCT GAATTCATCC ATACTAGATA CAGATTACGT ATGACATTTG ACTATAGGTT CTTTTGTTTG   
  
  
+ TTTGTATGAT TTGTAAAAGT ATGGCGAACA AATTCTTGTC TCGTGTGATA AGCTCAGTGT CGTAAGAGAC   
  
  
+ CTAGGACATT TTACCTAGTT TCGTTTTTTA CTTCGTTAGA TGGTCTTTGT TCCCCCTTAC CATATTAGTA   
  
  
+ TGTCGAGATG GGATGGGAAT CTCTTAGTGA TAATCGAGGT GTTGCACGAT AGAGAAAGTA GTAGTCGTCT   
  
  
+ CATGCGTTGA CACATATTGA AAAGTGAGAG CATCCGAAGT GTCTCGAATC GAATCTCTTT CGGTTAGACG   
  
  
+ TCCGTAATTC AAAGCGGAGT TCCTGAGAGG AACTTTAAAG ATGTCGGTTC TTTTGTCCTT GGGTCTTATC   
  
  
+ CCTTCGAATT ATCTCCAGTT TTTTAGTTTA GTGTAATCTT TCCTTTTGTT TTAATGTATT TATTTATTTA   
  
  
+ AGTTGGGTCC CGTACCGTTG ACTTGTACAT TGTTTCTTCT TAAGTTCTTT TAAAATTAAT TAATATTTTA   
  
  
+ TATAATACAC ATGATACCAA AGGAAGTTCA ACATCTTCCT CTATGGACAG AAGAAGTCTG ACTATTGGCA   
  
  
+ ATTAGGTATC AGGTAGTTGT CTCATATTAA CTACCTCGTT TGTTTACGAA CACAATCAAT TTACTTTACT   
  
  
+ AACAAACTCA TCATATGCTA AAGCGAATGG ACCGCCACGG TACAGTCTGG CTCCAAGCTT GACGATGTGT   
  
  
+ TTCGACTTCT AAAGTTTTAA CAATACCTTT GGTGGTTCTG CTTCCGCCAT TATCTTATTT TCCGCTGGTA   
  
  
+ GGTCGCTGCA GGATGAAGTA CTCCGAGAAC GGTGCTTGTA GAGAGAAACG GATGACGGAG AGGATCTATC   
  
  
+ AATTAAGTTA TCGGTTTCTT TGTGTTCTAA GAACGAGACA AGGGCTTTGA CTGTTCATGT TGAGAAAAAA   
  
  
+ GGAAAGTTCA TAGAGTCCTT CGCTGGTACT GTCGATCATT TAGTTCCAAG TTTCGAGTGA TCGGTAAAAA   
  
  
+ TTCATCGGTG AGAAATCACC TCCTAACGT  

- CAAATTTTCA ACCCATTGTA GGGTCATAAC CCCCTCTGAA GGCCAGGAAA TTGGTTCTGG AATGTTATTA   
  
  
- TTCATTTTAC CCCCACAGCA ATTCTCTTCA TTTGCTACTA GAGTTTCTAT ACAGAAAGGA TCCTTTCTGT   
  
  
- CAACAAGAAC AAGTTGAGTA TCTCCTGCCA CATACTTTTT GCTAGTGTCT CCTGCCACAT ATTTTTCACT   
  
  
- AGTCATGTGG CGAATGCCGT TAGTCATCCT TGTAACAGAT AGTTTCTGCT CATCATCCAA GTTATCAAGA   
  
  
- CACACTTCAT GTAGTGCGGG AAGAGCACGC TCTGAGTTTT GAGCCTGCAT AATCTAGTAA TCAGTAATAG   
  
  
- AAAACAATTC ATAATTGCGA AAAGTAAGCA ACTGTAAATC GCAACATTTA ACAAAATCAC CTCCAACTTG   
  
  
- TTAACTCAGA CTTAAGTAGG TATGATCTAT GTCTAATGCA TACTGTAAAC TGATATCCAA GAAAACAAAC   
  
  
- AAACATACTA AACATTTTCA TACCGCTTGT TTAAGAACAG AGCACACTAT TCGAGTCACA GCATTCTCTG   
  
  
- GATCCTGTAA AATGGATCAA AGCAAAAAAT GAAGCAATCT ACCAGAAACA AGGGGGAATG GTATAATCAT   
  
  
- ACAGCTCTAC CCTACCCTTA GAGAATCACT ATTAGCTCCA CAACGTGCTA TCTCTTTCAT CATCAGCAGA   
  
  
- GTACGCAACT GTGTATAACT TTTCACTCTC GTAGGCTTCA CAGAGCTTAG CTTAGAGAAA GCCAATCTGC   
  
  
- AGGCATTAAG TTTCGCCTCA AGGACTCTCC TTGAAATTTC TACAGCCAAG AAAACAGGAA CCCAGAATAG   
  
  
- GGAAGCTTAA TAGAGGTCAA AAAATCAAAT CACATTAGAA AGGAAAACAA AATTACATAA ATAAATAAAT   
  
  
- TCAACCCAGG GCATGGCAAC TGAACATGTA ACAAAGAAGA ATTCAAGAAA ATTTTAATTA ATTATAAAAT   
  
  
- ATATTATGTG TACTATGGTT TCCTTCAAGT TGTAGAAGGA GATACCTGTC TTCTTCAGAC TGATAACCGT   
  
  
- TAATCCATAG TCCATCAACA GAGTATAATT GATGGAGCAA ACAAATGCTT GTGTTAGTTA AATGAAATGA   
  
  
- TTGTTTGAGT AGTATACGAT TTCGCTTACC TGGCGGTGCC ATGTCAGACC GAGGTTCGAA CTGCTACACA   
  
  
- AAGCTGAAGA TTTCAAAATT GTTATGGAAA CCACCAAGAC GAAGGCGGTA ATAGAATAAA AGGCGACCAT   
  
  
- CCAGCGACGT CCTACTTCAT GAGGCTCTTG CCACGAACAT CTCTCTTTGC CTACTGCCTC TCCTAGATAG   
  
  
- TTAATTCAAT AGCCAAAGAA ACACAAGATT CTTGCTCTGT TCCCGAAACT GACAAGTACA ACTCTTTTTT   
  
  
- CCTTTCAAGT ATCTCAGGAA GCGACCATGA CAGCTAGTAA ATCAAGGTTC AAAGCTCACT AGCCATTTTT   
  
  
- AAGTAGCCAC TCTTTAGTGG AGGATTGCA

+     CCGTCC-box

| Site Name | Organism | Position | Strand | Matrix score. | sequence | function |
| --- | --- | --- | --- | --- | --- | --- |
| CCGTCC-box | Arabidopsis thaliana | 164 | - | 6 | CCGTCC | cis-acting regulatory element related to meristem specific activation |
| CCGTCC-box | Arabidopsis thaliana | 191 | - | 6 | CCGTCC | cis-acting regulatory element related to meristem specific activation |

> 2018/04/13 10:10:12  
+ GTTTAAAAGT TGGGTAACAT CCCAGTATTG GGGGAGACTT CCGGTCCTTT AACCAAGACC TTACAATAAT   
  
  
+ AAGTAAAATG GGGGTGTCGT TAAGAGAAGT AAACGATGAT CTCAAAGATA TGTCTTTCCT AGGAAAGACA   
  
  
+ GTTGTTCTTG TTCAACTCAT AGAGGACGGT GTATGAAAAA CGATCACAGA GGACGGTGTA TAAAAAGTGA   
  
  
+ TCAGTACACC GCTTACGGCA ATCAGTAGGA ACATTGTCTA TCAAAGACGA GTAGTAGGTT CAATAGTTCT   
  
  
+ GTGTGAAGTA CATCACGCCC TTCTCGTGCG AGACTCAAAA CTCGGACGTA TTAGATCATT AGTCATTATC   
  
  
+ TTTTGTTAAG TATTAACGCT TTTCATTCGT TGACATTTAG CGTTGTAAAT TGTTTTAGTG GAGGTTGAAC   
  
  
+ AATTGAGTCT GAATTCATCC ATACTAGATA CAGATTACGT ATGACATTTG ACTATAGGTT CTTTTGTTTG   
  
  
+ TTTGTATGAT TTGTAAAAGT ATGGCGAACA AATTCTTGTC TCGTGTGATA AGCTCAGTGT CGTAAGAGAC   
  
  
+ CTAGGACATT TTACCTAGTT TCGTTTTTTA CTTCGTTAGA TGGTCTTTGT TCCCCCTTAC CATATTAGTA   
  
  
+ TGTCGAGATG GGATGGGAAT CTCTTAGTGA TAATCGAGGT GTTGCACGAT AGAGAAAGTA GTAGTCGTCT   
  
  
+ CATGCGTTGA CACATATTGA AAAGTGAGAG CATCCGAAGT GTCTCGAATC GAATCTCTTT CGGTTAGACG   
  
  
+ TCCGTAATTC AAAGCGGAGT TCCTGAGAGG AACTTTAAAG ATGTCGGTTC TTTTGTCCTT GGGTCTTATC   
  
  
+ CCTTCGAATT ATCTCCAGTT TTTTAGTTTA GTGTAATCTT TCCTTTTGTT TTAATGTATT TATTTATTTA   
  
  
+ AGTTGGGTCC CGTACCGTTG ACTTGTACAT TGTTTCTTCT TAAGTTCTTT TAAAATTAAT TAATATTTTA   
  
  
+ TATAATACAC ATGATACCAA AGGAAGTTCA ACATCTTCCT CTATGGACAG AAGAAGTCTG ACTATTGGCA   
  
  
+ ATTAGGTATC AGGTAGTTGT CTCATATTAA CTACCTCGTT TGTTTACGAA CACAATCAAT TTACTTTACT   
  
  
+ AACAAACTCA TCATATGCTA AAGCGAATGG ACCGCCACGG TACAGTCTGG CTCCAAGCTT GACGATGTGT   
  
  
+ TTCGACTTCT AAAGTTTTAA CAATACCTTT GGTGGTTCTG CTTCCGCCAT TATCTTATTT TCCGCTGGTA   
  
  
+ GGTCGCTGCA GGATGAAGTA CTCCGAGAAC GGTGCTTGTA GAGAGAAACG GATGACGGAG AGGATCTATC   
  
  
+ AATTAAGTTA TCGGTTTCTT TGTGTTCTAA GAACGAGACA AGGGCTTTGA CTGTTCATGT TGAGAAAAAA   
  
  
+ GGAAAGTTCA TAGAGTCCTT CGCTGGTACT GTCGATCATT TAGTTCCAAG TTTCGAGTGA TCGGTAAAAA   
  
  
+ TTCATCGGTG AGAAATCACC TCCTAACGT  

- CAAATTTTCA ACCCATTGTA GGGTCATAAC CCCCTCTGAA GGCCAGGAAA TTGGTTCTGG AATGTTATTA   
  
  
- TTCATTTTAC CCCCACAGCA ATTCTCTTCA TTTGCTACTA GAGTTTCTAT ACAGAAAGGA TCCTTTCTGT   
  
  
- CAACAAGAAC AAGTTGAGTA TCTCCTGCCA CATACTTTTT GCTAGTGTCT CCTGCCACAT ATTTTTCACT   
  
  
- AGTCATGTGG CGAATGCCGT TAGTCATCCT TGTAACAGAT AGTTTCTGCT CATCATCCAA GTTATCAAGA   
  
  
- CACACTTCAT GTAGTGCGGG AAGAGCACGC TCTGAGTTTT GAGCCTGCAT AATCTAGTAA TCAGTAATAG   
  
  
- AAAACAATTC ATAATTGCGA AAAGTAAGCA ACTGTAAATC GCAACATTTA ACAAAATCAC CTCCAACTTG   
  
  
- TTAACTCAGA CTTAAGTAGG TATGATCTAT GTCTAATGCA TACTGTAAAC TGATATCCAA GAAAACAAAC   
  
  
- AAACATACTA AACATTTTCA TACCGCTTGT TTAAGAACAG AGCACACTAT TCGAGTCACA GCATTCTCTG   
  
  
- GATCCTGTAA AATGGATCAA AGCAAAAAAT GAAGCAATCT ACCAGAAACA AGGGGGAATG GTATAATCAT   
  
  
- ACAGCTCTAC CCTACCCTTA GAGAATCACT ATTAGCTCCA CAACGTGCTA TCTCTTTCAT CATCAGCAGA   
  
  
- GTACGCAACT GTGTATAACT TTTCACTCTC GTAGGCTTCA CAGAGCTTAG CTTAGAGAAA GCCAATCTGC   
  
  
- AGGCATTAAG TTTCGCCTCA AGGACTCTCC TTGAAATTTC TACAGCCAAG AAAACAGGAA CCCAGAATAG   
  
  
- GGAAGCTTAA TAGAGGTCAA AAAATCAAAT CACATTAGAA AGGAAAACAA AATTACATAA ATAAATAAAT   
  
  
- TCAACCCAGG GCATGGCAAC TGAACATGTA ACAAAGAAGA ATTCAAGAAA ATTTTAATTA ATTATAAAAT   
  
  
- ATATTATGTG TACTATGGTT TCCTTCAAGT TGTAGAAGGA GATACCTGTC TTCTTCAGAC TGATAACCGT   
  
  
- TAATCCATAG TCCATCAACA GAGTATAATT GATGGAGCAA ACAAATGCTT GTGTTAGTTA AATGAAATGA   
  
  
- TTGTTTGAGT AGTATACGAT TTCGCTTACC TGGCGGTGCC ATGTCAGACC GAGGTTCGAA CTGCTACACA   
  
  
- AAGCTGAAGA TTTCAAAATT GTTATGGAAA CCACCAAGAC GAAGGCGGTA ATAGAATAAA AGGCGACCAT   
  
  
- CCAGCGACGT CCTACTTCAT GAGGCTCTTG CCACGAACAT CTCTCTTTGC CTACTGCCTC TCCTAGATAG   
  
  
- TTAATTCAAT AGCCAAAGAA ACACAAGATT CTTGCTCTGT TCCCGAAACT GACAAGTACA ACTCTTTTTT   
  
  
- CCTTTCAAGT ATCTCAGGAA GCGACCATGA CAGCTAGTAA ATCAAGGTTC AAAGCTCACT AGCCATTTTT   
  
  
- AAGTAGCCAC TCTTTAGTGG AGGATTGCA

+     CGTCA-motif

| Site Name | Organism | Position | Strand | Matrix score. | sequence | function |
| --- | --- | --- | --- | --- | --- | --- |
| CGTCA-motif | Hordeum vulgare | 1313 | - | 5 | CGTCA | cis-acting regulatory element involved in the MeJA-responsiveness |
| CGTCA-motif | Hordeum vulgare | 1180 | - | 5 | CGTCA | cis-acting regulatory element involved in the MeJA-responsiveness |

> 2018/04/13 10:10:12  
+ GTTTAAAAGT TGGGTAACAT CCCAGTATTG GGGGAGACTT CCGGTCCTTT AACCAAGACC TTACAATAAT   
  
  
+ AAGTAAAATG GGGGTGTCGT TAAGAGAAGT AAACGATGAT CTCAAAGATA TGTCTTTCCT AGGAAAGACA   
  
  
+ GTTGTTCTTG TTCAACTCAT AGAGGACGGT GTATGAAAAA CGATCACAGA GGACGGTGTA TAAAAAGTGA   
  
  
+ TCAGTACACC GCTTACGGCA ATCAGTAGGA ACATTGTCTA TCAAAGACGA GTAGTAGGTT CAATAGTTCT   
  
  
+ GTGTGAAGTA CATCACGCCC TTCTCGTGCG AGACTCAAAA CTCGGACGTA TTAGATCATT AGTCATTATC   
  
  
+ TTTTGTTAAG TATTAACGCT TTTCATTCGT TGACATTTAG CGTTGTAAAT TGTTTTAGTG GAGGTTGAAC   
  
  
+ AATTGAGTCT GAATTCATCC ATACTAGATA CAGATTACGT ATGACATTTG ACTATAGGTT CTTTTGTTTG   
  
  
+ TTTGTATGAT TTGTAAAAGT ATGGCGAACA AATTCTTGTC TCGTGTGATA AGCTCAGTGT CGTAAGAGAC   
  
  
+ CTAGGACATT TTACCTAGTT TCGTTTTTTA CTTCGTTAGA TGGTCTTTGT TCCCCCTTAC CATATTAGTA   
  
  
+ TGTCGAGATG GGATGGGAAT CTCTTAGTGA TAATCGAGGT GTTGCACGAT AGAGAAAGTA GTAGTCGTCT   
  
  
+ CATGCGTTGA CACATATTGA AAAGTGAGAG CATCCGAAGT GTCTCGAATC GAATCTCTTT CGGTTAGACG   
  
  
+ TCCGTAATTC AAAGCGGAGT TCCTGAGAGG AACTTTAAAG ATGTCGGTTC TTTTGTCCTT GGGTCTTATC   
  
  
+ CCTTCGAATT ATCTCCAGTT TTTTAGTTTA GTGTAATCTT TCCTTTTGTT TTAATGTATT TATTTATTTA   
  
  
+ AGTTGGGTCC CGTACCGTTG ACTTGTACAT TGTTTCTTCT TAAGTTCTTT TAAAATTAAT TAATATTTTA   
  
  
+ TATAATACAC ATGATACCAA AGGAAGTTCA ACATCTTCCT CTATGGACAG AAGAAGTCTG ACTATTGGCA   
  
  
+ ATTAGGTATC AGGTAGTTGT CTCATATTAA CTACCTCGTT TGTTTACGAA CACAATCAAT TTACTTTACT   
  
  
+ AACAAACTCA TCATATGCTA AAGCGAATGG ACCGCCACGG TACAGTCTGG CTCCAAGCTT GACGATGTGT   
  
  
+ TTCGACTTCT AAAGTTTTAA CAATACCTTT GGTGGTTCTG CTTCCGCCAT TATCTTATTT TCCGCTGGTA   
  
  
+ GGTCGCTGCA GGATGAAGTA CTCCGAGAAC GGTGCTTGTA GAGAGAAACG GATGACGGAG AGGATCTATC   
  
  
+ AATTAAGTTA TCGGTTTCTT TGTGTTCTAA GAACGAGACA AGGGCTTTGA CTGTTCATGT TGAGAAAAAA   
  
  
+ GGAAAGTTCA TAGAGTCCTT CGCTGGTACT GTCGATCATT TAGTTCCAAG TTTCGAGTGA TCGGTAAAAA   
  
  
+ TTCATCGGTG AGAAATCACC TCCTAACGT  

- CAAATTTTCA ACCCATTGTA GGGTCATAAC CCCCTCTGAA GGCCAGGAAA TTGGTTCTGG AATGTTATTA   
  
  
- TTCATTTTAC CCCCACAGCA ATTCTCTTCA TTTGCTACTA GAGTTTCTAT ACAGAAAGGA TCCTTTCTGT   
  
  
- CAACAAGAAC AAGTTGAGTA TCTCCTGCCA CATACTTTTT GCTAGTGTCT CCTGCCACAT ATTTTTCACT   
  
  
- AGTCATGTGG CGAATGCCGT TAGTCATCCT TGTAACAGAT AGTTTCTGCT CATCATCCAA GTTATCAAGA   
  
  
- CACACTTCAT GTAGTGCGGG AAGAGCACGC TCTGAGTTTT GAGCCTGCAT AATCTAGTAA TCAGTAATAG   
  
  
- AAAACAATTC ATAATTGCGA AAAGTAAGCA ACTGTAAATC GCAACATTTA ACAAAATCAC CTCCAACTTG   
  
  
- TTAACTCAGA CTTAAGTAGG TATGATCTAT GTCTAATGCA TACTGTAAAC TGATATCCAA GAAAACAAAC   
  
  
- AAACATACTA AACATTTTCA TACCGCTTGT TTAAGAACAG AGCACACTAT TCGAGTCACA GCATTCTCTG   
  
  
- GATCCTGTAA AATGGATCAA AGCAAAAAAT GAAGCAATCT ACCAGAAACA AGGGGGAATG GTATAATCAT   
  
  
- ACAGCTCTAC CCTACCCTTA GAGAATCACT ATTAGCTCCA CAACGTGCTA TCTCTTTCAT CATCAGCAGA   
  
  
- GTACGCAACT GTGTATAACT TTTCACTCTC GTAGGCTTCA CAGAGCTTAG CTTAGAGAAA GCCAATCTGC   
  
  
- AGGCATTAAG TTTCGCCTCA AGGACTCTCC TTGAAATTTC TACAGCCAAG AAAACAGGAA CCCAGAATAG   
  
  
- GGAAGCTTAA TAGAGGTCAA AAAATCAAAT CACATTAGAA AGGAAAACAA AATTACATAA ATAAATAAAT   
  
  
- TCAACCCAGG GCATGGCAAC TGAACATGTA ACAAAGAAGA ATTCAAGAAA ATTTTAATTA ATTATAAAAT   
  
  
- ATATTATGTG TACTATGGTT TCCTTCAAGT TGTAGAAGGA GATACCTGTC TTCTTCAGAC TGATAACCGT   
  
  
- TAATCCATAG TCCATCAACA GAGTATAATT GATGGAGCAA ACAAATGCTT GTGTTAGTTA AATGAAATGA   
  
  
- TTGTTTGAGT AGTATACGAT TTCGCTTACC TGGCGGTGCC ATGTCAGACC GAGGTTCGAA CTGCTACACA   
  
  
- AAGCTGAAGA TTTCAAAATT GTTATGGAAA CCACCAAGAC GAAGGCGGTA ATAGAATAAA AGGCGACCAT   
  
  
- CCAGCGACGT CCTACTTCAT GAGGCTCTTG CCACGAACAT CTCTCTTTGC CTACTGCCTC TCCTAGATAG   
  
  
- TTAATTCAAT AGCCAAAGAA ACACAAGATT CTTGCTCTGT TCCCGAAACT GACAAGTACA ACTCTTTTTT   
  
  
- CCTTTCAAGT ATCTCAGGAA GCGACCATGA CAGCTAGTAA ATCAAGGTTC AAAGCTCACT AGCCATTTTT   
  
  
- AAGTAGCCAC TCTTTAGTGG AGGATTGCA

+     GCN4\_motif

| Site Name | Organism | Position | Strand | Matrix score. | sequence | function |
| --- | --- | --- | --- | --- | --- | --- |
| GCN4\_motif | Oryza sativa | 708 | - | 7 | TGTGTCA | cis-regulatory element involved in endosperm expression |

> 2018/04/13 10:10:12  
+ GTTTAAAAGT TGGGTAACAT CCCAGTATTG GGGGAGACTT CCGGTCCTTT AACCAAGACC TTACAATAAT   
  
  
+ AAGTAAAATG GGGGTGTCGT TAAGAGAAGT AAACGATGAT CTCAAAGATA TGTCTTTCCT AGGAAAGACA   
  
  
+ GTTGTTCTTG TTCAACTCAT AGAGGACGGT GTATGAAAAA CGATCACAGA GGACGGTGTA TAAAAAGTGA   
  
  
+ TCAGTACACC GCTTACGGCA ATCAGTAGGA ACATTGTCTA TCAAAGACGA GTAGTAGGTT CAATAGTTCT   
  
  
+ GTGTGAAGTA CATCACGCCC TTCTCGTGCG AGACTCAAAA CTCGGACGTA TTAGATCATT AGTCATTATC   
  
  
+ TTTTGTTAAG TATTAACGCT TTTCATTCGT TGACATTTAG CGTTGTAAAT TGTTTTAGTG GAGGTTGAAC   
  
  
+ AATTGAGTCT GAATTCATCC ATACTAGATA CAGATTACGT ATGACATTTG ACTATAGGTT CTTTTGTTTG   
  
  
+ TTTGTATGAT TTGTAAAAGT ATGGCGAACA AATTCTTGTC TCGTGTGATA AGCTCAGTGT CGTAAGAGAC   
  
  
+ CTAGGACATT TTACCTAGTT TCGTTTTTTA CTTCGTTAGA TGGTCTTTGT TCCCCCTTAC CATATTAGTA   
  
  
+ TGTCGAGATG GGATGGGAAT CTCTTAGTGA TAATCGAGGT GTTGCACGAT AGAGAAAGTA GTAGTCGTCT   
  
  
+ CATGCGTTGA CACATATTGA AAAGTGAGAG CATCCGAAGT GTCTCGAATC GAATCTCTTT CGGTTAGACG   
  
  
+ TCCGTAATTC AAAGCGGAGT TCCTGAGAGG AACTTTAAAG ATGTCGGTTC TTTTGTCCTT GGGTCTTATC   
  
  
+ CCTTCGAATT ATCTCCAGTT TTTTAGTTTA GTGTAATCTT TCCTTTTGTT TTAATGTATT TATTTATTTA   
  
  
+ AGTTGGGTCC CGTACCGTTG ACTTGTACAT TGTTTCTTCT TAAGTTCTTT TAAAATTAAT TAATATTTTA   
  
  
+ TATAATACAC ATGATACCAA AGGAAGTTCA ACATCTTCCT CTATGGACAG AAGAAGTCTG ACTATTGGCA   
  
  
+ ATTAGGTATC AGGTAGTTGT CTCATATTAA CTACCTCGTT TGTTTACGAA CACAATCAAT TTACTTTACT   
  
  
+ AACAAACTCA TCATATGCTA AAGCGAATGG ACCGCCACGG TACAGTCTGG CTCCAAGCTT GACGATGTGT   
  
  
+ TTCGACTTCT AAAGTTTTAA CAATACCTTT GGTGGTTCTG CTTCCGCCAT TATCTTATTT TCCGCTGGTA   
  
  
+ GGTCGCTGCA GGATGAAGTA CTCCGAGAAC GGTGCTTGTA GAGAGAAACG GATGACGGAG AGGATCTATC   
  
  
+ AATTAAGTTA TCGGTTTCTT TGTGTTCTAA GAACGAGACA AGGGCTTTGA CTGTTCATGT TGAGAAAAAA   
  
  
+ GGAAAGTTCA TAGAGTCCTT CGCTGGTACT GTCGATCATT TAGTTCCAAG TTTCGAGTGA TCGGTAAAAA   
  
  
+ TTCATCGGTG AGAAATCACC TCCTAACGT  

- CAAATTTTCA ACCCATTGTA GGGTCATAAC CCCCTCTGAA GGCCAGGAAA TTGGTTCTGG AATGTTATTA   
  
  
- TTCATTTTAC CCCCACAGCA ATTCTCTTCA TTTGCTACTA GAGTTTCTAT ACAGAAAGGA TCCTTTCTGT   
  
  
- CAACAAGAAC AAGTTGAGTA TCTCCTGCCA CATACTTTTT GCTAGTGTCT CCTGCCACAT ATTTTTCACT   
  
  
- AGTCATGTGG CGAATGCCGT TAGTCATCCT TGTAACAGAT AGTTTCTGCT CATCATCCAA GTTATCAAGA   
  
  
- CACACTTCAT GTAGTGCGGG AAGAGCACGC TCTGAGTTTT GAGCCTGCAT AATCTAGTAA TCAGTAATAG   
  
  
- AAAACAATTC ATAATTGCGA AAAGTAAGCA ACTGTAAATC GCAACATTTA ACAAAATCAC CTCCAACTTG   
  
  
- TTAACTCAGA CTTAAGTAGG TATGATCTAT GTCTAATGCA TACTGTAAAC TGATATCCAA GAAAACAAAC   
  
  
- AAACATACTA AACATTTTCA TACCGCTTGT TTAAGAACAG AGCACACTAT TCGAGTCACA GCATTCTCTG   
  
  
- GATCCTGTAA AATGGATCAA AGCAAAAAAT GAAGCAATCT ACCAGAAACA AGGGGGAATG GTATAATCAT   
  
  
- ACAGCTCTAC CCTACCCTTA GAGAATCACT ATTAGCTCCA CAACGTGCTA TCTCTTTCAT CATCAGCAGA   
  
  
- GTACGCAACT GTGTATAACT TTTCACTCTC GTAGGCTTCA CAGAGCTTAG CTTAGAGAAA GCCAATCTGC   
  
  
- AGGCATTAAG TTTCGCCTCA AGGACTCTCC TTGAAATTTC TACAGCCAAG AAAACAGGAA CCCAGAATAG   
  
  
- GGAAGCTTAA TAGAGGTCAA AAAATCAAAT CACATTAGAA AGGAAAACAA AATTACATAA ATAAATAAAT   
  
  
- TCAACCCAGG GCATGGCAAC TGAACATGTA ACAAAGAAGA ATTCAAGAAA ATTTTAATTA ATTATAAAAT   
  
  
- ATATTATGTG TACTATGGTT TCCTTCAAGT TGTAGAAGGA GATACCTGTC TTCTTCAGAC TGATAACCGT   
  
  
- TAATCCATAG TCCATCAACA GAGTATAATT GATGGAGCAA ACAAATGCTT GTGTTAGTTA AATGAAATGA   
  
  
- TTGTTTGAGT AGTATACGAT TTCGCTTACC TGGCGGTGCC ATGTCAGACC GAGGTTCGAA CTGCTACACA   
  
  
- AAGCTGAAGA TTTCAAAATT GTTATGGAAA CCACCAAGAC GAAGGCGGTA ATAGAATAAA AGGCGACCAT   
  
  
- CCAGCGACGT CCTACTTCAT GAGGCTCTTG CCACGAACAT CTCTCTTTGC CTACTGCCTC TCCTAGATAG   
  
  
- TTAATTCAAT AGCCAAAGAA ACACAAGATT CTTGCTCTGT TCCCGAAACT GACAAGTACA ACTCTTTTTT   
  
  
- CCTTTCAAGT ATCTCAGGAA GCGACCATGA CAGCTAGTAA ATCAAGGTTC AAAGCTCACT AGCCATTTTT   
  
  
- AAGTAGCCAC TCTTTAGTGG AGGATTGCA

+     GT1-motif

| Site Name | Organism | Position | Strand | Matrix score. | sequence | function |
| --- | --- | --- | --- | --- | --- | --- |
| GT1-motif | Arabidopsis thaliana | 49 | - | 6 | GGTTAA | light responsive element |

> 2018/04/13 10:10:12  
+ GTTTAAAAGT TGGGTAACAT CCCAGTATTG GGGGAGACTT CCGGTCCTTT AACCAAGACC TTACAATAAT   
  
  
+ AAGTAAAATG GGGGTGTCGT TAAGAGAAGT AAACGATGAT CTCAAAGATA TGTCTTTCCT AGGAAAGACA   
  
  
+ GTTGTTCTTG TTCAACTCAT AGAGGACGGT GTATGAAAAA CGATCACAGA GGACGGTGTA TAAAAAGTGA   
  
  
+ TCAGTACACC GCTTACGGCA ATCAGTAGGA ACATTGTCTA TCAAAGACGA GTAGTAGGTT CAATAGTTCT   
  
  
+ GTGTGAAGTA CATCACGCCC TTCTCGTGCG AGACTCAAAA CTCGGACGTA TTAGATCATT AGTCATTATC   
  
  
+ TTTTGTTAAG TATTAACGCT TTTCATTCGT TGACATTTAG CGTTGTAAAT TGTTTTAGTG GAGGTTGAAC   
  
  
+ AATTGAGTCT GAATTCATCC ATACTAGATA CAGATTACGT ATGACATTTG ACTATAGGTT CTTTTGTTTG   
  
  
+ TTTGTATGAT TTGTAAAAGT ATGGCGAACA AATTCTTGTC TCGTGTGATA AGCTCAGTGT CGTAAGAGAC   
  
  
+ CTAGGACATT TTACCTAGTT TCGTTTTTTA CTTCGTTAGA TGGTCTTTGT TCCCCCTTAC CATATTAGTA   
  
  
+ TGTCGAGATG GGATGGGAAT CTCTTAGTGA TAATCGAGGT GTTGCACGAT AGAGAAAGTA GTAGTCGTCT   
  
  
+ CATGCGTTGA CACATATTGA AAAGTGAGAG CATCCGAAGT GTCTCGAATC GAATCTCTTT CGGTTAGACG   
  
  
+ TCCGTAATTC AAAGCGGAGT TCCTGAGAGG AACTTTAAAG ATGTCGGTTC TTTTGTCCTT GGGTCTTATC   
  
  
+ CCTTCGAATT ATCTCCAGTT TTTTAGTTTA GTGTAATCTT TCCTTTTGTT TTAATGTATT TATTTATTTA   
  
  
+ AGTTGGGTCC CGTACCGTTG ACTTGTACAT TGTTTCTTCT TAAGTTCTTT TAAAATTAAT TAATATTTTA   
  
  
+ TATAATACAC ATGATACCAA AGGAAGTTCA ACATCTTCCT CTATGGACAG AAGAAGTCTG ACTATTGGCA   
  
  
+ ATTAGGTATC AGGTAGTTGT CTCATATTAA CTACCTCGTT TGTTTACGAA CACAATCAAT TTACTTTACT   
  
  
+ AACAAACTCA TCATATGCTA AAGCGAATGG ACCGCCACGG TACAGTCTGG CTCCAAGCTT GACGATGTGT   
  
  
+ TTCGACTTCT AAAGTTTTAA CAATACCTTT GGTGGTTCTG CTTCCGCCAT TATCTTATTT TCCGCTGGTA   
  
  
+ GGTCGCTGCA GGATGAAGTA CTCCGAGAAC GGTGCTTGTA GAGAGAAACG GATGACGGAG AGGATCTATC   
  
  
+ AATTAAGTTA TCGGTTTCTT TGTGTTCTAA GAACGAGACA AGGGCTTTGA CTGTTCATGT TGAGAAAAAA   
  
  
+ GGAAAGTTCA TAGAGTCCTT CGCTGGTACT GTCGATCATT TAGTTCCAAG TTTCGAGTGA TCGGTAAAAA   
  
  
+ TTCATCGGTG AGAAATCACC TCCTAACGT  

- CAAATTTTCA ACCCATTGTA GGGTCATAAC CCCCTCTGAA GGCCAGGAAA TTGGTTCTGG AATGTTATTA   
  
  
- TTCATTTTAC CCCCACAGCA ATTCTCTTCA TTTGCTACTA GAGTTTCTAT ACAGAAAGGA TCCTTTCTGT   
  
  
- CAACAAGAAC AAGTTGAGTA TCTCCTGCCA CATACTTTTT GCTAGTGTCT CCTGCCACAT ATTTTTCACT   
  
  
- AGTCATGTGG CGAATGCCGT TAGTCATCCT TGTAACAGAT AGTTTCTGCT CATCATCCAA GTTATCAAGA   
  
  
- CACACTTCAT GTAGTGCGGG AAGAGCACGC TCTGAGTTTT GAGCCTGCAT AATCTAGTAA TCAGTAATAG   
  
  
- AAAACAATTC ATAATTGCGA AAAGTAAGCA ACTGTAAATC GCAACATTTA ACAAAATCAC CTCCAACTTG   
  
  
- TTAACTCAGA CTTAAGTAGG TATGATCTAT GTCTAATGCA TACTGTAAAC TGATATCCAA GAAAACAAAC   
  
  
- AAACATACTA AACATTTTCA TACCGCTTGT TTAAGAACAG AGCACACTAT TCGAGTCACA GCATTCTCTG   
  
  
- GATCCTGTAA AATGGATCAA AGCAAAAAAT GAAGCAATCT ACCAGAAACA AGGGGGAATG GTATAATCAT   
  
  
- ACAGCTCTAC CCTACCCTTA GAGAATCACT ATTAGCTCCA CAACGTGCTA TCTCTTTCAT CATCAGCAGA   
  
  
- GTACGCAACT GTGTATAACT TTTCACTCTC GTAGGCTTCA CAGAGCTTAG CTTAGAGAAA GCCAATCTGC   
  
  
- AGGCATTAAG TTTCGCCTCA AGGACTCTCC TTGAAATTTC TACAGCCAAG AAAACAGGAA CCCAGAATAG   
  
  
- GGAAGCTTAA TAGAGGTCAA AAAATCAAAT CACATTAGAA AGGAAAACAA AATTACATAA ATAAATAAAT   
  
  
- TCAACCCAGG GCATGGCAAC TGAACATGTA ACAAAGAAGA ATTCAAGAAA ATTTTAATTA ATTATAAAAT   
  
  
- ATATTATGTG TACTATGGTT TCCTTCAAGT TGTAGAAGGA GATACCTGTC TTCTTCAGAC TGATAACCGT   
  
  
- TAATCCATAG TCCATCAACA GAGTATAATT GATGGAGCAA ACAAATGCTT GTGTTAGTTA AATGAAATGA   
  
  
- TTGTTTGAGT AGTATACGAT TTCGCTTACC TGGCGGTGCC ATGTCAGACC GAGGTTCGAA CTGCTACACA   
  
  
- AAGCTGAAGA TTTCAAAATT GTTATGGAAA CCACCAAGAC GAAGGCGGTA ATAGAATAAA AGGCGACCAT   
  
  
- CCAGCGACGT CCTACTTCAT GAGGCTCTTG CCACGAACAT CTCTCTTTGC CTACTGCCTC TCCTAGATAG   
  
  
- TTAATTCAAT AGCCAAAGAA ACACAAGATT CTTGCTCTGT TCCCGAAACT GACAAGTACA ACTCTTTTTT   
  
  
- CCTTTCAAGT ATCTCAGGAA GCGACCATGA CAGCTAGTAA ATCAAGGTTC AAAGCTCACT AGCCATTTTT   
  
  
- AAGTAGCCAC TCTTTAGTGG AGGATTGCA

+     HSE

| Site Name | Organism | Position | Strand | Matrix score. | sequence | function |
| --- | --- | --- | --- | --- | --- | --- |
| HSE | Brassica oleracea | 845 | - | 9 | AGAAAATTCG | cis-acting element involved in heat stress responsiveness |

> 2018/04/13 10:10:12  
+ GTTTAAAAGT TGGGTAACAT CCCAGTATTG GGGGAGACTT CCGGTCCTTT AACCAAGACC TTACAATAAT   
  
  
+ AAGTAAAATG GGGGTGTCGT TAAGAGAAGT AAACGATGAT CTCAAAGATA TGTCTTTCCT AGGAAAGACA   
  
  
+ GTTGTTCTTG TTCAACTCAT AGAGGACGGT GTATGAAAAA CGATCACAGA GGACGGTGTA TAAAAAGTGA   
  
  
+ TCAGTACACC GCTTACGGCA ATCAGTAGGA ACATTGTCTA TCAAAGACGA GTAGTAGGTT CAATAGTTCT   
  
  
+ GTGTGAAGTA CATCACGCCC TTCTCGTGCG AGACTCAAAA CTCGGACGTA TTAGATCATT AGTCATTATC   
  
  
+ TTTTGTTAAG TATTAACGCT TTTCATTCGT TGACATTTAG CGTTGTAAAT TGTTTTAGTG GAGGTTGAAC   
  
  
+ AATTGAGTCT GAATTCATCC ATACTAGATA CAGATTACGT ATGACATTTG ACTATAGGTT CTTTTGTTTG   
  
  
+ TTTGTATGAT TTGTAAAAGT ATGGCGAACA AATTCTTGTC TCGTGTGATA AGCTCAGTGT CGTAAGAGAC   
  
  
+ CTAGGACATT TTACCTAGTT TCGTTTTTTA CTTCGTTAGA TGGTCTTTGT TCCCCCTTAC CATATTAGTA   
  
  
+ TGTCGAGATG GGATGGGAAT CTCTTAGTGA TAATCGAGGT GTTGCACGAT AGAGAAAGTA GTAGTCGTCT   
  
  
+ CATGCGTTGA CACATATTGA AAAGTGAGAG CATCCGAAGT GTCTCGAATC GAATCTCTTT CGGTTAGACG   
  
  
+ TCCGTAATTC AAAGCGGAGT TCCTGAGAGG AACTTTAAAG ATGTCGGTTC TTTTGTCCTT GGGTCTTATC   
  
  
+ CCTTCGAATT ATCTCCAGTT TTTTAGTTTA GTGTAATCTT TCCTTTTGTT TTAATGTATT TATTTATTTA   
  
  
+ AGTTGGGTCC CGTACCGTTG ACTTGTACAT TGTTTCTTCT TAAGTTCTTT TAAAATTAAT TAATATTTTA   
  
  
+ TATAATACAC ATGATACCAA AGGAAGTTCA ACATCTTCCT CTATGGACAG AAGAAGTCTG ACTATTGGCA   
  
  
+ ATTAGGTATC AGGTAGTTGT CTCATATTAA CTACCTCGTT TGTTTACGAA CACAATCAAT TTACTTTACT   
  
  
+ AACAAACTCA TCATATGCTA AAGCGAATGG ACCGCCACGG TACAGTCTGG CTCCAAGCTT GACGATGTGT   
  
  
+ TTCGACTTCT AAAGTTTTAA CAATACCTTT GGTGGTTCTG CTTCCGCCAT TATCTTATTT TCCGCTGGTA   
  
  
+ GGTCGCTGCA GGATGAAGTA CTCCGAGAAC GGTGCTTGTA GAGAGAAACG GATGACGGAG AGGATCTATC   
  
  
+ AATTAAGTTA TCGGTTTCTT TGTGTTCTAA GAACGAGACA AGGGCTTTGA CTGTTCATGT TGAGAAAAAA   
  
  
+ GGAAAGTTCA TAGAGTCCTT CGCTGGTACT GTCGATCATT TAGTTCCAAG TTTCGAGTGA TCGGTAAAAA   
  
  
+ TTCATCGGTG AGAAATCACC TCCTAACGT  

- CAAATTTTCA ACCCATTGTA GGGTCATAAC CCCCTCTGAA GGCCAGGAAA TTGGTTCTGG AATGTTATTA   
  
  
- TTCATTTTAC CCCCACAGCA ATTCTCTTCA TTTGCTACTA GAGTTTCTAT ACAGAAAGGA TCCTTTCTGT   
  
  
- CAACAAGAAC AAGTTGAGTA TCTCCTGCCA CATACTTTTT GCTAGTGTCT CCTGCCACAT ATTTTTCACT   
  
  
- AGTCATGTGG CGAATGCCGT TAGTCATCCT TGTAACAGAT AGTTTCTGCT CATCATCCAA GTTATCAAGA   
  
  
- CACACTTCAT GTAGTGCGGG AAGAGCACGC TCTGAGTTTT GAGCCTGCAT AATCTAGTAA TCAGTAATAG   
  
  
- AAAACAATTC ATAATTGCGA AAAGTAAGCA ACTGTAAATC GCAACATTTA ACAAAATCAC CTCCAACTTG   
  
  
- TTAACTCAGA CTTAAGTAGG TATGATCTAT GTCTAATGCA TACTGTAAAC TGATATCCAA GAAAACAAAC   
  
  
- AAACATACTA AACATTTTCA TACCGCTTGT TTAAGAACAG AGCACACTAT TCGAGTCACA GCATTCTCTG   
  
  
- GATCCTGTAA AATGGATCAA AGCAAAAAAT GAAGCAATCT ACCAGAAACA AGGGGGAATG GTATAATCAT   
  
  
- ACAGCTCTAC CCTACCCTTA GAGAATCACT ATTAGCTCCA CAACGTGCTA TCTCTTTCAT CATCAGCAGA   
  
  
- GTACGCAACT GTGTATAACT TTTCACTCTC GTAGGCTTCA CAGAGCTTAG CTTAGAGAAA GCCAATCTGC   
  
  
- AGGCATTAAG TTTCGCCTCA AGGACTCTCC TTGAAATTTC TACAGCCAAG AAAACAGGAA CCCAGAATAG   
  
  
- GGAAGCTTAA TAGAGGTCAA AAAATCAAAT CACATTAGAA AGGAAAACAA AATTACATAA ATAAATAAAT   
  
  
- TCAACCCAGG GCATGGCAAC TGAACATGTA ACAAAGAAGA ATTCAAGAAA ATTTTAATTA ATTATAAAAT   
  
  
- ATATTATGTG TACTATGGTT TCCTTCAAGT TGTAGAAGGA GATACCTGTC TTCTTCAGAC TGATAACCGT   
  
  
- TAATCCATAG TCCATCAACA GAGTATAATT GATGGAGCAA ACAAATGCTT GTGTTAGTTA AATGAAATGA   
  
  
- TTGTTTGAGT AGTATACGAT TTCGCTTACC TGGCGGTGCC ATGTCAGACC GAGGTTCGAA CTGCTACACA   
  
  
- AAGCTGAAGA TTTCAAAATT GTTATGGAAA CCACCAAGAC GAAGGCGGTA ATAGAATAAA AGGCGACCAT   
  
  
- CCAGCGACGT CCTACTTCAT GAGGCTCTTG CCACGAACAT CTCTCTTTGC CTACTGCCTC TCCTAGATAG   
  
  
- TTAATTCAAT AGCCAAAGAA ACACAAGATT CTTGCTCTGT TCCCGAAACT GACAAGTACA ACTCTTTTTT   
  
  
- CCTTTCAAGT ATCTCAGGAA GCGACCATGA CAGCTAGTAA ATCAAGGTTC AAAGCTCACT AGCCATTTTT   
  
  
- AAGTAGCCAC TCTTTAGTGG AGGATTGCA

+     LTR

| Site Name | Organism | Position | Strand | Matrix score. | sequence | function |
| --- | --- | --- | --- | --- | --- | --- |
| LTR | Hordeum vulgare | 758 | - | 6 | CCGAAA | cis-acting element involved in low-temperature responsiveness |

> 2018/04/13 10:10:12  
+ GTTTAAAAGT TGGGTAACAT CCCAGTATTG GGGGAGACTT CCGGTCCTTT AACCAAGACC TTACAATAAT   
  
  
+ AAGTAAAATG GGGGTGTCGT TAAGAGAAGT AAACGATGAT CTCAAAGATA TGTCTTTCCT AGGAAAGACA   
  
  
+ GTTGTTCTTG TTCAACTCAT AGAGGACGGT GTATGAAAAA CGATCACAGA GGACGGTGTA TAAAAAGTGA   
  
  
+ TCAGTACACC GCTTACGGCA ATCAGTAGGA ACATTGTCTA TCAAAGACGA GTAGTAGGTT CAATAGTTCT   
  
  
+ GTGTGAAGTA CATCACGCCC TTCTCGTGCG AGACTCAAAA CTCGGACGTA TTAGATCATT AGTCATTATC   
  
  
+ TTTTGTTAAG TATTAACGCT TTTCATTCGT TGACATTTAG CGTTGTAAAT TGTTTTAGTG GAGGTTGAAC   
  
  
+ AATTGAGTCT GAATTCATCC ATACTAGATA CAGATTACGT ATGACATTTG ACTATAGGTT CTTTTGTTTG   
  
  
+ TTTGTATGAT TTGTAAAAGT ATGGCGAACA AATTCTTGTC TCGTGTGATA AGCTCAGTGT CGTAAGAGAC   
  
  
+ CTAGGACATT TTACCTAGTT TCGTTTTTTA CTTCGTTAGA TGGTCTTTGT TCCCCCTTAC CATATTAGTA   
  
  
+ TGTCGAGATG GGATGGGAAT CTCTTAGTGA TAATCGAGGT GTTGCACGAT AGAGAAAGTA GTAGTCGTCT   
  
  
+ CATGCGTTGA CACATATTGA AAAGTGAGAG CATCCGAAGT GTCTCGAATC GAATCTCTTT CGGTTAGACG   
  
  
+ TCCGTAATTC AAAGCGGAGT TCCTGAGAGG AACTTTAAAG ATGTCGGTTC TTTTGTCCTT GGGTCTTATC   
  
  
+ CCTTCGAATT ATCTCCAGTT TTTTAGTTTA GTGTAATCTT TCCTTTTGTT TTAATGTATT TATTTATTTA   
  
  
+ AGTTGGGTCC CGTACCGTTG ACTTGTACAT TGTTTCTTCT TAAGTTCTTT TAAAATTAAT TAATATTTTA   
  
  
+ TATAATACAC ATGATACCAA AGGAAGTTCA ACATCTTCCT CTATGGACAG AAGAAGTCTG ACTATTGGCA   
  
  
+ ATTAGGTATC AGGTAGTTGT CTCATATTAA CTACCTCGTT TGTTTACGAA CACAATCAAT TTACTTTACT   
  
  
+ AACAAACTCA TCATATGCTA AAGCGAATGG ACCGCCACGG TACAGTCTGG CTCCAAGCTT GACGATGTGT   
  
  
+ TTCGACTTCT AAAGTTTTAA CAATACCTTT GGTGGTTCTG CTTCCGCCAT TATCTTATTT TCCGCTGGTA   
  
  
+ GGTCGCTGCA GGATGAAGTA CTCCGAGAAC GGTGCTTGTA GAGAGAAACG GATGACGGAG AGGATCTATC   
  
  
+ AATTAAGTTA TCGGTTTCTT TGTGTTCTAA GAACGAGACA AGGGCTTTGA CTGTTCATGT TGAGAAAAAA   
  
  
+ GGAAAGTTCA TAGAGTCCTT CGCTGGTACT GTCGATCATT TAGTTCCAAG TTTCGAGTGA TCGGTAAAAA   
  
  
+ TTCATCGGTG AGAAATCACC TCCTAACGT  

- CAAATTTTCA ACCCATTGTA GGGTCATAAC CCCCTCTGAA GGCCAGGAAA TTGGTTCTGG AATGTTATTA   
  
  
- TTCATTTTAC CCCCACAGCA ATTCTCTTCA TTTGCTACTA GAGTTTCTAT ACAGAAAGGA TCCTTTCTGT   
  
  
- CAACAAGAAC AAGTTGAGTA TCTCCTGCCA CATACTTTTT GCTAGTGTCT CCTGCCACAT ATTTTTCACT   
  
  
- AGTCATGTGG CGAATGCCGT TAGTCATCCT TGTAACAGAT AGTTTCTGCT CATCATCCAA GTTATCAAGA   
  
  
- CACACTTCAT GTAGTGCGGG AAGAGCACGC TCTGAGTTTT GAGCCTGCAT AATCTAGTAA TCAGTAATAG   
  
  
- AAAACAATTC ATAATTGCGA AAAGTAAGCA ACTGTAAATC GCAACATTTA ACAAAATCAC CTCCAACTTG   
  
  
- TTAACTCAGA CTTAAGTAGG TATGATCTAT GTCTAATGCA TACTGTAAAC TGATATCCAA GAAAACAAAC   
  
  
- AAACATACTA AACATTTTCA TACCGCTTGT TTAAGAACAG AGCACACTAT TCGAGTCACA GCATTCTCTG   
  
  
- GATCCTGTAA AATGGATCAA AGCAAAAAAT GAAGCAATCT ACCAGAAACA AGGGGGAATG GTATAATCAT   
  
  
- ACAGCTCTAC CCTACCCTTA GAGAATCACT ATTAGCTCCA CAACGTGCTA TCTCTTTCAT CATCAGCAGA   
  
  
- GTACGCAACT GTGTATAACT TTTCACTCTC GTAGGCTTCA CAGAGCTTAG CTTAGAGAAA GCCAATCTGC   
  
  
- AGGCATTAAG TTTCGCCTCA AGGACTCTCC TTGAAATTTC TACAGCCAAG AAAACAGGAA CCCAGAATAG   
  
  
- GGAAGCTTAA TAGAGGTCAA AAAATCAAAT CACATTAGAA AGGAAAACAA AATTACATAA ATAAATAAAT   
  
  
- TCAACCCAGG GCATGGCAAC TGAACATGTA ACAAAGAAGA ATTCAAGAAA ATTTTAATTA ATTATAAAAT   
  
  
- ATATTATGTG TACTATGGTT TCCTTCAAGT TGTAGAAGGA GATACCTGTC TTCTTCAGAC TGATAACCGT   
  
  
- TAATCCATAG TCCATCAACA GAGTATAATT GATGGAGCAA ACAAATGCTT GTGTTAGTTA AATGAAATGA   
  
  
- TTGTTTGAGT AGTATACGAT TTCGCTTACC TGGCGGTGCC ATGTCAGACC GAGGTTCGAA CTGCTACACA   
  
  
- AAGCTGAAGA TTTCAAAATT GTTATGGAAA CCACCAAGAC GAAGGCGGTA ATAGAATAAA AGGCGACCAT   
  
  
- CCAGCGACGT CCTACTTCAT GAGGCTCTTG CCACGAACAT CTCTCTTTGC CTACTGCCTC TCCTAGATAG   
  
  
- TTAATTCAAT AGCCAAAGAA ACACAAGATT CTTGCTCTGT TCCCGAAACT GACAAGTACA ACTCTTTTTT   
  
  
- CCTTTCAAGT ATCTCAGGAA GCGACCATGA CAGCTAGTAA ATCAAGGTTC AAAGCTCACT AGCCATTTTT   
  
  
- AAGTAGCCAC TCTTTAGTGG AGGATTGCA

+     MBS

| Site Name | Organism | Position | Strand | Matrix score. | sequence | function |
| --- | --- | --- | --- | --- | --- | --- |
| MBS | Arabidopsis thaliana | 139 | - | 6 | CAACTG | MYB binding site involved in drought-inducibility |

> 2018/04/13 10:10:12  
+ GTTTAAAAGT TGGGTAACAT CCCAGTATTG GGGGAGACTT CCGGTCCTTT AACCAAGACC TTACAATAAT   
  
  
+ AAGTAAAATG GGGGTGTCGT TAAGAGAAGT AAACGATGAT CTCAAAGATA TGTCTTTCCT AGGAAAGACA   
  
  
+ GTTGTTCTTG TTCAACTCAT AGAGGACGGT GTATGAAAAA CGATCACAGA GGACGGTGTA TAAAAAGTGA   
  
  
+ TCAGTACACC GCTTACGGCA ATCAGTAGGA ACATTGTCTA TCAAAGACGA GTAGTAGGTT CAATAGTTCT   
  
  
+ GTGTGAAGTA CATCACGCCC TTCTCGTGCG AGACTCAAAA CTCGGACGTA TTAGATCATT AGTCATTATC   
  
  
+ TTTTGTTAAG TATTAACGCT TTTCATTCGT TGACATTTAG CGTTGTAAAT TGTTTTAGTG GAGGTTGAAC   
  
  
+ AATTGAGTCT GAATTCATCC ATACTAGATA CAGATTACGT ATGACATTTG ACTATAGGTT CTTTTGTTTG   
  
  
+ TTTGTATGAT TTGTAAAAGT ATGGCGAACA AATTCTTGTC TCGTGTGATA AGCTCAGTGT CGTAAGAGAC   
  
  
+ CTAGGACATT TTACCTAGTT TCGTTTTTTA CTTCGTTAGA TGGTCTTTGT TCCCCCTTAC CATATTAGTA   
  
  
+ TGTCGAGATG GGATGGGAAT CTCTTAGTGA TAATCGAGGT GTTGCACGAT AGAGAAAGTA GTAGTCGTCT   
  
  
+ CATGCGTTGA CACATATTGA AAAGTGAGAG CATCCGAAGT GTCTCGAATC GAATCTCTTT CGGTTAGACG   
  
  
+ TCCGTAATTC AAAGCGGAGT TCCTGAGAGG AACTTTAAAG ATGTCGGTTC TTTTGTCCTT GGGTCTTATC   
  
  
+ CCTTCGAATT ATCTCCAGTT TTTTAGTTTA GTGTAATCTT TCCTTTTGTT TTAATGTATT TATTTATTTA   
  
  
+ AGTTGGGTCC CGTACCGTTG ACTTGTACAT TGTTTCTTCT TAAGTTCTTT TAAAATTAAT TAATATTTTA   
  
  
+ TATAATACAC ATGATACCAA AGGAAGTTCA ACATCTTCCT CTATGGACAG AAGAAGTCTG ACTATTGGCA   
  
  
+ ATTAGGTATC AGGTAGTTGT CTCATATTAA CTACCTCGTT TGTTTACGAA CACAATCAAT TTACTTTACT   
  
  
+ AACAAACTCA TCATATGCTA AAGCGAATGG ACCGCCACGG TACAGTCTGG CTCCAAGCTT GACGATGTGT   
  
  
+ TTCGACTTCT AAAGTTTTAA CAATACCTTT GGTGGTTCTG CTTCCGCCAT TATCTTATTT TCCGCTGGTA   
  
  
+ GGTCGCTGCA GGATGAAGTA CTCCGAGAAC GGTGCTTGTA GAGAGAAACG GATGACGGAG AGGATCTATC   
  
  
+ AATTAAGTTA TCGGTTTCTT TGTGTTCTAA GAACGAGACA AGGGCTTTGA CTGTTCATGT TGAGAAAAAA   
  
  
+ GGAAAGTTCA TAGAGTCCTT CGCTGGTACT GTCGATCATT TAGTTCCAAG TTTCGAGTGA TCGGTAAAAA   
  
  
+ TTCATCGGTG AGAAATCACC TCCTAACGT  

- CAAATTTTCA ACCCATTGTA GGGTCATAAC CCCCTCTGAA GGCCAGGAAA TTGGTTCTGG AATGTTATTA   
  
  
- TTCATTTTAC CCCCACAGCA ATTCTCTTCA TTTGCTACTA GAGTTTCTAT ACAGAAAGGA TCCTTTCTGT   
  
  
- CAACAAGAAC AAGTTGAGTA TCTCCTGCCA CATACTTTTT GCTAGTGTCT CCTGCCACAT ATTTTTCACT   
  
  
- AGTCATGTGG CGAATGCCGT TAGTCATCCT TGTAACAGAT AGTTTCTGCT CATCATCCAA GTTATCAAGA   
  
  
- CACACTTCAT GTAGTGCGGG AAGAGCACGC TCTGAGTTTT GAGCCTGCAT AATCTAGTAA TCAGTAATAG   
  
  
- AAAACAATTC ATAATTGCGA AAAGTAAGCA ACTGTAAATC GCAACATTTA ACAAAATCAC CTCCAACTTG   
  
  
- TTAACTCAGA CTTAAGTAGG TATGATCTAT GTCTAATGCA TACTGTAAAC TGATATCCAA GAAAACAAAC   
  
  
- AAACATACTA AACATTTTCA TACCGCTTGT TTAAGAACAG AGCACACTAT TCGAGTCACA GCATTCTCTG   
  
  
- GATCCTGTAA AATGGATCAA AGCAAAAAAT GAAGCAATCT ACCAGAAACA AGGGGGAATG GTATAATCAT   
  
  
- ACAGCTCTAC CCTACCCTTA GAGAATCACT ATTAGCTCCA CAACGTGCTA TCTCTTTCAT CATCAGCAGA   
  
  
- GTACGCAACT GTGTATAACT TTTCACTCTC GTAGGCTTCA CAGAGCTTAG CTTAGAGAAA GCCAATCTGC   
  
  
- AGGCATTAAG TTTCGCCTCA AGGACTCTCC TTGAAATTTC TACAGCCAAG AAAACAGGAA CCCAGAATAG   
  
  
- GGAAGCTTAA TAGAGGTCAA AAAATCAAAT CACATTAGAA AGGAAAACAA AATTACATAA ATAAATAAAT   
  
  
- TCAACCCAGG GCATGGCAAC TGAACATGTA ACAAAGAAGA ATTCAAGAAA ATTTTAATTA ATTATAAAAT   
  
  
- ATATTATGTG TACTATGGTT TCCTTCAAGT TGTAGAAGGA GATACCTGTC TTCTTCAGAC TGATAACCGT   
  
  
- TAATCCATAG TCCATCAACA GAGTATAATT GATGGAGCAA ACAAATGCTT GTGTTAGTTA AATGAAATGA   
  
  
- TTGTTTGAGT AGTATACGAT TTCGCTTACC TGGCGGTGCC ATGTCAGACC GAGGTTCGAA CTGCTACACA   
  
  
- AAGCTGAAGA TTTCAAAATT GTTATGGAAA CCACCAAGAC GAAGGCGGTA ATAGAATAAA AGGCGACCAT   
  
  
- CCAGCGACGT CCTACTTCAT GAGGCTCTTG CCACGAACAT CTCTCTTTGC CTACTGCCTC TCCTAGATAG   
  
  
- TTAATTCAAT AGCCAAAGAA ACACAAGATT CTTGCTCTGT TCCCGAAACT GACAAGTACA ACTCTTTTTT   
  
  
- CCTTTCAAGT ATCTCAGGAA GCGACCATGA CAGCTAGTAA ATCAAGGTTC AAAGCTCACT AGCCATTTTT   
  
  
- AAGTAGCCAC TCTTTAGTGG AGGATTGCA

+     P-box

| Site Name | Organism | Position | Strand | Matrix score. | sequence | function |
| --- | --- | --- | --- | --- | --- | --- |
| P-box | Oryza sativa | 882 | + | 7 | CCTTTTG | gibberellin-responsive element |

> 2018/04/13 10:10:12  
+ GTTTAAAAGT TGGGTAACAT CCCAGTATTG GGGGAGACTT CCGGTCCTTT AACCAAGACC TTACAATAAT   
  
  
+ AAGTAAAATG GGGGTGTCGT TAAGAGAAGT AAACGATGAT CTCAAAGATA TGTCTTTCCT AGGAAAGACA   
  
  
+ GTTGTTCTTG TTCAACTCAT AGAGGACGGT GTATGAAAAA CGATCACAGA GGACGGTGTA TAAAAAGTGA   
  
  
+ TCAGTACACC GCTTACGGCA ATCAGTAGGA ACATTGTCTA TCAAAGACGA GTAGTAGGTT CAATAGTTCT   
  
  
+ GTGTGAAGTA CATCACGCCC TTCTCGTGCG AGACTCAAAA CTCGGACGTA TTAGATCATT AGTCATTATC   
  
  
+ TTTTGTTAAG TATTAACGCT TTTCATTCGT TGACATTTAG CGTTGTAAAT TGTTTTAGTG GAGGTTGAAC   
  
  
+ AATTGAGTCT GAATTCATCC ATACTAGATA CAGATTACGT ATGACATTTG ACTATAGGTT CTTTTGTTTG   
  
  
+ TTTGTATGAT TTGTAAAAGT ATGGCGAACA AATTCTTGTC TCGTGTGATA AGCTCAGTGT CGTAAGAGAC   
  
  
+ CTAGGACATT TTACCTAGTT TCGTTTTTTA CTTCGTTAGA TGGTCTTTGT TCCCCCTTAC CATATTAGTA   
  
  
+ TGTCGAGATG GGATGGGAAT CTCTTAGTGA TAATCGAGGT GTTGCACGAT AGAGAAAGTA GTAGTCGTCT   
  
  
+ CATGCGTTGA CACATATTGA AAAGTGAGAG CATCCGAAGT GTCTCGAATC GAATCTCTTT CGGTTAGACG   
  
  
+ TCCGTAATTC AAAGCGGAGT TCCTGAGAGG AACTTTAAAG ATGTCGGTTC TTTTGTCCTT GGGTCTTATC   
  
  
+ CCTTCGAATT ATCTCCAGTT TTTTAGTTTA GTGTAATCTT TCCTTTTGTT TTAATGTATT TATTTATTTA   
  
  
+ AGTTGGGTCC CGTACCGTTG ACTTGTACAT TGTTTCTTCT TAAGTTCTTT TAAAATTAAT TAATATTTTA   
  
  
+ TATAATACAC ATGATACCAA AGGAAGTTCA ACATCTTCCT CTATGGACAG AAGAAGTCTG ACTATTGGCA   
  
  
+ ATTAGGTATC AGGTAGTTGT CTCATATTAA CTACCTCGTT TGTTTACGAA CACAATCAAT TTACTTTACT   
  
  
+ AACAAACTCA TCATATGCTA AAGCGAATGG ACCGCCACGG TACAGTCTGG CTCCAAGCTT GACGATGTGT   
  
  
+ TTCGACTTCT AAAGTTTTAA CAATACCTTT GGTGGTTCTG CTTCCGCCAT TATCTTATTT TCCGCTGGTA   
  
  
+ GGTCGCTGCA GGATGAAGTA CTCCGAGAAC GGTGCTTGTA GAGAGAAACG GATGACGGAG AGGATCTATC   
  
  
+ AATTAAGTTA TCGGTTTCTT TGTGTTCTAA GAACGAGACA AGGGCTTTGA CTGTTCATGT TGAGAAAAAA   
  
  
+ GGAAAGTTCA TAGAGTCCTT CGCTGGTACT GTCGATCATT TAGTTCCAAG TTTCGAGTGA TCGGTAAAAA   
  
  
+ TTCATCGGTG AGAAATCACC TCCTAACGT  

- CAAATTTTCA ACCCATTGTA GGGTCATAAC CCCCTCTGAA GGCCAGGAAA TTGGTTCTGG AATGTTATTA   
  
  
- TTCATTTTAC CCCCACAGCA ATTCTCTTCA TTTGCTACTA GAGTTTCTAT ACAGAAAGGA TCCTTTCTGT   
  
  
- CAACAAGAAC AAGTTGAGTA TCTCCTGCCA CATACTTTTT GCTAGTGTCT CCTGCCACAT ATTTTTCACT   
  
  
- AGTCATGTGG CGAATGCCGT TAGTCATCCT TGTAACAGAT AGTTTCTGCT CATCATCCAA GTTATCAAGA   
  
  
- CACACTTCAT GTAGTGCGGG AAGAGCACGC TCTGAGTTTT GAGCCTGCAT AATCTAGTAA TCAGTAATAG   
  
  
- AAAACAATTC ATAATTGCGA AAAGTAAGCA ACTGTAAATC GCAACATTTA ACAAAATCAC CTCCAACTTG   
  
  
- TTAACTCAGA CTTAAGTAGG TATGATCTAT GTCTAATGCA TACTGTAAAC TGATATCCAA GAAAACAAAC   
  
  
- AAACATACTA AACATTTTCA TACCGCTTGT TTAAGAACAG AGCACACTAT TCGAGTCACA GCATTCTCTG   
  
  
- GATCCTGTAA AATGGATCAA AGCAAAAAAT GAAGCAATCT ACCAGAAACA AGGGGGAATG GTATAATCAT   
  
  
- ACAGCTCTAC CCTACCCTTA GAGAATCACT ATTAGCTCCA CAACGTGCTA TCTCTTTCAT CATCAGCAGA   
  
  
- GTACGCAACT GTGTATAACT TTTCACTCTC GTAGGCTTCA CAGAGCTTAG CTTAGAGAAA GCCAATCTGC   
  
  
- AGGCATTAAG TTTCGCCTCA AGGACTCTCC TTGAAATTTC TACAGCCAAG AAAACAGGAA CCCAGAATAG   
  
  
- GGAAGCTTAA TAGAGGTCAA AAAATCAAAT CACATTAGAA AGGAAAACAA AATTACATAA ATAAATAAAT   
  
  
- TCAACCCAGG GCATGGCAAC TGAACATGTA ACAAAGAAGA ATTCAAGAAA ATTTTAATTA ATTATAAAAT   
  
  
- ATATTATGTG TACTATGGTT TCCTTCAAGT TGTAGAAGGA GATACCTGTC TTCTTCAGAC TGATAACCGT   
  
  
- TAATCCATAG TCCATCAACA GAGTATAATT GATGGAGCAA ACAAATGCTT GTGTTAGTTA AATGAAATGA   
  
  
- TTGTTTGAGT AGTATACGAT TTCGCTTACC TGGCGGTGCC ATGTCAGACC GAGGTTCGAA CTGCTACACA   
  
  
- AAGCTGAAGA TTTCAAAATT GTTATGGAAA CCACCAAGAC GAAGGCGGTA ATAGAATAAA AGGCGACCAT   
  
  
- CCAGCGACGT CCTACTTCAT GAGGCTCTTG CCACGAACAT CTCTCTTTGC CTACTGCCTC TCCTAGATAG   
  
  
- TTAATTCAAT AGCCAAAGAA ACACAAGATT CTTGCTCTGT TCCCGAAACT GACAAGTACA ACTCTTTTTT   
  
  
- CCTTTCAAGT ATCTCAGGAA GCGACCATGA CAGCTAGTAA ATCAAGGTTC AAAGCTCACT AGCCATTTTT   
  
  
- AAGTAGCCAC TCTTTAGTGG AGGATTGCA

+     Pc-CMA2a

| Site Name | Organism | Position | Strand | Matrix score. | sequence | function |
| --- | --- | --- | --- | --- | --- | --- |
| Pc-CMA2a | Spinacia oleracea | 370 | - | 12 | CAACCAATGAAAA | part of a light responsive element |

> 2018/04/13 10:10:12  
+ GTTTAAAAGT TGGGTAACAT CCCAGTATTG GGGGAGACTT CCGGTCCTTT AACCAAGACC TTACAATAAT   
  
  
+ AAGTAAAATG GGGGTGTCGT TAAGAGAAGT AAACGATGAT CTCAAAGATA TGTCTTTCCT AGGAAAGACA   
  
  
+ GTTGTTCTTG TTCAACTCAT AGAGGACGGT GTATGAAAAA CGATCACAGA GGACGGTGTA TAAAAAGTGA   
  
  
+ TCAGTACACC GCTTACGGCA ATCAGTAGGA ACATTGTCTA TCAAAGACGA GTAGTAGGTT CAATAGTTCT   
  
  
+ GTGTGAAGTA CATCACGCCC TTCTCGTGCG AGACTCAAAA CTCGGACGTA TTAGATCATT AGTCATTATC   
  
  
+ TTTTGTTAAG TATTAACGCT TTTCATTCGT TGACATTTAG CGTTGTAAAT TGTTTTAGTG GAGGTTGAAC   
  
  
+ AATTGAGTCT GAATTCATCC ATACTAGATA CAGATTACGT ATGACATTTG ACTATAGGTT CTTTTGTTTG   
  
  
+ TTTGTATGAT TTGTAAAAGT ATGGCGAACA AATTCTTGTC TCGTGTGATA AGCTCAGTGT CGTAAGAGAC   
  
  
+ CTAGGACATT TTACCTAGTT TCGTTTTTTA CTTCGTTAGA TGGTCTTTGT TCCCCCTTAC CATATTAGTA   
  
  
+ TGTCGAGATG GGATGGGAAT CTCTTAGTGA TAATCGAGGT GTTGCACGAT AGAGAAAGTA GTAGTCGTCT   
  
  
+ CATGCGTTGA CACATATTGA AAAGTGAGAG CATCCGAAGT GTCTCGAATC GAATCTCTTT CGGTTAGACG   
  
  
+ TCCGTAATTC AAAGCGGAGT TCCTGAGAGG AACTTTAAAG ATGTCGGTTC TTTTGTCCTT GGGTCTTATC   
  
  
+ CCTTCGAATT ATCTCCAGTT TTTTAGTTTA GTGTAATCTT TCCTTTTGTT TTAATGTATT TATTTATTTA   
  
  
+ AGTTGGGTCC CGTACCGTTG ACTTGTACAT TGTTTCTTCT TAAGTTCTTT TAAAATTAAT TAATATTTTA   
  
  
+ TATAATACAC ATGATACCAA AGGAAGTTCA ACATCTTCCT CTATGGACAG AAGAAGTCTG ACTATTGGCA   
  
  
+ ATTAGGTATC AGGTAGTTGT CTCATATTAA CTACCTCGTT TGTTTACGAA CACAATCAAT TTACTTTACT   
  
  
+ AACAAACTCA TCATATGCTA AAGCGAATGG ACCGCCACGG TACAGTCTGG CTCCAAGCTT GACGATGTGT   
  
  
+ TTCGACTTCT AAAGTTTTAA CAATACCTTT GGTGGTTCTG CTTCCGCCAT TATCTTATTT TCCGCTGGTA   
  
  
+ GGTCGCTGCA GGATGAAGTA CTCCGAGAAC GGTGCTTGTA GAGAGAAACG GATGACGGAG AGGATCTATC   
  
  
+ AATTAAGTTA TCGGTTTCTT TGTGTTCTAA GAACGAGACA AGGGCTTTGA CTGTTCATGT TGAGAAAAAA   
  
  
+ GGAAAGTTCA TAGAGTCCTT CGCTGGTACT GTCGATCATT TAGTTCCAAG TTTCGAGTGA TCGGTAAAAA   
  
  
+ TTCATCGGTG AGAAATCACC TCCTAACGT  

- CAAATTTTCA ACCCATTGTA GGGTCATAAC CCCCTCTGAA GGCCAGGAAA TTGGTTCTGG AATGTTATTA   
  
  
- TTCATTTTAC CCCCACAGCA ATTCTCTTCA TTTGCTACTA GAGTTTCTAT ACAGAAAGGA TCCTTTCTGT   
  
  
- CAACAAGAAC AAGTTGAGTA TCTCCTGCCA CATACTTTTT GCTAGTGTCT CCTGCCACAT ATTTTTCACT   
  
  
- AGTCATGTGG CGAATGCCGT TAGTCATCCT TGTAACAGAT AGTTTCTGCT CATCATCCAA GTTATCAAGA   
  
  
- CACACTTCAT GTAGTGCGGG AAGAGCACGC TCTGAGTTTT GAGCCTGCAT AATCTAGTAA TCAGTAATAG   
  
  
- AAAACAATTC ATAATTGCGA AAAGTAAGCA ACTGTAAATC GCAACATTTA ACAAAATCAC CTCCAACTTG   
  
  
- TTAACTCAGA CTTAAGTAGG TATGATCTAT GTCTAATGCA TACTGTAAAC TGATATCCAA GAAAACAAAC   
  
  
- AAACATACTA AACATTTTCA TACCGCTTGT TTAAGAACAG AGCACACTAT TCGAGTCACA GCATTCTCTG   
  
  
- GATCCTGTAA AATGGATCAA AGCAAAAAAT GAAGCAATCT ACCAGAAACA AGGGGGAATG GTATAATCAT   
  
  
- ACAGCTCTAC CCTACCCTTA GAGAATCACT ATTAGCTCCA CAACGTGCTA TCTCTTTCAT CATCAGCAGA   
  
  
- GTACGCAACT GTGTATAACT TTTCACTCTC GTAGGCTTCA CAGAGCTTAG CTTAGAGAAA GCCAATCTGC   
  
  
- AGGCATTAAG TTTCGCCTCA AGGACTCTCC TTGAAATTTC TACAGCCAAG AAAACAGGAA CCCAGAATAG   
  
  
- GGAAGCTTAA TAGAGGTCAA AAAATCAAAT CACATTAGAA AGGAAAACAA AATTACATAA ATAAATAAAT   
  
  
- TCAACCCAGG GCATGGCAAC TGAACATGTA ACAAAGAAGA ATTCAAGAAA ATTTTAATTA ATTATAAAAT   
  
  
- ATATTATGTG TACTATGGTT TCCTTCAAGT TGTAGAAGGA GATACCTGTC TTCTTCAGAC TGATAACCGT   
  
  
- TAATCCATAG TCCATCAACA GAGTATAATT GATGGAGCAA ACAAATGCTT GTGTTAGTTA AATGAAATGA   
  
  
- TTGTTTGAGT AGTATACGAT TTCGCTTACC TGGCGGTGCC ATGTCAGACC GAGGTTCGAA CTGCTACACA   
  
  
- AAGCTGAAGA TTTCAAAATT GTTATGGAAA CCACCAAGAC GAAGGCGGTA ATAGAATAAA AGGCGACCAT   
  
  
- CCAGCGACGT CCTACTTCAT GAGGCTCTTG CCACGAACAT CTCTCTTTGC CTACTGCCTC TCCTAGATAG   
  
  
- TTAATTCAAT AGCCAAAGAA ACACAAGATT CTTGCTCTGT TCCCGAAACT GACAAGTACA ACTCTTTTTT   
  
  
- CCTTTCAAGT ATCTCAGGAA GCGACCATGA CAGCTAGTAA ATCAAGGTTC AAAGCTCACT AGCCATTTTT   
  
  
- AAGTAGCCAC TCTTTAGTGG AGGATTGCA

+     Skn-1\_motif

| Site Name | Organism | Position | Strand | Matrix score. | sequence | function |
| --- | --- | --- | --- | --- | --- | --- |
| Skn-1\_motif | Oryza sativa | 1312 | - | 5 | GTCAT | cis-acting regulatory element required for endosperm expression |
| Skn-1\_motif | Oryza sativa | 461 | - | 5 | GTCAT | cis-acting regulatory element required for endosperm expression |
| Skn-1\_motif | Oryza sativa | 342 | + | 5 | GTCAT | cis-acting regulatory element required for endosperm expression |

> 2018/04/13 10:10:12  
+ GTTTAAAAGT TGGGTAACAT CCCAGTATTG GGGGAGACTT CCGGTCCTTT AACCAAGACC TTACAATAAT   
  
  
+ AAGTAAAATG GGGGTGTCGT TAAGAGAAGT AAACGATGAT CTCAAAGATA TGTCTTTCCT AGGAAAGACA   
  
  
+ GTTGTTCTTG TTCAACTCAT AGAGGACGGT GTATGAAAAA CGATCACAGA GGACGGTGTA TAAAAAGTGA   
  
  
+ TCAGTACACC GCTTACGGCA ATCAGTAGGA ACATTGTCTA TCAAAGACGA GTAGTAGGTT CAATAGTTCT   
  
  
+ GTGTGAAGTA CATCACGCCC TTCTCGTGCG AGACTCAAAA CTCGGACGTA TTAGATCATT AGTCATTATC   
  
  
+ TTTTGTTAAG TATTAACGCT TTTCATTCGT TGACATTTAG CGTTGTAAAT TGTTTTAGTG GAGGTTGAAC   
  
  
+ AATTGAGTCT GAATTCATCC ATACTAGATA CAGATTACGT ATGACATTTG ACTATAGGTT CTTTTGTTTG   
  
  
+ TTTGTATGAT TTGTAAAAGT ATGGCGAACA AATTCTTGTC TCGTGTGATA AGCTCAGTGT CGTAAGAGAC   
  
  
+ CTAGGACATT TTACCTAGTT TCGTTTTTTA CTTCGTTAGA TGGTCTTTGT TCCCCCTTAC CATATTAGTA   
  
  
+ TGTCGAGATG GGATGGGAAT CTCTTAGTGA TAATCGAGGT GTTGCACGAT AGAGAAAGTA GTAGTCGTCT   
  
  
+ CATGCGTTGA CACATATTGA AAAGTGAGAG CATCCGAAGT GTCTCGAATC GAATCTCTTT CGGTTAGACG   
  
  
+ TCCGTAATTC AAAGCGGAGT TCCTGAGAGG AACTTTAAAG ATGTCGGTTC TTTTGTCCTT GGGTCTTATC   
  
  
+ CCTTCGAATT ATCTCCAGTT TTTTAGTTTA GTGTAATCTT TCCTTTTGTT TTAATGTATT TATTTATTTA   
  
  
+ AGTTGGGTCC CGTACCGTTG ACTTGTACAT TGTTTCTTCT TAAGTTCTTT TAAAATTAAT TAATATTTTA   
  
  
+ TATAATACAC ATGATACCAA AGGAAGTTCA ACATCTTCCT CTATGGACAG AAGAAGTCTG ACTATTGGCA   
  
  
+ ATTAGGTATC AGGTAGTTGT CTCATATTAA CTACCTCGTT TGTTTACGAA CACAATCAAT TTACTTTACT   
  
  
+ AACAAACTCA TCATATGCTA AAGCGAATGG ACCGCCACGG TACAGTCTGG CTCCAAGCTT GACGATGTGT   
  
  
+ TTCGACTTCT AAAGTTTTAA CAATACCTTT GGTGGTTCTG CTTCCGCCAT TATCTTATTT TCCGCTGGTA   
  
  
+ GGTCGCTGCA GGATGAAGTA CTCCGAGAAC GGTGCTTGTA GAGAGAAACG GATGACGGAG AGGATCTATC   
  
  
+ AATTAAGTTA TCGGTTTCTT TGTGTTCTAA GAACGAGACA AGGGCTTTGA CTGTTCATGT TGAGAAAAAA   
  
  
+ GGAAAGTTCA TAGAGTCCTT CGCTGGTACT GTCGATCATT TAGTTCCAAG TTTCGAGTGA TCGGTAAAAA   
  
  
+ TTCATCGGTG AGAAATCACC TCCTAACGT  

- CAAATTTTCA ACCCATTGTA GGGTCATAAC CCCCTCTGAA GGCCAGGAAA TTGGTTCTGG AATGTTATTA   
  
  
- TTCATTTTAC CCCCACAGCA ATTCTCTTCA TTTGCTACTA GAGTTTCTAT ACAGAAAGGA TCCTTTCTGT   
  
  
- CAACAAGAAC AAGTTGAGTA TCTCCTGCCA CATACTTTTT GCTAGTGTCT CCTGCCACAT ATTTTTCACT   
  
  
- AGTCATGTGG CGAATGCCGT TAGTCATCCT TGTAACAGAT AGTTTCTGCT CATCATCCAA GTTATCAAGA   
  
  
- CACACTTCAT GTAGTGCGGG AAGAGCACGC TCTGAGTTTT GAGCCTGCAT AATCTAGTAA TCAGTAATAG   
  
  
- AAAACAATTC ATAATTGCGA AAAGTAAGCA ACTGTAAATC GCAACATTTA ACAAAATCAC CTCCAACTTG   
  
  
- TTAACTCAGA CTTAAGTAGG TATGATCTAT GTCTAATGCA TACTGTAAAC TGATATCCAA GAAAACAAAC   
  
  
- AAACATACTA AACATTTTCA TACCGCTTGT TTAAGAACAG AGCACACTAT TCGAGTCACA GCATTCTCTG   
  
  
- GATCCTGTAA AATGGATCAA AGCAAAAAAT GAAGCAATCT ACCAGAAACA AGGGGGAATG GTATAATCAT   
  
  
- ACAGCTCTAC CCTACCCTTA GAGAATCACT ATTAGCTCCA CAACGTGCTA TCTCTTTCAT CATCAGCAGA   
  
  
- GTACGCAACT GTGTATAACT TTTCACTCTC GTAGGCTTCA CAGAGCTTAG CTTAGAGAAA GCCAATCTGC   
  
  
- AGGCATTAAG TTTCGCCTCA AGGACTCTCC TTGAAATTTC TACAGCCAAG AAAACAGGAA CCCAGAATAG   
  
  
- GGAAGCTTAA TAGAGGTCAA AAAATCAAAT CACATTAGAA AGGAAAACAA AATTACATAA ATAAATAAAT   
  
  
- TCAACCCAGG GCATGGCAAC TGAACATGTA ACAAAGAAGA ATTCAAGAAA ATTTTAATTA ATTATAAAAT   
  
  
- ATATTATGTG TACTATGGTT TCCTTCAAGT TGTAGAAGGA GATACCTGTC TTCTTCAGAC TGATAACCGT   
  
  
- TAATCCATAG TCCATCAACA GAGTATAATT GATGGAGCAA ACAAATGCTT GTGTTAGTTA AATGAAATGA   
  
  
- TTGTTTGAGT AGTATACGAT TTCGCTTACC TGGCGGTGCC ATGTCAGACC GAGGTTCGAA CTGCTACACA   
  
  
- AAGCTGAAGA TTTCAAAATT GTTATGGAAA CCACCAAGAC GAAGGCGGTA ATAGAATAAA AGGCGACCAT   
  
  
- CCAGCGACGT CCTACTTCAT GAGGCTCTTG CCACGAACAT CTCTCTTTGC CTACTGCCTC TCCTAGATAG   
  
  
- TTAATTCAAT AGCCAAAGAA ACACAAGATT CTTGCTCTGT TCCCGAAACT GACAAGTACA ACTCTTTTTT   
  
  
- CCTTTCAAGT ATCTCAGGAA GCGACCATGA CAGCTAGTAA ATCAAGGTTC AAAGCTCACT AGCCATTTTT   
  
  
- AAGTAGCCAC TCTTTAGTGG AGGATTGCA

+     TATA-box

| Site Name | Organism | Position | Strand | Matrix score. | sequence | function |
| --- | --- | --- | --- | --- | --- | --- |
| TATA-box | Arabidopsis thaliana | 979 | - | 4 | TATA | core promoter element around -30 of transcription start |
| TATA-box | Glycine max | 623 | - | 5 | TAATA | core promoter element around -30 of transcription start |
| TATA-box | Lycopersicon esculentum | 569 | + | 5 | TTTTA | core promoter element around -30 of transcription start |
| TATA-box | Brassica oleracea | 980 | + | 7 | ATATAAT | core promoter element around -30 of transcription start |
| TATA-box | Arabidopsis thaliana | 977 | - | 6 | TATAAA | core promoter element around -30 of transcription start |
| TATA-box | Lycopersicon esculentum | 961 | - | 5 | TTTTA | core promoter element around -30 of transcription start |
| TATA-box | Lycopersicon esculentum | 958 | + | 5 | TTTTA | core promoter element around -30 of transcription start |
| TATA-box | Lycopersicon esculentum | 403 | + | 5 | TTTTA | core promoter element around -30 of transcription start |
| TATA-box | Pisum sativum | 975 | - | 8 | TATAAAAT | core promoter element around -30 of transcription start |
| TATA-box | Lycopersicon esculentum | 504 | - | 5 | TTTTA | core promoter element around -30 of transcription start |
| TATA-box | Glycine max | 361 | - | 5 | TAATA | core promoter element around -30 of transcription start |
| TATA-box | Glycine max | 329 | - | 5 | TAATA | core promoter element around -30 of transcription start |
| TATA-box | Glycine max | 1075 | - | 5 | TAATA | core promoter element around -30 of transcription start |
| TATA-box | Lycopersicon esculentum | 861 | + | 5 | TTTTA | core promoter element around -30 of transcription start |
| TATA-box | Glycine max | 983 | + | 5 | TAATA | core promoter element around -30 of transcription start |
| TATA-box | Glycine max | 67 | + | 5 | TAATA | core promoter element around -30 of transcription start |
| TATA-box | Lycopersicon esculentum | 4 | - | 5 | TTTTA | core promoter element around -30 of transcription start |
| TATA-box | Lycopersicon esculentum | 1465 | - | 5 | TTTTA | core promoter element around -30 of transcription start |
| TATA-box | Lycopersicon esculentum | 1205 | + | 5 | TTTTA | core promoter element around -30 of transcription start |
| TATA-box | Arabidopsis thaliana | 473 | + | 4 | TATA | core promoter element around -30 of transcription start |
| TATA-box | Lycopersicon esculentum | 586 | + | 5 | TTTTA | core promoter element around -30 of transcription start |
| TATA-box | Arabidopsis thaliana | 976 | - | 7 | TATAAAA | core promoter element around -30 of transcription start |
| TATA-box | Arabidopsis thaliana | 199 | + | 6 | TATAAA | core promoter element around -30 of transcription start |
| TATA-box | Glycine max | 971 | + | 5 | TAATA | core promoter element around -30 of transcription start |
| TATA-box | Lycopersicon esculentum | 74 | - | 5 | TTTTA | core promoter element around -30 of transcription start |
| TATA-box | Arabidopsis thaliana | 981 | - | 4 | TATA | core promoter element around -30 of transcription start |
| TATA-box | Lycopersicon esculentum | 889 | + | 5 | TTTTA | core promoter element around -30 of transcription start |
| TATA-box | Arabidopsis thaliana | 978 | - | 7 | TATATAA | core promoter element around -30 of transcription start |
| TATA-box | Helianthus annuus | 197 | - | 6 | TATACA | core promoter element around -30 of transcription start |
| TATA-box | Lycopersicon esculentum | 201 | - | 5 | TTTTA | core promoter element around -30 of transcription start |

> 2018/04/13 10:10:12  
+ GTTTAAAAGT TGGGTAACAT CCCAGTATTG GGGGAGACTT CCGGTCCTTT AACCAAGACC TTACAATAAT   
  
  
+ AAGTAAAATG GGGGTGTCGT TAAGAGAAGT AAACGATGAT CTCAAAGATA TGTCTTTCCT AGGAAAGACA   
  
  
+ GTTGTTCTTG TTCAACTCAT AGAGGACGGT GTATGAAAAA CGATCACAGA GGACGGTGTA TAAAAAGTGA   
  
  
+ TCAGTACACC GCTTACGGCA ATCAGTAGGA ACATTGTCTA TCAAAGACGA GTAGTAGGTT CAATAGTTCT   
  
  
+ GTGTGAAGTA CATCACGCCC TTCTCGTGCG AGACTCAAAA CTCGGACGTA TTAGATCATT AGTCATTATC   
  
  
+ TTTTGTTAAG TATTAACGCT TTTCATTCGT TGACATTTAG CGTTGTAAAT TGTTTTAGTG GAGGTTGAAC   
  
  
+ AATTGAGTCT GAATTCATCC ATACTAGATA CAGATTACGT ATGACATTTG ACTATAGGTT CTTTTGTTTG   
  
  
+ TTTGTATGAT TTGTAAAAGT ATGGCGAACA AATTCTTGTC TCGTGTGATA AGCTCAGTGT CGTAAGAGAC   
  
  
+ CTAGGACATT TTACCTAGTT TCGTTTTTTA CTTCGTTAGA TGGTCTTTGT TCCCCCTTAC CATATTAGTA   
  
  
+ TGTCGAGATG GGATGGGAAT CTCTTAGTGA TAATCGAGGT GTTGCACGAT AGAGAAAGTA GTAGTCGTCT   
  
  
+ CATGCGTTGA CACATATTGA AAAGTGAGAG CATCCGAAGT GTCTCGAATC GAATCTCTTT CGGTTAGACG   
  
  
+ TCCGTAATTC AAAGCGGAGT TCCTGAGAGG AACTTTAAAG ATGTCGGTTC TTTTGTCCTT GGGTCTTATC   
  
  
+ CCTTCGAATT ATCTCCAGTT TTTTAGTTTA GTGTAATCTT TCCTTTTGTT TTAATGTATT TATTTATTTA   
  
  
+ AGTTGGGTCC CGTACCGTTG ACTTGTACAT TGTTTCTTCT TAAGTTCTTT TAAAATTAAT TAATATTTTA   
  
  
+ TATAATACAC ATGATACCAA AGGAAGTTCA ACATCTTCCT CTATGGACAG AAGAAGTCTG ACTATTGGCA   
  
  
+ ATTAGGTATC AGGTAGTTGT CTCATATTAA CTACCTCGTT TGTTTACGAA CACAATCAAT TTACTTTACT   
  
  
+ AACAAACTCA TCATATGCTA AAGCGAATGG ACCGCCACGG TACAGTCTGG CTCCAAGCTT GACGATGTGT   
  
  
+ TTCGACTTCT AAAGTTTTAA CAATACCTTT GGTGGTTCTG CTTCCGCCAT TATCTTATTT TCCGCTGGTA   
  
  
+ GGTCGCTGCA GGATGAAGTA CTCCGAGAAC GGTGCTTGTA GAGAGAAACG GATGACGGAG AGGATCTATC   
  
  
+ AATTAAGTTA TCGGTTTCTT TGTGTTCTAA GAACGAGACA AGGGCTTTGA CTGTTCATGT TGAGAAAAAA   
  
  
+ GGAAAGTTCA TAGAGTCCTT CGCTGGTACT GTCGATCATT TAGTTCCAAG TTTCGAGTGA TCGGTAAAAA   
  
  
+ TTCATCGGTG AGAAATCACC TCCTAACGT  

- CAAATTTTCA ACCCATTGTA GGGTCATAAC CCCCTCTGAA GGCCAGGAAA TTGGTTCTGG AATGTTATTA   
  
  
- TTCATTTTAC CCCCACAGCA ATTCTCTTCA TTTGCTACTA GAGTTTCTAT ACAGAAAGGA TCCTTTCTGT   
  
  
- CAACAAGAAC AAGTTGAGTA TCTCCTGCCA CATACTTTTT GCTAGTGTCT CCTGCCACAT ATTTTTCACT   
  
  
- AGTCATGTGG CGAATGCCGT TAGTCATCCT TGTAACAGAT AGTTTCTGCT CATCATCCAA GTTATCAAGA   
  
  
- CACACTTCAT GTAGTGCGGG AAGAGCACGC TCTGAGTTTT GAGCCTGCAT AATCTAGTAA TCAGTAATAG   
  
  
- AAAACAATTC ATAATTGCGA AAAGTAAGCA ACTGTAAATC GCAACATTTA ACAAAATCAC CTCCAACTTG   
  
  
- TTAACTCAGA CTTAAGTAGG TATGATCTAT GTCTAATGCA TACTGTAAAC TGATATCCAA GAAAACAAAC   
  
  
- AAACATACTA AACATTTTCA TACCGCTTGT TTAAGAACAG AGCACACTAT TCGAGTCACA GCATTCTCTG   
  
  
- GATCCTGTAA AATGGATCAA AGCAAAAAAT GAAGCAATCT ACCAGAAACA AGGGGGAATG GTATAATCAT   
  
  
- ACAGCTCTAC CCTACCCTTA GAGAATCACT ATTAGCTCCA CAACGTGCTA TCTCTTTCAT CATCAGCAGA   
  
  
- GTACGCAACT GTGTATAACT TTTCACTCTC GTAGGCTTCA CAGAGCTTAG CTTAGAGAAA GCCAATCTGC   
  
  
- AGGCATTAAG TTTCGCCTCA AGGACTCTCC TTGAAATTTC TACAGCCAAG AAAACAGGAA CCCAGAATAG   
  
  
- GGAAGCTTAA TAGAGGTCAA AAAATCAAAT CACATTAGAA AGGAAAACAA AATTACATAA ATAAATAAAT   
  
  
- TCAACCCAGG GCATGGCAAC TGAACATGTA ACAAAGAAGA ATTCAAGAAA ATTTTAATTA ATTATAAAAT   
  
  
- ATATTATGTG TACTATGGTT TCCTTCAAGT TGTAGAAGGA GATACCTGTC TTCTTCAGAC TGATAACCGT   
  
  
- TAATCCATAG TCCATCAACA GAGTATAATT GATGGAGCAA ACAAATGCTT GTGTTAGTTA AATGAAATGA   
  
  
- TTGTTTGAGT AGTATACGAT TTCGCTTACC TGGCGGTGCC ATGTCAGACC GAGGTTCGAA CTGCTACACA   
  
  
- AAGCTGAAGA TTTCAAAATT GTTATGGAAA CCACCAAGAC GAAGGCGGTA ATAGAATAAA AGGCGACCAT   
  
  
- CCAGCGACGT CCTACTTCAT GAGGCTCTTG CCACGAACAT CTCTCTTTGC CTACTGCCTC TCCTAGATAG   
  
  
- TTAATTCAAT AGCCAAAGAA ACACAAGATT CTTGCTCTGT TCCCGAAACT GACAAGTACA ACTCTTTTTT   
  
  
- CCTTTCAAGT ATCTCAGGAA GCGACCATGA CAGCTAGTAA ATCAAGGTTC AAAGCTCACT AGCCATTTTT   
  
  
- AAGTAGCCAC TCTTTAGTGG AGGATTGCA

+     TC-rich repeats

| Site Name | Organism | Position | Strand | Matrix score. | sequence | function |
| --- | --- | --- | --- | --- | --- | --- |
| TC-rich repeats | Nicotiana tabacum | 848 | + | 9 | ATTTTCTCCA | cis-acting element involved in defense and stress responsiveness |

> 2018/04/13 10:10:12  
+ GTTTAAAAGT TGGGTAACAT CCCAGTATTG GGGGAGACTT CCGGTCCTTT AACCAAGACC TTACAATAAT   
  
  
+ AAGTAAAATG GGGGTGTCGT TAAGAGAAGT AAACGATGAT CTCAAAGATA TGTCTTTCCT AGGAAAGACA   
  
  
+ GTTGTTCTTG TTCAACTCAT AGAGGACGGT GTATGAAAAA CGATCACAGA GGACGGTGTA TAAAAAGTGA   
  
  
+ TCAGTACACC GCTTACGGCA ATCAGTAGGA ACATTGTCTA TCAAAGACGA GTAGTAGGTT CAATAGTTCT   
  
  
+ GTGTGAAGTA CATCACGCCC TTCTCGTGCG AGACTCAAAA CTCGGACGTA TTAGATCATT AGTCATTATC   
  
  
+ TTTTGTTAAG TATTAACGCT TTTCATTCGT TGACATTTAG CGTTGTAAAT TGTTTTAGTG GAGGTTGAAC   
  
  
+ AATTGAGTCT GAATTCATCC ATACTAGATA CAGATTACGT ATGACATTTG ACTATAGGTT CTTTTGTTTG   
  
  
+ TTTGTATGAT TTGTAAAAGT ATGGCGAACA AATTCTTGTC TCGTGTGATA AGCTCAGTGT CGTAAGAGAC   
  
  
+ CTAGGACATT TTACCTAGTT TCGTTTTTTA CTTCGTTAGA TGGTCTTTGT TCCCCCTTAC CATATTAGTA   
  
  
+ TGTCGAGATG GGATGGGAAT CTCTTAGTGA TAATCGAGGT GTTGCACGAT AGAGAAAGTA GTAGTCGTCT   
  
  
+ CATGCGTTGA CACATATTGA AAAGTGAGAG CATCCGAAGT GTCTCGAATC GAATCTCTTT CGGTTAGACG   
  
  
+ TCCGTAATTC AAAGCGGAGT TCCTGAGAGG AACTTTAAAG ATGTCGGTTC TTTTGTCCTT GGGTCTTATC   
  
  
+ CCTTCGAATT ATCTCCAGTT TTTTAGTTTA GTGTAATCTT TCCTTTTGTT TTAATGTATT TATTTATTTA   
  
  
+ AGTTGGGTCC CGTACCGTTG ACTTGTACAT TGTTTCTTCT TAAGTTCTTT TAAAATTAAT TAATATTTTA   
  
  
+ TATAATACAC ATGATACCAA AGGAAGTTCA ACATCTTCCT CTATGGACAG AAGAAGTCTG ACTATTGGCA   
  
  
+ ATTAGGTATC AGGTAGTTGT CTCATATTAA CTACCTCGTT TGTTTACGAA CACAATCAAT TTACTTTACT   
  
  
+ AACAAACTCA TCATATGCTA AAGCGAATGG ACCGCCACGG TACAGTCTGG CTCCAAGCTT GACGATGTGT   
  
  
+ TTCGACTTCT AAAGTTTTAA CAATACCTTT GGTGGTTCTG CTTCCGCCAT TATCTTATTT TCCGCTGGTA   
  
  
+ GGTCGCTGCA GGATGAAGTA CTCCGAGAAC GGTGCTTGTA GAGAGAAACG GATGACGGAG AGGATCTATC   
  
  
+ AATTAAGTTA TCGGTTTCTT TGTGTTCTAA GAACGAGACA AGGGCTTTGA CTGTTCATGT TGAGAAAAAA   
  
  
+ GGAAAGTTCA TAGAGTCCTT CGCTGGTACT GTCGATCATT TAGTTCCAAG TTTCGAGTGA TCGGTAAAAA   
  
  
+ TTCATCGGTG AGAAATCACC TCCTAACGT  

- CAAATTTTCA ACCCATTGTA GGGTCATAAC CCCCTCTGAA GGCCAGGAAA TTGGTTCTGG AATGTTATTA   
  
  
- TTCATTTTAC CCCCACAGCA ATTCTCTTCA TTTGCTACTA GAGTTTCTAT ACAGAAAGGA TCCTTTCTGT   
  
  
- CAACAAGAAC AAGTTGAGTA TCTCCTGCCA CATACTTTTT GCTAGTGTCT CCTGCCACAT ATTTTTCACT   
  
  
- AGTCATGTGG CGAATGCCGT TAGTCATCCT TGTAACAGAT AGTTTCTGCT CATCATCCAA GTTATCAAGA   
  
  
- CACACTTCAT GTAGTGCGGG AAGAGCACGC TCTGAGTTTT GAGCCTGCAT AATCTAGTAA TCAGTAATAG   
  
  
- AAAACAATTC ATAATTGCGA AAAGTAAGCA ACTGTAAATC GCAACATTTA ACAAAATCAC CTCCAACTTG   
  
  
- TTAACTCAGA CTTAAGTAGG TATGATCTAT GTCTAATGCA TACTGTAAAC TGATATCCAA GAAAACAAAC   
  
  
- AAACATACTA AACATTTTCA TACCGCTTGT TTAAGAACAG AGCACACTAT TCGAGTCACA GCATTCTCTG   
  
  
- GATCCTGTAA AATGGATCAA AGCAAAAAAT GAAGCAATCT ACCAGAAACA AGGGGGAATG GTATAATCAT   
  
  
- ACAGCTCTAC CCTACCCTTA GAGAATCACT ATTAGCTCCA CAACGTGCTA TCTCTTTCAT CATCAGCAGA   
  
  
- GTACGCAACT GTGTATAACT TTTCACTCTC GTAGGCTTCA CAGAGCTTAG CTTAGAGAAA GCCAATCTGC   
  
  
- AGGCATTAAG TTTCGCCTCA AGGACTCTCC TTGAAATTTC TACAGCCAAG AAAACAGGAA CCCAGAATAG   
  
  
- GGAAGCTTAA TAGAGGTCAA AAAATCAAAT CACATTAGAA AGGAAAACAA AATTACATAA ATAAATAAAT   
  
  
- TCAACCCAGG GCATGGCAAC TGAACATGTA ACAAAGAAGA ATTCAAGAAA ATTTTAATTA ATTATAAAAT   
  
  
- ATATTATGTG TACTATGGTT TCCTTCAAGT TGTAGAAGGA GATACCTGTC TTCTTCAGAC TGATAACCGT   
  
  
- TAATCCATAG TCCATCAACA GAGTATAATT GATGGAGCAA ACAAATGCTT GTGTTAGTTA AATGAAATGA   
  
  
- TTGTTTGAGT AGTATACGAT TTCGCTTACC TGGCGGTGCC ATGTCAGACC GAGGTTCGAA CTGCTACACA   
  
  
- AAGCTGAAGA TTTCAAAATT GTTATGGAAA CCACCAAGAC GAAGGCGGTA ATAGAATAAA AGGCGACCAT   
  
  
- CCAGCGACGT CCTACTTCAT GAGGCTCTTG CCACGAACAT CTCTCTTTGC CTACTGCCTC TCCTAGATAG   
  
  
- TTAATTCAAT AGCCAAAGAA ACACAAGATT CTTGCTCTGT TCCCGAAACT GACAAGTACA ACTCTTTTTT   
  
  
- CCTTTCAAGT ATCTCAGGAA GCGACCATGA CAGCTAGTAA ATCAAGGTTC AAAGCTCACT AGCCATTTTT   
  
  
- AAGTAGCCAC TCTTTAGTGG AGGATTGCA

+     TCT-motif

| Site Name | Organism | Position | Strand | Matrix score. | sequence | function |
| --- | --- | --- | --- | --- | --- | --- |
| TCT-motif | Arabidopsis thaliana | 552 | - | 6 | TCTTAC | part of a light responsive element |

> 2018/04/13 10:10:12  
+ GTTTAAAAGT TGGGTAACAT CCCAGTATTG GGGGAGACTT CCGGTCCTTT AACCAAGACC TTACAATAAT   
  
  
+ AAGTAAAATG GGGGTGTCGT TAAGAGAAGT AAACGATGAT CTCAAAGATA TGTCTTTCCT AGGAAAGACA   
  
  
+ GTTGTTCTTG TTCAACTCAT AGAGGACGGT GTATGAAAAA CGATCACAGA GGACGGTGTA TAAAAAGTGA   
  
  
+ TCAGTACACC GCTTACGGCA ATCAGTAGGA ACATTGTCTA TCAAAGACGA GTAGTAGGTT CAATAGTTCT   
  
  
+ GTGTGAAGTA CATCACGCCC TTCTCGTGCG AGACTCAAAA CTCGGACGTA TTAGATCATT AGTCATTATC   
  
  
+ TTTTGTTAAG TATTAACGCT TTTCATTCGT TGACATTTAG CGTTGTAAAT TGTTTTAGTG GAGGTTGAAC   
  
  
+ AATTGAGTCT GAATTCATCC ATACTAGATA CAGATTACGT ATGACATTTG ACTATAGGTT CTTTTGTTTG   
  
  
+ TTTGTATGAT TTGTAAAAGT ATGGCGAACA AATTCTTGTC TCGTGTGATA AGCTCAGTGT CGTAAGAGAC   
  
  
+ CTAGGACATT TTACCTAGTT TCGTTTTTTA CTTCGTTAGA TGGTCTTTGT TCCCCCTTAC CATATTAGTA   
  
  
+ TGTCGAGATG GGATGGGAAT CTCTTAGTGA TAATCGAGGT GTTGCACGAT AGAGAAAGTA GTAGTCGTCT   
  
  
+ CATGCGTTGA CACATATTGA AAAGTGAGAG CATCCGAAGT GTCTCGAATC GAATCTCTTT CGGTTAGACG   
  
  
+ TCCGTAATTC AAAGCGGAGT TCCTGAGAGG AACTTTAAAG ATGTCGGTTC TTTTGTCCTT GGGTCTTATC   
  
  
+ CCTTCGAATT ATCTCCAGTT TTTTAGTTTA GTGTAATCTT TCCTTTTGTT TTAATGTATT TATTTATTTA   
  
  
+ AGTTGGGTCC CGTACCGTTG ACTTGTACAT TGTTTCTTCT TAAGTTCTTT TAAAATTAAT TAATATTTTA   
  
  
+ TATAATACAC ATGATACCAA AGGAAGTTCA ACATCTTCCT CTATGGACAG AAGAAGTCTG ACTATTGGCA   
  
  
+ ATTAGGTATC AGGTAGTTGT CTCATATTAA CTACCTCGTT TGTTTACGAA CACAATCAAT TTACTTTACT   
  
  
+ AACAAACTCA TCATATGCTA AAGCGAATGG ACCGCCACGG TACAGTCTGG CTCCAAGCTT GACGATGTGT   
  
  
+ TTCGACTTCT AAAGTTTTAA CAATACCTTT GGTGGTTCTG CTTCCGCCAT TATCTTATTT TCCGCTGGTA   
  
  
+ GGTCGCTGCA GGATGAAGTA CTCCGAGAAC GGTGCTTGTA GAGAGAAACG GATGACGGAG AGGATCTATC   
  
  
+ AATTAAGTTA TCGGTTTCTT TGTGTTCTAA GAACGAGACA AGGGCTTTGA CTGTTCATGT TGAGAAAAAA   
  
  
+ GGAAAGTTCA TAGAGTCCTT CGCTGGTACT GTCGATCATT TAGTTCCAAG TTTCGAGTGA TCGGTAAAAA   
  
  
+ TTCATCGGTG AGAAATCACC TCCTAACGT  

- CAAATTTTCA ACCCATTGTA GGGTCATAAC CCCCTCTGAA GGCCAGGAAA TTGGTTCTGG AATGTTATTA   
  
  
- TTCATTTTAC CCCCACAGCA ATTCTCTTCA TTTGCTACTA GAGTTTCTAT ACAGAAAGGA TCCTTTCTGT   
  
  
- CAACAAGAAC AAGTTGAGTA TCTCCTGCCA CATACTTTTT GCTAGTGTCT CCTGCCACAT ATTTTTCACT   
  
  
- AGTCATGTGG CGAATGCCGT TAGTCATCCT TGTAACAGAT AGTTTCTGCT CATCATCCAA GTTATCAAGA   
  
  
- CACACTTCAT GTAGTGCGGG AAGAGCACGC TCTGAGTTTT GAGCCTGCAT AATCTAGTAA TCAGTAATAG   
  
  
- AAAACAATTC ATAATTGCGA AAAGTAAGCA ACTGTAAATC GCAACATTTA ACAAAATCAC CTCCAACTTG   
  
  
- TTAACTCAGA CTTAAGTAGG TATGATCTAT GTCTAATGCA TACTGTAAAC TGATATCCAA GAAAACAAAC   
  
  
- AAACATACTA AACATTTTCA TACCGCTTGT TTAAGAACAG AGCACACTAT TCGAGTCACA GCATTCTCTG   
  
  
- GATCCTGTAA AATGGATCAA AGCAAAAAAT GAAGCAATCT ACCAGAAACA AGGGGGAATG GTATAATCAT   
  
  
- ACAGCTCTAC CCTACCCTTA GAGAATCACT ATTAGCTCCA CAACGTGCTA TCTCTTTCAT CATCAGCAGA   
  
  
- GTACGCAACT GTGTATAACT TTTCACTCTC GTAGGCTTCA CAGAGCTTAG CTTAGAGAAA GCCAATCTGC   
  
  
- AGGCATTAAG TTTCGCCTCA AGGACTCTCC TTGAAATTTC TACAGCCAAG AAAACAGGAA CCCAGAATAG   
  
  
- GGAAGCTTAA TAGAGGTCAA AAAATCAAAT CACATTAGAA AGGAAAACAA AATTACATAA ATAAATAAAT   
  
  
- TCAACCCAGG GCATGGCAAC TGAACATGTA ACAAAGAAGA ATTCAAGAAA ATTTTAATTA ATTATAAAAT   
  
  
- ATATTATGTG TACTATGGTT TCCTTCAAGT TGTAGAAGGA GATACCTGTC TTCTTCAGAC TGATAACCGT   
  
  
- TAATCCATAG TCCATCAACA GAGTATAATT GATGGAGCAA ACAAATGCTT GTGTTAGTTA AATGAAATGA   
  
  
- TTGTTTGAGT AGTATACGAT TTCGCTTACC TGGCGGTGCC ATGTCAGACC GAGGTTCGAA CTGCTACACA   
  
  
- AAGCTGAAGA TTTCAAAATT GTTATGGAAA CCACCAAGAC GAAGGCGGTA ATAGAATAAA AGGCGACCAT   
  
  
- CCAGCGACGT CCTACTTCAT GAGGCTCTTG CCACGAACAT CTCTCTTTGC CTACTGCCTC TCCTAGATAG   
  
  
- TTAATTCAAT AGCCAAAGAA ACACAAGATT CTTGCTCTGT TCCCGAAACT GACAAGTACA ACTCTTTTTT   
  
  
- CCTTTCAAGT ATCTCAGGAA GCGACCATGA CAGCTAGTAA ATCAAGGTTC AAAGCTCACT AGCCATTTTT   
  
  
- AAGTAGCCAC TCTTTAGTGG AGGATTGCA

+     TGA-element

| Site Name | Organism | Position | Strand | Matrix score. | sequence | function |
| --- | --- | --- | --- | --- | --- | --- |
| TGA-element | Brassica oleracea | 86 | - | 6 | AACGAC | auxin-responsive element |

> 2018/04/13 10:10:12  
+ GTTTAAAAGT TGGGTAACAT CCCAGTATTG GGGGAGACTT CCGGTCCTTT AACCAAGACC TTACAATAAT   
  
  
+ AAGTAAAATG GGGGTGTCGT TAAGAGAAGT AAACGATGAT CTCAAAGATA TGTCTTTCCT AGGAAAGACA   
  
  
+ GTTGTTCTTG TTCAACTCAT AGAGGACGGT GTATGAAAAA CGATCACAGA GGACGGTGTA TAAAAAGTGA   
  
  
+ TCAGTACACC GCTTACGGCA ATCAGTAGGA ACATTGTCTA TCAAAGACGA GTAGTAGGTT CAATAGTTCT   
  
  
+ GTGTGAAGTA CATCACGCCC TTCTCGTGCG AGACTCAAAA CTCGGACGTA TTAGATCATT AGTCATTATC   
  
  
+ TTTTGTTAAG TATTAACGCT TTTCATTCGT TGACATTTAG CGTTGTAAAT TGTTTTAGTG GAGGTTGAAC   
  
  
+ AATTGAGTCT GAATTCATCC ATACTAGATA CAGATTACGT ATGACATTTG ACTATAGGTT CTTTTGTTTG   
  
  
+ TTTGTATGAT TTGTAAAAGT ATGGCGAACA AATTCTTGTC TCGTGTGATA AGCTCAGTGT CGTAAGAGAC   
  
  
+ CTAGGACATT TTACCTAGTT TCGTTTTTTA CTTCGTTAGA TGGTCTTTGT TCCCCCTTAC CATATTAGTA   
  
  
+ TGTCGAGATG GGATGGGAAT CTCTTAGTGA TAATCGAGGT GTTGCACGAT AGAGAAAGTA GTAGTCGTCT   
  
  
+ CATGCGTTGA CACATATTGA AAAGTGAGAG CATCCGAAGT GTCTCGAATC GAATCTCTTT CGGTTAGACG   
  
  
+ TCCGTAATTC AAAGCGGAGT TCCTGAGAGG AACTTTAAAG ATGTCGGTTC TTTTGTCCTT GGGTCTTATC   
  
  
+ CCTTCGAATT ATCTCCAGTT TTTTAGTTTA GTGTAATCTT TCCTTTTGTT TTAATGTATT TATTTATTTA   
  
  
+ AGTTGGGTCC CGTACCGTTG ACTTGTACAT TGTTTCTTCT TAAGTTCTTT TAAAATTAAT TAATATTTTA   
  
  
+ TATAATACAC ATGATACCAA AGGAAGTTCA ACATCTTCCT CTATGGACAG AAGAAGTCTG ACTATTGGCA   
  
  
+ ATTAGGTATC AGGTAGTTGT CTCATATTAA CTACCTCGTT TGTTTACGAA CACAATCAAT TTACTTTACT   
  
  
+ AACAAACTCA TCATATGCTA AAGCGAATGG ACCGCCACGG TACAGTCTGG CTCCAAGCTT GACGATGTGT   
  
  
+ TTCGACTTCT AAAGTTTTAA CAATACCTTT GGTGGTTCTG CTTCCGCCAT TATCTTATTT TCCGCTGGTA   
  
  
+ GGTCGCTGCA GGATGAAGTA CTCCGAGAAC GGTGCTTGTA GAGAGAAACG GATGACGGAG AGGATCTATC   
  
  
+ AATTAAGTTA TCGGTTTCTT TGTGTTCTAA GAACGAGACA AGGGCTTTGA CTGTTCATGT TGAGAAAAAA   
  
  
+ GGAAAGTTCA TAGAGTCCTT CGCTGGTACT GTCGATCATT TAGTTCCAAG TTTCGAGTGA TCGGTAAAAA   
  
  
+ TTCATCGGTG AGAAATCACC TCCTAACGT  

- CAAATTTTCA ACCCATTGTA GGGTCATAAC CCCCTCTGAA GGCCAGGAAA TTGGTTCTGG AATGTTATTA   
  
  
- TTCATTTTAC CCCCACAGCA ATTCTCTTCA TTTGCTACTA GAGTTTCTAT ACAGAAAGGA TCCTTTCTGT   
  
  
- CAACAAGAAC AAGTTGAGTA TCTCCTGCCA CATACTTTTT GCTAGTGTCT CCTGCCACAT ATTTTTCACT   
  
  
- AGTCATGTGG CGAATGCCGT TAGTCATCCT TGTAACAGAT AGTTTCTGCT CATCATCCAA GTTATCAAGA   
  
  
- CACACTTCAT GTAGTGCGGG AAGAGCACGC TCTGAGTTTT GAGCCTGCAT AATCTAGTAA TCAGTAATAG   
  
  
- AAAACAATTC ATAATTGCGA AAAGTAAGCA ACTGTAAATC GCAACATTTA ACAAAATCAC CTCCAACTTG   
  
  
- TTAACTCAGA CTTAAGTAGG TATGATCTAT GTCTAATGCA TACTGTAAAC TGATATCCAA GAAAACAAAC   
  
  
- AAACATACTA AACATTTTCA TACCGCTTGT TTAAGAACAG AGCACACTAT TCGAGTCACA GCATTCTCTG   
  
  
- GATCCTGTAA AATGGATCAA AGCAAAAAAT GAAGCAATCT ACCAGAAACA AGGGGGAATG GTATAATCAT   
  
  
- ACAGCTCTAC CCTACCCTTA GAGAATCACT ATTAGCTCCA CAACGTGCTA TCTCTTTCAT CATCAGCAGA   
  
  
- GTACGCAACT GTGTATAACT TTTCACTCTC GTAGGCTTCA CAGAGCTTAG CTTAGAGAAA GCCAATCTGC   
  
  
- AGGCATTAAG TTTCGCCTCA AGGACTCTCC TTGAAATTTC TACAGCCAAG AAAACAGGAA CCCAGAATAG   
  
  
- GGAAGCTTAA TAGAGGTCAA AAAATCAAAT CACATTAGAA AGGAAAACAA AATTACATAA ATAAATAAAT   
  
  
- TCAACCCAGG GCATGGCAAC TGAACATGTA ACAAAGAAGA ATTCAAGAAA ATTTTAATTA ATTATAAAAT   
  
  
- ATATTATGTG TACTATGGTT TCCTTCAAGT TGTAGAAGGA GATACCTGTC TTCTTCAGAC TGATAACCGT   
  
  
- TAATCCATAG TCCATCAACA GAGTATAATT GATGGAGCAA ACAAATGCTT GTGTTAGTTA AATGAAATGA   
  
  
- TTGTTTGAGT AGTATACGAT TTCGCTTACC TGGCGGTGCC ATGTCAGACC GAGGTTCGAA CTGCTACACA   
  
  
- AAGCTGAAGA TTTCAAAATT GTTATGGAAA CCACCAAGAC GAAGGCGGTA ATAGAATAAA AGGCGACCAT   
  
  
- CCAGCGACGT CCTACTTCAT GAGGCTCTTG CCACGAACAT CTCTCTTTGC CTACTGCCTC TCCTAGATAG   
  
  
- TTAATTCAAT AGCCAAAGAA ACACAAGATT CTTGCTCTGT TCCCGAAACT GACAAGTACA ACTCTTTTTT   
  
  
- CCTTTCAAGT ATCTCAGGAA GCGACCATGA CAGCTAGTAA ATCAAGGTTC AAAGCTCACT AGCCATTTTT   
  
  
- AAGTAGCCAC TCTTTAGTGG AGGATTGCA

+     TGACG-motif

| Site Name | Organism | Position | Strand | Matrix score. | sequence | function |
| --- | --- | --- | --- | --- | --- | --- |
| TGACG-motif | Hordeum vulgare | 1180 | + | 5 | TGACG | cis-acting regulatory element involved in the MeJA-responsiveness |
| TGACG-motif | Hordeum vulgare | 1313 | + | 5 | TGACG | cis-acting regulatory element involved in the MeJA-responsiveness |

> 2018/04/13 10:10:12  
+ GTTTAAAAGT TGGGTAACAT CCCAGTATTG GGGGAGACTT CCGGTCCTTT AACCAAGACC TTACAATAAT   
  
  
+ AAGTAAAATG GGGGTGTCGT TAAGAGAAGT AAACGATGAT CTCAAAGATA TGTCTTTCCT AGGAAAGACA   
  
  
+ GTTGTTCTTG TTCAACTCAT AGAGGACGGT GTATGAAAAA CGATCACAGA GGACGGTGTA TAAAAAGTGA   
  
  
+ TCAGTACACC GCTTACGGCA ATCAGTAGGA ACATTGTCTA TCAAAGACGA GTAGTAGGTT CAATAGTTCT   
  
  
+ GTGTGAAGTA CATCACGCCC TTCTCGTGCG AGACTCAAAA CTCGGACGTA TTAGATCATT AGTCATTATC   
  
  
+ TTTTGTTAAG TATTAACGCT TTTCATTCGT TGACATTTAG CGTTGTAAAT TGTTTTAGTG GAGGTTGAAC   
  
  
+ AATTGAGTCT GAATTCATCC ATACTAGATA CAGATTACGT ATGACATTTG ACTATAGGTT CTTTTGTTTG   
  
  
+ TTTGTATGAT TTGTAAAAGT ATGGCGAACA AATTCTTGTC TCGTGTGATA AGCTCAGTGT CGTAAGAGAC   
  
  
+ CTAGGACATT TTACCTAGTT TCGTTTTTTA CTTCGTTAGA TGGTCTTTGT TCCCCCTTAC CATATTAGTA   
  
  
+ TGTCGAGATG GGATGGGAAT CTCTTAGTGA TAATCGAGGT GTTGCACGAT AGAGAAAGTA GTAGTCGTCT   
  
  
+ CATGCGTTGA CACATATTGA AAAGTGAGAG CATCCGAAGT GTCTCGAATC GAATCTCTTT CGGTTAGACG   
  
  
+ TCCGTAATTC AAAGCGGAGT TCCTGAGAGG AACTTTAAAG ATGTCGGTTC TTTTGTCCTT GGGTCTTATC   
  
  
+ CCTTCGAATT ATCTCCAGTT TTTTAGTTTA GTGTAATCTT TCCTTTTGTT TTAATGTATT TATTTATTTA   
  
  
+ AGTTGGGTCC CGTACCGTTG ACTTGTACAT TGTTTCTTCT TAAGTTCTTT TAAAATTAAT TAATATTTTA   
  
  
+ TATAATACAC ATGATACCAA AGGAAGTTCA ACATCTTCCT CTATGGACAG AAGAAGTCTG ACTATTGGCA   
  
  
+ ATTAGGTATC AGGTAGTTGT CTCATATTAA CTACCTCGTT TGTTTACGAA CACAATCAAT TTACTTTACT   
  
  
+ AACAAACTCA TCATATGCTA AAGCGAATGG ACCGCCACGG TACAGTCTGG CTCCAAGCTT GACGATGTGT   
  
  
+ TTCGACTTCT AAAGTTTTAA CAATACCTTT GGTGGTTCTG CTTCCGCCAT TATCTTATTT TCCGCTGGTA   
  
  
+ GGTCGCTGCA GGATGAAGTA CTCCGAGAAC GGTGCTTGTA GAGAGAAACG GATGACGGAG AGGATCTATC   
  
  
+ AATTAAGTTA TCGGTTTCTT TGTGTTCTAA GAACGAGACA AGGGCTTTGA CTGTTCATGT TGAGAAAAAA   
  
  
+ GGAAAGTTCA TAGAGTCCTT CGCTGGTACT GTCGATCATT TAGTTCCAAG TTTCGAGTGA TCGGTAAAAA   
  
  
+ TTCATCGGTG AGAAATCACC TCCTAACGT  

- CAAATTTTCA ACCCATTGTA GGGTCATAAC CCCCTCTGAA GGCCAGGAAA TTGGTTCTGG AATGTTATTA   
  
  
- TTCATTTTAC CCCCACAGCA ATTCTCTTCA TTTGCTACTA GAGTTTCTAT ACAGAAAGGA TCCTTTCTGT   
  
  
- CAACAAGAAC AAGTTGAGTA TCTCCTGCCA CATACTTTTT GCTAGTGTCT CCTGCCACAT ATTTTTCACT   
  
  
- AGTCATGTGG CGAATGCCGT TAGTCATCCT TGTAACAGAT AGTTTCTGCT CATCATCCAA GTTATCAAGA   
  
  
- CACACTTCAT GTAGTGCGGG AAGAGCACGC TCTGAGTTTT GAGCCTGCAT AATCTAGTAA TCAGTAATAG   
  
  
- AAAACAATTC ATAATTGCGA AAAGTAAGCA ACTGTAAATC GCAACATTTA ACAAAATCAC CTCCAACTTG   
  
  
- TTAACTCAGA CTTAAGTAGG TATGATCTAT GTCTAATGCA TACTGTAAAC TGATATCCAA GAAAACAAAC   
  
  
- AAACATACTA AACATTTTCA TACCGCTTGT TTAAGAACAG AGCACACTAT TCGAGTCACA GCATTCTCTG   
  
  
- GATCCTGTAA AATGGATCAA AGCAAAAAAT GAAGCAATCT ACCAGAAACA AGGGGGAATG GTATAATCAT   
  
  
- ACAGCTCTAC CCTACCCTTA GAGAATCACT ATTAGCTCCA CAACGTGCTA TCTCTTTCAT CATCAGCAGA   
  
  
- GTACGCAACT GTGTATAACT TTTCACTCTC GTAGGCTTCA CAGAGCTTAG CTTAGAGAAA GCCAATCTGC   
  
  
- AGGCATTAAG TTTCGCCTCA AGGACTCTCC TTGAAATTTC TACAGCCAAG AAAACAGGAA CCCAGAATAG   
  
  
- GGAAGCTTAA TAGAGGTCAA AAAATCAAAT CACATTAGAA AGGAAAACAA AATTACATAA ATAAATAAAT   
  
  
- TCAACCCAGG GCATGGCAAC TGAACATGTA ACAAAGAAGA ATTCAAGAAA ATTTTAATTA ATTATAAAAT   
  
  
- ATATTATGTG TACTATGGTT TCCTTCAAGT TGTAGAAGGA GATACCTGTC TTCTTCAGAC TGATAACCGT   
  
  
- TAATCCATAG TCCATCAACA GAGTATAATT GATGGAGCAA ACAAATGCTT GTGTTAGTTA AATGAAATGA   
  
  
- TTGTTTGAGT AGTATACGAT TTCGCTTACC TGGCGGTGCC ATGTCAGACC GAGGTTCGAA CTGCTACACA   
  
  
- AAGCTGAAGA TTTCAAAATT GTTATGGAAA CCACCAAGAC GAAGGCGGTA ATAGAATAAA AGGCGACCAT   
  
  
- CCAGCGACGT CCTACTTCAT GAGGCTCTTG CCACGAACAT CTCTCTTTGC CTACTGCCTC TCCTAGATAG   
  
  
- TTAATTCAAT AGCCAAAGAA ACACAAGATT CTTGCTCTGT TCCCGAAACT GACAAGTACA ACTCTTTTTT   
  
  
- CCTTTCAAGT ATCTCAGGAA GCGACCATGA CAGCTAGTAA ATCAAGGTTC AAAGCTCACT AGCCATTTTT   
  
  
- AAGTAGCCAC TCTTTAGTGG AGGATTGCA

+     Unnamed\_\_1

| Site Name | Organism | Position | Strand | Matrix score. | sequence | function |
| --- | --- | --- | --- | --- | --- | --- |
| Unnamed\_\_1 | Zea mays | 1155 | - | 5 | CGTGG |  |

> 2018/04/13 10:10:12  
+ GTTTAAAAGT TGGGTAACAT CCCAGTATTG GGGGAGACTT CCGGTCCTTT AACCAAGACC TTACAATAAT   
  
  
+ AAGTAAAATG GGGGTGTCGT TAAGAGAAGT AAACGATGAT CTCAAAGATA TGTCTTTCCT AGGAAAGACA   
  
  
+ GTTGTTCTTG TTCAACTCAT AGAGGACGGT GTATGAAAAA CGATCACAGA GGACGGTGTA TAAAAAGTGA   
  
  
+ TCAGTACACC GCTTACGGCA ATCAGTAGGA ACATTGTCTA TCAAAGACGA GTAGTAGGTT CAATAGTTCT   
  
  
+ GTGTGAAGTA CATCACGCCC TTCTCGTGCG AGACTCAAAA CTCGGACGTA TTAGATCATT AGTCATTATC   
  
  
+ TTTTGTTAAG TATTAACGCT TTTCATTCGT TGACATTTAG CGTTGTAAAT TGTTTTAGTG GAGGTTGAAC   
  
  
+ AATTGAGTCT GAATTCATCC ATACTAGATA CAGATTACGT ATGACATTTG ACTATAGGTT CTTTTGTTTG   
  
  
+ TTTGTATGAT TTGTAAAAGT ATGGCGAACA AATTCTTGTC TCGTGTGATA AGCTCAGTGT CGTAAGAGAC   
  
  
+ CTAGGACATT TTACCTAGTT TCGTTTTTTA CTTCGTTAGA TGGTCTTTGT TCCCCCTTAC CATATTAGTA   
  
  
+ TGTCGAGATG GGATGGGAAT CTCTTAGTGA TAATCGAGGT GTTGCACGAT AGAGAAAGTA GTAGTCGTCT   
  
  
+ CATGCGTTGA CACATATTGA AAAGTGAGAG CATCCGAAGT GTCTCGAATC GAATCTCTTT CGGTTAGACG   
  
  
+ TCCGTAATTC AAAGCGGAGT TCCTGAGAGG AACTTTAAAG ATGTCGGTTC TTTTGTCCTT GGGTCTTATC   
  
  
+ CCTTCGAATT ATCTCCAGTT TTTTAGTTTA GTGTAATCTT TCCTTTTGTT TTAATGTATT TATTTATTTA   
  
  
+ AGTTGGGTCC CGTACCGTTG ACTTGTACAT TGTTTCTTCT TAAGTTCTTT TAAAATTAAT TAATATTTTA   
  
  
+ TATAATACAC ATGATACCAA AGGAAGTTCA ACATCTTCCT CTATGGACAG AAGAAGTCTG ACTATTGGCA   
  
  
+ ATTAGGTATC AGGTAGTTGT CTCATATTAA CTACCTCGTT TGTTTACGAA CACAATCAAT TTACTTTACT   
  
  
+ AACAAACTCA TCATATGCTA AAGCGAATGG ACCGCCACGG TACAGTCTGG CTCCAAGCTT GACGATGTGT   
  
  
+ TTCGACTTCT AAAGTTTTAA CAATACCTTT GGTGGTTCTG CTTCCGCCAT TATCTTATTT TCCGCTGGTA   
  
  
+ GGTCGCTGCA GGATGAAGTA CTCCGAGAAC GGTGCTTGTA GAGAGAAACG GATGACGGAG AGGATCTATC   
  
  
+ AATTAAGTTA TCGGTTTCTT TGTGTTCTAA GAACGAGACA AGGGCTTTGA CTGTTCATGT TGAGAAAAAA   
  
  
+ GGAAAGTTCA TAGAGTCCTT CGCTGGTACT GTCGATCATT TAGTTCCAAG TTTCGAGTGA TCGGTAAAAA   
  
  
+ TTCATCGGTG AGAAATCACC TCCTAACGT  

- CAAATTTTCA ACCCATTGTA GGGTCATAAC CCCCTCTGAA GGCCAGGAAA TTGGTTCTGG AATGTTATTA   
  
  
- TTCATTTTAC CCCCACAGCA ATTCTCTTCA TTTGCTACTA GAGTTTCTAT ACAGAAAGGA TCCTTTCTGT   
  
  
- CAACAAGAAC AAGTTGAGTA TCTCCTGCCA CATACTTTTT GCTAGTGTCT CCTGCCACAT ATTTTTCACT   
  
  
- AGTCATGTGG CGAATGCCGT TAGTCATCCT TGTAACAGAT AGTTTCTGCT CATCATCCAA GTTATCAAGA   
  
  
- CACACTTCAT GTAGTGCGGG AAGAGCACGC TCTGAGTTTT GAGCCTGCAT AATCTAGTAA TCAGTAATAG   
  
  
- AAAACAATTC ATAATTGCGA AAAGTAAGCA ACTGTAAATC GCAACATTTA ACAAAATCAC CTCCAACTTG   
  
  
- TTAACTCAGA CTTAAGTAGG TATGATCTAT GTCTAATGCA TACTGTAAAC TGATATCCAA GAAAACAAAC   
  
  
- AAACATACTA AACATTTTCA TACCGCTTGT TTAAGAACAG AGCACACTAT TCGAGTCACA GCATTCTCTG   
  
  
- GATCCTGTAA AATGGATCAA AGCAAAAAAT GAAGCAATCT ACCAGAAACA AGGGGGAATG GTATAATCAT   
  
  
- ACAGCTCTAC CCTACCCTTA GAGAATCACT ATTAGCTCCA CAACGTGCTA TCTCTTTCAT CATCAGCAGA   
  
  
- GTACGCAACT GTGTATAACT TTTCACTCTC GTAGGCTTCA CAGAGCTTAG CTTAGAGAAA GCCAATCTGC   
  
  
- AGGCATTAAG TTTCGCCTCA AGGACTCTCC TTGAAATTTC TACAGCCAAG AAAACAGGAA CCCAGAATAG   
  
  
- GGAAGCTTAA TAGAGGTCAA AAAATCAAAT CACATTAGAA AGGAAAACAA AATTACATAA ATAAATAAAT   
  
  
- TCAACCCAGG GCATGGCAAC TGAACATGTA ACAAAGAAGA ATTCAAGAAA ATTTTAATTA ATTATAAAAT   
  
  
- ATATTATGTG TACTATGGTT TCCTTCAAGT TGTAGAAGGA GATACCTGTC TTCTTCAGAC TGATAACCGT   
  
  
- TAATCCATAG TCCATCAACA GAGTATAATT GATGGAGCAA ACAAATGCTT GTGTTAGTTA AATGAAATGA   
  
  
- TTGTTTGAGT AGTATACGAT TTCGCTTACC TGGCGGTGCC ATGTCAGACC GAGGTTCGAA CTGCTACACA   
  
  
- AAGCTGAAGA TTTCAAAATT GTTATGGAAA CCACCAAGAC GAAGGCGGTA ATAGAATAAA AGGCGACCAT   
  
  
- CCAGCGACGT CCTACTTCAT GAGGCTCTTG CCACGAACAT CTCTCTTTGC CTACTGCCTC TCCTAGATAG   
  
  
- TTAATTCAAT AGCCAAAGAA ACACAAGATT CTTGCTCTGT TCCCGAAACT GACAAGTACA ACTCTTTTTT   
  
  
- CCTTTCAAGT ATCTCAGGAA GCGACCATGA CAGCTAGTAA ATCAAGGTTC AAAGCTCACT AGCCATTTTT   
  
  
- AAGTAGCCAC TCTTTAGTGG AGGATTGCA

+     Unnamed\_\_3

| Site Name | Organism | Position | Strand | Matrix score. | sequence | function |
| --- | --- | --- | --- | --- | --- | --- |
| Unnamed\_\_3 | Zea mays | 1155 | - | 5 | CGTGG |  |

> 2018/04/13 10:10:12  
+ GTTTAAAAGT TGGGTAACAT CCCAGTATTG GGGGAGACTT CCGGTCCTTT AACCAAGACC TTACAATAAT   
  
  
+ AAGTAAAATG GGGGTGTCGT TAAGAGAAGT AAACGATGAT CTCAAAGATA TGTCTTTCCT AGGAAAGACA   
  
  
+ GTTGTTCTTG TTCAACTCAT AGAGGACGGT GTATGAAAAA CGATCACAGA GGACGGTGTA TAAAAAGTGA   
  
  
+ TCAGTACACC GCTTACGGCA ATCAGTAGGA ACATTGTCTA TCAAAGACGA GTAGTAGGTT CAATAGTTCT   
  
  
+ GTGTGAAGTA CATCACGCCC TTCTCGTGCG AGACTCAAAA CTCGGACGTA TTAGATCATT AGTCATTATC   
  
  
+ TTTTGTTAAG TATTAACGCT TTTCATTCGT TGACATTTAG CGTTGTAAAT TGTTTTAGTG GAGGTTGAAC   
  
  
+ AATTGAGTCT GAATTCATCC ATACTAGATA CAGATTACGT ATGACATTTG ACTATAGGTT CTTTTGTTTG   
  
  
+ TTTGTATGAT TTGTAAAAGT ATGGCGAACA AATTCTTGTC TCGTGTGATA AGCTCAGTGT CGTAAGAGAC   
  
  
+ CTAGGACATT TTACCTAGTT TCGTTTTTTA CTTCGTTAGA TGGTCTTTGT TCCCCCTTAC CATATTAGTA   
  
  
+ TGTCGAGATG GGATGGGAAT CTCTTAGTGA TAATCGAGGT GTTGCACGAT AGAGAAAGTA GTAGTCGTCT   
  
  
+ CATGCGTTGA CACATATTGA AAAGTGAGAG CATCCGAAGT GTCTCGAATC GAATCTCTTT CGGTTAGACG   
  
  
+ TCCGTAATTC AAAGCGGAGT TCCTGAGAGG AACTTTAAAG ATGTCGGTTC TTTTGTCCTT GGGTCTTATC   
  
  
+ CCTTCGAATT ATCTCCAGTT TTTTAGTTTA GTGTAATCTT TCCTTTTGTT TTAATGTATT TATTTATTTA   
  
  
+ AGTTGGGTCC CGTACCGTTG ACTTGTACAT TGTTTCTTCT TAAGTTCTTT TAAAATTAAT TAATATTTTA   
  
  
+ TATAATACAC ATGATACCAA AGGAAGTTCA ACATCTTCCT CTATGGACAG AAGAAGTCTG ACTATTGGCA   
  
  
+ ATTAGGTATC AGGTAGTTGT CTCATATTAA CTACCTCGTT TGTTTACGAA CACAATCAAT TTACTTTACT   
  
  
+ AACAAACTCA TCATATGCTA AAGCGAATGG ACCGCCACGG TACAGTCTGG CTCCAAGCTT GACGATGTGT   
  
  
+ TTCGACTTCT AAAGTTTTAA CAATACCTTT GGTGGTTCTG CTTCCGCCAT TATCTTATTT TCCGCTGGTA   
  
  
+ GGTCGCTGCA GGATGAAGTA CTCCGAGAAC GGTGCTTGTA GAGAGAAACG GATGACGGAG AGGATCTATC   
  
  
+ AATTAAGTTA TCGGTTTCTT TGTGTTCTAA GAACGAGACA AGGGCTTTGA CTGTTCATGT TGAGAAAAAA   
  
  
+ GGAAAGTTCA TAGAGTCCTT CGCTGGTACT GTCGATCATT TAGTTCCAAG TTTCGAGTGA TCGGTAAAAA   
  
  
+ TTCATCGGTG AGAAATCACC TCCTAACGT  

- CAAATTTTCA ACCCATTGTA GGGTCATAAC CCCCTCTGAA GGCCAGGAAA TTGGTTCTGG AATGTTATTA   
  
  
- TTCATTTTAC CCCCACAGCA ATTCTCTTCA TTTGCTACTA GAGTTTCTAT ACAGAAAGGA TCCTTTCTGT   
  
  
- CAACAAGAAC AAGTTGAGTA TCTCCTGCCA CATACTTTTT GCTAGTGTCT CCTGCCACAT ATTTTTCACT   
  
  
- AGTCATGTGG CGAATGCCGT TAGTCATCCT TGTAACAGAT AGTTTCTGCT CATCATCCAA GTTATCAAGA   
  
  
- CACACTTCAT GTAGTGCGGG AAGAGCACGC TCTGAGTTTT GAGCCTGCAT AATCTAGTAA TCAGTAATAG   
  
  
- AAAACAATTC ATAATTGCGA AAAGTAAGCA ACTGTAAATC GCAACATTTA ACAAAATCAC CTCCAACTTG   
  
  
- TTAACTCAGA CTTAAGTAGG TATGATCTAT GTCTAATGCA TACTGTAAAC TGATATCCAA GAAAACAAAC   
  
  
- AAACATACTA AACATTTTCA TACCGCTTGT TTAAGAACAG AGCACACTAT TCGAGTCACA GCATTCTCTG   
  
  
- GATCCTGTAA AATGGATCAA AGCAAAAAAT GAAGCAATCT ACCAGAAACA AGGGGGAATG GTATAATCAT   
  
  
- ACAGCTCTAC CCTACCCTTA GAGAATCACT ATTAGCTCCA CAACGTGCTA TCTCTTTCAT CATCAGCAGA   
  
  
- GTACGCAACT GTGTATAACT TTTCACTCTC GTAGGCTTCA CAGAGCTTAG CTTAGAGAAA GCCAATCTGC   
  
  
- AGGCATTAAG TTTCGCCTCA AGGACTCTCC TTGAAATTTC TACAGCCAAG AAAACAGGAA CCCAGAATAG   
  
  
- GGAAGCTTAA TAGAGGTCAA AAAATCAAAT CACATTAGAA AGGAAAACAA AATTACATAA ATAAATAAAT   
  
  
- TCAACCCAGG GCATGGCAAC TGAACATGTA ACAAAGAAGA ATTCAAGAAA ATTTTAATTA ATTATAAAAT   
  
  
- ATATTATGTG TACTATGGTT TCCTTCAAGT TGTAGAAGGA GATACCTGTC TTCTTCAGAC TGATAACCGT   
  
  
- TAATCCATAG TCCATCAACA GAGTATAATT GATGGAGCAA ACAAATGCTT GTGTTAGTTA AATGAAATGA   
  
  
- TTGTTTGAGT AGTATACGAT TTCGCTTACC TGGCGGTGCC ATGTCAGACC GAGGTTCGAA CTGCTACACA   
  
  
- AAGCTGAAGA TTTCAAAATT GTTATGGAAA CCACCAAGAC GAAGGCGGTA ATAGAATAAA AGGCGACCAT   
  
  
- CCAGCGACGT CCTACTTCAT GAGGCTCTTG CCACGAACAT CTCTCTTTGC CTACTGCCTC TCCTAGATAG   
  
  
- TTAATTCAAT AGCCAAAGAA ACACAAGATT CTTGCTCTGT TCCCGAAACT GACAAGTACA ACTCTTTTTT   
  
  
- CCTTTCAAGT ATCTCAGGAA GCGACCATGA CAGCTAGTAA ATCAAGGTTC AAAGCTCACT AGCCATTTTT   
  
  
- AAGTAGCCAC TCTTTAGTGG AGGATTGCA

+     Unnamed\_\_4

| Site Name | Organism | Position | Strand | Matrix score. | sequence | function |
| --- | --- | --- | --- | --- | --- | --- |
| Unnamed\_\_4 | Petroselinum hortense | 1490 | + | 4 | CTCC |  |
| Unnamed\_\_4 | Petroselinum hortense | 33 | - | 4 | CTCC |  |
| Unnamed\_\_4 | Petroselinum hortense | 1317 | - | 4 | CTCC |  |
| Unnamed\_\_4 | Petroselinum hortense | 410 | - | 4 | CTCC |  |
| Unnamed\_\_4 | Petroselinum hortense | 786 | - | 4 | CTCC |  |
| Unnamed\_\_4 | Petroselinum hortense | 1171 | + | 4 | CTCC |  |
| Unnamed\_\_4 | Petroselinum hortense | 1281 | + | 4 | CTCC |  |
| Unnamed\_\_4 | Petroselinum hortense | 853 | + | 4 | CTCC |  |

> 2018/04/13 10:10:12  
+ GTTTAAAAGT TGGGTAACAT CCCAGTATTG GGGGAGACTT CCGGTCCTTT AACCAAGACC TTACAATAAT   
  
  
+ AAGTAAAATG GGGGTGTCGT TAAGAGAAGT AAACGATGAT CTCAAAGATA TGTCTTTCCT AGGAAAGACA   
  
  
+ GTTGTTCTTG TTCAACTCAT AGAGGACGGT GTATGAAAAA CGATCACAGA GGACGGTGTA TAAAAAGTGA   
  
  
+ TCAGTACACC GCTTACGGCA ATCAGTAGGA ACATTGTCTA TCAAAGACGA GTAGTAGGTT CAATAGTTCT   
  
  
+ GTGTGAAGTA CATCACGCCC TTCTCGTGCG AGACTCAAAA CTCGGACGTA TTAGATCATT AGTCATTATC   
  
  
+ TTTTGTTAAG TATTAACGCT TTTCATTCGT TGACATTTAG CGTTGTAAAT TGTTTTAGTG GAGGTTGAAC   
  
  
+ AATTGAGTCT GAATTCATCC ATACTAGATA CAGATTACGT ATGACATTTG ACTATAGGTT CTTTTGTTTG   
  
  
+ TTTGTATGAT TTGTAAAAGT ATGGCGAACA AATTCTTGTC TCGTGTGATA AGCTCAGTGT CGTAAGAGAC   
  
  
+ CTAGGACATT TTACCTAGTT TCGTTTTTTA CTTCGTTAGA TGGTCTTTGT TCCCCCTTAC CATATTAGTA   
  
  
+ TGTCGAGATG GGATGGGAAT CTCTTAGTGA TAATCGAGGT GTTGCACGAT AGAGAAAGTA GTAGTCGTCT   
  
  
+ CATGCGTTGA CACATATTGA AAAGTGAGAG CATCCGAAGT GTCTCGAATC GAATCTCTTT CGGTTAGACG   
  
  
+ TCCGTAATTC AAAGCGGAGT TCCTGAGAGG AACTTTAAAG ATGTCGGTTC TTTTGTCCTT GGGTCTTATC   
  
  
+ CCTTCGAATT ATCTCCAGTT TTTTAGTTTA GTGTAATCTT TCCTTTTGTT TTAATGTATT TATTTATTTA   
  
  
+ AGTTGGGTCC CGTACCGTTG ACTTGTACAT TGTTTCTTCT TAAGTTCTTT TAAAATTAAT TAATATTTTA   
  
  
+ TATAATACAC ATGATACCAA AGGAAGTTCA ACATCTTCCT CTATGGACAG AAGAAGTCTG ACTATTGGCA   
  
  
+ ATTAGGTATC AGGTAGTTGT CTCATATTAA CTACCTCGTT TGTTTACGAA CACAATCAAT TTACTTTACT   
  
  
+ AACAAACTCA TCATATGCTA AAGCGAATGG ACCGCCACGG TACAGTCTGG CTCCAAGCTT GACGATGTGT   
  
  
+ TTCGACTTCT AAAGTTTTAA CAATACCTTT GGTGGTTCTG CTTCCGCCAT TATCTTATTT TCCGCTGGTA   
  
  
+ GGTCGCTGCA GGATGAAGTA CTCCGAGAAC GGTGCTTGTA GAGAGAAACG GATGACGGAG AGGATCTATC   
  
  
+ AATTAAGTTA TCGGTTTCTT TGTGTTCTAA GAACGAGACA AGGGCTTTGA CTGTTCATGT TGAGAAAAAA   
  
  
+ GGAAAGTTCA TAGAGTCCTT CGCTGGTACT GTCGATCATT TAGTTCCAAG TTTCGAGTGA TCGGTAAAAA   
  
  
+ TTCATCGGTG AGAAATCACC TCCTAACGT  

- CAAATTTTCA ACCCATTGTA GGGTCATAAC CCCCTCTGAA GGCCAGGAAA TTGGTTCTGG AATGTTATTA   
  
  
- TTCATTTTAC CCCCACAGCA ATTCTCTTCA TTTGCTACTA GAGTTTCTAT ACAGAAAGGA TCCTTTCTGT   
  
  
- CAACAAGAAC AAGTTGAGTA TCTCCTGCCA CATACTTTTT GCTAGTGTCT CCTGCCACAT ATTTTTCACT   
  
  
- AGTCATGTGG CGAATGCCGT TAGTCATCCT TGTAACAGAT AGTTTCTGCT CATCATCCAA GTTATCAAGA   
  
  
- CACACTTCAT GTAGTGCGGG AAGAGCACGC TCTGAGTTTT GAGCCTGCAT AATCTAGTAA TCAGTAATAG   
  
  
- AAAACAATTC ATAATTGCGA AAAGTAAGCA ACTGTAAATC GCAACATTTA ACAAAATCAC CTCCAACTTG   
  
  
- TTAACTCAGA CTTAAGTAGG TATGATCTAT GTCTAATGCA TACTGTAAAC TGATATCCAA GAAAACAAAC   
  
  
- AAACATACTA AACATTTTCA TACCGCTTGT TTAAGAACAG AGCACACTAT TCGAGTCACA GCATTCTCTG   
  
  
- GATCCTGTAA AATGGATCAA AGCAAAAAAT GAAGCAATCT ACCAGAAACA AGGGGGAATG GTATAATCAT   
  
  
- ACAGCTCTAC CCTACCCTTA GAGAATCACT ATTAGCTCCA CAACGTGCTA TCTCTTTCAT CATCAGCAGA   
  
  
- GTACGCAACT GTGTATAACT TTTCACTCTC GTAGGCTTCA CAGAGCTTAG CTTAGAGAAA GCCAATCTGC   
  
  
- AGGCATTAAG TTTCGCCTCA AGGACTCTCC TTGAAATTTC TACAGCCAAG AAAACAGGAA CCCAGAATAG   
  
  
- GGAAGCTTAA TAGAGGTCAA AAAATCAAAT CACATTAGAA AGGAAAACAA AATTACATAA ATAAATAAAT   
  
  
- TCAACCCAGG GCATGGCAAC TGAACATGTA ACAAAGAAGA ATTCAAGAAA ATTTTAATTA ATTATAAAAT   
  
  
- ATATTATGTG TACTATGGTT TCCTTCAAGT TGTAGAAGGA GATACCTGTC TTCTTCAGAC TGATAACCGT   
  
  
- TAATCCATAG TCCATCAACA GAGTATAATT GATGGAGCAA ACAAATGCTT GTGTTAGTTA AATGAAATGA   
  
  
- TTGTTTGAGT AGTATACGAT TTCGCTTACC TGGCGGTGCC ATGTCAGACC GAGGTTCGAA CTGCTACACA   
  
  
- AAGCTGAAGA TTTCAAAATT GTTATGGAAA CCACCAAGAC GAAGGCGGTA ATAGAATAAA AGGCGACCAT   
  
  
- CCAGCGACGT CCTACTTCAT GAGGCTCTTG CCACGAACAT CTCTCTTTGC CTACTGCCTC TCCTAGATAG   
  
  
- TTAATTCAAT AGCCAAAGAA ACACAAGATT CTTGCTCTGT TCCCGAAACT GACAAGTACA ACTCTTTTTT   
  
  
- CCTTTCAAGT ATCTCAGGAA GCGACCATGA CAGCTAGTAA ATCAAGGTTC AAAGCTCACT AGCCATTTTT   
  
  
- AAGTAGCCAC TCTTTAGTGG AGGATTGCA

+     circadian

| Site Name | Organism | Position | Strand | Matrix score. | sequence | function |
| --- | --- | --- | --- | --- | --- | --- |
| circadian | Lycopersicon esculentum | 1123 | + | 6 | CAANNNNATC | cis-acting regulatory element involved in circadian control |
| circadian | Lycopersicon esculentum | 113 | + | 9 | CAAAGATATC | cis-acting regulatory element involved in circadian control |

> 2018/04/13 10:10:12  
+ GTTTAAAAGT TGGGTAACAT CCCAGTATTG GGGGAGACTT CCGGTCCTTT AACCAAGACC TTACAATAAT   
  
  
+ AAGTAAAATG GGGGTGTCGT TAAGAGAAGT AAACGATGAT CTCAAAGATA TGTCTTTCCT AGGAAAGACA   
  
  
+ GTTGTTCTTG TTCAACTCAT AGAGGACGGT GTATGAAAAA CGATCACAGA GGACGGTGTA TAAAAAGTGA   
  
  
+ TCAGTACACC GCTTACGGCA ATCAGTAGGA ACATTGTCTA TCAAAGACGA GTAGTAGGTT CAATAGTTCT   
  
  
+ GTGTGAAGTA CATCACGCCC TTCTCGTGCG AGACTCAAAA CTCGGACGTA TTAGATCATT AGTCATTATC   
  
  
+ TTTTGTTAAG TATTAACGCT TTTCATTCGT TGACATTTAG CGTTGTAAAT TGTTTTAGTG GAGGTTGAAC   
  
  
+ AATTGAGTCT GAATTCATCC ATACTAGATA CAGATTACGT ATGACATTTG ACTATAGGTT CTTTTGTTTG   
  
  
+ TTTGTATGAT TTGTAAAAGT ATGGCGAACA AATTCTTGTC TCGTGTGATA AGCTCAGTGT CGTAAGAGAC   
  
  
+ CTAGGACATT TTACCTAGTT TCGTTTTTTA CTTCGTTAGA TGGTCTTTGT TCCCCCTTAC CATATTAGTA   
  
  
+ TGTCGAGATG GGATGGGAAT CTCTTAGTGA TAATCGAGGT GTTGCACGAT AGAGAAAGTA GTAGTCGTCT   
  
  
+ CATGCGTTGA CACATATTGA AAAGTGAGAG CATCCGAAGT GTCTCGAATC GAATCTCTTT CGGTTAGACG   
  
  
+ TCCGTAATTC AAAGCGGAGT TCCTGAGAGG AACTTTAAAG ATGTCGGTTC TTTTGTCCTT GGGTCTTATC   
  
  
+ CCTTCGAATT ATCTCCAGTT TTTTAGTTTA GTGTAATCTT TCCTTTTGTT TTAATGTATT TATTTATTTA   
  
  
+ AGTTGGGTCC CGTACCGTTG ACTTGTACAT TGTTTCTTCT TAAGTTCTTT TAAAATTAAT TAATATTTTA   
  
  
+ TATAATACAC ATGATACCAA AGGAAGTTCA ACATCTTCCT CTATGGACAG AAGAAGTCTG ACTATTGGCA   
  
  
+ ATTAGGTATC AGGTAGTTGT CTCATATTAA CTACCTCGTT TGTTTACGAA CACAATCAAT TTACTTTACT   
  
  
+ AACAAACTCA TCATATGCTA AAGCGAATGG ACCGCCACGG TACAGTCTGG CTCCAAGCTT GACGATGTGT   
  
  
+ TTCGACTTCT AAAGTTTTAA CAATACCTTT GGTGGTTCTG CTTCCGCCAT TATCTTATTT TCCGCTGGTA   
  
  
+ GGTCGCTGCA GGATGAAGTA CTCCGAGAAC GGTGCTTGTA GAGAGAAACG GATGACGGAG AGGATCTATC   
  
  
+ AATTAAGTTA TCGGTTTCTT TGTGTTCTAA GAACGAGACA AGGGCTTTGA CTGTTCATGT TGAGAAAAAA   
  
  
+ GGAAAGTTCA TAGAGTCCTT CGCTGGTACT GTCGATCATT TAGTTCCAAG TTTCGAGTGA TCGGTAAAAA   
  
  
+ TTCATCGGTG AGAAATCACC TCCTAACGT  

- CAAATTTTCA ACCCATTGTA GGGTCATAAC CCCCTCTGAA GGCCAGGAAA TTGGTTCTGG AATGTTATTA   
  
  
- TTCATTTTAC CCCCACAGCA ATTCTCTTCA TTTGCTACTA GAGTTTCTAT ACAGAAAGGA TCCTTTCTGT   
  
  
- CAACAAGAAC AAGTTGAGTA TCTCCTGCCA CATACTTTTT GCTAGTGTCT CCTGCCACAT ATTTTTCACT   
  
  
- AGTCATGTGG CGAATGCCGT TAGTCATCCT TGTAACAGAT AGTTTCTGCT CATCATCCAA GTTATCAAGA   
  
  
- CACACTTCAT GTAGTGCGGG AAGAGCACGC TCTGAGTTTT GAGCCTGCAT AATCTAGTAA TCAGTAATAG   
  
  
- AAAACAATTC ATAATTGCGA AAAGTAAGCA ACTGTAAATC GCAACATTTA ACAAAATCAC CTCCAACTTG   
  
  
- TTAACTCAGA CTTAAGTAGG TATGATCTAT GTCTAATGCA TACTGTAAAC TGATATCCAA GAAAACAAAC   
  
  
- AAACATACTA AACATTTTCA TACCGCTTGT TTAAGAACAG AGCACACTAT TCGAGTCACA GCATTCTCTG   
  
  
- GATCCTGTAA AATGGATCAA AGCAAAAAAT GAAGCAATCT ACCAGAAACA AGGGGGAATG GTATAATCAT   
  
  
- ACAGCTCTAC CCTACCCTTA GAGAATCACT ATTAGCTCCA CAACGTGCTA TCTCTTTCAT CATCAGCAGA   
  
  
- GTACGCAACT GTGTATAACT TTTCACTCTC GTAGGCTTCA CAGAGCTTAG CTTAGAGAAA GCCAATCTGC   
  
  
- AGGCATTAAG TTTCGCCTCA AGGACTCTCC TTGAAATTTC TACAGCCAAG AAAACAGGAA CCCAGAATAG   
  
  
- GGAAGCTTAA TAGAGGTCAA AAAATCAAAT CACATTAGAA AGGAAAACAA AATTACATAA ATAAATAAAT   
  
  
- TCAACCCAGG GCATGGCAAC TGAACATGTA ACAAAGAAGA ATTCAAGAAA ATTTTAATTA ATTATAAAAT   
  
  
- ATATTATGTG TACTATGGTT TCCTTCAAGT TGTAGAAGGA GATACCTGTC TTCTTCAGAC TGATAACCGT   
  
  
- TAATCCATAG TCCATCAACA GAGTATAATT GATGGAGCAA ACAAATGCTT GTGTTAGTTA AATGAAATGA   
  
  
- TTGTTTGAGT AGTATACGAT TTCGCTTACC TGGCGGTGCC ATGTCAGACC GAGGTTCGAA CTGCTACACA   
  
  
- AAGCTGAAGA TTTCAAAATT GTTATGGAAA CCACCAAGAC GAAGGCGGTA ATAGAATAAA AGGCGACCAT   
  
  
- CCAGCGACGT CCTACTTCAT GAGGCTCTTG CCACGAACAT CTCTCTTTGC CTACTGCCTC TCCTAGATAG   
  
  
- TTAATTCAAT AGCCAAAGAA ACACAAGATT CTTGCTCTGT TCCCGAAACT GACAAGTACA ACTCTTTTTT   
  
  
- CCTTTCAAGT ATCTCAGGAA GCGACCATGA CAGCTAGTAA ATCAAGGTTC AAAGCTCACT AGCCATTTTT   
  
  
- AAGTAGCCAC TCTTTAGTGG AGGATTGCA
