# Supplementary material for: Genome-wide analysis of the WRKY gene family in drumstick (Moringa oleifera Lam.)
Source: PeerJ. 2019 Jun 10;7:e7063. doi: 10.7717/peerj.7063 (PMC6563795; doi:10.7717/peerj.7063)
Supplement: Supplemental Information 1 [file peerj-07-7063-s003.gz › MoWRKY23_plantcare.html]

Content-Type: text/html; charset=ISO-8859-1


CallMat\_Firefox


Webmaster Firefox specific output  
To save the result:
click on the frame with the right mouse button and save the source code as a text file with extension .html  
REFERENCE:PlantCARE: a database of plant cis-acting regulatory elements and a portal to tools for in silico analysis of promoter sequences.  
Lescot, M., Déhais, P., Moreau, Y., De Moor, B., Rouzé ,P.,and Rombauts, S.  
Nucleic Acids Res., Database issue(2002), 30(1):325-327.   


---

> 2018/04/13 10:10:12  
+ CCGACCGGCT GCTGAATCTA GCTAGCTTTG GTTCTCTATG GGTCGTTTGT CTAACGGACT TTAACTGGAT   
  
  
+ TGTTATTTCG TCTGATTCAA GGAGATCAAA TAGCTTAGAA CGTTCGAAAC ACATCTTTGT CTTAAGTTCA   
  
  
+ ACCAGTTCGA CCGGATCGTA CACTTTACTT CTTTTCGCAC ACTATATATC TCGCCTTTCC ACGACAACCA   
  
  
+ CCCTTTGAAC CACCTTTACT CTGCCTGTGC CTATTCTACA ACGTCCGTGT TGAGATATAT ATTACTGAGA   
  
  
+ CTCAGATAGA TTTCTAGGTG CACCACCCTA AACAACATAT CAAATAATTA TTCTGTTAAT TGACAAAGGG   
  
  
+ TCAAACCACT CACAACCCTC CTCGGTACAC TTCTTCCTTC TTCAAAAGAA AAGAAAAAAA AAGTGGCTTA   
  
  
+ GACTTAATCA CAATTGAAAA AATTTTCATT TTCTTTTTTT TATATGTAAA AATATTAAAT AACATAATTA   
  
  
+ AATGATTTTA TTTATATGAC TTAATCACAA TTGAAAAAAT TTTCATTTTC TTTTTTTTAT ATGTAAAAAT   
  
  
+ ATTAAATAAC ATAATTAAAT GATTTTATTT ATATAATTTA ATATTAATAA TTAATTAAAA AGAAAAATAT   
  
  
+ AATTGTATTC TTTAATATTT TAATAAAAAA TAAAAATGAG TTTTTTAATT TTATTTAAGT TTATTTATCT   
  
  
+ TTTACAAGCA CTAATAAAAA ATTTTATTTT CGTATACAAA CTAATTATAA TTGTATTTTT TATGTAGAAT   
  
  
+ TATTAAACAA AATAATTTAG TAATACGTAA TAATAAAAAT AAATTTTTAT TATATTTAAT TTAATTTTAT   
  
  
+ TTTTTAATTA AGTTGGTATA TATATATATA AATAGGTAAG TTAATGTATA ATAATGCACA CGATCCCTGT   
  
  
+ ACGACCAGCC AGTACCCACA CGAATCAGTT TCAGTTCGAG TGGTATTTTC GGTTCTTTGT AACCCACCAC   
  
  
+ CAGAGTAAAA TAGATGATAA GGAGACACCT TAATTATTAA GTATATTATC TACAGATTAA GAATTAATTT   
  
  
+ AAAGTATAAC TTGGGTTAGA ATAGAAACAG AGAAGATGAC ATGGTTTACG AATTAATTTA ATTTTACTGG   
  
  
+ TTAATTATAA AAAATATTTT TTTTACTCAA ATGTCTCTAC AGTGCAAAAA CTTTCGTAAA AGGTGCAATG   
  
  
+ TACGTGGTTA AGCTAAGAAA ATGCAGAAAA GTTTTCCCTA AAAAAAAAAA AAAACAAAGA ATGTAACTTA   
  
  
+ ATTAAATAAG GTGCAATGTA CGTGGTTAAG CTAAGAAAAT GCAGAAAAGT TTTCCCTAAA AAAAAAAAAA   
  
  
+ AACAAAGAAT GTAACTTAAT TAAAAAACTT AGATCTGAGA ACGTACATTA AGTAACATGG TTCTTCCGAA   
  
  
+ TGAATAAGTA AGATTCATAA GTTTCTCCTT ACACTCGTTT TACCACCTCT ACATGGTATG ATAATAATTC   
  
  
+ AGAATTAGCA AAAGTTCTAA AAAAAACAG  

- GGCTGGCCGA CGACTTAGAT CGATCGAAAC CAAGAGATAC CCAGCAAACA GATTGCCTGA AATTGACCTA   
  
  
- ACAATAAAGC AGACTAAGTT CCTCTAGTTT ATCGAATCTT GCAAGCTTTG TGTAGAAACA GAATTCAAGT   
  
  
- TGGTCAAGCT GGCCTAGCAT GTGAAATGAA GAAAAGCGTG TGATATATAG AGCGGAAAGG TGCTGTTGGT   
  
  
- GGGAAACTTG GTGGAAATGA GACGGACACG GATAAGATGT TGCAGGCACA ACTCTATATA TAATGACTCT   
  
  
- GAGTCTATCT AAAGATCCAC GTGGTGGGAT TTGTTGTATA GTTTATTAAT AAGACAATTA ACTGTTTCCC   
  
  
- AGTTTGGTGA GTGTTGGGAG GAGCCATGTG AAGAAGGAAG AAGTTTTCTT TTCTTTTTTT TTCACCGAAT   
  
  
- CTGAATTAGT GTTAACTTTT TTAAAAGTAA AAGAAAAAAA ATATACATTT TTATAATTTA TTGTATTAAT   
  
  
- TTACTAAAAT AAATATACTG AATTAGTGTT AACTTTTTTA AAAGTAAAAG AAAAAAAATA TACATTTTTA   
  
  
- TAATTTATTG TATTAATTTA CTAAAATAAA TATATTAAAT TATAATTATT AATTAATTTT TCTTTTTATA   
  
  
- TTAACATAAG AAATTATAAA ATTATTTTTT ATTTTTACTC AAAAAATTAA AATAAATTCA AATAAATAGA   
  
  
- AAATGTTCGT GATTATTTTT TAAAATAAAA GCATATGTTT GATTAATATT AACATAAAAA ATACATCTTA   
  
  
- ATAATTTGTT TTATTAAATC ATTATGCATT ATTATTTTTA TTTAAAAATA ATATAAATTA AATTAAAATA   
  
  
- AAAAATTAAT TCAACCATAT ATATATATAT TTATCCATTC AATTACATAT TATTACGTGT GCTAGGGACA   
  
  
- TGCTGGTCGG TCATGGGTGT GCTTAGTCAA AGTCAAGCTC ACCATAAAAG CCAAGAAACA TTGGGTGGTG   
  
  
- GTCTCATTTT ATCTACTATT CCTCTGTGGA ATTAATAATT CATATAATAG ATGTCTAATT CTTAATTAAA   
  
  
- TTTCATATTG AACCCAATCT TATCTTTGTC TCTTCTACTG TACCAAATGC TTAATTAAAT TAAAATGACC   
  
  
- AATTAATATT TTTTATAAAA AAAATGAGTT TACAGAGATG TCACGTTTTT GAAAGCATTT TCCACGTTAC   
  
  
- ATGCACCAAT TCGATTCTTT TACGTCTTTT CAAAAGGGAT TTTTTTTTTT TTTTGTTTCT TACATTGAAT   
  
  
- TAATTTATTC CACGTTACAT GCACCAATTC GATTCTTTTA CGTCTTTTCA AAAGGGATTT TTTTTTTTTT   
  
  
- TTGTTTCTTA CATTGAATTA ATTTTTTGAA TCTAGACTCT TGCATGTAAT TCATTGTACC AAGAAGGCTT   
  
  
- ACTTATTCAT TCTAAGTATT CAAAGAGGAA TGTGAGCAAA ATGGTGGAGA TGTACCATAC TATTATTAAG   
  
  
- TCTTAATCGT TTTCAAGATT TTTTTTGTC

  
  
Motifs Found  

+     5UTR Py-rich stretch

| Site Name | Organism | Position | Strand | Matrix score. | sequence | function |
| --- | --- | --- | --- | --- | --- | --- |
| 5UTR Py-rich stretch | Lycopersicon esculentum | 397 | - | 9 | TTTCTTCTCT | cis-acting element conferring high transcription levels |

> 2018/04/13 10:10:12  
+ CCGACCGGCT GCTGAATCTA GCTAGCTTTG GTTCTCTATG GGTCGTTTGT CTAACGGACT TTAACTGGAT   
  
  
+ TGTTATTTCG TCTGATTCAA GGAGATCAAA TAGCTTAGAA CGTTCGAAAC ACATCTTTGT CTTAAGTTCA   
  
  
+ ACCAGTTCGA CCGGATCGTA CACTTTACTT CTTTTCGCAC ACTATATATC TCGCCTTTCC ACGACAACCA   
  
  
+ CCCTTTGAAC CACCTTTACT CTGCCTGTGC CTATTCTACA ACGTCCGTGT TGAGATATAT ATTACTGAGA   
  
  
+ CTCAGATAGA TTTCTAGGTG CACCACCCTA AACAACATAT CAAATAATTA TTCTGTTAAT TGACAAAGGG   
  
  
+ TCAAACCACT CACAACCCTC CTCGGTACAC TTCTTCCTTC TTCAAAAGAA AAGAAAAAAA AAGTGGCTTA   
  
  
+ GACTTAATCA CAATTGAAAA AATTTTCATT TTCTTTTTTT TATATGTAAA AATATTAAAT AACATAATTA   
  
  
+ AATGATTTTA TTTATATGAC TTAATCACAA TTGAAAAAAT TTTCATTTTC TTTTTTTTAT ATGTAAAAAT   
  
  
+ ATTAAATAAC ATAATTAAAT GATTTTATTT ATATAATTTA ATATTAATAA TTAATTAAAA AGAAAAATAT   
  
  
+ AATTGTATTC TTTAATATTT TAATAAAAAA TAAAAATGAG TTTTTTAATT TTATTTAAGT TTATTTATCT   
  
  
+ TTTACAAGCA CTAATAAAAA ATTTTATTTT CGTATACAAA CTAATTATAA TTGTATTTTT TATGTAGAAT   
  
  
+ TATTAAACAA AATAATTTAG TAATACGTAA TAATAAAAAT AAATTTTTAT TATATTTAAT TTAATTTTAT   
  
  
+ TTTTTAATTA AGTTGGTATA TATATATATA AATAGGTAAG TTAATGTATA ATAATGCACA CGATCCCTGT   
  
  
+ ACGACCAGCC AGTACCCACA CGAATCAGTT TCAGTTCGAG TGGTATTTTC GGTTCTTTGT AACCCACCAC   
  
  
+ CAGAGTAAAA TAGATGATAA GGAGACACCT TAATTATTAA GTATATTATC TACAGATTAA GAATTAATTT   
  
  
+ AAAGTATAAC TTGGGTTAGA ATAGAAACAG AGAAGATGAC ATGGTTTACG AATTAATTTA ATTTTACTGG   
  
  
+ TTAATTATAA AAAATATTTT TTTTACTCAA ATGTCTCTAC AGTGCAAAAA CTTTCGTAAA AGGTGCAATG   
  
  
+ TACGTGGTTA AGCTAAGAAA ATGCAGAAAA GTTTTCCCTA AAAAAAAAAA AAAACAAAGA ATGTAACTTA   
  
  
+ ATTAAATAAG GTGCAATGTA CGTGGTTAAG CTAAGAAAAT GCAGAAAAGT TTTCCCTAAA AAAAAAAAAA   
  
  
+ AACAAAGAAT GTAACTTAAT TAAAAAACTT AGATCTGAGA ACGTACATTA AGTAACATGG TTCTTCCGAA   
  
  
+ TGAATAAGTA AGATTCATAA GTTTCTCCTT ACACTCGTTT TACCACCTCT ACATGGTATG ATAATAATTC   
  
  
+ AGAATTAGCA AAAGTTCTAA AAAAAACAG  

- GGCTGGCCGA CGACTTAGAT CGATCGAAAC CAAGAGATAC CCAGCAAACA GATTGCCTGA AATTGACCTA   
  
  
- ACAATAAAGC AGACTAAGTT CCTCTAGTTT ATCGAATCTT GCAAGCTTTG TGTAGAAACA GAATTCAAGT   
  
  
- TGGTCAAGCT GGCCTAGCAT GTGAAATGAA GAAAAGCGTG TGATATATAG AGCGGAAAGG TGCTGTTGGT   
  
  
- GGGAAACTTG GTGGAAATGA GACGGACACG GATAAGATGT TGCAGGCACA ACTCTATATA TAATGACTCT   
  
  
- GAGTCTATCT AAAGATCCAC GTGGTGGGAT TTGTTGTATA GTTTATTAAT AAGACAATTA ACTGTTTCCC   
  
  
- AGTTTGGTGA GTGTTGGGAG GAGCCATGTG AAGAAGGAAG AAGTTTTCTT TTCTTTTTTT TTCACCGAAT   
  
  
- CTGAATTAGT GTTAACTTTT TTAAAAGTAA AAGAAAAAAA ATATACATTT TTATAATTTA TTGTATTAAT   
  
  
- TTACTAAAAT AAATATACTG AATTAGTGTT AACTTTTTTA AAAGTAAAAG AAAAAAAATA TACATTTTTA   
  
  
- TAATTTATTG TATTAATTTA CTAAAATAAA TATATTAAAT TATAATTATT AATTAATTTT TCTTTTTATA   
  
  
- TTAACATAAG AAATTATAAA ATTATTTTTT ATTTTTACTC AAAAAATTAA AATAAATTCA AATAAATAGA   
  
  
- AAATGTTCGT GATTATTTTT TAAAATAAAA GCATATGTTT GATTAATATT AACATAAAAA ATACATCTTA   
  
  
- ATAATTTGTT TTATTAAATC ATTATGCATT ATTATTTTTA TTTAAAAATA ATATAAATTA AATTAAAATA   
  
  
- AAAAATTAAT TCAACCATAT ATATATATAT TTATCCATTC AATTACATAT TATTACGTGT GCTAGGGACA   
  
  
- TGCTGGTCGG TCATGGGTGT GCTTAGTCAA AGTCAAGCTC ACCATAAAAG CCAAGAAACA TTGGGTGGTG   
  
  
- GTCTCATTTT ATCTACTATT CCTCTGTGGA ATTAATAATT CATATAATAG ATGTCTAATT CTTAATTAAA   
  
  
- TTTCATATTG AACCCAATCT TATCTTTGTC TCTTCTACTG TACCAAATGC TTAATTAAAT TAAAATGACC   
  
  
- AATTAATATT TTTTATAAAA AAAATGAGTT TACAGAGATG TCACGTTTTT GAAAGCATTT TCCACGTTAC   
  
  
- ATGCACCAAT TCGATTCTTT TACGTCTTTT CAAAAGGGAT TTTTTTTTTT TTTTGTTTCT TACATTGAAT   
  
  
- TAATTTATTC CACGTTACAT GCACCAATTC GATTCTTTTA CGTCTTTTCA AAAGGGATTT TTTTTTTTTT   
  
  
- TTGTTTCTTA CATTGAATTA ATTTTTTGAA TCTAGACTCT TGCATGTAAT TCATTGTACC AAGAAGGCTT   
  
  
- ACTTATTCAT TCTAAGTATT CAAAGAGGAA TGTGAGCAAA ATGGTGGAGA TGTACCATAC TATTATTAAG   
  
  
- TCTTAATCGT TTTCAAGATT TTTTTTGTC

+     ABRE

| Site Name | Organism | Position | Strand | Matrix score. | sequence | function |
| --- | --- | --- | --- | --- | --- | --- |
| ABRE | Hordeum vulgare | 1189 | - | 9 | GCCACGTACA | cis-acting element involved in the abscisic acid responsiveness |
| ABRE | Hordeum vulgare | 1277 | - | 9 | GCCACGTACA | cis-acting element involved in the abscisic acid responsiveness |
| ABRE | Arabidopsis thaliana | 1191 | + | 6 | TACGTG | cis-acting element involved in the abscisic acid responsiveness |
| ABRE | Arabidopsis thaliana | 1279 | + | 6 | TACGTG | cis-acting element involved in the abscisic acid responsiveness |

> 2018/04/13 10:10:12  
+ CCGACCGGCT GCTGAATCTA GCTAGCTTTG GTTCTCTATG GGTCGTTTGT CTAACGGACT TTAACTGGAT   
  
  
+ TGTTATTTCG TCTGATTCAA GGAGATCAAA TAGCTTAGAA CGTTCGAAAC ACATCTTTGT CTTAAGTTCA   
  
  
+ ACCAGTTCGA CCGGATCGTA CACTTTACTT CTTTTCGCAC ACTATATATC TCGCCTTTCC ACGACAACCA   
  
  
+ CCCTTTGAAC CACCTTTACT CTGCCTGTGC CTATTCTACA ACGTCCGTGT TGAGATATAT ATTACTGAGA   
  
  
+ CTCAGATAGA TTTCTAGGTG CACCACCCTA AACAACATAT CAAATAATTA TTCTGTTAAT TGACAAAGGG   
  
  
+ TCAAACCACT CACAACCCTC CTCGGTACAC TTCTTCCTTC TTCAAAAGAA AAGAAAAAAA AAGTGGCTTA   
  
  
+ GACTTAATCA CAATTGAAAA AATTTTCATT TTCTTTTTTT TATATGTAAA AATATTAAAT AACATAATTA   
  
  
+ AATGATTTTA TTTATATGAC TTAATCACAA TTGAAAAAAT TTTCATTTTC TTTTTTTTAT ATGTAAAAAT   
  
  
+ ATTAAATAAC ATAATTAAAT GATTTTATTT ATATAATTTA ATATTAATAA TTAATTAAAA AGAAAAATAT   
  
  
+ AATTGTATTC TTTAATATTT TAATAAAAAA TAAAAATGAG TTTTTTAATT TTATTTAAGT TTATTTATCT   
  
  
+ TTTACAAGCA CTAATAAAAA ATTTTATTTT CGTATACAAA CTAATTATAA TTGTATTTTT TATGTAGAAT   
  
  
+ TATTAAACAA AATAATTTAG TAATACGTAA TAATAAAAAT AAATTTTTAT TATATTTAAT TTAATTTTAT   
  
  
+ TTTTTAATTA AGTTGGTATA TATATATATA AATAGGTAAG TTAATGTATA ATAATGCACA CGATCCCTGT   
  
  
+ ACGACCAGCC AGTACCCACA CGAATCAGTT TCAGTTCGAG TGGTATTTTC GGTTCTTTGT AACCCACCAC   
  
  
+ CAGAGTAAAA TAGATGATAA GGAGACACCT TAATTATTAA GTATATTATC TACAGATTAA GAATTAATTT   
  
  
+ AAAGTATAAC TTGGGTTAGA ATAGAAACAG AGAAGATGAC ATGGTTTACG AATTAATTTA ATTTTACTGG   
  
  
+ TTAATTATAA AAAATATTTT TTTTACTCAA ATGTCTCTAC AGTGCAAAAA CTTTCGTAAA AGGTGCAATG   
  
  
+ TACGTGGTTA AGCTAAGAAA ATGCAGAAAA GTTTTCCCTA AAAAAAAAAA AAAACAAAGA ATGTAACTTA   
  
  
+ ATTAAATAAG GTGCAATGTA CGTGGTTAAG CTAAGAAAAT GCAGAAAAGT TTTCCCTAAA AAAAAAAAAA   
  
  
+ AACAAAGAAT GTAACTTAAT TAAAAAACTT AGATCTGAGA ACGTACATTA AGTAACATGG TTCTTCCGAA   
  
  
+ TGAATAAGTA AGATTCATAA GTTTCTCCTT ACACTCGTTT TACCACCTCT ACATGGTATG ATAATAATTC   
  
  
+ AGAATTAGCA AAAGTTCTAA AAAAAACAG  

- GGCTGGCCGA CGACTTAGAT CGATCGAAAC CAAGAGATAC CCAGCAAACA GATTGCCTGA AATTGACCTA   
  
  
- ACAATAAAGC AGACTAAGTT CCTCTAGTTT ATCGAATCTT GCAAGCTTTG TGTAGAAACA GAATTCAAGT   
  
  
- TGGTCAAGCT GGCCTAGCAT GTGAAATGAA GAAAAGCGTG TGATATATAG AGCGGAAAGG TGCTGTTGGT   
  
  
- GGGAAACTTG GTGGAAATGA GACGGACACG GATAAGATGT TGCAGGCACA ACTCTATATA TAATGACTCT   
  
  
- GAGTCTATCT AAAGATCCAC GTGGTGGGAT TTGTTGTATA GTTTATTAAT AAGACAATTA ACTGTTTCCC   
  
  
- AGTTTGGTGA GTGTTGGGAG GAGCCATGTG AAGAAGGAAG AAGTTTTCTT TTCTTTTTTT TTCACCGAAT   
  
  
- CTGAATTAGT GTTAACTTTT TTAAAAGTAA AAGAAAAAAA ATATACATTT TTATAATTTA TTGTATTAAT   
  
  
- TTACTAAAAT AAATATACTG AATTAGTGTT AACTTTTTTA AAAGTAAAAG AAAAAAAATA TACATTTTTA   
  
  
- TAATTTATTG TATTAATTTA CTAAAATAAA TATATTAAAT TATAATTATT AATTAATTTT TCTTTTTATA   
  
  
- TTAACATAAG AAATTATAAA ATTATTTTTT ATTTTTACTC AAAAAATTAA AATAAATTCA AATAAATAGA   
  
  
- AAATGTTCGT GATTATTTTT TAAAATAAAA GCATATGTTT GATTAATATT AACATAAAAA ATACATCTTA   
  
  
- ATAATTTGTT TTATTAAATC ATTATGCATT ATTATTTTTA TTTAAAAATA ATATAAATTA AATTAAAATA   
  
  
- AAAAATTAAT TCAACCATAT ATATATATAT TTATCCATTC AATTACATAT TATTACGTGT GCTAGGGACA   
  
  
- TGCTGGTCGG TCATGGGTGT GCTTAGTCAA AGTCAAGCTC ACCATAAAAG CCAAGAAACA TTGGGTGGTG   
  
  
- GTCTCATTTT ATCTACTATT CCTCTGTGGA ATTAATAATT CATATAATAG ATGTCTAATT CTTAATTAAA   
  
  
- TTTCATATTG AACCCAATCT TATCTTTGTC TCTTCTACTG TACCAAATGC TTAATTAAAT TAAAATGACC   
  
  
- AATTAATATT TTTTATAAAA AAAATGAGTT TACAGAGATG TCACGTTTTT GAAAGCATTT TCCACGTTAC   
  
  
- ATGCACCAAT TCGATTCTTT TACGTCTTTT CAAAAGGGAT TTTTTTTTTT TTTTGTTTCT TACATTGAAT   
  
  
- TAATTTATTC CACGTTACAT GCACCAATTC GATTCTTTTA CGTCTTTTCA AAAGGGATTT TTTTTTTTTT   
  
  
- TTGTTTCTTA CATTGAATTA ATTTTTTGAA TCTAGACTCT TGCATGTAAT TCATTGTACC AAGAAGGCTT   
  
  
- ACTTATTCAT TCTAAGTATT CAAAGAGGAA TGTGAGCAAA ATGGTGGAGA TGTACCATAC TATTATTAAG   
  
  
- TCTTAATCGT TTTCAAGATT TTTTTTGTC

+     AE-box

| Site Name | Organism | Position | Strand | Matrix score. | sequence | function |
| --- | --- | --- | --- | --- | --- | --- |
| AE-box | Arabidopsis thaliana | 1419 | - | 8 | AGAAACTT | part of a module for light response |

> 2018/04/13 10:10:12  
+ CCGACCGGCT GCTGAATCTA GCTAGCTTTG GTTCTCTATG GGTCGTTTGT CTAACGGACT TTAACTGGAT   
  
  
+ TGTTATTTCG TCTGATTCAA GGAGATCAAA TAGCTTAGAA CGTTCGAAAC ACATCTTTGT CTTAAGTTCA   
  
  
+ ACCAGTTCGA CCGGATCGTA CACTTTACTT CTTTTCGCAC ACTATATATC TCGCCTTTCC ACGACAACCA   
  
  
+ CCCTTTGAAC CACCTTTACT CTGCCTGTGC CTATTCTACA ACGTCCGTGT TGAGATATAT ATTACTGAGA   
  
  
+ CTCAGATAGA TTTCTAGGTG CACCACCCTA AACAACATAT CAAATAATTA TTCTGTTAAT TGACAAAGGG   
  
  
+ TCAAACCACT CACAACCCTC CTCGGTACAC TTCTTCCTTC TTCAAAAGAA AAGAAAAAAA AAGTGGCTTA   
  
  
+ GACTTAATCA CAATTGAAAA AATTTTCATT TTCTTTTTTT TATATGTAAA AATATTAAAT AACATAATTA   
  
  
+ AATGATTTTA TTTATATGAC TTAATCACAA TTGAAAAAAT TTTCATTTTC TTTTTTTTAT ATGTAAAAAT   
  
  
+ ATTAAATAAC ATAATTAAAT GATTTTATTT ATATAATTTA ATATTAATAA TTAATTAAAA AGAAAAATAT   
  
  
+ AATTGTATTC TTTAATATTT TAATAAAAAA TAAAAATGAG TTTTTTAATT TTATTTAAGT TTATTTATCT   
  
  
+ TTTACAAGCA CTAATAAAAA ATTTTATTTT CGTATACAAA CTAATTATAA TTGTATTTTT TATGTAGAAT   
  
  
+ TATTAAACAA AATAATTTAG TAATACGTAA TAATAAAAAT AAATTTTTAT TATATTTAAT TTAATTTTAT   
  
  
+ TTTTTAATTA AGTTGGTATA TATATATATA AATAGGTAAG TTAATGTATA ATAATGCACA CGATCCCTGT   
  
  
+ ACGACCAGCC AGTACCCACA CGAATCAGTT TCAGTTCGAG TGGTATTTTC GGTTCTTTGT AACCCACCAC   
  
  
+ CAGAGTAAAA TAGATGATAA GGAGACACCT TAATTATTAA GTATATTATC TACAGATTAA GAATTAATTT   
  
  
+ AAAGTATAAC TTGGGTTAGA ATAGAAACAG AGAAGATGAC ATGGTTTACG AATTAATTTA ATTTTACTGG   
  
  
+ TTAATTATAA AAAATATTTT TTTTACTCAA ATGTCTCTAC AGTGCAAAAA CTTTCGTAAA AGGTGCAATG   
  
  
+ TACGTGGTTA AGCTAAGAAA ATGCAGAAAA GTTTTCCCTA AAAAAAAAAA AAAACAAAGA ATGTAACTTA   
  
  
+ ATTAAATAAG GTGCAATGTA CGTGGTTAAG CTAAGAAAAT GCAGAAAAGT TTTCCCTAAA AAAAAAAAAA   
  
  
+ AACAAAGAAT GTAACTTAAT TAAAAAACTT AGATCTGAGA ACGTACATTA AGTAACATGG TTCTTCCGAA   
  
  
+ TGAATAAGTA AGATTCATAA GTTTCTCCTT ACACTCGTTT TACCACCTCT ACATGGTATG ATAATAATTC   
  
  
+ AGAATTAGCA AAAGTTCTAA AAAAAACAG  

- GGCTGGCCGA CGACTTAGAT CGATCGAAAC CAAGAGATAC CCAGCAAACA GATTGCCTGA AATTGACCTA   
  
  
- ACAATAAAGC AGACTAAGTT CCTCTAGTTT ATCGAATCTT GCAAGCTTTG TGTAGAAACA GAATTCAAGT   
  
  
- TGGTCAAGCT GGCCTAGCAT GTGAAATGAA GAAAAGCGTG TGATATATAG AGCGGAAAGG TGCTGTTGGT   
  
  
- GGGAAACTTG GTGGAAATGA GACGGACACG GATAAGATGT TGCAGGCACA ACTCTATATA TAATGACTCT   
  
  
- GAGTCTATCT AAAGATCCAC GTGGTGGGAT TTGTTGTATA GTTTATTAAT AAGACAATTA ACTGTTTCCC   
  
  
- AGTTTGGTGA GTGTTGGGAG GAGCCATGTG AAGAAGGAAG AAGTTTTCTT TTCTTTTTTT TTCACCGAAT   
  
  
- CTGAATTAGT GTTAACTTTT TTAAAAGTAA AAGAAAAAAA ATATACATTT TTATAATTTA TTGTATTAAT   
  
  
- TTACTAAAAT AAATATACTG AATTAGTGTT AACTTTTTTA AAAGTAAAAG AAAAAAAATA TACATTTTTA   
  
  
- TAATTTATTG TATTAATTTA CTAAAATAAA TATATTAAAT TATAATTATT AATTAATTTT TCTTTTTATA   
  
  
- TTAACATAAG AAATTATAAA ATTATTTTTT ATTTTTACTC AAAAAATTAA AATAAATTCA AATAAATAGA   
  
  
- AAATGTTCGT GATTATTTTT TAAAATAAAA GCATATGTTT GATTAATATT AACATAAAAA ATACATCTTA   
  
  
- ATAATTTGTT TTATTAAATC ATTATGCATT ATTATTTTTA TTTAAAAATA ATATAAATTA AATTAAAATA   
  
  
- AAAAATTAAT TCAACCATAT ATATATATAT TTATCCATTC AATTACATAT TATTACGTGT GCTAGGGACA   
  
  
- TGCTGGTCGG TCATGGGTGT GCTTAGTCAA AGTCAAGCTC ACCATAAAAG CCAAGAAACA TTGGGTGGTG   
  
  
- GTCTCATTTT ATCTACTATT CCTCTGTGGA ATTAATAATT CATATAATAG ATGTCTAATT CTTAATTAAA   
  
  
- TTTCATATTG AACCCAATCT TATCTTTGTC TCTTCTACTG TACCAAATGC TTAATTAAAT TAAAATGACC   
  
  
- AATTAATATT TTTTATAAAA AAAATGAGTT TACAGAGATG TCACGTTTTT GAAAGCATTT TCCACGTTAC   
  
  
- ATGCACCAAT TCGATTCTTT TACGTCTTTT CAAAAGGGAT TTTTTTTTTT TTTTGTTTCT TACATTGAAT   
  
  
- TAATTTATTC CACGTTACAT GCACCAATTC GATTCTTTTA CGTCTTTTCA AAAGGGATTT TTTTTTTTTT   
  
  
- TTGTTTCTTA CATTGAATTA ATTTTTTGAA TCTAGACTCT TGCATGTAAT TCATTGTACC AAGAAGGCTT   
  
  
- ACTTATTCAT TCTAAGTATT CAAAGAGGAA TGTGAGCAAA ATGGTGGAGA TGTACCATAC TATTATTAAG   
  
  
- TCTTAATCGT TTTCAAGATT TTTTTTGTC

+     ARE

| Site Name | Organism | Position | Strand | Matrix score. | sequence | function |
| --- | --- | --- | --- | --- | --- | --- |
| ARE | Zea mays | 353 | - | 6 | TGGTTT | cis-acting regulatory element essential for the anaerobic induction |
| ARE | Zea mays | 1092 | + | 6 | TGGTTT | cis-acting regulatory element essential for the anaerobic induction |

> 2018/04/13 10:10:12  
+ CCGACCGGCT GCTGAATCTA GCTAGCTTTG GTTCTCTATG GGTCGTTTGT CTAACGGACT TTAACTGGAT   
  
  
+ TGTTATTTCG TCTGATTCAA GGAGATCAAA TAGCTTAGAA CGTTCGAAAC ACATCTTTGT CTTAAGTTCA   
  
  
+ ACCAGTTCGA CCGGATCGTA CACTTTACTT CTTTTCGCAC ACTATATATC TCGCCTTTCC ACGACAACCA   
  
  
+ CCCTTTGAAC CACCTTTACT CTGCCTGTGC CTATTCTACA ACGTCCGTGT TGAGATATAT ATTACTGAGA   
  
  
+ CTCAGATAGA TTTCTAGGTG CACCACCCTA AACAACATAT CAAATAATTA TTCTGTTAAT TGACAAAGGG   
  
  
+ TCAAACCACT CACAACCCTC CTCGGTACAC TTCTTCCTTC TTCAAAAGAA AAGAAAAAAA AAGTGGCTTA   
  
  
+ GACTTAATCA CAATTGAAAA AATTTTCATT TTCTTTTTTT TATATGTAAA AATATTAAAT AACATAATTA   
  
  
+ AATGATTTTA TTTATATGAC TTAATCACAA TTGAAAAAAT TTTCATTTTC TTTTTTTTAT ATGTAAAAAT   
  
  
+ ATTAAATAAC ATAATTAAAT GATTTTATTT ATATAATTTA ATATTAATAA TTAATTAAAA AGAAAAATAT   
  
  
+ AATTGTATTC TTTAATATTT TAATAAAAAA TAAAAATGAG TTTTTTAATT TTATTTAAGT TTATTTATCT   
  
  
+ TTTACAAGCA CTAATAAAAA ATTTTATTTT CGTATACAAA CTAATTATAA TTGTATTTTT TATGTAGAAT   
  
  
+ TATTAAACAA AATAATTTAG TAATACGTAA TAATAAAAAT AAATTTTTAT TATATTTAAT TTAATTTTAT   
  
  
+ TTTTTAATTA AGTTGGTATA TATATATATA AATAGGTAAG TTAATGTATA ATAATGCACA CGATCCCTGT   
  
  
+ ACGACCAGCC AGTACCCACA CGAATCAGTT TCAGTTCGAG TGGTATTTTC GGTTCTTTGT AACCCACCAC   
  
  
+ CAGAGTAAAA TAGATGATAA GGAGACACCT TAATTATTAA GTATATTATC TACAGATTAA GAATTAATTT   
  
  
+ AAAGTATAAC TTGGGTTAGA ATAGAAACAG AGAAGATGAC ATGGTTTACG AATTAATTTA ATTTTACTGG   
  
  
+ TTAATTATAA AAAATATTTT TTTTACTCAA ATGTCTCTAC AGTGCAAAAA CTTTCGTAAA AGGTGCAATG   
  
  
+ TACGTGGTTA AGCTAAGAAA ATGCAGAAAA GTTTTCCCTA AAAAAAAAAA AAAACAAAGA ATGTAACTTA   
  
  
+ ATTAAATAAG GTGCAATGTA CGTGGTTAAG CTAAGAAAAT GCAGAAAAGT TTTCCCTAAA AAAAAAAAAA   
  
  
+ AACAAAGAAT GTAACTTAAT TAAAAAACTT AGATCTGAGA ACGTACATTA AGTAACATGG TTCTTCCGAA   
  
  
+ TGAATAAGTA AGATTCATAA GTTTCTCCTT ACACTCGTTT TACCACCTCT ACATGGTATG ATAATAATTC   
  
  
+ AGAATTAGCA AAAGTTCTAA AAAAAACAG  

- GGCTGGCCGA CGACTTAGAT CGATCGAAAC CAAGAGATAC CCAGCAAACA GATTGCCTGA AATTGACCTA   
  
  
- ACAATAAAGC AGACTAAGTT CCTCTAGTTT ATCGAATCTT GCAAGCTTTG TGTAGAAACA GAATTCAAGT   
  
  
- TGGTCAAGCT GGCCTAGCAT GTGAAATGAA GAAAAGCGTG TGATATATAG AGCGGAAAGG TGCTGTTGGT   
  
  
- GGGAAACTTG GTGGAAATGA GACGGACACG GATAAGATGT TGCAGGCACA ACTCTATATA TAATGACTCT   
  
  
- GAGTCTATCT AAAGATCCAC GTGGTGGGAT TTGTTGTATA GTTTATTAAT AAGACAATTA ACTGTTTCCC   
  
  
- AGTTTGGTGA GTGTTGGGAG GAGCCATGTG AAGAAGGAAG AAGTTTTCTT TTCTTTTTTT TTCACCGAAT   
  
  
- CTGAATTAGT GTTAACTTTT TTAAAAGTAA AAGAAAAAAA ATATACATTT TTATAATTTA TTGTATTAAT   
  
  
- TTACTAAAAT AAATATACTG AATTAGTGTT AACTTTTTTA AAAGTAAAAG AAAAAAAATA TACATTTTTA   
  
  
- TAATTTATTG TATTAATTTA CTAAAATAAA TATATTAAAT TATAATTATT AATTAATTTT TCTTTTTATA   
  
  
- TTAACATAAG AAATTATAAA ATTATTTTTT ATTTTTACTC AAAAAATTAA AATAAATTCA AATAAATAGA   
  
  
- AAATGTTCGT GATTATTTTT TAAAATAAAA GCATATGTTT GATTAATATT AACATAAAAA ATACATCTTA   
  
  
- ATAATTTGTT TTATTAAATC ATTATGCATT ATTATTTTTA TTTAAAAATA ATATAAATTA AATTAAAATA   
  
  
- AAAAATTAAT TCAACCATAT ATATATATAT TTATCCATTC AATTACATAT TATTACGTGT GCTAGGGACA   
  
  
- TGCTGGTCGG TCATGGGTGT GCTTAGTCAA AGTCAAGCTC ACCATAAAAG CCAAGAAACA TTGGGTGGTG   
  
  
- GTCTCATTTT ATCTACTATT CCTCTGTGGA ATTAATAATT CATATAATAG ATGTCTAATT CTTAATTAAA   
  
  
- TTTCATATTG AACCCAATCT TATCTTTGTC TCTTCTACTG TACCAAATGC TTAATTAAAT TAAAATGACC   
  
  
- AATTAATATT TTTTATAAAA AAAATGAGTT TACAGAGATG TCACGTTTTT GAAAGCATTT TCCACGTTAC   
  
  
- ATGCACCAAT TCGATTCTTT TACGTCTTTT CAAAAGGGAT TTTTTTTTTT TTTTGTTTCT TACATTGAAT   
  
  
- TAATTTATTC CACGTTACAT GCACCAATTC GATTCTTTTA CGTCTTTTCA AAAGGGATTT TTTTTTTTTT   
  
  
- TTGTTTCTTA CATTGAATTA ATTTTTTGAA TCTAGACTCT TGCATGTAAT TCATTGTACC AAGAAGGCTT   
  
  
- ACTTATTCAT TCTAAGTATT CAAAGAGGAA TGTGAGCAAA ATGGTGGAGA TGTACCATAC TATTATTAAG   
  
  
- TCTTAATCGT TTTCAAGATT TTTTTTGTC

+     Box 4

| Site Name | Organism | Position | Strand | Matrix score. | sequence | function |
| --- | --- | --- | --- | --- | --- | --- |
| Box 4 | Petroselinum crispum | 603 | + | 6 | ATTAAT | part of a conserved DNA module involved in light responsiveness |
| Box 4 | Petroselinum crispum | 1043 | - | 6 | ATTAAT | part of a conserved DNA module involved in light responsiveness |
| Box 4 | Petroselinum crispum | 610 | + | 6 | ATTAAT | part of a conserved DNA module involved in light responsiveness |
| Box 4 | Petroselinum crispum | 1102 | - | 6 | ATTAAT | part of a conserved DNA module involved in light responsiveness |

> 2018/04/13 10:10:12  
+ CCGACCGGCT GCTGAATCTA GCTAGCTTTG GTTCTCTATG GGTCGTTTGT CTAACGGACT TTAACTGGAT   
  
  
+ TGTTATTTCG TCTGATTCAA GGAGATCAAA TAGCTTAGAA CGTTCGAAAC ACATCTTTGT CTTAAGTTCA   
  
  
+ ACCAGTTCGA CCGGATCGTA CACTTTACTT CTTTTCGCAC ACTATATATC TCGCCTTTCC ACGACAACCA   
  
  
+ CCCTTTGAAC CACCTTTACT CTGCCTGTGC CTATTCTACA ACGTCCGTGT TGAGATATAT ATTACTGAGA   
  
  
+ CTCAGATAGA TTTCTAGGTG CACCACCCTA AACAACATAT CAAATAATTA TTCTGTTAAT TGACAAAGGG   
  
  
+ TCAAACCACT CACAACCCTC CTCGGTACAC TTCTTCCTTC TTCAAAAGAA AAGAAAAAAA AAGTGGCTTA   
  
  
+ GACTTAATCA CAATTGAAAA AATTTTCATT TTCTTTTTTT TATATGTAAA AATATTAAAT AACATAATTA   
  
  
+ AATGATTTTA TTTATATGAC TTAATCACAA TTGAAAAAAT TTTCATTTTC TTTTTTTTAT ATGTAAAAAT   
  
  
+ ATTAAATAAC ATAATTAAAT GATTTTATTT ATATAATTTA ATATTAATAA TTAATTAAAA AGAAAAATAT   
  
  
+ AATTGTATTC TTTAATATTT TAATAAAAAA TAAAAATGAG TTTTTTAATT TTATTTAAGT TTATTTATCT   
  
  
+ TTTACAAGCA CTAATAAAAA ATTTTATTTT CGTATACAAA CTAATTATAA TTGTATTTTT TATGTAGAAT   
  
  
+ TATTAAACAA AATAATTTAG TAATACGTAA TAATAAAAAT AAATTTTTAT TATATTTAAT TTAATTTTAT   
  
  
+ TTTTTAATTA AGTTGGTATA TATATATATA AATAGGTAAG TTAATGTATA ATAATGCACA CGATCCCTGT   
  
  
+ ACGACCAGCC AGTACCCACA CGAATCAGTT TCAGTTCGAG TGGTATTTTC GGTTCTTTGT AACCCACCAC   
  
  
+ CAGAGTAAAA TAGATGATAA GGAGACACCT TAATTATTAA GTATATTATC TACAGATTAA GAATTAATTT   
  
  
+ AAAGTATAAC TTGGGTTAGA ATAGAAACAG AGAAGATGAC ATGGTTTACG AATTAATTTA ATTTTACTGG   
  
  
+ TTAATTATAA AAAATATTTT TTTTACTCAA ATGTCTCTAC AGTGCAAAAA CTTTCGTAAA AGGTGCAATG   
  
  
+ TACGTGGTTA AGCTAAGAAA ATGCAGAAAA GTTTTCCCTA AAAAAAAAAA AAAACAAAGA ATGTAACTTA   
  
  
+ ATTAAATAAG GTGCAATGTA CGTGGTTAAG CTAAGAAAAT GCAGAAAAGT TTTCCCTAAA AAAAAAAAAA   
  
  
+ AACAAAGAAT GTAACTTAAT TAAAAAACTT AGATCTGAGA ACGTACATTA AGTAACATGG TTCTTCCGAA   
  
  
+ TGAATAAGTA AGATTCATAA GTTTCTCCTT ACACTCGTTT TACCACCTCT ACATGGTATG ATAATAATTC   
  
  
+ AGAATTAGCA AAAGTTCTAA AAAAAACAG  

- GGCTGGCCGA CGACTTAGAT CGATCGAAAC CAAGAGATAC CCAGCAAACA GATTGCCTGA AATTGACCTA   
  
  
- ACAATAAAGC AGACTAAGTT CCTCTAGTTT ATCGAATCTT GCAAGCTTTG TGTAGAAACA GAATTCAAGT   
  
  
- TGGTCAAGCT GGCCTAGCAT GTGAAATGAA GAAAAGCGTG TGATATATAG AGCGGAAAGG TGCTGTTGGT   
  
  
- GGGAAACTTG GTGGAAATGA GACGGACACG GATAAGATGT TGCAGGCACA ACTCTATATA TAATGACTCT   
  
  
- GAGTCTATCT AAAGATCCAC GTGGTGGGAT TTGTTGTATA GTTTATTAAT AAGACAATTA ACTGTTTCCC   
  
  
- AGTTTGGTGA GTGTTGGGAG GAGCCATGTG AAGAAGGAAG AAGTTTTCTT TTCTTTTTTT TTCACCGAAT   
  
  
- CTGAATTAGT GTTAACTTTT TTAAAAGTAA AAGAAAAAAA ATATACATTT TTATAATTTA TTGTATTAAT   
  
  
- TTACTAAAAT AAATATACTG AATTAGTGTT AACTTTTTTA AAAGTAAAAG AAAAAAAATA TACATTTTTA   
  
  
- TAATTTATTG TATTAATTTA CTAAAATAAA TATATTAAAT TATAATTATT AATTAATTTT TCTTTTTATA   
  
  
- TTAACATAAG AAATTATAAA ATTATTTTTT ATTTTTACTC AAAAAATTAA AATAAATTCA AATAAATAGA   
  
  
- AAATGTTCGT GATTATTTTT TAAAATAAAA GCATATGTTT GATTAATATT AACATAAAAA ATACATCTTA   
  
  
- ATAATTTGTT TTATTAAATC ATTATGCATT ATTATTTTTA TTTAAAAATA ATATAAATTA AATTAAAATA   
  
  
- AAAAATTAAT TCAACCATAT ATATATATAT TTATCCATTC AATTACATAT TATTACGTGT GCTAGGGACA   
  
  
- TGCTGGTCGG TCATGGGTGT GCTTAGTCAA AGTCAAGCTC ACCATAAAAG CCAAGAAACA TTGGGTGGTG   
  
  
- GTCTCATTTT ATCTACTATT CCTCTGTGGA ATTAATAATT CATATAATAG ATGTCTAATT CTTAATTAAA   
  
  
- TTTCATATTG AACCCAATCT TATCTTTGTC TCTTCTACTG TACCAAATGC TTAATTAAAT TAAAATGACC   
  
  
- AATTAATATT TTTTATAAAA AAAATGAGTT TACAGAGATG TCACGTTTTT GAAAGCATTT TCCACGTTAC   
  
  
- ATGCACCAAT TCGATTCTTT TACGTCTTTT CAAAAGGGAT TTTTTTTTTT TTTTGTTTCT TACATTGAAT   
  
  
- TAATTTATTC CACGTTACAT GCACCAATTC GATTCTTTTA CGTCTTTTCA AAAGGGATTT TTTTTTTTTT   
  
  
- TTGTTTCTTA CATTGAATTA ATTTTTTGAA TCTAGACTCT TGCATGTAAT TCATTGTACC AAGAAGGCTT   
  
  
- ACTTATTCAT TCTAAGTATT CAAAGAGGAA TGTGAGCAAA ATGGTGGAGA TGTACCATAC TATTATTAAG   
  
  
- TCTTAATCGT TTTCAAGATT TTTTTTGTC

+     Box-W1

| Site Name | Organism | Position | Strand | Matrix score. | sequence | function |
| --- | --- | --- | --- | --- | --- | --- |
| Box-W1 | Petroselinum crispum | 349 | - | 6 | TTGACC | fungal elicitor responsive element |

> 2018/04/13 10:10:12  
+ CCGACCGGCT GCTGAATCTA GCTAGCTTTG GTTCTCTATG GGTCGTTTGT CTAACGGACT TTAACTGGAT   
  
  
+ TGTTATTTCG TCTGATTCAA GGAGATCAAA TAGCTTAGAA CGTTCGAAAC ACATCTTTGT CTTAAGTTCA   
  
  
+ ACCAGTTCGA CCGGATCGTA CACTTTACTT CTTTTCGCAC ACTATATATC TCGCCTTTCC ACGACAACCA   
  
  
+ CCCTTTGAAC CACCTTTACT CTGCCTGTGC CTATTCTACA ACGTCCGTGT TGAGATATAT ATTACTGAGA   
  
  
+ CTCAGATAGA TTTCTAGGTG CACCACCCTA AACAACATAT CAAATAATTA TTCTGTTAAT TGACAAAGGG   
  
  
+ TCAAACCACT CACAACCCTC CTCGGTACAC TTCTTCCTTC TTCAAAAGAA AAGAAAAAAA AAGTGGCTTA   
  
  
+ GACTTAATCA CAATTGAAAA AATTTTCATT TTCTTTTTTT TATATGTAAA AATATTAAAT AACATAATTA   
  
  
+ AATGATTTTA TTTATATGAC TTAATCACAA TTGAAAAAAT TTTCATTTTC TTTTTTTTAT ATGTAAAAAT   
  
  
+ ATTAAATAAC ATAATTAAAT GATTTTATTT ATATAATTTA ATATTAATAA TTAATTAAAA AGAAAAATAT   
  
  
+ AATTGTATTC TTTAATATTT TAATAAAAAA TAAAAATGAG TTTTTTAATT TTATTTAAGT TTATTTATCT   
  
  
+ TTTACAAGCA CTAATAAAAA ATTTTATTTT CGTATACAAA CTAATTATAA TTGTATTTTT TATGTAGAAT   
  
  
+ TATTAAACAA AATAATTTAG TAATACGTAA TAATAAAAAT AAATTTTTAT TATATTTAAT TTAATTTTAT   
  
  
+ TTTTTAATTA AGTTGGTATA TATATATATA AATAGGTAAG TTAATGTATA ATAATGCACA CGATCCCTGT   
  
  
+ ACGACCAGCC AGTACCCACA CGAATCAGTT TCAGTTCGAG TGGTATTTTC GGTTCTTTGT AACCCACCAC   
  
  
+ CAGAGTAAAA TAGATGATAA GGAGACACCT TAATTATTAA GTATATTATC TACAGATTAA GAATTAATTT   
  
  
+ AAAGTATAAC TTGGGTTAGA ATAGAAACAG AGAAGATGAC ATGGTTTACG AATTAATTTA ATTTTACTGG   
  
  
+ TTAATTATAA AAAATATTTT TTTTACTCAA ATGTCTCTAC AGTGCAAAAA CTTTCGTAAA AGGTGCAATG   
  
  
+ TACGTGGTTA AGCTAAGAAA ATGCAGAAAA GTTTTCCCTA AAAAAAAAAA AAAACAAAGA ATGTAACTTA   
  
  
+ ATTAAATAAG GTGCAATGTA CGTGGTTAAG CTAAGAAAAT GCAGAAAAGT TTTCCCTAAA AAAAAAAAAA   
  
  
+ AACAAAGAAT GTAACTTAAT TAAAAAACTT AGATCTGAGA ACGTACATTA AGTAACATGG TTCTTCCGAA   
  
  
+ TGAATAAGTA AGATTCATAA GTTTCTCCTT ACACTCGTTT TACCACCTCT ACATGGTATG ATAATAATTC   
  
  
+ AGAATTAGCA AAAGTTCTAA AAAAAACAG  

- GGCTGGCCGA CGACTTAGAT CGATCGAAAC CAAGAGATAC CCAGCAAACA GATTGCCTGA AATTGACCTA   
  
  
- ACAATAAAGC AGACTAAGTT CCTCTAGTTT ATCGAATCTT GCAAGCTTTG TGTAGAAACA GAATTCAAGT   
  
  
- TGGTCAAGCT GGCCTAGCAT GTGAAATGAA GAAAAGCGTG TGATATATAG AGCGGAAAGG TGCTGTTGGT   
  
  
- GGGAAACTTG GTGGAAATGA GACGGACACG GATAAGATGT TGCAGGCACA ACTCTATATA TAATGACTCT   
  
  
- GAGTCTATCT AAAGATCCAC GTGGTGGGAT TTGTTGTATA GTTTATTAAT AAGACAATTA ACTGTTTCCC   
  
  
- AGTTTGGTGA GTGTTGGGAG GAGCCATGTG AAGAAGGAAG AAGTTTTCTT TTCTTTTTTT TTCACCGAAT   
  
  
- CTGAATTAGT GTTAACTTTT TTAAAAGTAA AAGAAAAAAA ATATACATTT TTATAATTTA TTGTATTAAT   
  
  
- TTACTAAAAT AAATATACTG AATTAGTGTT AACTTTTTTA AAAGTAAAAG AAAAAAAATA TACATTTTTA   
  
  
- TAATTTATTG TATTAATTTA CTAAAATAAA TATATTAAAT TATAATTATT AATTAATTTT TCTTTTTATA   
  
  
- TTAACATAAG AAATTATAAA ATTATTTTTT ATTTTTACTC AAAAAATTAA AATAAATTCA AATAAATAGA   
  
  
- AAATGTTCGT GATTATTTTT TAAAATAAAA GCATATGTTT GATTAATATT AACATAAAAA ATACATCTTA   
  
  
- ATAATTTGTT TTATTAAATC ATTATGCATT ATTATTTTTA TTTAAAAATA ATATAAATTA AATTAAAATA   
  
  
- AAAAATTAAT TCAACCATAT ATATATATAT TTATCCATTC AATTACATAT TATTACGTGT GCTAGGGACA   
  
  
- TGCTGGTCGG TCATGGGTGT GCTTAGTCAA AGTCAAGCTC ACCATAAAAG CCAAGAAACA TTGGGTGGTG   
  
  
- GTCTCATTTT ATCTACTATT CCTCTGTGGA ATTAATAATT CATATAATAG ATGTCTAATT CTTAATTAAA   
  
  
- TTTCATATTG AACCCAATCT TATCTTTGTC TCTTCTACTG TACCAAATGC TTAATTAAAT TAAAATGACC   
  
  
- AATTAATATT TTTTATAAAA AAAATGAGTT TACAGAGATG TCACGTTTTT GAAAGCATTT TCCACGTTAC   
  
  
- ATGCACCAAT TCGATTCTTT TACGTCTTTT CAAAAGGGAT TTTTTTTTTT TTTTGTTTCT TACATTGAAT   
  
  
- TAATTTATTC CACGTTACAT GCACCAATTC GATTCTTTTA CGTCTTTTCA AAAGGGATTT TTTTTTTTTT   
  
  
- TTGTTTCTTA CATTGAATTA ATTTTTTGAA TCTAGACTCT TGCATGTAAT TCATTGTACC AAGAAGGCTT   
  
  
- ACTTATTCAT TCTAAGTATT CAAAGAGGAA TGTGAGCAAA ATGGTGGAGA TGTACCATAC TATTATTAAG   
  
  
- TCTTAATCGT TTTCAAGATT TTTTTTGTC

+     CAAT-box

| Site Name | Organism | Position | Strand | Matrix score. | sequence | function |
| --- | --- | --- | --- | --- | --- | --- |
| CAAT-box | Hordeum vulgare | 69 | - | 4 | CAAT | common cis-acting element in promoter and enhancer regions |
| CAAT-box | Brassica rapa | 97 | + | 5 | CAAAT | common cis-acting element in promoter and enhancer regions |
| CAAT-box | Brassica rapa | 321 | + | 5 | CAAAT | common cis-acting element in promoter and enhancer regions |
| CAAT-box | Glycine max | 338 | - | 5 | CAATT | common cis-acting element in promoter and enhancer regions |
| CAAT-box | Hordeum vulgare | 339 | - | 4 | CAAT | common cis-acting element in promoter and enhancer regions |
| CAAT-box | Glycine max | 431 | + | 5 | CAATT | common cis-acting element in promoter and enhancer regions |
| CAAT-box | Glycine max | 432 | - | 5 | CAATT | common cis-acting element in promoter and enhancer regions |
| CAAT-box | Hordeum vulgare | 433 | - | 4 | CAAT | common cis-acting element in promoter and enhancer regions |
| CAAT-box | Glycine max | 518 | + | 5 | CAATT | common cis-acting element in promoter and enhancer regions |
| CAAT-box | Glycine max | 519 | - | 5 | CAATT | common cis-acting element in promoter and enhancer regions |
| CAAT-box | Hordeum vulgare | 520 | - | 4 | CAAT | common cis-acting element in promoter and enhancer regions |
| CAAT-box | Glycine max | 631 | - | 5 | CAATT | common cis-acting element in promoter and enhancer regions |
| CAAT-box | Hordeum vulgare | 632 | - | 4 | CAAT | common cis-acting element in promoter and enhancer regions |
| CAAT-box | Glycine max | 749 | - | 5 | CAATT | common cis-acting element in promoter and enhancer regions |
| CAAT-box | Hordeum vulgare | 750 | - | 4 | CAAT | common cis-acting element in promoter and enhancer regions |
| CAAT-box | Brassica rapa | 1148 | + | 5 | CAAAT | common cis-acting element in promoter and enhancer regions |
| CAAT-box | Hordeum vulgare | 1186 | + | 4 | CAAT | common cis-acting element in promoter and enhancer regions |
| CAAT-box | Hordeum vulgare | 1274 | + | 4 | CAAT | common cis-acting element in promoter and enhancer regions |

> 2018/04/13 10:10:12  
+ CCGACCGGCT GCTGAATCTA GCTAGCTTTG GTTCTCTATG GGTCGTTTGT CTAACGGACT TTAACTGGAT   
  
  
+ TGTTATTTCG TCTGATTCAA GGAGATCAAA TAGCTTAGAA CGTTCGAAAC ACATCTTTGT CTTAAGTTCA   
  
  
+ ACCAGTTCGA CCGGATCGTA CACTTTACTT CTTTTCGCAC ACTATATATC TCGCCTTTCC ACGACAACCA   
  
  
+ CCCTTTGAAC CACCTTTACT CTGCCTGTGC CTATTCTACA ACGTCCGTGT TGAGATATAT ATTACTGAGA   
  
  
+ CTCAGATAGA TTTCTAGGTG CACCACCCTA AACAACATAT CAAATAATTA TTCTGTTAAT TGACAAAGGG   
  
  
+ TCAAACCACT CACAACCCTC CTCGGTACAC TTCTTCCTTC TTCAAAAGAA AAGAAAAAAA AAGTGGCTTA   
  
  
+ GACTTAATCA CAATTGAAAA AATTTTCATT TTCTTTTTTT TATATGTAAA AATATTAAAT AACATAATTA   
  
  
+ AATGATTTTA TTTATATGAC TTAATCACAA TTGAAAAAAT TTTCATTTTC TTTTTTTTAT ATGTAAAAAT   
  
  
+ ATTAAATAAC ATAATTAAAT GATTTTATTT ATATAATTTA ATATTAATAA TTAATTAAAA AGAAAAATAT   
  
  
+ AATTGTATTC TTTAATATTT TAATAAAAAA TAAAAATGAG TTTTTTAATT TTATTTAAGT TTATTTATCT   
  
  
+ TTTACAAGCA CTAATAAAAA ATTTTATTTT CGTATACAAA CTAATTATAA TTGTATTTTT TATGTAGAAT   
  
  
+ TATTAAACAA AATAATTTAG TAATACGTAA TAATAAAAAT AAATTTTTAT TATATTTAAT TTAATTTTAT   
  
  
+ TTTTTAATTA AGTTGGTATA TATATATATA AATAGGTAAG TTAATGTATA ATAATGCACA CGATCCCTGT   
  
  
+ ACGACCAGCC AGTACCCACA CGAATCAGTT TCAGTTCGAG TGGTATTTTC GGTTCTTTGT AACCCACCAC   
  
  
+ CAGAGTAAAA TAGATGATAA GGAGACACCT TAATTATTAA GTATATTATC TACAGATTAA GAATTAATTT   
  
  
+ AAAGTATAAC TTGGGTTAGA ATAGAAACAG AGAAGATGAC ATGGTTTACG AATTAATTTA ATTTTACTGG   
  
  
+ TTAATTATAA AAAATATTTT TTTTACTCAA ATGTCTCTAC AGTGCAAAAA CTTTCGTAAA AGGTGCAATG   
  
  
+ TACGTGGTTA AGCTAAGAAA ATGCAGAAAA GTTTTCCCTA AAAAAAAAAA AAAACAAAGA ATGTAACTTA   
  
  
+ ATTAAATAAG GTGCAATGTA CGTGGTTAAG CTAAGAAAAT GCAGAAAAGT TTTCCCTAAA AAAAAAAAAA   
  
  
+ AACAAAGAAT GTAACTTAAT TAAAAAACTT AGATCTGAGA ACGTACATTA AGTAACATGG TTCTTCCGAA   
  
  
+ TGAATAAGTA AGATTCATAA GTTTCTCCTT ACACTCGTTT TACCACCTCT ACATGGTATG ATAATAATTC   
  
  
+ AGAATTAGCA AAAGTTCTAA AAAAAACAG  

- GGCTGGCCGA CGACTTAGAT CGATCGAAAC CAAGAGATAC CCAGCAAACA GATTGCCTGA AATTGACCTA   
  
  
- ACAATAAAGC AGACTAAGTT CCTCTAGTTT ATCGAATCTT GCAAGCTTTG TGTAGAAACA GAATTCAAGT   
  
  
- TGGTCAAGCT GGCCTAGCAT GTGAAATGAA GAAAAGCGTG TGATATATAG AGCGGAAAGG TGCTGTTGGT   
  
  
- GGGAAACTTG GTGGAAATGA GACGGACACG GATAAGATGT TGCAGGCACA ACTCTATATA TAATGACTCT   
  
  
- GAGTCTATCT AAAGATCCAC GTGGTGGGAT TTGTTGTATA GTTTATTAAT AAGACAATTA ACTGTTTCCC   
  
  
- AGTTTGGTGA GTGTTGGGAG GAGCCATGTG AAGAAGGAAG AAGTTTTCTT TTCTTTTTTT TTCACCGAAT   
  
  
- CTGAATTAGT GTTAACTTTT TTAAAAGTAA AAGAAAAAAA ATATACATTT TTATAATTTA TTGTATTAAT   
  
  
- TTACTAAAAT AAATATACTG AATTAGTGTT AACTTTTTTA AAAGTAAAAG AAAAAAAATA TACATTTTTA   
  
  
- TAATTTATTG TATTAATTTA CTAAAATAAA TATATTAAAT TATAATTATT AATTAATTTT TCTTTTTATA   
  
  
- TTAACATAAG AAATTATAAA ATTATTTTTT ATTTTTACTC AAAAAATTAA AATAAATTCA AATAAATAGA   
  
  
- AAATGTTCGT GATTATTTTT TAAAATAAAA GCATATGTTT GATTAATATT AACATAAAAA ATACATCTTA   
  
  
- ATAATTTGTT TTATTAAATC ATTATGCATT ATTATTTTTA TTTAAAAATA ATATAAATTA AATTAAAATA   
  
  
- AAAAATTAAT TCAACCATAT ATATATATAT TTATCCATTC AATTACATAT TATTACGTGT GCTAGGGACA   
  
  
- TGCTGGTCGG TCATGGGTGT GCTTAGTCAA AGTCAAGCTC ACCATAAAAG CCAAGAAACA TTGGGTGGTG   
  
  
- GTCTCATTTT ATCTACTATT CCTCTGTGGA ATTAATAATT CATATAATAG ATGTCTAATT CTTAATTAAA   
  
  
- TTTCATATTG AACCCAATCT TATCTTTGTC TCTTCTACTG TACCAAATGC TTAATTAAAT TAAAATGACC   
  
  
- AATTAATATT TTTTATAAAA AAAATGAGTT TACAGAGATG TCACGTTTTT GAAAGCATTT TCCACGTTAC   
  
  
- ATGCACCAAT TCGATTCTTT TACGTCTTTT CAAAAGGGAT TTTTTTTTTT TTTTGTTTCT TACATTGAAT   
  
  
- TAATTTATTC CACGTTACAT GCACCAATTC GATTCTTTTA CGTCTTTTCA AAAGGGATTT TTTTTTTTTT   
  
  
- TTGTTTCTTA CATTGAATTA ATTTTTTGAA TCTAGACTCT TGCATGTAAT TCATTGTACC AAGAAGGCTT   
  
  
- ACTTATTCAT TCTAAGTATT CAAAGAGGAA TGTGAGCAAA ATGGTGGAGA TGTACCATAC TATTATTAAG   
  
  
- TCTTAATCGT TTTCAAGATT TTTTTTGTC

+     CAT-box

| Site Name | Organism | Position | Strand | Matrix score. | sequence | function |
| --- | --- | --- | --- | --- | --- | --- |
| CAT-box | Arabidopsis thaliana | 412 | - | 6 | GCCACT | cis-acting regulatory element related to meristem expression |

> 2018/04/13 10:10:12  
+ CCGACCGGCT GCTGAATCTA GCTAGCTTTG GTTCTCTATG GGTCGTTTGT CTAACGGACT TTAACTGGAT   
  
  
+ TGTTATTTCG TCTGATTCAA GGAGATCAAA TAGCTTAGAA CGTTCGAAAC ACATCTTTGT CTTAAGTTCA   
  
  
+ ACCAGTTCGA CCGGATCGTA CACTTTACTT CTTTTCGCAC ACTATATATC TCGCCTTTCC ACGACAACCA   
  
  
+ CCCTTTGAAC CACCTTTACT CTGCCTGTGC CTATTCTACA ACGTCCGTGT TGAGATATAT ATTACTGAGA   
  
  
+ CTCAGATAGA TTTCTAGGTG CACCACCCTA AACAACATAT CAAATAATTA TTCTGTTAAT TGACAAAGGG   
  
  
+ TCAAACCACT CACAACCCTC CTCGGTACAC TTCTTCCTTC TTCAAAAGAA AAGAAAAAAA AAGTGGCTTA   
  
  
+ GACTTAATCA CAATTGAAAA AATTTTCATT TTCTTTTTTT TATATGTAAA AATATTAAAT AACATAATTA   
  
  
+ AATGATTTTA TTTATATGAC TTAATCACAA TTGAAAAAAT TTTCATTTTC TTTTTTTTAT ATGTAAAAAT   
  
  
+ ATTAAATAAC ATAATTAAAT GATTTTATTT ATATAATTTA ATATTAATAA TTAATTAAAA AGAAAAATAT   
  
  
+ AATTGTATTC TTTAATATTT TAATAAAAAA TAAAAATGAG TTTTTTAATT TTATTTAAGT TTATTTATCT   
  
  
+ TTTACAAGCA CTAATAAAAA ATTTTATTTT CGTATACAAA CTAATTATAA TTGTATTTTT TATGTAGAAT   
  
  
+ TATTAAACAA AATAATTTAG TAATACGTAA TAATAAAAAT AAATTTTTAT TATATTTAAT TTAATTTTAT   
  
  
+ TTTTTAATTA AGTTGGTATA TATATATATA AATAGGTAAG TTAATGTATA ATAATGCACA CGATCCCTGT   
  
  
+ ACGACCAGCC AGTACCCACA CGAATCAGTT TCAGTTCGAG TGGTATTTTC GGTTCTTTGT AACCCACCAC   
  
  
+ CAGAGTAAAA TAGATGATAA GGAGACACCT TAATTATTAA GTATATTATC TACAGATTAA GAATTAATTT   
  
  
+ AAAGTATAAC TTGGGTTAGA ATAGAAACAG AGAAGATGAC ATGGTTTACG AATTAATTTA ATTTTACTGG   
  
  
+ TTAATTATAA AAAATATTTT TTTTACTCAA ATGTCTCTAC AGTGCAAAAA CTTTCGTAAA AGGTGCAATG   
  
  
+ TACGTGGTTA AGCTAAGAAA ATGCAGAAAA GTTTTCCCTA AAAAAAAAAA AAAACAAAGA ATGTAACTTA   
  
  
+ ATTAAATAAG GTGCAATGTA CGTGGTTAAG CTAAGAAAAT GCAGAAAAGT TTTCCCTAAA AAAAAAAAAA   
  
  
+ AACAAAGAAT GTAACTTAAT TAAAAAACTT AGATCTGAGA ACGTACATTA AGTAACATGG TTCTTCCGAA   
  
  
+ TGAATAAGTA AGATTCATAA GTTTCTCCTT ACACTCGTTT TACCACCTCT ACATGGTATG ATAATAATTC   
  
  
+ AGAATTAGCA AAAGTTCTAA AAAAAACAG  

- GGCTGGCCGA CGACTTAGAT CGATCGAAAC CAAGAGATAC CCAGCAAACA GATTGCCTGA AATTGACCTA   
  
  
- ACAATAAAGC AGACTAAGTT CCTCTAGTTT ATCGAATCTT GCAAGCTTTG TGTAGAAACA GAATTCAAGT   
  
  
- TGGTCAAGCT GGCCTAGCAT GTGAAATGAA GAAAAGCGTG TGATATATAG AGCGGAAAGG TGCTGTTGGT   
  
  
- GGGAAACTTG GTGGAAATGA GACGGACACG GATAAGATGT TGCAGGCACA ACTCTATATA TAATGACTCT   
  
  
- GAGTCTATCT AAAGATCCAC GTGGTGGGAT TTGTTGTATA GTTTATTAAT AAGACAATTA ACTGTTTCCC   
  
  
- AGTTTGGTGA GTGTTGGGAG GAGCCATGTG AAGAAGGAAG AAGTTTTCTT TTCTTTTTTT TTCACCGAAT   
  
  
- CTGAATTAGT GTTAACTTTT TTAAAAGTAA AAGAAAAAAA ATATACATTT TTATAATTTA TTGTATTAAT   
  
  
- TTACTAAAAT AAATATACTG AATTAGTGTT AACTTTTTTA AAAGTAAAAG AAAAAAAATA TACATTTTTA   
  
  
- TAATTTATTG TATTAATTTA CTAAAATAAA TATATTAAAT TATAATTATT AATTAATTTT TCTTTTTATA   
  
  
- TTAACATAAG AAATTATAAA ATTATTTTTT ATTTTTACTC AAAAAATTAA AATAAATTCA AATAAATAGA   
  
  
- AAATGTTCGT GATTATTTTT TAAAATAAAA GCATATGTTT GATTAATATT AACATAAAAA ATACATCTTA   
  
  
- ATAATTTGTT TTATTAAATC ATTATGCATT ATTATTTTTA TTTAAAAATA ATATAAATTA AATTAAAATA   
  
  
- AAAAATTAAT TCAACCATAT ATATATATAT TTATCCATTC AATTACATAT TATTACGTGT GCTAGGGACA   
  
  
- TGCTGGTCGG TCATGGGTGT GCTTAGTCAA AGTCAAGCTC ACCATAAAAG CCAAGAAACA TTGGGTGGTG   
  
  
- GTCTCATTTT ATCTACTATT CCTCTGTGGA ATTAATAATT CATATAATAG ATGTCTAATT CTTAATTAAA   
  
  
- TTTCATATTG AACCCAATCT TATCTTTGTC TCTTCTACTG TACCAAATGC TTAATTAAAT TAAAATGACC   
  
  
- AATTAATATT TTTTATAAAA AAAATGAGTT TACAGAGATG TCACGTTTTT GAAAGCATTT TCCACGTTAC   
  
  
- ATGCACCAAT TCGATTCTTT TACGTCTTTT CAAAAGGGAT TTTTTTTTTT TTTTGTTTCT TACATTGAAT   
  
  
- TAATTTATTC CACGTTACAT GCACCAATTC GATTCTTTTA CGTCTTTTCA AAAGGGATTT TTTTTTTTTT   
  
  
- TTGTTTCTTA CATTGAATTA ATTTTTTGAA TCTAGACTCT TGCATGTAAT TCATTGTACC AAGAAGGCTT   
  
  
- ACTTATTCAT TCTAAGTATT CAAAGAGGAA TGTGAGCAAA ATGGTGGAGA TGTACCATAC TATTATTAAG   
  
  
- TCTTAATCGT TTTCAAGATT TTTTTTGTC

+     EIRE

| Site Name | Organism | Position | Strand | Matrix score. | sequence | function |
| --- | --- | --- | --- | --- | --- | --- |
| EIRE | Nicotiana tabacum | 146 | + | 7 | TTCGACC | elicitor-responsive element |

> 2018/04/13 10:10:12  
+ CCGACCGGCT GCTGAATCTA GCTAGCTTTG GTTCTCTATG GGTCGTTTGT CTAACGGACT TTAACTGGAT   
  
  
+ TGTTATTTCG TCTGATTCAA GGAGATCAAA TAGCTTAGAA CGTTCGAAAC ACATCTTTGT CTTAAGTTCA   
  
  
+ ACCAGTTCGA CCGGATCGTA CACTTTACTT CTTTTCGCAC ACTATATATC TCGCCTTTCC ACGACAACCA   
  
  
+ CCCTTTGAAC CACCTTTACT CTGCCTGTGC CTATTCTACA ACGTCCGTGT TGAGATATAT ATTACTGAGA   
  
  
+ CTCAGATAGA TTTCTAGGTG CACCACCCTA AACAACATAT CAAATAATTA TTCTGTTAAT TGACAAAGGG   
  
  
+ TCAAACCACT CACAACCCTC CTCGGTACAC TTCTTCCTTC TTCAAAAGAA AAGAAAAAAA AAGTGGCTTA   
  
  
+ GACTTAATCA CAATTGAAAA AATTTTCATT TTCTTTTTTT TATATGTAAA AATATTAAAT AACATAATTA   
  
  
+ AATGATTTTA TTTATATGAC TTAATCACAA TTGAAAAAAT TTTCATTTTC TTTTTTTTAT ATGTAAAAAT   
  
  
+ ATTAAATAAC ATAATTAAAT GATTTTATTT ATATAATTTA ATATTAATAA TTAATTAAAA AGAAAAATAT   
  
  
+ AATTGTATTC TTTAATATTT TAATAAAAAA TAAAAATGAG TTTTTTAATT TTATTTAAGT TTATTTATCT   
  
  
+ TTTACAAGCA CTAATAAAAA ATTTTATTTT CGTATACAAA CTAATTATAA TTGTATTTTT TATGTAGAAT   
  
  
+ TATTAAACAA AATAATTTAG TAATACGTAA TAATAAAAAT AAATTTTTAT TATATTTAAT TTAATTTTAT   
  
  
+ TTTTTAATTA AGTTGGTATA TATATATATA AATAGGTAAG TTAATGTATA ATAATGCACA CGATCCCTGT   
  
  
+ ACGACCAGCC AGTACCCACA CGAATCAGTT TCAGTTCGAG TGGTATTTTC GGTTCTTTGT AACCCACCAC   
  
  
+ CAGAGTAAAA TAGATGATAA GGAGACACCT TAATTATTAA GTATATTATC TACAGATTAA GAATTAATTT   
  
  
+ AAAGTATAAC TTGGGTTAGA ATAGAAACAG AGAAGATGAC ATGGTTTACG AATTAATTTA ATTTTACTGG   
  
  
+ TTAATTATAA AAAATATTTT TTTTACTCAA ATGTCTCTAC AGTGCAAAAA CTTTCGTAAA AGGTGCAATG   
  
  
+ TACGTGGTTA AGCTAAGAAA ATGCAGAAAA GTTTTCCCTA AAAAAAAAAA AAAACAAAGA ATGTAACTTA   
  
  
+ ATTAAATAAG GTGCAATGTA CGTGGTTAAG CTAAGAAAAT GCAGAAAAGT TTTCCCTAAA AAAAAAAAAA   
  
  
+ AACAAAGAAT GTAACTTAAT TAAAAAACTT AGATCTGAGA ACGTACATTA AGTAACATGG TTCTTCCGAA   
  
  
+ TGAATAAGTA AGATTCATAA GTTTCTCCTT ACACTCGTTT TACCACCTCT ACATGGTATG ATAATAATTC   
  
  
+ AGAATTAGCA AAAGTTCTAA AAAAAACAG  

- GGCTGGCCGA CGACTTAGAT CGATCGAAAC CAAGAGATAC CCAGCAAACA GATTGCCTGA AATTGACCTA   
  
  
- ACAATAAAGC AGACTAAGTT CCTCTAGTTT ATCGAATCTT GCAAGCTTTG TGTAGAAACA GAATTCAAGT   
  
  
- TGGTCAAGCT GGCCTAGCAT GTGAAATGAA GAAAAGCGTG TGATATATAG AGCGGAAAGG TGCTGTTGGT   
  
  
- GGGAAACTTG GTGGAAATGA GACGGACACG GATAAGATGT TGCAGGCACA ACTCTATATA TAATGACTCT   
  
  
- GAGTCTATCT AAAGATCCAC GTGGTGGGAT TTGTTGTATA GTTTATTAAT AAGACAATTA ACTGTTTCCC   
  
  
- AGTTTGGTGA GTGTTGGGAG GAGCCATGTG AAGAAGGAAG AAGTTTTCTT TTCTTTTTTT TTCACCGAAT   
  
  
- CTGAATTAGT GTTAACTTTT TTAAAAGTAA AAGAAAAAAA ATATACATTT TTATAATTTA TTGTATTAAT   
  
  
- TTACTAAAAT AAATATACTG AATTAGTGTT AACTTTTTTA AAAGTAAAAG AAAAAAAATA TACATTTTTA   
  
  
- TAATTTATTG TATTAATTTA CTAAAATAAA TATATTAAAT TATAATTATT AATTAATTTT TCTTTTTATA   
  
  
- TTAACATAAG AAATTATAAA ATTATTTTTT ATTTTTACTC AAAAAATTAA AATAAATTCA AATAAATAGA   
  
  
- AAATGTTCGT GATTATTTTT TAAAATAAAA GCATATGTTT GATTAATATT AACATAAAAA ATACATCTTA   
  
  
- ATAATTTGTT TTATTAAATC ATTATGCATT ATTATTTTTA TTTAAAAATA ATATAAATTA AATTAAAATA   
  
  
- AAAAATTAAT TCAACCATAT ATATATATAT TTATCCATTC AATTACATAT TATTACGTGT GCTAGGGACA   
  
  
- TGCTGGTCGG TCATGGGTGT GCTTAGTCAA AGTCAAGCTC ACCATAAAAG CCAAGAAACA TTGGGTGGTG   
  
  
- GTCTCATTTT ATCTACTATT CCTCTGTGGA ATTAATAATT CATATAATAG ATGTCTAATT CTTAATTAAA   
  
  
- TTTCATATTG AACCCAATCT TATCTTTGTC TCTTCTACTG TACCAAATGC TTAATTAAAT TAAAATGACC   
  
  
- AATTAATATT TTTTATAAAA AAAATGAGTT TACAGAGATG TCACGTTTTT GAAAGCATTT TCCACGTTAC   
  
  
- ATGCACCAAT TCGATTCTTT TACGTCTTTT CAAAAGGGAT TTTTTTTTTT TTTTGTTTCT TACATTGAAT   
  
  
- TAATTTATTC CACGTTACAT GCACCAATTC GATTCTTTTA CGTCTTTTCA AAAGGGATTT TTTTTTTTTT   
  
  
- TTGTTTCTTA CATTGAATTA ATTTTTTGAA TCTAGACTCT TGCATGTAAT TCATTGTACC AAGAAGGCTT   
  
  
- ACTTATTCAT TCTAAGTATT CAAAGAGGAA TGTGAGCAAA ATGGTGGAGA TGTACCATAC TATTATTAAG   
  
  
- TCTTAATCGT TTTCAAGATT TTTTTTGTC

+     G-Box

| Site Name | Organism | Position | Strand | Matrix score. | sequence | function |
| --- | --- | --- | --- | --- | --- | --- |
| G-Box | Antirrhinum majus | 1191 | - | 6 | CACGTA | cis-acting regulatory element involved in light responsiveness |
| G-Box | Antirrhinum majus | 1279 | - | 6 | CACGTA | cis-acting regulatory element involved in light responsiveness |

> 2018/04/13 10:10:12  
+ CCGACCGGCT GCTGAATCTA GCTAGCTTTG GTTCTCTATG GGTCGTTTGT CTAACGGACT TTAACTGGAT   
  
  
+ TGTTATTTCG TCTGATTCAA GGAGATCAAA TAGCTTAGAA CGTTCGAAAC ACATCTTTGT CTTAAGTTCA   
  
  
+ ACCAGTTCGA CCGGATCGTA CACTTTACTT CTTTTCGCAC ACTATATATC TCGCCTTTCC ACGACAACCA   
  
  
+ CCCTTTGAAC CACCTTTACT CTGCCTGTGC CTATTCTACA ACGTCCGTGT TGAGATATAT ATTACTGAGA   
  
  
+ CTCAGATAGA TTTCTAGGTG CACCACCCTA AACAACATAT CAAATAATTA TTCTGTTAAT TGACAAAGGG   
  
  
+ TCAAACCACT CACAACCCTC CTCGGTACAC TTCTTCCTTC TTCAAAAGAA AAGAAAAAAA AAGTGGCTTA   
  
  
+ GACTTAATCA CAATTGAAAA AATTTTCATT TTCTTTTTTT TATATGTAAA AATATTAAAT AACATAATTA   
  
  
+ AATGATTTTA TTTATATGAC TTAATCACAA TTGAAAAAAT TTTCATTTTC TTTTTTTTAT ATGTAAAAAT   
  
  
+ ATTAAATAAC ATAATTAAAT GATTTTATTT ATATAATTTA ATATTAATAA TTAATTAAAA AGAAAAATAT   
  
  
+ AATTGTATTC TTTAATATTT TAATAAAAAA TAAAAATGAG TTTTTTAATT TTATTTAAGT TTATTTATCT   
  
  
+ TTTACAAGCA CTAATAAAAA ATTTTATTTT CGTATACAAA CTAATTATAA TTGTATTTTT TATGTAGAAT   
  
  
+ TATTAAACAA AATAATTTAG TAATACGTAA TAATAAAAAT AAATTTTTAT TATATTTAAT TTAATTTTAT   
  
  
+ TTTTTAATTA AGTTGGTATA TATATATATA AATAGGTAAG TTAATGTATA ATAATGCACA CGATCCCTGT   
  
  
+ ACGACCAGCC AGTACCCACA CGAATCAGTT TCAGTTCGAG TGGTATTTTC GGTTCTTTGT AACCCACCAC   
  
  
+ CAGAGTAAAA TAGATGATAA GGAGACACCT TAATTATTAA GTATATTATC TACAGATTAA GAATTAATTT   
  
  
+ AAAGTATAAC TTGGGTTAGA ATAGAAACAG AGAAGATGAC ATGGTTTACG AATTAATTTA ATTTTACTGG   
  
  
+ TTAATTATAA AAAATATTTT TTTTACTCAA ATGTCTCTAC AGTGCAAAAA CTTTCGTAAA AGGTGCAATG   
  
  
+ TACGTGGTTA AGCTAAGAAA ATGCAGAAAA GTTTTCCCTA AAAAAAAAAA AAAACAAAGA ATGTAACTTA   
  
  
+ ATTAAATAAG GTGCAATGTA CGTGGTTAAG CTAAGAAAAT GCAGAAAAGT TTTCCCTAAA AAAAAAAAAA   
  
  
+ AACAAAGAAT GTAACTTAAT TAAAAAACTT AGATCTGAGA ACGTACATTA AGTAACATGG TTCTTCCGAA   
  
  
+ TGAATAAGTA AGATTCATAA GTTTCTCCTT ACACTCGTTT TACCACCTCT ACATGGTATG ATAATAATTC   
  
  
+ AGAATTAGCA AAAGTTCTAA AAAAAACAG  

- GGCTGGCCGA CGACTTAGAT CGATCGAAAC CAAGAGATAC CCAGCAAACA GATTGCCTGA AATTGACCTA   
  
  
- ACAATAAAGC AGACTAAGTT CCTCTAGTTT ATCGAATCTT GCAAGCTTTG TGTAGAAACA GAATTCAAGT   
  
  
- TGGTCAAGCT GGCCTAGCAT GTGAAATGAA GAAAAGCGTG TGATATATAG AGCGGAAAGG TGCTGTTGGT   
  
  
- GGGAAACTTG GTGGAAATGA GACGGACACG GATAAGATGT TGCAGGCACA ACTCTATATA TAATGACTCT   
  
  
- GAGTCTATCT AAAGATCCAC GTGGTGGGAT TTGTTGTATA GTTTATTAAT AAGACAATTA ACTGTTTCCC   
  
  
- AGTTTGGTGA GTGTTGGGAG GAGCCATGTG AAGAAGGAAG AAGTTTTCTT TTCTTTTTTT TTCACCGAAT   
  
  
- CTGAATTAGT GTTAACTTTT TTAAAAGTAA AAGAAAAAAA ATATACATTT TTATAATTTA TTGTATTAAT   
  
  
- TTACTAAAAT AAATATACTG AATTAGTGTT AACTTTTTTA AAAGTAAAAG AAAAAAAATA TACATTTTTA   
  
  
- TAATTTATTG TATTAATTTA CTAAAATAAA TATATTAAAT TATAATTATT AATTAATTTT TCTTTTTATA   
  
  
- TTAACATAAG AAATTATAAA ATTATTTTTT ATTTTTACTC AAAAAATTAA AATAAATTCA AATAAATAGA   
  
  
- AAATGTTCGT GATTATTTTT TAAAATAAAA GCATATGTTT GATTAATATT AACATAAAAA ATACATCTTA   
  
  
- ATAATTTGTT TTATTAAATC ATTATGCATT ATTATTTTTA TTTAAAAATA ATATAAATTA AATTAAAATA   
  
  
- AAAAATTAAT TCAACCATAT ATATATATAT TTATCCATTC AATTACATAT TATTACGTGT GCTAGGGACA   
  
  
- TGCTGGTCGG TCATGGGTGT GCTTAGTCAA AGTCAAGCTC ACCATAAAAG CCAAGAAACA TTGGGTGGTG   
  
  
- GTCTCATTTT ATCTACTATT CCTCTGTGGA ATTAATAATT CATATAATAG ATGTCTAATT CTTAATTAAA   
  
  
- TTTCATATTG AACCCAATCT TATCTTTGTC TCTTCTACTG TACCAAATGC TTAATTAAAT TAAAATGACC   
  
  
- AATTAATATT TTTTATAAAA AAAATGAGTT TACAGAGATG TCACGTTTTT GAAAGCATTT TCCACGTTAC   
  
  
- ATGCACCAAT TCGATTCTTT TACGTCTTTT CAAAAGGGAT TTTTTTTTTT TTTTGTTTCT TACATTGAAT   
  
  
- TAATTTATTC CACGTTACAT GCACCAATTC GATTCTTTTA CGTCTTTTCA AAAGGGATTT TTTTTTTTTT   
  
  
- TTGTTTCTTA CATTGAATTA ATTTTTTGAA TCTAGACTCT TGCATGTAAT TCATTGTACC AAGAAGGCTT   
  
  
- ACTTATTCAT TCTAAGTATT CAAAGAGGAA TGTGAGCAAA ATGGTGGAGA TGTACCATAC TATTATTAAG   
  
  
- TCTTAATCGT TTTCAAGATT TTTTTTGTC

+     G-box

| Site Name | Organism | Position | Strand | Matrix score. | sequence | function |
| --- | --- | --- | --- | --- | --- | --- |
| G-box | Daucus carota | 1191 | + | 6 | TACGTG | cis-acting regulatory element involved in light responsiveness |
| G-box | Daucus carota | 1279 | + | 6 | TACGTG | cis-acting regulatory element involved in light responsiveness |
| G-box | Zea mays | 200 | + | 6 | CACGAC | cis-acting regulatory element involved in light responsiveness |
| G-box | Oryza sativa | 1278 | + | 7 | GTACGTG | cis-acting regulatory element involved in light responsiveness |
| G-box | Oryza sativa | 1190 | + | 7 | GTACGTG | cis-acting regulatory element involved in light responsiveness |

> 2018/04/13 10:10:12  
+ CCGACCGGCT GCTGAATCTA GCTAGCTTTG GTTCTCTATG GGTCGTTTGT CTAACGGACT TTAACTGGAT   
  
  
+ TGTTATTTCG TCTGATTCAA GGAGATCAAA TAGCTTAGAA CGTTCGAAAC ACATCTTTGT CTTAAGTTCA   
  
  
+ ACCAGTTCGA CCGGATCGTA CACTTTACTT CTTTTCGCAC ACTATATATC TCGCCTTTCC ACGACAACCA   
  
  
+ CCCTTTGAAC CACCTTTACT CTGCCTGTGC CTATTCTACA ACGTCCGTGT TGAGATATAT ATTACTGAGA   
  
  
+ CTCAGATAGA TTTCTAGGTG CACCACCCTA AACAACATAT CAAATAATTA TTCTGTTAAT TGACAAAGGG   
  
  
+ TCAAACCACT CACAACCCTC CTCGGTACAC TTCTTCCTTC TTCAAAAGAA AAGAAAAAAA AAGTGGCTTA   
  
  
+ GACTTAATCA CAATTGAAAA AATTTTCATT TTCTTTTTTT TATATGTAAA AATATTAAAT AACATAATTA   
  
  
+ AATGATTTTA TTTATATGAC TTAATCACAA TTGAAAAAAT TTTCATTTTC TTTTTTTTAT ATGTAAAAAT   
  
  
+ ATTAAATAAC ATAATTAAAT GATTTTATTT ATATAATTTA ATATTAATAA TTAATTAAAA AGAAAAATAT   
  
  
+ AATTGTATTC TTTAATATTT TAATAAAAAA TAAAAATGAG TTTTTTAATT TTATTTAAGT TTATTTATCT   
  
  
+ TTTACAAGCA CTAATAAAAA ATTTTATTTT CGTATACAAA CTAATTATAA TTGTATTTTT TATGTAGAAT   
  
  
+ TATTAAACAA AATAATTTAG TAATACGTAA TAATAAAAAT AAATTTTTAT TATATTTAAT TTAATTTTAT   
  
  
+ TTTTTAATTA AGTTGGTATA TATATATATA AATAGGTAAG TTAATGTATA ATAATGCACA CGATCCCTGT   
  
  
+ ACGACCAGCC AGTACCCACA CGAATCAGTT TCAGTTCGAG TGGTATTTTC GGTTCTTTGT AACCCACCAC   
  
  
+ CAGAGTAAAA TAGATGATAA GGAGACACCT TAATTATTAA GTATATTATC TACAGATTAA GAATTAATTT   
  
  
+ AAAGTATAAC TTGGGTTAGA ATAGAAACAG AGAAGATGAC ATGGTTTACG AATTAATTTA ATTTTACTGG   
  
  
+ TTAATTATAA AAAATATTTT TTTTACTCAA ATGTCTCTAC AGTGCAAAAA CTTTCGTAAA AGGTGCAATG   
  
  
+ TACGTGGTTA AGCTAAGAAA ATGCAGAAAA GTTTTCCCTA AAAAAAAAAA AAAACAAAGA ATGTAACTTA   
  
  
+ ATTAAATAAG GTGCAATGTA CGTGGTTAAG CTAAGAAAAT GCAGAAAAGT TTTCCCTAAA AAAAAAAAAA   
  
  
+ AACAAAGAAT GTAACTTAAT TAAAAAACTT AGATCTGAGA ACGTACATTA AGTAACATGG TTCTTCCGAA   
  
  
+ TGAATAAGTA AGATTCATAA GTTTCTCCTT ACACTCGTTT TACCACCTCT ACATGGTATG ATAATAATTC   
  
  
+ AGAATTAGCA AAAGTTCTAA AAAAAACAG  

- GGCTGGCCGA CGACTTAGAT CGATCGAAAC CAAGAGATAC CCAGCAAACA GATTGCCTGA AATTGACCTA   
  
  
- ACAATAAAGC AGACTAAGTT CCTCTAGTTT ATCGAATCTT GCAAGCTTTG TGTAGAAACA GAATTCAAGT   
  
  
- TGGTCAAGCT GGCCTAGCAT GTGAAATGAA GAAAAGCGTG TGATATATAG AGCGGAAAGG TGCTGTTGGT   
  
  
- GGGAAACTTG GTGGAAATGA GACGGACACG GATAAGATGT TGCAGGCACA ACTCTATATA TAATGACTCT   
  
  
- GAGTCTATCT AAAGATCCAC GTGGTGGGAT TTGTTGTATA GTTTATTAAT AAGACAATTA ACTGTTTCCC   
  
  
- AGTTTGGTGA GTGTTGGGAG GAGCCATGTG AAGAAGGAAG AAGTTTTCTT TTCTTTTTTT TTCACCGAAT   
  
  
- CTGAATTAGT GTTAACTTTT TTAAAAGTAA AAGAAAAAAA ATATACATTT TTATAATTTA TTGTATTAAT   
  
  
- TTACTAAAAT AAATATACTG AATTAGTGTT AACTTTTTTA AAAGTAAAAG AAAAAAAATA TACATTTTTA   
  
  
- TAATTTATTG TATTAATTTA CTAAAATAAA TATATTAAAT TATAATTATT AATTAATTTT TCTTTTTATA   
  
  
- TTAACATAAG AAATTATAAA ATTATTTTTT ATTTTTACTC AAAAAATTAA AATAAATTCA AATAAATAGA   
  
  
- AAATGTTCGT GATTATTTTT TAAAATAAAA GCATATGTTT GATTAATATT AACATAAAAA ATACATCTTA   
  
  
- ATAATTTGTT TTATTAAATC ATTATGCATT ATTATTTTTA TTTAAAAATA ATATAAATTA AATTAAAATA   
  
  
- AAAAATTAAT TCAACCATAT ATATATATAT TTATCCATTC AATTACATAT TATTACGTGT GCTAGGGACA   
  
  
- TGCTGGTCGG TCATGGGTGT GCTTAGTCAA AGTCAAGCTC ACCATAAAAG CCAAGAAACA TTGGGTGGTG   
  
  
- GTCTCATTTT ATCTACTATT CCTCTGTGGA ATTAATAATT CATATAATAG ATGTCTAATT CTTAATTAAA   
  
  
- TTTCATATTG AACCCAATCT TATCTTTGTC TCTTCTACTG TACCAAATGC TTAATTAAAT TAAAATGACC   
  
  
- AATTAATATT TTTTATAAAA AAAATGAGTT TACAGAGATG TCACGTTTTT GAAAGCATTT TCCACGTTAC   
  
  
- ATGCACCAAT TCGATTCTTT TACGTCTTTT CAAAAGGGAT TTTTTTTTTT TTTTGTTTCT TACATTGAAT   
  
  
- TAATTTATTC CACGTTACAT GCACCAATTC GATTCTTTTA CGTCTTTTCA AAAGGGATTT TTTTTTTTTT   
  
  
- TTGTTTCTTA CATTGAATTA ATTTTTTGAA TCTAGACTCT TGCATGTAAT TCATTGTACC AAGAAGGCTT   
  
  
- ACTTATTCAT TCTAAGTATT CAAAGAGGAA TGTGAGCAAA ATGGTGGAGA TGTACCATAC TATTATTAAG   
  
  
- TCTTAATCGT TTTCAAGATT TTTTTTGTC

+     GA-motif

| Site Name | Organism | Position | Strand | Matrix score. | sequence | function |
| --- | --- | --- | --- | --- | --- | --- |
| GA-motif | Glycine max | 382 | - | 8 | AAGGAAGA | part of a light responsive element |

> 2018/04/13 10:10:12  
+ CCGACCGGCT GCTGAATCTA GCTAGCTTTG GTTCTCTATG GGTCGTTTGT CTAACGGACT TTAACTGGAT   
  
  
+ TGTTATTTCG TCTGATTCAA GGAGATCAAA TAGCTTAGAA CGTTCGAAAC ACATCTTTGT CTTAAGTTCA   
  
  
+ ACCAGTTCGA CCGGATCGTA CACTTTACTT CTTTTCGCAC ACTATATATC TCGCCTTTCC ACGACAACCA   
  
  
+ CCCTTTGAAC CACCTTTACT CTGCCTGTGC CTATTCTACA ACGTCCGTGT TGAGATATAT ATTACTGAGA   
  
  
+ CTCAGATAGA TTTCTAGGTG CACCACCCTA AACAACATAT CAAATAATTA TTCTGTTAAT TGACAAAGGG   
  
  
+ TCAAACCACT CACAACCCTC CTCGGTACAC TTCTTCCTTC TTCAAAAGAA AAGAAAAAAA AAGTGGCTTA   
  
  
+ GACTTAATCA CAATTGAAAA AATTTTCATT TTCTTTTTTT TATATGTAAA AATATTAAAT AACATAATTA   
  
  
+ AATGATTTTA TTTATATGAC TTAATCACAA TTGAAAAAAT TTTCATTTTC TTTTTTTTAT ATGTAAAAAT   
  
  
+ ATTAAATAAC ATAATTAAAT GATTTTATTT ATATAATTTA ATATTAATAA TTAATTAAAA AGAAAAATAT   
  
  
+ AATTGTATTC TTTAATATTT TAATAAAAAA TAAAAATGAG TTTTTTAATT TTATTTAAGT TTATTTATCT   
  
  
+ TTTACAAGCA CTAATAAAAA ATTTTATTTT CGTATACAAA CTAATTATAA TTGTATTTTT TATGTAGAAT   
  
  
+ TATTAAACAA AATAATTTAG TAATACGTAA TAATAAAAAT AAATTTTTAT TATATTTAAT TTAATTTTAT   
  
  
+ TTTTTAATTA AGTTGGTATA TATATATATA AATAGGTAAG TTAATGTATA ATAATGCACA CGATCCCTGT   
  
  
+ ACGACCAGCC AGTACCCACA CGAATCAGTT TCAGTTCGAG TGGTATTTTC GGTTCTTTGT AACCCACCAC   
  
  
+ CAGAGTAAAA TAGATGATAA GGAGACACCT TAATTATTAA GTATATTATC TACAGATTAA GAATTAATTT   
  
  
+ AAAGTATAAC TTGGGTTAGA ATAGAAACAG AGAAGATGAC ATGGTTTACG AATTAATTTA ATTTTACTGG   
  
  
+ TTAATTATAA AAAATATTTT TTTTACTCAA ATGTCTCTAC AGTGCAAAAA CTTTCGTAAA AGGTGCAATG   
  
  
+ TACGTGGTTA AGCTAAGAAA ATGCAGAAAA GTTTTCCCTA AAAAAAAAAA AAAACAAAGA ATGTAACTTA   
  
  
+ ATTAAATAAG GTGCAATGTA CGTGGTTAAG CTAAGAAAAT GCAGAAAAGT TTTCCCTAAA AAAAAAAAAA   
  
  
+ AACAAAGAAT GTAACTTAAT TAAAAAACTT AGATCTGAGA ACGTACATTA AGTAACATGG TTCTTCCGAA   
  
  
+ TGAATAAGTA AGATTCATAA GTTTCTCCTT ACACTCGTTT TACCACCTCT ACATGGTATG ATAATAATTC   
  
  
+ AGAATTAGCA AAAGTTCTAA AAAAAACAG  

- GGCTGGCCGA CGACTTAGAT CGATCGAAAC CAAGAGATAC CCAGCAAACA GATTGCCTGA AATTGACCTA   
  
  
- ACAATAAAGC AGACTAAGTT CCTCTAGTTT ATCGAATCTT GCAAGCTTTG TGTAGAAACA GAATTCAAGT   
  
  
- TGGTCAAGCT GGCCTAGCAT GTGAAATGAA GAAAAGCGTG TGATATATAG AGCGGAAAGG TGCTGTTGGT   
  
  
- GGGAAACTTG GTGGAAATGA GACGGACACG GATAAGATGT TGCAGGCACA ACTCTATATA TAATGACTCT   
  
  
- GAGTCTATCT AAAGATCCAC GTGGTGGGAT TTGTTGTATA GTTTATTAAT AAGACAATTA ACTGTTTCCC   
  
  
- AGTTTGGTGA GTGTTGGGAG GAGCCATGTG AAGAAGGAAG AAGTTTTCTT TTCTTTTTTT TTCACCGAAT   
  
  
- CTGAATTAGT GTTAACTTTT TTAAAAGTAA AAGAAAAAAA ATATACATTT TTATAATTTA TTGTATTAAT   
  
  
- TTACTAAAAT AAATATACTG AATTAGTGTT AACTTTTTTA AAAGTAAAAG AAAAAAAATA TACATTTTTA   
  
  
- TAATTTATTG TATTAATTTA CTAAAATAAA TATATTAAAT TATAATTATT AATTAATTTT TCTTTTTATA   
  
  
- TTAACATAAG AAATTATAAA ATTATTTTTT ATTTTTACTC AAAAAATTAA AATAAATTCA AATAAATAGA   
  
  
- AAATGTTCGT GATTATTTTT TAAAATAAAA GCATATGTTT GATTAATATT AACATAAAAA ATACATCTTA   
  
  
- ATAATTTGTT TTATTAAATC ATTATGCATT ATTATTTTTA TTTAAAAATA ATATAAATTA AATTAAAATA   
  
  
- AAAAATTAAT TCAACCATAT ATATATATAT TTATCCATTC AATTACATAT TATTACGTGT GCTAGGGACA   
  
  
- TGCTGGTCGG TCATGGGTGT GCTTAGTCAA AGTCAAGCTC ACCATAAAAG CCAAGAAACA TTGGGTGGTG   
  
  
- GTCTCATTTT ATCTACTATT CCTCTGTGGA ATTAATAATT CATATAATAG ATGTCTAATT CTTAATTAAA   
  
  
- TTTCATATTG AACCCAATCT TATCTTTGTC TCTTCTACTG TACCAAATGC TTAATTAAAT TAAAATGACC   
  
  
- AATTAATATT TTTTATAAAA AAAATGAGTT TACAGAGATG TCACGTTTTT GAAAGCATTT TCCACGTTAC   
  
  
- ATGCACCAAT TCGATTCTTT TACGTCTTTT CAAAAGGGAT TTTTTTTTTT TTTTGTTTCT TACATTGAAT   
  
  
- TAATTTATTC CACGTTACAT GCACCAATTC GATTCTTTTA CGTCTTTTCA AAAGGGATTT TTTTTTTTTT   
  
  
- TTGTTTCTTA CATTGAATTA ATTTTTTGAA TCTAGACTCT TGCATGTAAT TCATTGTACC AAGAAGGCTT   
  
  
- ACTTATTCAT TCTAAGTATT CAAAGAGGAA TGTGAGCAAA ATGGTGGAGA TGTACCATAC TATTATTAAG   
  
  
- TCTTAATCGT TTTCAAGATT TTTTTTGTC

+     GARE-motif

| Site Name | Organism | Position | Strand | Matrix score. | sequence | function |
| --- | --- | --- | --- | --- | --- | --- |
| GARE-motif | Brassica oleracea | 1075 | + | 7 | AAACAGA | gibberellin-responsive element |

> 2018/04/13 10:10:12  
+ CCGACCGGCT GCTGAATCTA GCTAGCTTTG GTTCTCTATG GGTCGTTTGT CTAACGGACT TTAACTGGAT   
  
  
+ TGTTATTTCG TCTGATTCAA GGAGATCAAA TAGCTTAGAA CGTTCGAAAC ACATCTTTGT CTTAAGTTCA   
  
  
+ ACCAGTTCGA CCGGATCGTA CACTTTACTT CTTTTCGCAC ACTATATATC TCGCCTTTCC ACGACAACCA   
  
  
+ CCCTTTGAAC CACCTTTACT CTGCCTGTGC CTATTCTACA ACGTCCGTGT TGAGATATAT ATTACTGAGA   
  
  
+ CTCAGATAGA TTTCTAGGTG CACCACCCTA AACAACATAT CAAATAATTA TTCTGTTAAT TGACAAAGGG   
  
  
+ TCAAACCACT CACAACCCTC CTCGGTACAC TTCTTCCTTC TTCAAAAGAA AAGAAAAAAA AAGTGGCTTA   
  
  
+ GACTTAATCA CAATTGAAAA AATTTTCATT TTCTTTTTTT TATATGTAAA AATATTAAAT AACATAATTA   
  
  
+ AATGATTTTA TTTATATGAC TTAATCACAA TTGAAAAAAT TTTCATTTTC TTTTTTTTAT ATGTAAAAAT   
  
  
+ ATTAAATAAC ATAATTAAAT GATTTTATTT ATATAATTTA ATATTAATAA TTAATTAAAA AGAAAAATAT   
  
  
+ AATTGTATTC TTTAATATTT TAATAAAAAA TAAAAATGAG TTTTTTAATT TTATTTAAGT TTATTTATCT   
  
  
+ TTTACAAGCA CTAATAAAAA ATTTTATTTT CGTATACAAA CTAATTATAA TTGTATTTTT TATGTAGAAT   
  
  
+ TATTAAACAA AATAATTTAG TAATACGTAA TAATAAAAAT AAATTTTTAT TATATTTAAT TTAATTTTAT   
  
  
+ TTTTTAATTA AGTTGGTATA TATATATATA AATAGGTAAG TTAATGTATA ATAATGCACA CGATCCCTGT   
  
  
+ ACGACCAGCC AGTACCCACA CGAATCAGTT TCAGTTCGAG TGGTATTTTC GGTTCTTTGT AACCCACCAC   
  
  
+ CAGAGTAAAA TAGATGATAA GGAGACACCT TAATTATTAA GTATATTATC TACAGATTAA GAATTAATTT   
  
  
+ AAAGTATAAC TTGGGTTAGA ATAGAAACAG AGAAGATGAC ATGGTTTACG AATTAATTTA ATTTTACTGG   
  
  
+ TTAATTATAA AAAATATTTT TTTTACTCAA ATGTCTCTAC AGTGCAAAAA CTTTCGTAAA AGGTGCAATG   
  
  
+ TACGTGGTTA AGCTAAGAAA ATGCAGAAAA GTTTTCCCTA AAAAAAAAAA AAAACAAAGA ATGTAACTTA   
  
  
+ ATTAAATAAG GTGCAATGTA CGTGGTTAAG CTAAGAAAAT GCAGAAAAGT TTTCCCTAAA AAAAAAAAAA   
  
  
+ AACAAAGAAT GTAACTTAAT TAAAAAACTT AGATCTGAGA ACGTACATTA AGTAACATGG TTCTTCCGAA   
  
  
+ TGAATAAGTA AGATTCATAA GTTTCTCCTT ACACTCGTTT TACCACCTCT ACATGGTATG ATAATAATTC   
  
  
+ AGAATTAGCA AAAGTTCTAA AAAAAACAG  

- GGCTGGCCGA CGACTTAGAT CGATCGAAAC CAAGAGATAC CCAGCAAACA GATTGCCTGA AATTGACCTA   
  
  
- ACAATAAAGC AGACTAAGTT CCTCTAGTTT ATCGAATCTT GCAAGCTTTG TGTAGAAACA GAATTCAAGT   
  
  
- TGGTCAAGCT GGCCTAGCAT GTGAAATGAA GAAAAGCGTG TGATATATAG AGCGGAAAGG TGCTGTTGGT   
  
  
- GGGAAACTTG GTGGAAATGA GACGGACACG GATAAGATGT TGCAGGCACA ACTCTATATA TAATGACTCT   
  
  
- GAGTCTATCT AAAGATCCAC GTGGTGGGAT TTGTTGTATA GTTTATTAAT AAGACAATTA ACTGTTTCCC   
  
  
- AGTTTGGTGA GTGTTGGGAG GAGCCATGTG AAGAAGGAAG AAGTTTTCTT TTCTTTTTTT TTCACCGAAT   
  
  
- CTGAATTAGT GTTAACTTTT TTAAAAGTAA AAGAAAAAAA ATATACATTT TTATAATTTA TTGTATTAAT   
  
  
- TTACTAAAAT AAATATACTG AATTAGTGTT AACTTTTTTA AAAGTAAAAG AAAAAAAATA TACATTTTTA   
  
  
- TAATTTATTG TATTAATTTA CTAAAATAAA TATATTAAAT TATAATTATT AATTAATTTT TCTTTTTATA   
  
  
- TTAACATAAG AAATTATAAA ATTATTTTTT ATTTTTACTC AAAAAATTAA AATAAATTCA AATAAATAGA   
  
  
- AAATGTTCGT GATTATTTTT TAAAATAAAA GCATATGTTT GATTAATATT AACATAAAAA ATACATCTTA   
  
  
- ATAATTTGTT TTATTAAATC ATTATGCATT ATTATTTTTA TTTAAAAATA ATATAAATTA AATTAAAATA   
  
  
- AAAAATTAAT TCAACCATAT ATATATATAT TTATCCATTC AATTACATAT TATTACGTGT GCTAGGGACA   
  
  
- TGCTGGTCGG TCATGGGTGT GCTTAGTCAA AGTCAAGCTC ACCATAAAAG CCAAGAAACA TTGGGTGGTG   
  
  
- GTCTCATTTT ATCTACTATT CCTCTGTGGA ATTAATAATT CATATAATAG ATGTCTAATT CTTAATTAAA   
  
  
- TTTCATATTG AACCCAATCT TATCTTTGTC TCTTCTACTG TACCAAATGC TTAATTAAAT TAAAATGACC   
  
  
- AATTAATATT TTTTATAAAA AAAATGAGTT TACAGAGATG TCACGTTTTT GAAAGCATTT TCCACGTTAC   
  
  
- ATGCACCAAT TCGATTCTTT TACGTCTTTT CAAAAGGGAT TTTTTTTTTT TTTTGTTTCT TACATTGAAT   
  
  
- TAATTTATTC CACGTTACAT GCACCAATTC GATTCTTTTA CGTCTTTTCA AAAGGGATTT TTTTTTTTTT   
  
  
- TTGTTTCTTA CATTGAATTA ATTTTTTGAA TCTAGACTCT TGCATGTAAT TCATTGTACC AAGAAGGCTT   
  
  
- ACTTATTCAT TCTAAGTATT CAAAGAGGAA TGTGAGCAAA ATGGTGGAGA TGTACCATAC TATTATTAAG   
  
  
- TCTTAATCGT TTTCAAGATT TTTTTTGTC

+     GT1-motif

| Site Name | Organism | Position | Strand | Matrix score. | sequence | function |
| --- | --- | --- | --- | --- | --- | --- |
| GT1-motif | Avena sativa | 1119 | + | 7 | GGTTAAT | light responsive element |
| GT1-motif | Arabidopsis thaliana | 1196 | + | 6 | GGTTAA | light responsive element |
| GT1-motif | Arabidopsis thaliana | 1284 | + | 6 | GGTTAA | light responsive element |

> 2018/04/13 10:10:12  
+ CCGACCGGCT GCTGAATCTA GCTAGCTTTG GTTCTCTATG GGTCGTTTGT CTAACGGACT TTAACTGGAT   
  
  
+ TGTTATTTCG TCTGATTCAA GGAGATCAAA TAGCTTAGAA CGTTCGAAAC ACATCTTTGT CTTAAGTTCA   
  
  
+ ACCAGTTCGA CCGGATCGTA CACTTTACTT CTTTTCGCAC ACTATATATC TCGCCTTTCC ACGACAACCA   
  
  
+ CCCTTTGAAC CACCTTTACT CTGCCTGTGC CTATTCTACA ACGTCCGTGT TGAGATATAT ATTACTGAGA   
  
  
+ CTCAGATAGA TTTCTAGGTG CACCACCCTA AACAACATAT CAAATAATTA TTCTGTTAAT TGACAAAGGG   
  
  
+ TCAAACCACT CACAACCCTC CTCGGTACAC TTCTTCCTTC TTCAAAAGAA AAGAAAAAAA AAGTGGCTTA   
  
  
+ GACTTAATCA CAATTGAAAA AATTTTCATT TTCTTTTTTT TATATGTAAA AATATTAAAT AACATAATTA   
  
  
+ AATGATTTTA TTTATATGAC TTAATCACAA TTGAAAAAAT TTTCATTTTC TTTTTTTTAT ATGTAAAAAT   
  
  
+ ATTAAATAAC ATAATTAAAT GATTTTATTT ATATAATTTA ATATTAATAA TTAATTAAAA AGAAAAATAT   
  
  
+ AATTGTATTC TTTAATATTT TAATAAAAAA TAAAAATGAG TTTTTTAATT TTATTTAAGT TTATTTATCT   
  
  
+ TTTACAAGCA CTAATAAAAA ATTTTATTTT CGTATACAAA CTAATTATAA TTGTATTTTT TATGTAGAAT   
  
  
+ TATTAAACAA AATAATTTAG TAATACGTAA TAATAAAAAT AAATTTTTAT TATATTTAAT TTAATTTTAT   
  
  
+ TTTTTAATTA AGTTGGTATA TATATATATA AATAGGTAAG TTAATGTATA ATAATGCACA CGATCCCTGT   
  
  
+ ACGACCAGCC AGTACCCACA CGAATCAGTT TCAGTTCGAG TGGTATTTTC GGTTCTTTGT AACCCACCAC   
  
  
+ CAGAGTAAAA TAGATGATAA GGAGACACCT TAATTATTAA GTATATTATC TACAGATTAA GAATTAATTT   
  
  
+ AAAGTATAAC TTGGGTTAGA ATAGAAACAG AGAAGATGAC ATGGTTTACG AATTAATTTA ATTTTACTGG   
  
  
+ TTAATTATAA AAAATATTTT TTTTACTCAA ATGTCTCTAC AGTGCAAAAA CTTTCGTAAA AGGTGCAATG   
  
  
+ TACGTGGTTA AGCTAAGAAA ATGCAGAAAA GTTTTCCCTA AAAAAAAAAA AAAACAAAGA ATGTAACTTA   
  
  
+ ATTAAATAAG GTGCAATGTA CGTGGTTAAG CTAAGAAAAT GCAGAAAAGT TTTCCCTAAA AAAAAAAAAA   
  
  
+ AACAAAGAAT GTAACTTAAT TAAAAAACTT AGATCTGAGA ACGTACATTA AGTAACATGG TTCTTCCGAA   
  
  
+ TGAATAAGTA AGATTCATAA GTTTCTCCTT ACACTCGTTT TACCACCTCT ACATGGTATG ATAATAATTC   
  
  
+ AGAATTAGCA AAAGTTCTAA AAAAAACAG  

- GGCTGGCCGA CGACTTAGAT CGATCGAAAC CAAGAGATAC CCAGCAAACA GATTGCCTGA AATTGACCTA   
  
  
- ACAATAAAGC AGACTAAGTT CCTCTAGTTT ATCGAATCTT GCAAGCTTTG TGTAGAAACA GAATTCAAGT   
  
  
- TGGTCAAGCT GGCCTAGCAT GTGAAATGAA GAAAAGCGTG TGATATATAG AGCGGAAAGG TGCTGTTGGT   
  
  
- GGGAAACTTG GTGGAAATGA GACGGACACG GATAAGATGT TGCAGGCACA ACTCTATATA TAATGACTCT   
  
  
- GAGTCTATCT AAAGATCCAC GTGGTGGGAT TTGTTGTATA GTTTATTAAT AAGACAATTA ACTGTTTCCC   
  
  
- AGTTTGGTGA GTGTTGGGAG GAGCCATGTG AAGAAGGAAG AAGTTTTCTT TTCTTTTTTT TTCACCGAAT   
  
  
- CTGAATTAGT GTTAACTTTT TTAAAAGTAA AAGAAAAAAA ATATACATTT TTATAATTTA TTGTATTAAT   
  
  
- TTACTAAAAT AAATATACTG AATTAGTGTT AACTTTTTTA AAAGTAAAAG AAAAAAAATA TACATTTTTA   
  
  
- TAATTTATTG TATTAATTTA CTAAAATAAA TATATTAAAT TATAATTATT AATTAATTTT TCTTTTTATA   
  
  
- TTAACATAAG AAATTATAAA ATTATTTTTT ATTTTTACTC AAAAAATTAA AATAAATTCA AATAAATAGA   
  
  
- AAATGTTCGT GATTATTTTT TAAAATAAAA GCATATGTTT GATTAATATT AACATAAAAA ATACATCTTA   
  
  
- ATAATTTGTT TTATTAAATC ATTATGCATT ATTATTTTTA TTTAAAAATA ATATAAATTA AATTAAAATA   
  
  
- AAAAATTAAT TCAACCATAT ATATATATAT TTATCCATTC AATTACATAT TATTACGTGT GCTAGGGACA   
  
  
- TGCTGGTCGG TCATGGGTGT GCTTAGTCAA AGTCAAGCTC ACCATAAAAG CCAAGAAACA TTGGGTGGTG   
  
  
- GTCTCATTTT ATCTACTATT CCTCTGTGGA ATTAATAATT CATATAATAG ATGTCTAATT CTTAATTAAA   
  
  
- TTTCATATTG AACCCAATCT TATCTTTGTC TCTTCTACTG TACCAAATGC TTAATTAAAT TAAAATGACC   
  
  
- AATTAATATT TTTTATAAAA AAAATGAGTT TACAGAGATG TCACGTTTTT GAAAGCATTT TCCACGTTAC   
  
  
- ATGCACCAAT TCGATTCTTT TACGTCTTTT CAAAAGGGAT TTTTTTTTTT TTTTGTTTCT TACATTGAAT   
  
  
- TAATTTATTC CACGTTACAT GCACCAATTC GATTCTTTTA CGTCTTTTCA AAAGGGATTT TTTTTTTTTT   
  
  
- TTGTTTCTTA CATTGAATTA ATTTTTTGAA TCTAGACTCT TGCATGTAAT TCATTGTACC AAGAAGGCTT   
  
  
- ACTTATTCAT TCTAAGTATT CAAAGAGGAA TGTGAGCAAA ATGGTGGAGA TGTACCATAC TATTATTAAG   
  
  
- TCTTAATCGT TTTCAAGATT TTTTTTGTC

+     HSE

| Site Name | Organism | Position | Strand | Matrix score. | sequence | function |
| --- | --- | --- | --- | --- | --- | --- |
| HSE | Brassica oleracea | 437 | + | 9 | AAAAAATTTC | cis-acting element involved in heat stress responsiveness |
| HSE | Brassica oleracea | 716 | + | 9 | AAAAAATTTC | cis-acting element involved in heat stress responsiveness |
| HSE | Brassica oleracea | 438 | + | 9 | AAAAAATTTC | cis-acting element involved in heat stress responsiveness |
| HSE | Brassica oleracea | 1166 | + | 9 | AAAAAATTTC | cis-acting element involved in heat stress responsiveness |
| HSE | Brassica oleracea | 524 | + | 9 | AAAAAATTTC | cis-acting element involved in heat stress responsiveness |
| HSE | Brassica oleracea | 525 | + | 9 | AAAAAATTTC | cis-acting element involved in heat stress responsiveness |

> 2018/04/13 10:10:12  
+ CCGACCGGCT GCTGAATCTA GCTAGCTTTG GTTCTCTATG GGTCGTTTGT CTAACGGACT TTAACTGGAT   
  
  
+ TGTTATTTCG TCTGATTCAA GGAGATCAAA TAGCTTAGAA CGTTCGAAAC ACATCTTTGT CTTAAGTTCA   
  
  
+ ACCAGTTCGA CCGGATCGTA CACTTTACTT CTTTTCGCAC ACTATATATC TCGCCTTTCC ACGACAACCA   
  
  
+ CCCTTTGAAC CACCTTTACT CTGCCTGTGC CTATTCTACA ACGTCCGTGT TGAGATATAT ATTACTGAGA   
  
  
+ CTCAGATAGA TTTCTAGGTG CACCACCCTA AACAACATAT CAAATAATTA TTCTGTTAAT TGACAAAGGG   
  
  
+ TCAAACCACT CACAACCCTC CTCGGTACAC TTCTTCCTTC TTCAAAAGAA AAGAAAAAAA AAGTGGCTTA   
  
  
+ GACTTAATCA CAATTGAAAA AATTTTCATT TTCTTTTTTT TATATGTAAA AATATTAAAT AACATAATTA   
  
  
+ AATGATTTTA TTTATATGAC TTAATCACAA TTGAAAAAAT TTTCATTTTC TTTTTTTTAT ATGTAAAAAT   
  
  
+ ATTAAATAAC ATAATTAAAT GATTTTATTT ATATAATTTA ATATTAATAA TTAATTAAAA AGAAAAATAT   
  
  
+ AATTGTATTC TTTAATATTT TAATAAAAAA TAAAAATGAG TTTTTTAATT TTATTTAAGT TTATTTATCT   
  
  
+ TTTACAAGCA CTAATAAAAA ATTTTATTTT CGTATACAAA CTAATTATAA TTGTATTTTT TATGTAGAAT   
  
  
+ TATTAAACAA AATAATTTAG TAATACGTAA TAATAAAAAT AAATTTTTAT TATATTTAAT TTAATTTTAT   
  
  
+ TTTTTAATTA AGTTGGTATA TATATATATA AATAGGTAAG TTAATGTATA ATAATGCACA CGATCCCTGT   
  
  
+ ACGACCAGCC AGTACCCACA CGAATCAGTT TCAGTTCGAG TGGTATTTTC GGTTCTTTGT AACCCACCAC   
  
  
+ CAGAGTAAAA TAGATGATAA GGAGACACCT TAATTATTAA GTATATTATC TACAGATTAA GAATTAATTT   
  
  
+ AAAGTATAAC TTGGGTTAGA ATAGAAACAG AGAAGATGAC ATGGTTTACG AATTAATTTA ATTTTACTGG   
  
  
+ TTAATTATAA AAAATATTTT TTTTACTCAA ATGTCTCTAC AGTGCAAAAA CTTTCGTAAA AGGTGCAATG   
  
  
+ TACGTGGTTA AGCTAAGAAA ATGCAGAAAA GTTTTCCCTA AAAAAAAAAA AAAACAAAGA ATGTAACTTA   
  
  
+ ATTAAATAAG GTGCAATGTA CGTGGTTAAG CTAAGAAAAT GCAGAAAAGT TTTCCCTAAA AAAAAAAAAA   
  
  
+ AACAAAGAAT GTAACTTAAT TAAAAAACTT AGATCTGAGA ACGTACATTA AGTAACATGG TTCTTCCGAA   
  
  
+ TGAATAAGTA AGATTCATAA GTTTCTCCTT ACACTCGTTT TACCACCTCT ACATGGTATG ATAATAATTC   
  
  
+ AGAATTAGCA AAAGTTCTAA AAAAAACAG  

- GGCTGGCCGA CGACTTAGAT CGATCGAAAC CAAGAGATAC CCAGCAAACA GATTGCCTGA AATTGACCTA   
  
  
- ACAATAAAGC AGACTAAGTT CCTCTAGTTT ATCGAATCTT GCAAGCTTTG TGTAGAAACA GAATTCAAGT   
  
  
- TGGTCAAGCT GGCCTAGCAT GTGAAATGAA GAAAAGCGTG TGATATATAG AGCGGAAAGG TGCTGTTGGT   
  
  
- GGGAAACTTG GTGGAAATGA GACGGACACG GATAAGATGT TGCAGGCACA ACTCTATATA TAATGACTCT   
  
  
- GAGTCTATCT AAAGATCCAC GTGGTGGGAT TTGTTGTATA GTTTATTAAT AAGACAATTA ACTGTTTCCC   
  
  
- AGTTTGGTGA GTGTTGGGAG GAGCCATGTG AAGAAGGAAG AAGTTTTCTT TTCTTTTTTT TTCACCGAAT   
  
  
- CTGAATTAGT GTTAACTTTT TTAAAAGTAA AAGAAAAAAA ATATACATTT TTATAATTTA TTGTATTAAT   
  
  
- TTACTAAAAT AAATATACTG AATTAGTGTT AACTTTTTTA AAAGTAAAAG AAAAAAAATA TACATTTTTA   
  
  
- TAATTTATTG TATTAATTTA CTAAAATAAA TATATTAAAT TATAATTATT AATTAATTTT TCTTTTTATA   
  
  
- TTAACATAAG AAATTATAAA ATTATTTTTT ATTTTTACTC AAAAAATTAA AATAAATTCA AATAAATAGA   
  
  
- AAATGTTCGT GATTATTTTT TAAAATAAAA GCATATGTTT GATTAATATT AACATAAAAA ATACATCTTA   
  
  
- ATAATTTGTT TTATTAAATC ATTATGCATT ATTATTTTTA TTTAAAAATA ATATAAATTA AATTAAAATA   
  
  
- AAAAATTAAT TCAACCATAT ATATATATAT TTATCCATTC AATTACATAT TATTACGTGT GCTAGGGACA   
  
  
- TGCTGGTCGG TCATGGGTGT GCTTAGTCAA AGTCAAGCTC ACCATAAAAG CCAAGAAACA TTGGGTGGTG   
  
  
- GTCTCATTTT ATCTACTATT CCTCTGTGGA ATTAATAATT CATATAATAG ATGTCTAATT CTTAATTAAA   
  
  
- TTTCATATTG AACCCAATCT TATCTTTGTC TCTTCTACTG TACCAAATGC TTAATTAAAT TAAAATGACC   
  
  
- AATTAATATT TTTTATAAAA AAAATGAGTT TACAGAGATG TCACGTTTTT GAAAGCATTT TCCACGTTAC   
  
  
- ATGCACCAAT TCGATTCTTT TACGTCTTTT CAAAAGGGAT TTTTTTTTTT TTTTGTTTCT TACATTGAAT   
  
  
- TAATTTATTC CACGTTACAT GCACCAATTC GATTCTTTTA CGTCTTTTCA AAAGGGATTT TTTTTTTTTT   
  
  
- TTGTTTCTTA CATTGAATTA ATTTTTTGAA TCTAGACTCT TGCATGTAAT TCATTGTACC AAGAAGGCTT   
  
  
- ACTTATTCAT TCTAAGTATT CAAAGAGGAA TGTGAGCAAA ATGGTGGAGA TGTACCATAC TATTATTAAG   
  
  
- TCTTAATCGT TTTCAAGATT TTTTTTGTC

+     I-box

| Site Name | Organism | Position | Strand | Matrix score. | sequence | function |
| --- | --- | --- | --- | --- | --- | --- |
| I-box | Solanum tuberosum | 1024 | + | 10 | TATTATCTAGA | part of a light responsive element |

> 2018/04/13 10:10:12  
+ CCGACCGGCT GCTGAATCTA GCTAGCTTTG GTTCTCTATG GGTCGTTTGT CTAACGGACT TTAACTGGAT   
  
  
+ TGTTATTTCG TCTGATTCAA GGAGATCAAA TAGCTTAGAA CGTTCGAAAC ACATCTTTGT CTTAAGTTCA   
  
  
+ ACCAGTTCGA CCGGATCGTA CACTTTACTT CTTTTCGCAC ACTATATATC TCGCCTTTCC ACGACAACCA   
  
  
+ CCCTTTGAAC CACCTTTACT CTGCCTGTGC CTATTCTACA ACGTCCGTGT TGAGATATAT ATTACTGAGA   
  
  
+ CTCAGATAGA TTTCTAGGTG CACCACCCTA AACAACATAT CAAATAATTA TTCTGTTAAT TGACAAAGGG   
  
  
+ TCAAACCACT CACAACCCTC CTCGGTACAC TTCTTCCTTC TTCAAAAGAA AAGAAAAAAA AAGTGGCTTA   
  
  
+ GACTTAATCA CAATTGAAAA AATTTTCATT TTCTTTTTTT TATATGTAAA AATATTAAAT AACATAATTA   
  
  
+ AATGATTTTA TTTATATGAC TTAATCACAA TTGAAAAAAT TTTCATTTTC TTTTTTTTAT ATGTAAAAAT   
  
  
+ ATTAAATAAC ATAATTAAAT GATTTTATTT ATATAATTTA ATATTAATAA TTAATTAAAA AGAAAAATAT   
  
  
+ AATTGTATTC TTTAATATTT TAATAAAAAA TAAAAATGAG TTTTTTAATT TTATTTAAGT TTATTTATCT   
  
  
+ TTTACAAGCA CTAATAAAAA ATTTTATTTT CGTATACAAA CTAATTATAA TTGTATTTTT TATGTAGAAT   
  
  
+ TATTAAACAA AATAATTTAG TAATACGTAA TAATAAAAAT AAATTTTTAT TATATTTAAT TTAATTTTAT   
  
  
+ TTTTTAATTA AGTTGGTATA TATATATATA AATAGGTAAG TTAATGTATA ATAATGCACA CGATCCCTGT   
  
  
+ ACGACCAGCC AGTACCCACA CGAATCAGTT TCAGTTCGAG TGGTATTTTC GGTTCTTTGT AACCCACCAC   
  
  
+ CAGAGTAAAA TAGATGATAA GGAGACACCT TAATTATTAA GTATATTATC TACAGATTAA GAATTAATTT   
  
  
+ AAAGTATAAC TTGGGTTAGA ATAGAAACAG AGAAGATGAC ATGGTTTACG AATTAATTTA ATTTTACTGG   
  
  
+ TTAATTATAA AAAATATTTT TTTTACTCAA ATGTCTCTAC AGTGCAAAAA CTTTCGTAAA AGGTGCAATG   
  
  
+ TACGTGGTTA AGCTAAGAAA ATGCAGAAAA GTTTTCCCTA AAAAAAAAAA AAAACAAAGA ATGTAACTTA   
  
  
+ ATTAAATAAG GTGCAATGTA CGTGGTTAAG CTAAGAAAAT GCAGAAAAGT TTTCCCTAAA AAAAAAAAAA   
  
  
+ AACAAAGAAT GTAACTTAAT TAAAAAACTT AGATCTGAGA ACGTACATTA AGTAACATGG TTCTTCCGAA   
  
  
+ TGAATAAGTA AGATTCATAA GTTTCTCCTT ACACTCGTTT TACCACCTCT ACATGGTATG ATAATAATTC   
  
  
+ AGAATTAGCA AAAGTTCTAA AAAAAACAG  

- GGCTGGCCGA CGACTTAGAT CGATCGAAAC CAAGAGATAC CCAGCAAACA GATTGCCTGA AATTGACCTA   
  
  
- ACAATAAAGC AGACTAAGTT CCTCTAGTTT ATCGAATCTT GCAAGCTTTG TGTAGAAACA GAATTCAAGT   
  
  
- TGGTCAAGCT GGCCTAGCAT GTGAAATGAA GAAAAGCGTG TGATATATAG AGCGGAAAGG TGCTGTTGGT   
  
  
- GGGAAACTTG GTGGAAATGA GACGGACACG GATAAGATGT TGCAGGCACA ACTCTATATA TAATGACTCT   
  
  
- GAGTCTATCT AAAGATCCAC GTGGTGGGAT TTGTTGTATA GTTTATTAAT AAGACAATTA ACTGTTTCCC   
  
  
- AGTTTGGTGA GTGTTGGGAG GAGCCATGTG AAGAAGGAAG AAGTTTTCTT TTCTTTTTTT TTCACCGAAT   
  
  
- CTGAATTAGT GTTAACTTTT TTAAAAGTAA AAGAAAAAAA ATATACATTT TTATAATTTA TTGTATTAAT   
  
  
- TTACTAAAAT AAATATACTG AATTAGTGTT AACTTTTTTA AAAGTAAAAG AAAAAAAATA TACATTTTTA   
  
  
- TAATTTATTG TATTAATTTA CTAAAATAAA TATATTAAAT TATAATTATT AATTAATTTT TCTTTTTATA   
  
  
- TTAACATAAG AAATTATAAA ATTATTTTTT ATTTTTACTC AAAAAATTAA AATAAATTCA AATAAATAGA   
  
  
- AAATGTTCGT GATTATTTTT TAAAATAAAA GCATATGTTT GATTAATATT AACATAAAAA ATACATCTTA   
  
  
- ATAATTTGTT TTATTAAATC ATTATGCATT ATTATTTTTA TTTAAAAATA ATATAAATTA AATTAAAATA   
  
  
- AAAAATTAAT TCAACCATAT ATATATATAT TTATCCATTC AATTACATAT TATTACGTGT GCTAGGGACA   
  
  
- TGCTGGTCGG TCATGGGTGT GCTTAGTCAA AGTCAAGCTC ACCATAAAAG CCAAGAAACA TTGGGTGGTG   
  
  
- GTCTCATTTT ATCTACTATT CCTCTGTGGA ATTAATAATT CATATAATAG ATGTCTAATT CTTAATTAAA   
  
  
- TTTCATATTG AACCCAATCT TATCTTTGTC TCTTCTACTG TACCAAATGC TTAATTAAAT TAAAATGACC   
  
  
- AATTAATATT TTTTATAAAA AAAATGAGTT TACAGAGATG TCACGTTTTT GAAAGCATTT TCCACGTTAC   
  
  
- ATGCACCAAT TCGATTCTTT TACGTCTTTT CAAAAGGGAT TTTTTTTTTT TTTTGTTTCT TACATTGAAT   
  
  
- TAATTTATTC CACGTTACAT GCACCAATTC GATTCTTTTA CGTCTTTTCA AAAGGGATTT TTTTTTTTTT   
  
  
- TTGTTTCTTA CATTGAATTA ATTTTTTGAA TCTAGACTCT TGCATGTAAT TCATTGTACC AAGAAGGCTT   
  
  
- ACTTATTCAT TCTAAGTATT CAAAGAGGAA TGTGAGCAAA ATGGTGGAGA TGTACCATAC TATTATTAAG   
  
  
- TCTTAATCGT TTTCAAGATT TTTTTTGTC

+     LTR

| Site Name | Organism | Position | Strand | Matrix score. | sequence | function |
| --- | --- | --- | --- | --- | --- | --- |
| LTR | Hordeum vulgare | 957 | - | 6 | CCGAAA | cis-acting element involved in low-temperature responsiveness |

> 2018/04/13 10:10:12  
+ CCGACCGGCT GCTGAATCTA GCTAGCTTTG GTTCTCTATG GGTCGTTTGT CTAACGGACT TTAACTGGAT   
  
  
+ TGTTATTTCG TCTGATTCAA GGAGATCAAA TAGCTTAGAA CGTTCGAAAC ACATCTTTGT CTTAAGTTCA   
  
  
+ ACCAGTTCGA CCGGATCGTA CACTTTACTT CTTTTCGCAC ACTATATATC TCGCCTTTCC ACGACAACCA   
  
  
+ CCCTTTGAAC CACCTTTACT CTGCCTGTGC CTATTCTACA ACGTCCGTGT TGAGATATAT ATTACTGAGA   
  
  
+ CTCAGATAGA TTTCTAGGTG CACCACCCTA AACAACATAT CAAATAATTA TTCTGTTAAT TGACAAAGGG   
  
  
+ TCAAACCACT CACAACCCTC CTCGGTACAC TTCTTCCTTC TTCAAAAGAA AAGAAAAAAA AAGTGGCTTA   
  
  
+ GACTTAATCA CAATTGAAAA AATTTTCATT TTCTTTTTTT TATATGTAAA AATATTAAAT AACATAATTA   
  
  
+ AATGATTTTA TTTATATGAC TTAATCACAA TTGAAAAAAT TTTCATTTTC TTTTTTTTAT ATGTAAAAAT   
  
  
+ ATTAAATAAC ATAATTAAAT GATTTTATTT ATATAATTTA ATATTAATAA TTAATTAAAA AGAAAAATAT   
  
  
+ AATTGTATTC TTTAATATTT TAATAAAAAA TAAAAATGAG TTTTTTAATT TTATTTAAGT TTATTTATCT   
  
  
+ TTTACAAGCA CTAATAAAAA ATTTTATTTT CGTATACAAA CTAATTATAA TTGTATTTTT TATGTAGAAT   
  
  
+ TATTAAACAA AATAATTTAG TAATACGTAA TAATAAAAAT AAATTTTTAT TATATTTAAT TTAATTTTAT   
  
  
+ TTTTTAATTA AGTTGGTATA TATATATATA AATAGGTAAG TTAATGTATA ATAATGCACA CGATCCCTGT   
  
  
+ ACGACCAGCC AGTACCCACA CGAATCAGTT TCAGTTCGAG TGGTATTTTC GGTTCTTTGT AACCCACCAC   
  
  
+ CAGAGTAAAA TAGATGATAA GGAGACACCT TAATTATTAA GTATATTATC TACAGATTAA GAATTAATTT   
  
  
+ AAAGTATAAC TTGGGTTAGA ATAGAAACAG AGAAGATGAC ATGGTTTACG AATTAATTTA ATTTTACTGG   
  
  
+ TTAATTATAA AAAATATTTT TTTTACTCAA ATGTCTCTAC AGTGCAAAAA CTTTCGTAAA AGGTGCAATG   
  
  
+ TACGTGGTTA AGCTAAGAAA ATGCAGAAAA GTTTTCCCTA AAAAAAAAAA AAAACAAAGA ATGTAACTTA   
  
  
+ ATTAAATAAG GTGCAATGTA CGTGGTTAAG CTAAGAAAAT GCAGAAAAGT TTTCCCTAAA AAAAAAAAAA   
  
  
+ AACAAAGAAT GTAACTTAAT TAAAAAACTT AGATCTGAGA ACGTACATTA AGTAACATGG TTCTTCCGAA   
  
  
+ TGAATAAGTA AGATTCATAA GTTTCTCCTT ACACTCGTTT TACCACCTCT ACATGGTATG ATAATAATTC   
  
  
+ AGAATTAGCA AAAGTTCTAA AAAAAACAG  

- GGCTGGCCGA CGACTTAGAT CGATCGAAAC CAAGAGATAC CCAGCAAACA GATTGCCTGA AATTGACCTA   
  
  
- ACAATAAAGC AGACTAAGTT CCTCTAGTTT ATCGAATCTT GCAAGCTTTG TGTAGAAACA GAATTCAAGT   
  
  
- TGGTCAAGCT GGCCTAGCAT GTGAAATGAA GAAAAGCGTG TGATATATAG AGCGGAAAGG TGCTGTTGGT   
  
  
- GGGAAACTTG GTGGAAATGA GACGGACACG GATAAGATGT TGCAGGCACA ACTCTATATA TAATGACTCT   
  
  
- GAGTCTATCT AAAGATCCAC GTGGTGGGAT TTGTTGTATA GTTTATTAAT AAGACAATTA ACTGTTTCCC   
  
  
- AGTTTGGTGA GTGTTGGGAG GAGCCATGTG AAGAAGGAAG AAGTTTTCTT TTCTTTTTTT TTCACCGAAT   
  
  
- CTGAATTAGT GTTAACTTTT TTAAAAGTAA AAGAAAAAAA ATATACATTT TTATAATTTA TTGTATTAAT   
  
  
- TTACTAAAAT AAATATACTG AATTAGTGTT AACTTTTTTA AAAGTAAAAG AAAAAAAATA TACATTTTTA   
  
  
- TAATTTATTG TATTAATTTA CTAAAATAAA TATATTAAAT TATAATTATT AATTAATTTT TCTTTTTATA   
  
  
- TTAACATAAG AAATTATAAA ATTATTTTTT ATTTTTACTC AAAAAATTAA AATAAATTCA AATAAATAGA   
  
  
- AAATGTTCGT GATTATTTTT TAAAATAAAA GCATATGTTT GATTAATATT AACATAAAAA ATACATCTTA   
  
  
- ATAATTTGTT TTATTAAATC ATTATGCATT ATTATTTTTA TTTAAAAATA ATATAAATTA AATTAAAATA   
  
  
- AAAAATTAAT TCAACCATAT ATATATATAT TTATCCATTC AATTACATAT TATTACGTGT GCTAGGGACA   
  
  
- TGCTGGTCGG TCATGGGTGT GCTTAGTCAA AGTCAAGCTC ACCATAAAAG CCAAGAAACA TTGGGTGGTG   
  
  
- GTCTCATTTT ATCTACTATT CCTCTGTGGA ATTAATAATT CATATAATAG ATGTCTAATT CTTAATTAAA   
  
  
- TTTCATATTG AACCCAATCT TATCTTTGTC TCTTCTACTG TACCAAATGC TTAATTAAAT TAAAATGACC   
  
  
- AATTAATATT TTTTATAAAA AAAATGAGTT TACAGAGATG TCACGTTTTT GAAAGCATTT TCCACGTTAC   
  
  
- ATGCACCAAT TCGATTCTTT TACGTCTTTT CAAAAGGGAT TTTTTTTTTT TTTTGTTTCT TACATTGAAT   
  
  
- TAATTTATTC CACGTTACAT GCACCAATTC GATTCTTTTA CGTCTTTTCA AAAGGGATTT TTTTTTTTTT   
  
  
- TTGTTTCTTA CATTGAATTA ATTTTTTGAA TCTAGACTCT TGCATGTAAT TCATTGTACC AAGAAGGCTT   
  
  
- ACTTATTCAT TCTAAGTATT CAAAGAGGAA TGTGAGCAAA ATGGTGGAGA TGTACCATAC TATTATTAAG   
  
  
- TCTTAATCGT TTTCAAGATT TTTTTTGTC

+     MBS

| Site Name | Organism | Position | Strand | Matrix score. | sequence | function |
| --- | --- | --- | --- | --- | --- | --- |
| MBS | Arabidopsis thaliana | 62 | + | 6 | TAACTG | MYB binding site involved in drought-inducibility |

> 2018/04/13 10:10:12  
+ CCGACCGGCT GCTGAATCTA GCTAGCTTTG GTTCTCTATG GGTCGTTTGT CTAACGGACT TTAACTGGAT   
  
  
+ TGTTATTTCG TCTGATTCAA GGAGATCAAA TAGCTTAGAA CGTTCGAAAC ACATCTTTGT CTTAAGTTCA   
  
  
+ ACCAGTTCGA CCGGATCGTA CACTTTACTT CTTTTCGCAC ACTATATATC TCGCCTTTCC ACGACAACCA   
  
  
+ CCCTTTGAAC CACCTTTACT CTGCCTGTGC CTATTCTACA ACGTCCGTGT TGAGATATAT ATTACTGAGA   
  
  
+ CTCAGATAGA TTTCTAGGTG CACCACCCTA AACAACATAT CAAATAATTA TTCTGTTAAT TGACAAAGGG   
  
  
+ TCAAACCACT CACAACCCTC CTCGGTACAC TTCTTCCTTC TTCAAAAGAA AAGAAAAAAA AAGTGGCTTA   
  
  
+ GACTTAATCA CAATTGAAAA AATTTTCATT TTCTTTTTTT TATATGTAAA AATATTAAAT AACATAATTA   
  
  
+ AATGATTTTA TTTATATGAC TTAATCACAA TTGAAAAAAT TTTCATTTTC TTTTTTTTAT ATGTAAAAAT   
  
  
+ ATTAAATAAC ATAATTAAAT GATTTTATTT ATATAATTTA ATATTAATAA TTAATTAAAA AGAAAAATAT   
  
  
+ AATTGTATTC TTTAATATTT TAATAAAAAA TAAAAATGAG TTTTTTAATT TTATTTAAGT TTATTTATCT   
  
  
+ TTTACAAGCA CTAATAAAAA ATTTTATTTT CGTATACAAA CTAATTATAA TTGTATTTTT TATGTAGAAT   
  
  
+ TATTAAACAA AATAATTTAG TAATACGTAA TAATAAAAAT AAATTTTTAT TATATTTAAT TTAATTTTAT   
  
  
+ TTTTTAATTA AGTTGGTATA TATATATATA AATAGGTAAG TTAATGTATA ATAATGCACA CGATCCCTGT   
  
  
+ ACGACCAGCC AGTACCCACA CGAATCAGTT TCAGTTCGAG TGGTATTTTC GGTTCTTTGT AACCCACCAC   
  
  
+ CAGAGTAAAA TAGATGATAA GGAGACACCT TAATTATTAA GTATATTATC TACAGATTAA GAATTAATTT   
  
  
+ AAAGTATAAC TTGGGTTAGA ATAGAAACAG AGAAGATGAC ATGGTTTACG AATTAATTTA ATTTTACTGG   
  
  
+ TTAATTATAA AAAATATTTT TTTTACTCAA ATGTCTCTAC AGTGCAAAAA CTTTCGTAAA AGGTGCAATG   
  
  
+ TACGTGGTTA AGCTAAGAAA ATGCAGAAAA GTTTTCCCTA AAAAAAAAAA AAAACAAAGA ATGTAACTTA   
  
  
+ ATTAAATAAG GTGCAATGTA CGTGGTTAAG CTAAGAAAAT GCAGAAAAGT TTTCCCTAAA AAAAAAAAAA   
  
  
+ AACAAAGAAT GTAACTTAAT TAAAAAACTT AGATCTGAGA ACGTACATTA AGTAACATGG TTCTTCCGAA   
  
  
+ TGAATAAGTA AGATTCATAA GTTTCTCCTT ACACTCGTTT TACCACCTCT ACATGGTATG ATAATAATTC   
  
  
+ AGAATTAGCA AAAGTTCTAA AAAAAACAG  

- GGCTGGCCGA CGACTTAGAT CGATCGAAAC CAAGAGATAC CCAGCAAACA GATTGCCTGA AATTGACCTA   
  
  
- ACAATAAAGC AGACTAAGTT CCTCTAGTTT ATCGAATCTT GCAAGCTTTG TGTAGAAACA GAATTCAAGT   
  
  
- TGGTCAAGCT GGCCTAGCAT GTGAAATGAA GAAAAGCGTG TGATATATAG AGCGGAAAGG TGCTGTTGGT   
  
  
- GGGAAACTTG GTGGAAATGA GACGGACACG GATAAGATGT TGCAGGCACA ACTCTATATA TAATGACTCT   
  
  
- GAGTCTATCT AAAGATCCAC GTGGTGGGAT TTGTTGTATA GTTTATTAAT AAGACAATTA ACTGTTTCCC   
  
  
- AGTTTGGTGA GTGTTGGGAG GAGCCATGTG AAGAAGGAAG AAGTTTTCTT TTCTTTTTTT TTCACCGAAT   
  
  
- CTGAATTAGT GTTAACTTTT TTAAAAGTAA AAGAAAAAAA ATATACATTT TTATAATTTA TTGTATTAAT   
  
  
- TTACTAAAAT AAATATACTG AATTAGTGTT AACTTTTTTA AAAGTAAAAG AAAAAAAATA TACATTTTTA   
  
  
- TAATTTATTG TATTAATTTA CTAAAATAAA TATATTAAAT TATAATTATT AATTAATTTT TCTTTTTATA   
  
  
- TTAACATAAG AAATTATAAA ATTATTTTTT ATTTTTACTC AAAAAATTAA AATAAATTCA AATAAATAGA   
  
  
- AAATGTTCGT GATTATTTTT TAAAATAAAA GCATATGTTT GATTAATATT AACATAAAAA ATACATCTTA   
  
  
- ATAATTTGTT TTATTAAATC ATTATGCATT ATTATTTTTA TTTAAAAATA ATATAAATTA AATTAAAATA   
  
  
- AAAAATTAAT TCAACCATAT ATATATATAT TTATCCATTC AATTACATAT TATTACGTGT GCTAGGGACA   
  
  
- TGCTGGTCGG TCATGGGTGT GCTTAGTCAA AGTCAAGCTC ACCATAAAAG CCAAGAAACA TTGGGTGGTG   
  
  
- GTCTCATTTT ATCTACTATT CCTCTGTGGA ATTAATAATT CATATAATAG ATGTCTAATT CTTAATTAAA   
  
  
- TTTCATATTG AACCCAATCT TATCTTTGTC TCTTCTACTG TACCAAATGC TTAATTAAAT TAAAATGACC   
  
  
- AATTAATATT TTTTATAAAA AAAATGAGTT TACAGAGATG TCACGTTTTT GAAAGCATTT TCCACGTTAC   
  
  
- ATGCACCAAT TCGATTCTTT TACGTCTTTT CAAAAGGGAT TTTTTTTTTT TTTTGTTTCT TACATTGAAT   
  
  
- TAATTTATTC CACGTTACAT GCACCAATTC GATTCTTTTA CGTCTTTTCA AAAGGGATTT TTTTTTTTTT   
  
  
- TTGTTTCTTA CATTGAATTA ATTTTTTGAA TCTAGACTCT TGCATGTAAT TCATTGTACC AAGAAGGCTT   
  
  
- ACTTATTCAT TCTAAGTATT CAAAGAGGAA TGTGAGCAAA ATGGTGGAGA TGTACCATAC TATTATTAAG   
  
  
- TCTTAATCGT TTTCAAGATT TTTTTTGTC

+     O2-site

| Site Name | Organism | Position | Strand | Matrix score. | sequence | function |
| --- | --- | --- | --- | --- | --- | --- |
| O2-site | Zea mays | 993 | + | 9 | GATGATATGG | cis-acting regulatory element involved in zein metabolism regulation |
| O2-site | Zea mays | 1085 | + | 10 | GATGACATGG | cis-acting regulatory element involved in zein metabolism regulation |

> 2018/04/13 10:10:12  
+ CCGACCGGCT GCTGAATCTA GCTAGCTTTG GTTCTCTATG GGTCGTTTGT CTAACGGACT TTAACTGGAT   
  
  
+ TGTTATTTCG TCTGATTCAA GGAGATCAAA TAGCTTAGAA CGTTCGAAAC ACATCTTTGT CTTAAGTTCA   
  
  
+ ACCAGTTCGA CCGGATCGTA CACTTTACTT CTTTTCGCAC ACTATATATC TCGCCTTTCC ACGACAACCA   
  
  
+ CCCTTTGAAC CACCTTTACT CTGCCTGTGC CTATTCTACA ACGTCCGTGT TGAGATATAT ATTACTGAGA   
  
  
+ CTCAGATAGA TTTCTAGGTG CACCACCCTA AACAACATAT CAAATAATTA TTCTGTTAAT TGACAAAGGG   
  
  
+ TCAAACCACT CACAACCCTC CTCGGTACAC TTCTTCCTTC TTCAAAAGAA AAGAAAAAAA AAGTGGCTTA   
  
  
+ GACTTAATCA CAATTGAAAA AATTTTCATT TTCTTTTTTT TATATGTAAA AATATTAAAT AACATAATTA   
  
  
+ AATGATTTTA TTTATATGAC TTAATCACAA TTGAAAAAAT TTTCATTTTC TTTTTTTTAT ATGTAAAAAT   
  
  
+ ATTAAATAAC ATAATTAAAT GATTTTATTT ATATAATTTA ATATTAATAA TTAATTAAAA AGAAAAATAT   
  
  
+ AATTGTATTC TTTAATATTT TAATAAAAAA TAAAAATGAG TTTTTTAATT TTATTTAAGT TTATTTATCT   
  
  
+ TTTACAAGCA CTAATAAAAA ATTTTATTTT CGTATACAAA CTAATTATAA TTGTATTTTT TATGTAGAAT   
  
  
+ TATTAAACAA AATAATTTAG TAATACGTAA TAATAAAAAT AAATTTTTAT TATATTTAAT TTAATTTTAT   
  
  
+ TTTTTAATTA AGTTGGTATA TATATATATA AATAGGTAAG TTAATGTATA ATAATGCACA CGATCCCTGT   
  
  
+ ACGACCAGCC AGTACCCACA CGAATCAGTT TCAGTTCGAG TGGTATTTTC GGTTCTTTGT AACCCACCAC   
  
  
+ CAGAGTAAAA TAGATGATAA GGAGACACCT TAATTATTAA GTATATTATC TACAGATTAA GAATTAATTT   
  
  
+ AAAGTATAAC TTGGGTTAGA ATAGAAACAG AGAAGATGAC ATGGTTTACG AATTAATTTA ATTTTACTGG   
  
  
+ TTAATTATAA AAAATATTTT TTTTACTCAA ATGTCTCTAC AGTGCAAAAA CTTTCGTAAA AGGTGCAATG   
  
  
+ TACGTGGTTA AGCTAAGAAA ATGCAGAAAA GTTTTCCCTA AAAAAAAAAA AAAACAAAGA ATGTAACTTA   
  
  
+ ATTAAATAAG GTGCAATGTA CGTGGTTAAG CTAAGAAAAT GCAGAAAAGT TTTCCCTAAA AAAAAAAAAA   
  
  
+ AACAAAGAAT GTAACTTAAT TAAAAAACTT AGATCTGAGA ACGTACATTA AGTAACATGG TTCTTCCGAA   
  
  
+ TGAATAAGTA AGATTCATAA GTTTCTCCTT ACACTCGTTT TACCACCTCT ACATGGTATG ATAATAATTC   
  
  
+ AGAATTAGCA AAAGTTCTAA AAAAAACAG  

- GGCTGGCCGA CGACTTAGAT CGATCGAAAC CAAGAGATAC CCAGCAAACA GATTGCCTGA AATTGACCTA   
  
  
- ACAATAAAGC AGACTAAGTT CCTCTAGTTT ATCGAATCTT GCAAGCTTTG TGTAGAAACA GAATTCAAGT   
  
  
- TGGTCAAGCT GGCCTAGCAT GTGAAATGAA GAAAAGCGTG TGATATATAG AGCGGAAAGG TGCTGTTGGT   
  
  
- GGGAAACTTG GTGGAAATGA GACGGACACG GATAAGATGT TGCAGGCACA ACTCTATATA TAATGACTCT   
  
  
- GAGTCTATCT AAAGATCCAC GTGGTGGGAT TTGTTGTATA GTTTATTAAT AAGACAATTA ACTGTTTCCC   
  
  
- AGTTTGGTGA GTGTTGGGAG GAGCCATGTG AAGAAGGAAG AAGTTTTCTT TTCTTTTTTT TTCACCGAAT   
  
  
- CTGAATTAGT GTTAACTTTT TTAAAAGTAA AAGAAAAAAA ATATACATTT TTATAATTTA TTGTATTAAT   
  
  
- TTACTAAAAT AAATATACTG AATTAGTGTT AACTTTTTTA AAAGTAAAAG AAAAAAAATA TACATTTTTA   
  
  
- TAATTTATTG TATTAATTTA CTAAAATAAA TATATTAAAT TATAATTATT AATTAATTTT TCTTTTTATA   
  
  
- TTAACATAAG AAATTATAAA ATTATTTTTT ATTTTTACTC AAAAAATTAA AATAAATTCA AATAAATAGA   
  
  
- AAATGTTCGT GATTATTTTT TAAAATAAAA GCATATGTTT GATTAATATT AACATAAAAA ATACATCTTA   
  
  
- ATAATTTGTT TTATTAAATC ATTATGCATT ATTATTTTTA TTTAAAAATA ATATAAATTA AATTAAAATA   
  
  
- AAAAATTAAT TCAACCATAT ATATATATAT TTATCCATTC AATTACATAT TATTACGTGT GCTAGGGACA   
  
  
- TGCTGGTCGG TCATGGGTGT GCTTAGTCAA AGTCAAGCTC ACCATAAAAG CCAAGAAACA TTGGGTGGTG   
  
  
- GTCTCATTTT ATCTACTATT CCTCTGTGGA ATTAATAATT CATATAATAG ATGTCTAATT CTTAATTAAA   
  
  
- TTTCATATTG AACCCAATCT TATCTTTGTC TCTTCTACTG TACCAAATGC TTAATTAAAT TAAAATGACC   
  
  
- AATTAATATT TTTTATAAAA AAAATGAGTT TACAGAGATG TCACGTTTTT GAAAGCATTT TCCACGTTAC   
  
  
- ATGCACCAAT TCGATTCTTT TACGTCTTTT CAAAAGGGAT TTTTTTTTTT TTTTGTTTCT TACATTGAAT   
  
  
- TAATTTATTC CACGTTACAT GCACCAATTC GATTCTTTTA CGTCTTTTCA AAAGGGATTT TTTTTTTTTT   
  
  
- TTGTTTCTTA CATTGAATTA ATTTTTTGAA TCTAGACTCT TGCATGTAAT TCATTGTACC AAGAAGGCTT   
  
  
- ACTTATTCAT TCTAAGTATT CAAAGAGGAA TGTGAGCAAA ATGGTGGAGA TGTACCATAC TATTATTAAG   
  
  
- TCTTAATCGT TTTCAAGATT TTTTTTGTC

+     Skn-1\_motif

| Site Name | Organism | Position | Strand | Matrix score. | sequence | function |
| --- | --- | --- | --- | --- | --- | --- |
| Skn-1\_motif | Oryza sativa | 506 | - | 5 | GTCAT | cis-acting regulatory element required for endosperm expression |
| Skn-1\_motif | Oryza sativa | 1086 | - | 5 | GTCAT | cis-acting regulatory element required for endosperm expression |

> 2018/04/13 10:10:12  
+ CCGACCGGCT GCTGAATCTA GCTAGCTTTG GTTCTCTATG GGTCGTTTGT CTAACGGACT TTAACTGGAT   
  
  
+ TGTTATTTCG TCTGATTCAA GGAGATCAAA TAGCTTAGAA CGTTCGAAAC ACATCTTTGT CTTAAGTTCA   
  
  
+ ACCAGTTCGA CCGGATCGTA CACTTTACTT CTTTTCGCAC ACTATATATC TCGCCTTTCC ACGACAACCA   
  
  
+ CCCTTTGAAC CACCTTTACT CTGCCTGTGC CTATTCTACA ACGTCCGTGT TGAGATATAT ATTACTGAGA   
  
  
+ CTCAGATAGA TTTCTAGGTG CACCACCCTA AACAACATAT CAAATAATTA TTCTGTTAAT TGACAAAGGG   
  
  
+ TCAAACCACT CACAACCCTC CTCGGTACAC TTCTTCCTTC TTCAAAAGAA AAGAAAAAAA AAGTGGCTTA   
  
  
+ GACTTAATCA CAATTGAAAA AATTTTCATT TTCTTTTTTT TATATGTAAA AATATTAAAT AACATAATTA   
  
  
+ AATGATTTTA TTTATATGAC TTAATCACAA TTGAAAAAAT TTTCATTTTC TTTTTTTTAT ATGTAAAAAT   
  
  
+ ATTAAATAAC ATAATTAAAT GATTTTATTT ATATAATTTA ATATTAATAA TTAATTAAAA AGAAAAATAT   
  
  
+ AATTGTATTC TTTAATATTT TAATAAAAAA TAAAAATGAG TTTTTTAATT TTATTTAAGT TTATTTATCT   
  
  
+ TTTACAAGCA CTAATAAAAA ATTTTATTTT CGTATACAAA CTAATTATAA TTGTATTTTT TATGTAGAAT   
  
  
+ TATTAAACAA AATAATTTAG TAATACGTAA TAATAAAAAT AAATTTTTAT TATATTTAAT TTAATTTTAT   
  
  
+ TTTTTAATTA AGTTGGTATA TATATATATA AATAGGTAAG TTAATGTATA ATAATGCACA CGATCCCTGT   
  
  
+ ACGACCAGCC AGTACCCACA CGAATCAGTT TCAGTTCGAG TGGTATTTTC GGTTCTTTGT AACCCACCAC   
  
  
+ CAGAGTAAAA TAGATGATAA GGAGACACCT TAATTATTAA GTATATTATC TACAGATTAA GAATTAATTT   
  
  
+ AAAGTATAAC TTGGGTTAGA ATAGAAACAG AGAAGATGAC ATGGTTTACG AATTAATTTA ATTTTACTGG   
  
  
+ TTAATTATAA AAAATATTTT TTTTACTCAA ATGTCTCTAC AGTGCAAAAA CTTTCGTAAA AGGTGCAATG   
  
  
+ TACGTGGTTA AGCTAAGAAA ATGCAGAAAA GTTTTCCCTA AAAAAAAAAA AAAACAAAGA ATGTAACTTA   
  
  
+ ATTAAATAAG GTGCAATGTA CGTGGTTAAG CTAAGAAAAT GCAGAAAAGT TTTCCCTAAA AAAAAAAAAA   
  
  
+ AACAAAGAAT GTAACTTAAT TAAAAAACTT AGATCTGAGA ACGTACATTA AGTAACATGG TTCTTCCGAA   
  
  
+ TGAATAAGTA AGATTCATAA GTTTCTCCTT ACACTCGTTT TACCACCTCT ACATGGTATG ATAATAATTC   
  
  
+ AGAATTAGCA AAAGTTCTAA AAAAAACAG  

- GGCTGGCCGA CGACTTAGAT CGATCGAAAC CAAGAGATAC CCAGCAAACA GATTGCCTGA AATTGACCTA   
  
  
- ACAATAAAGC AGACTAAGTT CCTCTAGTTT ATCGAATCTT GCAAGCTTTG TGTAGAAACA GAATTCAAGT   
  
  
- TGGTCAAGCT GGCCTAGCAT GTGAAATGAA GAAAAGCGTG TGATATATAG AGCGGAAAGG TGCTGTTGGT   
  
  
- GGGAAACTTG GTGGAAATGA GACGGACACG GATAAGATGT TGCAGGCACA ACTCTATATA TAATGACTCT   
  
  
- GAGTCTATCT AAAGATCCAC GTGGTGGGAT TTGTTGTATA GTTTATTAAT AAGACAATTA ACTGTTTCCC   
  
  
- AGTTTGGTGA GTGTTGGGAG GAGCCATGTG AAGAAGGAAG AAGTTTTCTT TTCTTTTTTT TTCACCGAAT   
  
  
- CTGAATTAGT GTTAACTTTT TTAAAAGTAA AAGAAAAAAA ATATACATTT TTATAATTTA TTGTATTAAT   
  
  
- TTACTAAAAT AAATATACTG AATTAGTGTT AACTTTTTTA AAAGTAAAAG AAAAAAAATA TACATTTTTA   
  
  
- TAATTTATTG TATTAATTTA CTAAAATAAA TATATTAAAT TATAATTATT AATTAATTTT TCTTTTTATA   
  
  
- TTAACATAAG AAATTATAAA ATTATTTTTT ATTTTTACTC AAAAAATTAA AATAAATTCA AATAAATAGA   
  
  
- AAATGTTCGT GATTATTTTT TAAAATAAAA GCATATGTTT GATTAATATT AACATAAAAA ATACATCTTA   
  
  
- ATAATTTGTT TTATTAAATC ATTATGCATT ATTATTTTTA TTTAAAAATA ATATAAATTA AATTAAAATA   
  
  
- AAAAATTAAT TCAACCATAT ATATATATAT TTATCCATTC AATTACATAT TATTACGTGT GCTAGGGACA   
  
  
- TGCTGGTCGG TCATGGGTGT GCTTAGTCAA AGTCAAGCTC ACCATAAAAG CCAAGAAACA TTGGGTGGTG   
  
  
- GTCTCATTTT ATCTACTATT CCTCTGTGGA ATTAATAATT CATATAATAG ATGTCTAATT CTTAATTAAA   
  
  
- TTTCATATTG AACCCAATCT TATCTTTGTC TCTTCTACTG TACCAAATGC TTAATTAAAT TAAAATGACC   
  
  
- AATTAATATT TTTTATAAAA AAAATGAGTT TACAGAGATG TCACGTTTTT GAAAGCATTT TCCACGTTAC   
  
  
- ATGCACCAAT TCGATTCTTT TACGTCTTTT CAAAAGGGAT TTTTTTTTTT TTTTGTTTCT TACATTGAAT   
  
  
- TAATTTATTC CACGTTACAT GCACCAATTC GATTCTTTTA CGTCTTTTCA AAAGGGATTT TTTTTTTTTT   
  
  
- TTGTTTCTTA CATTGAATTA ATTTTTTGAA TCTAGACTCT TGCATGTAAT TCATTGTACC AAGAAGGCTT   
  
  
- ACTTATTCAT TCTAAGTATT CAAAGAGGAA TGTGAGCAAA ATGGTGGAGA TGTACCATAC TATTATTAAG   
  
  
- TCTTAATCGT TTTCAAGATT TTTTTTGTC

+     Sp1

| Site Name | Organism | Position | Strand | Matrix score. | sequence | function |
| --- | --- | --- | --- | --- | --- | --- |
| Sp1 | Zea mays | 208 | + | 5.5 | CC(G/A)CCC | light responsive element |
| Sp1 | Zea mays | 303 | + | 5.5 | CC(G/A)CCC | light responsive element |

> 2018/04/13 10:10:12  
+ CCGACCGGCT GCTGAATCTA GCTAGCTTTG GTTCTCTATG GGTCGTTTGT CTAACGGACT TTAACTGGAT   
  
  
+ TGTTATTTCG TCTGATTCAA GGAGATCAAA TAGCTTAGAA CGTTCGAAAC ACATCTTTGT CTTAAGTTCA   
  
  
+ ACCAGTTCGA CCGGATCGTA CACTTTACTT CTTTTCGCAC ACTATATATC TCGCCTTTCC ACGACAACCA   
  
  
+ CCCTTTGAAC CACCTTTACT CTGCCTGTGC CTATTCTACA ACGTCCGTGT TGAGATATAT ATTACTGAGA   
  
  
+ CTCAGATAGA TTTCTAGGTG CACCACCCTA AACAACATAT CAAATAATTA TTCTGTTAAT TGACAAAGGG   
  
  
+ TCAAACCACT CACAACCCTC CTCGGTACAC TTCTTCCTTC TTCAAAAGAA AAGAAAAAAA AAGTGGCTTA   
  
  
+ GACTTAATCA CAATTGAAAA AATTTTCATT TTCTTTTTTT TATATGTAAA AATATTAAAT AACATAATTA   
  
  
+ AATGATTTTA TTTATATGAC TTAATCACAA TTGAAAAAAT TTTCATTTTC TTTTTTTTAT ATGTAAAAAT   
  
  
+ ATTAAATAAC ATAATTAAAT GATTTTATTT ATATAATTTA ATATTAATAA TTAATTAAAA AGAAAAATAT   
  
  
+ AATTGTATTC TTTAATATTT TAATAAAAAA TAAAAATGAG TTTTTTAATT TTATTTAAGT TTATTTATCT   
  
  
+ TTTACAAGCA CTAATAAAAA ATTTTATTTT CGTATACAAA CTAATTATAA TTGTATTTTT TATGTAGAAT   
  
  
+ TATTAAACAA AATAATTTAG TAATACGTAA TAATAAAAAT AAATTTTTAT TATATTTAAT TTAATTTTAT   
  
  
+ TTTTTAATTA AGTTGGTATA TATATATATA AATAGGTAAG TTAATGTATA ATAATGCACA CGATCCCTGT   
  
  
+ ACGACCAGCC AGTACCCACA CGAATCAGTT TCAGTTCGAG TGGTATTTTC GGTTCTTTGT AACCCACCAC   
  
  
+ CAGAGTAAAA TAGATGATAA GGAGACACCT TAATTATTAA GTATATTATC TACAGATTAA GAATTAATTT   
  
  
+ AAAGTATAAC TTGGGTTAGA ATAGAAACAG AGAAGATGAC ATGGTTTACG AATTAATTTA ATTTTACTGG   
  
  
+ TTAATTATAA AAAATATTTT TTTTACTCAA ATGTCTCTAC AGTGCAAAAA CTTTCGTAAA AGGTGCAATG   
  
  
+ TACGTGGTTA AGCTAAGAAA ATGCAGAAAA GTTTTCCCTA AAAAAAAAAA AAAACAAAGA ATGTAACTTA   
  
  
+ ATTAAATAAG GTGCAATGTA CGTGGTTAAG CTAAGAAAAT GCAGAAAAGT TTTCCCTAAA AAAAAAAAAA   
  
  
+ AACAAAGAAT GTAACTTAAT TAAAAAACTT AGATCTGAGA ACGTACATTA AGTAACATGG TTCTTCCGAA   
  
  
+ TGAATAAGTA AGATTCATAA GTTTCTCCTT ACACTCGTTT TACCACCTCT ACATGGTATG ATAATAATTC   
  
  
+ AGAATTAGCA AAAGTTCTAA AAAAAACAG  

- GGCTGGCCGA CGACTTAGAT CGATCGAAAC CAAGAGATAC CCAGCAAACA GATTGCCTGA AATTGACCTA   
  
  
- ACAATAAAGC AGACTAAGTT CCTCTAGTTT ATCGAATCTT GCAAGCTTTG TGTAGAAACA GAATTCAAGT   
  
  
- TGGTCAAGCT GGCCTAGCAT GTGAAATGAA GAAAAGCGTG TGATATATAG AGCGGAAAGG TGCTGTTGGT   
  
  
- GGGAAACTTG GTGGAAATGA GACGGACACG GATAAGATGT TGCAGGCACA ACTCTATATA TAATGACTCT   
  
  
- GAGTCTATCT AAAGATCCAC GTGGTGGGAT TTGTTGTATA GTTTATTAAT AAGACAATTA ACTGTTTCCC   
  
  
- AGTTTGGTGA GTGTTGGGAG GAGCCATGTG AAGAAGGAAG AAGTTTTCTT TTCTTTTTTT TTCACCGAAT   
  
  
- CTGAATTAGT GTTAACTTTT TTAAAAGTAA AAGAAAAAAA ATATACATTT TTATAATTTA TTGTATTAAT   
  
  
- TTACTAAAAT AAATATACTG AATTAGTGTT AACTTTTTTA AAAGTAAAAG AAAAAAAATA TACATTTTTA   
  
  
- TAATTTATTG TATTAATTTA CTAAAATAAA TATATTAAAT TATAATTATT AATTAATTTT TCTTTTTATA   
  
  
- TTAACATAAG AAATTATAAA ATTATTTTTT ATTTTTACTC AAAAAATTAA AATAAATTCA AATAAATAGA   
  
  
- AAATGTTCGT GATTATTTTT TAAAATAAAA GCATATGTTT GATTAATATT AACATAAAAA ATACATCTTA   
  
  
- ATAATTTGTT TTATTAAATC ATTATGCATT ATTATTTTTA TTTAAAAATA ATATAAATTA AATTAAAATA   
  
  
- AAAAATTAAT TCAACCATAT ATATATATAT TTATCCATTC AATTACATAT TATTACGTGT GCTAGGGACA   
  
  
- TGCTGGTCGG TCATGGGTGT GCTTAGTCAA AGTCAAGCTC ACCATAAAAG CCAAGAAACA TTGGGTGGTG   
  
  
- GTCTCATTTT ATCTACTATT CCTCTGTGGA ATTAATAATT CATATAATAG ATGTCTAATT CTTAATTAAA   
  
  
- TTTCATATTG AACCCAATCT TATCTTTGTC TCTTCTACTG TACCAAATGC TTAATTAAAT TAAAATGACC   
  
  
- AATTAATATT TTTTATAAAA AAAATGAGTT TACAGAGATG TCACGTTTTT GAAAGCATTT TCCACGTTAC   
  
  
- ATGCACCAAT TCGATTCTTT TACGTCTTTT CAAAAGGGAT TTTTTTTTTT TTTTGTTTCT TACATTGAAT   
  
  
- TAATTTATTC CACGTTACAT GCACCAATTC GATTCTTTTA CGTCTTTTCA AAAGGGATTT TTTTTTTTTT   
  
  
- TTGTTTCTTA CATTGAATTA ATTTTTTGAA TCTAGACTCT TGCATGTAAT TCATTGTACC AAGAAGGCTT   
  
  
- ACTTATTCAT TCTAAGTATT CAAAGAGGAA TGTGAGCAAA ATGGTGGAGA TGTACCATAC TATTATTAAG   
  
  
- TCTTAATCGT TTTCAAGATT TTTTTTGTC

+     TATA-box

| Site Name | Organism | Position | Strand | Matrix score. | sequence | function |
| --- | --- | --- | --- | --- | --- | --- |
| TATA-box | Arabidopsis thaliana | 861 | + | 11 | TATAAATATAAA | core promoter element around -30 of transcription start |
| TATA-box | Arabidopsis thaliana | 820 | - | 5 | TATAA | core promoter element around -30 of transcription start |
| TATA-box | Brassica napus | 819 | + | 6 | ATTATA | core promoter element around -30 of transcription start |
| TATA-box | Lycopersicon esculentum | 1438 | + | 5 | TTTTA | core promoter element around -30 of transcription start |
| TATA-box | Lycopersicon esculentum | 1112 | + | 5 | TTTTA | core promoter element around -30 of transcription start |
| TATA-box | Arabidopsis thaliana | 586 | - | 9 | taTATAAAtc | core promoter element around -30 of transcription start |
| TATA-box | Arabidopsis thaliana | 547 | - | 5 | TATAA | core promoter element around -30 of transcription start |
| TATA-box | Arabidopsis thaliana | 501 | - | 6 | TATAAA | core promoter element around -30 of transcription start |
| TATA-box | Arabidopsis thaliana | 804 | + | 9 | TAAAAATAA | core promoter element around -30 of transcription start |
| TATA-box | Arabidopsis thaliana | 458 | - | 7 | TATAAAA | core promoter element around -30 of transcription start |
| TATA-box | Glycine max | 712 | + | 5 | TAATA | core promoter element around -30 of transcription start |
| TATA-box | Lycopersicon esculentum | 648 | + | 5 | TTTTA | core promoter element around -30 of transcription start |
| TATA-box | Brassica napus | 858 | - | 6 | ATATAT | core promoter element around -30 of transcription start |
| TATA-box | Brassica napus | 862 | - | 6 | ATATAT | core promoter element around -30 of transcription start |
| TATA-box | Arabidopsis thaliana | 183 | + | 4 | TATA | core promoter element around -30 of transcription start |
| TATA-box | Glycine max | 605 | + | 5 | TAATA | core promoter element around -30 of transcription start |
| TATA-box | Lycopersicon esculentum | 815 | + | 5 | TTTTA | core promoter element around -30 of transcription start |
| TATA-box | Arabidopsis thaliana | 502 | - | 5 | TATAA | core promoter element around -30 of transcription start |
| TATA-box | Helianthus annuus | 885 | - | 6 | TATACA | core promoter element around -30 of transcription start |
| TATA-box | Arabidopsis thaliana | 1125 | - | 5 | TATAA | core promoter element around -30 of transcription start |
| TATA-box | Glycine max | 599 | + | 5 | TAATA | core promoter element around -30 of transcription start |
| TATA-box | Arabidopsis thaliana | 503 | + | 4 | TATA | core promoter element around -30 of transcription start |
| TATA-box | Brassica oleracea | 591 | + | 7 | ATATAAT | core promoter element around -30 of transcription start |
| TATA-box | Lycopersicon esculentum | 715 | - | 5 | TTTTA | core promoter element around -30 of transcription start |
| TATA-box | Brassica napus | 267 | + | 6 | ATATAT | core promoter element around -30 of transcription start |
| TATA-box | Ac | 867 | + | 7 | TATAAAT | core promoter element around -30 of transcription start |
| TATA-box | Glycine max | 651 | + | 5 | TAATA | core promoter element around -30 of transcription start |
| TATA-box | Glycine max | 771 | - | 5 | TAATA | core promoter element around -30 of transcription start |
| TATA-box | Arabidopsis thaliana | 461 | + | 4 | TATA | core promoter element around -30 of transcription start |
| TATA-box | Brassica oleracea | 627 | + | 7 | ATATAAT | core promoter element around -30 of transcription start |
| TATA-box | Glycine max | 473 | - | 5 | TAATA | core promoter element around -30 of transcription start |
| TATA-box | Arabidopsis thaliana | 592 | + | 4 | TATA | core promoter element around -30 of transcription start |
| TATA-box | Arabidopsis thaliana | 821 | - | 4 | TATA | core promoter element around -30 of transcription start |
| TATA-box | Lycopersicon esculentum | 583 | + | 5 | TTTTA | core promoter element around -30 of transcription start |
| TATA-box | Glycine max | 1024 | - | 5 | TAATA | core promoter element around -30 of transcription start |
| TATA-box | Arabidopsis thaliana | 185 | + | 4 | TATA | core promoter element around -30 of transcription start |
| TATA-box | Lycopersicon esculentum | 679 | + | 5 | TTTTA | core promoter element around -30 of transcription start |
| TATA-box | Lycopersicon esculentum | 467 | - | 5 | TTTTA | core promoter element around -30 of transcription start |
| TATA-box | Arabidopsis thaliana | 545 | - | 7 | TATAAAA | core promoter element around -30 of transcription start |
| TATA-box | Arabidopsis thaliana | 546 | - | 6 | TATAAA | core promoter element around -30 of transcription start |
| TATA-box | Lycopersicon esculentum | 554 | - | 5 | TTTTA | core promoter element around -30 of transcription start |
| TATA-box | Lycopersicon esculentum | 722 | + | 5 | TTTTA | core promoter element around -30 of transcription start |
| TATA-box | Daucus carota | 499 | - | 8 | TATAAATA | core promoter element around -30 of transcription start |
| TATA-box | Glycine max | 1015 | - | 5 | TAATA | core promoter element around -30 of transcription start |
| TATA-box | Lycopersicon esculentum | 1317 | - | 5 | TTTTA | core promoter element around -30 of transcription start |
| TATA-box | Lycopersicon esculentum | 842 | + | 5 | TTTTA | core promoter element around -30 of transcription start |
| TATA-box | Brassica napus | 864 | - | 6 | ATATAT | core promoter element around -30 of transcription start |
| TATA-box | Glycine max | 643 | + | 5 | TAATA | core promoter element around -30 of transcription start |
| TATA-box | Arabidopsis thaliana | 548 | + | 4 | TATA | core promoter element around -30 of transcription start |
| TATA-box | Glycine max | 798 | + | 5 | TAATA | core promoter element around -30 of transcription start |
| TATA-box | Lycopersicon esculentum | 1488 | - | 5 | TTTTA | core promoter element around -30 of transcription start |
| TATA-box | Glycine max | 602 | - | 5 | TAATA | core promoter element around -30 of transcription start |
| TATA-box | Lycopersicon esculentum | 661 | - | 5 | TTTTA | core promoter element around -30 of transcription start |
| TATA-box | Glycine max | 818 | - | 5 | TAATA | core promoter element around -30 of transcription start |
| TATA-box | Arabidopsis thaliana | 268 | + | 4 | TATA | core promoter element around -30 of transcription start |
| TATA-box | Arabidopsis thaliana | 460 | - | 5 | TATAA | core promoter element around -30 of transcription start |
| TATA-box | Brassica napus | 184 | + | 6 | ATATAT | core promoter element around -30 of transcription start |
| TATA-box | Arabidopsis thaliana | 628 | + | 4 | TATA | core promoter element around -30 of transcription start |
| TATA-box | Lycopersicon esculentum | 700 | + | 5 | TTTTA | core promoter element around -30 of transcription start |
| TATA-box | Brassica napus | 744 | + | 6 | ATTATA | core promoter element around -30 of transcription start |
| TATA-box | Ac | 587 | - | 7 | TATAAAT | core promoter element around -30 of transcription start |
| TATA-box | Arabidopsis thaliana | 588 | - | 6 | TATAAA | core promoter element around -30 of transcription start |
| TATA-box | Lycopersicon esculentum | 616 | - | 5 | TTTTA | core promoter element around -30 of transcription start |
| TATA-box | Glycine max | 889 | + | 5 | TAATA | core promoter element around -30 of transcription start |
| TATA-box | Brassica napus | 860 | - | 6 | ATATAT | core promoter element around -30 of transcription start |
| TATA-box | Arabidopsis thaliana | 589 | - | 7 | TATATAA | core promoter element around -30 of transcription start |
| TATA-box | Brassica oleracea | 866 | + | 6 | ATATAA | core promoter element around -30 of transcription start |
| TATA-box | Lycopersicon esculentum | 496 | + | 5 | TTTTA | core promoter element around -30 of transcription start |
| TATA-box | Ac | 500 | - | 7 | TATAAAT | core promoter element around -30 of transcription start |
| TATA-box | Arabidopsis thaliana | 457 | - | 9 | ccTATAAAaa | core promoter element around -30 of transcription start |
| TATA-box | Glycine max | 801 | + | 5 | TAATA | core promoter element around -30 of transcription start |
| TATA-box | Glycine max | 560 | - | 5 | TAATA | core promoter element around -30 of transcription start |
| TATA-box | Arabidopsis thaliana | 1126 | + | 6 | TATAAA | core promoter element around -30 of transcription start |
| TATA-box | Lycopersicon esculentum | 654 | - | 5 | TTTTA | core promoter element around -30 of transcription start |
| TATA-box | Arabidopsis thaliana | 1022 | - | 4 | TATA | core promoter element around -30 of transcription start |
| TATA-box | Arabidopsis thaliana | 857 | - | 8 | TATATATA | core promoter element around -30 of transcription start |
| TATA-box | Arabidopsis thaliana | 266 | + | 4 | TATA | core promoter element around -30 of transcription start |
| TATA-box | Arabidopsis thaliana | 544 | - | 9 | ccTATAAAaa | core promoter element around -30 of transcription start |
| TATA-box | Arabidopsis thaliana | 590 | + | 4 | TATA | core promoter element around -30 of transcription start |
| TATA-box | Arabidopsis thaliana | 459 | - | 6 | TATAAA | core promoter element around -30 of transcription start |
| TATA-box | Lycopersicon esculentum | 673 | + | 5 | TTTTA | core promoter element around -30 of transcription start |
| TATA-box | Arabidopsis thaliana | 733 | + | 4 | TATA | core promoter element around -30 of transcription start |
| TATA-box | Brassica napus | 265 | + | 6 | ATATAT | core promoter element around -30 of transcription start |
| TATA-box | Arabidopsis thaliana | 1055 | - | 4 | TATA | core promoter element around -30 of transcription start |
| TATA-box | Lycopersicon esculentum | 835 | + | 5 | TTTTA | core promoter element around -30 of transcription start |
| TATA-box | Arabidopsis thaliana | 863 | - | 8 | TATATATA | core promoter element around -30 of transcription start |
| TATA-box | Glycine max | 270 | - | 5 | TAATA | core promoter element around -30 of transcription start |
| TATA-box | Oryza sativa | 759 | - | 8 | TACATAAA | core promoter element around -30 of transcription start |
| TATA-box | Lycopersicon esculentum | 1351 | - | 5 | TTTTA | core promoter element around -30 of transcription start |
| TATA-box | Arabidopsis thaliana | 859 | - | 8 | TATATATA | core promoter element around -30 of transcription start |
| TATA-box | Arabidopsis thaliana | 887 | - | 4 | TATA | core promoter element around -30 of transcription start |
| TATA-box | Arabidopsis thaliana | 746 | + | 4 | TATA | core promoter element around -30 of transcription start |
| TATA-box | Lycopersicon esculentum | 1128 | - | 5 | TTTTA | core promoter element around -30 of transcription start |
| TATA-box | Arabidopsis thaliana | 865 | + | 9 | taTATAAAtc | core promoter element around -30 of transcription start |
| TATA-box | Glycine max | 791 | + | 5 | TAATA | core promoter element around -30 of transcription start |
| TATA-box | Arabidopsis thaliana | 745 | - | 5 | TATAA | core promoter element around -30 of transcription start |
| TATA-box | Glycine max | 1462 | + | 5 | TAATA | core promoter element around -30 of transcription start |
| TATA-box | Lycopersicon esculentum | 758 | + | 5 | TTTTA | core promoter element around -30 of transcription start |
| TATA-box | Lycopersicon esculentum | 986 | - | 5 | TTTTA | core promoter element around -30 of transcription start |
| TATA-box | Brassica napus | 1124 | + | 6 | ATTATA | core promoter element around -30 of transcription start |
| TATA-box | Lycopersicon esculentum | 1141 | + | 5 | TTTTA | core promoter element around -30 of transcription start |
| TATA-box | Lycopersicon esculentum | 1177 | - | 5 | TTTTA | core promoter element around -30 of transcription start |
| TATA-box | Arabidopsis thaliana | 1227 | + | 9 | ccTATAAAaa | core promoter element around -30 of transcription start |
| TATA-box | Lycopersicon esculentum | 1229 | - | 5 | TTTTA | core promoter element around -30 of transcription start |
| TATA-box | Arabidopsis thaliana | 1315 | + | 9 | ccTATAAAaa | core promoter element around -30 of transcription start |

> 2018/04/13 10:10:12  
+ CCGACCGGCT GCTGAATCTA GCTAGCTTTG GTTCTCTATG GGTCGTTTGT CTAACGGACT TTAACTGGAT   
  
  
+ TGTTATTTCG TCTGATTCAA GGAGATCAAA TAGCTTAGAA CGTTCGAAAC ACATCTTTGT CTTAAGTTCA   
  
  
+ ACCAGTTCGA CCGGATCGTA CACTTTACTT CTTTTCGCAC ACTATATATC TCGCCTTTCC ACGACAACCA   
  
  
+ CCCTTTGAAC CACCTTTACT CTGCCTGTGC CTATTCTACA ACGTCCGTGT TGAGATATAT ATTACTGAGA   
  
  
+ CTCAGATAGA TTTCTAGGTG CACCACCCTA AACAACATAT CAAATAATTA TTCTGTTAAT TGACAAAGGG   
  
  
+ TCAAACCACT CACAACCCTC CTCGGTACAC TTCTTCCTTC TTCAAAAGAA AAGAAAAAAA AAGTGGCTTA   
  
  
+ GACTTAATCA CAATTGAAAA AATTTTCATT TTCTTTTTTT TATATGTAAA AATATTAAAT AACATAATTA   
  
  
+ AATGATTTTA TTTATATGAC TTAATCACAA TTGAAAAAAT TTTCATTTTC TTTTTTTTAT ATGTAAAAAT   
  
  
+ ATTAAATAAC ATAATTAAAT GATTTTATTT ATATAATTTA ATATTAATAA TTAATTAAAA AGAAAAATAT   
  
  
+ AATTGTATTC TTTAATATTT TAATAAAAAA TAAAAATGAG TTTTTTAATT TTATTTAAGT TTATTTATCT   
  
  
+ TTTACAAGCA CTAATAAAAA ATTTTATTTT CGTATACAAA CTAATTATAA TTGTATTTTT TATGTAGAAT   
  
  
+ TATTAAACAA AATAATTTAG TAATACGTAA TAATAAAAAT AAATTTTTAT TATATTTAAT TTAATTTTAT   
  
  
+ TTTTTAATTA AGTTGGTATA TATATATATA AATAGGTAAG TTAATGTATA ATAATGCACA CGATCCCTGT   
  
  
+ ACGACCAGCC AGTACCCACA CGAATCAGTT TCAGTTCGAG TGGTATTTTC GGTTCTTTGT AACCCACCAC   
  
  
+ CAGAGTAAAA TAGATGATAA GGAGACACCT TAATTATTAA GTATATTATC TACAGATTAA GAATTAATTT   
  
  
+ AAAGTATAAC TTGGGTTAGA ATAGAAACAG AGAAGATGAC ATGGTTTACG AATTAATTTA ATTTTACTGG   
  
  
+ TTAATTATAA AAAATATTTT TTTTACTCAA ATGTCTCTAC AGTGCAAAAA CTTTCGTAAA AGGTGCAATG   
  
  
+ TACGTGGTTA AGCTAAGAAA ATGCAGAAAA GTTTTCCCTA AAAAAAAAAA AAAACAAAGA ATGTAACTTA   
  
  
+ ATTAAATAAG GTGCAATGTA CGTGGTTAAG CTAAGAAAAT GCAGAAAAGT TTTCCCTAAA AAAAAAAAAA   
  
  
+ AACAAAGAAT GTAACTTAAT TAAAAAACTT AGATCTGAGA ACGTACATTA AGTAACATGG TTCTTCCGAA   
  
  
+ TGAATAAGTA AGATTCATAA GTTTCTCCTT ACACTCGTTT TACCACCTCT ACATGGTATG ATAATAATTC   
  
  
+ AGAATTAGCA AAAGTTCTAA AAAAAACAG  

- GGCTGGCCGA CGACTTAGAT CGATCGAAAC CAAGAGATAC CCAGCAAACA GATTGCCTGA AATTGACCTA   
  
  
- ACAATAAAGC AGACTAAGTT CCTCTAGTTT ATCGAATCTT GCAAGCTTTG TGTAGAAACA GAATTCAAGT   
  
  
- TGGTCAAGCT GGCCTAGCAT GTGAAATGAA GAAAAGCGTG TGATATATAG AGCGGAAAGG TGCTGTTGGT   
  
  
- GGGAAACTTG GTGGAAATGA GACGGACACG GATAAGATGT TGCAGGCACA ACTCTATATA TAATGACTCT   
  
  
- GAGTCTATCT AAAGATCCAC GTGGTGGGAT TTGTTGTATA GTTTATTAAT AAGACAATTA ACTGTTTCCC   
  
  
- AGTTTGGTGA GTGTTGGGAG GAGCCATGTG AAGAAGGAAG AAGTTTTCTT TTCTTTTTTT TTCACCGAAT   
  
  
- CTGAATTAGT GTTAACTTTT TTAAAAGTAA AAGAAAAAAA ATATACATTT TTATAATTTA TTGTATTAAT   
  
  
- TTACTAAAAT AAATATACTG AATTAGTGTT AACTTTTTTA AAAGTAAAAG AAAAAAAATA TACATTTTTA   
  
  
- TAATTTATTG TATTAATTTA CTAAAATAAA TATATTAAAT TATAATTATT AATTAATTTT TCTTTTTATA   
  
  
- TTAACATAAG AAATTATAAA ATTATTTTTT ATTTTTACTC AAAAAATTAA AATAAATTCA AATAAATAGA   
  
  
- AAATGTTCGT GATTATTTTT TAAAATAAAA GCATATGTTT GATTAATATT AACATAAAAA ATACATCTTA   
  
  
- ATAATTTGTT TTATTAAATC ATTATGCATT ATTATTTTTA TTTAAAAATA ATATAAATTA AATTAAAATA   
  
  
- AAAAATTAAT TCAACCATAT ATATATATAT TTATCCATTC AATTACATAT TATTACGTGT GCTAGGGACA   
  
  
- TGCTGGTCGG TCATGGGTGT GCTTAGTCAA AGTCAAGCTC ACCATAAAAG CCAAGAAACA TTGGGTGGTG   
  
  
- GTCTCATTTT ATCTACTATT CCTCTGTGGA ATTAATAATT CATATAATAG ATGTCTAATT CTTAATTAAA   
  
  
- TTTCATATTG AACCCAATCT TATCTTTGTC TCTTCTACTG TACCAAATGC TTAATTAAAT TAAAATGACC   
  
  
- AATTAATATT TTTTATAAAA AAAATGAGTT TACAGAGATG TCACGTTTTT GAAAGCATTT TCCACGTTAC   
  
  
- ATGCACCAAT TCGATTCTTT TACGTCTTTT CAAAAGGGAT TTTTTTTTTT TTTTGTTTCT TACATTGAAT   
  
  
- TAATTTATTC CACGTTACAT GCACCAATTC GATTCTTTTA CGTCTTTTCA AAAGGGATTT TTTTTTTTTT   
  
  
- TTGTTTCTTA CATTGAATTA ATTTTTTGAA TCTAGACTCT TGCATGTAAT TCATTGTACC AAGAAGGCTT   
  
  
- ACTTATTCAT TCTAAGTATT CAAAGAGGAA TGTGAGCAAA ATGGTGGAGA TGTACCATAC TATTATTAAG   
  
  
- TCTTAATCGT TTTCAAGATT TTTTTTGTC

+     TCT-motif

| Site Name | Organism | Position | Strand | Matrix score. | sequence | function |
| --- | --- | --- | --- | --- | --- | --- |
| TCT-motif | Arabidopsis thaliana | 1408 | - | 6 | TCTTAC | part of a light responsive element |

> 2018/04/13 10:10:12  
+ CCGACCGGCT GCTGAATCTA GCTAGCTTTG GTTCTCTATG GGTCGTTTGT CTAACGGACT TTAACTGGAT   
  
  
+ TGTTATTTCG TCTGATTCAA GGAGATCAAA TAGCTTAGAA CGTTCGAAAC ACATCTTTGT CTTAAGTTCA   
  
  
+ ACCAGTTCGA CCGGATCGTA CACTTTACTT CTTTTCGCAC ACTATATATC TCGCCTTTCC ACGACAACCA   
  
  
+ CCCTTTGAAC CACCTTTACT CTGCCTGTGC CTATTCTACA ACGTCCGTGT TGAGATATAT ATTACTGAGA   
  
  
+ CTCAGATAGA TTTCTAGGTG CACCACCCTA AACAACATAT CAAATAATTA TTCTGTTAAT TGACAAAGGG   
  
  
+ TCAAACCACT CACAACCCTC CTCGGTACAC TTCTTCCTTC TTCAAAAGAA AAGAAAAAAA AAGTGGCTTA   
  
  
+ GACTTAATCA CAATTGAAAA AATTTTCATT TTCTTTTTTT TATATGTAAA AATATTAAAT AACATAATTA   
  
  
+ AATGATTTTA TTTATATGAC TTAATCACAA TTGAAAAAAT TTTCATTTTC TTTTTTTTAT ATGTAAAAAT   
  
  
+ ATTAAATAAC ATAATTAAAT GATTTTATTT ATATAATTTA ATATTAATAA TTAATTAAAA AGAAAAATAT   
  
  
+ AATTGTATTC TTTAATATTT TAATAAAAAA TAAAAATGAG TTTTTTAATT TTATTTAAGT TTATTTATCT   
  
  
+ TTTACAAGCA CTAATAAAAA ATTTTATTTT CGTATACAAA CTAATTATAA TTGTATTTTT TATGTAGAAT   
  
  
+ TATTAAACAA AATAATTTAG TAATACGTAA TAATAAAAAT AAATTTTTAT TATATTTAAT TTAATTTTAT   
  
  
+ TTTTTAATTA AGTTGGTATA TATATATATA AATAGGTAAG TTAATGTATA ATAATGCACA CGATCCCTGT   
  
  
+ ACGACCAGCC AGTACCCACA CGAATCAGTT TCAGTTCGAG TGGTATTTTC GGTTCTTTGT AACCCACCAC   
  
  
+ CAGAGTAAAA TAGATGATAA GGAGACACCT TAATTATTAA GTATATTATC TACAGATTAA GAATTAATTT   
  
  
+ AAAGTATAAC TTGGGTTAGA ATAGAAACAG AGAAGATGAC ATGGTTTACG AATTAATTTA ATTTTACTGG   
  
  
+ TTAATTATAA AAAATATTTT TTTTACTCAA ATGTCTCTAC AGTGCAAAAA CTTTCGTAAA AGGTGCAATG   
  
  
+ TACGTGGTTA AGCTAAGAAA ATGCAGAAAA GTTTTCCCTA AAAAAAAAAA AAAACAAAGA ATGTAACTTA   
  
  
+ ATTAAATAAG GTGCAATGTA CGTGGTTAAG CTAAGAAAAT GCAGAAAAGT TTTCCCTAAA AAAAAAAAAA   
  
  
+ AACAAAGAAT GTAACTTAAT TAAAAAACTT AGATCTGAGA ACGTACATTA AGTAACATGG TTCTTCCGAA   
  
  
+ TGAATAAGTA AGATTCATAA GTTTCTCCTT ACACTCGTTT TACCACCTCT ACATGGTATG ATAATAATTC   
  
  
+ AGAATTAGCA AAAGTTCTAA AAAAAACAG  

- GGCTGGCCGA CGACTTAGAT CGATCGAAAC CAAGAGATAC CCAGCAAACA GATTGCCTGA AATTGACCTA   
  
  
- ACAATAAAGC AGACTAAGTT CCTCTAGTTT ATCGAATCTT GCAAGCTTTG TGTAGAAACA GAATTCAAGT   
  
  
- TGGTCAAGCT GGCCTAGCAT GTGAAATGAA GAAAAGCGTG TGATATATAG AGCGGAAAGG TGCTGTTGGT   
  
  
- GGGAAACTTG GTGGAAATGA GACGGACACG GATAAGATGT TGCAGGCACA ACTCTATATA TAATGACTCT   
  
  
- GAGTCTATCT AAAGATCCAC GTGGTGGGAT TTGTTGTATA GTTTATTAAT AAGACAATTA ACTGTTTCCC   
  
  
- AGTTTGGTGA GTGTTGGGAG GAGCCATGTG AAGAAGGAAG AAGTTTTCTT TTCTTTTTTT TTCACCGAAT   
  
  
- CTGAATTAGT GTTAACTTTT TTAAAAGTAA AAGAAAAAAA ATATACATTT TTATAATTTA TTGTATTAAT   
  
  
- TTACTAAAAT AAATATACTG AATTAGTGTT AACTTTTTTA AAAGTAAAAG AAAAAAAATA TACATTTTTA   
  
  
- TAATTTATTG TATTAATTTA CTAAAATAAA TATATTAAAT TATAATTATT AATTAATTTT TCTTTTTATA   
  
  
- TTAACATAAG AAATTATAAA ATTATTTTTT ATTTTTACTC AAAAAATTAA AATAAATTCA AATAAATAGA   
  
  
- AAATGTTCGT GATTATTTTT TAAAATAAAA GCATATGTTT GATTAATATT AACATAAAAA ATACATCTTA   
  
  
- ATAATTTGTT TTATTAAATC ATTATGCATT ATTATTTTTA TTTAAAAATA ATATAAATTA AATTAAAATA   
  
  
- AAAAATTAAT TCAACCATAT ATATATATAT TTATCCATTC AATTACATAT TATTACGTGT GCTAGGGACA   
  
  
- TGCTGGTCGG TCATGGGTGT GCTTAGTCAA AGTCAAGCTC ACCATAAAAG CCAAGAAACA TTGGGTGGTG   
  
  
- GTCTCATTTT ATCTACTATT CCTCTGTGGA ATTAATAATT CATATAATAG ATGTCTAATT CTTAATTAAA   
  
  
- TTTCATATTG AACCCAATCT TATCTTTGTC TCTTCTACTG TACCAAATGC TTAATTAAAT TAAAATGACC   
  
  
- AATTAATATT TTTTATAAAA AAAATGAGTT TACAGAGATG TCACGTTTTT GAAAGCATTT TCCACGTTAC   
  
  
- ATGCACCAAT TCGATTCTTT TACGTCTTTT CAAAAGGGAT TTTTTTTTTT TTTTGTTTCT TACATTGAAT   
  
  
- TAATTTATTC CACGTTACAT GCACCAATTC GATTCTTTTA CGTCTTTTCA AAAGGGATTT TTTTTTTTTT   
  
  
- TTGTTTCTTA CATTGAATTA ATTTTTTGAA TCTAGACTCT TGCATGTAAT TCATTGTACC AAGAAGGCTT   
  
  
- ACTTATTCAT TCTAAGTATT CAAAGAGGAA TGTGAGCAAA ATGGTGGAGA TGTACCATAC TATTATTAAG   
  
  
- TCTTAATCGT TTTCAAGATT TTTTTTGTC

+     TGA-element

| Site Name | Organism | Position | Strand | Matrix score. | sequence | function |
| --- | --- | --- | --- | --- | --- | --- |
| TGA-element | Brassica oleracea | 42 | - | 6 | AACGAC | auxin-responsive element |

> 2018/04/13 10:10:12  
+ CCGACCGGCT GCTGAATCTA GCTAGCTTTG GTTCTCTATG GGTCGTTTGT CTAACGGACT TTAACTGGAT   
  
  
+ TGTTATTTCG TCTGATTCAA GGAGATCAAA TAGCTTAGAA CGTTCGAAAC ACATCTTTGT CTTAAGTTCA   
  
  
+ ACCAGTTCGA CCGGATCGTA CACTTTACTT CTTTTCGCAC ACTATATATC TCGCCTTTCC ACGACAACCA   
  
  
+ CCCTTTGAAC CACCTTTACT CTGCCTGTGC CTATTCTACA ACGTCCGTGT TGAGATATAT ATTACTGAGA   
  
  
+ CTCAGATAGA TTTCTAGGTG CACCACCCTA AACAACATAT CAAATAATTA TTCTGTTAAT TGACAAAGGG   
  
  
+ TCAAACCACT CACAACCCTC CTCGGTACAC TTCTTCCTTC TTCAAAAGAA AAGAAAAAAA AAGTGGCTTA   
  
  
+ GACTTAATCA CAATTGAAAA AATTTTCATT TTCTTTTTTT TATATGTAAA AATATTAAAT AACATAATTA   
  
  
+ AATGATTTTA TTTATATGAC TTAATCACAA TTGAAAAAAT TTTCATTTTC TTTTTTTTAT ATGTAAAAAT   
  
  
+ ATTAAATAAC ATAATTAAAT GATTTTATTT ATATAATTTA ATATTAATAA TTAATTAAAA AGAAAAATAT   
  
  
+ AATTGTATTC TTTAATATTT TAATAAAAAA TAAAAATGAG TTTTTTAATT TTATTTAAGT TTATTTATCT   
  
  
+ TTTACAAGCA CTAATAAAAA ATTTTATTTT CGTATACAAA CTAATTATAA TTGTATTTTT TATGTAGAAT   
  
  
+ TATTAAACAA AATAATTTAG TAATACGTAA TAATAAAAAT AAATTTTTAT TATATTTAAT TTAATTTTAT   
  
  
+ TTTTTAATTA AGTTGGTATA TATATATATA AATAGGTAAG TTAATGTATA ATAATGCACA CGATCCCTGT   
  
  
+ ACGACCAGCC AGTACCCACA CGAATCAGTT TCAGTTCGAG TGGTATTTTC GGTTCTTTGT AACCCACCAC   
  
  
+ CAGAGTAAAA TAGATGATAA GGAGACACCT TAATTATTAA GTATATTATC TACAGATTAA GAATTAATTT   
  
  
+ AAAGTATAAC TTGGGTTAGA ATAGAAACAG AGAAGATGAC ATGGTTTACG AATTAATTTA ATTTTACTGG   
  
  
+ TTAATTATAA AAAATATTTT TTTTACTCAA ATGTCTCTAC AGTGCAAAAA CTTTCGTAAA AGGTGCAATG   
  
  
+ TACGTGGTTA AGCTAAGAAA ATGCAGAAAA GTTTTCCCTA AAAAAAAAAA AAAACAAAGA ATGTAACTTA   
  
  
+ ATTAAATAAG GTGCAATGTA CGTGGTTAAG CTAAGAAAAT GCAGAAAAGT TTTCCCTAAA AAAAAAAAAA   
  
  
+ AACAAAGAAT GTAACTTAAT TAAAAAACTT AGATCTGAGA ACGTACATTA AGTAACATGG TTCTTCCGAA   
  
  
+ TGAATAAGTA AGATTCATAA GTTTCTCCTT ACACTCGTTT TACCACCTCT ACATGGTATG ATAATAATTC   
  
  
+ AGAATTAGCA AAAGTTCTAA AAAAAACAG  

- GGCTGGCCGA CGACTTAGAT CGATCGAAAC CAAGAGATAC CCAGCAAACA GATTGCCTGA AATTGACCTA   
  
  
- ACAATAAAGC AGACTAAGTT CCTCTAGTTT ATCGAATCTT GCAAGCTTTG TGTAGAAACA GAATTCAAGT   
  
  
- TGGTCAAGCT GGCCTAGCAT GTGAAATGAA GAAAAGCGTG TGATATATAG AGCGGAAAGG TGCTGTTGGT   
  
  
- GGGAAACTTG GTGGAAATGA GACGGACACG GATAAGATGT TGCAGGCACA ACTCTATATA TAATGACTCT   
  
  
- GAGTCTATCT AAAGATCCAC GTGGTGGGAT TTGTTGTATA GTTTATTAAT AAGACAATTA ACTGTTTCCC   
  
  
- AGTTTGGTGA GTGTTGGGAG GAGCCATGTG AAGAAGGAAG AAGTTTTCTT TTCTTTTTTT TTCACCGAAT   
  
  
- CTGAATTAGT GTTAACTTTT TTAAAAGTAA AAGAAAAAAA ATATACATTT TTATAATTTA TTGTATTAAT   
  
  
- TTACTAAAAT AAATATACTG AATTAGTGTT AACTTTTTTA AAAGTAAAAG AAAAAAAATA TACATTTTTA   
  
  
- TAATTTATTG TATTAATTTA CTAAAATAAA TATATTAAAT TATAATTATT AATTAATTTT TCTTTTTATA   
  
  
- TTAACATAAG AAATTATAAA ATTATTTTTT ATTTTTACTC AAAAAATTAA AATAAATTCA AATAAATAGA   
  
  
- AAATGTTCGT GATTATTTTT TAAAATAAAA GCATATGTTT GATTAATATT AACATAAAAA ATACATCTTA   
  
  
- ATAATTTGTT TTATTAAATC ATTATGCATT ATTATTTTTA TTTAAAAATA ATATAAATTA AATTAAAATA   
  
  
- AAAAATTAAT TCAACCATAT ATATATATAT TTATCCATTC AATTACATAT TATTACGTGT GCTAGGGACA   
  
  
- TGCTGGTCGG TCATGGGTGT GCTTAGTCAA AGTCAAGCTC ACCATAAAAG CCAAGAAACA TTGGGTGGTG   
  
  
- GTCTCATTTT ATCTACTATT CCTCTGTGGA ATTAATAATT CATATAATAG ATGTCTAATT CTTAATTAAA   
  
  
- TTTCATATTG AACCCAATCT TATCTTTGTC TCTTCTACTG TACCAAATGC TTAATTAAAT TAAAATGACC   
  
  
- AATTAATATT TTTTATAAAA AAAATGAGTT TACAGAGATG TCACGTTTTT GAAAGCATTT TCCACGTTAC   
  
  
- ATGCACCAAT TCGATTCTTT TACGTCTTTT CAAAAGGGAT TTTTTTTTTT TTTTGTTTCT TACATTGAAT   
  
  
- TAATTTATTC CACGTTACAT GCACCAATTC GATTCTTTTA CGTCTTTTCA AAAGGGATTT TTTTTTTTTT   
  
  
- TTGTTTCTTA CATTGAATTA ATTTTTTGAA TCTAGACTCT TGCATGTAAT TCATTGTACC AAGAAGGCTT   
  
  
- ACTTATTCAT TCTAAGTATT CAAAGAGGAA TGTGAGCAAA ATGGTGGAGA TGTACCATAC TATTATTAAG   
  
  
- TCTTAATCGT TTTCAAGATT TTTTTTGTC

+     Unnamed\_\_1

| Site Name | Organism | Position | Strand | Matrix score. | sequence | function |
| --- | --- | --- | --- | --- | --- | --- |
| Unnamed\_\_1 | Zea mays | 199 | - | 5 | CGTGG |  |
| Unnamed\_\_1 | Zea mays | 1281 | + | 5 | CGTGG |  |
| Unnamed\_\_1 | Zea mays | 1193 | + | 5 | CGTGG |  |

> 2018/04/13 10:10:12  
+ CCGACCGGCT GCTGAATCTA GCTAGCTTTG GTTCTCTATG GGTCGTTTGT CTAACGGACT TTAACTGGAT   
  
  
+ TGTTATTTCG TCTGATTCAA GGAGATCAAA TAGCTTAGAA CGTTCGAAAC ACATCTTTGT CTTAAGTTCA   
  
  
+ ACCAGTTCGA CCGGATCGTA CACTTTACTT CTTTTCGCAC ACTATATATC TCGCCTTTCC ACGACAACCA   
  
  
+ CCCTTTGAAC CACCTTTACT CTGCCTGTGC CTATTCTACA ACGTCCGTGT TGAGATATAT ATTACTGAGA   
  
  
+ CTCAGATAGA TTTCTAGGTG CACCACCCTA AACAACATAT CAAATAATTA TTCTGTTAAT TGACAAAGGG   
  
  
+ TCAAACCACT CACAACCCTC CTCGGTACAC TTCTTCCTTC TTCAAAAGAA AAGAAAAAAA AAGTGGCTTA   
  
  
+ GACTTAATCA CAATTGAAAA AATTTTCATT TTCTTTTTTT TATATGTAAA AATATTAAAT AACATAATTA   
  
  
+ AATGATTTTA TTTATATGAC TTAATCACAA TTGAAAAAAT TTTCATTTTC TTTTTTTTAT ATGTAAAAAT   
  
  
+ ATTAAATAAC ATAATTAAAT GATTTTATTT ATATAATTTA ATATTAATAA TTAATTAAAA AGAAAAATAT   
  
  
+ AATTGTATTC TTTAATATTT TAATAAAAAA TAAAAATGAG TTTTTTAATT TTATTTAAGT TTATTTATCT   
  
  
+ TTTACAAGCA CTAATAAAAA ATTTTATTTT CGTATACAAA CTAATTATAA TTGTATTTTT TATGTAGAAT   
  
  
+ TATTAAACAA AATAATTTAG TAATACGTAA TAATAAAAAT AAATTTTTAT TATATTTAAT TTAATTTTAT   
  
  
+ TTTTTAATTA AGTTGGTATA TATATATATA AATAGGTAAG TTAATGTATA ATAATGCACA CGATCCCTGT   
  
  
+ ACGACCAGCC AGTACCCACA CGAATCAGTT TCAGTTCGAG TGGTATTTTC GGTTCTTTGT AACCCACCAC   
  
  
+ CAGAGTAAAA TAGATGATAA GGAGACACCT TAATTATTAA GTATATTATC TACAGATTAA GAATTAATTT   
  
  
+ AAAGTATAAC TTGGGTTAGA ATAGAAACAG AGAAGATGAC ATGGTTTACG AATTAATTTA ATTTTACTGG   
  
  
+ TTAATTATAA AAAATATTTT TTTTACTCAA ATGTCTCTAC AGTGCAAAAA CTTTCGTAAA AGGTGCAATG   
  
  
+ TACGTGGTTA AGCTAAGAAA ATGCAGAAAA GTTTTCCCTA AAAAAAAAAA AAAACAAAGA ATGTAACTTA   
  
  
+ ATTAAATAAG GTGCAATGTA CGTGGTTAAG CTAAGAAAAT GCAGAAAAGT TTTCCCTAAA AAAAAAAAAA   
  
  
+ AACAAAGAAT GTAACTTAAT TAAAAAACTT AGATCTGAGA ACGTACATTA AGTAACATGG TTCTTCCGAA   
  
  
+ TGAATAAGTA AGATTCATAA GTTTCTCCTT ACACTCGTTT TACCACCTCT ACATGGTATG ATAATAATTC   
  
  
+ AGAATTAGCA AAAGTTCTAA AAAAAACAG  

- GGCTGGCCGA CGACTTAGAT CGATCGAAAC CAAGAGATAC CCAGCAAACA GATTGCCTGA AATTGACCTA   
  
  
- ACAATAAAGC AGACTAAGTT CCTCTAGTTT ATCGAATCTT GCAAGCTTTG TGTAGAAACA GAATTCAAGT   
  
  
- TGGTCAAGCT GGCCTAGCAT GTGAAATGAA GAAAAGCGTG TGATATATAG AGCGGAAAGG TGCTGTTGGT   
  
  
- GGGAAACTTG GTGGAAATGA GACGGACACG GATAAGATGT TGCAGGCACA ACTCTATATA TAATGACTCT   
  
  
- GAGTCTATCT AAAGATCCAC GTGGTGGGAT TTGTTGTATA GTTTATTAAT AAGACAATTA ACTGTTTCCC   
  
  
- AGTTTGGTGA GTGTTGGGAG GAGCCATGTG AAGAAGGAAG AAGTTTTCTT TTCTTTTTTT TTCACCGAAT   
  
  
- CTGAATTAGT GTTAACTTTT TTAAAAGTAA AAGAAAAAAA ATATACATTT TTATAATTTA TTGTATTAAT   
  
  
- TTACTAAAAT AAATATACTG AATTAGTGTT AACTTTTTTA AAAGTAAAAG AAAAAAAATA TACATTTTTA   
  
  
- TAATTTATTG TATTAATTTA CTAAAATAAA TATATTAAAT TATAATTATT AATTAATTTT TCTTTTTATA   
  
  
- TTAACATAAG AAATTATAAA ATTATTTTTT ATTTTTACTC AAAAAATTAA AATAAATTCA AATAAATAGA   
  
  
- AAATGTTCGT GATTATTTTT TAAAATAAAA GCATATGTTT GATTAATATT AACATAAAAA ATACATCTTA   
  
  
- ATAATTTGTT TTATTAAATC ATTATGCATT ATTATTTTTA TTTAAAAATA ATATAAATTA AATTAAAATA   
  
  
- AAAAATTAAT TCAACCATAT ATATATATAT TTATCCATTC AATTACATAT TATTACGTGT GCTAGGGACA   
  
  
- TGCTGGTCGG TCATGGGTGT GCTTAGTCAA AGTCAAGCTC ACCATAAAAG CCAAGAAACA TTGGGTGGTG   
  
  
- GTCTCATTTT ATCTACTATT CCTCTGTGGA ATTAATAATT CATATAATAG ATGTCTAATT CTTAATTAAA   
  
  
- TTTCATATTG AACCCAATCT TATCTTTGTC TCTTCTACTG TACCAAATGC TTAATTAAAT TAAAATGACC   
  
  
- AATTAATATT TTTTATAAAA AAAATGAGTT TACAGAGATG TCACGTTTTT GAAAGCATTT TCCACGTTAC   
  
  
- ATGCACCAAT TCGATTCTTT TACGTCTTTT CAAAAGGGAT TTTTTTTTTT TTTTGTTTCT TACATTGAAT   
  
  
- TAATTTATTC CACGTTACAT GCACCAATTC GATTCTTTTA CGTCTTTTCA AAAGGGATTT TTTTTTTTTT   
  
  
- TTGTTTCTTA CATTGAATTA ATTTTTTGAA TCTAGACTCT TGCATGTAAT TCATTGTACC AAGAAGGCTT   
  
  
- ACTTATTCAT TCTAAGTATT CAAAGAGGAA TGTGAGCAAA ATGGTGGAGA TGTACCATAC TATTATTAAG   
  
  
- TCTTAATCGT TTTCAAGATT TTTTTTGTC

+     Unnamed\_\_3

| Site Name | Organism | Position | Strand | Matrix score. | sequence | function |
| --- | --- | --- | --- | --- | --- | --- |
| Unnamed\_\_3 | Zea mays | 199 | - | 5 | CGTGG |  |
| Unnamed\_\_3 | Zea mays | 1281 | + | 5 | CGTGG |  |
| Unnamed\_\_3 | Zea mays | 1193 | + | 5 | CGTGG |  |

> 2018/04/13 10:10:12  
+ CCGACCGGCT GCTGAATCTA GCTAGCTTTG GTTCTCTATG GGTCGTTTGT CTAACGGACT TTAACTGGAT   
  
  
+ TGTTATTTCG TCTGATTCAA GGAGATCAAA TAGCTTAGAA CGTTCGAAAC ACATCTTTGT CTTAAGTTCA   
  
  
+ ACCAGTTCGA CCGGATCGTA CACTTTACTT CTTTTCGCAC ACTATATATC TCGCCTTTCC ACGACAACCA   
  
  
+ CCCTTTGAAC CACCTTTACT CTGCCTGTGC CTATTCTACA ACGTCCGTGT TGAGATATAT ATTACTGAGA   
  
  
+ CTCAGATAGA TTTCTAGGTG CACCACCCTA AACAACATAT CAAATAATTA TTCTGTTAAT TGACAAAGGG   
  
  
+ TCAAACCACT CACAACCCTC CTCGGTACAC TTCTTCCTTC TTCAAAAGAA AAGAAAAAAA AAGTGGCTTA   
  
  
+ GACTTAATCA CAATTGAAAA AATTTTCATT TTCTTTTTTT TATATGTAAA AATATTAAAT AACATAATTA   
  
  
+ AATGATTTTA TTTATATGAC TTAATCACAA TTGAAAAAAT TTTCATTTTC TTTTTTTTAT ATGTAAAAAT   
  
  
+ ATTAAATAAC ATAATTAAAT GATTTTATTT ATATAATTTA ATATTAATAA TTAATTAAAA AGAAAAATAT   
  
  
+ AATTGTATTC TTTAATATTT TAATAAAAAA TAAAAATGAG TTTTTTAATT TTATTTAAGT TTATTTATCT   
  
  
+ TTTACAAGCA CTAATAAAAA ATTTTATTTT CGTATACAAA CTAATTATAA TTGTATTTTT TATGTAGAAT   
  
  
+ TATTAAACAA AATAATTTAG TAATACGTAA TAATAAAAAT AAATTTTTAT TATATTTAAT TTAATTTTAT   
  
  
+ TTTTTAATTA AGTTGGTATA TATATATATA AATAGGTAAG TTAATGTATA ATAATGCACA CGATCCCTGT   
  
  
+ ACGACCAGCC AGTACCCACA CGAATCAGTT TCAGTTCGAG TGGTATTTTC GGTTCTTTGT AACCCACCAC   
  
  
+ CAGAGTAAAA TAGATGATAA GGAGACACCT TAATTATTAA GTATATTATC TACAGATTAA GAATTAATTT   
  
  
+ AAAGTATAAC TTGGGTTAGA ATAGAAACAG AGAAGATGAC ATGGTTTACG AATTAATTTA ATTTTACTGG   
  
  
+ TTAATTATAA AAAATATTTT TTTTACTCAA ATGTCTCTAC AGTGCAAAAA CTTTCGTAAA AGGTGCAATG   
  
  
+ TACGTGGTTA AGCTAAGAAA ATGCAGAAAA GTTTTCCCTA AAAAAAAAAA AAAACAAAGA ATGTAACTTA   
  
  
+ ATTAAATAAG GTGCAATGTA CGTGGTTAAG CTAAGAAAAT GCAGAAAAGT TTTCCCTAAA AAAAAAAAAA   
  
  
+ AACAAAGAAT GTAACTTAAT TAAAAAACTT AGATCTGAGA ACGTACATTA AGTAACATGG TTCTTCCGAA   
  
  
+ TGAATAAGTA AGATTCATAA GTTTCTCCTT ACACTCGTTT TACCACCTCT ACATGGTATG ATAATAATTC   
  
  
+ AGAATTAGCA AAAGTTCTAA AAAAAACAG  

- GGCTGGCCGA CGACTTAGAT CGATCGAAAC CAAGAGATAC CCAGCAAACA GATTGCCTGA AATTGACCTA   
  
  
- ACAATAAAGC AGACTAAGTT CCTCTAGTTT ATCGAATCTT GCAAGCTTTG TGTAGAAACA GAATTCAAGT   
  
  
- TGGTCAAGCT GGCCTAGCAT GTGAAATGAA GAAAAGCGTG TGATATATAG AGCGGAAAGG TGCTGTTGGT   
  
  
- GGGAAACTTG GTGGAAATGA GACGGACACG GATAAGATGT TGCAGGCACA ACTCTATATA TAATGACTCT   
  
  
- GAGTCTATCT AAAGATCCAC GTGGTGGGAT TTGTTGTATA GTTTATTAAT AAGACAATTA ACTGTTTCCC   
  
  
- AGTTTGGTGA GTGTTGGGAG GAGCCATGTG AAGAAGGAAG AAGTTTTCTT TTCTTTTTTT TTCACCGAAT   
  
  
- CTGAATTAGT GTTAACTTTT TTAAAAGTAA AAGAAAAAAA ATATACATTT TTATAATTTA TTGTATTAAT   
  
  
- TTACTAAAAT AAATATACTG AATTAGTGTT AACTTTTTTA AAAGTAAAAG AAAAAAAATA TACATTTTTA   
  
  
- TAATTTATTG TATTAATTTA CTAAAATAAA TATATTAAAT TATAATTATT AATTAATTTT TCTTTTTATA   
  
  
- TTAACATAAG AAATTATAAA ATTATTTTTT ATTTTTACTC AAAAAATTAA AATAAATTCA AATAAATAGA   
  
  
- AAATGTTCGT GATTATTTTT TAAAATAAAA GCATATGTTT GATTAATATT AACATAAAAA ATACATCTTA   
  
  
- ATAATTTGTT TTATTAAATC ATTATGCATT ATTATTTTTA TTTAAAAATA ATATAAATTA AATTAAAATA   
  
  
- AAAAATTAAT TCAACCATAT ATATATATAT TTATCCATTC AATTACATAT TATTACGTGT GCTAGGGACA   
  
  
- TGCTGGTCGG TCATGGGTGT GCTTAGTCAA AGTCAAGCTC ACCATAAAAG CCAAGAAACA TTGGGTGGTG   
  
  
- GTCTCATTTT ATCTACTATT CCTCTGTGGA ATTAATAATT CATATAATAG ATGTCTAATT CTTAATTAAA   
  
  
- TTTCATATTG AACCCAATCT TATCTTTGTC TCTTCTACTG TACCAAATGC TTAATTAAAT TAAAATGACC   
  
  
- AATTAATATT TTTTATAAAA AAAATGAGTT TACAGAGATG TCACGTTTTT GAAAGCATTT TCCACGTTAC   
  
  
- ATGCACCAAT TCGATTCTTT TACGTCTTTT CAAAAGGGAT TTTTTTTTTT TTTTGTTTCT TACATTGAAT   
  
  
- TAATTTATTC CACGTTACAT GCACCAATTC GATTCTTTTA CGTCTTTTCA AAAGGGATTT TTTTTTTTTT   
  
  
- TTGTTTCTTA CATTGAATTA ATTTTTTGAA TCTAGACTCT TGCATGTAAT TCATTGTACC AAGAAGGCTT   
  
  
- ACTTATTCAT TCTAAGTATT CAAAGAGGAA TGTGAGCAAA ATGGTGGAGA TGTACCATAC TATTATTAAG   
  
  
- TCTTAATCGT TTTCAAGATT TTTTTTGTC

+     Unnamed\_\_4

| Site Name | Organism | Position | Strand | Matrix score. | sequence | function |
| --- | --- | --- | --- | --- | --- | --- |
| Unnamed\_\_4 | Petroselinum hortense | 91 | - | 4 | CTCC |  |
| Unnamed\_\_4 | Petroselinum hortense | 1001 | - | 4 | CTCC |  |
| Unnamed\_\_4 | Petroselinum hortense | 368 | + | 4 | CTCC |  |
| Unnamed\_\_4 | Petroselinum hortense | 1425 | + | 4 | CTCC |  |

> 2018/04/13 10:10:12  
+ CCGACCGGCT GCTGAATCTA GCTAGCTTTG GTTCTCTATG GGTCGTTTGT CTAACGGACT TTAACTGGAT   
  
  
+ TGTTATTTCG TCTGATTCAA GGAGATCAAA TAGCTTAGAA CGTTCGAAAC ACATCTTTGT CTTAAGTTCA   
  
  
+ ACCAGTTCGA CCGGATCGTA CACTTTACTT CTTTTCGCAC ACTATATATC TCGCCTTTCC ACGACAACCA   
  
  
+ CCCTTTGAAC CACCTTTACT CTGCCTGTGC CTATTCTACA ACGTCCGTGT TGAGATATAT ATTACTGAGA   
  
  
+ CTCAGATAGA TTTCTAGGTG CACCACCCTA AACAACATAT CAAATAATTA TTCTGTTAAT TGACAAAGGG   
  
  
+ TCAAACCACT CACAACCCTC CTCGGTACAC TTCTTCCTTC TTCAAAAGAA AAGAAAAAAA AAGTGGCTTA   
  
  
+ GACTTAATCA CAATTGAAAA AATTTTCATT TTCTTTTTTT TATATGTAAA AATATTAAAT AACATAATTA   
  
  
+ AATGATTTTA TTTATATGAC TTAATCACAA TTGAAAAAAT TTTCATTTTC TTTTTTTTAT ATGTAAAAAT   
  
  
+ ATTAAATAAC ATAATTAAAT GATTTTATTT ATATAATTTA ATATTAATAA TTAATTAAAA AGAAAAATAT   
  
  
+ AATTGTATTC TTTAATATTT TAATAAAAAA TAAAAATGAG TTTTTTAATT TTATTTAAGT TTATTTATCT   
  
  
+ TTTACAAGCA CTAATAAAAA ATTTTATTTT CGTATACAAA CTAATTATAA TTGTATTTTT TATGTAGAAT   
  
  
+ TATTAAACAA AATAATTTAG TAATACGTAA TAATAAAAAT AAATTTTTAT TATATTTAAT TTAATTTTAT   
  
  
+ TTTTTAATTA AGTTGGTATA TATATATATA AATAGGTAAG TTAATGTATA ATAATGCACA CGATCCCTGT   
  
  
+ ACGACCAGCC AGTACCCACA CGAATCAGTT TCAGTTCGAG TGGTATTTTC GGTTCTTTGT AACCCACCAC   
  
  
+ CAGAGTAAAA TAGATGATAA GGAGACACCT TAATTATTAA GTATATTATC TACAGATTAA GAATTAATTT   
  
  
+ AAAGTATAAC TTGGGTTAGA ATAGAAACAG AGAAGATGAC ATGGTTTACG AATTAATTTA ATTTTACTGG   
  
  
+ TTAATTATAA AAAATATTTT TTTTACTCAA ATGTCTCTAC AGTGCAAAAA CTTTCGTAAA AGGTGCAATG   
  
  
+ TACGTGGTTA AGCTAAGAAA ATGCAGAAAA GTTTTCCCTA AAAAAAAAAA AAAACAAAGA ATGTAACTTA   
  
  
+ ATTAAATAAG GTGCAATGTA CGTGGTTAAG CTAAGAAAAT GCAGAAAAGT TTTCCCTAAA AAAAAAAAAA   
  
  
+ AACAAAGAAT GTAACTTAAT TAAAAAACTT AGATCTGAGA ACGTACATTA AGTAACATGG TTCTTCCGAA   
  
  
+ TGAATAAGTA AGATTCATAA GTTTCTCCTT ACACTCGTTT TACCACCTCT ACATGGTATG ATAATAATTC   
  
  
+ AGAATTAGCA AAAGTTCTAA AAAAAACAG  

- GGCTGGCCGA CGACTTAGAT CGATCGAAAC CAAGAGATAC CCAGCAAACA GATTGCCTGA AATTGACCTA   
  
  
- ACAATAAAGC AGACTAAGTT CCTCTAGTTT ATCGAATCTT GCAAGCTTTG TGTAGAAACA GAATTCAAGT   
  
  
- TGGTCAAGCT GGCCTAGCAT GTGAAATGAA GAAAAGCGTG TGATATATAG AGCGGAAAGG TGCTGTTGGT   
  
  
- GGGAAACTTG GTGGAAATGA GACGGACACG GATAAGATGT TGCAGGCACA ACTCTATATA TAATGACTCT   
  
  
- GAGTCTATCT AAAGATCCAC GTGGTGGGAT TTGTTGTATA GTTTATTAAT AAGACAATTA ACTGTTTCCC   
  
  
- AGTTTGGTGA GTGTTGGGAG GAGCCATGTG AAGAAGGAAG AAGTTTTCTT TTCTTTTTTT TTCACCGAAT   
  
  
- CTGAATTAGT GTTAACTTTT TTAAAAGTAA AAGAAAAAAA ATATACATTT TTATAATTTA TTGTATTAAT   
  
  
- TTACTAAAAT AAATATACTG AATTAGTGTT AACTTTTTTA AAAGTAAAAG AAAAAAAATA TACATTTTTA   
  
  
- TAATTTATTG TATTAATTTA CTAAAATAAA TATATTAAAT TATAATTATT AATTAATTTT TCTTTTTATA   
  
  
- TTAACATAAG AAATTATAAA ATTATTTTTT ATTTTTACTC AAAAAATTAA AATAAATTCA AATAAATAGA   
  
  
- AAATGTTCGT GATTATTTTT TAAAATAAAA GCATATGTTT GATTAATATT AACATAAAAA ATACATCTTA   
  
  
- ATAATTTGTT TTATTAAATC ATTATGCATT ATTATTTTTA TTTAAAAATA ATATAAATTA AATTAAAATA   
  
  
- AAAAATTAAT TCAACCATAT ATATATATAT TTATCCATTC AATTACATAT TATTACGTGT GCTAGGGACA   
  
  
- TGCTGGTCGG TCATGGGTGT GCTTAGTCAA AGTCAAGCTC ACCATAAAAG CCAAGAAACA TTGGGTGGTG   
  
  
- GTCTCATTTT ATCTACTATT CCTCTGTGGA ATTAATAATT CATATAATAG ATGTCTAATT CTTAATTAAA   
  
  
- TTTCATATTG AACCCAATCT TATCTTTGTC TCTTCTACTG TACCAAATGC TTAATTAAAT TAAAATGACC   
  
  
- AATTAATATT TTTTATAAAA AAAATGAGTT TACAGAGATG TCACGTTTTT GAAAGCATTT TCCACGTTAC   
  
  
- ATGCACCAAT TCGATTCTTT TACGTCTTTT CAAAAGGGAT TTTTTTTTTT TTTTGTTTCT TACATTGAAT   
  
  
- TAATTTATTC CACGTTACAT GCACCAATTC GATTCTTTTA CGTCTTTTCA AAAGGGATTT TTTTTTTTTT   
  
  
- TTGTTTCTTA CATTGAATTA ATTTTTTGAA TCTAGACTCT TGCATGTAAT TCATTGTACC AAGAAGGCTT   
  
  
- ACTTATTCAT TCTAAGTATT CAAAGAGGAA TGTGAGCAAA ATGGTGGAGA TGTACCATAC TATTATTAAG   
  
  
- TCTTAATCGT TTTCAAGATT TTTTTTGTC

+     W box

| Site Name | Organism | Position | Strand | Matrix score. | sequence | function |
| --- | --- | --- | --- | --- | --- | --- |
| W box | Arabidopsis thaliana | 349 | - | 6 | TTGACC |  |

> 2018/04/13 10:10:12  
+ CCGACCGGCT GCTGAATCTA GCTAGCTTTG GTTCTCTATG GGTCGTTTGT CTAACGGACT TTAACTGGAT   
  
  
+ TGTTATTTCG TCTGATTCAA GGAGATCAAA TAGCTTAGAA CGTTCGAAAC ACATCTTTGT CTTAAGTTCA   
  
  
+ ACCAGTTCGA CCGGATCGTA CACTTTACTT CTTTTCGCAC ACTATATATC TCGCCTTTCC ACGACAACCA   
  
  
+ CCCTTTGAAC CACCTTTACT CTGCCTGTGC CTATTCTACA ACGTCCGTGT TGAGATATAT ATTACTGAGA   
  
  
+ CTCAGATAGA TTTCTAGGTG CACCACCCTA AACAACATAT CAAATAATTA TTCTGTTAAT TGACAAAGGG   
  
  
+ TCAAACCACT CACAACCCTC CTCGGTACAC TTCTTCCTTC TTCAAAAGAA AAGAAAAAAA AAGTGGCTTA   
  
  
+ GACTTAATCA CAATTGAAAA AATTTTCATT TTCTTTTTTT TATATGTAAA AATATTAAAT AACATAATTA   
  
  
+ AATGATTTTA TTTATATGAC TTAATCACAA TTGAAAAAAT TTTCATTTTC TTTTTTTTAT ATGTAAAAAT   
  
  
+ ATTAAATAAC ATAATTAAAT GATTTTATTT ATATAATTTA ATATTAATAA TTAATTAAAA AGAAAAATAT   
  
  
+ AATTGTATTC TTTAATATTT TAATAAAAAA TAAAAATGAG TTTTTTAATT TTATTTAAGT TTATTTATCT   
  
  
+ TTTACAAGCA CTAATAAAAA ATTTTATTTT CGTATACAAA CTAATTATAA TTGTATTTTT TATGTAGAAT   
  
  
+ TATTAAACAA AATAATTTAG TAATACGTAA TAATAAAAAT AAATTTTTAT TATATTTAAT TTAATTTTAT   
  
  
+ TTTTTAATTA AGTTGGTATA TATATATATA AATAGGTAAG TTAATGTATA ATAATGCACA CGATCCCTGT   
  
  
+ ACGACCAGCC AGTACCCACA CGAATCAGTT TCAGTTCGAG TGGTATTTTC GGTTCTTTGT AACCCACCAC   
  
  
+ CAGAGTAAAA TAGATGATAA GGAGACACCT TAATTATTAA GTATATTATC TACAGATTAA GAATTAATTT   
  
  
+ AAAGTATAAC TTGGGTTAGA ATAGAAACAG AGAAGATGAC ATGGTTTACG AATTAATTTA ATTTTACTGG   
  
  
+ TTAATTATAA AAAATATTTT TTTTACTCAA ATGTCTCTAC AGTGCAAAAA CTTTCGTAAA AGGTGCAATG   
  
  
+ TACGTGGTTA AGCTAAGAAA ATGCAGAAAA GTTTTCCCTA AAAAAAAAAA AAAACAAAGA ATGTAACTTA   
  
  
+ ATTAAATAAG GTGCAATGTA CGTGGTTAAG CTAAGAAAAT GCAGAAAAGT TTTCCCTAAA AAAAAAAAAA   
  
  
+ AACAAAGAAT GTAACTTAAT TAAAAAACTT AGATCTGAGA ACGTACATTA AGTAACATGG TTCTTCCGAA   
  
  
+ TGAATAAGTA AGATTCATAA GTTTCTCCTT ACACTCGTTT TACCACCTCT ACATGGTATG ATAATAATTC   
  
  
+ AGAATTAGCA AAAGTTCTAA AAAAAACAG  

- GGCTGGCCGA CGACTTAGAT CGATCGAAAC CAAGAGATAC CCAGCAAACA GATTGCCTGA AATTGACCTA   
  
  
- ACAATAAAGC AGACTAAGTT CCTCTAGTTT ATCGAATCTT GCAAGCTTTG TGTAGAAACA GAATTCAAGT   
  
  
- TGGTCAAGCT GGCCTAGCAT GTGAAATGAA GAAAAGCGTG TGATATATAG AGCGGAAAGG TGCTGTTGGT   
  
  
- GGGAAACTTG GTGGAAATGA GACGGACACG GATAAGATGT TGCAGGCACA ACTCTATATA TAATGACTCT   
  
  
- GAGTCTATCT AAAGATCCAC GTGGTGGGAT TTGTTGTATA GTTTATTAAT AAGACAATTA ACTGTTTCCC   
  
  
- AGTTTGGTGA GTGTTGGGAG GAGCCATGTG AAGAAGGAAG AAGTTTTCTT TTCTTTTTTT TTCACCGAAT   
  
  
- CTGAATTAGT GTTAACTTTT TTAAAAGTAA AAGAAAAAAA ATATACATTT TTATAATTTA TTGTATTAAT   
  
  
- TTACTAAAAT AAATATACTG AATTAGTGTT AACTTTTTTA AAAGTAAAAG AAAAAAAATA TACATTTTTA   
  
  
- TAATTTATTG TATTAATTTA CTAAAATAAA TATATTAAAT TATAATTATT AATTAATTTT TCTTTTTATA   
  
  
- TTAACATAAG AAATTATAAA ATTATTTTTT ATTTTTACTC AAAAAATTAA AATAAATTCA AATAAATAGA   
  
  
- AAATGTTCGT GATTATTTTT TAAAATAAAA GCATATGTTT GATTAATATT AACATAAAAA ATACATCTTA   
  
  
- ATAATTTGTT TTATTAAATC ATTATGCATT ATTATTTTTA TTTAAAAATA ATATAAATTA AATTAAAATA   
  
  
- AAAAATTAAT TCAACCATAT ATATATATAT TTATCCATTC AATTACATAT TATTACGTGT GCTAGGGACA   
  
  
- TGCTGGTCGG TCATGGGTGT GCTTAGTCAA AGTCAAGCTC ACCATAAAAG CCAAGAAACA TTGGGTGGTG   
  
  
- GTCTCATTTT ATCTACTATT CCTCTGTGGA ATTAATAATT CATATAATAG ATGTCTAATT CTTAATTAAA   
  
  
- TTTCATATTG AACCCAATCT TATCTTTGTC TCTTCTACTG TACCAAATGC TTAATTAAAT TAAAATGACC   
  
  
- AATTAATATT TTTTATAAAA AAAATGAGTT TACAGAGATG TCACGTTTTT GAAAGCATTT TCCACGTTAC   
  
  
- ATGCACCAAT TCGATTCTTT TACGTCTTTT CAAAAGGGAT TTTTTTTTTT TTTTGTTTCT TACATTGAAT   
  
  
- TAATTTATTC CACGTTACAT GCACCAATTC GATTCTTTTA CGTCTTTTCA AAAGGGATTT TTTTTTTTTT   
  
  
- TTGTTTCTTA CATTGAATTA ATTTTTTGAA TCTAGACTCT TGCATGTAAT TCATTGTACC AAGAAGGCTT   
  
  
- ACTTATTCAT TCTAAGTATT CAAAGAGGAA TGTGAGCAAA ATGGTGGAGA TGTACCATAC TATTATTAAG   
  
  
- TCTTAATCGT TTTCAAGATT TTTTTTGTC

+     chs-CMA1a

| Site Name | Organism | Position | Strand | Matrix score. | sequence | function |
| --- | --- | --- | --- | --- | --- | --- |
| chs-CMA1a | Daucus carota | 1378 | - | 8 | TTACTTAA | part of a light responsive element |

> 2018/04/13 10:10:12  
+ CCGACCGGCT GCTGAATCTA GCTAGCTTTG GTTCTCTATG GGTCGTTTGT CTAACGGACT TTAACTGGAT   
  
  
+ TGTTATTTCG TCTGATTCAA GGAGATCAAA TAGCTTAGAA CGTTCGAAAC ACATCTTTGT CTTAAGTTCA   
  
  
+ ACCAGTTCGA CCGGATCGTA CACTTTACTT CTTTTCGCAC ACTATATATC TCGCCTTTCC ACGACAACCA   
  
  
+ CCCTTTGAAC CACCTTTACT CTGCCTGTGC CTATTCTACA ACGTCCGTGT TGAGATATAT ATTACTGAGA   
  
  
+ CTCAGATAGA TTTCTAGGTG CACCACCCTA AACAACATAT CAAATAATTA TTCTGTTAAT TGACAAAGGG   
  
  
+ TCAAACCACT CACAACCCTC CTCGGTACAC TTCTTCCTTC TTCAAAAGAA AAGAAAAAAA AAGTGGCTTA   
  
  
+ GACTTAATCA CAATTGAAAA AATTTTCATT TTCTTTTTTT TATATGTAAA AATATTAAAT AACATAATTA   
  
  
+ AATGATTTTA TTTATATGAC TTAATCACAA TTGAAAAAAT TTTCATTTTC TTTTTTTTAT ATGTAAAAAT   
  
  
+ ATTAAATAAC ATAATTAAAT GATTTTATTT ATATAATTTA ATATTAATAA TTAATTAAAA AGAAAAATAT   
  
  
+ AATTGTATTC TTTAATATTT TAATAAAAAA TAAAAATGAG TTTTTTAATT TTATTTAAGT TTATTTATCT   
  
  
+ TTTACAAGCA CTAATAAAAA ATTTTATTTT CGTATACAAA CTAATTATAA TTGTATTTTT TATGTAGAAT   
  
  
+ TATTAAACAA AATAATTTAG TAATACGTAA TAATAAAAAT AAATTTTTAT TATATTTAAT TTAATTTTAT   
  
  
+ TTTTTAATTA AGTTGGTATA TATATATATA AATAGGTAAG TTAATGTATA ATAATGCACA CGATCCCTGT   
  
  
+ ACGACCAGCC AGTACCCACA CGAATCAGTT TCAGTTCGAG TGGTATTTTC GGTTCTTTGT AACCCACCAC   
  
  
+ CAGAGTAAAA TAGATGATAA GGAGACACCT TAATTATTAA GTATATTATC TACAGATTAA GAATTAATTT   
  
  
+ AAAGTATAAC TTGGGTTAGA ATAGAAACAG AGAAGATGAC ATGGTTTACG AATTAATTTA ATTTTACTGG   
  
  
+ TTAATTATAA AAAATATTTT TTTTACTCAA ATGTCTCTAC AGTGCAAAAA CTTTCGTAAA AGGTGCAATG   
  
  
+ TACGTGGTTA AGCTAAGAAA ATGCAGAAAA GTTTTCCCTA AAAAAAAAAA AAAACAAAGA ATGTAACTTA   
  
  
+ ATTAAATAAG GTGCAATGTA CGTGGTTAAG CTAAGAAAAT GCAGAAAAGT TTTCCCTAAA AAAAAAAAAA   
  
  
+ AACAAAGAAT GTAACTTAAT TAAAAAACTT AGATCTGAGA ACGTACATTA AGTAACATGG TTCTTCCGAA   
  
  
+ TGAATAAGTA AGATTCATAA GTTTCTCCTT ACACTCGTTT TACCACCTCT ACATGGTATG ATAATAATTC   
  
  
+ AGAATTAGCA AAAGTTCTAA AAAAAACAG  

- GGCTGGCCGA CGACTTAGAT CGATCGAAAC CAAGAGATAC CCAGCAAACA GATTGCCTGA AATTGACCTA   
  
  
- ACAATAAAGC AGACTAAGTT CCTCTAGTTT ATCGAATCTT GCAAGCTTTG TGTAGAAACA GAATTCAAGT   
  
  
- TGGTCAAGCT GGCCTAGCAT GTGAAATGAA GAAAAGCGTG TGATATATAG AGCGGAAAGG TGCTGTTGGT   
  
  
- GGGAAACTTG GTGGAAATGA GACGGACACG GATAAGATGT TGCAGGCACA ACTCTATATA TAATGACTCT   
  
  
- GAGTCTATCT AAAGATCCAC GTGGTGGGAT TTGTTGTATA GTTTATTAAT AAGACAATTA ACTGTTTCCC   
  
  
- AGTTTGGTGA GTGTTGGGAG GAGCCATGTG AAGAAGGAAG AAGTTTTCTT TTCTTTTTTT TTCACCGAAT   
  
  
- CTGAATTAGT GTTAACTTTT TTAAAAGTAA AAGAAAAAAA ATATACATTT TTATAATTTA TTGTATTAAT   
  
  
- TTACTAAAAT AAATATACTG AATTAGTGTT AACTTTTTTA AAAGTAAAAG AAAAAAAATA TACATTTTTA   
  
  
- TAATTTATTG TATTAATTTA CTAAAATAAA TATATTAAAT TATAATTATT AATTAATTTT TCTTTTTATA   
  
  
- TTAACATAAG AAATTATAAA ATTATTTTTT ATTTTTACTC AAAAAATTAA AATAAATTCA AATAAATAGA   
  
  
- AAATGTTCGT GATTATTTTT TAAAATAAAA GCATATGTTT GATTAATATT AACATAAAAA ATACATCTTA   
  
  
- ATAATTTGTT TTATTAAATC ATTATGCATT ATTATTTTTA TTTAAAAATA ATATAAATTA AATTAAAATA   
  
  
- AAAAATTAAT TCAACCATAT ATATATATAT TTATCCATTC AATTACATAT TATTACGTGT GCTAGGGACA   
  
  
- TGCTGGTCGG TCATGGGTGT GCTTAGTCAA AGTCAAGCTC ACCATAAAAG CCAAGAAACA TTGGGTGGTG   
  
  
- GTCTCATTTT ATCTACTATT CCTCTGTGGA ATTAATAATT CATATAATAG ATGTCTAATT CTTAATTAAA   
  
  
- TTTCATATTG AACCCAATCT TATCTTTGTC TCTTCTACTG TACCAAATGC TTAATTAAAT TAAAATGACC   
  
  
- AATTAATATT TTTTATAAAA AAAATGAGTT TACAGAGATG TCACGTTTTT GAAAGCATTT TCCACGTTAC   
  
  
- ATGCACCAAT TCGATTCTTT TACGTCTTTT CAAAAGGGAT TTTTTTTTTT TTTTGTTTCT TACATTGAAT   
  
  
- TAATTTATTC CACGTTACAT GCACCAATTC GATTCTTTTA CGTCTTTTCA AAAGGGATTT TTTTTTTTTT   
  
  
- TTGTTTCTTA CATTGAATTA ATTTTTTGAA TCTAGACTCT TGCATGTAAT TCATTGTACC AAGAAGGCTT   
  
  
- ACTTATTCAT TCTAAGTATT CAAAGAGGAA TGTGAGCAAA ATGGTGGAGA TGTACCATAC TATTATTAAG   
  
  
- TCTTAATCGT TTTCAAGATT TTTTTTGTC

+     circadian

| Site Name | Organism | Position | Strand | Matrix score. | sequence | function |
| --- | --- | --- | --- | --- | --- | --- |
| circadian | Lycopersicon esculentum | 88 | + | 6 | CAANNNNATC | cis-acting regulatory element involved in circadian control |

> 2018/04/13 10:10:12  
+ CCGACCGGCT GCTGAATCTA GCTAGCTTTG GTTCTCTATG GGTCGTTTGT CTAACGGACT TTAACTGGAT   
  
  
+ TGTTATTTCG TCTGATTCAA GGAGATCAAA TAGCTTAGAA CGTTCGAAAC ACATCTTTGT CTTAAGTTCA   
  
  
+ ACCAGTTCGA CCGGATCGTA CACTTTACTT CTTTTCGCAC ACTATATATC TCGCCTTTCC ACGACAACCA   
  
  
+ CCCTTTGAAC CACCTTTACT CTGCCTGTGC CTATTCTACA ACGTCCGTGT TGAGATATAT ATTACTGAGA   
  
  
+ CTCAGATAGA TTTCTAGGTG CACCACCCTA AACAACATAT CAAATAATTA TTCTGTTAAT TGACAAAGGG   
  
  
+ TCAAACCACT CACAACCCTC CTCGGTACAC TTCTTCCTTC TTCAAAAGAA AAGAAAAAAA AAGTGGCTTA   
  
  
+ GACTTAATCA CAATTGAAAA AATTTTCATT TTCTTTTTTT TATATGTAAA AATATTAAAT AACATAATTA   
  
  
+ AATGATTTTA TTTATATGAC TTAATCACAA TTGAAAAAAT TTTCATTTTC TTTTTTTTAT ATGTAAAAAT   
  
  
+ ATTAAATAAC ATAATTAAAT GATTTTATTT ATATAATTTA ATATTAATAA TTAATTAAAA AGAAAAATAT   
  
  
+ AATTGTATTC TTTAATATTT TAATAAAAAA TAAAAATGAG TTTTTTAATT TTATTTAAGT TTATTTATCT   
  
  
+ TTTACAAGCA CTAATAAAAA ATTTTATTTT CGTATACAAA CTAATTATAA TTGTATTTTT TATGTAGAAT   
  
  
+ TATTAAACAA AATAATTTAG TAATACGTAA TAATAAAAAT AAATTTTTAT TATATTTAAT TTAATTTTAT   
  
  
+ TTTTTAATTA AGTTGGTATA TATATATATA AATAGGTAAG TTAATGTATA ATAATGCACA CGATCCCTGT   
  
  
+ ACGACCAGCC AGTACCCACA CGAATCAGTT TCAGTTCGAG TGGTATTTTC GGTTCTTTGT AACCCACCAC   
  
  
+ CAGAGTAAAA TAGATGATAA GGAGACACCT TAATTATTAA GTATATTATC TACAGATTAA GAATTAATTT   
  
  
+ AAAGTATAAC TTGGGTTAGA ATAGAAACAG AGAAGATGAC ATGGTTTACG AATTAATTTA ATTTTACTGG   
  
  
+ TTAATTATAA AAAATATTTT TTTTACTCAA ATGTCTCTAC AGTGCAAAAA CTTTCGTAAA AGGTGCAATG   
  
  
+ TACGTGGTTA AGCTAAGAAA ATGCAGAAAA GTTTTCCCTA AAAAAAAAAA AAAACAAAGA ATGTAACTTA   
  
  
+ ATTAAATAAG GTGCAATGTA CGTGGTTAAG CTAAGAAAAT GCAGAAAAGT TTTCCCTAAA AAAAAAAAAA   
  
  
+ AACAAAGAAT GTAACTTAAT TAAAAAACTT AGATCTGAGA ACGTACATTA AGTAACATGG TTCTTCCGAA   
  
  
+ TGAATAAGTA AGATTCATAA GTTTCTCCTT ACACTCGTTT TACCACCTCT ACATGGTATG ATAATAATTC   
  
  
+ AGAATTAGCA AAAGTTCTAA AAAAAACAG  

- GGCTGGCCGA CGACTTAGAT CGATCGAAAC CAAGAGATAC CCAGCAAACA GATTGCCTGA AATTGACCTA   
  
  
- ACAATAAAGC AGACTAAGTT CCTCTAGTTT ATCGAATCTT GCAAGCTTTG TGTAGAAACA GAATTCAAGT   
  
  
- TGGTCAAGCT GGCCTAGCAT GTGAAATGAA GAAAAGCGTG TGATATATAG AGCGGAAAGG TGCTGTTGGT   
  
  
- GGGAAACTTG GTGGAAATGA GACGGACACG GATAAGATGT TGCAGGCACA ACTCTATATA TAATGACTCT   
  
  
- GAGTCTATCT AAAGATCCAC GTGGTGGGAT TTGTTGTATA GTTTATTAAT AAGACAATTA ACTGTTTCCC   
  
  
- AGTTTGGTGA GTGTTGGGAG GAGCCATGTG AAGAAGGAAG AAGTTTTCTT TTCTTTTTTT TTCACCGAAT   
  
  
- CTGAATTAGT GTTAACTTTT TTAAAAGTAA AAGAAAAAAA ATATACATTT TTATAATTTA TTGTATTAAT   
  
  
- TTACTAAAAT AAATATACTG AATTAGTGTT AACTTTTTTA AAAGTAAAAG AAAAAAAATA TACATTTTTA   
  
  
- TAATTTATTG TATTAATTTA CTAAAATAAA TATATTAAAT TATAATTATT AATTAATTTT TCTTTTTATA   
  
  
- TTAACATAAG AAATTATAAA ATTATTTTTT ATTTTTACTC AAAAAATTAA AATAAATTCA AATAAATAGA   
  
  
- AAATGTTCGT GATTATTTTT TAAAATAAAA GCATATGTTT GATTAATATT AACATAAAAA ATACATCTTA   
  
  
- ATAATTTGTT TTATTAAATC ATTATGCATT ATTATTTTTA TTTAAAAATA ATATAAATTA AATTAAAATA   
  
  
- AAAAATTAAT TCAACCATAT ATATATATAT TTATCCATTC AATTACATAT TATTACGTGT GCTAGGGACA   
  
  
- TGCTGGTCGG TCATGGGTGT GCTTAGTCAA AGTCAAGCTC ACCATAAAAG CCAAGAAACA TTGGGTGGTG   
  
  
- GTCTCATTTT ATCTACTATT CCTCTGTGGA ATTAATAATT CATATAATAG ATGTCTAATT CTTAATTAAA   
  
  
- TTTCATATTG AACCCAATCT TATCTTTGTC TCTTCTACTG TACCAAATGC TTAATTAAAT TAAAATGACC   
  
  
- AATTAATATT TTTTATAAAA AAAATGAGTT TACAGAGATG TCACGTTTTT GAAAGCATTT TCCACGTTAC   
  
  
- ATGCACCAAT TCGATTCTTT TACGTCTTTT CAAAAGGGAT TTTTTTTTTT TTTTGTTTCT TACATTGAAT   
  
  
- TAATTTATTC CACGTTACAT GCACCAATTC GATTCTTTTA CGTCTTTTCA AAAGGGATTT TTTTTTTTTT   
  
  
- TTGTTTCTTA CATTGAATTA ATTTTTTGAA TCTAGACTCT TGCATGTAAT TCATTGTACC AAGAAGGCTT   
  
  
- ACTTATTCAT TCTAAGTATT CAAAGAGGAA TGTGAGCAAA ATGGTGGAGA TGTACCATAC TATTATTAAG   
  
  
- TCTTAATCGT TTTCAAGATT TTTTTTGTC
