# Supplementary material for: Genome-wide analysis of the WRKY gene family in drumstick (Moringa oleifera Lam.)
Source: PeerJ. 2019 Jun 10;7:e7063. doi: 10.7717/peerj.7063 (PMC6563795; doi:10.7717/peerj.7063)
Supplement: Supplemental Information 1 [file peerj-07-7063-s003.gz › MoWRKY24_plantcare.html]

Content-Type: text/html; charset=ISO-8859-1


CallMat\_Firefox


Webmaster Firefox specific output  
To save the result:
click on the frame with the right mouse button and save the source code as a text file with extension .html  
REFERENCE:PlantCARE: a database of plant cis-acting regulatory elements and a portal to tools for in silico analysis of promoter sequences.  
Lescot, M., Déhais, P., Moreau, Y., De Moor, B., Rouzé ,P.,and Rombauts, S.  
Nucleic Acids Res., Database issue(2002), 30(1):325-327.   


---

> 2018/04/13 10:10:12  
+ AACGTCAGAA ACTCAAGAGG TAAGAAAGAA GAAAGAACCC CCTCCTTCGA AACCTAAACA GTTTTCTCGG   
  
  
+ ACACCCCCGG GAACGATAGT TAAGTCGAAG AATTACTGTT TCACTACAAT TAAAATAGAC AAGAGAGAAC   
  
  
+ TAAACCCTAC AAGATCAAAC ATACTGGTAT GCTTTTGATC AACTATGTTT CAGTTAGTTC ATTCGATTTG   
  
  
+ TTTCTTCCAT TGTACAGAAT TTCTATTCTG TTCTAATCTT ATTACTCGAG TATACACTTT TGTTTTCACG   
  
  
+ TTTTTTCGAA GTATTAGAAA CGTGTAAGTA CAAAAATAAA GTACAATTAG TTAATTAAGA AAAAGCGAGT   
  
  
+ AGATAATCTT GGATTAAATA TGGAAAGAAC CGTACGACAT CCACACGAAC CCCAATAGGC TATTTTGCCA   
  
  
+ CGCAAAACTA TTGTCGCCTT TTACGATGCG GAATTAAAAA CGCTCATCCC TACCGAGGCC GAGGCAAGAT   
  
  
+ CAGATCACTC GAAGGGAAGC AGAGAGCCAA CACTCGGTAC CACGCGTCCT TTCGTTCACG TGGGCGTCCT   
  
  
+ CCGCGGCTCT TTAGCGTTTT CGTCGCCGAA TCGCCGCGGC TGGCCAGCCG AATGAAACGG TCCTCCCTTC   
  
  
+ GCCGCTTCCA CAACGCTACG CAAGAGCTTC CGCTGCGAGC AGCCGTTCGG CAACCGAGAT AGTTCAGCGA   
  
  
+ GCAGATGTCG GGAAGGCACT CCTTGTTTTC CCGTTGAAAG CGAGAACAAA CAGTTTGAGA GTTCACATTC   
  
  
+ AATGGTTAAA ACATAAAGAC AGACAATCCA CATGAAAGCC AACGACAAAT CAACCGAAAA GACCCTCGAA   
  
  
+ AGACGTTCAT TGAATCAAAA GAAAACTTTA AGAAAACCCA AACCCTCGGA TCCCTCATTC TCGAACTGCT   
  
  
+ TTCTAAAACC CACATTTCAA CTTGAAAGGA ATACTAGACA AAAACCAGAA ACCACAAAAA AATCCGTCGA   
  
  
+ GTTTGAGTAT AGTGTCAAAA GCATGAATTT TTGGAACTAA AGACCCTACG TATGACGGTG ATCGTAGTAG   
  
  
+ ACTGAAACGG GAAGAGAGAG AGAAGGAAGG GAGTAACAAC ACATTTAAGA CAATAAACGT CATGGACTTG   
  
  
+ TTTGACATGT AAAATTCACT AATTGTAAAA GCAGAAAAAC CAAAGTGAAC AAAAGTCTAG AAGGATAATA   
  
  
+ TTTCCTACAG AACCTCCGAC GATTTTATGA CTTATCATCT ACCTTCCATA TTCGGTACGA GGCTGTCGAA   
  
  
+ TACTTGTAAT AAAAACGGGA AGAGTTCTCC TAATTCAAAA AAGTGAAAAG ATTTACTATA AAAACAGGTT   
  
  
+ CCGTCTAAAT CGATATCCCA CTTGTAACAA AACGACGAAC GAACTTACGA TCGAAAAGGG TCAACTTTAC   
  
  
+ AAAGGATTGC GAATTACCAA TAACGATAAC GTCTCCGTTC TCCGTAACTA GATGTATGAG ACAATACTGT   
  
  
+ AAGATAAACC CGTCCACTAT TGAAACATG  

- TTGCAGTCTT TGAGTTCTCC ATTCTTTCTT CTTTCTTGGG GGAGGAAGCT TTGGATTTGT CAAAAGAGCC   
  
  
- TGTGGGGGCC CTTGCTATCA ATTCAGCTTC TTAATGACAA AGTGATGTTA ATTTTATCTG TTCTCTCTTG   
  
  
- ATTTGGGATG TTCTAGTTTG TATGACCATA CGAAAACTAG TTGATACAAA GTCAATCAAG TAAGCTAAAC   
  
  
- AAAGAAGGTA ACATGTCTTA AAGATAAGAC AAGATTAGAA TAATGAGCTC ATATGTGAAA ACAAAAGTGC   
  
  
- AAAAAAGCTT CATAATCTTT GCACATTCAT GTTTTTATTT CATGTTAATC AATTAATTCT TTTTCGCTCA   
  
  
- TCTATTAGAA CCTAATTTAT ACCTTTCTTG GCATGCTGTA GGTGTGCTTG GGGTTATCCG ATAAAACGGT   
  
  
- GCGTTTTGAT AACAGCGGAA AATGCTACGC CTTAATTTTT GCGAGTAGGG ATGGCTCCGG CTCCGTTCTA   
  
  
- GTCTAGTGAG CTTCCCTTCG TCTCTCGGTT GTGAGCCATG GTGCGCAGGA AAGCAAGTGC ACCCGCAGGA   
  
  
- GGCGCCGAGA AATCGCAAAA GCAGCGGCTT AGCGGCGCCG ACCGGTCGGC TTACTTTGCC AGGAGGGAAG   
  
  
- CGGCGAAGGT GTTGCGATGC GTTCTCGAAG GCGACGCTCG TCGGCAAGCC GTTGGCTCTA TCAAGTCGCT   
  
  
- CGTCTACAGC CCTTCCGTGA GGAACAAAAG GGCAACTTTC GCTCTTGTTT GTCAAACTCT CAAGTGTAAG   
  
  
- TTACCAATTT TGTATTTCTG TCTGTTAGGT GTACTTTCGG TTGCTGTTTA GTTGGCTTTT CTGGGAGCTT   
  
  
- TCTGCAAGTA ACTTAGTTTT CTTTTGAAAT TCTTTTGGGT TTGGGAGCCT AGGGAGTAAG AGCTTGACGA   
  
  
- AAGATTTTGG GTGTAAAGTT GAACTTTCCT TATGATCTGT TTTTGGTCTT TGGTGTTTTT TTAGGCAGCT   
  
  
- CAAACTCATA TCACAGTTTT CGTACTTAAA AACCTTGATT TCTGGGATGC ATACTGCCAC TAGCATCATC   
  
  
- TGACTTTGCC CTTCTCTCTC TCTTCCTTCC CTCATTGTTG TGTAAATTCT GTTATTTGCA GTACCTGAAC   
  
  
- AAACTGTACA TTTTAAGTGA TTAACATTTT CGTCTTTTTG GTTTCACTTG TTTTCAGATC TTCCTATTAT   
  
  
- AAAGGATGTC TTGGAGGCTG CTAAAATACT GAATAGTAGA TGGAAGGTAT AAGCCATGCT CCGACAGCTT   
  
  
- ATGAACATTA TTTTTGCCCT TCTCAAGAGG ATTAAGTTTT TTCACTTTTC TAAATGATAT TTTTGTCCAA   
  
  
- GGCAGATTTA GCTATAGGGT GAACATTGTT TTGCTGCTTG CTTGAATGCT AGCTTTTCCC AGTTGAAATG   
  
  
- TTTCCTAACG CTTAATGGTT ATTGCTATTG CAGAGGCAAG AGGCATTGAT CTACATACTC TGTTATGACA   
  
  
- TTCTATTTGG GCAGGTGATA ACTTTGTAC

  
  
Motifs Found  

+     5UTR Py-rich stretch

| Site Name | Organism | Position | Strand | Matrix score. | sequence | function |
| --- | --- | --- | --- | --- | --- | --- |
| 5UTR Py-rich stretch | Lycopersicon esculentum | 25 | - | 9 | TTTCTTCTCT | cis-acting element conferring high transcription levels |
| 5UTR Py-rich stretch | Lycopersicon esculentum | 1069 | - | 9 | TTTCTTCTCT | cis-acting element conferring high transcription levels |

> 2018/04/13 10:10:12  
+ AACGTCAGAA ACTCAAGAGG TAAGAAAGAA GAAAGAACCC CCTCCTTCGA AACCTAAACA GTTTTCTCGG   
  
  
+ ACACCCCCGG GAACGATAGT TAAGTCGAAG AATTACTGTT TCACTACAAT TAAAATAGAC AAGAGAGAAC   
  
  
+ TAAACCCTAC AAGATCAAAC ATACTGGTAT GCTTTTGATC AACTATGTTT CAGTTAGTTC ATTCGATTTG   
  
  
+ TTTCTTCCAT TGTACAGAAT TTCTATTCTG TTCTAATCTT ATTACTCGAG TATACACTTT TGTTTTCACG   
  
  
+ TTTTTTCGAA GTATTAGAAA CGTGTAAGTA CAAAAATAAA GTACAATTAG TTAATTAAGA AAAAGCGAGT   
  
  
+ AGATAATCTT GGATTAAATA TGGAAAGAAC CGTACGACAT CCACACGAAC CCCAATAGGC TATTTTGCCA   
  
  
+ CGCAAAACTA TTGTCGCCTT TTACGATGCG GAATTAAAAA CGCTCATCCC TACCGAGGCC GAGGCAAGAT   
  
  
+ CAGATCACTC GAAGGGAAGC AGAGAGCCAA CACTCGGTAC CACGCGTCCT TTCGTTCACG TGGGCGTCCT   
  
  
+ CCGCGGCTCT TTAGCGTTTT CGTCGCCGAA TCGCCGCGGC TGGCCAGCCG AATGAAACGG TCCTCCCTTC   
  
  
+ GCCGCTTCCA CAACGCTACG CAAGAGCTTC CGCTGCGAGC AGCCGTTCGG CAACCGAGAT AGTTCAGCGA   
  
  
+ GCAGATGTCG GGAAGGCACT CCTTGTTTTC CCGTTGAAAG CGAGAACAAA CAGTTTGAGA GTTCACATTC   
  
  
+ AATGGTTAAA ACATAAAGAC AGACAATCCA CATGAAAGCC AACGACAAAT CAACCGAAAA GACCCTCGAA   
  
  
+ AGACGTTCAT TGAATCAAAA GAAAACTTTA AGAAAACCCA AACCCTCGGA TCCCTCATTC TCGAACTGCT   
  
  
+ TTCTAAAACC CACATTTCAA CTTGAAAGGA ATACTAGACA AAAACCAGAA ACCACAAAAA AATCCGTCGA   
  
  
+ GTTTGAGTAT AGTGTCAAAA GCATGAATTT TTGGAACTAA AGACCCTACG TATGACGGTG ATCGTAGTAG   
  
  
+ ACTGAAACGG GAAGAGAGAG AGAAGGAAGG GAGTAACAAC ACATTTAAGA CAATAAACGT CATGGACTTG   
  
  
+ TTTGACATGT AAAATTCACT AATTGTAAAA GCAGAAAAAC CAAAGTGAAC AAAAGTCTAG AAGGATAATA   
  
  
+ TTTCCTACAG AACCTCCGAC GATTTTATGA CTTATCATCT ACCTTCCATA TTCGGTACGA GGCTGTCGAA   
  
  
+ TACTTGTAAT AAAAACGGGA AGAGTTCTCC TAATTCAAAA AAGTGAAAAG ATTTACTATA AAAACAGGTT   
  
  
+ CCGTCTAAAT CGATATCCCA CTTGTAACAA AACGACGAAC GAACTTACGA TCGAAAAGGG TCAACTTTAC   
  
  
+ AAAGGATTGC GAATTACCAA TAACGATAAC GTCTCCGTTC TCCGTAACTA GATGTATGAG ACAATACTGT   
  
  
+ AAGATAAACC CGTCCACTAT TGAAACATG  

- TTGCAGTCTT TGAGTTCTCC ATTCTTTCTT CTTTCTTGGG GGAGGAAGCT TTGGATTTGT CAAAAGAGCC   
  
  
- TGTGGGGGCC CTTGCTATCA ATTCAGCTTC TTAATGACAA AGTGATGTTA ATTTTATCTG TTCTCTCTTG   
  
  
- ATTTGGGATG TTCTAGTTTG TATGACCATA CGAAAACTAG TTGATACAAA GTCAATCAAG TAAGCTAAAC   
  
  
- AAAGAAGGTA ACATGTCTTA AAGATAAGAC AAGATTAGAA TAATGAGCTC ATATGTGAAA ACAAAAGTGC   
  
  
- AAAAAAGCTT CATAATCTTT GCACATTCAT GTTTTTATTT CATGTTAATC AATTAATTCT TTTTCGCTCA   
  
  
- TCTATTAGAA CCTAATTTAT ACCTTTCTTG GCATGCTGTA GGTGTGCTTG GGGTTATCCG ATAAAACGGT   
  
  
- GCGTTTTGAT AACAGCGGAA AATGCTACGC CTTAATTTTT GCGAGTAGGG ATGGCTCCGG CTCCGTTCTA   
  
  
- GTCTAGTGAG CTTCCCTTCG TCTCTCGGTT GTGAGCCATG GTGCGCAGGA AAGCAAGTGC ACCCGCAGGA   
  
  
- GGCGCCGAGA AATCGCAAAA GCAGCGGCTT AGCGGCGCCG ACCGGTCGGC TTACTTTGCC AGGAGGGAAG   
  
  
- CGGCGAAGGT GTTGCGATGC GTTCTCGAAG GCGACGCTCG TCGGCAAGCC GTTGGCTCTA TCAAGTCGCT   
  
  
- CGTCTACAGC CCTTCCGTGA GGAACAAAAG GGCAACTTTC GCTCTTGTTT GTCAAACTCT CAAGTGTAAG   
  
  
- TTACCAATTT TGTATTTCTG TCTGTTAGGT GTACTTTCGG TTGCTGTTTA GTTGGCTTTT CTGGGAGCTT   
  
  
- TCTGCAAGTA ACTTAGTTTT CTTTTGAAAT TCTTTTGGGT TTGGGAGCCT AGGGAGTAAG AGCTTGACGA   
  
  
- AAGATTTTGG GTGTAAAGTT GAACTTTCCT TATGATCTGT TTTTGGTCTT TGGTGTTTTT TTAGGCAGCT   
  
  
- CAAACTCATA TCACAGTTTT CGTACTTAAA AACCTTGATT TCTGGGATGC ATACTGCCAC TAGCATCATC   
  
  
- TGACTTTGCC CTTCTCTCTC TCTTCCTTCC CTCATTGTTG TGTAAATTCT GTTATTTGCA GTACCTGAAC   
  
  
- AAACTGTACA TTTTAAGTGA TTAACATTTT CGTCTTTTTG GTTTCACTTG TTTTCAGATC TTCCTATTAT   
  
  
- AAAGGATGTC TTGGAGGCTG CTAAAATACT GAATAGTAGA TGGAAGGTAT AAGCCATGCT CCGACAGCTT   
  
  
- ATGAACATTA TTTTTGCCCT TCTCAAGAGG ATTAAGTTTT TTCACTTTTC TAAATGATAT TTTTGTCCAA   
  
  
- GGCAGATTTA GCTATAGGGT GAACATTGTT TTGCTGCTTG CTTGAATGCT AGCTTTTCCC AGTTGAAATG   
  
  
- TTTCCTAACG CTTAATGGTT ATTGCTATTG CAGAGGCAAG AGGCATTGAT CTACATACTC TGTTATGACA   
  
  
- TTCTATTTGG GCAGGTGATA ACTTTGTAC

+     A-box

| Site Name | Organism | Position | Strand | Matrix score. | sequence | function |
| --- | --- | --- | --- | --- | --- | --- |
| A-box | Petroselinum crispum | 1480 | + | 6 | CCGTCC | cis-acting regulatory element |

> 2018/04/13 10:10:12  
+ AACGTCAGAA ACTCAAGAGG TAAGAAAGAA GAAAGAACCC CCTCCTTCGA AACCTAAACA GTTTTCTCGG   
  
  
+ ACACCCCCGG GAACGATAGT TAAGTCGAAG AATTACTGTT TCACTACAAT TAAAATAGAC AAGAGAGAAC   
  
  
+ TAAACCCTAC AAGATCAAAC ATACTGGTAT GCTTTTGATC AACTATGTTT CAGTTAGTTC ATTCGATTTG   
  
  
+ TTTCTTCCAT TGTACAGAAT TTCTATTCTG TTCTAATCTT ATTACTCGAG TATACACTTT TGTTTTCACG   
  
  
+ TTTTTTCGAA GTATTAGAAA CGTGTAAGTA CAAAAATAAA GTACAATTAG TTAATTAAGA AAAAGCGAGT   
  
  
+ AGATAATCTT GGATTAAATA TGGAAAGAAC CGTACGACAT CCACACGAAC CCCAATAGGC TATTTTGCCA   
  
  
+ CGCAAAACTA TTGTCGCCTT TTACGATGCG GAATTAAAAA CGCTCATCCC TACCGAGGCC GAGGCAAGAT   
  
  
+ CAGATCACTC GAAGGGAAGC AGAGAGCCAA CACTCGGTAC CACGCGTCCT TTCGTTCACG TGGGCGTCCT   
  
  
+ CCGCGGCTCT TTAGCGTTTT CGTCGCCGAA TCGCCGCGGC TGGCCAGCCG AATGAAACGG TCCTCCCTTC   
  
  
+ GCCGCTTCCA CAACGCTACG CAAGAGCTTC CGCTGCGAGC AGCCGTTCGG CAACCGAGAT AGTTCAGCGA   
  
  
+ GCAGATGTCG GGAAGGCACT CCTTGTTTTC CCGTTGAAAG CGAGAACAAA CAGTTTGAGA GTTCACATTC   
  
  
+ AATGGTTAAA ACATAAAGAC AGACAATCCA CATGAAAGCC AACGACAAAT CAACCGAAAA GACCCTCGAA   
  
  
+ AGACGTTCAT TGAATCAAAA GAAAACTTTA AGAAAACCCA AACCCTCGGA TCCCTCATTC TCGAACTGCT   
  
  
+ TTCTAAAACC CACATTTCAA CTTGAAAGGA ATACTAGACA AAAACCAGAA ACCACAAAAA AATCCGTCGA   
  
  
+ GTTTGAGTAT AGTGTCAAAA GCATGAATTT TTGGAACTAA AGACCCTACG TATGACGGTG ATCGTAGTAG   
  
  
+ ACTGAAACGG GAAGAGAGAG AGAAGGAAGG GAGTAACAAC ACATTTAAGA CAATAAACGT CATGGACTTG   
  
  
+ TTTGACATGT AAAATTCACT AATTGTAAAA GCAGAAAAAC CAAAGTGAAC AAAAGTCTAG AAGGATAATA   
  
  
+ TTTCCTACAG AACCTCCGAC GATTTTATGA CTTATCATCT ACCTTCCATA TTCGGTACGA GGCTGTCGAA   
  
  
+ TACTTGTAAT AAAAACGGGA AGAGTTCTCC TAATTCAAAA AAGTGAAAAG ATTTACTATA AAAACAGGTT   
  
  
+ CCGTCTAAAT CGATATCCCA CTTGTAACAA AACGACGAAC GAACTTACGA TCGAAAAGGG TCAACTTTAC   
  
  
+ AAAGGATTGC GAATTACCAA TAACGATAAC GTCTCCGTTC TCCGTAACTA GATGTATGAG ACAATACTGT   
  
  
+ AAGATAAACC CGTCCACTAT TGAAACATG  

- TTGCAGTCTT TGAGTTCTCC ATTCTTTCTT CTTTCTTGGG GGAGGAAGCT TTGGATTTGT CAAAAGAGCC   
  
  
- TGTGGGGGCC CTTGCTATCA ATTCAGCTTC TTAATGACAA AGTGATGTTA ATTTTATCTG TTCTCTCTTG   
  
  
- ATTTGGGATG TTCTAGTTTG TATGACCATA CGAAAACTAG TTGATACAAA GTCAATCAAG TAAGCTAAAC   
  
  
- AAAGAAGGTA ACATGTCTTA AAGATAAGAC AAGATTAGAA TAATGAGCTC ATATGTGAAA ACAAAAGTGC   
  
  
- AAAAAAGCTT CATAATCTTT GCACATTCAT GTTTTTATTT CATGTTAATC AATTAATTCT TTTTCGCTCA   
  
  
- TCTATTAGAA CCTAATTTAT ACCTTTCTTG GCATGCTGTA GGTGTGCTTG GGGTTATCCG ATAAAACGGT   
  
  
- GCGTTTTGAT AACAGCGGAA AATGCTACGC CTTAATTTTT GCGAGTAGGG ATGGCTCCGG CTCCGTTCTA   
  
  
- GTCTAGTGAG CTTCCCTTCG TCTCTCGGTT GTGAGCCATG GTGCGCAGGA AAGCAAGTGC ACCCGCAGGA   
  
  
- GGCGCCGAGA AATCGCAAAA GCAGCGGCTT AGCGGCGCCG ACCGGTCGGC TTACTTTGCC AGGAGGGAAG   
  
  
- CGGCGAAGGT GTTGCGATGC GTTCTCGAAG GCGACGCTCG TCGGCAAGCC GTTGGCTCTA TCAAGTCGCT   
  
  
- CGTCTACAGC CCTTCCGTGA GGAACAAAAG GGCAACTTTC GCTCTTGTTT GTCAAACTCT CAAGTGTAAG   
  
  
- TTACCAATTT TGTATTTCTG TCTGTTAGGT GTACTTTCGG TTGCTGTTTA GTTGGCTTTT CTGGGAGCTT   
  
  
- TCTGCAAGTA ACTTAGTTTT CTTTTGAAAT TCTTTTGGGT TTGGGAGCCT AGGGAGTAAG AGCTTGACGA   
  
  
- AAGATTTTGG GTGTAAAGTT GAACTTTCCT TATGATCTGT TTTTGGTCTT TGGTGTTTTT TTAGGCAGCT   
  
  
- CAAACTCATA TCACAGTTTT CGTACTTAAA AACCTTGATT TCTGGGATGC ATACTGCCAC TAGCATCATC   
  
  
- TGACTTTGCC CTTCTCTCTC TCTTCCTTCC CTCATTGTTG TGTAAATTCT GTTATTTGCA GTACCTGAAC   
  
  
- AAACTGTACA TTTTAAGTGA TTAACATTTT CGTCTTTTTG GTTTCACTTG TTTTCAGATC TTCCTATTAT   
  
  
- AAAGGATGTC TTGGAGGCTG CTAAAATACT GAATAGTAGA TGGAAGGTAT AAGCCATGCT CCGACAGCTT   
  
  
- ATGAACATTA TTTTTGCCCT TCTCAAGAGG ATTAAGTTTT TTCACTTTTC TAAATGATAT TTTTGTCCAA   
  
  
- GGCAGATTTA GCTATAGGGT GAACATTGTT TTGCTGCTTG CTTGAATGCT AGCTTTTCCC AGTTGAAATG   
  
  
- TTTCCTAACG CTTAATGGTT ATTGCTATTG CAGAGGCAAG AGGCATTGAT CTACATACTC TGTTATGACA   
  
  
- TTCTATTTGG GCAGGTGATA ACTTTGTAC

+     AAGAA-motif

| Site Name | Organism | Position | Strand | Matrix score. | sequence | function |
| --- | --- | --- | --- | --- | --- | --- |
| AAGAA-motif | Avena sativa | 19 | + | 8 | gGTAAGAA |  |
| AAGAA-motif | Avena sativa | 31 | + | 7 | GAAAGAA |  |
| AAGAA-motif | Avena sativa | 24 | + | 7 | GAAAGAA |  |
| AAGAA-motif | Avena sativa | 373 | + | 7 | GAAAGAA |  |

> 2018/04/13 10:10:12  
+ AACGTCAGAA ACTCAAGAGG TAAGAAAGAA GAAAGAACCC CCTCCTTCGA AACCTAAACA GTTTTCTCGG   
  
  
+ ACACCCCCGG GAACGATAGT TAAGTCGAAG AATTACTGTT TCACTACAAT TAAAATAGAC AAGAGAGAAC   
  
  
+ TAAACCCTAC AAGATCAAAC ATACTGGTAT GCTTTTGATC AACTATGTTT CAGTTAGTTC ATTCGATTTG   
  
  
+ TTTCTTCCAT TGTACAGAAT TTCTATTCTG TTCTAATCTT ATTACTCGAG TATACACTTT TGTTTTCACG   
  
  
+ TTTTTTCGAA GTATTAGAAA CGTGTAAGTA CAAAAATAAA GTACAATTAG TTAATTAAGA AAAAGCGAGT   
  
  
+ AGATAATCTT GGATTAAATA TGGAAAGAAC CGTACGACAT CCACACGAAC CCCAATAGGC TATTTTGCCA   
  
  
+ CGCAAAACTA TTGTCGCCTT TTACGATGCG GAATTAAAAA CGCTCATCCC TACCGAGGCC GAGGCAAGAT   
  
  
+ CAGATCACTC GAAGGGAAGC AGAGAGCCAA CACTCGGTAC CACGCGTCCT TTCGTTCACG TGGGCGTCCT   
  
  
+ CCGCGGCTCT TTAGCGTTTT CGTCGCCGAA TCGCCGCGGC TGGCCAGCCG AATGAAACGG TCCTCCCTTC   
  
  
+ GCCGCTTCCA CAACGCTACG CAAGAGCTTC CGCTGCGAGC AGCCGTTCGG CAACCGAGAT AGTTCAGCGA   
  
  
+ GCAGATGTCG GGAAGGCACT CCTTGTTTTC CCGTTGAAAG CGAGAACAAA CAGTTTGAGA GTTCACATTC   
  
  
+ AATGGTTAAA ACATAAAGAC AGACAATCCA CATGAAAGCC AACGACAAAT CAACCGAAAA GACCCTCGAA   
  
  
+ AGACGTTCAT TGAATCAAAA GAAAACTTTA AGAAAACCCA AACCCTCGGA TCCCTCATTC TCGAACTGCT   
  
  
+ TTCTAAAACC CACATTTCAA CTTGAAAGGA ATACTAGACA AAAACCAGAA ACCACAAAAA AATCCGTCGA   
  
  
+ GTTTGAGTAT AGTGTCAAAA GCATGAATTT TTGGAACTAA AGACCCTACG TATGACGGTG ATCGTAGTAG   
  
  
+ ACTGAAACGG GAAGAGAGAG AGAAGGAAGG GAGTAACAAC ACATTTAAGA CAATAAACGT CATGGACTTG   
  
  
+ TTTGACATGT AAAATTCACT AATTGTAAAA GCAGAAAAAC CAAAGTGAAC AAAAGTCTAG AAGGATAATA   
  
  
+ TTTCCTACAG AACCTCCGAC GATTTTATGA CTTATCATCT ACCTTCCATA TTCGGTACGA GGCTGTCGAA   
  
  
+ TACTTGTAAT AAAAACGGGA AGAGTTCTCC TAATTCAAAA AAGTGAAAAG ATTTACTATA AAAACAGGTT   
  
  
+ CCGTCTAAAT CGATATCCCA CTTGTAACAA AACGACGAAC GAACTTACGA TCGAAAAGGG TCAACTTTAC   
  
  
+ AAAGGATTGC GAATTACCAA TAACGATAAC GTCTCCGTTC TCCGTAACTA GATGTATGAG ACAATACTGT   
  
  
+ AAGATAAACC CGTCCACTAT TGAAACATG  

- TTGCAGTCTT TGAGTTCTCC ATTCTTTCTT CTTTCTTGGG GGAGGAAGCT TTGGATTTGT CAAAAGAGCC   
  
  
- TGTGGGGGCC CTTGCTATCA ATTCAGCTTC TTAATGACAA AGTGATGTTA ATTTTATCTG TTCTCTCTTG   
  
  
- ATTTGGGATG TTCTAGTTTG TATGACCATA CGAAAACTAG TTGATACAAA GTCAATCAAG TAAGCTAAAC   
  
  
- AAAGAAGGTA ACATGTCTTA AAGATAAGAC AAGATTAGAA TAATGAGCTC ATATGTGAAA ACAAAAGTGC   
  
  
- AAAAAAGCTT CATAATCTTT GCACATTCAT GTTTTTATTT CATGTTAATC AATTAATTCT TTTTCGCTCA   
  
  
- TCTATTAGAA CCTAATTTAT ACCTTTCTTG GCATGCTGTA GGTGTGCTTG GGGTTATCCG ATAAAACGGT   
  
  
- GCGTTTTGAT AACAGCGGAA AATGCTACGC CTTAATTTTT GCGAGTAGGG ATGGCTCCGG CTCCGTTCTA   
  
  
- GTCTAGTGAG CTTCCCTTCG TCTCTCGGTT GTGAGCCATG GTGCGCAGGA AAGCAAGTGC ACCCGCAGGA   
  
  
- GGCGCCGAGA AATCGCAAAA GCAGCGGCTT AGCGGCGCCG ACCGGTCGGC TTACTTTGCC AGGAGGGAAG   
  
  
- CGGCGAAGGT GTTGCGATGC GTTCTCGAAG GCGACGCTCG TCGGCAAGCC GTTGGCTCTA TCAAGTCGCT   
  
  
- CGTCTACAGC CCTTCCGTGA GGAACAAAAG GGCAACTTTC GCTCTTGTTT GTCAAACTCT CAAGTGTAAG   
  
  
- TTACCAATTT TGTATTTCTG TCTGTTAGGT GTACTTTCGG TTGCTGTTTA GTTGGCTTTT CTGGGAGCTT   
  
  
- TCTGCAAGTA ACTTAGTTTT CTTTTGAAAT TCTTTTGGGT TTGGGAGCCT AGGGAGTAAG AGCTTGACGA   
  
  
- AAGATTTTGG GTGTAAAGTT GAACTTTCCT TATGATCTGT TTTTGGTCTT TGGTGTTTTT TTAGGCAGCT   
  
  
- CAAACTCATA TCACAGTTTT CGTACTTAAA AACCTTGATT TCTGGGATGC ATACTGCCAC TAGCATCATC   
  
  
- TGACTTTGCC CTTCTCTCTC TCTTCCTTCC CTCATTGTTG TGTAAATTCT GTTATTTGCA GTACCTGAAC   
  
  
- AAACTGTACA TTTTAAGTGA TTAACATTTT CGTCTTTTTG GTTTCACTTG TTTTCAGATC TTCCTATTAT   
  
  
- AAAGGATGTC TTGGAGGCTG CTAAAATACT GAATAGTAGA TGGAAGGTAT AAGCCATGCT CCGACAGCTT   
  
  
- ATGAACATTA TTTTTGCCCT TCTCAAGAGG ATTAAGTTTT TTCACTTTTC TAAATGATAT TTTTGTCCAA   
  
  
- GGCAGATTTA GCTATAGGGT GAACATTGTT TTGCTGCTTG CTTGAATGCT AGCTTTTCCC AGTTGAAATG   
  
  
- TTTCCTAACG CTTAATGGTT ATTGCTATTG CAGAGGCAAG AGGCATTGAT CTACATACTC TGTTATGACA   
  
  
- TTCTATTTGG GCAGGTGATA ACTTTGTAC

+     ABRE

| Site Name | Organism | Position | Strand | Matrix score. | sequence | function |
| --- | --- | --- | --- | --- | --- | --- |
| ABRE | Arabidopsis thaliana | 547 | + | 6 | CACGTG | cis-acting element involved in the abscisic acid responsiveness |

> 2018/04/13 10:10:12  
+ AACGTCAGAA ACTCAAGAGG TAAGAAAGAA GAAAGAACCC CCTCCTTCGA AACCTAAACA GTTTTCTCGG   
  
  
+ ACACCCCCGG GAACGATAGT TAAGTCGAAG AATTACTGTT TCACTACAAT TAAAATAGAC AAGAGAGAAC   
  
  
+ TAAACCCTAC AAGATCAAAC ATACTGGTAT GCTTTTGATC AACTATGTTT CAGTTAGTTC ATTCGATTTG   
  
  
+ TTTCTTCCAT TGTACAGAAT TTCTATTCTG TTCTAATCTT ATTACTCGAG TATACACTTT TGTTTTCACG   
  
  
+ TTTTTTCGAA GTATTAGAAA CGTGTAAGTA CAAAAATAAA GTACAATTAG TTAATTAAGA AAAAGCGAGT   
  
  
+ AGATAATCTT GGATTAAATA TGGAAAGAAC CGTACGACAT CCACACGAAC CCCAATAGGC TATTTTGCCA   
  
  
+ CGCAAAACTA TTGTCGCCTT TTACGATGCG GAATTAAAAA CGCTCATCCC TACCGAGGCC GAGGCAAGAT   
  
  
+ CAGATCACTC GAAGGGAAGC AGAGAGCCAA CACTCGGTAC CACGCGTCCT TTCGTTCACG TGGGCGTCCT   
  
  
+ CCGCGGCTCT TTAGCGTTTT CGTCGCCGAA TCGCCGCGGC TGGCCAGCCG AATGAAACGG TCCTCCCTTC   
  
  
+ GCCGCTTCCA CAACGCTACG CAAGAGCTTC CGCTGCGAGC AGCCGTTCGG CAACCGAGAT AGTTCAGCGA   
  
  
+ GCAGATGTCG GGAAGGCACT CCTTGTTTTC CCGTTGAAAG CGAGAACAAA CAGTTTGAGA GTTCACATTC   
  
  
+ AATGGTTAAA ACATAAAGAC AGACAATCCA CATGAAAGCC AACGACAAAT CAACCGAAAA GACCCTCGAA   
  
  
+ AGACGTTCAT TGAATCAAAA GAAAACTTTA AGAAAACCCA AACCCTCGGA TCCCTCATTC TCGAACTGCT   
  
  
+ TTCTAAAACC CACATTTCAA CTTGAAAGGA ATACTAGACA AAAACCAGAA ACCACAAAAA AATCCGTCGA   
  
  
+ GTTTGAGTAT AGTGTCAAAA GCATGAATTT TTGGAACTAA AGACCCTACG TATGACGGTG ATCGTAGTAG   
  
  
+ ACTGAAACGG GAAGAGAGAG AGAAGGAAGG GAGTAACAAC ACATTTAAGA CAATAAACGT CATGGACTTG   
  
  
+ TTTGACATGT AAAATTCACT AATTGTAAAA GCAGAAAAAC CAAAGTGAAC AAAAGTCTAG AAGGATAATA   
  
  
+ TTTCCTACAG AACCTCCGAC GATTTTATGA CTTATCATCT ACCTTCCATA TTCGGTACGA GGCTGTCGAA   
  
  
+ TACTTGTAAT AAAAACGGGA AGAGTTCTCC TAATTCAAAA AAGTGAAAAG ATTTACTATA AAAACAGGTT   
  
  
+ CCGTCTAAAT CGATATCCCA CTTGTAACAA AACGACGAAC GAACTTACGA TCGAAAAGGG TCAACTTTAC   
  
  
+ AAAGGATTGC GAATTACCAA TAACGATAAC GTCTCCGTTC TCCGTAACTA GATGTATGAG ACAATACTGT   
  
  
+ AAGATAAACC CGTCCACTAT TGAAACATG  

- TTGCAGTCTT TGAGTTCTCC ATTCTTTCTT CTTTCTTGGG GGAGGAAGCT TTGGATTTGT CAAAAGAGCC   
  
  
- TGTGGGGGCC CTTGCTATCA ATTCAGCTTC TTAATGACAA AGTGATGTTA ATTTTATCTG TTCTCTCTTG   
  
  
- ATTTGGGATG TTCTAGTTTG TATGACCATA CGAAAACTAG TTGATACAAA GTCAATCAAG TAAGCTAAAC   
  
  
- AAAGAAGGTA ACATGTCTTA AAGATAAGAC AAGATTAGAA TAATGAGCTC ATATGTGAAA ACAAAAGTGC   
  
  
- AAAAAAGCTT CATAATCTTT GCACATTCAT GTTTTTATTT CATGTTAATC AATTAATTCT TTTTCGCTCA   
  
  
- TCTATTAGAA CCTAATTTAT ACCTTTCTTG GCATGCTGTA GGTGTGCTTG GGGTTATCCG ATAAAACGGT   
  
  
- GCGTTTTGAT AACAGCGGAA AATGCTACGC CTTAATTTTT GCGAGTAGGG ATGGCTCCGG CTCCGTTCTA   
  
  
- GTCTAGTGAG CTTCCCTTCG TCTCTCGGTT GTGAGCCATG GTGCGCAGGA AAGCAAGTGC ACCCGCAGGA   
  
  
- GGCGCCGAGA AATCGCAAAA GCAGCGGCTT AGCGGCGCCG ACCGGTCGGC TTACTTTGCC AGGAGGGAAG   
  
  
- CGGCGAAGGT GTTGCGATGC GTTCTCGAAG GCGACGCTCG TCGGCAAGCC GTTGGCTCTA TCAAGTCGCT   
  
  
- CGTCTACAGC CCTTCCGTGA GGAACAAAAG GGCAACTTTC GCTCTTGTTT GTCAAACTCT CAAGTGTAAG   
  
  
- TTACCAATTT TGTATTTCTG TCTGTTAGGT GTACTTTCGG TTGCTGTTTA GTTGGCTTTT CTGGGAGCTT   
  
  
- TCTGCAAGTA ACTTAGTTTT CTTTTGAAAT TCTTTTGGGT TTGGGAGCCT AGGGAGTAAG AGCTTGACGA   
  
  
- AAGATTTTGG GTGTAAAGTT GAACTTTCCT TATGATCTGT TTTTGGTCTT TGGTGTTTTT TTAGGCAGCT   
  
  
- CAAACTCATA TCACAGTTTT CGTACTTAAA AACCTTGATT TCTGGGATGC ATACTGCCAC TAGCATCATC   
  
  
- TGACTTTGCC CTTCTCTCTC TCTTCCTTCC CTCATTGTTG TGTAAATTCT GTTATTTGCA GTACCTGAAC   
  
  
- AAACTGTACA TTTTAAGTGA TTAACATTTT CGTCTTTTTG GTTTCACTTG TTTTCAGATC TTCCTATTAT   
  
  
- AAAGGATGTC TTGGAGGCTG CTAAAATACT GAATAGTAGA TGGAAGGTAT AAGCCATGCT CCGACAGCTT   
  
  
- ATGAACATTA TTTTTGCCCT TCTCAAGAGG ATTAAGTTTT TTCACTTTTC TAAATGATAT TTTTGTCCAA   
  
  
- GGCAGATTTA GCTATAGGGT GAACATTGTT TTGCTGCTTG CTTGAATGCT AGCTTTTCCC AGTTGAAATG   
  
  
- TTTCCTAACG CTTAATGGTT ATTGCTATTG CAGAGGCAAG AGGCATTGAT CTACATACTC TGTTATGACA   
  
  
- TTCTATTTGG GCAGGTGATA ACTTTGTAC

+     AE-box

| Site Name | Organism | Position | Strand | Matrix score. | sequence | function |
| --- | --- | --- | --- | --- | --- | --- |
| AE-box | Arabidopsis thaliana | 208 | - | 8 | AGAAACAA | part of a module for light response |

> 2018/04/13 10:10:12  
+ AACGTCAGAA ACTCAAGAGG TAAGAAAGAA GAAAGAACCC CCTCCTTCGA AACCTAAACA GTTTTCTCGG   
  
  
+ ACACCCCCGG GAACGATAGT TAAGTCGAAG AATTACTGTT TCACTACAAT TAAAATAGAC AAGAGAGAAC   
  
  
+ TAAACCCTAC AAGATCAAAC ATACTGGTAT GCTTTTGATC AACTATGTTT CAGTTAGTTC ATTCGATTTG   
  
  
+ TTTCTTCCAT TGTACAGAAT TTCTATTCTG TTCTAATCTT ATTACTCGAG TATACACTTT TGTTTTCACG   
  
  
+ TTTTTTCGAA GTATTAGAAA CGTGTAAGTA CAAAAATAAA GTACAATTAG TTAATTAAGA AAAAGCGAGT   
  
  
+ AGATAATCTT GGATTAAATA TGGAAAGAAC CGTACGACAT CCACACGAAC CCCAATAGGC TATTTTGCCA   
  
  
+ CGCAAAACTA TTGTCGCCTT TTACGATGCG GAATTAAAAA CGCTCATCCC TACCGAGGCC GAGGCAAGAT   
  
  
+ CAGATCACTC GAAGGGAAGC AGAGAGCCAA CACTCGGTAC CACGCGTCCT TTCGTTCACG TGGGCGTCCT   
  
  
+ CCGCGGCTCT TTAGCGTTTT CGTCGCCGAA TCGCCGCGGC TGGCCAGCCG AATGAAACGG TCCTCCCTTC   
  
  
+ GCCGCTTCCA CAACGCTACG CAAGAGCTTC CGCTGCGAGC AGCCGTTCGG CAACCGAGAT AGTTCAGCGA   
  
  
+ GCAGATGTCG GGAAGGCACT CCTTGTTTTC CCGTTGAAAG CGAGAACAAA CAGTTTGAGA GTTCACATTC   
  
  
+ AATGGTTAAA ACATAAAGAC AGACAATCCA CATGAAAGCC AACGACAAAT CAACCGAAAA GACCCTCGAA   
  
  
+ AGACGTTCAT TGAATCAAAA GAAAACTTTA AGAAAACCCA AACCCTCGGA TCCCTCATTC TCGAACTGCT   
  
  
+ TTCTAAAACC CACATTTCAA CTTGAAAGGA ATACTAGACA AAAACCAGAA ACCACAAAAA AATCCGTCGA   
  
  
+ GTTTGAGTAT AGTGTCAAAA GCATGAATTT TTGGAACTAA AGACCCTACG TATGACGGTG ATCGTAGTAG   
  
  
+ ACTGAAACGG GAAGAGAGAG AGAAGGAAGG GAGTAACAAC ACATTTAAGA CAATAAACGT CATGGACTTG   
  
  
+ TTTGACATGT AAAATTCACT AATTGTAAAA GCAGAAAAAC CAAAGTGAAC AAAAGTCTAG AAGGATAATA   
  
  
+ TTTCCTACAG AACCTCCGAC GATTTTATGA CTTATCATCT ACCTTCCATA TTCGGTACGA GGCTGTCGAA   
  
  
+ TACTTGTAAT AAAAACGGGA AGAGTTCTCC TAATTCAAAA AAGTGAAAAG ATTTACTATA AAAACAGGTT   
  
  
+ CCGTCTAAAT CGATATCCCA CTTGTAACAA AACGACGAAC GAACTTACGA TCGAAAAGGG TCAACTTTAC   
  
  
+ AAAGGATTGC GAATTACCAA TAACGATAAC GTCTCCGTTC TCCGTAACTA GATGTATGAG ACAATACTGT   
  
  
+ AAGATAAACC CGTCCACTAT TGAAACATG  

- TTGCAGTCTT TGAGTTCTCC ATTCTTTCTT CTTTCTTGGG GGAGGAAGCT TTGGATTTGT CAAAAGAGCC   
  
  
- TGTGGGGGCC CTTGCTATCA ATTCAGCTTC TTAATGACAA AGTGATGTTA ATTTTATCTG TTCTCTCTTG   
  
  
- ATTTGGGATG TTCTAGTTTG TATGACCATA CGAAAACTAG TTGATACAAA GTCAATCAAG TAAGCTAAAC   
  
  
- AAAGAAGGTA ACATGTCTTA AAGATAAGAC AAGATTAGAA TAATGAGCTC ATATGTGAAA ACAAAAGTGC   
  
  
- AAAAAAGCTT CATAATCTTT GCACATTCAT GTTTTTATTT CATGTTAATC AATTAATTCT TTTTCGCTCA   
  
  
- TCTATTAGAA CCTAATTTAT ACCTTTCTTG GCATGCTGTA GGTGTGCTTG GGGTTATCCG ATAAAACGGT   
  
  
- GCGTTTTGAT AACAGCGGAA AATGCTACGC CTTAATTTTT GCGAGTAGGG ATGGCTCCGG CTCCGTTCTA   
  
  
- GTCTAGTGAG CTTCCCTTCG TCTCTCGGTT GTGAGCCATG GTGCGCAGGA AAGCAAGTGC ACCCGCAGGA   
  
  
- GGCGCCGAGA AATCGCAAAA GCAGCGGCTT AGCGGCGCCG ACCGGTCGGC TTACTTTGCC AGGAGGGAAG   
  
  
- CGGCGAAGGT GTTGCGATGC GTTCTCGAAG GCGACGCTCG TCGGCAAGCC GTTGGCTCTA TCAAGTCGCT   
  
  
- CGTCTACAGC CCTTCCGTGA GGAACAAAAG GGCAACTTTC GCTCTTGTTT GTCAAACTCT CAAGTGTAAG   
  
  
- TTACCAATTT TGTATTTCTG TCTGTTAGGT GTACTTTCGG TTGCTGTTTA GTTGGCTTTT CTGGGAGCTT   
  
  
- TCTGCAAGTA ACTTAGTTTT CTTTTGAAAT TCTTTTGGGT TTGGGAGCCT AGGGAGTAAG AGCTTGACGA   
  
  
- AAGATTTTGG GTGTAAAGTT GAACTTTCCT TATGATCTGT TTTTGGTCTT TGGTGTTTTT TTAGGCAGCT   
  
  
- CAAACTCATA TCACAGTTTT CGTACTTAAA AACCTTGATT TCTGGGATGC ATACTGCCAC TAGCATCATC   
  
  
- TGACTTTGCC CTTCTCTCTC TCTTCCTTCC CTCATTGTTG TGTAAATTCT GTTATTTGCA GTACCTGAAC   
  
  
- AAACTGTACA TTTTAAGTGA TTAACATTTT CGTCTTTTTG GTTTCACTTG TTTTCAGATC TTCCTATTAT   
  
  
- AAAGGATGTC TTGGAGGCTG CTAAAATACT GAATAGTAGA TGGAAGGTAT AAGCCATGCT CCGACAGCTT   
  
  
- ATGAACATTA TTTTTGCCCT TCTCAAGAGG ATTAAGTTTT TTCACTTTTC TAAATGATAT TTTTGTCCAA   
  
  
- GGCAGATTTA GCTATAGGGT GAACATTGTT TTGCTGCTTG CTTGAATGCT AGCTTTTCCC AGTTGAAATG   
  
  
- TTTCCTAACG CTTAATGGTT ATTGCTATTG CAGAGGCAAG AGGCATTGAT CTACATACTC TGTTATGACA   
  
  
- TTCTATTTGG GCAGGTGATA ACTTTGTAC

+     ARE

| Site Name | Organism | Position | Strand | Matrix score. | sequence | function |
| --- | --- | --- | --- | --- | --- | --- |
| ARE | Zea mays | 952 | - | 6 | TGGTTT | cis-acting regulatory element essential for the anaerobic induction |
| ARE | Zea mays | 1157 | - | 6 | TGGTTT | cis-acting regulatory element essential for the anaerobic induction |
| ARE | Zea mays | 959 | - | 6 | TGGTTT | cis-acting regulatory element essential for the anaerobic induction |

> 2018/04/13 10:10:12  
+ AACGTCAGAA ACTCAAGAGG TAAGAAAGAA GAAAGAACCC CCTCCTTCGA AACCTAAACA GTTTTCTCGG   
  
  
+ ACACCCCCGG GAACGATAGT TAAGTCGAAG AATTACTGTT TCACTACAAT TAAAATAGAC AAGAGAGAAC   
  
  
+ TAAACCCTAC AAGATCAAAC ATACTGGTAT GCTTTTGATC AACTATGTTT CAGTTAGTTC ATTCGATTTG   
  
  
+ TTTCTTCCAT TGTACAGAAT TTCTATTCTG TTCTAATCTT ATTACTCGAG TATACACTTT TGTTTTCACG   
  
  
+ TTTTTTCGAA GTATTAGAAA CGTGTAAGTA CAAAAATAAA GTACAATTAG TTAATTAAGA AAAAGCGAGT   
  
  
+ AGATAATCTT GGATTAAATA TGGAAAGAAC CGTACGACAT CCACACGAAC CCCAATAGGC TATTTTGCCA   
  
  
+ CGCAAAACTA TTGTCGCCTT TTACGATGCG GAATTAAAAA CGCTCATCCC TACCGAGGCC GAGGCAAGAT   
  
  
+ CAGATCACTC GAAGGGAAGC AGAGAGCCAA CACTCGGTAC CACGCGTCCT TTCGTTCACG TGGGCGTCCT   
  
  
+ CCGCGGCTCT TTAGCGTTTT CGTCGCCGAA TCGCCGCGGC TGGCCAGCCG AATGAAACGG TCCTCCCTTC   
  
  
+ GCCGCTTCCA CAACGCTACG CAAGAGCTTC CGCTGCGAGC AGCCGTTCGG CAACCGAGAT AGTTCAGCGA   
  
  
+ GCAGATGTCG GGAAGGCACT CCTTGTTTTC CCGTTGAAAG CGAGAACAAA CAGTTTGAGA GTTCACATTC   
  
  
+ AATGGTTAAA ACATAAAGAC AGACAATCCA CATGAAAGCC AACGACAAAT CAACCGAAAA GACCCTCGAA   
  
  
+ AGACGTTCAT TGAATCAAAA GAAAACTTTA AGAAAACCCA AACCCTCGGA TCCCTCATTC TCGAACTGCT   
  
  
+ TTCTAAAACC CACATTTCAA CTTGAAAGGA ATACTAGACA AAAACCAGAA ACCACAAAAA AATCCGTCGA   
  
  
+ GTTTGAGTAT AGTGTCAAAA GCATGAATTT TTGGAACTAA AGACCCTACG TATGACGGTG ATCGTAGTAG   
  
  
+ ACTGAAACGG GAAGAGAGAG AGAAGGAAGG GAGTAACAAC ACATTTAAGA CAATAAACGT CATGGACTTG   
  
  
+ TTTGACATGT AAAATTCACT AATTGTAAAA GCAGAAAAAC CAAAGTGAAC AAAAGTCTAG AAGGATAATA   
  
  
+ TTTCCTACAG AACCTCCGAC GATTTTATGA CTTATCATCT ACCTTCCATA TTCGGTACGA GGCTGTCGAA   
  
  
+ TACTTGTAAT AAAAACGGGA AGAGTTCTCC TAATTCAAAA AAGTGAAAAG ATTTACTATA AAAACAGGTT   
  
  
+ CCGTCTAAAT CGATATCCCA CTTGTAACAA AACGACGAAC GAACTTACGA TCGAAAAGGG TCAACTTTAC   
  
  
+ AAAGGATTGC GAATTACCAA TAACGATAAC GTCTCCGTTC TCCGTAACTA GATGTATGAG ACAATACTGT   
  
  
+ AAGATAAACC CGTCCACTAT TGAAACATG  

- TTGCAGTCTT TGAGTTCTCC ATTCTTTCTT CTTTCTTGGG GGAGGAAGCT TTGGATTTGT CAAAAGAGCC   
  
  
- TGTGGGGGCC CTTGCTATCA ATTCAGCTTC TTAATGACAA AGTGATGTTA ATTTTATCTG TTCTCTCTTG   
  
  
- ATTTGGGATG TTCTAGTTTG TATGACCATA CGAAAACTAG TTGATACAAA GTCAATCAAG TAAGCTAAAC   
  
  
- AAAGAAGGTA ACATGTCTTA AAGATAAGAC AAGATTAGAA TAATGAGCTC ATATGTGAAA ACAAAAGTGC   
  
  
- AAAAAAGCTT CATAATCTTT GCACATTCAT GTTTTTATTT CATGTTAATC AATTAATTCT TTTTCGCTCA   
  
  
- TCTATTAGAA CCTAATTTAT ACCTTTCTTG GCATGCTGTA GGTGTGCTTG GGGTTATCCG ATAAAACGGT   
  
  
- GCGTTTTGAT AACAGCGGAA AATGCTACGC CTTAATTTTT GCGAGTAGGG ATGGCTCCGG CTCCGTTCTA   
  
  
- GTCTAGTGAG CTTCCCTTCG TCTCTCGGTT GTGAGCCATG GTGCGCAGGA AAGCAAGTGC ACCCGCAGGA   
  
  
- GGCGCCGAGA AATCGCAAAA GCAGCGGCTT AGCGGCGCCG ACCGGTCGGC TTACTTTGCC AGGAGGGAAG   
  
  
- CGGCGAAGGT GTTGCGATGC GTTCTCGAAG GCGACGCTCG TCGGCAAGCC GTTGGCTCTA TCAAGTCGCT   
  
  
- CGTCTACAGC CCTTCCGTGA GGAACAAAAG GGCAACTTTC GCTCTTGTTT GTCAAACTCT CAAGTGTAAG   
  
  
- TTACCAATTT TGTATTTCTG TCTGTTAGGT GTACTTTCGG TTGCTGTTTA GTTGGCTTTT CTGGGAGCTT   
  
  
- TCTGCAAGTA ACTTAGTTTT CTTTTGAAAT TCTTTTGGGT TTGGGAGCCT AGGGAGTAAG AGCTTGACGA   
  
  
- AAGATTTTGG GTGTAAAGTT GAACTTTCCT TATGATCTGT TTTTGGTCTT TGGTGTTTTT TTAGGCAGCT   
  
  
- CAAACTCATA TCACAGTTTT CGTACTTAAA AACCTTGATT TCTGGGATGC ATACTGCCAC TAGCATCATC   
  
  
- TGACTTTGCC CTTCTCTCTC TCTTCCTTCC CTCATTGTTG TGTAAATTCT GTTATTTGCA GTACCTGAAC   
  
  
- AAACTGTACA TTTTAAGTGA TTAACATTTT CGTCTTTTTG GTTTCACTTG TTTTCAGATC TTCCTATTAT   
  
  
- AAAGGATGTC TTGGAGGCTG CTAAAATACT GAATAGTAGA TGGAAGGTAT AAGCCATGCT CCGACAGCTT   
  
  
- ATGAACATTA TTTTTGCCCT TCTCAAGAGG ATTAAGTTTT TTCACTTTTC TAAATGATAT TTTTGTCCAA   
  
  
- GGCAGATTTA GCTATAGGGT GAACATTGTT TTGCTGCTTG CTTGAATGCT AGCTTTTCCC AGTTGAAATG   
  
  
- TTTCCTAACG CTTAATGGTT ATTGCTATTG CAGAGGCAAG AGGCATTGAT CTACATACTC TGTTATGACA   
  
  
- TTCTATTTGG GCAGGTGATA ACTTTGTAC

+     Box-W1

| Site Name | Organism | Position | Strand | Matrix score. | sequence | function |
| --- | --- | --- | --- | --- | --- | --- |
| Box-W1 | Petroselinum crispum | 1389 | - | 6 | TTGACC | fungal elicitor responsive element |

> 2018/04/13 10:10:12  
+ AACGTCAGAA ACTCAAGAGG TAAGAAAGAA GAAAGAACCC CCTCCTTCGA AACCTAAACA GTTTTCTCGG   
  
  
+ ACACCCCCGG GAACGATAGT TAAGTCGAAG AATTACTGTT TCACTACAAT TAAAATAGAC AAGAGAGAAC   
  
  
+ TAAACCCTAC AAGATCAAAC ATACTGGTAT GCTTTTGATC AACTATGTTT CAGTTAGTTC ATTCGATTTG   
  
  
+ TTTCTTCCAT TGTACAGAAT TTCTATTCTG TTCTAATCTT ATTACTCGAG TATACACTTT TGTTTTCACG   
  
  
+ TTTTTTCGAA GTATTAGAAA CGTGTAAGTA CAAAAATAAA GTACAATTAG TTAATTAAGA AAAAGCGAGT   
  
  
+ AGATAATCTT GGATTAAATA TGGAAAGAAC CGTACGACAT CCACACGAAC CCCAATAGGC TATTTTGCCA   
  
  
+ CGCAAAACTA TTGTCGCCTT TTACGATGCG GAATTAAAAA CGCTCATCCC TACCGAGGCC GAGGCAAGAT   
  
  
+ CAGATCACTC GAAGGGAAGC AGAGAGCCAA CACTCGGTAC CACGCGTCCT TTCGTTCACG TGGGCGTCCT   
  
  
+ CCGCGGCTCT TTAGCGTTTT CGTCGCCGAA TCGCCGCGGC TGGCCAGCCG AATGAAACGG TCCTCCCTTC   
  
  
+ GCCGCTTCCA CAACGCTACG CAAGAGCTTC CGCTGCGAGC AGCCGTTCGG CAACCGAGAT AGTTCAGCGA   
  
  
+ GCAGATGTCG GGAAGGCACT CCTTGTTTTC CCGTTGAAAG CGAGAACAAA CAGTTTGAGA GTTCACATTC   
  
  
+ AATGGTTAAA ACATAAAGAC AGACAATCCA CATGAAAGCC AACGACAAAT CAACCGAAAA GACCCTCGAA   
  
  
+ AGACGTTCAT TGAATCAAAA GAAAACTTTA AGAAAACCCA AACCCTCGGA TCCCTCATTC TCGAACTGCT   
  
  
+ TTCTAAAACC CACATTTCAA CTTGAAAGGA ATACTAGACA AAAACCAGAA ACCACAAAAA AATCCGTCGA   
  
  
+ GTTTGAGTAT AGTGTCAAAA GCATGAATTT TTGGAACTAA AGACCCTACG TATGACGGTG ATCGTAGTAG   
  
  
+ ACTGAAACGG GAAGAGAGAG AGAAGGAAGG GAGTAACAAC ACATTTAAGA CAATAAACGT CATGGACTTG   
  
  
+ TTTGACATGT AAAATTCACT AATTGTAAAA GCAGAAAAAC CAAAGTGAAC AAAAGTCTAG AAGGATAATA   
  
  
+ TTTCCTACAG AACCTCCGAC GATTTTATGA CTTATCATCT ACCTTCCATA TTCGGTACGA GGCTGTCGAA   
  
  
+ TACTTGTAAT AAAAACGGGA AGAGTTCTCC TAATTCAAAA AAGTGAAAAG ATTTACTATA AAAACAGGTT   
  
  
+ CCGTCTAAAT CGATATCCCA CTTGTAACAA AACGACGAAC GAACTTACGA TCGAAAAGGG TCAACTTTAC   
  
  
+ AAAGGATTGC GAATTACCAA TAACGATAAC GTCTCCGTTC TCCGTAACTA GATGTATGAG ACAATACTGT   
  
  
+ AAGATAAACC CGTCCACTAT TGAAACATG  

- TTGCAGTCTT TGAGTTCTCC ATTCTTTCTT CTTTCTTGGG GGAGGAAGCT TTGGATTTGT CAAAAGAGCC   
  
  
- TGTGGGGGCC CTTGCTATCA ATTCAGCTTC TTAATGACAA AGTGATGTTA ATTTTATCTG TTCTCTCTTG   
  
  
- ATTTGGGATG TTCTAGTTTG TATGACCATA CGAAAACTAG TTGATACAAA GTCAATCAAG TAAGCTAAAC   
  
  
- AAAGAAGGTA ACATGTCTTA AAGATAAGAC AAGATTAGAA TAATGAGCTC ATATGTGAAA ACAAAAGTGC   
  
  
- AAAAAAGCTT CATAATCTTT GCACATTCAT GTTTTTATTT CATGTTAATC AATTAATTCT TTTTCGCTCA   
  
  
- TCTATTAGAA CCTAATTTAT ACCTTTCTTG GCATGCTGTA GGTGTGCTTG GGGTTATCCG ATAAAACGGT   
  
  
- GCGTTTTGAT AACAGCGGAA AATGCTACGC CTTAATTTTT GCGAGTAGGG ATGGCTCCGG CTCCGTTCTA   
  
  
- GTCTAGTGAG CTTCCCTTCG TCTCTCGGTT GTGAGCCATG GTGCGCAGGA AAGCAAGTGC ACCCGCAGGA   
  
  
- GGCGCCGAGA AATCGCAAAA GCAGCGGCTT AGCGGCGCCG ACCGGTCGGC TTACTTTGCC AGGAGGGAAG   
  
  
- CGGCGAAGGT GTTGCGATGC GTTCTCGAAG GCGACGCTCG TCGGCAAGCC GTTGGCTCTA TCAAGTCGCT   
  
  
- CGTCTACAGC CCTTCCGTGA GGAACAAAAG GGCAACTTTC GCTCTTGTTT GTCAAACTCT CAAGTGTAAG   
  
  
- TTACCAATTT TGTATTTCTG TCTGTTAGGT GTACTTTCGG TTGCTGTTTA GTTGGCTTTT CTGGGAGCTT   
  
  
- TCTGCAAGTA ACTTAGTTTT CTTTTGAAAT TCTTTTGGGT TTGGGAGCCT AGGGAGTAAG AGCTTGACGA   
  
  
- AAGATTTTGG GTGTAAAGTT GAACTTTCCT TATGATCTGT TTTTGGTCTT TGGTGTTTTT TTAGGCAGCT   
  
  
- CAAACTCATA TCACAGTTTT CGTACTTAAA AACCTTGATT TCTGGGATGC ATACTGCCAC TAGCATCATC   
  
  
- TGACTTTGCC CTTCTCTCTC TCTTCCTTCC CTCATTGTTG TGTAAATTCT GTTATTTGCA GTACCTGAAC   
  
  
- AAACTGTACA TTTTAAGTGA TTAACATTTT CGTCTTTTTG GTTTCACTTG TTTTCAGATC TTCCTATTAT   
  
  
- AAAGGATGTC TTGGAGGCTG CTAAAATACT GAATAGTAGA TGGAAGGTAT AAGCCATGCT CCGACAGCTT   
  
  
- ATGAACATTA TTTTTGCCCT TCTCAAGAGG ATTAAGTTTT TTCACTTTTC TAAATGATAT TTTTGTCCAA   
  
  
- GGCAGATTTA GCTATAGGGT GAACATTGTT TTGCTGCTTG CTTGAATGCT AGCTTTTCCC AGTTGAAATG   
  
  
- TTTCCTAACG CTTAATGGTT ATTGCTATTG CAGAGGCAAG AGGCATTGAT CTACATACTC TGTTATGACA   
  
  
- TTCTATTTGG GCAGGTGATA ACTTTGTAC

+     CAAT-box

| Site Name | Organism | Position | Strand | Matrix score. | sequence | function |
| --- | --- | --- | --- | --- | --- | --- |
| CAAT-box | Hordeum vulgare | 794 | + | 4 | CAAT | common cis-acting element in promoter and enhancer regions |
| CAAT-box | Hordeum vulgare | 1489 | - | 4 | CAAT | common cis-acting element in promoter and enhancer regions |
| CAAT-box | Hordeum vulgare | 770 | + | 4 | CAAT | common cis-acting element in promoter and enhancer regions |
| CAAT-box | Hordeum vulgare | 430 | - | 4 | CAAT | common cis-acting element in promoter and enhancer regions |
| CAAT-box | Hordeum vulgare | 1418 | + | 4 | CAAT | common cis-acting element in promoter and enhancer regions |
| CAAT-box | Hordeum vulgare | 1142 | - | 4 | CAAT | common cis-acting element in promoter and enhancer regions |
| CAAT-box | Arabidopsis thaliana | 402 | + | 5 | CCAAT | common cis-acting element in promoter and enhancer regions |
| CAAT-box | Glycine max | 324 | + | 5 | CAATT | common cis-acting element in promoter and enhancer regions |
| CAAT-box | Brassica rapa | 206 | - | 5 | CAAAT | common cis-acting element in promoter and enhancer regions |
| CAAT-box | Glycine max | 117 | + | 5 | CAATT | common cis-acting element in promoter and enhancer regions |
| CAAT-box | Hordeum vulgare | 1406 | - | 4 | CAAT | common cis-acting element in promoter and enhancer regions |
| CAAT-box | Brassica rapa | 816 | + | 5 | CAAAT | common cis-acting element in promoter and enhancer regions |
| CAAT-box | Hordeum vulgare | 1462 | + | 4 | CAAT | common cis-acting element in promoter and enhancer regions |
| CAAT-box | Hordeum vulgare | 403 | + | 4 | CAAT | common cis-acting element in promoter and enhancer regions |
| CAAT-box | Arabidopsis thaliana | 1417 | + | 5 | CCAAT | common cis-acting element in promoter and enhancer regions |
| CAAT-box | Hordeum vulgare | 219 | - | 4 | CAAT | common cis-acting element in promoter and enhancer regions |
| CAAT-box | Hordeum vulgare | 849 | - | 4 | CAAT | common cis-acting element in promoter and enhancer regions |
| CAAT-box | Hordeum vulgare | 1101 | + | 4 | CAAT | common cis-acting element in promoter and enhancer regions |
| CAAT-box | Glycine max | 1141 | - | 5 | CAATT | common cis-acting element in promoter and enhancer regions |

> 2018/04/13 10:10:12  
+ AACGTCAGAA ACTCAAGAGG TAAGAAAGAA GAAAGAACCC CCTCCTTCGA AACCTAAACA GTTTTCTCGG   
  
  
+ ACACCCCCGG GAACGATAGT TAAGTCGAAG AATTACTGTT TCACTACAAT TAAAATAGAC AAGAGAGAAC   
  
  
+ TAAACCCTAC AAGATCAAAC ATACTGGTAT GCTTTTGATC AACTATGTTT CAGTTAGTTC ATTCGATTTG   
  
  
+ TTTCTTCCAT TGTACAGAAT TTCTATTCTG TTCTAATCTT ATTACTCGAG TATACACTTT TGTTTTCACG   
  
  
+ TTTTTTCGAA GTATTAGAAA CGTGTAAGTA CAAAAATAAA GTACAATTAG TTAATTAAGA AAAAGCGAGT   
  
  
+ AGATAATCTT GGATTAAATA TGGAAAGAAC CGTACGACAT CCACACGAAC CCCAATAGGC TATTTTGCCA   
  
  
+ CGCAAAACTA TTGTCGCCTT TTACGATGCG GAATTAAAAA CGCTCATCCC TACCGAGGCC GAGGCAAGAT   
  
  
+ CAGATCACTC GAAGGGAAGC AGAGAGCCAA CACTCGGTAC CACGCGTCCT TTCGTTCACG TGGGCGTCCT   
  
  
+ CCGCGGCTCT TTAGCGTTTT CGTCGCCGAA TCGCCGCGGC TGGCCAGCCG AATGAAACGG TCCTCCCTTC   
  
  
+ GCCGCTTCCA CAACGCTACG CAAGAGCTTC CGCTGCGAGC AGCCGTTCGG CAACCGAGAT AGTTCAGCGA   
  
  
+ GCAGATGTCG GGAAGGCACT CCTTGTTTTC CCGTTGAAAG CGAGAACAAA CAGTTTGAGA GTTCACATTC   
  
  
+ AATGGTTAAA ACATAAAGAC AGACAATCCA CATGAAAGCC AACGACAAAT CAACCGAAAA GACCCTCGAA   
  
  
+ AGACGTTCAT TGAATCAAAA GAAAACTTTA AGAAAACCCA AACCCTCGGA TCCCTCATTC TCGAACTGCT   
  
  
+ TTCTAAAACC CACATTTCAA CTTGAAAGGA ATACTAGACA AAAACCAGAA ACCACAAAAA AATCCGTCGA   
  
  
+ GTTTGAGTAT AGTGTCAAAA GCATGAATTT TTGGAACTAA AGACCCTACG TATGACGGTG ATCGTAGTAG   
  
  
+ ACTGAAACGG GAAGAGAGAG AGAAGGAAGG GAGTAACAAC ACATTTAAGA CAATAAACGT CATGGACTTG   
  
  
+ TTTGACATGT AAAATTCACT AATTGTAAAA GCAGAAAAAC CAAAGTGAAC AAAAGTCTAG AAGGATAATA   
  
  
+ TTTCCTACAG AACCTCCGAC GATTTTATGA CTTATCATCT ACCTTCCATA TTCGGTACGA GGCTGTCGAA   
  
  
+ TACTTGTAAT AAAAACGGGA AGAGTTCTCC TAATTCAAAA AAGTGAAAAG ATTTACTATA AAAACAGGTT   
  
  
+ CCGTCTAAAT CGATATCCCA CTTGTAACAA AACGACGAAC GAACTTACGA TCGAAAAGGG TCAACTTTAC   
  
  
+ AAAGGATTGC GAATTACCAA TAACGATAAC GTCTCCGTTC TCCGTAACTA GATGTATGAG ACAATACTGT   
  
  
+ AAGATAAACC CGTCCACTAT TGAAACATG  

- TTGCAGTCTT TGAGTTCTCC ATTCTTTCTT CTTTCTTGGG GGAGGAAGCT TTGGATTTGT CAAAAGAGCC   
  
  
- TGTGGGGGCC CTTGCTATCA ATTCAGCTTC TTAATGACAA AGTGATGTTA ATTTTATCTG TTCTCTCTTG   
  
  
- ATTTGGGATG TTCTAGTTTG TATGACCATA CGAAAACTAG TTGATACAAA GTCAATCAAG TAAGCTAAAC   
  
  
- AAAGAAGGTA ACATGTCTTA AAGATAAGAC AAGATTAGAA TAATGAGCTC ATATGTGAAA ACAAAAGTGC   
  
  
- AAAAAAGCTT CATAATCTTT GCACATTCAT GTTTTTATTT CATGTTAATC AATTAATTCT TTTTCGCTCA   
  
  
- TCTATTAGAA CCTAATTTAT ACCTTTCTTG GCATGCTGTA GGTGTGCTTG GGGTTATCCG ATAAAACGGT   
  
  
- GCGTTTTGAT AACAGCGGAA AATGCTACGC CTTAATTTTT GCGAGTAGGG ATGGCTCCGG CTCCGTTCTA   
  
  
- GTCTAGTGAG CTTCCCTTCG TCTCTCGGTT GTGAGCCATG GTGCGCAGGA AAGCAAGTGC ACCCGCAGGA   
  
  
- GGCGCCGAGA AATCGCAAAA GCAGCGGCTT AGCGGCGCCG ACCGGTCGGC TTACTTTGCC AGGAGGGAAG   
  
  
- CGGCGAAGGT GTTGCGATGC GTTCTCGAAG GCGACGCTCG TCGGCAAGCC GTTGGCTCTA TCAAGTCGCT   
  
  
- CGTCTACAGC CCTTCCGTGA GGAACAAAAG GGCAACTTTC GCTCTTGTTT GTCAAACTCT CAAGTGTAAG   
  
  
- TTACCAATTT TGTATTTCTG TCTGTTAGGT GTACTTTCGG TTGCTGTTTA GTTGGCTTTT CTGGGAGCTT   
  
  
- TCTGCAAGTA ACTTAGTTTT CTTTTGAAAT TCTTTTGGGT TTGGGAGCCT AGGGAGTAAG AGCTTGACGA   
  
  
- AAGATTTTGG GTGTAAAGTT GAACTTTCCT TATGATCTGT TTTTGGTCTT TGGTGTTTTT TTAGGCAGCT   
  
  
- CAAACTCATA TCACAGTTTT CGTACTTAAA AACCTTGATT TCTGGGATGC ATACTGCCAC TAGCATCATC   
  
  
- TGACTTTGCC CTTCTCTCTC TCTTCCTTCC CTCATTGTTG TGTAAATTCT GTTATTTGCA GTACCTGAAC   
  
  
- AAACTGTACA TTTTAAGTGA TTAACATTTT CGTCTTTTTG GTTTCACTTG TTTTCAGATC TTCCTATTAT   
  
  
- AAAGGATGTC TTGGAGGCTG CTAAAATACT GAATAGTAGA TGGAAGGTAT AAGCCATGCT CCGACAGCTT   
  
  
- ATGAACATTA TTTTTGCCCT TCTCAAGAGG ATTAAGTTTT TTCACTTTTC TAAATGATAT TTTTGTCCAA   
  
  
- GGCAGATTTA GCTATAGGGT GAACATTGTT TTGCTGCTTG CTTGAATGCT AGCTTTTCCC AGTTGAAATG   
  
  
- TTTCCTAACG CTTAATGGTT ATTGCTATTG CAGAGGCAAG AGGCATTGAT CTACATACTC TGTTATGACA   
  
  
- TTCTATTTGG GCAGGTGATA ACTTTGTAC

+     CCAAT-box

| Site Name | Organism | Position | Strand | Matrix score. | sequence | function |
| --- | --- | --- | --- | --- | --- | --- |
| CCAAT-box | Hordeum vulgare | 731 | - | 6 | CAACGG | MYBHv1 binding site |

> 2018/04/13 10:10:12  
+ AACGTCAGAA ACTCAAGAGG TAAGAAAGAA GAAAGAACCC CCTCCTTCGA AACCTAAACA GTTTTCTCGG   
  
  
+ ACACCCCCGG GAACGATAGT TAAGTCGAAG AATTACTGTT TCACTACAAT TAAAATAGAC AAGAGAGAAC   
  
  
+ TAAACCCTAC AAGATCAAAC ATACTGGTAT GCTTTTGATC AACTATGTTT CAGTTAGTTC ATTCGATTTG   
  
  
+ TTTCTTCCAT TGTACAGAAT TTCTATTCTG TTCTAATCTT ATTACTCGAG TATACACTTT TGTTTTCACG   
  
  
+ TTTTTTCGAA GTATTAGAAA CGTGTAAGTA CAAAAATAAA GTACAATTAG TTAATTAAGA AAAAGCGAGT   
  
  
+ AGATAATCTT GGATTAAATA TGGAAAGAAC CGTACGACAT CCACACGAAC CCCAATAGGC TATTTTGCCA   
  
  
+ CGCAAAACTA TTGTCGCCTT TTACGATGCG GAATTAAAAA CGCTCATCCC TACCGAGGCC GAGGCAAGAT   
  
  
+ CAGATCACTC GAAGGGAAGC AGAGAGCCAA CACTCGGTAC CACGCGTCCT TTCGTTCACG TGGGCGTCCT   
  
  
+ CCGCGGCTCT TTAGCGTTTT CGTCGCCGAA TCGCCGCGGC TGGCCAGCCG AATGAAACGG TCCTCCCTTC   
  
  
+ GCCGCTTCCA CAACGCTACG CAAGAGCTTC CGCTGCGAGC AGCCGTTCGG CAACCGAGAT AGTTCAGCGA   
  
  
+ GCAGATGTCG GGAAGGCACT CCTTGTTTTC CCGTTGAAAG CGAGAACAAA CAGTTTGAGA GTTCACATTC   
  
  
+ AATGGTTAAA ACATAAAGAC AGACAATCCA CATGAAAGCC AACGACAAAT CAACCGAAAA GACCCTCGAA   
  
  
+ AGACGTTCAT TGAATCAAAA GAAAACTTTA AGAAAACCCA AACCCTCGGA TCCCTCATTC TCGAACTGCT   
  
  
+ TTCTAAAACC CACATTTCAA CTTGAAAGGA ATACTAGACA AAAACCAGAA ACCACAAAAA AATCCGTCGA   
  
  
+ GTTTGAGTAT AGTGTCAAAA GCATGAATTT TTGGAACTAA AGACCCTACG TATGACGGTG ATCGTAGTAG   
  
  
+ ACTGAAACGG GAAGAGAGAG AGAAGGAAGG GAGTAACAAC ACATTTAAGA CAATAAACGT CATGGACTTG   
  
  
+ TTTGACATGT AAAATTCACT AATTGTAAAA GCAGAAAAAC CAAAGTGAAC AAAAGTCTAG AAGGATAATA   
  
  
+ TTTCCTACAG AACCTCCGAC GATTTTATGA CTTATCATCT ACCTTCCATA TTCGGTACGA GGCTGTCGAA   
  
  
+ TACTTGTAAT AAAAACGGGA AGAGTTCTCC TAATTCAAAA AAGTGAAAAG ATTTACTATA AAAACAGGTT   
  
  
+ CCGTCTAAAT CGATATCCCA CTTGTAACAA AACGACGAAC GAACTTACGA TCGAAAAGGG TCAACTTTAC   
  
  
+ AAAGGATTGC GAATTACCAA TAACGATAAC GTCTCCGTTC TCCGTAACTA GATGTATGAG ACAATACTGT   
  
  
+ AAGATAAACC CGTCCACTAT TGAAACATG  

- TTGCAGTCTT TGAGTTCTCC ATTCTTTCTT CTTTCTTGGG GGAGGAAGCT TTGGATTTGT CAAAAGAGCC   
  
  
- TGTGGGGGCC CTTGCTATCA ATTCAGCTTC TTAATGACAA AGTGATGTTA ATTTTATCTG TTCTCTCTTG   
  
  
- ATTTGGGATG TTCTAGTTTG TATGACCATA CGAAAACTAG TTGATACAAA GTCAATCAAG TAAGCTAAAC   
  
  
- AAAGAAGGTA ACATGTCTTA AAGATAAGAC AAGATTAGAA TAATGAGCTC ATATGTGAAA ACAAAAGTGC   
  
  
- AAAAAAGCTT CATAATCTTT GCACATTCAT GTTTTTATTT CATGTTAATC AATTAATTCT TTTTCGCTCA   
  
  
- TCTATTAGAA CCTAATTTAT ACCTTTCTTG GCATGCTGTA GGTGTGCTTG GGGTTATCCG ATAAAACGGT   
  
  
- GCGTTTTGAT AACAGCGGAA AATGCTACGC CTTAATTTTT GCGAGTAGGG ATGGCTCCGG CTCCGTTCTA   
  
  
- GTCTAGTGAG CTTCCCTTCG TCTCTCGGTT GTGAGCCATG GTGCGCAGGA AAGCAAGTGC ACCCGCAGGA   
  
  
- GGCGCCGAGA AATCGCAAAA GCAGCGGCTT AGCGGCGCCG ACCGGTCGGC TTACTTTGCC AGGAGGGAAG   
  
  
- CGGCGAAGGT GTTGCGATGC GTTCTCGAAG GCGACGCTCG TCGGCAAGCC GTTGGCTCTA TCAAGTCGCT   
  
  
- CGTCTACAGC CCTTCCGTGA GGAACAAAAG GGCAACTTTC GCTCTTGTTT GTCAAACTCT CAAGTGTAAG   
  
  
- TTACCAATTT TGTATTTCTG TCTGTTAGGT GTACTTTCGG TTGCTGTTTA GTTGGCTTTT CTGGGAGCTT   
  
  
- TCTGCAAGTA ACTTAGTTTT CTTTTGAAAT TCTTTTGGGT TTGGGAGCCT AGGGAGTAAG AGCTTGACGA   
  
  
- AAGATTTTGG GTGTAAAGTT GAACTTTCCT TATGATCTGT TTTTGGTCTT TGGTGTTTTT TTAGGCAGCT   
  
  
- CAAACTCATA TCACAGTTTT CGTACTTAAA AACCTTGATT TCTGGGATGC ATACTGCCAC TAGCATCATC   
  
  
- TGACTTTGCC CTTCTCTCTC TCTTCCTTCC CTCATTGTTG TGTAAATTCT GTTATTTGCA GTACCTGAAC   
  
  
- AAACTGTACA TTTTAAGTGA TTAACATTTT CGTCTTTTTG GTTTCACTTG TTTTCAGATC TTCCTATTAT   
  
  
- AAAGGATGTC TTGGAGGCTG CTAAAATACT GAATAGTAGA TGGAAGGTAT AAGCCATGCT CCGACAGCTT   
  
  
- ATGAACATTA TTTTTGCCCT TCTCAAGAGG ATTAAGTTTT TTCACTTTTC TAAATGATAT TTTTGTCCAA   
  
  
- GGCAGATTTA GCTATAGGGT GAACATTGTT TTGCTGCTTG CTTGAATGCT AGCTTTTCCC AGTTGAAATG   
  
  
- TTTCCTAACG CTTAATGGTT ATTGCTATTG CAGAGGCAAG AGGCATTGAT CTACATACTC TGTTATGACA   
  
  
- TTCTATTTGG GCAGGTGATA ACTTTGTAC

+     CCGTCC-box

| Site Name | Organism | Position | Strand | Matrix score. | sequence | function |
| --- | --- | --- | --- | --- | --- | --- |
| CCGTCC-box | Arabidopsis thaliana | 1480 | + | 6 | CCGTCC | cis-acting regulatory element related to meristem specific activation |

> 2018/04/13 10:10:12  
+ AACGTCAGAA ACTCAAGAGG TAAGAAAGAA GAAAGAACCC CCTCCTTCGA AACCTAAACA GTTTTCTCGG   
  
  
+ ACACCCCCGG GAACGATAGT TAAGTCGAAG AATTACTGTT TCACTACAAT TAAAATAGAC AAGAGAGAAC   
  
  
+ TAAACCCTAC AAGATCAAAC ATACTGGTAT GCTTTTGATC AACTATGTTT CAGTTAGTTC ATTCGATTTG   
  
  
+ TTTCTTCCAT TGTACAGAAT TTCTATTCTG TTCTAATCTT ATTACTCGAG TATACACTTT TGTTTTCACG   
  
  
+ TTTTTTCGAA GTATTAGAAA CGTGTAAGTA CAAAAATAAA GTACAATTAG TTAATTAAGA AAAAGCGAGT   
  
  
+ AGATAATCTT GGATTAAATA TGGAAAGAAC CGTACGACAT CCACACGAAC CCCAATAGGC TATTTTGCCA   
  
  
+ CGCAAAACTA TTGTCGCCTT TTACGATGCG GAATTAAAAA CGCTCATCCC TACCGAGGCC GAGGCAAGAT   
  
  
+ CAGATCACTC GAAGGGAAGC AGAGAGCCAA CACTCGGTAC CACGCGTCCT TTCGTTCACG TGGGCGTCCT   
  
  
+ CCGCGGCTCT TTAGCGTTTT CGTCGCCGAA TCGCCGCGGC TGGCCAGCCG AATGAAACGG TCCTCCCTTC   
  
  
+ GCCGCTTCCA CAACGCTACG CAAGAGCTTC CGCTGCGAGC AGCCGTTCGG CAACCGAGAT AGTTCAGCGA   
  
  
+ GCAGATGTCG GGAAGGCACT CCTTGTTTTC CCGTTGAAAG CGAGAACAAA CAGTTTGAGA GTTCACATTC   
  
  
+ AATGGTTAAA ACATAAAGAC AGACAATCCA CATGAAAGCC AACGACAAAT CAACCGAAAA GACCCTCGAA   
  
  
+ AGACGTTCAT TGAATCAAAA GAAAACTTTA AGAAAACCCA AACCCTCGGA TCCCTCATTC TCGAACTGCT   
  
  
+ TTCTAAAACC CACATTTCAA CTTGAAAGGA ATACTAGACA AAAACCAGAA ACCACAAAAA AATCCGTCGA   
  
  
+ GTTTGAGTAT AGTGTCAAAA GCATGAATTT TTGGAACTAA AGACCCTACG TATGACGGTG ATCGTAGTAG   
  
  
+ ACTGAAACGG GAAGAGAGAG AGAAGGAAGG GAGTAACAAC ACATTTAAGA CAATAAACGT CATGGACTTG   
  
  
+ TTTGACATGT AAAATTCACT AATTGTAAAA GCAGAAAAAC CAAAGTGAAC AAAAGTCTAG AAGGATAATA   
  
  
+ TTTCCTACAG AACCTCCGAC GATTTTATGA CTTATCATCT ACCTTCCATA TTCGGTACGA GGCTGTCGAA   
  
  
+ TACTTGTAAT AAAAACGGGA AGAGTTCTCC TAATTCAAAA AAGTGAAAAG ATTTACTATA AAAACAGGTT   
  
  
+ CCGTCTAAAT CGATATCCCA CTTGTAACAA AACGACGAAC GAACTTACGA TCGAAAAGGG TCAACTTTAC   
  
  
+ AAAGGATTGC GAATTACCAA TAACGATAAC GTCTCCGTTC TCCGTAACTA GATGTATGAG ACAATACTGT   
  
  
+ AAGATAAACC CGTCCACTAT TGAAACATG  

- TTGCAGTCTT TGAGTTCTCC ATTCTTTCTT CTTTCTTGGG GGAGGAAGCT TTGGATTTGT CAAAAGAGCC   
  
  
- TGTGGGGGCC CTTGCTATCA ATTCAGCTTC TTAATGACAA AGTGATGTTA ATTTTATCTG TTCTCTCTTG   
  
  
- ATTTGGGATG TTCTAGTTTG TATGACCATA CGAAAACTAG TTGATACAAA GTCAATCAAG TAAGCTAAAC   
  
  
- AAAGAAGGTA ACATGTCTTA AAGATAAGAC AAGATTAGAA TAATGAGCTC ATATGTGAAA ACAAAAGTGC   
  
  
- AAAAAAGCTT CATAATCTTT GCACATTCAT GTTTTTATTT CATGTTAATC AATTAATTCT TTTTCGCTCA   
  
  
- TCTATTAGAA CCTAATTTAT ACCTTTCTTG GCATGCTGTA GGTGTGCTTG GGGTTATCCG ATAAAACGGT   
  
  
- GCGTTTTGAT AACAGCGGAA AATGCTACGC CTTAATTTTT GCGAGTAGGG ATGGCTCCGG CTCCGTTCTA   
  
  
- GTCTAGTGAG CTTCCCTTCG TCTCTCGGTT GTGAGCCATG GTGCGCAGGA AAGCAAGTGC ACCCGCAGGA   
  
  
- GGCGCCGAGA AATCGCAAAA GCAGCGGCTT AGCGGCGCCG ACCGGTCGGC TTACTTTGCC AGGAGGGAAG   
  
  
- CGGCGAAGGT GTTGCGATGC GTTCTCGAAG GCGACGCTCG TCGGCAAGCC GTTGGCTCTA TCAAGTCGCT   
  
  
- CGTCTACAGC CCTTCCGTGA GGAACAAAAG GGCAACTTTC GCTCTTGTTT GTCAAACTCT CAAGTGTAAG   
  
  
- TTACCAATTT TGTATTTCTG TCTGTTAGGT GTACTTTCGG TTGCTGTTTA GTTGGCTTTT CTGGGAGCTT   
  
  
- TCTGCAAGTA ACTTAGTTTT CTTTTGAAAT TCTTTTGGGT TTGGGAGCCT AGGGAGTAAG AGCTTGACGA   
  
  
- AAGATTTTGG GTGTAAAGTT GAACTTTCCT TATGATCTGT TTTTGGTCTT TGGTGTTTTT TTAGGCAGCT   
  
  
- CAAACTCATA TCACAGTTTT CGTACTTAAA AACCTTGATT TCTGGGATGC ATACTGCCAC TAGCATCATC   
  
  
- TGACTTTGCC CTTCTCTCTC TCTTCCTTCC CTCATTGTTG TGTAAATTCT GTTATTTGCA GTACCTGAAC   
  
  
- AAACTGTACA TTTTAAGTGA TTAACATTTT CGTCTTTTTG GTTTCACTTG TTTTCAGATC TTCCTATTAT   
  
  
- AAAGGATGTC TTGGAGGCTG CTAAAATACT GAATAGTAGA TGGAAGGTAT AAGCCATGCT CCGACAGCTT   
  
  
- ATGAACATTA TTTTTGCCCT TCTCAAGAGG ATTAAGTTTT TTCACTTTTC TAAATGATAT TTTTGTCCAA   
  
  
- GGCAGATTTA GCTATAGGGT GAACATTGTT TTGCTGCTTG CTTGAATGCT AGCTTTTCCC AGTTGAAATG   
  
  
- TTTCCTAACG CTTAATGGTT ATTGCTATTG CAGAGGCAAG AGGCATTGAT CTACATACTC TGTTATGACA   
  
  
- TTCTATTTGG GCAGGTGATA ACTTTGTAC

+     CGTCA-motif

| Site Name | Organism | Position | Strand | Matrix score. | sequence | function |
| --- | --- | --- | --- | --- | --- | --- |
| CGTCA-motif | Hordeum vulgare | 1108 | + | 5 | CGTCA | cis-acting regulatory element involved in the MeJA-responsiveness |
| CGTCA-motif | Hordeum vulgare | 3 | + | 5 | CGTCA | cis-acting regulatory element involved in the MeJA-responsiveness |
| CGTCA-motif | Hordeum vulgare | 1033 | - | 5 | CGTCA | cis-acting regulatory element involved in the MeJA-responsiveness |

> 2018/04/13 10:10:12  
+ AACGTCAGAA ACTCAAGAGG TAAGAAAGAA GAAAGAACCC CCTCCTTCGA AACCTAAACA GTTTTCTCGG   
  
  
+ ACACCCCCGG GAACGATAGT TAAGTCGAAG AATTACTGTT TCACTACAAT TAAAATAGAC AAGAGAGAAC   
  
  
+ TAAACCCTAC AAGATCAAAC ATACTGGTAT GCTTTTGATC AACTATGTTT CAGTTAGTTC ATTCGATTTG   
  
  
+ TTTCTTCCAT TGTACAGAAT TTCTATTCTG TTCTAATCTT ATTACTCGAG TATACACTTT TGTTTTCACG   
  
  
+ TTTTTTCGAA GTATTAGAAA CGTGTAAGTA CAAAAATAAA GTACAATTAG TTAATTAAGA AAAAGCGAGT   
  
  
+ AGATAATCTT GGATTAAATA TGGAAAGAAC CGTACGACAT CCACACGAAC CCCAATAGGC TATTTTGCCA   
  
  
+ CGCAAAACTA TTGTCGCCTT TTACGATGCG GAATTAAAAA CGCTCATCCC TACCGAGGCC GAGGCAAGAT   
  
  
+ CAGATCACTC GAAGGGAAGC AGAGAGCCAA CACTCGGTAC CACGCGTCCT TTCGTTCACG TGGGCGTCCT   
  
  
+ CCGCGGCTCT TTAGCGTTTT CGTCGCCGAA TCGCCGCGGC TGGCCAGCCG AATGAAACGG TCCTCCCTTC   
  
  
+ GCCGCTTCCA CAACGCTACG CAAGAGCTTC CGCTGCGAGC AGCCGTTCGG CAACCGAGAT AGTTCAGCGA   
  
  
+ GCAGATGTCG GGAAGGCACT CCTTGTTTTC CCGTTGAAAG CGAGAACAAA CAGTTTGAGA GTTCACATTC   
  
  
+ AATGGTTAAA ACATAAAGAC AGACAATCCA CATGAAAGCC AACGACAAAT CAACCGAAAA GACCCTCGAA   
  
  
+ AGACGTTCAT TGAATCAAAA GAAAACTTTA AGAAAACCCA AACCCTCGGA TCCCTCATTC TCGAACTGCT   
  
  
+ TTCTAAAACC CACATTTCAA CTTGAAAGGA ATACTAGACA AAAACCAGAA ACCACAAAAA AATCCGTCGA   
  
  
+ GTTTGAGTAT AGTGTCAAAA GCATGAATTT TTGGAACTAA AGACCCTACG TATGACGGTG ATCGTAGTAG   
  
  
+ ACTGAAACGG GAAGAGAGAG AGAAGGAAGG GAGTAACAAC ACATTTAAGA CAATAAACGT CATGGACTTG   
  
  
+ TTTGACATGT AAAATTCACT AATTGTAAAA GCAGAAAAAC CAAAGTGAAC AAAAGTCTAG AAGGATAATA   
  
  
+ TTTCCTACAG AACCTCCGAC GATTTTATGA CTTATCATCT ACCTTCCATA TTCGGTACGA GGCTGTCGAA   
  
  
+ TACTTGTAAT AAAAACGGGA AGAGTTCTCC TAATTCAAAA AAGTGAAAAG ATTTACTATA AAAACAGGTT   
  
  
+ CCGTCTAAAT CGATATCCCA CTTGTAACAA AACGACGAAC GAACTTACGA TCGAAAAGGG TCAACTTTAC   
  
  
+ AAAGGATTGC GAATTACCAA TAACGATAAC GTCTCCGTTC TCCGTAACTA GATGTATGAG ACAATACTGT   
  
  
+ AAGATAAACC CGTCCACTAT TGAAACATG  

- TTGCAGTCTT TGAGTTCTCC ATTCTTTCTT CTTTCTTGGG GGAGGAAGCT TTGGATTTGT CAAAAGAGCC   
  
  
- TGTGGGGGCC CTTGCTATCA ATTCAGCTTC TTAATGACAA AGTGATGTTA ATTTTATCTG TTCTCTCTTG   
  
  
- ATTTGGGATG TTCTAGTTTG TATGACCATA CGAAAACTAG TTGATACAAA GTCAATCAAG TAAGCTAAAC   
  
  
- AAAGAAGGTA ACATGTCTTA AAGATAAGAC AAGATTAGAA TAATGAGCTC ATATGTGAAA ACAAAAGTGC   
  
  
- AAAAAAGCTT CATAATCTTT GCACATTCAT GTTTTTATTT CATGTTAATC AATTAATTCT TTTTCGCTCA   
  
  
- TCTATTAGAA CCTAATTTAT ACCTTTCTTG GCATGCTGTA GGTGTGCTTG GGGTTATCCG ATAAAACGGT   
  
  
- GCGTTTTGAT AACAGCGGAA AATGCTACGC CTTAATTTTT GCGAGTAGGG ATGGCTCCGG CTCCGTTCTA   
  
  
- GTCTAGTGAG CTTCCCTTCG TCTCTCGGTT GTGAGCCATG GTGCGCAGGA AAGCAAGTGC ACCCGCAGGA   
  
  
- GGCGCCGAGA AATCGCAAAA GCAGCGGCTT AGCGGCGCCG ACCGGTCGGC TTACTTTGCC AGGAGGGAAG   
  
  
- CGGCGAAGGT GTTGCGATGC GTTCTCGAAG GCGACGCTCG TCGGCAAGCC GTTGGCTCTA TCAAGTCGCT   
  
  
- CGTCTACAGC CCTTCCGTGA GGAACAAAAG GGCAACTTTC GCTCTTGTTT GTCAAACTCT CAAGTGTAAG   
  
  
- TTACCAATTT TGTATTTCTG TCTGTTAGGT GTACTTTCGG TTGCTGTTTA GTTGGCTTTT CTGGGAGCTT   
  
  
- TCTGCAAGTA ACTTAGTTTT CTTTTGAAAT TCTTTTGGGT TTGGGAGCCT AGGGAGTAAG AGCTTGACGA   
  
  
- AAGATTTTGG GTGTAAAGTT GAACTTTCCT TATGATCTGT TTTTGGTCTT TGGTGTTTTT TTAGGCAGCT   
  
  
- CAAACTCATA TCACAGTTTT CGTACTTAAA AACCTTGATT TCTGGGATGC ATACTGCCAC TAGCATCATC   
  
  
- TGACTTTGCC CTTCTCTCTC TCTTCCTTCC CTCATTGTTG TGTAAATTCT GTTATTTGCA GTACCTGAAC   
  
  
- AAACTGTACA TTTTAAGTGA TTAACATTTT CGTCTTTTTG GTTTCACTTG TTTTCAGATC TTCCTATTAT   
  
  
- AAAGGATGTC TTGGAGGCTG CTAAAATACT GAATAGTAGA TGGAAGGTAT AAGCCATGCT CCGACAGCTT   
  
  
- ATGAACATTA TTTTTGCCCT TCTCAAGAGG ATTAAGTTTT TTCACTTTTC TAAATGATAT TTTTGTCCAA   
  
  
- GGCAGATTTA GCTATAGGGT GAACATTGTT TTGCTGCTTG CTTGAATGCT AGCTTTTCCC AGTTGAAATG   
  
  
- TTTCCTAACG CTTAATGGTT ATTGCTATTG CAGAGGCAAG AGGCATTGAT CTACATACTC TGTTATGACA   
  
  
- TTCTATTTGG GCAGGTGATA ACTTTGTAC

+     G-Box

| Site Name | Organism | Position | Strand | Matrix score. | sequence | function |
| --- | --- | --- | --- | --- | --- | --- |
| G-Box | Pisum sativum | 277 | + | 6 | CACGTT | cis-acting regulatory element involved in light responsiveness |
| G-Box | Pisum sativum | 299 | - | 6 | CACGTT | cis-acting regulatory element involved in light responsiveness |
| G-Box | Pisum sativum | 547 | + | 6 | CACGTG | cis-acting regulatory element involved in light responsiveness |

> 2018/04/13 10:10:12  
+ AACGTCAGAA ACTCAAGAGG TAAGAAAGAA GAAAGAACCC CCTCCTTCGA AACCTAAACA GTTTTCTCGG   
  
  
+ ACACCCCCGG GAACGATAGT TAAGTCGAAG AATTACTGTT TCACTACAAT TAAAATAGAC AAGAGAGAAC   
  
  
+ TAAACCCTAC AAGATCAAAC ATACTGGTAT GCTTTTGATC AACTATGTTT CAGTTAGTTC ATTCGATTTG   
  
  
+ TTTCTTCCAT TGTACAGAAT TTCTATTCTG TTCTAATCTT ATTACTCGAG TATACACTTT TGTTTTCACG   
  
  
+ TTTTTTCGAA GTATTAGAAA CGTGTAAGTA CAAAAATAAA GTACAATTAG TTAATTAAGA AAAAGCGAGT   
  
  
+ AGATAATCTT GGATTAAATA TGGAAAGAAC CGTACGACAT CCACACGAAC CCCAATAGGC TATTTTGCCA   
  
  
+ CGCAAAACTA TTGTCGCCTT TTACGATGCG GAATTAAAAA CGCTCATCCC TACCGAGGCC GAGGCAAGAT   
  
  
+ CAGATCACTC GAAGGGAAGC AGAGAGCCAA CACTCGGTAC CACGCGTCCT TTCGTTCACG TGGGCGTCCT   
  
  
+ CCGCGGCTCT TTAGCGTTTT CGTCGCCGAA TCGCCGCGGC TGGCCAGCCG AATGAAACGG TCCTCCCTTC   
  
  
+ GCCGCTTCCA CAACGCTACG CAAGAGCTTC CGCTGCGAGC AGCCGTTCGG CAACCGAGAT AGTTCAGCGA   
  
  
+ GCAGATGTCG GGAAGGCACT CCTTGTTTTC CCGTTGAAAG CGAGAACAAA CAGTTTGAGA GTTCACATTC   
  
  
+ AATGGTTAAA ACATAAAGAC AGACAATCCA CATGAAAGCC AACGACAAAT CAACCGAAAA GACCCTCGAA   
  
  
+ AGACGTTCAT TGAATCAAAA GAAAACTTTA AGAAAACCCA AACCCTCGGA TCCCTCATTC TCGAACTGCT   
  
  
+ TTCTAAAACC CACATTTCAA CTTGAAAGGA ATACTAGACA AAAACCAGAA ACCACAAAAA AATCCGTCGA   
  
  
+ GTTTGAGTAT AGTGTCAAAA GCATGAATTT TTGGAACTAA AGACCCTACG TATGACGGTG ATCGTAGTAG   
  
  
+ ACTGAAACGG GAAGAGAGAG AGAAGGAAGG GAGTAACAAC ACATTTAAGA CAATAAACGT CATGGACTTG   
  
  
+ TTTGACATGT AAAATTCACT AATTGTAAAA GCAGAAAAAC CAAAGTGAAC AAAAGTCTAG AAGGATAATA   
  
  
+ TTTCCTACAG AACCTCCGAC GATTTTATGA CTTATCATCT ACCTTCCATA TTCGGTACGA GGCTGTCGAA   
  
  
+ TACTTGTAAT AAAAACGGGA AGAGTTCTCC TAATTCAAAA AAGTGAAAAG ATTTACTATA AAAACAGGTT   
  
  
+ CCGTCTAAAT CGATATCCCA CTTGTAACAA AACGACGAAC GAACTTACGA TCGAAAAGGG TCAACTTTAC   
  
  
+ AAAGGATTGC GAATTACCAA TAACGATAAC GTCTCCGTTC TCCGTAACTA GATGTATGAG ACAATACTGT   
  
  
+ AAGATAAACC CGTCCACTAT TGAAACATG  

- TTGCAGTCTT TGAGTTCTCC ATTCTTTCTT CTTTCTTGGG GGAGGAAGCT TTGGATTTGT CAAAAGAGCC   
  
  
- TGTGGGGGCC CTTGCTATCA ATTCAGCTTC TTAATGACAA AGTGATGTTA ATTTTATCTG TTCTCTCTTG   
  
  
- ATTTGGGATG TTCTAGTTTG TATGACCATA CGAAAACTAG TTGATACAAA GTCAATCAAG TAAGCTAAAC   
  
  
- AAAGAAGGTA ACATGTCTTA AAGATAAGAC AAGATTAGAA TAATGAGCTC ATATGTGAAA ACAAAAGTGC   
  
  
- AAAAAAGCTT CATAATCTTT GCACATTCAT GTTTTTATTT CATGTTAATC AATTAATTCT TTTTCGCTCA   
  
  
- TCTATTAGAA CCTAATTTAT ACCTTTCTTG GCATGCTGTA GGTGTGCTTG GGGTTATCCG ATAAAACGGT   
  
  
- GCGTTTTGAT AACAGCGGAA AATGCTACGC CTTAATTTTT GCGAGTAGGG ATGGCTCCGG CTCCGTTCTA   
  
  
- GTCTAGTGAG CTTCCCTTCG TCTCTCGGTT GTGAGCCATG GTGCGCAGGA AAGCAAGTGC ACCCGCAGGA   
  
  
- GGCGCCGAGA AATCGCAAAA GCAGCGGCTT AGCGGCGCCG ACCGGTCGGC TTACTTTGCC AGGAGGGAAG   
  
  
- CGGCGAAGGT GTTGCGATGC GTTCTCGAAG GCGACGCTCG TCGGCAAGCC GTTGGCTCTA TCAAGTCGCT   
  
  
- CGTCTACAGC CCTTCCGTGA GGAACAAAAG GGCAACTTTC GCTCTTGTTT GTCAAACTCT CAAGTGTAAG   
  
  
- TTACCAATTT TGTATTTCTG TCTGTTAGGT GTACTTTCGG TTGCTGTTTA GTTGGCTTTT CTGGGAGCTT   
  
  
- TCTGCAAGTA ACTTAGTTTT CTTTTGAAAT TCTTTTGGGT TTGGGAGCCT AGGGAGTAAG AGCTTGACGA   
  
  
- AAGATTTTGG GTGTAAAGTT GAACTTTCCT TATGATCTGT TTTTGGTCTT TGGTGTTTTT TTAGGCAGCT   
  
  
- CAAACTCATA TCACAGTTTT CGTACTTAAA AACCTTGATT TCTGGGATGC ATACTGCCAC TAGCATCATC   
  
  
- TGACTTTGCC CTTCTCTCTC TCTTCCTTCC CTCATTGTTG TGTAAATTCT GTTATTTGCA GTACCTGAAC   
  
  
- AAACTGTACA TTTTAAGTGA TTAACATTTT CGTCTTTTTG GTTTCACTTG TTTTCAGATC TTCCTATTAT   
  
  
- AAAGGATGTC TTGGAGGCTG CTAAAATACT GAATAGTAGA TGGAAGGTAT AAGCCATGCT CCGACAGCTT   
  
  
- ATGAACATTA TTTTTGCCCT TCTCAAGAGG ATTAAGTTTT TTCACTTTTC TAAATGATAT TTTTGTCCAA   
  
  
- GGCAGATTTA GCTATAGGGT GAACATTGTT TTGCTGCTTG CTTGAATGCT AGCTTTTCCC AGTTGAAATG   
  
  
- TTTCCTAACG CTTAATGGTT ATTGCTATTG CAGAGGCAAG AGGCATTGAT CTACATACTC TGTTATGACA   
  
  
- TTCTATTTGG GCAGGTGATA ACTTTGTAC

+     G-box

| Site Name | Organism | Position | Strand | Matrix score. | sequence | function |
| --- | --- | --- | --- | --- | --- | --- |
| G-box | Arabidopsis thaliana | 547 | + | 6 | CACGTG | cis-acting regulatory element involved in light responsiveness |
| G-box | Zea mays | 277 | + | 6 | CACGTT | cis-acting regulatory element involved in light responsiveness |
| G-box | Zea mays | 299 | - | 6 | CACGTT | cis-acting regulatory element involved in light responsiveness |

> 2018/04/13 10:10:12  
+ AACGTCAGAA ACTCAAGAGG TAAGAAAGAA GAAAGAACCC CCTCCTTCGA AACCTAAACA GTTTTCTCGG   
  
  
+ ACACCCCCGG GAACGATAGT TAAGTCGAAG AATTACTGTT TCACTACAAT TAAAATAGAC AAGAGAGAAC   
  
  
+ TAAACCCTAC AAGATCAAAC ATACTGGTAT GCTTTTGATC AACTATGTTT CAGTTAGTTC ATTCGATTTG   
  
  
+ TTTCTTCCAT TGTACAGAAT TTCTATTCTG TTCTAATCTT ATTACTCGAG TATACACTTT TGTTTTCACG   
  
  
+ TTTTTTCGAA GTATTAGAAA CGTGTAAGTA CAAAAATAAA GTACAATTAG TTAATTAAGA AAAAGCGAGT   
  
  
+ AGATAATCTT GGATTAAATA TGGAAAGAAC CGTACGACAT CCACACGAAC CCCAATAGGC TATTTTGCCA   
  
  
+ CGCAAAACTA TTGTCGCCTT TTACGATGCG GAATTAAAAA CGCTCATCCC TACCGAGGCC GAGGCAAGAT   
  
  
+ CAGATCACTC GAAGGGAAGC AGAGAGCCAA CACTCGGTAC CACGCGTCCT TTCGTTCACG TGGGCGTCCT   
  
  
+ CCGCGGCTCT TTAGCGTTTT CGTCGCCGAA TCGCCGCGGC TGGCCAGCCG AATGAAACGG TCCTCCCTTC   
  
  
+ GCCGCTTCCA CAACGCTACG CAAGAGCTTC CGCTGCGAGC AGCCGTTCGG CAACCGAGAT AGTTCAGCGA   
  
  
+ GCAGATGTCG GGAAGGCACT CCTTGTTTTC CCGTTGAAAG CGAGAACAAA CAGTTTGAGA GTTCACATTC   
  
  
+ AATGGTTAAA ACATAAAGAC AGACAATCCA CATGAAAGCC AACGACAAAT CAACCGAAAA GACCCTCGAA   
  
  
+ AGACGTTCAT TGAATCAAAA GAAAACTTTA AGAAAACCCA AACCCTCGGA TCCCTCATTC TCGAACTGCT   
  
  
+ TTCTAAAACC CACATTTCAA CTTGAAAGGA ATACTAGACA AAAACCAGAA ACCACAAAAA AATCCGTCGA   
  
  
+ GTTTGAGTAT AGTGTCAAAA GCATGAATTT TTGGAACTAA AGACCCTACG TATGACGGTG ATCGTAGTAG   
  
  
+ ACTGAAACGG GAAGAGAGAG AGAAGGAAGG GAGTAACAAC ACATTTAAGA CAATAAACGT CATGGACTTG   
  
  
+ TTTGACATGT AAAATTCACT AATTGTAAAA GCAGAAAAAC CAAAGTGAAC AAAAGTCTAG AAGGATAATA   
  
  
+ TTTCCTACAG AACCTCCGAC GATTTTATGA CTTATCATCT ACCTTCCATA TTCGGTACGA GGCTGTCGAA   
  
  
+ TACTTGTAAT AAAAACGGGA AGAGTTCTCC TAATTCAAAA AAGTGAAAAG ATTTACTATA AAAACAGGTT   
  
  
+ CCGTCTAAAT CGATATCCCA CTTGTAACAA AACGACGAAC GAACTTACGA TCGAAAAGGG TCAACTTTAC   
  
  
+ AAAGGATTGC GAATTACCAA TAACGATAAC GTCTCCGTTC TCCGTAACTA GATGTATGAG ACAATACTGT   
  
  
+ AAGATAAACC CGTCCACTAT TGAAACATG  

- TTGCAGTCTT TGAGTTCTCC ATTCTTTCTT CTTTCTTGGG GGAGGAAGCT TTGGATTTGT CAAAAGAGCC   
  
  
- TGTGGGGGCC CTTGCTATCA ATTCAGCTTC TTAATGACAA AGTGATGTTA ATTTTATCTG TTCTCTCTTG   
  
  
- ATTTGGGATG TTCTAGTTTG TATGACCATA CGAAAACTAG TTGATACAAA GTCAATCAAG TAAGCTAAAC   
  
  
- AAAGAAGGTA ACATGTCTTA AAGATAAGAC AAGATTAGAA TAATGAGCTC ATATGTGAAA ACAAAAGTGC   
  
  
- AAAAAAGCTT CATAATCTTT GCACATTCAT GTTTTTATTT CATGTTAATC AATTAATTCT TTTTCGCTCA   
  
  
- TCTATTAGAA CCTAATTTAT ACCTTTCTTG GCATGCTGTA GGTGTGCTTG GGGTTATCCG ATAAAACGGT   
  
  
- GCGTTTTGAT AACAGCGGAA AATGCTACGC CTTAATTTTT GCGAGTAGGG ATGGCTCCGG CTCCGTTCTA   
  
  
- GTCTAGTGAG CTTCCCTTCG TCTCTCGGTT GTGAGCCATG GTGCGCAGGA AAGCAAGTGC ACCCGCAGGA   
  
  
- GGCGCCGAGA AATCGCAAAA GCAGCGGCTT AGCGGCGCCG ACCGGTCGGC TTACTTTGCC AGGAGGGAAG   
  
  
- CGGCGAAGGT GTTGCGATGC GTTCTCGAAG GCGACGCTCG TCGGCAAGCC GTTGGCTCTA TCAAGTCGCT   
  
  
- CGTCTACAGC CCTTCCGTGA GGAACAAAAG GGCAACTTTC GCTCTTGTTT GTCAAACTCT CAAGTGTAAG   
  
  
- TTACCAATTT TGTATTTCTG TCTGTTAGGT GTACTTTCGG TTGCTGTTTA GTTGGCTTTT CTGGGAGCTT   
  
  
- TCTGCAAGTA ACTTAGTTTT CTTTTGAAAT TCTTTTGGGT TTGGGAGCCT AGGGAGTAAG AGCTTGACGA   
  
  
- AAGATTTTGG GTGTAAAGTT GAACTTTCCT TATGATCTGT TTTTGGTCTT TGGTGTTTTT TTAGGCAGCT   
  
  
- CAAACTCATA TCACAGTTTT CGTACTTAAA AACCTTGATT TCTGGGATGC ATACTGCCAC TAGCATCATC   
  
  
- TGACTTTGCC CTTCTCTCTC TCTTCCTTCC CTCATTGTTG TGTAAATTCT GTTATTTGCA GTACCTGAAC   
  
  
- AAACTGTACA TTTTAAGTGA TTAACATTTT CGTCTTTTTG GTTTCACTTG TTTTCAGATC TTCCTATTAT   
  
  
- AAAGGATGTC TTGGAGGCTG CTAAAATACT GAATAGTAGA TGGAAGGTAT AAGCCATGCT CCGACAGCTT   
  
  
- ATGAACATTA TTTTTGCCCT TCTCAAGAGG ATTAAGTTTT TTCACTTTTC TAAATGATAT TTTTGTCCAA   
  
  
- GGCAGATTTA GCTATAGGGT GAACATTGTT TTGCTGCTTG CTTGAATGCT AGCTTTTCCC AGTTGAAATG   
  
  
- TTTCCTAACG CTTAATGGTT ATTGCTATTG CAGAGGCAAG AGGCATTGAT CTACATACTC TGTTATGACA   
  
  
- TTCTATTTGG GCAGGTGATA ACTTTGTAC

+     GC-motif

| Site Name | Organism | Position | Strand | Matrix score. | sequence | function |
| --- | --- | --- | --- | --- | --- | --- |
| GC-motif | Zea mays | 74 | + | 6 | CCCCCG | enhancer-like element involved in anoxic specific inducibility |

> 2018/04/13 10:10:12  
+ AACGTCAGAA ACTCAAGAGG TAAGAAAGAA GAAAGAACCC CCTCCTTCGA AACCTAAACA GTTTTCTCGG   
  
  
+ ACACCCCCGG GAACGATAGT TAAGTCGAAG AATTACTGTT TCACTACAAT TAAAATAGAC AAGAGAGAAC   
  
  
+ TAAACCCTAC AAGATCAAAC ATACTGGTAT GCTTTTGATC AACTATGTTT CAGTTAGTTC ATTCGATTTG   
  
  
+ TTTCTTCCAT TGTACAGAAT TTCTATTCTG TTCTAATCTT ATTACTCGAG TATACACTTT TGTTTTCACG   
  
  
+ TTTTTTCGAA GTATTAGAAA CGTGTAAGTA CAAAAATAAA GTACAATTAG TTAATTAAGA AAAAGCGAGT   
  
  
+ AGATAATCTT GGATTAAATA TGGAAAGAAC CGTACGACAT CCACACGAAC CCCAATAGGC TATTTTGCCA   
  
  
+ CGCAAAACTA TTGTCGCCTT TTACGATGCG GAATTAAAAA CGCTCATCCC TACCGAGGCC GAGGCAAGAT   
  
  
+ CAGATCACTC GAAGGGAAGC AGAGAGCCAA CACTCGGTAC CACGCGTCCT TTCGTTCACG TGGGCGTCCT   
  
  
+ CCGCGGCTCT TTAGCGTTTT CGTCGCCGAA TCGCCGCGGC TGGCCAGCCG AATGAAACGG TCCTCCCTTC   
  
  
+ GCCGCTTCCA CAACGCTACG CAAGAGCTTC CGCTGCGAGC AGCCGTTCGG CAACCGAGAT AGTTCAGCGA   
  
  
+ GCAGATGTCG GGAAGGCACT CCTTGTTTTC CCGTTGAAAG CGAGAACAAA CAGTTTGAGA GTTCACATTC   
  
  
+ AATGGTTAAA ACATAAAGAC AGACAATCCA CATGAAAGCC AACGACAAAT CAACCGAAAA GACCCTCGAA   
  
  
+ AGACGTTCAT TGAATCAAAA GAAAACTTTA AGAAAACCCA AACCCTCGGA TCCCTCATTC TCGAACTGCT   
  
  
+ TTCTAAAACC CACATTTCAA CTTGAAAGGA ATACTAGACA AAAACCAGAA ACCACAAAAA AATCCGTCGA   
  
  
+ GTTTGAGTAT AGTGTCAAAA GCATGAATTT TTGGAACTAA AGACCCTACG TATGACGGTG ATCGTAGTAG   
  
  
+ ACTGAAACGG GAAGAGAGAG AGAAGGAAGG GAGTAACAAC ACATTTAAGA CAATAAACGT CATGGACTTG   
  
  
+ TTTGACATGT AAAATTCACT AATTGTAAAA GCAGAAAAAC CAAAGTGAAC AAAAGTCTAG AAGGATAATA   
  
  
+ TTTCCTACAG AACCTCCGAC GATTTTATGA CTTATCATCT ACCTTCCATA TTCGGTACGA GGCTGTCGAA   
  
  
+ TACTTGTAAT AAAAACGGGA AGAGTTCTCC TAATTCAAAA AAGTGAAAAG ATTTACTATA AAAACAGGTT   
  
  
+ CCGTCTAAAT CGATATCCCA CTTGTAACAA AACGACGAAC GAACTTACGA TCGAAAAGGG TCAACTTTAC   
  
  
+ AAAGGATTGC GAATTACCAA TAACGATAAC GTCTCCGTTC TCCGTAACTA GATGTATGAG ACAATACTGT   
  
  
+ AAGATAAACC CGTCCACTAT TGAAACATG  

- TTGCAGTCTT TGAGTTCTCC ATTCTTTCTT CTTTCTTGGG GGAGGAAGCT TTGGATTTGT CAAAAGAGCC   
  
  
- TGTGGGGGCC CTTGCTATCA ATTCAGCTTC TTAATGACAA AGTGATGTTA ATTTTATCTG TTCTCTCTTG   
  
  
- ATTTGGGATG TTCTAGTTTG TATGACCATA CGAAAACTAG TTGATACAAA GTCAATCAAG TAAGCTAAAC   
  
  
- AAAGAAGGTA ACATGTCTTA AAGATAAGAC AAGATTAGAA TAATGAGCTC ATATGTGAAA ACAAAAGTGC   
  
  
- AAAAAAGCTT CATAATCTTT GCACATTCAT GTTTTTATTT CATGTTAATC AATTAATTCT TTTTCGCTCA   
  
  
- TCTATTAGAA CCTAATTTAT ACCTTTCTTG GCATGCTGTA GGTGTGCTTG GGGTTATCCG ATAAAACGGT   
  
  
- GCGTTTTGAT AACAGCGGAA AATGCTACGC CTTAATTTTT GCGAGTAGGG ATGGCTCCGG CTCCGTTCTA   
  
  
- GTCTAGTGAG CTTCCCTTCG TCTCTCGGTT GTGAGCCATG GTGCGCAGGA AAGCAAGTGC ACCCGCAGGA   
  
  
- GGCGCCGAGA AATCGCAAAA GCAGCGGCTT AGCGGCGCCG ACCGGTCGGC TTACTTTGCC AGGAGGGAAG   
  
  
- CGGCGAAGGT GTTGCGATGC GTTCTCGAAG GCGACGCTCG TCGGCAAGCC GTTGGCTCTA TCAAGTCGCT   
  
  
- CGTCTACAGC CCTTCCGTGA GGAACAAAAG GGCAACTTTC GCTCTTGTTT GTCAAACTCT CAAGTGTAAG   
  
  
- TTACCAATTT TGTATTTCTG TCTGTTAGGT GTACTTTCGG TTGCTGTTTA GTTGGCTTTT CTGGGAGCTT   
  
  
- TCTGCAAGTA ACTTAGTTTT CTTTTGAAAT TCTTTTGGGT TTGGGAGCCT AGGGAGTAAG AGCTTGACGA   
  
  
- AAGATTTTGG GTGTAAAGTT GAACTTTCCT TATGATCTGT TTTTGGTCTT TGGTGTTTTT TTAGGCAGCT   
  
  
- CAAACTCATA TCACAGTTTT CGTACTTAAA AACCTTGATT TCTGGGATGC ATACTGCCAC TAGCATCATC   
  
  
- TGACTTTGCC CTTCTCTCTC TCTTCCTTCC CTCATTGTTG TGTAAATTCT GTTATTTGCA GTACCTGAAC   
  
  
- AAACTGTACA TTTTAAGTGA TTAACATTTT CGTCTTTTTG GTTTCACTTG TTTTCAGATC TTCCTATTAT   
  
  
- AAAGGATGTC TTGGAGGCTG CTAAAATACT GAATAGTAGA TGGAAGGTAT AAGCCATGCT CCGACAGCTT   
  
  
- ATGAACATTA TTTTTGCCCT TCTCAAGAGG ATTAAGTTTT TTCACTTTTC TAAATGATAT TTTTGTCCAA   
  
  
- GGCAGATTTA GCTATAGGGT GAACATTGTT TTGCTGCTTG CTTGAATGCT AGCTTTTCCC AGTTGAAATG   
  
  
- TTTCCTAACG CTTAATGGTT ATTGCTATTG CAGAGGCAAG AGGCATTGAT CTACATACTC TGTTATGACA   
  
  
- TTCTATTTGG GCAGGTGATA ACTTTGTAC

+     GT1-motif

| Site Name | Organism | Position | Strand | Matrix score. | sequence | function |
| --- | --- | --- | --- | --- | --- | --- |
| GT1-motif | Arabidopsis thaliana | 774 | + | 6 | GGTTAA | light responsive element |
| GT1-motif | Solanum tuberosum | 795 | + | 8 | AATCCACA | light responsive element |

> 2018/04/13 10:10:12  
+ AACGTCAGAA ACTCAAGAGG TAAGAAAGAA GAAAGAACCC CCTCCTTCGA AACCTAAACA GTTTTCTCGG   
  
  
+ ACACCCCCGG GAACGATAGT TAAGTCGAAG AATTACTGTT TCACTACAAT TAAAATAGAC AAGAGAGAAC   
  
  
+ TAAACCCTAC AAGATCAAAC ATACTGGTAT GCTTTTGATC AACTATGTTT CAGTTAGTTC ATTCGATTTG   
  
  
+ TTTCTTCCAT TGTACAGAAT TTCTATTCTG TTCTAATCTT ATTACTCGAG TATACACTTT TGTTTTCACG   
  
  
+ TTTTTTCGAA GTATTAGAAA CGTGTAAGTA CAAAAATAAA GTACAATTAG TTAATTAAGA AAAAGCGAGT   
  
  
+ AGATAATCTT GGATTAAATA TGGAAAGAAC CGTACGACAT CCACACGAAC CCCAATAGGC TATTTTGCCA   
  
  
+ CGCAAAACTA TTGTCGCCTT TTACGATGCG GAATTAAAAA CGCTCATCCC TACCGAGGCC GAGGCAAGAT   
  
  
+ CAGATCACTC GAAGGGAAGC AGAGAGCCAA CACTCGGTAC CACGCGTCCT TTCGTTCACG TGGGCGTCCT   
  
  
+ CCGCGGCTCT TTAGCGTTTT CGTCGCCGAA TCGCCGCGGC TGGCCAGCCG AATGAAACGG TCCTCCCTTC   
  
  
+ GCCGCTTCCA CAACGCTACG CAAGAGCTTC CGCTGCGAGC AGCCGTTCGG CAACCGAGAT AGTTCAGCGA   
  
  
+ GCAGATGTCG GGAAGGCACT CCTTGTTTTC CCGTTGAAAG CGAGAACAAA CAGTTTGAGA GTTCACATTC   
  
  
+ AATGGTTAAA ACATAAAGAC AGACAATCCA CATGAAAGCC AACGACAAAT CAACCGAAAA GACCCTCGAA   
  
  
+ AGACGTTCAT TGAATCAAAA GAAAACTTTA AGAAAACCCA AACCCTCGGA TCCCTCATTC TCGAACTGCT   
  
  
+ TTCTAAAACC CACATTTCAA CTTGAAAGGA ATACTAGACA AAAACCAGAA ACCACAAAAA AATCCGTCGA   
  
  
+ GTTTGAGTAT AGTGTCAAAA GCATGAATTT TTGGAACTAA AGACCCTACG TATGACGGTG ATCGTAGTAG   
  
  
+ ACTGAAACGG GAAGAGAGAG AGAAGGAAGG GAGTAACAAC ACATTTAAGA CAATAAACGT CATGGACTTG   
  
  
+ TTTGACATGT AAAATTCACT AATTGTAAAA GCAGAAAAAC CAAAGTGAAC AAAAGTCTAG AAGGATAATA   
  
  
+ TTTCCTACAG AACCTCCGAC GATTTTATGA CTTATCATCT ACCTTCCATA TTCGGTACGA GGCTGTCGAA   
  
  
+ TACTTGTAAT AAAAACGGGA AGAGTTCTCC TAATTCAAAA AAGTGAAAAG ATTTACTATA AAAACAGGTT   
  
  
+ CCGTCTAAAT CGATATCCCA CTTGTAACAA AACGACGAAC GAACTTACGA TCGAAAAGGG TCAACTTTAC   
  
  
+ AAAGGATTGC GAATTACCAA TAACGATAAC GTCTCCGTTC TCCGTAACTA GATGTATGAG ACAATACTGT   
  
  
+ AAGATAAACC CGTCCACTAT TGAAACATG  

- TTGCAGTCTT TGAGTTCTCC ATTCTTTCTT CTTTCTTGGG GGAGGAAGCT TTGGATTTGT CAAAAGAGCC   
  
  
- TGTGGGGGCC CTTGCTATCA ATTCAGCTTC TTAATGACAA AGTGATGTTA ATTTTATCTG TTCTCTCTTG   
  
  
- ATTTGGGATG TTCTAGTTTG TATGACCATA CGAAAACTAG TTGATACAAA GTCAATCAAG TAAGCTAAAC   
  
  
- AAAGAAGGTA ACATGTCTTA AAGATAAGAC AAGATTAGAA TAATGAGCTC ATATGTGAAA ACAAAAGTGC   
  
  
- AAAAAAGCTT CATAATCTTT GCACATTCAT GTTTTTATTT CATGTTAATC AATTAATTCT TTTTCGCTCA   
  
  
- TCTATTAGAA CCTAATTTAT ACCTTTCTTG GCATGCTGTA GGTGTGCTTG GGGTTATCCG ATAAAACGGT   
  
  
- GCGTTTTGAT AACAGCGGAA AATGCTACGC CTTAATTTTT GCGAGTAGGG ATGGCTCCGG CTCCGTTCTA   
  
  
- GTCTAGTGAG CTTCCCTTCG TCTCTCGGTT GTGAGCCATG GTGCGCAGGA AAGCAAGTGC ACCCGCAGGA   
  
  
- GGCGCCGAGA AATCGCAAAA GCAGCGGCTT AGCGGCGCCG ACCGGTCGGC TTACTTTGCC AGGAGGGAAG   
  
  
- CGGCGAAGGT GTTGCGATGC GTTCTCGAAG GCGACGCTCG TCGGCAAGCC GTTGGCTCTA TCAAGTCGCT   
  
  
- CGTCTACAGC CCTTCCGTGA GGAACAAAAG GGCAACTTTC GCTCTTGTTT GTCAAACTCT CAAGTGTAAG   
  
  
- TTACCAATTT TGTATTTCTG TCTGTTAGGT GTACTTTCGG TTGCTGTTTA GTTGGCTTTT CTGGGAGCTT   
  
  
- TCTGCAAGTA ACTTAGTTTT CTTTTGAAAT TCTTTTGGGT TTGGGAGCCT AGGGAGTAAG AGCTTGACGA   
  
  
- AAGATTTTGG GTGTAAAGTT GAACTTTCCT TATGATCTGT TTTTGGTCTT TGGTGTTTTT TTAGGCAGCT   
  
  
- CAAACTCATA TCACAGTTTT CGTACTTAAA AACCTTGATT TCTGGGATGC ATACTGCCAC TAGCATCATC   
  
  
- TGACTTTGCC CTTCTCTCTC TCTTCCTTCC CTCATTGTTG TGTAAATTCT GTTATTTGCA GTACCTGAAC   
  
  
- AAACTGTACA TTTTAAGTGA TTAACATTTT CGTCTTTTTG GTTTCACTTG TTTTCAGATC TTCCTATTAT   
  
  
- AAAGGATGTC TTGGAGGCTG CTAAAATACT GAATAGTAGA TGGAAGGTAT AAGCCATGCT CCGACAGCTT   
  
  
- ATGAACATTA TTTTTGCCCT TCTCAAGAGG ATTAAGTTTT TTCACTTTTC TAAATGATAT TTTTGTCCAA   
  
  
- GGCAGATTTA GCTATAGGGT GAACATTGTT TTGCTGCTTG CTTGAATGCT AGCTTTTCCC AGTTGAAATG   
  
  
- TTTCCTAACG CTTAATGGTT ATTGCTATTG CAGAGGCAAG AGGCATTGAT CTACATACTC TGTTATGACA   
  
  
- TTCTATTTGG GCAGGTGATA ACTTTGTAC

+     LTR

| Site Name | Organism | Position | Strand | Matrix score. | sequence | function |
| --- | --- | --- | --- | --- | --- | --- |
| LTR | Hordeum vulgare | 824 | + | 6 | CCGAAA | cis-acting element involved in low-temperature responsiveness |

> 2018/04/13 10:10:12  
+ AACGTCAGAA ACTCAAGAGG TAAGAAAGAA GAAAGAACCC CCTCCTTCGA AACCTAAACA GTTTTCTCGG   
  
  
+ ACACCCCCGG GAACGATAGT TAAGTCGAAG AATTACTGTT TCACTACAAT TAAAATAGAC AAGAGAGAAC   
  
  
+ TAAACCCTAC AAGATCAAAC ATACTGGTAT GCTTTTGATC AACTATGTTT CAGTTAGTTC ATTCGATTTG   
  
  
+ TTTCTTCCAT TGTACAGAAT TTCTATTCTG TTCTAATCTT ATTACTCGAG TATACACTTT TGTTTTCACG   
  
  
+ TTTTTTCGAA GTATTAGAAA CGTGTAAGTA CAAAAATAAA GTACAATTAG TTAATTAAGA AAAAGCGAGT   
  
  
+ AGATAATCTT GGATTAAATA TGGAAAGAAC CGTACGACAT CCACACGAAC CCCAATAGGC TATTTTGCCA   
  
  
+ CGCAAAACTA TTGTCGCCTT TTACGATGCG GAATTAAAAA CGCTCATCCC TACCGAGGCC GAGGCAAGAT   
  
  
+ CAGATCACTC GAAGGGAAGC AGAGAGCCAA CACTCGGTAC CACGCGTCCT TTCGTTCACG TGGGCGTCCT   
  
  
+ CCGCGGCTCT TTAGCGTTTT CGTCGCCGAA TCGCCGCGGC TGGCCAGCCG AATGAAACGG TCCTCCCTTC   
  
  
+ GCCGCTTCCA CAACGCTACG CAAGAGCTTC CGCTGCGAGC AGCCGTTCGG CAACCGAGAT AGTTCAGCGA   
  
  
+ GCAGATGTCG GGAAGGCACT CCTTGTTTTC CCGTTGAAAG CGAGAACAAA CAGTTTGAGA GTTCACATTC   
  
  
+ AATGGTTAAA ACATAAAGAC AGACAATCCA CATGAAAGCC AACGACAAAT CAACCGAAAA GACCCTCGAA   
  
  
+ AGACGTTCAT TGAATCAAAA GAAAACTTTA AGAAAACCCA AACCCTCGGA TCCCTCATTC TCGAACTGCT   
  
  
+ TTCTAAAACC CACATTTCAA CTTGAAAGGA ATACTAGACA AAAACCAGAA ACCACAAAAA AATCCGTCGA   
  
  
+ GTTTGAGTAT AGTGTCAAAA GCATGAATTT TTGGAACTAA AGACCCTACG TATGACGGTG ATCGTAGTAG   
  
  
+ ACTGAAACGG GAAGAGAGAG AGAAGGAAGG GAGTAACAAC ACATTTAAGA CAATAAACGT CATGGACTTG   
  
  
+ TTTGACATGT AAAATTCACT AATTGTAAAA GCAGAAAAAC CAAAGTGAAC AAAAGTCTAG AAGGATAATA   
  
  
+ TTTCCTACAG AACCTCCGAC GATTTTATGA CTTATCATCT ACCTTCCATA TTCGGTACGA GGCTGTCGAA   
  
  
+ TACTTGTAAT AAAAACGGGA AGAGTTCTCC TAATTCAAAA AAGTGAAAAG ATTTACTATA AAAACAGGTT   
  
  
+ CCGTCTAAAT CGATATCCCA CTTGTAACAA AACGACGAAC GAACTTACGA TCGAAAAGGG TCAACTTTAC   
  
  
+ AAAGGATTGC GAATTACCAA TAACGATAAC GTCTCCGTTC TCCGTAACTA GATGTATGAG ACAATACTGT   
  
  
+ AAGATAAACC CGTCCACTAT TGAAACATG  

- TTGCAGTCTT TGAGTTCTCC ATTCTTTCTT CTTTCTTGGG GGAGGAAGCT TTGGATTTGT CAAAAGAGCC   
  
  
- TGTGGGGGCC CTTGCTATCA ATTCAGCTTC TTAATGACAA AGTGATGTTA ATTTTATCTG TTCTCTCTTG   
  
  
- ATTTGGGATG TTCTAGTTTG TATGACCATA CGAAAACTAG TTGATACAAA GTCAATCAAG TAAGCTAAAC   
  
  
- AAAGAAGGTA ACATGTCTTA AAGATAAGAC AAGATTAGAA TAATGAGCTC ATATGTGAAA ACAAAAGTGC   
  
  
- AAAAAAGCTT CATAATCTTT GCACATTCAT GTTTTTATTT CATGTTAATC AATTAATTCT TTTTCGCTCA   
  
  
- TCTATTAGAA CCTAATTTAT ACCTTTCTTG GCATGCTGTA GGTGTGCTTG GGGTTATCCG ATAAAACGGT   
  
  
- GCGTTTTGAT AACAGCGGAA AATGCTACGC CTTAATTTTT GCGAGTAGGG ATGGCTCCGG CTCCGTTCTA   
  
  
- GTCTAGTGAG CTTCCCTTCG TCTCTCGGTT GTGAGCCATG GTGCGCAGGA AAGCAAGTGC ACCCGCAGGA   
  
  
- GGCGCCGAGA AATCGCAAAA GCAGCGGCTT AGCGGCGCCG ACCGGTCGGC TTACTTTGCC AGGAGGGAAG   
  
  
- CGGCGAAGGT GTTGCGATGC GTTCTCGAAG GCGACGCTCG TCGGCAAGCC GTTGGCTCTA TCAAGTCGCT   
  
  
- CGTCTACAGC CCTTCCGTGA GGAACAAAAG GGCAACTTTC GCTCTTGTTT GTCAAACTCT CAAGTGTAAG   
  
  
- TTACCAATTT TGTATTTCTG TCTGTTAGGT GTACTTTCGG TTGCTGTTTA GTTGGCTTTT CTGGGAGCTT   
  
  
- TCTGCAAGTA ACTTAGTTTT CTTTTGAAAT TCTTTTGGGT TTGGGAGCCT AGGGAGTAAG AGCTTGACGA   
  
  
- AAGATTTTGG GTGTAAAGTT GAACTTTCCT TATGATCTGT TTTTGGTCTT TGGTGTTTTT TTAGGCAGCT   
  
  
- CAAACTCATA TCACAGTTTT CGTACTTAAA AACCTTGATT TCTGGGATGC ATACTGCCAC TAGCATCATC   
  
  
- TGACTTTGCC CTTCTCTCTC TCTTCCTTCC CTCATTGTTG TGTAAATTCT GTTATTTGCA GTACCTGAAC   
  
  
- AAACTGTACA TTTTAAGTGA TTAACATTTT CGTCTTTTTG GTTTCACTTG TTTTCAGATC TTCCTATTAT   
  
  
- AAAGGATGTC TTGGAGGCTG CTAAAATACT GAATAGTAGA TGGAAGGTAT AAGCCATGCT CCGACAGCTT   
  
  
- ATGAACATTA TTTTTGCCCT TCTCAAGAGG ATTAAGTTTT TTCACTTTTC TAAATGATAT TTTTGTCCAA   
  
  
- GGCAGATTTA GCTATAGGGT GAACATTGTT TTGCTGCTTG CTTGAATGCT AGCTTTTCCC AGTTGAAATG   
  
  
- TTTCCTAACG CTTAATGGTT ATTGCTATTG CAGAGGCAAG AGGCATTGAT CTACATACTC TGTTATGACA   
  
  
- TTCTATTTGG GCAGGTGATA ACTTTGTAC

+     MBS

| Site Name | Organism | Position | Strand | Matrix score. | sequence | function |
| --- | --- | --- | --- | --- | --- | --- |
| MBS | Arabidopsis thaliana | 191 | - | 6 | TAACTG | MYB binding site involved in drought-inducibility |

> 2018/04/13 10:10:12  
+ AACGTCAGAA ACTCAAGAGG TAAGAAAGAA GAAAGAACCC CCTCCTTCGA AACCTAAACA GTTTTCTCGG   
  
  
+ ACACCCCCGG GAACGATAGT TAAGTCGAAG AATTACTGTT TCACTACAAT TAAAATAGAC AAGAGAGAAC   
  
  
+ TAAACCCTAC AAGATCAAAC ATACTGGTAT GCTTTTGATC AACTATGTTT CAGTTAGTTC ATTCGATTTG   
  
  
+ TTTCTTCCAT TGTACAGAAT TTCTATTCTG TTCTAATCTT ATTACTCGAG TATACACTTT TGTTTTCACG   
  
  
+ TTTTTTCGAA GTATTAGAAA CGTGTAAGTA CAAAAATAAA GTACAATTAG TTAATTAAGA AAAAGCGAGT   
  
  
+ AGATAATCTT GGATTAAATA TGGAAAGAAC CGTACGACAT CCACACGAAC CCCAATAGGC TATTTTGCCA   
  
  
+ CGCAAAACTA TTGTCGCCTT TTACGATGCG GAATTAAAAA CGCTCATCCC TACCGAGGCC GAGGCAAGAT   
  
  
+ CAGATCACTC GAAGGGAAGC AGAGAGCCAA CACTCGGTAC CACGCGTCCT TTCGTTCACG TGGGCGTCCT   
  
  
+ CCGCGGCTCT TTAGCGTTTT CGTCGCCGAA TCGCCGCGGC TGGCCAGCCG AATGAAACGG TCCTCCCTTC   
  
  
+ GCCGCTTCCA CAACGCTACG CAAGAGCTTC CGCTGCGAGC AGCCGTTCGG CAACCGAGAT AGTTCAGCGA   
  
  
+ GCAGATGTCG GGAAGGCACT CCTTGTTTTC CCGTTGAAAG CGAGAACAAA CAGTTTGAGA GTTCACATTC   
  
  
+ AATGGTTAAA ACATAAAGAC AGACAATCCA CATGAAAGCC AACGACAAAT CAACCGAAAA GACCCTCGAA   
  
  
+ AGACGTTCAT TGAATCAAAA GAAAACTTTA AGAAAACCCA AACCCTCGGA TCCCTCATTC TCGAACTGCT   
  
  
+ TTCTAAAACC CACATTTCAA CTTGAAAGGA ATACTAGACA AAAACCAGAA ACCACAAAAA AATCCGTCGA   
  
  
+ GTTTGAGTAT AGTGTCAAAA GCATGAATTT TTGGAACTAA AGACCCTACG TATGACGGTG ATCGTAGTAG   
  
  
+ ACTGAAACGG GAAGAGAGAG AGAAGGAAGG GAGTAACAAC ACATTTAAGA CAATAAACGT CATGGACTTG   
  
  
+ TTTGACATGT AAAATTCACT AATTGTAAAA GCAGAAAAAC CAAAGTGAAC AAAAGTCTAG AAGGATAATA   
  
  
+ TTTCCTACAG AACCTCCGAC GATTTTATGA CTTATCATCT ACCTTCCATA TTCGGTACGA GGCTGTCGAA   
  
  
+ TACTTGTAAT AAAAACGGGA AGAGTTCTCC TAATTCAAAA AAGTGAAAAG ATTTACTATA AAAACAGGTT   
  
  
+ CCGTCTAAAT CGATATCCCA CTTGTAACAA AACGACGAAC GAACTTACGA TCGAAAAGGG TCAACTTTAC   
  
  
+ AAAGGATTGC GAATTACCAA TAACGATAAC GTCTCCGTTC TCCGTAACTA GATGTATGAG ACAATACTGT   
  
  
+ AAGATAAACC CGTCCACTAT TGAAACATG  

- TTGCAGTCTT TGAGTTCTCC ATTCTTTCTT CTTTCTTGGG GGAGGAAGCT TTGGATTTGT CAAAAGAGCC   
  
  
- TGTGGGGGCC CTTGCTATCA ATTCAGCTTC TTAATGACAA AGTGATGTTA ATTTTATCTG TTCTCTCTTG   
  
  
- ATTTGGGATG TTCTAGTTTG TATGACCATA CGAAAACTAG TTGATACAAA GTCAATCAAG TAAGCTAAAC   
  
  
- AAAGAAGGTA ACATGTCTTA AAGATAAGAC AAGATTAGAA TAATGAGCTC ATATGTGAAA ACAAAAGTGC   
  
  
- AAAAAAGCTT CATAATCTTT GCACATTCAT GTTTTTATTT CATGTTAATC AATTAATTCT TTTTCGCTCA   
  
  
- TCTATTAGAA CCTAATTTAT ACCTTTCTTG GCATGCTGTA GGTGTGCTTG GGGTTATCCG ATAAAACGGT   
  
  
- GCGTTTTGAT AACAGCGGAA AATGCTACGC CTTAATTTTT GCGAGTAGGG ATGGCTCCGG CTCCGTTCTA   
  
  
- GTCTAGTGAG CTTCCCTTCG TCTCTCGGTT GTGAGCCATG GTGCGCAGGA AAGCAAGTGC ACCCGCAGGA   
  
  
- GGCGCCGAGA AATCGCAAAA GCAGCGGCTT AGCGGCGCCG ACCGGTCGGC TTACTTTGCC AGGAGGGAAG   
  
  
- CGGCGAAGGT GTTGCGATGC GTTCTCGAAG GCGACGCTCG TCGGCAAGCC GTTGGCTCTA TCAAGTCGCT   
  
  
- CGTCTACAGC CCTTCCGTGA GGAACAAAAG GGCAACTTTC GCTCTTGTTT GTCAAACTCT CAAGTGTAAG   
  
  
- TTACCAATTT TGTATTTCTG TCTGTTAGGT GTACTTTCGG TTGCTGTTTA GTTGGCTTTT CTGGGAGCTT   
  
  
- TCTGCAAGTA ACTTAGTTTT CTTTTGAAAT TCTTTTGGGT TTGGGAGCCT AGGGAGTAAG AGCTTGACGA   
  
  
- AAGATTTTGG GTGTAAAGTT GAACTTTCCT TATGATCTGT TTTTGGTCTT TGGTGTTTTT TTAGGCAGCT   
  
  
- CAAACTCATA TCACAGTTTT CGTACTTAAA AACCTTGATT TCTGGGATGC ATACTGCCAC TAGCATCATC   
  
  
- TGACTTTGCC CTTCTCTCTC TCTTCCTTCC CTCATTGTTG TGTAAATTCT GTTATTTGCA GTACCTGAAC   
  
  
- AAACTGTACA TTTTAAGTGA TTAACATTTT CGTCTTTTTG GTTTCACTTG TTTTCAGATC TTCCTATTAT   
  
  
- AAAGGATGTC TTGGAGGCTG CTAAAATACT GAATAGTAGA TGGAAGGTAT AAGCCATGCT CCGACAGCTT   
  
  
- ATGAACATTA TTTTTGCCCT TCTCAAGAGG ATTAAGTTTT TTCACTTTTC TAAATGATAT TTTTGTCCAA   
  
  
- GGCAGATTTA GCTATAGGGT GAACATTGTT TTGCTGCTTG CTTGAATGCT AGCTTTTCCC AGTTGAAATG   
  
  
- TTTCCTAACG CTTAATGGTT ATTGCTATTG CAGAGGCAAG AGGCATTGAT CTACATACTC TGTTATGACA   
  
  
- TTCTATTTGG GCAGGTGATA ACTTTGTAC

+     MRE

| Site Name | Organism | Position | Strand | Matrix score. | sequence | function |
| --- | --- | --- | --- | --- | --- | --- |
| MRE | Petroselinum crispum | 51 | + | 7 | AACCTAA | MYB binding site involved in light responsiveness |

> 2018/04/13 10:10:12  
+ AACGTCAGAA ACTCAAGAGG TAAGAAAGAA GAAAGAACCC CCTCCTTCGA AACCTAAACA GTTTTCTCGG   
  
  
+ ACACCCCCGG GAACGATAGT TAAGTCGAAG AATTACTGTT TCACTACAAT TAAAATAGAC AAGAGAGAAC   
  
  
+ TAAACCCTAC AAGATCAAAC ATACTGGTAT GCTTTTGATC AACTATGTTT CAGTTAGTTC ATTCGATTTG   
  
  
+ TTTCTTCCAT TGTACAGAAT TTCTATTCTG TTCTAATCTT ATTACTCGAG TATACACTTT TGTTTTCACG   
  
  
+ TTTTTTCGAA GTATTAGAAA CGTGTAAGTA CAAAAATAAA GTACAATTAG TTAATTAAGA AAAAGCGAGT   
  
  
+ AGATAATCTT GGATTAAATA TGGAAAGAAC CGTACGACAT CCACACGAAC CCCAATAGGC TATTTTGCCA   
  
  
+ CGCAAAACTA TTGTCGCCTT TTACGATGCG GAATTAAAAA CGCTCATCCC TACCGAGGCC GAGGCAAGAT   
  
  
+ CAGATCACTC GAAGGGAAGC AGAGAGCCAA CACTCGGTAC CACGCGTCCT TTCGTTCACG TGGGCGTCCT   
  
  
+ CCGCGGCTCT TTAGCGTTTT CGTCGCCGAA TCGCCGCGGC TGGCCAGCCG AATGAAACGG TCCTCCCTTC   
  
  
+ GCCGCTTCCA CAACGCTACG CAAGAGCTTC CGCTGCGAGC AGCCGTTCGG CAACCGAGAT AGTTCAGCGA   
  
  
+ GCAGATGTCG GGAAGGCACT CCTTGTTTTC CCGTTGAAAG CGAGAACAAA CAGTTTGAGA GTTCACATTC   
  
  
+ AATGGTTAAA ACATAAAGAC AGACAATCCA CATGAAAGCC AACGACAAAT CAACCGAAAA GACCCTCGAA   
  
  
+ AGACGTTCAT TGAATCAAAA GAAAACTTTA AGAAAACCCA AACCCTCGGA TCCCTCATTC TCGAACTGCT   
  
  
+ TTCTAAAACC CACATTTCAA CTTGAAAGGA ATACTAGACA AAAACCAGAA ACCACAAAAA AATCCGTCGA   
  
  
+ GTTTGAGTAT AGTGTCAAAA GCATGAATTT TTGGAACTAA AGACCCTACG TATGACGGTG ATCGTAGTAG   
  
  
+ ACTGAAACGG GAAGAGAGAG AGAAGGAAGG GAGTAACAAC ACATTTAAGA CAATAAACGT CATGGACTTG   
  
  
+ TTTGACATGT AAAATTCACT AATTGTAAAA GCAGAAAAAC CAAAGTGAAC AAAAGTCTAG AAGGATAATA   
  
  
+ TTTCCTACAG AACCTCCGAC GATTTTATGA CTTATCATCT ACCTTCCATA TTCGGTACGA GGCTGTCGAA   
  
  
+ TACTTGTAAT AAAAACGGGA AGAGTTCTCC TAATTCAAAA AAGTGAAAAG ATTTACTATA AAAACAGGTT   
  
  
+ CCGTCTAAAT CGATATCCCA CTTGTAACAA AACGACGAAC GAACTTACGA TCGAAAAGGG TCAACTTTAC   
  
  
+ AAAGGATTGC GAATTACCAA TAACGATAAC GTCTCCGTTC TCCGTAACTA GATGTATGAG ACAATACTGT   
  
  
+ AAGATAAACC CGTCCACTAT TGAAACATG  

- TTGCAGTCTT TGAGTTCTCC ATTCTTTCTT CTTTCTTGGG GGAGGAAGCT TTGGATTTGT CAAAAGAGCC   
  
  
- TGTGGGGGCC CTTGCTATCA ATTCAGCTTC TTAATGACAA AGTGATGTTA ATTTTATCTG TTCTCTCTTG   
  
  
- ATTTGGGATG TTCTAGTTTG TATGACCATA CGAAAACTAG TTGATACAAA GTCAATCAAG TAAGCTAAAC   
  
  
- AAAGAAGGTA ACATGTCTTA AAGATAAGAC AAGATTAGAA TAATGAGCTC ATATGTGAAA ACAAAAGTGC   
  
  
- AAAAAAGCTT CATAATCTTT GCACATTCAT GTTTTTATTT CATGTTAATC AATTAATTCT TTTTCGCTCA   
  
  
- TCTATTAGAA CCTAATTTAT ACCTTTCTTG GCATGCTGTA GGTGTGCTTG GGGTTATCCG ATAAAACGGT   
  
  
- GCGTTTTGAT AACAGCGGAA AATGCTACGC CTTAATTTTT GCGAGTAGGG ATGGCTCCGG CTCCGTTCTA   
  
  
- GTCTAGTGAG CTTCCCTTCG TCTCTCGGTT GTGAGCCATG GTGCGCAGGA AAGCAAGTGC ACCCGCAGGA   
  
  
- GGCGCCGAGA AATCGCAAAA GCAGCGGCTT AGCGGCGCCG ACCGGTCGGC TTACTTTGCC AGGAGGGAAG   
  
  
- CGGCGAAGGT GTTGCGATGC GTTCTCGAAG GCGACGCTCG TCGGCAAGCC GTTGGCTCTA TCAAGTCGCT   
  
  
- CGTCTACAGC CCTTCCGTGA GGAACAAAAG GGCAACTTTC GCTCTTGTTT GTCAAACTCT CAAGTGTAAG   
  
  
- TTACCAATTT TGTATTTCTG TCTGTTAGGT GTACTTTCGG TTGCTGTTTA GTTGGCTTTT CTGGGAGCTT   
  
  
- TCTGCAAGTA ACTTAGTTTT CTTTTGAAAT TCTTTTGGGT TTGGGAGCCT AGGGAGTAAG AGCTTGACGA   
  
  
- AAGATTTTGG GTGTAAAGTT GAACTTTCCT TATGATCTGT TTTTGGTCTT TGGTGTTTTT TTAGGCAGCT   
  
  
- CAAACTCATA TCACAGTTTT CGTACTTAAA AACCTTGATT TCTGGGATGC ATACTGCCAC TAGCATCATC   
  
  
- TGACTTTGCC CTTCTCTCTC TCTTCCTTCC CTCATTGTTG TGTAAATTCT GTTATTTGCA GTACCTGAAC   
  
  
- AAACTGTACA TTTTAAGTGA TTAACATTTT CGTCTTTTTG GTTTCACTTG TTTTCAGATC TTCCTATTAT   
  
  
- AAAGGATGTC TTGGAGGCTG CTAAAATACT GAATAGTAGA TGGAAGGTAT AAGCCATGCT CCGACAGCTT   
  
  
- ATGAACATTA TTTTTGCCCT TCTCAAGAGG ATTAAGTTTT TTCACTTTTC TAAATGATAT TTTTGTCCAA   
  
  
- GGCAGATTTA GCTATAGGGT GAACATTGTT TTGCTGCTTG CTTGAATGCT AGCTTTTCCC AGTTGAAATG   
  
  
- TTTCCTAACG CTTAATGGTT ATTGCTATTG CAGAGGCAAG AGGCATTGAT CTACATACTC TGTTATGACA   
  
  
- TTCTATTTGG GCAGGTGATA ACTTTGTAC

+     OBP-1 site

| Site Name | Organism | Position | Strand | Matrix score. | sequence | function |
| --- | --- | --- | --- | --- | --- | --- |
| OBP-1 site | Arabidopsis thaliana | 263 | + | 10 | TACACTTTTGG | cis-acting regulatory element |

> 2018/04/13 10:10:12  
+ AACGTCAGAA ACTCAAGAGG TAAGAAAGAA GAAAGAACCC CCTCCTTCGA AACCTAAACA GTTTTCTCGG   
  
  
+ ACACCCCCGG GAACGATAGT TAAGTCGAAG AATTACTGTT TCACTACAAT TAAAATAGAC AAGAGAGAAC   
  
  
+ TAAACCCTAC AAGATCAAAC ATACTGGTAT GCTTTTGATC AACTATGTTT CAGTTAGTTC ATTCGATTTG   
  
  
+ TTTCTTCCAT TGTACAGAAT TTCTATTCTG TTCTAATCTT ATTACTCGAG TATACACTTT TGTTTTCACG   
  
  
+ TTTTTTCGAA GTATTAGAAA CGTGTAAGTA CAAAAATAAA GTACAATTAG TTAATTAAGA AAAAGCGAGT   
  
  
+ AGATAATCTT GGATTAAATA TGGAAAGAAC CGTACGACAT CCACACGAAC CCCAATAGGC TATTTTGCCA   
  
  
+ CGCAAAACTA TTGTCGCCTT TTACGATGCG GAATTAAAAA CGCTCATCCC TACCGAGGCC GAGGCAAGAT   
  
  
+ CAGATCACTC GAAGGGAAGC AGAGAGCCAA CACTCGGTAC CACGCGTCCT TTCGTTCACG TGGGCGTCCT   
  
  
+ CCGCGGCTCT TTAGCGTTTT CGTCGCCGAA TCGCCGCGGC TGGCCAGCCG AATGAAACGG TCCTCCCTTC   
  
  
+ GCCGCTTCCA CAACGCTACG CAAGAGCTTC CGCTGCGAGC AGCCGTTCGG CAACCGAGAT AGTTCAGCGA   
  
  
+ GCAGATGTCG GGAAGGCACT CCTTGTTTTC CCGTTGAAAG CGAGAACAAA CAGTTTGAGA GTTCACATTC   
  
  
+ AATGGTTAAA ACATAAAGAC AGACAATCCA CATGAAAGCC AACGACAAAT CAACCGAAAA GACCCTCGAA   
  
  
+ AGACGTTCAT TGAATCAAAA GAAAACTTTA AGAAAACCCA AACCCTCGGA TCCCTCATTC TCGAACTGCT   
  
  
+ TTCTAAAACC CACATTTCAA CTTGAAAGGA ATACTAGACA AAAACCAGAA ACCACAAAAA AATCCGTCGA   
  
  
+ GTTTGAGTAT AGTGTCAAAA GCATGAATTT TTGGAACTAA AGACCCTACG TATGACGGTG ATCGTAGTAG   
  
  
+ ACTGAAACGG GAAGAGAGAG AGAAGGAAGG GAGTAACAAC ACATTTAAGA CAATAAACGT CATGGACTTG   
  
  
+ TTTGACATGT AAAATTCACT AATTGTAAAA GCAGAAAAAC CAAAGTGAAC AAAAGTCTAG AAGGATAATA   
  
  
+ TTTCCTACAG AACCTCCGAC GATTTTATGA CTTATCATCT ACCTTCCATA TTCGGTACGA GGCTGTCGAA   
  
  
+ TACTTGTAAT AAAAACGGGA AGAGTTCTCC TAATTCAAAA AAGTGAAAAG ATTTACTATA AAAACAGGTT   
  
  
+ CCGTCTAAAT CGATATCCCA CTTGTAACAA AACGACGAAC GAACTTACGA TCGAAAAGGG TCAACTTTAC   
  
  
+ AAAGGATTGC GAATTACCAA TAACGATAAC GTCTCCGTTC TCCGTAACTA GATGTATGAG ACAATACTGT   
  
  
+ AAGATAAACC CGTCCACTAT TGAAACATG  

- TTGCAGTCTT TGAGTTCTCC ATTCTTTCTT CTTTCTTGGG GGAGGAAGCT TTGGATTTGT CAAAAGAGCC   
  
  
- TGTGGGGGCC CTTGCTATCA ATTCAGCTTC TTAATGACAA AGTGATGTTA ATTTTATCTG TTCTCTCTTG   
  
  
- ATTTGGGATG TTCTAGTTTG TATGACCATA CGAAAACTAG TTGATACAAA GTCAATCAAG TAAGCTAAAC   
  
  
- AAAGAAGGTA ACATGTCTTA AAGATAAGAC AAGATTAGAA TAATGAGCTC ATATGTGAAA ACAAAAGTGC   
  
  
- AAAAAAGCTT CATAATCTTT GCACATTCAT GTTTTTATTT CATGTTAATC AATTAATTCT TTTTCGCTCA   
  
  
- TCTATTAGAA CCTAATTTAT ACCTTTCTTG GCATGCTGTA GGTGTGCTTG GGGTTATCCG ATAAAACGGT   
  
  
- GCGTTTTGAT AACAGCGGAA AATGCTACGC CTTAATTTTT GCGAGTAGGG ATGGCTCCGG CTCCGTTCTA   
  
  
- GTCTAGTGAG CTTCCCTTCG TCTCTCGGTT GTGAGCCATG GTGCGCAGGA AAGCAAGTGC ACCCGCAGGA   
  
  
- GGCGCCGAGA AATCGCAAAA GCAGCGGCTT AGCGGCGCCG ACCGGTCGGC TTACTTTGCC AGGAGGGAAG   
  
  
- CGGCGAAGGT GTTGCGATGC GTTCTCGAAG GCGACGCTCG TCGGCAAGCC GTTGGCTCTA TCAAGTCGCT   
  
  
- CGTCTACAGC CCTTCCGTGA GGAACAAAAG GGCAACTTTC GCTCTTGTTT GTCAAACTCT CAAGTGTAAG   
  
  
- TTACCAATTT TGTATTTCTG TCTGTTAGGT GTACTTTCGG TTGCTGTTTA GTTGGCTTTT CTGGGAGCTT   
  
  
- TCTGCAAGTA ACTTAGTTTT CTTTTGAAAT TCTTTTGGGT TTGGGAGCCT AGGGAGTAAG AGCTTGACGA   
  
  
- AAGATTTTGG GTGTAAAGTT GAACTTTCCT TATGATCTGT TTTTGGTCTT TGGTGTTTTT TTAGGCAGCT   
  
  
- CAAACTCATA TCACAGTTTT CGTACTTAAA AACCTTGATT TCTGGGATGC ATACTGCCAC TAGCATCATC   
  
  
- TGACTTTGCC CTTCTCTCTC TCTTCCTTCC CTCATTGTTG TGTAAATTCT GTTATTTGCA GTACCTGAAC   
  
  
- AAACTGTACA TTTTAAGTGA TTAACATTTT CGTCTTTTTG GTTTCACTTG TTTTCAGATC TTCCTATTAT   
  
  
- AAAGGATGTC TTGGAGGCTG CTAAAATACT GAATAGTAGA TGGAAGGTAT AAGCCATGCT CCGACAGCTT   
  
  
- ATGAACATTA TTTTTGCCCT TCTCAAGAGG ATTAAGTTTT TTCACTTTTC TAAATGATAT TTTTGTCCAA   
  
  
- GGCAGATTTA GCTATAGGGT GAACATTGTT TTGCTGCTTG CTTGAATGCT AGCTTTTCCC AGTTGAAATG   
  
  
- TTTCCTAACG CTTAATGGTT ATTGCTATTG CAGAGGCAAG AGGCATTGAT CTACATACTC TGTTATGACA   
  
  
- TTCTATTTGG GCAGGTGATA ACTTTGTAC

+     Skn-1\_motif

| Site Name | Organism | Position | Strand | Matrix score. | sequence | function |
| --- | --- | --- | --- | --- | --- | --- |
| Skn-1\_motif | Oryza sativa | 1109 | + | 5 | GTCAT | cis-acting regulatory element required for endosperm expression |
| Skn-1\_motif | Oryza sativa | 1217 | - | 5 | GTCAT | cis-acting regulatory element required for endosperm expression |
| Skn-1\_motif | Oryza sativa | 1032 | - | 5 | GTCAT | cis-acting regulatory element required for endosperm expression |

> 2018/04/13 10:10:12  
+ AACGTCAGAA ACTCAAGAGG TAAGAAAGAA GAAAGAACCC CCTCCTTCGA AACCTAAACA GTTTTCTCGG   
  
  
+ ACACCCCCGG GAACGATAGT TAAGTCGAAG AATTACTGTT TCACTACAAT TAAAATAGAC AAGAGAGAAC   
  
  
+ TAAACCCTAC AAGATCAAAC ATACTGGTAT GCTTTTGATC AACTATGTTT CAGTTAGTTC ATTCGATTTG   
  
  
+ TTTCTTCCAT TGTACAGAAT TTCTATTCTG TTCTAATCTT ATTACTCGAG TATACACTTT TGTTTTCACG   
  
  
+ TTTTTTCGAA GTATTAGAAA CGTGTAAGTA CAAAAATAAA GTACAATTAG TTAATTAAGA AAAAGCGAGT   
  
  
+ AGATAATCTT GGATTAAATA TGGAAAGAAC CGTACGACAT CCACACGAAC CCCAATAGGC TATTTTGCCA   
  
  
+ CGCAAAACTA TTGTCGCCTT TTACGATGCG GAATTAAAAA CGCTCATCCC TACCGAGGCC GAGGCAAGAT   
  
  
+ CAGATCACTC GAAGGGAAGC AGAGAGCCAA CACTCGGTAC CACGCGTCCT TTCGTTCACG TGGGCGTCCT   
  
  
+ CCGCGGCTCT TTAGCGTTTT CGTCGCCGAA TCGCCGCGGC TGGCCAGCCG AATGAAACGG TCCTCCCTTC   
  
  
+ GCCGCTTCCA CAACGCTACG CAAGAGCTTC CGCTGCGAGC AGCCGTTCGG CAACCGAGAT AGTTCAGCGA   
  
  
+ GCAGATGTCG GGAAGGCACT CCTTGTTTTC CCGTTGAAAG CGAGAACAAA CAGTTTGAGA GTTCACATTC   
  
  
+ AATGGTTAAA ACATAAAGAC AGACAATCCA CATGAAAGCC AACGACAAAT CAACCGAAAA GACCCTCGAA   
  
  
+ AGACGTTCAT TGAATCAAAA GAAAACTTTA AGAAAACCCA AACCCTCGGA TCCCTCATTC TCGAACTGCT   
  
  
+ TTCTAAAACC CACATTTCAA CTTGAAAGGA ATACTAGACA AAAACCAGAA ACCACAAAAA AATCCGTCGA   
  
  
+ GTTTGAGTAT AGTGTCAAAA GCATGAATTT TTGGAACTAA AGACCCTACG TATGACGGTG ATCGTAGTAG   
  
  
+ ACTGAAACGG GAAGAGAGAG AGAAGGAAGG GAGTAACAAC ACATTTAAGA CAATAAACGT CATGGACTTG   
  
  
+ TTTGACATGT AAAATTCACT AATTGTAAAA GCAGAAAAAC CAAAGTGAAC AAAAGTCTAG AAGGATAATA   
  
  
+ TTTCCTACAG AACCTCCGAC GATTTTATGA CTTATCATCT ACCTTCCATA TTCGGTACGA GGCTGTCGAA   
  
  
+ TACTTGTAAT AAAAACGGGA AGAGTTCTCC TAATTCAAAA AAGTGAAAAG ATTTACTATA AAAACAGGTT   
  
  
+ CCGTCTAAAT CGATATCCCA CTTGTAACAA AACGACGAAC GAACTTACGA TCGAAAAGGG TCAACTTTAC   
  
  
+ AAAGGATTGC GAATTACCAA TAACGATAAC GTCTCCGTTC TCCGTAACTA GATGTATGAG ACAATACTGT   
  
  
+ AAGATAAACC CGTCCACTAT TGAAACATG  

- TTGCAGTCTT TGAGTTCTCC ATTCTTTCTT CTTTCTTGGG GGAGGAAGCT TTGGATTTGT CAAAAGAGCC   
  
  
- TGTGGGGGCC CTTGCTATCA ATTCAGCTTC TTAATGACAA AGTGATGTTA ATTTTATCTG TTCTCTCTTG   
  
  
- ATTTGGGATG TTCTAGTTTG TATGACCATA CGAAAACTAG TTGATACAAA GTCAATCAAG TAAGCTAAAC   
  
  
- AAAGAAGGTA ACATGTCTTA AAGATAAGAC AAGATTAGAA TAATGAGCTC ATATGTGAAA ACAAAAGTGC   
  
  
- AAAAAAGCTT CATAATCTTT GCACATTCAT GTTTTTATTT CATGTTAATC AATTAATTCT TTTTCGCTCA   
  
  
- TCTATTAGAA CCTAATTTAT ACCTTTCTTG GCATGCTGTA GGTGTGCTTG GGGTTATCCG ATAAAACGGT   
  
  
- GCGTTTTGAT AACAGCGGAA AATGCTACGC CTTAATTTTT GCGAGTAGGG ATGGCTCCGG CTCCGTTCTA   
  
  
- GTCTAGTGAG CTTCCCTTCG TCTCTCGGTT GTGAGCCATG GTGCGCAGGA AAGCAAGTGC ACCCGCAGGA   
  
  
- GGCGCCGAGA AATCGCAAAA GCAGCGGCTT AGCGGCGCCG ACCGGTCGGC TTACTTTGCC AGGAGGGAAG   
  
  
- CGGCGAAGGT GTTGCGATGC GTTCTCGAAG GCGACGCTCG TCGGCAAGCC GTTGGCTCTA TCAAGTCGCT   
  
  
- CGTCTACAGC CCTTCCGTGA GGAACAAAAG GGCAACTTTC GCTCTTGTTT GTCAAACTCT CAAGTGTAAG   
  
  
- TTACCAATTT TGTATTTCTG TCTGTTAGGT GTACTTTCGG TTGCTGTTTA GTTGGCTTTT CTGGGAGCTT   
  
  
- TCTGCAAGTA ACTTAGTTTT CTTTTGAAAT TCTTTTGGGT TTGGGAGCCT AGGGAGTAAG AGCTTGACGA   
  
  
- AAGATTTTGG GTGTAAAGTT GAACTTTCCT TATGATCTGT TTTTGGTCTT TGGTGTTTTT TTAGGCAGCT   
  
  
- CAAACTCATA TCACAGTTTT CGTACTTAAA AACCTTGATT TCTGGGATGC ATACTGCCAC TAGCATCATC   
  
  
- TGACTTTGCC CTTCTCTCTC TCTTCCTTCC CTCATTGTTG TGTAAATTCT GTTATTTGCA GTACCTGAAC   
  
  
- AAACTGTACA TTTTAAGTGA TTAACATTTT CGTCTTTTTG GTTTCACTTG TTTTCAGATC TTCCTATTAT   
  
  
- AAAGGATGTC TTGGAGGCTG CTAAAATACT GAATAGTAGA TGGAAGGTAT AAGCCATGCT CCGACAGCTT   
  
  
- ATGAACATTA TTTTTGCCCT TCTCAAGAGG ATTAAGTTTT TTCACTTTTC TAAATGATAT TTTTGTCCAA   
  
  
- GGCAGATTTA GCTATAGGGT GAACATTGTT TTGCTGCTTG CTTGAATGCT AGCTTTTCCC AGTTGAAATG   
  
  
- TTTCCTAACG CTTAATGGTT ATTGCTATTG CAGAGGCAAG AGGCATTGAT CTACATACTC TGTTATGACA   
  
  
- TTCTATTTGG GCAGGTGATA ACTTTGTAC

+     Sp1

| Site Name | Organism | Position | Strand | Matrix score. | sequence | function |
| --- | --- | --- | --- | --- | --- | --- |
| Sp1 | Zea mays | 622 | + | 5 | CC(G/A)CCC | light responsive element |

> 2018/04/13 10:10:12  
+ AACGTCAGAA ACTCAAGAGG TAAGAAAGAA GAAAGAACCC CCTCCTTCGA AACCTAAACA GTTTTCTCGG   
  
  
+ ACACCCCCGG GAACGATAGT TAAGTCGAAG AATTACTGTT TCACTACAAT TAAAATAGAC AAGAGAGAAC   
  
  
+ TAAACCCTAC AAGATCAAAC ATACTGGTAT GCTTTTGATC AACTATGTTT CAGTTAGTTC ATTCGATTTG   
  
  
+ TTTCTTCCAT TGTACAGAAT TTCTATTCTG TTCTAATCTT ATTACTCGAG TATACACTTT TGTTTTCACG   
  
  
+ TTTTTTCGAA GTATTAGAAA CGTGTAAGTA CAAAAATAAA GTACAATTAG TTAATTAAGA AAAAGCGAGT   
  
  
+ AGATAATCTT GGATTAAATA TGGAAAGAAC CGTACGACAT CCACACGAAC CCCAATAGGC TATTTTGCCA   
  
  
+ CGCAAAACTA TTGTCGCCTT TTACGATGCG GAATTAAAAA CGCTCATCCC TACCGAGGCC GAGGCAAGAT   
  
  
+ CAGATCACTC GAAGGGAAGC AGAGAGCCAA CACTCGGTAC CACGCGTCCT TTCGTTCACG TGGGCGTCCT   
  
  
+ CCGCGGCTCT TTAGCGTTTT CGTCGCCGAA TCGCCGCGGC TGGCCAGCCG AATGAAACGG TCCTCCCTTC   
  
  
+ GCCGCTTCCA CAACGCTACG CAAGAGCTTC CGCTGCGAGC AGCCGTTCGG CAACCGAGAT AGTTCAGCGA   
  
  
+ GCAGATGTCG GGAAGGCACT CCTTGTTTTC CCGTTGAAAG CGAGAACAAA CAGTTTGAGA GTTCACATTC   
  
  
+ AATGGTTAAA ACATAAAGAC AGACAATCCA CATGAAAGCC AACGACAAAT CAACCGAAAA GACCCTCGAA   
  
  
+ AGACGTTCAT TGAATCAAAA GAAAACTTTA AGAAAACCCA AACCCTCGGA TCCCTCATTC TCGAACTGCT   
  
  
+ TTCTAAAACC CACATTTCAA CTTGAAAGGA ATACTAGACA AAAACCAGAA ACCACAAAAA AATCCGTCGA   
  
  
+ GTTTGAGTAT AGTGTCAAAA GCATGAATTT TTGGAACTAA AGACCCTACG TATGACGGTG ATCGTAGTAG   
  
  
+ ACTGAAACGG GAAGAGAGAG AGAAGGAAGG GAGTAACAAC ACATTTAAGA CAATAAACGT CATGGACTTG   
  
  
+ TTTGACATGT AAAATTCACT AATTGTAAAA GCAGAAAAAC CAAAGTGAAC AAAAGTCTAG AAGGATAATA   
  
  
+ TTTCCTACAG AACCTCCGAC GATTTTATGA CTTATCATCT ACCTTCCATA TTCGGTACGA GGCTGTCGAA   
  
  
+ TACTTGTAAT AAAAACGGGA AGAGTTCTCC TAATTCAAAA AAGTGAAAAG ATTTACTATA AAAACAGGTT   
  
  
+ CCGTCTAAAT CGATATCCCA CTTGTAACAA AACGACGAAC GAACTTACGA TCGAAAAGGG TCAACTTTAC   
  
  
+ AAAGGATTGC GAATTACCAA TAACGATAAC GTCTCCGTTC TCCGTAACTA GATGTATGAG ACAATACTGT   
  
  
+ AAGATAAACC CGTCCACTAT TGAAACATG  

- TTGCAGTCTT TGAGTTCTCC ATTCTTTCTT CTTTCTTGGG GGAGGAAGCT TTGGATTTGT CAAAAGAGCC   
  
  
- TGTGGGGGCC CTTGCTATCA ATTCAGCTTC TTAATGACAA AGTGATGTTA ATTTTATCTG TTCTCTCTTG   
  
  
- ATTTGGGATG TTCTAGTTTG TATGACCATA CGAAAACTAG TTGATACAAA GTCAATCAAG TAAGCTAAAC   
  
  
- AAAGAAGGTA ACATGTCTTA AAGATAAGAC AAGATTAGAA TAATGAGCTC ATATGTGAAA ACAAAAGTGC   
  
  
- AAAAAAGCTT CATAATCTTT GCACATTCAT GTTTTTATTT CATGTTAATC AATTAATTCT TTTTCGCTCA   
  
  
- TCTATTAGAA CCTAATTTAT ACCTTTCTTG GCATGCTGTA GGTGTGCTTG GGGTTATCCG ATAAAACGGT   
  
  
- GCGTTTTGAT AACAGCGGAA AATGCTACGC CTTAATTTTT GCGAGTAGGG ATGGCTCCGG CTCCGTTCTA   
  
  
- GTCTAGTGAG CTTCCCTTCG TCTCTCGGTT GTGAGCCATG GTGCGCAGGA AAGCAAGTGC ACCCGCAGGA   
  
  
- GGCGCCGAGA AATCGCAAAA GCAGCGGCTT AGCGGCGCCG ACCGGTCGGC TTACTTTGCC AGGAGGGAAG   
  
  
- CGGCGAAGGT GTTGCGATGC GTTCTCGAAG GCGACGCTCG TCGGCAAGCC GTTGGCTCTA TCAAGTCGCT   
  
  
- CGTCTACAGC CCTTCCGTGA GGAACAAAAG GGCAACTTTC GCTCTTGTTT GTCAAACTCT CAAGTGTAAG   
  
  
- TTACCAATTT TGTATTTCTG TCTGTTAGGT GTACTTTCGG TTGCTGTTTA GTTGGCTTTT CTGGGAGCTT   
  
  
- TCTGCAAGTA ACTTAGTTTT CTTTTGAAAT TCTTTTGGGT TTGGGAGCCT AGGGAGTAAG AGCTTGACGA   
  
  
- AAGATTTTGG GTGTAAAGTT GAACTTTCCT TATGATCTGT TTTTGGTCTT TGGTGTTTTT TTAGGCAGCT   
  
  
- CAAACTCATA TCACAGTTTT CGTACTTAAA AACCTTGATT TCTGGGATGC ATACTGCCAC TAGCATCATC   
  
  
- TGACTTTGCC CTTCTCTCTC TCTTCCTTCC CTCATTGTTG TGTAAATTCT GTTATTTGCA GTACCTGAAC   
  
  
- AAACTGTACA TTTTAAGTGA TTAACATTTT CGTCTTTTTG GTTTCACTTG TTTTCAGATC TTCCTATTAT   
  
  
- AAAGGATGTC TTGGAGGCTG CTAAAATACT GAATAGTAGA TGGAAGGTAT AAGCCATGCT CCGACAGCTT   
  
  
- ATGAACATTA TTTTTGCCCT TCTCAAGAGG ATTAAGTTTT TTCACTTTTC TAAATGATAT TTTTGTCCAA   
  
  
- GGCAGATTTA GCTATAGGGT GAACATTGTT TTGCTGCTTG CTTGAATGCT AGCTTTTCCC AGTTGAAATG   
  
  
- TTTCCTAACG CTTAATGGTT ATTGCTATTG CAGAGGCAAG AGGCATTGAT CTACATACTC TGTTATGACA   
  
  
- TTCTATTTGG GCAGGTGATA ACTTTGTAC

+     TATA-box

| Site Name | Organism | Position | Strand | Matrix score. | sequence | function |
| --- | --- | --- | --- | --- | --- | --- |
| TATA-box | Lycopersicon esculentum | 1270 | - | 5 | TTTTA | core promoter element around -30 of transcription start |
| TATA-box | Lycopersicon esculentum | 1213 | + | 5 | TTTTA | core promoter element around -30 of transcription start |
| TATA-box | Arabidopsis thaliana | 261 | + | 4 | TATA | core promoter element around -30 of transcription start |
| TATA-box | Glycine max | 1186 | + | 5 | TAATA | core promoter element around -30 of transcription start |
| TATA-box | Lycopersicon esculentum | 455 | - | 5 | TTTTA | core promoter element around -30 of transcription start |
| TATA-box | Arabidopsis thaliana | 1317 | + | 6 | TATAAA | core promoter element around -30 of transcription start |
| TATA-box | Lycopersicon esculentum | 777 | - | 5 | TTTTA | core promoter element around -30 of transcription start |
| TATA-box | Oryza sativa | 309 | + | 7 | TACAAAA | core promoter element around -30 of transcription start |
| TATA-box | Arabidopsis thaliana | 1315 | + | 9 | ccTATAAAaa | core promoter element around -30 of transcription start |
| TATA-box | Glycine max | 1267 | + | 5 | TAATA | core promoter element around -30 of transcription start |
| TATA-box | Lycopersicon esculentum | 1146 | - | 5 | TTTTA | core promoter element around -30 of transcription start |
| TATA-box | Glycine max | 250 | - | 5 | TAATA | core promoter element around -30 of transcription start |
| TATA-box | Lycopersicon esculentum | 1130 | - | 5 | TTTTA | core promoter element around -30 of transcription start |
| TATA-box | Lycopersicon esculentum | 121 | - | 5 | TTTTA | core promoter element around -30 of transcription start |
| TATA-box | Lycopersicon esculentum | 439 | + | 5 | TTTTA | core promoter element around -30 of transcription start |
| TATA-box | Lycopersicon esculentum | 1319 | - | 5 | TTTTA | core promoter element around -30 of transcription start |
| TATA-box | Arabidopsis thaliana | 988 | - | 4 | TATA | core promoter element around -30 of transcription start |
| TATA-box | Glycine max | 292 | - | 5 | TAATA | core promoter element around -30 of transcription start |
| TATA-box | Lycopersicon esculentum | 914 | - | 5 | TTTTA | core promoter element around -30 of transcription start |

> 2018/04/13 10:10:12  
+ AACGTCAGAA ACTCAAGAGG TAAGAAAGAA GAAAGAACCC CCTCCTTCGA AACCTAAACA GTTTTCTCGG   
  
  
+ ACACCCCCGG GAACGATAGT TAAGTCGAAG AATTACTGTT TCACTACAAT TAAAATAGAC AAGAGAGAAC   
  
  
+ TAAACCCTAC AAGATCAAAC ATACTGGTAT GCTTTTGATC AACTATGTTT CAGTTAGTTC ATTCGATTTG   
  
  
+ TTTCTTCCAT TGTACAGAAT TTCTATTCTG TTCTAATCTT ATTACTCGAG TATACACTTT TGTTTTCACG   
  
  
+ TTTTTTCGAA GTATTAGAAA CGTGTAAGTA CAAAAATAAA GTACAATTAG TTAATTAAGA AAAAGCGAGT   
  
  
+ AGATAATCTT GGATTAAATA TGGAAAGAAC CGTACGACAT CCACACGAAC CCCAATAGGC TATTTTGCCA   
  
  
+ CGCAAAACTA TTGTCGCCTT TTACGATGCG GAATTAAAAA CGCTCATCCC TACCGAGGCC GAGGCAAGAT   
  
  
+ CAGATCACTC GAAGGGAAGC AGAGAGCCAA CACTCGGTAC CACGCGTCCT TTCGTTCACG TGGGCGTCCT   
  
  
+ CCGCGGCTCT TTAGCGTTTT CGTCGCCGAA TCGCCGCGGC TGGCCAGCCG AATGAAACGG TCCTCCCTTC   
  
  
+ GCCGCTTCCA CAACGCTACG CAAGAGCTTC CGCTGCGAGC AGCCGTTCGG CAACCGAGAT AGTTCAGCGA   
  
  
+ GCAGATGTCG GGAAGGCACT CCTTGTTTTC CCGTTGAAAG CGAGAACAAA CAGTTTGAGA GTTCACATTC   
  
  
+ AATGGTTAAA ACATAAAGAC AGACAATCCA CATGAAAGCC AACGACAAAT CAACCGAAAA GACCCTCGAA   
  
  
+ AGACGTTCAT TGAATCAAAA GAAAACTTTA AGAAAACCCA AACCCTCGGA TCCCTCATTC TCGAACTGCT   
  
  
+ TTCTAAAACC CACATTTCAA CTTGAAAGGA ATACTAGACA AAAACCAGAA ACCACAAAAA AATCCGTCGA   
  
  
+ GTTTGAGTAT AGTGTCAAAA GCATGAATTT TTGGAACTAA AGACCCTACG TATGACGGTG ATCGTAGTAG   
  
  
+ ACTGAAACGG GAAGAGAGAG AGAAGGAAGG GAGTAACAAC ACATTTAAGA CAATAAACGT CATGGACTTG   
  
  
+ TTTGACATGT AAAATTCACT AATTGTAAAA GCAGAAAAAC CAAAGTGAAC AAAAGTCTAG AAGGATAATA   
  
  
+ TTTCCTACAG AACCTCCGAC GATTTTATGA CTTATCATCT ACCTTCCATA TTCGGTACGA GGCTGTCGAA   
  
  
+ TACTTGTAAT AAAAACGGGA AGAGTTCTCC TAATTCAAAA AAGTGAAAAG ATTTACTATA AAAACAGGTT   
  
  
+ CCGTCTAAAT CGATATCCCA CTTGTAACAA AACGACGAAC GAACTTACGA TCGAAAAGGG TCAACTTTAC   
  
  
+ AAAGGATTGC GAATTACCAA TAACGATAAC GTCTCCGTTC TCCGTAACTA GATGTATGAG ACAATACTGT   
  
  
+ AAGATAAACC CGTCCACTAT TGAAACATG  

- TTGCAGTCTT TGAGTTCTCC ATTCTTTCTT CTTTCTTGGG GGAGGAAGCT TTGGATTTGT CAAAAGAGCC   
  
  
- TGTGGGGGCC CTTGCTATCA ATTCAGCTTC TTAATGACAA AGTGATGTTA ATTTTATCTG TTCTCTCTTG   
  
  
- ATTTGGGATG TTCTAGTTTG TATGACCATA CGAAAACTAG TTGATACAAA GTCAATCAAG TAAGCTAAAC   
  
  
- AAAGAAGGTA ACATGTCTTA AAGATAAGAC AAGATTAGAA TAATGAGCTC ATATGTGAAA ACAAAAGTGC   
  
  
- AAAAAAGCTT CATAATCTTT GCACATTCAT GTTTTTATTT CATGTTAATC AATTAATTCT TTTTCGCTCA   
  
  
- TCTATTAGAA CCTAATTTAT ACCTTTCTTG GCATGCTGTA GGTGTGCTTG GGGTTATCCG ATAAAACGGT   
  
  
- GCGTTTTGAT AACAGCGGAA AATGCTACGC CTTAATTTTT GCGAGTAGGG ATGGCTCCGG CTCCGTTCTA   
  
  
- GTCTAGTGAG CTTCCCTTCG TCTCTCGGTT GTGAGCCATG GTGCGCAGGA AAGCAAGTGC ACCCGCAGGA   
  
  
- GGCGCCGAGA AATCGCAAAA GCAGCGGCTT AGCGGCGCCG ACCGGTCGGC TTACTTTGCC AGGAGGGAAG   
  
  
- CGGCGAAGGT GTTGCGATGC GTTCTCGAAG GCGACGCTCG TCGGCAAGCC GTTGGCTCTA TCAAGTCGCT   
  
  
- CGTCTACAGC CCTTCCGTGA GGAACAAAAG GGCAACTTTC GCTCTTGTTT GTCAAACTCT CAAGTGTAAG   
  
  
- TTACCAATTT TGTATTTCTG TCTGTTAGGT GTACTTTCGG TTGCTGTTTA GTTGGCTTTT CTGGGAGCTT   
  
  
- TCTGCAAGTA ACTTAGTTTT CTTTTGAAAT TCTTTTGGGT TTGGGAGCCT AGGGAGTAAG AGCTTGACGA   
  
  
- AAGATTTTGG GTGTAAAGTT GAACTTTCCT TATGATCTGT TTTTGGTCTT TGGTGTTTTT TTAGGCAGCT   
  
  
- CAAACTCATA TCACAGTTTT CGTACTTAAA AACCTTGATT TCTGGGATGC ATACTGCCAC TAGCATCATC   
  
  
- TGACTTTGCC CTTCTCTCTC TCTTCCTTCC CTCATTGTTG TGTAAATTCT GTTATTTGCA GTACCTGAAC   
  
  
- AAACTGTACA TTTTAAGTGA TTAACATTTT CGTCTTTTTG GTTTCACTTG TTTTCAGATC TTCCTATTAT   
  
  
- AAAGGATGTC TTGGAGGCTG CTAAAATACT GAATAGTAGA TGGAAGGTAT AAGCCATGCT CCGACAGCTT   
  
  
- ATGAACATTA TTTTTGCCCT TCTCAAGAGG ATTAAGTTTT TTCACTTTTC TAAATGATAT TTTTGTCCAA   
  
  
- GGCAGATTTA GCTATAGGGT GAACATTGTT TTGCTGCTTG CTTGAATGCT AGCTTTTCCC AGTTGAAATG   
  
  
- TTTCCTAACG CTTAATGGTT ATTGCTATTG CAGAGGCAAG AGGCATTGAT CTACATACTC TGTTATGACA   
  
  
- TTCTATTTGG GCAGGTGATA ACTTTGTAC

+     TATC-box

| Site Name | Organism | Position | Strand | Matrix score. | sequence | function |
| --- | --- | --- | --- | --- | --- | --- |
| TATC-box | Oryza sativa | 1344 | + | 7 | TATCCCA | cis-acting element involved in gibberellin-responsiveness |

> 2018/04/13 10:10:12  
+ AACGTCAGAA ACTCAAGAGG TAAGAAAGAA GAAAGAACCC CCTCCTTCGA AACCTAAACA GTTTTCTCGG   
  
  
+ ACACCCCCGG GAACGATAGT TAAGTCGAAG AATTACTGTT TCACTACAAT TAAAATAGAC AAGAGAGAAC   
  
  
+ TAAACCCTAC AAGATCAAAC ATACTGGTAT GCTTTTGATC AACTATGTTT CAGTTAGTTC ATTCGATTTG   
  
  
+ TTTCTTCCAT TGTACAGAAT TTCTATTCTG TTCTAATCTT ATTACTCGAG TATACACTTT TGTTTTCACG   
  
  
+ TTTTTTCGAA GTATTAGAAA CGTGTAAGTA CAAAAATAAA GTACAATTAG TTAATTAAGA AAAAGCGAGT   
  
  
+ AGATAATCTT GGATTAAATA TGGAAAGAAC CGTACGACAT CCACACGAAC CCCAATAGGC TATTTTGCCA   
  
  
+ CGCAAAACTA TTGTCGCCTT TTACGATGCG GAATTAAAAA CGCTCATCCC TACCGAGGCC GAGGCAAGAT   
  
  
+ CAGATCACTC GAAGGGAAGC AGAGAGCCAA CACTCGGTAC CACGCGTCCT TTCGTTCACG TGGGCGTCCT   
  
  
+ CCGCGGCTCT TTAGCGTTTT CGTCGCCGAA TCGCCGCGGC TGGCCAGCCG AATGAAACGG TCCTCCCTTC   
  
  
+ GCCGCTTCCA CAACGCTACG CAAGAGCTTC CGCTGCGAGC AGCCGTTCGG CAACCGAGAT AGTTCAGCGA   
  
  
+ GCAGATGTCG GGAAGGCACT CCTTGTTTTC CCGTTGAAAG CGAGAACAAA CAGTTTGAGA GTTCACATTC   
  
  
+ AATGGTTAAA ACATAAAGAC AGACAATCCA CATGAAAGCC AACGACAAAT CAACCGAAAA GACCCTCGAA   
  
  
+ AGACGTTCAT TGAATCAAAA GAAAACTTTA AGAAAACCCA AACCCTCGGA TCCCTCATTC TCGAACTGCT   
  
  
+ TTCTAAAACC CACATTTCAA CTTGAAAGGA ATACTAGACA AAAACCAGAA ACCACAAAAA AATCCGTCGA   
  
  
+ GTTTGAGTAT AGTGTCAAAA GCATGAATTT TTGGAACTAA AGACCCTACG TATGACGGTG ATCGTAGTAG   
  
  
+ ACTGAAACGG GAAGAGAGAG AGAAGGAAGG GAGTAACAAC ACATTTAAGA CAATAAACGT CATGGACTTG   
  
  
+ TTTGACATGT AAAATTCACT AATTGTAAAA GCAGAAAAAC CAAAGTGAAC AAAAGTCTAG AAGGATAATA   
  
  
+ TTTCCTACAG AACCTCCGAC GATTTTATGA CTTATCATCT ACCTTCCATA TTCGGTACGA GGCTGTCGAA   
  
  
+ TACTTGTAAT AAAAACGGGA AGAGTTCTCC TAATTCAAAA AAGTGAAAAG ATTTACTATA AAAACAGGTT   
  
  
+ CCGTCTAAAT CGATATCCCA CTTGTAACAA AACGACGAAC GAACTTACGA TCGAAAAGGG TCAACTTTAC   
  
  
+ AAAGGATTGC GAATTACCAA TAACGATAAC GTCTCCGTTC TCCGTAACTA GATGTATGAG ACAATACTGT   
  
  
+ AAGATAAACC CGTCCACTAT TGAAACATG  

- TTGCAGTCTT TGAGTTCTCC ATTCTTTCTT CTTTCTTGGG GGAGGAAGCT TTGGATTTGT CAAAAGAGCC   
  
  
- TGTGGGGGCC CTTGCTATCA ATTCAGCTTC TTAATGACAA AGTGATGTTA ATTTTATCTG TTCTCTCTTG   
  
  
- ATTTGGGATG TTCTAGTTTG TATGACCATA CGAAAACTAG TTGATACAAA GTCAATCAAG TAAGCTAAAC   
  
  
- AAAGAAGGTA ACATGTCTTA AAGATAAGAC AAGATTAGAA TAATGAGCTC ATATGTGAAA ACAAAAGTGC   
  
  
- AAAAAAGCTT CATAATCTTT GCACATTCAT GTTTTTATTT CATGTTAATC AATTAATTCT TTTTCGCTCA   
  
  
- TCTATTAGAA CCTAATTTAT ACCTTTCTTG GCATGCTGTA GGTGTGCTTG GGGTTATCCG ATAAAACGGT   
  
  
- GCGTTTTGAT AACAGCGGAA AATGCTACGC CTTAATTTTT GCGAGTAGGG ATGGCTCCGG CTCCGTTCTA   
  
  
- GTCTAGTGAG CTTCCCTTCG TCTCTCGGTT GTGAGCCATG GTGCGCAGGA AAGCAAGTGC ACCCGCAGGA   
  
  
- GGCGCCGAGA AATCGCAAAA GCAGCGGCTT AGCGGCGCCG ACCGGTCGGC TTACTTTGCC AGGAGGGAAG   
  
  
- CGGCGAAGGT GTTGCGATGC GTTCTCGAAG GCGACGCTCG TCGGCAAGCC GTTGGCTCTA TCAAGTCGCT   
  
  
- CGTCTACAGC CCTTCCGTGA GGAACAAAAG GGCAACTTTC GCTCTTGTTT GTCAAACTCT CAAGTGTAAG   
  
  
- TTACCAATTT TGTATTTCTG TCTGTTAGGT GTACTTTCGG TTGCTGTTTA GTTGGCTTTT CTGGGAGCTT   
  
  
- TCTGCAAGTA ACTTAGTTTT CTTTTGAAAT TCTTTTGGGT TTGGGAGCCT AGGGAGTAAG AGCTTGACGA   
  
  
- AAGATTTTGG GTGTAAAGTT GAACTTTCCT TATGATCTGT TTTTGGTCTT TGGTGTTTTT TTAGGCAGCT   
  
  
- CAAACTCATA TCACAGTTTT CGTACTTAAA AACCTTGATT TCTGGGATGC ATACTGCCAC TAGCATCATC   
  
  
- TGACTTTGCC CTTCTCTCTC TCTTCCTTCC CTCATTGTTG TGTAAATTCT GTTATTTGCA GTACCTGAAC   
  
  
- AAACTGTACA TTTTAAGTGA TTAACATTTT CGTCTTTTTG GTTTCACTTG TTTTCAGATC TTCCTATTAT   
  
  
- AAAGGATGTC TTGGAGGCTG CTAAAATACT GAATAGTAGA TGGAAGGTAT AAGCCATGCT CCGACAGCTT   
  
  
- ATGAACATTA TTTTTGCCCT TCTCAAGAGG ATTAAGTTTT TTCACTTTTC TAAATGATAT TTTTGTCCAA   
  
  
- GGCAGATTTA GCTATAGGGT GAACATTGTT TTGCTGCTTG CTTGAATGCT AGCTTTTCCC AGTTGAAATG   
  
  
- TTTCCTAACG CTTAATGGTT ATTGCTATTG CAGAGGCAAG AGGCATTGAT CTACATACTC TGTTATGACA   
  
  
- TTCTATTTGG GCAGGTGATA ACTTTGTAC

+     TC-rich repeats

| Site Name | Organism | Position | Strand | Matrix score. | sequence | function |
| --- | --- | --- | --- | --- | --- | --- |
| TC-rich repeats | Nicotiana tabacum | 1354 | - | 9 | GTTTTCTTAC | cis-acting element involved in defense and stress responsiveness |
| TC-rich repeats | Nicotiana tabacum | 868 | - | 9 | GTTTTCTTAC | cis-acting element involved in defense and stress responsiveness |
| TC-rich repeats | Nicotiana tabacum | 897 | + | 9 | ATTCTCTAAC | cis-acting element involved in defense and stress responsiveness |

> 2018/04/13 10:10:12  
+ AACGTCAGAA ACTCAAGAGG TAAGAAAGAA GAAAGAACCC CCTCCTTCGA AACCTAAACA GTTTTCTCGG   
  
  
+ ACACCCCCGG GAACGATAGT TAAGTCGAAG AATTACTGTT TCACTACAAT TAAAATAGAC AAGAGAGAAC   
  
  
+ TAAACCCTAC AAGATCAAAC ATACTGGTAT GCTTTTGATC AACTATGTTT CAGTTAGTTC ATTCGATTTG   
  
  
+ TTTCTTCCAT TGTACAGAAT TTCTATTCTG TTCTAATCTT ATTACTCGAG TATACACTTT TGTTTTCACG   
  
  
+ TTTTTTCGAA GTATTAGAAA CGTGTAAGTA CAAAAATAAA GTACAATTAG TTAATTAAGA AAAAGCGAGT   
  
  
+ AGATAATCTT GGATTAAATA TGGAAAGAAC CGTACGACAT CCACACGAAC CCCAATAGGC TATTTTGCCA   
  
  
+ CGCAAAACTA TTGTCGCCTT TTACGATGCG GAATTAAAAA CGCTCATCCC TACCGAGGCC GAGGCAAGAT   
  
  
+ CAGATCACTC GAAGGGAAGC AGAGAGCCAA CACTCGGTAC CACGCGTCCT TTCGTTCACG TGGGCGTCCT   
  
  
+ CCGCGGCTCT TTAGCGTTTT CGTCGCCGAA TCGCCGCGGC TGGCCAGCCG AATGAAACGG TCCTCCCTTC   
  
  
+ GCCGCTTCCA CAACGCTACG CAAGAGCTTC CGCTGCGAGC AGCCGTTCGG CAACCGAGAT AGTTCAGCGA   
  
  
+ GCAGATGTCG GGAAGGCACT CCTTGTTTTC CCGTTGAAAG CGAGAACAAA CAGTTTGAGA GTTCACATTC   
  
  
+ AATGGTTAAA ACATAAAGAC AGACAATCCA CATGAAAGCC AACGACAAAT CAACCGAAAA GACCCTCGAA   
  
  
+ AGACGTTCAT TGAATCAAAA GAAAACTTTA AGAAAACCCA AACCCTCGGA TCCCTCATTC TCGAACTGCT   
  
  
+ TTCTAAAACC CACATTTCAA CTTGAAAGGA ATACTAGACA AAAACCAGAA ACCACAAAAA AATCCGTCGA   
  
  
+ GTTTGAGTAT AGTGTCAAAA GCATGAATTT TTGGAACTAA AGACCCTACG TATGACGGTG ATCGTAGTAG   
  
  
+ ACTGAAACGG GAAGAGAGAG AGAAGGAAGG GAGTAACAAC ACATTTAAGA CAATAAACGT CATGGACTTG   
  
  
+ TTTGACATGT AAAATTCACT AATTGTAAAA GCAGAAAAAC CAAAGTGAAC AAAAGTCTAG AAGGATAATA   
  
  
+ TTTCCTACAG AACCTCCGAC GATTTTATGA CTTATCATCT ACCTTCCATA TTCGGTACGA GGCTGTCGAA   
  
  
+ TACTTGTAAT AAAAACGGGA AGAGTTCTCC TAATTCAAAA AAGTGAAAAG ATTTACTATA AAAACAGGTT   
  
  
+ CCGTCTAAAT CGATATCCCA CTTGTAACAA AACGACGAAC GAACTTACGA TCGAAAAGGG TCAACTTTAC   
  
  
+ AAAGGATTGC GAATTACCAA TAACGATAAC GTCTCCGTTC TCCGTAACTA GATGTATGAG ACAATACTGT   
  
  
+ AAGATAAACC CGTCCACTAT TGAAACATG  

- TTGCAGTCTT TGAGTTCTCC ATTCTTTCTT CTTTCTTGGG GGAGGAAGCT TTGGATTTGT CAAAAGAGCC   
  
  
- TGTGGGGGCC CTTGCTATCA ATTCAGCTTC TTAATGACAA AGTGATGTTA ATTTTATCTG TTCTCTCTTG   
  
  
- ATTTGGGATG TTCTAGTTTG TATGACCATA CGAAAACTAG TTGATACAAA GTCAATCAAG TAAGCTAAAC   
  
  
- AAAGAAGGTA ACATGTCTTA AAGATAAGAC AAGATTAGAA TAATGAGCTC ATATGTGAAA ACAAAAGTGC   
  
  
- AAAAAAGCTT CATAATCTTT GCACATTCAT GTTTTTATTT CATGTTAATC AATTAATTCT TTTTCGCTCA   
  
  
- TCTATTAGAA CCTAATTTAT ACCTTTCTTG GCATGCTGTA GGTGTGCTTG GGGTTATCCG ATAAAACGGT   
  
  
- GCGTTTTGAT AACAGCGGAA AATGCTACGC CTTAATTTTT GCGAGTAGGG ATGGCTCCGG CTCCGTTCTA   
  
  
- GTCTAGTGAG CTTCCCTTCG TCTCTCGGTT GTGAGCCATG GTGCGCAGGA AAGCAAGTGC ACCCGCAGGA   
  
  
- GGCGCCGAGA AATCGCAAAA GCAGCGGCTT AGCGGCGCCG ACCGGTCGGC TTACTTTGCC AGGAGGGAAG   
  
  
- CGGCGAAGGT GTTGCGATGC GTTCTCGAAG GCGACGCTCG TCGGCAAGCC GTTGGCTCTA TCAAGTCGCT   
  
  
- CGTCTACAGC CCTTCCGTGA GGAACAAAAG GGCAACTTTC GCTCTTGTTT GTCAAACTCT CAAGTGTAAG   
  
  
- TTACCAATTT TGTATTTCTG TCTGTTAGGT GTACTTTCGG TTGCTGTTTA GTTGGCTTTT CTGGGAGCTT   
  
  
- TCTGCAAGTA ACTTAGTTTT CTTTTGAAAT TCTTTTGGGT TTGGGAGCCT AGGGAGTAAG AGCTTGACGA   
  
  
- AAGATTTTGG GTGTAAAGTT GAACTTTCCT TATGATCTGT TTTTGGTCTT TGGTGTTTTT TTAGGCAGCT   
  
  
- CAAACTCATA TCACAGTTTT CGTACTTAAA AACCTTGATT TCTGGGATGC ATACTGCCAC TAGCATCATC   
  
  
- TGACTTTGCC CTTCTCTCTC TCTTCCTTCC CTCATTGTTG TGTAAATTCT GTTATTTGCA GTACCTGAAC   
  
  
- AAACTGTACA TTTTAAGTGA TTAACATTTT CGTCTTTTTG GTTTCACTTG TTTTCAGATC TTCCTATTAT   
  
  
- AAAGGATGTC TTGGAGGCTG CTAAAATACT GAATAGTAGA TGGAAGGTAT AAGCCATGCT CCGACAGCTT   
  
  
- ATGAACATTA TTTTTGCCCT TCTCAAGAGG ATTAAGTTTT TTCACTTTTC TAAATGATAT TTTTGTCCAA   
  
  
- GGCAGATTTA GCTATAGGGT GAACATTGTT TTGCTGCTTG CTTGAATGCT AGCTTTTCCC AGTTGAAATG   
  
  
- TTTCCTAACG CTTAATGGTT ATTGCTATTG CAGAGGCAAG AGGCATTGAT CTACATACTC TGTTATGACA   
  
  
- TTCTATTTGG GCAGGTGATA ACTTTGTAC

+     TCT-motif

| Site Name | Organism | Position | Strand | Matrix score. | sequence | function |
| --- | --- | --- | --- | --- | --- | --- |
| TCT-motif | Arabidopsis thaliana | 20 | - | 6 | TCTTAC | part of a light responsive element |
| TCT-motif | Arabidopsis thaliana | 1469 | - | 6 | TCTTAC | part of a light responsive element |

> 2018/04/13 10:10:12  
+ AACGTCAGAA ACTCAAGAGG TAAGAAAGAA GAAAGAACCC CCTCCTTCGA AACCTAAACA GTTTTCTCGG   
  
  
+ ACACCCCCGG GAACGATAGT TAAGTCGAAG AATTACTGTT TCACTACAAT TAAAATAGAC AAGAGAGAAC   
  
  
+ TAAACCCTAC AAGATCAAAC ATACTGGTAT GCTTTTGATC AACTATGTTT CAGTTAGTTC ATTCGATTTG   
  
  
+ TTTCTTCCAT TGTACAGAAT TTCTATTCTG TTCTAATCTT ATTACTCGAG TATACACTTT TGTTTTCACG   
  
  
+ TTTTTTCGAA GTATTAGAAA CGTGTAAGTA CAAAAATAAA GTACAATTAG TTAATTAAGA AAAAGCGAGT   
  
  
+ AGATAATCTT GGATTAAATA TGGAAAGAAC CGTACGACAT CCACACGAAC CCCAATAGGC TATTTTGCCA   
  
  
+ CGCAAAACTA TTGTCGCCTT TTACGATGCG GAATTAAAAA CGCTCATCCC TACCGAGGCC GAGGCAAGAT   
  
  
+ CAGATCACTC GAAGGGAAGC AGAGAGCCAA CACTCGGTAC CACGCGTCCT TTCGTTCACG TGGGCGTCCT   
  
  
+ CCGCGGCTCT TTAGCGTTTT CGTCGCCGAA TCGCCGCGGC TGGCCAGCCG AATGAAACGG TCCTCCCTTC   
  
  
+ GCCGCTTCCA CAACGCTACG CAAGAGCTTC CGCTGCGAGC AGCCGTTCGG CAACCGAGAT AGTTCAGCGA   
  
  
+ GCAGATGTCG GGAAGGCACT CCTTGTTTTC CCGTTGAAAG CGAGAACAAA CAGTTTGAGA GTTCACATTC   
  
  
+ AATGGTTAAA ACATAAAGAC AGACAATCCA CATGAAAGCC AACGACAAAT CAACCGAAAA GACCCTCGAA   
  
  
+ AGACGTTCAT TGAATCAAAA GAAAACTTTA AGAAAACCCA AACCCTCGGA TCCCTCATTC TCGAACTGCT   
  
  
+ TTCTAAAACC CACATTTCAA CTTGAAAGGA ATACTAGACA AAAACCAGAA ACCACAAAAA AATCCGTCGA   
  
  
+ GTTTGAGTAT AGTGTCAAAA GCATGAATTT TTGGAACTAA AGACCCTACG TATGACGGTG ATCGTAGTAG   
  
  
+ ACTGAAACGG GAAGAGAGAG AGAAGGAAGG GAGTAACAAC ACATTTAAGA CAATAAACGT CATGGACTTG   
  
  
+ TTTGACATGT AAAATTCACT AATTGTAAAA GCAGAAAAAC CAAAGTGAAC AAAAGTCTAG AAGGATAATA   
  
  
+ TTTCCTACAG AACCTCCGAC GATTTTATGA CTTATCATCT ACCTTCCATA TTCGGTACGA GGCTGTCGAA   
  
  
+ TACTTGTAAT AAAAACGGGA AGAGTTCTCC TAATTCAAAA AAGTGAAAAG ATTTACTATA AAAACAGGTT   
  
  
+ CCGTCTAAAT CGATATCCCA CTTGTAACAA AACGACGAAC GAACTTACGA TCGAAAAGGG TCAACTTTAC   
  
  
+ AAAGGATTGC GAATTACCAA TAACGATAAC GTCTCCGTTC TCCGTAACTA GATGTATGAG ACAATACTGT   
  
  
+ AAGATAAACC CGTCCACTAT TGAAACATG  

- TTGCAGTCTT TGAGTTCTCC ATTCTTTCTT CTTTCTTGGG GGAGGAAGCT TTGGATTTGT CAAAAGAGCC   
  
  
- TGTGGGGGCC CTTGCTATCA ATTCAGCTTC TTAATGACAA AGTGATGTTA ATTTTATCTG TTCTCTCTTG   
  
  
- ATTTGGGATG TTCTAGTTTG TATGACCATA CGAAAACTAG TTGATACAAA GTCAATCAAG TAAGCTAAAC   
  
  
- AAAGAAGGTA ACATGTCTTA AAGATAAGAC AAGATTAGAA TAATGAGCTC ATATGTGAAA ACAAAAGTGC   
  
  
- AAAAAAGCTT CATAATCTTT GCACATTCAT GTTTTTATTT CATGTTAATC AATTAATTCT TTTTCGCTCA   
  
  
- TCTATTAGAA CCTAATTTAT ACCTTTCTTG GCATGCTGTA GGTGTGCTTG GGGTTATCCG ATAAAACGGT   
  
  
- GCGTTTTGAT AACAGCGGAA AATGCTACGC CTTAATTTTT GCGAGTAGGG ATGGCTCCGG CTCCGTTCTA   
  
  
- GTCTAGTGAG CTTCCCTTCG TCTCTCGGTT GTGAGCCATG GTGCGCAGGA AAGCAAGTGC ACCCGCAGGA   
  
  
- GGCGCCGAGA AATCGCAAAA GCAGCGGCTT AGCGGCGCCG ACCGGTCGGC TTACTTTGCC AGGAGGGAAG   
  
  
- CGGCGAAGGT GTTGCGATGC GTTCTCGAAG GCGACGCTCG TCGGCAAGCC GTTGGCTCTA TCAAGTCGCT   
  
  
- CGTCTACAGC CCTTCCGTGA GGAACAAAAG GGCAACTTTC GCTCTTGTTT GTCAAACTCT CAAGTGTAAG   
  
  
- TTACCAATTT TGTATTTCTG TCTGTTAGGT GTACTTTCGG TTGCTGTTTA GTTGGCTTTT CTGGGAGCTT   
  
  
- TCTGCAAGTA ACTTAGTTTT CTTTTGAAAT TCTTTTGGGT TTGGGAGCCT AGGGAGTAAG AGCTTGACGA   
  
  
- AAGATTTTGG GTGTAAAGTT GAACTTTCCT TATGATCTGT TTTTGGTCTT TGGTGTTTTT TTAGGCAGCT   
  
  
- CAAACTCATA TCACAGTTTT CGTACTTAAA AACCTTGATT TCTGGGATGC ATACTGCCAC TAGCATCATC   
  
  
- TGACTTTGCC CTTCTCTCTC TCTTCCTTCC CTCATTGTTG TGTAAATTCT GTTATTTGCA GTACCTGAAC   
  
  
- AAACTGTACA TTTTAAGTGA TTAACATTTT CGTCTTTTTG GTTTCACTTG TTTTCAGATC TTCCTATTAT   
  
  
- AAAGGATGTC TTGGAGGCTG CTAAAATACT GAATAGTAGA TGGAAGGTAT AAGCCATGCT CCGACAGCTT   
  
  
- ATGAACATTA TTTTTGCCCT TCTCAAGAGG ATTAAGTTTT TTCACTTTTC TAAATGATAT TTTTGTCCAA   
  
  
- GGCAGATTTA GCTATAGGGT GAACATTGTT TTGCTGCTTG CTTGAATGCT AGCTTTTCCC AGTTGAAATG   
  
  
- TTTCCTAACG CTTAATGGTT ATTGCTATTG CAGAGGCAAG AGGCATTGAT CTACATACTC TGTTATGACA   
  
  
- TTCTATTTGG GCAGGTGATA ACTTTGTAC

+     TGA-element

| Site Name | Organism | Position | Strand | Matrix score. | sequence | function |
| --- | --- | --- | --- | --- | --- | --- |
| TGA-element | Brassica oleracea | 1361 | + | 6 | AACGAC | auxin-responsive element |
| TGA-element | Brassica oleracea | 811 | + | 6 | AACGAC | auxin-responsive element |

> 2018/04/13 10:10:12  
+ AACGTCAGAA ACTCAAGAGG TAAGAAAGAA GAAAGAACCC CCTCCTTCGA AACCTAAACA GTTTTCTCGG   
  
  
+ ACACCCCCGG GAACGATAGT TAAGTCGAAG AATTACTGTT TCACTACAAT TAAAATAGAC AAGAGAGAAC   
  
  
+ TAAACCCTAC AAGATCAAAC ATACTGGTAT GCTTTTGATC AACTATGTTT CAGTTAGTTC ATTCGATTTG   
  
  
+ TTTCTTCCAT TGTACAGAAT TTCTATTCTG TTCTAATCTT ATTACTCGAG TATACACTTT TGTTTTCACG   
  
  
+ TTTTTTCGAA GTATTAGAAA CGTGTAAGTA CAAAAATAAA GTACAATTAG TTAATTAAGA AAAAGCGAGT   
  
  
+ AGATAATCTT GGATTAAATA TGGAAAGAAC CGTACGACAT CCACACGAAC CCCAATAGGC TATTTTGCCA   
  
  
+ CGCAAAACTA TTGTCGCCTT TTACGATGCG GAATTAAAAA CGCTCATCCC TACCGAGGCC GAGGCAAGAT   
  
  
+ CAGATCACTC GAAGGGAAGC AGAGAGCCAA CACTCGGTAC CACGCGTCCT TTCGTTCACG TGGGCGTCCT   
  
  
+ CCGCGGCTCT TTAGCGTTTT CGTCGCCGAA TCGCCGCGGC TGGCCAGCCG AATGAAACGG TCCTCCCTTC   
  
  
+ GCCGCTTCCA CAACGCTACG CAAGAGCTTC CGCTGCGAGC AGCCGTTCGG CAACCGAGAT AGTTCAGCGA   
  
  
+ GCAGATGTCG GGAAGGCACT CCTTGTTTTC CCGTTGAAAG CGAGAACAAA CAGTTTGAGA GTTCACATTC   
  
  
+ AATGGTTAAA ACATAAAGAC AGACAATCCA CATGAAAGCC AACGACAAAT CAACCGAAAA GACCCTCGAA   
  
  
+ AGACGTTCAT TGAATCAAAA GAAAACTTTA AGAAAACCCA AACCCTCGGA TCCCTCATTC TCGAACTGCT   
  
  
+ TTCTAAAACC CACATTTCAA CTTGAAAGGA ATACTAGACA AAAACCAGAA ACCACAAAAA AATCCGTCGA   
  
  
+ GTTTGAGTAT AGTGTCAAAA GCATGAATTT TTGGAACTAA AGACCCTACG TATGACGGTG ATCGTAGTAG   
  
  
+ ACTGAAACGG GAAGAGAGAG AGAAGGAAGG GAGTAACAAC ACATTTAAGA CAATAAACGT CATGGACTTG   
  
  
+ TTTGACATGT AAAATTCACT AATTGTAAAA GCAGAAAAAC CAAAGTGAAC AAAAGTCTAG AAGGATAATA   
  
  
+ TTTCCTACAG AACCTCCGAC GATTTTATGA CTTATCATCT ACCTTCCATA TTCGGTACGA GGCTGTCGAA   
  
  
+ TACTTGTAAT AAAAACGGGA AGAGTTCTCC TAATTCAAAA AAGTGAAAAG ATTTACTATA AAAACAGGTT   
  
  
+ CCGTCTAAAT CGATATCCCA CTTGTAACAA AACGACGAAC GAACTTACGA TCGAAAAGGG TCAACTTTAC   
  
  
+ AAAGGATTGC GAATTACCAA TAACGATAAC GTCTCCGTTC TCCGTAACTA GATGTATGAG ACAATACTGT   
  
  
+ AAGATAAACC CGTCCACTAT TGAAACATG  

- TTGCAGTCTT TGAGTTCTCC ATTCTTTCTT CTTTCTTGGG GGAGGAAGCT TTGGATTTGT CAAAAGAGCC   
  
  
- TGTGGGGGCC CTTGCTATCA ATTCAGCTTC TTAATGACAA AGTGATGTTA ATTTTATCTG TTCTCTCTTG   
  
  
- ATTTGGGATG TTCTAGTTTG TATGACCATA CGAAAACTAG TTGATACAAA GTCAATCAAG TAAGCTAAAC   
  
  
- AAAGAAGGTA ACATGTCTTA AAGATAAGAC AAGATTAGAA TAATGAGCTC ATATGTGAAA ACAAAAGTGC   
  
  
- AAAAAAGCTT CATAATCTTT GCACATTCAT GTTTTTATTT CATGTTAATC AATTAATTCT TTTTCGCTCA   
  
  
- TCTATTAGAA CCTAATTTAT ACCTTTCTTG GCATGCTGTA GGTGTGCTTG GGGTTATCCG ATAAAACGGT   
  
  
- GCGTTTTGAT AACAGCGGAA AATGCTACGC CTTAATTTTT GCGAGTAGGG ATGGCTCCGG CTCCGTTCTA   
  
  
- GTCTAGTGAG CTTCCCTTCG TCTCTCGGTT GTGAGCCATG GTGCGCAGGA AAGCAAGTGC ACCCGCAGGA   
  
  
- GGCGCCGAGA AATCGCAAAA GCAGCGGCTT AGCGGCGCCG ACCGGTCGGC TTACTTTGCC AGGAGGGAAG   
  
  
- CGGCGAAGGT GTTGCGATGC GTTCTCGAAG GCGACGCTCG TCGGCAAGCC GTTGGCTCTA TCAAGTCGCT   
  
  
- CGTCTACAGC CCTTCCGTGA GGAACAAAAG GGCAACTTTC GCTCTTGTTT GTCAAACTCT CAAGTGTAAG   
  
  
- TTACCAATTT TGTATTTCTG TCTGTTAGGT GTACTTTCGG TTGCTGTTTA GTTGGCTTTT CTGGGAGCTT   
  
  
- TCTGCAAGTA ACTTAGTTTT CTTTTGAAAT TCTTTTGGGT TTGGGAGCCT AGGGAGTAAG AGCTTGACGA   
  
  
- AAGATTTTGG GTGTAAAGTT GAACTTTCCT TATGATCTGT TTTTGGTCTT TGGTGTTTTT TTAGGCAGCT   
  
  
- CAAACTCATA TCACAGTTTT CGTACTTAAA AACCTTGATT TCTGGGATGC ATACTGCCAC TAGCATCATC   
  
  
- TGACTTTGCC CTTCTCTCTC TCTTCCTTCC CTCATTGTTG TGTAAATTCT GTTATTTGCA GTACCTGAAC   
  
  
- AAACTGTACA TTTTAAGTGA TTAACATTTT CGTCTTTTTG GTTTCACTTG TTTTCAGATC TTCCTATTAT   
  
  
- AAAGGATGTC TTGGAGGCTG CTAAAATACT GAATAGTAGA TGGAAGGTAT AAGCCATGCT CCGACAGCTT   
  
  
- ATGAACATTA TTTTTGCCCT TCTCAAGAGG ATTAAGTTTT TTCACTTTTC TAAATGATAT TTTTGTCCAA   
  
  
- GGCAGATTTA GCTATAGGGT GAACATTGTT TTGCTGCTTG CTTGAATGCT AGCTTTTCCC AGTTGAAATG   
  
  
- TTTCCTAACG CTTAATGGTT ATTGCTATTG CAGAGGCAAG AGGCATTGAT CTACATACTC TGTTATGACA   
  
  
- TTCTATTTGG GCAGGTGATA ACTTTGTAC

+     TGACG-motif

| Site Name | Organism | Position | Strand | Matrix score. | sequence | function |
| --- | --- | --- | --- | --- | --- | --- |
| TGACG-motif | Hordeum vulgare | 3 | - | 5 | TGACG | cis-acting regulatory element involved in the MeJA-responsiveness |
| TGACG-motif | Hordeum vulgare | 1108 | - | 5 | TGACG | cis-acting regulatory element involved in the MeJA-responsiveness |
| TGACG-motif | Hordeum vulgare | 1033 | + | 5 | TGACG | cis-acting regulatory element involved in the MeJA-responsiveness |

> 2018/04/13 10:10:12  
+ AACGTCAGAA ACTCAAGAGG TAAGAAAGAA GAAAGAACCC CCTCCTTCGA AACCTAAACA GTTTTCTCGG   
  
  
+ ACACCCCCGG GAACGATAGT TAAGTCGAAG AATTACTGTT TCACTACAAT TAAAATAGAC AAGAGAGAAC   
  
  
+ TAAACCCTAC AAGATCAAAC ATACTGGTAT GCTTTTGATC AACTATGTTT CAGTTAGTTC ATTCGATTTG   
  
  
+ TTTCTTCCAT TGTACAGAAT TTCTATTCTG TTCTAATCTT ATTACTCGAG TATACACTTT TGTTTTCACG   
  
  
+ TTTTTTCGAA GTATTAGAAA CGTGTAAGTA CAAAAATAAA GTACAATTAG TTAATTAAGA AAAAGCGAGT   
  
  
+ AGATAATCTT GGATTAAATA TGGAAAGAAC CGTACGACAT CCACACGAAC CCCAATAGGC TATTTTGCCA   
  
  
+ CGCAAAACTA TTGTCGCCTT TTACGATGCG GAATTAAAAA CGCTCATCCC TACCGAGGCC GAGGCAAGAT   
  
  
+ CAGATCACTC GAAGGGAAGC AGAGAGCCAA CACTCGGTAC CACGCGTCCT TTCGTTCACG TGGGCGTCCT   
  
  
+ CCGCGGCTCT TTAGCGTTTT CGTCGCCGAA TCGCCGCGGC TGGCCAGCCG AATGAAACGG TCCTCCCTTC   
  
  
+ GCCGCTTCCA CAACGCTACG CAAGAGCTTC CGCTGCGAGC AGCCGTTCGG CAACCGAGAT AGTTCAGCGA   
  
  
+ GCAGATGTCG GGAAGGCACT CCTTGTTTTC CCGTTGAAAG CGAGAACAAA CAGTTTGAGA GTTCACATTC   
  
  
+ AATGGTTAAA ACATAAAGAC AGACAATCCA CATGAAAGCC AACGACAAAT CAACCGAAAA GACCCTCGAA   
  
  
+ AGACGTTCAT TGAATCAAAA GAAAACTTTA AGAAAACCCA AACCCTCGGA TCCCTCATTC TCGAACTGCT   
  
  
+ TTCTAAAACC CACATTTCAA CTTGAAAGGA ATACTAGACA AAAACCAGAA ACCACAAAAA AATCCGTCGA   
  
  
+ GTTTGAGTAT AGTGTCAAAA GCATGAATTT TTGGAACTAA AGACCCTACG TATGACGGTG ATCGTAGTAG   
  
  
+ ACTGAAACGG GAAGAGAGAG AGAAGGAAGG GAGTAACAAC ACATTTAAGA CAATAAACGT CATGGACTTG   
  
  
+ TTTGACATGT AAAATTCACT AATTGTAAAA GCAGAAAAAC CAAAGTGAAC AAAAGTCTAG AAGGATAATA   
  
  
+ TTTCCTACAG AACCTCCGAC GATTTTATGA CTTATCATCT ACCTTCCATA TTCGGTACGA GGCTGTCGAA   
  
  
+ TACTTGTAAT AAAAACGGGA AGAGTTCTCC TAATTCAAAA AAGTGAAAAG ATTTACTATA AAAACAGGTT   
  
  
+ CCGTCTAAAT CGATATCCCA CTTGTAACAA AACGACGAAC GAACTTACGA TCGAAAAGGG TCAACTTTAC   
  
  
+ AAAGGATTGC GAATTACCAA TAACGATAAC GTCTCCGTTC TCCGTAACTA GATGTATGAG ACAATACTGT   
  
  
+ AAGATAAACC CGTCCACTAT TGAAACATG  

- TTGCAGTCTT TGAGTTCTCC ATTCTTTCTT CTTTCTTGGG GGAGGAAGCT TTGGATTTGT CAAAAGAGCC   
  
  
- TGTGGGGGCC CTTGCTATCA ATTCAGCTTC TTAATGACAA AGTGATGTTA ATTTTATCTG TTCTCTCTTG   
  
  
- ATTTGGGATG TTCTAGTTTG TATGACCATA CGAAAACTAG TTGATACAAA GTCAATCAAG TAAGCTAAAC   
  
  
- AAAGAAGGTA ACATGTCTTA AAGATAAGAC AAGATTAGAA TAATGAGCTC ATATGTGAAA ACAAAAGTGC   
  
  
- AAAAAAGCTT CATAATCTTT GCACATTCAT GTTTTTATTT CATGTTAATC AATTAATTCT TTTTCGCTCA   
  
  
- TCTATTAGAA CCTAATTTAT ACCTTTCTTG GCATGCTGTA GGTGTGCTTG GGGTTATCCG ATAAAACGGT   
  
  
- GCGTTTTGAT AACAGCGGAA AATGCTACGC CTTAATTTTT GCGAGTAGGG ATGGCTCCGG CTCCGTTCTA   
  
  
- GTCTAGTGAG CTTCCCTTCG TCTCTCGGTT GTGAGCCATG GTGCGCAGGA AAGCAAGTGC ACCCGCAGGA   
  
  
- GGCGCCGAGA AATCGCAAAA GCAGCGGCTT AGCGGCGCCG ACCGGTCGGC TTACTTTGCC AGGAGGGAAG   
  
  
- CGGCGAAGGT GTTGCGATGC GTTCTCGAAG GCGACGCTCG TCGGCAAGCC GTTGGCTCTA TCAAGTCGCT   
  
  
- CGTCTACAGC CCTTCCGTGA GGAACAAAAG GGCAACTTTC GCTCTTGTTT GTCAAACTCT CAAGTGTAAG   
  
  
- TTACCAATTT TGTATTTCTG TCTGTTAGGT GTACTTTCGG TTGCTGTTTA GTTGGCTTTT CTGGGAGCTT   
  
  
- TCTGCAAGTA ACTTAGTTTT CTTTTGAAAT TCTTTTGGGT TTGGGAGCCT AGGGAGTAAG AGCTTGACGA   
  
  
- AAGATTTTGG GTGTAAAGTT GAACTTTCCT TATGATCTGT TTTTGGTCTT TGGTGTTTTT TTAGGCAGCT   
  
  
- CAAACTCATA TCACAGTTTT CGTACTTAAA AACCTTGATT TCTGGGATGC ATACTGCCAC TAGCATCATC   
  
  
- TGACTTTGCC CTTCTCTCTC TCTTCCTTCC CTCATTGTTG TGTAAATTCT GTTATTTGCA GTACCTGAAC   
  
  
- AAACTGTACA TTTTAAGTGA TTAACATTTT CGTCTTTTTG GTTTCACTTG TTTTCAGATC TTCCTATTAT   
  
  
- AAAGGATGTC TTGGAGGCTG CTAAAATACT GAATAGTAGA TGGAAGGTAT AAGCCATGCT CCGACAGCTT   
  
  
- ATGAACATTA TTTTTGCCCT TCTCAAGAGG ATTAAGTTTT TTCACTTTTC TAAATGATAT TTTTGTCCAA   
  
  
- GGCAGATTTA GCTATAGGGT GAACATTGTT TTGCTGCTTG CTTGAATGCT AGCTTTTCCC AGTTGAAATG   
  
  
- TTTCCTAACG CTTAATGGTT ATTGCTATTG CAGAGGCAAG AGGCATTGAT CTACATACTC TGTTATGACA   
  
  
- TTCTATTTGG GCAGGTGATA ACTTTGTAC

+     Unnamed\_\_1

| Site Name | Organism | Position | Strand | Matrix score. | sequence | function |
| --- | --- | --- | --- | --- | --- | --- |
| Unnamed\_\_1 | Zea mays | 418 | - | 5 | CGTGG |  |
| Unnamed\_\_1 | Zea mays | 530 | - | 5 | CGTGG |  |
| Unnamed\_\_1 | Zea mays | 549 | + | 5 | CGTGG |  |

> 2018/04/13 10:10:12  
+ AACGTCAGAA ACTCAAGAGG TAAGAAAGAA GAAAGAACCC CCTCCTTCGA AACCTAAACA GTTTTCTCGG   
  
  
+ ACACCCCCGG GAACGATAGT TAAGTCGAAG AATTACTGTT TCACTACAAT TAAAATAGAC AAGAGAGAAC   
  
  
+ TAAACCCTAC AAGATCAAAC ATACTGGTAT GCTTTTGATC AACTATGTTT CAGTTAGTTC ATTCGATTTG   
  
  
+ TTTCTTCCAT TGTACAGAAT TTCTATTCTG TTCTAATCTT ATTACTCGAG TATACACTTT TGTTTTCACG   
  
  
+ TTTTTTCGAA GTATTAGAAA CGTGTAAGTA CAAAAATAAA GTACAATTAG TTAATTAAGA AAAAGCGAGT   
  
  
+ AGATAATCTT GGATTAAATA TGGAAAGAAC CGTACGACAT CCACACGAAC CCCAATAGGC TATTTTGCCA   
  
  
+ CGCAAAACTA TTGTCGCCTT TTACGATGCG GAATTAAAAA CGCTCATCCC TACCGAGGCC GAGGCAAGAT   
  
  
+ CAGATCACTC GAAGGGAAGC AGAGAGCCAA CACTCGGTAC CACGCGTCCT TTCGTTCACG TGGGCGTCCT   
  
  
+ CCGCGGCTCT TTAGCGTTTT CGTCGCCGAA TCGCCGCGGC TGGCCAGCCG AATGAAACGG TCCTCCCTTC   
  
  
+ GCCGCTTCCA CAACGCTACG CAAGAGCTTC CGCTGCGAGC AGCCGTTCGG CAACCGAGAT AGTTCAGCGA   
  
  
+ GCAGATGTCG GGAAGGCACT CCTTGTTTTC CCGTTGAAAG CGAGAACAAA CAGTTTGAGA GTTCACATTC   
  
  
+ AATGGTTAAA ACATAAAGAC AGACAATCCA CATGAAAGCC AACGACAAAT CAACCGAAAA GACCCTCGAA   
  
  
+ AGACGTTCAT TGAATCAAAA GAAAACTTTA AGAAAACCCA AACCCTCGGA TCCCTCATTC TCGAACTGCT   
  
  
+ TTCTAAAACC CACATTTCAA CTTGAAAGGA ATACTAGACA AAAACCAGAA ACCACAAAAA AATCCGTCGA   
  
  
+ GTTTGAGTAT AGTGTCAAAA GCATGAATTT TTGGAACTAA AGACCCTACG TATGACGGTG ATCGTAGTAG   
  
  
+ ACTGAAACGG GAAGAGAGAG AGAAGGAAGG GAGTAACAAC ACATTTAAGA CAATAAACGT CATGGACTTG   
  
  
+ TTTGACATGT AAAATTCACT AATTGTAAAA GCAGAAAAAC CAAAGTGAAC AAAAGTCTAG AAGGATAATA   
  
  
+ TTTCCTACAG AACCTCCGAC GATTTTATGA CTTATCATCT ACCTTCCATA TTCGGTACGA GGCTGTCGAA   
  
  
+ TACTTGTAAT AAAAACGGGA AGAGTTCTCC TAATTCAAAA AAGTGAAAAG ATTTACTATA AAAACAGGTT   
  
  
+ CCGTCTAAAT CGATATCCCA CTTGTAACAA AACGACGAAC GAACTTACGA TCGAAAAGGG TCAACTTTAC   
  
  
+ AAAGGATTGC GAATTACCAA TAACGATAAC GTCTCCGTTC TCCGTAACTA GATGTATGAG ACAATACTGT   
  
  
+ AAGATAAACC CGTCCACTAT TGAAACATG  

- TTGCAGTCTT TGAGTTCTCC ATTCTTTCTT CTTTCTTGGG GGAGGAAGCT TTGGATTTGT CAAAAGAGCC   
  
  
- TGTGGGGGCC CTTGCTATCA ATTCAGCTTC TTAATGACAA AGTGATGTTA ATTTTATCTG TTCTCTCTTG   
  
  
- ATTTGGGATG TTCTAGTTTG TATGACCATA CGAAAACTAG TTGATACAAA GTCAATCAAG TAAGCTAAAC   
  
  
- AAAGAAGGTA ACATGTCTTA AAGATAAGAC AAGATTAGAA TAATGAGCTC ATATGTGAAA ACAAAAGTGC   
  
  
- AAAAAAGCTT CATAATCTTT GCACATTCAT GTTTTTATTT CATGTTAATC AATTAATTCT TTTTCGCTCA   
  
  
- TCTATTAGAA CCTAATTTAT ACCTTTCTTG GCATGCTGTA GGTGTGCTTG GGGTTATCCG ATAAAACGGT   
  
  
- GCGTTTTGAT AACAGCGGAA AATGCTACGC CTTAATTTTT GCGAGTAGGG ATGGCTCCGG CTCCGTTCTA   
  
  
- GTCTAGTGAG CTTCCCTTCG TCTCTCGGTT GTGAGCCATG GTGCGCAGGA AAGCAAGTGC ACCCGCAGGA   
  
  
- GGCGCCGAGA AATCGCAAAA GCAGCGGCTT AGCGGCGCCG ACCGGTCGGC TTACTTTGCC AGGAGGGAAG   
  
  
- CGGCGAAGGT GTTGCGATGC GTTCTCGAAG GCGACGCTCG TCGGCAAGCC GTTGGCTCTA TCAAGTCGCT   
  
  
- CGTCTACAGC CCTTCCGTGA GGAACAAAAG GGCAACTTTC GCTCTTGTTT GTCAAACTCT CAAGTGTAAG   
  
  
- TTACCAATTT TGTATTTCTG TCTGTTAGGT GTACTTTCGG TTGCTGTTTA GTTGGCTTTT CTGGGAGCTT   
  
  
- TCTGCAAGTA ACTTAGTTTT CTTTTGAAAT TCTTTTGGGT TTGGGAGCCT AGGGAGTAAG AGCTTGACGA   
  
  
- AAGATTTTGG GTGTAAAGTT GAACTTTCCT TATGATCTGT TTTTGGTCTT TGGTGTTTTT TTAGGCAGCT   
  
  
- CAAACTCATA TCACAGTTTT CGTACTTAAA AACCTTGATT TCTGGGATGC ATACTGCCAC TAGCATCATC   
  
  
- TGACTTTGCC CTTCTCTCTC TCTTCCTTCC CTCATTGTTG TGTAAATTCT GTTATTTGCA GTACCTGAAC   
  
  
- AAACTGTACA TTTTAAGTGA TTAACATTTT CGTCTTTTTG GTTTCACTTG TTTTCAGATC TTCCTATTAT   
  
  
- AAAGGATGTC TTGGAGGCTG CTAAAATACT GAATAGTAGA TGGAAGGTAT AAGCCATGCT CCGACAGCTT   
  
  
- ATGAACATTA TTTTTGCCCT TCTCAAGAGG ATTAAGTTTT TTCACTTTTC TAAATGATAT TTTTGTCCAA   
  
  
- GGCAGATTTA GCTATAGGGT GAACATTGTT TTGCTGCTTG CTTGAATGCT AGCTTTTCCC AGTTGAAATG   
  
  
- TTTCCTAACG CTTAATGGTT ATTGCTATTG CAGAGGCAAG AGGCATTGAT CTACATACTC TGTTATGACA   
  
  
- TTCTATTTGG GCAGGTGATA ACTTTGTAC

+     Unnamed\_\_2

| Site Name | Organism | Position | Strand | Matrix score. | sequence | function |
| --- | --- | --- | --- | --- | --- | --- |
| Unnamed\_\_2 | Zea mays | 75 | + | 6 | CCCCGG |  |

> 2018/04/13 10:10:12  
+ AACGTCAGAA ACTCAAGAGG TAAGAAAGAA GAAAGAACCC CCTCCTTCGA AACCTAAACA GTTTTCTCGG   
  
  
+ ACACCCCCGG GAACGATAGT TAAGTCGAAG AATTACTGTT TCACTACAAT TAAAATAGAC AAGAGAGAAC   
  
  
+ TAAACCCTAC AAGATCAAAC ATACTGGTAT GCTTTTGATC AACTATGTTT CAGTTAGTTC ATTCGATTTG   
  
  
+ TTTCTTCCAT TGTACAGAAT TTCTATTCTG TTCTAATCTT ATTACTCGAG TATACACTTT TGTTTTCACG   
  
  
+ TTTTTTCGAA GTATTAGAAA CGTGTAAGTA CAAAAATAAA GTACAATTAG TTAATTAAGA AAAAGCGAGT   
  
  
+ AGATAATCTT GGATTAAATA TGGAAAGAAC CGTACGACAT CCACACGAAC CCCAATAGGC TATTTTGCCA   
  
  
+ CGCAAAACTA TTGTCGCCTT TTACGATGCG GAATTAAAAA CGCTCATCCC TACCGAGGCC GAGGCAAGAT   
  
  
+ CAGATCACTC GAAGGGAAGC AGAGAGCCAA CACTCGGTAC CACGCGTCCT TTCGTTCACG TGGGCGTCCT   
  
  
+ CCGCGGCTCT TTAGCGTTTT CGTCGCCGAA TCGCCGCGGC TGGCCAGCCG AATGAAACGG TCCTCCCTTC   
  
  
+ GCCGCTTCCA CAACGCTACG CAAGAGCTTC CGCTGCGAGC AGCCGTTCGG CAACCGAGAT AGTTCAGCGA   
  
  
+ GCAGATGTCG GGAAGGCACT CCTTGTTTTC CCGTTGAAAG CGAGAACAAA CAGTTTGAGA GTTCACATTC   
  
  
+ AATGGTTAAA ACATAAAGAC AGACAATCCA CATGAAAGCC AACGACAAAT CAACCGAAAA GACCCTCGAA   
  
  
+ AGACGTTCAT TGAATCAAAA GAAAACTTTA AGAAAACCCA AACCCTCGGA TCCCTCATTC TCGAACTGCT   
  
  
+ TTCTAAAACC CACATTTCAA CTTGAAAGGA ATACTAGACA AAAACCAGAA ACCACAAAAA AATCCGTCGA   
  
  
+ GTTTGAGTAT AGTGTCAAAA GCATGAATTT TTGGAACTAA AGACCCTACG TATGACGGTG ATCGTAGTAG   
  
  
+ ACTGAAACGG GAAGAGAGAG AGAAGGAAGG GAGTAACAAC ACATTTAAGA CAATAAACGT CATGGACTTG   
  
  
+ TTTGACATGT AAAATTCACT AATTGTAAAA GCAGAAAAAC CAAAGTGAAC AAAAGTCTAG AAGGATAATA   
  
  
+ TTTCCTACAG AACCTCCGAC GATTTTATGA CTTATCATCT ACCTTCCATA TTCGGTACGA GGCTGTCGAA   
  
  
+ TACTTGTAAT AAAAACGGGA AGAGTTCTCC TAATTCAAAA AAGTGAAAAG ATTTACTATA AAAACAGGTT   
  
  
+ CCGTCTAAAT CGATATCCCA CTTGTAACAA AACGACGAAC GAACTTACGA TCGAAAAGGG TCAACTTTAC   
  
  
+ AAAGGATTGC GAATTACCAA TAACGATAAC GTCTCCGTTC TCCGTAACTA GATGTATGAG ACAATACTGT   
  
  
+ AAGATAAACC CGTCCACTAT TGAAACATG  

- TTGCAGTCTT TGAGTTCTCC ATTCTTTCTT CTTTCTTGGG GGAGGAAGCT TTGGATTTGT CAAAAGAGCC   
  
  
- TGTGGGGGCC CTTGCTATCA ATTCAGCTTC TTAATGACAA AGTGATGTTA ATTTTATCTG TTCTCTCTTG   
  
  
- ATTTGGGATG TTCTAGTTTG TATGACCATA CGAAAACTAG TTGATACAAA GTCAATCAAG TAAGCTAAAC   
  
  
- AAAGAAGGTA ACATGTCTTA AAGATAAGAC AAGATTAGAA TAATGAGCTC ATATGTGAAA ACAAAAGTGC   
  
  
- AAAAAAGCTT CATAATCTTT GCACATTCAT GTTTTTATTT CATGTTAATC AATTAATTCT TTTTCGCTCA   
  
  
- TCTATTAGAA CCTAATTTAT ACCTTTCTTG GCATGCTGTA GGTGTGCTTG GGGTTATCCG ATAAAACGGT   
  
  
- GCGTTTTGAT AACAGCGGAA AATGCTACGC CTTAATTTTT GCGAGTAGGG ATGGCTCCGG CTCCGTTCTA   
  
  
- GTCTAGTGAG CTTCCCTTCG TCTCTCGGTT GTGAGCCATG GTGCGCAGGA AAGCAAGTGC ACCCGCAGGA   
  
  
- GGCGCCGAGA AATCGCAAAA GCAGCGGCTT AGCGGCGCCG ACCGGTCGGC TTACTTTGCC AGGAGGGAAG   
  
  
- CGGCGAAGGT GTTGCGATGC GTTCTCGAAG GCGACGCTCG TCGGCAAGCC GTTGGCTCTA TCAAGTCGCT   
  
  
- CGTCTACAGC CCTTCCGTGA GGAACAAAAG GGCAACTTTC GCTCTTGTTT GTCAAACTCT CAAGTGTAAG   
  
  
- TTACCAATTT TGTATTTCTG TCTGTTAGGT GTACTTTCGG TTGCTGTTTA GTTGGCTTTT CTGGGAGCTT   
  
  
- TCTGCAAGTA ACTTAGTTTT CTTTTGAAAT TCTTTTGGGT TTGGGAGCCT AGGGAGTAAG AGCTTGACGA   
  
  
- AAGATTTTGG GTGTAAAGTT GAACTTTCCT TATGATCTGT TTTTGGTCTT TGGTGTTTTT TTAGGCAGCT   
  
  
- CAAACTCATA TCACAGTTTT CGTACTTAAA AACCTTGATT TCTGGGATGC ATACTGCCAC TAGCATCATC   
  
  
- TGACTTTGCC CTTCTCTCTC TCTTCCTTCC CTCATTGTTG TGTAAATTCT GTTATTTGCA GTACCTGAAC   
  
  
- AAACTGTACA TTTTAAGTGA TTAACATTTT CGTCTTTTTG GTTTCACTTG TTTTCAGATC TTCCTATTAT   
  
  
- AAAGGATGTC TTGGAGGCTG CTAAAATACT GAATAGTAGA TGGAAGGTAT AAGCCATGCT CCGACAGCTT   
  
  
- ATGAACATTA TTTTTGCCCT TCTCAAGAGG ATTAAGTTTT TTCACTTTTC TAAATGATAT TTTTGTCCAA   
  
  
- GGCAGATTTA GCTATAGGGT GAACATTGTT TTGCTGCTTG CTTGAATGCT AGCTTTTCCC AGTTGAAATG   
  
  
- TTTCCTAACG CTTAATGGTT ATTGCTATTG CAGAGGCAAG AGGCATTGAT CTACATACTC TGTTATGACA   
  
  
- TTCTATTTGG GCAGGTGATA ACTTTGTAC

+     Unnamed\_\_3

| Site Name | Organism | Position | Strand | Matrix score. | sequence | function |
| --- | --- | --- | --- | --- | --- | --- |
| Unnamed\_\_3 | Zea mays | 549 | + | 5 | CGTGG |  |
| Unnamed\_\_3 | Zea mays | 530 | - | 5 | CGTGG |  |
| Unnamed\_\_3 | Zea mays | 418 | - | 5 | CGTGG |  |

> 2018/04/13 10:10:12  
+ AACGTCAGAA ACTCAAGAGG TAAGAAAGAA GAAAGAACCC CCTCCTTCGA AACCTAAACA GTTTTCTCGG   
  
  
+ ACACCCCCGG GAACGATAGT TAAGTCGAAG AATTACTGTT TCACTACAAT TAAAATAGAC AAGAGAGAAC   
  
  
+ TAAACCCTAC AAGATCAAAC ATACTGGTAT GCTTTTGATC AACTATGTTT CAGTTAGTTC ATTCGATTTG   
  
  
+ TTTCTTCCAT TGTACAGAAT TTCTATTCTG TTCTAATCTT ATTACTCGAG TATACACTTT TGTTTTCACG   
  
  
+ TTTTTTCGAA GTATTAGAAA CGTGTAAGTA CAAAAATAAA GTACAATTAG TTAATTAAGA AAAAGCGAGT   
  
  
+ AGATAATCTT GGATTAAATA TGGAAAGAAC CGTACGACAT CCACACGAAC CCCAATAGGC TATTTTGCCA   
  
  
+ CGCAAAACTA TTGTCGCCTT TTACGATGCG GAATTAAAAA CGCTCATCCC TACCGAGGCC GAGGCAAGAT   
  
  
+ CAGATCACTC GAAGGGAAGC AGAGAGCCAA CACTCGGTAC CACGCGTCCT TTCGTTCACG TGGGCGTCCT   
  
  
+ CCGCGGCTCT TTAGCGTTTT CGTCGCCGAA TCGCCGCGGC TGGCCAGCCG AATGAAACGG TCCTCCCTTC   
  
  
+ GCCGCTTCCA CAACGCTACG CAAGAGCTTC CGCTGCGAGC AGCCGTTCGG CAACCGAGAT AGTTCAGCGA   
  
  
+ GCAGATGTCG GGAAGGCACT CCTTGTTTTC CCGTTGAAAG CGAGAACAAA CAGTTTGAGA GTTCACATTC   
  
  
+ AATGGTTAAA ACATAAAGAC AGACAATCCA CATGAAAGCC AACGACAAAT CAACCGAAAA GACCCTCGAA   
  
  
+ AGACGTTCAT TGAATCAAAA GAAAACTTTA AGAAAACCCA AACCCTCGGA TCCCTCATTC TCGAACTGCT   
  
  
+ TTCTAAAACC CACATTTCAA CTTGAAAGGA ATACTAGACA AAAACCAGAA ACCACAAAAA AATCCGTCGA   
  
  
+ GTTTGAGTAT AGTGTCAAAA GCATGAATTT TTGGAACTAA AGACCCTACG TATGACGGTG ATCGTAGTAG   
  
  
+ ACTGAAACGG GAAGAGAGAG AGAAGGAAGG GAGTAACAAC ACATTTAAGA CAATAAACGT CATGGACTTG   
  
  
+ TTTGACATGT AAAATTCACT AATTGTAAAA GCAGAAAAAC CAAAGTGAAC AAAAGTCTAG AAGGATAATA   
  
  
+ TTTCCTACAG AACCTCCGAC GATTTTATGA CTTATCATCT ACCTTCCATA TTCGGTACGA GGCTGTCGAA   
  
  
+ TACTTGTAAT AAAAACGGGA AGAGTTCTCC TAATTCAAAA AAGTGAAAAG ATTTACTATA AAAACAGGTT   
  
  
+ CCGTCTAAAT CGATATCCCA CTTGTAACAA AACGACGAAC GAACTTACGA TCGAAAAGGG TCAACTTTAC   
  
  
+ AAAGGATTGC GAATTACCAA TAACGATAAC GTCTCCGTTC TCCGTAACTA GATGTATGAG ACAATACTGT   
  
  
+ AAGATAAACC CGTCCACTAT TGAAACATG  

- TTGCAGTCTT TGAGTTCTCC ATTCTTTCTT CTTTCTTGGG GGAGGAAGCT TTGGATTTGT CAAAAGAGCC   
  
  
- TGTGGGGGCC CTTGCTATCA ATTCAGCTTC TTAATGACAA AGTGATGTTA ATTTTATCTG TTCTCTCTTG   
  
  
- ATTTGGGATG TTCTAGTTTG TATGACCATA CGAAAACTAG TTGATACAAA GTCAATCAAG TAAGCTAAAC   
  
  
- AAAGAAGGTA ACATGTCTTA AAGATAAGAC AAGATTAGAA TAATGAGCTC ATATGTGAAA ACAAAAGTGC   
  
  
- AAAAAAGCTT CATAATCTTT GCACATTCAT GTTTTTATTT CATGTTAATC AATTAATTCT TTTTCGCTCA   
  
  
- TCTATTAGAA CCTAATTTAT ACCTTTCTTG GCATGCTGTA GGTGTGCTTG GGGTTATCCG ATAAAACGGT   
  
  
- GCGTTTTGAT AACAGCGGAA AATGCTACGC CTTAATTTTT GCGAGTAGGG ATGGCTCCGG CTCCGTTCTA   
  
  
- GTCTAGTGAG CTTCCCTTCG TCTCTCGGTT GTGAGCCATG GTGCGCAGGA AAGCAAGTGC ACCCGCAGGA   
  
  
- GGCGCCGAGA AATCGCAAAA GCAGCGGCTT AGCGGCGCCG ACCGGTCGGC TTACTTTGCC AGGAGGGAAG   
  
  
- CGGCGAAGGT GTTGCGATGC GTTCTCGAAG GCGACGCTCG TCGGCAAGCC GTTGGCTCTA TCAAGTCGCT   
  
  
- CGTCTACAGC CCTTCCGTGA GGAACAAAAG GGCAACTTTC GCTCTTGTTT GTCAAACTCT CAAGTGTAAG   
  
  
- TTACCAATTT TGTATTTCTG TCTGTTAGGT GTACTTTCGG TTGCTGTTTA GTTGGCTTTT CTGGGAGCTT   
  
  
- TCTGCAAGTA ACTTAGTTTT CTTTTGAAAT TCTTTTGGGT TTGGGAGCCT AGGGAGTAAG AGCTTGACGA   
  
  
- AAGATTTTGG GTGTAAAGTT GAACTTTCCT TATGATCTGT TTTTGGTCTT TGGTGTTTTT TTAGGCAGCT   
  
  
- CAAACTCATA TCACAGTTTT CGTACTTAAA AACCTTGATT TCTGGGATGC ATACTGCCAC TAGCATCATC   
  
  
- TGACTTTGCC CTTCTCTCTC TCTTCCTTCC CTCATTGTTG TGTAAATTCT GTTATTTGCA GTACCTGAAC   
  
  
- AAACTGTACA TTTTAAGTGA TTAACATTTT CGTCTTTTTG GTTTCACTTG TTTTCAGATC TTCCTATTAT   
  
  
- AAAGGATGTC TTGGAGGCTG CTAAAATACT GAATAGTAGA TGGAAGGTAT AAGCCATGCT CCGACAGCTT   
  
  
- ATGAACATTA TTTTTGCCCT TCTCAAGAGG ATTAAGTTTT TTCACTTTTC TAAATGATAT TTTTGTCCAA   
  
  
- GGCAGATTTA GCTATAGGGT GAACATTGTT TTGCTGCTTG CTTGAATGCT AGCTTTTCCC AGTTGAAATG   
  
  
- TTTCCTAACG CTTAATGGTT ATTGCTATTG CAGAGGCAAG AGGCATTGAT CTACATACTC TGTTATGACA   
  
  
- TTCTATTTGG GCAGGTGATA ACTTTGTAC

+     Unnamed\_\_4

| Site Name | Organism | Position | Strand | Matrix score. | sequence | function |
| --- | --- | --- | --- | --- | --- | --- |
| Unnamed\_\_4 | Petroselinum hortense | 1204 | + | 4 | CTCC |  |
| Unnamed\_\_4 | Petroselinum hortense | 1440 | + | 4 | CTCC |  |
| Unnamed\_\_4 | Petroselinum hortense | 1080 | - | 4 | CTCC |  |
| Unnamed\_\_4 | Petroselinum hortense | 1433 | + | 4 | CTCC |  |
| Unnamed\_\_4 | Petroselinum hortense | 1287 | + | 4 | CTCC |  |
| Unnamed\_\_4 | Petroselinum hortense | 623 | + | 4 | CTCC |  |
| Unnamed\_\_4 | Petroselinum hortense | 559 | + | 4 | CTCC |  |
| Unnamed\_\_4 | Petroselinum hortense | 42 | + | 4 | CTCC |  |
| Unnamed\_\_4 | Petroselinum hortense | 719 | + | 4 | CTCC |  |

> 2018/04/13 10:10:12  
+ AACGTCAGAA ACTCAAGAGG TAAGAAAGAA GAAAGAACCC CCTCCTTCGA AACCTAAACA GTTTTCTCGG   
  
  
+ ACACCCCCGG GAACGATAGT TAAGTCGAAG AATTACTGTT TCACTACAAT TAAAATAGAC AAGAGAGAAC   
  
  
+ TAAACCCTAC AAGATCAAAC ATACTGGTAT GCTTTTGATC AACTATGTTT CAGTTAGTTC ATTCGATTTG   
  
  
+ TTTCTTCCAT TGTACAGAAT TTCTATTCTG TTCTAATCTT ATTACTCGAG TATACACTTT TGTTTTCACG   
  
  
+ TTTTTTCGAA GTATTAGAAA CGTGTAAGTA CAAAAATAAA GTACAATTAG TTAATTAAGA AAAAGCGAGT   
  
  
+ AGATAATCTT GGATTAAATA TGGAAAGAAC CGTACGACAT CCACACGAAC CCCAATAGGC TATTTTGCCA   
  
  
+ CGCAAAACTA TTGTCGCCTT TTACGATGCG GAATTAAAAA CGCTCATCCC TACCGAGGCC GAGGCAAGAT   
  
  
+ CAGATCACTC GAAGGGAAGC AGAGAGCCAA CACTCGGTAC CACGCGTCCT TTCGTTCACG TGGGCGTCCT   
  
  
+ CCGCGGCTCT TTAGCGTTTT CGTCGCCGAA TCGCCGCGGC TGGCCAGCCG AATGAAACGG TCCTCCCTTC   
  
  
+ GCCGCTTCCA CAACGCTACG CAAGAGCTTC CGCTGCGAGC AGCCGTTCGG CAACCGAGAT AGTTCAGCGA   
  
  
+ GCAGATGTCG GGAAGGCACT CCTTGTTTTC CCGTTGAAAG CGAGAACAAA CAGTTTGAGA GTTCACATTC   
  
  
+ AATGGTTAAA ACATAAAGAC AGACAATCCA CATGAAAGCC AACGACAAAT CAACCGAAAA GACCCTCGAA   
  
  
+ AGACGTTCAT TGAATCAAAA GAAAACTTTA AGAAAACCCA AACCCTCGGA TCCCTCATTC TCGAACTGCT   
  
  
+ TTCTAAAACC CACATTTCAA CTTGAAAGGA ATACTAGACA AAAACCAGAA ACCACAAAAA AATCCGTCGA   
  
  
+ GTTTGAGTAT AGTGTCAAAA GCATGAATTT TTGGAACTAA AGACCCTACG TATGACGGTG ATCGTAGTAG   
  
  
+ ACTGAAACGG GAAGAGAGAG AGAAGGAAGG GAGTAACAAC ACATTTAAGA CAATAAACGT CATGGACTTG   
  
  
+ TTTGACATGT AAAATTCACT AATTGTAAAA GCAGAAAAAC CAAAGTGAAC AAAAGTCTAG AAGGATAATA   
  
  
+ TTTCCTACAG AACCTCCGAC GATTTTATGA CTTATCATCT ACCTTCCATA TTCGGTACGA GGCTGTCGAA   
  
  
+ TACTTGTAAT AAAAACGGGA AGAGTTCTCC TAATTCAAAA AAGTGAAAAG ATTTACTATA AAAACAGGTT   
  
  
+ CCGTCTAAAT CGATATCCCA CTTGTAACAA AACGACGAAC GAACTTACGA TCGAAAAGGG TCAACTTTAC   
  
  
+ AAAGGATTGC GAATTACCAA TAACGATAAC GTCTCCGTTC TCCGTAACTA GATGTATGAG ACAATACTGT   
  
  
+ AAGATAAACC CGTCCACTAT TGAAACATG  

- TTGCAGTCTT TGAGTTCTCC ATTCTTTCTT CTTTCTTGGG GGAGGAAGCT TTGGATTTGT CAAAAGAGCC   
  
  
- TGTGGGGGCC CTTGCTATCA ATTCAGCTTC TTAATGACAA AGTGATGTTA ATTTTATCTG TTCTCTCTTG   
  
  
- ATTTGGGATG TTCTAGTTTG TATGACCATA CGAAAACTAG TTGATACAAA GTCAATCAAG TAAGCTAAAC   
  
  
- AAAGAAGGTA ACATGTCTTA AAGATAAGAC AAGATTAGAA TAATGAGCTC ATATGTGAAA ACAAAAGTGC   
  
  
- AAAAAAGCTT CATAATCTTT GCACATTCAT GTTTTTATTT CATGTTAATC AATTAATTCT TTTTCGCTCA   
  
  
- TCTATTAGAA CCTAATTTAT ACCTTTCTTG GCATGCTGTA GGTGTGCTTG GGGTTATCCG ATAAAACGGT   
  
  
- GCGTTTTGAT AACAGCGGAA AATGCTACGC CTTAATTTTT GCGAGTAGGG ATGGCTCCGG CTCCGTTCTA   
  
  
- GTCTAGTGAG CTTCCCTTCG TCTCTCGGTT GTGAGCCATG GTGCGCAGGA AAGCAAGTGC ACCCGCAGGA   
  
  
- GGCGCCGAGA AATCGCAAAA GCAGCGGCTT AGCGGCGCCG ACCGGTCGGC TTACTTTGCC AGGAGGGAAG   
  
  
- CGGCGAAGGT GTTGCGATGC GTTCTCGAAG GCGACGCTCG TCGGCAAGCC GTTGGCTCTA TCAAGTCGCT   
  
  
- CGTCTACAGC CCTTCCGTGA GGAACAAAAG GGCAACTTTC GCTCTTGTTT GTCAAACTCT CAAGTGTAAG   
  
  
- TTACCAATTT TGTATTTCTG TCTGTTAGGT GTACTTTCGG TTGCTGTTTA GTTGGCTTTT CTGGGAGCTT   
  
  
- TCTGCAAGTA ACTTAGTTTT CTTTTGAAAT TCTTTTGGGT TTGGGAGCCT AGGGAGTAAG AGCTTGACGA   
  
  
- AAGATTTTGG GTGTAAAGTT GAACTTTCCT TATGATCTGT TTTTGGTCTT TGGTGTTTTT TTAGGCAGCT   
  
  
- CAAACTCATA TCACAGTTTT CGTACTTAAA AACCTTGATT TCTGGGATGC ATACTGCCAC TAGCATCATC   
  
  
- TGACTTTGCC CTTCTCTCTC TCTTCCTTCC CTCATTGTTG TGTAAATTCT GTTATTTGCA GTACCTGAAC   
  
  
- AAACTGTACA TTTTAAGTGA TTAACATTTT CGTCTTTTTG GTTTCACTTG TTTTCAGATC TTCCTATTAT   
  
  
- AAAGGATGTC TTGGAGGCTG CTAAAATACT GAATAGTAGA TGGAAGGTAT AAGCCATGCT CCGACAGCTT   
  
  
- ATGAACATTA TTTTTGCCCT TCTCAAGAGG ATTAAGTTTT TTCACTTTTC TAAATGATAT TTTTGTCCAA   
  
  
- GGCAGATTTA GCTATAGGGT GAACATTGTT TTGCTGCTTG CTTGAATGCT AGCTTTTCCC AGTTGAAATG   
  
  
- TTTCCTAACG CTTAATGGTT ATTGCTATTG CAGAGGCAAG AGGCATTGAT CTACATACTC TGTTATGACA   
  
  
- TTCTATTTGG GCAGGTGATA ACTTTGTAC

+     W box

| Site Name | Organism | Position | Strand | Matrix score. | sequence | function |
| --- | --- | --- | --- | --- | --- | --- |
| W box | Arabidopsis thaliana | 1389 | - | 6 | TTGACC |  |

> 2018/04/13 10:10:12  
+ AACGTCAGAA ACTCAAGAGG TAAGAAAGAA GAAAGAACCC CCTCCTTCGA AACCTAAACA GTTTTCTCGG   
  
  
+ ACACCCCCGG GAACGATAGT TAAGTCGAAG AATTACTGTT TCACTACAAT TAAAATAGAC AAGAGAGAAC   
  
  
+ TAAACCCTAC AAGATCAAAC ATACTGGTAT GCTTTTGATC AACTATGTTT CAGTTAGTTC ATTCGATTTG   
  
  
+ TTTCTTCCAT TGTACAGAAT TTCTATTCTG TTCTAATCTT ATTACTCGAG TATACACTTT TGTTTTCACG   
  
  
+ TTTTTTCGAA GTATTAGAAA CGTGTAAGTA CAAAAATAAA GTACAATTAG TTAATTAAGA AAAAGCGAGT   
  
  
+ AGATAATCTT GGATTAAATA TGGAAAGAAC CGTACGACAT CCACACGAAC CCCAATAGGC TATTTTGCCA   
  
  
+ CGCAAAACTA TTGTCGCCTT TTACGATGCG GAATTAAAAA CGCTCATCCC TACCGAGGCC GAGGCAAGAT   
  
  
+ CAGATCACTC GAAGGGAAGC AGAGAGCCAA CACTCGGTAC CACGCGTCCT TTCGTTCACG TGGGCGTCCT   
  
  
+ CCGCGGCTCT TTAGCGTTTT CGTCGCCGAA TCGCCGCGGC TGGCCAGCCG AATGAAACGG TCCTCCCTTC   
  
  
+ GCCGCTTCCA CAACGCTACG CAAGAGCTTC CGCTGCGAGC AGCCGTTCGG CAACCGAGAT AGTTCAGCGA   
  
  
+ GCAGATGTCG GGAAGGCACT CCTTGTTTTC CCGTTGAAAG CGAGAACAAA CAGTTTGAGA GTTCACATTC   
  
  
+ AATGGTTAAA ACATAAAGAC AGACAATCCA CATGAAAGCC AACGACAAAT CAACCGAAAA GACCCTCGAA   
  
  
+ AGACGTTCAT TGAATCAAAA GAAAACTTTA AGAAAACCCA AACCCTCGGA TCCCTCATTC TCGAACTGCT   
  
  
+ TTCTAAAACC CACATTTCAA CTTGAAAGGA ATACTAGACA AAAACCAGAA ACCACAAAAA AATCCGTCGA   
  
  
+ GTTTGAGTAT AGTGTCAAAA GCATGAATTT TTGGAACTAA AGACCCTACG TATGACGGTG ATCGTAGTAG   
  
  
+ ACTGAAACGG GAAGAGAGAG AGAAGGAAGG GAGTAACAAC ACATTTAAGA CAATAAACGT CATGGACTTG   
  
  
+ TTTGACATGT AAAATTCACT AATTGTAAAA GCAGAAAAAC CAAAGTGAAC AAAAGTCTAG AAGGATAATA   
  
  
+ TTTCCTACAG AACCTCCGAC GATTTTATGA CTTATCATCT ACCTTCCATA TTCGGTACGA GGCTGTCGAA   
  
  
+ TACTTGTAAT AAAAACGGGA AGAGTTCTCC TAATTCAAAA AAGTGAAAAG ATTTACTATA AAAACAGGTT   
  
  
+ CCGTCTAAAT CGATATCCCA CTTGTAACAA AACGACGAAC GAACTTACGA TCGAAAAGGG TCAACTTTAC   
  
  
+ AAAGGATTGC GAATTACCAA TAACGATAAC GTCTCCGTTC TCCGTAACTA GATGTATGAG ACAATACTGT   
  
  
+ AAGATAAACC CGTCCACTAT TGAAACATG  

- TTGCAGTCTT TGAGTTCTCC ATTCTTTCTT CTTTCTTGGG GGAGGAAGCT TTGGATTTGT CAAAAGAGCC   
  
  
- TGTGGGGGCC CTTGCTATCA ATTCAGCTTC TTAATGACAA AGTGATGTTA ATTTTATCTG TTCTCTCTTG   
  
  
- ATTTGGGATG TTCTAGTTTG TATGACCATA CGAAAACTAG TTGATACAAA GTCAATCAAG TAAGCTAAAC   
  
  
- AAAGAAGGTA ACATGTCTTA AAGATAAGAC AAGATTAGAA TAATGAGCTC ATATGTGAAA ACAAAAGTGC   
  
  
- AAAAAAGCTT CATAATCTTT GCACATTCAT GTTTTTATTT CATGTTAATC AATTAATTCT TTTTCGCTCA   
  
  
- TCTATTAGAA CCTAATTTAT ACCTTTCTTG GCATGCTGTA GGTGTGCTTG GGGTTATCCG ATAAAACGGT   
  
  
- GCGTTTTGAT AACAGCGGAA AATGCTACGC CTTAATTTTT GCGAGTAGGG ATGGCTCCGG CTCCGTTCTA   
  
  
- GTCTAGTGAG CTTCCCTTCG TCTCTCGGTT GTGAGCCATG GTGCGCAGGA AAGCAAGTGC ACCCGCAGGA   
  
  
- GGCGCCGAGA AATCGCAAAA GCAGCGGCTT AGCGGCGCCG ACCGGTCGGC TTACTTTGCC AGGAGGGAAG   
  
  
- CGGCGAAGGT GTTGCGATGC GTTCTCGAAG GCGACGCTCG TCGGCAAGCC GTTGGCTCTA TCAAGTCGCT   
  
  
- CGTCTACAGC CCTTCCGTGA GGAACAAAAG GGCAACTTTC GCTCTTGTTT GTCAAACTCT CAAGTGTAAG   
  
  
- TTACCAATTT TGTATTTCTG TCTGTTAGGT GTACTTTCGG TTGCTGTTTA GTTGGCTTTT CTGGGAGCTT   
  
  
- TCTGCAAGTA ACTTAGTTTT CTTTTGAAAT TCTTTTGGGT TTGGGAGCCT AGGGAGTAAG AGCTTGACGA   
  
  
- AAGATTTTGG GTGTAAAGTT GAACTTTCCT TATGATCTGT TTTTGGTCTT TGGTGTTTTT TTAGGCAGCT   
  
  
- CAAACTCATA TCACAGTTTT CGTACTTAAA AACCTTGATT TCTGGGATGC ATACTGCCAC TAGCATCATC   
  
  
- TGACTTTGCC CTTCTCTCTC TCTTCCTTCC CTCATTGTTG TGTAAATTCT GTTATTTGCA GTACCTGAAC   
  
  
- AAACTGTACA TTTTAAGTGA TTAACATTTT CGTCTTTTTG GTTTCACTTG TTTTCAGATC TTCCTATTAT   
  
  
- AAAGGATGTC TTGGAGGCTG CTAAAATACT GAATAGTAGA TGGAAGGTAT AAGCCATGCT CCGACAGCTT   
  
  
- ATGAACATTA TTTTTGCCCT TCTCAAGAGG ATTAAGTTTT TTCACTTTTC TAAATGATAT TTTTGTCCAA   
  
  
- GGCAGATTTA GCTATAGGGT GAACATTGTT TTGCTGCTTG CTTGAATGCT AGCTTTTCCC AGTTGAAATG   
  
  
- TTTCCTAACG CTTAATGGTT ATTGCTATTG CAGAGGCAAG AGGCATTGAT CTACATACTC TGTTATGACA   
  
  
- TTCTATTTGG GCAGGTGATA ACTTTGTAC

+     circadian

| Site Name | Organism | Position | Strand | Matrix score. | sequence | function |
| --- | --- | --- | --- | --- | --- | --- |
| circadian | Lycopersicon esculentum | 352 | - | 6 | CAANNNNATC | cis-acting regulatory element involved in circadian control |
| circadian | Lycopersicon esculentum | 965 | + | 6 | CAANNNNATC | cis-acting regulatory element involved in circadian control |

> 2018/04/13 10:10:12  
+ AACGTCAGAA ACTCAAGAGG TAAGAAAGAA GAAAGAACCC CCTCCTTCGA AACCTAAACA GTTTTCTCGG   
  
  
+ ACACCCCCGG GAACGATAGT TAAGTCGAAG AATTACTGTT TCACTACAAT TAAAATAGAC AAGAGAGAAC   
  
  
+ TAAACCCTAC AAGATCAAAC ATACTGGTAT GCTTTTGATC AACTATGTTT CAGTTAGTTC ATTCGATTTG   
  
  
+ TTTCTTCCAT TGTACAGAAT TTCTATTCTG TTCTAATCTT ATTACTCGAG TATACACTTT TGTTTTCACG   
  
  
+ TTTTTTCGAA GTATTAGAAA CGTGTAAGTA CAAAAATAAA GTACAATTAG TTAATTAAGA AAAAGCGAGT   
  
  
+ AGATAATCTT GGATTAAATA TGGAAAGAAC CGTACGACAT CCACACGAAC CCCAATAGGC TATTTTGCCA   
  
  
+ CGCAAAACTA TTGTCGCCTT TTACGATGCG GAATTAAAAA CGCTCATCCC TACCGAGGCC GAGGCAAGAT   
  
  
+ CAGATCACTC GAAGGGAAGC AGAGAGCCAA CACTCGGTAC CACGCGTCCT TTCGTTCACG TGGGCGTCCT   
  
  
+ CCGCGGCTCT TTAGCGTTTT CGTCGCCGAA TCGCCGCGGC TGGCCAGCCG AATGAAACGG TCCTCCCTTC   
  
  
+ GCCGCTTCCA CAACGCTACG CAAGAGCTTC CGCTGCGAGC AGCCGTTCGG CAACCGAGAT AGTTCAGCGA   
  
  
+ GCAGATGTCG GGAAGGCACT CCTTGTTTTC CCGTTGAAAG CGAGAACAAA CAGTTTGAGA GTTCACATTC   
  
  
+ AATGGTTAAA ACATAAAGAC AGACAATCCA CATGAAAGCC AACGACAAAT CAACCGAAAA GACCCTCGAA   
  
  
+ AGACGTTCAT TGAATCAAAA GAAAACTTTA AGAAAACCCA AACCCTCGGA TCCCTCATTC TCGAACTGCT   
  
  
+ TTCTAAAACC CACATTTCAA CTTGAAAGGA ATACTAGACA AAAACCAGAA ACCACAAAAA AATCCGTCGA   
  
  
+ GTTTGAGTAT AGTGTCAAAA GCATGAATTT TTGGAACTAA AGACCCTACG TATGACGGTG ATCGTAGTAG   
  
  
+ ACTGAAACGG GAAGAGAGAG AGAAGGAAGG GAGTAACAAC ACATTTAAGA CAATAAACGT CATGGACTTG   
  
  
+ TTTGACATGT AAAATTCACT AATTGTAAAA GCAGAAAAAC CAAAGTGAAC AAAAGTCTAG AAGGATAATA   
  
  
+ TTTCCTACAG AACCTCCGAC GATTTTATGA CTTATCATCT ACCTTCCATA TTCGGTACGA GGCTGTCGAA   
  
  
+ TACTTGTAAT AAAAACGGGA AGAGTTCTCC TAATTCAAAA AAGTGAAAAG ATTTACTATA AAAACAGGTT   
  
  
+ CCGTCTAAAT CGATATCCCA CTTGTAACAA AACGACGAAC GAACTTACGA TCGAAAAGGG TCAACTTTAC   
  
  
+ AAAGGATTGC GAATTACCAA TAACGATAAC GTCTCCGTTC TCCGTAACTA GATGTATGAG ACAATACTGT   
  
  
+ AAGATAAACC CGTCCACTAT TGAAACATG  

- TTGCAGTCTT TGAGTTCTCC ATTCTTTCTT CTTTCTTGGG GGAGGAAGCT TTGGATTTGT CAAAAGAGCC   
  
  
- TGTGGGGGCC CTTGCTATCA ATTCAGCTTC TTAATGACAA AGTGATGTTA ATTTTATCTG TTCTCTCTTG   
  
  
- ATTTGGGATG TTCTAGTTTG TATGACCATA CGAAAACTAG TTGATACAAA GTCAATCAAG TAAGCTAAAC   
  
  
- AAAGAAGGTA ACATGTCTTA AAGATAAGAC AAGATTAGAA TAATGAGCTC ATATGTGAAA ACAAAAGTGC   
  
  
- AAAAAAGCTT CATAATCTTT GCACATTCAT GTTTTTATTT CATGTTAATC AATTAATTCT TTTTCGCTCA   
  
  
- TCTATTAGAA CCTAATTTAT ACCTTTCTTG GCATGCTGTA GGTGTGCTTG GGGTTATCCG ATAAAACGGT   
  
  
- GCGTTTTGAT AACAGCGGAA AATGCTACGC CTTAATTTTT GCGAGTAGGG ATGGCTCCGG CTCCGTTCTA   
  
  
- GTCTAGTGAG CTTCCCTTCG TCTCTCGGTT GTGAGCCATG GTGCGCAGGA AAGCAAGTGC ACCCGCAGGA   
  
  
- GGCGCCGAGA AATCGCAAAA GCAGCGGCTT AGCGGCGCCG ACCGGTCGGC TTACTTTGCC AGGAGGGAAG   
  
  
- CGGCGAAGGT GTTGCGATGC GTTCTCGAAG GCGACGCTCG TCGGCAAGCC GTTGGCTCTA TCAAGTCGCT   
  
  
- CGTCTACAGC CCTTCCGTGA GGAACAAAAG GGCAACTTTC GCTCTTGTTT GTCAAACTCT CAAGTGTAAG   
  
  
- TTACCAATTT TGTATTTCTG TCTGTTAGGT GTACTTTCGG TTGCTGTTTA GTTGGCTTTT CTGGGAGCTT   
  
  
- TCTGCAAGTA ACTTAGTTTT CTTTTGAAAT TCTTTTGGGT TTGGGAGCCT AGGGAGTAAG AGCTTGACGA   
  
  
- AAGATTTTGG GTGTAAAGTT GAACTTTCCT TATGATCTGT TTTTGGTCTT TGGTGTTTTT TTAGGCAGCT   
  
  
- CAAACTCATA TCACAGTTTT CGTACTTAAA AACCTTGATT TCTGGGATGC ATACTGCCAC TAGCATCATC   
  
  
- TGACTTTGCC CTTCTCTCTC TCTTCCTTCC CTCATTGTTG TGTAAATTCT GTTATTTGCA GTACCTGAAC   
  
  
- AAACTGTACA TTTTAAGTGA TTAACATTTT CGTCTTTTTG GTTTCACTTG TTTTCAGATC TTCCTATTAT   
  
  
- AAAGGATGTC TTGGAGGCTG CTAAAATACT GAATAGTAGA TGGAAGGTAT AAGCCATGCT CCGACAGCTT   
  
  
- ATGAACATTA TTTTTGCCCT TCTCAAGAGG ATTAAGTTTT TTCACTTTTC TAAATGATAT TTTTGTCCAA   
  
  
- GGCAGATTTA GCTATAGGGT GAACATTGTT TTGCTGCTTG CTTGAATGCT AGCTTTTCCC AGTTGAAATG   
  
  
- TTTCCTAACG CTTAATGGTT ATTGCTATTG CAGAGGCAAG AGGCATTGAT CTACATACTC TGTTATGACA   
  
  
- TTCTATTTGG GCAGGTGATA ACTTTGTAC
