# Supplementary material for: Genome-wide analysis of the WRKY gene family in drumstick (Moringa oleifera Lam.)
Source: PeerJ. 2019 Jun 10;7:e7063. doi: 10.7717/peerj.7063 (PMC6563795; doi:10.7717/peerj.7063)
Supplement: Supplemental Information 1 [file peerj-07-7063-s003.gz › MoWRKY25_plantcare.html]

Content-Type: text/html; charset=ISO-8859-1


CallMat\_Firefox


Webmaster Firefox specific output  
To save the result:
click on the frame with the right mouse button and save the source code as a text file with extension .html  
REFERENCE:PlantCARE: a database of plant cis-acting regulatory elements and a portal to tools for in silico analysis of promoter sequences.  
Lescot, M., Déhais, P., Moreau, Y., De Moor, B., Rouzé ,P.,and Rombauts, S.  
Nucleic Acids Res., Database issue(2002), 30(1):325-327.   


---

> 2018/04/13 10:10:12  
+ TTTTATTAAA AATATTTTTG AAAAATAATA TATAAGTAAA CATCCCCCAG TTACCAATCA ACTGTACTTC   
  
  
+ ACCTATACTA GTACTAGTAC TTACTCGTGT AAGTACTTTT TAATTATCAT CACCATCCAA TCTATCACCG   
  
  
+ ACATCCTTAA GACTTTAGCT TCCTATTTAG ATTCACTATT CCCCCGAAGA CAAAGATTTA CGTGATTTCT   
  
  
+ CTTTCCCTGT TATAATACAA ACAAGACACC CATCAAACTC CCAAACGGAT CAATCTGATT TCCACAACCT   
  
  
+ AAGAACAGCA ATGCTATGCT AGATTTTGTC TGATTCGCTT CCTATCATCG GATCCTAATT GCACCGACCC   
  
  
+ GATAATTCAT CTCATTGACC ATAATTTCGG GTCATATCAG TACCCTTTTC TACTACTCAA TCGTTACTTA   
  
  
+ ACATCTAAAC TAGCTGGTGG GTATTAATAT ATATTTTAAA TGTTTAGAAT AATCTTTATT TAATATATCC   
  
  
+ AACTTGGGCC TCTTTTTTTT TTTTTAATTT AGGGGAAAAA TAAATCAACT TATAATAAGG CCAAGAGATT   
  
  
+ ATAGAAAGAA AATGAGGCCA GTTGGTGAAA CGAACTTTCG GAGGAAGTCA ACATTTAGGC AGCCAACTAC   
  
  
+ ATCATGTTTG TCTTCTACGT TCTTCATATC TTTGGGTCGT AGGTGTTCCT TCGGCTTTCC GTCTTTGACT   
  
  
+ TTTTGTCTGT AAGCAGTCAA CATTCGTATC AAATCCCGCA CGTGCCTCAA ACGCAATTAT GCTCCAACCA   
  
  
+ AATCAAGACT AGATCAAGTT CATTTAGTGA GACAATGTTT GTTAGGACAA CTGGGTCGTC GATCCAACCT   
  
  
+ CGTGACTCTC GGTTGGAGGA TTGCAGCTTA AACGACATCG GTTGGTTGAA GAGGATTGTT ATGGGGTTTC   
  
  
+ TAGGTAATAT GTAAATTTAG AGGAAGTGGG GGTAGAAATT GAATTTATTA TAACAGCGGG GGCATGTTAG   
  
  
+ GAAAAGTTTC TGCTAATCTG CCATATCTGG GTGTCTAAAG TCAACTGCAT CCTGCTGGCA CCAACCAAAA   
  
  
+ CACTCCTGTA CTTAAAAGCA CCCTGACGCA GATCAGACGG CTCAGCACGA AAGTTTGACC TGATCTCCTT   
  
  
+ CCATGTCCAC AACGTCCCGT GGACCGTCAT AACCCTTGTG GGTCCCGCAC GCCAGACGAG GTCAATGTCC   
  
  
+ TTATGTATAT CACCACAGCT TTAATAGAAA AAAGAAATAA AATATTAAAA AACAATTTAT GTGTGCACAG   
  
  
+ CAGCCAAAAA AGCACGATGT GGTTGGATCT TATTCGCACC AACATTTGAC GGCAAAGGAT AGTGTGACAG   
  
  
+ GTCAGTGACC TAATCGACCA ATAGCATCTC GTAGCCTGGT ACTCATCTTT TTTGACCGGT GTCCCTTCCC   
  
  
+ TTTATCAACA GCACCCCTCT CTCCATTTTC TTTTCACGCT GTTCTCTCTT TTCTCTCCCT TTCTCTTTCT   
  
  
+ TTTCTTTCAT GGTGGTGGTG TTGCAGAGA  

- AAAATAATTT TTATAAAAAC TTTTTATTAT ATATTCATTT GTAGGGGGTC AATGGTTAGT TGACATGAAG   
  
  
- TGGATATGAT CATGATCATG AATGAGCACA TTCATGAAAA ATTAATAGTA GTGGTAGGTT AGATAGTGGC   
  
  
- TGTAGGAATT CTGAAATCGA AGGATAAATC TAAGTGATAA GGGGGCTTCT GTTTCTAAAT GCACTAAAGA   
  
  
- GAAAGGGACA ATATTATGTT TGTTCTGTGG GTAGTTTGAG GGTTTGCCTA GTTAGACTAA AGGTGTTGGA   
  
  
- TTCTTGTCGT TACGATACGA TCTAAAACAG ACTAAGCGAA GGATAGTAGC CTAGGATTAA CGTGGCTGGG   
  
  
- CTATTAAGTA GAGTAACTGG TATTAAAGCC CAGTATAGTC ATGGGAAAAG ATGATGAGTT AGCAATGAAT   
  
  
- TGTAGATTTG ATCGACCACC CATAATTATA TATAAAATTT ACAAATCTTA TTAGAAATAA ATTATATAGG   
  
  
- TTGAACCCGG AGAAAAAAAA AAAAATTAAA TCCCCTTTTT ATTTAGTTGA ATATTATTCC GGTTCTCTAA   
  
  
- TATCTTTCTT TTACTCCGGT CAACCACTTT GCTTGAAAGC CTCCTTCAGT TGTAAATCCG TCGGTTGATG   
  
  
- TAGTACAAAC AGAAGATGCA AGAAGTATAG AAACCCAGCA TCCACAAGGA AGCCGAAAGG CAGAAACTGA   
  
  
- AAAACAGACA TTCGTCAGTT GTAAGCATAG TTTAGGGCGT GCACGGAGTT TGCGTTAATA CGAGGTTGGT   
  
  
- TTAGTTCTGA TCTAGTTCAA GTAAATCACT CTGTTACAAA CAATCCTGTT GACCCAGCAG CTAGGTTGGA   
  
  
- GCACTGAGAG CCAACCTCCT AACGTCGAAT TTGCTGTAGC CAACCAACTT CTCCTAACAA TACCCCAAAG   
  
  
- ATCCATTATA CATTTAAATC TCCTTCACCC CCATCTTTAA CTTAAATAAT ATTGTCGCCC CCGTACAATC   
  
  
- CTTTTCAAAG ACGATTAGAC GGTATAGACC CACAGATTTC AGTTGACGTA GGACGACCGT GGTTGGTTTT   
  
  
- GTGAGGACAT GAATTTTCGT GGGACTGCGT CTAGTCTGCC GAGTCGTGCT TTCAAACTGG ACTAGAGGAA   
  
  
- GGTACAGGTG TTGCAGGGCA CCTGGCAGTA TTGGGAACAC CCAGGGCGTG CGGTCTGCTC CAGTTACAGG   
  
  
- AATACATATA GTGGTGTCGA AATTATCTTT TTTCTTTATT TTATAATTTT TTGTTAAATA CACACGTGTC   
  
  
- GTCGGTTTTT TCGTGCTACA CCAACCTAGA ATAAGCGTGG TTGTAAACTG CCGTTTCCTA TCACACTGTC   
  
  
- CAGTCACTGG ATTAGCTGGT TATCGTAGAG CATCGGACCA TGAGTAGAAA AAACTGGCCA CAGGGAAGGG   
  
  
- AAATAGTTGT CGTGGGGAGA GAGGTAAAAG AAAAGTGCGA CAAGAGAGAA AAGAGAGGGA AAGAGAAAGA   
  
  
- AAAGAAAGTA CCACCACCAC AACGTCTCT

  
  
Motifs Found  

+     5UTR Py-rich stretch

| Site Name | Organism | Position | Strand | Matrix score. | sequence | function |
| --- | --- | --- | --- | --- | --- | --- |
| 5UTR Py-rich stretch | Lycopersicon esculentum | 1466 | + | 9 | TTTCTTCTCT | cis-acting element conferring high transcription levels |

> 2018/04/13 10:10:12  
+ TTTTATTAAA AATATTTTTG AAAAATAATA TATAAGTAAA CATCCCCCAG TTACCAATCA ACTGTACTTC   
  
  
+ ACCTATACTA GTACTAGTAC TTACTCGTGT AAGTACTTTT TAATTATCAT CACCATCCAA TCTATCACCG   
  
  
+ ACATCCTTAA GACTTTAGCT TCCTATTTAG ATTCACTATT CCCCCGAAGA CAAAGATTTA CGTGATTTCT   
  
  
+ CTTTCCCTGT TATAATACAA ACAAGACACC CATCAAACTC CCAAACGGAT CAATCTGATT TCCACAACCT   
  
  
+ AAGAACAGCA ATGCTATGCT AGATTTTGTC TGATTCGCTT CCTATCATCG GATCCTAATT GCACCGACCC   
  
  
+ GATAATTCAT CTCATTGACC ATAATTTCGG GTCATATCAG TACCCTTTTC TACTACTCAA TCGTTACTTA   
  
  
+ ACATCTAAAC TAGCTGGTGG GTATTAATAT ATATTTTAAA TGTTTAGAAT AATCTTTATT TAATATATCC   
  
  
+ AACTTGGGCC TCTTTTTTTT TTTTTAATTT AGGGGAAAAA TAAATCAACT TATAATAAGG CCAAGAGATT   
  
  
+ ATAGAAAGAA AATGAGGCCA GTTGGTGAAA CGAACTTTCG GAGGAAGTCA ACATTTAGGC AGCCAACTAC   
  
  
+ ATCATGTTTG TCTTCTACGT TCTTCATATC TTTGGGTCGT AGGTGTTCCT TCGGCTTTCC GTCTTTGACT   
  
  
+ TTTTGTCTGT AAGCAGTCAA CATTCGTATC AAATCCCGCA CGTGCCTCAA ACGCAATTAT GCTCCAACCA   
  
  
+ AATCAAGACT AGATCAAGTT CATTTAGTGA GACAATGTTT GTTAGGACAA CTGGGTCGTC GATCCAACCT   
  
  
+ CGTGACTCTC GGTTGGAGGA TTGCAGCTTA AACGACATCG GTTGGTTGAA GAGGATTGTT ATGGGGTTTC   
  
  
+ TAGGTAATAT GTAAATTTAG AGGAAGTGGG GGTAGAAATT GAATTTATTA TAACAGCGGG GGCATGTTAG   
  
  
+ GAAAAGTTTC TGCTAATCTG CCATATCTGG GTGTCTAAAG TCAACTGCAT CCTGCTGGCA CCAACCAAAA   
  
  
+ CACTCCTGTA CTTAAAAGCA CCCTGACGCA GATCAGACGG CTCAGCACGA AAGTTTGACC TGATCTCCTT   
  
  
+ CCATGTCCAC AACGTCCCGT GGACCGTCAT AACCCTTGTG GGTCCCGCAC GCCAGACGAG GTCAATGTCC   
  
  
+ TTATGTATAT CACCACAGCT TTAATAGAAA AAAGAAATAA AATATTAAAA AACAATTTAT GTGTGCACAG   
  
  
+ CAGCCAAAAA AGCACGATGT GGTTGGATCT TATTCGCACC AACATTTGAC GGCAAAGGAT AGTGTGACAG   
  
  
+ GTCAGTGACC TAATCGACCA ATAGCATCTC GTAGCCTGGT ACTCATCTTT TTTGACCGGT GTCCCTTCCC   
  
  
+ TTTATCAACA GCACCCCTCT CTCCATTTTC TTTTCACGCT GTTCTCTCTT TTCTCTCCCT TTCTCTTTCT   
  
  
+ TTTCTTTCAT GGTGGTGGTG TTGCAGAGA  

- AAAATAATTT TTATAAAAAC TTTTTATTAT ATATTCATTT GTAGGGGGTC AATGGTTAGT TGACATGAAG   
  
  
- TGGATATGAT CATGATCATG AATGAGCACA TTCATGAAAA ATTAATAGTA GTGGTAGGTT AGATAGTGGC   
  
  
- TGTAGGAATT CTGAAATCGA AGGATAAATC TAAGTGATAA GGGGGCTTCT GTTTCTAAAT GCACTAAAGA   
  
  
- GAAAGGGACA ATATTATGTT TGTTCTGTGG GTAGTTTGAG GGTTTGCCTA GTTAGACTAA AGGTGTTGGA   
  
  
- TTCTTGTCGT TACGATACGA TCTAAAACAG ACTAAGCGAA GGATAGTAGC CTAGGATTAA CGTGGCTGGG   
  
  
- CTATTAAGTA GAGTAACTGG TATTAAAGCC CAGTATAGTC ATGGGAAAAG ATGATGAGTT AGCAATGAAT   
  
  
- TGTAGATTTG ATCGACCACC CATAATTATA TATAAAATTT ACAAATCTTA TTAGAAATAA ATTATATAGG   
  
  
- TTGAACCCGG AGAAAAAAAA AAAAATTAAA TCCCCTTTTT ATTTAGTTGA ATATTATTCC GGTTCTCTAA   
  
  
- TATCTTTCTT TTACTCCGGT CAACCACTTT GCTTGAAAGC CTCCTTCAGT TGTAAATCCG TCGGTTGATG   
  
  
- TAGTACAAAC AGAAGATGCA AGAAGTATAG AAACCCAGCA TCCACAAGGA AGCCGAAAGG CAGAAACTGA   
  
  
- AAAACAGACA TTCGTCAGTT GTAAGCATAG TTTAGGGCGT GCACGGAGTT TGCGTTAATA CGAGGTTGGT   
  
  
- TTAGTTCTGA TCTAGTTCAA GTAAATCACT CTGTTACAAA CAATCCTGTT GACCCAGCAG CTAGGTTGGA   
  
  
- GCACTGAGAG CCAACCTCCT AACGTCGAAT TTGCTGTAGC CAACCAACTT CTCCTAACAA TACCCCAAAG   
  
  
- ATCCATTATA CATTTAAATC TCCTTCACCC CCATCTTTAA CTTAAATAAT ATTGTCGCCC CCGTACAATC   
  
  
- CTTTTCAAAG ACGATTAGAC GGTATAGACC CACAGATTTC AGTTGACGTA GGACGACCGT GGTTGGTTTT   
  
  
- GTGAGGACAT GAATTTTCGT GGGACTGCGT CTAGTCTGCC GAGTCGTGCT TTCAAACTGG ACTAGAGGAA   
  
  
- GGTACAGGTG TTGCAGGGCA CCTGGCAGTA TTGGGAACAC CCAGGGCGTG CGGTCTGCTC CAGTTACAGG   
  
  
- AATACATATA GTGGTGTCGA AATTATCTTT TTTCTTTATT TTATAATTTT TTGTTAAATA CACACGTGTC   
  
  
- GTCGGTTTTT TCGTGCTACA CCAACCTAGA ATAAGCGTGG TTGTAAACTG CCGTTTCCTA TCACACTGTC   
  
  
- CAGTCACTGG ATTAGCTGGT TATCGTAGAG CATCGGACCA TGAGTAGAAA AAACTGGCCA CAGGGAAGGG   
  
  
- AAATAGTTGT CGTGGGGAGA GAGGTAAAAG AAAAGTGCGA CAAGAGAGAA AAGAGAGGGA AAGAGAAAGA   
  
  
- AAAGAAAGTA CCACCACCAC AACGTCTCT

+     AAGAA-motif

| Site Name | Organism | Position | Strand | Matrix score. | sequence | function |
| --- | --- | --- | --- | --- | --- | --- |
| AAGAA-motif | Avena sativa | 1472 | - | 7 | GAAAGAA |  |
| AAGAA-motif | Avena sativa | 564 | + | 7 | GAAAGAA |  |

> 2018/04/13 10:10:12  
+ TTTTATTAAA AATATTTTTG AAAAATAATA TATAAGTAAA CATCCCCCAG TTACCAATCA ACTGTACTTC   
  
  
+ ACCTATACTA GTACTAGTAC TTACTCGTGT AAGTACTTTT TAATTATCAT CACCATCCAA TCTATCACCG   
  
  
+ ACATCCTTAA GACTTTAGCT TCCTATTTAG ATTCACTATT CCCCCGAAGA CAAAGATTTA CGTGATTTCT   
  
  
+ CTTTCCCTGT TATAATACAA ACAAGACACC CATCAAACTC CCAAACGGAT CAATCTGATT TCCACAACCT   
  
  
+ AAGAACAGCA ATGCTATGCT AGATTTTGTC TGATTCGCTT CCTATCATCG GATCCTAATT GCACCGACCC   
  
  
+ GATAATTCAT CTCATTGACC ATAATTTCGG GTCATATCAG TACCCTTTTC TACTACTCAA TCGTTACTTA   
  
  
+ ACATCTAAAC TAGCTGGTGG GTATTAATAT ATATTTTAAA TGTTTAGAAT AATCTTTATT TAATATATCC   
  
  
+ AACTTGGGCC TCTTTTTTTT TTTTTAATTT AGGGGAAAAA TAAATCAACT TATAATAAGG CCAAGAGATT   
  
  
+ ATAGAAAGAA AATGAGGCCA GTTGGTGAAA CGAACTTTCG GAGGAAGTCA ACATTTAGGC AGCCAACTAC   
  
  
+ ATCATGTTTG TCTTCTACGT TCTTCATATC TTTGGGTCGT AGGTGTTCCT TCGGCTTTCC GTCTTTGACT   
  
  
+ TTTTGTCTGT AAGCAGTCAA CATTCGTATC AAATCCCGCA CGTGCCTCAA ACGCAATTAT GCTCCAACCA   
  
  
+ AATCAAGACT AGATCAAGTT CATTTAGTGA GACAATGTTT GTTAGGACAA CTGGGTCGTC GATCCAACCT   
  
  
+ CGTGACTCTC GGTTGGAGGA TTGCAGCTTA AACGACATCG GTTGGTTGAA GAGGATTGTT ATGGGGTTTC   
  
  
+ TAGGTAATAT GTAAATTTAG AGGAAGTGGG GGTAGAAATT GAATTTATTA TAACAGCGGG GGCATGTTAG   
  
  
+ GAAAAGTTTC TGCTAATCTG CCATATCTGG GTGTCTAAAG TCAACTGCAT CCTGCTGGCA CCAACCAAAA   
  
  
+ CACTCCTGTA CTTAAAAGCA CCCTGACGCA GATCAGACGG CTCAGCACGA AAGTTTGACC TGATCTCCTT   
  
  
+ CCATGTCCAC AACGTCCCGT GGACCGTCAT AACCCTTGTG GGTCCCGCAC GCCAGACGAG GTCAATGTCC   
  
  
+ TTATGTATAT CACCACAGCT TTAATAGAAA AAAGAAATAA AATATTAAAA AACAATTTAT GTGTGCACAG   
  
  
+ CAGCCAAAAA AGCACGATGT GGTTGGATCT TATTCGCACC AACATTTGAC GGCAAAGGAT AGTGTGACAG   
  
  
+ GTCAGTGACC TAATCGACCA ATAGCATCTC GTAGCCTGGT ACTCATCTTT TTTGACCGGT GTCCCTTCCC   
  
  
+ TTTATCAACA GCACCCCTCT CTCCATTTTC TTTTCACGCT GTTCTCTCTT TTCTCTCCCT TTCTCTTTCT   
  
  
+ TTTCTTTCAT GGTGGTGGTG TTGCAGAGA  

- AAAATAATTT TTATAAAAAC TTTTTATTAT ATATTCATTT GTAGGGGGTC AATGGTTAGT TGACATGAAG   
  
  
- TGGATATGAT CATGATCATG AATGAGCACA TTCATGAAAA ATTAATAGTA GTGGTAGGTT AGATAGTGGC   
  
  
- TGTAGGAATT CTGAAATCGA AGGATAAATC TAAGTGATAA GGGGGCTTCT GTTTCTAAAT GCACTAAAGA   
  
  
- GAAAGGGACA ATATTATGTT TGTTCTGTGG GTAGTTTGAG GGTTTGCCTA GTTAGACTAA AGGTGTTGGA   
  
  
- TTCTTGTCGT TACGATACGA TCTAAAACAG ACTAAGCGAA GGATAGTAGC CTAGGATTAA CGTGGCTGGG   
  
  
- CTATTAAGTA GAGTAACTGG TATTAAAGCC CAGTATAGTC ATGGGAAAAG ATGATGAGTT AGCAATGAAT   
  
  
- TGTAGATTTG ATCGACCACC CATAATTATA TATAAAATTT ACAAATCTTA TTAGAAATAA ATTATATAGG   
  
  
- TTGAACCCGG AGAAAAAAAA AAAAATTAAA TCCCCTTTTT ATTTAGTTGA ATATTATTCC GGTTCTCTAA   
  
  
- TATCTTTCTT TTACTCCGGT CAACCACTTT GCTTGAAAGC CTCCTTCAGT TGTAAATCCG TCGGTTGATG   
  
  
- TAGTACAAAC AGAAGATGCA AGAAGTATAG AAACCCAGCA TCCACAAGGA AGCCGAAAGG CAGAAACTGA   
  
  
- AAAACAGACA TTCGTCAGTT GTAAGCATAG TTTAGGGCGT GCACGGAGTT TGCGTTAATA CGAGGTTGGT   
  
  
- TTAGTTCTGA TCTAGTTCAA GTAAATCACT CTGTTACAAA CAATCCTGTT GACCCAGCAG CTAGGTTGGA   
  
  
- GCACTGAGAG CCAACCTCCT AACGTCGAAT TTGCTGTAGC CAACCAACTT CTCCTAACAA TACCCCAAAG   
  
  
- ATCCATTATA CATTTAAATC TCCTTCACCC CCATCTTTAA CTTAAATAAT ATTGTCGCCC CCGTACAATC   
  
  
- CTTTTCAAAG ACGATTAGAC GGTATAGACC CACAGATTTC AGTTGACGTA GGACGACCGT GGTTGGTTTT   
  
  
- GTGAGGACAT GAATTTTCGT GGGACTGCGT CTAGTCTGCC GAGTCGTGCT TTCAAACTGG ACTAGAGGAA   
  
  
- GGTACAGGTG TTGCAGGGCA CCTGGCAGTA TTGGGAACAC CCAGGGCGTG CGGTCTGCTC CAGTTACAGG   
  
  
- AATACATATA GTGGTGTCGA AATTATCTTT TTTCTTTATT TTATAATTTT TTGTTAAATA CACACGTGTC   
  
  
- GTCGGTTTTT TCGTGCTACA CCAACCTAGA ATAAGCGTGG TTGTAAACTG CCGTTTCCTA TCACACTGTC   
  
  
- CAGTCACTGG ATTAGCTGGT TATCGTAGAG CATCGGACCA TGAGTAGAAA AAACTGGCCA CAGGGAAGGG   
  
  
- AAATAGTTGT CGTGGGGAGA GAGGTAAAAG AAAAGTGCGA CAAGAGAGAA AAGAGAGGGA AAGAGAAAGA   
  
  
- AAAGAAAGTA CCACCACCAC AACGTCTCT

+     ABRE

| Site Name | Organism | Position | Strand | Matrix score. | sequence | function |
| --- | --- | --- | --- | --- | --- | --- |
| ABRE | Hordeum vulgare | 737 | + | 9 | CGCACGTGTC | cis-acting element involved in the abscisic acid responsiveness |
| ABRE | Arabidopsis thaliana | 199 | + | 6 | TACGTG | cis-acting element involved in the abscisic acid responsiveness |
| ABRE | Arabidopsis thaliana | 739 | + | 6 | CACGTG | cis-acting element involved in the abscisic acid responsiveness |

> 2018/04/13 10:10:12  
+ TTTTATTAAA AATATTTTTG AAAAATAATA TATAAGTAAA CATCCCCCAG TTACCAATCA ACTGTACTTC   
  
  
+ ACCTATACTA GTACTAGTAC TTACTCGTGT AAGTACTTTT TAATTATCAT CACCATCCAA TCTATCACCG   
  
  
+ ACATCCTTAA GACTTTAGCT TCCTATTTAG ATTCACTATT CCCCCGAAGA CAAAGATTTA CGTGATTTCT   
  
  
+ CTTTCCCTGT TATAATACAA ACAAGACACC CATCAAACTC CCAAACGGAT CAATCTGATT TCCACAACCT   
  
  
+ AAGAACAGCA ATGCTATGCT AGATTTTGTC TGATTCGCTT CCTATCATCG GATCCTAATT GCACCGACCC   
  
  
+ GATAATTCAT CTCATTGACC ATAATTTCGG GTCATATCAG TACCCTTTTC TACTACTCAA TCGTTACTTA   
  
  
+ ACATCTAAAC TAGCTGGTGG GTATTAATAT ATATTTTAAA TGTTTAGAAT AATCTTTATT TAATATATCC   
  
  
+ AACTTGGGCC TCTTTTTTTT TTTTTAATTT AGGGGAAAAA TAAATCAACT TATAATAAGG CCAAGAGATT   
  
  
+ ATAGAAAGAA AATGAGGCCA GTTGGTGAAA CGAACTTTCG GAGGAAGTCA ACATTTAGGC AGCCAACTAC   
  
  
+ ATCATGTTTG TCTTCTACGT TCTTCATATC TTTGGGTCGT AGGTGTTCCT TCGGCTTTCC GTCTTTGACT   
  
  
+ TTTTGTCTGT AAGCAGTCAA CATTCGTATC AAATCCCGCA CGTGCCTCAA ACGCAATTAT GCTCCAACCA   
  
  
+ AATCAAGACT AGATCAAGTT CATTTAGTGA GACAATGTTT GTTAGGACAA CTGGGTCGTC GATCCAACCT   
  
  
+ CGTGACTCTC GGTTGGAGGA TTGCAGCTTA AACGACATCG GTTGGTTGAA GAGGATTGTT ATGGGGTTTC   
  
  
+ TAGGTAATAT GTAAATTTAG AGGAAGTGGG GGTAGAAATT GAATTTATTA TAACAGCGGG GGCATGTTAG   
  
  
+ GAAAAGTTTC TGCTAATCTG CCATATCTGG GTGTCTAAAG TCAACTGCAT CCTGCTGGCA CCAACCAAAA   
  
  
+ CACTCCTGTA CTTAAAAGCA CCCTGACGCA GATCAGACGG CTCAGCACGA AAGTTTGACC TGATCTCCTT   
  
  
+ CCATGTCCAC AACGTCCCGT GGACCGTCAT AACCCTTGTG GGTCCCGCAC GCCAGACGAG GTCAATGTCC   
  
  
+ TTATGTATAT CACCACAGCT TTAATAGAAA AAAGAAATAA AATATTAAAA AACAATTTAT GTGTGCACAG   
  
  
+ CAGCCAAAAA AGCACGATGT GGTTGGATCT TATTCGCACC AACATTTGAC GGCAAAGGAT AGTGTGACAG   
  
  
+ GTCAGTGACC TAATCGACCA ATAGCATCTC GTAGCCTGGT ACTCATCTTT TTTGACCGGT GTCCCTTCCC   
  
  
+ TTTATCAACA GCACCCCTCT CTCCATTTTC TTTTCACGCT GTTCTCTCTT TTCTCTCCCT TTCTCTTTCT   
  
  
+ TTTCTTTCAT GGTGGTGGTG TTGCAGAGA  

- AAAATAATTT TTATAAAAAC TTTTTATTAT ATATTCATTT GTAGGGGGTC AATGGTTAGT TGACATGAAG   
  
  
- TGGATATGAT CATGATCATG AATGAGCACA TTCATGAAAA ATTAATAGTA GTGGTAGGTT AGATAGTGGC   
  
  
- TGTAGGAATT CTGAAATCGA AGGATAAATC TAAGTGATAA GGGGGCTTCT GTTTCTAAAT GCACTAAAGA   
  
  
- GAAAGGGACA ATATTATGTT TGTTCTGTGG GTAGTTTGAG GGTTTGCCTA GTTAGACTAA AGGTGTTGGA   
  
  
- TTCTTGTCGT TACGATACGA TCTAAAACAG ACTAAGCGAA GGATAGTAGC CTAGGATTAA CGTGGCTGGG   
  
  
- CTATTAAGTA GAGTAACTGG TATTAAAGCC CAGTATAGTC ATGGGAAAAG ATGATGAGTT AGCAATGAAT   
  
  
- TGTAGATTTG ATCGACCACC CATAATTATA TATAAAATTT ACAAATCTTA TTAGAAATAA ATTATATAGG   
  
  
- TTGAACCCGG AGAAAAAAAA AAAAATTAAA TCCCCTTTTT ATTTAGTTGA ATATTATTCC GGTTCTCTAA   
  
  
- TATCTTTCTT TTACTCCGGT CAACCACTTT GCTTGAAAGC CTCCTTCAGT TGTAAATCCG TCGGTTGATG   
  
  
- TAGTACAAAC AGAAGATGCA AGAAGTATAG AAACCCAGCA TCCACAAGGA AGCCGAAAGG CAGAAACTGA   
  
  
- AAAACAGACA TTCGTCAGTT GTAAGCATAG TTTAGGGCGT GCACGGAGTT TGCGTTAATA CGAGGTTGGT   
  
  
- TTAGTTCTGA TCTAGTTCAA GTAAATCACT CTGTTACAAA CAATCCTGTT GACCCAGCAG CTAGGTTGGA   
  
  
- GCACTGAGAG CCAACCTCCT AACGTCGAAT TTGCTGTAGC CAACCAACTT CTCCTAACAA TACCCCAAAG   
  
  
- ATCCATTATA CATTTAAATC TCCTTCACCC CCATCTTTAA CTTAAATAAT ATTGTCGCCC CCGTACAATC   
  
  
- CTTTTCAAAG ACGATTAGAC GGTATAGACC CACAGATTTC AGTTGACGTA GGACGACCGT GGTTGGTTTT   
  
  
- GTGAGGACAT GAATTTTCGT GGGACTGCGT CTAGTCTGCC GAGTCGTGCT TTCAAACTGG ACTAGAGGAA   
  
  
- GGTACAGGTG TTGCAGGGCA CCTGGCAGTA TTGGGAACAC CCAGGGCGTG CGGTCTGCTC CAGTTACAGG   
  
  
- AATACATATA GTGGTGTCGA AATTATCTTT TTTCTTTATT TTATAATTTT TTGTTAAATA CACACGTGTC   
  
  
- GTCGGTTTTT TCGTGCTACA CCAACCTAGA ATAAGCGTGG TTGTAAACTG CCGTTTCCTA TCACACTGTC   
  
  
- CAGTCACTGG ATTAGCTGGT TATCGTAGAG CATCGGACCA TGAGTAGAAA AAACTGGCCA CAGGGAAGGG   
  
  
- AAATAGTTGT CGTGGGGAGA GAGGTAAAAG AAAAGTGCGA CAAGAGAGAA AAGAGAGGGA AAGAGAAAGA   
  
  
- AAAGAAAGTA CCACCACCAC AACGTCTCT

+     AE-box

| Site Name | Organism | Position | Strand | Matrix score. | sequence | function |
| --- | --- | --- | --- | --- | --- | --- |
| AE-box | Arabidopsis thaliana | 984 | - | 8 | AGAAACTT | part of a module for light response |

> 2018/04/13 10:10:12  
+ TTTTATTAAA AATATTTTTG AAAAATAATA TATAAGTAAA CATCCCCCAG TTACCAATCA ACTGTACTTC   
  
  
+ ACCTATACTA GTACTAGTAC TTACTCGTGT AAGTACTTTT TAATTATCAT CACCATCCAA TCTATCACCG   
  
  
+ ACATCCTTAA GACTTTAGCT TCCTATTTAG ATTCACTATT CCCCCGAAGA CAAAGATTTA CGTGATTTCT   
  
  
+ CTTTCCCTGT TATAATACAA ACAAGACACC CATCAAACTC CCAAACGGAT CAATCTGATT TCCACAACCT   
  
  
+ AAGAACAGCA ATGCTATGCT AGATTTTGTC TGATTCGCTT CCTATCATCG GATCCTAATT GCACCGACCC   
  
  
+ GATAATTCAT CTCATTGACC ATAATTTCGG GTCATATCAG TACCCTTTTC TACTACTCAA TCGTTACTTA   
  
  
+ ACATCTAAAC TAGCTGGTGG GTATTAATAT ATATTTTAAA TGTTTAGAAT AATCTTTATT TAATATATCC   
  
  
+ AACTTGGGCC TCTTTTTTTT TTTTTAATTT AGGGGAAAAA TAAATCAACT TATAATAAGG CCAAGAGATT   
  
  
+ ATAGAAAGAA AATGAGGCCA GTTGGTGAAA CGAACTTTCG GAGGAAGTCA ACATTTAGGC AGCCAACTAC   
  
  
+ ATCATGTTTG TCTTCTACGT TCTTCATATC TTTGGGTCGT AGGTGTTCCT TCGGCTTTCC GTCTTTGACT   
  
  
+ TTTTGTCTGT AAGCAGTCAA CATTCGTATC AAATCCCGCA CGTGCCTCAA ACGCAATTAT GCTCCAACCA   
  
  
+ AATCAAGACT AGATCAAGTT CATTTAGTGA GACAATGTTT GTTAGGACAA CTGGGTCGTC GATCCAACCT   
  
  
+ CGTGACTCTC GGTTGGAGGA TTGCAGCTTA AACGACATCG GTTGGTTGAA GAGGATTGTT ATGGGGTTTC   
  
  
+ TAGGTAATAT GTAAATTTAG AGGAAGTGGG GGTAGAAATT GAATTTATTA TAACAGCGGG GGCATGTTAG   
  
  
+ GAAAAGTTTC TGCTAATCTG CCATATCTGG GTGTCTAAAG TCAACTGCAT CCTGCTGGCA CCAACCAAAA   
  
  
+ CACTCCTGTA CTTAAAAGCA CCCTGACGCA GATCAGACGG CTCAGCACGA AAGTTTGACC TGATCTCCTT   
  
  
+ CCATGTCCAC AACGTCCCGT GGACCGTCAT AACCCTTGTG GGTCCCGCAC GCCAGACGAG GTCAATGTCC   
  
  
+ TTATGTATAT CACCACAGCT TTAATAGAAA AAAGAAATAA AATATTAAAA AACAATTTAT GTGTGCACAG   
  
  
+ CAGCCAAAAA AGCACGATGT GGTTGGATCT TATTCGCACC AACATTTGAC GGCAAAGGAT AGTGTGACAG   
  
  
+ GTCAGTGACC TAATCGACCA ATAGCATCTC GTAGCCTGGT ACTCATCTTT TTTGACCGGT GTCCCTTCCC   
  
  
+ TTTATCAACA GCACCCCTCT CTCCATTTTC TTTTCACGCT GTTCTCTCTT TTCTCTCCCT TTCTCTTTCT   
  
  
+ TTTCTTTCAT GGTGGTGGTG TTGCAGAGA  

- AAAATAATTT TTATAAAAAC TTTTTATTAT ATATTCATTT GTAGGGGGTC AATGGTTAGT TGACATGAAG   
  
  
- TGGATATGAT CATGATCATG AATGAGCACA TTCATGAAAA ATTAATAGTA GTGGTAGGTT AGATAGTGGC   
  
  
- TGTAGGAATT CTGAAATCGA AGGATAAATC TAAGTGATAA GGGGGCTTCT GTTTCTAAAT GCACTAAAGA   
  
  
- GAAAGGGACA ATATTATGTT TGTTCTGTGG GTAGTTTGAG GGTTTGCCTA GTTAGACTAA AGGTGTTGGA   
  
  
- TTCTTGTCGT TACGATACGA TCTAAAACAG ACTAAGCGAA GGATAGTAGC CTAGGATTAA CGTGGCTGGG   
  
  
- CTATTAAGTA GAGTAACTGG TATTAAAGCC CAGTATAGTC ATGGGAAAAG ATGATGAGTT AGCAATGAAT   
  
  
- TGTAGATTTG ATCGACCACC CATAATTATA TATAAAATTT ACAAATCTTA TTAGAAATAA ATTATATAGG   
  
  
- TTGAACCCGG AGAAAAAAAA AAAAATTAAA TCCCCTTTTT ATTTAGTTGA ATATTATTCC GGTTCTCTAA   
  
  
- TATCTTTCTT TTACTCCGGT CAACCACTTT GCTTGAAAGC CTCCTTCAGT TGTAAATCCG TCGGTTGATG   
  
  
- TAGTACAAAC AGAAGATGCA AGAAGTATAG AAACCCAGCA TCCACAAGGA AGCCGAAAGG CAGAAACTGA   
  
  
- AAAACAGACA TTCGTCAGTT GTAAGCATAG TTTAGGGCGT GCACGGAGTT TGCGTTAATA CGAGGTTGGT   
  
  
- TTAGTTCTGA TCTAGTTCAA GTAAATCACT CTGTTACAAA CAATCCTGTT GACCCAGCAG CTAGGTTGGA   
  
  
- GCACTGAGAG CCAACCTCCT AACGTCGAAT TTGCTGTAGC CAACCAACTT CTCCTAACAA TACCCCAAAG   
  
  
- ATCCATTATA CATTTAAATC TCCTTCACCC CCATCTTTAA CTTAAATAAT ATTGTCGCCC CCGTACAATC   
  
  
- CTTTTCAAAG ACGATTAGAC GGTATAGACC CACAGATTTC AGTTGACGTA GGACGACCGT GGTTGGTTTT   
  
  
- GTGAGGACAT GAATTTTCGT GGGACTGCGT CTAGTCTGCC GAGTCGTGCT TTCAAACTGG ACTAGAGGAA   
  
  
- GGTACAGGTG TTGCAGGGCA CCTGGCAGTA TTGGGAACAC CCAGGGCGTG CGGTCTGCTC CAGTTACAGG   
  
  
- AATACATATA GTGGTGTCGA AATTATCTTT TTTCTTTATT TTATAATTTT TTGTTAAATA CACACGTGTC   
  
  
- GTCGGTTTTT TCGTGCTACA CCAACCTAGA ATAAGCGTGG TTGTAAACTG CCGTTTCCTA TCACACTGTC   
  
  
- CAGTCACTGG ATTAGCTGGT TATCGTAGAG CATCGGACCA TGAGTAGAAA AAACTGGCCA CAGGGAAGGG   
  
  
- AAATAGTTGT CGTGGGGAGA GAGGTAAAAG AAAAGTGCGA CAAGAGAGAA AAGAGAGGGA AAGAGAAAGA   
  
  
- AAAGAAAGTA CCACCACCAC AACGTCTCT

+     ATCC-motif

| Site Name | Organism | Position | Strand | Matrix score. | sequence | function |
| --- | --- | --- | --- | --- | --- | --- |
| ATCC-motif | Pisum sativum | 891 | - | 8 | CAATCCTC | part of a conserved DNA module involved in light responsiveness |
| ATCC-motif | Pisum sativum | 856 | - | 8 | CAATCCTC | part of a conserved DNA module involved in light responsiveness |

> 2018/04/13 10:10:12  
+ TTTTATTAAA AATATTTTTG AAAAATAATA TATAAGTAAA CATCCCCCAG TTACCAATCA ACTGTACTTC   
  
  
+ ACCTATACTA GTACTAGTAC TTACTCGTGT AAGTACTTTT TAATTATCAT CACCATCCAA TCTATCACCG   
  
  
+ ACATCCTTAA GACTTTAGCT TCCTATTTAG ATTCACTATT CCCCCGAAGA CAAAGATTTA CGTGATTTCT   
  
  
+ CTTTCCCTGT TATAATACAA ACAAGACACC CATCAAACTC CCAAACGGAT CAATCTGATT TCCACAACCT   
  
  
+ AAGAACAGCA ATGCTATGCT AGATTTTGTC TGATTCGCTT CCTATCATCG GATCCTAATT GCACCGACCC   
  
  
+ GATAATTCAT CTCATTGACC ATAATTTCGG GTCATATCAG TACCCTTTTC TACTACTCAA TCGTTACTTA   
  
  
+ ACATCTAAAC TAGCTGGTGG GTATTAATAT ATATTTTAAA TGTTTAGAAT AATCTTTATT TAATATATCC   
  
  
+ AACTTGGGCC TCTTTTTTTT TTTTTAATTT AGGGGAAAAA TAAATCAACT TATAATAAGG CCAAGAGATT   
  
  
+ ATAGAAAGAA AATGAGGCCA GTTGGTGAAA CGAACTTTCG GAGGAAGTCA ACATTTAGGC AGCCAACTAC   
  
  
+ ATCATGTTTG TCTTCTACGT TCTTCATATC TTTGGGTCGT AGGTGTTCCT TCGGCTTTCC GTCTTTGACT   
  
  
+ TTTTGTCTGT AAGCAGTCAA CATTCGTATC AAATCCCGCA CGTGCCTCAA ACGCAATTAT GCTCCAACCA   
  
  
+ AATCAAGACT AGATCAAGTT CATTTAGTGA GACAATGTTT GTTAGGACAA CTGGGTCGTC GATCCAACCT   
  
  
+ CGTGACTCTC GGTTGGAGGA TTGCAGCTTA AACGACATCG GTTGGTTGAA GAGGATTGTT ATGGGGTTTC   
  
  
+ TAGGTAATAT GTAAATTTAG AGGAAGTGGG GGTAGAAATT GAATTTATTA TAACAGCGGG GGCATGTTAG   
  
  
+ GAAAAGTTTC TGCTAATCTG CCATATCTGG GTGTCTAAAG TCAACTGCAT CCTGCTGGCA CCAACCAAAA   
  
  
+ CACTCCTGTA CTTAAAAGCA CCCTGACGCA GATCAGACGG CTCAGCACGA AAGTTTGACC TGATCTCCTT   
  
  
+ CCATGTCCAC AACGTCCCGT GGACCGTCAT AACCCTTGTG GGTCCCGCAC GCCAGACGAG GTCAATGTCC   
  
  
+ TTATGTATAT CACCACAGCT TTAATAGAAA AAAGAAATAA AATATTAAAA AACAATTTAT GTGTGCACAG   
  
  
+ CAGCCAAAAA AGCACGATGT GGTTGGATCT TATTCGCACC AACATTTGAC GGCAAAGGAT AGTGTGACAG   
  
  
+ GTCAGTGACC TAATCGACCA ATAGCATCTC GTAGCCTGGT ACTCATCTTT TTTGACCGGT GTCCCTTCCC   
  
  
+ TTTATCAACA GCACCCCTCT CTCCATTTTC TTTTCACGCT GTTCTCTCTT TTCTCTCCCT TTCTCTTTCT   
  
  
+ TTTCTTTCAT GGTGGTGGTG TTGCAGAGA  

- AAAATAATTT TTATAAAAAC TTTTTATTAT ATATTCATTT GTAGGGGGTC AATGGTTAGT TGACATGAAG   
  
  
- TGGATATGAT CATGATCATG AATGAGCACA TTCATGAAAA ATTAATAGTA GTGGTAGGTT AGATAGTGGC   
  
  
- TGTAGGAATT CTGAAATCGA AGGATAAATC TAAGTGATAA GGGGGCTTCT GTTTCTAAAT GCACTAAAGA   
  
  
- GAAAGGGACA ATATTATGTT TGTTCTGTGG GTAGTTTGAG GGTTTGCCTA GTTAGACTAA AGGTGTTGGA   
  
  
- TTCTTGTCGT TACGATACGA TCTAAAACAG ACTAAGCGAA GGATAGTAGC CTAGGATTAA CGTGGCTGGG   
  
  
- CTATTAAGTA GAGTAACTGG TATTAAAGCC CAGTATAGTC ATGGGAAAAG ATGATGAGTT AGCAATGAAT   
  
  
- TGTAGATTTG ATCGACCACC CATAATTATA TATAAAATTT ACAAATCTTA TTAGAAATAA ATTATATAGG   
  
  
- TTGAACCCGG AGAAAAAAAA AAAAATTAAA TCCCCTTTTT ATTTAGTTGA ATATTATTCC GGTTCTCTAA   
  
  
- TATCTTTCTT TTACTCCGGT CAACCACTTT GCTTGAAAGC CTCCTTCAGT TGTAAATCCG TCGGTTGATG   
  
  
- TAGTACAAAC AGAAGATGCA AGAAGTATAG AAACCCAGCA TCCACAAGGA AGCCGAAAGG CAGAAACTGA   
  
  
- AAAACAGACA TTCGTCAGTT GTAAGCATAG TTTAGGGCGT GCACGGAGTT TGCGTTAATA CGAGGTTGGT   
  
  
- TTAGTTCTGA TCTAGTTCAA GTAAATCACT CTGTTACAAA CAATCCTGTT GACCCAGCAG CTAGGTTGGA   
  
  
- GCACTGAGAG CCAACCTCCT AACGTCGAAT TTGCTGTAGC CAACCAACTT CTCCTAACAA TACCCCAAAG   
  
  
- ATCCATTATA CATTTAAATC TCCTTCACCC CCATCTTTAA CTTAAATAAT ATTGTCGCCC CCGTACAATC   
  
  
- CTTTTCAAAG ACGATTAGAC GGTATAGACC CACAGATTTC AGTTGACGTA GGACGACCGT GGTTGGTTTT   
  
  
- GTGAGGACAT GAATTTTCGT GGGACTGCGT CTAGTCTGCC GAGTCGTGCT TTCAAACTGG ACTAGAGGAA   
  
  
- GGTACAGGTG TTGCAGGGCA CCTGGCAGTA TTGGGAACAC CCAGGGCGTG CGGTCTGCTC CAGTTACAGG   
  
  
- AATACATATA GTGGTGTCGA AATTATCTTT TTTCTTTATT TTATAATTTT TTGTTAAATA CACACGTGTC   
  
  
- GTCGGTTTTT TCGTGCTACA CCAACCTAGA ATAAGCGTGG TTGTAAACTG CCGTTTCCTA TCACACTGTC   
  
  
- CAGTCACTGG ATTAGCTGGT TATCGTAGAG CATCGGACCA TGAGTAGAAA AAACTGGCCA CAGGGAAGGG   
  
  
- AAATAGTTGT CGTGGGGAGA GAGGTAAAAG AAAAGTGCGA CAAGAGAGAA AAGAGAGGGA AAGAGAAAGA   
  
  
- AAAGAAAGTA CCACCACCAC AACGTCTCT

+     Box 4

| Site Name | Organism | Position | Strand | Matrix score. | sequence | function |
| --- | --- | --- | --- | --- | --- | --- |
| Box 4 | Petroselinum crispum | 443 | + | 6 | ATTAAT | part of a conserved DNA module involved in light responsiveness |

> 2018/04/13 10:10:12  
+ TTTTATTAAA AATATTTTTG AAAAATAATA TATAAGTAAA CATCCCCCAG TTACCAATCA ACTGTACTTC   
  
  
+ ACCTATACTA GTACTAGTAC TTACTCGTGT AAGTACTTTT TAATTATCAT CACCATCCAA TCTATCACCG   
  
  
+ ACATCCTTAA GACTTTAGCT TCCTATTTAG ATTCACTATT CCCCCGAAGA CAAAGATTTA CGTGATTTCT   
  
  
+ CTTTCCCTGT TATAATACAA ACAAGACACC CATCAAACTC CCAAACGGAT CAATCTGATT TCCACAACCT   
  
  
+ AAGAACAGCA ATGCTATGCT AGATTTTGTC TGATTCGCTT CCTATCATCG GATCCTAATT GCACCGACCC   
  
  
+ GATAATTCAT CTCATTGACC ATAATTTCGG GTCATATCAG TACCCTTTTC TACTACTCAA TCGTTACTTA   
  
  
+ ACATCTAAAC TAGCTGGTGG GTATTAATAT ATATTTTAAA TGTTTAGAAT AATCTTTATT TAATATATCC   
  
  
+ AACTTGGGCC TCTTTTTTTT TTTTTAATTT AGGGGAAAAA TAAATCAACT TATAATAAGG CCAAGAGATT   
  
  
+ ATAGAAAGAA AATGAGGCCA GTTGGTGAAA CGAACTTTCG GAGGAAGTCA ACATTTAGGC AGCCAACTAC   
  
  
+ ATCATGTTTG TCTTCTACGT TCTTCATATC TTTGGGTCGT AGGTGTTCCT TCGGCTTTCC GTCTTTGACT   
  
  
+ TTTTGTCTGT AAGCAGTCAA CATTCGTATC AAATCCCGCA CGTGCCTCAA ACGCAATTAT GCTCCAACCA   
  
  
+ AATCAAGACT AGATCAAGTT CATTTAGTGA GACAATGTTT GTTAGGACAA CTGGGTCGTC GATCCAACCT   
  
  
+ CGTGACTCTC GGTTGGAGGA TTGCAGCTTA AACGACATCG GTTGGTTGAA GAGGATTGTT ATGGGGTTTC   
  
  
+ TAGGTAATAT GTAAATTTAG AGGAAGTGGG GGTAGAAATT GAATTTATTA TAACAGCGGG GGCATGTTAG   
  
  
+ GAAAAGTTTC TGCTAATCTG CCATATCTGG GTGTCTAAAG TCAACTGCAT CCTGCTGGCA CCAACCAAAA   
  
  
+ CACTCCTGTA CTTAAAAGCA CCCTGACGCA GATCAGACGG CTCAGCACGA AAGTTTGACC TGATCTCCTT   
  
  
+ CCATGTCCAC AACGTCCCGT GGACCGTCAT AACCCTTGTG GGTCCCGCAC GCCAGACGAG GTCAATGTCC   
  
  
+ TTATGTATAT CACCACAGCT TTAATAGAAA AAAGAAATAA AATATTAAAA AACAATTTAT GTGTGCACAG   
  
  
+ CAGCCAAAAA AGCACGATGT GGTTGGATCT TATTCGCACC AACATTTGAC GGCAAAGGAT AGTGTGACAG   
  
  
+ GTCAGTGACC TAATCGACCA ATAGCATCTC GTAGCCTGGT ACTCATCTTT TTTGACCGGT GTCCCTTCCC   
  
  
+ TTTATCAACA GCACCCCTCT CTCCATTTTC TTTTCACGCT GTTCTCTCTT TTCTCTCCCT TTCTCTTTCT   
  
  
+ TTTCTTTCAT GGTGGTGGTG TTGCAGAGA  

- AAAATAATTT TTATAAAAAC TTTTTATTAT ATATTCATTT GTAGGGGGTC AATGGTTAGT TGACATGAAG   
  
  
- TGGATATGAT CATGATCATG AATGAGCACA TTCATGAAAA ATTAATAGTA GTGGTAGGTT AGATAGTGGC   
  
  
- TGTAGGAATT CTGAAATCGA AGGATAAATC TAAGTGATAA GGGGGCTTCT GTTTCTAAAT GCACTAAAGA   
  
  
- GAAAGGGACA ATATTATGTT TGTTCTGTGG GTAGTTTGAG GGTTTGCCTA GTTAGACTAA AGGTGTTGGA   
  
  
- TTCTTGTCGT TACGATACGA TCTAAAACAG ACTAAGCGAA GGATAGTAGC CTAGGATTAA CGTGGCTGGG   
  
  
- CTATTAAGTA GAGTAACTGG TATTAAAGCC CAGTATAGTC ATGGGAAAAG ATGATGAGTT AGCAATGAAT   
  
  
- TGTAGATTTG ATCGACCACC CATAATTATA TATAAAATTT ACAAATCTTA TTAGAAATAA ATTATATAGG   
  
  
- TTGAACCCGG AGAAAAAAAA AAAAATTAAA TCCCCTTTTT ATTTAGTTGA ATATTATTCC GGTTCTCTAA   
  
  
- TATCTTTCTT TTACTCCGGT CAACCACTTT GCTTGAAAGC CTCCTTCAGT TGTAAATCCG TCGGTTGATG   
  
  
- TAGTACAAAC AGAAGATGCA AGAAGTATAG AAACCCAGCA TCCACAAGGA AGCCGAAAGG CAGAAACTGA   
  
  
- AAAACAGACA TTCGTCAGTT GTAAGCATAG TTTAGGGCGT GCACGGAGTT TGCGTTAATA CGAGGTTGGT   
  
  
- TTAGTTCTGA TCTAGTTCAA GTAAATCACT CTGTTACAAA CAATCCTGTT GACCCAGCAG CTAGGTTGGA   
  
  
- GCACTGAGAG CCAACCTCCT AACGTCGAAT TTGCTGTAGC CAACCAACTT CTCCTAACAA TACCCCAAAG   
  
  
- ATCCATTATA CATTTAAATC TCCTTCACCC CCATCTTTAA CTTAAATAAT ATTGTCGCCC CCGTACAATC   
  
  
- CTTTTCAAAG ACGATTAGAC GGTATAGACC CACAGATTTC AGTTGACGTA GGACGACCGT GGTTGGTTTT   
  
  
- GTGAGGACAT GAATTTTCGT GGGACTGCGT CTAGTCTGCC GAGTCGTGCT TTCAAACTGG ACTAGAGGAA   
  
  
- GGTACAGGTG TTGCAGGGCA CCTGGCAGTA TTGGGAACAC CCAGGGCGTG CGGTCTGCTC CAGTTACAGG   
  
  
- AATACATATA GTGGTGTCGA AATTATCTTT TTTCTTTATT TTATAATTTT TTGTTAAATA CACACGTGTC   
  
  
- GTCGGTTTTT TCGTGCTACA CCAACCTAGA ATAAGCGTGG TTGTAAACTG CCGTTTCCTA TCACACTGTC   
  
  
- CAGTCACTGG ATTAGCTGGT TATCGTAGAG CATCGGACCA TGAGTAGAAA AAACTGGCCA CAGGGAAGGG   
  
  
- AAATAGTTGT CGTGGGGAGA GAGGTAAAAG AAAAGTGCGA CAAGAGAGAA AAGAGAGGGA AAGAGAAAGA   
  
  
- AAAGAAAGTA CCACCACCAC AACGTCTCT

+     Box I

| Site Name | Organism | Position | Strand | Matrix score. | sequence | function |
| --- | --- | --- | --- | --- | --- | --- |
| Box I | Pisum sativum | 17 | - | 7 | TTTCAAA | light responsive element |

> 2018/04/13 10:10:12  
+ TTTTATTAAA AATATTTTTG AAAAATAATA TATAAGTAAA CATCCCCCAG TTACCAATCA ACTGTACTTC   
  
  
+ ACCTATACTA GTACTAGTAC TTACTCGTGT AAGTACTTTT TAATTATCAT CACCATCCAA TCTATCACCG   
  
  
+ ACATCCTTAA GACTTTAGCT TCCTATTTAG ATTCACTATT CCCCCGAAGA CAAAGATTTA CGTGATTTCT   
  
  
+ CTTTCCCTGT TATAATACAA ACAAGACACC CATCAAACTC CCAAACGGAT CAATCTGATT TCCACAACCT   
  
  
+ AAGAACAGCA ATGCTATGCT AGATTTTGTC TGATTCGCTT CCTATCATCG GATCCTAATT GCACCGACCC   
  
  
+ GATAATTCAT CTCATTGACC ATAATTTCGG GTCATATCAG TACCCTTTTC TACTACTCAA TCGTTACTTA   
  
  
+ ACATCTAAAC TAGCTGGTGG GTATTAATAT ATATTTTAAA TGTTTAGAAT AATCTTTATT TAATATATCC   
  
  
+ AACTTGGGCC TCTTTTTTTT TTTTTAATTT AGGGGAAAAA TAAATCAACT TATAATAAGG CCAAGAGATT   
  
  
+ ATAGAAAGAA AATGAGGCCA GTTGGTGAAA CGAACTTTCG GAGGAAGTCA ACATTTAGGC AGCCAACTAC   
  
  
+ ATCATGTTTG TCTTCTACGT TCTTCATATC TTTGGGTCGT AGGTGTTCCT TCGGCTTTCC GTCTTTGACT   
  
  
+ TTTTGTCTGT AAGCAGTCAA CATTCGTATC AAATCCCGCA CGTGCCTCAA ACGCAATTAT GCTCCAACCA   
  
  
+ AATCAAGACT AGATCAAGTT CATTTAGTGA GACAATGTTT GTTAGGACAA CTGGGTCGTC GATCCAACCT   
  
  
+ CGTGACTCTC GGTTGGAGGA TTGCAGCTTA AACGACATCG GTTGGTTGAA GAGGATTGTT ATGGGGTTTC   
  
  
+ TAGGTAATAT GTAAATTTAG AGGAAGTGGG GGTAGAAATT GAATTTATTA TAACAGCGGG GGCATGTTAG   
  
  
+ GAAAAGTTTC TGCTAATCTG CCATATCTGG GTGTCTAAAG TCAACTGCAT CCTGCTGGCA CCAACCAAAA   
  
  
+ CACTCCTGTA CTTAAAAGCA CCCTGACGCA GATCAGACGG CTCAGCACGA AAGTTTGACC TGATCTCCTT   
  
  
+ CCATGTCCAC AACGTCCCGT GGACCGTCAT AACCCTTGTG GGTCCCGCAC GCCAGACGAG GTCAATGTCC   
  
  
+ TTATGTATAT CACCACAGCT TTAATAGAAA AAAGAAATAA AATATTAAAA AACAATTTAT GTGTGCACAG   
  
  
+ CAGCCAAAAA AGCACGATGT GGTTGGATCT TATTCGCACC AACATTTGAC GGCAAAGGAT AGTGTGACAG   
  
  
+ GTCAGTGACC TAATCGACCA ATAGCATCTC GTAGCCTGGT ACTCATCTTT TTTGACCGGT GTCCCTTCCC   
  
  
+ TTTATCAACA GCACCCCTCT CTCCATTTTC TTTTCACGCT GTTCTCTCTT TTCTCTCCCT TTCTCTTTCT   
  
  
+ TTTCTTTCAT GGTGGTGGTG TTGCAGAGA  

- AAAATAATTT TTATAAAAAC TTTTTATTAT ATATTCATTT GTAGGGGGTC AATGGTTAGT TGACATGAAG   
  
  
- TGGATATGAT CATGATCATG AATGAGCACA TTCATGAAAA ATTAATAGTA GTGGTAGGTT AGATAGTGGC   
  
  
- TGTAGGAATT CTGAAATCGA AGGATAAATC TAAGTGATAA GGGGGCTTCT GTTTCTAAAT GCACTAAAGA   
  
  
- GAAAGGGACA ATATTATGTT TGTTCTGTGG GTAGTTTGAG GGTTTGCCTA GTTAGACTAA AGGTGTTGGA   
  
  
- TTCTTGTCGT TACGATACGA TCTAAAACAG ACTAAGCGAA GGATAGTAGC CTAGGATTAA CGTGGCTGGG   
  
  
- CTATTAAGTA GAGTAACTGG TATTAAAGCC CAGTATAGTC ATGGGAAAAG ATGATGAGTT AGCAATGAAT   
  
  
- TGTAGATTTG ATCGACCACC CATAATTATA TATAAAATTT ACAAATCTTA TTAGAAATAA ATTATATAGG   
  
  
- TTGAACCCGG AGAAAAAAAA AAAAATTAAA TCCCCTTTTT ATTTAGTTGA ATATTATTCC GGTTCTCTAA   
  
  
- TATCTTTCTT TTACTCCGGT CAACCACTTT GCTTGAAAGC CTCCTTCAGT TGTAAATCCG TCGGTTGATG   
  
  
- TAGTACAAAC AGAAGATGCA AGAAGTATAG AAACCCAGCA TCCACAAGGA AGCCGAAAGG CAGAAACTGA   
  
  
- AAAACAGACA TTCGTCAGTT GTAAGCATAG TTTAGGGCGT GCACGGAGTT TGCGTTAATA CGAGGTTGGT   
  
  
- TTAGTTCTGA TCTAGTTCAA GTAAATCACT CTGTTACAAA CAATCCTGTT GACCCAGCAG CTAGGTTGGA   
  
  
- GCACTGAGAG CCAACCTCCT AACGTCGAAT TTGCTGTAGC CAACCAACTT CTCCTAACAA TACCCCAAAG   
  
  
- ATCCATTATA CATTTAAATC TCCTTCACCC CCATCTTTAA CTTAAATAAT ATTGTCGCCC CCGTACAATC   
  
  
- CTTTTCAAAG ACGATTAGAC GGTATAGACC CACAGATTTC AGTTGACGTA GGACGACCGT GGTTGGTTTT   
  
  
- GTGAGGACAT GAATTTTCGT GGGACTGCGT CTAGTCTGCC GAGTCGTGCT TTCAAACTGG ACTAGAGGAA   
  
  
- GGTACAGGTG TTGCAGGGCA CCTGGCAGTA TTGGGAACAC CCAGGGCGTG CGGTCTGCTC CAGTTACAGG   
  
  
- AATACATATA GTGGTGTCGA AATTATCTTT TTTCTTTATT TTATAATTTT TTGTTAAATA CACACGTGTC   
  
  
- GTCGGTTTTT TCGTGCTACA CCAACCTAGA ATAAGCGTGG TTGTAAACTG CCGTTTCCTA TCACACTGTC   
  
  
- CAGTCACTGG ATTAGCTGGT TATCGTAGAG CATCGGACCA TGAGTAGAAA AAACTGGCCA CAGGGAAGGG   
  
  
- AAATAGTTGT CGTGGGGAGA GAGGTAAAAG AAAAGTGCGA CAAGAGAGAA AAGAGAGGGA AAGAGAAAGA   
  
  
- AAAGAAAGTA CCACCACCAC AACGTCTCT

+     Box-W1

| Site Name | Organism | Position | Strand | Matrix score. | sequence | function |
| --- | --- | --- | --- | --- | --- | --- |
| Box-W1 | Petroselinum crispum | 1382 | + | 6 | TTGACC | fungal elicitor responsive element |
| Box-W1 | Petroselinum crispum | 1180 | - | 6 | TTGACC | fungal elicitor responsive element |
| Box-W1 | Petroselinum crispum | 1105 | + | 6 | TTGACC | fungal elicitor responsive element |
| Box-W1 | Petroselinum crispum | 365 | + | 6 | TTGACC | fungal elicitor responsive element |

> 2018/04/13 10:10:12  
+ TTTTATTAAA AATATTTTTG AAAAATAATA TATAAGTAAA CATCCCCCAG TTACCAATCA ACTGTACTTC   
  
  
+ ACCTATACTA GTACTAGTAC TTACTCGTGT AAGTACTTTT TAATTATCAT CACCATCCAA TCTATCACCG   
  
  
+ ACATCCTTAA GACTTTAGCT TCCTATTTAG ATTCACTATT CCCCCGAAGA CAAAGATTTA CGTGATTTCT   
  
  
+ CTTTCCCTGT TATAATACAA ACAAGACACC CATCAAACTC CCAAACGGAT CAATCTGATT TCCACAACCT   
  
  
+ AAGAACAGCA ATGCTATGCT AGATTTTGTC TGATTCGCTT CCTATCATCG GATCCTAATT GCACCGACCC   
  
  
+ GATAATTCAT CTCATTGACC ATAATTTCGG GTCATATCAG TACCCTTTTC TACTACTCAA TCGTTACTTA   
  
  
+ ACATCTAAAC TAGCTGGTGG GTATTAATAT ATATTTTAAA TGTTTAGAAT AATCTTTATT TAATATATCC   
  
  
+ AACTTGGGCC TCTTTTTTTT TTTTTAATTT AGGGGAAAAA TAAATCAACT TATAATAAGG CCAAGAGATT   
  
  
+ ATAGAAAGAA AATGAGGCCA GTTGGTGAAA CGAACTTTCG GAGGAAGTCA ACATTTAGGC AGCCAACTAC   
  
  
+ ATCATGTTTG TCTTCTACGT TCTTCATATC TTTGGGTCGT AGGTGTTCCT TCGGCTTTCC GTCTTTGACT   
  
  
+ TTTTGTCTGT AAGCAGTCAA CATTCGTATC AAATCCCGCA CGTGCCTCAA ACGCAATTAT GCTCCAACCA   
  
  
+ AATCAAGACT AGATCAAGTT CATTTAGTGA GACAATGTTT GTTAGGACAA CTGGGTCGTC GATCCAACCT   
  
  
+ CGTGACTCTC GGTTGGAGGA TTGCAGCTTA AACGACATCG GTTGGTTGAA GAGGATTGTT ATGGGGTTTC   
  
  
+ TAGGTAATAT GTAAATTTAG AGGAAGTGGG GGTAGAAATT GAATTTATTA TAACAGCGGG GGCATGTTAG   
  
  
+ GAAAAGTTTC TGCTAATCTG CCATATCTGG GTGTCTAAAG TCAACTGCAT CCTGCTGGCA CCAACCAAAA   
  
  
+ CACTCCTGTA CTTAAAAGCA CCCTGACGCA GATCAGACGG CTCAGCACGA AAGTTTGACC TGATCTCCTT   
  
  
+ CCATGTCCAC AACGTCCCGT GGACCGTCAT AACCCTTGTG GGTCCCGCAC GCCAGACGAG GTCAATGTCC   
  
  
+ TTATGTATAT CACCACAGCT TTAATAGAAA AAAGAAATAA AATATTAAAA AACAATTTAT GTGTGCACAG   
  
  
+ CAGCCAAAAA AGCACGATGT GGTTGGATCT TATTCGCACC AACATTTGAC GGCAAAGGAT AGTGTGACAG   
  
  
+ GTCAGTGACC TAATCGACCA ATAGCATCTC GTAGCCTGGT ACTCATCTTT TTTGACCGGT GTCCCTTCCC   
  
  
+ TTTATCAACA GCACCCCTCT CTCCATTTTC TTTTCACGCT GTTCTCTCTT TTCTCTCCCT TTCTCTTTCT   
  
  
+ TTTCTTTCAT GGTGGTGGTG TTGCAGAGA  

- AAAATAATTT TTATAAAAAC TTTTTATTAT ATATTCATTT GTAGGGGGTC AATGGTTAGT TGACATGAAG   
  
  
- TGGATATGAT CATGATCATG AATGAGCACA TTCATGAAAA ATTAATAGTA GTGGTAGGTT AGATAGTGGC   
  
  
- TGTAGGAATT CTGAAATCGA AGGATAAATC TAAGTGATAA GGGGGCTTCT GTTTCTAAAT GCACTAAAGA   
  
  
- GAAAGGGACA ATATTATGTT TGTTCTGTGG GTAGTTTGAG GGTTTGCCTA GTTAGACTAA AGGTGTTGGA   
  
  
- TTCTTGTCGT TACGATACGA TCTAAAACAG ACTAAGCGAA GGATAGTAGC CTAGGATTAA CGTGGCTGGG   
  
  
- CTATTAAGTA GAGTAACTGG TATTAAAGCC CAGTATAGTC ATGGGAAAAG ATGATGAGTT AGCAATGAAT   
  
  
- TGTAGATTTG ATCGACCACC CATAATTATA TATAAAATTT ACAAATCTTA TTAGAAATAA ATTATATAGG   
  
  
- TTGAACCCGG AGAAAAAAAA AAAAATTAAA TCCCCTTTTT ATTTAGTTGA ATATTATTCC GGTTCTCTAA   
  
  
- TATCTTTCTT TTACTCCGGT CAACCACTTT GCTTGAAAGC CTCCTTCAGT TGTAAATCCG TCGGTTGATG   
  
  
- TAGTACAAAC AGAAGATGCA AGAAGTATAG AAACCCAGCA TCCACAAGGA AGCCGAAAGG CAGAAACTGA   
  
  
- AAAACAGACA TTCGTCAGTT GTAAGCATAG TTTAGGGCGT GCACGGAGTT TGCGTTAATA CGAGGTTGGT   
  
  
- TTAGTTCTGA TCTAGTTCAA GTAAATCACT CTGTTACAAA CAATCCTGTT GACCCAGCAG CTAGGTTGGA   
  
  
- GCACTGAGAG CCAACCTCCT AACGTCGAAT TTGCTGTAGC CAACCAACTT CTCCTAACAA TACCCCAAAG   
  
  
- ATCCATTATA CATTTAAATC TCCTTCACCC CCATCTTTAA CTTAAATAAT ATTGTCGCCC CCGTACAATC   
  
  
- CTTTTCAAAG ACGATTAGAC GGTATAGACC CACAGATTTC AGTTGACGTA GGACGACCGT GGTTGGTTTT   
  
  
- GTGAGGACAT GAATTTTCGT GGGACTGCGT CTAGTCTGCC GAGTCGTGCT TTCAAACTGG ACTAGAGGAA   
  
  
- GGTACAGGTG TTGCAGGGCA CCTGGCAGTA TTGGGAACAC CCAGGGCGTG CGGTCTGCTC CAGTTACAGG   
  
  
- AATACATATA GTGGTGTCGA AATTATCTTT TTTCTTTATT TTATAATTTT TTGTTAAATA CACACGTGTC   
  
  
- GTCGGTTTTT TCGTGCTACA CCAACCTAGA ATAAGCGTGG TTGTAAACTG CCGTTTCCTA TCACACTGTC   
  
  
- CAGTCACTGG ATTAGCTGGT TATCGTAGAG CATCGGACCA TGAGTAGAAA AAACTGGCCA CAGGGAAGGG   
  
  
- AAATAGTTGT CGTGGGGAGA GAGGTAAAAG AAAAGTGCGA CAAGAGAGAA AAGAGAGGGA AAGAGAAAGA   
  
  
- AAAGAAAGTA CCACCACCAC AACGTCTCT

+     CAAT-box

| Site Name | Organism | Position | Strand | Matrix score. | sequence | function |
| --- | --- | --- | --- | --- | --- | --- |
| CAAT-box | Glycine max | 754 | + | 5 | CAATT | common cis-acting element in promoter and enhancer regions |
| CAAT-box | Hordeum vulgare | 261 | + | 4 | CAAT | common cis-acting element in promoter and enhancer regions |
| CAAT-box | Hordeum vulgare | 1183 | + | 4 | CAAT | common cis-acting element in promoter and enhancer regions |
| CAAT-box | Hordeum vulgare | 948 | - | 4 | CAAT | common cis-acting element in promoter and enhancer regions |
| CAAT-box | Hordeum vulgare | 1349 | + | 4 | CAAT | common cis-acting element in promoter and enhancer regions |
| CAAT-box | Glycine max | 947 | - | 5 | CAATT | common cis-acting element in promoter and enhancer regions |
| CAAT-box | Brassica rapa | 769 | + | 5 | CAAAT | common cis-acting element in promoter and enhancer regions |
| CAAT-box | Hordeum vulgare | 803 | + | 4 | CAAT | common cis-acting element in promoter and enhancer regions |
| CAAT-box | Hordeum vulgare | 895 | - | 4 | CAAT | common cis-acting element in promoter and enhancer regions |
| CAAT-box | Brassica rapa | 730 | + | 5 | CAAAT | common cis-acting element in promoter and enhancer regions |
| CAAT-box | Hordeum vulgare | 338 | - | 4 | CAAT | common cis-acting element in promoter and enhancer regions |
| CAAT-box | Hordeum vulgare | 408 | + | 4 | CAAT | common cis-acting element in promoter and enhancer regions |
| CAAT-box | Brassica rapa | 1304 | - | 5 | CAAAT | common cis-acting element in promoter and enhancer regions |
| CAAT-box | Hordeum vulgare | 860 | - | 4 | CAAT | common cis-acting element in promoter and enhancer regions |
| CAAT-box | Arabidopsis thaliana | 54 | + | 5 | CCAAT | common cis-acting element in promoter and enhancer regions |
| CAAT-box | Hordeum vulgare | 55 | + | 4 | CAAT | common cis-acting element in promoter and enhancer regions |
| CAAT-box | Hordeum vulgare | 289 | + | 4 | CAAT | common cis-acting element in promoter and enhancer regions |
| CAAT-box | Glycine max | 1243 | + | 5 | CAATT | common cis-acting element in promoter and enhancer regions |
| CAAT-box | Arabidopsis thaliana | 1348 | + | 5 | CCAAT | common cis-acting element in promoter and enhancer regions |
| CAAT-box | Arabidopsis thaliana | 127 | + | 5 | CCAAT | common cis-acting element in promoter and enhancer regions |
| CAAT-box | Glycine max | 337 | - | 5 | CAATT | common cis-acting element in promoter and enhancer regions |
| CAAT-box | Hordeum vulgare | 364 | - | 4 | CAAT | common cis-acting element in promoter and enhancer regions |
| CAAT-box | Hordeum vulgare | 128 | + | 4 | CAAT | common cis-acting element in promoter and enhancer regions |

> 2018/04/13 10:10:12  
+ TTTTATTAAA AATATTTTTG AAAAATAATA TATAAGTAAA CATCCCCCAG TTACCAATCA ACTGTACTTC   
  
  
+ ACCTATACTA GTACTAGTAC TTACTCGTGT AAGTACTTTT TAATTATCAT CACCATCCAA TCTATCACCG   
  
  
+ ACATCCTTAA GACTTTAGCT TCCTATTTAG ATTCACTATT CCCCCGAAGA CAAAGATTTA CGTGATTTCT   
  
  
+ CTTTCCCTGT TATAATACAA ACAAGACACC CATCAAACTC CCAAACGGAT CAATCTGATT TCCACAACCT   
  
  
+ AAGAACAGCA ATGCTATGCT AGATTTTGTC TGATTCGCTT CCTATCATCG GATCCTAATT GCACCGACCC   
  
  
+ GATAATTCAT CTCATTGACC ATAATTTCGG GTCATATCAG TACCCTTTTC TACTACTCAA TCGTTACTTA   
  
  
+ ACATCTAAAC TAGCTGGTGG GTATTAATAT ATATTTTAAA TGTTTAGAAT AATCTTTATT TAATATATCC   
  
  
+ AACTTGGGCC TCTTTTTTTT TTTTTAATTT AGGGGAAAAA TAAATCAACT TATAATAAGG CCAAGAGATT   
  
  
+ ATAGAAAGAA AATGAGGCCA GTTGGTGAAA CGAACTTTCG GAGGAAGTCA ACATTTAGGC AGCCAACTAC   
  
  
+ ATCATGTTTG TCTTCTACGT TCTTCATATC TTTGGGTCGT AGGTGTTCCT TCGGCTTTCC GTCTTTGACT   
  
  
+ TTTTGTCTGT AAGCAGTCAA CATTCGTATC AAATCCCGCA CGTGCCTCAA ACGCAATTAT GCTCCAACCA   
  
  
+ AATCAAGACT AGATCAAGTT CATTTAGTGA GACAATGTTT GTTAGGACAA CTGGGTCGTC GATCCAACCT   
  
  
+ CGTGACTCTC GGTTGGAGGA TTGCAGCTTA AACGACATCG GTTGGTTGAA GAGGATTGTT ATGGGGTTTC   
  
  
+ TAGGTAATAT GTAAATTTAG AGGAAGTGGG GGTAGAAATT GAATTTATTA TAACAGCGGG GGCATGTTAG   
  
  
+ GAAAAGTTTC TGCTAATCTG CCATATCTGG GTGTCTAAAG TCAACTGCAT CCTGCTGGCA CCAACCAAAA   
  
  
+ CACTCCTGTA CTTAAAAGCA CCCTGACGCA GATCAGACGG CTCAGCACGA AAGTTTGACC TGATCTCCTT   
  
  
+ CCATGTCCAC AACGTCCCGT GGACCGTCAT AACCCTTGTG GGTCCCGCAC GCCAGACGAG GTCAATGTCC   
  
  
+ TTATGTATAT CACCACAGCT TTAATAGAAA AAAGAAATAA AATATTAAAA AACAATTTAT GTGTGCACAG   
  
  
+ CAGCCAAAAA AGCACGATGT GGTTGGATCT TATTCGCACC AACATTTGAC GGCAAAGGAT AGTGTGACAG   
  
  
+ GTCAGTGACC TAATCGACCA ATAGCATCTC GTAGCCTGGT ACTCATCTTT TTTGACCGGT GTCCCTTCCC   
  
  
+ TTTATCAACA GCACCCCTCT CTCCATTTTC TTTTCACGCT GTTCTCTCTT TTCTCTCCCT TTCTCTTTCT   
  
  
+ TTTCTTTCAT GGTGGTGGTG TTGCAGAGA  

- AAAATAATTT TTATAAAAAC TTTTTATTAT ATATTCATTT GTAGGGGGTC AATGGTTAGT TGACATGAAG   
  
  
- TGGATATGAT CATGATCATG AATGAGCACA TTCATGAAAA ATTAATAGTA GTGGTAGGTT AGATAGTGGC   
  
  
- TGTAGGAATT CTGAAATCGA AGGATAAATC TAAGTGATAA GGGGGCTTCT GTTTCTAAAT GCACTAAAGA   
  
  
- GAAAGGGACA ATATTATGTT TGTTCTGTGG GTAGTTTGAG GGTTTGCCTA GTTAGACTAA AGGTGTTGGA   
  
  
- TTCTTGTCGT TACGATACGA TCTAAAACAG ACTAAGCGAA GGATAGTAGC CTAGGATTAA CGTGGCTGGG   
  
  
- CTATTAAGTA GAGTAACTGG TATTAAAGCC CAGTATAGTC ATGGGAAAAG ATGATGAGTT AGCAATGAAT   
  
  
- TGTAGATTTG ATCGACCACC CATAATTATA TATAAAATTT ACAAATCTTA TTAGAAATAA ATTATATAGG   
  
  
- TTGAACCCGG AGAAAAAAAA AAAAATTAAA TCCCCTTTTT ATTTAGTTGA ATATTATTCC GGTTCTCTAA   
  
  
- TATCTTTCTT TTACTCCGGT CAACCACTTT GCTTGAAAGC CTCCTTCAGT TGTAAATCCG TCGGTTGATG   
  
  
- TAGTACAAAC AGAAGATGCA AGAAGTATAG AAACCCAGCA TCCACAAGGA AGCCGAAAGG CAGAAACTGA   
  
  
- AAAACAGACA TTCGTCAGTT GTAAGCATAG TTTAGGGCGT GCACGGAGTT TGCGTTAATA CGAGGTTGGT   
  
  
- TTAGTTCTGA TCTAGTTCAA GTAAATCACT CTGTTACAAA CAATCCTGTT GACCCAGCAG CTAGGTTGGA   
  
  
- GCACTGAGAG CCAACCTCCT AACGTCGAAT TTGCTGTAGC CAACCAACTT CTCCTAACAA TACCCCAAAG   
  
  
- ATCCATTATA CATTTAAATC TCCTTCACCC CCATCTTTAA CTTAAATAAT ATTGTCGCCC CCGTACAATC   
  
  
- CTTTTCAAAG ACGATTAGAC GGTATAGACC CACAGATTTC AGTTGACGTA GGACGACCGT GGTTGGTTTT   
  
  
- GTGAGGACAT GAATTTTCGT GGGACTGCGT CTAGTCTGCC GAGTCGTGCT TTCAAACTGG ACTAGAGGAA   
  
  
- GGTACAGGTG TTGCAGGGCA CCTGGCAGTA TTGGGAACAC CCAGGGCGTG CGGTCTGCTC CAGTTACAGG   
  
  
- AATACATATA GTGGTGTCGA AATTATCTTT TTTCTTTATT TTATAATTTT TTGTTAAATA CACACGTGTC   
  
  
- GTCGGTTTTT TCGTGCTACA CCAACCTAGA ATAAGCGTGG TTGTAAACTG CCGTTTCCTA TCACACTGTC   
  
  
- CAGTCACTGG ATTAGCTGGT TATCGTAGAG CATCGGACCA TGAGTAGAAA AAACTGGCCA CAGGGAAGGG   
  
  
- AAATAGTTGT CGTGGGGAGA GAGGTAAAAG AAAAGTGCGA CAAGAGAGAA AAGAGAGGGA AAGAGAAAGA   
  
  
- AAAGAAAGTA CCACCACCAC AACGTCTCT

+     CGTCA-motif

| Site Name | Organism | Position | Strand | Matrix score. | sequence | function |
| --- | --- | --- | --- | --- | --- | --- |
| CGTCA-motif | Hordeum vulgare | 1145 | + | 5 | CGTCA | cis-acting regulatory element involved in the MeJA-responsiveness |
| CGTCA-motif | Hordeum vulgare | 1307 | - | 5 | CGTCA | cis-acting regulatory element involved in the MeJA-responsiveness |
| CGTCA-motif | Hordeum vulgare | 1074 | - | 5 | CGTCA | cis-acting regulatory element involved in the MeJA-responsiveness |

> 2018/04/13 10:10:12  
+ TTTTATTAAA AATATTTTTG AAAAATAATA TATAAGTAAA CATCCCCCAG TTACCAATCA ACTGTACTTC   
  
  
+ ACCTATACTA GTACTAGTAC TTACTCGTGT AAGTACTTTT TAATTATCAT CACCATCCAA TCTATCACCG   
  
  
+ ACATCCTTAA GACTTTAGCT TCCTATTTAG ATTCACTATT CCCCCGAAGA CAAAGATTTA CGTGATTTCT   
  
  
+ CTTTCCCTGT TATAATACAA ACAAGACACC CATCAAACTC CCAAACGGAT CAATCTGATT TCCACAACCT   
  
  
+ AAGAACAGCA ATGCTATGCT AGATTTTGTC TGATTCGCTT CCTATCATCG GATCCTAATT GCACCGACCC   
  
  
+ GATAATTCAT CTCATTGACC ATAATTTCGG GTCATATCAG TACCCTTTTC TACTACTCAA TCGTTACTTA   
  
  
+ ACATCTAAAC TAGCTGGTGG GTATTAATAT ATATTTTAAA TGTTTAGAAT AATCTTTATT TAATATATCC   
  
  
+ AACTTGGGCC TCTTTTTTTT TTTTTAATTT AGGGGAAAAA TAAATCAACT TATAATAAGG CCAAGAGATT   
  
  
+ ATAGAAAGAA AATGAGGCCA GTTGGTGAAA CGAACTTTCG GAGGAAGTCA ACATTTAGGC AGCCAACTAC   
  
  
+ ATCATGTTTG TCTTCTACGT TCTTCATATC TTTGGGTCGT AGGTGTTCCT TCGGCTTTCC GTCTTTGACT   
  
  
+ TTTTGTCTGT AAGCAGTCAA CATTCGTATC AAATCCCGCA CGTGCCTCAA ACGCAATTAT GCTCCAACCA   
  
  
+ AATCAAGACT AGATCAAGTT CATTTAGTGA GACAATGTTT GTTAGGACAA CTGGGTCGTC GATCCAACCT   
  
  
+ CGTGACTCTC GGTTGGAGGA TTGCAGCTTA AACGACATCG GTTGGTTGAA GAGGATTGTT ATGGGGTTTC   
  
  
+ TAGGTAATAT GTAAATTTAG AGGAAGTGGG GGTAGAAATT GAATTTATTA TAACAGCGGG GGCATGTTAG   
  
  
+ GAAAAGTTTC TGCTAATCTG CCATATCTGG GTGTCTAAAG TCAACTGCAT CCTGCTGGCA CCAACCAAAA   
  
  
+ CACTCCTGTA CTTAAAAGCA CCCTGACGCA GATCAGACGG CTCAGCACGA AAGTTTGACC TGATCTCCTT   
  
  
+ CCATGTCCAC AACGTCCCGT GGACCGTCAT AACCCTTGTG GGTCCCGCAC GCCAGACGAG GTCAATGTCC   
  
  
+ TTATGTATAT CACCACAGCT TTAATAGAAA AAAGAAATAA AATATTAAAA AACAATTTAT GTGTGCACAG   
  
  
+ CAGCCAAAAA AGCACGATGT GGTTGGATCT TATTCGCACC AACATTTGAC GGCAAAGGAT AGTGTGACAG   
  
  
+ GTCAGTGACC TAATCGACCA ATAGCATCTC GTAGCCTGGT ACTCATCTTT TTTGACCGGT GTCCCTTCCC   
  
  
+ TTTATCAACA GCACCCCTCT CTCCATTTTC TTTTCACGCT GTTCTCTCTT TTCTCTCCCT TTCTCTTTCT   
  
  
+ TTTCTTTCAT GGTGGTGGTG TTGCAGAGA  

- AAAATAATTT TTATAAAAAC TTTTTATTAT ATATTCATTT GTAGGGGGTC AATGGTTAGT TGACATGAAG   
  
  
- TGGATATGAT CATGATCATG AATGAGCACA TTCATGAAAA ATTAATAGTA GTGGTAGGTT AGATAGTGGC   
  
  
- TGTAGGAATT CTGAAATCGA AGGATAAATC TAAGTGATAA GGGGGCTTCT GTTTCTAAAT GCACTAAAGA   
  
  
- GAAAGGGACA ATATTATGTT TGTTCTGTGG GTAGTTTGAG GGTTTGCCTA GTTAGACTAA AGGTGTTGGA   
  
  
- TTCTTGTCGT TACGATACGA TCTAAAACAG ACTAAGCGAA GGATAGTAGC CTAGGATTAA CGTGGCTGGG   
  
  
- CTATTAAGTA GAGTAACTGG TATTAAAGCC CAGTATAGTC ATGGGAAAAG ATGATGAGTT AGCAATGAAT   
  
  
- TGTAGATTTG ATCGACCACC CATAATTATA TATAAAATTT ACAAATCTTA TTAGAAATAA ATTATATAGG   
  
  
- TTGAACCCGG AGAAAAAAAA AAAAATTAAA TCCCCTTTTT ATTTAGTTGA ATATTATTCC GGTTCTCTAA   
  
  
- TATCTTTCTT TTACTCCGGT CAACCACTTT GCTTGAAAGC CTCCTTCAGT TGTAAATCCG TCGGTTGATG   
  
  
- TAGTACAAAC AGAAGATGCA AGAAGTATAG AAACCCAGCA TCCACAAGGA AGCCGAAAGG CAGAAACTGA   
  
  
- AAAACAGACA TTCGTCAGTT GTAAGCATAG TTTAGGGCGT GCACGGAGTT TGCGTTAATA CGAGGTTGGT   
  
  
- TTAGTTCTGA TCTAGTTCAA GTAAATCACT CTGTTACAAA CAATCCTGTT GACCCAGCAG CTAGGTTGGA   
  
  
- GCACTGAGAG CCAACCTCCT AACGTCGAAT TTGCTGTAGC CAACCAACTT CTCCTAACAA TACCCCAAAG   
  
  
- ATCCATTATA CATTTAAATC TCCTTCACCC CCATCTTTAA CTTAAATAAT ATTGTCGCCC CCGTACAATC   
  
  
- CTTTTCAAAG ACGATTAGAC GGTATAGACC CACAGATTTC AGTTGACGTA GGACGACCGT GGTTGGTTTT   
  
  
- GTGAGGACAT GAATTTTCGT GGGACTGCGT CTAGTCTGCC GAGTCGTGCT TTCAAACTGG ACTAGAGGAA   
  
  
- GGTACAGGTG TTGCAGGGCA CCTGGCAGTA TTGGGAACAC CCAGGGCGTG CGGTCTGCTC CAGTTACAGG   
  
  
- AATACATATA GTGGTGTCGA AATTATCTTT TTTCTTTATT TTATAATTTT TTGTTAAATA CACACGTGTC   
  
  
- GTCGGTTTTT TCGTGCTACA CCAACCTAGA ATAAGCGTGG TTGTAAACTG CCGTTTCCTA TCACACTGTC   
  
  
- CAGTCACTGG ATTAGCTGGT TATCGTAGAG CATCGGACCA TGAGTAGAAA AAACTGGCCA CAGGGAAGGG   
  
  
- AAATAGTTGT CGTGGGGAGA GAGGTAAAAG AAAAGTGCGA CAAGAGAGAA AAGAGAGGGA AAGAGAAAGA   
  
  
- AAAGAAAGTA CCACCACCAC AACGTCTCT

+     CTAG-motif

| Site Name | Organism | Position | Strand | Matrix score. | sequence | function |
| --- | --- | --- | --- | --- | --- | --- |
| CTAG-motif | Avena sativa | 988 | - | 9 | ACTAGCAGAA |  |

> 2018/04/13 10:10:12  
+ TTTTATTAAA AATATTTTTG AAAAATAATA TATAAGTAAA CATCCCCCAG TTACCAATCA ACTGTACTTC   
  
  
+ ACCTATACTA GTACTAGTAC TTACTCGTGT AAGTACTTTT TAATTATCAT CACCATCCAA TCTATCACCG   
  
  
+ ACATCCTTAA GACTTTAGCT TCCTATTTAG ATTCACTATT CCCCCGAAGA CAAAGATTTA CGTGATTTCT   
  
  
+ CTTTCCCTGT TATAATACAA ACAAGACACC CATCAAACTC CCAAACGGAT CAATCTGATT TCCACAACCT   
  
  
+ AAGAACAGCA ATGCTATGCT AGATTTTGTC TGATTCGCTT CCTATCATCG GATCCTAATT GCACCGACCC   
  
  
+ GATAATTCAT CTCATTGACC ATAATTTCGG GTCATATCAG TACCCTTTTC TACTACTCAA TCGTTACTTA   
  
  
+ ACATCTAAAC TAGCTGGTGG GTATTAATAT ATATTTTAAA TGTTTAGAAT AATCTTTATT TAATATATCC   
  
  
+ AACTTGGGCC TCTTTTTTTT TTTTTAATTT AGGGGAAAAA TAAATCAACT TATAATAAGG CCAAGAGATT   
  
  
+ ATAGAAAGAA AATGAGGCCA GTTGGTGAAA CGAACTTTCG GAGGAAGTCA ACATTTAGGC AGCCAACTAC   
  
  
+ ATCATGTTTG TCTTCTACGT TCTTCATATC TTTGGGTCGT AGGTGTTCCT TCGGCTTTCC GTCTTTGACT   
  
  
+ TTTTGTCTGT AAGCAGTCAA CATTCGTATC AAATCCCGCA CGTGCCTCAA ACGCAATTAT GCTCCAACCA   
  
  
+ AATCAAGACT AGATCAAGTT CATTTAGTGA GACAATGTTT GTTAGGACAA CTGGGTCGTC GATCCAACCT   
  
  
+ CGTGACTCTC GGTTGGAGGA TTGCAGCTTA AACGACATCG GTTGGTTGAA GAGGATTGTT ATGGGGTTTC   
  
  
+ TAGGTAATAT GTAAATTTAG AGGAAGTGGG GGTAGAAATT GAATTTATTA TAACAGCGGG GGCATGTTAG   
  
  
+ GAAAAGTTTC TGCTAATCTG CCATATCTGG GTGTCTAAAG TCAACTGCAT CCTGCTGGCA CCAACCAAAA   
  
  
+ CACTCCTGTA CTTAAAAGCA CCCTGACGCA GATCAGACGG CTCAGCACGA AAGTTTGACC TGATCTCCTT   
  
  
+ CCATGTCCAC AACGTCCCGT GGACCGTCAT AACCCTTGTG GGTCCCGCAC GCCAGACGAG GTCAATGTCC   
  
  
+ TTATGTATAT CACCACAGCT TTAATAGAAA AAAGAAATAA AATATTAAAA AACAATTTAT GTGTGCACAG   
  
  
+ CAGCCAAAAA AGCACGATGT GGTTGGATCT TATTCGCACC AACATTTGAC GGCAAAGGAT AGTGTGACAG   
  
  
+ GTCAGTGACC TAATCGACCA ATAGCATCTC GTAGCCTGGT ACTCATCTTT TTTGACCGGT GTCCCTTCCC   
  
  
+ TTTATCAACA GCACCCCTCT CTCCATTTTC TTTTCACGCT GTTCTCTCTT TTCTCTCCCT TTCTCTTTCT   
  
  
+ TTTCTTTCAT GGTGGTGGTG TTGCAGAGA  

- AAAATAATTT TTATAAAAAC TTTTTATTAT ATATTCATTT GTAGGGGGTC AATGGTTAGT TGACATGAAG   
  
  
- TGGATATGAT CATGATCATG AATGAGCACA TTCATGAAAA ATTAATAGTA GTGGTAGGTT AGATAGTGGC   
  
  
- TGTAGGAATT CTGAAATCGA AGGATAAATC TAAGTGATAA GGGGGCTTCT GTTTCTAAAT GCACTAAAGA   
  
  
- GAAAGGGACA ATATTATGTT TGTTCTGTGG GTAGTTTGAG GGTTTGCCTA GTTAGACTAA AGGTGTTGGA   
  
  
- TTCTTGTCGT TACGATACGA TCTAAAACAG ACTAAGCGAA GGATAGTAGC CTAGGATTAA CGTGGCTGGG   
  
  
- CTATTAAGTA GAGTAACTGG TATTAAAGCC CAGTATAGTC ATGGGAAAAG ATGATGAGTT AGCAATGAAT   
  
  
- TGTAGATTTG ATCGACCACC CATAATTATA TATAAAATTT ACAAATCTTA TTAGAAATAA ATTATATAGG   
  
  
- TTGAACCCGG AGAAAAAAAA AAAAATTAAA TCCCCTTTTT ATTTAGTTGA ATATTATTCC GGTTCTCTAA   
  
  
- TATCTTTCTT TTACTCCGGT CAACCACTTT GCTTGAAAGC CTCCTTCAGT TGTAAATCCG TCGGTTGATG   
  
  
- TAGTACAAAC AGAAGATGCA AGAAGTATAG AAACCCAGCA TCCACAAGGA AGCCGAAAGG CAGAAACTGA   
  
  
- AAAACAGACA TTCGTCAGTT GTAAGCATAG TTTAGGGCGT GCACGGAGTT TGCGTTAATA CGAGGTTGGT   
  
  
- TTAGTTCTGA TCTAGTTCAA GTAAATCACT CTGTTACAAA CAATCCTGTT GACCCAGCAG CTAGGTTGGA   
  
  
- GCACTGAGAG CCAACCTCCT AACGTCGAAT TTGCTGTAGC CAACCAACTT CTCCTAACAA TACCCCAAAG   
  
  
- ATCCATTATA CATTTAAATC TCCTTCACCC CCATCTTTAA CTTAAATAAT ATTGTCGCCC CCGTACAATC   
  
  
- CTTTTCAAAG ACGATTAGAC GGTATAGACC CACAGATTTC AGTTGACGTA GGACGACCGT GGTTGGTTTT   
  
  
- GTGAGGACAT GAATTTTCGT GGGACTGCGT CTAGTCTGCC GAGTCGTGCT TTCAAACTGG ACTAGAGGAA   
  
  
- GGTACAGGTG TTGCAGGGCA CCTGGCAGTA TTGGGAACAC CCAGGGCGTG CGGTCTGCTC CAGTTACAGG   
  
  
- AATACATATA GTGGTGTCGA AATTATCTTT TTTCTTTATT TTATAATTTT TTGTTAAATA CACACGTGTC   
  
  
- GTCGGTTTTT TCGTGCTACA CCAACCTAGA ATAAGCGTGG TTGTAAACTG CCGTTTCCTA TCACACTGTC   
  
  
- CAGTCACTGG ATTAGCTGGT TATCGTAGAG CATCGGACCA TGAGTAGAAA AAACTGGCCA CAGGGAAGGG   
  
  
- AAATAGTTGT CGTGGGGAGA GAGGTAAAAG AAAAGTGCGA CAAGAGAGAA AAGAGAGGGA AAGAGAAAGA   
  
  
- AAAGAAAGTA CCACCACCAC AACGTCTCT

+     G-Box

| Site Name | Organism | Position | Strand | Matrix score. | sequence | function |
| --- | --- | --- | --- | --- | --- | --- |
| G-Box | Antirrhinum majus | 199 | - | 6 | CACGTA | cis-acting regulatory element involved in light responsiveness |
| G-Box | Pisum sativum | 739 | + | 6 | CACGTG | cis-acting regulatory element involved in light responsiveness |

> 2018/04/13 10:10:12  
+ TTTTATTAAA AATATTTTTG AAAAATAATA TATAAGTAAA CATCCCCCAG TTACCAATCA ACTGTACTTC   
  
  
+ ACCTATACTA GTACTAGTAC TTACTCGTGT AAGTACTTTT TAATTATCAT CACCATCCAA TCTATCACCG   
  
  
+ ACATCCTTAA GACTTTAGCT TCCTATTTAG ATTCACTATT CCCCCGAAGA CAAAGATTTA CGTGATTTCT   
  
  
+ CTTTCCCTGT TATAATACAA ACAAGACACC CATCAAACTC CCAAACGGAT CAATCTGATT TCCACAACCT   
  
  
+ AAGAACAGCA ATGCTATGCT AGATTTTGTC TGATTCGCTT CCTATCATCG GATCCTAATT GCACCGACCC   
  
  
+ GATAATTCAT CTCATTGACC ATAATTTCGG GTCATATCAG TACCCTTTTC TACTACTCAA TCGTTACTTA   
  
  
+ ACATCTAAAC TAGCTGGTGG GTATTAATAT ATATTTTAAA TGTTTAGAAT AATCTTTATT TAATATATCC   
  
  
+ AACTTGGGCC TCTTTTTTTT TTTTTAATTT AGGGGAAAAA TAAATCAACT TATAATAAGG CCAAGAGATT   
  
  
+ ATAGAAAGAA AATGAGGCCA GTTGGTGAAA CGAACTTTCG GAGGAAGTCA ACATTTAGGC AGCCAACTAC   
  
  
+ ATCATGTTTG TCTTCTACGT TCTTCATATC TTTGGGTCGT AGGTGTTCCT TCGGCTTTCC GTCTTTGACT   
  
  
+ TTTTGTCTGT AAGCAGTCAA CATTCGTATC AAATCCCGCA CGTGCCTCAA ACGCAATTAT GCTCCAACCA   
  
  
+ AATCAAGACT AGATCAAGTT CATTTAGTGA GACAATGTTT GTTAGGACAA CTGGGTCGTC GATCCAACCT   
  
  
+ CGTGACTCTC GGTTGGAGGA TTGCAGCTTA AACGACATCG GTTGGTTGAA GAGGATTGTT ATGGGGTTTC   
  
  
+ TAGGTAATAT GTAAATTTAG AGGAAGTGGG GGTAGAAATT GAATTTATTA TAACAGCGGG GGCATGTTAG   
  
  
+ GAAAAGTTTC TGCTAATCTG CCATATCTGG GTGTCTAAAG TCAACTGCAT CCTGCTGGCA CCAACCAAAA   
  
  
+ CACTCCTGTA CTTAAAAGCA CCCTGACGCA GATCAGACGG CTCAGCACGA AAGTTTGACC TGATCTCCTT   
  
  
+ CCATGTCCAC AACGTCCCGT GGACCGTCAT AACCCTTGTG GGTCCCGCAC GCCAGACGAG GTCAATGTCC   
  
  
+ TTATGTATAT CACCACAGCT TTAATAGAAA AAAGAAATAA AATATTAAAA AACAATTTAT GTGTGCACAG   
  
  
+ CAGCCAAAAA AGCACGATGT GGTTGGATCT TATTCGCACC AACATTTGAC GGCAAAGGAT AGTGTGACAG   
  
  
+ GTCAGTGACC TAATCGACCA ATAGCATCTC GTAGCCTGGT ACTCATCTTT TTTGACCGGT GTCCCTTCCC   
  
  
+ TTTATCAACA GCACCCCTCT CTCCATTTTC TTTTCACGCT GTTCTCTCTT TTCTCTCCCT TTCTCTTTCT   
  
  
+ TTTCTTTCAT GGTGGTGGTG TTGCAGAGA  

- AAAATAATTT TTATAAAAAC TTTTTATTAT ATATTCATTT GTAGGGGGTC AATGGTTAGT TGACATGAAG   
  
  
- TGGATATGAT CATGATCATG AATGAGCACA TTCATGAAAA ATTAATAGTA GTGGTAGGTT AGATAGTGGC   
  
  
- TGTAGGAATT CTGAAATCGA AGGATAAATC TAAGTGATAA GGGGGCTTCT GTTTCTAAAT GCACTAAAGA   
  
  
- GAAAGGGACA ATATTATGTT TGTTCTGTGG GTAGTTTGAG GGTTTGCCTA GTTAGACTAA AGGTGTTGGA   
  
  
- TTCTTGTCGT TACGATACGA TCTAAAACAG ACTAAGCGAA GGATAGTAGC CTAGGATTAA CGTGGCTGGG   
  
  
- CTATTAAGTA GAGTAACTGG TATTAAAGCC CAGTATAGTC ATGGGAAAAG ATGATGAGTT AGCAATGAAT   
  
  
- TGTAGATTTG ATCGACCACC CATAATTATA TATAAAATTT ACAAATCTTA TTAGAAATAA ATTATATAGG   
  
  
- TTGAACCCGG AGAAAAAAAA AAAAATTAAA TCCCCTTTTT ATTTAGTTGA ATATTATTCC GGTTCTCTAA   
  
  
- TATCTTTCTT TTACTCCGGT CAACCACTTT GCTTGAAAGC CTCCTTCAGT TGTAAATCCG TCGGTTGATG   
  
  
- TAGTACAAAC AGAAGATGCA AGAAGTATAG AAACCCAGCA TCCACAAGGA AGCCGAAAGG CAGAAACTGA   
  
  
- AAAACAGACA TTCGTCAGTT GTAAGCATAG TTTAGGGCGT GCACGGAGTT TGCGTTAATA CGAGGTTGGT   
  
  
- TTAGTTCTGA TCTAGTTCAA GTAAATCACT CTGTTACAAA CAATCCTGTT GACCCAGCAG CTAGGTTGGA   
  
  
- GCACTGAGAG CCAACCTCCT AACGTCGAAT TTGCTGTAGC CAACCAACTT CTCCTAACAA TACCCCAAAG   
  
  
- ATCCATTATA CATTTAAATC TCCTTCACCC CCATCTTTAA CTTAAATAAT ATTGTCGCCC CCGTACAATC   
  
  
- CTTTTCAAAG ACGATTAGAC GGTATAGACC CACAGATTTC AGTTGACGTA GGACGACCGT GGTTGGTTTT   
  
  
- GTGAGGACAT GAATTTTCGT GGGACTGCGT CTAGTCTGCC GAGTCGTGCT TTCAAACTGG ACTAGAGGAA   
  
  
- GGTACAGGTG TTGCAGGGCA CCTGGCAGTA TTGGGAACAC CCAGGGCGTG CGGTCTGCTC CAGTTACAGG   
  
  
- AATACATATA GTGGTGTCGA AATTATCTTT TTTCTTTATT TTATAATTTT TTGTTAAATA CACACGTGTC   
  
  
- GTCGGTTTTT TCGTGCTACA CCAACCTAGA ATAAGCGTGG TTGTAAACTG CCGTTTCCTA TCACACTGTC   
  
  
- CAGTCACTGG ATTAGCTGGT TATCGTAGAG CATCGGACCA TGAGTAGAAA AAACTGGCCA CAGGGAAGGG   
  
  
- AAATAGTTGT CGTGGGGAGA GAGGTAAAAG AAAAGTGCGA CAAGAGAGAA AAGAGAGGGA AAGAGAAAGA   
  
  
- AAAGAAAGTA CCACCACCAC AACGTCTCT

+     G-box

| Site Name | Organism | Position | Strand | Matrix score. | sequence | function |
| --- | --- | --- | --- | --- | --- | --- |
| G-box | Daucus carota | 199 | + | 6 | TACGTG | cis-acting regulatory element involved in light responsiveness |
| G-box | Arabidopsis thaliana | 739 | + | 6 | CACGTG | cis-acting regulatory element involved in light responsiveness |

> 2018/04/13 10:10:12  
+ TTTTATTAAA AATATTTTTG AAAAATAATA TATAAGTAAA CATCCCCCAG TTACCAATCA ACTGTACTTC   
  
  
+ ACCTATACTA GTACTAGTAC TTACTCGTGT AAGTACTTTT TAATTATCAT CACCATCCAA TCTATCACCG   
  
  
+ ACATCCTTAA GACTTTAGCT TCCTATTTAG ATTCACTATT CCCCCGAAGA CAAAGATTTA CGTGATTTCT   
  
  
+ CTTTCCCTGT TATAATACAA ACAAGACACC CATCAAACTC CCAAACGGAT CAATCTGATT TCCACAACCT   
  
  
+ AAGAACAGCA ATGCTATGCT AGATTTTGTC TGATTCGCTT CCTATCATCG GATCCTAATT GCACCGACCC   
  
  
+ GATAATTCAT CTCATTGACC ATAATTTCGG GTCATATCAG TACCCTTTTC TACTACTCAA TCGTTACTTA   
  
  
+ ACATCTAAAC TAGCTGGTGG GTATTAATAT ATATTTTAAA TGTTTAGAAT AATCTTTATT TAATATATCC   
  
  
+ AACTTGGGCC TCTTTTTTTT TTTTTAATTT AGGGGAAAAA TAAATCAACT TATAATAAGG CCAAGAGATT   
  
  
+ ATAGAAAGAA AATGAGGCCA GTTGGTGAAA CGAACTTTCG GAGGAAGTCA ACATTTAGGC AGCCAACTAC   
  
  
+ ATCATGTTTG TCTTCTACGT TCTTCATATC TTTGGGTCGT AGGTGTTCCT TCGGCTTTCC GTCTTTGACT   
  
  
+ TTTTGTCTGT AAGCAGTCAA CATTCGTATC AAATCCCGCA CGTGCCTCAA ACGCAATTAT GCTCCAACCA   
  
  
+ AATCAAGACT AGATCAAGTT CATTTAGTGA GACAATGTTT GTTAGGACAA CTGGGTCGTC GATCCAACCT   
  
  
+ CGTGACTCTC GGTTGGAGGA TTGCAGCTTA AACGACATCG GTTGGTTGAA GAGGATTGTT ATGGGGTTTC   
  
  
+ TAGGTAATAT GTAAATTTAG AGGAAGTGGG GGTAGAAATT GAATTTATTA TAACAGCGGG GGCATGTTAG   
  
  
+ GAAAAGTTTC TGCTAATCTG CCATATCTGG GTGTCTAAAG TCAACTGCAT CCTGCTGGCA CCAACCAAAA   
  
  
+ CACTCCTGTA CTTAAAAGCA CCCTGACGCA GATCAGACGG CTCAGCACGA AAGTTTGACC TGATCTCCTT   
  
  
+ CCATGTCCAC AACGTCCCGT GGACCGTCAT AACCCTTGTG GGTCCCGCAC GCCAGACGAG GTCAATGTCC   
  
  
+ TTATGTATAT CACCACAGCT TTAATAGAAA AAAGAAATAA AATATTAAAA AACAATTTAT GTGTGCACAG   
  
  
+ CAGCCAAAAA AGCACGATGT GGTTGGATCT TATTCGCACC AACATTTGAC GGCAAAGGAT AGTGTGACAG   
  
  
+ GTCAGTGACC TAATCGACCA ATAGCATCTC GTAGCCTGGT ACTCATCTTT TTTGACCGGT GTCCCTTCCC   
  
  
+ TTTATCAACA GCACCCCTCT CTCCATTTTC TTTTCACGCT GTTCTCTCTT TTCTCTCCCT TTCTCTTTCT   
  
  
+ TTTCTTTCAT GGTGGTGGTG TTGCAGAGA  

- AAAATAATTT TTATAAAAAC TTTTTATTAT ATATTCATTT GTAGGGGGTC AATGGTTAGT TGACATGAAG   
  
  
- TGGATATGAT CATGATCATG AATGAGCACA TTCATGAAAA ATTAATAGTA GTGGTAGGTT AGATAGTGGC   
  
  
- TGTAGGAATT CTGAAATCGA AGGATAAATC TAAGTGATAA GGGGGCTTCT GTTTCTAAAT GCACTAAAGA   
  
  
- GAAAGGGACA ATATTATGTT TGTTCTGTGG GTAGTTTGAG GGTTTGCCTA GTTAGACTAA AGGTGTTGGA   
  
  
- TTCTTGTCGT TACGATACGA TCTAAAACAG ACTAAGCGAA GGATAGTAGC CTAGGATTAA CGTGGCTGGG   
  
  
- CTATTAAGTA GAGTAACTGG TATTAAAGCC CAGTATAGTC ATGGGAAAAG ATGATGAGTT AGCAATGAAT   
  
  
- TGTAGATTTG ATCGACCACC CATAATTATA TATAAAATTT ACAAATCTTA TTAGAAATAA ATTATATAGG   
  
  
- TTGAACCCGG AGAAAAAAAA AAAAATTAAA TCCCCTTTTT ATTTAGTTGA ATATTATTCC GGTTCTCTAA   
  
  
- TATCTTTCTT TTACTCCGGT CAACCACTTT GCTTGAAAGC CTCCTTCAGT TGTAAATCCG TCGGTTGATG   
  
  
- TAGTACAAAC AGAAGATGCA AGAAGTATAG AAACCCAGCA TCCACAAGGA AGCCGAAAGG CAGAAACTGA   
  
  
- AAAACAGACA TTCGTCAGTT GTAAGCATAG TTTAGGGCGT GCACGGAGTT TGCGTTAATA CGAGGTTGGT   
  
  
- TTAGTTCTGA TCTAGTTCAA GTAAATCACT CTGTTACAAA CAATCCTGTT GACCCAGCAG CTAGGTTGGA   
  
  
- GCACTGAGAG CCAACCTCCT AACGTCGAAT TTGCTGTAGC CAACCAACTT CTCCTAACAA TACCCCAAAG   
  
  
- ATCCATTATA CATTTAAATC TCCTTCACCC CCATCTTTAA CTTAAATAAT ATTGTCGCCC CCGTACAATC   
  
  
- CTTTTCAAAG ACGATTAGAC GGTATAGACC CACAGATTTC AGTTGACGTA GGACGACCGT GGTTGGTTTT   
  
  
- GTGAGGACAT GAATTTTCGT GGGACTGCGT CTAGTCTGCC GAGTCGTGCT TTCAAACTGG ACTAGAGGAA   
  
  
- GGTACAGGTG TTGCAGGGCA CCTGGCAGTA TTGGGAACAC CCAGGGCGTG CGGTCTGCTC CAGTTACAGG   
  
  
- AATACATATA GTGGTGTCGA AATTATCTTT TTTCTTTATT TTATAATTTT TTGTTAAATA CACACGTGTC   
  
  
- GTCGGTTTTT TCGTGCTACA CCAACCTAGA ATAAGCGTGG TTGTAAACTG CCGTTTCCTA TCACACTGTC   
  
  
- CAGTCACTGG ATTAGCTGGT TATCGTAGAG CATCGGACCA TGAGTAGAAA AAACTGGCCA CAGGGAAGGG   
  
  
- AAATAGTTGT CGTGGGGAGA GAGGTAAAAG AAAAGTGCGA CAAGAGAGAA AAGAGAGGGA AAGAGAAAGA   
  
  
- AAAGAAAGTA CCACCACCAC AACGTCTCT

+     GA-motif

| Site Name | Organism | Position | Strand | Matrix score. | sequence | function |
| --- | --- | --- | --- | --- | --- | --- |
| GA-motif | Helianthus annuus | 1373 | - | 8 | AAAGATGA | part of a light responsive element |

> 2018/04/13 10:10:12  
+ TTTTATTAAA AATATTTTTG AAAAATAATA TATAAGTAAA CATCCCCCAG TTACCAATCA ACTGTACTTC   
  
  
+ ACCTATACTA GTACTAGTAC TTACTCGTGT AAGTACTTTT TAATTATCAT CACCATCCAA TCTATCACCG   
  
  
+ ACATCCTTAA GACTTTAGCT TCCTATTTAG ATTCACTATT CCCCCGAAGA CAAAGATTTA CGTGATTTCT   
  
  
+ CTTTCCCTGT TATAATACAA ACAAGACACC CATCAAACTC CCAAACGGAT CAATCTGATT TCCACAACCT   
  
  
+ AAGAACAGCA ATGCTATGCT AGATTTTGTC TGATTCGCTT CCTATCATCG GATCCTAATT GCACCGACCC   
  
  
+ GATAATTCAT CTCATTGACC ATAATTTCGG GTCATATCAG TACCCTTTTC TACTACTCAA TCGTTACTTA   
  
  
+ ACATCTAAAC TAGCTGGTGG GTATTAATAT ATATTTTAAA TGTTTAGAAT AATCTTTATT TAATATATCC   
  
  
+ AACTTGGGCC TCTTTTTTTT TTTTTAATTT AGGGGAAAAA TAAATCAACT TATAATAAGG CCAAGAGATT   
  
  
+ ATAGAAAGAA AATGAGGCCA GTTGGTGAAA CGAACTTTCG GAGGAAGTCA ACATTTAGGC AGCCAACTAC   
  
  
+ ATCATGTTTG TCTTCTACGT TCTTCATATC TTTGGGTCGT AGGTGTTCCT TCGGCTTTCC GTCTTTGACT   
  
  
+ TTTTGTCTGT AAGCAGTCAA CATTCGTATC AAATCCCGCA CGTGCCTCAA ACGCAATTAT GCTCCAACCA   
  
  
+ AATCAAGACT AGATCAAGTT CATTTAGTGA GACAATGTTT GTTAGGACAA CTGGGTCGTC GATCCAACCT   
  
  
+ CGTGACTCTC GGTTGGAGGA TTGCAGCTTA AACGACATCG GTTGGTTGAA GAGGATTGTT ATGGGGTTTC   
  
  
+ TAGGTAATAT GTAAATTTAG AGGAAGTGGG GGTAGAAATT GAATTTATTA TAACAGCGGG GGCATGTTAG   
  
  
+ GAAAAGTTTC TGCTAATCTG CCATATCTGG GTGTCTAAAG TCAACTGCAT CCTGCTGGCA CCAACCAAAA   
  
  
+ CACTCCTGTA CTTAAAAGCA CCCTGACGCA GATCAGACGG CTCAGCACGA AAGTTTGACC TGATCTCCTT   
  
  
+ CCATGTCCAC AACGTCCCGT GGACCGTCAT AACCCTTGTG GGTCCCGCAC GCCAGACGAG GTCAATGTCC   
  
  
+ TTATGTATAT CACCACAGCT TTAATAGAAA AAAGAAATAA AATATTAAAA AACAATTTAT GTGTGCACAG   
  
  
+ CAGCCAAAAA AGCACGATGT GGTTGGATCT TATTCGCACC AACATTTGAC GGCAAAGGAT AGTGTGACAG   
  
  
+ GTCAGTGACC TAATCGACCA ATAGCATCTC GTAGCCTGGT ACTCATCTTT TTTGACCGGT GTCCCTTCCC   
  
  
+ TTTATCAACA GCACCCCTCT CTCCATTTTC TTTTCACGCT GTTCTCTCTT TTCTCTCCCT TTCTCTTTCT   
  
  
+ TTTCTTTCAT GGTGGTGGTG TTGCAGAGA  

- AAAATAATTT TTATAAAAAC TTTTTATTAT ATATTCATTT GTAGGGGGTC AATGGTTAGT TGACATGAAG   
  
  
- TGGATATGAT CATGATCATG AATGAGCACA TTCATGAAAA ATTAATAGTA GTGGTAGGTT AGATAGTGGC   
  
  
- TGTAGGAATT CTGAAATCGA AGGATAAATC TAAGTGATAA GGGGGCTTCT GTTTCTAAAT GCACTAAAGA   
  
  
- GAAAGGGACA ATATTATGTT TGTTCTGTGG GTAGTTTGAG GGTTTGCCTA GTTAGACTAA AGGTGTTGGA   
  
  
- TTCTTGTCGT TACGATACGA TCTAAAACAG ACTAAGCGAA GGATAGTAGC CTAGGATTAA CGTGGCTGGG   
  
  
- CTATTAAGTA GAGTAACTGG TATTAAAGCC CAGTATAGTC ATGGGAAAAG ATGATGAGTT AGCAATGAAT   
  
  
- TGTAGATTTG ATCGACCACC CATAATTATA TATAAAATTT ACAAATCTTA TTAGAAATAA ATTATATAGG   
  
  
- TTGAACCCGG AGAAAAAAAA AAAAATTAAA TCCCCTTTTT ATTTAGTTGA ATATTATTCC GGTTCTCTAA   
  
  
- TATCTTTCTT TTACTCCGGT CAACCACTTT GCTTGAAAGC CTCCTTCAGT TGTAAATCCG TCGGTTGATG   
  
  
- TAGTACAAAC AGAAGATGCA AGAAGTATAG AAACCCAGCA TCCACAAGGA AGCCGAAAGG CAGAAACTGA   
  
  
- AAAACAGACA TTCGTCAGTT GTAAGCATAG TTTAGGGCGT GCACGGAGTT TGCGTTAATA CGAGGTTGGT   
  
  
- TTAGTTCTGA TCTAGTTCAA GTAAATCACT CTGTTACAAA CAATCCTGTT GACCCAGCAG CTAGGTTGGA   
  
  
- GCACTGAGAG CCAACCTCCT AACGTCGAAT TTGCTGTAGC CAACCAACTT CTCCTAACAA TACCCCAAAG   
  
  
- ATCCATTATA CATTTAAATC TCCTTCACCC CCATCTTTAA CTTAAATAAT ATTGTCGCCC CCGTACAATC   
  
  
- CTTTTCAAAG ACGATTAGAC GGTATAGACC CACAGATTTC AGTTGACGTA GGACGACCGT GGTTGGTTTT   
  
  
- GTGAGGACAT GAATTTTCGT GGGACTGCGT CTAGTCTGCC GAGTCGTGCT TTCAAACTGG ACTAGAGGAA   
  
  
- GGTACAGGTG TTGCAGGGCA CCTGGCAGTA TTGGGAACAC CCAGGGCGTG CGGTCTGCTC CAGTTACAGG   
  
  
- AATACATATA GTGGTGTCGA AATTATCTTT TTTCTTTATT TTATAATTTT TTGTTAAATA CACACGTGTC   
  
  
- GTCGGTTTTT TCGTGCTACA CCAACCTAGA ATAAGCGTGG TTGTAAACTG CCGTTTCCTA TCACACTGTC   
  
  
- CAGTCACTGG ATTAGCTGGT TATCGTAGAG CATCGGACCA TGAGTAGAAA AAACTGGCCA CAGGGAAGGG   
  
  
- AAATAGTTGT CGTGGGGAGA GAGGTAAAAG AAAAGTGCGA CAAGAGAGAA AAGAGAGGGA AAGAGAAAGA   
  
  
- AAAGAAAGTA CCACCACCAC AACGTCTCT

+     GATA-motif

| Site Name | Organism | Position | Strand | Matrix score. | sequence | function |
| --- | --- | --- | --- | --- | --- | --- |
| GATA-motif | Arabidopsis thaliana | 320 | - | 7 | GATAGGA | part of a light responsive element |

> 2018/04/13 10:10:12  
+ TTTTATTAAA AATATTTTTG AAAAATAATA TATAAGTAAA CATCCCCCAG TTACCAATCA ACTGTACTTC   
  
  
+ ACCTATACTA GTACTAGTAC TTACTCGTGT AAGTACTTTT TAATTATCAT CACCATCCAA TCTATCACCG   
  
  
+ ACATCCTTAA GACTTTAGCT TCCTATTTAG ATTCACTATT CCCCCGAAGA CAAAGATTTA CGTGATTTCT   
  
  
+ CTTTCCCTGT TATAATACAA ACAAGACACC CATCAAACTC CCAAACGGAT CAATCTGATT TCCACAACCT   
  
  
+ AAGAACAGCA ATGCTATGCT AGATTTTGTC TGATTCGCTT CCTATCATCG GATCCTAATT GCACCGACCC   
  
  
+ GATAATTCAT CTCATTGACC ATAATTTCGG GTCATATCAG TACCCTTTTC TACTACTCAA TCGTTACTTA   
  
  
+ ACATCTAAAC TAGCTGGTGG GTATTAATAT ATATTTTAAA TGTTTAGAAT AATCTTTATT TAATATATCC   
  
  
+ AACTTGGGCC TCTTTTTTTT TTTTTAATTT AGGGGAAAAA TAAATCAACT TATAATAAGG CCAAGAGATT   
  
  
+ ATAGAAAGAA AATGAGGCCA GTTGGTGAAA CGAACTTTCG GAGGAAGTCA ACATTTAGGC AGCCAACTAC   
  
  
+ ATCATGTTTG TCTTCTACGT TCTTCATATC TTTGGGTCGT AGGTGTTCCT TCGGCTTTCC GTCTTTGACT   
  
  
+ TTTTGTCTGT AAGCAGTCAA CATTCGTATC AAATCCCGCA CGTGCCTCAA ACGCAATTAT GCTCCAACCA   
  
  
+ AATCAAGACT AGATCAAGTT CATTTAGTGA GACAATGTTT GTTAGGACAA CTGGGTCGTC GATCCAACCT   
  
  
+ CGTGACTCTC GGTTGGAGGA TTGCAGCTTA AACGACATCG GTTGGTTGAA GAGGATTGTT ATGGGGTTTC   
  
  
+ TAGGTAATAT GTAAATTTAG AGGAAGTGGG GGTAGAAATT GAATTTATTA TAACAGCGGG GGCATGTTAG   
  
  
+ GAAAAGTTTC TGCTAATCTG CCATATCTGG GTGTCTAAAG TCAACTGCAT CCTGCTGGCA CCAACCAAAA   
  
  
+ CACTCCTGTA CTTAAAAGCA CCCTGACGCA GATCAGACGG CTCAGCACGA AAGTTTGACC TGATCTCCTT   
  
  
+ CCATGTCCAC AACGTCCCGT GGACCGTCAT AACCCTTGTG GGTCCCGCAC GCCAGACGAG GTCAATGTCC   
  
  
+ TTATGTATAT CACCACAGCT TTAATAGAAA AAAGAAATAA AATATTAAAA AACAATTTAT GTGTGCACAG   
  
  
+ CAGCCAAAAA AGCACGATGT GGTTGGATCT TATTCGCACC AACATTTGAC GGCAAAGGAT AGTGTGACAG   
  
  
+ GTCAGTGACC TAATCGACCA ATAGCATCTC GTAGCCTGGT ACTCATCTTT TTTGACCGGT GTCCCTTCCC   
  
  
+ TTTATCAACA GCACCCCTCT CTCCATTTTC TTTTCACGCT GTTCTCTCTT TTCTCTCCCT TTCTCTTTCT   
  
  
+ TTTCTTTCAT GGTGGTGGTG TTGCAGAGA  

- AAAATAATTT TTATAAAAAC TTTTTATTAT ATATTCATTT GTAGGGGGTC AATGGTTAGT TGACATGAAG   
  
  
- TGGATATGAT CATGATCATG AATGAGCACA TTCATGAAAA ATTAATAGTA GTGGTAGGTT AGATAGTGGC   
  
  
- TGTAGGAATT CTGAAATCGA AGGATAAATC TAAGTGATAA GGGGGCTTCT GTTTCTAAAT GCACTAAAGA   
  
  
- GAAAGGGACA ATATTATGTT TGTTCTGTGG GTAGTTTGAG GGTTTGCCTA GTTAGACTAA AGGTGTTGGA   
  
  
- TTCTTGTCGT TACGATACGA TCTAAAACAG ACTAAGCGAA GGATAGTAGC CTAGGATTAA CGTGGCTGGG   
  
  
- CTATTAAGTA GAGTAACTGG TATTAAAGCC CAGTATAGTC ATGGGAAAAG ATGATGAGTT AGCAATGAAT   
  
  
- TGTAGATTTG ATCGACCACC CATAATTATA TATAAAATTT ACAAATCTTA TTAGAAATAA ATTATATAGG   
  
  
- TTGAACCCGG AGAAAAAAAA AAAAATTAAA TCCCCTTTTT ATTTAGTTGA ATATTATTCC GGTTCTCTAA   
  
  
- TATCTTTCTT TTACTCCGGT CAACCACTTT GCTTGAAAGC CTCCTTCAGT TGTAAATCCG TCGGTTGATG   
  
  
- TAGTACAAAC AGAAGATGCA AGAAGTATAG AAACCCAGCA TCCACAAGGA AGCCGAAAGG CAGAAACTGA   
  
  
- AAAACAGACA TTCGTCAGTT GTAAGCATAG TTTAGGGCGT GCACGGAGTT TGCGTTAATA CGAGGTTGGT   
  
  
- TTAGTTCTGA TCTAGTTCAA GTAAATCACT CTGTTACAAA CAATCCTGTT GACCCAGCAG CTAGGTTGGA   
  
  
- GCACTGAGAG CCAACCTCCT AACGTCGAAT TTGCTGTAGC CAACCAACTT CTCCTAACAA TACCCCAAAG   
  
  
- ATCCATTATA CATTTAAATC TCCTTCACCC CCATCTTTAA CTTAAATAAT ATTGTCGCCC CCGTACAATC   
  
  
- CTTTTCAAAG ACGATTAGAC GGTATAGACC CACAGATTTC AGTTGACGTA GGACGACCGT GGTTGGTTTT   
  
  
- GTGAGGACAT GAATTTTCGT GGGACTGCGT CTAGTCTGCC GAGTCGTGCT TTCAAACTGG ACTAGAGGAA   
  
  
- GGTACAGGTG TTGCAGGGCA CCTGGCAGTA TTGGGAACAC CCAGGGCGTG CGGTCTGCTC CAGTTACAGG   
  
  
- AATACATATA GTGGTGTCGA AATTATCTTT TTTCTTTATT TTATAATTTT TTGTTAAATA CACACGTGTC   
  
  
- GTCGGTTTTT TCGTGCTACA CCAACCTAGA ATAAGCGTGG TTGTAAACTG CCGTTTCCTA TCACACTGTC   
  
  
- CAGTCACTGG ATTAGCTGGT TATCGTAGAG CATCGGACCA TGAGTAGAAA AAACTGGCCA CAGGGAAGGG   
  
  
- AAATAGTTGT CGTGGGGAGA GAGGTAAAAG AAAAGTGCGA CAAGAGAGAA AAGAGAGGGA AAGAGAAAGA   
  
  
- AAAGAAAGTA CCACCACCAC AACGTCTCT

+     GC-motif

| Site Name | Organism | Position | Strand | Matrix score. | sequence | function |
| --- | --- | --- | --- | --- | --- | --- |
| GC-motif | Zea mays | 967 | - | 6 | CCCCCG | enhancer-like element involved in anoxic specific inducibility |
| GC-motif | Zea mays | 181 | + | 6 | CCCCCG | enhancer-like element involved in anoxic specific inducibility |

> 2018/04/13 10:10:12  
+ TTTTATTAAA AATATTTTTG AAAAATAATA TATAAGTAAA CATCCCCCAG TTACCAATCA ACTGTACTTC   
  
  
+ ACCTATACTA GTACTAGTAC TTACTCGTGT AAGTACTTTT TAATTATCAT CACCATCCAA TCTATCACCG   
  
  
+ ACATCCTTAA GACTTTAGCT TCCTATTTAG ATTCACTATT CCCCCGAAGA CAAAGATTTA CGTGATTTCT   
  
  
+ CTTTCCCTGT TATAATACAA ACAAGACACC CATCAAACTC CCAAACGGAT CAATCTGATT TCCACAACCT   
  
  
+ AAGAACAGCA ATGCTATGCT AGATTTTGTC TGATTCGCTT CCTATCATCG GATCCTAATT GCACCGACCC   
  
  
+ GATAATTCAT CTCATTGACC ATAATTTCGG GTCATATCAG TACCCTTTTC TACTACTCAA TCGTTACTTA   
  
  
+ ACATCTAAAC TAGCTGGTGG GTATTAATAT ATATTTTAAA TGTTTAGAAT AATCTTTATT TAATATATCC   
  
  
+ AACTTGGGCC TCTTTTTTTT TTTTTAATTT AGGGGAAAAA TAAATCAACT TATAATAAGG CCAAGAGATT   
  
  
+ ATAGAAAGAA AATGAGGCCA GTTGGTGAAA CGAACTTTCG GAGGAAGTCA ACATTTAGGC AGCCAACTAC   
  
  
+ ATCATGTTTG TCTTCTACGT TCTTCATATC TTTGGGTCGT AGGTGTTCCT TCGGCTTTCC GTCTTTGACT   
  
  
+ TTTTGTCTGT AAGCAGTCAA CATTCGTATC AAATCCCGCA CGTGCCTCAA ACGCAATTAT GCTCCAACCA   
  
  
+ AATCAAGACT AGATCAAGTT CATTTAGTGA GACAATGTTT GTTAGGACAA CTGGGTCGTC GATCCAACCT   
  
  
+ CGTGACTCTC GGTTGGAGGA TTGCAGCTTA AACGACATCG GTTGGTTGAA GAGGATTGTT ATGGGGTTTC   
  
  
+ TAGGTAATAT GTAAATTTAG AGGAAGTGGG GGTAGAAATT GAATTTATTA TAACAGCGGG GGCATGTTAG   
  
  
+ GAAAAGTTTC TGCTAATCTG CCATATCTGG GTGTCTAAAG TCAACTGCAT CCTGCTGGCA CCAACCAAAA   
  
  
+ CACTCCTGTA CTTAAAAGCA CCCTGACGCA GATCAGACGG CTCAGCACGA AAGTTTGACC TGATCTCCTT   
  
  
+ CCATGTCCAC AACGTCCCGT GGACCGTCAT AACCCTTGTG GGTCCCGCAC GCCAGACGAG GTCAATGTCC   
  
  
+ TTATGTATAT CACCACAGCT TTAATAGAAA AAAGAAATAA AATATTAAAA AACAATTTAT GTGTGCACAG   
  
  
+ CAGCCAAAAA AGCACGATGT GGTTGGATCT TATTCGCACC AACATTTGAC GGCAAAGGAT AGTGTGACAG   
  
  
+ GTCAGTGACC TAATCGACCA ATAGCATCTC GTAGCCTGGT ACTCATCTTT TTTGACCGGT GTCCCTTCCC   
  
  
+ TTTATCAACA GCACCCCTCT CTCCATTTTC TTTTCACGCT GTTCTCTCTT TTCTCTCCCT TTCTCTTTCT   
  
  
+ TTTCTTTCAT GGTGGTGGTG TTGCAGAGA  

- AAAATAATTT TTATAAAAAC TTTTTATTAT ATATTCATTT GTAGGGGGTC AATGGTTAGT TGACATGAAG   
  
  
- TGGATATGAT CATGATCATG AATGAGCACA TTCATGAAAA ATTAATAGTA GTGGTAGGTT AGATAGTGGC   
  
  
- TGTAGGAATT CTGAAATCGA AGGATAAATC TAAGTGATAA GGGGGCTTCT GTTTCTAAAT GCACTAAAGA   
  
  
- GAAAGGGACA ATATTATGTT TGTTCTGTGG GTAGTTTGAG GGTTTGCCTA GTTAGACTAA AGGTGTTGGA   
  
  
- TTCTTGTCGT TACGATACGA TCTAAAACAG ACTAAGCGAA GGATAGTAGC CTAGGATTAA CGTGGCTGGG   
  
  
- CTATTAAGTA GAGTAACTGG TATTAAAGCC CAGTATAGTC ATGGGAAAAG ATGATGAGTT AGCAATGAAT   
  
  
- TGTAGATTTG ATCGACCACC CATAATTATA TATAAAATTT ACAAATCTTA TTAGAAATAA ATTATATAGG   
  
  
- TTGAACCCGG AGAAAAAAAA AAAAATTAAA TCCCCTTTTT ATTTAGTTGA ATATTATTCC GGTTCTCTAA   
  
  
- TATCTTTCTT TTACTCCGGT CAACCACTTT GCTTGAAAGC CTCCTTCAGT TGTAAATCCG TCGGTTGATG   
  
  
- TAGTACAAAC AGAAGATGCA AGAAGTATAG AAACCCAGCA TCCACAAGGA AGCCGAAAGG CAGAAACTGA   
  
  
- AAAACAGACA TTCGTCAGTT GTAAGCATAG TTTAGGGCGT GCACGGAGTT TGCGTTAATA CGAGGTTGGT   
  
  
- TTAGTTCTGA TCTAGTTCAA GTAAATCACT CTGTTACAAA CAATCCTGTT GACCCAGCAG CTAGGTTGGA   
  
  
- GCACTGAGAG CCAACCTCCT AACGTCGAAT TTGCTGTAGC CAACCAACTT CTCCTAACAA TACCCCAAAG   
  
  
- ATCCATTATA CATTTAAATC TCCTTCACCC CCATCTTTAA CTTAAATAAT ATTGTCGCCC CCGTACAATC   
  
  
- CTTTTCAAAG ACGATTAGAC GGTATAGACC CACAGATTTC AGTTGACGTA GGACGACCGT GGTTGGTTTT   
  
  
- GTGAGGACAT GAATTTTCGT GGGACTGCGT CTAGTCTGCC GAGTCGTGCT TTCAAACTGG ACTAGAGGAA   
  
  
- GGTACAGGTG TTGCAGGGCA CCTGGCAGTA TTGGGAACAC CCAGGGCGTG CGGTCTGCTC CAGTTACAGG   
  
  
- AATACATATA GTGGTGTCGA AATTATCTTT TTTCTTTATT TTATAATTTT TTGTTAAATA CACACGTGTC   
  
  
- GTCGGTTTTT TCGTGCTACA CCAACCTAGA ATAAGCGTGG TTGTAAACTG CCGTTTCCTA TCACACTGTC   
  
  
- CAGTCACTGG ATTAGCTGGT TATCGTAGAG CATCGGACCA TGAGTAGAAA AAACTGGCCA CAGGGAAGGG   
  
  
- AAATAGTTGT CGTGGGGAGA GAGGTAAAAG AAAAGTGCGA CAAGAGAGAA AAGAGAGGGA AAGAGAAAGA   
  
  
- AAAGAAAGTA CCACCACCAC AACGTCTCT

+     Gap-box

| Site Name | Organism | Position | Strand | Matrix score. | sequence | function |
| --- | --- | --- | --- | --- | --- | --- |
| Gap-box | Arabidopsis thaliana | 1420 | - | 9 | AAATGGAGA | part of a light responsive element |

> 2018/04/13 10:10:12  
+ TTTTATTAAA AATATTTTTG AAAAATAATA TATAAGTAAA CATCCCCCAG TTACCAATCA ACTGTACTTC   
  
  
+ ACCTATACTA GTACTAGTAC TTACTCGTGT AAGTACTTTT TAATTATCAT CACCATCCAA TCTATCACCG   
  
  
+ ACATCCTTAA GACTTTAGCT TCCTATTTAG ATTCACTATT CCCCCGAAGA CAAAGATTTA CGTGATTTCT   
  
  
+ CTTTCCCTGT TATAATACAA ACAAGACACC CATCAAACTC CCAAACGGAT CAATCTGATT TCCACAACCT   
  
  
+ AAGAACAGCA ATGCTATGCT AGATTTTGTC TGATTCGCTT CCTATCATCG GATCCTAATT GCACCGACCC   
  
  
+ GATAATTCAT CTCATTGACC ATAATTTCGG GTCATATCAG TACCCTTTTC TACTACTCAA TCGTTACTTA   
  
  
+ ACATCTAAAC TAGCTGGTGG GTATTAATAT ATATTTTAAA TGTTTAGAAT AATCTTTATT TAATATATCC   
  
  
+ AACTTGGGCC TCTTTTTTTT TTTTTAATTT AGGGGAAAAA TAAATCAACT TATAATAAGG CCAAGAGATT   
  
  
+ ATAGAAAGAA AATGAGGCCA GTTGGTGAAA CGAACTTTCG GAGGAAGTCA ACATTTAGGC AGCCAACTAC   
  
  
+ ATCATGTTTG TCTTCTACGT TCTTCATATC TTTGGGTCGT AGGTGTTCCT TCGGCTTTCC GTCTTTGACT   
  
  
+ TTTTGTCTGT AAGCAGTCAA CATTCGTATC AAATCCCGCA CGTGCCTCAA ACGCAATTAT GCTCCAACCA   
  
  
+ AATCAAGACT AGATCAAGTT CATTTAGTGA GACAATGTTT GTTAGGACAA CTGGGTCGTC GATCCAACCT   
  
  
+ CGTGACTCTC GGTTGGAGGA TTGCAGCTTA AACGACATCG GTTGGTTGAA GAGGATTGTT ATGGGGTTTC   
  
  
+ TAGGTAATAT GTAAATTTAG AGGAAGTGGG GGTAGAAATT GAATTTATTA TAACAGCGGG GGCATGTTAG   
  
  
+ GAAAAGTTTC TGCTAATCTG CCATATCTGG GTGTCTAAAG TCAACTGCAT CCTGCTGGCA CCAACCAAAA   
  
  
+ CACTCCTGTA CTTAAAAGCA CCCTGACGCA GATCAGACGG CTCAGCACGA AAGTTTGACC TGATCTCCTT   
  
  
+ CCATGTCCAC AACGTCCCGT GGACCGTCAT AACCCTTGTG GGTCCCGCAC GCCAGACGAG GTCAATGTCC   
  
  
+ TTATGTATAT CACCACAGCT TTAATAGAAA AAAGAAATAA AATATTAAAA AACAATTTAT GTGTGCACAG   
  
  
+ CAGCCAAAAA AGCACGATGT GGTTGGATCT TATTCGCACC AACATTTGAC GGCAAAGGAT AGTGTGACAG   
  
  
+ GTCAGTGACC TAATCGACCA ATAGCATCTC GTAGCCTGGT ACTCATCTTT TTTGACCGGT GTCCCTTCCC   
  
  
+ TTTATCAACA GCACCCCTCT CTCCATTTTC TTTTCACGCT GTTCTCTCTT TTCTCTCCCT TTCTCTTTCT   
  
  
+ TTTCTTTCAT GGTGGTGGTG TTGCAGAGA  

- AAAATAATTT TTATAAAAAC TTTTTATTAT ATATTCATTT GTAGGGGGTC AATGGTTAGT TGACATGAAG   
  
  
- TGGATATGAT CATGATCATG AATGAGCACA TTCATGAAAA ATTAATAGTA GTGGTAGGTT AGATAGTGGC   
  
  
- TGTAGGAATT CTGAAATCGA AGGATAAATC TAAGTGATAA GGGGGCTTCT GTTTCTAAAT GCACTAAAGA   
  
  
- GAAAGGGACA ATATTATGTT TGTTCTGTGG GTAGTTTGAG GGTTTGCCTA GTTAGACTAA AGGTGTTGGA   
  
  
- TTCTTGTCGT TACGATACGA TCTAAAACAG ACTAAGCGAA GGATAGTAGC CTAGGATTAA CGTGGCTGGG   
  
  
- CTATTAAGTA GAGTAACTGG TATTAAAGCC CAGTATAGTC ATGGGAAAAG ATGATGAGTT AGCAATGAAT   
  
  
- TGTAGATTTG ATCGACCACC CATAATTATA TATAAAATTT ACAAATCTTA TTAGAAATAA ATTATATAGG   
  
  
- TTGAACCCGG AGAAAAAAAA AAAAATTAAA TCCCCTTTTT ATTTAGTTGA ATATTATTCC GGTTCTCTAA   
  
  
- TATCTTTCTT TTACTCCGGT CAACCACTTT GCTTGAAAGC CTCCTTCAGT TGTAAATCCG TCGGTTGATG   
  
  
- TAGTACAAAC AGAAGATGCA AGAAGTATAG AAACCCAGCA TCCACAAGGA AGCCGAAAGG CAGAAACTGA   
  
  
- AAAACAGACA TTCGTCAGTT GTAAGCATAG TTTAGGGCGT GCACGGAGTT TGCGTTAATA CGAGGTTGGT   
  
  
- TTAGTTCTGA TCTAGTTCAA GTAAATCACT CTGTTACAAA CAATCCTGTT GACCCAGCAG CTAGGTTGGA   
  
  
- GCACTGAGAG CCAACCTCCT AACGTCGAAT TTGCTGTAGC CAACCAACTT CTCCTAACAA TACCCCAAAG   
  
  
- ATCCATTATA CATTTAAATC TCCTTCACCC CCATCTTTAA CTTAAATAAT ATTGTCGCCC CCGTACAATC   
  
  
- CTTTTCAAAG ACGATTAGAC GGTATAGACC CACAGATTTC AGTTGACGTA GGACGACCGT GGTTGGTTTT   
  
  
- GTGAGGACAT GAATTTTCGT GGGACTGCGT CTAGTCTGCC GAGTCGTGCT TTCAAACTGG ACTAGAGGAA   
  
  
- GGTACAGGTG TTGCAGGGCA CCTGGCAGTA TTGGGAACAC CCAGGGCGTG CGGTCTGCTC CAGTTACAGG   
  
  
- AATACATATA GTGGTGTCGA AATTATCTTT TTTCTTTATT TTATAATTTT TTGTTAAATA CACACGTGTC   
  
  
- GTCGGTTTTT TCGTGCTACA CCAACCTAGA ATAAGCGTGG TTGTAAACTG CCGTTTCCTA TCACACTGTC   
  
  
- CAGTCACTGG ATTAGCTGGT TATCGTAGAG CATCGGACCA TGAGTAGAAA AAACTGGCCA CAGGGAAGGG   
  
  
- AAATAGTTGT CGTGGGGAGA GAGGTAAAAG AAAAGTGCGA CAAGAGAGAA AAGAGAGGGA AAGAGAAAGA   
  
  
- AAAGAAAGTA CCACCACCAC AACGTCTCT

+     I-box

| Site Name | Organism | Position | Strand | Matrix score. | sequence | function |
| --- | --- | --- | --- | --- | --- | --- |
| I-box | Solanum tuberosum | 909 | - | 10 | TATTATCTAGA | part of a light responsive element |
| I-box | Flaveria trinervia | 1001 | - | 7 | GATATGG | part of a light responsive element |

> 2018/04/13 10:10:12  
+ TTTTATTAAA AATATTTTTG AAAAATAATA TATAAGTAAA CATCCCCCAG TTACCAATCA ACTGTACTTC   
  
  
+ ACCTATACTA GTACTAGTAC TTACTCGTGT AAGTACTTTT TAATTATCAT CACCATCCAA TCTATCACCG   
  
  
+ ACATCCTTAA GACTTTAGCT TCCTATTTAG ATTCACTATT CCCCCGAAGA CAAAGATTTA CGTGATTTCT   
  
  
+ CTTTCCCTGT TATAATACAA ACAAGACACC CATCAAACTC CCAAACGGAT CAATCTGATT TCCACAACCT   
  
  
+ AAGAACAGCA ATGCTATGCT AGATTTTGTC TGATTCGCTT CCTATCATCG GATCCTAATT GCACCGACCC   
  
  
+ GATAATTCAT CTCATTGACC ATAATTTCGG GTCATATCAG TACCCTTTTC TACTACTCAA TCGTTACTTA   
  
  
+ ACATCTAAAC TAGCTGGTGG GTATTAATAT ATATTTTAAA TGTTTAGAAT AATCTTTATT TAATATATCC   
  
  
+ AACTTGGGCC TCTTTTTTTT TTTTTAATTT AGGGGAAAAA TAAATCAACT TATAATAAGG CCAAGAGATT   
  
  
+ ATAGAAAGAA AATGAGGCCA GTTGGTGAAA CGAACTTTCG GAGGAAGTCA ACATTTAGGC AGCCAACTAC   
  
  
+ ATCATGTTTG TCTTCTACGT TCTTCATATC TTTGGGTCGT AGGTGTTCCT TCGGCTTTCC GTCTTTGACT   
  
  
+ TTTTGTCTGT AAGCAGTCAA CATTCGTATC AAATCCCGCA CGTGCCTCAA ACGCAATTAT GCTCCAACCA   
  
  
+ AATCAAGACT AGATCAAGTT CATTTAGTGA GACAATGTTT GTTAGGACAA CTGGGTCGTC GATCCAACCT   
  
  
+ CGTGACTCTC GGTTGGAGGA TTGCAGCTTA AACGACATCG GTTGGTTGAA GAGGATTGTT ATGGGGTTTC   
  
  
+ TAGGTAATAT GTAAATTTAG AGGAAGTGGG GGTAGAAATT GAATTTATTA TAACAGCGGG GGCATGTTAG   
  
  
+ GAAAAGTTTC TGCTAATCTG CCATATCTGG GTGTCTAAAG TCAACTGCAT CCTGCTGGCA CCAACCAAAA   
  
  
+ CACTCCTGTA CTTAAAAGCA CCCTGACGCA GATCAGACGG CTCAGCACGA AAGTTTGACC TGATCTCCTT   
  
  
+ CCATGTCCAC AACGTCCCGT GGACCGTCAT AACCCTTGTG GGTCCCGCAC GCCAGACGAG GTCAATGTCC   
  
  
+ TTATGTATAT CACCACAGCT TTAATAGAAA AAAGAAATAA AATATTAAAA AACAATTTAT GTGTGCACAG   
  
  
+ CAGCCAAAAA AGCACGATGT GGTTGGATCT TATTCGCACC AACATTTGAC GGCAAAGGAT AGTGTGACAG   
  
  
+ GTCAGTGACC TAATCGACCA ATAGCATCTC GTAGCCTGGT ACTCATCTTT TTTGACCGGT GTCCCTTCCC   
  
  
+ TTTATCAACA GCACCCCTCT CTCCATTTTC TTTTCACGCT GTTCTCTCTT TTCTCTCCCT TTCTCTTTCT   
  
  
+ TTTCTTTCAT GGTGGTGGTG TTGCAGAGA  

- AAAATAATTT TTATAAAAAC TTTTTATTAT ATATTCATTT GTAGGGGGTC AATGGTTAGT TGACATGAAG   
  
  
- TGGATATGAT CATGATCATG AATGAGCACA TTCATGAAAA ATTAATAGTA GTGGTAGGTT AGATAGTGGC   
  
  
- TGTAGGAATT CTGAAATCGA AGGATAAATC TAAGTGATAA GGGGGCTTCT GTTTCTAAAT GCACTAAAGA   
  
  
- GAAAGGGACA ATATTATGTT TGTTCTGTGG GTAGTTTGAG GGTTTGCCTA GTTAGACTAA AGGTGTTGGA   
  
  
- TTCTTGTCGT TACGATACGA TCTAAAACAG ACTAAGCGAA GGATAGTAGC CTAGGATTAA CGTGGCTGGG   
  
  
- CTATTAAGTA GAGTAACTGG TATTAAAGCC CAGTATAGTC ATGGGAAAAG ATGATGAGTT AGCAATGAAT   
  
  
- TGTAGATTTG ATCGACCACC CATAATTATA TATAAAATTT ACAAATCTTA TTAGAAATAA ATTATATAGG   
  
  
- TTGAACCCGG AGAAAAAAAA AAAAATTAAA TCCCCTTTTT ATTTAGTTGA ATATTATTCC GGTTCTCTAA   
  
  
- TATCTTTCTT TTACTCCGGT CAACCACTTT GCTTGAAAGC CTCCTTCAGT TGTAAATCCG TCGGTTGATG   
  
  
- TAGTACAAAC AGAAGATGCA AGAAGTATAG AAACCCAGCA TCCACAAGGA AGCCGAAAGG CAGAAACTGA   
  
  
- AAAACAGACA TTCGTCAGTT GTAAGCATAG TTTAGGGCGT GCACGGAGTT TGCGTTAATA CGAGGTTGGT   
  
  
- TTAGTTCTGA TCTAGTTCAA GTAAATCACT CTGTTACAAA CAATCCTGTT GACCCAGCAG CTAGGTTGGA   
  
  
- GCACTGAGAG CCAACCTCCT AACGTCGAAT TTGCTGTAGC CAACCAACTT CTCCTAACAA TACCCCAAAG   
  
  
- ATCCATTATA CATTTAAATC TCCTTCACCC CCATCTTTAA CTTAAATAAT ATTGTCGCCC CCGTACAATC   
  
  
- CTTTTCAAAG ACGATTAGAC GGTATAGACC CACAGATTTC AGTTGACGTA GGACGACCGT GGTTGGTTTT   
  
  
- GTGAGGACAT GAATTTTCGT GGGACTGCGT CTAGTCTGCC GAGTCGTGCT TTCAAACTGG ACTAGAGGAA   
  
  
- GGTACAGGTG TTGCAGGGCA CCTGGCAGTA TTGGGAACAC CCAGGGCGTG CGGTCTGCTC CAGTTACAGG   
  
  
- AATACATATA GTGGTGTCGA AATTATCTTT TTTCTTTATT TTATAATTTT TTGTTAAATA CACACGTGTC   
  
  
- GTCGGTTTTT TCGTGCTACA CCAACCTAGA ATAAGCGTGG TTGTAAACTG CCGTTTCCTA TCACACTGTC   
  
  
- CAGTCACTGG ATTAGCTGGT TATCGTAGAG CATCGGACCA TGAGTAGAAA AAACTGGCCA CAGGGAAGGG   
  
  
- AAATAGTTGT CGTGGGGAGA GAGGTAAAAG AAAAGTGCGA CAAGAGAGAA AAGAGAGGGA AAGAGAAAGA   
  
  
- AAAGAAAGTA CCACCACCAC AACGTCTCT

+     LAMP-element

| Site Name | Organism | Position | Strand | Matrix score. | sequence | function |
| --- | --- | --- | --- | --- | --- | --- |
| LAMP-element | Pisum sativum | 1400 | + | 8 | CTTTATCA | part of a light responsive element |

> 2018/04/13 10:10:12  
+ TTTTATTAAA AATATTTTTG AAAAATAATA TATAAGTAAA CATCCCCCAG TTACCAATCA ACTGTACTTC   
  
  
+ ACCTATACTA GTACTAGTAC TTACTCGTGT AAGTACTTTT TAATTATCAT CACCATCCAA TCTATCACCG   
  
  
+ ACATCCTTAA GACTTTAGCT TCCTATTTAG ATTCACTATT CCCCCGAAGA CAAAGATTTA CGTGATTTCT   
  
  
+ CTTTCCCTGT TATAATACAA ACAAGACACC CATCAAACTC CCAAACGGAT CAATCTGATT TCCACAACCT   
  
  
+ AAGAACAGCA ATGCTATGCT AGATTTTGTC TGATTCGCTT CCTATCATCG GATCCTAATT GCACCGACCC   
  
  
+ GATAATTCAT CTCATTGACC ATAATTTCGG GTCATATCAG TACCCTTTTC TACTACTCAA TCGTTACTTA   
  
  
+ ACATCTAAAC TAGCTGGTGG GTATTAATAT ATATTTTAAA TGTTTAGAAT AATCTTTATT TAATATATCC   
  
  
+ AACTTGGGCC TCTTTTTTTT TTTTTAATTT AGGGGAAAAA TAAATCAACT TATAATAAGG CCAAGAGATT   
  
  
+ ATAGAAAGAA AATGAGGCCA GTTGGTGAAA CGAACTTTCG GAGGAAGTCA ACATTTAGGC AGCCAACTAC   
  
  
+ ATCATGTTTG TCTTCTACGT TCTTCATATC TTTGGGTCGT AGGTGTTCCT TCGGCTTTCC GTCTTTGACT   
  
  
+ TTTTGTCTGT AAGCAGTCAA CATTCGTATC AAATCCCGCA CGTGCCTCAA ACGCAATTAT GCTCCAACCA   
  
  
+ AATCAAGACT AGATCAAGTT CATTTAGTGA GACAATGTTT GTTAGGACAA CTGGGTCGTC GATCCAACCT   
  
  
+ CGTGACTCTC GGTTGGAGGA TTGCAGCTTA AACGACATCG GTTGGTTGAA GAGGATTGTT ATGGGGTTTC   
  
  
+ TAGGTAATAT GTAAATTTAG AGGAAGTGGG GGTAGAAATT GAATTTATTA TAACAGCGGG GGCATGTTAG   
  
  
+ GAAAAGTTTC TGCTAATCTG CCATATCTGG GTGTCTAAAG TCAACTGCAT CCTGCTGGCA CCAACCAAAA   
  
  
+ CACTCCTGTA CTTAAAAGCA CCCTGACGCA GATCAGACGG CTCAGCACGA AAGTTTGACC TGATCTCCTT   
  
  
+ CCATGTCCAC AACGTCCCGT GGACCGTCAT AACCCTTGTG GGTCCCGCAC GCCAGACGAG GTCAATGTCC   
  
  
+ TTATGTATAT CACCACAGCT TTAATAGAAA AAAGAAATAA AATATTAAAA AACAATTTAT GTGTGCACAG   
  
  
+ CAGCCAAAAA AGCACGATGT GGTTGGATCT TATTCGCACC AACATTTGAC GGCAAAGGAT AGTGTGACAG   
  
  
+ GTCAGTGACC TAATCGACCA ATAGCATCTC GTAGCCTGGT ACTCATCTTT TTTGACCGGT GTCCCTTCCC   
  
  
+ TTTATCAACA GCACCCCTCT CTCCATTTTC TTTTCACGCT GTTCTCTCTT TTCTCTCCCT TTCTCTTTCT   
  
  
+ TTTCTTTCAT GGTGGTGGTG TTGCAGAGA  

- AAAATAATTT TTATAAAAAC TTTTTATTAT ATATTCATTT GTAGGGGGTC AATGGTTAGT TGACATGAAG   
  
  
- TGGATATGAT CATGATCATG AATGAGCACA TTCATGAAAA ATTAATAGTA GTGGTAGGTT AGATAGTGGC   
  
  
- TGTAGGAATT CTGAAATCGA AGGATAAATC TAAGTGATAA GGGGGCTTCT GTTTCTAAAT GCACTAAAGA   
  
  
- GAAAGGGACA ATATTATGTT TGTTCTGTGG GTAGTTTGAG GGTTTGCCTA GTTAGACTAA AGGTGTTGGA   
  
  
- TTCTTGTCGT TACGATACGA TCTAAAACAG ACTAAGCGAA GGATAGTAGC CTAGGATTAA CGTGGCTGGG   
  
  
- CTATTAAGTA GAGTAACTGG TATTAAAGCC CAGTATAGTC ATGGGAAAAG ATGATGAGTT AGCAATGAAT   
  
  
- TGTAGATTTG ATCGACCACC CATAATTATA TATAAAATTT ACAAATCTTA TTAGAAATAA ATTATATAGG   
  
  
- TTGAACCCGG AGAAAAAAAA AAAAATTAAA TCCCCTTTTT ATTTAGTTGA ATATTATTCC GGTTCTCTAA   
  
  
- TATCTTTCTT TTACTCCGGT CAACCACTTT GCTTGAAAGC CTCCTTCAGT TGTAAATCCG TCGGTTGATG   
  
  
- TAGTACAAAC AGAAGATGCA AGAAGTATAG AAACCCAGCA TCCACAAGGA AGCCGAAAGG CAGAAACTGA   
  
  
- AAAACAGACA TTCGTCAGTT GTAAGCATAG TTTAGGGCGT GCACGGAGTT TGCGTTAATA CGAGGTTGGT   
  
  
- TTAGTTCTGA TCTAGTTCAA GTAAATCACT CTGTTACAAA CAATCCTGTT GACCCAGCAG CTAGGTTGGA   
  
  
- GCACTGAGAG CCAACCTCCT AACGTCGAAT TTGCTGTAGC CAACCAACTT CTCCTAACAA TACCCCAAAG   
  
  
- ATCCATTATA CATTTAAATC TCCTTCACCC CCATCTTTAA CTTAAATAAT ATTGTCGCCC CCGTACAATC   
  
  
- CTTTTCAAAG ACGATTAGAC GGTATAGACC CACAGATTTC AGTTGACGTA GGACGACCGT GGTTGGTTTT   
  
  
- GTGAGGACAT GAATTTTCGT GGGACTGCGT CTAGTCTGCC GAGTCGTGCT TTCAAACTGG ACTAGAGGAA   
  
  
- GGTACAGGTG TTGCAGGGCA CCTGGCAGTA TTGGGAACAC CCAGGGCGTG CGGTCTGCTC CAGTTACAGG   
  
  
- AATACATATA GTGGTGTCGA AATTATCTTT TTTCTTTATT TTATAATTTT TTGTTAAATA CACACGTGTC   
  
  
- GTCGGTTTTT TCGTGCTACA CCAACCTAGA ATAAGCGTGG TTGTAAACTG CCGTTTCCTA TCACACTGTC   
  
  
- CAGTCACTGG ATTAGCTGGT TATCGTAGAG CATCGGACCA TGAGTAGAAA AAACTGGCCA CAGGGAAGGG   
  
  
- AAATAGTTGT CGTGGGGAGA GAGGTAAAAG AAAAGTGCGA CAAGAGAGAA AAGAGAGGGA AAGAGAAAGA   
  
  
- AAAGAAAGTA CCACCACCAC AACGTCTCT

+     LTR

| Site Name | Organism | Position | Strand | Matrix score. | sequence | function |
| --- | --- | --- | --- | --- | --- | --- |
| LTR | Hordeum vulgare | 375 | - | 6 | CCGAAA | cis-acting element involved in low-temperature responsiveness |
| LTR | Hordeum vulgare | 596 | - | 6 | CCGAAA | cis-acting element involved in low-temperature responsiveness |

> 2018/04/13 10:10:12  
+ TTTTATTAAA AATATTTTTG AAAAATAATA TATAAGTAAA CATCCCCCAG TTACCAATCA ACTGTACTTC   
  
  
+ ACCTATACTA GTACTAGTAC TTACTCGTGT AAGTACTTTT TAATTATCAT CACCATCCAA TCTATCACCG   
  
  
+ ACATCCTTAA GACTTTAGCT TCCTATTTAG ATTCACTATT CCCCCGAAGA CAAAGATTTA CGTGATTTCT   
  
  
+ CTTTCCCTGT TATAATACAA ACAAGACACC CATCAAACTC CCAAACGGAT CAATCTGATT TCCACAACCT   
  
  
+ AAGAACAGCA ATGCTATGCT AGATTTTGTC TGATTCGCTT CCTATCATCG GATCCTAATT GCACCGACCC   
  
  
+ GATAATTCAT CTCATTGACC ATAATTTCGG GTCATATCAG TACCCTTTTC TACTACTCAA TCGTTACTTA   
  
  
+ ACATCTAAAC TAGCTGGTGG GTATTAATAT ATATTTTAAA TGTTTAGAAT AATCTTTATT TAATATATCC   
  
  
+ AACTTGGGCC TCTTTTTTTT TTTTTAATTT AGGGGAAAAA TAAATCAACT TATAATAAGG CCAAGAGATT   
  
  
+ ATAGAAAGAA AATGAGGCCA GTTGGTGAAA CGAACTTTCG GAGGAAGTCA ACATTTAGGC AGCCAACTAC   
  
  
+ ATCATGTTTG TCTTCTACGT TCTTCATATC TTTGGGTCGT AGGTGTTCCT TCGGCTTTCC GTCTTTGACT   
  
  
+ TTTTGTCTGT AAGCAGTCAA CATTCGTATC AAATCCCGCA CGTGCCTCAA ACGCAATTAT GCTCCAACCA   
  
  
+ AATCAAGACT AGATCAAGTT CATTTAGTGA GACAATGTTT GTTAGGACAA CTGGGTCGTC GATCCAACCT   
  
  
+ CGTGACTCTC GGTTGGAGGA TTGCAGCTTA AACGACATCG GTTGGTTGAA GAGGATTGTT ATGGGGTTTC   
  
  
+ TAGGTAATAT GTAAATTTAG AGGAAGTGGG GGTAGAAATT GAATTTATTA TAACAGCGGG GGCATGTTAG   
  
  
+ GAAAAGTTTC TGCTAATCTG CCATATCTGG GTGTCTAAAG TCAACTGCAT CCTGCTGGCA CCAACCAAAA   
  
  
+ CACTCCTGTA CTTAAAAGCA CCCTGACGCA GATCAGACGG CTCAGCACGA AAGTTTGACC TGATCTCCTT   
  
  
+ CCATGTCCAC AACGTCCCGT GGACCGTCAT AACCCTTGTG GGTCCCGCAC GCCAGACGAG GTCAATGTCC   
  
  
+ TTATGTATAT CACCACAGCT TTAATAGAAA AAAGAAATAA AATATTAAAA AACAATTTAT GTGTGCACAG   
  
  
+ CAGCCAAAAA AGCACGATGT GGTTGGATCT TATTCGCACC AACATTTGAC GGCAAAGGAT AGTGTGACAG   
  
  
+ GTCAGTGACC TAATCGACCA ATAGCATCTC GTAGCCTGGT ACTCATCTTT TTTGACCGGT GTCCCTTCCC   
  
  
+ TTTATCAACA GCACCCCTCT CTCCATTTTC TTTTCACGCT GTTCTCTCTT TTCTCTCCCT TTCTCTTTCT   
  
  
+ TTTCTTTCAT GGTGGTGGTG TTGCAGAGA  

- AAAATAATTT TTATAAAAAC TTTTTATTAT ATATTCATTT GTAGGGGGTC AATGGTTAGT TGACATGAAG   
  
  
- TGGATATGAT CATGATCATG AATGAGCACA TTCATGAAAA ATTAATAGTA GTGGTAGGTT AGATAGTGGC   
  
  
- TGTAGGAATT CTGAAATCGA AGGATAAATC TAAGTGATAA GGGGGCTTCT GTTTCTAAAT GCACTAAAGA   
  
  
- GAAAGGGACA ATATTATGTT TGTTCTGTGG GTAGTTTGAG GGTTTGCCTA GTTAGACTAA AGGTGTTGGA   
  
  
- TTCTTGTCGT TACGATACGA TCTAAAACAG ACTAAGCGAA GGATAGTAGC CTAGGATTAA CGTGGCTGGG   
  
  
- CTATTAAGTA GAGTAACTGG TATTAAAGCC CAGTATAGTC ATGGGAAAAG ATGATGAGTT AGCAATGAAT   
  
  
- TGTAGATTTG ATCGACCACC CATAATTATA TATAAAATTT ACAAATCTTA TTAGAAATAA ATTATATAGG   
  
  
- TTGAACCCGG AGAAAAAAAA AAAAATTAAA TCCCCTTTTT ATTTAGTTGA ATATTATTCC GGTTCTCTAA   
  
  
- TATCTTTCTT TTACTCCGGT CAACCACTTT GCTTGAAAGC CTCCTTCAGT TGTAAATCCG TCGGTTGATG   
  
  
- TAGTACAAAC AGAAGATGCA AGAAGTATAG AAACCCAGCA TCCACAAGGA AGCCGAAAGG CAGAAACTGA   
  
  
- AAAACAGACA TTCGTCAGTT GTAAGCATAG TTTAGGGCGT GCACGGAGTT TGCGTTAATA CGAGGTTGGT   
  
  
- TTAGTTCTGA TCTAGTTCAA GTAAATCACT CTGTTACAAA CAATCCTGTT GACCCAGCAG CTAGGTTGGA   
  
  
- GCACTGAGAG CCAACCTCCT AACGTCGAAT TTGCTGTAGC CAACCAACTT CTCCTAACAA TACCCCAAAG   
  
  
- ATCCATTATA CATTTAAATC TCCTTCACCC CCATCTTTAA CTTAAATAAT ATTGTCGCCC CCGTACAATC   
  
  
- CTTTTCAAAG ACGATTAGAC GGTATAGACC CACAGATTTC AGTTGACGTA GGACGACCGT GGTTGGTTTT   
  
  
- GTGAGGACAT GAATTTTCGT GGGACTGCGT CTAGTCTGCC GAGTCGTGCT TTCAAACTGG ACTAGAGGAA   
  
  
- GGTACAGGTG TTGCAGGGCA CCTGGCAGTA TTGGGAACAC CCAGGGCGTG CGGTCTGCTC CAGTTACAGG   
  
  
- AATACATATA GTGGTGTCGA AATTATCTTT TTTCTTTATT TTATAATTTT TTGTTAAATA CACACGTGTC   
  
  
- GTCGGTTTTT TCGTGCTACA CCAACCTAGA ATAAGCGTGG TTGTAAACTG CCGTTTCCTA TCACACTGTC   
  
  
- CAGTCACTGG ATTAGCTGGT TATCGTAGAG CATCGGACCA TGAGTAGAAA AAACTGGCCA CAGGGAAGGG   
  
  
- AAATAGTTGT CGTGGGGAGA GAGGTAAAAG AAAAGTGCGA CAAGAGAGAA AAGAGAGGGA AAGAGAAAGA   
  
  
- AAAGAAAGTA CCACCACCAC AACGTCTCT

+     MBS

| Site Name | Organism | Position | Strand | Matrix score. | sequence | function |
| --- | --- | --- | --- | --- | --- | --- |
| MBS | Zea mays | 1383 | - | 6 | CGGTCA | MYB Binding Site |
| MBS | Arabidopsis thaliana | 1022 | + | 6 | CAACTG | MYB binding site involved in drought-inducibility |
| MBS | Arabidopsis thaliana | 59 | + | 6 | CAACTG | MYB binding site involved in drought-inducibility |
| MBS | Arabidopsis thaliana | 818 | + | 6 | CAACTG | MYB binding site involved in drought-inducibility |
| MBS | Arabidopsis thaliana | 48 | - | 6 | TAACTG | MYB binding site involved in drought-inducibility |
| MBS | Arabidopsis thaliana | 579 | - | 6 | CAACTG | MYB binding site involved in drought-inducibility |

> 2018/04/13 10:10:12  
+ TTTTATTAAA AATATTTTTG AAAAATAATA TATAAGTAAA CATCCCCCAG TTACCAATCA ACTGTACTTC   
  
  
+ ACCTATACTA GTACTAGTAC TTACTCGTGT AAGTACTTTT TAATTATCAT CACCATCCAA TCTATCACCG   
  
  
+ ACATCCTTAA GACTTTAGCT TCCTATTTAG ATTCACTATT CCCCCGAAGA CAAAGATTTA CGTGATTTCT   
  
  
+ CTTTCCCTGT TATAATACAA ACAAGACACC CATCAAACTC CCAAACGGAT CAATCTGATT TCCACAACCT   
  
  
+ AAGAACAGCA ATGCTATGCT AGATTTTGTC TGATTCGCTT CCTATCATCG GATCCTAATT GCACCGACCC   
  
  
+ GATAATTCAT CTCATTGACC ATAATTTCGG GTCATATCAG TACCCTTTTC TACTACTCAA TCGTTACTTA   
  
  
+ ACATCTAAAC TAGCTGGTGG GTATTAATAT ATATTTTAAA TGTTTAGAAT AATCTTTATT TAATATATCC   
  
  
+ AACTTGGGCC TCTTTTTTTT TTTTTAATTT AGGGGAAAAA TAAATCAACT TATAATAAGG CCAAGAGATT   
  
  
+ ATAGAAAGAA AATGAGGCCA GTTGGTGAAA CGAACTTTCG GAGGAAGTCA ACATTTAGGC AGCCAACTAC   
  
  
+ ATCATGTTTG TCTTCTACGT TCTTCATATC TTTGGGTCGT AGGTGTTCCT TCGGCTTTCC GTCTTTGACT   
  
  
+ TTTTGTCTGT AAGCAGTCAA CATTCGTATC AAATCCCGCA CGTGCCTCAA ACGCAATTAT GCTCCAACCA   
  
  
+ AATCAAGACT AGATCAAGTT CATTTAGTGA GACAATGTTT GTTAGGACAA CTGGGTCGTC GATCCAACCT   
  
  
+ CGTGACTCTC GGTTGGAGGA TTGCAGCTTA AACGACATCG GTTGGTTGAA GAGGATTGTT ATGGGGTTTC   
  
  
+ TAGGTAATAT GTAAATTTAG AGGAAGTGGG GGTAGAAATT GAATTTATTA TAACAGCGGG GGCATGTTAG   
  
  
+ GAAAAGTTTC TGCTAATCTG CCATATCTGG GTGTCTAAAG TCAACTGCAT CCTGCTGGCA CCAACCAAAA   
  
  
+ CACTCCTGTA CTTAAAAGCA CCCTGACGCA GATCAGACGG CTCAGCACGA AAGTTTGACC TGATCTCCTT   
  
  
+ CCATGTCCAC AACGTCCCGT GGACCGTCAT AACCCTTGTG GGTCCCGCAC GCCAGACGAG GTCAATGTCC   
  
  
+ TTATGTATAT CACCACAGCT TTAATAGAAA AAAGAAATAA AATATTAAAA AACAATTTAT GTGTGCACAG   
  
  
+ CAGCCAAAAA AGCACGATGT GGTTGGATCT TATTCGCACC AACATTTGAC GGCAAAGGAT AGTGTGACAG   
  
  
+ GTCAGTGACC TAATCGACCA ATAGCATCTC GTAGCCTGGT ACTCATCTTT TTTGACCGGT GTCCCTTCCC   
  
  
+ TTTATCAACA GCACCCCTCT CTCCATTTTC TTTTCACGCT GTTCTCTCTT TTCTCTCCCT TTCTCTTTCT   
  
  
+ TTTCTTTCAT GGTGGTGGTG TTGCAGAGA  

- AAAATAATTT TTATAAAAAC TTTTTATTAT ATATTCATTT GTAGGGGGTC AATGGTTAGT TGACATGAAG   
  
  
- TGGATATGAT CATGATCATG AATGAGCACA TTCATGAAAA ATTAATAGTA GTGGTAGGTT AGATAGTGGC   
  
  
- TGTAGGAATT CTGAAATCGA AGGATAAATC TAAGTGATAA GGGGGCTTCT GTTTCTAAAT GCACTAAAGA   
  
  
- GAAAGGGACA ATATTATGTT TGTTCTGTGG GTAGTTTGAG GGTTTGCCTA GTTAGACTAA AGGTGTTGGA   
  
  
- TTCTTGTCGT TACGATACGA TCTAAAACAG ACTAAGCGAA GGATAGTAGC CTAGGATTAA CGTGGCTGGG   
  
  
- CTATTAAGTA GAGTAACTGG TATTAAAGCC CAGTATAGTC ATGGGAAAAG ATGATGAGTT AGCAATGAAT   
  
  
- TGTAGATTTG ATCGACCACC CATAATTATA TATAAAATTT ACAAATCTTA TTAGAAATAA ATTATATAGG   
  
  
- TTGAACCCGG AGAAAAAAAA AAAAATTAAA TCCCCTTTTT ATTTAGTTGA ATATTATTCC GGTTCTCTAA   
  
  
- TATCTTTCTT TTACTCCGGT CAACCACTTT GCTTGAAAGC CTCCTTCAGT TGTAAATCCG TCGGTTGATG   
  
  
- TAGTACAAAC AGAAGATGCA AGAAGTATAG AAACCCAGCA TCCACAAGGA AGCCGAAAGG CAGAAACTGA   
  
  
- AAAACAGACA TTCGTCAGTT GTAAGCATAG TTTAGGGCGT GCACGGAGTT TGCGTTAATA CGAGGTTGGT   
  
  
- TTAGTTCTGA TCTAGTTCAA GTAAATCACT CTGTTACAAA CAATCCTGTT GACCCAGCAG CTAGGTTGGA   
  
  
- GCACTGAGAG CCAACCTCCT AACGTCGAAT TTGCTGTAGC CAACCAACTT CTCCTAACAA TACCCCAAAG   
  
  
- ATCCATTATA CATTTAAATC TCCTTCACCC CCATCTTTAA CTTAAATAAT ATTGTCGCCC CCGTACAATC   
  
  
- CTTTTCAAAG ACGATTAGAC GGTATAGACC CACAGATTTC AGTTGACGTA GGACGACCGT GGTTGGTTTT   
  
  
- GTGAGGACAT GAATTTTCGT GGGACTGCGT CTAGTCTGCC GAGTCGTGCT TTCAAACTGG ACTAGAGGAA   
  
  
- GGTACAGGTG TTGCAGGGCA CCTGGCAGTA TTGGGAACAC CCAGGGCGTG CGGTCTGCTC CAGTTACAGG   
  
  
- AATACATATA GTGGTGTCGA AATTATCTTT TTTCTTTATT TTATAATTTT TTGTTAAATA CACACGTGTC   
  
  
- GTCGGTTTTT TCGTGCTACA CCAACCTAGA ATAAGCGTGG TTGTAAACTG CCGTTTCCTA TCACACTGTC   
  
  
- CAGTCACTGG ATTAGCTGGT TATCGTAGAG CATCGGACCA TGAGTAGAAA AAACTGGCCA CAGGGAAGGG   
  
  
- AAATAGTTGT CGTGGGGAGA GAGGTAAAAG AAAAGTGCGA CAAGAGAGAA AAGAGAGGGA AAGAGAAAGA   
  
  
- AAAGAAAGTA CCACCACCAC AACGTCTCT

+     MRE

| Site Name | Organism | Position | Strand | Matrix score. | sequence | function |
| --- | --- | --- | --- | --- | --- | --- |
| MRE | Petroselinum crispum | 276 | + | 7 | AACCTAA | MYB binding site involved in light responsiveness |

> 2018/04/13 10:10:12  
+ TTTTATTAAA AATATTTTTG AAAAATAATA TATAAGTAAA CATCCCCCAG TTACCAATCA ACTGTACTTC   
  
  
+ ACCTATACTA GTACTAGTAC TTACTCGTGT AAGTACTTTT TAATTATCAT CACCATCCAA TCTATCACCG   
  
  
+ ACATCCTTAA GACTTTAGCT TCCTATTTAG ATTCACTATT CCCCCGAAGA CAAAGATTTA CGTGATTTCT   
  
  
+ CTTTCCCTGT TATAATACAA ACAAGACACC CATCAAACTC CCAAACGGAT CAATCTGATT TCCACAACCT   
  
  
+ AAGAACAGCA ATGCTATGCT AGATTTTGTC TGATTCGCTT CCTATCATCG GATCCTAATT GCACCGACCC   
  
  
+ GATAATTCAT CTCATTGACC ATAATTTCGG GTCATATCAG TACCCTTTTC TACTACTCAA TCGTTACTTA   
  
  
+ ACATCTAAAC TAGCTGGTGG GTATTAATAT ATATTTTAAA TGTTTAGAAT AATCTTTATT TAATATATCC   
  
  
+ AACTTGGGCC TCTTTTTTTT TTTTTAATTT AGGGGAAAAA TAAATCAACT TATAATAAGG CCAAGAGATT   
  
  
+ ATAGAAAGAA AATGAGGCCA GTTGGTGAAA CGAACTTTCG GAGGAAGTCA ACATTTAGGC AGCCAACTAC   
  
  
+ ATCATGTTTG TCTTCTACGT TCTTCATATC TTTGGGTCGT AGGTGTTCCT TCGGCTTTCC GTCTTTGACT   
  
  
+ TTTTGTCTGT AAGCAGTCAA CATTCGTATC AAATCCCGCA CGTGCCTCAA ACGCAATTAT GCTCCAACCA   
  
  
+ AATCAAGACT AGATCAAGTT CATTTAGTGA GACAATGTTT GTTAGGACAA CTGGGTCGTC GATCCAACCT   
  
  
+ CGTGACTCTC GGTTGGAGGA TTGCAGCTTA AACGACATCG GTTGGTTGAA GAGGATTGTT ATGGGGTTTC   
  
  
+ TAGGTAATAT GTAAATTTAG AGGAAGTGGG GGTAGAAATT GAATTTATTA TAACAGCGGG GGCATGTTAG   
  
  
+ GAAAAGTTTC TGCTAATCTG CCATATCTGG GTGTCTAAAG TCAACTGCAT CCTGCTGGCA CCAACCAAAA   
  
  
+ CACTCCTGTA CTTAAAAGCA CCCTGACGCA GATCAGACGG CTCAGCACGA AAGTTTGACC TGATCTCCTT   
  
  
+ CCATGTCCAC AACGTCCCGT GGACCGTCAT AACCCTTGTG GGTCCCGCAC GCCAGACGAG GTCAATGTCC   
  
  
+ TTATGTATAT CACCACAGCT TTAATAGAAA AAAGAAATAA AATATTAAAA AACAATTTAT GTGTGCACAG   
  
  
+ CAGCCAAAAA AGCACGATGT GGTTGGATCT TATTCGCACC AACATTTGAC GGCAAAGGAT AGTGTGACAG   
  
  
+ GTCAGTGACC TAATCGACCA ATAGCATCTC GTAGCCTGGT ACTCATCTTT TTTGACCGGT GTCCCTTCCC   
  
  
+ TTTATCAACA GCACCCCTCT CTCCATTTTC TTTTCACGCT GTTCTCTCTT TTCTCTCCCT TTCTCTTTCT   
  
  
+ TTTCTTTCAT GGTGGTGGTG TTGCAGAGA  

- AAAATAATTT TTATAAAAAC TTTTTATTAT ATATTCATTT GTAGGGGGTC AATGGTTAGT TGACATGAAG   
  
  
- TGGATATGAT CATGATCATG AATGAGCACA TTCATGAAAA ATTAATAGTA GTGGTAGGTT AGATAGTGGC   
  
  
- TGTAGGAATT CTGAAATCGA AGGATAAATC TAAGTGATAA GGGGGCTTCT GTTTCTAAAT GCACTAAAGA   
  
  
- GAAAGGGACA ATATTATGTT TGTTCTGTGG GTAGTTTGAG GGTTTGCCTA GTTAGACTAA AGGTGTTGGA   
  
  
- TTCTTGTCGT TACGATACGA TCTAAAACAG ACTAAGCGAA GGATAGTAGC CTAGGATTAA CGTGGCTGGG   
  
  
- CTATTAAGTA GAGTAACTGG TATTAAAGCC CAGTATAGTC ATGGGAAAAG ATGATGAGTT AGCAATGAAT   
  
  
- TGTAGATTTG ATCGACCACC CATAATTATA TATAAAATTT ACAAATCTTA TTAGAAATAA ATTATATAGG   
  
  
- TTGAACCCGG AGAAAAAAAA AAAAATTAAA TCCCCTTTTT ATTTAGTTGA ATATTATTCC GGTTCTCTAA   
  
  
- TATCTTTCTT TTACTCCGGT CAACCACTTT GCTTGAAAGC CTCCTTCAGT TGTAAATCCG TCGGTTGATG   
  
  
- TAGTACAAAC AGAAGATGCA AGAAGTATAG AAACCCAGCA TCCACAAGGA AGCCGAAAGG CAGAAACTGA   
  
  
- AAAACAGACA TTCGTCAGTT GTAAGCATAG TTTAGGGCGT GCACGGAGTT TGCGTTAATA CGAGGTTGGT   
  
  
- TTAGTTCTGA TCTAGTTCAA GTAAATCACT CTGTTACAAA CAATCCTGTT GACCCAGCAG CTAGGTTGGA   
  
  
- GCACTGAGAG CCAACCTCCT AACGTCGAAT TTGCTGTAGC CAACCAACTT CTCCTAACAA TACCCCAAAG   
  
  
- ATCCATTATA CATTTAAATC TCCTTCACCC CCATCTTTAA CTTAAATAAT ATTGTCGCCC CCGTACAATC   
  
  
- CTTTTCAAAG ACGATTAGAC GGTATAGACC CACAGATTTC AGTTGACGTA GGACGACCGT GGTTGGTTTT   
  
  
- GTGAGGACAT GAATTTTCGT GGGACTGCGT CTAGTCTGCC GAGTCGTGCT TTCAAACTGG ACTAGAGGAA   
  
  
- GGTACAGGTG TTGCAGGGCA CCTGGCAGTA TTGGGAACAC CCAGGGCGTG CGGTCTGCTC CAGTTACAGG   
  
  
- AATACATATA GTGGTGTCGA AATTATCTTT TTTCTTTATT TTATAATTTT TTGTTAAATA CACACGTGTC   
  
  
- GTCGGTTTTT TCGTGCTACA CCAACCTAGA ATAAGCGTGG TTGTAAACTG CCGTTTCCTA TCACACTGTC   
  
  
- CAGTCACTGG ATTAGCTGGT TATCGTAGAG CATCGGACCA TGAGTAGAAA AAACTGGCCA CAGGGAAGGG   
  
  
- AAATAGTTGT CGTGGGGAGA GAGGTAAAAG AAAAGTGCGA CAAGAGAGAA AAGAGAGGGA AAGAGAAAGA   
  
  
- AAAGAAAGTA CCACCACCAC AACGTCTCT

+     Skn-1\_motif

| Site Name | Organism | Position | Strand | Matrix score. | sequence | function |
| --- | --- | --- | --- | --- | --- | --- |
| Skn-1\_motif | Oryza sativa | 381 | + | 5 | GTCAT | cis-acting regulatory element required for endosperm expression |
| Skn-1\_motif | Oryza sativa | 1146 | + | 5 | GTCAT | cis-acting regulatory element required for endosperm expression |

> 2018/04/13 10:10:12  
+ TTTTATTAAA AATATTTTTG AAAAATAATA TATAAGTAAA CATCCCCCAG TTACCAATCA ACTGTACTTC   
  
  
+ ACCTATACTA GTACTAGTAC TTACTCGTGT AAGTACTTTT TAATTATCAT CACCATCCAA TCTATCACCG   
  
  
+ ACATCCTTAA GACTTTAGCT TCCTATTTAG ATTCACTATT CCCCCGAAGA CAAAGATTTA CGTGATTTCT   
  
  
+ CTTTCCCTGT TATAATACAA ACAAGACACC CATCAAACTC CCAAACGGAT CAATCTGATT TCCACAACCT   
  
  
+ AAGAACAGCA ATGCTATGCT AGATTTTGTC TGATTCGCTT CCTATCATCG GATCCTAATT GCACCGACCC   
  
  
+ GATAATTCAT CTCATTGACC ATAATTTCGG GTCATATCAG TACCCTTTTC TACTACTCAA TCGTTACTTA   
  
  
+ ACATCTAAAC TAGCTGGTGG GTATTAATAT ATATTTTAAA TGTTTAGAAT AATCTTTATT TAATATATCC   
  
  
+ AACTTGGGCC TCTTTTTTTT TTTTTAATTT AGGGGAAAAA TAAATCAACT TATAATAAGG CCAAGAGATT   
  
  
+ ATAGAAAGAA AATGAGGCCA GTTGGTGAAA CGAACTTTCG GAGGAAGTCA ACATTTAGGC AGCCAACTAC   
  
  
+ ATCATGTTTG TCTTCTACGT TCTTCATATC TTTGGGTCGT AGGTGTTCCT TCGGCTTTCC GTCTTTGACT   
  
  
+ TTTTGTCTGT AAGCAGTCAA CATTCGTATC AAATCCCGCA CGTGCCTCAA ACGCAATTAT GCTCCAACCA   
  
  
+ AATCAAGACT AGATCAAGTT CATTTAGTGA GACAATGTTT GTTAGGACAA CTGGGTCGTC GATCCAACCT   
  
  
+ CGTGACTCTC GGTTGGAGGA TTGCAGCTTA AACGACATCG GTTGGTTGAA GAGGATTGTT ATGGGGTTTC   
  
  
+ TAGGTAATAT GTAAATTTAG AGGAAGTGGG GGTAGAAATT GAATTTATTA TAACAGCGGG GGCATGTTAG   
  
  
+ GAAAAGTTTC TGCTAATCTG CCATATCTGG GTGTCTAAAG TCAACTGCAT CCTGCTGGCA CCAACCAAAA   
  
  
+ CACTCCTGTA CTTAAAAGCA CCCTGACGCA GATCAGACGG CTCAGCACGA AAGTTTGACC TGATCTCCTT   
  
  
+ CCATGTCCAC AACGTCCCGT GGACCGTCAT AACCCTTGTG GGTCCCGCAC GCCAGACGAG GTCAATGTCC   
  
  
+ TTATGTATAT CACCACAGCT TTAATAGAAA AAAGAAATAA AATATTAAAA AACAATTTAT GTGTGCACAG   
  
  
+ CAGCCAAAAA AGCACGATGT GGTTGGATCT TATTCGCACC AACATTTGAC GGCAAAGGAT AGTGTGACAG   
  
  
+ GTCAGTGACC TAATCGACCA ATAGCATCTC GTAGCCTGGT ACTCATCTTT TTTGACCGGT GTCCCTTCCC   
  
  
+ TTTATCAACA GCACCCCTCT CTCCATTTTC TTTTCACGCT GTTCTCTCTT TTCTCTCCCT TTCTCTTTCT   
  
  
+ TTTCTTTCAT GGTGGTGGTG TTGCAGAGA  

- AAAATAATTT TTATAAAAAC TTTTTATTAT ATATTCATTT GTAGGGGGTC AATGGTTAGT TGACATGAAG   
  
  
- TGGATATGAT CATGATCATG AATGAGCACA TTCATGAAAA ATTAATAGTA GTGGTAGGTT AGATAGTGGC   
  
  
- TGTAGGAATT CTGAAATCGA AGGATAAATC TAAGTGATAA GGGGGCTTCT GTTTCTAAAT GCACTAAAGA   
  
  
- GAAAGGGACA ATATTATGTT TGTTCTGTGG GTAGTTTGAG GGTTTGCCTA GTTAGACTAA AGGTGTTGGA   
  
  
- TTCTTGTCGT TACGATACGA TCTAAAACAG ACTAAGCGAA GGATAGTAGC CTAGGATTAA CGTGGCTGGG   
  
  
- CTATTAAGTA GAGTAACTGG TATTAAAGCC CAGTATAGTC ATGGGAAAAG ATGATGAGTT AGCAATGAAT   
  
  
- TGTAGATTTG ATCGACCACC CATAATTATA TATAAAATTT ACAAATCTTA TTAGAAATAA ATTATATAGG   
  
  
- TTGAACCCGG AGAAAAAAAA AAAAATTAAA TCCCCTTTTT ATTTAGTTGA ATATTATTCC GGTTCTCTAA   
  
  
- TATCTTTCTT TTACTCCGGT CAACCACTTT GCTTGAAAGC CTCCTTCAGT TGTAAATCCG TCGGTTGATG   
  
  
- TAGTACAAAC AGAAGATGCA AGAAGTATAG AAACCCAGCA TCCACAAGGA AGCCGAAAGG CAGAAACTGA   
  
  
- AAAACAGACA TTCGTCAGTT GTAAGCATAG TTTAGGGCGT GCACGGAGTT TGCGTTAATA CGAGGTTGGT   
  
  
- TTAGTTCTGA TCTAGTTCAA GTAAATCACT CTGTTACAAA CAATCCTGTT GACCCAGCAG CTAGGTTGGA   
  
  
- GCACTGAGAG CCAACCTCCT AACGTCGAAT TTGCTGTAGC CAACCAACTT CTCCTAACAA TACCCCAAAG   
  
  
- ATCCATTATA CATTTAAATC TCCTTCACCC CCATCTTTAA CTTAAATAAT ATTGTCGCCC CCGTACAATC   
  
  
- CTTTTCAAAG ACGATTAGAC GGTATAGACC CACAGATTTC AGTTGACGTA GGACGACCGT GGTTGGTTTT   
  
  
- GTGAGGACAT GAATTTTCGT GGGACTGCGT CTAGTCTGCC GAGTCGTGCT TTCAAACTGG ACTAGAGGAA   
  
  
- GGTACAGGTG TTGCAGGGCA CCTGGCAGTA TTGGGAACAC CCAGGGCGTG CGGTCTGCTC CAGTTACAGG   
  
  
- AATACATATA GTGGTGTCGA AATTATCTTT TTTCTTTATT TTATAATTTT TTGTTAAATA CACACGTGTC   
  
  
- GTCGGTTTTT TCGTGCTACA CCAACCTAGA ATAAGCGTGG TTGTAAACTG CCGTTTCCTA TCACACTGTC   
  
  
- CAGTCACTGG ATTAGCTGGT TATCGTAGAG CATCGGACCA TGAGTAGAAA AAACTGGCCA CAGGGAAGGG   
  
  
- AAATAGTTGT CGTGGGGAGA GAGGTAAAAG AAAAGTGCGA CAAGAGAGAA AAGAGAGGGA AAGAGAAAGA   
  
  
- AAAGAAAGTA CCACCACCAC AACGTCTCT

+     TATA-box

| Site Name | Organism | Position | Strand | Matrix score. | sequence | function |
| --- | --- | --- | --- | --- | --- | --- |
| TATA-box | Arabidopsis thaliana | 484 | + | 4 | TATA | core promoter element around -30 of transcription start |
| TATA-box | Brassica napus | 483 | + | 6 | ATATAT | core promoter element around -30 of transcription start |
| TATA-box | Helianthus annuus | 1194 | - | 6 | TATACA | core promoter element around -30 of transcription start |
| TATA-box | Arabidopsis thaliana | 471 | - | 8 | TAAAGATT | core promoter element around -30 of transcription start |
| TATA-box | Arabidopsis thaliana | 448 | + | 4 | TATA | core promoter element around -30 of transcription start |
| TATA-box | Arabidopsis thaliana | 541 | + | 4 | TATA | core promoter element around -30 of transcription start |
| TATA-box | Glycine max | 442 | - | 5 | TAATA | core promoter element around -30 of transcription start |
| TATA-box | Lycopersicon esculentum | 512 | + | 5 | TTTTA | core promoter element around -30 of transcription start |
| TATA-box | Glycine max | 543 | + | 5 | TAATA | core promoter element around -30 of transcription start |
| TATA-box | Arabidopsis thaliana | 560 | + | 4 | TATA | core promoter element around -30 of transcription start |
| TATA-box | Glycine max | 915 | + | 5 | TAATA | core promoter element around -30 of transcription start |
| TATA-box | Daucus carota | 924 | - | 9 | ccTATAAATT | core promoter element around -30 of transcription start |
| TATA-box | Glycine max | 223 | + | 5 | TAATA | core promoter element around -30 of transcription start |
| TATA-box | Lycopersicon esculentum | 1063 | - | 5 | TTTTA | core promoter element around -30 of transcription start |
| TATA-box | Lycopersicon esculentum | 454 | + | 5 | TTTTA | core promoter element around -30 of transcription start |
| TATA-box | Arabidopsis thaliana | 74 | + | 4 | TATA | core promoter element around -30 of transcription start |
| TATA-box | Brassica napus | 28 | + | 6 | ATATAT | core promoter element around -30 of transcription start |
| TATA-box | Arabidopsis thaliana | 540 | - | 5 | TATAA | core promoter element around -30 of transcription start |
| TATA-box | Arabidopsis thaliana | 559 | - | 5 | TATAA | core promoter element around -30 of transcription start |
| TATA-box | Brassica napus | 558 | + | 6 | ATTATA | core promoter element around -30 of transcription start |
| TATA-box | Brassica napus | 957 | + | 6 | ATTATA | core promoter element around -30 of transcription start |
| TATA-box | Lycopersicon esculentum | 1228 | - | 5 | TTTTA | core promoter element around -30 of transcription start |
| TATA-box | Glycine max | 1212 | + | 5 | TAATA | core promoter element around -30 of transcription start |
| TATA-box | Arabidopsis thaliana | 29 | + | 4 | TATA | core promoter element around -30 of transcription start |
| TATA-box | Arabidopsis thaliana | 220 | - | 5 | TATAA | core promoter element around -30 of transcription start |
| TATA-box | Arabidopsis thaliana | 221 | + | 4 | TATA | core promoter element around -30 of transcription start |
| TATA-box | Glycine max | 956 | - | 5 | TAATA | core promoter element around -30 of transcription start |
| TATA-box | Arabidopsis thaliana | 1196 | - | 4 | TATA | core promoter element around -30 of transcription start |
| TATA-box | Arabidopsis thaliana | 959 | - | 4 | TATA | core promoter element around -30 of transcription start |
| TATA-box | Lycopersicon esculentum | 7 | - | 5 | TTTTA | core promoter element around -30 of transcription start |
| TATA-box | Lycopersicon esculentum | 108 | + | 5 | TTTTA | core promoter element around -30 of transcription start |
| TATA-box | Lycopersicon esculentum | 1236 | - | 5 | TTTTA | core promoter element around -30 of transcription start |
| TATA-box | Brassica oleracea | 30 | + | 6 | ATATAA | core promoter element around -30 of transcription start |
| TATA-box | Arabidopsis thaliana | 958 | - | 5 | TATAA | core promoter element around -30 of transcription start |
| TATA-box | Lycopersicon esculentum | 1 | + | 5 | TTTTA | core promoter element around -30 of transcription start |
| TATA-box | Arabidopsis thaliana | 31 | + | 4 | TATA | core promoter element around -30 of transcription start |
| TATA-box | Glycine max | 4 | - | 5 | TAATA | core promoter element around -30 of transcription start |
| TATA-box | Glycine max | 26 | + | 5 | TAATA | core promoter element around -30 of transcription start |
| TATA-box | Glycine max | 445 | + | 5 | TAATA | core promoter element around -30 of transcription start |
| TATA-box | Arabidopsis thaliana | 450 | + | 4 | TATA | core promoter element around -30 of transcription start |
| TATA-box | Glycine max | 481 | + | 5 | TAATA | core promoter element around -30 of transcription start |
| TATA-box | Brassica napus | 449 | + | 6 | ATATAT | core promoter element around -30 of transcription start |
| TATA-box | Brassica napus | 447 | + | 6 | ATATAT | core promoter element around -30 of transcription start |
| TATA-box | Glycine max | 1233 | - | 5 | TAATA | core promoter element around -30 of transcription start |

> 2018/04/13 10:10:12  
+ TTTTATTAAA AATATTTTTG AAAAATAATA TATAAGTAAA CATCCCCCAG TTACCAATCA ACTGTACTTC   
  
  
+ ACCTATACTA GTACTAGTAC TTACTCGTGT AAGTACTTTT TAATTATCAT CACCATCCAA TCTATCACCG   
  
  
+ ACATCCTTAA GACTTTAGCT TCCTATTTAG ATTCACTATT CCCCCGAAGA CAAAGATTTA CGTGATTTCT   
  
  
+ CTTTCCCTGT TATAATACAA ACAAGACACC CATCAAACTC CCAAACGGAT CAATCTGATT TCCACAACCT   
  
  
+ AAGAACAGCA ATGCTATGCT AGATTTTGTC TGATTCGCTT CCTATCATCG GATCCTAATT GCACCGACCC   
  
  
+ GATAATTCAT CTCATTGACC ATAATTTCGG GTCATATCAG TACCCTTTTC TACTACTCAA TCGTTACTTA   
  
  
+ ACATCTAAAC TAGCTGGTGG GTATTAATAT ATATTTTAAA TGTTTAGAAT AATCTTTATT TAATATATCC   
  
  
+ AACTTGGGCC TCTTTTTTTT TTTTTAATTT AGGGGAAAAA TAAATCAACT TATAATAAGG CCAAGAGATT   
  
  
+ ATAGAAAGAA AATGAGGCCA GTTGGTGAAA CGAACTTTCG GAGGAAGTCA ACATTTAGGC AGCCAACTAC   
  
  
+ ATCATGTTTG TCTTCTACGT TCTTCATATC TTTGGGTCGT AGGTGTTCCT TCGGCTTTCC GTCTTTGACT   
  
  
+ TTTTGTCTGT AAGCAGTCAA CATTCGTATC AAATCCCGCA CGTGCCTCAA ACGCAATTAT GCTCCAACCA   
  
  
+ AATCAAGACT AGATCAAGTT CATTTAGTGA GACAATGTTT GTTAGGACAA CTGGGTCGTC GATCCAACCT   
  
  
+ CGTGACTCTC GGTTGGAGGA TTGCAGCTTA AACGACATCG GTTGGTTGAA GAGGATTGTT ATGGGGTTTC   
  
  
+ TAGGTAATAT GTAAATTTAG AGGAAGTGGG GGTAGAAATT GAATTTATTA TAACAGCGGG GGCATGTTAG   
  
  
+ GAAAAGTTTC TGCTAATCTG CCATATCTGG GTGTCTAAAG TCAACTGCAT CCTGCTGGCA CCAACCAAAA   
  
  
+ CACTCCTGTA CTTAAAAGCA CCCTGACGCA GATCAGACGG CTCAGCACGA AAGTTTGACC TGATCTCCTT   
  
  
+ CCATGTCCAC AACGTCCCGT GGACCGTCAT AACCCTTGTG GGTCCCGCAC GCCAGACGAG GTCAATGTCC   
  
  
+ TTATGTATAT CACCACAGCT TTAATAGAAA AAAGAAATAA AATATTAAAA AACAATTTAT GTGTGCACAG   
  
  
+ CAGCCAAAAA AGCACGATGT GGTTGGATCT TATTCGCACC AACATTTGAC GGCAAAGGAT AGTGTGACAG   
  
  
+ GTCAGTGACC TAATCGACCA ATAGCATCTC GTAGCCTGGT ACTCATCTTT TTTGACCGGT GTCCCTTCCC   
  
  
+ TTTATCAACA GCACCCCTCT CTCCATTTTC TTTTCACGCT GTTCTCTCTT TTCTCTCCCT TTCTCTTTCT   
  
  
+ TTTCTTTCAT GGTGGTGGTG TTGCAGAGA  

- AAAATAATTT TTATAAAAAC TTTTTATTAT ATATTCATTT GTAGGGGGTC AATGGTTAGT TGACATGAAG   
  
  
- TGGATATGAT CATGATCATG AATGAGCACA TTCATGAAAA ATTAATAGTA GTGGTAGGTT AGATAGTGGC   
  
  
- TGTAGGAATT CTGAAATCGA AGGATAAATC TAAGTGATAA GGGGGCTTCT GTTTCTAAAT GCACTAAAGA   
  
  
- GAAAGGGACA ATATTATGTT TGTTCTGTGG GTAGTTTGAG GGTTTGCCTA GTTAGACTAA AGGTGTTGGA   
  
  
- TTCTTGTCGT TACGATACGA TCTAAAACAG ACTAAGCGAA GGATAGTAGC CTAGGATTAA CGTGGCTGGG   
  
  
- CTATTAAGTA GAGTAACTGG TATTAAAGCC CAGTATAGTC ATGGGAAAAG ATGATGAGTT AGCAATGAAT   
  
  
- TGTAGATTTG ATCGACCACC CATAATTATA TATAAAATTT ACAAATCTTA TTAGAAATAA ATTATATAGG   
  
  
- TTGAACCCGG AGAAAAAAAA AAAAATTAAA TCCCCTTTTT ATTTAGTTGA ATATTATTCC GGTTCTCTAA   
  
  
- TATCTTTCTT TTACTCCGGT CAACCACTTT GCTTGAAAGC CTCCTTCAGT TGTAAATCCG TCGGTTGATG   
  
  
- TAGTACAAAC AGAAGATGCA AGAAGTATAG AAACCCAGCA TCCACAAGGA AGCCGAAAGG CAGAAACTGA   
  
  
- AAAACAGACA TTCGTCAGTT GTAAGCATAG TTTAGGGCGT GCACGGAGTT TGCGTTAATA CGAGGTTGGT   
  
  
- TTAGTTCTGA TCTAGTTCAA GTAAATCACT CTGTTACAAA CAATCCTGTT GACCCAGCAG CTAGGTTGGA   
  
  
- GCACTGAGAG CCAACCTCCT AACGTCGAAT TTGCTGTAGC CAACCAACTT CTCCTAACAA TACCCCAAAG   
  
  
- ATCCATTATA CATTTAAATC TCCTTCACCC CCATCTTTAA CTTAAATAAT ATTGTCGCCC CCGTACAATC   
  
  
- CTTTTCAAAG ACGATTAGAC GGTATAGACC CACAGATTTC AGTTGACGTA GGACGACCGT GGTTGGTTTT   
  
  
- GTGAGGACAT GAATTTTCGT GGGACTGCGT CTAGTCTGCC GAGTCGTGCT TTCAAACTGG ACTAGAGGAA   
  
  
- GGTACAGGTG TTGCAGGGCA CCTGGCAGTA TTGGGAACAC CCAGGGCGTG CGGTCTGCTC CAGTTACAGG   
  
  
- AATACATATA GTGGTGTCGA AATTATCTTT TTTCTTTATT TTATAATTTT TTGTTAAATA CACACGTGTC   
  
  
- GTCGGTTTTT TCGTGCTACA CCAACCTAGA ATAAGCGTGG TTGTAAACTG CCGTTTCCTA TCACACTGTC   
  
  
- CAGTCACTGG ATTAGCTGGT TATCGTAGAG CATCGGACCA TGAGTAGAAA AAACTGGCCA CAGGGAAGGG   
  
  
- AAATAGTTGT CGTGGGGAGA GAGGTAAAAG AAAAGTGCGA CAAGAGAGAA AAGAGAGGGA AAGAGAAAGA   
  
  
- AAAGAAAGTA CCACCACCAC AACGTCTCT

+     TCA-element

| Site Name | Organism | Position | Strand | Matrix score. | sequence | function |
| --- | --- | --- | --- | --- | --- | --- |
| TCA-element | Nicotiana tabacum | 1373 | + | 9 | CCATCTTTTT | cis-acting element involved in salicylic acid responsiveness |

> 2018/04/13 10:10:12  
+ TTTTATTAAA AATATTTTTG AAAAATAATA TATAAGTAAA CATCCCCCAG TTACCAATCA ACTGTACTTC   
  
  
+ ACCTATACTA GTACTAGTAC TTACTCGTGT AAGTACTTTT TAATTATCAT CACCATCCAA TCTATCACCG   
  
  
+ ACATCCTTAA GACTTTAGCT TCCTATTTAG ATTCACTATT CCCCCGAAGA CAAAGATTTA CGTGATTTCT   
  
  
+ CTTTCCCTGT TATAATACAA ACAAGACACC CATCAAACTC CCAAACGGAT CAATCTGATT TCCACAACCT   
  
  
+ AAGAACAGCA ATGCTATGCT AGATTTTGTC TGATTCGCTT CCTATCATCG GATCCTAATT GCACCGACCC   
  
  
+ GATAATTCAT CTCATTGACC ATAATTTCGG GTCATATCAG TACCCTTTTC TACTACTCAA TCGTTACTTA   
  
  
+ ACATCTAAAC TAGCTGGTGG GTATTAATAT ATATTTTAAA TGTTTAGAAT AATCTTTATT TAATATATCC   
  
  
+ AACTTGGGCC TCTTTTTTTT TTTTTAATTT AGGGGAAAAA TAAATCAACT TATAATAAGG CCAAGAGATT   
  
  
+ ATAGAAAGAA AATGAGGCCA GTTGGTGAAA CGAACTTTCG GAGGAAGTCA ACATTTAGGC AGCCAACTAC   
  
  
+ ATCATGTTTG TCTTCTACGT TCTTCATATC TTTGGGTCGT AGGTGTTCCT TCGGCTTTCC GTCTTTGACT   
  
  
+ TTTTGTCTGT AAGCAGTCAA CATTCGTATC AAATCCCGCA CGTGCCTCAA ACGCAATTAT GCTCCAACCA   
  
  
+ AATCAAGACT AGATCAAGTT CATTTAGTGA GACAATGTTT GTTAGGACAA CTGGGTCGTC GATCCAACCT   
  
  
+ CGTGACTCTC GGTTGGAGGA TTGCAGCTTA AACGACATCG GTTGGTTGAA GAGGATTGTT ATGGGGTTTC   
  
  
+ TAGGTAATAT GTAAATTTAG AGGAAGTGGG GGTAGAAATT GAATTTATTA TAACAGCGGG GGCATGTTAG   
  
  
+ GAAAAGTTTC TGCTAATCTG CCATATCTGG GTGTCTAAAG TCAACTGCAT CCTGCTGGCA CCAACCAAAA   
  
  
+ CACTCCTGTA CTTAAAAGCA CCCTGACGCA GATCAGACGG CTCAGCACGA AAGTTTGACC TGATCTCCTT   
  
  
+ CCATGTCCAC AACGTCCCGT GGACCGTCAT AACCCTTGTG GGTCCCGCAC GCCAGACGAG GTCAATGTCC   
  
  
+ TTATGTATAT CACCACAGCT TTAATAGAAA AAAGAAATAA AATATTAAAA AACAATTTAT GTGTGCACAG   
  
  
+ CAGCCAAAAA AGCACGATGT GGTTGGATCT TATTCGCACC AACATTTGAC GGCAAAGGAT AGTGTGACAG   
  
  
+ GTCAGTGACC TAATCGACCA ATAGCATCTC GTAGCCTGGT ACTCATCTTT TTTGACCGGT GTCCCTTCCC   
  
  
+ TTTATCAACA GCACCCCTCT CTCCATTTTC TTTTCACGCT GTTCTCTCTT TTCTCTCCCT TTCTCTTTCT   
  
  
+ TTTCTTTCAT GGTGGTGGTG TTGCAGAGA  

- AAAATAATTT TTATAAAAAC TTTTTATTAT ATATTCATTT GTAGGGGGTC AATGGTTAGT TGACATGAAG   
  
  
- TGGATATGAT CATGATCATG AATGAGCACA TTCATGAAAA ATTAATAGTA GTGGTAGGTT AGATAGTGGC   
  
  
- TGTAGGAATT CTGAAATCGA AGGATAAATC TAAGTGATAA GGGGGCTTCT GTTTCTAAAT GCACTAAAGA   
  
  
- GAAAGGGACA ATATTATGTT TGTTCTGTGG GTAGTTTGAG GGTTTGCCTA GTTAGACTAA AGGTGTTGGA   
  
  
- TTCTTGTCGT TACGATACGA TCTAAAACAG ACTAAGCGAA GGATAGTAGC CTAGGATTAA CGTGGCTGGG   
  
  
- CTATTAAGTA GAGTAACTGG TATTAAAGCC CAGTATAGTC ATGGGAAAAG ATGATGAGTT AGCAATGAAT   
  
  
- TGTAGATTTG ATCGACCACC CATAATTATA TATAAAATTT ACAAATCTTA TTAGAAATAA ATTATATAGG   
  
  
- TTGAACCCGG AGAAAAAAAA AAAAATTAAA TCCCCTTTTT ATTTAGTTGA ATATTATTCC GGTTCTCTAA   
  
  
- TATCTTTCTT TTACTCCGGT CAACCACTTT GCTTGAAAGC CTCCTTCAGT TGTAAATCCG TCGGTTGATG   
  
  
- TAGTACAAAC AGAAGATGCA AGAAGTATAG AAACCCAGCA TCCACAAGGA AGCCGAAAGG CAGAAACTGA   
  
  
- AAAACAGACA TTCGTCAGTT GTAAGCATAG TTTAGGGCGT GCACGGAGTT TGCGTTAATA CGAGGTTGGT   
  
  
- TTAGTTCTGA TCTAGTTCAA GTAAATCACT CTGTTACAAA CAATCCTGTT GACCCAGCAG CTAGGTTGGA   
  
  
- GCACTGAGAG CCAACCTCCT AACGTCGAAT TTGCTGTAGC CAACCAACTT CTCCTAACAA TACCCCAAAG   
  
  
- ATCCATTATA CATTTAAATC TCCTTCACCC CCATCTTTAA CTTAAATAAT ATTGTCGCCC CCGTACAATC   
  
  
- CTTTTCAAAG ACGATTAGAC GGTATAGACC CACAGATTTC AGTTGACGTA GGACGACCGT GGTTGGTTTT   
  
  
- GTGAGGACAT GAATTTTCGT GGGACTGCGT CTAGTCTGCC GAGTCGTGCT TTCAAACTGG ACTAGAGGAA   
  
  
- GGTACAGGTG TTGCAGGGCA CCTGGCAGTA TTGGGAACAC CCAGGGCGTG CGGTCTGCTC CAGTTACAGG   
  
  
- AATACATATA GTGGTGTCGA AATTATCTTT TTTCTTTATT TTATAATTTT TTGTTAAATA CACACGTGTC   
  
  
- GTCGGTTTTT TCGTGCTACA CCAACCTAGA ATAAGCGTGG TTGTAAACTG CCGTTTCCTA TCACACTGTC   
  
  
- CAGTCACTGG ATTAGCTGGT TATCGTAGAG CATCGGACCA TGAGTAGAAA AAACTGGCCA CAGGGAAGGG   
  
  
- AAATAGTTGT CGTGGGGAGA GAGGTAAAAG AAAAGTGCGA CAAGAGAGAA AAGAGAGGGA AAGAGAAAGA   
  
  
- AAAGAAAGTA CCACCACCAC AACGTCTCT

+     TCCC-motif

| Site Name | Organism | Position | Strand | Matrix score. | sequence | function |
| --- | --- | --- | --- | --- | --- | --- |
| TCCC-motif | Spinacia oleracea | 1454 | + | 7 | TCTCCCT | part of a light responsive element |

> 2018/04/13 10:10:12  
+ TTTTATTAAA AATATTTTTG AAAAATAATA TATAAGTAAA CATCCCCCAG TTACCAATCA ACTGTACTTC   
  
  
+ ACCTATACTA GTACTAGTAC TTACTCGTGT AAGTACTTTT TAATTATCAT CACCATCCAA TCTATCACCG   
  
  
+ ACATCCTTAA GACTTTAGCT TCCTATTTAG ATTCACTATT CCCCCGAAGA CAAAGATTTA CGTGATTTCT   
  
  
+ CTTTCCCTGT TATAATACAA ACAAGACACC CATCAAACTC CCAAACGGAT CAATCTGATT TCCACAACCT   
  
  
+ AAGAACAGCA ATGCTATGCT AGATTTTGTC TGATTCGCTT CCTATCATCG GATCCTAATT GCACCGACCC   
  
  
+ GATAATTCAT CTCATTGACC ATAATTTCGG GTCATATCAG TACCCTTTTC TACTACTCAA TCGTTACTTA   
  
  
+ ACATCTAAAC TAGCTGGTGG GTATTAATAT ATATTTTAAA TGTTTAGAAT AATCTTTATT TAATATATCC   
  
  
+ AACTTGGGCC TCTTTTTTTT TTTTTAATTT AGGGGAAAAA TAAATCAACT TATAATAAGG CCAAGAGATT   
  
  
+ ATAGAAAGAA AATGAGGCCA GTTGGTGAAA CGAACTTTCG GAGGAAGTCA ACATTTAGGC AGCCAACTAC   
  
  
+ ATCATGTTTG TCTTCTACGT TCTTCATATC TTTGGGTCGT AGGTGTTCCT TCGGCTTTCC GTCTTTGACT   
  
  
+ TTTTGTCTGT AAGCAGTCAA CATTCGTATC AAATCCCGCA CGTGCCTCAA ACGCAATTAT GCTCCAACCA   
  
  
+ AATCAAGACT AGATCAAGTT CATTTAGTGA GACAATGTTT GTTAGGACAA CTGGGTCGTC GATCCAACCT   
  
  
+ CGTGACTCTC GGTTGGAGGA TTGCAGCTTA AACGACATCG GTTGGTTGAA GAGGATTGTT ATGGGGTTTC   
  
  
+ TAGGTAATAT GTAAATTTAG AGGAAGTGGG GGTAGAAATT GAATTTATTA TAACAGCGGG GGCATGTTAG   
  
  
+ GAAAAGTTTC TGCTAATCTG CCATATCTGG GTGTCTAAAG TCAACTGCAT CCTGCTGGCA CCAACCAAAA   
  
  
+ CACTCCTGTA CTTAAAAGCA CCCTGACGCA GATCAGACGG CTCAGCACGA AAGTTTGACC TGATCTCCTT   
  
  
+ CCATGTCCAC AACGTCCCGT GGACCGTCAT AACCCTTGTG GGTCCCGCAC GCCAGACGAG GTCAATGTCC   
  
  
+ TTATGTATAT CACCACAGCT TTAATAGAAA AAAGAAATAA AATATTAAAA AACAATTTAT GTGTGCACAG   
  
  
+ CAGCCAAAAA AGCACGATGT GGTTGGATCT TATTCGCACC AACATTTGAC GGCAAAGGAT AGTGTGACAG   
  
  
+ GTCAGTGACC TAATCGACCA ATAGCATCTC GTAGCCTGGT ACTCATCTTT TTTGACCGGT GTCCCTTCCC   
  
  
+ TTTATCAACA GCACCCCTCT CTCCATTTTC TTTTCACGCT GTTCTCTCTT TTCTCTCCCT TTCTCTTTCT   
  
  
+ TTTCTTTCAT GGTGGTGGTG TTGCAGAGA  

- AAAATAATTT TTATAAAAAC TTTTTATTAT ATATTCATTT GTAGGGGGTC AATGGTTAGT TGACATGAAG   
  
  
- TGGATATGAT CATGATCATG AATGAGCACA TTCATGAAAA ATTAATAGTA GTGGTAGGTT AGATAGTGGC   
  
  
- TGTAGGAATT CTGAAATCGA AGGATAAATC TAAGTGATAA GGGGGCTTCT GTTTCTAAAT GCACTAAAGA   
  
  
- GAAAGGGACA ATATTATGTT TGTTCTGTGG GTAGTTTGAG GGTTTGCCTA GTTAGACTAA AGGTGTTGGA   
  
  
- TTCTTGTCGT TACGATACGA TCTAAAACAG ACTAAGCGAA GGATAGTAGC CTAGGATTAA CGTGGCTGGG   
  
  
- CTATTAAGTA GAGTAACTGG TATTAAAGCC CAGTATAGTC ATGGGAAAAG ATGATGAGTT AGCAATGAAT   
  
  
- TGTAGATTTG ATCGACCACC CATAATTATA TATAAAATTT ACAAATCTTA TTAGAAATAA ATTATATAGG   
  
  
- TTGAACCCGG AGAAAAAAAA AAAAATTAAA TCCCCTTTTT ATTTAGTTGA ATATTATTCC GGTTCTCTAA   
  
  
- TATCTTTCTT TTACTCCGGT CAACCACTTT GCTTGAAAGC CTCCTTCAGT TGTAAATCCG TCGGTTGATG   
  
  
- TAGTACAAAC AGAAGATGCA AGAAGTATAG AAACCCAGCA TCCACAAGGA AGCCGAAAGG CAGAAACTGA   
  
  
- AAAACAGACA TTCGTCAGTT GTAAGCATAG TTTAGGGCGT GCACGGAGTT TGCGTTAATA CGAGGTTGGT   
  
  
- TTAGTTCTGA TCTAGTTCAA GTAAATCACT CTGTTACAAA CAATCCTGTT GACCCAGCAG CTAGGTTGGA   
  
  
- GCACTGAGAG CCAACCTCCT AACGTCGAAT TTGCTGTAGC CAACCAACTT CTCCTAACAA TACCCCAAAG   
  
  
- ATCCATTATA CATTTAAATC TCCTTCACCC CCATCTTTAA CTTAAATAAT ATTGTCGCCC CCGTACAATC   
  
  
- CTTTTCAAAG ACGATTAGAC GGTATAGACC CACAGATTTC AGTTGACGTA GGACGACCGT GGTTGGTTTT   
  
  
- GTGAGGACAT GAATTTTCGT GGGACTGCGT CTAGTCTGCC GAGTCGTGCT TTCAAACTGG ACTAGAGGAA   
  
  
- GGTACAGGTG TTGCAGGGCA CCTGGCAGTA TTGGGAACAC CCAGGGCGTG CGGTCTGCTC CAGTTACAGG   
  
  
- AATACATATA GTGGTGTCGA AATTATCTTT TTTCTTTATT TTATAATTTT TTGTTAAATA CACACGTGTC   
  
  
- GTCGGTTTTT TCGTGCTACA CCAACCTAGA ATAAGCGTGG TTGTAAACTG CCGTTTCCTA TCACACTGTC   
  
  
- CAGTCACTGG ATTAGCTGGT TATCGTAGAG CATCGGACCA TGAGTAGAAA AAACTGGCCA CAGGGAAGGG   
  
  
- AAATAGTTGT CGTGGGGAGA GAGGTAAAAG AAAAGTGCGA CAAGAGAGAA AAGAGAGGGA AAGAGAAAGA   
  
  
- AAAGAAAGTA CCACCACCAC AACGTCTCT

+     TGA-element

| Site Name | Organism | Position | Strand | Matrix score. | sequence | function |
| --- | --- | --- | --- | --- | --- | --- |
| TGA-element | Brassica oleracea | 871 | + | 6 | AACGAC | auxin-responsive element |

> 2018/04/13 10:10:12  
+ TTTTATTAAA AATATTTTTG AAAAATAATA TATAAGTAAA CATCCCCCAG TTACCAATCA ACTGTACTTC   
  
  
+ ACCTATACTA GTACTAGTAC TTACTCGTGT AAGTACTTTT TAATTATCAT CACCATCCAA TCTATCACCG   
  
  
+ ACATCCTTAA GACTTTAGCT TCCTATTTAG ATTCACTATT CCCCCGAAGA CAAAGATTTA CGTGATTTCT   
  
  
+ CTTTCCCTGT TATAATACAA ACAAGACACC CATCAAACTC CCAAACGGAT CAATCTGATT TCCACAACCT   
  
  
+ AAGAACAGCA ATGCTATGCT AGATTTTGTC TGATTCGCTT CCTATCATCG GATCCTAATT GCACCGACCC   
  
  
+ GATAATTCAT CTCATTGACC ATAATTTCGG GTCATATCAG TACCCTTTTC TACTACTCAA TCGTTACTTA   
  
  
+ ACATCTAAAC TAGCTGGTGG GTATTAATAT ATATTTTAAA TGTTTAGAAT AATCTTTATT TAATATATCC   
  
  
+ AACTTGGGCC TCTTTTTTTT TTTTTAATTT AGGGGAAAAA TAAATCAACT TATAATAAGG CCAAGAGATT   
  
  
+ ATAGAAAGAA AATGAGGCCA GTTGGTGAAA CGAACTTTCG GAGGAAGTCA ACATTTAGGC AGCCAACTAC   
  
  
+ ATCATGTTTG TCTTCTACGT TCTTCATATC TTTGGGTCGT AGGTGTTCCT TCGGCTTTCC GTCTTTGACT   
  
  
+ TTTTGTCTGT AAGCAGTCAA CATTCGTATC AAATCCCGCA CGTGCCTCAA ACGCAATTAT GCTCCAACCA   
  
  
+ AATCAAGACT AGATCAAGTT CATTTAGTGA GACAATGTTT GTTAGGACAA CTGGGTCGTC GATCCAACCT   
  
  
+ CGTGACTCTC GGTTGGAGGA TTGCAGCTTA AACGACATCG GTTGGTTGAA GAGGATTGTT ATGGGGTTTC   
  
  
+ TAGGTAATAT GTAAATTTAG AGGAAGTGGG GGTAGAAATT GAATTTATTA TAACAGCGGG GGCATGTTAG   
  
  
+ GAAAAGTTTC TGCTAATCTG CCATATCTGG GTGTCTAAAG TCAACTGCAT CCTGCTGGCA CCAACCAAAA   
  
  
+ CACTCCTGTA CTTAAAAGCA CCCTGACGCA GATCAGACGG CTCAGCACGA AAGTTTGACC TGATCTCCTT   
  
  
+ CCATGTCCAC AACGTCCCGT GGACCGTCAT AACCCTTGTG GGTCCCGCAC GCCAGACGAG GTCAATGTCC   
  
  
+ TTATGTATAT CACCACAGCT TTAATAGAAA AAAGAAATAA AATATTAAAA AACAATTTAT GTGTGCACAG   
  
  
+ CAGCCAAAAA AGCACGATGT GGTTGGATCT TATTCGCACC AACATTTGAC GGCAAAGGAT AGTGTGACAG   
  
  
+ GTCAGTGACC TAATCGACCA ATAGCATCTC GTAGCCTGGT ACTCATCTTT TTTGACCGGT GTCCCTTCCC   
  
  
+ TTTATCAACA GCACCCCTCT CTCCATTTTC TTTTCACGCT GTTCTCTCTT TTCTCTCCCT TTCTCTTTCT   
  
  
+ TTTCTTTCAT GGTGGTGGTG TTGCAGAGA  

- AAAATAATTT TTATAAAAAC TTTTTATTAT ATATTCATTT GTAGGGGGTC AATGGTTAGT TGACATGAAG   
  
  
- TGGATATGAT CATGATCATG AATGAGCACA TTCATGAAAA ATTAATAGTA GTGGTAGGTT AGATAGTGGC   
  
  
- TGTAGGAATT CTGAAATCGA AGGATAAATC TAAGTGATAA GGGGGCTTCT GTTTCTAAAT GCACTAAAGA   
  
  
- GAAAGGGACA ATATTATGTT TGTTCTGTGG GTAGTTTGAG GGTTTGCCTA GTTAGACTAA AGGTGTTGGA   
  
  
- TTCTTGTCGT TACGATACGA TCTAAAACAG ACTAAGCGAA GGATAGTAGC CTAGGATTAA CGTGGCTGGG   
  
  
- CTATTAAGTA GAGTAACTGG TATTAAAGCC CAGTATAGTC ATGGGAAAAG ATGATGAGTT AGCAATGAAT   
  
  
- TGTAGATTTG ATCGACCACC CATAATTATA TATAAAATTT ACAAATCTTA TTAGAAATAA ATTATATAGG   
  
  
- TTGAACCCGG AGAAAAAAAA AAAAATTAAA TCCCCTTTTT ATTTAGTTGA ATATTATTCC GGTTCTCTAA   
  
  
- TATCTTTCTT TTACTCCGGT CAACCACTTT GCTTGAAAGC CTCCTTCAGT TGTAAATCCG TCGGTTGATG   
  
  
- TAGTACAAAC AGAAGATGCA AGAAGTATAG AAACCCAGCA TCCACAAGGA AGCCGAAAGG CAGAAACTGA   
  
  
- AAAACAGACA TTCGTCAGTT GTAAGCATAG TTTAGGGCGT GCACGGAGTT TGCGTTAATA CGAGGTTGGT   
  
  
- TTAGTTCTGA TCTAGTTCAA GTAAATCACT CTGTTACAAA CAATCCTGTT GACCCAGCAG CTAGGTTGGA   
  
  
- GCACTGAGAG CCAACCTCCT AACGTCGAAT TTGCTGTAGC CAACCAACTT CTCCTAACAA TACCCCAAAG   
  
  
- ATCCATTATA CATTTAAATC TCCTTCACCC CCATCTTTAA CTTAAATAAT ATTGTCGCCC CCGTACAATC   
  
  
- CTTTTCAAAG ACGATTAGAC GGTATAGACC CACAGATTTC AGTTGACGTA GGACGACCGT GGTTGGTTTT   
  
  
- GTGAGGACAT GAATTTTCGT GGGACTGCGT CTAGTCTGCC GAGTCGTGCT TTCAAACTGG ACTAGAGGAA   
  
  
- GGTACAGGTG TTGCAGGGCA CCTGGCAGTA TTGGGAACAC CCAGGGCGTG CGGTCTGCTC CAGTTACAGG   
  
  
- AATACATATA GTGGTGTCGA AATTATCTTT TTTCTTTATT TTATAATTTT TTGTTAAATA CACACGTGTC   
  
  
- GTCGGTTTTT TCGTGCTACA CCAACCTAGA ATAAGCGTGG TTGTAAACTG CCGTTTCCTA TCACACTGTC   
  
  
- CAGTCACTGG ATTAGCTGGT TATCGTAGAG CATCGGACCA TGAGTAGAAA AAACTGGCCA CAGGGAAGGG   
  
  
- AAATAGTTGT CGTGGGGAGA GAGGTAAAAG AAAAGTGCGA CAAGAGAGAA AAGAGAGGGA AAGAGAAAGA   
  
  
- AAAGAAAGTA CCACCACCAC AACGTCTCT

+     TGACG-motif

| Site Name | Organism | Position | Strand | Matrix score. | sequence | function |
| --- | --- | --- | --- | --- | --- | --- |
| TGACG-motif | Hordeum vulgare | 1307 | + | 5 | TGACG | cis-acting regulatory element involved in the MeJA-responsiveness |
| TGACG-motif | Hordeum vulgare | 1145 | - | 5 | TGACG | cis-acting regulatory element involved in the MeJA-responsiveness |
| TGACG-motif | Hordeum vulgare | 1074 | + | 5 | TGACG | cis-acting regulatory element involved in the MeJA-responsiveness |

> 2018/04/13 10:10:12  
+ TTTTATTAAA AATATTTTTG AAAAATAATA TATAAGTAAA CATCCCCCAG TTACCAATCA ACTGTACTTC   
  
  
+ ACCTATACTA GTACTAGTAC TTACTCGTGT AAGTACTTTT TAATTATCAT CACCATCCAA TCTATCACCG   
  
  
+ ACATCCTTAA GACTTTAGCT TCCTATTTAG ATTCACTATT CCCCCGAAGA CAAAGATTTA CGTGATTTCT   
  
  
+ CTTTCCCTGT TATAATACAA ACAAGACACC CATCAAACTC CCAAACGGAT CAATCTGATT TCCACAACCT   
  
  
+ AAGAACAGCA ATGCTATGCT AGATTTTGTC TGATTCGCTT CCTATCATCG GATCCTAATT GCACCGACCC   
  
  
+ GATAATTCAT CTCATTGACC ATAATTTCGG GTCATATCAG TACCCTTTTC TACTACTCAA TCGTTACTTA   
  
  
+ ACATCTAAAC TAGCTGGTGG GTATTAATAT ATATTTTAAA TGTTTAGAAT AATCTTTATT TAATATATCC   
  
  
+ AACTTGGGCC TCTTTTTTTT TTTTTAATTT AGGGGAAAAA TAAATCAACT TATAATAAGG CCAAGAGATT   
  
  
+ ATAGAAAGAA AATGAGGCCA GTTGGTGAAA CGAACTTTCG GAGGAAGTCA ACATTTAGGC AGCCAACTAC   
  
  
+ ATCATGTTTG TCTTCTACGT TCTTCATATC TTTGGGTCGT AGGTGTTCCT TCGGCTTTCC GTCTTTGACT   
  
  
+ TTTTGTCTGT AAGCAGTCAA CATTCGTATC AAATCCCGCA CGTGCCTCAA ACGCAATTAT GCTCCAACCA   
  
  
+ AATCAAGACT AGATCAAGTT CATTTAGTGA GACAATGTTT GTTAGGACAA CTGGGTCGTC GATCCAACCT   
  
  
+ CGTGACTCTC GGTTGGAGGA TTGCAGCTTA AACGACATCG GTTGGTTGAA GAGGATTGTT ATGGGGTTTC   
  
  
+ TAGGTAATAT GTAAATTTAG AGGAAGTGGG GGTAGAAATT GAATTTATTA TAACAGCGGG GGCATGTTAG   
  
  
+ GAAAAGTTTC TGCTAATCTG CCATATCTGG GTGTCTAAAG TCAACTGCAT CCTGCTGGCA CCAACCAAAA   
  
  
+ CACTCCTGTA CTTAAAAGCA CCCTGACGCA GATCAGACGG CTCAGCACGA AAGTTTGACC TGATCTCCTT   
  
  
+ CCATGTCCAC AACGTCCCGT GGACCGTCAT AACCCTTGTG GGTCCCGCAC GCCAGACGAG GTCAATGTCC   
  
  
+ TTATGTATAT CACCACAGCT TTAATAGAAA AAAGAAATAA AATATTAAAA AACAATTTAT GTGTGCACAG   
  
  
+ CAGCCAAAAA AGCACGATGT GGTTGGATCT TATTCGCACC AACATTTGAC GGCAAAGGAT AGTGTGACAG   
  
  
+ GTCAGTGACC TAATCGACCA ATAGCATCTC GTAGCCTGGT ACTCATCTTT TTTGACCGGT GTCCCTTCCC   
  
  
+ TTTATCAACA GCACCCCTCT CTCCATTTTC TTTTCACGCT GTTCTCTCTT TTCTCTCCCT TTCTCTTTCT   
  
  
+ TTTCTTTCAT GGTGGTGGTG TTGCAGAGA  

- AAAATAATTT TTATAAAAAC TTTTTATTAT ATATTCATTT GTAGGGGGTC AATGGTTAGT TGACATGAAG   
  
  
- TGGATATGAT CATGATCATG AATGAGCACA TTCATGAAAA ATTAATAGTA GTGGTAGGTT AGATAGTGGC   
  
  
- TGTAGGAATT CTGAAATCGA AGGATAAATC TAAGTGATAA GGGGGCTTCT GTTTCTAAAT GCACTAAAGA   
  
  
- GAAAGGGACA ATATTATGTT TGTTCTGTGG GTAGTTTGAG GGTTTGCCTA GTTAGACTAA AGGTGTTGGA   
  
  
- TTCTTGTCGT TACGATACGA TCTAAAACAG ACTAAGCGAA GGATAGTAGC CTAGGATTAA CGTGGCTGGG   
  
  
- CTATTAAGTA GAGTAACTGG TATTAAAGCC CAGTATAGTC ATGGGAAAAG ATGATGAGTT AGCAATGAAT   
  
  
- TGTAGATTTG ATCGACCACC CATAATTATA TATAAAATTT ACAAATCTTA TTAGAAATAA ATTATATAGG   
  
  
- TTGAACCCGG AGAAAAAAAA AAAAATTAAA TCCCCTTTTT ATTTAGTTGA ATATTATTCC GGTTCTCTAA   
  
  
- TATCTTTCTT TTACTCCGGT CAACCACTTT GCTTGAAAGC CTCCTTCAGT TGTAAATCCG TCGGTTGATG   
  
  
- TAGTACAAAC AGAAGATGCA AGAAGTATAG AAACCCAGCA TCCACAAGGA AGCCGAAAGG CAGAAACTGA   
  
  
- AAAACAGACA TTCGTCAGTT GTAAGCATAG TTTAGGGCGT GCACGGAGTT TGCGTTAATA CGAGGTTGGT   
  
  
- TTAGTTCTGA TCTAGTTCAA GTAAATCACT CTGTTACAAA CAATCCTGTT GACCCAGCAG CTAGGTTGGA   
  
  
- GCACTGAGAG CCAACCTCCT AACGTCGAAT TTGCTGTAGC CAACCAACTT CTCCTAACAA TACCCCAAAG   
  
  
- ATCCATTATA CATTTAAATC TCCTTCACCC CCATCTTTAA CTTAAATAAT ATTGTCGCCC CCGTACAATC   
  
  
- CTTTTCAAAG ACGATTAGAC GGTATAGACC CACAGATTTC AGTTGACGTA GGACGACCGT GGTTGGTTTT   
  
  
- GTGAGGACAT GAATTTTCGT GGGACTGCGT CTAGTCTGCC GAGTCGTGCT TTCAAACTGG ACTAGAGGAA   
  
  
- GGTACAGGTG TTGCAGGGCA CCTGGCAGTA TTGGGAACAC CCAGGGCGTG CGGTCTGCTC CAGTTACAGG   
  
  
- AATACATATA GTGGTGTCGA AATTATCTTT TTTCTTTATT TTATAATTTT TTGTTAAATA CACACGTGTC   
  
  
- GTCGGTTTTT TCGTGCTACA CCAACCTAGA ATAAGCGTGG TTGTAAACTG CCGTTTCCTA TCACACTGTC   
  
  
- CAGTCACTGG ATTAGCTGGT TATCGTAGAG CATCGGACCA TGAGTAGAAA AAACTGGCCA CAGGGAAGGG   
  
  
- AAATAGTTGT CGTGGGGAGA GAGGTAAAAG AAAAGTGCGA CAAGAGAGAA AAGAGAGGGA AAGAGAAAGA   
  
  
- AAAGAAAGTA CCACCACCAC AACGTCTCT

+     Unnamed\_\_1

| Site Name | Organism | Position | Strand | Matrix score. | sequence | function |
| --- | --- | --- | --- | --- | --- | --- |
| Unnamed\_\_1 | Zea mays | 1138 | + | 5 | CGTGG |  |

> 2018/04/13 10:10:12  
+ TTTTATTAAA AATATTTTTG AAAAATAATA TATAAGTAAA CATCCCCCAG TTACCAATCA ACTGTACTTC   
  
  
+ ACCTATACTA GTACTAGTAC TTACTCGTGT AAGTACTTTT TAATTATCAT CACCATCCAA TCTATCACCG   
  
  
+ ACATCCTTAA GACTTTAGCT TCCTATTTAG ATTCACTATT CCCCCGAAGA CAAAGATTTA CGTGATTTCT   
  
  
+ CTTTCCCTGT TATAATACAA ACAAGACACC CATCAAACTC CCAAACGGAT CAATCTGATT TCCACAACCT   
  
  
+ AAGAACAGCA ATGCTATGCT AGATTTTGTC TGATTCGCTT CCTATCATCG GATCCTAATT GCACCGACCC   
  
  
+ GATAATTCAT CTCATTGACC ATAATTTCGG GTCATATCAG TACCCTTTTC TACTACTCAA TCGTTACTTA   
  
  
+ ACATCTAAAC TAGCTGGTGG GTATTAATAT ATATTTTAAA TGTTTAGAAT AATCTTTATT TAATATATCC   
  
  
+ AACTTGGGCC TCTTTTTTTT TTTTTAATTT AGGGGAAAAA TAAATCAACT TATAATAAGG CCAAGAGATT   
  
  
+ ATAGAAAGAA AATGAGGCCA GTTGGTGAAA CGAACTTTCG GAGGAAGTCA ACATTTAGGC AGCCAACTAC   
  
  
+ ATCATGTTTG TCTTCTACGT TCTTCATATC TTTGGGTCGT AGGTGTTCCT TCGGCTTTCC GTCTTTGACT   
  
  
+ TTTTGTCTGT AAGCAGTCAA CATTCGTATC AAATCCCGCA CGTGCCTCAA ACGCAATTAT GCTCCAACCA   
  
  
+ AATCAAGACT AGATCAAGTT CATTTAGTGA GACAATGTTT GTTAGGACAA CTGGGTCGTC GATCCAACCT   
  
  
+ CGTGACTCTC GGTTGGAGGA TTGCAGCTTA AACGACATCG GTTGGTTGAA GAGGATTGTT ATGGGGTTTC   
  
  
+ TAGGTAATAT GTAAATTTAG AGGAAGTGGG GGTAGAAATT GAATTTATTA TAACAGCGGG GGCATGTTAG   
  
  
+ GAAAAGTTTC TGCTAATCTG CCATATCTGG GTGTCTAAAG TCAACTGCAT CCTGCTGGCA CCAACCAAAA   
  
  
+ CACTCCTGTA CTTAAAAGCA CCCTGACGCA GATCAGACGG CTCAGCACGA AAGTTTGACC TGATCTCCTT   
  
  
+ CCATGTCCAC AACGTCCCGT GGACCGTCAT AACCCTTGTG GGTCCCGCAC GCCAGACGAG GTCAATGTCC   
  
  
+ TTATGTATAT CACCACAGCT TTAATAGAAA AAAGAAATAA AATATTAAAA AACAATTTAT GTGTGCACAG   
  
  
+ CAGCCAAAAA AGCACGATGT GGTTGGATCT TATTCGCACC AACATTTGAC GGCAAAGGAT AGTGTGACAG   
  
  
+ GTCAGTGACC TAATCGACCA ATAGCATCTC GTAGCCTGGT ACTCATCTTT TTTGACCGGT GTCCCTTCCC   
  
  
+ TTTATCAACA GCACCCCTCT CTCCATTTTC TTTTCACGCT GTTCTCTCTT TTCTCTCCCT TTCTCTTTCT   
  
  
+ TTTCTTTCAT GGTGGTGGTG TTGCAGAGA  

- AAAATAATTT TTATAAAAAC TTTTTATTAT ATATTCATTT GTAGGGGGTC AATGGTTAGT TGACATGAAG   
  
  
- TGGATATGAT CATGATCATG AATGAGCACA TTCATGAAAA ATTAATAGTA GTGGTAGGTT AGATAGTGGC   
  
  
- TGTAGGAATT CTGAAATCGA AGGATAAATC TAAGTGATAA GGGGGCTTCT GTTTCTAAAT GCACTAAAGA   
  
  
- GAAAGGGACA ATATTATGTT TGTTCTGTGG GTAGTTTGAG GGTTTGCCTA GTTAGACTAA AGGTGTTGGA   
  
  
- TTCTTGTCGT TACGATACGA TCTAAAACAG ACTAAGCGAA GGATAGTAGC CTAGGATTAA CGTGGCTGGG   
  
  
- CTATTAAGTA GAGTAACTGG TATTAAAGCC CAGTATAGTC ATGGGAAAAG ATGATGAGTT AGCAATGAAT   
  
  
- TGTAGATTTG ATCGACCACC CATAATTATA TATAAAATTT ACAAATCTTA TTAGAAATAA ATTATATAGG   
  
  
- TTGAACCCGG AGAAAAAAAA AAAAATTAAA TCCCCTTTTT ATTTAGTTGA ATATTATTCC GGTTCTCTAA   
  
  
- TATCTTTCTT TTACTCCGGT CAACCACTTT GCTTGAAAGC CTCCTTCAGT TGTAAATCCG TCGGTTGATG   
  
  
- TAGTACAAAC AGAAGATGCA AGAAGTATAG AAACCCAGCA TCCACAAGGA AGCCGAAAGG CAGAAACTGA   
  
  
- AAAACAGACA TTCGTCAGTT GTAAGCATAG TTTAGGGCGT GCACGGAGTT TGCGTTAATA CGAGGTTGGT   
  
  
- TTAGTTCTGA TCTAGTTCAA GTAAATCACT CTGTTACAAA CAATCCTGTT GACCCAGCAG CTAGGTTGGA   
  
  
- GCACTGAGAG CCAACCTCCT AACGTCGAAT TTGCTGTAGC CAACCAACTT CTCCTAACAA TACCCCAAAG   
  
  
- ATCCATTATA CATTTAAATC TCCTTCACCC CCATCTTTAA CTTAAATAAT ATTGTCGCCC CCGTACAATC   
  
  
- CTTTTCAAAG ACGATTAGAC GGTATAGACC CACAGATTTC AGTTGACGTA GGACGACCGT GGTTGGTTTT   
  
  
- GTGAGGACAT GAATTTTCGT GGGACTGCGT CTAGTCTGCC GAGTCGTGCT TTCAAACTGG ACTAGAGGAA   
  
  
- GGTACAGGTG TTGCAGGGCA CCTGGCAGTA TTGGGAACAC CCAGGGCGTG CGGTCTGCTC CAGTTACAGG   
  
  
- AATACATATA GTGGTGTCGA AATTATCTTT TTTCTTTATT TTATAATTTT TTGTTAAATA CACACGTGTC   
  
  
- GTCGGTTTTT TCGTGCTACA CCAACCTAGA ATAAGCGTGG TTGTAAACTG CCGTTTCCTA TCACACTGTC   
  
  
- CAGTCACTGG ATTAGCTGGT TATCGTAGAG CATCGGACCA TGAGTAGAAA AAACTGGCCA CAGGGAAGGG   
  
  
- AAATAGTTGT CGTGGGGAGA GAGGTAAAAG AAAAGTGCGA CAAGAGAGAA AAGAGAGGGA AAGAGAAAGA   
  
  
- AAAGAAAGTA CCACCACCAC AACGTCTCT

+     Unnamed\_\_3

| Site Name | Organism | Position | Strand | Matrix score. | sequence | function |
| --- | --- | --- | --- | --- | --- | --- |
| Unnamed\_\_3 | Zea mays | 1138 | + | 5 | CGTGG |  |

> 2018/04/13 10:10:12  
+ TTTTATTAAA AATATTTTTG AAAAATAATA TATAAGTAAA CATCCCCCAG TTACCAATCA ACTGTACTTC   
  
  
+ ACCTATACTA GTACTAGTAC TTACTCGTGT AAGTACTTTT TAATTATCAT CACCATCCAA TCTATCACCG   
  
  
+ ACATCCTTAA GACTTTAGCT TCCTATTTAG ATTCACTATT CCCCCGAAGA CAAAGATTTA CGTGATTTCT   
  
  
+ CTTTCCCTGT TATAATACAA ACAAGACACC CATCAAACTC CCAAACGGAT CAATCTGATT TCCACAACCT   
  
  
+ AAGAACAGCA ATGCTATGCT AGATTTTGTC TGATTCGCTT CCTATCATCG GATCCTAATT GCACCGACCC   
  
  
+ GATAATTCAT CTCATTGACC ATAATTTCGG GTCATATCAG TACCCTTTTC TACTACTCAA TCGTTACTTA   
  
  
+ ACATCTAAAC TAGCTGGTGG GTATTAATAT ATATTTTAAA TGTTTAGAAT AATCTTTATT TAATATATCC   
  
  
+ AACTTGGGCC TCTTTTTTTT TTTTTAATTT AGGGGAAAAA TAAATCAACT TATAATAAGG CCAAGAGATT   
  
  
+ ATAGAAAGAA AATGAGGCCA GTTGGTGAAA CGAACTTTCG GAGGAAGTCA ACATTTAGGC AGCCAACTAC   
  
  
+ ATCATGTTTG TCTTCTACGT TCTTCATATC TTTGGGTCGT AGGTGTTCCT TCGGCTTTCC GTCTTTGACT   
  
  
+ TTTTGTCTGT AAGCAGTCAA CATTCGTATC AAATCCCGCA CGTGCCTCAA ACGCAATTAT GCTCCAACCA   
  
  
+ AATCAAGACT AGATCAAGTT CATTTAGTGA GACAATGTTT GTTAGGACAA CTGGGTCGTC GATCCAACCT   
  
  
+ CGTGACTCTC GGTTGGAGGA TTGCAGCTTA AACGACATCG GTTGGTTGAA GAGGATTGTT ATGGGGTTTC   
  
  
+ TAGGTAATAT GTAAATTTAG AGGAAGTGGG GGTAGAAATT GAATTTATTA TAACAGCGGG GGCATGTTAG   
  
  
+ GAAAAGTTTC TGCTAATCTG CCATATCTGG GTGTCTAAAG TCAACTGCAT CCTGCTGGCA CCAACCAAAA   
  
  
+ CACTCCTGTA CTTAAAAGCA CCCTGACGCA GATCAGACGG CTCAGCACGA AAGTTTGACC TGATCTCCTT   
  
  
+ CCATGTCCAC AACGTCCCGT GGACCGTCAT AACCCTTGTG GGTCCCGCAC GCCAGACGAG GTCAATGTCC   
  
  
+ TTATGTATAT CACCACAGCT TTAATAGAAA AAAGAAATAA AATATTAAAA AACAATTTAT GTGTGCACAG   
  
  
+ CAGCCAAAAA AGCACGATGT GGTTGGATCT TATTCGCACC AACATTTGAC GGCAAAGGAT AGTGTGACAG   
  
  
+ GTCAGTGACC TAATCGACCA ATAGCATCTC GTAGCCTGGT ACTCATCTTT TTTGACCGGT GTCCCTTCCC   
  
  
+ TTTATCAACA GCACCCCTCT CTCCATTTTC TTTTCACGCT GTTCTCTCTT TTCTCTCCCT TTCTCTTTCT   
  
  
+ TTTCTTTCAT GGTGGTGGTG TTGCAGAGA  

- AAAATAATTT TTATAAAAAC TTTTTATTAT ATATTCATTT GTAGGGGGTC AATGGTTAGT TGACATGAAG   
  
  
- TGGATATGAT CATGATCATG AATGAGCACA TTCATGAAAA ATTAATAGTA GTGGTAGGTT AGATAGTGGC   
  
  
- TGTAGGAATT CTGAAATCGA AGGATAAATC TAAGTGATAA GGGGGCTTCT GTTTCTAAAT GCACTAAAGA   
  
  
- GAAAGGGACA ATATTATGTT TGTTCTGTGG GTAGTTTGAG GGTTTGCCTA GTTAGACTAA AGGTGTTGGA   
  
  
- TTCTTGTCGT TACGATACGA TCTAAAACAG ACTAAGCGAA GGATAGTAGC CTAGGATTAA CGTGGCTGGG   
  
  
- CTATTAAGTA GAGTAACTGG TATTAAAGCC CAGTATAGTC ATGGGAAAAG ATGATGAGTT AGCAATGAAT   
  
  
- TGTAGATTTG ATCGACCACC CATAATTATA TATAAAATTT ACAAATCTTA TTAGAAATAA ATTATATAGG   
  
  
- TTGAACCCGG AGAAAAAAAA AAAAATTAAA TCCCCTTTTT ATTTAGTTGA ATATTATTCC GGTTCTCTAA   
  
  
- TATCTTTCTT TTACTCCGGT CAACCACTTT GCTTGAAAGC CTCCTTCAGT TGTAAATCCG TCGGTTGATG   
  
  
- TAGTACAAAC AGAAGATGCA AGAAGTATAG AAACCCAGCA TCCACAAGGA AGCCGAAAGG CAGAAACTGA   
  
  
- AAAACAGACA TTCGTCAGTT GTAAGCATAG TTTAGGGCGT GCACGGAGTT TGCGTTAATA CGAGGTTGGT   
  
  
- TTAGTTCTGA TCTAGTTCAA GTAAATCACT CTGTTACAAA CAATCCTGTT GACCCAGCAG CTAGGTTGGA   
  
  
- GCACTGAGAG CCAACCTCCT AACGTCGAAT TTGCTGTAGC CAACCAACTT CTCCTAACAA TACCCCAAAG   
  
  
- ATCCATTATA CATTTAAATC TCCTTCACCC CCATCTTTAA CTTAAATAAT ATTGTCGCCC CCGTACAATC   
  
  
- CTTTTCAAAG ACGATTAGAC GGTATAGACC CACAGATTTC AGTTGACGTA GGACGACCGT GGTTGGTTTT   
  
  
- GTGAGGACAT GAATTTTCGT GGGACTGCGT CTAGTCTGCC GAGTCGTGCT TTCAAACTGG ACTAGAGGAA   
  
  
- GGTACAGGTG TTGCAGGGCA CCTGGCAGTA TTGGGAACAC CCAGGGCGTG CGGTCTGCTC CAGTTACAGG   
  
  
- AATACATATA GTGGTGTCGA AATTATCTTT TTTCTTTATT TTATAATTTT TTGTTAAATA CACACGTGTC   
  
  
- GTCGGTTTTT TCGTGCTACA CCAACCTAGA ATAAGCGTGG TTGTAAACTG CCGTTTCCTA TCACACTGTC   
  
  
- CAGTCACTGG ATTAGCTGGT TATCGTAGAG CATCGGACCA TGAGTAGAAA AAACTGGCCA CAGGGAAGGG   
  
  
- AAATAGTTGT CGTGGGGAGA GAGGTAAAAG AAAAGTGCGA CAAGAGAGAA AAGAGAGGGA AAGAGAAAGA   
  
  
- AAAGAAAGTA CCACCACCAC AACGTCTCT

+     Unnamed\_\_4

| Site Name | Organism | Position | Strand | Matrix score. | sequence | function |
| --- | --- | --- | --- | --- | --- | --- |
| Unnamed\_\_4 | Petroselinum hortense | 1421 | + | 4 | CTCC |  |
| Unnamed\_\_4 | Petroselinum hortense | 600 | - | 4 | CTCC |  |
| Unnamed\_\_4 | Petroselinum hortense | 855 | - | 4 | CTCC |  |
| Unnamed\_\_4 | Petroselinum hortense | 1115 | + | 4 | CTCC |  |
| Unnamed\_\_4 | Petroselinum hortense | 762 | + | 4 | CTCC |  |
| Unnamed\_\_4 | Petroselinum hortense | 1053 | + | 4 | CTCC |  |
| Unnamed\_\_4 | Petroselinum hortense | 1455 | + | 4 | CTCC |  |
| Unnamed\_\_4 | Petroselinum hortense | 248 | + | 4 | CTCC |  |

> 2018/04/13 10:10:12  
+ TTTTATTAAA AATATTTTTG AAAAATAATA TATAAGTAAA CATCCCCCAG TTACCAATCA ACTGTACTTC   
  
  
+ ACCTATACTA GTACTAGTAC TTACTCGTGT AAGTACTTTT TAATTATCAT CACCATCCAA TCTATCACCG   
  
  
+ ACATCCTTAA GACTTTAGCT TCCTATTTAG ATTCACTATT CCCCCGAAGA CAAAGATTTA CGTGATTTCT   
  
  
+ CTTTCCCTGT TATAATACAA ACAAGACACC CATCAAACTC CCAAACGGAT CAATCTGATT TCCACAACCT   
  
  
+ AAGAACAGCA ATGCTATGCT AGATTTTGTC TGATTCGCTT CCTATCATCG GATCCTAATT GCACCGACCC   
  
  
+ GATAATTCAT CTCATTGACC ATAATTTCGG GTCATATCAG TACCCTTTTC TACTACTCAA TCGTTACTTA   
  
  
+ ACATCTAAAC TAGCTGGTGG GTATTAATAT ATATTTTAAA TGTTTAGAAT AATCTTTATT TAATATATCC   
  
  
+ AACTTGGGCC TCTTTTTTTT TTTTTAATTT AGGGGAAAAA TAAATCAACT TATAATAAGG CCAAGAGATT   
  
  
+ ATAGAAAGAA AATGAGGCCA GTTGGTGAAA CGAACTTTCG GAGGAAGTCA ACATTTAGGC AGCCAACTAC   
  
  
+ ATCATGTTTG TCTTCTACGT TCTTCATATC TTTGGGTCGT AGGTGTTCCT TCGGCTTTCC GTCTTTGACT   
  
  
+ TTTTGTCTGT AAGCAGTCAA CATTCGTATC AAATCCCGCA CGTGCCTCAA ACGCAATTAT GCTCCAACCA   
  
  
+ AATCAAGACT AGATCAAGTT CATTTAGTGA GACAATGTTT GTTAGGACAA CTGGGTCGTC GATCCAACCT   
  
  
+ CGTGACTCTC GGTTGGAGGA TTGCAGCTTA AACGACATCG GTTGGTTGAA GAGGATTGTT ATGGGGTTTC   
  
  
+ TAGGTAATAT GTAAATTTAG AGGAAGTGGG GGTAGAAATT GAATTTATTA TAACAGCGGG GGCATGTTAG   
  
  
+ GAAAAGTTTC TGCTAATCTG CCATATCTGG GTGTCTAAAG TCAACTGCAT CCTGCTGGCA CCAACCAAAA   
  
  
+ CACTCCTGTA CTTAAAAGCA CCCTGACGCA GATCAGACGG CTCAGCACGA AAGTTTGACC TGATCTCCTT   
  
  
+ CCATGTCCAC AACGTCCCGT GGACCGTCAT AACCCTTGTG GGTCCCGCAC GCCAGACGAG GTCAATGTCC   
  
  
+ TTATGTATAT CACCACAGCT TTAATAGAAA AAAGAAATAA AATATTAAAA AACAATTTAT GTGTGCACAG   
  
  
+ CAGCCAAAAA AGCACGATGT GGTTGGATCT TATTCGCACC AACATTTGAC GGCAAAGGAT AGTGTGACAG   
  
  
+ GTCAGTGACC TAATCGACCA ATAGCATCTC GTAGCCTGGT ACTCATCTTT TTTGACCGGT GTCCCTTCCC   
  
  
+ TTTATCAACA GCACCCCTCT CTCCATTTTC TTTTCACGCT GTTCTCTCTT TTCTCTCCCT TTCTCTTTCT   
  
  
+ TTTCTTTCAT GGTGGTGGTG TTGCAGAGA  

- AAAATAATTT TTATAAAAAC TTTTTATTAT ATATTCATTT GTAGGGGGTC AATGGTTAGT TGACATGAAG   
  
  
- TGGATATGAT CATGATCATG AATGAGCACA TTCATGAAAA ATTAATAGTA GTGGTAGGTT AGATAGTGGC   
  
  
- TGTAGGAATT CTGAAATCGA AGGATAAATC TAAGTGATAA GGGGGCTTCT GTTTCTAAAT GCACTAAAGA   
  
  
- GAAAGGGACA ATATTATGTT TGTTCTGTGG GTAGTTTGAG GGTTTGCCTA GTTAGACTAA AGGTGTTGGA   
  
  
- TTCTTGTCGT TACGATACGA TCTAAAACAG ACTAAGCGAA GGATAGTAGC CTAGGATTAA CGTGGCTGGG   
  
  
- CTATTAAGTA GAGTAACTGG TATTAAAGCC CAGTATAGTC ATGGGAAAAG ATGATGAGTT AGCAATGAAT   
  
  
- TGTAGATTTG ATCGACCACC CATAATTATA TATAAAATTT ACAAATCTTA TTAGAAATAA ATTATATAGG   
  
  
- TTGAACCCGG AGAAAAAAAA AAAAATTAAA TCCCCTTTTT ATTTAGTTGA ATATTATTCC GGTTCTCTAA   
  
  
- TATCTTTCTT TTACTCCGGT CAACCACTTT GCTTGAAAGC CTCCTTCAGT TGTAAATCCG TCGGTTGATG   
  
  
- TAGTACAAAC AGAAGATGCA AGAAGTATAG AAACCCAGCA TCCACAAGGA AGCCGAAAGG CAGAAACTGA   
  
  
- AAAACAGACA TTCGTCAGTT GTAAGCATAG TTTAGGGCGT GCACGGAGTT TGCGTTAATA CGAGGTTGGT   
  
  
- TTAGTTCTGA TCTAGTTCAA GTAAATCACT CTGTTACAAA CAATCCTGTT GACCCAGCAG CTAGGTTGGA   
  
  
- GCACTGAGAG CCAACCTCCT AACGTCGAAT TTGCTGTAGC CAACCAACTT CTCCTAACAA TACCCCAAAG   
  
  
- ATCCATTATA CATTTAAATC TCCTTCACCC CCATCTTTAA CTTAAATAAT ATTGTCGCCC CCGTACAATC   
  
  
- CTTTTCAAAG ACGATTAGAC GGTATAGACC CACAGATTTC AGTTGACGTA GGACGACCGT GGTTGGTTTT   
  
  
- GTGAGGACAT GAATTTTCGT GGGACTGCGT CTAGTCTGCC GAGTCGTGCT TTCAAACTGG ACTAGAGGAA   
  
  
- GGTACAGGTG TTGCAGGGCA CCTGGCAGTA TTGGGAACAC CCAGGGCGTG CGGTCTGCTC CAGTTACAGG   
  
  
- AATACATATA GTGGTGTCGA AATTATCTTT TTTCTTTATT TTATAATTTT TTGTTAAATA CACACGTGTC   
  
  
- GTCGGTTTTT TCGTGCTACA CCAACCTAGA ATAAGCGTGG TTGTAAACTG CCGTTTCCTA TCACACTGTC   
  
  
- CAGTCACTGG ATTAGCTGGT TATCGTAGAG CATCGGACCA TGAGTAGAAA AAACTGGCCA CAGGGAAGGG   
  
  
- AAATAGTTGT CGTGGGGAGA GAGGTAAAAG AAAAGTGCGA CAAGAGAGAA AAGAGAGGGA AAGAGAAAGA   
  
  
- AAAGAAAGTA CCACCACCAC AACGTCTCT

+     W box

| Site Name | Organism | Position | Strand | Matrix score. | sequence | function |
| --- | --- | --- | --- | --- | --- | --- |
| W box | Arabidopsis thaliana | 365 | + | 6 | TTGACC |  |
| W box | Arabidopsis thaliana | 1180 | - | 6 | TTGACC |  |
| W box | Arabidopsis thaliana | 1105 | + | 6 | TTGACC |  |
| W box | Arabidopsis thaliana | 1382 | + | 6 | TTGACC |  |

> 2018/04/13 10:10:12  
+ TTTTATTAAA AATATTTTTG AAAAATAATA TATAAGTAAA CATCCCCCAG TTACCAATCA ACTGTACTTC   
  
  
+ ACCTATACTA GTACTAGTAC TTACTCGTGT AAGTACTTTT TAATTATCAT CACCATCCAA TCTATCACCG   
  
  
+ ACATCCTTAA GACTTTAGCT TCCTATTTAG ATTCACTATT CCCCCGAAGA CAAAGATTTA CGTGATTTCT   
  
  
+ CTTTCCCTGT TATAATACAA ACAAGACACC CATCAAACTC CCAAACGGAT CAATCTGATT TCCACAACCT   
  
  
+ AAGAACAGCA ATGCTATGCT AGATTTTGTC TGATTCGCTT CCTATCATCG GATCCTAATT GCACCGACCC   
  
  
+ GATAATTCAT CTCATTGACC ATAATTTCGG GTCATATCAG TACCCTTTTC TACTACTCAA TCGTTACTTA   
  
  
+ ACATCTAAAC TAGCTGGTGG GTATTAATAT ATATTTTAAA TGTTTAGAAT AATCTTTATT TAATATATCC   
  
  
+ AACTTGGGCC TCTTTTTTTT TTTTTAATTT AGGGGAAAAA TAAATCAACT TATAATAAGG CCAAGAGATT   
  
  
+ ATAGAAAGAA AATGAGGCCA GTTGGTGAAA CGAACTTTCG GAGGAAGTCA ACATTTAGGC AGCCAACTAC   
  
  
+ ATCATGTTTG TCTTCTACGT TCTTCATATC TTTGGGTCGT AGGTGTTCCT TCGGCTTTCC GTCTTTGACT   
  
  
+ TTTTGTCTGT AAGCAGTCAA CATTCGTATC AAATCCCGCA CGTGCCTCAA ACGCAATTAT GCTCCAACCA   
  
  
+ AATCAAGACT AGATCAAGTT CATTTAGTGA GACAATGTTT GTTAGGACAA CTGGGTCGTC GATCCAACCT   
  
  
+ CGTGACTCTC GGTTGGAGGA TTGCAGCTTA AACGACATCG GTTGGTTGAA GAGGATTGTT ATGGGGTTTC   
  
  
+ TAGGTAATAT GTAAATTTAG AGGAAGTGGG GGTAGAAATT GAATTTATTA TAACAGCGGG GGCATGTTAG   
  
  
+ GAAAAGTTTC TGCTAATCTG CCATATCTGG GTGTCTAAAG TCAACTGCAT CCTGCTGGCA CCAACCAAAA   
  
  
+ CACTCCTGTA CTTAAAAGCA CCCTGACGCA GATCAGACGG CTCAGCACGA AAGTTTGACC TGATCTCCTT   
  
  
+ CCATGTCCAC AACGTCCCGT GGACCGTCAT AACCCTTGTG GGTCCCGCAC GCCAGACGAG GTCAATGTCC   
  
  
+ TTATGTATAT CACCACAGCT TTAATAGAAA AAAGAAATAA AATATTAAAA AACAATTTAT GTGTGCACAG   
  
  
+ CAGCCAAAAA AGCACGATGT GGTTGGATCT TATTCGCACC AACATTTGAC GGCAAAGGAT AGTGTGACAG   
  
  
+ GTCAGTGACC TAATCGACCA ATAGCATCTC GTAGCCTGGT ACTCATCTTT TTTGACCGGT GTCCCTTCCC   
  
  
+ TTTATCAACA GCACCCCTCT CTCCATTTTC TTTTCACGCT GTTCTCTCTT TTCTCTCCCT TTCTCTTTCT   
  
  
+ TTTCTTTCAT GGTGGTGGTG TTGCAGAGA  

- AAAATAATTT TTATAAAAAC TTTTTATTAT ATATTCATTT GTAGGGGGTC AATGGTTAGT TGACATGAAG   
  
  
- TGGATATGAT CATGATCATG AATGAGCACA TTCATGAAAA ATTAATAGTA GTGGTAGGTT AGATAGTGGC   
  
  
- TGTAGGAATT CTGAAATCGA AGGATAAATC TAAGTGATAA GGGGGCTTCT GTTTCTAAAT GCACTAAAGA   
  
  
- GAAAGGGACA ATATTATGTT TGTTCTGTGG GTAGTTTGAG GGTTTGCCTA GTTAGACTAA AGGTGTTGGA   
  
  
- TTCTTGTCGT TACGATACGA TCTAAAACAG ACTAAGCGAA GGATAGTAGC CTAGGATTAA CGTGGCTGGG   
  
  
- CTATTAAGTA GAGTAACTGG TATTAAAGCC CAGTATAGTC ATGGGAAAAG ATGATGAGTT AGCAATGAAT   
  
  
- TGTAGATTTG ATCGACCACC CATAATTATA TATAAAATTT ACAAATCTTA TTAGAAATAA ATTATATAGG   
  
  
- TTGAACCCGG AGAAAAAAAA AAAAATTAAA TCCCCTTTTT ATTTAGTTGA ATATTATTCC GGTTCTCTAA   
  
  
- TATCTTTCTT TTACTCCGGT CAACCACTTT GCTTGAAAGC CTCCTTCAGT TGTAAATCCG TCGGTTGATG   
  
  
- TAGTACAAAC AGAAGATGCA AGAAGTATAG AAACCCAGCA TCCACAAGGA AGCCGAAAGG CAGAAACTGA   
  
  
- AAAACAGACA TTCGTCAGTT GTAAGCATAG TTTAGGGCGT GCACGGAGTT TGCGTTAATA CGAGGTTGGT   
  
  
- TTAGTTCTGA TCTAGTTCAA GTAAATCACT CTGTTACAAA CAATCCTGTT GACCCAGCAG CTAGGTTGGA   
  
  
- GCACTGAGAG CCAACCTCCT AACGTCGAAT TTGCTGTAGC CAACCAACTT CTCCTAACAA TACCCCAAAG   
  
  
- ATCCATTATA CATTTAAATC TCCTTCACCC CCATCTTTAA CTTAAATAAT ATTGTCGCCC CCGTACAATC   
  
  
- CTTTTCAAAG ACGATTAGAC GGTATAGACC CACAGATTTC AGTTGACGTA GGACGACCGT GGTTGGTTTT   
  
  
- GTGAGGACAT GAATTTTCGT GGGACTGCGT CTAGTCTGCC GAGTCGTGCT TTCAAACTGG ACTAGAGGAA   
  
  
- GGTACAGGTG TTGCAGGGCA CCTGGCAGTA TTGGGAACAC CCAGGGCGTG CGGTCTGCTC CAGTTACAGG   
  
  
- AATACATATA GTGGTGTCGA AATTATCTTT TTTCTTTATT TTATAATTTT TTGTTAAATA CACACGTGTC   
  
  
- GTCGGTTTTT TCGTGCTACA CCAACCTAGA ATAAGCGTGG TTGTAAACTG CCGTTTCCTA TCACACTGTC   
  
  
- CAGTCACTGG ATTAGCTGGT TATCGTAGAG CATCGGACCA TGAGTAGAAA AAACTGGCCA CAGGGAAGGG   
  
  
- AAATAGTTGT CGTGGGGAGA GAGGTAAAAG AAAAGTGCGA CAAGAGAGAA AAGAGAGGGA AAGAGAAAGA   
  
  
- AAAGAAAGTA CCACCACCAC AACGTCTCT

+     box E

| Site Name | Organism | Position | Strand | Matrix score. | sequence | function |
| --- | --- | --- | --- | --- | --- | --- |
| box E | Petroselinum crispum | 238 | + | 9 | ACCCATCAAG |  |

> 2018/04/13 10:10:12  
+ TTTTATTAAA AATATTTTTG AAAAATAATA TATAAGTAAA CATCCCCCAG TTACCAATCA ACTGTACTTC   
  
  
+ ACCTATACTA GTACTAGTAC TTACTCGTGT AAGTACTTTT TAATTATCAT CACCATCCAA TCTATCACCG   
  
  
+ ACATCCTTAA GACTTTAGCT TCCTATTTAG ATTCACTATT CCCCCGAAGA CAAAGATTTA CGTGATTTCT   
  
  
+ CTTTCCCTGT TATAATACAA ACAAGACACC CATCAAACTC CCAAACGGAT CAATCTGATT TCCACAACCT   
  
  
+ AAGAACAGCA ATGCTATGCT AGATTTTGTC TGATTCGCTT CCTATCATCG GATCCTAATT GCACCGACCC   
  
  
+ GATAATTCAT CTCATTGACC ATAATTTCGG GTCATATCAG TACCCTTTTC TACTACTCAA TCGTTACTTA   
  
  
+ ACATCTAAAC TAGCTGGTGG GTATTAATAT ATATTTTAAA TGTTTAGAAT AATCTTTATT TAATATATCC   
  
  
+ AACTTGGGCC TCTTTTTTTT TTTTTAATTT AGGGGAAAAA TAAATCAACT TATAATAAGG CCAAGAGATT   
  
  
+ ATAGAAAGAA AATGAGGCCA GTTGGTGAAA CGAACTTTCG GAGGAAGTCA ACATTTAGGC AGCCAACTAC   
  
  
+ ATCATGTTTG TCTTCTACGT TCTTCATATC TTTGGGTCGT AGGTGTTCCT TCGGCTTTCC GTCTTTGACT   
  
  
+ TTTTGTCTGT AAGCAGTCAA CATTCGTATC AAATCCCGCA CGTGCCTCAA ACGCAATTAT GCTCCAACCA   
  
  
+ AATCAAGACT AGATCAAGTT CATTTAGTGA GACAATGTTT GTTAGGACAA CTGGGTCGTC GATCCAACCT   
  
  
+ CGTGACTCTC GGTTGGAGGA TTGCAGCTTA AACGACATCG GTTGGTTGAA GAGGATTGTT ATGGGGTTTC   
  
  
+ TAGGTAATAT GTAAATTTAG AGGAAGTGGG GGTAGAAATT GAATTTATTA TAACAGCGGG GGCATGTTAG   
  
  
+ GAAAAGTTTC TGCTAATCTG CCATATCTGG GTGTCTAAAG TCAACTGCAT CCTGCTGGCA CCAACCAAAA   
  
  
+ CACTCCTGTA CTTAAAAGCA CCCTGACGCA GATCAGACGG CTCAGCACGA AAGTTTGACC TGATCTCCTT   
  
  
+ CCATGTCCAC AACGTCCCGT GGACCGTCAT AACCCTTGTG GGTCCCGCAC GCCAGACGAG GTCAATGTCC   
  
  
+ TTATGTATAT CACCACAGCT TTAATAGAAA AAAGAAATAA AATATTAAAA AACAATTTAT GTGTGCACAG   
  
  
+ CAGCCAAAAA AGCACGATGT GGTTGGATCT TATTCGCACC AACATTTGAC GGCAAAGGAT AGTGTGACAG   
  
  
+ GTCAGTGACC TAATCGACCA ATAGCATCTC GTAGCCTGGT ACTCATCTTT TTTGACCGGT GTCCCTTCCC   
  
  
+ TTTATCAACA GCACCCCTCT CTCCATTTTC TTTTCACGCT GTTCTCTCTT TTCTCTCCCT TTCTCTTTCT   
  
  
+ TTTCTTTCAT GGTGGTGGTG TTGCAGAGA  

- AAAATAATTT TTATAAAAAC TTTTTATTAT ATATTCATTT GTAGGGGGTC AATGGTTAGT TGACATGAAG   
  
  
- TGGATATGAT CATGATCATG AATGAGCACA TTCATGAAAA ATTAATAGTA GTGGTAGGTT AGATAGTGGC   
  
  
- TGTAGGAATT CTGAAATCGA AGGATAAATC TAAGTGATAA GGGGGCTTCT GTTTCTAAAT GCACTAAAGA   
  
  
- GAAAGGGACA ATATTATGTT TGTTCTGTGG GTAGTTTGAG GGTTTGCCTA GTTAGACTAA AGGTGTTGGA   
  
  
- TTCTTGTCGT TACGATACGA TCTAAAACAG ACTAAGCGAA GGATAGTAGC CTAGGATTAA CGTGGCTGGG   
  
  
- CTATTAAGTA GAGTAACTGG TATTAAAGCC CAGTATAGTC ATGGGAAAAG ATGATGAGTT AGCAATGAAT   
  
  
- TGTAGATTTG ATCGACCACC CATAATTATA TATAAAATTT ACAAATCTTA TTAGAAATAA ATTATATAGG   
  
  
- TTGAACCCGG AGAAAAAAAA AAAAATTAAA TCCCCTTTTT ATTTAGTTGA ATATTATTCC GGTTCTCTAA   
  
  
- TATCTTTCTT TTACTCCGGT CAACCACTTT GCTTGAAAGC CTCCTTCAGT TGTAAATCCG TCGGTTGATG   
  
  
- TAGTACAAAC AGAAGATGCA AGAAGTATAG AAACCCAGCA TCCACAAGGA AGCCGAAAGG CAGAAACTGA   
  
  
- AAAACAGACA TTCGTCAGTT GTAAGCATAG TTTAGGGCGT GCACGGAGTT TGCGTTAATA CGAGGTTGGT   
  
  
- TTAGTTCTGA TCTAGTTCAA GTAAATCACT CTGTTACAAA CAATCCTGTT GACCCAGCAG CTAGGTTGGA   
  
  
- GCACTGAGAG CCAACCTCCT AACGTCGAAT TTGCTGTAGC CAACCAACTT CTCCTAACAA TACCCCAAAG   
  
  
- ATCCATTATA CATTTAAATC TCCTTCACCC CCATCTTTAA CTTAAATAAT ATTGTCGCCC CCGTACAATC   
  
  
- CTTTTCAAAG ACGATTAGAC GGTATAGACC CACAGATTTC AGTTGACGTA GGACGACCGT GGTTGGTTTT   
  
  
- GTGAGGACAT GAATTTTCGT GGGACTGCGT CTAGTCTGCC GAGTCGTGCT TTCAAACTGG ACTAGAGGAA   
  
  
- GGTACAGGTG TTGCAGGGCA CCTGGCAGTA TTGGGAACAC CCAGGGCGTG CGGTCTGCTC CAGTTACAGG   
  
  
- AATACATATA GTGGTGTCGA AATTATCTTT TTTCTTTATT TTATAATTTT TTGTTAAATA CACACGTGTC   
  
  
- GTCGGTTTTT TCGTGCTACA CCAACCTAGA ATAAGCGTGG TTGTAAACTG CCGTTTCCTA TCACACTGTC   
  
  
- CAGTCACTGG ATTAGCTGGT TATCGTAGAG CATCGGACCA TGAGTAGAAA AAACTGGCCA CAGGGAAGGG   
  
  
- AAATAGTTGT CGTGGGGAGA GAGGTAAAAG AAAAGTGCGA CAAGAGAGAA AAGAGAGGGA AAGAGAAAGA   
  
  
- AAAGAAAGTA CCACCACCAC AACGTCTCT

+     chs-CMA1a

| Site Name | Organism | Position | Strand | Matrix score. | sequence | function |
| --- | --- | --- | --- | --- | --- | --- |
| chs-CMA1a | Daucus carota | 414 | + | 8 | TTACTTAA | part of a light responsive element |

> 2018/04/13 10:10:12  
+ TTTTATTAAA AATATTTTTG AAAAATAATA TATAAGTAAA CATCCCCCAG TTACCAATCA ACTGTACTTC   
  
  
+ ACCTATACTA GTACTAGTAC TTACTCGTGT AAGTACTTTT TAATTATCAT CACCATCCAA TCTATCACCG   
  
  
+ ACATCCTTAA GACTTTAGCT TCCTATTTAG ATTCACTATT CCCCCGAAGA CAAAGATTTA CGTGATTTCT   
  
  
+ CTTTCCCTGT TATAATACAA ACAAGACACC CATCAAACTC CCAAACGGAT CAATCTGATT TCCACAACCT   
  
  
+ AAGAACAGCA ATGCTATGCT AGATTTTGTC TGATTCGCTT CCTATCATCG GATCCTAATT GCACCGACCC   
  
  
+ GATAATTCAT CTCATTGACC ATAATTTCGG GTCATATCAG TACCCTTTTC TACTACTCAA TCGTTACTTA   
  
  
+ ACATCTAAAC TAGCTGGTGG GTATTAATAT ATATTTTAAA TGTTTAGAAT AATCTTTATT TAATATATCC   
  
  
+ AACTTGGGCC TCTTTTTTTT TTTTTAATTT AGGGGAAAAA TAAATCAACT TATAATAAGG CCAAGAGATT   
  
  
+ ATAGAAAGAA AATGAGGCCA GTTGGTGAAA CGAACTTTCG GAGGAAGTCA ACATTTAGGC AGCCAACTAC   
  
  
+ ATCATGTTTG TCTTCTACGT TCTTCATATC TTTGGGTCGT AGGTGTTCCT TCGGCTTTCC GTCTTTGACT   
  
  
+ TTTTGTCTGT AAGCAGTCAA CATTCGTATC AAATCCCGCA CGTGCCTCAA ACGCAATTAT GCTCCAACCA   
  
  
+ AATCAAGACT AGATCAAGTT CATTTAGTGA GACAATGTTT GTTAGGACAA CTGGGTCGTC GATCCAACCT   
  
  
+ CGTGACTCTC GGTTGGAGGA TTGCAGCTTA AACGACATCG GTTGGTTGAA GAGGATTGTT ATGGGGTTTC   
  
  
+ TAGGTAATAT GTAAATTTAG AGGAAGTGGG GGTAGAAATT GAATTTATTA TAACAGCGGG GGCATGTTAG   
  
  
+ GAAAAGTTTC TGCTAATCTG CCATATCTGG GTGTCTAAAG TCAACTGCAT CCTGCTGGCA CCAACCAAAA   
  
  
+ CACTCCTGTA CTTAAAAGCA CCCTGACGCA GATCAGACGG CTCAGCACGA AAGTTTGACC TGATCTCCTT   
  
  
+ CCATGTCCAC AACGTCCCGT GGACCGTCAT AACCCTTGTG GGTCCCGCAC GCCAGACGAG GTCAATGTCC   
  
  
+ TTATGTATAT CACCACAGCT TTAATAGAAA AAAGAAATAA AATATTAAAA AACAATTTAT GTGTGCACAG   
  
  
+ CAGCCAAAAA AGCACGATGT GGTTGGATCT TATTCGCACC AACATTTGAC GGCAAAGGAT AGTGTGACAG   
  
  
+ GTCAGTGACC TAATCGACCA ATAGCATCTC GTAGCCTGGT ACTCATCTTT TTTGACCGGT GTCCCTTCCC   
  
  
+ TTTATCAACA GCACCCCTCT CTCCATTTTC TTTTCACGCT GTTCTCTCTT TTCTCTCCCT TTCTCTTTCT   
  
  
+ TTTCTTTCAT GGTGGTGGTG TTGCAGAGA  

- AAAATAATTT TTATAAAAAC TTTTTATTAT ATATTCATTT GTAGGGGGTC AATGGTTAGT TGACATGAAG   
  
  
- TGGATATGAT CATGATCATG AATGAGCACA TTCATGAAAA ATTAATAGTA GTGGTAGGTT AGATAGTGGC   
  
  
- TGTAGGAATT CTGAAATCGA AGGATAAATC TAAGTGATAA GGGGGCTTCT GTTTCTAAAT GCACTAAAGA   
  
  
- GAAAGGGACA ATATTATGTT TGTTCTGTGG GTAGTTTGAG GGTTTGCCTA GTTAGACTAA AGGTGTTGGA   
  
  
- TTCTTGTCGT TACGATACGA TCTAAAACAG ACTAAGCGAA GGATAGTAGC CTAGGATTAA CGTGGCTGGG   
  
  
- CTATTAAGTA GAGTAACTGG TATTAAAGCC CAGTATAGTC ATGGGAAAAG ATGATGAGTT AGCAATGAAT   
  
  
- TGTAGATTTG ATCGACCACC CATAATTATA TATAAAATTT ACAAATCTTA TTAGAAATAA ATTATATAGG   
  
  
- TTGAACCCGG AGAAAAAAAA AAAAATTAAA TCCCCTTTTT ATTTAGTTGA ATATTATTCC GGTTCTCTAA   
  
  
- TATCTTTCTT TTACTCCGGT CAACCACTTT GCTTGAAAGC CTCCTTCAGT TGTAAATCCG TCGGTTGATG   
  
  
- TAGTACAAAC AGAAGATGCA AGAAGTATAG AAACCCAGCA TCCACAAGGA AGCCGAAAGG CAGAAACTGA   
  
  
- AAAACAGACA TTCGTCAGTT GTAAGCATAG TTTAGGGCGT GCACGGAGTT TGCGTTAATA CGAGGTTGGT   
  
  
- TTAGTTCTGA TCTAGTTCAA GTAAATCACT CTGTTACAAA CAATCCTGTT GACCCAGCAG CTAGGTTGGA   
  
  
- GCACTGAGAG CCAACCTCCT AACGTCGAAT TTGCTGTAGC CAACCAACTT CTCCTAACAA TACCCCAAAG   
  
  
- ATCCATTATA CATTTAAATC TCCTTCACCC CCATCTTTAA CTTAAATAAT ATTGTCGCCC CCGTACAATC   
  
  
- CTTTTCAAAG ACGATTAGAC GGTATAGACC CACAGATTTC AGTTGACGTA GGACGACCGT GGTTGGTTTT   
  
  
- GTGAGGACAT GAATTTTCGT GGGACTGCGT CTAGTCTGCC GAGTCGTGCT TTCAAACTGG ACTAGAGGAA   
  
  
- GGTACAGGTG TTGCAGGGCA CCTGGCAGTA TTGGGAACAC CCAGGGCGTG CGGTCTGCTC CAGTTACAGG   
  
  
- AATACATATA GTGGTGTCGA AATTATCTTT TTTCTTTATT TTATAATTTT TTGTTAAATA CACACGTGTC   
  
  
- GTCGGTTTTT TCGTGCTACA CCAACCTAGA ATAAGCGTGG TTGTAAACTG CCGTTTCCTA TCACACTGTC   
  
  
- CAGTCACTGG ATTAGCTGGT TATCGTAGAG CATCGGACCA TGAGTAGAAA AAACTGGCCA CAGGGAAGGG   
  
  
- AAATAGTTGT CGTGGGGAGA GAGGTAAAAG AAAAGTGCGA CAAGAGAGAA AAGAGAGGGA AAGAGAAAGA   
  
  
- AAAGAAAGTA CCACCACCAC AACGTCTCT

+     circadian

| Site Name | Organism | Position | Strand | Matrix score. | sequence | function |
| --- | --- | --- | --- | --- | --- | --- |
| circadian | Lycopersicon esculentum | 1022 | + | 6 | CAANNNNATC | cis-acting regulatory element involved in circadian control |
| circadian | Lycopersicon esculentum | 1349 | + | 6 | CAANNNNATC | cis-acting regulatory element involved in circadian control |
| circadian | Lycopersicon esculentum | 655 | - | 9 | CAAAGATATC | cis-acting regulatory element involved in circadian control |
| circadian | Lycopersicon esculentum | 1276 | - | 6 | CAANNNNATC | cis-acting regulatory element involved in circadian control |
| circadian | Lycopersicon esculentum | 765 | + | 6 | CAANNNNATC | cis-acting regulatory element involved in circadian control |
| circadian | Lycopersicon esculentum | 252 | + | 6 | CAANNNNATC | cis-acting regulatory element involved in circadian control |
| circadian | Lycopersicon esculentum | 624 | + | 6 | CAANNNNATC | cis-acting regulatory element involved in circadian control |

> 2018/04/13 10:10:12  
+ TTTTATTAAA AATATTTTTG AAAAATAATA TATAAGTAAA CATCCCCCAG TTACCAATCA ACTGTACTTC   
  
  
+ ACCTATACTA GTACTAGTAC TTACTCGTGT AAGTACTTTT TAATTATCAT CACCATCCAA TCTATCACCG   
  
  
+ ACATCCTTAA GACTTTAGCT TCCTATTTAG ATTCACTATT CCCCCGAAGA CAAAGATTTA CGTGATTTCT   
  
  
+ CTTTCCCTGT TATAATACAA ACAAGACACC CATCAAACTC CCAAACGGAT CAATCTGATT TCCACAACCT   
  
  
+ AAGAACAGCA ATGCTATGCT AGATTTTGTC TGATTCGCTT CCTATCATCG GATCCTAATT GCACCGACCC   
  
  
+ GATAATTCAT CTCATTGACC ATAATTTCGG GTCATATCAG TACCCTTTTC TACTACTCAA TCGTTACTTA   
  
  
+ ACATCTAAAC TAGCTGGTGG GTATTAATAT ATATTTTAAA TGTTTAGAAT AATCTTTATT TAATATATCC   
  
  
+ AACTTGGGCC TCTTTTTTTT TTTTTAATTT AGGGGAAAAA TAAATCAACT TATAATAAGG CCAAGAGATT   
  
  
+ ATAGAAAGAA AATGAGGCCA GTTGGTGAAA CGAACTTTCG GAGGAAGTCA ACATTTAGGC AGCCAACTAC   
  
  
+ ATCATGTTTG TCTTCTACGT TCTTCATATC TTTGGGTCGT AGGTGTTCCT TCGGCTTTCC GTCTTTGACT   
  
  
+ TTTTGTCTGT AAGCAGTCAA CATTCGTATC AAATCCCGCA CGTGCCTCAA ACGCAATTAT GCTCCAACCA   
  
  
+ AATCAAGACT AGATCAAGTT CATTTAGTGA GACAATGTTT GTTAGGACAA CTGGGTCGTC GATCCAACCT   
  
  
+ CGTGACTCTC GGTTGGAGGA TTGCAGCTTA AACGACATCG GTTGGTTGAA GAGGATTGTT ATGGGGTTTC   
  
  
+ TAGGTAATAT GTAAATTTAG AGGAAGTGGG GGTAGAAATT GAATTTATTA TAACAGCGGG GGCATGTTAG   
  
  
+ GAAAAGTTTC TGCTAATCTG CCATATCTGG GTGTCTAAAG TCAACTGCAT CCTGCTGGCA CCAACCAAAA   
  
  
+ CACTCCTGTA CTTAAAAGCA CCCTGACGCA GATCAGACGG CTCAGCACGA AAGTTTGACC TGATCTCCTT   
  
  
+ CCATGTCCAC AACGTCCCGT GGACCGTCAT AACCCTTGTG GGTCCCGCAC GCCAGACGAG GTCAATGTCC   
  
  
+ TTATGTATAT CACCACAGCT TTAATAGAAA AAAGAAATAA AATATTAAAA AACAATTTAT GTGTGCACAG   
  
  
+ CAGCCAAAAA AGCACGATGT GGTTGGATCT TATTCGCACC AACATTTGAC GGCAAAGGAT AGTGTGACAG   
  
  
+ GTCAGTGACC TAATCGACCA ATAGCATCTC GTAGCCTGGT ACTCATCTTT TTTGACCGGT GTCCCTTCCC   
  
  
+ TTTATCAACA GCACCCCTCT CTCCATTTTC TTTTCACGCT GTTCTCTCTT TTCTCTCCCT TTCTCTTTCT   
  
  
+ TTTCTTTCAT GGTGGTGGTG TTGCAGAGA  

- AAAATAATTT TTATAAAAAC TTTTTATTAT ATATTCATTT GTAGGGGGTC AATGGTTAGT TGACATGAAG   
  
  
- TGGATATGAT CATGATCATG AATGAGCACA TTCATGAAAA ATTAATAGTA GTGGTAGGTT AGATAGTGGC   
  
  
- TGTAGGAATT CTGAAATCGA AGGATAAATC TAAGTGATAA GGGGGCTTCT GTTTCTAAAT GCACTAAAGA   
  
  
- GAAAGGGACA ATATTATGTT TGTTCTGTGG GTAGTTTGAG GGTTTGCCTA GTTAGACTAA AGGTGTTGGA   
  
  
- TTCTTGTCGT TACGATACGA TCTAAAACAG ACTAAGCGAA GGATAGTAGC CTAGGATTAA CGTGGCTGGG   
  
  
- CTATTAAGTA GAGTAACTGG TATTAAAGCC CAGTATAGTC ATGGGAAAAG ATGATGAGTT AGCAATGAAT   
  
  
- TGTAGATTTG ATCGACCACC CATAATTATA TATAAAATTT ACAAATCTTA TTAGAAATAA ATTATATAGG   
  
  
- TTGAACCCGG AGAAAAAAAA AAAAATTAAA TCCCCTTTTT ATTTAGTTGA ATATTATTCC GGTTCTCTAA   
  
  
- TATCTTTCTT TTACTCCGGT CAACCACTTT GCTTGAAAGC CTCCTTCAGT TGTAAATCCG TCGGTTGATG   
  
  
- TAGTACAAAC AGAAGATGCA AGAAGTATAG AAACCCAGCA TCCACAAGGA AGCCGAAAGG CAGAAACTGA   
  
  
- AAAACAGACA TTCGTCAGTT GTAAGCATAG TTTAGGGCGT GCACGGAGTT TGCGTTAATA CGAGGTTGGT   
  
  
- TTAGTTCTGA TCTAGTTCAA GTAAATCACT CTGTTACAAA CAATCCTGTT GACCCAGCAG CTAGGTTGGA   
  
  
- GCACTGAGAG CCAACCTCCT AACGTCGAAT TTGCTGTAGC CAACCAACTT CTCCTAACAA TACCCCAAAG   
  
  
- ATCCATTATA CATTTAAATC TCCTTCACCC CCATCTTTAA CTTAAATAAT ATTGTCGCCC CCGTACAATC   
  
  
- CTTTTCAAAG ACGATTAGAC GGTATAGACC CACAGATTTC AGTTGACGTA GGACGACCGT GGTTGGTTTT   
  
  
- GTGAGGACAT GAATTTTCGT GGGACTGCGT CTAGTCTGCC GAGTCGTGCT TTCAAACTGG ACTAGAGGAA   
  
  
- GGTACAGGTG TTGCAGGGCA CCTGGCAGTA TTGGGAACAC CCAGGGCGTG CGGTCTGCTC CAGTTACAGG   
  
  
- AATACATATA GTGGTGTCGA AATTATCTTT TTTCTTTATT TTATAATTTT TTGTTAAATA CACACGTGTC   
  
  
- GTCGGTTTTT TCGTGCTACA CCAACCTAGA ATAAGCGTGG TTGTAAACTG CCGTTTCCTA TCACACTGTC   
  
  
- CAGTCACTGG ATTAGCTGGT TATCGTAGAG CATCGGACCA TGAGTAGAAA AAACTGGCCA CAGGGAAGGG   
  
  
- AAATAGTTGT CGTGGGGAGA GAGGTAAAAG AAAAGTGCGA CAAGAGAGAA AAGAGAGGGA AAGAGAAAGA   
  
  
- AAAGAAAGTA CCACCACCAC AACGTCTCT

+     rbcS-CMA7a

| Site Name | Organism | Position | Strand | Matrix score. | sequence | function |
| --- | --- | --- | --- | --- | --- | --- |
| rbcS-CMA7a | Lemna gibba | 1339 | - | 9 | GTCGATAAGG | part of a light responsive element |

> 2018/04/13 10:10:12  
+ TTTTATTAAA AATATTTTTG AAAAATAATA TATAAGTAAA CATCCCCCAG TTACCAATCA ACTGTACTTC   
  
  
+ ACCTATACTA GTACTAGTAC TTACTCGTGT AAGTACTTTT TAATTATCAT CACCATCCAA TCTATCACCG   
  
  
+ ACATCCTTAA GACTTTAGCT TCCTATTTAG ATTCACTATT CCCCCGAAGA CAAAGATTTA CGTGATTTCT   
  
  
+ CTTTCCCTGT TATAATACAA ACAAGACACC CATCAAACTC CCAAACGGAT CAATCTGATT TCCACAACCT   
  
  
+ AAGAACAGCA ATGCTATGCT AGATTTTGTC TGATTCGCTT CCTATCATCG GATCCTAATT GCACCGACCC   
  
  
+ GATAATTCAT CTCATTGACC ATAATTTCGG GTCATATCAG TACCCTTTTC TACTACTCAA TCGTTACTTA   
  
  
+ ACATCTAAAC TAGCTGGTGG GTATTAATAT ATATTTTAAA TGTTTAGAAT AATCTTTATT TAATATATCC   
  
  
+ AACTTGGGCC TCTTTTTTTT TTTTTAATTT AGGGGAAAAA TAAATCAACT TATAATAAGG CCAAGAGATT   
  
  
+ ATAGAAAGAA AATGAGGCCA GTTGGTGAAA CGAACTTTCG GAGGAAGTCA ACATTTAGGC AGCCAACTAC   
  
  
+ ATCATGTTTG TCTTCTACGT TCTTCATATC TTTGGGTCGT AGGTGTTCCT TCGGCTTTCC GTCTTTGACT   
  
  
+ TTTTGTCTGT AAGCAGTCAA CATTCGTATC AAATCCCGCA CGTGCCTCAA ACGCAATTAT GCTCCAACCA   
  
  
+ AATCAAGACT AGATCAAGTT CATTTAGTGA GACAATGTTT GTTAGGACAA CTGGGTCGTC GATCCAACCT   
  
  
+ CGTGACTCTC GGTTGGAGGA TTGCAGCTTA AACGACATCG GTTGGTTGAA GAGGATTGTT ATGGGGTTTC   
  
  
+ TAGGTAATAT GTAAATTTAG AGGAAGTGGG GGTAGAAATT GAATTTATTA TAACAGCGGG GGCATGTTAG   
  
  
+ GAAAAGTTTC TGCTAATCTG CCATATCTGG GTGTCTAAAG TCAACTGCAT CCTGCTGGCA CCAACCAAAA   
  
  
+ CACTCCTGTA CTTAAAAGCA CCCTGACGCA GATCAGACGG CTCAGCACGA AAGTTTGACC TGATCTCCTT   
  
  
+ CCATGTCCAC AACGTCCCGT GGACCGTCAT AACCCTTGTG GGTCCCGCAC GCCAGACGAG GTCAATGTCC   
  
  
+ TTATGTATAT CACCACAGCT TTAATAGAAA AAAGAAATAA AATATTAAAA AACAATTTAT GTGTGCACAG   
  
  
+ CAGCCAAAAA AGCACGATGT GGTTGGATCT TATTCGCACC AACATTTGAC GGCAAAGGAT AGTGTGACAG   
  
  
+ GTCAGTGACC TAATCGACCA ATAGCATCTC GTAGCCTGGT ACTCATCTTT TTTGACCGGT GTCCCTTCCC   
  
  
+ TTTATCAACA GCACCCCTCT CTCCATTTTC TTTTCACGCT GTTCTCTCTT TTCTCTCCCT TTCTCTTTCT   
  
  
+ TTTCTTTCAT GGTGGTGGTG TTGCAGAGA  

- AAAATAATTT TTATAAAAAC TTTTTATTAT ATATTCATTT GTAGGGGGTC AATGGTTAGT TGACATGAAG   
  
  
- TGGATATGAT CATGATCATG AATGAGCACA TTCATGAAAA ATTAATAGTA GTGGTAGGTT AGATAGTGGC   
  
  
- TGTAGGAATT CTGAAATCGA AGGATAAATC TAAGTGATAA GGGGGCTTCT GTTTCTAAAT GCACTAAAGA   
  
  
- GAAAGGGACA ATATTATGTT TGTTCTGTGG GTAGTTTGAG GGTTTGCCTA GTTAGACTAA AGGTGTTGGA   
  
  
- TTCTTGTCGT TACGATACGA TCTAAAACAG ACTAAGCGAA GGATAGTAGC CTAGGATTAA CGTGGCTGGG   
  
  
- CTATTAAGTA GAGTAACTGG TATTAAAGCC CAGTATAGTC ATGGGAAAAG ATGATGAGTT AGCAATGAAT   
  
  
- TGTAGATTTG ATCGACCACC CATAATTATA TATAAAATTT ACAAATCTTA TTAGAAATAA ATTATATAGG   
  
  
- TTGAACCCGG AGAAAAAAAA AAAAATTAAA TCCCCTTTTT ATTTAGTTGA ATATTATTCC GGTTCTCTAA   
  
  
- TATCTTTCTT TTACTCCGGT CAACCACTTT GCTTGAAAGC CTCCTTCAGT TGTAAATCCG TCGGTTGATG   
  
  
- TAGTACAAAC AGAAGATGCA AGAAGTATAG AAACCCAGCA TCCACAAGGA AGCCGAAAGG CAGAAACTGA   
  
  
- AAAACAGACA TTCGTCAGTT GTAAGCATAG TTTAGGGCGT GCACGGAGTT TGCGTTAATA CGAGGTTGGT   
  
  
- TTAGTTCTGA TCTAGTTCAA GTAAATCACT CTGTTACAAA CAATCCTGTT GACCCAGCAG CTAGGTTGGA   
  
  
- GCACTGAGAG CCAACCTCCT AACGTCGAAT TTGCTGTAGC CAACCAACTT CTCCTAACAA TACCCCAAAG   
  
  
- ATCCATTATA CATTTAAATC TCCTTCACCC CCATCTTTAA CTTAAATAAT ATTGTCGCCC CCGTACAATC   
  
  
- CTTTTCAAAG ACGATTAGAC GGTATAGACC CACAGATTTC AGTTGACGTA GGACGACCGT GGTTGGTTTT   
  
  
- GTGAGGACAT GAATTTTCGT GGGACTGCGT CTAGTCTGCC GAGTCGTGCT TTCAAACTGG ACTAGAGGAA   
  
  
- GGTACAGGTG TTGCAGGGCA CCTGGCAGTA TTGGGAACAC CCAGGGCGTG CGGTCTGCTC CAGTTACAGG   
  
  
- AATACATATA GTGGTGTCGA AATTATCTTT TTTCTTTATT TTATAATTTT TTGTTAAATA CACACGTGTC   
  
  
- GTCGGTTTTT TCGTGCTACA CCAACCTAGA ATAAGCGTGG TTGTAAACTG CCGTTTCCTA TCACACTGTC   
  
  
- CAGTCACTGG ATTAGCTGGT TATCGTAGAG CATCGGACCA TGAGTAGAAA AAACTGGCCA CAGGGAAGGG   
  
  
- AAATAGTTGT CGTGGGGAGA GAGGTAAAAG AAAAGTGCGA CAAGAGAGAA AAGAGAGGGA AAGAGAAAGA   
  
  
- AAAGAAAGTA CCACCACCAC AACGTCTCT
