# Supplementary material for: Genome-wide analysis of the WRKY gene family in drumstick (Moringa oleifera Lam.)
Source: PeerJ. 2019 Jun 10;7:e7063. doi: 10.7717/peerj.7063 (PMC6563795; doi:10.7717/peerj.7063)
Supplement: Supplemental Information 1 [file peerj-07-7063-s003.gz › MoWRKY26_plantcare.html]

Content-Type: text/html; charset=ISO-8859-1


CallMat\_Firefox


Webmaster Firefox specific output  
To save the result:
click on the frame with the right mouse button and save the source code as a text file with extension .html  
REFERENCE:PlantCARE: a database of plant cis-acting regulatory elements and a portal to tools for in silico analysis of promoter sequences.  
Lescot, M., Déhais, P., Moreau, Y., De Moor, B., Rouzé ,P.,and Rombauts, S.  
Nucleic Acids Res., Database issue(2002), 30(1):325-327.   


---

> 2018/04/13 10:10:12  
+ GACTCGAAAC TGAAATTAAT TAAACTGAGA TTGTAATTGA TTTAGTTTGT GTGGAGAAAT AATGTGTTTT   
  
  
+ GGTAGTGAAG GGGTGAGGGT TAATTAAAGA GTAATTACGG TGGGGGAAAC GGTTTAGTAG ACAATAATAA   
  
  
+ TAGTGAATTA CAATTTGAAA AAGTTTTCCC TAAAACTATA AAACTATTAA ATTGTATAAT TGTTTTTTTA   
  
  
+ TTTTTTAAAA TTATTAATTA AATTATTTAA TAGTACACAG TAGTAGAAAA ATTTTCTAGT TGTGTTAAAT   
  
  
+ TTATTTAGTT TTTAAGTTTA AGTTAGTTTT TTTATAACCA CATGGTTTTT TAAACTAAGT TTGATATTTT   
  
  
+ TAAATTGTGC ATTATTAAAT TATTTTATTT ATTTATTAAA AAATAAAAAT ACATAGTAAT ATTTTAATAA   
  
  
+ TTTTTTTATG GAAAATTTGA AGTTAAATTT ACTTATGTTT AAAATAATAT ATATACTATT ATTTAAAACC   
  
  
+ CATTTTAGAG TAGTTTGGAA GTTCAAACCA ATATTTTTTA ATTTCTCTAA CAACAATAAC TTTACAAAGT   
  
  
+ TTTATTAAGT TTACTTAACA AAAAATAAAT TTAAAATAAA AAGTTTAAAT TTATAAAAAT TTATATTATA   
  
  
+ AATAAAAAAC ACTTAAAAAA TTAAAAAACT ATCATATTTT CAATTTTATA TATAAAAGTC ATGAGTAATT   
  
  
+ TCAAATTTTT TTTTTAATAC TACTATCGAA CTTATAAAAA AATGCATACA TTTTTAAAAA TTCAGTTTTA   
  
  
+ AGTAATATAT TCTAAAAGTT AAATTTATAG AGATTAAAAA TTTCAAAAAC TATTTTAAGT TAAATCTAAT   
  
  
+ GCCAAGTTAC AATTTAATAA AAACTTATAT GTTTAAATTA ATCATCGTAT TTTATTTATA GTTAAAAAAT   
  
  
+ ACCATAAATA TAAAATTAAA AATTTTTAAA TAAAAATTAA TTATATTATT TAATATTTTT TTAGTAAGGG   
  
  
+ GGAATATGGT AGTATTCAAG AAGTTTAATG AAAATTGTAA AAAGTTAATA CCTAGAAGTA TGTACGTACG   
  
  
+ TTTCGAACGT ATAGATACAT ACATGATATA TATATAAATT AGTTAATATG TACGTACGTA CGTGTACGTA   
  
  
+ TTGTATACAT ATATCGATAC GTAATGGCAT ACTCTTCTCT ATTAATTACT ATTTGTTAAA CTAATGTAGT   
  
  
+ AGAACGGGTG TACCTATAAT TATTCTCGCT CAGAATATGC TTTAGGCTAC TCTCCTTTCG GAGCCCTAGT   
  
  
+ GTAGAATCTT TAACAAAATG CCCGAACTGT ACCAGAAGGG GTGTACCATA GGCTCGGAGC ATGCTTTAGG   
  
  
+ GTACTCTTCT CTCAGAAAAC CCTAGTGTAG AACTTTAGCA GTTGAGGTTA CGGAGAGGAA TTAGTTAGTC   
  
  
+ ATCTAAATTA ATTAATTAGA GTTGAATCAA CAAATTAGGG GGTAGAGAAT ATATTCATGA CTTGGTGATT   
  
  
+ TAAATTGTTT TAATCTTATC AGGTTTATT  

- CTGAGCTTTG ACTTTAATTA ATTTGACTCT AACATTAACT AAATCAAACA CACCTCTTTA TTACACAAAA   
  
  
- CCATCACTTC CCCACTCCCA ATTAATTTCT CATTAATGCC ACCCCCTTTG CCAAATCATC TGTTATTATT   
  
  
- ATCACTTAAT GTTAAACTTT TTCAAAAGGG ATTTTGATAT TTTGATAATT TAACATATTA ACAAAAAAAT   
  
  
- AAAAAATTTT AATAATTAAT TTAATAAATT ATCATGTGTC ATCATCTTTT TAAAAGATCA ACACAATTTA   
  
  
- AATAAATCAA AAATTCAAAT TCAATCAAAA AAATATTGGT GTACCAAAAA ATTTGATTCA AACTATAAAA   
  
  
- ATTTAACACG TAATAATTTA ATAAAATAAA TAAATAATTT TTTATTTTTA TGTATCATTA TAAAATTATT   
  
  
- AAAAAAATAC CTTTTAAACT TCAATTTAAA TGAATACAAA TTTTATTATA TATATGATAA TAAATTTTGG   
  
  
- GTAAAATCTC ATCAAACCTT CAAGTTTGGT TATAAAAAAT TAAAGAGATT GTTGTTATTG AAATGTTTCA   
  
  
- AAATAATTCA AATGAATTGT TTTTTATTTA AATTTTATTT TTCAAATTTA AATATTTTTA AATATAATAT   
  
  
- TTATTTTTTG TGAATTTTTT AATTTTTTGA TAGTATAAAA GTTAAAATAT ATATTTTCAG TACTCATTAA   
  
  
- AGTTTAAAAA AAAAATTATG ATGATAGCTT GAATATTTTT TTACGTATGT AAAAATTTTT AAGTCAAAAT   
  
  
- TCATTATATA AGATTTTCAA TTTAAATATC TCTAATTTTT AAAGTTTTTG ATAAAATTCA ATTTAGATTA   
  
  
- CGGTTCAATG TTAAATTATT TTTGAATATA CAAATTTAAT TAGTAGCATA AAATAAATAT CAATTTTTTA   
  
  
- TGGTATTTAT ATTTTAATTT TTAAAAATTT ATTTTTAATT AATATAATAA ATTATAAAAA AATCATTCCC   
  
  
- CCTTATACCA TCATAAGTTC TTCAAATTAC TTTTAACATT TTTCAATTAT GGATCTTCAT ACATGCATGC   
  
  
- AAAGCTTGCA TATCTATGTA TGTACTATAT ATATATTTAA TCAATTATAC ATGCATGCAT GCACATGCAT   
  
  
- AACATATGTA TATAGCTATG CATTACCGTA TGAGAAGAGA TAATTAATGA TAAACAATTT GATTACATCA   
  
  
- TCTTGCCCAC ATGGATATTA ATAAGAGCGA GTCTTATACG AAATCCGATG AGAGGAAAGC CTCGGGATCA   
  
  
- CATCTTAGAA ATTGTTTTAC GGGCTTGACA TGGTCTTCCC CACATGGTAT CCGAGCCTCG TACGAAATCC   
  
  
- CATGAGAAGA GAGTCTTTTG GGATCACATC TTGAAATCGT CAACTCCAAT GCCTCTCCTT AATCAATCAG   
  
  
- TAGATTTAAT TAATTAATCT CAACTTAGTT GTTTAATCCC CCATCTCTTA TATAAGTACT GAACCACTAA   
  
  
- ATTTAACAAA ATTAGAATAG TCCAAATAA

  
  
Motifs Found  

+     ABRE

| Site Name | Organism | Position | Strand | Matrix score. | sequence | function |
| --- | --- | --- | --- | --- | --- | --- |
| ABRE | Arabidopsis thaliana | 1109 | + | 6 | TACGTG | cis-acting element involved in the abscisic acid responsiveness |
| ABRE | Hordeum vulgare | 1107 | + | 9 | CGTACGTGCA | cis-acting element involved in the abscisic acid responsiveness |
| ABRE | Hordeum vulgare | 1041 | - | 9 | CGTACGTGCA | cis-acting element involved in the abscisic acid responsiveness |
| ABRE | Hordeum vulgare | 1099 | - | 9 | CGTACGTGCA | cis-acting element involved in the abscisic acid responsiveness |
| ABRE | Oryza sativa | 1044 | - | 10 | GACACGTACGT | cis-acting element involved in the abscisic acid responsiveness |
| ABRE | Oryza sativa | 1106 | - | 10 | GACACGTACGT | cis-acting element involved in the abscisic acid responsiveness |

> 2018/04/13 10:10:12  
+ GACTCGAAAC TGAAATTAAT TAAACTGAGA TTGTAATTGA TTTAGTTTGT GTGGAGAAAT AATGTGTTTT   
  
  
+ GGTAGTGAAG GGGTGAGGGT TAATTAAAGA GTAATTACGG TGGGGGAAAC GGTTTAGTAG ACAATAATAA   
  
  
+ TAGTGAATTA CAATTTGAAA AAGTTTTCCC TAAAACTATA AAACTATTAA ATTGTATAAT TGTTTTTTTA   
  
  
+ TTTTTTAAAA TTATTAATTA AATTATTTAA TAGTACACAG TAGTAGAAAA ATTTTCTAGT TGTGTTAAAT   
  
  
+ TTATTTAGTT TTTAAGTTTA AGTTAGTTTT TTTATAACCA CATGGTTTTT TAAACTAAGT TTGATATTTT   
  
  
+ TAAATTGTGC ATTATTAAAT TATTTTATTT ATTTATTAAA AAATAAAAAT ACATAGTAAT ATTTTAATAA   
  
  
+ TTTTTTTATG GAAAATTTGA AGTTAAATTT ACTTATGTTT AAAATAATAT ATATACTATT ATTTAAAACC   
  
  
+ CATTTTAGAG TAGTTTGGAA GTTCAAACCA ATATTTTTTA ATTTCTCTAA CAACAATAAC TTTACAAAGT   
  
  
+ TTTATTAAGT TTACTTAACA AAAAATAAAT TTAAAATAAA AAGTTTAAAT TTATAAAAAT TTATATTATA   
  
  
+ AATAAAAAAC ACTTAAAAAA TTAAAAAACT ATCATATTTT CAATTTTATA TATAAAAGTC ATGAGTAATT   
  
  
+ TCAAATTTTT TTTTTAATAC TACTATCGAA CTTATAAAAA AATGCATACA TTTTTAAAAA TTCAGTTTTA   
  
  
+ AGTAATATAT TCTAAAAGTT AAATTTATAG AGATTAAAAA TTTCAAAAAC TATTTTAAGT TAAATCTAAT   
  
  
+ GCCAAGTTAC AATTTAATAA AAACTTATAT GTTTAAATTA ATCATCGTAT TTTATTTATA GTTAAAAAAT   
  
  
+ ACCATAAATA TAAAATTAAA AATTTTTAAA TAAAAATTAA TTATATTATT TAATATTTTT TTAGTAAGGG   
  
  
+ GGAATATGGT AGTATTCAAG AAGTTTAATG AAAATTGTAA AAAGTTAATA CCTAGAAGTA TGTACGTACG   
  
  
+ TTTCGAACGT ATAGATACAT ACATGATATA TATATAAATT AGTTAATATG TACGTACGTA CGTGTACGTA   
  
  
+ TTGTATACAT ATATCGATAC GTAATGGCAT ACTCTTCTCT ATTAATTACT ATTTGTTAAA CTAATGTAGT   
  
  
+ AGAACGGGTG TACCTATAAT TATTCTCGCT CAGAATATGC TTTAGGCTAC TCTCCTTTCG GAGCCCTAGT   
  
  
+ GTAGAATCTT TAACAAAATG CCCGAACTGT ACCAGAAGGG GTGTACCATA GGCTCGGAGC ATGCTTTAGG   
  
  
+ GTACTCTTCT CTCAGAAAAC CCTAGTGTAG AACTTTAGCA GTTGAGGTTA CGGAGAGGAA TTAGTTAGTC   
  
  
+ ATCTAAATTA ATTAATTAGA GTTGAATCAA CAAATTAGGG GGTAGAGAAT ATATTCATGA CTTGGTGATT   
  
  
+ TAAATTGTTT TAATCTTATC AGGTTTATT  

- CTGAGCTTTG ACTTTAATTA ATTTGACTCT AACATTAACT AAATCAAACA CACCTCTTTA TTACACAAAA   
  
  
- CCATCACTTC CCCACTCCCA ATTAATTTCT CATTAATGCC ACCCCCTTTG CCAAATCATC TGTTATTATT   
  
  
- ATCACTTAAT GTTAAACTTT TTCAAAAGGG ATTTTGATAT TTTGATAATT TAACATATTA ACAAAAAAAT   
  
  
- AAAAAATTTT AATAATTAAT TTAATAAATT ATCATGTGTC ATCATCTTTT TAAAAGATCA ACACAATTTA   
  
  
- AATAAATCAA AAATTCAAAT TCAATCAAAA AAATATTGGT GTACCAAAAA ATTTGATTCA AACTATAAAA   
  
  
- ATTTAACACG TAATAATTTA ATAAAATAAA TAAATAATTT TTTATTTTTA TGTATCATTA TAAAATTATT   
  
  
- AAAAAAATAC CTTTTAAACT TCAATTTAAA TGAATACAAA TTTTATTATA TATATGATAA TAAATTTTGG   
  
  
- GTAAAATCTC ATCAAACCTT CAAGTTTGGT TATAAAAAAT TAAAGAGATT GTTGTTATTG AAATGTTTCA   
  
  
- AAATAATTCA AATGAATTGT TTTTTATTTA AATTTTATTT TTCAAATTTA AATATTTTTA AATATAATAT   
  
  
- TTATTTTTTG TGAATTTTTT AATTTTTTGA TAGTATAAAA GTTAAAATAT ATATTTTCAG TACTCATTAA   
  
  
- AGTTTAAAAA AAAAATTATG ATGATAGCTT GAATATTTTT TTACGTATGT AAAAATTTTT AAGTCAAAAT   
  
  
- TCATTATATA AGATTTTCAA TTTAAATATC TCTAATTTTT AAAGTTTTTG ATAAAATTCA ATTTAGATTA   
  
  
- CGGTTCAATG TTAAATTATT TTTGAATATA CAAATTTAAT TAGTAGCATA AAATAAATAT CAATTTTTTA   
  
  
- TGGTATTTAT ATTTTAATTT TTAAAAATTT ATTTTTAATT AATATAATAA ATTATAAAAA AATCATTCCC   
  
  
- CCTTATACCA TCATAAGTTC TTCAAATTAC TTTTAACATT TTTCAATTAT GGATCTTCAT ACATGCATGC   
  
  
- AAAGCTTGCA TATCTATGTA TGTACTATAT ATATATTTAA TCAATTATAC ATGCATGCAT GCACATGCAT   
  
  
- AACATATGTA TATAGCTATG CATTACCGTA TGAGAAGAGA TAATTAATGA TAAACAATTT GATTACATCA   
  
  
- TCTTGCCCAC ATGGATATTA ATAAGAGCGA GTCTTATACG AAATCCGATG AGAGGAAAGC CTCGGGATCA   
  
  
- CATCTTAGAA ATTGTTTTAC GGGCTTGACA TGGTCTTCCC CACATGGTAT CCGAGCCTCG TACGAAATCC   
  
  
- CATGAGAAGA GAGTCTTTTG GGATCACATC TTGAAATCGT CAACTCCAAT GCCTCTCCTT AATCAATCAG   
  
  
- TAGATTTAAT TAATTAATCT CAACTTAGTT GTTTAATCCC CCATCTCTTA TATAAGTACT GAACCACTAA   
  
  
- ATTTAACAAA ATTAGAATAG TCCAAATAA

+     ACE

| Site Name | Organism | Position | Strand | Matrix score. | sequence | function |
| --- | --- | --- | --- | --- | --- | --- |
| ACE | Petroselinum crispum | 598 | + | 9 | AAAACGTTTA | cis-acting element involved in light responsiveness |

> 2018/04/13 10:10:12  
+ GACTCGAAAC TGAAATTAAT TAAACTGAGA TTGTAATTGA TTTAGTTTGT GTGGAGAAAT AATGTGTTTT   
  
  
+ GGTAGTGAAG GGGTGAGGGT TAATTAAAGA GTAATTACGG TGGGGGAAAC GGTTTAGTAG ACAATAATAA   
  
  
+ TAGTGAATTA CAATTTGAAA AAGTTTTCCC TAAAACTATA AAACTATTAA ATTGTATAAT TGTTTTTTTA   
  
  
+ TTTTTTAAAA TTATTAATTA AATTATTTAA TAGTACACAG TAGTAGAAAA ATTTTCTAGT TGTGTTAAAT   
  
  
+ TTATTTAGTT TTTAAGTTTA AGTTAGTTTT TTTATAACCA CATGGTTTTT TAAACTAAGT TTGATATTTT   
  
  
+ TAAATTGTGC ATTATTAAAT TATTTTATTT ATTTATTAAA AAATAAAAAT ACATAGTAAT ATTTTAATAA   
  
  
+ TTTTTTTATG GAAAATTTGA AGTTAAATTT ACTTATGTTT AAAATAATAT ATATACTATT ATTTAAAACC   
  
  
+ CATTTTAGAG TAGTTTGGAA GTTCAAACCA ATATTTTTTA ATTTCTCTAA CAACAATAAC TTTACAAAGT   
  
  
+ TTTATTAAGT TTACTTAACA AAAAATAAAT TTAAAATAAA AAGTTTAAAT TTATAAAAAT TTATATTATA   
  
  
+ AATAAAAAAC ACTTAAAAAA TTAAAAAACT ATCATATTTT CAATTTTATA TATAAAAGTC ATGAGTAATT   
  
  
+ TCAAATTTTT TTTTTAATAC TACTATCGAA CTTATAAAAA AATGCATACA TTTTTAAAAA TTCAGTTTTA   
  
  
+ AGTAATATAT TCTAAAAGTT AAATTTATAG AGATTAAAAA TTTCAAAAAC TATTTTAAGT TAAATCTAAT   
  
  
+ GCCAAGTTAC AATTTAATAA AAACTTATAT GTTTAAATTA ATCATCGTAT TTTATTTATA GTTAAAAAAT   
  
  
+ ACCATAAATA TAAAATTAAA AATTTTTAAA TAAAAATTAA TTATATTATT TAATATTTTT TTAGTAAGGG   
  
  
+ GGAATATGGT AGTATTCAAG AAGTTTAATG AAAATTGTAA AAAGTTAATA CCTAGAAGTA TGTACGTACG   
  
  
+ TTTCGAACGT ATAGATACAT ACATGATATA TATATAAATT AGTTAATATG TACGTACGTA CGTGTACGTA   
  
  
+ TTGTATACAT ATATCGATAC GTAATGGCAT ACTCTTCTCT ATTAATTACT ATTTGTTAAA CTAATGTAGT   
  
  
+ AGAACGGGTG TACCTATAAT TATTCTCGCT CAGAATATGC TTTAGGCTAC TCTCCTTTCG GAGCCCTAGT   
  
  
+ GTAGAATCTT TAACAAAATG CCCGAACTGT ACCAGAAGGG GTGTACCATA GGCTCGGAGC ATGCTTTAGG   
  
  
+ GTACTCTTCT CTCAGAAAAC CCTAGTGTAG AACTTTAGCA GTTGAGGTTA CGGAGAGGAA TTAGTTAGTC   
  
  
+ ATCTAAATTA ATTAATTAGA GTTGAATCAA CAAATTAGGG GGTAGAGAAT ATATTCATGA CTTGGTGATT   
  
  
+ TAAATTGTTT TAATCTTATC AGGTTTATT  

- CTGAGCTTTG ACTTTAATTA ATTTGACTCT AACATTAACT AAATCAAACA CACCTCTTTA TTACACAAAA   
  
  
- CCATCACTTC CCCACTCCCA ATTAATTTCT CATTAATGCC ACCCCCTTTG CCAAATCATC TGTTATTATT   
  
  
- ATCACTTAAT GTTAAACTTT TTCAAAAGGG ATTTTGATAT TTTGATAATT TAACATATTA ACAAAAAAAT   
  
  
- AAAAAATTTT AATAATTAAT TTAATAAATT ATCATGTGTC ATCATCTTTT TAAAAGATCA ACACAATTTA   
  
  
- AATAAATCAA AAATTCAAAT TCAATCAAAA AAATATTGGT GTACCAAAAA ATTTGATTCA AACTATAAAA   
  
  
- ATTTAACACG TAATAATTTA ATAAAATAAA TAAATAATTT TTTATTTTTA TGTATCATTA TAAAATTATT   
  
  
- AAAAAAATAC CTTTTAAACT TCAATTTAAA TGAATACAAA TTTTATTATA TATATGATAA TAAATTTTGG   
  
  
- GTAAAATCTC ATCAAACCTT CAAGTTTGGT TATAAAAAAT TAAAGAGATT GTTGTTATTG AAATGTTTCA   
  
  
- AAATAATTCA AATGAATTGT TTTTTATTTA AATTTTATTT TTCAAATTTA AATATTTTTA AATATAATAT   
  
  
- TTATTTTTTG TGAATTTTTT AATTTTTTGA TAGTATAAAA GTTAAAATAT ATATTTTCAG TACTCATTAA   
  
  
- AGTTTAAAAA AAAAATTATG ATGATAGCTT GAATATTTTT TTACGTATGT AAAAATTTTT AAGTCAAAAT   
  
  
- TCATTATATA AGATTTTCAA TTTAAATATC TCTAATTTTT AAAGTTTTTG ATAAAATTCA ATTTAGATTA   
  
  
- CGGTTCAATG TTAAATTATT TTTGAATATA CAAATTTAAT TAGTAGCATA AAATAAATAT CAATTTTTTA   
  
  
- TGGTATTTAT ATTTTAATTT TTAAAAATTT ATTTTTAATT AATATAATAA ATTATAAAAA AATCATTCCC   
  
  
- CCTTATACCA TCATAAGTTC TTCAAATTAC TTTTAACATT TTTCAATTAT GGATCTTCAT ACATGCATGC   
  
  
- AAAGCTTGCA TATCTATGTA TGTACTATAT ATATATTTAA TCAATTATAC ATGCATGCAT GCACATGCAT   
  
  
- AACATATGTA TATAGCTATG CATTACCGTA TGAGAAGAGA TAATTAATGA TAAACAATTT GATTACATCA   
  
  
- TCTTGCCCAC ATGGATATTA ATAAGAGCGA GTCTTATACG AAATCCGATG AGAGGAAAGC CTCGGGATCA   
  
  
- CATCTTAGAA ATTGTTTTAC GGGCTTGACA TGGTCTTCCC CACATGGTAT CCGAGCCTCG TACGAAATCC   
  
  
- CATGAGAAGA GAGTCTTTTG GGATCACATC TTGAAATCGT CAACTCCAAT GCCTCTCCTT AATCAATCAG   
  
  
- TAGATTTAAT TAATTAATCT CAACTTAGTT GTTTAATCCC CCATCTCTTA TATAAGTACT GAACCACTAA   
  
  
- ATTTAACAAA ATTAGAATAG TCCAAATAA

+     ARE

| Site Name | Organism | Position | Strand | Matrix score. | sequence | function |
| --- | --- | --- | --- | --- | --- | --- |
| ARE | Zea mays | 323 | + | 6 | TGGTTT | cis-acting regulatory element essential for the anaerobic induction |
| ARE | Zea mays | 515 | - | 6 | TGGTTT | cis-acting regulatory element essential for the anaerobic induction |

> 2018/04/13 10:10:12  
+ GACTCGAAAC TGAAATTAAT TAAACTGAGA TTGTAATTGA TTTAGTTTGT GTGGAGAAAT AATGTGTTTT   
  
  
+ GGTAGTGAAG GGGTGAGGGT TAATTAAAGA GTAATTACGG TGGGGGAAAC GGTTTAGTAG ACAATAATAA   
  
  
+ TAGTGAATTA CAATTTGAAA AAGTTTTCCC TAAAACTATA AAACTATTAA ATTGTATAAT TGTTTTTTTA   
  
  
+ TTTTTTAAAA TTATTAATTA AATTATTTAA TAGTACACAG TAGTAGAAAA ATTTTCTAGT TGTGTTAAAT   
  
  
+ TTATTTAGTT TTTAAGTTTA AGTTAGTTTT TTTATAACCA CATGGTTTTT TAAACTAAGT TTGATATTTT   
  
  
+ TAAATTGTGC ATTATTAAAT TATTTTATTT ATTTATTAAA AAATAAAAAT ACATAGTAAT ATTTTAATAA   
  
  
+ TTTTTTTATG GAAAATTTGA AGTTAAATTT ACTTATGTTT AAAATAATAT ATATACTATT ATTTAAAACC   
  
  
+ CATTTTAGAG TAGTTTGGAA GTTCAAACCA ATATTTTTTA ATTTCTCTAA CAACAATAAC TTTACAAAGT   
  
  
+ TTTATTAAGT TTACTTAACA AAAAATAAAT TTAAAATAAA AAGTTTAAAT TTATAAAAAT TTATATTATA   
  
  
+ AATAAAAAAC ACTTAAAAAA TTAAAAAACT ATCATATTTT CAATTTTATA TATAAAAGTC ATGAGTAATT   
  
  
+ TCAAATTTTT TTTTTAATAC TACTATCGAA CTTATAAAAA AATGCATACA TTTTTAAAAA TTCAGTTTTA   
  
  
+ AGTAATATAT TCTAAAAGTT AAATTTATAG AGATTAAAAA TTTCAAAAAC TATTTTAAGT TAAATCTAAT   
  
  
+ GCCAAGTTAC AATTTAATAA AAACTTATAT GTTTAAATTA ATCATCGTAT TTTATTTATA GTTAAAAAAT   
  
  
+ ACCATAAATA TAAAATTAAA AATTTTTAAA TAAAAATTAA TTATATTATT TAATATTTTT TTAGTAAGGG   
  
  
+ GGAATATGGT AGTATTCAAG AAGTTTAATG AAAATTGTAA AAAGTTAATA CCTAGAAGTA TGTACGTACG   
  
  
+ TTTCGAACGT ATAGATACAT ACATGATATA TATATAAATT AGTTAATATG TACGTACGTA CGTGTACGTA   
  
  
+ TTGTATACAT ATATCGATAC GTAATGGCAT ACTCTTCTCT ATTAATTACT ATTTGTTAAA CTAATGTAGT   
  
  
+ AGAACGGGTG TACCTATAAT TATTCTCGCT CAGAATATGC TTTAGGCTAC TCTCCTTTCG GAGCCCTAGT   
  
  
+ GTAGAATCTT TAACAAAATG CCCGAACTGT ACCAGAAGGG GTGTACCATA GGCTCGGAGC ATGCTTTAGG   
  
  
+ GTACTCTTCT CTCAGAAAAC CCTAGTGTAG AACTTTAGCA GTTGAGGTTA CGGAGAGGAA TTAGTTAGTC   
  
  
+ ATCTAAATTA ATTAATTAGA GTTGAATCAA CAAATTAGGG GGTAGAGAAT ATATTCATGA CTTGGTGATT   
  
  
+ TAAATTGTTT TAATCTTATC AGGTTTATT  

- CTGAGCTTTG ACTTTAATTA ATTTGACTCT AACATTAACT AAATCAAACA CACCTCTTTA TTACACAAAA   
  
  
- CCATCACTTC CCCACTCCCA ATTAATTTCT CATTAATGCC ACCCCCTTTG CCAAATCATC TGTTATTATT   
  
  
- ATCACTTAAT GTTAAACTTT TTCAAAAGGG ATTTTGATAT TTTGATAATT TAACATATTA ACAAAAAAAT   
  
  
- AAAAAATTTT AATAATTAAT TTAATAAATT ATCATGTGTC ATCATCTTTT TAAAAGATCA ACACAATTTA   
  
  
- AATAAATCAA AAATTCAAAT TCAATCAAAA AAATATTGGT GTACCAAAAA ATTTGATTCA AACTATAAAA   
  
  
- ATTTAACACG TAATAATTTA ATAAAATAAA TAAATAATTT TTTATTTTTA TGTATCATTA TAAAATTATT   
  
  
- AAAAAAATAC CTTTTAAACT TCAATTTAAA TGAATACAAA TTTTATTATA TATATGATAA TAAATTTTGG   
  
  
- GTAAAATCTC ATCAAACCTT CAAGTTTGGT TATAAAAAAT TAAAGAGATT GTTGTTATTG AAATGTTTCA   
  
  
- AAATAATTCA AATGAATTGT TTTTTATTTA AATTTTATTT TTCAAATTTA AATATTTTTA AATATAATAT   
  
  
- TTATTTTTTG TGAATTTTTT AATTTTTTGA TAGTATAAAA GTTAAAATAT ATATTTTCAG TACTCATTAA   
  
  
- AGTTTAAAAA AAAAATTATG ATGATAGCTT GAATATTTTT TTACGTATGT AAAAATTTTT AAGTCAAAAT   
  
  
- TCATTATATA AGATTTTCAA TTTAAATATC TCTAATTTTT AAAGTTTTTG ATAAAATTCA ATTTAGATTA   
  
  
- CGGTTCAATG TTAAATTATT TTTGAATATA CAAATTTAAT TAGTAGCATA AAATAAATAT CAATTTTTTA   
  
  
- TGGTATTTAT ATTTTAATTT TTAAAAATTT ATTTTTAATT AATATAATAA ATTATAAAAA AATCATTCCC   
  
  
- CCTTATACCA TCATAAGTTC TTCAAATTAC TTTTAACATT TTTCAATTAT GGATCTTCAT ACATGCATGC   
  
  
- AAAGCTTGCA TATCTATGTA TGTACTATAT ATATATTTAA TCAATTATAC ATGCATGCAT GCACATGCAT   
  
  
- AACATATGTA TATAGCTATG CATTACCGTA TGAGAAGAGA TAATTAATGA TAAACAATTT GATTACATCA   
  
  
- TCTTGCCCAC ATGGATATTA ATAAGAGCGA GTCTTATACG AAATCCGATG AGAGGAAAGC CTCGGGATCA   
  
  
- CATCTTAGAA ATTGTTTTAC GGGCTTGACA TGGTCTTCCC CACATGGTAT CCGAGCCTCG TACGAAATCC   
  
  
- CATGAGAAGA GAGTCTTTTG GGATCACATC TTGAAATCGT CAACTCCAAT GCCTCTCCTT AATCAATCAG   
  
  
- TAGATTTAAT TAATTAATCT CAACTTAGTT GTTTAATCCC CCATCTCTTA TATAAGTACT GAACCACTAA   
  
  
- ATTTAACAAA ATTAGAATAG TCCAAATAA

+     AT1-motif

| Site Name | Organism | Position | Strand | Matrix score. | sequence | function |
| --- | --- | --- | --- | --- | --- | --- |
| AT1-motif | Solanum tuberosum | 939 | - | 13 | AATTATTTTTTATT | part of a light responsive module |

> 2018/04/13 10:10:12  
+ GACTCGAAAC TGAAATTAAT TAAACTGAGA TTGTAATTGA TTTAGTTTGT GTGGAGAAAT AATGTGTTTT   
  
  
+ GGTAGTGAAG GGGTGAGGGT TAATTAAAGA GTAATTACGG TGGGGGAAAC GGTTTAGTAG ACAATAATAA   
  
  
+ TAGTGAATTA CAATTTGAAA AAGTTTTCCC TAAAACTATA AAACTATTAA ATTGTATAAT TGTTTTTTTA   
  
  
+ TTTTTTAAAA TTATTAATTA AATTATTTAA TAGTACACAG TAGTAGAAAA ATTTTCTAGT TGTGTTAAAT   
  
  
+ TTATTTAGTT TTTAAGTTTA AGTTAGTTTT TTTATAACCA CATGGTTTTT TAAACTAAGT TTGATATTTT   
  
  
+ TAAATTGTGC ATTATTAAAT TATTTTATTT ATTTATTAAA AAATAAAAAT ACATAGTAAT ATTTTAATAA   
  
  
+ TTTTTTTATG GAAAATTTGA AGTTAAATTT ACTTATGTTT AAAATAATAT ATATACTATT ATTTAAAACC   
  
  
+ CATTTTAGAG TAGTTTGGAA GTTCAAACCA ATATTTTTTA ATTTCTCTAA CAACAATAAC TTTACAAAGT   
  
  
+ TTTATTAAGT TTACTTAACA AAAAATAAAT TTAAAATAAA AAGTTTAAAT TTATAAAAAT TTATATTATA   
  
  
+ AATAAAAAAC ACTTAAAAAA TTAAAAAACT ATCATATTTT CAATTTTATA TATAAAAGTC ATGAGTAATT   
  
  
+ TCAAATTTTT TTTTTAATAC TACTATCGAA CTTATAAAAA AATGCATACA TTTTTAAAAA TTCAGTTTTA   
  
  
+ AGTAATATAT TCTAAAAGTT AAATTTATAG AGATTAAAAA TTTCAAAAAC TATTTTAAGT TAAATCTAAT   
  
  
+ GCCAAGTTAC AATTTAATAA AAACTTATAT GTTTAAATTA ATCATCGTAT TTTATTTATA GTTAAAAAAT   
  
  
+ ACCATAAATA TAAAATTAAA AATTTTTAAA TAAAAATTAA TTATATTATT TAATATTTTT TTAGTAAGGG   
  
  
+ GGAATATGGT AGTATTCAAG AAGTTTAATG AAAATTGTAA AAAGTTAATA CCTAGAAGTA TGTACGTACG   
  
  
+ TTTCGAACGT ATAGATACAT ACATGATATA TATATAAATT AGTTAATATG TACGTACGTA CGTGTACGTA   
  
  
+ TTGTATACAT ATATCGATAC GTAATGGCAT ACTCTTCTCT ATTAATTACT ATTTGTTAAA CTAATGTAGT   
  
  
+ AGAACGGGTG TACCTATAAT TATTCTCGCT CAGAATATGC TTTAGGCTAC TCTCCTTTCG GAGCCCTAGT   
  
  
+ GTAGAATCTT TAACAAAATG CCCGAACTGT ACCAGAAGGG GTGTACCATA GGCTCGGAGC ATGCTTTAGG   
  
  
+ GTACTCTTCT CTCAGAAAAC CCTAGTGTAG AACTTTAGCA GTTGAGGTTA CGGAGAGGAA TTAGTTAGTC   
  
  
+ ATCTAAATTA ATTAATTAGA GTTGAATCAA CAAATTAGGG GGTAGAGAAT ATATTCATGA CTTGGTGATT   
  
  
+ TAAATTGTTT TAATCTTATC AGGTTTATT  

- CTGAGCTTTG ACTTTAATTA ATTTGACTCT AACATTAACT AAATCAAACA CACCTCTTTA TTACACAAAA   
  
  
- CCATCACTTC CCCACTCCCA ATTAATTTCT CATTAATGCC ACCCCCTTTG CCAAATCATC TGTTATTATT   
  
  
- ATCACTTAAT GTTAAACTTT TTCAAAAGGG ATTTTGATAT TTTGATAATT TAACATATTA ACAAAAAAAT   
  
  
- AAAAAATTTT AATAATTAAT TTAATAAATT ATCATGTGTC ATCATCTTTT TAAAAGATCA ACACAATTTA   
  
  
- AATAAATCAA AAATTCAAAT TCAATCAAAA AAATATTGGT GTACCAAAAA ATTTGATTCA AACTATAAAA   
  
  
- ATTTAACACG TAATAATTTA ATAAAATAAA TAAATAATTT TTTATTTTTA TGTATCATTA TAAAATTATT   
  
  
- AAAAAAATAC CTTTTAAACT TCAATTTAAA TGAATACAAA TTTTATTATA TATATGATAA TAAATTTTGG   
  
  
- GTAAAATCTC ATCAAACCTT CAAGTTTGGT TATAAAAAAT TAAAGAGATT GTTGTTATTG AAATGTTTCA   
  
  
- AAATAATTCA AATGAATTGT TTTTTATTTA AATTTTATTT TTCAAATTTA AATATTTTTA AATATAATAT   
  
  
- TTATTTTTTG TGAATTTTTT AATTTTTTGA TAGTATAAAA GTTAAAATAT ATATTTTCAG TACTCATTAA   
  
  
- AGTTTAAAAA AAAAATTATG ATGATAGCTT GAATATTTTT TTACGTATGT AAAAATTTTT AAGTCAAAAT   
  
  
- TCATTATATA AGATTTTCAA TTTAAATATC TCTAATTTTT AAAGTTTTTG ATAAAATTCA ATTTAGATTA   
  
  
- CGGTTCAATG TTAAATTATT TTTGAATATA CAAATTTAAT TAGTAGCATA AAATAAATAT CAATTTTTTA   
  
  
- TGGTATTTAT ATTTTAATTT TTAAAAATTT ATTTTTAATT AATATAATAA ATTATAAAAA AATCATTCCC   
  
  
- CCTTATACCA TCATAAGTTC TTCAAATTAC TTTTAACATT TTTCAATTAT GGATCTTCAT ACATGCATGC   
  
  
- AAAGCTTGCA TATCTATGTA TGTACTATAT ATATATTTAA TCAATTATAC ATGCATGCAT GCACATGCAT   
  
  
- AACATATGTA TATAGCTATG CATTACCGTA TGAGAAGAGA TAATTAATGA TAAACAATTT GATTACATCA   
  
  
- TCTTGCCCAC ATGGATATTA ATAAGAGCGA GTCTTATACG AAATCCGATG AGAGGAAAGC CTCGGGATCA   
  
  
- CATCTTAGAA ATTGTTTTAC GGGCTTGACA TGGTCTTCCC CACATGGTAT CCGAGCCTCG TACGAAATCC   
  
  
- CATGAGAAGA GAGTCTTTTG GGATCACATC TTGAAATCGT CAACTCCAAT GCCTCTCCTT AATCAATCAG   
  
  
- TAGATTTAAT TAATTAATCT CAACTTAGTT GTTTAATCCC CCATCTCTTA TATAAGTACT GAACCACTAA   
  
  
- ATTTAACAAA ATTAGAATAG TCCAAATAA

+     ATCT-motif

| Site Name | Organism | Position | Strand | Matrix score. | sequence | function |
| --- | --- | --- | --- | --- | --- | --- |
| ATCT-motif | Pisum sativum | 833 | + | 9 | AATCTAATCC | part of a conserved DNA module involved in light responsiveness |

> 2018/04/13 10:10:12  
+ GACTCGAAAC TGAAATTAAT TAAACTGAGA TTGTAATTGA TTTAGTTTGT GTGGAGAAAT AATGTGTTTT   
  
  
+ GGTAGTGAAG GGGTGAGGGT TAATTAAAGA GTAATTACGG TGGGGGAAAC GGTTTAGTAG ACAATAATAA   
  
  
+ TAGTGAATTA CAATTTGAAA AAGTTTTCCC TAAAACTATA AAACTATTAA ATTGTATAAT TGTTTTTTTA   
  
  
+ TTTTTTAAAA TTATTAATTA AATTATTTAA TAGTACACAG TAGTAGAAAA ATTTTCTAGT TGTGTTAAAT   
  
  
+ TTATTTAGTT TTTAAGTTTA AGTTAGTTTT TTTATAACCA CATGGTTTTT TAAACTAAGT TTGATATTTT   
  
  
+ TAAATTGTGC ATTATTAAAT TATTTTATTT ATTTATTAAA AAATAAAAAT ACATAGTAAT ATTTTAATAA   
  
  
+ TTTTTTTATG GAAAATTTGA AGTTAAATTT ACTTATGTTT AAAATAATAT ATATACTATT ATTTAAAACC   
  
  
+ CATTTTAGAG TAGTTTGGAA GTTCAAACCA ATATTTTTTA ATTTCTCTAA CAACAATAAC TTTACAAAGT   
  
  
+ TTTATTAAGT TTACTTAACA AAAAATAAAT TTAAAATAAA AAGTTTAAAT TTATAAAAAT TTATATTATA   
  
  
+ AATAAAAAAC ACTTAAAAAA TTAAAAAACT ATCATATTTT CAATTTTATA TATAAAAGTC ATGAGTAATT   
  
  
+ TCAAATTTTT TTTTTAATAC TACTATCGAA CTTATAAAAA AATGCATACA TTTTTAAAAA TTCAGTTTTA   
  
  
+ AGTAATATAT TCTAAAAGTT AAATTTATAG AGATTAAAAA TTTCAAAAAC TATTTTAAGT TAAATCTAAT   
  
  
+ GCCAAGTTAC AATTTAATAA AAACTTATAT GTTTAAATTA ATCATCGTAT TTTATTTATA GTTAAAAAAT   
  
  
+ ACCATAAATA TAAAATTAAA AATTTTTAAA TAAAAATTAA TTATATTATT TAATATTTTT TTAGTAAGGG   
  
  
+ GGAATATGGT AGTATTCAAG AAGTTTAATG AAAATTGTAA AAAGTTAATA CCTAGAAGTA TGTACGTACG   
  
  
+ TTTCGAACGT ATAGATACAT ACATGATATA TATATAAATT AGTTAATATG TACGTACGTA CGTGTACGTA   
  
  
+ TTGTATACAT ATATCGATAC GTAATGGCAT ACTCTTCTCT ATTAATTACT ATTTGTTAAA CTAATGTAGT   
  
  
+ AGAACGGGTG TACCTATAAT TATTCTCGCT CAGAATATGC TTTAGGCTAC TCTCCTTTCG GAGCCCTAGT   
  
  
+ GTAGAATCTT TAACAAAATG CCCGAACTGT ACCAGAAGGG GTGTACCATA GGCTCGGAGC ATGCTTTAGG   
  
  
+ GTACTCTTCT CTCAGAAAAC CCTAGTGTAG AACTTTAGCA GTTGAGGTTA CGGAGAGGAA TTAGTTAGTC   
  
  
+ ATCTAAATTA ATTAATTAGA GTTGAATCAA CAAATTAGGG GGTAGAGAAT ATATTCATGA CTTGGTGATT   
  
  
+ TAAATTGTTT TAATCTTATC AGGTTTATT  

- CTGAGCTTTG ACTTTAATTA ATTTGACTCT AACATTAACT AAATCAAACA CACCTCTTTA TTACACAAAA   
  
  
- CCATCACTTC CCCACTCCCA ATTAATTTCT CATTAATGCC ACCCCCTTTG CCAAATCATC TGTTATTATT   
  
  
- ATCACTTAAT GTTAAACTTT TTCAAAAGGG ATTTTGATAT TTTGATAATT TAACATATTA ACAAAAAAAT   
  
  
- AAAAAATTTT AATAATTAAT TTAATAAATT ATCATGTGTC ATCATCTTTT TAAAAGATCA ACACAATTTA   
  
  
- AATAAATCAA AAATTCAAAT TCAATCAAAA AAATATTGGT GTACCAAAAA ATTTGATTCA AACTATAAAA   
  
  
- ATTTAACACG TAATAATTTA ATAAAATAAA TAAATAATTT TTTATTTTTA TGTATCATTA TAAAATTATT   
  
  
- AAAAAAATAC CTTTTAAACT TCAATTTAAA TGAATACAAA TTTTATTATA TATATGATAA TAAATTTTGG   
  
  
- GTAAAATCTC ATCAAACCTT CAAGTTTGGT TATAAAAAAT TAAAGAGATT GTTGTTATTG AAATGTTTCA   
  
  
- AAATAATTCA AATGAATTGT TTTTTATTTA AATTTTATTT TTCAAATTTA AATATTTTTA AATATAATAT   
  
  
- TTATTTTTTG TGAATTTTTT AATTTTTTGA TAGTATAAAA GTTAAAATAT ATATTTTCAG TACTCATTAA   
  
  
- AGTTTAAAAA AAAAATTATG ATGATAGCTT GAATATTTTT TTACGTATGT AAAAATTTTT AAGTCAAAAT   
  
  
- TCATTATATA AGATTTTCAA TTTAAATATC TCTAATTTTT AAAGTTTTTG ATAAAATTCA ATTTAGATTA   
  
  
- CGGTTCAATG TTAAATTATT TTTGAATATA CAAATTTAAT TAGTAGCATA AAATAAATAT CAATTTTTTA   
  
  
- TGGTATTTAT ATTTTAATTT TTAAAAATTT ATTTTTAATT AATATAATAA ATTATAAAAA AATCATTCCC   
  
  
- CCTTATACCA TCATAAGTTC TTCAAATTAC TTTTAACATT TTTCAATTAT GGATCTTCAT ACATGCATGC   
  
  
- AAAGCTTGCA TATCTATGTA TGTACTATAT ATATATTTAA TCAATTATAC ATGCATGCAT GCACATGCAT   
  
  
- AACATATGTA TATAGCTATG CATTACCGTA TGAGAAGAGA TAATTAATGA TAAACAATTT GATTACATCA   
  
  
- TCTTGCCCAC ATGGATATTA ATAAGAGCGA GTCTTATACG AAATCCGATG AGAGGAAAGC CTCGGGATCA   
  
  
- CATCTTAGAA ATTGTTTTAC GGGCTTGACA TGGTCTTCCC CACATGGTAT CCGAGCCTCG TACGAAATCC   
  
  
- CATGAGAAGA GAGTCTTTTG GGATCACATC TTGAAATCGT CAACTCCAAT GCCTCTCCTT AATCAATCAG   
  
  
- TAGATTTAAT TAATTAATCT CAACTTAGTT GTTTAATCCC CCATCTCTTA TATAAGTACT GAACCACTAA   
  
  
- ATTTAACAAA ATTAGAATAG TCCAAATAA

+     Box 4

| Site Name | Organism | Position | Strand | Matrix score. | sequence | function |
| --- | --- | --- | --- | --- | --- | --- |
| Box 4 | Petroselinum crispum | 946 | - | 6 | ATTAAT | part of a conserved DNA module involved in light responsiveness |
| Box 4 | Petroselinum crispum | 15 | + | 6 | ATTAAT | part of a conserved DNA module involved in light responsiveness |
| Box 4 | Petroselinum crispum | 1161 | - | 6 | ATTAAT | part of a conserved DNA module involved in light responsiveness |
| Box 4 | Petroselinum crispum | 1407 | - | 6 | ATTAAT | part of a conserved DNA module involved in light responsiveness |
| Box 4 | Petroselinum crispum | 1411 | - | 6 | ATTAAT | part of a conserved DNA module involved in light responsiveness |
| Box 4 | Petroselinum crispum | 223 | + | 6 | ATTAAT | part of a conserved DNA module involved in light responsiveness |
| Box 4 | Petroselinum crispum | 877 | - | 6 | ATTAAT | part of a conserved DNA module involved in light responsiveness |

> 2018/04/13 10:10:12  
+ GACTCGAAAC TGAAATTAAT TAAACTGAGA TTGTAATTGA TTTAGTTTGT GTGGAGAAAT AATGTGTTTT   
  
  
+ GGTAGTGAAG GGGTGAGGGT TAATTAAAGA GTAATTACGG TGGGGGAAAC GGTTTAGTAG ACAATAATAA   
  
  
+ TAGTGAATTA CAATTTGAAA AAGTTTTCCC TAAAACTATA AAACTATTAA ATTGTATAAT TGTTTTTTTA   
  
  
+ TTTTTTAAAA TTATTAATTA AATTATTTAA TAGTACACAG TAGTAGAAAA ATTTTCTAGT TGTGTTAAAT   
  
  
+ TTATTTAGTT TTTAAGTTTA AGTTAGTTTT TTTATAACCA CATGGTTTTT TAAACTAAGT TTGATATTTT   
  
  
+ TAAATTGTGC ATTATTAAAT TATTTTATTT ATTTATTAAA AAATAAAAAT ACATAGTAAT ATTTTAATAA   
  
  
+ TTTTTTTATG GAAAATTTGA AGTTAAATTT ACTTATGTTT AAAATAATAT ATATACTATT ATTTAAAACC   
  
  
+ CATTTTAGAG TAGTTTGGAA GTTCAAACCA ATATTTTTTA ATTTCTCTAA CAACAATAAC TTTACAAAGT   
  
  
+ TTTATTAAGT TTACTTAACA AAAAATAAAT TTAAAATAAA AAGTTTAAAT TTATAAAAAT TTATATTATA   
  
  
+ AATAAAAAAC ACTTAAAAAA TTAAAAAACT ATCATATTTT CAATTTTATA TATAAAAGTC ATGAGTAATT   
  
  
+ TCAAATTTTT TTTTTAATAC TACTATCGAA CTTATAAAAA AATGCATACA TTTTTAAAAA TTCAGTTTTA   
  
  
+ AGTAATATAT TCTAAAAGTT AAATTTATAG AGATTAAAAA TTTCAAAAAC TATTTTAAGT TAAATCTAAT   
  
  
+ GCCAAGTTAC AATTTAATAA AAACTTATAT GTTTAAATTA ATCATCGTAT TTTATTTATA GTTAAAAAAT   
  
  
+ ACCATAAATA TAAAATTAAA AATTTTTAAA TAAAAATTAA TTATATTATT TAATATTTTT TTAGTAAGGG   
  
  
+ GGAATATGGT AGTATTCAAG AAGTTTAATG AAAATTGTAA AAAGTTAATA CCTAGAAGTA TGTACGTACG   
  
  
+ TTTCGAACGT ATAGATACAT ACATGATATA TATATAAATT AGTTAATATG TACGTACGTA CGTGTACGTA   
  
  
+ TTGTATACAT ATATCGATAC GTAATGGCAT ACTCTTCTCT ATTAATTACT ATTTGTTAAA CTAATGTAGT   
  
  
+ AGAACGGGTG TACCTATAAT TATTCTCGCT CAGAATATGC TTTAGGCTAC TCTCCTTTCG GAGCCCTAGT   
  
  
+ GTAGAATCTT TAACAAAATG CCCGAACTGT ACCAGAAGGG GTGTACCATA GGCTCGGAGC ATGCTTTAGG   
  
  
+ GTACTCTTCT CTCAGAAAAC CCTAGTGTAG AACTTTAGCA GTTGAGGTTA CGGAGAGGAA TTAGTTAGTC   
  
  
+ ATCTAAATTA ATTAATTAGA GTTGAATCAA CAAATTAGGG GGTAGAGAAT ATATTCATGA CTTGGTGATT   
  
  
+ TAAATTGTTT TAATCTTATC AGGTTTATT  

- CTGAGCTTTG ACTTTAATTA ATTTGACTCT AACATTAACT AAATCAAACA CACCTCTTTA TTACACAAAA   
  
  
- CCATCACTTC CCCACTCCCA ATTAATTTCT CATTAATGCC ACCCCCTTTG CCAAATCATC TGTTATTATT   
  
  
- ATCACTTAAT GTTAAACTTT TTCAAAAGGG ATTTTGATAT TTTGATAATT TAACATATTA ACAAAAAAAT   
  
  
- AAAAAATTTT AATAATTAAT TTAATAAATT ATCATGTGTC ATCATCTTTT TAAAAGATCA ACACAATTTA   
  
  
- AATAAATCAA AAATTCAAAT TCAATCAAAA AAATATTGGT GTACCAAAAA ATTTGATTCA AACTATAAAA   
  
  
- ATTTAACACG TAATAATTTA ATAAAATAAA TAAATAATTT TTTATTTTTA TGTATCATTA TAAAATTATT   
  
  
- AAAAAAATAC CTTTTAAACT TCAATTTAAA TGAATACAAA TTTTATTATA TATATGATAA TAAATTTTGG   
  
  
- GTAAAATCTC ATCAAACCTT CAAGTTTGGT TATAAAAAAT TAAAGAGATT GTTGTTATTG AAATGTTTCA   
  
  
- AAATAATTCA AATGAATTGT TTTTTATTTA AATTTTATTT TTCAAATTTA AATATTTTTA AATATAATAT   
  
  
- TTATTTTTTG TGAATTTTTT AATTTTTTGA TAGTATAAAA GTTAAAATAT ATATTTTCAG TACTCATTAA   
  
  
- AGTTTAAAAA AAAAATTATG ATGATAGCTT GAATATTTTT TTACGTATGT AAAAATTTTT AAGTCAAAAT   
  
  
- TCATTATATA AGATTTTCAA TTTAAATATC TCTAATTTTT AAAGTTTTTG ATAAAATTCA ATTTAGATTA   
  
  
- CGGTTCAATG TTAAATTATT TTTGAATATA CAAATTTAAT TAGTAGCATA AAATAAATAT CAATTTTTTA   
  
  
- TGGTATTTAT ATTTTAATTT TTAAAAATTT ATTTTTAATT AATATAATAA ATTATAAAAA AATCATTCCC   
  
  
- CCTTATACCA TCATAAGTTC TTCAAATTAC TTTTAACATT TTTCAATTAT GGATCTTCAT ACATGCATGC   
  
  
- AAAGCTTGCA TATCTATGTA TGTACTATAT ATATATTTAA TCAATTATAC ATGCATGCAT GCACATGCAT   
  
  
- AACATATGTA TATAGCTATG CATTACCGTA TGAGAAGAGA TAATTAATGA TAAACAATTT GATTACATCA   
  
  
- TCTTGCCCAC ATGGATATTA ATAAGAGCGA GTCTTATACG AAATCCGATG AGAGGAAAGC CTCGGGATCA   
  
  
- CATCTTAGAA ATTGTTTTAC GGGCTTGACA TGGTCTTCCC CACATGGTAT CCGAGCCTCG TACGAAATCC   
  
  
- CATGAGAAGA GAGTCTTTTG GGATCACATC TTGAAATCGT CAACTCCAAT GCCTCTCCTT AATCAATCAG   
  
  
- TAGATTTAAT TAATTAATCT CAACTTAGTT GTTTAATCCC CCATCTCTTA TATAAGTACT GAACCACTAA   
  
  
- ATTTAACAAA ATTAGAATAG TCCAAATAA

+     Box I

| Site Name | Organism | Position | Strand | Matrix score. | sequence | function |
| --- | --- | --- | --- | --- | --- | --- |
| Box I | Pisum sativum | 154 | - | 7 | TTTCAAA | light responsive element |
| Box I | Pisum sativum | 811 | + | 7 | TTTCAAA | light responsive element |
| Box I | Pisum sativum | 699 | + | 7 | TTTCAAA | light responsive element |

> 2018/04/13 10:10:12  
+ GACTCGAAAC TGAAATTAAT TAAACTGAGA TTGTAATTGA TTTAGTTTGT GTGGAGAAAT AATGTGTTTT   
  
  
+ GGTAGTGAAG GGGTGAGGGT TAATTAAAGA GTAATTACGG TGGGGGAAAC GGTTTAGTAG ACAATAATAA   
  
  
+ TAGTGAATTA CAATTTGAAA AAGTTTTCCC TAAAACTATA AAACTATTAA ATTGTATAAT TGTTTTTTTA   
  
  
+ TTTTTTAAAA TTATTAATTA AATTATTTAA TAGTACACAG TAGTAGAAAA ATTTTCTAGT TGTGTTAAAT   
  
  
+ TTATTTAGTT TTTAAGTTTA AGTTAGTTTT TTTATAACCA CATGGTTTTT TAAACTAAGT TTGATATTTT   
  
  
+ TAAATTGTGC ATTATTAAAT TATTTTATTT ATTTATTAAA AAATAAAAAT ACATAGTAAT ATTTTAATAA   
  
  
+ TTTTTTTATG GAAAATTTGA AGTTAAATTT ACTTATGTTT AAAATAATAT ATATACTATT ATTTAAAACC   
  
  
+ CATTTTAGAG TAGTTTGGAA GTTCAAACCA ATATTTTTTA ATTTCTCTAA CAACAATAAC TTTACAAAGT   
  
  
+ TTTATTAAGT TTACTTAACA AAAAATAAAT TTAAAATAAA AAGTTTAAAT TTATAAAAAT TTATATTATA   
  
  
+ AATAAAAAAC ACTTAAAAAA TTAAAAAACT ATCATATTTT CAATTTTATA TATAAAAGTC ATGAGTAATT   
  
  
+ TCAAATTTTT TTTTTAATAC TACTATCGAA CTTATAAAAA AATGCATACA TTTTTAAAAA TTCAGTTTTA   
  
  
+ AGTAATATAT TCTAAAAGTT AAATTTATAG AGATTAAAAA TTTCAAAAAC TATTTTAAGT TAAATCTAAT   
  
  
+ GCCAAGTTAC AATTTAATAA AAACTTATAT GTTTAAATTA ATCATCGTAT TTTATTTATA GTTAAAAAAT   
  
  
+ ACCATAAATA TAAAATTAAA AATTTTTAAA TAAAAATTAA TTATATTATT TAATATTTTT TTAGTAAGGG   
  
  
+ GGAATATGGT AGTATTCAAG AAGTTTAATG AAAATTGTAA AAAGTTAATA CCTAGAAGTA TGTACGTACG   
  
  
+ TTTCGAACGT ATAGATACAT ACATGATATA TATATAAATT AGTTAATATG TACGTACGTA CGTGTACGTA   
  
  
+ TTGTATACAT ATATCGATAC GTAATGGCAT ACTCTTCTCT ATTAATTACT ATTTGTTAAA CTAATGTAGT   
  
  
+ AGAACGGGTG TACCTATAAT TATTCTCGCT CAGAATATGC TTTAGGCTAC TCTCCTTTCG GAGCCCTAGT   
  
  
+ GTAGAATCTT TAACAAAATG CCCGAACTGT ACCAGAAGGG GTGTACCATA GGCTCGGAGC ATGCTTTAGG   
  
  
+ GTACTCTTCT CTCAGAAAAC CCTAGTGTAG AACTTTAGCA GTTGAGGTTA CGGAGAGGAA TTAGTTAGTC   
  
  
+ ATCTAAATTA ATTAATTAGA GTTGAATCAA CAAATTAGGG GGTAGAGAAT ATATTCATGA CTTGGTGATT   
  
  
+ TAAATTGTTT TAATCTTATC AGGTTTATT  

- CTGAGCTTTG ACTTTAATTA ATTTGACTCT AACATTAACT AAATCAAACA CACCTCTTTA TTACACAAAA   
  
  
- CCATCACTTC CCCACTCCCA ATTAATTTCT CATTAATGCC ACCCCCTTTG CCAAATCATC TGTTATTATT   
  
  
- ATCACTTAAT GTTAAACTTT TTCAAAAGGG ATTTTGATAT TTTGATAATT TAACATATTA ACAAAAAAAT   
  
  
- AAAAAATTTT AATAATTAAT TTAATAAATT ATCATGTGTC ATCATCTTTT TAAAAGATCA ACACAATTTA   
  
  
- AATAAATCAA AAATTCAAAT TCAATCAAAA AAATATTGGT GTACCAAAAA ATTTGATTCA AACTATAAAA   
  
  
- ATTTAACACG TAATAATTTA ATAAAATAAA TAAATAATTT TTTATTTTTA TGTATCATTA TAAAATTATT   
  
  
- AAAAAAATAC CTTTTAAACT TCAATTTAAA TGAATACAAA TTTTATTATA TATATGATAA TAAATTTTGG   
  
  
- GTAAAATCTC ATCAAACCTT CAAGTTTGGT TATAAAAAAT TAAAGAGATT GTTGTTATTG AAATGTTTCA   
  
  
- AAATAATTCA AATGAATTGT TTTTTATTTA AATTTTATTT TTCAAATTTA AATATTTTTA AATATAATAT   
  
  
- TTATTTTTTG TGAATTTTTT AATTTTTTGA TAGTATAAAA GTTAAAATAT ATATTTTCAG TACTCATTAA   
  
  
- AGTTTAAAAA AAAAATTATG ATGATAGCTT GAATATTTTT TTACGTATGT AAAAATTTTT AAGTCAAAAT   
  
  
- TCATTATATA AGATTTTCAA TTTAAATATC TCTAATTTTT AAAGTTTTTG ATAAAATTCA ATTTAGATTA   
  
  
- CGGTTCAATG TTAAATTATT TTTGAATATA CAAATTTAAT TAGTAGCATA AAATAAATAT CAATTTTTTA   
  
  
- TGGTATTTAT ATTTTAATTT TTAAAAATTT ATTTTTAATT AATATAATAA ATTATAAAAA AATCATTCCC   
  
  
- CCTTATACCA TCATAAGTTC TTCAAATTAC TTTTAACATT TTTCAATTAT GGATCTTCAT ACATGCATGC   
  
  
- AAAGCTTGCA TATCTATGTA TGTACTATAT ATATATTTAA TCAATTATAC ATGCATGCAT GCACATGCAT   
  
  
- AACATATGTA TATAGCTATG CATTACCGTA TGAGAAGAGA TAATTAATGA TAAACAATTT GATTACATCA   
  
  
- TCTTGCCCAC ATGGATATTA ATAAGAGCGA GTCTTATACG AAATCCGATG AGAGGAAAGC CTCGGGATCA   
  
  
- CATCTTAGAA ATTGTTTTAC GGGCTTGACA TGGTCTTCCC CACATGGTAT CCGAGCCTCG TACGAAATCC   
  
  
- CATGAGAAGA GAGTCTTTTG GGATCACATC TTGAAATCGT CAACTCCAAT GCCTCTCCTT AATCAATCAG   
  
  
- TAGATTTAAT TAATTAATCT CAACTTAGTT GTTTAATCCC CCATCTCTTA TATAAGTACT GAACCACTAA   
  
  
- ATTTAACAAA ATTAGAATAG TCCAAATAA

+     CAAT-box

| Site Name | Organism | Position | Strand | Matrix score. | sequence | function |
| --- | --- | --- | --- | --- | --- | --- |
| CAAT-box | Brassica rapa | 1431 | + | 5 | CAAAT | common cis-acting element in promoter and enhancer regions |
| CAAT-box | Brassica rapa | 1171 | - | 5 | CAAAT | common cis-acting element in promoter and enhancer regions |
| CAAT-box | Hordeum vulgare | 1120 | - | 4 | CAAT | common cis-acting element in promoter and enhancer regions |
| CAAT-box | Hordeum vulgare | 1014 | - | 4 | CAAT | common cis-acting element in promoter and enhancer regions |
| CAAT-box | Hordeum vulgare | 199 | - | 4 | CAAT | common cis-acting element in promoter and enhancer regions |
| CAAT-box | Glycine max | 198 | - | 5 | CAATT | common cis-acting element in promoter and enhancer regions |
| CAAT-box | Brassica rapa | 702 | + | 5 | CAAAT | common cis-acting element in promoter and enhancer regions |
| CAAT-box | Hordeum vulgare | 354 | - | 4 | CAAT | common cis-acting element in promoter and enhancer regions |
| CAAT-box | Glycine max | 671 | + | 5 | CAATT | common cis-acting element in promoter and enhancer regions |
| CAAT-box | Hordeum vulgare | 544 | + | 4 | CAAT | common cis-acting element in promoter and enhancer regions |
| CAAT-box | Brassica rapa | 435 | - | 5 | CAAAT | common cis-acting element in promoter and enhancer regions |
| CAAT-box | Arabidopsis thaliana | 518 | + | 5 | CCAAT | common cis-acting element in promoter and enhancer regions |
| CAAT-box | Glycine max | 1473 | - | 5 | CAATT | common cis-acting element in promoter and enhancer regions |
| CAAT-box | Brassica rapa | 153 | - | 5 | CAAAT | common cis-acting element in promoter and enhancer regions |
| CAAT-box | Hordeum vulgare | 30 | - | 4 | CAAT | common cis-acting element in promoter and enhancer regions |
| CAAT-box | Hordeum vulgare | 519 | + | 4 | CAAT | common cis-acting element in promoter and enhancer regions |
| CAAT-box | Glycine max | 353 | - | 5 | CAATT | common cis-acting element in promoter and enhancer regions |
| CAAT-box | Glycine max | 850 | + | 5 | CAATT | common cis-acting element in promoter and enhancer regions |
| CAAT-box | Hordeum vulgare | 36 | - | 4 | CAAT | common cis-acting element in promoter and enhancer regions |
| CAAT-box | Hordeum vulgare | 132 | + | 4 | CAAT | common cis-acting element in promoter and enhancer regions |
| CAAT-box | Hordeum vulgare | 1474 | - | 4 | CAAT | common cis-acting element in promoter and enhancer regions |
| CAAT-box | Glycine max | 190 | - | 5 | CAATT | common cis-acting element in promoter and enhancer regions |
| CAAT-box | Glycine max | 1013 | - | 5 | CAATT | common cis-acting element in promoter and enhancer regions |
| CAAT-box | Glycine max | 35 | - | 5 | CAATT | common cis-acting element in promoter and enhancer regions |
| CAAT-box | Glycine max | 151 | + | 5 | CAATT | common cis-acting element in promoter and enhancer regions |
| CAAT-box | Hordeum vulgare | 191 | - | 4 | CAAT | common cis-acting element in promoter and enhancer regions |

> 2018/04/13 10:10:12  
+ GACTCGAAAC TGAAATTAAT TAAACTGAGA TTGTAATTGA TTTAGTTTGT GTGGAGAAAT AATGTGTTTT   
  
  
+ GGTAGTGAAG GGGTGAGGGT TAATTAAAGA GTAATTACGG TGGGGGAAAC GGTTTAGTAG ACAATAATAA   
  
  
+ TAGTGAATTA CAATTTGAAA AAGTTTTCCC TAAAACTATA AAACTATTAA ATTGTATAAT TGTTTTTTTA   
  
  
+ TTTTTTAAAA TTATTAATTA AATTATTTAA TAGTACACAG TAGTAGAAAA ATTTTCTAGT TGTGTTAAAT   
  
  
+ TTATTTAGTT TTTAAGTTTA AGTTAGTTTT TTTATAACCA CATGGTTTTT TAAACTAAGT TTGATATTTT   
  
  
+ TAAATTGTGC ATTATTAAAT TATTTTATTT ATTTATTAAA AAATAAAAAT ACATAGTAAT ATTTTAATAA   
  
  
+ TTTTTTTATG GAAAATTTGA AGTTAAATTT ACTTATGTTT AAAATAATAT ATATACTATT ATTTAAAACC   
  
  
+ CATTTTAGAG TAGTTTGGAA GTTCAAACCA ATATTTTTTA ATTTCTCTAA CAACAATAAC TTTACAAAGT   
  
  
+ TTTATTAAGT TTACTTAACA AAAAATAAAT TTAAAATAAA AAGTTTAAAT TTATAAAAAT TTATATTATA   
  
  
+ AATAAAAAAC ACTTAAAAAA TTAAAAAACT ATCATATTTT CAATTTTATA TATAAAAGTC ATGAGTAATT   
  
  
+ TCAAATTTTT TTTTTAATAC TACTATCGAA CTTATAAAAA AATGCATACA TTTTTAAAAA TTCAGTTTTA   
  
  
+ AGTAATATAT TCTAAAAGTT AAATTTATAG AGATTAAAAA TTTCAAAAAC TATTTTAAGT TAAATCTAAT   
  
  
+ GCCAAGTTAC AATTTAATAA AAACTTATAT GTTTAAATTA ATCATCGTAT TTTATTTATA GTTAAAAAAT   
  
  
+ ACCATAAATA TAAAATTAAA AATTTTTAAA TAAAAATTAA TTATATTATT TAATATTTTT TTAGTAAGGG   
  
  
+ GGAATATGGT AGTATTCAAG AAGTTTAATG AAAATTGTAA AAAGTTAATA CCTAGAAGTA TGTACGTACG   
  
  
+ TTTCGAACGT ATAGATACAT ACATGATATA TATATAAATT AGTTAATATG TACGTACGTA CGTGTACGTA   
  
  
+ TTGTATACAT ATATCGATAC GTAATGGCAT ACTCTTCTCT ATTAATTACT ATTTGTTAAA CTAATGTAGT   
  
  
+ AGAACGGGTG TACCTATAAT TATTCTCGCT CAGAATATGC TTTAGGCTAC TCTCCTTTCG GAGCCCTAGT   
  
  
+ GTAGAATCTT TAACAAAATG CCCGAACTGT ACCAGAAGGG GTGTACCATA GGCTCGGAGC ATGCTTTAGG   
  
  
+ GTACTCTTCT CTCAGAAAAC CCTAGTGTAG AACTTTAGCA GTTGAGGTTA CGGAGAGGAA TTAGTTAGTC   
  
  
+ ATCTAAATTA ATTAATTAGA GTTGAATCAA CAAATTAGGG GGTAGAGAAT ATATTCATGA CTTGGTGATT   
  
  
+ TAAATTGTTT TAATCTTATC AGGTTTATT  

- CTGAGCTTTG ACTTTAATTA ATTTGACTCT AACATTAACT AAATCAAACA CACCTCTTTA TTACACAAAA   
  
  
- CCATCACTTC CCCACTCCCA ATTAATTTCT CATTAATGCC ACCCCCTTTG CCAAATCATC TGTTATTATT   
  
  
- ATCACTTAAT GTTAAACTTT TTCAAAAGGG ATTTTGATAT TTTGATAATT TAACATATTA ACAAAAAAAT   
  
  
- AAAAAATTTT AATAATTAAT TTAATAAATT ATCATGTGTC ATCATCTTTT TAAAAGATCA ACACAATTTA   
  
  
- AATAAATCAA AAATTCAAAT TCAATCAAAA AAATATTGGT GTACCAAAAA ATTTGATTCA AACTATAAAA   
  
  
- ATTTAACACG TAATAATTTA ATAAAATAAA TAAATAATTT TTTATTTTTA TGTATCATTA TAAAATTATT   
  
  
- AAAAAAATAC CTTTTAAACT TCAATTTAAA TGAATACAAA TTTTATTATA TATATGATAA TAAATTTTGG   
  
  
- GTAAAATCTC ATCAAACCTT CAAGTTTGGT TATAAAAAAT TAAAGAGATT GTTGTTATTG AAATGTTTCA   
  
  
- AAATAATTCA AATGAATTGT TTTTTATTTA AATTTTATTT TTCAAATTTA AATATTTTTA AATATAATAT   
  
  
- TTATTTTTTG TGAATTTTTT AATTTTTTGA TAGTATAAAA GTTAAAATAT ATATTTTCAG TACTCATTAA   
  
  
- AGTTTAAAAA AAAAATTATG ATGATAGCTT GAATATTTTT TTACGTATGT AAAAATTTTT AAGTCAAAAT   
  
  
- TCATTATATA AGATTTTCAA TTTAAATATC TCTAATTTTT AAAGTTTTTG ATAAAATTCA ATTTAGATTA   
  
  
- CGGTTCAATG TTAAATTATT TTTGAATATA CAAATTTAAT TAGTAGCATA AAATAAATAT CAATTTTTTA   
  
  
- TGGTATTTAT ATTTTAATTT TTAAAAATTT ATTTTTAATT AATATAATAA ATTATAAAAA AATCATTCCC   
  
  
- CCTTATACCA TCATAAGTTC TTCAAATTAC TTTTAACATT TTTCAATTAT GGATCTTCAT ACATGCATGC   
  
  
- AAAGCTTGCA TATCTATGTA TGTACTATAT ATATATTTAA TCAATTATAC ATGCATGCAT GCACATGCAT   
  
  
- AACATATGTA TATAGCTATG CATTACCGTA TGAGAAGAGA TAATTAATGA TAAACAATTT GATTACATCA   
  
  
- TCTTGCCCAC ATGGATATTA ATAAGAGCGA GTCTTATACG AAATCCGATG AGAGGAAAGC CTCGGGATCA   
  
  
- CATCTTAGAA ATTGTTTTAC GGGCTTGACA TGGTCTTCCC CACATGGTAT CCGAGCCTCG TACGAAATCC   
  
  
- CATGAGAAGA GAGTCTTTTG GGATCACATC TTGAAATCGT CAACTCCAAT GCCTCTCCTT AATCAATCAG   
  
  
- TAGATTTAAT TAATTAATCT CAACTTAGTT GTTTAATCCC CCATCTCTTA TATAAGTACT GAACCACTAA   
  
  
- ATTTAACAAA ATTAGAATAG TCCAAATAA

+     ERE

| Site Name | Organism | Position | Strand | Matrix score. | sequence | function |
| --- | --- | --- | --- | --- | --- | --- |
| ERE | Dianthus caryophyllus | 698 | + | 8 | ATTTCAAA | ethylene-responsive element |
| ERE | Dianthus caryophyllus | 810 | + | 8 | ATTTCAAA | ethylene-responsive element |

> 2018/04/13 10:10:12  
+ GACTCGAAAC TGAAATTAAT TAAACTGAGA TTGTAATTGA TTTAGTTTGT GTGGAGAAAT AATGTGTTTT   
  
  
+ GGTAGTGAAG GGGTGAGGGT TAATTAAAGA GTAATTACGG TGGGGGAAAC GGTTTAGTAG ACAATAATAA   
  
  
+ TAGTGAATTA CAATTTGAAA AAGTTTTCCC TAAAACTATA AAACTATTAA ATTGTATAAT TGTTTTTTTA   
  
  
+ TTTTTTAAAA TTATTAATTA AATTATTTAA TAGTACACAG TAGTAGAAAA ATTTTCTAGT TGTGTTAAAT   
  
  
+ TTATTTAGTT TTTAAGTTTA AGTTAGTTTT TTTATAACCA CATGGTTTTT TAAACTAAGT TTGATATTTT   
  
  
+ TAAATTGTGC ATTATTAAAT TATTTTATTT ATTTATTAAA AAATAAAAAT ACATAGTAAT ATTTTAATAA   
  
  
+ TTTTTTTATG GAAAATTTGA AGTTAAATTT ACTTATGTTT AAAATAATAT ATATACTATT ATTTAAAACC   
  
  
+ CATTTTAGAG TAGTTTGGAA GTTCAAACCA ATATTTTTTA ATTTCTCTAA CAACAATAAC TTTACAAAGT   
  
  
+ TTTATTAAGT TTACTTAACA AAAAATAAAT TTAAAATAAA AAGTTTAAAT TTATAAAAAT TTATATTATA   
  
  
+ AATAAAAAAC ACTTAAAAAA TTAAAAAACT ATCATATTTT CAATTTTATA TATAAAAGTC ATGAGTAATT   
  
  
+ TCAAATTTTT TTTTTAATAC TACTATCGAA CTTATAAAAA AATGCATACA TTTTTAAAAA TTCAGTTTTA   
  
  
+ AGTAATATAT TCTAAAAGTT AAATTTATAG AGATTAAAAA TTTCAAAAAC TATTTTAAGT TAAATCTAAT   
  
  
+ GCCAAGTTAC AATTTAATAA AAACTTATAT GTTTAAATTA ATCATCGTAT TTTATTTATA GTTAAAAAAT   
  
  
+ ACCATAAATA TAAAATTAAA AATTTTTAAA TAAAAATTAA TTATATTATT TAATATTTTT TTAGTAAGGG   
  
  
+ GGAATATGGT AGTATTCAAG AAGTTTAATG AAAATTGTAA AAAGTTAATA CCTAGAAGTA TGTACGTACG   
  
  
+ TTTCGAACGT ATAGATACAT ACATGATATA TATATAAATT AGTTAATATG TACGTACGTA CGTGTACGTA   
  
  
+ TTGTATACAT ATATCGATAC GTAATGGCAT ACTCTTCTCT ATTAATTACT ATTTGTTAAA CTAATGTAGT   
  
  
+ AGAACGGGTG TACCTATAAT TATTCTCGCT CAGAATATGC TTTAGGCTAC TCTCCTTTCG GAGCCCTAGT   
  
  
+ GTAGAATCTT TAACAAAATG CCCGAACTGT ACCAGAAGGG GTGTACCATA GGCTCGGAGC ATGCTTTAGG   
  
  
+ GTACTCTTCT CTCAGAAAAC CCTAGTGTAG AACTTTAGCA GTTGAGGTTA CGGAGAGGAA TTAGTTAGTC   
  
  
+ ATCTAAATTA ATTAATTAGA GTTGAATCAA CAAATTAGGG GGTAGAGAAT ATATTCATGA CTTGGTGATT   
  
  
+ TAAATTGTTT TAATCTTATC AGGTTTATT  

- CTGAGCTTTG ACTTTAATTA ATTTGACTCT AACATTAACT AAATCAAACA CACCTCTTTA TTACACAAAA   
  
  
- CCATCACTTC CCCACTCCCA ATTAATTTCT CATTAATGCC ACCCCCTTTG CCAAATCATC TGTTATTATT   
  
  
- ATCACTTAAT GTTAAACTTT TTCAAAAGGG ATTTTGATAT TTTGATAATT TAACATATTA ACAAAAAAAT   
  
  
- AAAAAATTTT AATAATTAAT TTAATAAATT ATCATGTGTC ATCATCTTTT TAAAAGATCA ACACAATTTA   
  
  
- AATAAATCAA AAATTCAAAT TCAATCAAAA AAATATTGGT GTACCAAAAA ATTTGATTCA AACTATAAAA   
  
  
- ATTTAACACG TAATAATTTA ATAAAATAAA TAAATAATTT TTTATTTTTA TGTATCATTA TAAAATTATT   
  
  
- AAAAAAATAC CTTTTAAACT TCAATTTAAA TGAATACAAA TTTTATTATA TATATGATAA TAAATTTTGG   
  
  
- GTAAAATCTC ATCAAACCTT CAAGTTTGGT TATAAAAAAT TAAAGAGATT GTTGTTATTG AAATGTTTCA   
  
  
- AAATAATTCA AATGAATTGT TTTTTATTTA AATTTTATTT TTCAAATTTA AATATTTTTA AATATAATAT   
  
  
- TTATTTTTTG TGAATTTTTT AATTTTTTGA TAGTATAAAA GTTAAAATAT ATATTTTCAG TACTCATTAA   
  
  
- AGTTTAAAAA AAAAATTATG ATGATAGCTT GAATATTTTT TTACGTATGT AAAAATTTTT AAGTCAAAAT   
  
  
- TCATTATATA AGATTTTCAA TTTAAATATC TCTAATTTTT AAAGTTTTTG ATAAAATTCA ATTTAGATTA   
  
  
- CGGTTCAATG TTAAATTATT TTTGAATATA CAAATTTAAT TAGTAGCATA AAATAAATAT CAATTTTTTA   
  
  
- TGGTATTTAT ATTTTAATTT TTAAAAATTT ATTTTTAATT AATATAATAA ATTATAAAAA AATCATTCCC   
  
  
- CCTTATACCA TCATAAGTTC TTCAAATTAC TTTTAACATT TTTCAATTAT GGATCTTCAT ACATGCATGC   
  
  
- AAAGCTTGCA TATCTATGTA TGTACTATAT ATATATTTAA TCAATTATAC ATGCATGCAT GCACATGCAT   
  
  
- AACATATGTA TATAGCTATG CATTACCGTA TGAGAAGAGA TAATTAATGA TAAACAATTT GATTACATCA   
  
  
- TCTTGCCCAC ATGGATATTA ATAAGAGCGA GTCTTATACG AAATCCGATG AGAGGAAAGC CTCGGGATCA   
  
  
- CATCTTAGAA ATTGTTTTAC GGGCTTGACA TGGTCTTCCC CACATGGTAT CCGAGCCTCG TACGAAATCC   
  
  
- CATGAGAAGA GAGTCTTTTG GGATCACATC TTGAAATCGT CAACTCCAAT GCCTCTCCTT AATCAATCAG   
  
  
- TAGATTTAAT TAATTAATCT CAACTTAGTT GTTTAATCCC CCATCTCTTA TATAAGTACT GAACCACTAA   
  
  
- ATTTAACAAA ATTAGAATAG TCCAAATAA

+     G-Box

| Site Name | Organism | Position | Strand | Matrix score. | sequence | function |
| --- | --- | --- | --- | --- | --- | --- |
| G-Box | Antirrhinum majus | 1109 | - | 6 | CACGTA | cis-acting regulatory element involved in light responsiveness |

> 2018/04/13 10:10:12  
+ GACTCGAAAC TGAAATTAAT TAAACTGAGA TTGTAATTGA TTTAGTTTGT GTGGAGAAAT AATGTGTTTT   
  
  
+ GGTAGTGAAG GGGTGAGGGT TAATTAAAGA GTAATTACGG TGGGGGAAAC GGTTTAGTAG ACAATAATAA   
  
  
+ TAGTGAATTA CAATTTGAAA AAGTTTTCCC TAAAACTATA AAACTATTAA ATTGTATAAT TGTTTTTTTA   
  
  
+ TTTTTTAAAA TTATTAATTA AATTATTTAA TAGTACACAG TAGTAGAAAA ATTTTCTAGT TGTGTTAAAT   
  
  
+ TTATTTAGTT TTTAAGTTTA AGTTAGTTTT TTTATAACCA CATGGTTTTT TAAACTAAGT TTGATATTTT   
  
  
+ TAAATTGTGC ATTATTAAAT TATTTTATTT ATTTATTAAA AAATAAAAAT ACATAGTAAT ATTTTAATAA   
  
  
+ TTTTTTTATG GAAAATTTGA AGTTAAATTT ACTTATGTTT AAAATAATAT ATATACTATT ATTTAAAACC   
  
  
+ CATTTTAGAG TAGTTTGGAA GTTCAAACCA ATATTTTTTA ATTTCTCTAA CAACAATAAC TTTACAAAGT   
  
  
+ TTTATTAAGT TTACTTAACA AAAAATAAAT TTAAAATAAA AAGTTTAAAT TTATAAAAAT TTATATTATA   
  
  
+ AATAAAAAAC ACTTAAAAAA TTAAAAAACT ATCATATTTT CAATTTTATA TATAAAAGTC ATGAGTAATT   
  
  
+ TCAAATTTTT TTTTTAATAC TACTATCGAA CTTATAAAAA AATGCATACA TTTTTAAAAA TTCAGTTTTA   
  
  
+ AGTAATATAT TCTAAAAGTT AAATTTATAG AGATTAAAAA TTTCAAAAAC TATTTTAAGT TAAATCTAAT   
  
  
+ GCCAAGTTAC AATTTAATAA AAACTTATAT GTTTAAATTA ATCATCGTAT TTTATTTATA GTTAAAAAAT   
  
  
+ ACCATAAATA TAAAATTAAA AATTTTTAAA TAAAAATTAA TTATATTATT TAATATTTTT TTAGTAAGGG   
  
  
+ GGAATATGGT AGTATTCAAG AAGTTTAATG AAAATTGTAA AAAGTTAATA CCTAGAAGTA TGTACGTACG   
  
  
+ TTTCGAACGT ATAGATACAT ACATGATATA TATATAAATT AGTTAATATG TACGTACGTA CGTGTACGTA   
  
  
+ TTGTATACAT ATATCGATAC GTAATGGCAT ACTCTTCTCT ATTAATTACT ATTTGTTAAA CTAATGTAGT   
  
  
+ AGAACGGGTG TACCTATAAT TATTCTCGCT CAGAATATGC TTTAGGCTAC TCTCCTTTCG GAGCCCTAGT   
  
  
+ GTAGAATCTT TAACAAAATG CCCGAACTGT ACCAGAAGGG GTGTACCATA GGCTCGGAGC ATGCTTTAGG   
  
  
+ GTACTCTTCT CTCAGAAAAC CCTAGTGTAG AACTTTAGCA GTTGAGGTTA CGGAGAGGAA TTAGTTAGTC   
  
  
+ ATCTAAATTA ATTAATTAGA GTTGAATCAA CAAATTAGGG GGTAGAGAAT ATATTCATGA CTTGGTGATT   
  
  
+ TAAATTGTTT TAATCTTATC AGGTTTATT  

- CTGAGCTTTG ACTTTAATTA ATTTGACTCT AACATTAACT AAATCAAACA CACCTCTTTA TTACACAAAA   
  
  
- CCATCACTTC CCCACTCCCA ATTAATTTCT CATTAATGCC ACCCCCTTTG CCAAATCATC TGTTATTATT   
  
  
- ATCACTTAAT GTTAAACTTT TTCAAAAGGG ATTTTGATAT TTTGATAATT TAACATATTA ACAAAAAAAT   
  
  
- AAAAAATTTT AATAATTAAT TTAATAAATT ATCATGTGTC ATCATCTTTT TAAAAGATCA ACACAATTTA   
  
  
- AATAAATCAA AAATTCAAAT TCAATCAAAA AAATATTGGT GTACCAAAAA ATTTGATTCA AACTATAAAA   
  
  
- ATTTAACACG TAATAATTTA ATAAAATAAA TAAATAATTT TTTATTTTTA TGTATCATTA TAAAATTATT   
  
  
- AAAAAAATAC CTTTTAAACT TCAATTTAAA TGAATACAAA TTTTATTATA TATATGATAA TAAATTTTGG   
  
  
- GTAAAATCTC ATCAAACCTT CAAGTTTGGT TATAAAAAAT TAAAGAGATT GTTGTTATTG AAATGTTTCA   
  
  
- AAATAATTCA AATGAATTGT TTTTTATTTA AATTTTATTT TTCAAATTTA AATATTTTTA AATATAATAT   
  
  
- TTATTTTTTG TGAATTTTTT AATTTTTTGA TAGTATAAAA GTTAAAATAT ATATTTTCAG TACTCATTAA   
  
  
- AGTTTAAAAA AAAAATTATG ATGATAGCTT GAATATTTTT TTACGTATGT AAAAATTTTT AAGTCAAAAT   
  
  
- TCATTATATA AGATTTTCAA TTTAAATATC TCTAATTTTT AAAGTTTTTG ATAAAATTCA ATTTAGATTA   
  
  
- CGGTTCAATG TTAAATTATT TTTGAATATA CAAATTTAAT TAGTAGCATA AAATAAATAT CAATTTTTTA   
  
  
- TGGTATTTAT ATTTTAATTT TTAAAAATTT ATTTTTAATT AATATAATAA ATTATAAAAA AATCATTCCC   
  
  
- CCTTATACCA TCATAAGTTC TTCAAATTAC TTTTAACATT TTTCAATTAT GGATCTTCAT ACATGCATGC   
  
  
- AAAGCTTGCA TATCTATGTA TGTACTATAT ATATATTTAA TCAATTATAC ATGCATGCAT GCACATGCAT   
  
  
- AACATATGTA TATAGCTATG CATTACCGTA TGAGAAGAGA TAATTAATGA TAAACAATTT GATTACATCA   
  
  
- TCTTGCCCAC ATGGATATTA ATAAGAGCGA GTCTTATACG AAATCCGATG AGAGGAAAGC CTCGGGATCA   
  
  
- CATCTTAGAA ATTGTTTTAC GGGCTTGACA TGGTCTTCCC CACATGGTAT CCGAGCCTCG TACGAAATCC   
  
  
- CATGAGAAGA GAGTCTTTTG GGATCACATC TTGAAATCGT CAACTCCAAT GCCTCTCCTT AATCAATCAG   
  
  
- TAGATTTAAT TAATTAATCT CAACTTAGTT GTTTAATCCC CCATCTCTTA TATAAGTACT GAACCACTAA   
  
  
- ATTTAACAAA ATTAGAATAG TCCAAATAA

+     G-box

| Site Name | Organism | Position | Strand | Matrix score. | sequence | function |
| --- | --- | --- | --- | --- | --- | --- |
| G-box | Daucus carota | 1109 | + | 6 | TACGTG | cis-acting regulatory element involved in light responsiveness |
| G-box | Solanum tuberosum | 319 | + | 7 | CACATGG | cis-acting regulatory element involved in light responsiveness |
| G-box | Oryza sativa | 1108 | + | 7 | GTACGTG | cis-acting regulatory element involved in light responsiveness |

> 2018/04/13 10:10:12  
+ GACTCGAAAC TGAAATTAAT TAAACTGAGA TTGTAATTGA TTTAGTTTGT GTGGAGAAAT AATGTGTTTT   
  
  
+ GGTAGTGAAG GGGTGAGGGT TAATTAAAGA GTAATTACGG TGGGGGAAAC GGTTTAGTAG ACAATAATAA   
  
  
+ TAGTGAATTA CAATTTGAAA AAGTTTTCCC TAAAACTATA AAACTATTAA ATTGTATAAT TGTTTTTTTA   
  
  
+ TTTTTTAAAA TTATTAATTA AATTATTTAA TAGTACACAG TAGTAGAAAA ATTTTCTAGT TGTGTTAAAT   
  
  
+ TTATTTAGTT TTTAAGTTTA AGTTAGTTTT TTTATAACCA CATGGTTTTT TAAACTAAGT TTGATATTTT   
  
  
+ TAAATTGTGC ATTATTAAAT TATTTTATTT ATTTATTAAA AAATAAAAAT ACATAGTAAT ATTTTAATAA   
  
  
+ TTTTTTTATG GAAAATTTGA AGTTAAATTT ACTTATGTTT AAAATAATAT ATATACTATT ATTTAAAACC   
  
  
+ CATTTTAGAG TAGTTTGGAA GTTCAAACCA ATATTTTTTA ATTTCTCTAA CAACAATAAC TTTACAAAGT   
  
  
+ TTTATTAAGT TTACTTAACA AAAAATAAAT TTAAAATAAA AAGTTTAAAT TTATAAAAAT TTATATTATA   
  
  
+ AATAAAAAAC ACTTAAAAAA TTAAAAAACT ATCATATTTT CAATTTTATA TATAAAAGTC ATGAGTAATT   
  
  
+ TCAAATTTTT TTTTTAATAC TACTATCGAA CTTATAAAAA AATGCATACA TTTTTAAAAA TTCAGTTTTA   
  
  
+ AGTAATATAT TCTAAAAGTT AAATTTATAG AGATTAAAAA TTTCAAAAAC TATTTTAAGT TAAATCTAAT   
  
  
+ GCCAAGTTAC AATTTAATAA AAACTTATAT GTTTAAATTA ATCATCGTAT TTTATTTATA GTTAAAAAAT   
  
  
+ ACCATAAATA TAAAATTAAA AATTTTTAAA TAAAAATTAA TTATATTATT TAATATTTTT TTAGTAAGGG   
  
  
+ GGAATATGGT AGTATTCAAG AAGTTTAATG AAAATTGTAA AAAGTTAATA CCTAGAAGTA TGTACGTACG   
  
  
+ TTTCGAACGT ATAGATACAT ACATGATATA TATATAAATT AGTTAATATG TACGTACGTA CGTGTACGTA   
  
  
+ TTGTATACAT ATATCGATAC GTAATGGCAT ACTCTTCTCT ATTAATTACT ATTTGTTAAA CTAATGTAGT   
  
  
+ AGAACGGGTG TACCTATAAT TATTCTCGCT CAGAATATGC TTTAGGCTAC TCTCCTTTCG GAGCCCTAGT   
  
  
+ GTAGAATCTT TAACAAAATG CCCGAACTGT ACCAGAAGGG GTGTACCATA GGCTCGGAGC ATGCTTTAGG   
  
  
+ GTACTCTTCT CTCAGAAAAC CCTAGTGTAG AACTTTAGCA GTTGAGGTTA CGGAGAGGAA TTAGTTAGTC   
  
  
+ ATCTAAATTA ATTAATTAGA GTTGAATCAA CAAATTAGGG GGTAGAGAAT ATATTCATGA CTTGGTGATT   
  
  
+ TAAATTGTTT TAATCTTATC AGGTTTATT  

- CTGAGCTTTG ACTTTAATTA ATTTGACTCT AACATTAACT AAATCAAACA CACCTCTTTA TTACACAAAA   
  
  
- CCATCACTTC CCCACTCCCA ATTAATTTCT CATTAATGCC ACCCCCTTTG CCAAATCATC TGTTATTATT   
  
  
- ATCACTTAAT GTTAAACTTT TTCAAAAGGG ATTTTGATAT TTTGATAATT TAACATATTA ACAAAAAAAT   
  
  
- AAAAAATTTT AATAATTAAT TTAATAAATT ATCATGTGTC ATCATCTTTT TAAAAGATCA ACACAATTTA   
  
  
- AATAAATCAA AAATTCAAAT TCAATCAAAA AAATATTGGT GTACCAAAAA ATTTGATTCA AACTATAAAA   
  
  
- ATTTAACACG TAATAATTTA ATAAAATAAA TAAATAATTT TTTATTTTTA TGTATCATTA TAAAATTATT   
  
  
- AAAAAAATAC CTTTTAAACT TCAATTTAAA TGAATACAAA TTTTATTATA TATATGATAA TAAATTTTGG   
  
  
- GTAAAATCTC ATCAAACCTT CAAGTTTGGT TATAAAAAAT TAAAGAGATT GTTGTTATTG AAATGTTTCA   
  
  
- AAATAATTCA AATGAATTGT TTTTTATTTA AATTTTATTT TTCAAATTTA AATATTTTTA AATATAATAT   
  
  
- TTATTTTTTG TGAATTTTTT AATTTTTTGA TAGTATAAAA GTTAAAATAT ATATTTTCAG TACTCATTAA   
  
  
- AGTTTAAAAA AAAAATTATG ATGATAGCTT GAATATTTTT TTACGTATGT AAAAATTTTT AAGTCAAAAT   
  
  
- TCATTATATA AGATTTTCAA TTTAAATATC TCTAATTTTT AAAGTTTTTG ATAAAATTCA ATTTAGATTA   
  
  
- CGGTTCAATG TTAAATTATT TTTGAATATA CAAATTTAAT TAGTAGCATA AAATAAATAT CAATTTTTTA   
  
  
- TGGTATTTAT ATTTTAATTT TTAAAAATTT ATTTTTAATT AATATAATAA ATTATAAAAA AATCATTCCC   
  
  
- CCTTATACCA TCATAAGTTC TTCAAATTAC TTTTAACATT TTTCAATTAT GGATCTTCAT ACATGCATGC   
  
  
- AAAGCTTGCA TATCTATGTA TGTACTATAT ATATATTTAA TCAATTATAC ATGCATGCAT GCACATGCAT   
  
  
- AACATATGTA TATAGCTATG CATTACCGTA TGAGAAGAGA TAATTAATGA TAAACAATTT GATTACATCA   
  
  
- TCTTGCCCAC ATGGATATTA ATAAGAGCGA GTCTTATACG AAATCCGATG AGAGGAAAGC CTCGGGATCA   
  
  
- CATCTTAGAA ATTGTTTTAC GGGCTTGACA TGGTCTTCCC CACATGGTAT CCGAGCCTCG TACGAAATCC   
  
  
- CATGAGAAGA GAGTCTTTTG GGATCACATC TTGAAATCGT CAACTCCAAT GCCTCTCCTT AATCAATCAG   
  
  
- TAGATTTAAT TAATTAATCT CAACTTAGTT GTTTAATCCC CCATCTCTTA TATAAGTACT GAACCACTAA   
  
  
- ATTTAACAAA ATTAGAATAG TCCAAATAA

+     GT1-motif

| Site Name | Organism | Position | Strand | Matrix score. | sequence | function |
| --- | --- | --- | --- | --- | --- | --- |
| GT1-motif | Avena sativa | 88 | + | 7 | GGTTAAT | light responsive element |

> 2018/04/13 10:10:12  
+ GACTCGAAAC TGAAATTAAT TAAACTGAGA TTGTAATTGA TTTAGTTTGT GTGGAGAAAT AATGTGTTTT   
  
  
+ GGTAGTGAAG GGGTGAGGGT TAATTAAAGA GTAATTACGG TGGGGGAAAC GGTTTAGTAG ACAATAATAA   
  
  
+ TAGTGAATTA CAATTTGAAA AAGTTTTCCC TAAAACTATA AAACTATTAA ATTGTATAAT TGTTTTTTTA   
  
  
+ TTTTTTAAAA TTATTAATTA AATTATTTAA TAGTACACAG TAGTAGAAAA ATTTTCTAGT TGTGTTAAAT   
  
  
+ TTATTTAGTT TTTAAGTTTA AGTTAGTTTT TTTATAACCA CATGGTTTTT TAAACTAAGT TTGATATTTT   
  
  
+ TAAATTGTGC ATTATTAAAT TATTTTATTT ATTTATTAAA AAATAAAAAT ACATAGTAAT ATTTTAATAA   
  
  
+ TTTTTTTATG GAAAATTTGA AGTTAAATTT ACTTATGTTT AAAATAATAT ATATACTATT ATTTAAAACC   
  
  
+ CATTTTAGAG TAGTTTGGAA GTTCAAACCA ATATTTTTTA ATTTCTCTAA CAACAATAAC TTTACAAAGT   
  
  
+ TTTATTAAGT TTACTTAACA AAAAATAAAT TTAAAATAAA AAGTTTAAAT TTATAAAAAT TTATATTATA   
  
  
+ AATAAAAAAC ACTTAAAAAA TTAAAAAACT ATCATATTTT CAATTTTATA TATAAAAGTC ATGAGTAATT   
  
  
+ TCAAATTTTT TTTTTAATAC TACTATCGAA CTTATAAAAA AATGCATACA TTTTTAAAAA TTCAGTTTTA   
  
  
+ AGTAATATAT TCTAAAAGTT AAATTTATAG AGATTAAAAA TTTCAAAAAC TATTTTAAGT TAAATCTAAT   
  
  
+ GCCAAGTTAC AATTTAATAA AAACTTATAT GTTTAAATTA ATCATCGTAT TTTATTTATA GTTAAAAAAT   
  
  
+ ACCATAAATA TAAAATTAAA AATTTTTAAA TAAAAATTAA TTATATTATT TAATATTTTT TTAGTAAGGG   
  
  
+ GGAATATGGT AGTATTCAAG AAGTTTAATG AAAATTGTAA AAAGTTAATA CCTAGAAGTA TGTACGTACG   
  
  
+ TTTCGAACGT ATAGATACAT ACATGATATA TATATAAATT AGTTAATATG TACGTACGTA CGTGTACGTA   
  
  
+ TTGTATACAT ATATCGATAC GTAATGGCAT ACTCTTCTCT ATTAATTACT ATTTGTTAAA CTAATGTAGT   
  
  
+ AGAACGGGTG TACCTATAAT TATTCTCGCT CAGAATATGC TTTAGGCTAC TCTCCTTTCG GAGCCCTAGT   
  
  
+ GTAGAATCTT TAACAAAATG CCCGAACTGT ACCAGAAGGG GTGTACCATA GGCTCGGAGC ATGCTTTAGG   
  
  
+ GTACTCTTCT CTCAGAAAAC CCTAGTGTAG AACTTTAGCA GTTGAGGTTA CGGAGAGGAA TTAGTTAGTC   
  
  
+ ATCTAAATTA ATTAATTAGA GTTGAATCAA CAAATTAGGG GGTAGAGAAT ATATTCATGA CTTGGTGATT   
  
  
+ TAAATTGTTT TAATCTTATC AGGTTTATT  

- CTGAGCTTTG ACTTTAATTA ATTTGACTCT AACATTAACT AAATCAAACA CACCTCTTTA TTACACAAAA   
  
  
- CCATCACTTC CCCACTCCCA ATTAATTTCT CATTAATGCC ACCCCCTTTG CCAAATCATC TGTTATTATT   
  
  
- ATCACTTAAT GTTAAACTTT TTCAAAAGGG ATTTTGATAT TTTGATAATT TAACATATTA ACAAAAAAAT   
  
  
- AAAAAATTTT AATAATTAAT TTAATAAATT ATCATGTGTC ATCATCTTTT TAAAAGATCA ACACAATTTA   
  
  
- AATAAATCAA AAATTCAAAT TCAATCAAAA AAATATTGGT GTACCAAAAA ATTTGATTCA AACTATAAAA   
  
  
- ATTTAACACG TAATAATTTA ATAAAATAAA TAAATAATTT TTTATTTTTA TGTATCATTA TAAAATTATT   
  
  
- AAAAAAATAC CTTTTAAACT TCAATTTAAA TGAATACAAA TTTTATTATA TATATGATAA TAAATTTTGG   
  
  
- GTAAAATCTC ATCAAACCTT CAAGTTTGGT TATAAAAAAT TAAAGAGATT GTTGTTATTG AAATGTTTCA   
  
  
- AAATAATTCA AATGAATTGT TTTTTATTTA AATTTTATTT TTCAAATTTA AATATTTTTA AATATAATAT   
  
  
- TTATTTTTTG TGAATTTTTT AATTTTTTGA TAGTATAAAA GTTAAAATAT ATATTTTCAG TACTCATTAA   
  
  
- AGTTTAAAAA AAAAATTATG ATGATAGCTT GAATATTTTT TTACGTATGT AAAAATTTTT AAGTCAAAAT   
  
  
- TCATTATATA AGATTTTCAA TTTAAATATC TCTAATTTTT AAAGTTTTTG ATAAAATTCA ATTTAGATTA   
  
  
- CGGTTCAATG TTAAATTATT TTTGAATATA CAAATTTAAT TAGTAGCATA AAATAAATAT CAATTTTTTA   
  
  
- TGGTATTTAT ATTTTAATTT TTAAAAATTT ATTTTTAATT AATATAATAA ATTATAAAAA AATCATTCCC   
  
  
- CCTTATACCA TCATAAGTTC TTCAAATTAC TTTTAACATT TTTCAATTAT GGATCTTCAT ACATGCATGC   
  
  
- AAAGCTTGCA TATCTATGTA TGTACTATAT ATATATTTAA TCAATTATAC ATGCATGCAT GCACATGCAT   
  
  
- AACATATGTA TATAGCTATG CATTACCGTA TGAGAAGAGA TAATTAATGA TAAACAATTT GATTACATCA   
  
  
- TCTTGCCCAC ATGGATATTA ATAAGAGCGA GTCTTATACG AAATCCGATG AGAGGAAAGC CTCGGGATCA   
  
  
- CATCTTAGAA ATTGTTTTAC GGGCTTGACA TGGTCTTCCC CACATGGTAT CCGAGCCTCG TACGAAATCC   
  
  
- CATGAGAAGA GAGTCTTTTG GGATCACATC TTGAAATCGT CAACTCCAAT GCCTCTCCTT AATCAATCAG   
  
  
- TAGATTTAAT TAATTAATCT CAACTTAGTT GTTTAATCCC CCATCTCTTA TATAAGTACT GAACCACTAA   
  
  
- ATTTAACAAA ATTAGAATAG TCCAAATAA

+     HSE

| Site Name | Organism | Position | Strand | Matrix score. | sequence | function |
| --- | --- | --- | --- | --- | --- | --- |
| HSE | Brassica oleracea | 805 | + | 9 | AAAAAATTTC | cis-acting element involved in heat stress responsiveness |
| HSE | Brassica oleracea | 702 | - | 9 | AAAAAATTTC | cis-acting element involved in heat stress responsiveness |
| HSE | Brassica oleracea | 257 | + | 9 | AAAAAATTTC | cis-acting element involved in heat stress responsiveness |

> 2018/04/13 10:10:12  
+ GACTCGAAAC TGAAATTAAT TAAACTGAGA TTGTAATTGA TTTAGTTTGT GTGGAGAAAT AATGTGTTTT   
  
  
+ GGTAGTGAAG GGGTGAGGGT TAATTAAAGA GTAATTACGG TGGGGGAAAC GGTTTAGTAG ACAATAATAA   
  
  
+ TAGTGAATTA CAATTTGAAA AAGTTTTCCC TAAAACTATA AAACTATTAA ATTGTATAAT TGTTTTTTTA   
  
  
+ TTTTTTAAAA TTATTAATTA AATTATTTAA TAGTACACAG TAGTAGAAAA ATTTTCTAGT TGTGTTAAAT   
  
  
+ TTATTTAGTT TTTAAGTTTA AGTTAGTTTT TTTATAACCA CATGGTTTTT TAAACTAAGT TTGATATTTT   
  
  
+ TAAATTGTGC ATTATTAAAT TATTTTATTT ATTTATTAAA AAATAAAAAT ACATAGTAAT ATTTTAATAA   
  
  
+ TTTTTTTATG GAAAATTTGA AGTTAAATTT ACTTATGTTT AAAATAATAT ATATACTATT ATTTAAAACC   
  
  
+ CATTTTAGAG TAGTTTGGAA GTTCAAACCA ATATTTTTTA ATTTCTCTAA CAACAATAAC TTTACAAAGT   
  
  
+ TTTATTAAGT TTACTTAACA AAAAATAAAT TTAAAATAAA AAGTTTAAAT TTATAAAAAT TTATATTATA   
  
  
+ AATAAAAAAC ACTTAAAAAA TTAAAAAACT ATCATATTTT CAATTTTATA TATAAAAGTC ATGAGTAATT   
  
  
+ TCAAATTTTT TTTTTAATAC TACTATCGAA CTTATAAAAA AATGCATACA TTTTTAAAAA TTCAGTTTTA   
  
  
+ AGTAATATAT TCTAAAAGTT AAATTTATAG AGATTAAAAA TTTCAAAAAC TATTTTAAGT TAAATCTAAT   
  
  
+ GCCAAGTTAC AATTTAATAA AAACTTATAT GTTTAAATTA ATCATCGTAT TTTATTTATA GTTAAAAAAT   
  
  
+ ACCATAAATA TAAAATTAAA AATTTTTAAA TAAAAATTAA TTATATTATT TAATATTTTT TTAGTAAGGG   
  
  
+ GGAATATGGT AGTATTCAAG AAGTTTAATG AAAATTGTAA AAAGTTAATA CCTAGAAGTA TGTACGTACG   
  
  
+ TTTCGAACGT ATAGATACAT ACATGATATA TATATAAATT AGTTAATATG TACGTACGTA CGTGTACGTA   
  
  
+ TTGTATACAT ATATCGATAC GTAATGGCAT ACTCTTCTCT ATTAATTACT ATTTGTTAAA CTAATGTAGT   
  
  
+ AGAACGGGTG TACCTATAAT TATTCTCGCT CAGAATATGC TTTAGGCTAC TCTCCTTTCG GAGCCCTAGT   
  
  
+ GTAGAATCTT TAACAAAATG CCCGAACTGT ACCAGAAGGG GTGTACCATA GGCTCGGAGC ATGCTTTAGG   
  
  
+ GTACTCTTCT CTCAGAAAAC CCTAGTGTAG AACTTTAGCA GTTGAGGTTA CGGAGAGGAA TTAGTTAGTC   
  
  
+ ATCTAAATTA ATTAATTAGA GTTGAATCAA CAAATTAGGG GGTAGAGAAT ATATTCATGA CTTGGTGATT   
  
  
+ TAAATTGTTT TAATCTTATC AGGTTTATT  

- CTGAGCTTTG ACTTTAATTA ATTTGACTCT AACATTAACT AAATCAAACA CACCTCTTTA TTACACAAAA   
  
  
- CCATCACTTC CCCACTCCCA ATTAATTTCT CATTAATGCC ACCCCCTTTG CCAAATCATC TGTTATTATT   
  
  
- ATCACTTAAT GTTAAACTTT TTCAAAAGGG ATTTTGATAT TTTGATAATT TAACATATTA ACAAAAAAAT   
  
  
- AAAAAATTTT AATAATTAAT TTAATAAATT ATCATGTGTC ATCATCTTTT TAAAAGATCA ACACAATTTA   
  
  
- AATAAATCAA AAATTCAAAT TCAATCAAAA AAATATTGGT GTACCAAAAA ATTTGATTCA AACTATAAAA   
  
  
- ATTTAACACG TAATAATTTA ATAAAATAAA TAAATAATTT TTTATTTTTA TGTATCATTA TAAAATTATT   
  
  
- AAAAAAATAC CTTTTAAACT TCAATTTAAA TGAATACAAA TTTTATTATA TATATGATAA TAAATTTTGG   
  
  
- GTAAAATCTC ATCAAACCTT CAAGTTTGGT TATAAAAAAT TAAAGAGATT GTTGTTATTG AAATGTTTCA   
  
  
- AAATAATTCA AATGAATTGT TTTTTATTTA AATTTTATTT TTCAAATTTA AATATTTTTA AATATAATAT   
  
  
- TTATTTTTTG TGAATTTTTT AATTTTTTGA TAGTATAAAA GTTAAAATAT ATATTTTCAG TACTCATTAA   
  
  
- AGTTTAAAAA AAAAATTATG ATGATAGCTT GAATATTTTT TTACGTATGT AAAAATTTTT AAGTCAAAAT   
  
  
- TCATTATATA AGATTTTCAA TTTAAATATC TCTAATTTTT AAAGTTTTTG ATAAAATTCA ATTTAGATTA   
  
  
- CGGTTCAATG TTAAATTATT TTTGAATATA CAAATTTAAT TAGTAGCATA AAATAAATAT CAATTTTTTA   
  
  
- TGGTATTTAT ATTTTAATTT TTAAAAATTT ATTTTTAATT AATATAATAA ATTATAAAAA AATCATTCCC   
  
  
- CCTTATACCA TCATAAGTTC TTCAAATTAC TTTTAACATT TTTCAATTAT GGATCTTCAT ACATGCATGC   
  
  
- AAAGCTTGCA TATCTATGTA TGTACTATAT ATATATTTAA TCAATTATAC ATGCATGCAT GCACATGCAT   
  
  
- AACATATGTA TATAGCTATG CATTACCGTA TGAGAAGAGA TAATTAATGA TAAACAATTT GATTACATCA   
  
  
- TCTTGCCCAC ATGGATATTA ATAAGAGCGA GTCTTATACG AAATCCGATG AGAGGAAAGC CTCGGGATCA   
  
  
- CATCTTAGAA ATTGTTTTAC GGGCTTGACA TGGTCTTCCC CACATGGTAT CCGAGCCTCG TACGAAATCC   
  
  
- CATGAGAAGA GAGTCTTTTG GGATCACATC TTGAAATCGT CAACTCCAAT GCCTCTCCTT AATCAATCAG   
  
  
- TAGATTTAAT TAATTAATCT CAACTTAGTT GTTTAATCCC CCATCTCTTA TATAAGTACT GAACCACTAA   
  
  
- ATTTAACAAA ATTAGAATAG TCCAAATAA

+     I-box

| Site Name | Organism | Position | Strand | Matrix score. | sequence | function |
| --- | --- | --- | --- | --- | --- | --- |
| I-box | Arabidopsis thaliana | 1482 | - | 9 | GATAAGATT | part of a light responsive element |

> 2018/04/13 10:10:12  
+ GACTCGAAAC TGAAATTAAT TAAACTGAGA TTGTAATTGA TTTAGTTTGT GTGGAGAAAT AATGTGTTTT   
  
  
+ GGTAGTGAAG GGGTGAGGGT TAATTAAAGA GTAATTACGG TGGGGGAAAC GGTTTAGTAG ACAATAATAA   
  
  
+ TAGTGAATTA CAATTTGAAA AAGTTTTCCC TAAAACTATA AAACTATTAA ATTGTATAAT TGTTTTTTTA   
  
  
+ TTTTTTAAAA TTATTAATTA AATTATTTAA TAGTACACAG TAGTAGAAAA ATTTTCTAGT TGTGTTAAAT   
  
  
+ TTATTTAGTT TTTAAGTTTA AGTTAGTTTT TTTATAACCA CATGGTTTTT TAAACTAAGT TTGATATTTT   
  
  
+ TAAATTGTGC ATTATTAAAT TATTTTATTT ATTTATTAAA AAATAAAAAT ACATAGTAAT ATTTTAATAA   
  
  
+ TTTTTTTATG GAAAATTTGA AGTTAAATTT ACTTATGTTT AAAATAATAT ATATACTATT ATTTAAAACC   
  
  
+ CATTTTAGAG TAGTTTGGAA GTTCAAACCA ATATTTTTTA ATTTCTCTAA CAACAATAAC TTTACAAAGT   
  
  
+ TTTATTAAGT TTACTTAACA AAAAATAAAT TTAAAATAAA AAGTTTAAAT TTATAAAAAT TTATATTATA   
  
  
+ AATAAAAAAC ACTTAAAAAA TTAAAAAACT ATCATATTTT CAATTTTATA TATAAAAGTC ATGAGTAATT   
  
  
+ TCAAATTTTT TTTTTAATAC TACTATCGAA CTTATAAAAA AATGCATACA TTTTTAAAAA TTCAGTTTTA   
  
  
+ AGTAATATAT TCTAAAAGTT AAATTTATAG AGATTAAAAA TTTCAAAAAC TATTTTAAGT TAAATCTAAT   
  
  
+ GCCAAGTTAC AATTTAATAA AAACTTATAT GTTTAAATTA ATCATCGTAT TTTATTTATA GTTAAAAAAT   
  
  
+ ACCATAAATA TAAAATTAAA AATTTTTAAA TAAAAATTAA TTATATTATT TAATATTTTT TTAGTAAGGG   
  
  
+ GGAATATGGT AGTATTCAAG AAGTTTAATG AAAATTGTAA AAAGTTAATA CCTAGAAGTA TGTACGTACG   
  
  
+ TTTCGAACGT ATAGATACAT ACATGATATA TATATAAATT AGTTAATATG TACGTACGTA CGTGTACGTA   
  
  
+ TTGTATACAT ATATCGATAC GTAATGGCAT ACTCTTCTCT ATTAATTACT ATTTGTTAAA CTAATGTAGT   
  
  
+ AGAACGGGTG TACCTATAAT TATTCTCGCT CAGAATATGC TTTAGGCTAC TCTCCTTTCG GAGCCCTAGT   
  
  
+ GTAGAATCTT TAACAAAATG CCCGAACTGT ACCAGAAGGG GTGTACCATA GGCTCGGAGC ATGCTTTAGG   
  
  
+ GTACTCTTCT CTCAGAAAAC CCTAGTGTAG AACTTTAGCA GTTGAGGTTA CGGAGAGGAA TTAGTTAGTC   
  
  
+ ATCTAAATTA ATTAATTAGA GTTGAATCAA CAAATTAGGG GGTAGAGAAT ATATTCATGA CTTGGTGATT   
  
  
+ TAAATTGTTT TAATCTTATC AGGTTTATT  

- CTGAGCTTTG ACTTTAATTA ATTTGACTCT AACATTAACT AAATCAAACA CACCTCTTTA TTACACAAAA   
  
  
- CCATCACTTC CCCACTCCCA ATTAATTTCT CATTAATGCC ACCCCCTTTG CCAAATCATC TGTTATTATT   
  
  
- ATCACTTAAT GTTAAACTTT TTCAAAAGGG ATTTTGATAT TTTGATAATT TAACATATTA ACAAAAAAAT   
  
  
- AAAAAATTTT AATAATTAAT TTAATAAATT ATCATGTGTC ATCATCTTTT TAAAAGATCA ACACAATTTA   
  
  
- AATAAATCAA AAATTCAAAT TCAATCAAAA AAATATTGGT GTACCAAAAA ATTTGATTCA AACTATAAAA   
  
  
- ATTTAACACG TAATAATTTA ATAAAATAAA TAAATAATTT TTTATTTTTA TGTATCATTA TAAAATTATT   
  
  
- AAAAAAATAC CTTTTAAACT TCAATTTAAA TGAATACAAA TTTTATTATA TATATGATAA TAAATTTTGG   
  
  
- GTAAAATCTC ATCAAACCTT CAAGTTTGGT TATAAAAAAT TAAAGAGATT GTTGTTATTG AAATGTTTCA   
  
  
- AAATAATTCA AATGAATTGT TTTTTATTTA AATTTTATTT TTCAAATTTA AATATTTTTA AATATAATAT   
  
  
- TTATTTTTTG TGAATTTTTT AATTTTTTGA TAGTATAAAA GTTAAAATAT ATATTTTCAG TACTCATTAA   
  
  
- AGTTTAAAAA AAAAATTATG ATGATAGCTT GAATATTTTT TTACGTATGT AAAAATTTTT AAGTCAAAAT   
  
  
- TCATTATATA AGATTTTCAA TTTAAATATC TCTAATTTTT AAAGTTTTTG ATAAAATTCA ATTTAGATTA   
  
  
- CGGTTCAATG TTAAATTATT TTTGAATATA CAAATTTAAT TAGTAGCATA AAATAAATAT CAATTTTTTA   
  
  
- TGGTATTTAT ATTTTAATTT TTAAAAATTT ATTTTTAATT AATATAATAA ATTATAAAAA AATCATTCCC   
  
  
- CCTTATACCA TCATAAGTTC TTCAAATTAC TTTTAACATT TTTCAATTAT GGATCTTCAT ACATGCATGC   
  
  
- AAAGCTTGCA TATCTATGTA TGTACTATAT ATATATTTAA TCAATTATAC ATGCATGCAT GCACATGCAT   
  
  
- AACATATGTA TATAGCTATG CATTACCGTA TGAGAAGAGA TAATTAATGA TAAACAATTT GATTACATCA   
  
  
- TCTTGCCCAC ATGGATATTA ATAAGAGCGA GTCTTATACG AAATCCGATG AGAGGAAAGC CTCGGGATCA   
  
  
- CATCTTAGAA ATTGTTTTAC GGGCTTGACA TGGTCTTCCC CACATGGTAT CCGAGCCTCG TACGAAATCC   
  
  
- CATGAGAAGA GAGTCTTTTG GGATCACATC TTGAAATCGT CAACTCCAAT GCCTCTCCTT AATCAATCAG   
  
  
- TAGATTTAAT TAATTAATCT CAACTTAGTT GTTTAATCCC CCATCTCTTA TATAAGTACT GAACCACTAA   
  
  
- ATTTAACAAA ATTAGAATAG TCCAAATAA

+     LTR

| Site Name | Organism | Position | Strand | Matrix score. | sequence | function |
| --- | --- | --- | --- | --- | --- | --- |
| LTR | Hordeum vulgare | 1246 | - | 6 | CCGAAA | cis-acting element involved in low-temperature responsiveness |

> 2018/04/13 10:10:12  
+ GACTCGAAAC TGAAATTAAT TAAACTGAGA TTGTAATTGA TTTAGTTTGT GTGGAGAAAT AATGTGTTTT   
  
  
+ GGTAGTGAAG GGGTGAGGGT TAATTAAAGA GTAATTACGG TGGGGGAAAC GGTTTAGTAG ACAATAATAA   
  
  
+ TAGTGAATTA CAATTTGAAA AAGTTTTCCC TAAAACTATA AAACTATTAA ATTGTATAAT TGTTTTTTTA   
  
  
+ TTTTTTAAAA TTATTAATTA AATTATTTAA TAGTACACAG TAGTAGAAAA ATTTTCTAGT TGTGTTAAAT   
  
  
+ TTATTTAGTT TTTAAGTTTA AGTTAGTTTT TTTATAACCA CATGGTTTTT TAAACTAAGT TTGATATTTT   
  
  
+ TAAATTGTGC ATTATTAAAT TATTTTATTT ATTTATTAAA AAATAAAAAT ACATAGTAAT ATTTTAATAA   
  
  
+ TTTTTTTATG GAAAATTTGA AGTTAAATTT ACTTATGTTT AAAATAATAT ATATACTATT ATTTAAAACC   
  
  
+ CATTTTAGAG TAGTTTGGAA GTTCAAACCA ATATTTTTTA ATTTCTCTAA CAACAATAAC TTTACAAAGT   
  
  
+ TTTATTAAGT TTACTTAACA AAAAATAAAT TTAAAATAAA AAGTTTAAAT TTATAAAAAT TTATATTATA   
  
  
+ AATAAAAAAC ACTTAAAAAA TTAAAAAACT ATCATATTTT CAATTTTATA TATAAAAGTC ATGAGTAATT   
  
  
+ TCAAATTTTT TTTTTAATAC TACTATCGAA CTTATAAAAA AATGCATACA TTTTTAAAAA TTCAGTTTTA   
  
  
+ AGTAATATAT TCTAAAAGTT AAATTTATAG AGATTAAAAA TTTCAAAAAC TATTTTAAGT TAAATCTAAT   
  
  
+ GCCAAGTTAC AATTTAATAA AAACTTATAT GTTTAAATTA ATCATCGTAT TTTATTTATA GTTAAAAAAT   
  
  
+ ACCATAAATA TAAAATTAAA AATTTTTAAA TAAAAATTAA TTATATTATT TAATATTTTT TTAGTAAGGG   
  
  
+ GGAATATGGT AGTATTCAAG AAGTTTAATG AAAATTGTAA AAAGTTAATA CCTAGAAGTA TGTACGTACG   
  
  
+ TTTCGAACGT ATAGATACAT ACATGATATA TATATAAATT AGTTAATATG TACGTACGTA CGTGTACGTA   
  
  
+ TTGTATACAT ATATCGATAC GTAATGGCAT ACTCTTCTCT ATTAATTACT ATTTGTTAAA CTAATGTAGT   
  
  
+ AGAACGGGTG TACCTATAAT TATTCTCGCT CAGAATATGC TTTAGGCTAC TCTCCTTTCG GAGCCCTAGT   
  
  
+ GTAGAATCTT TAACAAAATG CCCGAACTGT ACCAGAAGGG GTGTACCATA GGCTCGGAGC ATGCTTTAGG   
  
  
+ GTACTCTTCT CTCAGAAAAC CCTAGTGTAG AACTTTAGCA GTTGAGGTTA CGGAGAGGAA TTAGTTAGTC   
  
  
+ ATCTAAATTA ATTAATTAGA GTTGAATCAA CAAATTAGGG GGTAGAGAAT ATATTCATGA CTTGGTGATT   
  
  
+ TAAATTGTTT TAATCTTATC AGGTTTATT  

- CTGAGCTTTG ACTTTAATTA ATTTGACTCT AACATTAACT AAATCAAACA CACCTCTTTA TTACACAAAA   
  
  
- CCATCACTTC CCCACTCCCA ATTAATTTCT CATTAATGCC ACCCCCTTTG CCAAATCATC TGTTATTATT   
  
  
- ATCACTTAAT GTTAAACTTT TTCAAAAGGG ATTTTGATAT TTTGATAATT TAACATATTA ACAAAAAAAT   
  
  
- AAAAAATTTT AATAATTAAT TTAATAAATT ATCATGTGTC ATCATCTTTT TAAAAGATCA ACACAATTTA   
  
  
- AATAAATCAA AAATTCAAAT TCAATCAAAA AAATATTGGT GTACCAAAAA ATTTGATTCA AACTATAAAA   
  
  
- ATTTAACACG TAATAATTTA ATAAAATAAA TAAATAATTT TTTATTTTTA TGTATCATTA TAAAATTATT   
  
  
- AAAAAAATAC CTTTTAAACT TCAATTTAAA TGAATACAAA TTTTATTATA TATATGATAA TAAATTTTGG   
  
  
- GTAAAATCTC ATCAAACCTT CAAGTTTGGT TATAAAAAAT TAAAGAGATT GTTGTTATTG AAATGTTTCA   
  
  
- AAATAATTCA AATGAATTGT TTTTTATTTA AATTTTATTT TTCAAATTTA AATATTTTTA AATATAATAT   
  
  
- TTATTTTTTG TGAATTTTTT AATTTTTTGA TAGTATAAAA GTTAAAATAT ATATTTTCAG TACTCATTAA   
  
  
- AGTTTAAAAA AAAAATTATG ATGATAGCTT GAATATTTTT TTACGTATGT AAAAATTTTT AAGTCAAAAT   
  
  
- TCATTATATA AGATTTTCAA TTTAAATATC TCTAATTTTT AAAGTTTTTG ATAAAATTCA ATTTAGATTA   
  
  
- CGGTTCAATG TTAAATTATT TTTGAATATA CAAATTTAAT TAGTAGCATA AAATAAATAT CAATTTTTTA   
  
  
- TGGTATTTAT ATTTTAATTT TTAAAAATTT ATTTTTAATT AATATAATAA ATTATAAAAA AATCATTCCC   
  
  
- CCTTATACCA TCATAAGTTC TTCAAATTAC TTTTAACATT TTTCAATTAT GGATCTTCAT ACATGCATGC   
  
  
- AAAGCTTGCA TATCTATGTA TGTACTATAT ATATATTTAA TCAATTATAC ATGCATGCAT GCACATGCAT   
  
  
- AACATATGTA TATAGCTATG CATTACCGTA TGAGAAGAGA TAATTAATGA TAAACAATTT GATTACATCA   
  
  
- TCTTGCCCAC ATGGATATTA ATAAGAGCGA GTCTTATACG AAATCCGATG AGAGGAAAGC CTCGGGATCA   
  
  
- CATCTTAGAA ATTGTTTTAC GGGCTTGACA TGGTCTTCCC CACATGGTAT CCGAGCCTCG TACGAAATCC   
  
  
- CATGAGAAGA GAGTCTTTTG GGATCACATC TTGAAATCGT CAACTCCAAT GCCTCTCCTT AATCAATCAG   
  
  
- TAGATTTAAT TAATTAATCT CAACTTAGTT GTTTAATCCC CCATCTCTTA TATAAGTACT GAACCACTAA   
  
  
- ATTTAACAAA ATTAGAATAG TCCAAATAA

+     MBS

| Site Name | Organism | Position | Strand | Matrix score. | sequence | function |
| --- | --- | --- | --- | --- | --- | --- |
| MBS | Arabidopsis thaliana | 1369 | - | 6 | CAACTG | MYB binding site involved in drought-inducibility |

> 2018/04/13 10:10:12  
+ GACTCGAAAC TGAAATTAAT TAAACTGAGA TTGTAATTGA TTTAGTTTGT GTGGAGAAAT AATGTGTTTT   
  
  
+ GGTAGTGAAG GGGTGAGGGT TAATTAAAGA GTAATTACGG TGGGGGAAAC GGTTTAGTAG ACAATAATAA   
  
  
+ TAGTGAATTA CAATTTGAAA AAGTTTTCCC TAAAACTATA AAACTATTAA ATTGTATAAT TGTTTTTTTA   
  
  
+ TTTTTTAAAA TTATTAATTA AATTATTTAA TAGTACACAG TAGTAGAAAA ATTTTCTAGT TGTGTTAAAT   
  
  
+ TTATTTAGTT TTTAAGTTTA AGTTAGTTTT TTTATAACCA CATGGTTTTT TAAACTAAGT TTGATATTTT   
  
  
+ TAAATTGTGC ATTATTAAAT TATTTTATTT ATTTATTAAA AAATAAAAAT ACATAGTAAT ATTTTAATAA   
  
  
+ TTTTTTTATG GAAAATTTGA AGTTAAATTT ACTTATGTTT AAAATAATAT ATATACTATT ATTTAAAACC   
  
  
+ CATTTTAGAG TAGTTTGGAA GTTCAAACCA ATATTTTTTA ATTTCTCTAA CAACAATAAC TTTACAAAGT   
  
  
+ TTTATTAAGT TTACTTAACA AAAAATAAAT TTAAAATAAA AAGTTTAAAT TTATAAAAAT TTATATTATA   
  
  
+ AATAAAAAAC ACTTAAAAAA TTAAAAAACT ATCATATTTT CAATTTTATA TATAAAAGTC ATGAGTAATT   
  
  
+ TCAAATTTTT TTTTTAATAC TACTATCGAA CTTATAAAAA AATGCATACA TTTTTAAAAA TTCAGTTTTA   
  
  
+ AGTAATATAT TCTAAAAGTT AAATTTATAG AGATTAAAAA TTTCAAAAAC TATTTTAAGT TAAATCTAAT   
  
  
+ GCCAAGTTAC AATTTAATAA AAACTTATAT GTTTAAATTA ATCATCGTAT TTTATTTATA GTTAAAAAAT   
  
  
+ ACCATAAATA TAAAATTAAA AATTTTTAAA TAAAAATTAA TTATATTATT TAATATTTTT TTAGTAAGGG   
  
  
+ GGAATATGGT AGTATTCAAG AAGTTTAATG AAAATTGTAA AAAGTTAATA CCTAGAAGTA TGTACGTACG   
  
  
+ TTTCGAACGT ATAGATACAT ACATGATATA TATATAAATT AGTTAATATG TACGTACGTA CGTGTACGTA   
  
  
+ TTGTATACAT ATATCGATAC GTAATGGCAT ACTCTTCTCT ATTAATTACT ATTTGTTAAA CTAATGTAGT   
  
  
+ AGAACGGGTG TACCTATAAT TATTCTCGCT CAGAATATGC TTTAGGCTAC TCTCCTTTCG GAGCCCTAGT   
  
  
+ GTAGAATCTT TAACAAAATG CCCGAACTGT ACCAGAAGGG GTGTACCATA GGCTCGGAGC ATGCTTTAGG   
  
  
+ GTACTCTTCT CTCAGAAAAC CCTAGTGTAG AACTTTAGCA GTTGAGGTTA CGGAGAGGAA TTAGTTAGTC   
  
  
+ ATCTAAATTA ATTAATTAGA GTTGAATCAA CAAATTAGGG GGTAGAGAAT ATATTCATGA CTTGGTGATT   
  
  
+ TAAATTGTTT TAATCTTATC AGGTTTATT  

- CTGAGCTTTG ACTTTAATTA ATTTGACTCT AACATTAACT AAATCAAACA CACCTCTTTA TTACACAAAA   
  
  
- CCATCACTTC CCCACTCCCA ATTAATTTCT CATTAATGCC ACCCCCTTTG CCAAATCATC TGTTATTATT   
  
  
- ATCACTTAAT GTTAAACTTT TTCAAAAGGG ATTTTGATAT TTTGATAATT TAACATATTA ACAAAAAAAT   
  
  
- AAAAAATTTT AATAATTAAT TTAATAAATT ATCATGTGTC ATCATCTTTT TAAAAGATCA ACACAATTTA   
  
  
- AATAAATCAA AAATTCAAAT TCAATCAAAA AAATATTGGT GTACCAAAAA ATTTGATTCA AACTATAAAA   
  
  
- ATTTAACACG TAATAATTTA ATAAAATAAA TAAATAATTT TTTATTTTTA TGTATCATTA TAAAATTATT   
  
  
- AAAAAAATAC CTTTTAAACT TCAATTTAAA TGAATACAAA TTTTATTATA TATATGATAA TAAATTTTGG   
  
  
- GTAAAATCTC ATCAAACCTT CAAGTTTGGT TATAAAAAAT TAAAGAGATT GTTGTTATTG AAATGTTTCA   
  
  
- AAATAATTCA AATGAATTGT TTTTTATTTA AATTTTATTT TTCAAATTTA AATATTTTTA AATATAATAT   
  
  
- TTATTTTTTG TGAATTTTTT AATTTTTTGA TAGTATAAAA GTTAAAATAT ATATTTTCAG TACTCATTAA   
  
  
- AGTTTAAAAA AAAAATTATG ATGATAGCTT GAATATTTTT TTACGTATGT AAAAATTTTT AAGTCAAAAT   
  
  
- TCATTATATA AGATTTTCAA TTTAAATATC TCTAATTTTT AAAGTTTTTG ATAAAATTCA ATTTAGATTA   
  
  
- CGGTTCAATG TTAAATTATT TTTGAATATA CAAATTTAAT TAGTAGCATA AAATAAATAT CAATTTTTTA   
  
  
- TGGTATTTAT ATTTTAATTT TTAAAAATTT ATTTTTAATT AATATAATAA ATTATAAAAA AATCATTCCC   
  
  
- CCTTATACCA TCATAAGTTC TTCAAATTAC TTTTAACATT TTTCAATTAT GGATCTTCAT ACATGCATGC   
  
  
- AAAGCTTGCA TATCTATGTA TGTACTATAT ATATATTTAA TCAATTATAC ATGCATGCAT GCACATGCAT   
  
  
- AACATATGTA TATAGCTATG CATTACCGTA TGAGAAGAGA TAATTAATGA TAAACAATTT GATTACATCA   
  
  
- TCTTGCCCAC ATGGATATTA ATAAGAGCGA GTCTTATACG AAATCCGATG AGAGGAAAGC CTCGGGATCA   
  
  
- CATCTTAGAA ATTGTTTTAC GGGCTTGACA TGGTCTTCCC CACATGGTAT CCGAGCCTCG TACGAAATCC   
  
  
- CATGAGAAGA GAGTCTTTTG GGATCACATC TTGAAATCGT CAACTCCAAT GCCTCTCCTT AATCAATCAG   
  
  
- TAGATTTAAT TAATTAATCT CAACTTAGTT GTTTAATCCC CCATCTCTTA TATAAGTACT GAACCACTAA   
  
  
- ATTTAACAAA ATTAGAATAG TCCAAATAA

+     Skn-1\_motif

| Site Name | Organism | Position | Strand | Matrix score. | sequence | function |
| --- | --- | --- | --- | --- | --- | --- |
| Skn-1\_motif | Oryza sativa | 1398 | + | 5 | GTCAT | cis-acting regulatory element required for endosperm expression |
| Skn-1\_motif | Oryza sativa | 1457 | - | 5 | GTCAT | cis-acting regulatory element required for endosperm expression |
| Skn-1\_motif | Oryza sativa | 688 | + | 5 | GTCAT | cis-acting regulatory element required for endosperm expression |

> 2018/04/13 10:10:12  
+ GACTCGAAAC TGAAATTAAT TAAACTGAGA TTGTAATTGA TTTAGTTTGT GTGGAGAAAT AATGTGTTTT   
  
  
+ GGTAGTGAAG GGGTGAGGGT TAATTAAAGA GTAATTACGG TGGGGGAAAC GGTTTAGTAG ACAATAATAA   
  
  
+ TAGTGAATTA CAATTTGAAA AAGTTTTCCC TAAAACTATA AAACTATTAA ATTGTATAAT TGTTTTTTTA   
  
  
+ TTTTTTAAAA TTATTAATTA AATTATTTAA TAGTACACAG TAGTAGAAAA ATTTTCTAGT TGTGTTAAAT   
  
  
+ TTATTTAGTT TTTAAGTTTA AGTTAGTTTT TTTATAACCA CATGGTTTTT TAAACTAAGT TTGATATTTT   
  
  
+ TAAATTGTGC ATTATTAAAT TATTTTATTT ATTTATTAAA AAATAAAAAT ACATAGTAAT ATTTTAATAA   
  
  
+ TTTTTTTATG GAAAATTTGA AGTTAAATTT ACTTATGTTT AAAATAATAT ATATACTATT ATTTAAAACC   
  
  
+ CATTTTAGAG TAGTTTGGAA GTTCAAACCA ATATTTTTTA ATTTCTCTAA CAACAATAAC TTTACAAAGT   
  
  
+ TTTATTAAGT TTACTTAACA AAAAATAAAT TTAAAATAAA AAGTTTAAAT TTATAAAAAT TTATATTATA   
  
  
+ AATAAAAAAC ACTTAAAAAA TTAAAAAACT ATCATATTTT CAATTTTATA TATAAAAGTC ATGAGTAATT   
  
  
+ TCAAATTTTT TTTTTAATAC TACTATCGAA CTTATAAAAA AATGCATACA TTTTTAAAAA TTCAGTTTTA   
  
  
+ AGTAATATAT TCTAAAAGTT AAATTTATAG AGATTAAAAA TTTCAAAAAC TATTTTAAGT TAAATCTAAT   
  
  
+ GCCAAGTTAC AATTTAATAA AAACTTATAT GTTTAAATTA ATCATCGTAT TTTATTTATA GTTAAAAAAT   
  
  
+ ACCATAAATA TAAAATTAAA AATTTTTAAA TAAAAATTAA TTATATTATT TAATATTTTT TTAGTAAGGG   
  
  
+ GGAATATGGT AGTATTCAAG AAGTTTAATG AAAATTGTAA AAAGTTAATA CCTAGAAGTA TGTACGTACG   
  
  
+ TTTCGAACGT ATAGATACAT ACATGATATA TATATAAATT AGTTAATATG TACGTACGTA CGTGTACGTA   
  
  
+ TTGTATACAT ATATCGATAC GTAATGGCAT ACTCTTCTCT ATTAATTACT ATTTGTTAAA CTAATGTAGT   
  
  
+ AGAACGGGTG TACCTATAAT TATTCTCGCT CAGAATATGC TTTAGGCTAC TCTCCTTTCG GAGCCCTAGT   
  
  
+ GTAGAATCTT TAACAAAATG CCCGAACTGT ACCAGAAGGG GTGTACCATA GGCTCGGAGC ATGCTTTAGG   
  
  
+ GTACTCTTCT CTCAGAAAAC CCTAGTGTAG AACTTTAGCA GTTGAGGTTA CGGAGAGGAA TTAGTTAGTC   
  
  
+ ATCTAAATTA ATTAATTAGA GTTGAATCAA CAAATTAGGG GGTAGAGAAT ATATTCATGA CTTGGTGATT   
  
  
+ TAAATTGTTT TAATCTTATC AGGTTTATT  

- CTGAGCTTTG ACTTTAATTA ATTTGACTCT AACATTAACT AAATCAAACA CACCTCTTTA TTACACAAAA   
  
  
- CCATCACTTC CCCACTCCCA ATTAATTTCT CATTAATGCC ACCCCCTTTG CCAAATCATC TGTTATTATT   
  
  
- ATCACTTAAT GTTAAACTTT TTCAAAAGGG ATTTTGATAT TTTGATAATT TAACATATTA ACAAAAAAAT   
  
  
- AAAAAATTTT AATAATTAAT TTAATAAATT ATCATGTGTC ATCATCTTTT TAAAAGATCA ACACAATTTA   
  
  
- AATAAATCAA AAATTCAAAT TCAATCAAAA AAATATTGGT GTACCAAAAA ATTTGATTCA AACTATAAAA   
  
  
- ATTTAACACG TAATAATTTA ATAAAATAAA TAAATAATTT TTTATTTTTA TGTATCATTA TAAAATTATT   
  
  
- AAAAAAATAC CTTTTAAACT TCAATTTAAA TGAATACAAA TTTTATTATA TATATGATAA TAAATTTTGG   
  
  
- GTAAAATCTC ATCAAACCTT CAAGTTTGGT TATAAAAAAT TAAAGAGATT GTTGTTATTG AAATGTTTCA   
  
  
- AAATAATTCA AATGAATTGT TTTTTATTTA AATTTTATTT TTCAAATTTA AATATTTTTA AATATAATAT   
  
  
- TTATTTTTTG TGAATTTTTT AATTTTTTGA TAGTATAAAA GTTAAAATAT ATATTTTCAG TACTCATTAA   
  
  
- AGTTTAAAAA AAAAATTATG ATGATAGCTT GAATATTTTT TTACGTATGT AAAAATTTTT AAGTCAAAAT   
  
  
- TCATTATATA AGATTTTCAA TTTAAATATC TCTAATTTTT AAAGTTTTTG ATAAAATTCA ATTTAGATTA   
  
  
- CGGTTCAATG TTAAATTATT TTTGAATATA CAAATTTAAT TAGTAGCATA AAATAAATAT CAATTTTTTA   
  
  
- TGGTATTTAT ATTTTAATTT TTAAAAATTT ATTTTTAATT AATATAATAA ATTATAAAAA AATCATTCCC   
  
  
- CCTTATACCA TCATAAGTTC TTCAAATTAC TTTTAACATT TTTCAATTAT GGATCTTCAT ACATGCATGC   
  
  
- AAAGCTTGCA TATCTATGTA TGTACTATAT ATATATTTAA TCAATTATAC ATGCATGCAT GCACATGCAT   
  
  
- AACATATGTA TATAGCTATG CATTACCGTA TGAGAAGAGA TAATTAATGA TAAACAATTT GATTACATCA   
  
  
- TCTTGCCCAC ATGGATATTA ATAAGAGCGA GTCTTATACG AAATCCGATG AGAGGAAAGC CTCGGGATCA   
  
  
- CATCTTAGAA ATTGTTTTAC GGGCTTGACA TGGTCTTCCC CACATGGTAT CCGAGCCTCG TACGAAATCC   
  
  
- CATGAGAAGA GAGTCTTTTG GGATCACATC TTGAAATCGT CAACTCCAAT GCCTCTCCTT AATCAATCAG   
  
  
- TAGATTTAAT TAATTAATCT CAACTTAGTT GTTTAATCCC CCATCTCTTA TATAAGTACT GAACCACTAA   
  
  
- ATTTAACAAA ATTAGAATAG TCCAAATAA

+     TATA-box

| Site Name | Organism | Position | Strand | Matrix score. | sequence | function |
| --- | --- | --- | --- | --- | --- | --- |
| TATA-box | Lycopersicon esculentum | 484 | - | 5 | TTTTA | core promoter element around -30 of transcription start |
| TATA-box | Arabidopsis thaliana | 468 | + | 8 | TATATATA | core promoter element around -30 of transcription start |
| TATA-box | Arabidopsis thaliana | 935 | - | 8 | TATTTAAA | core promoter element around -30 of transcription start |
| TATA-box | Brassica oleracea | 680 | + | 6 | ATATAA | core promoter element around -30 of transcription start |
| TATA-box | Brassica napus | 678 | + | 6 | ATATAT | core promoter element around -30 of transcription start |
| TATA-box | Arabidopsis thaliana | 466 | - | 9 | tcTATATAtt | core promoter element around -30 of transcription start |
| TATA-box | Arabidopsis thaliana | 1450 | - | 4 | TATA | core promoter element around -30 of transcription start |
| TATA-box | Arabidopsis thaliana | 913 | + | 11 | TATAAATATAAA | core promoter element around -30 of transcription start |
| TATA-box | Ac | 627 | + | 7 | TATAAAT | core promoter element around -30 of transcription start |
| TATA-box | Arabidopsis thaliana | 865 | - | 5 | TATAA | core promoter element around -30 of transcription start |
| TATA-box | Lycopersicon esculentum | 644 | - | 5 | TTTTA | core promoter element around -30 of transcription start |
| TATA-box | Lycopersicon esculentum | 805 | - | 5 | TTTTA | core promoter element around -30 of transcription start |
| TATA-box | Lycopersicon esculentum | 941 | - | 5 | TTTTA | core promoter element around -30 of transcription start |
| TATA-box | Lycopersicon esculentum | 934 | + | 5 | TTTTA | core promoter element around -30 of transcription start |
| TATA-box | Lycopersicon esculentum | 652 | - | 5 | TTTTA | core promoter element around -30 of transcription start |
| TATA-box | Brassica oleracea | 918 | + | 6 | ATATAA | core promoter element around -30 of transcription start |
| TATA-box | Brassica napus | 467 | + | 6 | ATATAT | core promoter element around -30 of transcription start |
| TATA-box | Glycine max | 1160 | - | 5 | TAATA | core promoter element around -30 of transcription start |
| TATA-box | Arabidopsis thaliana | 1130 | - | 4 | TATA | core promoter element around -30 of transcription start |
| TATA-box | Zea mays | 933 | - | 8 | TTTAAAAA | core promoter element around -30 of transcription start |
| TATA-box | Lycopersicon esculentum | 903 | - | 5 | TTTTA | core promoter element around -30 of transcription start |
| TATA-box | Brassica napus | 1129 | - | 6 | ATATAT | core promoter element around -30 of transcription start |
| TATA-box | Helianthus annuus | 1122 | - | 6 | TATACA | core promoter element around -30 of transcription start |
| TATA-box | Arabidopsis thaliana | 776 | - | 4 | TATA | core promoter element around -30 of transcription start |
| TATA-box | Arabidopsis thaliana | 1081 | + | 9 | taTATAAAtc | core promoter element around -30 of transcription start |
| TATA-box | Lycopersicon esculentum | 921 | - | 5 | TTTTA | core promoter element around -30 of transcription start |
| TATA-box | Arabidopsis thaliana | 951 | - | 5 | TATAA | core promoter element around -30 of transcription start |
| TATA-box | Pisum sativum | 673 | - | 8 | TATAAAAT | core promoter element around -30 of transcription start |
| TATA-box | Lycopersicon esculentum | 460 | - | 5 | TTTTA | core promoter element around -30 of transcription start |
| TATA-box | Arabidopsis thaliana | 622 | + | 4 | TATA | core promoter element around -30 of transcription start |
| TATA-box | Lycopersicon esculentum | 424 | + | 5 | TTTTA | core promoter element around -30 of transcription start |
| TATA-box | Lycopersicon esculentum | 412 | + | 5 | TTTTA | core promoter element around -30 of transcription start |
| TATA-box | Lycopersicon esculentum | 373 | + | 5 | TTTTA | core promoter element around -30 of transcription start |
| TATA-box | Pisum sativum | 1127 | - | 7 | TATATGT | core promoter element around -30 of transcription start |
| TATA-box | Brassica napus | 775 | - | 6 | ATATAT | core promoter element around -30 of transcription start |
| TATA-box | Lycopersicon esculentum | 614 | - | 5 | TTTTA | core promoter element around -30 of transcription start |
| TATA-box | Brassica napus | 1076 | - | 6 | ATATAT | core promoter element around -30 of transcription start |
| TATA-box | Glycine max | 415 | + | 5 | TAATA | core promoter element around -30 of transcription start |
| TATA-box | Lycopersicon esculentum | 328 | + | 5 | TTTTA | core promoter element around -30 of transcription start |
| TATA-box | Glycine max | 855 | + | 5 | TAATA | core promoter element around -30 of transcription start |
| TATA-box | Glycine max | 773 | + | 5 | TAATA | core promoter element around -30 of transcription start |
| TATA-box | Arabidopsis thaliana | 610 | - | 6 | TATAAA | core promoter element around -30 of transcription start |
| TATA-box | Brassica napus | 1449 | - | 6 | ATATAT | core promoter element around -30 of transcription start |
| TATA-box | Ac | 609 | - | 7 | TATAAAT | core promoter element around -30 of transcription start |
| TATA-box | Arabidopsis thaliana | 897 | - | 4 | TATA | core promoter element around -30 of transcription start |
| TATA-box | Brassica napus | 469 | + | 6 | ATATAT | core promoter element around -30 of transcription start |
| TATA-box | Arabidopsis thaliana | 1265 | - | 8 | TAAAGATT | core promoter element around -30 of transcription start |
| TATA-box | Lycopersicon esculentum | 927 | - | 5 | TTTTA | core promoter element around -30 of transcription start |
| TATA-box | Arabidopsis thaliana | 1060 | - | 4 | TATA | core promoter element around -30 of transcription start |
| TATA-box | Arabidopsis thaliana | 675 | - | 6 | TATAAA | core promoter element around -30 of transcription start |
| TATA-box | Ac | 1083 | + | 7 | TATAAAT | core promoter element around -30 of transcription start |
| TATA-box | Brassica napus | 1078 | - | 6 | ATATAT | core promoter element around -30 of transcription start |
| TATA-box | Glycine max | 1026 | + | 5 | TAATA | core promoter element around -30 of transcription start |
| TATA-box | Lycopersicon esculentum | 858 | - | 5 | TTTTA | core promoter element around -30 of transcription start |
| TATA-box | Lycopersicon esculentum | 526 | + | 5 | TTTTA | core promoter element around -30 of transcription start |
| TATA-box | Glycine max | 961 | + | 5 | TAATA | core promoter element around -30 of transcription start |
| TATA-box | Brassica napus | 1080 | - | 6 | ATATAT | core promoter element around -30 of transcription start |
| TATA-box | Lycopersicon esculentum | 1018 | - | 5 | TTTTA | core promoter element around -30 of transcription start |
| TATA-box | Lycopersicon esculentum | 755 | - | 5 | TTTTA | core promoter element around -30 of transcription start |
| TATA-box | Lycopersicon esculentum | 683 | - | 5 | TTTTA | core promoter element around -30 of transcription start |
| TATA-box | Arabidopsis thaliana | 472 | + | 4 | TATA | core promoter element around -30 of transcription start |
| TATA-box | Glycine max | 363 | - | 5 | TAATA | core promoter element around -30 of transcription start |
| TATA-box | Lycopersicon esculentum | 783 | - | 5 | TTTTA | core promoter element around -30 of transcription start |
| TATA-box | Lycopersicon esculentum | 493 | + | 5 | TTTTA | core promoter element around -30 of transcription start |
| TATA-box | Arabidopsis thaliana | 919 | + | 6 | TATAAA | core promoter element around -30 of transcription start |
| TATA-box | Arabidopsis thaliana | 896 | - | 5 | TATAA | core promoter element around -30 of transcription start |
| TATA-box | Arabidopsis thaliana | 470 | + | 4 | TATA | core promoter element around -30 of transcription start |
| TATA-box | Daucus carota | 893 | - | 8 | TATAAATA | core promoter element around -30 of transcription start |
| TATA-box | Arabidopsis thaliana | 795 | - | 5 | TATAA | core promoter element around -30 of transcription start |
| TATA-box | Arabidopsis thaliana | 1124 | - | 4 | TATA | core promoter element around -30 of transcription start |
| TATA-box | Arabidopsis thaliana | 952 | - | 4 | TATA | core promoter element around -30 of transcription start |
| TATA-box | Glycine max | 407 | + | 5 | TAATA | core promoter element around -30 of transcription start |
| TATA-box | Lycopersicon esculentum | 823 | + | 5 | TTTTA | core promoter element around -30 of transcription start |
| TATA-box | Brassica napus | 950 | + | 6 | ATTATA | core promoter element around -30 of transcription start |
| TATA-box | Arabidopsis thaliana | 677 | + | 8 | TATATATA | core promoter element around -30 of transcription start |
| TATA-box | Zea mays | 753 | + | 8 | TTTAAAAA | core promoter element around -30 of transcription start |
| TATA-box | Lycopersicon esculentum | 1478 | + | 5 | TTTTA | core promoter element around -30 of transcription start |
| TATA-box | Arabidopsis thaliana | 681 | + | 6 | TATAAA | core promoter element around -30 of transcription start |
| TATA-box | Arabidopsis thaliana | 679 | + | 4 | TATA | core promoter element around -30 of transcription start |
| TATA-box | Arabidopsis thaliana | 621 | - | 5 | TATAA | core promoter element around -30 of transcription start |
| TATA-box | Arabidopsis thaliana | 312 | - | 5 | TATAA | core promoter element around -30 of transcription start |
| TATA-box | Lycopersicon esculentum | 290 | + | 5 | TTTTA | core promoter element around -30 of transcription start |
| TATA-box | Glycine max | 185 | - | 5 | TAATA | core promoter element around -30 of transcription start |
| TATA-box | Glycine max | 135 | + | 5 | TAATA | core promoter element around -30 of transcription start |
| TATA-box | Ac | 894 | - | 7 | TATAAAT | core promoter element around -30 of transcription start |
| TATA-box | Arabidopsis thaliana | 611 | - | 5 | TATAA | core promoter element around -30 of transcription start |
| TATA-box | Brassica oleracea | 1082 | + | 6 | ATATAA | core promoter element around -30 of transcription start |
| TATA-box | Glycine max | 465 | + | 5 | TAATA | core promoter element around -30 of transcription start |
| TATA-box | Lycopersicon esculentum | 394 | - | 5 | TTTTA | core promoter element around -30 of transcription start |
| TATA-box | Arabidopsis thaliana | 731 | + | 9 | ccTATAAAaa | core promoter element around -30 of transcription start |
| TATA-box | Arabidopsis thaliana | 796 | - | 4 | TATA | core promoter element around -30 of transcription start |
| TATA-box | Glycine max | 563 | - | 5 | TAATA | core promoter element around -30 of transcription start |
| TATA-box | Lycopersicon esculentum | 171 | - | 5 | TTTTA | core promoter element around -30 of transcription start |
| TATA-box | Arabidopsis thaliana | 620 | - | 6 | TATAAA | core promoter element around -30 of transcription start |
| TATA-box | Lycopersicon esculentum | 735 | - | 5 | TTTTA | core promoter element around -30 of transcription start |
| TATA-box | Arabidopsis thaliana | 895 | - | 6 | TATAAA | core promoter element around -30 of transcription start |
| TATA-box | Glycine max | 384 | - | 5 | TAATA | core promoter element around -30 of transcription start |
| TATA-box | Glycine max | 477 | - | 5 | TAATA | core promoter element around -30 of transcription start |
| TATA-box | Arabidopsis thaliana | 1075 | - | 9 | taTATAAAtc | core promoter element around -30 of transcription start |
| TATA-box | Lycopersicon esculentum | 766 | + | 5 | TTTTA | core promoter element around -30 of transcription start |
| TATA-box | Arabidopsis thaliana | 311 | - | 6 | TATAAA | core promoter element around -30 of transcription start |
| TATA-box | Lycopersicon esculentum | 592 | - | 5 | TTTTA | core promoter element around -30 of transcription start |
| TATA-box | Glycine max | 954 | - | 5 | TAATA | core promoter element around -30 of transcription start |
| TATA-box | Lycopersicon esculentum | 206 | + | 5 | TTTTA | core promoter element around -30 of transcription start |
| TATA-box | Arabidopsis thaliana | 1077 | + | 11 | TATAAATATAAA | core promoter element around -30 of transcription start |
| TATA-box | Arabidopsis thaliana | 674 | - | 7 | TATAAAA | core promoter element around -30 of transcription start |
| TATA-box | Lycopersicon esculentum | 969 | + | 5 | TTTTA | core promoter element around -30 of transcription start |
| TATA-box | Lycopersicon esculentum | 387 | - | 5 | TTTTA | core promoter element around -30 of transcription start |
| TATA-box | Arabidopsis thaliana | 866 | - | 4 | TATA | core promoter element around -30 of transcription start |
| TATA-box | Lycopersicon esculentum | 890 | + | 5 | TTTTA | core promoter element around -30 of transcription start |
| TATA-box | Lycopersicon esculentum | 348 | + | 5 | TTTTA | core promoter element around -30 of transcription start |
| TATA-box | Arabidopsis thaliana | 676 | - | 7 | TATATAA | core promoter element around -30 of transcription start |
| TATA-box | Arabidopsis thaliana | 1205 | - | 4 | TATA | core promoter element around -30 of transcription start |
| TATA-box | Arabidopsis thaliana | 310 | - | 7 | TATAAAA | core promoter element around -30 of transcription start |
| TATA-box | Lycopersicon esculentum | 712 | + | 5 | TTTTA | core promoter element around -30 of transcription start |
| TATA-box | Glycine max | 222 | - | 5 | TAATA | core promoter element around -30 of transcription start |
| TATA-box | Glycine max | 1094 | + | 5 | TAATA | core promoter element around -30 of transcription start |
| TATA-box | Antirrhinum majus | 618 | - | 8 | TATAAATT | core promoter element around -30 of transcription start |
| TATA-box | Lycopersicon esculentum | 560 | + | 5 | TTTTA | core promoter element around -30 of transcription start |
| TATA-box | Helianthus annuus | 193 | - | 6 | TATACA | core promoter element around -30 of transcription start |
| TATA-box | Arabidopsis thaliana | 1079 | - | 8 | TATATATA | core promoter element around -30 of transcription start |
| TATA-box | Arabidopsis thaliana | 177 | + | 6 | TATAAA | core promoter element around -30 of transcription start |
| TATA-box | Antirrhinum majus | 792 | - | 8 | TATAAATT | core promoter element around -30 of transcription start |
| TATA-box | Ac | 793 | - | 7 | TATAAAT | core promoter element around -30 of transcription start |
| TATA-box | Lycopersicon esculentum | 633 | - | 5 | TTTTA | core promoter element around -30 of transcription start |
| TATA-box | Lycopersicon esculentum | 179 | - | 5 | TTTTA | core promoter element around -30 of transcription start |
| TATA-box | Zea mays | 751 | - | 8 | TTTAAAAA | core promoter element around -30 of transcription start |
| TATA-box | Arabidopsis thaliana | 732 | - | 5 | TATAA | core promoter element around -30 of transcription start |
| TATA-box | Glycine max | 138 | + | 5 | TAATA | core promoter element around -30 of transcription start |
| TATA-box | Arabidopsis thaliana | 480 | + | 8 | TATTTAAA | core promoter element around -30 of transcription start |
| TATA-box | Antirrhinum majus | 608 | - | 8 | TATAAATT | core promoter element around -30 of transcription start |
| TATA-box | Arabidopsis thaliana | 733 | + | 6 | TATAAA | core promoter element around -30 of transcription start |
| TATA-box | Brassica napus | 625 | + | 6 | ATTATA | core promoter element around -30 of transcription start |
| TATA-box | Arabidopsis thaliana | 604 | - | 11 | TATAAATATAAA | core promoter element around -30 of transcription start |
| TATA-box | Arabidopsis thaliana | 794 | - | 6 | TATAAA | core promoter element around -30 of transcription start |
| TATA-box | Zea mays | 347 | - | 8 | TTTAAAAA | core promoter element around -30 of transcription start |
| TATA-box | Arabidopsis thaliana | 195 | + | 4 | TATA | core promoter element around -30 of transcription start |
| TATA-box | Glycine max | 715 | + | 5 | TAATA | core promoter element around -30 of transcription start |
| TATA-box | Glycine max | 624 | - | 5 | TAATA | core promoter element around -30 of transcription start |
| TATA-box | Lycopersicon esculentum | 752 | + | 5 | TTTTA | core promoter element around -30 of transcription start |
| TATA-box | Zea mays | 327 | - | 8 | TTTAAAAA | core promoter element around -30 of transcription start |
| TATA-box | Lycopersicon esculentum | 597 | - | 5 | TTTTA | core promoter element around -30 of transcription start |
| TATA-box | Zea mays | 212 | - | 8 | TTTAAAAA | core promoter element around -30 of transcription start |
| TATA-box | Arabidopsis thaliana | 313 | + | 4 | TATA | core promoter element around -30 of transcription start |
| TATA-box | Lycopersicon esculentum | 213 | + | 5 | TTTTA | core promoter element around -30 of transcription start |
| TATA-box | Arabidopsis thaliana | 626 | - | 5 | TATAA | core promoter element around -30 of transcription start |
| TATA-box | Lycopersicon esculentum | 216 | - | 5 | TTTTA | core promoter element around -30 of transcription start |
| TATA-box | Arabidopsis thaliana | 612 | + | 6 | TATAAA | core promoter element around -30 of transcription start |
| TATA-box | Glycine max | 238 | + | 5 | TAATA | core promoter element around -30 of transcription start |
| TATA-box | Ac | 619 | - | 7 | TATAAAT | core promoter element around -30 of transcription start |

> 2018/04/13 10:10:12  
+ GACTCGAAAC TGAAATTAAT TAAACTGAGA TTGTAATTGA TTTAGTTTGT GTGGAGAAAT AATGTGTTTT   
  
  
+ GGTAGTGAAG GGGTGAGGGT TAATTAAAGA GTAATTACGG TGGGGGAAAC GGTTTAGTAG ACAATAATAA   
  
  
+ TAGTGAATTA CAATTTGAAA AAGTTTTCCC TAAAACTATA AAACTATTAA ATTGTATAAT TGTTTTTTTA   
  
  
+ TTTTTTAAAA TTATTAATTA AATTATTTAA TAGTACACAG TAGTAGAAAA ATTTTCTAGT TGTGTTAAAT   
  
  
+ TTATTTAGTT TTTAAGTTTA AGTTAGTTTT TTTATAACCA CATGGTTTTT TAAACTAAGT TTGATATTTT   
  
  
+ TAAATTGTGC ATTATTAAAT TATTTTATTT ATTTATTAAA AAATAAAAAT ACATAGTAAT ATTTTAATAA   
  
  
+ TTTTTTTATG GAAAATTTGA AGTTAAATTT ACTTATGTTT AAAATAATAT ATATACTATT ATTTAAAACC   
  
  
+ CATTTTAGAG TAGTTTGGAA GTTCAAACCA ATATTTTTTA ATTTCTCTAA CAACAATAAC TTTACAAAGT   
  
  
+ TTTATTAAGT TTACTTAACA AAAAATAAAT TTAAAATAAA AAGTTTAAAT TTATAAAAAT TTATATTATA   
  
  
+ AATAAAAAAC ACTTAAAAAA TTAAAAAACT ATCATATTTT CAATTTTATA TATAAAAGTC ATGAGTAATT   
  
  
+ TCAAATTTTT TTTTTAATAC TACTATCGAA CTTATAAAAA AATGCATACA TTTTTAAAAA TTCAGTTTTA   
  
  
+ AGTAATATAT TCTAAAAGTT AAATTTATAG AGATTAAAAA TTTCAAAAAC TATTTTAAGT TAAATCTAAT   
  
  
+ GCCAAGTTAC AATTTAATAA AAACTTATAT GTTTAAATTA ATCATCGTAT TTTATTTATA GTTAAAAAAT   
  
  
+ ACCATAAATA TAAAATTAAA AATTTTTAAA TAAAAATTAA TTATATTATT TAATATTTTT TTAGTAAGGG   
  
  
+ GGAATATGGT AGTATTCAAG AAGTTTAATG AAAATTGTAA AAAGTTAATA CCTAGAAGTA TGTACGTACG   
  
  
+ TTTCGAACGT ATAGATACAT ACATGATATA TATATAAATT AGTTAATATG TACGTACGTA CGTGTACGTA   
  
  
+ TTGTATACAT ATATCGATAC GTAATGGCAT ACTCTTCTCT ATTAATTACT ATTTGTTAAA CTAATGTAGT   
  
  
+ AGAACGGGTG TACCTATAAT TATTCTCGCT CAGAATATGC TTTAGGCTAC TCTCCTTTCG GAGCCCTAGT   
  
  
+ GTAGAATCTT TAACAAAATG CCCGAACTGT ACCAGAAGGG GTGTACCATA GGCTCGGAGC ATGCTTTAGG   
  
  
+ GTACTCTTCT CTCAGAAAAC CCTAGTGTAG AACTTTAGCA GTTGAGGTTA CGGAGAGGAA TTAGTTAGTC   
  
  
+ ATCTAAATTA ATTAATTAGA GTTGAATCAA CAAATTAGGG GGTAGAGAAT ATATTCATGA CTTGGTGATT   
  
  
+ TAAATTGTTT TAATCTTATC AGGTTTATT  

- CTGAGCTTTG ACTTTAATTA ATTTGACTCT AACATTAACT AAATCAAACA CACCTCTTTA TTACACAAAA   
  
  
- CCATCACTTC CCCACTCCCA ATTAATTTCT CATTAATGCC ACCCCCTTTG CCAAATCATC TGTTATTATT   
  
  
- ATCACTTAAT GTTAAACTTT TTCAAAAGGG ATTTTGATAT TTTGATAATT TAACATATTA ACAAAAAAAT   
  
  
- AAAAAATTTT AATAATTAAT TTAATAAATT ATCATGTGTC ATCATCTTTT TAAAAGATCA ACACAATTTA   
  
  
- AATAAATCAA AAATTCAAAT TCAATCAAAA AAATATTGGT GTACCAAAAA ATTTGATTCA AACTATAAAA   
  
  
- ATTTAACACG TAATAATTTA ATAAAATAAA TAAATAATTT TTTATTTTTA TGTATCATTA TAAAATTATT   
  
  
- AAAAAAATAC CTTTTAAACT TCAATTTAAA TGAATACAAA TTTTATTATA TATATGATAA TAAATTTTGG   
  
  
- GTAAAATCTC ATCAAACCTT CAAGTTTGGT TATAAAAAAT TAAAGAGATT GTTGTTATTG AAATGTTTCA   
  
  
- AAATAATTCA AATGAATTGT TTTTTATTTA AATTTTATTT TTCAAATTTA AATATTTTTA AATATAATAT   
  
  
- TTATTTTTTG TGAATTTTTT AATTTTTTGA TAGTATAAAA GTTAAAATAT ATATTTTCAG TACTCATTAA   
  
  
- AGTTTAAAAA AAAAATTATG ATGATAGCTT GAATATTTTT TTACGTATGT AAAAATTTTT AAGTCAAAAT   
  
  
- TCATTATATA AGATTTTCAA TTTAAATATC TCTAATTTTT AAAGTTTTTG ATAAAATTCA ATTTAGATTA   
  
  
- CGGTTCAATG TTAAATTATT TTTGAATATA CAAATTTAAT TAGTAGCATA AAATAAATAT CAATTTTTTA   
  
  
- TGGTATTTAT ATTTTAATTT TTAAAAATTT ATTTTTAATT AATATAATAA ATTATAAAAA AATCATTCCC   
  
  
- CCTTATACCA TCATAAGTTC TTCAAATTAC TTTTAACATT TTTCAATTAT GGATCTTCAT ACATGCATGC   
  
  
- AAAGCTTGCA TATCTATGTA TGTACTATAT ATATATTTAA TCAATTATAC ATGCATGCAT GCACATGCAT   
  
  
- AACATATGTA TATAGCTATG CATTACCGTA TGAGAAGAGA TAATTAATGA TAAACAATTT GATTACATCA   
  
  
- TCTTGCCCAC ATGGATATTA ATAAGAGCGA GTCTTATACG AAATCCGATG AGAGGAAAGC CTCGGGATCA   
  
  
- CATCTTAGAA ATTGTTTTAC GGGCTTGACA TGGTCTTCCC CACATGGTAT CCGAGCCTCG TACGAAATCC   
  
  
- CATGAGAAGA GAGTCTTTTG GGATCACATC TTGAAATCGT CAACTCCAAT GCCTCTCCTT AATCAATCAG   
  
  
- TAGATTTAAT TAATTAATCT CAACTTAGTT GTTTAATCCC CCATCTCTTA TATAAGTACT GAACCACTAA   
  
  
- ATTTAACAAA ATTAGAATAG TCCAAATAA

+     TC-rich repeats

| Site Name | Organism | Position | Strand | Matrix score. | sequence | function |
| --- | --- | --- | --- | --- | --- | --- |
| TC-rich repeats | Nicotiana tabacum | 532 | + | 9 | ATTCTCTAAC | cis-acting element involved in defense and stress responsiveness |
| TC-rich repeats | Nicotiana tabacum | 1441 | - | 9 | ATTCTCTAAC | cis-acting element involved in defense and stress responsiveness |

> 2018/04/13 10:10:12  
+ GACTCGAAAC TGAAATTAAT TAAACTGAGA TTGTAATTGA TTTAGTTTGT GTGGAGAAAT AATGTGTTTT   
  
  
+ GGTAGTGAAG GGGTGAGGGT TAATTAAAGA GTAATTACGG TGGGGGAAAC GGTTTAGTAG ACAATAATAA   
  
  
+ TAGTGAATTA CAATTTGAAA AAGTTTTCCC TAAAACTATA AAACTATTAA ATTGTATAAT TGTTTTTTTA   
  
  
+ TTTTTTAAAA TTATTAATTA AATTATTTAA TAGTACACAG TAGTAGAAAA ATTTTCTAGT TGTGTTAAAT   
  
  
+ TTATTTAGTT TTTAAGTTTA AGTTAGTTTT TTTATAACCA CATGGTTTTT TAAACTAAGT TTGATATTTT   
  
  
+ TAAATTGTGC ATTATTAAAT TATTTTATTT ATTTATTAAA AAATAAAAAT ACATAGTAAT ATTTTAATAA   
  
  
+ TTTTTTTATG GAAAATTTGA AGTTAAATTT ACTTATGTTT AAAATAATAT ATATACTATT ATTTAAAACC   
  
  
+ CATTTTAGAG TAGTTTGGAA GTTCAAACCA ATATTTTTTA ATTTCTCTAA CAACAATAAC TTTACAAAGT   
  
  
+ TTTATTAAGT TTACTTAACA AAAAATAAAT TTAAAATAAA AAGTTTAAAT TTATAAAAAT TTATATTATA   
  
  
+ AATAAAAAAC ACTTAAAAAA TTAAAAAACT ATCATATTTT CAATTTTATA TATAAAAGTC ATGAGTAATT   
  
  
+ TCAAATTTTT TTTTTAATAC TACTATCGAA CTTATAAAAA AATGCATACA TTTTTAAAAA TTCAGTTTTA   
  
  
+ AGTAATATAT TCTAAAAGTT AAATTTATAG AGATTAAAAA TTTCAAAAAC TATTTTAAGT TAAATCTAAT   
  
  
+ GCCAAGTTAC AATTTAATAA AAACTTATAT GTTTAAATTA ATCATCGTAT TTTATTTATA GTTAAAAAAT   
  
  
+ ACCATAAATA TAAAATTAAA AATTTTTAAA TAAAAATTAA TTATATTATT TAATATTTTT TTAGTAAGGG   
  
  
+ GGAATATGGT AGTATTCAAG AAGTTTAATG AAAATTGTAA AAAGTTAATA CCTAGAAGTA TGTACGTACG   
  
  
+ TTTCGAACGT ATAGATACAT ACATGATATA TATATAAATT AGTTAATATG TACGTACGTA CGTGTACGTA   
  
  
+ TTGTATACAT ATATCGATAC GTAATGGCAT ACTCTTCTCT ATTAATTACT ATTTGTTAAA CTAATGTAGT   
  
  
+ AGAACGGGTG TACCTATAAT TATTCTCGCT CAGAATATGC TTTAGGCTAC TCTCCTTTCG GAGCCCTAGT   
  
  
+ GTAGAATCTT TAACAAAATG CCCGAACTGT ACCAGAAGGG GTGTACCATA GGCTCGGAGC ATGCTTTAGG   
  
  
+ GTACTCTTCT CTCAGAAAAC CCTAGTGTAG AACTTTAGCA GTTGAGGTTA CGGAGAGGAA TTAGTTAGTC   
  
  
+ ATCTAAATTA ATTAATTAGA GTTGAATCAA CAAATTAGGG GGTAGAGAAT ATATTCATGA CTTGGTGATT   
  
  
+ TAAATTGTTT TAATCTTATC AGGTTTATT  

- CTGAGCTTTG ACTTTAATTA ATTTGACTCT AACATTAACT AAATCAAACA CACCTCTTTA TTACACAAAA   
  
  
- CCATCACTTC CCCACTCCCA ATTAATTTCT CATTAATGCC ACCCCCTTTG CCAAATCATC TGTTATTATT   
  
  
- ATCACTTAAT GTTAAACTTT TTCAAAAGGG ATTTTGATAT TTTGATAATT TAACATATTA ACAAAAAAAT   
  
  
- AAAAAATTTT AATAATTAAT TTAATAAATT ATCATGTGTC ATCATCTTTT TAAAAGATCA ACACAATTTA   
  
  
- AATAAATCAA AAATTCAAAT TCAATCAAAA AAATATTGGT GTACCAAAAA ATTTGATTCA AACTATAAAA   
  
  
- ATTTAACACG TAATAATTTA ATAAAATAAA TAAATAATTT TTTATTTTTA TGTATCATTA TAAAATTATT   
  
  
- AAAAAAATAC CTTTTAAACT TCAATTTAAA TGAATACAAA TTTTATTATA TATATGATAA TAAATTTTGG   
  
  
- GTAAAATCTC ATCAAACCTT CAAGTTTGGT TATAAAAAAT TAAAGAGATT GTTGTTATTG AAATGTTTCA   
  
  
- AAATAATTCA AATGAATTGT TTTTTATTTA AATTTTATTT TTCAAATTTA AATATTTTTA AATATAATAT   
  
  
- TTATTTTTTG TGAATTTTTT AATTTTTTGA TAGTATAAAA GTTAAAATAT ATATTTTCAG TACTCATTAA   
  
  
- AGTTTAAAAA AAAAATTATG ATGATAGCTT GAATATTTTT TTACGTATGT AAAAATTTTT AAGTCAAAAT   
  
  
- TCATTATATA AGATTTTCAA TTTAAATATC TCTAATTTTT AAAGTTTTTG ATAAAATTCA ATTTAGATTA   
  
  
- CGGTTCAATG TTAAATTATT TTTGAATATA CAAATTTAAT TAGTAGCATA AAATAAATAT CAATTTTTTA   
  
  
- TGGTATTTAT ATTTTAATTT TTAAAAATTT ATTTTTAATT AATATAATAA ATTATAAAAA AATCATTCCC   
  
  
- CCTTATACCA TCATAAGTTC TTCAAATTAC TTTTAACATT TTTCAATTAT GGATCTTCAT ACATGCATGC   
  
  
- AAAGCTTGCA TATCTATGTA TGTACTATAT ATATATTTAA TCAATTATAC ATGCATGCAT GCACATGCAT   
  
  
- AACATATGTA TATAGCTATG CATTACCGTA TGAGAAGAGA TAATTAATGA TAAACAATTT GATTACATCA   
  
  
- TCTTGCCCAC ATGGATATTA ATAAGAGCGA GTCTTATACG AAATCCGATG AGAGGAAAGC CTCGGGATCA   
  
  
- CATCTTAGAA ATTGTTTTAC GGGCTTGACA TGGTCTTCCC CACATGGTAT CCGAGCCTCG TACGAAATCC   
  
  
- CATGAGAAGA GAGTCTTTTG GGATCACATC TTGAAATCGT CAACTCCAAT GCCTCTCCTT AATCAATCAG   
  
  
- TAGATTTAAT TAATTAATCT CAACTTAGTT GTTTAATCCC CCATCTCTTA TATAAGTACT GAACCACTAA   
  
  
- ATTTAACAAA ATTAGAATAG TCCAAATAA

+     TCA-element

| Site Name | Organism | Position | Strand | Matrix score. | sequence | function |
| --- | --- | --- | --- | --- | --- | --- |
| TCA-element | Brassica oleracea | 1150 | - | 9 | GAGAAGAATA | cis-acting element involved in salicylic acid responsiveness |
| TCA-element | Brassica oleracea | 1332 | - | 9 | GAGAAGAATA | cis-acting element involved in salicylic acid responsiveness |

> 2018/04/13 10:10:12  
+ GACTCGAAAC TGAAATTAAT TAAACTGAGA TTGTAATTGA TTTAGTTTGT GTGGAGAAAT AATGTGTTTT   
  
  
+ GGTAGTGAAG GGGTGAGGGT TAATTAAAGA GTAATTACGG TGGGGGAAAC GGTTTAGTAG ACAATAATAA   
  
  
+ TAGTGAATTA CAATTTGAAA AAGTTTTCCC TAAAACTATA AAACTATTAA ATTGTATAAT TGTTTTTTTA   
  
  
+ TTTTTTAAAA TTATTAATTA AATTATTTAA TAGTACACAG TAGTAGAAAA ATTTTCTAGT TGTGTTAAAT   
  
  
+ TTATTTAGTT TTTAAGTTTA AGTTAGTTTT TTTATAACCA CATGGTTTTT TAAACTAAGT TTGATATTTT   
  
  
+ TAAATTGTGC ATTATTAAAT TATTTTATTT ATTTATTAAA AAATAAAAAT ACATAGTAAT ATTTTAATAA   
  
  
+ TTTTTTTATG GAAAATTTGA AGTTAAATTT ACTTATGTTT AAAATAATAT ATATACTATT ATTTAAAACC   
  
  
+ CATTTTAGAG TAGTTTGGAA GTTCAAACCA ATATTTTTTA ATTTCTCTAA CAACAATAAC TTTACAAAGT   
  
  
+ TTTATTAAGT TTACTTAACA AAAAATAAAT TTAAAATAAA AAGTTTAAAT TTATAAAAAT TTATATTATA   
  
  
+ AATAAAAAAC ACTTAAAAAA TTAAAAAACT ATCATATTTT CAATTTTATA TATAAAAGTC ATGAGTAATT   
  
  
+ TCAAATTTTT TTTTTAATAC TACTATCGAA CTTATAAAAA AATGCATACA TTTTTAAAAA TTCAGTTTTA   
  
  
+ AGTAATATAT TCTAAAAGTT AAATTTATAG AGATTAAAAA TTTCAAAAAC TATTTTAAGT TAAATCTAAT   
  
  
+ GCCAAGTTAC AATTTAATAA AAACTTATAT GTTTAAATTA ATCATCGTAT TTTATTTATA GTTAAAAAAT   
  
  
+ ACCATAAATA TAAAATTAAA AATTTTTAAA TAAAAATTAA TTATATTATT TAATATTTTT TTAGTAAGGG   
  
  
+ GGAATATGGT AGTATTCAAG AAGTTTAATG AAAATTGTAA AAAGTTAATA CCTAGAAGTA TGTACGTACG   
  
  
+ TTTCGAACGT ATAGATACAT ACATGATATA TATATAAATT AGTTAATATG TACGTACGTA CGTGTACGTA   
  
  
+ TTGTATACAT ATATCGATAC GTAATGGCAT ACTCTTCTCT ATTAATTACT ATTTGTTAAA CTAATGTAGT   
  
  
+ AGAACGGGTG TACCTATAAT TATTCTCGCT CAGAATATGC TTTAGGCTAC TCTCCTTTCG GAGCCCTAGT   
  
  
+ GTAGAATCTT TAACAAAATG CCCGAACTGT ACCAGAAGGG GTGTACCATA GGCTCGGAGC ATGCTTTAGG   
  
  
+ GTACTCTTCT CTCAGAAAAC CCTAGTGTAG AACTTTAGCA GTTGAGGTTA CGGAGAGGAA TTAGTTAGTC   
  
  
+ ATCTAAATTA ATTAATTAGA GTTGAATCAA CAAATTAGGG GGTAGAGAAT ATATTCATGA CTTGGTGATT   
  
  
+ TAAATTGTTT TAATCTTATC AGGTTTATT  

- CTGAGCTTTG ACTTTAATTA ATTTGACTCT AACATTAACT AAATCAAACA CACCTCTTTA TTACACAAAA   
  
  
- CCATCACTTC CCCACTCCCA ATTAATTTCT CATTAATGCC ACCCCCTTTG CCAAATCATC TGTTATTATT   
  
  
- ATCACTTAAT GTTAAACTTT TTCAAAAGGG ATTTTGATAT TTTGATAATT TAACATATTA ACAAAAAAAT   
  
  
- AAAAAATTTT AATAATTAAT TTAATAAATT ATCATGTGTC ATCATCTTTT TAAAAGATCA ACACAATTTA   
  
  
- AATAAATCAA AAATTCAAAT TCAATCAAAA AAATATTGGT GTACCAAAAA ATTTGATTCA AACTATAAAA   
  
  
- ATTTAACACG TAATAATTTA ATAAAATAAA TAAATAATTT TTTATTTTTA TGTATCATTA TAAAATTATT   
  
  
- AAAAAAATAC CTTTTAAACT TCAATTTAAA TGAATACAAA TTTTATTATA TATATGATAA TAAATTTTGG   
  
  
- GTAAAATCTC ATCAAACCTT CAAGTTTGGT TATAAAAAAT TAAAGAGATT GTTGTTATTG AAATGTTTCA   
  
  
- AAATAATTCA AATGAATTGT TTTTTATTTA AATTTTATTT TTCAAATTTA AATATTTTTA AATATAATAT   
  
  
- TTATTTTTTG TGAATTTTTT AATTTTTTGA TAGTATAAAA GTTAAAATAT ATATTTTCAG TACTCATTAA   
  
  
- AGTTTAAAAA AAAAATTATG ATGATAGCTT GAATATTTTT TTACGTATGT AAAAATTTTT AAGTCAAAAT   
  
  
- TCATTATATA AGATTTTCAA TTTAAATATC TCTAATTTTT AAAGTTTTTG ATAAAATTCA ATTTAGATTA   
  
  
- CGGTTCAATG TTAAATTATT TTTGAATATA CAAATTTAAT TAGTAGCATA AAATAAATAT CAATTTTTTA   
  
  
- TGGTATTTAT ATTTTAATTT TTAAAAATTT ATTTTTAATT AATATAATAA ATTATAAAAA AATCATTCCC   
  
  
- CCTTATACCA TCATAAGTTC TTCAAATTAC TTTTAACATT TTTCAATTAT GGATCTTCAT ACATGCATGC   
  
  
- AAAGCTTGCA TATCTATGTA TGTACTATAT ATATATTTAA TCAATTATAC ATGCATGCAT GCACATGCAT   
  
  
- AACATATGTA TATAGCTATG CATTACCGTA TGAGAAGAGA TAATTAATGA TAAACAATTT GATTACATCA   
  
  
- TCTTGCCCAC ATGGATATTA ATAAGAGCGA GTCTTATACG AAATCCGATG AGAGGAAAGC CTCGGGATCA   
  
  
- CATCTTAGAA ATTGTTTTAC GGGCTTGACA TGGTCTTCCC CACATGGTAT CCGAGCCTCG TACGAAATCC   
  
  
- CATGAGAAGA GAGTCTTTTG GGATCACATC TTGAAATCGT CAACTCCAAT GCCTCTCCTT AATCAATCAG   
  
  
- TAGATTTAAT TAATTAATCT CAACTTAGTT GTTTAATCCC CCATCTCTTA TATAAGTACT GAACCACTAA   
  
  
- ATTTAACAAA ATTAGAATAG TCCAAATAA

+     Unnamed\_\_1

| Site Name | Organism | Position | Strand | Matrix score. | sequence | function |
| --- | --- | --- | --- | --- | --- | --- |
| Unnamed\_\_1 | Glycine max | 224 | - | 11 | GAATTTAATTAA | 60K protein binding site |
| Unnamed\_\_1 | Glycine max | 12 | + | 11 | GAATTTAATTAA | 60K protein binding site |

> 2018/04/13 10:10:12  
+ GACTCGAAAC TGAAATTAAT TAAACTGAGA TTGTAATTGA TTTAGTTTGT GTGGAGAAAT AATGTGTTTT   
  
  
+ GGTAGTGAAG GGGTGAGGGT TAATTAAAGA GTAATTACGG TGGGGGAAAC GGTTTAGTAG ACAATAATAA   
  
  
+ TAGTGAATTA CAATTTGAAA AAGTTTTCCC TAAAACTATA AAACTATTAA ATTGTATAAT TGTTTTTTTA   
  
  
+ TTTTTTAAAA TTATTAATTA AATTATTTAA TAGTACACAG TAGTAGAAAA ATTTTCTAGT TGTGTTAAAT   
  
  
+ TTATTTAGTT TTTAAGTTTA AGTTAGTTTT TTTATAACCA CATGGTTTTT TAAACTAAGT TTGATATTTT   
  
  
+ TAAATTGTGC ATTATTAAAT TATTTTATTT ATTTATTAAA AAATAAAAAT ACATAGTAAT ATTTTAATAA   
  
  
+ TTTTTTTATG GAAAATTTGA AGTTAAATTT ACTTATGTTT AAAATAATAT ATATACTATT ATTTAAAACC   
  
  
+ CATTTTAGAG TAGTTTGGAA GTTCAAACCA ATATTTTTTA ATTTCTCTAA CAACAATAAC TTTACAAAGT   
  
  
+ TTTATTAAGT TTACTTAACA AAAAATAAAT TTAAAATAAA AAGTTTAAAT TTATAAAAAT TTATATTATA   
  
  
+ AATAAAAAAC ACTTAAAAAA TTAAAAAACT ATCATATTTT CAATTTTATA TATAAAAGTC ATGAGTAATT   
  
  
+ TCAAATTTTT TTTTTAATAC TACTATCGAA CTTATAAAAA AATGCATACA TTTTTAAAAA TTCAGTTTTA   
  
  
+ AGTAATATAT TCTAAAAGTT AAATTTATAG AGATTAAAAA TTTCAAAAAC TATTTTAAGT TAAATCTAAT   
  
  
+ GCCAAGTTAC AATTTAATAA AAACTTATAT GTTTAAATTA ATCATCGTAT TTTATTTATA GTTAAAAAAT   
  
  
+ ACCATAAATA TAAAATTAAA AATTTTTAAA TAAAAATTAA TTATATTATT TAATATTTTT TTAGTAAGGG   
  
  
+ GGAATATGGT AGTATTCAAG AAGTTTAATG AAAATTGTAA AAAGTTAATA CCTAGAAGTA TGTACGTACG   
  
  
+ TTTCGAACGT ATAGATACAT ACATGATATA TATATAAATT AGTTAATATG TACGTACGTA CGTGTACGTA   
  
  
+ TTGTATACAT ATATCGATAC GTAATGGCAT ACTCTTCTCT ATTAATTACT ATTTGTTAAA CTAATGTAGT   
  
  
+ AGAACGGGTG TACCTATAAT TATTCTCGCT CAGAATATGC TTTAGGCTAC TCTCCTTTCG GAGCCCTAGT   
  
  
+ GTAGAATCTT TAACAAAATG CCCGAACTGT ACCAGAAGGG GTGTACCATA GGCTCGGAGC ATGCTTTAGG   
  
  
+ GTACTCTTCT CTCAGAAAAC CCTAGTGTAG AACTTTAGCA GTTGAGGTTA CGGAGAGGAA TTAGTTAGTC   
  
  
+ ATCTAAATTA ATTAATTAGA GTTGAATCAA CAAATTAGGG GGTAGAGAAT ATATTCATGA CTTGGTGATT   
  
  
+ TAAATTGTTT TAATCTTATC AGGTTTATT  

- CTGAGCTTTG ACTTTAATTA ATTTGACTCT AACATTAACT AAATCAAACA CACCTCTTTA TTACACAAAA   
  
  
- CCATCACTTC CCCACTCCCA ATTAATTTCT CATTAATGCC ACCCCCTTTG CCAAATCATC TGTTATTATT   
  
  
- ATCACTTAAT GTTAAACTTT TTCAAAAGGG ATTTTGATAT TTTGATAATT TAACATATTA ACAAAAAAAT   
  
  
- AAAAAATTTT AATAATTAAT TTAATAAATT ATCATGTGTC ATCATCTTTT TAAAAGATCA ACACAATTTA   
  
  
- AATAAATCAA AAATTCAAAT TCAATCAAAA AAATATTGGT GTACCAAAAA ATTTGATTCA AACTATAAAA   
  
  
- ATTTAACACG TAATAATTTA ATAAAATAAA TAAATAATTT TTTATTTTTA TGTATCATTA TAAAATTATT   
  
  
- AAAAAAATAC CTTTTAAACT TCAATTTAAA TGAATACAAA TTTTATTATA TATATGATAA TAAATTTTGG   
  
  
- GTAAAATCTC ATCAAACCTT CAAGTTTGGT TATAAAAAAT TAAAGAGATT GTTGTTATTG AAATGTTTCA   
  
  
- AAATAATTCA AATGAATTGT TTTTTATTTA AATTTTATTT TTCAAATTTA AATATTTTTA AATATAATAT   
  
  
- TTATTTTTTG TGAATTTTTT AATTTTTTGA TAGTATAAAA GTTAAAATAT ATATTTTCAG TACTCATTAA   
  
  
- AGTTTAAAAA AAAAATTATG ATGATAGCTT GAATATTTTT TTACGTATGT AAAAATTTTT AAGTCAAAAT   
  
  
- TCATTATATA AGATTTTCAA TTTAAATATC TCTAATTTTT AAAGTTTTTG ATAAAATTCA ATTTAGATTA   
  
  
- CGGTTCAATG TTAAATTATT TTTGAATATA CAAATTTAAT TAGTAGCATA AAATAAATAT CAATTTTTTA   
  
  
- TGGTATTTAT ATTTTAATTT TTAAAAATTT ATTTTTAATT AATATAATAA ATTATAAAAA AATCATTCCC   
  
  
- CCTTATACCA TCATAAGTTC TTCAAATTAC TTTTAACATT TTTCAATTAT GGATCTTCAT ACATGCATGC   
  
  
- AAAGCTTGCA TATCTATGTA TGTACTATAT ATATATTTAA TCAATTATAC ATGCATGCAT GCACATGCAT   
  
  
- AACATATGTA TATAGCTATG CATTACCGTA TGAGAAGAGA TAATTAATGA TAAACAATTT GATTACATCA   
  
  
- TCTTGCCCAC ATGGATATTA ATAAGAGCGA GTCTTATACG AAATCCGATG AGAGGAAAGC CTCGGGATCA   
  
  
- CATCTTAGAA ATTGTTTTAC GGGCTTGACA TGGTCTTCCC CACATGGTAT CCGAGCCTCG TACGAAATCC   
  
  
- CATGAGAAGA GAGTCTTTTG GGATCACATC TTGAAATCGT CAACTCCAAT GCCTCTCCTT AATCAATCAG   
  
  
- TAGATTTAAT TAATTAATCT CAACTTAGTT GTTTAATCCC CCATCTCTTA TATAAGTACT GAACCACTAA   
  
  
- ATTTAACAAA ATTAGAATAG TCCAAATAA

+     Unnamed\_\_4

| Site Name | Organism | Position | Strand | Matrix score. | sequence | function |
| --- | --- | --- | --- | --- | --- | --- |
| Unnamed\_\_4 | Petroselinum hortense | 1242 | + | 4 | CTCC |  |
| Unnamed\_\_4 | Petroselinum hortense | 53 | - | 4 | CTCC |  |
| Unnamed\_\_4 | Petroselinum hortense | 1250 | - | 4 | CTCC |  |
| Unnamed\_\_4 | Petroselinum hortense | 1382 | - | 4 | CTCC |  |
| Unnamed\_\_4 | Petroselinum hortense | 1316 | - | 4 | CTCC |  |

> 2018/04/13 10:10:12  
+ GACTCGAAAC TGAAATTAAT TAAACTGAGA TTGTAATTGA TTTAGTTTGT GTGGAGAAAT AATGTGTTTT   
  
  
+ GGTAGTGAAG GGGTGAGGGT TAATTAAAGA GTAATTACGG TGGGGGAAAC GGTTTAGTAG ACAATAATAA   
  
  
+ TAGTGAATTA CAATTTGAAA AAGTTTTCCC TAAAACTATA AAACTATTAA ATTGTATAAT TGTTTTTTTA   
  
  
+ TTTTTTAAAA TTATTAATTA AATTATTTAA TAGTACACAG TAGTAGAAAA ATTTTCTAGT TGTGTTAAAT   
  
  
+ TTATTTAGTT TTTAAGTTTA AGTTAGTTTT TTTATAACCA CATGGTTTTT TAAACTAAGT TTGATATTTT   
  
  
+ TAAATTGTGC ATTATTAAAT TATTTTATTT ATTTATTAAA AAATAAAAAT ACATAGTAAT ATTTTAATAA   
  
  
+ TTTTTTTATG GAAAATTTGA AGTTAAATTT ACTTATGTTT AAAATAATAT ATATACTATT ATTTAAAACC   
  
  
+ CATTTTAGAG TAGTTTGGAA GTTCAAACCA ATATTTTTTA ATTTCTCTAA CAACAATAAC TTTACAAAGT   
  
  
+ TTTATTAAGT TTACTTAACA AAAAATAAAT TTAAAATAAA AAGTTTAAAT TTATAAAAAT TTATATTATA   
  
  
+ AATAAAAAAC ACTTAAAAAA TTAAAAAACT ATCATATTTT CAATTTTATA TATAAAAGTC ATGAGTAATT   
  
  
+ TCAAATTTTT TTTTTAATAC TACTATCGAA CTTATAAAAA AATGCATACA TTTTTAAAAA TTCAGTTTTA   
  
  
+ AGTAATATAT TCTAAAAGTT AAATTTATAG AGATTAAAAA TTTCAAAAAC TATTTTAAGT TAAATCTAAT   
  
  
+ GCCAAGTTAC AATTTAATAA AAACTTATAT GTTTAAATTA ATCATCGTAT TTTATTTATA GTTAAAAAAT   
  
  
+ ACCATAAATA TAAAATTAAA AATTTTTAAA TAAAAATTAA TTATATTATT TAATATTTTT TTAGTAAGGG   
  
  
+ GGAATATGGT AGTATTCAAG AAGTTTAATG AAAATTGTAA AAAGTTAATA CCTAGAAGTA TGTACGTACG   
  
  
+ TTTCGAACGT ATAGATACAT ACATGATATA TATATAAATT AGTTAATATG TACGTACGTA CGTGTACGTA   
  
  
+ TTGTATACAT ATATCGATAC GTAATGGCAT ACTCTTCTCT ATTAATTACT ATTTGTTAAA CTAATGTAGT   
  
  
+ AGAACGGGTG TACCTATAAT TATTCTCGCT CAGAATATGC TTTAGGCTAC TCTCCTTTCG GAGCCCTAGT   
  
  
+ GTAGAATCTT TAACAAAATG CCCGAACTGT ACCAGAAGGG GTGTACCATA GGCTCGGAGC ATGCTTTAGG   
  
  
+ GTACTCTTCT CTCAGAAAAC CCTAGTGTAG AACTTTAGCA GTTGAGGTTA CGGAGAGGAA TTAGTTAGTC   
  
  
+ ATCTAAATTA ATTAATTAGA GTTGAATCAA CAAATTAGGG GGTAGAGAAT ATATTCATGA CTTGGTGATT   
  
  
+ TAAATTGTTT TAATCTTATC AGGTTTATT  

- CTGAGCTTTG ACTTTAATTA ATTTGACTCT AACATTAACT AAATCAAACA CACCTCTTTA TTACACAAAA   
  
  
- CCATCACTTC CCCACTCCCA ATTAATTTCT CATTAATGCC ACCCCCTTTG CCAAATCATC TGTTATTATT   
  
  
- ATCACTTAAT GTTAAACTTT TTCAAAAGGG ATTTTGATAT TTTGATAATT TAACATATTA ACAAAAAAAT   
  
  
- AAAAAATTTT AATAATTAAT TTAATAAATT ATCATGTGTC ATCATCTTTT TAAAAGATCA ACACAATTTA   
  
  
- AATAAATCAA AAATTCAAAT TCAATCAAAA AAATATTGGT GTACCAAAAA ATTTGATTCA AACTATAAAA   
  
  
- ATTTAACACG TAATAATTTA ATAAAATAAA TAAATAATTT TTTATTTTTA TGTATCATTA TAAAATTATT   
  
  
- AAAAAAATAC CTTTTAAACT TCAATTTAAA TGAATACAAA TTTTATTATA TATATGATAA TAAATTTTGG   
  
  
- GTAAAATCTC ATCAAACCTT CAAGTTTGGT TATAAAAAAT TAAAGAGATT GTTGTTATTG AAATGTTTCA   
  
  
- AAATAATTCA AATGAATTGT TTTTTATTTA AATTTTATTT TTCAAATTTA AATATTTTTA AATATAATAT   
  
  
- TTATTTTTTG TGAATTTTTT AATTTTTTGA TAGTATAAAA GTTAAAATAT ATATTTTCAG TACTCATTAA   
  
  
- AGTTTAAAAA AAAAATTATG ATGATAGCTT GAATATTTTT TTACGTATGT AAAAATTTTT AAGTCAAAAT   
  
  
- TCATTATATA AGATTTTCAA TTTAAATATC TCTAATTTTT AAAGTTTTTG ATAAAATTCA ATTTAGATTA   
  
  
- CGGTTCAATG TTAAATTATT TTTGAATATA CAAATTTAAT TAGTAGCATA AAATAAATAT CAATTTTTTA   
  
  
- TGGTATTTAT ATTTTAATTT TTAAAAATTT ATTTTTAATT AATATAATAA ATTATAAAAA AATCATTCCC   
  
  
- CCTTATACCA TCATAAGTTC TTCAAATTAC TTTTAACATT TTTCAATTAT GGATCTTCAT ACATGCATGC   
  
  
- AAAGCTTGCA TATCTATGTA TGTACTATAT ATATATTTAA TCAATTATAC ATGCATGCAT GCACATGCAT   
  
  
- AACATATGTA TATAGCTATG CATTACCGTA TGAGAAGAGA TAATTAATGA TAAACAATTT GATTACATCA   
  
  
- TCTTGCCCAC ATGGATATTA ATAAGAGCGA GTCTTATACG AAATCCGATG AGAGGAAAGC CTCGGGATCA   
  
  
- CATCTTAGAA ATTGTTTTAC GGGCTTGACA TGGTCTTCCC CACATGGTAT CCGAGCCTCG TACGAAATCC   
  
  
- CATGAGAAGA GAGTCTTTTG GGATCACATC TTGAAATCGT CAACTCCAAT GCCTCTCCTT AATCAATCAG   
  
  
- TAGATTTAAT TAATTAATCT CAACTTAGTT GTTTAATCCC CCATCTCTTA TATAAGTACT GAACCACTAA   
  
  
- ATTTAACAAA ATTAGAATAG TCCAAATAA

+     chs-CMA1a

| Site Name | Organism | Position | Strand | Matrix score. | sequence | function |
| --- | --- | --- | --- | --- | --- | --- |
| chs-CMA1a | Daucus carota | 768 | - | 8 | TTACTTAA | part of a light responsive element |
| chs-CMA1a | Daucus carota | 571 | + | 8 | TTACTTAA | part of a light responsive element |

> 2018/04/13 10:10:12  
+ GACTCGAAAC TGAAATTAAT TAAACTGAGA TTGTAATTGA TTTAGTTTGT GTGGAGAAAT AATGTGTTTT   
  
  
+ GGTAGTGAAG GGGTGAGGGT TAATTAAAGA GTAATTACGG TGGGGGAAAC GGTTTAGTAG ACAATAATAA   
  
  
+ TAGTGAATTA CAATTTGAAA AAGTTTTCCC TAAAACTATA AAACTATTAA ATTGTATAAT TGTTTTTTTA   
  
  
+ TTTTTTAAAA TTATTAATTA AATTATTTAA TAGTACACAG TAGTAGAAAA ATTTTCTAGT TGTGTTAAAT   
  
  
+ TTATTTAGTT TTTAAGTTTA AGTTAGTTTT TTTATAACCA CATGGTTTTT TAAACTAAGT TTGATATTTT   
  
  
+ TAAATTGTGC ATTATTAAAT TATTTTATTT ATTTATTAAA AAATAAAAAT ACATAGTAAT ATTTTAATAA   
  
  
+ TTTTTTTATG GAAAATTTGA AGTTAAATTT ACTTATGTTT AAAATAATAT ATATACTATT ATTTAAAACC   
  
  
+ CATTTTAGAG TAGTTTGGAA GTTCAAACCA ATATTTTTTA ATTTCTCTAA CAACAATAAC TTTACAAAGT   
  
  
+ TTTATTAAGT TTACTTAACA AAAAATAAAT TTAAAATAAA AAGTTTAAAT TTATAAAAAT TTATATTATA   
  
  
+ AATAAAAAAC ACTTAAAAAA TTAAAAAACT ATCATATTTT CAATTTTATA TATAAAAGTC ATGAGTAATT   
  
  
+ TCAAATTTTT TTTTTAATAC TACTATCGAA CTTATAAAAA AATGCATACA TTTTTAAAAA TTCAGTTTTA   
  
  
+ AGTAATATAT TCTAAAAGTT AAATTTATAG AGATTAAAAA TTTCAAAAAC TATTTTAAGT TAAATCTAAT   
  
  
+ GCCAAGTTAC AATTTAATAA AAACTTATAT GTTTAAATTA ATCATCGTAT TTTATTTATA GTTAAAAAAT   
  
  
+ ACCATAAATA TAAAATTAAA AATTTTTAAA TAAAAATTAA TTATATTATT TAATATTTTT TTAGTAAGGG   
  
  
+ GGAATATGGT AGTATTCAAG AAGTTTAATG AAAATTGTAA AAAGTTAATA CCTAGAAGTA TGTACGTACG   
  
  
+ TTTCGAACGT ATAGATACAT ACATGATATA TATATAAATT AGTTAATATG TACGTACGTA CGTGTACGTA   
  
  
+ TTGTATACAT ATATCGATAC GTAATGGCAT ACTCTTCTCT ATTAATTACT ATTTGTTAAA CTAATGTAGT   
  
  
+ AGAACGGGTG TACCTATAAT TATTCTCGCT CAGAATATGC TTTAGGCTAC TCTCCTTTCG GAGCCCTAGT   
  
  
+ GTAGAATCTT TAACAAAATG CCCGAACTGT ACCAGAAGGG GTGTACCATA GGCTCGGAGC ATGCTTTAGG   
  
  
+ GTACTCTTCT CTCAGAAAAC CCTAGTGTAG AACTTTAGCA GTTGAGGTTA CGGAGAGGAA TTAGTTAGTC   
  
  
+ ATCTAAATTA ATTAATTAGA GTTGAATCAA CAAATTAGGG GGTAGAGAAT ATATTCATGA CTTGGTGATT   
  
  
+ TAAATTGTTT TAATCTTATC AGGTTTATT  

- CTGAGCTTTG ACTTTAATTA ATTTGACTCT AACATTAACT AAATCAAACA CACCTCTTTA TTACACAAAA   
  
  
- CCATCACTTC CCCACTCCCA ATTAATTTCT CATTAATGCC ACCCCCTTTG CCAAATCATC TGTTATTATT   
  
  
- ATCACTTAAT GTTAAACTTT TTCAAAAGGG ATTTTGATAT TTTGATAATT TAACATATTA ACAAAAAAAT   
  
  
- AAAAAATTTT AATAATTAAT TTAATAAATT ATCATGTGTC ATCATCTTTT TAAAAGATCA ACACAATTTA   
  
  
- AATAAATCAA AAATTCAAAT TCAATCAAAA AAATATTGGT GTACCAAAAA ATTTGATTCA AACTATAAAA   
  
  
- ATTTAACACG TAATAATTTA ATAAAATAAA TAAATAATTT TTTATTTTTA TGTATCATTA TAAAATTATT   
  
  
- AAAAAAATAC CTTTTAAACT TCAATTTAAA TGAATACAAA TTTTATTATA TATATGATAA TAAATTTTGG   
  
  
- GTAAAATCTC ATCAAACCTT CAAGTTTGGT TATAAAAAAT TAAAGAGATT GTTGTTATTG AAATGTTTCA   
  
  
- AAATAATTCA AATGAATTGT TTTTTATTTA AATTTTATTT TTCAAATTTA AATATTTTTA AATATAATAT   
  
  
- TTATTTTTTG TGAATTTTTT AATTTTTTGA TAGTATAAAA GTTAAAATAT ATATTTTCAG TACTCATTAA   
  
  
- AGTTTAAAAA AAAAATTATG ATGATAGCTT GAATATTTTT TTACGTATGT AAAAATTTTT AAGTCAAAAT   
  
  
- TCATTATATA AGATTTTCAA TTTAAATATC TCTAATTTTT AAAGTTTTTG ATAAAATTCA ATTTAGATTA   
  
  
- CGGTTCAATG TTAAATTATT TTTGAATATA CAAATTTAAT TAGTAGCATA AAATAAATAT CAATTTTTTA   
  
  
- TGGTATTTAT ATTTTAATTT TTAAAAATTT ATTTTTAATT AATATAATAA ATTATAAAAA AATCATTCCC   
  
  
- CCTTATACCA TCATAAGTTC TTCAAATTAC TTTTAACATT TTTCAATTAT GGATCTTCAT ACATGCATGC   
  
  
- AAAGCTTGCA TATCTATGTA TGTACTATAT ATATATTTAA TCAATTATAC ATGCATGCAT GCACATGCAT   
  
  
- AACATATGTA TATAGCTATG CATTACCGTA TGAGAAGAGA TAATTAATGA TAAACAATTT GATTACATCA   
  
  
- TCTTGCCCAC ATGGATATTA ATAAGAGCGA GTCTTATACG AAATCCGATG AGAGGAAAGC CTCGGGATCA   
  
  
- CATCTTAGAA ATTGTTTTAC GGGCTTGACA TGGTCTTCCC CACATGGTAT CCGAGCCTCG TACGAAATCC   
  
  
- CATGAGAAGA GAGTCTTTTG GGATCACATC TTGAAATCGT CAACTCCAAT GCCTCTCCTT AATCAATCAG   
  
  
- TAGATTTAAT TAATTAATCT CAACTTAGTT GTTTAATCCC CCATCTCTTA TATAAGTACT GAACCACTAA   
  
  
- ATTTAACAAA ATTAGAATAG TCCAAATAA
