# Supplementary material for: Genome-wide analysis of the WRKY gene family in drumstick (Moringa oleifera Lam.)
Source: PeerJ. 2019 Jun 10;7:e7063. doi: 10.7717/peerj.7063 (PMC6563795; doi:10.7717/peerj.7063)
Supplement: Supplemental Information 1 [file peerj-07-7063-s003.gz › MoWRKY27_plantcare.html]

Content-Type: text/html; charset=ISO-8859-1


CallMat\_Firefox


Webmaster Firefox specific output  
To save the result:
click on the frame with the right mouse button and save the source code as a text file with extension .html  
REFERENCE:PlantCARE: a database of plant cis-acting regulatory elements and a portal to tools for in silico analysis of promoter sequences.  
Lescot, M., Déhais, P., Moreau, Y., De Moor, B., Rouzé ,P.,and Rombauts, S.  
Nucleic Acids Res., Database issue(2002), 30(1):325-327.   


---

> 2018/04/13 10:10:12  
+ GACGTCGAAG AGATTTCTCT TTCAATTTGG AGAAGCACTC TCTACTTTCC CCCTATCTCT CTCTTTCTCT   
  
  
+ CTAAGCCTCG AGTCGTTCTG TAACTTGACT TTGAGTCTCT TGACGATTTT ACCAAGAAAC CCTCCCCCTC   
  
  
+ TCTTCGGTTC CCTTCTACCC CCTACTATAT TCCTCCCACC TAGTGGCCCA CTGGCCCATT CTGTATCCCC   
  
  
+ AAATCAGTTT CACCCAAAAA CTAGGTTGCC GACACTACTC AAACTTTTAA AGGTACCCTA CTACACTTAT   
  
  
+ TTCTTTAGTC TCCGACCAGA GGAGTTCTTC ATTCATAACC CTGGGTGGGA ACCTTTTCTA TCTTTATCTA   
  
  
+ TATTTTCATC CCTGAGCAAA GAAAAAGAAA AAGGAGCCAT TTTTATCAAT GTGGAGAATA AGACAAACTT   
  
  
+ ATAAAGGACC TTTGATCTCT GATGATCGTA AATCAGAAAT TTCAATTCAA ACTAATACAC GGTGTTGGCA   
  
  
+ ACTGCCAGTC TTAACCTAGG ATACTCATGT GCATCATAAA AGTACTAACG ATGAAGATCA TTGTAAATTG   
  
  
+ CCTCAATAAA CTGGCAATTA CACTTTTTAT TTCCTTTTTC CTTCTCGTTA CCATTATTAA CGAATTAATC   
  
  
+ CGTCATTTTA AAGAATTTTG AATGGATTGC GCACCAATGA GTACTCAACG ACAACAAGTG GGTTTTTTTC   
  
  
+ CGTTGTGCAT GTCATGAACC TTGATCCGCT CTTCCTTTCT TTCGACTGGT TTTCACCCAG AGTGCACCCC   
  
  
+ CGGTAAAGAA AAATATTAGA CTTTGACAAC TGGTCGAAAA GAATAAAGAA GGAATGGGGA GCAAAGGCCA   
  
  
+ TAGACAGAAG ACTTGGCAGG CTAGATAAGA GTAGTTAGGT TGCCAGCTTA GCGTGGCTCT TTCAACTGGT   
  
  
+ TCTGAAAATA TGAAACCTCT TGGGTCTTTT TCGATGTTCG GAACCCAAAA AGTGGTGCAC CGTATTCCGA   
  
  
+ CCACGGTTAA GTTTTGCTTT GTGTTCGTAT CTCTCTCTTT GTCTGTCTGG AATTTTTCAA ATAATTTCTA   
  
  
+ TTTTGCTTCA GCAAAATCGG ACGATTAAAT ACATCAGGAC ATTAAACAGT TGCCGAGTCT ACTAATTACA   
  
  
+ CCCCAACTGG GTTGGCCCAC TCTTCTGAGG TGACTAGTTA AAAGATCTTA AGACTATCGA CCTACGAACT   
  
  
+ TGTTCAGAAT TCACCATTCG TTTAATTATT TATTAATAAT TCTATTGTTT CGAATGTCAT GGAAGTGGTT   
  
  
+ GTAAATTGCG TAATTAATTG TCGAAAAACT GGTTTTTTGG TGGCATAAAA GAGTACGTAA GGACTACCCA   
  
  
+ TTTGCTATTC CGAGTACATA AGAATTAAAC CCATATTAAG CTTTTAAATC GACGATTAAC TCCTATTTTG   
  
  
+ ACATTAAATT CACGCCTATA TATTACAATA TTTATGTATT TCCGAACACC GTGCTGATCT AATGGACGAA   
  
  
+ TCAACTACGG AAGGAAGACC GTTAATTGG  

- CTGCAGCTTC TCTAAAGAGA AAGTTAAACC TCTTCGTGAG AGATGAAAGG GGGATAGAGA GAGAAAGAGA   
  
  
- GATTCGGAGC TCAGCAAGAC ATTGAACTGA AACTCAGAGA ACTGCTAAAA TGGTTCTTTG GGAGGGGGAG   
  
  
- AGAAGCCAAG GGAAGATGGG GGATGATATA AGGAGGGTGG ATCACCGGGT GACCGGGTAA GACATAGGGG   
  
  
- TTTAGTCAAA GTGGGTTTTT GATCCAACGG CTGTGATGAG TTTGAAAATT TCCATGGGAT GATGTGAATA   
  
  
- AAGAAATCAG AGGCTGGTCT CCTCAAGAAG TAAGTATTGG GACCCACCCT TGGAAAAGAT AGAAATAGAT   
  
  
- ATAAAAGTAG GGACTCGTTT CTTTTTCTTT TTCCTCGGTA AAAATAGTTA CACCTCTTAT TCTGTTTGAA   
  
  
- TATTTCCTGG AAACTAGAGA CTACTAGCAT TTAGTCTTTA AAGTTAAGTT TGATTATGTG CCACAACCGT   
  
  
- TGACGGTCAG AATTGGATCC TATGAGTACA CGTAGTATTT TCATGATTGC TACTTCTAGT AACATTTAAC   
  
  
- GGAGTTATTT GACCGTTAAT GTGAAAAATA AAGGAAAAAG GAAGAGCAAT GGTAATAATT GCTTAATTAG   
  
  
- GCAGTAAAAT TTCTTAAAAC TTACCTAACG CGTGGTTACT CATGAGTTGC TGTTGTTCAC CCAAAAAAAG   
  
  
- GCAACACGTA CAGTACTTGG AACTAGGCGA GAAGGAAAGA AAGCTGACCA AAAGTGGGTC TCACGTGGGG   
  
  
- GCCATTTCTT TTTATAATCT GAAACTGTTG ACCAGCTTTT CTTATTTCTT CCTTACCCCT CGTTTCCGGT   
  
  
- ATCTGTCTTC TGAACCGTCC GATCTATTCT CATCAATCCA ACGGTCGAAT CGCACCGAGA AAGTTGACCA   
  
  
- AGACTTTTAT ACTTTGGAGA ACCCAGAAAA AGCTACAAGC CTTGGGTTTT TCACCACGTG GCATAAGGCT   
  
  
- GGTGCCAATT CAAAACGAAA CACAAGCATA GAGAGAGAAA CAGACAGACC TTAAAAAGTT TATTAAAGAT   
  
  
- AAAACGAAGT CGTTTTAGCC TGCTAATTTA TGTAGTCCTG TAATTTGTCA ACGGCTCAGA TGATTAATGT   
  
  
- GGGGTTGACC CAACCGGGTG AGAAGACTCC ACTGATCAAT TTTCTAGAAT TCTGATAGCT GGATGCTTGA   
  
  
- ACAAGTCTTA AGTGGTAAGC AAATTAATAA ATAATTATTA AGATAACAAA GCTTACAGTA CCTTCACCAA   
  
  
- CATTTAACGC ATTAATTAAC AGCTTTTTGA CCAAAAAACC ACCGTATTTT CTCATGCATT CCTGATGGGT   
  
  
- AAACGATAAG GCTCATGTAT TCTTAATTTG GGTATAATTC GAAAATTTAG CTGCTAATTG AGGATAAAAC   
  
  
- TGTAATTTAA GTGCGGATAT ATAATGTTAT AAATACATAA AGGCTTGTGG CACGACTAGA TTACCTGCTT   
  
  
- AGTTGATGCC TTCCTTCTGG CAATTAACC

  
  
Motifs Found  

+     AAGAA-motif

| Site Name | Organism | Position | Strand | Matrix score. | sequence | function |
| --- | --- | --- | --- | --- | --- | --- |
| AAGAA-motif | Avena sativa | 772 | + | 10 | gGTAAAGAAA |  |
| AAGAA-motif | Avena sativa | 737 | - | 7 | GAAAGAA |  |

> 2018/04/13 10:10:12  
+ GACGTCGAAG AGATTTCTCT TTCAATTTGG AGAAGCACTC TCTACTTTCC CCCTATCTCT CTCTTTCTCT   
  
  
+ CTAAGCCTCG AGTCGTTCTG TAACTTGACT TTGAGTCTCT TGACGATTTT ACCAAGAAAC CCTCCCCCTC   
  
  
+ TCTTCGGTTC CCTTCTACCC CCTACTATAT TCCTCCCACC TAGTGGCCCA CTGGCCCATT CTGTATCCCC   
  
  
+ AAATCAGTTT CACCCAAAAA CTAGGTTGCC GACACTACTC AAACTTTTAA AGGTACCCTA CTACACTTAT   
  
  
+ TTCTTTAGTC TCCGACCAGA GGAGTTCTTC ATTCATAACC CTGGGTGGGA ACCTTTTCTA TCTTTATCTA   
  
  
+ TATTTTCATC CCTGAGCAAA GAAAAAGAAA AAGGAGCCAT TTTTATCAAT GTGGAGAATA AGACAAACTT   
  
  
+ ATAAAGGACC TTTGATCTCT GATGATCGTA AATCAGAAAT TTCAATTCAA ACTAATACAC GGTGTTGGCA   
  
  
+ ACTGCCAGTC TTAACCTAGG ATACTCATGT GCATCATAAA AGTACTAACG ATGAAGATCA TTGTAAATTG   
  
  
+ CCTCAATAAA CTGGCAATTA CACTTTTTAT TTCCTTTTTC CTTCTCGTTA CCATTATTAA CGAATTAATC   
  
  
+ CGTCATTTTA AAGAATTTTG AATGGATTGC GCACCAATGA GTACTCAACG ACAACAAGTG GGTTTTTTTC   
  
  
+ CGTTGTGCAT GTCATGAACC TTGATCCGCT CTTCCTTTCT TTCGACTGGT TTTCACCCAG AGTGCACCCC   
  
  
+ CGGTAAAGAA AAATATTAGA CTTTGACAAC TGGTCGAAAA GAATAAAGAA GGAATGGGGA GCAAAGGCCA   
  
  
+ TAGACAGAAG ACTTGGCAGG CTAGATAAGA GTAGTTAGGT TGCCAGCTTA GCGTGGCTCT TTCAACTGGT   
  
  
+ TCTGAAAATA TGAAACCTCT TGGGTCTTTT TCGATGTTCG GAACCCAAAA AGTGGTGCAC CGTATTCCGA   
  
  
+ CCACGGTTAA GTTTTGCTTT GTGTTCGTAT CTCTCTCTTT GTCTGTCTGG AATTTTTCAA ATAATTTCTA   
  
  
+ TTTTGCTTCA GCAAAATCGG ACGATTAAAT ACATCAGGAC ATTAAACAGT TGCCGAGTCT ACTAATTACA   
  
  
+ CCCCAACTGG GTTGGCCCAC TCTTCTGAGG TGACTAGTTA AAAGATCTTA AGACTATCGA CCTACGAACT   
  
  
+ TGTTCAGAAT TCACCATTCG TTTAATTATT TATTAATAAT TCTATTGTTT CGAATGTCAT GGAAGTGGTT   
  
  
+ GTAAATTGCG TAATTAATTG TCGAAAAACT GGTTTTTTGG TGGCATAAAA GAGTACGTAA GGACTACCCA   
  
  
+ TTTGCTATTC CGAGTACATA AGAATTAAAC CCATATTAAG CTTTTAAATC GACGATTAAC TCCTATTTTG   
  
  
+ ACATTAAATT CACGCCTATA TATTACAATA TTTATGTATT TCCGAACACC GTGCTGATCT AATGGACGAA   
  
  
+ TCAACTACGG AAGGAAGACC GTTAATTGG  

- CTGCAGCTTC TCTAAAGAGA AAGTTAAACC TCTTCGTGAG AGATGAAAGG GGGATAGAGA GAGAAAGAGA   
  
  
- GATTCGGAGC TCAGCAAGAC ATTGAACTGA AACTCAGAGA ACTGCTAAAA TGGTTCTTTG GGAGGGGGAG   
  
  
- AGAAGCCAAG GGAAGATGGG GGATGATATA AGGAGGGTGG ATCACCGGGT GACCGGGTAA GACATAGGGG   
  
  
- TTTAGTCAAA GTGGGTTTTT GATCCAACGG CTGTGATGAG TTTGAAAATT TCCATGGGAT GATGTGAATA   
  
  
- AAGAAATCAG AGGCTGGTCT CCTCAAGAAG TAAGTATTGG GACCCACCCT TGGAAAAGAT AGAAATAGAT   
  
  
- ATAAAAGTAG GGACTCGTTT CTTTTTCTTT TTCCTCGGTA AAAATAGTTA CACCTCTTAT TCTGTTTGAA   
  
  
- TATTTCCTGG AAACTAGAGA CTACTAGCAT TTAGTCTTTA AAGTTAAGTT TGATTATGTG CCACAACCGT   
  
  
- TGACGGTCAG AATTGGATCC TATGAGTACA CGTAGTATTT TCATGATTGC TACTTCTAGT AACATTTAAC   
  
  
- GGAGTTATTT GACCGTTAAT GTGAAAAATA AAGGAAAAAG GAAGAGCAAT GGTAATAATT GCTTAATTAG   
  
  
- GCAGTAAAAT TTCTTAAAAC TTACCTAACG CGTGGTTACT CATGAGTTGC TGTTGTTCAC CCAAAAAAAG   
  
  
- GCAACACGTA CAGTACTTGG AACTAGGCGA GAAGGAAAGA AAGCTGACCA AAAGTGGGTC TCACGTGGGG   
  
  
- GCCATTTCTT TTTATAATCT GAAACTGTTG ACCAGCTTTT CTTATTTCTT CCTTACCCCT CGTTTCCGGT   
  
  
- ATCTGTCTTC TGAACCGTCC GATCTATTCT CATCAATCCA ACGGTCGAAT CGCACCGAGA AAGTTGACCA   
  
  
- AGACTTTTAT ACTTTGGAGA ACCCAGAAAA AGCTACAAGC CTTGGGTTTT TCACCACGTG GCATAAGGCT   
  
  
- GGTGCCAATT CAAAACGAAA CACAAGCATA GAGAGAGAAA CAGACAGACC TTAAAAAGTT TATTAAAGAT   
  
  
- AAAACGAAGT CGTTTTAGCC TGCTAATTTA TGTAGTCCTG TAATTTGTCA ACGGCTCAGA TGATTAATGT   
  
  
- GGGGTTGACC CAACCGGGTG AGAAGACTCC ACTGATCAAT TTTCTAGAAT TCTGATAGCT GGATGCTTGA   
  
  
- ACAAGTCTTA AGTGGTAAGC AAATTAATAA ATAATTATTA AGATAACAAA GCTTACAGTA CCTTCACCAA   
  
  
- CATTTAACGC ATTAATTAAC AGCTTTTTGA CCAAAAAACC ACCGTATTTT CTCATGCATT CCTGATGGGT   
  
  
- AAACGATAAG GCTCATGTAT TCTTAATTTG GGTATAATTC GAAAATTTAG CTGCTAATTG AGGATAAAAC   
  
  
- TGTAATTTAA GTGCGGATAT ATAATGTTAT AAATACATAA AGGCTTGTGG CACGACTAGA TTACCTGCTT   
  
  
- AGTTGATGCC TTCCTTCTGG CAATTAACC

+     ARE

| Site Name | Organism | Position | Strand | Matrix score. | sequence | function |
| --- | --- | --- | --- | --- | --- | --- |
| ARE | Zea mays | 1290 | + | 6 | TGGTTT | cis-acting regulatory element essential for the anaerobic induction |
| ARE | Zea mays | 747 | + | 6 | TGGTTT | cis-acting regulatory element essential for the anaerobic induction |

> 2018/04/13 10:10:12  
+ GACGTCGAAG AGATTTCTCT TTCAATTTGG AGAAGCACTC TCTACTTTCC CCCTATCTCT CTCTTTCTCT   
  
  
+ CTAAGCCTCG AGTCGTTCTG TAACTTGACT TTGAGTCTCT TGACGATTTT ACCAAGAAAC CCTCCCCCTC   
  
  
+ TCTTCGGTTC CCTTCTACCC CCTACTATAT TCCTCCCACC TAGTGGCCCA CTGGCCCATT CTGTATCCCC   
  
  
+ AAATCAGTTT CACCCAAAAA CTAGGTTGCC GACACTACTC AAACTTTTAA AGGTACCCTA CTACACTTAT   
  
  
+ TTCTTTAGTC TCCGACCAGA GGAGTTCTTC ATTCATAACC CTGGGTGGGA ACCTTTTCTA TCTTTATCTA   
  
  
+ TATTTTCATC CCTGAGCAAA GAAAAAGAAA AAGGAGCCAT TTTTATCAAT GTGGAGAATA AGACAAACTT   
  
  
+ ATAAAGGACC TTTGATCTCT GATGATCGTA AATCAGAAAT TTCAATTCAA ACTAATACAC GGTGTTGGCA   
  
  
+ ACTGCCAGTC TTAACCTAGG ATACTCATGT GCATCATAAA AGTACTAACG ATGAAGATCA TTGTAAATTG   
  
  
+ CCTCAATAAA CTGGCAATTA CACTTTTTAT TTCCTTTTTC CTTCTCGTTA CCATTATTAA CGAATTAATC   
  
  
+ CGTCATTTTA AAGAATTTTG AATGGATTGC GCACCAATGA GTACTCAACG ACAACAAGTG GGTTTTTTTC   
  
  
+ CGTTGTGCAT GTCATGAACC TTGATCCGCT CTTCCTTTCT TTCGACTGGT TTTCACCCAG AGTGCACCCC   
  
  
+ CGGTAAAGAA AAATATTAGA CTTTGACAAC TGGTCGAAAA GAATAAAGAA GGAATGGGGA GCAAAGGCCA   
  
  
+ TAGACAGAAG ACTTGGCAGG CTAGATAAGA GTAGTTAGGT TGCCAGCTTA GCGTGGCTCT TTCAACTGGT   
  
  
+ TCTGAAAATA TGAAACCTCT TGGGTCTTTT TCGATGTTCG GAACCCAAAA AGTGGTGCAC CGTATTCCGA   
  
  
+ CCACGGTTAA GTTTTGCTTT GTGTTCGTAT CTCTCTCTTT GTCTGTCTGG AATTTTTCAA ATAATTTCTA   
  
  
+ TTTTGCTTCA GCAAAATCGG ACGATTAAAT ACATCAGGAC ATTAAACAGT TGCCGAGTCT ACTAATTACA   
  
  
+ CCCCAACTGG GTTGGCCCAC TCTTCTGAGG TGACTAGTTA AAAGATCTTA AGACTATCGA CCTACGAACT   
  
  
+ TGTTCAGAAT TCACCATTCG TTTAATTATT TATTAATAAT TCTATTGTTT CGAATGTCAT GGAAGTGGTT   
  
  
+ GTAAATTGCG TAATTAATTG TCGAAAAACT GGTTTTTTGG TGGCATAAAA GAGTACGTAA GGACTACCCA   
  
  
+ TTTGCTATTC CGAGTACATA AGAATTAAAC CCATATTAAG CTTTTAAATC GACGATTAAC TCCTATTTTG   
  
  
+ ACATTAAATT CACGCCTATA TATTACAATA TTTATGTATT TCCGAACACC GTGCTGATCT AATGGACGAA   
  
  
+ TCAACTACGG AAGGAAGACC GTTAATTGG  

- CTGCAGCTTC TCTAAAGAGA AAGTTAAACC TCTTCGTGAG AGATGAAAGG GGGATAGAGA GAGAAAGAGA   
  
  
- GATTCGGAGC TCAGCAAGAC ATTGAACTGA AACTCAGAGA ACTGCTAAAA TGGTTCTTTG GGAGGGGGAG   
  
  
- AGAAGCCAAG GGAAGATGGG GGATGATATA AGGAGGGTGG ATCACCGGGT GACCGGGTAA GACATAGGGG   
  
  
- TTTAGTCAAA GTGGGTTTTT GATCCAACGG CTGTGATGAG TTTGAAAATT TCCATGGGAT GATGTGAATA   
  
  
- AAGAAATCAG AGGCTGGTCT CCTCAAGAAG TAAGTATTGG GACCCACCCT TGGAAAAGAT AGAAATAGAT   
  
  
- ATAAAAGTAG GGACTCGTTT CTTTTTCTTT TTCCTCGGTA AAAATAGTTA CACCTCTTAT TCTGTTTGAA   
  
  
- TATTTCCTGG AAACTAGAGA CTACTAGCAT TTAGTCTTTA AAGTTAAGTT TGATTATGTG CCACAACCGT   
  
  
- TGACGGTCAG AATTGGATCC TATGAGTACA CGTAGTATTT TCATGATTGC TACTTCTAGT AACATTTAAC   
  
  
- GGAGTTATTT GACCGTTAAT GTGAAAAATA AAGGAAAAAG GAAGAGCAAT GGTAATAATT GCTTAATTAG   
  
  
- GCAGTAAAAT TTCTTAAAAC TTACCTAACG CGTGGTTACT CATGAGTTGC TGTTGTTCAC CCAAAAAAAG   
  
  
- GCAACACGTA CAGTACTTGG AACTAGGCGA GAAGGAAAGA AAGCTGACCA AAAGTGGGTC TCACGTGGGG   
  
  
- GCCATTTCTT TTTATAATCT GAAACTGTTG ACCAGCTTTT CTTATTTCTT CCTTACCCCT CGTTTCCGGT   
  
  
- ATCTGTCTTC TGAACCGTCC GATCTATTCT CATCAATCCA ACGGTCGAAT CGCACCGAGA AAGTTGACCA   
  
  
- AGACTTTTAT ACTTTGGAGA ACCCAGAAAA AGCTACAAGC CTTGGGTTTT TCACCACGTG GCATAAGGCT   
  
  
- GGTGCCAATT CAAAACGAAA CACAAGCATA GAGAGAGAAA CAGACAGACC TTAAAAAGTT TATTAAAGAT   
  
  
- AAAACGAAGT CGTTTTAGCC TGCTAATTTA TGTAGTCCTG TAATTTGTCA ACGGCTCAGA TGATTAATGT   
  
  
- GGGGTTGACC CAACCGGGTG AGAAGACTCC ACTGATCAAT TTTCTAGAAT TCTGATAGCT GGATGCTTGA   
  
  
- ACAAGTCTTA AGTGGTAAGC AAATTAATAA ATAATTATTA AGATAACAAA GCTTACAGTA CCTTCACCAA   
  
  
- CATTTAACGC ATTAATTAAC AGCTTTTTGA CCAAAAAACC ACCGTATTTT CTCATGCATT CCTGATGGGT   
  
  
- AAACGATAAG GCTCATGTAT TCTTAATTTG GGTATAATTC GAAAATTTAG CTGCTAATTG AGGATAAAAC   
  
  
- TGTAATTTAA GTGCGGATAT ATAATGTTAT AAATACATAA AGGCTTGTGG CACGACTAGA TTACCTGCTT   
  
  
- AGTTGATGCC TTCCTTCTGG CAATTAACC

+     Box 4

| Site Name | Organism | Position | Strand | Matrix score. | sequence | function |
| --- | --- | --- | --- | --- | --- | --- |
| Box 4 | Petroselinum crispum | 1273 | - | 6 | ATTAAT | part of a conserved DNA module involved in light responsiveness |
| Box 4 | Petroselinum crispum | 624 | + | 6 | ATTAAT | part of a conserved DNA module involved in light responsiveness |
| Box 4 | Petroselinum crispum | 1222 | - | 6 | ATTAAT | part of a conserved DNA module involved in light responsiveness |

> 2018/04/13 10:10:12  
+ GACGTCGAAG AGATTTCTCT TTCAATTTGG AGAAGCACTC TCTACTTTCC CCCTATCTCT CTCTTTCTCT   
  
  
+ CTAAGCCTCG AGTCGTTCTG TAACTTGACT TTGAGTCTCT TGACGATTTT ACCAAGAAAC CCTCCCCCTC   
  
  
+ TCTTCGGTTC CCTTCTACCC CCTACTATAT TCCTCCCACC TAGTGGCCCA CTGGCCCATT CTGTATCCCC   
  
  
+ AAATCAGTTT CACCCAAAAA CTAGGTTGCC GACACTACTC AAACTTTTAA AGGTACCCTA CTACACTTAT   
  
  
+ TTCTTTAGTC TCCGACCAGA GGAGTTCTTC ATTCATAACC CTGGGTGGGA ACCTTTTCTA TCTTTATCTA   
  
  
+ TATTTTCATC CCTGAGCAAA GAAAAAGAAA AAGGAGCCAT TTTTATCAAT GTGGAGAATA AGACAAACTT   
  
  
+ ATAAAGGACC TTTGATCTCT GATGATCGTA AATCAGAAAT TTCAATTCAA ACTAATACAC GGTGTTGGCA   
  
  
+ ACTGCCAGTC TTAACCTAGG ATACTCATGT GCATCATAAA AGTACTAACG ATGAAGATCA TTGTAAATTG   
  
  
+ CCTCAATAAA CTGGCAATTA CACTTTTTAT TTCCTTTTTC CTTCTCGTTA CCATTATTAA CGAATTAATC   
  
  
+ CGTCATTTTA AAGAATTTTG AATGGATTGC GCACCAATGA GTACTCAACG ACAACAAGTG GGTTTTTTTC   
  
  
+ CGTTGTGCAT GTCATGAACC TTGATCCGCT CTTCCTTTCT TTCGACTGGT TTTCACCCAG AGTGCACCCC   
  
  
+ CGGTAAAGAA AAATATTAGA CTTTGACAAC TGGTCGAAAA GAATAAAGAA GGAATGGGGA GCAAAGGCCA   
  
  
+ TAGACAGAAG ACTTGGCAGG CTAGATAAGA GTAGTTAGGT TGCCAGCTTA GCGTGGCTCT TTCAACTGGT   
  
  
+ TCTGAAAATA TGAAACCTCT TGGGTCTTTT TCGATGTTCG GAACCCAAAA AGTGGTGCAC CGTATTCCGA   
  
  
+ CCACGGTTAA GTTTTGCTTT GTGTTCGTAT CTCTCTCTTT GTCTGTCTGG AATTTTTCAA ATAATTTCTA   
  
  
+ TTTTGCTTCA GCAAAATCGG ACGATTAAAT ACATCAGGAC ATTAAACAGT TGCCGAGTCT ACTAATTACA   
  
  
+ CCCCAACTGG GTTGGCCCAC TCTTCTGAGG TGACTAGTTA AAAGATCTTA AGACTATCGA CCTACGAACT   
  
  
+ TGTTCAGAAT TCACCATTCG TTTAATTATT TATTAATAAT TCTATTGTTT CGAATGTCAT GGAAGTGGTT   
  
  
+ GTAAATTGCG TAATTAATTG TCGAAAAACT GGTTTTTTGG TGGCATAAAA GAGTACGTAA GGACTACCCA   
  
  
+ TTTGCTATTC CGAGTACATA AGAATTAAAC CCATATTAAG CTTTTAAATC GACGATTAAC TCCTATTTTG   
  
  
+ ACATTAAATT CACGCCTATA TATTACAATA TTTATGTATT TCCGAACACC GTGCTGATCT AATGGACGAA   
  
  
+ TCAACTACGG AAGGAAGACC GTTAATTGG  

- CTGCAGCTTC TCTAAAGAGA AAGTTAAACC TCTTCGTGAG AGATGAAAGG GGGATAGAGA GAGAAAGAGA   
  
  
- GATTCGGAGC TCAGCAAGAC ATTGAACTGA AACTCAGAGA ACTGCTAAAA TGGTTCTTTG GGAGGGGGAG   
  
  
- AGAAGCCAAG GGAAGATGGG GGATGATATA AGGAGGGTGG ATCACCGGGT GACCGGGTAA GACATAGGGG   
  
  
- TTTAGTCAAA GTGGGTTTTT GATCCAACGG CTGTGATGAG TTTGAAAATT TCCATGGGAT GATGTGAATA   
  
  
- AAGAAATCAG AGGCTGGTCT CCTCAAGAAG TAAGTATTGG GACCCACCCT TGGAAAAGAT AGAAATAGAT   
  
  
- ATAAAAGTAG GGACTCGTTT CTTTTTCTTT TTCCTCGGTA AAAATAGTTA CACCTCTTAT TCTGTTTGAA   
  
  
- TATTTCCTGG AAACTAGAGA CTACTAGCAT TTAGTCTTTA AAGTTAAGTT TGATTATGTG CCACAACCGT   
  
  
- TGACGGTCAG AATTGGATCC TATGAGTACA CGTAGTATTT TCATGATTGC TACTTCTAGT AACATTTAAC   
  
  
- GGAGTTATTT GACCGTTAAT GTGAAAAATA AAGGAAAAAG GAAGAGCAAT GGTAATAATT GCTTAATTAG   
  
  
- GCAGTAAAAT TTCTTAAAAC TTACCTAACG CGTGGTTACT CATGAGTTGC TGTTGTTCAC CCAAAAAAAG   
  
  
- GCAACACGTA CAGTACTTGG AACTAGGCGA GAAGGAAAGA AAGCTGACCA AAAGTGGGTC TCACGTGGGG   
  
  
- GCCATTTCTT TTTATAATCT GAAACTGTTG ACCAGCTTTT CTTATTTCTT CCTTACCCCT CGTTTCCGGT   
  
  
- ATCTGTCTTC TGAACCGTCC GATCTATTCT CATCAATCCA ACGGTCGAAT CGCACCGAGA AAGTTGACCA   
  
  
- AGACTTTTAT ACTTTGGAGA ACCCAGAAAA AGCTACAAGC CTTGGGTTTT TCACCACGTG GCATAAGGCT   
  
  
- GGTGCCAATT CAAAACGAAA CACAAGCATA GAGAGAGAAA CAGACAGACC TTAAAAAGTT TATTAAAGAT   
  
  
- AAAACGAAGT CGTTTTAGCC TGCTAATTTA TGTAGTCCTG TAATTTGTCA ACGGCTCAGA TGATTAATGT   
  
  
- GGGGTTGACC CAACCGGGTG AGAAGACTCC ACTGATCAAT TTTCTAGAAT TCTGATAGCT GGATGCTTGA   
  
  
- ACAAGTCTTA AGTGGTAAGC AAATTAATAA ATAATTATTA AGATAACAAA GCTTACAGTA CCTTCACCAA   
  
  
- CATTTAACGC ATTAATTAAC AGCTTTTTGA CCAAAAAACC ACCGTATTTT CTCATGCATT CCTGATGGGT   
  
  
- AAACGATAAG GCTCATGTAT TCTTAATTTG GGTATAATTC GAAAATTTAG CTGCTAATTG AGGATAAAAC   
  
  
- TGTAATTTAA GTGCGGATAT ATAATGTTAT AAATACATAA AGGCTTGTGG CACGACTAGA TTACCTGCTT   
  
  
- AGTTGATGCC TTCCTTCTGG CAATTAACC

+     Box I

| Site Name | Organism | Position | Strand | Matrix score. | sequence | function |
| --- | --- | --- | --- | --- | --- | --- |
| Box I | Pisum sativum | 1035 | + | 7 | TTTCAAA | light responsive element |

> 2018/04/13 10:10:12  
+ GACGTCGAAG AGATTTCTCT TTCAATTTGG AGAAGCACTC TCTACTTTCC CCCTATCTCT CTCTTTCTCT   
  
  
+ CTAAGCCTCG AGTCGTTCTG TAACTTGACT TTGAGTCTCT TGACGATTTT ACCAAGAAAC CCTCCCCCTC   
  
  
+ TCTTCGGTTC CCTTCTACCC CCTACTATAT TCCTCCCACC TAGTGGCCCA CTGGCCCATT CTGTATCCCC   
  
  
+ AAATCAGTTT CACCCAAAAA CTAGGTTGCC GACACTACTC AAACTTTTAA AGGTACCCTA CTACACTTAT   
  
  
+ TTCTTTAGTC TCCGACCAGA GGAGTTCTTC ATTCATAACC CTGGGTGGGA ACCTTTTCTA TCTTTATCTA   
  
  
+ TATTTTCATC CCTGAGCAAA GAAAAAGAAA AAGGAGCCAT TTTTATCAAT GTGGAGAATA AGACAAACTT   
  
  
+ ATAAAGGACC TTTGATCTCT GATGATCGTA AATCAGAAAT TTCAATTCAA ACTAATACAC GGTGTTGGCA   
  
  
+ ACTGCCAGTC TTAACCTAGG ATACTCATGT GCATCATAAA AGTACTAACG ATGAAGATCA TTGTAAATTG   
  
  
+ CCTCAATAAA CTGGCAATTA CACTTTTTAT TTCCTTTTTC CTTCTCGTTA CCATTATTAA CGAATTAATC   
  
  
+ CGTCATTTTA AAGAATTTTG AATGGATTGC GCACCAATGA GTACTCAACG ACAACAAGTG GGTTTTTTTC   
  
  
+ CGTTGTGCAT GTCATGAACC TTGATCCGCT CTTCCTTTCT TTCGACTGGT TTTCACCCAG AGTGCACCCC   
  
  
+ CGGTAAAGAA AAATATTAGA CTTTGACAAC TGGTCGAAAA GAATAAAGAA GGAATGGGGA GCAAAGGCCA   
  
  
+ TAGACAGAAG ACTTGGCAGG CTAGATAAGA GTAGTTAGGT TGCCAGCTTA GCGTGGCTCT TTCAACTGGT   
  
  
+ TCTGAAAATA TGAAACCTCT TGGGTCTTTT TCGATGTTCG GAACCCAAAA AGTGGTGCAC CGTATTCCGA   
  
  
+ CCACGGTTAA GTTTTGCTTT GTGTTCGTAT CTCTCTCTTT GTCTGTCTGG AATTTTTCAA ATAATTTCTA   
  
  
+ TTTTGCTTCA GCAAAATCGG ACGATTAAAT ACATCAGGAC ATTAAACAGT TGCCGAGTCT ACTAATTACA   
  
  
+ CCCCAACTGG GTTGGCCCAC TCTTCTGAGG TGACTAGTTA AAAGATCTTA AGACTATCGA CCTACGAACT   
  
  
+ TGTTCAGAAT TCACCATTCG TTTAATTATT TATTAATAAT TCTATTGTTT CGAATGTCAT GGAAGTGGTT   
  
  
+ GTAAATTGCG TAATTAATTG TCGAAAAACT GGTTTTTTGG TGGCATAAAA GAGTACGTAA GGACTACCCA   
  
  
+ TTTGCTATTC CGAGTACATA AGAATTAAAC CCATATTAAG CTTTTAAATC GACGATTAAC TCCTATTTTG   
  
  
+ ACATTAAATT CACGCCTATA TATTACAATA TTTATGTATT TCCGAACACC GTGCTGATCT AATGGACGAA   
  
  
+ TCAACTACGG AAGGAAGACC GTTAATTGG  

- CTGCAGCTTC TCTAAAGAGA AAGTTAAACC TCTTCGTGAG AGATGAAAGG GGGATAGAGA GAGAAAGAGA   
  
  
- GATTCGGAGC TCAGCAAGAC ATTGAACTGA AACTCAGAGA ACTGCTAAAA TGGTTCTTTG GGAGGGGGAG   
  
  
- AGAAGCCAAG GGAAGATGGG GGATGATATA AGGAGGGTGG ATCACCGGGT GACCGGGTAA GACATAGGGG   
  
  
- TTTAGTCAAA GTGGGTTTTT GATCCAACGG CTGTGATGAG TTTGAAAATT TCCATGGGAT GATGTGAATA   
  
  
- AAGAAATCAG AGGCTGGTCT CCTCAAGAAG TAAGTATTGG GACCCACCCT TGGAAAAGAT AGAAATAGAT   
  
  
- ATAAAAGTAG GGACTCGTTT CTTTTTCTTT TTCCTCGGTA AAAATAGTTA CACCTCTTAT TCTGTTTGAA   
  
  
- TATTTCCTGG AAACTAGAGA CTACTAGCAT TTAGTCTTTA AAGTTAAGTT TGATTATGTG CCACAACCGT   
  
  
- TGACGGTCAG AATTGGATCC TATGAGTACA CGTAGTATTT TCATGATTGC TACTTCTAGT AACATTTAAC   
  
  
- GGAGTTATTT GACCGTTAAT GTGAAAAATA AAGGAAAAAG GAAGAGCAAT GGTAATAATT GCTTAATTAG   
  
  
- GCAGTAAAAT TTCTTAAAAC TTACCTAACG CGTGGTTACT CATGAGTTGC TGTTGTTCAC CCAAAAAAAG   
  
  
- GCAACACGTA CAGTACTTGG AACTAGGCGA GAAGGAAAGA AAGCTGACCA AAAGTGGGTC TCACGTGGGG   
  
  
- GCCATTTCTT TTTATAATCT GAAACTGTTG ACCAGCTTTT CTTATTTCTT CCTTACCCCT CGTTTCCGGT   
  
  
- ATCTGTCTTC TGAACCGTCC GATCTATTCT CATCAATCCA ACGGTCGAAT CGCACCGAGA AAGTTGACCA   
  
  
- AGACTTTTAT ACTTTGGAGA ACCCAGAAAA AGCTACAAGC CTTGGGTTTT TCACCACGTG GCATAAGGCT   
  
  
- GGTGCCAATT CAAAACGAAA CACAAGCATA GAGAGAGAAA CAGACAGACC TTAAAAAGTT TATTAAAGAT   
  
  
- AAAACGAAGT CGTTTTAGCC TGCTAATTTA TGTAGTCCTG TAATTTGTCA ACGGCTCAGA TGATTAATGT   
  
  
- GGGGTTGACC CAACCGGGTG AGAAGACTCC ACTGATCAAT TTTCTAGAAT TCTGATAGCT GGATGCTTGA   
  
  
- ACAAGTCTTA AGTGGTAAGC AAATTAATAA ATAATTATTA AGATAACAAA GCTTACAGTA CCTTCACCAA   
  
  
- CATTTAACGC ATTAATTAAC AGCTTTTTGA CCAAAAAACC ACCGTATTTT CTCATGCATT CCTGATGGGT   
  
  
- AAACGATAAG GCTCATGTAT TCTTAATTTG GGTATAATTC GAAAATTTAG CTGCTAATTG AGGATAAAAC   
  
  
- TGTAATTTAA GTGCGGATAT ATAATGTTAT AAATACATAA AGGCTTGTGG CACGACTAGA TTACCTGCTT   
  
  
- AGTTGATGCC TTCCTTCTGG CAATTAACC

+     Box III

| Site Name | Organism | Position | Strand | Matrix score. | sequence | function |
| --- | --- | --- | --- | --- | --- | --- |
| Box III | Pisum sativum | 575 | + | 9 | CATTTACACT | protein binding site |

> 2018/04/13 10:10:12  
+ GACGTCGAAG AGATTTCTCT TTCAATTTGG AGAAGCACTC TCTACTTTCC CCCTATCTCT CTCTTTCTCT   
  
  
+ CTAAGCCTCG AGTCGTTCTG TAACTTGACT TTGAGTCTCT TGACGATTTT ACCAAGAAAC CCTCCCCCTC   
  
  
+ TCTTCGGTTC CCTTCTACCC CCTACTATAT TCCTCCCACC TAGTGGCCCA CTGGCCCATT CTGTATCCCC   
  
  
+ AAATCAGTTT CACCCAAAAA CTAGGTTGCC GACACTACTC AAACTTTTAA AGGTACCCTA CTACACTTAT   
  
  
+ TTCTTTAGTC TCCGACCAGA GGAGTTCTTC ATTCATAACC CTGGGTGGGA ACCTTTTCTA TCTTTATCTA   
  
  
+ TATTTTCATC CCTGAGCAAA GAAAAAGAAA AAGGAGCCAT TTTTATCAAT GTGGAGAATA AGACAAACTT   
  
  
+ ATAAAGGACC TTTGATCTCT GATGATCGTA AATCAGAAAT TTCAATTCAA ACTAATACAC GGTGTTGGCA   
  
  
+ ACTGCCAGTC TTAACCTAGG ATACTCATGT GCATCATAAA AGTACTAACG ATGAAGATCA TTGTAAATTG   
  
  
+ CCTCAATAAA CTGGCAATTA CACTTTTTAT TTCCTTTTTC CTTCTCGTTA CCATTATTAA CGAATTAATC   
  
  
+ CGTCATTTTA AAGAATTTTG AATGGATTGC GCACCAATGA GTACTCAACG ACAACAAGTG GGTTTTTTTC   
  
  
+ CGTTGTGCAT GTCATGAACC TTGATCCGCT CTTCCTTTCT TTCGACTGGT TTTCACCCAG AGTGCACCCC   
  
  
+ CGGTAAAGAA AAATATTAGA CTTTGACAAC TGGTCGAAAA GAATAAAGAA GGAATGGGGA GCAAAGGCCA   
  
  
+ TAGACAGAAG ACTTGGCAGG CTAGATAAGA GTAGTTAGGT TGCCAGCTTA GCGTGGCTCT TTCAACTGGT   
  
  
+ TCTGAAAATA TGAAACCTCT TGGGTCTTTT TCGATGTTCG GAACCCAAAA AGTGGTGCAC CGTATTCCGA   
  
  
+ CCACGGTTAA GTTTTGCTTT GTGTTCGTAT CTCTCTCTTT GTCTGTCTGG AATTTTTCAA ATAATTTCTA   
  
  
+ TTTTGCTTCA GCAAAATCGG ACGATTAAAT ACATCAGGAC ATTAAACAGT TGCCGAGTCT ACTAATTACA   
  
  
+ CCCCAACTGG GTTGGCCCAC TCTTCTGAGG TGACTAGTTA AAAGATCTTA AGACTATCGA CCTACGAACT   
  
  
+ TGTTCAGAAT TCACCATTCG TTTAATTATT TATTAATAAT TCTATTGTTT CGAATGTCAT GGAAGTGGTT   
  
  
+ GTAAATTGCG TAATTAATTG TCGAAAAACT GGTTTTTTGG TGGCATAAAA GAGTACGTAA GGACTACCCA   
  
  
+ TTTGCTATTC CGAGTACATA AGAATTAAAC CCATATTAAG CTTTTAAATC GACGATTAAC TCCTATTTTG   
  
  
+ ACATTAAATT CACGCCTATA TATTACAATA TTTATGTATT TCCGAACACC GTGCTGATCT AATGGACGAA   
  
  
+ TCAACTACGG AAGGAAGACC GTTAATTGG  

- CTGCAGCTTC TCTAAAGAGA AAGTTAAACC TCTTCGTGAG AGATGAAAGG GGGATAGAGA GAGAAAGAGA   
  
  
- GATTCGGAGC TCAGCAAGAC ATTGAACTGA AACTCAGAGA ACTGCTAAAA TGGTTCTTTG GGAGGGGGAG   
  
  
- AGAAGCCAAG GGAAGATGGG GGATGATATA AGGAGGGTGG ATCACCGGGT GACCGGGTAA GACATAGGGG   
  
  
- TTTAGTCAAA GTGGGTTTTT GATCCAACGG CTGTGATGAG TTTGAAAATT TCCATGGGAT GATGTGAATA   
  
  
- AAGAAATCAG AGGCTGGTCT CCTCAAGAAG TAAGTATTGG GACCCACCCT TGGAAAAGAT AGAAATAGAT   
  
  
- ATAAAAGTAG GGACTCGTTT CTTTTTCTTT TTCCTCGGTA AAAATAGTTA CACCTCTTAT TCTGTTTGAA   
  
  
- TATTTCCTGG AAACTAGAGA CTACTAGCAT TTAGTCTTTA AAGTTAAGTT TGATTATGTG CCACAACCGT   
  
  
- TGACGGTCAG AATTGGATCC TATGAGTACA CGTAGTATTT TCATGATTGC TACTTCTAGT AACATTTAAC   
  
  
- GGAGTTATTT GACCGTTAAT GTGAAAAATA AAGGAAAAAG GAAGAGCAAT GGTAATAATT GCTTAATTAG   
  
  
- GCAGTAAAAT TTCTTAAAAC TTACCTAACG CGTGGTTACT CATGAGTTGC TGTTGTTCAC CCAAAAAAAG   
  
  
- GCAACACGTA CAGTACTTGG AACTAGGCGA GAAGGAAAGA AAGCTGACCA AAAGTGGGTC TCACGTGGGG   
  
  
- GCCATTTCTT TTTATAATCT GAAACTGTTG ACCAGCTTTT CTTATTTCTT CCTTACCCCT CGTTTCCGGT   
  
  
- ATCTGTCTTC TGAACCGTCC GATCTATTCT CATCAATCCA ACGGTCGAAT CGCACCGAGA AAGTTGACCA   
  
  
- AGACTTTTAT ACTTTGGAGA ACCCAGAAAA AGCTACAAGC CTTGGGTTTT TCACCACGTG GCATAAGGCT   
  
  
- GGTGCCAATT CAAAACGAAA CACAAGCATA GAGAGAGAAA CAGACAGACC TTAAAAAGTT TATTAAAGAT   
  
  
- AAAACGAAGT CGTTTTAGCC TGCTAATTTA TGTAGTCCTG TAATTTGTCA ACGGCTCAGA TGATTAATGT   
  
  
- GGGGTTGACC CAACCGGGTG AGAAGACTCC ACTGATCAAT TTTCTAGAAT TCTGATAGCT GGATGCTTGA   
  
  
- ACAAGTCTTA AGTGGTAAGC AAATTAATAA ATAATTATTA AGATAACAAA GCTTACAGTA CCTTCACCAA   
  
  
- CATTTAACGC ATTAATTAAC AGCTTTTTGA CCAAAAAACC ACCGTATTTT CTCATGCATT CCTGATGGGT   
  
  
- AAACGATAAG GCTCATGTAT TCTTAATTTG GGTATAATTC GAAAATTTAG CTGCTAATTG AGGATAAAAC   
  
  
- TGTAATTTAA GTGCGGATAT ATAATGTTAT AAATACATAA AGGCTTGTGG CACGACTAGA TTACCTGCTT   
  
  
- AGTTGATGCC TTCCTTCTGG CAATTAACC

+     CAAT-box

| Site Name | Organism | Position | Strand | Matrix score. | sequence | function |
| --- | --- | --- | --- | --- | --- | --- |
| CAAT-box | Hordeum vulgare | 1277 | - | 4 | CAAT | common cis-acting element in promoter and enhancer regions |
| CAAT-box | Hordeum vulgare | 1265 | - | 4 | CAAT | common cis-acting element in promoter and enhancer regions |
| CAAT-box | Hordeum vulgare | 1234 | - | 4 | CAAT | common cis-acting element in promoter and enhancer regions |
| CAAT-box | Brassica rapa | 1038 | + | 5 | CAAAT | common cis-acting element in promoter and enhancer regions |
| CAAT-box | Hordeum vulgare | 656 | - | 4 | CAAT | common cis-acting element in promoter and enhancer regions |
| CAAT-box | Hordeum vulgare | 564 | + | 4 | CAAT | common cis-acting element in promoter and enhancer regions |
| CAAT-box | Hordeum vulgare | 550 | - | 4 | CAAT | common cis-acting element in promoter and enhancer regions |
| CAAT-box | Hordeum vulgare | 397 | + | 4 | CAAT | common cis-acting element in promoter and enhancer regions |
| CAAT-box | Glycine max | 1276 | - | 5 | CAATT | common cis-acting element in promoter and enhancer regions |
| CAAT-box | Hordeum vulgare | 665 | + | 4 | CAAT | common cis-acting element in promoter and enhancer regions |
| CAAT-box | Petunia hybrida | 484 | - | 7 | TGCCAAC | common cis-acting element in promoter and enhancer regions |
| CAAT-box | Brassica rapa | 210 | + | 5 | CAAAT | common cis-acting element in promoter and enhancer regions |
| CAAT-box | Glycine max | 463 | + | 5 | CAATT | common cis-acting element in promoter and enhancer regions |
| CAAT-box | Arabidopsis thaliana | 1495 | - | 5 | CCAAT | common cis-acting element in promoter and enhancer regions |
| CAAT-box | Arabidopsis thaliana | 573 | + | 6 | gGCAAT | common cis-acting element in promoter and enhancer regions |
| CAAT-box | Glycine max | 1264 | - | 5 | CAATT | common cis-acting element in promoter and enhancer regions |
| CAAT-box | Glycine max | 23 | + | 5 | CAATT | common cis-acting element in promoter and enhancer regions |
| CAAT-box | Hordeum vulgare | 1426 | + | 4 | CAAT | common cis-acting element in promoter and enhancer regions |
| CAAT-box | Arabidopsis thaliana | 664 | + | 5 | CCAAT | common cis-acting element in promoter and enhancer regions |
| CAAT-box | Brassica rapa | 1330 | - | 5 | CAAAT | common cis-acting element in promoter and enhancer regions |
| CAAT-box | Brassica rapa | 25 | - | 5 | CAAAT | common cis-acting element in promoter and enhancer regions |
| CAAT-box | Glycine max | 1494 | - | 5 | CAATT | common cis-acting element in promoter and enhancer regions |
| CAAT-box | Arabidopsis thaliana | 557 | - | 6 | gGCAAT | common cis-acting element in promoter and enhancer regions |
| CAAT-box | Glycine max | 556 | - | 5 | CAATT | common cis-acting element in promoter and enhancer regions |
| CAAT-box | Glycine max | 575 | + | 5 | CAATT | common cis-acting element in promoter and enhancer regions |

> 2018/04/13 10:10:12  
+ GACGTCGAAG AGATTTCTCT TTCAATTTGG AGAAGCACTC TCTACTTTCC CCCTATCTCT CTCTTTCTCT   
  
  
+ CTAAGCCTCG AGTCGTTCTG TAACTTGACT TTGAGTCTCT TGACGATTTT ACCAAGAAAC CCTCCCCCTC   
  
  
+ TCTTCGGTTC CCTTCTACCC CCTACTATAT TCCTCCCACC TAGTGGCCCA CTGGCCCATT CTGTATCCCC   
  
  
+ AAATCAGTTT CACCCAAAAA CTAGGTTGCC GACACTACTC AAACTTTTAA AGGTACCCTA CTACACTTAT   
  
  
+ TTCTTTAGTC TCCGACCAGA GGAGTTCTTC ATTCATAACC CTGGGTGGGA ACCTTTTCTA TCTTTATCTA   
  
  
+ TATTTTCATC CCTGAGCAAA GAAAAAGAAA AAGGAGCCAT TTTTATCAAT GTGGAGAATA AGACAAACTT   
  
  
+ ATAAAGGACC TTTGATCTCT GATGATCGTA AATCAGAAAT TTCAATTCAA ACTAATACAC GGTGTTGGCA   
  
  
+ ACTGCCAGTC TTAACCTAGG ATACTCATGT GCATCATAAA AGTACTAACG ATGAAGATCA TTGTAAATTG   
  
  
+ CCTCAATAAA CTGGCAATTA CACTTTTTAT TTCCTTTTTC CTTCTCGTTA CCATTATTAA CGAATTAATC   
  
  
+ CGTCATTTTA AAGAATTTTG AATGGATTGC GCACCAATGA GTACTCAACG ACAACAAGTG GGTTTTTTTC   
  
  
+ CGTTGTGCAT GTCATGAACC TTGATCCGCT CTTCCTTTCT TTCGACTGGT TTTCACCCAG AGTGCACCCC   
  
  
+ CGGTAAAGAA AAATATTAGA CTTTGACAAC TGGTCGAAAA GAATAAAGAA GGAATGGGGA GCAAAGGCCA   
  
  
+ TAGACAGAAG ACTTGGCAGG CTAGATAAGA GTAGTTAGGT TGCCAGCTTA GCGTGGCTCT TTCAACTGGT   
  
  
+ TCTGAAAATA TGAAACCTCT TGGGTCTTTT TCGATGTTCG GAACCCAAAA AGTGGTGCAC CGTATTCCGA   
  
  
+ CCACGGTTAA GTTTTGCTTT GTGTTCGTAT CTCTCTCTTT GTCTGTCTGG AATTTTTCAA ATAATTTCTA   
  
  
+ TTTTGCTTCA GCAAAATCGG ACGATTAAAT ACATCAGGAC ATTAAACAGT TGCCGAGTCT ACTAATTACA   
  
  
+ CCCCAACTGG GTTGGCCCAC TCTTCTGAGG TGACTAGTTA AAAGATCTTA AGACTATCGA CCTACGAACT   
  
  
+ TGTTCAGAAT TCACCATTCG TTTAATTATT TATTAATAAT TCTATTGTTT CGAATGTCAT GGAAGTGGTT   
  
  
+ GTAAATTGCG TAATTAATTG TCGAAAAACT GGTTTTTTGG TGGCATAAAA GAGTACGTAA GGACTACCCA   
  
  
+ TTTGCTATTC CGAGTACATA AGAATTAAAC CCATATTAAG CTTTTAAATC GACGATTAAC TCCTATTTTG   
  
  
+ ACATTAAATT CACGCCTATA TATTACAATA TTTATGTATT TCCGAACACC GTGCTGATCT AATGGACGAA   
  
  
+ TCAACTACGG AAGGAAGACC GTTAATTGG  

- CTGCAGCTTC TCTAAAGAGA AAGTTAAACC TCTTCGTGAG AGATGAAAGG GGGATAGAGA GAGAAAGAGA   
  
  
- GATTCGGAGC TCAGCAAGAC ATTGAACTGA AACTCAGAGA ACTGCTAAAA TGGTTCTTTG GGAGGGGGAG   
  
  
- AGAAGCCAAG GGAAGATGGG GGATGATATA AGGAGGGTGG ATCACCGGGT GACCGGGTAA GACATAGGGG   
  
  
- TTTAGTCAAA GTGGGTTTTT GATCCAACGG CTGTGATGAG TTTGAAAATT TCCATGGGAT GATGTGAATA   
  
  
- AAGAAATCAG AGGCTGGTCT CCTCAAGAAG TAAGTATTGG GACCCACCCT TGGAAAAGAT AGAAATAGAT   
  
  
- ATAAAAGTAG GGACTCGTTT CTTTTTCTTT TTCCTCGGTA AAAATAGTTA CACCTCTTAT TCTGTTTGAA   
  
  
- TATTTCCTGG AAACTAGAGA CTACTAGCAT TTAGTCTTTA AAGTTAAGTT TGATTATGTG CCACAACCGT   
  
  
- TGACGGTCAG AATTGGATCC TATGAGTACA CGTAGTATTT TCATGATTGC TACTTCTAGT AACATTTAAC   
  
  
- GGAGTTATTT GACCGTTAAT GTGAAAAATA AAGGAAAAAG GAAGAGCAAT GGTAATAATT GCTTAATTAG   
  
  
- GCAGTAAAAT TTCTTAAAAC TTACCTAACG CGTGGTTACT CATGAGTTGC TGTTGTTCAC CCAAAAAAAG   
  
  
- GCAACACGTA CAGTACTTGG AACTAGGCGA GAAGGAAAGA AAGCTGACCA AAAGTGGGTC TCACGTGGGG   
  
  
- GCCATTTCTT TTTATAATCT GAAACTGTTG ACCAGCTTTT CTTATTTCTT CCTTACCCCT CGTTTCCGGT   
  
  
- ATCTGTCTTC TGAACCGTCC GATCTATTCT CATCAATCCA ACGGTCGAAT CGCACCGAGA AAGTTGACCA   
  
  
- AGACTTTTAT ACTTTGGAGA ACCCAGAAAA AGCTACAAGC CTTGGGTTTT TCACCACGTG GCATAAGGCT   
  
  
- GGTGCCAATT CAAAACGAAA CACAAGCATA GAGAGAGAAA CAGACAGACC TTAAAAAGTT TATTAAAGAT   
  
  
- AAAACGAAGT CGTTTTAGCC TGCTAATTTA TGTAGTCCTG TAATTTGTCA ACGGCTCAGA TGATTAATGT   
  
  
- GGGGTTGACC CAACCGGGTG AGAAGACTCC ACTGATCAAT TTTCTAGAAT TCTGATAGCT GGATGCTTGA   
  
  
- ACAAGTCTTA AGTGGTAAGC AAATTAATAA ATAATTATTA AGATAACAAA GCTTACAGTA CCTTCACCAA   
  
  
- CATTTAACGC ATTAATTAAC AGCTTTTTGA CCAAAAAACC ACCGTATTTT CTCATGCATT CCTGATGGGT   
  
  
- AAACGATAAG GCTCATGTAT TCTTAATTTG GGTATAATTC GAAAATTTAG CTGCTAATTG AGGATAAAAC   
  
  
- TGTAATTTAA GTGCGGATAT ATAATGTTAT AAATACATAA AGGCTTGTGG CACGACTAGA TTACCTGCTT   
  
  
- AGTTGATGCC TTCCTTCTGG CAATTAACC

+     CAT-box

| Site Name | Organism | Position | Strand | Matrix score. | sequence | function |
| --- | --- | --- | --- | --- | --- | --- |
| CAT-box | Arabidopsis thaliana | 182 | - | 6 | GCCACT | cis-acting regulatory element related to meristem expression |

> 2018/04/13 10:10:12  
+ GACGTCGAAG AGATTTCTCT TTCAATTTGG AGAAGCACTC TCTACTTTCC CCCTATCTCT CTCTTTCTCT   
  
  
+ CTAAGCCTCG AGTCGTTCTG TAACTTGACT TTGAGTCTCT TGACGATTTT ACCAAGAAAC CCTCCCCCTC   
  
  
+ TCTTCGGTTC CCTTCTACCC CCTACTATAT TCCTCCCACC TAGTGGCCCA CTGGCCCATT CTGTATCCCC   
  
  
+ AAATCAGTTT CACCCAAAAA CTAGGTTGCC GACACTACTC AAACTTTTAA AGGTACCCTA CTACACTTAT   
  
  
+ TTCTTTAGTC TCCGACCAGA GGAGTTCTTC ATTCATAACC CTGGGTGGGA ACCTTTTCTA TCTTTATCTA   
  
  
+ TATTTTCATC CCTGAGCAAA GAAAAAGAAA AAGGAGCCAT TTTTATCAAT GTGGAGAATA AGACAAACTT   
  
  
+ ATAAAGGACC TTTGATCTCT GATGATCGTA AATCAGAAAT TTCAATTCAA ACTAATACAC GGTGTTGGCA   
  
  
+ ACTGCCAGTC TTAACCTAGG ATACTCATGT GCATCATAAA AGTACTAACG ATGAAGATCA TTGTAAATTG   
  
  
+ CCTCAATAAA CTGGCAATTA CACTTTTTAT TTCCTTTTTC CTTCTCGTTA CCATTATTAA CGAATTAATC   
  
  
+ CGTCATTTTA AAGAATTTTG AATGGATTGC GCACCAATGA GTACTCAACG ACAACAAGTG GGTTTTTTTC   
  
  
+ CGTTGTGCAT GTCATGAACC TTGATCCGCT CTTCCTTTCT TTCGACTGGT TTTCACCCAG AGTGCACCCC   
  
  
+ CGGTAAAGAA AAATATTAGA CTTTGACAAC TGGTCGAAAA GAATAAAGAA GGAATGGGGA GCAAAGGCCA   
  
  
+ TAGACAGAAG ACTTGGCAGG CTAGATAAGA GTAGTTAGGT TGCCAGCTTA GCGTGGCTCT TTCAACTGGT   
  
  
+ TCTGAAAATA TGAAACCTCT TGGGTCTTTT TCGATGTTCG GAACCCAAAA AGTGGTGCAC CGTATTCCGA   
  
  
+ CCACGGTTAA GTTTTGCTTT GTGTTCGTAT CTCTCTCTTT GTCTGTCTGG AATTTTTCAA ATAATTTCTA   
  
  
+ TTTTGCTTCA GCAAAATCGG ACGATTAAAT ACATCAGGAC ATTAAACAGT TGCCGAGTCT ACTAATTACA   
  
  
+ CCCCAACTGG GTTGGCCCAC TCTTCTGAGG TGACTAGTTA AAAGATCTTA AGACTATCGA CCTACGAACT   
  
  
+ TGTTCAGAAT TCACCATTCG TTTAATTATT TATTAATAAT TCTATTGTTT CGAATGTCAT GGAAGTGGTT   
  
  
+ GTAAATTGCG TAATTAATTG TCGAAAAACT GGTTTTTTGG TGGCATAAAA GAGTACGTAA GGACTACCCA   
  
  
+ TTTGCTATTC CGAGTACATA AGAATTAAAC CCATATTAAG CTTTTAAATC GACGATTAAC TCCTATTTTG   
  
  
+ ACATTAAATT CACGCCTATA TATTACAATA TTTATGTATT TCCGAACACC GTGCTGATCT AATGGACGAA   
  
  
+ TCAACTACGG AAGGAAGACC GTTAATTGG  

- CTGCAGCTTC TCTAAAGAGA AAGTTAAACC TCTTCGTGAG AGATGAAAGG GGGATAGAGA GAGAAAGAGA   
  
  
- GATTCGGAGC TCAGCAAGAC ATTGAACTGA AACTCAGAGA ACTGCTAAAA TGGTTCTTTG GGAGGGGGAG   
  
  
- AGAAGCCAAG GGAAGATGGG GGATGATATA AGGAGGGTGG ATCACCGGGT GACCGGGTAA GACATAGGGG   
  
  
- TTTAGTCAAA GTGGGTTTTT GATCCAACGG CTGTGATGAG TTTGAAAATT TCCATGGGAT GATGTGAATA   
  
  
- AAGAAATCAG AGGCTGGTCT CCTCAAGAAG TAAGTATTGG GACCCACCCT TGGAAAAGAT AGAAATAGAT   
  
  
- ATAAAAGTAG GGACTCGTTT CTTTTTCTTT TTCCTCGGTA AAAATAGTTA CACCTCTTAT TCTGTTTGAA   
  
  
- TATTTCCTGG AAACTAGAGA CTACTAGCAT TTAGTCTTTA AAGTTAAGTT TGATTATGTG CCACAACCGT   
  
  
- TGACGGTCAG AATTGGATCC TATGAGTACA CGTAGTATTT TCATGATTGC TACTTCTAGT AACATTTAAC   
  
  
- GGAGTTATTT GACCGTTAAT GTGAAAAATA AAGGAAAAAG GAAGAGCAAT GGTAATAATT GCTTAATTAG   
  
  
- GCAGTAAAAT TTCTTAAAAC TTACCTAACG CGTGGTTACT CATGAGTTGC TGTTGTTCAC CCAAAAAAAG   
  
  
- GCAACACGTA CAGTACTTGG AACTAGGCGA GAAGGAAAGA AAGCTGACCA AAAGTGGGTC TCACGTGGGG   
  
  
- GCCATTTCTT TTTATAATCT GAAACTGTTG ACCAGCTTTT CTTATTTCTT CCTTACCCCT CGTTTCCGGT   
  
  
- ATCTGTCTTC TGAACCGTCC GATCTATTCT CATCAATCCA ACGGTCGAAT CGCACCGAGA AAGTTGACCA   
  
  
- AGACTTTTAT ACTTTGGAGA ACCCAGAAAA AGCTACAAGC CTTGGGTTTT TCACCACGTG GCATAAGGCT   
  
  
- GGTGCCAATT CAAAACGAAA CACAAGCATA GAGAGAGAAA CAGACAGACC TTAAAAAGTT TATTAAAGAT   
  
  
- AAAACGAAGT CGTTTTAGCC TGCTAATTTA TGTAGTCCTG TAATTTGTCA ACGGCTCAGA TGATTAATGT   
  
  
- GGGGTTGACC CAACCGGGTG AGAAGACTCC ACTGATCAAT TTTCTAGAAT TCTGATAGCT GGATGCTTGA   
  
  
- ACAAGTCTTA AGTGGTAAGC AAATTAATAA ATAATTATTA AGATAACAAA GCTTACAGTA CCTTCACCAA   
  
  
- CATTTAACGC ATTAATTAAC AGCTTTTTGA CCAAAAAACC ACCGTATTTT CTCATGCATT CCTGATGGGT   
  
  
- AAACGATAAG GCTCATGTAT TCTTAATTTG GGTATAATTC GAAAATTTAG CTGCTAATTG AGGATAAAAC   
  
  
- TGTAATTTAA GTGCGGATAT ATAATGTTAT AAATACATAA AGGCTTGTGG CACGACTAGA TTACCTGCTT   
  
  
- AGTTGATGCC TTCCTTCTGG CAATTAACC

+     CCAAT-box

| Site Name | Organism | Position | Strand | Matrix score. | sequence | function |
| --- | --- | --- | --- | --- | --- | --- |
| CCAAT-box | Hordeum vulgare | 700 | - | 6 | CAACGG | MYBHv1 binding site |

> 2018/04/13 10:10:12  
+ GACGTCGAAG AGATTTCTCT TTCAATTTGG AGAAGCACTC TCTACTTTCC CCCTATCTCT CTCTTTCTCT   
  
  
+ CTAAGCCTCG AGTCGTTCTG TAACTTGACT TTGAGTCTCT TGACGATTTT ACCAAGAAAC CCTCCCCCTC   
  
  
+ TCTTCGGTTC CCTTCTACCC CCTACTATAT TCCTCCCACC TAGTGGCCCA CTGGCCCATT CTGTATCCCC   
  
  
+ AAATCAGTTT CACCCAAAAA CTAGGTTGCC GACACTACTC AAACTTTTAA AGGTACCCTA CTACACTTAT   
  
  
+ TTCTTTAGTC TCCGACCAGA GGAGTTCTTC ATTCATAACC CTGGGTGGGA ACCTTTTCTA TCTTTATCTA   
  
  
+ TATTTTCATC CCTGAGCAAA GAAAAAGAAA AAGGAGCCAT TTTTATCAAT GTGGAGAATA AGACAAACTT   
  
  
+ ATAAAGGACC TTTGATCTCT GATGATCGTA AATCAGAAAT TTCAATTCAA ACTAATACAC GGTGTTGGCA   
  
  
+ ACTGCCAGTC TTAACCTAGG ATACTCATGT GCATCATAAA AGTACTAACG ATGAAGATCA TTGTAAATTG   
  
  
+ CCTCAATAAA CTGGCAATTA CACTTTTTAT TTCCTTTTTC CTTCTCGTTA CCATTATTAA CGAATTAATC   
  
  
+ CGTCATTTTA AAGAATTTTG AATGGATTGC GCACCAATGA GTACTCAACG ACAACAAGTG GGTTTTTTTC   
  
  
+ CGTTGTGCAT GTCATGAACC TTGATCCGCT CTTCCTTTCT TTCGACTGGT TTTCACCCAG AGTGCACCCC   
  
  
+ CGGTAAAGAA AAATATTAGA CTTTGACAAC TGGTCGAAAA GAATAAAGAA GGAATGGGGA GCAAAGGCCA   
  
  
+ TAGACAGAAG ACTTGGCAGG CTAGATAAGA GTAGTTAGGT TGCCAGCTTA GCGTGGCTCT TTCAACTGGT   
  
  
+ TCTGAAAATA TGAAACCTCT TGGGTCTTTT TCGATGTTCG GAACCCAAAA AGTGGTGCAC CGTATTCCGA   
  
  
+ CCACGGTTAA GTTTTGCTTT GTGTTCGTAT CTCTCTCTTT GTCTGTCTGG AATTTTTCAA ATAATTTCTA   
  
  
+ TTTTGCTTCA GCAAAATCGG ACGATTAAAT ACATCAGGAC ATTAAACAGT TGCCGAGTCT ACTAATTACA   
  
  
+ CCCCAACTGG GTTGGCCCAC TCTTCTGAGG TGACTAGTTA AAAGATCTTA AGACTATCGA CCTACGAACT   
  
  
+ TGTTCAGAAT TCACCATTCG TTTAATTATT TATTAATAAT TCTATTGTTT CGAATGTCAT GGAAGTGGTT   
  
  
+ GTAAATTGCG TAATTAATTG TCGAAAAACT GGTTTTTTGG TGGCATAAAA GAGTACGTAA GGACTACCCA   
  
  
+ TTTGCTATTC CGAGTACATA AGAATTAAAC CCATATTAAG CTTTTAAATC GACGATTAAC TCCTATTTTG   
  
  
+ ACATTAAATT CACGCCTATA TATTACAATA TTTATGTATT TCCGAACACC GTGCTGATCT AATGGACGAA   
  
  
+ TCAACTACGG AAGGAAGACC GTTAATTGG  

- CTGCAGCTTC TCTAAAGAGA AAGTTAAACC TCTTCGTGAG AGATGAAAGG GGGATAGAGA GAGAAAGAGA   
  
  
- GATTCGGAGC TCAGCAAGAC ATTGAACTGA AACTCAGAGA ACTGCTAAAA TGGTTCTTTG GGAGGGGGAG   
  
  
- AGAAGCCAAG GGAAGATGGG GGATGATATA AGGAGGGTGG ATCACCGGGT GACCGGGTAA GACATAGGGG   
  
  
- TTTAGTCAAA GTGGGTTTTT GATCCAACGG CTGTGATGAG TTTGAAAATT TCCATGGGAT GATGTGAATA   
  
  
- AAGAAATCAG AGGCTGGTCT CCTCAAGAAG TAAGTATTGG GACCCACCCT TGGAAAAGAT AGAAATAGAT   
  
  
- ATAAAAGTAG GGACTCGTTT CTTTTTCTTT TTCCTCGGTA AAAATAGTTA CACCTCTTAT TCTGTTTGAA   
  
  
- TATTTCCTGG AAACTAGAGA CTACTAGCAT TTAGTCTTTA AAGTTAAGTT TGATTATGTG CCACAACCGT   
  
  
- TGACGGTCAG AATTGGATCC TATGAGTACA CGTAGTATTT TCATGATTGC TACTTCTAGT AACATTTAAC   
  
  
- GGAGTTATTT GACCGTTAAT GTGAAAAATA AAGGAAAAAG GAAGAGCAAT GGTAATAATT GCTTAATTAG   
  
  
- GCAGTAAAAT TTCTTAAAAC TTACCTAACG CGTGGTTACT CATGAGTTGC TGTTGTTCAC CCAAAAAAAG   
  
  
- GCAACACGTA CAGTACTTGG AACTAGGCGA GAAGGAAAGA AAGCTGACCA AAAGTGGGTC TCACGTGGGG   
  
  
- GCCATTTCTT TTTATAATCT GAAACTGTTG ACCAGCTTTT CTTATTTCTT CCTTACCCCT CGTTTCCGGT   
  
  
- ATCTGTCTTC TGAACCGTCC GATCTATTCT CATCAATCCA ACGGTCGAAT CGCACCGAGA AAGTTGACCA   
  
  
- AGACTTTTAT ACTTTGGAGA ACCCAGAAAA AGCTACAAGC CTTGGGTTTT TCACCACGTG GCATAAGGCT   
  
  
- GGTGCCAATT CAAAACGAAA CACAAGCATA GAGAGAGAAA CAGACAGACC TTAAAAAGTT TATTAAAGAT   
  
  
- AAAACGAAGT CGTTTTAGCC TGCTAATTTA TGTAGTCCTG TAATTTGTCA ACGGCTCAGA TGATTAATGT   
  
  
- GGGGTTGACC CAACCGGGTG AGAAGACTCC ACTGATCAAT TTTCTAGAAT TCTGATAGCT GGATGCTTGA   
  
  
- ACAAGTCTTA AGTGGTAAGC AAATTAATAA ATAATTATTA AGATAACAAA GCTTACAGTA CCTTCACCAA   
  
  
- CATTTAACGC ATTAATTAAC AGCTTTTTGA CCAAAAAACC ACCGTATTTT CTCATGCATT CCTGATGGGT   
  
  
- AAACGATAAG GCTCATGTAT TCTTAATTTG GGTATAATTC GAAAATTTAG CTGCTAATTG AGGATAAAAC   
  
  
- TGTAATTTAA GTGCGGATAT ATAATGTTAT AAATACATAA AGGCTTGTGG CACGACTAGA TTACCTGCTT   
  
  
- AGTTGATGCC TTCCTTCTGG CAATTAACC

+     CGTCA-motif

| Site Name | Organism | Position | Strand | Matrix score. | sequence | function |
| --- | --- | --- | --- | --- | --- | --- |
| CGTCA-motif | Hordeum vulgare | 631 | + | 5 | CGTCA | cis-acting regulatory element involved in the MeJA-responsiveness |
| CGTCA-motif | Hordeum vulgare | 111 | - | 5 | CGTCA | cis-acting regulatory element involved in the MeJA-responsiveness |

> 2018/04/13 10:10:12  
+ GACGTCGAAG AGATTTCTCT TTCAATTTGG AGAAGCACTC TCTACTTTCC CCCTATCTCT CTCTTTCTCT   
  
  
+ CTAAGCCTCG AGTCGTTCTG TAACTTGACT TTGAGTCTCT TGACGATTTT ACCAAGAAAC CCTCCCCCTC   
  
  
+ TCTTCGGTTC CCTTCTACCC CCTACTATAT TCCTCCCACC TAGTGGCCCA CTGGCCCATT CTGTATCCCC   
  
  
+ AAATCAGTTT CACCCAAAAA CTAGGTTGCC GACACTACTC AAACTTTTAA AGGTACCCTA CTACACTTAT   
  
  
+ TTCTTTAGTC TCCGACCAGA GGAGTTCTTC ATTCATAACC CTGGGTGGGA ACCTTTTCTA TCTTTATCTA   
  
  
+ TATTTTCATC CCTGAGCAAA GAAAAAGAAA AAGGAGCCAT TTTTATCAAT GTGGAGAATA AGACAAACTT   
  
  
+ ATAAAGGACC TTTGATCTCT GATGATCGTA AATCAGAAAT TTCAATTCAA ACTAATACAC GGTGTTGGCA   
  
  
+ ACTGCCAGTC TTAACCTAGG ATACTCATGT GCATCATAAA AGTACTAACG ATGAAGATCA TTGTAAATTG   
  
  
+ CCTCAATAAA CTGGCAATTA CACTTTTTAT TTCCTTTTTC CTTCTCGTTA CCATTATTAA CGAATTAATC   
  
  
+ CGTCATTTTA AAGAATTTTG AATGGATTGC GCACCAATGA GTACTCAACG ACAACAAGTG GGTTTTTTTC   
  
  
+ CGTTGTGCAT GTCATGAACC TTGATCCGCT CTTCCTTTCT TTCGACTGGT TTTCACCCAG AGTGCACCCC   
  
  
+ CGGTAAAGAA AAATATTAGA CTTTGACAAC TGGTCGAAAA GAATAAAGAA GGAATGGGGA GCAAAGGCCA   
  
  
+ TAGACAGAAG ACTTGGCAGG CTAGATAAGA GTAGTTAGGT TGCCAGCTTA GCGTGGCTCT TTCAACTGGT   
  
  
+ TCTGAAAATA TGAAACCTCT TGGGTCTTTT TCGATGTTCG GAACCCAAAA AGTGGTGCAC CGTATTCCGA   
  
  
+ CCACGGTTAA GTTTTGCTTT GTGTTCGTAT CTCTCTCTTT GTCTGTCTGG AATTTTTCAA ATAATTTCTA   
  
  
+ TTTTGCTTCA GCAAAATCGG ACGATTAAAT ACATCAGGAC ATTAAACAGT TGCCGAGTCT ACTAATTACA   
  
  
+ CCCCAACTGG GTTGGCCCAC TCTTCTGAGG TGACTAGTTA AAAGATCTTA AGACTATCGA CCTACGAACT   
  
  
+ TGTTCAGAAT TCACCATTCG TTTAATTATT TATTAATAAT TCTATTGTTT CGAATGTCAT GGAAGTGGTT   
  
  
+ GTAAATTGCG TAATTAATTG TCGAAAAACT GGTTTTTTGG TGGCATAAAA GAGTACGTAA GGACTACCCA   
  
  
+ TTTGCTATTC CGAGTACATA AGAATTAAAC CCATATTAAG CTTTTAAATC GACGATTAAC TCCTATTTTG   
  
  
+ ACATTAAATT CACGCCTATA TATTACAATA TTTATGTATT TCCGAACACC GTGCTGATCT AATGGACGAA   
  
  
+ TCAACTACGG AAGGAAGACC GTTAATTGG  

- CTGCAGCTTC TCTAAAGAGA AAGTTAAACC TCTTCGTGAG AGATGAAAGG GGGATAGAGA GAGAAAGAGA   
  
  
- GATTCGGAGC TCAGCAAGAC ATTGAACTGA AACTCAGAGA ACTGCTAAAA TGGTTCTTTG GGAGGGGGAG   
  
  
- AGAAGCCAAG GGAAGATGGG GGATGATATA AGGAGGGTGG ATCACCGGGT GACCGGGTAA GACATAGGGG   
  
  
- TTTAGTCAAA GTGGGTTTTT GATCCAACGG CTGTGATGAG TTTGAAAATT TCCATGGGAT GATGTGAATA   
  
  
- AAGAAATCAG AGGCTGGTCT CCTCAAGAAG TAAGTATTGG GACCCACCCT TGGAAAAGAT AGAAATAGAT   
  
  
- ATAAAAGTAG GGACTCGTTT CTTTTTCTTT TTCCTCGGTA AAAATAGTTA CACCTCTTAT TCTGTTTGAA   
  
  
- TATTTCCTGG AAACTAGAGA CTACTAGCAT TTAGTCTTTA AAGTTAAGTT TGATTATGTG CCACAACCGT   
  
  
- TGACGGTCAG AATTGGATCC TATGAGTACA CGTAGTATTT TCATGATTGC TACTTCTAGT AACATTTAAC   
  
  
- GGAGTTATTT GACCGTTAAT GTGAAAAATA AAGGAAAAAG GAAGAGCAAT GGTAATAATT GCTTAATTAG   
  
  
- GCAGTAAAAT TTCTTAAAAC TTACCTAACG CGTGGTTACT CATGAGTTGC TGTTGTTCAC CCAAAAAAAG   
  
  
- GCAACACGTA CAGTACTTGG AACTAGGCGA GAAGGAAAGA AAGCTGACCA AAAGTGGGTC TCACGTGGGG   
  
  
- GCCATTTCTT TTTATAATCT GAAACTGTTG ACCAGCTTTT CTTATTTCTT CCTTACCCCT CGTTTCCGGT   
  
  
- ATCTGTCTTC TGAACCGTCC GATCTATTCT CATCAATCCA ACGGTCGAAT CGCACCGAGA AAGTTGACCA   
  
  
- AGACTTTTAT ACTTTGGAGA ACCCAGAAAA AGCTACAAGC CTTGGGTTTT TCACCACGTG GCATAAGGCT   
  
  
- GGTGCCAATT CAAAACGAAA CACAAGCATA GAGAGAGAAA CAGACAGACC TTAAAAAGTT TATTAAAGAT   
  
  
- AAAACGAAGT CGTTTTAGCC TGCTAATTTA TGTAGTCCTG TAATTTGTCA ACGGCTCAGA TGATTAATGT   
  
  
- GGGGTTGACC CAACCGGGTG AGAAGACTCC ACTGATCAAT TTTCTAGAAT TCTGATAGCT GGATGCTTGA   
  
  
- ACAAGTCTTA AGTGGTAAGC AAATTAATAA ATAATTATTA AGATAACAAA GCTTACAGTA CCTTCACCAA   
  
  
- CATTTAACGC ATTAATTAAC AGCTTTTTGA CCAAAAAACC ACCGTATTTT CTCATGCATT CCTGATGGGT   
  
  
- AAACGATAAG GCTCATGTAT TCTTAATTTG GGTATAATTC GAAAATTTAG CTGCTAATTG AGGATAAAAC   
  
  
- TGTAATTTAA GTGCGGATAT ATAATGTTAT AAATACATAA AGGCTTGTGG CACGACTAGA TTACCTGCTT   
  
  
- AGTTGATGCC TTCCTTCTGG CAATTAACC

+     EIRE

| Site Name | Organism | Position | Strand | Matrix score. | sequence | function |
| --- | --- | --- | --- | --- | --- | --- |
| EIRE | Nicotiana tabacum | 802 | - | 7 | TTCGACC | elicitor-responsive element |

> 2018/04/13 10:10:12  
+ GACGTCGAAG AGATTTCTCT TTCAATTTGG AGAAGCACTC TCTACTTTCC CCCTATCTCT CTCTTTCTCT   
  
  
+ CTAAGCCTCG AGTCGTTCTG TAACTTGACT TTGAGTCTCT TGACGATTTT ACCAAGAAAC CCTCCCCCTC   
  
  
+ TCTTCGGTTC CCTTCTACCC CCTACTATAT TCCTCCCACC TAGTGGCCCA CTGGCCCATT CTGTATCCCC   
  
  
+ AAATCAGTTT CACCCAAAAA CTAGGTTGCC GACACTACTC AAACTTTTAA AGGTACCCTA CTACACTTAT   
  
  
+ TTCTTTAGTC TCCGACCAGA GGAGTTCTTC ATTCATAACC CTGGGTGGGA ACCTTTTCTA TCTTTATCTA   
  
  
+ TATTTTCATC CCTGAGCAAA GAAAAAGAAA AAGGAGCCAT TTTTATCAAT GTGGAGAATA AGACAAACTT   
  
  
+ ATAAAGGACC TTTGATCTCT GATGATCGTA AATCAGAAAT TTCAATTCAA ACTAATACAC GGTGTTGGCA   
  
  
+ ACTGCCAGTC TTAACCTAGG ATACTCATGT GCATCATAAA AGTACTAACG ATGAAGATCA TTGTAAATTG   
  
  
+ CCTCAATAAA CTGGCAATTA CACTTTTTAT TTCCTTTTTC CTTCTCGTTA CCATTATTAA CGAATTAATC   
  
  
+ CGTCATTTTA AAGAATTTTG AATGGATTGC GCACCAATGA GTACTCAACG ACAACAAGTG GGTTTTTTTC   
  
  
+ CGTTGTGCAT GTCATGAACC TTGATCCGCT CTTCCTTTCT TTCGACTGGT TTTCACCCAG AGTGCACCCC   
  
  
+ CGGTAAAGAA AAATATTAGA CTTTGACAAC TGGTCGAAAA GAATAAAGAA GGAATGGGGA GCAAAGGCCA   
  
  
+ TAGACAGAAG ACTTGGCAGG CTAGATAAGA GTAGTTAGGT TGCCAGCTTA GCGTGGCTCT TTCAACTGGT   
  
  
+ TCTGAAAATA TGAAACCTCT TGGGTCTTTT TCGATGTTCG GAACCCAAAA AGTGGTGCAC CGTATTCCGA   
  
  
+ CCACGGTTAA GTTTTGCTTT GTGTTCGTAT CTCTCTCTTT GTCTGTCTGG AATTTTTCAA ATAATTTCTA   
  
  
+ TTTTGCTTCA GCAAAATCGG ACGATTAAAT ACATCAGGAC ATTAAACAGT TGCCGAGTCT ACTAATTACA   
  
  
+ CCCCAACTGG GTTGGCCCAC TCTTCTGAGG TGACTAGTTA AAAGATCTTA AGACTATCGA CCTACGAACT   
  
  
+ TGTTCAGAAT TCACCATTCG TTTAATTATT TATTAATAAT TCTATTGTTT CGAATGTCAT GGAAGTGGTT   
  
  
+ GTAAATTGCG TAATTAATTG TCGAAAAACT GGTTTTTTGG TGGCATAAAA GAGTACGTAA GGACTACCCA   
  
  
+ TTTGCTATTC CGAGTACATA AGAATTAAAC CCATATTAAG CTTTTAAATC GACGATTAAC TCCTATTTTG   
  
  
+ ACATTAAATT CACGCCTATA TATTACAATA TTTATGTATT TCCGAACACC GTGCTGATCT AATGGACGAA   
  
  
+ TCAACTACGG AAGGAAGACC GTTAATTGG  

- CTGCAGCTTC TCTAAAGAGA AAGTTAAACC TCTTCGTGAG AGATGAAAGG GGGATAGAGA GAGAAAGAGA   
  
  
- GATTCGGAGC TCAGCAAGAC ATTGAACTGA AACTCAGAGA ACTGCTAAAA TGGTTCTTTG GGAGGGGGAG   
  
  
- AGAAGCCAAG GGAAGATGGG GGATGATATA AGGAGGGTGG ATCACCGGGT GACCGGGTAA GACATAGGGG   
  
  
- TTTAGTCAAA GTGGGTTTTT GATCCAACGG CTGTGATGAG TTTGAAAATT TCCATGGGAT GATGTGAATA   
  
  
- AAGAAATCAG AGGCTGGTCT CCTCAAGAAG TAAGTATTGG GACCCACCCT TGGAAAAGAT AGAAATAGAT   
  
  
- ATAAAAGTAG GGACTCGTTT CTTTTTCTTT TTCCTCGGTA AAAATAGTTA CACCTCTTAT TCTGTTTGAA   
  
  
- TATTTCCTGG AAACTAGAGA CTACTAGCAT TTAGTCTTTA AAGTTAAGTT TGATTATGTG CCACAACCGT   
  
  
- TGACGGTCAG AATTGGATCC TATGAGTACA CGTAGTATTT TCATGATTGC TACTTCTAGT AACATTTAAC   
  
  
- GGAGTTATTT GACCGTTAAT GTGAAAAATA AAGGAAAAAG GAAGAGCAAT GGTAATAATT GCTTAATTAG   
  
  
- GCAGTAAAAT TTCTTAAAAC TTACCTAACG CGTGGTTACT CATGAGTTGC TGTTGTTCAC CCAAAAAAAG   
  
  
- GCAACACGTA CAGTACTTGG AACTAGGCGA GAAGGAAAGA AAGCTGACCA AAAGTGGGTC TCACGTGGGG   
  
  
- GCCATTTCTT TTTATAATCT GAAACTGTTG ACCAGCTTTT CTTATTTCTT CCTTACCCCT CGTTTCCGGT   
  
  
- ATCTGTCTTC TGAACCGTCC GATCTATTCT CATCAATCCA ACGGTCGAAT CGCACCGAGA AAGTTGACCA   
  
  
- AGACTTTTAT ACTTTGGAGA ACCCAGAAAA AGCTACAAGC CTTGGGTTTT TCACCACGTG GCATAAGGCT   
  
  
- GGTGCCAATT CAAAACGAAA CACAAGCATA GAGAGAGAAA CAGACAGACC TTAAAAAGTT TATTAAAGAT   
  
  
- AAAACGAAGT CGTTTTAGCC TGCTAATTTA TGTAGTCCTG TAATTTGTCA ACGGCTCAGA TGATTAATGT   
  
  
- GGGGTTGACC CAACCGGGTG AGAAGACTCC ACTGATCAAT TTTCTAGAAT TCTGATAGCT GGATGCTTGA   
  
  
- ACAAGTCTTA AGTGGTAAGC AAATTAATAA ATAATTATTA AGATAACAAA GCTTACAGTA CCTTCACCAA   
  
  
- CATTTAACGC ATTAATTAAC AGCTTTTTGA CCAAAAAACC ACCGTATTTT CTCATGCATT CCTGATGGGT   
  
  
- AAACGATAAG GCTCATGTAT TCTTAATTTG GGTATAATTC GAAAATTTAG CTGCTAATTG AGGATAAAAC   
  
  
- TGTAATTTAA GTGCGGATAT ATAATGTTAT AAATACATAA AGGCTTGTGG CACGACTAGA TTACCTGCTT   
  
  
- AGTTGATGCC TTCCTTCTGG CAATTAACC

+     GA-motif

| Site Name | Organism | Position | Strand | Matrix score. | sequence | function |
| --- | --- | --- | --- | --- | --- | --- |
| GA-motif | Glycine max | 1481 | + | 8 | AAGGAAGA | part of a light responsive element |
| GA-motif | Arabidopsis thaliana | 344 | - | 8 | ATAGATAA | part of a light responsive element |
| GA-motif | Glycine max | 730 | - | 8 | AAGGAAGA | part of a light responsive element |

> 2018/04/13 10:10:12  
+ GACGTCGAAG AGATTTCTCT TTCAATTTGG AGAAGCACTC TCTACTTTCC CCCTATCTCT CTCTTTCTCT   
  
  
+ CTAAGCCTCG AGTCGTTCTG TAACTTGACT TTGAGTCTCT TGACGATTTT ACCAAGAAAC CCTCCCCCTC   
  
  
+ TCTTCGGTTC CCTTCTACCC CCTACTATAT TCCTCCCACC TAGTGGCCCA CTGGCCCATT CTGTATCCCC   
  
  
+ AAATCAGTTT CACCCAAAAA CTAGGTTGCC GACACTACTC AAACTTTTAA AGGTACCCTA CTACACTTAT   
  
  
+ TTCTTTAGTC TCCGACCAGA GGAGTTCTTC ATTCATAACC CTGGGTGGGA ACCTTTTCTA TCTTTATCTA   
  
  
+ TATTTTCATC CCTGAGCAAA GAAAAAGAAA AAGGAGCCAT TTTTATCAAT GTGGAGAATA AGACAAACTT   
  
  
+ ATAAAGGACC TTTGATCTCT GATGATCGTA AATCAGAAAT TTCAATTCAA ACTAATACAC GGTGTTGGCA   
  
  
+ ACTGCCAGTC TTAACCTAGG ATACTCATGT GCATCATAAA AGTACTAACG ATGAAGATCA TTGTAAATTG   
  
  
+ CCTCAATAAA CTGGCAATTA CACTTTTTAT TTCCTTTTTC CTTCTCGTTA CCATTATTAA CGAATTAATC   
  
  
+ CGTCATTTTA AAGAATTTTG AATGGATTGC GCACCAATGA GTACTCAACG ACAACAAGTG GGTTTTTTTC   
  
  
+ CGTTGTGCAT GTCATGAACC TTGATCCGCT CTTCCTTTCT TTCGACTGGT TTTCACCCAG AGTGCACCCC   
  
  
+ CGGTAAAGAA AAATATTAGA CTTTGACAAC TGGTCGAAAA GAATAAAGAA GGAATGGGGA GCAAAGGCCA   
  
  
+ TAGACAGAAG ACTTGGCAGG CTAGATAAGA GTAGTTAGGT TGCCAGCTTA GCGTGGCTCT TTCAACTGGT   
  
  
+ TCTGAAAATA TGAAACCTCT TGGGTCTTTT TCGATGTTCG GAACCCAAAA AGTGGTGCAC CGTATTCCGA   
  
  
+ CCACGGTTAA GTTTTGCTTT GTGTTCGTAT CTCTCTCTTT GTCTGTCTGG AATTTTTCAA ATAATTTCTA   
  
  
+ TTTTGCTTCA GCAAAATCGG ACGATTAAAT ACATCAGGAC ATTAAACAGT TGCCGAGTCT ACTAATTACA   
  
  
+ CCCCAACTGG GTTGGCCCAC TCTTCTGAGG TGACTAGTTA AAAGATCTTA AGACTATCGA CCTACGAACT   
  
  
+ TGTTCAGAAT TCACCATTCG TTTAATTATT TATTAATAAT TCTATTGTTT CGAATGTCAT GGAAGTGGTT   
  
  
+ GTAAATTGCG TAATTAATTG TCGAAAAACT GGTTTTTTGG TGGCATAAAA GAGTACGTAA GGACTACCCA   
  
  
+ TTTGCTATTC CGAGTACATA AGAATTAAAC CCATATTAAG CTTTTAAATC GACGATTAAC TCCTATTTTG   
  
  
+ ACATTAAATT CACGCCTATA TATTACAATA TTTATGTATT TCCGAACACC GTGCTGATCT AATGGACGAA   
  
  
+ TCAACTACGG AAGGAAGACC GTTAATTGG  

- CTGCAGCTTC TCTAAAGAGA AAGTTAAACC TCTTCGTGAG AGATGAAAGG GGGATAGAGA GAGAAAGAGA   
  
  
- GATTCGGAGC TCAGCAAGAC ATTGAACTGA AACTCAGAGA ACTGCTAAAA TGGTTCTTTG GGAGGGGGAG   
  
  
- AGAAGCCAAG GGAAGATGGG GGATGATATA AGGAGGGTGG ATCACCGGGT GACCGGGTAA GACATAGGGG   
  
  
- TTTAGTCAAA GTGGGTTTTT GATCCAACGG CTGTGATGAG TTTGAAAATT TCCATGGGAT GATGTGAATA   
  
  
- AAGAAATCAG AGGCTGGTCT CCTCAAGAAG TAAGTATTGG GACCCACCCT TGGAAAAGAT AGAAATAGAT   
  
  
- ATAAAAGTAG GGACTCGTTT CTTTTTCTTT TTCCTCGGTA AAAATAGTTA CACCTCTTAT TCTGTTTGAA   
  
  
- TATTTCCTGG AAACTAGAGA CTACTAGCAT TTAGTCTTTA AAGTTAAGTT TGATTATGTG CCACAACCGT   
  
  
- TGACGGTCAG AATTGGATCC TATGAGTACA CGTAGTATTT TCATGATTGC TACTTCTAGT AACATTTAAC   
  
  
- GGAGTTATTT GACCGTTAAT GTGAAAAATA AAGGAAAAAG GAAGAGCAAT GGTAATAATT GCTTAATTAG   
  
  
- GCAGTAAAAT TTCTTAAAAC TTACCTAACG CGTGGTTACT CATGAGTTGC TGTTGTTCAC CCAAAAAAAG   
  
  
- GCAACACGTA CAGTACTTGG AACTAGGCGA GAAGGAAAGA AAGCTGACCA AAAGTGGGTC TCACGTGGGG   
  
  
- GCCATTTCTT TTTATAATCT GAAACTGTTG ACCAGCTTTT CTTATTTCTT CCTTACCCCT CGTTTCCGGT   
  
  
- ATCTGTCTTC TGAACCGTCC GATCTATTCT CATCAATCCA ACGGTCGAAT CGCACCGAGA AAGTTGACCA   
  
  
- AGACTTTTAT ACTTTGGAGA ACCCAGAAAA AGCTACAAGC CTTGGGTTTT TCACCACGTG GCATAAGGCT   
  
  
- GGTGCCAATT CAAAACGAAA CACAAGCATA GAGAGAGAAA CAGACAGACC TTAAAAAGTT TATTAAAGAT   
  
  
- AAAACGAAGT CGTTTTAGCC TGCTAATTTA TGTAGTCCTG TAATTTGTCA ACGGCTCAGA TGATTAATGT   
  
  
- GGGGTTGACC CAACCGGGTG AGAAGACTCC ACTGATCAAT TTTCTAGAAT TCTGATAGCT GGATGCTTGA   
  
  
- ACAAGTCTTA AGTGGTAAGC AAATTAATAA ATAATTATTA AGATAACAAA GCTTACAGTA CCTTCACCAA   
  
  
- CATTTAACGC ATTAATTAAC AGCTTTTTGA CCAAAAAACC ACCGTATTTT CTCATGCATT CCTGATGGGT   
  
  
- AAACGATAAG GCTCATGTAT TCTTAATTTG GGTATAATTC GAAAATTTAG CTGCTAATTG AGGATAAAAC   
  
  
- TGTAATTTAA GTGCGGATAT ATAATGTTAT AAATACATAA AGGCTTGTGG CACGACTAGA TTACCTGCTT   
  
  
- AGTTGATGCC TTCCTTCTGG CAATTAACC

+     GAG-motif

| Site Name | Organism | Position | Strand | Matrix score. | sequence | function |
| --- | --- | --- | --- | --- | --- | --- |
| GAG-motif | Arabidopsis thaliana | 37 | - | 7 | AGAGAGT | part of a light responsive element |

> 2018/04/13 10:10:12  
+ GACGTCGAAG AGATTTCTCT TTCAATTTGG AGAAGCACTC TCTACTTTCC CCCTATCTCT CTCTTTCTCT   
  
  
+ CTAAGCCTCG AGTCGTTCTG TAACTTGACT TTGAGTCTCT TGACGATTTT ACCAAGAAAC CCTCCCCCTC   
  
  
+ TCTTCGGTTC CCTTCTACCC CCTACTATAT TCCTCCCACC TAGTGGCCCA CTGGCCCATT CTGTATCCCC   
  
  
+ AAATCAGTTT CACCCAAAAA CTAGGTTGCC GACACTACTC AAACTTTTAA AGGTACCCTA CTACACTTAT   
  
  
+ TTCTTTAGTC TCCGACCAGA GGAGTTCTTC ATTCATAACC CTGGGTGGGA ACCTTTTCTA TCTTTATCTA   
  
  
+ TATTTTCATC CCTGAGCAAA GAAAAAGAAA AAGGAGCCAT TTTTATCAAT GTGGAGAATA AGACAAACTT   
  
  
+ ATAAAGGACC TTTGATCTCT GATGATCGTA AATCAGAAAT TTCAATTCAA ACTAATACAC GGTGTTGGCA   
  
  
+ ACTGCCAGTC TTAACCTAGG ATACTCATGT GCATCATAAA AGTACTAACG ATGAAGATCA TTGTAAATTG   
  
  
+ CCTCAATAAA CTGGCAATTA CACTTTTTAT TTCCTTTTTC CTTCTCGTTA CCATTATTAA CGAATTAATC   
  
  
+ CGTCATTTTA AAGAATTTTG AATGGATTGC GCACCAATGA GTACTCAACG ACAACAAGTG GGTTTTTTTC   
  
  
+ CGTTGTGCAT GTCATGAACC TTGATCCGCT CTTCCTTTCT TTCGACTGGT TTTCACCCAG AGTGCACCCC   
  
  
+ CGGTAAAGAA AAATATTAGA CTTTGACAAC TGGTCGAAAA GAATAAAGAA GGAATGGGGA GCAAAGGCCA   
  
  
+ TAGACAGAAG ACTTGGCAGG CTAGATAAGA GTAGTTAGGT TGCCAGCTTA GCGTGGCTCT TTCAACTGGT   
  
  
+ TCTGAAAATA TGAAACCTCT TGGGTCTTTT TCGATGTTCG GAACCCAAAA AGTGGTGCAC CGTATTCCGA   
  
  
+ CCACGGTTAA GTTTTGCTTT GTGTTCGTAT CTCTCTCTTT GTCTGTCTGG AATTTTTCAA ATAATTTCTA   
  
  
+ TTTTGCTTCA GCAAAATCGG ACGATTAAAT ACATCAGGAC ATTAAACAGT TGCCGAGTCT ACTAATTACA   
  
  
+ CCCCAACTGG GTTGGCCCAC TCTTCTGAGG TGACTAGTTA AAAGATCTTA AGACTATCGA CCTACGAACT   
  
  
+ TGTTCAGAAT TCACCATTCG TTTAATTATT TATTAATAAT TCTATTGTTT CGAATGTCAT GGAAGTGGTT   
  
  
+ GTAAATTGCG TAATTAATTG TCGAAAAACT GGTTTTTTGG TGGCATAAAA GAGTACGTAA GGACTACCCA   
  
  
+ TTTGCTATTC CGAGTACATA AGAATTAAAC CCATATTAAG CTTTTAAATC GACGATTAAC TCCTATTTTG   
  
  
+ ACATTAAATT CACGCCTATA TATTACAATA TTTATGTATT TCCGAACACC GTGCTGATCT AATGGACGAA   
  
  
+ TCAACTACGG AAGGAAGACC GTTAATTGG  

- CTGCAGCTTC TCTAAAGAGA AAGTTAAACC TCTTCGTGAG AGATGAAAGG GGGATAGAGA GAGAAAGAGA   
  
  
- GATTCGGAGC TCAGCAAGAC ATTGAACTGA AACTCAGAGA ACTGCTAAAA TGGTTCTTTG GGAGGGGGAG   
  
  
- AGAAGCCAAG GGAAGATGGG GGATGATATA AGGAGGGTGG ATCACCGGGT GACCGGGTAA GACATAGGGG   
  
  
- TTTAGTCAAA GTGGGTTTTT GATCCAACGG CTGTGATGAG TTTGAAAATT TCCATGGGAT GATGTGAATA   
  
  
- AAGAAATCAG AGGCTGGTCT CCTCAAGAAG TAAGTATTGG GACCCACCCT TGGAAAAGAT AGAAATAGAT   
  
  
- ATAAAAGTAG GGACTCGTTT CTTTTTCTTT TTCCTCGGTA AAAATAGTTA CACCTCTTAT TCTGTTTGAA   
  
  
- TATTTCCTGG AAACTAGAGA CTACTAGCAT TTAGTCTTTA AAGTTAAGTT TGATTATGTG CCACAACCGT   
  
  
- TGACGGTCAG AATTGGATCC TATGAGTACA CGTAGTATTT TCATGATTGC TACTTCTAGT AACATTTAAC   
  
  
- GGAGTTATTT GACCGTTAAT GTGAAAAATA AAGGAAAAAG GAAGAGCAAT GGTAATAATT GCTTAATTAG   
  
  
- GCAGTAAAAT TTCTTAAAAC TTACCTAACG CGTGGTTACT CATGAGTTGC TGTTGTTCAC CCAAAAAAAG   
  
  
- GCAACACGTA CAGTACTTGG AACTAGGCGA GAAGGAAAGA AAGCTGACCA AAAGTGGGTC TCACGTGGGG   
  
  
- GCCATTTCTT TTTATAATCT GAAACTGTTG ACCAGCTTTT CTTATTTCTT CCTTACCCCT CGTTTCCGGT   
  
  
- ATCTGTCTTC TGAACCGTCC GATCTATTCT CATCAATCCA ACGGTCGAAT CGCACCGAGA AAGTTGACCA   
  
  
- AGACTTTTAT ACTTTGGAGA ACCCAGAAAA AGCTACAAGC CTTGGGTTTT TCACCACGTG GCATAAGGCT   
  
  
- GGTGCCAATT CAAAACGAAA CACAAGCATA GAGAGAGAAA CAGACAGACC TTAAAAAGTT TATTAAAGAT   
  
  
- AAAACGAAGT CGTTTTAGCC TGCTAATTTA TGTAGTCCTG TAATTTGTCA ACGGCTCAGA TGATTAATGT   
  
  
- GGGGTTGACC CAACCGGGTG AGAAGACTCC ACTGATCAAT TTTCTAGAAT TCTGATAGCT GGATGCTTGA   
  
  
- ACAAGTCTTA AGTGGTAAGC AAATTAATAA ATAATTATTA AGATAACAAA GCTTACAGTA CCTTCACCAA   
  
  
- CATTTAACGC ATTAATTAAC AGCTTTTTGA CCAAAAAACC ACCGTATTTT CTCATGCATT CCTGATGGGT   
  
  
- AAACGATAAG GCTCATGTAT TCTTAATTTG GGTATAATTC GAAAATTTAG CTGCTAATTG AGGATAAAAC   
  
  
- TGTAATTTAA GTGCGGATAT ATAATGTTAT AAATACATAA AGGCTTGTGG CACGACTAGA TTACCTGCTT   
  
  
- AGTTGATGCC TTCCTTCTGG CAATTAACC

+     GATA-motif

| Site Name | Organism | Position | Strand | Matrix score. | sequence | function |
| --- | --- | --- | --- | --- | --- | --- |
| GATA-motif | Pisum sativum | 51 | - | 7 | GATAGGG | part of a light responsive element |

> 2018/04/13 10:10:12  
+ GACGTCGAAG AGATTTCTCT TTCAATTTGG AGAAGCACTC TCTACTTTCC CCCTATCTCT CTCTTTCTCT   
  
  
+ CTAAGCCTCG AGTCGTTCTG TAACTTGACT TTGAGTCTCT TGACGATTTT ACCAAGAAAC CCTCCCCCTC   
  
  
+ TCTTCGGTTC CCTTCTACCC CCTACTATAT TCCTCCCACC TAGTGGCCCA CTGGCCCATT CTGTATCCCC   
  
  
+ AAATCAGTTT CACCCAAAAA CTAGGTTGCC GACACTACTC AAACTTTTAA AGGTACCCTA CTACACTTAT   
  
  
+ TTCTTTAGTC TCCGACCAGA GGAGTTCTTC ATTCATAACC CTGGGTGGGA ACCTTTTCTA TCTTTATCTA   
  
  
+ TATTTTCATC CCTGAGCAAA GAAAAAGAAA AAGGAGCCAT TTTTATCAAT GTGGAGAATA AGACAAACTT   
  
  
+ ATAAAGGACC TTTGATCTCT GATGATCGTA AATCAGAAAT TTCAATTCAA ACTAATACAC GGTGTTGGCA   
  
  
+ ACTGCCAGTC TTAACCTAGG ATACTCATGT GCATCATAAA AGTACTAACG ATGAAGATCA TTGTAAATTG   
  
  
+ CCTCAATAAA CTGGCAATTA CACTTTTTAT TTCCTTTTTC CTTCTCGTTA CCATTATTAA CGAATTAATC   
  
  
+ CGTCATTTTA AAGAATTTTG AATGGATTGC GCACCAATGA GTACTCAACG ACAACAAGTG GGTTTTTTTC   
  
  
+ CGTTGTGCAT GTCATGAACC TTGATCCGCT CTTCCTTTCT TTCGACTGGT TTTCACCCAG AGTGCACCCC   
  
  
+ CGGTAAAGAA AAATATTAGA CTTTGACAAC TGGTCGAAAA GAATAAAGAA GGAATGGGGA GCAAAGGCCA   
  
  
+ TAGACAGAAG ACTTGGCAGG CTAGATAAGA GTAGTTAGGT TGCCAGCTTA GCGTGGCTCT TTCAACTGGT   
  
  
+ TCTGAAAATA TGAAACCTCT TGGGTCTTTT TCGATGTTCG GAACCCAAAA AGTGGTGCAC CGTATTCCGA   
  
  
+ CCACGGTTAA GTTTTGCTTT GTGTTCGTAT CTCTCTCTTT GTCTGTCTGG AATTTTTCAA ATAATTTCTA   
  
  
+ TTTTGCTTCA GCAAAATCGG ACGATTAAAT ACATCAGGAC ATTAAACAGT TGCCGAGTCT ACTAATTACA   
  
  
+ CCCCAACTGG GTTGGCCCAC TCTTCTGAGG TGACTAGTTA AAAGATCTTA AGACTATCGA CCTACGAACT   
  
  
+ TGTTCAGAAT TCACCATTCG TTTAATTATT TATTAATAAT TCTATTGTTT CGAATGTCAT GGAAGTGGTT   
  
  
+ GTAAATTGCG TAATTAATTG TCGAAAAACT GGTTTTTTGG TGGCATAAAA GAGTACGTAA GGACTACCCA   
  
  
+ TTTGCTATTC CGAGTACATA AGAATTAAAC CCATATTAAG CTTTTAAATC GACGATTAAC TCCTATTTTG   
  
  
+ ACATTAAATT CACGCCTATA TATTACAATA TTTATGTATT TCCGAACACC GTGCTGATCT AATGGACGAA   
  
  
+ TCAACTACGG AAGGAAGACC GTTAATTGG  

- CTGCAGCTTC TCTAAAGAGA AAGTTAAACC TCTTCGTGAG AGATGAAAGG GGGATAGAGA GAGAAAGAGA   
  
  
- GATTCGGAGC TCAGCAAGAC ATTGAACTGA AACTCAGAGA ACTGCTAAAA TGGTTCTTTG GGAGGGGGAG   
  
  
- AGAAGCCAAG GGAAGATGGG GGATGATATA AGGAGGGTGG ATCACCGGGT GACCGGGTAA GACATAGGGG   
  
  
- TTTAGTCAAA GTGGGTTTTT GATCCAACGG CTGTGATGAG TTTGAAAATT TCCATGGGAT GATGTGAATA   
  
  
- AAGAAATCAG AGGCTGGTCT CCTCAAGAAG TAAGTATTGG GACCCACCCT TGGAAAAGAT AGAAATAGAT   
  
  
- ATAAAAGTAG GGACTCGTTT CTTTTTCTTT TTCCTCGGTA AAAATAGTTA CACCTCTTAT TCTGTTTGAA   
  
  
- TATTTCCTGG AAACTAGAGA CTACTAGCAT TTAGTCTTTA AAGTTAAGTT TGATTATGTG CCACAACCGT   
  
  
- TGACGGTCAG AATTGGATCC TATGAGTACA CGTAGTATTT TCATGATTGC TACTTCTAGT AACATTTAAC   
  
  
- GGAGTTATTT GACCGTTAAT GTGAAAAATA AAGGAAAAAG GAAGAGCAAT GGTAATAATT GCTTAATTAG   
  
  
- GCAGTAAAAT TTCTTAAAAC TTACCTAACG CGTGGTTACT CATGAGTTGC TGTTGTTCAC CCAAAAAAAG   
  
  
- GCAACACGTA CAGTACTTGG AACTAGGCGA GAAGGAAAGA AAGCTGACCA AAAGTGGGTC TCACGTGGGG   
  
  
- GCCATTTCTT TTTATAATCT GAAACTGTTG ACCAGCTTTT CTTATTTCTT CCTTACCCCT CGTTTCCGGT   
  
  
- ATCTGTCTTC TGAACCGTCC GATCTATTCT CATCAATCCA ACGGTCGAAT CGCACCGAGA AAGTTGACCA   
  
  
- AGACTTTTAT ACTTTGGAGA ACCCAGAAAA AGCTACAAGC CTTGGGTTTT TCACCACGTG GCATAAGGCT   
  
  
- GGTGCCAATT CAAAACGAAA CACAAGCATA GAGAGAGAAA CAGACAGACC TTAAAAAGTT TATTAAAGAT   
  
  
- AAAACGAAGT CGTTTTAGCC TGCTAATTTA TGTAGTCCTG TAATTTGTCA ACGGCTCAGA TGATTAATGT   
  
  
- GGGGTTGACC CAACCGGGTG AGAAGACTCC ACTGATCAAT TTTCTAGAAT TCTGATAGCT GGATGCTTGA   
  
  
- ACAAGTCTTA AGTGGTAAGC AAATTAATAA ATAATTATTA AGATAACAAA GCTTACAGTA CCTTCACCAA   
  
  
- CATTTAACGC ATTAATTAAC AGCTTTTTGA CCAAAAAACC ACCGTATTTT CTCATGCATT CCTGATGGGT   
  
  
- AAACGATAAG GCTCATGTAT TCTTAATTTG GGTATAATTC GAAAATTTAG CTGCTAATTG AGGATAAAAC   
  
  
- TGTAATTTAA GTGCGGATAT ATAATGTTAT AAATACATAA AGGCTTGTGG CACGACTAGA TTACCTGCTT   
  
  
- AGTTGATGCC TTCCTTCTGG CAATTAACC

+     GC-motif

| Site Name | Organism | Position | Strand | Matrix score. | sequence | function |
| --- | --- | --- | --- | --- | --- | --- |
| GC-motif | Zea mays | 767 | + | 6 | CCCCCG | enhancer-like element involved in anoxic specific inducibility |

> 2018/04/13 10:10:12  
+ GACGTCGAAG AGATTTCTCT TTCAATTTGG AGAAGCACTC TCTACTTTCC CCCTATCTCT CTCTTTCTCT   
  
  
+ CTAAGCCTCG AGTCGTTCTG TAACTTGACT TTGAGTCTCT TGACGATTTT ACCAAGAAAC CCTCCCCCTC   
  
  
+ TCTTCGGTTC CCTTCTACCC CCTACTATAT TCCTCCCACC TAGTGGCCCA CTGGCCCATT CTGTATCCCC   
  
  
+ AAATCAGTTT CACCCAAAAA CTAGGTTGCC GACACTACTC AAACTTTTAA AGGTACCCTA CTACACTTAT   
  
  
+ TTCTTTAGTC TCCGACCAGA GGAGTTCTTC ATTCATAACC CTGGGTGGGA ACCTTTTCTA TCTTTATCTA   
  
  
+ TATTTTCATC CCTGAGCAAA GAAAAAGAAA AAGGAGCCAT TTTTATCAAT GTGGAGAATA AGACAAACTT   
  
  
+ ATAAAGGACC TTTGATCTCT GATGATCGTA AATCAGAAAT TTCAATTCAA ACTAATACAC GGTGTTGGCA   
  
  
+ ACTGCCAGTC TTAACCTAGG ATACTCATGT GCATCATAAA AGTACTAACG ATGAAGATCA TTGTAAATTG   
  
  
+ CCTCAATAAA CTGGCAATTA CACTTTTTAT TTCCTTTTTC CTTCTCGTTA CCATTATTAA CGAATTAATC   
  
  
+ CGTCATTTTA AAGAATTTTG AATGGATTGC GCACCAATGA GTACTCAACG ACAACAAGTG GGTTTTTTTC   
  
  
+ CGTTGTGCAT GTCATGAACC TTGATCCGCT CTTCCTTTCT TTCGACTGGT TTTCACCCAG AGTGCACCCC   
  
  
+ CGGTAAAGAA AAATATTAGA CTTTGACAAC TGGTCGAAAA GAATAAAGAA GGAATGGGGA GCAAAGGCCA   
  
  
+ TAGACAGAAG ACTTGGCAGG CTAGATAAGA GTAGTTAGGT TGCCAGCTTA GCGTGGCTCT TTCAACTGGT   
  
  
+ TCTGAAAATA TGAAACCTCT TGGGTCTTTT TCGATGTTCG GAACCCAAAA AGTGGTGCAC CGTATTCCGA   
  
  
+ CCACGGTTAA GTTTTGCTTT GTGTTCGTAT CTCTCTCTTT GTCTGTCTGG AATTTTTCAA ATAATTTCTA   
  
  
+ TTTTGCTTCA GCAAAATCGG ACGATTAAAT ACATCAGGAC ATTAAACAGT TGCCGAGTCT ACTAATTACA   
  
  
+ CCCCAACTGG GTTGGCCCAC TCTTCTGAGG TGACTAGTTA AAAGATCTTA AGACTATCGA CCTACGAACT   
  
  
+ TGTTCAGAAT TCACCATTCG TTTAATTATT TATTAATAAT TCTATTGTTT CGAATGTCAT GGAAGTGGTT   
  
  
+ GTAAATTGCG TAATTAATTG TCGAAAAACT GGTTTTTTGG TGGCATAAAA GAGTACGTAA GGACTACCCA   
  
  
+ TTTGCTATTC CGAGTACATA AGAATTAAAC CCATATTAAG CTTTTAAATC GACGATTAAC TCCTATTTTG   
  
  
+ ACATTAAATT CACGCCTATA TATTACAATA TTTATGTATT TCCGAACACC GTGCTGATCT AATGGACGAA   
  
  
+ TCAACTACGG AAGGAAGACC GTTAATTGG  

- CTGCAGCTTC TCTAAAGAGA AAGTTAAACC TCTTCGTGAG AGATGAAAGG GGGATAGAGA GAGAAAGAGA   
  
  
- GATTCGGAGC TCAGCAAGAC ATTGAACTGA AACTCAGAGA ACTGCTAAAA TGGTTCTTTG GGAGGGGGAG   
  
  
- AGAAGCCAAG GGAAGATGGG GGATGATATA AGGAGGGTGG ATCACCGGGT GACCGGGTAA GACATAGGGG   
  
  
- TTTAGTCAAA GTGGGTTTTT GATCCAACGG CTGTGATGAG TTTGAAAATT TCCATGGGAT GATGTGAATA   
  
  
- AAGAAATCAG AGGCTGGTCT CCTCAAGAAG TAAGTATTGG GACCCACCCT TGGAAAAGAT AGAAATAGAT   
  
  
- ATAAAAGTAG GGACTCGTTT CTTTTTCTTT TTCCTCGGTA AAAATAGTTA CACCTCTTAT TCTGTTTGAA   
  
  
- TATTTCCTGG AAACTAGAGA CTACTAGCAT TTAGTCTTTA AAGTTAAGTT TGATTATGTG CCACAACCGT   
  
  
- TGACGGTCAG AATTGGATCC TATGAGTACA CGTAGTATTT TCATGATTGC TACTTCTAGT AACATTTAAC   
  
  
- GGAGTTATTT GACCGTTAAT GTGAAAAATA AAGGAAAAAG GAAGAGCAAT GGTAATAATT GCTTAATTAG   
  
  
- GCAGTAAAAT TTCTTAAAAC TTACCTAACG CGTGGTTACT CATGAGTTGC TGTTGTTCAC CCAAAAAAAG   
  
  
- GCAACACGTA CAGTACTTGG AACTAGGCGA GAAGGAAAGA AAGCTGACCA AAAGTGGGTC TCACGTGGGG   
  
  
- GCCATTTCTT TTTATAATCT GAAACTGTTG ACCAGCTTTT CTTATTTCTT CCTTACCCCT CGTTTCCGGT   
  
  
- ATCTGTCTTC TGAACCGTCC GATCTATTCT CATCAATCCA ACGGTCGAAT CGCACCGAGA AAGTTGACCA   
  
  
- AGACTTTTAT ACTTTGGAGA ACCCAGAAAA AGCTACAAGC CTTGGGTTTT TCACCACGTG GCATAAGGCT   
  
  
- GGTGCCAATT CAAAACGAAA CACAAGCATA GAGAGAGAAA CAGACAGACC TTAAAAAGTT TATTAAAGAT   
  
  
- AAAACGAAGT CGTTTTAGCC TGCTAATTTA TGTAGTCCTG TAATTTGTCA ACGGCTCAGA TGATTAATGT   
  
  
- GGGGTTGACC CAACCGGGTG AGAAGACTCC ACTGATCAAT TTTCTAGAAT TCTGATAGCT GGATGCTTGA   
  
  
- ACAAGTCTTA AGTGGTAAGC AAATTAATAA ATAATTATTA AGATAACAAA GCTTACAGTA CCTTCACCAA   
  
  
- CATTTAACGC ATTAATTAAC AGCTTTTTGA CCAAAAAACC ACCGTATTTT CTCATGCATT CCTGATGGGT   
  
  
- AAACGATAAG GCTCATGTAT TCTTAATTTG GGTATAATTC GAAAATTTAG CTGCTAATTG AGGATAAAAC   
  
  
- TGTAATTTAA GTGCGGATAT ATAATGTTAT AAATACATAA AGGCTTGTGG CACGACTAGA TTACCTGCTT   
  
  
- AGTTGATGCC TTCCTTCTGG CAATTAACC

+     GT1-motif

| Site Name | Organism | Position | Strand | Matrix score. | sequence | function |
| --- | --- | --- | --- | --- | --- | --- |
| GT1-motif | Arabidopsis thaliana | 501 | - | 6 | GGTTAA | light responsive element |
| GT1-motif | Arabidopsis thaliana | 985 | + | 6 | GGTTAA | light responsive element |

> 2018/04/13 10:10:12  
+ GACGTCGAAG AGATTTCTCT TTCAATTTGG AGAAGCACTC TCTACTTTCC CCCTATCTCT CTCTTTCTCT   
  
  
+ CTAAGCCTCG AGTCGTTCTG TAACTTGACT TTGAGTCTCT TGACGATTTT ACCAAGAAAC CCTCCCCCTC   
  
  
+ TCTTCGGTTC CCTTCTACCC CCTACTATAT TCCTCCCACC TAGTGGCCCA CTGGCCCATT CTGTATCCCC   
  
  
+ AAATCAGTTT CACCCAAAAA CTAGGTTGCC GACACTACTC AAACTTTTAA AGGTACCCTA CTACACTTAT   
  
  
+ TTCTTTAGTC TCCGACCAGA GGAGTTCTTC ATTCATAACC CTGGGTGGGA ACCTTTTCTA TCTTTATCTA   
  
  
+ TATTTTCATC CCTGAGCAAA GAAAAAGAAA AAGGAGCCAT TTTTATCAAT GTGGAGAATA AGACAAACTT   
  
  
+ ATAAAGGACC TTTGATCTCT GATGATCGTA AATCAGAAAT TTCAATTCAA ACTAATACAC GGTGTTGGCA   
  
  
+ ACTGCCAGTC TTAACCTAGG ATACTCATGT GCATCATAAA AGTACTAACG ATGAAGATCA TTGTAAATTG   
  
  
+ CCTCAATAAA CTGGCAATTA CACTTTTTAT TTCCTTTTTC CTTCTCGTTA CCATTATTAA CGAATTAATC   
  
  
+ CGTCATTTTA AAGAATTTTG AATGGATTGC GCACCAATGA GTACTCAACG ACAACAAGTG GGTTTTTTTC   
  
  
+ CGTTGTGCAT GTCATGAACC TTGATCCGCT CTTCCTTTCT TTCGACTGGT TTTCACCCAG AGTGCACCCC   
  
  
+ CGGTAAAGAA AAATATTAGA CTTTGACAAC TGGTCGAAAA GAATAAAGAA GGAATGGGGA GCAAAGGCCA   
  
  
+ TAGACAGAAG ACTTGGCAGG CTAGATAAGA GTAGTTAGGT TGCCAGCTTA GCGTGGCTCT TTCAACTGGT   
  
  
+ TCTGAAAATA TGAAACCTCT TGGGTCTTTT TCGATGTTCG GAACCCAAAA AGTGGTGCAC CGTATTCCGA   
  
  
+ CCACGGTTAA GTTTTGCTTT GTGTTCGTAT CTCTCTCTTT GTCTGTCTGG AATTTTTCAA ATAATTTCTA   
  
  
+ TTTTGCTTCA GCAAAATCGG ACGATTAAAT ACATCAGGAC ATTAAACAGT TGCCGAGTCT ACTAATTACA   
  
  
+ CCCCAACTGG GTTGGCCCAC TCTTCTGAGG TGACTAGTTA AAAGATCTTA AGACTATCGA CCTACGAACT   
  
  
+ TGTTCAGAAT TCACCATTCG TTTAATTATT TATTAATAAT TCTATTGTTT CGAATGTCAT GGAAGTGGTT   
  
  
+ GTAAATTGCG TAATTAATTG TCGAAAAACT GGTTTTTTGG TGGCATAAAA GAGTACGTAA GGACTACCCA   
  
  
+ TTTGCTATTC CGAGTACATA AGAATTAAAC CCATATTAAG CTTTTAAATC GACGATTAAC TCCTATTTTG   
  
  
+ ACATTAAATT CACGCCTATA TATTACAATA TTTATGTATT TCCGAACACC GTGCTGATCT AATGGACGAA   
  
  
+ TCAACTACGG AAGGAAGACC GTTAATTGG  

- CTGCAGCTTC TCTAAAGAGA AAGTTAAACC TCTTCGTGAG AGATGAAAGG GGGATAGAGA GAGAAAGAGA   
  
  
- GATTCGGAGC TCAGCAAGAC ATTGAACTGA AACTCAGAGA ACTGCTAAAA TGGTTCTTTG GGAGGGGGAG   
  
  
- AGAAGCCAAG GGAAGATGGG GGATGATATA AGGAGGGTGG ATCACCGGGT GACCGGGTAA GACATAGGGG   
  
  
- TTTAGTCAAA GTGGGTTTTT GATCCAACGG CTGTGATGAG TTTGAAAATT TCCATGGGAT GATGTGAATA   
  
  
- AAGAAATCAG AGGCTGGTCT CCTCAAGAAG TAAGTATTGG GACCCACCCT TGGAAAAGAT AGAAATAGAT   
  
  
- ATAAAAGTAG GGACTCGTTT CTTTTTCTTT TTCCTCGGTA AAAATAGTTA CACCTCTTAT TCTGTTTGAA   
  
  
- TATTTCCTGG AAACTAGAGA CTACTAGCAT TTAGTCTTTA AAGTTAAGTT TGATTATGTG CCACAACCGT   
  
  
- TGACGGTCAG AATTGGATCC TATGAGTACA CGTAGTATTT TCATGATTGC TACTTCTAGT AACATTTAAC   
  
  
- GGAGTTATTT GACCGTTAAT GTGAAAAATA AAGGAAAAAG GAAGAGCAAT GGTAATAATT GCTTAATTAG   
  
  
- GCAGTAAAAT TTCTTAAAAC TTACCTAACG CGTGGTTACT CATGAGTTGC TGTTGTTCAC CCAAAAAAAG   
  
  
- GCAACACGTA CAGTACTTGG AACTAGGCGA GAAGGAAAGA AAGCTGACCA AAAGTGGGTC TCACGTGGGG   
  
  
- GCCATTTCTT TTTATAATCT GAAACTGTTG ACCAGCTTTT CTTATTTCTT CCTTACCCCT CGTTTCCGGT   
  
  
- ATCTGTCTTC TGAACCGTCC GATCTATTCT CATCAATCCA ACGGTCGAAT CGCACCGAGA AAGTTGACCA   
  
  
- AGACTTTTAT ACTTTGGAGA ACCCAGAAAA AGCTACAAGC CTTGGGTTTT TCACCACGTG GCATAAGGCT   
  
  
- GGTGCCAATT CAAAACGAAA CACAAGCATA GAGAGAGAAA CAGACAGACC TTAAAAAGTT TATTAAAGAT   
  
  
- AAAACGAAGT CGTTTTAGCC TGCTAATTTA TGTAGTCCTG TAATTTGTCA ACGGCTCAGA TGATTAATGT   
  
  
- GGGGTTGACC CAACCGGGTG AGAAGACTCC ACTGATCAAT TTTCTAGAAT TCTGATAGCT GGATGCTTGA   
  
  
- ACAAGTCTTA AGTGGTAAGC AAATTAATAA ATAATTATTA AGATAACAAA GCTTACAGTA CCTTCACCAA   
  
  
- CATTTAACGC ATTAATTAAC AGCTTTTTGA CCAAAAAACC ACCGTATTTT CTCATGCATT CCTGATGGGT   
  
  
- AAACGATAAG GCTCATGTAT TCTTAATTTG GGTATAATTC GAAAATTTAG CTGCTAATTG AGGATAAAAC   
  
  
- TGTAATTTAA GTGCGGATAT ATAATGTTAT AAATACATAA AGGCTTGTGG CACGACTAGA TTACCTGCTT   
  
  
- AGTTGATGCC TTCCTTCTGG CAATTAACC

+     HSE

| Site Name | Organism | Position | Strand | Matrix score. | sequence | function |
| --- | --- | --- | --- | --- | --- | --- |
| HSE | Brassica oleracea | 1039 | + | 9 | AAAAAATTTC | cis-acting element involved in heat stress responsiveness |

> 2018/04/13 10:10:12  
+ GACGTCGAAG AGATTTCTCT TTCAATTTGG AGAAGCACTC TCTACTTTCC CCCTATCTCT CTCTTTCTCT   
  
  
+ CTAAGCCTCG AGTCGTTCTG TAACTTGACT TTGAGTCTCT TGACGATTTT ACCAAGAAAC CCTCCCCCTC   
  
  
+ TCTTCGGTTC CCTTCTACCC CCTACTATAT TCCTCCCACC TAGTGGCCCA CTGGCCCATT CTGTATCCCC   
  
  
+ AAATCAGTTT CACCCAAAAA CTAGGTTGCC GACACTACTC AAACTTTTAA AGGTACCCTA CTACACTTAT   
  
  
+ TTCTTTAGTC TCCGACCAGA GGAGTTCTTC ATTCATAACC CTGGGTGGGA ACCTTTTCTA TCTTTATCTA   
  
  
+ TATTTTCATC CCTGAGCAAA GAAAAAGAAA AAGGAGCCAT TTTTATCAAT GTGGAGAATA AGACAAACTT   
  
  
+ ATAAAGGACC TTTGATCTCT GATGATCGTA AATCAGAAAT TTCAATTCAA ACTAATACAC GGTGTTGGCA   
  
  
+ ACTGCCAGTC TTAACCTAGG ATACTCATGT GCATCATAAA AGTACTAACG ATGAAGATCA TTGTAAATTG   
  
  
+ CCTCAATAAA CTGGCAATTA CACTTTTTAT TTCCTTTTTC CTTCTCGTTA CCATTATTAA CGAATTAATC   
  
  
+ CGTCATTTTA AAGAATTTTG AATGGATTGC GCACCAATGA GTACTCAACG ACAACAAGTG GGTTTTTTTC   
  
  
+ CGTTGTGCAT GTCATGAACC TTGATCCGCT CTTCCTTTCT TTCGACTGGT TTTCACCCAG AGTGCACCCC   
  
  
+ CGGTAAAGAA AAATATTAGA CTTTGACAAC TGGTCGAAAA GAATAAAGAA GGAATGGGGA GCAAAGGCCA   
  
  
+ TAGACAGAAG ACTTGGCAGG CTAGATAAGA GTAGTTAGGT TGCCAGCTTA GCGTGGCTCT TTCAACTGGT   
  
  
+ TCTGAAAATA TGAAACCTCT TGGGTCTTTT TCGATGTTCG GAACCCAAAA AGTGGTGCAC CGTATTCCGA   
  
  
+ CCACGGTTAA GTTTTGCTTT GTGTTCGTAT CTCTCTCTTT GTCTGTCTGG AATTTTTCAA ATAATTTCTA   
  
  
+ TTTTGCTTCA GCAAAATCGG ACGATTAAAT ACATCAGGAC ATTAAACAGT TGCCGAGTCT ACTAATTACA   
  
  
+ CCCCAACTGG GTTGGCCCAC TCTTCTGAGG TGACTAGTTA AAAGATCTTA AGACTATCGA CCTACGAACT   
  
  
+ TGTTCAGAAT TCACCATTCG TTTAATTATT TATTAATAAT TCTATTGTTT CGAATGTCAT GGAAGTGGTT   
  
  
+ GTAAATTGCG TAATTAATTG TCGAAAAACT GGTTTTTTGG TGGCATAAAA GAGTACGTAA GGACTACCCA   
  
  
+ TTTGCTATTC CGAGTACATA AGAATTAAAC CCATATTAAG CTTTTAAATC GACGATTAAC TCCTATTTTG   
  
  
+ ACATTAAATT CACGCCTATA TATTACAATA TTTATGTATT TCCGAACACC GTGCTGATCT AATGGACGAA   
  
  
+ TCAACTACGG AAGGAAGACC GTTAATTGG  

- CTGCAGCTTC TCTAAAGAGA AAGTTAAACC TCTTCGTGAG AGATGAAAGG GGGATAGAGA GAGAAAGAGA   
  
  
- GATTCGGAGC TCAGCAAGAC ATTGAACTGA AACTCAGAGA ACTGCTAAAA TGGTTCTTTG GGAGGGGGAG   
  
  
- AGAAGCCAAG GGAAGATGGG GGATGATATA AGGAGGGTGG ATCACCGGGT GACCGGGTAA GACATAGGGG   
  
  
- TTTAGTCAAA GTGGGTTTTT GATCCAACGG CTGTGATGAG TTTGAAAATT TCCATGGGAT GATGTGAATA   
  
  
- AAGAAATCAG AGGCTGGTCT CCTCAAGAAG TAAGTATTGG GACCCACCCT TGGAAAAGAT AGAAATAGAT   
  
  
- ATAAAAGTAG GGACTCGTTT CTTTTTCTTT TTCCTCGGTA AAAATAGTTA CACCTCTTAT TCTGTTTGAA   
  
  
- TATTTCCTGG AAACTAGAGA CTACTAGCAT TTAGTCTTTA AAGTTAAGTT TGATTATGTG CCACAACCGT   
  
  
- TGACGGTCAG AATTGGATCC TATGAGTACA CGTAGTATTT TCATGATTGC TACTTCTAGT AACATTTAAC   
  
  
- GGAGTTATTT GACCGTTAAT GTGAAAAATA AAGGAAAAAG GAAGAGCAAT GGTAATAATT GCTTAATTAG   
  
  
- GCAGTAAAAT TTCTTAAAAC TTACCTAACG CGTGGTTACT CATGAGTTGC TGTTGTTCAC CCAAAAAAAG   
  
  
- GCAACACGTA CAGTACTTGG AACTAGGCGA GAAGGAAAGA AAGCTGACCA AAAGTGGGTC TCACGTGGGG   
  
  
- GCCATTTCTT TTTATAATCT GAAACTGTTG ACCAGCTTTT CTTATTTCTT CCTTACCCCT CGTTTCCGGT   
  
  
- ATCTGTCTTC TGAACCGTCC GATCTATTCT CATCAATCCA ACGGTCGAAT CGCACCGAGA AAGTTGACCA   
  
  
- AGACTTTTAT ACTTTGGAGA ACCCAGAAAA AGCTACAAGC CTTGGGTTTT TCACCACGTG GCATAAGGCT   
  
  
- GGTGCCAATT CAAAACGAAA CACAAGCATA GAGAGAGAAA CAGACAGACC TTAAAAAGTT TATTAAAGAT   
  
  
- AAAACGAAGT CGTTTTAGCC TGCTAATTTA TGTAGTCCTG TAATTTGTCA ACGGCTCAGA TGATTAATGT   
  
  
- GGGGTTGACC CAACCGGGTG AGAAGACTCC ACTGATCAAT TTTCTAGAAT TCTGATAGCT GGATGCTTGA   
  
  
- ACAAGTCTTA AGTGGTAAGC AAATTAATAA ATAATTATTA AGATAACAAA GCTTACAGTA CCTTCACCAA   
  
  
- CATTTAACGC ATTAATTAAC AGCTTTTTGA CCAAAAAACC ACCGTATTTT CTCATGCATT CCTGATGGGT   
  
  
- AAACGATAAG GCTCATGTAT TCTTAATTTG GGTATAATTC GAAAATTTAG CTGCTAATTG AGGATAAAAC   
  
  
- TGTAATTTAA GTGCGGATAT ATAATGTTAT AAATACATAA AGGCTTGTGG CACGACTAGA TTACCTGCTT   
  
  
- AGTTGATGCC TTCCTTCTGG CAATTAACC

+     I-box

| Site Name | Organism | Position | Strand | Matrix score. | sequence | function |
| --- | --- | --- | --- | --- | --- | --- |
| I-box | Zea mays | 51 | - | 7 | GATAGGG | part of a light responsive element |

> 2018/04/13 10:10:12  
+ GACGTCGAAG AGATTTCTCT TTCAATTTGG AGAAGCACTC TCTACTTTCC CCCTATCTCT CTCTTTCTCT   
  
  
+ CTAAGCCTCG AGTCGTTCTG TAACTTGACT TTGAGTCTCT TGACGATTTT ACCAAGAAAC CCTCCCCCTC   
  
  
+ TCTTCGGTTC CCTTCTACCC CCTACTATAT TCCTCCCACC TAGTGGCCCA CTGGCCCATT CTGTATCCCC   
  
  
+ AAATCAGTTT CACCCAAAAA CTAGGTTGCC GACACTACTC AAACTTTTAA AGGTACCCTA CTACACTTAT   
  
  
+ TTCTTTAGTC TCCGACCAGA GGAGTTCTTC ATTCATAACC CTGGGTGGGA ACCTTTTCTA TCTTTATCTA   
  
  
+ TATTTTCATC CCTGAGCAAA GAAAAAGAAA AAGGAGCCAT TTTTATCAAT GTGGAGAATA AGACAAACTT   
  
  
+ ATAAAGGACC TTTGATCTCT GATGATCGTA AATCAGAAAT TTCAATTCAA ACTAATACAC GGTGTTGGCA   
  
  
+ ACTGCCAGTC TTAACCTAGG ATACTCATGT GCATCATAAA AGTACTAACG ATGAAGATCA TTGTAAATTG   
  
  
+ CCTCAATAAA CTGGCAATTA CACTTTTTAT TTCCTTTTTC CTTCTCGTTA CCATTATTAA CGAATTAATC   
  
  
+ CGTCATTTTA AAGAATTTTG AATGGATTGC GCACCAATGA GTACTCAACG ACAACAAGTG GGTTTTTTTC   
  
  
+ CGTTGTGCAT GTCATGAACC TTGATCCGCT CTTCCTTTCT TTCGACTGGT TTTCACCCAG AGTGCACCCC   
  
  
+ CGGTAAAGAA AAATATTAGA CTTTGACAAC TGGTCGAAAA GAATAAAGAA GGAATGGGGA GCAAAGGCCA   
  
  
+ TAGACAGAAG ACTTGGCAGG CTAGATAAGA GTAGTTAGGT TGCCAGCTTA GCGTGGCTCT TTCAACTGGT   
  
  
+ TCTGAAAATA TGAAACCTCT TGGGTCTTTT TCGATGTTCG GAACCCAAAA AGTGGTGCAC CGTATTCCGA   
  
  
+ CCACGGTTAA GTTTTGCTTT GTGTTCGTAT CTCTCTCTTT GTCTGTCTGG AATTTTTCAA ATAATTTCTA   
  
  
+ TTTTGCTTCA GCAAAATCGG ACGATTAAAT ACATCAGGAC ATTAAACAGT TGCCGAGTCT ACTAATTACA   
  
  
+ CCCCAACTGG GTTGGCCCAC TCTTCTGAGG TGACTAGTTA AAAGATCTTA AGACTATCGA CCTACGAACT   
  
  
+ TGTTCAGAAT TCACCATTCG TTTAATTATT TATTAATAAT TCTATTGTTT CGAATGTCAT GGAAGTGGTT   
  
  
+ GTAAATTGCG TAATTAATTG TCGAAAAACT GGTTTTTTGG TGGCATAAAA GAGTACGTAA GGACTACCCA   
  
  
+ TTTGCTATTC CGAGTACATA AGAATTAAAC CCATATTAAG CTTTTAAATC GACGATTAAC TCCTATTTTG   
  
  
+ ACATTAAATT CACGCCTATA TATTACAATA TTTATGTATT TCCGAACACC GTGCTGATCT AATGGACGAA   
  
  
+ TCAACTACGG AAGGAAGACC GTTAATTGG  

- CTGCAGCTTC TCTAAAGAGA AAGTTAAACC TCTTCGTGAG AGATGAAAGG GGGATAGAGA GAGAAAGAGA   
  
  
- GATTCGGAGC TCAGCAAGAC ATTGAACTGA AACTCAGAGA ACTGCTAAAA TGGTTCTTTG GGAGGGGGAG   
  
  
- AGAAGCCAAG GGAAGATGGG GGATGATATA AGGAGGGTGG ATCACCGGGT GACCGGGTAA GACATAGGGG   
  
  
- TTTAGTCAAA GTGGGTTTTT GATCCAACGG CTGTGATGAG TTTGAAAATT TCCATGGGAT GATGTGAATA   
  
  
- AAGAAATCAG AGGCTGGTCT CCTCAAGAAG TAAGTATTGG GACCCACCCT TGGAAAAGAT AGAAATAGAT   
  
  
- ATAAAAGTAG GGACTCGTTT CTTTTTCTTT TTCCTCGGTA AAAATAGTTA CACCTCTTAT TCTGTTTGAA   
  
  
- TATTTCCTGG AAACTAGAGA CTACTAGCAT TTAGTCTTTA AAGTTAAGTT TGATTATGTG CCACAACCGT   
  
  
- TGACGGTCAG AATTGGATCC TATGAGTACA CGTAGTATTT TCATGATTGC TACTTCTAGT AACATTTAAC   
  
  
- GGAGTTATTT GACCGTTAAT GTGAAAAATA AAGGAAAAAG GAAGAGCAAT GGTAATAATT GCTTAATTAG   
  
  
- GCAGTAAAAT TTCTTAAAAC TTACCTAACG CGTGGTTACT CATGAGTTGC TGTTGTTCAC CCAAAAAAAG   
  
  
- GCAACACGTA CAGTACTTGG AACTAGGCGA GAAGGAAAGA AAGCTGACCA AAAGTGGGTC TCACGTGGGG   
  
  
- GCCATTTCTT TTTATAATCT GAAACTGTTG ACCAGCTTTT CTTATTTCTT CCTTACCCCT CGTTTCCGGT   
  
  
- ATCTGTCTTC TGAACCGTCC GATCTATTCT CATCAATCCA ACGGTCGAAT CGCACCGAGA AAGTTGACCA   
  
  
- AGACTTTTAT ACTTTGGAGA ACCCAGAAAA AGCTACAAGC CTTGGGTTTT TCACCACGTG GCATAAGGCT   
  
  
- GGTGCCAATT CAAAACGAAA CACAAGCATA GAGAGAGAAA CAGACAGACC TTAAAAAGTT TATTAAAGAT   
  
  
- AAAACGAAGT CGTTTTAGCC TGCTAATTTA TGTAGTCCTG TAATTTGTCA ACGGCTCAGA TGATTAATGT   
  
  
- GGGGTTGACC CAACCGGGTG AGAAGACTCC ACTGATCAAT TTTCTAGAAT TCTGATAGCT GGATGCTTGA   
  
  
- ACAAGTCTTA AGTGGTAAGC AAATTAATAA ATAATTATTA AGATAACAAA GCTTACAGTA CCTTCACCAA   
  
  
- CATTTAACGC ATTAATTAAC AGCTTTTTGA CCAAAAAACC ACCGTATTTT CTCATGCATT CCTGATGGGT   
  
  
- AAACGATAAG GCTCATGTAT TCTTAATTTG GGTATAATTC GAAAATTTAG CTGCTAATTG AGGATAAAAC   
  
  
- TGTAATTTAA GTGCGGATAT ATAATGTTAT AAATACATAA AGGCTTGTGG CACGACTAGA TTACCTGCTT   
  
  
- AGTTGATGCC TTCCTTCTGG CAATTAACC

+     MBS

| Site Name | Organism | Position | Strand | Matrix score. | sequence | function |
| --- | --- | --- | --- | --- | --- | --- |
| MBS | Arabidopsis thaliana | 903 | + | 6 | CAACTG | MYB binding site involved in drought-inducibility |
| MBS | Arabidopsis thaliana | 1097 | - | 6 | CAACTG | MYB binding site involved in drought-inducibility |
| MBS | Arabidopsis thaliana | 797 | + | 6 | CAACTG | MYB binding site involved in drought-inducibility |
| MBS | Arabidopsis thaliana | 1124 | + | 6 | CAACTG | MYB binding site involved in drought-inducibility |
| MBS | Arabidopsis thaliana | 489 | + | 6 | CAACTG | MYB binding site involved in drought-inducibility |

> 2018/04/13 10:10:12  
+ GACGTCGAAG AGATTTCTCT TTCAATTTGG AGAAGCACTC TCTACTTTCC CCCTATCTCT CTCTTTCTCT   
  
  
+ CTAAGCCTCG AGTCGTTCTG TAACTTGACT TTGAGTCTCT TGACGATTTT ACCAAGAAAC CCTCCCCCTC   
  
  
+ TCTTCGGTTC CCTTCTACCC CCTACTATAT TCCTCCCACC TAGTGGCCCA CTGGCCCATT CTGTATCCCC   
  
  
+ AAATCAGTTT CACCCAAAAA CTAGGTTGCC GACACTACTC AAACTTTTAA AGGTACCCTA CTACACTTAT   
  
  
+ TTCTTTAGTC TCCGACCAGA GGAGTTCTTC ATTCATAACC CTGGGTGGGA ACCTTTTCTA TCTTTATCTA   
  
  
+ TATTTTCATC CCTGAGCAAA GAAAAAGAAA AAGGAGCCAT TTTTATCAAT GTGGAGAATA AGACAAACTT   
  
  
+ ATAAAGGACC TTTGATCTCT GATGATCGTA AATCAGAAAT TTCAATTCAA ACTAATACAC GGTGTTGGCA   
  
  
+ ACTGCCAGTC TTAACCTAGG ATACTCATGT GCATCATAAA AGTACTAACG ATGAAGATCA TTGTAAATTG   
  
  
+ CCTCAATAAA CTGGCAATTA CACTTTTTAT TTCCTTTTTC CTTCTCGTTA CCATTATTAA CGAATTAATC   
  
  
+ CGTCATTTTA AAGAATTTTG AATGGATTGC GCACCAATGA GTACTCAACG ACAACAAGTG GGTTTTTTTC   
  
  
+ CGTTGTGCAT GTCATGAACC TTGATCCGCT CTTCCTTTCT TTCGACTGGT TTTCACCCAG AGTGCACCCC   
  
  
+ CGGTAAAGAA AAATATTAGA CTTTGACAAC TGGTCGAAAA GAATAAAGAA GGAATGGGGA GCAAAGGCCA   
  
  
+ TAGACAGAAG ACTTGGCAGG CTAGATAAGA GTAGTTAGGT TGCCAGCTTA GCGTGGCTCT TTCAACTGGT   
  
  
+ TCTGAAAATA TGAAACCTCT TGGGTCTTTT TCGATGTTCG GAACCCAAAA AGTGGTGCAC CGTATTCCGA   
  
  
+ CCACGGTTAA GTTTTGCTTT GTGTTCGTAT CTCTCTCTTT GTCTGTCTGG AATTTTTCAA ATAATTTCTA   
  
  
+ TTTTGCTTCA GCAAAATCGG ACGATTAAAT ACATCAGGAC ATTAAACAGT TGCCGAGTCT ACTAATTACA   
  
  
+ CCCCAACTGG GTTGGCCCAC TCTTCTGAGG TGACTAGTTA AAAGATCTTA AGACTATCGA CCTACGAACT   
  
  
+ TGTTCAGAAT TCACCATTCG TTTAATTATT TATTAATAAT TCTATTGTTT CGAATGTCAT GGAAGTGGTT   
  
  
+ GTAAATTGCG TAATTAATTG TCGAAAAACT GGTTTTTTGG TGGCATAAAA GAGTACGTAA GGACTACCCA   
  
  
+ TTTGCTATTC CGAGTACATA AGAATTAAAC CCATATTAAG CTTTTAAATC GACGATTAAC TCCTATTTTG   
  
  
+ ACATTAAATT CACGCCTATA TATTACAATA TTTATGTATT TCCGAACACC GTGCTGATCT AATGGACGAA   
  
  
+ TCAACTACGG AAGGAAGACC GTTAATTGG  

- CTGCAGCTTC TCTAAAGAGA AAGTTAAACC TCTTCGTGAG AGATGAAAGG GGGATAGAGA GAGAAAGAGA   
  
  
- GATTCGGAGC TCAGCAAGAC ATTGAACTGA AACTCAGAGA ACTGCTAAAA TGGTTCTTTG GGAGGGGGAG   
  
  
- AGAAGCCAAG GGAAGATGGG GGATGATATA AGGAGGGTGG ATCACCGGGT GACCGGGTAA GACATAGGGG   
  
  
- TTTAGTCAAA GTGGGTTTTT GATCCAACGG CTGTGATGAG TTTGAAAATT TCCATGGGAT GATGTGAATA   
  
  
- AAGAAATCAG AGGCTGGTCT CCTCAAGAAG TAAGTATTGG GACCCACCCT TGGAAAAGAT AGAAATAGAT   
  
  
- ATAAAAGTAG GGACTCGTTT CTTTTTCTTT TTCCTCGGTA AAAATAGTTA CACCTCTTAT TCTGTTTGAA   
  
  
- TATTTCCTGG AAACTAGAGA CTACTAGCAT TTAGTCTTTA AAGTTAAGTT TGATTATGTG CCACAACCGT   
  
  
- TGACGGTCAG AATTGGATCC TATGAGTACA CGTAGTATTT TCATGATTGC TACTTCTAGT AACATTTAAC   
  
  
- GGAGTTATTT GACCGTTAAT GTGAAAAATA AAGGAAAAAG GAAGAGCAAT GGTAATAATT GCTTAATTAG   
  
  
- GCAGTAAAAT TTCTTAAAAC TTACCTAACG CGTGGTTACT CATGAGTTGC TGTTGTTCAC CCAAAAAAAG   
  
  
- GCAACACGTA CAGTACTTGG AACTAGGCGA GAAGGAAAGA AAGCTGACCA AAAGTGGGTC TCACGTGGGG   
  
  
- GCCATTTCTT TTTATAATCT GAAACTGTTG ACCAGCTTTT CTTATTTCTT CCTTACCCCT CGTTTCCGGT   
  
  
- ATCTGTCTTC TGAACCGTCC GATCTATTCT CATCAATCCA ACGGTCGAAT CGCACCGAGA AAGTTGACCA   
  
  
- AGACTTTTAT ACTTTGGAGA ACCCAGAAAA AGCTACAAGC CTTGGGTTTT TCACCACGTG GCATAAGGCT   
  
  
- GGTGCCAATT CAAAACGAAA CACAAGCATA GAGAGAGAAA CAGACAGACC TTAAAAAGTT TATTAAAGAT   
  
  
- AAAACGAAGT CGTTTTAGCC TGCTAATTTA TGTAGTCCTG TAATTTGTCA ACGGCTCAGA TGATTAATGT   
  
  
- GGGGTTGACC CAACCGGGTG AGAAGACTCC ACTGATCAAT TTTCTAGAAT TCTGATAGCT GGATGCTTGA   
  
  
- ACAAGTCTTA AGTGGTAAGC AAATTAATAA ATAATTATTA AGATAACAAA GCTTACAGTA CCTTCACCAA   
  
  
- CATTTAACGC ATTAATTAAC AGCTTTTTGA CCAAAAAACC ACCGTATTTT CTCATGCATT CCTGATGGGT   
  
  
- AAACGATAAG GCTCATGTAT TCTTAATTTG GGTATAATTC GAAAATTTAG CTGCTAATTG AGGATAAAAC   
  
  
- TGTAATTTAA GTGCGGATAT ATAATGTTAT AAATACATAA AGGCTTGTGG CACGACTAGA TTACCTGCTT   
  
  
- AGTTGATGCC TTCCTTCTGG CAATTAACC

+     MRE

| Site Name | Organism | Position | Strand | Matrix score. | sequence | function |
| --- | --- | --- | --- | --- | --- | --- |
| MRE | Petroselinum crispum | 875 | - | 7 | AACCTAA | MYB binding site involved in light responsiveness |

> 2018/04/13 10:10:12  
+ GACGTCGAAG AGATTTCTCT TTCAATTTGG AGAAGCACTC TCTACTTTCC CCCTATCTCT CTCTTTCTCT   
  
  
+ CTAAGCCTCG AGTCGTTCTG TAACTTGACT TTGAGTCTCT TGACGATTTT ACCAAGAAAC CCTCCCCCTC   
  
  
+ TCTTCGGTTC CCTTCTACCC CCTACTATAT TCCTCCCACC TAGTGGCCCA CTGGCCCATT CTGTATCCCC   
  
  
+ AAATCAGTTT CACCCAAAAA CTAGGTTGCC GACACTACTC AAACTTTTAA AGGTACCCTA CTACACTTAT   
  
  
+ TTCTTTAGTC TCCGACCAGA GGAGTTCTTC ATTCATAACC CTGGGTGGGA ACCTTTTCTA TCTTTATCTA   
  
  
+ TATTTTCATC CCTGAGCAAA GAAAAAGAAA AAGGAGCCAT TTTTATCAAT GTGGAGAATA AGACAAACTT   
  
  
+ ATAAAGGACC TTTGATCTCT GATGATCGTA AATCAGAAAT TTCAATTCAA ACTAATACAC GGTGTTGGCA   
  
  
+ ACTGCCAGTC TTAACCTAGG ATACTCATGT GCATCATAAA AGTACTAACG ATGAAGATCA TTGTAAATTG   
  
  
+ CCTCAATAAA CTGGCAATTA CACTTTTTAT TTCCTTTTTC CTTCTCGTTA CCATTATTAA CGAATTAATC   
  
  
+ CGTCATTTTA AAGAATTTTG AATGGATTGC GCACCAATGA GTACTCAACG ACAACAAGTG GGTTTTTTTC   
  
  
+ CGTTGTGCAT GTCATGAACC TTGATCCGCT CTTCCTTTCT TTCGACTGGT TTTCACCCAG AGTGCACCCC   
  
  
+ CGGTAAAGAA AAATATTAGA CTTTGACAAC TGGTCGAAAA GAATAAAGAA GGAATGGGGA GCAAAGGCCA   
  
  
+ TAGACAGAAG ACTTGGCAGG CTAGATAAGA GTAGTTAGGT TGCCAGCTTA GCGTGGCTCT TTCAACTGGT   
  
  
+ TCTGAAAATA TGAAACCTCT TGGGTCTTTT TCGATGTTCG GAACCCAAAA AGTGGTGCAC CGTATTCCGA   
  
  
+ CCACGGTTAA GTTTTGCTTT GTGTTCGTAT CTCTCTCTTT GTCTGTCTGG AATTTTTCAA ATAATTTCTA   
  
  
+ TTTTGCTTCA GCAAAATCGG ACGATTAAAT ACATCAGGAC ATTAAACAGT TGCCGAGTCT ACTAATTACA   
  
  
+ CCCCAACTGG GTTGGCCCAC TCTTCTGAGG TGACTAGTTA AAAGATCTTA AGACTATCGA CCTACGAACT   
  
  
+ TGTTCAGAAT TCACCATTCG TTTAATTATT TATTAATAAT TCTATTGTTT CGAATGTCAT GGAAGTGGTT   
  
  
+ GTAAATTGCG TAATTAATTG TCGAAAAACT GGTTTTTTGG TGGCATAAAA GAGTACGTAA GGACTACCCA   
  
  
+ TTTGCTATTC CGAGTACATA AGAATTAAAC CCATATTAAG CTTTTAAATC GACGATTAAC TCCTATTTTG   
  
  
+ ACATTAAATT CACGCCTATA TATTACAATA TTTATGTATT TCCGAACACC GTGCTGATCT AATGGACGAA   
  
  
+ TCAACTACGG AAGGAAGACC GTTAATTGG  

- CTGCAGCTTC TCTAAAGAGA AAGTTAAACC TCTTCGTGAG AGATGAAAGG GGGATAGAGA GAGAAAGAGA   
  
  
- GATTCGGAGC TCAGCAAGAC ATTGAACTGA AACTCAGAGA ACTGCTAAAA TGGTTCTTTG GGAGGGGGAG   
  
  
- AGAAGCCAAG GGAAGATGGG GGATGATATA AGGAGGGTGG ATCACCGGGT GACCGGGTAA GACATAGGGG   
  
  
- TTTAGTCAAA GTGGGTTTTT GATCCAACGG CTGTGATGAG TTTGAAAATT TCCATGGGAT GATGTGAATA   
  
  
- AAGAAATCAG AGGCTGGTCT CCTCAAGAAG TAAGTATTGG GACCCACCCT TGGAAAAGAT AGAAATAGAT   
  
  
- ATAAAAGTAG GGACTCGTTT CTTTTTCTTT TTCCTCGGTA AAAATAGTTA CACCTCTTAT TCTGTTTGAA   
  
  
- TATTTCCTGG AAACTAGAGA CTACTAGCAT TTAGTCTTTA AAGTTAAGTT TGATTATGTG CCACAACCGT   
  
  
- TGACGGTCAG AATTGGATCC TATGAGTACA CGTAGTATTT TCATGATTGC TACTTCTAGT AACATTTAAC   
  
  
- GGAGTTATTT GACCGTTAAT GTGAAAAATA AAGGAAAAAG GAAGAGCAAT GGTAATAATT GCTTAATTAG   
  
  
- GCAGTAAAAT TTCTTAAAAC TTACCTAACG CGTGGTTACT CATGAGTTGC TGTTGTTCAC CCAAAAAAAG   
  
  
- GCAACACGTA CAGTACTTGG AACTAGGCGA GAAGGAAAGA AAGCTGACCA AAAGTGGGTC TCACGTGGGG   
  
  
- GCCATTTCTT TTTATAATCT GAAACTGTTG ACCAGCTTTT CTTATTTCTT CCTTACCCCT CGTTTCCGGT   
  
  
- ATCTGTCTTC TGAACCGTCC GATCTATTCT CATCAATCCA ACGGTCGAAT CGCACCGAGA AAGTTGACCA   
  
  
- AGACTTTTAT ACTTTGGAGA ACCCAGAAAA AGCTACAAGC CTTGGGTTTT TCACCACGTG GCATAAGGCT   
  
  
- GGTGCCAATT CAAAACGAAA CACAAGCATA GAGAGAGAAA CAGACAGACC TTAAAAAGTT TATTAAAGAT   
  
  
- AAAACGAAGT CGTTTTAGCC TGCTAATTTA TGTAGTCCTG TAATTTGTCA ACGGCTCAGA TGATTAATGT   
  
  
- GGGGTTGACC CAACCGGGTG AGAAGACTCC ACTGATCAAT TTTCTAGAAT TCTGATAGCT GGATGCTTGA   
  
  
- ACAAGTCTTA AGTGGTAAGC AAATTAATAA ATAATTATTA AGATAACAAA GCTTACAGTA CCTTCACCAA   
  
  
- CATTTAACGC ATTAATTAAC AGCTTTTTGA CCAAAAAACC ACCGTATTTT CTCATGCATT CCTGATGGGT   
  
  
- AAACGATAAG GCTCATGTAT TCTTAATTTG GGTATAATTC GAAAATTTAG CTGCTAATTG AGGATAAAAC   
  
  
- TGTAATTTAA GTGCGGATAT ATAATGTTAT AAATACATAA AGGCTTGTGG CACGACTAGA TTACCTGCTT   
  
  
- AGTTGATGCC TTCCTTCTGG CAATTAACC

+     Skn-1\_motif

| Site Name | Organism | Position | Strand | Matrix score. | sequence | function |
| --- | --- | --- | --- | --- | --- | --- |
| Skn-1\_motif | Oryza sativa | 1246 | + | 5 | GTCAT | cis-acting regulatory element required for endosperm expression |
| Skn-1\_motif | Oryza sativa | 632 | + | 5 | GTCAT | cis-acting regulatory element required for endosperm expression |
| Skn-1\_motif | Oryza sativa | 711 | + | 5 | GTCAT | cis-acting regulatory element required for endosperm expression |

> 2018/04/13 10:10:12  
+ GACGTCGAAG AGATTTCTCT TTCAATTTGG AGAAGCACTC TCTACTTTCC CCCTATCTCT CTCTTTCTCT   
  
  
+ CTAAGCCTCG AGTCGTTCTG TAACTTGACT TTGAGTCTCT TGACGATTTT ACCAAGAAAC CCTCCCCCTC   
  
  
+ TCTTCGGTTC CCTTCTACCC CCTACTATAT TCCTCCCACC TAGTGGCCCA CTGGCCCATT CTGTATCCCC   
  
  
+ AAATCAGTTT CACCCAAAAA CTAGGTTGCC GACACTACTC AAACTTTTAA AGGTACCCTA CTACACTTAT   
  
  
+ TTCTTTAGTC TCCGACCAGA GGAGTTCTTC ATTCATAACC CTGGGTGGGA ACCTTTTCTA TCTTTATCTA   
  
  
+ TATTTTCATC CCTGAGCAAA GAAAAAGAAA AAGGAGCCAT TTTTATCAAT GTGGAGAATA AGACAAACTT   
  
  
+ ATAAAGGACC TTTGATCTCT GATGATCGTA AATCAGAAAT TTCAATTCAA ACTAATACAC GGTGTTGGCA   
  
  
+ ACTGCCAGTC TTAACCTAGG ATACTCATGT GCATCATAAA AGTACTAACG ATGAAGATCA TTGTAAATTG   
  
  
+ CCTCAATAAA CTGGCAATTA CACTTTTTAT TTCCTTTTTC CTTCTCGTTA CCATTATTAA CGAATTAATC   
  
  
+ CGTCATTTTA AAGAATTTTG AATGGATTGC GCACCAATGA GTACTCAACG ACAACAAGTG GGTTTTTTTC   
  
  
+ CGTTGTGCAT GTCATGAACC TTGATCCGCT CTTCCTTTCT TTCGACTGGT TTTCACCCAG AGTGCACCCC   
  
  
+ CGGTAAAGAA AAATATTAGA CTTTGACAAC TGGTCGAAAA GAATAAAGAA GGAATGGGGA GCAAAGGCCA   
  
  
+ TAGACAGAAG ACTTGGCAGG CTAGATAAGA GTAGTTAGGT TGCCAGCTTA GCGTGGCTCT TTCAACTGGT   
  
  
+ TCTGAAAATA TGAAACCTCT TGGGTCTTTT TCGATGTTCG GAACCCAAAA AGTGGTGCAC CGTATTCCGA   
  
  
+ CCACGGTTAA GTTTTGCTTT GTGTTCGTAT CTCTCTCTTT GTCTGTCTGG AATTTTTCAA ATAATTTCTA   
  
  
+ TTTTGCTTCA GCAAAATCGG ACGATTAAAT ACATCAGGAC ATTAAACAGT TGCCGAGTCT ACTAATTACA   
  
  
+ CCCCAACTGG GTTGGCCCAC TCTTCTGAGG TGACTAGTTA AAAGATCTTA AGACTATCGA CCTACGAACT   
  
  
+ TGTTCAGAAT TCACCATTCG TTTAATTATT TATTAATAAT TCTATTGTTT CGAATGTCAT GGAAGTGGTT   
  
  
+ GTAAATTGCG TAATTAATTG TCGAAAAACT GGTTTTTTGG TGGCATAAAA GAGTACGTAA GGACTACCCA   
  
  
+ TTTGCTATTC CGAGTACATA AGAATTAAAC CCATATTAAG CTTTTAAATC GACGATTAAC TCCTATTTTG   
  
  
+ ACATTAAATT CACGCCTATA TATTACAATA TTTATGTATT TCCGAACACC GTGCTGATCT AATGGACGAA   
  
  
+ TCAACTACGG AAGGAAGACC GTTAATTGG  

- CTGCAGCTTC TCTAAAGAGA AAGTTAAACC TCTTCGTGAG AGATGAAAGG GGGATAGAGA GAGAAAGAGA   
  
  
- GATTCGGAGC TCAGCAAGAC ATTGAACTGA AACTCAGAGA ACTGCTAAAA TGGTTCTTTG GGAGGGGGAG   
  
  
- AGAAGCCAAG GGAAGATGGG GGATGATATA AGGAGGGTGG ATCACCGGGT GACCGGGTAA GACATAGGGG   
  
  
- TTTAGTCAAA GTGGGTTTTT GATCCAACGG CTGTGATGAG TTTGAAAATT TCCATGGGAT GATGTGAATA   
  
  
- AAGAAATCAG AGGCTGGTCT CCTCAAGAAG TAAGTATTGG GACCCACCCT TGGAAAAGAT AGAAATAGAT   
  
  
- ATAAAAGTAG GGACTCGTTT CTTTTTCTTT TTCCTCGGTA AAAATAGTTA CACCTCTTAT TCTGTTTGAA   
  
  
- TATTTCCTGG AAACTAGAGA CTACTAGCAT TTAGTCTTTA AAGTTAAGTT TGATTATGTG CCACAACCGT   
  
  
- TGACGGTCAG AATTGGATCC TATGAGTACA CGTAGTATTT TCATGATTGC TACTTCTAGT AACATTTAAC   
  
  
- GGAGTTATTT GACCGTTAAT GTGAAAAATA AAGGAAAAAG GAAGAGCAAT GGTAATAATT GCTTAATTAG   
  
  
- GCAGTAAAAT TTCTTAAAAC TTACCTAACG CGTGGTTACT CATGAGTTGC TGTTGTTCAC CCAAAAAAAG   
  
  
- GCAACACGTA CAGTACTTGG AACTAGGCGA GAAGGAAAGA AAGCTGACCA AAAGTGGGTC TCACGTGGGG   
  
  
- GCCATTTCTT TTTATAATCT GAAACTGTTG ACCAGCTTTT CTTATTTCTT CCTTACCCCT CGTTTCCGGT   
  
  
- ATCTGTCTTC TGAACCGTCC GATCTATTCT CATCAATCCA ACGGTCGAAT CGCACCGAGA AAGTTGACCA   
  
  
- AGACTTTTAT ACTTTGGAGA ACCCAGAAAA AGCTACAAGC CTTGGGTTTT TCACCACGTG GCATAAGGCT   
  
  
- GGTGCCAATT CAAAACGAAA CACAAGCATA GAGAGAGAAA CAGACAGACC TTAAAAAGTT TATTAAAGAT   
  
  
- AAAACGAAGT CGTTTTAGCC TGCTAATTTA TGTAGTCCTG TAATTTGTCA ACGGCTCAGA TGATTAATGT   
  
  
- GGGGTTGACC CAACCGGGTG AGAAGACTCC ACTGATCAAT TTTCTAGAAT TCTGATAGCT GGATGCTTGA   
  
  
- ACAAGTCTTA AGTGGTAAGC AAATTAATAA ATAATTATTA AGATAACAAA GCTTACAGTA CCTTCACCAA   
  
  
- CATTTAACGC ATTAATTAAC AGCTTTTTGA CCAAAAAACC ACCGTATTTT CTCATGCATT CCTGATGGGT   
  
  
- AAACGATAAG GCTCATGTAT TCTTAATTTG GGTATAATTC GAAAATTTAG CTGCTAATTG AGGATAAAAC   
  
  
- TGTAATTTAA GTGCGGATAT ATAATGTTAT AAATACATAA AGGCTTGTGG CACGACTAGA TTACCTGCTT   
  
  
- AGTTGATGCC TTCCTTCTGG CAATTAACC

+     Sp1

| Site Name | Organism | Position | Strand | Matrix score. | sequence | function |
| --- | --- | --- | --- | --- | --- | --- |
| Sp1 | Zea mays | 131 | + | 5 | CC(G/A)CCC | light responsive element |
| Sp1 | Zea mays | 323 | - | 5.5 | CC(G/A)CCC | light responsive element |
| Sp1 | Zea mays | 172 | + | 5 | CC(G/A)CCC | light responsive element |

> 2018/04/13 10:10:12  
+ GACGTCGAAG AGATTTCTCT TTCAATTTGG AGAAGCACTC TCTACTTTCC CCCTATCTCT CTCTTTCTCT   
  
  
+ CTAAGCCTCG AGTCGTTCTG TAACTTGACT TTGAGTCTCT TGACGATTTT ACCAAGAAAC CCTCCCCCTC   
  
  
+ TCTTCGGTTC CCTTCTACCC CCTACTATAT TCCTCCCACC TAGTGGCCCA CTGGCCCATT CTGTATCCCC   
  
  
+ AAATCAGTTT CACCCAAAAA CTAGGTTGCC GACACTACTC AAACTTTTAA AGGTACCCTA CTACACTTAT   
  
  
+ TTCTTTAGTC TCCGACCAGA GGAGTTCTTC ATTCATAACC CTGGGTGGGA ACCTTTTCTA TCTTTATCTA   
  
  
+ TATTTTCATC CCTGAGCAAA GAAAAAGAAA AAGGAGCCAT TTTTATCAAT GTGGAGAATA AGACAAACTT   
  
  
+ ATAAAGGACC TTTGATCTCT GATGATCGTA AATCAGAAAT TTCAATTCAA ACTAATACAC GGTGTTGGCA   
  
  
+ ACTGCCAGTC TTAACCTAGG ATACTCATGT GCATCATAAA AGTACTAACG ATGAAGATCA TTGTAAATTG   
  
  
+ CCTCAATAAA CTGGCAATTA CACTTTTTAT TTCCTTTTTC CTTCTCGTTA CCATTATTAA CGAATTAATC   
  
  
+ CGTCATTTTA AAGAATTTTG AATGGATTGC GCACCAATGA GTACTCAACG ACAACAAGTG GGTTTTTTTC   
  
  
+ CGTTGTGCAT GTCATGAACC TTGATCCGCT CTTCCTTTCT TTCGACTGGT TTTCACCCAG AGTGCACCCC   
  
  
+ CGGTAAAGAA AAATATTAGA CTTTGACAAC TGGTCGAAAA GAATAAAGAA GGAATGGGGA GCAAAGGCCA   
  
  
+ TAGACAGAAG ACTTGGCAGG CTAGATAAGA GTAGTTAGGT TGCCAGCTTA GCGTGGCTCT TTCAACTGGT   
  
  
+ TCTGAAAATA TGAAACCTCT TGGGTCTTTT TCGATGTTCG GAACCCAAAA AGTGGTGCAC CGTATTCCGA   
  
  
+ CCACGGTTAA GTTTTGCTTT GTGTTCGTAT CTCTCTCTTT GTCTGTCTGG AATTTTTCAA ATAATTTCTA   
  
  
+ TTTTGCTTCA GCAAAATCGG ACGATTAAAT ACATCAGGAC ATTAAACAGT TGCCGAGTCT ACTAATTACA   
  
  
+ CCCCAACTGG GTTGGCCCAC TCTTCTGAGG TGACTAGTTA AAAGATCTTA AGACTATCGA CCTACGAACT   
  
  
+ TGTTCAGAAT TCACCATTCG TTTAATTATT TATTAATAAT TCTATTGTTT CGAATGTCAT GGAAGTGGTT   
  
  
+ GTAAATTGCG TAATTAATTG TCGAAAAACT GGTTTTTTGG TGGCATAAAA GAGTACGTAA GGACTACCCA   
  
  
+ TTTGCTATTC CGAGTACATA AGAATTAAAC CCATATTAAG CTTTTAAATC GACGATTAAC TCCTATTTTG   
  
  
+ ACATTAAATT CACGCCTATA TATTACAATA TTTATGTATT TCCGAACACC GTGCTGATCT AATGGACGAA   
  
  
+ TCAACTACGG AAGGAAGACC GTTAATTGG  

- CTGCAGCTTC TCTAAAGAGA AAGTTAAACC TCTTCGTGAG AGATGAAAGG GGGATAGAGA GAGAAAGAGA   
  
  
- GATTCGGAGC TCAGCAAGAC ATTGAACTGA AACTCAGAGA ACTGCTAAAA TGGTTCTTTG GGAGGGGGAG   
  
  
- AGAAGCCAAG GGAAGATGGG GGATGATATA AGGAGGGTGG ATCACCGGGT GACCGGGTAA GACATAGGGG   
  
  
- TTTAGTCAAA GTGGGTTTTT GATCCAACGG CTGTGATGAG TTTGAAAATT TCCATGGGAT GATGTGAATA   
  
  
- AAGAAATCAG AGGCTGGTCT CCTCAAGAAG TAAGTATTGG GACCCACCCT TGGAAAAGAT AGAAATAGAT   
  
  
- ATAAAAGTAG GGACTCGTTT CTTTTTCTTT TTCCTCGGTA AAAATAGTTA CACCTCTTAT TCTGTTTGAA   
  
  
- TATTTCCTGG AAACTAGAGA CTACTAGCAT TTAGTCTTTA AAGTTAAGTT TGATTATGTG CCACAACCGT   
  
  
- TGACGGTCAG AATTGGATCC TATGAGTACA CGTAGTATTT TCATGATTGC TACTTCTAGT AACATTTAAC   
  
  
- GGAGTTATTT GACCGTTAAT GTGAAAAATA AAGGAAAAAG GAAGAGCAAT GGTAATAATT GCTTAATTAG   
  
  
- GCAGTAAAAT TTCTTAAAAC TTACCTAACG CGTGGTTACT CATGAGTTGC TGTTGTTCAC CCAAAAAAAG   
  
  
- GCAACACGTA CAGTACTTGG AACTAGGCGA GAAGGAAAGA AAGCTGACCA AAAGTGGGTC TCACGTGGGG   
  
  
- GCCATTTCTT TTTATAATCT GAAACTGTTG ACCAGCTTTT CTTATTTCTT CCTTACCCCT CGTTTCCGGT   
  
  
- ATCTGTCTTC TGAACCGTCC GATCTATTCT CATCAATCCA ACGGTCGAAT CGCACCGAGA AAGTTGACCA   
  
  
- AGACTTTTAT ACTTTGGAGA ACCCAGAAAA AGCTACAAGC CTTGGGTTTT TCACCACGTG GCATAAGGCT   
  
  
- GGTGCCAATT CAAAACGAAA CACAAGCATA GAGAGAGAAA CAGACAGACC TTAAAAAGTT TATTAAAGAT   
  
  
- AAAACGAAGT CGTTTTAGCC TGCTAATTTA TGTAGTCCTG TAATTTGTCA ACGGCTCAGA TGATTAATGT   
  
  
- GGGGTTGACC CAACCGGGTG AGAAGACTCC ACTGATCAAT TTTCTAGAAT TCTGATAGCT GGATGCTTGA   
  
  
- ACAAGTCTTA AGTGGTAAGC AAATTAATAA ATAATTATTA AGATAACAAA GCTTACAGTA CCTTCACCAA   
  
  
- CATTTAACGC ATTAATTAAC AGCTTTTTGA CCAAAAAACC ACCGTATTTT CTCATGCATT CCTGATGGGT   
  
  
- AAACGATAAG GCTCATGTAT TCTTAATTTG GGTATAATTC GAAAATTTAG CTGCTAATTG AGGATAAAAC   
  
  
- TGTAATTTAA GTGCGGATAT ATAATGTTAT AAATACATAA AGGCTTGTGG CACGACTAGA TTACCTGCTT   
  
  
- AGTTGATGCC TTCCTTCTGG CAATTAACC

+     TATA-box

| Site Name | Organism | Position | Strand | Matrix score. | sequence | function |
| --- | --- | --- | --- | --- | --- | --- |
| TATA-box | Oryza sativa | 1431 | - | 8 | TACATAAA | core promoter element around -30 of transcription start |
| TATA-box | Arabidopsis thaliana | 1415 | + | 9 | tcTATATAtt | core promoter element around -30 of transcription start |
| TATA-box | Lycopersicon esculentum | 1372 | + | 5 | TTTTA | core promoter element around -30 of transcription start |
| TATA-box | Lycopersicon esculentum | 1306 | - | 5 | TTTTA | core promoter element around -30 of transcription start |
| TATA-box | Glycine max | 1221 | - | 5 | TAATA | core promoter element around -30 of transcription start |
| TATA-box | Lycopersicon esculentum | 527 | - | 5 | TTTTA | core promoter element around -30 of transcription start |
| TATA-box | Glycine max | 473 | + | 5 | TAATA | core promoter element around -30 of transcription start |
| TATA-box | Lycopersicon esculentum | 1159 | - | 5 | TTTTA | core promoter element around -30 of transcription start |
| TATA-box | Brassica napus | 1418 | - | 6 | ATATAT | core promoter element around -30 of transcription start |
| TATA-box | Glycine max | 784 | - | 5 | TAATA | core promoter element around -30 of transcription start |
| TATA-box | Lycopersicon esculentum | 636 | + | 5 | TTTTA | core promoter element around -30 of transcription start |
| TATA-box | Lycopersicon esculentum | 391 | + | 5 | TTTTA | core promoter element around -30 of transcription start |
| TATA-box | Arabidopsis thaliana | 349 | + | 4 | TATA | core promoter element around -30 of transcription start |
| TATA-box | Glycine max | 1421 | - | 5 | TAATA | core promoter element around -30 of transcription start |
| TATA-box | Lycopersicon esculentum | 585 | + | 5 | TTTTA | core promoter element around -30 of transcription start |
| TATA-box | Glycine max | 615 | - | 5 | TAATA | core promoter element around -30 of transcription start |
| TATA-box | Glycine max | 1364 | - | 5 | TAATA | core promoter element around -30 of transcription start |
| TATA-box | Glycine max | 1224 | + | 5 | TAATA | core promoter element around -30 of transcription start |
| TATA-box | Lycopersicon esculentum | 117 | + | 5 | TTTTA | core promoter element around -30 of transcription start |
| TATA-box | Arabidopsis thaliana | 420 | + | 6 | TATAAA | core promoter element around -30 of transcription start |
| TATA-box | Arabidopsis thaliana | 419 | - | 5 | TATAA | core promoter element around -30 of transcription start |
| TATA-box | Lycopersicon esculentum | 255 | + | 5 | TTTTA | core promoter element around -30 of transcription start |
| TATA-box | Arabidopsis thaliana | 1417 | - | 4 | TATA | core promoter element around -30 of transcription start |
| TATA-box | Arabidopsis thaliana | 347 | + | 9 | tcTATATAtt | core promoter element around -30 of transcription start |
| TATA-box | Arabidopsis thaliana | 1419 | - | 4 | TATA | core promoter element around -30 of transcription start |
| TATA-box | Arabidopsis thaliana | 166 | + | 4 | TATA | core promoter element around -30 of transcription start |

> 2018/04/13 10:10:12  
+ GACGTCGAAG AGATTTCTCT TTCAATTTGG AGAAGCACTC TCTACTTTCC CCCTATCTCT CTCTTTCTCT   
  
  
+ CTAAGCCTCG AGTCGTTCTG TAACTTGACT TTGAGTCTCT TGACGATTTT ACCAAGAAAC CCTCCCCCTC   
  
  
+ TCTTCGGTTC CCTTCTACCC CCTACTATAT TCCTCCCACC TAGTGGCCCA CTGGCCCATT CTGTATCCCC   
  
  
+ AAATCAGTTT CACCCAAAAA CTAGGTTGCC GACACTACTC AAACTTTTAA AGGTACCCTA CTACACTTAT   
  
  
+ TTCTTTAGTC TCCGACCAGA GGAGTTCTTC ATTCATAACC CTGGGTGGGA ACCTTTTCTA TCTTTATCTA   
  
  
+ TATTTTCATC CCTGAGCAAA GAAAAAGAAA AAGGAGCCAT TTTTATCAAT GTGGAGAATA AGACAAACTT   
  
  
+ ATAAAGGACC TTTGATCTCT GATGATCGTA AATCAGAAAT TTCAATTCAA ACTAATACAC GGTGTTGGCA   
  
  
+ ACTGCCAGTC TTAACCTAGG ATACTCATGT GCATCATAAA AGTACTAACG ATGAAGATCA TTGTAAATTG   
  
  
+ CCTCAATAAA CTGGCAATTA CACTTTTTAT TTCCTTTTTC CTTCTCGTTA CCATTATTAA CGAATTAATC   
  
  
+ CGTCATTTTA AAGAATTTTG AATGGATTGC GCACCAATGA GTACTCAACG ACAACAAGTG GGTTTTTTTC   
  
  
+ CGTTGTGCAT GTCATGAACC TTGATCCGCT CTTCCTTTCT TTCGACTGGT TTTCACCCAG AGTGCACCCC   
  
  
+ CGGTAAAGAA AAATATTAGA CTTTGACAAC TGGTCGAAAA GAATAAAGAA GGAATGGGGA GCAAAGGCCA   
  
  
+ TAGACAGAAG ACTTGGCAGG CTAGATAAGA GTAGTTAGGT TGCCAGCTTA GCGTGGCTCT TTCAACTGGT   
  
  
+ TCTGAAAATA TGAAACCTCT TGGGTCTTTT TCGATGTTCG GAACCCAAAA AGTGGTGCAC CGTATTCCGA   
  
  
+ CCACGGTTAA GTTTTGCTTT GTGTTCGTAT CTCTCTCTTT GTCTGTCTGG AATTTTTCAA ATAATTTCTA   
  
  
+ TTTTGCTTCA GCAAAATCGG ACGATTAAAT ACATCAGGAC ATTAAACAGT TGCCGAGTCT ACTAATTACA   
  
  
+ CCCCAACTGG GTTGGCCCAC TCTTCTGAGG TGACTAGTTA AAAGATCTTA AGACTATCGA CCTACGAACT   
  
  
+ TGTTCAGAAT TCACCATTCG TTTAATTATT TATTAATAAT TCTATTGTTT CGAATGTCAT GGAAGTGGTT   
  
  
+ GTAAATTGCG TAATTAATTG TCGAAAAACT GGTTTTTTGG TGGCATAAAA GAGTACGTAA GGACTACCCA   
  
  
+ TTTGCTATTC CGAGTACATA AGAATTAAAC CCATATTAAG CTTTTAAATC GACGATTAAC TCCTATTTTG   
  
  
+ ACATTAAATT CACGCCTATA TATTACAATA TTTATGTATT TCCGAACACC GTGCTGATCT AATGGACGAA   
  
  
+ TCAACTACGG AAGGAAGACC GTTAATTGG  

- CTGCAGCTTC TCTAAAGAGA AAGTTAAACC TCTTCGTGAG AGATGAAAGG GGGATAGAGA GAGAAAGAGA   
  
  
- GATTCGGAGC TCAGCAAGAC ATTGAACTGA AACTCAGAGA ACTGCTAAAA TGGTTCTTTG GGAGGGGGAG   
  
  
- AGAAGCCAAG GGAAGATGGG GGATGATATA AGGAGGGTGG ATCACCGGGT GACCGGGTAA GACATAGGGG   
  
  
- TTTAGTCAAA GTGGGTTTTT GATCCAACGG CTGTGATGAG TTTGAAAATT TCCATGGGAT GATGTGAATA   
  
  
- AAGAAATCAG AGGCTGGTCT CCTCAAGAAG TAAGTATTGG GACCCACCCT TGGAAAAGAT AGAAATAGAT   
  
  
- ATAAAAGTAG GGACTCGTTT CTTTTTCTTT TTCCTCGGTA AAAATAGTTA CACCTCTTAT TCTGTTTGAA   
  
  
- TATTTCCTGG AAACTAGAGA CTACTAGCAT TTAGTCTTTA AAGTTAAGTT TGATTATGTG CCACAACCGT   
  
  
- TGACGGTCAG AATTGGATCC TATGAGTACA CGTAGTATTT TCATGATTGC TACTTCTAGT AACATTTAAC   
  
  
- GGAGTTATTT GACCGTTAAT GTGAAAAATA AAGGAAAAAG GAAGAGCAAT GGTAATAATT GCTTAATTAG   
  
  
- GCAGTAAAAT TTCTTAAAAC TTACCTAACG CGTGGTTACT CATGAGTTGC TGTTGTTCAC CCAAAAAAAG   
  
  
- GCAACACGTA CAGTACTTGG AACTAGGCGA GAAGGAAAGA AAGCTGACCA AAAGTGGGTC TCACGTGGGG   
  
  
- GCCATTTCTT TTTATAATCT GAAACTGTTG ACCAGCTTTT CTTATTTCTT CCTTACCCCT CGTTTCCGGT   
  
  
- ATCTGTCTTC TGAACCGTCC GATCTATTCT CATCAATCCA ACGGTCGAAT CGCACCGAGA AAGTTGACCA   
  
  
- AGACTTTTAT ACTTTGGAGA ACCCAGAAAA AGCTACAAGC CTTGGGTTTT TCACCACGTG GCATAAGGCT   
  
  
- GGTGCCAATT CAAAACGAAA CACAAGCATA GAGAGAGAAA CAGACAGACC TTAAAAAGTT TATTAAAGAT   
  
  
- AAAACGAAGT CGTTTTAGCC TGCTAATTTA TGTAGTCCTG TAATTTGTCA ACGGCTCAGA TGATTAATGT   
  
  
- GGGGTTGACC CAACCGGGTG AGAAGACTCC ACTGATCAAT TTTCTAGAAT TCTGATAGCT GGATGCTTGA   
  
  
- ACAAGTCTTA AGTGGTAAGC AAATTAATAA ATAATTATTA AGATAACAAA GCTTACAGTA CCTTCACCAA   
  
  
- CATTTAACGC ATTAATTAAC AGCTTTTTGA CCAAAAAACC ACCGTATTTT CTCATGCATT CCTGATGGGT   
  
  
- AAACGATAAG GCTCATGTAT TCTTAATTTG GGTATAATTC GAAAATTTAG CTGCTAATTG AGGATAAAAC   
  
  
- TGTAATTTAA GTGCGGATAT ATAATGTTAT AAATACATAA AGGCTTGTGG CACGACTAGA TTACCTGCTT   
  
  
- AGTTGATGCC TTCCTTCTGG CAATTAACC

+     TCA-element

| Site Name | Organism | Position | Strand | Matrix score. | sequence | function |
| --- | --- | --- | --- | --- | --- | --- |
| TCA-element | Brassica oleracea | 1139 | - | 9 | TCAGAAGAGG | cis-acting element involved in salicylic acid responsiveness |
| TCA-element | Brassica oleracea | 806 | + | 9 | GAGAAGAATA | cis-acting element involved in salicylic acid responsiveness |

> 2018/04/13 10:10:12  
+ GACGTCGAAG AGATTTCTCT TTCAATTTGG AGAAGCACTC TCTACTTTCC CCCTATCTCT CTCTTTCTCT   
  
  
+ CTAAGCCTCG AGTCGTTCTG TAACTTGACT TTGAGTCTCT TGACGATTTT ACCAAGAAAC CCTCCCCCTC   
  
  
+ TCTTCGGTTC CCTTCTACCC CCTACTATAT TCCTCCCACC TAGTGGCCCA CTGGCCCATT CTGTATCCCC   
  
  
+ AAATCAGTTT CACCCAAAAA CTAGGTTGCC GACACTACTC AAACTTTTAA AGGTACCCTA CTACACTTAT   
  
  
+ TTCTTTAGTC TCCGACCAGA GGAGTTCTTC ATTCATAACC CTGGGTGGGA ACCTTTTCTA TCTTTATCTA   
  
  
+ TATTTTCATC CCTGAGCAAA GAAAAAGAAA AAGGAGCCAT TTTTATCAAT GTGGAGAATA AGACAAACTT   
  
  
+ ATAAAGGACC TTTGATCTCT GATGATCGTA AATCAGAAAT TTCAATTCAA ACTAATACAC GGTGTTGGCA   
  
  
+ ACTGCCAGTC TTAACCTAGG ATACTCATGT GCATCATAAA AGTACTAACG ATGAAGATCA TTGTAAATTG   
  
  
+ CCTCAATAAA CTGGCAATTA CACTTTTTAT TTCCTTTTTC CTTCTCGTTA CCATTATTAA CGAATTAATC   
  
  
+ CGTCATTTTA AAGAATTTTG AATGGATTGC GCACCAATGA GTACTCAACG ACAACAAGTG GGTTTTTTTC   
  
  
+ CGTTGTGCAT GTCATGAACC TTGATCCGCT CTTCCTTTCT TTCGACTGGT TTTCACCCAG AGTGCACCCC   
  
  
+ CGGTAAAGAA AAATATTAGA CTTTGACAAC TGGTCGAAAA GAATAAAGAA GGAATGGGGA GCAAAGGCCA   
  
  
+ TAGACAGAAG ACTTGGCAGG CTAGATAAGA GTAGTTAGGT TGCCAGCTTA GCGTGGCTCT TTCAACTGGT   
  
  
+ TCTGAAAATA TGAAACCTCT TGGGTCTTTT TCGATGTTCG GAACCCAAAA AGTGGTGCAC CGTATTCCGA   
  
  
+ CCACGGTTAA GTTTTGCTTT GTGTTCGTAT CTCTCTCTTT GTCTGTCTGG AATTTTTCAA ATAATTTCTA   
  
  
+ TTTTGCTTCA GCAAAATCGG ACGATTAAAT ACATCAGGAC ATTAAACAGT TGCCGAGTCT ACTAATTACA   
  
  
+ CCCCAACTGG GTTGGCCCAC TCTTCTGAGG TGACTAGTTA AAAGATCTTA AGACTATCGA CCTACGAACT   
  
  
+ TGTTCAGAAT TCACCATTCG TTTAATTATT TATTAATAAT TCTATTGTTT CGAATGTCAT GGAAGTGGTT   
  
  
+ GTAAATTGCG TAATTAATTG TCGAAAAACT GGTTTTTTGG TGGCATAAAA GAGTACGTAA GGACTACCCA   
  
  
+ TTTGCTATTC CGAGTACATA AGAATTAAAC CCATATTAAG CTTTTAAATC GACGATTAAC TCCTATTTTG   
  
  
+ ACATTAAATT CACGCCTATA TATTACAATA TTTATGTATT TCCGAACACC GTGCTGATCT AATGGACGAA   
  
  
+ TCAACTACGG AAGGAAGACC GTTAATTGG  

- CTGCAGCTTC TCTAAAGAGA AAGTTAAACC TCTTCGTGAG AGATGAAAGG GGGATAGAGA GAGAAAGAGA   
  
  
- GATTCGGAGC TCAGCAAGAC ATTGAACTGA AACTCAGAGA ACTGCTAAAA TGGTTCTTTG GGAGGGGGAG   
  
  
- AGAAGCCAAG GGAAGATGGG GGATGATATA AGGAGGGTGG ATCACCGGGT GACCGGGTAA GACATAGGGG   
  
  
- TTTAGTCAAA GTGGGTTTTT GATCCAACGG CTGTGATGAG TTTGAAAATT TCCATGGGAT GATGTGAATA   
  
  
- AAGAAATCAG AGGCTGGTCT CCTCAAGAAG TAAGTATTGG GACCCACCCT TGGAAAAGAT AGAAATAGAT   
  
  
- ATAAAAGTAG GGACTCGTTT CTTTTTCTTT TTCCTCGGTA AAAATAGTTA CACCTCTTAT TCTGTTTGAA   
  
  
- TATTTCCTGG AAACTAGAGA CTACTAGCAT TTAGTCTTTA AAGTTAAGTT TGATTATGTG CCACAACCGT   
  
  
- TGACGGTCAG AATTGGATCC TATGAGTACA CGTAGTATTT TCATGATTGC TACTTCTAGT AACATTTAAC   
  
  
- GGAGTTATTT GACCGTTAAT GTGAAAAATA AAGGAAAAAG GAAGAGCAAT GGTAATAATT GCTTAATTAG   
  
  
- GCAGTAAAAT TTCTTAAAAC TTACCTAACG CGTGGTTACT CATGAGTTGC TGTTGTTCAC CCAAAAAAAG   
  
  
- GCAACACGTA CAGTACTTGG AACTAGGCGA GAAGGAAAGA AAGCTGACCA AAAGTGGGTC TCACGTGGGG   
  
  
- GCCATTTCTT TTTATAATCT GAAACTGTTG ACCAGCTTTT CTTATTTCTT CCTTACCCCT CGTTTCCGGT   
  
  
- ATCTGTCTTC TGAACCGTCC GATCTATTCT CATCAATCCA ACGGTCGAAT CGCACCGAGA AAGTTGACCA   
  
  
- AGACTTTTAT ACTTTGGAGA ACCCAGAAAA AGCTACAAGC CTTGGGTTTT TCACCACGTG GCATAAGGCT   
  
  
- GGTGCCAATT CAAAACGAAA CACAAGCATA GAGAGAGAAA CAGACAGACC TTAAAAAGTT TATTAAAGAT   
  
  
- AAAACGAAGT CGTTTTAGCC TGCTAATTTA TGTAGTCCTG TAATTTGTCA ACGGCTCAGA TGATTAATGT   
  
  
- GGGGTTGACC CAACCGGGTG AGAAGACTCC ACTGATCAAT TTTCTAGAAT TCTGATAGCT GGATGCTTGA   
  
  
- ACAAGTCTTA AGTGGTAAGC AAATTAATAA ATAATTATTA AGATAACAAA GCTTACAGTA CCTTCACCAA   
  
  
- CATTTAACGC ATTAATTAAC AGCTTTTTGA CCAAAAAACC ACCGTATTTT CTCATGCATT CCTGATGGGT   
  
  
- AAACGATAAG GCTCATGTAT TCTTAATTTG GGTATAATTC GAAAATTTAG CTGCTAATTG AGGATAAAAC   
  
  
- TGTAATTTAA GTGCGGATAT ATAATGTTAT AAATACATAA AGGCTTGTGG CACGACTAGA TTACCTGCTT   
  
  
- AGTTGATGCC TTCCTTCTGG CAATTAACC

+     TGA-element

| Site Name | Organism | Position | Strand | Matrix score. | sequence | function |
| --- | --- | --- | --- | --- | --- | --- |
| TGA-element | Brassica oleracea | 677 | + | 6 | AACGAC | auxin-responsive element |
| TGA-element | Brassica oleracea | 82 | - | 6 | AACGAC | auxin-responsive element |

> 2018/04/13 10:10:12  
+ GACGTCGAAG AGATTTCTCT TTCAATTTGG AGAAGCACTC TCTACTTTCC CCCTATCTCT CTCTTTCTCT   
  
  
+ CTAAGCCTCG AGTCGTTCTG TAACTTGACT TTGAGTCTCT TGACGATTTT ACCAAGAAAC CCTCCCCCTC   
  
  
+ TCTTCGGTTC CCTTCTACCC CCTACTATAT TCCTCCCACC TAGTGGCCCA CTGGCCCATT CTGTATCCCC   
  
  
+ AAATCAGTTT CACCCAAAAA CTAGGTTGCC GACACTACTC AAACTTTTAA AGGTACCCTA CTACACTTAT   
  
  
+ TTCTTTAGTC TCCGACCAGA GGAGTTCTTC ATTCATAACC CTGGGTGGGA ACCTTTTCTA TCTTTATCTA   
  
  
+ TATTTTCATC CCTGAGCAAA GAAAAAGAAA AAGGAGCCAT TTTTATCAAT GTGGAGAATA AGACAAACTT   
  
  
+ ATAAAGGACC TTTGATCTCT GATGATCGTA AATCAGAAAT TTCAATTCAA ACTAATACAC GGTGTTGGCA   
  
  
+ ACTGCCAGTC TTAACCTAGG ATACTCATGT GCATCATAAA AGTACTAACG ATGAAGATCA TTGTAAATTG   
  
  
+ CCTCAATAAA CTGGCAATTA CACTTTTTAT TTCCTTTTTC CTTCTCGTTA CCATTATTAA CGAATTAATC   
  
  
+ CGTCATTTTA AAGAATTTTG AATGGATTGC GCACCAATGA GTACTCAACG ACAACAAGTG GGTTTTTTTC   
  
  
+ CGTTGTGCAT GTCATGAACC TTGATCCGCT CTTCCTTTCT TTCGACTGGT TTTCACCCAG AGTGCACCCC   
  
  
+ CGGTAAAGAA AAATATTAGA CTTTGACAAC TGGTCGAAAA GAATAAAGAA GGAATGGGGA GCAAAGGCCA   
  
  
+ TAGACAGAAG ACTTGGCAGG CTAGATAAGA GTAGTTAGGT TGCCAGCTTA GCGTGGCTCT TTCAACTGGT   
  
  
+ TCTGAAAATA TGAAACCTCT TGGGTCTTTT TCGATGTTCG GAACCCAAAA AGTGGTGCAC CGTATTCCGA   
  
  
+ CCACGGTTAA GTTTTGCTTT GTGTTCGTAT CTCTCTCTTT GTCTGTCTGG AATTTTTCAA ATAATTTCTA   
  
  
+ TTTTGCTTCA GCAAAATCGG ACGATTAAAT ACATCAGGAC ATTAAACAGT TGCCGAGTCT ACTAATTACA   
  
  
+ CCCCAACTGG GTTGGCCCAC TCTTCTGAGG TGACTAGTTA AAAGATCTTA AGACTATCGA CCTACGAACT   
  
  
+ TGTTCAGAAT TCACCATTCG TTTAATTATT TATTAATAAT TCTATTGTTT CGAATGTCAT GGAAGTGGTT   
  
  
+ GTAAATTGCG TAATTAATTG TCGAAAAACT GGTTTTTTGG TGGCATAAAA GAGTACGTAA GGACTACCCA   
  
  
+ TTTGCTATTC CGAGTACATA AGAATTAAAC CCATATTAAG CTTTTAAATC GACGATTAAC TCCTATTTTG   
  
  
+ ACATTAAATT CACGCCTATA TATTACAATA TTTATGTATT TCCGAACACC GTGCTGATCT AATGGACGAA   
  
  
+ TCAACTACGG AAGGAAGACC GTTAATTGG  

- CTGCAGCTTC TCTAAAGAGA AAGTTAAACC TCTTCGTGAG AGATGAAAGG GGGATAGAGA GAGAAAGAGA   
  
  
- GATTCGGAGC TCAGCAAGAC ATTGAACTGA AACTCAGAGA ACTGCTAAAA TGGTTCTTTG GGAGGGGGAG   
  
  
- AGAAGCCAAG GGAAGATGGG GGATGATATA AGGAGGGTGG ATCACCGGGT GACCGGGTAA GACATAGGGG   
  
  
- TTTAGTCAAA GTGGGTTTTT GATCCAACGG CTGTGATGAG TTTGAAAATT TCCATGGGAT GATGTGAATA   
  
  
- AAGAAATCAG AGGCTGGTCT CCTCAAGAAG TAAGTATTGG GACCCACCCT TGGAAAAGAT AGAAATAGAT   
  
  
- ATAAAAGTAG GGACTCGTTT CTTTTTCTTT TTCCTCGGTA AAAATAGTTA CACCTCTTAT TCTGTTTGAA   
  
  
- TATTTCCTGG AAACTAGAGA CTACTAGCAT TTAGTCTTTA AAGTTAAGTT TGATTATGTG CCACAACCGT   
  
  
- TGACGGTCAG AATTGGATCC TATGAGTACA CGTAGTATTT TCATGATTGC TACTTCTAGT AACATTTAAC   
  
  
- GGAGTTATTT GACCGTTAAT GTGAAAAATA AAGGAAAAAG GAAGAGCAAT GGTAATAATT GCTTAATTAG   
  
  
- GCAGTAAAAT TTCTTAAAAC TTACCTAACG CGTGGTTACT CATGAGTTGC TGTTGTTCAC CCAAAAAAAG   
  
  
- GCAACACGTA CAGTACTTGG AACTAGGCGA GAAGGAAAGA AAGCTGACCA AAAGTGGGTC TCACGTGGGG   
  
  
- GCCATTTCTT TTTATAATCT GAAACTGTTG ACCAGCTTTT CTTATTTCTT CCTTACCCCT CGTTTCCGGT   
  
  
- ATCTGTCTTC TGAACCGTCC GATCTATTCT CATCAATCCA ACGGTCGAAT CGCACCGAGA AAGTTGACCA   
  
  
- AGACTTTTAT ACTTTGGAGA ACCCAGAAAA AGCTACAAGC CTTGGGTTTT TCACCACGTG GCATAAGGCT   
  
  
- GGTGCCAATT CAAAACGAAA CACAAGCATA GAGAGAGAAA CAGACAGACC TTAAAAAGTT TATTAAAGAT   
  
  
- AAAACGAAGT CGTTTTAGCC TGCTAATTTA TGTAGTCCTG TAATTTGTCA ACGGCTCAGA TGATTAATGT   
  
  
- GGGGTTGACC CAACCGGGTG AGAAGACTCC ACTGATCAAT TTTCTAGAAT TCTGATAGCT GGATGCTTGA   
  
  
- ACAAGTCTTA AGTGGTAAGC AAATTAATAA ATAATTATTA AGATAACAAA GCTTACAGTA CCTTCACCAA   
  
  
- CATTTAACGC ATTAATTAAC AGCTTTTTGA CCAAAAAACC ACCGTATTTT CTCATGCATT CCTGATGGGT   
  
  
- AAACGATAAG GCTCATGTAT TCTTAATTTG GGTATAATTC GAAAATTTAG CTGCTAATTG AGGATAAAAC   
  
  
- TGTAATTTAA GTGCGGATAT ATAATGTTAT AAATACATAA AGGCTTGTGG CACGACTAGA TTACCTGCTT   
  
  
- AGTTGATGCC TTCCTTCTGG CAATTAACC

+     TGACG-motif

| Site Name | Organism | Position | Strand | Matrix score. | sequence | function |
| --- | --- | --- | --- | --- | --- | --- |
| TGACG-motif | Hordeum vulgare | 631 | - | 5 | TGACG | cis-acting regulatory element involved in the MeJA-responsiveness |
| TGACG-motif | Hordeum vulgare | 111 | + | 5 | TGACG | cis-acting regulatory element involved in the MeJA-responsiveness |

> 2018/04/13 10:10:12  
+ GACGTCGAAG AGATTTCTCT TTCAATTTGG AGAAGCACTC TCTACTTTCC CCCTATCTCT CTCTTTCTCT   
  
  
+ CTAAGCCTCG AGTCGTTCTG TAACTTGACT TTGAGTCTCT TGACGATTTT ACCAAGAAAC CCTCCCCCTC   
  
  
+ TCTTCGGTTC CCTTCTACCC CCTACTATAT TCCTCCCACC TAGTGGCCCA CTGGCCCATT CTGTATCCCC   
  
  
+ AAATCAGTTT CACCCAAAAA CTAGGTTGCC GACACTACTC AAACTTTTAA AGGTACCCTA CTACACTTAT   
  
  
+ TTCTTTAGTC TCCGACCAGA GGAGTTCTTC ATTCATAACC CTGGGTGGGA ACCTTTTCTA TCTTTATCTA   
  
  
+ TATTTTCATC CCTGAGCAAA GAAAAAGAAA AAGGAGCCAT TTTTATCAAT GTGGAGAATA AGACAAACTT   
  
  
+ ATAAAGGACC TTTGATCTCT GATGATCGTA AATCAGAAAT TTCAATTCAA ACTAATACAC GGTGTTGGCA   
  
  
+ ACTGCCAGTC TTAACCTAGG ATACTCATGT GCATCATAAA AGTACTAACG ATGAAGATCA TTGTAAATTG   
  
  
+ CCTCAATAAA CTGGCAATTA CACTTTTTAT TTCCTTTTTC CTTCTCGTTA CCATTATTAA CGAATTAATC   
  
  
+ CGTCATTTTA AAGAATTTTG AATGGATTGC GCACCAATGA GTACTCAACG ACAACAAGTG GGTTTTTTTC   
  
  
+ CGTTGTGCAT GTCATGAACC TTGATCCGCT CTTCCTTTCT TTCGACTGGT TTTCACCCAG AGTGCACCCC   
  
  
+ CGGTAAAGAA AAATATTAGA CTTTGACAAC TGGTCGAAAA GAATAAAGAA GGAATGGGGA GCAAAGGCCA   
  
  
+ TAGACAGAAG ACTTGGCAGG CTAGATAAGA GTAGTTAGGT TGCCAGCTTA GCGTGGCTCT TTCAACTGGT   
  
  
+ TCTGAAAATA TGAAACCTCT TGGGTCTTTT TCGATGTTCG GAACCCAAAA AGTGGTGCAC CGTATTCCGA   
  
  
+ CCACGGTTAA GTTTTGCTTT GTGTTCGTAT CTCTCTCTTT GTCTGTCTGG AATTTTTCAA ATAATTTCTA   
  
  
+ TTTTGCTTCA GCAAAATCGG ACGATTAAAT ACATCAGGAC ATTAAACAGT TGCCGAGTCT ACTAATTACA   
  
  
+ CCCCAACTGG GTTGGCCCAC TCTTCTGAGG TGACTAGTTA AAAGATCTTA AGACTATCGA CCTACGAACT   
  
  
+ TGTTCAGAAT TCACCATTCG TTTAATTATT TATTAATAAT TCTATTGTTT CGAATGTCAT GGAAGTGGTT   
  
  
+ GTAAATTGCG TAATTAATTG TCGAAAAACT GGTTTTTTGG TGGCATAAAA GAGTACGTAA GGACTACCCA   
  
  
+ TTTGCTATTC CGAGTACATA AGAATTAAAC CCATATTAAG CTTTTAAATC GACGATTAAC TCCTATTTTG   
  
  
+ ACATTAAATT CACGCCTATA TATTACAATA TTTATGTATT TCCGAACACC GTGCTGATCT AATGGACGAA   
  
  
+ TCAACTACGG AAGGAAGACC GTTAATTGG  

- CTGCAGCTTC TCTAAAGAGA AAGTTAAACC TCTTCGTGAG AGATGAAAGG GGGATAGAGA GAGAAAGAGA   
  
  
- GATTCGGAGC TCAGCAAGAC ATTGAACTGA AACTCAGAGA ACTGCTAAAA TGGTTCTTTG GGAGGGGGAG   
  
  
- AGAAGCCAAG GGAAGATGGG GGATGATATA AGGAGGGTGG ATCACCGGGT GACCGGGTAA GACATAGGGG   
  
  
- TTTAGTCAAA GTGGGTTTTT GATCCAACGG CTGTGATGAG TTTGAAAATT TCCATGGGAT GATGTGAATA   
  
  
- AAGAAATCAG AGGCTGGTCT CCTCAAGAAG TAAGTATTGG GACCCACCCT TGGAAAAGAT AGAAATAGAT   
  
  
- ATAAAAGTAG GGACTCGTTT CTTTTTCTTT TTCCTCGGTA AAAATAGTTA CACCTCTTAT TCTGTTTGAA   
  
  
- TATTTCCTGG AAACTAGAGA CTACTAGCAT TTAGTCTTTA AAGTTAAGTT TGATTATGTG CCACAACCGT   
  
  
- TGACGGTCAG AATTGGATCC TATGAGTACA CGTAGTATTT TCATGATTGC TACTTCTAGT AACATTTAAC   
  
  
- GGAGTTATTT GACCGTTAAT GTGAAAAATA AAGGAAAAAG GAAGAGCAAT GGTAATAATT GCTTAATTAG   
  
  
- GCAGTAAAAT TTCTTAAAAC TTACCTAACG CGTGGTTACT CATGAGTTGC TGTTGTTCAC CCAAAAAAAG   
  
  
- GCAACACGTA CAGTACTTGG AACTAGGCGA GAAGGAAAGA AAGCTGACCA AAAGTGGGTC TCACGTGGGG   
  
  
- GCCATTTCTT TTTATAATCT GAAACTGTTG ACCAGCTTTT CTTATTTCTT CCTTACCCCT CGTTTCCGGT   
  
  
- ATCTGTCTTC TGAACCGTCC GATCTATTCT CATCAATCCA ACGGTCGAAT CGCACCGAGA AAGTTGACCA   
  
  
- AGACTTTTAT ACTTTGGAGA ACCCAGAAAA AGCTACAAGC CTTGGGTTTT TCACCACGTG GCATAAGGCT   
  
  
- GGTGCCAATT CAAAACGAAA CACAAGCATA GAGAGAGAAA CAGACAGACC TTAAAAAGTT TATTAAAGAT   
  
  
- AAAACGAAGT CGTTTTAGCC TGCTAATTTA TGTAGTCCTG TAATTTGTCA ACGGCTCAGA TGATTAATGT   
  
  
- GGGGTTGACC CAACCGGGTG AGAAGACTCC ACTGATCAAT TTTCTAGAAT TCTGATAGCT GGATGCTTGA   
  
  
- ACAAGTCTTA AGTGGTAAGC AAATTAATAA ATAATTATTA AGATAACAAA GCTTACAGTA CCTTCACCAA   
  
  
- CATTTAACGC ATTAATTAAC AGCTTTTTGA CCAAAAAACC ACCGTATTTT CTCATGCATT CCTGATGGGT   
  
  
- AAACGATAAG GCTCATGTAT TCTTAATTTG GGTATAATTC GAAAATTTAG CTGCTAATTG AGGATAAAAC   
  
  
- TGTAATTTAA GTGCGGATAT ATAATGTTAT AAATACATAA AGGCTTGTGG CACGACTAGA TTACCTGCTT   
  
  
- AGTTGATGCC TTCCTTCTGG CAATTAACC

+     TGG-motif

| Site Name | Organism | Position | Strand | Matrix score. | sequence | function |
| --- | --- | --- | --- | --- | --- | --- |
| TGG-motif | Gossypium hirsutum | 878 | + | 8 | GGTTGCCA | part of a light responsive element |

> 2018/04/13 10:10:12  
+ GACGTCGAAG AGATTTCTCT TTCAATTTGG AGAAGCACTC TCTACTTTCC CCCTATCTCT CTCTTTCTCT   
  
  
+ CTAAGCCTCG AGTCGTTCTG TAACTTGACT TTGAGTCTCT TGACGATTTT ACCAAGAAAC CCTCCCCCTC   
  
  
+ TCTTCGGTTC CCTTCTACCC CCTACTATAT TCCTCCCACC TAGTGGCCCA CTGGCCCATT CTGTATCCCC   
  
  
+ AAATCAGTTT CACCCAAAAA CTAGGTTGCC GACACTACTC AAACTTTTAA AGGTACCCTA CTACACTTAT   
  
  
+ TTCTTTAGTC TCCGACCAGA GGAGTTCTTC ATTCATAACC CTGGGTGGGA ACCTTTTCTA TCTTTATCTA   
  
  
+ TATTTTCATC CCTGAGCAAA GAAAAAGAAA AAGGAGCCAT TTTTATCAAT GTGGAGAATA AGACAAACTT   
  
  
+ ATAAAGGACC TTTGATCTCT GATGATCGTA AATCAGAAAT TTCAATTCAA ACTAATACAC GGTGTTGGCA   
  
  
+ ACTGCCAGTC TTAACCTAGG ATACTCATGT GCATCATAAA AGTACTAACG ATGAAGATCA TTGTAAATTG   
  
  
+ CCTCAATAAA CTGGCAATTA CACTTTTTAT TTCCTTTTTC CTTCTCGTTA CCATTATTAA CGAATTAATC   
  
  
+ CGTCATTTTA AAGAATTTTG AATGGATTGC GCACCAATGA GTACTCAACG ACAACAAGTG GGTTTTTTTC   
  
  
+ CGTTGTGCAT GTCATGAACC TTGATCCGCT CTTCCTTTCT TTCGACTGGT TTTCACCCAG AGTGCACCCC   
  
  
+ CGGTAAAGAA AAATATTAGA CTTTGACAAC TGGTCGAAAA GAATAAAGAA GGAATGGGGA GCAAAGGCCA   
  
  
+ TAGACAGAAG ACTTGGCAGG CTAGATAAGA GTAGTTAGGT TGCCAGCTTA GCGTGGCTCT TTCAACTGGT   
  
  
+ TCTGAAAATA TGAAACCTCT TGGGTCTTTT TCGATGTTCG GAACCCAAAA AGTGGTGCAC CGTATTCCGA   
  
  
+ CCACGGTTAA GTTTTGCTTT GTGTTCGTAT CTCTCTCTTT GTCTGTCTGG AATTTTTCAA ATAATTTCTA   
  
  
+ TTTTGCTTCA GCAAAATCGG ACGATTAAAT ACATCAGGAC ATTAAACAGT TGCCGAGTCT ACTAATTACA   
  
  
+ CCCCAACTGG GTTGGCCCAC TCTTCTGAGG TGACTAGTTA AAAGATCTTA AGACTATCGA CCTACGAACT   
  
  
+ TGTTCAGAAT TCACCATTCG TTTAATTATT TATTAATAAT TCTATTGTTT CGAATGTCAT GGAAGTGGTT   
  
  
+ GTAAATTGCG TAATTAATTG TCGAAAAACT GGTTTTTTGG TGGCATAAAA GAGTACGTAA GGACTACCCA   
  
  
+ TTTGCTATTC CGAGTACATA AGAATTAAAC CCATATTAAG CTTTTAAATC GACGATTAAC TCCTATTTTG   
  
  
+ ACATTAAATT CACGCCTATA TATTACAATA TTTATGTATT TCCGAACACC GTGCTGATCT AATGGACGAA   
  
  
+ TCAACTACGG AAGGAAGACC GTTAATTGG  

- CTGCAGCTTC TCTAAAGAGA AAGTTAAACC TCTTCGTGAG AGATGAAAGG GGGATAGAGA GAGAAAGAGA   
  
  
- GATTCGGAGC TCAGCAAGAC ATTGAACTGA AACTCAGAGA ACTGCTAAAA TGGTTCTTTG GGAGGGGGAG   
  
  
- AGAAGCCAAG GGAAGATGGG GGATGATATA AGGAGGGTGG ATCACCGGGT GACCGGGTAA GACATAGGGG   
  
  
- TTTAGTCAAA GTGGGTTTTT GATCCAACGG CTGTGATGAG TTTGAAAATT TCCATGGGAT GATGTGAATA   
  
  
- AAGAAATCAG AGGCTGGTCT CCTCAAGAAG TAAGTATTGG GACCCACCCT TGGAAAAGAT AGAAATAGAT   
  
  
- ATAAAAGTAG GGACTCGTTT CTTTTTCTTT TTCCTCGGTA AAAATAGTTA CACCTCTTAT TCTGTTTGAA   
  
  
- TATTTCCTGG AAACTAGAGA CTACTAGCAT TTAGTCTTTA AAGTTAAGTT TGATTATGTG CCACAACCGT   
  
  
- TGACGGTCAG AATTGGATCC TATGAGTACA CGTAGTATTT TCATGATTGC TACTTCTAGT AACATTTAAC   
  
  
- GGAGTTATTT GACCGTTAAT GTGAAAAATA AAGGAAAAAG GAAGAGCAAT GGTAATAATT GCTTAATTAG   
  
  
- GCAGTAAAAT TTCTTAAAAC TTACCTAACG CGTGGTTACT CATGAGTTGC TGTTGTTCAC CCAAAAAAAG   
  
  
- GCAACACGTA CAGTACTTGG AACTAGGCGA GAAGGAAAGA AAGCTGACCA AAAGTGGGTC TCACGTGGGG   
  
  
- GCCATTTCTT TTTATAATCT GAAACTGTTG ACCAGCTTTT CTTATTTCTT CCTTACCCCT CGTTTCCGGT   
  
  
- ATCTGTCTTC TGAACCGTCC GATCTATTCT CATCAATCCA ACGGTCGAAT CGCACCGAGA AAGTTGACCA   
  
  
- AGACTTTTAT ACTTTGGAGA ACCCAGAAAA AGCTACAAGC CTTGGGTTTT TCACCACGTG GCATAAGGCT   
  
  
- GGTGCCAATT CAAAACGAAA CACAAGCATA GAGAGAGAAA CAGACAGACC TTAAAAAGTT TATTAAAGAT   
  
  
- AAAACGAAGT CGTTTTAGCC TGCTAATTTA TGTAGTCCTG TAATTTGTCA ACGGCTCAGA TGATTAATGT   
  
  
- GGGGTTGACC CAACCGGGTG AGAAGACTCC ACTGATCAAT TTTCTAGAAT TCTGATAGCT GGATGCTTGA   
  
  
- ACAAGTCTTA AGTGGTAAGC AAATTAATAA ATAATTATTA AGATAACAAA GCTTACAGTA CCTTCACCAA   
  
  
- CATTTAACGC ATTAATTAAC AGCTTTTTGA CCAAAAAACC ACCGTATTTT CTCATGCATT CCTGATGGGT   
  
  
- AAACGATAAG GCTCATGTAT TCTTAATTTG GGTATAATTC GAAAATTTAG CTGCTAATTG AGGATAAAAC   
  
  
- TGTAATTTAA GTGCGGATAT ATAATGTTAT AAATACATAA AGGCTTGTGG CACGACTAGA TTACCTGCTT   
  
  
- AGTTGATGCC TTCCTTCTGG CAATTAACC

+     Unnamed\_\_1

| Site Name | Organism | Position | Strand | Matrix score. | sequence | function |
| --- | --- | --- | --- | --- | --- | --- |
| Unnamed\_\_1 | Zea mays | 981 | - | 5 | CGTGG |  |
| Unnamed\_\_1 | Zea mays | 892 | + | 5 | CGTGG |  |

> 2018/04/13 10:10:12  
+ GACGTCGAAG AGATTTCTCT TTCAATTTGG AGAAGCACTC TCTACTTTCC CCCTATCTCT CTCTTTCTCT   
  
  
+ CTAAGCCTCG AGTCGTTCTG TAACTTGACT TTGAGTCTCT TGACGATTTT ACCAAGAAAC CCTCCCCCTC   
  
  
+ TCTTCGGTTC CCTTCTACCC CCTACTATAT TCCTCCCACC TAGTGGCCCA CTGGCCCATT CTGTATCCCC   
  
  
+ AAATCAGTTT CACCCAAAAA CTAGGTTGCC GACACTACTC AAACTTTTAA AGGTACCCTA CTACACTTAT   
  
  
+ TTCTTTAGTC TCCGACCAGA GGAGTTCTTC ATTCATAACC CTGGGTGGGA ACCTTTTCTA TCTTTATCTA   
  
  
+ TATTTTCATC CCTGAGCAAA GAAAAAGAAA AAGGAGCCAT TTTTATCAAT GTGGAGAATA AGACAAACTT   
  
  
+ ATAAAGGACC TTTGATCTCT GATGATCGTA AATCAGAAAT TTCAATTCAA ACTAATACAC GGTGTTGGCA   
  
  
+ ACTGCCAGTC TTAACCTAGG ATACTCATGT GCATCATAAA AGTACTAACG ATGAAGATCA TTGTAAATTG   
  
  
+ CCTCAATAAA CTGGCAATTA CACTTTTTAT TTCCTTTTTC CTTCTCGTTA CCATTATTAA CGAATTAATC   
  
  
+ CGTCATTTTA AAGAATTTTG AATGGATTGC GCACCAATGA GTACTCAACG ACAACAAGTG GGTTTTTTTC   
  
  
+ CGTTGTGCAT GTCATGAACC TTGATCCGCT CTTCCTTTCT TTCGACTGGT TTTCACCCAG AGTGCACCCC   
  
  
+ CGGTAAAGAA AAATATTAGA CTTTGACAAC TGGTCGAAAA GAATAAAGAA GGAATGGGGA GCAAAGGCCA   
  
  
+ TAGACAGAAG ACTTGGCAGG CTAGATAAGA GTAGTTAGGT TGCCAGCTTA GCGTGGCTCT TTCAACTGGT   
  
  
+ TCTGAAAATA TGAAACCTCT TGGGTCTTTT TCGATGTTCG GAACCCAAAA AGTGGTGCAC CGTATTCCGA   
  
  
+ CCACGGTTAA GTTTTGCTTT GTGTTCGTAT CTCTCTCTTT GTCTGTCTGG AATTTTTCAA ATAATTTCTA   
  
  
+ TTTTGCTTCA GCAAAATCGG ACGATTAAAT ACATCAGGAC ATTAAACAGT TGCCGAGTCT ACTAATTACA   
  
  
+ CCCCAACTGG GTTGGCCCAC TCTTCTGAGG TGACTAGTTA AAAGATCTTA AGACTATCGA CCTACGAACT   
  
  
+ TGTTCAGAAT TCACCATTCG TTTAATTATT TATTAATAAT TCTATTGTTT CGAATGTCAT GGAAGTGGTT   
  
  
+ GTAAATTGCG TAATTAATTG TCGAAAAACT GGTTTTTTGG TGGCATAAAA GAGTACGTAA GGACTACCCA   
  
  
+ TTTGCTATTC CGAGTACATA AGAATTAAAC CCATATTAAG CTTTTAAATC GACGATTAAC TCCTATTTTG   
  
  
+ ACATTAAATT CACGCCTATA TATTACAATA TTTATGTATT TCCGAACACC GTGCTGATCT AATGGACGAA   
  
  
+ TCAACTACGG AAGGAAGACC GTTAATTGG  

- CTGCAGCTTC TCTAAAGAGA AAGTTAAACC TCTTCGTGAG AGATGAAAGG GGGATAGAGA GAGAAAGAGA   
  
  
- GATTCGGAGC TCAGCAAGAC ATTGAACTGA AACTCAGAGA ACTGCTAAAA TGGTTCTTTG GGAGGGGGAG   
  
  
- AGAAGCCAAG GGAAGATGGG GGATGATATA AGGAGGGTGG ATCACCGGGT GACCGGGTAA GACATAGGGG   
  
  
- TTTAGTCAAA GTGGGTTTTT GATCCAACGG CTGTGATGAG TTTGAAAATT TCCATGGGAT GATGTGAATA   
  
  
- AAGAAATCAG AGGCTGGTCT CCTCAAGAAG TAAGTATTGG GACCCACCCT TGGAAAAGAT AGAAATAGAT   
  
  
- ATAAAAGTAG GGACTCGTTT CTTTTTCTTT TTCCTCGGTA AAAATAGTTA CACCTCTTAT TCTGTTTGAA   
  
  
- TATTTCCTGG AAACTAGAGA CTACTAGCAT TTAGTCTTTA AAGTTAAGTT TGATTATGTG CCACAACCGT   
  
  
- TGACGGTCAG AATTGGATCC TATGAGTACA CGTAGTATTT TCATGATTGC TACTTCTAGT AACATTTAAC   
  
  
- GGAGTTATTT GACCGTTAAT GTGAAAAATA AAGGAAAAAG GAAGAGCAAT GGTAATAATT GCTTAATTAG   
  
  
- GCAGTAAAAT TTCTTAAAAC TTACCTAACG CGTGGTTACT CATGAGTTGC TGTTGTTCAC CCAAAAAAAG   
  
  
- GCAACACGTA CAGTACTTGG AACTAGGCGA GAAGGAAAGA AAGCTGACCA AAAGTGGGTC TCACGTGGGG   
  
  
- GCCATTTCTT TTTATAATCT GAAACTGTTG ACCAGCTTTT CTTATTTCTT CCTTACCCCT CGTTTCCGGT   
  
  
- ATCTGTCTTC TGAACCGTCC GATCTATTCT CATCAATCCA ACGGTCGAAT CGCACCGAGA AAGTTGACCA   
  
  
- AGACTTTTAT ACTTTGGAGA ACCCAGAAAA AGCTACAAGC CTTGGGTTTT TCACCACGTG GCATAAGGCT   
  
  
- GGTGCCAATT CAAAACGAAA CACAAGCATA GAGAGAGAAA CAGACAGACC TTAAAAAGTT TATTAAAGAT   
  
  
- AAAACGAAGT CGTTTTAGCC TGCTAATTTA TGTAGTCCTG TAATTTGTCA ACGGCTCAGA TGATTAATGT   
  
  
- GGGGTTGACC CAACCGGGTG AGAAGACTCC ACTGATCAAT TTTCTAGAAT TCTGATAGCT GGATGCTTGA   
  
  
- ACAAGTCTTA AGTGGTAAGC AAATTAATAA ATAATTATTA AGATAACAAA GCTTACAGTA CCTTCACCAA   
  
  
- CATTTAACGC ATTAATTAAC AGCTTTTTGA CCAAAAAACC ACCGTATTTT CTCATGCATT CCTGATGGGT   
  
  
- AAACGATAAG GCTCATGTAT TCTTAATTTG GGTATAATTC GAAAATTTAG CTGCTAATTG AGGATAAAAC   
  
  
- TGTAATTTAA GTGCGGATAT ATAATGTTAT AAATACATAA AGGCTTGTGG CACGACTAGA TTACCTGCTT   
  
  
- AGTTGATGCC TTCCTTCTGG CAATTAACC

+     Unnamed\_\_11

| Site Name | Organism | Position | Strand | Matrix score. | sequence | function |
| --- | --- | --- | --- | --- | --- | --- |
| Unnamed\_\_11 | Zea mays | 396 | - | 9 | TCCACATAGA |  |

> 2018/04/13 10:10:12  
+ GACGTCGAAG AGATTTCTCT TTCAATTTGG AGAAGCACTC TCTACTTTCC CCCTATCTCT CTCTTTCTCT   
  
  
+ CTAAGCCTCG AGTCGTTCTG TAACTTGACT TTGAGTCTCT TGACGATTTT ACCAAGAAAC CCTCCCCCTC   
  
  
+ TCTTCGGTTC CCTTCTACCC CCTACTATAT TCCTCCCACC TAGTGGCCCA CTGGCCCATT CTGTATCCCC   
  
  
+ AAATCAGTTT CACCCAAAAA CTAGGTTGCC GACACTACTC AAACTTTTAA AGGTACCCTA CTACACTTAT   
  
  
+ TTCTTTAGTC TCCGACCAGA GGAGTTCTTC ATTCATAACC CTGGGTGGGA ACCTTTTCTA TCTTTATCTA   
  
  
+ TATTTTCATC CCTGAGCAAA GAAAAAGAAA AAGGAGCCAT TTTTATCAAT GTGGAGAATA AGACAAACTT   
  
  
+ ATAAAGGACC TTTGATCTCT GATGATCGTA AATCAGAAAT TTCAATTCAA ACTAATACAC GGTGTTGGCA   
  
  
+ ACTGCCAGTC TTAACCTAGG ATACTCATGT GCATCATAAA AGTACTAACG ATGAAGATCA TTGTAAATTG   
  
  
+ CCTCAATAAA CTGGCAATTA CACTTTTTAT TTCCTTTTTC CTTCTCGTTA CCATTATTAA CGAATTAATC   
  
  
+ CGTCATTTTA AAGAATTTTG AATGGATTGC GCACCAATGA GTACTCAACG ACAACAAGTG GGTTTTTTTC   
  
  
+ CGTTGTGCAT GTCATGAACC TTGATCCGCT CTTCCTTTCT TTCGACTGGT TTTCACCCAG AGTGCACCCC   
  
  
+ CGGTAAAGAA AAATATTAGA CTTTGACAAC TGGTCGAAAA GAATAAAGAA GGAATGGGGA GCAAAGGCCA   
  
  
+ TAGACAGAAG ACTTGGCAGG CTAGATAAGA GTAGTTAGGT TGCCAGCTTA GCGTGGCTCT TTCAACTGGT   
  
  
+ TCTGAAAATA TGAAACCTCT TGGGTCTTTT TCGATGTTCG GAACCCAAAA AGTGGTGCAC CGTATTCCGA   
  
  
+ CCACGGTTAA GTTTTGCTTT GTGTTCGTAT CTCTCTCTTT GTCTGTCTGG AATTTTTCAA ATAATTTCTA   
  
  
+ TTTTGCTTCA GCAAAATCGG ACGATTAAAT ACATCAGGAC ATTAAACAGT TGCCGAGTCT ACTAATTACA   
  
  
+ CCCCAACTGG GTTGGCCCAC TCTTCTGAGG TGACTAGTTA AAAGATCTTA AGACTATCGA CCTACGAACT   
  
  
+ TGTTCAGAAT TCACCATTCG TTTAATTATT TATTAATAAT TCTATTGTTT CGAATGTCAT GGAAGTGGTT   
  
  
+ GTAAATTGCG TAATTAATTG TCGAAAAACT GGTTTTTTGG TGGCATAAAA GAGTACGTAA GGACTACCCA   
  
  
+ TTTGCTATTC CGAGTACATA AGAATTAAAC CCATATTAAG CTTTTAAATC GACGATTAAC TCCTATTTTG   
  
  
+ ACATTAAATT CACGCCTATA TATTACAATA TTTATGTATT TCCGAACACC GTGCTGATCT AATGGACGAA   
  
  
+ TCAACTACGG AAGGAAGACC GTTAATTGG  

- CTGCAGCTTC TCTAAAGAGA AAGTTAAACC TCTTCGTGAG AGATGAAAGG GGGATAGAGA GAGAAAGAGA   
  
  
- GATTCGGAGC TCAGCAAGAC ATTGAACTGA AACTCAGAGA ACTGCTAAAA TGGTTCTTTG GGAGGGGGAG   
  
  
- AGAAGCCAAG GGAAGATGGG GGATGATATA AGGAGGGTGG ATCACCGGGT GACCGGGTAA GACATAGGGG   
  
  
- TTTAGTCAAA GTGGGTTTTT GATCCAACGG CTGTGATGAG TTTGAAAATT TCCATGGGAT GATGTGAATA   
  
  
- AAGAAATCAG AGGCTGGTCT CCTCAAGAAG TAAGTATTGG GACCCACCCT TGGAAAAGAT AGAAATAGAT   
  
  
- ATAAAAGTAG GGACTCGTTT CTTTTTCTTT TTCCTCGGTA AAAATAGTTA CACCTCTTAT TCTGTTTGAA   
  
  
- TATTTCCTGG AAACTAGAGA CTACTAGCAT TTAGTCTTTA AAGTTAAGTT TGATTATGTG CCACAACCGT   
  
  
- TGACGGTCAG AATTGGATCC TATGAGTACA CGTAGTATTT TCATGATTGC TACTTCTAGT AACATTTAAC   
  
  
- GGAGTTATTT GACCGTTAAT GTGAAAAATA AAGGAAAAAG GAAGAGCAAT GGTAATAATT GCTTAATTAG   
  
  
- GCAGTAAAAT TTCTTAAAAC TTACCTAACG CGTGGTTACT CATGAGTTGC TGTTGTTCAC CCAAAAAAAG   
  
  
- GCAACACGTA CAGTACTTGG AACTAGGCGA GAAGGAAAGA AAGCTGACCA AAAGTGGGTC TCACGTGGGG   
  
  
- GCCATTTCTT TTTATAATCT GAAACTGTTG ACCAGCTTTT CTTATTTCTT CCTTACCCCT CGTTTCCGGT   
  
  
- ATCTGTCTTC TGAACCGTCC GATCTATTCT CATCAATCCA ACGGTCGAAT CGCACCGAGA AAGTTGACCA   
  
  
- AGACTTTTAT ACTTTGGAGA ACCCAGAAAA AGCTACAAGC CTTGGGTTTT TCACCACGTG GCATAAGGCT   
  
  
- GGTGCCAATT CAAAACGAAA CACAAGCATA GAGAGAGAAA CAGACAGACC TTAAAAAGTT TATTAAAGAT   
  
  
- AAAACGAAGT CGTTTTAGCC TGCTAATTTA TGTAGTCCTG TAATTTGTCA ACGGCTCAGA TGATTAATGT   
  
  
- GGGGTTGACC CAACCGGGTG AGAAGACTCC ACTGATCAAT TTTCTAGAAT TCTGATAGCT GGATGCTTGA   
  
  
- ACAAGTCTTA AGTGGTAAGC AAATTAATAA ATAATTATTA AGATAACAAA GCTTACAGTA CCTTCACCAA   
  
  
- CATTTAACGC ATTAATTAAC AGCTTTTTGA CCAAAAAACC ACCGTATTTT CTCATGCATT CCTGATGGGT   
  
  
- AAACGATAAG GCTCATGTAT TCTTAATTTG GGTATAATTC GAAAATTTAG CTGCTAATTG AGGATAAAAC   
  
  
- TGTAATTTAA GTGCGGATAT ATAATGTTAT AAATACATAA AGGCTTGTGG CACGACTAGA TTACCTGCTT   
  
  
- AGTTGATGCC TTCCTTCTGG CAATTAACC

+     Unnamed\_\_2

| Site Name | Organism | Position | Strand | Matrix score. | sequence | function |
| --- | --- | --- | --- | --- | --- | --- |
| Unnamed\_\_2 | Zea mays | 768 | + | 6 | CCCCGG |  |

> 2018/04/13 10:10:12  
+ GACGTCGAAG AGATTTCTCT TTCAATTTGG AGAAGCACTC TCTACTTTCC CCCTATCTCT CTCTTTCTCT   
  
  
+ CTAAGCCTCG AGTCGTTCTG TAACTTGACT TTGAGTCTCT TGACGATTTT ACCAAGAAAC CCTCCCCCTC   
  
  
+ TCTTCGGTTC CCTTCTACCC CCTACTATAT TCCTCCCACC TAGTGGCCCA CTGGCCCATT CTGTATCCCC   
  
  
+ AAATCAGTTT CACCCAAAAA CTAGGTTGCC GACACTACTC AAACTTTTAA AGGTACCCTA CTACACTTAT   
  
  
+ TTCTTTAGTC TCCGACCAGA GGAGTTCTTC ATTCATAACC CTGGGTGGGA ACCTTTTCTA TCTTTATCTA   
  
  
+ TATTTTCATC CCTGAGCAAA GAAAAAGAAA AAGGAGCCAT TTTTATCAAT GTGGAGAATA AGACAAACTT   
  
  
+ ATAAAGGACC TTTGATCTCT GATGATCGTA AATCAGAAAT TTCAATTCAA ACTAATACAC GGTGTTGGCA   
  
  
+ ACTGCCAGTC TTAACCTAGG ATACTCATGT GCATCATAAA AGTACTAACG ATGAAGATCA TTGTAAATTG   
  
  
+ CCTCAATAAA CTGGCAATTA CACTTTTTAT TTCCTTTTTC CTTCTCGTTA CCATTATTAA CGAATTAATC   
  
  
+ CGTCATTTTA AAGAATTTTG AATGGATTGC GCACCAATGA GTACTCAACG ACAACAAGTG GGTTTTTTTC   
  
  
+ CGTTGTGCAT GTCATGAACC TTGATCCGCT CTTCCTTTCT TTCGACTGGT TTTCACCCAG AGTGCACCCC   
  
  
+ CGGTAAAGAA AAATATTAGA CTTTGACAAC TGGTCGAAAA GAATAAAGAA GGAATGGGGA GCAAAGGCCA   
  
  
+ TAGACAGAAG ACTTGGCAGG CTAGATAAGA GTAGTTAGGT TGCCAGCTTA GCGTGGCTCT TTCAACTGGT   
  
  
+ TCTGAAAATA TGAAACCTCT TGGGTCTTTT TCGATGTTCG GAACCCAAAA AGTGGTGCAC CGTATTCCGA   
  
  
+ CCACGGTTAA GTTTTGCTTT GTGTTCGTAT CTCTCTCTTT GTCTGTCTGG AATTTTTCAA ATAATTTCTA   
  
  
+ TTTTGCTTCA GCAAAATCGG ACGATTAAAT ACATCAGGAC ATTAAACAGT TGCCGAGTCT ACTAATTACA   
  
  
+ CCCCAACTGG GTTGGCCCAC TCTTCTGAGG TGACTAGTTA AAAGATCTTA AGACTATCGA CCTACGAACT   
  
  
+ TGTTCAGAAT TCACCATTCG TTTAATTATT TATTAATAAT TCTATTGTTT CGAATGTCAT GGAAGTGGTT   
  
  
+ GTAAATTGCG TAATTAATTG TCGAAAAACT GGTTTTTTGG TGGCATAAAA GAGTACGTAA GGACTACCCA   
  
  
+ TTTGCTATTC CGAGTACATA AGAATTAAAC CCATATTAAG CTTTTAAATC GACGATTAAC TCCTATTTTG   
  
  
+ ACATTAAATT CACGCCTATA TATTACAATA TTTATGTATT TCCGAACACC GTGCTGATCT AATGGACGAA   
  
  
+ TCAACTACGG AAGGAAGACC GTTAATTGG  

- CTGCAGCTTC TCTAAAGAGA AAGTTAAACC TCTTCGTGAG AGATGAAAGG GGGATAGAGA GAGAAAGAGA   
  
  
- GATTCGGAGC TCAGCAAGAC ATTGAACTGA AACTCAGAGA ACTGCTAAAA TGGTTCTTTG GGAGGGGGAG   
  
  
- AGAAGCCAAG GGAAGATGGG GGATGATATA AGGAGGGTGG ATCACCGGGT GACCGGGTAA GACATAGGGG   
  
  
- TTTAGTCAAA GTGGGTTTTT GATCCAACGG CTGTGATGAG TTTGAAAATT TCCATGGGAT GATGTGAATA   
  
  
- AAGAAATCAG AGGCTGGTCT CCTCAAGAAG TAAGTATTGG GACCCACCCT TGGAAAAGAT AGAAATAGAT   
  
  
- ATAAAAGTAG GGACTCGTTT CTTTTTCTTT TTCCTCGGTA AAAATAGTTA CACCTCTTAT TCTGTTTGAA   
  
  
- TATTTCCTGG AAACTAGAGA CTACTAGCAT TTAGTCTTTA AAGTTAAGTT TGATTATGTG CCACAACCGT   
  
  
- TGACGGTCAG AATTGGATCC TATGAGTACA CGTAGTATTT TCATGATTGC TACTTCTAGT AACATTTAAC   
  
  
- GGAGTTATTT GACCGTTAAT GTGAAAAATA AAGGAAAAAG GAAGAGCAAT GGTAATAATT GCTTAATTAG   
  
  
- GCAGTAAAAT TTCTTAAAAC TTACCTAACG CGTGGTTACT CATGAGTTGC TGTTGTTCAC CCAAAAAAAG   
  
  
- GCAACACGTA CAGTACTTGG AACTAGGCGA GAAGGAAAGA AAGCTGACCA AAAGTGGGTC TCACGTGGGG   
  
  
- GCCATTTCTT TTTATAATCT GAAACTGTTG ACCAGCTTTT CTTATTTCTT CCTTACCCCT CGTTTCCGGT   
  
  
- ATCTGTCTTC TGAACCGTCC GATCTATTCT CATCAATCCA ACGGTCGAAT CGCACCGAGA AAGTTGACCA   
  
  
- AGACTTTTAT ACTTTGGAGA ACCCAGAAAA AGCTACAAGC CTTGGGTTTT TCACCACGTG GCATAAGGCT   
  
  
- GGTGCCAATT CAAAACGAAA CACAAGCATA GAGAGAGAAA CAGACAGACC TTAAAAAGTT TATTAAAGAT   
  
  
- AAAACGAAGT CGTTTTAGCC TGCTAATTTA TGTAGTCCTG TAATTTGTCA ACGGCTCAGA TGATTAATGT   
  
  
- GGGGTTGACC CAACCGGGTG AGAAGACTCC ACTGATCAAT TTTCTAGAAT TCTGATAGCT GGATGCTTGA   
  
  
- ACAAGTCTTA AGTGGTAAGC AAATTAATAA ATAATTATTA AGATAACAAA GCTTACAGTA CCTTCACCAA   
  
  
- CATTTAACGC ATTAATTAAC AGCTTTTTGA CCAAAAAACC ACCGTATTTT CTCATGCATT CCTGATGGGT   
  
  
- AAACGATAAG GCTCATGTAT TCTTAATTTG GGTATAATTC GAAAATTTAG CTGCTAATTG AGGATAAAAC   
  
  
- TGTAATTTAA GTGCGGATAT ATAATGTTAT AAATACATAA AGGCTTGTGG CACGACTAGA TTACCTGCTT   
  
  
- AGTTGATGCC TTCCTTCTGG CAATTAACC

+     Unnamed\_\_3

| Site Name | Organism | Position | Strand | Matrix score. | sequence | function |
| --- | --- | --- | --- | --- | --- | --- |
| Unnamed\_\_3 | Zea mays | 981 | - | 5 | CGTGG |  |
| Unnamed\_\_3 | Zea mays | 892 | + | 5 | CGTGG |  |

> 2018/04/13 10:10:12  
+ GACGTCGAAG AGATTTCTCT TTCAATTTGG AGAAGCACTC TCTACTTTCC CCCTATCTCT CTCTTTCTCT   
  
  
+ CTAAGCCTCG AGTCGTTCTG TAACTTGACT TTGAGTCTCT TGACGATTTT ACCAAGAAAC CCTCCCCCTC   
  
  
+ TCTTCGGTTC CCTTCTACCC CCTACTATAT TCCTCCCACC TAGTGGCCCA CTGGCCCATT CTGTATCCCC   
  
  
+ AAATCAGTTT CACCCAAAAA CTAGGTTGCC GACACTACTC AAACTTTTAA AGGTACCCTA CTACACTTAT   
  
  
+ TTCTTTAGTC TCCGACCAGA GGAGTTCTTC ATTCATAACC CTGGGTGGGA ACCTTTTCTA TCTTTATCTA   
  
  
+ TATTTTCATC CCTGAGCAAA GAAAAAGAAA AAGGAGCCAT TTTTATCAAT GTGGAGAATA AGACAAACTT   
  
  
+ ATAAAGGACC TTTGATCTCT GATGATCGTA AATCAGAAAT TTCAATTCAA ACTAATACAC GGTGTTGGCA   
  
  
+ ACTGCCAGTC TTAACCTAGG ATACTCATGT GCATCATAAA AGTACTAACG ATGAAGATCA TTGTAAATTG   
  
  
+ CCTCAATAAA CTGGCAATTA CACTTTTTAT TTCCTTTTTC CTTCTCGTTA CCATTATTAA CGAATTAATC   
  
  
+ CGTCATTTTA AAGAATTTTG AATGGATTGC GCACCAATGA GTACTCAACG ACAACAAGTG GGTTTTTTTC   
  
  
+ CGTTGTGCAT GTCATGAACC TTGATCCGCT CTTCCTTTCT TTCGACTGGT TTTCACCCAG AGTGCACCCC   
  
  
+ CGGTAAAGAA AAATATTAGA CTTTGACAAC TGGTCGAAAA GAATAAAGAA GGAATGGGGA GCAAAGGCCA   
  
  
+ TAGACAGAAG ACTTGGCAGG CTAGATAAGA GTAGTTAGGT TGCCAGCTTA GCGTGGCTCT TTCAACTGGT   
  
  
+ TCTGAAAATA TGAAACCTCT TGGGTCTTTT TCGATGTTCG GAACCCAAAA AGTGGTGCAC CGTATTCCGA   
  
  
+ CCACGGTTAA GTTTTGCTTT GTGTTCGTAT CTCTCTCTTT GTCTGTCTGG AATTTTTCAA ATAATTTCTA   
  
  
+ TTTTGCTTCA GCAAAATCGG ACGATTAAAT ACATCAGGAC ATTAAACAGT TGCCGAGTCT ACTAATTACA   
  
  
+ CCCCAACTGG GTTGGCCCAC TCTTCTGAGG TGACTAGTTA AAAGATCTTA AGACTATCGA CCTACGAACT   
  
  
+ TGTTCAGAAT TCACCATTCG TTTAATTATT TATTAATAAT TCTATTGTTT CGAATGTCAT GGAAGTGGTT   
  
  
+ GTAAATTGCG TAATTAATTG TCGAAAAACT GGTTTTTTGG TGGCATAAAA GAGTACGTAA GGACTACCCA   
  
  
+ TTTGCTATTC CGAGTACATA AGAATTAAAC CCATATTAAG CTTTTAAATC GACGATTAAC TCCTATTTTG   
  
  
+ ACATTAAATT CACGCCTATA TATTACAATA TTTATGTATT TCCGAACACC GTGCTGATCT AATGGACGAA   
  
  
+ TCAACTACGG AAGGAAGACC GTTAATTGG  

- CTGCAGCTTC TCTAAAGAGA AAGTTAAACC TCTTCGTGAG AGATGAAAGG GGGATAGAGA GAGAAAGAGA   
  
  
- GATTCGGAGC TCAGCAAGAC ATTGAACTGA AACTCAGAGA ACTGCTAAAA TGGTTCTTTG GGAGGGGGAG   
  
  
- AGAAGCCAAG GGAAGATGGG GGATGATATA AGGAGGGTGG ATCACCGGGT GACCGGGTAA GACATAGGGG   
  
  
- TTTAGTCAAA GTGGGTTTTT GATCCAACGG CTGTGATGAG TTTGAAAATT TCCATGGGAT GATGTGAATA   
  
  
- AAGAAATCAG AGGCTGGTCT CCTCAAGAAG TAAGTATTGG GACCCACCCT TGGAAAAGAT AGAAATAGAT   
  
  
- ATAAAAGTAG GGACTCGTTT CTTTTTCTTT TTCCTCGGTA AAAATAGTTA CACCTCTTAT TCTGTTTGAA   
  
  
- TATTTCCTGG AAACTAGAGA CTACTAGCAT TTAGTCTTTA AAGTTAAGTT TGATTATGTG CCACAACCGT   
  
  
- TGACGGTCAG AATTGGATCC TATGAGTACA CGTAGTATTT TCATGATTGC TACTTCTAGT AACATTTAAC   
  
  
- GGAGTTATTT GACCGTTAAT GTGAAAAATA AAGGAAAAAG GAAGAGCAAT GGTAATAATT GCTTAATTAG   
  
  
- GCAGTAAAAT TTCTTAAAAC TTACCTAACG CGTGGTTACT CATGAGTTGC TGTTGTTCAC CCAAAAAAAG   
  
  
- GCAACACGTA CAGTACTTGG AACTAGGCGA GAAGGAAAGA AAGCTGACCA AAAGTGGGTC TCACGTGGGG   
  
  
- GCCATTTCTT TTTATAATCT GAAACTGTTG ACCAGCTTTT CTTATTTCTT CCTTACCCCT CGTTTCCGGT   
  
  
- ATCTGTCTTC TGAACCGTCC GATCTATTCT CATCAATCCA ACGGTCGAAT CGCACCGAGA AAGTTGACCA   
  
  
- AGACTTTTAT ACTTTGGAGA ACCCAGAAAA AGCTACAAGC CTTGGGTTTT TCACCACGTG GCATAAGGCT   
  
  
- GGTGCCAATT CAAAACGAAA CACAAGCATA GAGAGAGAAA CAGACAGACC TTAAAAAGTT TATTAAAGAT   
  
  
- AAAACGAAGT CGTTTTAGCC TGCTAATTTA TGTAGTCCTG TAATTTGTCA ACGGCTCAGA TGATTAATGT   
  
  
- GGGGTTGACC CAACCGGGTG AGAAGACTCC ACTGATCAAT TTTCTAGAAT TCTGATAGCT GGATGCTTGA   
  
  
- ACAAGTCTTA AGTGGTAAGC AAATTAATAA ATAATTATTA AGATAACAAA GCTTACAGTA CCTTCACCAA   
  
  
- CATTTAACGC ATTAATTAAC AGCTTTTTGA CCAAAAAACC ACCGTATTTT CTCATGCATT CCTGATGGGT   
  
  
- AAACGATAAG GCTCATGTAT TCTTAATTTG GGTATAATTC GAAAATTTAG CTGCTAATTG AGGATAAAAC   
  
  
- TGTAATTTAA GTGCGGATAT ATAATGTTAT AAATACATAA AGGCTTGTGG CACGACTAGA TTACCTGCTT   
  
  
- AGTTGATGCC TTCCTTCTGG CAATTAACC

+     Unnamed\_\_4

| Site Name | Organism | Position | Strand | Matrix score. | sequence | function |
| --- | --- | --- | --- | --- | --- | --- |
| Unnamed\_\_4 | Petroselinum hortense | 403 | - | 4 | CTCC |  |
| Unnamed\_\_4 | Petroselinum hortense | 301 | - | 4 | CTCC |  |
| Unnamed\_\_4 | Petroselinum hortense | 1390 | + | 4 | CTCC |  |
| Unnamed\_\_4 | Petroselinum hortense | 29 | - | 4 | CTCC |  |
| Unnamed\_\_4 | Petroselinum hortense | 383 | - | 4 | CTCC |  |
| Unnamed\_\_4 | Petroselinum hortense | 828 | - | 4 | CTCC |  |
| Unnamed\_\_4 | Petroselinum hortense | 290 | + | 4 | CTCC |  |
| Unnamed\_\_4 | Petroselinum hortense | 173 | + | 4 | CTCC |  |
| Unnamed\_\_4 | Petroselinum hortense | 132 | + | 4 | CTCC |  |

> 2018/04/13 10:10:12  
+ GACGTCGAAG AGATTTCTCT TTCAATTTGG AGAAGCACTC TCTACTTTCC CCCTATCTCT CTCTTTCTCT   
  
  
+ CTAAGCCTCG AGTCGTTCTG TAACTTGACT TTGAGTCTCT TGACGATTTT ACCAAGAAAC CCTCCCCCTC   
  
  
+ TCTTCGGTTC CCTTCTACCC CCTACTATAT TCCTCCCACC TAGTGGCCCA CTGGCCCATT CTGTATCCCC   
  
  
+ AAATCAGTTT CACCCAAAAA CTAGGTTGCC GACACTACTC AAACTTTTAA AGGTACCCTA CTACACTTAT   
  
  
+ TTCTTTAGTC TCCGACCAGA GGAGTTCTTC ATTCATAACC CTGGGTGGGA ACCTTTTCTA TCTTTATCTA   
  
  
+ TATTTTCATC CCTGAGCAAA GAAAAAGAAA AAGGAGCCAT TTTTATCAAT GTGGAGAATA AGACAAACTT   
  
  
+ ATAAAGGACC TTTGATCTCT GATGATCGTA AATCAGAAAT TTCAATTCAA ACTAATACAC GGTGTTGGCA   
  
  
+ ACTGCCAGTC TTAACCTAGG ATACTCATGT GCATCATAAA AGTACTAACG ATGAAGATCA TTGTAAATTG   
  
  
+ CCTCAATAAA CTGGCAATTA CACTTTTTAT TTCCTTTTTC CTTCTCGTTA CCATTATTAA CGAATTAATC   
  
  
+ CGTCATTTTA AAGAATTTTG AATGGATTGC GCACCAATGA GTACTCAACG ACAACAAGTG GGTTTTTTTC   
  
  
+ CGTTGTGCAT GTCATGAACC TTGATCCGCT CTTCCTTTCT TTCGACTGGT TTTCACCCAG AGTGCACCCC   
  
  
+ CGGTAAAGAA AAATATTAGA CTTTGACAAC TGGTCGAAAA GAATAAAGAA GGAATGGGGA GCAAAGGCCA   
  
  
+ TAGACAGAAG ACTTGGCAGG CTAGATAAGA GTAGTTAGGT TGCCAGCTTA GCGTGGCTCT TTCAACTGGT   
  
  
+ TCTGAAAATA TGAAACCTCT TGGGTCTTTT TCGATGTTCG GAACCCAAAA AGTGGTGCAC CGTATTCCGA   
  
  
+ CCACGGTTAA GTTTTGCTTT GTGTTCGTAT CTCTCTCTTT GTCTGTCTGG AATTTTTCAA ATAATTTCTA   
  
  
+ TTTTGCTTCA GCAAAATCGG ACGATTAAAT ACATCAGGAC ATTAAACAGT TGCCGAGTCT ACTAATTACA   
  
  
+ CCCCAACTGG GTTGGCCCAC TCTTCTGAGG TGACTAGTTA AAAGATCTTA AGACTATCGA CCTACGAACT   
  
  
+ TGTTCAGAAT TCACCATTCG TTTAATTATT TATTAATAAT TCTATTGTTT CGAATGTCAT GGAAGTGGTT   
  
  
+ GTAAATTGCG TAATTAATTG TCGAAAAACT GGTTTTTTGG TGGCATAAAA GAGTACGTAA GGACTACCCA   
  
  
+ TTTGCTATTC CGAGTACATA AGAATTAAAC CCATATTAAG CTTTTAAATC GACGATTAAC TCCTATTTTG   
  
  
+ ACATTAAATT CACGCCTATA TATTACAATA TTTATGTATT TCCGAACACC GTGCTGATCT AATGGACGAA   
  
  
+ TCAACTACGG AAGGAAGACC GTTAATTGG  

- CTGCAGCTTC TCTAAAGAGA AAGTTAAACC TCTTCGTGAG AGATGAAAGG GGGATAGAGA GAGAAAGAGA   
  
  
- GATTCGGAGC TCAGCAAGAC ATTGAACTGA AACTCAGAGA ACTGCTAAAA TGGTTCTTTG GGAGGGGGAG   
  
  
- AGAAGCCAAG GGAAGATGGG GGATGATATA AGGAGGGTGG ATCACCGGGT GACCGGGTAA GACATAGGGG   
  
  
- TTTAGTCAAA GTGGGTTTTT GATCCAACGG CTGTGATGAG TTTGAAAATT TCCATGGGAT GATGTGAATA   
  
  
- AAGAAATCAG AGGCTGGTCT CCTCAAGAAG TAAGTATTGG GACCCACCCT TGGAAAAGAT AGAAATAGAT   
  
  
- ATAAAAGTAG GGACTCGTTT CTTTTTCTTT TTCCTCGGTA AAAATAGTTA CACCTCTTAT TCTGTTTGAA   
  
  
- TATTTCCTGG AAACTAGAGA CTACTAGCAT TTAGTCTTTA AAGTTAAGTT TGATTATGTG CCACAACCGT   
  
  
- TGACGGTCAG AATTGGATCC TATGAGTACA CGTAGTATTT TCATGATTGC TACTTCTAGT AACATTTAAC   
  
  
- GGAGTTATTT GACCGTTAAT GTGAAAAATA AAGGAAAAAG GAAGAGCAAT GGTAATAATT GCTTAATTAG   
  
  
- GCAGTAAAAT TTCTTAAAAC TTACCTAACG CGTGGTTACT CATGAGTTGC TGTTGTTCAC CCAAAAAAAG   
  
  
- GCAACACGTA CAGTACTTGG AACTAGGCGA GAAGGAAAGA AAGCTGACCA AAAGTGGGTC TCACGTGGGG   
  
  
- GCCATTTCTT TTTATAATCT GAAACTGTTG ACCAGCTTTT CTTATTTCTT CCTTACCCCT CGTTTCCGGT   
  
  
- ATCTGTCTTC TGAACCGTCC GATCTATTCT CATCAATCCA ACGGTCGAAT CGCACCGAGA AAGTTGACCA   
  
  
- AGACTTTTAT ACTTTGGAGA ACCCAGAAAA AGCTACAAGC CTTGGGTTTT TCACCACGTG GCATAAGGCT   
  
  
- GGTGCCAATT CAAAACGAAA CACAAGCATA GAGAGAGAAA CAGACAGACC TTAAAAAGTT TATTAAAGAT   
  
  
- AAAACGAAGT CGTTTTAGCC TGCTAATTTA TGTAGTCCTG TAATTTGTCA ACGGCTCAGA TGATTAATGT   
  
  
- GGGGTTGACC CAACCGGGTG AGAAGACTCC ACTGATCAAT TTTCTAGAAT TCTGATAGCT GGATGCTTGA   
  
  
- ACAAGTCTTA AGTGGTAAGC AAATTAATAA ATAATTATTA AGATAACAAA GCTTACAGTA CCTTCACCAA   
  
  
- CATTTAACGC ATTAATTAAC AGCTTTTTGA CCAAAAAACC ACCGTATTTT CTCATGCATT CCTGATGGGT   
  
  
- AAACGATAAG GCTCATGTAT TCTTAATTTG GGTATAATTC GAAAATTTAG CTGCTAATTG AGGATAAAAC   
  
  
- TGTAATTTAA GTGCGGATAT ATAATGTTAT AAATACATAA AGGCTTGTGG CACGACTAGA TTACCTGCTT   
  
  
- AGTTGATGCC TTCCTTCTGG CAATTAACC

+     Unnamed\_\_6

| Site Name | Organism | Position | Strand | Matrix score. | sequence | function |
| --- | --- | --- | --- | --- | --- | --- |
| Unnamed\_\_6 | Glycine max cv. Provar | 387 | + | 11 | GCATTTTTATCA | SEF4 factor binding site |

> 2018/04/13 10:10:12  
+ GACGTCGAAG AGATTTCTCT TTCAATTTGG AGAAGCACTC TCTACTTTCC CCCTATCTCT CTCTTTCTCT   
  
  
+ CTAAGCCTCG AGTCGTTCTG TAACTTGACT TTGAGTCTCT TGACGATTTT ACCAAGAAAC CCTCCCCCTC   
  
  
+ TCTTCGGTTC CCTTCTACCC CCTACTATAT TCCTCCCACC TAGTGGCCCA CTGGCCCATT CTGTATCCCC   
  
  
+ AAATCAGTTT CACCCAAAAA CTAGGTTGCC GACACTACTC AAACTTTTAA AGGTACCCTA CTACACTTAT   
  
  
+ TTCTTTAGTC TCCGACCAGA GGAGTTCTTC ATTCATAACC CTGGGTGGGA ACCTTTTCTA TCTTTATCTA   
  
  
+ TATTTTCATC CCTGAGCAAA GAAAAAGAAA AAGGAGCCAT TTTTATCAAT GTGGAGAATA AGACAAACTT   
  
  
+ ATAAAGGACC TTTGATCTCT GATGATCGTA AATCAGAAAT TTCAATTCAA ACTAATACAC GGTGTTGGCA   
  
  
+ ACTGCCAGTC TTAACCTAGG ATACTCATGT GCATCATAAA AGTACTAACG ATGAAGATCA TTGTAAATTG   
  
  
+ CCTCAATAAA CTGGCAATTA CACTTTTTAT TTCCTTTTTC CTTCTCGTTA CCATTATTAA CGAATTAATC   
  
  
+ CGTCATTTTA AAGAATTTTG AATGGATTGC GCACCAATGA GTACTCAACG ACAACAAGTG GGTTTTTTTC   
  
  
+ CGTTGTGCAT GTCATGAACC TTGATCCGCT CTTCCTTTCT TTCGACTGGT TTTCACCCAG AGTGCACCCC   
  
  
+ CGGTAAAGAA AAATATTAGA CTTTGACAAC TGGTCGAAAA GAATAAAGAA GGAATGGGGA GCAAAGGCCA   
  
  
+ TAGACAGAAG ACTTGGCAGG CTAGATAAGA GTAGTTAGGT TGCCAGCTTA GCGTGGCTCT TTCAACTGGT   
  
  
+ TCTGAAAATA TGAAACCTCT TGGGTCTTTT TCGATGTTCG GAACCCAAAA AGTGGTGCAC CGTATTCCGA   
  
  
+ CCACGGTTAA GTTTTGCTTT GTGTTCGTAT CTCTCTCTTT GTCTGTCTGG AATTTTTCAA ATAATTTCTA   
  
  
+ TTTTGCTTCA GCAAAATCGG ACGATTAAAT ACATCAGGAC ATTAAACAGT TGCCGAGTCT ACTAATTACA   
  
  
+ CCCCAACTGG GTTGGCCCAC TCTTCTGAGG TGACTAGTTA AAAGATCTTA AGACTATCGA CCTACGAACT   
  
  
+ TGTTCAGAAT TCACCATTCG TTTAATTATT TATTAATAAT TCTATTGTTT CGAATGTCAT GGAAGTGGTT   
  
  
+ GTAAATTGCG TAATTAATTG TCGAAAAACT GGTTTTTTGG TGGCATAAAA GAGTACGTAA GGACTACCCA   
  
  
+ TTTGCTATTC CGAGTACATA AGAATTAAAC CCATATTAAG CTTTTAAATC GACGATTAAC TCCTATTTTG   
  
  
+ ACATTAAATT CACGCCTATA TATTACAATA TTTATGTATT TCCGAACACC GTGCTGATCT AATGGACGAA   
  
  
+ TCAACTACGG AAGGAAGACC GTTAATTGG  

- CTGCAGCTTC TCTAAAGAGA AAGTTAAACC TCTTCGTGAG AGATGAAAGG GGGATAGAGA GAGAAAGAGA   
  
  
- GATTCGGAGC TCAGCAAGAC ATTGAACTGA AACTCAGAGA ACTGCTAAAA TGGTTCTTTG GGAGGGGGAG   
  
  
- AGAAGCCAAG GGAAGATGGG GGATGATATA AGGAGGGTGG ATCACCGGGT GACCGGGTAA GACATAGGGG   
  
  
- TTTAGTCAAA GTGGGTTTTT GATCCAACGG CTGTGATGAG TTTGAAAATT TCCATGGGAT GATGTGAATA   
  
  
- AAGAAATCAG AGGCTGGTCT CCTCAAGAAG TAAGTATTGG GACCCACCCT TGGAAAAGAT AGAAATAGAT   
  
  
- ATAAAAGTAG GGACTCGTTT CTTTTTCTTT TTCCTCGGTA AAAATAGTTA CACCTCTTAT TCTGTTTGAA   
  
  
- TATTTCCTGG AAACTAGAGA CTACTAGCAT TTAGTCTTTA AAGTTAAGTT TGATTATGTG CCACAACCGT   
  
  
- TGACGGTCAG AATTGGATCC TATGAGTACA CGTAGTATTT TCATGATTGC TACTTCTAGT AACATTTAAC   
  
  
- GGAGTTATTT GACCGTTAAT GTGAAAAATA AAGGAAAAAG GAAGAGCAAT GGTAATAATT GCTTAATTAG   
  
  
- GCAGTAAAAT TTCTTAAAAC TTACCTAACG CGTGGTTACT CATGAGTTGC TGTTGTTCAC CCAAAAAAAG   
  
  
- GCAACACGTA CAGTACTTGG AACTAGGCGA GAAGGAAAGA AAGCTGACCA AAAGTGGGTC TCACGTGGGG   
  
  
- GCCATTTCTT TTTATAATCT GAAACTGTTG ACCAGCTTTT CTTATTTCTT CCTTACCCCT CGTTTCCGGT   
  
  
- ATCTGTCTTC TGAACCGTCC GATCTATTCT CATCAATCCA ACGGTCGAAT CGCACCGAGA AAGTTGACCA   
  
  
- AGACTTTTAT ACTTTGGAGA ACCCAGAAAA AGCTACAAGC CTTGGGTTTT TCACCACGTG GCATAAGGCT   
  
  
- GGTGCCAATT CAAAACGAAA CACAAGCATA GAGAGAGAAA CAGACAGACC TTAAAAAGTT TATTAAAGAT   
  
  
- AAAACGAAGT CGTTTTAGCC TGCTAATTTA TGTAGTCCTG TAATTTGTCA ACGGCTCAGA TGATTAATGT   
  
  
- GGGGTTGACC CAACCGGGTG AGAAGACTCC ACTGATCAAT TTTCTAGAAT TCTGATAGCT GGATGCTTGA   
  
  
- ACAAGTCTTA AGTGGTAAGC AAATTAATAA ATAATTATTA AGATAACAAA GCTTACAGTA CCTTCACCAA   
  
  
- CATTTAACGC ATTAATTAAC AGCTTTTTGA CCAAAAAACC ACCGTATTTT CTCATGCATT CCTGATGGGT   
  
  
- AAACGATAAG GCTCATGTAT TCTTAATTTG GGTATAATTC GAAAATTTAG CTGCTAATTG AGGATAAAAC   
  
  
- TGTAATTTAA GTGCGGATAT ATAATGTTAT AAATACATAA AGGCTTGTGG CACGACTAGA TTACCTGCTT   
  
  
- AGTTGATGCC TTCCTTCTGG CAATTAACC
