# Supplementary material for: Genome-wide analysis of the WRKY gene family in drumstick (Moringa oleifera Lam.)
Source: PeerJ. 2019 Jun 10;7:e7063. doi: 10.7717/peerj.7063 (PMC6563795; doi:10.7717/peerj.7063)
Supplement: Supplemental Information 1 [file peerj-07-7063-s003.gz › MoWRKY28_plantcare.html]

Content-Type: text/html; charset=ISO-8859-1


CallMat\_Firefox


Webmaster Firefox specific output  
To save the result:
click on the frame with the right mouse button and save the source code as a text file with extension .html  
REFERENCE:PlantCARE: a database of plant cis-acting regulatory elements and a portal to tools for in silico analysis of promoter sequences.  
Lescot, M., Déhais, P., Moreau, Y., De Moor, B., Rouzé ,P.,and Rombauts, S.  
Nucleic Acids Res., Database issue(2002), 30(1):325-327.   


---

> 2018/04/13 10:10:12  
+ CGAGTGGGTT TTGGGGTTTT TTCTTTTGCT TTTTTTTTTG GTTTAGGGAT AAAGTGTCTT GGGAATCTTG   
  
  
+ GGTTTACTCT TTCGTAGTAT TTTTATTGTT CTTCATCTTG TTAAGGGTTT TCTAGTCCGA AAACTGTGGA   
  
  
+ GACTTTTGGT CTAGAATTAG GAATTTACGT GAATTTTGGG GTCTACTTTT ATTTAGTTTG TTCAGTAGGT   
  
  
+ GTTACCACCG ATTACCTGTT AAAACATTTG GGGGAACGGA TTTATGACTT TTAGAATACT AGGTTCTGTT   
  
  
+ TTCTATGTTG TCTCTCTACT GTTTTTGAGT CCGATGCTTC CTCTTATTTG TTTTGGGTAA AGGTATTTGT   
  
  
+ CTATGTGGGA ATGTCCCAAA ACGCGTTTTA AGTTTTGAAG GATAAATGAA ACTTTCACTT TCGTCTGTCT   
  
  
+ TTGTCTTACT CGAGTACTCG TTCCTGATAG GTCTTATTTT AGTCTGTCAA AGAAGGCCGA TTAAGAGGTT   
  
  
+ TCTTAGTTTT CCCATCTTAC TTCTTTTTTT AGTTCTGGTT TTGCTGTTTG GGTACAATTT TTGAACTCGT   
  
  
+ CCCCTGAGTT TCTAATAGGG TAACAACCTT AACTTTCCAC ACTTTCTTGC TGAAACGAAA GGGAGGCCCT   
  
  
+ GAAATAATTT TAGTGGGTTT TCTTTTGTTT CAGGGAGAAA ATTTAAGAAA CCGTTTTCTG TTCGTTTAGT   
  
  
+ TTTAGTATTA TCTTAACTTA AGGTGAGTTC TCACTGTGTC GTCTCCTCTT CTTCTTATGT TTAAGAAATA   
  
  
+ AATTTTTTTT TTTTTCCTTC TCTCTCTCTC TCTCTCCTCC GCCACGAGCG GTACCGTCAC CACCTGTGTT   
  
  
+ TCATCCCTTC TTTTTTGGTT GCAGAGACAG TTTCTAGGAT ACCAGCCAGG GTGGTAGTCT GGATCAATAA   
  
  
+ GGAAAAAAAG TTAAAAAATA AAAAAATAAA AAACAATTTC ATTTAGATAA ATAATAGCAA AAATACTCTT   
  
  
+ TCACCGAATC ACAGTTTTTA GTTTCACAGA CGGTTCGTTT TTTCACCCGG GTGCTGAGGT GACACCTGGC   
  
  
+ CCTCGTAGGG CTCATACGTT GTATGTCATT ACTTGCCAGA TATAAGTTGG TGCACCGTTA TTAGGAACAC   
  
  
+ TATATCCCTG TGAACAGGGA TCTTACAACT GATTTTCGTC CCGAAACAAC AGAAAAACCC GAAAATTTTT   
  
  
+ TTATTTTAAG ACTCGCACCC TGGGTGACCA GTCCTGTGTC AACTGGACTG TCTCTAAGAG AGACGGGGAA   
  
  
+ GATAAATACG TCGACGAACC TCGGAAACAA AAACTCGGTT GTATTTATTA TTCAGTATCT ATAACCTAAA   
  
  
+ GAATTTTACA ATTTATGATA AATAGAAATT TTAATATTTT TTATCACTTT TTTAATTAAA ATAATAAATA   
  
  
+ TATAATTAAC AATTACCTAA CAGTTGACCT AACTTTAATC GGGTTTAGAT AAGGTAACCG AGTAAAAAAT   
  
  
+ ACTTATTAAT ATTTTACTTT AACTCGAAC  

- GCTCACCCAA AACCCCAAAA AAGAAAACGA AAAAAAAAAC CAAATCCCTA TTTCACAGAA CCCTTAGAAC   
  
  
- CCAAATGAGA AAGCATCATA AAAATAACAA GAAGTAGAAC AATTCCCAAA AGATCAGGCT TTTGACACCT   
  
  
- CTGAAAACCA GATCTTAATC CTTAAATGCA CTTAAAACCC CAGATGAAAA TAAATCAAAC AAGTCATCCA   
  
  
- CAATGGTGGC TAATGGACAA TTTTGTAAAC CCCCTTGCCT AAATACTGAA AATCTTATGA TCCAAGACAA   
  
  
- AAGATACAAC AGAGAGATGA CAAAAACTCA GGCTACGAAG GAGAATAAAC AAAACCCATT TCCATAAACA   
  
  
- GATACACCCT TACAGGGTTT TGCGCAAAAT TCAAAACTTC CTATTTACTT TGAAAGTGAA AGCAGACAGA   
  
  
- AACAGAATGA GCTCATGAGC AAGGACTATC CAGAATAAAA TCAGACAGTT TCTTCCGGCT AATTCTCCAA   
  
  
- AGAATCAAAA GGGTAGAATG AAGAAAAAAA TCAAGACCAA AACGACAAAC CCATGTTAAA AACTTGAGCA   
  
  
- GGGGACTCAA AGATTATCCC ATTGTTGGAA TTGAAAGGTG TGAAAGAACG ACTTTGCTTT CCCTCCGGGA   
  
  
- CTTTATTAAA ATCACCCAAA AGAAAACAAA GTCCCTCTTT TAAATTCTTT GGCAAAAGAC AAGCAAATCA   
  
  
- AAATCATAAT AGAATTGAAT TCCACTCAAG AGTGACACAG CAGAGGAGAA GAAGAATACA AATTCTTTAT   
  
  
- TTAAAAAAAA AAAAAGGAAG AGAGAGAGAG AGAGAGGAGG CGGTGCTCGC CATGGCAGTG GTGGACACAA   
  
  
- AGTAGGGAAG AAAAAACCAA CGTCTCTGTC AAAGATCCTA TGGTCGGTCC CACCATCAGA CCTAGTTATT   
  
  
- CCTTTTTTTC AATTTTTTAT TTTTTTATTT TTTGTTAAAG TAAATCTATT TATTATCGTT TTTATGAGAA   
  
  
- AGTGGCTTAG TGTCAAAAAT CAAAGTGTCT GCCAAGCAAA AAAGTGGGCC CACGACTCCA CTGTGGACCG   
  
  
- GGAGCATCCC GAGTATGCAA CATACAGTAA TGAACGGTCT ATATTCAACC ACGTGGCAAT AATCCTTGTG   
  
  
- ATATAGGGAC ACTTGTCCCT AGAATGTTGA CTAAAAGCAG GGCTTTGTTG TCTTTTTGGG CTTTTAAAAA   
  
  
- AATAAAATTC TGAGCGTGGG ACCCACTGGT CAGGACACAG TTGACCTGAC AGAGATTCTC TCTGCCCCTT   
  
  
- CTATTTATGC AGCTGCTTGG AGCCTTTGTT TTTGAGCCAA CATAAATAAT AAGTCATAGA TATTGGATTT   
  
  
- CTTAAAATGT TAAATACTAT TTATCTTTAA AATTATAAAA AATAGTGAAA AAATTAATTT TATTATTTAT   
  
  
- ATATTAATTG TTAATGGATT GTCAACTGGA TTGAAATTAG CCCAAATCTA TTCCATTGGC TCATTTTTTA   
  
  
- TGAATAATTA TAAAATGAAA TTGAGCTTG

  
  
Motifs Found  

+     5UTR Py-rich stretch

| Site Name | Organism | Position | Strand | Matrix score. | sequence | function |
| --- | --- | --- | --- | --- | --- | --- |
| 5UTR Py-rich stretch | Lycopersicon esculentum | 791 | + | 13 | TTTCTCTCTCTCTC | cis-acting element conferring high transcription levels |
| 5UTR Py-rich stretch | Lycopersicon esculentum | 789 | + | 13 | TTTCTCTCTCTCTC | cis-acting element conferring high transcription levels |
| 5UTR Py-rich stretch | Lycopersicon esculentum | 787 | + | 13 | TTTCTCTCTCTCTC | cis-acting element conferring high transcription levels |
| 5UTR Py-rich stretch | Lycopersicon esculentum | 793 | + | 13 | TTTCTCTCTCTCTC | cis-acting element conferring high transcription levels |
| 5UTR Py-rich stretch | Lycopersicon esculentum | 784 | + | 9 | TTTCTTCTCT | cis-acting element conferring high transcription levels |

> 2018/04/13 10:10:12  
+ CGAGTGGGTT TTGGGGTTTT TTCTTTTGCT TTTTTTTTTG GTTTAGGGAT AAAGTGTCTT GGGAATCTTG   
  
  
+ GGTTTACTCT TTCGTAGTAT TTTTATTGTT CTTCATCTTG TTAAGGGTTT TCTAGTCCGA AAACTGTGGA   
  
  
+ GACTTTTGGT CTAGAATTAG GAATTTACGT GAATTTTGGG GTCTACTTTT ATTTAGTTTG TTCAGTAGGT   
  
  
+ GTTACCACCG ATTACCTGTT AAAACATTTG GGGGAACGGA TTTATGACTT TTAGAATACT AGGTTCTGTT   
  
  
+ TTCTATGTTG TCTCTCTACT GTTTTTGAGT CCGATGCTTC CTCTTATTTG TTTTGGGTAA AGGTATTTGT   
  
  
+ CTATGTGGGA ATGTCCCAAA ACGCGTTTTA AGTTTTGAAG GATAAATGAA ACTTTCACTT TCGTCTGTCT   
  
  
+ TTGTCTTACT CGAGTACTCG TTCCTGATAG GTCTTATTTT AGTCTGTCAA AGAAGGCCGA TTAAGAGGTT   
  
  
+ TCTTAGTTTT CCCATCTTAC TTCTTTTTTT AGTTCTGGTT TTGCTGTTTG GGTACAATTT TTGAACTCGT   
  
  
+ CCCCTGAGTT TCTAATAGGG TAACAACCTT AACTTTCCAC ACTTTCTTGC TGAAACGAAA GGGAGGCCCT   
  
  
+ GAAATAATTT TAGTGGGTTT TCTTTTGTTT CAGGGAGAAA ATTTAAGAAA CCGTTTTCTG TTCGTTTAGT   
  
  
+ TTTAGTATTA TCTTAACTTA AGGTGAGTTC TCACTGTGTC GTCTCCTCTT CTTCTTATGT TTAAGAAATA   
  
  
+ AATTTTTTTT TTTTTCCTTC TCTCTCTCTC TCTCTCCTCC GCCACGAGCG GTACCGTCAC CACCTGTGTT   
  
  
+ TCATCCCTTC TTTTTTGGTT GCAGAGACAG TTTCTAGGAT ACCAGCCAGG GTGGTAGTCT GGATCAATAA   
  
  
+ GGAAAAAAAG TTAAAAAATA AAAAAATAAA AAACAATTTC ATTTAGATAA ATAATAGCAA AAATACTCTT   
  
  
+ TCACCGAATC ACAGTTTTTA GTTTCACAGA CGGTTCGTTT TTTCACCCGG GTGCTGAGGT GACACCTGGC   
  
  
+ CCTCGTAGGG CTCATACGTT GTATGTCATT ACTTGCCAGA TATAAGTTGG TGCACCGTTA TTAGGAACAC   
  
  
+ TATATCCCTG TGAACAGGGA TCTTACAACT GATTTTCGTC CCGAAACAAC AGAAAAACCC GAAAATTTTT   
  
  
+ TTATTTTAAG ACTCGCACCC TGGGTGACCA GTCCTGTGTC AACTGGACTG TCTCTAAGAG AGACGGGGAA   
  
  
+ GATAAATACG TCGACGAACC TCGGAAACAA AAACTCGGTT GTATTTATTA TTCAGTATCT ATAACCTAAA   
  
  
+ GAATTTTACA ATTTATGATA AATAGAAATT TTAATATTTT TTATCACTTT TTTAATTAAA ATAATAAATA   
  
  
+ TATAATTAAC AATTACCTAA CAGTTGACCT AACTTTAATC GGGTTTAGAT AAGGTAACCG AGTAAAAAAT   
  
  
+ ACTTATTAAT ATTTTACTTT AACTCGAAC  

- GCTCACCCAA AACCCCAAAA AAGAAAACGA AAAAAAAAAC CAAATCCCTA TTTCACAGAA CCCTTAGAAC   
  
  
- CCAAATGAGA AAGCATCATA AAAATAACAA GAAGTAGAAC AATTCCCAAA AGATCAGGCT TTTGACACCT   
  
  
- CTGAAAACCA GATCTTAATC CTTAAATGCA CTTAAAACCC CAGATGAAAA TAAATCAAAC AAGTCATCCA   
  
  
- CAATGGTGGC TAATGGACAA TTTTGTAAAC CCCCTTGCCT AAATACTGAA AATCTTATGA TCCAAGACAA   
  
  
- AAGATACAAC AGAGAGATGA CAAAAACTCA GGCTACGAAG GAGAATAAAC AAAACCCATT TCCATAAACA   
  
  
- GATACACCCT TACAGGGTTT TGCGCAAAAT TCAAAACTTC CTATTTACTT TGAAAGTGAA AGCAGACAGA   
  
  
- AACAGAATGA GCTCATGAGC AAGGACTATC CAGAATAAAA TCAGACAGTT TCTTCCGGCT AATTCTCCAA   
  
  
- AGAATCAAAA GGGTAGAATG AAGAAAAAAA TCAAGACCAA AACGACAAAC CCATGTTAAA AACTTGAGCA   
  
  
- GGGGACTCAA AGATTATCCC ATTGTTGGAA TTGAAAGGTG TGAAAGAACG ACTTTGCTTT CCCTCCGGGA   
  
  
- CTTTATTAAA ATCACCCAAA AGAAAACAAA GTCCCTCTTT TAAATTCTTT GGCAAAAGAC AAGCAAATCA   
  
  
- AAATCATAAT AGAATTGAAT TCCACTCAAG AGTGACACAG CAGAGGAGAA GAAGAATACA AATTCTTTAT   
  
  
- TTAAAAAAAA AAAAAGGAAG AGAGAGAGAG AGAGAGGAGG CGGTGCTCGC CATGGCAGTG GTGGACACAA   
  
  
- AGTAGGGAAG AAAAAACCAA CGTCTCTGTC AAAGATCCTA TGGTCGGTCC CACCATCAGA CCTAGTTATT   
  
  
- CCTTTTTTTC AATTTTTTAT TTTTTTATTT TTTGTTAAAG TAAATCTATT TATTATCGTT TTTATGAGAA   
  
  
- AGTGGCTTAG TGTCAAAAAT CAAAGTGTCT GCCAAGCAAA AAAGTGGGCC CACGACTCCA CTGTGGACCG   
  
  
- GGAGCATCCC GAGTATGCAA CATACAGTAA TGAACGGTCT ATATTCAACC ACGTGGCAAT AATCCTTGTG   
  
  
- ATATAGGGAC ACTTGTCCCT AGAATGTTGA CTAAAAGCAG GGCTTTGTTG TCTTTTTGGG CTTTTAAAAA   
  
  
- AATAAAATTC TGAGCGTGGG ACCCACTGGT CAGGACACAG TTGACCTGAC AGAGATTCTC TCTGCCCCTT   
  
  
- CTATTTATGC AGCTGCTTGG AGCCTTTGTT TTTGAGCCAA CATAAATAAT AAGTCATAGA TATTGGATTT   
  
  
- CTTAAAATGT TAAATACTAT TTATCTTTAA AATTATAAAA AATAGTGAAA AAATTAATTT TATTATTTAT   
  
  
- ATATTAATTG TTAATGGATT GTCAACTGGA TTGAAATTAG CCCAAATCTA TTCCATTGGC TCATTTTTTA   
  
  
- TGAATAATTA TAAAATGAAA TTGAGCTTG

+     ABRE

| Site Name | Organism | Position | Strand | Matrix score. | sequence | function |
| --- | --- | --- | --- | --- | --- | --- |
| ABRE | Triticum aestivum | 1041 | + | 9 | GACACGTGGC | cis-acting element involved in the abscisic acid responsiveness |
| ABRE | Arabidopsis thaliana | 166 | + | 6 | TACGTG | cis-acting element involved in the abscisic acid responsiveness |

> 2018/04/13 10:10:12  
+ CGAGTGGGTT TTGGGGTTTT TTCTTTTGCT TTTTTTTTTG GTTTAGGGAT AAAGTGTCTT GGGAATCTTG   
  
  
+ GGTTTACTCT TTCGTAGTAT TTTTATTGTT CTTCATCTTG TTAAGGGTTT TCTAGTCCGA AAACTGTGGA   
  
  
+ GACTTTTGGT CTAGAATTAG GAATTTACGT GAATTTTGGG GTCTACTTTT ATTTAGTTTG TTCAGTAGGT   
  
  
+ GTTACCACCG ATTACCTGTT AAAACATTTG GGGGAACGGA TTTATGACTT TTAGAATACT AGGTTCTGTT   
  
  
+ TTCTATGTTG TCTCTCTACT GTTTTTGAGT CCGATGCTTC CTCTTATTTG TTTTGGGTAA AGGTATTTGT   
  
  
+ CTATGTGGGA ATGTCCCAAA ACGCGTTTTA AGTTTTGAAG GATAAATGAA ACTTTCACTT TCGTCTGTCT   
  
  
+ TTGTCTTACT CGAGTACTCG TTCCTGATAG GTCTTATTTT AGTCTGTCAA AGAAGGCCGA TTAAGAGGTT   
  
  
+ TCTTAGTTTT CCCATCTTAC TTCTTTTTTT AGTTCTGGTT TTGCTGTTTG GGTACAATTT TTGAACTCGT   
  
  
+ CCCCTGAGTT TCTAATAGGG TAACAACCTT AACTTTCCAC ACTTTCTTGC TGAAACGAAA GGGAGGCCCT   
  
  
+ GAAATAATTT TAGTGGGTTT TCTTTTGTTT CAGGGAGAAA ATTTAAGAAA CCGTTTTCTG TTCGTTTAGT   
  
  
+ TTTAGTATTA TCTTAACTTA AGGTGAGTTC TCACTGTGTC GTCTCCTCTT CTTCTTATGT TTAAGAAATA   
  
  
+ AATTTTTTTT TTTTTCCTTC TCTCTCTCTC TCTCTCCTCC GCCACGAGCG GTACCGTCAC CACCTGTGTT   
  
  
+ TCATCCCTTC TTTTTTGGTT GCAGAGACAG TTTCTAGGAT ACCAGCCAGG GTGGTAGTCT GGATCAATAA   
  
  
+ GGAAAAAAAG TTAAAAAATA AAAAAATAAA AAACAATTTC ATTTAGATAA ATAATAGCAA AAATACTCTT   
  
  
+ TCACCGAATC ACAGTTTTTA GTTTCACAGA CGGTTCGTTT TTTCACCCGG GTGCTGAGGT GACACCTGGC   
  
  
+ CCTCGTAGGG CTCATACGTT GTATGTCATT ACTTGCCAGA TATAAGTTGG TGCACCGTTA TTAGGAACAC   
  
  
+ TATATCCCTG TGAACAGGGA TCTTACAACT GATTTTCGTC CCGAAACAAC AGAAAAACCC GAAAATTTTT   
  
  
+ TTATTTTAAG ACTCGCACCC TGGGTGACCA GTCCTGTGTC AACTGGACTG TCTCTAAGAG AGACGGGGAA   
  
  
+ GATAAATACG TCGACGAACC TCGGAAACAA AAACTCGGTT GTATTTATTA TTCAGTATCT ATAACCTAAA   
  
  
+ GAATTTTACA ATTTATGATA AATAGAAATT TTAATATTTT TTATCACTTT TTTAATTAAA ATAATAAATA   
  
  
+ TATAATTAAC AATTACCTAA CAGTTGACCT AACTTTAATC GGGTTTAGAT AAGGTAACCG AGTAAAAAAT   
  
  
+ ACTTATTAAT ATTTTACTTT AACTCGAAC  

- GCTCACCCAA AACCCCAAAA AAGAAAACGA AAAAAAAAAC CAAATCCCTA TTTCACAGAA CCCTTAGAAC   
  
  
- CCAAATGAGA AAGCATCATA AAAATAACAA GAAGTAGAAC AATTCCCAAA AGATCAGGCT TTTGACACCT   
  
  
- CTGAAAACCA GATCTTAATC CTTAAATGCA CTTAAAACCC CAGATGAAAA TAAATCAAAC AAGTCATCCA   
  
  
- CAATGGTGGC TAATGGACAA TTTTGTAAAC CCCCTTGCCT AAATACTGAA AATCTTATGA TCCAAGACAA   
  
  
- AAGATACAAC AGAGAGATGA CAAAAACTCA GGCTACGAAG GAGAATAAAC AAAACCCATT TCCATAAACA   
  
  
- GATACACCCT TACAGGGTTT TGCGCAAAAT TCAAAACTTC CTATTTACTT TGAAAGTGAA AGCAGACAGA   
  
  
- AACAGAATGA GCTCATGAGC AAGGACTATC CAGAATAAAA TCAGACAGTT TCTTCCGGCT AATTCTCCAA   
  
  
- AGAATCAAAA GGGTAGAATG AAGAAAAAAA TCAAGACCAA AACGACAAAC CCATGTTAAA AACTTGAGCA   
  
  
- GGGGACTCAA AGATTATCCC ATTGTTGGAA TTGAAAGGTG TGAAAGAACG ACTTTGCTTT CCCTCCGGGA   
  
  
- CTTTATTAAA ATCACCCAAA AGAAAACAAA GTCCCTCTTT TAAATTCTTT GGCAAAAGAC AAGCAAATCA   
  
  
- AAATCATAAT AGAATTGAAT TCCACTCAAG AGTGACACAG CAGAGGAGAA GAAGAATACA AATTCTTTAT   
  
  
- TTAAAAAAAA AAAAAGGAAG AGAGAGAGAG AGAGAGGAGG CGGTGCTCGC CATGGCAGTG GTGGACACAA   
  
  
- AGTAGGGAAG AAAAAACCAA CGTCTCTGTC AAAGATCCTA TGGTCGGTCC CACCATCAGA CCTAGTTATT   
  
  
- CCTTTTTTTC AATTTTTTAT TTTTTTATTT TTTGTTAAAG TAAATCTATT TATTATCGTT TTTATGAGAA   
  
  
- AGTGGCTTAG TGTCAAAAAT CAAAGTGTCT GCCAAGCAAA AAAGTGGGCC CACGACTCCA CTGTGGACCG   
  
  
- GGAGCATCCC GAGTATGCAA CATACAGTAA TGAACGGTCT ATATTCAACC ACGTGGCAAT AATCCTTGTG   
  
  
- ATATAGGGAC ACTTGTCCCT AGAATGTTGA CTAAAAGCAG GGCTTTGTTG TCTTTTTGGG CTTTTAAAAA   
  
  
- AATAAAATTC TGAGCGTGGG ACCCACTGGT CAGGACACAG TTGACCTGAC AGAGATTCTC TCTGCCCCTT   
  
  
- CTATTTATGC AGCTGCTTGG AGCCTTTGTT TTTGAGCCAA CATAAATAAT AAGTCATAGA TATTGGATTT   
  
  
- CTTAAAATGT TAAATACTAT TTATCTTTAA AATTATAAAA AATAGTGAAA AAATTAATTT TATTATTTAT   
  
  
- ATATTAATTG TTAATGGATT GTCAACTGGA TTGAAATTAG CCCAAATCTA TTCCATTGGC TCATTTTTTA   
  
  
- TGAATAATTA TAAAATGAAA TTGAGCTTG

+     ARE

| Site Name | Organism | Position | Strand | Matrix score. | sequence | function |
| --- | --- | --- | --- | --- | --- | --- |
| ARE | Zea mays | 39 | + | 6 | TGGTTT | cis-acting regulatory element essential for the anaerobic induction |
| ARE | Zea mays | 526 | + | 6 | TGGTTT | cis-acting regulatory element essential for the anaerobic induction |

> 2018/04/13 10:10:12  
+ CGAGTGGGTT TTGGGGTTTT TTCTTTTGCT TTTTTTTTTG GTTTAGGGAT AAAGTGTCTT GGGAATCTTG   
  
  
+ GGTTTACTCT TTCGTAGTAT TTTTATTGTT CTTCATCTTG TTAAGGGTTT TCTAGTCCGA AAACTGTGGA   
  
  
+ GACTTTTGGT CTAGAATTAG GAATTTACGT GAATTTTGGG GTCTACTTTT ATTTAGTTTG TTCAGTAGGT   
  
  
+ GTTACCACCG ATTACCTGTT AAAACATTTG GGGGAACGGA TTTATGACTT TTAGAATACT AGGTTCTGTT   
  
  
+ TTCTATGTTG TCTCTCTACT GTTTTTGAGT CCGATGCTTC CTCTTATTTG TTTTGGGTAA AGGTATTTGT   
  
  
+ CTATGTGGGA ATGTCCCAAA ACGCGTTTTA AGTTTTGAAG GATAAATGAA ACTTTCACTT TCGTCTGTCT   
  
  
+ TTGTCTTACT CGAGTACTCG TTCCTGATAG GTCTTATTTT AGTCTGTCAA AGAAGGCCGA TTAAGAGGTT   
  
  
+ TCTTAGTTTT CCCATCTTAC TTCTTTTTTT AGTTCTGGTT TTGCTGTTTG GGTACAATTT TTGAACTCGT   
  
  
+ CCCCTGAGTT TCTAATAGGG TAACAACCTT AACTTTCCAC ACTTTCTTGC TGAAACGAAA GGGAGGCCCT   
  
  
+ GAAATAATTT TAGTGGGTTT TCTTTTGTTT CAGGGAGAAA ATTTAAGAAA CCGTTTTCTG TTCGTTTAGT   
  
  
+ TTTAGTATTA TCTTAACTTA AGGTGAGTTC TCACTGTGTC GTCTCCTCTT CTTCTTATGT TTAAGAAATA   
  
  
+ AATTTTTTTT TTTTTCCTTC TCTCTCTCTC TCTCTCCTCC GCCACGAGCG GTACCGTCAC CACCTGTGTT   
  
  
+ TCATCCCTTC TTTTTTGGTT GCAGAGACAG TTTCTAGGAT ACCAGCCAGG GTGGTAGTCT GGATCAATAA   
  
  
+ GGAAAAAAAG TTAAAAAATA AAAAAATAAA AAACAATTTC ATTTAGATAA ATAATAGCAA AAATACTCTT   
  
  
+ TCACCGAATC ACAGTTTTTA GTTTCACAGA CGGTTCGTTT TTTCACCCGG GTGCTGAGGT GACACCTGGC   
  
  
+ CCTCGTAGGG CTCATACGTT GTATGTCATT ACTTGCCAGA TATAAGTTGG TGCACCGTTA TTAGGAACAC   
  
  
+ TATATCCCTG TGAACAGGGA TCTTACAACT GATTTTCGTC CCGAAACAAC AGAAAAACCC GAAAATTTTT   
  
  
+ TTATTTTAAG ACTCGCACCC TGGGTGACCA GTCCTGTGTC AACTGGACTG TCTCTAAGAG AGACGGGGAA   
  
  
+ GATAAATACG TCGACGAACC TCGGAAACAA AAACTCGGTT GTATTTATTA TTCAGTATCT ATAACCTAAA   
  
  
+ GAATTTTACA ATTTATGATA AATAGAAATT TTAATATTTT TTATCACTTT TTTAATTAAA ATAATAAATA   
  
  
+ TATAATTAAC AATTACCTAA CAGTTGACCT AACTTTAATC GGGTTTAGAT AAGGTAACCG AGTAAAAAAT   
  
  
+ ACTTATTAAT ATTTTACTTT AACTCGAAC  

- GCTCACCCAA AACCCCAAAA AAGAAAACGA AAAAAAAAAC CAAATCCCTA TTTCACAGAA CCCTTAGAAC   
  
  
- CCAAATGAGA AAGCATCATA AAAATAACAA GAAGTAGAAC AATTCCCAAA AGATCAGGCT TTTGACACCT   
  
  
- CTGAAAACCA GATCTTAATC CTTAAATGCA CTTAAAACCC CAGATGAAAA TAAATCAAAC AAGTCATCCA   
  
  
- CAATGGTGGC TAATGGACAA TTTTGTAAAC CCCCTTGCCT AAATACTGAA AATCTTATGA TCCAAGACAA   
  
  
- AAGATACAAC AGAGAGATGA CAAAAACTCA GGCTACGAAG GAGAATAAAC AAAACCCATT TCCATAAACA   
  
  
- GATACACCCT TACAGGGTTT TGCGCAAAAT TCAAAACTTC CTATTTACTT TGAAAGTGAA AGCAGACAGA   
  
  
- AACAGAATGA GCTCATGAGC AAGGACTATC CAGAATAAAA TCAGACAGTT TCTTCCGGCT AATTCTCCAA   
  
  
- AGAATCAAAA GGGTAGAATG AAGAAAAAAA TCAAGACCAA AACGACAAAC CCATGTTAAA AACTTGAGCA   
  
  
- GGGGACTCAA AGATTATCCC ATTGTTGGAA TTGAAAGGTG TGAAAGAACG ACTTTGCTTT CCCTCCGGGA   
  
  
- CTTTATTAAA ATCACCCAAA AGAAAACAAA GTCCCTCTTT TAAATTCTTT GGCAAAAGAC AAGCAAATCA   
  
  
- AAATCATAAT AGAATTGAAT TCCACTCAAG AGTGACACAG CAGAGGAGAA GAAGAATACA AATTCTTTAT   
  
  
- TTAAAAAAAA AAAAAGGAAG AGAGAGAGAG AGAGAGGAGG CGGTGCTCGC CATGGCAGTG GTGGACACAA   
  
  
- AGTAGGGAAG AAAAAACCAA CGTCTCTGTC AAAGATCCTA TGGTCGGTCC CACCATCAGA CCTAGTTATT   
  
  
- CCTTTTTTTC AATTTTTTAT TTTTTTATTT TTTGTTAAAG TAAATCTATT TATTATCGTT TTTATGAGAA   
  
  
- AGTGGCTTAG TGTCAAAAAT CAAAGTGTCT GCCAAGCAAA AAAGTGGGCC CACGACTCCA CTGTGGACCG   
  
  
- GGAGCATCCC GAGTATGCAA CATACAGTAA TGAACGGTCT ATATTCAACC ACGTGGCAAT AATCCTTGTG   
  
  
- ATATAGGGAC ACTTGTCCCT AGAATGTTGA CTAAAAGCAG GGCTTTGTTG TCTTTTTGGG CTTTTAAAAA   
  
  
- AATAAAATTC TGAGCGTGGG ACCCACTGGT CAGGACACAG TTGACCTGAC AGAGATTCTC TCTGCCCCTT   
  
  
- CTATTTATGC AGCTGCTTGG AGCCTTTGTT TTTGAGCCAA CATAAATAAT AAGTCATAGA TATTGGATTT   
  
  
- CTTAAAATGT TAAATACTAT TTATCTTTAA AATTATAAAA AATAGTGAAA AAATTAATTT TATTATTTAT   
  
  
- ATATTAATTG TTAATGGATT GTCAACTGGA TTGAAATTAG CCCAAATCTA TTCCATTGGC TCATTTTTTA   
  
  
- TGAATAATTA TAAAATGAAA TTGAGCTTG

+     AT1-motif

| Site Name | Organism | Position | Strand | Matrix score. | sequence | function |
| --- | --- | --- | --- | --- | --- | --- |
| AT1-motif | Solanum tuberosum | 935 | - | 13 | AATTATTTTTTATT | part of a light responsive module |

> 2018/04/13 10:10:12  
+ CGAGTGGGTT TTGGGGTTTT TTCTTTTGCT TTTTTTTTTG GTTTAGGGAT AAAGTGTCTT GGGAATCTTG   
  
  
+ GGTTTACTCT TTCGTAGTAT TTTTATTGTT CTTCATCTTG TTAAGGGTTT TCTAGTCCGA AAACTGTGGA   
  
  
+ GACTTTTGGT CTAGAATTAG GAATTTACGT GAATTTTGGG GTCTACTTTT ATTTAGTTTG TTCAGTAGGT   
  
  
+ GTTACCACCG ATTACCTGTT AAAACATTTG GGGGAACGGA TTTATGACTT TTAGAATACT AGGTTCTGTT   
  
  
+ TTCTATGTTG TCTCTCTACT GTTTTTGAGT CCGATGCTTC CTCTTATTTG TTTTGGGTAA AGGTATTTGT   
  
  
+ CTATGTGGGA ATGTCCCAAA ACGCGTTTTA AGTTTTGAAG GATAAATGAA ACTTTCACTT TCGTCTGTCT   
  
  
+ TTGTCTTACT CGAGTACTCG TTCCTGATAG GTCTTATTTT AGTCTGTCAA AGAAGGCCGA TTAAGAGGTT   
  
  
+ TCTTAGTTTT CCCATCTTAC TTCTTTTTTT AGTTCTGGTT TTGCTGTTTG GGTACAATTT TTGAACTCGT   
  
  
+ CCCCTGAGTT TCTAATAGGG TAACAACCTT AACTTTCCAC ACTTTCTTGC TGAAACGAAA GGGAGGCCCT   
  
  
+ GAAATAATTT TAGTGGGTTT TCTTTTGTTT CAGGGAGAAA ATTTAAGAAA CCGTTTTCTG TTCGTTTAGT   
  
  
+ TTTAGTATTA TCTTAACTTA AGGTGAGTTC TCACTGTGTC GTCTCCTCTT CTTCTTATGT TTAAGAAATA   
  
  
+ AATTTTTTTT TTTTTCCTTC TCTCTCTCTC TCTCTCCTCC GCCACGAGCG GTACCGTCAC CACCTGTGTT   
  
  
+ TCATCCCTTC TTTTTTGGTT GCAGAGACAG TTTCTAGGAT ACCAGCCAGG GTGGTAGTCT GGATCAATAA   
  
  
+ GGAAAAAAAG TTAAAAAATA AAAAAATAAA AAACAATTTC ATTTAGATAA ATAATAGCAA AAATACTCTT   
  
  
+ TCACCGAATC ACAGTTTTTA GTTTCACAGA CGGTTCGTTT TTTCACCCGG GTGCTGAGGT GACACCTGGC   
  
  
+ CCTCGTAGGG CTCATACGTT GTATGTCATT ACTTGCCAGA TATAAGTTGG TGCACCGTTA TTAGGAACAC   
  
  
+ TATATCCCTG TGAACAGGGA TCTTACAACT GATTTTCGTC CCGAAACAAC AGAAAAACCC GAAAATTTTT   
  
  
+ TTATTTTAAG ACTCGCACCC TGGGTGACCA GTCCTGTGTC AACTGGACTG TCTCTAAGAG AGACGGGGAA   
  
  
+ GATAAATACG TCGACGAACC TCGGAAACAA AAACTCGGTT GTATTTATTA TTCAGTATCT ATAACCTAAA   
  
  
+ GAATTTTACA ATTTATGATA AATAGAAATT TTAATATTTT TTATCACTTT TTTAATTAAA ATAATAAATA   
  
  
+ TATAATTAAC AATTACCTAA CAGTTGACCT AACTTTAATC GGGTTTAGAT AAGGTAACCG AGTAAAAAAT   
  
  
+ ACTTATTAAT ATTTTACTTT AACTCGAAC  

- GCTCACCCAA AACCCCAAAA AAGAAAACGA AAAAAAAAAC CAAATCCCTA TTTCACAGAA CCCTTAGAAC   
  
  
- CCAAATGAGA AAGCATCATA AAAATAACAA GAAGTAGAAC AATTCCCAAA AGATCAGGCT TTTGACACCT   
  
  
- CTGAAAACCA GATCTTAATC CTTAAATGCA CTTAAAACCC CAGATGAAAA TAAATCAAAC AAGTCATCCA   
  
  
- CAATGGTGGC TAATGGACAA TTTTGTAAAC CCCCTTGCCT AAATACTGAA AATCTTATGA TCCAAGACAA   
  
  
- AAGATACAAC AGAGAGATGA CAAAAACTCA GGCTACGAAG GAGAATAAAC AAAACCCATT TCCATAAACA   
  
  
- GATACACCCT TACAGGGTTT TGCGCAAAAT TCAAAACTTC CTATTTACTT TGAAAGTGAA AGCAGACAGA   
  
  
- AACAGAATGA GCTCATGAGC AAGGACTATC CAGAATAAAA TCAGACAGTT TCTTCCGGCT AATTCTCCAA   
  
  
- AGAATCAAAA GGGTAGAATG AAGAAAAAAA TCAAGACCAA AACGACAAAC CCATGTTAAA AACTTGAGCA   
  
  
- GGGGACTCAA AGATTATCCC ATTGTTGGAA TTGAAAGGTG TGAAAGAACG ACTTTGCTTT CCCTCCGGGA   
  
  
- CTTTATTAAA ATCACCCAAA AGAAAACAAA GTCCCTCTTT TAAATTCTTT GGCAAAAGAC AAGCAAATCA   
  
  
- AAATCATAAT AGAATTGAAT TCCACTCAAG AGTGACACAG CAGAGGAGAA GAAGAATACA AATTCTTTAT   
  
  
- TTAAAAAAAA AAAAAGGAAG AGAGAGAGAG AGAGAGGAGG CGGTGCTCGC CATGGCAGTG GTGGACACAA   
  
  
- AGTAGGGAAG AAAAAACCAA CGTCTCTGTC AAAGATCCTA TGGTCGGTCC CACCATCAGA CCTAGTTATT   
  
  
- CCTTTTTTTC AATTTTTTAT TTTTTTATTT TTTGTTAAAG TAAATCTATT TATTATCGTT TTTATGAGAA   
  
  
- AGTGGCTTAG TGTCAAAAAT CAAAGTGTCT GCCAAGCAAA AAAGTGGGCC CACGACTCCA CTGTGGACCG   
  
  
- GGAGCATCCC GAGTATGCAA CATACAGTAA TGAACGGTCT ATATTCAACC ACGTGGCAAT AATCCTTGTG   
  
  
- ATATAGGGAC ACTTGTCCCT AGAATGTTGA CTAAAAGCAG GGCTTTGTTG TCTTTTTGGG CTTTTAAAAA   
  
  
- AATAAAATTC TGAGCGTGGG ACCCACTGGT CAGGACACAG TTGACCTGAC AGAGATTCTC TCTGCCCCTT   
  
  
- CTATTTATGC AGCTGCTTGG AGCCTTTGTT TTTGAGCCAA CATAAATAAT AAGTCATAGA TATTGGATTT   
  
  
- CTTAAAATGT TAAATACTAT TTATCTTTAA AATTATAAAA AATAGTGAAA AAATTAATTT TATTATTTAT   
  
  
- ATATTAATTG TTAATGGATT GTCAACTGGA TTGAAATTAG CCCAAATCTA TTCCATTGGC TCATTTTTTA   
  
  
- TGAATAATTA TAAAATGAAA TTGAGCTTG

+     Box 4

| Site Name | Organism | Position | Strand | Matrix score. | sequence | function |
| --- | --- | --- | --- | --- | --- | --- |
| Box 4 | Petroselinum crispum | 1475 | - | 6 | ATTAAT | part of a conserved DNA module involved in light responsiveness |

> 2018/04/13 10:10:12  
+ CGAGTGGGTT TTGGGGTTTT TTCTTTTGCT TTTTTTTTTG GTTTAGGGAT AAAGTGTCTT GGGAATCTTG   
  
  
+ GGTTTACTCT TTCGTAGTAT TTTTATTGTT CTTCATCTTG TTAAGGGTTT TCTAGTCCGA AAACTGTGGA   
  
  
+ GACTTTTGGT CTAGAATTAG GAATTTACGT GAATTTTGGG GTCTACTTTT ATTTAGTTTG TTCAGTAGGT   
  
  
+ GTTACCACCG ATTACCTGTT AAAACATTTG GGGGAACGGA TTTATGACTT TTAGAATACT AGGTTCTGTT   
  
  
+ TTCTATGTTG TCTCTCTACT GTTTTTGAGT CCGATGCTTC CTCTTATTTG TTTTGGGTAA AGGTATTTGT   
  
  
+ CTATGTGGGA ATGTCCCAAA ACGCGTTTTA AGTTTTGAAG GATAAATGAA ACTTTCACTT TCGTCTGTCT   
  
  
+ TTGTCTTACT CGAGTACTCG TTCCTGATAG GTCTTATTTT AGTCTGTCAA AGAAGGCCGA TTAAGAGGTT   
  
  
+ TCTTAGTTTT CCCATCTTAC TTCTTTTTTT AGTTCTGGTT TTGCTGTTTG GGTACAATTT TTGAACTCGT   
  
  
+ CCCCTGAGTT TCTAATAGGG TAACAACCTT AACTTTCCAC ACTTTCTTGC TGAAACGAAA GGGAGGCCCT   
  
  
+ GAAATAATTT TAGTGGGTTT TCTTTTGTTT CAGGGAGAAA ATTTAAGAAA CCGTTTTCTG TTCGTTTAGT   
  
  
+ TTTAGTATTA TCTTAACTTA AGGTGAGTTC TCACTGTGTC GTCTCCTCTT CTTCTTATGT TTAAGAAATA   
  
  
+ AATTTTTTTT TTTTTCCTTC TCTCTCTCTC TCTCTCCTCC GCCACGAGCG GTACCGTCAC CACCTGTGTT   
  
  
+ TCATCCCTTC TTTTTTGGTT GCAGAGACAG TTTCTAGGAT ACCAGCCAGG GTGGTAGTCT GGATCAATAA   
  
  
+ GGAAAAAAAG TTAAAAAATA AAAAAATAAA AAACAATTTC ATTTAGATAA ATAATAGCAA AAATACTCTT   
  
  
+ TCACCGAATC ACAGTTTTTA GTTTCACAGA CGGTTCGTTT TTTCACCCGG GTGCTGAGGT GACACCTGGC   
  
  
+ CCTCGTAGGG CTCATACGTT GTATGTCATT ACTTGCCAGA TATAAGTTGG TGCACCGTTA TTAGGAACAC   
  
  
+ TATATCCCTG TGAACAGGGA TCTTACAACT GATTTTCGTC CCGAAACAAC AGAAAAACCC GAAAATTTTT   
  
  
+ TTATTTTAAG ACTCGCACCC TGGGTGACCA GTCCTGTGTC AACTGGACTG TCTCTAAGAG AGACGGGGAA   
  
  
+ GATAAATACG TCGACGAACC TCGGAAACAA AAACTCGGTT GTATTTATTA TTCAGTATCT ATAACCTAAA   
  
  
+ GAATTTTACA ATTTATGATA AATAGAAATT TTAATATTTT TTATCACTTT TTTAATTAAA ATAATAAATA   
  
  
+ TATAATTAAC AATTACCTAA CAGTTGACCT AACTTTAATC GGGTTTAGAT AAGGTAACCG AGTAAAAAAT   
  
  
+ ACTTATTAAT ATTTTACTTT AACTCGAAC  

- GCTCACCCAA AACCCCAAAA AAGAAAACGA AAAAAAAAAC CAAATCCCTA TTTCACAGAA CCCTTAGAAC   
  
  
- CCAAATGAGA AAGCATCATA AAAATAACAA GAAGTAGAAC AATTCCCAAA AGATCAGGCT TTTGACACCT   
  
  
- CTGAAAACCA GATCTTAATC CTTAAATGCA CTTAAAACCC CAGATGAAAA TAAATCAAAC AAGTCATCCA   
  
  
- CAATGGTGGC TAATGGACAA TTTTGTAAAC CCCCTTGCCT AAATACTGAA AATCTTATGA TCCAAGACAA   
  
  
- AAGATACAAC AGAGAGATGA CAAAAACTCA GGCTACGAAG GAGAATAAAC AAAACCCATT TCCATAAACA   
  
  
- GATACACCCT TACAGGGTTT TGCGCAAAAT TCAAAACTTC CTATTTACTT TGAAAGTGAA AGCAGACAGA   
  
  
- AACAGAATGA GCTCATGAGC AAGGACTATC CAGAATAAAA TCAGACAGTT TCTTCCGGCT AATTCTCCAA   
  
  
- AGAATCAAAA GGGTAGAATG AAGAAAAAAA TCAAGACCAA AACGACAAAC CCATGTTAAA AACTTGAGCA   
  
  
- GGGGACTCAA AGATTATCCC ATTGTTGGAA TTGAAAGGTG TGAAAGAACG ACTTTGCTTT CCCTCCGGGA   
  
  
- CTTTATTAAA ATCACCCAAA AGAAAACAAA GTCCCTCTTT TAAATTCTTT GGCAAAAGAC AAGCAAATCA   
  
  
- AAATCATAAT AGAATTGAAT TCCACTCAAG AGTGACACAG CAGAGGAGAA GAAGAATACA AATTCTTTAT   
  
  
- TTAAAAAAAA AAAAAGGAAG AGAGAGAGAG AGAGAGGAGG CGGTGCTCGC CATGGCAGTG GTGGACACAA   
  
  
- AGTAGGGAAG AAAAAACCAA CGTCTCTGTC AAAGATCCTA TGGTCGGTCC CACCATCAGA CCTAGTTATT   
  
  
- CCTTTTTTTC AATTTTTTAT TTTTTTATTT TTTGTTAAAG TAAATCTATT TATTATCGTT TTTATGAGAA   
  
  
- AGTGGCTTAG TGTCAAAAAT CAAAGTGTCT GCCAAGCAAA AAAGTGGGCC CACGACTCCA CTGTGGACCG   
  
  
- GGAGCATCCC GAGTATGCAA CATACAGTAA TGAACGGTCT ATATTCAACC ACGTGGCAAT AATCCTTGTG   
  
  
- ATATAGGGAC ACTTGTCCCT AGAATGTTGA CTAAAAGCAG GGCTTTGTTG TCTTTTTGGG CTTTTAAAAA   
  
  
- AATAAAATTC TGAGCGTGGG ACCCACTGGT CAGGACACAG TTGACCTGAC AGAGATTCTC TCTGCCCCTT   
  
  
- CTATTTATGC AGCTGCTTGG AGCCTTTGTT TTTGAGCCAA CATAAATAAT AAGTCATAGA TATTGGATTT   
  
  
- CTTAAAATGT TAAATACTAT TTATCTTTAA AATTATAAAA AATAGTGAAA AAATTAATTT TATTATTTAT   
  
  
- ATATTAATTG TTAATGGATT GTCAACTGGA TTGAAATTAG CCCAAATCTA TTCCATTGGC TCATTTTTTA   
  
  
- TGAATAATTA TAAAATGAAA TTGAGCTTG

+     Box-W1

| Site Name | Organism | Position | Strand | Matrix score. | sequence | function |
| --- | --- | --- | --- | --- | --- | --- |
| Box-W1 | Petroselinum crispum | 1424 | + | 6 | TTGACC | fungal elicitor responsive element |

> 2018/04/13 10:10:12  
+ CGAGTGGGTT TTGGGGTTTT TTCTTTTGCT TTTTTTTTTG GTTTAGGGAT AAAGTGTCTT GGGAATCTTG   
  
  
+ GGTTTACTCT TTCGTAGTAT TTTTATTGTT CTTCATCTTG TTAAGGGTTT TCTAGTCCGA AAACTGTGGA   
  
  
+ GACTTTTGGT CTAGAATTAG GAATTTACGT GAATTTTGGG GTCTACTTTT ATTTAGTTTG TTCAGTAGGT   
  
  
+ GTTACCACCG ATTACCTGTT AAAACATTTG GGGGAACGGA TTTATGACTT TTAGAATACT AGGTTCTGTT   
  
  
+ TTCTATGTTG TCTCTCTACT GTTTTTGAGT CCGATGCTTC CTCTTATTTG TTTTGGGTAA AGGTATTTGT   
  
  
+ CTATGTGGGA ATGTCCCAAA ACGCGTTTTA AGTTTTGAAG GATAAATGAA ACTTTCACTT TCGTCTGTCT   
  
  
+ TTGTCTTACT CGAGTACTCG TTCCTGATAG GTCTTATTTT AGTCTGTCAA AGAAGGCCGA TTAAGAGGTT   
  
  
+ TCTTAGTTTT CCCATCTTAC TTCTTTTTTT AGTTCTGGTT TTGCTGTTTG GGTACAATTT TTGAACTCGT   
  
  
+ CCCCTGAGTT TCTAATAGGG TAACAACCTT AACTTTCCAC ACTTTCTTGC TGAAACGAAA GGGAGGCCCT   
  
  
+ GAAATAATTT TAGTGGGTTT TCTTTTGTTT CAGGGAGAAA ATTTAAGAAA CCGTTTTCTG TTCGTTTAGT   
  
  
+ TTTAGTATTA TCTTAACTTA AGGTGAGTTC TCACTGTGTC GTCTCCTCTT CTTCTTATGT TTAAGAAATA   
  
  
+ AATTTTTTTT TTTTTCCTTC TCTCTCTCTC TCTCTCCTCC GCCACGAGCG GTACCGTCAC CACCTGTGTT   
  
  
+ TCATCCCTTC TTTTTTGGTT GCAGAGACAG TTTCTAGGAT ACCAGCCAGG GTGGTAGTCT GGATCAATAA   
  
  
+ GGAAAAAAAG TTAAAAAATA AAAAAATAAA AAACAATTTC ATTTAGATAA ATAATAGCAA AAATACTCTT   
  
  
+ TCACCGAATC ACAGTTTTTA GTTTCACAGA CGGTTCGTTT TTTCACCCGG GTGCTGAGGT GACACCTGGC   
  
  
+ CCTCGTAGGG CTCATACGTT GTATGTCATT ACTTGCCAGA TATAAGTTGG TGCACCGTTA TTAGGAACAC   
  
  
+ TATATCCCTG TGAACAGGGA TCTTACAACT GATTTTCGTC CCGAAACAAC AGAAAAACCC GAAAATTTTT   
  
  
+ TTATTTTAAG ACTCGCACCC TGGGTGACCA GTCCTGTGTC AACTGGACTG TCTCTAAGAG AGACGGGGAA   
  
  
+ GATAAATACG TCGACGAACC TCGGAAACAA AAACTCGGTT GTATTTATTA TTCAGTATCT ATAACCTAAA   
  
  
+ GAATTTTACA ATTTATGATA AATAGAAATT TTAATATTTT TTATCACTTT TTTAATTAAA ATAATAAATA   
  
  
+ TATAATTAAC AATTACCTAA CAGTTGACCT AACTTTAATC GGGTTTAGAT AAGGTAACCG AGTAAAAAAT   
  
  
+ ACTTATTAAT ATTTTACTTT AACTCGAAC  

- GCTCACCCAA AACCCCAAAA AAGAAAACGA AAAAAAAAAC CAAATCCCTA TTTCACAGAA CCCTTAGAAC   
  
  
- CCAAATGAGA AAGCATCATA AAAATAACAA GAAGTAGAAC AATTCCCAAA AGATCAGGCT TTTGACACCT   
  
  
- CTGAAAACCA GATCTTAATC CTTAAATGCA CTTAAAACCC CAGATGAAAA TAAATCAAAC AAGTCATCCA   
  
  
- CAATGGTGGC TAATGGACAA TTTTGTAAAC CCCCTTGCCT AAATACTGAA AATCTTATGA TCCAAGACAA   
  
  
- AAGATACAAC AGAGAGATGA CAAAAACTCA GGCTACGAAG GAGAATAAAC AAAACCCATT TCCATAAACA   
  
  
- GATACACCCT TACAGGGTTT TGCGCAAAAT TCAAAACTTC CTATTTACTT TGAAAGTGAA AGCAGACAGA   
  
  
- AACAGAATGA GCTCATGAGC AAGGACTATC CAGAATAAAA TCAGACAGTT TCTTCCGGCT AATTCTCCAA   
  
  
- AGAATCAAAA GGGTAGAATG AAGAAAAAAA TCAAGACCAA AACGACAAAC CCATGTTAAA AACTTGAGCA   
  
  
- GGGGACTCAA AGATTATCCC ATTGTTGGAA TTGAAAGGTG TGAAAGAACG ACTTTGCTTT CCCTCCGGGA   
  
  
- CTTTATTAAA ATCACCCAAA AGAAAACAAA GTCCCTCTTT TAAATTCTTT GGCAAAAGAC AAGCAAATCA   
  
  
- AAATCATAAT AGAATTGAAT TCCACTCAAG AGTGACACAG CAGAGGAGAA GAAGAATACA AATTCTTTAT   
  
  
- TTAAAAAAAA AAAAAGGAAG AGAGAGAGAG AGAGAGGAGG CGGTGCTCGC CATGGCAGTG GTGGACACAA   
  
  
- AGTAGGGAAG AAAAAACCAA CGTCTCTGTC AAAGATCCTA TGGTCGGTCC CACCATCAGA CCTAGTTATT   
  
  
- CCTTTTTTTC AATTTTTTAT TTTTTTATTT TTTGTTAAAG TAAATCTATT TATTATCGTT TTTATGAGAA   
  
  
- AGTGGCTTAG TGTCAAAAAT CAAAGTGTCT GCCAAGCAAA AAAGTGGGCC CACGACTCCA CTGTGGACCG   
  
  
- GGAGCATCCC GAGTATGCAA CATACAGTAA TGAACGGTCT ATATTCAACC ACGTGGCAAT AATCCTTGTG   
  
  
- ATATAGGGAC ACTTGTCCCT AGAATGTTGA CTAAAAGCAG GGCTTTGTTG TCTTTTTGGG CTTTTAAAAA   
  
  
- AATAAAATTC TGAGCGTGGG ACCCACTGGT CAGGACACAG TTGACCTGAC AGAGATTCTC TCTGCCCCTT   
  
  
- CTATTTATGC AGCTGCTTGG AGCCTTTGTT TTTGAGCCAA CATAAATAAT AAGTCATAGA TATTGGATTT   
  
  
- CTTAAAATGT TAAATACTAT TTATCTTTAA AATTATAAAA AATAGTGAAA AAATTAATTT TATTATTTAT   
  
  
- ATATTAATTG TTAATGGATT GTCAACTGGA TTGAAATTAG CCCAAATCTA TTCCATTGGC TCATTTTTTA   
  
  
- TGAATAATTA TAAAATGAAA TTGAGCTTG

+     CAAT-box

| Site Name | Organism | Position | Strand | Matrix score. | sequence | function |
| --- | --- | --- | --- | --- | --- | --- |
| CAAT-box | Glycine max | 545 | + | 5 | CAATT | common cis-acting element in promoter and enhancer regions |
| CAAT-box | Glycine max | 1410 | + | 5 | CAATT | common cis-acting element in promoter and enhancer regions |
| CAAT-box | Glycine max | 1339 | + | 5 | CAATT | common cis-acting element in promoter and enhancer regions |
| CAAT-box | Glycine max | 944 | + | 5 | CAATT | common cis-acting element in promoter and enhancer regions |
| CAAT-box | Hordeum vulgare | 905 | + | 4 | CAAT | common cis-acting element in promoter and enhancer regions |
| CAAT-box | Brassica rapa | 345 | - | 5 | CAAAT | common cis-acting element in promoter and enhancer regions |
| CAAT-box | Hordeum vulgare | 95 | - | 4 | CAAT | common cis-acting element in promoter and enhancer regions |
| CAAT-box | Brassica rapa | 326 | - | 5 | CAAAT | common cis-acting element in promoter and enhancer regions |
| CAAT-box | Brassica rapa | 236 | - | 5 | CAAAT | common cis-acting element in promoter and enhancer regions |

> 2018/04/13 10:10:12  
+ CGAGTGGGTT TTGGGGTTTT TTCTTTTGCT TTTTTTTTTG GTTTAGGGAT AAAGTGTCTT GGGAATCTTG   
  
  
+ GGTTTACTCT TTCGTAGTAT TTTTATTGTT CTTCATCTTG TTAAGGGTTT TCTAGTCCGA AAACTGTGGA   
  
  
+ GACTTTTGGT CTAGAATTAG GAATTTACGT GAATTTTGGG GTCTACTTTT ATTTAGTTTG TTCAGTAGGT   
  
  
+ GTTACCACCG ATTACCTGTT AAAACATTTG GGGGAACGGA TTTATGACTT TTAGAATACT AGGTTCTGTT   
  
  
+ TTCTATGTTG TCTCTCTACT GTTTTTGAGT CCGATGCTTC CTCTTATTTG TTTTGGGTAA AGGTATTTGT   
  
  
+ CTATGTGGGA ATGTCCCAAA ACGCGTTTTA AGTTTTGAAG GATAAATGAA ACTTTCACTT TCGTCTGTCT   
  
  
+ TTGTCTTACT CGAGTACTCG TTCCTGATAG GTCTTATTTT AGTCTGTCAA AGAAGGCCGA TTAAGAGGTT   
  
  
+ TCTTAGTTTT CCCATCTTAC TTCTTTTTTT AGTTCTGGTT TTGCTGTTTG GGTACAATTT TTGAACTCGT   
  
  
+ CCCCTGAGTT TCTAATAGGG TAACAACCTT AACTTTCCAC ACTTTCTTGC TGAAACGAAA GGGAGGCCCT   
  
  
+ GAAATAATTT TAGTGGGTTT TCTTTTGTTT CAGGGAGAAA ATTTAAGAAA CCGTTTTCTG TTCGTTTAGT   
  
  
+ TTTAGTATTA TCTTAACTTA AGGTGAGTTC TCACTGTGTC GTCTCCTCTT CTTCTTATGT TTAAGAAATA   
  
  
+ AATTTTTTTT TTTTTCCTTC TCTCTCTCTC TCTCTCCTCC GCCACGAGCG GTACCGTCAC CACCTGTGTT   
  
  
+ TCATCCCTTC TTTTTTGGTT GCAGAGACAG TTTCTAGGAT ACCAGCCAGG GTGGTAGTCT GGATCAATAA   
  
  
+ GGAAAAAAAG TTAAAAAATA AAAAAATAAA AAACAATTTC ATTTAGATAA ATAATAGCAA AAATACTCTT   
  
  
+ TCACCGAATC ACAGTTTTTA GTTTCACAGA CGGTTCGTTT TTTCACCCGG GTGCTGAGGT GACACCTGGC   
  
  
+ CCTCGTAGGG CTCATACGTT GTATGTCATT ACTTGCCAGA TATAAGTTGG TGCACCGTTA TTAGGAACAC   
  
  
+ TATATCCCTG TGAACAGGGA TCTTACAACT GATTTTCGTC CCGAAACAAC AGAAAAACCC GAAAATTTTT   
  
  
+ TTATTTTAAG ACTCGCACCC TGGGTGACCA GTCCTGTGTC AACTGGACTG TCTCTAAGAG AGACGGGGAA   
  
  
+ GATAAATACG TCGACGAACC TCGGAAACAA AAACTCGGTT GTATTTATTA TTCAGTATCT ATAACCTAAA   
  
  
+ GAATTTTACA ATTTATGATA AATAGAAATT TTAATATTTT TTATCACTTT TTTAATTAAA ATAATAAATA   
  
  
+ TATAATTAAC AATTACCTAA CAGTTGACCT AACTTTAATC GGGTTTAGAT AAGGTAACCG AGTAAAAAAT   
  
  
+ ACTTATTAAT ATTTTACTTT AACTCGAAC  

- GCTCACCCAA AACCCCAAAA AAGAAAACGA AAAAAAAAAC CAAATCCCTA TTTCACAGAA CCCTTAGAAC   
  
  
- CCAAATGAGA AAGCATCATA AAAATAACAA GAAGTAGAAC AATTCCCAAA AGATCAGGCT TTTGACACCT   
  
  
- CTGAAAACCA GATCTTAATC CTTAAATGCA CTTAAAACCC CAGATGAAAA TAAATCAAAC AAGTCATCCA   
  
  
- CAATGGTGGC TAATGGACAA TTTTGTAAAC CCCCTTGCCT AAATACTGAA AATCTTATGA TCCAAGACAA   
  
  
- AAGATACAAC AGAGAGATGA CAAAAACTCA GGCTACGAAG GAGAATAAAC AAAACCCATT TCCATAAACA   
  
  
- GATACACCCT TACAGGGTTT TGCGCAAAAT TCAAAACTTC CTATTTACTT TGAAAGTGAA AGCAGACAGA   
  
  
- AACAGAATGA GCTCATGAGC AAGGACTATC CAGAATAAAA TCAGACAGTT TCTTCCGGCT AATTCTCCAA   
  
  
- AGAATCAAAA GGGTAGAATG AAGAAAAAAA TCAAGACCAA AACGACAAAC CCATGTTAAA AACTTGAGCA   
  
  
- GGGGACTCAA AGATTATCCC ATTGTTGGAA TTGAAAGGTG TGAAAGAACG ACTTTGCTTT CCCTCCGGGA   
  
  
- CTTTATTAAA ATCACCCAAA AGAAAACAAA GTCCCTCTTT TAAATTCTTT GGCAAAAGAC AAGCAAATCA   
  
  
- AAATCATAAT AGAATTGAAT TCCACTCAAG AGTGACACAG CAGAGGAGAA GAAGAATACA AATTCTTTAT   
  
  
- TTAAAAAAAA AAAAAGGAAG AGAGAGAGAG AGAGAGGAGG CGGTGCTCGC CATGGCAGTG GTGGACACAA   
  
  
- AGTAGGGAAG AAAAAACCAA CGTCTCTGTC AAAGATCCTA TGGTCGGTCC CACCATCAGA CCTAGTTATT   
  
  
- CCTTTTTTTC AATTTTTTAT TTTTTTATTT TTTGTTAAAG TAAATCTATT TATTATCGTT TTTATGAGAA   
  
  
- AGTGGCTTAG TGTCAAAAAT CAAAGTGTCT GCCAAGCAAA AAAGTGGGCC CACGACTCCA CTGTGGACCG   
  
  
- GGAGCATCCC GAGTATGCAA CATACAGTAA TGAACGGTCT ATATTCAACC ACGTGGCAAT AATCCTTGTG   
  
  
- ATATAGGGAC ACTTGTCCCT AGAATGTTGA CTAAAAGCAG GGCTTTGTTG TCTTTTTGGG CTTTTAAAAA   
  
  
- AATAAAATTC TGAGCGTGGG ACCCACTGGT CAGGACACAG TTGACCTGAC AGAGATTCTC TCTGCCCCTT   
  
  
- CTATTTATGC AGCTGCTTGG AGCCTTTGTT TTTGAGCCAA CATAAATAAT AAGTCATAGA TATTGGATTT   
  
  
- CTTAAAATGT TAAATACTAT TTATCTTTAA AATTATAAAA AATAGTGAAA AAATTAATTT TATTATTTAT   
  
  
- ATATTAATTG TTAATGGATT GTCAACTGGA TTGAAATTAG CCCAAATCTA TTCCATTGGC TCATTTTTTA   
  
  
- TGAATAATTA TAAAATGAAA TTGAGCTTG

+     CGTCA-motif

| Site Name | Organism | Position | Strand | Matrix score. | sequence | function |
| --- | --- | --- | --- | --- | --- | --- |
| CGTCA-motif | Hordeum vulgare | 825 | + | 5 | CGTCA | cis-acting regulatory element involved in the MeJA-responsiveness |

> 2018/04/13 10:10:12  
+ CGAGTGGGTT TTGGGGTTTT TTCTTTTGCT TTTTTTTTTG GTTTAGGGAT AAAGTGTCTT GGGAATCTTG   
  
  
+ GGTTTACTCT TTCGTAGTAT TTTTATTGTT CTTCATCTTG TTAAGGGTTT TCTAGTCCGA AAACTGTGGA   
  
  
+ GACTTTTGGT CTAGAATTAG GAATTTACGT GAATTTTGGG GTCTACTTTT ATTTAGTTTG TTCAGTAGGT   
  
  
+ GTTACCACCG ATTACCTGTT AAAACATTTG GGGGAACGGA TTTATGACTT TTAGAATACT AGGTTCTGTT   
  
  
+ TTCTATGTTG TCTCTCTACT GTTTTTGAGT CCGATGCTTC CTCTTATTTG TTTTGGGTAA AGGTATTTGT   
  
  
+ CTATGTGGGA ATGTCCCAAA ACGCGTTTTA AGTTTTGAAG GATAAATGAA ACTTTCACTT TCGTCTGTCT   
  
  
+ TTGTCTTACT CGAGTACTCG TTCCTGATAG GTCTTATTTT AGTCTGTCAA AGAAGGCCGA TTAAGAGGTT   
  
  
+ TCTTAGTTTT CCCATCTTAC TTCTTTTTTT AGTTCTGGTT TTGCTGTTTG GGTACAATTT TTGAACTCGT   
  
  
+ CCCCTGAGTT TCTAATAGGG TAACAACCTT AACTTTCCAC ACTTTCTTGC TGAAACGAAA GGGAGGCCCT   
  
  
+ GAAATAATTT TAGTGGGTTT TCTTTTGTTT CAGGGAGAAA ATTTAAGAAA CCGTTTTCTG TTCGTTTAGT   
  
  
+ TTTAGTATTA TCTTAACTTA AGGTGAGTTC TCACTGTGTC GTCTCCTCTT CTTCTTATGT TTAAGAAATA   
  
  
+ AATTTTTTTT TTTTTCCTTC TCTCTCTCTC TCTCTCCTCC GCCACGAGCG GTACCGTCAC CACCTGTGTT   
  
  
+ TCATCCCTTC TTTTTTGGTT GCAGAGACAG TTTCTAGGAT ACCAGCCAGG GTGGTAGTCT GGATCAATAA   
  
  
+ GGAAAAAAAG TTAAAAAATA AAAAAATAAA AAACAATTTC ATTTAGATAA ATAATAGCAA AAATACTCTT   
  
  
+ TCACCGAATC ACAGTTTTTA GTTTCACAGA CGGTTCGTTT TTTCACCCGG GTGCTGAGGT GACACCTGGC   
  
  
+ CCTCGTAGGG CTCATACGTT GTATGTCATT ACTTGCCAGA TATAAGTTGG TGCACCGTTA TTAGGAACAC   
  
  
+ TATATCCCTG TGAACAGGGA TCTTACAACT GATTTTCGTC CCGAAACAAC AGAAAAACCC GAAAATTTTT   
  
  
+ TTATTTTAAG ACTCGCACCC TGGGTGACCA GTCCTGTGTC AACTGGACTG TCTCTAAGAG AGACGGGGAA   
  
  
+ GATAAATACG TCGACGAACC TCGGAAACAA AAACTCGGTT GTATTTATTA TTCAGTATCT ATAACCTAAA   
  
  
+ GAATTTTACA ATTTATGATA AATAGAAATT TTAATATTTT TTATCACTTT TTTAATTAAA ATAATAAATA   
  
  
+ TATAATTAAC AATTACCTAA CAGTTGACCT AACTTTAATC GGGTTTAGAT AAGGTAACCG AGTAAAAAAT   
  
  
+ ACTTATTAAT ATTTTACTTT AACTCGAAC  

- GCTCACCCAA AACCCCAAAA AAGAAAACGA AAAAAAAAAC CAAATCCCTA TTTCACAGAA CCCTTAGAAC   
  
  
- CCAAATGAGA AAGCATCATA AAAATAACAA GAAGTAGAAC AATTCCCAAA AGATCAGGCT TTTGACACCT   
  
  
- CTGAAAACCA GATCTTAATC CTTAAATGCA CTTAAAACCC CAGATGAAAA TAAATCAAAC AAGTCATCCA   
  
  
- CAATGGTGGC TAATGGACAA TTTTGTAAAC CCCCTTGCCT AAATACTGAA AATCTTATGA TCCAAGACAA   
  
  
- AAGATACAAC AGAGAGATGA CAAAAACTCA GGCTACGAAG GAGAATAAAC AAAACCCATT TCCATAAACA   
  
  
- GATACACCCT TACAGGGTTT TGCGCAAAAT TCAAAACTTC CTATTTACTT TGAAAGTGAA AGCAGACAGA   
  
  
- AACAGAATGA GCTCATGAGC AAGGACTATC CAGAATAAAA TCAGACAGTT TCTTCCGGCT AATTCTCCAA   
  
  
- AGAATCAAAA GGGTAGAATG AAGAAAAAAA TCAAGACCAA AACGACAAAC CCATGTTAAA AACTTGAGCA   
  
  
- GGGGACTCAA AGATTATCCC ATTGTTGGAA TTGAAAGGTG TGAAAGAACG ACTTTGCTTT CCCTCCGGGA   
  
  
- CTTTATTAAA ATCACCCAAA AGAAAACAAA GTCCCTCTTT TAAATTCTTT GGCAAAAGAC AAGCAAATCA   
  
  
- AAATCATAAT AGAATTGAAT TCCACTCAAG AGTGACACAG CAGAGGAGAA GAAGAATACA AATTCTTTAT   
  
  
- TTAAAAAAAA AAAAAGGAAG AGAGAGAGAG AGAGAGGAGG CGGTGCTCGC CATGGCAGTG GTGGACACAA   
  
  
- AGTAGGGAAG AAAAAACCAA CGTCTCTGTC AAAGATCCTA TGGTCGGTCC CACCATCAGA CCTAGTTATT   
  
  
- CCTTTTTTTC AATTTTTTAT TTTTTTATTT TTTGTTAAAG TAAATCTATT TATTATCGTT TTTATGAGAA   
  
  
- AGTGGCTTAG TGTCAAAAAT CAAAGTGTCT GCCAAGCAAA AAAGTGGGCC CACGACTCCA CTGTGGACCG   
  
  
- GGAGCATCCC GAGTATGCAA CATACAGTAA TGAACGGTCT ATATTCAACC ACGTGGCAAT AATCCTTGTG   
  
  
- ATATAGGGAC ACTTGTCCCT AGAATGTTGA CTAAAAGCAG GGCTTTGTTG TCTTTTTGGG CTTTTAAAAA   
  
  
- AATAAAATTC TGAGCGTGGG ACCCACTGGT CAGGACACAG TTGACCTGAC AGAGATTCTC TCTGCCCCTT   
  
  
- CTATTTATGC AGCTGCTTGG AGCCTTTGTT TTTGAGCCAA CATAAATAAT AAGTCATAGA TATTGGATTT   
  
  
- CTTAAAATGT TAAATACTAT TTATCTTTAA AATTATAAAA AATAGTGAAA AAATTAATTT TATTATTTAT   
  
  
- ATATTAATTG TTAATGGATT GTCAACTGGA TTGAAATTAG CCCAAATCTA TTCCATTGGC TCATTTTTTA   
  
  
- TGAATAATTA TAAAATGAAA TTGAGCTTG

+     G-Box

| Site Name | Organism | Position | Strand | Matrix score. | sequence | function |
| --- | --- | --- | --- | --- | --- | --- |
| G-Box | Antirrhinum majus | 166 | - | 6 | CACGTA | cis-acting regulatory element involved in light responsiveness |

> 2018/04/13 10:10:12  
+ CGAGTGGGTT TTGGGGTTTT TTCTTTTGCT TTTTTTTTTG GTTTAGGGAT AAAGTGTCTT GGGAATCTTG   
  
  
+ GGTTTACTCT TTCGTAGTAT TTTTATTGTT CTTCATCTTG TTAAGGGTTT TCTAGTCCGA AAACTGTGGA   
  
  
+ GACTTTTGGT CTAGAATTAG GAATTTACGT GAATTTTGGG GTCTACTTTT ATTTAGTTTG TTCAGTAGGT   
  
  
+ GTTACCACCG ATTACCTGTT AAAACATTTG GGGGAACGGA TTTATGACTT TTAGAATACT AGGTTCTGTT   
  
  
+ TTCTATGTTG TCTCTCTACT GTTTTTGAGT CCGATGCTTC CTCTTATTTG TTTTGGGTAA AGGTATTTGT   
  
  
+ CTATGTGGGA ATGTCCCAAA ACGCGTTTTA AGTTTTGAAG GATAAATGAA ACTTTCACTT TCGTCTGTCT   
  
  
+ TTGTCTTACT CGAGTACTCG TTCCTGATAG GTCTTATTTT AGTCTGTCAA AGAAGGCCGA TTAAGAGGTT   
  
  
+ TCTTAGTTTT CCCATCTTAC TTCTTTTTTT AGTTCTGGTT TTGCTGTTTG GGTACAATTT TTGAACTCGT   
  
  
+ CCCCTGAGTT TCTAATAGGG TAACAACCTT AACTTTCCAC ACTTTCTTGC TGAAACGAAA GGGAGGCCCT   
  
  
+ GAAATAATTT TAGTGGGTTT TCTTTTGTTT CAGGGAGAAA ATTTAAGAAA CCGTTTTCTG TTCGTTTAGT   
  
  
+ TTTAGTATTA TCTTAACTTA AGGTGAGTTC TCACTGTGTC GTCTCCTCTT CTTCTTATGT TTAAGAAATA   
  
  
+ AATTTTTTTT TTTTTCCTTC TCTCTCTCTC TCTCTCCTCC GCCACGAGCG GTACCGTCAC CACCTGTGTT   
  
  
+ TCATCCCTTC TTTTTTGGTT GCAGAGACAG TTTCTAGGAT ACCAGCCAGG GTGGTAGTCT GGATCAATAA   
  
  
+ GGAAAAAAAG TTAAAAAATA AAAAAATAAA AAACAATTTC ATTTAGATAA ATAATAGCAA AAATACTCTT   
  
  
+ TCACCGAATC ACAGTTTTTA GTTTCACAGA CGGTTCGTTT TTTCACCCGG GTGCTGAGGT GACACCTGGC   
  
  
+ CCTCGTAGGG CTCATACGTT GTATGTCATT ACTTGCCAGA TATAAGTTGG TGCACCGTTA TTAGGAACAC   
  
  
+ TATATCCCTG TGAACAGGGA TCTTACAACT GATTTTCGTC CCGAAACAAC AGAAAAACCC GAAAATTTTT   
  
  
+ TTATTTTAAG ACTCGCACCC TGGGTGACCA GTCCTGTGTC AACTGGACTG TCTCTAAGAG AGACGGGGAA   
  
  
+ GATAAATACG TCGACGAACC TCGGAAACAA AAACTCGGTT GTATTTATTA TTCAGTATCT ATAACCTAAA   
  
  
+ GAATTTTACA ATTTATGATA AATAGAAATT TTAATATTTT TTATCACTTT TTTAATTAAA ATAATAAATA   
  
  
+ TATAATTAAC AATTACCTAA CAGTTGACCT AACTTTAATC GGGTTTAGAT AAGGTAACCG AGTAAAAAAT   
  
  
+ ACTTATTAAT ATTTTACTTT AACTCGAAC  

- GCTCACCCAA AACCCCAAAA AAGAAAACGA AAAAAAAAAC CAAATCCCTA TTTCACAGAA CCCTTAGAAC   
  
  
- CCAAATGAGA AAGCATCATA AAAATAACAA GAAGTAGAAC AATTCCCAAA AGATCAGGCT TTTGACACCT   
  
  
- CTGAAAACCA GATCTTAATC CTTAAATGCA CTTAAAACCC CAGATGAAAA TAAATCAAAC AAGTCATCCA   
  
  
- CAATGGTGGC TAATGGACAA TTTTGTAAAC CCCCTTGCCT AAATACTGAA AATCTTATGA TCCAAGACAA   
  
  
- AAGATACAAC AGAGAGATGA CAAAAACTCA GGCTACGAAG GAGAATAAAC AAAACCCATT TCCATAAACA   
  
  
- GATACACCCT TACAGGGTTT TGCGCAAAAT TCAAAACTTC CTATTTACTT TGAAAGTGAA AGCAGACAGA   
  
  
- AACAGAATGA GCTCATGAGC AAGGACTATC CAGAATAAAA TCAGACAGTT TCTTCCGGCT AATTCTCCAA   
  
  
- AGAATCAAAA GGGTAGAATG AAGAAAAAAA TCAAGACCAA AACGACAAAC CCATGTTAAA AACTTGAGCA   
  
  
- GGGGACTCAA AGATTATCCC ATTGTTGGAA TTGAAAGGTG TGAAAGAACG ACTTTGCTTT CCCTCCGGGA   
  
  
- CTTTATTAAA ATCACCCAAA AGAAAACAAA GTCCCTCTTT TAAATTCTTT GGCAAAAGAC AAGCAAATCA   
  
  
- AAATCATAAT AGAATTGAAT TCCACTCAAG AGTGACACAG CAGAGGAGAA GAAGAATACA AATTCTTTAT   
  
  
- TTAAAAAAAA AAAAAGGAAG AGAGAGAGAG AGAGAGGAGG CGGTGCTCGC CATGGCAGTG GTGGACACAA   
  
  
- AGTAGGGAAG AAAAAACCAA CGTCTCTGTC AAAGATCCTA TGGTCGGTCC CACCATCAGA CCTAGTTATT   
  
  
- CCTTTTTTTC AATTTTTTAT TTTTTTATTT TTTGTTAAAG TAAATCTATT TATTATCGTT TTTATGAGAA   
  
  
- AGTGGCTTAG TGTCAAAAAT CAAAGTGTCT GCCAAGCAAA AAAGTGGGCC CACGACTCCA CTGTGGACCG   
  
  
- GGAGCATCCC GAGTATGCAA CATACAGTAA TGAACGGTCT ATATTCAACC ACGTGGCAAT AATCCTTGTG   
  
  
- ATATAGGGAC ACTTGTCCCT AGAATGTTGA CTAAAAGCAG GGCTTTGTTG TCTTTTTGGG CTTTTAAAAA   
  
  
- AATAAAATTC TGAGCGTGGG ACCCACTGGT CAGGACACAG TTGACCTGAC AGAGATTCTC TCTGCCCCTT   
  
  
- CTATTTATGC AGCTGCTTGG AGCCTTTGTT TTTGAGCCAA CATAAATAAT AAGTCATAGA TATTGGATTT   
  
  
- CTTAAAATGT TAAATACTAT TTATCTTTAA AATTATAAAA AATAGTGAAA AAATTAATTT TATTATTTAT   
  
  
- ATATTAATTG TTAATGGATT GTCAACTGGA TTGAAATTAG CCCAAATCTA TTCCATTGGC TCATTTTTTA   
  
  
- TGAATAATTA TAAAATGAAA TTGAGCTTG

+     G-box

| Site Name | Organism | Position | Strand | Matrix score. | sequence | function |
| --- | --- | --- | --- | --- | --- | --- |
| G-box | Daucus carota | 166 | + | 6 | TACGTG | cis-acting regulatory element involved in light responsiveness |

> 2018/04/13 10:10:12  
+ CGAGTGGGTT TTGGGGTTTT TTCTTTTGCT TTTTTTTTTG GTTTAGGGAT AAAGTGTCTT GGGAATCTTG   
  
  
+ GGTTTACTCT TTCGTAGTAT TTTTATTGTT CTTCATCTTG TTAAGGGTTT TCTAGTCCGA AAACTGTGGA   
  
  
+ GACTTTTGGT CTAGAATTAG GAATTTACGT GAATTTTGGG GTCTACTTTT ATTTAGTTTG TTCAGTAGGT   
  
  
+ GTTACCACCG ATTACCTGTT AAAACATTTG GGGGAACGGA TTTATGACTT TTAGAATACT AGGTTCTGTT   
  
  
+ TTCTATGTTG TCTCTCTACT GTTTTTGAGT CCGATGCTTC CTCTTATTTG TTTTGGGTAA AGGTATTTGT   
  
  
+ CTATGTGGGA ATGTCCCAAA ACGCGTTTTA AGTTTTGAAG GATAAATGAA ACTTTCACTT TCGTCTGTCT   
  
  
+ TTGTCTTACT CGAGTACTCG TTCCTGATAG GTCTTATTTT AGTCTGTCAA AGAAGGCCGA TTAAGAGGTT   
  
  
+ TCTTAGTTTT CCCATCTTAC TTCTTTTTTT AGTTCTGGTT TTGCTGTTTG GGTACAATTT TTGAACTCGT   
  
  
+ CCCCTGAGTT TCTAATAGGG TAACAACCTT AACTTTCCAC ACTTTCTTGC TGAAACGAAA GGGAGGCCCT   
  
  
+ GAAATAATTT TAGTGGGTTT TCTTTTGTTT CAGGGAGAAA ATTTAAGAAA CCGTTTTCTG TTCGTTTAGT   
  
  
+ TTTAGTATTA TCTTAACTTA AGGTGAGTTC TCACTGTGTC GTCTCCTCTT CTTCTTATGT TTAAGAAATA   
  
  
+ AATTTTTTTT TTTTTCCTTC TCTCTCTCTC TCTCTCCTCC GCCACGAGCG GTACCGTCAC CACCTGTGTT   
  
  
+ TCATCCCTTC TTTTTTGGTT GCAGAGACAG TTTCTAGGAT ACCAGCCAGG GTGGTAGTCT GGATCAATAA   
  
  
+ GGAAAAAAAG TTAAAAAATA AAAAAATAAA AAACAATTTC ATTTAGATAA ATAATAGCAA AAATACTCTT   
  
  
+ TCACCGAATC ACAGTTTTTA GTTTCACAGA CGGTTCGTTT TTTCACCCGG GTGCTGAGGT GACACCTGGC   
  
  
+ CCTCGTAGGG CTCATACGTT GTATGTCATT ACTTGCCAGA TATAAGTTGG TGCACCGTTA TTAGGAACAC   
  
  
+ TATATCCCTG TGAACAGGGA TCTTACAACT GATTTTCGTC CCGAAACAAC AGAAAAACCC GAAAATTTTT   
  
  
+ TTATTTTAAG ACTCGCACCC TGGGTGACCA GTCCTGTGTC AACTGGACTG TCTCTAAGAG AGACGGGGAA   
  
  
+ GATAAATACG TCGACGAACC TCGGAAACAA AAACTCGGTT GTATTTATTA TTCAGTATCT ATAACCTAAA   
  
  
+ GAATTTTACA ATTTATGATA AATAGAAATT TTAATATTTT TTATCACTTT TTTAATTAAA ATAATAAATA   
  
  
+ TATAATTAAC AATTACCTAA CAGTTGACCT AACTTTAATC GGGTTTAGAT AAGGTAACCG AGTAAAAAAT   
  
  
+ ACTTATTAAT ATTTTACTTT AACTCGAAC  

- GCTCACCCAA AACCCCAAAA AAGAAAACGA AAAAAAAAAC CAAATCCCTA TTTCACAGAA CCCTTAGAAC   
  
  
- CCAAATGAGA AAGCATCATA AAAATAACAA GAAGTAGAAC AATTCCCAAA AGATCAGGCT TTTGACACCT   
  
  
- CTGAAAACCA GATCTTAATC CTTAAATGCA CTTAAAACCC CAGATGAAAA TAAATCAAAC AAGTCATCCA   
  
  
- CAATGGTGGC TAATGGACAA TTTTGTAAAC CCCCTTGCCT AAATACTGAA AATCTTATGA TCCAAGACAA   
  
  
- AAGATACAAC AGAGAGATGA CAAAAACTCA GGCTACGAAG GAGAATAAAC AAAACCCATT TCCATAAACA   
  
  
- GATACACCCT TACAGGGTTT TGCGCAAAAT TCAAAACTTC CTATTTACTT TGAAAGTGAA AGCAGACAGA   
  
  
- AACAGAATGA GCTCATGAGC AAGGACTATC CAGAATAAAA TCAGACAGTT TCTTCCGGCT AATTCTCCAA   
  
  
- AGAATCAAAA GGGTAGAATG AAGAAAAAAA TCAAGACCAA AACGACAAAC CCATGTTAAA AACTTGAGCA   
  
  
- GGGGACTCAA AGATTATCCC ATTGTTGGAA TTGAAAGGTG TGAAAGAACG ACTTTGCTTT CCCTCCGGGA   
  
  
- CTTTATTAAA ATCACCCAAA AGAAAACAAA GTCCCTCTTT TAAATTCTTT GGCAAAAGAC AAGCAAATCA   
  
  
- AAATCATAAT AGAATTGAAT TCCACTCAAG AGTGACACAG CAGAGGAGAA GAAGAATACA AATTCTTTAT   
  
  
- TTAAAAAAAA AAAAAGGAAG AGAGAGAGAG AGAGAGGAGG CGGTGCTCGC CATGGCAGTG GTGGACACAA   
  
  
- AGTAGGGAAG AAAAAACCAA CGTCTCTGTC AAAGATCCTA TGGTCGGTCC CACCATCAGA CCTAGTTATT   
  
  
- CCTTTTTTTC AATTTTTTAT TTTTTTATTT TTTGTTAAAG TAAATCTATT TATTATCGTT TTTATGAGAA   
  
  
- AGTGGCTTAG TGTCAAAAAT CAAAGTGTCT GCCAAGCAAA AAAGTGGGCC CACGACTCCA CTGTGGACCG   
  
  
- GGAGCATCCC GAGTATGCAA CATACAGTAA TGAACGGTCT ATATTCAACC ACGTGGCAAT AATCCTTGTG   
  
  
- ATATAGGGAC ACTTGTCCCT AGAATGTTGA CTAAAAGCAG GGCTTTGTTG TCTTTTTGGG CTTTTAAAAA   
  
  
- AATAAAATTC TGAGCGTGGG ACCCACTGGT CAGGACACAG TTGACCTGAC AGAGATTCTC TCTGCCCCTT   
  
  
- CTATTTATGC AGCTGCTTGG AGCCTTTGTT TTTGAGCCAA CATAAATAAT AAGTCATAGA TATTGGATTT   
  
  
- CTTAAAATGT TAAATACTAT TTATCTTTAA AATTATAAAA AATAGTGAAA AAATTAATTT TATTATTTAT   
  
  
- ATATTAATTG TTAATGGATT GTCAACTGGA TTGAAATTAG CCCAAATCTA TTCCATTGGC TCATTTTTTA   
  
  
- TGAATAATTA TAAAATGAAA TTGAGCTTG

+     GARE-motif

| Site Name | Organism | Position | Strand | Matrix score. | sequence | function |
| --- | --- | --- | --- | --- | --- | --- |
| GARE-motif | Brassica oleracea | 1167 | - | 7 | TCTGTTG | gibberellin-responsive element |
| GARE-motif | Brassica oleracea | 275 | - | 7 | AAACAGA | gibberellin-responsive element |

> 2018/04/13 10:10:12  
+ CGAGTGGGTT TTGGGGTTTT TTCTTTTGCT TTTTTTTTTG GTTTAGGGAT AAAGTGTCTT GGGAATCTTG   
  
  
+ GGTTTACTCT TTCGTAGTAT TTTTATTGTT CTTCATCTTG TTAAGGGTTT TCTAGTCCGA AAACTGTGGA   
  
  
+ GACTTTTGGT CTAGAATTAG GAATTTACGT GAATTTTGGG GTCTACTTTT ATTTAGTTTG TTCAGTAGGT   
  
  
+ GTTACCACCG ATTACCTGTT AAAACATTTG GGGGAACGGA TTTATGACTT TTAGAATACT AGGTTCTGTT   
  
  
+ TTCTATGTTG TCTCTCTACT GTTTTTGAGT CCGATGCTTC CTCTTATTTG TTTTGGGTAA AGGTATTTGT   
  
  
+ CTATGTGGGA ATGTCCCAAA ACGCGTTTTA AGTTTTGAAG GATAAATGAA ACTTTCACTT TCGTCTGTCT   
  
  
+ TTGTCTTACT CGAGTACTCG TTCCTGATAG GTCTTATTTT AGTCTGTCAA AGAAGGCCGA TTAAGAGGTT   
  
  
+ TCTTAGTTTT CCCATCTTAC TTCTTTTTTT AGTTCTGGTT TTGCTGTTTG GGTACAATTT TTGAACTCGT   
  
  
+ CCCCTGAGTT TCTAATAGGG TAACAACCTT AACTTTCCAC ACTTTCTTGC TGAAACGAAA GGGAGGCCCT   
  
  
+ GAAATAATTT TAGTGGGTTT TCTTTTGTTT CAGGGAGAAA ATTTAAGAAA CCGTTTTCTG TTCGTTTAGT   
  
  
+ TTTAGTATTA TCTTAACTTA AGGTGAGTTC TCACTGTGTC GTCTCCTCTT CTTCTTATGT TTAAGAAATA   
  
  
+ AATTTTTTTT TTTTTCCTTC TCTCTCTCTC TCTCTCCTCC GCCACGAGCG GTACCGTCAC CACCTGTGTT   
  
  
+ TCATCCCTTC TTTTTTGGTT GCAGAGACAG TTTCTAGGAT ACCAGCCAGG GTGGTAGTCT GGATCAATAA   
  
  
+ GGAAAAAAAG TTAAAAAATA AAAAAATAAA AAACAATTTC ATTTAGATAA ATAATAGCAA AAATACTCTT   
  
  
+ TCACCGAATC ACAGTTTTTA GTTTCACAGA CGGTTCGTTT TTTCACCCGG GTGCTGAGGT GACACCTGGC   
  
  
+ CCTCGTAGGG CTCATACGTT GTATGTCATT ACTTGCCAGA TATAAGTTGG TGCACCGTTA TTAGGAACAC   
  
  
+ TATATCCCTG TGAACAGGGA TCTTACAACT GATTTTCGTC CCGAAACAAC AGAAAAACCC GAAAATTTTT   
  
  
+ TTATTTTAAG ACTCGCACCC TGGGTGACCA GTCCTGTGTC AACTGGACTG TCTCTAAGAG AGACGGGGAA   
  
  
+ GATAAATACG TCGACGAACC TCGGAAACAA AAACTCGGTT GTATTTATTA TTCAGTATCT ATAACCTAAA   
  
  
+ GAATTTTACA ATTTATGATA AATAGAAATT TTAATATTTT TTATCACTTT TTTAATTAAA ATAATAAATA   
  
  
+ TATAATTAAC AATTACCTAA CAGTTGACCT AACTTTAATC GGGTTTAGAT AAGGTAACCG AGTAAAAAAT   
  
  
+ ACTTATTAAT ATTTTACTTT AACTCGAAC  

- GCTCACCCAA AACCCCAAAA AAGAAAACGA AAAAAAAAAC CAAATCCCTA TTTCACAGAA CCCTTAGAAC   
  
  
- CCAAATGAGA AAGCATCATA AAAATAACAA GAAGTAGAAC AATTCCCAAA AGATCAGGCT TTTGACACCT   
  
  
- CTGAAAACCA GATCTTAATC CTTAAATGCA CTTAAAACCC CAGATGAAAA TAAATCAAAC AAGTCATCCA   
  
  
- CAATGGTGGC TAATGGACAA TTTTGTAAAC CCCCTTGCCT AAATACTGAA AATCTTATGA TCCAAGACAA   
  
  
- AAGATACAAC AGAGAGATGA CAAAAACTCA GGCTACGAAG GAGAATAAAC AAAACCCATT TCCATAAACA   
  
  
- GATACACCCT TACAGGGTTT TGCGCAAAAT TCAAAACTTC CTATTTACTT TGAAAGTGAA AGCAGACAGA   
  
  
- AACAGAATGA GCTCATGAGC AAGGACTATC CAGAATAAAA TCAGACAGTT TCTTCCGGCT AATTCTCCAA   
  
  
- AGAATCAAAA GGGTAGAATG AAGAAAAAAA TCAAGACCAA AACGACAAAC CCATGTTAAA AACTTGAGCA   
  
  
- GGGGACTCAA AGATTATCCC ATTGTTGGAA TTGAAAGGTG TGAAAGAACG ACTTTGCTTT CCCTCCGGGA   
  
  
- CTTTATTAAA ATCACCCAAA AGAAAACAAA GTCCCTCTTT TAAATTCTTT GGCAAAAGAC AAGCAAATCA   
  
  
- AAATCATAAT AGAATTGAAT TCCACTCAAG AGTGACACAG CAGAGGAGAA GAAGAATACA AATTCTTTAT   
  
  
- TTAAAAAAAA AAAAAGGAAG AGAGAGAGAG AGAGAGGAGG CGGTGCTCGC CATGGCAGTG GTGGACACAA   
  
  
- AGTAGGGAAG AAAAAACCAA CGTCTCTGTC AAAGATCCTA TGGTCGGTCC CACCATCAGA CCTAGTTATT   
  
  
- CCTTTTTTTC AATTTTTTAT TTTTTTATTT TTTGTTAAAG TAAATCTATT TATTATCGTT TTTATGAGAA   
  
  
- AGTGGCTTAG TGTCAAAAAT CAAAGTGTCT GCCAAGCAAA AAAGTGGGCC CACGACTCCA CTGTGGACCG   
  
  
- GGAGCATCCC GAGTATGCAA CATACAGTAA TGAACGGTCT ATATTCAACC ACGTGGCAAT AATCCTTGTG   
  
  
- ATATAGGGAC ACTTGTCCCT AGAATGTTGA CTAAAAGCAG GGCTTTGTTG TCTTTTTGGG CTTTTAAAAA   
  
  
- AATAAAATTC TGAGCGTGGG ACCCACTGGT CAGGACACAG TTGACCTGAC AGAGATTCTC TCTGCCCCTT   
  
  
- CTATTTATGC AGCTGCTTGG AGCCTTTGTT TTTGAGCCAA CATAAATAAT AAGTCATAGA TATTGGATTT   
  
  
- CTTAAAATGT TAAATACTAT TTATCTTTAA AATTATAAAA AATAGTGAAA AAATTAATTT TATTATTTAT   
  
  
- ATATTAATTG TTAATGGATT GTCAACTGGA TTGAAATTAG CCCAAATCTA TTCCATTGGC TCATTTTTTA   
  
  
- TGAATAATTA TAAAATGAAA TTGAGCTTG

+     GCN4\_motif

| Site Name | Organism | Position | Strand | Matrix score. | sequence | function |
| --- | --- | --- | --- | --- | --- | --- |
| GCN4\_motif | Oryza sativa | 1225 | + | 7 | TGTGTCA | cis-regulatory element involved in endosperm expression |

> 2018/04/13 10:10:12  
+ CGAGTGGGTT TTGGGGTTTT TTCTTTTGCT TTTTTTTTTG GTTTAGGGAT AAAGTGTCTT GGGAATCTTG   
  
  
+ GGTTTACTCT TTCGTAGTAT TTTTATTGTT CTTCATCTTG TTAAGGGTTT TCTAGTCCGA AAACTGTGGA   
  
  
+ GACTTTTGGT CTAGAATTAG GAATTTACGT GAATTTTGGG GTCTACTTTT ATTTAGTTTG TTCAGTAGGT   
  
  
+ GTTACCACCG ATTACCTGTT AAAACATTTG GGGGAACGGA TTTATGACTT TTAGAATACT AGGTTCTGTT   
  
  
+ TTCTATGTTG TCTCTCTACT GTTTTTGAGT CCGATGCTTC CTCTTATTTG TTTTGGGTAA AGGTATTTGT   
  
  
+ CTATGTGGGA ATGTCCCAAA ACGCGTTTTA AGTTTTGAAG GATAAATGAA ACTTTCACTT TCGTCTGTCT   
  
  
+ TTGTCTTACT CGAGTACTCG TTCCTGATAG GTCTTATTTT AGTCTGTCAA AGAAGGCCGA TTAAGAGGTT   
  
  
+ TCTTAGTTTT CCCATCTTAC TTCTTTTTTT AGTTCTGGTT TTGCTGTTTG GGTACAATTT TTGAACTCGT   
  
  
+ CCCCTGAGTT TCTAATAGGG TAACAACCTT AACTTTCCAC ACTTTCTTGC TGAAACGAAA GGGAGGCCCT   
  
  
+ GAAATAATTT TAGTGGGTTT TCTTTTGTTT CAGGGAGAAA ATTTAAGAAA CCGTTTTCTG TTCGTTTAGT   
  
  
+ TTTAGTATTA TCTTAACTTA AGGTGAGTTC TCACTGTGTC GTCTCCTCTT CTTCTTATGT TTAAGAAATA   
  
  
+ AATTTTTTTT TTTTTCCTTC TCTCTCTCTC TCTCTCCTCC GCCACGAGCG GTACCGTCAC CACCTGTGTT   
  
  
+ TCATCCCTTC TTTTTTGGTT GCAGAGACAG TTTCTAGGAT ACCAGCCAGG GTGGTAGTCT GGATCAATAA   
  
  
+ GGAAAAAAAG TTAAAAAATA AAAAAATAAA AAACAATTTC ATTTAGATAA ATAATAGCAA AAATACTCTT   
  
  
+ TCACCGAATC ACAGTTTTTA GTTTCACAGA CGGTTCGTTT TTTCACCCGG GTGCTGAGGT GACACCTGGC   
  
  
+ CCTCGTAGGG CTCATACGTT GTATGTCATT ACTTGCCAGA TATAAGTTGG TGCACCGTTA TTAGGAACAC   
  
  
+ TATATCCCTG TGAACAGGGA TCTTACAACT GATTTTCGTC CCGAAACAAC AGAAAAACCC GAAAATTTTT   
  
  
+ TTATTTTAAG ACTCGCACCC TGGGTGACCA GTCCTGTGTC AACTGGACTG TCTCTAAGAG AGACGGGGAA   
  
  
+ GATAAATACG TCGACGAACC TCGGAAACAA AAACTCGGTT GTATTTATTA TTCAGTATCT ATAACCTAAA   
  
  
+ GAATTTTACA ATTTATGATA AATAGAAATT TTAATATTTT TTATCACTTT TTTAATTAAA ATAATAAATA   
  
  
+ TATAATTAAC AATTACCTAA CAGTTGACCT AACTTTAATC GGGTTTAGAT AAGGTAACCG AGTAAAAAAT   
  
  
+ ACTTATTAAT ATTTTACTTT AACTCGAAC  

- GCTCACCCAA AACCCCAAAA AAGAAAACGA AAAAAAAAAC CAAATCCCTA TTTCACAGAA CCCTTAGAAC   
  
  
- CCAAATGAGA AAGCATCATA AAAATAACAA GAAGTAGAAC AATTCCCAAA AGATCAGGCT TTTGACACCT   
  
  
- CTGAAAACCA GATCTTAATC CTTAAATGCA CTTAAAACCC CAGATGAAAA TAAATCAAAC AAGTCATCCA   
  
  
- CAATGGTGGC TAATGGACAA TTTTGTAAAC CCCCTTGCCT AAATACTGAA AATCTTATGA TCCAAGACAA   
  
  
- AAGATACAAC AGAGAGATGA CAAAAACTCA GGCTACGAAG GAGAATAAAC AAAACCCATT TCCATAAACA   
  
  
- GATACACCCT TACAGGGTTT TGCGCAAAAT TCAAAACTTC CTATTTACTT TGAAAGTGAA AGCAGACAGA   
  
  
- AACAGAATGA GCTCATGAGC AAGGACTATC CAGAATAAAA TCAGACAGTT TCTTCCGGCT AATTCTCCAA   
  
  
- AGAATCAAAA GGGTAGAATG AAGAAAAAAA TCAAGACCAA AACGACAAAC CCATGTTAAA AACTTGAGCA   
  
  
- GGGGACTCAA AGATTATCCC ATTGTTGGAA TTGAAAGGTG TGAAAGAACG ACTTTGCTTT CCCTCCGGGA   
  
  
- CTTTATTAAA ATCACCCAAA AGAAAACAAA GTCCCTCTTT TAAATTCTTT GGCAAAAGAC AAGCAAATCA   
  
  
- AAATCATAAT AGAATTGAAT TCCACTCAAG AGTGACACAG CAGAGGAGAA GAAGAATACA AATTCTTTAT   
  
  
- TTAAAAAAAA AAAAAGGAAG AGAGAGAGAG AGAGAGGAGG CGGTGCTCGC CATGGCAGTG GTGGACACAA   
  
  
- AGTAGGGAAG AAAAAACCAA CGTCTCTGTC AAAGATCCTA TGGTCGGTCC CACCATCAGA CCTAGTTATT   
  
  
- CCTTTTTTTC AATTTTTTAT TTTTTTATTT TTTGTTAAAG TAAATCTATT TATTATCGTT TTTATGAGAA   
  
  
- AGTGGCTTAG TGTCAAAAAT CAAAGTGTCT GCCAAGCAAA AAAGTGGGCC CACGACTCCA CTGTGGACCG   
  
  
- GGAGCATCCC GAGTATGCAA CATACAGTAA TGAACGGTCT ATATTCAACC ACGTGGCAAT AATCCTTGTG   
  
  
- ATATAGGGAC ACTTGTCCCT AGAATGTTGA CTAAAAGCAG GGCTTTGTTG TCTTTTTGGG CTTTTAAAAA   
  
  
- AATAAAATTC TGAGCGTGGG ACCCACTGGT CAGGACACAG TTGACCTGAC AGAGATTCTC TCTGCCCCTT   
  
  
- CTATTTATGC AGCTGCTTGG AGCCTTTGTT TTTGAGCCAA CATAAATAAT AAGTCATAGA TATTGGATTT   
  
  
- CTTAAAATGT TAAATACTAT TTATCTTTAA AATTATAAAA AATAGTGAAA AAATTAATTT TATTATTTAT   
  
  
- ATATTAATTG TTAATGGATT GTCAACTGGA TTGAAATTAG CCCAAATCTA TTCCATTGGC TCATTTTTTA   
  
  
- TGAATAATTA TAAAATGAAA TTGAGCTTG

+     HSE

| Site Name | Organism | Position | Strand | Matrix score. | sequence | function |
| --- | --- | --- | --- | --- | --- | --- |
| HSE | Brassica oleracea | 941 | + | 9 | AAAAAATTTC | cis-acting element involved in heat stress responsiveness |
| HSE | Brassica oleracea | 1181 | - | 9 | AAAAAATTTC | cis-acting element involved in heat stress responsiveness |
| HSE | Brassica oleracea | 769 | - | 9 | AAAAAATTTC | cis-acting element involved in heat stress responsiveness |
| HSE | Brassica oleracea | 1182 | - | 9 | AAAAAATTTC | cis-acting element involved in heat stress responsiveness |

> 2018/04/13 10:10:12  
+ CGAGTGGGTT TTGGGGTTTT TTCTTTTGCT TTTTTTTTTG GTTTAGGGAT AAAGTGTCTT GGGAATCTTG   
  
  
+ GGTTTACTCT TTCGTAGTAT TTTTATTGTT CTTCATCTTG TTAAGGGTTT TCTAGTCCGA AAACTGTGGA   
  
  
+ GACTTTTGGT CTAGAATTAG GAATTTACGT GAATTTTGGG GTCTACTTTT ATTTAGTTTG TTCAGTAGGT   
  
  
+ GTTACCACCG ATTACCTGTT AAAACATTTG GGGGAACGGA TTTATGACTT TTAGAATACT AGGTTCTGTT   
  
  
+ TTCTATGTTG TCTCTCTACT GTTTTTGAGT CCGATGCTTC CTCTTATTTG TTTTGGGTAA AGGTATTTGT   
  
  
+ CTATGTGGGA ATGTCCCAAA ACGCGTTTTA AGTTTTGAAG GATAAATGAA ACTTTCACTT TCGTCTGTCT   
  
  
+ TTGTCTTACT CGAGTACTCG TTCCTGATAG GTCTTATTTT AGTCTGTCAA AGAAGGCCGA TTAAGAGGTT   
  
  
+ TCTTAGTTTT CCCATCTTAC TTCTTTTTTT AGTTCTGGTT TTGCTGTTTG GGTACAATTT TTGAACTCGT   
  
  
+ CCCCTGAGTT TCTAATAGGG TAACAACCTT AACTTTCCAC ACTTTCTTGC TGAAACGAAA GGGAGGCCCT   
  
  
+ GAAATAATTT TAGTGGGTTT TCTTTTGTTT CAGGGAGAAA ATTTAAGAAA CCGTTTTCTG TTCGTTTAGT   
  
  
+ TTTAGTATTA TCTTAACTTA AGGTGAGTTC TCACTGTGTC GTCTCCTCTT CTTCTTATGT TTAAGAAATA   
  
  
+ AATTTTTTTT TTTTTCCTTC TCTCTCTCTC TCTCTCCTCC GCCACGAGCG GTACCGTCAC CACCTGTGTT   
  
  
+ TCATCCCTTC TTTTTTGGTT GCAGAGACAG TTTCTAGGAT ACCAGCCAGG GTGGTAGTCT GGATCAATAA   
  
  
+ GGAAAAAAAG TTAAAAAATA AAAAAATAAA AAACAATTTC ATTTAGATAA ATAATAGCAA AAATACTCTT   
  
  
+ TCACCGAATC ACAGTTTTTA GTTTCACAGA CGGTTCGTTT TTTCACCCGG GTGCTGAGGT GACACCTGGC   
  
  
+ CCTCGTAGGG CTCATACGTT GTATGTCATT ACTTGCCAGA TATAAGTTGG TGCACCGTTA TTAGGAACAC   
  
  
+ TATATCCCTG TGAACAGGGA TCTTACAACT GATTTTCGTC CCGAAACAAC AGAAAAACCC GAAAATTTTT   
  
  
+ TTATTTTAAG ACTCGCACCC TGGGTGACCA GTCCTGTGTC AACTGGACTG TCTCTAAGAG AGACGGGGAA   
  
  
+ GATAAATACG TCGACGAACC TCGGAAACAA AAACTCGGTT GTATTTATTA TTCAGTATCT ATAACCTAAA   
  
  
+ GAATTTTACA ATTTATGATA AATAGAAATT TTAATATTTT TTATCACTTT TTTAATTAAA ATAATAAATA   
  
  
+ TATAATTAAC AATTACCTAA CAGTTGACCT AACTTTAATC GGGTTTAGAT AAGGTAACCG AGTAAAAAAT   
  
  
+ ACTTATTAAT ATTTTACTTT AACTCGAAC  

- GCTCACCCAA AACCCCAAAA AAGAAAACGA AAAAAAAAAC CAAATCCCTA TTTCACAGAA CCCTTAGAAC   
  
  
- CCAAATGAGA AAGCATCATA AAAATAACAA GAAGTAGAAC AATTCCCAAA AGATCAGGCT TTTGACACCT   
  
  
- CTGAAAACCA GATCTTAATC CTTAAATGCA CTTAAAACCC CAGATGAAAA TAAATCAAAC AAGTCATCCA   
  
  
- CAATGGTGGC TAATGGACAA TTTTGTAAAC CCCCTTGCCT AAATACTGAA AATCTTATGA TCCAAGACAA   
  
  
- AAGATACAAC AGAGAGATGA CAAAAACTCA GGCTACGAAG GAGAATAAAC AAAACCCATT TCCATAAACA   
  
  
- GATACACCCT TACAGGGTTT TGCGCAAAAT TCAAAACTTC CTATTTACTT TGAAAGTGAA AGCAGACAGA   
  
  
- AACAGAATGA GCTCATGAGC AAGGACTATC CAGAATAAAA TCAGACAGTT TCTTCCGGCT AATTCTCCAA   
  
  
- AGAATCAAAA GGGTAGAATG AAGAAAAAAA TCAAGACCAA AACGACAAAC CCATGTTAAA AACTTGAGCA   
  
  
- GGGGACTCAA AGATTATCCC ATTGTTGGAA TTGAAAGGTG TGAAAGAACG ACTTTGCTTT CCCTCCGGGA   
  
  
- CTTTATTAAA ATCACCCAAA AGAAAACAAA GTCCCTCTTT TAAATTCTTT GGCAAAAGAC AAGCAAATCA   
  
  
- AAATCATAAT AGAATTGAAT TCCACTCAAG AGTGACACAG CAGAGGAGAA GAAGAATACA AATTCTTTAT   
  
  
- TTAAAAAAAA AAAAAGGAAG AGAGAGAGAG AGAGAGGAGG CGGTGCTCGC CATGGCAGTG GTGGACACAA   
  
  
- AGTAGGGAAG AAAAAACCAA CGTCTCTGTC AAAGATCCTA TGGTCGGTCC CACCATCAGA CCTAGTTATT   
  
  
- CCTTTTTTTC AATTTTTTAT TTTTTTATTT TTTGTTAAAG TAAATCTATT TATTATCGTT TTTATGAGAA   
  
  
- AGTGGCTTAG TGTCAAAAAT CAAAGTGTCT GCCAAGCAAA AAAGTGGGCC CACGACTCCA CTGTGGACCG   
  
  
- GGAGCATCCC GAGTATGCAA CATACAGTAA TGAACGGTCT ATATTCAACC ACGTGGCAAT AATCCTTGTG   
  
  
- ATATAGGGAC ACTTGTCCCT AGAATGTTGA CTAAAAGCAG GGCTTTGTTG TCTTTTTGGG CTTTTAAAAA   
  
  
- AATAAAATTC TGAGCGTGGG ACCCACTGGT CAGGACACAG TTGACCTGAC AGAGATTCTC TCTGCCCCTT   
  
  
- CTATTTATGC AGCTGCTTGG AGCCTTTGTT TTTGAGCCAA CATAAATAAT AAGTCATAGA TATTGGATTT   
  
  
- CTTAAAATGT TAAATACTAT TTATCTTTAA AATTATAAAA AATAGTGAAA AAATTAATTT TATTATTTAT   
  
  
- ATATTAATTG TTAATGGATT GTCAACTGGA TTGAAATTAG CCCAAATCTA TTCCATTGGC TCATTTTTTA   
  
  
- TGAATAATTA TAAAATGAAA TTGAGCTTG

+     I-box

| Site Name | Organism | Position | Strand | Matrix score. | sequence | function |
| --- | --- | --- | --- | --- | --- | --- |
| I-box | Triticum aestivum | 1447 | + | 8 | AGATAAGG | part of a light responsive element |
| I-box | Zea mays | 47 | + | 9 | gGATAAGGTG | part of a light responsive element |

> 2018/04/13 10:10:12  
+ CGAGTGGGTT TTGGGGTTTT TTCTTTTGCT TTTTTTTTTG GTTTAGGGAT AAAGTGTCTT GGGAATCTTG   
  
  
+ GGTTTACTCT TTCGTAGTAT TTTTATTGTT CTTCATCTTG TTAAGGGTTT TCTAGTCCGA AAACTGTGGA   
  
  
+ GACTTTTGGT CTAGAATTAG GAATTTACGT GAATTTTGGG GTCTACTTTT ATTTAGTTTG TTCAGTAGGT   
  
  
+ GTTACCACCG ATTACCTGTT AAAACATTTG GGGGAACGGA TTTATGACTT TTAGAATACT AGGTTCTGTT   
  
  
+ TTCTATGTTG TCTCTCTACT GTTTTTGAGT CCGATGCTTC CTCTTATTTG TTTTGGGTAA AGGTATTTGT   
  
  
+ CTATGTGGGA ATGTCCCAAA ACGCGTTTTA AGTTTTGAAG GATAAATGAA ACTTTCACTT TCGTCTGTCT   
  
  
+ TTGTCTTACT CGAGTACTCG TTCCTGATAG GTCTTATTTT AGTCTGTCAA AGAAGGCCGA TTAAGAGGTT   
  
  
+ TCTTAGTTTT CCCATCTTAC TTCTTTTTTT AGTTCTGGTT TTGCTGTTTG GGTACAATTT TTGAACTCGT   
  
  
+ CCCCTGAGTT TCTAATAGGG TAACAACCTT AACTTTCCAC ACTTTCTTGC TGAAACGAAA GGGAGGCCCT   
  
  
+ GAAATAATTT TAGTGGGTTT TCTTTTGTTT CAGGGAGAAA ATTTAAGAAA CCGTTTTCTG TTCGTTTAGT   
  
  
+ TTTAGTATTA TCTTAACTTA AGGTGAGTTC TCACTGTGTC GTCTCCTCTT CTTCTTATGT TTAAGAAATA   
  
  
+ AATTTTTTTT TTTTTCCTTC TCTCTCTCTC TCTCTCCTCC GCCACGAGCG GTACCGTCAC CACCTGTGTT   
  
  
+ TCATCCCTTC TTTTTTGGTT GCAGAGACAG TTTCTAGGAT ACCAGCCAGG GTGGTAGTCT GGATCAATAA   
  
  
+ GGAAAAAAAG TTAAAAAATA AAAAAATAAA AAACAATTTC ATTTAGATAA ATAATAGCAA AAATACTCTT   
  
  
+ TCACCGAATC ACAGTTTTTA GTTTCACAGA CGGTTCGTTT TTTCACCCGG GTGCTGAGGT GACACCTGGC   
  
  
+ CCTCGTAGGG CTCATACGTT GTATGTCATT ACTTGCCAGA TATAAGTTGG TGCACCGTTA TTAGGAACAC   
  
  
+ TATATCCCTG TGAACAGGGA TCTTACAACT GATTTTCGTC CCGAAACAAC AGAAAAACCC GAAAATTTTT   
  
  
+ TTATTTTAAG ACTCGCACCC TGGGTGACCA GTCCTGTGTC AACTGGACTG TCTCTAAGAG AGACGGGGAA   
  
  
+ GATAAATACG TCGACGAACC TCGGAAACAA AAACTCGGTT GTATTTATTA TTCAGTATCT ATAACCTAAA   
  
  
+ GAATTTTACA ATTTATGATA AATAGAAATT TTAATATTTT TTATCACTTT TTTAATTAAA ATAATAAATA   
  
  
+ TATAATTAAC AATTACCTAA CAGTTGACCT AACTTTAATC GGGTTTAGAT AAGGTAACCG AGTAAAAAAT   
  
  
+ ACTTATTAAT ATTTTACTTT AACTCGAAC  

- GCTCACCCAA AACCCCAAAA AAGAAAACGA AAAAAAAAAC CAAATCCCTA TTTCACAGAA CCCTTAGAAC   
  
  
- CCAAATGAGA AAGCATCATA AAAATAACAA GAAGTAGAAC AATTCCCAAA AGATCAGGCT TTTGACACCT   
  
  
- CTGAAAACCA GATCTTAATC CTTAAATGCA CTTAAAACCC CAGATGAAAA TAAATCAAAC AAGTCATCCA   
  
  
- CAATGGTGGC TAATGGACAA TTTTGTAAAC CCCCTTGCCT AAATACTGAA AATCTTATGA TCCAAGACAA   
  
  
- AAGATACAAC AGAGAGATGA CAAAAACTCA GGCTACGAAG GAGAATAAAC AAAACCCATT TCCATAAACA   
  
  
- GATACACCCT TACAGGGTTT TGCGCAAAAT TCAAAACTTC CTATTTACTT TGAAAGTGAA AGCAGACAGA   
  
  
- AACAGAATGA GCTCATGAGC AAGGACTATC CAGAATAAAA TCAGACAGTT TCTTCCGGCT AATTCTCCAA   
  
  
- AGAATCAAAA GGGTAGAATG AAGAAAAAAA TCAAGACCAA AACGACAAAC CCATGTTAAA AACTTGAGCA   
  
  
- GGGGACTCAA AGATTATCCC ATTGTTGGAA TTGAAAGGTG TGAAAGAACG ACTTTGCTTT CCCTCCGGGA   
  
  
- CTTTATTAAA ATCACCCAAA AGAAAACAAA GTCCCTCTTT TAAATTCTTT GGCAAAAGAC AAGCAAATCA   
  
  
- AAATCATAAT AGAATTGAAT TCCACTCAAG AGTGACACAG CAGAGGAGAA GAAGAATACA AATTCTTTAT   
  
  
- TTAAAAAAAA AAAAAGGAAG AGAGAGAGAG AGAGAGGAGG CGGTGCTCGC CATGGCAGTG GTGGACACAA   
  
  
- AGTAGGGAAG AAAAAACCAA CGTCTCTGTC AAAGATCCTA TGGTCGGTCC CACCATCAGA CCTAGTTATT   
  
  
- CCTTTTTTTC AATTTTTTAT TTTTTTATTT TTTGTTAAAG TAAATCTATT TATTATCGTT TTTATGAGAA   
  
  
- AGTGGCTTAG TGTCAAAAAT CAAAGTGTCT GCCAAGCAAA AAAGTGGGCC CACGACTCCA CTGTGGACCG   
  
  
- GGAGCATCCC GAGTATGCAA CATACAGTAA TGAACGGTCT ATATTCAACC ACGTGGCAAT AATCCTTGTG   
  
  
- ATATAGGGAC ACTTGTCCCT AGAATGTTGA CTAAAAGCAG GGCTTTGTTG TCTTTTTGGG CTTTTAAAAA   
  
  
- AATAAAATTC TGAGCGTGGG ACCCACTGGT CAGGACACAG TTGACCTGAC AGAGATTCTC TCTGCCCCTT   
  
  
- CTATTTATGC AGCTGCTTGG AGCCTTTGTT TTTGAGCCAA CATAAATAAT AAGTCATAGA TATTGGATTT   
  
  
- CTTAAAATGT TAAATACTAT TTATCTTTAA AATTATAAAA AATAGTGAAA AAATTAATTT TATTATTTAT   
  
  
- ATATTAATTG TTAATGGATT GTCAACTGGA TTGAAATTAG CCCAAATCTA TTCCATTGGC TCATTTTTTA   
  
  
- TGAATAATTA TAAAATGAAA TTGAGCTTG

+     LTR

| Site Name | Organism | Position | Strand | Matrix score. | sequence | function |
| --- | --- | --- | --- | --- | --- | --- |
| LTR | Hordeum vulgare | 1179 | + | 6 | CCGAAA | cis-acting element involved in low-temperature responsiveness |
| LTR | Hordeum vulgare | 1161 | + | 6 | CCGAAA | cis-acting element involved in low-temperature responsiveness |
| LTR | Hordeum vulgare | 127 | + | 6 | CCGAAA | cis-acting element involved in low-temperature responsiveness |

> 2018/04/13 10:10:12  
+ CGAGTGGGTT TTGGGGTTTT TTCTTTTGCT TTTTTTTTTG GTTTAGGGAT AAAGTGTCTT GGGAATCTTG   
  
  
+ GGTTTACTCT TTCGTAGTAT TTTTATTGTT CTTCATCTTG TTAAGGGTTT TCTAGTCCGA AAACTGTGGA   
  
  
+ GACTTTTGGT CTAGAATTAG GAATTTACGT GAATTTTGGG GTCTACTTTT ATTTAGTTTG TTCAGTAGGT   
  
  
+ GTTACCACCG ATTACCTGTT AAAACATTTG GGGGAACGGA TTTATGACTT TTAGAATACT AGGTTCTGTT   
  
  
+ TTCTATGTTG TCTCTCTACT GTTTTTGAGT CCGATGCTTC CTCTTATTTG TTTTGGGTAA AGGTATTTGT   
  
  
+ CTATGTGGGA ATGTCCCAAA ACGCGTTTTA AGTTTTGAAG GATAAATGAA ACTTTCACTT TCGTCTGTCT   
  
  
+ TTGTCTTACT CGAGTACTCG TTCCTGATAG GTCTTATTTT AGTCTGTCAA AGAAGGCCGA TTAAGAGGTT   
  
  
+ TCTTAGTTTT CCCATCTTAC TTCTTTTTTT AGTTCTGGTT TTGCTGTTTG GGTACAATTT TTGAACTCGT   
  
  
+ CCCCTGAGTT TCTAATAGGG TAACAACCTT AACTTTCCAC ACTTTCTTGC TGAAACGAAA GGGAGGCCCT   
  
  
+ GAAATAATTT TAGTGGGTTT TCTTTTGTTT CAGGGAGAAA ATTTAAGAAA CCGTTTTCTG TTCGTTTAGT   
  
  
+ TTTAGTATTA TCTTAACTTA AGGTGAGTTC TCACTGTGTC GTCTCCTCTT CTTCTTATGT TTAAGAAATA   
  
  
+ AATTTTTTTT TTTTTCCTTC TCTCTCTCTC TCTCTCCTCC GCCACGAGCG GTACCGTCAC CACCTGTGTT   
  
  
+ TCATCCCTTC TTTTTTGGTT GCAGAGACAG TTTCTAGGAT ACCAGCCAGG GTGGTAGTCT GGATCAATAA   
  
  
+ GGAAAAAAAG TTAAAAAATA AAAAAATAAA AAACAATTTC ATTTAGATAA ATAATAGCAA AAATACTCTT   
  
  
+ TCACCGAATC ACAGTTTTTA GTTTCACAGA CGGTTCGTTT TTTCACCCGG GTGCTGAGGT GACACCTGGC   
  
  
+ CCTCGTAGGG CTCATACGTT GTATGTCATT ACTTGCCAGA TATAAGTTGG TGCACCGTTA TTAGGAACAC   
  
  
+ TATATCCCTG TGAACAGGGA TCTTACAACT GATTTTCGTC CCGAAACAAC AGAAAAACCC GAAAATTTTT   
  
  
+ TTATTTTAAG ACTCGCACCC TGGGTGACCA GTCCTGTGTC AACTGGACTG TCTCTAAGAG AGACGGGGAA   
  
  
+ GATAAATACG TCGACGAACC TCGGAAACAA AAACTCGGTT GTATTTATTA TTCAGTATCT ATAACCTAAA   
  
  
+ GAATTTTACA ATTTATGATA AATAGAAATT TTAATATTTT TTATCACTTT TTTAATTAAA ATAATAAATA   
  
  
+ TATAATTAAC AATTACCTAA CAGTTGACCT AACTTTAATC GGGTTTAGAT AAGGTAACCG AGTAAAAAAT   
  
  
+ ACTTATTAAT ATTTTACTTT AACTCGAAC  

- GCTCACCCAA AACCCCAAAA AAGAAAACGA AAAAAAAAAC CAAATCCCTA TTTCACAGAA CCCTTAGAAC   
  
  
- CCAAATGAGA AAGCATCATA AAAATAACAA GAAGTAGAAC AATTCCCAAA AGATCAGGCT TTTGACACCT   
  
  
- CTGAAAACCA GATCTTAATC CTTAAATGCA CTTAAAACCC CAGATGAAAA TAAATCAAAC AAGTCATCCA   
  
  
- CAATGGTGGC TAATGGACAA TTTTGTAAAC CCCCTTGCCT AAATACTGAA AATCTTATGA TCCAAGACAA   
  
  
- AAGATACAAC AGAGAGATGA CAAAAACTCA GGCTACGAAG GAGAATAAAC AAAACCCATT TCCATAAACA   
  
  
- GATACACCCT TACAGGGTTT TGCGCAAAAT TCAAAACTTC CTATTTACTT TGAAAGTGAA AGCAGACAGA   
  
  
- AACAGAATGA GCTCATGAGC AAGGACTATC CAGAATAAAA TCAGACAGTT TCTTCCGGCT AATTCTCCAA   
  
  
- AGAATCAAAA GGGTAGAATG AAGAAAAAAA TCAAGACCAA AACGACAAAC CCATGTTAAA AACTTGAGCA   
  
  
- GGGGACTCAA AGATTATCCC ATTGTTGGAA TTGAAAGGTG TGAAAGAACG ACTTTGCTTT CCCTCCGGGA   
  
  
- CTTTATTAAA ATCACCCAAA AGAAAACAAA GTCCCTCTTT TAAATTCTTT GGCAAAAGAC AAGCAAATCA   
  
  
- AAATCATAAT AGAATTGAAT TCCACTCAAG AGTGACACAG CAGAGGAGAA GAAGAATACA AATTCTTTAT   
  
  
- TTAAAAAAAA AAAAAGGAAG AGAGAGAGAG AGAGAGGAGG CGGTGCTCGC CATGGCAGTG GTGGACACAA   
  
  
- AGTAGGGAAG AAAAAACCAA CGTCTCTGTC AAAGATCCTA TGGTCGGTCC CACCATCAGA CCTAGTTATT   
  
  
- CCTTTTTTTC AATTTTTTAT TTTTTTATTT TTTGTTAAAG TAAATCTATT TATTATCGTT TTTATGAGAA   
  
  
- AGTGGCTTAG TGTCAAAAAT CAAAGTGTCT GCCAAGCAAA AAAGTGGGCC CACGACTCCA CTGTGGACCG   
  
  
- GGAGCATCCC GAGTATGCAA CATACAGTAA TGAACGGTCT ATATTCAACC ACGTGGCAAT AATCCTTGTG   
  
  
- ATATAGGGAC ACTTGTCCCT AGAATGTTGA CTAAAAGCAG GGCTTTGTTG TCTTTTTGGG CTTTTAAAAA   
  
  
- AATAAAATTC TGAGCGTGGG ACCCACTGGT CAGGACACAG TTGACCTGAC AGAGATTCTC TCTGCCCCTT   
  
  
- CTATTTATGC AGCTGCTTGG AGCCTTTGTT TTTGAGCCAA CATAAATAAT AAGTCATAGA TATTGGATTT   
  
  
- CTTAAAATGT TAAATACTAT TTATCTTTAA AATTATAAAA AATAGTGAAA AAATTAATTT TATTATTTAT   
  
  
- ATATTAATTG TTAATGGATT GTCAACTGGA TTGAAATTAG CCCAAATCTA TTCCATTGGC TCATTTTTTA   
  
  
- TGAATAATTA TAAAATGAAA TTGAGCTTG

+     MBS

| Site Name | Organism | Position | Strand | Matrix score. | sequence | function |
| --- | --- | --- | --- | --- | --- | --- |
| MBS | Arabidopsis thaliana | 1146 | + | 6 | CAACTG | MYB binding site involved in drought-inducibility |
| MBS | Arabidopsis thaliana | 1421 | - | 6 | CAACTG | MYB binding site involved in drought-inducibility |
| MBS | Arabidopsis thaliana | 1230 | + | 6 | CAACTG | MYB binding site involved in drought-inducibility |

> 2018/04/13 10:10:12  
+ CGAGTGGGTT TTGGGGTTTT TTCTTTTGCT TTTTTTTTTG GTTTAGGGAT AAAGTGTCTT GGGAATCTTG   
  
  
+ GGTTTACTCT TTCGTAGTAT TTTTATTGTT CTTCATCTTG TTAAGGGTTT TCTAGTCCGA AAACTGTGGA   
  
  
+ GACTTTTGGT CTAGAATTAG GAATTTACGT GAATTTTGGG GTCTACTTTT ATTTAGTTTG TTCAGTAGGT   
  
  
+ GTTACCACCG ATTACCTGTT AAAACATTTG GGGGAACGGA TTTATGACTT TTAGAATACT AGGTTCTGTT   
  
  
+ TTCTATGTTG TCTCTCTACT GTTTTTGAGT CCGATGCTTC CTCTTATTTG TTTTGGGTAA AGGTATTTGT   
  
  
+ CTATGTGGGA ATGTCCCAAA ACGCGTTTTA AGTTTTGAAG GATAAATGAA ACTTTCACTT TCGTCTGTCT   
  
  
+ TTGTCTTACT CGAGTACTCG TTCCTGATAG GTCTTATTTT AGTCTGTCAA AGAAGGCCGA TTAAGAGGTT   
  
  
+ TCTTAGTTTT CCCATCTTAC TTCTTTTTTT AGTTCTGGTT TTGCTGTTTG GGTACAATTT TTGAACTCGT   
  
  
+ CCCCTGAGTT TCTAATAGGG TAACAACCTT AACTTTCCAC ACTTTCTTGC TGAAACGAAA GGGAGGCCCT   
  
  
+ GAAATAATTT TAGTGGGTTT TCTTTTGTTT CAGGGAGAAA ATTTAAGAAA CCGTTTTCTG TTCGTTTAGT   
  
  
+ TTTAGTATTA TCTTAACTTA AGGTGAGTTC TCACTGTGTC GTCTCCTCTT CTTCTTATGT TTAAGAAATA   
  
  
+ AATTTTTTTT TTTTTCCTTC TCTCTCTCTC TCTCTCCTCC GCCACGAGCG GTACCGTCAC CACCTGTGTT   
  
  
+ TCATCCCTTC TTTTTTGGTT GCAGAGACAG TTTCTAGGAT ACCAGCCAGG GTGGTAGTCT GGATCAATAA   
  
  
+ GGAAAAAAAG TTAAAAAATA AAAAAATAAA AAACAATTTC ATTTAGATAA ATAATAGCAA AAATACTCTT   
  
  
+ TCACCGAATC ACAGTTTTTA GTTTCACAGA CGGTTCGTTT TTTCACCCGG GTGCTGAGGT GACACCTGGC   
  
  
+ CCTCGTAGGG CTCATACGTT GTATGTCATT ACTTGCCAGA TATAAGTTGG TGCACCGTTA TTAGGAACAC   
  
  
+ TATATCCCTG TGAACAGGGA TCTTACAACT GATTTTCGTC CCGAAACAAC AGAAAAACCC GAAAATTTTT   
  
  
+ TTATTTTAAG ACTCGCACCC TGGGTGACCA GTCCTGTGTC AACTGGACTG TCTCTAAGAG AGACGGGGAA   
  
  
+ GATAAATACG TCGACGAACC TCGGAAACAA AAACTCGGTT GTATTTATTA TTCAGTATCT ATAACCTAAA   
  
  
+ GAATTTTACA ATTTATGATA AATAGAAATT TTAATATTTT TTATCACTTT TTTAATTAAA ATAATAAATA   
  
  
+ TATAATTAAC AATTACCTAA CAGTTGACCT AACTTTAATC GGGTTTAGAT AAGGTAACCG AGTAAAAAAT   
  
  
+ ACTTATTAAT ATTTTACTTT AACTCGAAC  

- GCTCACCCAA AACCCCAAAA AAGAAAACGA AAAAAAAAAC CAAATCCCTA TTTCACAGAA CCCTTAGAAC   
  
  
- CCAAATGAGA AAGCATCATA AAAATAACAA GAAGTAGAAC AATTCCCAAA AGATCAGGCT TTTGACACCT   
  
  
- CTGAAAACCA GATCTTAATC CTTAAATGCA CTTAAAACCC CAGATGAAAA TAAATCAAAC AAGTCATCCA   
  
  
- CAATGGTGGC TAATGGACAA TTTTGTAAAC CCCCTTGCCT AAATACTGAA AATCTTATGA TCCAAGACAA   
  
  
- AAGATACAAC AGAGAGATGA CAAAAACTCA GGCTACGAAG GAGAATAAAC AAAACCCATT TCCATAAACA   
  
  
- GATACACCCT TACAGGGTTT TGCGCAAAAT TCAAAACTTC CTATTTACTT TGAAAGTGAA AGCAGACAGA   
  
  
- AACAGAATGA GCTCATGAGC AAGGACTATC CAGAATAAAA TCAGACAGTT TCTTCCGGCT AATTCTCCAA   
  
  
- AGAATCAAAA GGGTAGAATG AAGAAAAAAA TCAAGACCAA AACGACAAAC CCATGTTAAA AACTTGAGCA   
  
  
- GGGGACTCAA AGATTATCCC ATTGTTGGAA TTGAAAGGTG TGAAAGAACG ACTTTGCTTT CCCTCCGGGA   
  
  
- CTTTATTAAA ATCACCCAAA AGAAAACAAA GTCCCTCTTT TAAATTCTTT GGCAAAAGAC AAGCAAATCA   
  
  
- AAATCATAAT AGAATTGAAT TCCACTCAAG AGTGACACAG CAGAGGAGAA GAAGAATACA AATTCTTTAT   
  
  
- TTAAAAAAAA AAAAAGGAAG AGAGAGAGAG AGAGAGGAGG CGGTGCTCGC CATGGCAGTG GTGGACACAA   
  
  
- AGTAGGGAAG AAAAAACCAA CGTCTCTGTC AAAGATCCTA TGGTCGGTCC CACCATCAGA CCTAGTTATT   
  
  
- CCTTTTTTTC AATTTTTTAT TTTTTTATTT TTTGTTAAAG TAAATCTATT TATTATCGTT TTTATGAGAA   
  
  
- AGTGGCTTAG TGTCAAAAAT CAAAGTGTCT GCCAAGCAAA AAAGTGGGCC CACGACTCCA CTGTGGACCG   
  
  
- GGAGCATCCC GAGTATGCAA CATACAGTAA TGAACGGTCT ATATTCAACC ACGTGGCAAT AATCCTTGTG   
  
  
- ATATAGGGAC ACTTGTCCCT AGAATGTTGA CTAAAAGCAG GGCTTTGTTG TCTTTTTGGG CTTTTAAAAA   
  
  
- AATAAAATTC TGAGCGTGGG ACCCACTGGT CAGGACACAG TTGACCTGAC AGAGATTCTC TCTGCCCCTT   
  
  
- CTATTTATGC AGCTGCTTGG AGCCTTTGTT TTTGAGCCAA CATAAATAAT AAGTCATAGA TATTGGATTT   
  
  
- CTTAAAATGT TAAATACTAT TTATCTTTAA AATTATAAAA AATAGTGAAA AAATTAATTT TATTATTTAT   
  
  
- ATATTAATTG TTAATGGATT GTCAACTGGA TTGAAATTAG CCCAAATCTA TTCCATTGGC TCATTTTTTA   
  
  
- TGAATAATTA TAAAATGAAA TTGAGCTTG

+     MRE

| Site Name | Organism | Position | Strand | Matrix score. | sequence | function |
| --- | --- | --- | --- | --- | --- | --- |
| MRE | Petroselinum crispum | 1323 | + | 7 | AACCTAA | MYB binding site involved in light responsiveness |

> 2018/04/13 10:10:12  
+ CGAGTGGGTT TTGGGGTTTT TTCTTTTGCT TTTTTTTTTG GTTTAGGGAT AAAGTGTCTT GGGAATCTTG   
  
  
+ GGTTTACTCT TTCGTAGTAT TTTTATTGTT CTTCATCTTG TTAAGGGTTT TCTAGTCCGA AAACTGTGGA   
  
  
+ GACTTTTGGT CTAGAATTAG GAATTTACGT GAATTTTGGG GTCTACTTTT ATTTAGTTTG TTCAGTAGGT   
  
  
+ GTTACCACCG ATTACCTGTT AAAACATTTG GGGGAACGGA TTTATGACTT TTAGAATACT AGGTTCTGTT   
  
  
+ TTCTATGTTG TCTCTCTACT GTTTTTGAGT CCGATGCTTC CTCTTATTTG TTTTGGGTAA AGGTATTTGT   
  
  
+ CTATGTGGGA ATGTCCCAAA ACGCGTTTTA AGTTTTGAAG GATAAATGAA ACTTTCACTT TCGTCTGTCT   
  
  
+ TTGTCTTACT CGAGTACTCG TTCCTGATAG GTCTTATTTT AGTCTGTCAA AGAAGGCCGA TTAAGAGGTT   
  
  
+ TCTTAGTTTT CCCATCTTAC TTCTTTTTTT AGTTCTGGTT TTGCTGTTTG GGTACAATTT TTGAACTCGT   
  
  
+ CCCCTGAGTT TCTAATAGGG TAACAACCTT AACTTTCCAC ACTTTCTTGC TGAAACGAAA GGGAGGCCCT   
  
  
+ GAAATAATTT TAGTGGGTTT TCTTTTGTTT CAGGGAGAAA ATTTAAGAAA CCGTTTTCTG TTCGTTTAGT   
  
  
+ TTTAGTATTA TCTTAACTTA AGGTGAGTTC TCACTGTGTC GTCTCCTCTT CTTCTTATGT TTAAGAAATA   
  
  
+ AATTTTTTTT TTTTTCCTTC TCTCTCTCTC TCTCTCCTCC GCCACGAGCG GTACCGTCAC CACCTGTGTT   
  
  
+ TCATCCCTTC TTTTTTGGTT GCAGAGACAG TTTCTAGGAT ACCAGCCAGG GTGGTAGTCT GGATCAATAA   
  
  
+ GGAAAAAAAG TTAAAAAATA AAAAAATAAA AAACAATTTC ATTTAGATAA ATAATAGCAA AAATACTCTT   
  
  
+ TCACCGAATC ACAGTTTTTA GTTTCACAGA CGGTTCGTTT TTTCACCCGG GTGCTGAGGT GACACCTGGC   
  
  
+ CCTCGTAGGG CTCATACGTT GTATGTCATT ACTTGCCAGA TATAAGTTGG TGCACCGTTA TTAGGAACAC   
  
  
+ TATATCCCTG TGAACAGGGA TCTTACAACT GATTTTCGTC CCGAAACAAC AGAAAAACCC GAAAATTTTT   
  
  
+ TTATTTTAAG ACTCGCACCC TGGGTGACCA GTCCTGTGTC AACTGGACTG TCTCTAAGAG AGACGGGGAA   
  
  
+ GATAAATACG TCGACGAACC TCGGAAACAA AAACTCGGTT GTATTTATTA TTCAGTATCT ATAACCTAAA   
  
  
+ GAATTTTACA ATTTATGATA AATAGAAATT TTAATATTTT TTATCACTTT TTTAATTAAA ATAATAAATA   
  
  
+ TATAATTAAC AATTACCTAA CAGTTGACCT AACTTTAATC GGGTTTAGAT AAGGTAACCG AGTAAAAAAT   
  
  
+ ACTTATTAAT ATTTTACTTT AACTCGAAC  

- GCTCACCCAA AACCCCAAAA AAGAAAACGA AAAAAAAAAC CAAATCCCTA TTTCACAGAA CCCTTAGAAC   
  
  
- CCAAATGAGA AAGCATCATA AAAATAACAA GAAGTAGAAC AATTCCCAAA AGATCAGGCT TTTGACACCT   
  
  
- CTGAAAACCA GATCTTAATC CTTAAATGCA CTTAAAACCC CAGATGAAAA TAAATCAAAC AAGTCATCCA   
  
  
- CAATGGTGGC TAATGGACAA TTTTGTAAAC CCCCTTGCCT AAATACTGAA AATCTTATGA TCCAAGACAA   
  
  
- AAGATACAAC AGAGAGATGA CAAAAACTCA GGCTACGAAG GAGAATAAAC AAAACCCATT TCCATAAACA   
  
  
- GATACACCCT TACAGGGTTT TGCGCAAAAT TCAAAACTTC CTATTTACTT TGAAAGTGAA AGCAGACAGA   
  
  
- AACAGAATGA GCTCATGAGC AAGGACTATC CAGAATAAAA TCAGACAGTT TCTTCCGGCT AATTCTCCAA   
  
  
- AGAATCAAAA GGGTAGAATG AAGAAAAAAA TCAAGACCAA AACGACAAAC CCATGTTAAA AACTTGAGCA   
  
  
- GGGGACTCAA AGATTATCCC ATTGTTGGAA TTGAAAGGTG TGAAAGAACG ACTTTGCTTT CCCTCCGGGA   
  
  
- CTTTATTAAA ATCACCCAAA AGAAAACAAA GTCCCTCTTT TAAATTCTTT GGCAAAAGAC AAGCAAATCA   
  
  
- AAATCATAAT AGAATTGAAT TCCACTCAAG AGTGACACAG CAGAGGAGAA GAAGAATACA AATTCTTTAT   
  
  
- TTAAAAAAAA AAAAAGGAAG AGAGAGAGAG AGAGAGGAGG CGGTGCTCGC CATGGCAGTG GTGGACACAA   
  
  
- AGTAGGGAAG AAAAAACCAA CGTCTCTGTC AAAGATCCTA TGGTCGGTCC CACCATCAGA CCTAGTTATT   
  
  
- CCTTTTTTTC AATTTTTTAT TTTTTTATTT TTTGTTAAAG TAAATCTATT TATTATCGTT TTTATGAGAA   
  
  
- AGTGGCTTAG TGTCAAAAAT CAAAGTGTCT GCCAAGCAAA AAAGTGGGCC CACGACTCCA CTGTGGACCG   
  
  
- GGAGCATCCC GAGTATGCAA CATACAGTAA TGAACGGTCT ATATTCAACC ACGTGGCAAT AATCCTTGTG   
  
  
- ATATAGGGAC ACTTGTCCCT AGAATGTTGA CTAAAAGCAG GGCTTTGTTG TCTTTTTGGG CTTTTAAAAA   
  
  
- AATAAAATTC TGAGCGTGGG ACCCACTGGT CAGGACACAG TTGACCTGAC AGAGATTCTC TCTGCCCCTT   
  
  
- CTATTTATGC AGCTGCTTGG AGCCTTTGTT TTTGAGCCAA CATAAATAAT AAGTCATAGA TATTGGATTT   
  
  
- CTTAAAATGT TAAATACTAT TTATCTTTAA AATTATAAAA AATAGTGAAA AAATTAATTT TATTATTTAT   
  
  
- ATATTAATTG TTAATGGATT GTCAACTGGA TTGAAATTAG CCCAAATCTA TTCCATTGGC TCATTTTTTA   
  
  
- TGAATAATTA TAAAATGAAA TTGAGCTTG

+     Skn-1\_motif

| Site Name | Organism | Position | Strand | Matrix score. | sequence | function |
| --- | --- | --- | --- | --- | --- | --- |
| Skn-1\_motif | Oryza sativa | 1075 | + | 5 | GTCAT | cis-acting regulatory element required for endosperm expression |
| Skn-1\_motif | Oryza sativa | 254 | - | 5 | GTCAT | cis-acting regulatory element required for endosperm expression |

> 2018/04/13 10:10:12  
+ CGAGTGGGTT TTGGGGTTTT TTCTTTTGCT TTTTTTTTTG GTTTAGGGAT AAAGTGTCTT GGGAATCTTG   
  
  
+ GGTTTACTCT TTCGTAGTAT TTTTATTGTT CTTCATCTTG TTAAGGGTTT TCTAGTCCGA AAACTGTGGA   
  
  
+ GACTTTTGGT CTAGAATTAG GAATTTACGT GAATTTTGGG GTCTACTTTT ATTTAGTTTG TTCAGTAGGT   
  
  
+ GTTACCACCG ATTACCTGTT AAAACATTTG GGGGAACGGA TTTATGACTT TTAGAATACT AGGTTCTGTT   
  
  
+ TTCTATGTTG TCTCTCTACT GTTTTTGAGT CCGATGCTTC CTCTTATTTG TTTTGGGTAA AGGTATTTGT   
  
  
+ CTATGTGGGA ATGTCCCAAA ACGCGTTTTA AGTTTTGAAG GATAAATGAA ACTTTCACTT TCGTCTGTCT   
  
  
+ TTGTCTTACT CGAGTACTCG TTCCTGATAG GTCTTATTTT AGTCTGTCAA AGAAGGCCGA TTAAGAGGTT   
  
  
+ TCTTAGTTTT CCCATCTTAC TTCTTTTTTT AGTTCTGGTT TTGCTGTTTG GGTACAATTT TTGAACTCGT   
  
  
+ CCCCTGAGTT TCTAATAGGG TAACAACCTT AACTTTCCAC ACTTTCTTGC TGAAACGAAA GGGAGGCCCT   
  
  
+ GAAATAATTT TAGTGGGTTT TCTTTTGTTT CAGGGAGAAA ATTTAAGAAA CCGTTTTCTG TTCGTTTAGT   
  
  
+ TTTAGTATTA TCTTAACTTA AGGTGAGTTC TCACTGTGTC GTCTCCTCTT CTTCTTATGT TTAAGAAATA   
  
  
+ AATTTTTTTT TTTTTCCTTC TCTCTCTCTC TCTCTCCTCC GCCACGAGCG GTACCGTCAC CACCTGTGTT   
  
  
+ TCATCCCTTC TTTTTTGGTT GCAGAGACAG TTTCTAGGAT ACCAGCCAGG GTGGTAGTCT GGATCAATAA   
  
  
+ GGAAAAAAAG TTAAAAAATA AAAAAATAAA AAACAATTTC ATTTAGATAA ATAATAGCAA AAATACTCTT   
  
  
+ TCACCGAATC ACAGTTTTTA GTTTCACAGA CGGTTCGTTT TTTCACCCGG GTGCTGAGGT GACACCTGGC   
  
  
+ CCTCGTAGGG CTCATACGTT GTATGTCATT ACTTGCCAGA TATAAGTTGG TGCACCGTTA TTAGGAACAC   
  
  
+ TATATCCCTG TGAACAGGGA TCTTACAACT GATTTTCGTC CCGAAACAAC AGAAAAACCC GAAAATTTTT   
  
  
+ TTATTTTAAG ACTCGCACCC TGGGTGACCA GTCCTGTGTC AACTGGACTG TCTCTAAGAG AGACGGGGAA   
  
  
+ GATAAATACG TCGACGAACC TCGGAAACAA AAACTCGGTT GTATTTATTA TTCAGTATCT ATAACCTAAA   
  
  
+ GAATTTTACA ATTTATGATA AATAGAAATT TTAATATTTT TTATCACTTT TTTAATTAAA ATAATAAATA   
  
  
+ TATAATTAAC AATTACCTAA CAGTTGACCT AACTTTAATC GGGTTTAGAT AAGGTAACCG AGTAAAAAAT   
  
  
+ ACTTATTAAT ATTTTACTTT AACTCGAAC  

- GCTCACCCAA AACCCCAAAA AAGAAAACGA AAAAAAAAAC CAAATCCCTA TTTCACAGAA CCCTTAGAAC   
  
  
- CCAAATGAGA AAGCATCATA AAAATAACAA GAAGTAGAAC AATTCCCAAA AGATCAGGCT TTTGACACCT   
  
  
- CTGAAAACCA GATCTTAATC CTTAAATGCA CTTAAAACCC CAGATGAAAA TAAATCAAAC AAGTCATCCA   
  
  
- CAATGGTGGC TAATGGACAA TTTTGTAAAC CCCCTTGCCT AAATACTGAA AATCTTATGA TCCAAGACAA   
  
  
- AAGATACAAC AGAGAGATGA CAAAAACTCA GGCTACGAAG GAGAATAAAC AAAACCCATT TCCATAAACA   
  
  
- GATACACCCT TACAGGGTTT TGCGCAAAAT TCAAAACTTC CTATTTACTT TGAAAGTGAA AGCAGACAGA   
  
  
- AACAGAATGA GCTCATGAGC AAGGACTATC CAGAATAAAA TCAGACAGTT TCTTCCGGCT AATTCTCCAA   
  
  
- AGAATCAAAA GGGTAGAATG AAGAAAAAAA TCAAGACCAA AACGACAAAC CCATGTTAAA AACTTGAGCA   
  
  
- GGGGACTCAA AGATTATCCC ATTGTTGGAA TTGAAAGGTG TGAAAGAACG ACTTTGCTTT CCCTCCGGGA   
  
  
- CTTTATTAAA ATCACCCAAA AGAAAACAAA GTCCCTCTTT TAAATTCTTT GGCAAAAGAC AAGCAAATCA   
  
  
- AAATCATAAT AGAATTGAAT TCCACTCAAG AGTGACACAG CAGAGGAGAA GAAGAATACA AATTCTTTAT   
  
  
- TTAAAAAAAA AAAAAGGAAG AGAGAGAGAG AGAGAGGAGG CGGTGCTCGC CATGGCAGTG GTGGACACAA   
  
  
- AGTAGGGAAG AAAAAACCAA CGTCTCTGTC AAAGATCCTA TGGTCGGTCC CACCATCAGA CCTAGTTATT   
  
  
- CCTTTTTTTC AATTTTTTAT TTTTTTATTT TTTGTTAAAG TAAATCTATT TATTATCGTT TTTATGAGAA   
  
  
- AGTGGCTTAG TGTCAAAAAT CAAAGTGTCT GCCAAGCAAA AAAGTGGGCC CACGACTCCA CTGTGGACCG   
  
  
- GGAGCATCCC GAGTATGCAA CATACAGTAA TGAACGGTCT ATATTCAACC ACGTGGCAAT AATCCTTGTG   
  
  
- ATATAGGGAC ACTTGTCCCT AGAATGTTGA CTAAAAGCAG GGCTTTGTTG TCTTTTTGGG CTTTTAAAAA   
  
  
- AATAAAATTC TGAGCGTGGG ACCCACTGGT CAGGACACAG TTGACCTGAC AGAGATTCTC TCTGCCCCTT   
  
  
- CTATTTATGC AGCTGCTTGG AGCCTTTGTT TTTGAGCCAA CATAAATAAT AAGTCATAGA TATTGGATTT   
  
  
- CTTAAAATGT TAAATACTAT TTATCTTTAA AATTATAAAA AATAGTGAAA AAATTAATTT TATTATTTAT   
  
  
- ATATTAATTG TTAATGGATT GTCAACTGGA TTGAAATTAG CCCAAATCTA TTCCATTGGC TCATTTTTTA   
  
  
- TGAATAATTA TAAAATGAAA TTGAGCTTG

+     Sp1

| Site Name | Organism | Position | Strand | Matrix score. | sequence | function |
| --- | --- | --- | --- | --- | --- | --- |
| Sp1 | Zea mays | 621 | - | 5 | CC(G/A)CCC | light responsive element |
| Sp1 | Zea mays | 889 | - | 5.5 | CC(G/A)CCC | light responsive element |

> 2018/04/13 10:10:12  
+ CGAGTGGGTT TTGGGGTTTT TTCTTTTGCT TTTTTTTTTG GTTTAGGGAT AAAGTGTCTT GGGAATCTTG   
  
  
+ GGTTTACTCT TTCGTAGTAT TTTTATTGTT CTTCATCTTG TTAAGGGTTT TCTAGTCCGA AAACTGTGGA   
  
  
+ GACTTTTGGT CTAGAATTAG GAATTTACGT GAATTTTGGG GTCTACTTTT ATTTAGTTTG TTCAGTAGGT   
  
  
+ GTTACCACCG ATTACCTGTT AAAACATTTG GGGGAACGGA TTTATGACTT TTAGAATACT AGGTTCTGTT   
  
  
+ TTCTATGTTG TCTCTCTACT GTTTTTGAGT CCGATGCTTC CTCTTATTTG TTTTGGGTAA AGGTATTTGT   
  
  
+ CTATGTGGGA ATGTCCCAAA ACGCGTTTTA AGTTTTGAAG GATAAATGAA ACTTTCACTT TCGTCTGTCT   
  
  
+ TTGTCTTACT CGAGTACTCG TTCCTGATAG GTCTTATTTT AGTCTGTCAA AGAAGGCCGA TTAAGAGGTT   
  
  
+ TCTTAGTTTT CCCATCTTAC TTCTTTTTTT AGTTCTGGTT TTGCTGTTTG GGTACAATTT TTGAACTCGT   
  
  
+ CCCCTGAGTT TCTAATAGGG TAACAACCTT AACTTTCCAC ACTTTCTTGC TGAAACGAAA GGGAGGCCCT   
  
  
+ GAAATAATTT TAGTGGGTTT TCTTTTGTTT CAGGGAGAAA ATTTAAGAAA CCGTTTTCTG TTCGTTTAGT   
  
  
+ TTTAGTATTA TCTTAACTTA AGGTGAGTTC TCACTGTGTC GTCTCCTCTT CTTCTTATGT TTAAGAAATA   
  
  
+ AATTTTTTTT TTTTTCCTTC TCTCTCTCTC TCTCTCCTCC GCCACGAGCG GTACCGTCAC CACCTGTGTT   
  
  
+ TCATCCCTTC TTTTTTGGTT GCAGAGACAG TTTCTAGGAT ACCAGCCAGG GTGGTAGTCT GGATCAATAA   
  
  
+ GGAAAAAAAG TTAAAAAATA AAAAAATAAA AAACAATTTC ATTTAGATAA ATAATAGCAA AAATACTCTT   
  
  
+ TCACCGAATC ACAGTTTTTA GTTTCACAGA CGGTTCGTTT TTTCACCCGG GTGCTGAGGT GACACCTGGC   
  
  
+ CCTCGTAGGG CTCATACGTT GTATGTCATT ACTTGCCAGA TATAAGTTGG TGCACCGTTA TTAGGAACAC   
  
  
+ TATATCCCTG TGAACAGGGA TCTTACAACT GATTTTCGTC CCGAAACAAC AGAAAAACCC GAAAATTTTT   
  
  
+ TTATTTTAAG ACTCGCACCC TGGGTGACCA GTCCTGTGTC AACTGGACTG TCTCTAAGAG AGACGGGGAA   
  
  
+ GATAAATACG TCGACGAACC TCGGAAACAA AAACTCGGTT GTATTTATTA TTCAGTATCT ATAACCTAAA   
  
  
+ GAATTTTACA ATTTATGATA AATAGAAATT TTAATATTTT TTATCACTTT TTTAATTAAA ATAATAAATA   
  
  
+ TATAATTAAC AATTACCTAA CAGTTGACCT AACTTTAATC GGGTTTAGAT AAGGTAACCG AGTAAAAAAT   
  
  
+ ACTTATTAAT ATTTTACTTT AACTCGAAC  

- GCTCACCCAA AACCCCAAAA AAGAAAACGA AAAAAAAAAC CAAATCCCTA TTTCACAGAA CCCTTAGAAC   
  
  
- CCAAATGAGA AAGCATCATA AAAATAACAA GAAGTAGAAC AATTCCCAAA AGATCAGGCT TTTGACACCT   
  
  
- CTGAAAACCA GATCTTAATC CTTAAATGCA CTTAAAACCC CAGATGAAAA TAAATCAAAC AAGTCATCCA   
  
  
- CAATGGTGGC TAATGGACAA TTTTGTAAAC CCCCTTGCCT AAATACTGAA AATCTTATGA TCCAAGACAA   
  
  
- AAGATACAAC AGAGAGATGA CAAAAACTCA GGCTACGAAG GAGAATAAAC AAAACCCATT TCCATAAACA   
  
  
- GATACACCCT TACAGGGTTT TGCGCAAAAT TCAAAACTTC CTATTTACTT TGAAAGTGAA AGCAGACAGA   
  
  
- AACAGAATGA GCTCATGAGC AAGGACTATC CAGAATAAAA TCAGACAGTT TCTTCCGGCT AATTCTCCAA   
  
  
- AGAATCAAAA GGGTAGAATG AAGAAAAAAA TCAAGACCAA AACGACAAAC CCATGTTAAA AACTTGAGCA   
  
  
- GGGGACTCAA AGATTATCCC ATTGTTGGAA TTGAAAGGTG TGAAAGAACG ACTTTGCTTT CCCTCCGGGA   
  
  
- CTTTATTAAA ATCACCCAAA AGAAAACAAA GTCCCTCTTT TAAATTCTTT GGCAAAAGAC AAGCAAATCA   
  
  
- AAATCATAAT AGAATTGAAT TCCACTCAAG AGTGACACAG CAGAGGAGAA GAAGAATACA AATTCTTTAT   
  
  
- TTAAAAAAAA AAAAAGGAAG AGAGAGAGAG AGAGAGGAGG CGGTGCTCGC CATGGCAGTG GTGGACACAA   
  
  
- AGTAGGGAAG AAAAAACCAA CGTCTCTGTC AAAGATCCTA TGGTCGGTCC CACCATCAGA CCTAGTTATT   
  
  
- CCTTTTTTTC AATTTTTTAT TTTTTTATTT TTTGTTAAAG TAAATCTATT TATTATCGTT TTTATGAGAA   
  
  
- AGTGGCTTAG TGTCAAAAAT CAAAGTGTCT GCCAAGCAAA AAAGTGGGCC CACGACTCCA CTGTGGACCG   
  
  
- GGAGCATCCC GAGTATGCAA CATACAGTAA TGAACGGTCT ATATTCAACC ACGTGGCAAT AATCCTTGTG   
  
  
- ATATAGGGAC ACTTGTCCCT AGAATGTTGA CTAAAAGCAG GGCTTTGTTG TCTTTTTGGG CTTTTAAAAA   
  
  
- AATAAAATTC TGAGCGTGGG ACCCACTGGT CAGGACACAG TTGACCTGAC AGAGATTCTC TCTGCCCCTT   
  
  
- CTATTTATGC AGCTGCTTGG AGCCTTTGTT TTTGAGCCAA CATAAATAAT AAGTCATAGA TATTGGATTT   
  
  
- CTTAAAATGT TAAATACTAT TTATCTTTAA AATTATAAAA AATAGTGAAA AAATTAATTT TATTATTTAT   
  
  
- ATATTAATTG TTAATGGATT GTCAACTGGA TTGAAATTAG CCCAAATCTA TTCCATTGGC TCATTTTTTA   
  
  
- TGAATAATTA TAAAATGAAA TTGAGCTTG

+     TATA-box

| Site Name | Organism | Position | Strand | Matrix score. | sequence | function |
| --- | --- | --- | --- | --- | --- | --- |
| TATA-box | Glycine max | 1474 | - | 5 | TAATA | core promoter element around -30 of transcription start |
| TATA-box | Lycopersicon esculentum | 1334 | + | 5 | TTTTA | core promoter element around -30 of transcription start |
| TATA-box | Lycopersicon esculentum | 700 | + | 5 | TTTTA | core promoter element around -30 of transcription start |
| TATA-box | Brassica oleracea | 1400 | + | 7 | ATATAAT | core promoter element around -30 of transcription start |
| TATA-box | Lycopersicon esculentum | 1194 | + | 5 | TTTTA | core promoter element around -30 of transcription start |
| TATA-box | Glycine max | 1109 | - | 5 | TAATA | core promoter element around -30 of transcription start |
| TATA-box | Arabidopsis thaliana | 1091 | - | 4 | TATA | core promoter element around -30 of transcription start |
| TATA-box | Lycopersicon esculentum | 996 | + | 5 | TTTTA | core promoter element around -30 of transcription start |
| TATA-box | Lycopersicon esculentum | 937 | - | 5 | TTTTA | core promoter element around -30 of transcription start |
| TATA-box | Lycopersicon esculentum | 638 | + | 5 | TTTTA | core promoter element around -30 of transcription start |
| TATA-box | Lycopersicon esculentum | 457 | + | 5 | TTTTA | core promoter element around -30 of transcription start |
| TATA-box | Glycine max | 573 | + | 5 | TAATA | core promoter element around -30 of transcription start |
| TATA-box | Lycopersicon esculentum | 1189 | + | 5 | TTTTA | core promoter element around -30 of transcription start |
| TATA-box | Lycopersicon esculentum | 1482 | + | 5 | TTTTA | core promoter element around -30 of transcription start |
| TATA-box | Lycopersicon esculentum | 929 | - | 5 | TTTTA | core promoter element around -30 of transcription start |
| TATA-box | Glycine max | 706 | - | 5 | TAATA | core promoter element around -30 of transcription start |
| TATA-box | Arabidopsis thaliana | 1399 | - | 4 | TATA | core promoter element around -30 of transcription start |
| TATA-box | Glycine max | 1392 | + | 5 | TAATA | core promoter element around -30 of transcription start |
| TATA-box | Lycopersicon esculentum | 376 | + | 5 | TTTTA | core promoter element around -30 of transcription start |
| TATA-box | Lycopersicon esculentum | 187 | + | 5 | TTTTA | core promoter element around -30 of transcription start |
| TATA-box | Glycine max | 1477 | + | 5 | TAATA | core promoter element around -30 of transcription start |
| TATA-box | Brassica oleracea | 1090 | + | 6 | ATATAA | core promoter element around -30 of transcription start |
| TATA-box | Lycopersicon esculentum | 91 | + | 5 | TTTTA | core promoter element around -30 of transcription start |
| TATA-box | Glycine max | 962 | + | 5 | TAATA | core promoter element around -30 of transcription start |
| TATA-box | Brassica napus | 1398 | - | 6 | ATATAT | core promoter element around -30 of transcription start |
| TATA-box | Arabidopsis thaliana | 1401 | - | 4 | TATA | core promoter element around -30 of transcription start |
| TATA-box | Glycine max | 1306 | - | 5 | TAATA | core promoter element around -30 of transcription start |
| TATA-box | Lycopersicon esculentum | 517 | + | 5 | TTTTA | core promoter element around -30 of transcription start |
| TATA-box | Lycopersicon esculentum | 1387 | - | 5 | TTTTA | core promoter element around -30 of transcription start |
| TATA-box | Arabidopsis thaliana | 1121 | - | 4 | TATA | core promoter element around -30 of transcription start |
| TATA-box | Lycopersicon esculentum | 259 | + | 5 | TTTTA | core promoter element around -30 of transcription start |
| TATA-box | Lycopersicon esculentum | 1463 | - | 5 | TTTTA | core promoter element around -30 of transcription start |
| TATA-box | Lycopersicon esculentum | 922 | - | 5 | TTTTA | core promoter element around -30 of transcription start |
| TATA-box | Lycopersicon esculentum | 1359 | + | 5 | TTTTA | core promoter element around -30 of transcription start |
| TATA-box | Lycopersicon esculentum | 230 | - | 5 | TTTTA | core promoter element around -30 of transcription start |
| TATA-box | Glycine max | 1362 | + | 5 | TAATA | core promoter element around -30 of transcription start |
| TATA-box | Arabidopsis thaliana | 1320 | - | 4 | TATA | core promoter element around -30 of transcription start |
| TATA-box | Lycopersicon esculentum | 1380 | + | 5 | TTTTA | core promoter element around -30 of transcription start |
| TATA-box | Lycopersicon esculentum | 1369 | + | 5 | TTTTA | core promoter element around -30 of transcription start |

> 2018/04/13 10:10:12  
+ CGAGTGGGTT TTGGGGTTTT TTCTTTTGCT TTTTTTTTTG GTTTAGGGAT AAAGTGTCTT GGGAATCTTG   
  
  
+ GGTTTACTCT TTCGTAGTAT TTTTATTGTT CTTCATCTTG TTAAGGGTTT TCTAGTCCGA AAACTGTGGA   
  
  
+ GACTTTTGGT CTAGAATTAG GAATTTACGT GAATTTTGGG GTCTACTTTT ATTTAGTTTG TTCAGTAGGT   
  
  
+ GTTACCACCG ATTACCTGTT AAAACATTTG GGGGAACGGA TTTATGACTT TTAGAATACT AGGTTCTGTT   
  
  
+ TTCTATGTTG TCTCTCTACT GTTTTTGAGT CCGATGCTTC CTCTTATTTG TTTTGGGTAA AGGTATTTGT   
  
  
+ CTATGTGGGA ATGTCCCAAA ACGCGTTTTA AGTTTTGAAG GATAAATGAA ACTTTCACTT TCGTCTGTCT   
  
  
+ TTGTCTTACT CGAGTACTCG TTCCTGATAG GTCTTATTTT AGTCTGTCAA AGAAGGCCGA TTAAGAGGTT   
  
  
+ TCTTAGTTTT CCCATCTTAC TTCTTTTTTT AGTTCTGGTT TTGCTGTTTG GGTACAATTT TTGAACTCGT   
  
  
+ CCCCTGAGTT TCTAATAGGG TAACAACCTT AACTTTCCAC ACTTTCTTGC TGAAACGAAA GGGAGGCCCT   
  
  
+ GAAATAATTT TAGTGGGTTT TCTTTTGTTT CAGGGAGAAA ATTTAAGAAA CCGTTTTCTG TTCGTTTAGT   
  
  
+ TTTAGTATTA TCTTAACTTA AGGTGAGTTC TCACTGTGTC GTCTCCTCTT CTTCTTATGT TTAAGAAATA   
  
  
+ AATTTTTTTT TTTTTCCTTC TCTCTCTCTC TCTCTCCTCC GCCACGAGCG GTACCGTCAC CACCTGTGTT   
  
  
+ TCATCCCTTC TTTTTTGGTT GCAGAGACAG TTTCTAGGAT ACCAGCCAGG GTGGTAGTCT GGATCAATAA   
  
  
+ GGAAAAAAAG TTAAAAAATA AAAAAATAAA AAACAATTTC ATTTAGATAA ATAATAGCAA AAATACTCTT   
  
  
+ TCACCGAATC ACAGTTTTTA GTTTCACAGA CGGTTCGTTT TTTCACCCGG GTGCTGAGGT GACACCTGGC   
  
  
+ CCTCGTAGGG CTCATACGTT GTATGTCATT ACTTGCCAGA TATAAGTTGG TGCACCGTTA TTAGGAACAC   
  
  
+ TATATCCCTG TGAACAGGGA TCTTACAACT GATTTTCGTC CCGAAACAAC AGAAAAACCC GAAAATTTTT   
  
  
+ TTATTTTAAG ACTCGCACCC TGGGTGACCA GTCCTGTGTC AACTGGACTG TCTCTAAGAG AGACGGGGAA   
  
  
+ GATAAATACG TCGACGAACC TCGGAAACAA AAACTCGGTT GTATTTATTA TTCAGTATCT ATAACCTAAA   
  
  
+ GAATTTTACA ATTTATGATA AATAGAAATT TTAATATTTT TTATCACTTT TTTAATTAAA ATAATAAATA   
  
  
+ TATAATTAAC AATTACCTAA CAGTTGACCT AACTTTAATC GGGTTTAGAT AAGGTAACCG AGTAAAAAAT   
  
  
+ ACTTATTAAT ATTTTACTTT AACTCGAAC  

- GCTCACCCAA AACCCCAAAA AAGAAAACGA AAAAAAAAAC CAAATCCCTA TTTCACAGAA CCCTTAGAAC   
  
  
- CCAAATGAGA AAGCATCATA AAAATAACAA GAAGTAGAAC AATTCCCAAA AGATCAGGCT TTTGACACCT   
  
  
- CTGAAAACCA GATCTTAATC CTTAAATGCA CTTAAAACCC CAGATGAAAA TAAATCAAAC AAGTCATCCA   
  
  
- CAATGGTGGC TAATGGACAA TTTTGTAAAC CCCCTTGCCT AAATACTGAA AATCTTATGA TCCAAGACAA   
  
  
- AAGATACAAC AGAGAGATGA CAAAAACTCA GGCTACGAAG GAGAATAAAC AAAACCCATT TCCATAAACA   
  
  
- GATACACCCT TACAGGGTTT TGCGCAAAAT TCAAAACTTC CTATTTACTT TGAAAGTGAA AGCAGACAGA   
  
  
- AACAGAATGA GCTCATGAGC AAGGACTATC CAGAATAAAA TCAGACAGTT TCTTCCGGCT AATTCTCCAA   
  
  
- AGAATCAAAA GGGTAGAATG AAGAAAAAAA TCAAGACCAA AACGACAAAC CCATGTTAAA AACTTGAGCA   
  
  
- GGGGACTCAA AGATTATCCC ATTGTTGGAA TTGAAAGGTG TGAAAGAACG ACTTTGCTTT CCCTCCGGGA   
  
  
- CTTTATTAAA ATCACCCAAA AGAAAACAAA GTCCCTCTTT TAAATTCTTT GGCAAAAGAC AAGCAAATCA   
  
  
- AAATCATAAT AGAATTGAAT TCCACTCAAG AGTGACACAG CAGAGGAGAA GAAGAATACA AATTCTTTAT   
  
  
- TTAAAAAAAA AAAAAGGAAG AGAGAGAGAG AGAGAGGAGG CGGTGCTCGC CATGGCAGTG GTGGACACAA   
  
  
- AGTAGGGAAG AAAAAACCAA CGTCTCTGTC AAAGATCCTA TGGTCGGTCC CACCATCAGA CCTAGTTATT   
  
  
- CCTTTTTTTC AATTTTTTAT TTTTTTATTT TTTGTTAAAG TAAATCTATT TATTATCGTT TTTATGAGAA   
  
  
- AGTGGCTTAG TGTCAAAAAT CAAAGTGTCT GCCAAGCAAA AAAGTGGGCC CACGACTCCA CTGTGGACCG   
  
  
- GGAGCATCCC GAGTATGCAA CATACAGTAA TGAACGGTCT ATATTCAACC ACGTGGCAAT AATCCTTGTG   
  
  
- ATATAGGGAC ACTTGTCCCT AGAATGTTGA CTAAAAGCAG GGCTTTGTTG TCTTTTTGGG CTTTTAAAAA   
  
  
- AATAAAATTC TGAGCGTGGG ACCCACTGGT CAGGACACAG TTGACCTGAC AGAGATTCTC TCTGCCCCTT   
  
  
- CTATTTATGC AGCTGCTTGG AGCCTTTGTT TTTGAGCCAA CATAAATAAT AAGTCATAGA TATTGGATTT   
  
  
- CTTAAAATGT TAAATACTAT TTATCTTTAA AATTATAAAA AATAGTGAAA AAATTAATTT TATTATTTAT   
  
  
- ATATTAATTG TTAATGGATT GTCAACTGGA TTGAAATTAG CCCAAATCTA TTCCATTGGC TCATTTTTTA   
  
  
- TGAATAATTA TAAAATGAAA TTGAGCTTG

+     TC-rich repeats

| Site Name | Organism | Position | Strand | Matrix score. | sequence | function |
| --- | --- | --- | --- | --- | --- | --- |
| TC-rich repeats | Nicotiana tabacum | 663 | - | 9 | ATTTTCTCCA | cis-acting element involved in defense and stress responsiveness |

> 2018/04/13 10:10:12  
+ CGAGTGGGTT TTGGGGTTTT TTCTTTTGCT TTTTTTTTTG GTTTAGGGAT AAAGTGTCTT GGGAATCTTG   
  
  
+ GGTTTACTCT TTCGTAGTAT TTTTATTGTT CTTCATCTTG TTAAGGGTTT TCTAGTCCGA AAACTGTGGA   
  
  
+ GACTTTTGGT CTAGAATTAG GAATTTACGT GAATTTTGGG GTCTACTTTT ATTTAGTTTG TTCAGTAGGT   
  
  
+ GTTACCACCG ATTACCTGTT AAAACATTTG GGGGAACGGA TTTATGACTT TTAGAATACT AGGTTCTGTT   
  
  
+ TTCTATGTTG TCTCTCTACT GTTTTTGAGT CCGATGCTTC CTCTTATTTG TTTTGGGTAA AGGTATTTGT   
  
  
+ CTATGTGGGA ATGTCCCAAA ACGCGTTTTA AGTTTTGAAG GATAAATGAA ACTTTCACTT TCGTCTGTCT   
  
  
+ TTGTCTTACT CGAGTACTCG TTCCTGATAG GTCTTATTTT AGTCTGTCAA AGAAGGCCGA TTAAGAGGTT   
  
  
+ TCTTAGTTTT CCCATCTTAC TTCTTTTTTT AGTTCTGGTT TTGCTGTTTG GGTACAATTT TTGAACTCGT   
  
  
+ CCCCTGAGTT TCTAATAGGG TAACAACCTT AACTTTCCAC ACTTTCTTGC TGAAACGAAA GGGAGGCCCT   
  
  
+ GAAATAATTT TAGTGGGTTT TCTTTTGTTT CAGGGAGAAA ATTTAAGAAA CCGTTTTCTG TTCGTTTAGT   
  
  
+ TTTAGTATTA TCTTAACTTA AGGTGAGTTC TCACTGTGTC GTCTCCTCTT CTTCTTATGT TTAAGAAATA   
  
  
+ AATTTTTTTT TTTTTCCTTC TCTCTCTCTC TCTCTCCTCC GCCACGAGCG GTACCGTCAC CACCTGTGTT   
  
  
+ TCATCCCTTC TTTTTTGGTT GCAGAGACAG TTTCTAGGAT ACCAGCCAGG GTGGTAGTCT GGATCAATAA   
  
  
+ GGAAAAAAAG TTAAAAAATA AAAAAATAAA AAACAATTTC ATTTAGATAA ATAATAGCAA AAATACTCTT   
  
  
+ TCACCGAATC ACAGTTTTTA GTTTCACAGA CGGTTCGTTT TTTCACCCGG GTGCTGAGGT GACACCTGGC   
  
  
+ CCTCGTAGGG CTCATACGTT GTATGTCATT ACTTGCCAGA TATAAGTTGG TGCACCGTTA TTAGGAACAC   
  
  
+ TATATCCCTG TGAACAGGGA TCTTACAACT GATTTTCGTC CCGAAACAAC AGAAAAACCC GAAAATTTTT   
  
  
+ TTATTTTAAG ACTCGCACCC TGGGTGACCA GTCCTGTGTC AACTGGACTG TCTCTAAGAG AGACGGGGAA   
  
  
+ GATAAATACG TCGACGAACC TCGGAAACAA AAACTCGGTT GTATTTATTA TTCAGTATCT ATAACCTAAA   
  
  
+ GAATTTTACA ATTTATGATA AATAGAAATT TTAATATTTT TTATCACTTT TTTAATTAAA ATAATAAATA   
  
  
+ TATAATTAAC AATTACCTAA CAGTTGACCT AACTTTAATC GGGTTTAGAT AAGGTAACCG AGTAAAAAAT   
  
  
+ ACTTATTAAT ATTTTACTTT AACTCGAAC  

- GCTCACCCAA AACCCCAAAA AAGAAAACGA AAAAAAAAAC CAAATCCCTA TTTCACAGAA CCCTTAGAAC   
  
  
- CCAAATGAGA AAGCATCATA AAAATAACAA GAAGTAGAAC AATTCCCAAA AGATCAGGCT TTTGACACCT   
  
  
- CTGAAAACCA GATCTTAATC CTTAAATGCA CTTAAAACCC CAGATGAAAA TAAATCAAAC AAGTCATCCA   
  
  
- CAATGGTGGC TAATGGACAA TTTTGTAAAC CCCCTTGCCT AAATACTGAA AATCTTATGA TCCAAGACAA   
  
  
- AAGATACAAC AGAGAGATGA CAAAAACTCA GGCTACGAAG GAGAATAAAC AAAACCCATT TCCATAAACA   
  
  
- GATACACCCT TACAGGGTTT TGCGCAAAAT TCAAAACTTC CTATTTACTT TGAAAGTGAA AGCAGACAGA   
  
  
- AACAGAATGA GCTCATGAGC AAGGACTATC CAGAATAAAA TCAGACAGTT TCTTCCGGCT AATTCTCCAA   
  
  
- AGAATCAAAA GGGTAGAATG AAGAAAAAAA TCAAGACCAA AACGACAAAC CCATGTTAAA AACTTGAGCA   
  
  
- GGGGACTCAA AGATTATCCC ATTGTTGGAA TTGAAAGGTG TGAAAGAACG ACTTTGCTTT CCCTCCGGGA   
  
  
- CTTTATTAAA ATCACCCAAA AGAAAACAAA GTCCCTCTTT TAAATTCTTT GGCAAAAGAC AAGCAAATCA   
  
  
- AAATCATAAT AGAATTGAAT TCCACTCAAG AGTGACACAG CAGAGGAGAA GAAGAATACA AATTCTTTAT   
  
  
- TTAAAAAAAA AAAAAGGAAG AGAGAGAGAG AGAGAGGAGG CGGTGCTCGC CATGGCAGTG GTGGACACAA   
  
  
- AGTAGGGAAG AAAAAACCAA CGTCTCTGTC AAAGATCCTA TGGTCGGTCC CACCATCAGA CCTAGTTATT   
  
  
- CCTTTTTTTC AATTTTTTAT TTTTTTATTT TTTGTTAAAG TAAATCTATT TATTATCGTT TTTATGAGAA   
  
  
- AGTGGCTTAG TGTCAAAAAT CAAAGTGTCT GCCAAGCAAA AAAGTGGGCC CACGACTCCA CTGTGGACCG   
  
  
- GGAGCATCCC GAGTATGCAA CATACAGTAA TGAACGGTCT ATATTCAACC ACGTGGCAAT AATCCTTGTG   
  
  
- ATATAGGGAC ACTTGTCCCT AGAATGTTGA CTAAAAGCAG GGCTTTGTTG TCTTTTTGGG CTTTTAAAAA   
  
  
- AATAAAATTC TGAGCGTGGG ACCCACTGGT CAGGACACAG TTGACCTGAC AGAGATTCTC TCTGCCCCTT   
  
  
- CTATTTATGC AGCTGCTTGG AGCCTTTGTT TTTGAGCCAA CATAAATAAT AAGTCATAGA TATTGGATTT   
  
  
- CTTAAAATGT TAAATACTAT TTATCTTTAA AATTATAAAA AATAGTGAAA AAATTAATTT TATTATTTAT   
  
  
- ATATTAATTG TTAATGGATT GTCAACTGGA TTGAAATTAG CCCAAATCTA TTCCATTGGC TCATTTTTTA   
  
  
- TGAATAATTA TAAAATGAAA TTGAGCTTG

+     TCA-element

| Site Name | Organism | Position | Strand | Matrix score. | sequence | function |
| --- | --- | --- | --- | --- | --- | --- |
| TCA-element | Nicotiana tabacum | 846 | + | 9 | CCATCTTTTT | cis-acting element involved in salicylic acid responsiveness |

> 2018/04/13 10:10:12  
+ CGAGTGGGTT TTGGGGTTTT TTCTTTTGCT TTTTTTTTTG GTTTAGGGAT AAAGTGTCTT GGGAATCTTG   
  
  
+ GGTTTACTCT TTCGTAGTAT TTTTATTGTT CTTCATCTTG TTAAGGGTTT TCTAGTCCGA AAACTGTGGA   
  
  
+ GACTTTTGGT CTAGAATTAG GAATTTACGT GAATTTTGGG GTCTACTTTT ATTTAGTTTG TTCAGTAGGT   
  
  
+ GTTACCACCG ATTACCTGTT AAAACATTTG GGGGAACGGA TTTATGACTT TTAGAATACT AGGTTCTGTT   
  
  
+ TTCTATGTTG TCTCTCTACT GTTTTTGAGT CCGATGCTTC CTCTTATTTG TTTTGGGTAA AGGTATTTGT   
  
  
+ CTATGTGGGA ATGTCCCAAA ACGCGTTTTA AGTTTTGAAG GATAAATGAA ACTTTCACTT TCGTCTGTCT   
  
  
+ TTGTCTTACT CGAGTACTCG TTCCTGATAG GTCTTATTTT AGTCTGTCAA AGAAGGCCGA TTAAGAGGTT   
  
  
+ TCTTAGTTTT CCCATCTTAC TTCTTTTTTT AGTTCTGGTT TTGCTGTTTG GGTACAATTT TTGAACTCGT   
  
  
+ CCCCTGAGTT TCTAATAGGG TAACAACCTT AACTTTCCAC ACTTTCTTGC TGAAACGAAA GGGAGGCCCT   
  
  
+ GAAATAATTT TAGTGGGTTT TCTTTTGTTT CAGGGAGAAA ATTTAAGAAA CCGTTTTCTG TTCGTTTAGT   
  
  
+ TTTAGTATTA TCTTAACTTA AGGTGAGTTC TCACTGTGTC GTCTCCTCTT CTTCTTATGT TTAAGAAATA   
  
  
+ AATTTTTTTT TTTTTCCTTC TCTCTCTCTC TCTCTCCTCC GCCACGAGCG GTACCGTCAC CACCTGTGTT   
  
  
+ TCATCCCTTC TTTTTTGGTT GCAGAGACAG TTTCTAGGAT ACCAGCCAGG GTGGTAGTCT GGATCAATAA   
  
  
+ GGAAAAAAAG TTAAAAAATA AAAAAATAAA AAACAATTTC ATTTAGATAA ATAATAGCAA AAATACTCTT   
  
  
+ TCACCGAATC ACAGTTTTTA GTTTCACAGA CGGTTCGTTT TTTCACCCGG GTGCTGAGGT GACACCTGGC   
  
  
+ CCTCGTAGGG CTCATACGTT GTATGTCATT ACTTGCCAGA TATAAGTTGG TGCACCGTTA TTAGGAACAC   
  
  
+ TATATCCCTG TGAACAGGGA TCTTACAACT GATTTTCGTC CCGAAACAAC AGAAAAACCC GAAAATTTTT   
  
  
+ TTATTTTAAG ACTCGCACCC TGGGTGACCA GTCCTGTGTC AACTGGACTG TCTCTAAGAG AGACGGGGAA   
  
  
+ GATAAATACG TCGACGAACC TCGGAAACAA AAACTCGGTT GTATTTATTA TTCAGTATCT ATAACCTAAA   
  
  
+ GAATTTTACA ATTTATGATA AATAGAAATT TTAATATTTT TTATCACTTT TTTAATTAAA ATAATAAATA   
  
  
+ TATAATTAAC AATTACCTAA CAGTTGACCT AACTTTAATC GGGTTTAGAT AAGGTAACCG AGTAAAAAAT   
  
  
+ ACTTATTAAT ATTTTACTTT AACTCGAAC  

- GCTCACCCAA AACCCCAAAA AAGAAAACGA AAAAAAAAAC CAAATCCCTA TTTCACAGAA CCCTTAGAAC   
  
  
- CCAAATGAGA AAGCATCATA AAAATAACAA GAAGTAGAAC AATTCCCAAA AGATCAGGCT TTTGACACCT   
  
  
- CTGAAAACCA GATCTTAATC CTTAAATGCA CTTAAAACCC CAGATGAAAA TAAATCAAAC AAGTCATCCA   
  
  
- CAATGGTGGC TAATGGACAA TTTTGTAAAC CCCCTTGCCT AAATACTGAA AATCTTATGA TCCAAGACAA   
  
  
- AAGATACAAC AGAGAGATGA CAAAAACTCA GGCTACGAAG GAGAATAAAC AAAACCCATT TCCATAAACA   
  
  
- GATACACCCT TACAGGGTTT TGCGCAAAAT TCAAAACTTC CTATTTACTT TGAAAGTGAA AGCAGACAGA   
  
  
- AACAGAATGA GCTCATGAGC AAGGACTATC CAGAATAAAA TCAGACAGTT TCTTCCGGCT AATTCTCCAA   
  
  
- AGAATCAAAA GGGTAGAATG AAGAAAAAAA TCAAGACCAA AACGACAAAC CCATGTTAAA AACTTGAGCA   
  
  
- GGGGACTCAA AGATTATCCC ATTGTTGGAA TTGAAAGGTG TGAAAGAACG ACTTTGCTTT CCCTCCGGGA   
  
  
- CTTTATTAAA ATCACCCAAA AGAAAACAAA GTCCCTCTTT TAAATTCTTT GGCAAAAGAC AAGCAAATCA   
  
  
- AAATCATAAT AGAATTGAAT TCCACTCAAG AGTGACACAG CAGAGGAGAA GAAGAATACA AATTCTTTAT   
  
  
- TTAAAAAAAA AAAAAGGAAG AGAGAGAGAG AGAGAGGAGG CGGTGCTCGC CATGGCAGTG GTGGACACAA   
  
  
- AGTAGGGAAG AAAAAACCAA CGTCTCTGTC AAAGATCCTA TGGTCGGTCC CACCATCAGA CCTAGTTATT   
  
  
- CCTTTTTTTC AATTTTTTAT TTTTTTATTT TTTGTTAAAG TAAATCTATT TATTATCGTT TTTATGAGAA   
  
  
- AGTGGCTTAG TGTCAAAAAT CAAAGTGTCT GCCAAGCAAA AAAGTGGGCC CACGACTCCA CTGTGGACCG   
  
  
- GGAGCATCCC GAGTATGCAA CATACAGTAA TGAACGGTCT ATATTCAACC ACGTGGCAAT AATCCTTGTG   
  
  
- ATATAGGGAC ACTTGTCCCT AGAATGTTGA CTAAAAGCAG GGCTTTGTTG TCTTTTTGGG CTTTTAAAAA   
  
  
- AATAAAATTC TGAGCGTGGG ACCCACTGGT CAGGACACAG TTGACCTGAC AGAGATTCTC TCTGCCCCTT   
  
  
- CTATTTATGC AGCTGCTTGG AGCCTTTGTT TTTGAGCCAA CATAAATAAT AAGTCATAGA TATTGGATTT   
  
  
- CTTAAAATGT TAAATACTAT TTATCTTTAA AATTATAAAA AATAGTGAAA AAATTAATTT TATTATTTAT   
  
  
- ATATTAATTG TTAATGGATT GTCAACTGGA TTGAAATTAG CCCAAATCTA TTCCATTGGC TCATTTTTTA   
  
  
- TGAATAATTA TAAAATGAAA TTGAGCTTG

+     TCCC-motif

| Site Name | Organism | Position | Strand | Matrix score. | sequence | function |
| --- | --- | --- | --- | --- | --- | --- |
| TCCC-motif | Spinacia oleracea | 662 | - | 7 | TCTCCCT | part of a light responsive element |

> 2018/04/13 10:10:12  
+ CGAGTGGGTT TTGGGGTTTT TTCTTTTGCT TTTTTTTTTG GTTTAGGGAT AAAGTGTCTT GGGAATCTTG   
  
  
+ GGTTTACTCT TTCGTAGTAT TTTTATTGTT CTTCATCTTG TTAAGGGTTT TCTAGTCCGA AAACTGTGGA   
  
  
+ GACTTTTGGT CTAGAATTAG GAATTTACGT GAATTTTGGG GTCTACTTTT ATTTAGTTTG TTCAGTAGGT   
  
  
+ GTTACCACCG ATTACCTGTT AAAACATTTG GGGGAACGGA TTTATGACTT TTAGAATACT AGGTTCTGTT   
  
  
+ TTCTATGTTG TCTCTCTACT GTTTTTGAGT CCGATGCTTC CTCTTATTTG TTTTGGGTAA AGGTATTTGT   
  
  
+ CTATGTGGGA ATGTCCCAAA ACGCGTTTTA AGTTTTGAAG GATAAATGAA ACTTTCACTT TCGTCTGTCT   
  
  
+ TTGTCTTACT CGAGTACTCG TTCCTGATAG GTCTTATTTT AGTCTGTCAA AGAAGGCCGA TTAAGAGGTT   
  
  
+ TCTTAGTTTT CCCATCTTAC TTCTTTTTTT AGTTCTGGTT TTGCTGTTTG GGTACAATTT TTGAACTCGT   
  
  
+ CCCCTGAGTT TCTAATAGGG TAACAACCTT AACTTTCCAC ACTTTCTTGC TGAAACGAAA GGGAGGCCCT   
  
  
+ GAAATAATTT TAGTGGGTTT TCTTTTGTTT CAGGGAGAAA ATTTAAGAAA CCGTTTTCTG TTCGTTTAGT   
  
  
+ TTTAGTATTA TCTTAACTTA AGGTGAGTTC TCACTGTGTC GTCTCCTCTT CTTCTTATGT TTAAGAAATA   
  
  
+ AATTTTTTTT TTTTTCCTTC TCTCTCTCTC TCTCTCCTCC GCCACGAGCG GTACCGTCAC CACCTGTGTT   
  
  
+ TCATCCCTTC TTTTTTGGTT GCAGAGACAG TTTCTAGGAT ACCAGCCAGG GTGGTAGTCT GGATCAATAA   
  
  
+ GGAAAAAAAG TTAAAAAATA AAAAAATAAA AAACAATTTC ATTTAGATAA ATAATAGCAA AAATACTCTT   
  
  
+ TCACCGAATC ACAGTTTTTA GTTTCACAGA CGGTTCGTTT TTTCACCCGG GTGCTGAGGT GACACCTGGC   
  
  
+ CCTCGTAGGG CTCATACGTT GTATGTCATT ACTTGCCAGA TATAAGTTGG TGCACCGTTA TTAGGAACAC   
  
  
+ TATATCCCTG TGAACAGGGA TCTTACAACT GATTTTCGTC CCGAAACAAC AGAAAAACCC GAAAATTTTT   
  
  
+ TTATTTTAAG ACTCGCACCC TGGGTGACCA GTCCTGTGTC AACTGGACTG TCTCTAAGAG AGACGGGGAA   
  
  
+ GATAAATACG TCGACGAACC TCGGAAACAA AAACTCGGTT GTATTTATTA TTCAGTATCT ATAACCTAAA   
  
  
+ GAATTTTACA ATTTATGATA AATAGAAATT TTAATATTTT TTATCACTTT TTTAATTAAA ATAATAAATA   
  
  
+ TATAATTAAC AATTACCTAA CAGTTGACCT AACTTTAATC GGGTTTAGAT AAGGTAACCG AGTAAAAAAT   
  
  
+ ACTTATTAAT ATTTTACTTT AACTCGAAC  

- GCTCACCCAA AACCCCAAAA AAGAAAACGA AAAAAAAAAC CAAATCCCTA TTTCACAGAA CCCTTAGAAC   
  
  
- CCAAATGAGA AAGCATCATA AAAATAACAA GAAGTAGAAC AATTCCCAAA AGATCAGGCT TTTGACACCT   
  
  
- CTGAAAACCA GATCTTAATC CTTAAATGCA CTTAAAACCC CAGATGAAAA TAAATCAAAC AAGTCATCCA   
  
  
- CAATGGTGGC TAATGGACAA TTTTGTAAAC CCCCTTGCCT AAATACTGAA AATCTTATGA TCCAAGACAA   
  
  
- AAGATACAAC AGAGAGATGA CAAAAACTCA GGCTACGAAG GAGAATAAAC AAAACCCATT TCCATAAACA   
  
  
- GATACACCCT TACAGGGTTT TGCGCAAAAT TCAAAACTTC CTATTTACTT TGAAAGTGAA AGCAGACAGA   
  
  
- AACAGAATGA GCTCATGAGC AAGGACTATC CAGAATAAAA TCAGACAGTT TCTTCCGGCT AATTCTCCAA   
  
  
- AGAATCAAAA GGGTAGAATG AAGAAAAAAA TCAAGACCAA AACGACAAAC CCATGTTAAA AACTTGAGCA   
  
  
- GGGGACTCAA AGATTATCCC ATTGTTGGAA TTGAAAGGTG TGAAAGAACG ACTTTGCTTT CCCTCCGGGA   
  
  
- CTTTATTAAA ATCACCCAAA AGAAAACAAA GTCCCTCTTT TAAATTCTTT GGCAAAAGAC AAGCAAATCA   
  
  
- AAATCATAAT AGAATTGAAT TCCACTCAAG AGTGACACAG CAGAGGAGAA GAAGAATACA AATTCTTTAT   
  
  
- TTAAAAAAAA AAAAAGGAAG AGAGAGAGAG AGAGAGGAGG CGGTGCTCGC CATGGCAGTG GTGGACACAA   
  
  
- AGTAGGGAAG AAAAAACCAA CGTCTCTGTC AAAGATCCTA TGGTCGGTCC CACCATCAGA CCTAGTTATT   
  
  
- CCTTTTTTTC AATTTTTTAT TTTTTTATTT TTTGTTAAAG TAAATCTATT TATTATCGTT TTTATGAGAA   
  
  
- AGTGGCTTAG TGTCAAAAAT CAAAGTGTCT GCCAAGCAAA AAAGTGGGCC CACGACTCCA CTGTGGACCG   
  
  
- GGAGCATCCC GAGTATGCAA CATACAGTAA TGAACGGTCT ATATTCAACC ACGTGGCAAT AATCCTTGTG   
  
  
- ATATAGGGAC ACTTGTCCCT AGAATGTTGA CTAAAAGCAG GGCTTTGTTG TCTTTTTGGG CTTTTAAAAA   
  
  
- AATAAAATTC TGAGCGTGGG ACCCACTGGT CAGGACACAG TTGACCTGAC AGAGATTCTC TCTGCCCCTT   
  
  
- CTATTTATGC AGCTGCTTGG AGCCTTTGTT TTTGAGCCAA CATAAATAAT AAGTCATAGA TATTGGATTT   
  
  
- CTTAAAATGT TAAATACTAT TTATCTTTAA AATTATAAAA AATAGTGAAA AAATTAATTT TATTATTTAT   
  
  
- ATATTAATTG TTAATGGATT GTCAACTGGA TTGAAATTAG CCCAAATCTA TTCCATTGGC TCATTTTTTA   
  
  
- TGAATAATTA TAAAATGAAA TTGAGCTTG

+     TCT-motif

| Site Name | Organism | Position | Strand | Matrix score. | sequence | function |
| --- | --- | --- | --- | --- | --- | --- |
| TCT-motif | Arabidopsis thaliana | 505 | + | 6 | TCTTAC | part of a light responsive element |
| TCT-motif | Arabidopsis thaliana | 424 | + | 6 | TCTTAC | part of a light responsive element |
| TCT-motif | Arabidopsis thaliana | 1141 | + | 6 | TCTTAC | part of a light responsive element |

> 2018/04/13 10:10:12  
+ CGAGTGGGTT TTGGGGTTTT TTCTTTTGCT TTTTTTTTTG GTTTAGGGAT AAAGTGTCTT GGGAATCTTG   
  
  
+ GGTTTACTCT TTCGTAGTAT TTTTATTGTT CTTCATCTTG TTAAGGGTTT TCTAGTCCGA AAACTGTGGA   
  
  
+ GACTTTTGGT CTAGAATTAG GAATTTACGT GAATTTTGGG GTCTACTTTT ATTTAGTTTG TTCAGTAGGT   
  
  
+ GTTACCACCG ATTACCTGTT AAAACATTTG GGGGAACGGA TTTATGACTT TTAGAATACT AGGTTCTGTT   
  
  
+ TTCTATGTTG TCTCTCTACT GTTTTTGAGT CCGATGCTTC CTCTTATTTG TTTTGGGTAA AGGTATTTGT   
  
  
+ CTATGTGGGA ATGTCCCAAA ACGCGTTTTA AGTTTTGAAG GATAAATGAA ACTTTCACTT TCGTCTGTCT   
  
  
+ TTGTCTTACT CGAGTACTCG TTCCTGATAG GTCTTATTTT AGTCTGTCAA AGAAGGCCGA TTAAGAGGTT   
  
  
+ TCTTAGTTTT CCCATCTTAC TTCTTTTTTT AGTTCTGGTT TTGCTGTTTG GGTACAATTT TTGAACTCGT   
  
  
+ CCCCTGAGTT TCTAATAGGG TAACAACCTT AACTTTCCAC ACTTTCTTGC TGAAACGAAA GGGAGGCCCT   
  
  
+ GAAATAATTT TAGTGGGTTT TCTTTTGTTT CAGGGAGAAA ATTTAAGAAA CCGTTTTCTG TTCGTTTAGT   
  
  
+ TTTAGTATTA TCTTAACTTA AGGTGAGTTC TCACTGTGTC GTCTCCTCTT CTTCTTATGT TTAAGAAATA   
  
  
+ AATTTTTTTT TTTTTCCTTC TCTCTCTCTC TCTCTCCTCC GCCACGAGCG GTACCGTCAC CACCTGTGTT   
  
  
+ TCATCCCTTC TTTTTTGGTT GCAGAGACAG TTTCTAGGAT ACCAGCCAGG GTGGTAGTCT GGATCAATAA   
  
  
+ GGAAAAAAAG TTAAAAAATA AAAAAATAAA AAACAATTTC ATTTAGATAA ATAATAGCAA AAATACTCTT   
  
  
+ TCACCGAATC ACAGTTTTTA GTTTCACAGA CGGTTCGTTT TTTCACCCGG GTGCTGAGGT GACACCTGGC   
  
  
+ CCTCGTAGGG CTCATACGTT GTATGTCATT ACTTGCCAGA TATAAGTTGG TGCACCGTTA TTAGGAACAC   
  
  
+ TATATCCCTG TGAACAGGGA TCTTACAACT GATTTTCGTC CCGAAACAAC AGAAAAACCC GAAAATTTTT   
  
  
+ TTATTTTAAG ACTCGCACCC TGGGTGACCA GTCCTGTGTC AACTGGACTG TCTCTAAGAG AGACGGGGAA   
  
  
+ GATAAATACG TCGACGAACC TCGGAAACAA AAACTCGGTT GTATTTATTA TTCAGTATCT ATAACCTAAA   
  
  
+ GAATTTTACA ATTTATGATA AATAGAAATT TTAATATTTT TTATCACTTT TTTAATTAAA ATAATAAATA   
  
  
+ TATAATTAAC AATTACCTAA CAGTTGACCT AACTTTAATC GGGTTTAGAT AAGGTAACCG AGTAAAAAAT   
  
  
+ ACTTATTAAT ATTTTACTTT AACTCGAAC  

- GCTCACCCAA AACCCCAAAA AAGAAAACGA AAAAAAAAAC CAAATCCCTA TTTCACAGAA CCCTTAGAAC   
  
  
- CCAAATGAGA AAGCATCATA AAAATAACAA GAAGTAGAAC AATTCCCAAA AGATCAGGCT TTTGACACCT   
  
  
- CTGAAAACCA GATCTTAATC CTTAAATGCA CTTAAAACCC CAGATGAAAA TAAATCAAAC AAGTCATCCA   
  
  
- CAATGGTGGC TAATGGACAA TTTTGTAAAC CCCCTTGCCT AAATACTGAA AATCTTATGA TCCAAGACAA   
  
  
- AAGATACAAC AGAGAGATGA CAAAAACTCA GGCTACGAAG GAGAATAAAC AAAACCCATT TCCATAAACA   
  
  
- GATACACCCT TACAGGGTTT TGCGCAAAAT TCAAAACTTC CTATTTACTT TGAAAGTGAA AGCAGACAGA   
  
  
- AACAGAATGA GCTCATGAGC AAGGACTATC CAGAATAAAA TCAGACAGTT TCTTCCGGCT AATTCTCCAA   
  
  
- AGAATCAAAA GGGTAGAATG AAGAAAAAAA TCAAGACCAA AACGACAAAC CCATGTTAAA AACTTGAGCA   
  
  
- GGGGACTCAA AGATTATCCC ATTGTTGGAA TTGAAAGGTG TGAAAGAACG ACTTTGCTTT CCCTCCGGGA   
  
  
- CTTTATTAAA ATCACCCAAA AGAAAACAAA GTCCCTCTTT TAAATTCTTT GGCAAAAGAC AAGCAAATCA   
  
  
- AAATCATAAT AGAATTGAAT TCCACTCAAG AGTGACACAG CAGAGGAGAA GAAGAATACA AATTCTTTAT   
  
  
- TTAAAAAAAA AAAAAGGAAG AGAGAGAGAG AGAGAGGAGG CGGTGCTCGC CATGGCAGTG GTGGACACAA   
  
  
- AGTAGGGAAG AAAAAACCAA CGTCTCTGTC AAAGATCCTA TGGTCGGTCC CACCATCAGA CCTAGTTATT   
  
  
- CCTTTTTTTC AATTTTTTAT TTTTTTATTT TTTGTTAAAG TAAATCTATT TATTATCGTT TTTATGAGAA   
  
  
- AGTGGCTTAG TGTCAAAAAT CAAAGTGTCT GCCAAGCAAA AAAGTGGGCC CACGACTCCA CTGTGGACCG   
  
  
- GGAGCATCCC GAGTATGCAA CATACAGTAA TGAACGGTCT ATATTCAACC ACGTGGCAAT AATCCTTGTG   
  
  
- ATATAGGGAC ACTTGTCCCT AGAATGTTGA CTAAAAGCAG GGCTTTGTTG TCTTTTTGGG CTTTTAAAAA   
  
  
- AATAAAATTC TGAGCGTGGG ACCCACTGGT CAGGACACAG TTGACCTGAC AGAGATTCTC TCTGCCCCTT   
  
  
- CTATTTATGC AGCTGCTTGG AGCCTTTGTT TTTGAGCCAA CATAAATAAT AAGTCATAGA TATTGGATTT   
  
  
- CTTAAAATGT TAAATACTAT TTATCTTTAA AATTATAAAA AATAGTGAAA AAATTAATTT TATTATTTAT   
  
  
- ATATTAATTG TTAATGGATT GTCAACTGGA TTGAAATTAG CCCAAATCTA TTCCATTGGC TCATTTTTTA   
  
  
- TGAATAATTA TAAAATGAAA TTGAGCTTG

+     TGACG-motif

| Site Name | Organism | Position | Strand | Matrix score. | sequence | function |
| --- | --- | --- | --- | --- | --- | --- |
| TGACG-motif | Hordeum vulgare | 825 | - | 5 | TGACG | cis-acting regulatory element involved in the MeJA-responsiveness |

> 2018/04/13 10:10:12  
+ CGAGTGGGTT TTGGGGTTTT TTCTTTTGCT TTTTTTTTTG GTTTAGGGAT AAAGTGTCTT GGGAATCTTG   
  
  
+ GGTTTACTCT TTCGTAGTAT TTTTATTGTT CTTCATCTTG TTAAGGGTTT TCTAGTCCGA AAACTGTGGA   
  
  
+ GACTTTTGGT CTAGAATTAG GAATTTACGT GAATTTTGGG GTCTACTTTT ATTTAGTTTG TTCAGTAGGT   
  
  
+ GTTACCACCG ATTACCTGTT AAAACATTTG GGGGAACGGA TTTATGACTT TTAGAATACT AGGTTCTGTT   
  
  
+ TTCTATGTTG TCTCTCTACT GTTTTTGAGT CCGATGCTTC CTCTTATTTG TTTTGGGTAA AGGTATTTGT   
  
  
+ CTATGTGGGA ATGTCCCAAA ACGCGTTTTA AGTTTTGAAG GATAAATGAA ACTTTCACTT TCGTCTGTCT   
  
  
+ TTGTCTTACT CGAGTACTCG TTCCTGATAG GTCTTATTTT AGTCTGTCAA AGAAGGCCGA TTAAGAGGTT   
  
  
+ TCTTAGTTTT CCCATCTTAC TTCTTTTTTT AGTTCTGGTT TTGCTGTTTG GGTACAATTT TTGAACTCGT   
  
  
+ CCCCTGAGTT TCTAATAGGG TAACAACCTT AACTTTCCAC ACTTTCTTGC TGAAACGAAA GGGAGGCCCT   
  
  
+ GAAATAATTT TAGTGGGTTT TCTTTTGTTT CAGGGAGAAA ATTTAAGAAA CCGTTTTCTG TTCGTTTAGT   
  
  
+ TTTAGTATTA TCTTAACTTA AGGTGAGTTC TCACTGTGTC GTCTCCTCTT CTTCTTATGT TTAAGAAATA   
  
  
+ AATTTTTTTT TTTTTCCTTC TCTCTCTCTC TCTCTCCTCC GCCACGAGCG GTACCGTCAC CACCTGTGTT   
  
  
+ TCATCCCTTC TTTTTTGGTT GCAGAGACAG TTTCTAGGAT ACCAGCCAGG GTGGTAGTCT GGATCAATAA   
  
  
+ GGAAAAAAAG TTAAAAAATA AAAAAATAAA AAACAATTTC ATTTAGATAA ATAATAGCAA AAATACTCTT   
  
  
+ TCACCGAATC ACAGTTTTTA GTTTCACAGA CGGTTCGTTT TTTCACCCGG GTGCTGAGGT GACACCTGGC   
  
  
+ CCTCGTAGGG CTCATACGTT GTATGTCATT ACTTGCCAGA TATAAGTTGG TGCACCGTTA TTAGGAACAC   
  
  
+ TATATCCCTG TGAACAGGGA TCTTACAACT GATTTTCGTC CCGAAACAAC AGAAAAACCC GAAAATTTTT   
  
  
+ TTATTTTAAG ACTCGCACCC TGGGTGACCA GTCCTGTGTC AACTGGACTG TCTCTAAGAG AGACGGGGAA   
  
  
+ GATAAATACG TCGACGAACC TCGGAAACAA AAACTCGGTT GTATTTATTA TTCAGTATCT ATAACCTAAA   
  
  
+ GAATTTTACA ATTTATGATA AATAGAAATT TTAATATTTT TTATCACTTT TTTAATTAAA ATAATAAATA   
  
  
+ TATAATTAAC AATTACCTAA CAGTTGACCT AACTTTAATC GGGTTTAGAT AAGGTAACCG AGTAAAAAAT   
  
  
+ ACTTATTAAT ATTTTACTTT AACTCGAAC  

- GCTCACCCAA AACCCCAAAA AAGAAAACGA AAAAAAAAAC CAAATCCCTA TTTCACAGAA CCCTTAGAAC   
  
  
- CCAAATGAGA AAGCATCATA AAAATAACAA GAAGTAGAAC AATTCCCAAA AGATCAGGCT TTTGACACCT   
  
  
- CTGAAAACCA GATCTTAATC CTTAAATGCA CTTAAAACCC CAGATGAAAA TAAATCAAAC AAGTCATCCA   
  
  
- CAATGGTGGC TAATGGACAA TTTTGTAAAC CCCCTTGCCT AAATACTGAA AATCTTATGA TCCAAGACAA   
  
  
- AAGATACAAC AGAGAGATGA CAAAAACTCA GGCTACGAAG GAGAATAAAC AAAACCCATT TCCATAAACA   
  
  
- GATACACCCT TACAGGGTTT TGCGCAAAAT TCAAAACTTC CTATTTACTT TGAAAGTGAA AGCAGACAGA   
  
  
- AACAGAATGA GCTCATGAGC AAGGACTATC CAGAATAAAA TCAGACAGTT TCTTCCGGCT AATTCTCCAA   
  
  
- AGAATCAAAA GGGTAGAATG AAGAAAAAAA TCAAGACCAA AACGACAAAC CCATGTTAAA AACTTGAGCA   
  
  
- GGGGACTCAA AGATTATCCC ATTGTTGGAA TTGAAAGGTG TGAAAGAACG ACTTTGCTTT CCCTCCGGGA   
  
  
- CTTTATTAAA ATCACCCAAA AGAAAACAAA GTCCCTCTTT TAAATTCTTT GGCAAAAGAC AAGCAAATCA   
  
  
- AAATCATAAT AGAATTGAAT TCCACTCAAG AGTGACACAG CAGAGGAGAA GAAGAATACA AATTCTTTAT   
  
  
- TTAAAAAAAA AAAAAGGAAG AGAGAGAGAG AGAGAGGAGG CGGTGCTCGC CATGGCAGTG GTGGACACAA   
  
  
- AGTAGGGAAG AAAAAACCAA CGTCTCTGTC AAAGATCCTA TGGTCGGTCC CACCATCAGA CCTAGTTATT   
  
  
- CCTTTTTTTC AATTTTTTAT TTTTTTATTT TTTGTTAAAG TAAATCTATT TATTATCGTT TTTATGAGAA   
  
  
- AGTGGCTTAG TGTCAAAAAT CAAAGTGTCT GCCAAGCAAA AAAGTGGGCC CACGACTCCA CTGTGGACCG   
  
  
- GGAGCATCCC GAGTATGCAA CATACAGTAA TGAACGGTCT ATATTCAACC ACGTGGCAAT AATCCTTGTG   
  
  
- ATATAGGGAC ACTTGTCCCT AGAATGTTGA CTAAAAGCAG GGCTTTGTTG TCTTTTTGGG CTTTTAAAAA   
  
  
- AATAAAATTC TGAGCGTGGG ACCCACTGGT CAGGACACAG TTGACCTGAC AGAGATTCTC TCTGCCCCTT   
  
  
- CTATTTATGC AGCTGCTTGG AGCCTTTGTT TTTGAGCCAA CATAAATAAT AAGTCATAGA TATTGGATTT   
  
  
- CTTAAAATGT TAAATACTAT TTATCTTTAA AATTATAAAA AATAGTGAAA AAATTAATTT TATTATTTAT   
  
  
- ATATTAATTG TTAATGGATT GTCAACTGGA TTGAAATTAG CCCAAATCTA TTCCATTGGC TCATTTTTTA   
  
  
- TGAATAATTA TAAAATGAAA TTGAGCTTG

+     Unnamed\_\_1

| Site Name | Organism | Position | Strand | Matrix score. | sequence | function |
| --- | --- | --- | --- | --- | --- | --- |
| Unnamed\_\_1 | Zea mays | 812 | - | 5 | CGTGG |  |

> 2018/04/13 10:10:12  
+ CGAGTGGGTT TTGGGGTTTT TTCTTTTGCT TTTTTTTTTG GTTTAGGGAT AAAGTGTCTT GGGAATCTTG   
  
  
+ GGTTTACTCT TTCGTAGTAT TTTTATTGTT CTTCATCTTG TTAAGGGTTT TCTAGTCCGA AAACTGTGGA   
  
  
+ GACTTTTGGT CTAGAATTAG GAATTTACGT GAATTTTGGG GTCTACTTTT ATTTAGTTTG TTCAGTAGGT   
  
  
+ GTTACCACCG ATTACCTGTT AAAACATTTG GGGGAACGGA TTTATGACTT TTAGAATACT AGGTTCTGTT   
  
  
+ TTCTATGTTG TCTCTCTACT GTTTTTGAGT CCGATGCTTC CTCTTATTTG TTTTGGGTAA AGGTATTTGT   
  
  
+ CTATGTGGGA ATGTCCCAAA ACGCGTTTTA AGTTTTGAAG GATAAATGAA ACTTTCACTT TCGTCTGTCT   
  
  
+ TTGTCTTACT CGAGTACTCG TTCCTGATAG GTCTTATTTT AGTCTGTCAA AGAAGGCCGA TTAAGAGGTT   
  
  
+ TCTTAGTTTT CCCATCTTAC TTCTTTTTTT AGTTCTGGTT TTGCTGTTTG GGTACAATTT TTGAACTCGT   
  
  
+ CCCCTGAGTT TCTAATAGGG TAACAACCTT AACTTTCCAC ACTTTCTTGC TGAAACGAAA GGGAGGCCCT   
  
  
+ GAAATAATTT TAGTGGGTTT TCTTTTGTTT CAGGGAGAAA ATTTAAGAAA CCGTTTTCTG TTCGTTTAGT   
  
  
+ TTTAGTATTA TCTTAACTTA AGGTGAGTTC TCACTGTGTC GTCTCCTCTT CTTCTTATGT TTAAGAAATA   
  
  
+ AATTTTTTTT TTTTTCCTTC TCTCTCTCTC TCTCTCCTCC GCCACGAGCG GTACCGTCAC CACCTGTGTT   
  
  
+ TCATCCCTTC TTTTTTGGTT GCAGAGACAG TTTCTAGGAT ACCAGCCAGG GTGGTAGTCT GGATCAATAA   
  
  
+ GGAAAAAAAG TTAAAAAATA AAAAAATAAA AAACAATTTC ATTTAGATAA ATAATAGCAA AAATACTCTT   
  
  
+ TCACCGAATC ACAGTTTTTA GTTTCACAGA CGGTTCGTTT TTTCACCCGG GTGCTGAGGT GACACCTGGC   
  
  
+ CCTCGTAGGG CTCATACGTT GTATGTCATT ACTTGCCAGA TATAAGTTGG TGCACCGTTA TTAGGAACAC   
  
  
+ TATATCCCTG TGAACAGGGA TCTTACAACT GATTTTCGTC CCGAAACAAC AGAAAAACCC GAAAATTTTT   
  
  
+ TTATTTTAAG ACTCGCACCC TGGGTGACCA GTCCTGTGTC AACTGGACTG TCTCTAAGAG AGACGGGGAA   
  
  
+ GATAAATACG TCGACGAACC TCGGAAACAA AAACTCGGTT GTATTTATTA TTCAGTATCT ATAACCTAAA   
  
  
+ GAATTTTACA ATTTATGATA AATAGAAATT TTAATATTTT TTATCACTTT TTTAATTAAA ATAATAAATA   
  
  
+ TATAATTAAC AATTACCTAA CAGTTGACCT AACTTTAATC GGGTTTAGAT AAGGTAACCG AGTAAAAAAT   
  
  
+ ACTTATTAAT ATTTTACTTT AACTCGAAC  

- GCTCACCCAA AACCCCAAAA AAGAAAACGA AAAAAAAAAC CAAATCCCTA TTTCACAGAA CCCTTAGAAC   
  
  
- CCAAATGAGA AAGCATCATA AAAATAACAA GAAGTAGAAC AATTCCCAAA AGATCAGGCT TTTGACACCT   
  
  
- CTGAAAACCA GATCTTAATC CTTAAATGCA CTTAAAACCC CAGATGAAAA TAAATCAAAC AAGTCATCCA   
  
  
- CAATGGTGGC TAATGGACAA TTTTGTAAAC CCCCTTGCCT AAATACTGAA AATCTTATGA TCCAAGACAA   
  
  
- AAGATACAAC AGAGAGATGA CAAAAACTCA GGCTACGAAG GAGAATAAAC AAAACCCATT TCCATAAACA   
  
  
- GATACACCCT TACAGGGTTT TGCGCAAAAT TCAAAACTTC CTATTTACTT TGAAAGTGAA AGCAGACAGA   
  
  
- AACAGAATGA GCTCATGAGC AAGGACTATC CAGAATAAAA TCAGACAGTT TCTTCCGGCT AATTCTCCAA   
  
  
- AGAATCAAAA GGGTAGAATG AAGAAAAAAA TCAAGACCAA AACGACAAAC CCATGTTAAA AACTTGAGCA   
  
  
- GGGGACTCAA AGATTATCCC ATTGTTGGAA TTGAAAGGTG TGAAAGAACG ACTTTGCTTT CCCTCCGGGA   
  
  
- CTTTATTAAA ATCACCCAAA AGAAAACAAA GTCCCTCTTT TAAATTCTTT GGCAAAAGAC AAGCAAATCA   
  
  
- AAATCATAAT AGAATTGAAT TCCACTCAAG AGTGACACAG CAGAGGAGAA GAAGAATACA AATTCTTTAT   
  
  
- TTAAAAAAAA AAAAAGGAAG AGAGAGAGAG AGAGAGGAGG CGGTGCTCGC CATGGCAGTG GTGGACACAA   
  
  
- AGTAGGGAAG AAAAAACCAA CGTCTCTGTC AAAGATCCTA TGGTCGGTCC CACCATCAGA CCTAGTTATT   
  
  
- CCTTTTTTTC AATTTTTTAT TTTTTTATTT TTTGTTAAAG TAAATCTATT TATTATCGTT TTTATGAGAA   
  
  
- AGTGGCTTAG TGTCAAAAAT CAAAGTGTCT GCCAAGCAAA AAAGTGGGCC CACGACTCCA CTGTGGACCG   
  
  
- GGAGCATCCC GAGTATGCAA CATACAGTAA TGAACGGTCT ATATTCAACC ACGTGGCAAT AATCCTTGTG   
  
  
- ATATAGGGAC ACTTGTCCCT AGAATGTTGA CTAAAAGCAG GGCTTTGTTG TCTTTTTGGG CTTTTAAAAA   
  
  
- AATAAAATTC TGAGCGTGGG ACCCACTGGT CAGGACACAG TTGACCTGAC AGAGATTCTC TCTGCCCCTT   
  
  
- CTATTTATGC AGCTGCTTGG AGCCTTTGTT TTTGAGCCAA CATAAATAAT AAGTCATAGA TATTGGATTT   
  
  
- CTTAAAATGT TAAATACTAT TTATCTTTAA AATTATAAAA AATAGTGAAA AAATTAATTT TATTATTTAT   
  
  
- ATATTAATTG TTAATGGATT GTCAACTGGA TTGAAATTAG CCCAAATCTA TTCCATTGGC TCATTTTTTA   
  
  
- TGAATAATTA TAAAATGAAA TTGAGCTTG

+     Unnamed\_\_11

| Site Name | Organism | Position | Strand | Matrix score. | sequence | function |
| --- | --- | --- | --- | --- | --- | --- |
| Unnamed\_\_11 | Zea mays | 350 | - | 9 | TCCACATAGA |  |

> 2018/04/13 10:10:12  
+ CGAGTGGGTT TTGGGGTTTT TTCTTTTGCT TTTTTTTTTG GTTTAGGGAT AAAGTGTCTT GGGAATCTTG   
  
  
+ GGTTTACTCT TTCGTAGTAT TTTTATTGTT CTTCATCTTG TTAAGGGTTT TCTAGTCCGA AAACTGTGGA   
  
  
+ GACTTTTGGT CTAGAATTAG GAATTTACGT GAATTTTGGG GTCTACTTTT ATTTAGTTTG TTCAGTAGGT   
  
  
+ GTTACCACCG ATTACCTGTT AAAACATTTG GGGGAACGGA TTTATGACTT TTAGAATACT AGGTTCTGTT   
  
  
+ TTCTATGTTG TCTCTCTACT GTTTTTGAGT CCGATGCTTC CTCTTATTTG TTTTGGGTAA AGGTATTTGT   
  
  
+ CTATGTGGGA ATGTCCCAAA ACGCGTTTTA AGTTTTGAAG GATAAATGAA ACTTTCACTT TCGTCTGTCT   
  
  
+ TTGTCTTACT CGAGTACTCG TTCCTGATAG GTCTTATTTT AGTCTGTCAA AGAAGGCCGA TTAAGAGGTT   
  
  
+ TCTTAGTTTT CCCATCTTAC TTCTTTTTTT AGTTCTGGTT TTGCTGTTTG GGTACAATTT TTGAACTCGT   
  
  
+ CCCCTGAGTT TCTAATAGGG TAACAACCTT AACTTTCCAC ACTTTCTTGC TGAAACGAAA GGGAGGCCCT   
  
  
+ GAAATAATTT TAGTGGGTTT TCTTTTGTTT CAGGGAGAAA ATTTAAGAAA CCGTTTTCTG TTCGTTTAGT   
  
  
+ TTTAGTATTA TCTTAACTTA AGGTGAGTTC TCACTGTGTC GTCTCCTCTT CTTCTTATGT TTAAGAAATA   
  
  
+ AATTTTTTTT TTTTTCCTTC TCTCTCTCTC TCTCTCCTCC GCCACGAGCG GTACCGTCAC CACCTGTGTT   
  
  
+ TCATCCCTTC TTTTTTGGTT GCAGAGACAG TTTCTAGGAT ACCAGCCAGG GTGGTAGTCT GGATCAATAA   
  
  
+ GGAAAAAAAG TTAAAAAATA AAAAAATAAA AAACAATTTC ATTTAGATAA ATAATAGCAA AAATACTCTT   
  
  
+ TCACCGAATC ACAGTTTTTA GTTTCACAGA CGGTTCGTTT TTTCACCCGG GTGCTGAGGT GACACCTGGC   
  
  
+ CCTCGTAGGG CTCATACGTT GTATGTCATT ACTTGCCAGA TATAAGTTGG TGCACCGTTA TTAGGAACAC   
  
  
+ TATATCCCTG TGAACAGGGA TCTTACAACT GATTTTCGTC CCGAAACAAC AGAAAAACCC GAAAATTTTT   
  
  
+ TTATTTTAAG ACTCGCACCC TGGGTGACCA GTCCTGTGTC AACTGGACTG TCTCTAAGAG AGACGGGGAA   
  
  
+ GATAAATACG TCGACGAACC TCGGAAACAA AAACTCGGTT GTATTTATTA TTCAGTATCT ATAACCTAAA   
  
  
+ GAATTTTACA ATTTATGATA AATAGAAATT TTAATATTTT TTATCACTTT TTTAATTAAA ATAATAAATA   
  
  
+ TATAATTAAC AATTACCTAA CAGTTGACCT AACTTTAATC GGGTTTAGAT AAGGTAACCG AGTAAAAAAT   
  
  
+ ACTTATTAAT ATTTTACTTT AACTCGAAC  

- GCTCACCCAA AACCCCAAAA AAGAAAACGA AAAAAAAAAC CAAATCCCTA TTTCACAGAA CCCTTAGAAC   
  
  
- CCAAATGAGA AAGCATCATA AAAATAACAA GAAGTAGAAC AATTCCCAAA AGATCAGGCT TTTGACACCT   
  
  
- CTGAAAACCA GATCTTAATC CTTAAATGCA CTTAAAACCC CAGATGAAAA TAAATCAAAC AAGTCATCCA   
  
  
- CAATGGTGGC TAATGGACAA TTTTGTAAAC CCCCTTGCCT AAATACTGAA AATCTTATGA TCCAAGACAA   
  
  
- AAGATACAAC AGAGAGATGA CAAAAACTCA GGCTACGAAG GAGAATAAAC AAAACCCATT TCCATAAACA   
  
  
- GATACACCCT TACAGGGTTT TGCGCAAAAT TCAAAACTTC CTATTTACTT TGAAAGTGAA AGCAGACAGA   
  
  
- AACAGAATGA GCTCATGAGC AAGGACTATC CAGAATAAAA TCAGACAGTT TCTTCCGGCT AATTCTCCAA   
  
  
- AGAATCAAAA GGGTAGAATG AAGAAAAAAA TCAAGACCAA AACGACAAAC CCATGTTAAA AACTTGAGCA   
  
  
- GGGGACTCAA AGATTATCCC ATTGTTGGAA TTGAAAGGTG TGAAAGAACG ACTTTGCTTT CCCTCCGGGA   
  
  
- CTTTATTAAA ATCACCCAAA AGAAAACAAA GTCCCTCTTT TAAATTCTTT GGCAAAAGAC AAGCAAATCA   
  
  
- AAATCATAAT AGAATTGAAT TCCACTCAAG AGTGACACAG CAGAGGAGAA GAAGAATACA AATTCTTTAT   
  
  
- TTAAAAAAAA AAAAAGGAAG AGAGAGAGAG AGAGAGGAGG CGGTGCTCGC CATGGCAGTG GTGGACACAA   
  
  
- AGTAGGGAAG AAAAAACCAA CGTCTCTGTC AAAGATCCTA TGGTCGGTCC CACCATCAGA CCTAGTTATT   
  
  
- CCTTTTTTTC AATTTTTTAT TTTTTTATTT TTTGTTAAAG TAAATCTATT TATTATCGTT TTTATGAGAA   
  
  
- AGTGGCTTAG TGTCAAAAAT CAAAGTGTCT GCCAAGCAAA AAAGTGGGCC CACGACTCCA CTGTGGACCG   
  
  
- GGAGCATCCC GAGTATGCAA CATACAGTAA TGAACGGTCT ATATTCAACC ACGTGGCAAT AATCCTTGTG   
  
  
- ATATAGGGAC ACTTGTCCCT AGAATGTTGA CTAAAAGCAG GGCTTTGTTG TCTTTTTGGG CTTTTAAAAA   
  
  
- AATAAAATTC TGAGCGTGGG ACCCACTGGT CAGGACACAG TTGACCTGAC AGAGATTCTC TCTGCCCCTT   
  
  
- CTATTTATGC AGCTGCTTGG AGCCTTTGTT TTTGAGCCAA CATAAATAAT AAGTCATAGA TATTGGATTT   
  
  
- CTTAAAATGT TAAATACTAT TTATCTTTAA AATTATAAAA AATAGTGAAA AAATTAATTT TATTATTTAT   
  
  
- ATATTAATTG TTAATGGATT GTCAACTGGA TTGAAATTAG CCCAAATCTA TTCCATTGGC TCATTTTTTA   
  
  
- TGAATAATTA TAAAATGAAA TTGAGCTTG

+     Unnamed\_\_3

| Site Name | Organism | Position | Strand | Matrix score. | sequence | function |
| --- | --- | --- | --- | --- | --- | --- |
| Unnamed\_\_3 | Zea mays | 812 | - | 5 | CGTGG |  |

> 2018/04/13 10:10:12  
+ CGAGTGGGTT TTGGGGTTTT TTCTTTTGCT TTTTTTTTTG GTTTAGGGAT AAAGTGTCTT GGGAATCTTG   
  
  
+ GGTTTACTCT TTCGTAGTAT TTTTATTGTT CTTCATCTTG TTAAGGGTTT TCTAGTCCGA AAACTGTGGA   
  
  
+ GACTTTTGGT CTAGAATTAG GAATTTACGT GAATTTTGGG GTCTACTTTT ATTTAGTTTG TTCAGTAGGT   
  
  
+ GTTACCACCG ATTACCTGTT AAAACATTTG GGGGAACGGA TTTATGACTT TTAGAATACT AGGTTCTGTT   
  
  
+ TTCTATGTTG TCTCTCTACT GTTTTTGAGT CCGATGCTTC CTCTTATTTG TTTTGGGTAA AGGTATTTGT   
  
  
+ CTATGTGGGA ATGTCCCAAA ACGCGTTTTA AGTTTTGAAG GATAAATGAA ACTTTCACTT TCGTCTGTCT   
  
  
+ TTGTCTTACT CGAGTACTCG TTCCTGATAG GTCTTATTTT AGTCTGTCAA AGAAGGCCGA TTAAGAGGTT   
  
  
+ TCTTAGTTTT CCCATCTTAC TTCTTTTTTT AGTTCTGGTT TTGCTGTTTG GGTACAATTT TTGAACTCGT   
  
  
+ CCCCTGAGTT TCTAATAGGG TAACAACCTT AACTTTCCAC ACTTTCTTGC TGAAACGAAA GGGAGGCCCT   
  
  
+ GAAATAATTT TAGTGGGTTT TCTTTTGTTT CAGGGAGAAA ATTTAAGAAA CCGTTTTCTG TTCGTTTAGT   
  
  
+ TTTAGTATTA TCTTAACTTA AGGTGAGTTC TCACTGTGTC GTCTCCTCTT CTTCTTATGT TTAAGAAATA   
  
  
+ AATTTTTTTT TTTTTCCTTC TCTCTCTCTC TCTCTCCTCC GCCACGAGCG GTACCGTCAC CACCTGTGTT   
  
  
+ TCATCCCTTC TTTTTTGGTT GCAGAGACAG TTTCTAGGAT ACCAGCCAGG GTGGTAGTCT GGATCAATAA   
  
  
+ GGAAAAAAAG TTAAAAAATA AAAAAATAAA AAACAATTTC ATTTAGATAA ATAATAGCAA AAATACTCTT   
  
  
+ TCACCGAATC ACAGTTTTTA GTTTCACAGA CGGTTCGTTT TTTCACCCGG GTGCTGAGGT GACACCTGGC   
  
  
+ CCTCGTAGGG CTCATACGTT GTATGTCATT ACTTGCCAGA TATAAGTTGG TGCACCGTTA TTAGGAACAC   
  
  
+ TATATCCCTG TGAACAGGGA TCTTACAACT GATTTTCGTC CCGAAACAAC AGAAAAACCC GAAAATTTTT   
  
  
+ TTATTTTAAG ACTCGCACCC TGGGTGACCA GTCCTGTGTC AACTGGACTG TCTCTAAGAG AGACGGGGAA   
  
  
+ GATAAATACG TCGACGAACC TCGGAAACAA AAACTCGGTT GTATTTATTA TTCAGTATCT ATAACCTAAA   
  
  
+ GAATTTTACA ATTTATGATA AATAGAAATT TTAATATTTT TTATCACTTT TTTAATTAAA ATAATAAATA   
  
  
+ TATAATTAAC AATTACCTAA CAGTTGACCT AACTTTAATC GGGTTTAGAT AAGGTAACCG AGTAAAAAAT   
  
  
+ ACTTATTAAT ATTTTACTTT AACTCGAAC  

- GCTCACCCAA AACCCCAAAA AAGAAAACGA AAAAAAAAAC CAAATCCCTA TTTCACAGAA CCCTTAGAAC   
  
  
- CCAAATGAGA AAGCATCATA AAAATAACAA GAAGTAGAAC AATTCCCAAA AGATCAGGCT TTTGACACCT   
  
  
- CTGAAAACCA GATCTTAATC CTTAAATGCA CTTAAAACCC CAGATGAAAA TAAATCAAAC AAGTCATCCA   
  
  
- CAATGGTGGC TAATGGACAA TTTTGTAAAC CCCCTTGCCT AAATACTGAA AATCTTATGA TCCAAGACAA   
  
  
- AAGATACAAC AGAGAGATGA CAAAAACTCA GGCTACGAAG GAGAATAAAC AAAACCCATT TCCATAAACA   
  
  
- GATACACCCT TACAGGGTTT TGCGCAAAAT TCAAAACTTC CTATTTACTT TGAAAGTGAA AGCAGACAGA   
  
  
- AACAGAATGA GCTCATGAGC AAGGACTATC CAGAATAAAA TCAGACAGTT TCTTCCGGCT AATTCTCCAA   
  
  
- AGAATCAAAA GGGTAGAATG AAGAAAAAAA TCAAGACCAA AACGACAAAC CCATGTTAAA AACTTGAGCA   
  
  
- GGGGACTCAA AGATTATCCC ATTGTTGGAA TTGAAAGGTG TGAAAGAACG ACTTTGCTTT CCCTCCGGGA   
  
  
- CTTTATTAAA ATCACCCAAA AGAAAACAAA GTCCCTCTTT TAAATTCTTT GGCAAAAGAC AAGCAAATCA   
  
  
- AAATCATAAT AGAATTGAAT TCCACTCAAG AGTGACACAG CAGAGGAGAA GAAGAATACA AATTCTTTAT   
  
  
- TTAAAAAAAA AAAAAGGAAG AGAGAGAGAG AGAGAGGAGG CGGTGCTCGC CATGGCAGTG GTGGACACAA   
  
  
- AGTAGGGAAG AAAAAACCAA CGTCTCTGTC AAAGATCCTA TGGTCGGTCC CACCATCAGA CCTAGTTATT   
  
  
- CCTTTTTTTC AATTTTTTAT TTTTTTATTT TTTGTTAAAG TAAATCTATT TATTATCGTT TTTATGAGAA   
  
  
- AGTGGCTTAG TGTCAAAAAT CAAAGTGTCT GCCAAGCAAA AAAGTGGGCC CACGACTCCA CTGTGGACCG   
  
  
- GGAGCATCCC GAGTATGCAA CATACAGTAA TGAACGGTCT ATATTCAACC ACGTGGCAAT AATCCTTGTG   
  
  
- ATATAGGGAC ACTTGTCCCT AGAATGTTGA CTAAAAGCAG GGCTTTGTTG TCTTTTTGGG CTTTTAAAAA   
  
  
- AATAAAATTC TGAGCGTGGG ACCCACTGGT CAGGACACAG TTGACCTGAC AGAGATTCTC TCTGCCCCTT   
  
  
- CTATTTATGC AGCTGCTTGG AGCCTTTGTT TTTGAGCCAA CATAAATAAT AAGTCATAGA TATTGGATTT   
  
  
- CTTAAAATGT TAAATACTAT TTATCTTTAA AATTATAAAA AATAGTGAAA AAATTAATTT TATTATTTAT   
  
  
- ATATTAATTG TTAATGGATT GTCAACTGGA TTGAAATTAG CCCAAATCTA TTCCATTGGC TCATTTTTTA   
  
  
- TGAATAATTA TAAAATGAAA TTGAGCTTG

+     Unnamed\_\_4

| Site Name | Organism | Position | Strand | Matrix score. | sequence | function |
| --- | --- | --- | --- | --- | --- | --- |
| Unnamed\_\_4 | Petroselinum hortense | 138 | - | 4 | CTCC |  |
| Unnamed\_\_4 | Petroselinum hortense | 664 | - | 4 | CTCC |  |
| Unnamed\_\_4 | Petroselinum hortense | 804 | + | 4 | CTCC |  |
| Unnamed\_\_4 | Petroselinum hortense | 807 | + | 4 | CTCC |  |
| Unnamed\_\_4 | Petroselinum hortense | 622 | - | 4 | CTCC |  |
| Unnamed\_\_4 | Petroselinum hortense | 743 | + | 4 | CTCC |  |

> 2018/04/13 10:10:12  
+ CGAGTGGGTT TTGGGGTTTT TTCTTTTGCT TTTTTTTTTG GTTTAGGGAT AAAGTGTCTT GGGAATCTTG   
  
  
+ GGTTTACTCT TTCGTAGTAT TTTTATTGTT CTTCATCTTG TTAAGGGTTT TCTAGTCCGA AAACTGTGGA   
  
  
+ GACTTTTGGT CTAGAATTAG GAATTTACGT GAATTTTGGG GTCTACTTTT ATTTAGTTTG TTCAGTAGGT   
  
  
+ GTTACCACCG ATTACCTGTT AAAACATTTG GGGGAACGGA TTTATGACTT TTAGAATACT AGGTTCTGTT   
  
  
+ TTCTATGTTG TCTCTCTACT GTTTTTGAGT CCGATGCTTC CTCTTATTTG TTTTGGGTAA AGGTATTTGT   
  
  
+ CTATGTGGGA ATGTCCCAAA ACGCGTTTTA AGTTTTGAAG GATAAATGAA ACTTTCACTT TCGTCTGTCT   
  
  
+ TTGTCTTACT CGAGTACTCG TTCCTGATAG GTCTTATTTT AGTCTGTCAA AGAAGGCCGA TTAAGAGGTT   
  
  
+ TCTTAGTTTT CCCATCTTAC TTCTTTTTTT AGTTCTGGTT TTGCTGTTTG GGTACAATTT TTGAACTCGT   
  
  
+ CCCCTGAGTT TCTAATAGGG TAACAACCTT AACTTTCCAC ACTTTCTTGC TGAAACGAAA GGGAGGCCCT   
  
  
+ GAAATAATTT TAGTGGGTTT TCTTTTGTTT CAGGGAGAAA ATTTAAGAAA CCGTTTTCTG TTCGTTTAGT   
  
  
+ TTTAGTATTA TCTTAACTTA AGGTGAGTTC TCACTGTGTC GTCTCCTCTT CTTCTTATGT TTAAGAAATA   
  
  
+ AATTTTTTTT TTTTTCCTTC TCTCTCTCTC TCTCTCCTCC GCCACGAGCG GTACCGTCAC CACCTGTGTT   
  
  
+ TCATCCCTTC TTTTTTGGTT GCAGAGACAG TTTCTAGGAT ACCAGCCAGG GTGGTAGTCT GGATCAATAA   
  
  
+ GGAAAAAAAG TTAAAAAATA AAAAAATAAA AAACAATTTC ATTTAGATAA ATAATAGCAA AAATACTCTT   
  
  
+ TCACCGAATC ACAGTTTTTA GTTTCACAGA CGGTTCGTTT TTTCACCCGG GTGCTGAGGT GACACCTGGC   
  
  
+ CCTCGTAGGG CTCATACGTT GTATGTCATT ACTTGCCAGA TATAAGTTGG TGCACCGTTA TTAGGAACAC   
  
  
+ TATATCCCTG TGAACAGGGA TCTTACAACT GATTTTCGTC CCGAAACAAC AGAAAAACCC GAAAATTTTT   
  
  
+ TTATTTTAAG ACTCGCACCC TGGGTGACCA GTCCTGTGTC AACTGGACTG TCTCTAAGAG AGACGGGGAA   
  
  
+ GATAAATACG TCGACGAACC TCGGAAACAA AAACTCGGTT GTATTTATTA TTCAGTATCT ATAACCTAAA   
  
  
+ GAATTTTACA ATTTATGATA AATAGAAATT TTAATATTTT TTATCACTTT TTTAATTAAA ATAATAAATA   
  
  
+ TATAATTAAC AATTACCTAA CAGTTGACCT AACTTTAATC GGGTTTAGAT AAGGTAACCG AGTAAAAAAT   
  
  
+ ACTTATTAAT ATTTTACTTT AACTCGAAC  

- GCTCACCCAA AACCCCAAAA AAGAAAACGA AAAAAAAAAC CAAATCCCTA TTTCACAGAA CCCTTAGAAC   
  
  
- CCAAATGAGA AAGCATCATA AAAATAACAA GAAGTAGAAC AATTCCCAAA AGATCAGGCT TTTGACACCT   
  
  
- CTGAAAACCA GATCTTAATC CTTAAATGCA CTTAAAACCC CAGATGAAAA TAAATCAAAC AAGTCATCCA   
  
  
- CAATGGTGGC TAATGGACAA TTTTGTAAAC CCCCTTGCCT AAATACTGAA AATCTTATGA TCCAAGACAA   
  
  
- AAGATACAAC AGAGAGATGA CAAAAACTCA GGCTACGAAG GAGAATAAAC AAAACCCATT TCCATAAACA   
  
  
- GATACACCCT TACAGGGTTT TGCGCAAAAT TCAAAACTTC CTATTTACTT TGAAAGTGAA AGCAGACAGA   
  
  
- AACAGAATGA GCTCATGAGC AAGGACTATC CAGAATAAAA TCAGACAGTT TCTTCCGGCT AATTCTCCAA   
  
  
- AGAATCAAAA GGGTAGAATG AAGAAAAAAA TCAAGACCAA AACGACAAAC CCATGTTAAA AACTTGAGCA   
  
  
- GGGGACTCAA AGATTATCCC ATTGTTGGAA TTGAAAGGTG TGAAAGAACG ACTTTGCTTT CCCTCCGGGA   
  
  
- CTTTATTAAA ATCACCCAAA AGAAAACAAA GTCCCTCTTT TAAATTCTTT GGCAAAAGAC AAGCAAATCA   
  
  
- AAATCATAAT AGAATTGAAT TCCACTCAAG AGTGACACAG CAGAGGAGAA GAAGAATACA AATTCTTTAT   
  
  
- TTAAAAAAAA AAAAAGGAAG AGAGAGAGAG AGAGAGGAGG CGGTGCTCGC CATGGCAGTG GTGGACACAA   
  
  
- AGTAGGGAAG AAAAAACCAA CGTCTCTGTC AAAGATCCTA TGGTCGGTCC CACCATCAGA CCTAGTTATT   
  
  
- CCTTTTTTTC AATTTTTTAT TTTTTTATTT TTTGTTAAAG TAAATCTATT TATTATCGTT TTTATGAGAA   
  
  
- AGTGGCTTAG TGTCAAAAAT CAAAGTGTCT GCCAAGCAAA AAAGTGGGCC CACGACTCCA CTGTGGACCG   
  
  
- GGAGCATCCC GAGTATGCAA CATACAGTAA TGAACGGTCT ATATTCAACC ACGTGGCAAT AATCCTTGTG   
  
  
- ATATAGGGAC ACTTGTCCCT AGAATGTTGA CTAAAAGCAG GGCTTTGTTG TCTTTTTGGG CTTTTAAAAA   
  
  
- AATAAAATTC TGAGCGTGGG ACCCACTGGT CAGGACACAG TTGACCTGAC AGAGATTCTC TCTGCCCCTT   
  
  
- CTATTTATGC AGCTGCTTGG AGCCTTTGTT TTTGAGCCAA CATAAATAAT AAGTCATAGA TATTGGATTT   
  
  
- CTTAAAATGT TAAATACTAT TTATCTTTAA AATTATAAAA AATAGTGAAA AAATTAATTT TATTATTTAT   
  
  
- ATATTAATTG TTAATGGATT GTCAACTGGA TTGAAATTAG CCCAAATCTA TTCCATTGGC TCATTTTTTA   
  
  
- TGAATAATTA TAAAATGAAA TTGAGCTTG

+     W box

| Site Name | Organism | Position | Strand | Matrix score. | sequence | function |
| --- | --- | --- | --- | --- | --- | --- |
| W box | Arabidopsis thaliana | 1424 | + | 6 | TTGACC |  |

> 2018/04/13 10:10:12  
+ CGAGTGGGTT TTGGGGTTTT TTCTTTTGCT TTTTTTTTTG GTTTAGGGAT AAAGTGTCTT GGGAATCTTG   
  
  
+ GGTTTACTCT TTCGTAGTAT TTTTATTGTT CTTCATCTTG TTAAGGGTTT TCTAGTCCGA AAACTGTGGA   
  
  
+ GACTTTTGGT CTAGAATTAG GAATTTACGT GAATTTTGGG GTCTACTTTT ATTTAGTTTG TTCAGTAGGT   
  
  
+ GTTACCACCG ATTACCTGTT AAAACATTTG GGGGAACGGA TTTATGACTT TTAGAATACT AGGTTCTGTT   
  
  
+ TTCTATGTTG TCTCTCTACT GTTTTTGAGT CCGATGCTTC CTCTTATTTG TTTTGGGTAA AGGTATTTGT   
  
  
+ CTATGTGGGA ATGTCCCAAA ACGCGTTTTA AGTTTTGAAG GATAAATGAA ACTTTCACTT TCGTCTGTCT   
  
  
+ TTGTCTTACT CGAGTACTCG TTCCTGATAG GTCTTATTTT AGTCTGTCAA AGAAGGCCGA TTAAGAGGTT   
  
  
+ TCTTAGTTTT CCCATCTTAC TTCTTTTTTT AGTTCTGGTT TTGCTGTTTG GGTACAATTT TTGAACTCGT   
  
  
+ CCCCTGAGTT TCTAATAGGG TAACAACCTT AACTTTCCAC ACTTTCTTGC TGAAACGAAA GGGAGGCCCT   
  
  
+ GAAATAATTT TAGTGGGTTT TCTTTTGTTT CAGGGAGAAA ATTTAAGAAA CCGTTTTCTG TTCGTTTAGT   
  
  
+ TTTAGTATTA TCTTAACTTA AGGTGAGTTC TCACTGTGTC GTCTCCTCTT CTTCTTATGT TTAAGAAATA   
  
  
+ AATTTTTTTT TTTTTCCTTC TCTCTCTCTC TCTCTCCTCC GCCACGAGCG GTACCGTCAC CACCTGTGTT   
  
  
+ TCATCCCTTC TTTTTTGGTT GCAGAGACAG TTTCTAGGAT ACCAGCCAGG GTGGTAGTCT GGATCAATAA   
  
  
+ GGAAAAAAAG TTAAAAAATA AAAAAATAAA AAACAATTTC ATTTAGATAA ATAATAGCAA AAATACTCTT   
  
  
+ TCACCGAATC ACAGTTTTTA GTTTCACAGA CGGTTCGTTT TTTCACCCGG GTGCTGAGGT GACACCTGGC   
  
  
+ CCTCGTAGGG CTCATACGTT GTATGTCATT ACTTGCCAGA TATAAGTTGG TGCACCGTTA TTAGGAACAC   
  
  
+ TATATCCCTG TGAACAGGGA TCTTACAACT GATTTTCGTC CCGAAACAAC AGAAAAACCC GAAAATTTTT   
  
  
+ TTATTTTAAG ACTCGCACCC TGGGTGACCA GTCCTGTGTC AACTGGACTG TCTCTAAGAG AGACGGGGAA   
  
  
+ GATAAATACG TCGACGAACC TCGGAAACAA AAACTCGGTT GTATTTATTA TTCAGTATCT ATAACCTAAA   
  
  
+ GAATTTTACA ATTTATGATA AATAGAAATT TTAATATTTT TTATCACTTT TTTAATTAAA ATAATAAATA   
  
  
+ TATAATTAAC AATTACCTAA CAGTTGACCT AACTTTAATC GGGTTTAGAT AAGGTAACCG AGTAAAAAAT   
  
  
+ ACTTATTAAT ATTTTACTTT AACTCGAAC  

- GCTCACCCAA AACCCCAAAA AAGAAAACGA AAAAAAAAAC CAAATCCCTA TTTCACAGAA CCCTTAGAAC   
  
  
- CCAAATGAGA AAGCATCATA AAAATAACAA GAAGTAGAAC AATTCCCAAA AGATCAGGCT TTTGACACCT   
  
  
- CTGAAAACCA GATCTTAATC CTTAAATGCA CTTAAAACCC CAGATGAAAA TAAATCAAAC AAGTCATCCA   
  
  
- CAATGGTGGC TAATGGACAA TTTTGTAAAC CCCCTTGCCT AAATACTGAA AATCTTATGA TCCAAGACAA   
  
  
- AAGATACAAC AGAGAGATGA CAAAAACTCA GGCTACGAAG GAGAATAAAC AAAACCCATT TCCATAAACA   
  
  
- GATACACCCT TACAGGGTTT TGCGCAAAAT TCAAAACTTC CTATTTACTT TGAAAGTGAA AGCAGACAGA   
  
  
- AACAGAATGA GCTCATGAGC AAGGACTATC CAGAATAAAA TCAGACAGTT TCTTCCGGCT AATTCTCCAA   
  
  
- AGAATCAAAA GGGTAGAATG AAGAAAAAAA TCAAGACCAA AACGACAAAC CCATGTTAAA AACTTGAGCA   
  
  
- GGGGACTCAA AGATTATCCC ATTGTTGGAA TTGAAAGGTG TGAAAGAACG ACTTTGCTTT CCCTCCGGGA   
  
  
- CTTTATTAAA ATCACCCAAA AGAAAACAAA GTCCCTCTTT TAAATTCTTT GGCAAAAGAC AAGCAAATCA   
  
  
- AAATCATAAT AGAATTGAAT TCCACTCAAG AGTGACACAG CAGAGGAGAA GAAGAATACA AATTCTTTAT   
  
  
- TTAAAAAAAA AAAAAGGAAG AGAGAGAGAG AGAGAGGAGG CGGTGCTCGC CATGGCAGTG GTGGACACAA   
  
  
- AGTAGGGAAG AAAAAACCAA CGTCTCTGTC AAAGATCCTA TGGTCGGTCC CACCATCAGA CCTAGTTATT   
  
  
- CCTTTTTTTC AATTTTTTAT TTTTTTATTT TTTGTTAAAG TAAATCTATT TATTATCGTT TTTATGAGAA   
  
  
- AGTGGCTTAG TGTCAAAAAT CAAAGTGTCT GCCAAGCAAA AAAGTGGGCC CACGACTCCA CTGTGGACCG   
  
  
- GGAGCATCCC GAGTATGCAA CATACAGTAA TGAACGGTCT ATATTCAACC ACGTGGCAAT AATCCTTGTG   
  
  
- ATATAGGGAC ACTTGTCCCT AGAATGTTGA CTAAAAGCAG GGCTTTGTTG TCTTTTTGGG CTTTTAAAAA   
  
  
- AATAAAATTC TGAGCGTGGG ACCCACTGGT CAGGACACAG TTGACCTGAC AGAGATTCTC TCTGCCCCTT   
  
  
- CTATTTATGC AGCTGCTTGG AGCCTTTGTT TTTGAGCCAA CATAAATAAT AAGTCATAGA TATTGGATTT   
  
  
- CTTAAAATGT TAAATACTAT TTATCTTTAA AATTATAAAA AATAGTGAAA AAATTAATTT TATTATTTAT   
  
  
- ATATTAATTG TTAATGGATT GTCAACTGGA TTGAAATTAG CCCAAATCTA TTCCATTGGC TCATTTTTTA   
  
  
- TGAATAATTA TAAAATGAAA TTGAGCTTG
