# Supplementary material for: Genome-wide analysis of the WRKY gene family in drumstick (Moringa oleifera Lam.)
Source: PeerJ. 2019 Jun 10;7:e7063. doi: 10.7717/peerj.7063 (PMC6563795; doi:10.7717/peerj.7063)
Supplement: Supplemental Information 1 [file peerj-07-7063-s003.gz › MoWRKY29_plantcare.html]

Content-Type: text/html; charset=ISO-8859-1


CallMat\_Firefox


Webmaster Firefox specific output  
To save the result:
click on the frame with the right mouse button and save the source code as a text file with extension .html  
REFERENCE:PlantCARE: a database of plant cis-acting regulatory elements and a portal to tools for in silico analysis of promoter sequences.  
Lescot, M., Déhais, P., Moreau, Y., De Moor, B., Rouzé ,P.,and Rombauts, S.  
Nucleic Acids Res., Database issue(2002), 30(1):325-327.   


---

> 2018/04/13 10:10:12  
+ ATTAACTTTC GCCGACGGAA TTCTTGACCC TCTTAAAGAC GTCTTATTCT TAGATGTTTC ATATAGTGAC   
  
  
+ CTGTGTTCGA CATAATGTTC ACTCGTCGCT AGAGGTTAAT CCAGTTCCCT TGCTCTCTAA GTATCGTTAT   
  
  
+ ATATCTTCTC TTTCTAGTGT ATTGAGTATT GCTCGACAGT TACCAGACAA CTTCCTTTCA GCGAGCTTTC   
  
  
+ TCCGGGTCCC TCTCTCTTTT TCTTTTTCTT TCAGACACAG GCACCCATGC AACTTATAAA CTTAAGGTTT   
  
  
+ CCTGCAGTTC CAGTTCGAAT CGTGCGCCTA TTTTAGGTGA AGCACATCAC CCATTTCGAA GTCTACGGTA   
  
  
+ AACTTTAAAG GGACACGTCT GATTTCTTCG AAGTACGACA GTCTCGAGAC TAAATGGAGC GGCCGAAAGG   
  
  
+ TACGAGAAAG GAGAGTAACC GGCGATACGT GTCTCTTATT GTGATGTGCT TTGCCGGTGC AAAGGTGTAT   
  
  
+ TAATCAAGTG CATTCTTGCT GACTTATAAG GCGTGAAGGA TCAGGAGAAA GAACAACTGA TGGGTTTTCG   
  
  
+ GGTTTTCGGC TGTTCGTCAC ACACAGGTGT GCCAAAACTA GAAACTTTGT AGCACGGCAA AGTAAGTTGT   
  
  
+ TTACGACCTG AAAGCCGTAG ATAGGTGAAA GAAGACAAGG AGTGGACCTT TTGTTTTGTT CAACGAGCAG   
  
  
+ GTAACCTATT AAGTACTAAT AAATAAAAAT TTTTTTATTT TTTAAACTTT AAATAATTTT ATTTTTAATA   
  
  
+ AAATTTTTAA AATTTTTTTA TTTTTATATA ATAGTGTTTT TTTATTAATT AATTAGTTTT TTATAAATCT   
  
  
+ AAGTTCCTTG TTTTAGTTTT ATTTTGCACT TTAAATAAAT TGATTAAAGA ATTCGTAAAG TTACTTTCGT   
  
  
+ AGCAATTAAA ACGTAGTAAC AAAACTAGTT TGAGATTAAA TTTTTTAATA TTTAATTTTA AATAAAATTT   
  
  
+ CATAAAATTA ATGTTTTTTA TTTTAATTTT AGGATTTACT ACACCAGAAC TAGAAATTAT TATTATATTA   
  
  
+ ATTAGTTTAT TAAACGAAAT AGACCACGAA TTAAAATATT TTAATTTTAG GATTTACTGC ACCAGAACTA   
  
  
+ GAAATTATTA TTATATTAAT TAGTTTATTA AACGAAATAG ACCACGAATT AAAAAATAAA CAAAGTAGTA   
  
  
+ ACTGCACCGG CAATTACTTT CCGGTTTGTT TAATTAATTC TATAAATTAG TTTGTTCATT AATAAAAGTA   
  
  
+ CATGTAACAA AGTAGTAACT GCACCGGCAA TTACTTTCCG GTTTGTTTAA TTAATTCTAT AAATTAGTTT   
  
  
+ GTTCATTAAT AAAAGTACAT GAAAGATACA AACAACCAGT ACCTTCTTCT ACTTTAAAAT TAAACTGTTT   
  
  
+ AATTTTCAGA TCGTCTTTAT ATTTTTAATT ACTGTCTGTA CGTTACTAAA TCTAATCATT AGAGACCCCA   
  
  
+ ACGTGCAGTG CACTACTTCA GTAATTGTT  

- TAATTGAAAG CGGCTGCCTT AAGAACTGGG AGAATTTCTG CAGAATAAGA ATCTACAAAG TATATCACTG   
  
  
- GACACAAGCT GTATTACAAG TGAGCAGCGA TCTCCAATTA GGTCAAGGGA ACGAGAGATT CATAGCAATA   
  
  
- TATAGAAGAG AAAGATCACA TAACTCATAA CGAGCTGTCA ATGGTCTGTT GAAGGAAAGT CGCTCGAAAG   
  
  
- AGGCCCAGGG AGAGAGAAAA AGAAAAAGAA AGTCTGTGTC CGTGGGTACG TTGAATATTT GAATTCCAAA   
  
  
- GGACGTCAAG GTCAAGCTTA GCACGCGGAT AAAATCCACT TCGTGTAGTG GGTAAAGCTT CAGATGCCAT   
  
  
- TTGAAATTTC CCTGTGCAGA CTAAAGAAGC TTCATGCTGT CAGAGCTCTG ATTTACCTCG CCGGCTTTCC   
  
  
- ATGCTCTTTC CTCTCATTGG CCGCTATGCA CAGAGAATAA CACTACACGA AACGGCCACG TTTCCACATA   
  
  
- ATTAGTTCAC GTAAGAACGA CTGAATATTC CGCACTTCCT AGTCCTCTTT CTTGTTGACT ACCCAAAAGC   
  
  
- CCAAAAGCCG ACAAGCAGTG TGTGTCCACA CGGTTTTGAT CTTTGAAACA TCGTGCCGTT TCATTCAACA   
  
  
- AATGCTGGAC TTTCGGCATC TATCCACTTT CTTCTGTTCC TCACCTGGAA AACAAAACAA GTTGCTCGTC   
  
  
- CATTGGATAA TTCATGATTA TTTATTTTTA AAAAAATAAA AAATTTGAAA TTTATTAAAA TAAAAATTAT   
  
  
- TTTAAAAATT TTAAAAAAAT AAAAATATAT TATCACAAAA AAATAATTAA TTAATCAAAA AATATTTAGA   
  
  
- TTCAAGGAAC AAAATCAAAA TAAAACGTGA AATTTATTTA ACTAATTTCT TAAGCATTTC AATGAAAGCA   
  
  
- TCGTTAATTT TGCATCATTG TTTTGATCAA ACTCTAATTT AAAAAATTAT AAATTAAAAT TTATTTTAAA   
  
  
- GTATTTTAAT TACAAAAAAT AAAATTAAAA TCCTAAATGA TGTGGTCTTG ATCTTTAATA ATAATATAAT   
  
  
- TAATCAAATA ATTTGCTTTA TCTGGTGCTT AATTTTATAA AATTAAAATC CTAAATGACG TGGTCTTGAT   
  
  
- CTTTAATAAT AATATAATTA ATCAAATAAT TTGCTTTATC TGGTGCTTAA TTTTTTATTT GTTTCATCAT   
  
  
- TGACGTGGCC GTTAATGAAA GGCCAAACAA ATTAATTAAG ATATTTAATC AAACAAGTAA TTATTTTCAT   
  
  
- GTACATTGTT TCATCATTGA CGTGGCCGTT AATGAAAGGC CAAACAAATT AATTAAGATA TTTAATCAAA   
  
  
- CAAGTAATTA TTTTCATGTA CTTTCTATGT TTGTTGGTCA TGGAAGAAGA TGAAATTTTA ATTTGACAAA   
  
  
- TTAAAAGTCT AGCAGAAATA TAAAAATTAA TGACAGACAT GCAATGATTT AGATTAGTAA TCTCTGGGGT   
  
  
- TGCACGTCAC GTGATGAAGT CATTAACAA

  
  
Motifs Found  

+     5UTR Py-rich stretch

| Site Name | Organism | Position | Strand | Matrix score. | sequence | function |
| --- | --- | --- | --- | --- | --- | --- |
| 5UTR Py-rich stretch | Lycopersicon esculentum | 142 | + | 9 | TTTCTTCTCT | cis-acting element conferring high transcription levels |

> 2018/04/13 10:10:12  
+ ATTAACTTTC GCCGACGGAA TTCTTGACCC TCTTAAAGAC GTCTTATTCT TAGATGTTTC ATATAGTGAC   
  
  
+ CTGTGTTCGA CATAATGTTC ACTCGTCGCT AGAGGTTAAT CCAGTTCCCT TGCTCTCTAA GTATCGTTAT   
  
  
+ ATATCTTCTC TTTCTAGTGT ATTGAGTATT GCTCGACAGT TACCAGACAA CTTCCTTTCA GCGAGCTTTC   
  
  
+ TCCGGGTCCC TCTCTCTTTT TCTTTTTCTT TCAGACACAG GCACCCATGC AACTTATAAA CTTAAGGTTT   
  
  
+ CCTGCAGTTC CAGTTCGAAT CGTGCGCCTA TTTTAGGTGA AGCACATCAC CCATTTCGAA GTCTACGGTA   
  
  
+ AACTTTAAAG GGACACGTCT GATTTCTTCG AAGTACGACA GTCTCGAGAC TAAATGGAGC GGCCGAAAGG   
  
  
+ TACGAGAAAG GAGAGTAACC GGCGATACGT GTCTCTTATT GTGATGTGCT TTGCCGGTGC AAAGGTGTAT   
  
  
+ TAATCAAGTG CATTCTTGCT GACTTATAAG GCGTGAAGGA TCAGGAGAAA GAACAACTGA TGGGTTTTCG   
  
  
+ GGTTTTCGGC TGTTCGTCAC ACACAGGTGT GCCAAAACTA GAAACTTTGT AGCACGGCAA AGTAAGTTGT   
  
  
+ TTACGACCTG AAAGCCGTAG ATAGGTGAAA GAAGACAAGG AGTGGACCTT TTGTTTTGTT CAACGAGCAG   
  
  
+ GTAACCTATT AAGTACTAAT AAATAAAAAT TTTTTTATTT TTTAAACTTT AAATAATTTT ATTTTTAATA   
  
  
+ AAATTTTTAA AATTTTTTTA TTTTTATATA ATAGTGTTTT TTTATTAATT AATTAGTTTT TTATAAATCT   
  
  
+ AAGTTCCTTG TTTTAGTTTT ATTTTGCACT TTAAATAAAT TGATTAAAGA ATTCGTAAAG TTACTTTCGT   
  
  
+ AGCAATTAAA ACGTAGTAAC AAAACTAGTT TGAGATTAAA TTTTTTAATA TTTAATTTTA AATAAAATTT   
  
  
+ CATAAAATTA ATGTTTTTTA TTTTAATTTT AGGATTTACT ACACCAGAAC TAGAAATTAT TATTATATTA   
  
  
+ ATTAGTTTAT TAAACGAAAT AGACCACGAA TTAAAATATT TTAATTTTAG GATTTACTGC ACCAGAACTA   
  
  
+ GAAATTATTA TTATATTAAT TAGTTTATTA AACGAAATAG ACCACGAATT AAAAAATAAA CAAAGTAGTA   
  
  
+ ACTGCACCGG CAATTACTTT CCGGTTTGTT TAATTAATTC TATAAATTAG TTTGTTCATT AATAAAAGTA   
  
  
+ CATGTAACAA AGTAGTAACT GCACCGGCAA TTACTTTCCG GTTTGTTTAA TTAATTCTAT AAATTAGTTT   
  
  
+ GTTCATTAAT AAAAGTACAT GAAAGATACA AACAACCAGT ACCTTCTTCT ACTTTAAAAT TAAACTGTTT   
  
  
+ AATTTTCAGA TCGTCTTTAT ATTTTTAATT ACTGTCTGTA CGTTACTAAA TCTAATCATT AGAGACCCCA   
  
  
+ ACGTGCAGTG CACTACTTCA GTAATTGTT  

- TAATTGAAAG CGGCTGCCTT AAGAACTGGG AGAATTTCTG CAGAATAAGA ATCTACAAAG TATATCACTG   
  
  
- GACACAAGCT GTATTACAAG TGAGCAGCGA TCTCCAATTA GGTCAAGGGA ACGAGAGATT CATAGCAATA   
  
  
- TATAGAAGAG AAAGATCACA TAACTCATAA CGAGCTGTCA ATGGTCTGTT GAAGGAAAGT CGCTCGAAAG   
  
  
- AGGCCCAGGG AGAGAGAAAA AGAAAAAGAA AGTCTGTGTC CGTGGGTACG TTGAATATTT GAATTCCAAA   
  
  
- GGACGTCAAG GTCAAGCTTA GCACGCGGAT AAAATCCACT TCGTGTAGTG GGTAAAGCTT CAGATGCCAT   
  
  
- TTGAAATTTC CCTGTGCAGA CTAAAGAAGC TTCATGCTGT CAGAGCTCTG ATTTACCTCG CCGGCTTTCC   
  
  
- ATGCTCTTTC CTCTCATTGG CCGCTATGCA CAGAGAATAA CACTACACGA AACGGCCACG TTTCCACATA   
  
  
- ATTAGTTCAC GTAAGAACGA CTGAATATTC CGCACTTCCT AGTCCTCTTT CTTGTTGACT ACCCAAAAGC   
  
  
- CCAAAAGCCG ACAAGCAGTG TGTGTCCACA CGGTTTTGAT CTTTGAAACA TCGTGCCGTT TCATTCAACA   
  
  
- AATGCTGGAC TTTCGGCATC TATCCACTTT CTTCTGTTCC TCACCTGGAA AACAAAACAA GTTGCTCGTC   
  
  
- CATTGGATAA TTCATGATTA TTTATTTTTA AAAAAATAAA AAATTTGAAA TTTATTAAAA TAAAAATTAT   
  
  
- TTTAAAAATT TTAAAAAAAT AAAAATATAT TATCACAAAA AAATAATTAA TTAATCAAAA AATATTTAGA   
  
  
- TTCAAGGAAC AAAATCAAAA TAAAACGTGA AATTTATTTA ACTAATTTCT TAAGCATTTC AATGAAAGCA   
  
  
- TCGTTAATTT TGCATCATTG TTTTGATCAA ACTCTAATTT AAAAAATTAT AAATTAAAAT TTATTTTAAA   
  
  
- GTATTTTAAT TACAAAAAAT AAAATTAAAA TCCTAAATGA TGTGGTCTTG ATCTTTAATA ATAATATAAT   
  
  
- TAATCAAATA ATTTGCTTTA TCTGGTGCTT AATTTTATAA AATTAAAATC CTAAATGACG TGGTCTTGAT   
  
  
- CTTTAATAAT AATATAATTA ATCAAATAAT TTGCTTTATC TGGTGCTTAA TTTTTTATTT GTTTCATCAT   
  
  
- TGACGTGGCC GTTAATGAAA GGCCAAACAA ATTAATTAAG ATATTTAATC AAACAAGTAA TTATTTTCAT   
  
  
- GTACATTGTT TCATCATTGA CGTGGCCGTT AATGAAAGGC CAAACAAATT AATTAAGATA TTTAATCAAA   
  
  
- CAAGTAATTA TTTTCATGTA CTTTCTATGT TTGTTGGTCA TGGAAGAAGA TGAAATTTTA ATTTGACAAA   
  
  
- TTAAAAGTCT AGCAGAAATA TAAAAATTAA TGACAGACAT GCAATGATTT AGATTAGTAA TCTCTGGGGT   
  
  
- TGCACGTCAC GTGATGAAGT CATTAACAA

+     AAGAA-motif

| Site Name | Organism | Position | Strand | Matrix score. | sequence | function |
| --- | --- | --- | --- | --- | --- | --- |
| AAGAA-motif | Avena sativa | 236 | - | 7 | GAAAGAA |  |
| AAGAA-motif | Avena sativa | 657 | + | 7 | GAAAGAA |  |
| AAGAA-motif | Avena sativa | 537 | + | 7 | GAAAGAA |  |

> 2018/04/13 10:10:12  
+ ATTAACTTTC GCCGACGGAA TTCTTGACCC TCTTAAAGAC GTCTTATTCT TAGATGTTTC ATATAGTGAC   
  
  
+ CTGTGTTCGA CATAATGTTC ACTCGTCGCT AGAGGTTAAT CCAGTTCCCT TGCTCTCTAA GTATCGTTAT   
  
  
+ ATATCTTCTC TTTCTAGTGT ATTGAGTATT GCTCGACAGT TACCAGACAA CTTCCTTTCA GCGAGCTTTC   
  
  
+ TCCGGGTCCC TCTCTCTTTT TCTTTTTCTT TCAGACACAG GCACCCATGC AACTTATAAA CTTAAGGTTT   
  
  
+ CCTGCAGTTC CAGTTCGAAT CGTGCGCCTA TTTTAGGTGA AGCACATCAC CCATTTCGAA GTCTACGGTA   
  
  
+ AACTTTAAAG GGACACGTCT GATTTCTTCG AAGTACGACA GTCTCGAGAC TAAATGGAGC GGCCGAAAGG   
  
  
+ TACGAGAAAG GAGAGTAACC GGCGATACGT GTCTCTTATT GTGATGTGCT TTGCCGGTGC AAAGGTGTAT   
  
  
+ TAATCAAGTG CATTCTTGCT GACTTATAAG GCGTGAAGGA TCAGGAGAAA GAACAACTGA TGGGTTTTCG   
  
  
+ GGTTTTCGGC TGTTCGTCAC ACACAGGTGT GCCAAAACTA GAAACTTTGT AGCACGGCAA AGTAAGTTGT   
  
  
+ TTACGACCTG AAAGCCGTAG ATAGGTGAAA GAAGACAAGG AGTGGACCTT TTGTTTTGTT CAACGAGCAG   
  
  
+ GTAACCTATT AAGTACTAAT AAATAAAAAT TTTTTTATTT TTTAAACTTT AAATAATTTT ATTTTTAATA   
  
  
+ AAATTTTTAA AATTTTTTTA TTTTTATATA ATAGTGTTTT TTTATTAATT AATTAGTTTT TTATAAATCT   
  
  
+ AAGTTCCTTG TTTTAGTTTT ATTTTGCACT TTAAATAAAT TGATTAAAGA ATTCGTAAAG TTACTTTCGT   
  
  
+ AGCAATTAAA ACGTAGTAAC AAAACTAGTT TGAGATTAAA TTTTTTAATA TTTAATTTTA AATAAAATTT   
  
  
+ CATAAAATTA ATGTTTTTTA TTTTAATTTT AGGATTTACT ACACCAGAAC TAGAAATTAT TATTATATTA   
  
  
+ ATTAGTTTAT TAAACGAAAT AGACCACGAA TTAAAATATT TTAATTTTAG GATTTACTGC ACCAGAACTA   
  
  
+ GAAATTATTA TTATATTAAT TAGTTTATTA AACGAAATAG ACCACGAATT AAAAAATAAA CAAAGTAGTA   
  
  
+ ACTGCACCGG CAATTACTTT CCGGTTTGTT TAATTAATTC TATAAATTAG TTTGTTCATT AATAAAAGTA   
  
  
+ CATGTAACAA AGTAGTAACT GCACCGGCAA TTACTTTCCG GTTTGTTTAA TTAATTCTAT AAATTAGTTT   
  
  
+ GTTCATTAAT AAAAGTACAT GAAAGATACA AACAACCAGT ACCTTCTTCT ACTTTAAAAT TAAACTGTTT   
  
  
+ AATTTTCAGA TCGTCTTTAT ATTTTTAATT ACTGTCTGTA CGTTACTAAA TCTAATCATT AGAGACCCCA   
  
  
+ ACGTGCAGTG CACTACTTCA GTAATTGTT  

- TAATTGAAAG CGGCTGCCTT AAGAACTGGG AGAATTTCTG CAGAATAAGA ATCTACAAAG TATATCACTG   
  
  
- GACACAAGCT GTATTACAAG TGAGCAGCGA TCTCCAATTA GGTCAAGGGA ACGAGAGATT CATAGCAATA   
  
  
- TATAGAAGAG AAAGATCACA TAACTCATAA CGAGCTGTCA ATGGTCTGTT GAAGGAAAGT CGCTCGAAAG   
  
  
- AGGCCCAGGG AGAGAGAAAA AGAAAAAGAA AGTCTGTGTC CGTGGGTACG TTGAATATTT GAATTCCAAA   
  
  
- GGACGTCAAG GTCAAGCTTA GCACGCGGAT AAAATCCACT TCGTGTAGTG GGTAAAGCTT CAGATGCCAT   
  
  
- TTGAAATTTC CCTGTGCAGA CTAAAGAAGC TTCATGCTGT CAGAGCTCTG ATTTACCTCG CCGGCTTTCC   
  
  
- ATGCTCTTTC CTCTCATTGG CCGCTATGCA CAGAGAATAA CACTACACGA AACGGCCACG TTTCCACATA   
  
  
- ATTAGTTCAC GTAAGAACGA CTGAATATTC CGCACTTCCT AGTCCTCTTT CTTGTTGACT ACCCAAAAGC   
  
  
- CCAAAAGCCG ACAAGCAGTG TGTGTCCACA CGGTTTTGAT CTTTGAAACA TCGTGCCGTT TCATTCAACA   
  
  
- AATGCTGGAC TTTCGGCATC TATCCACTTT CTTCTGTTCC TCACCTGGAA AACAAAACAA GTTGCTCGTC   
  
  
- CATTGGATAA TTCATGATTA TTTATTTTTA AAAAAATAAA AAATTTGAAA TTTATTAAAA TAAAAATTAT   
  
  
- TTTAAAAATT TTAAAAAAAT AAAAATATAT TATCACAAAA AAATAATTAA TTAATCAAAA AATATTTAGA   
  
  
- TTCAAGGAAC AAAATCAAAA TAAAACGTGA AATTTATTTA ACTAATTTCT TAAGCATTTC AATGAAAGCA   
  
  
- TCGTTAATTT TGCATCATTG TTTTGATCAA ACTCTAATTT AAAAAATTAT AAATTAAAAT TTATTTTAAA   
  
  
- GTATTTTAAT TACAAAAAAT AAAATTAAAA TCCTAAATGA TGTGGTCTTG ATCTTTAATA ATAATATAAT   
  
  
- TAATCAAATA ATTTGCTTTA TCTGGTGCTT AATTTTATAA AATTAAAATC CTAAATGACG TGGTCTTGAT   
  
  
- CTTTAATAAT AATATAATTA ATCAAATAAT TTGCTTTATC TGGTGCTTAA TTTTTTATTT GTTTCATCAT   
  
  
- TGACGTGGCC GTTAATGAAA GGCCAAACAA ATTAATTAAG ATATTTAATC AAACAAGTAA TTATTTTCAT   
  
  
- GTACATTGTT TCATCATTGA CGTGGCCGTT AATGAAAGGC CAAACAAATT AATTAAGATA TTTAATCAAA   
  
  
- CAAGTAATTA TTTTCATGTA CTTTCTATGT TTGTTGGTCA TGGAAGAAGA TGAAATTTTA ATTTGACAAA   
  
  
- TTAAAAGTCT AGCAGAAATA TAAAAATTAA TGACAGACAT GCAATGATTT AGATTAGTAA TCTCTGGGGT   
  
  
- TGCACGTCAC GTGATGAAGT CATTAACAA

+     ABRE

| Site Name | Organism | Position | Strand | Matrix score. | sequence | function |
| --- | --- | --- | --- | --- | --- | --- |
| ABRE | Arabidopsis thaliana | 446 | + | 6 | TACGTG | cis-acting element involved in the abscisic acid responsiveness |

> 2018/04/13 10:10:12  
+ ATTAACTTTC GCCGACGGAA TTCTTGACCC TCTTAAAGAC GTCTTATTCT TAGATGTTTC ATATAGTGAC   
  
  
+ CTGTGTTCGA CATAATGTTC ACTCGTCGCT AGAGGTTAAT CCAGTTCCCT TGCTCTCTAA GTATCGTTAT   
  
  
+ ATATCTTCTC TTTCTAGTGT ATTGAGTATT GCTCGACAGT TACCAGACAA CTTCCTTTCA GCGAGCTTTC   
  
  
+ TCCGGGTCCC TCTCTCTTTT TCTTTTTCTT TCAGACACAG GCACCCATGC AACTTATAAA CTTAAGGTTT   
  
  
+ CCTGCAGTTC CAGTTCGAAT CGTGCGCCTA TTTTAGGTGA AGCACATCAC CCATTTCGAA GTCTACGGTA   
  
  
+ AACTTTAAAG GGACACGTCT GATTTCTTCG AAGTACGACA GTCTCGAGAC TAAATGGAGC GGCCGAAAGG   
  
  
+ TACGAGAAAG GAGAGTAACC GGCGATACGT GTCTCTTATT GTGATGTGCT TTGCCGGTGC AAAGGTGTAT   
  
  
+ TAATCAAGTG CATTCTTGCT GACTTATAAG GCGTGAAGGA TCAGGAGAAA GAACAACTGA TGGGTTTTCG   
  
  
+ GGTTTTCGGC TGTTCGTCAC ACACAGGTGT GCCAAAACTA GAAACTTTGT AGCACGGCAA AGTAAGTTGT   
  
  
+ TTACGACCTG AAAGCCGTAG ATAGGTGAAA GAAGACAAGG AGTGGACCTT TTGTTTTGTT CAACGAGCAG   
  
  
+ GTAACCTATT AAGTACTAAT AAATAAAAAT TTTTTTATTT TTTAAACTTT AAATAATTTT ATTTTTAATA   
  
  
+ AAATTTTTAA AATTTTTTTA TTTTTATATA ATAGTGTTTT TTTATTAATT AATTAGTTTT TTATAAATCT   
  
  
+ AAGTTCCTTG TTTTAGTTTT ATTTTGCACT TTAAATAAAT TGATTAAAGA ATTCGTAAAG TTACTTTCGT   
  
  
+ AGCAATTAAA ACGTAGTAAC AAAACTAGTT TGAGATTAAA TTTTTTAATA TTTAATTTTA AATAAAATTT   
  
  
+ CATAAAATTA ATGTTTTTTA TTTTAATTTT AGGATTTACT ACACCAGAAC TAGAAATTAT TATTATATTA   
  
  
+ ATTAGTTTAT TAAACGAAAT AGACCACGAA TTAAAATATT TTAATTTTAG GATTTACTGC ACCAGAACTA   
  
  
+ GAAATTATTA TTATATTAAT TAGTTTATTA AACGAAATAG ACCACGAATT AAAAAATAAA CAAAGTAGTA   
  
  
+ ACTGCACCGG CAATTACTTT CCGGTTTGTT TAATTAATTC TATAAATTAG TTTGTTCATT AATAAAAGTA   
  
  
+ CATGTAACAA AGTAGTAACT GCACCGGCAA TTACTTTCCG GTTTGTTTAA TTAATTCTAT AAATTAGTTT   
  
  
+ GTTCATTAAT AAAAGTACAT GAAAGATACA AACAACCAGT ACCTTCTTCT ACTTTAAAAT TAAACTGTTT   
  
  
+ AATTTTCAGA TCGTCTTTAT ATTTTTAATT ACTGTCTGTA CGTTACTAAA TCTAATCATT AGAGACCCCA   
  
  
+ ACGTGCAGTG CACTACTTCA GTAATTGTT  

- TAATTGAAAG CGGCTGCCTT AAGAACTGGG AGAATTTCTG CAGAATAAGA ATCTACAAAG TATATCACTG   
  
  
- GACACAAGCT GTATTACAAG TGAGCAGCGA TCTCCAATTA GGTCAAGGGA ACGAGAGATT CATAGCAATA   
  
  
- TATAGAAGAG AAAGATCACA TAACTCATAA CGAGCTGTCA ATGGTCTGTT GAAGGAAAGT CGCTCGAAAG   
  
  
- AGGCCCAGGG AGAGAGAAAA AGAAAAAGAA AGTCTGTGTC CGTGGGTACG TTGAATATTT GAATTCCAAA   
  
  
- GGACGTCAAG GTCAAGCTTA GCACGCGGAT AAAATCCACT TCGTGTAGTG GGTAAAGCTT CAGATGCCAT   
  
  
- TTGAAATTTC CCTGTGCAGA CTAAAGAAGC TTCATGCTGT CAGAGCTCTG ATTTACCTCG CCGGCTTTCC   
  
  
- ATGCTCTTTC CTCTCATTGG CCGCTATGCA CAGAGAATAA CACTACACGA AACGGCCACG TTTCCACATA   
  
  
- ATTAGTTCAC GTAAGAACGA CTGAATATTC CGCACTTCCT AGTCCTCTTT CTTGTTGACT ACCCAAAAGC   
  
  
- CCAAAAGCCG ACAAGCAGTG TGTGTCCACA CGGTTTTGAT CTTTGAAACA TCGTGCCGTT TCATTCAACA   
  
  
- AATGCTGGAC TTTCGGCATC TATCCACTTT CTTCTGTTCC TCACCTGGAA AACAAAACAA GTTGCTCGTC   
  
  
- CATTGGATAA TTCATGATTA TTTATTTTTA AAAAAATAAA AAATTTGAAA TTTATTAAAA TAAAAATTAT   
  
  
- TTTAAAAATT TTAAAAAAAT AAAAATATAT TATCACAAAA AAATAATTAA TTAATCAAAA AATATTTAGA   
  
  
- TTCAAGGAAC AAAATCAAAA TAAAACGTGA AATTTATTTA ACTAATTTCT TAAGCATTTC AATGAAAGCA   
  
  
- TCGTTAATTT TGCATCATTG TTTTGATCAA ACTCTAATTT AAAAAATTAT AAATTAAAAT TTATTTTAAA   
  
  
- GTATTTTAAT TACAAAAAAT AAAATTAAAA TCCTAAATGA TGTGGTCTTG ATCTTTAATA ATAATATAAT   
  
  
- TAATCAAATA ATTTGCTTTA TCTGGTGCTT AATTTTATAA AATTAAAATC CTAAATGACG TGGTCTTGAT   
  
  
- CTTTAATAAT AATATAATTA ATCAAATAAT TTGCTTTATC TGGTGCTTAA TTTTTTATTT GTTTCATCAT   
  
  
- TGACGTGGCC GTTAATGAAA GGCCAAACAA ATTAATTAAG ATATTTAATC AAACAAGTAA TTATTTTCAT   
  
  
- GTACATTGTT TCATCATTGA CGTGGCCGTT AATGAAAGGC CAAACAAATT AATTAAGATA TTTAATCAAA   
  
  
- CAAGTAATTA TTTTCATGTA CTTTCTATGT TTGTTGGTCA TGGAAGAAGA TGAAATTTTA ATTTGACAAA   
  
  
- TTAAAAGTCT AGCAGAAATA TAAAAATTAA TGACAGACAT GCAATGATTT AGATTAGTAA TCTCTGGGGT   
  
  
- TGCACGTCAC GTGATGAAGT CATTAACAA

+     ACE

| Site Name | Organism | Position | Strand | Matrix score. | sequence | function |
| --- | --- | --- | --- | --- | --- | --- |
| ACE | Petroselinum crispum | 362 | + | 9 | GACACGTATG | cis-acting element involved in light responsiveness |
| ACE | Petroselinum crispum | 444 | - | 9 | GACACGTATG | cis-acting element involved in light responsiveness |

> 2018/04/13 10:10:12  
+ ATTAACTTTC GCCGACGGAA TTCTTGACCC TCTTAAAGAC GTCTTATTCT TAGATGTTTC ATATAGTGAC   
  
  
+ CTGTGTTCGA CATAATGTTC ACTCGTCGCT AGAGGTTAAT CCAGTTCCCT TGCTCTCTAA GTATCGTTAT   
  
  
+ ATATCTTCTC TTTCTAGTGT ATTGAGTATT GCTCGACAGT TACCAGACAA CTTCCTTTCA GCGAGCTTTC   
  
  
+ TCCGGGTCCC TCTCTCTTTT TCTTTTTCTT TCAGACACAG GCACCCATGC AACTTATAAA CTTAAGGTTT   
  
  
+ CCTGCAGTTC CAGTTCGAAT CGTGCGCCTA TTTTAGGTGA AGCACATCAC CCATTTCGAA GTCTACGGTA   
  
  
+ AACTTTAAAG GGACACGTCT GATTTCTTCG AAGTACGACA GTCTCGAGAC TAAATGGAGC GGCCGAAAGG   
  
  
+ TACGAGAAAG GAGAGTAACC GGCGATACGT GTCTCTTATT GTGATGTGCT TTGCCGGTGC AAAGGTGTAT   
  
  
+ TAATCAAGTG CATTCTTGCT GACTTATAAG GCGTGAAGGA TCAGGAGAAA GAACAACTGA TGGGTTTTCG   
  
  
+ GGTTTTCGGC TGTTCGTCAC ACACAGGTGT GCCAAAACTA GAAACTTTGT AGCACGGCAA AGTAAGTTGT   
  
  
+ TTACGACCTG AAAGCCGTAG ATAGGTGAAA GAAGACAAGG AGTGGACCTT TTGTTTTGTT CAACGAGCAG   
  
  
+ GTAACCTATT AAGTACTAAT AAATAAAAAT TTTTTTATTT TTTAAACTTT AAATAATTTT ATTTTTAATA   
  
  
+ AAATTTTTAA AATTTTTTTA TTTTTATATA ATAGTGTTTT TTTATTAATT AATTAGTTTT TTATAAATCT   
  
  
+ AAGTTCCTTG TTTTAGTTTT ATTTTGCACT TTAAATAAAT TGATTAAAGA ATTCGTAAAG TTACTTTCGT   
  
  
+ AGCAATTAAA ACGTAGTAAC AAAACTAGTT TGAGATTAAA TTTTTTAATA TTTAATTTTA AATAAAATTT   
  
  
+ CATAAAATTA ATGTTTTTTA TTTTAATTTT AGGATTTACT ACACCAGAAC TAGAAATTAT TATTATATTA   
  
  
+ ATTAGTTTAT TAAACGAAAT AGACCACGAA TTAAAATATT TTAATTTTAG GATTTACTGC ACCAGAACTA   
  
  
+ GAAATTATTA TTATATTAAT TAGTTTATTA AACGAAATAG ACCACGAATT AAAAAATAAA CAAAGTAGTA   
  
  
+ ACTGCACCGG CAATTACTTT CCGGTTTGTT TAATTAATTC TATAAATTAG TTTGTTCATT AATAAAAGTA   
  
  
+ CATGTAACAA AGTAGTAACT GCACCGGCAA TTACTTTCCG GTTTGTTTAA TTAATTCTAT AAATTAGTTT   
  
  
+ GTTCATTAAT AAAAGTACAT GAAAGATACA AACAACCAGT ACCTTCTTCT ACTTTAAAAT TAAACTGTTT   
  
  
+ AATTTTCAGA TCGTCTTTAT ATTTTTAATT ACTGTCTGTA CGTTACTAAA TCTAATCATT AGAGACCCCA   
  
  
+ ACGTGCAGTG CACTACTTCA GTAATTGTT  

- TAATTGAAAG CGGCTGCCTT AAGAACTGGG AGAATTTCTG CAGAATAAGA ATCTACAAAG TATATCACTG   
  
  
- GACACAAGCT GTATTACAAG TGAGCAGCGA TCTCCAATTA GGTCAAGGGA ACGAGAGATT CATAGCAATA   
  
  
- TATAGAAGAG AAAGATCACA TAACTCATAA CGAGCTGTCA ATGGTCTGTT GAAGGAAAGT CGCTCGAAAG   
  
  
- AGGCCCAGGG AGAGAGAAAA AGAAAAAGAA AGTCTGTGTC CGTGGGTACG TTGAATATTT GAATTCCAAA   
  
  
- GGACGTCAAG GTCAAGCTTA GCACGCGGAT AAAATCCACT TCGTGTAGTG GGTAAAGCTT CAGATGCCAT   
  
  
- TTGAAATTTC CCTGTGCAGA CTAAAGAAGC TTCATGCTGT CAGAGCTCTG ATTTACCTCG CCGGCTTTCC   
  
  
- ATGCTCTTTC CTCTCATTGG CCGCTATGCA CAGAGAATAA CACTACACGA AACGGCCACG TTTCCACATA   
  
  
- ATTAGTTCAC GTAAGAACGA CTGAATATTC CGCACTTCCT AGTCCTCTTT CTTGTTGACT ACCCAAAAGC   
  
  
- CCAAAAGCCG ACAAGCAGTG TGTGTCCACA CGGTTTTGAT CTTTGAAACA TCGTGCCGTT TCATTCAACA   
  
  
- AATGCTGGAC TTTCGGCATC TATCCACTTT CTTCTGTTCC TCACCTGGAA AACAAAACAA GTTGCTCGTC   
  
  
- CATTGGATAA TTCATGATTA TTTATTTTTA AAAAAATAAA AAATTTGAAA TTTATTAAAA TAAAAATTAT   
  
  
- TTTAAAAATT TTAAAAAAAT AAAAATATAT TATCACAAAA AAATAATTAA TTAATCAAAA AATATTTAGA   
  
  
- TTCAAGGAAC AAAATCAAAA TAAAACGTGA AATTTATTTA ACTAATTTCT TAAGCATTTC AATGAAAGCA   
  
  
- TCGTTAATTT TGCATCATTG TTTTGATCAA ACTCTAATTT AAAAAATTAT AAATTAAAAT TTATTTTAAA   
  
  
- GTATTTTAAT TACAAAAAAT AAAATTAAAA TCCTAAATGA TGTGGTCTTG ATCTTTAATA ATAATATAAT   
  
  
- TAATCAAATA ATTTGCTTTA TCTGGTGCTT AATTTTATAA AATTAAAATC CTAAATGACG TGGTCTTGAT   
  
  
- CTTTAATAAT AATATAATTA ATCAAATAAT TTGCTTTATC TGGTGCTTAA TTTTTTATTT GTTTCATCAT   
  
  
- TGACGTGGCC GTTAATGAAA GGCCAAACAA ATTAATTAAG ATATTTAATC AAACAAGTAA TTATTTTCAT   
  
  
- GTACATTGTT TCATCATTGA CGTGGCCGTT AATGAAAGGC CAAACAAATT AATTAAGATA TTTAATCAAA   
  
  
- CAAGTAATTA TTTTCATGTA CTTTCTATGT TTGTTGGTCA TGGAAGAAGA TGAAATTTTA ATTTGACAAA   
  
  
- TTAAAAGTCT AGCAGAAATA TAAAAATTAA TGACAGACAT GCAATGATTT AGATTAGTAA TCTCTGGGGT   
  
  
- TGCACGTCAC GTGATGAAGT CATTAACAA

+     AE-box

| Site Name | Organism | Position | Strand | Matrix score. | sequence | function |
| --- | --- | --- | --- | --- | --- | --- |
| AE-box | Arabidopsis thaliana | 600 | + | 8 | AGAAACTT | part of a module for light response |

> 2018/04/13 10:10:12  
+ ATTAACTTTC GCCGACGGAA TTCTTGACCC TCTTAAAGAC GTCTTATTCT TAGATGTTTC ATATAGTGAC   
  
  
+ CTGTGTTCGA CATAATGTTC ACTCGTCGCT AGAGGTTAAT CCAGTTCCCT TGCTCTCTAA GTATCGTTAT   
  
  
+ ATATCTTCTC TTTCTAGTGT ATTGAGTATT GCTCGACAGT TACCAGACAA CTTCCTTTCA GCGAGCTTTC   
  
  
+ TCCGGGTCCC TCTCTCTTTT TCTTTTTCTT TCAGACACAG GCACCCATGC AACTTATAAA CTTAAGGTTT   
  
  
+ CCTGCAGTTC CAGTTCGAAT CGTGCGCCTA TTTTAGGTGA AGCACATCAC CCATTTCGAA GTCTACGGTA   
  
  
+ AACTTTAAAG GGACACGTCT GATTTCTTCG AAGTACGACA GTCTCGAGAC TAAATGGAGC GGCCGAAAGG   
  
  
+ TACGAGAAAG GAGAGTAACC GGCGATACGT GTCTCTTATT GTGATGTGCT TTGCCGGTGC AAAGGTGTAT   
  
  
+ TAATCAAGTG CATTCTTGCT GACTTATAAG GCGTGAAGGA TCAGGAGAAA GAACAACTGA TGGGTTTTCG   
  
  
+ GGTTTTCGGC TGTTCGTCAC ACACAGGTGT GCCAAAACTA GAAACTTTGT AGCACGGCAA AGTAAGTTGT   
  
  
+ TTACGACCTG AAAGCCGTAG ATAGGTGAAA GAAGACAAGG AGTGGACCTT TTGTTTTGTT CAACGAGCAG   
  
  
+ GTAACCTATT AAGTACTAAT AAATAAAAAT TTTTTTATTT TTTAAACTTT AAATAATTTT ATTTTTAATA   
  
  
+ AAATTTTTAA AATTTTTTTA TTTTTATATA ATAGTGTTTT TTTATTAATT AATTAGTTTT TTATAAATCT   
  
  
+ AAGTTCCTTG TTTTAGTTTT ATTTTGCACT TTAAATAAAT TGATTAAAGA ATTCGTAAAG TTACTTTCGT   
  
  
+ AGCAATTAAA ACGTAGTAAC AAAACTAGTT TGAGATTAAA TTTTTTAATA TTTAATTTTA AATAAAATTT   
  
  
+ CATAAAATTA ATGTTTTTTA TTTTAATTTT AGGATTTACT ACACCAGAAC TAGAAATTAT TATTATATTA   
  
  
+ ATTAGTTTAT TAAACGAAAT AGACCACGAA TTAAAATATT TTAATTTTAG GATTTACTGC ACCAGAACTA   
  
  
+ GAAATTATTA TTATATTAAT TAGTTTATTA AACGAAATAG ACCACGAATT AAAAAATAAA CAAAGTAGTA   
  
  
+ ACTGCACCGG CAATTACTTT CCGGTTTGTT TAATTAATTC TATAAATTAG TTTGTTCATT AATAAAAGTA   
  
  
+ CATGTAACAA AGTAGTAACT GCACCGGCAA TTACTTTCCG GTTTGTTTAA TTAATTCTAT AAATTAGTTT   
  
  
+ GTTCATTAAT AAAAGTACAT GAAAGATACA AACAACCAGT ACCTTCTTCT ACTTTAAAAT TAAACTGTTT   
  
  
+ AATTTTCAGA TCGTCTTTAT ATTTTTAATT ACTGTCTGTA CGTTACTAAA TCTAATCATT AGAGACCCCA   
  
  
+ ACGTGCAGTG CACTACTTCA GTAATTGTT  

- TAATTGAAAG CGGCTGCCTT AAGAACTGGG AGAATTTCTG CAGAATAAGA ATCTACAAAG TATATCACTG   
  
  
- GACACAAGCT GTATTACAAG TGAGCAGCGA TCTCCAATTA GGTCAAGGGA ACGAGAGATT CATAGCAATA   
  
  
- TATAGAAGAG AAAGATCACA TAACTCATAA CGAGCTGTCA ATGGTCTGTT GAAGGAAAGT CGCTCGAAAG   
  
  
- AGGCCCAGGG AGAGAGAAAA AGAAAAAGAA AGTCTGTGTC CGTGGGTACG TTGAATATTT GAATTCCAAA   
  
  
- GGACGTCAAG GTCAAGCTTA GCACGCGGAT AAAATCCACT TCGTGTAGTG GGTAAAGCTT CAGATGCCAT   
  
  
- TTGAAATTTC CCTGTGCAGA CTAAAGAAGC TTCATGCTGT CAGAGCTCTG ATTTACCTCG CCGGCTTTCC   
  
  
- ATGCTCTTTC CTCTCATTGG CCGCTATGCA CAGAGAATAA CACTACACGA AACGGCCACG TTTCCACATA   
  
  
- ATTAGTTCAC GTAAGAACGA CTGAATATTC CGCACTTCCT AGTCCTCTTT CTTGTTGACT ACCCAAAAGC   
  
  
- CCAAAAGCCG ACAAGCAGTG TGTGTCCACA CGGTTTTGAT CTTTGAAACA TCGTGCCGTT TCATTCAACA   
  
  
- AATGCTGGAC TTTCGGCATC TATCCACTTT CTTCTGTTCC TCACCTGGAA AACAAAACAA GTTGCTCGTC   
  
  
- CATTGGATAA TTCATGATTA TTTATTTTTA AAAAAATAAA AAATTTGAAA TTTATTAAAA TAAAAATTAT   
  
  
- TTTAAAAATT TTAAAAAAAT AAAAATATAT TATCACAAAA AAATAATTAA TTAATCAAAA AATATTTAGA   
  
  
- TTCAAGGAAC AAAATCAAAA TAAAACGTGA AATTTATTTA ACTAATTTCT TAAGCATTTC AATGAAAGCA   
  
  
- TCGTTAATTT TGCATCATTG TTTTGATCAA ACTCTAATTT AAAAAATTAT AAATTAAAAT TTATTTTAAA   
  
  
- GTATTTTAAT TACAAAAAAT AAAATTAAAA TCCTAAATGA TGTGGTCTTG ATCTTTAATA ATAATATAAT   
  
  
- TAATCAAATA ATTTGCTTTA TCTGGTGCTT AATTTTATAA AATTAAAATC CTAAATGACG TGGTCTTGAT   
  
  
- CTTTAATAAT AATATAATTA ATCAAATAAT TTGCTTTATC TGGTGCTTAA TTTTTTATTT GTTTCATCAT   
  
  
- TGACGTGGCC GTTAATGAAA GGCCAAACAA ATTAATTAAG ATATTTAATC AAACAAGTAA TTATTTTCAT   
  
  
- GTACATTGTT TCATCATTGA CGTGGCCGTT AATGAAAGGC CAAACAAATT AATTAAGATA TTTAATCAAA   
  
  
- CAAGTAATTA TTTTCATGTA CTTTCTATGT TTGTTGGTCA TGGAAGAAGA TGAAATTTTA ATTTGACAAA   
  
  
- TTAAAAGTCT AGCAGAAATA TAAAAATTAA TGACAGACAT GCAATGATTT AGATTAGTAA TCTCTGGGGT   
  
  
- TGCACGTCAC GTGATGAAGT CATTAACAA

+     ATCT-motif

| Site Name | Organism | Position | Strand | Matrix score. | sequence | function |
| --- | --- | --- | --- | --- | --- | --- |
| ATCT-motif | Arabidopsis thaliana | 943 | - | 9 | AATCTAATCT | part of a conserved DNA module involved in light responsiveness |
| ATCT-motif | Arabidopsis thaliana | 1449 | + | 9 | AATCTAATCT | part of a conserved DNA module involved in light responsiveness |

> 2018/04/13 10:10:12  
+ ATTAACTTTC GCCGACGGAA TTCTTGACCC TCTTAAAGAC GTCTTATTCT TAGATGTTTC ATATAGTGAC   
  
  
+ CTGTGTTCGA CATAATGTTC ACTCGTCGCT AGAGGTTAAT CCAGTTCCCT TGCTCTCTAA GTATCGTTAT   
  
  
+ ATATCTTCTC TTTCTAGTGT ATTGAGTATT GCTCGACAGT TACCAGACAA CTTCCTTTCA GCGAGCTTTC   
  
  
+ TCCGGGTCCC TCTCTCTTTT TCTTTTTCTT TCAGACACAG GCACCCATGC AACTTATAAA CTTAAGGTTT   
  
  
+ CCTGCAGTTC CAGTTCGAAT CGTGCGCCTA TTTTAGGTGA AGCACATCAC CCATTTCGAA GTCTACGGTA   
  
  
+ AACTTTAAAG GGACACGTCT GATTTCTTCG AAGTACGACA GTCTCGAGAC TAAATGGAGC GGCCGAAAGG   
  
  
+ TACGAGAAAG GAGAGTAACC GGCGATACGT GTCTCTTATT GTGATGTGCT TTGCCGGTGC AAAGGTGTAT   
  
  
+ TAATCAAGTG CATTCTTGCT GACTTATAAG GCGTGAAGGA TCAGGAGAAA GAACAACTGA TGGGTTTTCG   
  
  
+ GGTTTTCGGC TGTTCGTCAC ACACAGGTGT GCCAAAACTA GAAACTTTGT AGCACGGCAA AGTAAGTTGT   
  
  
+ TTACGACCTG AAAGCCGTAG ATAGGTGAAA GAAGACAAGG AGTGGACCTT TTGTTTTGTT CAACGAGCAG   
  
  
+ GTAACCTATT AAGTACTAAT AAATAAAAAT TTTTTTATTT TTTAAACTTT AAATAATTTT ATTTTTAATA   
  
  
+ AAATTTTTAA AATTTTTTTA TTTTTATATA ATAGTGTTTT TTTATTAATT AATTAGTTTT TTATAAATCT   
  
  
+ AAGTTCCTTG TTTTAGTTTT ATTTTGCACT TTAAATAAAT TGATTAAAGA ATTCGTAAAG TTACTTTCGT   
  
  
+ AGCAATTAAA ACGTAGTAAC AAAACTAGTT TGAGATTAAA TTTTTTAATA TTTAATTTTA AATAAAATTT   
  
  
+ CATAAAATTA ATGTTTTTTA TTTTAATTTT AGGATTTACT ACACCAGAAC TAGAAATTAT TATTATATTA   
  
  
+ ATTAGTTTAT TAAACGAAAT AGACCACGAA TTAAAATATT TTAATTTTAG GATTTACTGC ACCAGAACTA   
  
  
+ GAAATTATTA TTATATTAAT TAGTTTATTA AACGAAATAG ACCACGAATT AAAAAATAAA CAAAGTAGTA   
  
  
+ ACTGCACCGG CAATTACTTT CCGGTTTGTT TAATTAATTC TATAAATTAG TTTGTTCATT AATAAAAGTA   
  
  
+ CATGTAACAA AGTAGTAACT GCACCGGCAA TTACTTTCCG GTTTGTTTAA TTAATTCTAT AAATTAGTTT   
  
  
+ GTTCATTAAT AAAAGTACAT GAAAGATACA AACAACCAGT ACCTTCTTCT ACTTTAAAAT TAAACTGTTT   
  
  
+ AATTTTCAGA TCGTCTTTAT ATTTTTAATT ACTGTCTGTA CGTTACTAAA TCTAATCATT AGAGACCCCA   
  
  
+ ACGTGCAGTG CACTACTTCA GTAATTGTT  

- TAATTGAAAG CGGCTGCCTT AAGAACTGGG AGAATTTCTG CAGAATAAGA ATCTACAAAG TATATCACTG   
  
  
- GACACAAGCT GTATTACAAG TGAGCAGCGA TCTCCAATTA GGTCAAGGGA ACGAGAGATT CATAGCAATA   
  
  
- TATAGAAGAG AAAGATCACA TAACTCATAA CGAGCTGTCA ATGGTCTGTT GAAGGAAAGT CGCTCGAAAG   
  
  
- AGGCCCAGGG AGAGAGAAAA AGAAAAAGAA AGTCTGTGTC CGTGGGTACG TTGAATATTT GAATTCCAAA   
  
  
- GGACGTCAAG GTCAAGCTTA GCACGCGGAT AAAATCCACT TCGTGTAGTG GGTAAAGCTT CAGATGCCAT   
  
  
- TTGAAATTTC CCTGTGCAGA CTAAAGAAGC TTCATGCTGT CAGAGCTCTG ATTTACCTCG CCGGCTTTCC   
  
  
- ATGCTCTTTC CTCTCATTGG CCGCTATGCA CAGAGAATAA CACTACACGA AACGGCCACG TTTCCACATA   
  
  
- ATTAGTTCAC GTAAGAACGA CTGAATATTC CGCACTTCCT AGTCCTCTTT CTTGTTGACT ACCCAAAAGC   
  
  
- CCAAAAGCCG ACAAGCAGTG TGTGTCCACA CGGTTTTGAT CTTTGAAACA TCGTGCCGTT TCATTCAACA   
  
  
- AATGCTGGAC TTTCGGCATC TATCCACTTT CTTCTGTTCC TCACCTGGAA AACAAAACAA GTTGCTCGTC   
  
  
- CATTGGATAA TTCATGATTA TTTATTTTTA AAAAAATAAA AAATTTGAAA TTTATTAAAA TAAAAATTAT   
  
  
- TTTAAAAATT TTAAAAAAAT AAAAATATAT TATCACAAAA AAATAATTAA TTAATCAAAA AATATTTAGA   
  
  
- TTCAAGGAAC AAAATCAAAA TAAAACGTGA AATTTATTTA ACTAATTTCT TAAGCATTTC AATGAAAGCA   
  
  
- TCGTTAATTT TGCATCATTG TTTTGATCAA ACTCTAATTT AAAAAATTAT AAATTAAAAT TTATTTTAAA   
  
  
- GTATTTTAAT TACAAAAAAT AAAATTAAAA TCCTAAATGA TGTGGTCTTG ATCTTTAATA ATAATATAAT   
  
  
- TAATCAAATA ATTTGCTTTA TCTGGTGCTT AATTTTATAA AATTAAAATC CTAAATGACG TGGTCTTGAT   
  
  
- CTTTAATAAT AATATAATTA ATCAAATAAT TTGCTTTATC TGGTGCTTAA TTTTTTATTT GTTTCATCAT   
  
  
- TGACGTGGCC GTTAATGAAA GGCCAAACAA ATTAATTAAG ATATTTAATC AAACAAGTAA TTATTTTCAT   
  
  
- GTACATTGTT TCATCATTGA CGTGGCCGTT AATGAAAGGC CAAACAAATT AATTAAGATA TTTAATCAAA   
  
  
- CAAGTAATTA TTTTCATGTA CTTTCTATGT TTGTTGGTCA TGGAAGAAGA TGAAATTTTA ATTTGACAAA   
  
  
- TTAAAAGTCT AGCAGAAATA TAAAAATTAA TGACAGACAT GCAATGATTT AGATTAGTAA TCTCTGGGGT   
  
  
- TGCACGTCAC GTGATGAAGT CATTAACAA

+     Box 4

| Site Name | Organism | Position | Strand | Matrix score. | sequence | function |
| --- | --- | --- | --- | --- | --- | --- |
| Box 4 | Petroselinum crispum | 1135 | - | 6 | ATTAAT | part of a conserved DNA module involved in light responsiveness |
| Box 4 | Petroselinum crispum | 987 | - | 6 | ATTAAT | part of a conserved DNA module involved in light responsiveness |
| Box 4 | Petroselinum crispum | 818 | - | 6 | ATTAAT | part of a conserved DNA module involved in light responsiveness |
| Box 4 | Petroselinum crispum | 489 | + | 6 | ATTAAT | part of a conserved DNA module involved in light responsiveness |
| Box 4 | Petroselinum crispum | 1335 | - | 6 | ATTAAT | part of a conserved DNA module involved in light responsiveness |
| Box 4 | Petroselinum crispum | 1047 | - | 6 | ATTAAT | part of a conserved DNA module involved in light responsiveness |
| Box 4 | Petroselinum crispum | 1310 | - | 6 | ATTAAT | part of a conserved DNA module involved in light responsiveness |
| Box 4 | Petroselinum crispum | 814 | - | 6 | ATTAAT | part of a conserved DNA module involved in light responsiveness |
| Box 4 | Petroselinum crispum | 1223 | - | 6 | ATTAAT | part of a conserved DNA module involved in light responsiveness |
| Box 4 | Petroselinum crispum | 1248 | - | 6 | ATTAAT | part of a conserved DNA module involved in light responsiveness |

> 2018/04/13 10:10:12  
+ ATTAACTTTC GCCGACGGAA TTCTTGACCC TCTTAAAGAC GTCTTATTCT TAGATGTTTC ATATAGTGAC   
  
  
+ CTGTGTTCGA CATAATGTTC ACTCGTCGCT AGAGGTTAAT CCAGTTCCCT TGCTCTCTAA GTATCGTTAT   
  
  
+ ATATCTTCTC TTTCTAGTGT ATTGAGTATT GCTCGACAGT TACCAGACAA CTTCCTTTCA GCGAGCTTTC   
  
  
+ TCCGGGTCCC TCTCTCTTTT TCTTTTTCTT TCAGACACAG GCACCCATGC AACTTATAAA CTTAAGGTTT   
  
  
+ CCTGCAGTTC CAGTTCGAAT CGTGCGCCTA TTTTAGGTGA AGCACATCAC CCATTTCGAA GTCTACGGTA   
  
  
+ AACTTTAAAG GGACACGTCT GATTTCTTCG AAGTACGACA GTCTCGAGAC TAAATGGAGC GGCCGAAAGG   
  
  
+ TACGAGAAAG GAGAGTAACC GGCGATACGT GTCTCTTATT GTGATGTGCT TTGCCGGTGC AAAGGTGTAT   
  
  
+ TAATCAAGTG CATTCTTGCT GACTTATAAG GCGTGAAGGA TCAGGAGAAA GAACAACTGA TGGGTTTTCG   
  
  
+ GGTTTTCGGC TGTTCGTCAC ACACAGGTGT GCCAAAACTA GAAACTTTGT AGCACGGCAA AGTAAGTTGT   
  
  
+ TTACGACCTG AAAGCCGTAG ATAGGTGAAA GAAGACAAGG AGTGGACCTT TTGTTTTGTT CAACGAGCAG   
  
  
+ GTAACCTATT AAGTACTAAT AAATAAAAAT TTTTTTATTT TTTAAACTTT AAATAATTTT ATTTTTAATA   
  
  
+ AAATTTTTAA AATTTTTTTA TTTTTATATA ATAGTGTTTT TTTATTAATT AATTAGTTTT TTATAAATCT   
  
  
+ AAGTTCCTTG TTTTAGTTTT ATTTTGCACT TTAAATAAAT TGATTAAAGA ATTCGTAAAG TTACTTTCGT   
  
  
+ AGCAATTAAA ACGTAGTAAC AAAACTAGTT TGAGATTAAA TTTTTTAATA TTTAATTTTA AATAAAATTT   
  
  
+ CATAAAATTA ATGTTTTTTA TTTTAATTTT AGGATTTACT ACACCAGAAC TAGAAATTAT TATTATATTA   
  
  
+ ATTAGTTTAT TAAACGAAAT AGACCACGAA TTAAAATATT TTAATTTTAG GATTTACTGC ACCAGAACTA   
  
  
+ GAAATTATTA TTATATTAAT TAGTTTATTA AACGAAATAG ACCACGAATT AAAAAATAAA CAAAGTAGTA   
  
  
+ ACTGCACCGG CAATTACTTT CCGGTTTGTT TAATTAATTC TATAAATTAG TTTGTTCATT AATAAAAGTA   
  
  
+ CATGTAACAA AGTAGTAACT GCACCGGCAA TTACTTTCCG GTTTGTTTAA TTAATTCTAT AAATTAGTTT   
  
  
+ GTTCATTAAT AAAAGTACAT GAAAGATACA AACAACCAGT ACCTTCTTCT ACTTTAAAAT TAAACTGTTT   
  
  
+ AATTTTCAGA TCGTCTTTAT ATTTTTAATT ACTGTCTGTA CGTTACTAAA TCTAATCATT AGAGACCCCA   
  
  
+ ACGTGCAGTG CACTACTTCA GTAATTGTT  

- TAATTGAAAG CGGCTGCCTT AAGAACTGGG AGAATTTCTG CAGAATAAGA ATCTACAAAG TATATCACTG   
  
  
- GACACAAGCT GTATTACAAG TGAGCAGCGA TCTCCAATTA GGTCAAGGGA ACGAGAGATT CATAGCAATA   
  
  
- TATAGAAGAG AAAGATCACA TAACTCATAA CGAGCTGTCA ATGGTCTGTT GAAGGAAAGT CGCTCGAAAG   
  
  
- AGGCCCAGGG AGAGAGAAAA AGAAAAAGAA AGTCTGTGTC CGTGGGTACG TTGAATATTT GAATTCCAAA   
  
  
- GGACGTCAAG GTCAAGCTTA GCACGCGGAT AAAATCCACT TCGTGTAGTG GGTAAAGCTT CAGATGCCAT   
  
  
- TTGAAATTTC CCTGTGCAGA CTAAAGAAGC TTCATGCTGT CAGAGCTCTG ATTTACCTCG CCGGCTTTCC   
  
  
- ATGCTCTTTC CTCTCATTGG CCGCTATGCA CAGAGAATAA CACTACACGA AACGGCCACG TTTCCACATA   
  
  
- ATTAGTTCAC GTAAGAACGA CTGAATATTC CGCACTTCCT AGTCCTCTTT CTTGTTGACT ACCCAAAAGC   
  
  
- CCAAAAGCCG ACAAGCAGTG TGTGTCCACA CGGTTTTGAT CTTTGAAACA TCGTGCCGTT TCATTCAACA   
  
  
- AATGCTGGAC TTTCGGCATC TATCCACTTT CTTCTGTTCC TCACCTGGAA AACAAAACAA GTTGCTCGTC   
  
  
- CATTGGATAA TTCATGATTA TTTATTTTTA AAAAAATAAA AAATTTGAAA TTTATTAAAA TAAAAATTAT   
  
  
- TTTAAAAATT TTAAAAAAAT AAAAATATAT TATCACAAAA AAATAATTAA TTAATCAAAA AATATTTAGA   
  
  
- TTCAAGGAAC AAAATCAAAA TAAAACGTGA AATTTATTTA ACTAATTTCT TAAGCATTTC AATGAAAGCA   
  
  
- TCGTTAATTT TGCATCATTG TTTTGATCAA ACTCTAATTT AAAAAATTAT AAATTAAAAT TTATTTTAAA   
  
  
- GTATTTTAAT TACAAAAAAT AAAATTAAAA TCCTAAATGA TGTGGTCTTG ATCTTTAATA ATAATATAAT   
  
  
- TAATCAAATA ATTTGCTTTA TCTGGTGCTT AATTTTATAA AATTAAAATC CTAAATGACG TGGTCTTGAT   
  
  
- CTTTAATAAT AATATAATTA ATCAAATAAT TTGCTTTATC TGGTGCTTAA TTTTTTATTT GTTTCATCAT   
  
  
- TGACGTGGCC GTTAATGAAA GGCCAAACAA ATTAATTAAG ATATTTAATC AAACAAGTAA TTATTTTCAT   
  
  
- GTACATTGTT TCATCATTGA CGTGGCCGTT AATGAAAGGC CAAACAAATT AATTAAGATA TTTAATCAAA   
  
  
- CAAGTAATTA TTTTCATGTA CTTTCTATGT TTGTTGGTCA TGGAAGAAGA TGAAATTTTA ATTTGACAAA   
  
  
- TTAAAAGTCT AGCAGAAATA TAAAAATTAA TGACAGACAT GCAATGATTT AGATTAGTAA TCTCTGGGGT   
  
  
- TGCACGTCAC GTGATGAAGT CATTAACAA

+     Box-W1

| Site Name | Organism | Position | Strand | Matrix score. | sequence | function |
| --- | --- | --- | --- | --- | --- | --- |
| Box-W1 | Petroselinum crispum | 24 | + | 6 | TTGACC | fungal elicitor responsive element |

> 2018/04/13 10:10:12  
+ ATTAACTTTC GCCGACGGAA TTCTTGACCC TCTTAAAGAC GTCTTATTCT TAGATGTTTC ATATAGTGAC   
  
  
+ CTGTGTTCGA CATAATGTTC ACTCGTCGCT AGAGGTTAAT CCAGTTCCCT TGCTCTCTAA GTATCGTTAT   
  
  
+ ATATCTTCTC TTTCTAGTGT ATTGAGTATT GCTCGACAGT TACCAGACAA CTTCCTTTCA GCGAGCTTTC   
  
  
+ TCCGGGTCCC TCTCTCTTTT TCTTTTTCTT TCAGACACAG GCACCCATGC AACTTATAAA CTTAAGGTTT   
  
  
+ CCTGCAGTTC CAGTTCGAAT CGTGCGCCTA TTTTAGGTGA AGCACATCAC CCATTTCGAA GTCTACGGTA   
  
  
+ AACTTTAAAG GGACACGTCT GATTTCTTCG AAGTACGACA GTCTCGAGAC TAAATGGAGC GGCCGAAAGG   
  
  
+ TACGAGAAAG GAGAGTAACC GGCGATACGT GTCTCTTATT GTGATGTGCT TTGCCGGTGC AAAGGTGTAT   
  
  
+ TAATCAAGTG CATTCTTGCT GACTTATAAG GCGTGAAGGA TCAGGAGAAA GAACAACTGA TGGGTTTTCG   
  
  
+ GGTTTTCGGC TGTTCGTCAC ACACAGGTGT GCCAAAACTA GAAACTTTGT AGCACGGCAA AGTAAGTTGT   
  
  
+ TTACGACCTG AAAGCCGTAG ATAGGTGAAA GAAGACAAGG AGTGGACCTT TTGTTTTGTT CAACGAGCAG   
  
  
+ GTAACCTATT AAGTACTAAT AAATAAAAAT TTTTTTATTT TTTAAACTTT AAATAATTTT ATTTTTAATA   
  
  
+ AAATTTTTAA AATTTTTTTA TTTTTATATA ATAGTGTTTT TTTATTAATT AATTAGTTTT TTATAAATCT   
  
  
+ AAGTTCCTTG TTTTAGTTTT ATTTTGCACT TTAAATAAAT TGATTAAAGA ATTCGTAAAG TTACTTTCGT   
  
  
+ AGCAATTAAA ACGTAGTAAC AAAACTAGTT TGAGATTAAA TTTTTTAATA TTTAATTTTA AATAAAATTT   
  
  
+ CATAAAATTA ATGTTTTTTA TTTTAATTTT AGGATTTACT ACACCAGAAC TAGAAATTAT TATTATATTA   
  
  
+ ATTAGTTTAT TAAACGAAAT AGACCACGAA TTAAAATATT TTAATTTTAG GATTTACTGC ACCAGAACTA   
  
  
+ GAAATTATTA TTATATTAAT TAGTTTATTA AACGAAATAG ACCACGAATT AAAAAATAAA CAAAGTAGTA   
  
  
+ ACTGCACCGG CAATTACTTT CCGGTTTGTT TAATTAATTC TATAAATTAG TTTGTTCATT AATAAAAGTA   
  
  
+ CATGTAACAA AGTAGTAACT GCACCGGCAA TTACTTTCCG GTTTGTTTAA TTAATTCTAT AAATTAGTTT   
  
  
+ GTTCATTAAT AAAAGTACAT GAAAGATACA AACAACCAGT ACCTTCTTCT ACTTTAAAAT TAAACTGTTT   
  
  
+ AATTTTCAGA TCGTCTTTAT ATTTTTAATT ACTGTCTGTA CGTTACTAAA TCTAATCATT AGAGACCCCA   
  
  
+ ACGTGCAGTG CACTACTTCA GTAATTGTT  

- TAATTGAAAG CGGCTGCCTT AAGAACTGGG AGAATTTCTG CAGAATAAGA ATCTACAAAG TATATCACTG   
  
  
- GACACAAGCT GTATTACAAG TGAGCAGCGA TCTCCAATTA GGTCAAGGGA ACGAGAGATT CATAGCAATA   
  
  
- TATAGAAGAG AAAGATCACA TAACTCATAA CGAGCTGTCA ATGGTCTGTT GAAGGAAAGT CGCTCGAAAG   
  
  
- AGGCCCAGGG AGAGAGAAAA AGAAAAAGAA AGTCTGTGTC CGTGGGTACG TTGAATATTT GAATTCCAAA   
  
  
- GGACGTCAAG GTCAAGCTTA GCACGCGGAT AAAATCCACT TCGTGTAGTG GGTAAAGCTT CAGATGCCAT   
  
  
- TTGAAATTTC CCTGTGCAGA CTAAAGAAGC TTCATGCTGT CAGAGCTCTG ATTTACCTCG CCGGCTTTCC   
  
  
- ATGCTCTTTC CTCTCATTGG CCGCTATGCA CAGAGAATAA CACTACACGA AACGGCCACG TTTCCACATA   
  
  
- ATTAGTTCAC GTAAGAACGA CTGAATATTC CGCACTTCCT AGTCCTCTTT CTTGTTGACT ACCCAAAAGC   
  
  
- CCAAAAGCCG ACAAGCAGTG TGTGTCCACA CGGTTTTGAT CTTTGAAACA TCGTGCCGTT TCATTCAACA   
  
  
- AATGCTGGAC TTTCGGCATC TATCCACTTT CTTCTGTTCC TCACCTGGAA AACAAAACAA GTTGCTCGTC   
  
  
- CATTGGATAA TTCATGATTA TTTATTTTTA AAAAAATAAA AAATTTGAAA TTTATTAAAA TAAAAATTAT   
  
  
- TTTAAAAATT TTAAAAAAAT AAAAATATAT TATCACAAAA AAATAATTAA TTAATCAAAA AATATTTAGA   
  
  
- TTCAAGGAAC AAAATCAAAA TAAAACGTGA AATTTATTTA ACTAATTTCT TAAGCATTTC AATGAAAGCA   
  
  
- TCGTTAATTT TGCATCATTG TTTTGATCAA ACTCTAATTT AAAAAATTAT AAATTAAAAT TTATTTTAAA   
  
  
- GTATTTTAAT TACAAAAAAT AAAATTAAAA TCCTAAATGA TGTGGTCTTG ATCTTTAATA ATAATATAAT   
  
  
- TAATCAAATA ATTTGCTTTA TCTGGTGCTT AATTTTATAA AATTAAAATC CTAAATGACG TGGTCTTGAT   
  
  
- CTTTAATAAT AATATAATTA ATCAAATAAT TTGCTTTATC TGGTGCTTAA TTTTTTATTT GTTTCATCAT   
  
  
- TGACGTGGCC GTTAATGAAA GGCCAAACAA ATTAATTAAG ATATTTAATC AAACAAGTAA TTATTTTCAT   
  
  
- GTACATTGTT TCATCATTGA CGTGGCCGTT AATGAAAGGC CAAACAAATT AATTAAGATA TTTAATCAAA   
  
  
- CAAGTAATTA TTTTCATGTA CTTTCTATGT TTGTTGGTCA TGGAAGAAGA TGAAATTTTA ATTTGACAAA   
  
  
- TTAAAAGTCT AGCAGAAATA TAAAAATTAA TGACAGACAT GCAATGATTT AGATTAGTAA TCTCTGGGGT   
  
  
- TGCACGTCAC GTGATGAAGT CATTAACAA

+     CAAT-box

| Site Name | Organism | Position | Strand | Matrix score. | sequence | function |
| --- | --- | --- | --- | --- | --- | --- |
| CAAT-box | Glycine max | 1493 | - | 5 | CAATT | common cis-acting element in promoter and enhancer regions |
| CAAT-box | Hordeum vulgare | 1494 | - | 4 | CAAT | common cis-acting element in promoter and enhancer regions |
| CAAT-box | Glycine max | 878 | - | 5 | CAATT | common cis-acting element in promoter and enhancer regions |
| CAAT-box | Hordeum vulgare | 879 | - | 4 | CAAT | common cis-acting element in promoter and enhancer regions |
| CAAT-box | Glycine max | 1201 | + | 5 | CAATT | common cis-acting element in promoter and enhancer regions |
| CAAT-box | Hordeum vulgare | 161 | - | 4 | CAAT | common cis-acting element in promoter and enhancer regions |
| CAAT-box | Arabidopsis thaliana | 1286 | + | 6 | gGCAAT | common cis-acting element in promoter and enhancer regions |
| CAAT-box | Arabidopsis thaliana | 1199 | + | 6 | gGCAAT | common cis-acting element in promoter and enhancer regions |
| CAAT-box | Glycine max | 913 | + | 5 | CAATT | common cis-acting element in promoter and enhancer regions |
| CAAT-box | Hordeum vulgare | 168 | - | 4 | CAAT | common cis-acting element in promoter and enhancer regions |
| CAAT-box | Glycine max | 1288 | + | 5 | CAATT | common cis-acting element in promoter and enhancer regions |
| CAAT-box | Hordeum vulgare | 458 | - | 4 | CAAT | common cis-acting element in promoter and enhancer regions |

> 2018/04/13 10:10:12  
+ ATTAACTTTC GCCGACGGAA TTCTTGACCC TCTTAAAGAC GTCTTATTCT TAGATGTTTC ATATAGTGAC   
  
  
+ CTGTGTTCGA CATAATGTTC ACTCGTCGCT AGAGGTTAAT CCAGTTCCCT TGCTCTCTAA GTATCGTTAT   
  
  
+ ATATCTTCTC TTTCTAGTGT ATTGAGTATT GCTCGACAGT TACCAGACAA CTTCCTTTCA GCGAGCTTTC   
  
  
+ TCCGGGTCCC TCTCTCTTTT TCTTTTTCTT TCAGACACAG GCACCCATGC AACTTATAAA CTTAAGGTTT   
  
  
+ CCTGCAGTTC CAGTTCGAAT CGTGCGCCTA TTTTAGGTGA AGCACATCAC CCATTTCGAA GTCTACGGTA   
  
  
+ AACTTTAAAG GGACACGTCT GATTTCTTCG AAGTACGACA GTCTCGAGAC TAAATGGAGC GGCCGAAAGG   
  
  
+ TACGAGAAAG GAGAGTAACC GGCGATACGT GTCTCTTATT GTGATGTGCT TTGCCGGTGC AAAGGTGTAT   
  
  
+ TAATCAAGTG CATTCTTGCT GACTTATAAG GCGTGAAGGA TCAGGAGAAA GAACAACTGA TGGGTTTTCG   
  
  
+ GGTTTTCGGC TGTTCGTCAC ACACAGGTGT GCCAAAACTA GAAACTTTGT AGCACGGCAA AGTAAGTTGT   
  
  
+ TTACGACCTG AAAGCCGTAG ATAGGTGAAA GAAGACAAGG AGTGGACCTT TTGTTTTGTT CAACGAGCAG   
  
  
+ GTAACCTATT AAGTACTAAT AAATAAAAAT TTTTTTATTT TTTAAACTTT AAATAATTTT ATTTTTAATA   
  
  
+ AAATTTTTAA AATTTTTTTA TTTTTATATA ATAGTGTTTT TTTATTAATT AATTAGTTTT TTATAAATCT   
  
  
+ AAGTTCCTTG TTTTAGTTTT ATTTTGCACT TTAAATAAAT TGATTAAAGA ATTCGTAAAG TTACTTTCGT   
  
  
+ AGCAATTAAA ACGTAGTAAC AAAACTAGTT TGAGATTAAA TTTTTTAATA TTTAATTTTA AATAAAATTT   
  
  
+ CATAAAATTA ATGTTTTTTA TTTTAATTTT AGGATTTACT ACACCAGAAC TAGAAATTAT TATTATATTA   
  
  
+ ATTAGTTTAT TAAACGAAAT AGACCACGAA TTAAAATATT TTAATTTTAG GATTTACTGC ACCAGAACTA   
  
  
+ GAAATTATTA TTATATTAAT TAGTTTATTA AACGAAATAG ACCACGAATT AAAAAATAAA CAAAGTAGTA   
  
  
+ ACTGCACCGG CAATTACTTT CCGGTTTGTT TAATTAATTC TATAAATTAG TTTGTTCATT AATAAAAGTA   
  
  
+ CATGTAACAA AGTAGTAACT GCACCGGCAA TTACTTTCCG GTTTGTTTAA TTAATTCTAT AAATTAGTTT   
  
  
+ GTTCATTAAT AAAAGTACAT GAAAGATACA AACAACCAGT ACCTTCTTCT ACTTTAAAAT TAAACTGTTT   
  
  
+ AATTTTCAGA TCGTCTTTAT ATTTTTAATT ACTGTCTGTA CGTTACTAAA TCTAATCATT AGAGACCCCA   
  
  
+ ACGTGCAGTG CACTACTTCA GTAATTGTT  

- TAATTGAAAG CGGCTGCCTT AAGAACTGGG AGAATTTCTG CAGAATAAGA ATCTACAAAG TATATCACTG   
  
  
- GACACAAGCT GTATTACAAG TGAGCAGCGA TCTCCAATTA GGTCAAGGGA ACGAGAGATT CATAGCAATA   
  
  
- TATAGAAGAG AAAGATCACA TAACTCATAA CGAGCTGTCA ATGGTCTGTT GAAGGAAAGT CGCTCGAAAG   
  
  
- AGGCCCAGGG AGAGAGAAAA AGAAAAAGAA AGTCTGTGTC CGTGGGTACG TTGAATATTT GAATTCCAAA   
  
  
- GGACGTCAAG GTCAAGCTTA GCACGCGGAT AAAATCCACT TCGTGTAGTG GGTAAAGCTT CAGATGCCAT   
  
  
- TTGAAATTTC CCTGTGCAGA CTAAAGAAGC TTCATGCTGT CAGAGCTCTG ATTTACCTCG CCGGCTTTCC   
  
  
- ATGCTCTTTC CTCTCATTGG CCGCTATGCA CAGAGAATAA CACTACACGA AACGGCCACG TTTCCACATA   
  
  
- ATTAGTTCAC GTAAGAACGA CTGAATATTC CGCACTTCCT AGTCCTCTTT CTTGTTGACT ACCCAAAAGC   
  
  
- CCAAAAGCCG ACAAGCAGTG TGTGTCCACA CGGTTTTGAT CTTTGAAACA TCGTGCCGTT TCATTCAACA   
  
  
- AATGCTGGAC TTTCGGCATC TATCCACTTT CTTCTGTTCC TCACCTGGAA AACAAAACAA GTTGCTCGTC   
  
  
- CATTGGATAA TTCATGATTA TTTATTTTTA AAAAAATAAA AAATTTGAAA TTTATTAAAA TAAAAATTAT   
  
  
- TTTAAAAATT TTAAAAAAAT AAAAATATAT TATCACAAAA AAATAATTAA TTAATCAAAA AATATTTAGA   
  
  
- TTCAAGGAAC AAAATCAAAA TAAAACGTGA AATTTATTTA ACTAATTTCT TAAGCATTTC AATGAAAGCA   
  
  
- TCGTTAATTT TGCATCATTG TTTTGATCAA ACTCTAATTT AAAAAATTAT AAATTAAAAT TTATTTTAAA   
  
  
- GTATTTTAAT TACAAAAAAT AAAATTAAAA TCCTAAATGA TGTGGTCTTG ATCTTTAATA ATAATATAAT   
  
  
- TAATCAAATA ATTTGCTTTA TCTGGTGCTT AATTTTATAA AATTAAAATC CTAAATGACG TGGTCTTGAT   
  
  
- CTTTAATAAT AATATAATTA ATCAAATAAT TTGCTTTATC TGGTGCTTAA TTTTTTATTT GTTTCATCAT   
  
  
- TGACGTGGCC GTTAATGAAA GGCCAAACAA ATTAATTAAG ATATTTAATC AAACAAGTAA TTATTTTCAT   
  
  
- GTACATTGTT TCATCATTGA CGTGGCCGTT AATGAAAGGC CAAACAAATT AATTAAGATA TTTAATCAAA   
  
  
- CAAGTAATTA TTTTCATGTA CTTTCTATGT TTGTTGGTCA TGGAAGAAGA TGAAATTTTA ATTTGACAAA   
  
  
- TTAAAAGTCT AGCAGAAATA TAAAAATTAA TGACAGACAT GCAATGATTT AGATTAGTAA TCTCTGGGGT   
  
  
- TGCACGTCAC GTGATGAAGT CATTAACAA

+     CATT-motif

| Site Name | Organism | Position | Strand | Matrix score. | sequence | function |
| --- | --- | --- | --- | --- | --- | --- |
| CATT-motif | Zea mays | 500 | + | 6 | GCATTC | part of a light responsive element |

> 2018/04/13 10:10:12  
+ ATTAACTTTC GCCGACGGAA TTCTTGACCC TCTTAAAGAC GTCTTATTCT TAGATGTTTC ATATAGTGAC   
  
  
+ CTGTGTTCGA CATAATGTTC ACTCGTCGCT AGAGGTTAAT CCAGTTCCCT TGCTCTCTAA GTATCGTTAT   
  
  
+ ATATCTTCTC TTTCTAGTGT ATTGAGTATT GCTCGACAGT TACCAGACAA CTTCCTTTCA GCGAGCTTTC   
  
  
+ TCCGGGTCCC TCTCTCTTTT TCTTTTTCTT TCAGACACAG GCACCCATGC AACTTATAAA CTTAAGGTTT   
  
  
+ CCTGCAGTTC CAGTTCGAAT CGTGCGCCTA TTTTAGGTGA AGCACATCAC CCATTTCGAA GTCTACGGTA   
  
  
+ AACTTTAAAG GGACACGTCT GATTTCTTCG AAGTACGACA GTCTCGAGAC TAAATGGAGC GGCCGAAAGG   
  
  
+ TACGAGAAAG GAGAGTAACC GGCGATACGT GTCTCTTATT GTGATGTGCT TTGCCGGTGC AAAGGTGTAT   
  
  
+ TAATCAAGTG CATTCTTGCT GACTTATAAG GCGTGAAGGA TCAGGAGAAA GAACAACTGA TGGGTTTTCG   
  
  
+ GGTTTTCGGC TGTTCGTCAC ACACAGGTGT GCCAAAACTA GAAACTTTGT AGCACGGCAA AGTAAGTTGT   
  
  
+ TTACGACCTG AAAGCCGTAG ATAGGTGAAA GAAGACAAGG AGTGGACCTT TTGTTTTGTT CAACGAGCAG   
  
  
+ GTAACCTATT AAGTACTAAT AAATAAAAAT TTTTTTATTT TTTAAACTTT AAATAATTTT ATTTTTAATA   
  
  
+ AAATTTTTAA AATTTTTTTA TTTTTATATA ATAGTGTTTT TTTATTAATT AATTAGTTTT TTATAAATCT   
  
  
+ AAGTTCCTTG TTTTAGTTTT ATTTTGCACT TTAAATAAAT TGATTAAAGA ATTCGTAAAG TTACTTTCGT   
  
  
+ AGCAATTAAA ACGTAGTAAC AAAACTAGTT TGAGATTAAA TTTTTTAATA TTTAATTTTA AATAAAATTT   
  
  
+ CATAAAATTA ATGTTTTTTA TTTTAATTTT AGGATTTACT ACACCAGAAC TAGAAATTAT TATTATATTA   
  
  
+ ATTAGTTTAT TAAACGAAAT AGACCACGAA TTAAAATATT TTAATTTTAG GATTTACTGC ACCAGAACTA   
  
  
+ GAAATTATTA TTATATTAAT TAGTTTATTA AACGAAATAG ACCACGAATT AAAAAATAAA CAAAGTAGTA   
  
  
+ ACTGCACCGG CAATTACTTT CCGGTTTGTT TAATTAATTC TATAAATTAG TTTGTTCATT AATAAAAGTA   
  
  
+ CATGTAACAA AGTAGTAACT GCACCGGCAA TTACTTTCCG GTTTGTTTAA TTAATTCTAT AAATTAGTTT   
  
  
+ GTTCATTAAT AAAAGTACAT GAAAGATACA AACAACCAGT ACCTTCTTCT ACTTTAAAAT TAAACTGTTT   
  
  
+ AATTTTCAGA TCGTCTTTAT ATTTTTAATT ACTGTCTGTA CGTTACTAAA TCTAATCATT AGAGACCCCA   
  
  
+ ACGTGCAGTG CACTACTTCA GTAATTGTT  

- TAATTGAAAG CGGCTGCCTT AAGAACTGGG AGAATTTCTG CAGAATAAGA ATCTACAAAG TATATCACTG   
  
  
- GACACAAGCT GTATTACAAG TGAGCAGCGA TCTCCAATTA GGTCAAGGGA ACGAGAGATT CATAGCAATA   
  
  
- TATAGAAGAG AAAGATCACA TAACTCATAA CGAGCTGTCA ATGGTCTGTT GAAGGAAAGT CGCTCGAAAG   
  
  
- AGGCCCAGGG AGAGAGAAAA AGAAAAAGAA AGTCTGTGTC CGTGGGTACG TTGAATATTT GAATTCCAAA   
  
  
- GGACGTCAAG GTCAAGCTTA GCACGCGGAT AAAATCCACT TCGTGTAGTG GGTAAAGCTT CAGATGCCAT   
  
  
- TTGAAATTTC CCTGTGCAGA CTAAAGAAGC TTCATGCTGT CAGAGCTCTG ATTTACCTCG CCGGCTTTCC   
  
  
- ATGCTCTTTC CTCTCATTGG CCGCTATGCA CAGAGAATAA CACTACACGA AACGGCCACG TTTCCACATA   
  
  
- ATTAGTTCAC GTAAGAACGA CTGAATATTC CGCACTTCCT AGTCCTCTTT CTTGTTGACT ACCCAAAAGC   
  
  
- CCAAAAGCCG ACAAGCAGTG TGTGTCCACA CGGTTTTGAT CTTTGAAACA TCGTGCCGTT TCATTCAACA   
  
  
- AATGCTGGAC TTTCGGCATC TATCCACTTT CTTCTGTTCC TCACCTGGAA AACAAAACAA GTTGCTCGTC   
  
  
- CATTGGATAA TTCATGATTA TTTATTTTTA AAAAAATAAA AAATTTGAAA TTTATTAAAA TAAAAATTAT   
  
  
- TTTAAAAATT TTAAAAAAAT AAAAATATAT TATCACAAAA AAATAATTAA TTAATCAAAA AATATTTAGA   
  
  
- TTCAAGGAAC AAAATCAAAA TAAAACGTGA AATTTATTTA ACTAATTTCT TAAGCATTTC AATGAAAGCA   
  
  
- TCGTTAATTT TGCATCATTG TTTTGATCAA ACTCTAATTT AAAAAATTAT AAATTAAAAT TTATTTTAAA   
  
  
- GTATTTTAAT TACAAAAAAT AAAATTAAAA TCCTAAATGA TGTGGTCTTG ATCTTTAATA ATAATATAAT   
  
  
- TAATCAAATA ATTTGCTTTA TCTGGTGCTT AATTTTATAA AATTAAAATC CTAAATGACG TGGTCTTGAT   
  
  
- CTTTAATAAT AATATAATTA ATCAAATAAT TTGCTTTATC TGGTGCTTAA TTTTTTATTT GTTTCATCAT   
  
  
- TGACGTGGCC GTTAATGAAA GGCCAAACAA ATTAATTAAG ATATTTAATC AAACAAGTAA TTATTTTCAT   
  
  
- GTACATTGTT TCATCATTGA CGTGGCCGTT AATGAAAGGC CAAACAAATT AATTAAGATA TTTAATCAAA   
  
  
- CAAGTAATTA TTTTCATGTA CTTTCTATGT TTGTTGGTCA TGGAAGAAGA TGAAATTTTA ATTTGACAAA   
  
  
- TTAAAAGTCT AGCAGAAATA TAAAAATTAA TGACAGACAT GCAATGATTT AGATTAGTAA TCTCTGGGGT   
  
  
- TGCACGTCAC GTGATGAAGT CATTAACAA

+     CGTCA-motif

| Site Name | Organism | Position | Strand | Matrix score. | sequence | function |
| --- | --- | --- | --- | --- | --- | --- |
| CGTCA-motif | Hordeum vulgare | 575 | + | 5 | CGTCA | cis-acting regulatory element involved in the MeJA-responsiveness |

> 2018/04/13 10:10:12  
+ ATTAACTTTC GCCGACGGAA TTCTTGACCC TCTTAAAGAC GTCTTATTCT TAGATGTTTC ATATAGTGAC   
  
  
+ CTGTGTTCGA CATAATGTTC ACTCGTCGCT AGAGGTTAAT CCAGTTCCCT TGCTCTCTAA GTATCGTTAT   
  
  
+ ATATCTTCTC TTTCTAGTGT ATTGAGTATT GCTCGACAGT TACCAGACAA CTTCCTTTCA GCGAGCTTTC   
  
  
+ TCCGGGTCCC TCTCTCTTTT TCTTTTTCTT TCAGACACAG GCACCCATGC AACTTATAAA CTTAAGGTTT   
  
  
+ CCTGCAGTTC CAGTTCGAAT CGTGCGCCTA TTTTAGGTGA AGCACATCAC CCATTTCGAA GTCTACGGTA   
  
  
+ AACTTTAAAG GGACACGTCT GATTTCTTCG AAGTACGACA GTCTCGAGAC TAAATGGAGC GGCCGAAAGG   
  
  
+ TACGAGAAAG GAGAGTAACC GGCGATACGT GTCTCTTATT GTGATGTGCT TTGCCGGTGC AAAGGTGTAT   
  
  
+ TAATCAAGTG CATTCTTGCT GACTTATAAG GCGTGAAGGA TCAGGAGAAA GAACAACTGA TGGGTTTTCG   
  
  
+ GGTTTTCGGC TGTTCGTCAC ACACAGGTGT GCCAAAACTA GAAACTTTGT AGCACGGCAA AGTAAGTTGT   
  
  
+ TTACGACCTG AAAGCCGTAG ATAGGTGAAA GAAGACAAGG AGTGGACCTT TTGTTTTGTT CAACGAGCAG   
  
  
+ GTAACCTATT AAGTACTAAT AAATAAAAAT TTTTTTATTT TTTAAACTTT AAATAATTTT ATTTTTAATA   
  
  
+ AAATTTTTAA AATTTTTTTA TTTTTATATA ATAGTGTTTT TTTATTAATT AATTAGTTTT TTATAAATCT   
  
  
+ AAGTTCCTTG TTTTAGTTTT ATTTTGCACT TTAAATAAAT TGATTAAAGA ATTCGTAAAG TTACTTTCGT   
  
  
+ AGCAATTAAA ACGTAGTAAC AAAACTAGTT TGAGATTAAA TTTTTTAATA TTTAATTTTA AATAAAATTT   
  
  
+ CATAAAATTA ATGTTTTTTA TTTTAATTTT AGGATTTACT ACACCAGAAC TAGAAATTAT TATTATATTA   
  
  
+ ATTAGTTTAT TAAACGAAAT AGACCACGAA TTAAAATATT TTAATTTTAG GATTTACTGC ACCAGAACTA   
  
  
+ GAAATTATTA TTATATTAAT TAGTTTATTA AACGAAATAG ACCACGAATT AAAAAATAAA CAAAGTAGTA   
  
  
+ ACTGCACCGG CAATTACTTT CCGGTTTGTT TAATTAATTC TATAAATTAG TTTGTTCATT AATAAAAGTA   
  
  
+ CATGTAACAA AGTAGTAACT GCACCGGCAA TTACTTTCCG GTTTGTTTAA TTAATTCTAT AAATTAGTTT   
  
  
+ GTTCATTAAT AAAAGTACAT GAAAGATACA AACAACCAGT ACCTTCTTCT ACTTTAAAAT TAAACTGTTT   
  
  
+ AATTTTCAGA TCGTCTTTAT ATTTTTAATT ACTGTCTGTA CGTTACTAAA TCTAATCATT AGAGACCCCA   
  
  
+ ACGTGCAGTG CACTACTTCA GTAATTGTT  

- TAATTGAAAG CGGCTGCCTT AAGAACTGGG AGAATTTCTG CAGAATAAGA ATCTACAAAG TATATCACTG   
  
  
- GACACAAGCT GTATTACAAG TGAGCAGCGA TCTCCAATTA GGTCAAGGGA ACGAGAGATT CATAGCAATA   
  
  
- TATAGAAGAG AAAGATCACA TAACTCATAA CGAGCTGTCA ATGGTCTGTT GAAGGAAAGT CGCTCGAAAG   
  
  
- AGGCCCAGGG AGAGAGAAAA AGAAAAAGAA AGTCTGTGTC CGTGGGTACG TTGAATATTT GAATTCCAAA   
  
  
- GGACGTCAAG GTCAAGCTTA GCACGCGGAT AAAATCCACT TCGTGTAGTG GGTAAAGCTT CAGATGCCAT   
  
  
- TTGAAATTTC CCTGTGCAGA CTAAAGAAGC TTCATGCTGT CAGAGCTCTG ATTTACCTCG CCGGCTTTCC   
  
  
- ATGCTCTTTC CTCTCATTGG CCGCTATGCA CAGAGAATAA CACTACACGA AACGGCCACG TTTCCACATA   
  
  
- ATTAGTTCAC GTAAGAACGA CTGAATATTC CGCACTTCCT AGTCCTCTTT CTTGTTGACT ACCCAAAAGC   
  
  
- CCAAAAGCCG ACAAGCAGTG TGTGTCCACA CGGTTTTGAT CTTTGAAACA TCGTGCCGTT TCATTCAACA   
  
  
- AATGCTGGAC TTTCGGCATC TATCCACTTT CTTCTGTTCC TCACCTGGAA AACAAAACAA GTTGCTCGTC   
  
  
- CATTGGATAA TTCATGATTA TTTATTTTTA AAAAAATAAA AAATTTGAAA TTTATTAAAA TAAAAATTAT   
  
  
- TTTAAAAATT TTAAAAAAAT AAAAATATAT TATCACAAAA AAATAATTAA TTAATCAAAA AATATTTAGA   
  
  
- TTCAAGGAAC AAAATCAAAA TAAAACGTGA AATTTATTTA ACTAATTTCT TAAGCATTTC AATGAAAGCA   
  
  
- TCGTTAATTT TGCATCATTG TTTTGATCAA ACTCTAATTT AAAAAATTAT AAATTAAAAT TTATTTTAAA   
  
  
- GTATTTTAAT TACAAAAAAT AAAATTAAAA TCCTAAATGA TGTGGTCTTG ATCTTTAATA ATAATATAAT   
  
  
- TAATCAAATA ATTTGCTTTA TCTGGTGCTT AATTTTATAA AATTAAAATC CTAAATGACG TGGTCTTGAT   
  
  
- CTTTAATAAT AATATAATTA ATCAAATAAT TTGCTTTATC TGGTGCTTAA TTTTTTATTT GTTTCATCAT   
  
  
- TGACGTGGCC GTTAATGAAA GGCCAAACAA ATTAATTAAG ATATTTAATC AAACAAGTAA TTATTTTCAT   
  
  
- GTACATTGTT TCATCATTGA CGTGGCCGTT AATGAAAGGC CAAACAAATT AATTAAGATA TTTAATCAAA   
  
  
- CAAGTAATTA TTTTCATGTA CTTTCTATGT TTGTTGGTCA TGGAAGAAGA TGAAATTTTA ATTTGACAAA   
  
  
- TTAAAAGTCT AGCAGAAATA TAAAAATTAA TGACAGACAT GCAATGATTT AGATTAGTAA TCTCTGGGGT   
  
  
- TGCACGTCAC GTGATGAAGT CATTAACAA

+     G-Box

| Site Name | Organism | Position | Strand | Matrix score. | sequence | function |
| --- | --- | --- | --- | --- | --- | --- |
| G-Box | Pisum sativum | 1470 | - | 6 | CACGTT | cis-acting regulatory element involved in light responsiveness |
| G-Box | Antirrhinum majus | 446 | - | 6 | CACGTA | cis-acting regulatory element involved in light responsiveness |

> 2018/04/13 10:10:12  
+ ATTAACTTTC GCCGACGGAA TTCTTGACCC TCTTAAAGAC GTCTTATTCT TAGATGTTTC ATATAGTGAC   
  
  
+ CTGTGTTCGA CATAATGTTC ACTCGTCGCT AGAGGTTAAT CCAGTTCCCT TGCTCTCTAA GTATCGTTAT   
  
  
+ ATATCTTCTC TTTCTAGTGT ATTGAGTATT GCTCGACAGT TACCAGACAA CTTCCTTTCA GCGAGCTTTC   
  
  
+ TCCGGGTCCC TCTCTCTTTT TCTTTTTCTT TCAGACACAG GCACCCATGC AACTTATAAA CTTAAGGTTT   
  
  
+ CCTGCAGTTC CAGTTCGAAT CGTGCGCCTA TTTTAGGTGA AGCACATCAC CCATTTCGAA GTCTACGGTA   
  
  
+ AACTTTAAAG GGACACGTCT GATTTCTTCG AAGTACGACA GTCTCGAGAC TAAATGGAGC GGCCGAAAGG   
  
  
+ TACGAGAAAG GAGAGTAACC GGCGATACGT GTCTCTTATT GTGATGTGCT TTGCCGGTGC AAAGGTGTAT   
  
  
+ TAATCAAGTG CATTCTTGCT GACTTATAAG GCGTGAAGGA TCAGGAGAAA GAACAACTGA TGGGTTTTCG   
  
  
+ GGTTTTCGGC TGTTCGTCAC ACACAGGTGT GCCAAAACTA GAAACTTTGT AGCACGGCAA AGTAAGTTGT   
  
  
+ TTACGACCTG AAAGCCGTAG ATAGGTGAAA GAAGACAAGG AGTGGACCTT TTGTTTTGTT CAACGAGCAG   
  
  
+ GTAACCTATT AAGTACTAAT AAATAAAAAT TTTTTTATTT TTTAAACTTT AAATAATTTT ATTTTTAATA   
  
  
+ AAATTTTTAA AATTTTTTTA TTTTTATATA ATAGTGTTTT TTTATTAATT AATTAGTTTT TTATAAATCT   
  
  
+ AAGTTCCTTG TTTTAGTTTT ATTTTGCACT TTAAATAAAT TGATTAAAGA ATTCGTAAAG TTACTTTCGT   
  
  
+ AGCAATTAAA ACGTAGTAAC AAAACTAGTT TGAGATTAAA TTTTTTAATA TTTAATTTTA AATAAAATTT   
  
  
+ CATAAAATTA ATGTTTTTTA TTTTAATTTT AGGATTTACT ACACCAGAAC TAGAAATTAT TATTATATTA   
  
  
+ ATTAGTTTAT TAAACGAAAT AGACCACGAA TTAAAATATT TTAATTTTAG GATTTACTGC ACCAGAACTA   
  
  
+ GAAATTATTA TTATATTAAT TAGTTTATTA AACGAAATAG ACCACGAATT AAAAAATAAA CAAAGTAGTA   
  
  
+ ACTGCACCGG CAATTACTTT CCGGTTTGTT TAATTAATTC TATAAATTAG TTTGTTCATT AATAAAAGTA   
  
  
+ CATGTAACAA AGTAGTAACT GCACCGGCAA TTACTTTCCG GTTTGTTTAA TTAATTCTAT AAATTAGTTT   
  
  
+ GTTCATTAAT AAAAGTACAT GAAAGATACA AACAACCAGT ACCTTCTTCT ACTTTAAAAT TAAACTGTTT   
  
  
+ AATTTTCAGA TCGTCTTTAT ATTTTTAATT ACTGTCTGTA CGTTACTAAA TCTAATCATT AGAGACCCCA   
  
  
+ ACGTGCAGTG CACTACTTCA GTAATTGTT  

- TAATTGAAAG CGGCTGCCTT AAGAACTGGG AGAATTTCTG CAGAATAAGA ATCTACAAAG TATATCACTG   
  
  
- GACACAAGCT GTATTACAAG TGAGCAGCGA TCTCCAATTA GGTCAAGGGA ACGAGAGATT CATAGCAATA   
  
  
- TATAGAAGAG AAAGATCACA TAACTCATAA CGAGCTGTCA ATGGTCTGTT GAAGGAAAGT CGCTCGAAAG   
  
  
- AGGCCCAGGG AGAGAGAAAA AGAAAAAGAA AGTCTGTGTC CGTGGGTACG TTGAATATTT GAATTCCAAA   
  
  
- GGACGTCAAG GTCAAGCTTA GCACGCGGAT AAAATCCACT TCGTGTAGTG GGTAAAGCTT CAGATGCCAT   
  
  
- TTGAAATTTC CCTGTGCAGA CTAAAGAAGC TTCATGCTGT CAGAGCTCTG ATTTACCTCG CCGGCTTTCC   
  
  
- ATGCTCTTTC CTCTCATTGG CCGCTATGCA CAGAGAATAA CACTACACGA AACGGCCACG TTTCCACATA   
  
  
- ATTAGTTCAC GTAAGAACGA CTGAATATTC CGCACTTCCT AGTCCTCTTT CTTGTTGACT ACCCAAAAGC   
  
  
- CCAAAAGCCG ACAAGCAGTG TGTGTCCACA CGGTTTTGAT CTTTGAAACA TCGTGCCGTT TCATTCAACA   
  
  
- AATGCTGGAC TTTCGGCATC TATCCACTTT CTTCTGTTCC TCACCTGGAA AACAAAACAA GTTGCTCGTC   
  
  
- CATTGGATAA TTCATGATTA TTTATTTTTA AAAAAATAAA AAATTTGAAA TTTATTAAAA TAAAAATTAT   
  
  
- TTTAAAAATT TTAAAAAAAT AAAAATATAT TATCACAAAA AAATAATTAA TTAATCAAAA AATATTTAGA   
  
  
- TTCAAGGAAC AAAATCAAAA TAAAACGTGA AATTTATTTA ACTAATTTCT TAAGCATTTC AATGAAAGCA   
  
  
- TCGTTAATTT TGCATCATTG TTTTGATCAA ACTCTAATTT AAAAAATTAT AAATTAAAAT TTATTTTAAA   
  
  
- GTATTTTAAT TACAAAAAAT AAAATTAAAA TCCTAAATGA TGTGGTCTTG ATCTTTAATA ATAATATAAT   
  
  
- TAATCAAATA ATTTGCTTTA TCTGGTGCTT AATTTTATAA AATTAAAATC CTAAATGACG TGGTCTTGAT   
  
  
- CTTTAATAAT AATATAATTA ATCAAATAAT TTGCTTTATC TGGTGCTTAA TTTTTTATTT GTTTCATCAT   
  
  
- TGACGTGGCC GTTAATGAAA GGCCAAACAA ATTAATTAAG ATATTTAATC AAACAAGTAA TTATTTTCAT   
  
  
- GTACATTGTT TCATCATTGA CGTGGCCGTT AATGAAAGGC CAAACAAATT AATTAAGATA TTTAATCAAA   
  
  
- CAAGTAATTA TTTTCATGTA CTTTCTATGT TTGTTGGTCA TGGAAGAAGA TGAAATTTTA ATTTGACAAA   
  
  
- TTAAAAGTCT AGCAGAAATA TAAAAATTAA TGACAGACAT GCAATGATTT AGATTAGTAA TCTCTGGGGT   
  
  
- TGCACGTCAC GTGATGAAGT CATTAACAA

+     G-box

| Site Name | Organism | Position | Strand | Matrix score. | sequence | function |
| --- | --- | --- | --- | --- | --- | --- |
| G-box | Brassica oleracea | 917 | + | 9 | TAACACGTAG | cis-acting regulatory element involved in light responsiveness |
| G-box | Zea mays | 1470 | - | 6 | CACGTT | cis-acting regulatory element involved in light responsiveness |
| G-box | Zea mays | 364 | + | 6 | CACGTC | cis-acting regulatory element involved in light responsiveness |
| G-box | Daucus carota | 446 | + | 6 | TACGTG | cis-acting regulatory element involved in light responsiveness |

> 2018/04/13 10:10:12  
+ ATTAACTTTC GCCGACGGAA TTCTTGACCC TCTTAAAGAC GTCTTATTCT TAGATGTTTC ATATAGTGAC   
  
  
+ CTGTGTTCGA CATAATGTTC ACTCGTCGCT AGAGGTTAAT CCAGTTCCCT TGCTCTCTAA GTATCGTTAT   
  
  
+ ATATCTTCTC TTTCTAGTGT ATTGAGTATT GCTCGACAGT TACCAGACAA CTTCCTTTCA GCGAGCTTTC   
  
  
+ TCCGGGTCCC TCTCTCTTTT TCTTTTTCTT TCAGACACAG GCACCCATGC AACTTATAAA CTTAAGGTTT   
  
  
+ CCTGCAGTTC CAGTTCGAAT CGTGCGCCTA TTTTAGGTGA AGCACATCAC CCATTTCGAA GTCTACGGTA   
  
  
+ AACTTTAAAG GGACACGTCT GATTTCTTCG AAGTACGACA GTCTCGAGAC TAAATGGAGC GGCCGAAAGG   
  
  
+ TACGAGAAAG GAGAGTAACC GGCGATACGT GTCTCTTATT GTGATGTGCT TTGCCGGTGC AAAGGTGTAT   
  
  
+ TAATCAAGTG CATTCTTGCT GACTTATAAG GCGTGAAGGA TCAGGAGAAA GAACAACTGA TGGGTTTTCG   
  
  
+ GGTTTTCGGC TGTTCGTCAC ACACAGGTGT GCCAAAACTA GAAACTTTGT AGCACGGCAA AGTAAGTTGT   
  
  
+ TTACGACCTG AAAGCCGTAG ATAGGTGAAA GAAGACAAGG AGTGGACCTT TTGTTTTGTT CAACGAGCAG   
  
  
+ GTAACCTATT AAGTACTAAT AAATAAAAAT TTTTTTATTT TTTAAACTTT AAATAATTTT ATTTTTAATA   
  
  
+ AAATTTTTAA AATTTTTTTA TTTTTATATA ATAGTGTTTT TTTATTAATT AATTAGTTTT TTATAAATCT   
  
  
+ AAGTTCCTTG TTTTAGTTTT ATTTTGCACT TTAAATAAAT TGATTAAAGA ATTCGTAAAG TTACTTTCGT   
  
  
+ AGCAATTAAA ACGTAGTAAC AAAACTAGTT TGAGATTAAA TTTTTTAATA TTTAATTTTA AATAAAATTT   
  
  
+ CATAAAATTA ATGTTTTTTA TTTTAATTTT AGGATTTACT ACACCAGAAC TAGAAATTAT TATTATATTA   
  
  
+ ATTAGTTTAT TAAACGAAAT AGACCACGAA TTAAAATATT TTAATTTTAG GATTTACTGC ACCAGAACTA   
  
  
+ GAAATTATTA TTATATTAAT TAGTTTATTA AACGAAATAG ACCACGAATT AAAAAATAAA CAAAGTAGTA   
  
  
+ ACTGCACCGG CAATTACTTT CCGGTTTGTT TAATTAATTC TATAAATTAG TTTGTTCATT AATAAAAGTA   
  
  
+ CATGTAACAA AGTAGTAACT GCACCGGCAA TTACTTTCCG GTTTGTTTAA TTAATTCTAT AAATTAGTTT   
  
  
+ GTTCATTAAT AAAAGTACAT GAAAGATACA AACAACCAGT ACCTTCTTCT ACTTTAAAAT TAAACTGTTT   
  
  
+ AATTTTCAGA TCGTCTTTAT ATTTTTAATT ACTGTCTGTA CGTTACTAAA TCTAATCATT AGAGACCCCA   
  
  
+ ACGTGCAGTG CACTACTTCA GTAATTGTT  

- TAATTGAAAG CGGCTGCCTT AAGAACTGGG AGAATTTCTG CAGAATAAGA ATCTACAAAG TATATCACTG   
  
  
- GACACAAGCT GTATTACAAG TGAGCAGCGA TCTCCAATTA GGTCAAGGGA ACGAGAGATT CATAGCAATA   
  
  
- TATAGAAGAG AAAGATCACA TAACTCATAA CGAGCTGTCA ATGGTCTGTT GAAGGAAAGT CGCTCGAAAG   
  
  
- AGGCCCAGGG AGAGAGAAAA AGAAAAAGAA AGTCTGTGTC CGTGGGTACG TTGAATATTT GAATTCCAAA   
  
  
- GGACGTCAAG GTCAAGCTTA GCACGCGGAT AAAATCCACT TCGTGTAGTG GGTAAAGCTT CAGATGCCAT   
  
  
- TTGAAATTTC CCTGTGCAGA CTAAAGAAGC TTCATGCTGT CAGAGCTCTG ATTTACCTCG CCGGCTTTCC   
  
  
- ATGCTCTTTC CTCTCATTGG CCGCTATGCA CAGAGAATAA CACTACACGA AACGGCCACG TTTCCACATA   
  
  
- ATTAGTTCAC GTAAGAACGA CTGAATATTC CGCACTTCCT AGTCCTCTTT CTTGTTGACT ACCCAAAAGC   
  
  
- CCAAAAGCCG ACAAGCAGTG TGTGTCCACA CGGTTTTGAT CTTTGAAACA TCGTGCCGTT TCATTCAACA   
  
  
- AATGCTGGAC TTTCGGCATC TATCCACTTT CTTCTGTTCC TCACCTGGAA AACAAAACAA GTTGCTCGTC   
  
  
- CATTGGATAA TTCATGATTA TTTATTTTTA AAAAAATAAA AAATTTGAAA TTTATTAAAA TAAAAATTAT   
  
  
- TTTAAAAATT TTAAAAAAAT AAAAATATAT TATCACAAAA AAATAATTAA TTAATCAAAA AATATTTAGA   
  
  
- TTCAAGGAAC AAAATCAAAA TAAAACGTGA AATTTATTTA ACTAATTTCT TAAGCATTTC AATGAAAGCA   
  
  
- TCGTTAATTT TGCATCATTG TTTTGATCAA ACTCTAATTT AAAAAATTAT AAATTAAAAT TTATTTTAAA   
  
  
- GTATTTTAAT TACAAAAAAT AAAATTAAAA TCCTAAATGA TGTGGTCTTG ATCTTTAATA ATAATATAAT   
  
  
- TAATCAAATA ATTTGCTTTA TCTGGTGCTT AATTTTATAA AATTAAAATC CTAAATGACG TGGTCTTGAT   
  
  
- CTTTAATAAT AATATAATTA ATCAAATAAT TTGCTTTATC TGGTGCTTAA TTTTTTATTT GTTTCATCAT   
  
  
- TGACGTGGCC GTTAATGAAA GGCCAAACAA ATTAATTAAG ATATTTAATC AAACAAGTAA TTATTTTCAT   
  
  
- GTACATTGTT TCATCATTGA CGTGGCCGTT AATGAAAGGC CAAACAAATT AATTAAGATA TTTAATCAAA   
  
  
- CAAGTAATTA TTTTCATGTA CTTTCTATGT TTGTTGGTCA TGGAAGAAGA TGAAATTTTA ATTTGACAAA   
  
  
- TTAAAAGTCT AGCAGAAATA TAAAAATTAA TGACAGACAT GCAATGATTT AGATTAGTAA TCTCTGGGGT   
  
  
- TGCACGTCAC GTGATGAAGT CATTAACAA

+     GATA-motif

| Site Name | Organism | Position | Strand | Matrix score. | sequence | function |
| --- | --- | --- | --- | --- | --- | --- |
| GATA-motif | Solanum tuberosum | 526 | + | 9 | AAGGATAAGG | part of a light responsive element |

> 2018/04/13 10:10:12  
+ ATTAACTTTC GCCGACGGAA TTCTTGACCC TCTTAAAGAC GTCTTATTCT TAGATGTTTC ATATAGTGAC   
  
  
+ CTGTGTTCGA CATAATGTTC ACTCGTCGCT AGAGGTTAAT CCAGTTCCCT TGCTCTCTAA GTATCGTTAT   
  
  
+ ATATCTTCTC TTTCTAGTGT ATTGAGTATT GCTCGACAGT TACCAGACAA CTTCCTTTCA GCGAGCTTTC   
  
  
+ TCCGGGTCCC TCTCTCTTTT TCTTTTTCTT TCAGACACAG GCACCCATGC AACTTATAAA CTTAAGGTTT   
  
  
+ CCTGCAGTTC CAGTTCGAAT CGTGCGCCTA TTTTAGGTGA AGCACATCAC CCATTTCGAA GTCTACGGTA   
  
  
+ AACTTTAAAG GGACACGTCT GATTTCTTCG AAGTACGACA GTCTCGAGAC TAAATGGAGC GGCCGAAAGG   
  
  
+ TACGAGAAAG GAGAGTAACC GGCGATACGT GTCTCTTATT GTGATGTGCT TTGCCGGTGC AAAGGTGTAT   
  
  
+ TAATCAAGTG CATTCTTGCT GACTTATAAG GCGTGAAGGA TCAGGAGAAA GAACAACTGA TGGGTTTTCG   
  
  
+ GGTTTTCGGC TGTTCGTCAC ACACAGGTGT GCCAAAACTA GAAACTTTGT AGCACGGCAA AGTAAGTTGT   
  
  
+ TTACGACCTG AAAGCCGTAG ATAGGTGAAA GAAGACAAGG AGTGGACCTT TTGTTTTGTT CAACGAGCAG   
  
  
+ GTAACCTATT AAGTACTAAT AAATAAAAAT TTTTTTATTT TTTAAACTTT AAATAATTTT ATTTTTAATA   
  
  
+ AAATTTTTAA AATTTTTTTA TTTTTATATA ATAGTGTTTT TTTATTAATT AATTAGTTTT TTATAAATCT   
  
  
+ AAGTTCCTTG TTTTAGTTTT ATTTTGCACT TTAAATAAAT TGATTAAAGA ATTCGTAAAG TTACTTTCGT   
  
  
+ AGCAATTAAA ACGTAGTAAC AAAACTAGTT TGAGATTAAA TTTTTTAATA TTTAATTTTA AATAAAATTT   
  
  
+ CATAAAATTA ATGTTTTTTA TTTTAATTTT AGGATTTACT ACACCAGAAC TAGAAATTAT TATTATATTA   
  
  
+ ATTAGTTTAT TAAACGAAAT AGACCACGAA TTAAAATATT TTAATTTTAG GATTTACTGC ACCAGAACTA   
  
  
+ GAAATTATTA TTATATTAAT TAGTTTATTA AACGAAATAG ACCACGAATT AAAAAATAAA CAAAGTAGTA   
  
  
+ ACTGCACCGG CAATTACTTT CCGGTTTGTT TAATTAATTC TATAAATTAG TTTGTTCATT AATAAAAGTA   
  
  
+ CATGTAACAA AGTAGTAACT GCACCGGCAA TTACTTTCCG GTTTGTTTAA TTAATTCTAT AAATTAGTTT   
  
  
+ GTTCATTAAT AAAAGTACAT GAAAGATACA AACAACCAGT ACCTTCTTCT ACTTTAAAAT TAAACTGTTT   
  
  
+ AATTTTCAGA TCGTCTTTAT ATTTTTAATT ACTGTCTGTA CGTTACTAAA TCTAATCATT AGAGACCCCA   
  
  
+ ACGTGCAGTG CACTACTTCA GTAATTGTT  

- TAATTGAAAG CGGCTGCCTT AAGAACTGGG AGAATTTCTG CAGAATAAGA ATCTACAAAG TATATCACTG   
  
  
- GACACAAGCT GTATTACAAG TGAGCAGCGA TCTCCAATTA GGTCAAGGGA ACGAGAGATT CATAGCAATA   
  
  
- TATAGAAGAG AAAGATCACA TAACTCATAA CGAGCTGTCA ATGGTCTGTT GAAGGAAAGT CGCTCGAAAG   
  
  
- AGGCCCAGGG AGAGAGAAAA AGAAAAAGAA AGTCTGTGTC CGTGGGTACG TTGAATATTT GAATTCCAAA   
  
  
- GGACGTCAAG GTCAAGCTTA GCACGCGGAT AAAATCCACT TCGTGTAGTG GGTAAAGCTT CAGATGCCAT   
  
  
- TTGAAATTTC CCTGTGCAGA CTAAAGAAGC TTCATGCTGT CAGAGCTCTG ATTTACCTCG CCGGCTTTCC   
  
  
- ATGCTCTTTC CTCTCATTGG CCGCTATGCA CAGAGAATAA CACTACACGA AACGGCCACG TTTCCACATA   
  
  
- ATTAGTTCAC GTAAGAACGA CTGAATATTC CGCACTTCCT AGTCCTCTTT CTTGTTGACT ACCCAAAAGC   
  
  
- CCAAAAGCCG ACAAGCAGTG TGTGTCCACA CGGTTTTGAT CTTTGAAACA TCGTGCCGTT TCATTCAACA   
  
  
- AATGCTGGAC TTTCGGCATC TATCCACTTT CTTCTGTTCC TCACCTGGAA AACAAAACAA GTTGCTCGTC   
  
  
- CATTGGATAA TTCATGATTA TTTATTTTTA AAAAAATAAA AAATTTGAAA TTTATTAAAA TAAAAATTAT   
  
  
- TTTAAAAATT TTAAAAAAAT AAAAATATAT TATCACAAAA AAATAATTAA TTAATCAAAA AATATTTAGA   
  
  
- TTCAAGGAAC AAAATCAAAA TAAAACGTGA AATTTATTTA ACTAATTTCT TAAGCATTTC AATGAAAGCA   
  
  
- TCGTTAATTT TGCATCATTG TTTTGATCAA ACTCTAATTT AAAAAATTAT AAATTAAAAT TTATTTTAAA   
  
  
- GTATTTTAAT TACAAAAAAT AAAATTAAAA TCCTAAATGA TGTGGTCTTG ATCTTTAATA ATAATATAAT   
  
  
- TAATCAAATA ATTTGCTTTA TCTGGTGCTT AATTTTATAA AATTAAAATC CTAAATGACG TGGTCTTGAT   
  
  
- CTTTAATAAT AATATAATTA ATCAAATAAT TTGCTTTATC TGGTGCTTAA TTTTTTATTT GTTTCATCAT   
  
  
- TGACGTGGCC GTTAATGAAA GGCCAAACAA ATTAATTAAG ATATTTAATC AAACAAGTAA TTATTTTCAT   
  
  
- GTACATTGTT TCATCATTGA CGTGGCCGTT AATGAAAGGC CAAACAAATT AATTAAGATA TTTAATCAAA   
  
  
- CAAGTAATTA TTTTCATGTA CTTTCTATGT TTGTTGGTCA TGGAAGAAGA TGAAATTTTA ATTTGACAAA   
  
  
- TTAAAAGTCT AGCAGAAATA TAAAAATTAA TGACAGACAT GCAATGATTT AGATTAGTAA TCTCTGGGGT   
  
  
- TGCACGTCAC GTGATGAAGT CATTAACAA

+     GT1-motif

| Site Name | Organism | Position | Strand | Matrix score. | sequence | function |
| --- | --- | --- | --- | --- | --- | --- |
| GT1-motif | Avena sativa | 104 | + | 7 | GGTTAAT | light responsive element |

> 2018/04/13 10:10:12  
+ ATTAACTTTC GCCGACGGAA TTCTTGACCC TCTTAAAGAC GTCTTATTCT TAGATGTTTC ATATAGTGAC   
  
  
+ CTGTGTTCGA CATAATGTTC ACTCGTCGCT AGAGGTTAAT CCAGTTCCCT TGCTCTCTAA GTATCGTTAT   
  
  
+ ATATCTTCTC TTTCTAGTGT ATTGAGTATT GCTCGACAGT TACCAGACAA CTTCCTTTCA GCGAGCTTTC   
  
  
+ TCCGGGTCCC TCTCTCTTTT TCTTTTTCTT TCAGACACAG GCACCCATGC AACTTATAAA CTTAAGGTTT   
  
  
+ CCTGCAGTTC CAGTTCGAAT CGTGCGCCTA TTTTAGGTGA AGCACATCAC CCATTTCGAA GTCTACGGTA   
  
  
+ AACTTTAAAG GGACACGTCT GATTTCTTCG AAGTACGACA GTCTCGAGAC TAAATGGAGC GGCCGAAAGG   
  
  
+ TACGAGAAAG GAGAGTAACC GGCGATACGT GTCTCTTATT GTGATGTGCT TTGCCGGTGC AAAGGTGTAT   
  
  
+ TAATCAAGTG CATTCTTGCT GACTTATAAG GCGTGAAGGA TCAGGAGAAA GAACAACTGA TGGGTTTTCG   
  
  
+ GGTTTTCGGC TGTTCGTCAC ACACAGGTGT GCCAAAACTA GAAACTTTGT AGCACGGCAA AGTAAGTTGT   
  
  
+ TTACGACCTG AAAGCCGTAG ATAGGTGAAA GAAGACAAGG AGTGGACCTT TTGTTTTGTT CAACGAGCAG   
  
  
+ GTAACCTATT AAGTACTAAT AAATAAAAAT TTTTTTATTT TTTAAACTTT AAATAATTTT ATTTTTAATA   
  
  
+ AAATTTTTAA AATTTTTTTA TTTTTATATA ATAGTGTTTT TTTATTAATT AATTAGTTTT TTATAAATCT   
  
  
+ AAGTTCCTTG TTTTAGTTTT ATTTTGCACT TTAAATAAAT TGATTAAAGA ATTCGTAAAG TTACTTTCGT   
  
  
+ AGCAATTAAA ACGTAGTAAC AAAACTAGTT TGAGATTAAA TTTTTTAATA TTTAATTTTA AATAAAATTT   
  
  
+ CATAAAATTA ATGTTTTTTA TTTTAATTTT AGGATTTACT ACACCAGAAC TAGAAATTAT TATTATATTA   
  
  
+ ATTAGTTTAT TAAACGAAAT AGACCACGAA TTAAAATATT TTAATTTTAG GATTTACTGC ACCAGAACTA   
  
  
+ GAAATTATTA TTATATTAAT TAGTTTATTA AACGAAATAG ACCACGAATT AAAAAATAAA CAAAGTAGTA   
  
  
+ ACTGCACCGG CAATTACTTT CCGGTTTGTT TAATTAATTC TATAAATTAG TTTGTTCATT AATAAAAGTA   
  
  
+ CATGTAACAA AGTAGTAACT GCACCGGCAA TTACTTTCCG GTTTGTTTAA TTAATTCTAT AAATTAGTTT   
  
  
+ GTTCATTAAT AAAAGTACAT GAAAGATACA AACAACCAGT ACCTTCTTCT ACTTTAAAAT TAAACTGTTT   
  
  
+ AATTTTCAGA TCGTCTTTAT ATTTTTAATT ACTGTCTGTA CGTTACTAAA TCTAATCATT AGAGACCCCA   
  
  
+ ACGTGCAGTG CACTACTTCA GTAATTGTT  

- TAATTGAAAG CGGCTGCCTT AAGAACTGGG AGAATTTCTG CAGAATAAGA ATCTACAAAG TATATCACTG   
  
  
- GACACAAGCT GTATTACAAG TGAGCAGCGA TCTCCAATTA GGTCAAGGGA ACGAGAGATT CATAGCAATA   
  
  
- TATAGAAGAG AAAGATCACA TAACTCATAA CGAGCTGTCA ATGGTCTGTT GAAGGAAAGT CGCTCGAAAG   
  
  
- AGGCCCAGGG AGAGAGAAAA AGAAAAAGAA AGTCTGTGTC CGTGGGTACG TTGAATATTT GAATTCCAAA   
  
  
- GGACGTCAAG GTCAAGCTTA GCACGCGGAT AAAATCCACT TCGTGTAGTG GGTAAAGCTT CAGATGCCAT   
  
  
- TTGAAATTTC CCTGTGCAGA CTAAAGAAGC TTCATGCTGT CAGAGCTCTG ATTTACCTCG CCGGCTTTCC   
  
  
- ATGCTCTTTC CTCTCATTGG CCGCTATGCA CAGAGAATAA CACTACACGA AACGGCCACG TTTCCACATA   
  
  
- ATTAGTTCAC GTAAGAACGA CTGAATATTC CGCACTTCCT AGTCCTCTTT CTTGTTGACT ACCCAAAAGC   
  
  
- CCAAAAGCCG ACAAGCAGTG TGTGTCCACA CGGTTTTGAT CTTTGAAACA TCGTGCCGTT TCATTCAACA   
  
  
- AATGCTGGAC TTTCGGCATC TATCCACTTT CTTCTGTTCC TCACCTGGAA AACAAAACAA GTTGCTCGTC   
  
  
- CATTGGATAA TTCATGATTA TTTATTTTTA AAAAAATAAA AAATTTGAAA TTTATTAAAA TAAAAATTAT   
  
  
- TTTAAAAATT TTAAAAAAAT AAAAATATAT TATCACAAAA AAATAATTAA TTAATCAAAA AATATTTAGA   
  
  
- TTCAAGGAAC AAAATCAAAA TAAAACGTGA AATTTATTTA ACTAATTTCT TAAGCATTTC AATGAAAGCA   
  
  
- TCGTTAATTT TGCATCATTG TTTTGATCAA ACTCTAATTT AAAAAATTAT AAATTAAAAT TTATTTTAAA   
  
  
- GTATTTTAAT TACAAAAAAT AAAATTAAAA TCCTAAATGA TGTGGTCTTG ATCTTTAATA ATAATATAAT   
  
  
- TAATCAAATA ATTTGCTTTA TCTGGTGCTT AATTTTATAA AATTAAAATC CTAAATGACG TGGTCTTGAT   
  
  
- CTTTAATAAT AATATAATTA ATCAAATAAT TTGCTTTATC TGGTGCTTAA TTTTTTATTT GTTTCATCAT   
  
  
- TGACGTGGCC GTTAATGAAA GGCCAAACAA ATTAATTAAG ATATTTAATC AAACAAGTAA TTATTTTCAT   
  
  
- GTACATTGTT TCATCATTGA CGTGGCCGTT AATGAAAGGC CAAACAAATT AATTAAGATA TTTAATCAAA   
  
  
- CAAGTAATTA TTTTCATGTA CTTTCTATGT TTGTTGGTCA TGGAAGAAGA TGAAATTTTA ATTTGACAAA   
  
  
- TTAAAAGTCT AGCAGAAATA TAAAAATTAA TGACAGACAT GCAATGATTT AGATTAGTAA TCTCTGGGGT   
  
  
- TGCACGTCAC GTGATGAAGT CATTAACAA

+     HSE

| Site Name | Organism | Position | Strand | Matrix score. | sequence | function |
| --- | --- | --- | --- | --- | --- | --- |
| HSE | Brassica oleracea | 947 | - | 9 | AAAAAATTTC | cis-acting element involved in heat stress responsiveness |
| HSE | Brassica oleracea | 972 | + | 9 | AAAAAATTTC | cis-acting element involved in heat stress responsiveness |
| HSE | Brassica oleracea | 726 | - | 9 | AAAAAATTTC | cis-acting element involved in heat stress responsiveness |
| HSE | Brassica oleracea | 779 | - | 9 | AAAAAATTTC | cis-acting element involved in heat stress responsiveness |

> 2018/04/13 10:10:12  
+ ATTAACTTTC GCCGACGGAA TTCTTGACCC TCTTAAAGAC GTCTTATTCT TAGATGTTTC ATATAGTGAC   
  
  
+ CTGTGTTCGA CATAATGTTC ACTCGTCGCT AGAGGTTAAT CCAGTTCCCT TGCTCTCTAA GTATCGTTAT   
  
  
+ ATATCTTCTC TTTCTAGTGT ATTGAGTATT GCTCGACAGT TACCAGACAA CTTCCTTTCA GCGAGCTTTC   
  
  
+ TCCGGGTCCC TCTCTCTTTT TCTTTTTCTT TCAGACACAG GCACCCATGC AACTTATAAA CTTAAGGTTT   
  
  
+ CCTGCAGTTC CAGTTCGAAT CGTGCGCCTA TTTTAGGTGA AGCACATCAC CCATTTCGAA GTCTACGGTA   
  
  
+ AACTTTAAAG GGACACGTCT GATTTCTTCG AAGTACGACA GTCTCGAGAC TAAATGGAGC GGCCGAAAGG   
  
  
+ TACGAGAAAG GAGAGTAACC GGCGATACGT GTCTCTTATT GTGATGTGCT TTGCCGGTGC AAAGGTGTAT   
  
  
+ TAATCAAGTG CATTCTTGCT GACTTATAAG GCGTGAAGGA TCAGGAGAAA GAACAACTGA TGGGTTTTCG   
  
  
+ GGTTTTCGGC TGTTCGTCAC ACACAGGTGT GCCAAAACTA GAAACTTTGT AGCACGGCAA AGTAAGTTGT   
  
  
+ TTACGACCTG AAAGCCGTAG ATAGGTGAAA GAAGACAAGG AGTGGACCTT TTGTTTTGTT CAACGAGCAG   
  
  
+ GTAACCTATT AAGTACTAAT AAATAAAAAT TTTTTTATTT TTTAAACTTT AAATAATTTT ATTTTTAATA   
  
  
+ AAATTTTTAA AATTTTTTTA TTTTTATATA ATAGTGTTTT TTTATTAATT AATTAGTTTT TTATAAATCT   
  
  
+ AAGTTCCTTG TTTTAGTTTT ATTTTGCACT TTAAATAAAT TGATTAAAGA ATTCGTAAAG TTACTTTCGT   
  
  
+ AGCAATTAAA ACGTAGTAAC AAAACTAGTT TGAGATTAAA TTTTTTAATA TTTAATTTTA AATAAAATTT   
  
  
+ CATAAAATTA ATGTTTTTTA TTTTAATTTT AGGATTTACT ACACCAGAAC TAGAAATTAT TATTATATTA   
  
  
+ ATTAGTTTAT TAAACGAAAT AGACCACGAA TTAAAATATT TTAATTTTAG GATTTACTGC ACCAGAACTA   
  
  
+ GAAATTATTA TTATATTAAT TAGTTTATTA AACGAAATAG ACCACGAATT AAAAAATAAA CAAAGTAGTA   
  
  
+ ACTGCACCGG CAATTACTTT CCGGTTTGTT TAATTAATTC TATAAATTAG TTTGTTCATT AATAAAAGTA   
  
  
+ CATGTAACAA AGTAGTAACT GCACCGGCAA TTACTTTCCG GTTTGTTTAA TTAATTCTAT AAATTAGTTT   
  
  
+ GTTCATTAAT AAAAGTACAT GAAAGATACA AACAACCAGT ACCTTCTTCT ACTTTAAAAT TAAACTGTTT   
  
  
+ AATTTTCAGA TCGTCTTTAT ATTTTTAATT ACTGTCTGTA CGTTACTAAA TCTAATCATT AGAGACCCCA   
  
  
+ ACGTGCAGTG CACTACTTCA GTAATTGTT  

- TAATTGAAAG CGGCTGCCTT AAGAACTGGG AGAATTTCTG CAGAATAAGA ATCTACAAAG TATATCACTG   
  
  
- GACACAAGCT GTATTACAAG TGAGCAGCGA TCTCCAATTA GGTCAAGGGA ACGAGAGATT CATAGCAATA   
  
  
- TATAGAAGAG AAAGATCACA TAACTCATAA CGAGCTGTCA ATGGTCTGTT GAAGGAAAGT CGCTCGAAAG   
  
  
- AGGCCCAGGG AGAGAGAAAA AGAAAAAGAA AGTCTGTGTC CGTGGGTACG TTGAATATTT GAATTCCAAA   
  
  
- GGACGTCAAG GTCAAGCTTA GCACGCGGAT AAAATCCACT TCGTGTAGTG GGTAAAGCTT CAGATGCCAT   
  
  
- TTGAAATTTC CCTGTGCAGA CTAAAGAAGC TTCATGCTGT CAGAGCTCTG ATTTACCTCG CCGGCTTTCC   
  
  
- ATGCTCTTTC CTCTCATTGG CCGCTATGCA CAGAGAATAA CACTACACGA AACGGCCACG TTTCCACATA   
  
  
- ATTAGTTCAC GTAAGAACGA CTGAATATTC CGCACTTCCT AGTCCTCTTT CTTGTTGACT ACCCAAAAGC   
  
  
- CCAAAAGCCG ACAAGCAGTG TGTGTCCACA CGGTTTTGAT CTTTGAAACA TCGTGCCGTT TCATTCAACA   
  
  
- AATGCTGGAC TTTCGGCATC TATCCACTTT CTTCTGTTCC TCACCTGGAA AACAAAACAA GTTGCTCGTC   
  
  
- CATTGGATAA TTCATGATTA TTTATTTTTA AAAAAATAAA AAATTTGAAA TTTATTAAAA TAAAAATTAT   
  
  
- TTTAAAAATT TTAAAAAAAT AAAAATATAT TATCACAAAA AAATAATTAA TTAATCAAAA AATATTTAGA   
  
  
- TTCAAGGAAC AAAATCAAAA TAAAACGTGA AATTTATTTA ACTAATTTCT TAAGCATTTC AATGAAAGCA   
  
  
- TCGTTAATTT TGCATCATTG TTTTGATCAA ACTCTAATTT AAAAAATTAT AAATTAAAAT TTATTTTAAA   
  
  
- GTATTTTAAT TACAAAAAAT AAAATTAAAA TCCTAAATGA TGTGGTCTTG ATCTTTAATA ATAATATAAT   
  
  
- TAATCAAATA ATTTGCTTTA TCTGGTGCTT AATTTTATAA AATTAAAATC CTAAATGACG TGGTCTTGAT   
  
  
- CTTTAATAAT AATATAATTA ATCAAATAAT TTGCTTTATC TGGTGCTTAA TTTTTTATTT GTTTCATCAT   
  
  
- TGACGTGGCC GTTAATGAAA GGCCAAACAA ATTAATTAAG ATATTTAATC AAACAAGTAA TTATTTTCAT   
  
  
- GTACATTGTT TCATCATTGA CGTGGCCGTT AATGAAAGGC CAAACAAATT AATTAAGATA TTTAATCAAA   
  
  
- CAAGTAATTA TTTTCATGTA CTTTCTATGT TTGTTGGTCA TGGAAGAAGA TGAAATTTTA ATTTGACAAA   
  
  
- TTAAAAGTCT AGCAGAAATA TAAAAATTAA TGACAGACAT GCAATGATTT AGATTAGTAA TCTCTGGGGT   
  
  
- TGCACGTCAC GTGATGAAGT CATTAACAA

+     LTR

| Site Name | Organism | Position | Strand | Matrix score. | sequence | function |
| --- | --- | --- | --- | --- | --- | --- |
| LTR | Hordeum vulgare | 564 | - | 6 | CCGAAA | cis-acting element involved in low-temperature responsiveness |
| LTR | Hordeum vulgare | 556 | - | 6 | CCGAAA | cis-acting element involved in low-temperature responsiveness |
| LTR | Hordeum vulgare | 413 | + | 6 | CCGAAA | cis-acting element involved in low-temperature responsiveness |

> 2018/04/13 10:10:12  
+ ATTAACTTTC GCCGACGGAA TTCTTGACCC TCTTAAAGAC GTCTTATTCT TAGATGTTTC ATATAGTGAC   
  
  
+ CTGTGTTCGA CATAATGTTC ACTCGTCGCT AGAGGTTAAT CCAGTTCCCT TGCTCTCTAA GTATCGTTAT   
  
  
+ ATATCTTCTC TTTCTAGTGT ATTGAGTATT GCTCGACAGT TACCAGACAA CTTCCTTTCA GCGAGCTTTC   
  
  
+ TCCGGGTCCC TCTCTCTTTT TCTTTTTCTT TCAGACACAG GCACCCATGC AACTTATAAA CTTAAGGTTT   
  
  
+ CCTGCAGTTC CAGTTCGAAT CGTGCGCCTA TTTTAGGTGA AGCACATCAC CCATTTCGAA GTCTACGGTA   
  
  
+ AACTTTAAAG GGACACGTCT GATTTCTTCG AAGTACGACA GTCTCGAGAC TAAATGGAGC GGCCGAAAGG   
  
  
+ TACGAGAAAG GAGAGTAACC GGCGATACGT GTCTCTTATT GTGATGTGCT TTGCCGGTGC AAAGGTGTAT   
  
  
+ TAATCAAGTG CATTCTTGCT GACTTATAAG GCGTGAAGGA TCAGGAGAAA GAACAACTGA TGGGTTTTCG   
  
  
+ GGTTTTCGGC TGTTCGTCAC ACACAGGTGT GCCAAAACTA GAAACTTTGT AGCACGGCAA AGTAAGTTGT   
  
  
+ TTACGACCTG AAAGCCGTAG ATAGGTGAAA GAAGACAAGG AGTGGACCTT TTGTTTTGTT CAACGAGCAG   
  
  
+ GTAACCTATT AAGTACTAAT AAATAAAAAT TTTTTTATTT TTTAAACTTT AAATAATTTT ATTTTTAATA   
  
  
+ AAATTTTTAA AATTTTTTTA TTTTTATATA ATAGTGTTTT TTTATTAATT AATTAGTTTT TTATAAATCT   
  
  
+ AAGTTCCTTG TTTTAGTTTT ATTTTGCACT TTAAATAAAT TGATTAAAGA ATTCGTAAAG TTACTTTCGT   
  
  
+ AGCAATTAAA ACGTAGTAAC AAAACTAGTT TGAGATTAAA TTTTTTAATA TTTAATTTTA AATAAAATTT   
  
  
+ CATAAAATTA ATGTTTTTTA TTTTAATTTT AGGATTTACT ACACCAGAAC TAGAAATTAT TATTATATTA   
  
  
+ ATTAGTTTAT TAAACGAAAT AGACCACGAA TTAAAATATT TTAATTTTAG GATTTACTGC ACCAGAACTA   
  
  
+ GAAATTATTA TTATATTAAT TAGTTTATTA AACGAAATAG ACCACGAATT AAAAAATAAA CAAAGTAGTA   
  
  
+ ACTGCACCGG CAATTACTTT CCGGTTTGTT TAATTAATTC TATAAATTAG TTTGTTCATT AATAAAAGTA   
  
  
+ CATGTAACAA AGTAGTAACT GCACCGGCAA TTACTTTCCG GTTTGTTTAA TTAATTCTAT AAATTAGTTT   
  
  
+ GTTCATTAAT AAAAGTACAT GAAAGATACA AACAACCAGT ACCTTCTTCT ACTTTAAAAT TAAACTGTTT   
  
  
+ AATTTTCAGA TCGTCTTTAT ATTTTTAATT ACTGTCTGTA CGTTACTAAA TCTAATCATT AGAGACCCCA   
  
  
+ ACGTGCAGTG CACTACTTCA GTAATTGTT  

- TAATTGAAAG CGGCTGCCTT AAGAACTGGG AGAATTTCTG CAGAATAAGA ATCTACAAAG TATATCACTG   
  
  
- GACACAAGCT GTATTACAAG TGAGCAGCGA TCTCCAATTA GGTCAAGGGA ACGAGAGATT CATAGCAATA   
  
  
- TATAGAAGAG AAAGATCACA TAACTCATAA CGAGCTGTCA ATGGTCTGTT GAAGGAAAGT CGCTCGAAAG   
  
  
- AGGCCCAGGG AGAGAGAAAA AGAAAAAGAA AGTCTGTGTC CGTGGGTACG TTGAATATTT GAATTCCAAA   
  
  
- GGACGTCAAG GTCAAGCTTA GCACGCGGAT AAAATCCACT TCGTGTAGTG GGTAAAGCTT CAGATGCCAT   
  
  
- TTGAAATTTC CCTGTGCAGA CTAAAGAAGC TTCATGCTGT CAGAGCTCTG ATTTACCTCG CCGGCTTTCC   
  
  
- ATGCTCTTTC CTCTCATTGG CCGCTATGCA CAGAGAATAA CACTACACGA AACGGCCACG TTTCCACATA   
  
  
- ATTAGTTCAC GTAAGAACGA CTGAATATTC CGCACTTCCT AGTCCTCTTT CTTGTTGACT ACCCAAAAGC   
  
  
- CCAAAAGCCG ACAAGCAGTG TGTGTCCACA CGGTTTTGAT CTTTGAAACA TCGTGCCGTT TCATTCAACA   
  
  
- AATGCTGGAC TTTCGGCATC TATCCACTTT CTTCTGTTCC TCACCTGGAA AACAAAACAA GTTGCTCGTC   
  
  
- CATTGGATAA TTCATGATTA TTTATTTTTA AAAAAATAAA AAATTTGAAA TTTATTAAAA TAAAAATTAT   
  
  
- TTTAAAAATT TTAAAAAAAT AAAAATATAT TATCACAAAA AAATAATTAA TTAATCAAAA AATATTTAGA   
  
  
- TTCAAGGAAC AAAATCAAAA TAAAACGTGA AATTTATTTA ACTAATTTCT TAAGCATTTC AATGAAAGCA   
  
  
- TCGTTAATTT TGCATCATTG TTTTGATCAA ACTCTAATTT AAAAAATTAT AAATTAAAAT TTATTTTAAA   
  
  
- GTATTTTAAT TACAAAAAAT AAAATTAAAA TCCTAAATGA TGTGGTCTTG ATCTTTAATA ATAATATAAT   
  
  
- TAATCAAATA ATTTGCTTTA TCTGGTGCTT AATTTTATAA AATTAAAATC CTAAATGACG TGGTCTTGAT   
  
  
- CTTTAATAAT AATATAATTA ATCAAATAAT TTGCTTTATC TGGTGCTTAA TTTTTTATTT GTTTCATCAT   
  
  
- TGACGTGGCC GTTAATGAAA GGCCAAACAA ATTAATTAAG ATATTTAATC AAACAAGTAA TTATTTTCAT   
  
  
- GTACATTGTT TCATCATTGA CGTGGCCGTT AATGAAAGGC CAAACAAATT AATTAAGATA TTTAATCAAA   
  
  
- CAAGTAATTA TTTTCATGTA CTTTCTATGT TTGTTGGTCA TGGAAGAAGA TGAAATTTTA ATTTGACAAA   
  
  
- TTAAAAGTCT AGCAGAAATA TAAAAATTAA TGACAGACAT GCAATGATTT AGATTAGTAA TCTCTGGGGT   
  
  
- TGCACGTCAC GTGATGAAGT CATTAACAA

+     MBS

| Site Name | Organism | Position | Strand | Matrix score. | sequence | function |
| --- | --- | --- | --- | --- | --- | --- |
| MBS | Arabidopsis thaliana | 1276 | + | 6 | TAACTG | MYB binding site involved in drought-inducibility |
| MBS | Arabidopsis thaliana | 177 | - | 6 | TAACTG | MYB binding site involved in drought-inducibility |
| MBS | Arabidopsis thaliana | 1189 | + | 6 | TAACTG | MYB binding site involved in drought-inducibility |
| MBS | Arabidopsis thaliana | 544 | + | 6 | CAACTG | MYB binding site involved in drought-inducibility |

> 2018/04/13 10:10:12  
+ ATTAACTTTC GCCGACGGAA TTCTTGACCC TCTTAAAGAC GTCTTATTCT TAGATGTTTC ATATAGTGAC   
  
  
+ CTGTGTTCGA CATAATGTTC ACTCGTCGCT AGAGGTTAAT CCAGTTCCCT TGCTCTCTAA GTATCGTTAT   
  
  
+ ATATCTTCTC TTTCTAGTGT ATTGAGTATT GCTCGACAGT TACCAGACAA CTTCCTTTCA GCGAGCTTTC   
  
  
+ TCCGGGTCCC TCTCTCTTTT TCTTTTTCTT TCAGACACAG GCACCCATGC AACTTATAAA CTTAAGGTTT   
  
  
+ CCTGCAGTTC CAGTTCGAAT CGTGCGCCTA TTTTAGGTGA AGCACATCAC CCATTTCGAA GTCTACGGTA   
  
  
+ AACTTTAAAG GGACACGTCT GATTTCTTCG AAGTACGACA GTCTCGAGAC TAAATGGAGC GGCCGAAAGG   
  
  
+ TACGAGAAAG GAGAGTAACC GGCGATACGT GTCTCTTATT GTGATGTGCT TTGCCGGTGC AAAGGTGTAT   
  
  
+ TAATCAAGTG CATTCTTGCT GACTTATAAG GCGTGAAGGA TCAGGAGAAA GAACAACTGA TGGGTTTTCG   
  
  
+ GGTTTTCGGC TGTTCGTCAC ACACAGGTGT GCCAAAACTA GAAACTTTGT AGCACGGCAA AGTAAGTTGT   
  
  
+ TTACGACCTG AAAGCCGTAG ATAGGTGAAA GAAGACAAGG AGTGGACCTT TTGTTTTGTT CAACGAGCAG   
  
  
+ GTAACCTATT AAGTACTAAT AAATAAAAAT TTTTTTATTT TTTAAACTTT AAATAATTTT ATTTTTAATA   
  
  
+ AAATTTTTAA AATTTTTTTA TTTTTATATA ATAGTGTTTT TTTATTAATT AATTAGTTTT TTATAAATCT   
  
  
+ AAGTTCCTTG TTTTAGTTTT ATTTTGCACT TTAAATAAAT TGATTAAAGA ATTCGTAAAG TTACTTTCGT   
  
  
+ AGCAATTAAA ACGTAGTAAC AAAACTAGTT TGAGATTAAA TTTTTTAATA TTTAATTTTA AATAAAATTT   
  
  
+ CATAAAATTA ATGTTTTTTA TTTTAATTTT AGGATTTACT ACACCAGAAC TAGAAATTAT TATTATATTA   
  
  
+ ATTAGTTTAT TAAACGAAAT AGACCACGAA TTAAAATATT TTAATTTTAG GATTTACTGC ACCAGAACTA   
  
  
+ GAAATTATTA TTATATTAAT TAGTTTATTA AACGAAATAG ACCACGAATT AAAAAATAAA CAAAGTAGTA   
  
  
+ ACTGCACCGG CAATTACTTT CCGGTTTGTT TAATTAATTC TATAAATTAG TTTGTTCATT AATAAAAGTA   
  
  
+ CATGTAACAA AGTAGTAACT GCACCGGCAA TTACTTTCCG GTTTGTTTAA TTAATTCTAT AAATTAGTTT   
  
  
+ GTTCATTAAT AAAAGTACAT GAAAGATACA AACAACCAGT ACCTTCTTCT ACTTTAAAAT TAAACTGTTT   
  
  
+ AATTTTCAGA TCGTCTTTAT ATTTTTAATT ACTGTCTGTA CGTTACTAAA TCTAATCATT AGAGACCCCA   
  
  
+ ACGTGCAGTG CACTACTTCA GTAATTGTT  

- TAATTGAAAG CGGCTGCCTT AAGAACTGGG AGAATTTCTG CAGAATAAGA ATCTACAAAG TATATCACTG   
  
  
- GACACAAGCT GTATTACAAG TGAGCAGCGA TCTCCAATTA GGTCAAGGGA ACGAGAGATT CATAGCAATA   
  
  
- TATAGAAGAG AAAGATCACA TAACTCATAA CGAGCTGTCA ATGGTCTGTT GAAGGAAAGT CGCTCGAAAG   
  
  
- AGGCCCAGGG AGAGAGAAAA AGAAAAAGAA AGTCTGTGTC CGTGGGTACG TTGAATATTT GAATTCCAAA   
  
  
- GGACGTCAAG GTCAAGCTTA GCACGCGGAT AAAATCCACT TCGTGTAGTG GGTAAAGCTT CAGATGCCAT   
  
  
- TTGAAATTTC CCTGTGCAGA CTAAAGAAGC TTCATGCTGT CAGAGCTCTG ATTTACCTCG CCGGCTTTCC   
  
  
- ATGCTCTTTC CTCTCATTGG CCGCTATGCA CAGAGAATAA CACTACACGA AACGGCCACG TTTCCACATA   
  
  
- ATTAGTTCAC GTAAGAACGA CTGAATATTC CGCACTTCCT AGTCCTCTTT CTTGTTGACT ACCCAAAAGC   
  
  
- CCAAAAGCCG ACAAGCAGTG TGTGTCCACA CGGTTTTGAT CTTTGAAACA TCGTGCCGTT TCATTCAACA   
  
  
- AATGCTGGAC TTTCGGCATC TATCCACTTT CTTCTGTTCC TCACCTGGAA AACAAAACAA GTTGCTCGTC   
  
  
- CATTGGATAA TTCATGATTA TTTATTTTTA AAAAAATAAA AAATTTGAAA TTTATTAAAA TAAAAATTAT   
  
  
- TTTAAAAATT TTAAAAAAAT AAAAATATAT TATCACAAAA AAATAATTAA TTAATCAAAA AATATTTAGA   
  
  
- TTCAAGGAAC AAAATCAAAA TAAAACGTGA AATTTATTTA ACTAATTTCT TAAGCATTTC AATGAAAGCA   
  
  
- TCGTTAATTT TGCATCATTG TTTTGATCAA ACTCTAATTT AAAAAATTAT AAATTAAAAT TTATTTTAAA   
  
  
- GTATTTTAAT TACAAAAAAT AAAATTAAAA TCCTAAATGA TGTGGTCTTG ATCTTTAATA ATAATATAAT   
  
  
- TAATCAAATA ATTTGCTTTA TCTGGTGCTT AATTTTATAA AATTAAAATC CTAAATGACG TGGTCTTGAT   
  
  
- CTTTAATAAT AATATAATTA ATCAAATAAT TTGCTTTATC TGGTGCTTAA TTTTTTATTT GTTTCATCAT   
  
  
- TGACGTGGCC GTTAATGAAA GGCCAAACAA ATTAATTAAG ATATTTAATC AAACAAGTAA TTATTTTCAT   
  
  
- GTACATTGTT TCATCATTGA CGTGGCCGTT AATGAAAGGC CAAACAAATT AATTAAGATA TTTAATCAAA   
  
  
- CAAGTAATTA TTTTCATGTA CTTTCTATGT TTGTTGGTCA TGGAAGAAGA TGAAATTTTA ATTTGACAAA   
  
  
- TTAAAAGTCT AGCAGAAATA TAAAAATTAA TGACAGACAT GCAATGATTT AGATTAGTAA TCTCTGGGGT   
  
  
- TGCACGTCAC GTGATGAAGT CATTAACAA

+     P-box

| Site Name | Organism | Position | Strand | Matrix score. | sequence | function |
| --- | --- | --- | --- | --- | --- | --- |
| P-box | Oryza sativa | 677 | + | 7 | CCTTTTG | gibberellin-responsive element |

> 2018/04/13 10:10:12  
+ ATTAACTTTC GCCGACGGAA TTCTTGACCC TCTTAAAGAC GTCTTATTCT TAGATGTTTC ATATAGTGAC   
  
  
+ CTGTGTTCGA CATAATGTTC ACTCGTCGCT AGAGGTTAAT CCAGTTCCCT TGCTCTCTAA GTATCGTTAT   
  
  
+ ATATCTTCTC TTTCTAGTGT ATTGAGTATT GCTCGACAGT TACCAGACAA CTTCCTTTCA GCGAGCTTTC   
  
  
+ TCCGGGTCCC TCTCTCTTTT TCTTTTTCTT TCAGACACAG GCACCCATGC AACTTATAAA CTTAAGGTTT   
  
  
+ CCTGCAGTTC CAGTTCGAAT CGTGCGCCTA TTTTAGGTGA AGCACATCAC CCATTTCGAA GTCTACGGTA   
  
  
+ AACTTTAAAG GGACACGTCT GATTTCTTCG AAGTACGACA GTCTCGAGAC TAAATGGAGC GGCCGAAAGG   
  
  
+ TACGAGAAAG GAGAGTAACC GGCGATACGT GTCTCTTATT GTGATGTGCT TTGCCGGTGC AAAGGTGTAT   
  
  
+ TAATCAAGTG CATTCTTGCT GACTTATAAG GCGTGAAGGA TCAGGAGAAA GAACAACTGA TGGGTTTTCG   
  
  
+ GGTTTTCGGC TGTTCGTCAC ACACAGGTGT GCCAAAACTA GAAACTTTGT AGCACGGCAA AGTAAGTTGT   
  
  
+ TTACGACCTG AAAGCCGTAG ATAGGTGAAA GAAGACAAGG AGTGGACCTT TTGTTTTGTT CAACGAGCAG   
  
  
+ GTAACCTATT AAGTACTAAT AAATAAAAAT TTTTTTATTT TTTAAACTTT AAATAATTTT ATTTTTAATA   
  
  
+ AAATTTTTAA AATTTTTTTA TTTTTATATA ATAGTGTTTT TTTATTAATT AATTAGTTTT TTATAAATCT   
  
  
+ AAGTTCCTTG TTTTAGTTTT ATTTTGCACT TTAAATAAAT TGATTAAAGA ATTCGTAAAG TTACTTTCGT   
  
  
+ AGCAATTAAA ACGTAGTAAC AAAACTAGTT TGAGATTAAA TTTTTTAATA TTTAATTTTA AATAAAATTT   
  
  
+ CATAAAATTA ATGTTTTTTA TTTTAATTTT AGGATTTACT ACACCAGAAC TAGAAATTAT TATTATATTA   
  
  
+ ATTAGTTTAT TAAACGAAAT AGACCACGAA TTAAAATATT TTAATTTTAG GATTTACTGC ACCAGAACTA   
  
  
+ GAAATTATTA TTATATTAAT TAGTTTATTA AACGAAATAG ACCACGAATT AAAAAATAAA CAAAGTAGTA   
  
  
+ ACTGCACCGG CAATTACTTT CCGGTTTGTT TAATTAATTC TATAAATTAG TTTGTTCATT AATAAAAGTA   
  
  
+ CATGTAACAA AGTAGTAACT GCACCGGCAA TTACTTTCCG GTTTGTTTAA TTAATTCTAT AAATTAGTTT   
  
  
+ GTTCATTAAT AAAAGTACAT GAAAGATACA AACAACCAGT ACCTTCTTCT ACTTTAAAAT TAAACTGTTT   
  
  
+ AATTTTCAGA TCGTCTTTAT ATTTTTAATT ACTGTCTGTA CGTTACTAAA TCTAATCATT AGAGACCCCA   
  
  
+ ACGTGCAGTG CACTACTTCA GTAATTGTT  

- TAATTGAAAG CGGCTGCCTT AAGAACTGGG AGAATTTCTG CAGAATAAGA ATCTACAAAG TATATCACTG   
  
  
- GACACAAGCT GTATTACAAG TGAGCAGCGA TCTCCAATTA GGTCAAGGGA ACGAGAGATT CATAGCAATA   
  
  
- TATAGAAGAG AAAGATCACA TAACTCATAA CGAGCTGTCA ATGGTCTGTT GAAGGAAAGT CGCTCGAAAG   
  
  
- AGGCCCAGGG AGAGAGAAAA AGAAAAAGAA AGTCTGTGTC CGTGGGTACG TTGAATATTT GAATTCCAAA   
  
  
- GGACGTCAAG GTCAAGCTTA GCACGCGGAT AAAATCCACT TCGTGTAGTG GGTAAAGCTT CAGATGCCAT   
  
  
- TTGAAATTTC CCTGTGCAGA CTAAAGAAGC TTCATGCTGT CAGAGCTCTG ATTTACCTCG CCGGCTTTCC   
  
  
- ATGCTCTTTC CTCTCATTGG CCGCTATGCA CAGAGAATAA CACTACACGA AACGGCCACG TTTCCACATA   
  
  
- ATTAGTTCAC GTAAGAACGA CTGAATATTC CGCACTTCCT AGTCCTCTTT CTTGTTGACT ACCCAAAAGC   
  
  
- CCAAAAGCCG ACAAGCAGTG TGTGTCCACA CGGTTTTGAT CTTTGAAACA TCGTGCCGTT TCATTCAACA   
  
  
- AATGCTGGAC TTTCGGCATC TATCCACTTT CTTCTGTTCC TCACCTGGAA AACAAAACAA GTTGCTCGTC   
  
  
- CATTGGATAA TTCATGATTA TTTATTTTTA AAAAAATAAA AAATTTGAAA TTTATTAAAA TAAAAATTAT   
  
  
- TTTAAAAATT TTAAAAAAAT AAAAATATAT TATCACAAAA AAATAATTAA TTAATCAAAA AATATTTAGA   
  
  
- TTCAAGGAAC AAAATCAAAA TAAAACGTGA AATTTATTTA ACTAATTTCT TAAGCATTTC AATGAAAGCA   
  
  
- TCGTTAATTT TGCATCATTG TTTTGATCAA ACTCTAATTT AAAAAATTAT AAATTAAAAT TTATTTTAAA   
  
  
- GTATTTTAAT TACAAAAAAT AAAATTAAAA TCCTAAATGA TGTGGTCTTG ATCTTTAATA ATAATATAAT   
  
  
- TAATCAAATA ATTTGCTTTA TCTGGTGCTT AATTTTATAA AATTAAAATC CTAAATGACG TGGTCTTGAT   
  
  
- CTTTAATAAT AATATAATTA ATCAAATAAT TTGCTTTATC TGGTGCTTAA TTTTTTATTT GTTTCATCAT   
  
  
- TGACGTGGCC GTTAATGAAA GGCCAAACAA ATTAATTAAG ATATTTAATC AAACAAGTAA TTATTTTCAT   
  
  
- GTACATTGTT TCATCATTGA CGTGGCCGTT AATGAAAGGC CAAACAAATT AATTAAGATA TTTAATCAAA   
  
  
- CAAGTAATTA TTTTCATGTA CTTTCTATGT TTGTTGGTCA TGGAAGAAGA TGAAATTTTA ATTTGACAAA   
  
  
- TTAAAAGTCT AGCAGAAATA TAAAAATTAA TGACAGACAT GCAATGATTT AGATTAGTAA TCTCTGGGGT   
  
  
- TGCACGTCAC GTGATGAAGT CATTAACAA

+     TATA-box

| Site Name | Organism | Position | Strand | Matrix score. | sequence | function |
| --- | --- | --- | --- | --- | --- | --- |
| TATA-box | Lycopersicon esculentum | 966 | + | 5 | TTTTA | core promoter element around -30 of transcription start |
| TATA-box | Glycine max | 956 | + | 5 | TAATA | core promoter element around -30 of transcription start |
| TATA-box | Lycopersicon esculentum | 775 | + | 5 | TTTTA | core promoter element around -30 of transcription start |
| TATA-box | Arabidopsis thaliana | 788 | - | 9 | TAAAAATAA | core promoter element around -30 of transcription start |
| TATA-box | Glycine max | 799 | + | 5 | TAATA | core promoter element around -30 of transcription start |
| TATA-box | Lycopersicon esculentum | 917 | - | 5 | TTTTA | core promoter element around -30 of transcription start |
| TATA-box | Lycopersicon esculentum | 778 | - | 5 | TTTTA | core promoter element around -30 of transcription start |
| TATA-box | Lycopersicon esculentum | 1089 | + | 5 | TTTTA | core promoter element around -30 of transcription start |
| TATA-box | Glycine max | 1046 | - | 5 | TAATA | core promoter element around -30 of transcription start |
| TATA-box | Arabidopsis thaliana | 1316 | + | 9 | tcTATATAtt | core promoter element around -30 of transcription start |
| TATA-box | Arabidopsis thaliana | 1044 | - | 4 | TATA | core promoter element around -30 of transcription start |
| TATA-box | Arabidopsis thaliana | 1043 | - | 5 | TATAA | core promoter element around -30 of transcription start |
| TATA-box | Arabidopsis thaliana | 967 | - | 8 | TATTTAAA | core promoter element around -30 of transcription start |
| TATA-box | Lycopersicon esculentum | 1082 | - | 5 | TTTTA | core promoter element around -30 of transcription start |
| TATA-box | Lycopersicon esculentum | 1170 | - | 5 | TTTTA | core promoter element around -30 of transcription start |
| TATA-box | Glycine max | 1041 | - | 5 | TAATA | core promoter element around -30 of transcription start |
| TATA-box | Glycine max | 1337 | + | 5 | TAATA | core promoter element around -30 of transcription start |
| TATA-box | Lycopersicon esculentum | 973 | - | 5 | TTTTA | core promoter element around -30 of transcription start |
| TATA-box | Arabidopsis thaliana | 870 | - | 8 | TATTTAAA | core promoter element around -30 of transcription start |
| TATA-box | Zea mays | 774 | - | 8 | TTTAAAAA | core promoter element around -30 of transcription start |
| TATA-box | Brassica oleracea | 796 | + | 7 | ATATAAT | core promoter element around -30 of transcription start |
| TATA-box | Glycine max | 1038 | - | 5 | TAATA | core promoter element around -30 of transcription start |
| TATA-box | Glycine max | 1126 | - | 5 | TAATA | core promoter element around -30 of transcription start |
| TATA-box | Arabidopsis thaliana | 1416 | - | 11 | TATAAATATAAA | core promoter element around -30 of transcription start |
| TATA-box | Glycine max | 1250 | + | 5 | TAATA | core promoter element around -30 of transcription start |
| TATA-box | Arabidopsis thaliana | 795 | - | 4 | TATA | core promoter element around -30 of transcription start |
| TATA-box | Glycine max | 707 | - | 5 | TAATA | core promoter element around -30 of transcription start |
| TATA-box | Lycopersicon esculentum | 953 | + | 5 | TTTTA | core promoter element around -30 of transcription start |
| TATA-box | Lycopersicon esculentum | 757 | + | 5 | TTTTA | core promoter element around -30 of transcription start |
| TATA-box | Lycopersicon esculentum | 996 | + | 5 | TTTTA | core promoter element around -30 of transcription start |
| TATA-box | Arabidopsis thaliana | 831 | - | 5 | TATAA | core promoter element around -30 of transcription start |
| TATA-box | Arabidopsis thaliana | 1414 | + | 9 | tcTATATAtt | core promoter element around -30 of transcription start |
| TATA-box | Arabidopsis thaliana | 793 | - | 6 | TATAAA | core promoter element around -30 of transcription start |
| TATA-box | Arabidopsis thaliana | 140 | + | 4 | TATA | core promoter element around -30 of transcription start |
| TATA-box | Brassica napus | 1042 | + | 6 | ATTATA | core promoter element around -30 of transcription start |
| TATA-box | Lycopersicon esculentum | 1253 | - | 5 | TTTTA | core promoter element around -30 of transcription start |
| TATA-box | Glycine max | 1134 | - | 5 | TAATA | core promoter element around -30 of transcription start |
| TATA-box | Glycine max | 1146 | - | 5 | TAATA | core promoter element around -30 of transcription start |
| TATA-box | Arabidopsis thaliana | 1131 | - | 5 | TATAA | core promoter element around -30 of transcription start |
| TATA-box | Lycopersicon esculentum | 983 | - | 5 | TTTTA | core promoter element around -30 of transcription start |
| TATA-box | Lycopersicon esculentum | 857 | + | 5 | TTTTA | core promoter element around -30 of transcription start |
| TATA-box | Lycopersicon esculentum | 1095 | + | 5 | TTTTA | core promoter element around -30 of transcription start |
| TATA-box | Glycine max | 1129 | - | 5 | TAATA | core promoter element around -30 of transcription start |
| TATA-box | Arabidopsis thaliana | 748 | - | 8 | TATTTAAA | core promoter element around -30 of transcription start |
| TATA-box | Lycopersicon esculentum | 724 | - | 5 | TTTTA | core promoter element around -30 of transcription start |
| TATA-box | Glycine max | 813 | - | 5 | TAATA | core promoter element around -30 of transcription start |
| TATA-box | Lycopersicon esculentum | 851 | + | 5 | TTTTA | core promoter element around -30 of transcription start |
| TATA-box | Lycopersicon esculentum | 1007 | + | 5 | TTTTA | core promoter element around -30 of transcription start |
| TATA-box | Arabidopsis thaliana | 787 | - | 11 | TATAAATATAAA | core promoter element around -30 of transcription start |
| TATA-box | Arabidopsis thaliana | 797 | - | 4 | TATA | core promoter element around -30 of transcription start |
| TATA-box | Glycine max | 717 | + | 5 | TAATA | core promoter element around -30 of transcription start |
| TATA-box | Lycopersicon esculentum | 769 | - | 5 | TTTTA | core promoter element around -30 of transcription start |
| TATA-box | Arabidopsis thaliana | 1417 | - | 5 | TATAA | core promoter element around -30 of transcription start |
| TATA-box | Lycopersicon esculentum | 763 | + | 5 | TTTTA | core promoter element around -30 of transcription start |
| TATA-box | Glycine max | 1058 | - | 5 | TAATA | core promoter element around -30 of transcription start |
| TATA-box | Zea mays | 739 | - | 8 | TTTAAAAA | core promoter element around -30 of transcription start |
| TATA-box | Ac | 1231 | + | 7 | TATAAAT | core promoter element around -30 of transcription start |
| TATA-box | Lycopersicon esculentum | 786 | + | 5 | TTTTA | core promoter element around -30 of transcription start |
| TATA-box | Lycopersicon esculentum | 1001 | + | 5 | TTTTA | core promoter element around -30 of transcription start |
| TATA-box | Lycopersicon esculentum | 733 | + | 5 | TTTTA | core promoter element around -30 of transcription start |
| TATA-box | Arabidopsis thaliana | 1132 | - | 4 | TATA | core promoter element around -30 of transcription start |
| TATA-box | Arabidopsis thaliana | 829 | - | 7 | TATAAAA | core promoter element around -30 of transcription start |
| TATA-box | Arabidopsis thaliana | 794 | - | 7 | TATATAA | core promoter element around -30 of transcription start |
| TATA-box | Arabidopsis thaliana | 264 | - | 5 | TATAA | core promoter element around -30 of transcription start |
| TATA-box | Brassica napus | 139 | + | 6 | ATATAT | core promoter element around -30 of transcription start |
| TATA-box | Ac | 1318 | + | 7 | TATAAAT | core promoter element around -30 of transcription start |
| TATA-box | Ac | 832 | + | 7 | TATAAAT | core promoter element around -30 of transcription start |
| TATA-box | Arabidopsis thaliana | 792 | - | 7 | TATAAAA | core promoter element around -30 of transcription start |
| TATA-box | Arabidopsis thaliana | 1229 | + | 9 | tcTATATAtt | core promoter element around -30 of transcription start |
| TATA-box | Lycopersicon esculentum | 810 | + | 5 | TTTTA | core promoter element around -30 of transcription start |
| TATA-box | Lycopersicon esculentum | 1423 | + | 5 | TTTTA | core promoter element around -30 of transcription start |
| TATA-box | Brassica napus | 1130 | + | 6 | ATTATA | core promoter element around -30 of transcription start |
| TATA-box | Arabidopsis thaliana | 759 | - | 9 | TAAAAATAA | core promoter element around -30 of transcription start |
| TATA-box | Lycopersicon esculentum | 1385 | - | 5 | TTTTA | core promoter element around -30 of transcription start |
| TATA-box | Lycopersicon esculentum | 1340 | - | 5 | TTTTA | core promoter element around -30 of transcription start |
| TATA-box | Arabidopsis thaliana | 138 | + | 4 | TATA | core promoter element around -30 of transcription start |
| TATA-box | Arabidopsis thaliana | 830 | - | 6 | TATAAA | core promoter element around -30 of transcription start |
| TATA-box | Arabidopsis thaliana | 515 | + | 4 | TATA | core promoter element around -30 of transcription start |
| TATA-box | Glycine max | 488 | - | 5 | TAATA | core promoter element around -30 of transcription start |
| TATA-box | Arabidopsis thaliana | 1418 | - | 4 | TATA | core promoter element around -30 of transcription start |
| TATA-box | Lycopersicon esculentum | 740 | + | 5 | TTTTA | core promoter element around -30 of transcription start |
| TATA-box | Glycine max | 766 | + | 5 | TAATA | core promoter element around -30 of transcription start |
| TATA-box | Arabidopsis thaliana | 62 | + | 4 | TATA | core promoter element around -30 of transcription start |
| TATA-box | Lycopersicon esculentum | 311 | + | 5 | TTTTA | core promoter element around -30 of transcription start |
| TATA-box | Arabidopsis thaliana | 265 | + | 6 | TATAAA | core promoter element around -30 of transcription start |
| TATA-box | Arabidopsis thaliana | 137 | - | 7 | TATATAA | core promoter element around -30 of transcription start |
| TATA-box | Arabidopsis thaliana | 514 | - | 5 | TATAA | core promoter element around -30 of transcription start |

> 2018/04/13 10:10:12  
+ ATTAACTTTC GCCGACGGAA TTCTTGACCC TCTTAAAGAC GTCTTATTCT TAGATGTTTC ATATAGTGAC   
  
  
+ CTGTGTTCGA CATAATGTTC ACTCGTCGCT AGAGGTTAAT CCAGTTCCCT TGCTCTCTAA GTATCGTTAT   
  
  
+ ATATCTTCTC TTTCTAGTGT ATTGAGTATT GCTCGACAGT TACCAGACAA CTTCCTTTCA GCGAGCTTTC   
  
  
+ TCCGGGTCCC TCTCTCTTTT TCTTTTTCTT TCAGACACAG GCACCCATGC AACTTATAAA CTTAAGGTTT   
  
  
+ CCTGCAGTTC CAGTTCGAAT CGTGCGCCTA TTTTAGGTGA AGCACATCAC CCATTTCGAA GTCTACGGTA   
  
  
+ AACTTTAAAG GGACACGTCT GATTTCTTCG AAGTACGACA GTCTCGAGAC TAAATGGAGC GGCCGAAAGG   
  
  
+ TACGAGAAAG GAGAGTAACC GGCGATACGT GTCTCTTATT GTGATGTGCT TTGCCGGTGC AAAGGTGTAT   
  
  
+ TAATCAAGTG CATTCTTGCT GACTTATAAG GCGTGAAGGA TCAGGAGAAA GAACAACTGA TGGGTTTTCG   
  
  
+ GGTTTTCGGC TGTTCGTCAC ACACAGGTGT GCCAAAACTA GAAACTTTGT AGCACGGCAA AGTAAGTTGT   
  
  
+ TTACGACCTG AAAGCCGTAG ATAGGTGAAA GAAGACAAGG AGTGGACCTT TTGTTTTGTT CAACGAGCAG   
  
  
+ GTAACCTATT AAGTACTAAT AAATAAAAAT TTTTTTATTT TTTAAACTTT AAATAATTTT ATTTTTAATA   
  
  
+ AAATTTTTAA AATTTTTTTA TTTTTATATA ATAGTGTTTT TTTATTAATT AATTAGTTTT TTATAAATCT   
  
  
+ AAGTTCCTTG TTTTAGTTTT ATTTTGCACT TTAAATAAAT TGATTAAAGA ATTCGTAAAG TTACTTTCGT   
  
  
+ AGCAATTAAA ACGTAGTAAC AAAACTAGTT TGAGATTAAA TTTTTTAATA TTTAATTTTA AATAAAATTT   
  
  
+ CATAAAATTA ATGTTTTTTA TTTTAATTTT AGGATTTACT ACACCAGAAC TAGAAATTAT TATTATATTA   
  
  
+ ATTAGTTTAT TAAACGAAAT AGACCACGAA TTAAAATATT TTAATTTTAG GATTTACTGC ACCAGAACTA   
  
  
+ GAAATTATTA TTATATTAAT TAGTTTATTA AACGAAATAG ACCACGAATT AAAAAATAAA CAAAGTAGTA   
  
  
+ ACTGCACCGG CAATTACTTT CCGGTTTGTT TAATTAATTC TATAAATTAG TTTGTTCATT AATAAAAGTA   
  
  
+ CATGTAACAA AGTAGTAACT GCACCGGCAA TTACTTTCCG GTTTGTTTAA TTAATTCTAT AAATTAGTTT   
  
  
+ GTTCATTAAT AAAAGTACAT GAAAGATACA AACAACCAGT ACCTTCTTCT ACTTTAAAAT TAAACTGTTT   
  
  
+ AATTTTCAGA TCGTCTTTAT ATTTTTAATT ACTGTCTGTA CGTTACTAAA TCTAATCATT AGAGACCCCA   
  
  
+ ACGTGCAGTG CACTACTTCA GTAATTGTT  

- TAATTGAAAG CGGCTGCCTT AAGAACTGGG AGAATTTCTG CAGAATAAGA ATCTACAAAG TATATCACTG   
  
  
- GACACAAGCT GTATTACAAG TGAGCAGCGA TCTCCAATTA GGTCAAGGGA ACGAGAGATT CATAGCAATA   
  
  
- TATAGAAGAG AAAGATCACA TAACTCATAA CGAGCTGTCA ATGGTCTGTT GAAGGAAAGT CGCTCGAAAG   
  
  
- AGGCCCAGGG AGAGAGAAAA AGAAAAAGAA AGTCTGTGTC CGTGGGTACG TTGAATATTT GAATTCCAAA   
  
  
- GGACGTCAAG GTCAAGCTTA GCACGCGGAT AAAATCCACT TCGTGTAGTG GGTAAAGCTT CAGATGCCAT   
  
  
- TTGAAATTTC CCTGTGCAGA CTAAAGAAGC TTCATGCTGT CAGAGCTCTG ATTTACCTCG CCGGCTTTCC   
  
  
- ATGCTCTTTC CTCTCATTGG CCGCTATGCA CAGAGAATAA CACTACACGA AACGGCCACG TTTCCACATA   
  
  
- ATTAGTTCAC GTAAGAACGA CTGAATATTC CGCACTTCCT AGTCCTCTTT CTTGTTGACT ACCCAAAAGC   
  
  
- CCAAAAGCCG ACAAGCAGTG TGTGTCCACA CGGTTTTGAT CTTTGAAACA TCGTGCCGTT TCATTCAACA   
  
  
- AATGCTGGAC TTTCGGCATC TATCCACTTT CTTCTGTTCC TCACCTGGAA AACAAAACAA GTTGCTCGTC   
  
  
- CATTGGATAA TTCATGATTA TTTATTTTTA AAAAAATAAA AAATTTGAAA TTTATTAAAA TAAAAATTAT   
  
  
- TTTAAAAATT TTAAAAAAAT AAAAATATAT TATCACAAAA AAATAATTAA TTAATCAAAA AATATTTAGA   
  
  
- TTCAAGGAAC AAAATCAAAA TAAAACGTGA AATTTATTTA ACTAATTTCT TAAGCATTTC AATGAAAGCA   
  
  
- TCGTTAATTT TGCATCATTG TTTTGATCAA ACTCTAATTT AAAAAATTAT AAATTAAAAT TTATTTTAAA   
  
  
- GTATTTTAAT TACAAAAAAT AAAATTAAAA TCCTAAATGA TGTGGTCTTG ATCTTTAATA ATAATATAAT   
  
  
- TAATCAAATA ATTTGCTTTA TCTGGTGCTT AATTTTATAA AATTAAAATC CTAAATGACG TGGTCTTGAT   
  
  
- CTTTAATAAT AATATAATTA ATCAAATAAT TTGCTTTATC TGGTGCTTAA TTTTTTATTT GTTTCATCAT   
  
  
- TGACGTGGCC GTTAATGAAA GGCCAAACAA ATTAATTAAG ATATTTAATC AAACAAGTAA TTATTTTCAT   
  
  
- GTACATTGTT TCATCATTGA CGTGGCCGTT AATGAAAGGC CAAACAAATT AATTAAGATA TTTAATCAAA   
  
  
- CAAGTAATTA TTTTCATGTA CTTTCTATGT TTGTTGGTCA TGGAAGAAGA TGAAATTTTA ATTTGACAAA   
  
  
- TTAAAAGTCT AGCAGAAATA TAAAAATTAA TGACAGACAT GCAATGATTT AGATTAGTAA TCTCTGGGGT   
  
  
- TGCACGTCAC GTGATGAAGT CATTAACAA

+     TC-rich repeats

| Site Name | Organism | Position | Strand | Matrix score. | sequence | function |
| --- | --- | --- | --- | --- | --- | --- |
| TC-rich repeats | Nicotiana tabacum | 926 | - | 9 | GTTTTCTTAC | cis-acting element involved in defense and stress responsiveness |

> 2018/04/13 10:10:12  
+ ATTAACTTTC GCCGACGGAA TTCTTGACCC TCTTAAAGAC GTCTTATTCT TAGATGTTTC ATATAGTGAC   
  
  
+ CTGTGTTCGA CATAATGTTC ACTCGTCGCT AGAGGTTAAT CCAGTTCCCT TGCTCTCTAA GTATCGTTAT   
  
  
+ ATATCTTCTC TTTCTAGTGT ATTGAGTATT GCTCGACAGT TACCAGACAA CTTCCTTTCA GCGAGCTTTC   
  
  
+ TCCGGGTCCC TCTCTCTTTT TCTTTTTCTT TCAGACACAG GCACCCATGC AACTTATAAA CTTAAGGTTT   
  
  
+ CCTGCAGTTC CAGTTCGAAT CGTGCGCCTA TTTTAGGTGA AGCACATCAC CCATTTCGAA GTCTACGGTA   
  
  
+ AACTTTAAAG GGACACGTCT GATTTCTTCG AAGTACGACA GTCTCGAGAC TAAATGGAGC GGCCGAAAGG   
  
  
+ TACGAGAAAG GAGAGTAACC GGCGATACGT GTCTCTTATT GTGATGTGCT TTGCCGGTGC AAAGGTGTAT   
  
  
+ TAATCAAGTG CATTCTTGCT GACTTATAAG GCGTGAAGGA TCAGGAGAAA GAACAACTGA TGGGTTTTCG   
  
  
+ GGTTTTCGGC TGTTCGTCAC ACACAGGTGT GCCAAAACTA GAAACTTTGT AGCACGGCAA AGTAAGTTGT   
  
  
+ TTACGACCTG AAAGCCGTAG ATAGGTGAAA GAAGACAAGG AGTGGACCTT TTGTTTTGTT CAACGAGCAG   
  
  
+ GTAACCTATT AAGTACTAAT AAATAAAAAT TTTTTTATTT TTTAAACTTT AAATAATTTT ATTTTTAATA   
  
  
+ AAATTTTTAA AATTTTTTTA TTTTTATATA ATAGTGTTTT TTTATTAATT AATTAGTTTT TTATAAATCT   
  
  
+ AAGTTCCTTG TTTTAGTTTT ATTTTGCACT TTAAATAAAT TGATTAAAGA ATTCGTAAAG TTACTTTCGT   
  
  
+ AGCAATTAAA ACGTAGTAAC AAAACTAGTT TGAGATTAAA TTTTTTAATA TTTAATTTTA AATAAAATTT   
  
  
+ CATAAAATTA ATGTTTTTTA TTTTAATTTT AGGATTTACT ACACCAGAAC TAGAAATTAT TATTATATTA   
  
  
+ ATTAGTTTAT TAAACGAAAT AGACCACGAA TTAAAATATT TTAATTTTAG GATTTACTGC ACCAGAACTA   
  
  
+ GAAATTATTA TTATATTAAT TAGTTTATTA AACGAAATAG ACCACGAATT AAAAAATAAA CAAAGTAGTA   
  
  
+ ACTGCACCGG CAATTACTTT CCGGTTTGTT TAATTAATTC TATAAATTAG TTTGTTCATT AATAAAAGTA   
  
  
+ CATGTAACAA AGTAGTAACT GCACCGGCAA TTACTTTCCG GTTTGTTTAA TTAATTCTAT AAATTAGTTT   
  
  
+ GTTCATTAAT AAAAGTACAT GAAAGATACA AACAACCAGT ACCTTCTTCT ACTTTAAAAT TAAACTGTTT   
  
  
+ AATTTTCAGA TCGTCTTTAT ATTTTTAATT ACTGTCTGTA CGTTACTAAA TCTAATCATT AGAGACCCCA   
  
  
+ ACGTGCAGTG CACTACTTCA GTAATTGTT  

- TAATTGAAAG CGGCTGCCTT AAGAACTGGG AGAATTTCTG CAGAATAAGA ATCTACAAAG TATATCACTG   
  
  
- GACACAAGCT GTATTACAAG TGAGCAGCGA TCTCCAATTA GGTCAAGGGA ACGAGAGATT CATAGCAATA   
  
  
- TATAGAAGAG AAAGATCACA TAACTCATAA CGAGCTGTCA ATGGTCTGTT GAAGGAAAGT CGCTCGAAAG   
  
  
- AGGCCCAGGG AGAGAGAAAA AGAAAAAGAA AGTCTGTGTC CGTGGGTACG TTGAATATTT GAATTCCAAA   
  
  
- GGACGTCAAG GTCAAGCTTA GCACGCGGAT AAAATCCACT TCGTGTAGTG GGTAAAGCTT CAGATGCCAT   
  
  
- TTGAAATTTC CCTGTGCAGA CTAAAGAAGC TTCATGCTGT CAGAGCTCTG ATTTACCTCG CCGGCTTTCC   
  
  
- ATGCTCTTTC CTCTCATTGG CCGCTATGCA CAGAGAATAA CACTACACGA AACGGCCACG TTTCCACATA   
  
  
- ATTAGTTCAC GTAAGAACGA CTGAATATTC CGCACTTCCT AGTCCTCTTT CTTGTTGACT ACCCAAAAGC   
  
  
- CCAAAAGCCG ACAAGCAGTG TGTGTCCACA CGGTTTTGAT CTTTGAAACA TCGTGCCGTT TCATTCAACA   
  
  
- AATGCTGGAC TTTCGGCATC TATCCACTTT CTTCTGTTCC TCACCTGGAA AACAAAACAA GTTGCTCGTC   
  
  
- CATTGGATAA TTCATGATTA TTTATTTTTA AAAAAATAAA AAATTTGAAA TTTATTAAAA TAAAAATTAT   
  
  
- TTTAAAAATT TTAAAAAAAT AAAAATATAT TATCACAAAA AAATAATTAA TTAATCAAAA AATATTTAGA   
  
  
- TTCAAGGAAC AAAATCAAAA TAAAACGTGA AATTTATTTA ACTAATTTCT TAAGCATTTC AATGAAAGCA   
  
  
- TCGTTAATTT TGCATCATTG TTTTGATCAA ACTCTAATTT AAAAAATTAT AAATTAAAAT TTATTTTAAA   
  
  
- GTATTTTAAT TACAAAAAAT AAAATTAAAA TCCTAAATGA TGTGGTCTTG ATCTTTAATA ATAATATAAT   
  
  
- TAATCAAATA ATTTGCTTTA TCTGGTGCTT AATTTTATAA AATTAAAATC CTAAATGACG TGGTCTTGAT   
  
  
- CTTTAATAAT AATATAATTA ATCAAATAAT TTGCTTTATC TGGTGCTTAA TTTTTTATTT GTTTCATCAT   
  
  
- TGACGTGGCC GTTAATGAAA GGCCAAACAA ATTAATTAAG ATATTTAATC AAACAAGTAA TTATTTTCAT   
  
  
- GTACATTGTT TCATCATTGA CGTGGCCGTT AATGAAAGGC CAAACAAATT AATTAAGATA TTTAATCAAA   
  
  
- CAAGTAATTA TTTTCATGTA CTTTCTATGT TTGTTGGTCA TGGAAGAAGA TGAAATTTTA ATTTGACAAA   
  
  
- TTAAAAGTCT AGCAGAAATA TAAAAATTAA TGACAGACAT GCAATGATTT AGATTAGTAA TCTCTGGGGT   
  
  
- TGCACGTCAC GTGATGAAGT CATTAACAA

+     TGACG-motif

| Site Name | Organism | Position | Strand | Matrix score. | sequence | function |
| --- | --- | --- | --- | --- | --- | --- |
| TGACG-motif | Hordeum vulgare | 575 | - | 5 | TGACG | cis-acting regulatory element involved in the MeJA-responsiveness |

> 2018/04/13 10:10:12  
+ ATTAACTTTC GCCGACGGAA TTCTTGACCC TCTTAAAGAC GTCTTATTCT TAGATGTTTC ATATAGTGAC   
  
  
+ CTGTGTTCGA CATAATGTTC ACTCGTCGCT AGAGGTTAAT CCAGTTCCCT TGCTCTCTAA GTATCGTTAT   
  
  
+ ATATCTTCTC TTTCTAGTGT ATTGAGTATT GCTCGACAGT TACCAGACAA CTTCCTTTCA GCGAGCTTTC   
  
  
+ TCCGGGTCCC TCTCTCTTTT TCTTTTTCTT TCAGACACAG GCACCCATGC AACTTATAAA CTTAAGGTTT   
  
  
+ CCTGCAGTTC CAGTTCGAAT CGTGCGCCTA TTTTAGGTGA AGCACATCAC CCATTTCGAA GTCTACGGTA   
  
  
+ AACTTTAAAG GGACACGTCT GATTTCTTCG AAGTACGACA GTCTCGAGAC TAAATGGAGC GGCCGAAAGG   
  
  
+ TACGAGAAAG GAGAGTAACC GGCGATACGT GTCTCTTATT GTGATGTGCT TTGCCGGTGC AAAGGTGTAT   
  
  
+ TAATCAAGTG CATTCTTGCT GACTTATAAG GCGTGAAGGA TCAGGAGAAA GAACAACTGA TGGGTTTTCG   
  
  
+ GGTTTTCGGC TGTTCGTCAC ACACAGGTGT GCCAAAACTA GAAACTTTGT AGCACGGCAA AGTAAGTTGT   
  
  
+ TTACGACCTG AAAGCCGTAG ATAGGTGAAA GAAGACAAGG AGTGGACCTT TTGTTTTGTT CAACGAGCAG   
  
  
+ GTAACCTATT AAGTACTAAT AAATAAAAAT TTTTTTATTT TTTAAACTTT AAATAATTTT ATTTTTAATA   
  
  
+ AAATTTTTAA AATTTTTTTA TTTTTATATA ATAGTGTTTT TTTATTAATT AATTAGTTTT TTATAAATCT   
  
  
+ AAGTTCCTTG TTTTAGTTTT ATTTTGCACT TTAAATAAAT TGATTAAAGA ATTCGTAAAG TTACTTTCGT   
  
  
+ AGCAATTAAA ACGTAGTAAC AAAACTAGTT TGAGATTAAA TTTTTTAATA TTTAATTTTA AATAAAATTT   
  
  
+ CATAAAATTA ATGTTTTTTA TTTTAATTTT AGGATTTACT ACACCAGAAC TAGAAATTAT TATTATATTA   
  
  
+ ATTAGTTTAT TAAACGAAAT AGACCACGAA TTAAAATATT TTAATTTTAG GATTTACTGC ACCAGAACTA   
  
  
+ GAAATTATTA TTATATTAAT TAGTTTATTA AACGAAATAG ACCACGAATT AAAAAATAAA CAAAGTAGTA   
  
  
+ ACTGCACCGG CAATTACTTT CCGGTTTGTT TAATTAATTC TATAAATTAG TTTGTTCATT AATAAAAGTA   
  
  
+ CATGTAACAA AGTAGTAACT GCACCGGCAA TTACTTTCCG GTTTGTTTAA TTAATTCTAT AAATTAGTTT   
  
  
+ GTTCATTAAT AAAAGTACAT GAAAGATACA AACAACCAGT ACCTTCTTCT ACTTTAAAAT TAAACTGTTT   
  
  
+ AATTTTCAGA TCGTCTTTAT ATTTTTAATT ACTGTCTGTA CGTTACTAAA TCTAATCATT AGAGACCCCA   
  
  
+ ACGTGCAGTG CACTACTTCA GTAATTGTT  

- TAATTGAAAG CGGCTGCCTT AAGAACTGGG AGAATTTCTG CAGAATAAGA ATCTACAAAG TATATCACTG   
  
  
- GACACAAGCT GTATTACAAG TGAGCAGCGA TCTCCAATTA GGTCAAGGGA ACGAGAGATT CATAGCAATA   
  
  
- TATAGAAGAG AAAGATCACA TAACTCATAA CGAGCTGTCA ATGGTCTGTT GAAGGAAAGT CGCTCGAAAG   
  
  
- AGGCCCAGGG AGAGAGAAAA AGAAAAAGAA AGTCTGTGTC CGTGGGTACG TTGAATATTT GAATTCCAAA   
  
  
- GGACGTCAAG GTCAAGCTTA GCACGCGGAT AAAATCCACT TCGTGTAGTG GGTAAAGCTT CAGATGCCAT   
  
  
- TTGAAATTTC CCTGTGCAGA CTAAAGAAGC TTCATGCTGT CAGAGCTCTG ATTTACCTCG CCGGCTTTCC   
  
  
- ATGCTCTTTC CTCTCATTGG CCGCTATGCA CAGAGAATAA CACTACACGA AACGGCCACG TTTCCACATA   
  
  
- ATTAGTTCAC GTAAGAACGA CTGAATATTC CGCACTTCCT AGTCCTCTTT CTTGTTGACT ACCCAAAAGC   
  
  
- CCAAAAGCCG ACAAGCAGTG TGTGTCCACA CGGTTTTGAT CTTTGAAACA TCGTGCCGTT TCATTCAACA   
  
  
- AATGCTGGAC TTTCGGCATC TATCCACTTT CTTCTGTTCC TCACCTGGAA AACAAAACAA GTTGCTCGTC   
  
  
- CATTGGATAA TTCATGATTA TTTATTTTTA AAAAAATAAA AAATTTGAAA TTTATTAAAA TAAAAATTAT   
  
  
- TTTAAAAATT TTAAAAAAAT AAAAATATAT TATCACAAAA AAATAATTAA TTAATCAAAA AATATTTAGA   
  
  
- TTCAAGGAAC AAAATCAAAA TAAAACGTGA AATTTATTTA ACTAATTTCT TAAGCATTTC AATGAAAGCA   
  
  
- TCGTTAATTT TGCATCATTG TTTTGATCAA ACTCTAATTT AAAAAATTAT AAATTAAAAT TTATTTTAAA   
  
  
- GTATTTTAAT TACAAAAAAT AAAATTAAAA TCCTAAATGA TGTGGTCTTG ATCTTTAATA ATAATATAAT   
  
  
- TAATCAAATA ATTTGCTTTA TCTGGTGCTT AATTTTATAA AATTAAAATC CTAAATGACG TGGTCTTGAT   
  
  
- CTTTAATAAT AATATAATTA ATCAAATAAT TTGCTTTATC TGGTGCTTAA TTTTTTATTT GTTTCATCAT   
  
  
- TGACGTGGCC GTTAATGAAA GGCCAAACAA ATTAATTAAG ATATTTAATC AAACAAGTAA TTATTTTCAT   
  
  
- GTACATTGTT TCATCATTGA CGTGGCCGTT AATGAAAGGC CAAACAAATT AATTAAGATA TTTAATCAAA   
  
  
- CAAGTAATTA TTTTCATGTA CTTTCTATGT TTGTTGGTCA TGGAAGAAGA TGAAATTTTA ATTTGACAAA   
  
  
- TTAAAAGTCT AGCAGAAATA TAAAAATTAA TGACAGACAT GCAATGATTT AGATTAGTAA TCTCTGGGGT   
  
  
- TGCACGTCAC GTGATGAAGT CATTAACAA

+     Unnamed\_\_1

| Site Name | Organism | Position | Strand | Matrix score. | sequence | function |
| --- | --- | --- | --- | --- | --- | --- |
| Unnamed\_\_1 | Zea mays | 1162 | - | 5 | CGTGG |  |
| Unnamed\_\_1 | Zea mays | 1074 | - | 5 | CGTGG |  |

> 2018/04/13 10:10:12  
+ ATTAACTTTC GCCGACGGAA TTCTTGACCC TCTTAAAGAC GTCTTATTCT TAGATGTTTC ATATAGTGAC   
  
  
+ CTGTGTTCGA CATAATGTTC ACTCGTCGCT AGAGGTTAAT CCAGTTCCCT TGCTCTCTAA GTATCGTTAT   
  
  
+ ATATCTTCTC TTTCTAGTGT ATTGAGTATT GCTCGACAGT TACCAGACAA CTTCCTTTCA GCGAGCTTTC   
  
  
+ TCCGGGTCCC TCTCTCTTTT TCTTTTTCTT TCAGACACAG GCACCCATGC AACTTATAAA CTTAAGGTTT   
  
  
+ CCTGCAGTTC CAGTTCGAAT CGTGCGCCTA TTTTAGGTGA AGCACATCAC CCATTTCGAA GTCTACGGTA   
  
  
+ AACTTTAAAG GGACACGTCT GATTTCTTCG AAGTACGACA GTCTCGAGAC TAAATGGAGC GGCCGAAAGG   
  
  
+ TACGAGAAAG GAGAGTAACC GGCGATACGT GTCTCTTATT GTGATGTGCT TTGCCGGTGC AAAGGTGTAT   
  
  
+ TAATCAAGTG CATTCTTGCT GACTTATAAG GCGTGAAGGA TCAGGAGAAA GAACAACTGA TGGGTTTTCG   
  
  
+ GGTTTTCGGC TGTTCGTCAC ACACAGGTGT GCCAAAACTA GAAACTTTGT AGCACGGCAA AGTAAGTTGT   
  
  
+ TTACGACCTG AAAGCCGTAG ATAGGTGAAA GAAGACAAGG AGTGGACCTT TTGTTTTGTT CAACGAGCAG   
  
  
+ GTAACCTATT AAGTACTAAT AAATAAAAAT TTTTTTATTT TTTAAACTTT AAATAATTTT ATTTTTAATA   
  
  
+ AAATTTTTAA AATTTTTTTA TTTTTATATA ATAGTGTTTT TTTATTAATT AATTAGTTTT TTATAAATCT   
  
  
+ AAGTTCCTTG TTTTAGTTTT ATTTTGCACT TTAAATAAAT TGATTAAAGA ATTCGTAAAG TTACTTTCGT   
  
  
+ AGCAATTAAA ACGTAGTAAC AAAACTAGTT TGAGATTAAA TTTTTTAATA TTTAATTTTA AATAAAATTT   
  
  
+ CATAAAATTA ATGTTTTTTA TTTTAATTTT AGGATTTACT ACACCAGAAC TAGAAATTAT TATTATATTA   
  
  
+ ATTAGTTTAT TAAACGAAAT AGACCACGAA TTAAAATATT TTAATTTTAG GATTTACTGC ACCAGAACTA   
  
  
+ GAAATTATTA TTATATTAAT TAGTTTATTA AACGAAATAG ACCACGAATT AAAAAATAAA CAAAGTAGTA   
  
  
+ ACTGCACCGG CAATTACTTT CCGGTTTGTT TAATTAATTC TATAAATTAG TTTGTTCATT AATAAAAGTA   
  
  
+ CATGTAACAA AGTAGTAACT GCACCGGCAA TTACTTTCCG GTTTGTTTAA TTAATTCTAT AAATTAGTTT   
  
  
+ GTTCATTAAT AAAAGTACAT GAAAGATACA AACAACCAGT ACCTTCTTCT ACTTTAAAAT TAAACTGTTT   
  
  
+ AATTTTCAGA TCGTCTTTAT ATTTTTAATT ACTGTCTGTA CGTTACTAAA TCTAATCATT AGAGACCCCA   
  
  
+ ACGTGCAGTG CACTACTTCA GTAATTGTT  

- TAATTGAAAG CGGCTGCCTT AAGAACTGGG AGAATTTCTG CAGAATAAGA ATCTACAAAG TATATCACTG   
  
  
- GACACAAGCT GTATTACAAG TGAGCAGCGA TCTCCAATTA GGTCAAGGGA ACGAGAGATT CATAGCAATA   
  
  
- TATAGAAGAG AAAGATCACA TAACTCATAA CGAGCTGTCA ATGGTCTGTT GAAGGAAAGT CGCTCGAAAG   
  
  
- AGGCCCAGGG AGAGAGAAAA AGAAAAAGAA AGTCTGTGTC CGTGGGTACG TTGAATATTT GAATTCCAAA   
  
  
- GGACGTCAAG GTCAAGCTTA GCACGCGGAT AAAATCCACT TCGTGTAGTG GGTAAAGCTT CAGATGCCAT   
  
  
- TTGAAATTTC CCTGTGCAGA CTAAAGAAGC TTCATGCTGT CAGAGCTCTG ATTTACCTCG CCGGCTTTCC   
  
  
- ATGCTCTTTC CTCTCATTGG CCGCTATGCA CAGAGAATAA CACTACACGA AACGGCCACG TTTCCACATA   
  
  
- ATTAGTTCAC GTAAGAACGA CTGAATATTC CGCACTTCCT AGTCCTCTTT CTTGTTGACT ACCCAAAAGC   
  
  
- CCAAAAGCCG ACAAGCAGTG TGTGTCCACA CGGTTTTGAT CTTTGAAACA TCGTGCCGTT TCATTCAACA   
  
  
- AATGCTGGAC TTTCGGCATC TATCCACTTT CTTCTGTTCC TCACCTGGAA AACAAAACAA GTTGCTCGTC   
  
  
- CATTGGATAA TTCATGATTA TTTATTTTTA AAAAAATAAA AAATTTGAAA TTTATTAAAA TAAAAATTAT   
  
  
- TTTAAAAATT TTAAAAAAAT AAAAATATAT TATCACAAAA AAATAATTAA TTAATCAAAA AATATTTAGA   
  
  
- TTCAAGGAAC AAAATCAAAA TAAAACGTGA AATTTATTTA ACTAATTTCT TAAGCATTTC AATGAAAGCA   
  
  
- TCGTTAATTT TGCATCATTG TTTTGATCAA ACTCTAATTT AAAAAATTAT AAATTAAAAT TTATTTTAAA   
  
  
- GTATTTTAAT TACAAAAAAT AAAATTAAAA TCCTAAATGA TGTGGTCTTG ATCTTTAATA ATAATATAAT   
  
  
- TAATCAAATA ATTTGCTTTA TCTGGTGCTT AATTTTATAA AATTAAAATC CTAAATGACG TGGTCTTGAT   
  
  
- CTTTAATAAT AATATAATTA ATCAAATAAT TTGCTTTATC TGGTGCTTAA TTTTTTATTT GTTTCATCAT   
  
  
- TGACGTGGCC GTTAATGAAA GGCCAAACAA ATTAATTAAG ATATTTAATC AAACAAGTAA TTATTTTCAT   
  
  
- GTACATTGTT TCATCATTGA CGTGGCCGTT AATGAAAGGC CAAACAAATT AATTAAGATA TTTAATCAAA   
  
  
- CAAGTAATTA TTTTCATGTA CTTTCTATGT TTGTTGGTCA TGGAAGAAGA TGAAATTTTA ATTTGACAAA   
  
  
- TTAAAAGTCT AGCAGAAATA TAAAAATTAA TGACAGACAT GCAATGATTT AGATTAGTAA TCTCTGGGGT   
  
  
- TGCACGTCAC GTGATGAAGT CATTAACAA

+     Unnamed\_\_3

| Site Name | Organism | Position | Strand | Matrix score. | sequence | function |
| --- | --- | --- | --- | --- | --- | --- |
| Unnamed\_\_3 | Zea mays | 1074 | - | 5 | CGTGG |  |
| Unnamed\_\_3 | Zea mays | 1162 | - | 5 | CGTGG |  |

> 2018/04/13 10:10:12  
+ ATTAACTTTC GCCGACGGAA TTCTTGACCC TCTTAAAGAC GTCTTATTCT TAGATGTTTC ATATAGTGAC   
  
  
+ CTGTGTTCGA CATAATGTTC ACTCGTCGCT AGAGGTTAAT CCAGTTCCCT TGCTCTCTAA GTATCGTTAT   
  
  
+ ATATCTTCTC TTTCTAGTGT ATTGAGTATT GCTCGACAGT TACCAGACAA CTTCCTTTCA GCGAGCTTTC   
  
  
+ TCCGGGTCCC TCTCTCTTTT TCTTTTTCTT TCAGACACAG GCACCCATGC AACTTATAAA CTTAAGGTTT   
  
  
+ CCTGCAGTTC CAGTTCGAAT CGTGCGCCTA TTTTAGGTGA AGCACATCAC CCATTTCGAA GTCTACGGTA   
  
  
+ AACTTTAAAG GGACACGTCT GATTTCTTCG AAGTACGACA GTCTCGAGAC TAAATGGAGC GGCCGAAAGG   
  
  
+ TACGAGAAAG GAGAGTAACC GGCGATACGT GTCTCTTATT GTGATGTGCT TTGCCGGTGC AAAGGTGTAT   
  
  
+ TAATCAAGTG CATTCTTGCT GACTTATAAG GCGTGAAGGA TCAGGAGAAA GAACAACTGA TGGGTTTTCG   
  
  
+ GGTTTTCGGC TGTTCGTCAC ACACAGGTGT GCCAAAACTA GAAACTTTGT AGCACGGCAA AGTAAGTTGT   
  
  
+ TTACGACCTG AAAGCCGTAG ATAGGTGAAA GAAGACAAGG AGTGGACCTT TTGTTTTGTT CAACGAGCAG   
  
  
+ GTAACCTATT AAGTACTAAT AAATAAAAAT TTTTTTATTT TTTAAACTTT AAATAATTTT ATTTTTAATA   
  
  
+ AAATTTTTAA AATTTTTTTA TTTTTATATA ATAGTGTTTT TTTATTAATT AATTAGTTTT TTATAAATCT   
  
  
+ AAGTTCCTTG TTTTAGTTTT ATTTTGCACT TTAAATAAAT TGATTAAAGA ATTCGTAAAG TTACTTTCGT   
  
  
+ AGCAATTAAA ACGTAGTAAC AAAACTAGTT TGAGATTAAA TTTTTTAATA TTTAATTTTA AATAAAATTT   
  
  
+ CATAAAATTA ATGTTTTTTA TTTTAATTTT AGGATTTACT ACACCAGAAC TAGAAATTAT TATTATATTA   
  
  
+ ATTAGTTTAT TAAACGAAAT AGACCACGAA TTAAAATATT TTAATTTTAG GATTTACTGC ACCAGAACTA   
  
  
+ GAAATTATTA TTATATTAAT TAGTTTATTA AACGAAATAG ACCACGAATT AAAAAATAAA CAAAGTAGTA   
  
  
+ ACTGCACCGG CAATTACTTT CCGGTTTGTT TAATTAATTC TATAAATTAG TTTGTTCATT AATAAAAGTA   
  
  
+ CATGTAACAA AGTAGTAACT GCACCGGCAA TTACTTTCCG GTTTGTTTAA TTAATTCTAT AAATTAGTTT   
  
  
+ GTTCATTAAT AAAAGTACAT GAAAGATACA AACAACCAGT ACCTTCTTCT ACTTTAAAAT TAAACTGTTT   
  
  
+ AATTTTCAGA TCGTCTTTAT ATTTTTAATT ACTGTCTGTA CGTTACTAAA TCTAATCATT AGAGACCCCA   
  
  
+ ACGTGCAGTG CACTACTTCA GTAATTGTT  

- TAATTGAAAG CGGCTGCCTT AAGAACTGGG AGAATTTCTG CAGAATAAGA ATCTACAAAG TATATCACTG   
  
  
- GACACAAGCT GTATTACAAG TGAGCAGCGA TCTCCAATTA GGTCAAGGGA ACGAGAGATT CATAGCAATA   
  
  
- TATAGAAGAG AAAGATCACA TAACTCATAA CGAGCTGTCA ATGGTCTGTT GAAGGAAAGT CGCTCGAAAG   
  
  
- AGGCCCAGGG AGAGAGAAAA AGAAAAAGAA AGTCTGTGTC CGTGGGTACG TTGAATATTT GAATTCCAAA   
  
  
- GGACGTCAAG GTCAAGCTTA GCACGCGGAT AAAATCCACT TCGTGTAGTG GGTAAAGCTT CAGATGCCAT   
  
  
- TTGAAATTTC CCTGTGCAGA CTAAAGAAGC TTCATGCTGT CAGAGCTCTG ATTTACCTCG CCGGCTTTCC   
  
  
- ATGCTCTTTC CTCTCATTGG CCGCTATGCA CAGAGAATAA CACTACACGA AACGGCCACG TTTCCACATA   
  
  
- ATTAGTTCAC GTAAGAACGA CTGAATATTC CGCACTTCCT AGTCCTCTTT CTTGTTGACT ACCCAAAAGC   
  
  
- CCAAAAGCCG ACAAGCAGTG TGTGTCCACA CGGTTTTGAT CTTTGAAACA TCGTGCCGTT TCATTCAACA   
  
  
- AATGCTGGAC TTTCGGCATC TATCCACTTT CTTCTGTTCC TCACCTGGAA AACAAAACAA GTTGCTCGTC   
  
  
- CATTGGATAA TTCATGATTA TTTATTTTTA AAAAAATAAA AAATTTGAAA TTTATTAAAA TAAAAATTAT   
  
  
- TTTAAAAATT TTAAAAAAAT AAAAATATAT TATCACAAAA AAATAATTAA TTAATCAAAA AATATTTAGA   
  
  
- TTCAAGGAAC AAAATCAAAA TAAAACGTGA AATTTATTTA ACTAATTTCT TAAGCATTTC AATGAAAGCA   
  
  
- TCGTTAATTT TGCATCATTG TTTTGATCAA ACTCTAATTT AAAAAATTAT AAATTAAAAT TTATTTTAAA   
  
  
- GTATTTTAAT TACAAAAAAT AAAATTAAAA TCCTAAATGA TGTGGTCTTG ATCTTTAATA ATAATATAAT   
  
  
- TAATCAAATA ATTTGCTTTA TCTGGTGCTT AATTTTATAA AATTAAAATC CTAAATGACG TGGTCTTGAT   
  
  
- CTTTAATAAT AATATAATTA ATCAAATAAT TTGCTTTATC TGGTGCTTAA TTTTTTATTT GTTTCATCAT   
  
  
- TGACGTGGCC GTTAATGAAA GGCCAAACAA ATTAATTAAG ATATTTAATC AAACAAGTAA TTATTTTCAT   
  
  
- GTACATTGTT TCATCATTGA CGTGGCCGTT AATGAAAGGC CAAACAAATT AATTAAGATA TTTAATCAAA   
  
  
- CAAGTAATTA TTTTCATGTA CTTTCTATGT TTGTTGGTCA TGGAAGAAGA TGAAATTTTA ATTTGACAAA   
  
  
- TTAAAAGTCT AGCAGAAATA TAAAAATTAA TGACAGACAT GCAATGATTT AGATTAGTAA TCTCTGGGGT   
  
  
- TGCACGTCAC GTGATGAAGT CATTAACAA

+     Unnamed\_\_4

| Site Name | Organism | Position | Strand | Matrix score. | sequence | function |
| --- | --- | --- | --- | --- | --- | --- |
| Unnamed\_\_4 | Petroselinum hortense | 534 | - | 4 | CTCC |  |
| Unnamed\_\_4 | Petroselinum hortense | 430 | - | 4 | CTCC |  |
| Unnamed\_\_4 | Petroselinum hortense | 406 | - | 4 | CTCC |  |
| Unnamed\_\_4 | Petroselinum hortense | 669 | - | 4 | CTCC |  |
| Unnamed\_\_4 | Petroselinum hortense | 210 | + | 4 | CTCC |  |

> 2018/04/13 10:10:12  
+ ATTAACTTTC GCCGACGGAA TTCTTGACCC TCTTAAAGAC GTCTTATTCT TAGATGTTTC ATATAGTGAC   
  
  
+ CTGTGTTCGA CATAATGTTC ACTCGTCGCT AGAGGTTAAT CCAGTTCCCT TGCTCTCTAA GTATCGTTAT   
  
  
+ ATATCTTCTC TTTCTAGTGT ATTGAGTATT GCTCGACAGT TACCAGACAA CTTCCTTTCA GCGAGCTTTC   
  
  
+ TCCGGGTCCC TCTCTCTTTT TCTTTTTCTT TCAGACACAG GCACCCATGC AACTTATAAA CTTAAGGTTT   
  
  
+ CCTGCAGTTC CAGTTCGAAT CGTGCGCCTA TTTTAGGTGA AGCACATCAC CCATTTCGAA GTCTACGGTA   
  
  
+ AACTTTAAAG GGACACGTCT GATTTCTTCG AAGTACGACA GTCTCGAGAC TAAATGGAGC GGCCGAAAGG   
  
  
+ TACGAGAAAG GAGAGTAACC GGCGATACGT GTCTCTTATT GTGATGTGCT TTGCCGGTGC AAAGGTGTAT   
  
  
+ TAATCAAGTG CATTCTTGCT GACTTATAAG GCGTGAAGGA TCAGGAGAAA GAACAACTGA TGGGTTTTCG   
  
  
+ GGTTTTCGGC TGTTCGTCAC ACACAGGTGT GCCAAAACTA GAAACTTTGT AGCACGGCAA AGTAAGTTGT   
  
  
+ TTACGACCTG AAAGCCGTAG ATAGGTGAAA GAAGACAAGG AGTGGACCTT TTGTTTTGTT CAACGAGCAG   
  
  
+ GTAACCTATT AAGTACTAAT AAATAAAAAT TTTTTTATTT TTTAAACTTT AAATAATTTT ATTTTTAATA   
  
  
+ AAATTTTTAA AATTTTTTTA TTTTTATATA ATAGTGTTTT TTTATTAATT AATTAGTTTT TTATAAATCT   
  
  
+ AAGTTCCTTG TTTTAGTTTT ATTTTGCACT TTAAATAAAT TGATTAAAGA ATTCGTAAAG TTACTTTCGT   
  
  
+ AGCAATTAAA ACGTAGTAAC AAAACTAGTT TGAGATTAAA TTTTTTAATA TTTAATTTTA AATAAAATTT   
  
  
+ CATAAAATTA ATGTTTTTTA TTTTAATTTT AGGATTTACT ACACCAGAAC TAGAAATTAT TATTATATTA   
  
  
+ ATTAGTTTAT TAAACGAAAT AGACCACGAA TTAAAATATT TTAATTTTAG GATTTACTGC ACCAGAACTA   
  
  
+ GAAATTATTA TTATATTAAT TAGTTTATTA AACGAAATAG ACCACGAATT AAAAAATAAA CAAAGTAGTA   
  
  
+ ACTGCACCGG CAATTACTTT CCGGTTTGTT TAATTAATTC TATAAATTAG TTTGTTCATT AATAAAAGTA   
  
  
+ CATGTAACAA AGTAGTAACT GCACCGGCAA TTACTTTCCG GTTTGTTTAA TTAATTCTAT AAATTAGTTT   
  
  
+ GTTCATTAAT AAAAGTACAT GAAAGATACA AACAACCAGT ACCTTCTTCT ACTTTAAAAT TAAACTGTTT   
  
  
+ AATTTTCAGA TCGTCTTTAT ATTTTTAATT ACTGTCTGTA CGTTACTAAA TCTAATCATT AGAGACCCCA   
  
  
+ ACGTGCAGTG CACTACTTCA GTAATTGTT  

- TAATTGAAAG CGGCTGCCTT AAGAACTGGG AGAATTTCTG CAGAATAAGA ATCTACAAAG TATATCACTG   
  
  
- GACACAAGCT GTATTACAAG TGAGCAGCGA TCTCCAATTA GGTCAAGGGA ACGAGAGATT CATAGCAATA   
  
  
- TATAGAAGAG AAAGATCACA TAACTCATAA CGAGCTGTCA ATGGTCTGTT GAAGGAAAGT CGCTCGAAAG   
  
  
- AGGCCCAGGG AGAGAGAAAA AGAAAAAGAA AGTCTGTGTC CGTGGGTACG TTGAATATTT GAATTCCAAA   
  
  
- GGACGTCAAG GTCAAGCTTA GCACGCGGAT AAAATCCACT TCGTGTAGTG GGTAAAGCTT CAGATGCCAT   
  
  
- TTGAAATTTC CCTGTGCAGA CTAAAGAAGC TTCATGCTGT CAGAGCTCTG ATTTACCTCG CCGGCTTTCC   
  
  
- ATGCTCTTTC CTCTCATTGG CCGCTATGCA CAGAGAATAA CACTACACGA AACGGCCACG TTTCCACATA   
  
  
- ATTAGTTCAC GTAAGAACGA CTGAATATTC CGCACTTCCT AGTCCTCTTT CTTGTTGACT ACCCAAAAGC   
  
  
- CCAAAAGCCG ACAAGCAGTG TGTGTCCACA CGGTTTTGAT CTTTGAAACA TCGTGCCGTT TCATTCAACA   
  
  
- AATGCTGGAC TTTCGGCATC TATCCACTTT CTTCTGTTCC TCACCTGGAA AACAAAACAA GTTGCTCGTC   
  
  
- CATTGGATAA TTCATGATTA TTTATTTTTA AAAAAATAAA AAATTTGAAA TTTATTAAAA TAAAAATTAT   
  
  
- TTTAAAAATT TTAAAAAAAT AAAAATATAT TATCACAAAA AAATAATTAA TTAATCAAAA AATATTTAGA   
  
  
- TTCAAGGAAC AAAATCAAAA TAAAACGTGA AATTTATTTA ACTAATTTCT TAAGCATTTC AATGAAAGCA   
  
  
- TCGTTAATTT TGCATCATTG TTTTGATCAA ACTCTAATTT AAAAAATTAT AAATTAAAAT TTATTTTAAA   
  
  
- GTATTTTAAT TACAAAAAAT AAAATTAAAA TCCTAAATGA TGTGGTCTTG ATCTTTAATA ATAATATAAT   
  
  
- TAATCAAATA ATTTGCTTTA TCTGGTGCTT AATTTTATAA AATTAAAATC CTAAATGACG TGGTCTTGAT   
  
  
- CTTTAATAAT AATATAATTA ATCAAATAAT TTGCTTTATC TGGTGCTTAA TTTTTTATTT GTTTCATCAT   
  
  
- TGACGTGGCC GTTAATGAAA GGCCAAACAA ATTAATTAAG ATATTTAATC AAACAAGTAA TTATTTTCAT   
  
  
- GTACATTGTT TCATCATTGA CGTGGCCGTT AATGAAAGGC CAAACAAATT AATTAAGATA TTTAATCAAA   
  
  
- CAAGTAATTA TTTTCATGTA CTTTCTATGT TTGTTGGTCA TGGAAGAAGA TGAAATTTTA ATTTGACAAA   
  
  
- TTAAAAGTCT AGCAGAAATA TAAAAATTAA TGACAGACAT GCAATGATTT AGATTAGTAA TCTCTGGGGT   
  
  
- TGCACGTCAC GTGATGAAGT CATTAACAA

+     W box

| Site Name | Organism | Position | Strand | Matrix score. | sequence | function |
| --- | --- | --- | --- | --- | --- | --- |
| W box | Arabidopsis thaliana | 24 | + | 6 | TTGACC |  |

> 2018/04/13 10:10:12  
+ ATTAACTTTC GCCGACGGAA TTCTTGACCC TCTTAAAGAC GTCTTATTCT TAGATGTTTC ATATAGTGAC   
  
  
+ CTGTGTTCGA CATAATGTTC ACTCGTCGCT AGAGGTTAAT CCAGTTCCCT TGCTCTCTAA GTATCGTTAT   
  
  
+ ATATCTTCTC TTTCTAGTGT ATTGAGTATT GCTCGACAGT TACCAGACAA CTTCCTTTCA GCGAGCTTTC   
  
  
+ TCCGGGTCCC TCTCTCTTTT TCTTTTTCTT TCAGACACAG GCACCCATGC AACTTATAAA CTTAAGGTTT   
  
  
+ CCTGCAGTTC CAGTTCGAAT CGTGCGCCTA TTTTAGGTGA AGCACATCAC CCATTTCGAA GTCTACGGTA   
  
  
+ AACTTTAAAG GGACACGTCT GATTTCTTCG AAGTACGACA GTCTCGAGAC TAAATGGAGC GGCCGAAAGG   
  
  
+ TACGAGAAAG GAGAGTAACC GGCGATACGT GTCTCTTATT GTGATGTGCT TTGCCGGTGC AAAGGTGTAT   
  
  
+ TAATCAAGTG CATTCTTGCT GACTTATAAG GCGTGAAGGA TCAGGAGAAA GAACAACTGA TGGGTTTTCG   
  
  
+ GGTTTTCGGC TGTTCGTCAC ACACAGGTGT GCCAAAACTA GAAACTTTGT AGCACGGCAA AGTAAGTTGT   
  
  
+ TTACGACCTG AAAGCCGTAG ATAGGTGAAA GAAGACAAGG AGTGGACCTT TTGTTTTGTT CAACGAGCAG   
  
  
+ GTAACCTATT AAGTACTAAT AAATAAAAAT TTTTTTATTT TTTAAACTTT AAATAATTTT ATTTTTAATA   
  
  
+ AAATTTTTAA AATTTTTTTA TTTTTATATA ATAGTGTTTT TTTATTAATT AATTAGTTTT TTATAAATCT   
  
  
+ AAGTTCCTTG TTTTAGTTTT ATTTTGCACT TTAAATAAAT TGATTAAAGA ATTCGTAAAG TTACTTTCGT   
  
  
+ AGCAATTAAA ACGTAGTAAC AAAACTAGTT TGAGATTAAA TTTTTTAATA TTTAATTTTA AATAAAATTT   
  
  
+ CATAAAATTA ATGTTTTTTA TTTTAATTTT AGGATTTACT ACACCAGAAC TAGAAATTAT TATTATATTA   
  
  
+ ATTAGTTTAT TAAACGAAAT AGACCACGAA TTAAAATATT TTAATTTTAG GATTTACTGC ACCAGAACTA   
  
  
+ GAAATTATTA TTATATTAAT TAGTTTATTA AACGAAATAG ACCACGAATT AAAAAATAAA CAAAGTAGTA   
  
  
+ ACTGCACCGG CAATTACTTT CCGGTTTGTT TAATTAATTC TATAAATTAG TTTGTTCATT AATAAAAGTA   
  
  
+ CATGTAACAA AGTAGTAACT GCACCGGCAA TTACTTTCCG GTTTGTTTAA TTAATTCTAT AAATTAGTTT   
  
  
+ GTTCATTAAT AAAAGTACAT GAAAGATACA AACAACCAGT ACCTTCTTCT ACTTTAAAAT TAAACTGTTT   
  
  
+ AATTTTCAGA TCGTCTTTAT ATTTTTAATT ACTGTCTGTA CGTTACTAAA TCTAATCATT AGAGACCCCA   
  
  
+ ACGTGCAGTG CACTACTTCA GTAATTGTT  

- TAATTGAAAG CGGCTGCCTT AAGAACTGGG AGAATTTCTG CAGAATAAGA ATCTACAAAG TATATCACTG   
  
  
- GACACAAGCT GTATTACAAG TGAGCAGCGA TCTCCAATTA GGTCAAGGGA ACGAGAGATT CATAGCAATA   
  
  
- TATAGAAGAG AAAGATCACA TAACTCATAA CGAGCTGTCA ATGGTCTGTT GAAGGAAAGT CGCTCGAAAG   
  
  
- AGGCCCAGGG AGAGAGAAAA AGAAAAAGAA AGTCTGTGTC CGTGGGTACG TTGAATATTT GAATTCCAAA   
  
  
- GGACGTCAAG GTCAAGCTTA GCACGCGGAT AAAATCCACT TCGTGTAGTG GGTAAAGCTT CAGATGCCAT   
  
  
- TTGAAATTTC CCTGTGCAGA CTAAAGAAGC TTCATGCTGT CAGAGCTCTG ATTTACCTCG CCGGCTTTCC   
  
  
- ATGCTCTTTC CTCTCATTGG CCGCTATGCA CAGAGAATAA CACTACACGA AACGGCCACG TTTCCACATA   
  
  
- ATTAGTTCAC GTAAGAACGA CTGAATATTC CGCACTTCCT AGTCCTCTTT CTTGTTGACT ACCCAAAAGC   
  
  
- CCAAAAGCCG ACAAGCAGTG TGTGTCCACA CGGTTTTGAT CTTTGAAACA TCGTGCCGTT TCATTCAACA   
  
  
- AATGCTGGAC TTTCGGCATC TATCCACTTT CTTCTGTTCC TCACCTGGAA AACAAAACAA GTTGCTCGTC   
  
  
- CATTGGATAA TTCATGATTA TTTATTTTTA AAAAAATAAA AAATTTGAAA TTTATTAAAA TAAAAATTAT   
  
  
- TTTAAAAATT TTAAAAAAAT AAAAATATAT TATCACAAAA AAATAATTAA TTAATCAAAA AATATTTAGA   
  
  
- TTCAAGGAAC AAAATCAAAA TAAAACGTGA AATTTATTTA ACTAATTTCT TAAGCATTTC AATGAAAGCA   
  
  
- TCGTTAATTT TGCATCATTG TTTTGATCAA ACTCTAATTT AAAAAATTAT AAATTAAAAT TTATTTTAAA   
  
  
- GTATTTTAAT TACAAAAAAT AAAATTAAAA TCCTAAATGA TGTGGTCTTG ATCTTTAATA ATAATATAAT   
  
  
- TAATCAAATA ATTTGCTTTA TCTGGTGCTT AATTTTATAA AATTAAAATC CTAAATGACG TGGTCTTGAT   
  
  
- CTTTAATAAT AATATAATTA ATCAAATAAT TTGCTTTATC TGGTGCTTAA TTTTTTATTT GTTTCATCAT   
  
  
- TGACGTGGCC GTTAATGAAA GGCCAAACAA ATTAATTAAG ATATTTAATC AAACAAGTAA TTATTTTCAT   
  
  
- GTACATTGTT TCATCATTGA CGTGGCCGTT AATGAAAGGC CAAACAAATT AATTAAGATA TTTAATCAAA   
  
  
- CAAGTAATTA TTTTCATGTA CTTTCTATGT TTGTTGGTCA TGGAAGAAGA TGAAATTTTA ATTTGACAAA   
  
  
- TTAAAAGTCT AGCAGAAATA TAAAAATTAA TGACAGACAT GCAATGATTT AGATTAGTAA TCTCTGGGGT   
  
  
- TGCACGTCAC GTGATGAAGT CATTAACAA
