# Supplementary material for: Genome-wide analysis of the WRKY gene family in drumstick (Moringa oleifera Lam.)
Source: PeerJ. 2019 Jun 10;7:e7063. doi: 10.7717/peerj.7063 (PMC6563795; doi:10.7717/peerj.7063)
Supplement: Supplemental Information 1 [file peerj-07-7063-s003.gz › MoWRKY2_plantcare.html]

Content-Type: text/html; charset=ISO-8859-1


CallMat\_Firefox


Webmaster Firefox specific output  
To save the result:
click on the frame with the right mouse button and save the source code as a text file with extension .html  
REFERENCE:PlantCARE: a database of plant cis-acting regulatory elements and a portal to tools for in silico analysis of promoter sequences.  
Lescot, M., Déhais, P., Moreau, Y., De Moor, B., Rouzé ,P.,and Rombauts, S.  
Nucleic Acids Res., Database issue(2002), 30(1):325-327.   


---

> 2018/04/13 10:10:12  
+ AATCATTGGG GTAAAATTTC AGTAAGGTTT GGTGGTTATA TGAAGTCTTT TAGTGTATGC TCAGAAAGGA   
  
  
+ ATTATTATTA TTTGTACTTC AATTTCAGTT TCATGATTTA GAGGTTCAGA TTCTTCCATG TGAAGAGATA   
  
  
+ CTTTTGTGCT ACTTCTTGTA AGTTGGGAAT CATGGATTTC ATTCGGGTCA TGAATTCTAC ACAATATTGC   
  
  
+ TCTTTCTGAG GCAATGGTTG AAAATAATCC ATCTAGTAAT TTCTTGCTGG TAAATGTTTC TTTCAAGAAG   
  
  
+ TTAGCAAAGT TATCTTTGTT CTTATTGAAG AAGAATATGG AGAGCTACCA AGAAGTATTT CTGCAACTTA   
  
  
+ AAAAAACATT TAGGAGTTGG CATGGATGGT AATGTAATAT TCGGTTATTG ATTAATAGGG CGTAAGCGAT   
  
  
+ AGTTCCCTGC TGGTGATGAG TGAACTGGGG GAGTAACCAA GTGAAAGTGA TAGAGGGGTC TATCTTGCAG   
  
  
+ CTCTCCTTTG ATAGAGAGTT AGTCTGTGAT GTGGTAGAAG TCTGAAGATT GTGCAGCTAT GATTTCTCTG   
  
  
+ CATTATTCTA AATAGTGAGT TATGCAACCA AATTTCAATC TTAATTGAAG CATACGGAGT TCTGGGGTTT   
  
  
+ CTCTAGAGAT TTGCATTTCT AAATTTTATG GCGAATTGGC TAGTTCTCTT AATTGCAATC TTTGATGACA   
  
  
+ GTTTATAGAC CAGTAGATTA TGAAAATTGC TTATGATCTT ATAAGGCAGC TTGAACTGTA TTCAAAATGG   
  
  
+ CAAGTACTAG TTGAAAAAAG AGCAGCCCCA GTGAAGGTTT ACAAACCTAA ACTGTAACCA GTATTTGGGC   
  
  
+ GAGAAGTACA AGGTCTCTGT GGGCAGGACG TTTCTGTTTG CATAAATCAA ACCTTTACCT CAGCTCATGA   
  
  
+ TTCCACATTC ATATTACATA TCTTCTATAA CAGTGACAAA TAAGATACCA GTGCATGAGA GCTTCTTGAG   
  
  
+ CTAGCTGAAG CAGACTGGTT TGAAGCTTCT TCTTTGCCAT ATTCTGCATA TTCATCTGTC CATTTCATCT   
  
  
+ CCAGGTTTAG TTCATGTTTG ATAGGTCTTG CGTTGTATCA ATTTCTGTGA GATGAACGAA TGCAAGATAC   
  
  
+ TTCCCAAAAT TTAAAAGTTA CAGCACATTT AGCACAGGTG TATTACAAAC TCTTTAGGGG CTACAGTTTC   
  
  
+ CTCTGTGATA TGATGCATGA GCCTTCACTT TCTTAAATTT ACTTTTCAAG TTTATATTAT TCATCAATAT   
  
  
+ AATCTCTGCA AAATACAGGT TTTTGATAAG TTACCATTGA GCTTGTTGCA CAGTTGGAGT CAACAACAAG   
  
  
+ AAGAAGTGGC TTTCCTAGAA TATGCTAACT CTGTCGGGCT TCCCTTCCTA ACAACTAATT TATTACTGTC   
  
  
+ CTGTGTTGAG GGCATAATAC AGTTGTGGTT CCCTCTTGTC CTTGCACTGA CACACTGTCC TCTACTGAGA   
  
  
+ TAAAATTGGC AGTTAATGTT TCCTTTGAC  

- TTAGTAACCC CATTTTAAAG TCATTCCAAA CCACCAATAT ACTTCAGAAA ATCACATACG AGTCTTTCCT   
  
  
- TAATAATAAT AAACATGAAG TTAAAGTCAA AGTACTAAAT CTCCAAGTCT AAGAAGGTAC ACTTCTCTAT   
  
  
- GAAAACACGA TGAAGAACAT TCAACCCTTA GTACCTAAAG TAAGCCCAGT ACTTAAGATG TGTTATAACG   
  
  
- AGAAAGACTC CGTTACCAAC TTTTATTAGG TAGATCATTA AAGAACGACC ATTTACAAAG AAAGTTCTTC   
  
  
- AATCGTTTCA ATAGAAACAA GAATAACTTC TTCTTATACC TCTCGATGGT TCTTCATAAA GACGTTGAAT   
  
  
- TTTTTTGTAA ATCCTCAACC GTACCTACCA TTACATTATA AGCCAATAAC TAATTATCCC GCATTCGCTA   
  
  
- TCAAGGGACG ACCACTACTC ACTTGACCCC CTCATTGGTT CACTTTCACT ATCTCCCCAG ATAGAACGTC   
  
  
- GAGAGGAAAC TATCTCTCAA TCAGACACTA CACCATCTTC AGACTTCTAA CACGTCGATA CTAAAGAGAC   
  
  
- GTAATAAGAT TTATCACTCA ATACGTTGGT TTAAAGTTAG AATTAACTTC GTATGCCTCA AGACCCCAAA   
  
  
- GAGATCTCTA AACGTAAAGA TTTAAAATAC CGCTTAACCG ATCAAGAGAA TTAACGTTAG AAACTACTGT   
  
  
- CAAATATCTG GTCATCTAAT ACTTTTAACG AATACTAGAA TATTCCGTCG AACTTGACAT AAGTTTTACC   
  
  
- GTTCATGATC AACTTTTTTC TCGTCGGGGT CACTTCCAAA TGTTTGGATT TGACATTGGT CATAAACCCG   
  
  
- CTCTTCATGT TCCAGAGACA CCCGTCCTGC AAAGACAAAC GTATTTAGTT TGGAAATGGA GTCGAGTACT   
  
  
- AAGGTGTAAG TATAATGTAT AGAAGATATT GTCACTGTTT ATTCTATGGT CACGTACTCT CGAAGAACTC   
  
  
- GATCGACTTC GTCTGACCAA ACTTCGAAGA AGAAACGGTA TAAGACGTAT AAGTAGACAG GTAAAGTAGA   
  
  
- GGTCCAAATC AAGTACAAAC TATCCAGAAC GCAACATAGT TAAAGACACT CTACTTGCTT ACGTTCTATG   
  
  
- AAGGGTTTTA AATTTTCAAT GTCGTGTAAA TCGTGTCCAC ATAATGTTTG AGAAATCCCC GATGTCAAAG   
  
  
- GAGACACTAT ACTACGTACT CGGAAGTGAA AGAATTTAAA TGAAAAGTTC AAATATAATA AGTAGTTATA   
  
  
- TTAGAGACGT TTTATGTCCA AAAACTATTC AATGGTAACT CGAACAACGT GTCAACCTCA GTTGTTGTTC   
  
  
- TTCTTCACCG AAAGGATCTT ATACGATTGA GACAGCCCGA AGGGAAGGAT TGTTGATTAA ATAATGACAG   
  
  
- GACACAACTC CCGTATTATG TCAACACCAA GGGAGAACAG GAACGTGACT GTGTGACAGG AGATGACTCT   
  
  
- ATTTTAACCG TCAATTACAA AGGAAACTG

  
  
Motifs Found  

+     3-AF1 binding site

| Site Name | Organism | Position | Strand | Matrix score. | sequence | function |
| --- | --- | --- | --- | --- | --- | --- |
| 3-AF1 binding site | Solanum tuberosum | 133 | + | 10 | AAGAGATATTT | light responsive element |

> 2018/04/13 10:10:12  
+ AATCATTGGG GTAAAATTTC AGTAAGGTTT GGTGGTTATA TGAAGTCTTT TAGTGTATGC TCAGAAAGGA   
  
  
+ ATTATTATTA TTTGTACTTC AATTTCAGTT TCATGATTTA GAGGTTCAGA TTCTTCCATG TGAAGAGATA   
  
  
+ CTTTTGTGCT ACTTCTTGTA AGTTGGGAAT CATGGATTTC ATTCGGGTCA TGAATTCTAC ACAATATTGC   
  
  
+ TCTTTCTGAG GCAATGGTTG AAAATAATCC ATCTAGTAAT TTCTTGCTGG TAAATGTTTC TTTCAAGAAG   
  
  
+ TTAGCAAAGT TATCTTTGTT CTTATTGAAG AAGAATATGG AGAGCTACCA AGAAGTATTT CTGCAACTTA   
  
  
+ AAAAAACATT TAGGAGTTGG CATGGATGGT AATGTAATAT TCGGTTATTG ATTAATAGGG CGTAAGCGAT   
  
  
+ AGTTCCCTGC TGGTGATGAG TGAACTGGGG GAGTAACCAA GTGAAAGTGA TAGAGGGGTC TATCTTGCAG   
  
  
+ CTCTCCTTTG ATAGAGAGTT AGTCTGTGAT GTGGTAGAAG TCTGAAGATT GTGCAGCTAT GATTTCTCTG   
  
  
+ CATTATTCTA AATAGTGAGT TATGCAACCA AATTTCAATC TTAATTGAAG CATACGGAGT TCTGGGGTTT   
  
  
+ CTCTAGAGAT TTGCATTTCT AAATTTTATG GCGAATTGGC TAGTTCTCTT AATTGCAATC TTTGATGACA   
  
  
+ GTTTATAGAC CAGTAGATTA TGAAAATTGC TTATGATCTT ATAAGGCAGC TTGAACTGTA TTCAAAATGG   
  
  
+ CAAGTACTAG TTGAAAAAAG AGCAGCCCCA GTGAAGGTTT ACAAACCTAA ACTGTAACCA GTATTTGGGC   
  
  
+ GAGAAGTACA AGGTCTCTGT GGGCAGGACG TTTCTGTTTG CATAAATCAA ACCTTTACCT CAGCTCATGA   
  
  
+ TTCCACATTC ATATTACATA TCTTCTATAA CAGTGACAAA TAAGATACCA GTGCATGAGA GCTTCTTGAG   
  
  
+ CTAGCTGAAG CAGACTGGTT TGAAGCTTCT TCTTTGCCAT ATTCTGCATA TTCATCTGTC CATTTCATCT   
  
  
+ CCAGGTTTAG TTCATGTTTG ATAGGTCTTG CGTTGTATCA ATTTCTGTGA GATGAACGAA TGCAAGATAC   
  
  
+ TTCCCAAAAT TTAAAAGTTA CAGCACATTT AGCACAGGTG TATTACAAAC TCTTTAGGGG CTACAGTTTC   
  
  
+ CTCTGTGATA TGATGCATGA GCCTTCACTT TCTTAAATTT ACTTTTCAAG TTTATATTAT TCATCAATAT   
  
  
+ AATCTCTGCA AAATACAGGT TTTTGATAAG TTACCATTGA GCTTGTTGCA CAGTTGGAGT CAACAACAAG   
  
  
+ AAGAAGTGGC TTTCCTAGAA TATGCTAACT CTGTCGGGCT TCCCTTCCTA ACAACTAATT TATTACTGTC   
  
  
+ CTGTGTTGAG GGCATAATAC AGTTGTGGTT CCCTCTTGTC CTTGCACTGA CACACTGTCC TCTACTGAGA   
  
  
+ TAAAATTGGC AGTTAATGTT TCCTTTGAC  

- TTAGTAACCC CATTTTAAAG TCATTCCAAA CCACCAATAT ACTTCAGAAA ATCACATACG AGTCTTTCCT   
  
  
- TAATAATAAT AAACATGAAG TTAAAGTCAA AGTACTAAAT CTCCAAGTCT AAGAAGGTAC ACTTCTCTAT   
  
  
- GAAAACACGA TGAAGAACAT TCAACCCTTA GTACCTAAAG TAAGCCCAGT ACTTAAGATG TGTTATAACG   
  
  
- AGAAAGACTC CGTTACCAAC TTTTATTAGG TAGATCATTA AAGAACGACC ATTTACAAAG AAAGTTCTTC   
  
  
- AATCGTTTCA ATAGAAACAA GAATAACTTC TTCTTATACC TCTCGATGGT TCTTCATAAA GACGTTGAAT   
  
  
- TTTTTTGTAA ATCCTCAACC GTACCTACCA TTACATTATA AGCCAATAAC TAATTATCCC GCATTCGCTA   
  
  
- TCAAGGGACG ACCACTACTC ACTTGACCCC CTCATTGGTT CACTTTCACT ATCTCCCCAG ATAGAACGTC   
  
  
- GAGAGGAAAC TATCTCTCAA TCAGACACTA CACCATCTTC AGACTTCTAA CACGTCGATA CTAAAGAGAC   
  
  
- GTAATAAGAT TTATCACTCA ATACGTTGGT TTAAAGTTAG AATTAACTTC GTATGCCTCA AGACCCCAAA   
  
  
- GAGATCTCTA AACGTAAAGA TTTAAAATAC CGCTTAACCG ATCAAGAGAA TTAACGTTAG AAACTACTGT   
  
  
- CAAATATCTG GTCATCTAAT ACTTTTAACG AATACTAGAA TATTCCGTCG AACTTGACAT AAGTTTTACC   
  
  
- GTTCATGATC AACTTTTTTC TCGTCGGGGT CACTTCCAAA TGTTTGGATT TGACATTGGT CATAAACCCG   
  
  
- CTCTTCATGT TCCAGAGACA CCCGTCCTGC AAAGACAAAC GTATTTAGTT TGGAAATGGA GTCGAGTACT   
  
  
- AAGGTGTAAG TATAATGTAT AGAAGATATT GTCACTGTTT ATTCTATGGT CACGTACTCT CGAAGAACTC   
  
  
- GATCGACTTC GTCTGACCAA ACTTCGAAGA AGAAACGGTA TAAGACGTAT AAGTAGACAG GTAAAGTAGA   
  
  
- GGTCCAAATC AAGTACAAAC TATCCAGAAC GCAACATAGT TAAAGACACT CTACTTGCTT ACGTTCTATG   
  
  
- AAGGGTTTTA AATTTTCAAT GTCGTGTAAA TCGTGTCCAC ATAATGTTTG AGAAATCCCC GATGTCAAAG   
  
  
- GAGACACTAT ACTACGTACT CGGAAGTGAA AGAATTTAAA TGAAAAGTTC AAATATAATA AGTAGTTATA   
  
  
- TTAGAGACGT TTTATGTCCA AAAACTATTC AATGGTAACT CGAACAACGT GTCAACCTCA GTTGTTGTTC   
  
  
- TTCTTCACCG AAAGGATCTT ATACGATTGA GACAGCCCGA AGGGAAGGAT TGTTGATTAA ATAATGACAG   
  
  
- GACACAACTC CCGTATTATG TCAACACCAA GGGAGAACAG GAACGTGACT GTGTGACAGG AGATGACTCT   
  
  
- ATTTTAACCG TCAATTACAA AGGAAACTG

+     AAGAA-motif

| Site Name | Organism | Position | Strand | Matrix score. | sequence | function |
| --- | --- | --- | --- | --- | --- | --- |
| AAGAA-motif | Avena sativa | 268 | - | 7 | GAAAGAA |  |

> 2018/04/13 10:10:12  
+ AATCATTGGG GTAAAATTTC AGTAAGGTTT GGTGGTTATA TGAAGTCTTT TAGTGTATGC TCAGAAAGGA   
  
  
+ ATTATTATTA TTTGTACTTC AATTTCAGTT TCATGATTTA GAGGTTCAGA TTCTTCCATG TGAAGAGATA   
  
  
+ CTTTTGTGCT ACTTCTTGTA AGTTGGGAAT CATGGATTTC ATTCGGGTCA TGAATTCTAC ACAATATTGC   
  
  
+ TCTTTCTGAG GCAATGGTTG AAAATAATCC ATCTAGTAAT TTCTTGCTGG TAAATGTTTC TTTCAAGAAG   
  
  
+ TTAGCAAAGT TATCTTTGTT CTTATTGAAG AAGAATATGG AGAGCTACCA AGAAGTATTT CTGCAACTTA   
  
  
+ AAAAAACATT TAGGAGTTGG CATGGATGGT AATGTAATAT TCGGTTATTG ATTAATAGGG CGTAAGCGAT   
  
  
+ AGTTCCCTGC TGGTGATGAG TGAACTGGGG GAGTAACCAA GTGAAAGTGA TAGAGGGGTC TATCTTGCAG   
  
  
+ CTCTCCTTTG ATAGAGAGTT AGTCTGTGAT GTGGTAGAAG TCTGAAGATT GTGCAGCTAT GATTTCTCTG   
  
  
+ CATTATTCTA AATAGTGAGT TATGCAACCA AATTTCAATC TTAATTGAAG CATACGGAGT TCTGGGGTTT   
  
  
+ CTCTAGAGAT TTGCATTTCT AAATTTTATG GCGAATTGGC TAGTTCTCTT AATTGCAATC TTTGATGACA   
  
  
+ GTTTATAGAC CAGTAGATTA TGAAAATTGC TTATGATCTT ATAAGGCAGC TTGAACTGTA TTCAAAATGG   
  
  
+ CAAGTACTAG TTGAAAAAAG AGCAGCCCCA GTGAAGGTTT ACAAACCTAA ACTGTAACCA GTATTTGGGC   
  
  
+ GAGAAGTACA AGGTCTCTGT GGGCAGGACG TTTCTGTTTG CATAAATCAA ACCTTTACCT CAGCTCATGA   
  
  
+ TTCCACATTC ATATTACATA TCTTCTATAA CAGTGACAAA TAAGATACCA GTGCATGAGA GCTTCTTGAG   
  
  
+ CTAGCTGAAG CAGACTGGTT TGAAGCTTCT TCTTTGCCAT ATTCTGCATA TTCATCTGTC CATTTCATCT   
  
  
+ CCAGGTTTAG TTCATGTTTG ATAGGTCTTG CGTTGTATCA ATTTCTGTGA GATGAACGAA TGCAAGATAC   
  
  
+ TTCCCAAAAT TTAAAAGTTA CAGCACATTT AGCACAGGTG TATTACAAAC TCTTTAGGGG CTACAGTTTC   
  
  
+ CTCTGTGATA TGATGCATGA GCCTTCACTT TCTTAAATTT ACTTTTCAAG TTTATATTAT TCATCAATAT   
  
  
+ AATCTCTGCA AAATACAGGT TTTTGATAAG TTACCATTGA GCTTGTTGCA CAGTTGGAGT CAACAACAAG   
  
  
+ AAGAAGTGGC TTTCCTAGAA TATGCTAACT CTGTCGGGCT TCCCTTCCTA ACAACTAATT TATTACTGTC   
  
  
+ CTGTGTTGAG GGCATAATAC AGTTGTGGTT CCCTCTTGTC CTTGCACTGA CACACTGTCC TCTACTGAGA   
  
  
+ TAAAATTGGC AGTTAATGTT TCCTTTGAC  

- TTAGTAACCC CATTTTAAAG TCATTCCAAA CCACCAATAT ACTTCAGAAA ATCACATACG AGTCTTTCCT   
  
  
- TAATAATAAT AAACATGAAG TTAAAGTCAA AGTACTAAAT CTCCAAGTCT AAGAAGGTAC ACTTCTCTAT   
  
  
- GAAAACACGA TGAAGAACAT TCAACCCTTA GTACCTAAAG TAAGCCCAGT ACTTAAGATG TGTTATAACG   
  
  
- AGAAAGACTC CGTTACCAAC TTTTATTAGG TAGATCATTA AAGAACGACC ATTTACAAAG AAAGTTCTTC   
  
  
- AATCGTTTCA ATAGAAACAA GAATAACTTC TTCTTATACC TCTCGATGGT TCTTCATAAA GACGTTGAAT   
  
  
- TTTTTTGTAA ATCCTCAACC GTACCTACCA TTACATTATA AGCCAATAAC TAATTATCCC GCATTCGCTA   
  
  
- TCAAGGGACG ACCACTACTC ACTTGACCCC CTCATTGGTT CACTTTCACT ATCTCCCCAG ATAGAACGTC   
  
  
- GAGAGGAAAC TATCTCTCAA TCAGACACTA CACCATCTTC AGACTTCTAA CACGTCGATA CTAAAGAGAC   
  
  
- GTAATAAGAT TTATCACTCA ATACGTTGGT TTAAAGTTAG AATTAACTTC GTATGCCTCA AGACCCCAAA   
  
  
- GAGATCTCTA AACGTAAAGA TTTAAAATAC CGCTTAACCG ATCAAGAGAA TTAACGTTAG AAACTACTGT   
  
  
- CAAATATCTG GTCATCTAAT ACTTTTAACG AATACTAGAA TATTCCGTCG AACTTGACAT AAGTTTTACC   
  
  
- GTTCATGATC AACTTTTTTC TCGTCGGGGT CACTTCCAAA TGTTTGGATT TGACATTGGT CATAAACCCG   
  
  
- CTCTTCATGT TCCAGAGACA CCCGTCCTGC AAAGACAAAC GTATTTAGTT TGGAAATGGA GTCGAGTACT   
  
  
- AAGGTGTAAG TATAATGTAT AGAAGATATT GTCACTGTTT ATTCTATGGT CACGTACTCT CGAAGAACTC   
  
  
- GATCGACTTC GTCTGACCAA ACTTCGAAGA AGAAACGGTA TAAGACGTAT AAGTAGACAG GTAAAGTAGA   
  
  
- GGTCCAAATC AAGTACAAAC TATCCAGAAC GCAACATAGT TAAAGACACT CTACTTGCTT ACGTTCTATG   
  
  
- AAGGGTTTTA AATTTTCAAT GTCGTGTAAA TCGTGTCCAC ATAATGTTTG AGAAATCCCC GATGTCAAAG   
  
  
- GAGACACTAT ACTACGTACT CGGAAGTGAA AGAATTTAAA TGAAAAGTTC AAATATAATA AGTAGTTATA   
  
  
- TTAGAGACGT TTTATGTCCA AAAACTATTC AATGGTAACT CGAACAACGT GTCAACCTCA GTTGTTGTTC   
  
  
- TTCTTCACCG AAAGGATCTT ATACGATTGA GACAGCCCGA AGGGAAGGAT TGTTGATTAA ATAATGACAG   
  
  
- GACACAACTC CCGTATTATG TCAACACCAA GGGAGAACAG GAACGTGACT GTGTGACAGG AGATGACTCT   
  
  
- ATTTTAACCG TCAATTACAA AGGAAACTG

+     ACE

| Site Name | Organism | Position | Strand | Matrix score. | sequence | function |
| --- | --- | --- | --- | --- | --- | --- |
| ACE | Petroselinum crispum | 353 | + | 9 | AAAACGTTTA | cis-acting element involved in light responsiveness |

> 2018/04/13 10:10:12  
+ AATCATTGGG GTAAAATTTC AGTAAGGTTT GGTGGTTATA TGAAGTCTTT TAGTGTATGC TCAGAAAGGA   
  
  
+ ATTATTATTA TTTGTACTTC AATTTCAGTT TCATGATTTA GAGGTTCAGA TTCTTCCATG TGAAGAGATA   
  
  
+ CTTTTGTGCT ACTTCTTGTA AGTTGGGAAT CATGGATTTC ATTCGGGTCA TGAATTCTAC ACAATATTGC   
  
  
+ TCTTTCTGAG GCAATGGTTG AAAATAATCC ATCTAGTAAT TTCTTGCTGG TAAATGTTTC TTTCAAGAAG   
  
  
+ TTAGCAAAGT TATCTTTGTT CTTATTGAAG AAGAATATGG AGAGCTACCA AGAAGTATTT CTGCAACTTA   
  
  
+ AAAAAACATT TAGGAGTTGG CATGGATGGT AATGTAATAT TCGGTTATTG ATTAATAGGG CGTAAGCGAT   
  
  
+ AGTTCCCTGC TGGTGATGAG TGAACTGGGG GAGTAACCAA GTGAAAGTGA TAGAGGGGTC TATCTTGCAG   
  
  
+ CTCTCCTTTG ATAGAGAGTT AGTCTGTGAT GTGGTAGAAG TCTGAAGATT GTGCAGCTAT GATTTCTCTG   
  
  
+ CATTATTCTA AATAGTGAGT TATGCAACCA AATTTCAATC TTAATTGAAG CATACGGAGT TCTGGGGTTT   
  
  
+ CTCTAGAGAT TTGCATTTCT AAATTTTATG GCGAATTGGC TAGTTCTCTT AATTGCAATC TTTGATGACA   
  
  
+ GTTTATAGAC CAGTAGATTA TGAAAATTGC TTATGATCTT ATAAGGCAGC TTGAACTGTA TTCAAAATGG   
  
  
+ CAAGTACTAG TTGAAAAAAG AGCAGCCCCA GTGAAGGTTT ACAAACCTAA ACTGTAACCA GTATTTGGGC   
  
  
+ GAGAAGTACA AGGTCTCTGT GGGCAGGACG TTTCTGTTTG CATAAATCAA ACCTTTACCT CAGCTCATGA   
  
  
+ TTCCACATTC ATATTACATA TCTTCTATAA CAGTGACAAA TAAGATACCA GTGCATGAGA GCTTCTTGAG   
  
  
+ CTAGCTGAAG CAGACTGGTT TGAAGCTTCT TCTTTGCCAT ATTCTGCATA TTCATCTGTC CATTTCATCT   
  
  
+ CCAGGTTTAG TTCATGTTTG ATAGGTCTTG CGTTGTATCA ATTTCTGTGA GATGAACGAA TGCAAGATAC   
  
  
+ TTCCCAAAAT TTAAAAGTTA CAGCACATTT AGCACAGGTG TATTACAAAC TCTTTAGGGG CTACAGTTTC   
  
  
+ CTCTGTGATA TGATGCATGA GCCTTCACTT TCTTAAATTT ACTTTTCAAG TTTATATTAT TCATCAATAT   
  
  
+ AATCTCTGCA AAATACAGGT TTTTGATAAG TTACCATTGA GCTTGTTGCA CAGTTGGAGT CAACAACAAG   
  
  
+ AAGAAGTGGC TTTCCTAGAA TATGCTAACT CTGTCGGGCT TCCCTTCCTA ACAACTAATT TATTACTGTC   
  
  
+ CTGTGTTGAG GGCATAATAC AGTTGTGGTT CCCTCTTGTC CTTGCACTGA CACACTGTCC TCTACTGAGA   
  
  
+ TAAAATTGGC AGTTAATGTT TCCTTTGAC  

- TTAGTAACCC CATTTTAAAG TCATTCCAAA CCACCAATAT ACTTCAGAAA ATCACATACG AGTCTTTCCT   
  
  
- TAATAATAAT AAACATGAAG TTAAAGTCAA AGTACTAAAT CTCCAAGTCT AAGAAGGTAC ACTTCTCTAT   
  
  
- GAAAACACGA TGAAGAACAT TCAACCCTTA GTACCTAAAG TAAGCCCAGT ACTTAAGATG TGTTATAACG   
  
  
- AGAAAGACTC CGTTACCAAC TTTTATTAGG TAGATCATTA AAGAACGACC ATTTACAAAG AAAGTTCTTC   
  
  
- AATCGTTTCA ATAGAAACAA GAATAACTTC TTCTTATACC TCTCGATGGT TCTTCATAAA GACGTTGAAT   
  
  
- TTTTTTGTAA ATCCTCAACC GTACCTACCA TTACATTATA AGCCAATAAC TAATTATCCC GCATTCGCTA   
  
  
- TCAAGGGACG ACCACTACTC ACTTGACCCC CTCATTGGTT CACTTTCACT ATCTCCCCAG ATAGAACGTC   
  
  
- GAGAGGAAAC TATCTCTCAA TCAGACACTA CACCATCTTC AGACTTCTAA CACGTCGATA CTAAAGAGAC   
  
  
- GTAATAAGAT TTATCACTCA ATACGTTGGT TTAAAGTTAG AATTAACTTC GTATGCCTCA AGACCCCAAA   
  
  
- GAGATCTCTA AACGTAAAGA TTTAAAATAC CGCTTAACCG ATCAAGAGAA TTAACGTTAG AAACTACTGT   
  
  
- CAAATATCTG GTCATCTAAT ACTTTTAACG AATACTAGAA TATTCCGTCG AACTTGACAT AAGTTTTACC   
  
  
- GTTCATGATC AACTTTTTTC TCGTCGGGGT CACTTCCAAA TGTTTGGATT TGACATTGGT CATAAACCCG   
  
  
- CTCTTCATGT TCCAGAGACA CCCGTCCTGC AAAGACAAAC GTATTTAGTT TGGAAATGGA GTCGAGTACT   
  
  
- AAGGTGTAAG TATAATGTAT AGAAGATATT GTCACTGTTT ATTCTATGGT CACGTACTCT CGAAGAACTC   
  
  
- GATCGACTTC GTCTGACCAA ACTTCGAAGA AGAAACGGTA TAAGACGTAT AAGTAGACAG GTAAAGTAGA   
  
  
- GGTCCAAATC AAGTACAAAC TATCCAGAAC GCAACATAGT TAAAGACACT CTACTTGCTT ACGTTCTATG   
  
  
- AAGGGTTTTA AATTTTCAAT GTCGTGTAAA TCGTGTCCAC ATAATGTTTG AGAAATCCCC GATGTCAAAG   
  
  
- GAGACACTAT ACTACGTACT CGGAAGTGAA AGAATTTAAA TGAAAAGTTC AAATATAATA AGTAGTTATA   
  
  
- TTAGAGACGT TTTATGTCCA AAAACTATTC AATGGTAACT CGAACAACGT GTCAACCTCA GTTGTTGTTC   
  
  
- TTCTTCACCG AAAGGATCTT ATACGATTGA GACAGCCCGA AGGGAAGGAT TGTTGATTAA ATAATGACAG   
  
  
- GACACAACTC CCGTATTATG TCAACACCAA GGGAGAACAG GAACGTGACT GTGTGACAGG AGATGACTCT   
  
  
- ATTTTAACCG TCAATTACAA AGGAAACTG

+     AE-box

| Site Name | Organism | Position | Strand | Matrix score. | sequence | function |
| --- | --- | --- | --- | --- | --- | --- |
| AE-box | Arabidopsis thaliana | 264 | - | 8 | AGAAACAT | part of a module for light response |

> 2018/04/13 10:10:12  
+ AATCATTGGG GTAAAATTTC AGTAAGGTTT GGTGGTTATA TGAAGTCTTT TAGTGTATGC TCAGAAAGGA   
  
  
+ ATTATTATTA TTTGTACTTC AATTTCAGTT TCATGATTTA GAGGTTCAGA TTCTTCCATG TGAAGAGATA   
  
  
+ CTTTTGTGCT ACTTCTTGTA AGTTGGGAAT CATGGATTTC ATTCGGGTCA TGAATTCTAC ACAATATTGC   
  
  
+ TCTTTCTGAG GCAATGGTTG AAAATAATCC ATCTAGTAAT TTCTTGCTGG TAAATGTTTC TTTCAAGAAG   
  
  
+ TTAGCAAAGT TATCTTTGTT CTTATTGAAG AAGAATATGG AGAGCTACCA AGAAGTATTT CTGCAACTTA   
  
  
+ AAAAAACATT TAGGAGTTGG CATGGATGGT AATGTAATAT TCGGTTATTG ATTAATAGGG CGTAAGCGAT   
  
  
+ AGTTCCCTGC TGGTGATGAG TGAACTGGGG GAGTAACCAA GTGAAAGTGA TAGAGGGGTC TATCTTGCAG   
  
  
+ CTCTCCTTTG ATAGAGAGTT AGTCTGTGAT GTGGTAGAAG TCTGAAGATT GTGCAGCTAT GATTTCTCTG   
  
  
+ CATTATTCTA AATAGTGAGT TATGCAACCA AATTTCAATC TTAATTGAAG CATACGGAGT TCTGGGGTTT   
  
  
+ CTCTAGAGAT TTGCATTTCT AAATTTTATG GCGAATTGGC TAGTTCTCTT AATTGCAATC TTTGATGACA   
  
  
+ GTTTATAGAC CAGTAGATTA TGAAAATTGC TTATGATCTT ATAAGGCAGC TTGAACTGTA TTCAAAATGG   
  
  
+ CAAGTACTAG TTGAAAAAAG AGCAGCCCCA GTGAAGGTTT ACAAACCTAA ACTGTAACCA GTATTTGGGC   
  
  
+ GAGAAGTACA AGGTCTCTGT GGGCAGGACG TTTCTGTTTG CATAAATCAA ACCTTTACCT CAGCTCATGA   
  
  
+ TTCCACATTC ATATTACATA TCTTCTATAA CAGTGACAAA TAAGATACCA GTGCATGAGA GCTTCTTGAG   
  
  
+ CTAGCTGAAG CAGACTGGTT TGAAGCTTCT TCTTTGCCAT ATTCTGCATA TTCATCTGTC CATTTCATCT   
  
  
+ CCAGGTTTAG TTCATGTTTG ATAGGTCTTG CGTTGTATCA ATTTCTGTGA GATGAACGAA TGCAAGATAC   
  
  
+ TTCCCAAAAT TTAAAAGTTA CAGCACATTT AGCACAGGTG TATTACAAAC TCTTTAGGGG CTACAGTTTC   
  
  
+ CTCTGTGATA TGATGCATGA GCCTTCACTT TCTTAAATTT ACTTTTCAAG TTTATATTAT TCATCAATAT   
  
  
+ AATCTCTGCA AAATACAGGT TTTTGATAAG TTACCATTGA GCTTGTTGCA CAGTTGGAGT CAACAACAAG   
  
  
+ AAGAAGTGGC TTTCCTAGAA TATGCTAACT CTGTCGGGCT TCCCTTCCTA ACAACTAATT TATTACTGTC   
  
  
+ CTGTGTTGAG GGCATAATAC AGTTGTGGTT CCCTCTTGTC CTTGCACTGA CACACTGTCC TCTACTGAGA   
  
  
+ TAAAATTGGC AGTTAATGTT TCCTTTGAC  

- TTAGTAACCC CATTTTAAAG TCATTCCAAA CCACCAATAT ACTTCAGAAA ATCACATACG AGTCTTTCCT   
  
  
- TAATAATAAT AAACATGAAG TTAAAGTCAA AGTACTAAAT CTCCAAGTCT AAGAAGGTAC ACTTCTCTAT   
  
  
- GAAAACACGA TGAAGAACAT TCAACCCTTA GTACCTAAAG TAAGCCCAGT ACTTAAGATG TGTTATAACG   
  
  
- AGAAAGACTC CGTTACCAAC TTTTATTAGG TAGATCATTA AAGAACGACC ATTTACAAAG AAAGTTCTTC   
  
  
- AATCGTTTCA ATAGAAACAA GAATAACTTC TTCTTATACC TCTCGATGGT TCTTCATAAA GACGTTGAAT   
  
  
- TTTTTTGTAA ATCCTCAACC GTACCTACCA TTACATTATA AGCCAATAAC TAATTATCCC GCATTCGCTA   
  
  
- TCAAGGGACG ACCACTACTC ACTTGACCCC CTCATTGGTT CACTTTCACT ATCTCCCCAG ATAGAACGTC   
  
  
- GAGAGGAAAC TATCTCTCAA TCAGACACTA CACCATCTTC AGACTTCTAA CACGTCGATA CTAAAGAGAC   
  
  
- GTAATAAGAT TTATCACTCA ATACGTTGGT TTAAAGTTAG AATTAACTTC GTATGCCTCA AGACCCCAAA   
  
  
- GAGATCTCTA AACGTAAAGA TTTAAAATAC CGCTTAACCG ATCAAGAGAA TTAACGTTAG AAACTACTGT   
  
  
- CAAATATCTG GTCATCTAAT ACTTTTAACG AATACTAGAA TATTCCGTCG AACTTGACAT AAGTTTTACC   
  
  
- GTTCATGATC AACTTTTTTC TCGTCGGGGT CACTTCCAAA TGTTTGGATT TGACATTGGT CATAAACCCG   
  
  
- CTCTTCATGT TCCAGAGACA CCCGTCCTGC AAAGACAAAC GTATTTAGTT TGGAAATGGA GTCGAGTACT   
  
  
- AAGGTGTAAG TATAATGTAT AGAAGATATT GTCACTGTTT ATTCTATGGT CACGTACTCT CGAAGAACTC   
  
  
- GATCGACTTC GTCTGACCAA ACTTCGAAGA AGAAACGGTA TAAGACGTAT AAGTAGACAG GTAAAGTAGA   
  
  
- GGTCCAAATC AAGTACAAAC TATCCAGAAC GCAACATAGT TAAAGACACT CTACTTGCTT ACGTTCTATG   
  
  
- AAGGGTTTTA AATTTTCAAT GTCGTGTAAA TCGTGTCCAC ATAATGTTTG AGAAATCCCC GATGTCAAAG   
  
  
- GAGACACTAT ACTACGTACT CGGAAGTGAA AGAATTTAAA TGAAAAGTTC AAATATAATA AGTAGTTATA   
  
  
- TTAGAGACGT TTTATGTCCA AAAACTATTC AATGGTAACT CGAACAACGT GTCAACCTCA GTTGTTGTTC   
  
  
- TTCTTCACCG AAAGGATCTT ATACGATTGA GACAGCCCGA AGGGAAGGAT TGTTGATTAA ATAATGACAG   
  
  
- GACACAACTC CCGTATTATG TCAACACCAA GGGAGAACAG GAACGTGACT GTGTGACAGG AGATGACTCT   
  
  
- ATTTTAACCG TCAATTACAA AGGAAACTG

+     ARE

| Site Name | Organism | Position | Strand | Matrix score. | sequence | function |
| --- | --- | --- | --- | --- | --- | --- |
| ARE | Zea mays | 996 | + | 6 | TGGTTT | cis-acting regulatory element essential for the anaerobic induction |

> 2018/04/13 10:10:12  
+ AATCATTGGG GTAAAATTTC AGTAAGGTTT GGTGGTTATA TGAAGTCTTT TAGTGTATGC TCAGAAAGGA   
  
  
+ ATTATTATTA TTTGTACTTC AATTTCAGTT TCATGATTTA GAGGTTCAGA TTCTTCCATG TGAAGAGATA   
  
  
+ CTTTTGTGCT ACTTCTTGTA AGTTGGGAAT CATGGATTTC ATTCGGGTCA TGAATTCTAC ACAATATTGC   
  
  
+ TCTTTCTGAG GCAATGGTTG AAAATAATCC ATCTAGTAAT TTCTTGCTGG TAAATGTTTC TTTCAAGAAG   
  
  
+ TTAGCAAAGT TATCTTTGTT CTTATTGAAG AAGAATATGG AGAGCTACCA AGAAGTATTT CTGCAACTTA   
  
  
+ AAAAAACATT TAGGAGTTGG CATGGATGGT AATGTAATAT TCGGTTATTG ATTAATAGGG CGTAAGCGAT   
  
  
+ AGTTCCCTGC TGGTGATGAG TGAACTGGGG GAGTAACCAA GTGAAAGTGA TAGAGGGGTC TATCTTGCAG   
  
  
+ CTCTCCTTTG ATAGAGAGTT AGTCTGTGAT GTGGTAGAAG TCTGAAGATT GTGCAGCTAT GATTTCTCTG   
  
  
+ CATTATTCTA AATAGTGAGT TATGCAACCA AATTTCAATC TTAATTGAAG CATACGGAGT TCTGGGGTTT   
  
  
+ CTCTAGAGAT TTGCATTTCT AAATTTTATG GCGAATTGGC TAGTTCTCTT AATTGCAATC TTTGATGACA   
  
  
+ GTTTATAGAC CAGTAGATTA TGAAAATTGC TTATGATCTT ATAAGGCAGC TTGAACTGTA TTCAAAATGG   
  
  
+ CAAGTACTAG TTGAAAAAAG AGCAGCCCCA GTGAAGGTTT ACAAACCTAA ACTGTAACCA GTATTTGGGC   
  
  
+ GAGAAGTACA AGGTCTCTGT GGGCAGGACG TTTCTGTTTG CATAAATCAA ACCTTTACCT CAGCTCATGA   
  
  
+ TTCCACATTC ATATTACATA TCTTCTATAA CAGTGACAAA TAAGATACCA GTGCATGAGA GCTTCTTGAG   
  
  
+ CTAGCTGAAG CAGACTGGTT TGAAGCTTCT TCTTTGCCAT ATTCTGCATA TTCATCTGTC CATTTCATCT   
  
  
+ CCAGGTTTAG TTCATGTTTG ATAGGTCTTG CGTTGTATCA ATTTCTGTGA GATGAACGAA TGCAAGATAC   
  
  
+ TTCCCAAAAT TTAAAAGTTA CAGCACATTT AGCACAGGTG TATTACAAAC TCTTTAGGGG CTACAGTTTC   
  
  
+ CTCTGTGATA TGATGCATGA GCCTTCACTT TCTTAAATTT ACTTTTCAAG TTTATATTAT TCATCAATAT   
  
  
+ AATCTCTGCA AAATACAGGT TTTTGATAAG TTACCATTGA GCTTGTTGCA CAGTTGGAGT CAACAACAAG   
  
  
+ AAGAAGTGGC TTTCCTAGAA TATGCTAACT CTGTCGGGCT TCCCTTCCTA ACAACTAATT TATTACTGTC   
  
  
+ CTGTGTTGAG GGCATAATAC AGTTGTGGTT CCCTCTTGTC CTTGCACTGA CACACTGTCC TCTACTGAGA   
  
  
+ TAAAATTGGC AGTTAATGTT TCCTTTGAC  

- TTAGTAACCC CATTTTAAAG TCATTCCAAA CCACCAATAT ACTTCAGAAA ATCACATACG AGTCTTTCCT   
  
  
- TAATAATAAT AAACATGAAG TTAAAGTCAA AGTACTAAAT CTCCAAGTCT AAGAAGGTAC ACTTCTCTAT   
  
  
- GAAAACACGA TGAAGAACAT TCAACCCTTA GTACCTAAAG TAAGCCCAGT ACTTAAGATG TGTTATAACG   
  
  
- AGAAAGACTC CGTTACCAAC TTTTATTAGG TAGATCATTA AAGAACGACC ATTTACAAAG AAAGTTCTTC   
  
  
- AATCGTTTCA ATAGAAACAA GAATAACTTC TTCTTATACC TCTCGATGGT TCTTCATAAA GACGTTGAAT   
  
  
- TTTTTTGTAA ATCCTCAACC GTACCTACCA TTACATTATA AGCCAATAAC TAATTATCCC GCATTCGCTA   
  
  
- TCAAGGGACG ACCACTACTC ACTTGACCCC CTCATTGGTT CACTTTCACT ATCTCCCCAG ATAGAACGTC   
  
  
- GAGAGGAAAC TATCTCTCAA TCAGACACTA CACCATCTTC AGACTTCTAA CACGTCGATA CTAAAGAGAC   
  
  
- GTAATAAGAT TTATCACTCA ATACGTTGGT TTAAAGTTAG AATTAACTTC GTATGCCTCA AGACCCCAAA   
  
  
- GAGATCTCTA AACGTAAAGA TTTAAAATAC CGCTTAACCG ATCAAGAGAA TTAACGTTAG AAACTACTGT   
  
  
- CAAATATCTG GTCATCTAAT ACTTTTAACG AATACTAGAA TATTCCGTCG AACTTGACAT AAGTTTTACC   
  
  
- GTTCATGATC AACTTTTTTC TCGTCGGGGT CACTTCCAAA TGTTTGGATT TGACATTGGT CATAAACCCG   
  
  
- CTCTTCATGT TCCAGAGACA CCCGTCCTGC AAAGACAAAC GTATTTAGTT TGGAAATGGA GTCGAGTACT   
  
  
- AAGGTGTAAG TATAATGTAT AGAAGATATT GTCACTGTTT ATTCTATGGT CACGTACTCT CGAAGAACTC   
  
  
- GATCGACTTC GTCTGACCAA ACTTCGAAGA AGAAACGGTA TAAGACGTAT AAGTAGACAG GTAAAGTAGA   
  
  
- GGTCCAAATC AAGTACAAAC TATCCAGAAC GCAACATAGT TAAAGACACT CTACTTGCTT ACGTTCTATG   
  
  
- AAGGGTTTTA AATTTTCAAT GTCGTGTAAA TCGTGTCCAC ATAATGTTTG AGAAATCCCC GATGTCAAAG   
  
  
- GAGACACTAT ACTACGTACT CGGAAGTGAA AGAATTTAAA TGAAAAGTTC AAATATAATA AGTAGTTATA   
  
  
- TTAGAGACGT TTTATGTCCA AAAACTATTC AATGGTAACT CGAACAACGT GTCAACCTCA GTTGTTGTTC   
  
  
- TTCTTCACCG AAAGGATCTT ATACGATTGA GACAGCCCGA AGGGAAGGAT TGTTGATTAA ATAATGACAG   
  
  
- GACACAACTC CCGTATTATG TCAACACCAA GGGAGAACAG GAACGTGACT GTGTGACAGG AGATGACTCT   
  
  
- ATTTTAACCG TCAATTACAA AGGAAACTG

+     ATCT-motif

| Site Name | Organism | Position | Strand | Matrix score. | sequence | function |
| --- | --- | --- | --- | --- | --- | --- |
| ATCT-motif | Arabidopsis thaliana | 1256 | + | 9 | AATCTAATCT | part of a conserved DNA module involved in light responsiveness |

> 2018/04/13 10:10:12  
+ AATCATTGGG GTAAAATTTC AGTAAGGTTT GGTGGTTATA TGAAGTCTTT TAGTGTATGC TCAGAAAGGA   
  
  
+ ATTATTATTA TTTGTACTTC AATTTCAGTT TCATGATTTA GAGGTTCAGA TTCTTCCATG TGAAGAGATA   
  
  
+ CTTTTGTGCT ACTTCTTGTA AGTTGGGAAT CATGGATTTC ATTCGGGTCA TGAATTCTAC ACAATATTGC   
  
  
+ TCTTTCTGAG GCAATGGTTG AAAATAATCC ATCTAGTAAT TTCTTGCTGG TAAATGTTTC TTTCAAGAAG   
  
  
+ TTAGCAAAGT TATCTTTGTT CTTATTGAAG AAGAATATGG AGAGCTACCA AGAAGTATTT CTGCAACTTA   
  
  
+ AAAAAACATT TAGGAGTTGG CATGGATGGT AATGTAATAT TCGGTTATTG ATTAATAGGG CGTAAGCGAT   
  
  
+ AGTTCCCTGC TGGTGATGAG TGAACTGGGG GAGTAACCAA GTGAAAGTGA TAGAGGGGTC TATCTTGCAG   
  
  
+ CTCTCCTTTG ATAGAGAGTT AGTCTGTGAT GTGGTAGAAG TCTGAAGATT GTGCAGCTAT GATTTCTCTG   
  
  
+ CATTATTCTA AATAGTGAGT TATGCAACCA AATTTCAATC TTAATTGAAG CATACGGAGT TCTGGGGTTT   
  
  
+ CTCTAGAGAT TTGCATTTCT AAATTTTATG GCGAATTGGC TAGTTCTCTT AATTGCAATC TTTGATGACA   
  
  
+ GTTTATAGAC CAGTAGATTA TGAAAATTGC TTATGATCTT ATAAGGCAGC TTGAACTGTA TTCAAAATGG   
  
  
+ CAAGTACTAG TTGAAAAAAG AGCAGCCCCA GTGAAGGTTT ACAAACCTAA ACTGTAACCA GTATTTGGGC   
  
  
+ GAGAAGTACA AGGTCTCTGT GGGCAGGACG TTTCTGTTTG CATAAATCAA ACCTTTACCT CAGCTCATGA   
  
  
+ TTCCACATTC ATATTACATA TCTTCTATAA CAGTGACAAA TAAGATACCA GTGCATGAGA GCTTCTTGAG   
  
  
+ CTAGCTGAAG CAGACTGGTT TGAAGCTTCT TCTTTGCCAT ATTCTGCATA TTCATCTGTC CATTTCATCT   
  
  
+ CCAGGTTTAG TTCATGTTTG ATAGGTCTTG CGTTGTATCA ATTTCTGTGA GATGAACGAA TGCAAGATAC   
  
  
+ TTCCCAAAAT TTAAAAGTTA CAGCACATTT AGCACAGGTG TATTACAAAC TCTTTAGGGG CTACAGTTTC   
  
  
+ CTCTGTGATA TGATGCATGA GCCTTCACTT TCTTAAATTT ACTTTTCAAG TTTATATTAT TCATCAATAT   
  
  
+ AATCTCTGCA AAATACAGGT TTTTGATAAG TTACCATTGA GCTTGTTGCA CAGTTGGAGT CAACAACAAG   
  
  
+ AAGAAGTGGC TTTCCTAGAA TATGCTAACT CTGTCGGGCT TCCCTTCCTA ACAACTAATT TATTACTGTC   
  
  
+ CTGTGTTGAG GGCATAATAC AGTTGTGGTT CCCTCTTGTC CTTGCACTGA CACACTGTCC TCTACTGAGA   
  
  
+ TAAAATTGGC AGTTAATGTT TCCTTTGAC  

- TTAGTAACCC CATTTTAAAG TCATTCCAAA CCACCAATAT ACTTCAGAAA ATCACATACG AGTCTTTCCT   
  
  
- TAATAATAAT AAACATGAAG TTAAAGTCAA AGTACTAAAT CTCCAAGTCT AAGAAGGTAC ACTTCTCTAT   
  
  
- GAAAACACGA TGAAGAACAT TCAACCCTTA GTACCTAAAG TAAGCCCAGT ACTTAAGATG TGTTATAACG   
  
  
- AGAAAGACTC CGTTACCAAC TTTTATTAGG TAGATCATTA AAGAACGACC ATTTACAAAG AAAGTTCTTC   
  
  
- AATCGTTTCA ATAGAAACAA GAATAACTTC TTCTTATACC TCTCGATGGT TCTTCATAAA GACGTTGAAT   
  
  
- TTTTTTGTAA ATCCTCAACC GTACCTACCA TTACATTATA AGCCAATAAC TAATTATCCC GCATTCGCTA   
  
  
- TCAAGGGACG ACCACTACTC ACTTGACCCC CTCATTGGTT CACTTTCACT ATCTCCCCAG ATAGAACGTC   
  
  
- GAGAGGAAAC TATCTCTCAA TCAGACACTA CACCATCTTC AGACTTCTAA CACGTCGATA CTAAAGAGAC   
  
  
- GTAATAAGAT TTATCACTCA ATACGTTGGT TTAAAGTTAG AATTAACTTC GTATGCCTCA AGACCCCAAA   
  
  
- GAGATCTCTA AACGTAAAGA TTTAAAATAC CGCTTAACCG ATCAAGAGAA TTAACGTTAG AAACTACTGT   
  
  
- CAAATATCTG GTCATCTAAT ACTTTTAACG AATACTAGAA TATTCCGTCG AACTTGACAT AAGTTTTACC   
  
  
- GTTCATGATC AACTTTTTTC TCGTCGGGGT CACTTCCAAA TGTTTGGATT TGACATTGGT CATAAACCCG   
  
  
- CTCTTCATGT TCCAGAGACA CCCGTCCTGC AAAGACAAAC GTATTTAGTT TGGAAATGGA GTCGAGTACT   
  
  
- AAGGTGTAAG TATAATGTAT AGAAGATATT GTCACTGTTT ATTCTATGGT CACGTACTCT CGAAGAACTC   
  
  
- GATCGACTTC GTCTGACCAA ACTTCGAAGA AGAAACGGTA TAAGACGTAT AAGTAGACAG GTAAAGTAGA   
  
  
- GGTCCAAATC AAGTACAAAC TATCCAGAAC GCAACATAGT TAAAGACACT CTACTTGCTT ACGTTCTATG   
  
  
- AAGGGTTTTA AATTTTCAAT GTCGTGTAAA TCGTGTCCAC ATAATGTTTG AGAAATCCCC GATGTCAAAG   
  
  
- GAGACACTAT ACTACGTACT CGGAAGTGAA AGAATTTAAA TGAAAAGTTC AAATATAATA AGTAGTTATA   
  
  
- TTAGAGACGT TTTATGTCCA AAAACTATTC AATGGTAACT CGAACAACGT GTCAACCTCA GTTGTTGTTC   
  
  
- TTCTTCACCG AAAGGATCTT ATACGATTGA GACAGCCCGA AGGGAAGGAT TGTTGATTAA ATAATGACAG   
  
  
- GACACAACTC CCGTATTATG TCAACACCAA GGGAGAACAG GAACGTGACT GTGTGACAGG AGATGACTCT   
  
  
- ATTTTAACCG TCAATTACAA AGGAAACTG

+     Box 4

| Site Name | Organism | Position | Strand | Matrix score. | sequence | function |
| --- | --- | --- | --- | --- | --- | --- |
| Box 4 | Petroselinum crispum | 401 | + | 6 | ATTAAT | part of a conserved DNA module involved in light responsiveness |

> 2018/04/13 10:10:12  
+ AATCATTGGG GTAAAATTTC AGTAAGGTTT GGTGGTTATA TGAAGTCTTT TAGTGTATGC TCAGAAAGGA   
  
  
+ ATTATTATTA TTTGTACTTC AATTTCAGTT TCATGATTTA GAGGTTCAGA TTCTTCCATG TGAAGAGATA   
  
  
+ CTTTTGTGCT ACTTCTTGTA AGTTGGGAAT CATGGATTTC ATTCGGGTCA TGAATTCTAC ACAATATTGC   
  
  
+ TCTTTCTGAG GCAATGGTTG AAAATAATCC ATCTAGTAAT TTCTTGCTGG TAAATGTTTC TTTCAAGAAG   
  
  
+ TTAGCAAAGT TATCTTTGTT CTTATTGAAG AAGAATATGG AGAGCTACCA AGAAGTATTT CTGCAACTTA   
  
  
+ AAAAAACATT TAGGAGTTGG CATGGATGGT AATGTAATAT TCGGTTATTG ATTAATAGGG CGTAAGCGAT   
  
  
+ AGTTCCCTGC TGGTGATGAG TGAACTGGGG GAGTAACCAA GTGAAAGTGA TAGAGGGGTC TATCTTGCAG   
  
  
+ CTCTCCTTTG ATAGAGAGTT AGTCTGTGAT GTGGTAGAAG TCTGAAGATT GTGCAGCTAT GATTTCTCTG   
  
  
+ CATTATTCTA AATAGTGAGT TATGCAACCA AATTTCAATC TTAATTGAAG CATACGGAGT TCTGGGGTTT   
  
  
+ CTCTAGAGAT TTGCATTTCT AAATTTTATG GCGAATTGGC TAGTTCTCTT AATTGCAATC TTTGATGACA   
  
  
+ GTTTATAGAC CAGTAGATTA TGAAAATTGC TTATGATCTT ATAAGGCAGC TTGAACTGTA TTCAAAATGG   
  
  
+ CAAGTACTAG TTGAAAAAAG AGCAGCCCCA GTGAAGGTTT ACAAACCTAA ACTGTAACCA GTATTTGGGC   
  
  
+ GAGAAGTACA AGGTCTCTGT GGGCAGGACG TTTCTGTTTG CATAAATCAA ACCTTTACCT CAGCTCATGA   
  
  
+ TTCCACATTC ATATTACATA TCTTCTATAA CAGTGACAAA TAAGATACCA GTGCATGAGA GCTTCTTGAG   
  
  
+ CTAGCTGAAG CAGACTGGTT TGAAGCTTCT TCTTTGCCAT ATTCTGCATA TTCATCTGTC CATTTCATCT   
  
  
+ CCAGGTTTAG TTCATGTTTG ATAGGTCTTG CGTTGTATCA ATTTCTGTGA GATGAACGAA TGCAAGATAC   
  
  
+ TTCCCAAAAT TTAAAAGTTA CAGCACATTT AGCACAGGTG TATTACAAAC TCTTTAGGGG CTACAGTTTC   
  
  
+ CTCTGTGATA TGATGCATGA GCCTTCACTT TCTTAAATTT ACTTTTCAAG TTTATATTAT TCATCAATAT   
  
  
+ AATCTCTGCA AAATACAGGT TTTTGATAAG TTACCATTGA GCTTGTTGCA CAGTTGGAGT CAACAACAAG   
  
  
+ AAGAAGTGGC TTTCCTAGAA TATGCTAACT CTGTCGGGCT TCCCTTCCTA ACAACTAATT TATTACTGTC   
  
  
+ CTGTGTTGAG GGCATAATAC AGTTGTGGTT CCCTCTTGTC CTTGCACTGA CACACTGTCC TCTACTGAGA   
  
  
+ TAAAATTGGC AGTTAATGTT TCCTTTGAC  

- TTAGTAACCC CATTTTAAAG TCATTCCAAA CCACCAATAT ACTTCAGAAA ATCACATACG AGTCTTTCCT   
  
  
- TAATAATAAT AAACATGAAG TTAAAGTCAA AGTACTAAAT CTCCAAGTCT AAGAAGGTAC ACTTCTCTAT   
  
  
- GAAAACACGA TGAAGAACAT TCAACCCTTA GTACCTAAAG TAAGCCCAGT ACTTAAGATG TGTTATAACG   
  
  
- AGAAAGACTC CGTTACCAAC TTTTATTAGG TAGATCATTA AAGAACGACC ATTTACAAAG AAAGTTCTTC   
  
  
- AATCGTTTCA ATAGAAACAA GAATAACTTC TTCTTATACC TCTCGATGGT TCTTCATAAA GACGTTGAAT   
  
  
- TTTTTTGTAA ATCCTCAACC GTACCTACCA TTACATTATA AGCCAATAAC TAATTATCCC GCATTCGCTA   
  
  
- TCAAGGGACG ACCACTACTC ACTTGACCCC CTCATTGGTT CACTTTCACT ATCTCCCCAG ATAGAACGTC   
  
  
- GAGAGGAAAC TATCTCTCAA TCAGACACTA CACCATCTTC AGACTTCTAA CACGTCGATA CTAAAGAGAC   
  
  
- GTAATAAGAT TTATCACTCA ATACGTTGGT TTAAAGTTAG AATTAACTTC GTATGCCTCA AGACCCCAAA   
  
  
- GAGATCTCTA AACGTAAAGA TTTAAAATAC CGCTTAACCG ATCAAGAGAA TTAACGTTAG AAACTACTGT   
  
  
- CAAATATCTG GTCATCTAAT ACTTTTAACG AATACTAGAA TATTCCGTCG AACTTGACAT AAGTTTTACC   
  
  
- GTTCATGATC AACTTTTTTC TCGTCGGGGT CACTTCCAAA TGTTTGGATT TGACATTGGT CATAAACCCG   
  
  
- CTCTTCATGT TCCAGAGACA CCCGTCCTGC AAAGACAAAC GTATTTAGTT TGGAAATGGA GTCGAGTACT   
  
  
- AAGGTGTAAG TATAATGTAT AGAAGATATT GTCACTGTTT ATTCTATGGT CACGTACTCT CGAAGAACTC   
  
  
- GATCGACTTC GTCTGACCAA ACTTCGAAGA AGAAACGGTA TAAGACGTAT AAGTAGACAG GTAAAGTAGA   
  
  
- GGTCCAAATC AAGTACAAAC TATCCAGAAC GCAACATAGT TAAAGACACT CTACTTGCTT ACGTTCTATG   
  
  
- AAGGGTTTTA AATTTTCAAT GTCGTGTAAA TCGTGTCCAC ATAATGTTTG AGAAATCCCC GATGTCAAAG   
  
  
- GAGACACTAT ACTACGTACT CGGAAGTGAA AGAATTTAAA TGAAAAGTTC AAATATAATA AGTAGTTATA   
  
  
- TTAGAGACGT TTTATGTCCA AAAACTATTC AATGGTAACT CGAACAACGT GTCAACCTCA GTTGTTGTTC   
  
  
- TTCTTCACCG AAAGGATCTT ATACGATTGA GACAGCCCGA AGGGAAGGAT TGTTGATTAA ATAATGACAG   
  
  
- GACACAACTC CCGTATTATG TCAACACCAA GGGAGAACAG GAACGTGACT GTGTGACAGG AGATGACTCT   
  
  
- ATTTTAACCG TCAATTACAA AGGAAACTG

+     Box III

| Site Name | Organism | Position | Strand | Matrix score. | sequence | function |
| --- | --- | --- | --- | --- | --- | --- |
| Box III | Pisum sativum | 460 | - | 11 | atCATTTTCACt | protein binding site |

> 2018/04/13 10:10:12  
+ AATCATTGGG GTAAAATTTC AGTAAGGTTT GGTGGTTATA TGAAGTCTTT TAGTGTATGC TCAGAAAGGA   
  
  
+ ATTATTATTA TTTGTACTTC AATTTCAGTT TCATGATTTA GAGGTTCAGA TTCTTCCATG TGAAGAGATA   
  
  
+ CTTTTGTGCT ACTTCTTGTA AGTTGGGAAT CATGGATTTC ATTCGGGTCA TGAATTCTAC ACAATATTGC   
  
  
+ TCTTTCTGAG GCAATGGTTG AAAATAATCC ATCTAGTAAT TTCTTGCTGG TAAATGTTTC TTTCAAGAAG   
  
  
+ TTAGCAAAGT TATCTTTGTT CTTATTGAAG AAGAATATGG AGAGCTACCA AGAAGTATTT CTGCAACTTA   
  
  
+ AAAAAACATT TAGGAGTTGG CATGGATGGT AATGTAATAT TCGGTTATTG ATTAATAGGG CGTAAGCGAT   
  
  
+ AGTTCCCTGC TGGTGATGAG TGAACTGGGG GAGTAACCAA GTGAAAGTGA TAGAGGGGTC TATCTTGCAG   
  
  
+ CTCTCCTTTG ATAGAGAGTT AGTCTGTGAT GTGGTAGAAG TCTGAAGATT GTGCAGCTAT GATTTCTCTG   
  
  
+ CATTATTCTA AATAGTGAGT TATGCAACCA AATTTCAATC TTAATTGAAG CATACGGAGT TCTGGGGTTT   
  
  
+ CTCTAGAGAT TTGCATTTCT AAATTTTATG GCGAATTGGC TAGTTCTCTT AATTGCAATC TTTGATGACA   
  
  
+ GTTTATAGAC CAGTAGATTA TGAAAATTGC TTATGATCTT ATAAGGCAGC TTGAACTGTA TTCAAAATGG   
  
  
+ CAAGTACTAG TTGAAAAAAG AGCAGCCCCA GTGAAGGTTT ACAAACCTAA ACTGTAACCA GTATTTGGGC   
  
  
+ GAGAAGTACA AGGTCTCTGT GGGCAGGACG TTTCTGTTTG CATAAATCAA ACCTTTACCT CAGCTCATGA   
  
  
+ TTCCACATTC ATATTACATA TCTTCTATAA CAGTGACAAA TAAGATACCA GTGCATGAGA GCTTCTTGAG   
  
  
+ CTAGCTGAAG CAGACTGGTT TGAAGCTTCT TCTTTGCCAT ATTCTGCATA TTCATCTGTC CATTTCATCT   
  
  
+ CCAGGTTTAG TTCATGTTTG ATAGGTCTTG CGTTGTATCA ATTTCTGTGA GATGAACGAA TGCAAGATAC   
  
  
+ TTCCCAAAAT TTAAAAGTTA CAGCACATTT AGCACAGGTG TATTACAAAC TCTTTAGGGG CTACAGTTTC   
  
  
+ CTCTGTGATA TGATGCATGA GCCTTCACTT TCTTAAATTT ACTTTTCAAG TTTATATTAT TCATCAATAT   
  
  
+ AATCTCTGCA AAATACAGGT TTTTGATAAG TTACCATTGA GCTTGTTGCA CAGTTGGAGT CAACAACAAG   
  
  
+ AAGAAGTGGC TTTCCTAGAA TATGCTAACT CTGTCGGGCT TCCCTTCCTA ACAACTAATT TATTACTGTC   
  
  
+ CTGTGTTGAG GGCATAATAC AGTTGTGGTT CCCTCTTGTC CTTGCACTGA CACACTGTCC TCTACTGAGA   
  
  
+ TAAAATTGGC AGTTAATGTT TCCTTTGAC  

- TTAGTAACCC CATTTTAAAG TCATTCCAAA CCACCAATAT ACTTCAGAAA ATCACATACG AGTCTTTCCT   
  
  
- TAATAATAAT AAACATGAAG TTAAAGTCAA AGTACTAAAT CTCCAAGTCT AAGAAGGTAC ACTTCTCTAT   
  
  
- GAAAACACGA TGAAGAACAT TCAACCCTTA GTACCTAAAG TAAGCCCAGT ACTTAAGATG TGTTATAACG   
  
  
- AGAAAGACTC CGTTACCAAC TTTTATTAGG TAGATCATTA AAGAACGACC ATTTACAAAG AAAGTTCTTC   
  
  
- AATCGTTTCA ATAGAAACAA GAATAACTTC TTCTTATACC TCTCGATGGT TCTTCATAAA GACGTTGAAT   
  
  
- TTTTTTGTAA ATCCTCAACC GTACCTACCA TTACATTATA AGCCAATAAC TAATTATCCC GCATTCGCTA   
  
  
- TCAAGGGACG ACCACTACTC ACTTGACCCC CTCATTGGTT CACTTTCACT ATCTCCCCAG ATAGAACGTC   
  
  
- GAGAGGAAAC TATCTCTCAA TCAGACACTA CACCATCTTC AGACTTCTAA CACGTCGATA CTAAAGAGAC   
  
  
- GTAATAAGAT TTATCACTCA ATACGTTGGT TTAAAGTTAG AATTAACTTC GTATGCCTCA AGACCCCAAA   
  
  
- GAGATCTCTA AACGTAAAGA TTTAAAATAC CGCTTAACCG ATCAAGAGAA TTAACGTTAG AAACTACTGT   
  
  
- CAAATATCTG GTCATCTAAT ACTTTTAACG AATACTAGAA TATTCCGTCG AACTTGACAT AAGTTTTACC   
  
  
- GTTCATGATC AACTTTTTTC TCGTCGGGGT CACTTCCAAA TGTTTGGATT TGACATTGGT CATAAACCCG   
  
  
- CTCTTCATGT TCCAGAGACA CCCGTCCTGC AAAGACAAAC GTATTTAGTT TGGAAATGGA GTCGAGTACT   
  
  
- AAGGTGTAAG TATAATGTAT AGAAGATATT GTCACTGTTT ATTCTATGGT CACGTACTCT CGAAGAACTC   
  
  
- GATCGACTTC GTCTGACCAA ACTTCGAAGA AGAAACGGTA TAAGACGTAT AAGTAGACAG GTAAAGTAGA   
  
  
- GGTCCAAATC AAGTACAAAC TATCCAGAAC GCAACATAGT TAAAGACACT CTACTTGCTT ACGTTCTATG   
  
  
- AAGGGTTTTA AATTTTCAAT GTCGTGTAAA TCGTGTCCAC ATAATGTTTG AGAAATCCCC GATGTCAAAG   
  
  
- GAGACACTAT ACTACGTACT CGGAAGTGAA AGAATTTAAA TGAAAAGTTC AAATATAATA AGTAGTTATA   
  
  
- TTAGAGACGT TTTATGTCCA AAAACTATTC AATGGTAACT CGAACAACGT GTCAACCTCA GTTGTTGTTC   
  
  
- TTCTTCACCG AAAGGATCTT ATACGATTGA GACAGCCCGA AGGGAAGGAT TGTTGATTAA ATAATGACAG   
  
  
- GACACAACTC CCGTATTATG TCAACACCAA GGGAGAACAG GAACGTGACT GTGTGACAGG AGATGACTCT   
  
  
- ATTTTAACCG TCAATTACAA AGGAAACTG

+     CAAT-box

| Site Name | Organism | Position | Strand | Matrix score. | sequence | function |
| --- | --- | --- | --- | --- | --- | --- |
| CAAT-box | Arabidopsis thaliana | 5 | - | 5 | CCAAT | common cis-acting element in promoter and enhancer regions |
| CAAT-box | Brassica rapa | 80 | - | 5 | CAAAT | common cis-acting element in promoter and enhancer regions |
| CAAT-box | Glycine max | 90 | + | 5 | CAATT | common cis-acting element in promoter and enhancer regions |
| CAAT-box | Hordeum vulgare | 202 | + | 4 | CAAT | common cis-acting element in promoter and enhancer regions |
| CAAT-box | Hordeum vulgare | 206 | - | 4 | CAAT | common cis-acting element in promoter and enhancer regions |
| CAAT-box | Arabidopsis thaliana | 220 | + | 6 | gGCAAT | common cis-acting element in promoter and enhancer regions |
| CAAT-box | Hordeum vulgare | 222 | + | 4 | CAAT | common cis-acting element in promoter and enhancer regions |
| CAAT-box | Hordeum vulgare | 304 | - | 4 | CAAT | common cis-acting element in promoter and enhancer regions |
| CAAT-box | Petunia hybrida | 366 | - | 7 | TGCCAAC | common cis-acting element in promoter and enhancer regions |
| CAAT-box | Hordeum vulgare | 397 | - | 4 | CAAT | common cis-acting element in promoter and enhancer regions |
| CAAT-box | Hordeum vulgare | 538 | - | 4 | CAAT | common cis-acting element in promoter and enhancer regions |
| CAAT-box | Brassica rapa | 589 | + | 5 | CAAAT | common cis-acting element in promoter and enhancer regions |
| CAAT-box | Hordeum vulgare | 596 | + | 4 | CAAT | common cis-acting element in promoter and enhancer regions |
| CAAT-box | Glycine max | 603 | - | 5 | CAATT | common cis-acting element in promoter and enhancer regions |
| CAAT-box | Hordeum vulgare | 604 | - | 4 | CAAT | common cis-acting element in promoter and enhancer regions |
| CAAT-box | Brassica juncea | 637 | - | 9 | TGCAAATCT | common cis-acting element in promoter and enhancer regions |
| CAAT-box | Brassica rapa | 639 | - | 5 | CAAAT | common cis-acting element in promoter and enhancer regions |
| CAAT-box | Glycine max | 664 | - | 5 | CAATT | common cis-acting element in promoter and enhancer regions |
| CAAT-box | Arabidopsis thaliana | 665 | - | 5 | CCAAT | common cis-acting element in promoter and enhancer regions |
| CAAT-box | Glycine max | 681 | - | 5 | CAATT | common cis-acting element in promoter and enhancer regions |
| CAAT-box | Hordeum vulgare | 682 | - | 4 | CAAT | common cis-acting element in promoter and enhancer regions |
| CAAT-box | Hordeum vulgare | 686 | + | 4 | CAAT | common cis-acting element in promoter and enhancer regions |
| CAAT-box | Glycine max | 725 | - | 5 | CAATT | common cis-acting element in promoter and enhancer regions |
| CAAT-box | Hordeum vulgare | 726 | - | 4 | CAAT | common cis-acting element in promoter and enhancer regions |
| CAAT-box | Brassica rapa | 833 | - | 5 | CAAAT | common cis-acting element in promoter and enhancer regions |
| CAAT-box | Brassica rapa | 947 | + | 5 | CAAAT | common cis-acting element in promoter and enhancer regions |
| CAAT-box | Glycine max | 1089 | + | 5 | CAATT | common cis-acting element in promoter and enhancer regions |
| CAAT-box | Hordeum vulgare | 1255 | + | 4 | CAAT | common cis-acting element in promoter and enhancer regions |
| CAAT-box | Hordeum vulgare | 1296 | - | 4 | CAAT | common cis-acting element in promoter and enhancer regions |
| CAAT-box | Glycine max | 1474 | - | 5 | CAATT | common cis-acting element in promoter and enhancer regions |
| CAAT-box | Arabidopsis thaliana | 1475 | - | 5 | CCAAT | common cis-acting element in promoter and enhancer regions |

> 2018/04/13 10:10:12  
+ AATCATTGGG GTAAAATTTC AGTAAGGTTT GGTGGTTATA TGAAGTCTTT TAGTGTATGC TCAGAAAGGA   
  
  
+ ATTATTATTA TTTGTACTTC AATTTCAGTT TCATGATTTA GAGGTTCAGA TTCTTCCATG TGAAGAGATA   
  
  
+ CTTTTGTGCT ACTTCTTGTA AGTTGGGAAT CATGGATTTC ATTCGGGTCA TGAATTCTAC ACAATATTGC   
  
  
+ TCTTTCTGAG GCAATGGTTG AAAATAATCC ATCTAGTAAT TTCTTGCTGG TAAATGTTTC TTTCAAGAAG   
  
  
+ TTAGCAAAGT TATCTTTGTT CTTATTGAAG AAGAATATGG AGAGCTACCA AGAAGTATTT CTGCAACTTA   
  
  
+ AAAAAACATT TAGGAGTTGG CATGGATGGT AATGTAATAT TCGGTTATTG ATTAATAGGG CGTAAGCGAT   
  
  
+ AGTTCCCTGC TGGTGATGAG TGAACTGGGG GAGTAACCAA GTGAAAGTGA TAGAGGGGTC TATCTTGCAG   
  
  
+ CTCTCCTTTG ATAGAGAGTT AGTCTGTGAT GTGGTAGAAG TCTGAAGATT GTGCAGCTAT GATTTCTCTG   
  
  
+ CATTATTCTA AATAGTGAGT TATGCAACCA AATTTCAATC TTAATTGAAG CATACGGAGT TCTGGGGTTT   
  
  
+ CTCTAGAGAT TTGCATTTCT AAATTTTATG GCGAATTGGC TAGTTCTCTT AATTGCAATC TTTGATGACA   
  
  
+ GTTTATAGAC CAGTAGATTA TGAAAATTGC TTATGATCTT ATAAGGCAGC TTGAACTGTA TTCAAAATGG   
  
  
+ CAAGTACTAG TTGAAAAAAG AGCAGCCCCA GTGAAGGTTT ACAAACCTAA ACTGTAACCA GTATTTGGGC   
  
  
+ GAGAAGTACA AGGTCTCTGT GGGCAGGACG TTTCTGTTTG CATAAATCAA ACCTTTACCT CAGCTCATGA   
  
  
+ TTCCACATTC ATATTACATA TCTTCTATAA CAGTGACAAA TAAGATACCA GTGCATGAGA GCTTCTTGAG   
  
  
+ CTAGCTGAAG CAGACTGGTT TGAAGCTTCT TCTTTGCCAT ATTCTGCATA TTCATCTGTC CATTTCATCT   
  
  
+ CCAGGTTTAG TTCATGTTTG ATAGGTCTTG CGTTGTATCA ATTTCTGTGA GATGAACGAA TGCAAGATAC   
  
  
+ TTCCCAAAAT TTAAAAGTTA CAGCACATTT AGCACAGGTG TATTACAAAC TCTTTAGGGG CTACAGTTTC   
  
  
+ CTCTGTGATA TGATGCATGA GCCTTCACTT TCTTAAATTT ACTTTTCAAG TTTATATTAT TCATCAATAT   
  
  
+ AATCTCTGCA AAATACAGGT TTTTGATAAG TTACCATTGA GCTTGTTGCA CAGTTGGAGT CAACAACAAG   
  
  
+ AAGAAGTGGC TTTCCTAGAA TATGCTAACT CTGTCGGGCT TCCCTTCCTA ACAACTAATT TATTACTGTC   
  
  
+ CTGTGTTGAG GGCATAATAC AGTTGTGGTT CCCTCTTGTC CTTGCACTGA CACACTGTCC TCTACTGAGA   
  
  
+ TAAAATTGGC AGTTAATGTT TCCTTTGAC  

- TTAGTAACCC CATTTTAAAG TCATTCCAAA CCACCAATAT ACTTCAGAAA ATCACATACG AGTCTTTCCT   
  
  
- TAATAATAAT AAACATGAAG TTAAAGTCAA AGTACTAAAT CTCCAAGTCT AAGAAGGTAC ACTTCTCTAT   
  
  
- GAAAACACGA TGAAGAACAT TCAACCCTTA GTACCTAAAG TAAGCCCAGT ACTTAAGATG TGTTATAACG   
  
  
- AGAAAGACTC CGTTACCAAC TTTTATTAGG TAGATCATTA AAGAACGACC ATTTACAAAG AAAGTTCTTC   
  
  
- AATCGTTTCA ATAGAAACAA GAATAACTTC TTCTTATACC TCTCGATGGT TCTTCATAAA GACGTTGAAT   
  
  
- TTTTTTGTAA ATCCTCAACC GTACCTACCA TTACATTATA AGCCAATAAC TAATTATCCC GCATTCGCTA   
  
  
- TCAAGGGACG ACCACTACTC ACTTGACCCC CTCATTGGTT CACTTTCACT ATCTCCCCAG ATAGAACGTC   
  
  
- GAGAGGAAAC TATCTCTCAA TCAGACACTA CACCATCTTC AGACTTCTAA CACGTCGATA CTAAAGAGAC   
  
  
- GTAATAAGAT TTATCACTCA ATACGTTGGT TTAAAGTTAG AATTAACTTC GTATGCCTCA AGACCCCAAA   
  
  
- GAGATCTCTA AACGTAAAGA TTTAAAATAC CGCTTAACCG ATCAAGAGAA TTAACGTTAG AAACTACTGT   
  
  
- CAAATATCTG GTCATCTAAT ACTTTTAACG AATACTAGAA TATTCCGTCG AACTTGACAT AAGTTTTACC   
  
  
- GTTCATGATC AACTTTTTTC TCGTCGGGGT CACTTCCAAA TGTTTGGATT TGACATTGGT CATAAACCCG   
  
  
- CTCTTCATGT TCCAGAGACA CCCGTCCTGC AAAGACAAAC GTATTTAGTT TGGAAATGGA GTCGAGTACT   
  
  
- AAGGTGTAAG TATAATGTAT AGAAGATATT GTCACTGTTT ATTCTATGGT CACGTACTCT CGAAGAACTC   
  
  
- GATCGACTTC GTCTGACCAA ACTTCGAAGA AGAAACGGTA TAAGACGTAT AAGTAGACAG GTAAAGTAGA   
  
  
- GGTCCAAATC AAGTACAAAC TATCCAGAAC GCAACATAGT TAAAGACACT CTACTTGCTT ACGTTCTATG   
  
  
- AAGGGTTTTA AATTTTCAAT GTCGTGTAAA TCGTGTCCAC ATAATGTTTG AGAAATCCCC GATGTCAAAG   
  
  
- GAGACACTAT ACTACGTACT CGGAAGTGAA AGAATTTAAA TGAAAAGTTC AAATATAATA AGTAGTTATA   
  
  
- TTAGAGACGT TTTATGTCCA AAAACTATTC AATGGTAACT CGAACAACGT GTCAACCTCA GTTGTTGTTC   
  
  
- TTCTTCACCG AAAGGATCTT ATACGATTGA GACAGCCCGA AGGGAAGGAT TGTTGATTAA ATAATGACAG   
  
  
- GACACAACTC CCGTATTATG TCAACACCAA GGGAGAACAG GAACGTGACT GTGTGACAGG AGATGACTCT   
  
  
- ATTTTAACCG TCAATTACAA AGGAAACTG

+     CAT-box

| Site Name | Organism | Position | Strand | Matrix score. | sequence | function |
| --- | --- | --- | --- | --- | --- | --- |
| CAT-box | Arabidopsis thaliana | 1335 | - | 6 | GCCACT | cis-acting regulatory element related to meristem expression |

> 2018/04/13 10:10:12  
+ AATCATTGGG GTAAAATTTC AGTAAGGTTT GGTGGTTATA TGAAGTCTTT TAGTGTATGC TCAGAAAGGA   
  
  
+ ATTATTATTA TTTGTACTTC AATTTCAGTT TCATGATTTA GAGGTTCAGA TTCTTCCATG TGAAGAGATA   
  
  
+ CTTTTGTGCT ACTTCTTGTA AGTTGGGAAT CATGGATTTC ATTCGGGTCA TGAATTCTAC ACAATATTGC   
  
  
+ TCTTTCTGAG GCAATGGTTG AAAATAATCC ATCTAGTAAT TTCTTGCTGG TAAATGTTTC TTTCAAGAAG   
  
  
+ TTAGCAAAGT TATCTTTGTT CTTATTGAAG AAGAATATGG AGAGCTACCA AGAAGTATTT CTGCAACTTA   
  
  
+ AAAAAACATT TAGGAGTTGG CATGGATGGT AATGTAATAT TCGGTTATTG ATTAATAGGG CGTAAGCGAT   
  
  
+ AGTTCCCTGC TGGTGATGAG TGAACTGGGG GAGTAACCAA GTGAAAGTGA TAGAGGGGTC TATCTTGCAG   
  
  
+ CTCTCCTTTG ATAGAGAGTT AGTCTGTGAT GTGGTAGAAG TCTGAAGATT GTGCAGCTAT GATTTCTCTG   
  
  
+ CATTATTCTA AATAGTGAGT TATGCAACCA AATTTCAATC TTAATTGAAG CATACGGAGT TCTGGGGTTT   
  
  
+ CTCTAGAGAT TTGCATTTCT AAATTTTATG GCGAATTGGC TAGTTCTCTT AATTGCAATC TTTGATGACA   
  
  
+ GTTTATAGAC CAGTAGATTA TGAAAATTGC TTATGATCTT ATAAGGCAGC TTGAACTGTA TTCAAAATGG   
  
  
+ CAAGTACTAG TTGAAAAAAG AGCAGCCCCA GTGAAGGTTT ACAAACCTAA ACTGTAACCA GTATTTGGGC   
  
  
+ GAGAAGTACA AGGTCTCTGT GGGCAGGACG TTTCTGTTTG CATAAATCAA ACCTTTACCT CAGCTCATGA   
  
  
+ TTCCACATTC ATATTACATA TCTTCTATAA CAGTGACAAA TAAGATACCA GTGCATGAGA GCTTCTTGAG   
  
  
+ CTAGCTGAAG CAGACTGGTT TGAAGCTTCT TCTTTGCCAT ATTCTGCATA TTCATCTGTC CATTTCATCT   
  
  
+ CCAGGTTTAG TTCATGTTTG ATAGGTCTTG CGTTGTATCA ATTTCTGTGA GATGAACGAA TGCAAGATAC   
  
  
+ TTCCCAAAAT TTAAAAGTTA CAGCACATTT AGCACAGGTG TATTACAAAC TCTTTAGGGG CTACAGTTTC   
  
  
+ CTCTGTGATA TGATGCATGA GCCTTCACTT TCTTAAATTT ACTTTTCAAG TTTATATTAT TCATCAATAT   
  
  
+ AATCTCTGCA AAATACAGGT TTTTGATAAG TTACCATTGA GCTTGTTGCA CAGTTGGAGT CAACAACAAG   
  
  
+ AAGAAGTGGC TTTCCTAGAA TATGCTAACT CTGTCGGGCT TCCCTTCCTA ACAACTAATT TATTACTGTC   
  
  
+ CTGTGTTGAG GGCATAATAC AGTTGTGGTT CCCTCTTGTC CTTGCACTGA CACACTGTCC TCTACTGAGA   
  
  
+ TAAAATTGGC AGTTAATGTT TCCTTTGAC  

- TTAGTAACCC CATTTTAAAG TCATTCCAAA CCACCAATAT ACTTCAGAAA ATCACATACG AGTCTTTCCT   
  
  
- TAATAATAAT AAACATGAAG TTAAAGTCAA AGTACTAAAT CTCCAAGTCT AAGAAGGTAC ACTTCTCTAT   
  
  
- GAAAACACGA TGAAGAACAT TCAACCCTTA GTACCTAAAG TAAGCCCAGT ACTTAAGATG TGTTATAACG   
  
  
- AGAAAGACTC CGTTACCAAC TTTTATTAGG TAGATCATTA AAGAACGACC ATTTACAAAG AAAGTTCTTC   
  
  
- AATCGTTTCA ATAGAAACAA GAATAACTTC TTCTTATACC TCTCGATGGT TCTTCATAAA GACGTTGAAT   
  
  
- TTTTTTGTAA ATCCTCAACC GTACCTACCA TTACATTATA AGCCAATAAC TAATTATCCC GCATTCGCTA   
  
  
- TCAAGGGACG ACCACTACTC ACTTGACCCC CTCATTGGTT CACTTTCACT ATCTCCCCAG ATAGAACGTC   
  
  
- GAGAGGAAAC TATCTCTCAA TCAGACACTA CACCATCTTC AGACTTCTAA CACGTCGATA CTAAAGAGAC   
  
  
- GTAATAAGAT TTATCACTCA ATACGTTGGT TTAAAGTTAG AATTAACTTC GTATGCCTCA AGACCCCAAA   
  
  
- GAGATCTCTA AACGTAAAGA TTTAAAATAC CGCTTAACCG ATCAAGAGAA TTAACGTTAG AAACTACTGT   
  
  
- CAAATATCTG GTCATCTAAT ACTTTTAACG AATACTAGAA TATTCCGTCG AACTTGACAT AAGTTTTACC   
  
  
- GTTCATGATC AACTTTTTTC TCGTCGGGGT CACTTCCAAA TGTTTGGATT TGACATTGGT CATAAACCCG   
  
  
- CTCTTCATGT TCCAGAGACA CCCGTCCTGC AAAGACAAAC GTATTTAGTT TGGAAATGGA GTCGAGTACT   
  
  
- AAGGTGTAAG TATAATGTAT AGAAGATATT GTCACTGTTT ATTCTATGGT CACGTACTCT CGAAGAACTC   
  
  
- GATCGACTTC GTCTGACCAA ACTTCGAAGA AGAAACGGTA TAAGACGTAT AAGTAGACAG GTAAAGTAGA   
  
  
- GGTCCAAATC AAGTACAAAC TATCCAGAAC GCAACATAGT TAAAGACACT CTACTTGCTT ACGTTCTATG   
  
  
- AAGGGTTTTA AATTTTCAAT GTCGTGTAAA TCGTGTCCAC ATAATGTTTG AGAAATCCCC GATGTCAAAG   
  
  
- GAGACACTAT ACTACGTACT CGGAAGTGAA AGAATTTAAA TGAAAAGTTC AAATATAATA AGTAGTTATA   
  
  
- TTAGAGACGT TTTATGTCCA AAAACTATTC AATGGTAACT CGAACAACGT GTCAACCTCA GTTGTTGTTC   
  
  
- TTCTTCACCG AAAGGATCTT ATACGATTGA GACAGCCCGA AGGGAAGGAT TGTTGATTAA ATAATGACAG   
  
  
- GACACAACTC CCGTATTATG TCAACACCAA GGGAGAACAG GAACGTGACT GTGTGACAGG AGATGACTCT   
  
  
- ATTTTAACCG TCAATTACAA AGGAAACTG

+     CATT-motif

| Site Name | Organism | Position | Strand | Matrix score. | sequence | function |
| --- | --- | --- | --- | --- | --- | --- |
| CATT-motif | Zea mays | 1108 | - | 6 | GCATTC | part of a light responsive element |

> 2018/04/13 10:10:12  
+ AATCATTGGG GTAAAATTTC AGTAAGGTTT GGTGGTTATA TGAAGTCTTT TAGTGTATGC TCAGAAAGGA   
  
  
+ ATTATTATTA TTTGTACTTC AATTTCAGTT TCATGATTTA GAGGTTCAGA TTCTTCCATG TGAAGAGATA   
  
  
+ CTTTTGTGCT ACTTCTTGTA AGTTGGGAAT CATGGATTTC ATTCGGGTCA TGAATTCTAC ACAATATTGC   
  
  
+ TCTTTCTGAG GCAATGGTTG AAAATAATCC ATCTAGTAAT TTCTTGCTGG TAAATGTTTC TTTCAAGAAG   
  
  
+ TTAGCAAAGT TATCTTTGTT CTTATTGAAG AAGAATATGG AGAGCTACCA AGAAGTATTT CTGCAACTTA   
  
  
+ AAAAAACATT TAGGAGTTGG CATGGATGGT AATGTAATAT TCGGTTATTG ATTAATAGGG CGTAAGCGAT   
  
  
+ AGTTCCCTGC TGGTGATGAG TGAACTGGGG GAGTAACCAA GTGAAAGTGA TAGAGGGGTC TATCTTGCAG   
  
  
+ CTCTCCTTTG ATAGAGAGTT AGTCTGTGAT GTGGTAGAAG TCTGAAGATT GTGCAGCTAT GATTTCTCTG   
  
  
+ CATTATTCTA AATAGTGAGT TATGCAACCA AATTTCAATC TTAATTGAAG CATACGGAGT TCTGGGGTTT   
  
  
+ CTCTAGAGAT TTGCATTTCT AAATTTTATG GCGAATTGGC TAGTTCTCTT AATTGCAATC TTTGATGACA   
  
  
+ GTTTATAGAC CAGTAGATTA TGAAAATTGC TTATGATCTT ATAAGGCAGC TTGAACTGTA TTCAAAATGG   
  
  
+ CAAGTACTAG TTGAAAAAAG AGCAGCCCCA GTGAAGGTTT ACAAACCTAA ACTGTAACCA GTATTTGGGC   
  
  
+ GAGAAGTACA AGGTCTCTGT GGGCAGGACG TTTCTGTTTG CATAAATCAA ACCTTTACCT CAGCTCATGA   
  
  
+ TTCCACATTC ATATTACATA TCTTCTATAA CAGTGACAAA TAAGATACCA GTGCATGAGA GCTTCTTGAG   
  
  
+ CTAGCTGAAG CAGACTGGTT TGAAGCTTCT TCTTTGCCAT ATTCTGCATA TTCATCTGTC CATTTCATCT   
  
  
+ CCAGGTTTAG TTCATGTTTG ATAGGTCTTG CGTTGTATCA ATTTCTGTGA GATGAACGAA TGCAAGATAC   
  
  
+ TTCCCAAAAT TTAAAAGTTA CAGCACATTT AGCACAGGTG TATTACAAAC TCTTTAGGGG CTACAGTTTC   
  
  
+ CTCTGTGATA TGATGCATGA GCCTTCACTT TCTTAAATTT ACTTTTCAAG TTTATATTAT TCATCAATAT   
  
  
+ AATCTCTGCA AAATACAGGT TTTTGATAAG TTACCATTGA GCTTGTTGCA CAGTTGGAGT CAACAACAAG   
  
  
+ AAGAAGTGGC TTTCCTAGAA TATGCTAACT CTGTCGGGCT TCCCTTCCTA ACAACTAATT TATTACTGTC   
  
  
+ CTGTGTTGAG GGCATAATAC AGTTGTGGTT CCCTCTTGTC CTTGCACTGA CACACTGTCC TCTACTGAGA   
  
  
+ TAAAATTGGC AGTTAATGTT TCCTTTGAC  

- TTAGTAACCC CATTTTAAAG TCATTCCAAA CCACCAATAT ACTTCAGAAA ATCACATACG AGTCTTTCCT   
  
  
- TAATAATAAT AAACATGAAG TTAAAGTCAA AGTACTAAAT CTCCAAGTCT AAGAAGGTAC ACTTCTCTAT   
  
  
- GAAAACACGA TGAAGAACAT TCAACCCTTA GTACCTAAAG TAAGCCCAGT ACTTAAGATG TGTTATAACG   
  
  
- AGAAAGACTC CGTTACCAAC TTTTATTAGG TAGATCATTA AAGAACGACC ATTTACAAAG AAAGTTCTTC   
  
  
- AATCGTTTCA ATAGAAACAA GAATAACTTC TTCTTATACC TCTCGATGGT TCTTCATAAA GACGTTGAAT   
  
  
- TTTTTTGTAA ATCCTCAACC GTACCTACCA TTACATTATA AGCCAATAAC TAATTATCCC GCATTCGCTA   
  
  
- TCAAGGGACG ACCACTACTC ACTTGACCCC CTCATTGGTT CACTTTCACT ATCTCCCCAG ATAGAACGTC   
  
  
- GAGAGGAAAC TATCTCTCAA TCAGACACTA CACCATCTTC AGACTTCTAA CACGTCGATA CTAAAGAGAC   
  
  
- GTAATAAGAT TTATCACTCA ATACGTTGGT TTAAAGTTAG AATTAACTTC GTATGCCTCA AGACCCCAAA   
  
  
- GAGATCTCTA AACGTAAAGA TTTAAAATAC CGCTTAACCG ATCAAGAGAA TTAACGTTAG AAACTACTGT   
  
  
- CAAATATCTG GTCATCTAAT ACTTTTAACG AATACTAGAA TATTCCGTCG AACTTGACAT AAGTTTTACC   
  
  
- GTTCATGATC AACTTTTTTC TCGTCGGGGT CACTTCCAAA TGTTTGGATT TGACATTGGT CATAAACCCG   
  
  
- CTCTTCATGT TCCAGAGACA CCCGTCCTGC AAAGACAAAC GTATTTAGTT TGGAAATGGA GTCGAGTACT   
  
  
- AAGGTGTAAG TATAATGTAT AGAAGATATT GTCACTGTTT ATTCTATGGT CACGTACTCT CGAAGAACTC   
  
  
- GATCGACTTC GTCTGACCAA ACTTCGAAGA AGAAACGGTA TAAGACGTAT AAGTAGACAG GTAAAGTAGA   
  
  
- GGTCCAAATC AAGTACAAAC TATCCAGAAC GCAACATAGT TAAAGACACT CTACTTGCTT ACGTTCTATG   
  
  
- AAGGGTTTTA AATTTTCAAT GTCGTGTAAA TCGTGTCCAC ATAATGTTTG AGAAATCCCC GATGTCAAAG   
  
  
- GAGACACTAT ACTACGTACT CGGAAGTGAA AGAATTTAAA TGAAAAGTTC AAATATAATA AGTAGTTATA   
  
  
- TTAGAGACGT TTTATGTCCA AAAACTATTC AATGGTAACT CGAACAACGT GTCAACCTCA GTTGTTGTTC   
  
  
- TTCTTCACCG AAAGGATCTT ATACGATTGA GACAGCCCGA AGGGAAGGAT TGTTGATTAA ATAATGACAG   
  
  
- GACACAACTC CCGTATTATG TCAACACCAA GGGAGAACAG GAACGTGACT GTGTGACAGG AGATGACTCT   
  
  
- ATTTTAACCG TCAATTACAA AGGAAACTG

+     G-box

| Site Name | Organism | Position | Strand | Matrix score. | sequence | function |
| --- | --- | --- | --- | --- | --- | --- |
| G-box | Solanum tuberosum | 126 | - | 7 | CACATGG | cis-acting regulatory element involved in light responsiveness |

> 2018/04/13 10:10:12  
+ AATCATTGGG GTAAAATTTC AGTAAGGTTT GGTGGTTATA TGAAGTCTTT TAGTGTATGC TCAGAAAGGA   
  
  
+ ATTATTATTA TTTGTACTTC AATTTCAGTT TCATGATTTA GAGGTTCAGA TTCTTCCATG TGAAGAGATA   
  
  
+ CTTTTGTGCT ACTTCTTGTA AGTTGGGAAT CATGGATTTC ATTCGGGTCA TGAATTCTAC ACAATATTGC   
  
  
+ TCTTTCTGAG GCAATGGTTG AAAATAATCC ATCTAGTAAT TTCTTGCTGG TAAATGTTTC TTTCAAGAAG   
  
  
+ TTAGCAAAGT TATCTTTGTT CTTATTGAAG AAGAATATGG AGAGCTACCA AGAAGTATTT CTGCAACTTA   
  
  
+ AAAAAACATT TAGGAGTTGG CATGGATGGT AATGTAATAT TCGGTTATTG ATTAATAGGG CGTAAGCGAT   
  
  
+ AGTTCCCTGC TGGTGATGAG TGAACTGGGG GAGTAACCAA GTGAAAGTGA TAGAGGGGTC TATCTTGCAG   
  
  
+ CTCTCCTTTG ATAGAGAGTT AGTCTGTGAT GTGGTAGAAG TCTGAAGATT GTGCAGCTAT GATTTCTCTG   
  
  
+ CATTATTCTA AATAGTGAGT TATGCAACCA AATTTCAATC TTAATTGAAG CATACGGAGT TCTGGGGTTT   
  
  
+ CTCTAGAGAT TTGCATTTCT AAATTTTATG GCGAATTGGC TAGTTCTCTT AATTGCAATC TTTGATGACA   
  
  
+ GTTTATAGAC CAGTAGATTA TGAAAATTGC TTATGATCTT ATAAGGCAGC TTGAACTGTA TTCAAAATGG   
  
  
+ CAAGTACTAG TTGAAAAAAG AGCAGCCCCA GTGAAGGTTT ACAAACCTAA ACTGTAACCA GTATTTGGGC   
  
  
+ GAGAAGTACA AGGTCTCTGT GGGCAGGACG TTTCTGTTTG CATAAATCAA ACCTTTACCT CAGCTCATGA   
  
  
+ TTCCACATTC ATATTACATA TCTTCTATAA CAGTGACAAA TAAGATACCA GTGCATGAGA GCTTCTTGAG   
  
  
+ CTAGCTGAAG CAGACTGGTT TGAAGCTTCT TCTTTGCCAT ATTCTGCATA TTCATCTGTC CATTTCATCT   
  
  
+ CCAGGTTTAG TTCATGTTTG ATAGGTCTTG CGTTGTATCA ATTTCTGTGA GATGAACGAA TGCAAGATAC   
  
  
+ TTCCCAAAAT TTAAAAGTTA CAGCACATTT AGCACAGGTG TATTACAAAC TCTTTAGGGG CTACAGTTTC   
  
  
+ CTCTGTGATA TGATGCATGA GCCTTCACTT TCTTAAATTT ACTTTTCAAG TTTATATTAT TCATCAATAT   
  
  
+ AATCTCTGCA AAATACAGGT TTTTGATAAG TTACCATTGA GCTTGTTGCA CAGTTGGAGT CAACAACAAG   
  
  
+ AAGAAGTGGC TTTCCTAGAA TATGCTAACT CTGTCGGGCT TCCCTTCCTA ACAACTAATT TATTACTGTC   
  
  
+ CTGTGTTGAG GGCATAATAC AGTTGTGGTT CCCTCTTGTC CTTGCACTGA CACACTGTCC TCTACTGAGA   
  
  
+ TAAAATTGGC AGTTAATGTT TCCTTTGAC  

- TTAGTAACCC CATTTTAAAG TCATTCCAAA CCACCAATAT ACTTCAGAAA ATCACATACG AGTCTTTCCT   
  
  
- TAATAATAAT AAACATGAAG TTAAAGTCAA AGTACTAAAT CTCCAAGTCT AAGAAGGTAC ACTTCTCTAT   
  
  
- GAAAACACGA TGAAGAACAT TCAACCCTTA GTACCTAAAG TAAGCCCAGT ACTTAAGATG TGTTATAACG   
  
  
- AGAAAGACTC CGTTACCAAC TTTTATTAGG TAGATCATTA AAGAACGACC ATTTACAAAG AAAGTTCTTC   
  
  
- AATCGTTTCA ATAGAAACAA GAATAACTTC TTCTTATACC TCTCGATGGT TCTTCATAAA GACGTTGAAT   
  
  
- TTTTTTGTAA ATCCTCAACC GTACCTACCA TTACATTATA AGCCAATAAC TAATTATCCC GCATTCGCTA   
  
  
- TCAAGGGACG ACCACTACTC ACTTGACCCC CTCATTGGTT CACTTTCACT ATCTCCCCAG ATAGAACGTC   
  
  
- GAGAGGAAAC TATCTCTCAA TCAGACACTA CACCATCTTC AGACTTCTAA CACGTCGATA CTAAAGAGAC   
  
  
- GTAATAAGAT TTATCACTCA ATACGTTGGT TTAAAGTTAG AATTAACTTC GTATGCCTCA AGACCCCAAA   
  
  
- GAGATCTCTA AACGTAAAGA TTTAAAATAC CGCTTAACCG ATCAAGAGAA TTAACGTTAG AAACTACTGT   
  
  
- CAAATATCTG GTCATCTAAT ACTTTTAACG AATACTAGAA TATTCCGTCG AACTTGACAT AAGTTTTACC   
  
  
- GTTCATGATC AACTTTTTTC TCGTCGGGGT CACTTCCAAA TGTTTGGATT TGACATTGGT CATAAACCCG   
  
  
- CTCTTCATGT TCCAGAGACA CCCGTCCTGC AAAGACAAAC GTATTTAGTT TGGAAATGGA GTCGAGTACT   
  
  
- AAGGTGTAAG TATAATGTAT AGAAGATATT GTCACTGTTT ATTCTATGGT CACGTACTCT CGAAGAACTC   
  
  
- GATCGACTTC GTCTGACCAA ACTTCGAAGA AGAAACGGTA TAAGACGTAT AAGTAGACAG GTAAAGTAGA   
  
  
- GGTCCAAATC AAGTACAAAC TATCCAGAAC GCAACATAGT TAAAGACACT CTACTTGCTT ACGTTCTATG   
  
  
- AAGGGTTTTA AATTTTCAAT GTCGTGTAAA TCGTGTCCAC ATAATGTTTG AGAAATCCCC GATGTCAAAG   
  
  
- GAGACACTAT ACTACGTACT CGGAAGTGAA AGAATTTAAA TGAAAAGTTC AAATATAATA AGTAGTTATA   
  
  
- TTAGAGACGT TTTATGTCCA AAAACTATTC AATGGTAACT CGAACAACGT GTCAACCTCA GTTGTTGTTC   
  
  
- TTCTTCACCG AAAGGATCTT ATACGATTGA GACAGCCCGA AGGGAAGGAT TGTTGATTAA ATAATGACAG   
  
  
- GACACAACTC CCGTATTATG TCAACACCAA GGGAGAACAG GAACGTGACT GTGTGACAGG AGATGACTCT   
  
  
- ATTTTAACCG TCAATTACAA AGGAAACTG

+     GAG-motif

| Site Name | Organism | Position | Strand | Matrix score. | sequence | function |
| --- | --- | --- | --- | --- | --- | --- |
| GAG-motif | Arabidopsis thaliana | 503 | + | 7 | AGAGAGT | part of a light responsive element |
| GAG-motif | Hordeum vulgare | 1046 | - | 7 | GGAGATG | part of a light responsive element |

> 2018/04/13 10:10:12  
+ AATCATTGGG GTAAAATTTC AGTAAGGTTT GGTGGTTATA TGAAGTCTTT TAGTGTATGC TCAGAAAGGA   
  
  
+ ATTATTATTA TTTGTACTTC AATTTCAGTT TCATGATTTA GAGGTTCAGA TTCTTCCATG TGAAGAGATA   
  
  
+ CTTTTGTGCT ACTTCTTGTA AGTTGGGAAT CATGGATTTC ATTCGGGTCA TGAATTCTAC ACAATATTGC   
  
  
+ TCTTTCTGAG GCAATGGTTG AAAATAATCC ATCTAGTAAT TTCTTGCTGG TAAATGTTTC TTTCAAGAAG   
  
  
+ TTAGCAAAGT TATCTTTGTT CTTATTGAAG AAGAATATGG AGAGCTACCA AGAAGTATTT CTGCAACTTA   
  
  
+ AAAAAACATT TAGGAGTTGG CATGGATGGT AATGTAATAT TCGGTTATTG ATTAATAGGG CGTAAGCGAT   
  
  
+ AGTTCCCTGC TGGTGATGAG TGAACTGGGG GAGTAACCAA GTGAAAGTGA TAGAGGGGTC TATCTTGCAG   
  
  
+ CTCTCCTTTG ATAGAGAGTT AGTCTGTGAT GTGGTAGAAG TCTGAAGATT GTGCAGCTAT GATTTCTCTG   
  
  
+ CATTATTCTA AATAGTGAGT TATGCAACCA AATTTCAATC TTAATTGAAG CATACGGAGT TCTGGGGTTT   
  
  
+ CTCTAGAGAT TTGCATTTCT AAATTTTATG GCGAATTGGC TAGTTCTCTT AATTGCAATC TTTGATGACA   
  
  
+ GTTTATAGAC CAGTAGATTA TGAAAATTGC TTATGATCTT ATAAGGCAGC TTGAACTGTA TTCAAAATGG   
  
  
+ CAAGTACTAG TTGAAAAAAG AGCAGCCCCA GTGAAGGTTT ACAAACCTAA ACTGTAACCA GTATTTGGGC   
  
  
+ GAGAAGTACA AGGTCTCTGT GGGCAGGACG TTTCTGTTTG CATAAATCAA ACCTTTACCT CAGCTCATGA   
  
  
+ TTCCACATTC ATATTACATA TCTTCTATAA CAGTGACAAA TAAGATACCA GTGCATGAGA GCTTCTTGAG   
  
  
+ CTAGCTGAAG CAGACTGGTT TGAAGCTTCT TCTTTGCCAT ATTCTGCATA TTCATCTGTC CATTTCATCT   
  
  
+ CCAGGTTTAG TTCATGTTTG ATAGGTCTTG CGTTGTATCA ATTTCTGTGA GATGAACGAA TGCAAGATAC   
  
  
+ TTCCCAAAAT TTAAAAGTTA CAGCACATTT AGCACAGGTG TATTACAAAC TCTTTAGGGG CTACAGTTTC   
  
  
+ CTCTGTGATA TGATGCATGA GCCTTCACTT TCTTAAATTT ACTTTTCAAG TTTATATTAT TCATCAATAT   
  
  
+ AATCTCTGCA AAATACAGGT TTTTGATAAG TTACCATTGA GCTTGTTGCA CAGTTGGAGT CAACAACAAG   
  
  
+ AAGAAGTGGC TTTCCTAGAA TATGCTAACT CTGTCGGGCT TCCCTTCCTA ACAACTAATT TATTACTGTC   
  
  
+ CTGTGTTGAG GGCATAATAC AGTTGTGGTT CCCTCTTGTC CTTGCACTGA CACACTGTCC TCTACTGAGA   
  
  
+ TAAAATTGGC AGTTAATGTT TCCTTTGAC  

- TTAGTAACCC CATTTTAAAG TCATTCCAAA CCACCAATAT ACTTCAGAAA ATCACATACG AGTCTTTCCT   
  
  
- TAATAATAAT AAACATGAAG TTAAAGTCAA AGTACTAAAT CTCCAAGTCT AAGAAGGTAC ACTTCTCTAT   
  
  
- GAAAACACGA TGAAGAACAT TCAACCCTTA GTACCTAAAG TAAGCCCAGT ACTTAAGATG TGTTATAACG   
  
  
- AGAAAGACTC CGTTACCAAC TTTTATTAGG TAGATCATTA AAGAACGACC ATTTACAAAG AAAGTTCTTC   
  
  
- AATCGTTTCA ATAGAAACAA GAATAACTTC TTCTTATACC TCTCGATGGT TCTTCATAAA GACGTTGAAT   
  
  
- TTTTTTGTAA ATCCTCAACC GTACCTACCA TTACATTATA AGCCAATAAC TAATTATCCC GCATTCGCTA   
  
  
- TCAAGGGACG ACCACTACTC ACTTGACCCC CTCATTGGTT CACTTTCACT ATCTCCCCAG ATAGAACGTC   
  
  
- GAGAGGAAAC TATCTCTCAA TCAGACACTA CACCATCTTC AGACTTCTAA CACGTCGATA CTAAAGAGAC   
  
  
- GTAATAAGAT TTATCACTCA ATACGTTGGT TTAAAGTTAG AATTAACTTC GTATGCCTCA AGACCCCAAA   
  
  
- GAGATCTCTA AACGTAAAGA TTTAAAATAC CGCTTAACCG ATCAAGAGAA TTAACGTTAG AAACTACTGT   
  
  
- CAAATATCTG GTCATCTAAT ACTTTTAACG AATACTAGAA TATTCCGTCG AACTTGACAT AAGTTTTACC   
  
  
- GTTCATGATC AACTTTTTTC TCGTCGGGGT CACTTCCAAA TGTTTGGATT TGACATTGGT CATAAACCCG   
  
  
- CTCTTCATGT TCCAGAGACA CCCGTCCTGC AAAGACAAAC GTATTTAGTT TGGAAATGGA GTCGAGTACT   
  
  
- AAGGTGTAAG TATAATGTAT AGAAGATATT GTCACTGTTT ATTCTATGGT CACGTACTCT CGAAGAACTC   
  
  
- GATCGACTTC GTCTGACCAA ACTTCGAAGA AGAAACGGTA TAAGACGTAT AAGTAGACAG GTAAAGTAGA   
  
  
- GGTCCAAATC AAGTACAAAC TATCCAGAAC GCAACATAGT TAAAGACACT CTACTTGCTT ACGTTCTATG   
  
  
- AAGGGTTTTA AATTTTCAAT GTCGTGTAAA TCGTGTCCAC ATAATGTTTG AGAAATCCCC GATGTCAAAG   
  
  
- GAGACACTAT ACTACGTACT CGGAAGTGAA AGAATTTAAA TGAAAAGTTC AAATATAATA AGTAGTTATA   
  
  
- TTAGAGACGT TTTATGTCCA AAAACTATTC AATGGTAACT CGAACAACGT GTCAACCTCA GTTGTTGTTC   
  
  
- TTCTTCACCG AAAGGATCTT ATACGATTGA GACAGCCCGA AGGGAAGGAT TGTTGATTAA ATAATGACAG   
  
  
- GACACAACTC CCGTATTATG TCAACACCAA GGGAGAACAG GAACGTGACT GTGTGACAGG AGATGACTCT   
  
  
- ATTTTAACCG TCAATTACAA AGGAAACTG

+     GARE-motif

| Site Name | Organism | Position | Strand | Matrix score. | sequence | function |
| --- | --- | --- | --- | --- | --- | --- |
| GARE-motif | Brassica oleracea | 873 | - | 7 | AAACAGA | gibberellin-responsive element |

> 2018/04/13 10:10:12  
+ AATCATTGGG GTAAAATTTC AGTAAGGTTT GGTGGTTATA TGAAGTCTTT TAGTGTATGC TCAGAAAGGA   
  
  
+ ATTATTATTA TTTGTACTTC AATTTCAGTT TCATGATTTA GAGGTTCAGA TTCTTCCATG TGAAGAGATA   
  
  
+ CTTTTGTGCT ACTTCTTGTA AGTTGGGAAT CATGGATTTC ATTCGGGTCA TGAATTCTAC ACAATATTGC   
  
  
+ TCTTTCTGAG GCAATGGTTG AAAATAATCC ATCTAGTAAT TTCTTGCTGG TAAATGTTTC TTTCAAGAAG   
  
  
+ TTAGCAAAGT TATCTTTGTT CTTATTGAAG AAGAATATGG AGAGCTACCA AGAAGTATTT CTGCAACTTA   
  
  
+ AAAAAACATT TAGGAGTTGG CATGGATGGT AATGTAATAT TCGGTTATTG ATTAATAGGG CGTAAGCGAT   
  
  
+ AGTTCCCTGC TGGTGATGAG TGAACTGGGG GAGTAACCAA GTGAAAGTGA TAGAGGGGTC TATCTTGCAG   
  
  
+ CTCTCCTTTG ATAGAGAGTT AGTCTGTGAT GTGGTAGAAG TCTGAAGATT GTGCAGCTAT GATTTCTCTG   
  
  
+ CATTATTCTA AATAGTGAGT TATGCAACCA AATTTCAATC TTAATTGAAG CATACGGAGT TCTGGGGTTT   
  
  
+ CTCTAGAGAT TTGCATTTCT AAATTTTATG GCGAATTGGC TAGTTCTCTT AATTGCAATC TTTGATGACA   
  
  
+ GTTTATAGAC CAGTAGATTA TGAAAATTGC TTATGATCTT ATAAGGCAGC TTGAACTGTA TTCAAAATGG   
  
  
+ CAAGTACTAG TTGAAAAAAG AGCAGCCCCA GTGAAGGTTT ACAAACCTAA ACTGTAACCA GTATTTGGGC   
  
  
+ GAGAAGTACA AGGTCTCTGT GGGCAGGACG TTTCTGTTTG CATAAATCAA ACCTTTACCT CAGCTCATGA   
  
  
+ TTCCACATTC ATATTACATA TCTTCTATAA CAGTGACAAA TAAGATACCA GTGCATGAGA GCTTCTTGAG   
  
  
+ CTAGCTGAAG CAGACTGGTT TGAAGCTTCT TCTTTGCCAT ATTCTGCATA TTCATCTGTC CATTTCATCT   
  
  
+ CCAGGTTTAG TTCATGTTTG ATAGGTCTTG CGTTGTATCA ATTTCTGTGA GATGAACGAA TGCAAGATAC   
  
  
+ TTCCCAAAAT TTAAAAGTTA CAGCACATTT AGCACAGGTG TATTACAAAC TCTTTAGGGG CTACAGTTTC   
  
  
+ CTCTGTGATA TGATGCATGA GCCTTCACTT TCTTAAATTT ACTTTTCAAG TTTATATTAT TCATCAATAT   
  
  
+ AATCTCTGCA AAATACAGGT TTTTGATAAG TTACCATTGA GCTTGTTGCA CAGTTGGAGT CAACAACAAG   
  
  
+ AAGAAGTGGC TTTCCTAGAA TATGCTAACT CTGTCGGGCT TCCCTTCCTA ACAACTAATT TATTACTGTC   
  
  
+ CTGTGTTGAG GGCATAATAC AGTTGTGGTT CCCTCTTGTC CTTGCACTGA CACACTGTCC TCTACTGAGA   
  
  
+ TAAAATTGGC AGTTAATGTT TCCTTTGAC  

- TTAGTAACCC CATTTTAAAG TCATTCCAAA CCACCAATAT ACTTCAGAAA ATCACATACG AGTCTTTCCT   
  
  
- TAATAATAAT AAACATGAAG TTAAAGTCAA AGTACTAAAT CTCCAAGTCT AAGAAGGTAC ACTTCTCTAT   
  
  
- GAAAACACGA TGAAGAACAT TCAACCCTTA GTACCTAAAG TAAGCCCAGT ACTTAAGATG TGTTATAACG   
  
  
- AGAAAGACTC CGTTACCAAC TTTTATTAGG TAGATCATTA AAGAACGACC ATTTACAAAG AAAGTTCTTC   
  
  
- AATCGTTTCA ATAGAAACAA GAATAACTTC TTCTTATACC TCTCGATGGT TCTTCATAAA GACGTTGAAT   
  
  
- TTTTTTGTAA ATCCTCAACC GTACCTACCA TTACATTATA AGCCAATAAC TAATTATCCC GCATTCGCTA   
  
  
- TCAAGGGACG ACCACTACTC ACTTGACCCC CTCATTGGTT CACTTTCACT ATCTCCCCAG ATAGAACGTC   
  
  
- GAGAGGAAAC TATCTCTCAA TCAGACACTA CACCATCTTC AGACTTCTAA CACGTCGATA CTAAAGAGAC   
  
  
- GTAATAAGAT TTATCACTCA ATACGTTGGT TTAAAGTTAG AATTAACTTC GTATGCCTCA AGACCCCAAA   
  
  
- GAGATCTCTA AACGTAAAGA TTTAAAATAC CGCTTAACCG ATCAAGAGAA TTAACGTTAG AAACTACTGT   
  
  
- CAAATATCTG GTCATCTAAT ACTTTTAACG AATACTAGAA TATTCCGTCG AACTTGACAT AAGTTTTACC   
  
  
- GTTCATGATC AACTTTTTTC TCGTCGGGGT CACTTCCAAA TGTTTGGATT TGACATTGGT CATAAACCCG   
  
  
- CTCTTCATGT TCCAGAGACA CCCGTCCTGC AAAGACAAAC GTATTTAGTT TGGAAATGGA GTCGAGTACT   
  
  
- AAGGTGTAAG TATAATGTAT AGAAGATATT GTCACTGTTT ATTCTATGGT CACGTACTCT CGAAGAACTC   
  
  
- GATCGACTTC GTCTGACCAA ACTTCGAAGA AGAAACGGTA TAAGACGTAT AAGTAGACAG GTAAAGTAGA   
  
  
- GGTCCAAATC AAGTACAAAC TATCCAGAAC GCAACATAGT TAAAGACACT CTACTTGCTT ACGTTCTATG   
  
  
- AAGGGTTTTA AATTTTCAAT GTCGTGTAAA TCGTGTCCAC ATAATGTTTG AGAAATCCCC GATGTCAAAG   
  
  
- GAGACACTAT ACTACGTACT CGGAAGTGAA AGAATTTAAA TGAAAAGTTC AAATATAATA AGTAGTTATA   
  
  
- TTAGAGACGT TTTATGTCCA AAAACTATTC AATGGTAACT CGAACAACGT GTCAACCTCA GTTGTTGTTC   
  
  
- TTCTTCACCG AAAGGATCTT ATACGATTGA GACAGCCCGA AGGGAAGGAT TGTTGATTAA ATAATGACAG   
  
  
- GACACAACTC CCGTATTATG TCAACACCAA GGGAGAACAG GAACGTGACT GTGTGACAGG AGATGACTCT   
  
  
- ATTTTAACCG TCAATTACAA AGGAAACTG

+     GCN4\_motif

| Site Name | Organism | Position | Strand | Matrix score. | sequence | function |
| --- | --- | --- | --- | --- | --- | --- |
| GCN4\_motif | Oryza sativa | 1448 | - | 7 | TGTGTCA | cis-regulatory element involved in endosperm expression |

> 2018/04/13 10:10:12  
+ AATCATTGGG GTAAAATTTC AGTAAGGTTT GGTGGTTATA TGAAGTCTTT TAGTGTATGC TCAGAAAGGA   
  
  
+ ATTATTATTA TTTGTACTTC AATTTCAGTT TCATGATTTA GAGGTTCAGA TTCTTCCATG TGAAGAGATA   
  
  
+ CTTTTGTGCT ACTTCTTGTA AGTTGGGAAT CATGGATTTC ATTCGGGTCA TGAATTCTAC ACAATATTGC   
  
  
+ TCTTTCTGAG GCAATGGTTG AAAATAATCC ATCTAGTAAT TTCTTGCTGG TAAATGTTTC TTTCAAGAAG   
  
  
+ TTAGCAAAGT TATCTTTGTT CTTATTGAAG AAGAATATGG AGAGCTACCA AGAAGTATTT CTGCAACTTA   
  
  
+ AAAAAACATT TAGGAGTTGG CATGGATGGT AATGTAATAT TCGGTTATTG ATTAATAGGG CGTAAGCGAT   
  
  
+ AGTTCCCTGC TGGTGATGAG TGAACTGGGG GAGTAACCAA GTGAAAGTGA TAGAGGGGTC TATCTTGCAG   
  
  
+ CTCTCCTTTG ATAGAGAGTT AGTCTGTGAT GTGGTAGAAG TCTGAAGATT GTGCAGCTAT GATTTCTCTG   
  
  
+ CATTATTCTA AATAGTGAGT TATGCAACCA AATTTCAATC TTAATTGAAG CATACGGAGT TCTGGGGTTT   
  
  
+ CTCTAGAGAT TTGCATTTCT AAATTTTATG GCGAATTGGC TAGTTCTCTT AATTGCAATC TTTGATGACA   
  
  
+ GTTTATAGAC CAGTAGATTA TGAAAATTGC TTATGATCTT ATAAGGCAGC TTGAACTGTA TTCAAAATGG   
  
  
+ CAAGTACTAG TTGAAAAAAG AGCAGCCCCA GTGAAGGTTT ACAAACCTAA ACTGTAACCA GTATTTGGGC   
  
  
+ GAGAAGTACA AGGTCTCTGT GGGCAGGACG TTTCTGTTTG CATAAATCAA ACCTTTACCT CAGCTCATGA   
  
  
+ TTCCACATTC ATATTACATA TCTTCTATAA CAGTGACAAA TAAGATACCA GTGCATGAGA GCTTCTTGAG   
  
  
+ CTAGCTGAAG CAGACTGGTT TGAAGCTTCT TCTTTGCCAT ATTCTGCATA TTCATCTGTC CATTTCATCT   
  
  
+ CCAGGTTTAG TTCATGTTTG ATAGGTCTTG CGTTGTATCA ATTTCTGTGA GATGAACGAA TGCAAGATAC   
  
  
+ TTCCCAAAAT TTAAAAGTTA CAGCACATTT AGCACAGGTG TATTACAAAC TCTTTAGGGG CTACAGTTTC   
  
  
+ CTCTGTGATA TGATGCATGA GCCTTCACTT TCTTAAATTT ACTTTTCAAG TTTATATTAT TCATCAATAT   
  
  
+ AATCTCTGCA AAATACAGGT TTTTGATAAG TTACCATTGA GCTTGTTGCA CAGTTGGAGT CAACAACAAG   
  
  
+ AAGAAGTGGC TTTCCTAGAA TATGCTAACT CTGTCGGGCT TCCCTTCCTA ACAACTAATT TATTACTGTC   
  
  
+ CTGTGTTGAG GGCATAATAC AGTTGTGGTT CCCTCTTGTC CTTGCACTGA CACACTGTCC TCTACTGAGA   
  
  
+ TAAAATTGGC AGTTAATGTT TCCTTTGAC  

- TTAGTAACCC CATTTTAAAG TCATTCCAAA CCACCAATAT ACTTCAGAAA ATCACATACG AGTCTTTCCT   
  
  
- TAATAATAAT AAACATGAAG TTAAAGTCAA AGTACTAAAT CTCCAAGTCT AAGAAGGTAC ACTTCTCTAT   
  
  
- GAAAACACGA TGAAGAACAT TCAACCCTTA GTACCTAAAG TAAGCCCAGT ACTTAAGATG TGTTATAACG   
  
  
- AGAAAGACTC CGTTACCAAC TTTTATTAGG TAGATCATTA AAGAACGACC ATTTACAAAG AAAGTTCTTC   
  
  
- AATCGTTTCA ATAGAAACAA GAATAACTTC TTCTTATACC TCTCGATGGT TCTTCATAAA GACGTTGAAT   
  
  
- TTTTTTGTAA ATCCTCAACC GTACCTACCA TTACATTATA AGCCAATAAC TAATTATCCC GCATTCGCTA   
  
  
- TCAAGGGACG ACCACTACTC ACTTGACCCC CTCATTGGTT CACTTTCACT ATCTCCCCAG ATAGAACGTC   
  
  
- GAGAGGAAAC TATCTCTCAA TCAGACACTA CACCATCTTC AGACTTCTAA CACGTCGATA CTAAAGAGAC   
  
  
- GTAATAAGAT TTATCACTCA ATACGTTGGT TTAAAGTTAG AATTAACTTC GTATGCCTCA AGACCCCAAA   
  
  
- GAGATCTCTA AACGTAAAGA TTTAAAATAC CGCTTAACCG ATCAAGAGAA TTAACGTTAG AAACTACTGT   
  
  
- CAAATATCTG GTCATCTAAT ACTTTTAACG AATACTAGAA TATTCCGTCG AACTTGACAT AAGTTTTACC   
  
  
- GTTCATGATC AACTTTTTTC TCGTCGGGGT CACTTCCAAA TGTTTGGATT TGACATTGGT CATAAACCCG   
  
  
- CTCTTCATGT TCCAGAGACA CCCGTCCTGC AAAGACAAAC GTATTTAGTT TGGAAATGGA GTCGAGTACT   
  
  
- AAGGTGTAAG TATAATGTAT AGAAGATATT GTCACTGTTT ATTCTATGGT CACGTACTCT CGAAGAACTC   
  
  
- GATCGACTTC GTCTGACCAA ACTTCGAAGA AGAAACGGTA TAAGACGTAT AAGTAGACAG GTAAAGTAGA   
  
  
- GGTCCAAATC AAGTACAAAC TATCCAGAAC GCAACATAGT TAAAGACACT CTACTTGCTT ACGTTCTATG   
  
  
- AAGGGTTTTA AATTTTCAAT GTCGTGTAAA TCGTGTCCAC ATAATGTTTG AGAAATCCCC GATGTCAAAG   
  
  
- GAGACACTAT ACTACGTACT CGGAAGTGAA AGAATTTAAA TGAAAAGTTC AAATATAATA AGTAGTTATA   
  
  
- TTAGAGACGT TTTATGTCCA AAAACTATTC AATGGTAACT CGAACAACGT GTCAACCTCA GTTGTTGTTC   
  
  
- TTCTTCACCG AAAGGATCTT ATACGATTGA GACAGCCCGA AGGGAAGGAT TGTTGATTAA ATAATGACAG   
  
  
- GACACAACTC CCGTATTATG TCAACACCAA GGGAGAACAG GAACGTGACT GTGTGACAGG AGATGACTCT   
  
  
- ATTTTAACCG TCAATTACAA AGGAAACTG

+     MBS

| Site Name | Organism | Position | Strand | Matrix score. | sequence | function |
| --- | --- | --- | --- | --- | --- | --- |
| MBS | Arabidopsis thaliana | 1311 | - | 6 | CAACTG | MYB binding site involved in drought-inducibility |
| MBS | Arabidopsis thaliana | 1480 | - | 6 | TAACTG | MYB binding site involved in drought-inducibility |
| MBS | Arabidopsis thaliana | 1420 | - | 6 | CAACTG | MYB binding site involved in drought-inducibility |

> 2018/04/13 10:10:12  
+ AATCATTGGG GTAAAATTTC AGTAAGGTTT GGTGGTTATA TGAAGTCTTT TAGTGTATGC TCAGAAAGGA   
  
  
+ ATTATTATTA TTTGTACTTC AATTTCAGTT TCATGATTTA GAGGTTCAGA TTCTTCCATG TGAAGAGATA   
  
  
+ CTTTTGTGCT ACTTCTTGTA AGTTGGGAAT CATGGATTTC ATTCGGGTCA TGAATTCTAC ACAATATTGC   
  
  
+ TCTTTCTGAG GCAATGGTTG AAAATAATCC ATCTAGTAAT TTCTTGCTGG TAAATGTTTC TTTCAAGAAG   
  
  
+ TTAGCAAAGT TATCTTTGTT CTTATTGAAG AAGAATATGG AGAGCTACCA AGAAGTATTT CTGCAACTTA   
  
  
+ AAAAAACATT TAGGAGTTGG CATGGATGGT AATGTAATAT TCGGTTATTG ATTAATAGGG CGTAAGCGAT   
  
  
+ AGTTCCCTGC TGGTGATGAG TGAACTGGGG GAGTAACCAA GTGAAAGTGA TAGAGGGGTC TATCTTGCAG   
  
  
+ CTCTCCTTTG ATAGAGAGTT AGTCTGTGAT GTGGTAGAAG TCTGAAGATT GTGCAGCTAT GATTTCTCTG   
  
  
+ CATTATTCTA AATAGTGAGT TATGCAACCA AATTTCAATC TTAATTGAAG CATACGGAGT TCTGGGGTTT   
  
  
+ CTCTAGAGAT TTGCATTTCT AAATTTTATG GCGAATTGGC TAGTTCTCTT AATTGCAATC TTTGATGACA   
  
  
+ GTTTATAGAC CAGTAGATTA TGAAAATTGC TTATGATCTT ATAAGGCAGC TTGAACTGTA TTCAAAATGG   
  
  
+ CAAGTACTAG TTGAAAAAAG AGCAGCCCCA GTGAAGGTTT ACAAACCTAA ACTGTAACCA GTATTTGGGC   
  
  
+ GAGAAGTACA AGGTCTCTGT GGGCAGGACG TTTCTGTTTG CATAAATCAA ACCTTTACCT CAGCTCATGA   
  
  
+ TTCCACATTC ATATTACATA TCTTCTATAA CAGTGACAAA TAAGATACCA GTGCATGAGA GCTTCTTGAG   
  
  
+ CTAGCTGAAG CAGACTGGTT TGAAGCTTCT TCTTTGCCAT ATTCTGCATA TTCATCTGTC CATTTCATCT   
  
  
+ CCAGGTTTAG TTCATGTTTG ATAGGTCTTG CGTTGTATCA ATTTCTGTGA GATGAACGAA TGCAAGATAC   
  
  
+ TTCCCAAAAT TTAAAAGTTA CAGCACATTT AGCACAGGTG TATTACAAAC TCTTTAGGGG CTACAGTTTC   
  
  
+ CTCTGTGATA TGATGCATGA GCCTTCACTT TCTTAAATTT ACTTTTCAAG TTTATATTAT TCATCAATAT   
  
  
+ AATCTCTGCA AAATACAGGT TTTTGATAAG TTACCATTGA GCTTGTTGCA CAGTTGGAGT CAACAACAAG   
  
  
+ AAGAAGTGGC TTTCCTAGAA TATGCTAACT CTGTCGGGCT TCCCTTCCTA ACAACTAATT TATTACTGTC   
  
  
+ CTGTGTTGAG GGCATAATAC AGTTGTGGTT CCCTCTTGTC CTTGCACTGA CACACTGTCC TCTACTGAGA   
  
  
+ TAAAATTGGC AGTTAATGTT TCCTTTGAC  

- TTAGTAACCC CATTTTAAAG TCATTCCAAA CCACCAATAT ACTTCAGAAA ATCACATACG AGTCTTTCCT   
  
  
- TAATAATAAT AAACATGAAG TTAAAGTCAA AGTACTAAAT CTCCAAGTCT AAGAAGGTAC ACTTCTCTAT   
  
  
- GAAAACACGA TGAAGAACAT TCAACCCTTA GTACCTAAAG TAAGCCCAGT ACTTAAGATG TGTTATAACG   
  
  
- AGAAAGACTC CGTTACCAAC TTTTATTAGG TAGATCATTA AAGAACGACC ATTTACAAAG AAAGTTCTTC   
  
  
- AATCGTTTCA ATAGAAACAA GAATAACTTC TTCTTATACC TCTCGATGGT TCTTCATAAA GACGTTGAAT   
  
  
- TTTTTTGTAA ATCCTCAACC GTACCTACCA TTACATTATA AGCCAATAAC TAATTATCCC GCATTCGCTA   
  
  
- TCAAGGGACG ACCACTACTC ACTTGACCCC CTCATTGGTT CACTTTCACT ATCTCCCCAG ATAGAACGTC   
  
  
- GAGAGGAAAC TATCTCTCAA TCAGACACTA CACCATCTTC AGACTTCTAA CACGTCGATA CTAAAGAGAC   
  
  
- GTAATAAGAT TTATCACTCA ATACGTTGGT TTAAAGTTAG AATTAACTTC GTATGCCTCA AGACCCCAAA   
  
  
- GAGATCTCTA AACGTAAAGA TTTAAAATAC CGCTTAACCG ATCAAGAGAA TTAACGTTAG AAACTACTGT   
  
  
- CAAATATCTG GTCATCTAAT ACTTTTAACG AATACTAGAA TATTCCGTCG AACTTGACAT AAGTTTTACC   
  
  
- GTTCATGATC AACTTTTTTC TCGTCGGGGT CACTTCCAAA TGTTTGGATT TGACATTGGT CATAAACCCG   
  
  
- CTCTTCATGT TCCAGAGACA CCCGTCCTGC AAAGACAAAC GTATTTAGTT TGGAAATGGA GTCGAGTACT   
  
  
- AAGGTGTAAG TATAATGTAT AGAAGATATT GTCACTGTTT ATTCTATGGT CACGTACTCT CGAAGAACTC   
  
  
- GATCGACTTC GTCTGACCAA ACTTCGAAGA AGAAACGGTA TAAGACGTAT AAGTAGACAG GTAAAGTAGA   
  
  
- GGTCCAAATC AAGTACAAAC TATCCAGAAC GCAACATAGT TAAAGACACT CTACTTGCTT ACGTTCTATG   
  
  
- AAGGGTTTTA AATTTTCAAT GTCGTGTAAA TCGTGTCCAC ATAATGTTTG AGAAATCCCC GATGTCAAAG   
  
  
- GAGACACTAT ACTACGTACT CGGAAGTGAA AGAATTTAAA TGAAAAGTTC AAATATAATA AGTAGTTATA   
  
  
- TTAGAGACGT TTTATGTCCA AAAACTATTC AATGGTAACT CGAACAACGT GTCAACCTCA GTTGTTGTTC   
  
  
- TTCTTCACCG AAAGGATCTT ATACGATTGA GACAGCCCGA AGGGAAGGAT TGTTGATTAA ATAATGACAG   
  
  
- GACACAACTC CCGTATTATG TCAACACCAA GGGAGAACAG GAACGTGACT GTGTGACAGG AGATGACTCT   
  
  
- ATTTTAACCG TCAATTACAA AGGAAACTG

+     MRE

| Site Name | Organism | Position | Strand | Matrix score. | sequence | function |
| --- | --- | --- | --- | --- | --- | --- |
| MRE | Petroselinum crispum | 814 | + | 7 | AACCTAA | MYB binding site involved in light responsiveness |

> 2018/04/13 10:10:12  
+ AATCATTGGG GTAAAATTTC AGTAAGGTTT GGTGGTTATA TGAAGTCTTT TAGTGTATGC TCAGAAAGGA   
  
  
+ ATTATTATTA TTTGTACTTC AATTTCAGTT TCATGATTTA GAGGTTCAGA TTCTTCCATG TGAAGAGATA   
  
  
+ CTTTTGTGCT ACTTCTTGTA AGTTGGGAAT CATGGATTTC ATTCGGGTCA TGAATTCTAC ACAATATTGC   
  
  
+ TCTTTCTGAG GCAATGGTTG AAAATAATCC ATCTAGTAAT TTCTTGCTGG TAAATGTTTC TTTCAAGAAG   
  
  
+ TTAGCAAAGT TATCTTTGTT CTTATTGAAG AAGAATATGG AGAGCTACCA AGAAGTATTT CTGCAACTTA   
  
  
+ AAAAAACATT TAGGAGTTGG CATGGATGGT AATGTAATAT TCGGTTATTG ATTAATAGGG CGTAAGCGAT   
  
  
+ AGTTCCCTGC TGGTGATGAG TGAACTGGGG GAGTAACCAA GTGAAAGTGA TAGAGGGGTC TATCTTGCAG   
  
  
+ CTCTCCTTTG ATAGAGAGTT AGTCTGTGAT GTGGTAGAAG TCTGAAGATT GTGCAGCTAT GATTTCTCTG   
  
  
+ CATTATTCTA AATAGTGAGT TATGCAACCA AATTTCAATC TTAATTGAAG CATACGGAGT TCTGGGGTTT   
  
  
+ CTCTAGAGAT TTGCATTTCT AAATTTTATG GCGAATTGGC TAGTTCTCTT AATTGCAATC TTTGATGACA   
  
  
+ GTTTATAGAC CAGTAGATTA TGAAAATTGC TTATGATCTT ATAAGGCAGC TTGAACTGTA TTCAAAATGG   
  
  
+ CAAGTACTAG TTGAAAAAAG AGCAGCCCCA GTGAAGGTTT ACAAACCTAA ACTGTAACCA GTATTTGGGC   
  
  
+ GAGAAGTACA AGGTCTCTGT GGGCAGGACG TTTCTGTTTG CATAAATCAA ACCTTTACCT CAGCTCATGA   
  
  
+ TTCCACATTC ATATTACATA TCTTCTATAA CAGTGACAAA TAAGATACCA GTGCATGAGA GCTTCTTGAG   
  
  
+ CTAGCTGAAG CAGACTGGTT TGAAGCTTCT TCTTTGCCAT ATTCTGCATA TTCATCTGTC CATTTCATCT   
  
  
+ CCAGGTTTAG TTCATGTTTG ATAGGTCTTG CGTTGTATCA ATTTCTGTGA GATGAACGAA TGCAAGATAC   
  
  
+ TTCCCAAAAT TTAAAAGTTA CAGCACATTT AGCACAGGTG TATTACAAAC TCTTTAGGGG CTACAGTTTC   
  
  
+ CTCTGTGATA TGATGCATGA GCCTTCACTT TCTTAAATTT ACTTTTCAAG TTTATATTAT TCATCAATAT   
  
  
+ AATCTCTGCA AAATACAGGT TTTTGATAAG TTACCATTGA GCTTGTTGCA CAGTTGGAGT CAACAACAAG   
  
  
+ AAGAAGTGGC TTTCCTAGAA TATGCTAACT CTGTCGGGCT TCCCTTCCTA ACAACTAATT TATTACTGTC   
  
  
+ CTGTGTTGAG GGCATAATAC AGTTGTGGTT CCCTCTTGTC CTTGCACTGA CACACTGTCC TCTACTGAGA   
  
  
+ TAAAATTGGC AGTTAATGTT TCCTTTGAC  

- TTAGTAACCC CATTTTAAAG TCATTCCAAA CCACCAATAT ACTTCAGAAA ATCACATACG AGTCTTTCCT   
  
  
- TAATAATAAT AAACATGAAG TTAAAGTCAA AGTACTAAAT CTCCAAGTCT AAGAAGGTAC ACTTCTCTAT   
  
  
- GAAAACACGA TGAAGAACAT TCAACCCTTA GTACCTAAAG TAAGCCCAGT ACTTAAGATG TGTTATAACG   
  
  
- AGAAAGACTC CGTTACCAAC TTTTATTAGG TAGATCATTA AAGAACGACC ATTTACAAAG AAAGTTCTTC   
  
  
- AATCGTTTCA ATAGAAACAA GAATAACTTC TTCTTATACC TCTCGATGGT TCTTCATAAA GACGTTGAAT   
  
  
- TTTTTTGTAA ATCCTCAACC GTACCTACCA TTACATTATA AGCCAATAAC TAATTATCCC GCATTCGCTA   
  
  
- TCAAGGGACG ACCACTACTC ACTTGACCCC CTCATTGGTT CACTTTCACT ATCTCCCCAG ATAGAACGTC   
  
  
- GAGAGGAAAC TATCTCTCAA TCAGACACTA CACCATCTTC AGACTTCTAA CACGTCGATA CTAAAGAGAC   
  
  
- GTAATAAGAT TTATCACTCA ATACGTTGGT TTAAAGTTAG AATTAACTTC GTATGCCTCA AGACCCCAAA   
  
  
- GAGATCTCTA AACGTAAAGA TTTAAAATAC CGCTTAACCG ATCAAGAGAA TTAACGTTAG AAACTACTGT   
  
  
- CAAATATCTG GTCATCTAAT ACTTTTAACG AATACTAGAA TATTCCGTCG AACTTGACAT AAGTTTTACC   
  
  
- GTTCATGATC AACTTTTTTC TCGTCGGGGT CACTTCCAAA TGTTTGGATT TGACATTGGT CATAAACCCG   
  
  
- CTCTTCATGT TCCAGAGACA CCCGTCCTGC AAAGACAAAC GTATTTAGTT TGGAAATGGA GTCGAGTACT   
  
  
- AAGGTGTAAG TATAATGTAT AGAAGATATT GTCACTGTTT ATTCTATGGT CACGTACTCT CGAAGAACTC   
  
  
- GATCGACTTC GTCTGACCAA ACTTCGAAGA AGAAACGGTA TAAGACGTAT AAGTAGACAG GTAAAGTAGA   
  
  
- GGTCCAAATC AAGTACAAAC TATCCAGAAC GCAACATAGT TAAAGACACT CTACTTGCTT ACGTTCTATG   
  
  
- AAGGGTTTTA AATTTTCAAT GTCGTGTAAA TCGTGTCCAC ATAATGTTTG AGAAATCCCC GATGTCAAAG   
  
  
- GAGACACTAT ACTACGTACT CGGAAGTGAA AGAATTTAAA TGAAAAGTTC AAATATAATA AGTAGTTATA   
  
  
- TTAGAGACGT TTTATGTCCA AAAACTATTC AATGGTAACT CGAACAACGT GTCAACCTCA GTTGTTGTTC   
  
  
- TTCTTCACCG AAAGGATCTT ATACGATTGA GACAGCCCGA AGGGAAGGAT TGTTGATTAA ATAATGACAG   
  
  
- GACACAACTC CCGTATTATG TCAACACCAA GGGAGAACAG GAACGTGACT GTGTGACAGG AGATGACTCT   
  
  
- ATTTTAACCG TCAATTACAA AGGAAACTG

+     O2-site

| Site Name | Organism | Position | Strand | Matrix score. | sequence | function |
| --- | --- | --- | --- | --- | --- | --- |
| O2-site | Zea mays | 1040 | - | 9 | GATGACATGG | cis-acting regulatory element involved in zein metabolism regulation |

> 2018/04/13 10:10:12  
+ AATCATTGGG GTAAAATTTC AGTAAGGTTT GGTGGTTATA TGAAGTCTTT TAGTGTATGC TCAGAAAGGA   
  
  
+ ATTATTATTA TTTGTACTTC AATTTCAGTT TCATGATTTA GAGGTTCAGA TTCTTCCATG TGAAGAGATA   
  
  
+ CTTTTGTGCT ACTTCTTGTA AGTTGGGAAT CATGGATTTC ATTCGGGTCA TGAATTCTAC ACAATATTGC   
  
  
+ TCTTTCTGAG GCAATGGTTG AAAATAATCC ATCTAGTAAT TTCTTGCTGG TAAATGTTTC TTTCAAGAAG   
  
  
+ TTAGCAAAGT TATCTTTGTT CTTATTGAAG AAGAATATGG AGAGCTACCA AGAAGTATTT CTGCAACTTA   
  
  
+ AAAAAACATT TAGGAGTTGG CATGGATGGT AATGTAATAT TCGGTTATTG ATTAATAGGG CGTAAGCGAT   
  
  
+ AGTTCCCTGC TGGTGATGAG TGAACTGGGG GAGTAACCAA GTGAAAGTGA TAGAGGGGTC TATCTTGCAG   
  
  
+ CTCTCCTTTG ATAGAGAGTT AGTCTGTGAT GTGGTAGAAG TCTGAAGATT GTGCAGCTAT GATTTCTCTG   
  
  
+ CATTATTCTA AATAGTGAGT TATGCAACCA AATTTCAATC TTAATTGAAG CATACGGAGT TCTGGGGTTT   
  
  
+ CTCTAGAGAT TTGCATTTCT AAATTTTATG GCGAATTGGC TAGTTCTCTT AATTGCAATC TTTGATGACA   
  
  
+ GTTTATAGAC CAGTAGATTA TGAAAATTGC TTATGATCTT ATAAGGCAGC TTGAACTGTA TTCAAAATGG   
  
  
+ CAAGTACTAG TTGAAAAAAG AGCAGCCCCA GTGAAGGTTT ACAAACCTAA ACTGTAACCA GTATTTGGGC   
  
  
+ GAGAAGTACA AGGTCTCTGT GGGCAGGACG TTTCTGTTTG CATAAATCAA ACCTTTACCT CAGCTCATGA   
  
  
+ TTCCACATTC ATATTACATA TCTTCTATAA CAGTGACAAA TAAGATACCA GTGCATGAGA GCTTCTTGAG   
  
  
+ CTAGCTGAAG CAGACTGGTT TGAAGCTTCT TCTTTGCCAT ATTCTGCATA TTCATCTGTC CATTTCATCT   
  
  
+ CCAGGTTTAG TTCATGTTTG ATAGGTCTTG CGTTGTATCA ATTTCTGTGA GATGAACGAA TGCAAGATAC   
  
  
+ TTCCCAAAAT TTAAAAGTTA CAGCACATTT AGCACAGGTG TATTACAAAC TCTTTAGGGG CTACAGTTTC   
  
  
+ CTCTGTGATA TGATGCATGA GCCTTCACTT TCTTAAATTT ACTTTTCAAG TTTATATTAT TCATCAATAT   
  
  
+ AATCTCTGCA AAATACAGGT TTTTGATAAG TTACCATTGA GCTTGTTGCA CAGTTGGAGT CAACAACAAG   
  
  
+ AAGAAGTGGC TTTCCTAGAA TATGCTAACT CTGTCGGGCT TCCCTTCCTA ACAACTAATT TATTACTGTC   
  
  
+ CTGTGTTGAG GGCATAATAC AGTTGTGGTT CCCTCTTGTC CTTGCACTGA CACACTGTCC TCTACTGAGA   
  
  
+ TAAAATTGGC AGTTAATGTT TCCTTTGAC  

- TTAGTAACCC CATTTTAAAG TCATTCCAAA CCACCAATAT ACTTCAGAAA ATCACATACG AGTCTTTCCT   
  
  
- TAATAATAAT AAACATGAAG TTAAAGTCAA AGTACTAAAT CTCCAAGTCT AAGAAGGTAC ACTTCTCTAT   
  
  
- GAAAACACGA TGAAGAACAT TCAACCCTTA GTACCTAAAG TAAGCCCAGT ACTTAAGATG TGTTATAACG   
  
  
- AGAAAGACTC CGTTACCAAC TTTTATTAGG TAGATCATTA AAGAACGACC ATTTACAAAG AAAGTTCTTC   
  
  
- AATCGTTTCA ATAGAAACAA GAATAACTTC TTCTTATACC TCTCGATGGT TCTTCATAAA GACGTTGAAT   
  
  
- TTTTTTGTAA ATCCTCAACC GTACCTACCA TTACATTATA AGCCAATAAC TAATTATCCC GCATTCGCTA   
  
  
- TCAAGGGACG ACCACTACTC ACTTGACCCC CTCATTGGTT CACTTTCACT ATCTCCCCAG ATAGAACGTC   
  
  
- GAGAGGAAAC TATCTCTCAA TCAGACACTA CACCATCTTC AGACTTCTAA CACGTCGATA CTAAAGAGAC   
  
  
- GTAATAAGAT TTATCACTCA ATACGTTGGT TTAAAGTTAG AATTAACTTC GTATGCCTCA AGACCCCAAA   
  
  
- GAGATCTCTA AACGTAAAGA TTTAAAATAC CGCTTAACCG ATCAAGAGAA TTAACGTTAG AAACTACTGT   
  
  
- CAAATATCTG GTCATCTAAT ACTTTTAACG AATACTAGAA TATTCCGTCG AACTTGACAT AAGTTTTACC   
  
  
- GTTCATGATC AACTTTTTTC TCGTCGGGGT CACTTCCAAA TGTTTGGATT TGACATTGGT CATAAACCCG   
  
  
- CTCTTCATGT TCCAGAGACA CCCGTCCTGC AAAGACAAAC GTATTTAGTT TGGAAATGGA GTCGAGTACT   
  
  
- AAGGTGTAAG TATAATGTAT AGAAGATATT GTCACTGTTT ATTCTATGGT CACGTACTCT CGAAGAACTC   
  
  
- GATCGACTTC GTCTGACCAA ACTTCGAAGA AGAAACGGTA TAAGACGTAT AAGTAGACAG GTAAAGTAGA   
  
  
- GGTCCAAATC AAGTACAAAC TATCCAGAAC GCAACATAGT TAAAGACACT CTACTTGCTT ACGTTCTATG   
  
  
- AAGGGTTTTA AATTTTCAAT GTCGTGTAAA TCGTGTCCAC ATAATGTTTG AGAAATCCCC GATGTCAAAG   
  
  
- GAGACACTAT ACTACGTACT CGGAAGTGAA AGAATTTAAA TGAAAAGTTC AAATATAATA AGTAGTTATA   
  
  
- TTAGAGACGT TTTATGTCCA AAAACTATTC AATGGTAACT CGAACAACGT GTCAACCTCA GTTGTTGTTC   
  
  
- TTCTTCACCG AAAGGATCTT ATACGATTGA GACAGCCCGA AGGGAAGGAT TGTTGATTAA ATAATGACAG   
  
  
- GACACAACTC CCGTATTATG TCAACACCAA GGGAGAACAG GAACGTGACT GTGTGACAGG AGATGACTCT   
  
  
- ATTTTAACCG TCAATTACAA AGGAAACTG

+     Skn-1\_motif

| Site Name | Organism | Position | Strand | Matrix score. | sequence | function |
| --- | --- | --- | --- | --- | --- | --- |
| Skn-1\_motif | Oryza sativa | 187 | + | 5 | GTCAT | cis-acting regulatory element required for endosperm expression |
| Skn-1\_motif | Oryza sativa | 695 | - | 5 | GTCAT | cis-acting regulatory element required for endosperm expression |

> 2018/04/13 10:10:12  
+ AATCATTGGG GTAAAATTTC AGTAAGGTTT GGTGGTTATA TGAAGTCTTT TAGTGTATGC TCAGAAAGGA   
  
  
+ ATTATTATTA TTTGTACTTC AATTTCAGTT TCATGATTTA GAGGTTCAGA TTCTTCCATG TGAAGAGATA   
  
  
+ CTTTTGTGCT ACTTCTTGTA AGTTGGGAAT CATGGATTTC ATTCGGGTCA TGAATTCTAC ACAATATTGC   
  
  
+ TCTTTCTGAG GCAATGGTTG AAAATAATCC ATCTAGTAAT TTCTTGCTGG TAAATGTTTC TTTCAAGAAG   
  
  
+ TTAGCAAAGT TATCTTTGTT CTTATTGAAG AAGAATATGG AGAGCTACCA AGAAGTATTT CTGCAACTTA   
  
  
+ AAAAAACATT TAGGAGTTGG CATGGATGGT AATGTAATAT TCGGTTATTG ATTAATAGGG CGTAAGCGAT   
  
  
+ AGTTCCCTGC TGGTGATGAG TGAACTGGGG GAGTAACCAA GTGAAAGTGA TAGAGGGGTC TATCTTGCAG   
  
  
+ CTCTCCTTTG ATAGAGAGTT AGTCTGTGAT GTGGTAGAAG TCTGAAGATT GTGCAGCTAT GATTTCTCTG   
  
  
+ CATTATTCTA AATAGTGAGT TATGCAACCA AATTTCAATC TTAATTGAAG CATACGGAGT TCTGGGGTTT   
  
  
+ CTCTAGAGAT TTGCATTTCT AAATTTTATG GCGAATTGGC TAGTTCTCTT AATTGCAATC TTTGATGACA   
  
  
+ GTTTATAGAC CAGTAGATTA TGAAAATTGC TTATGATCTT ATAAGGCAGC TTGAACTGTA TTCAAAATGG   
  
  
+ CAAGTACTAG TTGAAAAAAG AGCAGCCCCA GTGAAGGTTT ACAAACCTAA ACTGTAACCA GTATTTGGGC   
  
  
+ GAGAAGTACA AGGTCTCTGT GGGCAGGACG TTTCTGTTTG CATAAATCAA ACCTTTACCT CAGCTCATGA   
  
  
+ TTCCACATTC ATATTACATA TCTTCTATAA CAGTGACAAA TAAGATACCA GTGCATGAGA GCTTCTTGAG   
  
  
+ CTAGCTGAAG CAGACTGGTT TGAAGCTTCT TCTTTGCCAT ATTCTGCATA TTCATCTGTC CATTTCATCT   
  
  
+ CCAGGTTTAG TTCATGTTTG ATAGGTCTTG CGTTGTATCA ATTTCTGTGA GATGAACGAA TGCAAGATAC   
  
  
+ TTCCCAAAAT TTAAAAGTTA CAGCACATTT AGCACAGGTG TATTACAAAC TCTTTAGGGG CTACAGTTTC   
  
  
+ CTCTGTGATA TGATGCATGA GCCTTCACTT TCTTAAATTT ACTTTTCAAG TTTATATTAT TCATCAATAT   
  
  
+ AATCTCTGCA AAATACAGGT TTTTGATAAG TTACCATTGA GCTTGTTGCA CAGTTGGAGT CAACAACAAG   
  
  
+ AAGAAGTGGC TTTCCTAGAA TATGCTAACT CTGTCGGGCT TCCCTTCCTA ACAACTAATT TATTACTGTC   
  
  
+ CTGTGTTGAG GGCATAATAC AGTTGTGGTT CCCTCTTGTC CTTGCACTGA CACACTGTCC TCTACTGAGA   
  
  
+ TAAAATTGGC AGTTAATGTT TCCTTTGAC  

- TTAGTAACCC CATTTTAAAG TCATTCCAAA CCACCAATAT ACTTCAGAAA ATCACATACG AGTCTTTCCT   
  
  
- TAATAATAAT AAACATGAAG TTAAAGTCAA AGTACTAAAT CTCCAAGTCT AAGAAGGTAC ACTTCTCTAT   
  
  
- GAAAACACGA TGAAGAACAT TCAACCCTTA GTACCTAAAG TAAGCCCAGT ACTTAAGATG TGTTATAACG   
  
  
- AGAAAGACTC CGTTACCAAC TTTTATTAGG TAGATCATTA AAGAACGACC ATTTACAAAG AAAGTTCTTC   
  
  
- AATCGTTTCA ATAGAAACAA GAATAACTTC TTCTTATACC TCTCGATGGT TCTTCATAAA GACGTTGAAT   
  
  
- TTTTTTGTAA ATCCTCAACC GTACCTACCA TTACATTATA AGCCAATAAC TAATTATCCC GCATTCGCTA   
  
  
- TCAAGGGACG ACCACTACTC ACTTGACCCC CTCATTGGTT CACTTTCACT ATCTCCCCAG ATAGAACGTC   
  
  
- GAGAGGAAAC TATCTCTCAA TCAGACACTA CACCATCTTC AGACTTCTAA CACGTCGATA CTAAAGAGAC   
  
  
- GTAATAAGAT TTATCACTCA ATACGTTGGT TTAAAGTTAG AATTAACTTC GTATGCCTCA AGACCCCAAA   
  
  
- GAGATCTCTA AACGTAAAGA TTTAAAATAC CGCTTAACCG ATCAAGAGAA TTAACGTTAG AAACTACTGT   
  
  
- CAAATATCTG GTCATCTAAT ACTTTTAACG AATACTAGAA TATTCCGTCG AACTTGACAT AAGTTTTACC   
  
  
- GTTCATGATC AACTTTTTTC TCGTCGGGGT CACTTCCAAA TGTTTGGATT TGACATTGGT CATAAACCCG   
  
  
- CTCTTCATGT TCCAGAGACA CCCGTCCTGC AAAGACAAAC GTATTTAGTT TGGAAATGGA GTCGAGTACT   
  
  
- AAGGTGTAAG TATAATGTAT AGAAGATATT GTCACTGTTT ATTCTATGGT CACGTACTCT CGAAGAACTC   
  
  
- GATCGACTTC GTCTGACCAA ACTTCGAAGA AGAAACGGTA TAAGACGTAT AAGTAGACAG GTAAAGTAGA   
  
  
- GGTCCAAATC AAGTACAAAC TATCCAGAAC GCAACATAGT TAAAGACACT CTACTTGCTT ACGTTCTATG   
  
  
- AAGGGTTTTA AATTTTCAAT GTCGTGTAAA TCGTGTCCAC ATAATGTTTG AGAAATCCCC GATGTCAAAG   
  
  
- GAGACACTAT ACTACGTACT CGGAAGTGAA AGAATTTAAA TGAAAAGTTC AAATATAATA AGTAGTTATA   
  
  
- TTAGAGACGT TTTATGTCCA AAAACTATTC AATGGTAACT CGAACAACGT GTCAACCTCA GTTGTTGTTC   
  
  
- TTCTTCACCG AAAGGATCTT ATACGATTGA GACAGCCCGA AGGGAAGGAT TGTTGATTAA ATAATGACAG   
  
  
- GACACAACTC CCGTATTATG TCAACACCAA GGGAGAACAG GAACGTGACT GTGTGACAGG AGATGACTCT   
  
  
- ATTTTAACCG TCAATTACAA AGGAAACTG

+     TATA-box

| Site Name | Organism | Position | Strand | Matrix score. | sequence | function |
| --- | --- | --- | --- | --- | --- | --- |
| TATA-box | Arabidopsis thaliana | 37 | + | 4 | TATA | core promoter element around -30 of transcription start |
| TATA-box | Lycopersicon esculentum | 12 | - | 5 | TTTTA | core promoter element around -30 of transcription start |
| TATA-box | Arabidopsis thaliana | 36 | - | 5 | TATAA | core promoter element around -30 of transcription start |
| TATA-box | Lycopersicon esculentum | 48 | + | 5 | TTTTA | core promoter element around -30 of transcription start |
| TATA-box | Glycine max | 73 | - | 5 | TAATA | core promoter element around -30 of transcription start |
| TATA-box | Glycine max | 76 | - | 5 | TAATA | core promoter element around -30 of transcription start |
| TATA-box | Lycopersicon esculentum | 349 | - | 5 | TTTTA | core promoter element around -30 of transcription start |
| TATA-box | Glycine max | 385 | + | 5 | TAATA | core promoter element around -30 of transcription start |
| TATA-box | Glycine max | 403 | + | 5 | TAATA | core promoter element around -30 of transcription start |
| TATA-box | Lycopersicon esculentum | 654 | + | 5 | TTTTA | core promoter element around -30 of transcription start |
| TATA-box | Arabidopsis thaliana | 702 | - | 6 | TATAAA | core promoter element around -30 of transcription start |
| TATA-box | Arabidopsis thaliana | 703 | - | 5 | TATAA | core promoter element around -30 of transcription start |
| TATA-box | Arabidopsis thaliana | 704 | + | 4 | TATA | core promoter element around -30 of transcription start |
| TATA-box | Arabidopsis thaliana | 739 | - | 5 | TATAA | core promoter element around -30 of transcription start |
| TATA-box | Arabidopsis thaliana | 740 | + | 4 | TATA | core promoter element around -30 of transcription start |
| TATA-box | Glycine max | 922 | - | 5 | TAATA | core promoter element around -30 of transcription start |
| TATA-box | Arabidopsis thaliana | 936 | - | 4 | TATA | core promoter element around -30 of transcription start |
| TATA-box | Lycopersicon esculentum | 1132 | - | 5 | TTTTA | core promoter element around -30 of transcription start |
| TATA-box | Glycine max | 1161 | - | 5 | TAATA | core promoter element around -30 of transcription start |
| TATA-box | Arabidopsis thaliana | 1241 | - | 6 | TATAAA | core promoter element around -30 of transcription start |
| TATA-box | Arabidopsis thaliana | 1242 | - | 5 | TATAA | core promoter element around -30 of transcription start |
| TATA-box | Arabidopsis thaliana | 1243 | - | 4 | TATA | core promoter element around -30 of transcription start |
| TATA-box | Glycine max | 1245 | - | 5 | TAATA | core promoter element around -30 of transcription start |
| TATA-box | Brassica oleracea | 1257 | + | 7 | ATATAAT | core promoter element around -30 of transcription start |
| TATA-box | Arabidopsis thaliana | 1258 | - | 4 | TATA | core promoter element around -30 of transcription start |
| TATA-box | Glycine max | 1391 | - | 5 | TAATA | core promoter element around -30 of transcription start |
| TATA-box | Glycine max | 1415 | + | 5 | TAATA | core promoter element around -30 of transcription start |
| TATA-box | Lycopersicon esculentum | 1471 | - | 5 | TTTTA | core promoter element around -30 of transcription start |

> 2018/04/13 10:10:12  
+ AATCATTGGG GTAAAATTTC AGTAAGGTTT GGTGGTTATA TGAAGTCTTT TAGTGTATGC TCAGAAAGGA   
  
  
+ ATTATTATTA TTTGTACTTC AATTTCAGTT TCATGATTTA GAGGTTCAGA TTCTTCCATG TGAAGAGATA   
  
  
+ CTTTTGTGCT ACTTCTTGTA AGTTGGGAAT CATGGATTTC ATTCGGGTCA TGAATTCTAC ACAATATTGC   
  
  
+ TCTTTCTGAG GCAATGGTTG AAAATAATCC ATCTAGTAAT TTCTTGCTGG TAAATGTTTC TTTCAAGAAG   
  
  
+ TTAGCAAAGT TATCTTTGTT CTTATTGAAG AAGAATATGG AGAGCTACCA AGAAGTATTT CTGCAACTTA   
  
  
+ AAAAAACATT TAGGAGTTGG CATGGATGGT AATGTAATAT TCGGTTATTG ATTAATAGGG CGTAAGCGAT   
  
  
+ AGTTCCCTGC TGGTGATGAG TGAACTGGGG GAGTAACCAA GTGAAAGTGA TAGAGGGGTC TATCTTGCAG   
  
  
+ CTCTCCTTTG ATAGAGAGTT AGTCTGTGAT GTGGTAGAAG TCTGAAGATT GTGCAGCTAT GATTTCTCTG   
  
  
+ CATTATTCTA AATAGTGAGT TATGCAACCA AATTTCAATC TTAATTGAAG CATACGGAGT TCTGGGGTTT   
  
  
+ CTCTAGAGAT TTGCATTTCT AAATTTTATG GCGAATTGGC TAGTTCTCTT AATTGCAATC TTTGATGACA   
  
  
+ GTTTATAGAC CAGTAGATTA TGAAAATTGC TTATGATCTT ATAAGGCAGC TTGAACTGTA TTCAAAATGG   
  
  
+ CAAGTACTAG TTGAAAAAAG AGCAGCCCCA GTGAAGGTTT ACAAACCTAA ACTGTAACCA GTATTTGGGC   
  
  
+ GAGAAGTACA AGGTCTCTGT GGGCAGGACG TTTCTGTTTG CATAAATCAA ACCTTTACCT CAGCTCATGA   
  
  
+ TTCCACATTC ATATTACATA TCTTCTATAA CAGTGACAAA TAAGATACCA GTGCATGAGA GCTTCTTGAG   
  
  
+ CTAGCTGAAG CAGACTGGTT TGAAGCTTCT TCTTTGCCAT ATTCTGCATA TTCATCTGTC CATTTCATCT   
  
  
+ CCAGGTTTAG TTCATGTTTG ATAGGTCTTG CGTTGTATCA ATTTCTGTGA GATGAACGAA TGCAAGATAC   
  
  
+ TTCCCAAAAT TTAAAAGTTA CAGCACATTT AGCACAGGTG TATTACAAAC TCTTTAGGGG CTACAGTTTC   
  
  
+ CTCTGTGATA TGATGCATGA GCCTTCACTT TCTTAAATTT ACTTTTCAAG TTTATATTAT TCATCAATAT   
  
  
+ AATCTCTGCA AAATACAGGT TTTTGATAAG TTACCATTGA GCTTGTTGCA CAGTTGGAGT CAACAACAAG   
  
  
+ AAGAAGTGGC TTTCCTAGAA TATGCTAACT CTGTCGGGCT TCCCTTCCTA ACAACTAATT TATTACTGTC   
  
  
+ CTGTGTTGAG GGCATAATAC AGTTGTGGTT CCCTCTTGTC CTTGCACTGA CACACTGTCC TCTACTGAGA   
  
  
+ TAAAATTGGC AGTTAATGTT TCCTTTGAC  

- TTAGTAACCC CATTTTAAAG TCATTCCAAA CCACCAATAT ACTTCAGAAA ATCACATACG AGTCTTTCCT   
  
  
- TAATAATAAT AAACATGAAG TTAAAGTCAA AGTACTAAAT CTCCAAGTCT AAGAAGGTAC ACTTCTCTAT   
  
  
- GAAAACACGA TGAAGAACAT TCAACCCTTA GTACCTAAAG TAAGCCCAGT ACTTAAGATG TGTTATAACG   
  
  
- AGAAAGACTC CGTTACCAAC TTTTATTAGG TAGATCATTA AAGAACGACC ATTTACAAAG AAAGTTCTTC   
  
  
- AATCGTTTCA ATAGAAACAA GAATAACTTC TTCTTATACC TCTCGATGGT TCTTCATAAA GACGTTGAAT   
  
  
- TTTTTTGTAA ATCCTCAACC GTACCTACCA TTACATTATA AGCCAATAAC TAATTATCCC GCATTCGCTA   
  
  
- TCAAGGGACG ACCACTACTC ACTTGACCCC CTCATTGGTT CACTTTCACT ATCTCCCCAG ATAGAACGTC   
  
  
- GAGAGGAAAC TATCTCTCAA TCAGACACTA CACCATCTTC AGACTTCTAA CACGTCGATA CTAAAGAGAC   
  
  
- GTAATAAGAT TTATCACTCA ATACGTTGGT TTAAAGTTAG AATTAACTTC GTATGCCTCA AGACCCCAAA   
  
  
- GAGATCTCTA AACGTAAAGA TTTAAAATAC CGCTTAACCG ATCAAGAGAA TTAACGTTAG AAACTACTGT   
  
  
- CAAATATCTG GTCATCTAAT ACTTTTAACG AATACTAGAA TATTCCGTCG AACTTGACAT AAGTTTTACC   
  
  
- GTTCATGATC AACTTTTTTC TCGTCGGGGT CACTTCCAAA TGTTTGGATT TGACATTGGT CATAAACCCG   
  
  
- CTCTTCATGT TCCAGAGACA CCCGTCCTGC AAAGACAAAC GTATTTAGTT TGGAAATGGA GTCGAGTACT   
  
  
- AAGGTGTAAG TATAATGTAT AGAAGATATT GTCACTGTTT ATTCTATGGT CACGTACTCT CGAAGAACTC   
  
  
- GATCGACTTC GTCTGACCAA ACTTCGAAGA AGAAACGGTA TAAGACGTAT AAGTAGACAG GTAAAGTAGA   
  
  
- GGTCCAAATC AAGTACAAAC TATCCAGAAC GCAACATAGT TAAAGACACT CTACTTGCTT ACGTTCTATG   
  
  
- AAGGGTTTTA AATTTTCAAT GTCGTGTAAA TCGTGTCCAC ATAATGTTTG AGAAATCCCC GATGTCAAAG   
  
  
- GAGACACTAT ACTACGTACT CGGAAGTGAA AGAATTTAAA TGAAAAGTTC AAATATAATA AGTAGTTATA   
  
  
- TTAGAGACGT TTTATGTCCA AAAACTATTC AATGGTAACT CGAACAACGT GTCAACCTCA GTTGTTGTTC   
  
  
- TTCTTCACCG AAAGGATCTT ATACGATTGA GACAGCCCGA AGGGAAGGAT TGTTGATTAA ATAATGACAG   
  
  
- GACACAACTC CCGTATTATG TCAACACCAA GGGAGAACAG GAACGTGACT GTGTGACAGG AGATGACTCT   
  
  
- ATTTTAACCG TCAATTACAA AGGAAACTG

+     TCA-element

| Site Name | Organism | Position | Strand | Matrix score. | sequence | function |
| --- | --- | --- | --- | --- | --- | --- |
| TCA-element | Brassica oleracea | 308 | + | 9 | GAGAAGAATA | cis-acting element involved in salicylic acid responsiveness |
| TCA-element | Brassica oleracea | 1459 | - | 9 | TCAGAAGAGG | cis-acting element involved in salicylic acid responsiveness |

> 2018/04/13 10:10:12  
+ AATCATTGGG GTAAAATTTC AGTAAGGTTT GGTGGTTATA TGAAGTCTTT TAGTGTATGC TCAGAAAGGA   
  
  
+ ATTATTATTA TTTGTACTTC AATTTCAGTT TCATGATTTA GAGGTTCAGA TTCTTCCATG TGAAGAGATA   
  
  
+ CTTTTGTGCT ACTTCTTGTA AGTTGGGAAT CATGGATTTC ATTCGGGTCA TGAATTCTAC ACAATATTGC   
  
  
+ TCTTTCTGAG GCAATGGTTG AAAATAATCC ATCTAGTAAT TTCTTGCTGG TAAATGTTTC TTTCAAGAAG   
  
  
+ TTAGCAAAGT TATCTTTGTT CTTATTGAAG AAGAATATGG AGAGCTACCA AGAAGTATTT CTGCAACTTA   
  
  
+ AAAAAACATT TAGGAGTTGG CATGGATGGT AATGTAATAT TCGGTTATTG ATTAATAGGG CGTAAGCGAT   
  
  
+ AGTTCCCTGC TGGTGATGAG TGAACTGGGG GAGTAACCAA GTGAAAGTGA TAGAGGGGTC TATCTTGCAG   
  
  
+ CTCTCCTTTG ATAGAGAGTT AGTCTGTGAT GTGGTAGAAG TCTGAAGATT GTGCAGCTAT GATTTCTCTG   
  
  
+ CATTATTCTA AATAGTGAGT TATGCAACCA AATTTCAATC TTAATTGAAG CATACGGAGT TCTGGGGTTT   
  
  
+ CTCTAGAGAT TTGCATTTCT AAATTTTATG GCGAATTGGC TAGTTCTCTT AATTGCAATC TTTGATGACA   
  
  
+ GTTTATAGAC CAGTAGATTA TGAAAATTGC TTATGATCTT ATAAGGCAGC TTGAACTGTA TTCAAAATGG   
  
  
+ CAAGTACTAG TTGAAAAAAG AGCAGCCCCA GTGAAGGTTT ACAAACCTAA ACTGTAACCA GTATTTGGGC   
  
  
+ GAGAAGTACA AGGTCTCTGT GGGCAGGACG TTTCTGTTTG CATAAATCAA ACCTTTACCT CAGCTCATGA   
  
  
+ TTCCACATTC ATATTACATA TCTTCTATAA CAGTGACAAA TAAGATACCA GTGCATGAGA GCTTCTTGAG   
  
  
+ CTAGCTGAAG CAGACTGGTT TGAAGCTTCT TCTTTGCCAT ATTCTGCATA TTCATCTGTC CATTTCATCT   
  
  
+ CCAGGTTTAG TTCATGTTTG ATAGGTCTTG CGTTGTATCA ATTTCTGTGA GATGAACGAA TGCAAGATAC   
  
  
+ TTCCCAAAAT TTAAAAGTTA CAGCACATTT AGCACAGGTG TATTACAAAC TCTTTAGGGG CTACAGTTTC   
  
  
+ CTCTGTGATA TGATGCATGA GCCTTCACTT TCTTAAATTT ACTTTTCAAG TTTATATTAT TCATCAATAT   
  
  
+ AATCTCTGCA AAATACAGGT TTTTGATAAG TTACCATTGA GCTTGTTGCA CAGTTGGAGT CAACAACAAG   
  
  
+ AAGAAGTGGC TTTCCTAGAA TATGCTAACT CTGTCGGGCT TCCCTTCCTA ACAACTAATT TATTACTGTC   
  
  
+ CTGTGTTGAG GGCATAATAC AGTTGTGGTT CCCTCTTGTC CTTGCACTGA CACACTGTCC TCTACTGAGA   
  
  
+ TAAAATTGGC AGTTAATGTT TCCTTTGAC  

- TTAGTAACCC CATTTTAAAG TCATTCCAAA CCACCAATAT ACTTCAGAAA ATCACATACG AGTCTTTCCT   
  
  
- TAATAATAAT AAACATGAAG TTAAAGTCAA AGTACTAAAT CTCCAAGTCT AAGAAGGTAC ACTTCTCTAT   
  
  
- GAAAACACGA TGAAGAACAT TCAACCCTTA GTACCTAAAG TAAGCCCAGT ACTTAAGATG TGTTATAACG   
  
  
- AGAAAGACTC CGTTACCAAC TTTTATTAGG TAGATCATTA AAGAACGACC ATTTACAAAG AAAGTTCTTC   
  
  
- AATCGTTTCA ATAGAAACAA GAATAACTTC TTCTTATACC TCTCGATGGT TCTTCATAAA GACGTTGAAT   
  
  
- TTTTTTGTAA ATCCTCAACC GTACCTACCA TTACATTATA AGCCAATAAC TAATTATCCC GCATTCGCTA   
  
  
- TCAAGGGACG ACCACTACTC ACTTGACCCC CTCATTGGTT CACTTTCACT ATCTCCCCAG ATAGAACGTC   
  
  
- GAGAGGAAAC TATCTCTCAA TCAGACACTA CACCATCTTC AGACTTCTAA CACGTCGATA CTAAAGAGAC   
  
  
- GTAATAAGAT TTATCACTCA ATACGTTGGT TTAAAGTTAG AATTAACTTC GTATGCCTCA AGACCCCAAA   
  
  
- GAGATCTCTA AACGTAAAGA TTTAAAATAC CGCTTAACCG ATCAAGAGAA TTAACGTTAG AAACTACTGT   
  
  
- CAAATATCTG GTCATCTAAT ACTTTTAACG AATACTAGAA TATTCCGTCG AACTTGACAT AAGTTTTACC   
  
  
- GTTCATGATC AACTTTTTTC TCGTCGGGGT CACTTCCAAA TGTTTGGATT TGACATTGGT CATAAACCCG   
  
  
- CTCTTCATGT TCCAGAGACA CCCGTCCTGC AAAGACAAAC GTATTTAGTT TGGAAATGGA GTCGAGTACT   
  
  
- AAGGTGTAAG TATAATGTAT AGAAGATATT GTCACTGTTT ATTCTATGGT CACGTACTCT CGAAGAACTC   
  
  
- GATCGACTTC GTCTGACCAA ACTTCGAAGA AGAAACGGTA TAAGACGTAT AAGTAGACAG GTAAAGTAGA   
  
  
- GGTCCAAATC AAGTACAAAC TATCCAGAAC GCAACATAGT TAAAGACACT CTACTTGCTT ACGTTCTATG   
  
  
- AAGGGTTTTA AATTTTCAAT GTCGTGTAAA TCGTGTCCAC ATAATGTTTG AGAAATCCCC GATGTCAAAG   
  
  
- GAGACACTAT ACTACGTACT CGGAAGTGAA AGAATTTAAA TGAAAAGTTC AAATATAATA AGTAGTTATA   
  
  
- TTAGAGACGT TTTATGTCCA AAAACTATTC AATGGTAACT CGAACAACGT GTCAACCTCA GTTGTTGTTC   
  
  
- TTCTTCACCG AAAGGATCTT ATACGATTGA GACAGCCCGA AGGGAAGGAT TGTTGATTAA ATAATGACAG   
  
  
- GACACAACTC CCGTATTATG TCAACACCAA GGGAGAACAG GAACGTGACT GTGTGACAGG AGATGACTCT   
  
  
- ATTTTAACCG TCAATTACAA AGGAAACTG

+     Unnamed\_\_2

| Site Name | Organism | Position | Strand | Matrix score. | sequence | function |
| --- | --- | --- | --- | --- | --- | --- |
| Unnamed\_\_2 | Petroselinum hortense | 814 | + | 9 | AACCTAACCT |  |

> 2018/04/13 10:10:12  
+ AATCATTGGG GTAAAATTTC AGTAAGGTTT GGTGGTTATA TGAAGTCTTT TAGTGTATGC TCAGAAAGGA   
  
  
+ ATTATTATTA TTTGTACTTC AATTTCAGTT TCATGATTTA GAGGTTCAGA TTCTTCCATG TGAAGAGATA   
  
  
+ CTTTTGTGCT ACTTCTTGTA AGTTGGGAAT CATGGATTTC ATTCGGGTCA TGAATTCTAC ACAATATTGC   
  
  
+ TCTTTCTGAG GCAATGGTTG AAAATAATCC ATCTAGTAAT TTCTTGCTGG TAAATGTTTC TTTCAAGAAG   
  
  
+ TTAGCAAAGT TATCTTTGTT CTTATTGAAG AAGAATATGG AGAGCTACCA AGAAGTATTT CTGCAACTTA   
  
  
+ AAAAAACATT TAGGAGTTGG CATGGATGGT AATGTAATAT TCGGTTATTG ATTAATAGGG CGTAAGCGAT   
  
  
+ AGTTCCCTGC TGGTGATGAG TGAACTGGGG GAGTAACCAA GTGAAAGTGA TAGAGGGGTC TATCTTGCAG   
  
  
+ CTCTCCTTTG ATAGAGAGTT AGTCTGTGAT GTGGTAGAAG TCTGAAGATT GTGCAGCTAT GATTTCTCTG   
  
  
+ CATTATTCTA AATAGTGAGT TATGCAACCA AATTTCAATC TTAATTGAAG CATACGGAGT TCTGGGGTTT   
  
  
+ CTCTAGAGAT TTGCATTTCT AAATTTTATG GCGAATTGGC TAGTTCTCTT AATTGCAATC TTTGATGACA   
  
  
+ GTTTATAGAC CAGTAGATTA TGAAAATTGC TTATGATCTT ATAAGGCAGC TTGAACTGTA TTCAAAATGG   
  
  
+ CAAGTACTAG TTGAAAAAAG AGCAGCCCCA GTGAAGGTTT ACAAACCTAA ACTGTAACCA GTATTTGGGC   
  
  
+ GAGAAGTACA AGGTCTCTGT GGGCAGGACG TTTCTGTTTG CATAAATCAA ACCTTTACCT CAGCTCATGA   
  
  
+ TTCCACATTC ATATTACATA TCTTCTATAA CAGTGACAAA TAAGATACCA GTGCATGAGA GCTTCTTGAG   
  
  
+ CTAGCTGAAG CAGACTGGTT TGAAGCTTCT TCTTTGCCAT ATTCTGCATA TTCATCTGTC CATTTCATCT   
  
  
+ CCAGGTTTAG TTCATGTTTG ATAGGTCTTG CGTTGTATCA ATTTCTGTGA GATGAACGAA TGCAAGATAC   
  
  
+ TTCCCAAAAT TTAAAAGTTA CAGCACATTT AGCACAGGTG TATTACAAAC TCTTTAGGGG CTACAGTTTC   
  
  
+ CTCTGTGATA TGATGCATGA GCCTTCACTT TCTTAAATTT ACTTTTCAAG TTTATATTAT TCATCAATAT   
  
  
+ AATCTCTGCA AAATACAGGT TTTTGATAAG TTACCATTGA GCTTGTTGCA CAGTTGGAGT CAACAACAAG   
  
  
+ AAGAAGTGGC TTTCCTAGAA TATGCTAACT CTGTCGGGCT TCCCTTCCTA ACAACTAATT TATTACTGTC   
  
  
+ CTGTGTTGAG GGCATAATAC AGTTGTGGTT CCCTCTTGTC CTTGCACTGA CACACTGTCC TCTACTGAGA   
  
  
+ TAAAATTGGC AGTTAATGTT TCCTTTGAC  

- TTAGTAACCC CATTTTAAAG TCATTCCAAA CCACCAATAT ACTTCAGAAA ATCACATACG AGTCTTTCCT   
  
  
- TAATAATAAT AAACATGAAG TTAAAGTCAA AGTACTAAAT CTCCAAGTCT AAGAAGGTAC ACTTCTCTAT   
  
  
- GAAAACACGA TGAAGAACAT TCAACCCTTA GTACCTAAAG TAAGCCCAGT ACTTAAGATG TGTTATAACG   
  
  
- AGAAAGACTC CGTTACCAAC TTTTATTAGG TAGATCATTA AAGAACGACC ATTTACAAAG AAAGTTCTTC   
  
  
- AATCGTTTCA ATAGAAACAA GAATAACTTC TTCTTATACC TCTCGATGGT TCTTCATAAA GACGTTGAAT   
  
  
- TTTTTTGTAA ATCCTCAACC GTACCTACCA TTACATTATA AGCCAATAAC TAATTATCCC GCATTCGCTA   
  
  
- TCAAGGGACG ACCACTACTC ACTTGACCCC CTCATTGGTT CACTTTCACT ATCTCCCCAG ATAGAACGTC   
  
  
- GAGAGGAAAC TATCTCTCAA TCAGACACTA CACCATCTTC AGACTTCTAA CACGTCGATA CTAAAGAGAC   
  
  
- GTAATAAGAT TTATCACTCA ATACGTTGGT TTAAAGTTAG AATTAACTTC GTATGCCTCA AGACCCCAAA   
  
  
- GAGATCTCTA AACGTAAAGA TTTAAAATAC CGCTTAACCG ATCAAGAGAA TTAACGTTAG AAACTACTGT   
  
  
- CAAATATCTG GTCATCTAAT ACTTTTAACG AATACTAGAA TATTCCGTCG AACTTGACAT AAGTTTTACC   
  
  
- GTTCATGATC AACTTTTTTC TCGTCGGGGT CACTTCCAAA TGTTTGGATT TGACATTGGT CATAAACCCG   
  
  
- CTCTTCATGT TCCAGAGACA CCCGTCCTGC AAAGACAAAC GTATTTAGTT TGGAAATGGA GTCGAGTACT   
  
  
- AAGGTGTAAG TATAATGTAT AGAAGATATT GTCACTGTTT ATTCTATGGT CACGTACTCT CGAAGAACTC   
  
  
- GATCGACTTC GTCTGACCAA ACTTCGAAGA AGAAACGGTA TAAGACGTAT AAGTAGACAG GTAAAGTAGA   
  
  
- GGTCCAAATC AAGTACAAAC TATCCAGAAC GCAACATAGT TAAAGACACT CTACTTGCTT ACGTTCTATG   
  
  
- AAGGGTTTTA AATTTTCAAT GTCGTGTAAA TCGTGTCCAC ATAATGTTTG AGAAATCCCC GATGTCAAAG   
  
  
- GAGACACTAT ACTACGTACT CGGAAGTGAA AGAATTTAAA TGAAAAGTTC AAATATAATA AGTAGTTATA   
  
  
- TTAGAGACGT TTTATGTCCA AAAACTATTC AATGGTAACT CGAACAACGT GTCAACCTCA GTTGTTGTTC   
  
  
- TTCTTCACCG AAAGGATCTT ATACGATTGA GACAGCCCGA AGGGAAGGAT TGTTGATTAA ATAATGACAG   
  
  
- GACACAACTC CCGTATTATG TCAACACCAA GGGAGAACAG GAACGTGACT GTGTGACAGG AGATGACTCT   
  
  
- ATTTTAACCG TCAATTACAA AGGAAACTG

+     Unnamed\_\_4

| Site Name | Organism | Position | Strand | Matrix score. | sequence | function |
| --- | --- | --- | --- | --- | --- | --- |
| Unnamed\_\_4 | Petroselinum hortense | 319 | - | 4 | CTCC |  |
| Unnamed\_\_4 | Petroselinum hortense | 616 | - | 4 | CTCC |  |
| Unnamed\_\_4 | Petroselinum hortense | 450 | - | 4 | CTCC |  |
| Unnamed\_\_4 | Petroselinum hortense | 1316 | - | 4 | CTCC |  |
| Unnamed\_\_4 | Petroselinum hortense | 363 | - | 4 | CTCC |  |
| Unnamed\_\_4 | Petroselinum hortense | 1049 | + | 4 | CTCC |  |
| Unnamed\_\_4 | Petroselinum hortense | 493 | + | 4 | CTCC |  |

> 2018/04/13 10:10:12  
+ AATCATTGGG GTAAAATTTC AGTAAGGTTT GGTGGTTATA TGAAGTCTTT TAGTGTATGC TCAGAAAGGA   
  
  
+ ATTATTATTA TTTGTACTTC AATTTCAGTT TCATGATTTA GAGGTTCAGA TTCTTCCATG TGAAGAGATA   
  
  
+ CTTTTGTGCT ACTTCTTGTA AGTTGGGAAT CATGGATTTC ATTCGGGTCA TGAATTCTAC ACAATATTGC   
  
  
+ TCTTTCTGAG GCAATGGTTG AAAATAATCC ATCTAGTAAT TTCTTGCTGG TAAATGTTTC TTTCAAGAAG   
  
  
+ TTAGCAAAGT TATCTTTGTT CTTATTGAAG AAGAATATGG AGAGCTACCA AGAAGTATTT CTGCAACTTA   
  
  
+ AAAAAACATT TAGGAGTTGG CATGGATGGT AATGTAATAT TCGGTTATTG ATTAATAGGG CGTAAGCGAT   
  
  
+ AGTTCCCTGC TGGTGATGAG TGAACTGGGG GAGTAACCAA GTGAAAGTGA TAGAGGGGTC TATCTTGCAG   
  
  
+ CTCTCCTTTG ATAGAGAGTT AGTCTGTGAT GTGGTAGAAG TCTGAAGATT GTGCAGCTAT GATTTCTCTG   
  
  
+ CATTATTCTA AATAGTGAGT TATGCAACCA AATTTCAATC TTAATTGAAG CATACGGAGT TCTGGGGTTT   
  
  
+ CTCTAGAGAT TTGCATTTCT AAATTTTATG GCGAATTGGC TAGTTCTCTT AATTGCAATC TTTGATGACA   
  
  
+ GTTTATAGAC CAGTAGATTA TGAAAATTGC TTATGATCTT ATAAGGCAGC TTGAACTGTA TTCAAAATGG   
  
  
+ CAAGTACTAG TTGAAAAAAG AGCAGCCCCA GTGAAGGTTT ACAAACCTAA ACTGTAACCA GTATTTGGGC   
  
  
+ GAGAAGTACA AGGTCTCTGT GGGCAGGACG TTTCTGTTTG CATAAATCAA ACCTTTACCT CAGCTCATGA   
  
  
+ TTCCACATTC ATATTACATA TCTTCTATAA CAGTGACAAA TAAGATACCA GTGCATGAGA GCTTCTTGAG   
  
  
+ CTAGCTGAAG CAGACTGGTT TGAAGCTTCT TCTTTGCCAT ATTCTGCATA TTCATCTGTC CATTTCATCT   
  
  
+ CCAGGTTTAG TTCATGTTTG ATAGGTCTTG CGTTGTATCA ATTTCTGTGA GATGAACGAA TGCAAGATAC   
  
  
+ TTCCCAAAAT TTAAAAGTTA CAGCACATTT AGCACAGGTG TATTACAAAC TCTTTAGGGG CTACAGTTTC   
  
  
+ CTCTGTGATA TGATGCATGA GCCTTCACTT TCTTAAATTT ACTTTTCAAG TTTATATTAT TCATCAATAT   
  
  
+ AATCTCTGCA AAATACAGGT TTTTGATAAG TTACCATTGA GCTTGTTGCA CAGTTGGAGT CAACAACAAG   
  
  
+ AAGAAGTGGC TTTCCTAGAA TATGCTAACT CTGTCGGGCT TCCCTTCCTA ACAACTAATT TATTACTGTC   
  
  
+ CTGTGTTGAG GGCATAATAC AGTTGTGGTT CCCTCTTGTC CTTGCACTGA CACACTGTCC TCTACTGAGA   
  
  
+ TAAAATTGGC AGTTAATGTT TCCTTTGAC  

- TTAGTAACCC CATTTTAAAG TCATTCCAAA CCACCAATAT ACTTCAGAAA ATCACATACG AGTCTTTCCT   
  
  
- TAATAATAAT AAACATGAAG TTAAAGTCAA AGTACTAAAT CTCCAAGTCT AAGAAGGTAC ACTTCTCTAT   
  
  
- GAAAACACGA TGAAGAACAT TCAACCCTTA GTACCTAAAG TAAGCCCAGT ACTTAAGATG TGTTATAACG   
  
  
- AGAAAGACTC CGTTACCAAC TTTTATTAGG TAGATCATTA AAGAACGACC ATTTACAAAG AAAGTTCTTC   
  
  
- AATCGTTTCA ATAGAAACAA GAATAACTTC TTCTTATACC TCTCGATGGT TCTTCATAAA GACGTTGAAT   
  
  
- TTTTTTGTAA ATCCTCAACC GTACCTACCA TTACATTATA AGCCAATAAC TAATTATCCC GCATTCGCTA   
  
  
- TCAAGGGACG ACCACTACTC ACTTGACCCC CTCATTGGTT CACTTTCACT ATCTCCCCAG ATAGAACGTC   
  
  
- GAGAGGAAAC TATCTCTCAA TCAGACACTA CACCATCTTC AGACTTCTAA CACGTCGATA CTAAAGAGAC   
  
  
- GTAATAAGAT TTATCACTCA ATACGTTGGT TTAAAGTTAG AATTAACTTC GTATGCCTCA AGACCCCAAA   
  
  
- GAGATCTCTA AACGTAAAGA TTTAAAATAC CGCTTAACCG ATCAAGAGAA TTAACGTTAG AAACTACTGT   
  
  
- CAAATATCTG GTCATCTAAT ACTTTTAACG AATACTAGAA TATTCCGTCG AACTTGACAT AAGTTTTACC   
  
  
- GTTCATGATC AACTTTTTTC TCGTCGGGGT CACTTCCAAA TGTTTGGATT TGACATTGGT CATAAACCCG   
  
  
- CTCTTCATGT TCCAGAGACA CCCGTCCTGC AAAGACAAAC GTATTTAGTT TGGAAATGGA GTCGAGTACT   
  
  
- AAGGTGTAAG TATAATGTAT AGAAGATATT GTCACTGTTT ATTCTATGGT CACGTACTCT CGAAGAACTC   
  
  
- GATCGACTTC GTCTGACCAA ACTTCGAAGA AGAAACGGTA TAAGACGTAT AAGTAGACAG GTAAAGTAGA   
  
  
- GGTCCAAATC AAGTACAAAC TATCCAGAAC GCAACATAGT TAAAGACACT CTACTTGCTT ACGTTCTATG   
  
  
- AAGGGTTTTA AATTTTCAAT GTCGTGTAAA TCGTGTCCAC ATAATGTTTG AGAAATCCCC GATGTCAAAG   
  
  
- GAGACACTAT ACTACGTACT CGGAAGTGAA AGAATTTAAA TGAAAAGTTC AAATATAATA AGTAGTTATA   
  
  
- TTAGAGACGT TTTATGTCCA AAAACTATTC AATGGTAACT CGAACAACGT GTCAACCTCA GTTGTTGTTC   
  
  
- TTCTTCACCG AAAGGATCTT ATACGATTGA GACAGCCCGA AGGGAAGGAT TGTTGATTAA ATAATGACAG   
  
  
- GACACAACTC CCGTATTATG TCAACACCAA GGGAGAACAG GAACGTGACT GTGTGACAGG AGATGACTCT   
  
  
- ATTTTAACCG TCAATTACAA AGGAAACTG

+     circadian

| Site Name | Organism | Position | Strand | Matrix score. | sequence | function |
| --- | --- | --- | --- | --- | --- | --- |
| circadian | Lycopersicon esculentum | 137 | - | 6 | CAANNNNATC | cis-acting regulatory element involved in circadian control |
| circadian | Lycopersicon esculentum | 1469 | - | 6 | CAANNNNATC | cis-acting regulatory element involved in circadian control |
| circadian | Lycopersicon esculentum | 289 | - | 9 | CAAAGATATC | cis-acting regulatory element involved in circadian control |
| circadian | Lycopersicon esculentum | 285 | + | 9 | CAAAGATATC | cis-acting regulatory element involved in circadian control |
| circadian | Lycopersicon esculentum | 1255 | + | 6 | CAANNNNATC | cis-acting regulatory element involved in circadian control |

> 2018/04/13 10:10:12  
+ AATCATTGGG GTAAAATTTC AGTAAGGTTT GGTGGTTATA TGAAGTCTTT TAGTGTATGC TCAGAAAGGA   
  
  
+ ATTATTATTA TTTGTACTTC AATTTCAGTT TCATGATTTA GAGGTTCAGA TTCTTCCATG TGAAGAGATA   
  
  
+ CTTTTGTGCT ACTTCTTGTA AGTTGGGAAT CATGGATTTC ATTCGGGTCA TGAATTCTAC ACAATATTGC   
  
  
+ TCTTTCTGAG GCAATGGTTG AAAATAATCC ATCTAGTAAT TTCTTGCTGG TAAATGTTTC TTTCAAGAAG   
  
  
+ TTAGCAAAGT TATCTTTGTT CTTATTGAAG AAGAATATGG AGAGCTACCA AGAAGTATTT CTGCAACTTA   
  
  
+ AAAAAACATT TAGGAGTTGG CATGGATGGT AATGTAATAT TCGGTTATTG ATTAATAGGG CGTAAGCGAT   
  
  
+ AGTTCCCTGC TGGTGATGAG TGAACTGGGG GAGTAACCAA GTGAAAGTGA TAGAGGGGTC TATCTTGCAG   
  
  
+ CTCTCCTTTG ATAGAGAGTT AGTCTGTGAT GTGGTAGAAG TCTGAAGATT GTGCAGCTAT GATTTCTCTG   
  
  
+ CATTATTCTA AATAGTGAGT TATGCAACCA AATTTCAATC TTAATTGAAG CATACGGAGT TCTGGGGTTT   
  
  
+ CTCTAGAGAT TTGCATTTCT AAATTTTATG GCGAATTGGC TAGTTCTCTT AATTGCAATC TTTGATGACA   
  
  
+ GTTTATAGAC CAGTAGATTA TGAAAATTGC TTATGATCTT ATAAGGCAGC TTGAACTGTA TTCAAAATGG   
  
  
+ CAAGTACTAG TTGAAAAAAG AGCAGCCCCA GTGAAGGTTT ACAAACCTAA ACTGTAACCA GTATTTGGGC   
  
  
+ GAGAAGTACA AGGTCTCTGT GGGCAGGACG TTTCTGTTTG CATAAATCAA ACCTTTACCT CAGCTCATGA   
  
  
+ TTCCACATTC ATATTACATA TCTTCTATAA CAGTGACAAA TAAGATACCA GTGCATGAGA GCTTCTTGAG   
  
  
+ CTAGCTGAAG CAGACTGGTT TGAAGCTTCT TCTTTGCCAT ATTCTGCATA TTCATCTGTC CATTTCATCT   
  
  
+ CCAGGTTTAG TTCATGTTTG ATAGGTCTTG CGTTGTATCA ATTTCTGTGA GATGAACGAA TGCAAGATAC   
  
  
+ TTCCCAAAAT TTAAAAGTTA CAGCACATTT AGCACAGGTG TATTACAAAC TCTTTAGGGG CTACAGTTTC   
  
  
+ CTCTGTGATA TGATGCATGA GCCTTCACTT TCTTAAATTT ACTTTTCAAG TTTATATTAT TCATCAATAT   
  
  
+ AATCTCTGCA AAATACAGGT TTTTGATAAG TTACCATTGA GCTTGTTGCA CAGTTGGAGT CAACAACAAG   
  
  
+ AAGAAGTGGC TTTCCTAGAA TATGCTAACT CTGTCGGGCT TCCCTTCCTA ACAACTAATT TATTACTGTC   
  
  
+ CTGTGTTGAG GGCATAATAC AGTTGTGGTT CCCTCTTGTC CTTGCACTGA CACACTGTCC TCTACTGAGA   
  
  
+ TAAAATTGGC AGTTAATGTT TCCTTTGAC  

- TTAGTAACCC CATTTTAAAG TCATTCCAAA CCACCAATAT ACTTCAGAAA ATCACATACG AGTCTTTCCT   
  
  
- TAATAATAAT AAACATGAAG TTAAAGTCAA AGTACTAAAT CTCCAAGTCT AAGAAGGTAC ACTTCTCTAT   
  
  
- GAAAACACGA TGAAGAACAT TCAACCCTTA GTACCTAAAG TAAGCCCAGT ACTTAAGATG TGTTATAACG   
  
  
- AGAAAGACTC CGTTACCAAC TTTTATTAGG TAGATCATTA AAGAACGACC ATTTACAAAG AAAGTTCTTC   
  
  
- AATCGTTTCA ATAGAAACAA GAATAACTTC TTCTTATACC TCTCGATGGT TCTTCATAAA GACGTTGAAT   
  
  
- TTTTTTGTAA ATCCTCAACC GTACCTACCA TTACATTATA AGCCAATAAC TAATTATCCC GCATTCGCTA   
  
  
- TCAAGGGACG ACCACTACTC ACTTGACCCC CTCATTGGTT CACTTTCACT ATCTCCCCAG ATAGAACGTC   
  
  
- GAGAGGAAAC TATCTCTCAA TCAGACACTA CACCATCTTC AGACTTCTAA CACGTCGATA CTAAAGAGAC   
  
  
- GTAATAAGAT TTATCACTCA ATACGTTGGT TTAAAGTTAG AATTAACTTC GTATGCCTCA AGACCCCAAA   
  
  
- GAGATCTCTA AACGTAAAGA TTTAAAATAC CGCTTAACCG ATCAAGAGAA TTAACGTTAG AAACTACTGT   
  
  
- CAAATATCTG GTCATCTAAT ACTTTTAACG AATACTAGAA TATTCCGTCG AACTTGACAT AAGTTTTACC   
  
  
- GTTCATGATC AACTTTTTTC TCGTCGGGGT CACTTCCAAA TGTTTGGATT TGACATTGGT CATAAACCCG   
  
  
- CTCTTCATGT TCCAGAGACA CCCGTCCTGC AAAGACAAAC GTATTTAGTT TGGAAATGGA GTCGAGTACT   
  
  
- AAGGTGTAAG TATAATGTAT AGAAGATATT GTCACTGTTT ATTCTATGGT CACGTACTCT CGAAGAACTC   
  
  
- GATCGACTTC GTCTGACCAA ACTTCGAAGA AGAAACGGTA TAAGACGTAT AAGTAGACAG GTAAAGTAGA   
  
  
- GGTCCAAATC AAGTACAAAC TATCCAGAAC GCAACATAGT TAAAGACACT CTACTTGCTT ACGTTCTATG   
  
  
- AAGGGTTTTA AATTTTCAAT GTCGTGTAAA TCGTGTCCAC ATAATGTTTG AGAAATCCCC GATGTCAAAG   
  
  
- GAGACACTAT ACTACGTACT CGGAAGTGAA AGAATTTAAA TGAAAAGTTC AAATATAATA AGTAGTTATA   
  
  
- TTAGAGACGT TTTATGTCCA AAAACTATTC AATGGTAACT CGAACAACGT GTCAACCTCA GTTGTTGTTC   
  
  
- TTCTTCACCG AAAGGATCTT ATACGATTGA GACAGCCCGA AGGGAAGGAT TGTTGATTAA ATAATGACAG   
  
  
- GACACAACTC CCGTATTATG TCAACACCAA GGGAGAACAG GAACGTGACT GTGTGACAGG AGATGACTCT   
  
  
- ATTTTAACCG TCAATTACAA AGGAAACTG
