# Supplementary material for: Genome-wide analysis of the WRKY gene family in drumstick (Moringa oleifera Lam.)
Source: PeerJ. 2019 Jun 10;7:e7063. doi: 10.7717/peerj.7063 (PMC6563795; doi:10.7717/peerj.7063)
Supplement: Supplemental Information 1 [file peerj-07-7063-s003.gz › MoWRKY30_plantcare.html]

Content-Type: text/html; charset=ISO-8859-1


CallMat\_Firefox


Webmaster Firefox specific output  
To save the result:
click on the frame with the right mouse button and save the source code as a text file with extension .html  
REFERENCE:PlantCARE: a database of plant cis-acting regulatory elements and a portal to tools for in silico analysis of promoter sequences.  
Lescot, M., Déhais, P., Moreau, Y., De Moor, B., Rouzé ,P.,and Rombauts, S.  
Nucleic Acids Res., Database issue(2002), 30(1):325-327.   


---

> 2018/04/13 10:10:12  
+ CAACTATATA GCACGAAGGT GCATTACGTC TCTGCTAATA GGACCAACAT ATGTTTCTAC CCGTTGTCAA   
  
  
+ AAGAACATTC TTTTCATACA TAGATCAACC TTATACTTAG TCATAGGTGC CTCGATTTCA GGAGAAGGAG   
  
  
+ CAGGAGGAAA CAGGTATTTA TTTCGGCTCT CTCTCTCTCT CTCTCTCTCT CTCTCTCTCT CTCTCTCTCT   
  
  
+ ACAATAAATC TTTCTCGTCC TGAGGAAACA GGTATTTATT TCGGCTCTCT CTCTCTCTCT CTCTCTCTCT   
  
  
+ CTCTCTCTCT CTCTCTCTAC AATAAATCTT TCTCGTCCCA CTCCGACGTT ACCTGAGCCG TAATTTTTCC   
  
  
+ CAAAACCTGA CTTCTGGTTT ACACTTTGCT CACTACGAAT CCCTAAAACT ACTCTCAGAT ACTCCATTCC   
  
  
+ ATCACGCAGT TCTTTTGTCC ACCGGTACTT TCAGAATACT CTCTCTCTCT GTCTCCGTCT CTTTCTTCTC   
  
  
+ AGAATGACGA CGACAGTATT CTCTCTTTCT CTCTCTCTCT CTCTCTCTCT CTCTCTTTCT CAAACTTACA   
  
  
+ AACTTTCCTA GGGCGATCGT GTTTTTGTGA ATTAATGAAT TAATTTAATT TAATTTAATT TAATAAGTAA   
  
  
+ TAATTAATAG AGTGGTGTTT TTAGTAATTA AAAACAATGT GTAAAAAGTT GGGAAATAAT AGAGTACATT   
  
  
+ TTTAGTATAA GGTTTATTAA ATATTTTTAA ATTGAAAAAT ATTAAAGTAA GACTATTTAT ATTTAACTTT   
  
  
+ ACTACTTTTT ATTAAATTTA TTATTTAGAT TTTTTTATTT TTATCATACT TTTATTTAGT TTTGGTGAAC   
  
  
+ TTTAAATATA AATTTATTTT TATAAAATAA AATAAATAAA AATTCTAGAA TGTTCTCTTT TGTACAATGT   
  
  
+ ACTTCACACC CTCAAACTGA TCAAGGTCTT CATGTCGCCA AACTAACCGA CGGCTTCGCG GTGCACTCCC   
  
  
+ GATGCCCTAT TATCAGTTGT TTTACCTGCC CGGCCGAAAA TAGTGTCGAA GTACCTGCCC GGCACCAGAA   
  
  
+ ACTGGCGTGT TGAACAAGGA AGTTAAATAG AAACTAAAAA GTAGAAGAAA AGGAGAGTTA AAGCTTTTTA   
  
  
+ ATTATTTTCT TTTTCTTTAA GGGTTTATTC AGTAAGTATG AGAGGGAATT TAAGTACTTT AAAGGAAATA   
  
  
+ ATACTGCCGG TAGAGATGTA TAAAAGAAAA ACCCCTAAAA AAATGTTTAA TTTAATATCC ATTAAATGAA   
  
  
+ AAAAACCAAA ATTTTAGATA AAGTTGTTTA TAAAAATTTT TGTTTTTTAA ACTAAAAAAA TTTTTTATTT   
  
  
+ AATACACTAT TTTATACTTG AGCTCCAGTT TTGAGGAAAG ATTGAATTAG TTAAGCCGAT TCGATTCTTC   
  
  
+ TAATCGGGTT GTTTAATCCG AAAGTATTTC AATGATCTCT ATGGAGTATC TAATATCCGA CGCTGAAAAA   
  
  
+ GTTAGAGAGG TTTCAAATTA AACGTATAC  

- GTTGATATAT CGTGCTTCCA CGTAATGCAG AGACGATTAT CCTGGTTGTA TACAAAGATG GGCAACAGTT   
  
  
- TTCTTGTAAG AAAAGTATGT ATCTAGTTGG AATATGAATC AGTATCCACG GAGCTAAAGT CCTCTTCCTC   
  
  
- GTCCTCCTTT GTCCATAAAT AAAGCCGAGA GAGAGAGAGA GAGAGAGAGA GAGAGAGAGA GAGAGAGAGA   
  
  
- TGTTATTTAG AAAGAGCAGG ACTCCTTTGT CCATAAATAA AGCCGAGAGA GAGAGAGAGA GAGAGAGAGA   
  
  
- GAGAGAGAGA GAGAGAGATG TTATTTAGAA AGAGCAGGGT GAGGCTGCAA TGGACTCGGC ATTAAAAAGG   
  
  
- GTTTTGGACT GAAGACCAAA TGTGAAACGA GTGATGCTTA GGGATTTTGA TGAGAGTCTA TGAGGTAAGG   
  
  
- TAGTGCGTCA AGAAAACAGG TGGCCATGAA AGTCTTATGA GAGAGAGAGA CAGAGGCAGA GAAAGAAGAG   
  
  
- TCTTACTGCT GCTGTCATAA GAGAGAAAGA GAGAGAGAGA GAGAGAGAGA GAGAGAAAGA GTTTGAATGT   
  
  
- TTGAAAGGAT CCCGCTAGCA CAAAAACACT TAATTACTTA ATTAAATTAA ATTAAATTAA ATTATTCATT   
  
  
- ATTAATTATC TCACCACAAA AATCATTAAT TTTTGTTACA CATTTTTCAA CCCTTTATTA TCTCATGTAA   
  
  
- AAATCATATT CCAAATAATT TATAAAAATT TAACTTTTTA TAATTTCATT CTGATAAATA TAAATTGAAA   
  
  
- TGATGAAAAA TAATTTAAAT AATAAATCTA AAAAAATAAA AATAGTATGA AAATAAATCA AAACCACTTG   
  
  
- AAATTTATAT TTAAATAAAA ATATTTTATT TTATTTATTT TTAAGATCTT ACAAGAGAAA ACATGTTACA   
  
  
- TGAAGTGTGG GAGTTTGACT AGTTCCAGAA GTACAGCGGT TTGATTGGCT GCCGAAGCGC CACGTGAGGG   
  
  
- CTACGGGATA ATAGTCAACA AAATGGACGG GCCGGCTTTT ATCACAGCTT CATGGACGGG CCGTGGTCTT   
  
  
- TGACCGCACA ACTTGTTCCT TCAATTTATC TTTGATTTTT CATCTTCTTT TCCTCTCAAT TTCGAAAAAT   
  
  
- TAATAAAAGA AAAAGAAATT CCCAAATAAG TCATTCATAC TCTCCCTTAA ATTCATGAAA TTTCCTTTAT   
  
  
- TATGACGGCC ATCTCTACAT ATTTTCTTTT TGGGGATTTT TTTACAAATT AAATTATAGG TAATTTACTT   
  
  
- TTTTTGGTTT TAAAATCTAT TTCAACAAAT ATTTTTAAAA ACAAAAAATT TGATTTTTTT AAAAAATAAA   
  
  
- TTATGTGATA AAATATGAAC TCGAGGTCAA AACTCCTTTC TAACTTAATC AATTCGGCTA AGCTAAGAAG   
  
  
- ATTAGCCCAA CAAATTAGGC TTTCATAAAG TTACTAGAGA TACCTCATAG ATTATAGGCT GCGACTTTTT   
  
  
- CAATCTCTCC AAAGTTTAAT TTGCATATG

  
  
Motifs Found  

+     5UTR Py-rich stretch

| Site Name | Organism | Position | Strand | Matrix score. | sequence | function |
| --- | --- | --- | --- | --- | --- | --- |
| 5UTR Py-rich stretch | Lycopersicon esculentum | 530 | + | 13 | TTTCTCTCTCTCTC | cis-acting element conferring high transcription levels |
| 5UTR Py-rich stretch | Lycopersicon esculentum | 182 | + | 13 | TTTCTCTCTCTCTC | cis-acting element conferring high transcription levels |
| 5UTR Py-rich stretch | Lycopersicon esculentum | 172 | + | 13 | TTTCTCTCTCTCTC | cis-acting element conferring high transcription levels |
| 5UTR Py-rich stretch | Lycopersicon esculentum | 258 | + | 13 | TTTCTCTCTCTCTC | cis-acting element conferring high transcription levels |
| 5UTR Py-rich stretch | Lycopersicon esculentum | 174 | + | 13 | TTTCTCTCTCTCTC | cis-acting element conferring high transcription levels |
| 5UTR Py-rich stretch | Lycopersicon esculentum | 170 | + | 13 | TTTCTCTCTCTCTC | cis-acting element conferring high transcription levels |
| 5UTR Py-rich stretch | Lycopersicon esculentum | 196 | + | 13 | TTTCTCTCTCTCTC | cis-acting element conferring high transcription levels |
| 5UTR Py-rich stretch | Lycopersicon esculentum | 526 | + | 13 | TTTCTCTCTCTCTC | cis-acting element conferring high transcription levels |
| 5UTR Py-rich stretch | Lycopersicon esculentum | 284 | + | 13 | TTTCTCTCTCTCTC | cis-acting element conferring high transcription levels |
| 5UTR Py-rich stretch | Lycopersicon esculentum | 272 | + | 13 | TTTCTCTCTCTCTC | cis-acting element conferring high transcription levels |
| 5UTR Py-rich stretch | Lycopersicon esculentum | 278 | + | 13 | TTTCTCTCTCTCTC | cis-acting element conferring high transcription levels |
| 5UTR Py-rich stretch | Lycopersicon esculentum | 532 | + | 13 | TTTCTCTCTCTCTC | cis-acting element conferring high transcription levels |
| 5UTR Py-rich stretch | Lycopersicon esculentum | 282 | + | 13 | TTTCTCTCTCTCTC | cis-acting element conferring high transcription levels |
| 5UTR Py-rich stretch | Lycopersicon esculentum | 280 | + | 13 | TTTCTCTCTCTCTC | cis-acting element conferring high transcription levels |
| 5UTR Py-rich stretch | Lycopersicon esculentum | 266 | + | 13 | TTTCTCTCTCTCTC | cis-acting element conferring high transcription levels |
| 5UTR Py-rich stretch | Lycopersicon esculentum | 520 | + | 13 | TTTCTCTCTCTCTC | cis-acting element conferring high transcription levels |
| 5UTR Py-rich stretch | Lycopersicon esculentum | 256 | + | 13 | TTTCTCTCTCTCTC | cis-acting element conferring high transcription levels |
| 5UTR Py-rich stretch | Lycopersicon esculentum | 194 | + | 13 | TTTCTCTCTCTCTC | cis-acting element conferring high transcription levels |
| 5UTR Py-rich stretch | Lycopersicon esculentum | 528 | + | 13 | TTTCTCTCTCTCTC | cis-acting element conferring high transcription levels |
| 5UTR Py-rich stretch | Lycopersicon esculentum | 184 | + | 13 | TTTCTCTCTCTCTC | cis-acting element conferring high transcription levels |
| 5UTR Py-rich stretch | Lycopersicon esculentum | 268 | + | 13 | TTTCTCTCTCTCTC | cis-acting element conferring high transcription levels |
| 5UTR Py-rich stretch | Lycopersicon esculentum | 270 | + | 13 | TTTCTCTCTCTCTC | cis-acting element conferring high transcription levels |
| 5UTR Py-rich stretch | Lycopersicon esculentum | 176 | + | 13 | TTTCTCTCTCTCTC | cis-acting element conferring high transcription levels |
| 5UTR Py-rich stretch | Lycopersicon esculentum | 276 | + | 13 | TTTCTCTCTCTCTC | cis-acting element conferring high transcription levels |
| 5UTR Py-rich stretch | Lycopersicon esculentum | 178 | + | 13 | TTTCTCTCTCTCTC | cis-acting element conferring high transcription levels |
| 5UTR Py-rich stretch | Lycopersicon esculentum | 516 | + | 14 | TTTCTCTCTCTCTC | cis-acting element conferring high transcription levels |
| 5UTR Py-rich stretch | Lycopersicon esculentum | 522 | + | 13 | TTTCTCTCTCTCTC | cis-acting element conferring high transcription levels |
| 5UTR Py-rich stretch | Lycopersicon esculentum | 274 | + | 13 | TTTCTCTCTCTCTC | cis-acting element conferring high transcription levels |
| 5UTR Py-rich stretch | Lycopersicon esculentum | 524 | + | 13 | TTTCTCTCTCTCTC | cis-acting element conferring high transcription levels |
| 5UTR Py-rich stretch | Lycopersicon esculentum | 262 | + | 13 | TTTCTCTCTCTCTC | cis-acting element conferring high transcription levels |
| 5UTR Py-rich stretch | Lycopersicon esculentum | 180 | + | 13 | TTTCTCTCTCTCTC | cis-acting element conferring high transcription levels |
| 5UTR Py-rich stretch | Lycopersicon esculentum | 186 | + | 13 | TTTCTCTCTCTCTC | cis-acting element conferring high transcription levels |
| 5UTR Py-rich stretch | Lycopersicon esculentum | 190 | + | 13 | TTTCTCTCTCTCTC | cis-acting element conferring high transcription levels |
| 5UTR Py-rich stretch | Lycopersicon esculentum | 188 | + | 13 | TTTCTCTCTCTCTC | cis-acting element conferring high transcription levels |
| 5UTR Py-rich stretch | Lycopersicon esculentum | 518 | + | 13 | TTTCTCTCTCTCTC | cis-acting element conferring high transcription levels |
| 5UTR Py-rich stretch | Lycopersicon esculentum | 482 | + | 9 | TTTCTTCTCT | cis-acting element conferring high transcription levels |
| 5UTR Py-rich stretch | Lycopersicon esculentum | 168 | + | 13 | TTTCTCTCTCTCTC | cis-acting element conferring high transcription levels |
| 5UTR Py-rich stretch | Lycopersicon esculentum | 260 | + | 13 | TTTCTCTCTCTCTC | cis-acting element conferring high transcription levels |
| 5UTR Py-rich stretch | Lycopersicon esculentum | 192 | + | 13 | TTTCTCTCTCTCTC | cis-acting element conferring high transcription levels |
| 5UTR Py-rich stretch | Lycopersicon esculentum | 264 | + | 13 | TTTCTCTCTCTCTC | cis-acting element conferring high transcription levels |

> 2018/04/13 10:10:12  
+ CAACTATATA GCACGAAGGT GCATTACGTC TCTGCTAATA GGACCAACAT ATGTTTCTAC CCGTTGTCAA   
  
  
+ AAGAACATTC TTTTCATACA TAGATCAACC TTATACTTAG TCATAGGTGC CTCGATTTCA GGAGAAGGAG   
  
  
+ CAGGAGGAAA CAGGTATTTA TTTCGGCTCT CTCTCTCTCT CTCTCTCTCT CTCTCTCTCT CTCTCTCTCT   
  
  
+ ACAATAAATC TTTCTCGTCC TGAGGAAACA GGTATTTATT TCGGCTCTCT CTCTCTCTCT CTCTCTCTCT   
  
  
+ CTCTCTCTCT CTCTCTCTAC AATAAATCTT TCTCGTCCCA CTCCGACGTT ACCTGAGCCG TAATTTTTCC   
  
  
+ CAAAACCTGA CTTCTGGTTT ACACTTTGCT CACTACGAAT CCCTAAAACT ACTCTCAGAT ACTCCATTCC   
  
  
+ ATCACGCAGT TCTTTTGTCC ACCGGTACTT TCAGAATACT CTCTCTCTCT GTCTCCGTCT CTTTCTTCTC   
  
  
+ AGAATGACGA CGACAGTATT CTCTCTTTCT CTCTCTCTCT CTCTCTCTCT CTCTCTTTCT CAAACTTACA   
  
  
+ AACTTTCCTA GGGCGATCGT GTTTTTGTGA ATTAATGAAT TAATTTAATT TAATTTAATT TAATAAGTAA   
  
  
+ TAATTAATAG AGTGGTGTTT TTAGTAATTA AAAACAATGT GTAAAAAGTT GGGAAATAAT AGAGTACATT   
  
  
+ TTTAGTATAA GGTTTATTAA ATATTTTTAA ATTGAAAAAT ATTAAAGTAA GACTATTTAT ATTTAACTTT   
  
  
+ ACTACTTTTT ATTAAATTTA TTATTTAGAT TTTTTTATTT TTATCATACT TTTATTTAGT TTTGGTGAAC   
  
  
+ TTTAAATATA AATTTATTTT TATAAAATAA AATAAATAAA AATTCTAGAA TGTTCTCTTT TGTACAATGT   
  
  
+ ACTTCACACC CTCAAACTGA TCAAGGTCTT CATGTCGCCA AACTAACCGA CGGCTTCGCG GTGCACTCCC   
  
  
+ GATGCCCTAT TATCAGTTGT TTTACCTGCC CGGCCGAAAA TAGTGTCGAA GTACCTGCCC GGCACCAGAA   
  
  
+ ACTGGCGTGT TGAACAAGGA AGTTAAATAG AAACTAAAAA GTAGAAGAAA AGGAGAGTTA AAGCTTTTTA   
  
  
+ ATTATTTTCT TTTTCTTTAA GGGTTTATTC AGTAAGTATG AGAGGGAATT TAAGTACTTT AAAGGAAATA   
  
  
+ ATACTGCCGG TAGAGATGTA TAAAAGAAAA ACCCCTAAAA AAATGTTTAA TTTAATATCC ATTAAATGAA   
  
  
+ AAAAACCAAA ATTTTAGATA AAGTTGTTTA TAAAAATTTT TGTTTTTTAA ACTAAAAAAA TTTTTTATTT   
  
  
+ AATACACTAT TTTATACTTG AGCTCCAGTT TTGAGGAAAG ATTGAATTAG TTAAGCCGAT TCGATTCTTC   
  
  
+ TAATCGGGTT GTTTAATCCG AAAGTATTTC AATGATCTCT ATGGAGTATC TAATATCCGA CGCTGAAAAA   
  
  
+ GTTAGAGAGG TTTCAAATTA AACGTATAC  

- GTTGATATAT CGTGCTTCCA CGTAATGCAG AGACGATTAT CCTGGTTGTA TACAAAGATG GGCAACAGTT   
  
  
- TTCTTGTAAG AAAAGTATGT ATCTAGTTGG AATATGAATC AGTATCCACG GAGCTAAAGT CCTCTTCCTC   
  
  
- GTCCTCCTTT GTCCATAAAT AAAGCCGAGA GAGAGAGAGA GAGAGAGAGA GAGAGAGAGA GAGAGAGAGA   
  
  
- TGTTATTTAG AAAGAGCAGG ACTCCTTTGT CCATAAATAA AGCCGAGAGA GAGAGAGAGA GAGAGAGAGA   
  
  
- GAGAGAGAGA GAGAGAGATG TTATTTAGAA AGAGCAGGGT GAGGCTGCAA TGGACTCGGC ATTAAAAAGG   
  
  
- GTTTTGGACT GAAGACCAAA TGTGAAACGA GTGATGCTTA GGGATTTTGA TGAGAGTCTA TGAGGTAAGG   
  
  
- TAGTGCGTCA AGAAAACAGG TGGCCATGAA AGTCTTATGA GAGAGAGAGA CAGAGGCAGA GAAAGAAGAG   
  
  
- TCTTACTGCT GCTGTCATAA GAGAGAAAGA GAGAGAGAGA GAGAGAGAGA GAGAGAAAGA GTTTGAATGT   
  
  
- TTGAAAGGAT CCCGCTAGCA CAAAAACACT TAATTACTTA ATTAAATTAA ATTAAATTAA ATTATTCATT   
  
  
- ATTAATTATC TCACCACAAA AATCATTAAT TTTTGTTACA CATTTTTCAA CCCTTTATTA TCTCATGTAA   
  
  
- AAATCATATT CCAAATAATT TATAAAAATT TAACTTTTTA TAATTTCATT CTGATAAATA TAAATTGAAA   
  
  
- TGATGAAAAA TAATTTAAAT AATAAATCTA AAAAAATAAA AATAGTATGA AAATAAATCA AAACCACTTG   
  
  
- AAATTTATAT TTAAATAAAA ATATTTTATT TTATTTATTT TTAAGATCTT ACAAGAGAAA ACATGTTACA   
  
  
- TGAAGTGTGG GAGTTTGACT AGTTCCAGAA GTACAGCGGT TTGATTGGCT GCCGAAGCGC CACGTGAGGG   
  
  
- CTACGGGATA ATAGTCAACA AAATGGACGG GCCGGCTTTT ATCACAGCTT CATGGACGGG CCGTGGTCTT   
  
  
- TGACCGCACA ACTTGTTCCT TCAATTTATC TTTGATTTTT CATCTTCTTT TCCTCTCAAT TTCGAAAAAT   
  
  
- TAATAAAAGA AAAAGAAATT CCCAAATAAG TCATTCATAC TCTCCCTTAA ATTCATGAAA TTTCCTTTAT   
  
  
- TATGACGGCC ATCTCTACAT ATTTTCTTTT TGGGGATTTT TTTACAAATT AAATTATAGG TAATTTACTT   
  
  
- TTTTTGGTTT TAAAATCTAT TTCAACAAAT ATTTTTAAAA ACAAAAAATT TGATTTTTTT AAAAAATAAA   
  
  
- TTATGTGATA AAATATGAAC TCGAGGTCAA AACTCCTTTC TAACTTAATC AATTCGGCTA AGCTAAGAAG   
  
  
- ATTAGCCCAA CAAATTAGGC TTTCATAAAG TTACTAGAGA TACCTCATAG ATTATAGGCT GCGACTTTTT   
  
  
- CAATCTCTCC AAAGTTTAAT TTGCATATG

+     ACE

| Site Name | Organism | Position | Strand | Matrix score. | sequence | function |
| --- | --- | --- | --- | --- | --- | --- |
| ACE | Petroselinum crispum | 1230 | + | 9 | AAAACGTTTA | cis-acting element involved in light responsiveness |

> 2018/04/13 10:10:12  
+ CAACTATATA GCACGAAGGT GCATTACGTC TCTGCTAATA GGACCAACAT ATGTTTCTAC CCGTTGTCAA   
  
  
+ AAGAACATTC TTTTCATACA TAGATCAACC TTATACTTAG TCATAGGTGC CTCGATTTCA GGAGAAGGAG   
  
  
+ CAGGAGGAAA CAGGTATTTA TTTCGGCTCT CTCTCTCTCT CTCTCTCTCT CTCTCTCTCT CTCTCTCTCT   
  
  
+ ACAATAAATC TTTCTCGTCC TGAGGAAACA GGTATTTATT TCGGCTCTCT CTCTCTCTCT CTCTCTCTCT   
  
  
+ CTCTCTCTCT CTCTCTCTAC AATAAATCTT TCTCGTCCCA CTCCGACGTT ACCTGAGCCG TAATTTTTCC   
  
  
+ CAAAACCTGA CTTCTGGTTT ACACTTTGCT CACTACGAAT CCCTAAAACT ACTCTCAGAT ACTCCATTCC   
  
  
+ ATCACGCAGT TCTTTTGTCC ACCGGTACTT TCAGAATACT CTCTCTCTCT GTCTCCGTCT CTTTCTTCTC   
  
  
+ AGAATGACGA CGACAGTATT CTCTCTTTCT CTCTCTCTCT CTCTCTCTCT CTCTCTTTCT CAAACTTACA   
  
  
+ AACTTTCCTA GGGCGATCGT GTTTTTGTGA ATTAATGAAT TAATTTAATT TAATTTAATT TAATAAGTAA   
  
  
+ TAATTAATAG AGTGGTGTTT TTAGTAATTA AAAACAATGT GTAAAAAGTT GGGAAATAAT AGAGTACATT   
  
  
+ TTTAGTATAA GGTTTATTAA ATATTTTTAA ATTGAAAAAT ATTAAAGTAA GACTATTTAT ATTTAACTTT   
  
  
+ ACTACTTTTT ATTAAATTTA TTATTTAGAT TTTTTTATTT TTATCATACT TTTATTTAGT TTTGGTGAAC   
  
  
+ TTTAAATATA AATTTATTTT TATAAAATAA AATAAATAAA AATTCTAGAA TGTTCTCTTT TGTACAATGT   
  
  
+ ACTTCACACC CTCAAACTGA TCAAGGTCTT CATGTCGCCA AACTAACCGA CGGCTTCGCG GTGCACTCCC   
  
  
+ GATGCCCTAT TATCAGTTGT TTTACCTGCC CGGCCGAAAA TAGTGTCGAA GTACCTGCCC GGCACCAGAA   
  
  
+ ACTGGCGTGT TGAACAAGGA AGTTAAATAG AAACTAAAAA GTAGAAGAAA AGGAGAGTTA AAGCTTTTTA   
  
  
+ ATTATTTTCT TTTTCTTTAA GGGTTTATTC AGTAAGTATG AGAGGGAATT TAAGTACTTT AAAGGAAATA   
  
  
+ ATACTGCCGG TAGAGATGTA TAAAAGAAAA ACCCCTAAAA AAATGTTTAA TTTAATATCC ATTAAATGAA   
  
  
+ AAAAACCAAA ATTTTAGATA AAGTTGTTTA TAAAAATTTT TGTTTTTTAA ACTAAAAAAA TTTTTTATTT   
  
  
+ AATACACTAT TTTATACTTG AGCTCCAGTT TTGAGGAAAG ATTGAATTAG TTAAGCCGAT TCGATTCTTC   
  
  
+ TAATCGGGTT GTTTAATCCG AAAGTATTTC AATGATCTCT ATGGAGTATC TAATATCCGA CGCTGAAAAA   
  
  
+ GTTAGAGAGG TTTCAAATTA AACGTATAC  

- GTTGATATAT CGTGCTTCCA CGTAATGCAG AGACGATTAT CCTGGTTGTA TACAAAGATG GGCAACAGTT   
  
  
- TTCTTGTAAG AAAAGTATGT ATCTAGTTGG AATATGAATC AGTATCCACG GAGCTAAAGT CCTCTTCCTC   
  
  
- GTCCTCCTTT GTCCATAAAT AAAGCCGAGA GAGAGAGAGA GAGAGAGAGA GAGAGAGAGA GAGAGAGAGA   
  
  
- TGTTATTTAG AAAGAGCAGG ACTCCTTTGT CCATAAATAA AGCCGAGAGA GAGAGAGAGA GAGAGAGAGA   
  
  
- GAGAGAGAGA GAGAGAGATG TTATTTAGAA AGAGCAGGGT GAGGCTGCAA TGGACTCGGC ATTAAAAAGG   
  
  
- GTTTTGGACT GAAGACCAAA TGTGAAACGA GTGATGCTTA GGGATTTTGA TGAGAGTCTA TGAGGTAAGG   
  
  
- TAGTGCGTCA AGAAAACAGG TGGCCATGAA AGTCTTATGA GAGAGAGAGA CAGAGGCAGA GAAAGAAGAG   
  
  
- TCTTACTGCT GCTGTCATAA GAGAGAAAGA GAGAGAGAGA GAGAGAGAGA GAGAGAAAGA GTTTGAATGT   
  
  
- TTGAAAGGAT CCCGCTAGCA CAAAAACACT TAATTACTTA ATTAAATTAA ATTAAATTAA ATTATTCATT   
  
  
- ATTAATTATC TCACCACAAA AATCATTAAT TTTTGTTACA CATTTTTCAA CCCTTTATTA TCTCATGTAA   
  
  
- AAATCATATT CCAAATAATT TATAAAAATT TAACTTTTTA TAATTTCATT CTGATAAATA TAAATTGAAA   
  
  
- TGATGAAAAA TAATTTAAAT AATAAATCTA AAAAAATAAA AATAGTATGA AAATAAATCA AAACCACTTG   
  
  
- AAATTTATAT TTAAATAAAA ATATTTTATT TTATTTATTT TTAAGATCTT ACAAGAGAAA ACATGTTACA   
  
  
- TGAAGTGTGG GAGTTTGACT AGTTCCAGAA GTACAGCGGT TTGATTGGCT GCCGAAGCGC CACGTGAGGG   
  
  
- CTACGGGATA ATAGTCAACA AAATGGACGG GCCGGCTTTT ATCACAGCTT CATGGACGGG CCGTGGTCTT   
  
  
- TGACCGCACA ACTTGTTCCT TCAATTTATC TTTGATTTTT CATCTTCTTT TCCTCTCAAT TTCGAAAAAT   
  
  
- TAATAAAAGA AAAAGAAATT CCCAAATAAG TCATTCATAC TCTCCCTTAA ATTCATGAAA TTTCCTTTAT   
  
  
- TATGACGGCC ATCTCTACAT ATTTTCTTTT TGGGGATTTT TTTACAAATT AAATTATAGG TAATTTACTT   
  
  
- TTTTTGGTTT TAAAATCTAT TTCAACAAAT ATTTTTAAAA ACAAAAAATT TGATTTTTTT AAAAAATAAA   
  
  
- TTATGTGATA AAATATGAAC TCGAGGTCAA AACTCCTTTC TAACTTAATC AATTCGGCTA AGCTAAGAAG   
  
  
- ATTAGCCCAA CAAATTAGGC TTTCATAAAG TTACTAGAGA TACCTCATAG ATTATAGGCT GCGACTTTTT   
  
  
- CAATCTCTCC AAAGTTTAAT TTGCATATG

+     AE-box

| Site Name | Organism | Position | Strand | Matrix score. | sequence | function |
| --- | --- | --- | --- | --- | --- | --- |
| AE-box | Arabidopsis thaliana | 51 | - | 8 | AGAAACAT | part of a module for light response |

> 2018/04/13 10:10:12  
+ CAACTATATA GCACGAAGGT GCATTACGTC TCTGCTAATA GGACCAACAT ATGTTTCTAC CCGTTGTCAA   
  
  
+ AAGAACATTC TTTTCATACA TAGATCAACC TTATACTTAG TCATAGGTGC CTCGATTTCA GGAGAAGGAG   
  
  
+ CAGGAGGAAA CAGGTATTTA TTTCGGCTCT CTCTCTCTCT CTCTCTCTCT CTCTCTCTCT CTCTCTCTCT   
  
  
+ ACAATAAATC TTTCTCGTCC TGAGGAAACA GGTATTTATT TCGGCTCTCT CTCTCTCTCT CTCTCTCTCT   
  
  
+ CTCTCTCTCT CTCTCTCTAC AATAAATCTT TCTCGTCCCA CTCCGACGTT ACCTGAGCCG TAATTTTTCC   
  
  
+ CAAAACCTGA CTTCTGGTTT ACACTTTGCT CACTACGAAT CCCTAAAACT ACTCTCAGAT ACTCCATTCC   
  
  
+ ATCACGCAGT TCTTTTGTCC ACCGGTACTT TCAGAATACT CTCTCTCTCT GTCTCCGTCT CTTTCTTCTC   
  
  
+ AGAATGACGA CGACAGTATT CTCTCTTTCT CTCTCTCTCT CTCTCTCTCT CTCTCTTTCT CAAACTTACA   
  
  
+ AACTTTCCTA GGGCGATCGT GTTTTTGTGA ATTAATGAAT TAATTTAATT TAATTTAATT TAATAAGTAA   
  
  
+ TAATTAATAG AGTGGTGTTT TTAGTAATTA AAAACAATGT GTAAAAAGTT GGGAAATAAT AGAGTACATT   
  
  
+ TTTAGTATAA GGTTTATTAA ATATTTTTAA ATTGAAAAAT ATTAAAGTAA GACTATTTAT ATTTAACTTT   
  
  
+ ACTACTTTTT ATTAAATTTA TTATTTAGAT TTTTTTATTT TTATCATACT TTTATTTAGT TTTGGTGAAC   
  
  
+ TTTAAATATA AATTTATTTT TATAAAATAA AATAAATAAA AATTCTAGAA TGTTCTCTTT TGTACAATGT   
  
  
+ ACTTCACACC CTCAAACTGA TCAAGGTCTT CATGTCGCCA AACTAACCGA CGGCTTCGCG GTGCACTCCC   
  
  
+ GATGCCCTAT TATCAGTTGT TTTACCTGCC CGGCCGAAAA TAGTGTCGAA GTACCTGCCC GGCACCAGAA   
  
  
+ ACTGGCGTGT TGAACAAGGA AGTTAAATAG AAACTAAAAA GTAGAAGAAA AGGAGAGTTA AAGCTTTTTA   
  
  
+ ATTATTTTCT TTTTCTTTAA GGGTTTATTC AGTAAGTATG AGAGGGAATT TAAGTACTTT AAAGGAAATA   
  
  
+ ATACTGCCGG TAGAGATGTA TAAAAGAAAA ACCCCTAAAA AAATGTTTAA TTTAATATCC ATTAAATGAA   
  
  
+ AAAAACCAAA ATTTTAGATA AAGTTGTTTA TAAAAATTTT TGTTTTTTAA ACTAAAAAAA TTTTTTATTT   
  
  
+ AATACACTAT TTTATACTTG AGCTCCAGTT TTGAGGAAAG ATTGAATTAG TTAAGCCGAT TCGATTCTTC   
  
  
+ TAATCGGGTT GTTTAATCCG AAAGTATTTC AATGATCTCT ATGGAGTATC TAATATCCGA CGCTGAAAAA   
  
  
+ GTTAGAGAGG TTTCAAATTA AACGTATAC  

- GTTGATATAT CGTGCTTCCA CGTAATGCAG AGACGATTAT CCTGGTTGTA TACAAAGATG GGCAACAGTT   
  
  
- TTCTTGTAAG AAAAGTATGT ATCTAGTTGG AATATGAATC AGTATCCACG GAGCTAAAGT CCTCTTCCTC   
  
  
- GTCCTCCTTT GTCCATAAAT AAAGCCGAGA GAGAGAGAGA GAGAGAGAGA GAGAGAGAGA GAGAGAGAGA   
  
  
- TGTTATTTAG AAAGAGCAGG ACTCCTTTGT CCATAAATAA AGCCGAGAGA GAGAGAGAGA GAGAGAGAGA   
  
  
- GAGAGAGAGA GAGAGAGATG TTATTTAGAA AGAGCAGGGT GAGGCTGCAA TGGACTCGGC ATTAAAAAGG   
  
  
- GTTTTGGACT GAAGACCAAA TGTGAAACGA GTGATGCTTA GGGATTTTGA TGAGAGTCTA TGAGGTAAGG   
  
  
- TAGTGCGTCA AGAAAACAGG TGGCCATGAA AGTCTTATGA GAGAGAGAGA CAGAGGCAGA GAAAGAAGAG   
  
  
- TCTTACTGCT GCTGTCATAA GAGAGAAAGA GAGAGAGAGA GAGAGAGAGA GAGAGAAAGA GTTTGAATGT   
  
  
- TTGAAAGGAT CCCGCTAGCA CAAAAACACT TAATTACTTA ATTAAATTAA ATTAAATTAA ATTATTCATT   
  
  
- ATTAATTATC TCACCACAAA AATCATTAAT TTTTGTTACA CATTTTTCAA CCCTTTATTA TCTCATGTAA   
  
  
- AAATCATATT CCAAATAATT TATAAAAATT TAACTTTTTA TAATTTCATT CTGATAAATA TAAATTGAAA   
  
  
- TGATGAAAAA TAATTTAAAT AATAAATCTA AAAAAATAAA AATAGTATGA AAATAAATCA AAACCACTTG   
  
  
- AAATTTATAT TTAAATAAAA ATATTTTATT TTATTTATTT TTAAGATCTT ACAAGAGAAA ACATGTTACA   
  
  
- TGAAGTGTGG GAGTTTGACT AGTTCCAGAA GTACAGCGGT TTGATTGGCT GCCGAAGCGC CACGTGAGGG   
  
  
- CTACGGGATA ATAGTCAACA AAATGGACGG GCCGGCTTTT ATCACAGCTT CATGGACGGG CCGTGGTCTT   
  
  
- TGACCGCACA ACTTGTTCCT TCAATTTATC TTTGATTTTT CATCTTCTTT TCCTCTCAAT TTCGAAAAAT   
  
  
- TAATAAAAGA AAAAGAAATT CCCAAATAAG TCATTCATAC TCTCCCTTAA ATTCATGAAA TTTCCTTTAT   
  
  
- TATGACGGCC ATCTCTACAT ATTTTCTTTT TGGGGATTTT TTTACAAATT AAATTATAGG TAATTTACTT   
  
  
- TTTTTGGTTT TAAAATCTAT TTCAACAAAT ATTTTTAAAA ACAAAAAATT TGATTTTTTT AAAAAATAAA   
  
  
- TTATGTGATA AAATATGAAC TCGAGGTCAA AACTCCTTTC TAACTTAATC AATTCGGCTA AGCTAAGAAG   
  
  
- ATTAGCCCAA CAAATTAGGC TTTCATAAAG TTACTAGAGA TACCTCATAG ATTATAGGCT GCGACTTTTT   
  
  
- CAATCTCTCC AAAGTTTAAT TTGCATATG

+     ARE

| Site Name | Organism | Position | Strand | Matrix score. | sequence | function |
| --- | --- | --- | --- | --- | --- | --- |
| ARE | Zea mays | 1263 | - | 6 | TGGTTT | cis-acting regulatory element essential for the anaerobic induction |
| ARE | Zea mays | 365 | + | 6 | TGGTTT | cis-acting regulatory element essential for the anaerobic induction |

> 2018/04/13 10:10:12  
+ CAACTATATA GCACGAAGGT GCATTACGTC TCTGCTAATA GGACCAACAT ATGTTTCTAC CCGTTGTCAA   
  
  
+ AAGAACATTC TTTTCATACA TAGATCAACC TTATACTTAG TCATAGGTGC CTCGATTTCA GGAGAAGGAG   
  
  
+ CAGGAGGAAA CAGGTATTTA TTTCGGCTCT CTCTCTCTCT CTCTCTCTCT CTCTCTCTCT CTCTCTCTCT   
  
  
+ ACAATAAATC TTTCTCGTCC TGAGGAAACA GGTATTTATT TCGGCTCTCT CTCTCTCTCT CTCTCTCTCT   
  
  
+ CTCTCTCTCT CTCTCTCTAC AATAAATCTT TCTCGTCCCA CTCCGACGTT ACCTGAGCCG TAATTTTTCC   
  
  
+ CAAAACCTGA CTTCTGGTTT ACACTTTGCT CACTACGAAT CCCTAAAACT ACTCTCAGAT ACTCCATTCC   
  
  
+ ATCACGCAGT TCTTTTGTCC ACCGGTACTT TCAGAATACT CTCTCTCTCT GTCTCCGTCT CTTTCTTCTC   
  
  
+ AGAATGACGA CGACAGTATT CTCTCTTTCT CTCTCTCTCT CTCTCTCTCT CTCTCTTTCT CAAACTTACA   
  
  
+ AACTTTCCTA GGGCGATCGT GTTTTTGTGA ATTAATGAAT TAATTTAATT TAATTTAATT TAATAAGTAA   
  
  
+ TAATTAATAG AGTGGTGTTT TTAGTAATTA AAAACAATGT GTAAAAAGTT GGGAAATAAT AGAGTACATT   
  
  
+ TTTAGTATAA GGTTTATTAA ATATTTTTAA ATTGAAAAAT ATTAAAGTAA GACTATTTAT ATTTAACTTT   
  
  
+ ACTACTTTTT ATTAAATTTA TTATTTAGAT TTTTTTATTT TTATCATACT TTTATTTAGT TTTGGTGAAC   
  
  
+ TTTAAATATA AATTTATTTT TATAAAATAA AATAAATAAA AATTCTAGAA TGTTCTCTTT TGTACAATGT   
  
  
+ ACTTCACACC CTCAAACTGA TCAAGGTCTT CATGTCGCCA AACTAACCGA CGGCTTCGCG GTGCACTCCC   
  
  
+ GATGCCCTAT TATCAGTTGT TTTACCTGCC CGGCCGAAAA TAGTGTCGAA GTACCTGCCC GGCACCAGAA   
  
  
+ ACTGGCGTGT TGAACAAGGA AGTTAAATAG AAACTAAAAA GTAGAAGAAA AGGAGAGTTA AAGCTTTTTA   
  
  
+ ATTATTTTCT TTTTCTTTAA GGGTTTATTC AGTAAGTATG AGAGGGAATT TAAGTACTTT AAAGGAAATA   
  
  
+ ATACTGCCGG TAGAGATGTA TAAAAGAAAA ACCCCTAAAA AAATGTTTAA TTTAATATCC ATTAAATGAA   
  
  
+ AAAAACCAAA ATTTTAGATA AAGTTGTTTA TAAAAATTTT TGTTTTTTAA ACTAAAAAAA TTTTTTATTT   
  
  
+ AATACACTAT TTTATACTTG AGCTCCAGTT TTGAGGAAAG ATTGAATTAG TTAAGCCGAT TCGATTCTTC   
  
  
+ TAATCGGGTT GTTTAATCCG AAAGTATTTC AATGATCTCT ATGGAGTATC TAATATCCGA CGCTGAAAAA   
  
  
+ GTTAGAGAGG TTTCAAATTA AACGTATAC  

- GTTGATATAT CGTGCTTCCA CGTAATGCAG AGACGATTAT CCTGGTTGTA TACAAAGATG GGCAACAGTT   
  
  
- TTCTTGTAAG AAAAGTATGT ATCTAGTTGG AATATGAATC AGTATCCACG GAGCTAAAGT CCTCTTCCTC   
  
  
- GTCCTCCTTT GTCCATAAAT AAAGCCGAGA GAGAGAGAGA GAGAGAGAGA GAGAGAGAGA GAGAGAGAGA   
  
  
- TGTTATTTAG AAAGAGCAGG ACTCCTTTGT CCATAAATAA AGCCGAGAGA GAGAGAGAGA GAGAGAGAGA   
  
  
- GAGAGAGAGA GAGAGAGATG TTATTTAGAA AGAGCAGGGT GAGGCTGCAA TGGACTCGGC ATTAAAAAGG   
  
  
- GTTTTGGACT GAAGACCAAA TGTGAAACGA GTGATGCTTA GGGATTTTGA TGAGAGTCTA TGAGGTAAGG   
  
  
- TAGTGCGTCA AGAAAACAGG TGGCCATGAA AGTCTTATGA GAGAGAGAGA CAGAGGCAGA GAAAGAAGAG   
  
  
- TCTTACTGCT GCTGTCATAA GAGAGAAAGA GAGAGAGAGA GAGAGAGAGA GAGAGAAAGA GTTTGAATGT   
  
  
- TTGAAAGGAT CCCGCTAGCA CAAAAACACT TAATTACTTA ATTAAATTAA ATTAAATTAA ATTATTCATT   
  
  
- ATTAATTATC TCACCACAAA AATCATTAAT TTTTGTTACA CATTTTTCAA CCCTTTATTA TCTCATGTAA   
  
  
- AAATCATATT CCAAATAATT TATAAAAATT TAACTTTTTA TAATTTCATT CTGATAAATA TAAATTGAAA   
  
  
- TGATGAAAAA TAATTTAAAT AATAAATCTA AAAAAATAAA AATAGTATGA AAATAAATCA AAACCACTTG   
  
  
- AAATTTATAT TTAAATAAAA ATATTTTATT TTATTTATTT TTAAGATCTT ACAAGAGAAA ACATGTTACA   
  
  
- TGAAGTGTGG GAGTTTGACT AGTTCCAGAA GTACAGCGGT TTGATTGGCT GCCGAAGCGC CACGTGAGGG   
  
  
- CTACGGGATA ATAGTCAACA AAATGGACGG GCCGGCTTTT ATCACAGCTT CATGGACGGG CCGTGGTCTT   
  
  
- TGACCGCACA ACTTGTTCCT TCAATTTATC TTTGATTTTT CATCTTCTTT TCCTCTCAAT TTCGAAAAAT   
  
  
- TAATAAAAGA AAAAGAAATT CCCAAATAAG TCATTCATAC TCTCCCTTAA ATTCATGAAA TTTCCTTTAT   
  
  
- TATGACGGCC ATCTCTACAT ATTTTCTTTT TGGGGATTTT TTTACAAATT AAATTATAGG TAATTTACTT   
  
  
- TTTTTGGTTT TAAAATCTAT TTCAACAAAT ATTTTTAAAA ACAAAAAATT TGATTTTTTT AAAAAATAAA   
  
  
- TTATGTGATA AAATATGAAC TCGAGGTCAA AACTCCTTTC TAACTTAATC AATTCGGCTA AGCTAAGAAG   
  
  
- ATTAGCCCAA CAAATTAGGC TTTCATAAAG TTACTAGAGA TACCTCATAG ATTATAGGCT GCGACTTTTT   
  
  
- CAATCTCTCC AAAGTTTAAT TTGCATATG

+     Box 4

| Site Name | Organism | Position | Strand | Matrix score. | sequence | function |
| --- | --- | --- | --- | --- | --- | --- |
| Box 4 | Petroselinum crispum | 633 | + | 6 | ATTAAT | part of a conserved DNA module involved in light responsiveness |
| Box 4 | Petroselinum crispum | 599 | + | 6 | ATTAAT | part of a conserved DNA module involved in light responsiveness |
| Box 4 | Petroselinum crispum | 591 | + | 6 | ATTAAT | part of a conserved DNA module involved in light responsiveness |

> 2018/04/13 10:10:12  
+ CAACTATATA GCACGAAGGT GCATTACGTC TCTGCTAATA GGACCAACAT ATGTTTCTAC CCGTTGTCAA   
  
  
+ AAGAACATTC TTTTCATACA TAGATCAACC TTATACTTAG TCATAGGTGC CTCGATTTCA GGAGAAGGAG   
  
  
+ CAGGAGGAAA CAGGTATTTA TTTCGGCTCT CTCTCTCTCT CTCTCTCTCT CTCTCTCTCT CTCTCTCTCT   
  
  
+ ACAATAAATC TTTCTCGTCC TGAGGAAACA GGTATTTATT TCGGCTCTCT CTCTCTCTCT CTCTCTCTCT   
  
  
+ CTCTCTCTCT CTCTCTCTAC AATAAATCTT TCTCGTCCCA CTCCGACGTT ACCTGAGCCG TAATTTTTCC   
  
  
+ CAAAACCTGA CTTCTGGTTT ACACTTTGCT CACTACGAAT CCCTAAAACT ACTCTCAGAT ACTCCATTCC   
  
  
+ ATCACGCAGT TCTTTTGTCC ACCGGTACTT TCAGAATACT CTCTCTCTCT GTCTCCGTCT CTTTCTTCTC   
  
  
+ AGAATGACGA CGACAGTATT CTCTCTTTCT CTCTCTCTCT CTCTCTCTCT CTCTCTTTCT CAAACTTACA   
  
  
+ AACTTTCCTA GGGCGATCGT GTTTTTGTGA ATTAATGAAT TAATTTAATT TAATTTAATT TAATAAGTAA   
  
  
+ TAATTAATAG AGTGGTGTTT TTAGTAATTA AAAACAATGT GTAAAAAGTT GGGAAATAAT AGAGTACATT   
  
  
+ TTTAGTATAA GGTTTATTAA ATATTTTTAA ATTGAAAAAT ATTAAAGTAA GACTATTTAT ATTTAACTTT   
  
  
+ ACTACTTTTT ATTAAATTTA TTATTTAGAT TTTTTTATTT TTATCATACT TTTATTTAGT TTTGGTGAAC   
  
  
+ TTTAAATATA AATTTATTTT TATAAAATAA AATAAATAAA AATTCTAGAA TGTTCTCTTT TGTACAATGT   
  
  
+ ACTTCACACC CTCAAACTGA TCAAGGTCTT CATGTCGCCA AACTAACCGA CGGCTTCGCG GTGCACTCCC   
  
  
+ GATGCCCTAT TATCAGTTGT TTTACCTGCC CGGCCGAAAA TAGTGTCGAA GTACCTGCCC GGCACCAGAA   
  
  
+ ACTGGCGTGT TGAACAAGGA AGTTAAATAG AAACTAAAAA GTAGAAGAAA AGGAGAGTTA AAGCTTTTTA   
  
  
+ ATTATTTTCT TTTTCTTTAA GGGTTTATTC AGTAAGTATG AGAGGGAATT TAAGTACTTT AAAGGAAATA   
  
  
+ ATACTGCCGG TAGAGATGTA TAAAAGAAAA ACCCCTAAAA AAATGTTTAA TTTAATATCC ATTAAATGAA   
  
  
+ AAAAACCAAA ATTTTAGATA AAGTTGTTTA TAAAAATTTT TGTTTTTTAA ACTAAAAAAA TTTTTTATTT   
  
  
+ AATACACTAT TTTATACTTG AGCTCCAGTT TTGAGGAAAG ATTGAATTAG TTAAGCCGAT TCGATTCTTC   
  
  
+ TAATCGGGTT GTTTAATCCG AAAGTATTTC AATGATCTCT ATGGAGTATC TAATATCCGA CGCTGAAAAA   
  
  
+ GTTAGAGAGG TTTCAAATTA AACGTATAC  

- GTTGATATAT CGTGCTTCCA CGTAATGCAG AGACGATTAT CCTGGTTGTA TACAAAGATG GGCAACAGTT   
  
  
- TTCTTGTAAG AAAAGTATGT ATCTAGTTGG AATATGAATC AGTATCCACG GAGCTAAAGT CCTCTTCCTC   
  
  
- GTCCTCCTTT GTCCATAAAT AAAGCCGAGA GAGAGAGAGA GAGAGAGAGA GAGAGAGAGA GAGAGAGAGA   
  
  
- TGTTATTTAG AAAGAGCAGG ACTCCTTTGT CCATAAATAA AGCCGAGAGA GAGAGAGAGA GAGAGAGAGA   
  
  
- GAGAGAGAGA GAGAGAGATG TTATTTAGAA AGAGCAGGGT GAGGCTGCAA TGGACTCGGC ATTAAAAAGG   
  
  
- GTTTTGGACT GAAGACCAAA TGTGAAACGA GTGATGCTTA GGGATTTTGA TGAGAGTCTA TGAGGTAAGG   
  
  
- TAGTGCGTCA AGAAAACAGG TGGCCATGAA AGTCTTATGA GAGAGAGAGA CAGAGGCAGA GAAAGAAGAG   
  
  
- TCTTACTGCT GCTGTCATAA GAGAGAAAGA GAGAGAGAGA GAGAGAGAGA GAGAGAAAGA GTTTGAATGT   
  
  
- TTGAAAGGAT CCCGCTAGCA CAAAAACACT TAATTACTTA ATTAAATTAA ATTAAATTAA ATTATTCATT   
  
  
- ATTAATTATC TCACCACAAA AATCATTAAT TTTTGTTACA CATTTTTCAA CCCTTTATTA TCTCATGTAA   
  
  
- AAATCATATT CCAAATAATT TATAAAAATT TAACTTTTTA TAATTTCATT CTGATAAATA TAAATTGAAA   
  
  
- TGATGAAAAA TAATTTAAAT AATAAATCTA AAAAAATAAA AATAGTATGA AAATAAATCA AAACCACTTG   
  
  
- AAATTTATAT TTAAATAAAA ATATTTTATT TTATTTATTT TTAAGATCTT ACAAGAGAAA ACATGTTACA   
  
  
- TGAAGTGTGG GAGTTTGACT AGTTCCAGAA GTACAGCGGT TTGATTGGCT GCCGAAGCGC CACGTGAGGG   
  
  
- CTACGGGATA ATAGTCAACA AAATGGACGG GCCGGCTTTT ATCACAGCTT CATGGACGGG CCGTGGTCTT   
  
  
- TGACCGCACA ACTTGTTCCT TCAATTTATC TTTGATTTTT CATCTTCTTT TCCTCTCAAT TTCGAAAAAT   
  
  
- TAATAAAAGA AAAAGAAATT CCCAAATAAG TCATTCATAC TCTCCCTTAA ATTCATGAAA TTTCCTTTAT   
  
  
- TATGACGGCC ATCTCTACAT ATTTTCTTTT TGGGGATTTT TTTACAAATT AAATTATAGG TAATTTACTT   
  
  
- TTTTTGGTTT TAAAATCTAT TTCAACAAAT ATTTTTAAAA ACAAAAAATT TGATTTTTTT AAAAAATAAA   
  
  
- TTATGTGATA AAATATGAAC TCGAGGTCAA AACTCCTTTC TAACTTAATC AATTCGGCTA AGCTAAGAAG   
  
  
- ATTAGCCCAA CAAATTAGGC TTTCATAAAG TTACTAGAGA TACCTCATAG ATTATAGGCT GCGACTTTTT   
  
  
- CAATCTCTCC AAAGTTTAAT TTGCATATG

+     Box I

| Site Name | Organism | Position | Strand | Matrix score. | sequence | function |
| --- | --- | --- | --- | --- | --- | --- |
| Box I | Pisum sativum | 1481 | + | 7 | TTTCAAA | light responsive element |

> 2018/04/13 10:10:12  
+ CAACTATATA GCACGAAGGT GCATTACGTC TCTGCTAATA GGACCAACAT ATGTTTCTAC CCGTTGTCAA   
  
  
+ AAGAACATTC TTTTCATACA TAGATCAACC TTATACTTAG TCATAGGTGC CTCGATTTCA GGAGAAGGAG   
  
  
+ CAGGAGGAAA CAGGTATTTA TTTCGGCTCT CTCTCTCTCT CTCTCTCTCT CTCTCTCTCT CTCTCTCTCT   
  
  
+ ACAATAAATC TTTCTCGTCC TGAGGAAACA GGTATTTATT TCGGCTCTCT CTCTCTCTCT CTCTCTCTCT   
  
  
+ CTCTCTCTCT CTCTCTCTAC AATAAATCTT TCTCGTCCCA CTCCGACGTT ACCTGAGCCG TAATTTTTCC   
  
  
+ CAAAACCTGA CTTCTGGTTT ACACTTTGCT CACTACGAAT CCCTAAAACT ACTCTCAGAT ACTCCATTCC   
  
  
+ ATCACGCAGT TCTTTTGTCC ACCGGTACTT TCAGAATACT CTCTCTCTCT GTCTCCGTCT CTTTCTTCTC   
  
  
+ AGAATGACGA CGACAGTATT CTCTCTTTCT CTCTCTCTCT CTCTCTCTCT CTCTCTTTCT CAAACTTACA   
  
  
+ AACTTTCCTA GGGCGATCGT GTTTTTGTGA ATTAATGAAT TAATTTAATT TAATTTAATT TAATAAGTAA   
  
  
+ TAATTAATAG AGTGGTGTTT TTAGTAATTA AAAACAATGT GTAAAAAGTT GGGAAATAAT AGAGTACATT   
  
  
+ TTTAGTATAA GGTTTATTAA ATATTTTTAA ATTGAAAAAT ATTAAAGTAA GACTATTTAT ATTTAACTTT   
  
  
+ ACTACTTTTT ATTAAATTTA TTATTTAGAT TTTTTTATTT TTATCATACT TTTATTTAGT TTTGGTGAAC   
  
  
+ TTTAAATATA AATTTATTTT TATAAAATAA AATAAATAAA AATTCTAGAA TGTTCTCTTT TGTACAATGT   
  
  
+ ACTTCACACC CTCAAACTGA TCAAGGTCTT CATGTCGCCA AACTAACCGA CGGCTTCGCG GTGCACTCCC   
  
  
+ GATGCCCTAT TATCAGTTGT TTTACCTGCC CGGCCGAAAA TAGTGTCGAA GTACCTGCCC GGCACCAGAA   
  
  
+ ACTGGCGTGT TGAACAAGGA AGTTAAATAG AAACTAAAAA GTAGAAGAAA AGGAGAGTTA AAGCTTTTTA   
  
  
+ ATTATTTTCT TTTTCTTTAA GGGTTTATTC AGTAAGTATG AGAGGGAATT TAAGTACTTT AAAGGAAATA   
  
  
+ ATACTGCCGG TAGAGATGTA TAAAAGAAAA ACCCCTAAAA AAATGTTTAA TTTAATATCC ATTAAATGAA   
  
  
+ AAAAACCAAA ATTTTAGATA AAGTTGTTTA TAAAAATTTT TGTTTTTTAA ACTAAAAAAA TTTTTTATTT   
  
  
+ AATACACTAT TTTATACTTG AGCTCCAGTT TTGAGGAAAG ATTGAATTAG TTAAGCCGAT TCGATTCTTC   
  
  
+ TAATCGGGTT GTTTAATCCG AAAGTATTTC AATGATCTCT ATGGAGTATC TAATATCCGA CGCTGAAAAA   
  
  
+ GTTAGAGAGG TTTCAAATTA AACGTATAC  

- GTTGATATAT CGTGCTTCCA CGTAATGCAG AGACGATTAT CCTGGTTGTA TACAAAGATG GGCAACAGTT   
  
  
- TTCTTGTAAG AAAAGTATGT ATCTAGTTGG AATATGAATC AGTATCCACG GAGCTAAAGT CCTCTTCCTC   
  
  
- GTCCTCCTTT GTCCATAAAT AAAGCCGAGA GAGAGAGAGA GAGAGAGAGA GAGAGAGAGA GAGAGAGAGA   
  
  
- TGTTATTTAG AAAGAGCAGG ACTCCTTTGT CCATAAATAA AGCCGAGAGA GAGAGAGAGA GAGAGAGAGA   
  
  
- GAGAGAGAGA GAGAGAGATG TTATTTAGAA AGAGCAGGGT GAGGCTGCAA TGGACTCGGC ATTAAAAAGG   
  
  
- GTTTTGGACT GAAGACCAAA TGTGAAACGA GTGATGCTTA GGGATTTTGA TGAGAGTCTA TGAGGTAAGG   
  
  
- TAGTGCGTCA AGAAAACAGG TGGCCATGAA AGTCTTATGA GAGAGAGAGA CAGAGGCAGA GAAAGAAGAG   
  
  
- TCTTACTGCT GCTGTCATAA GAGAGAAAGA GAGAGAGAGA GAGAGAGAGA GAGAGAAAGA GTTTGAATGT   
  
  
- TTGAAAGGAT CCCGCTAGCA CAAAAACACT TAATTACTTA ATTAAATTAA ATTAAATTAA ATTATTCATT   
  
  
- ATTAATTATC TCACCACAAA AATCATTAAT TTTTGTTACA CATTTTTCAA CCCTTTATTA TCTCATGTAA   
  
  
- AAATCATATT CCAAATAATT TATAAAAATT TAACTTTTTA TAATTTCATT CTGATAAATA TAAATTGAAA   
  
  
- TGATGAAAAA TAATTTAAAT AATAAATCTA AAAAAATAAA AATAGTATGA AAATAAATCA AAACCACTTG   
  
  
- AAATTTATAT TTAAATAAAA ATATTTTATT TTATTTATTT TTAAGATCTT ACAAGAGAAA ACATGTTACA   
  
  
- TGAAGTGTGG GAGTTTGACT AGTTCCAGAA GTACAGCGGT TTGATTGGCT GCCGAAGCGC CACGTGAGGG   
  
  
- CTACGGGATA ATAGTCAACA AAATGGACGG GCCGGCTTTT ATCACAGCTT CATGGACGGG CCGTGGTCTT   
  
  
- TGACCGCACA ACTTGTTCCT TCAATTTATC TTTGATTTTT CATCTTCTTT TCCTCTCAAT TTCGAAAAAT   
  
  
- TAATAAAAGA AAAAGAAATT CCCAAATAAG TCATTCATAC TCTCCCTTAA ATTCATGAAA TTTCCTTTAT   
  
  
- TATGACGGCC ATCTCTACAT ATTTTCTTTT TGGGGATTTT TTTACAAATT AAATTATAGG TAATTTACTT   
  
  
- TTTTTGGTTT TAAAATCTAT TTCAACAAAT ATTTTTAAAA ACAAAAAATT TGATTTTTTT AAAAAATAAA   
  
  
- TTATGTGATA AAATATGAAC TCGAGGTCAA AACTCCTTTC TAACTTAATC AATTCGGCTA AGCTAAGAAG   
  
  
- ATTAGCCCAA CAAATTAGGC TTTCATAAAG TTACTAGAGA TACCTCATAG ATTATAGGCT GCGACTTTTT   
  
  
- CAATCTCTCC AAAGTTTAAT TTGCATATG

+     CAAT-box

| Site Name | Organism | Position | Strand | Matrix score. | sequence | function |
| --- | --- | --- | --- | --- | --- | --- |
| CAAT-box | Hordeum vulgare | 905 | + | 4 | CAAT | common cis-acting element in promoter and enhancer regions |
| CAAT-box | Hordeum vulgare | 665 | + | 4 | CAAT | common cis-acting element in promoter and enhancer regions |
| CAAT-box | Hordeum vulgare | 300 | + | 4 | CAAT | common cis-acting element in promoter and enhancer regions |
| CAAT-box | Hordeum vulgare | 212 | + | 4 | CAAT | common cis-acting element in promoter and enhancer regions |
| CAAT-box | Hordeum vulgare | 1371 | - | 4 | CAAT | common cis-acting element in promoter and enhancer regions |
| CAAT-box | Glycine max | 730 | - | 5 | CAATT | common cis-acting element in promoter and enhancer regions |
| CAAT-box | Brassica rapa | 1484 | + | 5 | CAAAT | common cis-acting element in promoter and enhancer regions |
| CAAT-box | Hordeum vulgare | 731 | - | 4 | CAAT | common cis-acting element in promoter and enhancer regions |
| CAAT-box | Hordeum vulgare | 1430 | + | 4 | CAAT | common cis-acting element in promoter and enhancer regions |

> 2018/04/13 10:10:12  
+ CAACTATATA GCACGAAGGT GCATTACGTC TCTGCTAATA GGACCAACAT ATGTTTCTAC CCGTTGTCAA   
  
  
+ AAGAACATTC TTTTCATACA TAGATCAACC TTATACTTAG TCATAGGTGC CTCGATTTCA GGAGAAGGAG   
  
  
+ CAGGAGGAAA CAGGTATTTA TTTCGGCTCT CTCTCTCTCT CTCTCTCTCT CTCTCTCTCT CTCTCTCTCT   
  
  
+ ACAATAAATC TTTCTCGTCC TGAGGAAACA GGTATTTATT TCGGCTCTCT CTCTCTCTCT CTCTCTCTCT   
  
  
+ CTCTCTCTCT CTCTCTCTAC AATAAATCTT TCTCGTCCCA CTCCGACGTT ACCTGAGCCG TAATTTTTCC   
  
  
+ CAAAACCTGA CTTCTGGTTT ACACTTTGCT CACTACGAAT CCCTAAAACT ACTCTCAGAT ACTCCATTCC   
  
  
+ ATCACGCAGT TCTTTTGTCC ACCGGTACTT TCAGAATACT CTCTCTCTCT GTCTCCGTCT CTTTCTTCTC   
  
  
+ AGAATGACGA CGACAGTATT CTCTCTTTCT CTCTCTCTCT CTCTCTCTCT CTCTCTTTCT CAAACTTACA   
  
  
+ AACTTTCCTA GGGCGATCGT GTTTTTGTGA ATTAATGAAT TAATTTAATT TAATTTAATT TAATAAGTAA   
  
  
+ TAATTAATAG AGTGGTGTTT TTAGTAATTA AAAACAATGT GTAAAAAGTT GGGAAATAAT AGAGTACATT   
  
  
+ TTTAGTATAA GGTTTATTAA ATATTTTTAA ATTGAAAAAT ATTAAAGTAA GACTATTTAT ATTTAACTTT   
  
  
+ ACTACTTTTT ATTAAATTTA TTATTTAGAT TTTTTTATTT TTATCATACT TTTATTTAGT TTTGGTGAAC   
  
  
+ TTTAAATATA AATTTATTTT TATAAAATAA AATAAATAAA AATTCTAGAA TGTTCTCTTT TGTACAATGT   
  
  
+ ACTTCACACC CTCAAACTGA TCAAGGTCTT CATGTCGCCA AACTAACCGA CGGCTTCGCG GTGCACTCCC   
  
  
+ GATGCCCTAT TATCAGTTGT TTTACCTGCC CGGCCGAAAA TAGTGTCGAA GTACCTGCCC GGCACCAGAA   
  
  
+ ACTGGCGTGT TGAACAAGGA AGTTAAATAG AAACTAAAAA GTAGAAGAAA AGGAGAGTTA AAGCTTTTTA   
  
  
+ ATTATTTTCT TTTTCTTTAA GGGTTTATTC AGTAAGTATG AGAGGGAATT TAAGTACTTT AAAGGAAATA   
  
  
+ ATACTGCCGG TAGAGATGTA TAAAAGAAAA ACCCCTAAAA AAATGTTTAA TTTAATATCC ATTAAATGAA   
  
  
+ AAAAACCAAA ATTTTAGATA AAGTTGTTTA TAAAAATTTT TGTTTTTTAA ACTAAAAAAA TTTTTTATTT   
  
  
+ AATACACTAT TTTATACTTG AGCTCCAGTT TTGAGGAAAG ATTGAATTAG TTAAGCCGAT TCGATTCTTC   
  
  
+ TAATCGGGTT GTTTAATCCG AAAGTATTTC AATGATCTCT ATGGAGTATC TAATATCCGA CGCTGAAAAA   
  
  
+ GTTAGAGAGG TTTCAAATTA AACGTATAC  

- GTTGATATAT CGTGCTTCCA CGTAATGCAG AGACGATTAT CCTGGTTGTA TACAAAGATG GGCAACAGTT   
  
  
- TTCTTGTAAG AAAAGTATGT ATCTAGTTGG AATATGAATC AGTATCCACG GAGCTAAAGT CCTCTTCCTC   
  
  
- GTCCTCCTTT GTCCATAAAT AAAGCCGAGA GAGAGAGAGA GAGAGAGAGA GAGAGAGAGA GAGAGAGAGA   
  
  
- TGTTATTTAG AAAGAGCAGG ACTCCTTTGT CCATAAATAA AGCCGAGAGA GAGAGAGAGA GAGAGAGAGA   
  
  
- GAGAGAGAGA GAGAGAGATG TTATTTAGAA AGAGCAGGGT GAGGCTGCAA TGGACTCGGC ATTAAAAAGG   
  
  
- GTTTTGGACT GAAGACCAAA TGTGAAACGA GTGATGCTTA GGGATTTTGA TGAGAGTCTA TGAGGTAAGG   
  
  
- TAGTGCGTCA AGAAAACAGG TGGCCATGAA AGTCTTATGA GAGAGAGAGA CAGAGGCAGA GAAAGAAGAG   
  
  
- TCTTACTGCT GCTGTCATAA GAGAGAAAGA GAGAGAGAGA GAGAGAGAGA GAGAGAAAGA GTTTGAATGT   
  
  
- TTGAAAGGAT CCCGCTAGCA CAAAAACACT TAATTACTTA ATTAAATTAA ATTAAATTAA ATTATTCATT   
  
  
- ATTAATTATC TCACCACAAA AATCATTAAT TTTTGTTACA CATTTTTCAA CCCTTTATTA TCTCATGTAA   
  
  
- AAATCATATT CCAAATAATT TATAAAAATT TAACTTTTTA TAATTTCATT CTGATAAATA TAAATTGAAA   
  
  
- TGATGAAAAA TAATTTAAAT AATAAATCTA AAAAAATAAA AATAGTATGA AAATAAATCA AAACCACTTG   
  
  
- AAATTTATAT TTAAATAAAA ATATTTTATT TTATTTATTT TTAAGATCTT ACAAGAGAAA ACATGTTACA   
  
  
- TGAAGTGTGG GAGTTTGACT AGTTCCAGAA GTACAGCGGT TTGATTGGCT GCCGAAGCGC CACGTGAGGG   
  
  
- CTACGGGATA ATAGTCAACA AAATGGACGG GCCGGCTTTT ATCACAGCTT CATGGACGGG CCGTGGTCTT   
  
  
- TGACCGCACA ACTTGTTCCT TCAATTTATC TTTGATTTTT CATCTTCTTT TCCTCTCAAT TTCGAAAAAT   
  
  
- TAATAAAAGA AAAAGAAATT CCCAAATAAG TCATTCATAC TCTCCCTTAA ATTCATGAAA TTTCCTTTAT   
  
  
- TATGACGGCC ATCTCTACAT ATTTTCTTTT TGGGGATTTT TTTACAAATT AAATTATAGG TAATTTACTT   
  
  
- TTTTTGGTTT TAAAATCTAT TTCAACAAAT ATTTTTAAAA ACAAAAAATT TGATTTTTTT AAAAAATAAA   
  
  
- TTATGTGATA AAATATGAAC TCGAGGTCAA AACTCCTTTC TAACTTAATC AATTCGGCTA AGCTAAGAAG   
  
  
- ATTAGCCCAA CAAATTAGGC TTTCATAAAG TTACTAGAGA TACCTCATAG ATTATAGGCT GCGACTTTTT   
  
  
- CAATCTCTCC AAAGTTTAAT TTGCATATG

+     CCAAT-box

| Site Name | Organism | Position | Strand | Matrix score. | sequence | function |
| --- | --- | --- | --- | --- | --- | --- |
| CCAAT-box | Hordeum vulgare | 61 | - | 6 | CAACGG | MYBHv1 binding site |

> 2018/04/13 10:10:12  
+ CAACTATATA GCACGAAGGT GCATTACGTC TCTGCTAATA GGACCAACAT ATGTTTCTAC CCGTTGTCAA   
  
  
+ AAGAACATTC TTTTCATACA TAGATCAACC TTATACTTAG TCATAGGTGC CTCGATTTCA GGAGAAGGAG   
  
  
+ CAGGAGGAAA CAGGTATTTA TTTCGGCTCT CTCTCTCTCT CTCTCTCTCT CTCTCTCTCT CTCTCTCTCT   
  
  
+ ACAATAAATC TTTCTCGTCC TGAGGAAACA GGTATTTATT TCGGCTCTCT CTCTCTCTCT CTCTCTCTCT   
  
  
+ CTCTCTCTCT CTCTCTCTAC AATAAATCTT TCTCGTCCCA CTCCGACGTT ACCTGAGCCG TAATTTTTCC   
  
  
+ CAAAACCTGA CTTCTGGTTT ACACTTTGCT CACTACGAAT CCCTAAAACT ACTCTCAGAT ACTCCATTCC   
  
  
+ ATCACGCAGT TCTTTTGTCC ACCGGTACTT TCAGAATACT CTCTCTCTCT GTCTCCGTCT CTTTCTTCTC   
  
  
+ AGAATGACGA CGACAGTATT CTCTCTTTCT CTCTCTCTCT CTCTCTCTCT CTCTCTTTCT CAAACTTACA   
  
  
+ AACTTTCCTA GGGCGATCGT GTTTTTGTGA ATTAATGAAT TAATTTAATT TAATTTAATT TAATAAGTAA   
  
  
+ TAATTAATAG AGTGGTGTTT TTAGTAATTA AAAACAATGT GTAAAAAGTT GGGAAATAAT AGAGTACATT   
  
  
+ TTTAGTATAA GGTTTATTAA ATATTTTTAA ATTGAAAAAT ATTAAAGTAA GACTATTTAT ATTTAACTTT   
  
  
+ ACTACTTTTT ATTAAATTTA TTATTTAGAT TTTTTTATTT TTATCATACT TTTATTTAGT TTTGGTGAAC   
  
  
+ TTTAAATATA AATTTATTTT TATAAAATAA AATAAATAAA AATTCTAGAA TGTTCTCTTT TGTACAATGT   
  
  
+ ACTTCACACC CTCAAACTGA TCAAGGTCTT CATGTCGCCA AACTAACCGA CGGCTTCGCG GTGCACTCCC   
  
  
+ GATGCCCTAT TATCAGTTGT TTTACCTGCC CGGCCGAAAA TAGTGTCGAA GTACCTGCCC GGCACCAGAA   
  
  
+ ACTGGCGTGT TGAACAAGGA AGTTAAATAG AAACTAAAAA GTAGAAGAAA AGGAGAGTTA AAGCTTTTTA   
  
  
+ ATTATTTTCT TTTTCTTTAA GGGTTTATTC AGTAAGTATG AGAGGGAATT TAAGTACTTT AAAGGAAATA   
  
  
+ ATACTGCCGG TAGAGATGTA TAAAAGAAAA ACCCCTAAAA AAATGTTTAA TTTAATATCC ATTAAATGAA   
  
  
+ AAAAACCAAA ATTTTAGATA AAGTTGTTTA TAAAAATTTT TGTTTTTTAA ACTAAAAAAA TTTTTTATTT   
  
  
+ AATACACTAT TTTATACTTG AGCTCCAGTT TTGAGGAAAG ATTGAATTAG TTAAGCCGAT TCGATTCTTC   
  
  
+ TAATCGGGTT GTTTAATCCG AAAGTATTTC AATGATCTCT ATGGAGTATC TAATATCCGA CGCTGAAAAA   
  
  
+ GTTAGAGAGG TTTCAAATTA AACGTATAC  

- GTTGATATAT CGTGCTTCCA CGTAATGCAG AGACGATTAT CCTGGTTGTA TACAAAGATG GGCAACAGTT   
  
  
- TTCTTGTAAG AAAAGTATGT ATCTAGTTGG AATATGAATC AGTATCCACG GAGCTAAAGT CCTCTTCCTC   
  
  
- GTCCTCCTTT GTCCATAAAT AAAGCCGAGA GAGAGAGAGA GAGAGAGAGA GAGAGAGAGA GAGAGAGAGA   
  
  
- TGTTATTTAG AAAGAGCAGG ACTCCTTTGT CCATAAATAA AGCCGAGAGA GAGAGAGAGA GAGAGAGAGA   
  
  
- GAGAGAGAGA GAGAGAGATG TTATTTAGAA AGAGCAGGGT GAGGCTGCAA TGGACTCGGC ATTAAAAAGG   
  
  
- GTTTTGGACT GAAGACCAAA TGTGAAACGA GTGATGCTTA GGGATTTTGA TGAGAGTCTA TGAGGTAAGG   
  
  
- TAGTGCGTCA AGAAAACAGG TGGCCATGAA AGTCTTATGA GAGAGAGAGA CAGAGGCAGA GAAAGAAGAG   
  
  
- TCTTACTGCT GCTGTCATAA GAGAGAAAGA GAGAGAGAGA GAGAGAGAGA GAGAGAAAGA GTTTGAATGT   
  
  
- TTGAAAGGAT CCCGCTAGCA CAAAAACACT TAATTACTTA ATTAAATTAA ATTAAATTAA ATTATTCATT   
  
  
- ATTAATTATC TCACCACAAA AATCATTAAT TTTTGTTACA CATTTTTCAA CCCTTTATTA TCTCATGTAA   
  
  
- AAATCATATT CCAAATAATT TATAAAAATT TAACTTTTTA TAATTTCATT CTGATAAATA TAAATTGAAA   
  
  
- TGATGAAAAA TAATTTAAAT AATAAATCTA AAAAAATAAA AATAGTATGA AAATAAATCA AAACCACTTG   
  
  
- AAATTTATAT TTAAATAAAA ATATTTTATT TTATTTATTT TTAAGATCTT ACAAGAGAAA ACATGTTACA   
  
  
- TGAAGTGTGG GAGTTTGACT AGTTCCAGAA GTACAGCGGT TTGATTGGCT GCCGAAGCGC CACGTGAGGG   
  
  
- CTACGGGATA ATAGTCAACA AAATGGACGG GCCGGCTTTT ATCACAGCTT CATGGACGGG CCGTGGTCTT   
  
  
- TGACCGCACA ACTTGTTCCT TCAATTTATC TTTGATTTTT CATCTTCTTT TCCTCTCAAT TTCGAAAAAT   
  
  
- TAATAAAAGA AAAAGAAATT CCCAAATAAG TCATTCATAC TCTCCCTTAA ATTCATGAAA TTTCCTTTAT   
  
  
- TATGACGGCC ATCTCTACAT ATTTTCTTTT TGGGGATTTT TTTACAAATT AAATTATAGG TAATTTACTT   
  
  
- TTTTTGGTTT TAAAATCTAT TTCAACAAAT ATTTTTAAAA ACAAAAAATT TGATTTTTTT AAAAAATAAA   
  
  
- TTATGTGATA AAATATGAAC TCGAGGTCAA AACTCCTTTC TAACTTAATC AATTCGGCTA AGCTAAGAAG   
  
  
- ATTAGCCCAA CAAATTAGGC TTTCATAAAG TTACTAGAGA TACCTCATAG ATTATAGGCT GCGACTTTTT   
  
  
- CAATCTCTCC AAAGTTTAAT TTGCATATG

+     CGTCA-motif

| Site Name | Organism | Position | Strand | Matrix score. | sequence | function |
| --- | --- | --- | --- | --- | --- | --- |
| CGTCA-motif | Hordeum vulgare | 495 | - | 5 | CGTCA | cis-acting regulatory element involved in the MeJA-responsiveness |

> 2018/04/13 10:10:12  
+ CAACTATATA GCACGAAGGT GCATTACGTC TCTGCTAATA GGACCAACAT ATGTTTCTAC CCGTTGTCAA   
  
  
+ AAGAACATTC TTTTCATACA TAGATCAACC TTATACTTAG TCATAGGTGC CTCGATTTCA GGAGAAGGAG   
  
  
+ CAGGAGGAAA CAGGTATTTA TTTCGGCTCT CTCTCTCTCT CTCTCTCTCT CTCTCTCTCT CTCTCTCTCT   
  
  
+ ACAATAAATC TTTCTCGTCC TGAGGAAACA GGTATTTATT TCGGCTCTCT CTCTCTCTCT CTCTCTCTCT   
  
  
+ CTCTCTCTCT CTCTCTCTAC AATAAATCTT TCTCGTCCCA CTCCGACGTT ACCTGAGCCG TAATTTTTCC   
  
  
+ CAAAACCTGA CTTCTGGTTT ACACTTTGCT CACTACGAAT CCCTAAAACT ACTCTCAGAT ACTCCATTCC   
  
  
+ ATCACGCAGT TCTTTTGTCC ACCGGTACTT TCAGAATACT CTCTCTCTCT GTCTCCGTCT CTTTCTTCTC   
  
  
+ AGAATGACGA CGACAGTATT CTCTCTTTCT CTCTCTCTCT CTCTCTCTCT CTCTCTTTCT CAAACTTACA   
  
  
+ AACTTTCCTA GGGCGATCGT GTTTTTGTGA ATTAATGAAT TAATTTAATT TAATTTAATT TAATAAGTAA   
  
  
+ TAATTAATAG AGTGGTGTTT TTAGTAATTA AAAACAATGT GTAAAAAGTT GGGAAATAAT AGAGTACATT   
  
  
+ TTTAGTATAA GGTTTATTAA ATATTTTTAA ATTGAAAAAT ATTAAAGTAA GACTATTTAT ATTTAACTTT   
  
  
+ ACTACTTTTT ATTAAATTTA TTATTTAGAT TTTTTTATTT TTATCATACT TTTATTTAGT TTTGGTGAAC   
  
  
+ TTTAAATATA AATTTATTTT TATAAAATAA AATAAATAAA AATTCTAGAA TGTTCTCTTT TGTACAATGT   
  
  
+ ACTTCACACC CTCAAACTGA TCAAGGTCTT CATGTCGCCA AACTAACCGA CGGCTTCGCG GTGCACTCCC   
  
  
+ GATGCCCTAT TATCAGTTGT TTTACCTGCC CGGCCGAAAA TAGTGTCGAA GTACCTGCCC GGCACCAGAA   
  
  
+ ACTGGCGTGT TGAACAAGGA AGTTAAATAG AAACTAAAAA GTAGAAGAAA AGGAGAGTTA AAGCTTTTTA   
  
  
+ ATTATTTTCT TTTTCTTTAA GGGTTTATTC AGTAAGTATG AGAGGGAATT TAAGTACTTT AAAGGAAATA   
  
  
+ ATACTGCCGG TAGAGATGTA TAAAAGAAAA ACCCCTAAAA AAATGTTTAA TTTAATATCC ATTAAATGAA   
  
  
+ AAAAACCAAA ATTTTAGATA AAGTTGTTTA TAAAAATTTT TGTTTTTTAA ACTAAAAAAA TTTTTTATTT   
  
  
+ AATACACTAT TTTATACTTG AGCTCCAGTT TTGAGGAAAG ATTGAATTAG TTAAGCCGAT TCGATTCTTC   
  
  
+ TAATCGGGTT GTTTAATCCG AAAGTATTTC AATGATCTCT ATGGAGTATC TAATATCCGA CGCTGAAAAA   
  
  
+ GTTAGAGAGG TTTCAAATTA AACGTATAC  

- GTTGATATAT CGTGCTTCCA CGTAATGCAG AGACGATTAT CCTGGTTGTA TACAAAGATG GGCAACAGTT   
  
  
- TTCTTGTAAG AAAAGTATGT ATCTAGTTGG AATATGAATC AGTATCCACG GAGCTAAAGT CCTCTTCCTC   
  
  
- GTCCTCCTTT GTCCATAAAT AAAGCCGAGA GAGAGAGAGA GAGAGAGAGA GAGAGAGAGA GAGAGAGAGA   
  
  
- TGTTATTTAG AAAGAGCAGG ACTCCTTTGT CCATAAATAA AGCCGAGAGA GAGAGAGAGA GAGAGAGAGA   
  
  
- GAGAGAGAGA GAGAGAGATG TTATTTAGAA AGAGCAGGGT GAGGCTGCAA TGGACTCGGC ATTAAAAAGG   
  
  
- GTTTTGGACT GAAGACCAAA TGTGAAACGA GTGATGCTTA GGGATTTTGA TGAGAGTCTA TGAGGTAAGG   
  
  
- TAGTGCGTCA AGAAAACAGG TGGCCATGAA AGTCTTATGA GAGAGAGAGA CAGAGGCAGA GAAAGAAGAG   
  
  
- TCTTACTGCT GCTGTCATAA GAGAGAAAGA GAGAGAGAGA GAGAGAGAGA GAGAGAAAGA GTTTGAATGT   
  
  
- TTGAAAGGAT CCCGCTAGCA CAAAAACACT TAATTACTTA ATTAAATTAA ATTAAATTAA ATTATTCATT   
  
  
- ATTAATTATC TCACCACAAA AATCATTAAT TTTTGTTACA CATTTTTCAA CCCTTTATTA TCTCATGTAA   
  
  
- AAATCATATT CCAAATAATT TATAAAAATT TAACTTTTTA TAATTTCATT CTGATAAATA TAAATTGAAA   
  
  
- TGATGAAAAA TAATTTAAAT AATAAATCTA AAAAAATAAA AATAGTATGA AAATAAATCA AAACCACTTG   
  
  
- AAATTTATAT TTAAATAAAA ATATTTTATT TTATTTATTT TTAAGATCTT ACAAGAGAAA ACATGTTACA   
  
  
- TGAAGTGTGG GAGTTTGACT AGTTCCAGAA GTACAGCGGT TTGATTGGCT GCCGAAGCGC CACGTGAGGG   
  
  
- CTACGGGATA ATAGTCAACA AAATGGACGG GCCGGCTTTT ATCACAGCTT CATGGACGGG CCGTGGTCTT   
  
  
- TGACCGCACA ACTTGTTCCT TCAATTTATC TTTGATTTTT CATCTTCTTT TCCTCTCAAT TTCGAAAAAT   
  
  
- TAATAAAAGA AAAAGAAATT CCCAAATAAG TCATTCATAC TCTCCCTTAA ATTCATGAAA TTTCCTTTAT   
  
  
- TATGACGGCC ATCTCTACAT ATTTTCTTTT TGGGGATTTT TTTACAAATT AAATTATAGG TAATTTACTT   
  
  
- TTTTTGGTTT TAAAATCTAT TTCAACAAAT ATTTTTAAAA ACAAAAAATT TGATTTTTTT AAAAAATAAA   
  
  
- TTATGTGATA AAATATGAAC TCGAGGTCAA AACTCCTTTC TAACTTAATC AATTCGGCTA AGCTAAGAAG   
  
  
- ATTAGCCCAA CAAATTAGGC TTTCATAAAG TTACTAGAGA TACCTCATAG ATTATAGGCT GCGACTTTTT   
  
  
- CAATCTCTCC AAAGTTTAAT TTGCATATG

+     GAG-motif

| Site Name | Organism | Position | Strand | Matrix score. | sequence | function |
| --- | --- | --- | --- | --- | --- | --- |
| GAG-motif | Arabidopsis thaliana | 458 | - | 7 | AGAGAGT | part of a light responsive element |
| GAG-motif | Spinacia oleracea | 1202 | + | 7 | AGAGATG | part of a light responsive element |

> 2018/04/13 10:10:12  
+ CAACTATATA GCACGAAGGT GCATTACGTC TCTGCTAATA GGACCAACAT ATGTTTCTAC CCGTTGTCAA   
  
  
+ AAGAACATTC TTTTCATACA TAGATCAACC TTATACTTAG TCATAGGTGC CTCGATTTCA GGAGAAGGAG   
  
  
+ CAGGAGGAAA CAGGTATTTA TTTCGGCTCT CTCTCTCTCT CTCTCTCTCT CTCTCTCTCT CTCTCTCTCT   
  
  
+ ACAATAAATC TTTCTCGTCC TGAGGAAACA GGTATTTATT TCGGCTCTCT CTCTCTCTCT CTCTCTCTCT   
  
  
+ CTCTCTCTCT CTCTCTCTAC AATAAATCTT TCTCGTCCCA CTCCGACGTT ACCTGAGCCG TAATTTTTCC   
  
  
+ CAAAACCTGA CTTCTGGTTT ACACTTTGCT CACTACGAAT CCCTAAAACT ACTCTCAGAT ACTCCATTCC   
  
  
+ ATCACGCAGT TCTTTTGTCC ACCGGTACTT TCAGAATACT CTCTCTCTCT GTCTCCGTCT CTTTCTTCTC   
  
  
+ AGAATGACGA CGACAGTATT CTCTCTTTCT CTCTCTCTCT CTCTCTCTCT CTCTCTTTCT CAAACTTACA   
  
  
+ AACTTTCCTA GGGCGATCGT GTTTTTGTGA ATTAATGAAT TAATTTAATT TAATTTAATT TAATAAGTAA   
  
  
+ TAATTAATAG AGTGGTGTTT TTAGTAATTA AAAACAATGT GTAAAAAGTT GGGAAATAAT AGAGTACATT   
  
  
+ TTTAGTATAA GGTTTATTAA ATATTTTTAA ATTGAAAAAT ATTAAAGTAA GACTATTTAT ATTTAACTTT   
  
  
+ ACTACTTTTT ATTAAATTTA TTATTTAGAT TTTTTTATTT TTATCATACT TTTATTTAGT TTTGGTGAAC   
  
  
+ TTTAAATATA AATTTATTTT TATAAAATAA AATAAATAAA AATTCTAGAA TGTTCTCTTT TGTACAATGT   
  
  
+ ACTTCACACC CTCAAACTGA TCAAGGTCTT CATGTCGCCA AACTAACCGA CGGCTTCGCG GTGCACTCCC   
  
  
+ GATGCCCTAT TATCAGTTGT TTTACCTGCC CGGCCGAAAA TAGTGTCGAA GTACCTGCCC GGCACCAGAA   
  
  
+ ACTGGCGTGT TGAACAAGGA AGTTAAATAG AAACTAAAAA GTAGAAGAAA AGGAGAGTTA AAGCTTTTTA   
  
  
+ ATTATTTTCT TTTTCTTTAA GGGTTTATTC AGTAAGTATG AGAGGGAATT TAAGTACTTT AAAGGAAATA   
  
  
+ ATACTGCCGG TAGAGATGTA TAAAAGAAAA ACCCCTAAAA AAATGTTTAA TTTAATATCC ATTAAATGAA   
  
  
+ AAAAACCAAA ATTTTAGATA AAGTTGTTTA TAAAAATTTT TGTTTTTTAA ACTAAAAAAA TTTTTTATTT   
  
  
+ AATACACTAT TTTATACTTG AGCTCCAGTT TTGAGGAAAG ATTGAATTAG TTAAGCCGAT TCGATTCTTC   
  
  
+ TAATCGGGTT GTTTAATCCG AAAGTATTTC AATGATCTCT ATGGAGTATC TAATATCCGA CGCTGAAAAA   
  
  
+ GTTAGAGAGG TTTCAAATTA AACGTATAC  

- GTTGATATAT CGTGCTTCCA CGTAATGCAG AGACGATTAT CCTGGTTGTA TACAAAGATG GGCAACAGTT   
  
  
- TTCTTGTAAG AAAAGTATGT ATCTAGTTGG AATATGAATC AGTATCCACG GAGCTAAAGT CCTCTTCCTC   
  
  
- GTCCTCCTTT GTCCATAAAT AAAGCCGAGA GAGAGAGAGA GAGAGAGAGA GAGAGAGAGA GAGAGAGAGA   
  
  
- TGTTATTTAG AAAGAGCAGG ACTCCTTTGT CCATAAATAA AGCCGAGAGA GAGAGAGAGA GAGAGAGAGA   
  
  
- GAGAGAGAGA GAGAGAGATG TTATTTAGAA AGAGCAGGGT GAGGCTGCAA TGGACTCGGC ATTAAAAAGG   
  
  
- GTTTTGGACT GAAGACCAAA TGTGAAACGA GTGATGCTTA GGGATTTTGA TGAGAGTCTA TGAGGTAAGG   
  
  
- TAGTGCGTCA AGAAAACAGG TGGCCATGAA AGTCTTATGA GAGAGAGAGA CAGAGGCAGA GAAAGAAGAG   
  
  
- TCTTACTGCT GCTGTCATAA GAGAGAAAGA GAGAGAGAGA GAGAGAGAGA GAGAGAAAGA GTTTGAATGT   
  
  
- TTGAAAGGAT CCCGCTAGCA CAAAAACACT TAATTACTTA ATTAAATTAA ATTAAATTAA ATTATTCATT   
  
  
- ATTAATTATC TCACCACAAA AATCATTAAT TTTTGTTACA CATTTTTCAA CCCTTTATTA TCTCATGTAA   
  
  
- AAATCATATT CCAAATAATT TATAAAAATT TAACTTTTTA TAATTTCATT CTGATAAATA TAAATTGAAA   
  
  
- TGATGAAAAA TAATTTAAAT AATAAATCTA AAAAAATAAA AATAGTATGA AAATAAATCA AAACCACTTG   
  
  
- AAATTTATAT TTAAATAAAA ATATTTTATT TTATTTATTT TTAAGATCTT ACAAGAGAAA ACATGTTACA   
  
  
- TGAAGTGTGG GAGTTTGACT AGTTCCAGAA GTACAGCGGT TTGATTGGCT GCCGAAGCGC CACGTGAGGG   
  
  
- CTACGGGATA ATAGTCAACA AAATGGACGG GCCGGCTTTT ATCACAGCTT CATGGACGGG CCGTGGTCTT   
  
  
- TGACCGCACA ACTTGTTCCT TCAATTTATC TTTGATTTTT CATCTTCTTT TCCTCTCAAT TTCGAAAAAT   
  
  
- TAATAAAAGA AAAAGAAATT CCCAAATAAG TCATTCATAC TCTCCCTTAA ATTCATGAAA TTTCCTTTAT   
  
  
- TATGACGGCC ATCTCTACAT ATTTTCTTTT TGGGGATTTT TTTACAAATT AAATTATAGG TAATTTACTT   
  
  
- TTTTTGGTTT TAAAATCTAT TTCAACAAAT ATTTTTAAAA ACAAAAAATT TGATTTTTTT AAAAAATAAA   
  
  
- TTATGTGATA AAATATGAAC TCGAGGTCAA AACTCCTTTC TAACTTAATC AATTCGGCTA AGCTAAGAAG   
  
  
- ATTAGCCCAA CAAATTAGGC TTTCATAAAG TTACTAGAGA TACCTCATAG ATTATAGGCT GCGACTTTTT   
  
  
- CAATCTCTCC AAAGTTTAAT TTGCATATG

+     GATA-motif

| Site Name | Organism | Position | Strand | Matrix score. | sequence | function |
| --- | --- | --- | --- | --- | --- | --- |
| GATA-motif | Solanum tuberosum | 99 | - | 9 | AAGGATAAGG | part of a light responsive element |

> 2018/04/13 10:10:12  
+ CAACTATATA GCACGAAGGT GCATTACGTC TCTGCTAATA GGACCAACAT ATGTTTCTAC CCGTTGTCAA   
  
  
+ AAGAACATTC TTTTCATACA TAGATCAACC TTATACTTAG TCATAGGTGC CTCGATTTCA GGAGAAGGAG   
  
  
+ CAGGAGGAAA CAGGTATTTA TTTCGGCTCT CTCTCTCTCT CTCTCTCTCT CTCTCTCTCT CTCTCTCTCT   
  
  
+ ACAATAAATC TTTCTCGTCC TGAGGAAACA GGTATTTATT TCGGCTCTCT CTCTCTCTCT CTCTCTCTCT   
  
  
+ CTCTCTCTCT CTCTCTCTAC AATAAATCTT TCTCGTCCCA CTCCGACGTT ACCTGAGCCG TAATTTTTCC   
  
  
+ CAAAACCTGA CTTCTGGTTT ACACTTTGCT CACTACGAAT CCCTAAAACT ACTCTCAGAT ACTCCATTCC   
  
  
+ ATCACGCAGT TCTTTTGTCC ACCGGTACTT TCAGAATACT CTCTCTCTCT GTCTCCGTCT CTTTCTTCTC   
  
  
+ AGAATGACGA CGACAGTATT CTCTCTTTCT CTCTCTCTCT CTCTCTCTCT CTCTCTTTCT CAAACTTACA   
  
  
+ AACTTTCCTA GGGCGATCGT GTTTTTGTGA ATTAATGAAT TAATTTAATT TAATTTAATT TAATAAGTAA   
  
  
+ TAATTAATAG AGTGGTGTTT TTAGTAATTA AAAACAATGT GTAAAAAGTT GGGAAATAAT AGAGTACATT   
  
  
+ TTTAGTATAA GGTTTATTAA ATATTTTTAA ATTGAAAAAT ATTAAAGTAA GACTATTTAT ATTTAACTTT   
  
  
+ ACTACTTTTT ATTAAATTTA TTATTTAGAT TTTTTTATTT TTATCATACT TTTATTTAGT TTTGGTGAAC   
  
  
+ TTTAAATATA AATTTATTTT TATAAAATAA AATAAATAAA AATTCTAGAA TGTTCTCTTT TGTACAATGT   
  
  
+ ACTTCACACC CTCAAACTGA TCAAGGTCTT CATGTCGCCA AACTAACCGA CGGCTTCGCG GTGCACTCCC   
  
  
+ GATGCCCTAT TATCAGTTGT TTTACCTGCC CGGCCGAAAA TAGTGTCGAA GTACCTGCCC GGCACCAGAA   
  
  
+ ACTGGCGTGT TGAACAAGGA AGTTAAATAG AAACTAAAAA GTAGAAGAAA AGGAGAGTTA AAGCTTTTTA   
  
  
+ ATTATTTTCT TTTTCTTTAA GGGTTTATTC AGTAAGTATG AGAGGGAATT TAAGTACTTT AAAGGAAATA   
  
  
+ ATACTGCCGG TAGAGATGTA TAAAAGAAAA ACCCCTAAAA AAATGTTTAA TTTAATATCC ATTAAATGAA   
  
  
+ AAAAACCAAA ATTTTAGATA AAGTTGTTTA TAAAAATTTT TGTTTTTTAA ACTAAAAAAA TTTTTTATTT   
  
  
+ AATACACTAT TTTATACTTG AGCTCCAGTT TTGAGGAAAG ATTGAATTAG TTAAGCCGAT TCGATTCTTC   
  
  
+ TAATCGGGTT GTTTAATCCG AAAGTATTTC AATGATCTCT ATGGAGTATC TAATATCCGA CGCTGAAAAA   
  
  
+ GTTAGAGAGG TTTCAAATTA AACGTATAC  

- GTTGATATAT CGTGCTTCCA CGTAATGCAG AGACGATTAT CCTGGTTGTA TACAAAGATG GGCAACAGTT   
  
  
- TTCTTGTAAG AAAAGTATGT ATCTAGTTGG AATATGAATC AGTATCCACG GAGCTAAAGT CCTCTTCCTC   
  
  
- GTCCTCCTTT GTCCATAAAT AAAGCCGAGA GAGAGAGAGA GAGAGAGAGA GAGAGAGAGA GAGAGAGAGA   
  
  
- TGTTATTTAG AAAGAGCAGG ACTCCTTTGT CCATAAATAA AGCCGAGAGA GAGAGAGAGA GAGAGAGAGA   
  
  
- GAGAGAGAGA GAGAGAGATG TTATTTAGAA AGAGCAGGGT GAGGCTGCAA TGGACTCGGC ATTAAAAAGG   
  
  
- GTTTTGGACT GAAGACCAAA TGTGAAACGA GTGATGCTTA GGGATTTTGA TGAGAGTCTA TGAGGTAAGG   
  
  
- TAGTGCGTCA AGAAAACAGG TGGCCATGAA AGTCTTATGA GAGAGAGAGA CAGAGGCAGA GAAAGAAGAG   
  
  
- TCTTACTGCT GCTGTCATAA GAGAGAAAGA GAGAGAGAGA GAGAGAGAGA GAGAGAAAGA GTTTGAATGT   
  
  
- TTGAAAGGAT CCCGCTAGCA CAAAAACACT TAATTACTTA ATTAAATTAA ATTAAATTAA ATTATTCATT   
  
  
- ATTAATTATC TCACCACAAA AATCATTAAT TTTTGTTACA CATTTTTCAA CCCTTTATTA TCTCATGTAA   
  
  
- AAATCATATT CCAAATAATT TATAAAAATT TAACTTTTTA TAATTTCATT CTGATAAATA TAAATTGAAA   
  
  
- TGATGAAAAA TAATTTAAAT AATAAATCTA AAAAAATAAA AATAGTATGA AAATAAATCA AAACCACTTG   
  
  
- AAATTTATAT TTAAATAAAA ATATTTTATT TTATTTATTT TTAAGATCTT ACAAGAGAAA ACATGTTACA   
  
  
- TGAAGTGTGG GAGTTTGACT AGTTCCAGAA GTACAGCGGT TTGATTGGCT GCCGAAGCGC CACGTGAGGG   
  
  
- CTACGGGATA ATAGTCAACA AAATGGACGG GCCGGCTTTT ATCACAGCTT CATGGACGGG CCGTGGTCTT   
  
  
- TGACCGCACA ACTTGTTCCT TCAATTTATC TTTGATTTTT CATCTTCTTT TCCTCTCAAT TTCGAAAAAT   
  
  
- TAATAAAAGA AAAAGAAATT CCCAAATAAG TCATTCATAC TCTCCCTTAA ATTCATGAAA TTTCCTTTAT   
  
  
- TATGACGGCC ATCTCTACAT ATTTTCTTTT TGGGGATTTT TTTACAAATT AAATTATAGG TAATTTACTT   
  
  
- TTTTTGGTTT TAAAATCTAT TTCAACAAAT ATTTTTAAAA ACAAAAAATT TGATTTTTTT AAAAAATAAA   
  
  
- TTATGTGATA AAATATGAAC TCGAGGTCAA AACTCCTTTC TAACTTAATC AATTCGGCTA AGCTAAGAAG   
  
  
- ATTAGCCCAA CAAATTAGGC TTTCATAAAG TTACTAGAGA TACCTCATAG ATTATAGGCT GCGACTTTTT   
  
  
- CAATCTCTCC AAAGTTTAAT TTGCATATG

+     HSE

| Site Name | Organism | Position | Strand | Matrix score. | sequence | function |
| --- | --- | --- | --- | --- | --- | --- |
| HSE | Brassica oleracea | 1315 | + | 9 | AAAAAATTTC | cis-acting element involved in heat stress responsiveness |
| HSE | Brassica oleracea | 1317 | - | 9 | AAAAAATTTC | cis-acting element involved in heat stress responsiveness |

> 2018/04/13 10:10:12  
+ CAACTATATA GCACGAAGGT GCATTACGTC TCTGCTAATA GGACCAACAT ATGTTTCTAC CCGTTGTCAA   
  
  
+ AAGAACATTC TTTTCATACA TAGATCAACC TTATACTTAG TCATAGGTGC CTCGATTTCA GGAGAAGGAG   
  
  
+ CAGGAGGAAA CAGGTATTTA TTTCGGCTCT CTCTCTCTCT CTCTCTCTCT CTCTCTCTCT CTCTCTCTCT   
  
  
+ ACAATAAATC TTTCTCGTCC TGAGGAAACA GGTATTTATT TCGGCTCTCT CTCTCTCTCT CTCTCTCTCT   
  
  
+ CTCTCTCTCT CTCTCTCTAC AATAAATCTT TCTCGTCCCA CTCCGACGTT ACCTGAGCCG TAATTTTTCC   
  
  
+ CAAAACCTGA CTTCTGGTTT ACACTTTGCT CACTACGAAT CCCTAAAACT ACTCTCAGAT ACTCCATTCC   
  
  
+ ATCACGCAGT TCTTTTGTCC ACCGGTACTT TCAGAATACT CTCTCTCTCT GTCTCCGTCT CTTTCTTCTC   
  
  
+ AGAATGACGA CGACAGTATT CTCTCTTTCT CTCTCTCTCT CTCTCTCTCT CTCTCTTTCT CAAACTTACA   
  
  
+ AACTTTCCTA GGGCGATCGT GTTTTTGTGA ATTAATGAAT TAATTTAATT TAATTTAATT TAATAAGTAA   
  
  
+ TAATTAATAG AGTGGTGTTT TTAGTAATTA AAAACAATGT GTAAAAAGTT GGGAAATAAT AGAGTACATT   
  
  
+ TTTAGTATAA GGTTTATTAA ATATTTTTAA ATTGAAAAAT ATTAAAGTAA GACTATTTAT ATTTAACTTT   
  
  
+ ACTACTTTTT ATTAAATTTA TTATTTAGAT TTTTTTATTT TTATCATACT TTTATTTAGT TTTGGTGAAC   
  
  
+ TTTAAATATA AATTTATTTT TATAAAATAA AATAAATAAA AATTCTAGAA TGTTCTCTTT TGTACAATGT   
  
  
+ ACTTCACACC CTCAAACTGA TCAAGGTCTT CATGTCGCCA AACTAACCGA CGGCTTCGCG GTGCACTCCC   
  
  
+ GATGCCCTAT TATCAGTTGT TTTACCTGCC CGGCCGAAAA TAGTGTCGAA GTACCTGCCC GGCACCAGAA   
  
  
+ ACTGGCGTGT TGAACAAGGA AGTTAAATAG AAACTAAAAA GTAGAAGAAA AGGAGAGTTA AAGCTTTTTA   
  
  
+ ATTATTTTCT TTTTCTTTAA GGGTTTATTC AGTAAGTATG AGAGGGAATT TAAGTACTTT AAAGGAAATA   
  
  
+ ATACTGCCGG TAGAGATGTA TAAAAGAAAA ACCCCTAAAA AAATGTTTAA TTTAATATCC ATTAAATGAA   
  
  
+ AAAAACCAAA ATTTTAGATA AAGTTGTTTA TAAAAATTTT TGTTTTTTAA ACTAAAAAAA TTTTTTATTT   
  
  
+ AATACACTAT TTTATACTTG AGCTCCAGTT TTGAGGAAAG ATTGAATTAG TTAAGCCGAT TCGATTCTTC   
  
  
+ TAATCGGGTT GTTTAATCCG AAAGTATTTC AATGATCTCT ATGGAGTATC TAATATCCGA CGCTGAAAAA   
  
  
+ GTTAGAGAGG TTTCAAATTA AACGTATAC  

- GTTGATATAT CGTGCTTCCA CGTAATGCAG AGACGATTAT CCTGGTTGTA TACAAAGATG GGCAACAGTT   
  
  
- TTCTTGTAAG AAAAGTATGT ATCTAGTTGG AATATGAATC AGTATCCACG GAGCTAAAGT CCTCTTCCTC   
  
  
- GTCCTCCTTT GTCCATAAAT AAAGCCGAGA GAGAGAGAGA GAGAGAGAGA GAGAGAGAGA GAGAGAGAGA   
  
  
- TGTTATTTAG AAAGAGCAGG ACTCCTTTGT CCATAAATAA AGCCGAGAGA GAGAGAGAGA GAGAGAGAGA   
  
  
- GAGAGAGAGA GAGAGAGATG TTATTTAGAA AGAGCAGGGT GAGGCTGCAA TGGACTCGGC ATTAAAAAGG   
  
  
- GTTTTGGACT GAAGACCAAA TGTGAAACGA GTGATGCTTA GGGATTTTGA TGAGAGTCTA TGAGGTAAGG   
  
  
- TAGTGCGTCA AGAAAACAGG TGGCCATGAA AGTCTTATGA GAGAGAGAGA CAGAGGCAGA GAAAGAAGAG   
  
  
- TCTTACTGCT GCTGTCATAA GAGAGAAAGA GAGAGAGAGA GAGAGAGAGA GAGAGAAAGA GTTTGAATGT   
  
  
- TTGAAAGGAT CCCGCTAGCA CAAAAACACT TAATTACTTA ATTAAATTAA ATTAAATTAA ATTATTCATT   
  
  
- ATTAATTATC TCACCACAAA AATCATTAAT TTTTGTTACA CATTTTTCAA CCCTTTATTA TCTCATGTAA   
  
  
- AAATCATATT CCAAATAATT TATAAAAATT TAACTTTTTA TAATTTCATT CTGATAAATA TAAATTGAAA   
  
  
- TGATGAAAAA TAATTTAAAT AATAAATCTA AAAAAATAAA AATAGTATGA AAATAAATCA AAACCACTTG   
  
  
- AAATTTATAT TTAAATAAAA ATATTTTATT TTATTTATTT TTAAGATCTT ACAAGAGAAA ACATGTTACA   
  
  
- TGAAGTGTGG GAGTTTGACT AGTTCCAGAA GTACAGCGGT TTGATTGGCT GCCGAAGCGC CACGTGAGGG   
  
  
- CTACGGGATA ATAGTCAACA AAATGGACGG GCCGGCTTTT ATCACAGCTT CATGGACGGG CCGTGGTCTT   
  
  
- TGACCGCACA ACTTGTTCCT TCAATTTATC TTTGATTTTT CATCTTCTTT TCCTCTCAAT TTCGAAAAAT   
  
  
- TAATAAAAGA AAAAGAAATT CCCAAATAAG TCATTCATAC TCTCCCTTAA ATTCATGAAA TTTCCTTTAT   
  
  
- TATGACGGCC ATCTCTACAT ATTTTCTTTT TGGGGATTTT TTTACAAATT AAATTATAGG TAATTTACTT   
  
  
- TTTTTGGTTT TAAAATCTAT TTCAACAAAT ATTTTTAAAA ACAAAAAATT TGATTTTTTT AAAAAATAAA   
  
  
- TTATGTGATA AAATATGAAC TCGAGGTCAA AACTCCTTTC TAACTTAATC AATTCGGCTA AGCTAAGAAG   
  
  
- ATTAGCCCAA CAAATTAGGC TTTCATAAAG TTACTAGAGA TACCTCATAG ATTATAGGCT GCGACTTTTT   
  
  
- CAATCTCTCC AAAGTTTAAT TTGCATATG

+     I-box

| Site Name | Organism | Position | Strand | Matrix score. | sequence | function |
| --- | --- | --- | --- | --- | --- | --- |
| I-box | Solanum tuberosum | 789 | + | 10 | TATTATCTAGA | part of a light responsive element |

> 2018/04/13 10:10:12  
+ CAACTATATA GCACGAAGGT GCATTACGTC TCTGCTAATA GGACCAACAT ATGTTTCTAC CCGTTGTCAA   
  
  
+ AAGAACATTC TTTTCATACA TAGATCAACC TTATACTTAG TCATAGGTGC CTCGATTTCA GGAGAAGGAG   
  
  
+ CAGGAGGAAA CAGGTATTTA TTTCGGCTCT CTCTCTCTCT CTCTCTCTCT CTCTCTCTCT CTCTCTCTCT   
  
  
+ ACAATAAATC TTTCTCGTCC TGAGGAAACA GGTATTTATT TCGGCTCTCT CTCTCTCTCT CTCTCTCTCT   
  
  
+ CTCTCTCTCT CTCTCTCTAC AATAAATCTT TCTCGTCCCA CTCCGACGTT ACCTGAGCCG TAATTTTTCC   
  
  
+ CAAAACCTGA CTTCTGGTTT ACACTTTGCT CACTACGAAT CCCTAAAACT ACTCTCAGAT ACTCCATTCC   
  
  
+ ATCACGCAGT TCTTTTGTCC ACCGGTACTT TCAGAATACT CTCTCTCTCT GTCTCCGTCT CTTTCTTCTC   
  
  
+ AGAATGACGA CGACAGTATT CTCTCTTTCT CTCTCTCTCT CTCTCTCTCT CTCTCTTTCT CAAACTTACA   
  
  
+ AACTTTCCTA GGGCGATCGT GTTTTTGTGA ATTAATGAAT TAATTTAATT TAATTTAATT TAATAAGTAA   
  
  
+ TAATTAATAG AGTGGTGTTT TTAGTAATTA AAAACAATGT GTAAAAAGTT GGGAAATAAT AGAGTACATT   
  
  
+ TTTAGTATAA GGTTTATTAA ATATTTTTAA ATTGAAAAAT ATTAAAGTAA GACTATTTAT ATTTAACTTT   
  
  
+ ACTACTTTTT ATTAAATTTA TTATTTAGAT TTTTTTATTT TTATCATACT TTTATTTAGT TTTGGTGAAC   
  
  
+ TTTAAATATA AATTTATTTT TATAAAATAA AATAAATAAA AATTCTAGAA TGTTCTCTTT TGTACAATGT   
  
  
+ ACTTCACACC CTCAAACTGA TCAAGGTCTT CATGTCGCCA AACTAACCGA CGGCTTCGCG GTGCACTCCC   
  
  
+ GATGCCCTAT TATCAGTTGT TTTACCTGCC CGGCCGAAAA TAGTGTCGAA GTACCTGCCC GGCACCAGAA   
  
  
+ ACTGGCGTGT TGAACAAGGA AGTTAAATAG AAACTAAAAA GTAGAAGAAA AGGAGAGTTA AAGCTTTTTA   
  
  
+ ATTATTTTCT TTTTCTTTAA GGGTTTATTC AGTAAGTATG AGAGGGAATT TAAGTACTTT AAAGGAAATA   
  
  
+ ATACTGCCGG TAGAGATGTA TAAAAGAAAA ACCCCTAAAA AAATGTTTAA TTTAATATCC ATTAAATGAA   
  
  
+ AAAAACCAAA ATTTTAGATA AAGTTGTTTA TAAAAATTTT TGTTTTTTAA ACTAAAAAAA TTTTTTATTT   
  
  
+ AATACACTAT TTTATACTTG AGCTCCAGTT TTGAGGAAAG ATTGAATTAG TTAAGCCGAT TCGATTCTTC   
  
  
+ TAATCGGGTT GTTTAATCCG AAAGTATTTC AATGATCTCT ATGGAGTATC TAATATCCGA CGCTGAAAAA   
  
  
+ GTTAGAGAGG TTTCAAATTA AACGTATAC  

- GTTGATATAT CGTGCTTCCA CGTAATGCAG AGACGATTAT CCTGGTTGTA TACAAAGATG GGCAACAGTT   
  
  
- TTCTTGTAAG AAAAGTATGT ATCTAGTTGG AATATGAATC AGTATCCACG GAGCTAAAGT CCTCTTCCTC   
  
  
- GTCCTCCTTT GTCCATAAAT AAAGCCGAGA GAGAGAGAGA GAGAGAGAGA GAGAGAGAGA GAGAGAGAGA   
  
  
- TGTTATTTAG AAAGAGCAGG ACTCCTTTGT CCATAAATAA AGCCGAGAGA GAGAGAGAGA GAGAGAGAGA   
  
  
- GAGAGAGAGA GAGAGAGATG TTATTTAGAA AGAGCAGGGT GAGGCTGCAA TGGACTCGGC ATTAAAAAGG   
  
  
- GTTTTGGACT GAAGACCAAA TGTGAAACGA GTGATGCTTA GGGATTTTGA TGAGAGTCTA TGAGGTAAGG   
  
  
- TAGTGCGTCA AGAAAACAGG TGGCCATGAA AGTCTTATGA GAGAGAGAGA CAGAGGCAGA GAAAGAAGAG   
  
  
- TCTTACTGCT GCTGTCATAA GAGAGAAAGA GAGAGAGAGA GAGAGAGAGA GAGAGAAAGA GTTTGAATGT   
  
  
- TTGAAAGGAT CCCGCTAGCA CAAAAACACT TAATTACTTA ATTAAATTAA ATTAAATTAA ATTATTCATT   
  
  
- ATTAATTATC TCACCACAAA AATCATTAAT TTTTGTTACA CATTTTTCAA CCCTTTATTA TCTCATGTAA   
  
  
- AAATCATATT CCAAATAATT TATAAAAATT TAACTTTTTA TAATTTCATT CTGATAAATA TAAATTGAAA   
  
  
- TGATGAAAAA TAATTTAAAT AATAAATCTA AAAAAATAAA AATAGTATGA AAATAAATCA AAACCACTTG   
  
  
- AAATTTATAT TTAAATAAAA ATATTTTATT TTATTTATTT TTAAGATCTT ACAAGAGAAA ACATGTTACA   
  
  
- TGAAGTGTGG GAGTTTGACT AGTTCCAGAA GTACAGCGGT TTGATTGGCT GCCGAAGCGC CACGTGAGGG   
  
  
- CTACGGGATA ATAGTCAACA AAATGGACGG GCCGGCTTTT ATCACAGCTT CATGGACGGG CCGTGGTCTT   
  
  
- TGACCGCACA ACTTGTTCCT TCAATTTATC TTTGATTTTT CATCTTCTTT TCCTCTCAAT TTCGAAAAAT   
  
  
- TAATAAAAGA AAAAGAAATT CCCAAATAAG TCATTCATAC TCTCCCTTAA ATTCATGAAA TTTCCTTTAT   
  
  
- TATGACGGCC ATCTCTACAT ATTTTCTTTT TGGGGATTTT TTTACAAATT AAATTATAGG TAATTTACTT   
  
  
- TTTTTGGTTT TAAAATCTAT TTCAACAAAT ATTTTTAAAA ACAAAAAATT TGATTTTTTT AAAAAATAAA   
  
  
- TTATGTGATA AAATATGAAC TCGAGGTCAA AACTCCTTTC TAACTTAATC AATTCGGCTA AGCTAAGAAG   
  
  
- ATTAGCCCAA CAAATTAGGC TTTCATAAAG TTACTAGAGA TACCTCATAG ATTATAGGCT GCGACTTTTT   
  
  
- CAATCTCTCC AAAGTTTAAT TTGCATATG

+     LTR

| Site Name | Organism | Position | Strand | Matrix score. | sequence | function |
| --- | --- | --- | --- | --- | --- | --- |
| LTR | Hordeum vulgare | 1418 | + | 6 | CCGAAA | cis-acting element involved in low-temperature responsiveness |
| LTR | Hordeum vulgare | 161 | - | 6 | CCGAAA | cis-acting element involved in low-temperature responsiveness |
| LTR | Hordeum vulgare | 249 | - | 6 | CCGAAA | cis-acting element involved in low-temperature responsiveness |
| LTR | Hordeum vulgare | 1014 | + | 6 | CCGAAA | cis-acting element involved in low-temperature responsiveness |

> 2018/04/13 10:10:12  
+ CAACTATATA GCACGAAGGT GCATTACGTC TCTGCTAATA GGACCAACAT ATGTTTCTAC CCGTTGTCAA   
  
  
+ AAGAACATTC TTTTCATACA TAGATCAACC TTATACTTAG TCATAGGTGC CTCGATTTCA GGAGAAGGAG   
  
  
+ CAGGAGGAAA CAGGTATTTA TTTCGGCTCT CTCTCTCTCT CTCTCTCTCT CTCTCTCTCT CTCTCTCTCT   
  
  
+ ACAATAAATC TTTCTCGTCC TGAGGAAACA GGTATTTATT TCGGCTCTCT CTCTCTCTCT CTCTCTCTCT   
  
  
+ CTCTCTCTCT CTCTCTCTAC AATAAATCTT TCTCGTCCCA CTCCGACGTT ACCTGAGCCG TAATTTTTCC   
  
  
+ CAAAACCTGA CTTCTGGTTT ACACTTTGCT CACTACGAAT CCCTAAAACT ACTCTCAGAT ACTCCATTCC   
  
  
+ ATCACGCAGT TCTTTTGTCC ACCGGTACTT TCAGAATACT CTCTCTCTCT GTCTCCGTCT CTTTCTTCTC   
  
  
+ AGAATGACGA CGACAGTATT CTCTCTTTCT CTCTCTCTCT CTCTCTCTCT CTCTCTTTCT CAAACTTACA   
  
  
+ AACTTTCCTA GGGCGATCGT GTTTTTGTGA ATTAATGAAT TAATTTAATT TAATTTAATT TAATAAGTAA   
  
  
+ TAATTAATAG AGTGGTGTTT TTAGTAATTA AAAACAATGT GTAAAAAGTT GGGAAATAAT AGAGTACATT   
  
  
+ TTTAGTATAA GGTTTATTAA ATATTTTTAA ATTGAAAAAT ATTAAAGTAA GACTATTTAT ATTTAACTTT   
  
  
+ ACTACTTTTT ATTAAATTTA TTATTTAGAT TTTTTTATTT TTATCATACT TTTATTTAGT TTTGGTGAAC   
  
  
+ TTTAAATATA AATTTATTTT TATAAAATAA AATAAATAAA AATTCTAGAA TGTTCTCTTT TGTACAATGT   
  
  
+ ACTTCACACC CTCAAACTGA TCAAGGTCTT CATGTCGCCA AACTAACCGA CGGCTTCGCG GTGCACTCCC   
  
  
+ GATGCCCTAT TATCAGTTGT TTTACCTGCC CGGCCGAAAA TAGTGTCGAA GTACCTGCCC GGCACCAGAA   
  
  
+ ACTGGCGTGT TGAACAAGGA AGTTAAATAG AAACTAAAAA GTAGAAGAAA AGGAGAGTTA AAGCTTTTTA   
  
  
+ ATTATTTTCT TTTTCTTTAA GGGTTTATTC AGTAAGTATG AGAGGGAATT TAAGTACTTT AAAGGAAATA   
  
  
+ ATACTGCCGG TAGAGATGTA TAAAAGAAAA ACCCCTAAAA AAATGTTTAA TTTAATATCC ATTAAATGAA   
  
  
+ AAAAACCAAA ATTTTAGATA AAGTTGTTTA TAAAAATTTT TGTTTTTTAA ACTAAAAAAA TTTTTTATTT   
  
  
+ AATACACTAT TTTATACTTG AGCTCCAGTT TTGAGGAAAG ATTGAATTAG TTAAGCCGAT TCGATTCTTC   
  
  
+ TAATCGGGTT GTTTAATCCG AAAGTATTTC AATGATCTCT ATGGAGTATC TAATATCCGA CGCTGAAAAA   
  
  
+ GTTAGAGAGG TTTCAAATTA AACGTATAC  

- GTTGATATAT CGTGCTTCCA CGTAATGCAG AGACGATTAT CCTGGTTGTA TACAAAGATG GGCAACAGTT   
  
  
- TTCTTGTAAG AAAAGTATGT ATCTAGTTGG AATATGAATC AGTATCCACG GAGCTAAAGT CCTCTTCCTC   
  
  
- GTCCTCCTTT GTCCATAAAT AAAGCCGAGA GAGAGAGAGA GAGAGAGAGA GAGAGAGAGA GAGAGAGAGA   
  
  
- TGTTATTTAG AAAGAGCAGG ACTCCTTTGT CCATAAATAA AGCCGAGAGA GAGAGAGAGA GAGAGAGAGA   
  
  
- GAGAGAGAGA GAGAGAGATG TTATTTAGAA AGAGCAGGGT GAGGCTGCAA TGGACTCGGC ATTAAAAAGG   
  
  
- GTTTTGGACT GAAGACCAAA TGTGAAACGA GTGATGCTTA GGGATTTTGA TGAGAGTCTA TGAGGTAAGG   
  
  
- TAGTGCGTCA AGAAAACAGG TGGCCATGAA AGTCTTATGA GAGAGAGAGA CAGAGGCAGA GAAAGAAGAG   
  
  
- TCTTACTGCT GCTGTCATAA GAGAGAAAGA GAGAGAGAGA GAGAGAGAGA GAGAGAAAGA GTTTGAATGT   
  
  
- TTGAAAGGAT CCCGCTAGCA CAAAAACACT TAATTACTTA ATTAAATTAA ATTAAATTAA ATTATTCATT   
  
  
- ATTAATTATC TCACCACAAA AATCATTAAT TTTTGTTACA CATTTTTCAA CCCTTTATTA TCTCATGTAA   
  
  
- AAATCATATT CCAAATAATT TATAAAAATT TAACTTTTTA TAATTTCATT CTGATAAATA TAAATTGAAA   
  
  
- TGATGAAAAA TAATTTAAAT AATAAATCTA AAAAAATAAA AATAGTATGA AAATAAATCA AAACCACTTG   
  
  
- AAATTTATAT TTAAATAAAA ATATTTTATT TTATTTATTT TTAAGATCTT ACAAGAGAAA ACATGTTACA   
  
  
- TGAAGTGTGG GAGTTTGACT AGTTCCAGAA GTACAGCGGT TTGATTGGCT GCCGAAGCGC CACGTGAGGG   
  
  
- CTACGGGATA ATAGTCAACA AAATGGACGG GCCGGCTTTT ATCACAGCTT CATGGACGGG CCGTGGTCTT   
  
  
- TGACCGCACA ACTTGTTCCT TCAATTTATC TTTGATTTTT CATCTTCTTT TCCTCTCAAT TTCGAAAAAT   
  
  
- TAATAAAAGA AAAAGAAATT CCCAAATAAG TCATTCATAC TCTCCCTTAA ATTCATGAAA TTTCCTTTAT   
  
  
- TATGACGGCC ATCTCTACAT ATTTTCTTTT TGGGGATTTT TTTACAAATT AAATTATAGG TAATTTACTT   
  
  
- TTTTTGGTTT TAAAATCTAT TTCAACAAAT ATTTTTAAAA ACAAAAAATT TGATTTTTTT AAAAAATAAA   
  
  
- TTATGTGATA AAATATGAAC TCGAGGTCAA AACTCCTTTC TAACTTAATC AATTCGGCTA AGCTAAGAAG   
  
  
- ATTAGCCCAA CAAATTAGGC TTTCATAAAG TTACTAGAGA TACCTCATAG ATTATAGGCT GCGACTTTTT   
  
  
- CAATCTCTCC AAAGTTTAAT TTGCATATG

+     MBS

| Site Name | Organism | Position | Strand | Matrix score. | sequence | function |
| --- | --- | --- | --- | --- | --- | --- |
| MBS | Arabidopsis thaliana | 994 | - | 6 | CAACTG | MYB binding site involved in drought-inducibility |

> 2018/04/13 10:10:12  
+ CAACTATATA GCACGAAGGT GCATTACGTC TCTGCTAATA GGACCAACAT ATGTTTCTAC CCGTTGTCAA   
  
  
+ AAGAACATTC TTTTCATACA TAGATCAACC TTATACTTAG TCATAGGTGC CTCGATTTCA GGAGAAGGAG   
  
  
+ CAGGAGGAAA CAGGTATTTA TTTCGGCTCT CTCTCTCTCT CTCTCTCTCT CTCTCTCTCT CTCTCTCTCT   
  
  
+ ACAATAAATC TTTCTCGTCC TGAGGAAACA GGTATTTATT TCGGCTCTCT CTCTCTCTCT CTCTCTCTCT   
  
  
+ CTCTCTCTCT CTCTCTCTAC AATAAATCTT TCTCGTCCCA CTCCGACGTT ACCTGAGCCG TAATTTTTCC   
  
  
+ CAAAACCTGA CTTCTGGTTT ACACTTTGCT CACTACGAAT CCCTAAAACT ACTCTCAGAT ACTCCATTCC   
  
  
+ ATCACGCAGT TCTTTTGTCC ACCGGTACTT TCAGAATACT CTCTCTCTCT GTCTCCGTCT CTTTCTTCTC   
  
  
+ AGAATGACGA CGACAGTATT CTCTCTTTCT CTCTCTCTCT CTCTCTCTCT CTCTCTTTCT CAAACTTACA   
  
  
+ AACTTTCCTA GGGCGATCGT GTTTTTGTGA ATTAATGAAT TAATTTAATT TAATTTAATT TAATAAGTAA   
  
  
+ TAATTAATAG AGTGGTGTTT TTAGTAATTA AAAACAATGT GTAAAAAGTT GGGAAATAAT AGAGTACATT   
  
  
+ TTTAGTATAA GGTTTATTAA ATATTTTTAA ATTGAAAAAT ATTAAAGTAA GACTATTTAT ATTTAACTTT   
  
  
+ ACTACTTTTT ATTAAATTTA TTATTTAGAT TTTTTTATTT TTATCATACT TTTATTTAGT TTTGGTGAAC   
  
  
+ TTTAAATATA AATTTATTTT TATAAAATAA AATAAATAAA AATTCTAGAA TGTTCTCTTT TGTACAATGT   
  
  
+ ACTTCACACC CTCAAACTGA TCAAGGTCTT CATGTCGCCA AACTAACCGA CGGCTTCGCG GTGCACTCCC   
  
  
+ GATGCCCTAT TATCAGTTGT TTTACCTGCC CGGCCGAAAA TAGTGTCGAA GTACCTGCCC GGCACCAGAA   
  
  
+ ACTGGCGTGT TGAACAAGGA AGTTAAATAG AAACTAAAAA GTAGAAGAAA AGGAGAGTTA AAGCTTTTTA   
  
  
+ ATTATTTTCT TTTTCTTTAA GGGTTTATTC AGTAAGTATG AGAGGGAATT TAAGTACTTT AAAGGAAATA   
  
  
+ ATACTGCCGG TAGAGATGTA TAAAAGAAAA ACCCCTAAAA AAATGTTTAA TTTAATATCC ATTAAATGAA   
  
  
+ AAAAACCAAA ATTTTAGATA AAGTTGTTTA TAAAAATTTT TGTTTTTTAA ACTAAAAAAA TTTTTTATTT   
  
  
+ AATACACTAT TTTATACTTG AGCTCCAGTT TTGAGGAAAG ATTGAATTAG TTAAGCCGAT TCGATTCTTC   
  
  
+ TAATCGGGTT GTTTAATCCG AAAGTATTTC AATGATCTCT ATGGAGTATC TAATATCCGA CGCTGAAAAA   
  
  
+ GTTAGAGAGG TTTCAAATTA AACGTATAC  

- GTTGATATAT CGTGCTTCCA CGTAATGCAG AGACGATTAT CCTGGTTGTA TACAAAGATG GGCAACAGTT   
  
  
- TTCTTGTAAG AAAAGTATGT ATCTAGTTGG AATATGAATC AGTATCCACG GAGCTAAAGT CCTCTTCCTC   
  
  
- GTCCTCCTTT GTCCATAAAT AAAGCCGAGA GAGAGAGAGA GAGAGAGAGA GAGAGAGAGA GAGAGAGAGA   
  
  
- TGTTATTTAG AAAGAGCAGG ACTCCTTTGT CCATAAATAA AGCCGAGAGA GAGAGAGAGA GAGAGAGAGA   
  
  
- GAGAGAGAGA GAGAGAGATG TTATTTAGAA AGAGCAGGGT GAGGCTGCAA TGGACTCGGC ATTAAAAAGG   
  
  
- GTTTTGGACT GAAGACCAAA TGTGAAACGA GTGATGCTTA GGGATTTTGA TGAGAGTCTA TGAGGTAAGG   
  
  
- TAGTGCGTCA AGAAAACAGG TGGCCATGAA AGTCTTATGA GAGAGAGAGA CAGAGGCAGA GAAAGAAGAG   
  
  
- TCTTACTGCT GCTGTCATAA GAGAGAAAGA GAGAGAGAGA GAGAGAGAGA GAGAGAAAGA GTTTGAATGT   
  
  
- TTGAAAGGAT CCCGCTAGCA CAAAAACACT TAATTACTTA ATTAAATTAA ATTAAATTAA ATTATTCATT   
  
  
- ATTAATTATC TCACCACAAA AATCATTAAT TTTTGTTACA CATTTTTCAA CCCTTTATTA TCTCATGTAA   
  
  
- AAATCATATT CCAAATAATT TATAAAAATT TAACTTTTTA TAATTTCATT CTGATAAATA TAAATTGAAA   
  
  
- TGATGAAAAA TAATTTAAAT AATAAATCTA AAAAAATAAA AATAGTATGA AAATAAATCA AAACCACTTG   
  
  
- AAATTTATAT TTAAATAAAA ATATTTTATT TTATTTATTT TTAAGATCTT ACAAGAGAAA ACATGTTACA   
  
  
- TGAAGTGTGG GAGTTTGACT AGTTCCAGAA GTACAGCGGT TTGATTGGCT GCCGAAGCGC CACGTGAGGG   
  
  
- CTACGGGATA ATAGTCAACA AAATGGACGG GCCGGCTTTT ATCACAGCTT CATGGACGGG CCGTGGTCTT   
  
  
- TGACCGCACA ACTTGTTCCT TCAATTTATC TTTGATTTTT CATCTTCTTT TCCTCTCAAT TTCGAAAAAT   
  
  
- TAATAAAAGA AAAAGAAATT CCCAAATAAG TCATTCATAC TCTCCCTTAA ATTCATGAAA TTTCCTTTAT   
  
  
- TATGACGGCC ATCTCTACAT ATTTTCTTTT TGGGGATTTT TTTACAAATT AAATTATAGG TAATTTACTT   
  
  
- TTTTTGGTTT TAAAATCTAT TTCAACAAAT ATTTTTAAAA ACAAAAAATT TGATTTTTTT AAAAAATAAA   
  
  
- TTATGTGATA AAATATGAAC TCGAGGTCAA AACTCCTTTC TAACTTAATC AATTCGGCTA AGCTAAGAAG   
  
  
- ATTAGCCCAA CAAATTAGGC TTTCATAAAG TTACTAGAGA TACCTCATAG ATTATAGGCT GCGACTTTTT   
  
  
- CAATCTCTCC AAAGTTTAAT TTGCATATG

+     Skn-1\_motif

| Site Name | Organism | Position | Strand | Matrix score. | sequence | function |
| --- | --- | --- | --- | --- | --- | --- |
| Skn-1\_motif | Oryza sativa | 494 | - | 5 | GTCAT | cis-acting regulatory element required for endosperm expression |
| Skn-1\_motif | Oryza sativa | 110 | + | 5 | GTCAT | cis-acting regulatory element required for endosperm expression |

> 2018/04/13 10:10:12  
+ CAACTATATA GCACGAAGGT GCATTACGTC TCTGCTAATA GGACCAACAT ATGTTTCTAC CCGTTGTCAA   
  
  
+ AAGAACATTC TTTTCATACA TAGATCAACC TTATACTTAG TCATAGGTGC CTCGATTTCA GGAGAAGGAG   
  
  
+ CAGGAGGAAA CAGGTATTTA TTTCGGCTCT CTCTCTCTCT CTCTCTCTCT CTCTCTCTCT CTCTCTCTCT   
  
  
+ ACAATAAATC TTTCTCGTCC TGAGGAAACA GGTATTTATT TCGGCTCTCT CTCTCTCTCT CTCTCTCTCT   
  
  
+ CTCTCTCTCT CTCTCTCTAC AATAAATCTT TCTCGTCCCA CTCCGACGTT ACCTGAGCCG TAATTTTTCC   
  
  
+ CAAAACCTGA CTTCTGGTTT ACACTTTGCT CACTACGAAT CCCTAAAACT ACTCTCAGAT ACTCCATTCC   
  
  
+ ATCACGCAGT TCTTTTGTCC ACCGGTACTT TCAGAATACT CTCTCTCTCT GTCTCCGTCT CTTTCTTCTC   
  
  
+ AGAATGACGA CGACAGTATT CTCTCTTTCT CTCTCTCTCT CTCTCTCTCT CTCTCTTTCT CAAACTTACA   
  
  
+ AACTTTCCTA GGGCGATCGT GTTTTTGTGA ATTAATGAAT TAATTTAATT TAATTTAATT TAATAAGTAA   
  
  
+ TAATTAATAG AGTGGTGTTT TTAGTAATTA AAAACAATGT GTAAAAAGTT GGGAAATAAT AGAGTACATT   
  
  
+ TTTAGTATAA GGTTTATTAA ATATTTTTAA ATTGAAAAAT ATTAAAGTAA GACTATTTAT ATTTAACTTT   
  
  
+ ACTACTTTTT ATTAAATTTA TTATTTAGAT TTTTTTATTT TTATCATACT TTTATTTAGT TTTGGTGAAC   
  
  
+ TTTAAATATA AATTTATTTT TATAAAATAA AATAAATAAA AATTCTAGAA TGTTCTCTTT TGTACAATGT   
  
  
+ ACTTCACACC CTCAAACTGA TCAAGGTCTT CATGTCGCCA AACTAACCGA CGGCTTCGCG GTGCACTCCC   
  
  
+ GATGCCCTAT TATCAGTTGT TTTACCTGCC CGGCCGAAAA TAGTGTCGAA GTACCTGCCC GGCACCAGAA   
  
  
+ ACTGGCGTGT TGAACAAGGA AGTTAAATAG AAACTAAAAA GTAGAAGAAA AGGAGAGTTA AAGCTTTTTA   
  
  
+ ATTATTTTCT TTTTCTTTAA GGGTTTATTC AGTAAGTATG AGAGGGAATT TAAGTACTTT AAAGGAAATA   
  
  
+ ATACTGCCGG TAGAGATGTA TAAAAGAAAA ACCCCTAAAA AAATGTTTAA TTTAATATCC ATTAAATGAA   
  
  
+ AAAAACCAAA ATTTTAGATA AAGTTGTTTA TAAAAATTTT TGTTTTTTAA ACTAAAAAAA TTTTTTATTT   
  
  
+ AATACACTAT TTTATACTTG AGCTCCAGTT TTGAGGAAAG ATTGAATTAG TTAAGCCGAT TCGATTCTTC   
  
  
+ TAATCGGGTT GTTTAATCCG AAAGTATTTC AATGATCTCT ATGGAGTATC TAATATCCGA CGCTGAAAAA   
  
  
+ GTTAGAGAGG TTTCAAATTA AACGTATAC  

- GTTGATATAT CGTGCTTCCA CGTAATGCAG AGACGATTAT CCTGGTTGTA TACAAAGATG GGCAACAGTT   
  
  
- TTCTTGTAAG AAAAGTATGT ATCTAGTTGG AATATGAATC AGTATCCACG GAGCTAAAGT CCTCTTCCTC   
  
  
- GTCCTCCTTT GTCCATAAAT AAAGCCGAGA GAGAGAGAGA GAGAGAGAGA GAGAGAGAGA GAGAGAGAGA   
  
  
- TGTTATTTAG AAAGAGCAGG ACTCCTTTGT CCATAAATAA AGCCGAGAGA GAGAGAGAGA GAGAGAGAGA   
  
  
- GAGAGAGAGA GAGAGAGATG TTATTTAGAA AGAGCAGGGT GAGGCTGCAA TGGACTCGGC ATTAAAAAGG   
  
  
- GTTTTGGACT GAAGACCAAA TGTGAAACGA GTGATGCTTA GGGATTTTGA TGAGAGTCTA TGAGGTAAGG   
  
  
- TAGTGCGTCA AGAAAACAGG TGGCCATGAA AGTCTTATGA GAGAGAGAGA CAGAGGCAGA GAAAGAAGAG   
  
  
- TCTTACTGCT GCTGTCATAA GAGAGAAAGA GAGAGAGAGA GAGAGAGAGA GAGAGAAAGA GTTTGAATGT   
  
  
- TTGAAAGGAT CCCGCTAGCA CAAAAACACT TAATTACTTA ATTAAATTAA ATTAAATTAA ATTATTCATT   
  
  
- ATTAATTATC TCACCACAAA AATCATTAAT TTTTGTTACA CATTTTTCAA CCCTTTATTA TCTCATGTAA   
  
  
- AAATCATATT CCAAATAATT TATAAAAATT TAACTTTTTA TAATTTCATT CTGATAAATA TAAATTGAAA   
  
  
- TGATGAAAAA TAATTTAAAT AATAAATCTA AAAAAATAAA AATAGTATGA AAATAAATCA AAACCACTTG   
  
  
- AAATTTATAT TTAAATAAAA ATATTTTATT TTATTTATTT TTAAGATCTT ACAAGAGAAA ACATGTTACA   
  
  
- TGAAGTGTGG GAGTTTGACT AGTTCCAGAA GTACAGCGGT TTGATTGGCT GCCGAAGCGC CACGTGAGGG   
  
  
- CTACGGGATA ATAGTCAACA AAATGGACGG GCCGGCTTTT ATCACAGCTT CATGGACGGG CCGTGGTCTT   
  
  
- TGACCGCACA ACTTGTTCCT TCAATTTATC TTTGATTTTT CATCTTCTTT TCCTCTCAAT TTCGAAAAAT   
  
  
- TAATAAAAGA AAAAGAAATT CCCAAATAAG TCATTCATAC TCTCCCTTAA ATTCATGAAA TTTCCTTTAT   
  
  
- TATGACGGCC ATCTCTACAT ATTTTCTTTT TGGGGATTTT TTTACAAATT AAATTATAGG TAATTTACTT   
  
  
- TTTTTGGTTT TAAAATCTAT TTCAACAAAT ATTTTTAAAA ACAAAAAATT TGATTTTTTT AAAAAATAAA   
  
  
- TTATGTGATA AAATATGAAC TCGAGGTCAA AACTCCTTTC TAACTTAATC AATTCGGCTA AGCTAAGAAG   
  
  
- ATTAGCCCAA CAAATTAGGC TTTCATAAAG TTACTAGAGA TACCTCATAG ATTATAGGCT GCGACTTTTT   
  
  
- CAATCTCTCC AAAGTTTAAT TTGCATATG

+     TATA-box

| Site Name | Organism | Position | Strand | Matrix score. | sequence | function |
| --- | --- | --- | --- | --- | --- | --- |
| TATA-box | Arabidopsis thaliana | 1343 | - | 4 | TATA | core promoter element around -30 of transcription start |
| TATA-box | Lycopersicon esculentum | 1313 | - | 5 | TTTTA | core promoter element around -30 of transcription start |
| TATA-box | Arabidopsis thaliana | 1342 | - | 5 | TATAA | core promoter element around -30 of transcription start |
| TATA-box | Arabidopsis thaliana | 1341 | - | 6 | TATAAA | core promoter element around -30 of transcription start |
| TATA-box | Lycopersicon esculentum | 1085 | - | 5 | TTTTA | core promoter element around -30 of transcription start |
| TATA-box | Zea mays | 724 | - | 8 | TTTAAAAA | core promoter element around -30 of transcription start |
| TATA-box | Arabidopsis thaliana | 853 | - | 11 | TATAAATATAAA | core promoter element around -30 of transcription start |
| TATA-box | Arabidopsis thaliana | 758 | - | 4 | TATA | core promoter element around -30 of transcription start |
| TATA-box | Lycopersicon esculentum | 1323 | + | 5 | TTTTA | core promoter element around -30 of transcription start |
| TATA-box | Lycopersicon esculentum | 1000 | + | 5 | TTTTA | core promoter element around -30 of transcription start |
| TATA-box | Lycopersicon esculentum | 863 | - | 5 | TTTTA | core promoter element around -30 of transcription start |
| TATA-box | Lycopersicon esculentum | 1226 | - | 5 | TTTTA | core promoter element around -30 of transcription start |
| TATA-box | Oryza sativa | 898 | - | 7 | TACAAAA | core promoter element around -30 of transcription start |
| TATA-box | Arabidopsis thaliana | 859 | - | 6 | TATAAA | core promoter element around -30 of transcription start |
| TATA-box | Glycine max | 621 | + | 5 | TAATA | core promoter element around -30 of transcription start |
| TATA-box | Glycine max | 1451 | + | 5 | TAATA | core promoter element around -30 of transcription start |
| TATA-box | Arabidopsis thaliana | 757 | - | 5 | TATAA | core promoter element around -30 of transcription start |
| TATA-box | Ac | 755 | - | 7 | TATAAAT | core promoter element around -30 of transcription start |
| TATA-box | Lycopersicon esculentum | 725 | + | 5 | TTTTA | core promoter element around -30 of transcription start |
| TATA-box | Arabidopsis thaliana | 756 | - | 6 | TATAAA | core promoter element around -30 of transcription start |
| TATA-box | Daucus carota | 754 | - | 8 | TATAAATA | core promoter element around -30 of transcription start |
| TATA-box | Glycine max | 628 | + | 5 | TAATA | core promoter element around -30 of transcription start |
| TATA-box | Lycopersicon esculentum | 1211 | - | 5 | TTTTA | core promoter element around -30 of transcription start |
| TATA-box | Lycopersicon esculentum | 394 | - | 5 | TTTTA | core promoter element around -30 of transcription start |
| TATA-box | Arabidopsis thaliana | 1495 | - | 4 | TATA | core promoter element around -30 of transcription start |
| TATA-box | Helianthus annuus | 1207 | - | 6 | TATACA | core promoter element around -30 of transcription start |
| TATA-box | Glycine max | 780 | - | 5 | TAATA | core promoter element around -30 of transcription start |
| TATA-box | Arabidopsis thaliana | 1209 | + | 6 | TATAAA | core promoter element around -30 of transcription start |
| TATA-box | Lycopersicon esculentum | 777 | + | 5 | TTTTA | core promoter element around -30 of transcription start |
| TATA-box | Glycine max | 740 | - | 5 | TAATA | core promoter element around -30 of transcription start |
| TATA-box | Pisum sativum | 1339 | - | 8 | TATAAAAT | core promoter element around -30 of transcription start |
| TATA-box | Lycopersicon esculentum | 1305 | + | 5 | TTTTA | core promoter element around -30 of transcription start |
| TATA-box | Glycine max | 988 | - | 5 | TAATA | core promoter element around -30 of transcription start |
| TATA-box | Lycopersicon esculentum | 700 | + | 5 | TTTTA | core promoter element around -30 of transcription start |
| TATA-box | Lycopersicon esculentum | 868 | - | 5 | TTTTA | core promoter element around -30 of transcription start |
| TATA-box | Arabidopsis thaliana | 706 | + | 4 | TATA | core promoter element around -30 of transcription start |
| TATA-box | Lycopersicon esculentum | 659 | - | 5 | TTTTA | core promoter element around -30 of transcription start |
| TATA-box | Glycine max | 1330 | + | 5 | TAATA | core promoter element around -30 of transcription start |
| TATA-box | Arabidopsis thaliana | 101 | - | 5 | TATAA | core promoter element around -30 of transcription start |
| TATA-box | Glycine max | 635 | + | 5 | TAATA | core promoter element around -30 of transcription start |
| TATA-box | Zea mays | 1304 | - | 8 | TTTAAAAA | core promoter element around -30 of transcription start |
| TATA-box | Arabidopsis thaliana | 102 | + | 4 | TATA | core promoter element around -30 of transcription start |
| TATA-box | Arabidopsis thaliana | 1340 | - | 7 | TATAAAA | core promoter element around -30 of transcription start |
| TATA-box | Lycopersicon esculentum | 1272 | + | 5 | TTTTA | core promoter element around -30 of transcription start |
| TATA-box | Ac | 847 | + | 7 | TATAAAT | core promoter element around -30 of transcription start |
| TATA-box | Glycine max | 687 | + | 5 | TAATA | core promoter element around -30 of transcription start |
| TATA-box | Arabidopsis thaliana | 860 | - | 5 | TATAA | core promoter element around -30 of transcription start |
| TATA-box | Lycopersicon esculentum | 649 | + | 5 | TTTTA | core promoter element around -30 of transcription start |
| TATA-box | Glycine max | 1189 | + | 5 | TAATA | core promoter element around -30 of transcription start |
| TATA-box | Lycopersicon esculentum | 803 | + | 5 | TTTTA | core promoter element around -30 of transcription start |
| TATA-box | Arabidopsis thaliana | 1288 | - | 5 | TATAA | core promoter element around -30 of transcription start |
| TATA-box | Arabidopsis thaliana | 7 | + | 4 | TATA | core promoter element around -30 of transcription start |
| TATA-box | Arabidopsis thaliana | 5 | + | 4 | TATA | core promoter element around -30 of transcription start |
| TATA-box | Arabidopsis thaliana | 841 | + | 11 | TATAAATATAAA | core promoter element around -30 of transcription start |
| TATA-box | Lycopersicon esculentum | 672 | - | 5 | TTTTA | core promoter element around -30 of transcription start |
| TATA-box | Lycopersicon esculentum | 877 | - | 5 | TTTTA | core promoter element around -30 of transcription start |
| TATA-box | Glycine max | 715 | - | 5 | TAATA | core promoter element around -30 of transcription start |
| TATA-box | Lycopersicon esculentum | 820 | + | 5 | TTTTA | core promoter element around -30 of transcription start |
| TATA-box | Arabidopsis thaliana | 1287 | - | 6 | TATAAA | core promoter element around -30 of transcription start |
| TATA-box | Lycopersicon esculentum | 1291 | - | 5 | TTTTA | core promoter element around -30 of transcription start |
| TATA-box | Glycine max | 1243 | + | 5 | TAATA | core promoter element around -30 of transcription start |
| TATA-box | Arabidopsis thaliana | 1224 | + | 9 | ccTATAAAaa | core promoter element around -30 of transcription start |
| TATA-box | Lycopersicon esculentum | 1116 | + | 5 | TTTTA | core promoter element around -30 of transcription start |
| TATA-box | Arabidopsis thaliana | 805 | - | 9 | TAAAAATAA | core promoter element around -30 of transcription start |
| TATA-box | Glycine max | 36 | + | 5 | TAATA | core promoter element around -30 of transcription start |
| TATA-box | Arabidopsis thaliana | 1289 | + | 6 | TATAAA | core promoter element around -30 of transcription start |
| TATA-box | Glycine max | 789 | - | 5 | TAATA | core promoter element around -30 of transcription start |
| TATA-box | Arabidopsis thaliana | 861 | + | 6 | TATAAA | core promoter element around -30 of transcription start |
| TATA-box | Lycopersicon esculentum | 809 | + | 5 | TTTTA | core promoter element around -30 of transcription start |
| TATA-box | Avena sativa | 844 | - | 12 | TATATTTATATTT | core promoter element around -30 of transcription start |
| TATA-box | Arabidopsis thaliana | 858 | - | 7 | TATAAAA | core promoter element around -30 of transcription start |
| TATA-box | Brassica oleracea | 846 | + | 6 | ATATAA | core promoter element around -30 of transcription start |
| TATA-box | Arabidopsis thaliana | 854 | - | 9 | TAAAAATAA | core promoter element around -30 of transcription start |

> 2018/04/13 10:10:12  
+ CAACTATATA GCACGAAGGT GCATTACGTC TCTGCTAATA GGACCAACAT ATGTTTCTAC CCGTTGTCAA   
  
  
+ AAGAACATTC TTTTCATACA TAGATCAACC TTATACTTAG TCATAGGTGC CTCGATTTCA GGAGAAGGAG   
  
  
+ CAGGAGGAAA CAGGTATTTA TTTCGGCTCT CTCTCTCTCT CTCTCTCTCT CTCTCTCTCT CTCTCTCTCT   
  
  
+ ACAATAAATC TTTCTCGTCC TGAGGAAACA GGTATTTATT TCGGCTCTCT CTCTCTCTCT CTCTCTCTCT   
  
  
+ CTCTCTCTCT CTCTCTCTAC AATAAATCTT TCTCGTCCCA CTCCGACGTT ACCTGAGCCG TAATTTTTCC   
  
  
+ CAAAACCTGA CTTCTGGTTT ACACTTTGCT CACTACGAAT CCCTAAAACT ACTCTCAGAT ACTCCATTCC   
  
  
+ ATCACGCAGT TCTTTTGTCC ACCGGTACTT TCAGAATACT CTCTCTCTCT GTCTCCGTCT CTTTCTTCTC   
  
  
+ AGAATGACGA CGACAGTATT CTCTCTTTCT CTCTCTCTCT CTCTCTCTCT CTCTCTTTCT CAAACTTACA   
  
  
+ AACTTTCCTA GGGCGATCGT GTTTTTGTGA ATTAATGAAT TAATTTAATT TAATTTAATT TAATAAGTAA   
  
  
+ TAATTAATAG AGTGGTGTTT TTAGTAATTA AAAACAATGT GTAAAAAGTT GGGAAATAAT AGAGTACATT   
  
  
+ TTTAGTATAA GGTTTATTAA ATATTTTTAA ATTGAAAAAT ATTAAAGTAA GACTATTTAT ATTTAACTTT   
  
  
+ ACTACTTTTT ATTAAATTTA TTATTTAGAT TTTTTTATTT TTATCATACT TTTATTTAGT TTTGGTGAAC   
  
  
+ TTTAAATATA AATTTATTTT TATAAAATAA AATAAATAAA AATTCTAGAA TGTTCTCTTT TGTACAATGT   
  
  
+ ACTTCACACC CTCAAACTGA TCAAGGTCTT CATGTCGCCA AACTAACCGA CGGCTTCGCG GTGCACTCCC   
  
  
+ GATGCCCTAT TATCAGTTGT TTTACCTGCC CGGCCGAAAA TAGTGTCGAA GTACCTGCCC GGCACCAGAA   
  
  
+ ACTGGCGTGT TGAACAAGGA AGTTAAATAG AAACTAAAAA GTAGAAGAAA AGGAGAGTTA AAGCTTTTTA   
  
  
+ ATTATTTTCT TTTTCTTTAA GGGTTTATTC AGTAAGTATG AGAGGGAATT TAAGTACTTT AAAGGAAATA   
  
  
+ ATACTGCCGG TAGAGATGTA TAAAAGAAAA ACCCCTAAAA AAATGTTTAA TTTAATATCC ATTAAATGAA   
  
  
+ AAAAACCAAA ATTTTAGATA AAGTTGTTTA TAAAAATTTT TGTTTTTTAA ACTAAAAAAA TTTTTTATTT   
  
  
+ AATACACTAT TTTATACTTG AGCTCCAGTT TTGAGGAAAG ATTGAATTAG TTAAGCCGAT TCGATTCTTC   
  
  
+ TAATCGGGTT GTTTAATCCG AAAGTATTTC AATGATCTCT ATGGAGTATC TAATATCCGA CGCTGAAAAA   
  
  
+ GTTAGAGAGG TTTCAAATTA AACGTATAC  

- GTTGATATAT CGTGCTTCCA CGTAATGCAG AGACGATTAT CCTGGTTGTA TACAAAGATG GGCAACAGTT   
  
  
- TTCTTGTAAG AAAAGTATGT ATCTAGTTGG AATATGAATC AGTATCCACG GAGCTAAAGT CCTCTTCCTC   
  
  
- GTCCTCCTTT GTCCATAAAT AAAGCCGAGA GAGAGAGAGA GAGAGAGAGA GAGAGAGAGA GAGAGAGAGA   
  
  
- TGTTATTTAG AAAGAGCAGG ACTCCTTTGT CCATAAATAA AGCCGAGAGA GAGAGAGAGA GAGAGAGAGA   
  
  
- GAGAGAGAGA GAGAGAGATG TTATTTAGAA AGAGCAGGGT GAGGCTGCAA TGGACTCGGC ATTAAAAAGG   
  
  
- GTTTTGGACT GAAGACCAAA TGTGAAACGA GTGATGCTTA GGGATTTTGA TGAGAGTCTA TGAGGTAAGG   
  
  
- TAGTGCGTCA AGAAAACAGG TGGCCATGAA AGTCTTATGA GAGAGAGAGA CAGAGGCAGA GAAAGAAGAG   
  
  
- TCTTACTGCT GCTGTCATAA GAGAGAAAGA GAGAGAGAGA GAGAGAGAGA GAGAGAAAGA GTTTGAATGT   
  
  
- TTGAAAGGAT CCCGCTAGCA CAAAAACACT TAATTACTTA ATTAAATTAA ATTAAATTAA ATTATTCATT   
  
  
- ATTAATTATC TCACCACAAA AATCATTAAT TTTTGTTACA CATTTTTCAA CCCTTTATTA TCTCATGTAA   
  
  
- AAATCATATT CCAAATAATT TATAAAAATT TAACTTTTTA TAATTTCATT CTGATAAATA TAAATTGAAA   
  
  
- TGATGAAAAA TAATTTAAAT AATAAATCTA AAAAAATAAA AATAGTATGA AAATAAATCA AAACCACTTG   
  
  
- AAATTTATAT TTAAATAAAA ATATTTTATT TTATTTATTT TTAAGATCTT ACAAGAGAAA ACATGTTACA   
  
  
- TGAAGTGTGG GAGTTTGACT AGTTCCAGAA GTACAGCGGT TTGATTGGCT GCCGAAGCGC CACGTGAGGG   
  
  
- CTACGGGATA ATAGTCAACA AAATGGACGG GCCGGCTTTT ATCACAGCTT CATGGACGGG CCGTGGTCTT   
  
  
- TGACCGCACA ACTTGTTCCT TCAATTTATC TTTGATTTTT CATCTTCTTT TCCTCTCAAT TTCGAAAAAT   
  
  
- TAATAAAAGA AAAAGAAATT CCCAAATAAG TCATTCATAC TCTCCCTTAA ATTCATGAAA TTTCCTTTAT   
  
  
- TATGACGGCC ATCTCTACAT ATTTTCTTTT TGGGGATTTT TTTACAAATT AAATTATAGG TAATTTACTT   
  
  
- TTTTTGGTTT TAAAATCTAT TTCAACAAAT ATTTTTAAAA ACAAAAAATT TGATTTTTTT AAAAAATAAA   
  
  
- TTATGTGATA AAATATGAAC TCGAGGTCAA AACTCCTTTC TAACTTAATC AATTCGGCTA AGCTAAGAAG   
  
  
- ATTAGCCCAA CAAATTAGGC TTTCATAAAG TTACTAGAGA TACCTCATAG ATTATAGGCT GCGACTTTTT   
  
  
- CAATCTCTCC AAAGTTTAAT TTGCATATG

+     TATCCAT/C-motif

| Site Name | Organism | Position | Strand | Matrix score. | sequence | function |
| --- | --- | --- | --- | --- | --- | --- |
| TATCCAT/C-motif | Oryza sativa | 1246 | + | 7 | TATCCAT |  |

> 2018/04/13 10:10:12  
+ CAACTATATA GCACGAAGGT GCATTACGTC TCTGCTAATA GGACCAACAT ATGTTTCTAC CCGTTGTCAA   
  
  
+ AAGAACATTC TTTTCATACA TAGATCAACC TTATACTTAG TCATAGGTGC CTCGATTTCA GGAGAAGGAG   
  
  
+ CAGGAGGAAA CAGGTATTTA TTTCGGCTCT CTCTCTCTCT CTCTCTCTCT CTCTCTCTCT CTCTCTCTCT   
  
  
+ ACAATAAATC TTTCTCGTCC TGAGGAAACA GGTATTTATT TCGGCTCTCT CTCTCTCTCT CTCTCTCTCT   
  
  
+ CTCTCTCTCT CTCTCTCTAC AATAAATCTT TCTCGTCCCA CTCCGACGTT ACCTGAGCCG TAATTTTTCC   
  
  
+ CAAAACCTGA CTTCTGGTTT ACACTTTGCT CACTACGAAT CCCTAAAACT ACTCTCAGAT ACTCCATTCC   
  
  
+ ATCACGCAGT TCTTTTGTCC ACCGGTACTT TCAGAATACT CTCTCTCTCT GTCTCCGTCT CTTTCTTCTC   
  
  
+ AGAATGACGA CGACAGTATT CTCTCTTTCT CTCTCTCTCT CTCTCTCTCT CTCTCTTTCT CAAACTTACA   
  
  
+ AACTTTCCTA GGGCGATCGT GTTTTTGTGA ATTAATGAAT TAATTTAATT TAATTTAATT TAATAAGTAA   
  
  
+ TAATTAATAG AGTGGTGTTT TTAGTAATTA AAAACAATGT GTAAAAAGTT GGGAAATAAT AGAGTACATT   
  
  
+ TTTAGTATAA GGTTTATTAA ATATTTTTAA ATTGAAAAAT ATTAAAGTAA GACTATTTAT ATTTAACTTT   
  
  
+ ACTACTTTTT ATTAAATTTA TTATTTAGAT TTTTTTATTT TTATCATACT TTTATTTAGT TTTGGTGAAC   
  
  
+ TTTAAATATA AATTTATTTT TATAAAATAA AATAAATAAA AATTCTAGAA TGTTCTCTTT TGTACAATGT   
  
  
+ ACTTCACACC CTCAAACTGA TCAAGGTCTT CATGTCGCCA AACTAACCGA CGGCTTCGCG GTGCACTCCC   
  
  
+ GATGCCCTAT TATCAGTTGT TTTACCTGCC CGGCCGAAAA TAGTGTCGAA GTACCTGCCC GGCACCAGAA   
  
  
+ ACTGGCGTGT TGAACAAGGA AGTTAAATAG AAACTAAAAA GTAGAAGAAA AGGAGAGTTA AAGCTTTTTA   
  
  
+ ATTATTTTCT TTTTCTTTAA GGGTTTATTC AGTAAGTATG AGAGGGAATT TAAGTACTTT AAAGGAAATA   
  
  
+ ATACTGCCGG TAGAGATGTA TAAAAGAAAA ACCCCTAAAA AAATGTTTAA TTTAATATCC ATTAAATGAA   
  
  
+ AAAAACCAAA ATTTTAGATA AAGTTGTTTA TAAAAATTTT TGTTTTTTAA ACTAAAAAAA TTTTTTATTT   
  
  
+ AATACACTAT TTTATACTTG AGCTCCAGTT TTGAGGAAAG ATTGAATTAG TTAAGCCGAT TCGATTCTTC   
  
  
+ TAATCGGGTT GTTTAATCCG AAAGTATTTC AATGATCTCT ATGGAGTATC TAATATCCGA CGCTGAAAAA   
  
  
+ GTTAGAGAGG TTTCAAATTA AACGTATAC  

- GTTGATATAT CGTGCTTCCA CGTAATGCAG AGACGATTAT CCTGGTTGTA TACAAAGATG GGCAACAGTT   
  
  
- TTCTTGTAAG AAAAGTATGT ATCTAGTTGG AATATGAATC AGTATCCACG GAGCTAAAGT CCTCTTCCTC   
  
  
- GTCCTCCTTT GTCCATAAAT AAAGCCGAGA GAGAGAGAGA GAGAGAGAGA GAGAGAGAGA GAGAGAGAGA   
  
  
- TGTTATTTAG AAAGAGCAGG ACTCCTTTGT CCATAAATAA AGCCGAGAGA GAGAGAGAGA GAGAGAGAGA   
  
  
- GAGAGAGAGA GAGAGAGATG TTATTTAGAA AGAGCAGGGT GAGGCTGCAA TGGACTCGGC ATTAAAAAGG   
  
  
- GTTTTGGACT GAAGACCAAA TGTGAAACGA GTGATGCTTA GGGATTTTGA TGAGAGTCTA TGAGGTAAGG   
  
  
- TAGTGCGTCA AGAAAACAGG TGGCCATGAA AGTCTTATGA GAGAGAGAGA CAGAGGCAGA GAAAGAAGAG   
  
  
- TCTTACTGCT GCTGTCATAA GAGAGAAAGA GAGAGAGAGA GAGAGAGAGA GAGAGAAAGA GTTTGAATGT   
  
  
- TTGAAAGGAT CCCGCTAGCA CAAAAACACT TAATTACTTA ATTAAATTAA ATTAAATTAA ATTATTCATT   
  
  
- ATTAATTATC TCACCACAAA AATCATTAAT TTTTGTTACA CATTTTTCAA CCCTTTATTA TCTCATGTAA   
  
  
- AAATCATATT CCAAATAATT TATAAAAATT TAACTTTTTA TAATTTCATT CTGATAAATA TAAATTGAAA   
  
  
- TGATGAAAAA TAATTTAAAT AATAAATCTA AAAAAATAAA AATAGTATGA AAATAAATCA AAACCACTTG   
  
  
- AAATTTATAT TTAAATAAAA ATATTTTATT TTATTTATTT TTAAGATCTT ACAAGAGAAA ACATGTTACA   
  
  
- TGAAGTGTGG GAGTTTGACT AGTTCCAGAA GTACAGCGGT TTGATTGGCT GCCGAAGCGC CACGTGAGGG   
  
  
- CTACGGGATA ATAGTCAACA AAATGGACGG GCCGGCTTTT ATCACAGCTT CATGGACGGG CCGTGGTCTT   
  
  
- TGACCGCACA ACTTGTTCCT TCAATTTATC TTTGATTTTT CATCTTCTTT TCCTCTCAAT TTCGAAAAAT   
  
  
- TAATAAAAGA AAAAGAAATT CCCAAATAAG TCATTCATAC TCTCCCTTAA ATTCATGAAA TTTCCTTTAT   
  
  
- TATGACGGCC ATCTCTACAT ATTTTCTTTT TGGGGATTTT TTTACAAATT AAATTATAGG TAATTTACTT   
  
  
- TTTTTGGTTT TAAAATCTAT TTCAACAAAT ATTTTTAAAA ACAAAAAATT TGATTTTTTT AAAAAATAAA   
  
  
- TTATGTGATA AAATATGAAC TCGAGGTCAA AACTCCTTTC TAACTTAATC AATTCGGCTA AGCTAAGAAG   
  
  
- ATTAGCCCAA CAAATTAGGC TTTCATAAAG TTACTAGAGA TACCTCATAG ATTATAGGCT GCGACTTTTT   
  
  
- CAATCTCTCC AAAGTTTAAT TTGCATATG

+     TCA-element

| Site Name | Organism | Position | Strand | Matrix score. | sequence | function |
| --- | --- | --- | --- | --- | --- | --- |
| TCA-element | Brassica oleracea | 1095 | + | 9 | CAGAAAAGGA | cis-acting element involved in salicylic acid responsiveness |
| TCA-element | Nicotiana tabacum | 673 | - | 9 | CCATCTTTTT | cis-acting element involved in salicylic acid responsiveness |

> 2018/04/13 10:10:12  
+ CAACTATATA GCACGAAGGT GCATTACGTC TCTGCTAATA GGACCAACAT ATGTTTCTAC CCGTTGTCAA   
  
  
+ AAGAACATTC TTTTCATACA TAGATCAACC TTATACTTAG TCATAGGTGC CTCGATTTCA GGAGAAGGAG   
  
  
+ CAGGAGGAAA CAGGTATTTA TTTCGGCTCT CTCTCTCTCT CTCTCTCTCT CTCTCTCTCT CTCTCTCTCT   
  
  
+ ACAATAAATC TTTCTCGTCC TGAGGAAACA GGTATTTATT TCGGCTCTCT CTCTCTCTCT CTCTCTCTCT   
  
  
+ CTCTCTCTCT CTCTCTCTAC AATAAATCTT TCTCGTCCCA CTCCGACGTT ACCTGAGCCG TAATTTTTCC   
  
  
+ CAAAACCTGA CTTCTGGTTT ACACTTTGCT CACTACGAAT CCCTAAAACT ACTCTCAGAT ACTCCATTCC   
  
  
+ ATCACGCAGT TCTTTTGTCC ACCGGTACTT TCAGAATACT CTCTCTCTCT GTCTCCGTCT CTTTCTTCTC   
  
  
+ AGAATGACGA CGACAGTATT CTCTCTTTCT CTCTCTCTCT CTCTCTCTCT CTCTCTTTCT CAAACTTACA   
  
  
+ AACTTTCCTA GGGCGATCGT GTTTTTGTGA ATTAATGAAT TAATTTAATT TAATTTAATT TAATAAGTAA   
  
  
+ TAATTAATAG AGTGGTGTTT TTAGTAATTA AAAACAATGT GTAAAAAGTT GGGAAATAAT AGAGTACATT   
  
  
+ TTTAGTATAA GGTTTATTAA ATATTTTTAA ATTGAAAAAT ATTAAAGTAA GACTATTTAT ATTTAACTTT   
  
  
+ ACTACTTTTT ATTAAATTTA TTATTTAGAT TTTTTTATTT TTATCATACT TTTATTTAGT TTTGGTGAAC   
  
  
+ TTTAAATATA AATTTATTTT TATAAAATAA AATAAATAAA AATTCTAGAA TGTTCTCTTT TGTACAATGT   
  
  
+ ACTTCACACC CTCAAACTGA TCAAGGTCTT CATGTCGCCA AACTAACCGA CGGCTTCGCG GTGCACTCCC   
  
  
+ GATGCCCTAT TATCAGTTGT TTTACCTGCC CGGCCGAAAA TAGTGTCGAA GTACCTGCCC GGCACCAGAA   
  
  
+ ACTGGCGTGT TGAACAAGGA AGTTAAATAG AAACTAAAAA GTAGAAGAAA AGGAGAGTTA AAGCTTTTTA   
  
  
+ ATTATTTTCT TTTTCTTTAA GGGTTTATTC AGTAAGTATG AGAGGGAATT TAAGTACTTT AAAGGAAATA   
  
  
+ ATACTGCCGG TAGAGATGTA TAAAAGAAAA ACCCCTAAAA AAATGTTTAA TTTAATATCC ATTAAATGAA   
  
  
+ AAAAACCAAA ATTTTAGATA AAGTTGTTTA TAAAAATTTT TGTTTTTTAA ACTAAAAAAA TTTTTTATTT   
  
  
+ AATACACTAT TTTATACTTG AGCTCCAGTT TTGAGGAAAG ATTGAATTAG TTAAGCCGAT TCGATTCTTC   
  
  
+ TAATCGGGTT GTTTAATCCG AAAGTATTTC AATGATCTCT ATGGAGTATC TAATATCCGA CGCTGAAAAA   
  
  
+ GTTAGAGAGG TTTCAAATTA AACGTATAC  

- GTTGATATAT CGTGCTTCCA CGTAATGCAG AGACGATTAT CCTGGTTGTA TACAAAGATG GGCAACAGTT   
  
  
- TTCTTGTAAG AAAAGTATGT ATCTAGTTGG AATATGAATC AGTATCCACG GAGCTAAAGT CCTCTTCCTC   
  
  
- GTCCTCCTTT GTCCATAAAT AAAGCCGAGA GAGAGAGAGA GAGAGAGAGA GAGAGAGAGA GAGAGAGAGA   
  
  
- TGTTATTTAG AAAGAGCAGG ACTCCTTTGT CCATAAATAA AGCCGAGAGA GAGAGAGAGA GAGAGAGAGA   
  
  
- GAGAGAGAGA GAGAGAGATG TTATTTAGAA AGAGCAGGGT GAGGCTGCAA TGGACTCGGC ATTAAAAAGG   
  
  
- GTTTTGGACT GAAGACCAAA TGTGAAACGA GTGATGCTTA GGGATTTTGA TGAGAGTCTA TGAGGTAAGG   
  
  
- TAGTGCGTCA AGAAAACAGG TGGCCATGAA AGTCTTATGA GAGAGAGAGA CAGAGGCAGA GAAAGAAGAG   
  
  
- TCTTACTGCT GCTGTCATAA GAGAGAAAGA GAGAGAGAGA GAGAGAGAGA GAGAGAAAGA GTTTGAATGT   
  
  
- TTGAAAGGAT CCCGCTAGCA CAAAAACACT TAATTACTTA ATTAAATTAA ATTAAATTAA ATTATTCATT   
  
  
- ATTAATTATC TCACCACAAA AATCATTAAT TTTTGTTACA CATTTTTCAA CCCTTTATTA TCTCATGTAA   
  
  
- AAATCATATT CCAAATAATT TATAAAAATT TAACTTTTTA TAATTTCATT CTGATAAATA TAAATTGAAA   
  
  
- TGATGAAAAA TAATTTAAAT AATAAATCTA AAAAAATAAA AATAGTATGA AAATAAATCA AAACCACTTG   
  
  
- AAATTTATAT TTAAATAAAA ATATTTTATT TTATTTATTT TTAAGATCTT ACAAGAGAAA ACATGTTACA   
  
  
- TGAAGTGTGG GAGTTTGACT AGTTCCAGAA GTACAGCGGT TTGATTGGCT GCCGAAGCGC CACGTGAGGG   
  
  
- CTACGGGATA ATAGTCAACA AAATGGACGG GCCGGCTTTT ATCACAGCTT CATGGACGGG CCGTGGTCTT   
  
  
- TGACCGCACA ACTTGTTCCT TCAATTTATC TTTGATTTTT CATCTTCTTT TCCTCTCAAT TTCGAAAAAT   
  
  
- TAATAAAAGA AAAAGAAATT CCCAAATAAG TCATTCATAC TCTCCCTTAA ATTCATGAAA TTTCCTTTAT   
  
  
- TATGACGGCC ATCTCTACAT ATTTTCTTTT TGGGGATTTT TTTACAAATT AAATTATAGG TAATTTACTT   
  
  
- TTTTTGGTTT TAAAATCTAT TTCAACAAAT ATTTTTAAAA ACAAAAAATT TGATTTTTTT AAAAAATAAA   
  
  
- TTATGTGATA AAATATGAAC TCGAGGTCAA AACTCCTTTC TAACTTAATC AATTCGGCTA AGCTAAGAAG   
  
  
- ATTAGCCCAA CAAATTAGGC TTTCATAAAG TTACTAGAGA TACCTCATAG ATTATAGGCT GCGACTTTTT   
  
  
- CAATCTCTCC AAAGTTTAAT TTGCATATG

+     TCT-motif

| Site Name | Organism | Position | Strand | Matrix score. | sequence | function |
| --- | --- | --- | --- | --- | --- | --- |
| TCT-motif | Arabidopsis thaliana | 747 | - | 6 | TCTTAC | part of a light responsive element |

> 2018/04/13 10:10:12  
+ CAACTATATA GCACGAAGGT GCATTACGTC TCTGCTAATA GGACCAACAT ATGTTTCTAC CCGTTGTCAA   
  
  
+ AAGAACATTC TTTTCATACA TAGATCAACC TTATACTTAG TCATAGGTGC CTCGATTTCA GGAGAAGGAG   
  
  
+ CAGGAGGAAA CAGGTATTTA TTTCGGCTCT CTCTCTCTCT CTCTCTCTCT CTCTCTCTCT CTCTCTCTCT   
  
  
+ ACAATAAATC TTTCTCGTCC TGAGGAAACA GGTATTTATT TCGGCTCTCT CTCTCTCTCT CTCTCTCTCT   
  
  
+ CTCTCTCTCT CTCTCTCTAC AATAAATCTT TCTCGTCCCA CTCCGACGTT ACCTGAGCCG TAATTTTTCC   
  
  
+ CAAAACCTGA CTTCTGGTTT ACACTTTGCT CACTACGAAT CCCTAAAACT ACTCTCAGAT ACTCCATTCC   
  
  
+ ATCACGCAGT TCTTTTGTCC ACCGGTACTT TCAGAATACT CTCTCTCTCT GTCTCCGTCT CTTTCTTCTC   
  
  
+ AGAATGACGA CGACAGTATT CTCTCTTTCT CTCTCTCTCT CTCTCTCTCT CTCTCTTTCT CAAACTTACA   
  
  
+ AACTTTCCTA GGGCGATCGT GTTTTTGTGA ATTAATGAAT TAATTTAATT TAATTTAATT TAATAAGTAA   
  
  
+ TAATTAATAG AGTGGTGTTT TTAGTAATTA AAAACAATGT GTAAAAAGTT GGGAAATAAT AGAGTACATT   
  
  
+ TTTAGTATAA GGTTTATTAA ATATTTTTAA ATTGAAAAAT ATTAAAGTAA GACTATTTAT ATTTAACTTT   
  
  
+ ACTACTTTTT ATTAAATTTA TTATTTAGAT TTTTTTATTT TTATCATACT TTTATTTAGT TTTGGTGAAC   
  
  
+ TTTAAATATA AATTTATTTT TATAAAATAA AATAAATAAA AATTCTAGAA TGTTCTCTTT TGTACAATGT   
  
  
+ ACTTCACACC CTCAAACTGA TCAAGGTCTT CATGTCGCCA AACTAACCGA CGGCTTCGCG GTGCACTCCC   
  
  
+ GATGCCCTAT TATCAGTTGT TTTACCTGCC CGGCCGAAAA TAGTGTCGAA GTACCTGCCC GGCACCAGAA   
  
  
+ ACTGGCGTGT TGAACAAGGA AGTTAAATAG AAACTAAAAA GTAGAAGAAA AGGAGAGTTA AAGCTTTTTA   
  
  
+ ATTATTTTCT TTTTCTTTAA GGGTTTATTC AGTAAGTATG AGAGGGAATT TAAGTACTTT AAAGGAAATA   
  
  
+ ATACTGCCGG TAGAGATGTA TAAAAGAAAA ACCCCTAAAA AAATGTTTAA TTTAATATCC ATTAAATGAA   
  
  
+ AAAAACCAAA ATTTTAGATA AAGTTGTTTA TAAAAATTTT TGTTTTTTAA ACTAAAAAAA TTTTTTATTT   
  
  
+ AATACACTAT TTTATACTTG AGCTCCAGTT TTGAGGAAAG ATTGAATTAG TTAAGCCGAT TCGATTCTTC   
  
  
+ TAATCGGGTT GTTTAATCCG AAAGTATTTC AATGATCTCT ATGGAGTATC TAATATCCGA CGCTGAAAAA   
  
  
+ GTTAGAGAGG TTTCAAATTA AACGTATAC  

- GTTGATATAT CGTGCTTCCA CGTAATGCAG AGACGATTAT CCTGGTTGTA TACAAAGATG GGCAACAGTT   
  
  
- TTCTTGTAAG AAAAGTATGT ATCTAGTTGG AATATGAATC AGTATCCACG GAGCTAAAGT CCTCTTCCTC   
  
  
- GTCCTCCTTT GTCCATAAAT AAAGCCGAGA GAGAGAGAGA GAGAGAGAGA GAGAGAGAGA GAGAGAGAGA   
  
  
- TGTTATTTAG AAAGAGCAGG ACTCCTTTGT CCATAAATAA AGCCGAGAGA GAGAGAGAGA GAGAGAGAGA   
  
  
- GAGAGAGAGA GAGAGAGATG TTATTTAGAA AGAGCAGGGT GAGGCTGCAA TGGACTCGGC ATTAAAAAGG   
  
  
- GTTTTGGACT GAAGACCAAA TGTGAAACGA GTGATGCTTA GGGATTTTGA TGAGAGTCTA TGAGGTAAGG   
  
  
- TAGTGCGTCA AGAAAACAGG TGGCCATGAA AGTCTTATGA GAGAGAGAGA CAGAGGCAGA GAAAGAAGAG   
  
  
- TCTTACTGCT GCTGTCATAA GAGAGAAAGA GAGAGAGAGA GAGAGAGAGA GAGAGAAAGA GTTTGAATGT   
  
  
- TTGAAAGGAT CCCGCTAGCA CAAAAACACT TAATTACTTA ATTAAATTAA ATTAAATTAA ATTATTCATT   
  
  
- ATTAATTATC TCACCACAAA AATCATTAAT TTTTGTTACA CATTTTTCAA CCCTTTATTA TCTCATGTAA   
  
  
- AAATCATATT CCAAATAATT TATAAAAATT TAACTTTTTA TAATTTCATT CTGATAAATA TAAATTGAAA   
  
  
- TGATGAAAAA TAATTTAAAT AATAAATCTA AAAAAATAAA AATAGTATGA AAATAAATCA AAACCACTTG   
  
  
- AAATTTATAT TTAAATAAAA ATATTTTATT TTATTTATTT TTAAGATCTT ACAAGAGAAA ACATGTTACA   
  
  
- TGAAGTGTGG GAGTTTGACT AGTTCCAGAA GTACAGCGGT TTGATTGGCT GCCGAAGCGC CACGTGAGGG   
  
  
- CTACGGGATA ATAGTCAACA AAATGGACGG GCCGGCTTTT ATCACAGCTT CATGGACGGG CCGTGGTCTT   
  
  
- TGACCGCACA ACTTGTTCCT TCAATTTATC TTTGATTTTT CATCTTCTTT TCCTCTCAAT TTCGAAAAAT   
  
  
- TAATAAAAGA AAAAGAAATT CCCAAATAAG TCATTCATAC TCTCCCTTAA ATTCATGAAA TTTCCTTTAT   
  
  
- TATGACGGCC ATCTCTACAT ATTTTCTTTT TGGGGATTTT TTTACAAATT AAATTATAGG TAATTTACTT   
  
  
- TTTTTGGTTT TAAAATCTAT TTCAACAAAT ATTTTTAAAA ACAAAAAATT TGATTTTTTT AAAAAATAAA   
  
  
- TTATGTGATA AAATATGAAC TCGAGGTCAA AACTCCTTTC TAACTTAATC AATTCGGCTA AGCTAAGAAG   
  
  
- ATTAGCCCAA CAAATTAGGC TTTCATAAAG TTACTAGAGA TACCTCATAG ATTATAGGCT GCGACTTTTT   
  
  
- CAATCTCTCC AAAGTTTAAT TTGCATATG

+     TGACG-motif

| Site Name | Organism | Position | Strand | Matrix score. | sequence | function |
| --- | --- | --- | --- | --- | --- | --- |
| TGACG-motif | Hordeum vulgare | 495 | + | 5 | TGACG | cis-acting regulatory element involved in the MeJA-responsiveness |

> 2018/04/13 10:10:12  
+ CAACTATATA GCACGAAGGT GCATTACGTC TCTGCTAATA GGACCAACAT ATGTTTCTAC CCGTTGTCAA   
  
  
+ AAGAACATTC TTTTCATACA TAGATCAACC TTATACTTAG TCATAGGTGC CTCGATTTCA GGAGAAGGAG   
  
  
+ CAGGAGGAAA CAGGTATTTA TTTCGGCTCT CTCTCTCTCT CTCTCTCTCT CTCTCTCTCT CTCTCTCTCT   
  
  
+ ACAATAAATC TTTCTCGTCC TGAGGAAACA GGTATTTATT TCGGCTCTCT CTCTCTCTCT CTCTCTCTCT   
  
  
+ CTCTCTCTCT CTCTCTCTAC AATAAATCTT TCTCGTCCCA CTCCGACGTT ACCTGAGCCG TAATTTTTCC   
  
  
+ CAAAACCTGA CTTCTGGTTT ACACTTTGCT CACTACGAAT CCCTAAAACT ACTCTCAGAT ACTCCATTCC   
  
  
+ ATCACGCAGT TCTTTTGTCC ACCGGTACTT TCAGAATACT CTCTCTCTCT GTCTCCGTCT CTTTCTTCTC   
  
  
+ AGAATGACGA CGACAGTATT CTCTCTTTCT CTCTCTCTCT CTCTCTCTCT CTCTCTTTCT CAAACTTACA   
  
  
+ AACTTTCCTA GGGCGATCGT GTTTTTGTGA ATTAATGAAT TAATTTAATT TAATTTAATT TAATAAGTAA   
  
  
+ TAATTAATAG AGTGGTGTTT TTAGTAATTA AAAACAATGT GTAAAAAGTT GGGAAATAAT AGAGTACATT   
  
  
+ TTTAGTATAA GGTTTATTAA ATATTTTTAA ATTGAAAAAT ATTAAAGTAA GACTATTTAT ATTTAACTTT   
  
  
+ ACTACTTTTT ATTAAATTTA TTATTTAGAT TTTTTTATTT TTATCATACT TTTATTTAGT TTTGGTGAAC   
  
  
+ TTTAAATATA AATTTATTTT TATAAAATAA AATAAATAAA AATTCTAGAA TGTTCTCTTT TGTACAATGT   
  
  
+ ACTTCACACC CTCAAACTGA TCAAGGTCTT CATGTCGCCA AACTAACCGA CGGCTTCGCG GTGCACTCCC   
  
  
+ GATGCCCTAT TATCAGTTGT TTTACCTGCC CGGCCGAAAA TAGTGTCGAA GTACCTGCCC GGCACCAGAA   
  
  
+ ACTGGCGTGT TGAACAAGGA AGTTAAATAG AAACTAAAAA GTAGAAGAAA AGGAGAGTTA AAGCTTTTTA   
  
  
+ ATTATTTTCT TTTTCTTTAA GGGTTTATTC AGTAAGTATG AGAGGGAATT TAAGTACTTT AAAGGAAATA   
  
  
+ ATACTGCCGG TAGAGATGTA TAAAAGAAAA ACCCCTAAAA AAATGTTTAA TTTAATATCC ATTAAATGAA   
  
  
+ AAAAACCAAA ATTTTAGATA AAGTTGTTTA TAAAAATTTT TGTTTTTTAA ACTAAAAAAA TTTTTTATTT   
  
  
+ AATACACTAT TTTATACTTG AGCTCCAGTT TTGAGGAAAG ATTGAATTAG TTAAGCCGAT TCGATTCTTC   
  
  
+ TAATCGGGTT GTTTAATCCG AAAGTATTTC AATGATCTCT ATGGAGTATC TAATATCCGA CGCTGAAAAA   
  
  
+ GTTAGAGAGG TTTCAAATTA AACGTATAC  

- GTTGATATAT CGTGCTTCCA CGTAATGCAG AGACGATTAT CCTGGTTGTA TACAAAGATG GGCAACAGTT   
  
  
- TTCTTGTAAG AAAAGTATGT ATCTAGTTGG AATATGAATC AGTATCCACG GAGCTAAAGT CCTCTTCCTC   
  
  
- GTCCTCCTTT GTCCATAAAT AAAGCCGAGA GAGAGAGAGA GAGAGAGAGA GAGAGAGAGA GAGAGAGAGA   
  
  
- TGTTATTTAG AAAGAGCAGG ACTCCTTTGT CCATAAATAA AGCCGAGAGA GAGAGAGAGA GAGAGAGAGA   
  
  
- GAGAGAGAGA GAGAGAGATG TTATTTAGAA AGAGCAGGGT GAGGCTGCAA TGGACTCGGC ATTAAAAAGG   
  
  
- GTTTTGGACT GAAGACCAAA TGTGAAACGA GTGATGCTTA GGGATTTTGA TGAGAGTCTA TGAGGTAAGG   
  
  
- TAGTGCGTCA AGAAAACAGG TGGCCATGAA AGTCTTATGA GAGAGAGAGA CAGAGGCAGA GAAAGAAGAG   
  
  
- TCTTACTGCT GCTGTCATAA GAGAGAAAGA GAGAGAGAGA GAGAGAGAGA GAGAGAAAGA GTTTGAATGT   
  
  
- TTGAAAGGAT CCCGCTAGCA CAAAAACACT TAATTACTTA ATTAAATTAA ATTAAATTAA ATTATTCATT   
  
  
- ATTAATTATC TCACCACAAA AATCATTAAT TTTTGTTACA CATTTTTCAA CCCTTTATTA TCTCATGTAA   
  
  
- AAATCATATT CCAAATAATT TATAAAAATT TAACTTTTTA TAATTTCATT CTGATAAATA TAAATTGAAA   
  
  
- TGATGAAAAA TAATTTAAAT AATAAATCTA AAAAAATAAA AATAGTATGA AAATAAATCA AAACCACTTG   
  
  
- AAATTTATAT TTAAATAAAA ATATTTTATT TTATTTATTT TTAAGATCTT ACAAGAGAAA ACATGTTACA   
  
  
- TGAAGTGTGG GAGTTTGACT AGTTCCAGAA GTACAGCGGT TTGATTGGCT GCCGAAGCGC CACGTGAGGG   
  
  
- CTACGGGATA ATAGTCAACA AAATGGACGG GCCGGCTTTT ATCACAGCTT CATGGACGGG CCGTGGTCTT   
  
  
- TGACCGCACA ACTTGTTCCT TCAATTTATC TTTGATTTTT CATCTTCTTT TCCTCTCAAT TTCGAAAAAT   
  
  
- TAATAAAAGA AAAAGAAATT CCCAAATAAG TCATTCATAC TCTCCCTTAA ATTCATGAAA TTTCCTTTAT   
  
  
- TATGACGGCC ATCTCTACAT ATTTTCTTTT TGGGGATTTT TTTACAAATT AAATTATAGG TAATTTACTT   
  
  
- TTTTTGGTTT TAAAATCTAT TTCAACAAAT ATTTTTAAAA ACAAAAAATT TGATTTTTTT AAAAAATAAA   
  
  
- TTATGTGATA AAATATGAAC TCGAGGTCAA AACTCCTTTC TAACTTAATC AATTCGGCTA AGCTAAGAAG   
  
  
- ATTAGCCCAA CAAATTAGGC TTTCATAAAG TTACTAGAGA TACCTCATAG ATTATAGGCT GCGACTTTTT   
  
  
- CAATCTCTCC AAAGTTTAAT TTGCATATG

+     Unnamed\_\_4

| Site Name | Organism | Position | Strand | Matrix score. | sequence | function |
| --- | --- | --- | --- | --- | --- | --- |
| Unnamed\_\_4 | Petroselinum hortense | 1443 | - | 4 | CTCC |  |
| Unnamed\_\_4 | Petroselinum hortense | 473 | + | 4 | CTCC |  |
| Unnamed\_\_4 | Petroselinum hortense | 321 | + | 4 | CTCC |  |
| Unnamed\_\_4 | Petroselinum hortense | 976 | + | 4 | CTCC |  |
| Unnamed\_\_4 | Petroselinum hortense | 1102 | - | 4 | CTCC |  |
| Unnamed\_\_4 | Petroselinum hortense | 412 | + | 4 | CTCC |  |
| Unnamed\_\_4 | Petroselinum hortense | 143 | - | 4 | CTCC |  |
| Unnamed\_\_4 | Petroselinum hortense | 131 | - | 4 | CTCC |  |
| Unnamed\_\_4 | Petroselinum hortense | 1353 | + | 4 | CTCC |  |
| Unnamed\_\_4 | Petroselinum hortense | 137 | - | 4 | CTCC |  |

> 2018/04/13 10:10:12  
+ CAACTATATA GCACGAAGGT GCATTACGTC TCTGCTAATA GGACCAACAT ATGTTTCTAC CCGTTGTCAA   
  
  
+ AAGAACATTC TTTTCATACA TAGATCAACC TTATACTTAG TCATAGGTGC CTCGATTTCA GGAGAAGGAG   
  
  
+ CAGGAGGAAA CAGGTATTTA TTTCGGCTCT CTCTCTCTCT CTCTCTCTCT CTCTCTCTCT CTCTCTCTCT   
  
  
+ ACAATAAATC TTTCTCGTCC TGAGGAAACA GGTATTTATT TCGGCTCTCT CTCTCTCTCT CTCTCTCTCT   
  
  
+ CTCTCTCTCT CTCTCTCTAC AATAAATCTT TCTCGTCCCA CTCCGACGTT ACCTGAGCCG TAATTTTTCC   
  
  
+ CAAAACCTGA CTTCTGGTTT ACACTTTGCT CACTACGAAT CCCTAAAACT ACTCTCAGAT ACTCCATTCC   
  
  
+ ATCACGCAGT TCTTTTGTCC ACCGGTACTT TCAGAATACT CTCTCTCTCT GTCTCCGTCT CTTTCTTCTC   
  
  
+ AGAATGACGA CGACAGTATT CTCTCTTTCT CTCTCTCTCT CTCTCTCTCT CTCTCTTTCT CAAACTTACA   
  
  
+ AACTTTCCTA GGGCGATCGT GTTTTTGTGA ATTAATGAAT TAATTTAATT TAATTTAATT TAATAAGTAA   
  
  
+ TAATTAATAG AGTGGTGTTT TTAGTAATTA AAAACAATGT GTAAAAAGTT GGGAAATAAT AGAGTACATT   
  
  
+ TTTAGTATAA GGTTTATTAA ATATTTTTAA ATTGAAAAAT ATTAAAGTAA GACTATTTAT ATTTAACTTT   
  
  
+ ACTACTTTTT ATTAAATTTA TTATTTAGAT TTTTTTATTT TTATCATACT TTTATTTAGT TTTGGTGAAC   
  
  
+ TTTAAATATA AATTTATTTT TATAAAATAA AATAAATAAA AATTCTAGAA TGTTCTCTTT TGTACAATGT   
  
  
+ ACTTCACACC CTCAAACTGA TCAAGGTCTT CATGTCGCCA AACTAACCGA CGGCTTCGCG GTGCACTCCC   
  
  
+ GATGCCCTAT TATCAGTTGT TTTACCTGCC CGGCCGAAAA TAGTGTCGAA GTACCTGCCC GGCACCAGAA   
  
  
+ ACTGGCGTGT TGAACAAGGA AGTTAAATAG AAACTAAAAA GTAGAAGAAA AGGAGAGTTA AAGCTTTTTA   
  
  
+ ATTATTTTCT TTTTCTTTAA GGGTTTATTC AGTAAGTATG AGAGGGAATT TAAGTACTTT AAAGGAAATA   
  
  
+ ATACTGCCGG TAGAGATGTA TAAAAGAAAA ACCCCTAAAA AAATGTTTAA TTTAATATCC ATTAAATGAA   
  
  
+ AAAAACCAAA ATTTTAGATA AAGTTGTTTA TAAAAATTTT TGTTTTTTAA ACTAAAAAAA TTTTTTATTT   
  
  
+ AATACACTAT TTTATACTTG AGCTCCAGTT TTGAGGAAAG ATTGAATTAG TTAAGCCGAT TCGATTCTTC   
  
  
+ TAATCGGGTT GTTTAATCCG AAAGTATTTC AATGATCTCT ATGGAGTATC TAATATCCGA CGCTGAAAAA   
  
  
+ GTTAGAGAGG TTTCAAATTA AACGTATAC  

- GTTGATATAT CGTGCTTCCA CGTAATGCAG AGACGATTAT CCTGGTTGTA TACAAAGATG GGCAACAGTT   
  
  
- TTCTTGTAAG AAAAGTATGT ATCTAGTTGG AATATGAATC AGTATCCACG GAGCTAAAGT CCTCTTCCTC   
  
  
- GTCCTCCTTT GTCCATAAAT AAAGCCGAGA GAGAGAGAGA GAGAGAGAGA GAGAGAGAGA GAGAGAGAGA   
  
  
- TGTTATTTAG AAAGAGCAGG ACTCCTTTGT CCATAAATAA AGCCGAGAGA GAGAGAGAGA GAGAGAGAGA   
  
  
- GAGAGAGAGA GAGAGAGATG TTATTTAGAA AGAGCAGGGT GAGGCTGCAA TGGACTCGGC ATTAAAAAGG   
  
  
- GTTTTGGACT GAAGACCAAA TGTGAAACGA GTGATGCTTA GGGATTTTGA TGAGAGTCTA TGAGGTAAGG   
  
  
- TAGTGCGTCA AGAAAACAGG TGGCCATGAA AGTCTTATGA GAGAGAGAGA CAGAGGCAGA GAAAGAAGAG   
  
  
- TCTTACTGCT GCTGTCATAA GAGAGAAAGA GAGAGAGAGA GAGAGAGAGA GAGAGAAAGA GTTTGAATGT   
  
  
- TTGAAAGGAT CCCGCTAGCA CAAAAACACT TAATTACTTA ATTAAATTAA ATTAAATTAA ATTATTCATT   
  
  
- ATTAATTATC TCACCACAAA AATCATTAAT TTTTGTTACA CATTTTTCAA CCCTTTATTA TCTCATGTAA   
  
  
- AAATCATATT CCAAATAATT TATAAAAATT TAACTTTTTA TAATTTCATT CTGATAAATA TAAATTGAAA   
  
  
- TGATGAAAAA TAATTTAAAT AATAAATCTA AAAAAATAAA AATAGTATGA AAATAAATCA AAACCACTTG   
  
  
- AAATTTATAT TTAAATAAAA ATATTTTATT TTATTTATTT TTAAGATCTT ACAAGAGAAA ACATGTTACA   
  
  
- TGAAGTGTGG GAGTTTGACT AGTTCCAGAA GTACAGCGGT TTGATTGGCT GCCGAAGCGC CACGTGAGGG   
  
  
- CTACGGGATA ATAGTCAACA AAATGGACGG GCCGGCTTTT ATCACAGCTT CATGGACGGG CCGTGGTCTT   
  
  
- TGACCGCACA ACTTGTTCCT TCAATTTATC TTTGATTTTT CATCTTCTTT TCCTCTCAAT TTCGAAAAAT   
  
  
- TAATAAAAGA AAAAGAAATT CCCAAATAAG TCATTCATAC TCTCCCTTAA ATTCATGAAA TTTCCTTTAT   
  
  
- TATGACGGCC ATCTCTACAT ATTTTCTTTT TGGGGATTTT TTTACAAATT AAATTATAGG TAATTTACTT   
  
  
- TTTTTGGTTT TAAAATCTAT TTCAACAAAT ATTTTTAAAA ACAAAAAATT TGATTTTTTT AAAAAATAAA   
  
  
- TTATGTGATA AAATATGAAC TCGAGGTCAA AACTCCTTTC TAACTTAATC AATTCGGCTA AGCTAAGAAG   
  
  
- ATTAGCCCAA CAAATTAGGC TTTCATAAAG TTACTAGAGA TACCTCATAG ATTATAGGCT GCGACTTTTT   
  
  
- CAATCTCTCC AAAGTTTAAT TTGCATATG

+     WUN-motif

| Site Name | Organism | Position | Strand | Matrix score. | sequence | function |
| --- | --- | --- | --- | --- | --- | --- |
| WUN-motif | Brassica oleracea | 380 | + | 9 | TCATTACGAA | wound-responsive element |

> 2018/04/13 10:10:12  
+ CAACTATATA GCACGAAGGT GCATTACGTC TCTGCTAATA GGACCAACAT ATGTTTCTAC CCGTTGTCAA   
  
  
+ AAGAACATTC TTTTCATACA TAGATCAACC TTATACTTAG TCATAGGTGC CTCGATTTCA GGAGAAGGAG   
  
  
+ CAGGAGGAAA CAGGTATTTA TTTCGGCTCT CTCTCTCTCT CTCTCTCTCT CTCTCTCTCT CTCTCTCTCT   
  
  
+ ACAATAAATC TTTCTCGTCC TGAGGAAACA GGTATTTATT TCGGCTCTCT CTCTCTCTCT CTCTCTCTCT   
  
  
+ CTCTCTCTCT CTCTCTCTAC AATAAATCTT TCTCGTCCCA CTCCGACGTT ACCTGAGCCG TAATTTTTCC   
  
  
+ CAAAACCTGA CTTCTGGTTT ACACTTTGCT CACTACGAAT CCCTAAAACT ACTCTCAGAT ACTCCATTCC   
  
  
+ ATCACGCAGT TCTTTTGTCC ACCGGTACTT TCAGAATACT CTCTCTCTCT GTCTCCGTCT CTTTCTTCTC   
  
  
+ AGAATGACGA CGACAGTATT CTCTCTTTCT CTCTCTCTCT CTCTCTCTCT CTCTCTTTCT CAAACTTACA   
  
  
+ AACTTTCCTA GGGCGATCGT GTTTTTGTGA ATTAATGAAT TAATTTAATT TAATTTAATT TAATAAGTAA   
  
  
+ TAATTAATAG AGTGGTGTTT TTAGTAATTA AAAACAATGT GTAAAAAGTT GGGAAATAAT AGAGTACATT   
  
  
+ TTTAGTATAA GGTTTATTAA ATATTTTTAA ATTGAAAAAT ATTAAAGTAA GACTATTTAT ATTTAACTTT   
  
  
+ ACTACTTTTT ATTAAATTTA TTATTTAGAT TTTTTTATTT TTATCATACT TTTATTTAGT TTTGGTGAAC   
  
  
+ TTTAAATATA AATTTATTTT TATAAAATAA AATAAATAAA AATTCTAGAA TGTTCTCTTT TGTACAATGT   
  
  
+ ACTTCACACC CTCAAACTGA TCAAGGTCTT CATGTCGCCA AACTAACCGA CGGCTTCGCG GTGCACTCCC   
  
  
+ GATGCCCTAT TATCAGTTGT TTTACCTGCC CGGCCGAAAA TAGTGTCGAA GTACCTGCCC GGCACCAGAA   
  
  
+ ACTGGCGTGT TGAACAAGGA AGTTAAATAG AAACTAAAAA GTAGAAGAAA AGGAGAGTTA AAGCTTTTTA   
  
  
+ ATTATTTTCT TTTTCTTTAA GGGTTTATTC AGTAAGTATG AGAGGGAATT TAAGTACTTT AAAGGAAATA   
  
  
+ ATACTGCCGG TAGAGATGTA TAAAAGAAAA ACCCCTAAAA AAATGTTTAA TTTAATATCC ATTAAATGAA   
  
  
+ AAAAACCAAA ATTTTAGATA AAGTTGTTTA TAAAAATTTT TGTTTTTTAA ACTAAAAAAA TTTTTTATTT   
  
  
+ AATACACTAT TTTATACTTG AGCTCCAGTT TTGAGGAAAG ATTGAATTAG TTAAGCCGAT TCGATTCTTC   
  
  
+ TAATCGGGTT GTTTAATCCG AAAGTATTTC AATGATCTCT ATGGAGTATC TAATATCCGA CGCTGAAAAA   
  
  
+ GTTAGAGAGG TTTCAAATTA AACGTATAC  

- GTTGATATAT CGTGCTTCCA CGTAATGCAG AGACGATTAT CCTGGTTGTA TACAAAGATG GGCAACAGTT   
  
  
- TTCTTGTAAG AAAAGTATGT ATCTAGTTGG AATATGAATC AGTATCCACG GAGCTAAAGT CCTCTTCCTC   
  
  
- GTCCTCCTTT GTCCATAAAT AAAGCCGAGA GAGAGAGAGA GAGAGAGAGA GAGAGAGAGA GAGAGAGAGA   
  
  
- TGTTATTTAG AAAGAGCAGG ACTCCTTTGT CCATAAATAA AGCCGAGAGA GAGAGAGAGA GAGAGAGAGA   
  
  
- GAGAGAGAGA GAGAGAGATG TTATTTAGAA AGAGCAGGGT GAGGCTGCAA TGGACTCGGC ATTAAAAAGG   
  
  
- GTTTTGGACT GAAGACCAAA TGTGAAACGA GTGATGCTTA GGGATTTTGA TGAGAGTCTA TGAGGTAAGG   
  
  
- TAGTGCGTCA AGAAAACAGG TGGCCATGAA AGTCTTATGA GAGAGAGAGA CAGAGGCAGA GAAAGAAGAG   
  
  
- TCTTACTGCT GCTGTCATAA GAGAGAAAGA GAGAGAGAGA GAGAGAGAGA GAGAGAAAGA GTTTGAATGT   
  
  
- TTGAAAGGAT CCCGCTAGCA CAAAAACACT TAATTACTTA ATTAAATTAA ATTAAATTAA ATTATTCATT   
  
  
- ATTAATTATC TCACCACAAA AATCATTAAT TTTTGTTACA CATTTTTCAA CCCTTTATTA TCTCATGTAA   
  
  
- AAATCATATT CCAAATAATT TATAAAAATT TAACTTTTTA TAATTTCATT CTGATAAATA TAAATTGAAA   
  
  
- TGATGAAAAA TAATTTAAAT AATAAATCTA AAAAAATAAA AATAGTATGA AAATAAATCA AAACCACTTG   
  
  
- AAATTTATAT TTAAATAAAA ATATTTTATT TTATTTATTT TTAAGATCTT ACAAGAGAAA ACATGTTACA   
  
  
- TGAAGTGTGG GAGTTTGACT AGTTCCAGAA GTACAGCGGT TTGATTGGCT GCCGAAGCGC CACGTGAGGG   
  
  
- CTACGGGATA ATAGTCAACA AAATGGACGG GCCGGCTTTT ATCACAGCTT CATGGACGGG CCGTGGTCTT   
  
  
- TGACCGCACA ACTTGTTCCT TCAATTTATC TTTGATTTTT CATCTTCTTT TCCTCTCAAT TTCGAAAAAT   
  
  
- TAATAAAAGA AAAAGAAATT CCCAAATAAG TCATTCATAC TCTCCCTTAA ATTCATGAAA TTTCCTTTAT   
  
  
- TATGACGGCC ATCTCTACAT ATTTTCTTTT TGGGGATTTT TTTACAAATT AAATTATAGG TAATTTACTT   
  
  
- TTTTTGGTTT TAAAATCTAT TTCAACAAAT ATTTTTAAAA ACAAAAAATT TGATTTTTTT AAAAAATAAA   
  
  
- TTATGTGATA AAATATGAAC TCGAGGTCAA AACTCCTTTC TAACTTAATC AATTCGGCTA AGCTAAGAAG   
  
  
- ATTAGCCCAA CAAATTAGGC TTTCATAAAG TTACTAGAGA TACCTCATAG ATTATAGGCT GCGACTTTTT   
  
  
- CAATCTCTCC AAAGTTTAAT TTGCATATG

+     circadian

| Site Name | Organism | Position | Strand | Matrix score. | sequence | function |
| --- | --- | --- | --- | --- | --- | --- |
| circadian | Lycopersicon esculentum | 1277 | - | 6 | CAANNNNATC | cis-acting regulatory element involved in circadian control |
| circadian | Lycopersicon esculentum | 923 | + | 6 | CAANNNNATC | cis-acting regulatory element involved in circadian control |

> 2018/04/13 10:10:12  
+ CAACTATATA GCACGAAGGT GCATTACGTC TCTGCTAATA GGACCAACAT ATGTTTCTAC CCGTTGTCAA   
  
  
+ AAGAACATTC TTTTCATACA TAGATCAACC TTATACTTAG TCATAGGTGC CTCGATTTCA GGAGAAGGAG   
  
  
+ CAGGAGGAAA CAGGTATTTA TTTCGGCTCT CTCTCTCTCT CTCTCTCTCT CTCTCTCTCT CTCTCTCTCT   
  
  
+ ACAATAAATC TTTCTCGTCC TGAGGAAACA GGTATTTATT TCGGCTCTCT CTCTCTCTCT CTCTCTCTCT   
  
  
+ CTCTCTCTCT CTCTCTCTAC AATAAATCTT TCTCGTCCCA CTCCGACGTT ACCTGAGCCG TAATTTTTCC   
  
  
+ CAAAACCTGA CTTCTGGTTT ACACTTTGCT CACTACGAAT CCCTAAAACT ACTCTCAGAT ACTCCATTCC   
  
  
+ ATCACGCAGT TCTTTTGTCC ACCGGTACTT TCAGAATACT CTCTCTCTCT GTCTCCGTCT CTTTCTTCTC   
  
  
+ AGAATGACGA CGACAGTATT CTCTCTTTCT CTCTCTCTCT CTCTCTCTCT CTCTCTTTCT CAAACTTACA   
  
  
+ AACTTTCCTA GGGCGATCGT GTTTTTGTGA ATTAATGAAT TAATTTAATT TAATTTAATT TAATAAGTAA   
  
  
+ TAATTAATAG AGTGGTGTTT TTAGTAATTA AAAACAATGT GTAAAAAGTT GGGAAATAAT AGAGTACATT   
  
  
+ TTTAGTATAA GGTTTATTAA ATATTTTTAA ATTGAAAAAT ATTAAAGTAA GACTATTTAT ATTTAACTTT   
  
  
+ ACTACTTTTT ATTAAATTTA TTATTTAGAT TTTTTTATTT TTATCATACT TTTATTTAGT TTTGGTGAAC   
  
  
+ TTTAAATATA AATTTATTTT TATAAAATAA AATAAATAAA AATTCTAGAA TGTTCTCTTT TGTACAATGT   
  
  
+ ACTTCACACC CTCAAACTGA TCAAGGTCTT CATGTCGCCA AACTAACCGA CGGCTTCGCG GTGCACTCCC   
  
  
+ GATGCCCTAT TATCAGTTGT TTTACCTGCC CGGCCGAAAA TAGTGTCGAA GTACCTGCCC GGCACCAGAA   
  
  
+ ACTGGCGTGT TGAACAAGGA AGTTAAATAG AAACTAAAAA GTAGAAGAAA AGGAGAGTTA AAGCTTTTTA   
  
  
+ ATTATTTTCT TTTTCTTTAA GGGTTTATTC AGTAAGTATG AGAGGGAATT TAAGTACTTT AAAGGAAATA   
  
  
+ ATACTGCCGG TAGAGATGTA TAAAAGAAAA ACCCCTAAAA AAATGTTTAA TTTAATATCC ATTAAATGAA   
  
  
+ AAAAACCAAA ATTTTAGATA AAGTTGTTTA TAAAAATTTT TGTTTTTTAA ACTAAAAAAA TTTTTTATTT   
  
  
+ AATACACTAT TTTATACTTG AGCTCCAGTT TTGAGGAAAG ATTGAATTAG TTAAGCCGAT TCGATTCTTC   
  
  
+ TAATCGGGTT GTTTAATCCG AAAGTATTTC AATGATCTCT ATGGAGTATC TAATATCCGA CGCTGAAAAA   
  
  
+ GTTAGAGAGG TTTCAAATTA AACGTATAC  

- GTTGATATAT CGTGCTTCCA CGTAATGCAG AGACGATTAT CCTGGTTGTA TACAAAGATG GGCAACAGTT   
  
  
- TTCTTGTAAG AAAAGTATGT ATCTAGTTGG AATATGAATC AGTATCCACG GAGCTAAAGT CCTCTTCCTC   
  
  
- GTCCTCCTTT GTCCATAAAT AAAGCCGAGA GAGAGAGAGA GAGAGAGAGA GAGAGAGAGA GAGAGAGAGA   
  
  
- TGTTATTTAG AAAGAGCAGG ACTCCTTTGT CCATAAATAA AGCCGAGAGA GAGAGAGAGA GAGAGAGAGA   
  
  
- GAGAGAGAGA GAGAGAGATG TTATTTAGAA AGAGCAGGGT GAGGCTGCAA TGGACTCGGC ATTAAAAAGG   
  
  
- GTTTTGGACT GAAGACCAAA TGTGAAACGA GTGATGCTTA GGGATTTTGA TGAGAGTCTA TGAGGTAAGG   
  
  
- TAGTGCGTCA AGAAAACAGG TGGCCATGAA AGTCTTATGA GAGAGAGAGA CAGAGGCAGA GAAAGAAGAG   
  
  
- TCTTACTGCT GCTGTCATAA GAGAGAAAGA GAGAGAGAGA GAGAGAGAGA GAGAGAAAGA GTTTGAATGT   
  
  
- TTGAAAGGAT CCCGCTAGCA CAAAAACACT TAATTACTTA ATTAAATTAA ATTAAATTAA ATTATTCATT   
  
  
- ATTAATTATC TCACCACAAA AATCATTAAT TTTTGTTACA CATTTTTCAA CCCTTTATTA TCTCATGTAA   
  
  
- AAATCATATT CCAAATAATT TATAAAAATT TAACTTTTTA TAATTTCATT CTGATAAATA TAAATTGAAA   
  
  
- TGATGAAAAA TAATTTAAAT AATAAATCTA AAAAAATAAA AATAGTATGA AAATAAATCA AAACCACTTG   
  
  
- AAATTTATAT TTAAATAAAA ATATTTTATT TTATTTATTT TTAAGATCTT ACAAGAGAAA ACATGTTACA   
  
  
- TGAAGTGTGG GAGTTTGACT AGTTCCAGAA GTACAGCGGT TTGATTGGCT GCCGAAGCGC CACGTGAGGG   
  
  
- CTACGGGATA ATAGTCAACA AAATGGACGG GCCGGCTTTT ATCACAGCTT CATGGACGGG CCGTGGTCTT   
  
  
- TGACCGCACA ACTTGTTCCT TCAATTTATC TTTGATTTTT CATCTTCTTT TCCTCTCAAT TTCGAAAAAT   
  
  
- TAATAAAAGA AAAAGAAATT CCCAAATAAG TCATTCATAC TCTCCCTTAA ATTCATGAAA TTTCCTTTAT   
  
  
- TATGACGGCC ATCTCTACAT ATTTTCTTTT TGGGGATTTT TTTACAAATT AAATTATAGG TAATTTACTT   
  
  
- TTTTTGGTTT TAAAATCTAT TTCAACAAAT ATTTTTAAAA ACAAAAAATT TGATTTTTTT AAAAAATAAA   
  
  
- TTATGTGATA AAATATGAAC TCGAGGTCAA AACTCCTTTC TAACTTAATC AATTCGGCTA AGCTAAGAAG   
  
  
- ATTAGCCCAA CAAATTAGGC TTTCATAAAG TTACTAGAGA TACCTCATAG ATTATAGGCT GCGACTTTTT   
  
  
- CAATCTCTCC AAAGTTTAAT TTGCATATG
