# Supplementary material for: Genome-wide analysis of the WRKY gene family in drumstick (Moringa oleifera Lam.)
Source: PeerJ. 2019 Jun 10;7:e7063. doi: 10.7717/peerj.7063 (PMC6563795; doi:10.7717/peerj.7063)
Supplement: Supplemental Information 1 [file peerj-07-7063-s003.gz › MoWRKY31_plantcare.html]

Content-Type: text/html; charset=ISO-8859-1


CallMat\_Firefox


Webmaster Firefox specific output  
To save the result:
click on the frame with the right mouse button and save the source code as a text file with extension .html  
REFERENCE:PlantCARE: a database of plant cis-acting regulatory elements and a portal to tools for in silico analysis of promoter sequences.  
Lescot, M., Déhais, P., Moreau, Y., De Moor, B., Rouzé ,P.,and Rombauts, S.  
Nucleic Acids Res., Database issue(2002), 30(1):325-327.   


---

> 2018/04/13 10:10:12  
+ GGAGGAAGTG AAGTGAAGTC AACGGCAGTG GGGCCAATCC TGGACCCATG CAGCTGTCAG TCACCGACTT   
  
  
+ AACAGTGTCT CCCCGGCTCA AGATAGCAGC AGCCCCCCAG CAGGAGTTTG TTGCGGTCAA AGCACATTTT   
  
  
+ GGAATACGTG TCAGGCTCCT ATGCAACTCT TTGTACACGT GGCATCATCC AGCTGGTAAT TTTTCTGGCA   
  
  
+ ACGGCTCTAT ATCTATAGGG AACCGGCTCT GCAGTCTCCA GTACCGGGTG GTGGGTCCCA CTTTTGGCGG   
  
  
+ ACATTTTGAT AAGCCCTTCA CAGATACTTT AAAAAAAAAA GGGCAGAGCA AAGTAAACAA CCAGTATTCT   
  
  
+ CTCTGTCATT TTTTTTTTAA AAAAGAAAAT ATGTTTTATA AAATTAATTT AATTTTCATA AAATTTATCT   
  
  
+ ATTTAATATT TTAAAAAGTA TTAAAATGCA GAAAAGAGAC AGTACATTAG AGGATGTGTT ATTGTTTCAT   
  
  
+ ATTATTATAA TAATTCACAA AATATTCTTT AAAATAAACT TTGAGACCAC CTCAGATCTG TTGAAGATCA   
  
  
+ TTGTCCTACA TAAATCCAGT TGGTGTAATA AATCTGGGGC TGTATGAAGA GGGACTGAGG ATGCAGCAGC   
  
  
+ GAGACAGGAG CTTAATTGGG AGGAGGAGAC AAAAGCCATG GCAATGGAAC ATCTGGGTGT GCAGTAAAAC   
  
  
+ TGAAGCTGAA AAAGGGGTGG TTCATTAATG GGGGAGGGGA GGGAGAAAAA GAATAACCAA GGTCTTTTCG   
  
  
+ TGGGACCCAC ACCTCAAATC TGTGACAGAG ACGGCAGTTT CATTTCTCAG CTCTTCCACC TTCACAATCC   
  
  
+ CTTATTTAAA TCTTTCTCTC TCTCACTTGA ACATTGTTGT TTCTCTGTTC TCTCTCTTTC TATCCTTTGT   
  
  
+ TTTTTGTTCT CCTGTTGAAG TTCTGACTCT CTCTTACTCT CTCTCATATG TTTTCTCTTT TAGAAAAATA   
  
  
+ TTATTGAATA AAGAAATGGG ACCTTTGTAT TCTTATGGCC GTATGTGAAA TCTCTAGCGA AGCAAAGTCT   
  
  
+ TCTGTTTCAC ACCTTTCAAT CTCAAAACAA CGGAAGAAGA AGCTAGGGTT TGACAGCACC ACCAAGCCCA   
  
  
+ AGGTTTGTTC TTTGGTACTT TTCCTCTTTG CTTCTATTGC TATTTTTTTT TATCGAATTT TTCTTATCAT   
  
  
+ AGCCGTAAGT TTATTTTCTC TTTGCTTTCA CTCAGGTTTC AAAGACCCGC TTGAATCTGA TTTTGTGGGT   
  
  
+ ATCCCTGTTT TTAAAAATTA AACCTTCTGA GAGGTTGAAC GTTTCGAATT TTTGATGAAA AGAACCACAC   
  
  
+ TTGGGATTGT GGTTTATCCA GAAATGGAAT TTTGACTACG TGGGTCGTCT TTCCTTTTTA CCTCGAGGAT   
  
  
+ CTGTTATCGT GGTAGATTTT TCCGAGTTTT TATGGTCTGT TTTGGTCTAG GTGTCCCGGG GTTGGAGAAT   
  
  
+ TAGGGTTCTT TTGGGGTTTT GGGGGCTAA  

- CCTCCTTCAC TTCACTTCAG TTGCCGTCAC CCCGGTTAGG ACCTGGGTAC GTCGACAGTC AGTGGCTGAA   
  
  
- TTGTCACAGA GGGGCCGAGT TCTATCGTCG TCGGGGGGTC GTCCTCAAAC AACGCCAGTT TCGTGTAAAA   
  
  
- CCTTATGCAC AGTCCGAGGA TACGTTGAGA AACATGTGCA CCGTAGTAGG TCGACCATTA AAAAGACCGT   
  
  
- TGCCGAGATA TAGATATCCC TTGGCCGAGA CGTCAGAGGT CATGGCCCAC CACCCAGGGT GAAAACCGCC   
  
  
- TGTAAAACTA TTCGGGAAGT GTCTATGAAA TTTTTTTTTT CCCGTCTCGT TTCATTTGTT GGTCATAAGA   
  
  
- GAGACAGTAA AAAAAAAATT TTTTCTTTTA TACAAAATAT TTTAATTAAA TTAAAAGTAT TTTAAATAGA   
  
  
- TAAATTATAA AATTTTTCAT AATTTTACGT CTTTTCTCTG TCATGTAATC TCCTACACAA TAACAAAGTA   
  
  
- TAATAATATT ATTAAGTGTT TTATAAGAAA TTTTATTTGA AACTCTGGTG GAGTCTAGAC AACTTCTAGT   
  
  
- AACAGGATGT ATTTAGGTCA ACCACATTAT TTAGACCCCG ACATACTTCT CCCTGACTCC TACGTCGTCG   
  
  
- CTCTGTCCTC GAATTAACCC TCCTCCTCTG TTTTCGGTAC CGTTACCTTG TAGACCCACA CGTCATTTTG   
  
  
- ACTTCGACTT TTTCCCCACC AAGTAATTAC CCCCTCCCCT CCCTCTTTTT CTTATTGGTT CCAGAAAAGC   
  
  
- ACCCTGGGTG TGGAGTTTAG ACACTGTCTC TGCCGTCAAA GTAAAGAGTC GAGAAGGTGG AAGTGTTAGG   
  
  
- GAATAAATTT AGAAAGAGAG AGAGTGAACT TGTAACAACA AAGAGACAAG AGAGAGAAAG ATAGGAAACA   
  
  
- AAAAACAAGA GGACAACTTC AAGACTGAGA GAGAATGAGA GAGAGTATAC AAAAGAGAAA ATCTTTTTAT   
  
  
- AATAACTTAT TTCTTTACCC TGGAAACATA AGAATACCGG CATACACTTT AGAGATCGCT TCGTTTCAGA   
  
  
- AGACAAAGTG TGGAAAGTTA GAGTTTTGTT GCCTTCTTCT TCGATCCCAA ACTGTCGTGG TGGTTCGGGT   
  
  
- TCCAAACAAG AAACCATGAA AAGGAGAAAC GAAGATAACG ATAAAAAAAA ATAGCTTAAA AAGAATAGTA   
  
  
- TCGGCATTCA AATAAAAGAG AAACGAAAGT GAGTCCAAAG TTTCTGGGCG AACTTAGACT AAAACACCCA   
  
  
- TAGGGACAAA AATTTTTAAT TTGGAAGACT CTCCAACTTG CAAAGCTTAA AAACTACTTT TCTTGGTGTG   
  
  
- AACCCTAACA CCAAATAGGT CTTTACCTTA AAACTGATGC ACCCAGCAGA AAGGAAAAAT GGAGCTCCTA   
  
  
- GACAATAGCA CCATCTAAAA AGGCTCAAAA ATACCAGACA AAACCAGATC CACAGGGCCC CAACCTCTTA   
  
  
- ATCCCAAGAA AACCCCAAAA CCCCCGATT

  
  
Motifs Found  

+     5UTR Py-rich stretch

| Site Name | Organism | Position | Strand | Matrix score. | sequence | function |
| --- | --- | --- | --- | --- | --- | --- |
| 5UTR Py-rich stretch | Lycopersicon esculentum | 853 | + | 13 | TTTCTCTCTCTCTC | cis-acting element conferring high transcription levels |

> 2018/04/13 10:10:12  
+ GGAGGAAGTG AAGTGAAGTC AACGGCAGTG GGGCCAATCC TGGACCCATG CAGCTGTCAG TCACCGACTT   
  
  
+ AACAGTGTCT CCCCGGCTCA AGATAGCAGC AGCCCCCCAG CAGGAGTTTG TTGCGGTCAA AGCACATTTT   
  
  
+ GGAATACGTG TCAGGCTCCT ATGCAACTCT TTGTACACGT GGCATCATCC AGCTGGTAAT TTTTCTGGCA   
  
  
+ ACGGCTCTAT ATCTATAGGG AACCGGCTCT GCAGTCTCCA GTACCGGGTG GTGGGTCCCA CTTTTGGCGG   
  
  
+ ACATTTTGAT AAGCCCTTCA CAGATACTTT AAAAAAAAAA GGGCAGAGCA AAGTAAACAA CCAGTATTCT   
  
  
+ CTCTGTCATT TTTTTTTTAA AAAAGAAAAT ATGTTTTATA AAATTAATTT AATTTTCATA AAATTTATCT   
  
  
+ ATTTAATATT TTAAAAAGTA TTAAAATGCA GAAAAGAGAC AGTACATTAG AGGATGTGTT ATTGTTTCAT   
  
  
+ ATTATTATAA TAATTCACAA AATATTCTTT AAAATAAACT TTGAGACCAC CTCAGATCTG TTGAAGATCA   
  
  
+ TTGTCCTACA TAAATCCAGT TGGTGTAATA AATCTGGGGC TGTATGAAGA GGGACTGAGG ATGCAGCAGC   
  
  
+ GAGACAGGAG CTTAATTGGG AGGAGGAGAC AAAAGCCATG GCAATGGAAC ATCTGGGTGT GCAGTAAAAC   
  
  
+ TGAAGCTGAA AAAGGGGTGG TTCATTAATG GGGGAGGGGA GGGAGAAAAA GAATAACCAA GGTCTTTTCG   
  
  
+ TGGGACCCAC ACCTCAAATC TGTGACAGAG ACGGCAGTTT CATTTCTCAG CTCTTCCACC TTCACAATCC   
  
  
+ CTTATTTAAA TCTTTCTCTC TCTCACTTGA ACATTGTTGT TTCTCTGTTC TCTCTCTTTC TATCCTTTGT   
  
  
+ TTTTTGTTCT CCTGTTGAAG TTCTGACTCT CTCTTACTCT CTCTCATATG TTTTCTCTTT TAGAAAAATA   
  
  
+ TTATTGAATA AAGAAATGGG ACCTTTGTAT TCTTATGGCC GTATGTGAAA TCTCTAGCGA AGCAAAGTCT   
  
  
+ TCTGTTTCAC ACCTTTCAAT CTCAAAACAA CGGAAGAAGA AGCTAGGGTT TGACAGCACC ACCAAGCCCA   
  
  
+ AGGTTTGTTC TTTGGTACTT TTCCTCTTTG CTTCTATTGC TATTTTTTTT TATCGAATTT TTCTTATCAT   
  
  
+ AGCCGTAAGT TTATTTTCTC TTTGCTTTCA CTCAGGTTTC AAAGACCCGC TTGAATCTGA TTTTGTGGGT   
  
  
+ ATCCCTGTTT TTAAAAATTA AACCTTCTGA GAGGTTGAAC GTTTCGAATT TTTGATGAAA AGAACCACAC   
  
  
+ TTGGGATTGT GGTTTATCCA GAAATGGAAT TTTGACTACG TGGGTCGTCT TTCCTTTTTA CCTCGAGGAT   
  
  
+ CTGTTATCGT GGTAGATTTT TCCGAGTTTT TATGGTCTGT TTTGGTCTAG GTGTCCCGGG GTTGGAGAAT   
  
  
+ TAGGGTTCTT TTGGGGTTTT GGGGGCTAA  

- CCTCCTTCAC TTCACTTCAG TTGCCGTCAC CCCGGTTAGG ACCTGGGTAC GTCGACAGTC AGTGGCTGAA   
  
  
- TTGTCACAGA GGGGCCGAGT TCTATCGTCG TCGGGGGGTC GTCCTCAAAC AACGCCAGTT TCGTGTAAAA   
  
  
- CCTTATGCAC AGTCCGAGGA TACGTTGAGA AACATGTGCA CCGTAGTAGG TCGACCATTA AAAAGACCGT   
  
  
- TGCCGAGATA TAGATATCCC TTGGCCGAGA CGTCAGAGGT CATGGCCCAC CACCCAGGGT GAAAACCGCC   
  
  
- TGTAAAACTA TTCGGGAAGT GTCTATGAAA TTTTTTTTTT CCCGTCTCGT TTCATTTGTT GGTCATAAGA   
  
  
- GAGACAGTAA AAAAAAAATT TTTTCTTTTA TACAAAATAT TTTAATTAAA TTAAAAGTAT TTTAAATAGA   
  
  
- TAAATTATAA AATTTTTCAT AATTTTACGT CTTTTCTCTG TCATGTAATC TCCTACACAA TAACAAAGTA   
  
  
- TAATAATATT ATTAAGTGTT TTATAAGAAA TTTTATTTGA AACTCTGGTG GAGTCTAGAC AACTTCTAGT   
  
  
- AACAGGATGT ATTTAGGTCA ACCACATTAT TTAGACCCCG ACATACTTCT CCCTGACTCC TACGTCGTCG   
  
  
- CTCTGTCCTC GAATTAACCC TCCTCCTCTG TTTTCGGTAC CGTTACCTTG TAGACCCACA CGTCATTTTG   
  
  
- ACTTCGACTT TTTCCCCACC AAGTAATTAC CCCCTCCCCT CCCTCTTTTT CTTATTGGTT CCAGAAAAGC   
  
  
- ACCCTGGGTG TGGAGTTTAG ACACTGTCTC TGCCGTCAAA GTAAAGAGTC GAGAAGGTGG AAGTGTTAGG   
  
  
- GAATAAATTT AGAAAGAGAG AGAGTGAACT TGTAACAACA AAGAGACAAG AGAGAGAAAG ATAGGAAACA   
  
  
- AAAAACAAGA GGACAACTTC AAGACTGAGA GAGAATGAGA GAGAGTATAC AAAAGAGAAA ATCTTTTTAT   
  
  
- AATAACTTAT TTCTTTACCC TGGAAACATA AGAATACCGG CATACACTTT AGAGATCGCT TCGTTTCAGA   
  
  
- AGACAAAGTG TGGAAAGTTA GAGTTTTGTT GCCTTCTTCT TCGATCCCAA ACTGTCGTGG TGGTTCGGGT   
  
  
- TCCAAACAAG AAACCATGAA AAGGAGAAAC GAAGATAACG ATAAAAAAAA ATAGCTTAAA AAGAATAGTA   
  
  
- TCGGCATTCA AATAAAAGAG AAACGAAAGT GAGTCCAAAG TTTCTGGGCG AACTTAGACT AAAACACCCA   
  
  
- TAGGGACAAA AATTTTTAAT TTGGAAGACT CTCCAACTTG CAAAGCTTAA AAACTACTTT TCTTGGTGTG   
  
  
- AACCCTAACA CCAAATAGGT CTTTACCTTA AAACTGATGC ACCCAGCAGA AAGGAAAAAT GGAGCTCCTA   
  
  
- GACAATAGCA CCATCTAAAA AGGCTCAAAA ATACCAGACA AAACCAGATC CACAGGGCCC CAACCTCTTA   
  
  
- ATCCCAAGAA AACCCCAAAA CCCCCGATT

+     ABRE

| Site Name | Organism | Position | Strand | Matrix score. | sequence | function |
| --- | --- | --- | --- | --- | --- | --- |
| ABRE | Triticum aestivum | 173 | + | 10 | GGACACGTGGC | cis-acting element involved in the abscisic acid responsiveness |
| ABRE | Arabidopsis thaliana | 145 | + | 6 | TACGTG | cis-acting element involved in the abscisic acid responsiveness |
| ABRE | Arabidopsis thaliana | 177 | + | 7 | ACGTGGC | cis-acting element involved in the abscisic acid responsiveness |
| ABRE | Triticum aestivum | 174 | + | 9 | GACACGTGGC | cis-acting element involved in the abscisic acid responsiveness |
| ABRE | Arabidopsis thaliana | 176 | + | 6 | CACGTG | cis-acting element involved in the abscisic acid responsiveness |
| ABRE | Brassica napus | 175 | + | 9 | ACACGTGGC | cis-acting element involved in the abscisic acid responsiveness |
| ABRE | Arabidopsis thaliana | 1367 | + | 6 | TACGTG | cis-acting element involved in the abscisic acid responsiveness |

> 2018/04/13 10:10:12  
+ GGAGGAAGTG AAGTGAAGTC AACGGCAGTG GGGCCAATCC TGGACCCATG CAGCTGTCAG TCACCGACTT   
  
  
+ AACAGTGTCT CCCCGGCTCA AGATAGCAGC AGCCCCCCAG CAGGAGTTTG TTGCGGTCAA AGCACATTTT   
  
  
+ GGAATACGTG TCAGGCTCCT ATGCAACTCT TTGTACACGT GGCATCATCC AGCTGGTAAT TTTTCTGGCA   
  
  
+ ACGGCTCTAT ATCTATAGGG AACCGGCTCT GCAGTCTCCA GTACCGGGTG GTGGGTCCCA CTTTTGGCGG   
  
  
+ ACATTTTGAT AAGCCCTTCA CAGATACTTT AAAAAAAAAA GGGCAGAGCA AAGTAAACAA CCAGTATTCT   
  
  
+ CTCTGTCATT TTTTTTTTAA AAAAGAAAAT ATGTTTTATA AAATTAATTT AATTTTCATA AAATTTATCT   
  
  
+ ATTTAATATT TTAAAAAGTA TTAAAATGCA GAAAAGAGAC AGTACATTAG AGGATGTGTT ATTGTTTCAT   
  
  
+ ATTATTATAA TAATTCACAA AATATTCTTT AAAATAAACT TTGAGACCAC CTCAGATCTG TTGAAGATCA   
  
  
+ TTGTCCTACA TAAATCCAGT TGGTGTAATA AATCTGGGGC TGTATGAAGA GGGACTGAGG ATGCAGCAGC   
  
  
+ GAGACAGGAG CTTAATTGGG AGGAGGAGAC AAAAGCCATG GCAATGGAAC ATCTGGGTGT GCAGTAAAAC   
  
  
+ TGAAGCTGAA AAAGGGGTGG TTCATTAATG GGGGAGGGGA GGGAGAAAAA GAATAACCAA GGTCTTTTCG   
  
  
+ TGGGACCCAC ACCTCAAATC TGTGACAGAG ACGGCAGTTT CATTTCTCAG CTCTTCCACC TTCACAATCC   
  
  
+ CTTATTTAAA TCTTTCTCTC TCTCACTTGA ACATTGTTGT TTCTCTGTTC TCTCTCTTTC TATCCTTTGT   
  
  
+ TTTTTGTTCT CCTGTTGAAG TTCTGACTCT CTCTTACTCT CTCTCATATG TTTTCTCTTT TAGAAAAATA   
  
  
+ TTATTGAATA AAGAAATGGG ACCTTTGTAT TCTTATGGCC GTATGTGAAA TCTCTAGCGA AGCAAAGTCT   
  
  
+ TCTGTTTCAC ACCTTTCAAT CTCAAAACAA CGGAAGAAGA AGCTAGGGTT TGACAGCACC ACCAAGCCCA   
  
  
+ AGGTTTGTTC TTTGGTACTT TTCCTCTTTG CTTCTATTGC TATTTTTTTT TATCGAATTT TTCTTATCAT   
  
  
+ AGCCGTAAGT TTATTTTCTC TTTGCTTTCA CTCAGGTTTC AAAGACCCGC TTGAATCTGA TTTTGTGGGT   
  
  
+ ATCCCTGTTT TTAAAAATTA AACCTTCTGA GAGGTTGAAC GTTTCGAATT TTTGATGAAA AGAACCACAC   
  
  
+ TTGGGATTGT GGTTTATCCA GAAATGGAAT TTTGACTACG TGGGTCGTCT TTCCTTTTTA CCTCGAGGAT   
  
  
+ CTGTTATCGT GGTAGATTTT TCCGAGTTTT TATGGTCTGT TTTGGTCTAG GTGTCCCGGG GTTGGAGAAT   
  
  
+ TAGGGTTCTT TTGGGGTTTT GGGGGCTAA  

- CCTCCTTCAC TTCACTTCAG TTGCCGTCAC CCCGGTTAGG ACCTGGGTAC GTCGACAGTC AGTGGCTGAA   
  
  
- TTGTCACAGA GGGGCCGAGT TCTATCGTCG TCGGGGGGTC GTCCTCAAAC AACGCCAGTT TCGTGTAAAA   
  
  
- CCTTATGCAC AGTCCGAGGA TACGTTGAGA AACATGTGCA CCGTAGTAGG TCGACCATTA AAAAGACCGT   
  
  
- TGCCGAGATA TAGATATCCC TTGGCCGAGA CGTCAGAGGT CATGGCCCAC CACCCAGGGT GAAAACCGCC   
  
  
- TGTAAAACTA TTCGGGAAGT GTCTATGAAA TTTTTTTTTT CCCGTCTCGT TTCATTTGTT GGTCATAAGA   
  
  
- GAGACAGTAA AAAAAAAATT TTTTCTTTTA TACAAAATAT TTTAATTAAA TTAAAAGTAT TTTAAATAGA   
  
  
- TAAATTATAA AATTTTTCAT AATTTTACGT CTTTTCTCTG TCATGTAATC TCCTACACAA TAACAAAGTA   
  
  
- TAATAATATT ATTAAGTGTT TTATAAGAAA TTTTATTTGA AACTCTGGTG GAGTCTAGAC AACTTCTAGT   
  
  
- AACAGGATGT ATTTAGGTCA ACCACATTAT TTAGACCCCG ACATACTTCT CCCTGACTCC TACGTCGTCG   
  
  
- CTCTGTCCTC GAATTAACCC TCCTCCTCTG TTTTCGGTAC CGTTACCTTG TAGACCCACA CGTCATTTTG   
  
  
- ACTTCGACTT TTTCCCCACC AAGTAATTAC CCCCTCCCCT CCCTCTTTTT CTTATTGGTT CCAGAAAAGC   
  
  
- ACCCTGGGTG TGGAGTTTAG ACACTGTCTC TGCCGTCAAA GTAAAGAGTC GAGAAGGTGG AAGTGTTAGG   
  
  
- GAATAAATTT AGAAAGAGAG AGAGTGAACT TGTAACAACA AAGAGACAAG AGAGAGAAAG ATAGGAAACA   
  
  
- AAAAACAAGA GGACAACTTC AAGACTGAGA GAGAATGAGA GAGAGTATAC AAAAGAGAAA ATCTTTTTAT   
  
  
- AATAACTTAT TTCTTTACCC TGGAAACATA AGAATACCGG CATACACTTT AGAGATCGCT TCGTTTCAGA   
  
  
- AGACAAAGTG TGGAAAGTTA GAGTTTTGTT GCCTTCTTCT TCGATCCCAA ACTGTCGTGG TGGTTCGGGT   
  
  
- TCCAAACAAG AAACCATGAA AAGGAGAAAC GAAGATAACG ATAAAAAAAA ATAGCTTAAA AAGAATAGTA   
  
  
- TCGGCATTCA AATAAAAGAG AAACGAAAGT GAGTCCAAAG TTTCTGGGCG AACTTAGACT AAAACACCCA   
  
  
- TAGGGACAAA AATTTTTAAT TTGGAAGACT CTCCAACTTG CAAAGCTTAA AAACTACTTT TCTTGGTGTG   
  
  
- AACCCTAACA CCAAATAGGT CTTTACCTTA AAACTGATGC ACCCAGCAGA AAGGAAAAAT GGAGCTCCTA   
  
  
- GACAATAGCA CCATCTAAAA AGGCTCAAAA ATACCAGACA AAACCAGATC CACAGGGCCC CAACCTCTTA   
  
  
- ATCCCAAGAA AACCCCAAAA CCCCCGATT

+     ACE

| Site Name | Organism | Position | Strand | Matrix score. | sequence | function |
| --- | --- | --- | --- | --- | --- | --- |
| ACE | Petroselinum crispum | 143 | - | 9 | GACACGTATG | cis-acting element involved in light responsiveness |
| ACE | Petroselinum crispum | 1365 | + | 9 | ACTACGTTGG | cis-acting element involved in light responsiveness |

> 2018/04/13 10:10:12  
+ GGAGGAAGTG AAGTGAAGTC AACGGCAGTG GGGCCAATCC TGGACCCATG CAGCTGTCAG TCACCGACTT   
  
  
+ AACAGTGTCT CCCCGGCTCA AGATAGCAGC AGCCCCCCAG CAGGAGTTTG TTGCGGTCAA AGCACATTTT   
  
  
+ GGAATACGTG TCAGGCTCCT ATGCAACTCT TTGTACACGT GGCATCATCC AGCTGGTAAT TTTTCTGGCA   
  
  
+ ACGGCTCTAT ATCTATAGGG AACCGGCTCT GCAGTCTCCA GTACCGGGTG GTGGGTCCCA CTTTTGGCGG   
  
  
+ ACATTTTGAT AAGCCCTTCA CAGATACTTT AAAAAAAAAA GGGCAGAGCA AAGTAAACAA CCAGTATTCT   
  
  
+ CTCTGTCATT TTTTTTTTAA AAAAGAAAAT ATGTTTTATA AAATTAATTT AATTTTCATA AAATTTATCT   
  
  
+ ATTTAATATT TTAAAAAGTA TTAAAATGCA GAAAAGAGAC AGTACATTAG AGGATGTGTT ATTGTTTCAT   
  
  
+ ATTATTATAA TAATTCACAA AATATTCTTT AAAATAAACT TTGAGACCAC CTCAGATCTG TTGAAGATCA   
  
  
+ TTGTCCTACA TAAATCCAGT TGGTGTAATA AATCTGGGGC TGTATGAAGA GGGACTGAGG ATGCAGCAGC   
  
  
+ GAGACAGGAG CTTAATTGGG AGGAGGAGAC AAAAGCCATG GCAATGGAAC ATCTGGGTGT GCAGTAAAAC   
  
  
+ TGAAGCTGAA AAAGGGGTGG TTCATTAATG GGGGAGGGGA GGGAGAAAAA GAATAACCAA GGTCTTTTCG   
  
  
+ TGGGACCCAC ACCTCAAATC TGTGACAGAG ACGGCAGTTT CATTTCTCAG CTCTTCCACC TTCACAATCC   
  
  
+ CTTATTTAAA TCTTTCTCTC TCTCACTTGA ACATTGTTGT TTCTCTGTTC TCTCTCTTTC TATCCTTTGT   
  
  
+ TTTTTGTTCT CCTGTTGAAG TTCTGACTCT CTCTTACTCT CTCTCATATG TTTTCTCTTT TAGAAAAATA   
  
  
+ TTATTGAATA AAGAAATGGG ACCTTTGTAT TCTTATGGCC GTATGTGAAA TCTCTAGCGA AGCAAAGTCT   
  
  
+ TCTGTTTCAC ACCTTTCAAT CTCAAAACAA CGGAAGAAGA AGCTAGGGTT TGACAGCACC ACCAAGCCCA   
  
  
+ AGGTTTGTTC TTTGGTACTT TTCCTCTTTG CTTCTATTGC TATTTTTTTT TATCGAATTT TTCTTATCAT   
  
  
+ AGCCGTAAGT TTATTTTCTC TTTGCTTTCA CTCAGGTTTC AAAGACCCGC TTGAATCTGA TTTTGTGGGT   
  
  
+ ATCCCTGTTT TTAAAAATTA AACCTTCTGA GAGGTTGAAC GTTTCGAATT TTTGATGAAA AGAACCACAC   
  
  
+ TTGGGATTGT GGTTTATCCA GAAATGGAAT TTTGACTACG TGGGTCGTCT TTCCTTTTTA CCTCGAGGAT   
  
  
+ CTGTTATCGT GGTAGATTTT TCCGAGTTTT TATGGTCTGT TTTGGTCTAG GTGTCCCGGG GTTGGAGAAT   
  
  
+ TAGGGTTCTT TTGGGGTTTT GGGGGCTAA  

- CCTCCTTCAC TTCACTTCAG TTGCCGTCAC CCCGGTTAGG ACCTGGGTAC GTCGACAGTC AGTGGCTGAA   
  
  
- TTGTCACAGA GGGGCCGAGT TCTATCGTCG TCGGGGGGTC GTCCTCAAAC AACGCCAGTT TCGTGTAAAA   
  
  
- CCTTATGCAC AGTCCGAGGA TACGTTGAGA AACATGTGCA CCGTAGTAGG TCGACCATTA AAAAGACCGT   
  
  
- TGCCGAGATA TAGATATCCC TTGGCCGAGA CGTCAGAGGT CATGGCCCAC CACCCAGGGT GAAAACCGCC   
  
  
- TGTAAAACTA TTCGGGAAGT GTCTATGAAA TTTTTTTTTT CCCGTCTCGT TTCATTTGTT GGTCATAAGA   
  
  
- GAGACAGTAA AAAAAAAATT TTTTCTTTTA TACAAAATAT TTTAATTAAA TTAAAAGTAT TTTAAATAGA   
  
  
- TAAATTATAA AATTTTTCAT AATTTTACGT CTTTTCTCTG TCATGTAATC TCCTACACAA TAACAAAGTA   
  
  
- TAATAATATT ATTAAGTGTT TTATAAGAAA TTTTATTTGA AACTCTGGTG GAGTCTAGAC AACTTCTAGT   
  
  
- AACAGGATGT ATTTAGGTCA ACCACATTAT TTAGACCCCG ACATACTTCT CCCTGACTCC TACGTCGTCG   
  
  
- CTCTGTCCTC GAATTAACCC TCCTCCTCTG TTTTCGGTAC CGTTACCTTG TAGACCCACA CGTCATTTTG   
  
  
- ACTTCGACTT TTTCCCCACC AAGTAATTAC CCCCTCCCCT CCCTCTTTTT CTTATTGGTT CCAGAAAAGC   
  
  
- ACCCTGGGTG TGGAGTTTAG ACACTGTCTC TGCCGTCAAA GTAAAGAGTC GAGAAGGTGG AAGTGTTAGG   
  
  
- GAATAAATTT AGAAAGAGAG AGAGTGAACT TGTAACAACA AAGAGACAAG AGAGAGAAAG ATAGGAAACA   
  
  
- AAAAACAAGA GGACAACTTC AAGACTGAGA GAGAATGAGA GAGAGTATAC AAAAGAGAAA ATCTTTTTAT   
  
  
- AATAACTTAT TTCTTTACCC TGGAAACATA AGAATACCGG CATACACTTT AGAGATCGCT TCGTTTCAGA   
  
  
- AGACAAAGTG TGGAAAGTTA GAGTTTTGTT GCCTTCTTCT TCGATCCCAA ACTGTCGTGG TGGTTCGGGT   
  
  
- TCCAAACAAG AAACCATGAA AAGGAGAAAC GAAGATAACG ATAAAAAAAA ATAGCTTAAA AAGAATAGTA   
  
  
- TCGGCATTCA AATAAAAGAG AAACGAAAGT GAGTCCAAAG TTTCTGGGCG AACTTAGACT AAAACACCCA   
  
  
- TAGGGACAAA AATTTTTAAT TTGGAAGACT CTCCAACTTG CAAAGCTTAA AAACTACTTT TCTTGGTGTG   
  
  
- AACCCTAACA CCAAATAGGT CTTTACCTTA AAACTGATGC ACCCAGCAGA AAGGAAAAAT GGAGCTCCTA   
  
  
- GACAATAGCA CCATCTAAAA AGGCTCAAAA ATACCAGACA AAACCAGATC CACAGGGCCC CAACCTCTTA   
  
  
- ATCCCAAGAA AACCCCAAAA CCCCCGATT

+     AE-box

| Site Name | Organism | Position | Strand | Matrix score. | sequence | function |
| --- | --- | --- | --- | --- | --- | --- |
| AE-box | Arabidopsis thaliana | 877 | - | 8 | AGAAACAA | part of a module for light response |

> 2018/04/13 10:10:12  
+ GGAGGAAGTG AAGTGAAGTC AACGGCAGTG GGGCCAATCC TGGACCCATG CAGCTGTCAG TCACCGACTT   
  
  
+ AACAGTGTCT CCCCGGCTCA AGATAGCAGC AGCCCCCCAG CAGGAGTTTG TTGCGGTCAA AGCACATTTT   
  
  
+ GGAATACGTG TCAGGCTCCT ATGCAACTCT TTGTACACGT GGCATCATCC AGCTGGTAAT TTTTCTGGCA   
  
  
+ ACGGCTCTAT ATCTATAGGG AACCGGCTCT GCAGTCTCCA GTACCGGGTG GTGGGTCCCA CTTTTGGCGG   
  
  
+ ACATTTTGAT AAGCCCTTCA CAGATACTTT AAAAAAAAAA GGGCAGAGCA AAGTAAACAA CCAGTATTCT   
  
  
+ CTCTGTCATT TTTTTTTTAA AAAAGAAAAT ATGTTTTATA AAATTAATTT AATTTTCATA AAATTTATCT   
  
  
+ ATTTAATATT TTAAAAAGTA TTAAAATGCA GAAAAGAGAC AGTACATTAG AGGATGTGTT ATTGTTTCAT   
  
  
+ ATTATTATAA TAATTCACAA AATATTCTTT AAAATAAACT TTGAGACCAC CTCAGATCTG TTGAAGATCA   
  
  
+ TTGTCCTACA TAAATCCAGT TGGTGTAATA AATCTGGGGC TGTATGAAGA GGGACTGAGG ATGCAGCAGC   
  
  
+ GAGACAGGAG CTTAATTGGG AGGAGGAGAC AAAAGCCATG GCAATGGAAC ATCTGGGTGT GCAGTAAAAC   
  
  
+ TGAAGCTGAA AAAGGGGTGG TTCATTAATG GGGGAGGGGA GGGAGAAAAA GAATAACCAA GGTCTTTTCG   
  
  
+ TGGGACCCAC ACCTCAAATC TGTGACAGAG ACGGCAGTTT CATTTCTCAG CTCTTCCACC TTCACAATCC   
  
  
+ CTTATTTAAA TCTTTCTCTC TCTCACTTGA ACATTGTTGT TTCTCTGTTC TCTCTCTTTC TATCCTTTGT   
  
  
+ TTTTTGTTCT CCTGTTGAAG TTCTGACTCT CTCTTACTCT CTCTCATATG TTTTCTCTTT TAGAAAAATA   
  
  
+ TTATTGAATA AAGAAATGGG ACCTTTGTAT TCTTATGGCC GTATGTGAAA TCTCTAGCGA AGCAAAGTCT   
  
  
+ TCTGTTTCAC ACCTTTCAAT CTCAAAACAA CGGAAGAAGA AGCTAGGGTT TGACAGCACC ACCAAGCCCA   
  
  
+ AGGTTTGTTC TTTGGTACTT TTCCTCTTTG CTTCTATTGC TATTTTTTTT TATCGAATTT TTCTTATCAT   
  
  
+ AGCCGTAAGT TTATTTTCTC TTTGCTTTCA CTCAGGTTTC AAAGACCCGC TTGAATCTGA TTTTGTGGGT   
  
  
+ ATCCCTGTTT TTAAAAATTA AACCTTCTGA GAGGTTGAAC GTTTCGAATT TTTGATGAAA AGAACCACAC   
  
  
+ TTGGGATTGT GGTTTATCCA GAAATGGAAT TTTGACTACG TGGGTCGTCT TTCCTTTTTA CCTCGAGGAT   
  
  
+ CTGTTATCGT GGTAGATTTT TCCGAGTTTT TATGGTCTGT TTTGGTCTAG GTGTCCCGGG GTTGGAGAAT   
  
  
+ TAGGGTTCTT TTGGGGTTTT GGGGGCTAA  

- CCTCCTTCAC TTCACTTCAG TTGCCGTCAC CCCGGTTAGG ACCTGGGTAC GTCGACAGTC AGTGGCTGAA   
  
  
- TTGTCACAGA GGGGCCGAGT TCTATCGTCG TCGGGGGGTC GTCCTCAAAC AACGCCAGTT TCGTGTAAAA   
  
  
- CCTTATGCAC AGTCCGAGGA TACGTTGAGA AACATGTGCA CCGTAGTAGG TCGACCATTA AAAAGACCGT   
  
  
- TGCCGAGATA TAGATATCCC TTGGCCGAGA CGTCAGAGGT CATGGCCCAC CACCCAGGGT GAAAACCGCC   
  
  
- TGTAAAACTA TTCGGGAAGT GTCTATGAAA TTTTTTTTTT CCCGTCTCGT TTCATTTGTT GGTCATAAGA   
  
  
- GAGACAGTAA AAAAAAAATT TTTTCTTTTA TACAAAATAT TTTAATTAAA TTAAAAGTAT TTTAAATAGA   
  
  
- TAAATTATAA AATTTTTCAT AATTTTACGT CTTTTCTCTG TCATGTAATC TCCTACACAA TAACAAAGTA   
  
  
- TAATAATATT ATTAAGTGTT TTATAAGAAA TTTTATTTGA AACTCTGGTG GAGTCTAGAC AACTTCTAGT   
  
  
- AACAGGATGT ATTTAGGTCA ACCACATTAT TTAGACCCCG ACATACTTCT CCCTGACTCC TACGTCGTCG   
  
  
- CTCTGTCCTC GAATTAACCC TCCTCCTCTG TTTTCGGTAC CGTTACCTTG TAGACCCACA CGTCATTTTG   
  
  
- ACTTCGACTT TTTCCCCACC AAGTAATTAC CCCCTCCCCT CCCTCTTTTT CTTATTGGTT CCAGAAAAGC   
  
  
- ACCCTGGGTG TGGAGTTTAG ACACTGTCTC TGCCGTCAAA GTAAAGAGTC GAGAAGGTGG AAGTGTTAGG   
  
  
- GAATAAATTT AGAAAGAGAG AGAGTGAACT TGTAACAACA AAGAGACAAG AGAGAGAAAG ATAGGAAACA   
  
  
- AAAAACAAGA GGACAACTTC AAGACTGAGA GAGAATGAGA GAGAGTATAC AAAAGAGAAA ATCTTTTTAT   
  
  
- AATAACTTAT TTCTTTACCC TGGAAACATA AGAATACCGG CATACACTTT AGAGATCGCT TCGTTTCAGA   
  
  
- AGACAAAGTG TGGAAAGTTA GAGTTTTGTT GCCTTCTTCT TCGATCCCAA ACTGTCGTGG TGGTTCGGGT   
  
  
- TCCAAACAAG AAACCATGAA AAGGAGAAAC GAAGATAACG ATAAAAAAAA ATAGCTTAAA AAGAATAGTA   
  
  
- TCGGCATTCA AATAAAAGAG AAACGAAAGT GAGTCCAAAG TTTCTGGGCG AACTTAGACT AAAACACCCA   
  
  
- TAGGGACAAA AATTTTTAAT TTGGAAGACT CTCCAACTTG CAAAGCTTAA AAACTACTTT TCTTGGTGTG   
  
  
- AACCCTAACA CCAAATAGGT CTTTACCTTA AAACTGATGC ACCCAGCAGA AAGGAAAAAT GGAGCTCCTA   
  
  
- GACAATAGCA CCATCTAAAA AGGCTCAAAA ATACCAGACA AAACCAGATC CACAGGGCCC CAACCTCTTA   
  
  
- ATCCCAAGAA AACCCCAAAA CCCCCGATT

+     ARE

| Site Name | Organism | Position | Strand | Matrix score. | sequence | function |
| --- | --- | --- | --- | --- | --- | --- |
| ARE | Zea mays | 1340 | + | 6 | TGGTTT | cis-acting regulatory element essential for the anaerobic induction |

> 2018/04/13 10:10:12  
+ GGAGGAAGTG AAGTGAAGTC AACGGCAGTG GGGCCAATCC TGGACCCATG CAGCTGTCAG TCACCGACTT   
  
  
+ AACAGTGTCT CCCCGGCTCA AGATAGCAGC AGCCCCCCAG CAGGAGTTTG TTGCGGTCAA AGCACATTTT   
  
  
+ GGAATACGTG TCAGGCTCCT ATGCAACTCT TTGTACACGT GGCATCATCC AGCTGGTAAT TTTTCTGGCA   
  
  
+ ACGGCTCTAT ATCTATAGGG AACCGGCTCT GCAGTCTCCA GTACCGGGTG GTGGGTCCCA CTTTTGGCGG   
  
  
+ ACATTTTGAT AAGCCCTTCA CAGATACTTT AAAAAAAAAA GGGCAGAGCA AAGTAAACAA CCAGTATTCT   
  
  
+ CTCTGTCATT TTTTTTTTAA AAAAGAAAAT ATGTTTTATA AAATTAATTT AATTTTCATA AAATTTATCT   
  
  
+ ATTTAATATT TTAAAAAGTA TTAAAATGCA GAAAAGAGAC AGTACATTAG AGGATGTGTT ATTGTTTCAT   
  
  
+ ATTATTATAA TAATTCACAA AATATTCTTT AAAATAAACT TTGAGACCAC CTCAGATCTG TTGAAGATCA   
  
  
+ TTGTCCTACA TAAATCCAGT TGGTGTAATA AATCTGGGGC TGTATGAAGA GGGACTGAGG ATGCAGCAGC   
  
  
+ GAGACAGGAG CTTAATTGGG AGGAGGAGAC AAAAGCCATG GCAATGGAAC ATCTGGGTGT GCAGTAAAAC   
  
  
+ TGAAGCTGAA AAAGGGGTGG TTCATTAATG GGGGAGGGGA GGGAGAAAAA GAATAACCAA GGTCTTTTCG   
  
  
+ TGGGACCCAC ACCTCAAATC TGTGACAGAG ACGGCAGTTT CATTTCTCAG CTCTTCCACC TTCACAATCC   
  
  
+ CTTATTTAAA TCTTTCTCTC TCTCACTTGA ACATTGTTGT TTCTCTGTTC TCTCTCTTTC TATCCTTTGT   
  
  
+ TTTTTGTTCT CCTGTTGAAG TTCTGACTCT CTCTTACTCT CTCTCATATG TTTTCTCTTT TAGAAAAATA   
  
  
+ TTATTGAATA AAGAAATGGG ACCTTTGTAT TCTTATGGCC GTATGTGAAA TCTCTAGCGA AGCAAAGTCT   
  
  
+ TCTGTTTCAC ACCTTTCAAT CTCAAAACAA CGGAAGAAGA AGCTAGGGTT TGACAGCACC ACCAAGCCCA   
  
  
+ AGGTTTGTTC TTTGGTACTT TTCCTCTTTG CTTCTATTGC TATTTTTTTT TATCGAATTT TTCTTATCAT   
  
  
+ AGCCGTAAGT TTATTTTCTC TTTGCTTTCA CTCAGGTTTC AAAGACCCGC TTGAATCTGA TTTTGTGGGT   
  
  
+ ATCCCTGTTT TTAAAAATTA AACCTTCTGA GAGGTTGAAC GTTTCGAATT TTTGATGAAA AGAACCACAC   
  
  
+ TTGGGATTGT GGTTTATCCA GAAATGGAAT TTTGACTACG TGGGTCGTCT TTCCTTTTTA CCTCGAGGAT   
  
  
+ CTGTTATCGT GGTAGATTTT TCCGAGTTTT TATGGTCTGT TTTGGTCTAG GTGTCCCGGG GTTGGAGAAT   
  
  
+ TAGGGTTCTT TTGGGGTTTT GGGGGCTAA  

- CCTCCTTCAC TTCACTTCAG TTGCCGTCAC CCCGGTTAGG ACCTGGGTAC GTCGACAGTC AGTGGCTGAA   
  
  
- TTGTCACAGA GGGGCCGAGT TCTATCGTCG TCGGGGGGTC GTCCTCAAAC AACGCCAGTT TCGTGTAAAA   
  
  
- CCTTATGCAC AGTCCGAGGA TACGTTGAGA AACATGTGCA CCGTAGTAGG TCGACCATTA AAAAGACCGT   
  
  
- TGCCGAGATA TAGATATCCC TTGGCCGAGA CGTCAGAGGT CATGGCCCAC CACCCAGGGT GAAAACCGCC   
  
  
- TGTAAAACTA TTCGGGAAGT GTCTATGAAA TTTTTTTTTT CCCGTCTCGT TTCATTTGTT GGTCATAAGA   
  
  
- GAGACAGTAA AAAAAAAATT TTTTCTTTTA TACAAAATAT TTTAATTAAA TTAAAAGTAT TTTAAATAGA   
  
  
- TAAATTATAA AATTTTTCAT AATTTTACGT CTTTTCTCTG TCATGTAATC TCCTACACAA TAACAAAGTA   
  
  
- TAATAATATT ATTAAGTGTT TTATAAGAAA TTTTATTTGA AACTCTGGTG GAGTCTAGAC AACTTCTAGT   
  
  
- AACAGGATGT ATTTAGGTCA ACCACATTAT TTAGACCCCG ACATACTTCT CCCTGACTCC TACGTCGTCG   
  
  
- CTCTGTCCTC GAATTAACCC TCCTCCTCTG TTTTCGGTAC CGTTACCTTG TAGACCCACA CGTCATTTTG   
  
  
- ACTTCGACTT TTTCCCCACC AAGTAATTAC CCCCTCCCCT CCCTCTTTTT CTTATTGGTT CCAGAAAAGC   
  
  
- ACCCTGGGTG TGGAGTTTAG ACACTGTCTC TGCCGTCAAA GTAAAGAGTC GAGAAGGTGG AAGTGTTAGG   
  
  
- GAATAAATTT AGAAAGAGAG AGAGTGAACT TGTAACAACA AAGAGACAAG AGAGAGAAAG ATAGGAAACA   
  
  
- AAAAACAAGA GGACAACTTC AAGACTGAGA GAGAATGAGA GAGAGTATAC AAAAGAGAAA ATCTTTTTAT   
  
  
- AATAACTTAT TTCTTTACCC TGGAAACATA AGAATACCGG CATACACTTT AGAGATCGCT TCGTTTCAGA   
  
  
- AGACAAAGTG TGGAAAGTTA GAGTTTTGTT GCCTTCTTCT TCGATCCCAA ACTGTCGTGG TGGTTCGGGT   
  
  
- TCCAAACAAG AAACCATGAA AAGGAGAAAC GAAGATAACG ATAAAAAAAA ATAGCTTAAA AAGAATAGTA   
  
  
- TCGGCATTCA AATAAAAGAG AAACGAAAGT GAGTCCAAAG TTTCTGGGCG AACTTAGACT AAAACACCCA   
  
  
- TAGGGACAAA AATTTTTAAT TTGGAAGACT CTCCAACTTG CAAAGCTTAA AAACTACTTT TCTTGGTGTG   
  
  
- AACCCTAACA CCAAATAGGT CTTTACCTTA AAACTGATGC ACCCAGCAGA AAGGAAAAAT GGAGCTCCTA   
  
  
- GACAATAGCA CCATCTAAAA AGGCTCAAAA ATACCAGACA AAACCAGATC CACAGGGCCC CAACCTCTTA   
  
  
- ATCCCAAGAA AACCCCAAAA CCCCCGATT

+     AT1-motif

| Site Name | Organism | Position | Strand | Matrix score. | sequence | function |
| --- | --- | --- | --- | --- | --- | --- |
| AT1-motif | Solanum tuberosum | 387 | - | 11 | ATTAATTTTACA | part of a light responsive module |

> 2018/04/13 10:10:12  
+ GGAGGAAGTG AAGTGAAGTC AACGGCAGTG GGGCCAATCC TGGACCCATG CAGCTGTCAG TCACCGACTT   
  
  
+ AACAGTGTCT CCCCGGCTCA AGATAGCAGC AGCCCCCCAG CAGGAGTTTG TTGCGGTCAA AGCACATTTT   
  
  
+ GGAATACGTG TCAGGCTCCT ATGCAACTCT TTGTACACGT GGCATCATCC AGCTGGTAAT TTTTCTGGCA   
  
  
+ ACGGCTCTAT ATCTATAGGG AACCGGCTCT GCAGTCTCCA GTACCGGGTG GTGGGTCCCA CTTTTGGCGG   
  
  
+ ACATTTTGAT AAGCCCTTCA CAGATACTTT AAAAAAAAAA GGGCAGAGCA AAGTAAACAA CCAGTATTCT   
  
  
+ CTCTGTCATT TTTTTTTTAA AAAAGAAAAT ATGTTTTATA AAATTAATTT AATTTTCATA AAATTTATCT   
  
  
+ ATTTAATATT TTAAAAAGTA TTAAAATGCA GAAAAGAGAC AGTACATTAG AGGATGTGTT ATTGTTTCAT   
  
  
+ ATTATTATAA TAATTCACAA AATATTCTTT AAAATAAACT TTGAGACCAC CTCAGATCTG TTGAAGATCA   
  
  
+ TTGTCCTACA TAAATCCAGT TGGTGTAATA AATCTGGGGC TGTATGAAGA GGGACTGAGG ATGCAGCAGC   
  
  
+ GAGACAGGAG CTTAATTGGG AGGAGGAGAC AAAAGCCATG GCAATGGAAC ATCTGGGTGT GCAGTAAAAC   
  
  
+ TGAAGCTGAA AAAGGGGTGG TTCATTAATG GGGGAGGGGA GGGAGAAAAA GAATAACCAA GGTCTTTTCG   
  
  
+ TGGGACCCAC ACCTCAAATC TGTGACAGAG ACGGCAGTTT CATTTCTCAG CTCTTCCACC TTCACAATCC   
  
  
+ CTTATTTAAA TCTTTCTCTC TCTCACTTGA ACATTGTTGT TTCTCTGTTC TCTCTCTTTC TATCCTTTGT   
  
  
+ TTTTTGTTCT CCTGTTGAAG TTCTGACTCT CTCTTACTCT CTCTCATATG TTTTCTCTTT TAGAAAAATA   
  
  
+ TTATTGAATA AAGAAATGGG ACCTTTGTAT TCTTATGGCC GTATGTGAAA TCTCTAGCGA AGCAAAGTCT   
  
  
+ TCTGTTTCAC ACCTTTCAAT CTCAAAACAA CGGAAGAAGA AGCTAGGGTT TGACAGCACC ACCAAGCCCA   
  
  
+ AGGTTTGTTC TTTGGTACTT TTCCTCTTTG CTTCTATTGC TATTTTTTTT TATCGAATTT TTCTTATCAT   
  
  
+ AGCCGTAAGT TTATTTTCTC TTTGCTTTCA CTCAGGTTTC AAAGACCCGC TTGAATCTGA TTTTGTGGGT   
  
  
+ ATCCCTGTTT TTAAAAATTA AACCTTCTGA GAGGTTGAAC GTTTCGAATT TTTGATGAAA AGAACCACAC   
  
  
+ TTGGGATTGT GGTTTATCCA GAAATGGAAT TTTGACTACG TGGGTCGTCT TTCCTTTTTA CCTCGAGGAT   
  
  
+ CTGTTATCGT GGTAGATTTT TCCGAGTTTT TATGGTCTGT TTTGGTCTAG GTGTCCCGGG GTTGGAGAAT   
  
  
+ TAGGGTTCTT TTGGGGTTTT GGGGGCTAA  

- CCTCCTTCAC TTCACTTCAG TTGCCGTCAC CCCGGTTAGG ACCTGGGTAC GTCGACAGTC AGTGGCTGAA   
  
  
- TTGTCACAGA GGGGCCGAGT TCTATCGTCG TCGGGGGGTC GTCCTCAAAC AACGCCAGTT TCGTGTAAAA   
  
  
- CCTTATGCAC AGTCCGAGGA TACGTTGAGA AACATGTGCA CCGTAGTAGG TCGACCATTA AAAAGACCGT   
  
  
- TGCCGAGATA TAGATATCCC TTGGCCGAGA CGTCAGAGGT CATGGCCCAC CACCCAGGGT GAAAACCGCC   
  
  
- TGTAAAACTA TTCGGGAAGT GTCTATGAAA TTTTTTTTTT CCCGTCTCGT TTCATTTGTT GGTCATAAGA   
  
  
- GAGACAGTAA AAAAAAAATT TTTTCTTTTA TACAAAATAT TTTAATTAAA TTAAAAGTAT TTTAAATAGA   
  
  
- TAAATTATAA AATTTTTCAT AATTTTACGT CTTTTCTCTG TCATGTAATC TCCTACACAA TAACAAAGTA   
  
  
- TAATAATATT ATTAAGTGTT TTATAAGAAA TTTTATTTGA AACTCTGGTG GAGTCTAGAC AACTTCTAGT   
  
  
- AACAGGATGT ATTTAGGTCA ACCACATTAT TTAGACCCCG ACATACTTCT CCCTGACTCC TACGTCGTCG   
  
  
- CTCTGTCCTC GAATTAACCC TCCTCCTCTG TTTTCGGTAC CGTTACCTTG TAGACCCACA CGTCATTTTG   
  
  
- ACTTCGACTT TTTCCCCACC AAGTAATTAC CCCCTCCCCT CCCTCTTTTT CTTATTGGTT CCAGAAAAGC   
  
  
- ACCCTGGGTG TGGAGTTTAG ACACTGTCTC TGCCGTCAAA GTAAAGAGTC GAGAAGGTGG AAGTGTTAGG   
  
  
- GAATAAATTT AGAAAGAGAG AGAGTGAACT TGTAACAACA AAGAGACAAG AGAGAGAAAG ATAGGAAACA   
  
  
- AAAAACAAGA GGACAACTTC AAGACTGAGA GAGAATGAGA GAGAGTATAC AAAAGAGAAA ATCTTTTTAT   
  
  
- AATAACTTAT TTCTTTACCC TGGAAACATA AGAATACCGG CATACACTTT AGAGATCGCT TCGTTTCAGA   
  
  
- AGACAAAGTG TGGAAAGTTA GAGTTTTGTT GCCTTCTTCT TCGATCCCAA ACTGTCGTGG TGGTTCGGGT   
  
  
- TCCAAACAAG AAACCATGAA AAGGAGAAAC GAAGATAACG ATAAAAAAAA ATAGCTTAAA AAGAATAGTA   
  
  
- TCGGCATTCA AATAAAAGAG AAACGAAAGT GAGTCCAAAG TTTCTGGGCG AACTTAGACT AAAACACCCA   
  
  
- TAGGGACAAA AATTTTTAAT TTGGAAGACT CTCCAACTTG CAAAGCTTAA AAACTACTTT TCTTGGTGTG   
  
  
- AACCCTAACA CCAAATAGGT CTTTACCTTA AAACTGATGC ACCCAGCAGA AAGGAAAAAT GGAGCTCCTA   
  
  
- GACAATAGCA CCATCTAAAA AGGCTCAAAA ATACCAGACA AAACCAGATC CACAGGGCCC CAACCTCTTA   
  
  
- ATCCCAAGAA AACCCCAAAA CCCCCGATT

+     ATC-motif

| Site Name | Organism | Position | Strand | Matrix score. | sequence | function |
| --- | --- | --- | --- | --- | --- | --- |
| ATC-motif | Arabidopsis thaliana | 33 | + | 8 | GCCAATCC | part of a conserved DNA module involved in light responsiveness |

> 2018/04/13 10:10:12  
+ GGAGGAAGTG AAGTGAAGTC AACGGCAGTG GGGCCAATCC TGGACCCATG CAGCTGTCAG TCACCGACTT   
  
  
+ AACAGTGTCT CCCCGGCTCA AGATAGCAGC AGCCCCCCAG CAGGAGTTTG TTGCGGTCAA AGCACATTTT   
  
  
+ GGAATACGTG TCAGGCTCCT ATGCAACTCT TTGTACACGT GGCATCATCC AGCTGGTAAT TTTTCTGGCA   
  
  
+ ACGGCTCTAT ATCTATAGGG AACCGGCTCT GCAGTCTCCA GTACCGGGTG GTGGGTCCCA CTTTTGGCGG   
  
  
+ ACATTTTGAT AAGCCCTTCA CAGATACTTT AAAAAAAAAA GGGCAGAGCA AAGTAAACAA CCAGTATTCT   
  
  
+ CTCTGTCATT TTTTTTTTAA AAAAGAAAAT ATGTTTTATA AAATTAATTT AATTTTCATA AAATTTATCT   
  
  
+ ATTTAATATT TTAAAAAGTA TTAAAATGCA GAAAAGAGAC AGTACATTAG AGGATGTGTT ATTGTTTCAT   
  
  
+ ATTATTATAA TAATTCACAA AATATTCTTT AAAATAAACT TTGAGACCAC CTCAGATCTG TTGAAGATCA   
  
  
+ TTGTCCTACA TAAATCCAGT TGGTGTAATA AATCTGGGGC TGTATGAAGA GGGACTGAGG ATGCAGCAGC   
  
  
+ GAGACAGGAG CTTAATTGGG AGGAGGAGAC AAAAGCCATG GCAATGGAAC ATCTGGGTGT GCAGTAAAAC   
  
  
+ TGAAGCTGAA AAAGGGGTGG TTCATTAATG GGGGAGGGGA GGGAGAAAAA GAATAACCAA GGTCTTTTCG   
  
  
+ TGGGACCCAC ACCTCAAATC TGTGACAGAG ACGGCAGTTT CATTTCTCAG CTCTTCCACC TTCACAATCC   
  
  
+ CTTATTTAAA TCTTTCTCTC TCTCACTTGA ACATTGTTGT TTCTCTGTTC TCTCTCTTTC TATCCTTTGT   
  
  
+ TTTTTGTTCT CCTGTTGAAG TTCTGACTCT CTCTTACTCT CTCTCATATG TTTTCTCTTT TAGAAAAATA   
  
  
+ TTATTGAATA AAGAAATGGG ACCTTTGTAT TCTTATGGCC GTATGTGAAA TCTCTAGCGA AGCAAAGTCT   
  
  
+ TCTGTTTCAC ACCTTTCAAT CTCAAAACAA CGGAAGAAGA AGCTAGGGTT TGACAGCACC ACCAAGCCCA   
  
  
+ AGGTTTGTTC TTTGGTACTT TTCCTCTTTG CTTCTATTGC TATTTTTTTT TATCGAATTT TTCTTATCAT   
  
  
+ AGCCGTAAGT TTATTTTCTC TTTGCTTTCA CTCAGGTTTC AAAGACCCGC TTGAATCTGA TTTTGTGGGT   
  
  
+ ATCCCTGTTT TTAAAAATTA AACCTTCTGA GAGGTTGAAC GTTTCGAATT TTTGATGAAA AGAACCACAC   
  
  
+ TTGGGATTGT GGTTTATCCA GAAATGGAAT TTTGACTACG TGGGTCGTCT TTCCTTTTTA CCTCGAGGAT   
  
  
+ CTGTTATCGT GGTAGATTTT TCCGAGTTTT TATGGTCTGT TTTGGTCTAG GTGTCCCGGG GTTGGAGAAT   
  
  
+ TAGGGTTCTT TTGGGGTTTT GGGGGCTAA  

- CCTCCTTCAC TTCACTTCAG TTGCCGTCAC CCCGGTTAGG ACCTGGGTAC GTCGACAGTC AGTGGCTGAA   
  
  
- TTGTCACAGA GGGGCCGAGT TCTATCGTCG TCGGGGGGTC GTCCTCAAAC AACGCCAGTT TCGTGTAAAA   
  
  
- CCTTATGCAC AGTCCGAGGA TACGTTGAGA AACATGTGCA CCGTAGTAGG TCGACCATTA AAAAGACCGT   
  
  
- TGCCGAGATA TAGATATCCC TTGGCCGAGA CGTCAGAGGT CATGGCCCAC CACCCAGGGT GAAAACCGCC   
  
  
- TGTAAAACTA TTCGGGAAGT GTCTATGAAA TTTTTTTTTT CCCGTCTCGT TTCATTTGTT GGTCATAAGA   
  
  
- GAGACAGTAA AAAAAAAATT TTTTCTTTTA TACAAAATAT TTTAATTAAA TTAAAAGTAT TTTAAATAGA   
  
  
- TAAATTATAA AATTTTTCAT AATTTTACGT CTTTTCTCTG TCATGTAATC TCCTACACAA TAACAAAGTA   
  
  
- TAATAATATT ATTAAGTGTT TTATAAGAAA TTTTATTTGA AACTCTGGTG GAGTCTAGAC AACTTCTAGT   
  
  
- AACAGGATGT ATTTAGGTCA ACCACATTAT TTAGACCCCG ACATACTTCT CCCTGACTCC TACGTCGTCG   
  
  
- CTCTGTCCTC GAATTAACCC TCCTCCTCTG TTTTCGGTAC CGTTACCTTG TAGACCCACA CGTCATTTTG   
  
  
- ACTTCGACTT TTTCCCCACC AAGTAATTAC CCCCTCCCCT CCCTCTTTTT CTTATTGGTT CCAGAAAAGC   
  
  
- ACCCTGGGTG TGGAGTTTAG ACACTGTCTC TGCCGTCAAA GTAAAGAGTC GAGAAGGTGG AAGTGTTAGG   
  
  
- GAATAAATTT AGAAAGAGAG AGAGTGAACT TGTAACAACA AAGAGACAAG AGAGAGAAAG ATAGGAAACA   
  
  
- AAAAACAAGA GGACAACTTC AAGACTGAGA GAGAATGAGA GAGAGTATAC AAAAGAGAAA ATCTTTTTAT   
  
  
- AATAACTTAT TTCTTTACCC TGGAAACATA AGAATACCGG CATACACTTT AGAGATCGCT TCGTTTCAGA   
  
  
- AGACAAAGTG TGGAAAGTTA GAGTTTTGTT GCCTTCTTCT TCGATCCCAA ACTGTCGTGG TGGTTCGGGT   
  
  
- TCCAAACAAG AAACCATGAA AAGGAGAAAC GAAGATAACG ATAAAAAAAA ATAGCTTAAA AAGAATAGTA   
  
  
- TCGGCATTCA AATAAAAGAG AAACGAAAGT GAGTCCAAAG TTTCTGGGCG AACTTAGACT AAAACACCCA   
  
  
- TAGGGACAAA AATTTTTAAT TTGGAAGACT CTCCAACTTG CAAAGCTTAA AAACTACTTT TCTTGGTGTG   
  
  
- AACCCTAACA CCAAATAGGT CTTTACCTTA AAACTGATGC ACCCAGCAGA AAGGAAAAAT GGAGCTCCTA   
  
  
- GACAATAGCA CCATCTAAAA AGGCTCAAAA ATACCAGACA AAACCAGATC CACAGGGCCC CAACCTCTTA   
  
  
- ATCCCAAGAA AACCCCAAAA CCCCCGATT

+     Box 4

| Site Name | Organism | Position | Strand | Matrix score. | sequence | function |
| --- | --- | --- | --- | --- | --- | --- |
| Box 4 | Petroselinum crispum | 393 | + | 6 | ATTAAT | part of a conserved DNA module involved in light responsiveness |
| Box 4 | Petroselinum crispum | 724 | + | 6 | ATTAAT | part of a conserved DNA module involved in light responsiveness |

> 2018/04/13 10:10:12  
+ GGAGGAAGTG AAGTGAAGTC AACGGCAGTG GGGCCAATCC TGGACCCATG CAGCTGTCAG TCACCGACTT   
  
  
+ AACAGTGTCT CCCCGGCTCA AGATAGCAGC AGCCCCCCAG CAGGAGTTTG TTGCGGTCAA AGCACATTTT   
  
  
+ GGAATACGTG TCAGGCTCCT ATGCAACTCT TTGTACACGT GGCATCATCC AGCTGGTAAT TTTTCTGGCA   
  
  
+ ACGGCTCTAT ATCTATAGGG AACCGGCTCT GCAGTCTCCA GTACCGGGTG GTGGGTCCCA CTTTTGGCGG   
  
  
+ ACATTTTGAT AAGCCCTTCA CAGATACTTT AAAAAAAAAA GGGCAGAGCA AAGTAAACAA CCAGTATTCT   
  
  
+ CTCTGTCATT TTTTTTTTAA AAAAGAAAAT ATGTTTTATA AAATTAATTT AATTTTCATA AAATTTATCT   
  
  
+ ATTTAATATT TTAAAAAGTA TTAAAATGCA GAAAAGAGAC AGTACATTAG AGGATGTGTT ATTGTTTCAT   
  
  
+ ATTATTATAA TAATTCACAA AATATTCTTT AAAATAAACT TTGAGACCAC CTCAGATCTG TTGAAGATCA   
  
  
+ TTGTCCTACA TAAATCCAGT TGGTGTAATA AATCTGGGGC TGTATGAAGA GGGACTGAGG ATGCAGCAGC   
  
  
+ GAGACAGGAG CTTAATTGGG AGGAGGAGAC AAAAGCCATG GCAATGGAAC ATCTGGGTGT GCAGTAAAAC   
  
  
+ TGAAGCTGAA AAAGGGGTGG TTCATTAATG GGGGAGGGGA GGGAGAAAAA GAATAACCAA GGTCTTTTCG   
  
  
+ TGGGACCCAC ACCTCAAATC TGTGACAGAG ACGGCAGTTT CATTTCTCAG CTCTTCCACC TTCACAATCC   
  
  
+ CTTATTTAAA TCTTTCTCTC TCTCACTTGA ACATTGTTGT TTCTCTGTTC TCTCTCTTTC TATCCTTTGT   
  
  
+ TTTTTGTTCT CCTGTTGAAG TTCTGACTCT CTCTTACTCT CTCTCATATG TTTTCTCTTT TAGAAAAATA   
  
  
+ TTATTGAATA AAGAAATGGG ACCTTTGTAT TCTTATGGCC GTATGTGAAA TCTCTAGCGA AGCAAAGTCT   
  
  
+ TCTGTTTCAC ACCTTTCAAT CTCAAAACAA CGGAAGAAGA AGCTAGGGTT TGACAGCACC ACCAAGCCCA   
  
  
+ AGGTTTGTTC TTTGGTACTT TTCCTCTTTG CTTCTATTGC TATTTTTTTT TATCGAATTT TTCTTATCAT   
  
  
+ AGCCGTAAGT TTATTTTCTC TTTGCTTTCA CTCAGGTTTC AAAGACCCGC TTGAATCTGA TTTTGTGGGT   
  
  
+ ATCCCTGTTT TTAAAAATTA AACCTTCTGA GAGGTTGAAC GTTTCGAATT TTTGATGAAA AGAACCACAC   
  
  
+ TTGGGATTGT GGTTTATCCA GAAATGGAAT TTTGACTACG TGGGTCGTCT TTCCTTTTTA CCTCGAGGAT   
  
  
+ CTGTTATCGT GGTAGATTTT TCCGAGTTTT TATGGTCTGT TTTGGTCTAG GTGTCCCGGG GTTGGAGAAT   
  
  
+ TAGGGTTCTT TTGGGGTTTT GGGGGCTAA  

- CCTCCTTCAC TTCACTTCAG TTGCCGTCAC CCCGGTTAGG ACCTGGGTAC GTCGACAGTC AGTGGCTGAA   
  
  
- TTGTCACAGA GGGGCCGAGT TCTATCGTCG TCGGGGGGTC GTCCTCAAAC AACGCCAGTT TCGTGTAAAA   
  
  
- CCTTATGCAC AGTCCGAGGA TACGTTGAGA AACATGTGCA CCGTAGTAGG TCGACCATTA AAAAGACCGT   
  
  
- TGCCGAGATA TAGATATCCC TTGGCCGAGA CGTCAGAGGT CATGGCCCAC CACCCAGGGT GAAAACCGCC   
  
  
- TGTAAAACTA TTCGGGAAGT GTCTATGAAA TTTTTTTTTT CCCGTCTCGT TTCATTTGTT GGTCATAAGA   
  
  
- GAGACAGTAA AAAAAAAATT TTTTCTTTTA TACAAAATAT TTTAATTAAA TTAAAAGTAT TTTAAATAGA   
  
  
- TAAATTATAA AATTTTTCAT AATTTTACGT CTTTTCTCTG TCATGTAATC TCCTACACAA TAACAAAGTA   
  
  
- TAATAATATT ATTAAGTGTT TTATAAGAAA TTTTATTTGA AACTCTGGTG GAGTCTAGAC AACTTCTAGT   
  
  
- AACAGGATGT ATTTAGGTCA ACCACATTAT TTAGACCCCG ACATACTTCT CCCTGACTCC TACGTCGTCG   
  
  
- CTCTGTCCTC GAATTAACCC TCCTCCTCTG TTTTCGGTAC CGTTACCTTG TAGACCCACA CGTCATTTTG   
  
  
- ACTTCGACTT TTTCCCCACC AAGTAATTAC CCCCTCCCCT CCCTCTTTTT CTTATTGGTT CCAGAAAAGC   
  
  
- ACCCTGGGTG TGGAGTTTAG ACACTGTCTC TGCCGTCAAA GTAAAGAGTC GAGAAGGTGG AAGTGTTAGG   
  
  
- GAATAAATTT AGAAAGAGAG AGAGTGAACT TGTAACAACA AAGAGACAAG AGAGAGAAAG ATAGGAAACA   
  
  
- AAAAACAAGA GGACAACTTC AAGACTGAGA GAGAATGAGA GAGAGTATAC AAAAGAGAAA ATCTTTTTAT   
  
  
- AATAACTTAT TTCTTTACCC TGGAAACATA AGAATACCGG CATACACTTT AGAGATCGCT TCGTTTCAGA   
  
  
- AGACAAAGTG TGGAAAGTTA GAGTTTTGTT GCCTTCTTCT TCGATCCCAA ACTGTCGTGG TGGTTCGGGT   
  
  
- TCCAAACAAG AAACCATGAA AAGGAGAAAC GAAGATAACG ATAAAAAAAA ATAGCTTAAA AAGAATAGTA   
  
  
- TCGGCATTCA AATAAAAGAG AAACGAAAGT GAGTCCAAAG TTTCTGGGCG AACTTAGACT AAAACACCCA   
  
  
- TAGGGACAAA AATTTTTAAT TTGGAAGACT CTCCAACTTG CAAAGCTTAA AAACTACTTT TCTTGGTGTG   
  
  
- AACCCTAACA CCAAATAGGT CTTTACCTTA AAACTGATGC ACCCAGCAGA AAGGAAAAAT GGAGCTCCTA   
  
  
- GACAATAGCA CCATCTAAAA AGGCTCAAAA ATACCAGACA AAACCAGATC CACAGGGCCC CAACCTCTTA   
  
  
- ATCCCAAGAA AACCCCAAAA CCCCCGATT

+     Box I

| Site Name | Organism | Position | Strand | Matrix score. | sequence | function |
| --- | --- | --- | --- | --- | --- | --- |
| Box I | Pisum sativum | 1227 | + | 7 | TTTCAAA | light responsive element |

> 2018/04/13 10:10:12  
+ GGAGGAAGTG AAGTGAAGTC AACGGCAGTG GGGCCAATCC TGGACCCATG CAGCTGTCAG TCACCGACTT   
  
  
+ AACAGTGTCT CCCCGGCTCA AGATAGCAGC AGCCCCCCAG CAGGAGTTTG TTGCGGTCAA AGCACATTTT   
  
  
+ GGAATACGTG TCAGGCTCCT ATGCAACTCT TTGTACACGT GGCATCATCC AGCTGGTAAT TTTTCTGGCA   
  
  
+ ACGGCTCTAT ATCTATAGGG AACCGGCTCT GCAGTCTCCA GTACCGGGTG GTGGGTCCCA CTTTTGGCGG   
  
  
+ ACATTTTGAT AAGCCCTTCA CAGATACTTT AAAAAAAAAA GGGCAGAGCA AAGTAAACAA CCAGTATTCT   
  
  
+ CTCTGTCATT TTTTTTTTAA AAAAGAAAAT ATGTTTTATA AAATTAATTT AATTTTCATA AAATTTATCT   
  
  
+ ATTTAATATT TTAAAAAGTA TTAAAATGCA GAAAAGAGAC AGTACATTAG AGGATGTGTT ATTGTTTCAT   
  
  
+ ATTATTATAA TAATTCACAA AATATTCTTT AAAATAAACT TTGAGACCAC CTCAGATCTG TTGAAGATCA   
  
  
+ TTGTCCTACA TAAATCCAGT TGGTGTAATA AATCTGGGGC TGTATGAAGA GGGACTGAGG ATGCAGCAGC   
  
  
+ GAGACAGGAG CTTAATTGGG AGGAGGAGAC AAAAGCCATG GCAATGGAAC ATCTGGGTGT GCAGTAAAAC   
  
  
+ TGAAGCTGAA AAAGGGGTGG TTCATTAATG GGGGAGGGGA GGGAGAAAAA GAATAACCAA GGTCTTTTCG   
  
  
+ TGGGACCCAC ACCTCAAATC TGTGACAGAG ACGGCAGTTT CATTTCTCAG CTCTTCCACC TTCACAATCC   
  
  
+ CTTATTTAAA TCTTTCTCTC TCTCACTTGA ACATTGTTGT TTCTCTGTTC TCTCTCTTTC TATCCTTTGT   
  
  
+ TTTTTGTTCT CCTGTTGAAG TTCTGACTCT CTCTTACTCT CTCTCATATG TTTTCTCTTT TAGAAAAATA   
  
  
+ TTATTGAATA AAGAAATGGG ACCTTTGTAT TCTTATGGCC GTATGTGAAA TCTCTAGCGA AGCAAAGTCT   
  
  
+ TCTGTTTCAC ACCTTTCAAT CTCAAAACAA CGGAAGAAGA AGCTAGGGTT TGACAGCACC ACCAAGCCCA   
  
  
+ AGGTTTGTTC TTTGGTACTT TTCCTCTTTG CTTCTATTGC TATTTTTTTT TATCGAATTT TTCTTATCAT   
  
  
+ AGCCGTAAGT TTATTTTCTC TTTGCTTTCA CTCAGGTTTC AAAGACCCGC TTGAATCTGA TTTTGTGGGT   
  
  
+ ATCCCTGTTT TTAAAAATTA AACCTTCTGA GAGGTTGAAC GTTTCGAATT TTTGATGAAA AGAACCACAC   
  
  
+ TTGGGATTGT GGTTTATCCA GAAATGGAAT TTTGACTACG TGGGTCGTCT TTCCTTTTTA CCTCGAGGAT   
  
  
+ CTGTTATCGT GGTAGATTTT TCCGAGTTTT TATGGTCTGT TTTGGTCTAG GTGTCCCGGG GTTGGAGAAT   
  
  
+ TAGGGTTCTT TTGGGGTTTT GGGGGCTAA  

- CCTCCTTCAC TTCACTTCAG TTGCCGTCAC CCCGGTTAGG ACCTGGGTAC GTCGACAGTC AGTGGCTGAA   
  
  
- TTGTCACAGA GGGGCCGAGT TCTATCGTCG TCGGGGGGTC GTCCTCAAAC AACGCCAGTT TCGTGTAAAA   
  
  
- CCTTATGCAC AGTCCGAGGA TACGTTGAGA AACATGTGCA CCGTAGTAGG TCGACCATTA AAAAGACCGT   
  
  
- TGCCGAGATA TAGATATCCC TTGGCCGAGA CGTCAGAGGT CATGGCCCAC CACCCAGGGT GAAAACCGCC   
  
  
- TGTAAAACTA TTCGGGAAGT GTCTATGAAA TTTTTTTTTT CCCGTCTCGT TTCATTTGTT GGTCATAAGA   
  
  
- GAGACAGTAA AAAAAAAATT TTTTCTTTTA TACAAAATAT TTTAATTAAA TTAAAAGTAT TTTAAATAGA   
  
  
- TAAATTATAA AATTTTTCAT AATTTTACGT CTTTTCTCTG TCATGTAATC TCCTACACAA TAACAAAGTA   
  
  
- TAATAATATT ATTAAGTGTT TTATAAGAAA TTTTATTTGA AACTCTGGTG GAGTCTAGAC AACTTCTAGT   
  
  
- AACAGGATGT ATTTAGGTCA ACCACATTAT TTAGACCCCG ACATACTTCT CCCTGACTCC TACGTCGTCG   
  
  
- CTCTGTCCTC GAATTAACCC TCCTCCTCTG TTTTCGGTAC CGTTACCTTG TAGACCCACA CGTCATTTTG   
  
  
- ACTTCGACTT TTTCCCCACC AAGTAATTAC CCCCTCCCCT CCCTCTTTTT CTTATTGGTT CCAGAAAAGC   
  
  
- ACCCTGGGTG TGGAGTTTAG ACACTGTCTC TGCCGTCAAA GTAAAGAGTC GAGAAGGTGG AAGTGTTAGG   
  
  
- GAATAAATTT AGAAAGAGAG AGAGTGAACT TGTAACAACA AAGAGACAAG AGAGAGAAAG ATAGGAAACA   
  
  
- AAAAACAAGA GGACAACTTC AAGACTGAGA GAGAATGAGA GAGAGTATAC AAAAGAGAAA ATCTTTTTAT   
  
  
- AATAACTTAT TTCTTTACCC TGGAAACATA AGAATACCGG CATACACTTT AGAGATCGCT TCGTTTCAGA   
  
  
- AGACAAAGTG TGGAAAGTTA GAGTTTTGTT GCCTTCTTCT TCGATCCCAA ACTGTCGTGG TGGTTCGGGT   
  
  
- TCCAAACAAG AAACCATGAA AAGGAGAAAC GAAGATAACG ATAAAAAAAA ATAGCTTAAA AAGAATAGTA   
  
  
- TCGGCATTCA AATAAAAGAG AAACGAAAGT GAGTCCAAAG TTTCTGGGCG AACTTAGACT AAAACACCCA   
  
  
- TAGGGACAAA AATTTTTAAT TTGGAAGACT CTCCAACTTG CAAAGCTTAA AAACTACTTT TCTTGGTGTG   
  
  
- AACCCTAACA CCAAATAGGT CTTTACCTTA AAACTGATGC ACCCAGCAGA AAGGAAAAAT GGAGCTCCTA   
  
  
- GACAATAGCA CCATCTAAAA AGGCTCAAAA ATACCAGACA AAACCAGATC CACAGGGCCC CAACCTCTTA   
  
  
- ATCCCAAGAA AACCCCAAAA CCCCCGATT

+     Box-W1

| Site Name | Organism | Position | Strand | Matrix score. | sequence | function |
| --- | --- | --- | --- | --- | --- | --- |
| Box-W1 | Petroselinum crispum | 125 | - | 6 | TTGACC | fungal elicitor responsive element |

> 2018/04/13 10:10:12  
+ GGAGGAAGTG AAGTGAAGTC AACGGCAGTG GGGCCAATCC TGGACCCATG CAGCTGTCAG TCACCGACTT   
  
  
+ AACAGTGTCT CCCCGGCTCA AGATAGCAGC AGCCCCCCAG CAGGAGTTTG TTGCGGTCAA AGCACATTTT   
  
  
+ GGAATACGTG TCAGGCTCCT ATGCAACTCT TTGTACACGT GGCATCATCC AGCTGGTAAT TTTTCTGGCA   
  
  
+ ACGGCTCTAT ATCTATAGGG AACCGGCTCT GCAGTCTCCA GTACCGGGTG GTGGGTCCCA CTTTTGGCGG   
  
  
+ ACATTTTGAT AAGCCCTTCA CAGATACTTT AAAAAAAAAA GGGCAGAGCA AAGTAAACAA CCAGTATTCT   
  
  
+ CTCTGTCATT TTTTTTTTAA AAAAGAAAAT ATGTTTTATA AAATTAATTT AATTTTCATA AAATTTATCT   
  
  
+ ATTTAATATT TTAAAAAGTA TTAAAATGCA GAAAAGAGAC AGTACATTAG AGGATGTGTT ATTGTTTCAT   
  
  
+ ATTATTATAA TAATTCACAA AATATTCTTT AAAATAAACT TTGAGACCAC CTCAGATCTG TTGAAGATCA   
  
  
+ TTGTCCTACA TAAATCCAGT TGGTGTAATA AATCTGGGGC TGTATGAAGA GGGACTGAGG ATGCAGCAGC   
  
  
+ GAGACAGGAG CTTAATTGGG AGGAGGAGAC AAAAGCCATG GCAATGGAAC ATCTGGGTGT GCAGTAAAAC   
  
  
+ TGAAGCTGAA AAAGGGGTGG TTCATTAATG GGGGAGGGGA GGGAGAAAAA GAATAACCAA GGTCTTTTCG   
  
  
+ TGGGACCCAC ACCTCAAATC TGTGACAGAG ACGGCAGTTT CATTTCTCAG CTCTTCCACC TTCACAATCC   
  
  
+ CTTATTTAAA TCTTTCTCTC TCTCACTTGA ACATTGTTGT TTCTCTGTTC TCTCTCTTTC TATCCTTTGT   
  
  
+ TTTTTGTTCT CCTGTTGAAG TTCTGACTCT CTCTTACTCT CTCTCATATG TTTTCTCTTT TAGAAAAATA   
  
  
+ TTATTGAATA AAGAAATGGG ACCTTTGTAT TCTTATGGCC GTATGTGAAA TCTCTAGCGA AGCAAAGTCT   
  
  
+ TCTGTTTCAC ACCTTTCAAT CTCAAAACAA CGGAAGAAGA AGCTAGGGTT TGACAGCACC ACCAAGCCCA   
  
  
+ AGGTTTGTTC TTTGGTACTT TTCCTCTTTG CTTCTATTGC TATTTTTTTT TATCGAATTT TTCTTATCAT   
  
  
+ AGCCGTAAGT TTATTTTCTC TTTGCTTTCA CTCAGGTTTC AAAGACCCGC TTGAATCTGA TTTTGTGGGT   
  
  
+ ATCCCTGTTT TTAAAAATTA AACCTTCTGA GAGGTTGAAC GTTTCGAATT TTTGATGAAA AGAACCACAC   
  
  
+ TTGGGATTGT GGTTTATCCA GAAATGGAAT TTTGACTACG TGGGTCGTCT TTCCTTTTTA CCTCGAGGAT   
  
  
+ CTGTTATCGT GGTAGATTTT TCCGAGTTTT TATGGTCTGT TTTGGTCTAG GTGTCCCGGG GTTGGAGAAT   
  
  
+ TAGGGTTCTT TTGGGGTTTT GGGGGCTAA  

- CCTCCTTCAC TTCACTTCAG TTGCCGTCAC CCCGGTTAGG ACCTGGGTAC GTCGACAGTC AGTGGCTGAA   
  
  
- TTGTCACAGA GGGGCCGAGT TCTATCGTCG TCGGGGGGTC GTCCTCAAAC AACGCCAGTT TCGTGTAAAA   
  
  
- CCTTATGCAC AGTCCGAGGA TACGTTGAGA AACATGTGCA CCGTAGTAGG TCGACCATTA AAAAGACCGT   
  
  
- TGCCGAGATA TAGATATCCC TTGGCCGAGA CGTCAGAGGT CATGGCCCAC CACCCAGGGT GAAAACCGCC   
  
  
- TGTAAAACTA TTCGGGAAGT GTCTATGAAA TTTTTTTTTT CCCGTCTCGT TTCATTTGTT GGTCATAAGA   
  
  
- GAGACAGTAA AAAAAAAATT TTTTCTTTTA TACAAAATAT TTTAATTAAA TTAAAAGTAT TTTAAATAGA   
  
  
- TAAATTATAA AATTTTTCAT AATTTTACGT CTTTTCTCTG TCATGTAATC TCCTACACAA TAACAAAGTA   
  
  
- TAATAATATT ATTAAGTGTT TTATAAGAAA TTTTATTTGA AACTCTGGTG GAGTCTAGAC AACTTCTAGT   
  
  
- AACAGGATGT ATTTAGGTCA ACCACATTAT TTAGACCCCG ACATACTTCT CCCTGACTCC TACGTCGTCG   
  
  
- CTCTGTCCTC GAATTAACCC TCCTCCTCTG TTTTCGGTAC CGTTACCTTG TAGACCCACA CGTCATTTTG   
  
  
- ACTTCGACTT TTTCCCCACC AAGTAATTAC CCCCTCCCCT CCCTCTTTTT CTTATTGGTT CCAGAAAAGC   
  
  
- ACCCTGGGTG TGGAGTTTAG ACACTGTCTC TGCCGTCAAA GTAAAGAGTC GAGAAGGTGG AAGTGTTAGG   
  
  
- GAATAAATTT AGAAAGAGAG AGAGTGAACT TGTAACAACA AAGAGACAAG AGAGAGAAAG ATAGGAAACA   
  
  
- AAAAACAAGA GGACAACTTC AAGACTGAGA GAGAATGAGA GAGAGTATAC AAAAGAGAAA ATCTTTTTAT   
  
  
- AATAACTTAT TTCTTTACCC TGGAAACATA AGAATACCGG CATACACTTT AGAGATCGCT TCGTTTCAGA   
  
  
- AGACAAAGTG TGGAAAGTTA GAGTTTTGTT GCCTTCTTCT TCGATCCCAA ACTGTCGTGG TGGTTCGGGT   
  
  
- TCCAAACAAG AAACCATGAA AAGGAGAAAC GAAGATAACG ATAAAAAAAA ATAGCTTAAA AAGAATAGTA   
  
  
- TCGGCATTCA AATAAAAGAG AAACGAAAGT GAGTCCAAAG TTTCTGGGCG AACTTAGACT AAAACACCCA   
  
  
- TAGGGACAAA AATTTTTAAT TTGGAAGACT CTCCAACTTG CAAAGCTTAA AAACTACTTT TCTTGGTGTG   
  
  
- AACCCTAACA CCAAATAGGT CTTTACCTTA AAACTGATGC ACCCAGCAGA AAGGAAAAAT GGAGCTCCTA   
  
  
- GACAATAGCA CCATCTAAAA AGGCTCAAAA ATACCAGACA AAACCAGATC CACAGGGCCC CAACCTCTTA   
  
  
- ATCCCAAGAA AACCCCAAAA CCCCCGATT

+     CAAT-box

| Site Name | Organism | Position | Strand | Matrix score. | sequence | function |
| --- | --- | --- | --- | --- | --- | --- |
| CAAT-box | Hordeum vulgare | 1336 | - | 4 | CAAT | common cis-acting element in promoter and enhancer regions |
| CAAT-box | Arabidopsis thaliana | 113 | - | 10 | CAACCAACTCC | common cis-acting element in promoter and enhancer regions |
| CAAT-box | Brassica rapa | 785 | + | 5 | CAAAT | common cis-acting element in promoter and enhancer regions |
| CAAT-box | Hordeum vulgare | 672 | + | 4 | CAAT | common cis-acting element in promoter and enhancer regions |
| CAAT-box | Hordeum vulgare | 873 | - | 4 | CAAT | common cis-acting element in promoter and enhancer regions |
| CAAT-box | Hordeum vulgare | 835 | + | 4 | CAAT | common cis-acting element in promoter and enhancer regions |
| CAAT-box | Hordeum vulgare | 1156 | - | 4 | CAAT | common cis-acting element in promoter and enhancer regions |
| CAAT-box | Hordeum vulgare | 1067 | + | 4 | CAAT | common cis-acting element in promoter and enhancer regions |
| CAAT-box | Hordeum vulgare | 983 | - | 4 | CAAT | common cis-acting element in promoter and enhancer regions |
| CAAT-box | Hordeum vulgare | 35 | + | 4 | CAAT | common cis-acting element in promoter and enhancer regions |
| CAAT-box | Arabidopsis thaliana | 34 | + | 5 | CCAAT | common cis-acting element in promoter and enhancer regions |
| CAAT-box | Hordeum vulgare | 560 | - | 4 | CAAT | common cis-acting element in promoter and enhancer regions |
| CAAT-box | Hordeum vulgare | 481 | - | 4 | CAAT | common cis-acting element in promoter and enhancer regions |
| CAAT-box | Arabidopsis thaliana | 670 | + | 6 | gGCAAT | common cis-acting element in promoter and enhancer regions |
| CAAT-box | Arabidopsis thaliana | 645 | - | 5 | CCAAT | common cis-acting element in promoter and enhancer regions |
| CAAT-box | Glycine max | 644 | - | 5 | CAATT | common cis-acting element in promoter and enhancer regions |

> 2018/04/13 10:10:12  
+ GGAGGAAGTG AAGTGAAGTC AACGGCAGTG GGGCCAATCC TGGACCCATG CAGCTGTCAG TCACCGACTT   
  
  
+ AACAGTGTCT CCCCGGCTCA AGATAGCAGC AGCCCCCCAG CAGGAGTTTG TTGCGGTCAA AGCACATTTT   
  
  
+ GGAATACGTG TCAGGCTCCT ATGCAACTCT TTGTACACGT GGCATCATCC AGCTGGTAAT TTTTCTGGCA   
  
  
+ ACGGCTCTAT ATCTATAGGG AACCGGCTCT GCAGTCTCCA GTACCGGGTG GTGGGTCCCA CTTTTGGCGG   
  
  
+ ACATTTTGAT AAGCCCTTCA CAGATACTTT AAAAAAAAAA GGGCAGAGCA AAGTAAACAA CCAGTATTCT   
  
  
+ CTCTGTCATT TTTTTTTTAA AAAAGAAAAT ATGTTTTATA AAATTAATTT AATTTTCATA AAATTTATCT   
  
  
+ ATTTAATATT TTAAAAAGTA TTAAAATGCA GAAAAGAGAC AGTACATTAG AGGATGTGTT ATTGTTTCAT   
  
  
+ ATTATTATAA TAATTCACAA AATATTCTTT AAAATAAACT TTGAGACCAC CTCAGATCTG TTGAAGATCA   
  
  
+ TTGTCCTACA TAAATCCAGT TGGTGTAATA AATCTGGGGC TGTATGAAGA GGGACTGAGG ATGCAGCAGC   
  
  
+ GAGACAGGAG CTTAATTGGG AGGAGGAGAC AAAAGCCATG GCAATGGAAC ATCTGGGTGT GCAGTAAAAC   
  
  
+ TGAAGCTGAA AAAGGGGTGG TTCATTAATG GGGGAGGGGA GGGAGAAAAA GAATAACCAA GGTCTTTTCG   
  
  
+ TGGGACCCAC ACCTCAAATC TGTGACAGAG ACGGCAGTTT CATTTCTCAG CTCTTCCACC TTCACAATCC   
  
  
+ CTTATTTAAA TCTTTCTCTC TCTCACTTGA ACATTGTTGT TTCTCTGTTC TCTCTCTTTC TATCCTTTGT   
  
  
+ TTTTTGTTCT CCTGTTGAAG TTCTGACTCT CTCTTACTCT CTCTCATATG TTTTCTCTTT TAGAAAAATA   
  
  
+ TTATTGAATA AAGAAATGGG ACCTTTGTAT TCTTATGGCC GTATGTGAAA TCTCTAGCGA AGCAAAGTCT   
  
  
+ TCTGTTTCAC ACCTTTCAAT CTCAAAACAA CGGAAGAAGA AGCTAGGGTT TGACAGCACC ACCAAGCCCA   
  
  
+ AGGTTTGTTC TTTGGTACTT TTCCTCTTTG CTTCTATTGC TATTTTTTTT TATCGAATTT TTCTTATCAT   
  
  
+ AGCCGTAAGT TTATTTTCTC TTTGCTTTCA CTCAGGTTTC AAAGACCCGC TTGAATCTGA TTTTGTGGGT   
  
  
+ ATCCCTGTTT TTAAAAATTA AACCTTCTGA GAGGTTGAAC GTTTCGAATT TTTGATGAAA AGAACCACAC   
  
  
+ TTGGGATTGT GGTTTATCCA GAAATGGAAT TTTGACTACG TGGGTCGTCT TTCCTTTTTA CCTCGAGGAT   
  
  
+ CTGTTATCGT GGTAGATTTT TCCGAGTTTT TATGGTCTGT TTTGGTCTAG GTGTCCCGGG GTTGGAGAAT   
  
  
+ TAGGGTTCTT TTGGGGTTTT GGGGGCTAA  

- CCTCCTTCAC TTCACTTCAG TTGCCGTCAC CCCGGTTAGG ACCTGGGTAC GTCGACAGTC AGTGGCTGAA   
  
  
- TTGTCACAGA GGGGCCGAGT TCTATCGTCG TCGGGGGGTC GTCCTCAAAC AACGCCAGTT TCGTGTAAAA   
  
  
- CCTTATGCAC AGTCCGAGGA TACGTTGAGA AACATGTGCA CCGTAGTAGG TCGACCATTA AAAAGACCGT   
  
  
- TGCCGAGATA TAGATATCCC TTGGCCGAGA CGTCAGAGGT CATGGCCCAC CACCCAGGGT GAAAACCGCC   
  
  
- TGTAAAACTA TTCGGGAAGT GTCTATGAAA TTTTTTTTTT CCCGTCTCGT TTCATTTGTT GGTCATAAGA   
  
  
- GAGACAGTAA AAAAAAAATT TTTTCTTTTA TACAAAATAT TTTAATTAAA TTAAAAGTAT TTTAAATAGA   
  
  
- TAAATTATAA AATTTTTCAT AATTTTACGT CTTTTCTCTG TCATGTAATC TCCTACACAA TAACAAAGTA   
  
  
- TAATAATATT ATTAAGTGTT TTATAAGAAA TTTTATTTGA AACTCTGGTG GAGTCTAGAC AACTTCTAGT   
  
  
- AACAGGATGT ATTTAGGTCA ACCACATTAT TTAGACCCCG ACATACTTCT CCCTGACTCC TACGTCGTCG   
  
  
- CTCTGTCCTC GAATTAACCC TCCTCCTCTG TTTTCGGTAC CGTTACCTTG TAGACCCACA CGTCATTTTG   
  
  
- ACTTCGACTT TTTCCCCACC AAGTAATTAC CCCCTCCCCT CCCTCTTTTT CTTATTGGTT CCAGAAAAGC   
  
  
- ACCCTGGGTG TGGAGTTTAG ACACTGTCTC TGCCGTCAAA GTAAAGAGTC GAGAAGGTGG AAGTGTTAGG   
  
  
- GAATAAATTT AGAAAGAGAG AGAGTGAACT TGTAACAACA AAGAGACAAG AGAGAGAAAG ATAGGAAACA   
  
  
- AAAAACAAGA GGACAACTTC AAGACTGAGA GAGAATGAGA GAGAGTATAC AAAAGAGAAA ATCTTTTTAT   
  
  
- AATAACTTAT TTCTTTACCC TGGAAACATA AGAATACCGG CATACACTTT AGAGATCGCT TCGTTTCAGA   
  
  
- AGACAAAGTG TGGAAAGTTA GAGTTTTGTT GCCTTCTTCT TCGATCCCAA ACTGTCGTGG TGGTTCGGGT   
  
  
- TCCAAACAAG AAACCATGAA AAGGAGAAAC GAAGATAACG ATAAAAAAAA ATAGCTTAAA AAGAATAGTA   
  
  
- TCGGCATTCA AATAAAAGAG AAACGAAAGT GAGTCCAAAG TTTCTGGGCG AACTTAGACT AAAACACCCA   
  
  
- TAGGGACAAA AATTTTTAAT TTGGAAGACT CTCCAACTTG CAAAGCTTAA AAACTACTTT TCTTGGTGTG   
  
  
- AACCCTAACA CCAAATAGGT CTTTACCTTA AAACTGATGC ACCCAGCAGA AAGGAAAAAT GGAGCTCCTA   
  
  
- GACAATAGCA CCATCTAAAA AGGCTCAAAA ATACCAGACA AAACCAGATC CACAGGGCCC CAACCTCTTA   
  
  
- ATCCCAAGAA AACCCCAAAA CCCCCGATT

+     CCAAT-box

| Site Name | Organism | Position | Strand | Matrix score. | sequence | function |
| --- | --- | --- | --- | --- | --- | --- |
| CCAAT-box | Hordeum vulgare | 1078 | + | 6 | CAACGG | MYBHv1 binding site |
| CCAAT-box | Hordeum vulgare | 20 | + | 6 | CAACGG | MYBHv1 binding site |
| CCAAT-box | Hordeum vulgare | 209 | + | 6 | CAACGG | MYBHv1 binding site |

> 2018/04/13 10:10:12  
+ GGAGGAAGTG AAGTGAAGTC AACGGCAGTG GGGCCAATCC TGGACCCATG CAGCTGTCAG TCACCGACTT   
  
  
+ AACAGTGTCT CCCCGGCTCA AGATAGCAGC AGCCCCCCAG CAGGAGTTTG TTGCGGTCAA AGCACATTTT   
  
  
+ GGAATACGTG TCAGGCTCCT ATGCAACTCT TTGTACACGT GGCATCATCC AGCTGGTAAT TTTTCTGGCA   
  
  
+ ACGGCTCTAT ATCTATAGGG AACCGGCTCT GCAGTCTCCA GTACCGGGTG GTGGGTCCCA CTTTTGGCGG   
  
  
+ ACATTTTGAT AAGCCCTTCA CAGATACTTT AAAAAAAAAA GGGCAGAGCA AAGTAAACAA CCAGTATTCT   
  
  
+ CTCTGTCATT TTTTTTTTAA AAAAGAAAAT ATGTTTTATA AAATTAATTT AATTTTCATA AAATTTATCT   
  
  
+ ATTTAATATT TTAAAAAGTA TTAAAATGCA GAAAAGAGAC AGTACATTAG AGGATGTGTT ATTGTTTCAT   
  
  
+ ATTATTATAA TAATTCACAA AATATTCTTT AAAATAAACT TTGAGACCAC CTCAGATCTG TTGAAGATCA   
  
  
+ TTGTCCTACA TAAATCCAGT TGGTGTAATA AATCTGGGGC TGTATGAAGA GGGACTGAGG ATGCAGCAGC   
  
  
+ GAGACAGGAG CTTAATTGGG AGGAGGAGAC AAAAGCCATG GCAATGGAAC ATCTGGGTGT GCAGTAAAAC   
  
  
+ TGAAGCTGAA AAAGGGGTGG TTCATTAATG GGGGAGGGGA GGGAGAAAAA GAATAACCAA GGTCTTTTCG   
  
  
+ TGGGACCCAC ACCTCAAATC TGTGACAGAG ACGGCAGTTT CATTTCTCAG CTCTTCCACC TTCACAATCC   
  
  
+ CTTATTTAAA TCTTTCTCTC TCTCACTTGA ACATTGTTGT TTCTCTGTTC TCTCTCTTTC TATCCTTTGT   
  
  
+ TTTTTGTTCT CCTGTTGAAG TTCTGACTCT CTCTTACTCT CTCTCATATG TTTTCTCTTT TAGAAAAATA   
  
  
+ TTATTGAATA AAGAAATGGG ACCTTTGTAT TCTTATGGCC GTATGTGAAA TCTCTAGCGA AGCAAAGTCT   
  
  
+ TCTGTTTCAC ACCTTTCAAT CTCAAAACAA CGGAAGAAGA AGCTAGGGTT TGACAGCACC ACCAAGCCCA   
  
  
+ AGGTTTGTTC TTTGGTACTT TTCCTCTTTG CTTCTATTGC TATTTTTTTT TATCGAATTT TTCTTATCAT   
  
  
+ AGCCGTAAGT TTATTTTCTC TTTGCTTTCA CTCAGGTTTC AAAGACCCGC TTGAATCTGA TTTTGTGGGT   
  
  
+ ATCCCTGTTT TTAAAAATTA AACCTTCTGA GAGGTTGAAC GTTTCGAATT TTTGATGAAA AGAACCACAC   
  
  
+ TTGGGATTGT GGTTTATCCA GAAATGGAAT TTTGACTACG TGGGTCGTCT TTCCTTTTTA CCTCGAGGAT   
  
  
+ CTGTTATCGT GGTAGATTTT TCCGAGTTTT TATGGTCTGT TTTGGTCTAG GTGTCCCGGG GTTGGAGAAT   
  
  
+ TAGGGTTCTT TTGGGGTTTT GGGGGCTAA  

- CCTCCTTCAC TTCACTTCAG TTGCCGTCAC CCCGGTTAGG ACCTGGGTAC GTCGACAGTC AGTGGCTGAA   
  
  
- TTGTCACAGA GGGGCCGAGT TCTATCGTCG TCGGGGGGTC GTCCTCAAAC AACGCCAGTT TCGTGTAAAA   
  
  
- CCTTATGCAC AGTCCGAGGA TACGTTGAGA AACATGTGCA CCGTAGTAGG TCGACCATTA AAAAGACCGT   
  
  
- TGCCGAGATA TAGATATCCC TTGGCCGAGA CGTCAGAGGT CATGGCCCAC CACCCAGGGT GAAAACCGCC   
  
  
- TGTAAAACTA TTCGGGAAGT GTCTATGAAA TTTTTTTTTT CCCGTCTCGT TTCATTTGTT GGTCATAAGA   
  
  
- GAGACAGTAA AAAAAAAATT TTTTCTTTTA TACAAAATAT TTTAATTAAA TTAAAAGTAT TTTAAATAGA   
  
  
- TAAATTATAA AATTTTTCAT AATTTTACGT CTTTTCTCTG TCATGTAATC TCCTACACAA TAACAAAGTA   
  
  
- TAATAATATT ATTAAGTGTT TTATAAGAAA TTTTATTTGA AACTCTGGTG GAGTCTAGAC AACTTCTAGT   
  
  
- AACAGGATGT ATTTAGGTCA ACCACATTAT TTAGACCCCG ACATACTTCT CCCTGACTCC TACGTCGTCG   
  
  
- CTCTGTCCTC GAATTAACCC TCCTCCTCTG TTTTCGGTAC CGTTACCTTG TAGACCCACA CGTCATTTTG   
  
  
- ACTTCGACTT TTTCCCCACC AAGTAATTAC CCCCTCCCCT CCCTCTTTTT CTTATTGGTT CCAGAAAAGC   
  
  
- ACCCTGGGTG TGGAGTTTAG ACACTGTCTC TGCCGTCAAA GTAAAGAGTC GAGAAGGTGG AAGTGTTAGG   
  
  
- GAATAAATTT AGAAAGAGAG AGAGTGAACT TGTAACAACA AAGAGACAAG AGAGAGAAAG ATAGGAAACA   
  
  
- AAAAACAAGA GGACAACTTC AAGACTGAGA GAGAATGAGA GAGAGTATAC AAAAGAGAAA ATCTTTTTAT   
  
  
- AATAACTTAT TTCTTTACCC TGGAAACATA AGAATACCGG CATACACTTT AGAGATCGCT TCGTTTCAGA   
  
  
- AGACAAAGTG TGGAAAGTTA GAGTTTTGTT GCCTTCTTCT TCGATCCCAA ACTGTCGTGG TGGTTCGGGT   
  
  
- TCCAAACAAG AAACCATGAA AAGGAGAAAC GAAGATAACG ATAAAAAAAA ATAGCTTAAA AAGAATAGTA   
  
  
- TCGGCATTCA AATAAAAGAG AAACGAAAGT GAGTCCAAAG TTTCTGGGCG AACTTAGACT AAAACACCCA   
  
  
- TAGGGACAAA AATTTTTAAT TTGGAAGACT CTCCAACTTG CAAAGCTTAA AAACTACTTT TCTTGGTGTG   
  
  
- AACCCTAACA CCAAATAGGT CTTTACCTTA AAACTGATGC ACCCAGCAGA AAGGAAAAAT GGAGCTCCTA   
  
  
- GACAATAGCA CCATCTAAAA AGGCTCAAAA ATACCAGACA AAACCAGATC CACAGGGCCC CAACCTCTTA   
  
  
- ATCCCAAGAA AACCCCAAAA CCCCCGATT

+     G-Box

| Site Name | Organism | Position | Strand | Matrix score. | sequence | function |
| --- | --- | --- | --- | --- | --- | --- |
| G-Box | Antirrhinum majus | 1367 | - | 6 | CACGTA | cis-acting regulatory element involved in light responsiveness |
| G-Box | Antirrhinum majus | 145 | - | 6 | CACGTA | cis-acting regulatory element involved in light responsiveness |
| G-Box | Pisum sativum | 176 | + | 6 | CACGTG | cis-acting regulatory element involved in light responsiveness |

> 2018/04/13 10:10:12  
+ GGAGGAAGTG AAGTGAAGTC AACGGCAGTG GGGCCAATCC TGGACCCATG CAGCTGTCAG TCACCGACTT   
  
  
+ AACAGTGTCT CCCCGGCTCA AGATAGCAGC AGCCCCCCAG CAGGAGTTTG TTGCGGTCAA AGCACATTTT   
  
  
+ GGAATACGTG TCAGGCTCCT ATGCAACTCT TTGTACACGT GGCATCATCC AGCTGGTAAT TTTTCTGGCA   
  
  
+ ACGGCTCTAT ATCTATAGGG AACCGGCTCT GCAGTCTCCA GTACCGGGTG GTGGGTCCCA CTTTTGGCGG   
  
  
+ ACATTTTGAT AAGCCCTTCA CAGATACTTT AAAAAAAAAA GGGCAGAGCA AAGTAAACAA CCAGTATTCT   
  
  
+ CTCTGTCATT TTTTTTTTAA AAAAGAAAAT ATGTTTTATA AAATTAATTT AATTTTCATA AAATTTATCT   
  
  
+ ATTTAATATT TTAAAAAGTA TTAAAATGCA GAAAAGAGAC AGTACATTAG AGGATGTGTT ATTGTTTCAT   
  
  
+ ATTATTATAA TAATTCACAA AATATTCTTT AAAATAAACT TTGAGACCAC CTCAGATCTG TTGAAGATCA   
  
  
+ TTGTCCTACA TAAATCCAGT TGGTGTAATA AATCTGGGGC TGTATGAAGA GGGACTGAGG ATGCAGCAGC   
  
  
+ GAGACAGGAG CTTAATTGGG AGGAGGAGAC AAAAGCCATG GCAATGGAAC ATCTGGGTGT GCAGTAAAAC   
  
  
+ TGAAGCTGAA AAAGGGGTGG TTCATTAATG GGGGAGGGGA GGGAGAAAAA GAATAACCAA GGTCTTTTCG   
  
  
+ TGGGACCCAC ACCTCAAATC TGTGACAGAG ACGGCAGTTT CATTTCTCAG CTCTTCCACC TTCACAATCC   
  
  
+ CTTATTTAAA TCTTTCTCTC TCTCACTTGA ACATTGTTGT TTCTCTGTTC TCTCTCTTTC TATCCTTTGT   
  
  
+ TTTTTGTTCT CCTGTTGAAG TTCTGACTCT CTCTTACTCT CTCTCATATG TTTTCTCTTT TAGAAAAATA   
  
  
+ TTATTGAATA AAGAAATGGG ACCTTTGTAT TCTTATGGCC GTATGTGAAA TCTCTAGCGA AGCAAAGTCT   
  
  
+ TCTGTTTCAC ACCTTTCAAT CTCAAAACAA CGGAAGAAGA AGCTAGGGTT TGACAGCACC ACCAAGCCCA   
  
  
+ AGGTTTGTTC TTTGGTACTT TTCCTCTTTG CTTCTATTGC TATTTTTTTT TATCGAATTT TTCTTATCAT   
  
  
+ AGCCGTAAGT TTATTTTCTC TTTGCTTTCA CTCAGGTTTC AAAGACCCGC TTGAATCTGA TTTTGTGGGT   
  
  
+ ATCCCTGTTT TTAAAAATTA AACCTTCTGA GAGGTTGAAC GTTTCGAATT TTTGATGAAA AGAACCACAC   
  
  
+ TTGGGATTGT GGTTTATCCA GAAATGGAAT TTTGACTACG TGGGTCGTCT TTCCTTTTTA CCTCGAGGAT   
  
  
+ CTGTTATCGT GGTAGATTTT TCCGAGTTTT TATGGTCTGT TTTGGTCTAG GTGTCCCGGG GTTGGAGAAT   
  
  
+ TAGGGTTCTT TTGGGGTTTT GGGGGCTAA  

- CCTCCTTCAC TTCACTTCAG TTGCCGTCAC CCCGGTTAGG ACCTGGGTAC GTCGACAGTC AGTGGCTGAA   
  
  
- TTGTCACAGA GGGGCCGAGT TCTATCGTCG TCGGGGGGTC GTCCTCAAAC AACGCCAGTT TCGTGTAAAA   
  
  
- CCTTATGCAC AGTCCGAGGA TACGTTGAGA AACATGTGCA CCGTAGTAGG TCGACCATTA AAAAGACCGT   
  
  
- TGCCGAGATA TAGATATCCC TTGGCCGAGA CGTCAGAGGT CATGGCCCAC CACCCAGGGT GAAAACCGCC   
  
  
- TGTAAAACTA TTCGGGAAGT GTCTATGAAA TTTTTTTTTT CCCGTCTCGT TTCATTTGTT GGTCATAAGA   
  
  
- GAGACAGTAA AAAAAAAATT TTTTCTTTTA TACAAAATAT TTTAATTAAA TTAAAAGTAT TTTAAATAGA   
  
  
- TAAATTATAA AATTTTTCAT AATTTTACGT CTTTTCTCTG TCATGTAATC TCCTACACAA TAACAAAGTA   
  
  
- TAATAATATT ATTAAGTGTT TTATAAGAAA TTTTATTTGA AACTCTGGTG GAGTCTAGAC AACTTCTAGT   
  
  
- AACAGGATGT ATTTAGGTCA ACCACATTAT TTAGACCCCG ACATACTTCT CCCTGACTCC TACGTCGTCG   
  
  
- CTCTGTCCTC GAATTAACCC TCCTCCTCTG TTTTCGGTAC CGTTACCTTG TAGACCCACA CGTCATTTTG   
  
  
- ACTTCGACTT TTTCCCCACC AAGTAATTAC CCCCTCCCCT CCCTCTTTTT CTTATTGGTT CCAGAAAAGC   
  
  
- ACCCTGGGTG TGGAGTTTAG ACACTGTCTC TGCCGTCAAA GTAAAGAGTC GAGAAGGTGG AAGTGTTAGG   
  
  
- GAATAAATTT AGAAAGAGAG AGAGTGAACT TGTAACAACA AAGAGACAAG AGAGAGAAAG ATAGGAAACA   
  
  
- AAAAACAAGA GGACAACTTC AAGACTGAGA GAGAATGAGA GAGAGTATAC AAAAGAGAAA ATCTTTTTAT   
  
  
- AATAACTTAT TTCTTTACCC TGGAAACATA AGAATACCGG CATACACTTT AGAGATCGCT TCGTTTCAGA   
  
  
- AGACAAAGTG TGGAAAGTTA GAGTTTTGTT GCCTTCTTCT TCGATCCCAA ACTGTCGTGG TGGTTCGGGT   
  
  
- TCCAAACAAG AAACCATGAA AAGGAGAAAC GAAGATAACG ATAAAAAAAA ATAGCTTAAA AAGAATAGTA   
  
  
- TCGGCATTCA AATAAAAGAG AAACGAAAGT GAGTCCAAAG TTTCTGGGCG AACTTAGACT AAAACACCCA   
  
  
- TAGGGACAAA AATTTTTAAT TTGGAAGACT CTCCAACTTG CAAAGCTTAA AAACTACTTT TCTTGGTGTG   
  
  
- AACCCTAACA CCAAATAGGT CTTTACCTTA AAACTGATGC ACCCAGCAGA AAGGAAAAAT GGAGCTCCTA   
  
  
- GACAATAGCA CCATCTAAAA AGGCTCAAAA ATACCAGACA AAACCAGATC CACAGGGCCC CAACCTCTTA   
  
  
- ATCCCAAGAA AACCCCAAAA CCCCCGATT

+     G-box

| Site Name | Organism | Position | Strand | Matrix score. | sequence | function |
| --- | --- | --- | --- | --- | --- | --- |
| G-box | Daucus carota | 1367 | + | 6 | TACGTG | cis-acting regulatory element involved in light responsiveness |
| G-box | Arabidopsis thaliana | 174 | - | 9 | GCCACGTGGA | cis-acting regulatory element involved in light responsiveness |
| G-box | Brassica napus | 175 | + | 9 | ACACGTGGC | cis-acting regulatory element involved in light responsiveness |
| G-box | Arabidopsis thaliana | 176 | + | 6 | CACGTG | cis-acting regulatory element involved in light responsiveness |
| G-box | Larix laricina | 143 | - | 9 | GACACGTAGT | cis-acting regulatory element involved in light responsiveness |
| G-box | Daucus carota | 145 | + | 6 | TACGTG | cis-acting regulatory element involved in light responsiveness |

> 2018/04/13 10:10:12  
+ GGAGGAAGTG AAGTGAAGTC AACGGCAGTG GGGCCAATCC TGGACCCATG CAGCTGTCAG TCACCGACTT   
  
  
+ AACAGTGTCT CCCCGGCTCA AGATAGCAGC AGCCCCCCAG CAGGAGTTTG TTGCGGTCAA AGCACATTTT   
  
  
+ GGAATACGTG TCAGGCTCCT ATGCAACTCT TTGTACACGT GGCATCATCC AGCTGGTAAT TTTTCTGGCA   
  
  
+ ACGGCTCTAT ATCTATAGGG AACCGGCTCT GCAGTCTCCA GTACCGGGTG GTGGGTCCCA CTTTTGGCGG   
  
  
+ ACATTTTGAT AAGCCCTTCA CAGATACTTT AAAAAAAAAA GGGCAGAGCA AAGTAAACAA CCAGTATTCT   
  
  
+ CTCTGTCATT TTTTTTTTAA AAAAGAAAAT ATGTTTTATA AAATTAATTT AATTTTCATA AAATTTATCT   
  
  
+ ATTTAATATT TTAAAAAGTA TTAAAATGCA GAAAAGAGAC AGTACATTAG AGGATGTGTT ATTGTTTCAT   
  
  
+ ATTATTATAA TAATTCACAA AATATTCTTT AAAATAAACT TTGAGACCAC CTCAGATCTG TTGAAGATCA   
  
  
+ TTGTCCTACA TAAATCCAGT TGGTGTAATA AATCTGGGGC TGTATGAAGA GGGACTGAGG ATGCAGCAGC   
  
  
+ GAGACAGGAG CTTAATTGGG AGGAGGAGAC AAAAGCCATG GCAATGGAAC ATCTGGGTGT GCAGTAAAAC   
  
  
+ TGAAGCTGAA AAAGGGGTGG TTCATTAATG GGGGAGGGGA GGGAGAAAAA GAATAACCAA GGTCTTTTCG   
  
  
+ TGGGACCCAC ACCTCAAATC TGTGACAGAG ACGGCAGTTT CATTTCTCAG CTCTTCCACC TTCACAATCC   
  
  
+ CTTATTTAAA TCTTTCTCTC TCTCACTTGA ACATTGTTGT TTCTCTGTTC TCTCTCTTTC TATCCTTTGT   
  
  
+ TTTTTGTTCT CCTGTTGAAG TTCTGACTCT CTCTTACTCT CTCTCATATG TTTTCTCTTT TAGAAAAATA   
  
  
+ TTATTGAATA AAGAAATGGG ACCTTTGTAT TCTTATGGCC GTATGTGAAA TCTCTAGCGA AGCAAAGTCT   
  
  
+ TCTGTTTCAC ACCTTTCAAT CTCAAAACAA CGGAAGAAGA AGCTAGGGTT TGACAGCACC ACCAAGCCCA   
  
  
+ AGGTTTGTTC TTTGGTACTT TTCCTCTTTG CTTCTATTGC TATTTTTTTT TATCGAATTT TTCTTATCAT   
  
  
+ AGCCGTAAGT TTATTTTCTC TTTGCTTTCA CTCAGGTTTC AAAGACCCGC TTGAATCTGA TTTTGTGGGT   
  
  
+ ATCCCTGTTT TTAAAAATTA AACCTTCTGA GAGGTTGAAC GTTTCGAATT TTTGATGAAA AGAACCACAC   
  
  
+ TTGGGATTGT GGTTTATCCA GAAATGGAAT TTTGACTACG TGGGTCGTCT TTCCTTTTTA CCTCGAGGAT   
  
  
+ CTGTTATCGT GGTAGATTTT TCCGAGTTTT TATGGTCTGT TTTGGTCTAG GTGTCCCGGG GTTGGAGAAT   
  
  
+ TAGGGTTCTT TTGGGGTTTT GGGGGCTAA  

- CCTCCTTCAC TTCACTTCAG TTGCCGTCAC CCCGGTTAGG ACCTGGGTAC GTCGACAGTC AGTGGCTGAA   
  
  
- TTGTCACAGA GGGGCCGAGT TCTATCGTCG TCGGGGGGTC GTCCTCAAAC AACGCCAGTT TCGTGTAAAA   
  
  
- CCTTATGCAC AGTCCGAGGA TACGTTGAGA AACATGTGCA CCGTAGTAGG TCGACCATTA AAAAGACCGT   
  
  
- TGCCGAGATA TAGATATCCC TTGGCCGAGA CGTCAGAGGT CATGGCCCAC CACCCAGGGT GAAAACCGCC   
  
  
- TGTAAAACTA TTCGGGAAGT GTCTATGAAA TTTTTTTTTT CCCGTCTCGT TTCATTTGTT GGTCATAAGA   
  
  
- GAGACAGTAA AAAAAAAATT TTTTCTTTTA TACAAAATAT TTTAATTAAA TTAAAAGTAT TTTAAATAGA   
  
  
- TAAATTATAA AATTTTTCAT AATTTTACGT CTTTTCTCTG TCATGTAATC TCCTACACAA TAACAAAGTA   
  
  
- TAATAATATT ATTAAGTGTT TTATAAGAAA TTTTATTTGA AACTCTGGTG GAGTCTAGAC AACTTCTAGT   
  
  
- AACAGGATGT ATTTAGGTCA ACCACATTAT TTAGACCCCG ACATACTTCT CCCTGACTCC TACGTCGTCG   
  
  
- CTCTGTCCTC GAATTAACCC TCCTCCTCTG TTTTCGGTAC CGTTACCTTG TAGACCCACA CGTCATTTTG   
  
  
- ACTTCGACTT TTTCCCCACC AAGTAATTAC CCCCTCCCCT CCCTCTTTTT CTTATTGGTT CCAGAAAAGC   
  
  
- ACCCTGGGTG TGGAGTTTAG ACACTGTCTC TGCCGTCAAA GTAAAGAGTC GAGAAGGTGG AAGTGTTAGG   
  
  
- GAATAAATTT AGAAAGAGAG AGAGTGAACT TGTAACAACA AAGAGACAAG AGAGAGAAAG ATAGGAAACA   
  
  
- AAAAACAAGA GGACAACTTC AAGACTGAGA GAGAATGAGA GAGAGTATAC AAAAGAGAAA ATCTTTTTAT   
  
  
- AATAACTTAT TTCTTTACCC TGGAAACATA AGAATACCGG CATACACTTT AGAGATCGCT TCGTTTCAGA   
  
  
- AGACAAAGTG TGGAAAGTTA GAGTTTTGTT GCCTTCTTCT TCGATCCCAA ACTGTCGTGG TGGTTCGGGT   
  
  
- TCCAAACAAG AAACCATGAA AAGGAGAAAC GAAGATAACG ATAAAAAAAA ATAGCTTAAA AAGAATAGTA   
  
  
- TCGGCATTCA AATAAAAGAG AAACGAAAGT GAGTCCAAAG TTTCTGGGCG AACTTAGACT AAAACACCCA   
  
  
- TAGGGACAAA AATTTTTAAT TTGGAAGACT CTCCAACTTG CAAAGCTTAA AAACTACTTT TCTTGGTGTG   
  
  
- AACCCTAACA CCAAATAGGT CTTTACCTTA AAACTGATGC ACCCAGCAGA AAGGAAAAAT GGAGCTCCTA   
  
  
- GACAATAGCA CCATCTAAAA AGGCTCAAAA ATACCAGACA AAACCAGATC CACAGGGCCC CAACCTCTTA   
  
  
- ATCCCAAGAA AACCCCAAAA CCCCCGATT

+     GA-motif

| Site Name | Organism | Position | Strand | Matrix score. | sequence | function |
| --- | --- | --- | --- | --- | --- | --- |
| GA-motif | Arabidopsis thaliana | 415 | - | 8 | ATAGATAA | part of a light responsive element |

> 2018/04/13 10:10:12  
+ GGAGGAAGTG AAGTGAAGTC AACGGCAGTG GGGCCAATCC TGGACCCATG CAGCTGTCAG TCACCGACTT   
  
  
+ AACAGTGTCT CCCCGGCTCA AGATAGCAGC AGCCCCCCAG CAGGAGTTTG TTGCGGTCAA AGCACATTTT   
  
  
+ GGAATACGTG TCAGGCTCCT ATGCAACTCT TTGTACACGT GGCATCATCC AGCTGGTAAT TTTTCTGGCA   
  
  
+ ACGGCTCTAT ATCTATAGGG AACCGGCTCT GCAGTCTCCA GTACCGGGTG GTGGGTCCCA CTTTTGGCGG   
  
  
+ ACATTTTGAT AAGCCCTTCA CAGATACTTT AAAAAAAAAA GGGCAGAGCA AAGTAAACAA CCAGTATTCT   
  
  
+ CTCTGTCATT TTTTTTTTAA AAAAGAAAAT ATGTTTTATA AAATTAATTT AATTTTCATA AAATTTATCT   
  
  
+ ATTTAATATT TTAAAAAGTA TTAAAATGCA GAAAAGAGAC AGTACATTAG AGGATGTGTT ATTGTTTCAT   
  
  
+ ATTATTATAA TAATTCACAA AATATTCTTT AAAATAAACT TTGAGACCAC CTCAGATCTG TTGAAGATCA   
  
  
+ TTGTCCTACA TAAATCCAGT TGGTGTAATA AATCTGGGGC TGTATGAAGA GGGACTGAGG ATGCAGCAGC   
  
  
+ GAGACAGGAG CTTAATTGGG AGGAGGAGAC AAAAGCCATG GCAATGGAAC ATCTGGGTGT GCAGTAAAAC   
  
  
+ TGAAGCTGAA AAAGGGGTGG TTCATTAATG GGGGAGGGGA GGGAGAAAAA GAATAACCAA GGTCTTTTCG   
  
  
+ TGGGACCCAC ACCTCAAATC TGTGACAGAG ACGGCAGTTT CATTTCTCAG CTCTTCCACC TTCACAATCC   
  
  
+ CTTATTTAAA TCTTTCTCTC TCTCACTTGA ACATTGTTGT TTCTCTGTTC TCTCTCTTTC TATCCTTTGT   
  
  
+ TTTTTGTTCT CCTGTTGAAG TTCTGACTCT CTCTTACTCT CTCTCATATG TTTTCTCTTT TAGAAAAATA   
  
  
+ TTATTGAATA AAGAAATGGG ACCTTTGTAT TCTTATGGCC GTATGTGAAA TCTCTAGCGA AGCAAAGTCT   
  
  
+ TCTGTTTCAC ACCTTTCAAT CTCAAAACAA CGGAAGAAGA AGCTAGGGTT TGACAGCACC ACCAAGCCCA   
  
  
+ AGGTTTGTTC TTTGGTACTT TTCCTCTTTG CTTCTATTGC TATTTTTTTT TATCGAATTT TTCTTATCAT   
  
  
+ AGCCGTAAGT TTATTTTCTC TTTGCTTTCA CTCAGGTTTC AAAGACCCGC TTGAATCTGA TTTTGTGGGT   
  
  
+ ATCCCTGTTT TTAAAAATTA AACCTTCTGA GAGGTTGAAC GTTTCGAATT TTTGATGAAA AGAACCACAC   
  
  
+ TTGGGATTGT GGTTTATCCA GAAATGGAAT TTTGACTACG TGGGTCGTCT TTCCTTTTTA CCTCGAGGAT   
  
  
+ CTGTTATCGT GGTAGATTTT TCCGAGTTTT TATGGTCTGT TTTGGTCTAG GTGTCCCGGG GTTGGAGAAT   
  
  
+ TAGGGTTCTT TTGGGGTTTT GGGGGCTAA  

- CCTCCTTCAC TTCACTTCAG TTGCCGTCAC CCCGGTTAGG ACCTGGGTAC GTCGACAGTC AGTGGCTGAA   
  
  
- TTGTCACAGA GGGGCCGAGT TCTATCGTCG TCGGGGGGTC GTCCTCAAAC AACGCCAGTT TCGTGTAAAA   
  
  
- CCTTATGCAC AGTCCGAGGA TACGTTGAGA AACATGTGCA CCGTAGTAGG TCGACCATTA AAAAGACCGT   
  
  
- TGCCGAGATA TAGATATCCC TTGGCCGAGA CGTCAGAGGT CATGGCCCAC CACCCAGGGT GAAAACCGCC   
  
  
- TGTAAAACTA TTCGGGAAGT GTCTATGAAA TTTTTTTTTT CCCGTCTCGT TTCATTTGTT GGTCATAAGA   
  
  
- GAGACAGTAA AAAAAAAATT TTTTCTTTTA TACAAAATAT TTTAATTAAA TTAAAAGTAT TTTAAATAGA   
  
  
- TAAATTATAA AATTTTTCAT AATTTTACGT CTTTTCTCTG TCATGTAATC TCCTACACAA TAACAAAGTA   
  
  
- TAATAATATT ATTAAGTGTT TTATAAGAAA TTTTATTTGA AACTCTGGTG GAGTCTAGAC AACTTCTAGT   
  
  
- AACAGGATGT ATTTAGGTCA ACCACATTAT TTAGACCCCG ACATACTTCT CCCTGACTCC TACGTCGTCG   
  
  
- CTCTGTCCTC GAATTAACCC TCCTCCTCTG TTTTCGGTAC CGTTACCTTG TAGACCCACA CGTCATTTTG   
  
  
- ACTTCGACTT TTTCCCCACC AAGTAATTAC CCCCTCCCCT CCCTCTTTTT CTTATTGGTT CCAGAAAAGC   
  
  
- ACCCTGGGTG TGGAGTTTAG ACACTGTCTC TGCCGTCAAA GTAAAGAGTC GAGAAGGTGG AAGTGTTAGG   
  
  
- GAATAAATTT AGAAAGAGAG AGAGTGAACT TGTAACAACA AAGAGACAAG AGAGAGAAAG ATAGGAAACA   
  
  
- AAAAACAAGA GGACAACTTC AAGACTGAGA GAGAATGAGA GAGAGTATAC AAAAGAGAAA ATCTTTTTAT   
  
  
- AATAACTTAT TTCTTTACCC TGGAAACATA AGAATACCGG CATACACTTT AGAGATCGCT TCGTTTCAGA   
  
  
- AGACAAAGTG TGGAAAGTTA GAGTTTTGTT GCCTTCTTCT TCGATCCCAA ACTGTCGTGG TGGTTCGGGT   
  
  
- TCCAAACAAG AAACCATGAA AAGGAGAAAC GAAGATAACG ATAAAAAAAA ATAGCTTAAA AAGAATAGTA   
  
  
- TCGGCATTCA AATAAAAGAG AAACGAAAGT GAGTCCAAAG TTTCTGGGCG AACTTAGACT AAAACACCCA   
  
  
- TAGGGACAAA AATTTTTAAT TTGGAAGACT CTCCAACTTG CAAAGCTTAA AAACTACTTT TCTTGGTGTG   
  
  
- AACCCTAACA CCAAATAGGT CTTTACCTTA AAACTGATGC ACCCAGCAGA AAGGAAAAAT GGAGCTCCTA   
  
  
- GACAATAGCA CCATCTAAAA AGGCTCAAAA ATACCAGACA AAACCAGATC CACAGGGCCC CAACCTCTTA   
  
  
- ATCCCAAGAA AACCCCAAAA CCCCCGATT

+     GAG-motif

| Site Name | Organism | Position | Strand | Matrix score. | sequence | function |
| --- | --- | --- | --- | --- | --- | --- |
| GAG-motif | Arabidopsis thaliana | 936 | - | 7 | AGAGAGT | part of a light responsive element |
| GAG-motif | Arabidopsis thaliana | 946 | - | 7 | AGAGAGT | part of a light responsive element |

> 2018/04/13 10:10:12  
+ GGAGGAAGTG AAGTGAAGTC AACGGCAGTG GGGCCAATCC TGGACCCATG CAGCTGTCAG TCACCGACTT   
  
  
+ AACAGTGTCT CCCCGGCTCA AGATAGCAGC AGCCCCCCAG CAGGAGTTTG TTGCGGTCAA AGCACATTTT   
  
  
+ GGAATACGTG TCAGGCTCCT ATGCAACTCT TTGTACACGT GGCATCATCC AGCTGGTAAT TTTTCTGGCA   
  
  
+ ACGGCTCTAT ATCTATAGGG AACCGGCTCT GCAGTCTCCA GTACCGGGTG GTGGGTCCCA CTTTTGGCGG   
  
  
+ ACATTTTGAT AAGCCCTTCA CAGATACTTT AAAAAAAAAA GGGCAGAGCA AAGTAAACAA CCAGTATTCT   
  
  
+ CTCTGTCATT TTTTTTTTAA AAAAGAAAAT ATGTTTTATA AAATTAATTT AATTTTCATA AAATTTATCT   
  
  
+ ATTTAATATT TTAAAAAGTA TTAAAATGCA GAAAAGAGAC AGTACATTAG AGGATGTGTT ATTGTTTCAT   
  
  
+ ATTATTATAA TAATTCACAA AATATTCTTT AAAATAAACT TTGAGACCAC CTCAGATCTG TTGAAGATCA   
  
  
+ TTGTCCTACA TAAATCCAGT TGGTGTAATA AATCTGGGGC TGTATGAAGA GGGACTGAGG ATGCAGCAGC   
  
  
+ GAGACAGGAG CTTAATTGGG AGGAGGAGAC AAAAGCCATG GCAATGGAAC ATCTGGGTGT GCAGTAAAAC   
  
  
+ TGAAGCTGAA AAAGGGGTGG TTCATTAATG GGGGAGGGGA GGGAGAAAAA GAATAACCAA GGTCTTTTCG   
  
  
+ TGGGACCCAC ACCTCAAATC TGTGACAGAG ACGGCAGTTT CATTTCTCAG CTCTTCCACC TTCACAATCC   
  
  
+ CTTATTTAAA TCTTTCTCTC TCTCACTTGA ACATTGTTGT TTCTCTGTTC TCTCTCTTTC TATCCTTTGT   
  
  
+ TTTTTGTTCT CCTGTTGAAG TTCTGACTCT CTCTTACTCT CTCTCATATG TTTTCTCTTT TAGAAAAATA   
  
  
+ TTATTGAATA AAGAAATGGG ACCTTTGTAT TCTTATGGCC GTATGTGAAA TCTCTAGCGA AGCAAAGTCT   
  
  
+ TCTGTTTCAC ACCTTTCAAT CTCAAAACAA CGGAAGAAGA AGCTAGGGTT TGACAGCACC ACCAAGCCCA   
  
  
+ AGGTTTGTTC TTTGGTACTT TTCCTCTTTG CTTCTATTGC TATTTTTTTT TATCGAATTT TTCTTATCAT   
  
  
+ AGCCGTAAGT TTATTTTCTC TTTGCTTTCA CTCAGGTTTC AAAGACCCGC TTGAATCTGA TTTTGTGGGT   
  
  
+ ATCCCTGTTT TTAAAAATTA AACCTTCTGA GAGGTTGAAC GTTTCGAATT TTTGATGAAA AGAACCACAC   
  
  
+ TTGGGATTGT GGTTTATCCA GAAATGGAAT TTTGACTACG TGGGTCGTCT TTCCTTTTTA CCTCGAGGAT   
  
  
+ CTGTTATCGT GGTAGATTTT TCCGAGTTTT TATGGTCTGT TTTGGTCTAG GTGTCCCGGG GTTGGAGAAT   
  
  
+ TAGGGTTCTT TTGGGGTTTT GGGGGCTAA  

- CCTCCTTCAC TTCACTTCAG TTGCCGTCAC CCCGGTTAGG ACCTGGGTAC GTCGACAGTC AGTGGCTGAA   
  
  
- TTGTCACAGA GGGGCCGAGT TCTATCGTCG TCGGGGGGTC GTCCTCAAAC AACGCCAGTT TCGTGTAAAA   
  
  
- CCTTATGCAC AGTCCGAGGA TACGTTGAGA AACATGTGCA CCGTAGTAGG TCGACCATTA AAAAGACCGT   
  
  
- TGCCGAGATA TAGATATCCC TTGGCCGAGA CGTCAGAGGT CATGGCCCAC CACCCAGGGT GAAAACCGCC   
  
  
- TGTAAAACTA TTCGGGAAGT GTCTATGAAA TTTTTTTTTT CCCGTCTCGT TTCATTTGTT GGTCATAAGA   
  
  
- GAGACAGTAA AAAAAAAATT TTTTCTTTTA TACAAAATAT TTTAATTAAA TTAAAAGTAT TTTAAATAGA   
  
  
- TAAATTATAA AATTTTTCAT AATTTTACGT CTTTTCTCTG TCATGTAATC TCCTACACAA TAACAAAGTA   
  
  
- TAATAATATT ATTAAGTGTT TTATAAGAAA TTTTATTTGA AACTCTGGTG GAGTCTAGAC AACTTCTAGT   
  
  
- AACAGGATGT ATTTAGGTCA ACCACATTAT TTAGACCCCG ACATACTTCT CCCTGACTCC TACGTCGTCG   
  
  
- CTCTGTCCTC GAATTAACCC TCCTCCTCTG TTTTCGGTAC CGTTACCTTG TAGACCCACA CGTCATTTTG   
  
  
- ACTTCGACTT TTTCCCCACC AAGTAATTAC CCCCTCCCCT CCCTCTTTTT CTTATTGGTT CCAGAAAAGC   
  
  
- ACCCTGGGTG TGGAGTTTAG ACACTGTCTC TGCCGTCAAA GTAAAGAGTC GAGAAGGTGG AAGTGTTAGG   
  
  
- GAATAAATTT AGAAAGAGAG AGAGTGAACT TGTAACAACA AAGAGACAAG AGAGAGAAAG ATAGGAAACA   
  
  
- AAAAACAAGA GGACAACTTC AAGACTGAGA GAGAATGAGA GAGAGTATAC AAAAGAGAAA ATCTTTTTAT   
  
  
- AATAACTTAT TTCTTTACCC TGGAAACATA AGAATACCGG CATACACTTT AGAGATCGCT TCGTTTCAGA   
  
  
- AGACAAAGTG TGGAAAGTTA GAGTTTTGTT GCCTTCTTCT TCGATCCCAA ACTGTCGTGG TGGTTCGGGT   
  
  
- TCCAAACAAG AAACCATGAA AAGGAGAAAC GAAGATAACG ATAAAAAAAA ATAGCTTAAA AAGAATAGTA   
  
  
- TCGGCATTCA AATAAAAGAG AAACGAAAGT GAGTCCAAAG TTTCTGGGCG AACTTAGACT AAAACACCCA   
  
  
- TAGGGACAAA AATTTTTAAT TTGGAAGACT CTCCAACTTG CAAAGCTTAA AAACTACTTT TCTTGGTGTG   
  
  
- AACCCTAACA CCAAATAGGT CTTTACCTTA AAACTGATGC ACCCAGCAGA AAGGAAAAAT GGAGCTCCTA   
  
  
- GACAATAGCA CCATCTAAAA AGGCTCAAAA ATACCAGACA AAACCAGATC CACAGGGCCC CAACCTCTTA   
  
  
- ATCCCAAGAA AACCCCAAAA CCCCCGATT

+     GARE-motif

| Site Name | Organism | Position | Strand | Matrix score. | sequence | function |
| --- | --- | --- | --- | --- | --- | --- |
| GARE-motif | Brassica oleracea | 1436 | - | 7 | AAACAGA | gibberellin-responsive element |
| GARE-motif | Brassica oleracea | 1051 | - | 7 | AAACAGA | gibberellin-responsive element |
| GARE-motif | Brassica oleracea | 547 | + | 7 | TCTGTTG | gibberellin-responsive element |

> 2018/04/13 10:10:12  
+ GGAGGAAGTG AAGTGAAGTC AACGGCAGTG GGGCCAATCC TGGACCCATG CAGCTGTCAG TCACCGACTT   
  
  
+ AACAGTGTCT CCCCGGCTCA AGATAGCAGC AGCCCCCCAG CAGGAGTTTG TTGCGGTCAA AGCACATTTT   
  
  
+ GGAATACGTG TCAGGCTCCT ATGCAACTCT TTGTACACGT GGCATCATCC AGCTGGTAAT TTTTCTGGCA   
  
  
+ ACGGCTCTAT ATCTATAGGG AACCGGCTCT GCAGTCTCCA GTACCGGGTG GTGGGTCCCA CTTTTGGCGG   
  
  
+ ACATTTTGAT AAGCCCTTCA CAGATACTTT AAAAAAAAAA GGGCAGAGCA AAGTAAACAA CCAGTATTCT   
  
  
+ CTCTGTCATT TTTTTTTTAA AAAAGAAAAT ATGTTTTATA AAATTAATTT AATTTTCATA AAATTTATCT   
  
  
+ ATTTAATATT TTAAAAAGTA TTAAAATGCA GAAAAGAGAC AGTACATTAG AGGATGTGTT ATTGTTTCAT   
  
  
+ ATTATTATAA TAATTCACAA AATATTCTTT AAAATAAACT TTGAGACCAC CTCAGATCTG TTGAAGATCA   
  
  
+ TTGTCCTACA TAAATCCAGT TGGTGTAATA AATCTGGGGC TGTATGAAGA GGGACTGAGG ATGCAGCAGC   
  
  
+ GAGACAGGAG CTTAATTGGG AGGAGGAGAC AAAAGCCATG GCAATGGAAC ATCTGGGTGT GCAGTAAAAC   
  
  
+ TGAAGCTGAA AAAGGGGTGG TTCATTAATG GGGGAGGGGA GGGAGAAAAA GAATAACCAA GGTCTTTTCG   
  
  
+ TGGGACCCAC ACCTCAAATC TGTGACAGAG ACGGCAGTTT CATTTCTCAG CTCTTCCACC TTCACAATCC   
  
  
+ CTTATTTAAA TCTTTCTCTC TCTCACTTGA ACATTGTTGT TTCTCTGTTC TCTCTCTTTC TATCCTTTGT   
  
  
+ TTTTTGTTCT CCTGTTGAAG TTCTGACTCT CTCTTACTCT CTCTCATATG TTTTCTCTTT TAGAAAAATA   
  
  
+ TTATTGAATA AAGAAATGGG ACCTTTGTAT TCTTATGGCC GTATGTGAAA TCTCTAGCGA AGCAAAGTCT   
  
  
+ TCTGTTTCAC ACCTTTCAAT CTCAAAACAA CGGAAGAAGA AGCTAGGGTT TGACAGCACC ACCAAGCCCA   
  
  
+ AGGTTTGTTC TTTGGTACTT TTCCTCTTTG CTTCTATTGC TATTTTTTTT TATCGAATTT TTCTTATCAT   
  
  
+ AGCCGTAAGT TTATTTTCTC TTTGCTTTCA CTCAGGTTTC AAAGACCCGC TTGAATCTGA TTTTGTGGGT   
  
  
+ ATCCCTGTTT TTAAAAATTA AACCTTCTGA GAGGTTGAAC GTTTCGAATT TTTGATGAAA AGAACCACAC   
  
  
+ TTGGGATTGT GGTTTATCCA GAAATGGAAT TTTGACTACG TGGGTCGTCT TTCCTTTTTA CCTCGAGGAT   
  
  
+ CTGTTATCGT GGTAGATTTT TCCGAGTTTT TATGGTCTGT TTTGGTCTAG GTGTCCCGGG GTTGGAGAAT   
  
  
+ TAGGGTTCTT TTGGGGTTTT GGGGGCTAA  

- CCTCCTTCAC TTCACTTCAG TTGCCGTCAC CCCGGTTAGG ACCTGGGTAC GTCGACAGTC AGTGGCTGAA   
  
  
- TTGTCACAGA GGGGCCGAGT TCTATCGTCG TCGGGGGGTC GTCCTCAAAC AACGCCAGTT TCGTGTAAAA   
  
  
- CCTTATGCAC AGTCCGAGGA TACGTTGAGA AACATGTGCA CCGTAGTAGG TCGACCATTA AAAAGACCGT   
  
  
- TGCCGAGATA TAGATATCCC TTGGCCGAGA CGTCAGAGGT CATGGCCCAC CACCCAGGGT GAAAACCGCC   
  
  
- TGTAAAACTA TTCGGGAAGT GTCTATGAAA TTTTTTTTTT CCCGTCTCGT TTCATTTGTT GGTCATAAGA   
  
  
- GAGACAGTAA AAAAAAAATT TTTTCTTTTA TACAAAATAT TTTAATTAAA TTAAAAGTAT TTTAAATAGA   
  
  
- TAAATTATAA AATTTTTCAT AATTTTACGT CTTTTCTCTG TCATGTAATC TCCTACACAA TAACAAAGTA   
  
  
- TAATAATATT ATTAAGTGTT TTATAAGAAA TTTTATTTGA AACTCTGGTG GAGTCTAGAC AACTTCTAGT   
  
  
- AACAGGATGT ATTTAGGTCA ACCACATTAT TTAGACCCCG ACATACTTCT CCCTGACTCC TACGTCGTCG   
  
  
- CTCTGTCCTC GAATTAACCC TCCTCCTCTG TTTTCGGTAC CGTTACCTTG TAGACCCACA CGTCATTTTG   
  
  
- ACTTCGACTT TTTCCCCACC AAGTAATTAC CCCCTCCCCT CCCTCTTTTT CTTATTGGTT CCAGAAAAGC   
  
  
- ACCCTGGGTG TGGAGTTTAG ACACTGTCTC TGCCGTCAAA GTAAAGAGTC GAGAAGGTGG AAGTGTTAGG   
  
  
- GAATAAATTT AGAAAGAGAG AGAGTGAACT TGTAACAACA AAGAGACAAG AGAGAGAAAG ATAGGAAACA   
  
  
- AAAAACAAGA GGACAACTTC AAGACTGAGA GAGAATGAGA GAGAGTATAC AAAAGAGAAA ATCTTTTTAT   
  
  
- AATAACTTAT TTCTTTACCC TGGAAACATA AGAATACCGG CATACACTTT AGAGATCGCT TCGTTTCAGA   
  
  
- AGACAAAGTG TGGAAAGTTA GAGTTTTGTT GCCTTCTTCT TCGATCCCAA ACTGTCGTGG TGGTTCGGGT   
  
  
- TCCAAACAAG AAACCATGAA AAGGAGAAAC GAAGATAACG ATAAAAAAAA ATAGCTTAAA AAGAATAGTA   
  
  
- TCGGCATTCA AATAAAAGAG AAACGAAAGT GAGTCCAAAG TTTCTGGGCG AACTTAGACT AAAACACCCA   
  
  
- TAGGGACAAA AATTTTTAAT TTGGAAGACT CTCCAACTTG CAAAGCTTAA AAACTACTTT TCTTGGTGTG   
  
  
- AACCCTAACA CCAAATAGGT CTTTACCTTA AAACTGATGC ACCCAGCAGA AAGGAAAAAT GGAGCTCCTA   
  
  
- GACAATAGCA CCATCTAAAA AGGCTCAAAA ATACCAGACA AAACCAGATC CACAGGGCCC CAACCTCTTA   
  
  
- ATCCCAAGAA AACCCCAAAA CCCCCGATT

+     MBS

| Site Name | Organism | Position | Strand | Matrix score. | sequence | function |
| --- | --- | --- | --- | --- | --- | --- |
| MBS | Arabidopsis thaliana | 577 | - | 6 | CAACTG | MYB binding site involved in drought-inducibility |
| MBS | Zea mays | 124 | + | 6 | CGGTCA | MYB Binding Site |

> 2018/04/13 10:10:12  
+ GGAGGAAGTG AAGTGAAGTC AACGGCAGTG GGGCCAATCC TGGACCCATG CAGCTGTCAG TCACCGACTT   
  
  
+ AACAGTGTCT CCCCGGCTCA AGATAGCAGC AGCCCCCCAG CAGGAGTTTG TTGCGGTCAA AGCACATTTT   
  
  
+ GGAATACGTG TCAGGCTCCT ATGCAACTCT TTGTACACGT GGCATCATCC AGCTGGTAAT TTTTCTGGCA   
  
  
+ ACGGCTCTAT ATCTATAGGG AACCGGCTCT GCAGTCTCCA GTACCGGGTG GTGGGTCCCA CTTTTGGCGG   
  
  
+ ACATTTTGAT AAGCCCTTCA CAGATACTTT AAAAAAAAAA GGGCAGAGCA AAGTAAACAA CCAGTATTCT   
  
  
+ CTCTGTCATT TTTTTTTTAA AAAAGAAAAT ATGTTTTATA AAATTAATTT AATTTTCATA AAATTTATCT   
  
  
+ ATTTAATATT TTAAAAAGTA TTAAAATGCA GAAAAGAGAC AGTACATTAG AGGATGTGTT ATTGTTTCAT   
  
  
+ ATTATTATAA TAATTCACAA AATATTCTTT AAAATAAACT TTGAGACCAC CTCAGATCTG TTGAAGATCA   
  
  
+ TTGTCCTACA TAAATCCAGT TGGTGTAATA AATCTGGGGC TGTATGAAGA GGGACTGAGG ATGCAGCAGC   
  
  
+ GAGACAGGAG CTTAATTGGG AGGAGGAGAC AAAAGCCATG GCAATGGAAC ATCTGGGTGT GCAGTAAAAC   
  
  
+ TGAAGCTGAA AAAGGGGTGG TTCATTAATG GGGGAGGGGA GGGAGAAAAA GAATAACCAA GGTCTTTTCG   
  
  
+ TGGGACCCAC ACCTCAAATC TGTGACAGAG ACGGCAGTTT CATTTCTCAG CTCTTCCACC TTCACAATCC   
  
  
+ CTTATTTAAA TCTTTCTCTC TCTCACTTGA ACATTGTTGT TTCTCTGTTC TCTCTCTTTC TATCCTTTGT   
  
  
+ TTTTTGTTCT CCTGTTGAAG TTCTGACTCT CTCTTACTCT CTCTCATATG TTTTCTCTTT TAGAAAAATA   
  
  
+ TTATTGAATA AAGAAATGGG ACCTTTGTAT TCTTATGGCC GTATGTGAAA TCTCTAGCGA AGCAAAGTCT   
  
  
+ TCTGTTTCAC ACCTTTCAAT CTCAAAACAA CGGAAGAAGA AGCTAGGGTT TGACAGCACC ACCAAGCCCA   
  
  
+ AGGTTTGTTC TTTGGTACTT TTCCTCTTTG CTTCTATTGC TATTTTTTTT TATCGAATTT TTCTTATCAT   
  
  
+ AGCCGTAAGT TTATTTTCTC TTTGCTTTCA CTCAGGTTTC AAAGACCCGC TTGAATCTGA TTTTGTGGGT   
  
  
+ ATCCCTGTTT TTAAAAATTA AACCTTCTGA GAGGTTGAAC GTTTCGAATT TTTGATGAAA AGAACCACAC   
  
  
+ TTGGGATTGT GGTTTATCCA GAAATGGAAT TTTGACTACG TGGGTCGTCT TTCCTTTTTA CCTCGAGGAT   
  
  
+ CTGTTATCGT GGTAGATTTT TCCGAGTTTT TATGGTCTGT TTTGGTCTAG GTGTCCCGGG GTTGGAGAAT   
  
  
+ TAGGGTTCTT TTGGGGTTTT GGGGGCTAA  

- CCTCCTTCAC TTCACTTCAG TTGCCGTCAC CCCGGTTAGG ACCTGGGTAC GTCGACAGTC AGTGGCTGAA   
  
  
- TTGTCACAGA GGGGCCGAGT TCTATCGTCG TCGGGGGGTC GTCCTCAAAC AACGCCAGTT TCGTGTAAAA   
  
  
- CCTTATGCAC AGTCCGAGGA TACGTTGAGA AACATGTGCA CCGTAGTAGG TCGACCATTA AAAAGACCGT   
  
  
- TGCCGAGATA TAGATATCCC TTGGCCGAGA CGTCAGAGGT CATGGCCCAC CACCCAGGGT GAAAACCGCC   
  
  
- TGTAAAACTA TTCGGGAAGT GTCTATGAAA TTTTTTTTTT CCCGTCTCGT TTCATTTGTT GGTCATAAGA   
  
  
- GAGACAGTAA AAAAAAAATT TTTTCTTTTA TACAAAATAT TTTAATTAAA TTAAAAGTAT TTTAAATAGA   
  
  
- TAAATTATAA AATTTTTCAT AATTTTACGT CTTTTCTCTG TCATGTAATC TCCTACACAA TAACAAAGTA   
  
  
- TAATAATATT ATTAAGTGTT TTATAAGAAA TTTTATTTGA AACTCTGGTG GAGTCTAGAC AACTTCTAGT   
  
  
- AACAGGATGT ATTTAGGTCA ACCACATTAT TTAGACCCCG ACATACTTCT CCCTGACTCC TACGTCGTCG   
  
  
- CTCTGTCCTC GAATTAACCC TCCTCCTCTG TTTTCGGTAC CGTTACCTTG TAGACCCACA CGTCATTTTG   
  
  
- ACTTCGACTT TTTCCCCACC AAGTAATTAC CCCCTCCCCT CCCTCTTTTT CTTATTGGTT CCAGAAAAGC   
  
  
- ACCCTGGGTG TGGAGTTTAG ACACTGTCTC TGCCGTCAAA GTAAAGAGTC GAGAAGGTGG AAGTGTTAGG   
  
  
- GAATAAATTT AGAAAGAGAG AGAGTGAACT TGTAACAACA AAGAGACAAG AGAGAGAAAG ATAGGAAACA   
  
  
- AAAAACAAGA GGACAACTTC AAGACTGAGA GAGAATGAGA GAGAGTATAC AAAAGAGAAA ATCTTTTTAT   
  
  
- AATAACTTAT TTCTTTACCC TGGAAACATA AGAATACCGG CATACACTTT AGAGATCGCT TCGTTTCAGA   
  
  
- AGACAAAGTG TGGAAAGTTA GAGTTTTGTT GCCTTCTTCT TCGATCCCAA ACTGTCGTGG TGGTTCGGGT   
  
  
- TCCAAACAAG AAACCATGAA AAGGAGAAAC GAAGATAACG ATAAAAAAAA ATAGCTTAAA AAGAATAGTA   
  
  
- TCGGCATTCA AATAAAAGAG AAACGAAAGT GAGTCCAAAG TTTCTGGGCG AACTTAGACT AAAACACCCA   
  
  
- TAGGGACAAA AATTTTTAAT TTGGAAGACT CTCCAACTTG CAAAGCTTAA AAACTACTTT TCTTGGTGTG   
  
  
- AACCCTAACA CCAAATAGGT CTTTACCTTA AAACTGATGC ACCCAGCAGA AAGGAAAAAT GGAGCTCCTA   
  
  
- GACAATAGCA CCATCTAAAA AGGCTCAAAA ATACCAGACA AAACCAGATC CACAGGGCCC CAACCTCTTA   
  
  
- ATCCCAAGAA AACCCCAAAA CCCCCGATT

+     Skn-1\_motif

| Site Name | Organism | Position | Strand | Matrix score. | sequence | function |
| --- | --- | --- | --- | --- | --- | --- |
| Skn-1\_motif | Oryza sativa | 355 | + | 5 | GTCAT | cis-acting regulatory element required for endosperm expression |

> 2018/04/13 10:10:12  
+ GGAGGAAGTG AAGTGAAGTC AACGGCAGTG GGGCCAATCC TGGACCCATG CAGCTGTCAG TCACCGACTT   
  
  
+ AACAGTGTCT CCCCGGCTCA AGATAGCAGC AGCCCCCCAG CAGGAGTTTG TTGCGGTCAA AGCACATTTT   
  
  
+ GGAATACGTG TCAGGCTCCT ATGCAACTCT TTGTACACGT GGCATCATCC AGCTGGTAAT TTTTCTGGCA   
  
  
+ ACGGCTCTAT ATCTATAGGG AACCGGCTCT GCAGTCTCCA GTACCGGGTG GTGGGTCCCA CTTTTGGCGG   
  
  
+ ACATTTTGAT AAGCCCTTCA CAGATACTTT AAAAAAAAAA GGGCAGAGCA AAGTAAACAA CCAGTATTCT   
  
  
+ CTCTGTCATT TTTTTTTTAA AAAAGAAAAT ATGTTTTATA AAATTAATTT AATTTTCATA AAATTTATCT   
  
  
+ ATTTAATATT TTAAAAAGTA TTAAAATGCA GAAAAGAGAC AGTACATTAG AGGATGTGTT ATTGTTTCAT   
  
  
+ ATTATTATAA TAATTCACAA AATATTCTTT AAAATAAACT TTGAGACCAC CTCAGATCTG TTGAAGATCA   
  
  
+ TTGTCCTACA TAAATCCAGT TGGTGTAATA AATCTGGGGC TGTATGAAGA GGGACTGAGG ATGCAGCAGC   
  
  
+ GAGACAGGAG CTTAATTGGG AGGAGGAGAC AAAAGCCATG GCAATGGAAC ATCTGGGTGT GCAGTAAAAC   
  
  
+ TGAAGCTGAA AAAGGGGTGG TTCATTAATG GGGGAGGGGA GGGAGAAAAA GAATAACCAA GGTCTTTTCG   
  
  
+ TGGGACCCAC ACCTCAAATC TGTGACAGAG ACGGCAGTTT CATTTCTCAG CTCTTCCACC TTCACAATCC   
  
  
+ CTTATTTAAA TCTTTCTCTC TCTCACTTGA ACATTGTTGT TTCTCTGTTC TCTCTCTTTC TATCCTTTGT   
  
  
+ TTTTTGTTCT CCTGTTGAAG TTCTGACTCT CTCTTACTCT CTCTCATATG TTTTCTCTTT TAGAAAAATA   
  
  
+ TTATTGAATA AAGAAATGGG ACCTTTGTAT TCTTATGGCC GTATGTGAAA TCTCTAGCGA AGCAAAGTCT   
  
  
+ TCTGTTTCAC ACCTTTCAAT CTCAAAACAA CGGAAGAAGA AGCTAGGGTT TGACAGCACC ACCAAGCCCA   
  
  
+ AGGTTTGTTC TTTGGTACTT TTCCTCTTTG CTTCTATTGC TATTTTTTTT TATCGAATTT TTCTTATCAT   
  
  
+ AGCCGTAAGT TTATTTTCTC TTTGCTTTCA CTCAGGTTTC AAAGACCCGC TTGAATCTGA TTTTGTGGGT   
  
  
+ ATCCCTGTTT TTAAAAATTA AACCTTCTGA GAGGTTGAAC GTTTCGAATT TTTGATGAAA AGAACCACAC   
  
  
+ TTGGGATTGT GGTTTATCCA GAAATGGAAT TTTGACTACG TGGGTCGTCT TTCCTTTTTA CCTCGAGGAT   
  
  
+ CTGTTATCGT GGTAGATTTT TCCGAGTTTT TATGGTCTGT TTTGGTCTAG GTGTCCCGGG GTTGGAGAAT   
  
  
+ TAGGGTTCTT TTGGGGTTTT GGGGGCTAA  

- CCTCCTTCAC TTCACTTCAG TTGCCGTCAC CCCGGTTAGG ACCTGGGTAC GTCGACAGTC AGTGGCTGAA   
  
  
- TTGTCACAGA GGGGCCGAGT TCTATCGTCG TCGGGGGGTC GTCCTCAAAC AACGCCAGTT TCGTGTAAAA   
  
  
- CCTTATGCAC AGTCCGAGGA TACGTTGAGA AACATGTGCA CCGTAGTAGG TCGACCATTA AAAAGACCGT   
  
  
- TGCCGAGATA TAGATATCCC TTGGCCGAGA CGTCAGAGGT CATGGCCCAC CACCCAGGGT GAAAACCGCC   
  
  
- TGTAAAACTA TTCGGGAAGT GTCTATGAAA TTTTTTTTTT CCCGTCTCGT TTCATTTGTT GGTCATAAGA   
  
  
- GAGACAGTAA AAAAAAAATT TTTTCTTTTA TACAAAATAT TTTAATTAAA TTAAAAGTAT TTTAAATAGA   
  
  
- TAAATTATAA AATTTTTCAT AATTTTACGT CTTTTCTCTG TCATGTAATC TCCTACACAA TAACAAAGTA   
  
  
- TAATAATATT ATTAAGTGTT TTATAAGAAA TTTTATTTGA AACTCTGGTG GAGTCTAGAC AACTTCTAGT   
  
  
- AACAGGATGT ATTTAGGTCA ACCACATTAT TTAGACCCCG ACATACTTCT CCCTGACTCC TACGTCGTCG   
  
  
- CTCTGTCCTC GAATTAACCC TCCTCCTCTG TTTTCGGTAC CGTTACCTTG TAGACCCACA CGTCATTTTG   
  
  
- ACTTCGACTT TTTCCCCACC AAGTAATTAC CCCCTCCCCT CCCTCTTTTT CTTATTGGTT CCAGAAAAGC   
  
  
- ACCCTGGGTG TGGAGTTTAG ACACTGTCTC TGCCGTCAAA GTAAAGAGTC GAGAAGGTGG AAGTGTTAGG   
  
  
- GAATAAATTT AGAAAGAGAG AGAGTGAACT TGTAACAACA AAGAGACAAG AGAGAGAAAG ATAGGAAACA   
  
  
- AAAAACAAGA GGACAACTTC AAGACTGAGA GAGAATGAGA GAGAGTATAC AAAAGAGAAA ATCTTTTTAT   
  
  
- AATAACTTAT TTCTTTACCC TGGAAACATA AGAATACCGG CATACACTTT AGAGATCGCT TCGTTTCAGA   
  
  
- AGACAAAGTG TGGAAAGTTA GAGTTTTGTT GCCTTCTTCT TCGATCCCAA ACTGTCGTGG TGGTTCGGGT   
  
  
- TCCAAACAAG AAACCATGAA AAGGAGAAAC GAAGATAACG ATAAAAAAAA ATAGCTTAAA AAGAATAGTA   
  
  
- TCGGCATTCA AATAAAAGAG AAACGAAAGT GAGTCCAAAG TTTCTGGGCG AACTTAGACT AAAACACCCA   
  
  
- TAGGGACAAA AATTTTTAAT TTGGAAGACT CTCCAACTTG CAAAGCTTAA AAACTACTTT TCTTGGTGTG   
  
  
- AACCCTAACA CCAAATAGGT CTTTACCTTA AAACTGATGC ACCCAGCAGA AAGGAAAAAT GGAGCTCCTA   
  
  
- GACAATAGCA CCATCTAAAA AGGCTCAAAA ATACCAGACA AAACCAGATC CACAGGGCCC CAACCTCTTA   
  
  
- ATCCCAAGAA AACCCCAAAA CCCCCGATT

+     Sp1

| Site Name | Organism | Position | Strand | Matrix score. | sequence | function |
| --- | --- | --- | --- | --- | --- | --- |
| Sp1 | Zea mays | 103 | + | 5 | CC(G/A)CCC | light responsive element |
| Sp1 | Zea mays | 732 | - | 5 | CC(G/A)CCC | light responsive element |
| Sp1 | Zea mays | 737 | - | 5 | CC(G/A)CCC | light responsive element |
| Sp1 | Zea mays | 648 | - | 5 | CC(G/A)CCC | light responsive element |
| Sp1 | Zea mays | 256 | - | 5.5 | CC(G/A)CCC | light responsive element |
| Sp1 | Zea mays | 715 | - | 5.5 | CC(G/A)CCC | light responsive element |

> 2018/04/13 10:10:12  
+ GGAGGAAGTG AAGTGAAGTC AACGGCAGTG GGGCCAATCC TGGACCCATG CAGCTGTCAG TCACCGACTT   
  
  
+ AACAGTGTCT CCCCGGCTCA AGATAGCAGC AGCCCCCCAG CAGGAGTTTG TTGCGGTCAA AGCACATTTT   
  
  
+ GGAATACGTG TCAGGCTCCT ATGCAACTCT TTGTACACGT GGCATCATCC AGCTGGTAAT TTTTCTGGCA   
  
  
+ ACGGCTCTAT ATCTATAGGG AACCGGCTCT GCAGTCTCCA GTACCGGGTG GTGGGTCCCA CTTTTGGCGG   
  
  
+ ACATTTTGAT AAGCCCTTCA CAGATACTTT AAAAAAAAAA GGGCAGAGCA AAGTAAACAA CCAGTATTCT   
  
  
+ CTCTGTCATT TTTTTTTTAA AAAAGAAAAT ATGTTTTATA AAATTAATTT AATTTTCATA AAATTTATCT   
  
  
+ ATTTAATATT TTAAAAAGTA TTAAAATGCA GAAAAGAGAC AGTACATTAG AGGATGTGTT ATTGTTTCAT   
  
  
+ ATTATTATAA TAATTCACAA AATATTCTTT AAAATAAACT TTGAGACCAC CTCAGATCTG TTGAAGATCA   
  
  
+ TTGTCCTACA TAAATCCAGT TGGTGTAATA AATCTGGGGC TGTATGAAGA GGGACTGAGG ATGCAGCAGC   
  
  
+ GAGACAGGAG CTTAATTGGG AGGAGGAGAC AAAAGCCATG GCAATGGAAC ATCTGGGTGT GCAGTAAAAC   
  
  
+ TGAAGCTGAA AAAGGGGTGG TTCATTAATG GGGGAGGGGA GGGAGAAAAA GAATAACCAA GGTCTTTTCG   
  
  
+ TGGGACCCAC ACCTCAAATC TGTGACAGAG ACGGCAGTTT CATTTCTCAG CTCTTCCACC TTCACAATCC   
  
  
+ CTTATTTAAA TCTTTCTCTC TCTCACTTGA ACATTGTTGT TTCTCTGTTC TCTCTCTTTC TATCCTTTGT   
  
  
+ TTTTTGTTCT CCTGTTGAAG TTCTGACTCT CTCTTACTCT CTCTCATATG TTTTCTCTTT TAGAAAAATA   
  
  
+ TTATTGAATA AAGAAATGGG ACCTTTGTAT TCTTATGGCC GTATGTGAAA TCTCTAGCGA AGCAAAGTCT   
  
  
+ TCTGTTTCAC ACCTTTCAAT CTCAAAACAA CGGAAGAAGA AGCTAGGGTT TGACAGCACC ACCAAGCCCA   
  
  
+ AGGTTTGTTC TTTGGTACTT TTCCTCTTTG CTTCTATTGC TATTTTTTTT TATCGAATTT TTCTTATCAT   
  
  
+ AGCCGTAAGT TTATTTTCTC TTTGCTTTCA CTCAGGTTTC AAAGACCCGC TTGAATCTGA TTTTGTGGGT   
  
  
+ ATCCCTGTTT TTAAAAATTA AACCTTCTGA GAGGTTGAAC GTTTCGAATT TTTGATGAAA AGAACCACAC   
  
  
+ TTGGGATTGT GGTTTATCCA GAAATGGAAT TTTGACTACG TGGGTCGTCT TTCCTTTTTA CCTCGAGGAT   
  
  
+ CTGTTATCGT GGTAGATTTT TCCGAGTTTT TATGGTCTGT TTTGGTCTAG GTGTCCCGGG GTTGGAGAAT   
  
  
+ TAGGGTTCTT TTGGGGTTTT GGGGGCTAA  

- CCTCCTTCAC TTCACTTCAG TTGCCGTCAC CCCGGTTAGG ACCTGGGTAC GTCGACAGTC AGTGGCTGAA   
  
  
- TTGTCACAGA GGGGCCGAGT TCTATCGTCG TCGGGGGGTC GTCCTCAAAC AACGCCAGTT TCGTGTAAAA   
  
  
- CCTTATGCAC AGTCCGAGGA TACGTTGAGA AACATGTGCA CCGTAGTAGG TCGACCATTA AAAAGACCGT   
  
  
- TGCCGAGATA TAGATATCCC TTGGCCGAGA CGTCAGAGGT CATGGCCCAC CACCCAGGGT GAAAACCGCC   
  
  
- TGTAAAACTA TTCGGGAAGT GTCTATGAAA TTTTTTTTTT CCCGTCTCGT TTCATTTGTT GGTCATAAGA   
  
  
- GAGACAGTAA AAAAAAAATT TTTTCTTTTA TACAAAATAT TTTAATTAAA TTAAAAGTAT TTTAAATAGA   
  
  
- TAAATTATAA AATTTTTCAT AATTTTACGT CTTTTCTCTG TCATGTAATC TCCTACACAA TAACAAAGTA   
  
  
- TAATAATATT ATTAAGTGTT TTATAAGAAA TTTTATTTGA AACTCTGGTG GAGTCTAGAC AACTTCTAGT   
  
  
- AACAGGATGT ATTTAGGTCA ACCACATTAT TTAGACCCCG ACATACTTCT CCCTGACTCC TACGTCGTCG   
  
  
- CTCTGTCCTC GAATTAACCC TCCTCCTCTG TTTTCGGTAC CGTTACCTTG TAGACCCACA CGTCATTTTG   
  
  
- ACTTCGACTT TTTCCCCACC AAGTAATTAC CCCCTCCCCT CCCTCTTTTT CTTATTGGTT CCAGAAAAGC   
  
  
- ACCCTGGGTG TGGAGTTTAG ACACTGTCTC TGCCGTCAAA GTAAAGAGTC GAGAAGGTGG AAGTGTTAGG   
  
  
- GAATAAATTT AGAAAGAGAG AGAGTGAACT TGTAACAACA AAGAGACAAG AGAGAGAAAG ATAGGAAACA   
  
  
- AAAAACAAGA GGACAACTTC AAGACTGAGA GAGAATGAGA GAGAGTATAC AAAAGAGAAA ATCTTTTTAT   
  
  
- AATAACTTAT TTCTTTACCC TGGAAACATA AGAATACCGG CATACACTTT AGAGATCGCT TCGTTTCAGA   
  
  
- AGACAAAGTG TGGAAAGTTA GAGTTTTGTT GCCTTCTTCT TCGATCCCAA ACTGTCGTGG TGGTTCGGGT   
  
  
- TCCAAACAAG AAACCATGAA AAGGAGAAAC GAAGATAACG ATAAAAAAAA ATAGCTTAAA AAGAATAGTA   
  
  
- TCGGCATTCA AATAAAAGAG AAACGAAAGT GAGTCCAAAG TTTCTGGGCG AACTTAGACT AAAACACCCA   
  
  
- TAGGGACAAA AATTTTTAAT TTGGAAGACT CTCCAACTTG CAAAGCTTAA AAACTACTTT TCTTGGTGTG   
  
  
- AACCCTAACA CCAAATAGGT CTTTACCTTA AAACTGATGC ACCCAGCAGA AAGGAAAAAT GGAGCTCCTA   
  
  
- GACAATAGCA CCATCTAAAA AGGCTCAAAA ATACCAGACA AAACCAGATC CACAGGGCCC CAACCTCTTA   
  
  
- ATCCCAAGAA AACCCCAAAA CCCCCGATT

+     TATA-box

| Site Name | Organism | Position | Strand | Matrix score. | sequence | function |
| --- | --- | --- | --- | --- | --- | --- |
| TATA-box | Zea mays | 1268 | - | 8 | TTTAAAAA | core promoter element around -30 of transcription start |
| TATA-box | Lycopersicon esculentum | 1168 | + | 5 | TTTTA | core promoter element around -30 of transcription start |
| TATA-box | Glycine max | 979 | - | 5 | TAATA | core promoter element around -30 of transcription start |
| TATA-box | Arabidopsis thaliana | 843 | + | 8 | TATTTAAA | core promoter element around -30 of transcription start |
| TATA-box | Lycopersicon esculentum | 968 | + | 5 | TTTTA | core promoter element around -30 of transcription start |
| TATA-box | Lycopersicon esculentum | 389 | - | 5 | TTTTA | core promoter element around -30 of transcription start |
| TATA-box | Arabidopsis thaliana | 385 | - | 6 | TATAAA | core promoter element around -30 of transcription start |
| TATA-box | Lycopersicon esculentum | 365 | + | 5 | TTTTA | core promoter element around -30 of transcription start |
| TATA-box | Arabidopsis thaliana | 224 | + | 4 | TATA | core promoter element around -30 of transcription start |
| TATA-box | Arabidopsis thaliana | 218 | + | 4 | TATA | core promoter element around -30 of transcription start |
| TATA-box | Lycopersicon esculentum | 1272 | - | 5 | TTTTA | core promoter element around -30 of transcription start |
| TATA-box | Lycopersicon esculentum | 1428 | + | 5 | TTTTA | core promoter element around -30 of transcription start |
| TATA-box | Lycopersicon esculentum | 1269 | + | 5 | TTTTA | core promoter element around -30 of transcription start |
| TATA-box | Arabidopsis thaliana | 567 | + | 9 | taTATAAAtc | core promoter element around -30 of transcription start |
| TATA-box | Glycine max | 498 | + | 5 | TAATA | core promoter element around -30 of transcription start |
| TATA-box | Brassica napus | 494 | + | 6 | ATTATA | core promoter element around -30 of transcription start |
| TATA-box | Lycopersicon esculentum | 432 | - | 5 | TTTTA | core promoter element around -30 of transcription start |
| TATA-box | Zea mays | 1270 | + | 8 | TTTAAAAA | core promoter element around -30 of transcription start |
| TATA-box | Glycine max | 493 | - | 5 | TAATA | core promoter element around -30 of transcription start |
| TATA-box | Zea mays | 430 | + | 8 | TTTAAAAA | core promoter element around -30 of transcription start |
| TATA-box | Lycopersicon esculentum | 429 | + | 5 | TTTTA | core promoter element around -30 of transcription start |
| TATA-box | Lycopersicon esculentum | 1386 | + | 5 | TTTTA | core promoter element around -30 of transcription start |
| TATA-box | Zea mays | 366 | + | 8 | TTTAAAAA | core promoter element around -30 of transcription start |
| TATA-box | Glycine max | 586 | + | 5 | TAATA | core promoter element around -30 of transcription start |
| TATA-box | Zea mays | 308 | + | 8 | TTTAAAAA | core promoter element around -30 of transcription start |
| TATA-box | Lycopersicon esculentum | 310 | - | 5 | TTTTA | core promoter element around -30 of transcription start |
| TATA-box | Glycine max | 490 | - | 5 | TAATA | core promoter element around -30 of transcription start |
| TATA-box | Arabidopsis thaliana | 496 | + | 4 | TATA | core promoter element around -30 of transcription start |
| TATA-box | Arabidopsis thaliana | 495 | - | 5 | TATAA | core promoter element around -30 of transcription start |
| TATA-box | Lycopersicon esculentum | 409 | - | 5 | TTTTA | core promoter element around -30 of transcription start |
| TATA-box | Zea mays | 364 | - | 8 | TTTAAAAA | core promoter element around -30 of transcription start |
| TATA-box | Lycopersicon esculentum | 520 | - | 5 | TTTTA | core promoter element around -30 of transcription start |
| TATA-box | Lycopersicon esculentum | 442 | - | 5 | TTTTA | core promoter element around -30 of transcription start |
| TATA-box | Lycopersicon esculentum | 695 | - | 5 | TTTTA | core promoter element around -30 of transcription start |
| TATA-box | Glycine max | 439 | - | 5 | TAATA | core promoter element around -30 of transcription start |
| TATA-box | Arabidopsis thaliana | 384 | - | 7 | TATAAAA | core promoter element around -30 of transcription start |
| TATA-box | Lycopersicon esculentum | 368 | - | 5 | TTTTA | core promoter element around -30 of transcription start |
| TATA-box | Glycine max | 424 | + | 5 | TAATA | core promoter element around -30 of transcription start |
| TATA-box | Arabidopsis thaliana | 386 | - | 5 | TATAA | core promoter element around -30 of transcription start |
| TATA-box | Arabidopsis thaliana | 387 | + | 6 | TATAAA | core promoter element around -30 of transcription start |

> 2018/04/13 10:10:12  
+ GGAGGAAGTG AAGTGAAGTC AACGGCAGTG GGGCCAATCC TGGACCCATG CAGCTGTCAG TCACCGACTT   
  
  
+ AACAGTGTCT CCCCGGCTCA AGATAGCAGC AGCCCCCCAG CAGGAGTTTG TTGCGGTCAA AGCACATTTT   
  
  
+ GGAATACGTG TCAGGCTCCT ATGCAACTCT TTGTACACGT GGCATCATCC AGCTGGTAAT TTTTCTGGCA   
  
  
+ ACGGCTCTAT ATCTATAGGG AACCGGCTCT GCAGTCTCCA GTACCGGGTG GTGGGTCCCA CTTTTGGCGG   
  
  
+ ACATTTTGAT AAGCCCTTCA CAGATACTTT AAAAAAAAAA GGGCAGAGCA AAGTAAACAA CCAGTATTCT   
  
  
+ CTCTGTCATT TTTTTTTTAA AAAAGAAAAT ATGTTTTATA AAATTAATTT AATTTTCATA AAATTTATCT   
  
  
+ ATTTAATATT TTAAAAAGTA TTAAAATGCA GAAAAGAGAC AGTACATTAG AGGATGTGTT ATTGTTTCAT   
  
  
+ ATTATTATAA TAATTCACAA AATATTCTTT AAAATAAACT TTGAGACCAC CTCAGATCTG TTGAAGATCA   
  
  
+ TTGTCCTACA TAAATCCAGT TGGTGTAATA AATCTGGGGC TGTATGAAGA GGGACTGAGG ATGCAGCAGC   
  
  
+ GAGACAGGAG CTTAATTGGG AGGAGGAGAC AAAAGCCATG GCAATGGAAC ATCTGGGTGT GCAGTAAAAC   
  
  
+ TGAAGCTGAA AAAGGGGTGG TTCATTAATG GGGGAGGGGA GGGAGAAAAA GAATAACCAA GGTCTTTTCG   
  
  
+ TGGGACCCAC ACCTCAAATC TGTGACAGAG ACGGCAGTTT CATTTCTCAG CTCTTCCACC TTCACAATCC   
  
  
+ CTTATTTAAA TCTTTCTCTC TCTCACTTGA ACATTGTTGT TTCTCTGTTC TCTCTCTTTC TATCCTTTGT   
  
  
+ TTTTTGTTCT CCTGTTGAAG TTCTGACTCT CTCTTACTCT CTCTCATATG TTTTCTCTTT TAGAAAAATA   
  
  
+ TTATTGAATA AAGAAATGGG ACCTTTGTAT TCTTATGGCC GTATGTGAAA TCTCTAGCGA AGCAAAGTCT   
  
  
+ TCTGTTTCAC ACCTTTCAAT CTCAAAACAA CGGAAGAAGA AGCTAGGGTT TGACAGCACC ACCAAGCCCA   
  
  
+ AGGTTTGTTC TTTGGTACTT TTCCTCTTTG CTTCTATTGC TATTTTTTTT TATCGAATTT TTCTTATCAT   
  
  
+ AGCCGTAAGT TTATTTTCTC TTTGCTTTCA CTCAGGTTTC AAAGACCCGC TTGAATCTGA TTTTGTGGGT   
  
  
+ ATCCCTGTTT TTAAAAATTA AACCTTCTGA GAGGTTGAAC GTTTCGAATT TTTGATGAAA AGAACCACAC   
  
  
+ TTGGGATTGT GGTTTATCCA GAAATGGAAT TTTGACTACG TGGGTCGTCT TTCCTTTTTA CCTCGAGGAT   
  
  
+ CTGTTATCGT GGTAGATTTT TCCGAGTTTT TATGGTCTGT TTTGGTCTAG GTGTCCCGGG GTTGGAGAAT   
  
  
+ TAGGGTTCTT TTGGGGTTTT GGGGGCTAA  

- CCTCCTTCAC TTCACTTCAG TTGCCGTCAC CCCGGTTAGG ACCTGGGTAC GTCGACAGTC AGTGGCTGAA   
  
  
- TTGTCACAGA GGGGCCGAGT TCTATCGTCG TCGGGGGGTC GTCCTCAAAC AACGCCAGTT TCGTGTAAAA   
  
  
- CCTTATGCAC AGTCCGAGGA TACGTTGAGA AACATGTGCA CCGTAGTAGG TCGACCATTA AAAAGACCGT   
  
  
- TGCCGAGATA TAGATATCCC TTGGCCGAGA CGTCAGAGGT CATGGCCCAC CACCCAGGGT GAAAACCGCC   
  
  
- TGTAAAACTA TTCGGGAAGT GTCTATGAAA TTTTTTTTTT CCCGTCTCGT TTCATTTGTT GGTCATAAGA   
  
  
- GAGACAGTAA AAAAAAAATT TTTTCTTTTA TACAAAATAT TTTAATTAAA TTAAAAGTAT TTTAAATAGA   
  
  
- TAAATTATAA AATTTTTCAT AATTTTACGT CTTTTCTCTG TCATGTAATC TCCTACACAA TAACAAAGTA   
  
  
- TAATAATATT ATTAAGTGTT TTATAAGAAA TTTTATTTGA AACTCTGGTG GAGTCTAGAC AACTTCTAGT   
  
  
- AACAGGATGT ATTTAGGTCA ACCACATTAT TTAGACCCCG ACATACTTCT CCCTGACTCC TACGTCGTCG   
  
  
- CTCTGTCCTC GAATTAACCC TCCTCCTCTG TTTTCGGTAC CGTTACCTTG TAGACCCACA CGTCATTTTG   
  
  
- ACTTCGACTT TTTCCCCACC AAGTAATTAC CCCCTCCCCT CCCTCTTTTT CTTATTGGTT CCAGAAAAGC   
  
  
- ACCCTGGGTG TGGAGTTTAG ACACTGTCTC TGCCGTCAAA GTAAAGAGTC GAGAAGGTGG AAGTGTTAGG   
  
  
- GAATAAATTT AGAAAGAGAG AGAGTGAACT TGTAACAACA AAGAGACAAG AGAGAGAAAG ATAGGAAACA   
  
  
- AAAAACAAGA GGACAACTTC AAGACTGAGA GAGAATGAGA GAGAGTATAC AAAAGAGAAA ATCTTTTTAT   
  
  
- AATAACTTAT TTCTTTACCC TGGAAACATA AGAATACCGG CATACACTTT AGAGATCGCT TCGTTTCAGA   
  
  
- AGACAAAGTG TGGAAAGTTA GAGTTTTGTT GCCTTCTTCT TCGATCCCAA ACTGTCGTGG TGGTTCGGGT   
  
  
- TCCAAACAAG AAACCATGAA AAGGAGAAAC GAAGATAACG ATAAAAAAAA ATAGCTTAAA AAGAATAGTA   
  
  
- TCGGCATTCA AATAAAAGAG AAACGAAAGT GAGTCCAAAG TTTCTGGGCG AACTTAGACT AAAACACCCA   
  
  
- TAGGGACAAA AATTTTTAAT TTGGAAGACT CTCCAACTTG CAAAGCTTAA AAACTACTTT TCTTGGTGTG   
  
  
- AACCCTAACA CCAAATAGGT CTTTACCTTA AAACTGATGC ACCCAGCAGA AAGGAAAAAT GGAGCTCCTA   
  
  
- GACAATAGCA CCATCTAAAA AGGCTCAAAA ATACCAGACA AAACCAGATC CACAGGGCCC CAACCTCTTA   
  
  
- ATCCCAAGAA AACCCCAAAA CCCCCGATT

+     TC-rich repeats

| Site Name | Organism | Position | Strand | Matrix score. | sequence | function |
| --- | --- | --- | --- | --- | --- | --- |
| TC-rich repeats | Nicotiana tabacum | 1461 | - | 9 | ATTCTCTAAC | cis-acting element involved in defense and stress responsiveness |

> 2018/04/13 10:10:12  
+ GGAGGAAGTG AAGTGAAGTC AACGGCAGTG GGGCCAATCC TGGACCCATG CAGCTGTCAG TCACCGACTT   
  
  
+ AACAGTGTCT CCCCGGCTCA AGATAGCAGC AGCCCCCCAG CAGGAGTTTG TTGCGGTCAA AGCACATTTT   
  
  
+ GGAATACGTG TCAGGCTCCT ATGCAACTCT TTGTACACGT GGCATCATCC AGCTGGTAAT TTTTCTGGCA   
  
  
+ ACGGCTCTAT ATCTATAGGG AACCGGCTCT GCAGTCTCCA GTACCGGGTG GTGGGTCCCA CTTTTGGCGG   
  
  
+ ACATTTTGAT AAGCCCTTCA CAGATACTTT AAAAAAAAAA GGGCAGAGCA AAGTAAACAA CCAGTATTCT   
  
  
+ CTCTGTCATT TTTTTTTTAA AAAAGAAAAT ATGTTTTATA AAATTAATTT AATTTTCATA AAATTTATCT   
  
  
+ ATTTAATATT TTAAAAAGTA TTAAAATGCA GAAAAGAGAC AGTACATTAG AGGATGTGTT ATTGTTTCAT   
  
  
+ ATTATTATAA TAATTCACAA AATATTCTTT AAAATAAACT TTGAGACCAC CTCAGATCTG TTGAAGATCA   
  
  
+ TTGTCCTACA TAAATCCAGT TGGTGTAATA AATCTGGGGC TGTATGAAGA GGGACTGAGG ATGCAGCAGC   
  
  
+ GAGACAGGAG CTTAATTGGG AGGAGGAGAC AAAAGCCATG GCAATGGAAC ATCTGGGTGT GCAGTAAAAC   
  
  
+ TGAAGCTGAA AAAGGGGTGG TTCATTAATG GGGGAGGGGA GGGAGAAAAA GAATAACCAA GGTCTTTTCG   
  
  
+ TGGGACCCAC ACCTCAAATC TGTGACAGAG ACGGCAGTTT CATTTCTCAG CTCTTCCACC TTCACAATCC   
  
  
+ CTTATTTAAA TCTTTCTCTC TCTCACTTGA ACATTGTTGT TTCTCTGTTC TCTCTCTTTC TATCCTTTGT   
  
  
+ TTTTTGTTCT CCTGTTGAAG TTCTGACTCT CTCTTACTCT CTCTCATATG TTTTCTCTTT TAGAAAAATA   
  
  
+ TTATTGAATA AAGAAATGGG ACCTTTGTAT TCTTATGGCC GTATGTGAAA TCTCTAGCGA AGCAAAGTCT   
  
  
+ TCTGTTTCAC ACCTTTCAAT CTCAAAACAA CGGAAGAAGA AGCTAGGGTT TGACAGCACC ACCAAGCCCA   
  
  
+ AGGTTTGTTC TTTGGTACTT TTCCTCTTTG CTTCTATTGC TATTTTTTTT TATCGAATTT TTCTTATCAT   
  
  
+ AGCCGTAAGT TTATTTTCTC TTTGCTTTCA CTCAGGTTTC AAAGACCCGC TTGAATCTGA TTTTGTGGGT   
  
  
+ ATCCCTGTTT TTAAAAATTA AACCTTCTGA GAGGTTGAAC GTTTCGAATT TTTGATGAAA AGAACCACAC   
  
  
+ TTGGGATTGT GGTTTATCCA GAAATGGAAT TTTGACTACG TGGGTCGTCT TTCCTTTTTA CCTCGAGGAT   
  
  
+ CTGTTATCGT GGTAGATTTT TCCGAGTTTT TATGGTCTGT TTTGGTCTAG GTGTCCCGGG GTTGGAGAAT   
  
  
+ TAGGGTTCTT TTGGGGTTTT GGGGGCTAA  

- CCTCCTTCAC TTCACTTCAG TTGCCGTCAC CCCGGTTAGG ACCTGGGTAC GTCGACAGTC AGTGGCTGAA   
  
  
- TTGTCACAGA GGGGCCGAGT TCTATCGTCG TCGGGGGGTC GTCCTCAAAC AACGCCAGTT TCGTGTAAAA   
  
  
- CCTTATGCAC AGTCCGAGGA TACGTTGAGA AACATGTGCA CCGTAGTAGG TCGACCATTA AAAAGACCGT   
  
  
- TGCCGAGATA TAGATATCCC TTGGCCGAGA CGTCAGAGGT CATGGCCCAC CACCCAGGGT GAAAACCGCC   
  
  
- TGTAAAACTA TTCGGGAAGT GTCTATGAAA TTTTTTTTTT CCCGTCTCGT TTCATTTGTT GGTCATAAGA   
  
  
- GAGACAGTAA AAAAAAAATT TTTTCTTTTA TACAAAATAT TTTAATTAAA TTAAAAGTAT TTTAAATAGA   
  
  
- TAAATTATAA AATTTTTCAT AATTTTACGT CTTTTCTCTG TCATGTAATC TCCTACACAA TAACAAAGTA   
  
  
- TAATAATATT ATTAAGTGTT TTATAAGAAA TTTTATTTGA AACTCTGGTG GAGTCTAGAC AACTTCTAGT   
  
  
- AACAGGATGT ATTTAGGTCA ACCACATTAT TTAGACCCCG ACATACTTCT CCCTGACTCC TACGTCGTCG   
  
  
- CTCTGTCCTC GAATTAACCC TCCTCCTCTG TTTTCGGTAC CGTTACCTTG TAGACCCACA CGTCATTTTG   
  
  
- ACTTCGACTT TTTCCCCACC AAGTAATTAC CCCCTCCCCT CCCTCTTTTT CTTATTGGTT CCAGAAAAGC   
  
  
- ACCCTGGGTG TGGAGTTTAG ACACTGTCTC TGCCGTCAAA GTAAAGAGTC GAGAAGGTGG AAGTGTTAGG   
  
  
- GAATAAATTT AGAAAGAGAG AGAGTGAACT TGTAACAACA AAGAGACAAG AGAGAGAAAG ATAGGAAACA   
  
  
- AAAAACAAGA GGACAACTTC AAGACTGAGA GAGAATGAGA GAGAGTATAC AAAAGAGAAA ATCTTTTTAT   
  
  
- AATAACTTAT TTCTTTACCC TGGAAACATA AGAATACCGG CATACACTTT AGAGATCGCT TCGTTTCAGA   
  
  
- AGACAAAGTG TGGAAAGTTA GAGTTTTGTT GCCTTCTTCT TCGATCCCAA ACTGTCGTGG TGGTTCGGGT   
  
  
- TCCAAACAAG AAACCATGAA AAGGAGAAAC GAAGATAACG ATAAAAAAAA ATAGCTTAAA AAGAATAGTA   
  
  
- TCGGCATTCA AATAAAAGAG AAACGAAAGT GAGTCCAAAG TTTCTGGGCG AACTTAGACT AAAACACCCA   
  
  
- TAGGGACAAA AATTTTTAAT TTGGAAGACT CTCCAACTTG CAAAGCTTAA AAACTACTTT TCTTGGTGTG   
  
  
- AACCCTAACA CCAAATAGGT CTTTACCTTA AAACTGATGC ACCCAGCAGA AAGGAAAAAT GGAGCTCCTA   
  
  
- GACAATAGCA CCATCTAAAA AGGCTCAAAA ATACCAGACA AAACCAGATC CACAGGGCCC CAACCTCTTA   
  
  
- ATCCCAAGAA AACCCCAAAA CCCCCGATT

+     TCA-element

| Site Name | Organism | Position | Strand | Matrix score. | sequence | function |
| --- | --- | --- | --- | --- | --- | --- |
| TCA-element | Brassica oleracea | 1349 | + | 9 | CAGAAAAGGA | cis-acting element involved in salicylic acid responsiveness |

> 2018/04/13 10:10:12  
+ GGAGGAAGTG AAGTGAAGTC AACGGCAGTG GGGCCAATCC TGGACCCATG CAGCTGTCAG TCACCGACTT   
  
  
+ AACAGTGTCT CCCCGGCTCA AGATAGCAGC AGCCCCCCAG CAGGAGTTTG TTGCGGTCAA AGCACATTTT   
  
  
+ GGAATACGTG TCAGGCTCCT ATGCAACTCT TTGTACACGT GGCATCATCC AGCTGGTAAT TTTTCTGGCA   
  
  
+ ACGGCTCTAT ATCTATAGGG AACCGGCTCT GCAGTCTCCA GTACCGGGTG GTGGGTCCCA CTTTTGGCGG   
  
  
+ ACATTTTGAT AAGCCCTTCA CAGATACTTT AAAAAAAAAA GGGCAGAGCA AAGTAAACAA CCAGTATTCT   
  
  
+ CTCTGTCATT TTTTTTTTAA AAAAGAAAAT ATGTTTTATA AAATTAATTT AATTTTCATA AAATTTATCT   
  
  
+ ATTTAATATT TTAAAAAGTA TTAAAATGCA GAAAAGAGAC AGTACATTAG AGGATGTGTT ATTGTTTCAT   
  
  
+ ATTATTATAA TAATTCACAA AATATTCTTT AAAATAAACT TTGAGACCAC CTCAGATCTG TTGAAGATCA   
  
  
+ TTGTCCTACA TAAATCCAGT TGGTGTAATA AATCTGGGGC TGTATGAAGA GGGACTGAGG ATGCAGCAGC   
  
  
+ GAGACAGGAG CTTAATTGGG AGGAGGAGAC AAAAGCCATG GCAATGGAAC ATCTGGGTGT GCAGTAAAAC   
  
  
+ TGAAGCTGAA AAAGGGGTGG TTCATTAATG GGGGAGGGGA GGGAGAAAAA GAATAACCAA GGTCTTTTCG   
  
  
+ TGGGACCCAC ACCTCAAATC TGTGACAGAG ACGGCAGTTT CATTTCTCAG CTCTTCCACC TTCACAATCC   
  
  
+ CTTATTTAAA TCTTTCTCTC TCTCACTTGA ACATTGTTGT TTCTCTGTTC TCTCTCTTTC TATCCTTTGT   
  
  
+ TTTTTGTTCT CCTGTTGAAG TTCTGACTCT CTCTTACTCT CTCTCATATG TTTTCTCTTT TAGAAAAATA   
  
  
+ TTATTGAATA AAGAAATGGG ACCTTTGTAT TCTTATGGCC GTATGTGAAA TCTCTAGCGA AGCAAAGTCT   
  
  
+ TCTGTTTCAC ACCTTTCAAT CTCAAAACAA CGGAAGAAGA AGCTAGGGTT TGACAGCACC ACCAAGCCCA   
  
  
+ AGGTTTGTTC TTTGGTACTT TTCCTCTTTG CTTCTATTGC TATTTTTTTT TATCGAATTT TTCTTATCAT   
  
  
+ AGCCGTAAGT TTATTTTCTC TTTGCTTTCA CTCAGGTTTC AAAGACCCGC TTGAATCTGA TTTTGTGGGT   
  
  
+ ATCCCTGTTT TTAAAAATTA AACCTTCTGA GAGGTTGAAC GTTTCGAATT TTTGATGAAA AGAACCACAC   
  
  
+ TTGGGATTGT GGTTTATCCA GAAATGGAAT TTTGACTACG TGGGTCGTCT TTCCTTTTTA CCTCGAGGAT   
  
  
+ CTGTTATCGT GGTAGATTTT TCCGAGTTTT TATGGTCTGT TTTGGTCTAG GTGTCCCGGG GTTGGAGAAT   
  
  
+ TAGGGTTCTT TTGGGGTTTT GGGGGCTAA  

- CCTCCTTCAC TTCACTTCAG TTGCCGTCAC CCCGGTTAGG ACCTGGGTAC GTCGACAGTC AGTGGCTGAA   
  
  
- TTGTCACAGA GGGGCCGAGT TCTATCGTCG TCGGGGGGTC GTCCTCAAAC AACGCCAGTT TCGTGTAAAA   
  
  
- CCTTATGCAC AGTCCGAGGA TACGTTGAGA AACATGTGCA CCGTAGTAGG TCGACCATTA AAAAGACCGT   
  
  
- TGCCGAGATA TAGATATCCC TTGGCCGAGA CGTCAGAGGT CATGGCCCAC CACCCAGGGT GAAAACCGCC   
  
  
- TGTAAAACTA TTCGGGAAGT GTCTATGAAA TTTTTTTTTT CCCGTCTCGT TTCATTTGTT GGTCATAAGA   
  
  
- GAGACAGTAA AAAAAAAATT TTTTCTTTTA TACAAAATAT TTTAATTAAA TTAAAAGTAT TTTAAATAGA   
  
  
- TAAATTATAA AATTTTTCAT AATTTTACGT CTTTTCTCTG TCATGTAATC TCCTACACAA TAACAAAGTA   
  
  
- TAATAATATT ATTAAGTGTT TTATAAGAAA TTTTATTTGA AACTCTGGTG GAGTCTAGAC AACTTCTAGT   
  
  
- AACAGGATGT ATTTAGGTCA ACCACATTAT TTAGACCCCG ACATACTTCT CCCTGACTCC TACGTCGTCG   
  
  
- CTCTGTCCTC GAATTAACCC TCCTCCTCTG TTTTCGGTAC CGTTACCTTG TAGACCCACA CGTCATTTTG   
  
  
- ACTTCGACTT TTTCCCCACC AAGTAATTAC CCCCTCCCCT CCCTCTTTTT CTTATTGGTT CCAGAAAAGC   
  
  
- ACCCTGGGTG TGGAGTTTAG ACACTGTCTC TGCCGTCAAA GTAAAGAGTC GAGAAGGTGG AAGTGTTAGG   
  
  
- GAATAAATTT AGAAAGAGAG AGAGTGAACT TGTAACAACA AAGAGACAAG AGAGAGAAAG ATAGGAAACA   
  
  
- AAAAACAAGA GGACAACTTC AAGACTGAGA GAGAATGAGA GAGAGTATAC AAAAGAGAAA ATCTTTTTAT   
  
  
- AATAACTTAT TTCTTTACCC TGGAAACATA AGAATACCGG CATACACTTT AGAGATCGCT TCGTTTCAGA   
  
  
- AGACAAAGTG TGGAAAGTTA GAGTTTTGTT GCCTTCTTCT TCGATCCCAA ACTGTCGTGG TGGTTCGGGT   
  
  
- TCCAAACAAG AAACCATGAA AAGGAGAAAC GAAGATAACG ATAAAAAAAA ATAGCTTAAA AAGAATAGTA   
  
  
- TCGGCATTCA AATAAAAGAG AAACGAAAGT GAGTCCAAAG TTTCTGGGCG AACTTAGACT AAAACACCCA   
  
  
- TAGGGACAAA AATTTTTAAT TTGGAAGACT CTCCAACTTG CAAAGCTTAA AAACTACTTT TCTTGGTGTG   
  
  
- AACCCTAACA CCAAATAGGT CTTTACCTTA AAACTGATGC ACCCAGCAGA AAGGAAAAAT GGAGCTCCTA   
  
  
- GACAATAGCA CCATCTAAAA AGGCTCAAAA ATACCAGACA AAACCAGATC CACAGGGCCC CAACCTCTTA   
  
  
- ATCCCAAGAA AACCCCAAAA CCCCCGATT

+     TCCACCT-motif

| Site Name | Organism | Position | Strand | Matrix score. | sequence | function |
| --- | --- | --- | --- | --- | --- | --- |
| TCCACCT-motif | Petroselinum hortense | 825 | + | 7 | TCCACCT |  |

> 2018/04/13 10:10:12  
+ GGAGGAAGTG AAGTGAAGTC AACGGCAGTG GGGCCAATCC TGGACCCATG CAGCTGTCAG TCACCGACTT   
  
  
+ AACAGTGTCT CCCCGGCTCA AGATAGCAGC AGCCCCCCAG CAGGAGTTTG TTGCGGTCAA AGCACATTTT   
  
  
+ GGAATACGTG TCAGGCTCCT ATGCAACTCT TTGTACACGT GGCATCATCC AGCTGGTAAT TTTTCTGGCA   
  
  
+ ACGGCTCTAT ATCTATAGGG AACCGGCTCT GCAGTCTCCA GTACCGGGTG GTGGGTCCCA CTTTTGGCGG   
  
  
+ ACATTTTGAT AAGCCCTTCA CAGATACTTT AAAAAAAAAA GGGCAGAGCA AAGTAAACAA CCAGTATTCT   
  
  
+ CTCTGTCATT TTTTTTTTAA AAAAGAAAAT ATGTTTTATA AAATTAATTT AATTTTCATA AAATTTATCT   
  
  
+ ATTTAATATT TTAAAAAGTA TTAAAATGCA GAAAAGAGAC AGTACATTAG AGGATGTGTT ATTGTTTCAT   
  
  
+ ATTATTATAA TAATTCACAA AATATTCTTT AAAATAAACT TTGAGACCAC CTCAGATCTG TTGAAGATCA   
  
  
+ TTGTCCTACA TAAATCCAGT TGGTGTAATA AATCTGGGGC TGTATGAAGA GGGACTGAGG ATGCAGCAGC   
  
  
+ GAGACAGGAG CTTAATTGGG AGGAGGAGAC AAAAGCCATG GCAATGGAAC ATCTGGGTGT GCAGTAAAAC   
  
  
+ TGAAGCTGAA AAAGGGGTGG TTCATTAATG GGGGAGGGGA GGGAGAAAAA GAATAACCAA GGTCTTTTCG   
  
  
+ TGGGACCCAC ACCTCAAATC TGTGACAGAG ACGGCAGTTT CATTTCTCAG CTCTTCCACC TTCACAATCC   
  
  
+ CTTATTTAAA TCTTTCTCTC TCTCACTTGA ACATTGTTGT TTCTCTGTTC TCTCTCTTTC TATCCTTTGT   
  
  
+ TTTTTGTTCT CCTGTTGAAG TTCTGACTCT CTCTTACTCT CTCTCATATG TTTTCTCTTT TAGAAAAATA   
  
  
+ TTATTGAATA AAGAAATGGG ACCTTTGTAT TCTTATGGCC GTATGTGAAA TCTCTAGCGA AGCAAAGTCT   
  
  
+ TCTGTTTCAC ACCTTTCAAT CTCAAAACAA CGGAAGAAGA AGCTAGGGTT TGACAGCACC ACCAAGCCCA   
  
  
+ AGGTTTGTTC TTTGGTACTT TTCCTCTTTG CTTCTATTGC TATTTTTTTT TATCGAATTT TTCTTATCAT   
  
  
+ AGCCGTAAGT TTATTTTCTC TTTGCTTTCA CTCAGGTTTC AAAGACCCGC TTGAATCTGA TTTTGTGGGT   
  
  
+ ATCCCTGTTT TTAAAAATTA AACCTTCTGA GAGGTTGAAC GTTTCGAATT TTTGATGAAA AGAACCACAC   
  
  
+ TTGGGATTGT GGTTTATCCA GAAATGGAAT TTTGACTACG TGGGTCGTCT TTCCTTTTTA CCTCGAGGAT   
  
  
+ CTGTTATCGT GGTAGATTTT TCCGAGTTTT TATGGTCTGT TTTGGTCTAG GTGTCCCGGG GTTGGAGAAT   
  
  
+ TAGGGTTCTT TTGGGGTTTT GGGGGCTAA  

- CCTCCTTCAC TTCACTTCAG TTGCCGTCAC CCCGGTTAGG ACCTGGGTAC GTCGACAGTC AGTGGCTGAA   
  
  
- TTGTCACAGA GGGGCCGAGT TCTATCGTCG TCGGGGGGTC GTCCTCAAAC AACGCCAGTT TCGTGTAAAA   
  
  
- CCTTATGCAC AGTCCGAGGA TACGTTGAGA AACATGTGCA CCGTAGTAGG TCGACCATTA AAAAGACCGT   
  
  
- TGCCGAGATA TAGATATCCC TTGGCCGAGA CGTCAGAGGT CATGGCCCAC CACCCAGGGT GAAAACCGCC   
  
  
- TGTAAAACTA TTCGGGAAGT GTCTATGAAA TTTTTTTTTT CCCGTCTCGT TTCATTTGTT GGTCATAAGA   
  
  
- GAGACAGTAA AAAAAAAATT TTTTCTTTTA TACAAAATAT TTTAATTAAA TTAAAAGTAT TTTAAATAGA   
  
  
- TAAATTATAA AATTTTTCAT AATTTTACGT CTTTTCTCTG TCATGTAATC TCCTACACAA TAACAAAGTA   
  
  
- TAATAATATT ATTAAGTGTT TTATAAGAAA TTTTATTTGA AACTCTGGTG GAGTCTAGAC AACTTCTAGT   
  
  
- AACAGGATGT ATTTAGGTCA ACCACATTAT TTAGACCCCG ACATACTTCT CCCTGACTCC TACGTCGTCG   
  
  
- CTCTGTCCTC GAATTAACCC TCCTCCTCTG TTTTCGGTAC CGTTACCTTG TAGACCCACA CGTCATTTTG   
  
  
- ACTTCGACTT TTTCCCCACC AAGTAATTAC CCCCTCCCCT CCCTCTTTTT CTTATTGGTT CCAGAAAAGC   
  
  
- ACCCTGGGTG TGGAGTTTAG ACACTGTCTC TGCCGTCAAA GTAAAGAGTC GAGAAGGTGG AAGTGTTAGG   
  
  
- GAATAAATTT AGAAAGAGAG AGAGTGAACT TGTAACAACA AAGAGACAAG AGAGAGAAAG ATAGGAAACA   
  
  
- AAAAACAAGA GGACAACTTC AAGACTGAGA GAGAATGAGA GAGAGTATAC AAAAGAGAAA ATCTTTTTAT   
  
  
- AATAACTTAT TTCTTTACCC TGGAAACATA AGAATACCGG CATACACTTT AGAGATCGCT TCGTTTCAGA   
  
  
- AGACAAAGTG TGGAAAGTTA GAGTTTTGTT GCCTTCTTCT TCGATCCCAA ACTGTCGTGG TGGTTCGGGT   
  
  
- TCCAAACAAG AAACCATGAA AAGGAGAAAC GAAGATAACG ATAAAAAAAA ATAGCTTAAA AAGAATAGTA   
  
  
- TCGGCATTCA AATAAAAGAG AAACGAAAGT GAGTCCAAAG TTTCTGGGCG AACTTAGACT AAAACACCCA   
  
  
- TAGGGACAAA AATTTTTAAT TTGGAAGACT CTCCAACTTG CAAAGCTTAA AAACTACTTT TCTTGGTGTG   
  
  
- AACCCTAACA CCAAATAGGT CTTTACCTTA AAACTGATGC ACCCAGCAGA AAGGAAAAAT GGAGCTCCTA   
  
  
- GACAATAGCA CCATCTAAAA AGGCTCAAAA ATACCAGACA AAACCAGATC CACAGGGCCC CAACCTCTTA   
  
  
- ATCCCAAGAA AACCCCAAAA CCCCCGATT

+     TCCC-motif

| Site Name | Organism | Position | Strand | Matrix score. | sequence | function |
| --- | --- | --- | --- | --- | --- | --- |
| TCCC-motif | Spinacia oleracea | 740 | - | 7 | TCTCCCT | part of a light responsive element |

> 2018/04/13 10:10:12  
+ GGAGGAAGTG AAGTGAAGTC AACGGCAGTG GGGCCAATCC TGGACCCATG CAGCTGTCAG TCACCGACTT   
  
  
+ AACAGTGTCT CCCCGGCTCA AGATAGCAGC AGCCCCCCAG CAGGAGTTTG TTGCGGTCAA AGCACATTTT   
  
  
+ GGAATACGTG TCAGGCTCCT ATGCAACTCT TTGTACACGT GGCATCATCC AGCTGGTAAT TTTTCTGGCA   
  
  
+ ACGGCTCTAT ATCTATAGGG AACCGGCTCT GCAGTCTCCA GTACCGGGTG GTGGGTCCCA CTTTTGGCGG   
  
  
+ ACATTTTGAT AAGCCCTTCA CAGATACTTT AAAAAAAAAA GGGCAGAGCA AAGTAAACAA CCAGTATTCT   
  
  
+ CTCTGTCATT TTTTTTTTAA AAAAGAAAAT ATGTTTTATA AAATTAATTT AATTTTCATA AAATTTATCT   
  
  
+ ATTTAATATT TTAAAAAGTA TTAAAATGCA GAAAAGAGAC AGTACATTAG AGGATGTGTT ATTGTTTCAT   
  
  
+ ATTATTATAA TAATTCACAA AATATTCTTT AAAATAAACT TTGAGACCAC CTCAGATCTG TTGAAGATCA   
  
  
+ TTGTCCTACA TAAATCCAGT TGGTGTAATA AATCTGGGGC TGTATGAAGA GGGACTGAGG ATGCAGCAGC   
  
  
+ GAGACAGGAG CTTAATTGGG AGGAGGAGAC AAAAGCCATG GCAATGGAAC ATCTGGGTGT GCAGTAAAAC   
  
  
+ TGAAGCTGAA AAAGGGGTGG TTCATTAATG GGGGAGGGGA GGGAGAAAAA GAATAACCAA GGTCTTTTCG   
  
  
+ TGGGACCCAC ACCTCAAATC TGTGACAGAG ACGGCAGTTT CATTTCTCAG CTCTTCCACC TTCACAATCC   
  
  
+ CTTATTTAAA TCTTTCTCTC TCTCACTTGA ACATTGTTGT TTCTCTGTTC TCTCTCTTTC TATCCTTTGT   
  
  
+ TTTTTGTTCT CCTGTTGAAG TTCTGACTCT CTCTTACTCT CTCTCATATG TTTTCTCTTT TAGAAAAATA   
  
  
+ TTATTGAATA AAGAAATGGG ACCTTTGTAT TCTTATGGCC GTATGTGAAA TCTCTAGCGA AGCAAAGTCT   
  
  
+ TCTGTTTCAC ACCTTTCAAT CTCAAAACAA CGGAAGAAGA AGCTAGGGTT TGACAGCACC ACCAAGCCCA   
  
  
+ AGGTTTGTTC TTTGGTACTT TTCCTCTTTG CTTCTATTGC TATTTTTTTT TATCGAATTT TTCTTATCAT   
  
  
+ AGCCGTAAGT TTATTTTCTC TTTGCTTTCA CTCAGGTTTC AAAGACCCGC TTGAATCTGA TTTTGTGGGT   
  
  
+ ATCCCTGTTT TTAAAAATTA AACCTTCTGA GAGGTTGAAC GTTTCGAATT TTTGATGAAA AGAACCACAC   
  
  
+ TTGGGATTGT GGTTTATCCA GAAATGGAAT TTTGACTACG TGGGTCGTCT TTCCTTTTTA CCTCGAGGAT   
  
  
+ CTGTTATCGT GGTAGATTTT TCCGAGTTTT TATGGTCTGT TTTGGTCTAG GTGTCCCGGG GTTGGAGAAT   
  
  
+ TAGGGTTCTT TTGGGGTTTT GGGGGCTAA  

- CCTCCTTCAC TTCACTTCAG TTGCCGTCAC CCCGGTTAGG ACCTGGGTAC GTCGACAGTC AGTGGCTGAA   
  
  
- TTGTCACAGA GGGGCCGAGT TCTATCGTCG TCGGGGGGTC GTCCTCAAAC AACGCCAGTT TCGTGTAAAA   
  
  
- CCTTATGCAC AGTCCGAGGA TACGTTGAGA AACATGTGCA CCGTAGTAGG TCGACCATTA AAAAGACCGT   
  
  
- TGCCGAGATA TAGATATCCC TTGGCCGAGA CGTCAGAGGT CATGGCCCAC CACCCAGGGT GAAAACCGCC   
  
  
- TGTAAAACTA TTCGGGAAGT GTCTATGAAA TTTTTTTTTT CCCGTCTCGT TTCATTTGTT GGTCATAAGA   
  
  
- GAGACAGTAA AAAAAAAATT TTTTCTTTTA TACAAAATAT TTTAATTAAA TTAAAAGTAT TTTAAATAGA   
  
  
- TAAATTATAA AATTTTTCAT AATTTTACGT CTTTTCTCTG TCATGTAATC TCCTACACAA TAACAAAGTA   
  
  
- TAATAATATT ATTAAGTGTT TTATAAGAAA TTTTATTTGA AACTCTGGTG GAGTCTAGAC AACTTCTAGT   
  
  
- AACAGGATGT ATTTAGGTCA ACCACATTAT TTAGACCCCG ACATACTTCT CCCTGACTCC TACGTCGTCG   
  
  
- CTCTGTCCTC GAATTAACCC TCCTCCTCTG TTTTCGGTAC CGTTACCTTG TAGACCCACA CGTCATTTTG   
  
  
- ACTTCGACTT TTTCCCCACC AAGTAATTAC CCCCTCCCCT CCCTCTTTTT CTTATTGGTT CCAGAAAAGC   
  
  
- ACCCTGGGTG TGGAGTTTAG ACACTGTCTC TGCCGTCAAA GTAAAGAGTC GAGAAGGTGG AAGTGTTAGG   
  
  
- GAATAAATTT AGAAAGAGAG AGAGTGAACT TGTAACAACA AAGAGACAAG AGAGAGAAAG ATAGGAAACA   
  
  
- AAAAACAAGA GGACAACTTC AAGACTGAGA GAGAATGAGA GAGAGTATAC AAAAGAGAAA ATCTTTTTAT   
  
  
- AATAACTTAT TTCTTTACCC TGGAAACATA AGAATACCGG CATACACTTT AGAGATCGCT TCGTTTCAGA   
  
  
- AGACAAAGTG TGGAAAGTTA GAGTTTTGTT GCCTTCTTCT TCGATCCCAA ACTGTCGTGG TGGTTCGGGT   
  
  
- TCCAAACAAG AAACCATGAA AAGGAGAAAC GAAGATAACG ATAAAAAAAA ATAGCTTAAA AAGAATAGTA   
  
  
- TCGGCATTCA AATAAAAGAG AAACGAAAGT GAGTCCAAAG TTTCTGGGCG AACTTAGACT AAAACACCCA   
  
  
- TAGGGACAAA AATTTTTAAT TTGGAAGACT CTCCAACTTG CAAAGCTTAA AAACTACTTT TCTTGGTGTG   
  
  
- AACCCTAACA CCAAATAGGT CTTTACCTTA AAACTGATGC ACCCAGCAGA AAGGAAAAAT GGAGCTCCTA   
  
  
- GACAATAGCA CCATCTAAAA AGGCTCAAAA ATACCAGACA AAACCAGATC CACAGGGCCC CAACCTCTTA   
  
  
- ATCCCAAGAA AACCCCAAAA CCCCCGATT

+     TCT-motif

| Site Name | Organism | Position | Strand | Matrix score. | sequence | function |
| --- | --- | --- | --- | --- | --- | --- |
| TCT-motif | Arabidopsis thaliana | 942 | + | 6 | TCTTAC | part of a light responsive element |

> 2018/04/13 10:10:12  
+ GGAGGAAGTG AAGTGAAGTC AACGGCAGTG GGGCCAATCC TGGACCCATG CAGCTGTCAG TCACCGACTT   
  
  
+ AACAGTGTCT CCCCGGCTCA AGATAGCAGC AGCCCCCCAG CAGGAGTTTG TTGCGGTCAA AGCACATTTT   
  
  
+ GGAATACGTG TCAGGCTCCT ATGCAACTCT TTGTACACGT GGCATCATCC AGCTGGTAAT TTTTCTGGCA   
  
  
+ ACGGCTCTAT ATCTATAGGG AACCGGCTCT GCAGTCTCCA GTACCGGGTG GTGGGTCCCA CTTTTGGCGG   
  
  
+ ACATTTTGAT AAGCCCTTCA CAGATACTTT AAAAAAAAAA GGGCAGAGCA AAGTAAACAA CCAGTATTCT   
  
  
+ CTCTGTCATT TTTTTTTTAA AAAAGAAAAT ATGTTTTATA AAATTAATTT AATTTTCATA AAATTTATCT   
  
  
+ ATTTAATATT TTAAAAAGTA TTAAAATGCA GAAAAGAGAC AGTACATTAG AGGATGTGTT ATTGTTTCAT   
  
  
+ ATTATTATAA TAATTCACAA AATATTCTTT AAAATAAACT TTGAGACCAC CTCAGATCTG TTGAAGATCA   
  
  
+ TTGTCCTACA TAAATCCAGT TGGTGTAATA AATCTGGGGC TGTATGAAGA GGGACTGAGG ATGCAGCAGC   
  
  
+ GAGACAGGAG CTTAATTGGG AGGAGGAGAC AAAAGCCATG GCAATGGAAC ATCTGGGTGT GCAGTAAAAC   
  
  
+ TGAAGCTGAA AAAGGGGTGG TTCATTAATG GGGGAGGGGA GGGAGAAAAA GAATAACCAA GGTCTTTTCG   
  
  
+ TGGGACCCAC ACCTCAAATC TGTGACAGAG ACGGCAGTTT CATTTCTCAG CTCTTCCACC TTCACAATCC   
  
  
+ CTTATTTAAA TCTTTCTCTC TCTCACTTGA ACATTGTTGT TTCTCTGTTC TCTCTCTTTC TATCCTTTGT   
  
  
+ TTTTTGTTCT CCTGTTGAAG TTCTGACTCT CTCTTACTCT CTCTCATATG TTTTCTCTTT TAGAAAAATA   
  
  
+ TTATTGAATA AAGAAATGGG ACCTTTGTAT TCTTATGGCC GTATGTGAAA TCTCTAGCGA AGCAAAGTCT   
  
  
+ TCTGTTTCAC ACCTTTCAAT CTCAAAACAA CGGAAGAAGA AGCTAGGGTT TGACAGCACC ACCAAGCCCA   
  
  
+ AGGTTTGTTC TTTGGTACTT TTCCTCTTTG CTTCTATTGC TATTTTTTTT TATCGAATTT TTCTTATCAT   
  
  
+ AGCCGTAAGT TTATTTTCTC TTTGCTTTCA CTCAGGTTTC AAAGACCCGC TTGAATCTGA TTTTGTGGGT   
  
  
+ ATCCCTGTTT TTAAAAATTA AACCTTCTGA GAGGTTGAAC GTTTCGAATT TTTGATGAAA AGAACCACAC   
  
  
+ TTGGGATTGT GGTTTATCCA GAAATGGAAT TTTGACTACG TGGGTCGTCT TTCCTTTTTA CCTCGAGGAT   
  
  
+ CTGTTATCGT GGTAGATTTT TCCGAGTTTT TATGGTCTGT TTTGGTCTAG GTGTCCCGGG GTTGGAGAAT   
  
  
+ TAGGGTTCTT TTGGGGTTTT GGGGGCTAA  

- CCTCCTTCAC TTCACTTCAG TTGCCGTCAC CCCGGTTAGG ACCTGGGTAC GTCGACAGTC AGTGGCTGAA   
  
  
- TTGTCACAGA GGGGCCGAGT TCTATCGTCG TCGGGGGGTC GTCCTCAAAC AACGCCAGTT TCGTGTAAAA   
  
  
- CCTTATGCAC AGTCCGAGGA TACGTTGAGA AACATGTGCA CCGTAGTAGG TCGACCATTA AAAAGACCGT   
  
  
- TGCCGAGATA TAGATATCCC TTGGCCGAGA CGTCAGAGGT CATGGCCCAC CACCCAGGGT GAAAACCGCC   
  
  
- TGTAAAACTA TTCGGGAAGT GTCTATGAAA TTTTTTTTTT CCCGTCTCGT TTCATTTGTT GGTCATAAGA   
  
  
- GAGACAGTAA AAAAAAAATT TTTTCTTTTA TACAAAATAT TTTAATTAAA TTAAAAGTAT TTTAAATAGA   
  
  
- TAAATTATAA AATTTTTCAT AATTTTACGT CTTTTCTCTG TCATGTAATC TCCTACACAA TAACAAAGTA   
  
  
- TAATAATATT ATTAAGTGTT TTATAAGAAA TTTTATTTGA AACTCTGGTG GAGTCTAGAC AACTTCTAGT   
  
  
- AACAGGATGT ATTTAGGTCA ACCACATTAT TTAGACCCCG ACATACTTCT CCCTGACTCC TACGTCGTCG   
  
  
- CTCTGTCCTC GAATTAACCC TCCTCCTCTG TTTTCGGTAC CGTTACCTTG TAGACCCACA CGTCATTTTG   
  
  
- ACTTCGACTT TTTCCCCACC AAGTAATTAC CCCCTCCCCT CCCTCTTTTT CTTATTGGTT CCAGAAAAGC   
  
  
- ACCCTGGGTG TGGAGTTTAG ACACTGTCTC TGCCGTCAAA GTAAAGAGTC GAGAAGGTGG AAGTGTTAGG   
  
  
- GAATAAATTT AGAAAGAGAG AGAGTGAACT TGTAACAACA AAGAGACAAG AGAGAGAAAG ATAGGAAACA   
  
  
- AAAAACAAGA GGACAACTTC AAGACTGAGA GAGAATGAGA GAGAGTATAC AAAAGAGAAA ATCTTTTTAT   
  
  
- AATAACTTAT TTCTTTACCC TGGAAACATA AGAATACCGG CATACACTTT AGAGATCGCT TCGTTTCAGA   
  
  
- AGACAAAGTG TGGAAAGTTA GAGTTTTGTT GCCTTCTTCT TCGATCCCAA ACTGTCGTGG TGGTTCGGGT   
  
  
- TCCAAACAAG AAACCATGAA AAGGAGAAAC GAAGATAACG ATAAAAAAAA ATAGCTTAAA AAGAATAGTA   
  
  
- TCGGCATTCA AATAAAAGAG AAACGAAAGT GAGTCCAAAG TTTCTGGGCG AACTTAGACT AAAACACCCA   
  
  
- TAGGGACAAA AATTTTTAAT TTGGAAGACT CTCCAACTTG CAAAGCTTAA AAACTACTTT TCTTGGTGTG   
  
  
- AACCCTAACA CCAAATAGGT CTTTACCTTA AAACTGATGC ACCCAGCAGA AAGGAAAAAT GGAGCTCCTA   
  
  
- GACAATAGCA CCATCTAAAA AGGCTCAAAA ATACCAGACA AAACCAGATC CACAGGGCCC CAACCTCTTA   
  
  
- ATCCCAAGAA AACCCCAAAA CCCCCGATT

+     Unnamed\_\_1

| Site Name | Organism | Position | Strand | Matrix score. | sequence | function |
| --- | --- | --- | --- | --- | --- | --- |
| Unnamed\_\_1 | Zea mays | 1408 | + | 5 | CGTGG |  |
| Unnamed\_\_1 | Zea mays | 1369 | + | 5 | CGTGG |  |
| Unnamed\_\_1 | Zea mays | 178 | + | 5 | CGTGG |  |
| Unnamed\_\_1 | Zea mays | 769 | + | 5 | CGTGG |  |

> 2018/04/13 10:10:12  
+ GGAGGAAGTG AAGTGAAGTC AACGGCAGTG GGGCCAATCC TGGACCCATG CAGCTGTCAG TCACCGACTT   
  
  
+ AACAGTGTCT CCCCGGCTCA AGATAGCAGC AGCCCCCCAG CAGGAGTTTG TTGCGGTCAA AGCACATTTT   
  
  
+ GGAATACGTG TCAGGCTCCT ATGCAACTCT TTGTACACGT GGCATCATCC AGCTGGTAAT TTTTCTGGCA   
  
  
+ ACGGCTCTAT ATCTATAGGG AACCGGCTCT GCAGTCTCCA GTACCGGGTG GTGGGTCCCA CTTTTGGCGG   
  
  
+ ACATTTTGAT AAGCCCTTCA CAGATACTTT AAAAAAAAAA GGGCAGAGCA AAGTAAACAA CCAGTATTCT   
  
  
+ CTCTGTCATT TTTTTTTTAA AAAAGAAAAT ATGTTTTATA AAATTAATTT AATTTTCATA AAATTTATCT   
  
  
+ ATTTAATATT TTAAAAAGTA TTAAAATGCA GAAAAGAGAC AGTACATTAG AGGATGTGTT ATTGTTTCAT   
  
  
+ ATTATTATAA TAATTCACAA AATATTCTTT AAAATAAACT TTGAGACCAC CTCAGATCTG TTGAAGATCA   
  
  
+ TTGTCCTACA TAAATCCAGT TGGTGTAATA AATCTGGGGC TGTATGAAGA GGGACTGAGG ATGCAGCAGC   
  
  
+ GAGACAGGAG CTTAATTGGG AGGAGGAGAC AAAAGCCATG GCAATGGAAC ATCTGGGTGT GCAGTAAAAC   
  
  
+ TGAAGCTGAA AAAGGGGTGG TTCATTAATG GGGGAGGGGA GGGAGAAAAA GAATAACCAA GGTCTTTTCG   
  
  
+ TGGGACCCAC ACCTCAAATC TGTGACAGAG ACGGCAGTTT CATTTCTCAG CTCTTCCACC TTCACAATCC   
  
  
+ CTTATTTAAA TCTTTCTCTC TCTCACTTGA ACATTGTTGT TTCTCTGTTC TCTCTCTTTC TATCCTTTGT   
  
  
+ TTTTTGTTCT CCTGTTGAAG TTCTGACTCT CTCTTACTCT CTCTCATATG TTTTCTCTTT TAGAAAAATA   
  
  
+ TTATTGAATA AAGAAATGGG ACCTTTGTAT TCTTATGGCC GTATGTGAAA TCTCTAGCGA AGCAAAGTCT   
  
  
+ TCTGTTTCAC ACCTTTCAAT CTCAAAACAA CGGAAGAAGA AGCTAGGGTT TGACAGCACC ACCAAGCCCA   
  
  
+ AGGTTTGTTC TTTGGTACTT TTCCTCTTTG CTTCTATTGC TATTTTTTTT TATCGAATTT TTCTTATCAT   
  
  
+ AGCCGTAAGT TTATTTTCTC TTTGCTTTCA CTCAGGTTTC AAAGACCCGC TTGAATCTGA TTTTGTGGGT   
  
  
+ ATCCCTGTTT TTAAAAATTA AACCTTCTGA GAGGTTGAAC GTTTCGAATT TTTGATGAAA AGAACCACAC   
  
  
+ TTGGGATTGT GGTTTATCCA GAAATGGAAT TTTGACTACG TGGGTCGTCT TTCCTTTTTA CCTCGAGGAT   
  
  
+ CTGTTATCGT GGTAGATTTT TCCGAGTTTT TATGGTCTGT TTTGGTCTAG GTGTCCCGGG GTTGGAGAAT   
  
  
+ TAGGGTTCTT TTGGGGTTTT GGGGGCTAA  

- CCTCCTTCAC TTCACTTCAG TTGCCGTCAC CCCGGTTAGG ACCTGGGTAC GTCGACAGTC AGTGGCTGAA   
  
  
- TTGTCACAGA GGGGCCGAGT TCTATCGTCG TCGGGGGGTC GTCCTCAAAC AACGCCAGTT TCGTGTAAAA   
  
  
- CCTTATGCAC AGTCCGAGGA TACGTTGAGA AACATGTGCA CCGTAGTAGG TCGACCATTA AAAAGACCGT   
  
  
- TGCCGAGATA TAGATATCCC TTGGCCGAGA CGTCAGAGGT CATGGCCCAC CACCCAGGGT GAAAACCGCC   
  
  
- TGTAAAACTA TTCGGGAAGT GTCTATGAAA TTTTTTTTTT CCCGTCTCGT TTCATTTGTT GGTCATAAGA   
  
  
- GAGACAGTAA AAAAAAAATT TTTTCTTTTA TACAAAATAT TTTAATTAAA TTAAAAGTAT TTTAAATAGA   
  
  
- TAAATTATAA AATTTTTCAT AATTTTACGT CTTTTCTCTG TCATGTAATC TCCTACACAA TAACAAAGTA   
  
  
- TAATAATATT ATTAAGTGTT TTATAAGAAA TTTTATTTGA AACTCTGGTG GAGTCTAGAC AACTTCTAGT   
  
  
- AACAGGATGT ATTTAGGTCA ACCACATTAT TTAGACCCCG ACATACTTCT CCCTGACTCC TACGTCGTCG   
  
  
- CTCTGTCCTC GAATTAACCC TCCTCCTCTG TTTTCGGTAC CGTTACCTTG TAGACCCACA CGTCATTTTG   
  
  
- ACTTCGACTT TTTCCCCACC AAGTAATTAC CCCCTCCCCT CCCTCTTTTT CTTATTGGTT CCAGAAAAGC   
  
  
- ACCCTGGGTG TGGAGTTTAG ACACTGTCTC TGCCGTCAAA GTAAAGAGTC GAGAAGGTGG AAGTGTTAGG   
  
  
- GAATAAATTT AGAAAGAGAG AGAGTGAACT TGTAACAACA AAGAGACAAG AGAGAGAAAG ATAGGAAACA   
  
  
- AAAAACAAGA GGACAACTTC AAGACTGAGA GAGAATGAGA GAGAGTATAC AAAAGAGAAA ATCTTTTTAT   
  
  
- AATAACTTAT TTCTTTACCC TGGAAACATA AGAATACCGG CATACACTTT AGAGATCGCT TCGTTTCAGA   
  
  
- AGACAAAGTG TGGAAAGTTA GAGTTTTGTT GCCTTCTTCT TCGATCCCAA ACTGTCGTGG TGGTTCGGGT   
  
  
- TCCAAACAAG AAACCATGAA AAGGAGAAAC GAAGATAACG ATAAAAAAAA ATAGCTTAAA AAGAATAGTA   
  
  
- TCGGCATTCA AATAAAAGAG AAACGAAAGT GAGTCCAAAG TTTCTGGGCG AACTTAGACT AAAACACCCA   
  
  
- TAGGGACAAA AATTTTTAAT TTGGAAGACT CTCCAACTTG CAAAGCTTAA AAACTACTTT TCTTGGTGTG   
  
  
- AACCCTAACA CCAAATAGGT CTTTACCTTA AAACTGATGC ACCCAGCAGA AAGGAAAAAT GGAGCTCCTA   
  
  
- GACAATAGCA CCATCTAAAA AGGCTCAAAA ATACCAGACA AAACCAGATC CACAGGGCCC CAACCTCTTA   
  
  
- ATCCCAAGAA AACCCCAAAA CCCCCGATT

+     Unnamed\_\_2

| Site Name | Organism | Position | Strand | Matrix score. | sequence | function |
| --- | --- | --- | --- | --- | --- | --- |
| Unnamed\_\_2 | Zea mays | 81 | + | 6 | CCCCGG |  |
| Unnamed\_\_2 | Zea mays | 1456 | - | 6 | CCCCGG |  |

> 2018/04/13 10:10:12  
+ GGAGGAAGTG AAGTGAAGTC AACGGCAGTG GGGCCAATCC TGGACCCATG CAGCTGTCAG TCACCGACTT   
  
  
+ AACAGTGTCT CCCCGGCTCA AGATAGCAGC AGCCCCCCAG CAGGAGTTTG TTGCGGTCAA AGCACATTTT   
  
  
+ GGAATACGTG TCAGGCTCCT ATGCAACTCT TTGTACACGT GGCATCATCC AGCTGGTAAT TTTTCTGGCA   
  
  
+ ACGGCTCTAT ATCTATAGGG AACCGGCTCT GCAGTCTCCA GTACCGGGTG GTGGGTCCCA CTTTTGGCGG   
  
  
+ ACATTTTGAT AAGCCCTTCA CAGATACTTT AAAAAAAAAA GGGCAGAGCA AAGTAAACAA CCAGTATTCT   
  
  
+ CTCTGTCATT TTTTTTTTAA AAAAGAAAAT ATGTTTTATA AAATTAATTT AATTTTCATA AAATTTATCT   
  
  
+ ATTTAATATT TTAAAAAGTA TTAAAATGCA GAAAAGAGAC AGTACATTAG AGGATGTGTT ATTGTTTCAT   
  
  
+ ATTATTATAA TAATTCACAA AATATTCTTT AAAATAAACT TTGAGACCAC CTCAGATCTG TTGAAGATCA   
  
  
+ TTGTCCTACA TAAATCCAGT TGGTGTAATA AATCTGGGGC TGTATGAAGA GGGACTGAGG ATGCAGCAGC   
  
  
+ GAGACAGGAG CTTAATTGGG AGGAGGAGAC AAAAGCCATG GCAATGGAAC ATCTGGGTGT GCAGTAAAAC   
  
  
+ TGAAGCTGAA AAAGGGGTGG TTCATTAATG GGGGAGGGGA GGGAGAAAAA GAATAACCAA GGTCTTTTCG   
  
  
+ TGGGACCCAC ACCTCAAATC TGTGACAGAG ACGGCAGTTT CATTTCTCAG CTCTTCCACC TTCACAATCC   
  
  
+ CTTATTTAAA TCTTTCTCTC TCTCACTTGA ACATTGTTGT TTCTCTGTTC TCTCTCTTTC TATCCTTTGT   
  
  
+ TTTTTGTTCT CCTGTTGAAG TTCTGACTCT CTCTTACTCT CTCTCATATG TTTTCTCTTT TAGAAAAATA   
  
  
+ TTATTGAATA AAGAAATGGG ACCTTTGTAT TCTTATGGCC GTATGTGAAA TCTCTAGCGA AGCAAAGTCT   
  
  
+ TCTGTTTCAC ACCTTTCAAT CTCAAAACAA CGGAAGAAGA AGCTAGGGTT TGACAGCACC ACCAAGCCCA   
  
  
+ AGGTTTGTTC TTTGGTACTT TTCCTCTTTG CTTCTATTGC TATTTTTTTT TATCGAATTT TTCTTATCAT   
  
  
+ AGCCGTAAGT TTATTTTCTC TTTGCTTTCA CTCAGGTTTC AAAGACCCGC TTGAATCTGA TTTTGTGGGT   
  
  
+ ATCCCTGTTT TTAAAAATTA AACCTTCTGA GAGGTTGAAC GTTTCGAATT TTTGATGAAA AGAACCACAC   
  
  
+ TTGGGATTGT GGTTTATCCA GAAATGGAAT TTTGACTACG TGGGTCGTCT TTCCTTTTTA CCTCGAGGAT   
  
  
+ CTGTTATCGT GGTAGATTTT TCCGAGTTTT TATGGTCTGT TTTGGTCTAG GTGTCCCGGG GTTGGAGAAT   
  
  
+ TAGGGTTCTT TTGGGGTTTT GGGGGCTAA  

- CCTCCTTCAC TTCACTTCAG TTGCCGTCAC CCCGGTTAGG ACCTGGGTAC GTCGACAGTC AGTGGCTGAA   
  
  
- TTGTCACAGA GGGGCCGAGT TCTATCGTCG TCGGGGGGTC GTCCTCAAAC AACGCCAGTT TCGTGTAAAA   
  
  
- CCTTATGCAC AGTCCGAGGA TACGTTGAGA AACATGTGCA CCGTAGTAGG TCGACCATTA AAAAGACCGT   
  
  
- TGCCGAGATA TAGATATCCC TTGGCCGAGA CGTCAGAGGT CATGGCCCAC CACCCAGGGT GAAAACCGCC   
  
  
- TGTAAAACTA TTCGGGAAGT GTCTATGAAA TTTTTTTTTT CCCGTCTCGT TTCATTTGTT GGTCATAAGA   
  
  
- GAGACAGTAA AAAAAAAATT TTTTCTTTTA TACAAAATAT TTTAATTAAA TTAAAAGTAT TTTAAATAGA   
  
  
- TAAATTATAA AATTTTTCAT AATTTTACGT CTTTTCTCTG TCATGTAATC TCCTACACAA TAACAAAGTA   
  
  
- TAATAATATT ATTAAGTGTT TTATAAGAAA TTTTATTTGA AACTCTGGTG GAGTCTAGAC AACTTCTAGT   
  
  
- AACAGGATGT ATTTAGGTCA ACCACATTAT TTAGACCCCG ACATACTTCT CCCTGACTCC TACGTCGTCG   
  
  
- CTCTGTCCTC GAATTAACCC TCCTCCTCTG TTTTCGGTAC CGTTACCTTG TAGACCCACA CGTCATTTTG   
  
  
- ACTTCGACTT TTTCCCCACC AAGTAATTAC CCCCTCCCCT CCCTCTTTTT CTTATTGGTT CCAGAAAAGC   
  
  
- ACCCTGGGTG TGGAGTTTAG ACACTGTCTC TGCCGTCAAA GTAAAGAGTC GAGAAGGTGG AAGTGTTAGG   
  
  
- GAATAAATTT AGAAAGAGAG AGAGTGAACT TGTAACAACA AAGAGACAAG AGAGAGAAAG ATAGGAAACA   
  
  
- AAAAACAAGA GGACAACTTC AAGACTGAGA GAGAATGAGA GAGAGTATAC AAAAGAGAAA ATCTTTTTAT   
  
  
- AATAACTTAT TTCTTTACCC TGGAAACATA AGAATACCGG CATACACTTT AGAGATCGCT TCGTTTCAGA   
  
  
- AGACAAAGTG TGGAAAGTTA GAGTTTTGTT GCCTTCTTCT TCGATCCCAA ACTGTCGTGG TGGTTCGGGT   
  
  
- TCCAAACAAG AAACCATGAA AAGGAGAAAC GAAGATAACG ATAAAAAAAA ATAGCTTAAA AAGAATAGTA   
  
  
- TCGGCATTCA AATAAAAGAG AAACGAAAGT GAGTCCAAAG TTTCTGGGCG AACTTAGACT AAAACACCCA   
  
  
- TAGGGACAAA AATTTTTAAT TTGGAAGACT CTCCAACTTG CAAAGCTTAA AAACTACTTT TCTTGGTGTG   
  
  
- AACCCTAACA CCAAATAGGT CTTTACCTTA AAACTGATGC ACCCAGCAGA AAGGAAAAAT GGAGCTCCTA   
  
  
- GACAATAGCA CCATCTAAAA AGGCTCAAAA ATACCAGACA AAACCAGATC CACAGGGCCC CAACCTCTTA   
  
  
- ATCCCAAGAA AACCCCAAAA CCCCCGATT

+     Unnamed\_\_3

| Site Name | Organism | Position | Strand | Matrix score. | sequence | function |
| --- | --- | --- | --- | --- | --- | --- |
| Unnamed\_\_3 | Zea mays | 1369 | + | 5 | CGTGG |  |
| Unnamed\_\_3 | Zea mays | 178 | + | 5 | CGTGG |  |
| Unnamed\_\_3 | Zea mays | 1408 | + | 5 | CGTGG |  |
| Unnamed\_\_3 | Zea mays | 769 | + | 5 | CGTGG |  |

> 2018/04/13 10:10:12  
+ GGAGGAAGTG AAGTGAAGTC AACGGCAGTG GGGCCAATCC TGGACCCATG CAGCTGTCAG TCACCGACTT   
  
  
+ AACAGTGTCT CCCCGGCTCA AGATAGCAGC AGCCCCCCAG CAGGAGTTTG TTGCGGTCAA AGCACATTTT   
  
  
+ GGAATACGTG TCAGGCTCCT ATGCAACTCT TTGTACACGT GGCATCATCC AGCTGGTAAT TTTTCTGGCA   
  
  
+ ACGGCTCTAT ATCTATAGGG AACCGGCTCT GCAGTCTCCA GTACCGGGTG GTGGGTCCCA CTTTTGGCGG   
  
  
+ ACATTTTGAT AAGCCCTTCA CAGATACTTT AAAAAAAAAA GGGCAGAGCA AAGTAAACAA CCAGTATTCT   
  
  
+ CTCTGTCATT TTTTTTTTAA AAAAGAAAAT ATGTTTTATA AAATTAATTT AATTTTCATA AAATTTATCT   
  
  
+ ATTTAATATT TTAAAAAGTA TTAAAATGCA GAAAAGAGAC AGTACATTAG AGGATGTGTT ATTGTTTCAT   
  
  
+ ATTATTATAA TAATTCACAA AATATTCTTT AAAATAAACT TTGAGACCAC CTCAGATCTG TTGAAGATCA   
  
  
+ TTGTCCTACA TAAATCCAGT TGGTGTAATA AATCTGGGGC TGTATGAAGA GGGACTGAGG ATGCAGCAGC   
  
  
+ GAGACAGGAG CTTAATTGGG AGGAGGAGAC AAAAGCCATG GCAATGGAAC ATCTGGGTGT GCAGTAAAAC   
  
  
+ TGAAGCTGAA AAAGGGGTGG TTCATTAATG GGGGAGGGGA GGGAGAAAAA GAATAACCAA GGTCTTTTCG   
  
  
+ TGGGACCCAC ACCTCAAATC TGTGACAGAG ACGGCAGTTT CATTTCTCAG CTCTTCCACC TTCACAATCC   
  
  
+ CTTATTTAAA TCTTTCTCTC TCTCACTTGA ACATTGTTGT TTCTCTGTTC TCTCTCTTTC TATCCTTTGT   
  
  
+ TTTTTGTTCT CCTGTTGAAG TTCTGACTCT CTCTTACTCT CTCTCATATG TTTTCTCTTT TAGAAAAATA   
  
  
+ TTATTGAATA AAGAAATGGG ACCTTTGTAT TCTTATGGCC GTATGTGAAA TCTCTAGCGA AGCAAAGTCT   
  
  
+ TCTGTTTCAC ACCTTTCAAT CTCAAAACAA CGGAAGAAGA AGCTAGGGTT TGACAGCACC ACCAAGCCCA   
  
  
+ AGGTTTGTTC TTTGGTACTT TTCCTCTTTG CTTCTATTGC TATTTTTTTT TATCGAATTT TTCTTATCAT   
  
  
+ AGCCGTAAGT TTATTTTCTC TTTGCTTTCA CTCAGGTTTC AAAGACCCGC TTGAATCTGA TTTTGTGGGT   
  
  
+ ATCCCTGTTT TTAAAAATTA AACCTTCTGA GAGGTTGAAC GTTTCGAATT TTTGATGAAA AGAACCACAC   
  
  
+ TTGGGATTGT GGTTTATCCA GAAATGGAAT TTTGACTACG TGGGTCGTCT TTCCTTTTTA CCTCGAGGAT   
  
  
+ CTGTTATCGT GGTAGATTTT TCCGAGTTTT TATGGTCTGT TTTGGTCTAG GTGTCCCGGG GTTGGAGAAT   
  
  
+ TAGGGTTCTT TTGGGGTTTT GGGGGCTAA  

- CCTCCTTCAC TTCACTTCAG TTGCCGTCAC CCCGGTTAGG ACCTGGGTAC GTCGACAGTC AGTGGCTGAA   
  
  
- TTGTCACAGA GGGGCCGAGT TCTATCGTCG TCGGGGGGTC GTCCTCAAAC AACGCCAGTT TCGTGTAAAA   
  
  
- CCTTATGCAC AGTCCGAGGA TACGTTGAGA AACATGTGCA CCGTAGTAGG TCGACCATTA AAAAGACCGT   
  
  
- TGCCGAGATA TAGATATCCC TTGGCCGAGA CGTCAGAGGT CATGGCCCAC CACCCAGGGT GAAAACCGCC   
  
  
- TGTAAAACTA TTCGGGAAGT GTCTATGAAA TTTTTTTTTT CCCGTCTCGT TTCATTTGTT GGTCATAAGA   
  
  
- GAGACAGTAA AAAAAAAATT TTTTCTTTTA TACAAAATAT TTTAATTAAA TTAAAAGTAT TTTAAATAGA   
  
  
- TAAATTATAA AATTTTTCAT AATTTTACGT CTTTTCTCTG TCATGTAATC TCCTACACAA TAACAAAGTA   
  
  
- TAATAATATT ATTAAGTGTT TTATAAGAAA TTTTATTTGA AACTCTGGTG GAGTCTAGAC AACTTCTAGT   
  
  
- AACAGGATGT ATTTAGGTCA ACCACATTAT TTAGACCCCG ACATACTTCT CCCTGACTCC TACGTCGTCG   
  
  
- CTCTGTCCTC GAATTAACCC TCCTCCTCTG TTTTCGGTAC CGTTACCTTG TAGACCCACA CGTCATTTTG   
  
  
- ACTTCGACTT TTTCCCCACC AAGTAATTAC CCCCTCCCCT CCCTCTTTTT CTTATTGGTT CCAGAAAAGC   
  
  
- ACCCTGGGTG TGGAGTTTAG ACACTGTCTC TGCCGTCAAA GTAAAGAGTC GAGAAGGTGG AAGTGTTAGG   
  
  
- GAATAAATTT AGAAAGAGAG AGAGTGAACT TGTAACAACA AAGAGACAAG AGAGAGAAAG ATAGGAAACA   
  
  
- AAAAACAAGA GGACAACTTC AAGACTGAGA GAGAATGAGA GAGAGTATAC AAAAGAGAAA ATCTTTTTAT   
  
  
- AATAACTTAT TTCTTTACCC TGGAAACATA AGAATACCGG CATACACTTT AGAGATCGCT TCGTTTCAGA   
  
  
- AGACAAAGTG TGGAAAGTTA GAGTTTTGTT GCCTTCTTCT TCGATCCCAA ACTGTCGTGG TGGTTCGGGT   
  
  
- TCCAAACAAG AAACCATGAA AAGGAGAAAC GAAGATAACG ATAAAAAAAA ATAGCTTAAA AAGAATAGTA   
  
  
- TCGGCATTCA AATAAAAGAG AAACGAAAGT GAGTCCAAAG TTTCTGGGCG AACTTAGACT AAAACACCCA   
  
  
- TAGGGACAAA AATTTTTAAT TTGGAAGACT CTCCAACTTG CAAAGCTTAA AAACTACTTT TCTTGGTGTG   
  
  
- AACCCTAACA CCAAATAGGT CTTTACCTTA AAACTGATGC ACCCAGCAGA AAGGAAAAAT GGAGCTCCTA   
  
  
- GACAATAGCA CCATCTAAAA AGGCTCAAAA ATACCAGACA AAACCAGATC CACAGGGCCC CAACCTCTTA   
  
  
- ATCCCAAGAA AACCCCAAAA CCCCCGATT

+     Unnamed\_\_4

| Site Name | Organism | Position | Strand | Matrix score. | sequence | function |
| --- | --- | --- | --- | --- | --- | --- |
| Unnamed\_\_4 | Petroselinum hortense | 742 | - | 4 | CTCC |  |
| Unnamed\_\_4 | Petroselinum hortense | 655 | - | 4 | CTCC |  |
| Unnamed\_\_4 | Petroselinum hortense | 1 | - | 4 | CTCC |  |
| Unnamed\_\_4 | Petroselinum hortense | 649 | - | 4 | CTCC |  |
| Unnamed\_\_4 | Petroselinum hortense | 1464 | - | 4 | CTCC |  |
| Unnamed\_\_4 | Petroselinum hortense | 156 | + | 4 | CTCC |  |
| Unnamed\_\_4 | Petroselinum hortense | 246 | + | 4 | CTCC |  |
| Unnamed\_\_4 | Petroselinum hortense | 733 | - | 4 | CTCC |  |
| Unnamed\_\_4 | Petroselinum hortense | 79 | + | 4 | CTCC |  |
| Unnamed\_\_4 | Petroselinum hortense | 637 | - | 4 | CTCC |  |
| Unnamed\_\_4 | Petroselinum hortense | 919 | + | 4 | CTCC |  |
| Unnamed\_\_4 | Petroselinum hortense | 738 | - | 4 | CTCC |  |
| Unnamed\_\_4 | Petroselinum hortense | 652 | - | 4 | CTCC |  |
| Unnamed\_\_4 | Petroselinum hortense | 113 | - | 4 | CTCC |  |

> 2018/04/13 10:10:12  
+ GGAGGAAGTG AAGTGAAGTC AACGGCAGTG GGGCCAATCC TGGACCCATG CAGCTGTCAG TCACCGACTT   
  
  
+ AACAGTGTCT CCCCGGCTCA AGATAGCAGC AGCCCCCCAG CAGGAGTTTG TTGCGGTCAA AGCACATTTT   
  
  
+ GGAATACGTG TCAGGCTCCT ATGCAACTCT TTGTACACGT GGCATCATCC AGCTGGTAAT TTTTCTGGCA   
  
  
+ ACGGCTCTAT ATCTATAGGG AACCGGCTCT GCAGTCTCCA GTACCGGGTG GTGGGTCCCA CTTTTGGCGG   
  
  
+ ACATTTTGAT AAGCCCTTCA CAGATACTTT AAAAAAAAAA GGGCAGAGCA AAGTAAACAA CCAGTATTCT   
  
  
+ CTCTGTCATT TTTTTTTTAA AAAAGAAAAT ATGTTTTATA AAATTAATTT AATTTTCATA AAATTTATCT   
  
  
+ ATTTAATATT TTAAAAAGTA TTAAAATGCA GAAAAGAGAC AGTACATTAG AGGATGTGTT ATTGTTTCAT   
  
  
+ ATTATTATAA TAATTCACAA AATATTCTTT AAAATAAACT TTGAGACCAC CTCAGATCTG TTGAAGATCA   
  
  
+ TTGTCCTACA TAAATCCAGT TGGTGTAATA AATCTGGGGC TGTATGAAGA GGGACTGAGG ATGCAGCAGC   
  
  
+ GAGACAGGAG CTTAATTGGG AGGAGGAGAC AAAAGCCATG GCAATGGAAC ATCTGGGTGT GCAGTAAAAC   
  
  
+ TGAAGCTGAA AAAGGGGTGG TTCATTAATG GGGGAGGGGA GGGAGAAAAA GAATAACCAA GGTCTTTTCG   
  
  
+ TGGGACCCAC ACCTCAAATC TGTGACAGAG ACGGCAGTTT CATTTCTCAG CTCTTCCACC TTCACAATCC   
  
  
+ CTTATTTAAA TCTTTCTCTC TCTCACTTGA ACATTGTTGT TTCTCTGTTC TCTCTCTTTC TATCCTTTGT   
  
  
+ TTTTTGTTCT CCTGTTGAAG TTCTGACTCT CTCTTACTCT CTCTCATATG TTTTCTCTTT TAGAAAAATA   
  
  
+ TTATTGAATA AAGAAATGGG ACCTTTGTAT TCTTATGGCC GTATGTGAAA TCTCTAGCGA AGCAAAGTCT   
  
  
+ TCTGTTTCAC ACCTTTCAAT CTCAAAACAA CGGAAGAAGA AGCTAGGGTT TGACAGCACC ACCAAGCCCA   
  
  
+ AGGTTTGTTC TTTGGTACTT TTCCTCTTTG CTTCTATTGC TATTTTTTTT TATCGAATTT TTCTTATCAT   
  
  
+ AGCCGTAAGT TTATTTTCTC TTTGCTTTCA CTCAGGTTTC AAAGACCCGC TTGAATCTGA TTTTGTGGGT   
  
  
+ ATCCCTGTTT TTAAAAATTA AACCTTCTGA GAGGTTGAAC GTTTCGAATT TTTGATGAAA AGAACCACAC   
  
  
+ TTGGGATTGT GGTTTATCCA GAAATGGAAT TTTGACTACG TGGGTCGTCT TTCCTTTTTA CCTCGAGGAT   
  
  
+ CTGTTATCGT GGTAGATTTT TCCGAGTTTT TATGGTCTGT TTTGGTCTAG GTGTCCCGGG GTTGGAGAAT   
  
  
+ TAGGGTTCTT TTGGGGTTTT GGGGGCTAA  

- CCTCCTTCAC TTCACTTCAG TTGCCGTCAC CCCGGTTAGG ACCTGGGTAC GTCGACAGTC AGTGGCTGAA   
  
  
- TTGTCACAGA GGGGCCGAGT TCTATCGTCG TCGGGGGGTC GTCCTCAAAC AACGCCAGTT TCGTGTAAAA   
  
  
- CCTTATGCAC AGTCCGAGGA TACGTTGAGA AACATGTGCA CCGTAGTAGG TCGACCATTA AAAAGACCGT   
  
  
- TGCCGAGATA TAGATATCCC TTGGCCGAGA CGTCAGAGGT CATGGCCCAC CACCCAGGGT GAAAACCGCC   
  
  
- TGTAAAACTA TTCGGGAAGT GTCTATGAAA TTTTTTTTTT CCCGTCTCGT TTCATTTGTT GGTCATAAGA   
  
  
- GAGACAGTAA AAAAAAAATT TTTTCTTTTA TACAAAATAT TTTAATTAAA TTAAAAGTAT TTTAAATAGA   
  
  
- TAAATTATAA AATTTTTCAT AATTTTACGT CTTTTCTCTG TCATGTAATC TCCTACACAA TAACAAAGTA   
  
  
- TAATAATATT ATTAAGTGTT TTATAAGAAA TTTTATTTGA AACTCTGGTG GAGTCTAGAC AACTTCTAGT   
  
  
- AACAGGATGT ATTTAGGTCA ACCACATTAT TTAGACCCCG ACATACTTCT CCCTGACTCC TACGTCGTCG   
  
  
- CTCTGTCCTC GAATTAACCC TCCTCCTCTG TTTTCGGTAC CGTTACCTTG TAGACCCACA CGTCATTTTG   
  
  
- ACTTCGACTT TTTCCCCACC AAGTAATTAC CCCCTCCCCT CCCTCTTTTT CTTATTGGTT CCAGAAAAGC   
  
  
- ACCCTGGGTG TGGAGTTTAG ACACTGTCTC TGCCGTCAAA GTAAAGAGTC GAGAAGGTGG AAGTGTTAGG   
  
  
- GAATAAATTT AGAAAGAGAG AGAGTGAACT TGTAACAACA AAGAGACAAG AGAGAGAAAG ATAGGAAACA   
  
  
- AAAAACAAGA GGACAACTTC AAGACTGAGA GAGAATGAGA GAGAGTATAC AAAAGAGAAA ATCTTTTTAT   
  
  
- AATAACTTAT TTCTTTACCC TGGAAACATA AGAATACCGG CATACACTTT AGAGATCGCT TCGTTTCAGA   
  
  
- AGACAAAGTG TGGAAAGTTA GAGTTTTGTT GCCTTCTTCT TCGATCCCAA ACTGTCGTGG TGGTTCGGGT   
  
  
- TCCAAACAAG AAACCATGAA AAGGAGAAAC GAAGATAACG ATAAAAAAAA ATAGCTTAAA AAGAATAGTA   
  
  
- TCGGCATTCA AATAAAAGAG AAACGAAAGT GAGTCCAAAG TTTCTGGGCG AACTTAGACT AAAACACCCA   
  
  
- TAGGGACAAA AATTTTTAAT TTGGAAGACT CTCCAACTTG CAAAGCTTAA AAACTACTTT TCTTGGTGTG   
  
  
- AACCCTAACA CCAAATAGGT CTTTACCTTA AAACTGATGC ACCCAGCAGA AAGGAAAAAT GGAGCTCCTA   
  
  
- GACAATAGCA CCATCTAAAA AGGCTCAAAA ATACCAGACA AAACCAGATC CACAGGGCCC CAACCTCTTA   
  
  
- ATCCCAAGAA AACCCCAAAA CCCCCGATT

+     W box

| Site Name | Organism | Position | Strand | Matrix score. | sequence | function |
| --- | --- | --- | --- | --- | --- | --- |
| W box | Arabidopsis thaliana | 125 | - | 6 | TTGACC |  |

> 2018/04/13 10:10:12  
+ GGAGGAAGTG AAGTGAAGTC AACGGCAGTG GGGCCAATCC TGGACCCATG CAGCTGTCAG TCACCGACTT   
  
  
+ AACAGTGTCT CCCCGGCTCA AGATAGCAGC AGCCCCCCAG CAGGAGTTTG TTGCGGTCAA AGCACATTTT   
  
  
+ GGAATACGTG TCAGGCTCCT ATGCAACTCT TTGTACACGT GGCATCATCC AGCTGGTAAT TTTTCTGGCA   
  
  
+ ACGGCTCTAT ATCTATAGGG AACCGGCTCT GCAGTCTCCA GTACCGGGTG GTGGGTCCCA CTTTTGGCGG   
  
  
+ ACATTTTGAT AAGCCCTTCA CAGATACTTT AAAAAAAAAA GGGCAGAGCA AAGTAAACAA CCAGTATTCT   
  
  
+ CTCTGTCATT TTTTTTTTAA AAAAGAAAAT ATGTTTTATA AAATTAATTT AATTTTCATA AAATTTATCT   
  
  
+ ATTTAATATT TTAAAAAGTA TTAAAATGCA GAAAAGAGAC AGTACATTAG AGGATGTGTT ATTGTTTCAT   
  
  
+ ATTATTATAA TAATTCACAA AATATTCTTT AAAATAAACT TTGAGACCAC CTCAGATCTG TTGAAGATCA   
  
  
+ TTGTCCTACA TAAATCCAGT TGGTGTAATA AATCTGGGGC TGTATGAAGA GGGACTGAGG ATGCAGCAGC   
  
  
+ GAGACAGGAG CTTAATTGGG AGGAGGAGAC AAAAGCCATG GCAATGGAAC ATCTGGGTGT GCAGTAAAAC   
  
  
+ TGAAGCTGAA AAAGGGGTGG TTCATTAATG GGGGAGGGGA GGGAGAAAAA GAATAACCAA GGTCTTTTCG   
  
  
+ TGGGACCCAC ACCTCAAATC TGTGACAGAG ACGGCAGTTT CATTTCTCAG CTCTTCCACC TTCACAATCC   
  
  
+ CTTATTTAAA TCTTTCTCTC TCTCACTTGA ACATTGTTGT TTCTCTGTTC TCTCTCTTTC TATCCTTTGT   
  
  
+ TTTTTGTTCT CCTGTTGAAG TTCTGACTCT CTCTTACTCT CTCTCATATG TTTTCTCTTT TAGAAAAATA   
  
  
+ TTATTGAATA AAGAAATGGG ACCTTTGTAT TCTTATGGCC GTATGTGAAA TCTCTAGCGA AGCAAAGTCT   
  
  
+ TCTGTTTCAC ACCTTTCAAT CTCAAAACAA CGGAAGAAGA AGCTAGGGTT TGACAGCACC ACCAAGCCCA   
  
  
+ AGGTTTGTTC TTTGGTACTT TTCCTCTTTG CTTCTATTGC TATTTTTTTT TATCGAATTT TTCTTATCAT   
  
  
+ AGCCGTAAGT TTATTTTCTC TTTGCTTTCA CTCAGGTTTC AAAGACCCGC TTGAATCTGA TTTTGTGGGT   
  
  
+ ATCCCTGTTT TTAAAAATTA AACCTTCTGA GAGGTTGAAC GTTTCGAATT TTTGATGAAA AGAACCACAC   
  
  
+ TTGGGATTGT GGTTTATCCA GAAATGGAAT TTTGACTACG TGGGTCGTCT TTCCTTTTTA CCTCGAGGAT   
  
  
+ CTGTTATCGT GGTAGATTTT TCCGAGTTTT TATGGTCTGT TTTGGTCTAG GTGTCCCGGG GTTGGAGAAT   
  
  
+ TAGGGTTCTT TTGGGGTTTT GGGGGCTAA  

- CCTCCTTCAC TTCACTTCAG TTGCCGTCAC CCCGGTTAGG ACCTGGGTAC GTCGACAGTC AGTGGCTGAA   
  
  
- TTGTCACAGA GGGGCCGAGT TCTATCGTCG TCGGGGGGTC GTCCTCAAAC AACGCCAGTT TCGTGTAAAA   
  
  
- CCTTATGCAC AGTCCGAGGA TACGTTGAGA AACATGTGCA CCGTAGTAGG TCGACCATTA AAAAGACCGT   
  
  
- TGCCGAGATA TAGATATCCC TTGGCCGAGA CGTCAGAGGT CATGGCCCAC CACCCAGGGT GAAAACCGCC   
  
  
- TGTAAAACTA TTCGGGAAGT GTCTATGAAA TTTTTTTTTT CCCGTCTCGT TTCATTTGTT GGTCATAAGA   
  
  
- GAGACAGTAA AAAAAAAATT TTTTCTTTTA TACAAAATAT TTTAATTAAA TTAAAAGTAT TTTAAATAGA   
  
  
- TAAATTATAA AATTTTTCAT AATTTTACGT CTTTTCTCTG TCATGTAATC TCCTACACAA TAACAAAGTA   
  
  
- TAATAATATT ATTAAGTGTT TTATAAGAAA TTTTATTTGA AACTCTGGTG GAGTCTAGAC AACTTCTAGT   
  
  
- AACAGGATGT ATTTAGGTCA ACCACATTAT TTAGACCCCG ACATACTTCT CCCTGACTCC TACGTCGTCG   
  
  
- CTCTGTCCTC GAATTAACCC TCCTCCTCTG TTTTCGGTAC CGTTACCTTG TAGACCCACA CGTCATTTTG   
  
  
- ACTTCGACTT TTTCCCCACC AAGTAATTAC CCCCTCCCCT CCCTCTTTTT CTTATTGGTT CCAGAAAAGC   
  
  
- ACCCTGGGTG TGGAGTTTAG ACACTGTCTC TGCCGTCAAA GTAAAGAGTC GAGAAGGTGG AAGTGTTAGG   
  
  
- GAATAAATTT AGAAAGAGAG AGAGTGAACT TGTAACAACA AAGAGACAAG AGAGAGAAAG ATAGGAAACA   
  
  
- AAAAACAAGA GGACAACTTC AAGACTGAGA GAGAATGAGA GAGAGTATAC AAAAGAGAAA ATCTTTTTAT   
  
  
- AATAACTTAT TTCTTTACCC TGGAAACATA AGAATACCGG CATACACTTT AGAGATCGCT TCGTTTCAGA   
  
  
- AGACAAAGTG TGGAAAGTTA GAGTTTTGTT GCCTTCTTCT TCGATCCCAA ACTGTCGTGG TGGTTCGGGT   
  
  
- TCCAAACAAG AAACCATGAA AAGGAGAAAC GAAGATAACG ATAAAAAAAA ATAGCTTAAA AAGAATAGTA   
  
  
- TCGGCATTCA AATAAAAGAG AAACGAAAGT GAGTCCAAAG TTTCTGGGCG AACTTAGACT AAAACACCCA   
  
  
- TAGGGACAAA AATTTTTAAT TTGGAAGACT CTCCAACTTG CAAAGCTTAA AAACTACTTT TCTTGGTGTG   
  
  
- AACCCTAACA CCAAATAGGT CTTTACCTTA AAACTGATGC ACCCAGCAGA AAGGAAAAAT GGAGCTCCTA   
  
  
- GACAATAGCA CCATCTAAAA AGGCTCAAAA ATACCAGACA AAACCAGATC CACAGGGCCC CAACCTCTTA   
  
  
- ATCCCAAGAA AACCCCAAAA CCCCCGATT

+     box II

| Site Name | Organism | Position | Strand | Matrix score. | sequence | function |
| --- | --- | --- | --- | --- | --- | --- |
| box II | Petroselinum hortense | 174 | + | 9 | TCCACGTGGC | part of a light responsive element |

> 2018/04/13 10:10:12  
+ GGAGGAAGTG AAGTGAAGTC AACGGCAGTG GGGCCAATCC TGGACCCATG CAGCTGTCAG TCACCGACTT   
  
  
+ AACAGTGTCT CCCCGGCTCA AGATAGCAGC AGCCCCCCAG CAGGAGTTTG TTGCGGTCAA AGCACATTTT   
  
  
+ GGAATACGTG TCAGGCTCCT ATGCAACTCT TTGTACACGT GGCATCATCC AGCTGGTAAT TTTTCTGGCA   
  
  
+ ACGGCTCTAT ATCTATAGGG AACCGGCTCT GCAGTCTCCA GTACCGGGTG GTGGGTCCCA CTTTTGGCGG   
  
  
+ ACATTTTGAT AAGCCCTTCA CAGATACTTT AAAAAAAAAA GGGCAGAGCA AAGTAAACAA CCAGTATTCT   
  
  
+ CTCTGTCATT TTTTTTTTAA AAAAGAAAAT ATGTTTTATA AAATTAATTT AATTTTCATA AAATTTATCT   
  
  
+ ATTTAATATT TTAAAAAGTA TTAAAATGCA GAAAAGAGAC AGTACATTAG AGGATGTGTT ATTGTTTCAT   
  
  
+ ATTATTATAA TAATTCACAA AATATTCTTT AAAATAAACT TTGAGACCAC CTCAGATCTG TTGAAGATCA   
  
  
+ TTGTCCTACA TAAATCCAGT TGGTGTAATA AATCTGGGGC TGTATGAAGA GGGACTGAGG ATGCAGCAGC   
  
  
+ GAGACAGGAG CTTAATTGGG AGGAGGAGAC AAAAGCCATG GCAATGGAAC ATCTGGGTGT GCAGTAAAAC   
  
  
+ TGAAGCTGAA AAAGGGGTGG TTCATTAATG GGGGAGGGGA GGGAGAAAAA GAATAACCAA GGTCTTTTCG   
  
  
+ TGGGACCCAC ACCTCAAATC TGTGACAGAG ACGGCAGTTT CATTTCTCAG CTCTTCCACC TTCACAATCC   
  
  
+ CTTATTTAAA TCTTTCTCTC TCTCACTTGA ACATTGTTGT TTCTCTGTTC TCTCTCTTTC TATCCTTTGT   
  
  
+ TTTTTGTTCT CCTGTTGAAG TTCTGACTCT CTCTTACTCT CTCTCATATG TTTTCTCTTT TAGAAAAATA   
  
  
+ TTATTGAATA AAGAAATGGG ACCTTTGTAT TCTTATGGCC GTATGTGAAA TCTCTAGCGA AGCAAAGTCT   
  
  
+ TCTGTTTCAC ACCTTTCAAT CTCAAAACAA CGGAAGAAGA AGCTAGGGTT TGACAGCACC ACCAAGCCCA   
  
  
+ AGGTTTGTTC TTTGGTACTT TTCCTCTTTG CTTCTATTGC TATTTTTTTT TATCGAATTT TTCTTATCAT   
  
  
+ AGCCGTAAGT TTATTTTCTC TTTGCTTTCA CTCAGGTTTC AAAGACCCGC TTGAATCTGA TTTTGTGGGT   
  
  
+ ATCCCTGTTT TTAAAAATTA AACCTTCTGA GAGGTTGAAC GTTTCGAATT TTTGATGAAA AGAACCACAC   
  
  
+ TTGGGATTGT GGTTTATCCA GAAATGGAAT TTTGACTACG TGGGTCGTCT TTCCTTTTTA CCTCGAGGAT   
  
  
+ CTGTTATCGT GGTAGATTTT TCCGAGTTTT TATGGTCTGT TTTGGTCTAG GTGTCCCGGG GTTGGAGAAT   
  
  
+ TAGGGTTCTT TTGGGGTTTT GGGGGCTAA  

- CCTCCTTCAC TTCACTTCAG TTGCCGTCAC CCCGGTTAGG ACCTGGGTAC GTCGACAGTC AGTGGCTGAA   
  
  
- TTGTCACAGA GGGGCCGAGT TCTATCGTCG TCGGGGGGTC GTCCTCAAAC AACGCCAGTT TCGTGTAAAA   
  
  
- CCTTATGCAC AGTCCGAGGA TACGTTGAGA AACATGTGCA CCGTAGTAGG TCGACCATTA AAAAGACCGT   
  
  
- TGCCGAGATA TAGATATCCC TTGGCCGAGA CGTCAGAGGT CATGGCCCAC CACCCAGGGT GAAAACCGCC   
  
  
- TGTAAAACTA TTCGGGAAGT GTCTATGAAA TTTTTTTTTT CCCGTCTCGT TTCATTTGTT GGTCATAAGA   
  
  
- GAGACAGTAA AAAAAAAATT TTTTCTTTTA TACAAAATAT TTTAATTAAA TTAAAAGTAT TTTAAATAGA   
  
  
- TAAATTATAA AATTTTTCAT AATTTTACGT CTTTTCTCTG TCATGTAATC TCCTACACAA TAACAAAGTA   
  
  
- TAATAATATT ATTAAGTGTT TTATAAGAAA TTTTATTTGA AACTCTGGTG GAGTCTAGAC AACTTCTAGT   
  
  
- AACAGGATGT ATTTAGGTCA ACCACATTAT TTAGACCCCG ACATACTTCT CCCTGACTCC TACGTCGTCG   
  
  
- CTCTGTCCTC GAATTAACCC TCCTCCTCTG TTTTCGGTAC CGTTACCTTG TAGACCCACA CGTCATTTTG   
  
  
- ACTTCGACTT TTTCCCCACC AAGTAATTAC CCCCTCCCCT CCCTCTTTTT CTTATTGGTT CCAGAAAAGC   
  
  
- ACCCTGGGTG TGGAGTTTAG ACACTGTCTC TGCCGTCAAA GTAAAGAGTC GAGAAGGTGG AAGTGTTAGG   
  
  
- GAATAAATTT AGAAAGAGAG AGAGTGAACT TGTAACAACA AAGAGACAAG AGAGAGAAAG ATAGGAAACA   
  
  
- AAAAACAAGA GGACAACTTC AAGACTGAGA GAGAATGAGA GAGAGTATAC AAAAGAGAAA ATCTTTTTAT   
  
  
- AATAACTTAT TTCTTTACCC TGGAAACATA AGAATACCGG CATACACTTT AGAGATCGCT TCGTTTCAGA   
  
  
- AGACAAAGTG TGGAAAGTTA GAGTTTTGTT GCCTTCTTCT TCGATCCCAA ACTGTCGTGG TGGTTCGGGT   
  
  
- TCCAAACAAG AAACCATGAA AAGGAGAAAC GAAGATAACG ATAAAAAAAA ATAGCTTAAA AAGAATAGTA   
  
  
- TCGGCATTCA AATAAAAGAG AAACGAAAGT GAGTCCAAAG TTTCTGGGCG AACTTAGACT AAAACACCCA   
  
  
- TAGGGACAAA AATTTTTAAT TTGGAAGACT CTCCAACTTG CAAAGCTTAA AAACTACTTT TCTTGGTGTG   
  
  
- AACCCTAACA CCAAATAGGT CTTTACCTTA AAACTGATGC ACCCAGCAGA AAGGAAAAAT GGAGCTCCTA   
  
  
- GACAATAGCA CCATCTAAAA AGGCTCAAAA ATACCAGACA AAACCAGATC CACAGGGCCC CAACCTCTTA   
  
  
- ATCCCAAGAA AACCCCAAAA CCCCCGATT

+     chs-CMA2a

| Site Name | Organism | Position | Strand | Matrix score. | sequence | function |
| --- | --- | --- | --- | --- | --- | --- |
| chs-CMA2a | Petroselinum crispum | 863 | + | 8 | TCACTTGA | part of a light responsive element |

> 2018/04/13 10:10:12  
+ GGAGGAAGTG AAGTGAAGTC AACGGCAGTG GGGCCAATCC TGGACCCATG CAGCTGTCAG TCACCGACTT   
  
  
+ AACAGTGTCT CCCCGGCTCA AGATAGCAGC AGCCCCCCAG CAGGAGTTTG TTGCGGTCAA AGCACATTTT   
  
  
+ GGAATACGTG TCAGGCTCCT ATGCAACTCT TTGTACACGT GGCATCATCC AGCTGGTAAT TTTTCTGGCA   
  
  
+ ACGGCTCTAT ATCTATAGGG AACCGGCTCT GCAGTCTCCA GTACCGGGTG GTGGGTCCCA CTTTTGGCGG   
  
  
+ ACATTTTGAT AAGCCCTTCA CAGATACTTT AAAAAAAAAA GGGCAGAGCA AAGTAAACAA CCAGTATTCT   
  
  
+ CTCTGTCATT TTTTTTTTAA AAAAGAAAAT ATGTTTTATA AAATTAATTT AATTTTCATA AAATTTATCT   
  
  
+ ATTTAATATT TTAAAAAGTA TTAAAATGCA GAAAAGAGAC AGTACATTAG AGGATGTGTT ATTGTTTCAT   
  
  
+ ATTATTATAA TAATTCACAA AATATTCTTT AAAATAAACT TTGAGACCAC CTCAGATCTG TTGAAGATCA   
  
  
+ TTGTCCTACA TAAATCCAGT TGGTGTAATA AATCTGGGGC TGTATGAAGA GGGACTGAGG ATGCAGCAGC   
  
  
+ GAGACAGGAG CTTAATTGGG AGGAGGAGAC AAAAGCCATG GCAATGGAAC ATCTGGGTGT GCAGTAAAAC   
  
  
+ TGAAGCTGAA AAAGGGGTGG TTCATTAATG GGGGAGGGGA GGGAGAAAAA GAATAACCAA GGTCTTTTCG   
  
  
+ TGGGACCCAC ACCTCAAATC TGTGACAGAG ACGGCAGTTT CATTTCTCAG CTCTTCCACC TTCACAATCC   
  
  
+ CTTATTTAAA TCTTTCTCTC TCTCACTTGA ACATTGTTGT TTCTCTGTTC TCTCTCTTTC TATCCTTTGT   
  
  
+ TTTTTGTTCT CCTGTTGAAG TTCTGACTCT CTCTTACTCT CTCTCATATG TTTTCTCTTT TAGAAAAATA   
  
  
+ TTATTGAATA AAGAAATGGG ACCTTTGTAT TCTTATGGCC GTATGTGAAA TCTCTAGCGA AGCAAAGTCT   
  
  
+ TCTGTTTCAC ACCTTTCAAT CTCAAAACAA CGGAAGAAGA AGCTAGGGTT TGACAGCACC ACCAAGCCCA   
  
  
+ AGGTTTGTTC TTTGGTACTT TTCCTCTTTG CTTCTATTGC TATTTTTTTT TATCGAATTT TTCTTATCAT   
  
  
+ AGCCGTAAGT TTATTTTCTC TTTGCTTTCA CTCAGGTTTC AAAGACCCGC TTGAATCTGA TTTTGTGGGT   
  
  
+ ATCCCTGTTT TTAAAAATTA AACCTTCTGA GAGGTTGAAC GTTTCGAATT TTTGATGAAA AGAACCACAC   
  
  
+ TTGGGATTGT GGTTTATCCA GAAATGGAAT TTTGACTACG TGGGTCGTCT TTCCTTTTTA CCTCGAGGAT   
  
  
+ CTGTTATCGT GGTAGATTTT TCCGAGTTTT TATGGTCTGT TTTGGTCTAG GTGTCCCGGG GTTGGAGAAT   
  
  
+ TAGGGTTCTT TTGGGGTTTT GGGGGCTAA  

- CCTCCTTCAC TTCACTTCAG TTGCCGTCAC CCCGGTTAGG ACCTGGGTAC GTCGACAGTC AGTGGCTGAA   
  
  
- TTGTCACAGA GGGGCCGAGT TCTATCGTCG TCGGGGGGTC GTCCTCAAAC AACGCCAGTT TCGTGTAAAA   
  
  
- CCTTATGCAC AGTCCGAGGA TACGTTGAGA AACATGTGCA CCGTAGTAGG TCGACCATTA AAAAGACCGT   
  
  
- TGCCGAGATA TAGATATCCC TTGGCCGAGA CGTCAGAGGT CATGGCCCAC CACCCAGGGT GAAAACCGCC   
  
  
- TGTAAAACTA TTCGGGAAGT GTCTATGAAA TTTTTTTTTT CCCGTCTCGT TTCATTTGTT GGTCATAAGA   
  
  
- GAGACAGTAA AAAAAAAATT TTTTCTTTTA TACAAAATAT TTTAATTAAA TTAAAAGTAT TTTAAATAGA   
  
  
- TAAATTATAA AATTTTTCAT AATTTTACGT CTTTTCTCTG TCATGTAATC TCCTACACAA TAACAAAGTA   
  
  
- TAATAATATT ATTAAGTGTT TTATAAGAAA TTTTATTTGA AACTCTGGTG GAGTCTAGAC AACTTCTAGT   
  
  
- AACAGGATGT ATTTAGGTCA ACCACATTAT TTAGACCCCG ACATACTTCT CCCTGACTCC TACGTCGTCG   
  
  
- CTCTGTCCTC GAATTAACCC TCCTCCTCTG TTTTCGGTAC CGTTACCTTG TAGACCCACA CGTCATTTTG   
  
  
- ACTTCGACTT TTTCCCCACC AAGTAATTAC CCCCTCCCCT CCCTCTTTTT CTTATTGGTT CCAGAAAAGC   
  
  
- ACCCTGGGTG TGGAGTTTAG ACACTGTCTC TGCCGTCAAA GTAAAGAGTC GAGAAGGTGG AAGTGTTAGG   
  
  
- GAATAAATTT AGAAAGAGAG AGAGTGAACT TGTAACAACA AAGAGACAAG AGAGAGAAAG ATAGGAAACA   
  
  
- AAAAACAAGA GGACAACTTC AAGACTGAGA GAGAATGAGA GAGAGTATAC AAAAGAGAAA ATCTTTTTAT   
  
  
- AATAACTTAT TTCTTTACCC TGGAAACATA AGAATACCGG CATACACTTT AGAGATCGCT TCGTTTCAGA   
  
  
- AGACAAAGTG TGGAAAGTTA GAGTTTTGTT GCCTTCTTCT TCGATCCCAA ACTGTCGTGG TGGTTCGGGT   
  
  
- TCCAAACAAG AAACCATGAA AAGGAGAAAC GAAGATAACG ATAAAAAAAA ATAGCTTAAA AAGAATAGTA   
  
  
- TCGGCATTCA AATAAAAGAG AAACGAAAGT GAGTCCAAAG TTTCTGGGCG AACTTAGACT AAAACACCCA   
  
  
- TAGGGACAAA AATTTTTAAT TTGGAAGACT CTCCAACTTG CAAAGCTTAA AAACTACTTT TCTTGGTGTG   
  
  
- AACCCTAACA CCAAATAGGT CTTTACCTTA AAACTGATGC ACCCAGCAGA AAGGAAAAAT GGAGCTCCTA   
  
  
- GACAATAGCA CCATCTAAAA AGGCTCAAAA ATACCAGACA AAACCAGATC CACAGGGCCC CAACCTCTTA   
  
  
- ATCCCAAGAA AACCCCAAAA CCCCCGATT
