# Supplementary material for: Genome-wide analysis of the WRKY gene family in drumstick (Moringa oleifera Lam.)
Source: PeerJ. 2019 Jun 10;7:e7063. doi: 10.7717/peerj.7063 (PMC6563795; doi:10.7717/peerj.7063)
Supplement: Supplemental Information 1 [file peerj-07-7063-s003.gz › MoWRKY32_plantcare.html]

Content-Type: text/html; charset=ISO-8859-1


CallMat\_Firefox


Webmaster Firefox specific output  
To save the result:
click on the frame with the right mouse button and save the source code as a text file with extension .html  
REFERENCE:PlantCARE: a database of plant cis-acting regulatory elements and a portal to tools for in silico analysis of promoter sequences.  
Lescot, M., Déhais, P., Moreau, Y., De Moor, B., Rouzé ,P.,and Rombauts, S.  
Nucleic Acids Res., Database issue(2002), 30(1):325-327.   


---

> 2018/04/13 10:10:12  
+ GTTCTAAAGA CAAATGATTT GTTCTAGCTA GACTCTCTTT TTCCTATTGA CAACCCGACT GAAAGTTCTG   
  
  
+ AAAAGTCTCT CTTAGCACTT CTCTATCTGT AAACGATCTC CTTCCGTTCC AAACAAATAT ATATAACCTC   
  
  
+ TCTCTGAGTC TAAAGAAATG CCGTTCATAG GTATAAGTTG TGGAGAAGAG GAAGGTACAC TTGGTCCTAT   
  
  
+ ACGTCGTAAG CAACTCAACT GAACAGTATT TCAGTTGGGT TTTTACTATT AAGTATGGTT TTCAAATTAG   
  
  
+ AATCTCATGA CTTTTGAAAT AACATAATCC TGAACGTGTA TTTAGAAGTA ATTAGTAGTG GCAACTGATA   
  
  
+ AAGAAATGTT TTTAAAATGT TAAAAATTAT TAATATTTTA GAGTTAGTAA GTAGTAAATT TTTTATGATT   
  
  
+ CAAAAAAAAT AGTTTACCCT TTCTAATTCT TTCTTTTAAC ACCTAAACCA CTACTTTCTT CCCGTACTAA   
  
  
+ CCAAGGAAAC TGAAAGTGTG AGAATTAATC TCACACTTAA ATTAAAAAAA TAAATAGACT TAATACAATT   
  
  
+ AAAAAACTAT TTTATTATTA TTATTTATTA AATTATTTTA ATTACATTAA TTAAAAAAAT AAAAATATAA   
  
  
+ TTATATTATT TAATAATTTT ATAAAAAATT TATAAAATTT TTCTGAATTG CAATTAAATC TACTTCTCTT   
  
  
+ CTTGAACTAG CGATCCCTCG TATTAAACGA GCTCTCAGAA TGGAGCAGAG ATAAACAAAC AGCCTCTCGA   
  
  
+ GGACAAACCG GTTACTCTAT TCTTCCTTTT AACGTAACCG TCTTTCCTAA AGTTTCAGTT TCTTTGTTTT   
  
  
+ ACTGCTTTTT CAGTCGGTGG CGTACGACGT TAAACGTTCC GGGGTTGATC GAAAAAGGTT AGTCTAAAAC   
  
  
+ AGTAGAGTAT ATATATATAT ATATATCTTT ATTAAACAAA GTTTTATTAG TTCAAATATA TAATTTTTAT   
  
  
+ CTTATTTTTT AATATTTAAT ATAAAATAAA AATAAAATGT AAAATTTTAT ATTATATATA TAAATTTATT   
  
  
+ AAAAATTTTT TAATAAAAAA ATATTAATAA ACTTTTTTTA GATTAAATTT AGTTTAATTT TCTAATTTAT   
  
  
+ GTGTTATTTA ATTAATTATT TCATTTTAGA TATAATTTTA TTAAATTTAA AAATAATACA AATGAAGTGT   
  
  
+ AATTATCTAT ATACTAAGAA CTGGTTATAT TTTAATATAG TTTTTTTACA TTACAGTTTA AAAATTATTA   
  
  
+ TTATATTAAA ACTGAAACTT TGCACATCGA TAGTTAACGA GACTCTTTTT AAAATAATAT TTAAGCAAGT   
  
  
+ ATTATTATAA ATTTTATAGT TTGAAGTATA TAGTATTTTA ATAAAAATTA GATATATTTG AAATTATTTT   
  
  
+ TATATATTGT AGTATTAAAA TTTTCAACTT AATGCTTGAA ATAATATATA TATGTTTCTA GAGTATTTTA   
  
  
+ AAATTATAAA TGAAATTTAA TGATTAGTT  

- CAAGATTTCT GTTTACTAAA CAAGATCGAT CTGAGAGAAA AAGGATAACT GTTGGGCTGA CTTTCAAGAC   
  
  
- TTTTCAGAGA GAATCGTGAA GAGATAGACA TTTGCTAGAG GAAGGCAAGG TTTGTTTATA TATATTGGAG   
  
  
- AGAGACTCAG ATTTCTTTAC GGCAAGTATC CATATTCAAC ACCTCTTCTC CTTCCATGTG AACCAGGATA   
  
  
- TGCAGCATTC GTTGAGTTGA CTTGTCATAA AGTCAACCCA AAAATGATAA TTCATACCAA AAGTTTAATC   
  
  
- TTAGAGTACT GAAAACTTTA TTGTATTAGG ACTTGCACAT AAATCTTCAT TAATCATCAC CGTTGACTAT   
  
  
- TTCTTTACAA AAATTTTACA ATTTTTAATA ATTATAAAAT CTCAATCATT CATCATTTAA AAAATACTAA   
  
  
- GTTTTTTTTA TCAAATGGGA AAGATTAAGA AAGAAAATTG TGGATTTGGT GATGAAAGAA GGGCATGATT   
  
  
- GGTTCCTTTG ACTTTCACAC TCTTAATTAG AGTGTGAATT TAATTTTTTT ATTTATCTGA ATTATGTTAA   
  
  
- TTTTTTGATA AAATAATAAT AATAAATAAT TTAATAAAAT TAATGTAATT AATTTTTTTA TTTTTATATT   
  
  
- AATATAATAA ATTATTAAAA TATTTTTTAA ATATTTTAAA AAGACTTAAC GTTAATTTAG ATGAAGAGAA   
  
  
- GAACTTGATC GCTAGGGAGC ATAATTTGCT CGAGAGTCTT ACCTCGTCTC TATTTGTTTG TCGGAGAGCT   
  
  
- CCTGTTTGGC CAATGAGATA AGAAGGAAAA TTGCATTGGC AGAAAGGATT TCAAAGTCAA AGAAACAAAA   
  
  
- TGACGAAAAA GTCAGCCACC GCATGCTGCA ATTTGCAAGG CCCCAACTAG CTTTTTCCAA TCAGATTTTG   
  
  
- TCATCTCATA TATATATATA TATATAGAAA TAATTTGTTT CAAAATAATC AAGTTTATAT ATTAAAAATA   
  
  
- GAATAAAAAA TTATAAATTA TATTTTATTT TTATTTTACA TTTTAAAATA TAATATATAT ATTTAAATAA   
  
  
- TTTTTAAAAA ATTATTTTTT TATAATTATT TGAAAAAAAT CTAATTTAAA TCAAATTAAA AGATTAAATA   
  
  
- CACAATAAAT TAATTAATAA AGTAAAATCT ATATTAAAAT AATTTAAATT TTTATTATGT TTACTTCACA   
  
  
- TTAATAGATA TATGATTCTT GACCAATATA AAATTATATC AAAAAAATGT AATGTCAAAT TTTTAATAAT   
  
  
- AATATAATTT TGACTTTGAA ACGTGTAGCT ATCAATTGCT CTGAGAAAAA TTTTATTATA AATTCGTTCA   
  
  
- TAATAATATT TAAAATATCA AACTTCATAT ATCATAAAAT TATTTTTAAT CTATATAAAC TTTAATAAAA   
  
  
- ATATATAACA TCATAATTTT AAAAGTTGAA TTACGAACTT TATTATATAT ATACAAAGAT CTCATAAAAT   
  
  
- TTTAATATTT ACTTTAAATT ACTAATCAA

  
  
Motifs Found  

+     AAGAA-motif

| Site Name | Organism | Position | Strand | Matrix score. | sequence | function |
| --- | --- | --- | --- | --- | --- | --- |
| AAGAA-motif | Avena sativa | 447 | - | 7 | GAAAGAA |  |
| AAGAA-motif | Avena sativa | 347 | + | 9 | gGTAAAGAAA |  |

> 2018/04/13 10:10:12  
+ GTTCTAAAGA CAAATGATTT GTTCTAGCTA GACTCTCTTT TTCCTATTGA CAACCCGACT GAAAGTTCTG   
  
  
+ AAAAGTCTCT CTTAGCACTT CTCTATCTGT AAACGATCTC CTTCCGTTCC AAACAAATAT ATATAACCTC   
  
  
+ TCTCTGAGTC TAAAGAAATG CCGTTCATAG GTATAAGTTG TGGAGAAGAG GAAGGTACAC TTGGTCCTAT   
  
  
+ ACGTCGTAAG CAACTCAACT GAACAGTATT TCAGTTGGGT TTTTACTATT AAGTATGGTT TTCAAATTAG   
  
  
+ AATCTCATGA CTTTTGAAAT AACATAATCC TGAACGTGTA TTTAGAAGTA ATTAGTAGTG GCAACTGATA   
  
  
+ AAGAAATGTT TTTAAAATGT TAAAAATTAT TAATATTTTA GAGTTAGTAA GTAGTAAATT TTTTATGATT   
  
  
+ CAAAAAAAAT AGTTTACCCT TTCTAATTCT TTCTTTTAAC ACCTAAACCA CTACTTTCTT CCCGTACTAA   
  
  
+ CCAAGGAAAC TGAAAGTGTG AGAATTAATC TCACACTTAA ATTAAAAAAA TAAATAGACT TAATACAATT   
  
  
+ AAAAAACTAT TTTATTATTA TTATTTATTA AATTATTTTA ATTACATTAA TTAAAAAAAT AAAAATATAA   
  
  
+ TTATATTATT TAATAATTTT ATAAAAAATT TATAAAATTT TTCTGAATTG CAATTAAATC TACTTCTCTT   
  
  
+ CTTGAACTAG CGATCCCTCG TATTAAACGA GCTCTCAGAA TGGAGCAGAG ATAAACAAAC AGCCTCTCGA   
  
  
+ GGACAAACCG GTTACTCTAT TCTTCCTTTT AACGTAACCG TCTTTCCTAA AGTTTCAGTT TCTTTGTTTT   
  
  
+ ACTGCTTTTT CAGTCGGTGG CGTACGACGT TAAACGTTCC GGGGTTGATC GAAAAAGGTT AGTCTAAAAC   
  
  
+ AGTAGAGTAT ATATATATAT ATATATCTTT ATTAAACAAA GTTTTATTAG TTCAAATATA TAATTTTTAT   
  
  
+ CTTATTTTTT AATATTTAAT ATAAAATAAA AATAAAATGT AAAATTTTAT ATTATATATA TAAATTTATT   
  
  
+ AAAAATTTTT TAATAAAAAA ATATTAATAA ACTTTTTTTA GATTAAATTT AGTTTAATTT TCTAATTTAT   
  
  
+ GTGTTATTTA ATTAATTATT TCATTTTAGA TATAATTTTA TTAAATTTAA AAATAATACA AATGAAGTGT   
  
  
+ AATTATCTAT ATACTAAGAA CTGGTTATAT TTTAATATAG TTTTTTTACA TTACAGTTTA AAAATTATTA   
  
  
+ TTATATTAAA ACTGAAACTT TGCACATCGA TAGTTAACGA GACTCTTTTT AAAATAATAT TTAAGCAAGT   
  
  
+ ATTATTATAA ATTTTATAGT TTGAAGTATA TAGTATTTTA ATAAAAATTA GATATATTTG AAATTATTTT   
  
  
+ TATATATTGT AGTATTAAAA TTTTCAACTT AATGCTTGAA ATAATATATA TATGTTTCTA GAGTATTTTA   
  
  
+ AAATTATAAA TGAAATTTAA TGATTAGTT  

- CAAGATTTCT GTTTACTAAA CAAGATCGAT CTGAGAGAAA AAGGATAACT GTTGGGCTGA CTTTCAAGAC   
  
  
- TTTTCAGAGA GAATCGTGAA GAGATAGACA TTTGCTAGAG GAAGGCAAGG TTTGTTTATA TATATTGGAG   
  
  
- AGAGACTCAG ATTTCTTTAC GGCAAGTATC CATATTCAAC ACCTCTTCTC CTTCCATGTG AACCAGGATA   
  
  
- TGCAGCATTC GTTGAGTTGA CTTGTCATAA AGTCAACCCA AAAATGATAA TTCATACCAA AAGTTTAATC   
  
  
- TTAGAGTACT GAAAACTTTA TTGTATTAGG ACTTGCACAT AAATCTTCAT TAATCATCAC CGTTGACTAT   
  
  
- TTCTTTACAA AAATTTTACA ATTTTTAATA ATTATAAAAT CTCAATCATT CATCATTTAA AAAATACTAA   
  
  
- GTTTTTTTTA TCAAATGGGA AAGATTAAGA AAGAAAATTG TGGATTTGGT GATGAAAGAA GGGCATGATT   
  
  
- GGTTCCTTTG ACTTTCACAC TCTTAATTAG AGTGTGAATT TAATTTTTTT ATTTATCTGA ATTATGTTAA   
  
  
- TTTTTTGATA AAATAATAAT AATAAATAAT TTAATAAAAT TAATGTAATT AATTTTTTTA TTTTTATATT   
  
  
- AATATAATAA ATTATTAAAA TATTTTTTAA ATATTTTAAA AAGACTTAAC GTTAATTTAG ATGAAGAGAA   
  
  
- GAACTTGATC GCTAGGGAGC ATAATTTGCT CGAGAGTCTT ACCTCGTCTC TATTTGTTTG TCGGAGAGCT   
  
  
- CCTGTTTGGC CAATGAGATA AGAAGGAAAA TTGCATTGGC AGAAAGGATT TCAAAGTCAA AGAAACAAAA   
  
  
- TGACGAAAAA GTCAGCCACC GCATGCTGCA ATTTGCAAGG CCCCAACTAG CTTTTTCCAA TCAGATTTTG   
  
  
- TCATCTCATA TATATATATA TATATAGAAA TAATTTGTTT CAAAATAATC AAGTTTATAT ATTAAAAATA   
  
  
- GAATAAAAAA TTATAAATTA TATTTTATTT TTATTTTACA TTTTAAAATA TAATATATAT ATTTAAATAA   
  
  
- TTTTTAAAAA ATTATTTTTT TATAATTATT TGAAAAAAAT CTAATTTAAA TCAAATTAAA AGATTAAATA   
  
  
- CACAATAAAT TAATTAATAA AGTAAAATCT ATATTAAAAT AATTTAAATT TTTATTATGT TTACTTCACA   
  
  
- TTAATAGATA TATGATTCTT GACCAATATA AAATTATATC AAAAAAATGT AATGTCAAAT TTTTAATAAT   
  
  
- AATATAATTT TGACTTTGAA ACGTGTAGCT ATCAATTGCT CTGAGAAAAA TTTTATTATA AATTCGTTCA   
  
  
- TAATAATATT TAAAATATCA AACTTCATAT ATCATAAAAT TATTTTTAAT CTATATAAAC TTTAATAAAA   
  
  
- ATATATAACA TCATAATTTT AAAAGTTGAA TTACGAACTT TATTATATAT ATACAAAGAT CTCATAAAAT   
  
  
- TTTAATATTT ACTTTAAATT ACTAATCAA

+     ACE

| Site Name | Organism | Position | Strand | Matrix score. | sequence | function |
| --- | --- | --- | --- | --- | --- | --- |
| ACE | Petroselinum crispum | 1078 | - | 9 | AAAACGTTTA | cis-acting element involved in light responsiveness |

> 2018/04/13 10:10:12  
+ GTTCTAAAGA CAAATGATTT GTTCTAGCTA GACTCTCTTT TTCCTATTGA CAACCCGACT GAAAGTTCTG   
  
  
+ AAAAGTCTCT CTTAGCACTT CTCTATCTGT AAACGATCTC CTTCCGTTCC AAACAAATAT ATATAACCTC   
  
  
+ TCTCTGAGTC TAAAGAAATG CCGTTCATAG GTATAAGTTG TGGAGAAGAG GAAGGTACAC TTGGTCCTAT   
  
  
+ ACGTCGTAAG CAACTCAACT GAACAGTATT TCAGTTGGGT TTTTACTATT AAGTATGGTT TTCAAATTAG   
  
  
+ AATCTCATGA CTTTTGAAAT AACATAATCC TGAACGTGTA TTTAGAAGTA ATTAGTAGTG GCAACTGATA   
  
  
+ AAGAAATGTT TTTAAAATGT TAAAAATTAT TAATATTTTA GAGTTAGTAA GTAGTAAATT TTTTATGATT   
  
  
+ CAAAAAAAAT AGTTTACCCT TTCTAATTCT TTCTTTTAAC ACCTAAACCA CTACTTTCTT CCCGTACTAA   
  
  
+ CCAAGGAAAC TGAAAGTGTG AGAATTAATC TCACACTTAA ATTAAAAAAA TAAATAGACT TAATACAATT   
  
  
+ AAAAAACTAT TTTATTATTA TTATTTATTA AATTATTTTA ATTACATTAA TTAAAAAAAT AAAAATATAA   
  
  
+ TTATATTATT TAATAATTTT ATAAAAAATT TATAAAATTT TTCTGAATTG CAATTAAATC TACTTCTCTT   
  
  
+ CTTGAACTAG CGATCCCTCG TATTAAACGA GCTCTCAGAA TGGAGCAGAG ATAAACAAAC AGCCTCTCGA   
  
  
+ GGACAAACCG GTTACTCTAT TCTTCCTTTT AACGTAACCG TCTTTCCTAA AGTTTCAGTT TCTTTGTTTT   
  
  
+ ACTGCTTTTT CAGTCGGTGG CGTACGACGT TAAACGTTCC GGGGTTGATC GAAAAAGGTT AGTCTAAAAC   
  
  
+ AGTAGAGTAT ATATATATAT ATATATCTTT ATTAAACAAA GTTTTATTAG TTCAAATATA TAATTTTTAT   
  
  
+ CTTATTTTTT AATATTTAAT ATAAAATAAA AATAAAATGT AAAATTTTAT ATTATATATA TAAATTTATT   
  
  
+ AAAAATTTTT TAATAAAAAA ATATTAATAA ACTTTTTTTA GATTAAATTT AGTTTAATTT TCTAATTTAT   
  
  
+ GTGTTATTTA ATTAATTATT TCATTTTAGA TATAATTTTA TTAAATTTAA AAATAATACA AATGAAGTGT   
  
  
+ AATTATCTAT ATACTAAGAA CTGGTTATAT TTTAATATAG TTTTTTTACA TTACAGTTTA AAAATTATTA   
  
  
+ TTATATTAAA ACTGAAACTT TGCACATCGA TAGTTAACGA GACTCTTTTT AAAATAATAT TTAAGCAAGT   
  
  
+ ATTATTATAA ATTTTATAGT TTGAAGTATA TAGTATTTTA ATAAAAATTA GATATATTTG AAATTATTTT   
  
  
+ TATATATTGT AGTATTAAAA TTTTCAACTT AATGCTTGAA ATAATATATA TATGTTTCTA GAGTATTTTA   
  
  
+ AAATTATAAA TGAAATTTAA TGATTAGTT  

- CAAGATTTCT GTTTACTAAA CAAGATCGAT CTGAGAGAAA AAGGATAACT GTTGGGCTGA CTTTCAAGAC   
  
  
- TTTTCAGAGA GAATCGTGAA GAGATAGACA TTTGCTAGAG GAAGGCAAGG TTTGTTTATA TATATTGGAG   
  
  
- AGAGACTCAG ATTTCTTTAC GGCAAGTATC CATATTCAAC ACCTCTTCTC CTTCCATGTG AACCAGGATA   
  
  
- TGCAGCATTC GTTGAGTTGA CTTGTCATAA AGTCAACCCA AAAATGATAA TTCATACCAA AAGTTTAATC   
  
  
- TTAGAGTACT GAAAACTTTA TTGTATTAGG ACTTGCACAT AAATCTTCAT TAATCATCAC CGTTGACTAT   
  
  
- TTCTTTACAA AAATTTTACA ATTTTTAATA ATTATAAAAT CTCAATCATT CATCATTTAA AAAATACTAA   
  
  
- GTTTTTTTTA TCAAATGGGA AAGATTAAGA AAGAAAATTG TGGATTTGGT GATGAAAGAA GGGCATGATT   
  
  
- GGTTCCTTTG ACTTTCACAC TCTTAATTAG AGTGTGAATT TAATTTTTTT ATTTATCTGA ATTATGTTAA   
  
  
- TTTTTTGATA AAATAATAAT AATAAATAAT TTAATAAAAT TAATGTAATT AATTTTTTTA TTTTTATATT   
  
  
- AATATAATAA ATTATTAAAA TATTTTTTAA ATATTTTAAA AAGACTTAAC GTTAATTTAG ATGAAGAGAA   
  
  
- GAACTTGATC GCTAGGGAGC ATAATTTGCT CGAGAGTCTT ACCTCGTCTC TATTTGTTTG TCGGAGAGCT   
  
  
- CCTGTTTGGC CAATGAGATA AGAAGGAAAA TTGCATTGGC AGAAAGGATT TCAAAGTCAA AGAAACAAAA   
  
  
- TGACGAAAAA GTCAGCCACC GCATGCTGCA ATTTGCAAGG CCCCAACTAG CTTTTTCCAA TCAGATTTTG   
  
  
- TCATCTCATA TATATATATA TATATAGAAA TAATTTGTTT CAAAATAATC AAGTTTATAT ATTAAAAATA   
  
  
- GAATAAAAAA TTATAAATTA TATTTTATTT TTATTTTACA TTTTAAAATA TAATATATAT ATTTAAATAA   
  
  
- TTTTTAAAAA ATTATTTTTT TATAATTATT TGAAAAAAAT CTAATTTAAA TCAAATTAAA AGATTAAATA   
  
  
- CACAATAAAT TAATTAATAA AGTAAAATCT ATATTAAAAT AATTTAAATT TTTATTATGT TTACTTCACA   
  
  
- TTAATAGATA TATGATTCTT GACCAATATA AAATTATATC AAAAAAATGT AATGTCAAAT TTTTAATAAT   
  
  
- AATATAATTT TGACTTTGAA ACGTGTAGCT ATCAATTGCT CTGAGAAAAA TTTTATTATA AATTCGTTCA   
  
  
- TAATAATATT TAAAATATCA AACTTCATAT ATCATAAAAT TATTTTTAAT CTATATAAAC TTTAATAAAA   
  
  
- ATATATAACA TCATAATTTT AAAAGTTGAA TTACGAACTT TATTATATAT ATACAAAGAT CTCATAAAAT   
  
  
- TTTAATATTT ACTTTAAATT ACTAATCAA

+     AE-box

| Site Name | Organism | Position | Strand | Matrix score. | sequence | function |
| --- | --- | --- | --- | --- | --- | --- |
| AE-box | Arabidopsis thaliana | 1452 | - | 8 | AGAAACAT | part of a module for light response |

> 2018/04/13 10:10:12  
+ GTTCTAAAGA CAAATGATTT GTTCTAGCTA GACTCTCTTT TTCCTATTGA CAACCCGACT GAAAGTTCTG   
  
  
+ AAAAGTCTCT CTTAGCACTT CTCTATCTGT AAACGATCTC CTTCCGTTCC AAACAAATAT ATATAACCTC   
  
  
+ TCTCTGAGTC TAAAGAAATG CCGTTCATAG GTATAAGTTG TGGAGAAGAG GAAGGTACAC TTGGTCCTAT   
  
  
+ ACGTCGTAAG CAACTCAACT GAACAGTATT TCAGTTGGGT TTTTACTATT AAGTATGGTT TTCAAATTAG   
  
  
+ AATCTCATGA CTTTTGAAAT AACATAATCC TGAACGTGTA TTTAGAAGTA ATTAGTAGTG GCAACTGATA   
  
  
+ AAGAAATGTT TTTAAAATGT TAAAAATTAT TAATATTTTA GAGTTAGTAA GTAGTAAATT TTTTATGATT   
  
  
+ CAAAAAAAAT AGTTTACCCT TTCTAATTCT TTCTTTTAAC ACCTAAACCA CTACTTTCTT CCCGTACTAA   
  
  
+ CCAAGGAAAC TGAAAGTGTG AGAATTAATC TCACACTTAA ATTAAAAAAA TAAATAGACT TAATACAATT   
  
  
+ AAAAAACTAT TTTATTATTA TTATTTATTA AATTATTTTA ATTACATTAA TTAAAAAAAT AAAAATATAA   
  
  
+ TTATATTATT TAATAATTTT ATAAAAAATT TATAAAATTT TTCTGAATTG CAATTAAATC TACTTCTCTT   
  
  
+ CTTGAACTAG CGATCCCTCG TATTAAACGA GCTCTCAGAA TGGAGCAGAG ATAAACAAAC AGCCTCTCGA   
  
  
+ GGACAAACCG GTTACTCTAT TCTTCCTTTT AACGTAACCG TCTTTCCTAA AGTTTCAGTT TCTTTGTTTT   
  
  
+ ACTGCTTTTT CAGTCGGTGG CGTACGACGT TAAACGTTCC GGGGTTGATC GAAAAAGGTT AGTCTAAAAC   
  
  
+ AGTAGAGTAT ATATATATAT ATATATCTTT ATTAAACAAA GTTTTATTAG TTCAAATATA TAATTTTTAT   
  
  
+ CTTATTTTTT AATATTTAAT ATAAAATAAA AATAAAATGT AAAATTTTAT ATTATATATA TAAATTTATT   
  
  
+ AAAAATTTTT TAATAAAAAA ATATTAATAA ACTTTTTTTA GATTAAATTT AGTTTAATTT TCTAATTTAT   
  
  
+ GTGTTATTTA ATTAATTATT TCATTTTAGA TATAATTTTA TTAAATTTAA AAATAATACA AATGAAGTGT   
  
  
+ AATTATCTAT ATACTAAGAA CTGGTTATAT TTTAATATAG TTTTTTTACA TTACAGTTTA AAAATTATTA   
  
  
+ TTATATTAAA ACTGAAACTT TGCACATCGA TAGTTAACGA GACTCTTTTT AAAATAATAT TTAAGCAAGT   
  
  
+ ATTATTATAA ATTTTATAGT TTGAAGTATA TAGTATTTTA ATAAAAATTA GATATATTTG AAATTATTTT   
  
  
+ TATATATTGT AGTATTAAAA TTTTCAACTT AATGCTTGAA ATAATATATA TATGTTTCTA GAGTATTTTA   
  
  
+ AAATTATAAA TGAAATTTAA TGATTAGTT  

- CAAGATTTCT GTTTACTAAA CAAGATCGAT CTGAGAGAAA AAGGATAACT GTTGGGCTGA CTTTCAAGAC   
  
  
- TTTTCAGAGA GAATCGTGAA GAGATAGACA TTTGCTAGAG GAAGGCAAGG TTTGTTTATA TATATTGGAG   
  
  
- AGAGACTCAG ATTTCTTTAC GGCAAGTATC CATATTCAAC ACCTCTTCTC CTTCCATGTG AACCAGGATA   
  
  
- TGCAGCATTC GTTGAGTTGA CTTGTCATAA AGTCAACCCA AAAATGATAA TTCATACCAA AAGTTTAATC   
  
  
- TTAGAGTACT GAAAACTTTA TTGTATTAGG ACTTGCACAT AAATCTTCAT TAATCATCAC CGTTGACTAT   
  
  
- TTCTTTACAA AAATTTTACA ATTTTTAATA ATTATAAAAT CTCAATCATT CATCATTTAA AAAATACTAA   
  
  
- GTTTTTTTTA TCAAATGGGA AAGATTAAGA AAGAAAATTG TGGATTTGGT GATGAAAGAA GGGCATGATT   
  
  
- GGTTCCTTTG ACTTTCACAC TCTTAATTAG AGTGTGAATT TAATTTTTTT ATTTATCTGA ATTATGTTAA   
  
  
- TTTTTTGATA AAATAATAAT AATAAATAAT TTAATAAAAT TAATGTAATT AATTTTTTTA TTTTTATATT   
  
  
- AATATAATAA ATTATTAAAA TATTTTTTAA ATATTTTAAA AAGACTTAAC GTTAATTTAG ATGAAGAGAA   
  
  
- GAACTTGATC GCTAGGGAGC ATAATTTGCT CGAGAGTCTT ACCTCGTCTC TATTTGTTTG TCGGAGAGCT   
  
  
- CCTGTTTGGC CAATGAGATA AGAAGGAAAA TTGCATTGGC AGAAAGGATT TCAAAGTCAA AGAAACAAAA   
  
  
- TGACGAAAAA GTCAGCCACC GCATGCTGCA ATTTGCAAGG CCCCAACTAG CTTTTTCCAA TCAGATTTTG   
  
  
- TCATCTCATA TATATATATA TATATAGAAA TAATTTGTTT CAAAATAATC AAGTTTATAT ATTAAAAATA   
  
  
- GAATAAAAAA TTATAAATTA TATTTTATTT TTATTTTACA TTTTAAAATA TAATATATAT ATTTAAATAA   
  
  
- TTTTTAAAAA ATTATTTTTT TATAATTATT TGAAAAAAAT CTAATTTAAA TCAAATTAAA AGATTAAATA   
  
  
- CACAATAAAT TAATTAATAA AGTAAAATCT ATATTAAAAT AATTTAAATT TTTATTATGT TTACTTCACA   
  
  
- TTAATAGATA TATGATTCTT GACCAATATA AAATTATATC AAAAAAATGT AATGTCAAAT TTTTAATAAT   
  
  
- AATATAATTT TGACTTTGAA ACGTGTAGCT ATCAATTGCT CTGAGAAAAA TTTTATTATA AATTCGTTCA   
  
  
- TAATAATATT TAAAATATCA AACTTCATAT ATCATAAAAT TATTTTTAAT CTATATAAAC TTTAATAAAA   
  
  
- ATATATAACA TCATAATTTT AAAAGTTGAA TTACGAACTT TATTATATAT ATACAAAGAT CTCATAAAAT   
  
  
- TTTAATATTT ACTTTAAATT ACTAATCAA

+     ARE

| Site Name | Organism | Position | Strand | Matrix score. | sequence | function |
| --- | --- | --- | --- | --- | --- | --- |
| ARE | Zea mays | 266 | + | 6 | TGGTTT | cis-acting regulatory element essential for the anaerobic induction |
| ARE | Zea mays | 465 | - | 6 | TGGTTT | cis-acting regulatory element essential for the anaerobic induction |

> 2018/04/13 10:10:12  
+ GTTCTAAAGA CAAATGATTT GTTCTAGCTA GACTCTCTTT TTCCTATTGA CAACCCGACT GAAAGTTCTG   
  
  
+ AAAAGTCTCT CTTAGCACTT CTCTATCTGT AAACGATCTC CTTCCGTTCC AAACAAATAT ATATAACCTC   
  
  
+ TCTCTGAGTC TAAAGAAATG CCGTTCATAG GTATAAGTTG TGGAGAAGAG GAAGGTACAC TTGGTCCTAT   
  
  
+ ACGTCGTAAG CAACTCAACT GAACAGTATT TCAGTTGGGT TTTTACTATT AAGTATGGTT TTCAAATTAG   
  
  
+ AATCTCATGA CTTTTGAAAT AACATAATCC TGAACGTGTA TTTAGAAGTA ATTAGTAGTG GCAACTGATA   
  
  
+ AAGAAATGTT TTTAAAATGT TAAAAATTAT TAATATTTTA GAGTTAGTAA GTAGTAAATT TTTTATGATT   
  
  
+ CAAAAAAAAT AGTTTACCCT TTCTAATTCT TTCTTTTAAC ACCTAAACCA CTACTTTCTT CCCGTACTAA   
  
  
+ CCAAGGAAAC TGAAAGTGTG AGAATTAATC TCACACTTAA ATTAAAAAAA TAAATAGACT TAATACAATT   
  
  
+ AAAAAACTAT TTTATTATTA TTATTTATTA AATTATTTTA ATTACATTAA TTAAAAAAAT AAAAATATAA   
  
  
+ TTATATTATT TAATAATTTT ATAAAAAATT TATAAAATTT TTCTGAATTG CAATTAAATC TACTTCTCTT   
  
  
+ CTTGAACTAG CGATCCCTCG TATTAAACGA GCTCTCAGAA TGGAGCAGAG ATAAACAAAC AGCCTCTCGA   
  
  
+ GGACAAACCG GTTACTCTAT TCTTCCTTTT AACGTAACCG TCTTTCCTAA AGTTTCAGTT TCTTTGTTTT   
  
  
+ ACTGCTTTTT CAGTCGGTGG CGTACGACGT TAAACGTTCC GGGGTTGATC GAAAAAGGTT AGTCTAAAAC   
  
  
+ AGTAGAGTAT ATATATATAT ATATATCTTT ATTAAACAAA GTTTTATTAG TTCAAATATA TAATTTTTAT   
  
  
+ CTTATTTTTT AATATTTAAT ATAAAATAAA AATAAAATGT AAAATTTTAT ATTATATATA TAAATTTATT   
  
  
+ AAAAATTTTT TAATAAAAAA ATATTAATAA ACTTTTTTTA GATTAAATTT AGTTTAATTT TCTAATTTAT   
  
  
+ GTGTTATTTA ATTAATTATT TCATTTTAGA TATAATTTTA TTAAATTTAA AAATAATACA AATGAAGTGT   
  
  
+ AATTATCTAT ATACTAAGAA CTGGTTATAT TTTAATATAG TTTTTTTACA TTACAGTTTA AAAATTATTA   
  
  
+ TTATATTAAA ACTGAAACTT TGCACATCGA TAGTTAACGA GACTCTTTTT AAAATAATAT TTAAGCAAGT   
  
  
+ ATTATTATAA ATTTTATAGT TTGAAGTATA TAGTATTTTA ATAAAAATTA GATATATTTG AAATTATTTT   
  
  
+ TATATATTGT AGTATTAAAA TTTTCAACTT AATGCTTGAA ATAATATATA TATGTTTCTA GAGTATTTTA   
  
  
+ AAATTATAAA TGAAATTTAA TGATTAGTT  

- CAAGATTTCT GTTTACTAAA CAAGATCGAT CTGAGAGAAA AAGGATAACT GTTGGGCTGA CTTTCAAGAC   
  
  
- TTTTCAGAGA GAATCGTGAA GAGATAGACA TTTGCTAGAG GAAGGCAAGG TTTGTTTATA TATATTGGAG   
  
  
- AGAGACTCAG ATTTCTTTAC GGCAAGTATC CATATTCAAC ACCTCTTCTC CTTCCATGTG AACCAGGATA   
  
  
- TGCAGCATTC GTTGAGTTGA CTTGTCATAA AGTCAACCCA AAAATGATAA TTCATACCAA AAGTTTAATC   
  
  
- TTAGAGTACT GAAAACTTTA TTGTATTAGG ACTTGCACAT AAATCTTCAT TAATCATCAC CGTTGACTAT   
  
  
- TTCTTTACAA AAATTTTACA ATTTTTAATA ATTATAAAAT CTCAATCATT CATCATTTAA AAAATACTAA   
  
  
- GTTTTTTTTA TCAAATGGGA AAGATTAAGA AAGAAAATTG TGGATTTGGT GATGAAAGAA GGGCATGATT   
  
  
- GGTTCCTTTG ACTTTCACAC TCTTAATTAG AGTGTGAATT TAATTTTTTT ATTTATCTGA ATTATGTTAA   
  
  
- TTTTTTGATA AAATAATAAT AATAAATAAT TTAATAAAAT TAATGTAATT AATTTTTTTA TTTTTATATT   
  
  
- AATATAATAA ATTATTAAAA TATTTTTTAA ATATTTTAAA AAGACTTAAC GTTAATTTAG ATGAAGAGAA   
  
  
- GAACTTGATC GCTAGGGAGC ATAATTTGCT CGAGAGTCTT ACCTCGTCTC TATTTGTTTG TCGGAGAGCT   
  
  
- CCTGTTTGGC CAATGAGATA AGAAGGAAAA TTGCATTGGC AGAAAGGATT TCAAAGTCAA AGAAACAAAA   
  
  
- TGACGAAAAA GTCAGCCACC GCATGCTGCA ATTTGCAAGG CCCCAACTAG CTTTTTCCAA TCAGATTTTG   
  
  
- TCATCTCATA TATATATATA TATATAGAAA TAATTTGTTT CAAAATAATC AAGTTTATAT ATTAAAAATA   
  
  
- GAATAAAAAA TTATAAATTA TATTTTATTT TTATTTTACA TTTTAAAATA TAATATATAT ATTTAAATAA   
  
  
- TTTTTAAAAA ATTATTTTTT TATAATTATT TGAAAAAAAT CTAATTTAAA TCAAATTAAA AGATTAAATA   
  
  
- CACAATAAAT TAATTAATAA AGTAAAATCT ATATTAAAAT AATTTAAATT TTTATTATGT TTACTTCACA   
  
  
- TTAATAGATA TATGATTCTT GACCAATATA AAATTATATC AAAAAAATGT AATGTCAAAT TTTTAATAAT   
  
  
- AATATAATTT TGACTTTGAA ACGTGTAGCT ATCAATTGCT CTGAGAAAAA TTTTATTATA AATTCGTTCA   
  
  
- TAATAATATT TAAAATATCA AACTTCATAT ATCATAAAAT TATTTTTAAT CTATATAAAC TTTAATAAAA   
  
  
- ATATATAACA TCATAATTTT AAAAGTTGAA TTACGAACTT TATTATATAT ATACAAAGAT CTCATAAAAT   
  
  
- TTTAATATTT ACTTTAAATT ACTAATCAA

+     AT-rich sequence

| Site Name | Organism | Position | Strand | Matrix score. | sequence | function |
| --- | --- | --- | --- | --- | --- | --- |
| AT-rich sequence | Pisum sativum | 1462 | - | 9 | TAAAATACT | element for maximal elicitor-mediated activation (2copies) |
| AT-rich sequence | Pisum sativum | 1362 | - | 9 | TAAAATACT | element for maximal elicitor-mediated activation (2copies) |

> 2018/04/13 10:10:12  
+ GTTCTAAAGA CAAATGATTT GTTCTAGCTA GACTCTCTTT TTCCTATTGA CAACCCGACT GAAAGTTCTG   
  
  
+ AAAAGTCTCT CTTAGCACTT CTCTATCTGT AAACGATCTC CTTCCGTTCC AAACAAATAT ATATAACCTC   
  
  
+ TCTCTGAGTC TAAAGAAATG CCGTTCATAG GTATAAGTTG TGGAGAAGAG GAAGGTACAC TTGGTCCTAT   
  
  
+ ACGTCGTAAG CAACTCAACT GAACAGTATT TCAGTTGGGT TTTTACTATT AAGTATGGTT TTCAAATTAG   
  
  
+ AATCTCATGA CTTTTGAAAT AACATAATCC TGAACGTGTA TTTAGAAGTA ATTAGTAGTG GCAACTGATA   
  
  
+ AAGAAATGTT TTTAAAATGT TAAAAATTAT TAATATTTTA GAGTTAGTAA GTAGTAAATT TTTTATGATT   
  
  
+ CAAAAAAAAT AGTTTACCCT TTCTAATTCT TTCTTTTAAC ACCTAAACCA CTACTTTCTT CCCGTACTAA   
  
  
+ CCAAGGAAAC TGAAAGTGTG AGAATTAATC TCACACTTAA ATTAAAAAAA TAAATAGACT TAATACAATT   
  
  
+ AAAAAACTAT TTTATTATTA TTATTTATTA AATTATTTTA ATTACATTAA TTAAAAAAAT AAAAATATAA   
  
  
+ TTATATTATT TAATAATTTT ATAAAAAATT TATAAAATTT TTCTGAATTG CAATTAAATC TACTTCTCTT   
  
  
+ CTTGAACTAG CGATCCCTCG TATTAAACGA GCTCTCAGAA TGGAGCAGAG ATAAACAAAC AGCCTCTCGA   
  
  
+ GGACAAACCG GTTACTCTAT TCTTCCTTTT AACGTAACCG TCTTTCCTAA AGTTTCAGTT TCTTTGTTTT   
  
  
+ ACTGCTTTTT CAGTCGGTGG CGTACGACGT TAAACGTTCC GGGGTTGATC GAAAAAGGTT AGTCTAAAAC   
  
  
+ AGTAGAGTAT ATATATATAT ATATATCTTT ATTAAACAAA GTTTTATTAG TTCAAATATA TAATTTTTAT   
  
  
+ CTTATTTTTT AATATTTAAT ATAAAATAAA AATAAAATGT AAAATTTTAT ATTATATATA TAAATTTATT   
  
  
+ AAAAATTTTT TAATAAAAAA ATATTAATAA ACTTTTTTTA GATTAAATTT AGTTTAATTT TCTAATTTAT   
  
  
+ GTGTTATTTA ATTAATTATT TCATTTTAGA TATAATTTTA TTAAATTTAA AAATAATACA AATGAAGTGT   
  
  
+ AATTATCTAT ATACTAAGAA CTGGTTATAT TTTAATATAG TTTTTTTACA TTACAGTTTA AAAATTATTA   
  
  
+ TTATATTAAA ACTGAAACTT TGCACATCGA TAGTTAACGA GACTCTTTTT AAAATAATAT TTAAGCAAGT   
  
  
+ ATTATTATAA ATTTTATAGT TTGAAGTATA TAGTATTTTA ATAAAAATTA GATATATTTG AAATTATTTT   
  
  
+ TATATATTGT AGTATTAAAA TTTTCAACTT AATGCTTGAA ATAATATATA TATGTTTCTA GAGTATTTTA   
  
  
+ AAATTATAAA TGAAATTTAA TGATTAGTT  

- CAAGATTTCT GTTTACTAAA CAAGATCGAT CTGAGAGAAA AAGGATAACT GTTGGGCTGA CTTTCAAGAC   
  
  
- TTTTCAGAGA GAATCGTGAA GAGATAGACA TTTGCTAGAG GAAGGCAAGG TTTGTTTATA TATATTGGAG   
  
  
- AGAGACTCAG ATTTCTTTAC GGCAAGTATC CATATTCAAC ACCTCTTCTC CTTCCATGTG AACCAGGATA   
  
  
- TGCAGCATTC GTTGAGTTGA CTTGTCATAA AGTCAACCCA AAAATGATAA TTCATACCAA AAGTTTAATC   
  
  
- TTAGAGTACT GAAAACTTTA TTGTATTAGG ACTTGCACAT AAATCTTCAT TAATCATCAC CGTTGACTAT   
  
  
- TTCTTTACAA AAATTTTACA ATTTTTAATA ATTATAAAAT CTCAATCATT CATCATTTAA AAAATACTAA   
  
  
- GTTTTTTTTA TCAAATGGGA AAGATTAAGA AAGAAAATTG TGGATTTGGT GATGAAAGAA GGGCATGATT   
  
  
- GGTTCCTTTG ACTTTCACAC TCTTAATTAG AGTGTGAATT TAATTTTTTT ATTTATCTGA ATTATGTTAA   
  
  
- TTTTTTGATA AAATAATAAT AATAAATAAT TTAATAAAAT TAATGTAATT AATTTTTTTA TTTTTATATT   
  
  
- AATATAATAA ATTATTAAAA TATTTTTTAA ATATTTTAAA AAGACTTAAC GTTAATTTAG ATGAAGAGAA   
  
  
- GAACTTGATC GCTAGGGAGC ATAATTTGCT CGAGAGTCTT ACCTCGTCTC TATTTGTTTG TCGGAGAGCT   
  
  
- CCTGTTTGGC CAATGAGATA AGAAGGAAAA TTGCATTGGC AGAAAGGATT TCAAAGTCAA AGAAACAAAA   
  
  
- TGACGAAAAA GTCAGCCACC GCATGCTGCA ATTTGCAAGG CCCCAACTAG CTTTTTCCAA TCAGATTTTG   
  
  
- TCATCTCATA TATATATATA TATATAGAAA TAATTTGTTT CAAAATAATC AAGTTTATAT ATTAAAAATA   
  
  
- GAATAAAAAA TTATAAATTA TATTTTATTT TTATTTTACA TTTTAAAATA TAATATATAT ATTTAAATAA   
  
  
- TTTTTAAAAA ATTATTTTTT TATAATTATT TGAAAAAAAT CTAATTTAAA TCAAATTAAA AGATTAAATA   
  
  
- CACAATAAAT TAATTAATAA AGTAAAATCT ATATTAAAAT AATTTAAATT TTTATTATGT TTACTTCACA   
  
  
- TTAATAGATA TATGATTCTT GACCAATATA AAATTATATC AAAAAAATGT AATGTCAAAT TTTTAATAAT   
  
  
- AATATAATTT TGACTTTGAA ACGTGTAGCT ATCAATTGCT CTGAGAAAAA TTTTATTATA AATTCGTTCA   
  
  
- TAATAATATT TAAAATATCA AACTTCATAT ATCATAAAAT TATTTTTAAT CTATATAAAC TTTAATAAAA   
  
  
- ATATATAACA TCATAATTTT AAAAGTTGAA TTACGAACTT TATTATATAT ATACAAAGAT CTCATAAAAT   
  
  
- TTTAATATTT ACTTTAAATT ACTAATCAA

+     ATCT-motif

| Site Name | Organism | Position | Strand | Matrix score. | sequence | function |
| --- | --- | --- | --- | --- | --- | --- |
| ATCT-motif | Arabidopsis thaliana | 1090 | - | 9 | AATCTAATCT | part of a conserved DNA module involved in light responsiveness |

> 2018/04/13 10:10:12  
+ GTTCTAAAGA CAAATGATTT GTTCTAGCTA GACTCTCTTT TTCCTATTGA CAACCCGACT GAAAGTTCTG   
  
  
+ AAAAGTCTCT CTTAGCACTT CTCTATCTGT AAACGATCTC CTTCCGTTCC AAACAAATAT ATATAACCTC   
  
  
+ TCTCTGAGTC TAAAGAAATG CCGTTCATAG GTATAAGTTG TGGAGAAGAG GAAGGTACAC TTGGTCCTAT   
  
  
+ ACGTCGTAAG CAACTCAACT GAACAGTATT TCAGTTGGGT TTTTACTATT AAGTATGGTT TTCAAATTAG   
  
  
+ AATCTCATGA CTTTTGAAAT AACATAATCC TGAACGTGTA TTTAGAAGTA ATTAGTAGTG GCAACTGATA   
  
  
+ AAGAAATGTT TTTAAAATGT TAAAAATTAT TAATATTTTA GAGTTAGTAA GTAGTAAATT TTTTATGATT   
  
  
+ CAAAAAAAAT AGTTTACCCT TTCTAATTCT TTCTTTTAAC ACCTAAACCA CTACTTTCTT CCCGTACTAA   
  
  
+ CCAAGGAAAC TGAAAGTGTG AGAATTAATC TCACACTTAA ATTAAAAAAA TAAATAGACT TAATACAATT   
  
  
+ AAAAAACTAT TTTATTATTA TTATTTATTA AATTATTTTA ATTACATTAA TTAAAAAAAT AAAAATATAA   
  
  
+ TTATATTATT TAATAATTTT ATAAAAAATT TATAAAATTT TTCTGAATTG CAATTAAATC TACTTCTCTT   
  
  
+ CTTGAACTAG CGATCCCTCG TATTAAACGA GCTCTCAGAA TGGAGCAGAG ATAAACAAAC AGCCTCTCGA   
  
  
+ GGACAAACCG GTTACTCTAT TCTTCCTTTT AACGTAACCG TCTTTCCTAA AGTTTCAGTT TCTTTGTTTT   
  
  
+ ACTGCTTTTT CAGTCGGTGG CGTACGACGT TAAACGTTCC GGGGTTGATC GAAAAAGGTT AGTCTAAAAC   
  
  
+ AGTAGAGTAT ATATATATAT ATATATCTTT ATTAAACAAA GTTTTATTAG TTCAAATATA TAATTTTTAT   
  
  
+ CTTATTTTTT AATATTTAAT ATAAAATAAA AATAAAATGT AAAATTTTAT ATTATATATA TAAATTTATT   
  
  
+ AAAAATTTTT TAATAAAAAA ATATTAATAA ACTTTTTTTA GATTAAATTT AGTTTAATTT TCTAATTTAT   
  
  
+ GTGTTATTTA ATTAATTATT TCATTTTAGA TATAATTTTA TTAAATTTAA AAATAATACA AATGAAGTGT   
  
  
+ AATTATCTAT ATACTAAGAA CTGGTTATAT TTTAATATAG TTTTTTTACA TTACAGTTTA AAAATTATTA   
  
  
+ TTATATTAAA ACTGAAACTT TGCACATCGA TAGTTAACGA GACTCTTTTT AAAATAATAT TTAAGCAAGT   
  
  
+ ATTATTATAA ATTTTATAGT TTGAAGTATA TAGTATTTTA ATAAAAATTA GATATATTTG AAATTATTTT   
  
  
+ TATATATTGT AGTATTAAAA TTTTCAACTT AATGCTTGAA ATAATATATA TATGTTTCTA GAGTATTTTA   
  
  
+ AAATTATAAA TGAAATTTAA TGATTAGTT  

- CAAGATTTCT GTTTACTAAA CAAGATCGAT CTGAGAGAAA AAGGATAACT GTTGGGCTGA CTTTCAAGAC   
  
  
- TTTTCAGAGA GAATCGTGAA GAGATAGACA TTTGCTAGAG GAAGGCAAGG TTTGTTTATA TATATTGGAG   
  
  
- AGAGACTCAG ATTTCTTTAC GGCAAGTATC CATATTCAAC ACCTCTTCTC CTTCCATGTG AACCAGGATA   
  
  
- TGCAGCATTC GTTGAGTTGA CTTGTCATAA AGTCAACCCA AAAATGATAA TTCATACCAA AAGTTTAATC   
  
  
- TTAGAGTACT GAAAACTTTA TTGTATTAGG ACTTGCACAT AAATCTTCAT TAATCATCAC CGTTGACTAT   
  
  
- TTCTTTACAA AAATTTTACA ATTTTTAATA ATTATAAAAT CTCAATCATT CATCATTTAA AAAATACTAA   
  
  
- GTTTTTTTTA TCAAATGGGA AAGATTAAGA AAGAAAATTG TGGATTTGGT GATGAAAGAA GGGCATGATT   
  
  
- GGTTCCTTTG ACTTTCACAC TCTTAATTAG AGTGTGAATT TAATTTTTTT ATTTATCTGA ATTATGTTAA   
  
  
- TTTTTTGATA AAATAATAAT AATAAATAAT TTAATAAAAT TAATGTAATT AATTTTTTTA TTTTTATATT   
  
  
- AATATAATAA ATTATTAAAA TATTTTTTAA ATATTTTAAA AAGACTTAAC GTTAATTTAG ATGAAGAGAA   
  
  
- GAACTTGATC GCTAGGGAGC ATAATTTGCT CGAGAGTCTT ACCTCGTCTC TATTTGTTTG TCGGAGAGCT   
  
  
- CCTGTTTGGC CAATGAGATA AGAAGGAAAA TTGCATTGGC AGAAAGGATT TCAAAGTCAA AGAAACAAAA   
  
  
- TGACGAAAAA GTCAGCCACC GCATGCTGCA ATTTGCAAGG CCCCAACTAG CTTTTTCCAA TCAGATTTTG   
  
  
- TCATCTCATA TATATATATA TATATAGAAA TAATTTGTTT CAAAATAATC AAGTTTATAT ATTAAAAATA   
  
  
- GAATAAAAAA TTATAAATTA TATTTTATTT TTATTTTACA TTTTAAAATA TAATATATAT ATTTAAATAA   
  
  
- TTTTTAAAAA ATTATTTTTT TATAATTATT TGAAAAAAAT CTAATTTAAA TCAAATTAAA AGATTAAATA   
  
  
- CACAATAAAT TAATTAATAA AGTAAAATCT ATATTAAAAT AATTTAAATT TTTATTATGT TTACTTCACA   
  
  
- TTAATAGATA TATGATTCTT GACCAATATA AAATTATATC AAAAAAATGT AATGTCAAAT TTTTAATAAT   
  
  
- AATATAATTT TGACTTTGAA ACGTGTAGCT ATCAATTGCT CTGAGAAAAA TTTTATTATA AATTCGTTCA   
  
  
- TAATAATATT TAAAATATCA AACTTCATAT ATCATAAAAT TATTTTTAAT CTATATAAAC TTTAATAAAA   
  
  
- ATATATAACA TCATAATTTT AAAAGTTGAA TTACGAACTT TATTATATAT ATACAAAGAT CTCATAAAAT   
  
  
- TTTAATATTT ACTTTAAATT ACTAATCAA

+     ATGCAAAT motif

| Site Name | Organism | Position | Strand | Matrix score. | sequence | function |
| --- | --- | --- | --- | --- | --- | --- |
| ATGCAAAT motif | Oryza sativa | 1176 | + | 8 | ATACAAAT | cis-acting regulatory element associated to the TGAGTCA motif |

> 2018/04/13 10:10:12  
+ GTTCTAAAGA CAAATGATTT GTTCTAGCTA GACTCTCTTT TTCCTATTGA CAACCCGACT GAAAGTTCTG   
  
  
+ AAAAGTCTCT CTTAGCACTT CTCTATCTGT AAACGATCTC CTTCCGTTCC AAACAAATAT ATATAACCTC   
  
  
+ TCTCTGAGTC TAAAGAAATG CCGTTCATAG GTATAAGTTG TGGAGAAGAG GAAGGTACAC TTGGTCCTAT   
  
  
+ ACGTCGTAAG CAACTCAACT GAACAGTATT TCAGTTGGGT TTTTACTATT AAGTATGGTT TTCAAATTAG   
  
  
+ AATCTCATGA CTTTTGAAAT AACATAATCC TGAACGTGTA TTTAGAAGTA ATTAGTAGTG GCAACTGATA   
  
  
+ AAGAAATGTT TTTAAAATGT TAAAAATTAT TAATATTTTA GAGTTAGTAA GTAGTAAATT TTTTATGATT   
  
  
+ CAAAAAAAAT AGTTTACCCT TTCTAATTCT TTCTTTTAAC ACCTAAACCA CTACTTTCTT CCCGTACTAA   
  
  
+ CCAAGGAAAC TGAAAGTGTG AGAATTAATC TCACACTTAA ATTAAAAAAA TAAATAGACT TAATACAATT   
  
  
+ AAAAAACTAT TTTATTATTA TTATTTATTA AATTATTTTA ATTACATTAA TTAAAAAAAT AAAAATATAA   
  
  
+ TTATATTATT TAATAATTTT ATAAAAAATT TATAAAATTT TTCTGAATTG CAATTAAATC TACTTCTCTT   
  
  
+ CTTGAACTAG CGATCCCTCG TATTAAACGA GCTCTCAGAA TGGAGCAGAG ATAAACAAAC AGCCTCTCGA   
  
  
+ GGACAAACCG GTTACTCTAT TCTTCCTTTT AACGTAACCG TCTTTCCTAA AGTTTCAGTT TCTTTGTTTT   
  
  
+ ACTGCTTTTT CAGTCGGTGG CGTACGACGT TAAACGTTCC GGGGTTGATC GAAAAAGGTT AGTCTAAAAC   
  
  
+ AGTAGAGTAT ATATATATAT ATATATCTTT ATTAAACAAA GTTTTATTAG TTCAAATATA TAATTTTTAT   
  
  
+ CTTATTTTTT AATATTTAAT ATAAAATAAA AATAAAATGT AAAATTTTAT ATTATATATA TAAATTTATT   
  
  
+ AAAAATTTTT TAATAAAAAA ATATTAATAA ACTTTTTTTA GATTAAATTT AGTTTAATTT TCTAATTTAT   
  
  
+ GTGTTATTTA ATTAATTATT TCATTTTAGA TATAATTTTA TTAAATTTAA AAATAATACA AATGAAGTGT   
  
  
+ AATTATCTAT ATACTAAGAA CTGGTTATAT TTTAATATAG TTTTTTTACA TTACAGTTTA AAAATTATTA   
  
  
+ TTATATTAAA ACTGAAACTT TGCACATCGA TAGTTAACGA GACTCTTTTT AAAATAATAT TTAAGCAAGT   
  
  
+ ATTATTATAA ATTTTATAGT TTGAAGTATA TAGTATTTTA ATAAAAATTA GATATATTTG AAATTATTTT   
  
  
+ TATATATTGT AGTATTAAAA TTTTCAACTT AATGCTTGAA ATAATATATA TATGTTTCTA GAGTATTTTA   
  
  
+ AAATTATAAA TGAAATTTAA TGATTAGTT  

- CAAGATTTCT GTTTACTAAA CAAGATCGAT CTGAGAGAAA AAGGATAACT GTTGGGCTGA CTTTCAAGAC   
  
  
- TTTTCAGAGA GAATCGTGAA GAGATAGACA TTTGCTAGAG GAAGGCAAGG TTTGTTTATA TATATTGGAG   
  
  
- AGAGACTCAG ATTTCTTTAC GGCAAGTATC CATATTCAAC ACCTCTTCTC CTTCCATGTG AACCAGGATA   
  
  
- TGCAGCATTC GTTGAGTTGA CTTGTCATAA AGTCAACCCA AAAATGATAA TTCATACCAA AAGTTTAATC   
  
  
- TTAGAGTACT GAAAACTTTA TTGTATTAGG ACTTGCACAT AAATCTTCAT TAATCATCAC CGTTGACTAT   
  
  
- TTCTTTACAA AAATTTTACA ATTTTTAATA ATTATAAAAT CTCAATCATT CATCATTTAA AAAATACTAA   
  
  
- GTTTTTTTTA TCAAATGGGA AAGATTAAGA AAGAAAATTG TGGATTTGGT GATGAAAGAA GGGCATGATT   
  
  
- GGTTCCTTTG ACTTTCACAC TCTTAATTAG AGTGTGAATT TAATTTTTTT ATTTATCTGA ATTATGTTAA   
  
  
- TTTTTTGATA AAATAATAAT AATAAATAAT TTAATAAAAT TAATGTAATT AATTTTTTTA TTTTTATATT   
  
  
- AATATAATAA ATTATTAAAA TATTTTTTAA ATATTTTAAA AAGACTTAAC GTTAATTTAG ATGAAGAGAA   
  
  
- GAACTTGATC GCTAGGGAGC ATAATTTGCT CGAGAGTCTT ACCTCGTCTC TATTTGTTTG TCGGAGAGCT   
  
  
- CCTGTTTGGC CAATGAGATA AGAAGGAAAA TTGCATTGGC AGAAAGGATT TCAAAGTCAA AGAAACAAAA   
  
  
- TGACGAAAAA GTCAGCCACC GCATGCTGCA ATTTGCAAGG CCCCAACTAG CTTTTTCCAA TCAGATTTTG   
  
  
- TCATCTCATA TATATATATA TATATAGAAA TAATTTGTTT CAAAATAATC AAGTTTATAT ATTAAAAATA   
  
  
- GAATAAAAAA TTATAAATTA TATTTTATTT TTATTTTACA TTTTAAAATA TAATATATAT ATTTAAATAA   
  
  
- TTTTTAAAAA ATTATTTTTT TATAATTATT TGAAAAAAAT CTAATTTAAA TCAAATTAAA AGATTAAATA   
  
  
- CACAATAAAT TAATTAATAA AGTAAAATCT ATATTAAAAT AATTTAAATT TTTATTATGT TTACTTCACA   
  
  
- TTAATAGATA TATGATTCTT GACCAATATA AAATTATATC AAAAAAATGT AATGTCAAAT TTTTAATAAT   
  
  
- AATATAATTT TGACTTTGAA ACGTGTAGCT ATCAATTGCT CTGAGAAAAA TTTTATTATA AATTCGTTCA   
  
  
- TAATAATATT TAAAATATCA AACTTCATAT ATCATAAAAT TATTTTTAAT CTATATAAAC TTTAATAAAA   
  
  
- ATATATAACA TCATAATTTT AAAAGTTGAA TTACGAACTT TATTATATAT ATACAAAGAT CTCATAAAAT   
  
  
- TTTAATATTT ACTTTAAATT ACTAATCAA

+     Box 4

| Site Name | Organism | Position | Strand | Matrix score. | sequence | function |
| --- | --- | --- | --- | --- | --- | --- |
| Box 4 | Petroselinum crispum | 1131 | - | 6 | ATTAAT | part of a conserved DNA module involved in light responsiveness |
| Box 4 | Petroselinum crispum | 514 | + | 6 | ATTAAT | part of a conserved DNA module involved in light responsiveness |
| Box 4 | Petroselinum crispum | 1073 | - | 6 | ATTAAT | part of a conserved DNA module involved in light responsiveness |
| Box 4 | Petroselinum crispum | 379 | + | 6 | ATTAAT | part of a conserved DNA module involved in light responsiveness |
| Box 4 | Petroselinum crispum | 606 | + | 6 | ATTAAT | part of a conserved DNA module involved in light responsiveness |

> 2018/04/13 10:10:12  
+ GTTCTAAAGA CAAATGATTT GTTCTAGCTA GACTCTCTTT TTCCTATTGA CAACCCGACT GAAAGTTCTG   
  
  
+ AAAAGTCTCT CTTAGCACTT CTCTATCTGT AAACGATCTC CTTCCGTTCC AAACAAATAT ATATAACCTC   
  
  
+ TCTCTGAGTC TAAAGAAATG CCGTTCATAG GTATAAGTTG TGGAGAAGAG GAAGGTACAC TTGGTCCTAT   
  
  
+ ACGTCGTAAG CAACTCAACT GAACAGTATT TCAGTTGGGT TTTTACTATT AAGTATGGTT TTCAAATTAG   
  
  
+ AATCTCATGA CTTTTGAAAT AACATAATCC TGAACGTGTA TTTAGAAGTA ATTAGTAGTG GCAACTGATA   
  
  
+ AAGAAATGTT TTTAAAATGT TAAAAATTAT TAATATTTTA GAGTTAGTAA GTAGTAAATT TTTTATGATT   
  
  
+ CAAAAAAAAT AGTTTACCCT TTCTAATTCT TTCTTTTAAC ACCTAAACCA CTACTTTCTT CCCGTACTAA   
  
  
+ CCAAGGAAAC TGAAAGTGTG AGAATTAATC TCACACTTAA ATTAAAAAAA TAAATAGACT TAATACAATT   
  
  
+ AAAAAACTAT TTTATTATTA TTATTTATTA AATTATTTTA ATTACATTAA TTAAAAAAAT AAAAATATAA   
  
  
+ TTATATTATT TAATAATTTT ATAAAAAATT TATAAAATTT TTCTGAATTG CAATTAAATC TACTTCTCTT   
  
  
+ CTTGAACTAG CGATCCCTCG TATTAAACGA GCTCTCAGAA TGGAGCAGAG ATAAACAAAC AGCCTCTCGA   
  
  
+ GGACAAACCG GTTACTCTAT TCTTCCTTTT AACGTAACCG TCTTTCCTAA AGTTTCAGTT TCTTTGTTTT   
  
  
+ ACTGCTTTTT CAGTCGGTGG CGTACGACGT TAAACGTTCC GGGGTTGATC GAAAAAGGTT AGTCTAAAAC   
  
  
+ AGTAGAGTAT ATATATATAT ATATATCTTT ATTAAACAAA GTTTTATTAG TTCAAATATA TAATTTTTAT   
  
  
+ CTTATTTTTT AATATTTAAT ATAAAATAAA AATAAAATGT AAAATTTTAT ATTATATATA TAAATTTATT   
  
  
+ AAAAATTTTT TAATAAAAAA ATATTAATAA ACTTTTTTTA GATTAAATTT AGTTTAATTT TCTAATTTAT   
  
  
+ GTGTTATTTA ATTAATTATT TCATTTTAGA TATAATTTTA TTAAATTTAA AAATAATACA AATGAAGTGT   
  
  
+ AATTATCTAT ATACTAAGAA CTGGTTATAT TTTAATATAG TTTTTTTACA TTACAGTTTA AAAATTATTA   
  
  
+ TTATATTAAA ACTGAAACTT TGCACATCGA TAGTTAACGA GACTCTTTTT AAAATAATAT TTAAGCAAGT   
  
  
+ ATTATTATAA ATTTTATAGT TTGAAGTATA TAGTATTTTA ATAAAAATTA GATATATTTG AAATTATTTT   
  
  
+ TATATATTGT AGTATTAAAA TTTTCAACTT AATGCTTGAA ATAATATATA TATGTTTCTA GAGTATTTTA   
  
  
+ AAATTATAAA TGAAATTTAA TGATTAGTT  

- CAAGATTTCT GTTTACTAAA CAAGATCGAT CTGAGAGAAA AAGGATAACT GTTGGGCTGA CTTTCAAGAC   
  
  
- TTTTCAGAGA GAATCGTGAA GAGATAGACA TTTGCTAGAG GAAGGCAAGG TTTGTTTATA TATATTGGAG   
  
  
- AGAGACTCAG ATTTCTTTAC GGCAAGTATC CATATTCAAC ACCTCTTCTC CTTCCATGTG AACCAGGATA   
  
  
- TGCAGCATTC GTTGAGTTGA CTTGTCATAA AGTCAACCCA AAAATGATAA TTCATACCAA AAGTTTAATC   
  
  
- TTAGAGTACT GAAAACTTTA TTGTATTAGG ACTTGCACAT AAATCTTCAT TAATCATCAC CGTTGACTAT   
  
  
- TTCTTTACAA AAATTTTACA ATTTTTAATA ATTATAAAAT CTCAATCATT CATCATTTAA AAAATACTAA   
  
  
- GTTTTTTTTA TCAAATGGGA AAGATTAAGA AAGAAAATTG TGGATTTGGT GATGAAAGAA GGGCATGATT   
  
  
- GGTTCCTTTG ACTTTCACAC TCTTAATTAG AGTGTGAATT TAATTTTTTT ATTTATCTGA ATTATGTTAA   
  
  
- TTTTTTGATA AAATAATAAT AATAAATAAT TTAATAAAAT TAATGTAATT AATTTTTTTA TTTTTATATT   
  
  
- AATATAATAA ATTATTAAAA TATTTTTTAA ATATTTTAAA AAGACTTAAC GTTAATTTAG ATGAAGAGAA   
  
  
- GAACTTGATC GCTAGGGAGC ATAATTTGCT CGAGAGTCTT ACCTCGTCTC TATTTGTTTG TCGGAGAGCT   
  
  
- CCTGTTTGGC CAATGAGATA AGAAGGAAAA TTGCATTGGC AGAAAGGATT TCAAAGTCAA AGAAACAAAA   
  
  
- TGACGAAAAA GTCAGCCACC GCATGCTGCA ATTTGCAAGG CCCCAACTAG CTTTTTCCAA TCAGATTTTG   
  
  
- TCATCTCATA TATATATATA TATATAGAAA TAATTTGTTT CAAAATAATC AAGTTTATAT ATTAAAAATA   
  
  
- GAATAAAAAA TTATAAATTA TATTTTATTT TTATTTTACA TTTTAAAATA TAATATATAT ATTTAAATAA   
  
  
- TTTTTAAAAA ATTATTTTTT TATAATTATT TGAAAAAAAT CTAATTTAAA TCAAATTAAA AGATTAAATA   
  
  
- CACAATAAAT TAATTAATAA AGTAAAATCT ATATTAAAAT AATTTAAATT TTTATTATGT TTACTTCACA   
  
  
- TTAATAGATA TATGATTCTT GACCAATATA AAATTATATC AAAAAAATGT AATGTCAAAT TTTTAATAAT   
  
  
- AATATAATTT TGACTTTGAA ACGTGTAGCT ATCAATTGCT CTGAGAAAAA TTTTATTATA AATTCGTTCA   
  
  
- TAATAATATT TAAAATATCA AACTTCATAT ATCATAAAAT TATTTTTAAT CTATATAAAC TTTAATAAAA   
  
  
- ATATATAACA TCATAATTTT AAAAGTTGAA TTACGAACTT TATTATATAT ATACAAAGAT CTCATAAAAT   
  
  
- TTTAATATTT ACTTTAAATT ACTAATCAA

+     Box I

| Site Name | Organism | Position | Strand | Matrix score. | sequence | function |
| --- | --- | --- | --- | --- | --- | --- |
| Box I | Pisum sativum | 293 | - | 7 | TTTCAAA | light responsive element |
| Box I | Pisum sativum | 1387 | - | 7 | TTTCAAA | light responsive element |
| Box I | Pisum sativum | 270 | + | 7 | TTTCAAA | light responsive element |

> 2018/04/13 10:10:12  
+ GTTCTAAAGA CAAATGATTT GTTCTAGCTA GACTCTCTTT TTCCTATTGA CAACCCGACT GAAAGTTCTG   
  
  
+ AAAAGTCTCT CTTAGCACTT CTCTATCTGT AAACGATCTC CTTCCGTTCC AAACAAATAT ATATAACCTC   
  
  
+ TCTCTGAGTC TAAAGAAATG CCGTTCATAG GTATAAGTTG TGGAGAAGAG GAAGGTACAC TTGGTCCTAT   
  
  
+ ACGTCGTAAG CAACTCAACT GAACAGTATT TCAGTTGGGT TTTTACTATT AAGTATGGTT TTCAAATTAG   
  
  
+ AATCTCATGA CTTTTGAAAT AACATAATCC TGAACGTGTA TTTAGAAGTA ATTAGTAGTG GCAACTGATA   
  
  
+ AAGAAATGTT TTTAAAATGT TAAAAATTAT TAATATTTTA GAGTTAGTAA GTAGTAAATT TTTTATGATT   
  
  
+ CAAAAAAAAT AGTTTACCCT TTCTAATTCT TTCTTTTAAC ACCTAAACCA CTACTTTCTT CCCGTACTAA   
  
  
+ CCAAGGAAAC TGAAAGTGTG AGAATTAATC TCACACTTAA ATTAAAAAAA TAAATAGACT TAATACAATT   
  
  
+ AAAAAACTAT TTTATTATTA TTATTTATTA AATTATTTTA ATTACATTAA TTAAAAAAAT AAAAATATAA   
  
  
+ TTATATTATT TAATAATTTT ATAAAAAATT TATAAAATTT TTCTGAATTG CAATTAAATC TACTTCTCTT   
  
  
+ CTTGAACTAG CGATCCCTCG TATTAAACGA GCTCTCAGAA TGGAGCAGAG ATAAACAAAC AGCCTCTCGA   
  
  
+ GGACAAACCG GTTACTCTAT TCTTCCTTTT AACGTAACCG TCTTTCCTAA AGTTTCAGTT TCTTTGTTTT   
  
  
+ ACTGCTTTTT CAGTCGGTGG CGTACGACGT TAAACGTTCC GGGGTTGATC GAAAAAGGTT AGTCTAAAAC   
  
  
+ AGTAGAGTAT ATATATATAT ATATATCTTT ATTAAACAAA GTTTTATTAG TTCAAATATA TAATTTTTAT   
  
  
+ CTTATTTTTT AATATTTAAT ATAAAATAAA AATAAAATGT AAAATTTTAT ATTATATATA TAAATTTATT   
  
  
+ AAAAATTTTT TAATAAAAAA ATATTAATAA ACTTTTTTTA GATTAAATTT AGTTTAATTT TCTAATTTAT   
  
  
+ GTGTTATTTA ATTAATTATT TCATTTTAGA TATAATTTTA TTAAATTTAA AAATAATACA AATGAAGTGT   
  
  
+ AATTATCTAT ATACTAAGAA CTGGTTATAT TTTAATATAG TTTTTTTACA TTACAGTTTA AAAATTATTA   
  
  
+ TTATATTAAA ACTGAAACTT TGCACATCGA TAGTTAACGA GACTCTTTTT AAAATAATAT TTAAGCAAGT   
  
  
+ ATTATTATAA ATTTTATAGT TTGAAGTATA TAGTATTTTA ATAAAAATTA GATATATTTG AAATTATTTT   
  
  
+ TATATATTGT AGTATTAAAA TTTTCAACTT AATGCTTGAA ATAATATATA TATGTTTCTA GAGTATTTTA   
  
  
+ AAATTATAAA TGAAATTTAA TGATTAGTT  

- CAAGATTTCT GTTTACTAAA CAAGATCGAT CTGAGAGAAA AAGGATAACT GTTGGGCTGA CTTTCAAGAC   
  
  
- TTTTCAGAGA GAATCGTGAA GAGATAGACA TTTGCTAGAG GAAGGCAAGG TTTGTTTATA TATATTGGAG   
  
  
- AGAGACTCAG ATTTCTTTAC GGCAAGTATC CATATTCAAC ACCTCTTCTC CTTCCATGTG AACCAGGATA   
  
  
- TGCAGCATTC GTTGAGTTGA CTTGTCATAA AGTCAACCCA AAAATGATAA TTCATACCAA AAGTTTAATC   
  
  
- TTAGAGTACT GAAAACTTTA TTGTATTAGG ACTTGCACAT AAATCTTCAT TAATCATCAC CGTTGACTAT   
  
  
- TTCTTTACAA AAATTTTACA ATTTTTAATA ATTATAAAAT CTCAATCATT CATCATTTAA AAAATACTAA   
  
  
- GTTTTTTTTA TCAAATGGGA AAGATTAAGA AAGAAAATTG TGGATTTGGT GATGAAAGAA GGGCATGATT   
  
  
- GGTTCCTTTG ACTTTCACAC TCTTAATTAG AGTGTGAATT TAATTTTTTT ATTTATCTGA ATTATGTTAA   
  
  
- TTTTTTGATA AAATAATAAT AATAAATAAT TTAATAAAAT TAATGTAATT AATTTTTTTA TTTTTATATT   
  
  
- AATATAATAA ATTATTAAAA TATTTTTTAA ATATTTTAAA AAGACTTAAC GTTAATTTAG ATGAAGAGAA   
  
  
- GAACTTGATC GCTAGGGAGC ATAATTTGCT CGAGAGTCTT ACCTCGTCTC TATTTGTTTG TCGGAGAGCT   
  
  
- CCTGTTTGGC CAATGAGATA AGAAGGAAAA TTGCATTGGC AGAAAGGATT TCAAAGTCAA AGAAACAAAA   
  
  
- TGACGAAAAA GTCAGCCACC GCATGCTGCA ATTTGCAAGG CCCCAACTAG CTTTTTCCAA TCAGATTTTG   
  
  
- TCATCTCATA TATATATATA TATATAGAAA TAATTTGTTT CAAAATAATC AAGTTTATAT ATTAAAAATA   
  
  
- GAATAAAAAA TTATAAATTA TATTTTATTT TTATTTTACA TTTTAAAATA TAATATATAT ATTTAAATAA   
  
  
- TTTTTAAAAA ATTATTTTTT TATAATTATT TGAAAAAAAT CTAATTTAAA TCAAATTAAA AGATTAAATA   
  
  
- CACAATAAAT TAATTAATAA AGTAAAATCT ATATTAAAAT AATTTAAATT TTTATTATGT TTACTTCACA   
  
  
- TTAATAGATA TATGATTCTT GACCAATATA AAATTATATC AAAAAAATGT AATGTCAAAT TTTTAATAAT   
  
  
- AATATAATTT TGACTTTGAA ACGTGTAGCT ATCAATTGCT CTGAGAAAAA TTTTATTATA AATTCGTTCA   
  
  
- TAATAATATT TAAAATATCA AACTTCATAT ATCATAAAAT TATTTTTAAT CTATATAAAC TTTAATAAAA   
  
  
- ATATATAACA TCATAATTTT AAAAGTTGAA TTACGAACTT TATTATATAT ATACAAAGAT CTCATAAAAT   
  
  
- TTTAATATTT ACTTTAAATT ACTAATCAA

+     CAAT-box

| Site Name | Organism | Position | Strand | Matrix score. | sequence | function |
| --- | --- | --- | --- | --- | --- | --- |
| CAAT-box | Brassica rapa | 963 | + | 5 | CAAAT | common cis-acting element in promoter and enhancer regions |
| CAAT-box | Hordeum vulgare | 1406 | - | 4 | CAAT | common cis-acting element in promoter and enhancer regions |
| CAAT-box | Hordeum vulgare | 46 | - | 4 | CAAT | common cis-acting element in promoter and enhancer regions |
| CAAT-box | Glycine max | 556 | + | 5 | CAATT | common cis-acting element in promoter and enhancer regions |
| CAAT-box | Brassica rapa | 273 | + | 5 | CAAAT | common cis-acting element in promoter and enhancer regions |
| CAAT-box | Brassica rapa | 124 | + | 5 | CAAAT | common cis-acting element in promoter and enhancer regions |
| CAAT-box | Brassica rapa | 17 | - | 5 | CAAAT | common cis-acting element in promoter and enhancer regions |
| CAAT-box | Brassica rapa | 11 | + | 5 | CAAAT | common cis-acting element in promoter and enhancer regions |
| CAAT-box | Brassica rapa | 1179 | + | 5 | CAAAT | common cis-acting element in promoter and enhancer regions |
| CAAT-box | Brassica rapa | 1386 | - | 5 | CAAAT | common cis-acting element in promoter and enhancer regions |
| CAAT-box | Hordeum vulgare | 677 | - | 4 | CAAT | common cis-acting element in promoter and enhancer regions |
| CAAT-box | Glycine max | 676 | - | 5 | CAATT | common cis-acting element in promoter and enhancer regions |
| CAAT-box | Glycine max | 681 | + | 5 | CAATT | common cis-acting element in promoter and enhancer regions |

> 2018/04/13 10:10:12  
+ GTTCTAAAGA CAAATGATTT GTTCTAGCTA GACTCTCTTT TTCCTATTGA CAACCCGACT GAAAGTTCTG   
  
  
+ AAAAGTCTCT CTTAGCACTT CTCTATCTGT AAACGATCTC CTTCCGTTCC AAACAAATAT ATATAACCTC   
  
  
+ TCTCTGAGTC TAAAGAAATG CCGTTCATAG GTATAAGTTG TGGAGAAGAG GAAGGTACAC TTGGTCCTAT   
  
  
+ ACGTCGTAAG CAACTCAACT GAACAGTATT TCAGTTGGGT TTTTACTATT AAGTATGGTT TTCAAATTAG   
  
  
+ AATCTCATGA CTTTTGAAAT AACATAATCC TGAACGTGTA TTTAGAAGTA ATTAGTAGTG GCAACTGATA   
  
  
+ AAGAAATGTT TTTAAAATGT TAAAAATTAT TAATATTTTA GAGTTAGTAA GTAGTAAATT TTTTATGATT   
  
  
+ CAAAAAAAAT AGTTTACCCT TTCTAATTCT TTCTTTTAAC ACCTAAACCA CTACTTTCTT CCCGTACTAA   
  
  
+ CCAAGGAAAC TGAAAGTGTG AGAATTAATC TCACACTTAA ATTAAAAAAA TAAATAGACT TAATACAATT   
  
  
+ AAAAAACTAT TTTATTATTA TTATTTATTA AATTATTTTA ATTACATTAA TTAAAAAAAT AAAAATATAA   
  
  
+ TTATATTATT TAATAATTTT ATAAAAAATT TATAAAATTT TTCTGAATTG CAATTAAATC TACTTCTCTT   
  
  
+ CTTGAACTAG CGATCCCTCG TATTAAACGA GCTCTCAGAA TGGAGCAGAG ATAAACAAAC AGCCTCTCGA   
  
  
+ GGACAAACCG GTTACTCTAT TCTTCCTTTT AACGTAACCG TCTTTCCTAA AGTTTCAGTT TCTTTGTTTT   
  
  
+ ACTGCTTTTT CAGTCGGTGG CGTACGACGT TAAACGTTCC GGGGTTGATC GAAAAAGGTT AGTCTAAAAC   
  
  
+ AGTAGAGTAT ATATATATAT ATATATCTTT ATTAAACAAA GTTTTATTAG TTCAAATATA TAATTTTTAT   
  
  
+ CTTATTTTTT AATATTTAAT ATAAAATAAA AATAAAATGT AAAATTTTAT ATTATATATA TAAATTTATT   
  
  
+ AAAAATTTTT TAATAAAAAA ATATTAATAA ACTTTTTTTA GATTAAATTT AGTTTAATTT TCTAATTTAT   
  
  
+ GTGTTATTTA ATTAATTATT TCATTTTAGA TATAATTTTA TTAAATTTAA AAATAATACA AATGAAGTGT   
  
  
+ AATTATCTAT ATACTAAGAA CTGGTTATAT TTTAATATAG TTTTTTTACA TTACAGTTTA AAAATTATTA   
  
  
+ TTATATTAAA ACTGAAACTT TGCACATCGA TAGTTAACGA GACTCTTTTT AAAATAATAT TTAAGCAAGT   
  
  
+ ATTATTATAA ATTTTATAGT TTGAAGTATA TAGTATTTTA ATAAAAATTA GATATATTTG AAATTATTTT   
  
  
+ TATATATTGT AGTATTAAAA TTTTCAACTT AATGCTTGAA ATAATATATA TATGTTTCTA GAGTATTTTA   
  
  
+ AAATTATAAA TGAAATTTAA TGATTAGTT  

- CAAGATTTCT GTTTACTAAA CAAGATCGAT CTGAGAGAAA AAGGATAACT GTTGGGCTGA CTTTCAAGAC   
  
  
- TTTTCAGAGA GAATCGTGAA GAGATAGACA TTTGCTAGAG GAAGGCAAGG TTTGTTTATA TATATTGGAG   
  
  
- AGAGACTCAG ATTTCTTTAC GGCAAGTATC CATATTCAAC ACCTCTTCTC CTTCCATGTG AACCAGGATA   
  
  
- TGCAGCATTC GTTGAGTTGA CTTGTCATAA AGTCAACCCA AAAATGATAA TTCATACCAA AAGTTTAATC   
  
  
- TTAGAGTACT GAAAACTTTA TTGTATTAGG ACTTGCACAT AAATCTTCAT TAATCATCAC CGTTGACTAT   
  
  
- TTCTTTACAA AAATTTTACA ATTTTTAATA ATTATAAAAT CTCAATCATT CATCATTTAA AAAATACTAA   
  
  
- GTTTTTTTTA TCAAATGGGA AAGATTAAGA AAGAAAATTG TGGATTTGGT GATGAAAGAA GGGCATGATT   
  
  
- GGTTCCTTTG ACTTTCACAC TCTTAATTAG AGTGTGAATT TAATTTTTTT ATTTATCTGA ATTATGTTAA   
  
  
- TTTTTTGATA AAATAATAAT AATAAATAAT TTAATAAAAT TAATGTAATT AATTTTTTTA TTTTTATATT   
  
  
- AATATAATAA ATTATTAAAA TATTTTTTAA ATATTTTAAA AAGACTTAAC GTTAATTTAG ATGAAGAGAA   
  
  
- GAACTTGATC GCTAGGGAGC ATAATTTGCT CGAGAGTCTT ACCTCGTCTC TATTTGTTTG TCGGAGAGCT   
  
  
- CCTGTTTGGC CAATGAGATA AGAAGGAAAA TTGCATTGGC AGAAAGGATT TCAAAGTCAA AGAAACAAAA   
  
  
- TGACGAAAAA GTCAGCCACC GCATGCTGCA ATTTGCAAGG CCCCAACTAG CTTTTTCCAA TCAGATTTTG   
  
  
- TCATCTCATA TATATATATA TATATAGAAA TAATTTGTTT CAAAATAATC AAGTTTATAT ATTAAAAATA   
  
  
- GAATAAAAAA TTATAAATTA TATTTTATTT TTATTTTACA TTTTAAAATA TAATATATAT ATTTAAATAA   
  
  
- TTTTTAAAAA ATTATTTTTT TATAATTATT TGAAAAAAAT CTAATTTAAA TCAAATTAAA AGATTAAATA   
  
  
- CACAATAAAT TAATTAATAA AGTAAAATCT ATATTAAAAT AATTTAAATT TTTATTATGT TTACTTCACA   
  
  
- TTAATAGATA TATGATTCTT GACCAATATA AAATTATATC AAAAAAATGT AATGTCAAAT TTTTAATAAT   
  
  
- AATATAATTT TGACTTTGAA ACGTGTAGCT ATCAATTGCT CTGAGAAAAA TTTTATTATA AATTCGTTCA   
  
  
- TAATAATATT TAAAATATCA AACTTCATAT ATCATAAAAT TATTTTTAAT CTATATAAAC TTTAATAAAA   
  
  
- ATATATAACA TCATAATTTT AAAAGTTGAA TTACGAACTT TATTATATAT ATACAAAGAT CTCATAAAAT   
  
  
- TTTAATATTT ACTTTAAATT ACTAATCAA

+     CAT-box

| Site Name | Organism | Position | Strand | Matrix score. | sequence | function |
| --- | --- | --- | --- | --- | --- | --- |
| CAT-box | Arabidopsis thaliana | 337 | - | 6 | GCCACT | cis-acting regulatory element related to meristem expression |

> 2018/04/13 10:10:12  
+ GTTCTAAAGA CAAATGATTT GTTCTAGCTA GACTCTCTTT TTCCTATTGA CAACCCGACT GAAAGTTCTG   
  
  
+ AAAAGTCTCT CTTAGCACTT CTCTATCTGT AAACGATCTC CTTCCGTTCC AAACAAATAT ATATAACCTC   
  
  
+ TCTCTGAGTC TAAAGAAATG CCGTTCATAG GTATAAGTTG TGGAGAAGAG GAAGGTACAC TTGGTCCTAT   
  
  
+ ACGTCGTAAG CAACTCAACT GAACAGTATT TCAGTTGGGT TTTTACTATT AAGTATGGTT TTCAAATTAG   
  
  
+ AATCTCATGA CTTTTGAAAT AACATAATCC TGAACGTGTA TTTAGAAGTA ATTAGTAGTG GCAACTGATA   
  
  
+ AAGAAATGTT TTTAAAATGT TAAAAATTAT TAATATTTTA GAGTTAGTAA GTAGTAAATT TTTTATGATT   
  
  
+ CAAAAAAAAT AGTTTACCCT TTCTAATTCT TTCTTTTAAC ACCTAAACCA CTACTTTCTT CCCGTACTAA   
  
  
+ CCAAGGAAAC TGAAAGTGTG AGAATTAATC TCACACTTAA ATTAAAAAAA TAAATAGACT TAATACAATT   
  
  
+ AAAAAACTAT TTTATTATTA TTATTTATTA AATTATTTTA ATTACATTAA TTAAAAAAAT AAAAATATAA   
  
  
+ TTATATTATT TAATAATTTT ATAAAAAATT TATAAAATTT TTCTGAATTG CAATTAAATC TACTTCTCTT   
  
  
+ CTTGAACTAG CGATCCCTCG TATTAAACGA GCTCTCAGAA TGGAGCAGAG ATAAACAAAC AGCCTCTCGA   
  
  
+ GGACAAACCG GTTACTCTAT TCTTCCTTTT AACGTAACCG TCTTTCCTAA AGTTTCAGTT TCTTTGTTTT   
  
  
+ ACTGCTTTTT CAGTCGGTGG CGTACGACGT TAAACGTTCC GGGGTTGATC GAAAAAGGTT AGTCTAAAAC   
  
  
+ AGTAGAGTAT ATATATATAT ATATATCTTT ATTAAACAAA GTTTTATTAG TTCAAATATA TAATTTTTAT   
  
  
+ CTTATTTTTT AATATTTAAT ATAAAATAAA AATAAAATGT AAAATTTTAT ATTATATATA TAAATTTATT   
  
  
+ AAAAATTTTT TAATAAAAAA ATATTAATAA ACTTTTTTTA GATTAAATTT AGTTTAATTT TCTAATTTAT   
  
  
+ GTGTTATTTA ATTAATTATT TCATTTTAGA TATAATTTTA TTAAATTTAA AAATAATACA AATGAAGTGT   
  
  
+ AATTATCTAT ATACTAAGAA CTGGTTATAT TTTAATATAG TTTTTTTACA TTACAGTTTA AAAATTATTA   
  
  
+ TTATATTAAA ACTGAAACTT TGCACATCGA TAGTTAACGA GACTCTTTTT AAAATAATAT TTAAGCAAGT   
  
  
+ ATTATTATAA ATTTTATAGT TTGAAGTATA TAGTATTTTA ATAAAAATTA GATATATTTG AAATTATTTT   
  
  
+ TATATATTGT AGTATTAAAA TTTTCAACTT AATGCTTGAA ATAATATATA TATGTTTCTA GAGTATTTTA   
  
  
+ AAATTATAAA TGAAATTTAA TGATTAGTT  

- CAAGATTTCT GTTTACTAAA CAAGATCGAT CTGAGAGAAA AAGGATAACT GTTGGGCTGA CTTTCAAGAC   
  
  
- TTTTCAGAGA GAATCGTGAA GAGATAGACA TTTGCTAGAG GAAGGCAAGG TTTGTTTATA TATATTGGAG   
  
  
- AGAGACTCAG ATTTCTTTAC GGCAAGTATC CATATTCAAC ACCTCTTCTC CTTCCATGTG AACCAGGATA   
  
  
- TGCAGCATTC GTTGAGTTGA CTTGTCATAA AGTCAACCCA AAAATGATAA TTCATACCAA AAGTTTAATC   
  
  
- TTAGAGTACT GAAAACTTTA TTGTATTAGG ACTTGCACAT AAATCTTCAT TAATCATCAC CGTTGACTAT   
  
  
- TTCTTTACAA AAATTTTACA ATTTTTAATA ATTATAAAAT CTCAATCATT CATCATTTAA AAAATACTAA   
  
  
- GTTTTTTTTA TCAAATGGGA AAGATTAAGA AAGAAAATTG TGGATTTGGT GATGAAAGAA GGGCATGATT   
  
  
- GGTTCCTTTG ACTTTCACAC TCTTAATTAG AGTGTGAATT TAATTTTTTT ATTTATCTGA ATTATGTTAA   
  
  
- TTTTTTGATA AAATAATAAT AATAAATAAT TTAATAAAAT TAATGTAATT AATTTTTTTA TTTTTATATT   
  
  
- AATATAATAA ATTATTAAAA TATTTTTTAA ATATTTTAAA AAGACTTAAC GTTAATTTAG ATGAAGAGAA   
  
  
- GAACTTGATC GCTAGGGAGC ATAATTTGCT CGAGAGTCTT ACCTCGTCTC TATTTGTTTG TCGGAGAGCT   
  
  
- CCTGTTTGGC CAATGAGATA AGAAGGAAAA TTGCATTGGC AGAAAGGATT TCAAAGTCAA AGAAACAAAA   
  
  
- TGACGAAAAA GTCAGCCACC GCATGCTGCA ATTTGCAAGG CCCCAACTAG CTTTTTCCAA TCAGATTTTG   
  
  
- TCATCTCATA TATATATATA TATATAGAAA TAATTTGTTT CAAAATAATC AAGTTTATAT ATTAAAAATA   
  
  
- GAATAAAAAA TTATAAATTA TATTTTATTT TTATTTTACA TTTTAAAATA TAATATATAT ATTTAAATAA   
  
  
- TTTTTAAAAA ATTATTTTTT TATAATTATT TGAAAAAAAT CTAATTTAAA TCAAATTAAA AGATTAAATA   
  
  
- CACAATAAAT TAATTAATAA AGTAAAATCT ATATTAAAAT AATTTAAATT TTTATTATGT TTACTTCACA   
  
  
- TTAATAGATA TATGATTCTT GACCAATATA AAATTATATC AAAAAAATGT AATGTCAAAT TTTTAATAAT   
  
  
- AATATAATTT TGACTTTGAA ACGTGTAGCT ATCAATTGCT CTGAGAAAAA TTTTATTATA AATTCGTTCA   
  
  
- TAATAATATT TAAAATATCA AACTTCATAT ATCATAAAAT TATTTTTAAT CTATATAAAC TTTAATAAAA   
  
  
- ATATATAACA TCATAATTTT AAAAGTTGAA TTACGAACTT TATTATATAT ATACAAAGAT CTCATAAAAT   
  
  
- TTTAATATTT ACTTTAAATT ACTAATCAA

+     ERE

| Site Name | Organism | Position | Strand | Matrix score. | sequence | function |
| --- | --- | --- | --- | --- | --- | --- |
| ERE | Dianthus caryophyllus | 293 | - | 8 | ATTTCAAA | ethylene-responsive element |
| ERE | Dianthus caryophyllus | 1387 | - | 8 | ATTTCAAA | ethylene-responsive element |

> 2018/04/13 10:10:12  
+ GTTCTAAAGA CAAATGATTT GTTCTAGCTA GACTCTCTTT TTCCTATTGA CAACCCGACT GAAAGTTCTG   
  
  
+ AAAAGTCTCT CTTAGCACTT CTCTATCTGT AAACGATCTC CTTCCGTTCC AAACAAATAT ATATAACCTC   
  
  
+ TCTCTGAGTC TAAAGAAATG CCGTTCATAG GTATAAGTTG TGGAGAAGAG GAAGGTACAC TTGGTCCTAT   
  
  
+ ACGTCGTAAG CAACTCAACT GAACAGTATT TCAGTTGGGT TTTTACTATT AAGTATGGTT TTCAAATTAG   
  
  
+ AATCTCATGA CTTTTGAAAT AACATAATCC TGAACGTGTA TTTAGAAGTA ATTAGTAGTG GCAACTGATA   
  
  
+ AAGAAATGTT TTTAAAATGT TAAAAATTAT TAATATTTTA GAGTTAGTAA GTAGTAAATT TTTTATGATT   
  
  
+ CAAAAAAAAT AGTTTACCCT TTCTAATTCT TTCTTTTAAC ACCTAAACCA CTACTTTCTT CCCGTACTAA   
  
  
+ CCAAGGAAAC TGAAAGTGTG AGAATTAATC TCACACTTAA ATTAAAAAAA TAAATAGACT TAATACAATT   
  
  
+ AAAAAACTAT TTTATTATTA TTATTTATTA AATTATTTTA ATTACATTAA TTAAAAAAAT AAAAATATAA   
  
  
+ TTATATTATT TAATAATTTT ATAAAAAATT TATAAAATTT TTCTGAATTG CAATTAAATC TACTTCTCTT   
  
  
+ CTTGAACTAG CGATCCCTCG TATTAAACGA GCTCTCAGAA TGGAGCAGAG ATAAACAAAC AGCCTCTCGA   
  
  
+ GGACAAACCG GTTACTCTAT TCTTCCTTTT AACGTAACCG TCTTTCCTAA AGTTTCAGTT TCTTTGTTTT   
  
  
+ ACTGCTTTTT CAGTCGGTGG CGTACGACGT TAAACGTTCC GGGGTTGATC GAAAAAGGTT AGTCTAAAAC   
  
  
+ AGTAGAGTAT ATATATATAT ATATATCTTT ATTAAACAAA GTTTTATTAG TTCAAATATA TAATTTTTAT   
  
  
+ CTTATTTTTT AATATTTAAT ATAAAATAAA AATAAAATGT AAAATTTTAT ATTATATATA TAAATTTATT   
  
  
+ AAAAATTTTT TAATAAAAAA ATATTAATAA ACTTTTTTTA GATTAAATTT AGTTTAATTT TCTAATTTAT   
  
  
+ GTGTTATTTA ATTAATTATT TCATTTTAGA TATAATTTTA TTAAATTTAA AAATAATACA AATGAAGTGT   
  
  
+ AATTATCTAT ATACTAAGAA CTGGTTATAT TTTAATATAG TTTTTTTACA TTACAGTTTA AAAATTATTA   
  
  
+ TTATATTAAA ACTGAAACTT TGCACATCGA TAGTTAACGA GACTCTTTTT AAAATAATAT TTAAGCAAGT   
  
  
+ ATTATTATAA ATTTTATAGT TTGAAGTATA TAGTATTTTA ATAAAAATTA GATATATTTG AAATTATTTT   
  
  
+ TATATATTGT AGTATTAAAA TTTTCAACTT AATGCTTGAA ATAATATATA TATGTTTCTA GAGTATTTTA   
  
  
+ AAATTATAAA TGAAATTTAA TGATTAGTT  

- CAAGATTTCT GTTTACTAAA CAAGATCGAT CTGAGAGAAA AAGGATAACT GTTGGGCTGA CTTTCAAGAC   
  
  
- TTTTCAGAGA GAATCGTGAA GAGATAGACA TTTGCTAGAG GAAGGCAAGG TTTGTTTATA TATATTGGAG   
  
  
- AGAGACTCAG ATTTCTTTAC GGCAAGTATC CATATTCAAC ACCTCTTCTC CTTCCATGTG AACCAGGATA   
  
  
- TGCAGCATTC GTTGAGTTGA CTTGTCATAA AGTCAACCCA AAAATGATAA TTCATACCAA AAGTTTAATC   
  
  
- TTAGAGTACT GAAAACTTTA TTGTATTAGG ACTTGCACAT AAATCTTCAT TAATCATCAC CGTTGACTAT   
  
  
- TTCTTTACAA AAATTTTACA ATTTTTAATA ATTATAAAAT CTCAATCATT CATCATTTAA AAAATACTAA   
  
  
- GTTTTTTTTA TCAAATGGGA AAGATTAAGA AAGAAAATTG TGGATTTGGT GATGAAAGAA GGGCATGATT   
  
  
- GGTTCCTTTG ACTTTCACAC TCTTAATTAG AGTGTGAATT TAATTTTTTT ATTTATCTGA ATTATGTTAA   
  
  
- TTTTTTGATA AAATAATAAT AATAAATAAT TTAATAAAAT TAATGTAATT AATTTTTTTA TTTTTATATT   
  
  
- AATATAATAA ATTATTAAAA TATTTTTTAA ATATTTTAAA AAGACTTAAC GTTAATTTAG ATGAAGAGAA   
  
  
- GAACTTGATC GCTAGGGAGC ATAATTTGCT CGAGAGTCTT ACCTCGTCTC TATTTGTTTG TCGGAGAGCT   
  
  
- CCTGTTTGGC CAATGAGATA AGAAGGAAAA TTGCATTGGC AGAAAGGATT TCAAAGTCAA AGAAACAAAA   
  
  
- TGACGAAAAA GTCAGCCACC GCATGCTGCA ATTTGCAAGG CCCCAACTAG CTTTTTCCAA TCAGATTTTG   
  
  
- TCATCTCATA TATATATATA TATATAGAAA TAATTTGTTT CAAAATAATC AAGTTTATAT ATTAAAAATA   
  
  
- GAATAAAAAA TTATAAATTA TATTTTATTT TTATTTTACA TTTTAAAATA TAATATATAT ATTTAAATAA   
  
  
- TTTTTAAAAA ATTATTTTTT TATAATTATT TGAAAAAAAT CTAATTTAAA TCAAATTAAA AGATTAAATA   
  
  
- CACAATAAAT TAATTAATAA AGTAAAATCT ATATTAAAAT AATTTAAATT TTTATTATGT TTACTTCACA   
  
  
- TTAATAGATA TATGATTCTT GACCAATATA AAATTATATC AAAAAAATGT AATGTCAAAT TTTTAATAAT   
  
  
- AATATAATTT TGACTTTGAA ACGTGTAGCT ATCAATTGCT CTGAGAAAAA TTTTATTATA AATTCGTTCA   
  
  
- TAATAATATT TAAAATATCA AACTTCATAT ATCATAAAAT TATTTTTAAT CTATATAAAC TTTAATAAAA   
  
  
- ATATATAACA TCATAATTTT AAAAGTTGAA TTACGAACTT TATTATATAT ATACAAAGAT CTCATAAAAT   
  
  
- TTTAATATTT ACTTTAAATT ACTAATCAA

+     G-Box

| Site Name | Organism | Position | Strand | Matrix score. | sequence | function |
| --- | --- | --- | --- | --- | --- | --- |
| G-Box | Pisum sativum | 313 | - | 6 | CACGTT | cis-acting regulatory element involved in light responsiveness |

> 2018/04/13 10:10:12  
+ GTTCTAAAGA CAAATGATTT GTTCTAGCTA GACTCTCTTT TTCCTATTGA CAACCCGACT GAAAGTTCTG   
  
  
+ AAAAGTCTCT CTTAGCACTT CTCTATCTGT AAACGATCTC CTTCCGTTCC AAACAAATAT ATATAACCTC   
  
  
+ TCTCTGAGTC TAAAGAAATG CCGTTCATAG GTATAAGTTG TGGAGAAGAG GAAGGTACAC TTGGTCCTAT   
  
  
+ ACGTCGTAAG CAACTCAACT GAACAGTATT TCAGTTGGGT TTTTACTATT AAGTATGGTT TTCAAATTAG   
  
  
+ AATCTCATGA CTTTTGAAAT AACATAATCC TGAACGTGTA TTTAGAAGTA ATTAGTAGTG GCAACTGATA   
  
  
+ AAGAAATGTT TTTAAAATGT TAAAAATTAT TAATATTTTA GAGTTAGTAA GTAGTAAATT TTTTATGATT   
  
  
+ CAAAAAAAAT AGTTTACCCT TTCTAATTCT TTCTTTTAAC ACCTAAACCA CTACTTTCTT CCCGTACTAA   
  
  
+ CCAAGGAAAC TGAAAGTGTG AGAATTAATC TCACACTTAA ATTAAAAAAA TAAATAGACT TAATACAATT   
  
  
+ AAAAAACTAT TTTATTATTA TTATTTATTA AATTATTTTA ATTACATTAA TTAAAAAAAT AAAAATATAA   
  
  
+ TTATATTATT TAATAATTTT ATAAAAAATT TATAAAATTT TTCTGAATTG CAATTAAATC TACTTCTCTT   
  
  
+ CTTGAACTAG CGATCCCTCG TATTAAACGA GCTCTCAGAA TGGAGCAGAG ATAAACAAAC AGCCTCTCGA   
  
  
+ GGACAAACCG GTTACTCTAT TCTTCCTTTT AACGTAACCG TCTTTCCTAA AGTTTCAGTT TCTTTGTTTT   
  
  
+ ACTGCTTTTT CAGTCGGTGG CGTACGACGT TAAACGTTCC GGGGTTGATC GAAAAAGGTT AGTCTAAAAC   
  
  
+ AGTAGAGTAT ATATATATAT ATATATCTTT ATTAAACAAA GTTTTATTAG TTCAAATATA TAATTTTTAT   
  
  
+ CTTATTTTTT AATATTTAAT ATAAAATAAA AATAAAATGT AAAATTTTAT ATTATATATA TAAATTTATT   
  
  
+ AAAAATTTTT TAATAAAAAA ATATTAATAA ACTTTTTTTA GATTAAATTT AGTTTAATTT TCTAATTTAT   
  
  
+ GTGTTATTTA ATTAATTATT TCATTTTAGA TATAATTTTA TTAAATTTAA AAATAATACA AATGAAGTGT   
  
  
+ AATTATCTAT ATACTAAGAA CTGGTTATAT TTTAATATAG TTTTTTTACA TTACAGTTTA AAAATTATTA   
  
  
+ TTATATTAAA ACTGAAACTT TGCACATCGA TAGTTAACGA GACTCTTTTT AAAATAATAT TTAAGCAAGT   
  
  
+ ATTATTATAA ATTTTATAGT TTGAAGTATA TAGTATTTTA ATAAAAATTA GATATATTTG AAATTATTTT   
  
  
+ TATATATTGT AGTATTAAAA TTTTCAACTT AATGCTTGAA ATAATATATA TATGTTTCTA GAGTATTTTA   
  
  
+ AAATTATAAA TGAAATTTAA TGATTAGTT  

- CAAGATTTCT GTTTACTAAA CAAGATCGAT CTGAGAGAAA AAGGATAACT GTTGGGCTGA CTTTCAAGAC   
  
  
- TTTTCAGAGA GAATCGTGAA GAGATAGACA TTTGCTAGAG GAAGGCAAGG TTTGTTTATA TATATTGGAG   
  
  
- AGAGACTCAG ATTTCTTTAC GGCAAGTATC CATATTCAAC ACCTCTTCTC CTTCCATGTG AACCAGGATA   
  
  
- TGCAGCATTC GTTGAGTTGA CTTGTCATAA AGTCAACCCA AAAATGATAA TTCATACCAA AAGTTTAATC   
  
  
- TTAGAGTACT GAAAACTTTA TTGTATTAGG ACTTGCACAT AAATCTTCAT TAATCATCAC CGTTGACTAT   
  
  
- TTCTTTACAA AAATTTTACA ATTTTTAATA ATTATAAAAT CTCAATCATT CATCATTTAA AAAATACTAA   
  
  
- GTTTTTTTTA TCAAATGGGA AAGATTAAGA AAGAAAATTG TGGATTTGGT GATGAAAGAA GGGCATGATT   
  
  
- GGTTCCTTTG ACTTTCACAC TCTTAATTAG AGTGTGAATT TAATTTTTTT ATTTATCTGA ATTATGTTAA   
  
  
- TTTTTTGATA AAATAATAAT AATAAATAAT TTAATAAAAT TAATGTAATT AATTTTTTTA TTTTTATATT   
  
  
- AATATAATAA ATTATTAAAA TATTTTTTAA ATATTTTAAA AAGACTTAAC GTTAATTTAG ATGAAGAGAA   
  
  
- GAACTTGATC GCTAGGGAGC ATAATTTGCT CGAGAGTCTT ACCTCGTCTC TATTTGTTTG TCGGAGAGCT   
  
  
- CCTGTTTGGC CAATGAGATA AGAAGGAAAA TTGCATTGGC AGAAAGGATT TCAAAGTCAA AGAAACAAAA   
  
  
- TGACGAAAAA GTCAGCCACC GCATGCTGCA ATTTGCAAGG CCCCAACTAG CTTTTTCCAA TCAGATTTTG   
  
  
- TCATCTCATA TATATATATA TATATAGAAA TAATTTGTTT CAAAATAATC AAGTTTATAT ATTAAAAATA   
  
  
- GAATAAAAAA TTATAAATTA TATTTTATTT TTATTTTACA TTTTAAAATA TAATATATAT ATTTAAATAA   
  
  
- TTTTTAAAAA ATTATTTTTT TATAATTATT TGAAAAAAAT CTAATTTAAA TCAAATTAAA AGATTAAATA   
  
  
- CACAATAAAT TAATTAATAA AGTAAAATCT ATATTAAAAT AATTTAAATT TTTATTATGT TTACTTCACA   
  
  
- TTAATAGATA TATGATTCTT GACCAATATA AAATTATATC AAAAAAATGT AATGTCAAAT TTTTAATAAT   
  
  
- AATATAATTT TGACTTTGAA ACGTGTAGCT ATCAATTGCT CTGAGAAAAA TTTTATTATA AATTCGTTCA   
  
  
- TAATAATATT TAAAATATCA AACTTCATAT ATCATAAAAT TATTTTTAAT CTATATAAAC TTTAATAAAA   
  
  
- ATATATAACA TCATAATTTT AAAAGTTGAA TTACGAACTT TATTATATAT ATACAAAGAT CTCATAAAAT   
  
  
- TTTAATATTT ACTTTAAATT ACTAATCAA

+     G-box

| Site Name | Organism | Position | Strand | Matrix score. | sequence | function |
| --- | --- | --- | --- | --- | --- | --- |
| G-box | Zea mays | 313 | - | 6 | CACGTT | cis-acting regulatory element involved in light responsiveness |

> 2018/04/13 10:10:12  
+ GTTCTAAAGA CAAATGATTT GTTCTAGCTA GACTCTCTTT TTCCTATTGA CAACCCGACT GAAAGTTCTG   
  
  
+ AAAAGTCTCT CTTAGCACTT CTCTATCTGT AAACGATCTC CTTCCGTTCC AAACAAATAT ATATAACCTC   
  
  
+ TCTCTGAGTC TAAAGAAATG CCGTTCATAG GTATAAGTTG TGGAGAAGAG GAAGGTACAC TTGGTCCTAT   
  
  
+ ACGTCGTAAG CAACTCAACT GAACAGTATT TCAGTTGGGT TTTTACTATT AAGTATGGTT TTCAAATTAG   
  
  
+ AATCTCATGA CTTTTGAAAT AACATAATCC TGAACGTGTA TTTAGAAGTA ATTAGTAGTG GCAACTGATA   
  
  
+ AAGAAATGTT TTTAAAATGT TAAAAATTAT TAATATTTTA GAGTTAGTAA GTAGTAAATT TTTTATGATT   
  
  
+ CAAAAAAAAT AGTTTACCCT TTCTAATTCT TTCTTTTAAC ACCTAAACCA CTACTTTCTT CCCGTACTAA   
  
  
+ CCAAGGAAAC TGAAAGTGTG AGAATTAATC TCACACTTAA ATTAAAAAAA TAAATAGACT TAATACAATT   
  
  
+ AAAAAACTAT TTTATTATTA TTATTTATTA AATTATTTTA ATTACATTAA TTAAAAAAAT AAAAATATAA   
  
  
+ TTATATTATT TAATAATTTT ATAAAAAATT TATAAAATTT TTCTGAATTG CAATTAAATC TACTTCTCTT   
  
  
+ CTTGAACTAG CGATCCCTCG TATTAAACGA GCTCTCAGAA TGGAGCAGAG ATAAACAAAC AGCCTCTCGA   
  
  
+ GGACAAACCG GTTACTCTAT TCTTCCTTTT AACGTAACCG TCTTTCCTAA AGTTTCAGTT TCTTTGTTTT   
  
  
+ ACTGCTTTTT CAGTCGGTGG CGTACGACGT TAAACGTTCC GGGGTTGATC GAAAAAGGTT AGTCTAAAAC   
  
  
+ AGTAGAGTAT ATATATATAT ATATATCTTT ATTAAACAAA GTTTTATTAG TTCAAATATA TAATTTTTAT   
  
  
+ CTTATTTTTT AATATTTAAT ATAAAATAAA AATAAAATGT AAAATTTTAT ATTATATATA TAAATTTATT   
  
  
+ AAAAATTTTT TAATAAAAAA ATATTAATAA ACTTTTTTTA GATTAAATTT AGTTTAATTT TCTAATTTAT   
  
  
+ GTGTTATTTA ATTAATTATT TCATTTTAGA TATAATTTTA TTAAATTTAA AAATAATACA AATGAAGTGT   
  
  
+ AATTATCTAT ATACTAAGAA CTGGTTATAT TTTAATATAG TTTTTTTACA TTACAGTTTA AAAATTATTA   
  
  
+ TTATATTAAA ACTGAAACTT TGCACATCGA TAGTTAACGA GACTCTTTTT AAAATAATAT TTAAGCAAGT   
  
  
+ ATTATTATAA ATTTTATAGT TTGAAGTATA TAGTATTTTA ATAAAAATTA GATATATTTG AAATTATTTT   
  
  
+ TATATATTGT AGTATTAAAA TTTTCAACTT AATGCTTGAA ATAATATATA TATGTTTCTA GAGTATTTTA   
  
  
+ AAATTATAAA TGAAATTTAA TGATTAGTT  

- CAAGATTTCT GTTTACTAAA CAAGATCGAT CTGAGAGAAA AAGGATAACT GTTGGGCTGA CTTTCAAGAC   
  
  
- TTTTCAGAGA GAATCGTGAA GAGATAGACA TTTGCTAGAG GAAGGCAAGG TTTGTTTATA TATATTGGAG   
  
  
- AGAGACTCAG ATTTCTTTAC GGCAAGTATC CATATTCAAC ACCTCTTCTC CTTCCATGTG AACCAGGATA   
  
  
- TGCAGCATTC GTTGAGTTGA CTTGTCATAA AGTCAACCCA AAAATGATAA TTCATACCAA AAGTTTAATC   
  
  
- TTAGAGTACT GAAAACTTTA TTGTATTAGG ACTTGCACAT AAATCTTCAT TAATCATCAC CGTTGACTAT   
  
  
- TTCTTTACAA AAATTTTACA ATTTTTAATA ATTATAAAAT CTCAATCATT CATCATTTAA AAAATACTAA   
  
  
- GTTTTTTTTA TCAAATGGGA AAGATTAAGA AAGAAAATTG TGGATTTGGT GATGAAAGAA GGGCATGATT   
  
  
- GGTTCCTTTG ACTTTCACAC TCTTAATTAG AGTGTGAATT TAATTTTTTT ATTTATCTGA ATTATGTTAA   
  
  
- TTTTTTGATA AAATAATAAT AATAAATAAT TTAATAAAAT TAATGTAATT AATTTTTTTA TTTTTATATT   
  
  
- AATATAATAA ATTATTAAAA TATTTTTTAA ATATTTTAAA AAGACTTAAC GTTAATTTAG ATGAAGAGAA   
  
  
- GAACTTGATC GCTAGGGAGC ATAATTTGCT CGAGAGTCTT ACCTCGTCTC TATTTGTTTG TCGGAGAGCT   
  
  
- CCTGTTTGGC CAATGAGATA AGAAGGAAAA TTGCATTGGC AGAAAGGATT TCAAAGTCAA AGAAACAAAA   
  
  
- TGACGAAAAA GTCAGCCACC GCATGCTGCA ATTTGCAAGG CCCCAACTAG CTTTTTCCAA TCAGATTTTG   
  
  
- TCATCTCATA TATATATATA TATATAGAAA TAATTTGTTT CAAAATAATC AAGTTTATAT ATTAAAAATA   
  
  
- GAATAAAAAA TTATAAATTA TATTTTATTT TTATTTTACA TTTTAAAATA TAATATATAT ATTTAAATAA   
  
  
- TTTTTAAAAA ATTATTTTTT TATAATTATT TGAAAAAAAT CTAATTTAAA TCAAATTAAA AGATTAAATA   
  
  
- CACAATAAAT TAATTAATAA AGTAAAATCT ATATTAAAAT AATTTAAATT TTTATTATGT TTACTTCACA   
  
  
- TTAATAGATA TATGATTCTT GACCAATATA AAATTATATC AAAAAAATGT AATGTCAAAT TTTTAATAAT   
  
  
- AATATAATTT TGACTTTGAA ACGTGTAGCT ATCAATTGCT CTGAGAAAAA TTTTATTATA AATTCGTTCA   
  
  
- TAATAATATT TAAAATATCA AACTTCATAT ATCATAAAAT TATTTTTAAT CTATATAAAC TTTAATAAAA   
  
  
- ATATATAACA TCATAATTTT AAAAGTTGAA TTACGAACTT TATTATATAT ATACAAAGAT CTCATAAAAT   
  
  
- TTTAATATTT ACTTTAAATT ACTAATCAA

+     GA-motif

| Site Name | Organism | Position | Strand | Matrix score. | sequence | function |
| --- | --- | --- | --- | --- | --- | --- |
| GA-motif | Glycine max | 791 | - | 8 | AAGGAAGA | part of a light responsive element |
| GA-motif | Arabidopsis thaliana | 1193 | - | 8 | ATAGATAA | part of a light responsive element |

> 2018/04/13 10:10:12  
+ GTTCTAAAGA CAAATGATTT GTTCTAGCTA GACTCTCTTT TTCCTATTGA CAACCCGACT GAAAGTTCTG   
  
  
+ AAAAGTCTCT CTTAGCACTT CTCTATCTGT AAACGATCTC CTTCCGTTCC AAACAAATAT ATATAACCTC   
  
  
+ TCTCTGAGTC TAAAGAAATG CCGTTCATAG GTATAAGTTG TGGAGAAGAG GAAGGTACAC TTGGTCCTAT   
  
  
+ ACGTCGTAAG CAACTCAACT GAACAGTATT TCAGTTGGGT TTTTACTATT AAGTATGGTT TTCAAATTAG   
  
  
+ AATCTCATGA CTTTTGAAAT AACATAATCC TGAACGTGTA TTTAGAAGTA ATTAGTAGTG GCAACTGATA   
  
  
+ AAGAAATGTT TTTAAAATGT TAAAAATTAT TAATATTTTA GAGTTAGTAA GTAGTAAATT TTTTATGATT   
  
  
+ CAAAAAAAAT AGTTTACCCT TTCTAATTCT TTCTTTTAAC ACCTAAACCA CTACTTTCTT CCCGTACTAA   
  
  
+ CCAAGGAAAC TGAAAGTGTG AGAATTAATC TCACACTTAA ATTAAAAAAA TAAATAGACT TAATACAATT   
  
  
+ AAAAAACTAT TTTATTATTA TTATTTATTA AATTATTTTA ATTACATTAA TTAAAAAAAT AAAAATATAA   
  
  
+ TTATATTATT TAATAATTTT ATAAAAAATT TATAAAATTT TTCTGAATTG CAATTAAATC TACTTCTCTT   
  
  
+ CTTGAACTAG CGATCCCTCG TATTAAACGA GCTCTCAGAA TGGAGCAGAG ATAAACAAAC AGCCTCTCGA   
  
  
+ GGACAAACCG GTTACTCTAT TCTTCCTTTT AACGTAACCG TCTTTCCTAA AGTTTCAGTT TCTTTGTTTT   
  
  
+ ACTGCTTTTT CAGTCGGTGG CGTACGACGT TAAACGTTCC GGGGTTGATC GAAAAAGGTT AGTCTAAAAC   
  
  
+ AGTAGAGTAT ATATATATAT ATATATCTTT ATTAAACAAA GTTTTATTAG TTCAAATATA TAATTTTTAT   
  
  
+ CTTATTTTTT AATATTTAAT ATAAAATAAA AATAAAATGT AAAATTTTAT ATTATATATA TAAATTTATT   
  
  
+ AAAAATTTTT TAATAAAAAA ATATTAATAA ACTTTTTTTA GATTAAATTT AGTTTAATTT TCTAATTTAT   
  
  
+ GTGTTATTTA ATTAATTATT TCATTTTAGA TATAATTTTA TTAAATTTAA AAATAATACA AATGAAGTGT   
  
  
+ AATTATCTAT ATACTAAGAA CTGGTTATAT TTTAATATAG TTTTTTTACA TTACAGTTTA AAAATTATTA   
  
  
+ TTATATTAAA ACTGAAACTT TGCACATCGA TAGTTAACGA GACTCTTTTT AAAATAATAT TTAAGCAAGT   
  
  
+ ATTATTATAA ATTTTATAGT TTGAAGTATA TAGTATTTTA ATAAAAATTA GATATATTTG AAATTATTTT   
  
  
+ TATATATTGT AGTATTAAAA TTTTCAACTT AATGCTTGAA ATAATATATA TATGTTTCTA GAGTATTTTA   
  
  
+ AAATTATAAA TGAAATTTAA TGATTAGTT  

- CAAGATTTCT GTTTACTAAA CAAGATCGAT CTGAGAGAAA AAGGATAACT GTTGGGCTGA CTTTCAAGAC   
  
  
- TTTTCAGAGA GAATCGTGAA GAGATAGACA TTTGCTAGAG GAAGGCAAGG TTTGTTTATA TATATTGGAG   
  
  
- AGAGACTCAG ATTTCTTTAC GGCAAGTATC CATATTCAAC ACCTCTTCTC CTTCCATGTG AACCAGGATA   
  
  
- TGCAGCATTC GTTGAGTTGA CTTGTCATAA AGTCAACCCA AAAATGATAA TTCATACCAA AAGTTTAATC   
  
  
- TTAGAGTACT GAAAACTTTA TTGTATTAGG ACTTGCACAT AAATCTTCAT TAATCATCAC CGTTGACTAT   
  
  
- TTCTTTACAA AAATTTTACA ATTTTTAATA ATTATAAAAT CTCAATCATT CATCATTTAA AAAATACTAA   
  
  
- GTTTTTTTTA TCAAATGGGA AAGATTAAGA AAGAAAATTG TGGATTTGGT GATGAAAGAA GGGCATGATT   
  
  
- GGTTCCTTTG ACTTTCACAC TCTTAATTAG AGTGTGAATT TAATTTTTTT ATTTATCTGA ATTATGTTAA   
  
  
- TTTTTTGATA AAATAATAAT AATAAATAAT TTAATAAAAT TAATGTAATT AATTTTTTTA TTTTTATATT   
  
  
- AATATAATAA ATTATTAAAA TATTTTTTAA ATATTTTAAA AAGACTTAAC GTTAATTTAG ATGAAGAGAA   
  
  
- GAACTTGATC GCTAGGGAGC ATAATTTGCT CGAGAGTCTT ACCTCGTCTC TATTTGTTTG TCGGAGAGCT   
  
  
- CCTGTTTGGC CAATGAGATA AGAAGGAAAA TTGCATTGGC AGAAAGGATT TCAAAGTCAA AGAAACAAAA   
  
  
- TGACGAAAAA GTCAGCCACC GCATGCTGCA ATTTGCAAGG CCCCAACTAG CTTTTTCCAA TCAGATTTTG   
  
  
- TCATCTCATA TATATATATA TATATAGAAA TAATTTGTTT CAAAATAATC AAGTTTATAT ATTAAAAATA   
  
  
- GAATAAAAAA TTATAAATTA TATTTTATTT TTATTTTACA TTTTAAAATA TAATATATAT ATTTAAATAA   
  
  
- TTTTTAAAAA ATTATTTTTT TATAATTATT TGAAAAAAAT CTAATTTAAA TCAAATTAAA AGATTAAATA   
  
  
- CACAATAAAT TAATTAATAA AGTAAAATCT ATATTAAAAT AATTTAAATT TTTATTATGT TTACTTCACA   
  
  
- TTAATAGATA TATGATTCTT GACCAATATA AAATTATATC AAAAAAATGT AATGTCAAAT TTTTAATAAT   
  
  
- AATATAATTT TGACTTTGAA ACGTGTAGCT ATCAATTGCT CTGAGAAAAA TTTTATTATA AATTCGTTCA   
  
  
- TAATAATATT TAAAATATCA AACTTCATAT ATCATAAAAT TATTTTTAAT CTATATAAAC TTTAATAAAA   
  
  
- ATATATAACA TCATAATTTT AAAAGTTGAA TTACGAACTT TATTATATAT ATACAAAGAT CTCATAAAAT   
  
  
- TTTAATATTT ACTTTAAATT ACTAATCAA

+     GAG-motif

| Site Name | Organism | Position | Strand | Matrix score. | sequence | function |
| --- | --- | --- | --- | --- | --- | --- |
| GAG-motif | Arabidopsis thaliana | 32 | - | 7 | AGAGAGT | part of a light responsive element |

> 2018/04/13 10:10:12  
+ GTTCTAAAGA CAAATGATTT GTTCTAGCTA GACTCTCTTT TTCCTATTGA CAACCCGACT GAAAGTTCTG   
  
  
+ AAAAGTCTCT CTTAGCACTT CTCTATCTGT AAACGATCTC CTTCCGTTCC AAACAAATAT ATATAACCTC   
  
  
+ TCTCTGAGTC TAAAGAAATG CCGTTCATAG GTATAAGTTG TGGAGAAGAG GAAGGTACAC TTGGTCCTAT   
  
  
+ ACGTCGTAAG CAACTCAACT GAACAGTATT TCAGTTGGGT TTTTACTATT AAGTATGGTT TTCAAATTAG   
  
  
+ AATCTCATGA CTTTTGAAAT AACATAATCC TGAACGTGTA TTTAGAAGTA ATTAGTAGTG GCAACTGATA   
  
  
+ AAGAAATGTT TTTAAAATGT TAAAAATTAT TAATATTTTA GAGTTAGTAA GTAGTAAATT TTTTATGATT   
  
  
+ CAAAAAAAAT AGTTTACCCT TTCTAATTCT TTCTTTTAAC ACCTAAACCA CTACTTTCTT CCCGTACTAA   
  
  
+ CCAAGGAAAC TGAAAGTGTG AGAATTAATC TCACACTTAA ATTAAAAAAA TAAATAGACT TAATACAATT   
  
  
+ AAAAAACTAT TTTATTATTA TTATTTATTA AATTATTTTA ATTACATTAA TTAAAAAAAT AAAAATATAA   
  
  
+ TTATATTATT TAATAATTTT ATAAAAAATT TATAAAATTT TTCTGAATTG CAATTAAATC TACTTCTCTT   
  
  
+ CTTGAACTAG CGATCCCTCG TATTAAACGA GCTCTCAGAA TGGAGCAGAG ATAAACAAAC AGCCTCTCGA   
  
  
+ GGACAAACCG GTTACTCTAT TCTTCCTTTT AACGTAACCG TCTTTCCTAA AGTTTCAGTT TCTTTGTTTT   
  
  
+ ACTGCTTTTT CAGTCGGTGG CGTACGACGT TAAACGTTCC GGGGTTGATC GAAAAAGGTT AGTCTAAAAC   
  
  
+ AGTAGAGTAT ATATATATAT ATATATCTTT ATTAAACAAA GTTTTATTAG TTCAAATATA TAATTTTTAT   
  
  
+ CTTATTTTTT AATATTTAAT ATAAAATAAA AATAAAATGT AAAATTTTAT ATTATATATA TAAATTTATT   
  
  
+ AAAAATTTTT TAATAAAAAA ATATTAATAA ACTTTTTTTA GATTAAATTT AGTTTAATTT TCTAATTTAT   
  
  
+ GTGTTATTTA ATTAATTATT TCATTTTAGA TATAATTTTA TTAAATTTAA AAATAATACA AATGAAGTGT   
  
  
+ AATTATCTAT ATACTAAGAA CTGGTTATAT TTTAATATAG TTTTTTTACA TTACAGTTTA AAAATTATTA   
  
  
+ TTATATTAAA ACTGAAACTT TGCACATCGA TAGTTAACGA GACTCTTTTT AAAATAATAT TTAAGCAAGT   
  
  
+ ATTATTATAA ATTTTATAGT TTGAAGTATA TAGTATTTTA ATAAAAATTA GATATATTTG AAATTATTTT   
  
  
+ TATATATTGT AGTATTAAAA TTTTCAACTT AATGCTTGAA ATAATATATA TATGTTTCTA GAGTATTTTA   
  
  
+ AAATTATAAA TGAAATTTAA TGATTAGTT  

- CAAGATTTCT GTTTACTAAA CAAGATCGAT CTGAGAGAAA AAGGATAACT GTTGGGCTGA CTTTCAAGAC   
  
  
- TTTTCAGAGA GAATCGTGAA GAGATAGACA TTTGCTAGAG GAAGGCAAGG TTTGTTTATA TATATTGGAG   
  
  
- AGAGACTCAG ATTTCTTTAC GGCAAGTATC CATATTCAAC ACCTCTTCTC CTTCCATGTG AACCAGGATA   
  
  
- TGCAGCATTC GTTGAGTTGA CTTGTCATAA AGTCAACCCA AAAATGATAA TTCATACCAA AAGTTTAATC   
  
  
- TTAGAGTACT GAAAACTTTA TTGTATTAGG ACTTGCACAT AAATCTTCAT TAATCATCAC CGTTGACTAT   
  
  
- TTCTTTACAA AAATTTTACA ATTTTTAATA ATTATAAAAT CTCAATCATT CATCATTTAA AAAATACTAA   
  
  
- GTTTTTTTTA TCAAATGGGA AAGATTAAGA AAGAAAATTG TGGATTTGGT GATGAAAGAA GGGCATGATT   
  
  
- GGTTCCTTTG ACTTTCACAC TCTTAATTAG AGTGTGAATT TAATTTTTTT ATTTATCTGA ATTATGTTAA   
  
  
- TTTTTTGATA AAATAATAAT AATAAATAAT TTAATAAAAT TAATGTAATT AATTTTTTTA TTTTTATATT   
  
  
- AATATAATAA ATTATTAAAA TATTTTTTAA ATATTTTAAA AAGACTTAAC GTTAATTTAG ATGAAGAGAA   
  
  
- GAACTTGATC GCTAGGGAGC ATAATTTGCT CGAGAGTCTT ACCTCGTCTC TATTTGTTTG TCGGAGAGCT   
  
  
- CCTGTTTGGC CAATGAGATA AGAAGGAAAA TTGCATTGGC AGAAAGGATT TCAAAGTCAA AGAAACAAAA   
  
  
- TGACGAAAAA GTCAGCCACC GCATGCTGCA ATTTGCAAGG CCCCAACTAG CTTTTTCCAA TCAGATTTTG   
  
  
- TCATCTCATA TATATATATA TATATAGAAA TAATTTGTTT CAAAATAATC AAGTTTATAT ATTAAAAATA   
  
  
- GAATAAAAAA TTATAAATTA TATTTTATTT TTATTTTACA TTTTAAAATA TAATATATAT ATTTAAATAA   
  
  
- TTTTTAAAAA ATTATTTTTT TATAATTATT TGAAAAAAAT CTAATTTAAA TCAAATTAAA AGATTAAATA   
  
  
- CACAATAAAT TAATTAATAA AGTAAAATCT ATATTAAAAT AATTTAAATT TTTATTATGT TTACTTCACA   
  
  
- TTAATAGATA TATGATTCTT GACCAATATA AAATTATATC AAAAAAATGT AATGTCAAAT TTTTAATAAT   
  
  
- AATATAATTT TGACTTTGAA ACGTGTAGCT ATCAATTGCT CTGAGAAAAA TTTTATTATA AATTCGTTCA   
  
  
- TAATAATATT TAAAATATCA AACTTCATAT ATCATAAAAT TATTTTTAAT CTATATAAAC TTTAATAAAA   
  
  
- ATATATAACA TCATAATTTT AAAAGTTGAA TTACGAACTT TATTATATAT ATACAAAGAT CTCATAAAAT   
  
  
- TTTAATATTT ACTTTAAATT ACTAATCAA

+     HSE

| Site Name | Organism | Position | Strand | Matrix score. | sequence | function |
| --- | --- | --- | --- | --- | --- | --- |
| HSE | Brassica oleracea | 405 | - | 9 | AAAAAATTTC | cis-acting element involved in heat stress responsiveness |
| HSE | Brassica oleracea | 1390 | - | 9 | AAAAAATTTC | cis-acting element involved in heat stress responsiveness |
| HSE | Brassica oleracea | 353 | - | 9 | AAAAAATTTC | cis-acting element involved in heat stress responsiveness |
| HSE | Brassica oleracea | 1052 | - | 9 | AAAAAATTTC | cis-acting element involved in heat stress responsiveness |
| HSE | Brassica oleracea | 653 | + | 9 | AAAAAATTTC | cis-acting element involved in heat stress responsiveness |

> 2018/04/13 10:10:12  
+ GTTCTAAAGA CAAATGATTT GTTCTAGCTA GACTCTCTTT TTCCTATTGA CAACCCGACT GAAAGTTCTG   
  
  
+ AAAAGTCTCT CTTAGCACTT CTCTATCTGT AAACGATCTC CTTCCGTTCC AAACAAATAT ATATAACCTC   
  
  
+ TCTCTGAGTC TAAAGAAATG CCGTTCATAG GTATAAGTTG TGGAGAAGAG GAAGGTACAC TTGGTCCTAT   
  
  
+ ACGTCGTAAG CAACTCAACT GAACAGTATT TCAGTTGGGT TTTTACTATT AAGTATGGTT TTCAAATTAG   
  
  
+ AATCTCATGA CTTTTGAAAT AACATAATCC TGAACGTGTA TTTAGAAGTA ATTAGTAGTG GCAACTGATA   
  
  
+ AAGAAATGTT TTTAAAATGT TAAAAATTAT TAATATTTTA GAGTTAGTAA GTAGTAAATT TTTTATGATT   
  
  
+ CAAAAAAAAT AGTTTACCCT TTCTAATTCT TTCTTTTAAC ACCTAAACCA CTACTTTCTT CCCGTACTAA   
  
  
+ CCAAGGAAAC TGAAAGTGTG AGAATTAATC TCACACTTAA ATTAAAAAAA TAAATAGACT TAATACAATT   
  
  
+ AAAAAACTAT TTTATTATTA TTATTTATTA AATTATTTTA ATTACATTAA TTAAAAAAAT AAAAATATAA   
  
  
+ TTATATTATT TAATAATTTT ATAAAAAATT TATAAAATTT TTCTGAATTG CAATTAAATC TACTTCTCTT   
  
  
+ CTTGAACTAG CGATCCCTCG TATTAAACGA GCTCTCAGAA TGGAGCAGAG ATAAACAAAC AGCCTCTCGA   
  
  
+ GGACAAACCG GTTACTCTAT TCTTCCTTTT AACGTAACCG TCTTTCCTAA AGTTTCAGTT TCTTTGTTTT   
  
  
+ ACTGCTTTTT CAGTCGGTGG CGTACGACGT TAAACGTTCC GGGGTTGATC GAAAAAGGTT AGTCTAAAAC   
  
  
+ AGTAGAGTAT ATATATATAT ATATATCTTT ATTAAACAAA GTTTTATTAG TTCAAATATA TAATTTTTAT   
  
  
+ CTTATTTTTT AATATTTAAT ATAAAATAAA AATAAAATGT AAAATTTTAT ATTATATATA TAAATTTATT   
  
  
+ AAAAATTTTT TAATAAAAAA ATATTAATAA ACTTTTTTTA GATTAAATTT AGTTTAATTT TCTAATTTAT   
  
  
+ GTGTTATTTA ATTAATTATT TCATTTTAGA TATAATTTTA TTAAATTTAA AAATAATACA AATGAAGTGT   
  
  
+ AATTATCTAT ATACTAAGAA CTGGTTATAT TTTAATATAG TTTTTTTACA TTACAGTTTA AAAATTATTA   
  
  
+ TTATATTAAA ACTGAAACTT TGCACATCGA TAGTTAACGA GACTCTTTTT AAAATAATAT TTAAGCAAGT   
  
  
+ ATTATTATAA ATTTTATAGT TTGAAGTATA TAGTATTTTA ATAAAAATTA GATATATTTG AAATTATTTT   
  
  
+ TATATATTGT AGTATTAAAA TTTTCAACTT AATGCTTGAA ATAATATATA TATGTTTCTA GAGTATTTTA   
  
  
+ AAATTATAAA TGAAATTTAA TGATTAGTT  

- CAAGATTTCT GTTTACTAAA CAAGATCGAT CTGAGAGAAA AAGGATAACT GTTGGGCTGA CTTTCAAGAC   
  
  
- TTTTCAGAGA GAATCGTGAA GAGATAGACA TTTGCTAGAG GAAGGCAAGG TTTGTTTATA TATATTGGAG   
  
  
- AGAGACTCAG ATTTCTTTAC GGCAAGTATC CATATTCAAC ACCTCTTCTC CTTCCATGTG AACCAGGATA   
  
  
- TGCAGCATTC GTTGAGTTGA CTTGTCATAA AGTCAACCCA AAAATGATAA TTCATACCAA AAGTTTAATC   
  
  
- TTAGAGTACT GAAAACTTTA TTGTATTAGG ACTTGCACAT AAATCTTCAT TAATCATCAC CGTTGACTAT   
  
  
- TTCTTTACAA AAATTTTACA ATTTTTAATA ATTATAAAAT CTCAATCATT CATCATTTAA AAAATACTAA   
  
  
- GTTTTTTTTA TCAAATGGGA AAGATTAAGA AAGAAAATTG TGGATTTGGT GATGAAAGAA GGGCATGATT   
  
  
- GGTTCCTTTG ACTTTCACAC TCTTAATTAG AGTGTGAATT TAATTTTTTT ATTTATCTGA ATTATGTTAA   
  
  
- TTTTTTGATA AAATAATAAT AATAAATAAT TTAATAAAAT TAATGTAATT AATTTTTTTA TTTTTATATT   
  
  
- AATATAATAA ATTATTAAAA TATTTTTTAA ATATTTTAAA AAGACTTAAC GTTAATTTAG ATGAAGAGAA   
  
  
- GAACTTGATC GCTAGGGAGC ATAATTTGCT CGAGAGTCTT ACCTCGTCTC TATTTGTTTG TCGGAGAGCT   
  
  
- CCTGTTTGGC CAATGAGATA AGAAGGAAAA TTGCATTGGC AGAAAGGATT TCAAAGTCAA AGAAACAAAA   
  
  
- TGACGAAAAA GTCAGCCACC GCATGCTGCA ATTTGCAAGG CCCCAACTAG CTTTTTCCAA TCAGATTTTG   
  
  
- TCATCTCATA TATATATATA TATATAGAAA TAATTTGTTT CAAAATAATC AAGTTTATAT ATTAAAAATA   
  
  
- GAATAAAAAA TTATAAATTA TATTTTATTT TTATTTTACA TTTTAAAATA TAATATATAT ATTTAAATAA   
  
  
- TTTTTAAAAA ATTATTTTTT TATAATTATT TGAAAAAAAT CTAATTTAAA TCAAATTAAA AGATTAAATA   
  
  
- CACAATAAAT TAATTAATAA AGTAAAATCT ATATTAAAAT AATTTAAATT TTTATTATGT TTACTTCACA   
  
  
- TTAATAGATA TATGATTCTT GACCAATATA AAATTATATC AAAAAAATGT AATGTCAAAT TTTTAATAAT   
  
  
- AATATAATTT TGACTTTGAA ACGTGTAGCT ATCAATTGCT CTGAGAAAAA TTTTATTATA AATTCGTTCA   
  
  
- TAATAATATT TAAAATATCA AACTTCATAT ATCATAAAAT TATTTTTAAT CTATATAAAC TTTAATAAAA   
  
  
- ATATATAACA TCATAATTTT AAAAGTTGAA TTACGAACTT TATTATATAT ATACAAAGAT CTCATAAAAT   
  
  
- TTTAATATTT ACTTTAAATT ACTAATCAA

+     LAMP-element

| Site Name | Organism | Position | Strand | Matrix score. | sequence | function |
| --- | --- | --- | --- | --- | --- | --- |
| LAMP-element | Pisum sativum | 346 | - | 8 | CTTTATCA | part of a light responsive element |

> 2018/04/13 10:10:12  
+ GTTCTAAAGA CAAATGATTT GTTCTAGCTA GACTCTCTTT TTCCTATTGA CAACCCGACT GAAAGTTCTG   
  
  
+ AAAAGTCTCT CTTAGCACTT CTCTATCTGT AAACGATCTC CTTCCGTTCC AAACAAATAT ATATAACCTC   
  
  
+ TCTCTGAGTC TAAAGAAATG CCGTTCATAG GTATAAGTTG TGGAGAAGAG GAAGGTACAC TTGGTCCTAT   
  
  
+ ACGTCGTAAG CAACTCAACT GAACAGTATT TCAGTTGGGT TTTTACTATT AAGTATGGTT TTCAAATTAG   
  
  
+ AATCTCATGA CTTTTGAAAT AACATAATCC TGAACGTGTA TTTAGAAGTA ATTAGTAGTG GCAACTGATA   
  
  
+ AAGAAATGTT TTTAAAATGT TAAAAATTAT TAATATTTTA GAGTTAGTAA GTAGTAAATT TTTTATGATT   
  
  
+ CAAAAAAAAT AGTTTACCCT TTCTAATTCT TTCTTTTAAC ACCTAAACCA CTACTTTCTT CCCGTACTAA   
  
  
+ CCAAGGAAAC TGAAAGTGTG AGAATTAATC TCACACTTAA ATTAAAAAAA TAAATAGACT TAATACAATT   
  
  
+ AAAAAACTAT TTTATTATTA TTATTTATTA AATTATTTTA ATTACATTAA TTAAAAAAAT AAAAATATAA   
  
  
+ TTATATTATT TAATAATTTT ATAAAAAATT TATAAAATTT TTCTGAATTG CAATTAAATC TACTTCTCTT   
  
  
+ CTTGAACTAG CGATCCCTCG TATTAAACGA GCTCTCAGAA TGGAGCAGAG ATAAACAAAC AGCCTCTCGA   
  
  
+ GGACAAACCG GTTACTCTAT TCTTCCTTTT AACGTAACCG TCTTTCCTAA AGTTTCAGTT TCTTTGTTTT   
  
  
+ ACTGCTTTTT CAGTCGGTGG CGTACGACGT TAAACGTTCC GGGGTTGATC GAAAAAGGTT AGTCTAAAAC   
  
  
+ AGTAGAGTAT ATATATATAT ATATATCTTT ATTAAACAAA GTTTTATTAG TTCAAATATA TAATTTTTAT   
  
  
+ CTTATTTTTT AATATTTAAT ATAAAATAAA AATAAAATGT AAAATTTTAT ATTATATATA TAAATTTATT   
  
  
+ AAAAATTTTT TAATAAAAAA ATATTAATAA ACTTTTTTTA GATTAAATTT AGTTTAATTT TCTAATTTAT   
  
  
+ GTGTTATTTA ATTAATTATT TCATTTTAGA TATAATTTTA TTAAATTTAA AAATAATACA AATGAAGTGT   
  
  
+ AATTATCTAT ATACTAAGAA CTGGTTATAT TTTAATATAG TTTTTTTACA TTACAGTTTA AAAATTATTA   
  
  
+ TTATATTAAA ACTGAAACTT TGCACATCGA TAGTTAACGA GACTCTTTTT AAAATAATAT TTAAGCAAGT   
  
  
+ ATTATTATAA ATTTTATAGT TTGAAGTATA TAGTATTTTA ATAAAAATTA GATATATTTG AAATTATTTT   
  
  
+ TATATATTGT AGTATTAAAA TTTTCAACTT AATGCTTGAA ATAATATATA TATGTTTCTA GAGTATTTTA   
  
  
+ AAATTATAAA TGAAATTTAA TGATTAGTT  

- CAAGATTTCT GTTTACTAAA CAAGATCGAT CTGAGAGAAA AAGGATAACT GTTGGGCTGA CTTTCAAGAC   
  
  
- TTTTCAGAGA GAATCGTGAA GAGATAGACA TTTGCTAGAG GAAGGCAAGG TTTGTTTATA TATATTGGAG   
  
  
- AGAGACTCAG ATTTCTTTAC GGCAAGTATC CATATTCAAC ACCTCTTCTC CTTCCATGTG AACCAGGATA   
  
  
- TGCAGCATTC GTTGAGTTGA CTTGTCATAA AGTCAACCCA AAAATGATAA TTCATACCAA AAGTTTAATC   
  
  
- TTAGAGTACT GAAAACTTTA TTGTATTAGG ACTTGCACAT AAATCTTCAT TAATCATCAC CGTTGACTAT   
  
  
- TTCTTTACAA AAATTTTACA ATTTTTAATA ATTATAAAAT CTCAATCATT CATCATTTAA AAAATACTAA   
  
  
- GTTTTTTTTA TCAAATGGGA AAGATTAAGA AAGAAAATTG TGGATTTGGT GATGAAAGAA GGGCATGATT   
  
  
- GGTTCCTTTG ACTTTCACAC TCTTAATTAG AGTGTGAATT TAATTTTTTT ATTTATCTGA ATTATGTTAA   
  
  
- TTTTTTGATA AAATAATAAT AATAAATAAT TTAATAAAAT TAATGTAATT AATTTTTTTA TTTTTATATT   
  
  
- AATATAATAA ATTATTAAAA TATTTTTTAA ATATTTTAAA AAGACTTAAC GTTAATTTAG ATGAAGAGAA   
  
  
- GAACTTGATC GCTAGGGAGC ATAATTTGCT CGAGAGTCTT ACCTCGTCTC TATTTGTTTG TCGGAGAGCT   
  
  
- CCTGTTTGGC CAATGAGATA AGAAGGAAAA TTGCATTGGC AGAAAGGATT TCAAAGTCAA AGAAACAAAA   
  
  
- TGACGAAAAA GTCAGCCACC GCATGCTGCA ATTTGCAAGG CCCCAACTAG CTTTTTCCAA TCAGATTTTG   
  
  
- TCATCTCATA TATATATATA TATATAGAAA TAATTTGTTT CAAAATAATC AAGTTTATAT ATTAAAAATA   
  
  
- GAATAAAAAA TTATAAATTA TATTTTATTT TTATTTTACA TTTTAAAATA TAATATATAT ATTTAAATAA   
  
  
- TTTTTAAAAA ATTATTTTTT TATAATTATT TGAAAAAAAT CTAATTTAAA TCAAATTAAA AGATTAAATA   
  
  
- CACAATAAAT TAATTAATAA AGTAAAATCT ATATTAAAAT AATTTAAATT TTTATTATGT TTACTTCACA   
  
  
- TTAATAGATA TATGATTCTT GACCAATATA AAATTATATC AAAAAAATGT AATGTCAAAT TTTTAATAAT   
  
  
- AATATAATTT TGACTTTGAA ACGTGTAGCT ATCAATTGCT CTGAGAAAAA TTTTATTATA AATTCGTTCA   
  
  
- TAATAATATT TAAAATATCA AACTTCATAT ATCATAAAAT TATTTTTAAT CTATATAAAC TTTAATAAAA   
  
  
- ATATATAACA TCATAATTTT AAAAGTTGAA TTACGAACTT TATTATATAT ATACAAAGAT CTCATAAAAT   
  
  
- TTTAATATTT ACTTTAAATT ACTAATCAA

+     MBS

| Site Name | Organism | Position | Strand | Matrix score. | sequence | function |
| --- | --- | --- | --- | --- | --- | --- |
| MBS | Arabidopsis thaliana | 226 | + | 6 | CAACTG | MYB binding site involved in drought-inducibility |
| MBS | Arabidopsis thaliana | 342 | + | 6 | CAACTG | MYB binding site involved in drought-inducibility |
| MBS | Arabidopsis thaliana | 242 | - | 6 | CAACTG | MYB binding site involved in drought-inducibility |

> 2018/04/13 10:10:12  
+ GTTCTAAAGA CAAATGATTT GTTCTAGCTA GACTCTCTTT TTCCTATTGA CAACCCGACT GAAAGTTCTG   
  
  
+ AAAAGTCTCT CTTAGCACTT CTCTATCTGT AAACGATCTC CTTCCGTTCC AAACAAATAT ATATAACCTC   
  
  
+ TCTCTGAGTC TAAAGAAATG CCGTTCATAG GTATAAGTTG TGGAGAAGAG GAAGGTACAC TTGGTCCTAT   
  
  
+ ACGTCGTAAG CAACTCAACT GAACAGTATT TCAGTTGGGT TTTTACTATT AAGTATGGTT TTCAAATTAG   
  
  
+ AATCTCATGA CTTTTGAAAT AACATAATCC TGAACGTGTA TTTAGAAGTA ATTAGTAGTG GCAACTGATA   
  
  
+ AAGAAATGTT TTTAAAATGT TAAAAATTAT TAATATTTTA GAGTTAGTAA GTAGTAAATT TTTTATGATT   
  
  
+ CAAAAAAAAT AGTTTACCCT TTCTAATTCT TTCTTTTAAC ACCTAAACCA CTACTTTCTT CCCGTACTAA   
  
  
+ CCAAGGAAAC TGAAAGTGTG AGAATTAATC TCACACTTAA ATTAAAAAAA TAAATAGACT TAATACAATT   
  
  
+ AAAAAACTAT TTTATTATTA TTATTTATTA AATTATTTTA ATTACATTAA TTAAAAAAAT AAAAATATAA   
  
  
+ TTATATTATT TAATAATTTT ATAAAAAATT TATAAAATTT TTCTGAATTG CAATTAAATC TACTTCTCTT   
  
  
+ CTTGAACTAG CGATCCCTCG TATTAAACGA GCTCTCAGAA TGGAGCAGAG ATAAACAAAC AGCCTCTCGA   
  
  
+ GGACAAACCG GTTACTCTAT TCTTCCTTTT AACGTAACCG TCTTTCCTAA AGTTTCAGTT TCTTTGTTTT   
  
  
+ ACTGCTTTTT CAGTCGGTGG CGTACGACGT TAAACGTTCC GGGGTTGATC GAAAAAGGTT AGTCTAAAAC   
  
  
+ AGTAGAGTAT ATATATATAT ATATATCTTT ATTAAACAAA GTTTTATTAG TTCAAATATA TAATTTTTAT   
  
  
+ CTTATTTTTT AATATTTAAT ATAAAATAAA AATAAAATGT AAAATTTTAT ATTATATATA TAAATTTATT   
  
  
+ AAAAATTTTT TAATAAAAAA ATATTAATAA ACTTTTTTTA GATTAAATTT AGTTTAATTT TCTAATTTAT   
  
  
+ GTGTTATTTA ATTAATTATT TCATTTTAGA TATAATTTTA TTAAATTTAA AAATAATACA AATGAAGTGT   
  
  
+ AATTATCTAT ATACTAAGAA CTGGTTATAT TTTAATATAG TTTTTTTACA TTACAGTTTA AAAATTATTA   
  
  
+ TTATATTAAA ACTGAAACTT TGCACATCGA TAGTTAACGA GACTCTTTTT AAAATAATAT TTAAGCAAGT   
  
  
+ ATTATTATAA ATTTTATAGT TTGAAGTATA TAGTATTTTA ATAAAAATTA GATATATTTG AAATTATTTT   
  
  
+ TATATATTGT AGTATTAAAA TTTTCAACTT AATGCTTGAA ATAATATATA TATGTTTCTA GAGTATTTTA   
  
  
+ AAATTATAAA TGAAATTTAA TGATTAGTT  

- CAAGATTTCT GTTTACTAAA CAAGATCGAT CTGAGAGAAA AAGGATAACT GTTGGGCTGA CTTTCAAGAC   
  
  
- TTTTCAGAGA GAATCGTGAA GAGATAGACA TTTGCTAGAG GAAGGCAAGG TTTGTTTATA TATATTGGAG   
  
  
- AGAGACTCAG ATTTCTTTAC GGCAAGTATC CATATTCAAC ACCTCTTCTC CTTCCATGTG AACCAGGATA   
  
  
- TGCAGCATTC GTTGAGTTGA CTTGTCATAA AGTCAACCCA AAAATGATAA TTCATACCAA AAGTTTAATC   
  
  
- TTAGAGTACT GAAAACTTTA TTGTATTAGG ACTTGCACAT AAATCTTCAT TAATCATCAC CGTTGACTAT   
  
  
- TTCTTTACAA AAATTTTACA ATTTTTAATA ATTATAAAAT CTCAATCATT CATCATTTAA AAAATACTAA   
  
  
- GTTTTTTTTA TCAAATGGGA AAGATTAAGA AAGAAAATTG TGGATTTGGT GATGAAAGAA GGGCATGATT   
  
  
- GGTTCCTTTG ACTTTCACAC TCTTAATTAG AGTGTGAATT TAATTTTTTT ATTTATCTGA ATTATGTTAA   
  
  
- TTTTTTGATA AAATAATAAT AATAAATAAT TTAATAAAAT TAATGTAATT AATTTTTTTA TTTTTATATT   
  
  
- AATATAATAA ATTATTAAAA TATTTTTTAA ATATTTTAAA AAGACTTAAC GTTAATTTAG ATGAAGAGAA   
  
  
- GAACTTGATC GCTAGGGAGC ATAATTTGCT CGAGAGTCTT ACCTCGTCTC TATTTGTTTG TCGGAGAGCT   
  
  
- CCTGTTTGGC CAATGAGATA AGAAGGAAAA TTGCATTGGC AGAAAGGATT TCAAAGTCAA AGAAACAAAA   
  
  
- TGACGAAAAA GTCAGCCACC GCATGCTGCA ATTTGCAAGG CCCCAACTAG CTTTTTCCAA TCAGATTTTG   
  
  
- TCATCTCATA TATATATATA TATATAGAAA TAATTTGTTT CAAAATAATC AAGTTTATAT ATTAAAAATA   
  
  
- GAATAAAAAA TTATAAATTA TATTTTATTT TTATTTTACA TTTTAAAATA TAATATATAT ATTTAAATAA   
  
  
- TTTTTAAAAA ATTATTTTTT TATAATTATT TGAAAAAAAT CTAATTTAAA TCAAATTAAA AGATTAAATA   
  
  
- CACAATAAAT TAATTAATAA AGTAAAATCT ATATTAAAAT AATTTAAATT TTTATTATGT TTACTTCACA   
  
  
- TTAATAGATA TATGATTCTT GACCAATATA AAATTATATC AAAAAAATGT AATGTCAAAT TTTTAATAAT   
  
  
- AATATAATTT TGACTTTGAA ACGTGTAGCT ATCAATTGCT CTGAGAAAAA TTTTATTATA AATTCGTTCA   
  
  
- TAATAATATT TAAAATATCA AACTTCATAT ATCATAAAAT TATTTTTAAT CTATATAAAC TTTAATAAAA   
  
  
- ATATATAACA TCATAATTTT AAAAGTTGAA TTACGAACTT TATTATATAT ATACAAAGAT CTCATAAAAT   
  
  
- TTTAATATTT ACTTTAAATT ACTAATCAA

+     Skn-1\_motif

| Site Name | Organism | Position | Strand | Matrix score. | sequence | function |
| --- | --- | --- | --- | --- | --- | --- |
| Skn-1\_motif | Oryza sativa | 287 | - | 5 | GTCAT | cis-acting regulatory element required for endosperm expression |

> 2018/04/13 10:10:12  
+ GTTCTAAAGA CAAATGATTT GTTCTAGCTA GACTCTCTTT TTCCTATTGA CAACCCGACT GAAAGTTCTG   
  
  
+ AAAAGTCTCT CTTAGCACTT CTCTATCTGT AAACGATCTC CTTCCGTTCC AAACAAATAT ATATAACCTC   
  
  
+ TCTCTGAGTC TAAAGAAATG CCGTTCATAG GTATAAGTTG TGGAGAAGAG GAAGGTACAC TTGGTCCTAT   
  
  
+ ACGTCGTAAG CAACTCAACT GAACAGTATT TCAGTTGGGT TTTTACTATT AAGTATGGTT TTCAAATTAG   
  
  
+ AATCTCATGA CTTTTGAAAT AACATAATCC TGAACGTGTA TTTAGAAGTA ATTAGTAGTG GCAACTGATA   
  
  
+ AAGAAATGTT TTTAAAATGT TAAAAATTAT TAATATTTTA GAGTTAGTAA GTAGTAAATT TTTTATGATT   
  
  
+ CAAAAAAAAT AGTTTACCCT TTCTAATTCT TTCTTTTAAC ACCTAAACCA CTACTTTCTT CCCGTACTAA   
  
  
+ CCAAGGAAAC TGAAAGTGTG AGAATTAATC TCACACTTAA ATTAAAAAAA TAAATAGACT TAATACAATT   
  
  
+ AAAAAACTAT TTTATTATTA TTATTTATTA AATTATTTTA ATTACATTAA TTAAAAAAAT AAAAATATAA   
  
  
+ TTATATTATT TAATAATTTT ATAAAAAATT TATAAAATTT TTCTGAATTG CAATTAAATC TACTTCTCTT   
  
  
+ CTTGAACTAG CGATCCCTCG TATTAAACGA GCTCTCAGAA TGGAGCAGAG ATAAACAAAC AGCCTCTCGA   
  
  
+ GGACAAACCG GTTACTCTAT TCTTCCTTTT AACGTAACCG TCTTTCCTAA AGTTTCAGTT TCTTTGTTTT   
  
  
+ ACTGCTTTTT CAGTCGGTGG CGTACGACGT TAAACGTTCC GGGGTTGATC GAAAAAGGTT AGTCTAAAAC   
  
  
+ AGTAGAGTAT ATATATATAT ATATATCTTT ATTAAACAAA GTTTTATTAG TTCAAATATA TAATTTTTAT   
  
  
+ CTTATTTTTT AATATTTAAT ATAAAATAAA AATAAAATGT AAAATTTTAT ATTATATATA TAAATTTATT   
  
  
+ AAAAATTTTT TAATAAAAAA ATATTAATAA ACTTTTTTTA GATTAAATTT AGTTTAATTT TCTAATTTAT   
  
  
+ GTGTTATTTA ATTAATTATT TCATTTTAGA TATAATTTTA TTAAATTTAA AAATAATACA AATGAAGTGT   
  
  
+ AATTATCTAT ATACTAAGAA CTGGTTATAT TTTAATATAG TTTTTTTACA TTACAGTTTA AAAATTATTA   
  
  
+ TTATATTAAA ACTGAAACTT TGCACATCGA TAGTTAACGA GACTCTTTTT AAAATAATAT TTAAGCAAGT   
  
  
+ ATTATTATAA ATTTTATAGT TTGAAGTATA TAGTATTTTA ATAAAAATTA GATATATTTG AAATTATTTT   
  
  
+ TATATATTGT AGTATTAAAA TTTTCAACTT AATGCTTGAA ATAATATATA TATGTTTCTA GAGTATTTTA   
  
  
+ AAATTATAAA TGAAATTTAA TGATTAGTT  

- CAAGATTTCT GTTTACTAAA CAAGATCGAT CTGAGAGAAA AAGGATAACT GTTGGGCTGA CTTTCAAGAC   
  
  
- TTTTCAGAGA GAATCGTGAA GAGATAGACA TTTGCTAGAG GAAGGCAAGG TTTGTTTATA TATATTGGAG   
  
  
- AGAGACTCAG ATTTCTTTAC GGCAAGTATC CATATTCAAC ACCTCTTCTC CTTCCATGTG AACCAGGATA   
  
  
- TGCAGCATTC GTTGAGTTGA CTTGTCATAA AGTCAACCCA AAAATGATAA TTCATACCAA AAGTTTAATC   
  
  
- TTAGAGTACT GAAAACTTTA TTGTATTAGG ACTTGCACAT AAATCTTCAT TAATCATCAC CGTTGACTAT   
  
  
- TTCTTTACAA AAATTTTACA ATTTTTAATA ATTATAAAAT CTCAATCATT CATCATTTAA AAAATACTAA   
  
  
- GTTTTTTTTA TCAAATGGGA AAGATTAAGA AAGAAAATTG TGGATTTGGT GATGAAAGAA GGGCATGATT   
  
  
- GGTTCCTTTG ACTTTCACAC TCTTAATTAG AGTGTGAATT TAATTTTTTT ATTTATCTGA ATTATGTTAA   
  
  
- TTTTTTGATA AAATAATAAT AATAAATAAT TTAATAAAAT TAATGTAATT AATTTTTTTA TTTTTATATT   
  
  
- AATATAATAA ATTATTAAAA TATTTTTTAA ATATTTTAAA AAGACTTAAC GTTAATTTAG ATGAAGAGAA   
  
  
- GAACTTGATC GCTAGGGAGC ATAATTTGCT CGAGAGTCTT ACCTCGTCTC TATTTGTTTG TCGGAGAGCT   
  
  
- CCTGTTTGGC CAATGAGATA AGAAGGAAAA TTGCATTGGC AGAAAGGATT TCAAAGTCAA AGAAACAAAA   
  
  
- TGACGAAAAA GTCAGCCACC GCATGCTGCA ATTTGCAAGG CCCCAACTAG CTTTTTCCAA TCAGATTTTG   
  
  
- TCATCTCATA TATATATATA TATATAGAAA TAATTTGTTT CAAAATAATC AAGTTTATAT ATTAAAAATA   
  
  
- GAATAAAAAA TTATAAATTA TATTTTATTT TTATTTTACA TTTTAAAATA TAATATATAT ATTTAAATAA   
  
  
- TTTTTAAAAA ATTATTTTTT TATAATTATT TGAAAAAAAT CTAATTTAAA TCAAATTAAA AGATTAAATA   
  
  
- CACAATAAAT TAATTAATAA AGTAAAATCT ATATTAAAAT AATTTAAATT TTTATTATGT TTACTTCACA   
  
  
- TTAATAGATA TATGATTCTT GACCAATATA AAATTATATC AAAAAAATGT AATGTCAAAT TTTTAATAAT   
  
  
- AATATAATTT TGACTTTGAA ACGTGTAGCT ATCAATTGCT CTGAGAAAAA TTTTATTATA AATTCGTTCA   
  
  
- TAATAATATT TAAAATATCA AACTTCATAT ATCATAAAAT TATTTTTAAT CTATATAAAC TTTAATAAAA   
  
  
- ATATATAACA TCATAATTTT AAAAGTTGAA TTACGAACTT TATTATATAT ATACAAAGAT CTCATAAAAT   
  
  
- TTTAATATTT ACTTTAAATT ACTAATCAA

+     TA-rich region

| Site Name | Organism | Position | Strand | Matrix score. | sequence | function |
| --- | --- | --- | --- | --- | --- | --- |
| TA-rich region | Nicotiana tabacum | 920 | - | 20 | TATATATATATATATATATATA | enhancer |
| TA-rich region | Nicotiana tabacum | 918 | - | 20 | TATATATATATATATATATATA | enhancer |

> 2018/04/13 10:10:12  
+ GTTCTAAAGA CAAATGATTT GTTCTAGCTA GACTCTCTTT TTCCTATTGA CAACCCGACT GAAAGTTCTG   
  
  
+ AAAAGTCTCT CTTAGCACTT CTCTATCTGT AAACGATCTC CTTCCGTTCC AAACAAATAT ATATAACCTC   
  
  
+ TCTCTGAGTC TAAAGAAATG CCGTTCATAG GTATAAGTTG TGGAGAAGAG GAAGGTACAC TTGGTCCTAT   
  
  
+ ACGTCGTAAG CAACTCAACT GAACAGTATT TCAGTTGGGT TTTTACTATT AAGTATGGTT TTCAAATTAG   
  
  
+ AATCTCATGA CTTTTGAAAT AACATAATCC TGAACGTGTA TTTAGAAGTA ATTAGTAGTG GCAACTGATA   
  
  
+ AAGAAATGTT TTTAAAATGT TAAAAATTAT TAATATTTTA GAGTTAGTAA GTAGTAAATT TTTTATGATT   
  
  
+ CAAAAAAAAT AGTTTACCCT TTCTAATTCT TTCTTTTAAC ACCTAAACCA CTACTTTCTT CCCGTACTAA   
  
  
+ CCAAGGAAAC TGAAAGTGTG AGAATTAATC TCACACTTAA ATTAAAAAAA TAAATAGACT TAATACAATT   
  
  
+ AAAAAACTAT TTTATTATTA TTATTTATTA AATTATTTTA ATTACATTAA TTAAAAAAAT AAAAATATAA   
  
  
+ TTATATTATT TAATAATTTT ATAAAAAATT TATAAAATTT TTCTGAATTG CAATTAAATC TACTTCTCTT   
  
  
+ CTTGAACTAG CGATCCCTCG TATTAAACGA GCTCTCAGAA TGGAGCAGAG ATAAACAAAC AGCCTCTCGA   
  
  
+ GGACAAACCG GTTACTCTAT TCTTCCTTTT AACGTAACCG TCTTTCCTAA AGTTTCAGTT TCTTTGTTTT   
  
  
+ ACTGCTTTTT CAGTCGGTGG CGTACGACGT TAAACGTTCC GGGGTTGATC GAAAAAGGTT AGTCTAAAAC   
  
  
+ AGTAGAGTAT ATATATATAT ATATATCTTT ATTAAACAAA GTTTTATTAG TTCAAATATA TAATTTTTAT   
  
  
+ CTTATTTTTT AATATTTAAT ATAAAATAAA AATAAAATGT AAAATTTTAT ATTATATATA TAAATTTATT   
  
  
+ AAAAATTTTT TAATAAAAAA ATATTAATAA ACTTTTTTTA GATTAAATTT AGTTTAATTT TCTAATTTAT   
  
  
+ GTGTTATTTA ATTAATTATT TCATTTTAGA TATAATTTTA TTAAATTTAA AAATAATACA AATGAAGTGT   
  
  
+ AATTATCTAT ATACTAAGAA CTGGTTATAT TTTAATATAG TTTTTTTACA TTACAGTTTA AAAATTATTA   
  
  
+ TTATATTAAA ACTGAAACTT TGCACATCGA TAGTTAACGA GACTCTTTTT AAAATAATAT TTAAGCAAGT   
  
  
+ ATTATTATAA ATTTTATAGT TTGAAGTATA TAGTATTTTA ATAAAAATTA GATATATTTG AAATTATTTT   
  
  
+ TATATATTGT AGTATTAAAA TTTTCAACTT AATGCTTGAA ATAATATATA TATGTTTCTA GAGTATTTTA   
  
  
+ AAATTATAAA TGAAATTTAA TGATTAGTT  

- CAAGATTTCT GTTTACTAAA CAAGATCGAT CTGAGAGAAA AAGGATAACT GTTGGGCTGA CTTTCAAGAC   
  
  
- TTTTCAGAGA GAATCGTGAA GAGATAGACA TTTGCTAGAG GAAGGCAAGG TTTGTTTATA TATATTGGAG   
  
  
- AGAGACTCAG ATTTCTTTAC GGCAAGTATC CATATTCAAC ACCTCTTCTC CTTCCATGTG AACCAGGATA   
  
  
- TGCAGCATTC GTTGAGTTGA CTTGTCATAA AGTCAACCCA AAAATGATAA TTCATACCAA AAGTTTAATC   
  
  
- TTAGAGTACT GAAAACTTTA TTGTATTAGG ACTTGCACAT AAATCTTCAT TAATCATCAC CGTTGACTAT   
  
  
- TTCTTTACAA AAATTTTACA ATTTTTAATA ATTATAAAAT CTCAATCATT CATCATTTAA AAAATACTAA   
  
  
- GTTTTTTTTA TCAAATGGGA AAGATTAAGA AAGAAAATTG TGGATTTGGT GATGAAAGAA GGGCATGATT   
  
  
- GGTTCCTTTG ACTTTCACAC TCTTAATTAG AGTGTGAATT TAATTTTTTT ATTTATCTGA ATTATGTTAA   
  
  
- TTTTTTGATA AAATAATAAT AATAAATAAT TTAATAAAAT TAATGTAATT AATTTTTTTA TTTTTATATT   
  
  
- AATATAATAA ATTATTAAAA TATTTTTTAA ATATTTTAAA AAGACTTAAC GTTAATTTAG ATGAAGAGAA   
  
  
- GAACTTGATC GCTAGGGAGC ATAATTTGCT CGAGAGTCTT ACCTCGTCTC TATTTGTTTG TCGGAGAGCT   
  
  
- CCTGTTTGGC CAATGAGATA AGAAGGAAAA TTGCATTGGC AGAAAGGATT TCAAAGTCAA AGAAACAAAA   
  
  
- TGACGAAAAA GTCAGCCACC GCATGCTGCA ATTTGCAAGG CCCCAACTAG CTTTTTCCAA TCAGATTTTG   
  
  
- TCATCTCATA TATATATATA TATATAGAAA TAATTTGTTT CAAAATAATC AAGTTTATAT ATTAAAAATA   
  
  
- GAATAAAAAA TTATAAATTA TATTTTATTT TTATTTTACA TTTTAAAATA TAATATATAT ATTTAAATAA   
  
  
- TTTTTAAAAA ATTATTTTTT TATAATTATT TGAAAAAAAT CTAATTTAAA TCAAATTAAA AGATTAAATA   
  
  
- CACAATAAAT TAATTAATAA AGTAAAATCT ATATTAAAAT AATTTAAATT TTTATTATGT TTACTTCACA   
  
  
- TTAATAGATA TATGATTCTT GACCAATATA AAATTATATC AAAAAAATGT AATGTCAAAT TTTTAATAAT   
  
  
- AATATAATTT TGACTTTGAA ACGTGTAGCT ATCAATTGCT CTGAGAAAAA TTTTATTATA AATTCGTTCA   
  
  
- TAATAATATT TAAAATATCA AACTTCATAT ATCATAAAAT TATTTTTAAT CTATATAAAC TTTAATAAAA   
  
  
- ATATATAACA TCATAATTTT AAAAGTTGAA TTACGAACTT TATTATATAT ATACAAAGAT CTCATAAAAT   
  
  
- TTTAATATTT ACTTTAAATT ACTAATCAA

+     TATA-box

| Site Name | Organism | Position | Strand | Matrix score. | sequence | function |
| --- | --- | --- | --- | --- | --- | --- |
| TATA-box | Arabidopsis thaliana | 1025 | - | 7 | TATAAAA | core promoter element around -30 of transcription start |
| TATA-box | Brassica napus | 1260 | + | 6 | ATTATA | core promoter element around -30 of transcription start |
| TATA-box | Glycine max | 1259 | - | 5 | TAATA | core promoter element around -30 of transcription start |
| TATA-box | Glycine max | 721 | - | 5 | TAATA | core promoter element around -30 of transcription start |
| TATA-box | Glycine max | 990 | + | 5 | TAATA | core promoter element around -30 of transcription start |
| TATA-box | Arabidopsis thaliana | 1000 | + | 6 | TATAAA | core promoter element around -30 of transcription start |
| TATA-box | Glycine max | 955 | - | 5 | TAATA | core promoter element around -30 of transcription start |
| TATA-box | Glycine max | 940 | - | 5 | TAATA | core promoter element around -30 of transcription start |
| TATA-box | Glycine max | 1413 | - | 5 | TAATA | core promoter element around -30 of transcription start |
| TATA-box | Brassica napus | 1382 | - | 6 | ATATAT | core promoter element around -30 of transcription start |
| TATA-box | Arabidopsis thaliana | 661 | + | 6 | TATAAA | core promoter element around -30 of transcription start |
| TATA-box | Pisum sativum | 1024 | - | 8 | TATAAAAT | core promoter element around -30 of transcription start |
| TATA-box | Brassica oleracea | 999 | + | 6 | ATATAA | core promoter element around -30 of transcription start |
| TATA-box | Glycine max | 997 | + | 5 | TAATA | core promoter element around -30 of transcription start |
| TATA-box | Arabidopsis thaliana | 926 | - | 8 | TATATATA | core promoter element around -30 of transcription start |
| TATA-box | Brassica napus | 931 | - | 6 | ATATAT | core promoter element around -30 of transcription start |
| TATA-box | Glycine max | 551 | + | 5 | TAATA | core promoter element around -30 of transcription start |
| TATA-box | Brassica napus | 925 | - | 6 | ATATAT | core promoter element around -30 of transcription start |
| TATA-box | Arabidopsis thaliana | 967 | - | 4 | TATA | core promoter element around -30 of transcription start |
| TATA-box | Arabidopsis thaliana | 660 | - | 5 | TATAA | core promoter element around -30 of transcription start |
| TATA-box | Glycine max | 381 | + | 5 | TAATA | core promoter element around -30 of transcription start |
| TATA-box | Glycine max | 1264 | - | 5 | TAATA | core promoter element around -30 of transcription start |
| TATA-box | Arabidopsis thaliana | 1261 | - | 5 | TATAA | core promoter element around -30 of transcription start |
| TATA-box | Glycine max | 1256 | - | 5 | TAATA | core promoter element around -30 of transcription start |
| TATA-box | Glycine max | 1223 | + | 5 | TAATA | core promoter element around -30 of transcription start |
| TATA-box | Glycine max | 378 | - | 5 | TAATA | core promoter element around -30 of transcription start |
| TATA-box | Arabidopsis thaliana | 132 | + | 4 | TATA | core promoter element around -30 of transcription start |
| TATA-box | Lycopersicon esculentum | 905 | - | 5 | TTTTA | core promoter element around -30 of transcription start |
| TATA-box | Brassica oleracea | 1150 | + | 7 | ATATAAT | core promoter element around -30 of transcription start |
| TATA-box | Glycine max | 1174 | + | 5 | TAATA | core promoter element around -30 of transcription start |
| TATA-box | Glycine max | 1159 | - | 5 | TAATA | core promoter element around -30 of transcription start |
| TATA-box | Glycine max | 1072 | - | 5 | TAATA | core promoter element around -30 of transcription start |
| TATA-box | Glycine max | 1075 | + | 5 | TAATA | core promoter element around -30 of transcription start |
| TATA-box | Brassica napus | 1031 | + | 6 | ATTATA | core promoter element around -30 of transcription start |
| TATA-box | Brassica napus | 919 | - | 6 | ATATAT | core promoter element around -30 of transcription start |
| TATA-box | Brassica napus | 966 | - | 6 | ATATAT | core promoter element around -30 of transcription start |
| TATA-box | Brassica oleracea | 968 | + | 7 | ATATAAT | core promoter element around -30 of transcription start |
| TATA-box | Arabidopsis thaliana | 647 | - | 7 | TATAAAA | core promoter element around -30 of transcription start |
| TATA-box | Glycine max | 641 | + | 5 | TAATA | core promoter element around -30 of transcription start |
| TATA-box | Lycopersicon esculentum | 1234 | + | 5 | TTTTA | core promoter element around -30 of transcription start |
| TATA-box | Glycine max | 1047 | - | 5 | TAATA | core promoter element around -30 of transcription start |
| TATA-box | Lycopersicon esculentum | 652 | - | 5 | TTTTA | core promoter element around -30 of transcription start |
| TATA-box | Brassica napus | 923 | - | 6 | ATATAT | core promoter element around -30 of transcription start |
| TATA-box | Brassica oleracea | 625 | + | 7 | ATATAAT | core promoter element around -30 of transcription start |
| TATA-box | Glycine max | 586 | - | 5 | TAATA | core promoter element around -30 of transcription start |
| TATA-box | Glycine max | 579 | - | 5 | TAATA | core promoter element around -30 of transcription start |
| TATA-box | Arabidopsis thaliana | 649 | - | 5 | TATAA | core promoter element around -30 of transcription start |
| TATA-box | Brassica napus | 127 | + | 6 | ATATAT | core promoter element around -30 of transcription start |
| TATA-box | Arabidopsis thaliana | 1200 | - | 4 | TATA | core promoter element around -30 of transcription start |
| TATA-box | Glycine max | 1030 | - | 5 | TAATA | core promoter element around -30 of transcription start |
| TATA-box | Arabidopsis thaliana | 1344 | - | 5 | TATAA | core promoter element around -30 of transcription start |
| TATA-box | Arabidopsis thaliana | 1342 | - | 7 | TATAAAA | core promoter element around -30 of transcription start |
| TATA-box | Ac | 1336 | + | 7 | TATAAAT | core promoter element around -30 of transcription start |
| TATA-box | Arabidopsis thaliana | 1032 | - | 7 | TATATAA | core promoter element around -30 of transcription start |
| TATA-box | Lycopersicon esculentum | 1020 | - | 5 | TTTTA | core promoter element around -30 of transcription start |
| TATA-box | Lycopersicon esculentum | 411 | + | 5 | TTTTA | core promoter element around -30 of transcription start |
| TATA-box | Glycine max | 573 | - | 5 | TAATA | core promoter element around -30 of transcription start |
| TATA-box | Arabidopsis thaliana | 1262 | - | 4 | TATA | core promoter element around -30 of transcription start |
| TATA-box | Brassica napus | 1036 | - | 6 | ATATAT | core promoter element around -30 of transcription start |
| TATA-box | Glycine max | 1369 | + | 5 | TAATA | core promoter element around -30 of transcription start |
| TATA-box | Brassica napus | 1444 | - | 6 | ATATAT | core promoter element around -30 of transcription start |
| TATA-box | Brassica napus | 1402 | - | 6 | ATATAT | core promoter element around -30 of transcription start |
| TATA-box | Arabidopsis thaliana | 1443 | - | 9 | tcTATATAtt | core promoter element around -30 of transcription start |
| TATA-box | Lycopersicon esculentum | 797 | + | 5 | TTTTA | core promoter element around -30 of transcription start |
| TATA-box | Pisum sativum | 646 | - | 8 | TATAAAAT | core promoter element around -30 of transcription start |
| TATA-box | Arabidopsis thaliana | 650 | + | 6 | TATAAA | core promoter element around -30 of transcription start |
| TATA-box | Arabidopsis thaliana | 1196 | + | 9 | tcTATATAtt | core promoter element around -30 of transcription start |
| TATA-box | Arabidopsis thaliana | 932 | - | 4 | TATA | core promoter element around -30 of transcription start |
| TATA-box | Lycopersicon esculentum | 1058 | + | 5 | TTTTA | core promoter element around -30 of transcription start |
| TATA-box | Brassica napus | 1034 | - | 6 | ATATAT | core promoter element around -30 of transcription start |
| TATA-box | Arabidopsis thaliana | 1400 | - | 7 | TATATAA | core promoter element around -30 of transcription start |
| TATA-box | Arabidopsis thaliana | 1398 | - | 7 | TATAAAA | core promoter element around -30 of transcription start |
| TATA-box | Arabidopsis thaliana | 1399 | - | 6 | TATAAA | core promoter element around -30 of transcription start |
| TATA-box | Zea mays | 1247 | + | 8 | TTTAAAAA | core promoter element around -30 of transcription start |
| TATA-box | Arabidopsis thaliana | 130 | + | 4 | TATA | core promoter element around -30 of transcription start |
| TATA-box | Arabidopsis thaliana | 631 | - | 5 | TATAA | core promoter element around -30 of transcription start |
| TATA-box | Brassica napus | 630 | + | 6 | ATTATA | core promoter element around -30 of transcription start |
| TATA-box | Arabidopsis thaliana | 1151 | - | 4 | TATA | core promoter element around -30 of transcription start |
| TATA-box | Brassica oleracea | 1038 | + | 6 | ATATAA | core promoter element around -30 of transcription start |
| TATA-box | Zea mays | 1306 | - | 8 | TTTAAAAA | core promoter element around -30 of transcription start |
| TATA-box | Arabidopsis thaliana | 1028 | - | 4 | TATA | core promoter element around -30 of transcription start |
| TATA-box | Brassica napus | 929 | - | 6 | ATATAT | core promoter element around -30 of transcription start |
| TATA-box | Arabidopsis thaliana | 1394 | - | 9 | TAAAAATAA | core promoter element around -30 of transcription start |
| TATA-box | Arabidopsis thaliana | 1215 | - | 5 | TATAA | core promoter element around -30 of transcription start |
| TATA-box | Ac | 1039 | + | 7 | TATAAAT | core promoter element around -30 of transcription start |
| TATA-box | Glycine max | 1333 | - | 5 | TAATA | core promoter element around -30 of transcription start |
| TATA-box | Brassica napus | 921 | - | 6 | ATATAT | core promoter element around -30 of transcription start |
| TATA-box | Arabidopsis thaliana | 1007 | + | 9 | TAAAAATAA | core promoter element around -30 of transcription start |
| TATA-box | Lycopersicon esculentum | 1267 | - | 5 | TTTTA | core promoter element around -30 of transcription start |
| TATA-box | Lycopersicon esculentum | 360 | + | 5 | TTTTA | core promoter element around -30 of transcription start |
| TATA-box | Glycine max | 257 | - | 5 | TAATA | core promoter element around -30 of transcription start |
| TATA-box | Antirrhinum majus | 657 | - | 8 | TATAAATT | core promoter element around -30 of transcription start |
| TATA-box | Arabidopsis thaliana | 659 | - | 6 | TATAAA | core promoter element around -30 of transcription start |
| TATA-box | Lycopersicon esculentum | 612 | - | 5 | TTTTA | core promoter element around -30 of transcription start |
| TATA-box | Ac | 1475 | + | 7 | TATAAAT | core promoter element around -30 of transcription start |
| TATA-box | Arabidopsis thaliana | 1474 | - | 5 | TATAA | core promoter element around -30 of transcription start |
| TATA-box | Brassica napus | 927 | - | 6 | ATATAT | core promoter element around -30 of transcription start |
| TATA-box | Lycopersicon esculentum | 1064 | - | 5 | TTTTA | core promoter element around -30 of transcription start |
| TATA-box | Arabidopsis thaliana | 1027 | - | 5 | TATAA | core promoter element around -30 of transcription start |
| TATA-box | Lycopersicon esculentum | 533 | - | 5 | TTTTA | core promoter element around -30 of transcription start |
| TATA-box | Lycopersicon esculentum | 596 | + | 5 | TTTTA | core promoter element around -30 of transcription start |
| TATA-box | Lycopersicon esculentum | 987 | + | 5 | TTTTA | core promoter element around -30 of transcription start |
| TATA-box | Arabidopsis thaliana | 128 | + | 8 | TATATATA | core promoter element around -30 of transcription start |
| TATA-box | Arabidopsis thaliana | 1357 | - | 4 | TATA | core promoter element around -30 of transcription start |
| TATA-box | Zea mays | 1166 | + | 8 | TTTAAAAA | core promoter element around -30 of transcription start |
| TATA-box | Arabidopsis thaliana | 1335 | - | 5 | TATAA | core promoter element around -30 of transcription start |
| TATA-box | Lycopersicon esculentum | 1086 | + | 5 | TTTTA | core promoter element around -30 of transcription start |
| TATA-box | Arabidopsis thaliana | 1403 | - | 4 | TATA | core promoter element around -30 of transcription start |
| TATA-box | Brassica napus | 1473 | + | 6 | ATTATA | core promoter element around -30 of transcription start |
| TATA-box | Lycopersicon esculentum | 1249 | - | 5 | TTTTA | core promoter element around -30 of transcription start |
| TATA-box | Arabidopsis thaliana | 918 | - | 8 | TATATATA | core promoter element around -30 of transcription start |
| TATA-box | Brassica napus | 129 | + | 6 | ATATAT | core promoter element around -30 of transcription start |
| TATA-box | Arabidopsis thaliana | 1026 | - | 6 | TATAAA | core promoter element around -30 of transcription start |
| TATA-box | Lycopersicon esculentum | 386 | + | 5 | TTTTA | core promoter element around -30 of transcription start |
| TATA-box | Brassica oleracea | 131 | + | 6 | ATATAA | core promoter element around -30 of transcription start |
| TATA-box | Arabidopsis thaliana | 924 | - | 8 | TATATATA | core promoter element around -30 of transcription start |
| TATA-box | Lycopersicon esculentum | 620 | - | 5 | TTTTA | core promoter element around -30 of transcription start |
| TATA-box | Lycopersicon esculentum | 952 | + | 5 | TTTTA | core promoter element around -30 of transcription start |
| TATA-box | Lycopersicon esculentum | 560 | - | 5 | TTTTA | core promoter element around -30 of transcription start |
| TATA-box | Ac | 658 | - | 7 | TATAAAT | core promoter element around -30 of transcription start |
| TATA-box | Arabidopsis thaliana | 928 | - | 8 | TATATATA | core promoter element around -30 of transcription start |
| TATA-box | Arabidopsis thaliana | 648 | - | 6 | TATAAA | core promoter element around -30 of transcription start |
| TATA-box | Arabidopsis thaliana | 208 | + | 4 | TATA | core promoter element around -30 of transcription start |
| TATA-box | Arabidopsis thaliana | 1033 | + | 11 | TATAAATATAAA | core promoter element around -30 of transcription start |
| TATA-box | Lycopersicon esculentum | 1002 | - | 5 | TTTTA | core promoter element around -30 of transcription start |
| TATA-box | Glycine max | 576 | - | 5 | TAATA | core promoter element around -30 of transcription start |
| TATA-box | Lycopersicon esculentum | 663 | - | 5 | TTTTA | core promoter element around -30 of transcription start |
| TATA-box | Lycopersicon esculentum | 363 | - | 5 | TTTTA | core promoter element around -30 of transcription start |
| TATA-box | Lycopersicon esculentum | 1372 | - | 5 | TTTTA | core promoter element around -30 of transcription start |
| TATA-box | Arabidopsis thaliana | 1226 | - | 4 | TATA | core promoter element around -30 of transcription start |
| TATA-box | Lycopersicon esculentum | 1310 | - | 5 | TTTTA | core promoter element around -30 of transcription start |
| TATA-box | Lycopersicon esculentum | 1050 | - | 5 | TTTTA | core promoter element around -30 of transcription start |
| TATA-box | Arabidopsis thaliana | 1445 | - | 8 | TATATATA | core promoter element around -30 of transcription start |
| TATA-box | Brassica napus | 1448 | - | 6 | ATATAT | core promoter element around -30 of transcription start |
| TATA-box | Arabidopsis thaliana | 969 | - | 4 | TATA | core promoter element around -30 of transcription start |
| TATA-box | Arabidopsis thaliana | 1198 | - | 4 | TATA | core promoter element around -30 of transcription start |
| TATA-box | Arabidopsis thaliana | 1035 | - | 8 | TATATATA | core promoter element around -30 of transcription start |
| TATA-box | Lycopersicon esculentum | 1366 | + | 5 | TTTTA | core promoter element around -30 of transcription start |
| TATA-box | Glycine max | 1330 | - | 5 | TAATA | core promoter element around -30 of transcription start |
| TATA-box | Arabidopsis thaliana | 632 | + | 4 | TATA | core promoter element around -30 of transcription start |
| TATA-box | Arabidopsis thaliana | 1216 | - | 4 | TATA | core promoter element around -30 of transcription start |
| TATA-box | Lycopersicon esculentum | 1156 | + | 5 | TTTTA | core promoter element around -30 of transcription start |
| TATA-box | Arabidopsis thaliana | 1401 | - | 4 | TATA | core promoter element around -30 of transcription start |
| TATA-box | Arabidopsis thaliana | 1168 | + | 9 | TAAAAATAA | core promoter element around -30 of transcription start |
| TATA-box | Glycine max | 1061 | + | 5 | TAATA | core promoter element around -30 of transcription start |
| TATA-box | Arabidopsis thaliana | 1383 | - | 4 | TATA | core promoter element around -30 of transcription start |
| TATA-box | Lycopersicon esculentum | 1220 | + | 5 | TTTTA | core promoter element around -30 of transcription start |
| TATA-box | Lycopersicon esculentum | 1416 | - | 5 | TTTTA | core promoter element around -30 of transcription start |
| TATA-box | Arabidopsis thaliana | 1037 | + | 9 | taTATAAAtc | core promoter element around -30 of transcription start |
| TATA-box | Arabidopsis thaliana | 1343 | - | 6 | TATAAA | core promoter element around -30 of transcription start |
| TATA-box | Lycopersicon esculentum | 1144 | + | 5 | TTTTA | core promoter element around -30 of transcription start |
| TATA-box | Lycopersicon esculentum | 1466 | + | 5 | TTTTA | core promoter element around -30 of transcription start |
| TATA-box | Arabidopsis thaliana | 1345 | - | 4 | TATA | core promoter element around -30 of transcription start |
| TATA-box | Arabidopsis thaliana | 1449 | - | 4 | TATA | core promoter element around -30 of transcription start |
| TATA-box | Lycopersicon esculentum | 1307 | + | 5 | TTTTA | core promoter element around -30 of transcription start |
| TATA-box | Glycine max | 1442 | + | 5 | TAATA | core promoter element around -30 of transcription start |
| TATA-box | Arabidopsis thaliana | 1359 | - | 4 | TATA | core promoter element around -30 of transcription start |
| TATA-box | Lycopersicon esculentum | 1469 | - | 5 | TTTTA | core promoter element around -30 of transcription start |
| TATA-box | Brassica napus | 1446 | - | 6 | ATATAT | core promoter element around -30 of transcription start |
| TATA-box | Brassica napus | 1334 | + | 6 | ATTATA | core promoter element around -30 of transcription start |
| TATA-box | Glycine max | 1315 | + | 5 | TAATA | core promoter element around -30 of transcription start |
| TATA-box | Arabidopsis thaliana | 1447 | - | 4 | TATA | core promoter element around -30 of transcription start |
| TATA-box | Pisum sativum | 1341 | - | 8 | TATAAAAT | core promoter element around -30 of transcription start |
| TATA-box | Glycine max | 634 | - | 5 | TAATA | core promoter element around -30 of transcription start |
| TATA-box | Arabidopsis thaliana | 920 | - | 8 | TATATATA | core promoter element around -30 of transcription start |
| TATA-box | Arabidopsis thaliana | 126 | - | 9 | tcTATATAtt | core promoter element around -30 of transcription start |
| TATA-box | Lycopersicon esculentum | 1013 | - | 5 | TTTTA | core promoter element around -30 of transcription start |
| TATA-box | Lycopersicon esculentum | 454 | + | 5 | TTTTA | core promoter element around -30 of transcription start |
| TATA-box | Lycopersicon esculentum | 837 | + | 5 | TTTTA | core promoter element around -30 of transcription start |
| TATA-box | Arabidopsis thaliana | 626 | + | 4 | TATA | core promoter element around -30 of transcription start |
| TATA-box | Lycopersicon esculentum | 975 | + | 5 | TTTTA | core promoter element around -30 of transcription start |
| TATA-box | Zea mays | 359 | - | 8 | TTTAAAAA | core promoter element around -30 of transcription start |
| TATA-box | Arabidopsis thaliana | 930 | - | 4 | TATA | core promoter element around -30 of transcription start |
| TATA-box | Lycopersicon esculentum | 570 | + | 5 | TTTTA | core promoter element around -30 of transcription start |
| TATA-box | Arabidopsis thaliana | 922 | - | 8 | TATATATA | core promoter element around -30 of transcription start |
| TATA-box | Lycopersicon esculentum | 371 | - | 5 | TTTTA | core promoter element around -30 of transcription start |
| TATA-box | Avena sativa | 623 | - | 12 | TATATTTATATTT | core promoter element around -30 of transcription start |
| TATA-box | Arabidopsis thaliana | 172 | + | 4 | TATA | core promoter element around -30 of transcription start |
| TATA-box | Avena sativa | 617 | - | 12 | TATATTTATATTT | core promoter element around -30 of transcription start |
| TATA-box | Arabidopsis thaliana | 539 | - | 9 | tcTATATAtt | core promoter element around -30 of transcription start |
| TATA-box | Lycopersicon esculentum | 251 | + | 5 | TTTTA | core promoter element around -30 of transcription start |

> 2018/04/13 10:10:12  
+ GTTCTAAAGA CAAATGATTT GTTCTAGCTA GACTCTCTTT TTCCTATTGA CAACCCGACT GAAAGTTCTG   
  
  
+ AAAAGTCTCT CTTAGCACTT CTCTATCTGT AAACGATCTC CTTCCGTTCC AAACAAATAT ATATAACCTC   
  
  
+ TCTCTGAGTC TAAAGAAATG CCGTTCATAG GTATAAGTTG TGGAGAAGAG GAAGGTACAC TTGGTCCTAT   
  
  
+ ACGTCGTAAG CAACTCAACT GAACAGTATT TCAGTTGGGT TTTTACTATT AAGTATGGTT TTCAAATTAG   
  
  
+ AATCTCATGA CTTTTGAAAT AACATAATCC TGAACGTGTA TTTAGAAGTA ATTAGTAGTG GCAACTGATA   
  
  
+ AAGAAATGTT TTTAAAATGT TAAAAATTAT TAATATTTTA GAGTTAGTAA GTAGTAAATT TTTTATGATT   
  
  
+ CAAAAAAAAT AGTTTACCCT TTCTAATTCT TTCTTTTAAC ACCTAAACCA CTACTTTCTT CCCGTACTAA   
  
  
+ CCAAGGAAAC TGAAAGTGTG AGAATTAATC TCACACTTAA ATTAAAAAAA TAAATAGACT TAATACAATT   
  
  
+ AAAAAACTAT TTTATTATTA TTATTTATTA AATTATTTTA ATTACATTAA TTAAAAAAAT AAAAATATAA   
  
  
+ TTATATTATT TAATAATTTT ATAAAAAATT TATAAAATTT TTCTGAATTG CAATTAAATC TACTTCTCTT   
  
  
+ CTTGAACTAG CGATCCCTCG TATTAAACGA GCTCTCAGAA TGGAGCAGAG ATAAACAAAC AGCCTCTCGA   
  
  
+ GGACAAACCG GTTACTCTAT TCTTCCTTTT AACGTAACCG TCTTTCCTAA AGTTTCAGTT TCTTTGTTTT   
  
  
+ ACTGCTTTTT CAGTCGGTGG CGTACGACGT TAAACGTTCC GGGGTTGATC GAAAAAGGTT AGTCTAAAAC   
  
  
+ AGTAGAGTAT ATATATATAT ATATATCTTT ATTAAACAAA GTTTTATTAG TTCAAATATA TAATTTTTAT   
  
  
+ CTTATTTTTT AATATTTAAT ATAAAATAAA AATAAAATGT AAAATTTTAT ATTATATATA TAAATTTATT   
  
  
+ AAAAATTTTT TAATAAAAAA ATATTAATAA ACTTTTTTTA GATTAAATTT AGTTTAATTT TCTAATTTAT   
  
  
+ GTGTTATTTA ATTAATTATT TCATTTTAGA TATAATTTTA TTAAATTTAA AAATAATACA AATGAAGTGT   
  
  
+ AATTATCTAT ATACTAAGAA CTGGTTATAT TTTAATATAG TTTTTTTACA TTACAGTTTA AAAATTATTA   
  
  
+ TTATATTAAA ACTGAAACTT TGCACATCGA TAGTTAACGA GACTCTTTTT AAAATAATAT TTAAGCAAGT   
  
  
+ ATTATTATAA ATTTTATAGT TTGAAGTATA TAGTATTTTA ATAAAAATTA GATATATTTG AAATTATTTT   
  
  
+ TATATATTGT AGTATTAAAA TTTTCAACTT AATGCTTGAA ATAATATATA TATGTTTCTA GAGTATTTTA   
  
  
+ AAATTATAAA TGAAATTTAA TGATTAGTT  

- CAAGATTTCT GTTTACTAAA CAAGATCGAT CTGAGAGAAA AAGGATAACT GTTGGGCTGA CTTTCAAGAC   
  
  
- TTTTCAGAGA GAATCGTGAA GAGATAGACA TTTGCTAGAG GAAGGCAAGG TTTGTTTATA TATATTGGAG   
  
  
- AGAGACTCAG ATTTCTTTAC GGCAAGTATC CATATTCAAC ACCTCTTCTC CTTCCATGTG AACCAGGATA   
  
  
- TGCAGCATTC GTTGAGTTGA CTTGTCATAA AGTCAACCCA AAAATGATAA TTCATACCAA AAGTTTAATC   
  
  
- TTAGAGTACT GAAAACTTTA TTGTATTAGG ACTTGCACAT AAATCTTCAT TAATCATCAC CGTTGACTAT   
  
  
- TTCTTTACAA AAATTTTACA ATTTTTAATA ATTATAAAAT CTCAATCATT CATCATTTAA AAAATACTAA   
  
  
- GTTTTTTTTA TCAAATGGGA AAGATTAAGA AAGAAAATTG TGGATTTGGT GATGAAAGAA GGGCATGATT   
  
  
- GGTTCCTTTG ACTTTCACAC TCTTAATTAG AGTGTGAATT TAATTTTTTT ATTTATCTGA ATTATGTTAA   
  
  
- TTTTTTGATA AAATAATAAT AATAAATAAT TTAATAAAAT TAATGTAATT AATTTTTTTA TTTTTATATT   
  
  
- AATATAATAA ATTATTAAAA TATTTTTTAA ATATTTTAAA AAGACTTAAC GTTAATTTAG ATGAAGAGAA   
  
  
- GAACTTGATC GCTAGGGAGC ATAATTTGCT CGAGAGTCTT ACCTCGTCTC TATTTGTTTG TCGGAGAGCT   
  
  
- CCTGTTTGGC CAATGAGATA AGAAGGAAAA TTGCATTGGC AGAAAGGATT TCAAAGTCAA AGAAACAAAA   
  
  
- TGACGAAAAA GTCAGCCACC GCATGCTGCA ATTTGCAAGG CCCCAACTAG CTTTTTCCAA TCAGATTTTG   
  
  
- TCATCTCATA TATATATATA TATATAGAAA TAATTTGTTT CAAAATAATC AAGTTTATAT ATTAAAAATA   
  
  
- GAATAAAAAA TTATAAATTA TATTTTATTT TTATTTTACA TTTTAAAATA TAATATATAT ATTTAAATAA   
  
  
- TTTTTAAAAA ATTATTTTTT TATAATTATT TGAAAAAAAT CTAATTTAAA TCAAATTAAA AGATTAAATA   
  
  
- CACAATAAAT TAATTAATAA AGTAAAATCT ATATTAAAAT AATTTAAATT TTTATTATGT TTACTTCACA   
  
  
- TTAATAGATA TATGATTCTT GACCAATATA AAATTATATC AAAAAAATGT AATGTCAAAT TTTTAATAAT   
  
  
- AATATAATTT TGACTTTGAA ACGTGTAGCT ATCAATTGCT CTGAGAAAAA TTTTATTATA AATTCGTTCA   
  
  
- TAATAATATT TAAAATATCA AACTTCATAT ATCATAAAAT TATTTTTAAT CTATATAAAC TTTAATAAAA   
  
  
- ATATATAACA TCATAATTTT AAAAGTTGAA TTACGAACTT TATTATATAT ATACAAAGAT CTCATAAAAT   
  
  
- TTTAATATTT ACTTTAAATT ACTAATCAA

+     TC-rich repeats

| Site Name | Organism | Position | Strand | Matrix score. | sequence | function |
| --- | --- | --- | --- | --- | --- | --- |
| TC-rich repeats | Nicotiana tabacum | 1230 | + | 9 | GTTTTCTTAC | cis-acting element involved in defense and stress responsiveness |

> 2018/04/13 10:10:12  
+ GTTCTAAAGA CAAATGATTT GTTCTAGCTA GACTCTCTTT TTCCTATTGA CAACCCGACT GAAAGTTCTG   
  
  
+ AAAAGTCTCT CTTAGCACTT CTCTATCTGT AAACGATCTC CTTCCGTTCC AAACAAATAT ATATAACCTC   
  
  
+ TCTCTGAGTC TAAAGAAATG CCGTTCATAG GTATAAGTTG TGGAGAAGAG GAAGGTACAC TTGGTCCTAT   
  
  
+ ACGTCGTAAG CAACTCAACT GAACAGTATT TCAGTTGGGT TTTTACTATT AAGTATGGTT TTCAAATTAG   
  
  
+ AATCTCATGA CTTTTGAAAT AACATAATCC TGAACGTGTA TTTAGAAGTA ATTAGTAGTG GCAACTGATA   
  
  
+ AAGAAATGTT TTTAAAATGT TAAAAATTAT TAATATTTTA GAGTTAGTAA GTAGTAAATT TTTTATGATT   
  
  
+ CAAAAAAAAT AGTTTACCCT TTCTAATTCT TTCTTTTAAC ACCTAAACCA CTACTTTCTT CCCGTACTAA   
  
  
+ CCAAGGAAAC TGAAAGTGTG AGAATTAATC TCACACTTAA ATTAAAAAAA TAAATAGACT TAATACAATT   
  
  
+ AAAAAACTAT TTTATTATTA TTATTTATTA AATTATTTTA ATTACATTAA TTAAAAAAAT AAAAATATAA   
  
  
+ TTATATTATT TAATAATTTT ATAAAAAATT TATAAAATTT TTCTGAATTG CAATTAAATC TACTTCTCTT   
  
  
+ CTTGAACTAG CGATCCCTCG TATTAAACGA GCTCTCAGAA TGGAGCAGAG ATAAACAAAC AGCCTCTCGA   
  
  
+ GGACAAACCG GTTACTCTAT TCTTCCTTTT AACGTAACCG TCTTTCCTAA AGTTTCAGTT TCTTTGTTTT   
  
  
+ ACTGCTTTTT CAGTCGGTGG CGTACGACGT TAAACGTTCC GGGGTTGATC GAAAAAGGTT AGTCTAAAAC   
  
  
+ AGTAGAGTAT ATATATATAT ATATATCTTT ATTAAACAAA GTTTTATTAG TTCAAATATA TAATTTTTAT   
  
  
+ CTTATTTTTT AATATTTAAT ATAAAATAAA AATAAAATGT AAAATTTTAT ATTATATATA TAAATTTATT   
  
  
+ AAAAATTTTT TAATAAAAAA ATATTAATAA ACTTTTTTTA GATTAAATTT AGTTTAATTT TCTAATTTAT   
  
  
+ GTGTTATTTA ATTAATTATT TCATTTTAGA TATAATTTTA TTAAATTTAA AAATAATACA AATGAAGTGT   
  
  
+ AATTATCTAT ATACTAAGAA CTGGTTATAT TTTAATATAG TTTTTTTACA TTACAGTTTA AAAATTATTA   
  
  
+ TTATATTAAA ACTGAAACTT TGCACATCGA TAGTTAACGA GACTCTTTTT AAAATAATAT TTAAGCAAGT   
  
  
+ ATTATTATAA ATTTTATAGT TTGAAGTATA TAGTATTTTA ATAAAAATTA GATATATTTG AAATTATTTT   
  
  
+ TATATATTGT AGTATTAAAA TTTTCAACTT AATGCTTGAA ATAATATATA TATGTTTCTA GAGTATTTTA   
  
  
+ AAATTATAAA TGAAATTTAA TGATTAGTT  

- CAAGATTTCT GTTTACTAAA CAAGATCGAT CTGAGAGAAA AAGGATAACT GTTGGGCTGA CTTTCAAGAC   
  
  
- TTTTCAGAGA GAATCGTGAA GAGATAGACA TTTGCTAGAG GAAGGCAAGG TTTGTTTATA TATATTGGAG   
  
  
- AGAGACTCAG ATTTCTTTAC GGCAAGTATC CATATTCAAC ACCTCTTCTC CTTCCATGTG AACCAGGATA   
  
  
- TGCAGCATTC GTTGAGTTGA CTTGTCATAA AGTCAACCCA AAAATGATAA TTCATACCAA AAGTTTAATC   
  
  
- TTAGAGTACT GAAAACTTTA TTGTATTAGG ACTTGCACAT AAATCTTCAT TAATCATCAC CGTTGACTAT   
  
  
- TTCTTTACAA AAATTTTACA ATTTTTAATA ATTATAAAAT CTCAATCATT CATCATTTAA AAAATACTAA   
  
  
- GTTTTTTTTA TCAAATGGGA AAGATTAAGA AAGAAAATTG TGGATTTGGT GATGAAAGAA GGGCATGATT   
  
  
- GGTTCCTTTG ACTTTCACAC TCTTAATTAG AGTGTGAATT TAATTTTTTT ATTTATCTGA ATTATGTTAA   
  
  
- TTTTTTGATA AAATAATAAT AATAAATAAT TTAATAAAAT TAATGTAATT AATTTTTTTA TTTTTATATT   
  
  
- AATATAATAA ATTATTAAAA TATTTTTTAA ATATTTTAAA AAGACTTAAC GTTAATTTAG ATGAAGAGAA   
  
  
- GAACTTGATC GCTAGGGAGC ATAATTTGCT CGAGAGTCTT ACCTCGTCTC TATTTGTTTG TCGGAGAGCT   
  
  
- CCTGTTTGGC CAATGAGATA AGAAGGAAAA TTGCATTGGC AGAAAGGATT TCAAAGTCAA AGAAACAAAA   
  
  
- TGACGAAAAA GTCAGCCACC GCATGCTGCA ATTTGCAAGG CCCCAACTAG CTTTTTCCAA TCAGATTTTG   
  
  
- TCATCTCATA TATATATATA TATATAGAAA TAATTTGTTT CAAAATAATC AAGTTTATAT ATTAAAAATA   
  
  
- GAATAAAAAA TTATAAATTA TATTTTATTT TTATTTTACA TTTTAAAATA TAATATATAT ATTTAAATAA   
  
  
- TTTTTAAAAA ATTATTTTTT TATAATTATT TGAAAAAAAT CTAATTTAAA TCAAATTAAA AGATTAAATA   
  
  
- CACAATAAAT TAATTAATAA AGTAAAATCT ATATTAAAAT AATTTAAATT TTTATTATGT TTACTTCACA   
  
  
- TTAATAGATA TATGATTCTT GACCAATATA AAATTATATC AAAAAAATGT AATGTCAAAT TTTTAATAAT   
  
  
- AATATAATTT TGACTTTGAA ACGTGTAGCT ATCAATTGCT CTGAGAAAAA TTTTATTATA AATTCGTTCA   
  
  
- TAATAATATT TAAAATATCA AACTTCATAT ATCATAAAAT TATTTTTAAT CTATATAAAC TTTAATAAAA   
  
  
- ATATATAACA TCATAATTTT AAAAGTTGAA TTACGAACTT TATTATATAT ATACAAAGAT CTCATAAAAT   
  
  
- TTTAATATTT ACTTTAAATT ACTAATCAA

+     Unnamed\_\_2

| Site Name | Organism | Position | Strand | Matrix score. | sequence | function |
| --- | --- | --- | --- | --- | --- | --- |
| Unnamed\_\_2 | Zea mays | 879 | - | 6 | CCCCGG |  |

> 2018/04/13 10:10:12  
+ GTTCTAAAGA CAAATGATTT GTTCTAGCTA GACTCTCTTT TTCCTATTGA CAACCCGACT GAAAGTTCTG   
  
  
+ AAAAGTCTCT CTTAGCACTT CTCTATCTGT AAACGATCTC CTTCCGTTCC AAACAAATAT ATATAACCTC   
  
  
+ TCTCTGAGTC TAAAGAAATG CCGTTCATAG GTATAAGTTG TGGAGAAGAG GAAGGTACAC TTGGTCCTAT   
  
  
+ ACGTCGTAAG CAACTCAACT GAACAGTATT TCAGTTGGGT TTTTACTATT AAGTATGGTT TTCAAATTAG   
  
  
+ AATCTCATGA CTTTTGAAAT AACATAATCC TGAACGTGTA TTTAGAAGTA ATTAGTAGTG GCAACTGATA   
  
  
+ AAGAAATGTT TTTAAAATGT TAAAAATTAT TAATATTTTA GAGTTAGTAA GTAGTAAATT TTTTATGATT   
  
  
+ CAAAAAAAAT AGTTTACCCT TTCTAATTCT TTCTTTTAAC ACCTAAACCA CTACTTTCTT CCCGTACTAA   
  
  
+ CCAAGGAAAC TGAAAGTGTG AGAATTAATC TCACACTTAA ATTAAAAAAA TAAATAGACT TAATACAATT   
  
  
+ AAAAAACTAT TTTATTATTA TTATTTATTA AATTATTTTA ATTACATTAA TTAAAAAAAT AAAAATATAA   
  
  
+ TTATATTATT TAATAATTTT ATAAAAAATT TATAAAATTT TTCTGAATTG CAATTAAATC TACTTCTCTT   
  
  
+ CTTGAACTAG CGATCCCTCG TATTAAACGA GCTCTCAGAA TGGAGCAGAG ATAAACAAAC AGCCTCTCGA   
  
  
+ GGACAAACCG GTTACTCTAT TCTTCCTTTT AACGTAACCG TCTTTCCTAA AGTTTCAGTT TCTTTGTTTT   
  
  
+ ACTGCTTTTT CAGTCGGTGG CGTACGACGT TAAACGTTCC GGGGTTGATC GAAAAAGGTT AGTCTAAAAC   
  
  
+ AGTAGAGTAT ATATATATAT ATATATCTTT ATTAAACAAA GTTTTATTAG TTCAAATATA TAATTTTTAT   
  
  
+ CTTATTTTTT AATATTTAAT ATAAAATAAA AATAAAATGT AAAATTTTAT ATTATATATA TAAATTTATT   
  
  
+ AAAAATTTTT TAATAAAAAA ATATTAATAA ACTTTTTTTA GATTAAATTT AGTTTAATTT TCTAATTTAT   
  
  
+ GTGTTATTTA ATTAATTATT TCATTTTAGA TATAATTTTA TTAAATTTAA AAATAATACA AATGAAGTGT   
  
  
+ AATTATCTAT ATACTAAGAA CTGGTTATAT TTTAATATAG TTTTTTTACA TTACAGTTTA AAAATTATTA   
  
  
+ TTATATTAAA ACTGAAACTT TGCACATCGA TAGTTAACGA GACTCTTTTT AAAATAATAT TTAAGCAAGT   
  
  
+ ATTATTATAA ATTTTATAGT TTGAAGTATA TAGTATTTTA ATAAAAATTA GATATATTTG AAATTATTTT   
  
  
+ TATATATTGT AGTATTAAAA TTTTCAACTT AATGCTTGAA ATAATATATA TATGTTTCTA GAGTATTTTA   
  
  
+ AAATTATAAA TGAAATTTAA TGATTAGTT  

- CAAGATTTCT GTTTACTAAA CAAGATCGAT CTGAGAGAAA AAGGATAACT GTTGGGCTGA CTTTCAAGAC   
  
  
- TTTTCAGAGA GAATCGTGAA GAGATAGACA TTTGCTAGAG GAAGGCAAGG TTTGTTTATA TATATTGGAG   
  
  
- AGAGACTCAG ATTTCTTTAC GGCAAGTATC CATATTCAAC ACCTCTTCTC CTTCCATGTG AACCAGGATA   
  
  
- TGCAGCATTC GTTGAGTTGA CTTGTCATAA AGTCAACCCA AAAATGATAA TTCATACCAA AAGTTTAATC   
  
  
- TTAGAGTACT GAAAACTTTA TTGTATTAGG ACTTGCACAT AAATCTTCAT TAATCATCAC CGTTGACTAT   
  
  
- TTCTTTACAA AAATTTTACA ATTTTTAATA ATTATAAAAT CTCAATCATT CATCATTTAA AAAATACTAA   
  
  
- GTTTTTTTTA TCAAATGGGA AAGATTAAGA AAGAAAATTG TGGATTTGGT GATGAAAGAA GGGCATGATT   
  
  
- GGTTCCTTTG ACTTTCACAC TCTTAATTAG AGTGTGAATT TAATTTTTTT ATTTATCTGA ATTATGTTAA   
  
  
- TTTTTTGATA AAATAATAAT AATAAATAAT TTAATAAAAT TAATGTAATT AATTTTTTTA TTTTTATATT   
  
  
- AATATAATAA ATTATTAAAA TATTTTTTAA ATATTTTAAA AAGACTTAAC GTTAATTTAG ATGAAGAGAA   
  
  
- GAACTTGATC GCTAGGGAGC ATAATTTGCT CGAGAGTCTT ACCTCGTCTC TATTTGTTTG TCGGAGAGCT   
  
  
- CCTGTTTGGC CAATGAGATA AGAAGGAAAA TTGCATTGGC AGAAAGGATT TCAAAGTCAA AGAAACAAAA   
  
  
- TGACGAAAAA GTCAGCCACC GCATGCTGCA ATTTGCAAGG CCCCAACTAG CTTTTTCCAA TCAGATTTTG   
  
  
- TCATCTCATA TATATATATA TATATAGAAA TAATTTGTTT CAAAATAATC AAGTTTATAT ATTAAAAATA   
  
  
- GAATAAAAAA TTATAAATTA TATTTTATTT TTATTTTACA TTTTAAAATA TAATATATAT ATTTAAATAA   
  
  
- TTTTTAAAAA ATTATTTTTT TATAATTATT TGAAAAAAAT CTAATTTAAA TCAAATTAAA AGATTAAATA   
  
  
- CACAATAAAT TAATTAATAA AGTAAAATCT ATATTAAAAT AATTTAAATT TTTATTATGT TTACTTCACA   
  
  
- TTAATAGATA TATGATTCTT GACCAATATA AAATTATATC AAAAAAATGT AATGTCAAAT TTTTAATAAT   
  
  
- AATATAATTT TGACTTTGAA ACGTGTAGCT ATCAATTGCT CTGAGAAAAA TTTTATTATA AATTCGTTCA   
  
  
- TAATAATATT TAAAATATCA AACTTCATAT ATCATAAAAT TATTTTTAAT CTATATAAAC TTTAATAAAA   
  
  
- ATATATAACA TCATAATTTT AAAAGTTGAA TTACGAACTT TATTATATAT ATACAAAGAT CTCATAAAAT   
  
  
- TTTAATATTT ACTTTAAATT ACTAATCAA

+     Unnamed\_\_4

| Site Name | Organism | Position | Strand | Matrix score. | sequence | function |
| --- | --- | --- | --- | --- | --- | --- |
| Unnamed\_\_4 | Petroselinum hortense | 182 | - | 4 | CTCC |  |
| Unnamed\_\_4 | Petroselinum hortense | 108 | + | 4 | CTCC |  |
| Unnamed\_\_4 | Petroselinum hortense | 742 | - | 4 | CTCC |  |

> 2018/04/13 10:10:12  
+ GTTCTAAAGA CAAATGATTT GTTCTAGCTA GACTCTCTTT TTCCTATTGA CAACCCGACT GAAAGTTCTG   
  
  
+ AAAAGTCTCT CTTAGCACTT CTCTATCTGT AAACGATCTC CTTCCGTTCC AAACAAATAT ATATAACCTC   
  
  
+ TCTCTGAGTC TAAAGAAATG CCGTTCATAG GTATAAGTTG TGGAGAAGAG GAAGGTACAC TTGGTCCTAT   
  
  
+ ACGTCGTAAG CAACTCAACT GAACAGTATT TCAGTTGGGT TTTTACTATT AAGTATGGTT TTCAAATTAG   
  
  
+ AATCTCATGA CTTTTGAAAT AACATAATCC TGAACGTGTA TTTAGAAGTA ATTAGTAGTG GCAACTGATA   
  
  
+ AAGAAATGTT TTTAAAATGT TAAAAATTAT TAATATTTTA GAGTTAGTAA GTAGTAAATT TTTTATGATT   
  
  
+ CAAAAAAAAT AGTTTACCCT TTCTAATTCT TTCTTTTAAC ACCTAAACCA CTACTTTCTT CCCGTACTAA   
  
  
+ CCAAGGAAAC TGAAAGTGTG AGAATTAATC TCACACTTAA ATTAAAAAAA TAAATAGACT TAATACAATT   
  
  
+ AAAAAACTAT TTTATTATTA TTATTTATTA AATTATTTTA ATTACATTAA TTAAAAAAAT AAAAATATAA   
  
  
+ TTATATTATT TAATAATTTT ATAAAAAATT TATAAAATTT TTCTGAATTG CAATTAAATC TACTTCTCTT   
  
  
+ CTTGAACTAG CGATCCCTCG TATTAAACGA GCTCTCAGAA TGGAGCAGAG ATAAACAAAC AGCCTCTCGA   
  
  
+ GGACAAACCG GTTACTCTAT TCTTCCTTTT AACGTAACCG TCTTTCCTAA AGTTTCAGTT TCTTTGTTTT   
  
  
+ ACTGCTTTTT CAGTCGGTGG CGTACGACGT TAAACGTTCC GGGGTTGATC GAAAAAGGTT AGTCTAAAAC   
  
  
+ AGTAGAGTAT ATATATATAT ATATATCTTT ATTAAACAAA GTTTTATTAG TTCAAATATA TAATTTTTAT   
  
  
+ CTTATTTTTT AATATTTAAT ATAAAATAAA AATAAAATGT AAAATTTTAT ATTATATATA TAAATTTATT   
  
  
+ AAAAATTTTT TAATAAAAAA ATATTAATAA ACTTTTTTTA GATTAAATTT AGTTTAATTT TCTAATTTAT   
  
  
+ GTGTTATTTA ATTAATTATT TCATTTTAGA TATAATTTTA TTAAATTTAA AAATAATACA AATGAAGTGT   
  
  
+ AATTATCTAT ATACTAAGAA CTGGTTATAT TTTAATATAG TTTTTTTACA TTACAGTTTA AAAATTATTA   
  
  
+ TTATATTAAA ACTGAAACTT TGCACATCGA TAGTTAACGA GACTCTTTTT AAAATAATAT TTAAGCAAGT   
  
  
+ ATTATTATAA ATTTTATAGT TTGAAGTATA TAGTATTTTA ATAAAAATTA GATATATTTG AAATTATTTT   
  
  
+ TATATATTGT AGTATTAAAA TTTTCAACTT AATGCTTGAA ATAATATATA TATGTTTCTA GAGTATTTTA   
  
  
+ AAATTATAAA TGAAATTTAA TGATTAGTT  

- CAAGATTTCT GTTTACTAAA CAAGATCGAT CTGAGAGAAA AAGGATAACT GTTGGGCTGA CTTTCAAGAC   
  
  
- TTTTCAGAGA GAATCGTGAA GAGATAGACA TTTGCTAGAG GAAGGCAAGG TTTGTTTATA TATATTGGAG   
  
  
- AGAGACTCAG ATTTCTTTAC GGCAAGTATC CATATTCAAC ACCTCTTCTC CTTCCATGTG AACCAGGATA   
  
  
- TGCAGCATTC GTTGAGTTGA CTTGTCATAA AGTCAACCCA AAAATGATAA TTCATACCAA AAGTTTAATC   
  
  
- TTAGAGTACT GAAAACTTTA TTGTATTAGG ACTTGCACAT AAATCTTCAT TAATCATCAC CGTTGACTAT   
  
  
- TTCTTTACAA AAATTTTACA ATTTTTAATA ATTATAAAAT CTCAATCATT CATCATTTAA AAAATACTAA   
  
  
- GTTTTTTTTA TCAAATGGGA AAGATTAAGA AAGAAAATTG TGGATTTGGT GATGAAAGAA GGGCATGATT   
  
  
- GGTTCCTTTG ACTTTCACAC TCTTAATTAG AGTGTGAATT TAATTTTTTT ATTTATCTGA ATTATGTTAA   
  
  
- TTTTTTGATA AAATAATAAT AATAAATAAT TTAATAAAAT TAATGTAATT AATTTTTTTA TTTTTATATT   
  
  
- AATATAATAA ATTATTAAAA TATTTTTTAA ATATTTTAAA AAGACTTAAC GTTAATTTAG ATGAAGAGAA   
  
  
- GAACTTGATC GCTAGGGAGC ATAATTTGCT CGAGAGTCTT ACCTCGTCTC TATTTGTTTG TCGGAGAGCT   
  
  
- CCTGTTTGGC CAATGAGATA AGAAGGAAAA TTGCATTGGC AGAAAGGATT TCAAAGTCAA AGAAACAAAA   
  
  
- TGACGAAAAA GTCAGCCACC GCATGCTGCA ATTTGCAAGG CCCCAACTAG CTTTTTCCAA TCAGATTTTG   
  
  
- TCATCTCATA TATATATATA TATATAGAAA TAATTTGTTT CAAAATAATC AAGTTTATAT ATTAAAAATA   
  
  
- GAATAAAAAA TTATAAATTA TATTTTATTT TTATTTTACA TTTTAAAATA TAATATATAT ATTTAAATAA   
  
  
- TTTTTAAAAA ATTATTTTTT TATAATTATT TGAAAAAAAT CTAATTTAAA TCAAATTAAA AGATTAAATA   
  
  
- CACAATAAAT TAATTAATAA AGTAAAATCT ATATTAAAAT AATTTAAATT TTTATTATGT TTACTTCACA   
  
  
- TTAATAGATA TATGATTCTT GACCAATATA AAATTATATC AAAAAAATGT AATGTCAAAT TTTTAATAAT   
  
  
- AATATAATTT TGACTTTGAA ACGTGTAGCT ATCAATTGCT CTGAGAAAAA TTTTATTATA AATTCGTTCA   
  
  
- TAATAATATT TAAAATATCA AACTTCATAT ATCATAAAAT TATTTTTAAT CTATATAAAC TTTAATAAAA   
  
  
- ATATATAACA TCATAATTTT AAAAGTTGAA TTACGAACTT TATTATATAT ATACAAAGAT CTCATAAAAT   
  
  
- TTTAATATTT ACTTTAAATT ACTAATCAA

+     Unnamed\_\_6

| Site Name | Organism | Position | Strand | Matrix score. | sequence | function |
| --- | --- | --- | --- | --- | --- | --- |
| Unnamed\_\_6 | Zea mays | 928 | + | 10 | taTAAATATct |  |

> 2018/04/13 10:10:12  
+ GTTCTAAAGA CAAATGATTT GTTCTAGCTA GACTCTCTTT TTCCTATTGA CAACCCGACT GAAAGTTCTG   
  
  
+ AAAAGTCTCT CTTAGCACTT CTCTATCTGT AAACGATCTC CTTCCGTTCC AAACAAATAT ATATAACCTC   
  
  
+ TCTCTGAGTC TAAAGAAATG CCGTTCATAG GTATAAGTTG TGGAGAAGAG GAAGGTACAC TTGGTCCTAT   
  
  
+ ACGTCGTAAG CAACTCAACT GAACAGTATT TCAGTTGGGT TTTTACTATT AAGTATGGTT TTCAAATTAG   
  
  
+ AATCTCATGA CTTTTGAAAT AACATAATCC TGAACGTGTA TTTAGAAGTA ATTAGTAGTG GCAACTGATA   
  
  
+ AAGAAATGTT TTTAAAATGT TAAAAATTAT TAATATTTTA GAGTTAGTAA GTAGTAAATT TTTTATGATT   
  
  
+ CAAAAAAAAT AGTTTACCCT TTCTAATTCT TTCTTTTAAC ACCTAAACCA CTACTTTCTT CCCGTACTAA   
  
  
+ CCAAGGAAAC TGAAAGTGTG AGAATTAATC TCACACTTAA ATTAAAAAAA TAAATAGACT TAATACAATT   
  
  
+ AAAAAACTAT TTTATTATTA TTATTTATTA AATTATTTTA ATTACATTAA TTAAAAAAAT AAAAATATAA   
  
  
+ TTATATTATT TAATAATTTT ATAAAAAATT TATAAAATTT TTCTGAATTG CAATTAAATC TACTTCTCTT   
  
  
+ CTTGAACTAG CGATCCCTCG TATTAAACGA GCTCTCAGAA TGGAGCAGAG ATAAACAAAC AGCCTCTCGA   
  
  
+ GGACAAACCG GTTACTCTAT TCTTCCTTTT AACGTAACCG TCTTTCCTAA AGTTTCAGTT TCTTTGTTTT   
  
  
+ ACTGCTTTTT CAGTCGGTGG CGTACGACGT TAAACGTTCC GGGGTTGATC GAAAAAGGTT AGTCTAAAAC   
  
  
+ AGTAGAGTAT ATATATATAT ATATATCTTT ATTAAACAAA GTTTTATTAG TTCAAATATA TAATTTTTAT   
  
  
+ CTTATTTTTT AATATTTAAT ATAAAATAAA AATAAAATGT AAAATTTTAT ATTATATATA TAAATTTATT   
  
  
+ AAAAATTTTT TAATAAAAAA ATATTAATAA ACTTTTTTTA GATTAAATTT AGTTTAATTT TCTAATTTAT   
  
  
+ GTGTTATTTA ATTAATTATT TCATTTTAGA TATAATTTTA TTAAATTTAA AAATAATACA AATGAAGTGT   
  
  
+ AATTATCTAT ATACTAAGAA CTGGTTATAT TTTAATATAG TTTTTTTACA TTACAGTTTA AAAATTATTA   
  
  
+ TTATATTAAA ACTGAAACTT TGCACATCGA TAGTTAACGA GACTCTTTTT AAAATAATAT TTAAGCAAGT   
  
  
+ ATTATTATAA ATTTTATAGT TTGAAGTATA TAGTATTTTA ATAAAAATTA GATATATTTG AAATTATTTT   
  
  
+ TATATATTGT AGTATTAAAA TTTTCAACTT AATGCTTGAA ATAATATATA TATGTTTCTA GAGTATTTTA   
  
  
+ AAATTATAAA TGAAATTTAA TGATTAGTT  

- CAAGATTTCT GTTTACTAAA CAAGATCGAT CTGAGAGAAA AAGGATAACT GTTGGGCTGA CTTTCAAGAC   
  
  
- TTTTCAGAGA GAATCGTGAA GAGATAGACA TTTGCTAGAG GAAGGCAAGG TTTGTTTATA TATATTGGAG   
  
  
- AGAGACTCAG ATTTCTTTAC GGCAAGTATC CATATTCAAC ACCTCTTCTC CTTCCATGTG AACCAGGATA   
  
  
- TGCAGCATTC GTTGAGTTGA CTTGTCATAA AGTCAACCCA AAAATGATAA TTCATACCAA AAGTTTAATC   
  
  
- TTAGAGTACT GAAAACTTTA TTGTATTAGG ACTTGCACAT AAATCTTCAT TAATCATCAC CGTTGACTAT   
  
  
- TTCTTTACAA AAATTTTACA ATTTTTAATA ATTATAAAAT CTCAATCATT CATCATTTAA AAAATACTAA   
  
  
- GTTTTTTTTA TCAAATGGGA AAGATTAAGA AAGAAAATTG TGGATTTGGT GATGAAAGAA GGGCATGATT   
  
  
- GGTTCCTTTG ACTTTCACAC TCTTAATTAG AGTGTGAATT TAATTTTTTT ATTTATCTGA ATTATGTTAA   
  
  
- TTTTTTGATA AAATAATAAT AATAAATAAT TTAATAAAAT TAATGTAATT AATTTTTTTA TTTTTATATT   
  
  
- AATATAATAA ATTATTAAAA TATTTTTTAA ATATTTTAAA AAGACTTAAC GTTAATTTAG ATGAAGAGAA   
  
  
- GAACTTGATC GCTAGGGAGC ATAATTTGCT CGAGAGTCTT ACCTCGTCTC TATTTGTTTG TCGGAGAGCT   
  
  
- CCTGTTTGGC CAATGAGATA AGAAGGAAAA TTGCATTGGC AGAAAGGATT TCAAAGTCAA AGAAACAAAA   
  
  
- TGACGAAAAA GTCAGCCACC GCATGCTGCA ATTTGCAAGG CCCCAACTAG CTTTTTCCAA TCAGATTTTG   
  
  
- TCATCTCATA TATATATATA TATATAGAAA TAATTTGTTT CAAAATAATC AAGTTTATAT ATTAAAAATA   
  
  
- GAATAAAAAA TTATAAATTA TATTTTATTT TTATTTTACA TTTTAAAATA TAATATATAT ATTTAAATAA   
  
  
- TTTTTAAAAA ATTATTTTTT TATAATTATT TGAAAAAAAT CTAATTTAAA TCAAATTAAA AGATTAAATA   
  
  
- CACAATAAAT TAATTAATAA AGTAAAATCT ATATTAAAAT AATTTAAATT TTTATTATGT TTACTTCACA   
  
  
- TTAATAGATA TATGATTCTT GACCAATATA AAATTATATC AAAAAAATGT AATGTCAAAT TTTTAATAAT   
  
  
- AATATAATTT TGACTTTGAA ACGTGTAGCT ATCAATTGCT CTGAGAAAAA TTTTATTATA AATTCGTTCA   
  
  
- TAATAATATT TAAAATATCA AACTTCATAT ATCATAAAAT TATTTTTAAT CTATATAAAC TTTAATAAAA   
  
  
- ATATATAACA TCATAATTTT AAAAGTTGAA TTACGAACTT TATTATATAT ATACAAAGAT CTCATAAAAT   
  
  
- TTTAATATTT ACTTTAAATT ACTAATCAA

+     circadian

| Site Name | Organism | Position | Strand | Matrix score. | sequence | function |
| --- | --- | --- | --- | --- | --- | --- |
| circadian | Lycopersicon esculentum | 681 | + | 6 | CAANNNNATC | cis-acting regulatory element involved in circadian control |
| circadian | Lycopersicon esculentum | 1381 | - | 9 | CAAAGATATC | cis-acting regulatory element involved in circadian control |

> 2018/04/13 10:10:12  
+ GTTCTAAAGA CAAATGATTT GTTCTAGCTA GACTCTCTTT TTCCTATTGA CAACCCGACT GAAAGTTCTG   
  
  
+ AAAAGTCTCT CTTAGCACTT CTCTATCTGT AAACGATCTC CTTCCGTTCC AAACAAATAT ATATAACCTC   
  
  
+ TCTCTGAGTC TAAAGAAATG CCGTTCATAG GTATAAGTTG TGGAGAAGAG GAAGGTACAC TTGGTCCTAT   
  
  
+ ACGTCGTAAG CAACTCAACT GAACAGTATT TCAGTTGGGT TTTTACTATT AAGTATGGTT TTCAAATTAG   
  
  
+ AATCTCATGA CTTTTGAAAT AACATAATCC TGAACGTGTA TTTAGAAGTA ATTAGTAGTG GCAACTGATA   
  
  
+ AAGAAATGTT TTTAAAATGT TAAAAATTAT TAATATTTTA GAGTTAGTAA GTAGTAAATT TTTTATGATT   
  
  
+ CAAAAAAAAT AGTTTACCCT TTCTAATTCT TTCTTTTAAC ACCTAAACCA CTACTTTCTT CCCGTACTAA   
  
  
+ CCAAGGAAAC TGAAAGTGTG AGAATTAATC TCACACTTAA ATTAAAAAAA TAAATAGACT TAATACAATT   
  
  
+ AAAAAACTAT TTTATTATTA TTATTTATTA AATTATTTTA ATTACATTAA TTAAAAAAAT AAAAATATAA   
  
  
+ TTATATTATT TAATAATTTT ATAAAAAATT TATAAAATTT TTCTGAATTG CAATTAAATC TACTTCTCTT   
  
  
+ CTTGAACTAG CGATCCCTCG TATTAAACGA GCTCTCAGAA TGGAGCAGAG ATAAACAAAC AGCCTCTCGA   
  
  
+ GGACAAACCG GTTACTCTAT TCTTCCTTTT AACGTAACCG TCTTTCCTAA AGTTTCAGTT TCTTTGTTTT   
  
  
+ ACTGCTTTTT CAGTCGGTGG CGTACGACGT TAAACGTTCC GGGGTTGATC GAAAAAGGTT AGTCTAAAAC   
  
  
+ AGTAGAGTAT ATATATATAT ATATATCTTT ATTAAACAAA GTTTTATTAG TTCAAATATA TAATTTTTAT   
  
  
+ CTTATTTTTT AATATTTAAT ATAAAATAAA AATAAAATGT AAAATTTTAT ATTATATATA TAAATTTATT   
  
  
+ AAAAATTTTT TAATAAAAAA ATATTAATAA ACTTTTTTTA GATTAAATTT AGTTTAATTT TCTAATTTAT   
  
  
+ GTGTTATTTA ATTAATTATT TCATTTTAGA TATAATTTTA TTAAATTTAA AAATAATACA AATGAAGTGT   
  
  
+ AATTATCTAT ATACTAAGAA CTGGTTATAT TTTAATATAG TTTTTTTACA TTACAGTTTA AAAATTATTA   
  
  
+ TTATATTAAA ACTGAAACTT TGCACATCGA TAGTTAACGA GACTCTTTTT AAAATAATAT TTAAGCAAGT   
  
  
+ ATTATTATAA ATTTTATAGT TTGAAGTATA TAGTATTTTA ATAAAAATTA GATATATTTG AAATTATTTT   
  
  
+ TATATATTGT AGTATTAAAA TTTTCAACTT AATGCTTGAA ATAATATATA TATGTTTCTA GAGTATTTTA   
  
  
+ AAATTATAAA TGAAATTTAA TGATTAGTT  

- CAAGATTTCT GTTTACTAAA CAAGATCGAT CTGAGAGAAA AAGGATAACT GTTGGGCTGA CTTTCAAGAC   
  
  
- TTTTCAGAGA GAATCGTGAA GAGATAGACA TTTGCTAGAG GAAGGCAAGG TTTGTTTATA TATATTGGAG   
  
  
- AGAGACTCAG ATTTCTTTAC GGCAAGTATC CATATTCAAC ACCTCTTCTC CTTCCATGTG AACCAGGATA   
  
  
- TGCAGCATTC GTTGAGTTGA CTTGTCATAA AGTCAACCCA AAAATGATAA TTCATACCAA AAGTTTAATC   
  
  
- TTAGAGTACT GAAAACTTTA TTGTATTAGG ACTTGCACAT AAATCTTCAT TAATCATCAC CGTTGACTAT   
  
  
- TTCTTTACAA AAATTTTACA ATTTTTAATA ATTATAAAAT CTCAATCATT CATCATTTAA AAAATACTAA   
  
  
- GTTTTTTTTA TCAAATGGGA AAGATTAAGA AAGAAAATTG TGGATTTGGT GATGAAAGAA GGGCATGATT   
  
  
- GGTTCCTTTG ACTTTCACAC TCTTAATTAG AGTGTGAATT TAATTTTTTT ATTTATCTGA ATTATGTTAA   
  
  
- TTTTTTGATA AAATAATAAT AATAAATAAT TTAATAAAAT TAATGTAATT AATTTTTTTA TTTTTATATT   
  
  
- AATATAATAA ATTATTAAAA TATTTTTTAA ATATTTTAAA AAGACTTAAC GTTAATTTAG ATGAAGAGAA   
  
  
- GAACTTGATC GCTAGGGAGC ATAATTTGCT CGAGAGTCTT ACCTCGTCTC TATTTGTTTG TCGGAGAGCT   
  
  
- CCTGTTTGGC CAATGAGATA AGAAGGAAAA TTGCATTGGC AGAAAGGATT TCAAAGTCAA AGAAACAAAA   
  
  
- TGACGAAAAA GTCAGCCACC GCATGCTGCA ATTTGCAAGG CCCCAACTAG CTTTTTCCAA TCAGATTTTG   
  
  
- TCATCTCATA TATATATATA TATATAGAAA TAATTTGTTT CAAAATAATC AAGTTTATAT ATTAAAAATA   
  
  
- GAATAAAAAA TTATAAATTA TATTTTATTT TTATTTTACA TTTTAAAATA TAATATATAT ATTTAAATAA   
  
  
- TTTTTAAAAA ATTATTTTTT TATAATTATT TGAAAAAAAT CTAATTTAAA TCAAATTAAA AGATTAAATA   
  
  
- CACAATAAAT TAATTAATAA AGTAAAATCT ATATTAAAAT AATTTAAATT TTTATTATGT TTACTTCACA   
  
  
- TTAATAGATA TATGATTCTT GACCAATATA AAATTATATC AAAAAAATGT AATGTCAAAT TTTTAATAAT   
  
  
- AATATAATTT TGACTTTGAA ACGTGTAGCT ATCAATTGCT CTGAGAAAAA TTTTATTATA AATTCGTTCA   
  
  
- TAATAATATT TAAAATATCA AACTTCATAT ATCATAAAAT TATTTTTAAT CTATATAAAC TTTAATAAAA   
  
  
- ATATATAACA TCATAATTTT AAAAGTTGAA TTACGAACTT TATTATATAT ATACAAAGAT CTCATAAAAT   
  
  
- TTTAATATTT ACTTTAAATT ACTAATCAA
