# Supplementary material for: Genome-wide analysis of the WRKY gene family in drumstick (Moringa oleifera Lam.)
Source: PeerJ. 2019 Jun 10;7:e7063. doi: 10.7717/peerj.7063 (PMC6563795; doi:10.7717/peerj.7063)
Supplement: Supplemental Information 1 [file peerj-07-7063-s003.gz › MoWRKY33_plantcare.html]

Content-Type: text/html; charset=ISO-8859-1


CallMat\_Firefox


Webmaster Firefox specific output  
To save the result:
click on the frame with the right mouse button and save the source code as a text file with extension .html  
REFERENCE:PlantCARE: a database of plant cis-acting regulatory elements and a portal to tools for in silico analysis of promoter sequences.  
Lescot, M., Déhais, P., Moreau, Y., De Moor, B., Rouzé ,P.,and Rombauts, S.  
Nucleic Acids Res., Database issue(2002), 30(1):325-327.   


---

> 2018/04/13 10:10:12  
+ TTTACCTCTC CTTCTCCTCT CCAAAACCCA CCTTCAGAAC TTTGCCACGT TATCTGGGCC GTAATTTTTC   
  
  
+ CCTCTCGACT CTGAAATCTT CTTCTGGTTT GTCATCATCA ATCACGAAGA CACAAAATTA ATAAAATGTT   
  
  
+ TGGGGAAAAA GAGTAAAATG TAGCTATATA GTATTAACTT TTCCTCCGCT CCTGTGAGAG AACCAGGATT   
  
  
+ AAGTGAAGGA AAGGGAAAAG AAAAGAAAAA AAAAAGTTTA TAATGAATAA TTAATTTAAA TAAAATGAAT   
  
  
+ TTTAATATAT AATAGTATAG ATAAAAAATT TTTAGACAAA GATATAGTTA GAATTTTTAT GAATCTCTAT   
  
  
+ ATAATAGTTT TTGTTAGAGA GGAAATAACA TTTTATAAGT AGTTTATAAT GAATTTTTAA CTTGGAGTTA   
  
  
+ TTTTGGTAGT ACGTTTAGTT AAACTACACC GAGACCAAAC TCGTAAATTC GTATAATCAT AAAAAAAAGA   
  
  
+ CTTTTTTTTT ACAAGAATTC TTTCTTTTAT TTTTTTATTT TGTCTACTAA AATATAAGTT AAAATTAAAA   
  
  
+ ATTCCTGAAG ACATTACCGA TGAATACACC ACATCACTGA TGACCGATTC TAACAGTATC ATTCTCTCTC   
  
  
+ TCTCCAACAA CACCCCGGGT TGTTTCCGTT CCCCCTTCAG AAGGCTAAAG AGAGGCATCT AACTGCGCGG   
  
  
+ GGTTTTAAAG TAGCGGACCT AACCGGGCCG CGTAGAGCCC GAGCACTTTT ACCCTCCAGA AACCAGTTGC   
  
  
+ TAGAACTGTT CGGACGACCC GGGGTGTTCC GTAGGCCAGA TTAAACACCC GGGGCGCGGA AGTATAACCT   
  
  
+ GAATAACGGT GCAGCCTACC CACAGGAGCC TGGGTTGGAT TTGCCCAACT GAGGCAGCAG GAAAGAAGTG   
  
  
+ GGGTAGATAG GGAGCGGAGC GGAGCGGAGT ATCGAACTTA ACAAATGAAT AATACAATGA GCTAAATAAA   
  
  
+ TAATAATAAA ATTTTTTTAA TTTTCATTTT ATATATAATG AAAATTAATT ATAAATTATT ATTATTTAAT   
  
  
+ AATTAATATT ATTTCATTTG TTTTGCCTTC AGCTAGACGG GGATGTCATC GTGTTGAACG TTCGTGTCCG   
  
  
+ TTGAACGTAT GCACTAAACT GTTCATTTCC ACCAATCATC TACTAACATG TGTGCTTATA AACTAAAATA   
  
  
+ TCATTAAATA TGTATACACA AATTTTTTAG TTTATAATTA AGAATGTAAT TAAATATAAA AATATATTAA   
  
  
+ TAAAAAATAT TTTTTGTTTA TTTTTAAACT TATAGAGTTT TAAATTATAA AAATTCGTCA CATCTTTTCC   
  
  
+ TGTCCACCTG GCGTAACAAA CTTACTCAGT TTCTGGAAAA ATGGGAAGTA TATAAGTTGT TAAAGAAGTG   
  
  
+ GTGGAGTGCA CTAAACGGTA GTTACAGAAT GTGAGGAAGG TGTGCCTTCC TAAGAGGAAG AAGGGTAAGA   
  
  
+ AGTCTTTAAT TCTGCCTTTA CCATTAACG  

- AAATGGAGAG GAAGAGGAGA GGTTTTGGGT GGAAGTCTTG AAACGGTGCA ATAGACCCGG CATTAAAAAG   
  
  
- GGAGAGCTGA GACTTTAGAA GAAGACCAAA CAGTAGTAGT TAGTGCTTCT GTGTTTTAAT TATTTTACAA   
  
  
- ACCCCTTTTT CTCATTTTAC ATCGATATAT CATAATTGAA AAGGAGGCGA GGACACTCTC TTGGTCCTAA   
  
  
- TTCACTTCCT TTCCCTTTTC TTTTCTTTTT TTTTTCAAAT ATTACTTATT AATTAAATTT ATTTTACTTA   
  
  
- AAATTATATA TTATCATATC TATTTTTTAA AAATCTGTTT CTATATCAAT CTTAAAAATA CTTAGAGATA   
  
  
- TATTATCAAA AACAATCTCT CCTTTATTGT AAAATATTCA TCAAATATTA CTTAAAAATT GAACCTCAAT   
  
  
- AAAACCATCA TGCAAATCAA TTTGATGTGG CTCTGGTTTG AGCATTTAAG CATATTAGTA TTTTTTTTCT   
  
  
- GAAAAAAAAA TGTTCTTAAG AAAGAAAATA AAAAAATAAA ACAGATGATT TTATATTCAA TTTTAATTTT   
  
  
- TAAGGACTTC TGTAATGGCT ACTTATGTGG TGTAGTGACT ACTGGCTAAG ATTGTCATAG TAAGAGAGAG   
  
  
- AGAGGTTGTT GTGGGGCCCA ACAAAGGCAA GGGGGAAGTC TTCCGATTTC TCTCCGTAGA TTGACGCGCC   
  
  
- CCAAAATTTC ATCGCCTGGA TTGGCCCGGC GCATCTCGGG CTCGTGAAAA TGGGAGGTCT TTGGTCAACG   
  
  
- ATCTTGACAA GCCTGCTGGG CCCCACAAGG CATCCGGTCT AATTTGTGGG CCCCGCGCCT TCATATTGGA   
  
  
- CTTATTGCCA CGTCGGATGG GTGTCCTCGG ACCCAACCTA AACGGGTTGA CTCCGTCGTC CTTTCTTCAC   
  
  
- CCCATCTATC CCTCGCCTCG CCTCGCCTCA TAGCTTGAAT TGTTTACTTA TTATGTTACT CGATTTATTT   
  
  
- ATTATTATTT TAAAAAAATT AAAAGTAAAA TATATATTAC TTTTAATTAA TATTTAATAA TAATAAATTA   
  
  
- TTAATTATAA TAAAGTAAAC AAAACGGAAG TCGATCTGCC CCTACAGTAG CACAACTTGC AAGCACAGGC   
  
  
- AACTTGCATA CGTGATTTGA CAAGTAAAGG TGGTTAGTAG ATGATTGTAC ACACGAATAT TTGATTTTAT   
  
  
- AGTAATTTAT ACATATGTGT TTAAAAAATC AAATATTAAT TCTTACATTA ATTTATATTT TTATATAATT   
  
  
- ATTTTTTATA AAAAACAAAT AAAAATTTGA ATATCTCAAA ATTTAATATT TTTAAGCAGT GTAGAAAAGG   
  
  
- ACAGGTGGAC CGCATTGTTT GAATGAGTCA AAGACCTTTT TACCCTTCAT ATATTCAACA ATTTCTTCAC   
  
  
- CACCTCACGT GATTTGCCAT CAATGTCTTA CACTCCTTCC ACACGGAAGG ATTCTCCTTC TTCCCATTCT   
  
  
- TCAGAAATTA AGACGGAAAT GGTAATTGC

  
  
Motifs Found  

+     3-AF1 binding site

| Site Name | Organism | Position | Strand | Matrix score. | sequence | function |
| --- | --- | --- | --- | --- | --- | --- |
| 3-AF1 binding site | Solanum tuberosum | 364 | + | 10 | TAAGAGAGGAA | light responsive element |

> 2018/04/13 10:10:12  
+ TTTACCTCTC CTTCTCCTCT CCAAAACCCA CCTTCAGAAC TTTGCCACGT TATCTGGGCC GTAATTTTTC   
  
  
+ CCTCTCGACT CTGAAATCTT CTTCTGGTTT GTCATCATCA ATCACGAAGA CACAAAATTA ATAAAATGTT   
  
  
+ TGGGGAAAAA GAGTAAAATG TAGCTATATA GTATTAACTT TTCCTCCGCT CCTGTGAGAG AACCAGGATT   
  
  
+ AAGTGAAGGA AAGGGAAAAG AAAAGAAAAA AAAAAGTTTA TAATGAATAA TTAATTTAAA TAAAATGAAT   
  
  
+ TTTAATATAT AATAGTATAG ATAAAAAATT TTTAGACAAA GATATAGTTA GAATTTTTAT GAATCTCTAT   
  
  
+ ATAATAGTTT TTGTTAGAGA GGAAATAACA TTTTATAAGT AGTTTATAAT GAATTTTTAA CTTGGAGTTA   
  
  
+ TTTTGGTAGT ACGTTTAGTT AAACTACACC GAGACCAAAC TCGTAAATTC GTATAATCAT AAAAAAAAGA   
  
  
+ CTTTTTTTTT ACAAGAATTC TTTCTTTTAT TTTTTTATTT TGTCTACTAA AATATAAGTT AAAATTAAAA   
  
  
+ ATTCCTGAAG ACATTACCGA TGAATACACC ACATCACTGA TGACCGATTC TAACAGTATC ATTCTCTCTC   
  
  
+ TCTCCAACAA CACCCCGGGT TGTTTCCGTT CCCCCTTCAG AAGGCTAAAG AGAGGCATCT AACTGCGCGG   
  
  
+ GGTTTTAAAG TAGCGGACCT AACCGGGCCG CGTAGAGCCC GAGCACTTTT ACCCTCCAGA AACCAGTTGC   
  
  
+ TAGAACTGTT CGGACGACCC GGGGTGTTCC GTAGGCCAGA TTAAACACCC GGGGCGCGGA AGTATAACCT   
  
  
+ GAATAACGGT GCAGCCTACC CACAGGAGCC TGGGTTGGAT TTGCCCAACT GAGGCAGCAG GAAAGAAGTG   
  
  
+ GGGTAGATAG GGAGCGGAGC GGAGCGGAGT ATCGAACTTA ACAAATGAAT AATACAATGA GCTAAATAAA   
  
  
+ TAATAATAAA ATTTTTTTAA TTTTCATTTT ATATATAATG AAAATTAATT ATAAATTATT ATTATTTAAT   
  
  
+ AATTAATATT ATTTCATTTG TTTTGCCTTC AGCTAGACGG GGATGTCATC GTGTTGAACG TTCGTGTCCG   
  
  
+ TTGAACGTAT GCACTAAACT GTTCATTTCC ACCAATCATC TACTAACATG TGTGCTTATA AACTAAAATA   
  
  
+ TCATTAAATA TGTATACACA AATTTTTTAG TTTATAATTA AGAATGTAAT TAAATATAAA AATATATTAA   
  
  
+ TAAAAAATAT TTTTTGTTTA TTTTTAAACT TATAGAGTTT TAAATTATAA AAATTCGTCA CATCTTTTCC   
  
  
+ TGTCCACCTG GCGTAACAAA CTTACTCAGT TTCTGGAAAA ATGGGAAGTA TATAAGTTGT TAAAGAAGTG   
  
  
+ GTGGAGTGCA CTAAACGGTA GTTACAGAAT GTGAGGAAGG TGTGCCTTCC TAAGAGGAAG AAGGGTAAGA   
  
  
+ AGTCTTTAAT TCTGCCTTTA CCATTAACG  

- AAATGGAGAG GAAGAGGAGA GGTTTTGGGT GGAAGTCTTG AAACGGTGCA ATAGACCCGG CATTAAAAAG   
  
  
- GGAGAGCTGA GACTTTAGAA GAAGACCAAA CAGTAGTAGT TAGTGCTTCT GTGTTTTAAT TATTTTACAA   
  
  
- ACCCCTTTTT CTCATTTTAC ATCGATATAT CATAATTGAA AAGGAGGCGA GGACACTCTC TTGGTCCTAA   
  
  
- TTCACTTCCT TTCCCTTTTC TTTTCTTTTT TTTTTCAAAT ATTACTTATT AATTAAATTT ATTTTACTTA   
  
  
- AAATTATATA TTATCATATC TATTTTTTAA AAATCTGTTT CTATATCAAT CTTAAAAATA CTTAGAGATA   
  
  
- TATTATCAAA AACAATCTCT CCTTTATTGT AAAATATTCA TCAAATATTA CTTAAAAATT GAACCTCAAT   
  
  
- AAAACCATCA TGCAAATCAA TTTGATGTGG CTCTGGTTTG AGCATTTAAG CATATTAGTA TTTTTTTTCT   
  
  
- GAAAAAAAAA TGTTCTTAAG AAAGAAAATA AAAAAATAAA ACAGATGATT TTATATTCAA TTTTAATTTT   
  
  
- TAAGGACTTC TGTAATGGCT ACTTATGTGG TGTAGTGACT ACTGGCTAAG ATTGTCATAG TAAGAGAGAG   
  
  
- AGAGGTTGTT GTGGGGCCCA ACAAAGGCAA GGGGGAAGTC TTCCGATTTC TCTCCGTAGA TTGACGCGCC   
  
  
- CCAAAATTTC ATCGCCTGGA TTGGCCCGGC GCATCTCGGG CTCGTGAAAA TGGGAGGTCT TTGGTCAACG   
  
  
- ATCTTGACAA GCCTGCTGGG CCCCACAAGG CATCCGGTCT AATTTGTGGG CCCCGCGCCT TCATATTGGA   
  
  
- CTTATTGCCA CGTCGGATGG GTGTCCTCGG ACCCAACCTA AACGGGTTGA CTCCGTCGTC CTTTCTTCAC   
  
  
- CCCATCTATC CCTCGCCTCG CCTCGCCTCA TAGCTTGAAT TGTTTACTTA TTATGTTACT CGATTTATTT   
  
  
- ATTATTATTT TAAAAAAATT AAAAGTAAAA TATATATTAC TTTTAATTAA TATTTAATAA TAATAAATTA   
  
  
- TTAATTATAA TAAAGTAAAC AAAACGGAAG TCGATCTGCC CCTACAGTAG CACAACTTGC AAGCACAGGC   
  
  
- AACTTGCATA CGTGATTTGA CAAGTAAAGG TGGTTAGTAG ATGATTGTAC ACACGAATAT TTGATTTTAT   
  
  
- AGTAATTTAT ACATATGTGT TTAAAAAATC AAATATTAAT TCTTACATTA ATTTATATTT TTATATAATT   
  
  
- ATTTTTTATA AAAAACAAAT AAAAATTTGA ATATCTCAAA ATTTAATATT TTTAAGCAGT GTAGAAAAGG   
  
  
- ACAGGTGGAC CGCATTGTTT GAATGAGTCA AAGACCTTTT TACCCTTCAT ATATTCAACA ATTTCTTCAC   
  
  
- CACCTCACGT GATTTGCCAT CAATGTCTTA CACTCCTTCC ACACGGAAGG ATTCTCCTTC TTCCCATTCT   
  
  
- TCAGAAATTA AGACGGAAAT GGTAATTGC

+     5UTR Py-rich stretch

| Site Name | Organism | Position | Strand | Matrix score. | sequence | function |
| --- | --- | --- | --- | --- | --- | --- |
| 5UTR Py-rich stretch | Lycopersicon esculentum | 621 | + | 13 | TTTCTCTCTCTCTC | cis-acting element conferring high transcription levels |
| 5UTR Py-rich stretch | Lycopersicon esculentum | 366 | - | 9 | TTTCTTCTCT | cis-acting element conferring high transcription levels |
| 5UTR Py-rich stretch | Lycopersicon esculentum | 229 | - | 9 | TTTCTTCTCT | cis-acting element conferring high transcription levels |

> 2018/04/13 10:10:12  
+ TTTACCTCTC CTTCTCCTCT CCAAAACCCA CCTTCAGAAC TTTGCCACGT TATCTGGGCC GTAATTTTTC   
  
  
+ CCTCTCGACT CTGAAATCTT CTTCTGGTTT GTCATCATCA ATCACGAAGA CACAAAATTA ATAAAATGTT   
  
  
+ TGGGGAAAAA GAGTAAAATG TAGCTATATA GTATTAACTT TTCCTCCGCT CCTGTGAGAG AACCAGGATT   
  
  
+ AAGTGAAGGA AAGGGAAAAG AAAAGAAAAA AAAAAGTTTA TAATGAATAA TTAATTTAAA TAAAATGAAT   
  
  
+ TTTAATATAT AATAGTATAG ATAAAAAATT TTTAGACAAA GATATAGTTA GAATTTTTAT GAATCTCTAT   
  
  
+ ATAATAGTTT TTGTTAGAGA GGAAATAACA TTTTATAAGT AGTTTATAAT GAATTTTTAA CTTGGAGTTA   
  
  
+ TTTTGGTAGT ACGTTTAGTT AAACTACACC GAGACCAAAC TCGTAAATTC GTATAATCAT AAAAAAAAGA   
  
  
+ CTTTTTTTTT ACAAGAATTC TTTCTTTTAT TTTTTTATTT TGTCTACTAA AATATAAGTT AAAATTAAAA   
  
  
+ ATTCCTGAAG ACATTACCGA TGAATACACC ACATCACTGA TGACCGATTC TAACAGTATC ATTCTCTCTC   
  
  
+ TCTCCAACAA CACCCCGGGT TGTTTCCGTT CCCCCTTCAG AAGGCTAAAG AGAGGCATCT AACTGCGCGG   
  
  
+ GGTTTTAAAG TAGCGGACCT AACCGGGCCG CGTAGAGCCC GAGCACTTTT ACCCTCCAGA AACCAGTTGC   
  
  
+ TAGAACTGTT CGGACGACCC GGGGTGTTCC GTAGGCCAGA TTAAACACCC GGGGCGCGGA AGTATAACCT   
  
  
+ GAATAACGGT GCAGCCTACC CACAGGAGCC TGGGTTGGAT TTGCCCAACT GAGGCAGCAG GAAAGAAGTG   
  
  
+ GGGTAGATAG GGAGCGGAGC GGAGCGGAGT ATCGAACTTA ACAAATGAAT AATACAATGA GCTAAATAAA   
  
  
+ TAATAATAAA ATTTTTTTAA TTTTCATTTT ATATATAATG AAAATTAATT ATAAATTATT ATTATTTAAT   
  
  
+ AATTAATATT ATTTCATTTG TTTTGCCTTC AGCTAGACGG GGATGTCATC GTGTTGAACG TTCGTGTCCG   
  
  
+ TTGAACGTAT GCACTAAACT GTTCATTTCC ACCAATCATC TACTAACATG TGTGCTTATA AACTAAAATA   
  
  
+ TCATTAAATA TGTATACACA AATTTTTTAG TTTATAATTA AGAATGTAAT TAAATATAAA AATATATTAA   
  
  
+ TAAAAAATAT TTTTTGTTTA TTTTTAAACT TATAGAGTTT TAAATTATAA AAATTCGTCA CATCTTTTCC   
  
  
+ TGTCCACCTG GCGTAACAAA CTTACTCAGT TTCTGGAAAA ATGGGAAGTA TATAAGTTGT TAAAGAAGTG   
  
  
+ GTGGAGTGCA CTAAACGGTA GTTACAGAAT GTGAGGAAGG TGTGCCTTCC TAAGAGGAAG AAGGGTAAGA   
  
  
+ AGTCTTTAAT TCTGCCTTTA CCATTAACG  

- AAATGGAGAG GAAGAGGAGA GGTTTTGGGT GGAAGTCTTG AAACGGTGCA ATAGACCCGG CATTAAAAAG   
  
  
- GGAGAGCTGA GACTTTAGAA GAAGACCAAA CAGTAGTAGT TAGTGCTTCT GTGTTTTAAT TATTTTACAA   
  
  
- ACCCCTTTTT CTCATTTTAC ATCGATATAT CATAATTGAA AAGGAGGCGA GGACACTCTC TTGGTCCTAA   
  
  
- TTCACTTCCT TTCCCTTTTC TTTTCTTTTT TTTTTCAAAT ATTACTTATT AATTAAATTT ATTTTACTTA   
  
  
- AAATTATATA TTATCATATC TATTTTTTAA AAATCTGTTT CTATATCAAT CTTAAAAATA CTTAGAGATA   
  
  
- TATTATCAAA AACAATCTCT CCTTTATTGT AAAATATTCA TCAAATATTA CTTAAAAATT GAACCTCAAT   
  
  
- AAAACCATCA TGCAAATCAA TTTGATGTGG CTCTGGTTTG AGCATTTAAG CATATTAGTA TTTTTTTTCT   
  
  
- GAAAAAAAAA TGTTCTTAAG AAAGAAAATA AAAAAATAAA ACAGATGATT TTATATTCAA TTTTAATTTT   
  
  
- TAAGGACTTC TGTAATGGCT ACTTATGTGG TGTAGTGACT ACTGGCTAAG ATTGTCATAG TAAGAGAGAG   
  
  
- AGAGGTTGTT GTGGGGCCCA ACAAAGGCAA GGGGGAAGTC TTCCGATTTC TCTCCGTAGA TTGACGCGCC   
  
  
- CCAAAATTTC ATCGCCTGGA TTGGCCCGGC GCATCTCGGG CTCGTGAAAA TGGGAGGTCT TTGGTCAACG   
  
  
- ATCTTGACAA GCCTGCTGGG CCCCACAAGG CATCCGGTCT AATTTGTGGG CCCCGCGCCT TCATATTGGA   
  
  
- CTTATTGCCA CGTCGGATGG GTGTCCTCGG ACCCAACCTA AACGGGTTGA CTCCGTCGTC CTTTCTTCAC   
  
  
- CCCATCTATC CCTCGCCTCG CCTCGCCTCA TAGCTTGAAT TGTTTACTTA TTATGTTACT CGATTTATTT   
  
  
- ATTATTATTT TAAAAAAATT AAAAGTAAAA TATATATTAC TTTTAATTAA TATTTAATAA TAATAAATTA   
  
  
- TTAATTATAA TAAAGTAAAC AAAACGGAAG TCGATCTGCC CCTACAGTAG CACAACTTGC AAGCACAGGC   
  
  
- AACTTGCATA CGTGATTTGA CAAGTAAAGG TGGTTAGTAG ATGATTGTAC ACACGAATAT TTGATTTTAT   
  
  
- AGTAATTTAT ACATATGTGT TTAAAAAATC AAATATTAAT TCTTACATTA ATTTATATTT TTATATAATT   
  
  
- ATTTTTTATA AAAAACAAAT AAAAATTTGA ATATCTCAAA ATTTAATATT TTTAAGCAGT GTAGAAAAGG   
  
  
- ACAGGTGGAC CGCATTGTTT GAATGAGTCA AAGACCTTTT TACCCTTCAT ATATTCAACA ATTTCTTCAC   
  
  
- CACCTCACGT GATTTGCCAT CAATGTCTTA CACTCCTTCC ACACGGAAGG ATTCTCCTTC TTCCCATTCT   
  
  
- TCAGAAATTA AGACGGAAAT GGTAATTGC

+     AAGAA-motif

| Site Name | Organism | Position | Strand | Matrix score. | sequence | function |
| --- | --- | --- | --- | --- | --- | --- |
| AAGAA-motif | Avena sativa | 1464 | + | 8 | gGTAAGAA |  |
| AAGAA-motif | Avena sativa | 224 | + | 9 | gGTAAAGAAA |  |
| AAGAA-motif | Avena sativa | 901 | + | 7 | GAAAGAA |  |
| AAGAA-motif | Avena sativa | 508 | - | 7 | GAAAGAA |  |

> 2018/04/13 10:10:12  
+ TTTACCTCTC CTTCTCCTCT CCAAAACCCA CCTTCAGAAC TTTGCCACGT TATCTGGGCC GTAATTTTTC   
  
  
+ CCTCTCGACT CTGAAATCTT CTTCTGGTTT GTCATCATCA ATCACGAAGA CACAAAATTA ATAAAATGTT   
  
  
+ TGGGGAAAAA GAGTAAAATG TAGCTATATA GTATTAACTT TTCCTCCGCT CCTGTGAGAG AACCAGGATT   
  
  
+ AAGTGAAGGA AAGGGAAAAG AAAAGAAAAA AAAAAGTTTA TAATGAATAA TTAATTTAAA TAAAATGAAT   
  
  
+ TTTAATATAT AATAGTATAG ATAAAAAATT TTTAGACAAA GATATAGTTA GAATTTTTAT GAATCTCTAT   
  
  
+ ATAATAGTTT TTGTTAGAGA GGAAATAACA TTTTATAAGT AGTTTATAAT GAATTTTTAA CTTGGAGTTA   
  
  
+ TTTTGGTAGT ACGTTTAGTT AAACTACACC GAGACCAAAC TCGTAAATTC GTATAATCAT AAAAAAAAGA   
  
  
+ CTTTTTTTTT ACAAGAATTC TTTCTTTTAT TTTTTTATTT TGTCTACTAA AATATAAGTT AAAATTAAAA   
  
  
+ ATTCCTGAAG ACATTACCGA TGAATACACC ACATCACTGA TGACCGATTC TAACAGTATC ATTCTCTCTC   
  
  
+ TCTCCAACAA CACCCCGGGT TGTTTCCGTT CCCCCTTCAG AAGGCTAAAG AGAGGCATCT AACTGCGCGG   
  
  
+ GGTTTTAAAG TAGCGGACCT AACCGGGCCG CGTAGAGCCC GAGCACTTTT ACCCTCCAGA AACCAGTTGC   
  
  
+ TAGAACTGTT CGGACGACCC GGGGTGTTCC GTAGGCCAGA TTAAACACCC GGGGCGCGGA AGTATAACCT   
  
  
+ GAATAACGGT GCAGCCTACC CACAGGAGCC TGGGTTGGAT TTGCCCAACT GAGGCAGCAG GAAAGAAGTG   
  
  
+ GGGTAGATAG GGAGCGGAGC GGAGCGGAGT ATCGAACTTA ACAAATGAAT AATACAATGA GCTAAATAAA   
  
  
+ TAATAATAAA ATTTTTTTAA TTTTCATTTT ATATATAATG AAAATTAATT ATAAATTATT ATTATTTAAT   
  
  
+ AATTAATATT ATTTCATTTG TTTTGCCTTC AGCTAGACGG GGATGTCATC GTGTTGAACG TTCGTGTCCG   
  
  
+ TTGAACGTAT GCACTAAACT GTTCATTTCC ACCAATCATC TACTAACATG TGTGCTTATA AACTAAAATA   
  
  
+ TCATTAAATA TGTATACACA AATTTTTTAG TTTATAATTA AGAATGTAAT TAAATATAAA AATATATTAA   
  
  
+ TAAAAAATAT TTTTTGTTTA TTTTTAAACT TATAGAGTTT TAAATTATAA AAATTCGTCA CATCTTTTCC   
  
  
+ TGTCCACCTG GCGTAACAAA CTTACTCAGT TTCTGGAAAA ATGGGAAGTA TATAAGTTGT TAAAGAAGTG   
  
  
+ GTGGAGTGCA CTAAACGGTA GTTACAGAAT GTGAGGAAGG TGTGCCTTCC TAAGAGGAAG AAGGGTAAGA   
  
  
+ AGTCTTTAAT TCTGCCTTTA CCATTAACG  

- AAATGGAGAG GAAGAGGAGA GGTTTTGGGT GGAAGTCTTG AAACGGTGCA ATAGACCCGG CATTAAAAAG   
  
  
- GGAGAGCTGA GACTTTAGAA GAAGACCAAA CAGTAGTAGT TAGTGCTTCT GTGTTTTAAT TATTTTACAA   
  
  
- ACCCCTTTTT CTCATTTTAC ATCGATATAT CATAATTGAA AAGGAGGCGA GGACACTCTC TTGGTCCTAA   
  
  
- TTCACTTCCT TTCCCTTTTC TTTTCTTTTT TTTTTCAAAT ATTACTTATT AATTAAATTT ATTTTACTTA   
  
  
- AAATTATATA TTATCATATC TATTTTTTAA AAATCTGTTT CTATATCAAT CTTAAAAATA CTTAGAGATA   
  
  
- TATTATCAAA AACAATCTCT CCTTTATTGT AAAATATTCA TCAAATATTA CTTAAAAATT GAACCTCAAT   
  
  
- AAAACCATCA TGCAAATCAA TTTGATGTGG CTCTGGTTTG AGCATTTAAG CATATTAGTA TTTTTTTTCT   
  
  
- GAAAAAAAAA TGTTCTTAAG AAAGAAAATA AAAAAATAAA ACAGATGATT TTATATTCAA TTTTAATTTT   
  
  
- TAAGGACTTC TGTAATGGCT ACTTATGTGG TGTAGTGACT ACTGGCTAAG ATTGTCATAG TAAGAGAGAG   
  
  
- AGAGGTTGTT GTGGGGCCCA ACAAAGGCAA GGGGGAAGTC TTCCGATTTC TCTCCGTAGA TTGACGCGCC   
  
  
- CCAAAATTTC ATCGCCTGGA TTGGCCCGGC GCATCTCGGG CTCGTGAAAA TGGGAGGTCT TTGGTCAACG   
  
  
- ATCTTGACAA GCCTGCTGGG CCCCACAAGG CATCCGGTCT AATTTGTGGG CCCCGCGCCT TCATATTGGA   
  
  
- CTTATTGCCA CGTCGGATGG GTGTCCTCGG ACCCAACCTA AACGGGTTGA CTCCGTCGTC CTTTCTTCAC   
  
  
- CCCATCTATC CCTCGCCTCG CCTCGCCTCA TAGCTTGAAT TGTTTACTTA TTATGTTACT CGATTTATTT   
  
  
- ATTATTATTT TAAAAAAATT AAAAGTAAAA TATATATTAC TTTTAATTAA TATTTAATAA TAATAAATTA   
  
  
- TTAATTATAA TAAAGTAAAC AAAACGGAAG TCGATCTGCC CCTACAGTAG CACAACTTGC AAGCACAGGC   
  
  
- AACTTGCATA CGTGATTTGA CAAGTAAAGG TGGTTAGTAG ATGATTGTAC ACACGAATAT TTGATTTTAT   
  
  
- AGTAATTTAT ACATATGTGT TTAAAAAATC AAATATTAAT TCTTACATTA ATTTATATTT TTATATAATT   
  
  
- ATTTTTTATA AAAAACAAAT AAAAATTTGA ATATCTCAAA ATTTAATATT TTTAAGCAGT GTAGAAAAGG   
  
  
- ACAGGTGGAC CGCATTGTTT GAATGAGTCA AAGACCTTTT TACCCTTCAT ATATTCAACA ATTTCTTCAC   
  
  
- CACCTCACGT GATTTGCCAT CAATGTCTTA CACTCCTTCC ACACGGAAGG ATTCTCCTTC TTCCCATTCT   
  
  
- TCAGAAATTA AGACGGAAAT GGTAATTGC

+     ABRE

| Site Name | Organism | Position | Strand | Matrix score. | sequence | function |
| --- | --- | --- | --- | --- | --- | --- |
| ABRE | Oryza sativa | 1108 | - | 10 | GACACGTACGT | cis-acting element involved in the abscisic acid responsiveness |
| ABRE | Arabidopsis thaliana | 44 | - | 7 | ACGTGGC | cis-acting element involved in the abscisic acid responsiveness |

> 2018/04/13 10:10:12  
+ TTTACCTCTC CTTCTCCTCT CCAAAACCCA CCTTCAGAAC TTTGCCACGT TATCTGGGCC GTAATTTTTC   
  
  
+ CCTCTCGACT CTGAAATCTT CTTCTGGTTT GTCATCATCA ATCACGAAGA CACAAAATTA ATAAAATGTT   
  
  
+ TGGGGAAAAA GAGTAAAATG TAGCTATATA GTATTAACTT TTCCTCCGCT CCTGTGAGAG AACCAGGATT   
  
  
+ AAGTGAAGGA AAGGGAAAAG AAAAGAAAAA AAAAAGTTTA TAATGAATAA TTAATTTAAA TAAAATGAAT   
  
  
+ TTTAATATAT AATAGTATAG ATAAAAAATT TTTAGACAAA GATATAGTTA GAATTTTTAT GAATCTCTAT   
  
  
+ ATAATAGTTT TTGTTAGAGA GGAAATAACA TTTTATAAGT AGTTTATAAT GAATTTTTAA CTTGGAGTTA   
  
  
+ TTTTGGTAGT ACGTTTAGTT AAACTACACC GAGACCAAAC TCGTAAATTC GTATAATCAT AAAAAAAAGA   
  
  
+ CTTTTTTTTT ACAAGAATTC TTTCTTTTAT TTTTTTATTT TGTCTACTAA AATATAAGTT AAAATTAAAA   
  
  
+ ATTCCTGAAG ACATTACCGA TGAATACACC ACATCACTGA TGACCGATTC TAACAGTATC ATTCTCTCTC   
  
  
+ TCTCCAACAA CACCCCGGGT TGTTTCCGTT CCCCCTTCAG AAGGCTAAAG AGAGGCATCT AACTGCGCGG   
  
  
+ GGTTTTAAAG TAGCGGACCT AACCGGGCCG CGTAGAGCCC GAGCACTTTT ACCCTCCAGA AACCAGTTGC   
  
  
+ TAGAACTGTT CGGACGACCC GGGGTGTTCC GTAGGCCAGA TTAAACACCC GGGGCGCGGA AGTATAACCT   
  
  
+ GAATAACGGT GCAGCCTACC CACAGGAGCC TGGGTTGGAT TTGCCCAACT GAGGCAGCAG GAAAGAAGTG   
  
  
+ GGGTAGATAG GGAGCGGAGC GGAGCGGAGT ATCGAACTTA ACAAATGAAT AATACAATGA GCTAAATAAA   
  
  
+ TAATAATAAA ATTTTTTTAA TTTTCATTTT ATATATAATG AAAATTAATT ATAAATTATT ATTATTTAAT   
  
  
+ AATTAATATT ATTTCATTTG TTTTGCCTTC AGCTAGACGG GGATGTCATC GTGTTGAACG TTCGTGTCCG   
  
  
+ TTGAACGTAT GCACTAAACT GTTCATTTCC ACCAATCATC TACTAACATG TGTGCTTATA AACTAAAATA   
  
  
+ TCATTAAATA TGTATACACA AATTTTTTAG TTTATAATTA AGAATGTAAT TAAATATAAA AATATATTAA   
  
  
+ TAAAAAATAT TTTTTGTTTA TTTTTAAACT TATAGAGTTT TAAATTATAA AAATTCGTCA CATCTTTTCC   
  
  
+ TGTCCACCTG GCGTAACAAA CTTACTCAGT TTCTGGAAAA ATGGGAAGTA TATAAGTTGT TAAAGAAGTG   
  
  
+ GTGGAGTGCA CTAAACGGTA GTTACAGAAT GTGAGGAAGG TGTGCCTTCC TAAGAGGAAG AAGGGTAAGA   
  
  
+ AGTCTTTAAT TCTGCCTTTA CCATTAACG  

- AAATGGAGAG GAAGAGGAGA GGTTTTGGGT GGAAGTCTTG AAACGGTGCA ATAGACCCGG CATTAAAAAG   
  
  
- GGAGAGCTGA GACTTTAGAA GAAGACCAAA CAGTAGTAGT TAGTGCTTCT GTGTTTTAAT TATTTTACAA   
  
  
- ACCCCTTTTT CTCATTTTAC ATCGATATAT CATAATTGAA AAGGAGGCGA GGACACTCTC TTGGTCCTAA   
  
  
- TTCACTTCCT TTCCCTTTTC TTTTCTTTTT TTTTTCAAAT ATTACTTATT AATTAAATTT ATTTTACTTA   
  
  
- AAATTATATA TTATCATATC TATTTTTTAA AAATCTGTTT CTATATCAAT CTTAAAAATA CTTAGAGATA   
  
  
- TATTATCAAA AACAATCTCT CCTTTATTGT AAAATATTCA TCAAATATTA CTTAAAAATT GAACCTCAAT   
  
  
- AAAACCATCA TGCAAATCAA TTTGATGTGG CTCTGGTTTG AGCATTTAAG CATATTAGTA TTTTTTTTCT   
  
  
- GAAAAAAAAA TGTTCTTAAG AAAGAAAATA AAAAAATAAA ACAGATGATT TTATATTCAA TTTTAATTTT   
  
  
- TAAGGACTTC TGTAATGGCT ACTTATGTGG TGTAGTGACT ACTGGCTAAG ATTGTCATAG TAAGAGAGAG   
  
  
- AGAGGTTGTT GTGGGGCCCA ACAAAGGCAA GGGGGAAGTC TTCCGATTTC TCTCCGTAGA TTGACGCGCC   
  
  
- CCAAAATTTC ATCGCCTGGA TTGGCCCGGC GCATCTCGGG CTCGTGAAAA TGGGAGGTCT TTGGTCAACG   
  
  
- ATCTTGACAA GCCTGCTGGG CCCCACAAGG CATCCGGTCT AATTTGTGGG CCCCGCGCCT TCATATTGGA   
  
  
- CTTATTGCCA CGTCGGATGG GTGTCCTCGG ACCCAACCTA AACGGGTTGA CTCCGTCGTC CTTTCTTCAC   
  
  
- CCCATCTATC CCTCGCCTCG CCTCGCCTCA TAGCTTGAAT TGTTTACTTA TTATGTTACT CGATTTATTT   
  
  
- ATTATTATTT TAAAAAAATT AAAAGTAAAA TATATATTAC TTTTAATTAA TATTTAATAA TAATAAATTA   
  
  
- TTAATTATAA TAAAGTAAAC AAAACGGAAG TCGATCTGCC CCTACAGTAG CACAACTTGC AAGCACAGGC   
  
  
- AACTTGCATA CGTGATTTGA CAAGTAAAGG TGGTTAGTAG ATGATTGTAC ACACGAATAT TTGATTTTAT   
  
  
- AGTAATTTAT ACATATGTGT TTAAAAAATC AAATATTAAT TCTTACATTA ATTTATATTT TTATATAATT   
  
  
- ATTTTTTATA AAAAACAAAT AAAAATTTGA ATATCTCAAA ATTTAATATT TTTAAGCAGT GTAGAAAAGG   
  
  
- ACAGGTGGAC CGCATTGTTT GAATGAGTCA AAGACCTTTT TACCCTTCAT ATATTCAACA ATTTCTTCAC   
  
  
- CACCTCACGT GATTTGCCAT CAATGTCTTA CACTCCTTCC ACACGGAAGG ATTCTCCTTC TTCCCATTCT   
  
  
- TCAGAAATTA AGACGGAAAT GGTAATTGC

+     AC-II

| Site Name | Organism | Position | Strand | Matrix score. | sequence | function |
| --- | --- | --- | --- | --- | --- | --- |
| AC-II | Phaseolus vulgaris | 628 | + | 9 | (C/T)T(T/C)(C/T)(A/C)(A/C)C(A/C)A(A/C)C(C/A)(C/A)C |  |

> 2018/04/13 10:10:12  
+ TTTACCTCTC CTTCTCCTCT CCAAAACCCA CCTTCAGAAC TTTGCCACGT TATCTGGGCC GTAATTTTTC   
  
  
+ CCTCTCGACT CTGAAATCTT CTTCTGGTTT GTCATCATCA ATCACGAAGA CACAAAATTA ATAAAATGTT   
  
  
+ TGGGGAAAAA GAGTAAAATG TAGCTATATA GTATTAACTT TTCCTCCGCT CCTGTGAGAG AACCAGGATT   
  
  
+ AAGTGAAGGA AAGGGAAAAG AAAAGAAAAA AAAAAGTTTA TAATGAATAA TTAATTTAAA TAAAATGAAT   
  
  
+ TTTAATATAT AATAGTATAG ATAAAAAATT TTTAGACAAA GATATAGTTA GAATTTTTAT GAATCTCTAT   
  
  
+ ATAATAGTTT TTGTTAGAGA GGAAATAACA TTTTATAAGT AGTTTATAAT GAATTTTTAA CTTGGAGTTA   
  
  
+ TTTTGGTAGT ACGTTTAGTT AAACTACACC GAGACCAAAC TCGTAAATTC GTATAATCAT AAAAAAAAGA   
  
  
+ CTTTTTTTTT ACAAGAATTC TTTCTTTTAT TTTTTTATTT TGTCTACTAA AATATAAGTT AAAATTAAAA   
  
  
+ ATTCCTGAAG ACATTACCGA TGAATACACC ACATCACTGA TGACCGATTC TAACAGTATC ATTCTCTCTC   
  
  
+ TCTCCAACAA CACCCCGGGT TGTTTCCGTT CCCCCTTCAG AAGGCTAAAG AGAGGCATCT AACTGCGCGG   
  
  
+ GGTTTTAAAG TAGCGGACCT AACCGGGCCG CGTAGAGCCC GAGCACTTTT ACCCTCCAGA AACCAGTTGC   
  
  
+ TAGAACTGTT CGGACGACCC GGGGTGTTCC GTAGGCCAGA TTAAACACCC GGGGCGCGGA AGTATAACCT   
  
  
+ GAATAACGGT GCAGCCTACC CACAGGAGCC TGGGTTGGAT TTGCCCAACT GAGGCAGCAG GAAAGAAGTG   
  
  
+ GGGTAGATAG GGAGCGGAGC GGAGCGGAGT ATCGAACTTA ACAAATGAAT AATACAATGA GCTAAATAAA   
  
  
+ TAATAATAAA ATTTTTTTAA TTTTCATTTT ATATATAATG AAAATTAATT ATAAATTATT ATTATTTAAT   
  
  
+ AATTAATATT ATTTCATTTG TTTTGCCTTC AGCTAGACGG GGATGTCATC GTGTTGAACG TTCGTGTCCG   
  
  
+ TTGAACGTAT GCACTAAACT GTTCATTTCC ACCAATCATC TACTAACATG TGTGCTTATA AACTAAAATA   
  
  
+ TCATTAAATA TGTATACACA AATTTTTTAG TTTATAATTA AGAATGTAAT TAAATATAAA AATATATTAA   
  
  
+ TAAAAAATAT TTTTTGTTTA TTTTTAAACT TATAGAGTTT TAAATTATAA AAATTCGTCA CATCTTTTCC   
  
  
+ TGTCCACCTG GCGTAACAAA CTTACTCAGT TTCTGGAAAA ATGGGAAGTA TATAAGTTGT TAAAGAAGTG   
  
  
+ GTGGAGTGCA CTAAACGGTA GTTACAGAAT GTGAGGAAGG TGTGCCTTCC TAAGAGGAAG AAGGGTAAGA   
  
  
+ AGTCTTTAAT TCTGCCTTTA CCATTAACG  

- AAATGGAGAG GAAGAGGAGA GGTTTTGGGT GGAAGTCTTG AAACGGTGCA ATAGACCCGG CATTAAAAAG   
  
  
- GGAGAGCTGA GACTTTAGAA GAAGACCAAA CAGTAGTAGT TAGTGCTTCT GTGTTTTAAT TATTTTACAA   
  
  
- ACCCCTTTTT CTCATTTTAC ATCGATATAT CATAATTGAA AAGGAGGCGA GGACACTCTC TTGGTCCTAA   
  
  
- TTCACTTCCT TTCCCTTTTC TTTTCTTTTT TTTTTCAAAT ATTACTTATT AATTAAATTT ATTTTACTTA   
  
  
- AAATTATATA TTATCATATC TATTTTTTAA AAATCTGTTT CTATATCAAT CTTAAAAATA CTTAGAGATA   
  
  
- TATTATCAAA AACAATCTCT CCTTTATTGT AAAATATTCA TCAAATATTA CTTAAAAATT GAACCTCAAT   
  
  
- AAAACCATCA TGCAAATCAA TTTGATGTGG CTCTGGTTTG AGCATTTAAG CATATTAGTA TTTTTTTTCT   
  
  
- GAAAAAAAAA TGTTCTTAAG AAAGAAAATA AAAAAATAAA ACAGATGATT TTATATTCAA TTTTAATTTT   
  
  
- TAAGGACTTC TGTAATGGCT ACTTATGTGG TGTAGTGACT ACTGGCTAAG ATTGTCATAG TAAGAGAGAG   
  
  
- AGAGGTTGTT GTGGGGCCCA ACAAAGGCAA GGGGGAAGTC TTCCGATTTC TCTCCGTAGA TTGACGCGCC   
  
  
- CCAAAATTTC ATCGCCTGGA TTGGCCCGGC GCATCTCGGG CTCGTGAAAA TGGGAGGTCT TTGGTCAACG   
  
  
- ATCTTGACAA GCCTGCTGGG CCCCACAAGG CATCCGGTCT AATTTGTGGG CCCCGCGCCT TCATATTGGA   
  
  
- CTTATTGCCA CGTCGGATGG GTGTCCTCGG ACCCAACCTA AACGGGTTGA CTCCGTCGTC CTTTCTTCAC   
  
  
- CCCATCTATC CCTCGCCTCG CCTCGCCTCA TAGCTTGAAT TGTTTACTTA TTATGTTACT CGATTTATTT   
  
  
- ATTATTATTT TAAAAAAATT AAAAGTAAAA TATATATTAC TTTTAATTAA TATTTAATAA TAATAAATTA   
  
  
- TTAATTATAA TAAAGTAAAC AAAACGGAAG TCGATCTGCC CCTACAGTAG CACAACTTGC AAGCACAGGC   
  
  
- AACTTGCATA CGTGATTTGA CAAGTAAAGG TGGTTAGTAG ATGATTGTAC ACACGAATAT TTGATTTTAT   
  
  
- AGTAATTTAT ACATATGTGT TTAAAAAATC AAATATTAAT TCTTACATTA ATTTATATTT TTATATAATT   
  
  
- ATTTTTTATA AAAAACAAAT AAAAATTTGA ATATCTCAAA ATTTAATATT TTTAAGCAGT GTAGAAAAGG   
  
  
- ACAGGTGGAC CGCATTGTTT GAATGAGTCA AAGACCTTTT TACCCTTCAT ATATTCAACA ATTTCTTCAC   
  
  
- CACCTCACGT GATTTGCCAT CAATGTCTTA CACTCCTTCC ACACGGAAGG ATTCTCCTTC TTCCCATTCT   
  
  
- TCAGAAATTA AGACGGAAAT GGTAATTGC

+     ACE

| Site Name | Organism | Position | Strand | Matrix score. | sequence | function |
| --- | --- | --- | --- | --- | --- | --- |
| ACE | Petroselinum crispum | 241 | + | 9 | AAAACGTTTA | cis-acting element involved in light responsiveness |

> 2018/04/13 10:10:12  
+ TTTACCTCTC CTTCTCCTCT CCAAAACCCA CCTTCAGAAC TTTGCCACGT TATCTGGGCC GTAATTTTTC   
  
  
+ CCTCTCGACT CTGAAATCTT CTTCTGGTTT GTCATCATCA ATCACGAAGA CACAAAATTA ATAAAATGTT   
  
  
+ TGGGGAAAAA GAGTAAAATG TAGCTATATA GTATTAACTT TTCCTCCGCT CCTGTGAGAG AACCAGGATT   
  
  
+ AAGTGAAGGA AAGGGAAAAG AAAAGAAAAA AAAAAGTTTA TAATGAATAA TTAATTTAAA TAAAATGAAT   
  
  
+ TTTAATATAT AATAGTATAG ATAAAAAATT TTTAGACAAA GATATAGTTA GAATTTTTAT GAATCTCTAT   
  
  
+ ATAATAGTTT TTGTTAGAGA GGAAATAACA TTTTATAAGT AGTTTATAAT GAATTTTTAA CTTGGAGTTA   
  
  
+ TTTTGGTAGT ACGTTTAGTT AAACTACACC GAGACCAAAC TCGTAAATTC GTATAATCAT AAAAAAAAGA   
  
  
+ CTTTTTTTTT ACAAGAATTC TTTCTTTTAT TTTTTTATTT TGTCTACTAA AATATAAGTT AAAATTAAAA   
  
  
+ ATTCCTGAAG ACATTACCGA TGAATACACC ACATCACTGA TGACCGATTC TAACAGTATC ATTCTCTCTC   
  
  
+ TCTCCAACAA CACCCCGGGT TGTTTCCGTT CCCCCTTCAG AAGGCTAAAG AGAGGCATCT AACTGCGCGG   
  
  
+ GGTTTTAAAG TAGCGGACCT AACCGGGCCG CGTAGAGCCC GAGCACTTTT ACCCTCCAGA AACCAGTTGC   
  
  
+ TAGAACTGTT CGGACGACCC GGGGTGTTCC GTAGGCCAGA TTAAACACCC GGGGCGCGGA AGTATAACCT   
  
  
+ GAATAACGGT GCAGCCTACC CACAGGAGCC TGGGTTGGAT TTGCCCAACT GAGGCAGCAG GAAAGAAGTG   
  
  
+ GGGTAGATAG GGAGCGGAGC GGAGCGGAGT ATCGAACTTA ACAAATGAAT AATACAATGA GCTAAATAAA   
  
  
+ TAATAATAAA ATTTTTTTAA TTTTCATTTT ATATATAATG AAAATTAATT ATAAATTATT ATTATTTAAT   
  
  
+ AATTAATATT ATTTCATTTG TTTTGCCTTC AGCTAGACGG GGATGTCATC GTGTTGAACG TTCGTGTCCG   
  
  
+ TTGAACGTAT GCACTAAACT GTTCATTTCC ACCAATCATC TACTAACATG TGTGCTTATA AACTAAAATA   
  
  
+ TCATTAAATA TGTATACACA AATTTTTTAG TTTATAATTA AGAATGTAAT TAAATATAAA AATATATTAA   
  
  
+ TAAAAAATAT TTTTTGTTTA TTTTTAAACT TATAGAGTTT TAAATTATAA AAATTCGTCA CATCTTTTCC   
  
  
+ TGTCCACCTG GCGTAACAAA CTTACTCAGT TTCTGGAAAA ATGGGAAGTA TATAAGTTGT TAAAGAAGTG   
  
  
+ GTGGAGTGCA CTAAACGGTA GTTACAGAAT GTGAGGAAGG TGTGCCTTCC TAAGAGGAAG AAGGGTAAGA   
  
  
+ AGTCTTTAAT TCTGCCTTTA CCATTAACG  

- AAATGGAGAG GAAGAGGAGA GGTTTTGGGT GGAAGTCTTG AAACGGTGCA ATAGACCCGG CATTAAAAAG   
  
  
- GGAGAGCTGA GACTTTAGAA GAAGACCAAA CAGTAGTAGT TAGTGCTTCT GTGTTTTAAT TATTTTACAA   
  
  
- ACCCCTTTTT CTCATTTTAC ATCGATATAT CATAATTGAA AAGGAGGCGA GGACACTCTC TTGGTCCTAA   
  
  
- TTCACTTCCT TTCCCTTTTC TTTTCTTTTT TTTTTCAAAT ATTACTTATT AATTAAATTT ATTTTACTTA   
  
  
- AAATTATATA TTATCATATC TATTTTTTAA AAATCTGTTT CTATATCAAT CTTAAAAATA CTTAGAGATA   
  
  
- TATTATCAAA AACAATCTCT CCTTTATTGT AAAATATTCA TCAAATATTA CTTAAAAATT GAACCTCAAT   
  
  
- AAAACCATCA TGCAAATCAA TTTGATGTGG CTCTGGTTTG AGCATTTAAG CATATTAGTA TTTTTTTTCT   
  
  
- GAAAAAAAAA TGTTCTTAAG AAAGAAAATA AAAAAATAAA ACAGATGATT TTATATTCAA TTTTAATTTT   
  
  
- TAAGGACTTC TGTAATGGCT ACTTATGTGG TGTAGTGACT ACTGGCTAAG ATTGTCATAG TAAGAGAGAG   
  
  
- AGAGGTTGTT GTGGGGCCCA ACAAAGGCAA GGGGGAAGTC TTCCGATTTC TCTCCGTAGA TTGACGCGCC   
  
  
- CCAAAATTTC ATCGCCTGGA TTGGCCCGGC GCATCTCGGG CTCGTGAAAA TGGGAGGTCT TTGGTCAACG   
  
  
- ATCTTGACAA GCCTGCTGGG CCCCACAAGG CATCCGGTCT AATTTGTGGG CCCCGCGCCT TCATATTGGA   
  
  
- CTTATTGCCA CGTCGGATGG GTGTCCTCGG ACCCAACCTA AACGGGTTGA CTCCGTCGTC CTTTCTTCAC   
  
  
- CCCATCTATC CCTCGCCTCG CCTCGCCTCA TAGCTTGAAT TGTTTACTTA TTATGTTACT CGATTTATTT   
  
  
- ATTATTATTT TAAAAAAATT AAAAGTAAAA TATATATTAC TTTTAATTAA TATTTAATAA TAATAAATTA   
  
  
- TTAATTATAA TAAAGTAAAC AAAACGGAAG TCGATCTGCC CCTACAGTAG CACAACTTGC AAGCACAGGC   
  
  
- AACTTGCATA CGTGATTTGA CAAGTAAAGG TGGTTAGTAG ATGATTGTAC ACACGAATAT TTGATTTTAT   
  
  
- AGTAATTTAT ACATATGTGT TTAAAAAATC AAATATTAAT TCTTACATTA ATTTATATTT TTATATAATT   
  
  
- ATTTTTTATA AAAAACAAAT AAAAATTTGA ATATCTCAAA ATTTAATATT TTTAAGCAGT GTAGAAAAGG   
  
  
- ACAGGTGGAC CGCATTGTTT GAATGAGTCA AAGACCTTTT TACCCTTCAT ATATTCAACA ATTTCTTCAC   
  
  
- CACCTCACGT GATTTGCCAT CAATGTCTTA CACTCCTTCC ACACGGAAGG ATTCTCCTTC TTCCCATTCT   
  
  
- TCAGAAATTA AGACGGAAAT GGTAATTGC

+     ARE

| Site Name | Organism | Position | Strand | Matrix score. | sequence | function |
| --- | --- | --- | --- | --- | --- | --- |
| ARE | Zea mays | 95 | + | 6 | TGGTTT | cis-acting regulatory element essential for the anaerobic induction |
| ARE | Zea mays | 760 | - | 6 | TGGTTT | cis-acting regulatory element essential for the anaerobic induction |

> 2018/04/13 10:10:12  
+ TTTACCTCTC CTTCTCCTCT CCAAAACCCA CCTTCAGAAC TTTGCCACGT TATCTGGGCC GTAATTTTTC   
  
  
+ CCTCTCGACT CTGAAATCTT CTTCTGGTTT GTCATCATCA ATCACGAAGA CACAAAATTA ATAAAATGTT   
  
  
+ TGGGGAAAAA GAGTAAAATG TAGCTATATA GTATTAACTT TTCCTCCGCT CCTGTGAGAG AACCAGGATT   
  
  
+ AAGTGAAGGA AAGGGAAAAG AAAAGAAAAA AAAAAGTTTA TAATGAATAA TTAATTTAAA TAAAATGAAT   
  
  
+ TTTAATATAT AATAGTATAG ATAAAAAATT TTTAGACAAA GATATAGTTA GAATTTTTAT GAATCTCTAT   
  
  
+ ATAATAGTTT TTGTTAGAGA GGAAATAACA TTTTATAAGT AGTTTATAAT GAATTTTTAA CTTGGAGTTA   
  
  
+ TTTTGGTAGT ACGTTTAGTT AAACTACACC GAGACCAAAC TCGTAAATTC GTATAATCAT AAAAAAAAGA   
  
  
+ CTTTTTTTTT ACAAGAATTC TTTCTTTTAT TTTTTTATTT TGTCTACTAA AATATAAGTT AAAATTAAAA   
  
  
+ ATTCCTGAAG ACATTACCGA TGAATACACC ACATCACTGA TGACCGATTC TAACAGTATC ATTCTCTCTC   
  
  
+ TCTCCAACAA CACCCCGGGT TGTTTCCGTT CCCCCTTCAG AAGGCTAAAG AGAGGCATCT AACTGCGCGG   
  
  
+ GGTTTTAAAG TAGCGGACCT AACCGGGCCG CGTAGAGCCC GAGCACTTTT ACCCTCCAGA AACCAGTTGC   
  
  
+ TAGAACTGTT CGGACGACCC GGGGTGTTCC GTAGGCCAGA TTAAACACCC GGGGCGCGGA AGTATAACCT   
  
  
+ GAATAACGGT GCAGCCTACC CACAGGAGCC TGGGTTGGAT TTGCCCAACT GAGGCAGCAG GAAAGAAGTG   
  
  
+ GGGTAGATAG GGAGCGGAGC GGAGCGGAGT ATCGAACTTA ACAAATGAAT AATACAATGA GCTAAATAAA   
  
  
+ TAATAATAAA ATTTTTTTAA TTTTCATTTT ATATATAATG AAAATTAATT ATAAATTATT ATTATTTAAT   
  
  
+ AATTAATATT ATTTCATTTG TTTTGCCTTC AGCTAGACGG GGATGTCATC GTGTTGAACG TTCGTGTCCG   
  
  
+ TTGAACGTAT GCACTAAACT GTTCATTTCC ACCAATCATC TACTAACATG TGTGCTTATA AACTAAAATA   
  
  
+ TCATTAAATA TGTATACACA AATTTTTTAG TTTATAATTA AGAATGTAAT TAAATATAAA AATATATTAA   
  
  
+ TAAAAAATAT TTTTTGTTTA TTTTTAAACT TATAGAGTTT TAAATTATAA AAATTCGTCA CATCTTTTCC   
  
  
+ TGTCCACCTG GCGTAACAAA CTTACTCAGT TTCTGGAAAA ATGGGAAGTA TATAAGTTGT TAAAGAAGTG   
  
  
+ GTGGAGTGCA CTAAACGGTA GTTACAGAAT GTGAGGAAGG TGTGCCTTCC TAAGAGGAAG AAGGGTAAGA   
  
  
+ AGTCTTTAAT TCTGCCTTTA CCATTAACG  

- AAATGGAGAG GAAGAGGAGA GGTTTTGGGT GGAAGTCTTG AAACGGTGCA ATAGACCCGG CATTAAAAAG   
  
  
- GGAGAGCTGA GACTTTAGAA GAAGACCAAA CAGTAGTAGT TAGTGCTTCT GTGTTTTAAT TATTTTACAA   
  
  
- ACCCCTTTTT CTCATTTTAC ATCGATATAT CATAATTGAA AAGGAGGCGA GGACACTCTC TTGGTCCTAA   
  
  
- TTCACTTCCT TTCCCTTTTC TTTTCTTTTT TTTTTCAAAT ATTACTTATT AATTAAATTT ATTTTACTTA   
  
  
- AAATTATATA TTATCATATC TATTTTTTAA AAATCTGTTT CTATATCAAT CTTAAAAATA CTTAGAGATA   
  
  
- TATTATCAAA AACAATCTCT CCTTTATTGT AAAATATTCA TCAAATATTA CTTAAAAATT GAACCTCAAT   
  
  
- AAAACCATCA TGCAAATCAA TTTGATGTGG CTCTGGTTTG AGCATTTAAG CATATTAGTA TTTTTTTTCT   
  
  
- GAAAAAAAAA TGTTCTTAAG AAAGAAAATA AAAAAATAAA ACAGATGATT TTATATTCAA TTTTAATTTT   
  
  
- TAAGGACTTC TGTAATGGCT ACTTATGTGG TGTAGTGACT ACTGGCTAAG ATTGTCATAG TAAGAGAGAG   
  
  
- AGAGGTTGTT GTGGGGCCCA ACAAAGGCAA GGGGGAAGTC TTCCGATTTC TCTCCGTAGA TTGACGCGCC   
  
  
- CCAAAATTTC ATCGCCTGGA TTGGCCCGGC GCATCTCGGG CTCGTGAAAA TGGGAGGTCT TTGGTCAACG   
  
  
- ATCTTGACAA GCCTGCTGGG CCCCACAAGG CATCCGGTCT AATTTGTGGG CCCCGCGCCT TCATATTGGA   
  
  
- CTTATTGCCA CGTCGGATGG GTGTCCTCGG ACCCAACCTA AACGGGTTGA CTCCGTCGTC CTTTCTTCAC   
  
  
- CCCATCTATC CCTCGCCTCG CCTCGCCTCA TAGCTTGAAT TGTTTACTTA TTATGTTACT CGATTTATTT   
  
  
- ATTATTATTT TAAAAAAATT AAAAGTAAAA TATATATTAC TTTTAATTAA TATTTAATAA TAATAAATTA   
  
  
- TTAATTATAA TAAAGTAAAC AAAACGGAAG TCGATCTGCC CCTACAGTAG CACAACTTGC AAGCACAGGC   
  
  
- AACTTGCATA CGTGATTTGA CAAGTAAAGG TGGTTAGTAG ATGATTGTAC ACACGAATAT TTGATTTTAT   
  
  
- AGTAATTTAT ACATATGTGT TTAAAAAATC AAATATTAAT TCTTACATTA ATTTATATTT TTATATAATT   
  
  
- ATTTTTTATA AAAAACAAAT AAAAATTTGA ATATCTCAAA ATTTAATATT TTTAAGCAGT GTAGAAAAGG   
  
  
- ACAGGTGGAC CGCATTGTTT GAATGAGTCA AAGACCTTTT TACCCTTCAT ATATTCAACA ATTTCTTCAC   
  
  
- CACCTCACGT GATTTGCCAT CAATGTCTTA CACTCCTTCC ACACGGAAGG ATTCTCCTTC TTCCCATTCT   
  
  
- TCAGAAATTA AGACGGAAAT GGTAATTGC

+     AT1-motif

| Site Name | Organism | Position | Strand | Matrix score. | sequence | function |
| --- | --- | --- | --- | --- | --- | --- |
| AT1-motif | Solanum tuberosum | 1259 | - | 13 | AATTATTTTTTATT | part of a light responsive module |

> 2018/04/13 10:10:12  
+ TTTACCTCTC CTTCTCCTCT CCAAAACCCA CCTTCAGAAC TTTGCCACGT TATCTGGGCC GTAATTTTTC   
  
  
+ CCTCTCGACT CTGAAATCTT CTTCTGGTTT GTCATCATCA ATCACGAAGA CACAAAATTA ATAAAATGTT   
  
  
+ TGGGGAAAAA GAGTAAAATG TAGCTATATA GTATTAACTT TTCCTCCGCT CCTGTGAGAG AACCAGGATT   
  
  
+ AAGTGAAGGA AAGGGAAAAG AAAAGAAAAA AAAAAGTTTA TAATGAATAA TTAATTTAAA TAAAATGAAT   
  
  
+ TTTAATATAT AATAGTATAG ATAAAAAATT TTTAGACAAA GATATAGTTA GAATTTTTAT GAATCTCTAT   
  
  
+ ATAATAGTTT TTGTTAGAGA GGAAATAACA TTTTATAAGT AGTTTATAAT GAATTTTTAA CTTGGAGTTA   
  
  
+ TTTTGGTAGT ACGTTTAGTT AAACTACACC GAGACCAAAC TCGTAAATTC GTATAATCAT AAAAAAAAGA   
  
  
+ CTTTTTTTTT ACAAGAATTC TTTCTTTTAT TTTTTTATTT TGTCTACTAA AATATAAGTT AAAATTAAAA   
  
  
+ ATTCCTGAAG ACATTACCGA TGAATACACC ACATCACTGA TGACCGATTC TAACAGTATC ATTCTCTCTC   
  
  
+ TCTCCAACAA CACCCCGGGT TGTTTCCGTT CCCCCTTCAG AAGGCTAAAG AGAGGCATCT AACTGCGCGG   
  
  
+ GGTTTTAAAG TAGCGGACCT AACCGGGCCG CGTAGAGCCC GAGCACTTTT ACCCTCCAGA AACCAGTTGC   
  
  
+ TAGAACTGTT CGGACGACCC GGGGTGTTCC GTAGGCCAGA TTAAACACCC GGGGCGCGGA AGTATAACCT   
  
  
+ GAATAACGGT GCAGCCTACC CACAGGAGCC TGGGTTGGAT TTGCCCAACT GAGGCAGCAG GAAAGAAGTG   
  
  
+ GGGTAGATAG GGAGCGGAGC GGAGCGGAGT ATCGAACTTA ACAAATGAAT AATACAATGA GCTAAATAAA   
  
  
+ TAATAATAAA ATTTTTTTAA TTTTCATTTT ATATATAATG AAAATTAATT ATAAATTATT ATTATTTAAT   
  
  
+ AATTAATATT ATTTCATTTG TTTTGCCTTC AGCTAGACGG GGATGTCATC GTGTTGAACG TTCGTGTCCG   
  
  
+ TTGAACGTAT GCACTAAACT GTTCATTTCC ACCAATCATC TACTAACATG TGTGCTTATA AACTAAAATA   
  
  
+ TCATTAAATA TGTATACACA AATTTTTTAG TTTATAATTA AGAATGTAAT TAAATATAAA AATATATTAA   
  
  
+ TAAAAAATAT TTTTTGTTTA TTTTTAAACT TATAGAGTTT TAAATTATAA AAATTCGTCA CATCTTTTCC   
  
  
+ TGTCCACCTG GCGTAACAAA CTTACTCAGT TTCTGGAAAA ATGGGAAGTA TATAAGTTGT TAAAGAAGTG   
  
  
+ GTGGAGTGCA CTAAACGGTA GTTACAGAAT GTGAGGAAGG TGTGCCTTCC TAAGAGGAAG AAGGGTAAGA   
  
  
+ AGTCTTTAAT TCTGCCTTTA CCATTAACG  

- AAATGGAGAG GAAGAGGAGA GGTTTTGGGT GGAAGTCTTG AAACGGTGCA ATAGACCCGG CATTAAAAAG   
  
  
- GGAGAGCTGA GACTTTAGAA GAAGACCAAA CAGTAGTAGT TAGTGCTTCT GTGTTTTAAT TATTTTACAA   
  
  
- ACCCCTTTTT CTCATTTTAC ATCGATATAT CATAATTGAA AAGGAGGCGA GGACACTCTC TTGGTCCTAA   
  
  
- TTCACTTCCT TTCCCTTTTC TTTTCTTTTT TTTTTCAAAT ATTACTTATT AATTAAATTT ATTTTACTTA   
  
  
- AAATTATATA TTATCATATC TATTTTTTAA AAATCTGTTT CTATATCAAT CTTAAAAATA CTTAGAGATA   
  
  
- TATTATCAAA AACAATCTCT CCTTTATTGT AAAATATTCA TCAAATATTA CTTAAAAATT GAACCTCAAT   
  
  
- AAAACCATCA TGCAAATCAA TTTGATGTGG CTCTGGTTTG AGCATTTAAG CATATTAGTA TTTTTTTTCT   
  
  
- GAAAAAAAAA TGTTCTTAAG AAAGAAAATA AAAAAATAAA ACAGATGATT TTATATTCAA TTTTAATTTT   
  
  
- TAAGGACTTC TGTAATGGCT ACTTATGTGG TGTAGTGACT ACTGGCTAAG ATTGTCATAG TAAGAGAGAG   
  
  
- AGAGGTTGTT GTGGGGCCCA ACAAAGGCAA GGGGGAAGTC TTCCGATTTC TCTCCGTAGA TTGACGCGCC   
  
  
- CCAAAATTTC ATCGCCTGGA TTGGCCCGGC GCATCTCGGG CTCGTGAAAA TGGGAGGTCT TTGGTCAACG   
  
  
- ATCTTGACAA GCCTGCTGGG CCCCACAAGG CATCCGGTCT AATTTGTGGG CCCCGCGCCT TCATATTGGA   
  
  
- CTTATTGCCA CGTCGGATGG GTGTCCTCGG ACCCAACCTA AACGGGTTGA CTCCGTCGTC CTTTCTTCAC   
  
  
- CCCATCTATC CCTCGCCTCG CCTCGCCTCA TAGCTTGAAT TGTTTACTTA TTATGTTACT CGATTTATTT   
  
  
- ATTATTATTT TAAAAAAATT AAAAGTAAAA TATATATTAC TTTTAATTAA TATTTAATAA TAATAAATTA   
  
  
- TTAATTATAA TAAAGTAAAC AAAACGGAAG TCGATCTGCC CCTACAGTAG CACAACTTGC AAGCACAGGC   
  
  
- AACTTGCATA CGTGATTTGA CAAGTAAAGG TGGTTAGTAG ATGATTGTAC ACACGAATAT TTGATTTTAT   
  
  
- AGTAATTTAT ACATATGTGT TTAAAAAATC AAATATTAAT TCTTACATTA ATTTATATTT TTATATAATT   
  
  
- ATTTTTTATA AAAAACAAAT AAAAATTTGA ATATCTCAAA ATTTAATATT TTTAAGCAGT GTAGAAAAGG   
  
  
- ACAGGTGGAC CGCATTGTTT GAATGAGTCA AAGACCTTTT TACCCTTCAT ATATTCAACA ATTTCTTCAC   
  
  
- CACCTCACGT GATTTGCCAT CAATGTCTTA CACTCCTTCC ACACGGAAGG ATTCTCCTTC TTCCCATTCT   
  
  
- TCAGAAATTA AGACGGAAAT GGTAATTGC

+     Box 4

| Site Name | Organism | Position | Strand | Matrix score. | sequence | function |
| --- | --- | --- | --- | --- | --- | --- |
| Box 4 | Petroselinum crispum | 1024 | - | 6 | ATTAAT | part of a conserved DNA module involved in light responsiveness |
| Box 4 | Petroselinum crispum | 1052 | - | 6 | ATTAAT | part of a conserved DNA module involved in light responsiveness |
| Box 4 | Petroselinum crispum | 260 | + | 6 | ATTAAT | part of a conserved DNA module involved in light responsiveness |
| Box 4 | Petroselinum crispum | 127 | + | 6 | ATTAAT | part of a conserved DNA module involved in light responsiveness |
| Box 4 | Petroselinum crispum | 1256 | - | 6 | ATTAAT | part of a conserved DNA module involved in light responsiveness |

> 2018/04/13 10:10:12  
+ TTTACCTCTC CTTCTCCTCT CCAAAACCCA CCTTCAGAAC TTTGCCACGT TATCTGGGCC GTAATTTTTC   
  
  
+ CCTCTCGACT CTGAAATCTT CTTCTGGTTT GTCATCATCA ATCACGAAGA CACAAAATTA ATAAAATGTT   
  
  
+ TGGGGAAAAA GAGTAAAATG TAGCTATATA GTATTAACTT TTCCTCCGCT CCTGTGAGAG AACCAGGATT   
  
  
+ AAGTGAAGGA AAGGGAAAAG AAAAGAAAAA AAAAAGTTTA TAATGAATAA TTAATTTAAA TAAAATGAAT   
  
  
+ TTTAATATAT AATAGTATAG ATAAAAAATT TTTAGACAAA GATATAGTTA GAATTTTTAT GAATCTCTAT   
  
  
+ ATAATAGTTT TTGTTAGAGA GGAAATAACA TTTTATAAGT AGTTTATAAT GAATTTTTAA CTTGGAGTTA   
  
  
+ TTTTGGTAGT ACGTTTAGTT AAACTACACC GAGACCAAAC TCGTAAATTC GTATAATCAT AAAAAAAAGA   
  
  
+ CTTTTTTTTT ACAAGAATTC TTTCTTTTAT TTTTTTATTT TGTCTACTAA AATATAAGTT AAAATTAAAA   
  
  
+ ATTCCTGAAG ACATTACCGA TGAATACACC ACATCACTGA TGACCGATTC TAACAGTATC ATTCTCTCTC   
  
  
+ TCTCCAACAA CACCCCGGGT TGTTTCCGTT CCCCCTTCAG AAGGCTAAAG AGAGGCATCT AACTGCGCGG   
  
  
+ GGTTTTAAAG TAGCGGACCT AACCGGGCCG CGTAGAGCCC GAGCACTTTT ACCCTCCAGA AACCAGTTGC   
  
  
+ TAGAACTGTT CGGACGACCC GGGGTGTTCC GTAGGCCAGA TTAAACACCC GGGGCGCGGA AGTATAACCT   
  
  
+ GAATAACGGT GCAGCCTACC CACAGGAGCC TGGGTTGGAT TTGCCCAACT GAGGCAGCAG GAAAGAAGTG   
  
  
+ GGGTAGATAG GGAGCGGAGC GGAGCGGAGT ATCGAACTTA ACAAATGAAT AATACAATGA GCTAAATAAA   
  
  
+ TAATAATAAA ATTTTTTTAA TTTTCATTTT ATATATAATG AAAATTAATT ATAAATTATT ATTATTTAAT   
  
  
+ AATTAATATT ATTTCATTTG TTTTGCCTTC AGCTAGACGG GGATGTCATC GTGTTGAACG TTCGTGTCCG   
  
  
+ TTGAACGTAT GCACTAAACT GTTCATTTCC ACCAATCATC TACTAACATG TGTGCTTATA AACTAAAATA   
  
  
+ TCATTAAATA TGTATACACA AATTTTTTAG TTTATAATTA AGAATGTAAT TAAATATAAA AATATATTAA   
  
  
+ TAAAAAATAT TTTTTGTTTA TTTTTAAACT TATAGAGTTT TAAATTATAA AAATTCGTCA CATCTTTTCC   
  
  
+ TGTCCACCTG GCGTAACAAA CTTACTCAGT TTCTGGAAAA ATGGGAAGTA TATAAGTTGT TAAAGAAGTG   
  
  
+ GTGGAGTGCA CTAAACGGTA GTTACAGAAT GTGAGGAAGG TGTGCCTTCC TAAGAGGAAG AAGGGTAAGA   
  
  
+ AGTCTTTAAT TCTGCCTTTA CCATTAACG  

- AAATGGAGAG GAAGAGGAGA GGTTTTGGGT GGAAGTCTTG AAACGGTGCA ATAGACCCGG CATTAAAAAG   
  
  
- GGAGAGCTGA GACTTTAGAA GAAGACCAAA CAGTAGTAGT TAGTGCTTCT GTGTTTTAAT TATTTTACAA   
  
  
- ACCCCTTTTT CTCATTTTAC ATCGATATAT CATAATTGAA AAGGAGGCGA GGACACTCTC TTGGTCCTAA   
  
  
- TTCACTTCCT TTCCCTTTTC TTTTCTTTTT TTTTTCAAAT ATTACTTATT AATTAAATTT ATTTTACTTA   
  
  
- AAATTATATA TTATCATATC TATTTTTTAA AAATCTGTTT CTATATCAAT CTTAAAAATA CTTAGAGATA   
  
  
- TATTATCAAA AACAATCTCT CCTTTATTGT AAAATATTCA TCAAATATTA CTTAAAAATT GAACCTCAAT   
  
  
- AAAACCATCA TGCAAATCAA TTTGATGTGG CTCTGGTTTG AGCATTTAAG CATATTAGTA TTTTTTTTCT   
  
  
- GAAAAAAAAA TGTTCTTAAG AAAGAAAATA AAAAAATAAA ACAGATGATT TTATATTCAA TTTTAATTTT   
  
  
- TAAGGACTTC TGTAATGGCT ACTTATGTGG TGTAGTGACT ACTGGCTAAG ATTGTCATAG TAAGAGAGAG   
  
  
- AGAGGTTGTT GTGGGGCCCA ACAAAGGCAA GGGGGAAGTC TTCCGATTTC TCTCCGTAGA TTGACGCGCC   
  
  
- CCAAAATTTC ATCGCCTGGA TTGGCCCGGC GCATCTCGGG CTCGTGAAAA TGGGAGGTCT TTGGTCAACG   
  
  
- ATCTTGACAA GCCTGCTGGG CCCCACAAGG CATCCGGTCT AATTTGTGGG CCCCGCGCCT TCATATTGGA   
  
  
- CTTATTGCCA CGTCGGATGG GTGTCCTCGG ACCCAACCTA AACGGGTTGA CTCCGTCGTC CTTTCTTCAC   
  
  
- CCCATCTATC CCTCGCCTCG CCTCGCCTCA TAGCTTGAAT TGTTTACTTA TTATGTTACT CGATTTATTT   
  
  
- ATTATTATTT TAAAAAAATT AAAAGTAAAA TATATATTAC TTTTAATTAA TATTTAATAA TAATAAATTA   
  
  
- TTAATTATAA TAAAGTAAAC AAAACGGAAG TCGATCTGCC CCTACAGTAG CACAACTTGC AAGCACAGGC   
  
  
- AACTTGCATA CGTGATTTGA CAAGTAAAGG TGGTTAGTAG ATGATTGTAC ACACGAATAT TTGATTTTAT   
  
  
- AGTAATTTAT ACATATGTGT TTAAAAAATC AAATATTAAT TCTTACATTA ATTTATATTT TTATATAATT   
  
  
- ATTTTTTATA AAAAACAAAT AAAAATTTGA ATATCTCAAA ATTTAATATT TTTAAGCAGT GTAGAAAAGG   
  
  
- ACAGGTGGAC CGCATTGTTT GAATGAGTCA AAGACCTTTT TACCCTTCAT ATATTCAACA ATTTCTTCAC   
  
  
- CACCTCACGT GATTTGCCAT CAATGTCTTA CACTCCTTCC ACACGGAAGG ATTCTCCTTC TTCCCATTCT   
  
  
- TCAGAAATTA AGACGGAAAT GGTAATTGC

+     CAAT-box

| Site Name | Organism | Position | Strand | Matrix score. | sequence | function |
| --- | --- | --- | --- | --- | --- | --- |
| CAAT-box | Brassica rapa | 1209 | + | 5 | CAAAT | common cis-acting element in promoter and enhancer regions |
| CAAT-box | Hordeum vulgare | 1153 | + | 4 | CAAT | common cis-acting element in promoter and enhancer regions |
| CAAT-box | Brassica rapa | 952 | + | 5 | CAAAT | common cis-acting element in promoter and enhancer regions |
| CAAT-box | Hordeum vulgare | 965 | + | 4 | CAAT | common cis-acting element in promoter and enhancer regions |
| CAAT-box | Brassica rapa | 879 | - | 5 | CAAAT | common cis-acting element in promoter and enhancer regions |
| CAAT-box | Hordeum vulgare | 109 | + | 4 | CAAT | common cis-acting element in promoter and enhancer regions |
| CAAT-box | Arabidopsis thaliana | 1152 | + | 5 | CCAAT | common cis-acting element in promoter and enhancer regions |
| CAAT-box | Brassica rapa | 1066 | - | 5 | CAAAT | common cis-acting element in promoter and enhancer regions |

> 2018/04/13 10:10:12  
+ TTTACCTCTC CTTCTCCTCT CCAAAACCCA CCTTCAGAAC TTTGCCACGT TATCTGGGCC GTAATTTTTC   
  
  
+ CCTCTCGACT CTGAAATCTT CTTCTGGTTT GTCATCATCA ATCACGAAGA CACAAAATTA ATAAAATGTT   
  
  
+ TGGGGAAAAA GAGTAAAATG TAGCTATATA GTATTAACTT TTCCTCCGCT CCTGTGAGAG AACCAGGATT   
  
  
+ AAGTGAAGGA AAGGGAAAAG AAAAGAAAAA AAAAAGTTTA TAATGAATAA TTAATTTAAA TAAAATGAAT   
  
  
+ TTTAATATAT AATAGTATAG ATAAAAAATT TTTAGACAAA GATATAGTTA GAATTTTTAT GAATCTCTAT   
  
  
+ ATAATAGTTT TTGTTAGAGA GGAAATAACA TTTTATAAGT AGTTTATAAT GAATTTTTAA CTTGGAGTTA   
  
  
+ TTTTGGTAGT ACGTTTAGTT AAACTACACC GAGACCAAAC TCGTAAATTC GTATAATCAT AAAAAAAAGA   
  
  
+ CTTTTTTTTT ACAAGAATTC TTTCTTTTAT TTTTTTATTT TGTCTACTAA AATATAAGTT AAAATTAAAA   
  
  
+ ATTCCTGAAG ACATTACCGA TGAATACACC ACATCACTGA TGACCGATTC TAACAGTATC ATTCTCTCTC   
  
  
+ TCTCCAACAA CACCCCGGGT TGTTTCCGTT CCCCCTTCAG AAGGCTAAAG AGAGGCATCT AACTGCGCGG   
  
  
+ GGTTTTAAAG TAGCGGACCT AACCGGGCCG CGTAGAGCCC GAGCACTTTT ACCCTCCAGA AACCAGTTGC   
  
  
+ TAGAACTGTT CGGACGACCC GGGGTGTTCC GTAGGCCAGA TTAAACACCC GGGGCGCGGA AGTATAACCT   
  
  
+ GAATAACGGT GCAGCCTACC CACAGGAGCC TGGGTTGGAT TTGCCCAACT GAGGCAGCAG GAAAGAAGTG   
  
  
+ GGGTAGATAG GGAGCGGAGC GGAGCGGAGT ATCGAACTTA ACAAATGAAT AATACAATGA GCTAAATAAA   
  
  
+ TAATAATAAA ATTTTTTTAA TTTTCATTTT ATATATAATG AAAATTAATT ATAAATTATT ATTATTTAAT   
  
  
+ AATTAATATT ATTTCATTTG TTTTGCCTTC AGCTAGACGG GGATGTCATC GTGTTGAACG TTCGTGTCCG   
  
  
+ TTGAACGTAT GCACTAAACT GTTCATTTCC ACCAATCATC TACTAACATG TGTGCTTATA AACTAAAATA   
  
  
+ TCATTAAATA TGTATACACA AATTTTTTAG TTTATAATTA AGAATGTAAT TAAATATAAA AATATATTAA   
  
  
+ TAAAAAATAT TTTTTGTTTA TTTTTAAACT TATAGAGTTT TAAATTATAA AAATTCGTCA CATCTTTTCC   
  
  
+ TGTCCACCTG GCGTAACAAA CTTACTCAGT TTCTGGAAAA ATGGGAAGTA TATAAGTTGT TAAAGAAGTG   
  
  
+ GTGGAGTGCA CTAAACGGTA GTTACAGAAT GTGAGGAAGG TGTGCCTTCC TAAGAGGAAG AAGGGTAAGA   
  
  
+ AGTCTTTAAT TCTGCCTTTA CCATTAACG  

- AAATGGAGAG GAAGAGGAGA GGTTTTGGGT GGAAGTCTTG AAACGGTGCA ATAGACCCGG CATTAAAAAG   
  
  
- GGAGAGCTGA GACTTTAGAA GAAGACCAAA CAGTAGTAGT TAGTGCTTCT GTGTTTTAAT TATTTTACAA   
  
  
- ACCCCTTTTT CTCATTTTAC ATCGATATAT CATAATTGAA AAGGAGGCGA GGACACTCTC TTGGTCCTAA   
  
  
- TTCACTTCCT TTCCCTTTTC TTTTCTTTTT TTTTTCAAAT ATTACTTATT AATTAAATTT ATTTTACTTA   
  
  
- AAATTATATA TTATCATATC TATTTTTTAA AAATCTGTTT CTATATCAAT CTTAAAAATA CTTAGAGATA   
  
  
- TATTATCAAA AACAATCTCT CCTTTATTGT AAAATATTCA TCAAATATTA CTTAAAAATT GAACCTCAAT   
  
  
- AAAACCATCA TGCAAATCAA TTTGATGTGG CTCTGGTTTG AGCATTTAAG CATATTAGTA TTTTTTTTCT   
  
  
- GAAAAAAAAA TGTTCTTAAG AAAGAAAATA AAAAAATAAA ACAGATGATT TTATATTCAA TTTTAATTTT   
  
  
- TAAGGACTTC TGTAATGGCT ACTTATGTGG TGTAGTGACT ACTGGCTAAG ATTGTCATAG TAAGAGAGAG   
  
  
- AGAGGTTGTT GTGGGGCCCA ACAAAGGCAA GGGGGAAGTC TTCCGATTTC TCTCCGTAGA TTGACGCGCC   
  
  
- CCAAAATTTC ATCGCCTGGA TTGGCCCGGC GCATCTCGGG CTCGTGAAAA TGGGAGGTCT TTGGTCAACG   
  
  
- ATCTTGACAA GCCTGCTGGG CCCCACAAGG CATCCGGTCT AATTTGTGGG CCCCGCGCCT TCATATTGGA   
  
  
- CTTATTGCCA CGTCGGATGG GTGTCCTCGG ACCCAACCTA AACGGGTTGA CTCCGTCGTC CTTTCTTCAC   
  
  
- CCCATCTATC CCTCGCCTCG CCTCGCCTCA TAGCTTGAAT TGTTTACTTA TTATGTTACT CGATTTATTT   
  
  
- ATTATTATTT TAAAAAAATT AAAAGTAAAA TATATATTAC TTTTAATTAA TATTTAATAA TAATAAATTA   
  
  
- TTAATTATAA TAAAGTAAAC AAAACGGAAG TCGATCTGCC CCTACAGTAG CACAACTTGC AAGCACAGGC   
  
  
- AACTTGCATA CGTGATTTGA CAAGTAAAGG TGGTTAGTAG ATGATTGTAC ACACGAATAT TTGATTTTAT   
  
  
- AGTAATTTAT ACATATGTGT TTAAAAAATC AAATATTAAT TCTTACATTA ATTTATATTT TTATATAATT   
  
  
- ATTTTTTATA AAAAACAAAT AAAAATTTGA ATATCTCAAA ATTTAATATT TTTAAGCAGT GTAGAAAAGG   
  
  
- ACAGGTGGAC CGCATTGTTT GAATGAGTCA AAGACCTTTT TACCCTTCAT ATATTCAACA ATTTCTTCAC   
  
  
- CACCTCACGT GATTTGCCAT CAATGTCTTA CACTCCTTCC ACACGGAAGG ATTCTCCTTC TTCCCATTCT   
  
  
- TCAGAAATTA AGACGGAAAT GGTAATTGC

+     CCAAT-box

| Site Name | Organism | Position | Strand | Matrix score. | sequence | function |
| --- | --- | --- | --- | --- | --- | --- |
| CCAAT-box | Hordeum vulgare | 1118 | - | 6 | CAACGG | MYBHv1 binding site |

> 2018/04/13 10:10:12  
+ TTTACCTCTC CTTCTCCTCT CCAAAACCCA CCTTCAGAAC TTTGCCACGT TATCTGGGCC GTAATTTTTC   
  
  
+ CCTCTCGACT CTGAAATCTT CTTCTGGTTT GTCATCATCA ATCACGAAGA CACAAAATTA ATAAAATGTT   
  
  
+ TGGGGAAAAA GAGTAAAATG TAGCTATATA GTATTAACTT TTCCTCCGCT CCTGTGAGAG AACCAGGATT   
  
  
+ AAGTGAAGGA AAGGGAAAAG AAAAGAAAAA AAAAAGTTTA TAATGAATAA TTAATTTAAA TAAAATGAAT   
  
  
+ TTTAATATAT AATAGTATAG ATAAAAAATT TTTAGACAAA GATATAGTTA GAATTTTTAT GAATCTCTAT   
  
  
+ ATAATAGTTT TTGTTAGAGA GGAAATAACA TTTTATAAGT AGTTTATAAT GAATTTTTAA CTTGGAGTTA   
  
  
+ TTTTGGTAGT ACGTTTAGTT AAACTACACC GAGACCAAAC TCGTAAATTC GTATAATCAT AAAAAAAAGA   
  
  
+ CTTTTTTTTT ACAAGAATTC TTTCTTTTAT TTTTTTATTT TGTCTACTAA AATATAAGTT AAAATTAAAA   
  
  
+ ATTCCTGAAG ACATTACCGA TGAATACACC ACATCACTGA TGACCGATTC TAACAGTATC ATTCTCTCTC   
  
  
+ TCTCCAACAA CACCCCGGGT TGTTTCCGTT CCCCCTTCAG AAGGCTAAAG AGAGGCATCT AACTGCGCGG   
  
  
+ GGTTTTAAAG TAGCGGACCT AACCGGGCCG CGTAGAGCCC GAGCACTTTT ACCCTCCAGA AACCAGTTGC   
  
  
+ TAGAACTGTT CGGACGACCC GGGGTGTTCC GTAGGCCAGA TTAAACACCC GGGGCGCGGA AGTATAACCT   
  
  
+ GAATAACGGT GCAGCCTACC CACAGGAGCC TGGGTTGGAT TTGCCCAACT GAGGCAGCAG GAAAGAAGTG   
  
  
+ GGGTAGATAG GGAGCGGAGC GGAGCGGAGT ATCGAACTTA ACAAATGAAT AATACAATGA GCTAAATAAA   
  
  
+ TAATAATAAA ATTTTTTTAA TTTTCATTTT ATATATAATG AAAATTAATT ATAAATTATT ATTATTTAAT   
  
  
+ AATTAATATT ATTTCATTTG TTTTGCCTTC AGCTAGACGG GGATGTCATC GTGTTGAACG TTCGTGTCCG   
  
  
+ TTGAACGTAT GCACTAAACT GTTCATTTCC ACCAATCATC TACTAACATG TGTGCTTATA AACTAAAATA   
  
  
+ TCATTAAATA TGTATACACA AATTTTTTAG TTTATAATTA AGAATGTAAT TAAATATAAA AATATATTAA   
  
  
+ TAAAAAATAT TTTTTGTTTA TTTTTAAACT TATAGAGTTT TAAATTATAA AAATTCGTCA CATCTTTTCC   
  
  
+ TGTCCACCTG GCGTAACAAA CTTACTCAGT TTCTGGAAAA ATGGGAAGTA TATAAGTTGT TAAAGAAGTG   
  
  
+ GTGGAGTGCA CTAAACGGTA GTTACAGAAT GTGAGGAAGG TGTGCCTTCC TAAGAGGAAG AAGGGTAAGA   
  
  
+ AGTCTTTAAT TCTGCCTTTA CCATTAACG  

- AAATGGAGAG GAAGAGGAGA GGTTTTGGGT GGAAGTCTTG AAACGGTGCA ATAGACCCGG CATTAAAAAG   
  
  
- GGAGAGCTGA GACTTTAGAA GAAGACCAAA CAGTAGTAGT TAGTGCTTCT GTGTTTTAAT TATTTTACAA   
  
  
- ACCCCTTTTT CTCATTTTAC ATCGATATAT CATAATTGAA AAGGAGGCGA GGACACTCTC TTGGTCCTAA   
  
  
- TTCACTTCCT TTCCCTTTTC TTTTCTTTTT TTTTTCAAAT ATTACTTATT AATTAAATTT ATTTTACTTA   
  
  
- AAATTATATA TTATCATATC TATTTTTTAA AAATCTGTTT CTATATCAAT CTTAAAAATA CTTAGAGATA   
  
  
- TATTATCAAA AACAATCTCT CCTTTATTGT AAAATATTCA TCAAATATTA CTTAAAAATT GAACCTCAAT   
  
  
- AAAACCATCA TGCAAATCAA TTTGATGTGG CTCTGGTTTG AGCATTTAAG CATATTAGTA TTTTTTTTCT   
  
  
- GAAAAAAAAA TGTTCTTAAG AAAGAAAATA AAAAAATAAA ACAGATGATT TTATATTCAA TTTTAATTTT   
  
  
- TAAGGACTTC TGTAATGGCT ACTTATGTGG TGTAGTGACT ACTGGCTAAG ATTGTCATAG TAAGAGAGAG   
  
  
- AGAGGTTGTT GTGGGGCCCA ACAAAGGCAA GGGGGAAGTC TTCCGATTTC TCTCCGTAGA TTGACGCGCC   
  
  
- CCAAAATTTC ATCGCCTGGA TTGGCCCGGC GCATCTCGGG CTCGTGAAAA TGGGAGGTCT TTGGTCAACG   
  
  
- ATCTTGACAA GCCTGCTGGG CCCCACAAGG CATCCGGTCT AATTTGTGGG CCCCGCGCCT TCATATTGGA   
  
  
- CTTATTGCCA CGTCGGATGG GTGTCCTCGG ACCCAACCTA AACGGGTTGA CTCCGTCGTC CTTTCTTCAC   
  
  
- CCCATCTATC CCTCGCCTCG CCTCGCCTCA TAGCTTGAAT TGTTTACTTA TTATGTTACT CGATTTATTT   
  
  
- ATTATTATTT TAAAAAAATT AAAAGTAAAA TATATATTAC TTTTAATTAA TATTTAATAA TAATAAATTA   
  
  
- TTAATTATAA TAAAGTAAAC AAAACGGAAG TCGATCTGCC CCTACAGTAG CACAACTTGC AAGCACAGGC   
  
  
- AACTTGCATA CGTGATTTGA CAAGTAAAGG TGGTTAGTAG ATGATTGTAC ACACGAATAT TTGATTTTAT   
  
  
- AGTAATTTAT ACATATGTGT TTAAAAAATC AAATATTAAT TCTTACATTA ATTTATATTT TTATATAATT   
  
  
- ATTTTTTATA AAAAACAAAT AAAAATTTGA ATATCTCAAA ATTTAATATT TTTAAGCAGT GTAGAAAAGG   
  
  
- ACAGGTGGAC CGCATTGTTT GAATGAGTCA AAGACCTTTT TACCCTTCAT ATATTCAACA ATTTCTTCAC   
  
  
- CACCTCACGT GATTTGCCAT CAATGTCTTA CACTCCTTCC ACACGGAAGG ATTCTCCTTC TTCCCATTCT   
  
  
- TCAGAAATTA AGACGGAAAT GGTAATTGC

+     CGTCA-motif

| Site Name | Organism | Position | Strand | Matrix score. | sequence | function |
| --- | --- | --- | --- | --- | --- | --- |
| CGTCA-motif | Hordeum vulgare | 1316 | + | 5 | CGTCA | cis-acting regulatory element involved in the MeJA-responsiveness |

> 2018/04/13 10:10:12  
+ TTTACCTCTC CTTCTCCTCT CCAAAACCCA CCTTCAGAAC TTTGCCACGT TATCTGGGCC GTAATTTTTC   
  
  
+ CCTCTCGACT CTGAAATCTT CTTCTGGTTT GTCATCATCA ATCACGAAGA CACAAAATTA ATAAAATGTT   
  
  
+ TGGGGAAAAA GAGTAAAATG TAGCTATATA GTATTAACTT TTCCTCCGCT CCTGTGAGAG AACCAGGATT   
  
  
+ AAGTGAAGGA AAGGGAAAAG AAAAGAAAAA AAAAAGTTTA TAATGAATAA TTAATTTAAA TAAAATGAAT   
  
  
+ TTTAATATAT AATAGTATAG ATAAAAAATT TTTAGACAAA GATATAGTTA GAATTTTTAT GAATCTCTAT   
  
  
+ ATAATAGTTT TTGTTAGAGA GGAAATAACA TTTTATAAGT AGTTTATAAT GAATTTTTAA CTTGGAGTTA   
  
  
+ TTTTGGTAGT ACGTTTAGTT AAACTACACC GAGACCAAAC TCGTAAATTC GTATAATCAT AAAAAAAAGA   
  
  
+ CTTTTTTTTT ACAAGAATTC TTTCTTTTAT TTTTTTATTT TGTCTACTAA AATATAAGTT AAAATTAAAA   
  
  
+ ATTCCTGAAG ACATTACCGA TGAATACACC ACATCACTGA TGACCGATTC TAACAGTATC ATTCTCTCTC   
  
  
+ TCTCCAACAA CACCCCGGGT TGTTTCCGTT CCCCCTTCAG AAGGCTAAAG AGAGGCATCT AACTGCGCGG   
  
  
+ GGTTTTAAAG TAGCGGACCT AACCGGGCCG CGTAGAGCCC GAGCACTTTT ACCCTCCAGA AACCAGTTGC   
  
  
+ TAGAACTGTT CGGACGACCC GGGGTGTTCC GTAGGCCAGA TTAAACACCC GGGGCGCGGA AGTATAACCT   
  
  
+ GAATAACGGT GCAGCCTACC CACAGGAGCC TGGGTTGGAT TTGCCCAACT GAGGCAGCAG GAAAGAAGTG   
  
  
+ GGGTAGATAG GGAGCGGAGC GGAGCGGAGT ATCGAACTTA ACAAATGAAT AATACAATGA GCTAAATAAA   
  
  
+ TAATAATAAA ATTTTTTTAA TTTTCATTTT ATATATAATG AAAATTAATT ATAAATTATT ATTATTTAAT   
  
  
+ AATTAATATT ATTTCATTTG TTTTGCCTTC AGCTAGACGG GGATGTCATC GTGTTGAACG TTCGTGTCCG   
  
  
+ TTGAACGTAT GCACTAAACT GTTCATTTCC ACCAATCATC TACTAACATG TGTGCTTATA AACTAAAATA   
  
  
+ TCATTAAATA TGTATACACA AATTTTTTAG TTTATAATTA AGAATGTAAT TAAATATAAA AATATATTAA   
  
  
+ TAAAAAATAT TTTTTGTTTA TTTTTAAACT TATAGAGTTT TAAATTATAA AAATTCGTCA CATCTTTTCC   
  
  
+ TGTCCACCTG GCGTAACAAA CTTACTCAGT TTCTGGAAAA ATGGGAAGTA TATAAGTTGT TAAAGAAGTG   
  
  
+ GTGGAGTGCA CTAAACGGTA GTTACAGAAT GTGAGGAAGG TGTGCCTTCC TAAGAGGAAG AAGGGTAAGA   
  
  
+ AGTCTTTAAT TCTGCCTTTA CCATTAACG  

- AAATGGAGAG GAAGAGGAGA GGTTTTGGGT GGAAGTCTTG AAACGGTGCA ATAGACCCGG CATTAAAAAG   
  
  
- GGAGAGCTGA GACTTTAGAA GAAGACCAAA CAGTAGTAGT TAGTGCTTCT GTGTTTTAAT TATTTTACAA   
  
  
- ACCCCTTTTT CTCATTTTAC ATCGATATAT CATAATTGAA AAGGAGGCGA GGACACTCTC TTGGTCCTAA   
  
  
- TTCACTTCCT TTCCCTTTTC TTTTCTTTTT TTTTTCAAAT ATTACTTATT AATTAAATTT ATTTTACTTA   
  
  
- AAATTATATA TTATCATATC TATTTTTTAA AAATCTGTTT CTATATCAAT CTTAAAAATA CTTAGAGATA   
  
  
- TATTATCAAA AACAATCTCT CCTTTATTGT AAAATATTCA TCAAATATTA CTTAAAAATT GAACCTCAAT   
  
  
- AAAACCATCA TGCAAATCAA TTTGATGTGG CTCTGGTTTG AGCATTTAAG CATATTAGTA TTTTTTTTCT   
  
  
- GAAAAAAAAA TGTTCTTAAG AAAGAAAATA AAAAAATAAA ACAGATGATT TTATATTCAA TTTTAATTTT   
  
  
- TAAGGACTTC TGTAATGGCT ACTTATGTGG TGTAGTGACT ACTGGCTAAG ATTGTCATAG TAAGAGAGAG   
  
  
- AGAGGTTGTT GTGGGGCCCA ACAAAGGCAA GGGGGAAGTC TTCCGATTTC TCTCCGTAGA TTGACGCGCC   
  
  
- CCAAAATTTC ATCGCCTGGA TTGGCCCGGC GCATCTCGGG CTCGTGAAAA TGGGAGGTCT TTGGTCAACG   
  
  
- ATCTTGACAA GCCTGCTGGG CCCCACAAGG CATCCGGTCT AATTTGTGGG CCCCGCGCCT TCATATTGGA   
  
  
- CTTATTGCCA CGTCGGATGG GTGTCCTCGG ACCCAACCTA AACGGGTTGA CTCCGTCGTC CTTTCTTCAC   
  
  
- CCCATCTATC CCTCGCCTCG CCTCGCCTCA TAGCTTGAAT TGTTTACTTA TTATGTTACT CGATTTATTT   
  
  
- ATTATTATTT TAAAAAAATT AAAAGTAAAA TATATATTAC TTTTAATTAA TATTTAATAA TAATAAATTA   
  
  
- TTAATTATAA TAAAGTAAAC AAAACGGAAG TCGATCTGCC CCTACAGTAG CACAACTTGC AAGCACAGGC   
  
  
- AACTTGCATA CGTGATTTGA CAAGTAAAGG TGGTTAGTAG ATGATTGTAC ACACGAATAT TTGATTTTAT   
  
  
- AGTAATTTAT ACATATGTGT TTAAAAAATC AAATATTAAT TCTTACATTA ATTTATATTT TTATATAATT   
  
  
- ATTTTTTATA AAAAACAAAT AAAAATTTGA ATATCTCAAA ATTTAATATT TTTAAGCAGT GTAGAAAAGG   
  
  
- ACAGGTGGAC CGCATTGTTT GAATGAGTCA AAGACCTTTT TACCCTTCAT ATATTCAACA ATTTCTTCAC   
  
  
- CACCTCACGT GATTTGCCAT CAATGTCTTA CACTCCTTCC ACACGGAAGG ATTCTCCTTC TTCCCATTCT   
  
  
- TCAGAAATTA AGACGGAAAT GGTAATTGC

+     G-Box

| Site Name | Organism | Position | Strand | Matrix score. | sequence | function |
| --- | --- | --- | --- | --- | --- | --- |
| G-Box | Pisum sativum | 46 | + | 6 | CACGTT | cis-acting regulatory element involved in light responsiveness |

> 2018/04/13 10:10:12  
+ TTTACCTCTC CTTCTCCTCT CCAAAACCCA CCTTCAGAAC TTTGCCACGT TATCTGGGCC GTAATTTTTC   
  
  
+ CCTCTCGACT CTGAAATCTT CTTCTGGTTT GTCATCATCA ATCACGAAGA CACAAAATTA ATAAAATGTT   
  
  
+ TGGGGAAAAA GAGTAAAATG TAGCTATATA GTATTAACTT TTCCTCCGCT CCTGTGAGAG AACCAGGATT   
  
  
+ AAGTGAAGGA AAGGGAAAAG AAAAGAAAAA AAAAAGTTTA TAATGAATAA TTAATTTAAA TAAAATGAAT   
  
  
+ TTTAATATAT AATAGTATAG ATAAAAAATT TTTAGACAAA GATATAGTTA GAATTTTTAT GAATCTCTAT   
  
  
+ ATAATAGTTT TTGTTAGAGA GGAAATAACA TTTTATAAGT AGTTTATAAT GAATTTTTAA CTTGGAGTTA   
  
  
+ TTTTGGTAGT ACGTTTAGTT AAACTACACC GAGACCAAAC TCGTAAATTC GTATAATCAT AAAAAAAAGA   
  
  
+ CTTTTTTTTT ACAAGAATTC TTTCTTTTAT TTTTTTATTT TGTCTACTAA AATATAAGTT AAAATTAAAA   
  
  
+ ATTCCTGAAG ACATTACCGA TGAATACACC ACATCACTGA TGACCGATTC TAACAGTATC ATTCTCTCTC   
  
  
+ TCTCCAACAA CACCCCGGGT TGTTTCCGTT CCCCCTTCAG AAGGCTAAAG AGAGGCATCT AACTGCGCGG   
  
  
+ GGTTTTAAAG TAGCGGACCT AACCGGGCCG CGTAGAGCCC GAGCACTTTT ACCCTCCAGA AACCAGTTGC   
  
  
+ TAGAACTGTT CGGACGACCC GGGGTGTTCC GTAGGCCAGA TTAAACACCC GGGGCGCGGA AGTATAACCT   
  
  
+ GAATAACGGT GCAGCCTACC CACAGGAGCC TGGGTTGGAT TTGCCCAACT GAGGCAGCAG GAAAGAAGTG   
  
  
+ GGGTAGATAG GGAGCGGAGC GGAGCGGAGT ATCGAACTTA ACAAATGAAT AATACAATGA GCTAAATAAA   
  
  
+ TAATAATAAA ATTTTTTTAA TTTTCATTTT ATATATAATG AAAATTAATT ATAAATTATT ATTATTTAAT   
  
  
+ AATTAATATT ATTTCATTTG TTTTGCCTTC AGCTAGACGG GGATGTCATC GTGTTGAACG TTCGTGTCCG   
  
  
+ TTGAACGTAT GCACTAAACT GTTCATTTCC ACCAATCATC TACTAACATG TGTGCTTATA AACTAAAATA   
  
  
+ TCATTAAATA TGTATACACA AATTTTTTAG TTTATAATTA AGAATGTAAT TAAATATAAA AATATATTAA   
  
  
+ TAAAAAATAT TTTTTGTTTA TTTTTAAACT TATAGAGTTT TAAATTATAA AAATTCGTCA CATCTTTTCC   
  
  
+ TGTCCACCTG GCGTAACAAA CTTACTCAGT TTCTGGAAAA ATGGGAAGTA TATAAGTTGT TAAAGAAGTG   
  
  
+ GTGGAGTGCA CTAAACGGTA GTTACAGAAT GTGAGGAAGG TGTGCCTTCC TAAGAGGAAG AAGGGTAAGA   
  
  
+ AGTCTTTAAT TCTGCCTTTA CCATTAACG  

- AAATGGAGAG GAAGAGGAGA GGTTTTGGGT GGAAGTCTTG AAACGGTGCA ATAGACCCGG CATTAAAAAG   
  
  
- GGAGAGCTGA GACTTTAGAA GAAGACCAAA CAGTAGTAGT TAGTGCTTCT GTGTTTTAAT TATTTTACAA   
  
  
- ACCCCTTTTT CTCATTTTAC ATCGATATAT CATAATTGAA AAGGAGGCGA GGACACTCTC TTGGTCCTAA   
  
  
- TTCACTTCCT TTCCCTTTTC TTTTCTTTTT TTTTTCAAAT ATTACTTATT AATTAAATTT ATTTTACTTA   
  
  
- AAATTATATA TTATCATATC TATTTTTTAA AAATCTGTTT CTATATCAAT CTTAAAAATA CTTAGAGATA   
  
  
- TATTATCAAA AACAATCTCT CCTTTATTGT AAAATATTCA TCAAATATTA CTTAAAAATT GAACCTCAAT   
  
  
- AAAACCATCA TGCAAATCAA TTTGATGTGG CTCTGGTTTG AGCATTTAAG CATATTAGTA TTTTTTTTCT   
  
  
- GAAAAAAAAA TGTTCTTAAG AAAGAAAATA AAAAAATAAA ACAGATGATT TTATATTCAA TTTTAATTTT   
  
  
- TAAGGACTTC TGTAATGGCT ACTTATGTGG TGTAGTGACT ACTGGCTAAG ATTGTCATAG TAAGAGAGAG   
  
  
- AGAGGTTGTT GTGGGGCCCA ACAAAGGCAA GGGGGAAGTC TTCCGATTTC TCTCCGTAGA TTGACGCGCC   
  
  
- CCAAAATTTC ATCGCCTGGA TTGGCCCGGC GCATCTCGGG CTCGTGAAAA TGGGAGGTCT TTGGTCAACG   
  
  
- ATCTTGACAA GCCTGCTGGG CCCCACAAGG CATCCGGTCT AATTTGTGGG CCCCGCGCCT TCATATTGGA   
  
  
- CTTATTGCCA CGTCGGATGG GTGTCCTCGG ACCCAACCTA AACGGGTTGA CTCCGTCGTC CTTTCTTCAC   
  
  
- CCCATCTATC CCTCGCCTCG CCTCGCCTCA TAGCTTGAAT TGTTTACTTA TTATGTTACT CGATTTATTT   
  
  
- ATTATTATTT TAAAAAAATT AAAAGTAAAA TATATATTAC TTTTAATTAA TATTTAATAA TAATAAATTA   
  
  
- TTAATTATAA TAAAGTAAAC AAAACGGAAG TCGATCTGCC CCTACAGTAG CACAACTTGC AAGCACAGGC   
  
  
- AACTTGCATA CGTGATTTGA CAAGTAAAGG TGGTTAGTAG ATGATTGTAC ACACGAATAT TTGATTTTAT   
  
  
- AGTAATTTAT ACATATGTGT TTAAAAAATC AAATATTAAT TCTTACATTA ATTTATATTT TTATATAATT   
  
  
- ATTTTTTATA AAAAACAAAT AAAAATTTGA ATATCTCAAA ATTTAATATT TTTAAGCAGT GTAGAAAAGG   
  
  
- ACAGGTGGAC CGCATTGTTT GAATGAGTCA AAGACCTTTT TACCCTTCAT ATATTCAACA ATTTCTTCAC   
  
  
- CACCTCACGT GATTTGCCAT CAATGTCTTA CACTCCTTCC ACACGGAAGG ATTCTCCTTC TTCCCATTCT   
  
  
- TCAGAAATTA AGACGGAAAT GGTAATTGC

+     G-box

| Site Name | Organism | Position | Strand | Matrix score. | sequence | function |
| --- | --- | --- | --- | --- | --- | --- |
| G-box | Arabidopsis thaliana | 1333 | - | 9 | GCCACGTGGA | cis-acting regulatory element involved in light responsiveness |
| G-box | Zea mays | 46 | + | 6 | CACGTT | cis-acting regulatory element involved in light responsiveness |

> 2018/04/13 10:10:12  
+ TTTACCTCTC CTTCTCCTCT CCAAAACCCA CCTTCAGAAC TTTGCCACGT TATCTGGGCC GTAATTTTTC   
  
  
+ CCTCTCGACT CTGAAATCTT CTTCTGGTTT GTCATCATCA ATCACGAAGA CACAAAATTA ATAAAATGTT   
  
  
+ TGGGGAAAAA GAGTAAAATG TAGCTATATA GTATTAACTT TTCCTCCGCT CCTGTGAGAG AACCAGGATT   
  
  
+ AAGTGAAGGA AAGGGAAAAG AAAAGAAAAA AAAAAGTTTA TAATGAATAA TTAATTTAAA TAAAATGAAT   
  
  
+ TTTAATATAT AATAGTATAG ATAAAAAATT TTTAGACAAA GATATAGTTA GAATTTTTAT GAATCTCTAT   
  
  
+ ATAATAGTTT TTGTTAGAGA GGAAATAACA TTTTATAAGT AGTTTATAAT GAATTTTTAA CTTGGAGTTA   
  
  
+ TTTTGGTAGT ACGTTTAGTT AAACTACACC GAGACCAAAC TCGTAAATTC GTATAATCAT AAAAAAAAGA   
  
  
+ CTTTTTTTTT ACAAGAATTC TTTCTTTTAT TTTTTTATTT TGTCTACTAA AATATAAGTT AAAATTAAAA   
  
  
+ ATTCCTGAAG ACATTACCGA TGAATACACC ACATCACTGA TGACCGATTC TAACAGTATC ATTCTCTCTC   
  
  
+ TCTCCAACAA CACCCCGGGT TGTTTCCGTT CCCCCTTCAG AAGGCTAAAG AGAGGCATCT AACTGCGCGG   
  
  
+ GGTTTTAAAG TAGCGGACCT AACCGGGCCG CGTAGAGCCC GAGCACTTTT ACCCTCCAGA AACCAGTTGC   
  
  
+ TAGAACTGTT CGGACGACCC GGGGTGTTCC GTAGGCCAGA TTAAACACCC GGGGCGCGGA AGTATAACCT   
  
  
+ GAATAACGGT GCAGCCTACC CACAGGAGCC TGGGTTGGAT TTGCCCAACT GAGGCAGCAG GAAAGAAGTG   
  
  
+ GGGTAGATAG GGAGCGGAGC GGAGCGGAGT ATCGAACTTA ACAAATGAAT AATACAATGA GCTAAATAAA   
  
  
+ TAATAATAAA ATTTTTTTAA TTTTCATTTT ATATATAATG AAAATTAATT ATAAATTATT ATTATTTAAT   
  
  
+ AATTAATATT ATTTCATTTG TTTTGCCTTC AGCTAGACGG GGATGTCATC GTGTTGAACG TTCGTGTCCG   
  
  
+ TTGAACGTAT GCACTAAACT GTTCATTTCC ACCAATCATC TACTAACATG TGTGCTTATA AACTAAAATA   
  
  
+ TCATTAAATA TGTATACACA AATTTTTTAG TTTATAATTA AGAATGTAAT TAAATATAAA AATATATTAA   
  
  
+ TAAAAAATAT TTTTTGTTTA TTTTTAAACT TATAGAGTTT TAAATTATAA AAATTCGTCA CATCTTTTCC   
  
  
+ TGTCCACCTG GCGTAACAAA CTTACTCAGT TTCTGGAAAA ATGGGAAGTA TATAAGTTGT TAAAGAAGTG   
  
  
+ GTGGAGTGCA CTAAACGGTA GTTACAGAAT GTGAGGAAGG TGTGCCTTCC TAAGAGGAAG AAGGGTAAGA   
  
  
+ AGTCTTTAAT TCTGCCTTTA CCATTAACG  

- AAATGGAGAG GAAGAGGAGA GGTTTTGGGT GGAAGTCTTG AAACGGTGCA ATAGACCCGG CATTAAAAAG   
  
  
- GGAGAGCTGA GACTTTAGAA GAAGACCAAA CAGTAGTAGT TAGTGCTTCT GTGTTTTAAT TATTTTACAA   
  
  
- ACCCCTTTTT CTCATTTTAC ATCGATATAT CATAATTGAA AAGGAGGCGA GGACACTCTC TTGGTCCTAA   
  
  
- TTCACTTCCT TTCCCTTTTC TTTTCTTTTT TTTTTCAAAT ATTACTTATT AATTAAATTT ATTTTACTTA   
  
  
- AAATTATATA TTATCATATC TATTTTTTAA AAATCTGTTT CTATATCAAT CTTAAAAATA CTTAGAGATA   
  
  
- TATTATCAAA AACAATCTCT CCTTTATTGT AAAATATTCA TCAAATATTA CTTAAAAATT GAACCTCAAT   
  
  
- AAAACCATCA TGCAAATCAA TTTGATGTGG CTCTGGTTTG AGCATTTAAG CATATTAGTA TTTTTTTTCT   
  
  
- GAAAAAAAAA TGTTCTTAAG AAAGAAAATA AAAAAATAAA ACAGATGATT TTATATTCAA TTTTAATTTT   
  
  
- TAAGGACTTC TGTAATGGCT ACTTATGTGG TGTAGTGACT ACTGGCTAAG ATTGTCATAG TAAGAGAGAG   
  
  
- AGAGGTTGTT GTGGGGCCCA ACAAAGGCAA GGGGGAAGTC TTCCGATTTC TCTCCGTAGA TTGACGCGCC   
  
  
- CCAAAATTTC ATCGCCTGGA TTGGCCCGGC GCATCTCGGG CTCGTGAAAA TGGGAGGTCT TTGGTCAACG   
  
  
- ATCTTGACAA GCCTGCTGGG CCCCACAAGG CATCCGGTCT AATTTGTGGG CCCCGCGCCT TCATATTGGA   
  
  
- CTTATTGCCA CGTCGGATGG GTGTCCTCGG ACCCAACCTA AACGGGTTGA CTCCGTCGTC CTTTCTTCAC   
  
  
- CCCATCTATC CCTCGCCTCG CCTCGCCTCA TAGCTTGAAT TGTTTACTTA TTATGTTACT CGATTTATTT   
  
  
- ATTATTATTT TAAAAAAATT AAAAGTAAAA TATATATTAC TTTTAATTAA TATTTAATAA TAATAAATTA   
  
  
- TTAATTATAA TAAAGTAAAC AAAACGGAAG TCGATCTGCC CCTACAGTAG CACAACTTGC AAGCACAGGC   
  
  
- AACTTGCATA CGTGATTTGA CAAGTAAAGG TGGTTAGTAG ATGATTGTAC ACACGAATAT TTGATTTTAT   
  
  
- AGTAATTTAT ACATATGTGT TTAAAAAATC AAATATTAAT TCTTACATTA ATTTATATTT TTATATAATT   
  
  
- ATTTTTTATA AAAAACAAAT AAAAATTTGA ATATCTCAAA ATTTAATATT TTTAAGCAGT GTAGAAAAGG   
  
  
- ACAGGTGGAC CGCATTGTTT GAATGAGTCA AAGACCTTTT TACCCTTCAT ATATTCAACA ATTTCTTCAC   
  
  
- CACCTCACGT GATTTGCCAT CAATGTCTTA CACTCCTTCC ACACGGAAGG ATTCTCCTTC TTCCCATTCT   
  
  
- TCAGAAATTA AGACGGAAAT GGTAATTGC

+     GA-motif

| Site Name | Organism | Position | Strand | Matrix score. | sequence | function |
| --- | --- | --- | --- | --- | --- | --- |
| GA-motif | Arabidopsis thaliana | 297 | + | 8 | ATAGATAA | part of a light responsive element |

> 2018/04/13 10:10:12  
+ TTTACCTCTC CTTCTCCTCT CCAAAACCCA CCTTCAGAAC TTTGCCACGT TATCTGGGCC GTAATTTTTC   
  
  
+ CCTCTCGACT CTGAAATCTT CTTCTGGTTT GTCATCATCA ATCACGAAGA CACAAAATTA ATAAAATGTT   
  
  
+ TGGGGAAAAA GAGTAAAATG TAGCTATATA GTATTAACTT TTCCTCCGCT CCTGTGAGAG AACCAGGATT   
  
  
+ AAGTGAAGGA AAGGGAAAAG AAAAGAAAAA AAAAAGTTTA TAATGAATAA TTAATTTAAA TAAAATGAAT   
  
  
+ TTTAATATAT AATAGTATAG ATAAAAAATT TTTAGACAAA GATATAGTTA GAATTTTTAT GAATCTCTAT   
  
  
+ ATAATAGTTT TTGTTAGAGA GGAAATAACA TTTTATAAGT AGTTTATAAT GAATTTTTAA CTTGGAGTTA   
  
  
+ TTTTGGTAGT ACGTTTAGTT AAACTACACC GAGACCAAAC TCGTAAATTC GTATAATCAT AAAAAAAAGA   
  
  
+ CTTTTTTTTT ACAAGAATTC TTTCTTTTAT TTTTTTATTT TGTCTACTAA AATATAAGTT AAAATTAAAA   
  
  
+ ATTCCTGAAG ACATTACCGA TGAATACACC ACATCACTGA TGACCGATTC TAACAGTATC ATTCTCTCTC   
  
  
+ TCTCCAACAA CACCCCGGGT TGTTTCCGTT CCCCCTTCAG AAGGCTAAAG AGAGGCATCT AACTGCGCGG   
  
  
+ GGTTTTAAAG TAGCGGACCT AACCGGGCCG CGTAGAGCCC GAGCACTTTT ACCCTCCAGA AACCAGTTGC   
  
  
+ TAGAACTGTT CGGACGACCC GGGGTGTTCC GTAGGCCAGA TTAAACACCC GGGGCGCGGA AGTATAACCT   
  
  
+ GAATAACGGT GCAGCCTACC CACAGGAGCC TGGGTTGGAT TTGCCCAACT GAGGCAGCAG GAAAGAAGTG   
  
  
+ GGGTAGATAG GGAGCGGAGC GGAGCGGAGT ATCGAACTTA ACAAATGAAT AATACAATGA GCTAAATAAA   
  
  
+ TAATAATAAA ATTTTTTTAA TTTTCATTTT ATATATAATG AAAATTAATT ATAAATTATT ATTATTTAAT   
  
  
+ AATTAATATT ATTTCATTTG TTTTGCCTTC AGCTAGACGG GGATGTCATC GTGTTGAACG TTCGTGTCCG   
  
  
+ TTGAACGTAT GCACTAAACT GTTCATTTCC ACCAATCATC TACTAACATG TGTGCTTATA AACTAAAATA   
  
  
+ TCATTAAATA TGTATACACA AATTTTTTAG TTTATAATTA AGAATGTAAT TAAATATAAA AATATATTAA   
  
  
+ TAAAAAATAT TTTTTGTTTA TTTTTAAACT TATAGAGTTT TAAATTATAA AAATTCGTCA CATCTTTTCC   
  
  
+ TGTCCACCTG GCGTAACAAA CTTACTCAGT TTCTGGAAAA ATGGGAAGTA TATAAGTTGT TAAAGAAGTG   
  
  
+ GTGGAGTGCA CTAAACGGTA GTTACAGAAT GTGAGGAAGG TGTGCCTTCC TAAGAGGAAG AAGGGTAAGA   
  
  
+ AGTCTTTAAT TCTGCCTTTA CCATTAACG  

- AAATGGAGAG GAAGAGGAGA GGTTTTGGGT GGAAGTCTTG AAACGGTGCA ATAGACCCGG CATTAAAAAG   
  
  
- GGAGAGCTGA GACTTTAGAA GAAGACCAAA CAGTAGTAGT TAGTGCTTCT GTGTTTTAAT TATTTTACAA   
  
  
- ACCCCTTTTT CTCATTTTAC ATCGATATAT CATAATTGAA AAGGAGGCGA GGACACTCTC TTGGTCCTAA   
  
  
- TTCACTTCCT TTCCCTTTTC TTTTCTTTTT TTTTTCAAAT ATTACTTATT AATTAAATTT ATTTTACTTA   
  
  
- AAATTATATA TTATCATATC TATTTTTTAA AAATCTGTTT CTATATCAAT CTTAAAAATA CTTAGAGATA   
  
  
- TATTATCAAA AACAATCTCT CCTTTATTGT AAAATATTCA TCAAATATTA CTTAAAAATT GAACCTCAAT   
  
  
- AAAACCATCA TGCAAATCAA TTTGATGTGG CTCTGGTTTG AGCATTTAAG CATATTAGTA TTTTTTTTCT   
  
  
- GAAAAAAAAA TGTTCTTAAG AAAGAAAATA AAAAAATAAA ACAGATGATT TTATATTCAA TTTTAATTTT   
  
  
- TAAGGACTTC TGTAATGGCT ACTTATGTGG TGTAGTGACT ACTGGCTAAG ATTGTCATAG TAAGAGAGAG   
  
  
- AGAGGTTGTT GTGGGGCCCA ACAAAGGCAA GGGGGAAGTC TTCCGATTTC TCTCCGTAGA TTGACGCGCC   
  
  
- CCAAAATTTC ATCGCCTGGA TTGGCCCGGC GCATCTCGGG CTCGTGAAAA TGGGAGGTCT TTGGTCAACG   
  
  
- ATCTTGACAA GCCTGCTGGG CCCCACAAGG CATCCGGTCT AATTTGTGGG CCCCGCGCCT TCATATTGGA   
  
  
- CTTATTGCCA CGTCGGATGG GTGTCCTCGG ACCCAACCTA AACGGGTTGA CTCCGTCGTC CTTTCTTCAC   
  
  
- CCCATCTATC CCTCGCCTCG CCTCGCCTCA TAGCTTGAAT TGTTTACTTA TTATGTTACT CGATTTATTT   
  
  
- ATTATTATTT TAAAAAAATT AAAAGTAAAA TATATATTAC TTTTAATTAA TATTTAATAA TAATAAATTA   
  
  
- TTAATTATAA TAAAGTAAAC AAAACGGAAG TCGATCTGCC CCTACAGTAG CACAACTTGC AAGCACAGGC   
  
  
- AACTTGCATA CGTGATTTGA CAAGTAAAGG TGGTTAGTAG ATGATTGTAC ACACGAATAT TTGATTTTAT   
  
  
- AGTAATTTAT ACATATGTGT TTAAAAAATC AAATATTAAT TCTTACATTA ATTTATATTT TTATATAATT   
  
  
- ATTTTTTATA AAAAACAAAT AAAAATTTGA ATATCTCAAA ATTTAATATT TTTAAGCAGT GTAGAAAAGG   
  
  
- ACAGGTGGAC CGCATTGTTT GAATGAGTCA AAGACCTTTT TACCCTTCAT ATATTCAACA ATTTCTTCAC   
  
  
- CACCTCACGT GATTTGCCAT CAATGTCTTA CACTCCTTCC ACACGGAAGG ATTCTCCTTC TTCCCATTCT   
  
  
- TCAGAAATTA AGACGGAAAT GGTAATTGC

+     GATA-motif

| Site Name | Organism | Position | Strand | Matrix score. | sequence | function |
| --- | --- | --- | --- | --- | --- | --- |
| GATA-motif | Pisum sativum | 916 | + | 7 | GATAGGG | part of a light responsive element |

> 2018/04/13 10:10:12  
+ TTTACCTCTC CTTCTCCTCT CCAAAACCCA CCTTCAGAAC TTTGCCACGT TATCTGGGCC GTAATTTTTC   
  
  
+ CCTCTCGACT CTGAAATCTT CTTCTGGTTT GTCATCATCA ATCACGAAGA CACAAAATTA ATAAAATGTT   
  
  
+ TGGGGAAAAA GAGTAAAATG TAGCTATATA GTATTAACTT TTCCTCCGCT CCTGTGAGAG AACCAGGATT   
  
  
+ AAGTGAAGGA AAGGGAAAAG AAAAGAAAAA AAAAAGTTTA TAATGAATAA TTAATTTAAA TAAAATGAAT   
  
  
+ TTTAATATAT AATAGTATAG ATAAAAAATT TTTAGACAAA GATATAGTTA GAATTTTTAT GAATCTCTAT   
  
  
+ ATAATAGTTT TTGTTAGAGA GGAAATAACA TTTTATAAGT AGTTTATAAT GAATTTTTAA CTTGGAGTTA   
  
  
+ TTTTGGTAGT ACGTTTAGTT AAACTACACC GAGACCAAAC TCGTAAATTC GTATAATCAT AAAAAAAAGA   
  
  
+ CTTTTTTTTT ACAAGAATTC TTTCTTTTAT TTTTTTATTT TGTCTACTAA AATATAAGTT AAAATTAAAA   
  
  
+ ATTCCTGAAG ACATTACCGA TGAATACACC ACATCACTGA TGACCGATTC TAACAGTATC ATTCTCTCTC   
  
  
+ TCTCCAACAA CACCCCGGGT TGTTTCCGTT CCCCCTTCAG AAGGCTAAAG AGAGGCATCT AACTGCGCGG   
  
  
+ GGTTTTAAAG TAGCGGACCT AACCGGGCCG CGTAGAGCCC GAGCACTTTT ACCCTCCAGA AACCAGTTGC   
  
  
+ TAGAACTGTT CGGACGACCC GGGGTGTTCC GTAGGCCAGA TTAAACACCC GGGGCGCGGA AGTATAACCT   
  
  
+ GAATAACGGT GCAGCCTACC CACAGGAGCC TGGGTTGGAT TTGCCCAACT GAGGCAGCAG GAAAGAAGTG   
  
  
+ GGGTAGATAG GGAGCGGAGC GGAGCGGAGT ATCGAACTTA ACAAATGAAT AATACAATGA GCTAAATAAA   
  
  
+ TAATAATAAA ATTTTTTTAA TTTTCATTTT ATATATAATG AAAATTAATT ATAAATTATT ATTATTTAAT   
  
  
+ AATTAATATT ATTTCATTTG TTTTGCCTTC AGCTAGACGG GGATGTCATC GTGTTGAACG TTCGTGTCCG   
  
  
+ TTGAACGTAT GCACTAAACT GTTCATTTCC ACCAATCATC TACTAACATG TGTGCTTATA AACTAAAATA   
  
  
+ TCATTAAATA TGTATACACA AATTTTTTAG TTTATAATTA AGAATGTAAT TAAATATAAA AATATATTAA   
  
  
+ TAAAAAATAT TTTTTGTTTA TTTTTAAACT TATAGAGTTT TAAATTATAA AAATTCGTCA CATCTTTTCC   
  
  
+ TGTCCACCTG GCGTAACAAA CTTACTCAGT TTCTGGAAAA ATGGGAAGTA TATAAGTTGT TAAAGAAGTG   
  
  
+ GTGGAGTGCA CTAAACGGTA GTTACAGAAT GTGAGGAAGG TGTGCCTTCC TAAGAGGAAG AAGGGTAAGA   
  
  
+ AGTCTTTAAT TCTGCCTTTA CCATTAACG  

- AAATGGAGAG GAAGAGGAGA GGTTTTGGGT GGAAGTCTTG AAACGGTGCA ATAGACCCGG CATTAAAAAG   
  
  
- GGAGAGCTGA GACTTTAGAA GAAGACCAAA CAGTAGTAGT TAGTGCTTCT GTGTTTTAAT TATTTTACAA   
  
  
- ACCCCTTTTT CTCATTTTAC ATCGATATAT CATAATTGAA AAGGAGGCGA GGACACTCTC TTGGTCCTAA   
  
  
- TTCACTTCCT TTCCCTTTTC TTTTCTTTTT TTTTTCAAAT ATTACTTATT AATTAAATTT ATTTTACTTA   
  
  
- AAATTATATA TTATCATATC TATTTTTTAA AAATCTGTTT CTATATCAAT CTTAAAAATA CTTAGAGATA   
  
  
- TATTATCAAA AACAATCTCT CCTTTATTGT AAAATATTCA TCAAATATTA CTTAAAAATT GAACCTCAAT   
  
  
- AAAACCATCA TGCAAATCAA TTTGATGTGG CTCTGGTTTG AGCATTTAAG CATATTAGTA TTTTTTTTCT   
  
  
- GAAAAAAAAA TGTTCTTAAG AAAGAAAATA AAAAAATAAA ACAGATGATT TTATATTCAA TTTTAATTTT   
  
  
- TAAGGACTTC TGTAATGGCT ACTTATGTGG TGTAGTGACT ACTGGCTAAG ATTGTCATAG TAAGAGAGAG   
  
  
- AGAGGTTGTT GTGGGGCCCA ACAAAGGCAA GGGGGAAGTC TTCCGATTTC TCTCCGTAGA TTGACGCGCC   
  
  
- CCAAAATTTC ATCGCCTGGA TTGGCCCGGC GCATCTCGGG CTCGTGAAAA TGGGAGGTCT TTGGTCAACG   
  
  
- ATCTTGACAA GCCTGCTGGG CCCCACAAGG CATCCGGTCT AATTTGTGGG CCCCGCGCCT TCATATTGGA   
  
  
- CTTATTGCCA CGTCGGATGG GTGTCCTCGG ACCCAACCTA AACGGGTTGA CTCCGTCGTC CTTTCTTCAC   
  
  
- CCCATCTATC CCTCGCCTCG CCTCGCCTCA TAGCTTGAAT TGTTTACTTA TTATGTTACT CGATTTATTT   
  
  
- ATTATTATTT TAAAAAAATT AAAAGTAAAA TATATATTAC TTTTAATTAA TATTTAATAA TAATAAATTA   
  
  
- TTAATTATAA TAAAGTAAAC AAAACGGAAG TCGATCTGCC CCTACAGTAG CACAACTTGC AAGCACAGGC   
  
  
- AACTTGCATA CGTGATTTGA CAAGTAAAGG TGGTTAGTAG ATGATTGTAC ACACGAATAT TTGATTTTAT   
  
  
- AGTAATTTAT ACATATGTGT TTAAAAAATC AAATATTAAT TCTTACATTA ATTTATATTT TTATATAATT   
  
  
- ATTTTTTATA AAAAACAAAT AAAAATTTGA ATATCTCAAA ATTTAATATT TTTAAGCAGT GTAGAAAAGG   
  
  
- ACAGGTGGAC CGCATTGTTT GAATGAGTCA AAGACCTTTT TACCCTTCAT ATATTCAACA ATTTCTTCAC   
  
  
- CACCTCACGT GATTTGCCAT CAATGTCTTA CACTCCTTCC ACACGGAAGG ATTCTCCTTC TTCCCATTCT   
  
  
- TCAGAAATTA AGACGGAAAT GGTAATTGC

+     GC-motif

| Site Name | Organism | Position | Strand | Matrix score. | sequence | function |
| --- | --- | --- | --- | --- | --- | --- |
| GC-motif | Zea mays | 819 | - | 7 | GCCCCGG | enhancer-like element involved in anoxic specific inducibility |

> 2018/04/13 10:10:12  
+ TTTACCTCTC CTTCTCCTCT CCAAAACCCA CCTTCAGAAC TTTGCCACGT TATCTGGGCC GTAATTTTTC   
  
  
+ CCTCTCGACT CTGAAATCTT CTTCTGGTTT GTCATCATCA ATCACGAAGA CACAAAATTA ATAAAATGTT   
  
  
+ TGGGGAAAAA GAGTAAAATG TAGCTATATA GTATTAACTT TTCCTCCGCT CCTGTGAGAG AACCAGGATT   
  
  
+ AAGTGAAGGA AAGGGAAAAG AAAAGAAAAA AAAAAGTTTA TAATGAATAA TTAATTTAAA TAAAATGAAT   
  
  
+ TTTAATATAT AATAGTATAG ATAAAAAATT TTTAGACAAA GATATAGTTA GAATTTTTAT GAATCTCTAT   
  
  
+ ATAATAGTTT TTGTTAGAGA GGAAATAACA TTTTATAAGT AGTTTATAAT GAATTTTTAA CTTGGAGTTA   
  
  
+ TTTTGGTAGT ACGTTTAGTT AAACTACACC GAGACCAAAC TCGTAAATTC GTATAATCAT AAAAAAAAGA   
  
  
+ CTTTTTTTTT ACAAGAATTC TTTCTTTTAT TTTTTTATTT TGTCTACTAA AATATAAGTT AAAATTAAAA   
  
  
+ ATTCCTGAAG ACATTACCGA TGAATACACC ACATCACTGA TGACCGATTC TAACAGTATC ATTCTCTCTC   
  
  
+ TCTCCAACAA CACCCCGGGT TGTTTCCGTT CCCCCTTCAG AAGGCTAAAG AGAGGCATCT AACTGCGCGG   
  
  
+ GGTTTTAAAG TAGCGGACCT AACCGGGCCG CGTAGAGCCC GAGCACTTTT ACCCTCCAGA AACCAGTTGC   
  
  
+ TAGAACTGTT CGGACGACCC GGGGTGTTCC GTAGGCCAGA TTAAACACCC GGGGCGCGGA AGTATAACCT   
  
  
+ GAATAACGGT GCAGCCTACC CACAGGAGCC TGGGTTGGAT TTGCCCAACT GAGGCAGCAG GAAAGAAGTG   
  
  
+ GGGTAGATAG GGAGCGGAGC GGAGCGGAGT ATCGAACTTA ACAAATGAAT AATACAATGA GCTAAATAAA   
  
  
+ TAATAATAAA ATTTTTTTAA TTTTCATTTT ATATATAATG AAAATTAATT ATAAATTATT ATTATTTAAT   
  
  
+ AATTAATATT ATTTCATTTG TTTTGCCTTC AGCTAGACGG GGATGTCATC GTGTTGAACG TTCGTGTCCG   
  
  
+ TTGAACGTAT GCACTAAACT GTTCATTTCC ACCAATCATC TACTAACATG TGTGCTTATA AACTAAAATA   
  
  
+ TCATTAAATA TGTATACACA AATTTTTTAG TTTATAATTA AGAATGTAAT TAAATATAAA AATATATTAA   
  
  
+ TAAAAAATAT TTTTTGTTTA TTTTTAAACT TATAGAGTTT TAAATTATAA AAATTCGTCA CATCTTTTCC   
  
  
+ TGTCCACCTG GCGTAACAAA CTTACTCAGT TTCTGGAAAA ATGGGAAGTA TATAAGTTGT TAAAGAAGTG   
  
  
+ GTGGAGTGCA CTAAACGGTA GTTACAGAAT GTGAGGAAGG TGTGCCTTCC TAAGAGGAAG AAGGGTAAGA   
  
  
+ AGTCTTTAAT TCTGCCTTTA CCATTAACG  

- AAATGGAGAG GAAGAGGAGA GGTTTTGGGT GGAAGTCTTG AAACGGTGCA ATAGACCCGG CATTAAAAAG   
  
  
- GGAGAGCTGA GACTTTAGAA GAAGACCAAA CAGTAGTAGT TAGTGCTTCT GTGTTTTAAT TATTTTACAA   
  
  
- ACCCCTTTTT CTCATTTTAC ATCGATATAT CATAATTGAA AAGGAGGCGA GGACACTCTC TTGGTCCTAA   
  
  
- TTCACTTCCT TTCCCTTTTC TTTTCTTTTT TTTTTCAAAT ATTACTTATT AATTAAATTT ATTTTACTTA   
  
  
- AAATTATATA TTATCATATC TATTTTTTAA AAATCTGTTT CTATATCAAT CTTAAAAATA CTTAGAGATA   
  
  
- TATTATCAAA AACAATCTCT CCTTTATTGT AAAATATTCA TCAAATATTA CTTAAAAATT GAACCTCAAT   
  
  
- AAAACCATCA TGCAAATCAA TTTGATGTGG CTCTGGTTTG AGCATTTAAG CATATTAGTA TTTTTTTTCT   
  
  
- GAAAAAAAAA TGTTCTTAAG AAAGAAAATA AAAAAATAAA ACAGATGATT TTATATTCAA TTTTAATTTT   
  
  
- TAAGGACTTC TGTAATGGCT ACTTATGTGG TGTAGTGACT ACTGGCTAAG ATTGTCATAG TAAGAGAGAG   
  
  
- AGAGGTTGTT GTGGGGCCCA ACAAAGGCAA GGGGGAAGTC TTCCGATTTC TCTCCGTAGA TTGACGCGCC   
  
  
- CCAAAATTTC ATCGCCTGGA TTGGCCCGGC GCATCTCGGG CTCGTGAAAA TGGGAGGTCT TTGGTCAACG   
  
  
- ATCTTGACAA GCCTGCTGGG CCCCACAAGG CATCCGGTCT AATTTGTGGG CCCCGCGCCT TCATATTGGA   
  
  
- CTTATTGCCA CGTCGGATGG GTGTCCTCGG ACCCAACCTA AACGGGTTGA CTCCGTCGTC CTTTCTTCAC   
  
  
- CCCATCTATC CCTCGCCTCG CCTCGCCTCA TAGCTTGAAT TGTTTACTTA TTATGTTACT CGATTTATTT   
  
  
- ATTATTATTT TAAAAAAATT AAAAGTAAAA TATATATTAC TTTTAATTAA TATTTAATAA TAATAAATTA   
  
  
- TTAATTATAA TAAAGTAAAC AAAACGGAAG TCGATCTGCC CCTACAGTAG CACAACTTGC AAGCACAGGC   
  
  
- AACTTGCATA CGTGATTTGA CAAGTAAAGG TGGTTAGTAG ATGATTGTAC ACACGAATAT TTGATTTTAT   
  
  
- AGTAATTTAT ACATATGTGT TTAAAAAATC AAATATTAAT TCTTACATTA ATTTATATTT TTATATAATT   
  
  
- ATTTTTTATA AAAAACAAAT AAAAATTTGA ATATCTCAAA ATTTAATATT TTTAAGCAGT GTAGAAAAGG   
  
  
- ACAGGTGGAC CGCATTGTTT GAATGAGTCA AAGACCTTTT TACCCTTCAT ATATTCAACA ATTTCTTCAC   
  
  
- CACCTCACGT GATTTGCCAT CAATGTCTTA CACTCCTTCC ACACGGAAGG ATTCTCCTTC TTCCCATTCT   
  
  
- TCAGAAATTA AGACGGAAAT GGTAATTGC

+     Gap-box

| Site Name | Organism | Position | Strand | Matrix score. | sequence | function |
| --- | --- | --- | --- | --- | --- | --- |
| Gap-box | Arabidopsis thaliana | 952 | + | 9 | CAAATGAA(A/G)A | part of a light responsive element |

> 2018/04/13 10:10:12  
+ TTTACCTCTC CTTCTCCTCT CCAAAACCCA CCTTCAGAAC TTTGCCACGT TATCTGGGCC GTAATTTTTC   
  
  
+ CCTCTCGACT CTGAAATCTT CTTCTGGTTT GTCATCATCA ATCACGAAGA CACAAAATTA ATAAAATGTT   
  
  
+ TGGGGAAAAA GAGTAAAATG TAGCTATATA GTATTAACTT TTCCTCCGCT CCTGTGAGAG AACCAGGATT   
  
  
+ AAGTGAAGGA AAGGGAAAAG AAAAGAAAAA AAAAAGTTTA TAATGAATAA TTAATTTAAA TAAAATGAAT   
  
  
+ TTTAATATAT AATAGTATAG ATAAAAAATT TTTAGACAAA GATATAGTTA GAATTTTTAT GAATCTCTAT   
  
  
+ ATAATAGTTT TTGTTAGAGA GGAAATAACA TTTTATAAGT AGTTTATAAT GAATTTTTAA CTTGGAGTTA   
  
  
+ TTTTGGTAGT ACGTTTAGTT AAACTACACC GAGACCAAAC TCGTAAATTC GTATAATCAT AAAAAAAAGA   
  
  
+ CTTTTTTTTT ACAAGAATTC TTTCTTTTAT TTTTTTATTT TGTCTACTAA AATATAAGTT AAAATTAAAA   
  
  
+ ATTCCTGAAG ACATTACCGA TGAATACACC ACATCACTGA TGACCGATTC TAACAGTATC ATTCTCTCTC   
  
  
+ TCTCCAACAA CACCCCGGGT TGTTTCCGTT CCCCCTTCAG AAGGCTAAAG AGAGGCATCT AACTGCGCGG   
  
  
+ GGTTTTAAAG TAGCGGACCT AACCGGGCCG CGTAGAGCCC GAGCACTTTT ACCCTCCAGA AACCAGTTGC   
  
  
+ TAGAACTGTT CGGACGACCC GGGGTGTTCC GTAGGCCAGA TTAAACACCC GGGGCGCGGA AGTATAACCT   
  
  
+ GAATAACGGT GCAGCCTACC CACAGGAGCC TGGGTTGGAT TTGCCCAACT GAGGCAGCAG GAAAGAAGTG   
  
  
+ GGGTAGATAG GGAGCGGAGC GGAGCGGAGT ATCGAACTTA ACAAATGAAT AATACAATGA GCTAAATAAA   
  
  
+ TAATAATAAA ATTTTTTTAA TTTTCATTTT ATATATAATG AAAATTAATT ATAAATTATT ATTATTTAAT   
  
  
+ AATTAATATT ATTTCATTTG TTTTGCCTTC AGCTAGACGG GGATGTCATC GTGTTGAACG TTCGTGTCCG   
  
  
+ TTGAACGTAT GCACTAAACT GTTCATTTCC ACCAATCATC TACTAACATG TGTGCTTATA AACTAAAATA   
  
  
+ TCATTAAATA TGTATACACA AATTTTTTAG TTTATAATTA AGAATGTAAT TAAATATAAA AATATATTAA   
  
  
+ TAAAAAATAT TTTTTGTTTA TTTTTAAACT TATAGAGTTT TAAATTATAA AAATTCGTCA CATCTTTTCC   
  
  
+ TGTCCACCTG GCGTAACAAA CTTACTCAGT TTCTGGAAAA ATGGGAAGTA TATAAGTTGT TAAAGAAGTG   
  
  
+ GTGGAGTGCA CTAAACGGTA GTTACAGAAT GTGAGGAAGG TGTGCCTTCC TAAGAGGAAG AAGGGTAAGA   
  
  
+ AGTCTTTAAT TCTGCCTTTA CCATTAACG  

- AAATGGAGAG GAAGAGGAGA GGTTTTGGGT GGAAGTCTTG AAACGGTGCA ATAGACCCGG CATTAAAAAG   
  
  
- GGAGAGCTGA GACTTTAGAA GAAGACCAAA CAGTAGTAGT TAGTGCTTCT GTGTTTTAAT TATTTTACAA   
  
  
- ACCCCTTTTT CTCATTTTAC ATCGATATAT CATAATTGAA AAGGAGGCGA GGACACTCTC TTGGTCCTAA   
  
  
- TTCACTTCCT TTCCCTTTTC TTTTCTTTTT TTTTTCAAAT ATTACTTATT AATTAAATTT ATTTTACTTA   
  
  
- AAATTATATA TTATCATATC TATTTTTTAA AAATCTGTTT CTATATCAAT CTTAAAAATA CTTAGAGATA   
  
  
- TATTATCAAA AACAATCTCT CCTTTATTGT AAAATATTCA TCAAATATTA CTTAAAAATT GAACCTCAAT   
  
  
- AAAACCATCA TGCAAATCAA TTTGATGTGG CTCTGGTTTG AGCATTTAAG CATATTAGTA TTTTTTTTCT   
  
  
- GAAAAAAAAA TGTTCTTAAG AAAGAAAATA AAAAAATAAA ACAGATGATT TTATATTCAA TTTTAATTTT   
  
  
- TAAGGACTTC TGTAATGGCT ACTTATGTGG TGTAGTGACT ACTGGCTAAG ATTGTCATAG TAAGAGAGAG   
  
  
- AGAGGTTGTT GTGGGGCCCA ACAAAGGCAA GGGGGAAGTC TTCCGATTTC TCTCCGTAGA TTGACGCGCC   
  
  
- CCAAAATTTC ATCGCCTGGA TTGGCCCGGC GCATCTCGGG CTCGTGAAAA TGGGAGGTCT TTGGTCAACG   
  
  
- ATCTTGACAA GCCTGCTGGG CCCCACAAGG CATCCGGTCT AATTTGTGGG CCCCGCGCCT TCATATTGGA   
  
  
- CTTATTGCCA CGTCGGATGG GTGTCCTCGG ACCCAACCTA AACGGGTTGA CTCCGTCGTC CTTTCTTCAC   
  
  
- CCCATCTATC CCTCGCCTCG CCTCGCCTCA TAGCTTGAAT TGTTTACTTA TTATGTTACT CGATTTATTT   
  
  
- ATTATTATTT TAAAAAAATT AAAAGTAAAA TATATATTAC TTTTAATTAA TATTTAATAA TAATAAATTA   
  
  
- TTAATTATAA TAAAGTAAAC AAAACGGAAG TCGATCTGCC CCTACAGTAG CACAACTTGC AAGCACAGGC   
  
  
- AACTTGCATA CGTGATTTGA CAAGTAAAGG TGGTTAGTAG ATGATTGTAC ACACGAATAT TTGATTTTAT   
  
  
- AGTAATTTAT ACATATGTGT TTAAAAAATC AAATATTAAT TCTTACATTA ATTTATATTT TTATATAATT   
  
  
- ATTTTTTATA AAAAACAAAT AAAAATTTGA ATATCTCAAA ATTTAATATT TTTAAGCAGT GTAGAAAAGG   
  
  
- ACAGGTGGAC CGCATTGTTT GAATGAGTCA AAGACCTTTT TACCCTTCAT ATATTCAACA ATTTCTTCAC   
  
  
- CACCTCACGT GATTTGCCAT CAATGTCTTA CACTCCTTCC ACACGGAAGG ATTCTCCTTC TTCCCATTCT   
  
  
- TCAGAAATTA AGACGGAAAT GGTAATTGC

+     HSE

| Site Name | Organism | Position | Strand | Matrix score. | sequence | function |
| --- | --- | --- | --- | --- | --- | --- |
| HSE | Brassica oleracea | 303 | + | 9 | AAAAAATTTC | cis-acting element involved in heat stress responsiveness |
| HSE | Brassica oleracea | 1209 | - | 9 | AAAAAATTTC | cis-acting element involved in heat stress responsiveness |
| HSE | Brassica oleracea | 988 | - | 9 | AAAAAATTTC | cis-acting element involved in heat stress responsiveness |

> 2018/04/13 10:10:12  
+ TTTACCTCTC CTTCTCCTCT CCAAAACCCA CCTTCAGAAC TTTGCCACGT TATCTGGGCC GTAATTTTTC   
  
  
+ CCTCTCGACT CTGAAATCTT CTTCTGGTTT GTCATCATCA ATCACGAAGA CACAAAATTA ATAAAATGTT   
  
  
+ TGGGGAAAAA GAGTAAAATG TAGCTATATA GTATTAACTT TTCCTCCGCT CCTGTGAGAG AACCAGGATT   
  
  
+ AAGTGAAGGA AAGGGAAAAG AAAAGAAAAA AAAAAGTTTA TAATGAATAA TTAATTTAAA TAAAATGAAT   
  
  
+ TTTAATATAT AATAGTATAG ATAAAAAATT TTTAGACAAA GATATAGTTA GAATTTTTAT GAATCTCTAT   
  
  
+ ATAATAGTTT TTGTTAGAGA GGAAATAACA TTTTATAAGT AGTTTATAAT GAATTTTTAA CTTGGAGTTA   
  
  
+ TTTTGGTAGT ACGTTTAGTT AAACTACACC GAGACCAAAC TCGTAAATTC GTATAATCAT AAAAAAAAGA   
  
  
+ CTTTTTTTTT ACAAGAATTC TTTCTTTTAT TTTTTTATTT TGTCTACTAA AATATAAGTT AAAATTAAAA   
  
  
+ ATTCCTGAAG ACATTACCGA TGAATACACC ACATCACTGA TGACCGATTC TAACAGTATC ATTCTCTCTC   
  
  
+ TCTCCAACAA CACCCCGGGT TGTTTCCGTT CCCCCTTCAG AAGGCTAAAG AGAGGCATCT AACTGCGCGG   
  
  
+ GGTTTTAAAG TAGCGGACCT AACCGGGCCG CGTAGAGCCC GAGCACTTTT ACCCTCCAGA AACCAGTTGC   
  
  
+ TAGAACTGTT CGGACGACCC GGGGTGTTCC GTAGGCCAGA TTAAACACCC GGGGCGCGGA AGTATAACCT   
  
  
+ GAATAACGGT GCAGCCTACC CACAGGAGCC TGGGTTGGAT TTGCCCAACT GAGGCAGCAG GAAAGAAGTG   
  
  
+ GGGTAGATAG GGAGCGGAGC GGAGCGGAGT ATCGAACTTA ACAAATGAAT AATACAATGA GCTAAATAAA   
  
  
+ TAATAATAAA ATTTTTTTAA TTTTCATTTT ATATATAATG AAAATTAATT ATAAATTATT ATTATTTAAT   
  
  
+ AATTAATATT ATTTCATTTG TTTTGCCTTC AGCTAGACGG GGATGTCATC GTGTTGAACG TTCGTGTCCG   
  
  
+ TTGAACGTAT GCACTAAACT GTTCATTTCC ACCAATCATC TACTAACATG TGTGCTTATA AACTAAAATA   
  
  
+ TCATTAAATA TGTATACACA AATTTTTTAG TTTATAATTA AGAATGTAAT TAAATATAAA AATATATTAA   
  
  
+ TAAAAAATAT TTTTTGTTTA TTTTTAAACT TATAGAGTTT TAAATTATAA AAATTCGTCA CATCTTTTCC   
  
  
+ TGTCCACCTG GCGTAACAAA CTTACTCAGT TTCTGGAAAA ATGGGAAGTA TATAAGTTGT TAAAGAAGTG   
  
  
+ GTGGAGTGCA CTAAACGGTA GTTACAGAAT GTGAGGAAGG TGTGCCTTCC TAAGAGGAAG AAGGGTAAGA   
  
  
+ AGTCTTTAAT TCTGCCTTTA CCATTAACG  

- AAATGGAGAG GAAGAGGAGA GGTTTTGGGT GGAAGTCTTG AAACGGTGCA ATAGACCCGG CATTAAAAAG   
  
  
- GGAGAGCTGA GACTTTAGAA GAAGACCAAA CAGTAGTAGT TAGTGCTTCT GTGTTTTAAT TATTTTACAA   
  
  
- ACCCCTTTTT CTCATTTTAC ATCGATATAT CATAATTGAA AAGGAGGCGA GGACACTCTC TTGGTCCTAA   
  
  
- TTCACTTCCT TTCCCTTTTC TTTTCTTTTT TTTTTCAAAT ATTACTTATT AATTAAATTT ATTTTACTTA   
  
  
- AAATTATATA TTATCATATC TATTTTTTAA AAATCTGTTT CTATATCAAT CTTAAAAATA CTTAGAGATA   
  
  
- TATTATCAAA AACAATCTCT CCTTTATTGT AAAATATTCA TCAAATATTA CTTAAAAATT GAACCTCAAT   
  
  
- AAAACCATCA TGCAAATCAA TTTGATGTGG CTCTGGTTTG AGCATTTAAG CATATTAGTA TTTTTTTTCT   
  
  
- GAAAAAAAAA TGTTCTTAAG AAAGAAAATA AAAAAATAAA ACAGATGATT TTATATTCAA TTTTAATTTT   
  
  
- TAAGGACTTC TGTAATGGCT ACTTATGTGG TGTAGTGACT ACTGGCTAAG ATTGTCATAG TAAGAGAGAG   
  
  
- AGAGGTTGTT GTGGGGCCCA ACAAAGGCAA GGGGGAAGTC TTCCGATTTC TCTCCGTAGA TTGACGCGCC   
  
  
- CCAAAATTTC ATCGCCTGGA TTGGCCCGGC GCATCTCGGG CTCGTGAAAA TGGGAGGTCT TTGGTCAACG   
  
  
- ATCTTGACAA GCCTGCTGGG CCCCACAAGG CATCCGGTCT AATTTGTGGG CCCCGCGCCT TCATATTGGA   
  
  
- CTTATTGCCA CGTCGGATGG GTGTCCTCGG ACCCAACCTA AACGGGTTGA CTCCGTCGTC CTTTCTTCAC   
  
  
- CCCATCTATC CCTCGCCTCG CCTCGCCTCA TAGCTTGAAT TGTTTACTTA TTATGTTACT CGATTTATTT   
  
  
- ATTATTATTT TAAAAAAATT AAAAGTAAAA TATATATTAC TTTTAATTAA TATTTAATAA TAATAAATTA   
  
  
- TTAATTATAA TAAAGTAAAC AAAACGGAAG TCGATCTGCC CCTACAGTAG CACAACTTGC AAGCACAGGC   
  
  
- AACTTGCATA CGTGATTTGA CAAGTAAAGG TGGTTAGTAG ATGATTGTAC ACACGAATAT TTGATTTTAT   
  
  
- AGTAATTTAT ACATATGTGT TTAAAAAATC AAATATTAAT TCTTACATTA ATTTATATTT TTATATAATT   
  
  
- ATTTTTTATA AAAAACAAAT AAAAATTTGA ATATCTCAAA ATTTAATATT TTTAAGCAGT GTAGAAAAGG   
  
  
- ACAGGTGGAC CGCATTGTTT GAATGAGTCA AAGACCTTTT TACCCTTCAT ATATTCAACA ATTTCTTCAC   
  
  
- CACCTCACGT GATTTGCCAT CAATGTCTTA CACTCCTTCC ACACGGAAGG ATTCTCCTTC TTCCCATTCT   
  
  
- TCAGAAATTA AGACGGAAAT GGTAATTGC

+     I-box

| Site Name | Organism | Position | Strand | Matrix score. | sequence | function |
| --- | --- | --- | --- | --- | --- | --- |
| I-box | Solanum tuberosum | 346 | - | 10 | TATTATCTAGA | part of a light responsive element |
| I-box | Zea mays | 916 | + | 7 | GATAGGG | part of a light responsive element |

> 2018/04/13 10:10:12  
+ TTTACCTCTC CTTCTCCTCT CCAAAACCCA CCTTCAGAAC TTTGCCACGT TATCTGGGCC GTAATTTTTC   
  
  
+ CCTCTCGACT CTGAAATCTT CTTCTGGTTT GTCATCATCA ATCACGAAGA CACAAAATTA ATAAAATGTT   
  
  
+ TGGGGAAAAA GAGTAAAATG TAGCTATATA GTATTAACTT TTCCTCCGCT CCTGTGAGAG AACCAGGATT   
  
  
+ AAGTGAAGGA AAGGGAAAAG AAAAGAAAAA AAAAAGTTTA TAATGAATAA TTAATTTAAA TAAAATGAAT   
  
  
+ TTTAATATAT AATAGTATAG ATAAAAAATT TTTAGACAAA GATATAGTTA GAATTTTTAT GAATCTCTAT   
  
  
+ ATAATAGTTT TTGTTAGAGA GGAAATAACA TTTTATAAGT AGTTTATAAT GAATTTTTAA CTTGGAGTTA   
  
  
+ TTTTGGTAGT ACGTTTAGTT AAACTACACC GAGACCAAAC TCGTAAATTC GTATAATCAT AAAAAAAAGA   
  
  
+ CTTTTTTTTT ACAAGAATTC TTTCTTTTAT TTTTTTATTT TGTCTACTAA AATATAAGTT AAAATTAAAA   
  
  
+ ATTCCTGAAG ACATTACCGA TGAATACACC ACATCACTGA TGACCGATTC TAACAGTATC ATTCTCTCTC   
  
  
+ TCTCCAACAA CACCCCGGGT TGTTTCCGTT CCCCCTTCAG AAGGCTAAAG AGAGGCATCT AACTGCGCGG   
  
  
+ GGTTTTAAAG TAGCGGACCT AACCGGGCCG CGTAGAGCCC GAGCACTTTT ACCCTCCAGA AACCAGTTGC   
  
  
+ TAGAACTGTT CGGACGACCC GGGGTGTTCC GTAGGCCAGA TTAAACACCC GGGGCGCGGA AGTATAACCT   
  
  
+ GAATAACGGT GCAGCCTACC CACAGGAGCC TGGGTTGGAT TTGCCCAACT GAGGCAGCAG GAAAGAAGTG   
  
  
+ GGGTAGATAG GGAGCGGAGC GGAGCGGAGT ATCGAACTTA ACAAATGAAT AATACAATGA GCTAAATAAA   
  
  
+ TAATAATAAA ATTTTTTTAA TTTTCATTTT ATATATAATG AAAATTAATT ATAAATTATT ATTATTTAAT   
  
  
+ AATTAATATT ATTTCATTTG TTTTGCCTTC AGCTAGACGG GGATGTCATC GTGTTGAACG TTCGTGTCCG   
  
  
+ TTGAACGTAT GCACTAAACT GTTCATTTCC ACCAATCATC TACTAACATG TGTGCTTATA AACTAAAATA   
  
  
+ TCATTAAATA TGTATACACA AATTTTTTAG TTTATAATTA AGAATGTAAT TAAATATAAA AATATATTAA   
  
  
+ TAAAAAATAT TTTTTGTTTA TTTTTAAACT TATAGAGTTT TAAATTATAA AAATTCGTCA CATCTTTTCC   
  
  
+ TGTCCACCTG GCGTAACAAA CTTACTCAGT TTCTGGAAAA ATGGGAAGTA TATAAGTTGT TAAAGAAGTG   
  
  
+ GTGGAGTGCA CTAAACGGTA GTTACAGAAT GTGAGGAAGG TGTGCCTTCC TAAGAGGAAG AAGGGTAAGA   
  
  
+ AGTCTTTAAT TCTGCCTTTA CCATTAACG  

- AAATGGAGAG GAAGAGGAGA GGTTTTGGGT GGAAGTCTTG AAACGGTGCA ATAGACCCGG CATTAAAAAG   
  
  
- GGAGAGCTGA GACTTTAGAA GAAGACCAAA CAGTAGTAGT TAGTGCTTCT GTGTTTTAAT TATTTTACAA   
  
  
- ACCCCTTTTT CTCATTTTAC ATCGATATAT CATAATTGAA AAGGAGGCGA GGACACTCTC TTGGTCCTAA   
  
  
- TTCACTTCCT TTCCCTTTTC TTTTCTTTTT TTTTTCAAAT ATTACTTATT AATTAAATTT ATTTTACTTA   
  
  
- AAATTATATA TTATCATATC TATTTTTTAA AAATCTGTTT CTATATCAAT CTTAAAAATA CTTAGAGATA   
  
  
- TATTATCAAA AACAATCTCT CCTTTATTGT AAAATATTCA TCAAATATTA CTTAAAAATT GAACCTCAAT   
  
  
- AAAACCATCA TGCAAATCAA TTTGATGTGG CTCTGGTTTG AGCATTTAAG CATATTAGTA TTTTTTTTCT   
  
  
- GAAAAAAAAA TGTTCTTAAG AAAGAAAATA AAAAAATAAA ACAGATGATT TTATATTCAA TTTTAATTTT   
  
  
- TAAGGACTTC TGTAATGGCT ACTTATGTGG TGTAGTGACT ACTGGCTAAG ATTGTCATAG TAAGAGAGAG   
  
  
- AGAGGTTGTT GTGGGGCCCA ACAAAGGCAA GGGGGAAGTC TTCCGATTTC TCTCCGTAGA TTGACGCGCC   
  
  
- CCAAAATTTC ATCGCCTGGA TTGGCCCGGC GCATCTCGGG CTCGTGAAAA TGGGAGGTCT TTGGTCAACG   
  
  
- ATCTTGACAA GCCTGCTGGG CCCCACAAGG CATCCGGTCT AATTTGTGGG CCCCGCGCCT TCATATTGGA   
  
  
- CTTATTGCCA CGTCGGATGG GTGTCCTCGG ACCCAACCTA AACGGGTTGA CTCCGTCGTC CTTTCTTCAC   
  
  
- CCCATCTATC CCTCGCCTCG CCTCGCCTCA TAGCTTGAAT TGTTTACTTA TTATGTTACT CGATTTATTT   
  
  
- ATTATTATTT TAAAAAAATT AAAAGTAAAA TATATATTAC TTTTAATTAA TATTTAATAA TAATAAATTA   
  
  
- TTAATTATAA TAAAGTAAAC AAAACGGAAG TCGATCTGCC CCTACAGTAG CACAACTTGC AAGCACAGGC   
  
  
- AACTTGCATA CGTGATTTGA CAAGTAAAGG TGGTTAGTAG ATGATTGTAC ACACGAATAT TTGATTTTAT   
  
  
- AGTAATTTAT ACATATGTGT TTAAAAAATC AAATATTAAT TCTTACATTA ATTTATATTT TTATATAATT   
  
  
- ATTTTTTATA AAAAACAAAT AAAAATTTGA ATATCTCAAA ATTTAATATT TTTAAGCAGT GTAGAAAAGG   
  
  
- ACAGGTGGAC CGCATTGTTT GAATGAGTCA AAGACCTTTT TACCCTTCAT ATATTCAACA ATTTCTTCAC   
  
  
- CACCTCACGT GATTTGCCAT CAATGTCTTA CACTCCTTCC ACACGGAAGG ATTCTCCTTC TTCCCATTCT   
  
  
- TCAGAAATTA AGACGGAAAT GGTAATTGC

+     MBS

| Site Name | Organism | Position | Strand | Matrix score. | sequence | function |
| --- | --- | --- | --- | --- | --- | --- |
| MBS | Arabidopsis thaliana | 886 | + | 6 | CAACTG | MYB binding site involved in drought-inducibility |
| MBS | Zea mays | 601 | - | 6 | CGGTCA | MYB Binding Site |
| MBS | Arabidopsis thaliana | 764 | - | 6 | CAACTG | MYB binding site involved in drought-inducibility |
| MBS | Arabidopsis thaliana | 690 | + | 6 | TAACTG | MYB binding site involved in drought-inducibility |

> 2018/04/13 10:10:12  
+ TTTACCTCTC CTTCTCCTCT CCAAAACCCA CCTTCAGAAC TTTGCCACGT TATCTGGGCC GTAATTTTTC   
  
  
+ CCTCTCGACT CTGAAATCTT CTTCTGGTTT GTCATCATCA ATCACGAAGA CACAAAATTA ATAAAATGTT   
  
  
+ TGGGGAAAAA GAGTAAAATG TAGCTATATA GTATTAACTT TTCCTCCGCT CCTGTGAGAG AACCAGGATT   
  
  
+ AAGTGAAGGA AAGGGAAAAG AAAAGAAAAA AAAAAGTTTA TAATGAATAA TTAATTTAAA TAAAATGAAT   
  
  
+ TTTAATATAT AATAGTATAG ATAAAAAATT TTTAGACAAA GATATAGTTA GAATTTTTAT GAATCTCTAT   
  
  
+ ATAATAGTTT TTGTTAGAGA GGAAATAACA TTTTATAAGT AGTTTATAAT GAATTTTTAA CTTGGAGTTA   
  
  
+ TTTTGGTAGT ACGTTTAGTT AAACTACACC GAGACCAAAC TCGTAAATTC GTATAATCAT AAAAAAAAGA   
  
  
+ CTTTTTTTTT ACAAGAATTC TTTCTTTTAT TTTTTTATTT TGTCTACTAA AATATAAGTT AAAATTAAAA   
  
  
+ ATTCCTGAAG ACATTACCGA TGAATACACC ACATCACTGA TGACCGATTC TAACAGTATC ATTCTCTCTC   
  
  
+ TCTCCAACAA CACCCCGGGT TGTTTCCGTT CCCCCTTCAG AAGGCTAAAG AGAGGCATCT AACTGCGCGG   
  
  
+ GGTTTTAAAG TAGCGGACCT AACCGGGCCG CGTAGAGCCC GAGCACTTTT ACCCTCCAGA AACCAGTTGC   
  
  
+ TAGAACTGTT CGGACGACCC GGGGTGTTCC GTAGGCCAGA TTAAACACCC GGGGCGCGGA AGTATAACCT   
  
  
+ GAATAACGGT GCAGCCTACC CACAGGAGCC TGGGTTGGAT TTGCCCAACT GAGGCAGCAG GAAAGAAGTG   
  
  
+ GGGTAGATAG GGAGCGGAGC GGAGCGGAGT ATCGAACTTA ACAAATGAAT AATACAATGA GCTAAATAAA   
  
  
+ TAATAATAAA ATTTTTTTAA TTTTCATTTT ATATATAATG AAAATTAATT ATAAATTATT ATTATTTAAT   
  
  
+ AATTAATATT ATTTCATTTG TTTTGCCTTC AGCTAGACGG GGATGTCATC GTGTTGAACG TTCGTGTCCG   
  
  
+ TTGAACGTAT GCACTAAACT GTTCATTTCC ACCAATCATC TACTAACATG TGTGCTTATA AACTAAAATA   
  
  
+ TCATTAAATA TGTATACACA AATTTTTTAG TTTATAATTA AGAATGTAAT TAAATATAAA AATATATTAA   
  
  
+ TAAAAAATAT TTTTTGTTTA TTTTTAAACT TATAGAGTTT TAAATTATAA AAATTCGTCA CATCTTTTCC   
  
  
+ TGTCCACCTG GCGTAACAAA CTTACTCAGT TTCTGGAAAA ATGGGAAGTA TATAAGTTGT TAAAGAAGTG   
  
  
+ GTGGAGTGCA CTAAACGGTA GTTACAGAAT GTGAGGAAGG TGTGCCTTCC TAAGAGGAAG AAGGGTAAGA   
  
  
+ AGTCTTTAAT TCTGCCTTTA CCATTAACG  

- AAATGGAGAG GAAGAGGAGA GGTTTTGGGT GGAAGTCTTG AAACGGTGCA ATAGACCCGG CATTAAAAAG   
  
  
- GGAGAGCTGA GACTTTAGAA GAAGACCAAA CAGTAGTAGT TAGTGCTTCT GTGTTTTAAT TATTTTACAA   
  
  
- ACCCCTTTTT CTCATTTTAC ATCGATATAT CATAATTGAA AAGGAGGCGA GGACACTCTC TTGGTCCTAA   
  
  
- TTCACTTCCT TTCCCTTTTC TTTTCTTTTT TTTTTCAAAT ATTACTTATT AATTAAATTT ATTTTACTTA   
  
  
- AAATTATATA TTATCATATC TATTTTTTAA AAATCTGTTT CTATATCAAT CTTAAAAATA CTTAGAGATA   
  
  
- TATTATCAAA AACAATCTCT CCTTTATTGT AAAATATTCA TCAAATATTA CTTAAAAATT GAACCTCAAT   
  
  
- AAAACCATCA TGCAAATCAA TTTGATGTGG CTCTGGTTTG AGCATTTAAG CATATTAGTA TTTTTTTTCT   
  
  
- GAAAAAAAAA TGTTCTTAAG AAAGAAAATA AAAAAATAAA ACAGATGATT TTATATTCAA TTTTAATTTT   
  
  
- TAAGGACTTC TGTAATGGCT ACTTATGTGG TGTAGTGACT ACTGGCTAAG ATTGTCATAG TAAGAGAGAG   
  
  
- AGAGGTTGTT GTGGGGCCCA ACAAAGGCAA GGGGGAAGTC TTCCGATTTC TCTCCGTAGA TTGACGCGCC   
  
  
- CCAAAATTTC ATCGCCTGGA TTGGCCCGGC GCATCTCGGG CTCGTGAAAA TGGGAGGTCT TTGGTCAACG   
  
  
- ATCTTGACAA GCCTGCTGGG CCCCACAAGG CATCCGGTCT AATTTGTGGG CCCCGCGCCT TCATATTGGA   
  
  
- CTTATTGCCA CGTCGGATGG GTGTCCTCGG ACCCAACCTA AACGGGTTGA CTCCGTCGTC CTTTCTTCAC   
  
  
- CCCATCTATC CCTCGCCTCG CCTCGCCTCA TAGCTTGAAT TGTTTACTTA TTATGTTACT CGATTTATTT   
  
  
- ATTATTATTT TAAAAAAATT AAAAGTAAAA TATATATTAC TTTTAATTAA TATTTAATAA TAATAAATTA   
  
  
- TTAATTATAA TAAAGTAAAC AAAACGGAAG TCGATCTGCC CCTACAGTAG CACAACTTGC AAGCACAGGC   
  
  
- AACTTGCATA CGTGATTTGA CAAGTAAAGG TGGTTAGTAG ATGATTGTAC ACACGAATAT TTGATTTTAT   
  
  
- AGTAATTTAT ACATATGTGT TTAAAAAATC AAATATTAAT TCTTACATTA ATTTATATTT TTATATAATT   
  
  
- ATTTTTTATA AAAAACAAAT AAAAATTTGA ATATCTCAAA ATTTAATATT TTTAAGCAGT GTAGAAAAGG   
  
  
- ACAGGTGGAC CGCATTGTTT GAATGAGTCA AAGACCTTTT TACCCTTCAT ATATTCAACA ATTTCTTCAC   
  
  
- CACCTCACGT GATTTGCCAT CAATGTCTTA CACTCCTTCC ACACGGAAGG ATTCTCCTTC TTCCCATTCT   
  
  
- TCAGAAATTA AGACGGAAAT GGTAATTGC

+     P-box

| Site Name | Organism | Position | Strand | Matrix score. | sequence | function |
| --- | --- | --- | --- | --- | --- | --- |
| P-box | Pisum sativum | 453 | + | 12 | GACCAAACTCGT | gibberellin-responsive element |

> 2018/04/13 10:10:12  
+ TTTACCTCTC CTTCTCCTCT CCAAAACCCA CCTTCAGAAC TTTGCCACGT TATCTGGGCC GTAATTTTTC   
  
  
+ CCTCTCGACT CTGAAATCTT CTTCTGGTTT GTCATCATCA ATCACGAAGA CACAAAATTA ATAAAATGTT   
  
  
+ TGGGGAAAAA GAGTAAAATG TAGCTATATA GTATTAACTT TTCCTCCGCT CCTGTGAGAG AACCAGGATT   
  
  
+ AAGTGAAGGA AAGGGAAAAG AAAAGAAAAA AAAAAGTTTA TAATGAATAA TTAATTTAAA TAAAATGAAT   
  
  
+ TTTAATATAT AATAGTATAG ATAAAAAATT TTTAGACAAA GATATAGTTA GAATTTTTAT GAATCTCTAT   
  
  
+ ATAATAGTTT TTGTTAGAGA GGAAATAACA TTTTATAAGT AGTTTATAAT GAATTTTTAA CTTGGAGTTA   
  
  
+ TTTTGGTAGT ACGTTTAGTT AAACTACACC GAGACCAAAC TCGTAAATTC GTATAATCAT AAAAAAAAGA   
  
  
+ CTTTTTTTTT ACAAGAATTC TTTCTTTTAT TTTTTTATTT TGTCTACTAA AATATAAGTT AAAATTAAAA   
  
  
+ ATTCCTGAAG ACATTACCGA TGAATACACC ACATCACTGA TGACCGATTC TAACAGTATC ATTCTCTCTC   
  
  
+ TCTCCAACAA CACCCCGGGT TGTTTCCGTT CCCCCTTCAG AAGGCTAAAG AGAGGCATCT AACTGCGCGG   
  
  
+ GGTTTTAAAG TAGCGGACCT AACCGGGCCG CGTAGAGCCC GAGCACTTTT ACCCTCCAGA AACCAGTTGC   
  
  
+ TAGAACTGTT CGGACGACCC GGGGTGTTCC GTAGGCCAGA TTAAACACCC GGGGCGCGGA AGTATAACCT   
  
  
+ GAATAACGGT GCAGCCTACC CACAGGAGCC TGGGTTGGAT TTGCCCAACT GAGGCAGCAG GAAAGAAGTG   
  
  
+ GGGTAGATAG GGAGCGGAGC GGAGCGGAGT ATCGAACTTA ACAAATGAAT AATACAATGA GCTAAATAAA   
  
  
+ TAATAATAAA ATTTTTTTAA TTTTCATTTT ATATATAATG AAAATTAATT ATAAATTATT ATTATTTAAT   
  
  
+ AATTAATATT ATTTCATTTG TTTTGCCTTC AGCTAGACGG GGATGTCATC GTGTTGAACG TTCGTGTCCG   
  
  
+ TTGAACGTAT GCACTAAACT GTTCATTTCC ACCAATCATC TACTAACATG TGTGCTTATA AACTAAAATA   
  
  
+ TCATTAAATA TGTATACACA AATTTTTTAG TTTATAATTA AGAATGTAAT TAAATATAAA AATATATTAA   
  
  
+ TAAAAAATAT TTTTTGTTTA TTTTTAAACT TATAGAGTTT TAAATTATAA AAATTCGTCA CATCTTTTCC   
  
  
+ TGTCCACCTG GCGTAACAAA CTTACTCAGT TTCTGGAAAA ATGGGAAGTA TATAAGTTGT TAAAGAAGTG   
  
  
+ GTGGAGTGCA CTAAACGGTA GTTACAGAAT GTGAGGAAGG TGTGCCTTCC TAAGAGGAAG AAGGGTAAGA   
  
  
+ AGTCTTTAAT TCTGCCTTTA CCATTAACG  

- AAATGGAGAG GAAGAGGAGA GGTTTTGGGT GGAAGTCTTG AAACGGTGCA ATAGACCCGG CATTAAAAAG   
  
  
- GGAGAGCTGA GACTTTAGAA GAAGACCAAA CAGTAGTAGT TAGTGCTTCT GTGTTTTAAT TATTTTACAA   
  
  
- ACCCCTTTTT CTCATTTTAC ATCGATATAT CATAATTGAA AAGGAGGCGA GGACACTCTC TTGGTCCTAA   
  
  
- TTCACTTCCT TTCCCTTTTC TTTTCTTTTT TTTTTCAAAT ATTACTTATT AATTAAATTT ATTTTACTTA   
  
  
- AAATTATATA TTATCATATC TATTTTTTAA AAATCTGTTT CTATATCAAT CTTAAAAATA CTTAGAGATA   
  
  
- TATTATCAAA AACAATCTCT CCTTTATTGT AAAATATTCA TCAAATATTA CTTAAAAATT GAACCTCAAT   
  
  
- AAAACCATCA TGCAAATCAA TTTGATGTGG CTCTGGTTTG AGCATTTAAG CATATTAGTA TTTTTTTTCT   
  
  
- GAAAAAAAAA TGTTCTTAAG AAAGAAAATA AAAAAATAAA ACAGATGATT TTATATTCAA TTTTAATTTT   
  
  
- TAAGGACTTC TGTAATGGCT ACTTATGTGG TGTAGTGACT ACTGGCTAAG ATTGTCATAG TAAGAGAGAG   
  
  
- AGAGGTTGTT GTGGGGCCCA ACAAAGGCAA GGGGGAAGTC TTCCGATTTC TCTCCGTAGA TTGACGCGCC   
  
  
- CCAAAATTTC ATCGCCTGGA TTGGCCCGGC GCATCTCGGG CTCGTGAAAA TGGGAGGTCT TTGGTCAACG   
  
  
- ATCTTGACAA GCCTGCTGGG CCCCACAAGG CATCCGGTCT AATTTGTGGG CCCCGCGCCT TCATATTGGA   
  
  
- CTTATTGCCA CGTCGGATGG GTGTCCTCGG ACCCAACCTA AACGGGTTGA CTCCGTCGTC CTTTCTTCAC   
  
  
- CCCATCTATC CCTCGCCTCG CCTCGCCTCA TAGCTTGAAT TGTTTACTTA TTATGTTACT CGATTTATTT   
  
  
- ATTATTATTT TAAAAAAATT AAAAGTAAAA TATATATTAC TTTTAATTAA TATTTAATAA TAATAAATTA   
  
  
- TTAATTATAA TAAAGTAAAC AAAACGGAAG TCGATCTGCC CCTACAGTAG CACAACTTGC AAGCACAGGC   
  
  
- AACTTGCATA CGTGATTTGA CAAGTAAAGG TGGTTAGTAG ATGATTGTAC ACACGAATAT TTGATTTTAT   
  
  
- AGTAATTTAT ACATATGTGT TTAAAAAATC AAATATTAAT TCTTACATTA ATTTATATTT TTATATAATT   
  
  
- ATTTTTTATA AAAAACAAAT AAAAATTTGA ATATCTCAAA ATTTAATATT TTTAAGCAGT GTAGAAAAGG   
  
  
- ACAGGTGGAC CGCATTGTTT GAATGAGTCA AAGACCTTTT TACCCTTCAT ATATTCAACA ATTTCTTCAC   
  
  
- CACCTCACGT GATTTGCCAT CAATGTCTTA CACTCCTTCC ACACGGAAGG ATTCTCCTTC TTCCCATTCT   
  
  
- TCAGAAATTA AGACGGAAAT GGTAATTGC

+     Skn-1\_motif

| Site Name | Organism | Position | Strand | Matrix score. | sequence | function |
| --- | --- | --- | --- | --- | --- | --- |
| Skn-1\_motif | Oryza sativa | 600 | - | 5 | GTCAT | cis-acting regulatory element required for endosperm expression |
| Skn-1\_motif | Oryza sativa | 101 | + | 5 | GTCAT | cis-acting regulatory element required for endosperm expression |
| Skn-1\_motif | Oryza sativa | 1095 | + | 5 | GTCAT | cis-acting regulatory element required for endosperm expression |

> 2018/04/13 10:10:12  
+ TTTACCTCTC CTTCTCCTCT CCAAAACCCA CCTTCAGAAC TTTGCCACGT TATCTGGGCC GTAATTTTTC   
  
  
+ CCTCTCGACT CTGAAATCTT CTTCTGGTTT GTCATCATCA ATCACGAAGA CACAAAATTA ATAAAATGTT   
  
  
+ TGGGGAAAAA GAGTAAAATG TAGCTATATA GTATTAACTT TTCCTCCGCT CCTGTGAGAG AACCAGGATT   
  
  
+ AAGTGAAGGA AAGGGAAAAG AAAAGAAAAA AAAAAGTTTA TAATGAATAA TTAATTTAAA TAAAATGAAT   
  
  
+ TTTAATATAT AATAGTATAG ATAAAAAATT TTTAGACAAA GATATAGTTA GAATTTTTAT GAATCTCTAT   
  
  
+ ATAATAGTTT TTGTTAGAGA GGAAATAACA TTTTATAAGT AGTTTATAAT GAATTTTTAA CTTGGAGTTA   
  
  
+ TTTTGGTAGT ACGTTTAGTT AAACTACACC GAGACCAAAC TCGTAAATTC GTATAATCAT AAAAAAAAGA   
  
  
+ CTTTTTTTTT ACAAGAATTC TTTCTTTTAT TTTTTTATTT TGTCTACTAA AATATAAGTT AAAATTAAAA   
  
  
+ ATTCCTGAAG ACATTACCGA TGAATACACC ACATCACTGA TGACCGATTC TAACAGTATC ATTCTCTCTC   
  
  
+ TCTCCAACAA CACCCCGGGT TGTTTCCGTT CCCCCTTCAG AAGGCTAAAG AGAGGCATCT AACTGCGCGG   
  
  
+ GGTTTTAAAG TAGCGGACCT AACCGGGCCG CGTAGAGCCC GAGCACTTTT ACCCTCCAGA AACCAGTTGC   
  
  
+ TAGAACTGTT CGGACGACCC GGGGTGTTCC GTAGGCCAGA TTAAACACCC GGGGCGCGGA AGTATAACCT   
  
  
+ GAATAACGGT GCAGCCTACC CACAGGAGCC TGGGTTGGAT TTGCCCAACT GAGGCAGCAG GAAAGAAGTG   
  
  
+ GGGTAGATAG GGAGCGGAGC GGAGCGGAGT ATCGAACTTA ACAAATGAAT AATACAATGA GCTAAATAAA   
  
  
+ TAATAATAAA ATTTTTTTAA TTTTCATTTT ATATATAATG AAAATTAATT ATAAATTATT ATTATTTAAT   
  
  
+ AATTAATATT ATTTCATTTG TTTTGCCTTC AGCTAGACGG GGATGTCATC GTGTTGAACG TTCGTGTCCG   
  
  
+ TTGAACGTAT GCACTAAACT GTTCATTTCC ACCAATCATC TACTAACATG TGTGCTTATA AACTAAAATA   
  
  
+ TCATTAAATA TGTATACACA AATTTTTTAG TTTATAATTA AGAATGTAAT TAAATATAAA AATATATTAA   
  
  
+ TAAAAAATAT TTTTTGTTTA TTTTTAAACT TATAGAGTTT TAAATTATAA AAATTCGTCA CATCTTTTCC   
  
  
+ TGTCCACCTG GCGTAACAAA CTTACTCAGT TTCTGGAAAA ATGGGAAGTA TATAAGTTGT TAAAGAAGTG   
  
  
+ GTGGAGTGCA CTAAACGGTA GTTACAGAAT GTGAGGAAGG TGTGCCTTCC TAAGAGGAAG AAGGGTAAGA   
  
  
+ AGTCTTTAAT TCTGCCTTTA CCATTAACG  

- AAATGGAGAG GAAGAGGAGA GGTTTTGGGT GGAAGTCTTG AAACGGTGCA ATAGACCCGG CATTAAAAAG   
  
  
- GGAGAGCTGA GACTTTAGAA GAAGACCAAA CAGTAGTAGT TAGTGCTTCT GTGTTTTAAT TATTTTACAA   
  
  
- ACCCCTTTTT CTCATTTTAC ATCGATATAT CATAATTGAA AAGGAGGCGA GGACACTCTC TTGGTCCTAA   
  
  
- TTCACTTCCT TTCCCTTTTC TTTTCTTTTT TTTTTCAAAT ATTACTTATT AATTAAATTT ATTTTACTTA   
  
  
- AAATTATATA TTATCATATC TATTTTTTAA AAATCTGTTT CTATATCAAT CTTAAAAATA CTTAGAGATA   
  
  
- TATTATCAAA AACAATCTCT CCTTTATTGT AAAATATTCA TCAAATATTA CTTAAAAATT GAACCTCAAT   
  
  
- AAAACCATCA TGCAAATCAA TTTGATGTGG CTCTGGTTTG AGCATTTAAG CATATTAGTA TTTTTTTTCT   
  
  
- GAAAAAAAAA TGTTCTTAAG AAAGAAAATA AAAAAATAAA ACAGATGATT TTATATTCAA TTTTAATTTT   
  
  
- TAAGGACTTC TGTAATGGCT ACTTATGTGG TGTAGTGACT ACTGGCTAAG ATTGTCATAG TAAGAGAGAG   
  
  
- AGAGGTTGTT GTGGGGCCCA ACAAAGGCAA GGGGGAAGTC TTCCGATTTC TCTCCGTAGA TTGACGCGCC   
  
  
- CCAAAATTTC ATCGCCTGGA TTGGCCCGGC GCATCTCGGG CTCGTGAAAA TGGGAGGTCT TTGGTCAACG   
  
  
- ATCTTGACAA GCCTGCTGGG CCCCACAAGG CATCCGGTCT AATTTGTGGG CCCCGCGCCT TCATATTGGA   
  
  
- CTTATTGCCA CGTCGGATGG GTGTCCTCGG ACCCAACCTA AACGGGTTGA CTCCGTCGTC CTTTCTTCAC   
  
  
- CCCATCTATC CCTCGCCTCG CCTCGCCTCA TAGCTTGAAT TGTTTACTTA TTATGTTACT CGATTTATTT   
  
  
- ATTATTATTT TAAAAAAATT AAAAGTAAAA TATATATTAC TTTTAATTAA TATTTAATAA TAATAAATTA   
  
  
- TTAATTATAA TAAAGTAAAC AAAACGGAAG TCGATCTGCC CCTACAGTAG CACAACTTGC AAGCACAGGC   
  
  
- AACTTGCATA CGTGATTTGA CAAGTAAAGG TGGTTAGTAG ATGATTGTAC ACACGAATAT TTGATTTTAT   
  
  
- AGTAATTTAT ACATATGTGT TTAAAAAATC AAATATTAAT TCTTACATTA ATTTATATTT TTATATAATT   
  
  
- ATTTTTTATA AAAAACAAAT AAAAATTTGA ATATCTCAAA ATTTAATATT TTTAAGCAGT GTAGAAAAGG   
  
  
- ACAGGTGGAC CGCATTGTTT GAATGAGTCA AAGACCTTTT TACCCTTCAT ATATTCAACA ATTTCTTCAC   
  
  
- CACCTCACGT GATTTGCCAT CAATGTCTTA CACTCCTTCC ACACGGAAGG ATTCTCCTTC TTCCCATTCT   
  
  
- TCAGAAATTA AGACGGAAAT GGTAATTGC

+     TATA-box

| Site Name | Organism | Position | Strand | Matrix score. | sequence | function |
| --- | --- | --- | --- | --- | --- | --- |
| TATA-box | Arabidopsis thaliana | 1203 | - | 4 | TATA | core promoter element around -30 of transcription start |
| TATA-box | Arabidopsis thaliana | 1014 | - | 4 | TATA | core promoter element around -30 of transcription start |
| TATA-box | Arabidopsis thaliana | 1278 | - | 9 | TAAAAATAA | core promoter element around -30 of transcription start |
| TATA-box | Lycopersicon esculentum | 1184 | - | 5 | TTTTA | core promoter element around -30 of transcription start |
| TATA-box | Glycine max | 1057 | - | 5 | TAATA | core promoter element around -30 of transcription start |
| TATA-box | Arabidopsis thaliana | 1012 | - | 4 | TATA | core promoter element around -30 of transcription start |
| TATA-box | Lycopersicon esculentum | 550 | - | 5 | TTTTA | core promoter element around -30 of transcription start |
| TATA-box | Glycine max | 1047 | + | 5 | TAATA | core promoter element around -30 of transcription start |
| TATA-box | Brassica napus | 1011 | - | 6 | ATATAT | core promoter element around -30 of transcription start |
| TATA-box | Pisum sativum | 1006 | - | 8 | TATAAAAT | core promoter element around -30 of transcription start |
| TATA-box | Lycopersicon esculentum | 995 | + | 5 | TTTTA | core promoter element around -30 of transcription start |
| TATA-box | Helianthus annuus | 1201 | - | 6 | TATACA | core promoter element around -30 of transcription start |
| TATA-box | Arabidopsis thaliana | 1177 | + | 6 | TATAAA | core promoter element around -30 of transcription start |
| TATA-box | Lycopersicon esculentum | 747 | + | 5 | TTTTA | core promoter element around -30 of transcription start |
| TATA-box | Arabidopsis thaliana | 384 | + | 4 | TATA | core promoter element around -30 of transcription start |
| TATA-box | Glycine max | 984 | + | 5 | TAATA | core promoter element around -30 of transcription start |
| TATA-box | Arabidopsis thaliana | 167 | + | 4 | TATA | core promoter element around -30 of transcription start |
| TATA-box | Glycine max | 1054 | + | 5 | TAATA | core promoter element around -30 of transcription start |
| TATA-box | Glycine max | 1040 | - | 5 | TAATA | core promoter element around -30 of transcription start |
| TATA-box | Ac | 1030 | + | 7 | TATAAAT | core promoter element around -30 of transcription start |
| TATA-box | Arabidopsis thaliana | 249 | + | 4 | TATA | core promoter element around -30 of transcription start |
| TATA-box | Lycopersicon esculentum | 302 | - | 5 | TTTTA | core promoter element around -30 of transcription start |
| TATA-box | Arabidopsis thaliana | 296 | + | 4 | TATA | core promoter element around -30 of transcription start |
| TATA-box | Glycine max | 1037 | - | 5 | TAATA | core promoter element around -30 of transcription start |
| TATA-box | Lycopersicon esculentum | 987 | - | 5 | TTTTA | core promoter element around -30 of transcription start |
| TATA-box | Arabidopsis thaliana | 286 | + | 4 | TATA | core promoter element around -30 of transcription start |
| TATA-box | Glycine max | 981 | + | 5 | TAATA | core promoter element around -30 of transcription start |
| TATA-box | Glycine max | 960 | + | 5 | TAATA | core promoter element around -30 of transcription start |
| TATA-box | Arabidopsis thaliana | 265 | - | 8 | TATTTAAA | core promoter element around -30 of transcription start |
| TATA-box | Lycopersicon esculentum | 1282 | + | 5 | TTTTA | core promoter element around -30 of transcription start |
| TATA-box | Arabidopsis thaliana | 1379 | - | 4 | TATA | core promoter element around -30 of transcription start |
| TATA-box | Arabidopsis thaliana | 1222 | - | 5 | TATAA | core promoter element around -30 of transcription start |
| TATA-box | Glycine max | 1258 | + | 5 | TAATA | core promoter element around -30 of transcription start |
| TATA-box | Arabidopsis thaliana | 1009 | - | 7 | TATATAA | core promoter element around -30 of transcription start |
| TATA-box | Glycine max | 129 | + | 5 | TAATA | core promoter element around -30 of transcription start |
| TATA-box | Lycopersicon esculentum | 280 | + | 5 | TTTTA | core promoter element around -30 of transcription start |
| TATA-box | Brassica oleracea | 349 | + | 7 | ATATAAT | core promoter element around -30 of transcription start |
| TATA-box | Lycopersicon esculentum | 271 | - | 5 | TTTTA | core promoter element around -30 of transcription start |
| TATA-box | Glycine max | 172 | - | 5 | TAATA | core promoter element around -30 of transcription start |
| TATA-box | Lycopersicon esculentum | 1215 | + | 5 | TTTTA | core promoter element around -30 of transcription start |
| TATA-box | Lycopersicon esculentum | 497 | + | 5 | TTTTA | core promoter element around -30 of transcription start |
| TATA-box | Arabidopsis thaliana | 472 | + | 4 | TATA | core promoter element around -30 of transcription start |
| TATA-box | Lycopersicon esculentum | 480 | - | 5 | TTTTA | core promoter element around -30 of transcription start |
| TATA-box | Arabidopsis thaliana | 395 | + | 4 | TATA | core promoter element around -30 of transcription start |
| TATA-box | Arabidopsis thaliana | 1305 | - | 5 | TATAA | core promoter element around -30 of transcription start |
| TATA-box | Brassica napus | 1304 | + | 6 | ATTATA | core promoter element around -30 of transcription start |
| TATA-box | Arabidopsis thaliana | 1176 | - | 5 | TATAA | core promoter element around -30 of transcription start |
| TATA-box | Arabidopsis thaliana | 1008 | - | 6 | TATAAA | core promoter element around -30 of transcription start |
| TATA-box | Arabidopsis thaliana | 165 | + | 4 | TATA | core promoter element around -30 of transcription start |
| TATA-box | Lycopersicon esculentum | 1298 | + | 5 | TTTTA | core promoter element around -30 of transcription start |
| TATA-box | Arabidopsis thaliana | 1291 | - | 4 | TATA | core promoter element around -30 of transcription start |
| TATA-box | Lycopersicon esculentum | 1308 | - | 5 | TTTTA | core promoter element around -30 of transcription start |
| TATA-box | Arabidopsis thaliana | 348 | + | 4 | TATA | core promoter element around -30 of transcription start |
| TATA-box | Arabidopsis thaliana | 394 | - | 5 | TATAA | core promoter element around -30 of transcription start |
| TATA-box | Arabidopsis thaliana | 383 | - | 5 | TATAA | core promoter element around -30 of transcription start |
| TATA-box | Lycopersicon esculentum | 132 | - | 5 | TTTTA | core promoter element around -30 of transcription start |
| TATA-box | Brassica napus | 1028 | + | 6 | ATTATA | core promoter element around -30 of transcription start |
| TATA-box | Lycopersicon esculentum | 154 | - | 5 | TTTTA | core promoter element around -30 of transcription start |
| TATA-box | Arabidopsis thaliana | 346 | + | 9 | tcTATATAtt | core promoter element around -30 of transcription start |
| TATA-box | Lycopersicon esculentum | 335 | + | 5 | TTTTA | core promoter element around -30 of transcription start |
| TATA-box | Arabidopsis thaliana | 323 | + | 4 | TATA | core promoter element around -30 of transcription start |
| TATA-box | Arabidopsis thaliana | 833 | - | 4 | TATA | core promoter element around -30 of transcription start |
| TATA-box | Brassica oleracea | 542 | + | 6 | ATATAA | core promoter element around -30 of transcription start |
| TATA-box | Glycine max | 1255 | - | 5 | TAATA | core promoter element around -30 of transcription start |
| TATA-box | Arabidopsis thaliana | 1029 | - | 5 | TATAA | core promoter element around -30 of transcription start |
| TATA-box | Arabidopsis thaliana | 1007 | - | 7 | TATAAAA | core promoter element around -30 of transcription start |
| TATA-box | Brassica napus | 285 | + | 6 | ATATAT | core promoter element around -30 of transcription start |
| TATA-box | Arabidopsis thaliana | 1010 | - | 8 | TATATATA | core promoter element around -30 of transcription start |
| TATA-box | Glycine max | 283 | + | 5 | TAATA | core promoter element around -30 of transcription start |
| TATA-box | Pisum sativum | 380 | - | 8 | TATAAAAT | core promoter element around -30 of transcription start |
| TATA-box | Lycopersicon esculentum | 703 | + | 5 | TTTTA | core promoter element around -30 of transcription start |
| TATA-box | Lycopersicon esculentum | 556 | - | 5 | TTTTA | core promoter element around -30 of transcription start |
| TATA-box | Glycine max | 352 | + | 5 | TAATA | core promoter element around -30 of transcription start |
| TATA-box | Arabidopsis thaliana | 350 | + | 4 | TATA | core promoter element around -30 of transcription start |
| TATA-box | Brassica oleracea | 1013 | + | 7 | ATATAAT | core promoter element around -30 of transcription start |
| TATA-box | Arabidopsis thaliana | 1306 | + | 6 | TATAAA | core promoter element around -30 of transcription start |
| TATA-box | Arabidopsis thaliana | 1221 | - | 6 | TATAAA | core promoter element around -30 of transcription start |
| TATA-box | Brassica oleracea | 1380 | + | 6 | ATATAA | core promoter element around -30 of transcription start |
| TATA-box | Brassica oleracea | 287 | + | 7 | ATATAAT | core promoter element around -30 of transcription start |
| TATA-box | Glycine max | 290 | + | 5 | TAATA | core promoter element around -30 of transcription start |
| TATA-box | Arabidopsis thaliana | 248 | - | 5 | TATAA | core promoter element around -30 of transcription start |
| TATA-box | Zea mays | 1281 | - | 8 | TTTAAAAA | core promoter element around -30 of transcription start |
| TATA-box | Arabidopsis thaliana | 1290 | - | 5 | TATAA | core promoter element around -30 of transcription start |
| TATA-box | Brassica napus | 1252 | - | 6 | ATATAT | core promoter element around -30 of transcription start |
| TATA-box | Lycopersicon esculentum | 1247 | - | 5 | TTTTA | core promoter element around -30 of transcription start |
| TATA-box | Brassica oleracea | 1244 | + | 6 | ATATAA | core promoter element around -30 of transcription start |
| TATA-box | Arabidopsis thaliana | 543 | + | 4 | TATA | core promoter element around -30 of transcription start |
| TATA-box | Lycopersicon esculentum | 515 | + | 5 | TTTTA | core promoter element around -30 of transcription start |
| TATA-box | Lycopersicon esculentum | 310 | + | 5 | TTTTA | core promoter element around -30 of transcription start |
| TATA-box | Arabidopsis thaliana | 288 | + | 4 | TATA | core promoter element around -30 of transcription start |
| TATA-box | Lycopersicon esculentum | 523 | + | 5 | TTTTA | core promoter element around -30 of transcription start |
| TATA-box | Arabidopsis thaliana | 1381 | - | 4 | TATA | core promoter element around -30 of transcription start |
| TATA-box | Arabidopsis thaliana | 247 | - | 6 | TATAAA | core promoter element around -30 of transcription start |
| TATA-box | Lycopersicon esculentum | 1261 | - | 5 | TTTTA | core promoter element around -30 of transcription start |
| TATA-box | Lycopersicon esculentum | 538 | - | 5 | TTTTA | core promoter element around -30 of transcription start |
| TATA-box | Arabidopsis thaliana | 1223 | - | 4 | TATA | core promoter element around -30 of transcription start |
| TATA-box | Lycopersicon esculentum | 405 | + | 5 | TTTTA | core promoter element around -30 of transcription start |
| TATA-box | Arabidopsis thaliana | 1253 | - | 4 | TATA | core promoter element around -30 of transcription start |
| TATA-box | Arabidopsis thaliana | 381 | - | 7 | TATAAAA | core promoter element around -30 of transcription start |
| TATA-box | Avena sativa | 1242 | - | 12 | TATATTTATATTT | core promoter element around -30 of transcription start |
| TATA-box | Arabidopsis thaliana | 382 | - | 6 | TATAAA | core promoter element around -30 of transcription start |
| TATA-box | Arabidopsis thaliana | 1245 | + | 6 | TATAAA | core promoter element around -30 of transcription start |
| TATA-box | Arabidopsis thaliana | 393 | - | 6 | TATAAA | core promoter element around -30 of transcription start |

> 2018/04/13 10:10:12  
+ TTTACCTCTC CTTCTCCTCT CCAAAACCCA CCTTCAGAAC TTTGCCACGT TATCTGGGCC GTAATTTTTC   
  
  
+ CCTCTCGACT CTGAAATCTT CTTCTGGTTT GTCATCATCA ATCACGAAGA CACAAAATTA ATAAAATGTT   
  
  
+ TGGGGAAAAA GAGTAAAATG TAGCTATATA GTATTAACTT TTCCTCCGCT CCTGTGAGAG AACCAGGATT   
  
  
+ AAGTGAAGGA AAGGGAAAAG AAAAGAAAAA AAAAAGTTTA TAATGAATAA TTAATTTAAA TAAAATGAAT   
  
  
+ TTTAATATAT AATAGTATAG ATAAAAAATT TTTAGACAAA GATATAGTTA GAATTTTTAT GAATCTCTAT   
  
  
+ ATAATAGTTT TTGTTAGAGA GGAAATAACA TTTTATAAGT AGTTTATAAT GAATTTTTAA CTTGGAGTTA   
  
  
+ TTTTGGTAGT ACGTTTAGTT AAACTACACC GAGACCAAAC TCGTAAATTC GTATAATCAT AAAAAAAAGA   
  
  
+ CTTTTTTTTT ACAAGAATTC TTTCTTTTAT TTTTTTATTT TGTCTACTAA AATATAAGTT AAAATTAAAA   
  
  
+ ATTCCTGAAG ACATTACCGA TGAATACACC ACATCACTGA TGACCGATTC TAACAGTATC ATTCTCTCTC   
  
  
+ TCTCCAACAA CACCCCGGGT TGTTTCCGTT CCCCCTTCAG AAGGCTAAAG AGAGGCATCT AACTGCGCGG   
  
  
+ GGTTTTAAAG TAGCGGACCT AACCGGGCCG CGTAGAGCCC GAGCACTTTT ACCCTCCAGA AACCAGTTGC   
  
  
+ TAGAACTGTT CGGACGACCC GGGGTGTTCC GTAGGCCAGA TTAAACACCC GGGGCGCGGA AGTATAACCT   
  
  
+ GAATAACGGT GCAGCCTACC CACAGGAGCC TGGGTTGGAT TTGCCCAACT GAGGCAGCAG GAAAGAAGTG   
  
  
+ GGGTAGATAG GGAGCGGAGC GGAGCGGAGT ATCGAACTTA ACAAATGAAT AATACAATGA GCTAAATAAA   
  
  
+ TAATAATAAA ATTTTTTTAA TTTTCATTTT ATATATAATG AAAATTAATT ATAAATTATT ATTATTTAAT   
  
  
+ AATTAATATT ATTTCATTTG TTTTGCCTTC AGCTAGACGG GGATGTCATC GTGTTGAACG TTCGTGTCCG   
  
  
+ TTGAACGTAT GCACTAAACT GTTCATTTCC ACCAATCATC TACTAACATG TGTGCTTATA AACTAAAATA   
  
  
+ TCATTAAATA TGTATACACA AATTTTTTAG TTTATAATTA AGAATGTAAT TAAATATAAA AATATATTAA   
  
  
+ TAAAAAATAT TTTTTGTTTA TTTTTAAACT TATAGAGTTT TAAATTATAA AAATTCGTCA CATCTTTTCC   
  
  
+ TGTCCACCTG GCGTAACAAA CTTACTCAGT TTCTGGAAAA ATGGGAAGTA TATAAGTTGT TAAAGAAGTG   
  
  
+ GTGGAGTGCA CTAAACGGTA GTTACAGAAT GTGAGGAAGG TGTGCCTTCC TAAGAGGAAG AAGGGTAAGA   
  
  
+ AGTCTTTAAT TCTGCCTTTA CCATTAACG  

- AAATGGAGAG GAAGAGGAGA GGTTTTGGGT GGAAGTCTTG AAACGGTGCA ATAGACCCGG CATTAAAAAG   
  
  
- GGAGAGCTGA GACTTTAGAA GAAGACCAAA CAGTAGTAGT TAGTGCTTCT GTGTTTTAAT TATTTTACAA   
  
  
- ACCCCTTTTT CTCATTTTAC ATCGATATAT CATAATTGAA AAGGAGGCGA GGACACTCTC TTGGTCCTAA   
  
  
- TTCACTTCCT TTCCCTTTTC TTTTCTTTTT TTTTTCAAAT ATTACTTATT AATTAAATTT ATTTTACTTA   
  
  
- AAATTATATA TTATCATATC TATTTTTTAA AAATCTGTTT CTATATCAAT CTTAAAAATA CTTAGAGATA   
  
  
- TATTATCAAA AACAATCTCT CCTTTATTGT AAAATATTCA TCAAATATTA CTTAAAAATT GAACCTCAAT   
  
  
- AAAACCATCA TGCAAATCAA TTTGATGTGG CTCTGGTTTG AGCATTTAAG CATATTAGTA TTTTTTTTCT   
  
  
- GAAAAAAAAA TGTTCTTAAG AAAGAAAATA AAAAAATAAA ACAGATGATT TTATATTCAA TTTTAATTTT   
  
  
- TAAGGACTTC TGTAATGGCT ACTTATGTGG TGTAGTGACT ACTGGCTAAG ATTGTCATAG TAAGAGAGAG   
  
  
- AGAGGTTGTT GTGGGGCCCA ACAAAGGCAA GGGGGAAGTC TTCCGATTTC TCTCCGTAGA TTGACGCGCC   
  
  
- CCAAAATTTC ATCGCCTGGA TTGGCCCGGC GCATCTCGGG CTCGTGAAAA TGGGAGGTCT TTGGTCAACG   
  
  
- ATCTTGACAA GCCTGCTGGG CCCCACAAGG CATCCGGTCT AATTTGTGGG CCCCGCGCCT TCATATTGGA   
  
  
- CTTATTGCCA CGTCGGATGG GTGTCCTCGG ACCCAACCTA AACGGGTTGA CTCCGTCGTC CTTTCTTCAC   
  
  
- CCCATCTATC CCTCGCCTCG CCTCGCCTCA TAGCTTGAAT TGTTTACTTA TTATGTTACT CGATTTATTT   
  
  
- ATTATTATTT TAAAAAAATT AAAAGTAAAA TATATATTAC TTTTAATTAA TATTTAATAA TAATAAATTA   
  
  
- TTAATTATAA TAAAGTAAAC AAAACGGAAG TCGATCTGCC CCTACAGTAG CACAACTTGC AAGCACAGGC   
  
  
- AACTTGCATA CGTGATTTGA CAAGTAAAGG TGGTTAGTAG ATGATTGTAC ACACGAATAT TTGATTTTAT   
  
  
- AGTAATTTAT ACATATGTGT TTAAAAAATC AAATATTAAT TCTTACATTA ATTTATATTT TTATATAATT   
  
  
- ATTTTTTATA AAAAACAAAT AAAAATTTGA ATATCTCAAA ATTTAATATT TTTAAGCAGT GTAGAAAAGG   
  
  
- ACAGGTGGAC CGCATTGTTT GAATGAGTCA AAGACCTTTT TACCCTTCAT ATATTCAACA ATTTCTTCAC   
  
  
- CACCTCACGT GATTTGCCAT CAATGTCTTA CACTCCTTCC ACACGGAAGG ATTCTCCTTC TTCCCATTCT   
  
  
- TCAGAAATTA AGACGGAAAT GGTAATTGC

+     TC-rich repeats

| Site Name | Organism | Position | Strand | Matrix score. | sequence | function |
| --- | --- | --- | --- | --- | --- | --- |
| TC-rich repeats | Nicotiana tabacum | 1421 | - | 9 | ATTCTCTAAC | cis-acting element involved in defense and stress responsiveness |

> 2018/04/13 10:10:12  
+ TTTACCTCTC CTTCTCCTCT CCAAAACCCA CCTTCAGAAC TTTGCCACGT TATCTGGGCC GTAATTTTTC   
  
  
+ CCTCTCGACT CTGAAATCTT CTTCTGGTTT GTCATCATCA ATCACGAAGA CACAAAATTA ATAAAATGTT   
  
  
+ TGGGGAAAAA GAGTAAAATG TAGCTATATA GTATTAACTT TTCCTCCGCT CCTGTGAGAG AACCAGGATT   
  
  
+ AAGTGAAGGA AAGGGAAAAG AAAAGAAAAA AAAAAGTTTA TAATGAATAA TTAATTTAAA TAAAATGAAT   
  
  
+ TTTAATATAT AATAGTATAG ATAAAAAATT TTTAGACAAA GATATAGTTA GAATTTTTAT GAATCTCTAT   
  
  
+ ATAATAGTTT TTGTTAGAGA GGAAATAACA TTTTATAAGT AGTTTATAAT GAATTTTTAA CTTGGAGTTA   
  
  
+ TTTTGGTAGT ACGTTTAGTT AAACTACACC GAGACCAAAC TCGTAAATTC GTATAATCAT AAAAAAAAGA   
  
  
+ CTTTTTTTTT ACAAGAATTC TTTCTTTTAT TTTTTTATTT TGTCTACTAA AATATAAGTT AAAATTAAAA   
  
  
+ ATTCCTGAAG ACATTACCGA TGAATACACC ACATCACTGA TGACCGATTC TAACAGTATC ATTCTCTCTC   
  
  
+ TCTCCAACAA CACCCCGGGT TGTTTCCGTT CCCCCTTCAG AAGGCTAAAG AGAGGCATCT AACTGCGCGG   
  
  
+ GGTTTTAAAG TAGCGGACCT AACCGGGCCG CGTAGAGCCC GAGCACTTTT ACCCTCCAGA AACCAGTTGC   
  
  
+ TAGAACTGTT CGGACGACCC GGGGTGTTCC GTAGGCCAGA TTAAACACCC GGGGCGCGGA AGTATAACCT   
  
  
+ GAATAACGGT GCAGCCTACC CACAGGAGCC TGGGTTGGAT TTGCCCAACT GAGGCAGCAG GAAAGAAGTG   
  
  
+ GGGTAGATAG GGAGCGGAGC GGAGCGGAGT ATCGAACTTA ACAAATGAAT AATACAATGA GCTAAATAAA   
  
  
+ TAATAATAAA ATTTTTTTAA TTTTCATTTT ATATATAATG AAAATTAATT ATAAATTATT ATTATTTAAT   
  
  
+ AATTAATATT ATTTCATTTG TTTTGCCTTC AGCTAGACGG GGATGTCATC GTGTTGAACG TTCGTGTCCG   
  
  
+ TTGAACGTAT GCACTAAACT GTTCATTTCC ACCAATCATC TACTAACATG TGTGCTTATA AACTAAAATA   
  
  
+ TCATTAAATA TGTATACACA AATTTTTTAG TTTATAATTA AGAATGTAAT TAAATATAAA AATATATTAA   
  
  
+ TAAAAAATAT TTTTTGTTTA TTTTTAAACT TATAGAGTTT TAAATTATAA AAATTCGTCA CATCTTTTCC   
  
  
+ TGTCCACCTG GCGTAACAAA CTTACTCAGT TTCTGGAAAA ATGGGAAGTA TATAAGTTGT TAAAGAAGTG   
  
  
+ GTGGAGTGCA CTAAACGGTA GTTACAGAAT GTGAGGAAGG TGTGCCTTCC TAAGAGGAAG AAGGGTAAGA   
  
  
+ AGTCTTTAAT TCTGCCTTTA CCATTAACG  

- AAATGGAGAG GAAGAGGAGA GGTTTTGGGT GGAAGTCTTG AAACGGTGCA ATAGACCCGG CATTAAAAAG   
  
  
- GGAGAGCTGA GACTTTAGAA GAAGACCAAA CAGTAGTAGT TAGTGCTTCT GTGTTTTAAT TATTTTACAA   
  
  
- ACCCCTTTTT CTCATTTTAC ATCGATATAT CATAATTGAA AAGGAGGCGA GGACACTCTC TTGGTCCTAA   
  
  
- TTCACTTCCT TTCCCTTTTC TTTTCTTTTT TTTTTCAAAT ATTACTTATT AATTAAATTT ATTTTACTTA   
  
  
- AAATTATATA TTATCATATC TATTTTTTAA AAATCTGTTT CTATATCAAT CTTAAAAATA CTTAGAGATA   
  
  
- TATTATCAAA AACAATCTCT CCTTTATTGT AAAATATTCA TCAAATATTA CTTAAAAATT GAACCTCAAT   
  
  
- AAAACCATCA TGCAAATCAA TTTGATGTGG CTCTGGTTTG AGCATTTAAG CATATTAGTA TTTTTTTTCT   
  
  
- GAAAAAAAAA TGTTCTTAAG AAAGAAAATA AAAAAATAAA ACAGATGATT TTATATTCAA TTTTAATTTT   
  
  
- TAAGGACTTC TGTAATGGCT ACTTATGTGG TGTAGTGACT ACTGGCTAAG ATTGTCATAG TAAGAGAGAG   
  
  
- AGAGGTTGTT GTGGGGCCCA ACAAAGGCAA GGGGGAAGTC TTCCGATTTC TCTCCGTAGA TTGACGCGCC   
  
  
- CCAAAATTTC ATCGCCTGGA TTGGCCCGGC GCATCTCGGG CTCGTGAAAA TGGGAGGTCT TTGGTCAACG   
  
  
- ATCTTGACAA GCCTGCTGGG CCCCACAAGG CATCCGGTCT AATTTGTGGG CCCCGCGCCT TCATATTGGA   
  
  
- CTTATTGCCA CGTCGGATGG GTGTCCTCGG ACCCAACCTA AACGGGTTGA CTCCGTCGTC CTTTCTTCAC   
  
  
- CCCATCTATC CCTCGCCTCG CCTCGCCTCA TAGCTTGAAT TGTTTACTTA TTATGTTACT CGATTTATTT   
  
  
- ATTATTATTT TAAAAAAATT AAAAGTAAAA TATATATTAC TTTTAATTAA TATTTAATAA TAATAAATTA   
  
  
- TTAATTATAA TAAAGTAAAC AAAACGGAAG TCGATCTGCC CCTACAGTAG CACAACTTGC AAGCACAGGC   
  
  
- AACTTGCATA CGTGATTTGA CAAGTAAAGG TGGTTAGTAG ATGATTGTAC ACACGAATAT TTGATTTTAT   
  
  
- AGTAATTTAT ACATATGTGT TTAAAAAATC AAATATTAAT TCTTACATTA ATTTATATTT TTATATAATT   
  
  
- ATTTTTTATA AAAAACAAAT AAAAATTTGA ATATCTCAAA ATTTAATATT TTTAAGCAGT GTAGAAAAGG   
  
  
- ACAGGTGGAC CGCATTGTTT GAATGAGTCA AAGACCTTTT TACCCTTCAT ATATTCAACA ATTTCTTCAC   
  
  
- CACCTCACGT GATTTGCCAT CAATGTCTTA CACTCCTTCC ACACGGAAGG ATTCTCCTTC TTCCCATTCT   
  
  
- TCAGAAATTA AGACGGAAAT GGTAATTGC

+     TCCACCT-motif

| Site Name | Organism | Position | Strand | Matrix score. | sequence | function |
| --- | --- | --- | --- | --- | --- | --- |
| TCCACCT-motif | Petroselinum hortense | 1333 | + | 7 | TCCACCT |  |

> 2018/04/13 10:10:12  
+ TTTACCTCTC CTTCTCCTCT CCAAAACCCA CCTTCAGAAC TTTGCCACGT TATCTGGGCC GTAATTTTTC   
  
  
+ CCTCTCGACT CTGAAATCTT CTTCTGGTTT GTCATCATCA ATCACGAAGA CACAAAATTA ATAAAATGTT   
  
  
+ TGGGGAAAAA GAGTAAAATG TAGCTATATA GTATTAACTT TTCCTCCGCT CCTGTGAGAG AACCAGGATT   
  
  
+ AAGTGAAGGA AAGGGAAAAG AAAAGAAAAA AAAAAGTTTA TAATGAATAA TTAATTTAAA TAAAATGAAT   
  
  
+ TTTAATATAT AATAGTATAG ATAAAAAATT TTTAGACAAA GATATAGTTA GAATTTTTAT GAATCTCTAT   
  
  
+ ATAATAGTTT TTGTTAGAGA GGAAATAACA TTTTATAAGT AGTTTATAAT GAATTTTTAA CTTGGAGTTA   
  
  
+ TTTTGGTAGT ACGTTTAGTT AAACTACACC GAGACCAAAC TCGTAAATTC GTATAATCAT AAAAAAAAGA   
  
  
+ CTTTTTTTTT ACAAGAATTC TTTCTTTTAT TTTTTTATTT TGTCTACTAA AATATAAGTT AAAATTAAAA   
  
  
+ ATTCCTGAAG ACATTACCGA TGAATACACC ACATCACTGA TGACCGATTC TAACAGTATC ATTCTCTCTC   
  
  
+ TCTCCAACAA CACCCCGGGT TGTTTCCGTT CCCCCTTCAG AAGGCTAAAG AGAGGCATCT AACTGCGCGG   
  
  
+ GGTTTTAAAG TAGCGGACCT AACCGGGCCG CGTAGAGCCC GAGCACTTTT ACCCTCCAGA AACCAGTTGC   
  
  
+ TAGAACTGTT CGGACGACCC GGGGTGTTCC GTAGGCCAGA TTAAACACCC GGGGCGCGGA AGTATAACCT   
  
  
+ GAATAACGGT GCAGCCTACC CACAGGAGCC TGGGTTGGAT TTGCCCAACT GAGGCAGCAG GAAAGAAGTG   
  
  
+ GGGTAGATAG GGAGCGGAGC GGAGCGGAGT ATCGAACTTA ACAAATGAAT AATACAATGA GCTAAATAAA   
  
  
+ TAATAATAAA ATTTTTTTAA TTTTCATTTT ATATATAATG AAAATTAATT ATAAATTATT ATTATTTAAT   
  
  
+ AATTAATATT ATTTCATTTG TTTTGCCTTC AGCTAGACGG GGATGTCATC GTGTTGAACG TTCGTGTCCG   
  
  
+ TTGAACGTAT GCACTAAACT GTTCATTTCC ACCAATCATC TACTAACATG TGTGCTTATA AACTAAAATA   
  
  
+ TCATTAAATA TGTATACACA AATTTTTTAG TTTATAATTA AGAATGTAAT TAAATATAAA AATATATTAA   
  
  
+ TAAAAAATAT TTTTTGTTTA TTTTTAAACT TATAGAGTTT TAAATTATAA AAATTCGTCA CATCTTTTCC   
  
  
+ TGTCCACCTG GCGTAACAAA CTTACTCAGT TTCTGGAAAA ATGGGAAGTA TATAAGTTGT TAAAGAAGTG   
  
  
+ GTGGAGTGCA CTAAACGGTA GTTACAGAAT GTGAGGAAGG TGTGCCTTCC TAAGAGGAAG AAGGGTAAGA   
  
  
+ AGTCTTTAAT TCTGCCTTTA CCATTAACG  

- AAATGGAGAG GAAGAGGAGA GGTTTTGGGT GGAAGTCTTG AAACGGTGCA ATAGACCCGG CATTAAAAAG   
  
  
- GGAGAGCTGA GACTTTAGAA GAAGACCAAA CAGTAGTAGT TAGTGCTTCT GTGTTTTAAT TATTTTACAA   
  
  
- ACCCCTTTTT CTCATTTTAC ATCGATATAT CATAATTGAA AAGGAGGCGA GGACACTCTC TTGGTCCTAA   
  
  
- TTCACTTCCT TTCCCTTTTC TTTTCTTTTT TTTTTCAAAT ATTACTTATT AATTAAATTT ATTTTACTTA   
  
  
- AAATTATATA TTATCATATC TATTTTTTAA AAATCTGTTT CTATATCAAT CTTAAAAATA CTTAGAGATA   
  
  
- TATTATCAAA AACAATCTCT CCTTTATTGT AAAATATTCA TCAAATATTA CTTAAAAATT GAACCTCAAT   
  
  
- AAAACCATCA TGCAAATCAA TTTGATGTGG CTCTGGTTTG AGCATTTAAG CATATTAGTA TTTTTTTTCT   
  
  
- GAAAAAAAAA TGTTCTTAAG AAAGAAAATA AAAAAATAAA ACAGATGATT TTATATTCAA TTTTAATTTT   
  
  
- TAAGGACTTC TGTAATGGCT ACTTATGTGG TGTAGTGACT ACTGGCTAAG ATTGTCATAG TAAGAGAGAG   
  
  
- AGAGGTTGTT GTGGGGCCCA ACAAAGGCAA GGGGGAAGTC TTCCGATTTC TCTCCGTAGA TTGACGCGCC   
  
  
- CCAAAATTTC ATCGCCTGGA TTGGCCCGGC GCATCTCGGG CTCGTGAAAA TGGGAGGTCT TTGGTCAACG   
  
  
- ATCTTGACAA GCCTGCTGGG CCCCACAAGG CATCCGGTCT AATTTGTGGG CCCCGCGCCT TCATATTGGA   
  
  
- CTTATTGCCA CGTCGGATGG GTGTCCTCGG ACCCAACCTA AACGGGTTGA CTCCGTCGTC CTTTCTTCAC   
  
  
- CCCATCTATC CCTCGCCTCG CCTCGCCTCA TAGCTTGAAT TGTTTACTTA TTATGTTACT CGATTTATTT   
  
  
- ATTATTATTT TAAAAAAATT AAAAGTAAAA TATATATTAC TTTTAATTAA TATTTAATAA TAATAAATTA   
  
  
- TTAATTATAA TAAAGTAAAC AAAACGGAAG TCGATCTGCC CCTACAGTAG CACAACTTGC AAGCACAGGC   
  
  
- AACTTGCATA CGTGATTTGA CAAGTAAAGG TGGTTAGTAG ATGATTGTAC ACACGAATAT TTGATTTTAT   
  
  
- AGTAATTTAT ACATATGTGT TTAAAAAATC AAATATTAAT TCTTACATTA ATTTATATTT TTATATAATT   
  
  
- ATTTTTTATA AAAAACAAAT AAAAATTTGA ATATCTCAAA ATTTAATATT TTTAAGCAGT GTAGAAAAGG   
  
  
- ACAGGTGGAC CGCATTGTTT GAATGAGTCA AAGACCTTTT TACCCTTCAT ATATTCAACA ATTTCTTCAC   
  
  
- CACCTCACGT GATTTGCCAT CAATGTCTTA CACTCCTTCC ACACGGAAGG ATTCTCCTTC TTCCCATTCT   
  
  
- TCAGAAATTA AGACGGAAAT GGTAATTGC

+     TCT-motif

| Site Name | Organism | Position | Strand | Matrix score. | sequence | function |
| --- | --- | --- | --- | --- | --- | --- |
| TCT-motif | Arabidopsis thaliana | 1465 | - | 6 | TCTTAC | part of a light responsive element |

> 2018/04/13 10:10:12  
+ TTTACCTCTC CTTCTCCTCT CCAAAACCCA CCTTCAGAAC TTTGCCACGT TATCTGGGCC GTAATTTTTC   
  
  
+ CCTCTCGACT CTGAAATCTT CTTCTGGTTT GTCATCATCA ATCACGAAGA CACAAAATTA ATAAAATGTT   
  
  
+ TGGGGAAAAA GAGTAAAATG TAGCTATATA GTATTAACTT TTCCTCCGCT CCTGTGAGAG AACCAGGATT   
  
  
+ AAGTGAAGGA AAGGGAAAAG AAAAGAAAAA AAAAAGTTTA TAATGAATAA TTAATTTAAA TAAAATGAAT   
  
  
+ TTTAATATAT AATAGTATAG ATAAAAAATT TTTAGACAAA GATATAGTTA GAATTTTTAT GAATCTCTAT   
  
  
+ ATAATAGTTT TTGTTAGAGA GGAAATAACA TTTTATAAGT AGTTTATAAT GAATTTTTAA CTTGGAGTTA   
  
  
+ TTTTGGTAGT ACGTTTAGTT AAACTACACC GAGACCAAAC TCGTAAATTC GTATAATCAT AAAAAAAAGA   
  
  
+ CTTTTTTTTT ACAAGAATTC TTTCTTTTAT TTTTTTATTT TGTCTACTAA AATATAAGTT AAAATTAAAA   
  
  
+ ATTCCTGAAG ACATTACCGA TGAATACACC ACATCACTGA TGACCGATTC TAACAGTATC ATTCTCTCTC   
  
  
+ TCTCCAACAA CACCCCGGGT TGTTTCCGTT CCCCCTTCAG AAGGCTAAAG AGAGGCATCT AACTGCGCGG   
  
  
+ GGTTTTAAAG TAGCGGACCT AACCGGGCCG CGTAGAGCCC GAGCACTTTT ACCCTCCAGA AACCAGTTGC   
  
  
+ TAGAACTGTT CGGACGACCC GGGGTGTTCC GTAGGCCAGA TTAAACACCC GGGGCGCGGA AGTATAACCT   
  
  
+ GAATAACGGT GCAGCCTACC CACAGGAGCC TGGGTTGGAT TTGCCCAACT GAGGCAGCAG GAAAGAAGTG   
  
  
+ GGGTAGATAG GGAGCGGAGC GGAGCGGAGT ATCGAACTTA ACAAATGAAT AATACAATGA GCTAAATAAA   
  
  
+ TAATAATAAA ATTTTTTTAA TTTTCATTTT ATATATAATG AAAATTAATT ATAAATTATT ATTATTTAAT   
  
  
+ AATTAATATT ATTTCATTTG TTTTGCCTTC AGCTAGACGG GGATGTCATC GTGTTGAACG TTCGTGTCCG   
  
  
+ TTGAACGTAT GCACTAAACT GTTCATTTCC ACCAATCATC TACTAACATG TGTGCTTATA AACTAAAATA   
  
  
+ TCATTAAATA TGTATACACA AATTTTTTAG TTTATAATTA AGAATGTAAT TAAATATAAA AATATATTAA   
  
  
+ TAAAAAATAT TTTTTGTTTA TTTTTAAACT TATAGAGTTT TAAATTATAA AAATTCGTCA CATCTTTTCC   
  
  
+ TGTCCACCTG GCGTAACAAA CTTACTCAGT TTCTGGAAAA ATGGGAAGTA TATAAGTTGT TAAAGAAGTG   
  
  
+ GTGGAGTGCA CTAAACGGTA GTTACAGAAT GTGAGGAAGG TGTGCCTTCC TAAGAGGAAG AAGGGTAAGA   
  
  
+ AGTCTTTAAT TCTGCCTTTA CCATTAACG  

- AAATGGAGAG GAAGAGGAGA GGTTTTGGGT GGAAGTCTTG AAACGGTGCA ATAGACCCGG CATTAAAAAG   
  
  
- GGAGAGCTGA GACTTTAGAA GAAGACCAAA CAGTAGTAGT TAGTGCTTCT GTGTTTTAAT TATTTTACAA   
  
  
- ACCCCTTTTT CTCATTTTAC ATCGATATAT CATAATTGAA AAGGAGGCGA GGACACTCTC TTGGTCCTAA   
  
  
- TTCACTTCCT TTCCCTTTTC TTTTCTTTTT TTTTTCAAAT ATTACTTATT AATTAAATTT ATTTTACTTA   
  
  
- AAATTATATA TTATCATATC TATTTTTTAA AAATCTGTTT CTATATCAAT CTTAAAAATA CTTAGAGATA   
  
  
- TATTATCAAA AACAATCTCT CCTTTATTGT AAAATATTCA TCAAATATTA CTTAAAAATT GAACCTCAAT   
  
  
- AAAACCATCA TGCAAATCAA TTTGATGTGG CTCTGGTTTG AGCATTTAAG CATATTAGTA TTTTTTTTCT   
  
  
- GAAAAAAAAA TGTTCTTAAG AAAGAAAATA AAAAAATAAA ACAGATGATT TTATATTCAA TTTTAATTTT   
  
  
- TAAGGACTTC TGTAATGGCT ACTTATGTGG TGTAGTGACT ACTGGCTAAG ATTGTCATAG TAAGAGAGAG   
  
  
- AGAGGTTGTT GTGGGGCCCA ACAAAGGCAA GGGGGAAGTC TTCCGATTTC TCTCCGTAGA TTGACGCGCC   
  
  
- CCAAAATTTC ATCGCCTGGA TTGGCCCGGC GCATCTCGGG CTCGTGAAAA TGGGAGGTCT TTGGTCAACG   
  
  
- ATCTTGACAA GCCTGCTGGG CCCCACAAGG CATCCGGTCT AATTTGTGGG CCCCGCGCCT TCATATTGGA   
  
  
- CTTATTGCCA CGTCGGATGG GTGTCCTCGG ACCCAACCTA AACGGGTTGA CTCCGTCGTC CTTTCTTCAC   
  
  
- CCCATCTATC CCTCGCCTCG CCTCGCCTCA TAGCTTGAAT TGTTTACTTA TTATGTTACT CGATTTATTT   
  
  
- ATTATTATTT TAAAAAAATT AAAAGTAAAA TATATATTAC TTTTAATTAA TATTTAATAA TAATAAATTA   
  
  
- TTAATTATAA TAAAGTAAAC AAAACGGAAG TCGATCTGCC CCTACAGTAG CACAACTTGC AAGCACAGGC   
  
  
- AACTTGCATA CGTGATTTGA CAAGTAAAGG TGGTTAGTAG ATGATTGTAC ACACGAATAT TTGATTTTAT   
  
  
- AGTAATTTAT ACATATGTGT TTAAAAAATC AAATATTAAT TCTTACATTA ATTTATATTT TTATATAATT   
  
  
- ATTTTTTATA AAAAACAAAT AAAAATTTGA ATATCTCAAA ATTTAATATT TTTAAGCAGT GTAGAAAAGG   
  
  
- ACAGGTGGAC CGCATTGTTT GAATGAGTCA AAGACCTTTT TACCCTTCAT ATATTCAACA ATTTCTTCAC   
  
  
- CACCTCACGT GATTTGCCAT CAATGTCTTA CACTCCTTCC ACACGGAAGG ATTCTCCTTC TTCCCATTCT   
  
  
- TCAGAAATTA AGACGGAAAT GGTAATTGC

+     TGACG-motif

| Site Name | Organism | Position | Strand | Matrix score. | sequence | function |
| --- | --- | --- | --- | --- | --- | --- |
| TGACG-motif | Hordeum vulgare | 1316 | - | 5 | TGACG | cis-acting regulatory element involved in the MeJA-responsiveness |

> 2018/04/13 10:10:12  
+ TTTACCTCTC CTTCTCCTCT CCAAAACCCA CCTTCAGAAC TTTGCCACGT TATCTGGGCC GTAATTTTTC   
  
  
+ CCTCTCGACT CTGAAATCTT CTTCTGGTTT GTCATCATCA ATCACGAAGA CACAAAATTA ATAAAATGTT   
  
  
+ TGGGGAAAAA GAGTAAAATG TAGCTATATA GTATTAACTT TTCCTCCGCT CCTGTGAGAG AACCAGGATT   
  
  
+ AAGTGAAGGA AAGGGAAAAG AAAAGAAAAA AAAAAGTTTA TAATGAATAA TTAATTTAAA TAAAATGAAT   
  
  
+ TTTAATATAT AATAGTATAG ATAAAAAATT TTTAGACAAA GATATAGTTA GAATTTTTAT GAATCTCTAT   
  
  
+ ATAATAGTTT TTGTTAGAGA GGAAATAACA TTTTATAAGT AGTTTATAAT GAATTTTTAA CTTGGAGTTA   
  
  
+ TTTTGGTAGT ACGTTTAGTT AAACTACACC GAGACCAAAC TCGTAAATTC GTATAATCAT AAAAAAAAGA   
  
  
+ CTTTTTTTTT ACAAGAATTC TTTCTTTTAT TTTTTTATTT TGTCTACTAA AATATAAGTT AAAATTAAAA   
  
  
+ ATTCCTGAAG ACATTACCGA TGAATACACC ACATCACTGA TGACCGATTC TAACAGTATC ATTCTCTCTC   
  
  
+ TCTCCAACAA CACCCCGGGT TGTTTCCGTT CCCCCTTCAG AAGGCTAAAG AGAGGCATCT AACTGCGCGG   
  
  
+ GGTTTTAAAG TAGCGGACCT AACCGGGCCG CGTAGAGCCC GAGCACTTTT ACCCTCCAGA AACCAGTTGC   
  
  
+ TAGAACTGTT CGGACGACCC GGGGTGTTCC GTAGGCCAGA TTAAACACCC GGGGCGCGGA AGTATAACCT   
  
  
+ GAATAACGGT GCAGCCTACC CACAGGAGCC TGGGTTGGAT TTGCCCAACT GAGGCAGCAG GAAAGAAGTG   
  
  
+ GGGTAGATAG GGAGCGGAGC GGAGCGGAGT ATCGAACTTA ACAAATGAAT AATACAATGA GCTAAATAAA   
  
  
+ TAATAATAAA ATTTTTTTAA TTTTCATTTT ATATATAATG AAAATTAATT ATAAATTATT ATTATTTAAT   
  
  
+ AATTAATATT ATTTCATTTG TTTTGCCTTC AGCTAGACGG GGATGTCATC GTGTTGAACG TTCGTGTCCG   
  
  
+ TTGAACGTAT GCACTAAACT GTTCATTTCC ACCAATCATC TACTAACATG TGTGCTTATA AACTAAAATA   
  
  
+ TCATTAAATA TGTATACACA AATTTTTTAG TTTATAATTA AGAATGTAAT TAAATATAAA AATATATTAA   
  
  
+ TAAAAAATAT TTTTTGTTTA TTTTTAAACT TATAGAGTTT TAAATTATAA AAATTCGTCA CATCTTTTCC   
  
  
+ TGTCCACCTG GCGTAACAAA CTTACTCAGT TTCTGGAAAA ATGGGAAGTA TATAAGTTGT TAAAGAAGTG   
  
  
+ GTGGAGTGCA CTAAACGGTA GTTACAGAAT GTGAGGAAGG TGTGCCTTCC TAAGAGGAAG AAGGGTAAGA   
  
  
+ AGTCTTTAAT TCTGCCTTTA CCATTAACG  

- AAATGGAGAG GAAGAGGAGA GGTTTTGGGT GGAAGTCTTG AAACGGTGCA ATAGACCCGG CATTAAAAAG   
  
  
- GGAGAGCTGA GACTTTAGAA GAAGACCAAA CAGTAGTAGT TAGTGCTTCT GTGTTTTAAT TATTTTACAA   
  
  
- ACCCCTTTTT CTCATTTTAC ATCGATATAT CATAATTGAA AAGGAGGCGA GGACACTCTC TTGGTCCTAA   
  
  
- TTCACTTCCT TTCCCTTTTC TTTTCTTTTT TTTTTCAAAT ATTACTTATT AATTAAATTT ATTTTACTTA   
  
  
- AAATTATATA TTATCATATC TATTTTTTAA AAATCTGTTT CTATATCAAT CTTAAAAATA CTTAGAGATA   
  
  
- TATTATCAAA AACAATCTCT CCTTTATTGT AAAATATTCA TCAAATATTA CTTAAAAATT GAACCTCAAT   
  
  
- AAAACCATCA TGCAAATCAA TTTGATGTGG CTCTGGTTTG AGCATTTAAG CATATTAGTA TTTTTTTTCT   
  
  
- GAAAAAAAAA TGTTCTTAAG AAAGAAAATA AAAAAATAAA ACAGATGATT TTATATTCAA TTTTAATTTT   
  
  
- TAAGGACTTC TGTAATGGCT ACTTATGTGG TGTAGTGACT ACTGGCTAAG ATTGTCATAG TAAGAGAGAG   
  
  
- AGAGGTTGTT GTGGGGCCCA ACAAAGGCAA GGGGGAAGTC TTCCGATTTC TCTCCGTAGA TTGACGCGCC   
  
  
- CCAAAATTTC ATCGCCTGGA TTGGCCCGGC GCATCTCGGG CTCGTGAAAA TGGGAGGTCT TTGGTCAACG   
  
  
- ATCTTGACAA GCCTGCTGGG CCCCACAAGG CATCCGGTCT AATTTGTGGG CCCCGCGCCT TCATATTGGA   
  
  
- CTTATTGCCA CGTCGGATGG GTGTCCTCGG ACCCAACCTA AACGGGTTGA CTCCGTCGTC CTTTCTTCAC   
  
  
- CCCATCTATC CCTCGCCTCG CCTCGCCTCA TAGCTTGAAT TGTTTACTTA TTATGTTACT CGATTTATTT   
  
  
- ATTATTATTT TAAAAAAATT AAAAGTAAAA TATATATTAC TTTTAATTAA TATTTAATAA TAATAAATTA   
  
  
- TTAATTATAA TAAAGTAAAC AAAACGGAAG TCGATCTGCC CCTACAGTAG CACAACTTGC AAGCACAGGC   
  
  
- AACTTGCATA CGTGATTTGA CAAGTAAAGG TGGTTAGTAG ATGATTGTAC ACACGAATAT TTGATTTTAT   
  
  
- AGTAATTTAT ACATATGTGT TTAAAAAATC AAATATTAAT TCTTACATTA ATTTATATTT TTATATAATT   
  
  
- ATTTTTTATA AAAAACAAAT AAAAATTTGA ATATCTCAAA ATTTAATATT TTTAAGCAGT GTAGAAAAGG   
  
  
- ACAGGTGGAC CGCATTGTTT GAATGAGTCA AAGACCTTTT TACCCTTCAT ATATTCAACA ATTTCTTCAC   
  
  
- CACCTCACGT GATTTGCCAT CAATGTCTTA CACTCCTTCC ACACGGAAGG ATTCTCCTTC TTCCCATTCT   
  
  
- TCAGAAATTA AGACGGAAAT GGTAATTGC

+     Unnamed\_\_1

| Site Name | Organism | Position | Strand | Matrix score. | sequence | function |
| --- | --- | --- | --- | --- | --- | --- |
| Unnamed\_\_1 | Glycine max | 1232 | + | 11 | GAATTTAATTAA | 60K protein binding site |
| Unnamed\_\_1 | Zea mays | 45 | - | 5 | CGTGG |  |

> 2018/04/13 10:10:12  
+ TTTACCTCTC CTTCTCCTCT CCAAAACCCA CCTTCAGAAC TTTGCCACGT TATCTGGGCC GTAATTTTTC   
  
  
+ CCTCTCGACT CTGAAATCTT CTTCTGGTTT GTCATCATCA ATCACGAAGA CACAAAATTA ATAAAATGTT   
  
  
+ TGGGGAAAAA GAGTAAAATG TAGCTATATA GTATTAACTT TTCCTCCGCT CCTGTGAGAG AACCAGGATT   
  
  
+ AAGTGAAGGA AAGGGAAAAG AAAAGAAAAA AAAAAGTTTA TAATGAATAA TTAATTTAAA TAAAATGAAT   
  
  
+ TTTAATATAT AATAGTATAG ATAAAAAATT TTTAGACAAA GATATAGTTA GAATTTTTAT GAATCTCTAT   
  
  
+ ATAATAGTTT TTGTTAGAGA GGAAATAACA TTTTATAAGT AGTTTATAAT GAATTTTTAA CTTGGAGTTA   
  
  
+ TTTTGGTAGT ACGTTTAGTT AAACTACACC GAGACCAAAC TCGTAAATTC GTATAATCAT AAAAAAAAGA   
  
  
+ CTTTTTTTTT ACAAGAATTC TTTCTTTTAT TTTTTTATTT TGTCTACTAA AATATAAGTT AAAATTAAAA   
  
  
+ ATTCCTGAAG ACATTACCGA TGAATACACC ACATCACTGA TGACCGATTC TAACAGTATC ATTCTCTCTC   
  
  
+ TCTCCAACAA CACCCCGGGT TGTTTCCGTT CCCCCTTCAG AAGGCTAAAG AGAGGCATCT AACTGCGCGG   
  
  
+ GGTTTTAAAG TAGCGGACCT AACCGGGCCG CGTAGAGCCC GAGCACTTTT ACCCTCCAGA AACCAGTTGC   
  
  
+ TAGAACTGTT CGGACGACCC GGGGTGTTCC GTAGGCCAGA TTAAACACCC GGGGCGCGGA AGTATAACCT   
  
  
+ GAATAACGGT GCAGCCTACC CACAGGAGCC TGGGTTGGAT TTGCCCAACT GAGGCAGCAG GAAAGAAGTG   
  
  
+ GGGTAGATAG GGAGCGGAGC GGAGCGGAGT ATCGAACTTA ACAAATGAAT AATACAATGA GCTAAATAAA   
  
  
+ TAATAATAAA ATTTTTTTAA TTTTCATTTT ATATATAATG AAAATTAATT ATAAATTATT ATTATTTAAT   
  
  
+ AATTAATATT ATTTCATTTG TTTTGCCTTC AGCTAGACGG GGATGTCATC GTGTTGAACG TTCGTGTCCG   
  
  
+ TTGAACGTAT GCACTAAACT GTTCATTTCC ACCAATCATC TACTAACATG TGTGCTTATA AACTAAAATA   
  
  
+ TCATTAAATA TGTATACACA AATTTTTTAG TTTATAATTA AGAATGTAAT TAAATATAAA AATATATTAA   
  
  
+ TAAAAAATAT TTTTTGTTTA TTTTTAAACT TATAGAGTTT TAAATTATAA AAATTCGTCA CATCTTTTCC   
  
  
+ TGTCCACCTG GCGTAACAAA CTTACTCAGT TTCTGGAAAA ATGGGAAGTA TATAAGTTGT TAAAGAAGTG   
  
  
+ GTGGAGTGCA CTAAACGGTA GTTACAGAAT GTGAGGAAGG TGTGCCTTCC TAAGAGGAAG AAGGGTAAGA   
  
  
+ AGTCTTTAAT TCTGCCTTTA CCATTAACG  

- AAATGGAGAG GAAGAGGAGA GGTTTTGGGT GGAAGTCTTG AAACGGTGCA ATAGACCCGG CATTAAAAAG   
  
  
- GGAGAGCTGA GACTTTAGAA GAAGACCAAA CAGTAGTAGT TAGTGCTTCT GTGTTTTAAT TATTTTACAA   
  
  
- ACCCCTTTTT CTCATTTTAC ATCGATATAT CATAATTGAA AAGGAGGCGA GGACACTCTC TTGGTCCTAA   
  
  
- TTCACTTCCT TTCCCTTTTC TTTTCTTTTT TTTTTCAAAT ATTACTTATT AATTAAATTT ATTTTACTTA   
  
  
- AAATTATATA TTATCATATC TATTTTTTAA AAATCTGTTT CTATATCAAT CTTAAAAATA CTTAGAGATA   
  
  
- TATTATCAAA AACAATCTCT CCTTTATTGT AAAATATTCA TCAAATATTA CTTAAAAATT GAACCTCAAT   
  
  
- AAAACCATCA TGCAAATCAA TTTGATGTGG CTCTGGTTTG AGCATTTAAG CATATTAGTA TTTTTTTTCT   
  
  
- GAAAAAAAAA TGTTCTTAAG AAAGAAAATA AAAAAATAAA ACAGATGATT TTATATTCAA TTTTAATTTT   
  
  
- TAAGGACTTC TGTAATGGCT ACTTATGTGG TGTAGTGACT ACTGGCTAAG ATTGTCATAG TAAGAGAGAG   
  
  
- AGAGGTTGTT GTGGGGCCCA ACAAAGGCAA GGGGGAAGTC TTCCGATTTC TCTCCGTAGA TTGACGCGCC   
  
  
- CCAAAATTTC ATCGCCTGGA TTGGCCCGGC GCATCTCGGG CTCGTGAAAA TGGGAGGTCT TTGGTCAACG   
  
  
- ATCTTGACAA GCCTGCTGGG CCCCACAAGG CATCCGGTCT AATTTGTGGG CCCCGCGCCT TCATATTGGA   
  
  
- CTTATTGCCA CGTCGGATGG GTGTCCTCGG ACCCAACCTA AACGGGTTGA CTCCGTCGTC CTTTCTTCAC   
  
  
- CCCATCTATC CCTCGCCTCG CCTCGCCTCA TAGCTTGAAT TGTTTACTTA TTATGTTACT CGATTTATTT   
  
  
- ATTATTATTT TAAAAAAATT AAAAGTAAAA TATATATTAC TTTTAATTAA TATTTAATAA TAATAAATTA   
  
  
- TTAATTATAA TAAAGTAAAC AAAACGGAAG TCGATCTGCC CCTACAGTAG CACAACTTGC AAGCACAGGC   
  
  
- AACTTGCATA CGTGATTTGA CAAGTAAAGG TGGTTAGTAG ATGATTGTAC ACACGAATAT TTGATTTTAT   
  
  
- AGTAATTTAT ACATATGTGT TTAAAAAATC AAATATTAAT TCTTACATTA ATTTATATTT TTATATAATT   
  
  
- ATTTTTTATA AAAAACAAAT AAAAATTTGA ATATCTCAAA ATTTAATATT TTTAAGCAGT GTAGAAAAGG   
  
  
- ACAGGTGGAC CGCATTGTTT GAATGAGTCA AAGACCTTTT TACCCTTCAT ATATTCAACA ATTTCTTCAC   
  
  
- CACCTCACGT GATTTGCCAT CAATGTCTTA CACTCCTTCC ACACGGAAGG ATTCTCCTTC TTCCCATTCT   
  
  
- TCAGAAATTA AGACGGAAAT GGTAATTGC

+     Unnamed\_\_2

| Site Name | Organism | Position | Strand | Matrix score. | sequence | function |
| --- | --- | --- | --- | --- | --- | --- |
| Unnamed\_\_2 | Zea mays | 789 | - | 6 | CCCCGG |  |
| Unnamed\_\_2 | Zea mays | 819 | - | 6 | CCCCGG |  |
| Unnamed\_\_2 | Zea mays | 643 | + | 6 | CCCCGG |  |

> 2018/04/13 10:10:12  
+ TTTACCTCTC CTTCTCCTCT CCAAAACCCA CCTTCAGAAC TTTGCCACGT TATCTGGGCC GTAATTTTTC   
  
  
+ CCTCTCGACT CTGAAATCTT CTTCTGGTTT GTCATCATCA ATCACGAAGA CACAAAATTA ATAAAATGTT   
  
  
+ TGGGGAAAAA GAGTAAAATG TAGCTATATA GTATTAACTT TTCCTCCGCT CCTGTGAGAG AACCAGGATT   
  
  
+ AAGTGAAGGA AAGGGAAAAG AAAAGAAAAA AAAAAGTTTA TAATGAATAA TTAATTTAAA TAAAATGAAT   
  
  
+ TTTAATATAT AATAGTATAG ATAAAAAATT TTTAGACAAA GATATAGTTA GAATTTTTAT GAATCTCTAT   
  
  
+ ATAATAGTTT TTGTTAGAGA GGAAATAACA TTTTATAAGT AGTTTATAAT GAATTTTTAA CTTGGAGTTA   
  
  
+ TTTTGGTAGT ACGTTTAGTT AAACTACACC GAGACCAAAC TCGTAAATTC GTATAATCAT AAAAAAAAGA   
  
  
+ CTTTTTTTTT ACAAGAATTC TTTCTTTTAT TTTTTTATTT TGTCTACTAA AATATAAGTT AAAATTAAAA   
  
  
+ ATTCCTGAAG ACATTACCGA TGAATACACC ACATCACTGA TGACCGATTC TAACAGTATC ATTCTCTCTC   
  
  
+ TCTCCAACAA CACCCCGGGT TGTTTCCGTT CCCCCTTCAG AAGGCTAAAG AGAGGCATCT AACTGCGCGG   
  
  
+ GGTTTTAAAG TAGCGGACCT AACCGGGCCG CGTAGAGCCC GAGCACTTTT ACCCTCCAGA AACCAGTTGC   
  
  
+ TAGAACTGTT CGGACGACCC GGGGTGTTCC GTAGGCCAGA TTAAACACCC GGGGCGCGGA AGTATAACCT   
  
  
+ GAATAACGGT GCAGCCTACC CACAGGAGCC TGGGTTGGAT TTGCCCAACT GAGGCAGCAG GAAAGAAGTG   
  
  
+ GGGTAGATAG GGAGCGGAGC GGAGCGGAGT ATCGAACTTA ACAAATGAAT AATACAATGA GCTAAATAAA   
  
  
+ TAATAATAAA ATTTTTTTAA TTTTCATTTT ATATATAATG AAAATTAATT ATAAATTATT ATTATTTAAT   
  
  
+ AATTAATATT ATTTCATTTG TTTTGCCTTC AGCTAGACGG GGATGTCATC GTGTTGAACG TTCGTGTCCG   
  
  
+ TTGAACGTAT GCACTAAACT GTTCATTTCC ACCAATCATC TACTAACATG TGTGCTTATA AACTAAAATA   
  
  
+ TCATTAAATA TGTATACACA AATTTTTTAG TTTATAATTA AGAATGTAAT TAAATATAAA AATATATTAA   
  
  
+ TAAAAAATAT TTTTTGTTTA TTTTTAAACT TATAGAGTTT TAAATTATAA AAATTCGTCA CATCTTTTCC   
  
  
+ TGTCCACCTG GCGTAACAAA CTTACTCAGT TTCTGGAAAA ATGGGAAGTA TATAAGTTGT TAAAGAAGTG   
  
  
+ GTGGAGTGCA CTAAACGGTA GTTACAGAAT GTGAGGAAGG TGTGCCTTCC TAAGAGGAAG AAGGGTAAGA   
  
  
+ AGTCTTTAAT TCTGCCTTTA CCATTAACG  

- AAATGGAGAG GAAGAGGAGA GGTTTTGGGT GGAAGTCTTG AAACGGTGCA ATAGACCCGG CATTAAAAAG   
  
  
- GGAGAGCTGA GACTTTAGAA GAAGACCAAA CAGTAGTAGT TAGTGCTTCT GTGTTTTAAT TATTTTACAA   
  
  
- ACCCCTTTTT CTCATTTTAC ATCGATATAT CATAATTGAA AAGGAGGCGA GGACACTCTC TTGGTCCTAA   
  
  
- TTCACTTCCT TTCCCTTTTC TTTTCTTTTT TTTTTCAAAT ATTACTTATT AATTAAATTT ATTTTACTTA   
  
  
- AAATTATATA TTATCATATC TATTTTTTAA AAATCTGTTT CTATATCAAT CTTAAAAATA CTTAGAGATA   
  
  
- TATTATCAAA AACAATCTCT CCTTTATTGT AAAATATTCA TCAAATATTA CTTAAAAATT GAACCTCAAT   
  
  
- AAAACCATCA TGCAAATCAA TTTGATGTGG CTCTGGTTTG AGCATTTAAG CATATTAGTA TTTTTTTTCT   
  
  
- GAAAAAAAAA TGTTCTTAAG AAAGAAAATA AAAAAATAAA ACAGATGATT TTATATTCAA TTTTAATTTT   
  
  
- TAAGGACTTC TGTAATGGCT ACTTATGTGG TGTAGTGACT ACTGGCTAAG ATTGTCATAG TAAGAGAGAG   
  
  
- AGAGGTTGTT GTGGGGCCCA ACAAAGGCAA GGGGGAAGTC TTCCGATTTC TCTCCGTAGA TTGACGCGCC   
  
  
- CCAAAATTTC ATCGCCTGGA TTGGCCCGGC GCATCTCGGG CTCGTGAAAA TGGGAGGTCT TTGGTCAACG   
  
  
- ATCTTGACAA GCCTGCTGGG CCCCACAAGG CATCCGGTCT AATTTGTGGG CCCCGCGCCT TCATATTGGA   
  
  
- CTTATTGCCA CGTCGGATGG GTGTCCTCGG ACCCAACCTA AACGGGTTGA CTCCGTCGTC CTTTCTTCAC   
  
  
- CCCATCTATC CCTCGCCTCG CCTCGCCTCA TAGCTTGAAT TGTTTACTTA TTATGTTACT CGATTTATTT   
  
  
- ATTATTATTT TAAAAAAATT AAAAGTAAAA TATATATTAC TTTTAATTAA TATTTAATAA TAATAAATTA   
  
  
- TTAATTATAA TAAAGTAAAC AAAACGGAAG TCGATCTGCC CCTACAGTAG CACAACTTGC AAGCACAGGC   
  
  
- AACTTGCATA CGTGATTTGA CAAGTAAAGG TGGTTAGTAG ATGATTGTAC ACACGAATAT TTGATTTTAT   
  
  
- AGTAATTTAT ACATATGTGT TTAAAAAATC AAATATTAAT TCTTACATTA ATTTATATTT TTATATAATT   
  
  
- ATTTTTTATA AAAAACAAAT AAAAATTTGA ATATCTCAAA ATTTAATATT TTTAAGCAGT GTAGAAAAGG   
  
  
- ACAGGTGGAC CGCATTGTTT GAATGAGTCA AAGACCTTTT TACCCTTCAT ATATTCAACA ATTTCTTCAC   
  
  
- CACCTCACGT GATTTGCCAT CAATGTCTTA CACTCCTTCC ACACGGAAGG ATTCTCCTTC TTCCCATTCT   
  
  
- TCAGAAATTA AGACGGAAAT GGTAATTGC

+     Unnamed\_\_3

| Site Name | Organism | Position | Strand | Matrix score. | sequence | function |
| --- | --- | --- | --- | --- | --- | --- |
| Unnamed\_\_3 | Zea mays | 45 | - | 5 | CGTGG |  |

> 2018/04/13 10:10:12  
+ TTTACCTCTC CTTCTCCTCT CCAAAACCCA CCTTCAGAAC TTTGCCACGT TATCTGGGCC GTAATTTTTC   
  
  
+ CCTCTCGACT CTGAAATCTT CTTCTGGTTT GTCATCATCA ATCACGAAGA CACAAAATTA ATAAAATGTT   
  
  
+ TGGGGAAAAA GAGTAAAATG TAGCTATATA GTATTAACTT TTCCTCCGCT CCTGTGAGAG AACCAGGATT   
  
  
+ AAGTGAAGGA AAGGGAAAAG AAAAGAAAAA AAAAAGTTTA TAATGAATAA TTAATTTAAA TAAAATGAAT   
  
  
+ TTTAATATAT AATAGTATAG ATAAAAAATT TTTAGACAAA GATATAGTTA GAATTTTTAT GAATCTCTAT   
  
  
+ ATAATAGTTT TTGTTAGAGA GGAAATAACA TTTTATAAGT AGTTTATAAT GAATTTTTAA CTTGGAGTTA   
  
  
+ TTTTGGTAGT ACGTTTAGTT AAACTACACC GAGACCAAAC TCGTAAATTC GTATAATCAT AAAAAAAAGA   
  
  
+ CTTTTTTTTT ACAAGAATTC TTTCTTTTAT TTTTTTATTT TGTCTACTAA AATATAAGTT AAAATTAAAA   
  
  
+ ATTCCTGAAG ACATTACCGA TGAATACACC ACATCACTGA TGACCGATTC TAACAGTATC ATTCTCTCTC   
  
  
+ TCTCCAACAA CACCCCGGGT TGTTTCCGTT CCCCCTTCAG AAGGCTAAAG AGAGGCATCT AACTGCGCGG   
  
  
+ GGTTTTAAAG TAGCGGACCT AACCGGGCCG CGTAGAGCCC GAGCACTTTT ACCCTCCAGA AACCAGTTGC   
  
  
+ TAGAACTGTT CGGACGACCC GGGGTGTTCC GTAGGCCAGA TTAAACACCC GGGGCGCGGA AGTATAACCT   
  
  
+ GAATAACGGT GCAGCCTACC CACAGGAGCC TGGGTTGGAT TTGCCCAACT GAGGCAGCAG GAAAGAAGTG   
  
  
+ GGGTAGATAG GGAGCGGAGC GGAGCGGAGT ATCGAACTTA ACAAATGAAT AATACAATGA GCTAAATAAA   
  
  
+ TAATAATAAA ATTTTTTTAA TTTTCATTTT ATATATAATG AAAATTAATT ATAAATTATT ATTATTTAAT   
  
  
+ AATTAATATT ATTTCATTTG TTTTGCCTTC AGCTAGACGG GGATGTCATC GTGTTGAACG TTCGTGTCCG   
  
  
+ TTGAACGTAT GCACTAAACT GTTCATTTCC ACCAATCATC TACTAACATG TGTGCTTATA AACTAAAATA   
  
  
+ TCATTAAATA TGTATACACA AATTTTTTAG TTTATAATTA AGAATGTAAT TAAATATAAA AATATATTAA   
  
  
+ TAAAAAATAT TTTTTGTTTA TTTTTAAACT TATAGAGTTT TAAATTATAA AAATTCGTCA CATCTTTTCC   
  
  
+ TGTCCACCTG GCGTAACAAA CTTACTCAGT TTCTGGAAAA ATGGGAAGTA TATAAGTTGT TAAAGAAGTG   
  
  
+ GTGGAGTGCA CTAAACGGTA GTTACAGAAT GTGAGGAAGG TGTGCCTTCC TAAGAGGAAG AAGGGTAAGA   
  
  
+ AGTCTTTAAT TCTGCCTTTA CCATTAACG  

- AAATGGAGAG GAAGAGGAGA GGTTTTGGGT GGAAGTCTTG AAACGGTGCA ATAGACCCGG CATTAAAAAG   
  
  
- GGAGAGCTGA GACTTTAGAA GAAGACCAAA CAGTAGTAGT TAGTGCTTCT GTGTTTTAAT TATTTTACAA   
  
  
- ACCCCTTTTT CTCATTTTAC ATCGATATAT CATAATTGAA AAGGAGGCGA GGACACTCTC TTGGTCCTAA   
  
  
- TTCACTTCCT TTCCCTTTTC TTTTCTTTTT TTTTTCAAAT ATTACTTATT AATTAAATTT ATTTTACTTA   
  
  
- AAATTATATA TTATCATATC TATTTTTTAA AAATCTGTTT CTATATCAAT CTTAAAAATA CTTAGAGATA   
  
  
- TATTATCAAA AACAATCTCT CCTTTATTGT AAAATATTCA TCAAATATTA CTTAAAAATT GAACCTCAAT   
  
  
- AAAACCATCA TGCAAATCAA TTTGATGTGG CTCTGGTTTG AGCATTTAAG CATATTAGTA TTTTTTTTCT   
  
  
- GAAAAAAAAA TGTTCTTAAG AAAGAAAATA AAAAAATAAA ACAGATGATT TTATATTCAA TTTTAATTTT   
  
  
- TAAGGACTTC TGTAATGGCT ACTTATGTGG TGTAGTGACT ACTGGCTAAG ATTGTCATAG TAAGAGAGAG   
  
  
- AGAGGTTGTT GTGGGGCCCA ACAAAGGCAA GGGGGAAGTC TTCCGATTTC TCTCCGTAGA TTGACGCGCC   
  
  
- CCAAAATTTC ATCGCCTGGA TTGGCCCGGC GCATCTCGGG CTCGTGAAAA TGGGAGGTCT TTGGTCAACG   
  
  
- ATCTTGACAA GCCTGCTGGG CCCCACAAGG CATCCGGTCT AATTTGTGGG CCCCGCGCCT TCATATTGGA   
  
  
- CTTATTGCCA CGTCGGATGG GTGTCCTCGG ACCCAACCTA AACGGGTTGA CTCCGTCGTC CTTTCTTCAC   
  
  
- CCCATCTATC CCTCGCCTCG CCTCGCCTCA TAGCTTGAAT TGTTTACTTA TTATGTTACT CGATTTATTT   
  
  
- ATTATTATTT TAAAAAAATT AAAAGTAAAA TATATATTAC TTTTAATTAA TATTTAATAA TAATAAATTA   
  
  
- TTAATTATAA TAAAGTAAAC AAAACGGAAG TCGATCTGCC CCTACAGTAG CACAACTTGC AAGCACAGGC   
  
  
- AACTTGCATA CGTGATTTGA CAAGTAAAGG TGGTTAGTAG ATGATTGTAC ACACGAATAT TTGATTTTAT   
  
  
- AGTAATTTAT ACATATGTGT TTAAAAAATC AAATATTAAT TCTTACATTA ATTTATATTT TTATATAATT   
  
  
- ATTTTTTATA AAAAACAAAT AAAAATTTGA ATATCTCAAA ATTTAATATT TTTAAGCAGT GTAGAAAAGG   
  
  
- ACAGGTGGAC CGCATTGTTT GAATGAGTCA AAGACCTTTT TACCCTTCAT ATATTCAACA ATTTCTTCAC   
  
  
- CACCTCACGT GATTTGCCAT CAATGTCTTA CACTCCTTCC ACACGGAAGG ATTCTCCTTC TTCCCATTCT   
  
  
- TCAGAAATTA AGACGGAAAT GGTAATTGC

+     Unnamed\_\_4

| Site Name | Organism | Position | Strand | Matrix score. | sequence | function |
| --- | --- | --- | --- | --- | --- | --- |
| Unnamed\_\_4 | Petroselinum hortense | 189 | + | 4 | CTCC |  |
| Unnamed\_\_4 | Petroselinum hortense | 19 | + | 4 | CTCC |  |
| Unnamed\_\_4 | Petroselinum hortense | 14 | + | 4 | CTCC |  |
| Unnamed\_\_4 | Petroselinum hortense | 8 | + | 4 | CTCC |  |
| Unnamed\_\_4 | Petroselinum hortense | 414 | - | 4 | CTCC |  |
| Unnamed\_\_4 | Petroselinum hortense | 184 | + | 4 | CTCC |  |
| Unnamed\_\_4 | Petroselinum hortense | 632 | + | 4 | CTCC |  |
| Unnamed\_\_4 | Petroselinum hortense | 936 | - | 4 | CTCC |  |
| Unnamed\_\_4 | Petroselinum hortense | 926 | - | 4 | CTCC |  |
| Unnamed\_\_4 | Petroselinum hortense | 931 | - | 4 | CTCC |  |
| Unnamed\_\_4 | Petroselinum hortense | 754 | + | 4 | CTCC |  |
| Unnamed\_\_4 | Petroselinum hortense | 1403 | - | 4 | CTCC |  |
| Unnamed\_\_4 | Petroselinum hortense | 865 | - | 4 | CTCC |  |
| Unnamed\_\_4 | Petroselinum hortense | 921 | - | 4 | CTCC |  |

> 2018/04/13 10:10:12  
+ TTTACCTCTC CTTCTCCTCT CCAAAACCCA CCTTCAGAAC TTTGCCACGT TATCTGGGCC GTAATTTTTC   
  
  
+ CCTCTCGACT CTGAAATCTT CTTCTGGTTT GTCATCATCA ATCACGAAGA CACAAAATTA ATAAAATGTT   
  
  
+ TGGGGAAAAA GAGTAAAATG TAGCTATATA GTATTAACTT TTCCTCCGCT CCTGTGAGAG AACCAGGATT   
  
  
+ AAGTGAAGGA AAGGGAAAAG AAAAGAAAAA AAAAAGTTTA TAATGAATAA TTAATTTAAA TAAAATGAAT   
  
  
+ TTTAATATAT AATAGTATAG ATAAAAAATT TTTAGACAAA GATATAGTTA GAATTTTTAT GAATCTCTAT   
  
  
+ ATAATAGTTT TTGTTAGAGA GGAAATAACA TTTTATAAGT AGTTTATAAT GAATTTTTAA CTTGGAGTTA   
  
  
+ TTTTGGTAGT ACGTTTAGTT AAACTACACC GAGACCAAAC TCGTAAATTC GTATAATCAT AAAAAAAAGA   
  
  
+ CTTTTTTTTT ACAAGAATTC TTTCTTTTAT TTTTTTATTT TGTCTACTAA AATATAAGTT AAAATTAAAA   
  
  
+ ATTCCTGAAG ACATTACCGA TGAATACACC ACATCACTGA TGACCGATTC TAACAGTATC ATTCTCTCTC   
  
  
+ TCTCCAACAA CACCCCGGGT TGTTTCCGTT CCCCCTTCAG AAGGCTAAAG AGAGGCATCT AACTGCGCGG   
  
  
+ GGTTTTAAAG TAGCGGACCT AACCGGGCCG CGTAGAGCCC GAGCACTTTT ACCCTCCAGA AACCAGTTGC   
  
  
+ TAGAACTGTT CGGACGACCC GGGGTGTTCC GTAGGCCAGA TTAAACACCC GGGGCGCGGA AGTATAACCT   
  
  
+ GAATAACGGT GCAGCCTACC CACAGGAGCC TGGGTTGGAT TTGCCCAACT GAGGCAGCAG GAAAGAAGTG   
  
  
+ GGGTAGATAG GGAGCGGAGC GGAGCGGAGT ATCGAACTTA ACAAATGAAT AATACAATGA GCTAAATAAA   
  
  
+ TAATAATAAA ATTTTTTTAA TTTTCATTTT ATATATAATG AAAATTAATT ATAAATTATT ATTATTTAAT   
  
  
+ AATTAATATT ATTTCATTTG TTTTGCCTTC AGCTAGACGG GGATGTCATC GTGTTGAACG TTCGTGTCCG   
  
  
+ TTGAACGTAT GCACTAAACT GTTCATTTCC ACCAATCATC TACTAACATG TGTGCTTATA AACTAAAATA   
  
  
+ TCATTAAATA TGTATACACA AATTTTTTAG TTTATAATTA AGAATGTAAT TAAATATAAA AATATATTAA   
  
  
+ TAAAAAATAT TTTTTGTTTA TTTTTAAACT TATAGAGTTT TAAATTATAA AAATTCGTCA CATCTTTTCC   
  
  
+ TGTCCACCTG GCGTAACAAA CTTACTCAGT TTCTGGAAAA ATGGGAAGTA TATAAGTTGT TAAAGAAGTG   
  
  
+ GTGGAGTGCA CTAAACGGTA GTTACAGAAT GTGAGGAAGG TGTGCCTTCC TAAGAGGAAG AAGGGTAAGA   
  
  
+ AGTCTTTAAT TCTGCCTTTA CCATTAACG  

- AAATGGAGAG GAAGAGGAGA GGTTTTGGGT GGAAGTCTTG AAACGGTGCA ATAGACCCGG CATTAAAAAG   
  
  
- GGAGAGCTGA GACTTTAGAA GAAGACCAAA CAGTAGTAGT TAGTGCTTCT GTGTTTTAAT TATTTTACAA   
  
  
- ACCCCTTTTT CTCATTTTAC ATCGATATAT CATAATTGAA AAGGAGGCGA GGACACTCTC TTGGTCCTAA   
  
  
- TTCACTTCCT TTCCCTTTTC TTTTCTTTTT TTTTTCAAAT ATTACTTATT AATTAAATTT ATTTTACTTA   
  
  
- AAATTATATA TTATCATATC TATTTTTTAA AAATCTGTTT CTATATCAAT CTTAAAAATA CTTAGAGATA   
  
  
- TATTATCAAA AACAATCTCT CCTTTATTGT AAAATATTCA TCAAATATTA CTTAAAAATT GAACCTCAAT   
  
  
- AAAACCATCA TGCAAATCAA TTTGATGTGG CTCTGGTTTG AGCATTTAAG CATATTAGTA TTTTTTTTCT   
  
  
- GAAAAAAAAA TGTTCTTAAG AAAGAAAATA AAAAAATAAA ACAGATGATT TTATATTCAA TTTTAATTTT   
  
  
- TAAGGACTTC TGTAATGGCT ACTTATGTGG TGTAGTGACT ACTGGCTAAG ATTGTCATAG TAAGAGAGAG   
  
  
- AGAGGTTGTT GTGGGGCCCA ACAAAGGCAA GGGGGAAGTC TTCCGATTTC TCTCCGTAGA TTGACGCGCC   
  
  
- CCAAAATTTC ATCGCCTGGA TTGGCCCGGC GCATCTCGGG CTCGTGAAAA TGGGAGGTCT TTGGTCAACG   
  
  
- ATCTTGACAA GCCTGCTGGG CCCCACAAGG CATCCGGTCT AATTTGTGGG CCCCGCGCCT TCATATTGGA   
  
  
- CTTATTGCCA CGTCGGATGG GTGTCCTCGG ACCCAACCTA AACGGGTTGA CTCCGTCGTC CTTTCTTCAC   
  
  
- CCCATCTATC CCTCGCCTCG CCTCGCCTCA TAGCTTGAAT TGTTTACTTA TTATGTTACT CGATTTATTT   
  
  
- ATTATTATTT TAAAAAAATT AAAAGTAAAA TATATATTAC TTTTAATTAA TATTTAATAA TAATAAATTA   
  
  
- TTAATTATAA TAAAGTAAAC AAAACGGAAG TCGATCTGCC CCTACAGTAG CACAACTTGC AAGCACAGGC   
  
  
- AACTTGCATA CGTGATTTGA CAAGTAAAGG TGGTTAGTAG ATGATTGTAC ACACGAATAT TTGATTTTAT   
  
  
- AGTAATTTAT ACATATGTGT TTAAAAAATC AAATATTAAT TCTTACATTA ATTTATATTT TTATATAATT   
  
  
- ATTTTTTATA AAAAACAAAT AAAAATTTGA ATATCTCAAA ATTTAATATT TTTAAGCAGT GTAGAAAAGG   
  
  
- ACAGGTGGAC CGCATTGTTT GAATGAGTCA AAGACCTTTT TACCCTTCAT ATATTCAACA ATTTCTTCAC   
  
  
- CACCTCACGT GATTTGCCAT CAATGTCTTA CACTCCTTCC ACACGGAAGG ATTCTCCTTC TTCCCATTCT   
  
  
- TCAGAAATTA AGACGGAAAT GGTAATTGC

+     box II

| Site Name | Organism | Position | Strand | Matrix score. | sequence | function |
| --- | --- | --- | --- | --- | --- | --- |
| box II | Petroselinum hortense | 1333 | + | 9 | TCCACGTGGC | part of a light responsive element |

> 2018/04/13 10:10:12  
+ TTTACCTCTC CTTCTCCTCT CCAAAACCCA CCTTCAGAAC TTTGCCACGT TATCTGGGCC GTAATTTTTC   
  
  
+ CCTCTCGACT CTGAAATCTT CTTCTGGTTT GTCATCATCA ATCACGAAGA CACAAAATTA ATAAAATGTT   
  
  
+ TGGGGAAAAA GAGTAAAATG TAGCTATATA GTATTAACTT TTCCTCCGCT CCTGTGAGAG AACCAGGATT   
  
  
+ AAGTGAAGGA AAGGGAAAAG AAAAGAAAAA AAAAAGTTTA TAATGAATAA TTAATTTAAA TAAAATGAAT   
  
  
+ TTTAATATAT AATAGTATAG ATAAAAAATT TTTAGACAAA GATATAGTTA GAATTTTTAT GAATCTCTAT   
  
  
+ ATAATAGTTT TTGTTAGAGA GGAAATAACA TTTTATAAGT AGTTTATAAT GAATTTTTAA CTTGGAGTTA   
  
  
+ TTTTGGTAGT ACGTTTAGTT AAACTACACC GAGACCAAAC TCGTAAATTC GTATAATCAT AAAAAAAAGA   
  
  
+ CTTTTTTTTT ACAAGAATTC TTTCTTTTAT TTTTTTATTT TGTCTACTAA AATATAAGTT AAAATTAAAA   
  
  
+ ATTCCTGAAG ACATTACCGA TGAATACACC ACATCACTGA TGACCGATTC TAACAGTATC ATTCTCTCTC   
  
  
+ TCTCCAACAA CACCCCGGGT TGTTTCCGTT CCCCCTTCAG AAGGCTAAAG AGAGGCATCT AACTGCGCGG   
  
  
+ GGTTTTAAAG TAGCGGACCT AACCGGGCCG CGTAGAGCCC GAGCACTTTT ACCCTCCAGA AACCAGTTGC   
  
  
+ TAGAACTGTT CGGACGACCC GGGGTGTTCC GTAGGCCAGA TTAAACACCC GGGGCGCGGA AGTATAACCT   
  
  
+ GAATAACGGT GCAGCCTACC CACAGGAGCC TGGGTTGGAT TTGCCCAACT GAGGCAGCAG GAAAGAAGTG   
  
  
+ GGGTAGATAG GGAGCGGAGC GGAGCGGAGT ATCGAACTTA ACAAATGAAT AATACAATGA GCTAAATAAA   
  
  
+ TAATAATAAA ATTTTTTTAA TTTTCATTTT ATATATAATG AAAATTAATT ATAAATTATT ATTATTTAAT   
  
  
+ AATTAATATT ATTTCATTTG TTTTGCCTTC AGCTAGACGG GGATGTCATC GTGTTGAACG TTCGTGTCCG   
  
  
+ TTGAACGTAT GCACTAAACT GTTCATTTCC ACCAATCATC TACTAACATG TGTGCTTATA AACTAAAATA   
  
  
+ TCATTAAATA TGTATACACA AATTTTTTAG TTTATAATTA AGAATGTAAT TAAATATAAA AATATATTAA   
  
  
+ TAAAAAATAT TTTTTGTTTA TTTTTAAACT TATAGAGTTT TAAATTATAA AAATTCGTCA CATCTTTTCC   
  
  
+ TGTCCACCTG GCGTAACAAA CTTACTCAGT TTCTGGAAAA ATGGGAAGTA TATAAGTTGT TAAAGAAGTG   
  
  
+ GTGGAGTGCA CTAAACGGTA GTTACAGAAT GTGAGGAAGG TGTGCCTTCC TAAGAGGAAG AAGGGTAAGA   
  
  
+ AGTCTTTAAT TCTGCCTTTA CCATTAACG  

- AAATGGAGAG GAAGAGGAGA GGTTTTGGGT GGAAGTCTTG AAACGGTGCA ATAGACCCGG CATTAAAAAG   
  
  
- GGAGAGCTGA GACTTTAGAA GAAGACCAAA CAGTAGTAGT TAGTGCTTCT GTGTTTTAAT TATTTTACAA   
  
  
- ACCCCTTTTT CTCATTTTAC ATCGATATAT CATAATTGAA AAGGAGGCGA GGACACTCTC TTGGTCCTAA   
  
  
- TTCACTTCCT TTCCCTTTTC TTTTCTTTTT TTTTTCAAAT ATTACTTATT AATTAAATTT ATTTTACTTA   
  
  
- AAATTATATA TTATCATATC TATTTTTTAA AAATCTGTTT CTATATCAAT CTTAAAAATA CTTAGAGATA   
  
  
- TATTATCAAA AACAATCTCT CCTTTATTGT AAAATATTCA TCAAATATTA CTTAAAAATT GAACCTCAAT   
  
  
- AAAACCATCA TGCAAATCAA TTTGATGTGG CTCTGGTTTG AGCATTTAAG CATATTAGTA TTTTTTTTCT   
  
  
- GAAAAAAAAA TGTTCTTAAG AAAGAAAATA AAAAAATAAA ACAGATGATT TTATATTCAA TTTTAATTTT   
  
  
- TAAGGACTTC TGTAATGGCT ACTTATGTGG TGTAGTGACT ACTGGCTAAG ATTGTCATAG TAAGAGAGAG   
  
  
- AGAGGTTGTT GTGGGGCCCA ACAAAGGCAA GGGGGAAGTC TTCCGATTTC TCTCCGTAGA TTGACGCGCC   
  
  
- CCAAAATTTC ATCGCCTGGA TTGGCCCGGC GCATCTCGGG CTCGTGAAAA TGGGAGGTCT TTGGTCAACG   
  
  
- ATCTTGACAA GCCTGCTGGG CCCCACAAGG CATCCGGTCT AATTTGTGGG CCCCGCGCCT TCATATTGGA   
  
  
- CTTATTGCCA CGTCGGATGG GTGTCCTCGG ACCCAACCTA AACGGGTTGA CTCCGTCGTC CTTTCTTCAC   
  
  
- CCCATCTATC CCTCGCCTCG CCTCGCCTCA TAGCTTGAAT TGTTTACTTA TTATGTTACT CGATTTATTT   
  
  
- ATTATTATTT TAAAAAAATT AAAAGTAAAA TATATATTAC TTTTAATTAA TATTTAATAA TAATAAATTA   
  
  
- TTAATTATAA TAAAGTAAAC AAAACGGAAG TCGATCTGCC CCTACAGTAG CACAACTTGC AAGCACAGGC   
  
  
- AACTTGCATA CGTGATTTGA CAAGTAAAGG TGGTTAGTAG ATGATTGTAC ACACGAATAT TTGATTTTAT   
  
  
- AGTAATTTAT ACATATGTGT TTAAAAAATC AAATATTAAT TCTTACATTA ATTTATATTT TTATATAATT   
  
  
- ATTTTTTATA AAAAACAAAT AAAAATTTGA ATATCTCAAA ATTTAATATT TTTAAGCAGT GTAGAAAAGG   
  
  
- ACAGGTGGAC CGCATTGTTT GAATGAGTCA AAGACCTTTT TACCCTTCAT ATATTCAACA ATTTCTTCAC   
  
  
- CACCTCACGT GATTTGCCAT CAATGTCTTA CACTCCTTCC ACACGGAAGG ATTCTCCTTC TTCCCATTCT   
  
  
- TCAGAAATTA AGACGGAAAT GGTAATTGC

+     chs-Unit 1 m1

| Site Name | Organism | Position | Strand | Matrix score. | sequence | function |
| --- | --- | --- | --- | --- | --- | --- |
| chs-Unit 1 m1 | Zea mays | 717 | + | 10 | ACCTAACCCGG | part of a light responsive element |

> 2018/04/13 10:10:12  
+ TTTACCTCTC CTTCTCCTCT CCAAAACCCA CCTTCAGAAC TTTGCCACGT TATCTGGGCC GTAATTTTTC   
  
  
+ CCTCTCGACT CTGAAATCTT CTTCTGGTTT GTCATCATCA ATCACGAAGA CACAAAATTA ATAAAATGTT   
  
  
+ TGGGGAAAAA GAGTAAAATG TAGCTATATA GTATTAACTT TTCCTCCGCT CCTGTGAGAG AACCAGGATT   
  
  
+ AAGTGAAGGA AAGGGAAAAG AAAAGAAAAA AAAAAGTTTA TAATGAATAA TTAATTTAAA TAAAATGAAT   
  
  
+ TTTAATATAT AATAGTATAG ATAAAAAATT TTTAGACAAA GATATAGTTA GAATTTTTAT GAATCTCTAT   
  
  
+ ATAATAGTTT TTGTTAGAGA GGAAATAACA TTTTATAAGT AGTTTATAAT GAATTTTTAA CTTGGAGTTA   
  
  
+ TTTTGGTAGT ACGTTTAGTT AAACTACACC GAGACCAAAC TCGTAAATTC GTATAATCAT AAAAAAAAGA   
  
  
+ CTTTTTTTTT ACAAGAATTC TTTCTTTTAT TTTTTTATTT TGTCTACTAA AATATAAGTT AAAATTAAAA   
  
  
+ ATTCCTGAAG ACATTACCGA TGAATACACC ACATCACTGA TGACCGATTC TAACAGTATC ATTCTCTCTC   
  
  
+ TCTCCAACAA CACCCCGGGT TGTTTCCGTT CCCCCTTCAG AAGGCTAAAG AGAGGCATCT AACTGCGCGG   
  
  
+ GGTTTTAAAG TAGCGGACCT AACCGGGCCG CGTAGAGCCC GAGCACTTTT ACCCTCCAGA AACCAGTTGC   
  
  
+ TAGAACTGTT CGGACGACCC GGGGTGTTCC GTAGGCCAGA TTAAACACCC GGGGCGCGGA AGTATAACCT   
  
  
+ GAATAACGGT GCAGCCTACC CACAGGAGCC TGGGTTGGAT TTGCCCAACT GAGGCAGCAG GAAAGAAGTG   
  
  
+ GGGTAGATAG GGAGCGGAGC GGAGCGGAGT ATCGAACTTA ACAAATGAAT AATACAATGA GCTAAATAAA   
  
  
+ TAATAATAAA ATTTTTTTAA TTTTCATTTT ATATATAATG AAAATTAATT ATAAATTATT ATTATTTAAT   
  
  
+ AATTAATATT ATTTCATTTG TTTTGCCTTC AGCTAGACGG GGATGTCATC GTGTTGAACG TTCGTGTCCG   
  
  
+ TTGAACGTAT GCACTAAACT GTTCATTTCC ACCAATCATC TACTAACATG TGTGCTTATA AACTAAAATA   
  
  
+ TCATTAAATA TGTATACACA AATTTTTTAG TTTATAATTA AGAATGTAAT TAAATATAAA AATATATTAA   
  
  
+ TAAAAAATAT TTTTTGTTTA TTTTTAAACT TATAGAGTTT TAAATTATAA AAATTCGTCA CATCTTTTCC   
  
  
+ TGTCCACCTG GCGTAACAAA CTTACTCAGT TTCTGGAAAA ATGGGAAGTA TATAAGTTGT TAAAGAAGTG   
  
  
+ GTGGAGTGCA CTAAACGGTA GTTACAGAAT GTGAGGAAGG TGTGCCTTCC TAAGAGGAAG AAGGGTAAGA   
  
  
+ AGTCTTTAAT TCTGCCTTTA CCATTAACG  

- AAATGGAGAG GAAGAGGAGA GGTTTTGGGT GGAAGTCTTG AAACGGTGCA ATAGACCCGG CATTAAAAAG   
  
  
- GGAGAGCTGA GACTTTAGAA GAAGACCAAA CAGTAGTAGT TAGTGCTTCT GTGTTTTAAT TATTTTACAA   
  
  
- ACCCCTTTTT CTCATTTTAC ATCGATATAT CATAATTGAA AAGGAGGCGA GGACACTCTC TTGGTCCTAA   
  
  
- TTCACTTCCT TTCCCTTTTC TTTTCTTTTT TTTTTCAAAT ATTACTTATT AATTAAATTT ATTTTACTTA   
  
  
- AAATTATATA TTATCATATC TATTTTTTAA AAATCTGTTT CTATATCAAT CTTAAAAATA CTTAGAGATA   
  
  
- TATTATCAAA AACAATCTCT CCTTTATTGT AAAATATTCA TCAAATATTA CTTAAAAATT GAACCTCAAT   
  
  
- AAAACCATCA TGCAAATCAA TTTGATGTGG CTCTGGTTTG AGCATTTAAG CATATTAGTA TTTTTTTTCT   
  
  
- GAAAAAAAAA TGTTCTTAAG AAAGAAAATA AAAAAATAAA ACAGATGATT TTATATTCAA TTTTAATTTT   
  
  
- TAAGGACTTC TGTAATGGCT ACTTATGTGG TGTAGTGACT ACTGGCTAAG ATTGTCATAG TAAGAGAGAG   
  
  
- AGAGGTTGTT GTGGGGCCCA ACAAAGGCAA GGGGGAAGTC TTCCGATTTC TCTCCGTAGA TTGACGCGCC   
  
  
- CCAAAATTTC ATCGCCTGGA TTGGCCCGGC GCATCTCGGG CTCGTGAAAA TGGGAGGTCT TTGGTCAACG   
  
  
- ATCTTGACAA GCCTGCTGGG CCCCACAAGG CATCCGGTCT AATTTGTGGG CCCCGCGCCT TCATATTGGA   
  
  
- CTTATTGCCA CGTCGGATGG GTGTCCTCGG ACCCAACCTA AACGGGTTGA CTCCGTCGTC CTTTCTTCAC   
  
  
- CCCATCTATC CCTCGCCTCG CCTCGCCTCA TAGCTTGAAT TGTTTACTTA TTATGTTACT CGATTTATTT   
  
  
- ATTATTATTT TAAAAAAATT AAAAGTAAAA TATATATTAC TTTTAATTAA TATTTAATAA TAATAAATTA   
  
  
- TTAATTATAA TAAAGTAAAC AAAACGGAAG TCGATCTGCC CCTACAGTAG CACAACTTGC AAGCACAGGC   
  
  
- AACTTGCATA CGTGATTTGA CAAGTAAAGG TGGTTAGTAG ATGATTGTAC ACACGAATAT TTGATTTTAT   
  
  
- AGTAATTTAT ACATATGTGT TTAAAAAATC AAATATTAAT TCTTACATTA ATTTATATTT TTATATAATT   
  
  
- ATTTTTTATA AAAAACAAAT AAAAATTTGA ATATCTCAAA ATTTAATATT TTTAAGCAGT GTAGAAAAGG   
  
  
- ACAGGTGGAC CGCATTGTTT GAATGAGTCA AAGACCTTTT TACCCTTCAT ATATTCAACA ATTTCTTCAC   
  
  
- CACCTCACGT GATTTGCCAT CAATGTCTTA CACTCCTTCC ACACGGAAGG ATTCTCCTTC TTCCCATTCT   
  
  
- TCAGAAATTA AGACGGAAAT GGTAATTGC

+     circadian

| Site Name | Organism | Position | Strand | Matrix score. | sequence | function |
| --- | --- | --- | --- | --- | --- | --- |
| circadian | Lycopersicon esculentum | 317 | + | 9 | CAAAGATATC | cis-acting regulatory element involved in circadian control |

> 2018/04/13 10:10:12  
+ TTTACCTCTC CTTCTCCTCT CCAAAACCCA CCTTCAGAAC TTTGCCACGT TATCTGGGCC GTAATTTTTC   
  
  
+ CCTCTCGACT CTGAAATCTT CTTCTGGTTT GTCATCATCA ATCACGAAGA CACAAAATTA ATAAAATGTT   
  
  
+ TGGGGAAAAA GAGTAAAATG TAGCTATATA GTATTAACTT TTCCTCCGCT CCTGTGAGAG AACCAGGATT   
  
  
+ AAGTGAAGGA AAGGGAAAAG AAAAGAAAAA AAAAAGTTTA TAATGAATAA TTAATTTAAA TAAAATGAAT   
  
  
+ TTTAATATAT AATAGTATAG ATAAAAAATT TTTAGACAAA GATATAGTTA GAATTTTTAT GAATCTCTAT   
  
  
+ ATAATAGTTT TTGTTAGAGA GGAAATAACA TTTTATAAGT AGTTTATAAT GAATTTTTAA CTTGGAGTTA   
  
  
+ TTTTGGTAGT ACGTTTAGTT AAACTACACC GAGACCAAAC TCGTAAATTC GTATAATCAT AAAAAAAAGA   
  
  
+ CTTTTTTTTT ACAAGAATTC TTTCTTTTAT TTTTTTATTT TGTCTACTAA AATATAAGTT AAAATTAAAA   
  
  
+ ATTCCTGAAG ACATTACCGA TGAATACACC ACATCACTGA TGACCGATTC TAACAGTATC ATTCTCTCTC   
  
  
+ TCTCCAACAA CACCCCGGGT TGTTTCCGTT CCCCCTTCAG AAGGCTAAAG AGAGGCATCT AACTGCGCGG   
  
  
+ GGTTTTAAAG TAGCGGACCT AACCGGGCCG CGTAGAGCCC GAGCACTTTT ACCCTCCAGA AACCAGTTGC   
  
  
+ TAGAACTGTT CGGACGACCC GGGGTGTTCC GTAGGCCAGA TTAAACACCC GGGGCGCGGA AGTATAACCT   
  
  
+ GAATAACGGT GCAGCCTACC CACAGGAGCC TGGGTTGGAT TTGCCCAACT GAGGCAGCAG GAAAGAAGTG   
  
  
+ GGGTAGATAG GGAGCGGAGC GGAGCGGAGT ATCGAACTTA ACAAATGAAT AATACAATGA GCTAAATAAA   
  
  
+ TAATAATAAA ATTTTTTTAA TTTTCATTTT ATATATAATG AAAATTAATT ATAAATTATT ATTATTTAAT   
  
  
+ AATTAATATT ATTTCATTTG TTTTGCCTTC AGCTAGACGG GGATGTCATC GTGTTGAACG TTCGTGTCCG   
  
  
+ TTGAACGTAT GCACTAAACT GTTCATTTCC ACCAATCATC TACTAACATG TGTGCTTATA AACTAAAATA   
  
  
+ TCATTAAATA TGTATACACA AATTTTTTAG TTTATAATTA AGAATGTAAT TAAATATAAA AATATATTAA   
  
  
+ TAAAAAATAT TTTTTGTTTA TTTTTAAACT TATAGAGTTT TAAATTATAA AAATTCGTCA CATCTTTTCC   
  
  
+ TGTCCACCTG GCGTAACAAA CTTACTCAGT TTCTGGAAAA ATGGGAAGTA TATAAGTTGT TAAAGAAGTG   
  
  
+ GTGGAGTGCA CTAAACGGTA GTTACAGAAT GTGAGGAAGG TGTGCCTTCC TAAGAGGAAG AAGGGTAAGA   
  
  
+ AGTCTTTAAT TCTGCCTTTA CCATTAACG  

- AAATGGAGAG GAAGAGGAGA GGTTTTGGGT GGAAGTCTTG AAACGGTGCA ATAGACCCGG CATTAAAAAG   
  
  
- GGAGAGCTGA GACTTTAGAA GAAGACCAAA CAGTAGTAGT TAGTGCTTCT GTGTTTTAAT TATTTTACAA   
  
  
- ACCCCTTTTT CTCATTTTAC ATCGATATAT CATAATTGAA AAGGAGGCGA GGACACTCTC TTGGTCCTAA   
  
  
- TTCACTTCCT TTCCCTTTTC TTTTCTTTTT TTTTTCAAAT ATTACTTATT AATTAAATTT ATTTTACTTA   
  
  
- AAATTATATA TTATCATATC TATTTTTTAA AAATCTGTTT CTATATCAAT CTTAAAAATA CTTAGAGATA   
  
  
- TATTATCAAA AACAATCTCT CCTTTATTGT AAAATATTCA TCAAATATTA CTTAAAAATT GAACCTCAAT   
  
  
- AAAACCATCA TGCAAATCAA TTTGATGTGG CTCTGGTTTG AGCATTTAAG CATATTAGTA TTTTTTTTCT   
  
  
- GAAAAAAAAA TGTTCTTAAG AAAGAAAATA AAAAAATAAA ACAGATGATT TTATATTCAA TTTTAATTTT   
  
  
- TAAGGACTTC TGTAATGGCT ACTTATGTGG TGTAGTGACT ACTGGCTAAG ATTGTCATAG TAAGAGAGAG   
  
  
- AGAGGTTGTT GTGGGGCCCA ACAAAGGCAA GGGGGAAGTC TTCCGATTTC TCTCCGTAGA TTGACGCGCC   
  
  
- CCAAAATTTC ATCGCCTGGA TTGGCCCGGC GCATCTCGGG CTCGTGAAAA TGGGAGGTCT TTGGTCAACG   
  
  
- ATCTTGACAA GCCTGCTGGG CCCCACAAGG CATCCGGTCT AATTTGTGGG CCCCGCGCCT TCATATTGGA   
  
  
- CTTATTGCCA CGTCGGATGG GTGTCCTCGG ACCCAACCTA AACGGGTTGA CTCCGTCGTC CTTTCTTCAC   
  
  
- CCCATCTATC CCTCGCCTCG CCTCGCCTCA TAGCTTGAAT TGTTTACTTA TTATGTTACT CGATTTATTT   
  
  
- ATTATTATTT TAAAAAAATT AAAAGTAAAA TATATATTAC TTTTAATTAA TATTTAATAA TAATAAATTA   
  
  
- TTAATTATAA TAAAGTAAAC AAAACGGAAG TCGATCTGCC CCTACAGTAG CACAACTTGC AAGCACAGGC   
  
  
- AACTTGCATA CGTGATTTGA CAAGTAAAGG TGGTTAGTAG ATGATTGTAC ACACGAATAT TTGATTTTAT   
  
  
- AGTAATTTAT ACATATGTGT TTAAAAAATC AAATATTAAT TCTTACATTA ATTTATATTT TTATATAATT   
  
  
- ATTTTTTATA AAAAACAAAT AAAAATTTGA ATATCTCAAA ATTTAATATT TTTAAGCAGT GTAGAAAAGG   
  
  
- ACAGGTGGAC CGCATTGTTT GAATGAGTCA AAGACCTTTT TACCCTTCAT ATATTCAACA ATTTCTTCAC   
  
  
- CACCTCACGT GATTTGCCAT CAATGTCTTA CACTCCTTCC ACACGGAAGG ATTCTCCTTC TTCCCATTCT   
  
  
- TCAGAAATTA AGACGGAAAT GGTAATTGC
