# Supplementary material for: Genome-wide analysis of the WRKY gene family in drumstick (Moringa oleifera Lam.)
Source: PeerJ. 2019 Jun 10;7:e7063. doi: 10.7717/peerj.7063 (PMC6563795; doi:10.7717/peerj.7063)
Supplement: Supplemental Information 1 [file peerj-07-7063-s003.gz › MoWRKY34_plantcare.html]

Content-Type: text/html; charset=ISO-8859-1


CallMat\_Firefox


Webmaster Firefox specific output  
To save the result:
click on the frame with the right mouse button and save the source code as a text file with extension .html  
REFERENCE:PlantCARE: a database of plant cis-acting regulatory elements and a portal to tools for in silico analysis of promoter sequences.  
Lescot, M., Déhais, P., Moreau, Y., De Moor, B., Rouzé ,P.,and Rombauts, S.  
Nucleic Acids Res., Database issue(2002), 30(1):325-327.   


---

> 2018/04/13 10:10:12  
+ TTCGATTTTG TAAAAAAGTT AAATTCTTAT TTTTTTAATA ATTTATCCAT AAAAATAAAG TGAAAAATTA   
  
  
+ AAACTATTCA GTGCTGATAT TTTGTAAAAC AAATAGATAT CCAGTCAATA TGAAACATCT ATACTGTGCT   
  
  
+ TAGTCATGGA CACGTGTTTT AATCTAATGA TTATGTAAAA CGATCACTGA TAAGTCAAAC ATCATTATAT   
  
  
+ TACGAAAAAT GGAACATGAA TAAAGACAAA TGGAACTGCA CATGTTAACT CGTAAACTGC GAACCATCTG   
  
  
+ TCTAACCAGG CTCAAGAGTT AAGAAAGAGA ACGCTGTCGT TTCGGAAGAA TACCAGGAGA GAAAAAAAAA   
  
  
+ AAAAAAACTC TTGAGTTTGT TAATATCGAC ACAAATGTAC GTATGTCGCC CAGAAAAGAA GATGAGAATA   
  
  
+ AAAACCAATA AACTTTCTTA GGATTGCACA TGTAAGGTTA ATTATGTAAA TTAATGAATC ATTAACACTG   
  
  
+ CGGCTTTCTA GACGAGCAGC ATGCAGTACA AAGCATACGT GTTACGCGAG TCACATGGGT GATCCATCGC   
  
  
+ CATCAGCGTA AGCAAGGGGT TTGACATGAC GTCATCATCT TACGTAAGCT ACCCCAATCT CTGCGCCAGA   
  
  
+ CGAGTAGTGA GTAGCTACGC GGAAGAAGTT GGGAAGATTC TCCCGTCTCT TCCTCTGGCC CTCTCTTTTT   
  
  
+ CTTCTTGTCT TCCTTGAATT TGTGTTTCCA CTCTTCCAGT CTTCAAATTG ATTCAACACT GCCCGAGAGA   
  
  
+ GAGAGAGAGA GAGTTTTGAA TTGGTCAGCA GTTGGACCTT TCTCACTCTC TTTTATTTTC TTTTTCAATT   
  
  
+ TCTTTTTCTT CTGTAAGCCT TATGTATATA TAATAGGAAA GAGATCCTGA TCCTCAGCGA GGAAACAATC   
  
  
+ TGAGTTTTTC TTTTGTCCCG GGTCAAAACG CCTCTTTTTC CCTATCATTT CTCTTGCATA TTCTCCTCTC   
  
  
+ CTTCTTCCCA ACTTCTTCAT TAAGACAGGC ATAGGCCTTT TCACTTTCCT TGCATTCAAA ACTTTACCAT   
  
  
+ TTCTCTCTCT CTCTCTCTCT CTCTCTCTCT TCAGGCTTAT ATTTCCCTTT ATGTACGCAT ATTGCCCATC   
  
  
+ AACTCTCCAC TTCCAGTATT TGGTGCTGGT GGAGGCTCTT GATCTCCCTT TTTGGTAATT ACTTGTCCAA   
  
  
+ GCCACTGATC TCAGTGAGCT ATTCTTCTTA TCGATCTTGA TCTCTTTGAT CAAACTCACT TTTGATTTCA   
  
  
+ ACACCCTTGC TCTTGATTCT TCATCTCTCA GCTTTTTACG CTATTCATCC CCTTATGCAG TTCCCCTTAA   
  
  
+ TTTCTCCTCT AGGCCTTAAA GGGTTTCTTC TCGACATGGT CTAGTGTGCA TTCGATCCAT TAGTCTGCTT   
  
  
+ TGTAAGTTAG TTCGCACGTA CCCACATAAG CTACAACTGT GTCGCTGCAA TTCTAAGCAT TGTTGGGTTC   
  
  
+ TCTCTTGCGT ACTGCATATT AGAGGAGGC  

- AAGCTAAAAC ATTTTTTCAA TTTAAGAATA AAAAAATTAT TAAATAGGTA TTTTTATTTC ACTTTTTAAT   
  
  
- TTTGATAAGT CACGACTATA AAACATTTTG TTTATCTATA GGTCAGTTAT ACTTTGTAGA TATGACACGA   
  
  
- ATCAGTACCT GTGCACAAAA TTAGATTACT AATACATTTT GCTAGTGACT ATTCAGTTTG TAGTAATATA   
  
  
- ATGCTTTTTA CCTTGTACTT ATTTCTGTTT ACCTTGACGT GTACAATTGA GCATTTGACG CTTGGTAGAC   
  
  
- AGATTGGTCC GAGTTCTCAA TTCTTTCTCT TGCGACAGCA AAGCCTTCTT ATGGTCCTCT CTTTTTTTTT   
  
  
- TTTTTTTGAG AACTCAAACA ATTATAGCTG TGTTTACATG CATACAGCGG GTCTTTTCTT CTACTCTTAT   
  
  
- TTTTGGTTAT TTGAAAGAAT CCTAACGTGT ACATTCCAAT TAATACATTT AATTACTTAG TAATTGTGAC   
  
  
- GCCGAAAGAT CTGCTCGTCG TACGTCATGT TTCGTATGCA CAATGCGCTC AGTGTACCCA CTAGGTAGCG   
  
  
- GTAGTCGCAT TCGTTCCCCA AACTGTACTG CAGTAGTAGA ATGCATTCGA TGGGGTTAGA GACGCGGTCT   
  
  
- GCTCATCACT CATCGATGCG CCTTCTTCAA CCCTTCTAAG AGGGCAGAGA AGGAGACCGG GAGAGAAAAA   
  
  
- GAAGAACAGA AGGAACTTAA ACACAAAGGT GAGAAGGTCA GAAGTTTAAC TAAGTTGTGA CGGGCTCTCT   
  
  
- CTCTCTCTCT CTCAAAACTT AACCAGTCGT CAACCTGGAA AGAGTGAGAG AAAATAAAAG AAAAAGTTAA   
  
  
- AGAAAAAGAA GACATTCGGA ATACATATAT ATTATCCTTT CTCTAGGACT AGGAGTCGCT CCTTTGTTAG   
  
  
- ACTCAAAAAG AAAACAGGGC CCAGTTTTGC GGAGAAAAAG GGATAGTAAA GAGAACGTAT AAGAGGAGAG   
  
  
- GAAGAAGGGT TGAAGAAGTA ATTCTGTCCG TATCCGGAAA AGTGAAAGGA ACGTAAGTTT TGAAATGGTA   
  
  
- AAGAGAGAGA GAGAGAGAGA GAGAGAGAGA AGTCCGAATA TAAAGGGAAA TACATGCGTA TAACGGGTAG   
  
  
- TTGAGAGGTG AAGGTCATAA ACCACGACCA CCTCCGAGAA CTAGAGGGAA AAACCATTAA TGAACAGGTT   
  
  
- CGGTGACTAG AGTCACTCGA TAAGAAGAAT AGCTAGAACT AGAGAAACTA GTTTGAGTGA AAACTAAAGT   
  
  
- TGTGGGAACG AGAACTAAGA AGTAGAGAGT CGAAAAATGC GATAAGTAGG GGAATACGTC AAGGGGAATT   
  
  
- AAAGAGGAGA TCCGGAATTT CCCAAAGAAG AGCTGTACCA GATCACACGT AAGCTAGGTA ATCAGACGAA   
  
  
- ACATTCAATC AAGCGTGCAT GGGTGTATTC GATGTTGACA CAGCGACGTT AAGATTCGTA ACAACCCAAG   
  
  
- AGAGAACGCA TGACGTATAA TCTCCTCCG

  
  
Motifs Found  

+     5UTR Py-rich stretch

| Site Name | Organism | Position | Strand | Matrix score. | sequence | function |
| --- | --- | --- | --- | --- | --- | --- |
| 5UTR Py-rich stretch | Lycopersicon esculentum | 1050 | + | 14 | TTTCTCTCTCTCTC | cis-acting element conferring high transcription levels |
| 5UTR Py-rich stretch | Lycopersicon esculentum | 845 | + | 9 | TTTCTTCTCT | cis-acting element conferring high transcription levels |
| 5UTR Py-rich stretch | Lycopersicon esculentum | 1056 | + | 13 | TTTCTCTCTCTCTC | cis-acting element conferring high transcription levels |
| 5UTR Py-rich stretch | Lycopersicon esculentum | 1058 | + | 13 | TTTCTCTCTCTCTC | cis-acting element conferring high transcription levels |
| 5UTR Py-rich stretch | Lycopersicon esculentum | 1054 | + | 13 | TTTCTCTCTCTCTC | cis-acting element conferring high transcription levels |
| 5UTR Py-rich stretch | Lycopersicon esculentum | 1064 | + | 13 | TTTCTCTCTCTCTC | cis-acting element conferring high transcription levels |
| 5UTR Py-rich stretch | Lycopersicon esculentum | 338 | - | 9 | TTTCTTCTCT | cis-acting element conferring high transcription levels |
| 5UTR Py-rich stretch | Lycopersicon esculentum | 765 | - | 13 | TTTCTCTCTCTCTC | cis-acting element conferring high transcription levels |
| 5UTR Py-rich stretch | Lycopersicon esculentum | 1052 | + | 13 | TTTCTCTCTCTCTC | cis-acting element conferring high transcription levels |
| 5UTR Py-rich stretch | Lycopersicon esculentum | 1060 | + | 13 | TTTCTCTCTCTCTC | cis-acting element conferring high transcription levels |
| 5UTR Py-rich stretch | Lycopersicon esculentum | 767 | - | 13 | TTTCTCTCTCTCTC | cis-acting element conferring high transcription levels |
| 5UTR Py-rich stretch | Lycopersicon esculentum | 1354 | + | 9 | TTTCTTCTCT | cis-acting element conferring high transcription levels |
| 5UTR Py-rich stretch | Lycopersicon esculentum | 1331 | + | 9 | TTTCTTCTCT | cis-acting element conferring high transcription levels |
| 5UTR Py-rich stretch | Lycopersicon esculentum | 769 | - | 13 | TTTCTCTCTCTCTC | cis-acting element conferring high transcription levels |
| 5UTR Py-rich stretch | Lycopersicon esculentum | 1066 | + | 13 | TTTCTCTCTCTCTC | cis-acting element conferring high transcription levels |
| 5UTR Py-rich stretch | Lycopersicon esculentum | 1062 | + | 13 | TTTCTCTCTCTCTC | cis-acting element conferring high transcription levels |

> 2018/04/13 10:10:12  
+ TTCGATTTTG TAAAAAAGTT AAATTCTTAT TTTTTTAATA ATTTATCCAT AAAAATAAAG TGAAAAATTA   
  
  
+ AAACTATTCA GTGCTGATAT TTTGTAAAAC AAATAGATAT CCAGTCAATA TGAAACATCT ATACTGTGCT   
  
  
+ TAGTCATGGA CACGTGTTTT AATCTAATGA TTATGTAAAA CGATCACTGA TAAGTCAAAC ATCATTATAT   
  
  
+ TACGAAAAAT GGAACATGAA TAAAGACAAA TGGAACTGCA CATGTTAACT CGTAAACTGC GAACCATCTG   
  
  
+ TCTAACCAGG CTCAAGAGTT AAGAAAGAGA ACGCTGTCGT TTCGGAAGAA TACCAGGAGA GAAAAAAAAA   
  
  
+ AAAAAAACTC TTGAGTTTGT TAATATCGAC ACAAATGTAC GTATGTCGCC CAGAAAAGAA GATGAGAATA   
  
  
+ AAAACCAATA AACTTTCTTA GGATTGCACA TGTAAGGTTA ATTATGTAAA TTAATGAATC ATTAACACTG   
  
  
+ CGGCTTTCTA GACGAGCAGC ATGCAGTACA AAGCATACGT GTTACGCGAG TCACATGGGT GATCCATCGC   
  
  
+ CATCAGCGTA AGCAAGGGGT TTGACATGAC GTCATCATCT TACGTAAGCT ACCCCAATCT CTGCGCCAGA   
  
  
+ CGAGTAGTGA GTAGCTACGC GGAAGAAGTT GGGAAGATTC TCCCGTCTCT TCCTCTGGCC CTCTCTTTTT   
  
  
+ CTTCTTGTCT TCCTTGAATT TGTGTTTCCA CTCTTCCAGT CTTCAAATTG ATTCAACACT GCCCGAGAGA   
  
  
+ GAGAGAGAGA GAGTTTTGAA TTGGTCAGCA GTTGGACCTT TCTCACTCTC TTTTATTTTC TTTTTCAATT   
  
  
+ TCTTTTTCTT CTGTAAGCCT TATGTATATA TAATAGGAAA GAGATCCTGA TCCTCAGCGA GGAAACAATC   
  
  
+ TGAGTTTTTC TTTTGTCCCG GGTCAAAACG CCTCTTTTTC CCTATCATTT CTCTTGCATA TTCTCCTCTC   
  
  
+ CTTCTTCCCA ACTTCTTCAT TAAGACAGGC ATAGGCCTTT TCACTTTCCT TGCATTCAAA ACTTTACCAT   
  
  
+ TTCTCTCTCT CTCTCTCTCT CTCTCTCTCT TCAGGCTTAT ATTTCCCTTT ATGTACGCAT ATTGCCCATC   
  
  
+ AACTCTCCAC TTCCAGTATT TGGTGCTGGT GGAGGCTCTT GATCTCCCTT TTTGGTAATT ACTTGTCCAA   
  
  
+ GCCACTGATC TCAGTGAGCT ATTCTTCTTA TCGATCTTGA TCTCTTTGAT CAAACTCACT TTTGATTTCA   
  
  
+ ACACCCTTGC TCTTGATTCT TCATCTCTCA GCTTTTTACG CTATTCATCC CCTTATGCAG TTCCCCTTAA   
  
  
+ TTTCTCCTCT AGGCCTTAAA GGGTTTCTTC TCGACATGGT CTAGTGTGCA TTCGATCCAT TAGTCTGCTT   
  
  
+ TGTAAGTTAG TTCGCACGTA CCCACATAAG CTACAACTGT GTCGCTGCAA TTCTAAGCAT TGTTGGGTTC   
  
  
+ TCTCTTGCGT ACTGCATATT AGAGGAGGC  

- AAGCTAAAAC ATTTTTTCAA TTTAAGAATA AAAAAATTAT TAAATAGGTA TTTTTATTTC ACTTTTTAAT   
  
  
- TTTGATAAGT CACGACTATA AAACATTTTG TTTATCTATA GGTCAGTTAT ACTTTGTAGA TATGACACGA   
  
  
- ATCAGTACCT GTGCACAAAA TTAGATTACT AATACATTTT GCTAGTGACT ATTCAGTTTG TAGTAATATA   
  
  
- ATGCTTTTTA CCTTGTACTT ATTTCTGTTT ACCTTGACGT GTACAATTGA GCATTTGACG CTTGGTAGAC   
  
  
- AGATTGGTCC GAGTTCTCAA TTCTTTCTCT TGCGACAGCA AAGCCTTCTT ATGGTCCTCT CTTTTTTTTT   
  
  
- TTTTTTTGAG AACTCAAACA ATTATAGCTG TGTTTACATG CATACAGCGG GTCTTTTCTT CTACTCTTAT   
  
  
- TTTTGGTTAT TTGAAAGAAT CCTAACGTGT ACATTCCAAT TAATACATTT AATTACTTAG TAATTGTGAC   
  
  
- GCCGAAAGAT CTGCTCGTCG TACGTCATGT TTCGTATGCA CAATGCGCTC AGTGTACCCA CTAGGTAGCG   
  
  
- GTAGTCGCAT TCGTTCCCCA AACTGTACTG CAGTAGTAGA ATGCATTCGA TGGGGTTAGA GACGCGGTCT   
  
  
- GCTCATCACT CATCGATGCG CCTTCTTCAA CCCTTCTAAG AGGGCAGAGA AGGAGACCGG GAGAGAAAAA   
  
  
- GAAGAACAGA AGGAACTTAA ACACAAAGGT GAGAAGGTCA GAAGTTTAAC TAAGTTGTGA CGGGCTCTCT   
  
  
- CTCTCTCTCT CTCAAAACTT AACCAGTCGT CAACCTGGAA AGAGTGAGAG AAAATAAAAG AAAAAGTTAA   
  
  
- AGAAAAAGAA GACATTCGGA ATACATATAT ATTATCCTTT CTCTAGGACT AGGAGTCGCT CCTTTGTTAG   
  
  
- ACTCAAAAAG AAAACAGGGC CCAGTTTTGC GGAGAAAAAG GGATAGTAAA GAGAACGTAT AAGAGGAGAG   
  
  
- GAAGAAGGGT TGAAGAAGTA ATTCTGTCCG TATCCGGAAA AGTGAAAGGA ACGTAAGTTT TGAAATGGTA   
  
  
- AAGAGAGAGA GAGAGAGAGA GAGAGAGAGA AGTCCGAATA TAAAGGGAAA TACATGCGTA TAACGGGTAG   
  
  
- TTGAGAGGTG AAGGTCATAA ACCACGACCA CCTCCGAGAA CTAGAGGGAA AAACCATTAA TGAACAGGTT   
  
  
- CGGTGACTAG AGTCACTCGA TAAGAAGAAT AGCTAGAACT AGAGAAACTA GTTTGAGTGA AAACTAAAGT   
  
  
- TGTGGGAACG AGAACTAAGA AGTAGAGAGT CGAAAAATGC GATAAGTAGG GGAATACGTC AAGGGGAATT   
  
  
- AAAGAGGAGA TCCGGAATTT CCCAAAGAAG AGCTGTACCA GATCACACGT AAGCTAGGTA ATCAGACGAA   
  
  
- ACATTCAATC AAGCGTGCAT GGGTGTATTC GATGTTGACA CAGCGACGTT AAGATTCGTA ACAACCCAAG   
  
  
- AGAGAACGCA TGACGTATAA TCTCCTCCG

+     AACA\_motif

| Site Name | Organism | Position | Strand | Matrix score. | sequence | function |
| --- | --- | --- | --- | --- | --- | --- |
| AACA\_motif | Oryza sativa | 361 | - | 11 | TAACAAACTCCA | involved in endosperm-specific negative expression |

> 2018/04/13 10:10:12  
+ TTCGATTTTG TAAAAAAGTT AAATTCTTAT TTTTTTAATA ATTTATCCAT AAAAATAAAG TGAAAAATTA   
  
  
+ AAACTATTCA GTGCTGATAT TTTGTAAAAC AAATAGATAT CCAGTCAATA TGAAACATCT ATACTGTGCT   
  
  
+ TAGTCATGGA CACGTGTTTT AATCTAATGA TTATGTAAAA CGATCACTGA TAAGTCAAAC ATCATTATAT   
  
  
+ TACGAAAAAT GGAACATGAA TAAAGACAAA TGGAACTGCA CATGTTAACT CGTAAACTGC GAACCATCTG   
  
  
+ TCTAACCAGG CTCAAGAGTT AAGAAAGAGA ACGCTGTCGT TTCGGAAGAA TACCAGGAGA GAAAAAAAAA   
  
  
+ AAAAAAACTC TTGAGTTTGT TAATATCGAC ACAAATGTAC GTATGTCGCC CAGAAAAGAA GATGAGAATA   
  
  
+ AAAACCAATA AACTTTCTTA GGATTGCACA TGTAAGGTTA ATTATGTAAA TTAATGAATC ATTAACACTG   
  
  
+ CGGCTTTCTA GACGAGCAGC ATGCAGTACA AAGCATACGT GTTACGCGAG TCACATGGGT GATCCATCGC   
  
  
+ CATCAGCGTA AGCAAGGGGT TTGACATGAC GTCATCATCT TACGTAAGCT ACCCCAATCT CTGCGCCAGA   
  
  
+ CGAGTAGTGA GTAGCTACGC GGAAGAAGTT GGGAAGATTC TCCCGTCTCT TCCTCTGGCC CTCTCTTTTT   
  
  
+ CTTCTTGTCT TCCTTGAATT TGTGTTTCCA CTCTTCCAGT CTTCAAATTG ATTCAACACT GCCCGAGAGA   
  
  
+ GAGAGAGAGA GAGTTTTGAA TTGGTCAGCA GTTGGACCTT TCTCACTCTC TTTTATTTTC TTTTTCAATT   
  
  
+ TCTTTTTCTT CTGTAAGCCT TATGTATATA TAATAGGAAA GAGATCCTGA TCCTCAGCGA GGAAACAATC   
  
  
+ TGAGTTTTTC TTTTGTCCCG GGTCAAAACG CCTCTTTTTC CCTATCATTT CTCTTGCATA TTCTCCTCTC   
  
  
+ CTTCTTCCCA ACTTCTTCAT TAAGACAGGC ATAGGCCTTT TCACTTTCCT TGCATTCAAA ACTTTACCAT   
  
  
+ TTCTCTCTCT CTCTCTCTCT CTCTCTCTCT TCAGGCTTAT ATTTCCCTTT ATGTACGCAT ATTGCCCATC   
  
  
+ AACTCTCCAC TTCCAGTATT TGGTGCTGGT GGAGGCTCTT GATCTCCCTT TTTGGTAATT ACTTGTCCAA   
  
  
+ GCCACTGATC TCAGTGAGCT ATTCTTCTTA TCGATCTTGA TCTCTTTGAT CAAACTCACT TTTGATTTCA   
  
  
+ ACACCCTTGC TCTTGATTCT TCATCTCTCA GCTTTTTACG CTATTCATCC CCTTATGCAG TTCCCCTTAA   
  
  
+ TTTCTCCTCT AGGCCTTAAA GGGTTTCTTC TCGACATGGT CTAGTGTGCA TTCGATCCAT TAGTCTGCTT   
  
  
+ TGTAAGTTAG TTCGCACGTA CCCACATAAG CTACAACTGT GTCGCTGCAA TTCTAAGCAT TGTTGGGTTC   
  
  
+ TCTCTTGCGT ACTGCATATT AGAGGAGGC  

- AAGCTAAAAC ATTTTTTCAA TTTAAGAATA AAAAAATTAT TAAATAGGTA TTTTTATTTC ACTTTTTAAT   
  
  
- TTTGATAAGT CACGACTATA AAACATTTTG TTTATCTATA GGTCAGTTAT ACTTTGTAGA TATGACACGA   
  
  
- ATCAGTACCT GTGCACAAAA TTAGATTACT AATACATTTT GCTAGTGACT ATTCAGTTTG TAGTAATATA   
  
  
- ATGCTTTTTA CCTTGTACTT ATTTCTGTTT ACCTTGACGT GTACAATTGA GCATTTGACG CTTGGTAGAC   
  
  
- AGATTGGTCC GAGTTCTCAA TTCTTTCTCT TGCGACAGCA AAGCCTTCTT ATGGTCCTCT CTTTTTTTTT   
  
  
- TTTTTTTGAG AACTCAAACA ATTATAGCTG TGTTTACATG CATACAGCGG GTCTTTTCTT CTACTCTTAT   
  
  
- TTTTGGTTAT TTGAAAGAAT CCTAACGTGT ACATTCCAAT TAATACATTT AATTACTTAG TAATTGTGAC   
  
  
- GCCGAAAGAT CTGCTCGTCG TACGTCATGT TTCGTATGCA CAATGCGCTC AGTGTACCCA CTAGGTAGCG   
  
  
- GTAGTCGCAT TCGTTCCCCA AACTGTACTG CAGTAGTAGA ATGCATTCGA TGGGGTTAGA GACGCGGTCT   
  
  
- GCTCATCACT CATCGATGCG CCTTCTTCAA CCCTTCTAAG AGGGCAGAGA AGGAGACCGG GAGAGAAAAA   
  
  
- GAAGAACAGA AGGAACTTAA ACACAAAGGT GAGAAGGTCA GAAGTTTAAC TAAGTTGTGA CGGGCTCTCT   
  
  
- CTCTCTCTCT CTCAAAACTT AACCAGTCGT CAACCTGGAA AGAGTGAGAG AAAATAAAAG AAAAAGTTAA   
  
  
- AGAAAAAGAA GACATTCGGA ATACATATAT ATTATCCTTT CTCTAGGACT AGGAGTCGCT CCTTTGTTAG   
  
  
- ACTCAAAAAG AAAACAGGGC CCAGTTTTGC GGAGAAAAAG GGATAGTAAA GAGAACGTAT AAGAGGAGAG   
  
  
- GAAGAAGGGT TGAAGAAGTA ATTCTGTCCG TATCCGGAAA AGTGAAAGGA ACGTAAGTTT TGAAATGGTA   
  
  
- AAGAGAGAGA GAGAGAGAGA GAGAGAGAGA AGTCCGAATA TAAAGGGAAA TACATGCGTA TAACGGGTAG   
  
  
- TTGAGAGGTG AAGGTCATAA ACCACGACCA CCTCCGAGAA CTAGAGGGAA AAACCATTAA TGAACAGGTT   
  
  
- CGGTGACTAG AGTCACTCGA TAAGAAGAAT AGCTAGAACT AGAGAAACTA GTTTGAGTGA AAACTAAAGT   
  
  
- TGTGGGAACG AGAACTAAGA AGTAGAGAGT CGAAAAATGC GATAAGTAGG GGAATACGTC AAGGGGAATT   
  
  
- AAAGAGGAGA TCCGGAATTT CCCAAAGAAG AGCTGTACCA GATCACACGT AAGCTAGGTA ATCAGACGAA   
  
  
- ACATTCAATC AAGCGTGCAT GGGTGTATTC GATGTTGACA CAGCGACGTT AAGATTCGTA ACAACCCAAG   
  
  
- AGAGAACGCA TGACGTATAA TCTCCTCCG

+     ABRE

| Site Name | Organism | Position | Strand | Matrix score. | sequence | function |
| --- | --- | --- | --- | --- | --- | --- |
| ABRE | Arabidopsis thaliana | 526 | + | 6 | TACGTG | cis-acting element involved in the abscisic acid responsiveness |
| ABRE | Arabidopsis thaliana | 1415 | - | 6 | TACGTG | cis-acting element involved in the abscisic acid responsiveness |
| ABRE | Arabidopsis thaliana | 151 | + | 6 | CACGTG | cis-acting element involved in the abscisic acid responsiveness |

> 2018/04/13 10:10:12  
+ TTCGATTTTG TAAAAAAGTT AAATTCTTAT TTTTTTAATA ATTTATCCAT AAAAATAAAG TGAAAAATTA   
  
  
+ AAACTATTCA GTGCTGATAT TTTGTAAAAC AAATAGATAT CCAGTCAATA TGAAACATCT ATACTGTGCT   
  
  
+ TAGTCATGGA CACGTGTTTT AATCTAATGA TTATGTAAAA CGATCACTGA TAAGTCAAAC ATCATTATAT   
  
  
+ TACGAAAAAT GGAACATGAA TAAAGACAAA TGGAACTGCA CATGTTAACT CGTAAACTGC GAACCATCTG   
  
  
+ TCTAACCAGG CTCAAGAGTT AAGAAAGAGA ACGCTGTCGT TTCGGAAGAA TACCAGGAGA GAAAAAAAAA   
  
  
+ AAAAAAACTC TTGAGTTTGT TAATATCGAC ACAAATGTAC GTATGTCGCC CAGAAAAGAA GATGAGAATA   
  
  
+ AAAACCAATA AACTTTCTTA GGATTGCACA TGTAAGGTTA ATTATGTAAA TTAATGAATC ATTAACACTG   
  
  
+ CGGCTTTCTA GACGAGCAGC ATGCAGTACA AAGCATACGT GTTACGCGAG TCACATGGGT GATCCATCGC   
  
  
+ CATCAGCGTA AGCAAGGGGT TTGACATGAC GTCATCATCT TACGTAAGCT ACCCCAATCT CTGCGCCAGA   
  
  
+ CGAGTAGTGA GTAGCTACGC GGAAGAAGTT GGGAAGATTC TCCCGTCTCT TCCTCTGGCC CTCTCTTTTT   
  
  
+ CTTCTTGTCT TCCTTGAATT TGTGTTTCCA CTCTTCCAGT CTTCAAATTG ATTCAACACT GCCCGAGAGA   
  
  
+ GAGAGAGAGA GAGTTTTGAA TTGGTCAGCA GTTGGACCTT TCTCACTCTC TTTTATTTTC TTTTTCAATT   
  
  
+ TCTTTTTCTT CTGTAAGCCT TATGTATATA TAATAGGAAA GAGATCCTGA TCCTCAGCGA GGAAACAATC   
  
  
+ TGAGTTTTTC TTTTGTCCCG GGTCAAAACG CCTCTTTTTC CCTATCATTT CTCTTGCATA TTCTCCTCTC   
  
  
+ CTTCTTCCCA ACTTCTTCAT TAAGACAGGC ATAGGCCTTT TCACTTTCCT TGCATTCAAA ACTTTACCAT   
  
  
+ TTCTCTCTCT CTCTCTCTCT CTCTCTCTCT TCAGGCTTAT ATTTCCCTTT ATGTACGCAT ATTGCCCATC   
  
  
+ AACTCTCCAC TTCCAGTATT TGGTGCTGGT GGAGGCTCTT GATCTCCCTT TTTGGTAATT ACTTGTCCAA   
  
  
+ GCCACTGATC TCAGTGAGCT ATTCTTCTTA TCGATCTTGA TCTCTTTGAT CAAACTCACT TTTGATTTCA   
  
  
+ ACACCCTTGC TCTTGATTCT TCATCTCTCA GCTTTTTACG CTATTCATCC CCTTATGCAG TTCCCCTTAA   
  
  
+ TTTCTCCTCT AGGCCTTAAA GGGTTTCTTC TCGACATGGT CTAGTGTGCA TTCGATCCAT TAGTCTGCTT   
  
  
+ TGTAAGTTAG TTCGCACGTA CCCACATAAG CTACAACTGT GTCGCTGCAA TTCTAAGCAT TGTTGGGTTC   
  
  
+ TCTCTTGCGT ACTGCATATT AGAGGAGGC  

- AAGCTAAAAC ATTTTTTCAA TTTAAGAATA AAAAAATTAT TAAATAGGTA TTTTTATTTC ACTTTTTAAT   
  
  
- TTTGATAAGT CACGACTATA AAACATTTTG TTTATCTATA GGTCAGTTAT ACTTTGTAGA TATGACACGA   
  
  
- ATCAGTACCT GTGCACAAAA TTAGATTACT AATACATTTT GCTAGTGACT ATTCAGTTTG TAGTAATATA   
  
  
- ATGCTTTTTA CCTTGTACTT ATTTCTGTTT ACCTTGACGT GTACAATTGA GCATTTGACG CTTGGTAGAC   
  
  
- AGATTGGTCC GAGTTCTCAA TTCTTTCTCT TGCGACAGCA AAGCCTTCTT ATGGTCCTCT CTTTTTTTTT   
  
  
- TTTTTTTGAG AACTCAAACA ATTATAGCTG TGTTTACATG CATACAGCGG GTCTTTTCTT CTACTCTTAT   
  
  
- TTTTGGTTAT TTGAAAGAAT CCTAACGTGT ACATTCCAAT TAATACATTT AATTACTTAG TAATTGTGAC   
  
  
- GCCGAAAGAT CTGCTCGTCG TACGTCATGT TTCGTATGCA CAATGCGCTC AGTGTACCCA CTAGGTAGCG   
  
  
- GTAGTCGCAT TCGTTCCCCA AACTGTACTG CAGTAGTAGA ATGCATTCGA TGGGGTTAGA GACGCGGTCT   
  
  
- GCTCATCACT CATCGATGCG CCTTCTTCAA CCCTTCTAAG AGGGCAGAGA AGGAGACCGG GAGAGAAAAA   
  
  
- GAAGAACAGA AGGAACTTAA ACACAAAGGT GAGAAGGTCA GAAGTTTAAC TAAGTTGTGA CGGGCTCTCT   
  
  
- CTCTCTCTCT CTCAAAACTT AACCAGTCGT CAACCTGGAA AGAGTGAGAG AAAATAAAAG AAAAAGTTAA   
  
  
- AGAAAAAGAA GACATTCGGA ATACATATAT ATTATCCTTT CTCTAGGACT AGGAGTCGCT CCTTTGTTAG   
  
  
- ACTCAAAAAG AAAACAGGGC CCAGTTTTGC GGAGAAAAAG GGATAGTAAA GAGAACGTAT AAGAGGAGAG   
  
  
- GAAGAAGGGT TGAAGAAGTA ATTCTGTCCG TATCCGGAAA AGTGAAAGGA ACGTAAGTTT TGAAATGGTA   
  
  
- AAGAGAGAGA GAGAGAGAGA GAGAGAGAGA AGTCCGAATA TAAAGGGAAA TACATGCGTA TAACGGGTAG   
  
  
- TTGAGAGGTG AAGGTCATAA ACCACGACCA CCTCCGAGAA CTAGAGGGAA AAACCATTAA TGAACAGGTT   
  
  
- CGGTGACTAG AGTCACTCGA TAAGAAGAAT AGCTAGAACT AGAGAAACTA GTTTGAGTGA AAACTAAAGT   
  
  
- TGTGGGAACG AGAACTAAGA AGTAGAGAGT CGAAAAATGC GATAAGTAGG GGAATACGTC AAGGGGAATT   
  
  
- AAAGAGGAGA TCCGGAATTT CCCAAAGAAG AGCTGTACCA GATCACACGT AAGCTAGGTA ATCAGACGAA   
  
  
- ACATTCAATC AAGCGTGCAT GGGTGTATTC GATGTTGACA CAGCGACGTT AAGATTCGTA ACAACCCAAG   
  
  
- AGAGAACGCA TGACGTATAA TCTCCTCCG

+     ACE

| Site Name | Organism | Position | Strand | Matrix score. | sequence | function |
| --- | --- | --- | --- | --- | --- | --- |
| ACE | Petroselinum crispum | 524 | - | 9 | GACACGTATG | cis-acting element involved in light responsiveness |

> 2018/04/13 10:10:12  
+ TTCGATTTTG TAAAAAAGTT AAATTCTTAT TTTTTTAATA ATTTATCCAT AAAAATAAAG TGAAAAATTA   
  
  
+ AAACTATTCA GTGCTGATAT TTTGTAAAAC AAATAGATAT CCAGTCAATA TGAAACATCT ATACTGTGCT   
  
  
+ TAGTCATGGA CACGTGTTTT AATCTAATGA TTATGTAAAA CGATCACTGA TAAGTCAAAC ATCATTATAT   
  
  
+ TACGAAAAAT GGAACATGAA TAAAGACAAA TGGAACTGCA CATGTTAACT CGTAAACTGC GAACCATCTG   
  
  
+ TCTAACCAGG CTCAAGAGTT AAGAAAGAGA ACGCTGTCGT TTCGGAAGAA TACCAGGAGA GAAAAAAAAA   
  
  
+ AAAAAAACTC TTGAGTTTGT TAATATCGAC ACAAATGTAC GTATGTCGCC CAGAAAAGAA GATGAGAATA   
  
  
+ AAAACCAATA AACTTTCTTA GGATTGCACA TGTAAGGTTA ATTATGTAAA TTAATGAATC ATTAACACTG   
  
  
+ CGGCTTTCTA GACGAGCAGC ATGCAGTACA AAGCATACGT GTTACGCGAG TCACATGGGT GATCCATCGC   
  
  
+ CATCAGCGTA AGCAAGGGGT TTGACATGAC GTCATCATCT TACGTAAGCT ACCCCAATCT CTGCGCCAGA   
  
  
+ CGAGTAGTGA GTAGCTACGC GGAAGAAGTT GGGAAGATTC TCCCGTCTCT TCCTCTGGCC CTCTCTTTTT   
  
  
+ CTTCTTGTCT TCCTTGAATT TGTGTTTCCA CTCTTCCAGT CTTCAAATTG ATTCAACACT GCCCGAGAGA   
  
  
+ GAGAGAGAGA GAGTTTTGAA TTGGTCAGCA GTTGGACCTT TCTCACTCTC TTTTATTTTC TTTTTCAATT   
  
  
+ TCTTTTTCTT CTGTAAGCCT TATGTATATA TAATAGGAAA GAGATCCTGA TCCTCAGCGA GGAAACAATC   
  
  
+ TGAGTTTTTC TTTTGTCCCG GGTCAAAACG CCTCTTTTTC CCTATCATTT CTCTTGCATA TTCTCCTCTC   
  
  
+ CTTCTTCCCA ACTTCTTCAT TAAGACAGGC ATAGGCCTTT TCACTTTCCT TGCATTCAAA ACTTTACCAT   
  
  
+ TTCTCTCTCT CTCTCTCTCT CTCTCTCTCT TCAGGCTTAT ATTTCCCTTT ATGTACGCAT ATTGCCCATC   
  
  
+ AACTCTCCAC TTCCAGTATT TGGTGCTGGT GGAGGCTCTT GATCTCCCTT TTTGGTAATT ACTTGTCCAA   
  
  
+ GCCACTGATC TCAGTGAGCT ATTCTTCTTA TCGATCTTGA TCTCTTTGAT CAAACTCACT TTTGATTTCA   
  
  
+ ACACCCTTGC TCTTGATTCT TCATCTCTCA GCTTTTTACG CTATTCATCC CCTTATGCAG TTCCCCTTAA   
  
  
+ TTTCTCCTCT AGGCCTTAAA GGGTTTCTTC TCGACATGGT CTAGTGTGCA TTCGATCCAT TAGTCTGCTT   
  
  
+ TGTAAGTTAG TTCGCACGTA CCCACATAAG CTACAACTGT GTCGCTGCAA TTCTAAGCAT TGTTGGGTTC   
  
  
+ TCTCTTGCGT ACTGCATATT AGAGGAGGC  

- AAGCTAAAAC ATTTTTTCAA TTTAAGAATA AAAAAATTAT TAAATAGGTA TTTTTATTTC ACTTTTTAAT   
  
  
- TTTGATAAGT CACGACTATA AAACATTTTG TTTATCTATA GGTCAGTTAT ACTTTGTAGA TATGACACGA   
  
  
- ATCAGTACCT GTGCACAAAA TTAGATTACT AATACATTTT GCTAGTGACT ATTCAGTTTG TAGTAATATA   
  
  
- ATGCTTTTTA CCTTGTACTT ATTTCTGTTT ACCTTGACGT GTACAATTGA GCATTTGACG CTTGGTAGAC   
  
  
- AGATTGGTCC GAGTTCTCAA TTCTTTCTCT TGCGACAGCA AAGCCTTCTT ATGGTCCTCT CTTTTTTTTT   
  
  
- TTTTTTTGAG AACTCAAACA ATTATAGCTG TGTTTACATG CATACAGCGG GTCTTTTCTT CTACTCTTAT   
  
  
- TTTTGGTTAT TTGAAAGAAT CCTAACGTGT ACATTCCAAT TAATACATTT AATTACTTAG TAATTGTGAC   
  
  
- GCCGAAAGAT CTGCTCGTCG TACGTCATGT TTCGTATGCA CAATGCGCTC AGTGTACCCA CTAGGTAGCG   
  
  
- GTAGTCGCAT TCGTTCCCCA AACTGTACTG CAGTAGTAGA ATGCATTCGA TGGGGTTAGA GACGCGGTCT   
  
  
- GCTCATCACT CATCGATGCG CCTTCTTCAA CCCTTCTAAG AGGGCAGAGA AGGAGACCGG GAGAGAAAAA   
  
  
- GAAGAACAGA AGGAACTTAA ACACAAAGGT GAGAAGGTCA GAAGTTTAAC TAAGTTGTGA CGGGCTCTCT   
  
  
- CTCTCTCTCT CTCAAAACTT AACCAGTCGT CAACCTGGAA AGAGTGAGAG AAAATAAAAG AAAAAGTTAA   
  
  
- AGAAAAAGAA GACATTCGGA ATACATATAT ATTATCCTTT CTCTAGGACT AGGAGTCGCT CCTTTGTTAG   
  
  
- ACTCAAAAAG AAAACAGGGC CCAGTTTTGC GGAGAAAAAG GGATAGTAAA GAGAACGTAT AAGAGGAGAG   
  
  
- GAAGAAGGGT TGAAGAAGTA ATTCTGTCCG TATCCGGAAA AGTGAAAGGA ACGTAAGTTT TGAAATGGTA   
  
  
- AAGAGAGAGA GAGAGAGAGA GAGAGAGAGA AGTCCGAATA TAAAGGGAAA TACATGCGTA TAACGGGTAG   
  
  
- TTGAGAGGTG AAGGTCATAA ACCACGACCA CCTCCGAGAA CTAGAGGGAA AAACCATTAA TGAACAGGTT   
  
  
- CGGTGACTAG AGTCACTCGA TAAGAAGAAT AGCTAGAACT AGAGAAACTA GTTTGAGTGA AAACTAAAGT   
  
  
- TGTGGGAACG AGAACTAAGA AGTAGAGAGT CGAAAAATGC GATAAGTAGG GGAATACGTC AAGGGGAATT   
  
  
- AAAGAGGAGA TCCGGAATTT CCCAAAGAAG AGCTGTACCA GATCACACGT AAGCTAGGTA ATCAGACGAA   
  
  
- ACATTCAATC AAGCGTGCAT GGGTGTATTC GATGTTGACA CAGCGACGTT AAGATTCGTA ACAACCCAAG   
  
  
- AGAGAACGCA TGACGTATAA TCTCCTCCG

+     ARE

| Site Name | Organism | Position | Strand | Matrix score. | sequence | function |
| --- | --- | --- | --- | --- | --- | --- |
| ARE | Zea mays | 422 | - | 6 | TGGTTT | cis-acting regulatory element essential for the anaerobic induction |

> 2018/04/13 10:10:12  
+ TTCGATTTTG TAAAAAAGTT AAATTCTTAT TTTTTTAATA ATTTATCCAT AAAAATAAAG TGAAAAATTA   
  
  
+ AAACTATTCA GTGCTGATAT TTTGTAAAAC AAATAGATAT CCAGTCAATA TGAAACATCT ATACTGTGCT   
  
  
+ TAGTCATGGA CACGTGTTTT AATCTAATGA TTATGTAAAA CGATCACTGA TAAGTCAAAC ATCATTATAT   
  
  
+ TACGAAAAAT GGAACATGAA TAAAGACAAA TGGAACTGCA CATGTTAACT CGTAAACTGC GAACCATCTG   
  
  
+ TCTAACCAGG CTCAAGAGTT AAGAAAGAGA ACGCTGTCGT TTCGGAAGAA TACCAGGAGA GAAAAAAAAA   
  
  
+ AAAAAAACTC TTGAGTTTGT TAATATCGAC ACAAATGTAC GTATGTCGCC CAGAAAAGAA GATGAGAATA   
  
  
+ AAAACCAATA AACTTTCTTA GGATTGCACA TGTAAGGTTA ATTATGTAAA TTAATGAATC ATTAACACTG   
  
  
+ CGGCTTTCTA GACGAGCAGC ATGCAGTACA AAGCATACGT GTTACGCGAG TCACATGGGT GATCCATCGC   
  
  
+ CATCAGCGTA AGCAAGGGGT TTGACATGAC GTCATCATCT TACGTAAGCT ACCCCAATCT CTGCGCCAGA   
  
  
+ CGAGTAGTGA GTAGCTACGC GGAAGAAGTT GGGAAGATTC TCCCGTCTCT TCCTCTGGCC CTCTCTTTTT   
  
  
+ CTTCTTGTCT TCCTTGAATT TGTGTTTCCA CTCTTCCAGT CTTCAAATTG ATTCAACACT GCCCGAGAGA   
  
  
+ GAGAGAGAGA GAGTTTTGAA TTGGTCAGCA GTTGGACCTT TCTCACTCTC TTTTATTTTC TTTTTCAATT   
  
  
+ TCTTTTTCTT CTGTAAGCCT TATGTATATA TAATAGGAAA GAGATCCTGA TCCTCAGCGA GGAAACAATC   
  
  
+ TGAGTTTTTC TTTTGTCCCG GGTCAAAACG CCTCTTTTTC CCTATCATTT CTCTTGCATA TTCTCCTCTC   
  
  
+ CTTCTTCCCA ACTTCTTCAT TAAGACAGGC ATAGGCCTTT TCACTTTCCT TGCATTCAAA ACTTTACCAT   
  
  
+ TTCTCTCTCT CTCTCTCTCT CTCTCTCTCT TCAGGCTTAT ATTTCCCTTT ATGTACGCAT ATTGCCCATC   
  
  
+ AACTCTCCAC TTCCAGTATT TGGTGCTGGT GGAGGCTCTT GATCTCCCTT TTTGGTAATT ACTTGTCCAA   
  
  
+ GCCACTGATC TCAGTGAGCT ATTCTTCTTA TCGATCTTGA TCTCTTTGAT CAAACTCACT TTTGATTTCA   
  
  
+ ACACCCTTGC TCTTGATTCT TCATCTCTCA GCTTTTTACG CTATTCATCC CCTTATGCAG TTCCCCTTAA   
  
  
+ TTTCTCCTCT AGGCCTTAAA GGGTTTCTTC TCGACATGGT CTAGTGTGCA TTCGATCCAT TAGTCTGCTT   
  
  
+ TGTAAGTTAG TTCGCACGTA CCCACATAAG CTACAACTGT GTCGCTGCAA TTCTAAGCAT TGTTGGGTTC   
  
  
+ TCTCTTGCGT ACTGCATATT AGAGGAGGC  

- AAGCTAAAAC ATTTTTTCAA TTTAAGAATA AAAAAATTAT TAAATAGGTA TTTTTATTTC ACTTTTTAAT   
  
  
- TTTGATAAGT CACGACTATA AAACATTTTG TTTATCTATA GGTCAGTTAT ACTTTGTAGA TATGACACGA   
  
  
- ATCAGTACCT GTGCACAAAA TTAGATTACT AATACATTTT GCTAGTGACT ATTCAGTTTG TAGTAATATA   
  
  
- ATGCTTTTTA CCTTGTACTT ATTTCTGTTT ACCTTGACGT GTACAATTGA GCATTTGACG CTTGGTAGAC   
  
  
- AGATTGGTCC GAGTTCTCAA TTCTTTCTCT TGCGACAGCA AAGCCTTCTT ATGGTCCTCT CTTTTTTTTT   
  
  
- TTTTTTTGAG AACTCAAACA ATTATAGCTG TGTTTACATG CATACAGCGG GTCTTTTCTT CTACTCTTAT   
  
  
- TTTTGGTTAT TTGAAAGAAT CCTAACGTGT ACATTCCAAT TAATACATTT AATTACTTAG TAATTGTGAC   
  
  
- GCCGAAAGAT CTGCTCGTCG TACGTCATGT TTCGTATGCA CAATGCGCTC AGTGTACCCA CTAGGTAGCG   
  
  
- GTAGTCGCAT TCGTTCCCCA AACTGTACTG CAGTAGTAGA ATGCATTCGA TGGGGTTAGA GACGCGGTCT   
  
  
- GCTCATCACT CATCGATGCG CCTTCTTCAA CCCTTCTAAG AGGGCAGAGA AGGAGACCGG GAGAGAAAAA   
  
  
- GAAGAACAGA AGGAACTTAA ACACAAAGGT GAGAAGGTCA GAAGTTTAAC TAAGTTGTGA CGGGCTCTCT   
  
  
- CTCTCTCTCT CTCAAAACTT AACCAGTCGT CAACCTGGAA AGAGTGAGAG AAAATAAAAG AAAAAGTTAA   
  
  
- AGAAAAAGAA GACATTCGGA ATACATATAT ATTATCCTTT CTCTAGGACT AGGAGTCGCT CCTTTGTTAG   
  
  
- ACTCAAAAAG AAAACAGGGC CCAGTTTTGC GGAGAAAAAG GGATAGTAAA GAGAACGTAT AAGAGGAGAG   
  
  
- GAAGAAGGGT TGAAGAAGTA ATTCTGTCCG TATCCGGAAA AGTGAAAGGA ACGTAAGTTT TGAAATGGTA   
  
  
- AAGAGAGAGA GAGAGAGAGA GAGAGAGAGA AGTCCGAATA TAAAGGGAAA TACATGCGTA TAACGGGTAG   
  
  
- TTGAGAGGTG AAGGTCATAA ACCACGACCA CCTCCGAGAA CTAGAGGGAA AAACCATTAA TGAACAGGTT   
  
  
- CGGTGACTAG AGTCACTCGA TAAGAAGAAT AGCTAGAACT AGAGAAACTA GTTTGAGTGA AAACTAAAGT   
  
  
- TGTGGGAACG AGAACTAAGA AGTAGAGAGT CGAAAAATGC GATAAGTAGG GGAATACGTC AAGGGGAATT   
  
  
- AAAGAGGAGA TCCGGAATTT CCCAAAGAAG AGCTGTACCA GATCACACGT AAGCTAGGTA ATCAGACGAA   
  
  
- ACATTCAATC AAGCGTGCAT GGGTGTATTC GATGTTGACA CAGCGACGTT AAGATTCGTA ACAACCCAAG   
  
  
- AGAGAACGCA TGACGTATAA TCTCCTCCG

+     Box 4

| Site Name | Organism | Position | Strand | Matrix score. | sequence | function |
| --- | --- | --- | --- | --- | --- | --- |
| Box 4 | Petroselinum crispum | 470 | + | 6 | ATTAAT | part of a conserved DNA module involved in light responsiveness |

> 2018/04/13 10:10:12  
+ TTCGATTTTG TAAAAAAGTT AAATTCTTAT TTTTTTAATA ATTTATCCAT AAAAATAAAG TGAAAAATTA   
  
  
+ AAACTATTCA GTGCTGATAT TTTGTAAAAC AAATAGATAT CCAGTCAATA TGAAACATCT ATACTGTGCT   
  
  
+ TAGTCATGGA CACGTGTTTT AATCTAATGA TTATGTAAAA CGATCACTGA TAAGTCAAAC ATCATTATAT   
  
  
+ TACGAAAAAT GGAACATGAA TAAAGACAAA TGGAACTGCA CATGTTAACT CGTAAACTGC GAACCATCTG   
  
  
+ TCTAACCAGG CTCAAGAGTT AAGAAAGAGA ACGCTGTCGT TTCGGAAGAA TACCAGGAGA GAAAAAAAAA   
  
  
+ AAAAAAACTC TTGAGTTTGT TAATATCGAC ACAAATGTAC GTATGTCGCC CAGAAAAGAA GATGAGAATA   
  
  
+ AAAACCAATA AACTTTCTTA GGATTGCACA TGTAAGGTTA ATTATGTAAA TTAATGAATC ATTAACACTG   
  
  
+ CGGCTTTCTA GACGAGCAGC ATGCAGTACA AAGCATACGT GTTACGCGAG TCACATGGGT GATCCATCGC   
  
  
+ CATCAGCGTA AGCAAGGGGT TTGACATGAC GTCATCATCT TACGTAAGCT ACCCCAATCT CTGCGCCAGA   
  
  
+ CGAGTAGTGA GTAGCTACGC GGAAGAAGTT GGGAAGATTC TCCCGTCTCT TCCTCTGGCC CTCTCTTTTT   
  
  
+ CTTCTTGTCT TCCTTGAATT TGTGTTTCCA CTCTTCCAGT CTTCAAATTG ATTCAACACT GCCCGAGAGA   
  
  
+ GAGAGAGAGA GAGTTTTGAA TTGGTCAGCA GTTGGACCTT TCTCACTCTC TTTTATTTTC TTTTTCAATT   
  
  
+ TCTTTTTCTT CTGTAAGCCT TATGTATATA TAATAGGAAA GAGATCCTGA TCCTCAGCGA GGAAACAATC   
  
  
+ TGAGTTTTTC TTTTGTCCCG GGTCAAAACG CCTCTTTTTC CCTATCATTT CTCTTGCATA TTCTCCTCTC   
  
  
+ CTTCTTCCCA ACTTCTTCAT TAAGACAGGC ATAGGCCTTT TCACTTTCCT TGCATTCAAA ACTTTACCAT   
  
  
+ TTCTCTCTCT CTCTCTCTCT CTCTCTCTCT TCAGGCTTAT ATTTCCCTTT ATGTACGCAT ATTGCCCATC   
  
  
+ AACTCTCCAC TTCCAGTATT TGGTGCTGGT GGAGGCTCTT GATCTCCCTT TTTGGTAATT ACTTGTCCAA   
  
  
+ GCCACTGATC TCAGTGAGCT ATTCTTCTTA TCGATCTTGA TCTCTTTGAT CAAACTCACT TTTGATTTCA   
  
  
+ ACACCCTTGC TCTTGATTCT TCATCTCTCA GCTTTTTACG CTATTCATCC CCTTATGCAG TTCCCCTTAA   
  
  
+ TTTCTCCTCT AGGCCTTAAA GGGTTTCTTC TCGACATGGT CTAGTGTGCA TTCGATCCAT TAGTCTGCTT   
  
  
+ TGTAAGTTAG TTCGCACGTA CCCACATAAG CTACAACTGT GTCGCTGCAA TTCTAAGCAT TGTTGGGTTC   
  
  
+ TCTCTTGCGT ACTGCATATT AGAGGAGGC  

- AAGCTAAAAC ATTTTTTCAA TTTAAGAATA AAAAAATTAT TAAATAGGTA TTTTTATTTC ACTTTTTAAT   
  
  
- TTTGATAAGT CACGACTATA AAACATTTTG TTTATCTATA GGTCAGTTAT ACTTTGTAGA TATGACACGA   
  
  
- ATCAGTACCT GTGCACAAAA TTAGATTACT AATACATTTT GCTAGTGACT ATTCAGTTTG TAGTAATATA   
  
  
- ATGCTTTTTA CCTTGTACTT ATTTCTGTTT ACCTTGACGT GTACAATTGA GCATTTGACG CTTGGTAGAC   
  
  
- AGATTGGTCC GAGTTCTCAA TTCTTTCTCT TGCGACAGCA AAGCCTTCTT ATGGTCCTCT CTTTTTTTTT   
  
  
- TTTTTTTGAG AACTCAAACA ATTATAGCTG TGTTTACATG CATACAGCGG GTCTTTTCTT CTACTCTTAT   
  
  
- TTTTGGTTAT TTGAAAGAAT CCTAACGTGT ACATTCCAAT TAATACATTT AATTACTTAG TAATTGTGAC   
  
  
- GCCGAAAGAT CTGCTCGTCG TACGTCATGT TTCGTATGCA CAATGCGCTC AGTGTACCCA CTAGGTAGCG   
  
  
- GTAGTCGCAT TCGTTCCCCA AACTGTACTG CAGTAGTAGA ATGCATTCGA TGGGGTTAGA GACGCGGTCT   
  
  
- GCTCATCACT CATCGATGCG CCTTCTTCAA CCCTTCTAAG AGGGCAGAGA AGGAGACCGG GAGAGAAAAA   
  
  
- GAAGAACAGA AGGAACTTAA ACACAAAGGT GAGAAGGTCA GAAGTTTAAC TAAGTTGTGA CGGGCTCTCT   
  
  
- CTCTCTCTCT CTCAAAACTT AACCAGTCGT CAACCTGGAA AGAGTGAGAG AAAATAAAAG AAAAAGTTAA   
  
  
- AGAAAAAGAA GACATTCGGA ATACATATAT ATTATCCTTT CTCTAGGACT AGGAGTCGCT CCTTTGTTAG   
  
  
- ACTCAAAAAG AAAACAGGGC CCAGTTTTGC GGAGAAAAAG GGATAGTAAA GAGAACGTAT AAGAGGAGAG   
  
  
- GAAGAAGGGT TGAAGAAGTA ATTCTGTCCG TATCCGGAAA AGTGAAAGGA ACGTAAGTTT TGAAATGGTA   
  
  
- AAGAGAGAGA GAGAGAGAGA GAGAGAGAGA AGTCCGAATA TAAAGGGAAA TACATGCGTA TAACGGGTAG   
  
  
- TTGAGAGGTG AAGGTCATAA ACCACGACCA CCTCCGAGAA CTAGAGGGAA AAACCATTAA TGAACAGGTT   
  
  
- CGGTGACTAG AGTCACTCGA TAAGAAGAAT AGCTAGAACT AGAGAAACTA GTTTGAGTGA AAACTAAAGT   
  
  
- TGTGGGAACG AGAACTAAGA AGTAGAGAGT CGAAAAATGC GATAAGTAGG GGAATACGTC AAGGGGAATT   
  
  
- AAAGAGGAGA TCCGGAATTT CCCAAAGAAG AGCTGTACCA GATCACACGT AAGCTAGGTA ATCAGACGAA   
  
  
- ACATTCAATC AAGCGTGCAT GGGTGTATTC GATGTTGACA CAGCGACGTT AAGATTCGTA ACAACCCAAG   
  
  
- AGAGAACGCA TGACGTATAA TCTCCTCCG

+     Box III

| Site Name | Organism | Position | Strand | Matrix score. | sequence | function |
| --- | --- | --- | --- | --- | --- | --- |
| Box III | Pisum sativum | 480 | + | 9 | CATTTACACT | protein binding site |

> 2018/04/13 10:10:12  
+ TTCGATTTTG TAAAAAAGTT AAATTCTTAT TTTTTTAATA ATTTATCCAT AAAAATAAAG TGAAAAATTA   
  
  
+ AAACTATTCA GTGCTGATAT TTTGTAAAAC AAATAGATAT CCAGTCAATA TGAAACATCT ATACTGTGCT   
  
  
+ TAGTCATGGA CACGTGTTTT AATCTAATGA TTATGTAAAA CGATCACTGA TAAGTCAAAC ATCATTATAT   
  
  
+ TACGAAAAAT GGAACATGAA TAAAGACAAA TGGAACTGCA CATGTTAACT CGTAAACTGC GAACCATCTG   
  
  
+ TCTAACCAGG CTCAAGAGTT AAGAAAGAGA ACGCTGTCGT TTCGGAAGAA TACCAGGAGA GAAAAAAAAA   
  
  
+ AAAAAAACTC TTGAGTTTGT TAATATCGAC ACAAATGTAC GTATGTCGCC CAGAAAAGAA GATGAGAATA   
  
  
+ AAAACCAATA AACTTTCTTA GGATTGCACA TGTAAGGTTA ATTATGTAAA TTAATGAATC ATTAACACTG   
  
  
+ CGGCTTTCTA GACGAGCAGC ATGCAGTACA AAGCATACGT GTTACGCGAG TCACATGGGT GATCCATCGC   
  
  
+ CATCAGCGTA AGCAAGGGGT TTGACATGAC GTCATCATCT TACGTAAGCT ACCCCAATCT CTGCGCCAGA   
  
  
+ CGAGTAGTGA GTAGCTACGC GGAAGAAGTT GGGAAGATTC TCCCGTCTCT TCCTCTGGCC CTCTCTTTTT   
  
  
+ CTTCTTGTCT TCCTTGAATT TGTGTTTCCA CTCTTCCAGT CTTCAAATTG ATTCAACACT GCCCGAGAGA   
  
  
+ GAGAGAGAGA GAGTTTTGAA TTGGTCAGCA GTTGGACCTT TCTCACTCTC TTTTATTTTC TTTTTCAATT   
  
  
+ TCTTTTTCTT CTGTAAGCCT TATGTATATA TAATAGGAAA GAGATCCTGA TCCTCAGCGA GGAAACAATC   
  
  
+ TGAGTTTTTC TTTTGTCCCG GGTCAAAACG CCTCTTTTTC CCTATCATTT CTCTTGCATA TTCTCCTCTC   
  
  
+ CTTCTTCCCA ACTTCTTCAT TAAGACAGGC ATAGGCCTTT TCACTTTCCT TGCATTCAAA ACTTTACCAT   
  
  
+ TTCTCTCTCT CTCTCTCTCT CTCTCTCTCT TCAGGCTTAT ATTTCCCTTT ATGTACGCAT ATTGCCCATC   
  
  
+ AACTCTCCAC TTCCAGTATT TGGTGCTGGT GGAGGCTCTT GATCTCCCTT TTTGGTAATT ACTTGTCCAA   
  
  
+ GCCACTGATC TCAGTGAGCT ATTCTTCTTA TCGATCTTGA TCTCTTTGAT CAAACTCACT TTTGATTTCA   
  
  
+ ACACCCTTGC TCTTGATTCT TCATCTCTCA GCTTTTTACG CTATTCATCC CCTTATGCAG TTCCCCTTAA   
  
  
+ TTTCTCCTCT AGGCCTTAAA GGGTTTCTTC TCGACATGGT CTAGTGTGCA TTCGATCCAT TAGTCTGCTT   
  
  
+ TGTAAGTTAG TTCGCACGTA CCCACATAAG CTACAACTGT GTCGCTGCAA TTCTAAGCAT TGTTGGGTTC   
  
  
+ TCTCTTGCGT ACTGCATATT AGAGGAGGC  

- AAGCTAAAAC ATTTTTTCAA TTTAAGAATA AAAAAATTAT TAAATAGGTA TTTTTATTTC ACTTTTTAAT   
  
  
- TTTGATAAGT CACGACTATA AAACATTTTG TTTATCTATA GGTCAGTTAT ACTTTGTAGA TATGACACGA   
  
  
- ATCAGTACCT GTGCACAAAA TTAGATTACT AATACATTTT GCTAGTGACT ATTCAGTTTG TAGTAATATA   
  
  
- ATGCTTTTTA CCTTGTACTT ATTTCTGTTT ACCTTGACGT GTACAATTGA GCATTTGACG CTTGGTAGAC   
  
  
- AGATTGGTCC GAGTTCTCAA TTCTTTCTCT TGCGACAGCA AAGCCTTCTT ATGGTCCTCT CTTTTTTTTT   
  
  
- TTTTTTTGAG AACTCAAACA ATTATAGCTG TGTTTACATG CATACAGCGG GTCTTTTCTT CTACTCTTAT   
  
  
- TTTTGGTTAT TTGAAAGAAT CCTAACGTGT ACATTCCAAT TAATACATTT AATTACTTAG TAATTGTGAC   
  
  
- GCCGAAAGAT CTGCTCGTCG TACGTCATGT TTCGTATGCA CAATGCGCTC AGTGTACCCA CTAGGTAGCG   
  
  
- GTAGTCGCAT TCGTTCCCCA AACTGTACTG CAGTAGTAGA ATGCATTCGA TGGGGTTAGA GACGCGGTCT   
  
  
- GCTCATCACT CATCGATGCG CCTTCTTCAA CCCTTCTAAG AGGGCAGAGA AGGAGACCGG GAGAGAAAAA   
  
  
- GAAGAACAGA AGGAACTTAA ACACAAAGGT GAGAAGGTCA GAAGTTTAAC TAAGTTGTGA CGGGCTCTCT   
  
  
- CTCTCTCTCT CTCAAAACTT AACCAGTCGT CAACCTGGAA AGAGTGAGAG AAAATAAAAG AAAAAGTTAA   
  
  
- AGAAAAAGAA GACATTCGGA ATACATATAT ATTATCCTTT CTCTAGGACT AGGAGTCGCT CCTTTGTTAG   
  
  
- ACTCAAAAAG AAAACAGGGC CCAGTTTTGC GGAGAAAAAG GGATAGTAAA GAGAACGTAT AAGAGGAGAG   
  
  
- GAAGAAGGGT TGAAGAAGTA ATTCTGTCCG TATCCGGAAA AGTGAAAGGA ACGTAAGTTT TGAAATGGTA   
  
  
- AAGAGAGAGA GAGAGAGAGA GAGAGAGAGA AGTCCGAATA TAAAGGGAAA TACATGCGTA TAACGGGTAG   
  
  
- TTGAGAGGTG AAGGTCATAA ACCACGACCA CCTCCGAGAA CTAGAGGGAA AAACCATTAA TGAACAGGTT   
  
  
- CGGTGACTAG AGTCACTCGA TAAGAAGAAT AGCTAGAACT AGAGAAACTA GTTTGAGTGA AAACTAAAGT   
  
  
- TGTGGGAACG AGAACTAAGA AGTAGAGAGT CGAAAAATGC GATAAGTAGG GGAATACGTC AAGGGGAATT   
  
  
- AAAGAGGAGA TCCGGAATTT CCCAAAGAAG AGCTGTACCA GATCACACGT AAGCTAGGTA ATCAGACGAA   
  
  
- ACATTCAATC AAGCGTGCAT GGGTGTATTC GATGTTGACA CAGCGACGTT AAGATTCGTA ACAACCCAAG   
  
  
- AGAGAACGCA TGACGTATAA TCTCCTCCG

+     Box-W1

| Site Name | Organism | Position | Strand | Matrix score. | sequence | function |
| --- | --- | --- | --- | --- | --- | --- |
| Box-W1 | Petroselinum crispum | 931 | - | 6 | TTGACC | fungal elicitor responsive element |

> 2018/04/13 10:10:12  
+ TTCGATTTTG TAAAAAAGTT AAATTCTTAT TTTTTTAATA ATTTATCCAT AAAAATAAAG TGAAAAATTA   
  
  
+ AAACTATTCA GTGCTGATAT TTTGTAAAAC AAATAGATAT CCAGTCAATA TGAAACATCT ATACTGTGCT   
  
  
+ TAGTCATGGA CACGTGTTTT AATCTAATGA TTATGTAAAA CGATCACTGA TAAGTCAAAC ATCATTATAT   
  
  
+ TACGAAAAAT GGAACATGAA TAAAGACAAA TGGAACTGCA CATGTTAACT CGTAAACTGC GAACCATCTG   
  
  
+ TCTAACCAGG CTCAAGAGTT AAGAAAGAGA ACGCTGTCGT TTCGGAAGAA TACCAGGAGA GAAAAAAAAA   
  
  
+ AAAAAAACTC TTGAGTTTGT TAATATCGAC ACAAATGTAC GTATGTCGCC CAGAAAAGAA GATGAGAATA   
  
  
+ AAAACCAATA AACTTTCTTA GGATTGCACA TGTAAGGTTA ATTATGTAAA TTAATGAATC ATTAACACTG   
  
  
+ CGGCTTTCTA GACGAGCAGC ATGCAGTACA AAGCATACGT GTTACGCGAG TCACATGGGT GATCCATCGC   
  
  
+ CATCAGCGTA AGCAAGGGGT TTGACATGAC GTCATCATCT TACGTAAGCT ACCCCAATCT CTGCGCCAGA   
  
  
+ CGAGTAGTGA GTAGCTACGC GGAAGAAGTT GGGAAGATTC TCCCGTCTCT TCCTCTGGCC CTCTCTTTTT   
  
  
+ CTTCTTGTCT TCCTTGAATT TGTGTTTCCA CTCTTCCAGT CTTCAAATTG ATTCAACACT GCCCGAGAGA   
  
  
+ GAGAGAGAGA GAGTTTTGAA TTGGTCAGCA GTTGGACCTT TCTCACTCTC TTTTATTTTC TTTTTCAATT   
  
  
+ TCTTTTTCTT CTGTAAGCCT TATGTATATA TAATAGGAAA GAGATCCTGA TCCTCAGCGA GGAAACAATC   
  
  
+ TGAGTTTTTC TTTTGTCCCG GGTCAAAACG CCTCTTTTTC CCTATCATTT CTCTTGCATA TTCTCCTCTC   
  
  
+ CTTCTTCCCA ACTTCTTCAT TAAGACAGGC ATAGGCCTTT TCACTTTCCT TGCATTCAAA ACTTTACCAT   
  
  
+ TTCTCTCTCT CTCTCTCTCT CTCTCTCTCT TCAGGCTTAT ATTTCCCTTT ATGTACGCAT ATTGCCCATC   
  
  
+ AACTCTCCAC TTCCAGTATT TGGTGCTGGT GGAGGCTCTT GATCTCCCTT TTTGGTAATT ACTTGTCCAA   
  
  
+ GCCACTGATC TCAGTGAGCT ATTCTTCTTA TCGATCTTGA TCTCTTTGAT CAAACTCACT TTTGATTTCA   
  
  
+ ACACCCTTGC TCTTGATTCT TCATCTCTCA GCTTTTTACG CTATTCATCC CCTTATGCAG TTCCCCTTAA   
  
  
+ TTTCTCCTCT AGGCCTTAAA GGGTTTCTTC TCGACATGGT CTAGTGTGCA TTCGATCCAT TAGTCTGCTT   
  
  
+ TGTAAGTTAG TTCGCACGTA CCCACATAAG CTACAACTGT GTCGCTGCAA TTCTAAGCAT TGTTGGGTTC   
  
  
+ TCTCTTGCGT ACTGCATATT AGAGGAGGC  

- AAGCTAAAAC ATTTTTTCAA TTTAAGAATA AAAAAATTAT TAAATAGGTA TTTTTATTTC ACTTTTTAAT   
  
  
- TTTGATAAGT CACGACTATA AAACATTTTG TTTATCTATA GGTCAGTTAT ACTTTGTAGA TATGACACGA   
  
  
- ATCAGTACCT GTGCACAAAA TTAGATTACT AATACATTTT GCTAGTGACT ATTCAGTTTG TAGTAATATA   
  
  
- ATGCTTTTTA CCTTGTACTT ATTTCTGTTT ACCTTGACGT GTACAATTGA GCATTTGACG CTTGGTAGAC   
  
  
- AGATTGGTCC GAGTTCTCAA TTCTTTCTCT TGCGACAGCA AAGCCTTCTT ATGGTCCTCT CTTTTTTTTT   
  
  
- TTTTTTTGAG AACTCAAACA ATTATAGCTG TGTTTACATG CATACAGCGG GTCTTTTCTT CTACTCTTAT   
  
  
- TTTTGGTTAT TTGAAAGAAT CCTAACGTGT ACATTCCAAT TAATACATTT AATTACTTAG TAATTGTGAC   
  
  
- GCCGAAAGAT CTGCTCGTCG TACGTCATGT TTCGTATGCA CAATGCGCTC AGTGTACCCA CTAGGTAGCG   
  
  
- GTAGTCGCAT TCGTTCCCCA AACTGTACTG CAGTAGTAGA ATGCATTCGA TGGGGTTAGA GACGCGGTCT   
  
  
- GCTCATCACT CATCGATGCG CCTTCTTCAA CCCTTCTAAG AGGGCAGAGA AGGAGACCGG GAGAGAAAAA   
  
  
- GAAGAACAGA AGGAACTTAA ACACAAAGGT GAGAAGGTCA GAAGTTTAAC TAAGTTGTGA CGGGCTCTCT   
  
  
- CTCTCTCTCT CTCAAAACTT AACCAGTCGT CAACCTGGAA AGAGTGAGAG AAAATAAAAG AAAAAGTTAA   
  
  
- AGAAAAAGAA GACATTCGGA ATACATATAT ATTATCCTTT CTCTAGGACT AGGAGTCGCT CCTTTGTTAG   
  
  
- ACTCAAAAAG AAAACAGGGC CCAGTTTTGC GGAGAAAAAG GGATAGTAAA GAGAACGTAT AAGAGGAGAG   
  
  
- GAAGAAGGGT TGAAGAAGTA ATTCTGTCCG TATCCGGAAA AGTGAAAGGA ACGTAAGTTT TGAAATGGTA   
  
  
- AAGAGAGAGA GAGAGAGAGA GAGAGAGAGA AGTCCGAATA TAAAGGGAAA TACATGCGTA TAACGGGTAG   
  
  
- TTGAGAGGTG AAGGTCATAA ACCACGACCA CCTCCGAGAA CTAGAGGGAA AAACCATTAA TGAACAGGTT   
  
  
- CGGTGACTAG AGTCACTCGA TAAGAAGAAT AGCTAGAACT AGAGAAACTA GTTTGAGTGA AAACTAAAGT   
  
  
- TGTGGGAACG AGAACTAAGA AGTAGAGAGT CGAAAAATGC GATAAGTAGG GGAATACGTC AAGGGGAATT   
  
  
- AAAGAGGAGA TCCGGAATTT CCCAAAGAAG AGCTGTACCA GATCACACGT AAGCTAGGTA ATCAGACGAA   
  
  
- ACATTCAATC AAGCGTGCAT GGGTGTATTC GATGTTGACA CAGCGACGTT AAGATTCGTA ACAACCCAAG   
  
  
- AGAGAACGCA TGACGTATAA TCTCCTCCG

+     CAAT-box

| Site Name | Organism | Position | Strand | Matrix score. | sequence | function |
| --- | --- | --- | --- | --- | --- | --- |
| CAAT-box | Hordeum vulgare | 906 | + | 4 | CAAT | common cis-acting element in promoter and enhancer regions |
| CAAT-box | Hordeum vulgare | 747 | - | 4 | CAAT | common cis-acting element in promoter and enhancer regions |
| CAAT-box | Brassica rapa | 382 | + | 5 | CAAAT | common cis-acting element in promoter and enhancer regions |
| CAAT-box | Hordeum vulgare | 615 | + | 4 | CAAT | common cis-acting element in promoter and enhancer regions |
| CAAT-box | Brassica rapa | 1138 | - | 5 | CAAAT | common cis-acting element in promoter and enhancer regions |
| CAAT-box | Hordeum vulgare | 426 | + | 4 | CAAT | common cis-acting element in promoter and enhancer regions |
| CAAT-box | Arabidopsis thaliana | 790 | - | 5 | CCAAT | common cis-acting element in promoter and enhancer regions |
| CAAT-box | Brassica rapa | 744 | + | 5 | CAAAT | common cis-acting element in promoter and enhancer regions |
| CAAT-box | Brassica rapa | 718 | - | 5 | CAAAT | common cis-acting element in promoter and enhancer regions |
| CAAT-box | Hordeum vulgare | 1459 | - | 4 | CAAT | common cis-acting element in promoter and enhancer regions |
| CAAT-box | Hordeum vulgare | 443 | - | 4 | CAAT | common cis-acting element in promoter and enhancer regions |
| CAAT-box | Arabidopsis thaliana | 614 | + | 5 | CCAAT | common cis-acting element in promoter and enhancer regions |
| CAAT-box | Glycine max | 789 | - | 5 | CAATT | common cis-acting element in promoter and enhancer regions |
| CAAT-box | Arabidopsis thaliana | 1111 | - | 6 | gGCAAT | common cis-acting element in promoter and enhancer regions |
| CAAT-box | Brassica rapa | 237 | + | 5 | CAAAT | common cis-acting element in promoter and enhancer regions |
| CAAT-box | Glycine max | 746 | - | 5 | CAATT | common cis-acting element in promoter and enhancer regions |
| CAAT-box | Glycine max | 1448 | + | 5 | CAATT | common cis-acting element in promoter and enhancer regions |
| CAAT-box | Arabidopsis thaliana | 425 | + | 5 | CCAAT | common cis-acting element in promoter and enhancer regions |
| CAAT-box | Hordeum vulgare | 116 | + | 4 | CAAT | common cis-acting element in promoter and enhancer regions |
| CAAT-box | Brassica rapa | 100 | + | 5 | CAAAT | common cis-acting element in promoter and enhancer regions |
| CAAT-box | Glycine max | 836 | + | 5 | CAATT | common cis-acting element in promoter and enhancer regions |

> 2018/04/13 10:10:12  
+ TTCGATTTTG TAAAAAAGTT AAATTCTTAT TTTTTTAATA ATTTATCCAT AAAAATAAAG TGAAAAATTA   
  
  
+ AAACTATTCA GTGCTGATAT TTTGTAAAAC AAATAGATAT CCAGTCAATA TGAAACATCT ATACTGTGCT   
  
  
+ TAGTCATGGA CACGTGTTTT AATCTAATGA TTATGTAAAA CGATCACTGA TAAGTCAAAC ATCATTATAT   
  
  
+ TACGAAAAAT GGAACATGAA TAAAGACAAA TGGAACTGCA CATGTTAACT CGTAAACTGC GAACCATCTG   
  
  
+ TCTAACCAGG CTCAAGAGTT AAGAAAGAGA ACGCTGTCGT TTCGGAAGAA TACCAGGAGA GAAAAAAAAA   
  
  
+ AAAAAAACTC TTGAGTTTGT TAATATCGAC ACAAATGTAC GTATGTCGCC CAGAAAAGAA GATGAGAATA   
  
  
+ AAAACCAATA AACTTTCTTA GGATTGCACA TGTAAGGTTA ATTATGTAAA TTAATGAATC ATTAACACTG   
  
  
+ CGGCTTTCTA GACGAGCAGC ATGCAGTACA AAGCATACGT GTTACGCGAG TCACATGGGT GATCCATCGC   
  
  
+ CATCAGCGTA AGCAAGGGGT TTGACATGAC GTCATCATCT TACGTAAGCT ACCCCAATCT CTGCGCCAGA   
  
  
+ CGAGTAGTGA GTAGCTACGC GGAAGAAGTT GGGAAGATTC TCCCGTCTCT TCCTCTGGCC CTCTCTTTTT   
  
  
+ CTTCTTGTCT TCCTTGAATT TGTGTTTCCA CTCTTCCAGT CTTCAAATTG ATTCAACACT GCCCGAGAGA   
  
  
+ GAGAGAGAGA GAGTTTTGAA TTGGTCAGCA GTTGGACCTT TCTCACTCTC TTTTATTTTC TTTTTCAATT   
  
  
+ TCTTTTTCTT CTGTAAGCCT TATGTATATA TAATAGGAAA GAGATCCTGA TCCTCAGCGA GGAAACAATC   
  
  
+ TGAGTTTTTC TTTTGTCCCG GGTCAAAACG CCTCTTTTTC CCTATCATTT CTCTTGCATA TTCTCCTCTC   
  
  
+ CTTCTTCCCA ACTTCTTCAT TAAGACAGGC ATAGGCCTTT TCACTTTCCT TGCATTCAAA ACTTTACCAT   
  
  
+ TTCTCTCTCT CTCTCTCTCT CTCTCTCTCT TCAGGCTTAT ATTTCCCTTT ATGTACGCAT ATTGCCCATC   
  
  
+ AACTCTCCAC TTCCAGTATT TGGTGCTGGT GGAGGCTCTT GATCTCCCTT TTTGGTAATT ACTTGTCCAA   
  
  
+ GCCACTGATC TCAGTGAGCT ATTCTTCTTA TCGATCTTGA TCTCTTTGAT CAAACTCACT TTTGATTTCA   
  
  
+ ACACCCTTGC TCTTGATTCT TCATCTCTCA GCTTTTTACG CTATTCATCC CCTTATGCAG TTCCCCTTAA   
  
  
+ TTTCTCCTCT AGGCCTTAAA GGGTTTCTTC TCGACATGGT CTAGTGTGCA TTCGATCCAT TAGTCTGCTT   
  
  
+ TGTAAGTTAG TTCGCACGTA CCCACATAAG CTACAACTGT GTCGCTGCAA TTCTAAGCAT TGTTGGGTTC   
  
  
+ TCTCTTGCGT ACTGCATATT AGAGGAGGC  

- AAGCTAAAAC ATTTTTTCAA TTTAAGAATA AAAAAATTAT TAAATAGGTA TTTTTATTTC ACTTTTTAAT   
  
  
- TTTGATAAGT CACGACTATA AAACATTTTG TTTATCTATA GGTCAGTTAT ACTTTGTAGA TATGACACGA   
  
  
- ATCAGTACCT GTGCACAAAA TTAGATTACT AATACATTTT GCTAGTGACT ATTCAGTTTG TAGTAATATA   
  
  
- ATGCTTTTTA CCTTGTACTT ATTTCTGTTT ACCTTGACGT GTACAATTGA GCATTTGACG CTTGGTAGAC   
  
  
- AGATTGGTCC GAGTTCTCAA TTCTTTCTCT TGCGACAGCA AAGCCTTCTT ATGGTCCTCT CTTTTTTTTT   
  
  
- TTTTTTTGAG AACTCAAACA ATTATAGCTG TGTTTACATG CATACAGCGG GTCTTTTCTT CTACTCTTAT   
  
  
- TTTTGGTTAT TTGAAAGAAT CCTAACGTGT ACATTCCAAT TAATACATTT AATTACTTAG TAATTGTGAC   
  
  
- GCCGAAAGAT CTGCTCGTCG TACGTCATGT TTCGTATGCA CAATGCGCTC AGTGTACCCA CTAGGTAGCG   
  
  
- GTAGTCGCAT TCGTTCCCCA AACTGTACTG CAGTAGTAGA ATGCATTCGA TGGGGTTAGA GACGCGGTCT   
  
  
- GCTCATCACT CATCGATGCG CCTTCTTCAA CCCTTCTAAG AGGGCAGAGA AGGAGACCGG GAGAGAAAAA   
  
  
- GAAGAACAGA AGGAACTTAA ACACAAAGGT GAGAAGGTCA GAAGTTTAAC TAAGTTGTGA CGGGCTCTCT   
  
  
- CTCTCTCTCT CTCAAAACTT AACCAGTCGT CAACCTGGAA AGAGTGAGAG AAAATAAAAG AAAAAGTTAA   
  
  
- AGAAAAAGAA GACATTCGGA ATACATATAT ATTATCCTTT CTCTAGGACT AGGAGTCGCT CCTTTGTTAG   
  
  
- ACTCAAAAAG AAAACAGGGC CCAGTTTTGC GGAGAAAAAG GGATAGTAAA GAGAACGTAT AAGAGGAGAG   
  
  
- GAAGAAGGGT TGAAGAAGTA ATTCTGTCCG TATCCGGAAA AGTGAAAGGA ACGTAAGTTT TGAAATGGTA   
  
  
- AAGAGAGAGA GAGAGAGAGA GAGAGAGAGA AGTCCGAATA TAAAGGGAAA TACATGCGTA TAACGGGTAG   
  
  
- TTGAGAGGTG AAGGTCATAA ACCACGACCA CCTCCGAGAA CTAGAGGGAA AAACCATTAA TGAACAGGTT   
  
  
- CGGTGACTAG AGTCACTCGA TAAGAAGAAT AGCTAGAACT AGAGAAACTA GTTTGAGTGA AAACTAAAGT   
  
  
- TGTGGGAACG AGAACTAAGA AGTAGAGAGT CGAAAAATGC GATAAGTAGG GGAATACGTC AAGGGGAATT   
  
  
- AAAGAGGAGA TCCGGAATTT CCCAAAGAAG AGCTGTACCA GATCACACGT AAGCTAGGTA ATCAGACGAA   
  
  
- ACATTCAATC AAGCGTGCAT GGGTGTATTC GATGTTGACA CAGCGACGTT AAGATTCGTA ACAACCCAAG   
  
  
- AGAGAACGCA TGACGTATAA TCTCCTCCG

+     CAT-box

| Site Name | Organism | Position | Strand | Matrix score. | sequence | function |
| --- | --- | --- | --- | --- | --- | --- |
| CAT-box | Arabidopsis thaliana | 1191 | + | 6 | GCCACT | cis-acting regulatory element related to meristem expression |

> 2018/04/13 10:10:12  
+ TTCGATTTTG TAAAAAAGTT AAATTCTTAT TTTTTTAATA ATTTATCCAT AAAAATAAAG TGAAAAATTA   
  
  
+ AAACTATTCA GTGCTGATAT TTTGTAAAAC AAATAGATAT CCAGTCAATA TGAAACATCT ATACTGTGCT   
  
  
+ TAGTCATGGA CACGTGTTTT AATCTAATGA TTATGTAAAA CGATCACTGA TAAGTCAAAC ATCATTATAT   
  
  
+ TACGAAAAAT GGAACATGAA TAAAGACAAA TGGAACTGCA CATGTTAACT CGTAAACTGC GAACCATCTG   
  
  
+ TCTAACCAGG CTCAAGAGTT AAGAAAGAGA ACGCTGTCGT TTCGGAAGAA TACCAGGAGA GAAAAAAAAA   
  
  
+ AAAAAAACTC TTGAGTTTGT TAATATCGAC ACAAATGTAC GTATGTCGCC CAGAAAAGAA GATGAGAATA   
  
  
+ AAAACCAATA AACTTTCTTA GGATTGCACA TGTAAGGTTA ATTATGTAAA TTAATGAATC ATTAACACTG   
  
  
+ CGGCTTTCTA GACGAGCAGC ATGCAGTACA AAGCATACGT GTTACGCGAG TCACATGGGT GATCCATCGC   
  
  
+ CATCAGCGTA AGCAAGGGGT TTGACATGAC GTCATCATCT TACGTAAGCT ACCCCAATCT CTGCGCCAGA   
  
  
+ CGAGTAGTGA GTAGCTACGC GGAAGAAGTT GGGAAGATTC TCCCGTCTCT TCCTCTGGCC CTCTCTTTTT   
  
  
+ CTTCTTGTCT TCCTTGAATT TGTGTTTCCA CTCTTCCAGT CTTCAAATTG ATTCAACACT GCCCGAGAGA   
  
  
+ GAGAGAGAGA GAGTTTTGAA TTGGTCAGCA GTTGGACCTT TCTCACTCTC TTTTATTTTC TTTTTCAATT   
  
  
+ TCTTTTTCTT CTGTAAGCCT TATGTATATA TAATAGGAAA GAGATCCTGA TCCTCAGCGA GGAAACAATC   
  
  
+ TGAGTTTTTC TTTTGTCCCG GGTCAAAACG CCTCTTTTTC CCTATCATTT CTCTTGCATA TTCTCCTCTC   
  
  
+ CTTCTTCCCA ACTTCTTCAT TAAGACAGGC ATAGGCCTTT TCACTTTCCT TGCATTCAAA ACTTTACCAT   
  
  
+ TTCTCTCTCT CTCTCTCTCT CTCTCTCTCT TCAGGCTTAT ATTTCCCTTT ATGTACGCAT ATTGCCCATC   
  
  
+ AACTCTCCAC TTCCAGTATT TGGTGCTGGT GGAGGCTCTT GATCTCCCTT TTTGGTAATT ACTTGTCCAA   
  
  
+ GCCACTGATC TCAGTGAGCT ATTCTTCTTA TCGATCTTGA TCTCTTTGAT CAAACTCACT TTTGATTTCA   
  
  
+ ACACCCTTGC TCTTGATTCT TCATCTCTCA GCTTTTTACG CTATTCATCC CCTTATGCAG TTCCCCTTAA   
  
  
+ TTTCTCCTCT AGGCCTTAAA GGGTTTCTTC TCGACATGGT CTAGTGTGCA TTCGATCCAT TAGTCTGCTT   
  
  
+ TGTAAGTTAG TTCGCACGTA CCCACATAAG CTACAACTGT GTCGCTGCAA TTCTAAGCAT TGTTGGGTTC   
  
  
+ TCTCTTGCGT ACTGCATATT AGAGGAGGC  

- AAGCTAAAAC ATTTTTTCAA TTTAAGAATA AAAAAATTAT TAAATAGGTA TTTTTATTTC ACTTTTTAAT   
  
  
- TTTGATAAGT CACGACTATA AAACATTTTG TTTATCTATA GGTCAGTTAT ACTTTGTAGA TATGACACGA   
  
  
- ATCAGTACCT GTGCACAAAA TTAGATTACT AATACATTTT GCTAGTGACT ATTCAGTTTG TAGTAATATA   
  
  
- ATGCTTTTTA CCTTGTACTT ATTTCTGTTT ACCTTGACGT GTACAATTGA GCATTTGACG CTTGGTAGAC   
  
  
- AGATTGGTCC GAGTTCTCAA TTCTTTCTCT TGCGACAGCA AAGCCTTCTT ATGGTCCTCT CTTTTTTTTT   
  
  
- TTTTTTTGAG AACTCAAACA ATTATAGCTG TGTTTACATG CATACAGCGG GTCTTTTCTT CTACTCTTAT   
  
  
- TTTTGGTTAT TTGAAAGAAT CCTAACGTGT ACATTCCAAT TAATACATTT AATTACTTAG TAATTGTGAC   
  
  
- GCCGAAAGAT CTGCTCGTCG TACGTCATGT TTCGTATGCA CAATGCGCTC AGTGTACCCA CTAGGTAGCG   
  
  
- GTAGTCGCAT TCGTTCCCCA AACTGTACTG CAGTAGTAGA ATGCATTCGA TGGGGTTAGA GACGCGGTCT   
  
  
- GCTCATCACT CATCGATGCG CCTTCTTCAA CCCTTCTAAG AGGGCAGAGA AGGAGACCGG GAGAGAAAAA   
  
  
- GAAGAACAGA AGGAACTTAA ACACAAAGGT GAGAAGGTCA GAAGTTTAAC TAAGTTGTGA CGGGCTCTCT   
  
  
- CTCTCTCTCT CTCAAAACTT AACCAGTCGT CAACCTGGAA AGAGTGAGAG AAAATAAAAG AAAAAGTTAA   
  
  
- AGAAAAAGAA GACATTCGGA ATACATATAT ATTATCCTTT CTCTAGGACT AGGAGTCGCT CCTTTGTTAG   
  
  
- ACTCAAAAAG AAAACAGGGC CCAGTTTTGC GGAGAAAAAG GGATAGTAAA GAGAACGTAT AAGAGGAGAG   
  
  
- GAAGAAGGGT TGAAGAAGTA ATTCTGTCCG TATCCGGAAA AGTGAAAGGA ACGTAAGTTT TGAAATGGTA   
  
  
- AAGAGAGAGA GAGAGAGAGA GAGAGAGAGA AGTCCGAATA TAAAGGGAAA TACATGCGTA TAACGGGTAG   
  
  
- TTGAGAGGTG AAGGTCATAA ACCACGACCA CCTCCGAGAA CTAGAGGGAA AAACCATTAA TGAACAGGTT   
  
  
- CGGTGACTAG AGTCACTCGA TAAGAAGAAT AGCTAGAACT AGAGAAACTA GTTTGAGTGA AAACTAAAGT   
  
  
- TGTGGGAACG AGAACTAAGA AGTAGAGAGT CGAAAAATGC GATAAGTAGG GGAATACGTC AAGGGGAATT   
  
  
- AAAGAGGAGA TCCGGAATTT CCCAAAGAAG AGCTGTACCA GATCACACGT AAGCTAGGTA ATCAGACGAA   
  
  
- ACATTCAATC AAGCGTGCAT GGGTGTATTC GATGTTGACA CAGCGACGTT AAGATTCGTA ACAACCCAAG   
  
  
- AGAGAACGCA TGACGTATAA TCTCCTCCG

+     CATT-motif

| Site Name | Organism | Position | Strand | Matrix score. | sequence | function |
| --- | --- | --- | --- | --- | --- | --- |
| CATT-motif | Zea mays | 1378 | + | 6 | GCATTC | part of a light responsive element |
| CATT-motif | Zea mays | 1032 | + | 6 | GCATTC | part of a light responsive element |

> 2018/04/13 10:10:12  
+ TTCGATTTTG TAAAAAAGTT AAATTCTTAT TTTTTTAATA ATTTATCCAT AAAAATAAAG TGAAAAATTA   
  
  
+ AAACTATTCA GTGCTGATAT TTTGTAAAAC AAATAGATAT CCAGTCAATA TGAAACATCT ATACTGTGCT   
  
  
+ TAGTCATGGA CACGTGTTTT AATCTAATGA TTATGTAAAA CGATCACTGA TAAGTCAAAC ATCATTATAT   
  
  
+ TACGAAAAAT GGAACATGAA TAAAGACAAA TGGAACTGCA CATGTTAACT CGTAAACTGC GAACCATCTG   
  
  
+ TCTAACCAGG CTCAAGAGTT AAGAAAGAGA ACGCTGTCGT TTCGGAAGAA TACCAGGAGA GAAAAAAAAA   
  
  
+ AAAAAAACTC TTGAGTTTGT TAATATCGAC ACAAATGTAC GTATGTCGCC CAGAAAAGAA GATGAGAATA   
  
  
+ AAAACCAATA AACTTTCTTA GGATTGCACA TGTAAGGTTA ATTATGTAAA TTAATGAATC ATTAACACTG   
  
  
+ CGGCTTTCTA GACGAGCAGC ATGCAGTACA AAGCATACGT GTTACGCGAG TCACATGGGT GATCCATCGC   
  
  
+ CATCAGCGTA AGCAAGGGGT TTGACATGAC GTCATCATCT TACGTAAGCT ACCCCAATCT CTGCGCCAGA   
  
  
+ CGAGTAGTGA GTAGCTACGC GGAAGAAGTT GGGAAGATTC TCCCGTCTCT TCCTCTGGCC CTCTCTTTTT   
  
  
+ CTTCTTGTCT TCCTTGAATT TGTGTTTCCA CTCTTCCAGT CTTCAAATTG ATTCAACACT GCCCGAGAGA   
  
  
+ GAGAGAGAGA GAGTTTTGAA TTGGTCAGCA GTTGGACCTT TCTCACTCTC TTTTATTTTC TTTTTCAATT   
  
  
+ TCTTTTTCTT CTGTAAGCCT TATGTATATA TAATAGGAAA GAGATCCTGA TCCTCAGCGA GGAAACAATC   
  
  
+ TGAGTTTTTC TTTTGTCCCG GGTCAAAACG CCTCTTTTTC CCTATCATTT CTCTTGCATA TTCTCCTCTC   
  
  
+ CTTCTTCCCA ACTTCTTCAT TAAGACAGGC ATAGGCCTTT TCACTTTCCT TGCATTCAAA ACTTTACCAT   
  
  
+ TTCTCTCTCT CTCTCTCTCT CTCTCTCTCT TCAGGCTTAT ATTTCCCTTT ATGTACGCAT ATTGCCCATC   
  
  
+ AACTCTCCAC TTCCAGTATT TGGTGCTGGT GGAGGCTCTT GATCTCCCTT TTTGGTAATT ACTTGTCCAA   
  
  
+ GCCACTGATC TCAGTGAGCT ATTCTTCTTA TCGATCTTGA TCTCTTTGAT CAAACTCACT TTTGATTTCA   
  
  
+ ACACCCTTGC TCTTGATTCT TCATCTCTCA GCTTTTTACG CTATTCATCC CCTTATGCAG TTCCCCTTAA   
  
  
+ TTTCTCCTCT AGGCCTTAAA GGGTTTCTTC TCGACATGGT CTAGTGTGCA TTCGATCCAT TAGTCTGCTT   
  
  
+ TGTAAGTTAG TTCGCACGTA CCCACATAAG CTACAACTGT GTCGCTGCAA TTCTAAGCAT TGTTGGGTTC   
  
  
+ TCTCTTGCGT ACTGCATATT AGAGGAGGC  

- AAGCTAAAAC ATTTTTTCAA TTTAAGAATA AAAAAATTAT TAAATAGGTA TTTTTATTTC ACTTTTTAAT   
  
  
- TTTGATAAGT CACGACTATA AAACATTTTG TTTATCTATA GGTCAGTTAT ACTTTGTAGA TATGACACGA   
  
  
- ATCAGTACCT GTGCACAAAA TTAGATTACT AATACATTTT GCTAGTGACT ATTCAGTTTG TAGTAATATA   
  
  
- ATGCTTTTTA CCTTGTACTT ATTTCTGTTT ACCTTGACGT GTACAATTGA GCATTTGACG CTTGGTAGAC   
  
  
- AGATTGGTCC GAGTTCTCAA TTCTTTCTCT TGCGACAGCA AAGCCTTCTT ATGGTCCTCT CTTTTTTTTT   
  
  
- TTTTTTTGAG AACTCAAACA ATTATAGCTG TGTTTACATG CATACAGCGG GTCTTTTCTT CTACTCTTAT   
  
  
- TTTTGGTTAT TTGAAAGAAT CCTAACGTGT ACATTCCAAT TAATACATTT AATTACTTAG TAATTGTGAC   
  
  
- GCCGAAAGAT CTGCTCGTCG TACGTCATGT TTCGTATGCA CAATGCGCTC AGTGTACCCA CTAGGTAGCG   
  
  
- GTAGTCGCAT TCGTTCCCCA AACTGTACTG CAGTAGTAGA ATGCATTCGA TGGGGTTAGA GACGCGGTCT   
  
  
- GCTCATCACT CATCGATGCG CCTTCTTCAA CCCTTCTAAG AGGGCAGAGA AGGAGACCGG GAGAGAAAAA   
  
  
- GAAGAACAGA AGGAACTTAA ACACAAAGGT GAGAAGGTCA GAAGTTTAAC TAAGTTGTGA CGGGCTCTCT   
  
  
- CTCTCTCTCT CTCAAAACTT AACCAGTCGT CAACCTGGAA AGAGTGAGAG AAAATAAAAG AAAAAGTTAA   
  
  
- AGAAAAAGAA GACATTCGGA ATACATATAT ATTATCCTTT CTCTAGGACT AGGAGTCGCT CCTTTGTTAG   
  
  
- ACTCAAAAAG AAAACAGGGC CCAGTTTTGC GGAGAAAAAG GGATAGTAAA GAGAACGTAT AAGAGGAGAG   
  
  
- GAAGAAGGGT TGAAGAAGTA ATTCTGTCCG TATCCGGAAA AGTGAAAGGA ACGTAAGTTT TGAAATGGTA   
  
  
- AAGAGAGAGA GAGAGAGAGA GAGAGAGAGA AGTCCGAATA TAAAGGGAAA TACATGCGTA TAACGGGTAG   
  
  
- TTGAGAGGTG AAGGTCATAA ACCACGACCA CCTCCGAGAA CTAGAGGGAA AAACCATTAA TGAACAGGTT   
  
  
- CGGTGACTAG AGTCACTCGA TAAGAAGAAT AGCTAGAACT AGAGAAACTA GTTTGAGTGA AAACTAAAGT   
  
  
- TGTGGGAACG AGAACTAAGA AGTAGAGAGT CGAAAAATGC GATAAGTAGG GGAATACGTC AAGGGGAATT   
  
  
- AAAGAGGAGA TCCGGAATTT CCCAAAGAAG AGCTGTACCA GATCACACGT AAGCTAGGTA ATCAGACGAA   
  
  
- ACATTCAATC AAGCGTGCAT GGGTGTATTC GATGTTGACA CAGCGACGTT AAGATTCGTA ACAACCCAAG   
  
  
- AGAGAACGCA TGACGTATAA TCTCCTCCG

+     CGTCA-motif

| Site Name | Organism | Position | Strand | Matrix score. | sequence | function |
| --- | --- | --- | --- | --- | --- | --- |
| CGTCA-motif | Hordeum vulgare | 587 | - | 5 | CGTCA | cis-acting regulatory element involved in the MeJA-responsiveness |
| CGTCA-motif | Hordeum vulgare | 590 | + | 5 | CGTCA | cis-acting regulatory element involved in the MeJA-responsiveness |

> 2018/04/13 10:10:12  
+ TTCGATTTTG TAAAAAAGTT AAATTCTTAT TTTTTTAATA ATTTATCCAT AAAAATAAAG TGAAAAATTA   
  
  
+ AAACTATTCA GTGCTGATAT TTTGTAAAAC AAATAGATAT CCAGTCAATA TGAAACATCT ATACTGTGCT   
  
  
+ TAGTCATGGA CACGTGTTTT AATCTAATGA TTATGTAAAA CGATCACTGA TAAGTCAAAC ATCATTATAT   
  
  
+ TACGAAAAAT GGAACATGAA TAAAGACAAA TGGAACTGCA CATGTTAACT CGTAAACTGC GAACCATCTG   
  
  
+ TCTAACCAGG CTCAAGAGTT AAGAAAGAGA ACGCTGTCGT TTCGGAAGAA TACCAGGAGA GAAAAAAAAA   
  
  
+ AAAAAAACTC TTGAGTTTGT TAATATCGAC ACAAATGTAC GTATGTCGCC CAGAAAAGAA GATGAGAATA   
  
  
+ AAAACCAATA AACTTTCTTA GGATTGCACA TGTAAGGTTA ATTATGTAAA TTAATGAATC ATTAACACTG   
  
  
+ CGGCTTTCTA GACGAGCAGC ATGCAGTACA AAGCATACGT GTTACGCGAG TCACATGGGT GATCCATCGC   
  
  
+ CATCAGCGTA AGCAAGGGGT TTGACATGAC GTCATCATCT TACGTAAGCT ACCCCAATCT CTGCGCCAGA   
  
  
+ CGAGTAGTGA GTAGCTACGC GGAAGAAGTT GGGAAGATTC TCCCGTCTCT TCCTCTGGCC CTCTCTTTTT   
  
  
+ CTTCTTGTCT TCCTTGAATT TGTGTTTCCA CTCTTCCAGT CTTCAAATTG ATTCAACACT GCCCGAGAGA   
  
  
+ GAGAGAGAGA GAGTTTTGAA TTGGTCAGCA GTTGGACCTT TCTCACTCTC TTTTATTTTC TTTTTCAATT   
  
  
+ TCTTTTTCTT CTGTAAGCCT TATGTATATA TAATAGGAAA GAGATCCTGA TCCTCAGCGA GGAAACAATC   
  
  
+ TGAGTTTTTC TTTTGTCCCG GGTCAAAACG CCTCTTTTTC CCTATCATTT CTCTTGCATA TTCTCCTCTC   
  
  
+ CTTCTTCCCA ACTTCTTCAT TAAGACAGGC ATAGGCCTTT TCACTTTCCT TGCATTCAAA ACTTTACCAT   
  
  
+ TTCTCTCTCT CTCTCTCTCT CTCTCTCTCT TCAGGCTTAT ATTTCCCTTT ATGTACGCAT ATTGCCCATC   
  
  
+ AACTCTCCAC TTCCAGTATT TGGTGCTGGT GGAGGCTCTT GATCTCCCTT TTTGGTAATT ACTTGTCCAA   
  
  
+ GCCACTGATC TCAGTGAGCT ATTCTTCTTA TCGATCTTGA TCTCTTTGAT CAAACTCACT TTTGATTTCA   
  
  
+ ACACCCTTGC TCTTGATTCT TCATCTCTCA GCTTTTTACG CTATTCATCC CCTTATGCAG TTCCCCTTAA   
  
  
+ TTTCTCCTCT AGGCCTTAAA GGGTTTCTTC TCGACATGGT CTAGTGTGCA TTCGATCCAT TAGTCTGCTT   
  
  
+ TGTAAGTTAG TTCGCACGTA CCCACATAAG CTACAACTGT GTCGCTGCAA TTCTAAGCAT TGTTGGGTTC   
  
  
+ TCTCTTGCGT ACTGCATATT AGAGGAGGC  

- AAGCTAAAAC ATTTTTTCAA TTTAAGAATA AAAAAATTAT TAAATAGGTA TTTTTATTTC ACTTTTTAAT   
  
  
- TTTGATAAGT CACGACTATA AAACATTTTG TTTATCTATA GGTCAGTTAT ACTTTGTAGA TATGACACGA   
  
  
- ATCAGTACCT GTGCACAAAA TTAGATTACT AATACATTTT GCTAGTGACT ATTCAGTTTG TAGTAATATA   
  
  
- ATGCTTTTTA CCTTGTACTT ATTTCTGTTT ACCTTGACGT GTACAATTGA GCATTTGACG CTTGGTAGAC   
  
  
- AGATTGGTCC GAGTTCTCAA TTCTTTCTCT TGCGACAGCA AAGCCTTCTT ATGGTCCTCT CTTTTTTTTT   
  
  
- TTTTTTTGAG AACTCAAACA ATTATAGCTG TGTTTACATG CATACAGCGG GTCTTTTCTT CTACTCTTAT   
  
  
- TTTTGGTTAT TTGAAAGAAT CCTAACGTGT ACATTCCAAT TAATACATTT AATTACTTAG TAATTGTGAC   
  
  
- GCCGAAAGAT CTGCTCGTCG TACGTCATGT TTCGTATGCA CAATGCGCTC AGTGTACCCA CTAGGTAGCG   
  
  
- GTAGTCGCAT TCGTTCCCCA AACTGTACTG CAGTAGTAGA ATGCATTCGA TGGGGTTAGA GACGCGGTCT   
  
  
- GCTCATCACT CATCGATGCG CCTTCTTCAA CCCTTCTAAG AGGGCAGAGA AGGAGACCGG GAGAGAAAAA   
  
  
- GAAGAACAGA AGGAACTTAA ACACAAAGGT GAGAAGGTCA GAAGTTTAAC TAAGTTGTGA CGGGCTCTCT   
  
  
- CTCTCTCTCT CTCAAAACTT AACCAGTCGT CAACCTGGAA AGAGTGAGAG AAAATAAAAG AAAAAGTTAA   
  
  
- AGAAAAAGAA GACATTCGGA ATACATATAT ATTATCCTTT CTCTAGGACT AGGAGTCGCT CCTTTGTTAG   
  
  
- ACTCAAAAAG AAAACAGGGC CCAGTTTTGC GGAGAAAAAG GGATAGTAAA GAGAACGTAT AAGAGGAGAG   
  
  
- GAAGAAGGGT TGAAGAAGTA ATTCTGTCCG TATCCGGAAA AGTGAAAGGA ACGTAAGTTT TGAAATGGTA   
  
  
- AAGAGAGAGA GAGAGAGAGA GAGAGAGAGA AGTCCGAATA TAAAGGGAAA TACATGCGTA TAACGGGTAG   
  
  
- TTGAGAGGTG AAGGTCATAA ACCACGACCA CCTCCGAGAA CTAGAGGGAA AAACCATTAA TGAACAGGTT   
  
  
- CGGTGACTAG AGTCACTCGA TAAGAAGAAT AGCTAGAACT AGAGAAACTA GTTTGAGTGA AAACTAAAGT   
  
  
- TGTGGGAACG AGAACTAAGA AGTAGAGAGT CGAAAAATGC GATAAGTAGG GGAATACGTC AAGGGGAATT   
  
  
- AAAGAGGAGA TCCGGAATTT CCCAAAGAAG AGCTGTACCA GATCACACGT AAGCTAGGTA ATCAGACGAA   
  
  
- ACATTCAATC AAGCGTGCAT GGGTGTATTC GATGTTGACA CAGCGACGTT AAGATTCGTA ACAACCCAAG   
  
  
- AGAGAACGCA TGACGTATAA TCTCCTCCG

+     G-Box

| Site Name | Organism | Position | Strand | Matrix score. | sequence | function |
| --- | --- | --- | --- | --- | --- | --- |
| G-Box | Antirrhinum majus | 1415 | + | 6 | CACGTA | cis-acting regulatory element involved in light responsiveness |
| G-Box | Pisum sativum | 151 | + | 6 | CACGTG | cis-acting regulatory element involved in light responsiveness |
| G-Box | Antirrhinum majus | 526 | - | 6 | CACGTA | cis-acting regulatory element involved in light responsiveness |

> 2018/04/13 10:10:12  
+ TTCGATTTTG TAAAAAAGTT AAATTCTTAT TTTTTTAATA ATTTATCCAT AAAAATAAAG TGAAAAATTA   
  
  
+ AAACTATTCA GTGCTGATAT TTTGTAAAAC AAATAGATAT CCAGTCAATA TGAAACATCT ATACTGTGCT   
  
  
+ TAGTCATGGA CACGTGTTTT AATCTAATGA TTATGTAAAA CGATCACTGA TAAGTCAAAC ATCATTATAT   
  
  
+ TACGAAAAAT GGAACATGAA TAAAGACAAA TGGAACTGCA CATGTTAACT CGTAAACTGC GAACCATCTG   
  
  
+ TCTAACCAGG CTCAAGAGTT AAGAAAGAGA ACGCTGTCGT TTCGGAAGAA TACCAGGAGA GAAAAAAAAA   
  
  
+ AAAAAAACTC TTGAGTTTGT TAATATCGAC ACAAATGTAC GTATGTCGCC CAGAAAAGAA GATGAGAATA   
  
  
+ AAAACCAATA AACTTTCTTA GGATTGCACA TGTAAGGTTA ATTATGTAAA TTAATGAATC ATTAACACTG   
  
  
+ CGGCTTTCTA GACGAGCAGC ATGCAGTACA AAGCATACGT GTTACGCGAG TCACATGGGT GATCCATCGC   
  
  
+ CATCAGCGTA AGCAAGGGGT TTGACATGAC GTCATCATCT TACGTAAGCT ACCCCAATCT CTGCGCCAGA   
  
  
+ CGAGTAGTGA GTAGCTACGC GGAAGAAGTT GGGAAGATTC TCCCGTCTCT TCCTCTGGCC CTCTCTTTTT   
  
  
+ CTTCTTGTCT TCCTTGAATT TGTGTTTCCA CTCTTCCAGT CTTCAAATTG ATTCAACACT GCCCGAGAGA   
  
  
+ GAGAGAGAGA GAGTTTTGAA TTGGTCAGCA GTTGGACCTT TCTCACTCTC TTTTATTTTC TTTTTCAATT   
  
  
+ TCTTTTTCTT CTGTAAGCCT TATGTATATA TAATAGGAAA GAGATCCTGA TCCTCAGCGA GGAAACAATC   
  
  
+ TGAGTTTTTC TTTTGTCCCG GGTCAAAACG CCTCTTTTTC CCTATCATTT CTCTTGCATA TTCTCCTCTC   
  
  
+ CTTCTTCCCA ACTTCTTCAT TAAGACAGGC ATAGGCCTTT TCACTTTCCT TGCATTCAAA ACTTTACCAT   
  
  
+ TTCTCTCTCT CTCTCTCTCT CTCTCTCTCT TCAGGCTTAT ATTTCCCTTT ATGTACGCAT ATTGCCCATC   
  
  
+ AACTCTCCAC TTCCAGTATT TGGTGCTGGT GGAGGCTCTT GATCTCCCTT TTTGGTAATT ACTTGTCCAA   
  
  
+ GCCACTGATC TCAGTGAGCT ATTCTTCTTA TCGATCTTGA TCTCTTTGAT CAAACTCACT TTTGATTTCA   
  
  
+ ACACCCTTGC TCTTGATTCT TCATCTCTCA GCTTTTTACG CTATTCATCC CCTTATGCAG TTCCCCTTAA   
  
  
+ TTTCTCCTCT AGGCCTTAAA GGGTTTCTTC TCGACATGGT CTAGTGTGCA TTCGATCCAT TAGTCTGCTT   
  
  
+ TGTAAGTTAG TTCGCACGTA CCCACATAAG CTACAACTGT GTCGCTGCAA TTCTAAGCAT TGTTGGGTTC   
  
  
+ TCTCTTGCGT ACTGCATATT AGAGGAGGC  

- AAGCTAAAAC ATTTTTTCAA TTTAAGAATA AAAAAATTAT TAAATAGGTA TTTTTATTTC ACTTTTTAAT   
  
  
- TTTGATAAGT CACGACTATA AAACATTTTG TTTATCTATA GGTCAGTTAT ACTTTGTAGA TATGACACGA   
  
  
- ATCAGTACCT GTGCACAAAA TTAGATTACT AATACATTTT GCTAGTGACT ATTCAGTTTG TAGTAATATA   
  
  
- ATGCTTTTTA CCTTGTACTT ATTTCTGTTT ACCTTGACGT GTACAATTGA GCATTTGACG CTTGGTAGAC   
  
  
- AGATTGGTCC GAGTTCTCAA TTCTTTCTCT TGCGACAGCA AAGCCTTCTT ATGGTCCTCT CTTTTTTTTT   
  
  
- TTTTTTTGAG AACTCAAACA ATTATAGCTG TGTTTACATG CATACAGCGG GTCTTTTCTT CTACTCTTAT   
  
  
- TTTTGGTTAT TTGAAAGAAT CCTAACGTGT ACATTCCAAT TAATACATTT AATTACTTAG TAATTGTGAC   
  
  
- GCCGAAAGAT CTGCTCGTCG TACGTCATGT TTCGTATGCA CAATGCGCTC AGTGTACCCA CTAGGTAGCG   
  
  
- GTAGTCGCAT TCGTTCCCCA AACTGTACTG CAGTAGTAGA ATGCATTCGA TGGGGTTAGA GACGCGGTCT   
  
  
- GCTCATCACT CATCGATGCG CCTTCTTCAA CCCTTCTAAG AGGGCAGAGA AGGAGACCGG GAGAGAAAAA   
  
  
- GAAGAACAGA AGGAACTTAA ACACAAAGGT GAGAAGGTCA GAAGTTTAAC TAAGTTGTGA CGGGCTCTCT   
  
  
- CTCTCTCTCT CTCAAAACTT AACCAGTCGT CAACCTGGAA AGAGTGAGAG AAAATAAAAG AAAAAGTTAA   
  
  
- AGAAAAAGAA GACATTCGGA ATACATATAT ATTATCCTTT CTCTAGGACT AGGAGTCGCT CCTTTGTTAG   
  
  
- ACTCAAAAAG AAAACAGGGC CCAGTTTTGC GGAGAAAAAG GGATAGTAAA GAGAACGTAT AAGAGGAGAG   
  
  
- GAAGAAGGGT TGAAGAAGTA ATTCTGTCCG TATCCGGAAA AGTGAAAGGA ACGTAAGTTT TGAAATGGTA   
  
  
- AAGAGAGAGA GAGAGAGAGA GAGAGAGAGA AGTCCGAATA TAAAGGGAAA TACATGCGTA TAACGGGTAG   
  
  
- TTGAGAGGTG AAGGTCATAA ACCACGACCA CCTCCGAGAA CTAGAGGGAA AAACCATTAA TGAACAGGTT   
  
  
- CGGTGACTAG AGTCACTCGA TAAGAAGAAT AGCTAGAACT AGAGAAACTA GTTTGAGTGA AAACTAAAGT   
  
  
- TGTGGGAACG AGAACTAAGA AGTAGAGAGT CGAAAAATGC GATAAGTAGG GGAATACGTC AAGGGGAATT   
  
  
- AAAGAGGAGA TCCGGAATTT CCCAAAGAAG AGCTGTACCA GATCACACGT AAGCTAGGTA ATCAGACGAA   
  
  
- ACATTCAATC AAGCGTGCAT GGGTGTATTC GATGTTGACA CAGCGACGTT AAGATTCGTA ACAACCCAAG   
  
  
- AGAGAACGCA TGACGTATAA TCTCCTCCG

+     G-box

| Site Name | Organism | Position | Strand | Matrix score. | sequence | function |
| --- | --- | --- | --- | --- | --- | --- |
| G-box | Brassica oleracea | 525 | - | 9 | TAACACGTAG | cis-acting regulatory element involved in light responsiveness |
| G-box | Solanum tuberosum | 542 | + | 7 | CACATGG | cis-acting regulatory element involved in light responsiveness |
| G-box | Daucus carota | 526 | + | 6 | TACGTG | cis-acting regulatory element involved in light responsiveness |
| G-box | Daucus carota | 1415 | - | 6 | TACGTG | cis-acting regulatory element involved in light responsiveness |
| G-box | Arabidopsis thaliana | 151 | + | 6 | CACGTG | cis-acting regulatory element involved in light responsiveness |
| G-box | Brassica napus | 150 | + | 8 | ACACGTGT | cis-acting regulatory element involved in light responsiveness |

> 2018/04/13 10:10:12  
+ TTCGATTTTG TAAAAAAGTT AAATTCTTAT TTTTTTAATA ATTTATCCAT AAAAATAAAG TGAAAAATTA   
  
  
+ AAACTATTCA GTGCTGATAT TTTGTAAAAC AAATAGATAT CCAGTCAATA TGAAACATCT ATACTGTGCT   
  
  
+ TAGTCATGGA CACGTGTTTT AATCTAATGA TTATGTAAAA CGATCACTGA TAAGTCAAAC ATCATTATAT   
  
  
+ TACGAAAAAT GGAACATGAA TAAAGACAAA TGGAACTGCA CATGTTAACT CGTAAACTGC GAACCATCTG   
  
  
+ TCTAACCAGG CTCAAGAGTT AAGAAAGAGA ACGCTGTCGT TTCGGAAGAA TACCAGGAGA GAAAAAAAAA   
  
  
+ AAAAAAACTC TTGAGTTTGT TAATATCGAC ACAAATGTAC GTATGTCGCC CAGAAAAGAA GATGAGAATA   
  
  
+ AAAACCAATA AACTTTCTTA GGATTGCACA TGTAAGGTTA ATTATGTAAA TTAATGAATC ATTAACACTG   
  
  
+ CGGCTTTCTA GACGAGCAGC ATGCAGTACA AAGCATACGT GTTACGCGAG TCACATGGGT GATCCATCGC   
  
  
+ CATCAGCGTA AGCAAGGGGT TTGACATGAC GTCATCATCT TACGTAAGCT ACCCCAATCT CTGCGCCAGA   
  
  
+ CGAGTAGTGA GTAGCTACGC GGAAGAAGTT GGGAAGATTC TCCCGTCTCT TCCTCTGGCC CTCTCTTTTT   
  
  
+ CTTCTTGTCT TCCTTGAATT TGTGTTTCCA CTCTTCCAGT CTTCAAATTG ATTCAACACT GCCCGAGAGA   
  
  
+ GAGAGAGAGA GAGTTTTGAA TTGGTCAGCA GTTGGACCTT TCTCACTCTC TTTTATTTTC TTTTTCAATT   
  
  
+ TCTTTTTCTT CTGTAAGCCT TATGTATATA TAATAGGAAA GAGATCCTGA TCCTCAGCGA GGAAACAATC   
  
  
+ TGAGTTTTTC TTTTGTCCCG GGTCAAAACG CCTCTTTTTC CCTATCATTT CTCTTGCATA TTCTCCTCTC   
  
  
+ CTTCTTCCCA ACTTCTTCAT TAAGACAGGC ATAGGCCTTT TCACTTTCCT TGCATTCAAA ACTTTACCAT   
  
  
+ TTCTCTCTCT CTCTCTCTCT CTCTCTCTCT TCAGGCTTAT ATTTCCCTTT ATGTACGCAT ATTGCCCATC   
  
  
+ AACTCTCCAC TTCCAGTATT TGGTGCTGGT GGAGGCTCTT GATCTCCCTT TTTGGTAATT ACTTGTCCAA   
  
  
+ GCCACTGATC TCAGTGAGCT ATTCTTCTTA TCGATCTTGA TCTCTTTGAT CAAACTCACT TTTGATTTCA   
  
  
+ ACACCCTTGC TCTTGATTCT TCATCTCTCA GCTTTTTACG CTATTCATCC CCTTATGCAG TTCCCCTTAA   
  
  
+ TTTCTCCTCT AGGCCTTAAA GGGTTTCTTC TCGACATGGT CTAGTGTGCA TTCGATCCAT TAGTCTGCTT   
  
  
+ TGTAAGTTAG TTCGCACGTA CCCACATAAG CTACAACTGT GTCGCTGCAA TTCTAAGCAT TGTTGGGTTC   
  
  
+ TCTCTTGCGT ACTGCATATT AGAGGAGGC  

- AAGCTAAAAC ATTTTTTCAA TTTAAGAATA AAAAAATTAT TAAATAGGTA TTTTTATTTC ACTTTTTAAT   
  
  
- TTTGATAAGT CACGACTATA AAACATTTTG TTTATCTATA GGTCAGTTAT ACTTTGTAGA TATGACACGA   
  
  
- ATCAGTACCT GTGCACAAAA TTAGATTACT AATACATTTT GCTAGTGACT ATTCAGTTTG TAGTAATATA   
  
  
- ATGCTTTTTA CCTTGTACTT ATTTCTGTTT ACCTTGACGT GTACAATTGA GCATTTGACG CTTGGTAGAC   
  
  
- AGATTGGTCC GAGTTCTCAA TTCTTTCTCT TGCGACAGCA AAGCCTTCTT ATGGTCCTCT CTTTTTTTTT   
  
  
- TTTTTTTGAG AACTCAAACA ATTATAGCTG TGTTTACATG CATACAGCGG GTCTTTTCTT CTACTCTTAT   
  
  
- TTTTGGTTAT TTGAAAGAAT CCTAACGTGT ACATTCCAAT TAATACATTT AATTACTTAG TAATTGTGAC   
  
  
- GCCGAAAGAT CTGCTCGTCG TACGTCATGT TTCGTATGCA CAATGCGCTC AGTGTACCCA CTAGGTAGCG   
  
  
- GTAGTCGCAT TCGTTCCCCA AACTGTACTG CAGTAGTAGA ATGCATTCGA TGGGGTTAGA GACGCGGTCT   
  
  
- GCTCATCACT CATCGATGCG CCTTCTTCAA CCCTTCTAAG AGGGCAGAGA AGGAGACCGG GAGAGAAAAA   
  
  
- GAAGAACAGA AGGAACTTAA ACACAAAGGT GAGAAGGTCA GAAGTTTAAC TAAGTTGTGA CGGGCTCTCT   
  
  
- CTCTCTCTCT CTCAAAACTT AACCAGTCGT CAACCTGGAA AGAGTGAGAG AAAATAAAAG AAAAAGTTAA   
  
  
- AGAAAAAGAA GACATTCGGA ATACATATAT ATTATCCTTT CTCTAGGACT AGGAGTCGCT CCTTTGTTAG   
  
  
- ACTCAAAAAG AAAACAGGGC CCAGTTTTGC GGAGAAAAAG GGATAGTAAA GAGAACGTAT AAGAGGAGAG   
  
  
- GAAGAAGGGT TGAAGAAGTA ATTCTGTCCG TATCCGGAAA AGTGAAAGGA ACGTAAGTTT TGAAATGGTA   
  
  
- AAGAGAGAGA GAGAGAGAGA GAGAGAGAGA AGTCCGAATA TAAAGGGAAA TACATGCGTA TAACGGGTAG   
  
  
- TTGAGAGGTG AAGGTCATAA ACCACGACCA CCTCCGAGAA CTAGAGGGAA AAACCATTAA TGAACAGGTT   
  
  
- CGGTGACTAG AGTCACTCGA TAAGAAGAAT AGCTAGAACT AGAGAAACTA GTTTGAGTGA AAACTAAAGT   
  
  
- TGTGGGAACG AGAACTAAGA AGTAGAGAGT CGAAAAATGC GATAAGTAGG GGAATACGTC AAGGGGAATT   
  
  
- AAAGAGGAGA TCCGGAATTT CCCAAAGAAG AGCTGTACCA GATCACACGT AAGCTAGGTA ATCAGACGAA   
  
  
- ACATTCAATC AAGCGTGCAT GGGTGTATTC GATGTTGACA CAGCGACGTT AAGATTCGTA ACAACCCAAG   
  
  
- AGAGAACGCA TGACGTATAA TCTCCTCCG

+     GA-motif

| Site Name | Organism | Position | Strand | Matrix score. | sequence | function |
| --- | --- | --- | --- | --- | --- | --- |
| GA-motif | Glycine max | 708 | - | 8 | AAGGAAGA | part of a light responsive element |

> 2018/04/13 10:10:12  
+ TTCGATTTTG TAAAAAAGTT AAATTCTTAT TTTTTTAATA ATTTATCCAT AAAAATAAAG TGAAAAATTA   
  
  
+ AAACTATTCA GTGCTGATAT TTTGTAAAAC AAATAGATAT CCAGTCAATA TGAAACATCT ATACTGTGCT   
  
  
+ TAGTCATGGA CACGTGTTTT AATCTAATGA TTATGTAAAA CGATCACTGA TAAGTCAAAC ATCATTATAT   
  
  
+ TACGAAAAAT GGAACATGAA TAAAGACAAA TGGAACTGCA CATGTTAACT CGTAAACTGC GAACCATCTG   
  
  
+ TCTAACCAGG CTCAAGAGTT AAGAAAGAGA ACGCTGTCGT TTCGGAAGAA TACCAGGAGA GAAAAAAAAA   
  
  
+ AAAAAAACTC TTGAGTTTGT TAATATCGAC ACAAATGTAC GTATGTCGCC CAGAAAAGAA GATGAGAATA   
  
  
+ AAAACCAATA AACTTTCTTA GGATTGCACA TGTAAGGTTA ATTATGTAAA TTAATGAATC ATTAACACTG   
  
  
+ CGGCTTTCTA GACGAGCAGC ATGCAGTACA AAGCATACGT GTTACGCGAG TCACATGGGT GATCCATCGC   
  
  
+ CATCAGCGTA AGCAAGGGGT TTGACATGAC GTCATCATCT TACGTAAGCT ACCCCAATCT CTGCGCCAGA   
  
  
+ CGAGTAGTGA GTAGCTACGC GGAAGAAGTT GGGAAGATTC TCCCGTCTCT TCCTCTGGCC CTCTCTTTTT   
  
  
+ CTTCTTGTCT TCCTTGAATT TGTGTTTCCA CTCTTCCAGT CTTCAAATTG ATTCAACACT GCCCGAGAGA   
  
  
+ GAGAGAGAGA GAGTTTTGAA TTGGTCAGCA GTTGGACCTT TCTCACTCTC TTTTATTTTC TTTTTCAATT   
  
  
+ TCTTTTTCTT CTGTAAGCCT TATGTATATA TAATAGGAAA GAGATCCTGA TCCTCAGCGA GGAAACAATC   
  
  
+ TGAGTTTTTC TTTTGTCCCG GGTCAAAACG CCTCTTTTTC CCTATCATTT CTCTTGCATA TTCTCCTCTC   
  
  
+ CTTCTTCCCA ACTTCTTCAT TAAGACAGGC ATAGGCCTTT TCACTTTCCT TGCATTCAAA ACTTTACCAT   
  
  
+ TTCTCTCTCT CTCTCTCTCT CTCTCTCTCT TCAGGCTTAT ATTTCCCTTT ATGTACGCAT ATTGCCCATC   
  
  
+ AACTCTCCAC TTCCAGTATT TGGTGCTGGT GGAGGCTCTT GATCTCCCTT TTTGGTAATT ACTTGTCCAA   
  
  
+ GCCACTGATC TCAGTGAGCT ATTCTTCTTA TCGATCTTGA TCTCTTTGAT CAAACTCACT TTTGATTTCA   
  
  
+ ACACCCTTGC TCTTGATTCT TCATCTCTCA GCTTTTTACG CTATTCATCC CCTTATGCAG TTCCCCTTAA   
  
  
+ TTTCTCCTCT AGGCCTTAAA GGGTTTCTTC TCGACATGGT CTAGTGTGCA TTCGATCCAT TAGTCTGCTT   
  
  
+ TGTAAGTTAG TTCGCACGTA CCCACATAAG CTACAACTGT GTCGCTGCAA TTCTAAGCAT TGTTGGGTTC   
  
  
+ TCTCTTGCGT ACTGCATATT AGAGGAGGC  

- AAGCTAAAAC ATTTTTTCAA TTTAAGAATA AAAAAATTAT TAAATAGGTA TTTTTATTTC ACTTTTTAAT   
  
  
- TTTGATAAGT CACGACTATA AAACATTTTG TTTATCTATA GGTCAGTTAT ACTTTGTAGA TATGACACGA   
  
  
- ATCAGTACCT GTGCACAAAA TTAGATTACT AATACATTTT GCTAGTGACT ATTCAGTTTG TAGTAATATA   
  
  
- ATGCTTTTTA CCTTGTACTT ATTTCTGTTT ACCTTGACGT GTACAATTGA GCATTTGACG CTTGGTAGAC   
  
  
- AGATTGGTCC GAGTTCTCAA TTCTTTCTCT TGCGACAGCA AAGCCTTCTT ATGGTCCTCT CTTTTTTTTT   
  
  
- TTTTTTTGAG AACTCAAACA ATTATAGCTG TGTTTACATG CATACAGCGG GTCTTTTCTT CTACTCTTAT   
  
  
- TTTTGGTTAT TTGAAAGAAT CCTAACGTGT ACATTCCAAT TAATACATTT AATTACTTAG TAATTGTGAC   
  
  
- GCCGAAAGAT CTGCTCGTCG TACGTCATGT TTCGTATGCA CAATGCGCTC AGTGTACCCA CTAGGTAGCG   
  
  
- GTAGTCGCAT TCGTTCCCCA AACTGTACTG CAGTAGTAGA ATGCATTCGA TGGGGTTAGA GACGCGGTCT   
  
  
- GCTCATCACT CATCGATGCG CCTTCTTCAA CCCTTCTAAG AGGGCAGAGA AGGAGACCGG GAGAGAAAAA   
  
  
- GAAGAACAGA AGGAACTTAA ACACAAAGGT GAGAAGGTCA GAAGTTTAAC TAAGTTGTGA CGGGCTCTCT   
  
  
- CTCTCTCTCT CTCAAAACTT AACCAGTCGT CAACCTGGAA AGAGTGAGAG AAAATAAAAG AAAAAGTTAA   
  
  
- AGAAAAAGAA GACATTCGGA ATACATATAT ATTATCCTTT CTCTAGGACT AGGAGTCGCT CCTTTGTTAG   
  
  
- ACTCAAAAAG AAAACAGGGC CCAGTTTTGC GGAGAAAAAG GGATAGTAAA GAGAACGTAT AAGAGGAGAG   
  
  
- GAAGAAGGGT TGAAGAAGTA ATTCTGTCCG TATCCGGAAA AGTGAAAGGA ACGTAAGTTT TGAAATGGTA   
  
  
- AAGAGAGAGA GAGAGAGAGA GAGAGAGAGA AGTCCGAATA TAAAGGGAAA TACATGCGTA TAACGGGTAG   
  
  
- TTGAGAGGTG AAGGTCATAA ACCACGACCA CCTCCGAGAA CTAGAGGGAA AAACCATTAA TGAACAGGTT   
  
  
- CGGTGACTAG AGTCACTCGA TAAGAAGAAT AGCTAGAACT AGAGAAACTA GTTTGAGTGA AAACTAAAGT   
  
  
- TGTGGGAACG AGAACTAAGA AGTAGAGAGT CGAAAAATGC GATAAGTAGG GGAATACGTC AAGGGGAATT   
  
  
- AAAGAGGAGA TCCGGAATTT CCCAAAGAAG AGCTGTACCA GATCACACGT AAGCTAGGTA ATCAGACGAA   
  
  
- ACATTCAATC AAGCGTGCAT GGGTGTATTC GATGTTGACA CAGCGACGTT AAGATTCGTA ACAACCCAAG   
  
  
- AGAGAACGCA TGACGTATAA TCTCCTCCG

+     GAG-motif

| Site Name | Organism | Position | Strand | Matrix score. | sequence | function |
| --- | --- | --- | --- | --- | --- | --- |
| GAG-motif | Arabidopsis thaliana | 815 | - | 7 | AGAGAGT | part of a light responsive element |
| GAG-motif | Spinacia oleracea | 1282 | - | 7 | AGAGATG | part of a light responsive element |
| GAG-motif | Arabidopsis thaliana | 778 | + | 7 | AGAGAGT | part of a light responsive element |

> 2018/04/13 10:10:12  
+ TTCGATTTTG TAAAAAAGTT AAATTCTTAT TTTTTTAATA ATTTATCCAT AAAAATAAAG TGAAAAATTA   
  
  
+ AAACTATTCA GTGCTGATAT TTTGTAAAAC AAATAGATAT CCAGTCAATA TGAAACATCT ATACTGTGCT   
  
  
+ TAGTCATGGA CACGTGTTTT AATCTAATGA TTATGTAAAA CGATCACTGA TAAGTCAAAC ATCATTATAT   
  
  
+ TACGAAAAAT GGAACATGAA TAAAGACAAA TGGAACTGCA CATGTTAACT CGTAAACTGC GAACCATCTG   
  
  
+ TCTAACCAGG CTCAAGAGTT AAGAAAGAGA ACGCTGTCGT TTCGGAAGAA TACCAGGAGA GAAAAAAAAA   
  
  
+ AAAAAAACTC TTGAGTTTGT TAATATCGAC ACAAATGTAC GTATGTCGCC CAGAAAAGAA GATGAGAATA   
  
  
+ AAAACCAATA AACTTTCTTA GGATTGCACA TGTAAGGTTA ATTATGTAAA TTAATGAATC ATTAACACTG   
  
  
+ CGGCTTTCTA GACGAGCAGC ATGCAGTACA AAGCATACGT GTTACGCGAG TCACATGGGT GATCCATCGC   
  
  
+ CATCAGCGTA AGCAAGGGGT TTGACATGAC GTCATCATCT TACGTAAGCT ACCCCAATCT CTGCGCCAGA   
  
  
+ CGAGTAGTGA GTAGCTACGC GGAAGAAGTT GGGAAGATTC TCCCGTCTCT TCCTCTGGCC CTCTCTTTTT   
  
  
+ CTTCTTGTCT TCCTTGAATT TGTGTTTCCA CTCTTCCAGT CTTCAAATTG ATTCAACACT GCCCGAGAGA   
  
  
+ GAGAGAGAGA GAGTTTTGAA TTGGTCAGCA GTTGGACCTT TCTCACTCTC TTTTATTTTC TTTTTCAATT   
  
  
+ TCTTTTTCTT CTGTAAGCCT TATGTATATA TAATAGGAAA GAGATCCTGA TCCTCAGCGA GGAAACAATC   
  
  
+ TGAGTTTTTC TTTTGTCCCG GGTCAAAACG CCTCTTTTTC CCTATCATTT CTCTTGCATA TTCTCCTCTC   
  
  
+ CTTCTTCCCA ACTTCTTCAT TAAGACAGGC ATAGGCCTTT TCACTTTCCT TGCATTCAAA ACTTTACCAT   
  
  
+ TTCTCTCTCT CTCTCTCTCT CTCTCTCTCT TCAGGCTTAT ATTTCCCTTT ATGTACGCAT ATTGCCCATC   
  
  
+ AACTCTCCAC TTCCAGTATT TGGTGCTGGT GGAGGCTCTT GATCTCCCTT TTTGGTAATT ACTTGTCCAA   
  
  
+ GCCACTGATC TCAGTGAGCT ATTCTTCTTA TCGATCTTGA TCTCTTTGAT CAAACTCACT TTTGATTTCA   
  
  
+ ACACCCTTGC TCTTGATTCT TCATCTCTCA GCTTTTTACG CTATTCATCC CCTTATGCAG TTCCCCTTAA   
  
  
+ TTTCTCCTCT AGGCCTTAAA GGGTTTCTTC TCGACATGGT CTAGTGTGCA TTCGATCCAT TAGTCTGCTT   
  
  
+ TGTAAGTTAG TTCGCACGTA CCCACATAAG CTACAACTGT GTCGCTGCAA TTCTAAGCAT TGTTGGGTTC   
  
  
+ TCTCTTGCGT ACTGCATATT AGAGGAGGC  

- AAGCTAAAAC ATTTTTTCAA TTTAAGAATA AAAAAATTAT TAAATAGGTA TTTTTATTTC ACTTTTTAAT   
  
  
- TTTGATAAGT CACGACTATA AAACATTTTG TTTATCTATA GGTCAGTTAT ACTTTGTAGA TATGACACGA   
  
  
- ATCAGTACCT GTGCACAAAA TTAGATTACT AATACATTTT GCTAGTGACT ATTCAGTTTG TAGTAATATA   
  
  
- ATGCTTTTTA CCTTGTACTT ATTTCTGTTT ACCTTGACGT GTACAATTGA GCATTTGACG CTTGGTAGAC   
  
  
- AGATTGGTCC GAGTTCTCAA TTCTTTCTCT TGCGACAGCA AAGCCTTCTT ATGGTCCTCT CTTTTTTTTT   
  
  
- TTTTTTTGAG AACTCAAACA ATTATAGCTG TGTTTACATG CATACAGCGG GTCTTTTCTT CTACTCTTAT   
  
  
- TTTTGGTTAT TTGAAAGAAT CCTAACGTGT ACATTCCAAT TAATACATTT AATTACTTAG TAATTGTGAC   
  
  
- GCCGAAAGAT CTGCTCGTCG TACGTCATGT TTCGTATGCA CAATGCGCTC AGTGTACCCA CTAGGTAGCG   
  
  
- GTAGTCGCAT TCGTTCCCCA AACTGTACTG CAGTAGTAGA ATGCATTCGA TGGGGTTAGA GACGCGGTCT   
  
  
- GCTCATCACT CATCGATGCG CCTTCTTCAA CCCTTCTAAG AGGGCAGAGA AGGAGACCGG GAGAGAAAAA   
  
  
- GAAGAACAGA AGGAACTTAA ACACAAAGGT GAGAAGGTCA GAAGTTTAAC TAAGTTGTGA CGGGCTCTCT   
  
  
- CTCTCTCTCT CTCAAAACTT AACCAGTCGT CAACCTGGAA AGAGTGAGAG AAAATAAAAG AAAAAGTTAA   
  
  
- AGAAAAAGAA GACATTCGGA ATACATATAT ATTATCCTTT CTCTAGGACT AGGAGTCGCT CCTTTGTTAG   
  
  
- ACTCAAAAAG AAAACAGGGC CCAGTTTTGC GGAGAAAAAG GGATAGTAAA GAGAACGTAT AAGAGGAGAG   
  
  
- GAAGAAGGGT TGAAGAAGTA ATTCTGTCCG TATCCGGAAA AGTGAAAGGA ACGTAAGTTT TGAAATGGTA   
  
  
- AAGAGAGAGA GAGAGAGAGA GAGAGAGAGA AGTCCGAATA TAAAGGGAAA TACATGCGTA TAACGGGTAG   
  
  
- TTGAGAGGTG AAGGTCATAA ACCACGACCA CCTCCGAGAA CTAGAGGGAA AAACCATTAA TGAACAGGTT   
  
  
- CGGTGACTAG AGTCACTCGA TAAGAAGAAT AGCTAGAACT AGAGAAACTA GTTTGAGTGA AAACTAAAGT   
  
  
- TGTGGGAACG AGAACTAAGA AGTAGAGAGT CGAAAAATGC GATAAGTAGG GGAATACGTC AAGGGGAATT   
  
  
- AAAGAGGAGA TCCGGAATTT CCCAAAGAAG AGCTGTACCA GATCACACGT AAGCTAGGTA ATCAGACGAA   
  
  
- ACATTCAATC AAGCGTGCAT GGGTGTATTC GATGTTGACA CAGCGACGTT AAGATTCGTA ACAACCCAAG   
  
  
- AGAGAACGCA TGACGTATAA TCTCCTCCG

+     GATA-motif

| Site Name | Organism | Position | Strand | Matrix score. | sequence | function |
| --- | --- | --- | --- | --- | --- | --- |
| GATA-motif | Pisum sativum | 950 | - | 7 | GATAGGG | part of a light responsive element |

> 2018/04/13 10:10:12  
+ TTCGATTTTG TAAAAAAGTT AAATTCTTAT TTTTTTAATA ATTTATCCAT AAAAATAAAG TGAAAAATTA   
  
  
+ AAACTATTCA GTGCTGATAT TTTGTAAAAC AAATAGATAT CCAGTCAATA TGAAACATCT ATACTGTGCT   
  
  
+ TAGTCATGGA CACGTGTTTT AATCTAATGA TTATGTAAAA CGATCACTGA TAAGTCAAAC ATCATTATAT   
  
  
+ TACGAAAAAT GGAACATGAA TAAAGACAAA TGGAACTGCA CATGTTAACT CGTAAACTGC GAACCATCTG   
  
  
+ TCTAACCAGG CTCAAGAGTT AAGAAAGAGA ACGCTGTCGT TTCGGAAGAA TACCAGGAGA GAAAAAAAAA   
  
  
+ AAAAAAACTC TTGAGTTTGT TAATATCGAC ACAAATGTAC GTATGTCGCC CAGAAAAGAA GATGAGAATA   
  
  
+ AAAACCAATA AACTTTCTTA GGATTGCACA TGTAAGGTTA ATTATGTAAA TTAATGAATC ATTAACACTG   
  
  
+ CGGCTTTCTA GACGAGCAGC ATGCAGTACA AAGCATACGT GTTACGCGAG TCACATGGGT GATCCATCGC   
  
  
+ CATCAGCGTA AGCAAGGGGT TTGACATGAC GTCATCATCT TACGTAAGCT ACCCCAATCT CTGCGCCAGA   
  
  
+ CGAGTAGTGA GTAGCTACGC GGAAGAAGTT GGGAAGATTC TCCCGTCTCT TCCTCTGGCC CTCTCTTTTT   
  
  
+ CTTCTTGTCT TCCTTGAATT TGTGTTTCCA CTCTTCCAGT CTTCAAATTG ATTCAACACT GCCCGAGAGA   
  
  
+ GAGAGAGAGA GAGTTTTGAA TTGGTCAGCA GTTGGACCTT TCTCACTCTC TTTTATTTTC TTTTTCAATT   
  
  
+ TCTTTTTCTT CTGTAAGCCT TATGTATATA TAATAGGAAA GAGATCCTGA TCCTCAGCGA GGAAACAATC   
  
  
+ TGAGTTTTTC TTTTGTCCCG GGTCAAAACG CCTCTTTTTC CCTATCATTT CTCTTGCATA TTCTCCTCTC   
  
  
+ CTTCTTCCCA ACTTCTTCAT TAAGACAGGC ATAGGCCTTT TCACTTTCCT TGCATTCAAA ACTTTACCAT   
  
  
+ TTCTCTCTCT CTCTCTCTCT CTCTCTCTCT TCAGGCTTAT ATTTCCCTTT ATGTACGCAT ATTGCCCATC   
  
  
+ AACTCTCCAC TTCCAGTATT TGGTGCTGGT GGAGGCTCTT GATCTCCCTT TTTGGTAATT ACTTGTCCAA   
  
  
+ GCCACTGATC TCAGTGAGCT ATTCTTCTTA TCGATCTTGA TCTCTTTGAT CAAACTCACT TTTGATTTCA   
  
  
+ ACACCCTTGC TCTTGATTCT TCATCTCTCA GCTTTTTACG CTATTCATCC CCTTATGCAG TTCCCCTTAA   
  
  
+ TTTCTCCTCT AGGCCTTAAA GGGTTTCTTC TCGACATGGT CTAGTGTGCA TTCGATCCAT TAGTCTGCTT   
  
  
+ TGTAAGTTAG TTCGCACGTA CCCACATAAG CTACAACTGT GTCGCTGCAA TTCTAAGCAT TGTTGGGTTC   
  
  
+ TCTCTTGCGT ACTGCATATT AGAGGAGGC  

- AAGCTAAAAC ATTTTTTCAA TTTAAGAATA AAAAAATTAT TAAATAGGTA TTTTTATTTC ACTTTTTAAT   
  
  
- TTTGATAAGT CACGACTATA AAACATTTTG TTTATCTATA GGTCAGTTAT ACTTTGTAGA TATGACACGA   
  
  
- ATCAGTACCT GTGCACAAAA TTAGATTACT AATACATTTT GCTAGTGACT ATTCAGTTTG TAGTAATATA   
  
  
- ATGCTTTTTA CCTTGTACTT ATTTCTGTTT ACCTTGACGT GTACAATTGA GCATTTGACG CTTGGTAGAC   
  
  
- AGATTGGTCC GAGTTCTCAA TTCTTTCTCT TGCGACAGCA AAGCCTTCTT ATGGTCCTCT CTTTTTTTTT   
  
  
- TTTTTTTGAG AACTCAAACA ATTATAGCTG TGTTTACATG CATACAGCGG GTCTTTTCTT CTACTCTTAT   
  
  
- TTTTGGTTAT TTGAAAGAAT CCTAACGTGT ACATTCCAAT TAATACATTT AATTACTTAG TAATTGTGAC   
  
  
- GCCGAAAGAT CTGCTCGTCG TACGTCATGT TTCGTATGCA CAATGCGCTC AGTGTACCCA CTAGGTAGCG   
  
  
- GTAGTCGCAT TCGTTCCCCA AACTGTACTG CAGTAGTAGA ATGCATTCGA TGGGGTTAGA GACGCGGTCT   
  
  
- GCTCATCACT CATCGATGCG CCTTCTTCAA CCCTTCTAAG AGGGCAGAGA AGGAGACCGG GAGAGAAAAA   
  
  
- GAAGAACAGA AGGAACTTAA ACACAAAGGT GAGAAGGTCA GAAGTTTAAC TAAGTTGTGA CGGGCTCTCT   
  
  
- CTCTCTCTCT CTCAAAACTT AACCAGTCGT CAACCTGGAA AGAGTGAGAG AAAATAAAAG AAAAAGTTAA   
  
  
- AGAAAAAGAA GACATTCGGA ATACATATAT ATTATCCTTT CTCTAGGACT AGGAGTCGCT CCTTTGTTAG   
  
  
- ACTCAAAAAG AAAACAGGGC CCAGTTTTGC GGAGAAAAAG GGATAGTAAA GAGAACGTAT AAGAGGAGAG   
  
  
- GAAGAAGGGT TGAAGAAGTA ATTCTGTCCG TATCCGGAAA AGTGAAAGGA ACGTAAGTTT TGAAATGGTA   
  
  
- AAGAGAGAGA GAGAGAGAGA GAGAGAGAGA AGTCCGAATA TAAAGGGAAA TACATGCGTA TAACGGGTAG   
  
  
- TTGAGAGGTG AAGGTCATAA ACCACGACCA CCTCCGAGAA CTAGAGGGAA AAACCATTAA TGAACAGGTT   
  
  
- CGGTGACTAG AGTCACTCGA TAAGAAGAAT AGCTAGAACT AGAGAAACTA GTTTGAGTGA AAACTAAAGT   
  
  
- TGTGGGAACG AGAACTAAGA AGTAGAGAGT CGAAAAATGC GATAAGTAGG GGAATACGTC AAGGGGAATT   
  
  
- AAAGAGGAGA TCCGGAATTT CCCAAAGAAG AGCTGTACCA GATCACACGT AAGCTAGGTA ATCAGACGAA   
  
  
- ACATTCAATC AAGCGTGCAT GGGTGTATTC GATGTTGACA CAGCGACGTT AAGATTCGTA ACAACCCAAG   
  
  
- AGAGAACGCA TGACGTATAA TCTCCTCCG

+     GCN4\_motif

| Site Name | Organism | Position | Strand | Matrix score. | sequence | function |
| --- | --- | --- | --- | --- | --- | --- |
| GCN4\_motif | Oryza sativa | 1188 | + | 7 | CAAGCCA | cis-regulatory element involved in endosperm expression |

> 2018/04/13 10:10:12  
+ TTCGATTTTG TAAAAAAGTT AAATTCTTAT TTTTTTAATA ATTTATCCAT AAAAATAAAG TGAAAAATTA   
  
  
+ AAACTATTCA GTGCTGATAT TTTGTAAAAC AAATAGATAT CCAGTCAATA TGAAACATCT ATACTGTGCT   
  
  
+ TAGTCATGGA CACGTGTTTT AATCTAATGA TTATGTAAAA CGATCACTGA TAAGTCAAAC ATCATTATAT   
  
  
+ TACGAAAAAT GGAACATGAA TAAAGACAAA TGGAACTGCA CATGTTAACT CGTAAACTGC GAACCATCTG   
  
  
+ TCTAACCAGG CTCAAGAGTT AAGAAAGAGA ACGCTGTCGT TTCGGAAGAA TACCAGGAGA GAAAAAAAAA   
  
  
+ AAAAAAACTC TTGAGTTTGT TAATATCGAC ACAAATGTAC GTATGTCGCC CAGAAAAGAA GATGAGAATA   
  
  
+ AAAACCAATA AACTTTCTTA GGATTGCACA TGTAAGGTTA ATTATGTAAA TTAATGAATC ATTAACACTG   
  
  
+ CGGCTTTCTA GACGAGCAGC ATGCAGTACA AAGCATACGT GTTACGCGAG TCACATGGGT GATCCATCGC   
  
  
+ CATCAGCGTA AGCAAGGGGT TTGACATGAC GTCATCATCT TACGTAAGCT ACCCCAATCT CTGCGCCAGA   
  
  
+ CGAGTAGTGA GTAGCTACGC GGAAGAAGTT GGGAAGATTC TCCCGTCTCT TCCTCTGGCC CTCTCTTTTT   
  
  
+ CTTCTTGTCT TCCTTGAATT TGTGTTTCCA CTCTTCCAGT CTTCAAATTG ATTCAACACT GCCCGAGAGA   
  
  
+ GAGAGAGAGA GAGTTTTGAA TTGGTCAGCA GTTGGACCTT TCTCACTCTC TTTTATTTTC TTTTTCAATT   
  
  
+ TCTTTTTCTT CTGTAAGCCT TATGTATATA TAATAGGAAA GAGATCCTGA TCCTCAGCGA GGAAACAATC   
  
  
+ TGAGTTTTTC TTTTGTCCCG GGTCAAAACG CCTCTTTTTC CCTATCATTT CTCTTGCATA TTCTCCTCTC   
  
  
+ CTTCTTCCCA ACTTCTTCAT TAAGACAGGC ATAGGCCTTT TCACTTTCCT TGCATTCAAA ACTTTACCAT   
  
  
+ TTCTCTCTCT CTCTCTCTCT CTCTCTCTCT TCAGGCTTAT ATTTCCCTTT ATGTACGCAT ATTGCCCATC   
  
  
+ AACTCTCCAC TTCCAGTATT TGGTGCTGGT GGAGGCTCTT GATCTCCCTT TTTGGTAATT ACTTGTCCAA   
  
  
+ GCCACTGATC TCAGTGAGCT ATTCTTCTTA TCGATCTTGA TCTCTTTGAT CAAACTCACT TTTGATTTCA   
  
  
+ ACACCCTTGC TCTTGATTCT TCATCTCTCA GCTTTTTACG CTATTCATCC CCTTATGCAG TTCCCCTTAA   
  
  
+ TTTCTCCTCT AGGCCTTAAA GGGTTTCTTC TCGACATGGT CTAGTGTGCA TTCGATCCAT TAGTCTGCTT   
  
  
+ TGTAAGTTAG TTCGCACGTA CCCACATAAG CTACAACTGT GTCGCTGCAA TTCTAAGCAT TGTTGGGTTC   
  
  
+ TCTCTTGCGT ACTGCATATT AGAGGAGGC  

- AAGCTAAAAC ATTTTTTCAA TTTAAGAATA AAAAAATTAT TAAATAGGTA TTTTTATTTC ACTTTTTAAT   
  
  
- TTTGATAAGT CACGACTATA AAACATTTTG TTTATCTATA GGTCAGTTAT ACTTTGTAGA TATGACACGA   
  
  
- ATCAGTACCT GTGCACAAAA TTAGATTACT AATACATTTT GCTAGTGACT ATTCAGTTTG TAGTAATATA   
  
  
- ATGCTTTTTA CCTTGTACTT ATTTCTGTTT ACCTTGACGT GTACAATTGA GCATTTGACG CTTGGTAGAC   
  
  
- AGATTGGTCC GAGTTCTCAA TTCTTTCTCT TGCGACAGCA AAGCCTTCTT ATGGTCCTCT CTTTTTTTTT   
  
  
- TTTTTTTGAG AACTCAAACA ATTATAGCTG TGTTTACATG CATACAGCGG GTCTTTTCTT CTACTCTTAT   
  
  
- TTTTGGTTAT TTGAAAGAAT CCTAACGTGT ACATTCCAAT TAATACATTT AATTACTTAG TAATTGTGAC   
  
  
- GCCGAAAGAT CTGCTCGTCG TACGTCATGT TTCGTATGCA CAATGCGCTC AGTGTACCCA CTAGGTAGCG   
  
  
- GTAGTCGCAT TCGTTCCCCA AACTGTACTG CAGTAGTAGA ATGCATTCGA TGGGGTTAGA GACGCGGTCT   
  
  
- GCTCATCACT CATCGATGCG CCTTCTTCAA CCCTTCTAAG AGGGCAGAGA AGGAGACCGG GAGAGAAAAA   
  
  
- GAAGAACAGA AGGAACTTAA ACACAAAGGT GAGAAGGTCA GAAGTTTAAC TAAGTTGTGA CGGGCTCTCT   
  
  
- CTCTCTCTCT CTCAAAACTT AACCAGTCGT CAACCTGGAA AGAGTGAGAG AAAATAAAAG AAAAAGTTAA   
  
  
- AGAAAAAGAA GACATTCGGA ATACATATAT ATTATCCTTT CTCTAGGACT AGGAGTCGCT CCTTTGTTAG   
  
  
- ACTCAAAAAG AAAACAGGGC CCAGTTTTGC GGAGAAAAAG GGATAGTAAA GAGAACGTAT AAGAGGAGAG   
  
  
- GAAGAAGGGT TGAAGAAGTA ATTCTGTCCG TATCCGGAAA AGTGAAAGGA ACGTAAGTTT TGAAATGGTA   
  
  
- AAGAGAGAGA GAGAGAGAGA GAGAGAGAGA AGTCCGAATA TAAAGGGAAA TACATGCGTA TAACGGGTAG   
  
  
- TTGAGAGGTG AAGGTCATAA ACCACGACCA CCTCCGAGAA CTAGAGGGAA AAACCATTAA TGAACAGGTT   
  
  
- CGGTGACTAG AGTCACTCGA TAAGAAGAAT AGCTAGAACT AGAGAAACTA GTTTGAGTGA AAACTAAAGT   
  
  
- TGTGGGAACG AGAACTAAGA AGTAGAGAGT CGAAAAATGC GATAAGTAGG GGAATACGTC AAGGGGAATT   
  
  
- AAAGAGGAGA TCCGGAATTT CCCAAAGAAG AGCTGTACCA GATCACACGT AAGCTAGGTA ATCAGACGAA   
  
  
- ACATTCAATC AAGCGTGCAT GGGTGTATTC GATGTTGACA CAGCGACGTT AAGATTCGTA ACAACCCAAG   
  
  
- AGAGAACGCA TGACGTATAA TCTCCTCCG

+     GT1-motif

| Site Name | Organism | Position | Strand | Matrix score. | sequence | function |
| --- | --- | --- | --- | --- | --- | --- |
| GT1-motif | Avena sativa | 456 | + | 7 | GGTTAAT | light responsive element |

> 2018/04/13 10:10:12  
+ TTCGATTTTG TAAAAAAGTT AAATTCTTAT TTTTTTAATA ATTTATCCAT AAAAATAAAG TGAAAAATTA   
  
  
+ AAACTATTCA GTGCTGATAT TTTGTAAAAC AAATAGATAT CCAGTCAATA TGAAACATCT ATACTGTGCT   
  
  
+ TAGTCATGGA CACGTGTTTT AATCTAATGA TTATGTAAAA CGATCACTGA TAAGTCAAAC ATCATTATAT   
  
  
+ TACGAAAAAT GGAACATGAA TAAAGACAAA TGGAACTGCA CATGTTAACT CGTAAACTGC GAACCATCTG   
  
  
+ TCTAACCAGG CTCAAGAGTT AAGAAAGAGA ACGCTGTCGT TTCGGAAGAA TACCAGGAGA GAAAAAAAAA   
  
  
+ AAAAAAACTC TTGAGTTTGT TAATATCGAC ACAAATGTAC GTATGTCGCC CAGAAAAGAA GATGAGAATA   
  
  
+ AAAACCAATA AACTTTCTTA GGATTGCACA TGTAAGGTTA ATTATGTAAA TTAATGAATC ATTAACACTG   
  
  
+ CGGCTTTCTA GACGAGCAGC ATGCAGTACA AAGCATACGT GTTACGCGAG TCACATGGGT GATCCATCGC   
  
  
+ CATCAGCGTA AGCAAGGGGT TTGACATGAC GTCATCATCT TACGTAAGCT ACCCCAATCT CTGCGCCAGA   
  
  
+ CGAGTAGTGA GTAGCTACGC GGAAGAAGTT GGGAAGATTC TCCCGTCTCT TCCTCTGGCC CTCTCTTTTT   
  
  
+ CTTCTTGTCT TCCTTGAATT TGTGTTTCCA CTCTTCCAGT CTTCAAATTG ATTCAACACT GCCCGAGAGA   
  
  
+ GAGAGAGAGA GAGTTTTGAA TTGGTCAGCA GTTGGACCTT TCTCACTCTC TTTTATTTTC TTTTTCAATT   
  
  
+ TCTTTTTCTT CTGTAAGCCT TATGTATATA TAATAGGAAA GAGATCCTGA TCCTCAGCGA GGAAACAATC   
  
  
+ TGAGTTTTTC TTTTGTCCCG GGTCAAAACG CCTCTTTTTC CCTATCATTT CTCTTGCATA TTCTCCTCTC   
  
  
+ CTTCTTCCCA ACTTCTTCAT TAAGACAGGC ATAGGCCTTT TCACTTTCCT TGCATTCAAA ACTTTACCAT   
  
  
+ TTCTCTCTCT CTCTCTCTCT CTCTCTCTCT TCAGGCTTAT ATTTCCCTTT ATGTACGCAT ATTGCCCATC   
  
  
+ AACTCTCCAC TTCCAGTATT TGGTGCTGGT GGAGGCTCTT GATCTCCCTT TTTGGTAATT ACTTGTCCAA   
  
  
+ GCCACTGATC TCAGTGAGCT ATTCTTCTTA TCGATCTTGA TCTCTTTGAT CAAACTCACT TTTGATTTCA   
  
  
+ ACACCCTTGC TCTTGATTCT TCATCTCTCA GCTTTTTACG CTATTCATCC CCTTATGCAG TTCCCCTTAA   
  
  
+ TTTCTCCTCT AGGCCTTAAA GGGTTTCTTC TCGACATGGT CTAGTGTGCA TTCGATCCAT TAGTCTGCTT   
  
  
+ TGTAAGTTAG TTCGCACGTA CCCACATAAG CTACAACTGT GTCGCTGCAA TTCTAAGCAT TGTTGGGTTC   
  
  
+ TCTCTTGCGT ACTGCATATT AGAGGAGGC  

- AAGCTAAAAC ATTTTTTCAA TTTAAGAATA AAAAAATTAT TAAATAGGTA TTTTTATTTC ACTTTTTAAT   
  
  
- TTTGATAAGT CACGACTATA AAACATTTTG TTTATCTATA GGTCAGTTAT ACTTTGTAGA TATGACACGA   
  
  
- ATCAGTACCT GTGCACAAAA TTAGATTACT AATACATTTT GCTAGTGACT ATTCAGTTTG TAGTAATATA   
  
  
- ATGCTTTTTA CCTTGTACTT ATTTCTGTTT ACCTTGACGT GTACAATTGA GCATTTGACG CTTGGTAGAC   
  
  
- AGATTGGTCC GAGTTCTCAA TTCTTTCTCT TGCGACAGCA AAGCCTTCTT ATGGTCCTCT CTTTTTTTTT   
  
  
- TTTTTTTGAG AACTCAAACA ATTATAGCTG TGTTTACATG CATACAGCGG GTCTTTTCTT CTACTCTTAT   
  
  
- TTTTGGTTAT TTGAAAGAAT CCTAACGTGT ACATTCCAAT TAATACATTT AATTACTTAG TAATTGTGAC   
  
  
- GCCGAAAGAT CTGCTCGTCG TACGTCATGT TTCGTATGCA CAATGCGCTC AGTGTACCCA CTAGGTAGCG   
  
  
- GTAGTCGCAT TCGTTCCCCA AACTGTACTG CAGTAGTAGA ATGCATTCGA TGGGGTTAGA GACGCGGTCT   
  
  
- GCTCATCACT CATCGATGCG CCTTCTTCAA CCCTTCTAAG AGGGCAGAGA AGGAGACCGG GAGAGAAAAA   
  
  
- GAAGAACAGA AGGAACTTAA ACACAAAGGT GAGAAGGTCA GAAGTTTAAC TAAGTTGTGA CGGGCTCTCT   
  
  
- CTCTCTCTCT CTCAAAACTT AACCAGTCGT CAACCTGGAA AGAGTGAGAG AAAATAAAAG AAAAAGTTAA   
  
  
- AGAAAAAGAA GACATTCGGA ATACATATAT ATTATCCTTT CTCTAGGACT AGGAGTCGCT CCTTTGTTAG   
  
  
- ACTCAAAAAG AAAACAGGGC CCAGTTTTGC GGAGAAAAAG GGATAGTAAA GAGAACGTAT AAGAGGAGAG   
  
  
- GAAGAAGGGT TGAAGAAGTA ATTCTGTCCG TATCCGGAAA AGTGAAAGGA ACGTAAGTTT TGAAATGGTA   
  
  
- AAGAGAGAGA GAGAGAGAGA GAGAGAGAGA AGTCCGAATA TAAAGGGAAA TACATGCGTA TAACGGGTAG   
  
  
- TTGAGAGGTG AAGGTCATAA ACCACGACCA CCTCCGAGAA CTAGAGGGAA AAACCATTAA TGAACAGGTT   
  
  
- CGGTGACTAG AGTCACTCGA TAAGAAGAAT AGCTAGAACT AGAGAAACTA GTTTGAGTGA AAACTAAAGT   
  
  
- TGTGGGAACG AGAACTAAGA AGTAGAGAGT CGAAAAATGC GATAAGTAGG GGAATACGTC AAGGGGAATT   
  
  
- AAAGAGGAGA TCCGGAATTT CCCAAAGAAG AGCTGTACCA GATCACACGT AAGCTAGGTA ATCAGACGAA   
  
  
- ACATTCAATC AAGCGTGCAT GGGTGTATTC GATGTTGACA CAGCGACGTT AAGATTCGTA ACAACCCAAG   
  
  
- AGAGAACGCA TGACGTATAA TCTCCTCCG

+     I-box

| Site Name | Organism | Position | Strand | Matrix score. | sequence | function |
| --- | --- | --- | --- | --- | --- | --- |
| I-box | Zea mays | 950 | - | 7 | GATAGGG | part of a light responsive element |

> 2018/04/13 10:10:12  
+ TTCGATTTTG TAAAAAAGTT AAATTCTTAT TTTTTTAATA ATTTATCCAT AAAAATAAAG TGAAAAATTA   
  
  
+ AAACTATTCA GTGCTGATAT TTTGTAAAAC AAATAGATAT CCAGTCAATA TGAAACATCT ATACTGTGCT   
  
  
+ TAGTCATGGA CACGTGTTTT AATCTAATGA TTATGTAAAA CGATCACTGA TAAGTCAAAC ATCATTATAT   
  
  
+ TACGAAAAAT GGAACATGAA TAAAGACAAA TGGAACTGCA CATGTTAACT CGTAAACTGC GAACCATCTG   
  
  
+ TCTAACCAGG CTCAAGAGTT AAGAAAGAGA ACGCTGTCGT TTCGGAAGAA TACCAGGAGA GAAAAAAAAA   
  
  
+ AAAAAAACTC TTGAGTTTGT TAATATCGAC ACAAATGTAC GTATGTCGCC CAGAAAAGAA GATGAGAATA   
  
  
+ AAAACCAATA AACTTTCTTA GGATTGCACA TGTAAGGTTA ATTATGTAAA TTAATGAATC ATTAACACTG   
  
  
+ CGGCTTTCTA GACGAGCAGC ATGCAGTACA AAGCATACGT GTTACGCGAG TCACATGGGT GATCCATCGC   
  
  
+ CATCAGCGTA AGCAAGGGGT TTGACATGAC GTCATCATCT TACGTAAGCT ACCCCAATCT CTGCGCCAGA   
  
  
+ CGAGTAGTGA GTAGCTACGC GGAAGAAGTT GGGAAGATTC TCCCGTCTCT TCCTCTGGCC CTCTCTTTTT   
  
  
+ CTTCTTGTCT TCCTTGAATT TGTGTTTCCA CTCTTCCAGT CTTCAAATTG ATTCAACACT GCCCGAGAGA   
  
  
+ GAGAGAGAGA GAGTTTTGAA TTGGTCAGCA GTTGGACCTT TCTCACTCTC TTTTATTTTC TTTTTCAATT   
  
  
+ TCTTTTTCTT CTGTAAGCCT TATGTATATA TAATAGGAAA GAGATCCTGA TCCTCAGCGA GGAAACAATC   
  
  
+ TGAGTTTTTC TTTTGTCCCG GGTCAAAACG CCTCTTTTTC CCTATCATTT CTCTTGCATA TTCTCCTCTC   
  
  
+ CTTCTTCCCA ACTTCTTCAT TAAGACAGGC ATAGGCCTTT TCACTTTCCT TGCATTCAAA ACTTTACCAT   
  
  
+ TTCTCTCTCT CTCTCTCTCT CTCTCTCTCT TCAGGCTTAT ATTTCCCTTT ATGTACGCAT ATTGCCCATC   
  
  
+ AACTCTCCAC TTCCAGTATT TGGTGCTGGT GGAGGCTCTT GATCTCCCTT TTTGGTAATT ACTTGTCCAA   
  
  
+ GCCACTGATC TCAGTGAGCT ATTCTTCTTA TCGATCTTGA TCTCTTTGAT CAAACTCACT TTTGATTTCA   
  
  
+ ACACCCTTGC TCTTGATTCT TCATCTCTCA GCTTTTTACG CTATTCATCC CCTTATGCAG TTCCCCTTAA   
  
  
+ TTTCTCCTCT AGGCCTTAAA GGGTTTCTTC TCGACATGGT CTAGTGTGCA TTCGATCCAT TAGTCTGCTT   
  
  
+ TGTAAGTTAG TTCGCACGTA CCCACATAAG CTACAACTGT GTCGCTGCAA TTCTAAGCAT TGTTGGGTTC   
  
  
+ TCTCTTGCGT ACTGCATATT AGAGGAGGC  

- AAGCTAAAAC ATTTTTTCAA TTTAAGAATA AAAAAATTAT TAAATAGGTA TTTTTATTTC ACTTTTTAAT   
  
  
- TTTGATAAGT CACGACTATA AAACATTTTG TTTATCTATA GGTCAGTTAT ACTTTGTAGA TATGACACGA   
  
  
- ATCAGTACCT GTGCACAAAA TTAGATTACT AATACATTTT GCTAGTGACT ATTCAGTTTG TAGTAATATA   
  
  
- ATGCTTTTTA CCTTGTACTT ATTTCTGTTT ACCTTGACGT GTACAATTGA GCATTTGACG CTTGGTAGAC   
  
  
- AGATTGGTCC GAGTTCTCAA TTCTTTCTCT TGCGACAGCA AAGCCTTCTT ATGGTCCTCT CTTTTTTTTT   
  
  
- TTTTTTTGAG AACTCAAACA ATTATAGCTG TGTTTACATG CATACAGCGG GTCTTTTCTT CTACTCTTAT   
  
  
- TTTTGGTTAT TTGAAAGAAT CCTAACGTGT ACATTCCAAT TAATACATTT AATTACTTAG TAATTGTGAC   
  
  
- GCCGAAAGAT CTGCTCGTCG TACGTCATGT TTCGTATGCA CAATGCGCTC AGTGTACCCA CTAGGTAGCG   
  
  
- GTAGTCGCAT TCGTTCCCCA AACTGTACTG CAGTAGTAGA ATGCATTCGA TGGGGTTAGA GACGCGGTCT   
  
  
- GCTCATCACT CATCGATGCG CCTTCTTCAA CCCTTCTAAG AGGGCAGAGA AGGAGACCGG GAGAGAAAAA   
  
  
- GAAGAACAGA AGGAACTTAA ACACAAAGGT GAGAAGGTCA GAAGTTTAAC TAAGTTGTGA CGGGCTCTCT   
  
  
- CTCTCTCTCT CTCAAAACTT AACCAGTCGT CAACCTGGAA AGAGTGAGAG AAAATAAAAG AAAAAGTTAA   
  
  
- AGAAAAAGAA GACATTCGGA ATACATATAT ATTATCCTTT CTCTAGGACT AGGAGTCGCT CCTTTGTTAG   
  
  
- ACTCAAAAAG AAAACAGGGC CCAGTTTTGC GGAGAAAAAG GGATAGTAAA GAGAACGTAT AAGAGGAGAG   
  
  
- GAAGAAGGGT TGAAGAAGTA ATTCTGTCCG TATCCGGAAA AGTGAAAGGA ACGTAAGTTT TGAAATGGTA   
  
  
- AAGAGAGAGA GAGAGAGAGA GAGAGAGAGA AGTCCGAATA TAAAGGGAAA TACATGCGTA TAACGGGTAG   
  
  
- TTGAGAGGTG AAGGTCATAA ACCACGACCA CCTCCGAGAA CTAGAGGGAA AAACCATTAA TGAACAGGTT   
  
  
- CGGTGACTAG AGTCACTCGA TAAGAAGAAT AGCTAGAACT AGAGAAACTA GTTTGAGTGA AAACTAAAGT   
  
  
- TGTGGGAACG AGAACTAAGA AGTAGAGAGT CGAAAAATGC GATAAGTAGG GGAATACGTC AAGGGGAATT   
  
  
- AAAGAGGAGA TCCGGAATTT CCCAAAGAAG AGCTGTACCA GATCACACGT AAGCTAGGTA ATCAGACGAA   
  
  
- ACATTCAATC AAGCGTGCAT GGGTGTATTC GATGTTGACA CAGCGACGTT AAGATTCGTA ACAACCCAAG   
  
  
- AGAGAACGCA TGACGTATAA TCTCCTCCG

+     LTR

| Site Name | Organism | Position | Strand | Matrix score. | sequence | function |
| --- | --- | --- | --- | --- | --- | --- |
| LTR | Hordeum vulgare | 320 | - | 6 | CCGAAA | cis-acting element involved in low-temperature responsiveness |

> 2018/04/13 10:10:12  
+ TTCGATTTTG TAAAAAAGTT AAATTCTTAT TTTTTTAATA ATTTATCCAT AAAAATAAAG TGAAAAATTA   
  
  
+ AAACTATTCA GTGCTGATAT TTTGTAAAAC AAATAGATAT CCAGTCAATA TGAAACATCT ATACTGTGCT   
  
  
+ TAGTCATGGA CACGTGTTTT AATCTAATGA TTATGTAAAA CGATCACTGA TAAGTCAAAC ATCATTATAT   
  
  
+ TACGAAAAAT GGAACATGAA TAAAGACAAA TGGAACTGCA CATGTTAACT CGTAAACTGC GAACCATCTG   
  
  
+ TCTAACCAGG CTCAAGAGTT AAGAAAGAGA ACGCTGTCGT TTCGGAAGAA TACCAGGAGA GAAAAAAAAA   
  
  
+ AAAAAAACTC TTGAGTTTGT TAATATCGAC ACAAATGTAC GTATGTCGCC CAGAAAAGAA GATGAGAATA   
  
  
+ AAAACCAATA AACTTTCTTA GGATTGCACA TGTAAGGTTA ATTATGTAAA TTAATGAATC ATTAACACTG   
  
  
+ CGGCTTTCTA GACGAGCAGC ATGCAGTACA AAGCATACGT GTTACGCGAG TCACATGGGT GATCCATCGC   
  
  
+ CATCAGCGTA AGCAAGGGGT TTGACATGAC GTCATCATCT TACGTAAGCT ACCCCAATCT CTGCGCCAGA   
  
  
+ CGAGTAGTGA GTAGCTACGC GGAAGAAGTT GGGAAGATTC TCCCGTCTCT TCCTCTGGCC CTCTCTTTTT   
  
  
+ CTTCTTGTCT TCCTTGAATT TGTGTTTCCA CTCTTCCAGT CTTCAAATTG ATTCAACACT GCCCGAGAGA   
  
  
+ GAGAGAGAGA GAGTTTTGAA TTGGTCAGCA GTTGGACCTT TCTCACTCTC TTTTATTTTC TTTTTCAATT   
  
  
+ TCTTTTTCTT CTGTAAGCCT TATGTATATA TAATAGGAAA GAGATCCTGA TCCTCAGCGA GGAAACAATC   
  
  
+ TGAGTTTTTC TTTTGTCCCG GGTCAAAACG CCTCTTTTTC CCTATCATTT CTCTTGCATA TTCTCCTCTC   
  
  
+ CTTCTTCCCA ACTTCTTCAT TAAGACAGGC ATAGGCCTTT TCACTTTCCT TGCATTCAAA ACTTTACCAT   
  
  
+ TTCTCTCTCT CTCTCTCTCT CTCTCTCTCT TCAGGCTTAT ATTTCCCTTT ATGTACGCAT ATTGCCCATC   
  
  
+ AACTCTCCAC TTCCAGTATT TGGTGCTGGT GGAGGCTCTT GATCTCCCTT TTTGGTAATT ACTTGTCCAA   
  
  
+ GCCACTGATC TCAGTGAGCT ATTCTTCTTA TCGATCTTGA TCTCTTTGAT CAAACTCACT TTTGATTTCA   
  
  
+ ACACCCTTGC TCTTGATTCT TCATCTCTCA GCTTTTTACG CTATTCATCC CCTTATGCAG TTCCCCTTAA   
  
  
+ TTTCTCCTCT AGGCCTTAAA GGGTTTCTTC TCGACATGGT CTAGTGTGCA TTCGATCCAT TAGTCTGCTT   
  
  
+ TGTAAGTTAG TTCGCACGTA CCCACATAAG CTACAACTGT GTCGCTGCAA TTCTAAGCAT TGTTGGGTTC   
  
  
+ TCTCTTGCGT ACTGCATATT AGAGGAGGC  

- AAGCTAAAAC ATTTTTTCAA TTTAAGAATA AAAAAATTAT TAAATAGGTA TTTTTATTTC ACTTTTTAAT   
  
  
- TTTGATAAGT CACGACTATA AAACATTTTG TTTATCTATA GGTCAGTTAT ACTTTGTAGA TATGACACGA   
  
  
- ATCAGTACCT GTGCACAAAA TTAGATTACT AATACATTTT GCTAGTGACT ATTCAGTTTG TAGTAATATA   
  
  
- ATGCTTTTTA CCTTGTACTT ATTTCTGTTT ACCTTGACGT GTACAATTGA GCATTTGACG CTTGGTAGAC   
  
  
- AGATTGGTCC GAGTTCTCAA TTCTTTCTCT TGCGACAGCA AAGCCTTCTT ATGGTCCTCT CTTTTTTTTT   
  
  
- TTTTTTTGAG AACTCAAACA ATTATAGCTG TGTTTACATG CATACAGCGG GTCTTTTCTT CTACTCTTAT   
  
  
- TTTTGGTTAT TTGAAAGAAT CCTAACGTGT ACATTCCAAT TAATACATTT AATTACTTAG TAATTGTGAC   
  
  
- GCCGAAAGAT CTGCTCGTCG TACGTCATGT TTCGTATGCA CAATGCGCTC AGTGTACCCA CTAGGTAGCG   
  
  
- GTAGTCGCAT TCGTTCCCCA AACTGTACTG CAGTAGTAGA ATGCATTCGA TGGGGTTAGA GACGCGGTCT   
  
  
- GCTCATCACT CATCGATGCG CCTTCTTCAA CCCTTCTAAG AGGGCAGAGA AGGAGACCGG GAGAGAAAAA   
  
  
- GAAGAACAGA AGGAACTTAA ACACAAAGGT GAGAAGGTCA GAAGTTTAAC TAAGTTGTGA CGGGCTCTCT   
  
  
- CTCTCTCTCT CTCAAAACTT AACCAGTCGT CAACCTGGAA AGAGTGAGAG AAAATAAAAG AAAAAGTTAA   
  
  
- AGAAAAAGAA GACATTCGGA ATACATATAT ATTATCCTTT CTCTAGGACT AGGAGTCGCT CCTTTGTTAG   
  
  
- ACTCAAAAAG AAAACAGGGC CCAGTTTTGC GGAGAAAAAG GGATAGTAAA GAGAACGTAT AAGAGGAGAG   
  
  
- GAAGAAGGGT TGAAGAAGTA ATTCTGTCCG TATCCGGAAA AGTGAAAGGA ACGTAAGTTT TGAAATGGTA   
  
  
- AAGAGAGAGA GAGAGAGAGA GAGAGAGAGA AGTCCGAATA TAAAGGGAAA TACATGCGTA TAACGGGTAG   
  
  
- TTGAGAGGTG AAGGTCATAA ACCACGACCA CCTCCGAGAA CTAGAGGGAA AAACCATTAA TGAACAGGTT   
  
  
- CGGTGACTAG AGTCACTCGA TAAGAAGAAT AGCTAGAACT AGAGAAACTA GTTTGAGTGA AAACTAAAGT   
  
  
- TGTGGGAACG AGAACTAAGA AGTAGAGAGT CGAAAAATGC GATAAGTAGG GGAATACGTC AAGGGGAATT   
  
  
- AAAGAGGAGA TCCGGAATTT CCCAAAGAAG AGCTGTACCA GATCACACGT AAGCTAGGTA ATCAGACGAA   
  
  
- ACATTCAATC AAGCGTGCAT GGGTGTATTC GATGTTGACA CAGCGACGTT AAGATTCGTA ACAACCCAAG   
  
  
- AGAGAACGCA TGACGTATAA TCTCCTCCG

+     MBS

| Site Name | Organism | Position | Strand | Matrix score. | sequence | function |
| --- | --- | --- | --- | --- | --- | --- |
| MBS | Arabidopsis thaliana | 799 | - | 6 | CAACTG | MYB binding site involved in drought-inducibility |
| MBS | Arabidopsis thaliana | 1434 | + | 6 | CAACTG | MYB binding site involved in drought-inducibility |

> 2018/04/13 10:10:12  
+ TTCGATTTTG TAAAAAAGTT AAATTCTTAT TTTTTTAATA ATTTATCCAT AAAAATAAAG TGAAAAATTA   
  
  
+ AAACTATTCA GTGCTGATAT TTTGTAAAAC AAATAGATAT CCAGTCAATA TGAAACATCT ATACTGTGCT   
  
  
+ TAGTCATGGA CACGTGTTTT AATCTAATGA TTATGTAAAA CGATCACTGA TAAGTCAAAC ATCATTATAT   
  
  
+ TACGAAAAAT GGAACATGAA TAAAGACAAA TGGAACTGCA CATGTTAACT CGTAAACTGC GAACCATCTG   
  
  
+ TCTAACCAGG CTCAAGAGTT AAGAAAGAGA ACGCTGTCGT TTCGGAAGAA TACCAGGAGA GAAAAAAAAA   
  
  
+ AAAAAAACTC TTGAGTTTGT TAATATCGAC ACAAATGTAC GTATGTCGCC CAGAAAAGAA GATGAGAATA   
  
  
+ AAAACCAATA AACTTTCTTA GGATTGCACA TGTAAGGTTA ATTATGTAAA TTAATGAATC ATTAACACTG   
  
  
+ CGGCTTTCTA GACGAGCAGC ATGCAGTACA AAGCATACGT GTTACGCGAG TCACATGGGT GATCCATCGC   
  
  
+ CATCAGCGTA AGCAAGGGGT TTGACATGAC GTCATCATCT TACGTAAGCT ACCCCAATCT CTGCGCCAGA   
  
  
+ CGAGTAGTGA GTAGCTACGC GGAAGAAGTT GGGAAGATTC TCCCGTCTCT TCCTCTGGCC CTCTCTTTTT   
  
  
+ CTTCTTGTCT TCCTTGAATT TGTGTTTCCA CTCTTCCAGT CTTCAAATTG ATTCAACACT GCCCGAGAGA   
  
  
+ GAGAGAGAGA GAGTTTTGAA TTGGTCAGCA GTTGGACCTT TCTCACTCTC TTTTATTTTC TTTTTCAATT   
  
  
+ TCTTTTTCTT CTGTAAGCCT TATGTATATA TAATAGGAAA GAGATCCTGA TCCTCAGCGA GGAAACAATC   
  
  
+ TGAGTTTTTC TTTTGTCCCG GGTCAAAACG CCTCTTTTTC CCTATCATTT CTCTTGCATA TTCTCCTCTC   
  
  
+ CTTCTTCCCA ACTTCTTCAT TAAGACAGGC ATAGGCCTTT TCACTTTCCT TGCATTCAAA ACTTTACCAT   
  
  
+ TTCTCTCTCT CTCTCTCTCT CTCTCTCTCT TCAGGCTTAT ATTTCCCTTT ATGTACGCAT ATTGCCCATC   
  
  
+ AACTCTCCAC TTCCAGTATT TGGTGCTGGT GGAGGCTCTT GATCTCCCTT TTTGGTAATT ACTTGTCCAA   
  
  
+ GCCACTGATC TCAGTGAGCT ATTCTTCTTA TCGATCTTGA TCTCTTTGAT CAAACTCACT TTTGATTTCA   
  
  
+ ACACCCTTGC TCTTGATTCT TCATCTCTCA GCTTTTTACG CTATTCATCC CCTTATGCAG TTCCCCTTAA   
  
  
+ TTTCTCCTCT AGGCCTTAAA GGGTTTCTTC TCGACATGGT CTAGTGTGCA TTCGATCCAT TAGTCTGCTT   
  
  
+ TGTAAGTTAG TTCGCACGTA CCCACATAAG CTACAACTGT GTCGCTGCAA TTCTAAGCAT TGTTGGGTTC   
  
  
+ TCTCTTGCGT ACTGCATATT AGAGGAGGC  

- AAGCTAAAAC ATTTTTTCAA TTTAAGAATA AAAAAATTAT TAAATAGGTA TTTTTATTTC ACTTTTTAAT   
  
  
- TTTGATAAGT CACGACTATA AAACATTTTG TTTATCTATA GGTCAGTTAT ACTTTGTAGA TATGACACGA   
  
  
- ATCAGTACCT GTGCACAAAA TTAGATTACT AATACATTTT GCTAGTGACT ATTCAGTTTG TAGTAATATA   
  
  
- ATGCTTTTTA CCTTGTACTT ATTTCTGTTT ACCTTGACGT GTACAATTGA GCATTTGACG CTTGGTAGAC   
  
  
- AGATTGGTCC GAGTTCTCAA TTCTTTCTCT TGCGACAGCA AAGCCTTCTT ATGGTCCTCT CTTTTTTTTT   
  
  
- TTTTTTTGAG AACTCAAACA ATTATAGCTG TGTTTACATG CATACAGCGG GTCTTTTCTT CTACTCTTAT   
  
  
- TTTTGGTTAT TTGAAAGAAT CCTAACGTGT ACATTCCAAT TAATACATTT AATTACTTAG TAATTGTGAC   
  
  
- GCCGAAAGAT CTGCTCGTCG TACGTCATGT TTCGTATGCA CAATGCGCTC AGTGTACCCA CTAGGTAGCG   
  
  
- GTAGTCGCAT TCGTTCCCCA AACTGTACTG CAGTAGTAGA ATGCATTCGA TGGGGTTAGA GACGCGGTCT   
  
  
- GCTCATCACT CATCGATGCG CCTTCTTCAA CCCTTCTAAG AGGGCAGAGA AGGAGACCGG GAGAGAAAAA   
  
  
- GAAGAACAGA AGGAACTTAA ACACAAAGGT GAGAAGGTCA GAAGTTTAAC TAAGTTGTGA CGGGCTCTCT   
  
  
- CTCTCTCTCT CTCAAAACTT AACCAGTCGT CAACCTGGAA AGAGTGAGAG AAAATAAAAG AAAAAGTTAA   
  
  
- AGAAAAAGAA GACATTCGGA ATACATATAT ATTATCCTTT CTCTAGGACT AGGAGTCGCT CCTTTGTTAG   
  
  
- ACTCAAAAAG AAAACAGGGC CCAGTTTTGC GGAGAAAAAG GGATAGTAAA GAGAACGTAT AAGAGGAGAG   
  
  
- GAAGAAGGGT TGAAGAAGTA ATTCTGTCCG TATCCGGAAA AGTGAAAGGA ACGTAAGTTT TGAAATGGTA   
  
  
- AAGAGAGAGA GAGAGAGAGA GAGAGAGAGA AGTCCGAATA TAAAGGGAAA TACATGCGTA TAACGGGTAG   
  
  
- TTGAGAGGTG AAGGTCATAA ACCACGACCA CCTCCGAGAA CTAGAGGGAA AAACCATTAA TGAACAGGTT   
  
  
- CGGTGACTAG AGTCACTCGA TAAGAAGAAT AGCTAGAACT AGAGAAACTA GTTTGAGTGA AAACTAAAGT   
  
  
- TGTGGGAACG AGAACTAAGA AGTAGAGAGT CGAAAAATGC GATAAGTAGG GGAATACGTC AAGGGGAATT   
  
  
- AAAGAGGAGA TCCGGAATTT CCCAAAGAAG AGCTGTACCA GATCACACGT AAGCTAGGTA ATCAGACGAA   
  
  
- ACATTCAATC AAGCGTGCAT GGGTGTATTC GATGTTGACA CAGCGACGTT AAGATTCGTA ACAACCCAAG   
  
  
- AGAGAACGCA TGACGTATAA TCTCCTCCG

+     Skn-1\_motif

| Site Name | Organism | Position | Strand | Matrix score. | sequence | function |
| --- | --- | --- | --- | --- | --- | --- |
| Skn-1\_motif | Oryza sativa | 586 | - | 5 | GTCAT | cis-acting regulatory element required for endosperm expression |
| Skn-1\_motif | Oryza sativa | 591 | + | 5 | GTCAT | cis-acting regulatory element required for endosperm expression |
| Skn-1\_motif | Oryza sativa | 143 | + | 5 | GTCAT | cis-acting regulatory element required for endosperm expression |

> 2018/04/13 10:10:12  
+ TTCGATTTTG TAAAAAAGTT AAATTCTTAT TTTTTTAATA ATTTATCCAT AAAAATAAAG TGAAAAATTA   
  
  
+ AAACTATTCA GTGCTGATAT TTTGTAAAAC AAATAGATAT CCAGTCAATA TGAAACATCT ATACTGTGCT   
  
  
+ TAGTCATGGA CACGTGTTTT AATCTAATGA TTATGTAAAA CGATCACTGA TAAGTCAAAC ATCATTATAT   
  
  
+ TACGAAAAAT GGAACATGAA TAAAGACAAA TGGAACTGCA CATGTTAACT CGTAAACTGC GAACCATCTG   
  
  
+ TCTAACCAGG CTCAAGAGTT AAGAAAGAGA ACGCTGTCGT TTCGGAAGAA TACCAGGAGA GAAAAAAAAA   
  
  
+ AAAAAAACTC TTGAGTTTGT TAATATCGAC ACAAATGTAC GTATGTCGCC CAGAAAAGAA GATGAGAATA   
  
  
+ AAAACCAATA AACTTTCTTA GGATTGCACA TGTAAGGTTA ATTATGTAAA TTAATGAATC ATTAACACTG   
  
  
+ CGGCTTTCTA GACGAGCAGC ATGCAGTACA AAGCATACGT GTTACGCGAG TCACATGGGT GATCCATCGC   
  
  
+ CATCAGCGTA AGCAAGGGGT TTGACATGAC GTCATCATCT TACGTAAGCT ACCCCAATCT CTGCGCCAGA   
  
  
+ CGAGTAGTGA GTAGCTACGC GGAAGAAGTT GGGAAGATTC TCCCGTCTCT TCCTCTGGCC CTCTCTTTTT   
  
  
+ CTTCTTGTCT TCCTTGAATT TGTGTTTCCA CTCTTCCAGT CTTCAAATTG ATTCAACACT GCCCGAGAGA   
  
  
+ GAGAGAGAGA GAGTTTTGAA TTGGTCAGCA GTTGGACCTT TCTCACTCTC TTTTATTTTC TTTTTCAATT   
  
  
+ TCTTTTTCTT CTGTAAGCCT TATGTATATA TAATAGGAAA GAGATCCTGA TCCTCAGCGA GGAAACAATC   
  
  
+ TGAGTTTTTC TTTTGTCCCG GGTCAAAACG CCTCTTTTTC CCTATCATTT CTCTTGCATA TTCTCCTCTC   
  
  
+ CTTCTTCCCA ACTTCTTCAT TAAGACAGGC ATAGGCCTTT TCACTTTCCT TGCATTCAAA ACTTTACCAT   
  
  
+ TTCTCTCTCT CTCTCTCTCT CTCTCTCTCT TCAGGCTTAT ATTTCCCTTT ATGTACGCAT ATTGCCCATC   
  
  
+ AACTCTCCAC TTCCAGTATT TGGTGCTGGT GGAGGCTCTT GATCTCCCTT TTTGGTAATT ACTTGTCCAA   
  
  
+ GCCACTGATC TCAGTGAGCT ATTCTTCTTA TCGATCTTGA TCTCTTTGAT CAAACTCACT TTTGATTTCA   
  
  
+ ACACCCTTGC TCTTGATTCT TCATCTCTCA GCTTTTTACG CTATTCATCC CCTTATGCAG TTCCCCTTAA   
  
  
+ TTTCTCCTCT AGGCCTTAAA GGGTTTCTTC TCGACATGGT CTAGTGTGCA TTCGATCCAT TAGTCTGCTT   
  
  
+ TGTAAGTTAG TTCGCACGTA CCCACATAAG CTACAACTGT GTCGCTGCAA TTCTAAGCAT TGTTGGGTTC   
  
  
+ TCTCTTGCGT ACTGCATATT AGAGGAGGC  

- AAGCTAAAAC ATTTTTTCAA TTTAAGAATA AAAAAATTAT TAAATAGGTA TTTTTATTTC ACTTTTTAAT   
  
  
- TTTGATAAGT CACGACTATA AAACATTTTG TTTATCTATA GGTCAGTTAT ACTTTGTAGA TATGACACGA   
  
  
- ATCAGTACCT GTGCACAAAA TTAGATTACT AATACATTTT GCTAGTGACT ATTCAGTTTG TAGTAATATA   
  
  
- ATGCTTTTTA CCTTGTACTT ATTTCTGTTT ACCTTGACGT GTACAATTGA GCATTTGACG CTTGGTAGAC   
  
  
- AGATTGGTCC GAGTTCTCAA TTCTTTCTCT TGCGACAGCA AAGCCTTCTT ATGGTCCTCT CTTTTTTTTT   
  
  
- TTTTTTTGAG AACTCAAACA ATTATAGCTG TGTTTACATG CATACAGCGG GTCTTTTCTT CTACTCTTAT   
  
  
- TTTTGGTTAT TTGAAAGAAT CCTAACGTGT ACATTCCAAT TAATACATTT AATTACTTAG TAATTGTGAC   
  
  
- GCCGAAAGAT CTGCTCGTCG TACGTCATGT TTCGTATGCA CAATGCGCTC AGTGTACCCA CTAGGTAGCG   
  
  
- GTAGTCGCAT TCGTTCCCCA AACTGTACTG CAGTAGTAGA ATGCATTCGA TGGGGTTAGA GACGCGGTCT   
  
  
- GCTCATCACT CATCGATGCG CCTTCTTCAA CCCTTCTAAG AGGGCAGAGA AGGAGACCGG GAGAGAAAAA   
  
  
- GAAGAACAGA AGGAACTTAA ACACAAAGGT GAGAAGGTCA GAAGTTTAAC TAAGTTGTGA CGGGCTCTCT   
  
  
- CTCTCTCTCT CTCAAAACTT AACCAGTCGT CAACCTGGAA AGAGTGAGAG AAAATAAAAG AAAAAGTTAA   
  
  
- AGAAAAAGAA GACATTCGGA ATACATATAT ATTATCCTTT CTCTAGGACT AGGAGTCGCT CCTTTGTTAG   
  
  
- ACTCAAAAAG AAAACAGGGC CCAGTTTTGC GGAGAAAAAG GGATAGTAAA GAGAACGTAT AAGAGGAGAG   
  
  
- GAAGAAGGGT TGAAGAAGTA ATTCTGTCCG TATCCGGAAA AGTGAAAGGA ACGTAAGTTT TGAAATGGTA   
  
  
- AAGAGAGAGA GAGAGAGAGA GAGAGAGAGA AGTCCGAATA TAAAGGGAAA TACATGCGTA TAACGGGTAG   
  
  
- TTGAGAGGTG AAGGTCATAA ACCACGACCA CCTCCGAGAA CTAGAGGGAA AAACCATTAA TGAACAGGTT   
  
  
- CGGTGACTAG AGTCACTCGA TAAGAAGAAT AGCTAGAACT AGAGAAACTA GTTTGAGTGA AAACTAAAGT   
  
  
- TGTGGGAACG AGAACTAAGA AGTAGAGAGT CGAAAAATGC GATAAGTAGG GGAATACGTC AAGGGGAATT   
  
  
- AAAGAGGAGA TCCGGAATTT CCCAAAGAAG AGCTGTACCA GATCACACGT AAGCTAGGTA ATCAGACGAA   
  
  
- ACATTCAATC AAGCGTGCAT GGGTGTATTC GATGTTGACA CAGCGACGTT AAGATTCGTA ACAACCCAAG   
  
  
- AGAGAACGCA TGACGTATAA TCTCCTCCG

+     TATA-box

| Site Name | Organism | Position | Strand | Matrix score. | sequence | function |
| --- | --- | --- | --- | --- | --- | --- |
| TATA-box | Arabidopsis thaliana | 1088 | - | 4 | TATA | core promoter element around -30 of transcription start |
| TATA-box | Lycopersicon esculentum | 11 | - | 5 | TTTTA | core promoter element around -30 of transcription start |
| TATA-box | Arabidopsis thaliana | 869 | - | 4 | TATA | core promoter element around -30 of transcription start |
| TATA-box | Arabidopsis thaliana | 206 | + | 4 | TATA | core promoter element around -30 of transcription start |
| TATA-box | Arabidopsis thaliana | 205 | - | 5 | TATAA | core promoter element around -30 of transcription start |
| TATA-box | Oryza sativa | 6 | - | 7 | TACAAAA | core promoter element around -30 of transcription start |
| TATA-box | Lycopersicon esculentum | 33 | + | 5 | TTTTA | core promoter element around -30 of transcription start |
| TATA-box | Lycopersicon esculentum | 419 | - | 5 | TTTTA | core promoter element around -30 of transcription start |
| TATA-box | Lycopersicon esculentum | 69 | - | 5 | TTTTA | core promoter element around -30 of transcription start |
| TATA-box | Lycopersicon esculentum | 1294 | + | 5 | TTTTA | core promoter element around -30 of transcription start |
| TATA-box | Lycopersicon esculentum | 821 | + | 5 | TTTTA | core promoter element around -30 of transcription start |
| TATA-box | Glycine max | 208 | - | 5 | TAATA | core promoter element around -30 of transcription start |
| TATA-box | Brassica napus | 866 | - | 6 | ATATAT | core promoter element around -30 of transcription start |
| TATA-box | Arabidopsis thaliana | 1087 | - | 5 | TATAA | core promoter element around -30 of transcription start |
| TATA-box | Glycine max | 371 | + | 5 | TAATA | core promoter element around -30 of transcription start |
| TATA-box | Lycopersicon esculentum | 176 | - | 5 | TTTTA | core promoter element around -30 of transcription start |
| TATA-box | Glycine max | 1487 | - | 5 | TAATA | core promoter element around -30 of transcription start |
| TATA-box | Arabidopsis thaliana | 867 | - | 4 | TATA | core promoter element around -30 of transcription start |
| TATA-box | Arabidopsis thaliana | 130 | + | 4 | TATA | core promoter element around -30 of transcription start |
| TATA-box | Lycopersicon esculentum | 157 | + | 5 | TTTTA | core promoter element around -30 of transcription start |
| TATA-box | Arabidopsis thaliana | 1096 | - | 9 | taTATAAAgg | core promoter element around -30 of transcription start |
| TATA-box | Oryza sativa | 90 | - | 7 | TACAAAA | core promoter element around -30 of transcription start |
| TATA-box | Arabidopsis thaliana | 50 | + | 9 | TAAAAATAA | core promoter element around -30 of transcription start |
| TATA-box | Glycine max | 36 | + | 5 | TAATA | core promoter element around -30 of transcription start |
| TATA-box | Helianthus annuus | 863 | - | 6 | TATACA | core promoter element around -30 of transcription start |
| TATA-box | Brassica napus | 204 | + | 6 | ATTATA | core promoter element around -30 of transcription start |
| TATA-box | Lycopersicon esculentum | 95 | - | 5 | TTTTA | core promoter element around -30 of transcription start |
| TATA-box | Brassica oleracea | 868 | + | 7 | ATATAAT | core promoter element around -30 of transcription start |
| TATA-box | Oryza sativa | 1098 | - | 8 | TACATAAA | core promoter element around -30 of transcription start |
| TATA-box | Arabidopsis thaliana | 865 | - | 8 | TATATATA | core promoter element around -30 of transcription start |
| TATA-box | Glycine max | 871 | + | 5 | TAATA | core promoter element around -30 of transcription start |

> 2018/04/13 10:10:12  
+ TTCGATTTTG TAAAAAAGTT AAATTCTTAT TTTTTTAATA ATTTATCCAT AAAAATAAAG TGAAAAATTA   
  
  
+ AAACTATTCA GTGCTGATAT TTTGTAAAAC AAATAGATAT CCAGTCAATA TGAAACATCT ATACTGTGCT   
  
  
+ TAGTCATGGA CACGTGTTTT AATCTAATGA TTATGTAAAA CGATCACTGA TAAGTCAAAC ATCATTATAT   
  
  
+ TACGAAAAAT GGAACATGAA TAAAGACAAA TGGAACTGCA CATGTTAACT CGTAAACTGC GAACCATCTG   
  
  
+ TCTAACCAGG CTCAAGAGTT AAGAAAGAGA ACGCTGTCGT TTCGGAAGAA TACCAGGAGA GAAAAAAAAA   
  
  
+ AAAAAAACTC TTGAGTTTGT TAATATCGAC ACAAATGTAC GTATGTCGCC CAGAAAAGAA GATGAGAATA   
  
  
+ AAAACCAATA AACTTTCTTA GGATTGCACA TGTAAGGTTA ATTATGTAAA TTAATGAATC ATTAACACTG   
  
  
+ CGGCTTTCTA GACGAGCAGC ATGCAGTACA AAGCATACGT GTTACGCGAG TCACATGGGT GATCCATCGC   
  
  
+ CATCAGCGTA AGCAAGGGGT TTGACATGAC GTCATCATCT TACGTAAGCT ACCCCAATCT CTGCGCCAGA   
  
  
+ CGAGTAGTGA GTAGCTACGC GGAAGAAGTT GGGAAGATTC TCCCGTCTCT TCCTCTGGCC CTCTCTTTTT   
  
  
+ CTTCTTGTCT TCCTTGAATT TGTGTTTCCA CTCTTCCAGT CTTCAAATTG ATTCAACACT GCCCGAGAGA   
  
  
+ GAGAGAGAGA GAGTTTTGAA TTGGTCAGCA GTTGGACCTT TCTCACTCTC TTTTATTTTC TTTTTCAATT   
  
  
+ TCTTTTTCTT CTGTAAGCCT TATGTATATA TAATAGGAAA GAGATCCTGA TCCTCAGCGA GGAAACAATC   
  
  
+ TGAGTTTTTC TTTTGTCCCG GGTCAAAACG CCTCTTTTTC CCTATCATTT CTCTTGCATA TTCTCCTCTC   
  
  
+ CTTCTTCCCA ACTTCTTCAT TAAGACAGGC ATAGGCCTTT TCACTTTCCT TGCATTCAAA ACTTTACCAT   
  
  
+ TTCTCTCTCT CTCTCTCTCT CTCTCTCTCT TCAGGCTTAT ATTTCCCTTT ATGTACGCAT ATTGCCCATC   
  
  
+ AACTCTCCAC TTCCAGTATT TGGTGCTGGT GGAGGCTCTT GATCTCCCTT TTTGGTAATT ACTTGTCCAA   
  
  
+ GCCACTGATC TCAGTGAGCT ATTCTTCTTA TCGATCTTGA TCTCTTTGAT CAAACTCACT TTTGATTTCA   
  
  
+ ACACCCTTGC TCTTGATTCT TCATCTCTCA GCTTTTTACG CTATTCATCC CCTTATGCAG TTCCCCTTAA   
  
  
+ TTTCTCCTCT AGGCCTTAAA GGGTTTCTTC TCGACATGGT CTAGTGTGCA TTCGATCCAT TAGTCTGCTT   
  
  
+ TGTAAGTTAG TTCGCACGTA CCCACATAAG CTACAACTGT GTCGCTGCAA TTCTAAGCAT TGTTGGGTTC   
  
  
+ TCTCTTGCGT ACTGCATATT AGAGGAGGC  

- AAGCTAAAAC ATTTTTTCAA TTTAAGAATA AAAAAATTAT TAAATAGGTA TTTTTATTTC ACTTTTTAAT   
  
  
- TTTGATAAGT CACGACTATA AAACATTTTG TTTATCTATA GGTCAGTTAT ACTTTGTAGA TATGACACGA   
  
  
- ATCAGTACCT GTGCACAAAA TTAGATTACT AATACATTTT GCTAGTGACT ATTCAGTTTG TAGTAATATA   
  
  
- ATGCTTTTTA CCTTGTACTT ATTTCTGTTT ACCTTGACGT GTACAATTGA GCATTTGACG CTTGGTAGAC   
  
  
- AGATTGGTCC GAGTTCTCAA TTCTTTCTCT TGCGACAGCA AAGCCTTCTT ATGGTCCTCT CTTTTTTTTT   
  
  
- TTTTTTTGAG AACTCAAACA ATTATAGCTG TGTTTACATG CATACAGCGG GTCTTTTCTT CTACTCTTAT   
  
  
- TTTTGGTTAT TTGAAAGAAT CCTAACGTGT ACATTCCAAT TAATACATTT AATTACTTAG TAATTGTGAC   
  
  
- GCCGAAAGAT CTGCTCGTCG TACGTCATGT TTCGTATGCA CAATGCGCTC AGTGTACCCA CTAGGTAGCG   
  
  
- GTAGTCGCAT TCGTTCCCCA AACTGTACTG CAGTAGTAGA ATGCATTCGA TGGGGTTAGA GACGCGGTCT   
  
  
- GCTCATCACT CATCGATGCG CCTTCTTCAA CCCTTCTAAG AGGGCAGAGA AGGAGACCGG GAGAGAAAAA   
  
  
- GAAGAACAGA AGGAACTTAA ACACAAAGGT GAGAAGGTCA GAAGTTTAAC TAAGTTGTGA CGGGCTCTCT   
  
  
- CTCTCTCTCT CTCAAAACTT AACCAGTCGT CAACCTGGAA AGAGTGAGAG AAAATAAAAG AAAAAGTTAA   
  
  
- AGAAAAAGAA GACATTCGGA ATACATATAT ATTATCCTTT CTCTAGGACT AGGAGTCGCT CCTTTGTTAG   
  
  
- ACTCAAAAAG AAAACAGGGC CCAGTTTTGC GGAGAAAAAG GGATAGTAAA GAGAACGTAT AAGAGGAGAG   
  
  
- GAAGAAGGGT TGAAGAAGTA ATTCTGTCCG TATCCGGAAA AGTGAAAGGA ACGTAAGTTT TGAAATGGTA   
  
  
- AAGAGAGAGA GAGAGAGAGA GAGAGAGAGA AGTCCGAATA TAAAGGGAAA TACATGCGTA TAACGGGTAG   
  
  
- TTGAGAGGTG AAGGTCATAA ACCACGACCA CCTCCGAGAA CTAGAGGGAA AAACCATTAA TGAACAGGTT   
  
  
- CGGTGACTAG AGTCACTCGA TAAGAAGAAT AGCTAGAACT AGAGAAACTA GTTTGAGTGA AAACTAAAGT   
  
  
- TGTGGGAACG AGAACTAAGA AGTAGAGAGT CGAAAAATGC GATAAGTAGG GGAATACGTC AAGGGGAATT   
  
  
- AAAGAGGAGA TCCGGAATTT CCCAAAGAAG AGCTGTACCA GATCACACGT AAGCTAGGTA ATCAGACGAA   
  
  
- ACATTCAATC AAGCGTGCAT GGGTGTATTC GATGTTGACA CAGCGACGTT AAGATTCGTA ACAACCCAAG   
  
  
- AGAGAACGCA TGACGTATAA TCTCCTCCG

+     TATCCAT/C-motif

| Site Name | Organism | Position | Strand | Matrix score. | sequence | function |
| --- | --- | --- | --- | --- | --- | --- |
| TATCCAT/C-motif | Oryza sativa | 44 | + | 7 | TATCCAT |  |

> 2018/04/13 10:10:12  
+ TTCGATTTTG TAAAAAAGTT AAATTCTTAT TTTTTTAATA ATTTATCCAT AAAAATAAAG TGAAAAATTA   
  
  
+ AAACTATTCA GTGCTGATAT TTTGTAAAAC AAATAGATAT CCAGTCAATA TGAAACATCT ATACTGTGCT   
  
  
+ TAGTCATGGA CACGTGTTTT AATCTAATGA TTATGTAAAA CGATCACTGA TAAGTCAAAC ATCATTATAT   
  
  
+ TACGAAAAAT GGAACATGAA TAAAGACAAA TGGAACTGCA CATGTTAACT CGTAAACTGC GAACCATCTG   
  
  
+ TCTAACCAGG CTCAAGAGTT AAGAAAGAGA ACGCTGTCGT TTCGGAAGAA TACCAGGAGA GAAAAAAAAA   
  
  
+ AAAAAAACTC TTGAGTTTGT TAATATCGAC ACAAATGTAC GTATGTCGCC CAGAAAAGAA GATGAGAATA   
  
  
+ AAAACCAATA AACTTTCTTA GGATTGCACA TGTAAGGTTA ATTATGTAAA TTAATGAATC ATTAACACTG   
  
  
+ CGGCTTTCTA GACGAGCAGC ATGCAGTACA AAGCATACGT GTTACGCGAG TCACATGGGT GATCCATCGC   
  
  
+ CATCAGCGTA AGCAAGGGGT TTGACATGAC GTCATCATCT TACGTAAGCT ACCCCAATCT CTGCGCCAGA   
  
  
+ CGAGTAGTGA GTAGCTACGC GGAAGAAGTT GGGAAGATTC TCCCGTCTCT TCCTCTGGCC CTCTCTTTTT   
  
  
+ CTTCTTGTCT TCCTTGAATT TGTGTTTCCA CTCTTCCAGT CTTCAAATTG ATTCAACACT GCCCGAGAGA   
  
  
+ GAGAGAGAGA GAGTTTTGAA TTGGTCAGCA GTTGGACCTT TCTCACTCTC TTTTATTTTC TTTTTCAATT   
  
  
+ TCTTTTTCTT CTGTAAGCCT TATGTATATA TAATAGGAAA GAGATCCTGA TCCTCAGCGA GGAAACAATC   
  
  
+ TGAGTTTTTC TTTTGTCCCG GGTCAAAACG CCTCTTTTTC CCTATCATTT CTCTTGCATA TTCTCCTCTC   
  
  
+ CTTCTTCCCA ACTTCTTCAT TAAGACAGGC ATAGGCCTTT TCACTTTCCT TGCATTCAAA ACTTTACCAT   
  
  
+ TTCTCTCTCT CTCTCTCTCT CTCTCTCTCT TCAGGCTTAT ATTTCCCTTT ATGTACGCAT ATTGCCCATC   
  
  
+ AACTCTCCAC TTCCAGTATT TGGTGCTGGT GGAGGCTCTT GATCTCCCTT TTTGGTAATT ACTTGTCCAA   
  
  
+ GCCACTGATC TCAGTGAGCT ATTCTTCTTA TCGATCTTGA TCTCTTTGAT CAAACTCACT TTTGATTTCA   
  
  
+ ACACCCTTGC TCTTGATTCT TCATCTCTCA GCTTTTTACG CTATTCATCC CCTTATGCAG TTCCCCTTAA   
  
  
+ TTTCTCCTCT AGGCCTTAAA GGGTTTCTTC TCGACATGGT CTAGTGTGCA TTCGATCCAT TAGTCTGCTT   
  
  
+ TGTAAGTTAG TTCGCACGTA CCCACATAAG CTACAACTGT GTCGCTGCAA TTCTAAGCAT TGTTGGGTTC   
  
  
+ TCTCTTGCGT ACTGCATATT AGAGGAGGC  

- AAGCTAAAAC ATTTTTTCAA TTTAAGAATA AAAAAATTAT TAAATAGGTA TTTTTATTTC ACTTTTTAAT   
  
  
- TTTGATAAGT CACGACTATA AAACATTTTG TTTATCTATA GGTCAGTTAT ACTTTGTAGA TATGACACGA   
  
  
- ATCAGTACCT GTGCACAAAA TTAGATTACT AATACATTTT GCTAGTGACT ATTCAGTTTG TAGTAATATA   
  
  
- ATGCTTTTTA CCTTGTACTT ATTTCTGTTT ACCTTGACGT GTACAATTGA GCATTTGACG CTTGGTAGAC   
  
  
- AGATTGGTCC GAGTTCTCAA TTCTTTCTCT TGCGACAGCA AAGCCTTCTT ATGGTCCTCT CTTTTTTTTT   
  
  
- TTTTTTTGAG AACTCAAACA ATTATAGCTG TGTTTACATG CATACAGCGG GTCTTTTCTT CTACTCTTAT   
  
  
- TTTTGGTTAT TTGAAAGAAT CCTAACGTGT ACATTCCAAT TAATACATTT AATTACTTAG TAATTGTGAC   
  
  
- GCCGAAAGAT CTGCTCGTCG TACGTCATGT TTCGTATGCA CAATGCGCTC AGTGTACCCA CTAGGTAGCG   
  
  
- GTAGTCGCAT TCGTTCCCCA AACTGTACTG CAGTAGTAGA ATGCATTCGA TGGGGTTAGA GACGCGGTCT   
  
  
- GCTCATCACT CATCGATGCG CCTTCTTCAA CCCTTCTAAG AGGGCAGAGA AGGAGACCGG GAGAGAAAAA   
  
  
- GAAGAACAGA AGGAACTTAA ACACAAAGGT GAGAAGGTCA GAAGTTTAAC TAAGTTGTGA CGGGCTCTCT   
  
  
- CTCTCTCTCT CTCAAAACTT AACCAGTCGT CAACCTGGAA AGAGTGAGAG AAAATAAAAG AAAAAGTTAA   
  
  
- AGAAAAAGAA GACATTCGGA ATACATATAT ATTATCCTTT CTCTAGGACT AGGAGTCGCT CCTTTGTTAG   
  
  
- ACTCAAAAAG AAAACAGGGC CCAGTTTTGC GGAGAAAAAG GGATAGTAAA GAGAACGTAT AAGAGGAGAG   
  
  
- GAAGAAGGGT TGAAGAAGTA ATTCTGTCCG TATCCGGAAA AGTGAAAGGA ACGTAAGTTT TGAAATGGTA   
  
  
- AAGAGAGAGA GAGAGAGAGA GAGAGAGAGA AGTCCGAATA TAAAGGGAAA TACATGCGTA TAACGGGTAG   
  
  
- TTGAGAGGTG AAGGTCATAA ACCACGACCA CCTCCGAGAA CTAGAGGGAA AAACCATTAA TGAACAGGTT   
  
  
- CGGTGACTAG AGTCACTCGA TAAGAAGAAT AGCTAGAACT AGAGAAACTA GTTTGAGTGA AAACTAAAGT   
  
  
- TGTGGGAACG AGAACTAAGA AGTAGAGAGT CGAAAAATGC GATAAGTAGG GGAATACGTC AAGGGGAATT   
  
  
- AAAGAGGAGA TCCGGAATTT CCCAAAGAAG AGCTGTACCA GATCACACGT AAGCTAGGTA ATCAGACGAA   
  
  
- ACATTCAATC AAGCGTGCAT GGGTGTATTC GATGTTGACA CAGCGACGTT AAGATTCGTA ACAACCCAAG   
  
  
- AGAGAACGCA TGACGTATAA TCTCCTCCG

+     TCA-element

| Site Name | Organism | Position | Strand | Matrix score. | sequence | function |
| --- | --- | --- | --- | --- | --- | --- |
| TCA-element | Brassica oleracea | 1210 | - | 9 | GAGAAGAATA | cis-acting element involved in salicylic acid responsiveness |
| TCA-element | Brassica oleracea | 401 | + | 9 | CAGAAAAGGA | cis-acting element involved in salicylic acid responsiveness |
| TCA-element | Brassica oleracea | 969 | - | 9 | GAGAAGAATA | cis-acting element involved in salicylic acid responsiveness |

> 2018/04/13 10:10:12  
+ TTCGATTTTG TAAAAAAGTT AAATTCTTAT TTTTTTAATA ATTTATCCAT AAAAATAAAG TGAAAAATTA   
  
  
+ AAACTATTCA GTGCTGATAT TTTGTAAAAC AAATAGATAT CCAGTCAATA TGAAACATCT ATACTGTGCT   
  
  
+ TAGTCATGGA CACGTGTTTT AATCTAATGA TTATGTAAAA CGATCACTGA TAAGTCAAAC ATCATTATAT   
  
  
+ TACGAAAAAT GGAACATGAA TAAAGACAAA TGGAACTGCA CATGTTAACT CGTAAACTGC GAACCATCTG   
  
  
+ TCTAACCAGG CTCAAGAGTT AAGAAAGAGA ACGCTGTCGT TTCGGAAGAA TACCAGGAGA GAAAAAAAAA   
  
  
+ AAAAAAACTC TTGAGTTTGT TAATATCGAC ACAAATGTAC GTATGTCGCC CAGAAAAGAA GATGAGAATA   
  
  
+ AAAACCAATA AACTTTCTTA GGATTGCACA TGTAAGGTTA ATTATGTAAA TTAATGAATC ATTAACACTG   
  
  
+ CGGCTTTCTA GACGAGCAGC ATGCAGTACA AAGCATACGT GTTACGCGAG TCACATGGGT GATCCATCGC   
  
  
+ CATCAGCGTA AGCAAGGGGT TTGACATGAC GTCATCATCT TACGTAAGCT ACCCCAATCT CTGCGCCAGA   
  
  
+ CGAGTAGTGA GTAGCTACGC GGAAGAAGTT GGGAAGATTC TCCCGTCTCT TCCTCTGGCC CTCTCTTTTT   
  
  
+ CTTCTTGTCT TCCTTGAATT TGTGTTTCCA CTCTTCCAGT CTTCAAATTG ATTCAACACT GCCCGAGAGA   
  
  
+ GAGAGAGAGA GAGTTTTGAA TTGGTCAGCA GTTGGACCTT TCTCACTCTC TTTTATTTTC TTTTTCAATT   
  
  
+ TCTTTTTCTT CTGTAAGCCT TATGTATATA TAATAGGAAA GAGATCCTGA TCCTCAGCGA GGAAACAATC   
  
  
+ TGAGTTTTTC TTTTGTCCCG GGTCAAAACG CCTCTTTTTC CCTATCATTT CTCTTGCATA TTCTCCTCTC   
  
  
+ CTTCTTCCCA ACTTCTTCAT TAAGACAGGC ATAGGCCTTT TCACTTTCCT TGCATTCAAA ACTTTACCAT   
  
  
+ TTCTCTCTCT CTCTCTCTCT CTCTCTCTCT TCAGGCTTAT ATTTCCCTTT ATGTACGCAT ATTGCCCATC   
  
  
+ AACTCTCCAC TTCCAGTATT TGGTGCTGGT GGAGGCTCTT GATCTCCCTT TTTGGTAATT ACTTGTCCAA   
  
  
+ GCCACTGATC TCAGTGAGCT ATTCTTCTTA TCGATCTTGA TCTCTTTGAT CAAACTCACT TTTGATTTCA   
  
  
+ ACACCCTTGC TCTTGATTCT TCATCTCTCA GCTTTTTACG CTATTCATCC CCTTATGCAG TTCCCCTTAA   
  
  
+ TTTCTCCTCT AGGCCTTAAA GGGTTTCTTC TCGACATGGT CTAGTGTGCA TTCGATCCAT TAGTCTGCTT   
  
  
+ TGTAAGTTAG TTCGCACGTA CCCACATAAG CTACAACTGT GTCGCTGCAA TTCTAAGCAT TGTTGGGTTC   
  
  
+ TCTCTTGCGT ACTGCATATT AGAGGAGGC  

- AAGCTAAAAC ATTTTTTCAA TTTAAGAATA AAAAAATTAT TAAATAGGTA TTTTTATTTC ACTTTTTAAT   
  
  
- TTTGATAAGT CACGACTATA AAACATTTTG TTTATCTATA GGTCAGTTAT ACTTTGTAGA TATGACACGA   
  
  
- ATCAGTACCT GTGCACAAAA TTAGATTACT AATACATTTT GCTAGTGACT ATTCAGTTTG TAGTAATATA   
  
  
- ATGCTTTTTA CCTTGTACTT ATTTCTGTTT ACCTTGACGT GTACAATTGA GCATTTGACG CTTGGTAGAC   
  
  
- AGATTGGTCC GAGTTCTCAA TTCTTTCTCT TGCGACAGCA AAGCCTTCTT ATGGTCCTCT CTTTTTTTTT   
  
  
- TTTTTTTGAG AACTCAAACA ATTATAGCTG TGTTTACATG CATACAGCGG GTCTTTTCTT CTACTCTTAT   
  
  
- TTTTGGTTAT TTGAAAGAAT CCTAACGTGT ACATTCCAAT TAATACATTT AATTACTTAG TAATTGTGAC   
  
  
- GCCGAAAGAT CTGCTCGTCG TACGTCATGT TTCGTATGCA CAATGCGCTC AGTGTACCCA CTAGGTAGCG   
  
  
- GTAGTCGCAT TCGTTCCCCA AACTGTACTG CAGTAGTAGA ATGCATTCGA TGGGGTTAGA GACGCGGTCT   
  
  
- GCTCATCACT CATCGATGCG CCTTCTTCAA CCCTTCTAAG AGGGCAGAGA AGGAGACCGG GAGAGAAAAA   
  
  
- GAAGAACAGA AGGAACTTAA ACACAAAGGT GAGAAGGTCA GAAGTTTAAC TAAGTTGTGA CGGGCTCTCT   
  
  
- CTCTCTCTCT CTCAAAACTT AACCAGTCGT CAACCTGGAA AGAGTGAGAG AAAATAAAAG AAAAAGTTAA   
  
  
- AGAAAAAGAA GACATTCGGA ATACATATAT ATTATCCTTT CTCTAGGACT AGGAGTCGCT CCTTTGTTAG   
  
  
- ACTCAAAAAG AAAACAGGGC CCAGTTTTGC GGAGAAAAAG GGATAGTAAA GAGAACGTAT AAGAGGAGAG   
  
  
- GAAGAAGGGT TGAAGAAGTA ATTCTGTCCG TATCCGGAAA AGTGAAAGGA ACGTAAGTTT TGAAATGGTA   
  
  
- AAGAGAGAGA GAGAGAGAGA GAGAGAGAGA AGTCCGAATA TAAAGGGAAA TACATGCGTA TAACGGGTAG   
  
  
- TTGAGAGGTG AAGGTCATAA ACCACGACCA CCTCCGAGAA CTAGAGGGAA AAACCATTAA TGAACAGGTT   
  
  
- CGGTGACTAG AGTCACTCGA TAAGAAGAAT AGCTAGAACT AGAGAAACTA GTTTGAGTGA AAACTAAAGT   
  
  
- TGTGGGAACG AGAACTAAGA AGTAGAGAGT CGAAAAATGC GATAAGTAGG GGAATACGTC AAGGGGAATT   
  
  
- AAAGAGGAGA TCCGGAATTT CCCAAAGAAG AGCTGTACCA GATCACACGT AAGCTAGGTA ATCAGACGAA   
  
  
- ACATTCAATC AAGCGTGCAT GGGTGTATTC GATGTTGACA CAGCGACGTT AAGATTCGTA ACAACCCAAG   
  
  
- AGAGAACGCA TGACGTATAA TCTCCTCCG

+     TCCC-motif

| Site Name | Organism | Position | Strand | Matrix score. | sequence | function |
| --- | --- | --- | --- | --- | --- | --- |
| TCCC-motif | Spinacia oleracea | 1163 | + | 7 | TCTCCCT | part of a light responsive element |

> 2018/04/13 10:10:12  
+ TTCGATTTTG TAAAAAAGTT AAATTCTTAT TTTTTTAATA ATTTATCCAT AAAAATAAAG TGAAAAATTA   
  
  
+ AAACTATTCA GTGCTGATAT TTTGTAAAAC AAATAGATAT CCAGTCAATA TGAAACATCT ATACTGTGCT   
  
  
+ TAGTCATGGA CACGTGTTTT AATCTAATGA TTATGTAAAA CGATCACTGA TAAGTCAAAC ATCATTATAT   
  
  
+ TACGAAAAAT GGAACATGAA TAAAGACAAA TGGAACTGCA CATGTTAACT CGTAAACTGC GAACCATCTG   
  
  
+ TCTAACCAGG CTCAAGAGTT AAGAAAGAGA ACGCTGTCGT TTCGGAAGAA TACCAGGAGA GAAAAAAAAA   
  
  
+ AAAAAAACTC TTGAGTTTGT TAATATCGAC ACAAATGTAC GTATGTCGCC CAGAAAAGAA GATGAGAATA   
  
  
+ AAAACCAATA AACTTTCTTA GGATTGCACA TGTAAGGTTA ATTATGTAAA TTAATGAATC ATTAACACTG   
  
  
+ CGGCTTTCTA GACGAGCAGC ATGCAGTACA AAGCATACGT GTTACGCGAG TCACATGGGT GATCCATCGC   
  
  
+ CATCAGCGTA AGCAAGGGGT TTGACATGAC GTCATCATCT TACGTAAGCT ACCCCAATCT CTGCGCCAGA   
  
  
+ CGAGTAGTGA GTAGCTACGC GGAAGAAGTT GGGAAGATTC TCCCGTCTCT TCCTCTGGCC CTCTCTTTTT   
  
  
+ CTTCTTGTCT TCCTTGAATT TGTGTTTCCA CTCTTCCAGT CTTCAAATTG ATTCAACACT GCCCGAGAGA   
  
  
+ GAGAGAGAGA GAGTTTTGAA TTGGTCAGCA GTTGGACCTT TCTCACTCTC TTTTATTTTC TTTTTCAATT   
  
  
+ TCTTTTTCTT CTGTAAGCCT TATGTATATA TAATAGGAAA GAGATCCTGA TCCTCAGCGA GGAAACAATC   
  
  
+ TGAGTTTTTC TTTTGTCCCG GGTCAAAACG CCTCTTTTTC CCTATCATTT CTCTTGCATA TTCTCCTCTC   
  
  
+ CTTCTTCCCA ACTTCTTCAT TAAGACAGGC ATAGGCCTTT TCACTTTCCT TGCATTCAAA ACTTTACCAT   
  
  
+ TTCTCTCTCT CTCTCTCTCT CTCTCTCTCT TCAGGCTTAT ATTTCCCTTT ATGTACGCAT ATTGCCCATC   
  
  
+ AACTCTCCAC TTCCAGTATT TGGTGCTGGT GGAGGCTCTT GATCTCCCTT TTTGGTAATT ACTTGTCCAA   
  
  
+ GCCACTGATC TCAGTGAGCT ATTCTTCTTA TCGATCTTGA TCTCTTTGAT CAAACTCACT TTTGATTTCA   
  
  
+ ACACCCTTGC TCTTGATTCT TCATCTCTCA GCTTTTTACG CTATTCATCC CCTTATGCAG TTCCCCTTAA   
  
  
+ TTTCTCCTCT AGGCCTTAAA GGGTTTCTTC TCGACATGGT CTAGTGTGCA TTCGATCCAT TAGTCTGCTT   
  
  
+ TGTAAGTTAG TTCGCACGTA CCCACATAAG CTACAACTGT GTCGCTGCAA TTCTAAGCAT TGTTGGGTTC   
  
  
+ TCTCTTGCGT ACTGCATATT AGAGGAGGC  

- AAGCTAAAAC ATTTTTTCAA TTTAAGAATA AAAAAATTAT TAAATAGGTA TTTTTATTTC ACTTTTTAAT   
  
  
- TTTGATAAGT CACGACTATA AAACATTTTG TTTATCTATA GGTCAGTTAT ACTTTGTAGA TATGACACGA   
  
  
- ATCAGTACCT GTGCACAAAA TTAGATTACT AATACATTTT GCTAGTGACT ATTCAGTTTG TAGTAATATA   
  
  
- ATGCTTTTTA CCTTGTACTT ATTTCTGTTT ACCTTGACGT GTACAATTGA GCATTTGACG CTTGGTAGAC   
  
  
- AGATTGGTCC GAGTTCTCAA TTCTTTCTCT TGCGACAGCA AAGCCTTCTT ATGGTCCTCT CTTTTTTTTT   
  
  
- TTTTTTTGAG AACTCAAACA ATTATAGCTG TGTTTACATG CATACAGCGG GTCTTTTCTT CTACTCTTAT   
  
  
- TTTTGGTTAT TTGAAAGAAT CCTAACGTGT ACATTCCAAT TAATACATTT AATTACTTAG TAATTGTGAC   
  
  
- GCCGAAAGAT CTGCTCGTCG TACGTCATGT TTCGTATGCA CAATGCGCTC AGTGTACCCA CTAGGTAGCG   
  
  
- GTAGTCGCAT TCGTTCCCCA AACTGTACTG CAGTAGTAGA ATGCATTCGA TGGGGTTAGA GACGCGGTCT   
  
  
- GCTCATCACT CATCGATGCG CCTTCTTCAA CCCTTCTAAG AGGGCAGAGA AGGAGACCGG GAGAGAAAAA   
  
  
- GAAGAACAGA AGGAACTTAA ACACAAAGGT GAGAAGGTCA GAAGTTTAAC TAAGTTGTGA CGGGCTCTCT   
  
  
- CTCTCTCTCT CTCAAAACTT AACCAGTCGT CAACCTGGAA AGAGTGAGAG AAAATAAAAG AAAAAGTTAA   
  
  
- AGAAAAAGAA GACATTCGGA ATACATATAT ATTATCCTTT CTCTAGGACT AGGAGTCGCT CCTTTGTTAG   
  
  
- ACTCAAAAAG AAAACAGGGC CCAGTTTTGC GGAGAAAAAG GGATAGTAAA GAGAACGTAT AAGAGGAGAG   
  
  
- GAAGAAGGGT TGAAGAAGTA ATTCTGTCCG TATCCGGAAA AGTGAAAGGA ACGTAAGTTT TGAAATGGTA   
  
  
- AAGAGAGAGA GAGAGAGAGA GAGAGAGAGA AGTCCGAATA TAAAGGGAAA TACATGCGTA TAACGGGTAG   
  
  
- TTGAGAGGTG AAGGTCATAA ACCACGACCA CCTCCGAGAA CTAGAGGGAA AAACCATTAA TGAACAGGTT   
  
  
- CGGTGACTAG AGTCACTCGA TAAGAAGAAT AGCTAGAACT AGAGAAACTA GTTTGAGTGA AAACTAAAGT   
  
  
- TGTGGGAACG AGAACTAAGA AGTAGAGAGT CGAAAAATGC GATAAGTAGG GGAATACGTC AAGGGGAATT   
  
  
- AAAGAGGAGA TCCGGAATTT CCCAAAGAAG AGCTGTACCA GATCACACGT AAGCTAGGTA ATCAGACGAA   
  
  
- ACATTCAATC AAGCGTGCAT GGGTGTATTC GATGTTGACA CAGCGACGTT AAGATTCGTA ACAACCCAAG   
  
  
- AGAGAACGCA TGACGTATAA TCTCCTCCG

+     TCT-motif

| Site Name | Organism | Position | Strand | Matrix score. | sequence | function |
| --- | --- | --- | --- | --- | --- | --- |
| TCT-motif | Arabidopsis thaliana | 598 | + | 6 | TCTTAC | part of a light responsive element |

> 2018/04/13 10:10:12  
+ TTCGATTTTG TAAAAAAGTT AAATTCTTAT TTTTTTAATA ATTTATCCAT AAAAATAAAG TGAAAAATTA   
  
  
+ AAACTATTCA GTGCTGATAT TTTGTAAAAC AAATAGATAT CCAGTCAATA TGAAACATCT ATACTGTGCT   
  
  
+ TAGTCATGGA CACGTGTTTT AATCTAATGA TTATGTAAAA CGATCACTGA TAAGTCAAAC ATCATTATAT   
  
  
+ TACGAAAAAT GGAACATGAA TAAAGACAAA TGGAACTGCA CATGTTAACT CGTAAACTGC GAACCATCTG   
  
  
+ TCTAACCAGG CTCAAGAGTT AAGAAAGAGA ACGCTGTCGT TTCGGAAGAA TACCAGGAGA GAAAAAAAAA   
  
  
+ AAAAAAACTC TTGAGTTTGT TAATATCGAC ACAAATGTAC GTATGTCGCC CAGAAAAGAA GATGAGAATA   
  
  
+ AAAACCAATA AACTTTCTTA GGATTGCACA TGTAAGGTTA ATTATGTAAA TTAATGAATC ATTAACACTG   
  
  
+ CGGCTTTCTA GACGAGCAGC ATGCAGTACA AAGCATACGT GTTACGCGAG TCACATGGGT GATCCATCGC   
  
  
+ CATCAGCGTA AGCAAGGGGT TTGACATGAC GTCATCATCT TACGTAAGCT ACCCCAATCT CTGCGCCAGA   
  
  
+ CGAGTAGTGA GTAGCTACGC GGAAGAAGTT GGGAAGATTC TCCCGTCTCT TCCTCTGGCC CTCTCTTTTT   
  
  
+ CTTCTTGTCT TCCTTGAATT TGTGTTTCCA CTCTTCCAGT CTTCAAATTG ATTCAACACT GCCCGAGAGA   
  
  
+ GAGAGAGAGA GAGTTTTGAA TTGGTCAGCA GTTGGACCTT TCTCACTCTC TTTTATTTTC TTTTTCAATT   
  
  
+ TCTTTTTCTT CTGTAAGCCT TATGTATATA TAATAGGAAA GAGATCCTGA TCCTCAGCGA GGAAACAATC   
  
  
+ TGAGTTTTTC TTTTGTCCCG GGTCAAAACG CCTCTTTTTC CCTATCATTT CTCTTGCATA TTCTCCTCTC   
  
  
+ CTTCTTCCCA ACTTCTTCAT TAAGACAGGC ATAGGCCTTT TCACTTTCCT TGCATTCAAA ACTTTACCAT   
  
  
+ TTCTCTCTCT CTCTCTCTCT CTCTCTCTCT TCAGGCTTAT ATTTCCCTTT ATGTACGCAT ATTGCCCATC   
  
  
+ AACTCTCCAC TTCCAGTATT TGGTGCTGGT GGAGGCTCTT GATCTCCCTT TTTGGTAATT ACTTGTCCAA   
  
  
+ GCCACTGATC TCAGTGAGCT ATTCTTCTTA TCGATCTTGA TCTCTTTGAT CAAACTCACT TTTGATTTCA   
  
  
+ ACACCCTTGC TCTTGATTCT TCATCTCTCA GCTTTTTACG CTATTCATCC CCTTATGCAG TTCCCCTTAA   
  
  
+ TTTCTCCTCT AGGCCTTAAA GGGTTTCTTC TCGACATGGT CTAGTGTGCA TTCGATCCAT TAGTCTGCTT   
  
  
+ TGTAAGTTAG TTCGCACGTA CCCACATAAG CTACAACTGT GTCGCTGCAA TTCTAAGCAT TGTTGGGTTC   
  
  
+ TCTCTTGCGT ACTGCATATT AGAGGAGGC  

- AAGCTAAAAC ATTTTTTCAA TTTAAGAATA AAAAAATTAT TAAATAGGTA TTTTTATTTC ACTTTTTAAT   
  
  
- TTTGATAAGT CACGACTATA AAACATTTTG TTTATCTATA GGTCAGTTAT ACTTTGTAGA TATGACACGA   
  
  
- ATCAGTACCT GTGCACAAAA TTAGATTACT AATACATTTT GCTAGTGACT ATTCAGTTTG TAGTAATATA   
  
  
- ATGCTTTTTA CCTTGTACTT ATTTCTGTTT ACCTTGACGT GTACAATTGA GCATTTGACG CTTGGTAGAC   
  
  
- AGATTGGTCC GAGTTCTCAA TTCTTTCTCT TGCGACAGCA AAGCCTTCTT ATGGTCCTCT CTTTTTTTTT   
  
  
- TTTTTTTGAG AACTCAAACA ATTATAGCTG TGTTTACATG CATACAGCGG GTCTTTTCTT CTACTCTTAT   
  
  
- TTTTGGTTAT TTGAAAGAAT CCTAACGTGT ACATTCCAAT TAATACATTT AATTACTTAG TAATTGTGAC   
  
  
- GCCGAAAGAT CTGCTCGTCG TACGTCATGT TTCGTATGCA CAATGCGCTC AGTGTACCCA CTAGGTAGCG   
  
  
- GTAGTCGCAT TCGTTCCCCA AACTGTACTG CAGTAGTAGA ATGCATTCGA TGGGGTTAGA GACGCGGTCT   
  
  
- GCTCATCACT CATCGATGCG CCTTCTTCAA CCCTTCTAAG AGGGCAGAGA AGGAGACCGG GAGAGAAAAA   
  
  
- GAAGAACAGA AGGAACTTAA ACACAAAGGT GAGAAGGTCA GAAGTTTAAC TAAGTTGTGA CGGGCTCTCT   
  
  
- CTCTCTCTCT CTCAAAACTT AACCAGTCGT CAACCTGGAA AGAGTGAGAG AAAATAAAAG AAAAAGTTAA   
  
  
- AGAAAAAGAA GACATTCGGA ATACATATAT ATTATCCTTT CTCTAGGACT AGGAGTCGCT CCTTTGTTAG   
  
  
- ACTCAAAAAG AAAACAGGGC CCAGTTTTGC GGAGAAAAAG GGATAGTAAA GAGAACGTAT AAGAGGAGAG   
  
  
- GAAGAAGGGT TGAAGAAGTA ATTCTGTCCG TATCCGGAAA AGTGAAAGGA ACGTAAGTTT TGAAATGGTA   
  
  
- AAGAGAGAGA GAGAGAGAGA GAGAGAGAGA AGTCCGAATA TAAAGGGAAA TACATGCGTA TAACGGGTAG   
  
  
- TTGAGAGGTG AAGGTCATAA ACCACGACCA CCTCCGAGAA CTAGAGGGAA AAACCATTAA TGAACAGGTT   
  
  
- CGGTGACTAG AGTCACTCGA TAAGAAGAAT AGCTAGAACT AGAGAAACTA GTTTGAGTGA AAACTAAAGT   
  
  
- TGTGGGAACG AGAACTAAGA AGTAGAGAGT CGAAAAATGC GATAAGTAGG GGAATACGTC AAGGGGAATT   
  
  
- AAAGAGGAGA TCCGGAATTT CCCAAAGAAG AGCTGTACCA GATCACACGT AAGCTAGGTA ATCAGACGAA   
  
  
- ACATTCAATC AAGCGTGCAT GGGTGTATTC GATGTTGACA CAGCGACGTT AAGATTCGTA ACAACCCAAG   
  
  
- AGAGAACGCA TGACGTATAA TCTCCTCCG

+     TGA-element

| Site Name | Organism | Position | Strand | Matrix score. | sequence | function |
| --- | --- | --- | --- | --- | --- | --- |
| TGA-element | Brassica oleracea | 316 | - | 6 | AACGAC | auxin-responsive element |

> 2018/04/13 10:10:12  
+ TTCGATTTTG TAAAAAAGTT AAATTCTTAT TTTTTTAATA ATTTATCCAT AAAAATAAAG TGAAAAATTA   
  
  
+ AAACTATTCA GTGCTGATAT TTTGTAAAAC AAATAGATAT CCAGTCAATA TGAAACATCT ATACTGTGCT   
  
  
+ TAGTCATGGA CACGTGTTTT AATCTAATGA TTATGTAAAA CGATCACTGA TAAGTCAAAC ATCATTATAT   
  
  
+ TACGAAAAAT GGAACATGAA TAAAGACAAA TGGAACTGCA CATGTTAACT CGTAAACTGC GAACCATCTG   
  
  
+ TCTAACCAGG CTCAAGAGTT AAGAAAGAGA ACGCTGTCGT TTCGGAAGAA TACCAGGAGA GAAAAAAAAA   
  
  
+ AAAAAAACTC TTGAGTTTGT TAATATCGAC ACAAATGTAC GTATGTCGCC CAGAAAAGAA GATGAGAATA   
  
  
+ AAAACCAATA AACTTTCTTA GGATTGCACA TGTAAGGTTA ATTATGTAAA TTAATGAATC ATTAACACTG   
  
  
+ CGGCTTTCTA GACGAGCAGC ATGCAGTACA AAGCATACGT GTTACGCGAG TCACATGGGT GATCCATCGC   
  
  
+ CATCAGCGTA AGCAAGGGGT TTGACATGAC GTCATCATCT TACGTAAGCT ACCCCAATCT CTGCGCCAGA   
  
  
+ CGAGTAGTGA GTAGCTACGC GGAAGAAGTT GGGAAGATTC TCCCGTCTCT TCCTCTGGCC CTCTCTTTTT   
  
  
+ CTTCTTGTCT TCCTTGAATT TGTGTTTCCA CTCTTCCAGT CTTCAAATTG ATTCAACACT GCCCGAGAGA   
  
  
+ GAGAGAGAGA GAGTTTTGAA TTGGTCAGCA GTTGGACCTT TCTCACTCTC TTTTATTTTC TTTTTCAATT   
  
  
+ TCTTTTTCTT CTGTAAGCCT TATGTATATA TAATAGGAAA GAGATCCTGA TCCTCAGCGA GGAAACAATC   
  
  
+ TGAGTTTTTC TTTTGTCCCG GGTCAAAACG CCTCTTTTTC CCTATCATTT CTCTTGCATA TTCTCCTCTC   
  
  
+ CTTCTTCCCA ACTTCTTCAT TAAGACAGGC ATAGGCCTTT TCACTTTCCT TGCATTCAAA ACTTTACCAT   
  
  
+ TTCTCTCTCT CTCTCTCTCT CTCTCTCTCT TCAGGCTTAT ATTTCCCTTT ATGTACGCAT ATTGCCCATC   
  
  
+ AACTCTCCAC TTCCAGTATT TGGTGCTGGT GGAGGCTCTT GATCTCCCTT TTTGGTAATT ACTTGTCCAA   
  
  
+ GCCACTGATC TCAGTGAGCT ATTCTTCTTA TCGATCTTGA TCTCTTTGAT CAAACTCACT TTTGATTTCA   
  
  
+ ACACCCTTGC TCTTGATTCT TCATCTCTCA GCTTTTTACG CTATTCATCC CCTTATGCAG TTCCCCTTAA   
  
  
+ TTTCTCCTCT AGGCCTTAAA GGGTTTCTTC TCGACATGGT CTAGTGTGCA TTCGATCCAT TAGTCTGCTT   
  
  
+ TGTAAGTTAG TTCGCACGTA CCCACATAAG CTACAACTGT GTCGCTGCAA TTCTAAGCAT TGTTGGGTTC   
  
  
+ TCTCTTGCGT ACTGCATATT AGAGGAGGC  

- AAGCTAAAAC ATTTTTTCAA TTTAAGAATA AAAAAATTAT TAAATAGGTA TTTTTATTTC ACTTTTTAAT   
  
  
- TTTGATAAGT CACGACTATA AAACATTTTG TTTATCTATA GGTCAGTTAT ACTTTGTAGA TATGACACGA   
  
  
- ATCAGTACCT GTGCACAAAA TTAGATTACT AATACATTTT GCTAGTGACT ATTCAGTTTG TAGTAATATA   
  
  
- ATGCTTTTTA CCTTGTACTT ATTTCTGTTT ACCTTGACGT GTACAATTGA GCATTTGACG CTTGGTAGAC   
  
  
- AGATTGGTCC GAGTTCTCAA TTCTTTCTCT TGCGACAGCA AAGCCTTCTT ATGGTCCTCT CTTTTTTTTT   
  
  
- TTTTTTTGAG AACTCAAACA ATTATAGCTG TGTTTACATG CATACAGCGG GTCTTTTCTT CTACTCTTAT   
  
  
- TTTTGGTTAT TTGAAAGAAT CCTAACGTGT ACATTCCAAT TAATACATTT AATTACTTAG TAATTGTGAC   
  
  
- GCCGAAAGAT CTGCTCGTCG TACGTCATGT TTCGTATGCA CAATGCGCTC AGTGTACCCA CTAGGTAGCG   
  
  
- GTAGTCGCAT TCGTTCCCCA AACTGTACTG CAGTAGTAGA ATGCATTCGA TGGGGTTAGA GACGCGGTCT   
  
  
- GCTCATCACT CATCGATGCG CCTTCTTCAA CCCTTCTAAG AGGGCAGAGA AGGAGACCGG GAGAGAAAAA   
  
  
- GAAGAACAGA AGGAACTTAA ACACAAAGGT GAGAAGGTCA GAAGTTTAAC TAAGTTGTGA CGGGCTCTCT   
  
  
- CTCTCTCTCT CTCAAAACTT AACCAGTCGT CAACCTGGAA AGAGTGAGAG AAAATAAAAG AAAAAGTTAA   
  
  
- AGAAAAAGAA GACATTCGGA ATACATATAT ATTATCCTTT CTCTAGGACT AGGAGTCGCT CCTTTGTTAG   
  
  
- ACTCAAAAAG AAAACAGGGC CCAGTTTTGC GGAGAAAAAG GGATAGTAAA GAGAACGTAT AAGAGGAGAG   
  
  
- GAAGAAGGGT TGAAGAAGTA ATTCTGTCCG TATCCGGAAA AGTGAAAGGA ACGTAAGTTT TGAAATGGTA   
  
  
- AAGAGAGAGA GAGAGAGAGA GAGAGAGAGA AGTCCGAATA TAAAGGGAAA TACATGCGTA TAACGGGTAG   
  
  
- TTGAGAGGTG AAGGTCATAA ACCACGACCA CCTCCGAGAA CTAGAGGGAA AAACCATTAA TGAACAGGTT   
  
  
- CGGTGACTAG AGTCACTCGA TAAGAAGAAT AGCTAGAACT AGAGAAACTA GTTTGAGTGA AAACTAAAGT   
  
  
- TGTGGGAACG AGAACTAAGA AGTAGAGAGT CGAAAAATGC GATAAGTAGG GGAATACGTC AAGGGGAATT   
  
  
- AAAGAGGAGA TCCGGAATTT CCCAAAGAAG AGCTGTACCA GATCACACGT AAGCTAGGTA ATCAGACGAA   
  
  
- ACATTCAATC AAGCGTGCAT GGGTGTATTC GATGTTGACA CAGCGACGTT AAGATTCGTA ACAACCCAAG   
  
  
- AGAGAACGCA TGACGTATAA TCTCCTCCG

+     TGACG-motif

| Site Name | Organism | Position | Strand | Matrix score. | sequence | function |
| --- | --- | --- | --- | --- | --- | --- |
| TGACG-motif | Hordeum vulgare | 587 | + | 5 | TGACG | cis-acting regulatory element involved in the MeJA-responsiveness |
| TGACG-motif | Hordeum vulgare | 590 | - | 5 | TGACG | cis-acting regulatory element involved in the MeJA-responsiveness |

> 2018/04/13 10:10:12  
+ TTCGATTTTG TAAAAAAGTT AAATTCTTAT TTTTTTAATA ATTTATCCAT AAAAATAAAG TGAAAAATTA   
  
  
+ AAACTATTCA GTGCTGATAT TTTGTAAAAC AAATAGATAT CCAGTCAATA TGAAACATCT ATACTGTGCT   
  
  
+ TAGTCATGGA CACGTGTTTT AATCTAATGA TTATGTAAAA CGATCACTGA TAAGTCAAAC ATCATTATAT   
  
  
+ TACGAAAAAT GGAACATGAA TAAAGACAAA TGGAACTGCA CATGTTAACT CGTAAACTGC GAACCATCTG   
  
  
+ TCTAACCAGG CTCAAGAGTT AAGAAAGAGA ACGCTGTCGT TTCGGAAGAA TACCAGGAGA GAAAAAAAAA   
  
  
+ AAAAAAACTC TTGAGTTTGT TAATATCGAC ACAAATGTAC GTATGTCGCC CAGAAAAGAA GATGAGAATA   
  
  
+ AAAACCAATA AACTTTCTTA GGATTGCACA TGTAAGGTTA ATTATGTAAA TTAATGAATC ATTAACACTG   
  
  
+ CGGCTTTCTA GACGAGCAGC ATGCAGTACA AAGCATACGT GTTACGCGAG TCACATGGGT GATCCATCGC   
  
  
+ CATCAGCGTA AGCAAGGGGT TTGACATGAC GTCATCATCT TACGTAAGCT ACCCCAATCT CTGCGCCAGA   
  
  
+ CGAGTAGTGA GTAGCTACGC GGAAGAAGTT GGGAAGATTC TCCCGTCTCT TCCTCTGGCC CTCTCTTTTT   
  
  
+ CTTCTTGTCT TCCTTGAATT TGTGTTTCCA CTCTTCCAGT CTTCAAATTG ATTCAACACT GCCCGAGAGA   
  
  
+ GAGAGAGAGA GAGTTTTGAA TTGGTCAGCA GTTGGACCTT TCTCACTCTC TTTTATTTTC TTTTTCAATT   
  
  
+ TCTTTTTCTT CTGTAAGCCT TATGTATATA TAATAGGAAA GAGATCCTGA TCCTCAGCGA GGAAACAATC   
  
  
+ TGAGTTTTTC TTTTGTCCCG GGTCAAAACG CCTCTTTTTC CCTATCATTT CTCTTGCATA TTCTCCTCTC   
  
  
+ CTTCTTCCCA ACTTCTTCAT TAAGACAGGC ATAGGCCTTT TCACTTTCCT TGCATTCAAA ACTTTACCAT   
  
  
+ TTCTCTCTCT CTCTCTCTCT CTCTCTCTCT TCAGGCTTAT ATTTCCCTTT ATGTACGCAT ATTGCCCATC   
  
  
+ AACTCTCCAC TTCCAGTATT TGGTGCTGGT GGAGGCTCTT GATCTCCCTT TTTGGTAATT ACTTGTCCAA   
  
  
+ GCCACTGATC TCAGTGAGCT ATTCTTCTTA TCGATCTTGA TCTCTTTGAT CAAACTCACT TTTGATTTCA   
  
  
+ ACACCCTTGC TCTTGATTCT TCATCTCTCA GCTTTTTACG CTATTCATCC CCTTATGCAG TTCCCCTTAA   
  
  
+ TTTCTCCTCT AGGCCTTAAA GGGTTTCTTC TCGACATGGT CTAGTGTGCA TTCGATCCAT TAGTCTGCTT   
  
  
+ TGTAAGTTAG TTCGCACGTA CCCACATAAG CTACAACTGT GTCGCTGCAA TTCTAAGCAT TGTTGGGTTC   
  
  
+ TCTCTTGCGT ACTGCATATT AGAGGAGGC  

- AAGCTAAAAC ATTTTTTCAA TTTAAGAATA AAAAAATTAT TAAATAGGTA TTTTTATTTC ACTTTTTAAT   
  
  
- TTTGATAAGT CACGACTATA AAACATTTTG TTTATCTATA GGTCAGTTAT ACTTTGTAGA TATGACACGA   
  
  
- ATCAGTACCT GTGCACAAAA TTAGATTACT AATACATTTT GCTAGTGACT ATTCAGTTTG TAGTAATATA   
  
  
- ATGCTTTTTA CCTTGTACTT ATTTCTGTTT ACCTTGACGT GTACAATTGA GCATTTGACG CTTGGTAGAC   
  
  
- AGATTGGTCC GAGTTCTCAA TTCTTTCTCT TGCGACAGCA AAGCCTTCTT ATGGTCCTCT CTTTTTTTTT   
  
  
- TTTTTTTGAG AACTCAAACA ATTATAGCTG TGTTTACATG CATACAGCGG GTCTTTTCTT CTACTCTTAT   
  
  
- TTTTGGTTAT TTGAAAGAAT CCTAACGTGT ACATTCCAAT TAATACATTT AATTACTTAG TAATTGTGAC   
  
  
- GCCGAAAGAT CTGCTCGTCG TACGTCATGT TTCGTATGCA CAATGCGCTC AGTGTACCCA CTAGGTAGCG   
  
  
- GTAGTCGCAT TCGTTCCCCA AACTGTACTG CAGTAGTAGA ATGCATTCGA TGGGGTTAGA GACGCGGTCT   
  
  
- GCTCATCACT CATCGATGCG CCTTCTTCAA CCCTTCTAAG AGGGCAGAGA AGGAGACCGG GAGAGAAAAA   
  
  
- GAAGAACAGA AGGAACTTAA ACACAAAGGT GAGAAGGTCA GAAGTTTAAC TAAGTTGTGA CGGGCTCTCT   
  
  
- CTCTCTCTCT CTCAAAACTT AACCAGTCGT CAACCTGGAA AGAGTGAGAG AAAATAAAAG AAAAAGTTAA   
  
  
- AGAAAAAGAA GACATTCGGA ATACATATAT ATTATCCTTT CTCTAGGACT AGGAGTCGCT CCTTTGTTAG   
  
  
- ACTCAAAAAG AAAACAGGGC CCAGTTTTGC GGAGAAAAAG GGATAGTAAA GAGAACGTAT AAGAGGAGAG   
  
  
- GAAGAAGGGT TGAAGAAGTA ATTCTGTCCG TATCCGGAAA AGTGAAAGGA ACGTAAGTTT TGAAATGGTA   
  
  
- AAGAGAGAGA GAGAGAGAGA GAGAGAGAGA AGTCCGAATA TAAAGGGAAA TACATGCGTA TAACGGGTAG   
  
  
- TTGAGAGGTG AAGGTCATAA ACCACGACCA CCTCCGAGAA CTAGAGGGAA AAACCATTAA TGAACAGGTT   
  
  
- CGGTGACTAG AGTCACTCGA TAAGAAGAAT AGCTAGAACT AGAGAAACTA GTTTGAGTGA AAACTAAAGT   
  
  
- TGTGGGAACG AGAACTAAGA AGTAGAGAGT CGAAAAATGC GATAAGTAGG GGAATACGTC AAGGGGAATT   
  
  
- AAAGAGGAGA TCCGGAATTT CCCAAAGAAG AGCTGTACCA GATCACACGT AAGCTAGGTA ATCAGACGAA   
  
  
- ACATTCAATC AAGCGTGCAT GGGTGTATTC GATGTTGACA CAGCGACGTT AAGATTCGTA ACAACCCAAG   
  
  
- AGAGAACGCA TGACGTATAA TCTCCTCCG

+     Unnamed\_\_16

| Site Name | Organism | Position | Strand | Matrix score. | sequence | function |
| --- | --- | --- | --- | --- | --- | --- |
| Unnamed\_\_16 | Zea mays | 501 | - | 9 | GCTGCCCGTC |  |

> 2018/04/13 10:10:12  
+ TTCGATTTTG TAAAAAAGTT AAATTCTTAT TTTTTTAATA ATTTATCCAT AAAAATAAAG TGAAAAATTA   
  
  
+ AAACTATTCA GTGCTGATAT TTTGTAAAAC AAATAGATAT CCAGTCAATA TGAAACATCT ATACTGTGCT   
  
  
+ TAGTCATGGA CACGTGTTTT AATCTAATGA TTATGTAAAA CGATCACTGA TAAGTCAAAC ATCATTATAT   
  
  
+ TACGAAAAAT GGAACATGAA TAAAGACAAA TGGAACTGCA CATGTTAACT CGTAAACTGC GAACCATCTG   
  
  
+ TCTAACCAGG CTCAAGAGTT AAGAAAGAGA ACGCTGTCGT TTCGGAAGAA TACCAGGAGA GAAAAAAAAA   
  
  
+ AAAAAAACTC TTGAGTTTGT TAATATCGAC ACAAATGTAC GTATGTCGCC CAGAAAAGAA GATGAGAATA   
  
  
+ AAAACCAATA AACTTTCTTA GGATTGCACA TGTAAGGTTA ATTATGTAAA TTAATGAATC ATTAACACTG   
  
  
+ CGGCTTTCTA GACGAGCAGC ATGCAGTACA AAGCATACGT GTTACGCGAG TCACATGGGT GATCCATCGC   
  
  
+ CATCAGCGTA AGCAAGGGGT TTGACATGAC GTCATCATCT TACGTAAGCT ACCCCAATCT CTGCGCCAGA   
  
  
+ CGAGTAGTGA GTAGCTACGC GGAAGAAGTT GGGAAGATTC TCCCGTCTCT TCCTCTGGCC CTCTCTTTTT   
  
  
+ CTTCTTGTCT TCCTTGAATT TGTGTTTCCA CTCTTCCAGT CTTCAAATTG ATTCAACACT GCCCGAGAGA   
  
  
+ GAGAGAGAGA GAGTTTTGAA TTGGTCAGCA GTTGGACCTT TCTCACTCTC TTTTATTTTC TTTTTCAATT   
  
  
+ TCTTTTTCTT CTGTAAGCCT TATGTATATA TAATAGGAAA GAGATCCTGA TCCTCAGCGA GGAAACAATC   
  
  
+ TGAGTTTTTC TTTTGTCCCG GGTCAAAACG CCTCTTTTTC CCTATCATTT CTCTTGCATA TTCTCCTCTC   
  
  
+ CTTCTTCCCA ACTTCTTCAT TAAGACAGGC ATAGGCCTTT TCACTTTCCT TGCATTCAAA ACTTTACCAT   
  
  
+ TTCTCTCTCT CTCTCTCTCT CTCTCTCTCT TCAGGCTTAT ATTTCCCTTT ATGTACGCAT ATTGCCCATC   
  
  
+ AACTCTCCAC TTCCAGTATT TGGTGCTGGT GGAGGCTCTT GATCTCCCTT TTTGGTAATT ACTTGTCCAA   
  
  
+ GCCACTGATC TCAGTGAGCT ATTCTTCTTA TCGATCTTGA TCTCTTTGAT CAAACTCACT TTTGATTTCA   
  
  
+ ACACCCTTGC TCTTGATTCT TCATCTCTCA GCTTTTTACG CTATTCATCC CCTTATGCAG TTCCCCTTAA   
  
  
+ TTTCTCCTCT AGGCCTTAAA GGGTTTCTTC TCGACATGGT CTAGTGTGCA TTCGATCCAT TAGTCTGCTT   
  
  
+ TGTAAGTTAG TTCGCACGTA CCCACATAAG CTACAACTGT GTCGCTGCAA TTCTAAGCAT TGTTGGGTTC   
  
  
+ TCTCTTGCGT ACTGCATATT AGAGGAGGC  

- AAGCTAAAAC ATTTTTTCAA TTTAAGAATA AAAAAATTAT TAAATAGGTA TTTTTATTTC ACTTTTTAAT   
  
  
- TTTGATAAGT CACGACTATA AAACATTTTG TTTATCTATA GGTCAGTTAT ACTTTGTAGA TATGACACGA   
  
  
- ATCAGTACCT GTGCACAAAA TTAGATTACT AATACATTTT GCTAGTGACT ATTCAGTTTG TAGTAATATA   
  
  
- ATGCTTTTTA CCTTGTACTT ATTTCTGTTT ACCTTGACGT GTACAATTGA GCATTTGACG CTTGGTAGAC   
  
  
- AGATTGGTCC GAGTTCTCAA TTCTTTCTCT TGCGACAGCA AAGCCTTCTT ATGGTCCTCT CTTTTTTTTT   
  
  
- TTTTTTTGAG AACTCAAACA ATTATAGCTG TGTTTACATG CATACAGCGG GTCTTTTCTT CTACTCTTAT   
  
  
- TTTTGGTTAT TTGAAAGAAT CCTAACGTGT ACATTCCAAT TAATACATTT AATTACTTAG TAATTGTGAC   
  
  
- GCCGAAAGAT CTGCTCGTCG TACGTCATGT TTCGTATGCA CAATGCGCTC AGTGTACCCA CTAGGTAGCG   
  
  
- GTAGTCGCAT TCGTTCCCCA AACTGTACTG CAGTAGTAGA ATGCATTCGA TGGGGTTAGA GACGCGGTCT   
  
  
- GCTCATCACT CATCGATGCG CCTTCTTCAA CCCTTCTAAG AGGGCAGAGA AGGAGACCGG GAGAGAAAAA   
  
  
- GAAGAACAGA AGGAACTTAA ACACAAAGGT GAGAAGGTCA GAAGTTTAAC TAAGTTGTGA CGGGCTCTCT   
  
  
- CTCTCTCTCT CTCAAAACTT AACCAGTCGT CAACCTGGAA AGAGTGAGAG AAAATAAAAG AAAAAGTTAA   
  
  
- AGAAAAAGAA GACATTCGGA ATACATATAT ATTATCCTTT CTCTAGGACT AGGAGTCGCT CCTTTGTTAG   
  
  
- ACTCAAAAAG AAAACAGGGC CCAGTTTTGC GGAGAAAAAG GGATAGTAAA GAGAACGTAT AAGAGGAGAG   
  
  
- GAAGAAGGGT TGAAGAAGTA ATTCTGTCCG TATCCGGAAA AGTGAAAGGA ACGTAAGTTT TGAAATGGTA   
  
  
- AAGAGAGAGA GAGAGAGAGA GAGAGAGAGA AGTCCGAATA TAAAGGGAAA TACATGCGTA TAACGGGTAG   
  
  
- TTGAGAGGTG AAGGTCATAA ACCACGACCA CCTCCGAGAA CTAGAGGGAA AAACCATTAA TGAACAGGTT   
  
  
- CGGTGACTAG AGTCACTCGA TAAGAAGAAT AGCTAGAACT AGAGAAACTA GTTTGAGTGA AAACTAAAGT   
  
  
- TGTGGGAACG AGAACTAAGA AGTAGAGAGT CGAAAAATGC GATAAGTAGG GGAATACGTC AAGGGGAATT   
  
  
- AAAGAGGAGA TCCGGAATTT CCCAAAGAAG AGCTGTACCA GATCACACGT AAGCTAGGTA ATCAGACGAA   
  
  
- ACATTCAATC AAGCGTGCAT GGGTGTATTC GATGTTGACA CAGCGACGTT AAGATTCGTA ACAACCCAAG   
  
  
- AGAGAACGCA TGACGTATAA TCTCCTCCG

+     Unnamed\_\_4

| Site Name | Organism | Position | Strand | Matrix score. | sequence | function |
| --- | --- | --- | --- | --- | --- | --- |
| Unnamed\_\_4 | Petroselinum hortense | 1334 | + | 4 | CTCC |  |
| Unnamed\_\_4 | Petroselinum hortense | 1151 | - | 4 | CTCC |  |
| Unnamed\_\_4 | Petroselinum hortense | 1125 | + | 4 | CTCC |  |
| Unnamed\_\_4 | Petroselinum hortense | 978 | + | 4 | CTCC |  |
| Unnamed\_\_4 | Petroselinum hortense | 1494 | - | 4 | CTCC |  |
| Unnamed\_\_4 | Petroselinum hortense | 973 | + | 4 | CTCC |  |
| Unnamed\_\_4 | Petroselinum hortense | 1164 | + | 4 | CTCC |  |
| Unnamed\_\_4 | Petroselinum hortense | 670 | + | 4 | CTCC |  |
| Unnamed\_\_4 | Petroselinum hortense | 336 | - | 4 | CTCC |  |

> 2018/04/13 10:10:12  
+ TTCGATTTTG TAAAAAAGTT AAATTCTTAT TTTTTTAATA ATTTATCCAT AAAAATAAAG TGAAAAATTA   
  
  
+ AAACTATTCA GTGCTGATAT TTTGTAAAAC AAATAGATAT CCAGTCAATA TGAAACATCT ATACTGTGCT   
  
  
+ TAGTCATGGA CACGTGTTTT AATCTAATGA TTATGTAAAA CGATCACTGA TAAGTCAAAC ATCATTATAT   
  
  
+ TACGAAAAAT GGAACATGAA TAAAGACAAA TGGAACTGCA CATGTTAACT CGTAAACTGC GAACCATCTG   
  
  
+ TCTAACCAGG CTCAAGAGTT AAGAAAGAGA ACGCTGTCGT TTCGGAAGAA TACCAGGAGA GAAAAAAAAA   
  
  
+ AAAAAAACTC TTGAGTTTGT TAATATCGAC ACAAATGTAC GTATGTCGCC CAGAAAAGAA GATGAGAATA   
  
  
+ AAAACCAATA AACTTTCTTA GGATTGCACA TGTAAGGTTA ATTATGTAAA TTAATGAATC ATTAACACTG   
  
  
+ CGGCTTTCTA GACGAGCAGC ATGCAGTACA AAGCATACGT GTTACGCGAG TCACATGGGT GATCCATCGC   
  
  
+ CATCAGCGTA AGCAAGGGGT TTGACATGAC GTCATCATCT TACGTAAGCT ACCCCAATCT CTGCGCCAGA   
  
  
+ CGAGTAGTGA GTAGCTACGC GGAAGAAGTT GGGAAGATTC TCCCGTCTCT TCCTCTGGCC CTCTCTTTTT   
  
  
+ CTTCTTGTCT TCCTTGAATT TGTGTTTCCA CTCTTCCAGT CTTCAAATTG ATTCAACACT GCCCGAGAGA   
  
  
+ GAGAGAGAGA GAGTTTTGAA TTGGTCAGCA GTTGGACCTT TCTCACTCTC TTTTATTTTC TTTTTCAATT   
  
  
+ TCTTTTTCTT CTGTAAGCCT TATGTATATA TAATAGGAAA GAGATCCTGA TCCTCAGCGA GGAAACAATC   
  
  
+ TGAGTTTTTC TTTTGTCCCG GGTCAAAACG CCTCTTTTTC CCTATCATTT CTCTTGCATA TTCTCCTCTC   
  
  
+ CTTCTTCCCA ACTTCTTCAT TAAGACAGGC ATAGGCCTTT TCACTTTCCT TGCATTCAAA ACTTTACCAT   
  
  
+ TTCTCTCTCT CTCTCTCTCT CTCTCTCTCT TCAGGCTTAT ATTTCCCTTT ATGTACGCAT ATTGCCCATC   
  
  
+ AACTCTCCAC TTCCAGTATT TGGTGCTGGT GGAGGCTCTT GATCTCCCTT TTTGGTAATT ACTTGTCCAA   
  
  
+ GCCACTGATC TCAGTGAGCT ATTCTTCTTA TCGATCTTGA TCTCTTTGAT CAAACTCACT TTTGATTTCA   
  
  
+ ACACCCTTGC TCTTGATTCT TCATCTCTCA GCTTTTTACG CTATTCATCC CCTTATGCAG TTCCCCTTAA   
  
  
+ TTTCTCCTCT AGGCCTTAAA GGGTTTCTTC TCGACATGGT CTAGTGTGCA TTCGATCCAT TAGTCTGCTT   
  
  
+ TGTAAGTTAG TTCGCACGTA CCCACATAAG CTACAACTGT GTCGCTGCAA TTCTAAGCAT TGTTGGGTTC   
  
  
+ TCTCTTGCGT ACTGCATATT AGAGGAGGC  

- AAGCTAAAAC ATTTTTTCAA TTTAAGAATA AAAAAATTAT TAAATAGGTA TTTTTATTTC ACTTTTTAAT   
  
  
- TTTGATAAGT CACGACTATA AAACATTTTG TTTATCTATA GGTCAGTTAT ACTTTGTAGA TATGACACGA   
  
  
- ATCAGTACCT GTGCACAAAA TTAGATTACT AATACATTTT GCTAGTGACT ATTCAGTTTG TAGTAATATA   
  
  
- ATGCTTTTTA CCTTGTACTT ATTTCTGTTT ACCTTGACGT GTACAATTGA GCATTTGACG CTTGGTAGAC   
  
  
- AGATTGGTCC GAGTTCTCAA TTCTTTCTCT TGCGACAGCA AAGCCTTCTT ATGGTCCTCT CTTTTTTTTT   
  
  
- TTTTTTTGAG AACTCAAACA ATTATAGCTG TGTTTACATG CATACAGCGG GTCTTTTCTT CTACTCTTAT   
  
  
- TTTTGGTTAT TTGAAAGAAT CCTAACGTGT ACATTCCAAT TAATACATTT AATTACTTAG TAATTGTGAC   
  
  
- GCCGAAAGAT CTGCTCGTCG TACGTCATGT TTCGTATGCA CAATGCGCTC AGTGTACCCA CTAGGTAGCG   
  
  
- GTAGTCGCAT TCGTTCCCCA AACTGTACTG CAGTAGTAGA ATGCATTCGA TGGGGTTAGA GACGCGGTCT   
  
  
- GCTCATCACT CATCGATGCG CCTTCTTCAA CCCTTCTAAG AGGGCAGAGA AGGAGACCGG GAGAGAAAAA   
  
  
- GAAGAACAGA AGGAACTTAA ACACAAAGGT GAGAAGGTCA GAAGTTTAAC TAAGTTGTGA CGGGCTCTCT   
  
  
- CTCTCTCTCT CTCAAAACTT AACCAGTCGT CAACCTGGAA AGAGTGAGAG AAAATAAAAG AAAAAGTTAA   
  
  
- AGAAAAAGAA GACATTCGGA ATACATATAT ATTATCCTTT CTCTAGGACT AGGAGTCGCT CCTTTGTTAG   
  
  
- ACTCAAAAAG AAAACAGGGC CCAGTTTTGC GGAGAAAAAG GGATAGTAAA GAGAACGTAT AAGAGGAGAG   
  
  
- GAAGAAGGGT TGAAGAAGTA ATTCTGTCCG TATCCGGAAA AGTGAAAGGA ACGTAAGTTT TGAAATGGTA   
  
  
- AAGAGAGAGA GAGAGAGAGA GAGAGAGAGA AGTCCGAATA TAAAGGGAAA TACATGCGTA TAACGGGTAG   
  
  
- TTGAGAGGTG AAGGTCATAA ACCACGACCA CCTCCGAGAA CTAGAGGGAA AAACCATTAA TGAACAGGTT   
  
  
- CGGTGACTAG AGTCACTCGA TAAGAAGAAT AGCTAGAACT AGAGAAACTA GTTTGAGTGA AAACTAAAGT   
  
  
- TGTGGGAACG AGAACTAAGA AGTAGAGAGT CGAAAAATGC GATAAGTAGG GGAATACGTC AAGGGGAATT   
  
  
- AAAGAGGAGA TCCGGAATTT CCCAAAGAAG AGCTGTACCA GATCACACGT AAGCTAGGTA ATCAGACGAA   
  
  
- ACATTCAATC AAGCGTGCAT GGGTGTATTC GATGTTGACA CAGCGACGTT AAGATTCGTA ACAACCCAAG   
  
  
- AGAGAACGCA TGACGTATAA TCTCCTCCG

+     W box

| Site Name | Organism | Position | Strand | Matrix score. | sequence | function |
| --- | --- | --- | --- | --- | --- | --- |
| W box | Arabidopsis thaliana | 931 | - | 6 | TTGACC |  |

> 2018/04/13 10:10:12  
+ TTCGATTTTG TAAAAAAGTT AAATTCTTAT TTTTTTAATA ATTTATCCAT AAAAATAAAG TGAAAAATTA   
  
  
+ AAACTATTCA GTGCTGATAT TTTGTAAAAC AAATAGATAT CCAGTCAATA TGAAACATCT ATACTGTGCT   
  
  
+ TAGTCATGGA CACGTGTTTT AATCTAATGA TTATGTAAAA CGATCACTGA TAAGTCAAAC ATCATTATAT   
  
  
+ TACGAAAAAT GGAACATGAA TAAAGACAAA TGGAACTGCA CATGTTAACT CGTAAACTGC GAACCATCTG   
  
  
+ TCTAACCAGG CTCAAGAGTT AAGAAAGAGA ACGCTGTCGT TTCGGAAGAA TACCAGGAGA GAAAAAAAAA   
  
  
+ AAAAAAACTC TTGAGTTTGT TAATATCGAC ACAAATGTAC GTATGTCGCC CAGAAAAGAA GATGAGAATA   
  
  
+ AAAACCAATA AACTTTCTTA GGATTGCACA TGTAAGGTTA ATTATGTAAA TTAATGAATC ATTAACACTG   
  
  
+ CGGCTTTCTA GACGAGCAGC ATGCAGTACA AAGCATACGT GTTACGCGAG TCACATGGGT GATCCATCGC   
  
  
+ CATCAGCGTA AGCAAGGGGT TTGACATGAC GTCATCATCT TACGTAAGCT ACCCCAATCT CTGCGCCAGA   
  
  
+ CGAGTAGTGA GTAGCTACGC GGAAGAAGTT GGGAAGATTC TCCCGTCTCT TCCTCTGGCC CTCTCTTTTT   
  
  
+ CTTCTTGTCT TCCTTGAATT TGTGTTTCCA CTCTTCCAGT CTTCAAATTG ATTCAACACT GCCCGAGAGA   
  
  
+ GAGAGAGAGA GAGTTTTGAA TTGGTCAGCA GTTGGACCTT TCTCACTCTC TTTTATTTTC TTTTTCAATT   
  
  
+ TCTTTTTCTT CTGTAAGCCT TATGTATATA TAATAGGAAA GAGATCCTGA TCCTCAGCGA GGAAACAATC   
  
  
+ TGAGTTTTTC TTTTGTCCCG GGTCAAAACG CCTCTTTTTC CCTATCATTT CTCTTGCATA TTCTCCTCTC   
  
  
+ CTTCTTCCCA ACTTCTTCAT TAAGACAGGC ATAGGCCTTT TCACTTTCCT TGCATTCAAA ACTTTACCAT   
  
  
+ TTCTCTCTCT CTCTCTCTCT CTCTCTCTCT TCAGGCTTAT ATTTCCCTTT ATGTACGCAT ATTGCCCATC   
  
  
+ AACTCTCCAC TTCCAGTATT TGGTGCTGGT GGAGGCTCTT GATCTCCCTT TTTGGTAATT ACTTGTCCAA   
  
  
+ GCCACTGATC TCAGTGAGCT ATTCTTCTTA TCGATCTTGA TCTCTTTGAT CAAACTCACT TTTGATTTCA   
  
  
+ ACACCCTTGC TCTTGATTCT TCATCTCTCA GCTTTTTACG CTATTCATCC CCTTATGCAG TTCCCCTTAA   
  
  
+ TTTCTCCTCT AGGCCTTAAA GGGTTTCTTC TCGACATGGT CTAGTGTGCA TTCGATCCAT TAGTCTGCTT   
  
  
+ TGTAAGTTAG TTCGCACGTA CCCACATAAG CTACAACTGT GTCGCTGCAA TTCTAAGCAT TGTTGGGTTC   
  
  
+ TCTCTTGCGT ACTGCATATT AGAGGAGGC  

- AAGCTAAAAC ATTTTTTCAA TTTAAGAATA AAAAAATTAT TAAATAGGTA TTTTTATTTC ACTTTTTAAT   
  
  
- TTTGATAAGT CACGACTATA AAACATTTTG TTTATCTATA GGTCAGTTAT ACTTTGTAGA TATGACACGA   
  
  
- ATCAGTACCT GTGCACAAAA TTAGATTACT AATACATTTT GCTAGTGACT ATTCAGTTTG TAGTAATATA   
  
  
- ATGCTTTTTA CCTTGTACTT ATTTCTGTTT ACCTTGACGT GTACAATTGA GCATTTGACG CTTGGTAGAC   
  
  
- AGATTGGTCC GAGTTCTCAA TTCTTTCTCT TGCGACAGCA AAGCCTTCTT ATGGTCCTCT CTTTTTTTTT   
  
  
- TTTTTTTGAG AACTCAAACA ATTATAGCTG TGTTTACATG CATACAGCGG GTCTTTTCTT CTACTCTTAT   
  
  
- TTTTGGTTAT TTGAAAGAAT CCTAACGTGT ACATTCCAAT TAATACATTT AATTACTTAG TAATTGTGAC   
  
  
- GCCGAAAGAT CTGCTCGTCG TACGTCATGT TTCGTATGCA CAATGCGCTC AGTGTACCCA CTAGGTAGCG   
  
  
- GTAGTCGCAT TCGTTCCCCA AACTGTACTG CAGTAGTAGA ATGCATTCGA TGGGGTTAGA GACGCGGTCT   
  
  
- GCTCATCACT CATCGATGCG CCTTCTTCAA CCCTTCTAAG AGGGCAGAGA AGGAGACCGG GAGAGAAAAA   
  
  
- GAAGAACAGA AGGAACTTAA ACACAAAGGT GAGAAGGTCA GAAGTTTAAC TAAGTTGTGA CGGGCTCTCT   
  
  
- CTCTCTCTCT CTCAAAACTT AACCAGTCGT CAACCTGGAA AGAGTGAGAG AAAATAAAAG AAAAAGTTAA   
  
  
- AGAAAAAGAA GACATTCGGA ATACATATAT ATTATCCTTT CTCTAGGACT AGGAGTCGCT CCTTTGTTAG   
  
  
- ACTCAAAAAG AAAACAGGGC CCAGTTTTGC GGAGAAAAAG GGATAGTAAA GAGAACGTAT AAGAGGAGAG   
  
  
- GAAGAAGGGT TGAAGAAGTA ATTCTGTCCG TATCCGGAAA AGTGAAAGGA ACGTAAGTTT TGAAATGGTA   
  
  
- AAGAGAGAGA GAGAGAGAGA GAGAGAGAGA AGTCCGAATA TAAAGGGAAA TACATGCGTA TAACGGGTAG   
  
  
- TTGAGAGGTG AAGGTCATAA ACCACGACCA CCTCCGAGAA CTAGAGGGAA AAACCATTAA TGAACAGGTT   
  
  
- CGGTGACTAG AGTCACTCGA TAAGAAGAAT AGCTAGAACT AGAGAAACTA GTTTGAGTGA AAACTAAAGT   
  
  
- TGTGGGAACG AGAACTAAGA AGTAGAGAGT CGAAAAATGC GATAAGTAGG GGAATACGTC AAGGGGAATT   
  
  
- AAAGAGGAGA TCCGGAATTT CCCAAAGAAG AGCTGTACCA GATCACACGT AAGCTAGGTA ATCAGACGAA   
  
  
- ACATTCAATC AAGCGTGCAT GGGTGTATTC GATGTTGACA CAGCGACGTT AAGATTCGTA ACAACCCAAG   
  
  
- AGAGAACGCA TGACGTATAA TCTCCTCCG

+     as-2-box

| Site Name | Organism | Position | Strand | Matrix score. | sequence | function |
| --- | --- | --- | --- | --- | --- | --- |
| as-2-box | Nicotiana tabacum | 200 | - | 9 | GATAatGATG | involved in shoot-specific expression and light responsiveness |

> 2018/04/13 10:10:12  
+ TTCGATTTTG TAAAAAAGTT AAATTCTTAT TTTTTTAATA ATTTATCCAT AAAAATAAAG TGAAAAATTA   
  
  
+ AAACTATTCA GTGCTGATAT TTTGTAAAAC AAATAGATAT CCAGTCAATA TGAAACATCT ATACTGTGCT   
  
  
+ TAGTCATGGA CACGTGTTTT AATCTAATGA TTATGTAAAA CGATCACTGA TAAGTCAAAC ATCATTATAT   
  
  
+ TACGAAAAAT GGAACATGAA TAAAGACAAA TGGAACTGCA CATGTTAACT CGTAAACTGC GAACCATCTG   
  
  
+ TCTAACCAGG CTCAAGAGTT AAGAAAGAGA ACGCTGTCGT TTCGGAAGAA TACCAGGAGA GAAAAAAAAA   
  
  
+ AAAAAAACTC TTGAGTTTGT TAATATCGAC ACAAATGTAC GTATGTCGCC CAGAAAAGAA GATGAGAATA   
  
  
+ AAAACCAATA AACTTTCTTA GGATTGCACA TGTAAGGTTA ATTATGTAAA TTAATGAATC ATTAACACTG   
  
  
+ CGGCTTTCTA GACGAGCAGC ATGCAGTACA AAGCATACGT GTTACGCGAG TCACATGGGT GATCCATCGC   
  
  
+ CATCAGCGTA AGCAAGGGGT TTGACATGAC GTCATCATCT TACGTAAGCT ACCCCAATCT CTGCGCCAGA   
  
  
+ CGAGTAGTGA GTAGCTACGC GGAAGAAGTT GGGAAGATTC TCCCGTCTCT TCCTCTGGCC CTCTCTTTTT   
  
  
+ CTTCTTGTCT TCCTTGAATT TGTGTTTCCA CTCTTCCAGT CTTCAAATTG ATTCAACACT GCCCGAGAGA   
  
  
+ GAGAGAGAGA GAGTTTTGAA TTGGTCAGCA GTTGGACCTT TCTCACTCTC TTTTATTTTC TTTTTCAATT   
  
  
+ TCTTTTTCTT CTGTAAGCCT TATGTATATA TAATAGGAAA GAGATCCTGA TCCTCAGCGA GGAAACAATC   
  
  
+ TGAGTTTTTC TTTTGTCCCG GGTCAAAACG CCTCTTTTTC CCTATCATTT CTCTTGCATA TTCTCCTCTC   
  
  
+ CTTCTTCCCA ACTTCTTCAT TAAGACAGGC ATAGGCCTTT TCACTTTCCT TGCATTCAAA ACTTTACCAT   
  
  
+ TTCTCTCTCT CTCTCTCTCT CTCTCTCTCT TCAGGCTTAT ATTTCCCTTT ATGTACGCAT ATTGCCCATC   
  
  
+ AACTCTCCAC TTCCAGTATT TGGTGCTGGT GGAGGCTCTT GATCTCCCTT TTTGGTAATT ACTTGTCCAA   
  
  
+ GCCACTGATC TCAGTGAGCT ATTCTTCTTA TCGATCTTGA TCTCTTTGAT CAAACTCACT TTTGATTTCA   
  
  
+ ACACCCTTGC TCTTGATTCT TCATCTCTCA GCTTTTTACG CTATTCATCC CCTTATGCAG TTCCCCTTAA   
  
  
+ TTTCTCCTCT AGGCCTTAAA GGGTTTCTTC TCGACATGGT CTAGTGTGCA TTCGATCCAT TAGTCTGCTT   
  
  
+ TGTAAGTTAG TTCGCACGTA CCCACATAAG CTACAACTGT GTCGCTGCAA TTCTAAGCAT TGTTGGGTTC   
  
  
+ TCTCTTGCGT ACTGCATATT AGAGGAGGC  

- AAGCTAAAAC ATTTTTTCAA TTTAAGAATA AAAAAATTAT TAAATAGGTA TTTTTATTTC ACTTTTTAAT   
  
  
- TTTGATAAGT CACGACTATA AAACATTTTG TTTATCTATA GGTCAGTTAT ACTTTGTAGA TATGACACGA   
  
  
- ATCAGTACCT GTGCACAAAA TTAGATTACT AATACATTTT GCTAGTGACT ATTCAGTTTG TAGTAATATA   
  
  
- ATGCTTTTTA CCTTGTACTT ATTTCTGTTT ACCTTGACGT GTACAATTGA GCATTTGACG CTTGGTAGAC   
  
  
- AGATTGGTCC GAGTTCTCAA TTCTTTCTCT TGCGACAGCA AAGCCTTCTT ATGGTCCTCT CTTTTTTTTT   
  
  
- TTTTTTTGAG AACTCAAACA ATTATAGCTG TGTTTACATG CATACAGCGG GTCTTTTCTT CTACTCTTAT   
  
  
- TTTTGGTTAT TTGAAAGAAT CCTAACGTGT ACATTCCAAT TAATACATTT AATTACTTAG TAATTGTGAC   
  
  
- GCCGAAAGAT CTGCTCGTCG TACGTCATGT TTCGTATGCA CAATGCGCTC AGTGTACCCA CTAGGTAGCG   
  
  
- GTAGTCGCAT TCGTTCCCCA AACTGTACTG CAGTAGTAGA ATGCATTCGA TGGGGTTAGA GACGCGGTCT   
  
  
- GCTCATCACT CATCGATGCG CCTTCTTCAA CCCTTCTAAG AGGGCAGAGA AGGAGACCGG GAGAGAAAAA   
  
  
- GAAGAACAGA AGGAACTTAA ACACAAAGGT GAGAAGGTCA GAAGTTTAAC TAAGTTGTGA CGGGCTCTCT   
  
  
- CTCTCTCTCT CTCAAAACTT AACCAGTCGT CAACCTGGAA AGAGTGAGAG AAAATAAAAG AAAAAGTTAA   
  
  
- AGAAAAAGAA GACATTCGGA ATACATATAT ATTATCCTTT CTCTAGGACT AGGAGTCGCT CCTTTGTTAG   
  
  
- ACTCAAAAAG AAAACAGGGC CCAGTTTTGC GGAGAAAAAG GGATAGTAAA GAGAACGTAT AAGAGGAGAG   
  
  
- GAAGAAGGGT TGAAGAAGTA ATTCTGTCCG TATCCGGAAA AGTGAAAGGA ACGTAAGTTT TGAAATGGTA   
  
  
- AAGAGAGAGA GAGAGAGAGA GAGAGAGAGA AGTCCGAATA TAAAGGGAAA TACATGCGTA TAACGGGTAG   
  
  
- TTGAGAGGTG AAGGTCATAA ACCACGACCA CCTCCGAGAA CTAGAGGGAA AAACCATTAA TGAACAGGTT   
  
  
- CGGTGACTAG AGTCACTCGA TAAGAAGAAT AGCTAGAACT AGAGAAACTA GTTTGAGTGA AAACTAAAGT   
  
  
- TGTGGGAACG AGAACTAAGA AGTAGAGAGT CGAAAAATGC GATAAGTAGG GGAATACGTC AAGGGGAATT   
  
  
- AAAGAGGAGA TCCGGAATTT CCCAAAGAAG AGCTGTACCA GATCACACGT AAGCTAGGTA ATCAGACGAA   
  
  
- ACATTCAATC AAGCGTGCAT GGGTGTATTC GATGTTGACA CAGCGACGTT AAGATTCGTA ACAACCCAAG   
  
  
- AGAGAACGCA TGACGTATAA TCTCCTCCG

+     as1

| Site Name | Organism | Position | Strand | Matrix score. | sequence | function |
| --- | --- | --- | --- | --- | --- | --- |
| as1 | Arabidopsis thaliana | 587 | + | 8 | TGACGTCA | cis-acting regulatory element involved in the root-specific expression |

> 2018/04/13 10:10:12  
+ TTCGATTTTG TAAAAAAGTT AAATTCTTAT TTTTTTAATA ATTTATCCAT AAAAATAAAG TGAAAAATTA   
  
  
+ AAACTATTCA GTGCTGATAT TTTGTAAAAC AAATAGATAT CCAGTCAATA TGAAACATCT ATACTGTGCT   
  
  
+ TAGTCATGGA CACGTGTTTT AATCTAATGA TTATGTAAAA CGATCACTGA TAAGTCAAAC ATCATTATAT   
  
  
+ TACGAAAAAT GGAACATGAA TAAAGACAAA TGGAACTGCA CATGTTAACT CGTAAACTGC GAACCATCTG   
  
  
+ TCTAACCAGG CTCAAGAGTT AAGAAAGAGA ACGCTGTCGT TTCGGAAGAA TACCAGGAGA GAAAAAAAAA   
  
  
+ AAAAAAACTC TTGAGTTTGT TAATATCGAC ACAAATGTAC GTATGTCGCC CAGAAAAGAA GATGAGAATA   
  
  
+ AAAACCAATA AACTTTCTTA GGATTGCACA TGTAAGGTTA ATTATGTAAA TTAATGAATC ATTAACACTG   
  
  
+ CGGCTTTCTA GACGAGCAGC ATGCAGTACA AAGCATACGT GTTACGCGAG TCACATGGGT GATCCATCGC   
  
  
+ CATCAGCGTA AGCAAGGGGT TTGACATGAC GTCATCATCT TACGTAAGCT ACCCCAATCT CTGCGCCAGA   
  
  
+ CGAGTAGTGA GTAGCTACGC GGAAGAAGTT GGGAAGATTC TCCCGTCTCT TCCTCTGGCC CTCTCTTTTT   
  
  
+ CTTCTTGTCT TCCTTGAATT TGTGTTTCCA CTCTTCCAGT CTTCAAATTG ATTCAACACT GCCCGAGAGA   
  
  
+ GAGAGAGAGA GAGTTTTGAA TTGGTCAGCA GTTGGACCTT TCTCACTCTC TTTTATTTTC TTTTTCAATT   
  
  
+ TCTTTTTCTT CTGTAAGCCT TATGTATATA TAATAGGAAA GAGATCCTGA TCCTCAGCGA GGAAACAATC   
  
  
+ TGAGTTTTTC TTTTGTCCCG GGTCAAAACG CCTCTTTTTC CCTATCATTT CTCTTGCATA TTCTCCTCTC   
  
  
+ CTTCTTCCCA ACTTCTTCAT TAAGACAGGC ATAGGCCTTT TCACTTTCCT TGCATTCAAA ACTTTACCAT   
  
  
+ TTCTCTCTCT CTCTCTCTCT CTCTCTCTCT TCAGGCTTAT ATTTCCCTTT ATGTACGCAT ATTGCCCATC   
  
  
+ AACTCTCCAC TTCCAGTATT TGGTGCTGGT GGAGGCTCTT GATCTCCCTT TTTGGTAATT ACTTGTCCAA   
  
  
+ GCCACTGATC TCAGTGAGCT ATTCTTCTTA TCGATCTTGA TCTCTTTGAT CAAACTCACT TTTGATTTCA   
  
  
+ ACACCCTTGC TCTTGATTCT TCATCTCTCA GCTTTTTACG CTATTCATCC CCTTATGCAG TTCCCCTTAA   
  
  
+ TTTCTCCTCT AGGCCTTAAA GGGTTTCTTC TCGACATGGT CTAGTGTGCA TTCGATCCAT TAGTCTGCTT   
  
  
+ TGTAAGTTAG TTCGCACGTA CCCACATAAG CTACAACTGT GTCGCTGCAA TTCTAAGCAT TGTTGGGTTC   
  
  
+ TCTCTTGCGT ACTGCATATT AGAGGAGGC  

- AAGCTAAAAC ATTTTTTCAA TTTAAGAATA AAAAAATTAT TAAATAGGTA TTTTTATTTC ACTTTTTAAT   
  
  
- TTTGATAAGT CACGACTATA AAACATTTTG TTTATCTATA GGTCAGTTAT ACTTTGTAGA TATGACACGA   
  
  
- ATCAGTACCT GTGCACAAAA TTAGATTACT AATACATTTT GCTAGTGACT ATTCAGTTTG TAGTAATATA   
  
  
- ATGCTTTTTA CCTTGTACTT ATTTCTGTTT ACCTTGACGT GTACAATTGA GCATTTGACG CTTGGTAGAC   
  
  
- AGATTGGTCC GAGTTCTCAA TTCTTTCTCT TGCGACAGCA AAGCCTTCTT ATGGTCCTCT CTTTTTTTTT   
  
  
- TTTTTTTGAG AACTCAAACA ATTATAGCTG TGTTTACATG CATACAGCGG GTCTTTTCTT CTACTCTTAT   
  
  
- TTTTGGTTAT TTGAAAGAAT CCTAACGTGT ACATTCCAAT TAATACATTT AATTACTTAG TAATTGTGAC   
  
  
- GCCGAAAGAT CTGCTCGTCG TACGTCATGT TTCGTATGCA CAATGCGCTC AGTGTACCCA CTAGGTAGCG   
  
  
- GTAGTCGCAT TCGTTCCCCA AACTGTACTG CAGTAGTAGA ATGCATTCGA TGGGGTTAGA GACGCGGTCT   
  
  
- GCTCATCACT CATCGATGCG CCTTCTTCAA CCCTTCTAAG AGGGCAGAGA AGGAGACCGG GAGAGAAAAA   
  
  
- GAAGAACAGA AGGAACTTAA ACACAAAGGT GAGAAGGTCA GAAGTTTAAC TAAGTTGTGA CGGGCTCTCT   
  
  
- CTCTCTCTCT CTCAAAACTT AACCAGTCGT CAACCTGGAA AGAGTGAGAG AAAATAAAAG AAAAAGTTAA   
  
  
- AGAAAAAGAA GACATTCGGA ATACATATAT ATTATCCTTT CTCTAGGACT AGGAGTCGCT CCTTTGTTAG   
  
  
- ACTCAAAAAG AAAACAGGGC CCAGTTTTGC GGAGAAAAAG GGATAGTAAA GAGAACGTAT AAGAGGAGAG   
  
  
- GAAGAAGGGT TGAAGAAGTA ATTCTGTCCG TATCCGGAAA AGTGAAAGGA ACGTAAGTTT TGAAATGGTA   
  
  
- AAGAGAGAGA GAGAGAGAGA GAGAGAGAGA AGTCCGAATA TAAAGGGAAA TACATGCGTA TAACGGGTAG   
  
  
- TTGAGAGGTG AAGGTCATAA ACCACGACCA CCTCCGAGAA CTAGAGGGAA AAACCATTAA TGAACAGGTT   
  
  
- CGGTGACTAG AGTCACTCGA TAAGAAGAAT AGCTAGAACT AGAGAAACTA GTTTGAGTGA AAACTAAAGT   
  
  
- TGTGGGAACG AGAACTAAGA AGTAGAGAGT CGAAAAATGC GATAAGTAGG GGAATACGTC AAGGGGAATT   
  
  
- AAAGAGGAGA TCCGGAATTT CCCAAAGAAG AGCTGTACCA GATCACACGT AAGCTAGGTA ATCAGACGAA   
  
  
- ACATTCAATC AAGCGTGCAT GGGTGTATTC GATGTTGACA CAGCGACGTT AAGATTCGTA ACAACCCAAG   
  
  
- AGAGAACGCA TGACGTATAA TCTCCTCCG

+     chs-CMA2b

| Site Name | Organism | Position | Strand | Matrix score. | sequence | function |
| --- | --- | --- | --- | --- | --- | --- |
| chs-CMA2b | Petroselinum crispum | 637 | - | 11 | GTATCTACTCAC | part of a light responsive element |

> 2018/04/13 10:10:12  
+ TTCGATTTTG TAAAAAAGTT AAATTCTTAT TTTTTTAATA ATTTATCCAT AAAAATAAAG TGAAAAATTA   
  
  
+ AAACTATTCA GTGCTGATAT TTTGTAAAAC AAATAGATAT CCAGTCAATA TGAAACATCT ATACTGTGCT   
  
  
+ TAGTCATGGA CACGTGTTTT AATCTAATGA TTATGTAAAA CGATCACTGA TAAGTCAAAC ATCATTATAT   
  
  
+ TACGAAAAAT GGAACATGAA TAAAGACAAA TGGAACTGCA CATGTTAACT CGTAAACTGC GAACCATCTG   
  
  
+ TCTAACCAGG CTCAAGAGTT AAGAAAGAGA ACGCTGTCGT TTCGGAAGAA TACCAGGAGA GAAAAAAAAA   
  
  
+ AAAAAAACTC TTGAGTTTGT TAATATCGAC ACAAATGTAC GTATGTCGCC CAGAAAAGAA GATGAGAATA   
  
  
+ AAAACCAATA AACTTTCTTA GGATTGCACA TGTAAGGTTA ATTATGTAAA TTAATGAATC ATTAACACTG   
  
  
+ CGGCTTTCTA GACGAGCAGC ATGCAGTACA AAGCATACGT GTTACGCGAG TCACATGGGT GATCCATCGC   
  
  
+ CATCAGCGTA AGCAAGGGGT TTGACATGAC GTCATCATCT TACGTAAGCT ACCCCAATCT CTGCGCCAGA   
  
  
+ CGAGTAGTGA GTAGCTACGC GGAAGAAGTT GGGAAGATTC TCCCGTCTCT TCCTCTGGCC CTCTCTTTTT   
  
  
+ CTTCTTGTCT TCCTTGAATT TGTGTTTCCA CTCTTCCAGT CTTCAAATTG ATTCAACACT GCCCGAGAGA   
  
  
+ GAGAGAGAGA GAGTTTTGAA TTGGTCAGCA GTTGGACCTT TCTCACTCTC TTTTATTTTC TTTTTCAATT   
  
  
+ TCTTTTTCTT CTGTAAGCCT TATGTATATA TAATAGGAAA GAGATCCTGA TCCTCAGCGA GGAAACAATC   
  
  
+ TGAGTTTTTC TTTTGTCCCG GGTCAAAACG CCTCTTTTTC CCTATCATTT CTCTTGCATA TTCTCCTCTC   
  
  
+ CTTCTTCCCA ACTTCTTCAT TAAGACAGGC ATAGGCCTTT TCACTTTCCT TGCATTCAAA ACTTTACCAT   
  
  
+ TTCTCTCTCT CTCTCTCTCT CTCTCTCTCT TCAGGCTTAT ATTTCCCTTT ATGTACGCAT ATTGCCCATC   
  
  
+ AACTCTCCAC TTCCAGTATT TGGTGCTGGT GGAGGCTCTT GATCTCCCTT TTTGGTAATT ACTTGTCCAA   
  
  
+ GCCACTGATC TCAGTGAGCT ATTCTTCTTA TCGATCTTGA TCTCTTTGAT CAAACTCACT TTTGATTTCA   
  
  
+ ACACCCTTGC TCTTGATTCT TCATCTCTCA GCTTTTTACG CTATTCATCC CCTTATGCAG TTCCCCTTAA   
  
  
+ TTTCTCCTCT AGGCCTTAAA GGGTTTCTTC TCGACATGGT CTAGTGTGCA TTCGATCCAT TAGTCTGCTT   
  
  
+ TGTAAGTTAG TTCGCACGTA CCCACATAAG CTACAACTGT GTCGCTGCAA TTCTAAGCAT TGTTGGGTTC   
  
  
+ TCTCTTGCGT ACTGCATATT AGAGGAGGC  

- AAGCTAAAAC ATTTTTTCAA TTTAAGAATA AAAAAATTAT TAAATAGGTA TTTTTATTTC ACTTTTTAAT   
  
  
- TTTGATAAGT CACGACTATA AAACATTTTG TTTATCTATA GGTCAGTTAT ACTTTGTAGA TATGACACGA   
  
  
- ATCAGTACCT GTGCACAAAA TTAGATTACT AATACATTTT GCTAGTGACT ATTCAGTTTG TAGTAATATA   
  
  
- ATGCTTTTTA CCTTGTACTT ATTTCTGTTT ACCTTGACGT GTACAATTGA GCATTTGACG CTTGGTAGAC   
  
  
- AGATTGGTCC GAGTTCTCAA TTCTTTCTCT TGCGACAGCA AAGCCTTCTT ATGGTCCTCT CTTTTTTTTT   
  
  
- TTTTTTTGAG AACTCAAACA ATTATAGCTG TGTTTACATG CATACAGCGG GTCTTTTCTT CTACTCTTAT   
  
  
- TTTTGGTTAT TTGAAAGAAT CCTAACGTGT ACATTCCAAT TAATACATTT AATTACTTAG TAATTGTGAC   
  
  
- GCCGAAAGAT CTGCTCGTCG TACGTCATGT TTCGTATGCA CAATGCGCTC AGTGTACCCA CTAGGTAGCG   
  
  
- GTAGTCGCAT TCGTTCCCCA AACTGTACTG CAGTAGTAGA ATGCATTCGA TGGGGTTAGA GACGCGGTCT   
  
  
- GCTCATCACT CATCGATGCG CCTTCTTCAA CCCTTCTAAG AGGGCAGAGA AGGAGACCGG GAGAGAAAAA   
  
  
- GAAGAACAGA AGGAACTTAA ACACAAAGGT GAGAAGGTCA GAAGTTTAAC TAAGTTGTGA CGGGCTCTCT   
  
  
- CTCTCTCTCT CTCAAAACTT AACCAGTCGT CAACCTGGAA AGAGTGAGAG AAAATAAAAG AAAAAGTTAA   
  
  
- AGAAAAAGAA GACATTCGGA ATACATATAT ATTATCCTTT CTCTAGGACT AGGAGTCGCT CCTTTGTTAG   
  
  
- ACTCAAAAAG AAAACAGGGC CCAGTTTTGC GGAGAAAAAG GGATAGTAAA GAGAACGTAT AAGAGGAGAG   
  
  
- GAAGAAGGGT TGAAGAAGTA ATTCTGTCCG TATCCGGAAA AGTGAAAGGA ACGTAAGTTT TGAAATGGTA   
  
  
- AAGAGAGAGA GAGAGAGAGA GAGAGAGAGA AGTCCGAATA TAAAGGGAAA TACATGCGTA TAACGGGTAG   
  
  
- TTGAGAGGTG AAGGTCATAA ACCACGACCA CCTCCGAGAA CTAGAGGGAA AAACCATTAA TGAACAGGTT   
  
  
- CGGTGACTAG AGTCACTCGA TAAGAAGAAT AGCTAGAACT AGAGAAACTA GTTTGAGTGA AAACTAAAGT   
  
  
- TGTGGGAACG AGAACTAAGA AGTAGAGAGT CGAAAAATGC GATAAGTAGG GGAATACGTC AAGGGGAATT   
  
  
- AAAGAGGAGA TCCGGAATTT CCCAAAGAAG AGCTGTACCA GATCACACGT AAGCTAGGTA ATCAGACGAA   
  
  
- ACATTCAATC AAGCGTGCAT GGGTGTATTC GATGTTGACA CAGCGACGTT AAGATTCGTA ACAACCCAAG   
  
  
- AGAGAACGCA TGACGTATAA TCTCCTCCG

+     circadian

| Site Name | Organism | Position | Strand | Matrix score. | sequence | function |
| --- | --- | --- | --- | --- | --- | --- |
| circadian | Lycopersicon esculentum | 1229 | - | 9 | CAAAGATATC | cis-acting regulatory element involved in circadian control |

> 2018/04/13 10:10:12  
+ TTCGATTTTG TAAAAAAGTT AAATTCTTAT TTTTTTAATA ATTTATCCAT AAAAATAAAG TGAAAAATTA   
  
  
+ AAACTATTCA GTGCTGATAT TTTGTAAAAC AAATAGATAT CCAGTCAATA TGAAACATCT ATACTGTGCT   
  
  
+ TAGTCATGGA CACGTGTTTT AATCTAATGA TTATGTAAAA CGATCACTGA TAAGTCAAAC ATCATTATAT   
  
  
+ TACGAAAAAT GGAACATGAA TAAAGACAAA TGGAACTGCA CATGTTAACT CGTAAACTGC GAACCATCTG   
  
  
+ TCTAACCAGG CTCAAGAGTT AAGAAAGAGA ACGCTGTCGT TTCGGAAGAA TACCAGGAGA GAAAAAAAAA   
  
  
+ AAAAAAACTC TTGAGTTTGT TAATATCGAC ACAAATGTAC GTATGTCGCC CAGAAAAGAA GATGAGAATA   
  
  
+ AAAACCAATA AACTTTCTTA GGATTGCACA TGTAAGGTTA ATTATGTAAA TTAATGAATC ATTAACACTG   
  
  
+ CGGCTTTCTA GACGAGCAGC ATGCAGTACA AAGCATACGT GTTACGCGAG TCACATGGGT GATCCATCGC   
  
  
+ CATCAGCGTA AGCAAGGGGT TTGACATGAC GTCATCATCT TACGTAAGCT ACCCCAATCT CTGCGCCAGA   
  
  
+ CGAGTAGTGA GTAGCTACGC GGAAGAAGTT GGGAAGATTC TCCCGTCTCT TCCTCTGGCC CTCTCTTTTT   
  
  
+ CTTCTTGTCT TCCTTGAATT TGTGTTTCCA CTCTTCCAGT CTTCAAATTG ATTCAACACT GCCCGAGAGA   
  
  
+ GAGAGAGAGA GAGTTTTGAA TTGGTCAGCA GTTGGACCTT TCTCACTCTC TTTTATTTTC TTTTTCAATT   
  
  
+ TCTTTTTCTT CTGTAAGCCT TATGTATATA TAATAGGAAA GAGATCCTGA TCCTCAGCGA GGAAACAATC   
  
  
+ TGAGTTTTTC TTTTGTCCCG GGTCAAAACG CCTCTTTTTC CCTATCATTT CTCTTGCATA TTCTCCTCTC   
  
  
+ CTTCTTCCCA ACTTCTTCAT TAAGACAGGC ATAGGCCTTT TCACTTTCCT TGCATTCAAA ACTTTACCAT   
  
  
+ TTCTCTCTCT CTCTCTCTCT CTCTCTCTCT TCAGGCTTAT ATTTCCCTTT ATGTACGCAT ATTGCCCATC   
  
  
+ AACTCTCCAC TTCCAGTATT TGGTGCTGGT GGAGGCTCTT GATCTCCCTT TTTGGTAATT ACTTGTCCAA   
  
  
+ GCCACTGATC TCAGTGAGCT ATTCTTCTTA TCGATCTTGA TCTCTTTGAT CAAACTCACT TTTGATTTCA   
  
  
+ ACACCCTTGC TCTTGATTCT TCATCTCTCA GCTTTTTACG CTATTCATCC CCTTATGCAG TTCCCCTTAA   
  
  
+ TTTCTCCTCT AGGCCTTAAA GGGTTTCTTC TCGACATGGT CTAGTGTGCA TTCGATCCAT TAGTCTGCTT   
  
  
+ TGTAAGTTAG TTCGCACGTA CCCACATAAG CTACAACTGT GTCGCTGCAA TTCTAAGCAT TGTTGGGTTC   
  
  
+ TCTCTTGCGT ACTGCATATT AGAGGAGGC  

- AAGCTAAAAC ATTTTTTCAA TTTAAGAATA AAAAAATTAT TAAATAGGTA TTTTTATTTC ACTTTTTAAT   
  
  
- TTTGATAAGT CACGACTATA AAACATTTTG TTTATCTATA GGTCAGTTAT ACTTTGTAGA TATGACACGA   
  
  
- ATCAGTACCT GTGCACAAAA TTAGATTACT AATACATTTT GCTAGTGACT ATTCAGTTTG TAGTAATATA   
  
  
- ATGCTTTTTA CCTTGTACTT ATTTCTGTTT ACCTTGACGT GTACAATTGA GCATTTGACG CTTGGTAGAC   
  
  
- AGATTGGTCC GAGTTCTCAA TTCTTTCTCT TGCGACAGCA AAGCCTTCTT ATGGTCCTCT CTTTTTTTTT   
  
  
- TTTTTTTGAG AACTCAAACA ATTATAGCTG TGTTTACATG CATACAGCGG GTCTTTTCTT CTACTCTTAT   
  
  
- TTTTGGTTAT TTGAAAGAAT CCTAACGTGT ACATTCCAAT TAATACATTT AATTACTTAG TAATTGTGAC   
  
  
- GCCGAAAGAT CTGCTCGTCG TACGTCATGT TTCGTATGCA CAATGCGCTC AGTGTACCCA CTAGGTAGCG   
  
  
- GTAGTCGCAT TCGTTCCCCA AACTGTACTG CAGTAGTAGA ATGCATTCGA TGGGGTTAGA GACGCGGTCT   
  
  
- GCTCATCACT CATCGATGCG CCTTCTTCAA CCCTTCTAAG AGGGCAGAGA AGGAGACCGG GAGAGAAAAA   
  
  
- GAAGAACAGA AGGAACTTAA ACACAAAGGT GAGAAGGTCA GAAGTTTAAC TAAGTTGTGA CGGGCTCTCT   
  
  
- CTCTCTCTCT CTCAAAACTT AACCAGTCGT CAACCTGGAA AGAGTGAGAG AAAATAAAAG AAAAAGTTAA   
  
  
- AGAAAAAGAA GACATTCGGA ATACATATAT ATTATCCTTT CTCTAGGACT AGGAGTCGCT CCTTTGTTAG   
  
  
- ACTCAAAAAG AAAACAGGGC CCAGTTTTGC GGAGAAAAAG GGATAGTAAA GAGAACGTAT AAGAGGAGAG   
  
  
- GAAGAAGGGT TGAAGAAGTA ATTCTGTCCG TATCCGGAAA AGTGAAAGGA ACGTAAGTTT TGAAATGGTA   
  
  
- AAGAGAGAGA GAGAGAGAGA GAGAGAGAGA AGTCCGAATA TAAAGGGAAA TACATGCGTA TAACGGGTAG   
  
  
- TTGAGAGGTG AAGGTCATAA ACCACGACCA CCTCCGAGAA CTAGAGGGAA AAACCATTAA TGAACAGGTT   
  
  
- CGGTGACTAG AGTCACTCGA TAAGAAGAAT AGCTAGAACT AGAGAAACTA GTTTGAGTGA AAACTAAAGT   
  
  
- TGTGGGAACG AGAACTAAGA AGTAGAGAGT CGAAAAATGC GATAAGTAGG GGAATACGTC AAGGGGAATT   
  
  
- AAAGAGGAGA TCCGGAATTT CCCAAAGAAG AGCTGTACCA GATCACACGT AAGCTAGGTA ATCAGACGAA   
  
  
- ACATTCAATC AAGCGTGCAT GGGTGTATTC GATGTTGACA CAGCGACGTT AAGATTCGTA ACAACCCAAG   
  
  
- AGAGAACGCA TGACGTATAA TCTCCTCCG
