# Supplementary material for: Genome-wide analysis of the WRKY gene family in drumstick (Moringa oleifera Lam.)
Source: PeerJ. 2019 Jun 10;7:e7063. doi: 10.7717/peerj.7063 (PMC6563795; doi:10.7717/peerj.7063)
Supplement: Supplemental Information 1 [file peerj-07-7063-s003.gz › MoWRKY35_plantcare.html]

Content-Type: text/html; charset=ISO-8859-1


CallMat\_Firefox


Webmaster Firefox specific output  
To save the result:
click on the frame with the right mouse button and save the source code as a text file with extension .html  
REFERENCE:PlantCARE: a database of plant cis-acting regulatory elements and a portal to tools for in silico analysis of promoter sequences.  
Lescot, M., Déhais, P., Moreau, Y., De Moor, B., Rouzé ,P.,and Rombauts, S.  
Nucleic Acids Res., Database issue(2002), 30(1):325-327.   


---

> 2018/04/13 10:10:12  
+ AGAAGGAAAG AAATAGGTGT AATGATAGTG GGGGGACAGA GTTTGCAACT ACCATTCCTT TATTTTTTCA   
  
  
+ TGGTGTTTTG GATTGAATGT CAAATTTAAG AATAAATTTT CTCAAATTTT CCTGATGATG ATGCATATTG   
  
  
+ TATCTGAAGT AAAGATTTTT TTTTTTTAAA ATATCCAAAG TAGTTAACAT TAAGTCATCT ACGGATCAGA   
  
  
+ TTTAATTCGG TCCATTCATA ATTTGTAAAA TTTTAGTTTA TTTAAATAAA ATTGTTGATA TTATTTTAAA   
  
  
+ AATAAAAATT ATTTAACACT TCAAACTATA TTTTATATAT TAAATCAAAG TTTAGTGTTT AAAAGTAATA   
  
  
+ AATCAAACTT TTAATTATTA ATTTGATTTA AATTAAATTT TGATTTAATA TTTTTTGTTA TTAAAAATAT   
  
  
+ TTTAAAAAAT AATTCAAATA AATTGTATAC TTTATTTTAA ATCAATTCTT TAATTTCAAA TAATTTTTGG   
  
  
+ ATTTATGAGT TATTTATTAT AAATTTTTAT AATAATTTTT CTAAAAACAT TTTAGTCAAA AAAACTTATA   
  
  
+ CTTTTTTATA ATGATAGTTT GAATATTTTT AAAAATTTTT AATATGATTT AATTTGATAT AAATTAATAA   
  
  
+ ATCAGATTTT AATTTGTTAC GTTTTAAACC TTAAGGTTAA ATTTGACAAA TCATATACTT AAAAGTTTTA   
  
  
+ ATTAATCTTT ACAATTAGTT TAATTAGATT TGGTACTATA AATAAAAATA CTCATAGTTT TATTTGAATT   
  
  
+ TATGAAACCT AAAATAAGTT TATATCTTTT TATTTTTTCT CAACAAATAT ATATCCAAAA TAGAAAAATA   
  
  
+ AATTTCAAAA TTTTCTTAAT ATACCATAAC CGTTTTATGT CATTAATAAA TAATGTCACA ATAAAAATAA   
  
  
+ TAAAATAAAA TCTCATTACC ACCTACAACT TTTAGCCTTT TACTATAGTA CAAAATTCTT TCATCTTTAA   
  
  
+ TAATAATTAC TTTCTTTCAT TCAGTGGTGT CATAAATTAA AGACCCTACA GTATACCATA TGCCATGAAA   
  
  
+ TTAATGAAAT CTAACACAAA TCATTATGAT TTTATCACTT ATAAAAAAAT TATATTTTTC AACCGCACCA   
  
  
+ TATAATATAA CATATATATT AGATTAATAT AGCAACCAAA ATGAATCATT TGGAGGGTAC ACGAGTCCTT   
  
  
+ AACATGGACC TGACTTCGTC GAATCATTCC CAATCGTTGT GACTAAAGAA ATATTAGAAT TGCGTAATTG   
  
  
+ CTTACACGTC CCCATATAAA CAAAACAACT GCCAATCTGT CATTACCTGA CGGTCCCATA TTAAACCCTC   
  
  
+ CTTAAGCACC CACTAATCAT ATCCACTTCT CTTTCACGTT TCTCGCCGGC CTCAGTCTGC CTTGCCGGTG   
  
  
+ ATCCTACGCC TCCATGATCA TCGCTACCAT TGTCGTCGTC GCCGTTTCAG TACGAAGCCG ACGGTCTTGA   
  
  
+ TTAACTTACC ACCTGATCCA GCACCATAT  

- TCTTCCTTTC TTTATCCACA TTACTATCAC CCCCCTGTCT CAAACGTTGA TGGTAAGGAA ATAAAAAAGT   
  
  
- ACCACAAAAC CTAACTTACA GTTTAAATTC TTATTTAAAA GAGTTTAAAA GGACTACTAC TACGTATAAC   
  
  
- ATAGACTTCA TTTCTAAAAA AAAAAAATTT TATAGGTTTC ATCAATTGTA ATTCAGTAGA TGCCTAGTCT   
  
  
- AAATTAAGCC AGGTAAGTAT TAAACATTTT AAAATCAAAT AAATTTATTT TAACAACTAT AATAAAATTT   
  
  
- TTATTTTTAA TAAATTGTGA AGTTTGATAT AAAATATATA ATTTAGTTTC AAATCACAAA TTTTCATTAT   
  
  
- TTAGTTTGAA AATTAATAAT TAAACTAAAT TTAATTTAAA ACTAAATTAT AAAAAACAAT AATTTTTATA   
  
  
- AAATTTTTTA TTAAGTTTAT TTAACATATG AAATAAAATT TAGTTAAGAA ATTAAAGTTT ATTAAAAACC   
  
  
- TAAATACTCA ATAAATAATA TTTAAAAATA TTATTAAAAA GATTTTTGTA AAATCAGTTT TTTTGAATAT   
  
  
- GAAAAAATAT TACTATCAAA CTTATAAAAA TTTTTAAAAA TTATACTAAA TTAAACTATA TTTAATTATT   
  
  
- TAGTCTAAAA TTAAACAATG CAAAATTTGG AATTCCAATT TAAACTGTTT AGTATATGAA TTTTCAAAAT   
  
  
- TAATTAGAAA TGTTAATCAA ATTAATCTAA ACCATGATAT TTATTTTTAT GAGTATCAAA ATAAACTTAA   
  
  
- ATACTTTGGA TTTTATTCAA ATATAGAAAA ATAAAAAAGA GTTGTTTATA TATAGGTTTT ATCTTTTTAT   
  
  
- TTAAAGTTTT AAAAGAATTA TATGGTATTG GCAAAATACA GTAATTATTT ATTACAGTGT TATTTTTATT   
  
  
- ATTTTATTTT AGAGTAATGG TGGATGTTGA AAATCGGAAA ATGATATCAT GTTTTAAGAA AGTAGAAATT   
  
  
- ATTATTAATG AAAGAAAGTA AGTCACCACA GTATTTAATT TCTGGGATGT CATATGGTAT ACGGTACTTT   
  
  
- AATTACTTTA GATTGTGTTT AGTAATACTA AAATAGTGAA TATTTTTTTA ATATAAAAAG TTGGCGTGGT   
  
  
- ATATTATATT GTATATATAA TCTAATTATA TCGTTGGTTT TACTTAGTAA ACCTCCCATG TGCTCAGGAA   
  
  
- TTGTACCTGG ACTGAAGCAG CTTAGTAAGG GTTAGCAACA CTGATTTCTT TATAATCTTA ACGCATTAAC   
  
  
- GAATGTGCAG GGGTATATTT GTTTTGTTGA CGGTTAGACA GTAATGGACT GCCAGGGTAT AATTTGGGAG   
  
  
- GAATTCGTGG GTGATTAGTA TAGGTGAAGA GAAAGTGCAA AGAGCGGCCG GAGTCAGACG GAACGGCCAC   
  
  
- TAGGATGCGG AGGTACTAGT AGCGATGGTA ACAGCAGCAG CGGCAAAGTC ATGCTTCGGC TGCCAGAACT   
  
  
- AATTGAATGG TGGACTAGGT CGTGGTATA

  
  
Motifs Found  

+     AAGAA-motif

| Site Name | Organism | Position | Strand | Matrix score. | sequence | function |
| --- | --- | --- | --- | --- | --- | --- |
| AAGAA-motif | Avena sativa | 992 | - | 7 | GAAAGAA |  |
| AAGAA-motif | Avena sativa | 966 | - | 7 | GAAAGAA |  |
| AAGAA-motif | Avena sativa | 6 | + | 7 | GAAAGAA |  |

> 2018/04/13 10:10:12  
+ AGAAGGAAAG AAATAGGTGT AATGATAGTG GGGGGACAGA GTTTGCAACT ACCATTCCTT TATTTTTTCA   
  
  
+ TGGTGTTTTG GATTGAATGT CAAATTTAAG AATAAATTTT CTCAAATTTT CCTGATGATG ATGCATATTG   
  
  
+ TATCTGAAGT AAAGATTTTT TTTTTTTAAA ATATCCAAAG TAGTTAACAT TAAGTCATCT ACGGATCAGA   
  
  
+ TTTAATTCGG TCCATTCATA ATTTGTAAAA TTTTAGTTTA TTTAAATAAA ATTGTTGATA TTATTTTAAA   
  
  
+ AATAAAAATT ATTTAACACT TCAAACTATA TTTTATATAT TAAATCAAAG TTTAGTGTTT AAAAGTAATA   
  
  
+ AATCAAACTT TTAATTATTA ATTTGATTTA AATTAAATTT TGATTTAATA TTTTTTGTTA TTAAAAATAT   
  
  
+ TTTAAAAAAT AATTCAAATA AATTGTATAC TTTATTTTAA ATCAATTCTT TAATTTCAAA TAATTTTTGG   
  
  
+ ATTTATGAGT TATTTATTAT AAATTTTTAT AATAATTTTT CTAAAAACAT TTTAGTCAAA AAAACTTATA   
  
  
+ CTTTTTTATA ATGATAGTTT GAATATTTTT AAAAATTTTT AATATGATTT AATTTGATAT AAATTAATAA   
  
  
+ ATCAGATTTT AATTTGTTAC GTTTTAAACC TTAAGGTTAA ATTTGACAAA TCATATACTT AAAAGTTTTA   
  
  
+ ATTAATCTTT ACAATTAGTT TAATTAGATT TGGTACTATA AATAAAAATA CTCATAGTTT TATTTGAATT   
  
  
+ TATGAAACCT AAAATAAGTT TATATCTTTT TATTTTTTCT CAACAAATAT ATATCCAAAA TAGAAAAATA   
  
  
+ AATTTCAAAA TTTTCTTAAT ATACCATAAC CGTTTTATGT CATTAATAAA TAATGTCACA ATAAAAATAA   
  
  
+ TAAAATAAAA TCTCATTACC ACCTACAACT TTTAGCCTTT TACTATAGTA CAAAATTCTT TCATCTTTAA   
  
  
+ TAATAATTAC TTTCTTTCAT TCAGTGGTGT CATAAATTAA AGACCCTACA GTATACCATA TGCCATGAAA   
  
  
+ TTAATGAAAT CTAACACAAA TCATTATGAT TTTATCACTT ATAAAAAAAT TATATTTTTC AACCGCACCA   
  
  
+ TATAATATAA CATATATATT AGATTAATAT AGCAACCAAA ATGAATCATT TGGAGGGTAC ACGAGTCCTT   
  
  
+ AACATGGACC TGACTTCGTC GAATCATTCC CAATCGTTGT GACTAAAGAA ATATTAGAAT TGCGTAATTG   
  
  
+ CTTACACGTC CCCATATAAA CAAAACAACT GCCAATCTGT CATTACCTGA CGGTCCCATA TTAAACCCTC   
  
  
+ CTTAAGCACC CACTAATCAT ATCCACTTCT CTTTCACGTT TCTCGCCGGC CTCAGTCTGC CTTGCCGGTG   
  
  
+ ATCCTACGCC TCCATGATCA TCGCTACCAT TGTCGTCGTC GCCGTTTCAG TACGAAGCCG ACGGTCTTGA   
  
  
+ TTAACTTACC ACCTGATCCA GCACCATAT  

- TCTTCCTTTC TTTATCCACA TTACTATCAC CCCCCTGTCT CAAACGTTGA TGGTAAGGAA ATAAAAAAGT   
  
  
- ACCACAAAAC CTAACTTACA GTTTAAATTC TTATTTAAAA GAGTTTAAAA GGACTACTAC TACGTATAAC   
  
  
- ATAGACTTCA TTTCTAAAAA AAAAAAATTT TATAGGTTTC ATCAATTGTA ATTCAGTAGA TGCCTAGTCT   
  
  
- AAATTAAGCC AGGTAAGTAT TAAACATTTT AAAATCAAAT AAATTTATTT TAACAACTAT AATAAAATTT   
  
  
- TTATTTTTAA TAAATTGTGA AGTTTGATAT AAAATATATA ATTTAGTTTC AAATCACAAA TTTTCATTAT   
  
  
- TTAGTTTGAA AATTAATAAT TAAACTAAAT TTAATTTAAA ACTAAATTAT AAAAAACAAT AATTTTTATA   
  
  
- AAATTTTTTA TTAAGTTTAT TTAACATATG AAATAAAATT TAGTTAAGAA ATTAAAGTTT ATTAAAAACC   
  
  
- TAAATACTCA ATAAATAATA TTTAAAAATA TTATTAAAAA GATTTTTGTA AAATCAGTTT TTTTGAATAT   
  
  
- GAAAAAATAT TACTATCAAA CTTATAAAAA TTTTTAAAAA TTATACTAAA TTAAACTATA TTTAATTATT   
  
  
- TAGTCTAAAA TTAAACAATG CAAAATTTGG AATTCCAATT TAAACTGTTT AGTATATGAA TTTTCAAAAT   
  
  
- TAATTAGAAA TGTTAATCAA ATTAATCTAA ACCATGATAT TTATTTTTAT GAGTATCAAA ATAAACTTAA   
  
  
- ATACTTTGGA TTTTATTCAA ATATAGAAAA ATAAAAAAGA GTTGTTTATA TATAGGTTTT ATCTTTTTAT   
  
  
- TTAAAGTTTT AAAAGAATTA TATGGTATTG GCAAAATACA GTAATTATTT ATTACAGTGT TATTTTTATT   
  
  
- ATTTTATTTT AGAGTAATGG TGGATGTTGA AAATCGGAAA ATGATATCAT GTTTTAAGAA AGTAGAAATT   
  
  
- ATTATTAATG AAAGAAAGTA AGTCACCACA GTATTTAATT TCTGGGATGT CATATGGTAT ACGGTACTTT   
  
  
- AATTACTTTA GATTGTGTTT AGTAATACTA AAATAGTGAA TATTTTTTTA ATATAAAAAG TTGGCGTGGT   
  
  
- ATATTATATT GTATATATAA TCTAATTATA TCGTTGGTTT TACTTAGTAA ACCTCCCATG TGCTCAGGAA   
  
  
- TTGTACCTGG ACTGAAGCAG CTTAGTAAGG GTTAGCAACA CTGATTTCTT TATAATCTTA ACGCATTAAC   
  
  
- GAATGTGCAG GGGTATATTT GTTTTGTTGA CGGTTAGACA GTAATGGACT GCCAGGGTAT AATTTGGGAG   
  
  
- GAATTCGTGG GTGATTAGTA TAGGTGAAGA GAAAGTGCAA AGAGCGGCCG GAGTCAGACG GAACGGCCAC   
  
  
- TAGGATGCGG AGGTACTAGT AGCGATGGTA ACAGCAGCAG CGGCAAAGTC ATGCTTCGGC TGCCAGAACT   
  
  
- AATTGAATGG TGGACTAGGT CGTGGTATA

+     ACE

| Site Name | Organism | Position | Strand | Matrix score. | sequence | function |
| --- | --- | --- | --- | --- | --- | --- |
| ACE | Petroselinum crispum | 175 | - | 9 | ACTACGTTGG | cis-acting element involved in light responsiveness |
| ACE | Petroselinum crispum | 867 | - | 9 | AAAACGTTTA | cis-acting element involved in light responsiveness |
| ACE | Petroselinum crispum | 690 | - | 9 | AAAACGTTTA | cis-acting element involved in light responsiveness |

> 2018/04/13 10:10:12  
+ AGAAGGAAAG AAATAGGTGT AATGATAGTG GGGGGACAGA GTTTGCAACT ACCATTCCTT TATTTTTTCA   
  
  
+ TGGTGTTTTG GATTGAATGT CAAATTTAAG AATAAATTTT CTCAAATTTT CCTGATGATG ATGCATATTG   
  
  
+ TATCTGAAGT AAAGATTTTT TTTTTTTAAA ATATCCAAAG TAGTTAACAT TAAGTCATCT ACGGATCAGA   
  
  
+ TTTAATTCGG TCCATTCATA ATTTGTAAAA TTTTAGTTTA TTTAAATAAA ATTGTTGATA TTATTTTAAA   
  
  
+ AATAAAAATT ATTTAACACT TCAAACTATA TTTTATATAT TAAATCAAAG TTTAGTGTTT AAAAGTAATA   
  
  
+ AATCAAACTT TTAATTATTA ATTTGATTTA AATTAAATTT TGATTTAATA TTTTTTGTTA TTAAAAATAT   
  
  
+ TTTAAAAAAT AATTCAAATA AATTGTATAC TTTATTTTAA ATCAATTCTT TAATTTCAAA TAATTTTTGG   
  
  
+ ATTTATGAGT TATTTATTAT AAATTTTTAT AATAATTTTT CTAAAAACAT TTTAGTCAAA AAAACTTATA   
  
  
+ CTTTTTTATA ATGATAGTTT GAATATTTTT AAAAATTTTT AATATGATTT AATTTGATAT AAATTAATAA   
  
  
+ ATCAGATTTT AATTTGTTAC GTTTTAAACC TTAAGGTTAA ATTTGACAAA TCATATACTT AAAAGTTTTA   
  
  
+ ATTAATCTTT ACAATTAGTT TAATTAGATT TGGTACTATA AATAAAAATA CTCATAGTTT TATTTGAATT   
  
  
+ TATGAAACCT AAAATAAGTT TATATCTTTT TATTTTTTCT CAACAAATAT ATATCCAAAA TAGAAAAATA   
  
  
+ AATTTCAAAA TTTTCTTAAT ATACCATAAC CGTTTTATGT CATTAATAAA TAATGTCACA ATAAAAATAA   
  
  
+ TAAAATAAAA TCTCATTACC ACCTACAACT TTTAGCCTTT TACTATAGTA CAAAATTCTT TCATCTTTAA   
  
  
+ TAATAATTAC TTTCTTTCAT TCAGTGGTGT CATAAATTAA AGACCCTACA GTATACCATA TGCCATGAAA   
  
  
+ TTAATGAAAT CTAACACAAA TCATTATGAT TTTATCACTT ATAAAAAAAT TATATTTTTC AACCGCACCA   
  
  
+ TATAATATAA CATATATATT AGATTAATAT AGCAACCAAA ATGAATCATT TGGAGGGTAC ACGAGTCCTT   
  
  
+ AACATGGACC TGACTTCGTC GAATCATTCC CAATCGTTGT GACTAAAGAA ATATTAGAAT TGCGTAATTG   
  
  
+ CTTACACGTC CCCATATAAA CAAAACAACT GCCAATCTGT CATTACCTGA CGGTCCCATA TTAAACCCTC   
  
  
+ CTTAAGCACC CACTAATCAT ATCCACTTCT CTTTCACGTT TCTCGCCGGC CTCAGTCTGC CTTGCCGGTG   
  
  
+ ATCCTACGCC TCCATGATCA TCGCTACCAT TGTCGTCGTC GCCGTTTCAG TACGAAGCCG ACGGTCTTGA   
  
  
+ TTAACTTACC ACCTGATCCA GCACCATAT  

- TCTTCCTTTC TTTATCCACA TTACTATCAC CCCCCTGTCT CAAACGTTGA TGGTAAGGAA ATAAAAAAGT   
  
  
- ACCACAAAAC CTAACTTACA GTTTAAATTC TTATTTAAAA GAGTTTAAAA GGACTACTAC TACGTATAAC   
  
  
- ATAGACTTCA TTTCTAAAAA AAAAAAATTT TATAGGTTTC ATCAATTGTA ATTCAGTAGA TGCCTAGTCT   
  
  
- AAATTAAGCC AGGTAAGTAT TAAACATTTT AAAATCAAAT AAATTTATTT TAACAACTAT AATAAAATTT   
  
  
- TTATTTTTAA TAAATTGTGA AGTTTGATAT AAAATATATA ATTTAGTTTC AAATCACAAA TTTTCATTAT   
  
  
- TTAGTTTGAA AATTAATAAT TAAACTAAAT TTAATTTAAA ACTAAATTAT AAAAAACAAT AATTTTTATA   
  
  
- AAATTTTTTA TTAAGTTTAT TTAACATATG AAATAAAATT TAGTTAAGAA ATTAAAGTTT ATTAAAAACC   
  
  
- TAAATACTCA ATAAATAATA TTTAAAAATA TTATTAAAAA GATTTTTGTA AAATCAGTTT TTTTGAATAT   
  
  
- GAAAAAATAT TACTATCAAA CTTATAAAAA TTTTTAAAAA TTATACTAAA TTAAACTATA TTTAATTATT   
  
  
- TAGTCTAAAA TTAAACAATG CAAAATTTGG AATTCCAATT TAAACTGTTT AGTATATGAA TTTTCAAAAT   
  
  
- TAATTAGAAA TGTTAATCAA ATTAATCTAA ACCATGATAT TTATTTTTAT GAGTATCAAA ATAAACTTAA   
  
  
- ATACTTTGGA TTTTATTCAA ATATAGAAAA ATAAAAAAGA GTTGTTTATA TATAGGTTTT ATCTTTTTAT   
  
  
- TTAAAGTTTT AAAAGAATTA TATGGTATTG GCAAAATACA GTAATTATTT ATTACAGTGT TATTTTTATT   
  
  
- ATTTTATTTT AGAGTAATGG TGGATGTTGA AAATCGGAAA ATGATATCAT GTTTTAAGAA AGTAGAAATT   
  
  
- ATTATTAATG AAAGAAAGTA AGTCACCACA GTATTTAATT TCTGGGATGT CATATGGTAT ACGGTACTTT   
  
  
- AATTACTTTA GATTGTGTTT AGTAATACTA AAATAGTGAA TATTTTTTTA ATATAAAAAG TTGGCGTGGT   
  
  
- ATATTATATT GTATATATAA TCTAATTATA TCGTTGGTTT TACTTAGTAA ACCTCCCATG TGCTCAGGAA   
  
  
- TTGTACCTGG ACTGAAGCAG CTTAGTAAGG GTTAGCAACA CTGATTTCTT TATAATCTTA ACGCATTAAC   
  
  
- GAATGTGCAG GGGTATATTT GTTTTGTTGA CGGTTAGACA GTAATGGACT GCCAGGGTAT AATTTGGGAG   
  
  
- GAATTCGTGG GTGATTAGTA TAGGTGAAGA GAAAGTGCAA AGAGCGGCCG GAGTCAGACG GAACGGCCAC   
  
  
- TAGGATGCGG AGGTACTAGT AGCGATGGTA ACAGCAGCAG CGGCAAAGTC ATGCTTCGGC TGCCAGAACT   
  
  
- AATTGAATGG TGGACTAGGT CGTGGTATA

+     ATCT-motif

| Site Name | Organism | Position | Strand | Matrix score. | sequence | function |
| --- | --- | --- | --- | --- | --- | --- |
| ATCT-motif | Pisum sativum | 203 | - | 9 | AATCTAATCC | part of a conserved DNA module involved in light responsiveness |
| ATCT-motif | Arabidopsis thaliana | 1136 | - | 9 | AATCTAATCT | part of a conserved DNA module involved in light responsiveness |

> 2018/04/13 10:10:12  
+ AGAAGGAAAG AAATAGGTGT AATGATAGTG GGGGGACAGA GTTTGCAACT ACCATTCCTT TATTTTTTCA   
  
  
+ TGGTGTTTTG GATTGAATGT CAAATTTAAG AATAAATTTT CTCAAATTTT CCTGATGATG ATGCATATTG   
  
  
+ TATCTGAAGT AAAGATTTTT TTTTTTTAAA ATATCCAAAG TAGTTAACAT TAAGTCATCT ACGGATCAGA   
  
  
+ TTTAATTCGG TCCATTCATA ATTTGTAAAA TTTTAGTTTA TTTAAATAAA ATTGTTGATA TTATTTTAAA   
  
  
+ AATAAAAATT ATTTAACACT TCAAACTATA TTTTATATAT TAAATCAAAG TTTAGTGTTT AAAAGTAATA   
  
  
+ AATCAAACTT TTAATTATTA ATTTGATTTA AATTAAATTT TGATTTAATA TTTTTTGTTA TTAAAAATAT   
  
  
+ TTTAAAAAAT AATTCAAATA AATTGTATAC TTTATTTTAA ATCAATTCTT TAATTTCAAA TAATTTTTGG   
  
  
+ ATTTATGAGT TATTTATTAT AAATTTTTAT AATAATTTTT CTAAAAACAT TTTAGTCAAA AAAACTTATA   
  
  
+ CTTTTTTATA ATGATAGTTT GAATATTTTT AAAAATTTTT AATATGATTT AATTTGATAT AAATTAATAA   
  
  
+ ATCAGATTTT AATTTGTTAC GTTTTAAACC TTAAGGTTAA ATTTGACAAA TCATATACTT AAAAGTTTTA   
  
  
+ ATTAATCTTT ACAATTAGTT TAATTAGATT TGGTACTATA AATAAAAATA CTCATAGTTT TATTTGAATT   
  
  
+ TATGAAACCT AAAATAAGTT TATATCTTTT TATTTTTTCT CAACAAATAT ATATCCAAAA TAGAAAAATA   
  
  
+ AATTTCAAAA TTTTCTTAAT ATACCATAAC CGTTTTATGT CATTAATAAA TAATGTCACA ATAAAAATAA   
  
  
+ TAAAATAAAA TCTCATTACC ACCTACAACT TTTAGCCTTT TACTATAGTA CAAAATTCTT TCATCTTTAA   
  
  
+ TAATAATTAC TTTCTTTCAT TCAGTGGTGT CATAAATTAA AGACCCTACA GTATACCATA TGCCATGAAA   
  
  
+ TTAATGAAAT CTAACACAAA TCATTATGAT TTTATCACTT ATAAAAAAAT TATATTTTTC AACCGCACCA   
  
  
+ TATAATATAA CATATATATT AGATTAATAT AGCAACCAAA ATGAATCATT TGGAGGGTAC ACGAGTCCTT   
  
  
+ AACATGGACC TGACTTCGTC GAATCATTCC CAATCGTTGT GACTAAAGAA ATATTAGAAT TGCGTAATTG   
  
  
+ CTTACACGTC CCCATATAAA CAAAACAACT GCCAATCTGT CATTACCTGA CGGTCCCATA TTAAACCCTC   
  
  
+ CTTAAGCACC CACTAATCAT ATCCACTTCT CTTTCACGTT TCTCGCCGGC CTCAGTCTGC CTTGCCGGTG   
  
  
+ ATCCTACGCC TCCATGATCA TCGCTACCAT TGTCGTCGTC GCCGTTTCAG TACGAAGCCG ACGGTCTTGA   
  
  
+ TTAACTTACC ACCTGATCCA GCACCATAT  

- TCTTCCTTTC TTTATCCACA TTACTATCAC CCCCCTGTCT CAAACGTTGA TGGTAAGGAA ATAAAAAAGT   
  
  
- ACCACAAAAC CTAACTTACA GTTTAAATTC TTATTTAAAA GAGTTTAAAA GGACTACTAC TACGTATAAC   
  
  
- ATAGACTTCA TTTCTAAAAA AAAAAAATTT TATAGGTTTC ATCAATTGTA ATTCAGTAGA TGCCTAGTCT   
  
  
- AAATTAAGCC AGGTAAGTAT TAAACATTTT AAAATCAAAT AAATTTATTT TAACAACTAT AATAAAATTT   
  
  
- TTATTTTTAA TAAATTGTGA AGTTTGATAT AAAATATATA ATTTAGTTTC AAATCACAAA TTTTCATTAT   
  
  
- TTAGTTTGAA AATTAATAAT TAAACTAAAT TTAATTTAAA ACTAAATTAT AAAAAACAAT AATTTTTATA   
  
  
- AAATTTTTTA TTAAGTTTAT TTAACATATG AAATAAAATT TAGTTAAGAA ATTAAAGTTT ATTAAAAACC   
  
  
- TAAATACTCA ATAAATAATA TTTAAAAATA TTATTAAAAA GATTTTTGTA AAATCAGTTT TTTTGAATAT   
  
  
- GAAAAAATAT TACTATCAAA CTTATAAAAA TTTTTAAAAA TTATACTAAA TTAAACTATA TTTAATTATT   
  
  
- TAGTCTAAAA TTAAACAATG CAAAATTTGG AATTCCAATT TAAACTGTTT AGTATATGAA TTTTCAAAAT   
  
  
- TAATTAGAAA TGTTAATCAA ATTAATCTAA ACCATGATAT TTATTTTTAT GAGTATCAAA ATAAACTTAA   
  
  
- ATACTTTGGA TTTTATTCAA ATATAGAAAA ATAAAAAAGA GTTGTTTATA TATAGGTTTT ATCTTTTTAT   
  
  
- TTAAAGTTTT AAAAGAATTA TATGGTATTG GCAAAATACA GTAATTATTT ATTACAGTGT TATTTTTATT   
  
  
- ATTTTATTTT AGAGTAATGG TGGATGTTGA AAATCGGAAA ATGATATCAT GTTTTAAGAA AGTAGAAATT   
  
  
- ATTATTAATG AAAGAAAGTA AGTCACCACA GTATTTAATT TCTGGGATGT CATATGGTAT ACGGTACTTT   
  
  
- AATTACTTTA GATTGTGTTT AGTAATACTA AAATAGTGAA TATTTTTTTA ATATAAAAAG TTGGCGTGGT   
  
  
- ATATTATATT GTATATATAA TCTAATTATA TCGTTGGTTT TACTTAGTAA ACCTCCCATG TGCTCAGGAA   
  
  
- TTGTACCTGG ACTGAAGCAG CTTAGTAAGG GTTAGCAACA CTGATTTCTT TATAATCTTA ACGCATTAAC   
  
  
- GAATGTGCAG GGGTATATTT GTTTTGTTGA CGGTTAGACA GTAATGGACT GCCAGGGTAT AATTTGGGAG   
  
  
- GAATTCGTGG GTGATTAGTA TAGGTGAAGA GAAAGTGCAA AGAGCGGCCG GAGTCAGACG GAACGGCCAC   
  
  
- TAGGATGCGG AGGTACTAGT AGCGATGGTA ACAGCAGCAG CGGCAAAGTC ATGCTTCGGC TGCCAGAACT   
  
  
- AATTGAATGG TGGACTAGGT CGTGGTATA

+     AuxRR-core

| Site Name | Organism | Position | Strand | Matrix score. | sequence | function |
| --- | --- | --- | --- | --- | --- | --- |
| AuxRR-core | Nicotiana tabacum | 1194 | - | 7 | GGTCCAT | cis-acting regulatory element involved in auxin responsiveness |
| AuxRR-core | Nicotiana tabacum | 219 | + | 7 | GGTCCAT | cis-acting regulatory element involved in auxin responsiveness |

> 2018/04/13 10:10:12  
+ AGAAGGAAAG AAATAGGTGT AATGATAGTG GGGGGACAGA GTTTGCAACT ACCATTCCTT TATTTTTTCA   
  
  
+ TGGTGTTTTG GATTGAATGT CAAATTTAAG AATAAATTTT CTCAAATTTT CCTGATGATG ATGCATATTG   
  
  
+ TATCTGAAGT AAAGATTTTT TTTTTTTAAA ATATCCAAAG TAGTTAACAT TAAGTCATCT ACGGATCAGA   
  
  
+ TTTAATTCGG TCCATTCATA ATTTGTAAAA TTTTAGTTTA TTTAAATAAA ATTGTTGATA TTATTTTAAA   
  
  
+ AATAAAAATT ATTTAACACT TCAAACTATA TTTTATATAT TAAATCAAAG TTTAGTGTTT AAAAGTAATA   
  
  
+ AATCAAACTT TTAATTATTA ATTTGATTTA AATTAAATTT TGATTTAATA TTTTTTGTTA TTAAAAATAT   
  
  
+ TTTAAAAAAT AATTCAAATA AATTGTATAC TTTATTTTAA ATCAATTCTT TAATTTCAAA TAATTTTTGG   
  
  
+ ATTTATGAGT TATTTATTAT AAATTTTTAT AATAATTTTT CTAAAAACAT TTTAGTCAAA AAAACTTATA   
  
  
+ CTTTTTTATA ATGATAGTTT GAATATTTTT AAAAATTTTT AATATGATTT AATTTGATAT AAATTAATAA   
  
  
+ ATCAGATTTT AATTTGTTAC GTTTTAAACC TTAAGGTTAA ATTTGACAAA TCATATACTT AAAAGTTTTA   
  
  
+ ATTAATCTTT ACAATTAGTT TAATTAGATT TGGTACTATA AATAAAAATA CTCATAGTTT TATTTGAATT   
  
  
+ TATGAAACCT AAAATAAGTT TATATCTTTT TATTTTTTCT CAACAAATAT ATATCCAAAA TAGAAAAATA   
  
  
+ AATTTCAAAA TTTTCTTAAT ATACCATAAC CGTTTTATGT CATTAATAAA TAATGTCACA ATAAAAATAA   
  
  
+ TAAAATAAAA TCTCATTACC ACCTACAACT TTTAGCCTTT TACTATAGTA CAAAATTCTT TCATCTTTAA   
  
  
+ TAATAATTAC TTTCTTTCAT TCAGTGGTGT CATAAATTAA AGACCCTACA GTATACCATA TGCCATGAAA   
  
  
+ TTAATGAAAT CTAACACAAA TCATTATGAT TTTATCACTT ATAAAAAAAT TATATTTTTC AACCGCACCA   
  
  
+ TATAATATAA CATATATATT AGATTAATAT AGCAACCAAA ATGAATCATT TGGAGGGTAC ACGAGTCCTT   
  
  
+ AACATGGACC TGACTTCGTC GAATCATTCC CAATCGTTGT GACTAAAGAA ATATTAGAAT TGCGTAATTG   
  
  
+ CTTACACGTC CCCATATAAA CAAAACAACT GCCAATCTGT CATTACCTGA CGGTCCCATA TTAAACCCTC   
  
  
+ CTTAAGCACC CACTAATCAT ATCCACTTCT CTTTCACGTT TCTCGCCGGC CTCAGTCTGC CTTGCCGGTG   
  
  
+ ATCCTACGCC TCCATGATCA TCGCTACCAT TGTCGTCGTC GCCGTTTCAG TACGAAGCCG ACGGTCTTGA   
  
  
+ TTAACTTACC ACCTGATCCA GCACCATAT  

- TCTTCCTTTC TTTATCCACA TTACTATCAC CCCCCTGTCT CAAACGTTGA TGGTAAGGAA ATAAAAAAGT   
  
  
- ACCACAAAAC CTAACTTACA GTTTAAATTC TTATTTAAAA GAGTTTAAAA GGACTACTAC TACGTATAAC   
  
  
- ATAGACTTCA TTTCTAAAAA AAAAAAATTT TATAGGTTTC ATCAATTGTA ATTCAGTAGA TGCCTAGTCT   
  
  
- AAATTAAGCC AGGTAAGTAT TAAACATTTT AAAATCAAAT AAATTTATTT TAACAACTAT AATAAAATTT   
  
  
- TTATTTTTAA TAAATTGTGA AGTTTGATAT AAAATATATA ATTTAGTTTC AAATCACAAA TTTTCATTAT   
  
  
- TTAGTTTGAA AATTAATAAT TAAACTAAAT TTAATTTAAA ACTAAATTAT AAAAAACAAT AATTTTTATA   
  
  
- AAATTTTTTA TTAAGTTTAT TTAACATATG AAATAAAATT TAGTTAAGAA ATTAAAGTTT ATTAAAAACC   
  
  
- TAAATACTCA ATAAATAATA TTTAAAAATA TTATTAAAAA GATTTTTGTA AAATCAGTTT TTTTGAATAT   
  
  
- GAAAAAATAT TACTATCAAA CTTATAAAAA TTTTTAAAAA TTATACTAAA TTAAACTATA TTTAATTATT   
  
  
- TAGTCTAAAA TTAAACAATG CAAAATTTGG AATTCCAATT TAAACTGTTT AGTATATGAA TTTTCAAAAT   
  
  
- TAATTAGAAA TGTTAATCAA ATTAATCTAA ACCATGATAT TTATTTTTAT GAGTATCAAA ATAAACTTAA   
  
  
- ATACTTTGGA TTTTATTCAA ATATAGAAAA ATAAAAAAGA GTTGTTTATA TATAGGTTTT ATCTTTTTAT   
  
  
- TTAAAGTTTT AAAAGAATTA TATGGTATTG GCAAAATACA GTAATTATTT ATTACAGTGT TATTTTTATT   
  
  
- ATTTTATTTT AGAGTAATGG TGGATGTTGA AAATCGGAAA ATGATATCAT GTTTTAAGAA AGTAGAAATT   
  
  
- ATTATTAATG AAAGAAAGTA AGTCACCACA GTATTTAATT TCTGGGATGT CATATGGTAT ACGGTACTTT   
  
  
- AATTACTTTA GATTGTGTTT AGTAATACTA AAATAGTGAA TATTTTTTTA ATATAAAAAG TTGGCGTGGT   
  
  
- ATATTATATT GTATATATAA TCTAATTATA TCGTTGGTTT TACTTAGTAA ACCTCCCATG TGCTCAGGAA   
  
  
- TTGTACCTGG ACTGAAGCAG CTTAGTAAGG GTTAGCAACA CTGATTTCTT TATAATCTTA ACGCATTAAC   
  
  
- GAATGTGCAG GGGTATATTT GTTTTGTTGA CGGTTAGACA GTAATGGACT GCCAGGGTAT AATTTGGGAG   
  
  
- GAATTCGTGG GTGATTAGTA TAGGTGAAGA GAAAGTGCAA AGAGCGGCCG GAGTCAGACG GAACGGCCAC   
  
  
- TAGGATGCGG AGGTACTAGT AGCGATGGTA ACAGCAGCAG CGGCAAAGTC ATGCTTCGGC TGCCAGAACT   
  
  
- AATTGAATGG TGGACTAGGT CGTGGTATA

+     Box 4

| Site Name | Organism | Position | Strand | Matrix score. | sequence | function |
| --- | --- | --- | --- | --- | --- | --- |
| Box 4 | Petroselinum crispum | 1050 | - | 6 | ATTAAT | part of a conserved DNA module involved in light responsiveness |
| Box 4 | Petroselinum crispum | 701 | + | 6 | ATTAAT | part of a conserved DNA module involved in light responsiveness |
| Box 4 | Petroselinum crispum | 1143 | - | 6 | ATTAAT | part of a conserved DNA module involved in light responsiveness |
| Box 4 | Petroselinum crispum | 882 | - | 6 | ATTAAT | part of a conserved DNA module involved in light responsiveness |
| Box 4 | Petroselinum crispum | 367 | + | 6 | ATTAAT | part of a conserved DNA module involved in light responsiveness |
| Box 4 | Petroselinum crispum | 623 | + | 6 | ATTAAT | part of a conserved DNA module involved in light responsiveness |

> 2018/04/13 10:10:12  
+ AGAAGGAAAG AAATAGGTGT AATGATAGTG GGGGGACAGA GTTTGCAACT ACCATTCCTT TATTTTTTCA   
  
  
+ TGGTGTTTTG GATTGAATGT CAAATTTAAG AATAAATTTT CTCAAATTTT CCTGATGATG ATGCATATTG   
  
  
+ TATCTGAAGT AAAGATTTTT TTTTTTTAAA ATATCCAAAG TAGTTAACAT TAAGTCATCT ACGGATCAGA   
  
  
+ TTTAATTCGG TCCATTCATA ATTTGTAAAA TTTTAGTTTA TTTAAATAAA ATTGTTGATA TTATTTTAAA   
  
  
+ AATAAAAATT ATTTAACACT TCAAACTATA TTTTATATAT TAAATCAAAG TTTAGTGTTT AAAAGTAATA   
  
  
+ AATCAAACTT TTAATTATTA ATTTGATTTA AATTAAATTT TGATTTAATA TTTTTTGTTA TTAAAAATAT   
  
  
+ TTTAAAAAAT AATTCAAATA AATTGTATAC TTTATTTTAA ATCAATTCTT TAATTTCAAA TAATTTTTGG   
  
  
+ ATTTATGAGT TATTTATTAT AAATTTTTAT AATAATTTTT CTAAAAACAT TTTAGTCAAA AAAACTTATA   
  
  
+ CTTTTTTATA ATGATAGTTT GAATATTTTT AAAAATTTTT AATATGATTT AATTTGATAT AAATTAATAA   
  
  
+ ATCAGATTTT AATTTGTTAC GTTTTAAACC TTAAGGTTAA ATTTGACAAA TCATATACTT AAAAGTTTTA   
  
  
+ ATTAATCTTT ACAATTAGTT TAATTAGATT TGGTACTATA AATAAAAATA CTCATAGTTT TATTTGAATT   
  
  
+ TATGAAACCT AAAATAAGTT TATATCTTTT TATTTTTTCT CAACAAATAT ATATCCAAAA TAGAAAAATA   
  
  
+ AATTTCAAAA TTTTCTTAAT ATACCATAAC CGTTTTATGT CATTAATAAA TAATGTCACA ATAAAAATAA   
  
  
+ TAAAATAAAA TCTCATTACC ACCTACAACT TTTAGCCTTT TACTATAGTA CAAAATTCTT TCATCTTTAA   
  
  
+ TAATAATTAC TTTCTTTCAT TCAGTGGTGT CATAAATTAA AGACCCTACA GTATACCATA TGCCATGAAA   
  
  
+ TTAATGAAAT CTAACACAAA TCATTATGAT TTTATCACTT ATAAAAAAAT TATATTTTTC AACCGCACCA   
  
  
+ TATAATATAA CATATATATT AGATTAATAT AGCAACCAAA ATGAATCATT TGGAGGGTAC ACGAGTCCTT   
  
  
+ AACATGGACC TGACTTCGTC GAATCATTCC CAATCGTTGT GACTAAAGAA ATATTAGAAT TGCGTAATTG   
  
  
+ CTTACACGTC CCCATATAAA CAAAACAACT GCCAATCTGT CATTACCTGA CGGTCCCATA TTAAACCCTC   
  
  
+ CTTAAGCACC CACTAATCAT ATCCACTTCT CTTTCACGTT TCTCGCCGGC CTCAGTCTGC CTTGCCGGTG   
  
  
+ ATCCTACGCC TCCATGATCA TCGCTACCAT TGTCGTCGTC GCCGTTTCAG TACGAAGCCG ACGGTCTTGA   
  
  
+ TTAACTTACC ACCTGATCCA GCACCATAT  

- TCTTCCTTTC TTTATCCACA TTACTATCAC CCCCCTGTCT CAAACGTTGA TGGTAAGGAA ATAAAAAAGT   
  
  
- ACCACAAAAC CTAACTTACA GTTTAAATTC TTATTTAAAA GAGTTTAAAA GGACTACTAC TACGTATAAC   
  
  
- ATAGACTTCA TTTCTAAAAA AAAAAAATTT TATAGGTTTC ATCAATTGTA ATTCAGTAGA TGCCTAGTCT   
  
  
- AAATTAAGCC AGGTAAGTAT TAAACATTTT AAAATCAAAT AAATTTATTT TAACAACTAT AATAAAATTT   
  
  
- TTATTTTTAA TAAATTGTGA AGTTTGATAT AAAATATATA ATTTAGTTTC AAATCACAAA TTTTCATTAT   
  
  
- TTAGTTTGAA AATTAATAAT TAAACTAAAT TTAATTTAAA ACTAAATTAT AAAAAACAAT AATTTTTATA   
  
  
- AAATTTTTTA TTAAGTTTAT TTAACATATG AAATAAAATT TAGTTAAGAA ATTAAAGTTT ATTAAAAACC   
  
  
- TAAATACTCA ATAAATAATA TTTAAAAATA TTATTAAAAA GATTTTTGTA AAATCAGTTT TTTTGAATAT   
  
  
- GAAAAAATAT TACTATCAAA CTTATAAAAA TTTTTAAAAA TTATACTAAA TTAAACTATA TTTAATTATT   
  
  
- TAGTCTAAAA TTAAACAATG CAAAATTTGG AATTCCAATT TAAACTGTTT AGTATATGAA TTTTCAAAAT   
  
  
- TAATTAGAAA TGTTAATCAA ATTAATCTAA ACCATGATAT TTATTTTTAT GAGTATCAAA ATAAACTTAA   
  
  
- ATACTTTGGA TTTTATTCAA ATATAGAAAA ATAAAAAAGA GTTGTTTATA TATAGGTTTT ATCTTTTTAT   
  
  
- TTAAAGTTTT AAAAGAATTA TATGGTATTG GCAAAATACA GTAATTATTT ATTACAGTGT TATTTTTATT   
  
  
- ATTTTATTTT AGAGTAATGG TGGATGTTGA AAATCGGAAA ATGATATCAT GTTTTAAGAA AGTAGAAATT   
  
  
- ATTATTAATG AAAGAAAGTA AGTCACCACA GTATTTAATT TCTGGGATGT CATATGGTAT ACGGTACTTT   
  
  
- AATTACTTTA GATTGTGTTT AGTAATACTA AAATAGTGAA TATTTTTTTA ATATAAAAAG TTGGCGTGGT   
  
  
- ATATTATATT GTATATATAA TCTAATTATA TCGTTGGTTT TACTTAGTAA ACCTCCCATG TGCTCAGGAA   
  
  
- TTGTACCTGG ACTGAAGCAG CTTAGTAAGG GTTAGCAACA CTGATTTCTT TATAATCTTA ACGCATTAAC   
  
  
- GAATGTGCAG GGGTATATTT GTTTTGTTGA CGGTTAGACA GTAATGGACT GCCAGGGTAT AATTTGGGAG   
  
  
- GAATTCGTGG GTGATTAGTA TAGGTGAAGA GAAAGTGCAA AGAGCGGCCG GAGTCAGACG GAACGGCCAC   
  
  
- TAGGATGCGG AGGTACTAGT AGCGATGGTA ACAGCAGCAG CGGCAAAGTC ATGCTTCGGC TGCCAGAACT   
  
  
- AATTGAATGG TGGACTAGGT CGTGGTATA

+     Box I

| Site Name | Organism | Position | Strand | Matrix score. | sequence | function |
| --- | --- | --- | --- | --- | --- | --- |
| Box I | Pisum sativum | 843 | + | 7 | TTTCAAA | light responsive element |
| Box I | Pisum sativum | 474 | + | 7 | TTTCAAA | light responsive element |

> 2018/04/13 10:10:12  
+ AGAAGGAAAG AAATAGGTGT AATGATAGTG GGGGGACAGA GTTTGCAACT ACCATTCCTT TATTTTTTCA   
  
  
+ TGGTGTTTTG GATTGAATGT CAAATTTAAG AATAAATTTT CTCAAATTTT CCTGATGATG ATGCATATTG   
  
  
+ TATCTGAAGT AAAGATTTTT TTTTTTTAAA ATATCCAAAG TAGTTAACAT TAAGTCATCT ACGGATCAGA   
  
  
+ TTTAATTCGG TCCATTCATA ATTTGTAAAA TTTTAGTTTA TTTAAATAAA ATTGTTGATA TTATTTTAAA   
  
  
+ AATAAAAATT ATTTAACACT TCAAACTATA TTTTATATAT TAAATCAAAG TTTAGTGTTT AAAAGTAATA   
  
  
+ AATCAAACTT TTAATTATTA ATTTGATTTA AATTAAATTT TGATTTAATA TTTTTTGTTA TTAAAAATAT   
  
  
+ TTTAAAAAAT AATTCAAATA AATTGTATAC TTTATTTTAA ATCAATTCTT TAATTTCAAA TAATTTTTGG   
  
  
+ ATTTATGAGT TATTTATTAT AAATTTTTAT AATAATTTTT CTAAAAACAT TTTAGTCAAA AAAACTTATA   
  
  
+ CTTTTTTATA ATGATAGTTT GAATATTTTT AAAAATTTTT AATATGATTT AATTTGATAT AAATTAATAA   
  
  
+ ATCAGATTTT AATTTGTTAC GTTTTAAACC TTAAGGTTAA ATTTGACAAA TCATATACTT AAAAGTTTTA   
  
  
+ ATTAATCTTT ACAATTAGTT TAATTAGATT TGGTACTATA AATAAAAATA CTCATAGTTT TATTTGAATT   
  
  
+ TATGAAACCT AAAATAAGTT TATATCTTTT TATTTTTTCT CAACAAATAT ATATCCAAAA TAGAAAAATA   
  
  
+ AATTTCAAAA TTTTCTTAAT ATACCATAAC CGTTTTATGT CATTAATAAA TAATGTCACA ATAAAAATAA   
  
  
+ TAAAATAAAA TCTCATTACC ACCTACAACT TTTAGCCTTT TACTATAGTA CAAAATTCTT TCATCTTTAA   
  
  
+ TAATAATTAC TTTCTTTCAT TCAGTGGTGT CATAAATTAA AGACCCTACA GTATACCATA TGCCATGAAA   
  
  
+ TTAATGAAAT CTAACACAAA TCATTATGAT TTTATCACTT ATAAAAAAAT TATATTTTTC AACCGCACCA   
  
  
+ TATAATATAA CATATATATT AGATTAATAT AGCAACCAAA ATGAATCATT TGGAGGGTAC ACGAGTCCTT   
  
  
+ AACATGGACC TGACTTCGTC GAATCATTCC CAATCGTTGT GACTAAAGAA ATATTAGAAT TGCGTAATTG   
  
  
+ CTTACACGTC CCCATATAAA CAAAACAACT GCCAATCTGT CATTACCTGA CGGTCCCATA TTAAACCCTC   
  
  
+ CTTAAGCACC CACTAATCAT ATCCACTTCT CTTTCACGTT TCTCGCCGGC CTCAGTCTGC CTTGCCGGTG   
  
  
+ ATCCTACGCC TCCATGATCA TCGCTACCAT TGTCGTCGTC GCCGTTTCAG TACGAAGCCG ACGGTCTTGA   
  
  
+ TTAACTTACC ACCTGATCCA GCACCATAT  

- TCTTCCTTTC TTTATCCACA TTACTATCAC CCCCCTGTCT CAAACGTTGA TGGTAAGGAA ATAAAAAAGT   
  
  
- ACCACAAAAC CTAACTTACA GTTTAAATTC TTATTTAAAA GAGTTTAAAA GGACTACTAC TACGTATAAC   
  
  
- ATAGACTTCA TTTCTAAAAA AAAAAAATTT TATAGGTTTC ATCAATTGTA ATTCAGTAGA TGCCTAGTCT   
  
  
- AAATTAAGCC AGGTAAGTAT TAAACATTTT AAAATCAAAT AAATTTATTT TAACAACTAT AATAAAATTT   
  
  
- TTATTTTTAA TAAATTGTGA AGTTTGATAT AAAATATATA ATTTAGTTTC AAATCACAAA TTTTCATTAT   
  
  
- TTAGTTTGAA AATTAATAAT TAAACTAAAT TTAATTTAAA ACTAAATTAT AAAAAACAAT AATTTTTATA   
  
  
- AAATTTTTTA TTAAGTTTAT TTAACATATG AAATAAAATT TAGTTAAGAA ATTAAAGTTT ATTAAAAACC   
  
  
- TAAATACTCA ATAAATAATA TTTAAAAATA TTATTAAAAA GATTTTTGTA AAATCAGTTT TTTTGAATAT   
  
  
- GAAAAAATAT TACTATCAAA CTTATAAAAA TTTTTAAAAA TTATACTAAA TTAAACTATA TTTAATTATT   
  
  
- TAGTCTAAAA TTAAACAATG CAAAATTTGG AATTCCAATT TAAACTGTTT AGTATATGAA TTTTCAAAAT   
  
  
- TAATTAGAAA TGTTAATCAA ATTAATCTAA ACCATGATAT TTATTTTTAT GAGTATCAAA ATAAACTTAA   
  
  
- ATACTTTGGA TTTTATTCAA ATATAGAAAA ATAAAAAAGA GTTGTTTATA TATAGGTTTT ATCTTTTTAT   
  
  
- TTAAAGTTTT AAAAGAATTA TATGGTATTG GCAAAATACA GTAATTATTT ATTACAGTGT TATTTTTATT   
  
  
- ATTTTATTTT AGAGTAATGG TGGATGTTGA AAATCGGAAA ATGATATCAT GTTTTAAGAA AGTAGAAATT   
  
  
- ATTATTAATG AAAGAAAGTA AGTCACCACA GTATTTAATT TCTGGGATGT CATATGGTAT ACGGTACTTT   
  
  
- AATTACTTTA GATTGTGTTT AGTAATACTA AAATAGTGAA TATTTTTTTA ATATAAAAAG TTGGCGTGGT   
  
  
- ATATTATATT GTATATATAA TCTAATTATA TCGTTGGTTT TACTTAGTAA ACCTCCCATG TGCTCAGGAA   
  
  
- TTGTACCTGG ACTGAAGCAG CTTAGTAAGG GTTAGCAACA CTGATTTCTT TATAATCTTA ACGCATTAAC   
  
  
- GAATGTGCAG GGGTATATTT GTTTTGTTGA CGGTTAGACA GTAATGGACT GCCAGGGTAT AATTTGGGAG   
  
  
- GAATTCGTGG GTGATTAGTA TAGGTGAAGA GAAAGTGCAA AGAGCGGCCG GAGTCAGACG GAACGGCCAC   
  
  
- TAGGATGCGG AGGTACTAGT AGCGATGGTA ACAGCAGCAG CGGCAAAGTC ATGCTTCGGC TGCCAGAACT   
  
  
- AATTGAATGG TGGACTAGGT CGTGGTATA

+     CAAT-box

| Site Name | Organism | Position | Strand | Matrix score. | sequence | function |
| --- | --- | --- | --- | --- | --- | --- |
| CAAT-box | Hordeum vulgare | 1429 | - | 4 | CAAT | common cis-acting element in promoter and enhancer regions |
| CAAT-box | Brassica rapa | 814 | + | 5 | CAAAT | common cis-acting element in promoter and enhancer regions |
| CAAT-box | Brassica rapa | 671 | - | 5 | CAAAT | common cis-acting element in promoter and enhancer regions |
| CAAT-box | Arabidopsis thaliana | 1292 | + | 5 | CCAAT | common cis-acting element in promoter and enhancer regions |
| CAAT-box | Hordeum vulgare | 1221 | + | 4 | CAAT | common cis-acting element in promoter and enhancer regions |
| CAAT-box | Hordeum vulgare | 1257 | - | 4 | CAAT | common cis-acting element in promoter and enhancer regions |
| CAAT-box | Brassica rapa | 1168 | - | 5 | CAAAT | common cis-acting element in promoter and enhancer regions |
| CAAT-box | Brassica rapa | 477 | + | 5 | CAAAT | common cis-acting element in promoter and enhancer regions |
| CAAT-box | Brassica rapa | 371 | - | 5 | CAAAT | common cis-acting element in promoter and enhancer regions |
| CAAT-box | Hordeum vulgare | 1249 | - | 4 | CAAT | common cis-acting element in promoter and enhancer regions |
| CAAT-box | Brassica rapa | 1067 | + | 5 | CAAAT | common cis-acting element in promoter and enhancer regions |
| CAAT-box | Brassica rapa | 231 | - | 5 | CAAAT | common cis-acting element in promoter and enhancer regions |
| CAAT-box | Hordeum vulgare | 899 | + | 4 | CAAT | common cis-acting element in promoter and enhancer regions |
| CAAT-box | Brassica rapa | 762 | - | 5 | CAAAT | common cis-acting element in promoter and enhancer regions |
| CAAT-box | Brassica rapa | 677 | + | 5 | CAAAT | common cis-acting element in promoter and enhancer regions |
| CAAT-box | Glycine max | 463 | + | 5 | CAATT | common cis-acting element in promoter and enhancer regions |
| CAAT-box | Brassica rapa | 91 | + | 5 | CAAAT | common cis-acting element in promoter and enhancer regions |
| CAAT-box | Glycine max | 1248 | - | 5 | CAATT | common cis-acting element in promoter and enhancer regions |
| CAAT-box | Arabidopsis thaliana | 1220 | + | 5 | CCAAT | common cis-acting element in promoter and enhancer regions |
| CAAT-box | Brassica rapa | 728 | - | 5 | CAAAT | common cis-acting element in promoter and enhancer regions |
| CAAT-box | Glycine max | 260 | - | 5 | CAATT | common cis-acting element in promoter and enhancer regions |
| CAAT-box | Brassica rapa | 642 | - | 5 | CAAAT | common cis-acting element in promoter and enhancer regions |
| CAAT-box | Hordeum vulgare | 1293 | + | 4 | CAAT | common cis-acting element in promoter and enhancer regions |
| CAAT-box | Glycine max | 1256 | - | 5 | CAATT | common cis-acting element in promoter and enhancer regions |
| CAAT-box | Brassica rapa | 612 | - | 5 | CAAAT | common cis-acting element in promoter and enhancer regions |
| CAAT-box | Brassica rapa | 435 | + | 5 | CAAAT | common cis-acting element in promoter and enhancer regions |
| CAAT-box | Glycine max | 441 | - | 5 | CAATT | common cis-acting element in promoter and enhancer regions |
| CAAT-box | Hordeum vulgare | 442 | - | 4 | CAAT | common cis-acting element in promoter and enhancer regions |
| CAAT-box | Brassica rapa | 113 | + | 5 | CAAAT | common cis-acting element in promoter and enhancer regions |
| CAAT-box | Glycine max | 712 | + | 5 | CAATT | common cis-acting element in promoter and enhancer regions |
| CAAT-box | Hordeum vulgare | 261 | - | 4 | CAAT | common cis-acting element in promoter and enhancer regions |
| CAAT-box | Hordeum vulgare | 137 | - | 4 | CAAT | common cis-acting element in promoter and enhancer regions |
| CAAT-box | Hordeum vulgare | 82 | - | 4 | CAAT | common cis-acting element in promoter and enhancer regions |

> 2018/04/13 10:10:12  
+ AGAAGGAAAG AAATAGGTGT AATGATAGTG GGGGGACAGA GTTTGCAACT ACCATTCCTT TATTTTTTCA   
  
  
+ TGGTGTTTTG GATTGAATGT CAAATTTAAG AATAAATTTT CTCAAATTTT CCTGATGATG ATGCATATTG   
  
  
+ TATCTGAAGT AAAGATTTTT TTTTTTTAAA ATATCCAAAG TAGTTAACAT TAAGTCATCT ACGGATCAGA   
  
  
+ TTTAATTCGG TCCATTCATA ATTTGTAAAA TTTTAGTTTA TTTAAATAAA ATTGTTGATA TTATTTTAAA   
  
  
+ AATAAAAATT ATTTAACACT TCAAACTATA TTTTATATAT TAAATCAAAG TTTAGTGTTT AAAAGTAATA   
  
  
+ AATCAAACTT TTAATTATTA ATTTGATTTA AATTAAATTT TGATTTAATA TTTTTTGTTA TTAAAAATAT   
  
  
+ TTTAAAAAAT AATTCAAATA AATTGTATAC TTTATTTTAA ATCAATTCTT TAATTTCAAA TAATTTTTGG   
  
  
+ ATTTATGAGT TATTTATTAT AAATTTTTAT AATAATTTTT CTAAAAACAT TTTAGTCAAA AAAACTTATA   
  
  
+ CTTTTTTATA ATGATAGTTT GAATATTTTT AAAAATTTTT AATATGATTT AATTTGATAT AAATTAATAA   
  
  
+ ATCAGATTTT AATTTGTTAC GTTTTAAACC TTAAGGTTAA ATTTGACAAA TCATATACTT AAAAGTTTTA   
  
  
+ ATTAATCTTT ACAATTAGTT TAATTAGATT TGGTACTATA AATAAAAATA CTCATAGTTT TATTTGAATT   
  
  
+ TATGAAACCT AAAATAAGTT TATATCTTTT TATTTTTTCT CAACAAATAT ATATCCAAAA TAGAAAAATA   
  
  
+ AATTTCAAAA TTTTCTTAAT ATACCATAAC CGTTTTATGT CATTAATAAA TAATGTCACA ATAAAAATAA   
  
  
+ TAAAATAAAA TCTCATTACC ACCTACAACT TTTAGCCTTT TACTATAGTA CAAAATTCTT TCATCTTTAA   
  
  
+ TAATAATTAC TTTCTTTCAT TCAGTGGTGT CATAAATTAA AGACCCTACA GTATACCATA TGCCATGAAA   
  
  
+ TTAATGAAAT CTAACACAAA TCATTATGAT TTTATCACTT ATAAAAAAAT TATATTTTTC AACCGCACCA   
  
  
+ TATAATATAA CATATATATT AGATTAATAT AGCAACCAAA ATGAATCATT TGGAGGGTAC ACGAGTCCTT   
  
  
+ AACATGGACC TGACTTCGTC GAATCATTCC CAATCGTTGT GACTAAAGAA ATATTAGAAT TGCGTAATTG   
  
  
+ CTTACACGTC CCCATATAAA CAAAACAACT GCCAATCTGT CATTACCTGA CGGTCCCATA TTAAACCCTC   
  
  
+ CTTAAGCACC CACTAATCAT ATCCACTTCT CTTTCACGTT TCTCGCCGGC CTCAGTCTGC CTTGCCGGTG   
  
  
+ ATCCTACGCC TCCATGATCA TCGCTACCAT TGTCGTCGTC GCCGTTTCAG TACGAAGCCG ACGGTCTTGA   
  
  
+ TTAACTTACC ACCTGATCCA GCACCATAT  

- TCTTCCTTTC TTTATCCACA TTACTATCAC CCCCCTGTCT CAAACGTTGA TGGTAAGGAA ATAAAAAAGT   
  
  
- ACCACAAAAC CTAACTTACA GTTTAAATTC TTATTTAAAA GAGTTTAAAA GGACTACTAC TACGTATAAC   
  
  
- ATAGACTTCA TTTCTAAAAA AAAAAAATTT TATAGGTTTC ATCAATTGTA ATTCAGTAGA TGCCTAGTCT   
  
  
- AAATTAAGCC AGGTAAGTAT TAAACATTTT AAAATCAAAT AAATTTATTT TAACAACTAT AATAAAATTT   
  
  
- TTATTTTTAA TAAATTGTGA AGTTTGATAT AAAATATATA ATTTAGTTTC AAATCACAAA TTTTCATTAT   
  
  
- TTAGTTTGAA AATTAATAAT TAAACTAAAT TTAATTTAAA ACTAAATTAT AAAAAACAAT AATTTTTATA   
  
  
- AAATTTTTTA TTAAGTTTAT TTAACATATG AAATAAAATT TAGTTAAGAA ATTAAAGTTT ATTAAAAACC   
  
  
- TAAATACTCA ATAAATAATA TTTAAAAATA TTATTAAAAA GATTTTTGTA AAATCAGTTT TTTTGAATAT   
  
  
- GAAAAAATAT TACTATCAAA CTTATAAAAA TTTTTAAAAA TTATACTAAA TTAAACTATA TTTAATTATT   
  
  
- TAGTCTAAAA TTAAACAATG CAAAATTTGG AATTCCAATT TAAACTGTTT AGTATATGAA TTTTCAAAAT   
  
  
- TAATTAGAAA TGTTAATCAA ATTAATCTAA ACCATGATAT TTATTTTTAT GAGTATCAAA ATAAACTTAA   
  
  
- ATACTTTGGA TTTTATTCAA ATATAGAAAA ATAAAAAAGA GTTGTTTATA TATAGGTTTT ATCTTTTTAT   
  
  
- TTAAAGTTTT AAAAGAATTA TATGGTATTG GCAAAATACA GTAATTATTT ATTACAGTGT TATTTTTATT   
  
  
- ATTTTATTTT AGAGTAATGG TGGATGTTGA AAATCGGAAA ATGATATCAT GTTTTAAGAA AGTAGAAATT   
  
  
- ATTATTAATG AAAGAAAGTA AGTCACCACA GTATTTAATT TCTGGGATGT CATATGGTAT ACGGTACTTT   
  
  
- AATTACTTTA GATTGTGTTT AGTAATACTA AAATAGTGAA TATTTTTTTA ATATAAAAAG TTGGCGTGGT   
  
  
- ATATTATATT GTATATATAA TCTAATTATA TCGTTGGTTT TACTTAGTAA ACCTCCCATG TGCTCAGGAA   
  
  
- TTGTACCTGG ACTGAAGCAG CTTAGTAAGG GTTAGCAACA CTGATTTCTT TATAATCTTA ACGCATTAAC   
  
  
- GAATGTGCAG GGGTATATTT GTTTTGTTGA CGGTTAGACA GTAATGGACT GCCAGGGTAT AATTTGGGAG   
  
  
- GAATTCGTGG GTGATTAGTA TAGGTGAAGA GAAAGTGCAA AGAGCGGCCG GAGTCAGACG GAACGGCCAC   
  
  
- TAGGATGCGG AGGTACTAGT AGCGATGGTA ACAGCAGCAG CGGCAAAGTC ATGCTTCGGC TGCCAGAACT   
  
  
- AATTGAATGG TGGACTAGGT CGTGGTATA

+     CAG-motif

| Site Name | Organism | Position | Strand | Matrix score. | sequence | function |
| --- | --- | --- | --- | --- | --- | --- |
| CAG-motif | Arabidopsis thaliana | 1385 | - | 10 | GAAAGGCAGAC | part of a light response element |

> 2018/04/13 10:10:12  
+ AGAAGGAAAG AAATAGGTGT AATGATAGTG GGGGGACAGA GTTTGCAACT ACCATTCCTT TATTTTTTCA   
  
  
+ TGGTGTTTTG GATTGAATGT CAAATTTAAG AATAAATTTT CTCAAATTTT CCTGATGATG ATGCATATTG   
  
  
+ TATCTGAAGT AAAGATTTTT TTTTTTTAAA ATATCCAAAG TAGTTAACAT TAAGTCATCT ACGGATCAGA   
  
  
+ TTTAATTCGG TCCATTCATA ATTTGTAAAA TTTTAGTTTA TTTAAATAAA ATTGTTGATA TTATTTTAAA   
  
  
+ AATAAAAATT ATTTAACACT TCAAACTATA TTTTATATAT TAAATCAAAG TTTAGTGTTT AAAAGTAATA   
  
  
+ AATCAAACTT TTAATTATTA ATTTGATTTA AATTAAATTT TGATTTAATA TTTTTTGTTA TTAAAAATAT   
  
  
+ TTTAAAAAAT AATTCAAATA AATTGTATAC TTTATTTTAA ATCAATTCTT TAATTTCAAA TAATTTTTGG   
  
  
+ ATTTATGAGT TATTTATTAT AAATTTTTAT AATAATTTTT CTAAAAACAT TTTAGTCAAA AAAACTTATA   
  
  
+ CTTTTTTATA ATGATAGTTT GAATATTTTT AAAAATTTTT AATATGATTT AATTTGATAT AAATTAATAA   
  
  
+ ATCAGATTTT AATTTGTTAC GTTTTAAACC TTAAGGTTAA ATTTGACAAA TCATATACTT AAAAGTTTTA   
  
  
+ ATTAATCTTT ACAATTAGTT TAATTAGATT TGGTACTATA AATAAAAATA CTCATAGTTT TATTTGAATT   
  
  
+ TATGAAACCT AAAATAAGTT TATATCTTTT TATTTTTTCT CAACAAATAT ATATCCAAAA TAGAAAAATA   
  
  
+ AATTTCAAAA TTTTCTTAAT ATACCATAAC CGTTTTATGT CATTAATAAA TAATGTCACA ATAAAAATAA   
  
  
+ TAAAATAAAA TCTCATTACC ACCTACAACT TTTAGCCTTT TACTATAGTA CAAAATTCTT TCATCTTTAA   
  
  
+ TAATAATTAC TTTCTTTCAT TCAGTGGTGT CATAAATTAA AGACCCTACA GTATACCATA TGCCATGAAA   
  
  
+ TTAATGAAAT CTAACACAAA TCATTATGAT TTTATCACTT ATAAAAAAAT TATATTTTTC AACCGCACCA   
  
  
+ TATAATATAA CATATATATT AGATTAATAT AGCAACCAAA ATGAATCATT TGGAGGGTAC ACGAGTCCTT   
  
  
+ AACATGGACC TGACTTCGTC GAATCATTCC CAATCGTTGT GACTAAAGAA ATATTAGAAT TGCGTAATTG   
  
  
+ CTTACACGTC CCCATATAAA CAAAACAACT GCCAATCTGT CATTACCTGA CGGTCCCATA TTAAACCCTC   
  
  
+ CTTAAGCACC CACTAATCAT ATCCACTTCT CTTTCACGTT TCTCGCCGGC CTCAGTCTGC CTTGCCGGTG   
  
  
+ ATCCTACGCC TCCATGATCA TCGCTACCAT TGTCGTCGTC GCCGTTTCAG TACGAAGCCG ACGGTCTTGA   
  
  
+ TTAACTTACC ACCTGATCCA GCACCATAT  

- TCTTCCTTTC TTTATCCACA TTACTATCAC CCCCCTGTCT CAAACGTTGA TGGTAAGGAA ATAAAAAAGT   
  
  
- ACCACAAAAC CTAACTTACA GTTTAAATTC TTATTTAAAA GAGTTTAAAA GGACTACTAC TACGTATAAC   
  
  
- ATAGACTTCA TTTCTAAAAA AAAAAAATTT TATAGGTTTC ATCAATTGTA ATTCAGTAGA TGCCTAGTCT   
  
  
- AAATTAAGCC AGGTAAGTAT TAAACATTTT AAAATCAAAT AAATTTATTT TAACAACTAT AATAAAATTT   
  
  
- TTATTTTTAA TAAATTGTGA AGTTTGATAT AAAATATATA ATTTAGTTTC AAATCACAAA TTTTCATTAT   
  
  
- TTAGTTTGAA AATTAATAAT TAAACTAAAT TTAATTTAAA ACTAAATTAT AAAAAACAAT AATTTTTATA   
  
  
- AAATTTTTTA TTAAGTTTAT TTAACATATG AAATAAAATT TAGTTAAGAA ATTAAAGTTT ATTAAAAACC   
  
  
- TAAATACTCA ATAAATAATA TTTAAAAATA TTATTAAAAA GATTTTTGTA AAATCAGTTT TTTTGAATAT   
  
  
- GAAAAAATAT TACTATCAAA CTTATAAAAA TTTTTAAAAA TTATACTAAA TTAAACTATA TTTAATTATT   
  
  
- TAGTCTAAAA TTAAACAATG CAAAATTTGG AATTCCAATT TAAACTGTTT AGTATATGAA TTTTCAAAAT   
  
  
- TAATTAGAAA TGTTAATCAA ATTAATCTAA ACCATGATAT TTATTTTTAT GAGTATCAAA ATAAACTTAA   
  
  
- ATACTTTGGA TTTTATTCAA ATATAGAAAA ATAAAAAAGA GTTGTTTATA TATAGGTTTT ATCTTTTTAT   
  
  
- TTAAAGTTTT AAAAGAATTA TATGGTATTG GCAAAATACA GTAATTATTT ATTACAGTGT TATTTTTATT   
  
  
- ATTTTATTTT AGAGTAATGG TGGATGTTGA AAATCGGAAA ATGATATCAT GTTTTAAGAA AGTAGAAATT   
  
  
- ATTATTAATG AAAGAAAGTA AGTCACCACA GTATTTAATT TCTGGGATGT CATATGGTAT ACGGTACTTT   
  
  
- AATTACTTTA GATTGTGTTT AGTAATACTA AAATAGTGAA TATTTTTTTA ATATAAAAAG TTGGCGTGGT   
  
  
- ATATTATATT GTATATATAA TCTAATTATA TCGTTGGTTT TACTTAGTAA ACCTCCCATG TGCTCAGGAA   
  
  
- TTGTACCTGG ACTGAAGCAG CTTAGTAAGG GTTAGCAACA CTGATTTCTT TATAATCTTA ACGCATTAAC   
  
  
- GAATGTGCAG GGGTATATTT GTTTTGTTGA CGGTTAGACA GTAATGGACT GCCAGGGTAT AATTTGGGAG   
  
  
- GAATTCGTGG GTGATTAGTA TAGGTGAAGA GAAAGTGCAA AGAGCGGCCG GAGTCAGACG GAACGGCCAC   
  
  
- TAGGATGCGG AGGTACTAGT AGCGATGGTA ACAGCAGCAG CGGCAAAGTC ATGCTTCGGC TGCCAGAACT   
  
  
- AATTGAATGG TGGACTAGGT CGTGGTATA

+     CGTCA-motif

| Site Name | Organism | Position | Strand | Matrix score. | sequence | function |
| --- | --- | --- | --- | --- | --- | --- |
| CGTCA-motif | Hordeum vulgare | 1308 | - | 5 | CGTCA | cis-acting regulatory element involved in the MeJA-responsiveness |

> 2018/04/13 10:10:12  
+ AGAAGGAAAG AAATAGGTGT AATGATAGTG GGGGGACAGA GTTTGCAACT ACCATTCCTT TATTTTTTCA   
  
  
+ TGGTGTTTTG GATTGAATGT CAAATTTAAG AATAAATTTT CTCAAATTTT CCTGATGATG ATGCATATTG   
  
  
+ TATCTGAAGT AAAGATTTTT TTTTTTTAAA ATATCCAAAG TAGTTAACAT TAAGTCATCT ACGGATCAGA   
  
  
+ TTTAATTCGG TCCATTCATA ATTTGTAAAA TTTTAGTTTA TTTAAATAAA ATTGTTGATA TTATTTTAAA   
  
  
+ AATAAAAATT ATTTAACACT TCAAACTATA TTTTATATAT TAAATCAAAG TTTAGTGTTT AAAAGTAATA   
  
  
+ AATCAAACTT TTAATTATTA ATTTGATTTA AATTAAATTT TGATTTAATA TTTTTTGTTA TTAAAAATAT   
  
  
+ TTTAAAAAAT AATTCAAATA AATTGTATAC TTTATTTTAA ATCAATTCTT TAATTTCAAA TAATTTTTGG   
  
  
+ ATTTATGAGT TATTTATTAT AAATTTTTAT AATAATTTTT CTAAAAACAT TTTAGTCAAA AAAACTTATA   
  
  
+ CTTTTTTATA ATGATAGTTT GAATATTTTT AAAAATTTTT AATATGATTT AATTTGATAT AAATTAATAA   
  
  
+ ATCAGATTTT AATTTGTTAC GTTTTAAACC TTAAGGTTAA ATTTGACAAA TCATATACTT AAAAGTTTTA   
  
  
+ ATTAATCTTT ACAATTAGTT TAATTAGATT TGGTACTATA AATAAAAATA CTCATAGTTT TATTTGAATT   
  
  
+ TATGAAACCT AAAATAAGTT TATATCTTTT TATTTTTTCT CAACAAATAT ATATCCAAAA TAGAAAAATA   
  
  
+ AATTTCAAAA TTTTCTTAAT ATACCATAAC CGTTTTATGT CATTAATAAA TAATGTCACA ATAAAAATAA   
  
  
+ TAAAATAAAA TCTCATTACC ACCTACAACT TTTAGCCTTT TACTATAGTA CAAAATTCTT TCATCTTTAA   
  
  
+ TAATAATTAC TTTCTTTCAT TCAGTGGTGT CATAAATTAA AGACCCTACA GTATACCATA TGCCATGAAA   
  
  
+ TTAATGAAAT CTAACACAAA TCATTATGAT TTTATCACTT ATAAAAAAAT TATATTTTTC AACCGCACCA   
  
  
+ TATAATATAA CATATATATT AGATTAATAT AGCAACCAAA ATGAATCATT TGGAGGGTAC ACGAGTCCTT   
  
  
+ AACATGGACC TGACTTCGTC GAATCATTCC CAATCGTTGT GACTAAAGAA ATATTAGAAT TGCGTAATTG   
  
  
+ CTTACACGTC CCCATATAAA CAAAACAACT GCCAATCTGT CATTACCTGA CGGTCCCATA TTAAACCCTC   
  
  
+ CTTAAGCACC CACTAATCAT ATCCACTTCT CTTTCACGTT TCTCGCCGGC CTCAGTCTGC CTTGCCGGTG   
  
  
+ ATCCTACGCC TCCATGATCA TCGCTACCAT TGTCGTCGTC GCCGTTTCAG TACGAAGCCG ACGGTCTTGA   
  
  
+ TTAACTTACC ACCTGATCCA GCACCATAT  

- TCTTCCTTTC TTTATCCACA TTACTATCAC CCCCCTGTCT CAAACGTTGA TGGTAAGGAA ATAAAAAAGT   
  
  
- ACCACAAAAC CTAACTTACA GTTTAAATTC TTATTTAAAA GAGTTTAAAA GGACTACTAC TACGTATAAC   
  
  
- ATAGACTTCA TTTCTAAAAA AAAAAAATTT TATAGGTTTC ATCAATTGTA ATTCAGTAGA TGCCTAGTCT   
  
  
- AAATTAAGCC AGGTAAGTAT TAAACATTTT AAAATCAAAT AAATTTATTT TAACAACTAT AATAAAATTT   
  
  
- TTATTTTTAA TAAATTGTGA AGTTTGATAT AAAATATATA ATTTAGTTTC AAATCACAAA TTTTCATTAT   
  
  
- TTAGTTTGAA AATTAATAAT TAAACTAAAT TTAATTTAAA ACTAAATTAT AAAAAACAAT AATTTTTATA   
  
  
- AAATTTTTTA TTAAGTTTAT TTAACATATG AAATAAAATT TAGTTAAGAA ATTAAAGTTT ATTAAAAACC   
  
  
- TAAATACTCA ATAAATAATA TTTAAAAATA TTATTAAAAA GATTTTTGTA AAATCAGTTT TTTTGAATAT   
  
  
- GAAAAAATAT TACTATCAAA CTTATAAAAA TTTTTAAAAA TTATACTAAA TTAAACTATA TTTAATTATT   
  
  
- TAGTCTAAAA TTAAACAATG CAAAATTTGG AATTCCAATT TAAACTGTTT AGTATATGAA TTTTCAAAAT   
  
  
- TAATTAGAAA TGTTAATCAA ATTAATCTAA ACCATGATAT TTATTTTTAT GAGTATCAAA ATAAACTTAA   
  
  
- ATACTTTGGA TTTTATTCAA ATATAGAAAA ATAAAAAAGA GTTGTTTATA TATAGGTTTT ATCTTTTTAT   
  
  
- TTAAAGTTTT AAAAGAATTA TATGGTATTG GCAAAATACA GTAATTATTT ATTACAGTGT TATTTTTATT   
  
  
- ATTTTATTTT AGAGTAATGG TGGATGTTGA AAATCGGAAA ATGATATCAT GTTTTAAGAA AGTAGAAATT   
  
  
- ATTATTAATG AAAGAAAGTA AGTCACCACA GTATTTAATT TCTGGGATGT CATATGGTAT ACGGTACTTT   
  
  
- AATTACTTTA GATTGTGTTT AGTAATACTA AAATAGTGAA TATTTTTTTA ATATAAAAAG TTGGCGTGGT   
  
  
- ATATTATATT GTATATATAA TCTAATTATA TCGTTGGTTT TACTTAGTAA ACCTCCCATG TGCTCAGGAA   
  
  
- TTGTACCTGG ACTGAAGCAG CTTAGTAAGG GTTAGCAACA CTGATTTCTT TATAATCTTA ACGCATTAAC   
  
  
- GAATGTGCAG GGGTATATTT GTTTTGTTGA CGGTTAGACA GTAATGGACT GCCAGGGTAT AATTTGGGAG   
  
  
- GAATTCGTGG GTGATTAGTA TAGGTGAAGA GAAAGTGCAA AGAGCGGCCG GAGTCAGACG GAACGGCCAC   
  
  
- TAGGATGCGG AGGTACTAGT AGCGATGGTA ACAGCAGCAG CGGCAAAGTC ATGCTTCGGC TGCCAGAACT   
  
  
- AATTGAATGG TGGACTAGGT CGTGGTATA

+     ERE

| Site Name | Organism | Position | Strand | Matrix score. | sequence | function |
| --- | --- | --- | --- | --- | --- | --- |
| ERE | Dianthus caryophyllus | 842 | + | 8 | ATTTCAAA | ethylene-responsive element |
| ERE | Dianthus caryophyllus | 473 | + | 8 | ATTTCAAA | ethylene-responsive element |

> 2018/04/13 10:10:12  
+ AGAAGGAAAG AAATAGGTGT AATGATAGTG GGGGGACAGA GTTTGCAACT ACCATTCCTT TATTTTTTCA   
  
  
+ TGGTGTTTTG GATTGAATGT CAAATTTAAG AATAAATTTT CTCAAATTTT CCTGATGATG ATGCATATTG   
  
  
+ TATCTGAAGT AAAGATTTTT TTTTTTTAAA ATATCCAAAG TAGTTAACAT TAAGTCATCT ACGGATCAGA   
  
  
+ TTTAATTCGG TCCATTCATA ATTTGTAAAA TTTTAGTTTA TTTAAATAAA ATTGTTGATA TTATTTTAAA   
  
  
+ AATAAAAATT ATTTAACACT TCAAACTATA TTTTATATAT TAAATCAAAG TTTAGTGTTT AAAAGTAATA   
  
  
+ AATCAAACTT TTAATTATTA ATTTGATTTA AATTAAATTT TGATTTAATA TTTTTTGTTA TTAAAAATAT   
  
  
+ TTTAAAAAAT AATTCAAATA AATTGTATAC TTTATTTTAA ATCAATTCTT TAATTTCAAA TAATTTTTGG   
  
  
+ ATTTATGAGT TATTTATTAT AAATTTTTAT AATAATTTTT CTAAAAACAT TTTAGTCAAA AAAACTTATA   
  
  
+ CTTTTTTATA ATGATAGTTT GAATATTTTT AAAAATTTTT AATATGATTT AATTTGATAT AAATTAATAA   
  
  
+ ATCAGATTTT AATTTGTTAC GTTTTAAACC TTAAGGTTAA ATTTGACAAA TCATATACTT AAAAGTTTTA   
  
  
+ ATTAATCTTT ACAATTAGTT TAATTAGATT TGGTACTATA AATAAAAATA CTCATAGTTT TATTTGAATT   
  
  
+ TATGAAACCT AAAATAAGTT TATATCTTTT TATTTTTTCT CAACAAATAT ATATCCAAAA TAGAAAAATA   
  
  
+ AATTTCAAAA TTTTCTTAAT ATACCATAAC CGTTTTATGT CATTAATAAA TAATGTCACA ATAAAAATAA   
  
  
+ TAAAATAAAA TCTCATTACC ACCTACAACT TTTAGCCTTT TACTATAGTA CAAAATTCTT TCATCTTTAA   
  
  
+ TAATAATTAC TTTCTTTCAT TCAGTGGTGT CATAAATTAA AGACCCTACA GTATACCATA TGCCATGAAA   
  
  
+ TTAATGAAAT CTAACACAAA TCATTATGAT TTTATCACTT ATAAAAAAAT TATATTTTTC AACCGCACCA   
  
  
+ TATAATATAA CATATATATT AGATTAATAT AGCAACCAAA ATGAATCATT TGGAGGGTAC ACGAGTCCTT   
  
  
+ AACATGGACC TGACTTCGTC GAATCATTCC CAATCGTTGT GACTAAAGAA ATATTAGAAT TGCGTAATTG   
  
  
+ CTTACACGTC CCCATATAAA CAAAACAACT GCCAATCTGT CATTACCTGA CGGTCCCATA TTAAACCCTC   
  
  
+ CTTAAGCACC CACTAATCAT ATCCACTTCT CTTTCACGTT TCTCGCCGGC CTCAGTCTGC CTTGCCGGTG   
  
  
+ ATCCTACGCC TCCATGATCA TCGCTACCAT TGTCGTCGTC GCCGTTTCAG TACGAAGCCG ACGGTCTTGA   
  
  
+ TTAACTTACC ACCTGATCCA GCACCATAT  

- TCTTCCTTTC TTTATCCACA TTACTATCAC CCCCCTGTCT CAAACGTTGA TGGTAAGGAA ATAAAAAAGT   
  
  
- ACCACAAAAC CTAACTTACA GTTTAAATTC TTATTTAAAA GAGTTTAAAA GGACTACTAC TACGTATAAC   
  
  
- ATAGACTTCA TTTCTAAAAA AAAAAAATTT TATAGGTTTC ATCAATTGTA ATTCAGTAGA TGCCTAGTCT   
  
  
- AAATTAAGCC AGGTAAGTAT TAAACATTTT AAAATCAAAT AAATTTATTT TAACAACTAT AATAAAATTT   
  
  
- TTATTTTTAA TAAATTGTGA AGTTTGATAT AAAATATATA ATTTAGTTTC AAATCACAAA TTTTCATTAT   
  
  
- TTAGTTTGAA AATTAATAAT TAAACTAAAT TTAATTTAAA ACTAAATTAT AAAAAACAAT AATTTTTATA   
  
  
- AAATTTTTTA TTAAGTTTAT TTAACATATG AAATAAAATT TAGTTAAGAA ATTAAAGTTT ATTAAAAACC   
  
  
- TAAATACTCA ATAAATAATA TTTAAAAATA TTATTAAAAA GATTTTTGTA AAATCAGTTT TTTTGAATAT   
  
  
- GAAAAAATAT TACTATCAAA CTTATAAAAA TTTTTAAAAA TTATACTAAA TTAAACTATA TTTAATTATT   
  
  
- TAGTCTAAAA TTAAACAATG CAAAATTTGG AATTCCAATT TAAACTGTTT AGTATATGAA TTTTCAAAAT   
  
  
- TAATTAGAAA TGTTAATCAA ATTAATCTAA ACCATGATAT TTATTTTTAT GAGTATCAAA ATAAACTTAA   
  
  
- ATACTTTGGA TTTTATTCAA ATATAGAAAA ATAAAAAAGA GTTGTTTATA TATAGGTTTT ATCTTTTTAT   
  
  
- TTAAAGTTTT AAAAGAATTA TATGGTATTG GCAAAATACA GTAATTATTT ATTACAGTGT TATTTTTATT   
  
  
- ATTTTATTTT AGAGTAATGG TGGATGTTGA AAATCGGAAA ATGATATCAT GTTTTAAGAA AGTAGAAATT   
  
  
- ATTATTAATG AAAGAAAGTA AGTCACCACA GTATTTAATT TCTGGGATGT CATATGGTAT ACGGTACTTT   
  
  
- AATTACTTTA GATTGTGTTT AGTAATACTA AAATAGTGAA TATTTTTTTA ATATAAAAAG TTGGCGTGGT   
  
  
- ATATTATATT GTATATATAA TCTAATTATA TCGTTGGTTT TACTTAGTAA ACCTCCCATG TGCTCAGGAA   
  
  
- TTGTACCTGG ACTGAAGCAG CTTAGTAAGG GTTAGCAACA CTGATTTCTT TATAATCTTA ACGCATTAAC   
  
  
- GAATGTGCAG GGGTATATTT GTTTTGTTGA CGGTTAGACA GTAATGGACT GCCAGGGTAT AATTTGGGAG   
  
  
- GAATTCGTGG GTGATTAGTA TAGGTGAAGA GAAAGTGCAA AGAGCGGCCG GAGTCAGACG GAACGGCCAC   
  
  
- TAGGATGCGG AGGTACTAGT AGCGATGGTA ACAGCAGCAG CGGCAAAGTC ATGCTTCGGC TGCCAGAACT   
  
  
- AATTGAATGG TGGACTAGGT CGTGGTATA

+     G-Box

| Site Name | Organism | Position | Strand | Matrix score. | sequence | function |
| --- | --- | --- | --- | --- | --- | --- |
| G-Box | Pisum sativum | 1365 | + | 6 | CACGTT | cis-acting regulatory element involved in light responsiveness |

> 2018/04/13 10:10:12  
+ AGAAGGAAAG AAATAGGTGT AATGATAGTG GGGGGACAGA GTTTGCAACT ACCATTCCTT TATTTTTTCA   
  
  
+ TGGTGTTTTG GATTGAATGT CAAATTTAAG AATAAATTTT CTCAAATTTT CCTGATGATG ATGCATATTG   
  
  
+ TATCTGAAGT AAAGATTTTT TTTTTTTAAA ATATCCAAAG TAGTTAACAT TAAGTCATCT ACGGATCAGA   
  
  
+ TTTAATTCGG TCCATTCATA ATTTGTAAAA TTTTAGTTTA TTTAAATAAA ATTGTTGATA TTATTTTAAA   
  
  
+ AATAAAAATT ATTTAACACT TCAAACTATA TTTTATATAT TAAATCAAAG TTTAGTGTTT AAAAGTAATA   
  
  
+ AATCAAACTT TTAATTATTA ATTTGATTTA AATTAAATTT TGATTTAATA TTTTTTGTTA TTAAAAATAT   
  
  
+ TTTAAAAAAT AATTCAAATA AATTGTATAC TTTATTTTAA ATCAATTCTT TAATTTCAAA TAATTTTTGG   
  
  
+ ATTTATGAGT TATTTATTAT AAATTTTTAT AATAATTTTT CTAAAAACAT TTTAGTCAAA AAAACTTATA   
  
  
+ CTTTTTTATA ATGATAGTTT GAATATTTTT AAAAATTTTT AATATGATTT AATTTGATAT AAATTAATAA   
  
  
+ ATCAGATTTT AATTTGTTAC GTTTTAAACC TTAAGGTTAA ATTTGACAAA TCATATACTT AAAAGTTTTA   
  
  
+ ATTAATCTTT ACAATTAGTT TAATTAGATT TGGTACTATA AATAAAAATA CTCATAGTTT TATTTGAATT   
  
  
+ TATGAAACCT AAAATAAGTT TATATCTTTT TATTTTTTCT CAACAAATAT ATATCCAAAA TAGAAAAATA   
  
  
+ AATTTCAAAA TTTTCTTAAT ATACCATAAC CGTTTTATGT CATTAATAAA TAATGTCACA ATAAAAATAA   
  
  
+ TAAAATAAAA TCTCATTACC ACCTACAACT TTTAGCCTTT TACTATAGTA CAAAATTCTT TCATCTTTAA   
  
  
+ TAATAATTAC TTTCTTTCAT TCAGTGGTGT CATAAATTAA AGACCCTACA GTATACCATA TGCCATGAAA   
  
  
+ TTAATGAAAT CTAACACAAA TCATTATGAT TTTATCACTT ATAAAAAAAT TATATTTTTC AACCGCACCA   
  
  
+ TATAATATAA CATATATATT AGATTAATAT AGCAACCAAA ATGAATCATT TGGAGGGTAC ACGAGTCCTT   
  
  
+ AACATGGACC TGACTTCGTC GAATCATTCC CAATCGTTGT GACTAAAGAA ATATTAGAAT TGCGTAATTG   
  
  
+ CTTACACGTC CCCATATAAA CAAAACAACT GCCAATCTGT CATTACCTGA CGGTCCCATA TTAAACCCTC   
  
  
+ CTTAAGCACC CACTAATCAT ATCCACTTCT CTTTCACGTT TCTCGCCGGC CTCAGTCTGC CTTGCCGGTG   
  
  
+ ATCCTACGCC TCCATGATCA TCGCTACCAT TGTCGTCGTC GCCGTTTCAG TACGAAGCCG ACGGTCTTGA   
  
  
+ TTAACTTACC ACCTGATCCA GCACCATAT  

- TCTTCCTTTC TTTATCCACA TTACTATCAC CCCCCTGTCT CAAACGTTGA TGGTAAGGAA ATAAAAAAGT   
  
  
- ACCACAAAAC CTAACTTACA GTTTAAATTC TTATTTAAAA GAGTTTAAAA GGACTACTAC TACGTATAAC   
  
  
- ATAGACTTCA TTTCTAAAAA AAAAAAATTT TATAGGTTTC ATCAATTGTA ATTCAGTAGA TGCCTAGTCT   
  
  
- AAATTAAGCC AGGTAAGTAT TAAACATTTT AAAATCAAAT AAATTTATTT TAACAACTAT AATAAAATTT   
  
  
- TTATTTTTAA TAAATTGTGA AGTTTGATAT AAAATATATA ATTTAGTTTC AAATCACAAA TTTTCATTAT   
  
  
- TTAGTTTGAA AATTAATAAT TAAACTAAAT TTAATTTAAA ACTAAATTAT AAAAAACAAT AATTTTTATA   
  
  
- AAATTTTTTA TTAAGTTTAT TTAACATATG AAATAAAATT TAGTTAAGAA ATTAAAGTTT ATTAAAAACC   
  
  
- TAAATACTCA ATAAATAATA TTTAAAAATA TTATTAAAAA GATTTTTGTA AAATCAGTTT TTTTGAATAT   
  
  
- GAAAAAATAT TACTATCAAA CTTATAAAAA TTTTTAAAAA TTATACTAAA TTAAACTATA TTTAATTATT   
  
  
- TAGTCTAAAA TTAAACAATG CAAAATTTGG AATTCCAATT TAAACTGTTT AGTATATGAA TTTTCAAAAT   
  
  
- TAATTAGAAA TGTTAATCAA ATTAATCTAA ACCATGATAT TTATTTTTAT GAGTATCAAA ATAAACTTAA   
  
  
- ATACTTTGGA TTTTATTCAA ATATAGAAAA ATAAAAAAGA GTTGTTTATA TATAGGTTTT ATCTTTTTAT   
  
  
- TTAAAGTTTT AAAAGAATTA TATGGTATTG GCAAAATACA GTAATTATTT ATTACAGTGT TATTTTTATT   
  
  
- ATTTTATTTT AGAGTAATGG TGGATGTTGA AAATCGGAAA ATGATATCAT GTTTTAAGAA AGTAGAAATT   
  
  
- ATTATTAATG AAAGAAAGTA AGTCACCACA GTATTTAATT TCTGGGATGT CATATGGTAT ACGGTACTTT   
  
  
- AATTACTTTA GATTGTGTTT AGTAATACTA AAATAGTGAA TATTTTTTTA ATATAAAAAG TTGGCGTGGT   
  
  
- ATATTATATT GTATATATAA TCTAATTATA TCGTTGGTTT TACTTAGTAA ACCTCCCATG TGCTCAGGAA   
  
  
- TTGTACCTGG ACTGAAGCAG CTTAGTAAGG GTTAGCAACA CTGATTTCTT TATAATCTTA ACGCATTAAC   
  
  
- GAATGTGCAG GGGTATATTT GTTTTGTTGA CGGTTAGACA GTAATGGACT GCCAGGGTAT AATTTGGGAG   
  
  
- GAATTCGTGG GTGATTAGTA TAGGTGAAGA GAAAGTGCAA AGAGCGGCCG GAGTCAGACG GAACGGCCAC   
  
  
- TAGGATGCGG AGGTACTAGT AGCGATGGTA ACAGCAGCAG CGGCAAAGTC ATGCTTCGGC TGCCAGAACT   
  
  
- AATTGAATGG TGGACTAGGT CGTGGTATA

+     G-box

| Site Name | Organism | Position | Strand | Matrix score. | sequence | function |
| --- | --- | --- | --- | --- | --- | --- |
| G-box | Zea mays | 1265 | + | 6 | CACGTC | cis-acting regulatory element involved in light responsiveness |
| G-box | Zea mays | 1365 | + | 6 | CACGTT | cis-acting regulatory element involved in light responsiveness |

> 2018/04/13 10:10:12  
+ AGAAGGAAAG AAATAGGTGT AATGATAGTG GGGGGACAGA GTTTGCAACT ACCATTCCTT TATTTTTTCA   
  
  
+ TGGTGTTTTG GATTGAATGT CAAATTTAAG AATAAATTTT CTCAAATTTT CCTGATGATG ATGCATATTG   
  
  
+ TATCTGAAGT AAAGATTTTT TTTTTTTAAA ATATCCAAAG TAGTTAACAT TAAGTCATCT ACGGATCAGA   
  
  
+ TTTAATTCGG TCCATTCATA ATTTGTAAAA TTTTAGTTTA TTTAAATAAA ATTGTTGATA TTATTTTAAA   
  
  
+ AATAAAAATT ATTTAACACT TCAAACTATA TTTTATATAT TAAATCAAAG TTTAGTGTTT AAAAGTAATA   
  
  
+ AATCAAACTT TTAATTATTA ATTTGATTTA AATTAAATTT TGATTTAATA TTTTTTGTTA TTAAAAATAT   
  
  
+ TTTAAAAAAT AATTCAAATA AATTGTATAC TTTATTTTAA ATCAATTCTT TAATTTCAAA TAATTTTTGG   
  
  
+ ATTTATGAGT TATTTATTAT AAATTTTTAT AATAATTTTT CTAAAAACAT TTTAGTCAAA AAAACTTATA   
  
  
+ CTTTTTTATA ATGATAGTTT GAATATTTTT AAAAATTTTT AATATGATTT AATTTGATAT AAATTAATAA   
  
  
+ ATCAGATTTT AATTTGTTAC GTTTTAAACC TTAAGGTTAA ATTTGACAAA TCATATACTT AAAAGTTTTA   
  
  
+ ATTAATCTTT ACAATTAGTT TAATTAGATT TGGTACTATA AATAAAAATA CTCATAGTTT TATTTGAATT   
  
  
+ TATGAAACCT AAAATAAGTT TATATCTTTT TATTTTTTCT CAACAAATAT ATATCCAAAA TAGAAAAATA   
  
  
+ AATTTCAAAA TTTTCTTAAT ATACCATAAC CGTTTTATGT CATTAATAAA TAATGTCACA ATAAAAATAA   
  
  
+ TAAAATAAAA TCTCATTACC ACCTACAACT TTTAGCCTTT TACTATAGTA CAAAATTCTT TCATCTTTAA   
  
  
+ TAATAATTAC TTTCTTTCAT TCAGTGGTGT CATAAATTAA AGACCCTACA GTATACCATA TGCCATGAAA   
  
  
+ TTAATGAAAT CTAACACAAA TCATTATGAT TTTATCACTT ATAAAAAAAT TATATTTTTC AACCGCACCA   
  
  
+ TATAATATAA CATATATATT AGATTAATAT AGCAACCAAA ATGAATCATT TGGAGGGTAC ACGAGTCCTT   
  
  
+ AACATGGACC TGACTTCGTC GAATCATTCC CAATCGTTGT GACTAAAGAA ATATTAGAAT TGCGTAATTG   
  
  
+ CTTACACGTC CCCATATAAA CAAAACAACT GCCAATCTGT CATTACCTGA CGGTCCCATA TTAAACCCTC   
  
  
+ CTTAAGCACC CACTAATCAT ATCCACTTCT CTTTCACGTT TCTCGCCGGC CTCAGTCTGC CTTGCCGGTG   
  
  
+ ATCCTACGCC TCCATGATCA TCGCTACCAT TGTCGTCGTC GCCGTTTCAG TACGAAGCCG ACGGTCTTGA   
  
  
+ TTAACTTACC ACCTGATCCA GCACCATAT  

- TCTTCCTTTC TTTATCCACA TTACTATCAC CCCCCTGTCT CAAACGTTGA TGGTAAGGAA ATAAAAAAGT   
  
  
- ACCACAAAAC CTAACTTACA GTTTAAATTC TTATTTAAAA GAGTTTAAAA GGACTACTAC TACGTATAAC   
  
  
- ATAGACTTCA TTTCTAAAAA AAAAAAATTT TATAGGTTTC ATCAATTGTA ATTCAGTAGA TGCCTAGTCT   
  
  
- AAATTAAGCC AGGTAAGTAT TAAACATTTT AAAATCAAAT AAATTTATTT TAACAACTAT AATAAAATTT   
  
  
- TTATTTTTAA TAAATTGTGA AGTTTGATAT AAAATATATA ATTTAGTTTC AAATCACAAA TTTTCATTAT   
  
  
- TTAGTTTGAA AATTAATAAT TAAACTAAAT TTAATTTAAA ACTAAATTAT AAAAAACAAT AATTTTTATA   
  
  
- AAATTTTTTA TTAAGTTTAT TTAACATATG AAATAAAATT TAGTTAAGAA ATTAAAGTTT ATTAAAAACC   
  
  
- TAAATACTCA ATAAATAATA TTTAAAAATA TTATTAAAAA GATTTTTGTA AAATCAGTTT TTTTGAATAT   
  
  
- GAAAAAATAT TACTATCAAA CTTATAAAAA TTTTTAAAAA TTATACTAAA TTAAACTATA TTTAATTATT   
  
  
- TAGTCTAAAA TTAAACAATG CAAAATTTGG AATTCCAATT TAAACTGTTT AGTATATGAA TTTTCAAAAT   
  
  
- TAATTAGAAA TGTTAATCAA ATTAATCTAA ACCATGATAT TTATTTTTAT GAGTATCAAA ATAAACTTAA   
  
  
- ATACTTTGGA TTTTATTCAA ATATAGAAAA ATAAAAAAGA GTTGTTTATA TATAGGTTTT ATCTTTTTAT   
  
  
- TTAAAGTTTT AAAAGAATTA TATGGTATTG GCAAAATACA GTAATTATTT ATTACAGTGT TATTTTTATT   
  
  
- ATTTTATTTT AGAGTAATGG TGGATGTTGA AAATCGGAAA ATGATATCAT GTTTTAAGAA AGTAGAAATT   
  
  
- ATTATTAATG AAAGAAAGTA AGTCACCACA GTATTTAATT TCTGGGATGT CATATGGTAT ACGGTACTTT   
  
  
- AATTACTTTA GATTGTGTTT AGTAATACTA AAATAGTGAA TATTTTTTTA ATATAAAAAG TTGGCGTGGT   
  
  
- ATATTATATT GTATATATAA TCTAATTATA TCGTTGGTTT TACTTAGTAA ACCTCCCATG TGCTCAGGAA   
  
  
- TTGTACCTGG ACTGAAGCAG CTTAGTAAGG GTTAGCAACA CTGATTTCTT TATAATCTTA ACGCATTAAC   
  
  
- GAATGTGCAG GGGTATATTT GTTTTGTTGA CGGTTAGACA GTAATGGACT GCCAGGGTAT AATTTGGGAG   
  
  
- GAATTCGTGG GTGATTAGTA TAGGTGAAGA GAAAGTGCAA AGAGCGGCCG GAGTCAGACG GAACGGCCAC   
  
  
- TAGGATGCGG AGGTACTAGT AGCGATGGTA ACAGCAGCAG CGGCAAAGTC ATGCTTCGGC TGCCAGAACT   
  
  
- AATTGAATGG TGGACTAGGT CGTGGTATA

+     GA-motif

| Site Name | Organism | Position | Strand | Matrix score. | sequence | function |
| --- | --- | --- | --- | --- | --- | --- |
| GA-motif | Helianthus annuus | 971 | - | 8 | AAAGATGA | part of a light responsive element |

> 2018/04/13 10:10:12  
+ AGAAGGAAAG AAATAGGTGT AATGATAGTG GGGGGACAGA GTTTGCAACT ACCATTCCTT TATTTTTTCA   
  
  
+ TGGTGTTTTG GATTGAATGT CAAATTTAAG AATAAATTTT CTCAAATTTT CCTGATGATG ATGCATATTG   
  
  
+ TATCTGAAGT AAAGATTTTT TTTTTTTAAA ATATCCAAAG TAGTTAACAT TAAGTCATCT ACGGATCAGA   
  
  
+ TTTAATTCGG TCCATTCATA ATTTGTAAAA TTTTAGTTTA TTTAAATAAA ATTGTTGATA TTATTTTAAA   
  
  
+ AATAAAAATT ATTTAACACT TCAAACTATA TTTTATATAT TAAATCAAAG TTTAGTGTTT AAAAGTAATA   
  
  
+ AATCAAACTT TTAATTATTA ATTTGATTTA AATTAAATTT TGATTTAATA TTTTTTGTTA TTAAAAATAT   
  
  
+ TTTAAAAAAT AATTCAAATA AATTGTATAC TTTATTTTAA ATCAATTCTT TAATTTCAAA TAATTTTTGG   
  
  
+ ATTTATGAGT TATTTATTAT AAATTTTTAT AATAATTTTT CTAAAAACAT TTTAGTCAAA AAAACTTATA   
  
  
+ CTTTTTTATA ATGATAGTTT GAATATTTTT AAAAATTTTT AATATGATTT AATTTGATAT AAATTAATAA   
  
  
+ ATCAGATTTT AATTTGTTAC GTTTTAAACC TTAAGGTTAA ATTTGACAAA TCATATACTT AAAAGTTTTA   
  
  
+ ATTAATCTTT ACAATTAGTT TAATTAGATT TGGTACTATA AATAAAAATA CTCATAGTTT TATTTGAATT   
  
  
+ TATGAAACCT AAAATAAGTT TATATCTTTT TATTTTTTCT CAACAAATAT ATATCCAAAA TAGAAAAATA   
  
  
+ AATTTCAAAA TTTTCTTAAT ATACCATAAC CGTTTTATGT CATTAATAAA TAATGTCACA ATAAAAATAA   
  
  
+ TAAAATAAAA TCTCATTACC ACCTACAACT TTTAGCCTTT TACTATAGTA CAAAATTCTT TCATCTTTAA   
  
  
+ TAATAATTAC TTTCTTTCAT TCAGTGGTGT CATAAATTAA AGACCCTACA GTATACCATA TGCCATGAAA   
  
  
+ TTAATGAAAT CTAACACAAA TCATTATGAT TTTATCACTT ATAAAAAAAT TATATTTTTC AACCGCACCA   
  
  
+ TATAATATAA CATATATATT AGATTAATAT AGCAACCAAA ATGAATCATT TGGAGGGTAC ACGAGTCCTT   
  
  
+ AACATGGACC TGACTTCGTC GAATCATTCC CAATCGTTGT GACTAAAGAA ATATTAGAAT TGCGTAATTG   
  
  
+ CTTACACGTC CCCATATAAA CAAAACAACT GCCAATCTGT CATTACCTGA CGGTCCCATA TTAAACCCTC   
  
  
+ CTTAAGCACC CACTAATCAT ATCCACTTCT CTTTCACGTT TCTCGCCGGC CTCAGTCTGC CTTGCCGGTG   
  
  
+ ATCCTACGCC TCCATGATCA TCGCTACCAT TGTCGTCGTC GCCGTTTCAG TACGAAGCCG ACGGTCTTGA   
  
  
+ TTAACTTACC ACCTGATCCA GCACCATAT  

- TCTTCCTTTC TTTATCCACA TTACTATCAC CCCCCTGTCT CAAACGTTGA TGGTAAGGAA ATAAAAAAGT   
  
  
- ACCACAAAAC CTAACTTACA GTTTAAATTC TTATTTAAAA GAGTTTAAAA GGACTACTAC TACGTATAAC   
  
  
- ATAGACTTCA TTTCTAAAAA AAAAAAATTT TATAGGTTTC ATCAATTGTA ATTCAGTAGA TGCCTAGTCT   
  
  
- AAATTAAGCC AGGTAAGTAT TAAACATTTT AAAATCAAAT AAATTTATTT TAACAACTAT AATAAAATTT   
  
  
- TTATTTTTAA TAAATTGTGA AGTTTGATAT AAAATATATA ATTTAGTTTC AAATCACAAA TTTTCATTAT   
  
  
- TTAGTTTGAA AATTAATAAT TAAACTAAAT TTAATTTAAA ACTAAATTAT AAAAAACAAT AATTTTTATA   
  
  
- AAATTTTTTA TTAAGTTTAT TTAACATATG AAATAAAATT TAGTTAAGAA ATTAAAGTTT ATTAAAAACC   
  
  
- TAAATACTCA ATAAATAATA TTTAAAAATA TTATTAAAAA GATTTTTGTA AAATCAGTTT TTTTGAATAT   
  
  
- GAAAAAATAT TACTATCAAA CTTATAAAAA TTTTTAAAAA TTATACTAAA TTAAACTATA TTTAATTATT   
  
  
- TAGTCTAAAA TTAAACAATG CAAAATTTGG AATTCCAATT TAAACTGTTT AGTATATGAA TTTTCAAAAT   
  
  
- TAATTAGAAA TGTTAATCAA ATTAATCTAA ACCATGATAT TTATTTTTAT GAGTATCAAA ATAAACTTAA   
  
  
- ATACTTTGGA TTTTATTCAA ATATAGAAAA ATAAAAAAGA GTTGTTTATA TATAGGTTTT ATCTTTTTAT   
  
  
- TTAAAGTTTT AAAAGAATTA TATGGTATTG GCAAAATACA GTAATTATTT ATTACAGTGT TATTTTTATT   
  
  
- ATTTTATTTT AGAGTAATGG TGGATGTTGA AAATCGGAAA ATGATATCAT GTTTTAAGAA AGTAGAAATT   
  
  
- ATTATTAATG AAAGAAAGTA AGTCACCACA GTATTTAATT TCTGGGATGT CATATGGTAT ACGGTACTTT   
  
  
- AATTACTTTA GATTGTGTTT AGTAATACTA AAATAGTGAA TATTTTTTTA ATATAAAAAG TTGGCGTGGT   
  
  
- ATATTATATT GTATATATAA TCTAATTATA TCGTTGGTTT TACTTAGTAA ACCTCCCATG TGCTCAGGAA   
  
  
- TTGTACCTGG ACTGAAGCAG CTTAGTAAGG GTTAGCAACA CTGATTTCTT TATAATCTTA ACGCATTAAC   
  
  
- GAATGTGCAG GGGTATATTT GTTTTGTTGA CGGTTAGACA GTAATGGACT GCCAGGGTAT AATTTGGGAG   
  
  
- GAATTCGTGG GTGATTAGTA TAGGTGAAGA GAAAGTGCAA AGAGCGGCCG GAGTCAGACG GAACGGCCAC   
  
  
- TAGGATGCGG AGGTACTAGT AGCGATGGTA ACAGCAGCAG CGGCAAAGTC ATGCTTCGGC TGCCAGAACT   
  
  
- AATTGAATGG TGGACTAGGT CGTGGTATA

+     GT1-motif

| Site Name | Organism | Position | Strand | Matrix score. | sequence | function |
| --- | --- | --- | --- | --- | --- | --- |
| GT1-motif | Arabidopsis thaliana | 665 | + | 6 | GGTTAA | light responsive element |

> 2018/04/13 10:10:12  
+ AGAAGGAAAG AAATAGGTGT AATGATAGTG GGGGGACAGA GTTTGCAACT ACCATTCCTT TATTTTTTCA   
  
  
+ TGGTGTTTTG GATTGAATGT CAAATTTAAG AATAAATTTT CTCAAATTTT CCTGATGATG ATGCATATTG   
  
  
+ TATCTGAAGT AAAGATTTTT TTTTTTTAAA ATATCCAAAG TAGTTAACAT TAAGTCATCT ACGGATCAGA   
  
  
+ TTTAATTCGG TCCATTCATA ATTTGTAAAA TTTTAGTTTA TTTAAATAAA ATTGTTGATA TTATTTTAAA   
  
  
+ AATAAAAATT ATTTAACACT TCAAACTATA TTTTATATAT TAAATCAAAG TTTAGTGTTT AAAAGTAATA   
  
  
+ AATCAAACTT TTAATTATTA ATTTGATTTA AATTAAATTT TGATTTAATA TTTTTTGTTA TTAAAAATAT   
  
  
+ TTTAAAAAAT AATTCAAATA AATTGTATAC TTTATTTTAA ATCAATTCTT TAATTTCAAA TAATTTTTGG   
  
  
+ ATTTATGAGT TATTTATTAT AAATTTTTAT AATAATTTTT CTAAAAACAT TTTAGTCAAA AAAACTTATA   
  
  
+ CTTTTTTATA ATGATAGTTT GAATATTTTT AAAAATTTTT AATATGATTT AATTTGATAT AAATTAATAA   
  
  
+ ATCAGATTTT AATTTGTTAC GTTTTAAACC TTAAGGTTAA ATTTGACAAA TCATATACTT AAAAGTTTTA   
  
  
+ ATTAATCTTT ACAATTAGTT TAATTAGATT TGGTACTATA AATAAAAATA CTCATAGTTT TATTTGAATT   
  
  
+ TATGAAACCT AAAATAAGTT TATATCTTTT TATTTTTTCT CAACAAATAT ATATCCAAAA TAGAAAAATA   
  
  
+ AATTTCAAAA TTTTCTTAAT ATACCATAAC CGTTTTATGT CATTAATAAA TAATGTCACA ATAAAAATAA   
  
  
+ TAAAATAAAA TCTCATTACC ACCTACAACT TTTAGCCTTT TACTATAGTA CAAAATTCTT TCATCTTTAA   
  
  
+ TAATAATTAC TTTCTTTCAT TCAGTGGTGT CATAAATTAA AGACCCTACA GTATACCATA TGCCATGAAA   
  
  
+ TTAATGAAAT CTAACACAAA TCATTATGAT TTTATCACTT ATAAAAAAAT TATATTTTTC AACCGCACCA   
  
  
+ TATAATATAA CATATATATT AGATTAATAT AGCAACCAAA ATGAATCATT TGGAGGGTAC ACGAGTCCTT   
  
  
+ AACATGGACC TGACTTCGTC GAATCATTCC CAATCGTTGT GACTAAAGAA ATATTAGAAT TGCGTAATTG   
  
  
+ CTTACACGTC CCCATATAAA CAAAACAACT GCCAATCTGT CATTACCTGA CGGTCCCATA TTAAACCCTC   
  
  
+ CTTAAGCACC CACTAATCAT ATCCACTTCT CTTTCACGTT TCTCGCCGGC CTCAGTCTGC CTTGCCGGTG   
  
  
+ ATCCTACGCC TCCATGATCA TCGCTACCAT TGTCGTCGTC GCCGTTTCAG TACGAAGCCG ACGGTCTTGA   
  
  
+ TTAACTTACC ACCTGATCCA GCACCATAT  

- TCTTCCTTTC TTTATCCACA TTACTATCAC CCCCCTGTCT CAAACGTTGA TGGTAAGGAA ATAAAAAAGT   
  
  
- ACCACAAAAC CTAACTTACA GTTTAAATTC TTATTTAAAA GAGTTTAAAA GGACTACTAC TACGTATAAC   
  
  
- ATAGACTTCA TTTCTAAAAA AAAAAAATTT TATAGGTTTC ATCAATTGTA ATTCAGTAGA TGCCTAGTCT   
  
  
- AAATTAAGCC AGGTAAGTAT TAAACATTTT AAAATCAAAT AAATTTATTT TAACAACTAT AATAAAATTT   
  
  
- TTATTTTTAA TAAATTGTGA AGTTTGATAT AAAATATATA ATTTAGTTTC AAATCACAAA TTTTCATTAT   
  
  
- TTAGTTTGAA AATTAATAAT TAAACTAAAT TTAATTTAAA ACTAAATTAT AAAAAACAAT AATTTTTATA   
  
  
- AAATTTTTTA TTAAGTTTAT TTAACATATG AAATAAAATT TAGTTAAGAA ATTAAAGTTT ATTAAAAACC   
  
  
- TAAATACTCA ATAAATAATA TTTAAAAATA TTATTAAAAA GATTTTTGTA AAATCAGTTT TTTTGAATAT   
  
  
- GAAAAAATAT TACTATCAAA CTTATAAAAA TTTTTAAAAA TTATACTAAA TTAAACTATA TTTAATTATT   
  
  
- TAGTCTAAAA TTAAACAATG CAAAATTTGG AATTCCAATT TAAACTGTTT AGTATATGAA TTTTCAAAAT   
  
  
- TAATTAGAAA TGTTAATCAA ATTAATCTAA ACCATGATAT TTATTTTTAT GAGTATCAAA ATAAACTTAA   
  
  
- ATACTTTGGA TTTTATTCAA ATATAGAAAA ATAAAAAAGA GTTGTTTATA TATAGGTTTT ATCTTTTTAT   
  
  
- TTAAAGTTTT AAAAGAATTA TATGGTATTG GCAAAATACA GTAATTATTT ATTACAGTGT TATTTTTATT   
  
  
- ATTTTATTTT AGAGTAATGG TGGATGTTGA AAATCGGAAA ATGATATCAT GTTTTAAGAA AGTAGAAATT   
  
  
- ATTATTAATG AAAGAAAGTA AGTCACCACA GTATTTAATT TCTGGGATGT CATATGGTAT ACGGTACTTT   
  
  
- AATTACTTTA GATTGTGTTT AGTAATACTA AAATAGTGAA TATTTTTTTA ATATAAAAAG TTGGCGTGGT   
  
  
- ATATTATATT GTATATATAA TCTAATTATA TCGTTGGTTT TACTTAGTAA ACCTCCCATG TGCTCAGGAA   
  
  
- TTGTACCTGG ACTGAAGCAG CTTAGTAAGG GTTAGCAACA CTGATTTCTT TATAATCTTA ACGCATTAAC   
  
  
- GAATGTGCAG GGGTATATTT GTTTTGTTGA CGGTTAGACA GTAATGGACT GCCAGGGTAT AATTTGGGAG   
  
  
- GAATTCGTGG GTGATTAGTA TAGGTGAAGA GAAAGTGCAA AGAGCGGCCG GAGTCAGACG GAACGGCCAC   
  
  
- TAGGATGCGG AGGTACTAGT AGCGATGGTA ACAGCAGCAG CGGCAAAGTC ATGCTTCGGC TGCCAGAACT   
  
  
- AATTGAATGG TGGACTAGGT CGTGGTATA

+     HSE

| Site Name | Organism | Position | Strand | Matrix score. | sequence | function |
| --- | --- | --- | --- | --- | --- | --- |
| HSE | Brassica oleracea | 837 | + | 9 | AAAAAATTTC | cis-acting element involved in heat stress responsiveness |

> 2018/04/13 10:10:12  
+ AGAAGGAAAG AAATAGGTGT AATGATAGTG GGGGGACAGA GTTTGCAACT ACCATTCCTT TATTTTTTCA   
  
  
+ TGGTGTTTTG GATTGAATGT CAAATTTAAG AATAAATTTT CTCAAATTTT CCTGATGATG ATGCATATTG   
  
  
+ TATCTGAAGT AAAGATTTTT TTTTTTTAAA ATATCCAAAG TAGTTAACAT TAAGTCATCT ACGGATCAGA   
  
  
+ TTTAATTCGG TCCATTCATA ATTTGTAAAA TTTTAGTTTA TTTAAATAAA ATTGTTGATA TTATTTTAAA   
  
  
+ AATAAAAATT ATTTAACACT TCAAACTATA TTTTATATAT TAAATCAAAG TTTAGTGTTT AAAAGTAATA   
  
  
+ AATCAAACTT TTAATTATTA ATTTGATTTA AATTAAATTT TGATTTAATA TTTTTTGTTA TTAAAAATAT   
  
  
+ TTTAAAAAAT AATTCAAATA AATTGTATAC TTTATTTTAA ATCAATTCTT TAATTTCAAA TAATTTTTGG   
  
  
+ ATTTATGAGT TATTTATTAT AAATTTTTAT AATAATTTTT CTAAAAACAT TTTAGTCAAA AAAACTTATA   
  
  
+ CTTTTTTATA ATGATAGTTT GAATATTTTT AAAAATTTTT AATATGATTT AATTTGATAT AAATTAATAA   
  
  
+ ATCAGATTTT AATTTGTTAC GTTTTAAACC TTAAGGTTAA ATTTGACAAA TCATATACTT AAAAGTTTTA   
  
  
+ ATTAATCTTT ACAATTAGTT TAATTAGATT TGGTACTATA AATAAAAATA CTCATAGTTT TATTTGAATT   
  
  
+ TATGAAACCT AAAATAAGTT TATATCTTTT TATTTTTTCT CAACAAATAT ATATCCAAAA TAGAAAAATA   
  
  
+ AATTTCAAAA TTTTCTTAAT ATACCATAAC CGTTTTATGT CATTAATAAA TAATGTCACA ATAAAAATAA   
  
  
+ TAAAATAAAA TCTCATTACC ACCTACAACT TTTAGCCTTT TACTATAGTA CAAAATTCTT TCATCTTTAA   
  
  
+ TAATAATTAC TTTCTTTCAT TCAGTGGTGT CATAAATTAA AGACCCTACA GTATACCATA TGCCATGAAA   
  
  
+ TTAATGAAAT CTAACACAAA TCATTATGAT TTTATCACTT ATAAAAAAAT TATATTTTTC AACCGCACCA   
  
  
+ TATAATATAA CATATATATT AGATTAATAT AGCAACCAAA ATGAATCATT TGGAGGGTAC ACGAGTCCTT   
  
  
+ AACATGGACC TGACTTCGTC GAATCATTCC CAATCGTTGT GACTAAAGAA ATATTAGAAT TGCGTAATTG   
  
  
+ CTTACACGTC CCCATATAAA CAAAACAACT GCCAATCTGT CATTACCTGA CGGTCCCATA TTAAACCCTC   
  
  
+ CTTAAGCACC CACTAATCAT ATCCACTTCT CTTTCACGTT TCTCGCCGGC CTCAGTCTGC CTTGCCGGTG   
  
  
+ ATCCTACGCC TCCATGATCA TCGCTACCAT TGTCGTCGTC GCCGTTTCAG TACGAAGCCG ACGGTCTTGA   
  
  
+ TTAACTTACC ACCTGATCCA GCACCATAT  

- TCTTCCTTTC TTTATCCACA TTACTATCAC CCCCCTGTCT CAAACGTTGA TGGTAAGGAA ATAAAAAAGT   
  
  
- ACCACAAAAC CTAACTTACA GTTTAAATTC TTATTTAAAA GAGTTTAAAA GGACTACTAC TACGTATAAC   
  
  
- ATAGACTTCA TTTCTAAAAA AAAAAAATTT TATAGGTTTC ATCAATTGTA ATTCAGTAGA TGCCTAGTCT   
  
  
- AAATTAAGCC AGGTAAGTAT TAAACATTTT AAAATCAAAT AAATTTATTT TAACAACTAT AATAAAATTT   
  
  
- TTATTTTTAA TAAATTGTGA AGTTTGATAT AAAATATATA ATTTAGTTTC AAATCACAAA TTTTCATTAT   
  
  
- TTAGTTTGAA AATTAATAAT TAAACTAAAT TTAATTTAAA ACTAAATTAT AAAAAACAAT AATTTTTATA   
  
  
- AAATTTTTTA TTAAGTTTAT TTAACATATG AAATAAAATT TAGTTAAGAA ATTAAAGTTT ATTAAAAACC   
  
  
- TAAATACTCA ATAAATAATA TTTAAAAATA TTATTAAAAA GATTTTTGTA AAATCAGTTT TTTTGAATAT   
  
  
- GAAAAAATAT TACTATCAAA CTTATAAAAA TTTTTAAAAA TTATACTAAA TTAAACTATA TTTAATTATT   
  
  
- TAGTCTAAAA TTAAACAATG CAAAATTTGG AATTCCAATT TAAACTGTTT AGTATATGAA TTTTCAAAAT   
  
  
- TAATTAGAAA TGTTAATCAA ATTAATCTAA ACCATGATAT TTATTTTTAT GAGTATCAAA ATAAACTTAA   
  
  
- ATACTTTGGA TTTTATTCAA ATATAGAAAA ATAAAAAAGA GTTGTTTATA TATAGGTTTT ATCTTTTTAT   
  
  
- TTAAAGTTTT AAAAGAATTA TATGGTATTG GCAAAATACA GTAATTATTT ATTACAGTGT TATTTTTATT   
  
  
- ATTTTATTTT AGAGTAATGG TGGATGTTGA AAATCGGAAA ATGATATCAT GTTTTAAGAA AGTAGAAATT   
  
  
- ATTATTAATG AAAGAAAGTA AGTCACCACA GTATTTAATT TCTGGGATGT CATATGGTAT ACGGTACTTT   
  
  
- AATTACTTTA GATTGTGTTT AGTAATACTA AAATAGTGAA TATTTTTTTA ATATAAAAAG TTGGCGTGGT   
  
  
- ATATTATATT GTATATATAA TCTAATTATA TCGTTGGTTT TACTTAGTAA ACCTCCCATG TGCTCAGGAA   
  
  
- TTGTACCTGG ACTGAAGCAG CTTAGTAAGG GTTAGCAACA CTGATTTCTT TATAATCTTA ACGCATTAAC   
  
  
- GAATGTGCAG GGGTATATTT GTTTTGTTGA CGGTTAGACA GTAATGGACT GCCAGGGTAT AATTTGGGAG   
  
  
- GAATTCGTGG GTGATTAGTA TAGGTGAAGA GAAAGTGCAA AGAGCGGCCG GAGTCAGACG GAACGGCCAC   
  
  
- TAGGATGCGG AGGTACTAGT AGCGATGGTA ACAGCAGCAG CGGCAAAGTC ATGCTTCGGC TGCCAGAACT   
  
  
- AATTGAATGG TGGACTAGGT CGTGGTATA

+     I-box

| Site Name | Organism | Position | Strand | Matrix score. | sequence | function |
| --- | --- | --- | --- | --- | --- | --- |
| I-box | Zea mays | 1480 | - | 9 | gGATAAGGTG | part of a light responsive element |
| I-box | Flaveria trinervia | 1494 | - | 7 | GATATGG | part of a light responsive element |

> 2018/04/13 10:10:12  
+ AGAAGGAAAG AAATAGGTGT AATGATAGTG GGGGGACAGA GTTTGCAACT ACCATTCCTT TATTTTTTCA   
  
  
+ TGGTGTTTTG GATTGAATGT CAAATTTAAG AATAAATTTT CTCAAATTTT CCTGATGATG ATGCATATTG   
  
  
+ TATCTGAAGT AAAGATTTTT TTTTTTTAAA ATATCCAAAG TAGTTAACAT TAAGTCATCT ACGGATCAGA   
  
  
+ TTTAATTCGG TCCATTCATA ATTTGTAAAA TTTTAGTTTA TTTAAATAAA ATTGTTGATA TTATTTTAAA   
  
  
+ AATAAAAATT ATTTAACACT TCAAACTATA TTTTATATAT TAAATCAAAG TTTAGTGTTT AAAAGTAATA   
  
  
+ AATCAAACTT TTAATTATTA ATTTGATTTA AATTAAATTT TGATTTAATA TTTTTTGTTA TTAAAAATAT   
  
  
+ TTTAAAAAAT AATTCAAATA AATTGTATAC TTTATTTTAA ATCAATTCTT TAATTTCAAA TAATTTTTGG   
  
  
+ ATTTATGAGT TATTTATTAT AAATTTTTAT AATAATTTTT CTAAAAACAT TTTAGTCAAA AAAACTTATA   
  
  
+ CTTTTTTATA ATGATAGTTT GAATATTTTT AAAAATTTTT AATATGATTT AATTTGATAT AAATTAATAA   
  
  
+ ATCAGATTTT AATTTGTTAC GTTTTAAACC TTAAGGTTAA ATTTGACAAA TCATATACTT AAAAGTTTTA   
  
  
+ ATTAATCTTT ACAATTAGTT TAATTAGATT TGGTACTATA AATAAAAATA CTCATAGTTT TATTTGAATT   
  
  
+ TATGAAACCT AAAATAAGTT TATATCTTTT TATTTTTTCT CAACAAATAT ATATCCAAAA TAGAAAAATA   
  
  
+ AATTTCAAAA TTTTCTTAAT ATACCATAAC CGTTTTATGT CATTAATAAA TAATGTCACA ATAAAAATAA   
  
  
+ TAAAATAAAA TCTCATTACC ACCTACAACT TTTAGCCTTT TACTATAGTA CAAAATTCTT TCATCTTTAA   
  
  
+ TAATAATTAC TTTCTTTCAT TCAGTGGTGT CATAAATTAA AGACCCTACA GTATACCATA TGCCATGAAA   
  
  
+ TTAATGAAAT CTAACACAAA TCATTATGAT TTTATCACTT ATAAAAAAAT TATATTTTTC AACCGCACCA   
  
  
+ TATAATATAA CATATATATT AGATTAATAT AGCAACCAAA ATGAATCATT TGGAGGGTAC ACGAGTCCTT   
  
  
+ AACATGGACC TGACTTCGTC GAATCATTCC CAATCGTTGT GACTAAAGAA ATATTAGAAT TGCGTAATTG   
  
  
+ CTTACACGTC CCCATATAAA CAAAACAACT GCCAATCTGT CATTACCTGA CGGTCCCATA TTAAACCCTC   
  
  
+ CTTAAGCACC CACTAATCAT ATCCACTTCT CTTTCACGTT TCTCGCCGGC CTCAGTCTGC CTTGCCGGTG   
  
  
+ ATCCTACGCC TCCATGATCA TCGCTACCAT TGTCGTCGTC GCCGTTTCAG TACGAAGCCG ACGGTCTTGA   
  
  
+ TTAACTTACC ACCTGATCCA GCACCATAT  

- TCTTCCTTTC TTTATCCACA TTACTATCAC CCCCCTGTCT CAAACGTTGA TGGTAAGGAA ATAAAAAAGT   
  
  
- ACCACAAAAC CTAACTTACA GTTTAAATTC TTATTTAAAA GAGTTTAAAA GGACTACTAC TACGTATAAC   
  
  
- ATAGACTTCA TTTCTAAAAA AAAAAAATTT TATAGGTTTC ATCAATTGTA ATTCAGTAGA TGCCTAGTCT   
  
  
- AAATTAAGCC AGGTAAGTAT TAAACATTTT AAAATCAAAT AAATTTATTT TAACAACTAT AATAAAATTT   
  
  
- TTATTTTTAA TAAATTGTGA AGTTTGATAT AAAATATATA ATTTAGTTTC AAATCACAAA TTTTCATTAT   
  
  
- TTAGTTTGAA AATTAATAAT TAAACTAAAT TTAATTTAAA ACTAAATTAT AAAAAACAAT AATTTTTATA   
  
  
- AAATTTTTTA TTAAGTTTAT TTAACATATG AAATAAAATT TAGTTAAGAA ATTAAAGTTT ATTAAAAACC   
  
  
- TAAATACTCA ATAAATAATA TTTAAAAATA TTATTAAAAA GATTTTTGTA AAATCAGTTT TTTTGAATAT   
  
  
- GAAAAAATAT TACTATCAAA CTTATAAAAA TTTTTAAAAA TTATACTAAA TTAAACTATA TTTAATTATT   
  
  
- TAGTCTAAAA TTAAACAATG CAAAATTTGG AATTCCAATT TAAACTGTTT AGTATATGAA TTTTCAAAAT   
  
  
- TAATTAGAAA TGTTAATCAA ATTAATCTAA ACCATGATAT TTATTTTTAT GAGTATCAAA ATAAACTTAA   
  
  
- ATACTTTGGA TTTTATTCAA ATATAGAAAA ATAAAAAAGA GTTGTTTATA TATAGGTTTT ATCTTTTTAT   
  
  
- TTAAAGTTTT AAAAGAATTA TATGGTATTG GCAAAATACA GTAATTATTT ATTACAGTGT TATTTTTATT   
  
  
- ATTTTATTTT AGAGTAATGG TGGATGTTGA AAATCGGAAA ATGATATCAT GTTTTAAGAA AGTAGAAATT   
  
  
- ATTATTAATG AAAGAAAGTA AGTCACCACA GTATTTAATT TCTGGGATGT CATATGGTAT ACGGTACTTT   
  
  
- AATTACTTTA GATTGTGTTT AGTAATACTA AAATAGTGAA TATTTTTTTA ATATAAAAAG TTGGCGTGGT   
  
  
- ATATTATATT GTATATATAA TCTAATTATA TCGTTGGTTT TACTTAGTAA ACCTCCCATG TGCTCAGGAA   
  
  
- TTGTACCTGG ACTGAAGCAG CTTAGTAAGG GTTAGCAACA CTGATTTCTT TATAATCTTA ACGCATTAAC   
  
  
- GAATGTGCAG GGGTATATTT GTTTTGTTGA CGGTTAGACA GTAATGGACT GCCAGGGTAT AATTTGGGAG   
  
  
- GAATTCGTGG GTGATTAGTA TAGGTGAAGA GAAAGTGCAA AGAGCGGCCG GAGTCAGACG GAACGGCCAC   
  
  
- TAGGATGCGG AGGTACTAGT AGCGATGGTA ACAGCAGCAG CGGCAAAGTC ATGCTTCGGC TGCCAGAACT   
  
  
- AATTGAATGG TGGACTAGGT CGTGGTATAG

+     MBS

| Site Name | Organism | Position | Strand | Matrix score. | sequence | function |
| --- | --- | --- | --- | --- | --- | --- |
| MBS | Arabidopsis thaliana | 1286 | + | 6 | CAACTG | MYB binding site involved in drought-inducibility |

> 2018/04/13 10:10:12  
+ AGAAGGAAAG AAATAGGTGT AATGATAGTG GGGGGACAGA GTTTGCAACT ACCATTCCTT TATTTTTTCA   
  
  
+ TGGTGTTTTG GATTGAATGT CAAATTTAAG AATAAATTTT CTCAAATTTT CCTGATGATG ATGCATATTG   
  
  
+ TATCTGAAGT AAAGATTTTT TTTTTTTAAA ATATCCAAAG TAGTTAACAT TAAGTCATCT ACGGATCAGA   
  
  
+ TTTAATTCGG TCCATTCATA ATTTGTAAAA TTTTAGTTTA TTTAAATAAA ATTGTTGATA TTATTTTAAA   
  
  
+ AATAAAAATT ATTTAACACT TCAAACTATA TTTTATATAT TAAATCAAAG TTTAGTGTTT AAAAGTAATA   
  
  
+ AATCAAACTT TTAATTATTA ATTTGATTTA AATTAAATTT TGATTTAATA TTTTTTGTTA TTAAAAATAT   
  
  
+ TTTAAAAAAT AATTCAAATA AATTGTATAC TTTATTTTAA ATCAATTCTT TAATTTCAAA TAATTTTTGG   
  
  
+ ATTTATGAGT TATTTATTAT AAATTTTTAT AATAATTTTT CTAAAAACAT TTTAGTCAAA AAAACTTATA   
  
  
+ CTTTTTTATA ATGATAGTTT GAATATTTTT AAAAATTTTT AATATGATTT AATTTGATAT AAATTAATAA   
  
  
+ ATCAGATTTT AATTTGTTAC GTTTTAAACC TTAAGGTTAA ATTTGACAAA TCATATACTT AAAAGTTTTA   
  
  
+ ATTAATCTTT ACAATTAGTT TAATTAGATT TGGTACTATA AATAAAAATA CTCATAGTTT TATTTGAATT   
  
  
+ TATGAAACCT AAAATAAGTT TATATCTTTT TATTTTTTCT CAACAAATAT ATATCCAAAA TAGAAAAATA   
  
  
+ AATTTCAAAA TTTTCTTAAT ATACCATAAC CGTTTTATGT CATTAATAAA TAATGTCACA ATAAAAATAA   
  
  
+ TAAAATAAAA TCTCATTACC ACCTACAACT TTTAGCCTTT TACTATAGTA CAAAATTCTT TCATCTTTAA   
  
  
+ TAATAATTAC TTTCTTTCAT TCAGTGGTGT CATAAATTAA AGACCCTACA GTATACCATA TGCCATGAAA   
  
  
+ TTAATGAAAT CTAACACAAA TCATTATGAT TTTATCACTT ATAAAAAAAT TATATTTTTC AACCGCACCA   
  
  
+ TATAATATAA CATATATATT AGATTAATAT AGCAACCAAA ATGAATCATT TGGAGGGTAC ACGAGTCCTT   
  
  
+ AACATGGACC TGACTTCGTC GAATCATTCC CAATCGTTGT GACTAAAGAA ATATTAGAAT TGCGTAATTG   
  
  
+ CTTACACGTC CCCATATAAA CAAAACAACT GCCAATCTGT CATTACCTGA CGGTCCCATA TTAAACCCTC   
  
  
+ CTTAAGCACC CACTAATCAT ATCCACTTCT CTTTCACGTT TCTCGCCGGC CTCAGTCTGC CTTGCCGGTG   
  
  
+ ATCCTACGCC TCCATGATCA TCGCTACCAT TGTCGTCGTC GCCGTTTCAG TACGAAGCCG ACGGTCTTGA   
  
  
+ TTAACTTACC ACCTGATCCA GCACCATAT  

- TCTTCCTTTC TTTATCCACA TTACTATCAC CCCCCTGTCT CAAACGTTGA TGGTAAGGAA ATAAAAAAGT   
  
  
- ACCACAAAAC CTAACTTACA GTTTAAATTC TTATTTAAAA GAGTTTAAAA GGACTACTAC TACGTATAAC   
  
  
- ATAGACTTCA TTTCTAAAAA AAAAAAATTT TATAGGTTTC ATCAATTGTA ATTCAGTAGA TGCCTAGTCT   
  
  
- AAATTAAGCC AGGTAAGTAT TAAACATTTT AAAATCAAAT AAATTTATTT TAACAACTAT AATAAAATTT   
  
  
- TTATTTTTAA TAAATTGTGA AGTTTGATAT AAAATATATA ATTTAGTTTC AAATCACAAA TTTTCATTAT   
  
  
- TTAGTTTGAA AATTAATAAT TAAACTAAAT TTAATTTAAA ACTAAATTAT AAAAAACAAT AATTTTTATA   
  
  
- AAATTTTTTA TTAAGTTTAT TTAACATATG AAATAAAATT TAGTTAAGAA ATTAAAGTTT ATTAAAAACC   
  
  
- TAAATACTCA ATAAATAATA TTTAAAAATA TTATTAAAAA GATTTTTGTA AAATCAGTTT TTTTGAATAT   
  
  
- GAAAAAATAT TACTATCAAA CTTATAAAAA TTTTTAAAAA TTATACTAAA TTAAACTATA TTTAATTATT   
  
  
- TAGTCTAAAA TTAAACAATG CAAAATTTGG AATTCCAATT TAAACTGTTT AGTATATGAA TTTTCAAAAT   
  
  
- TAATTAGAAA TGTTAATCAA ATTAATCTAA ACCATGATAT TTATTTTTAT GAGTATCAAA ATAAACTTAA   
  
  
- ATACTTTGGA TTTTATTCAA ATATAGAAAA ATAAAAAAGA GTTGTTTATA TATAGGTTTT ATCTTTTTAT   
  
  
- TTAAAGTTTT AAAAGAATTA TATGGTATTG GCAAAATACA GTAATTATTT ATTACAGTGT TATTTTTATT   
  
  
- ATTTTATTTT AGAGTAATGG TGGATGTTGA AAATCGGAAA ATGATATCAT GTTTTAAGAA AGTAGAAATT   
  
  
- ATTATTAATG AAAGAAAGTA AGTCACCACA GTATTTAATT TCTGGGATGT CATATGGTAT ACGGTACTTT   
  
  
- AATTACTTTA GATTGTGTTT AGTAATACTA AAATAGTGAA TATTTTTTTA ATATAAAAAG TTGGCGTGGT   
  
  
- ATATTATATT GTATATATAA TCTAATTATA TCGTTGGTTT TACTTAGTAA ACCTCCCATG TGCTCAGGAA   
  
  
- TTGTACCTGG ACTGAAGCAG CTTAGTAAGG GTTAGCAACA CTGATTTCTT TATAATCTTA ACGCATTAAC   
  
  
- GAATGTGCAG GGGTATATTT GTTTTGTTGA CGGTTAGACA GTAATGGACT GCCAGGGTAT AATTTGGGAG   
  
  
- GAATTCGTGG GTGATTAGTA TAGGTGAAGA GAAAGTGCAA AGAGCGGCCG GAGTCAGACG GAACGGCCAC   
  
  
- TAGGATGCGG AGGTACTAGT AGCGATGGTA ACAGCAGCAG CGGCAAAGTC ATGCTTCGGC TGCCAGAACT   
  
  
- AATTGAATGG TGGACTAGGT CGTGGTATA

+     MBSI

| Site Name | Organism | Position | Strand | Matrix score. | sequence | function |
| --- | --- | --- | --- | --- | --- | --- |
| MBSI | Petunia hybrida | 867 | - | 10.5 | aaaAaaC(G/C)GTTA | MYB binding site involved in flavonoid biosynthetic genes regulation |

> 2018/04/13 10:10:12  
+ AGAAGGAAAG AAATAGGTGT AATGATAGTG GGGGGACAGA GTTTGCAACT ACCATTCCTT TATTTTTTCA   
  
  
+ TGGTGTTTTG GATTGAATGT CAAATTTAAG AATAAATTTT CTCAAATTTT CCTGATGATG ATGCATATTG   
  
  
+ TATCTGAAGT AAAGATTTTT TTTTTTTAAA ATATCCAAAG TAGTTAACAT TAAGTCATCT ACGGATCAGA   
  
  
+ TTTAATTCGG TCCATTCATA ATTTGTAAAA TTTTAGTTTA TTTAAATAAA ATTGTTGATA TTATTTTAAA   
  
  
+ AATAAAAATT ATTTAACACT TCAAACTATA TTTTATATAT TAAATCAAAG TTTAGTGTTT AAAAGTAATA   
  
  
+ AATCAAACTT TTAATTATTA ATTTGATTTA AATTAAATTT TGATTTAATA TTTTTTGTTA TTAAAAATAT   
  
  
+ TTTAAAAAAT AATTCAAATA AATTGTATAC TTTATTTTAA ATCAATTCTT TAATTTCAAA TAATTTTTGG   
  
  
+ ATTTATGAGT TATTTATTAT AAATTTTTAT AATAATTTTT CTAAAAACAT TTTAGTCAAA AAAACTTATA   
  
  
+ CTTTTTTATA ATGATAGTTT GAATATTTTT AAAAATTTTT AATATGATTT AATTTGATAT AAATTAATAA   
  
  
+ ATCAGATTTT AATTTGTTAC GTTTTAAACC TTAAGGTTAA ATTTGACAAA TCATATACTT AAAAGTTTTA   
  
  
+ ATTAATCTTT ACAATTAGTT TAATTAGATT TGGTACTATA AATAAAAATA CTCATAGTTT TATTTGAATT   
  
  
+ TATGAAACCT AAAATAAGTT TATATCTTTT TATTTTTTCT CAACAAATAT ATATCCAAAA TAGAAAAATA   
  
  
+ AATTTCAAAA TTTTCTTAAT ATACCATAAC CGTTTTATGT CATTAATAAA TAATGTCACA ATAAAAATAA   
  
  
+ TAAAATAAAA TCTCATTACC ACCTACAACT TTTAGCCTTT TACTATAGTA CAAAATTCTT TCATCTTTAA   
  
  
+ TAATAATTAC TTTCTTTCAT TCAGTGGTGT CATAAATTAA AGACCCTACA GTATACCATA TGCCATGAAA   
  
  
+ TTAATGAAAT CTAACACAAA TCATTATGAT TTTATCACTT ATAAAAAAAT TATATTTTTC AACCGCACCA   
  
  
+ TATAATATAA CATATATATT AGATTAATAT AGCAACCAAA ATGAATCATT TGGAGGGTAC ACGAGTCCTT   
  
  
+ AACATGGACC TGACTTCGTC GAATCATTCC CAATCGTTGT GACTAAAGAA ATATTAGAAT TGCGTAATTG   
  
  
+ CTTACACGTC CCCATATAAA CAAAACAACT GCCAATCTGT CATTACCTGA CGGTCCCATA TTAAACCCTC   
  
  
+ CTTAAGCACC CACTAATCAT ATCCACTTCT CTTTCACGTT TCTCGCCGGC CTCAGTCTGC CTTGCCGGTG   
  
  
+ ATCCTACGCC TCCATGATCA TCGCTACCAT TGTCGTCGTC GCCGTTTCAG TACGAAGCCG ACGGTCTTGA   
  
  
+ TTAACTTACC ACCTGATCCA GCACCATAT  

- TCTTCCTTTC TTTATCCACA TTACTATCAC CCCCCTGTCT CAAACGTTGA TGGTAAGGAA ATAAAAAAGT   
  
  
- ACCACAAAAC CTAACTTACA GTTTAAATTC TTATTTAAAA GAGTTTAAAA GGACTACTAC TACGTATAAC   
  
  
- ATAGACTTCA TTTCTAAAAA AAAAAAATTT TATAGGTTTC ATCAATTGTA ATTCAGTAGA TGCCTAGTCT   
  
  
- AAATTAAGCC AGGTAAGTAT TAAACATTTT AAAATCAAAT AAATTTATTT TAACAACTAT AATAAAATTT   
  
  
- TTATTTTTAA TAAATTGTGA AGTTTGATAT AAAATATATA ATTTAGTTTC AAATCACAAA TTTTCATTAT   
  
  
- TTAGTTTGAA AATTAATAAT TAAACTAAAT TTAATTTAAA ACTAAATTAT AAAAAACAAT AATTTTTATA   
  
  
- AAATTTTTTA TTAAGTTTAT TTAACATATG AAATAAAATT TAGTTAAGAA ATTAAAGTTT ATTAAAAACC   
  
  
- TAAATACTCA ATAAATAATA TTTAAAAATA TTATTAAAAA GATTTTTGTA AAATCAGTTT TTTTGAATAT   
  
  
- GAAAAAATAT TACTATCAAA CTTATAAAAA TTTTTAAAAA TTATACTAAA TTAAACTATA TTTAATTATT   
  
  
- TAGTCTAAAA TTAAACAATG CAAAATTTGG AATTCCAATT TAAACTGTTT AGTATATGAA TTTTCAAAAT   
  
  
- TAATTAGAAA TGTTAATCAA ATTAATCTAA ACCATGATAT TTATTTTTAT GAGTATCAAA ATAAACTTAA   
  
  
- ATACTTTGGA TTTTATTCAA ATATAGAAAA ATAAAAAAGA GTTGTTTATA TATAGGTTTT ATCTTTTTAT   
  
  
- TTAAAGTTTT AAAAGAATTA TATGGTATTG GCAAAATACA GTAATTATTT ATTACAGTGT TATTTTTATT   
  
  
- ATTTTATTTT AGAGTAATGG TGGATGTTGA AAATCGGAAA ATGATATCAT GTTTTAAGAA AGTAGAAATT   
  
  
- ATTATTAATG AAAGAAAGTA AGTCACCACA GTATTTAATT TCTGGGATGT CATATGGTAT ACGGTACTTT   
  
  
- AATTACTTTA GATTGTGTTT AGTAATACTA AAATAGTGAA TATTTTTTTA ATATAAAAAG TTGGCGTGGT   
  
  
- ATATTATATT GTATATATAA TCTAATTATA TCGTTGGTTT TACTTAGTAA ACCTCCCATG TGCTCAGGAA   
  
  
- TTGTACCTGG ACTGAAGCAG CTTAGTAAGG GTTAGCAACA CTGATTTCTT TATAATCTTA ACGCATTAAC   
  
  
- GAATGTGCAG GGGTATATTT GTTTTGTTGA CGGTTAGACA GTAATGGACT GCCAGGGTAT AATTTGGGAG   
  
  
- GAATTCGTGG GTGATTAGTA TAGGTGAAGA GAAAGTGCAA AGAGCGGCCG GAGTCAGACG GAACGGCCAC   
  
  
- TAGGATGCGG AGGTACTAGT AGCGATGGTA ACAGCAGCAG CGGCAAAGTC ATGCTTCGGC TGCCAGAACT   
  
  
- AATTGAATGG TGGACTAGGT CGTGGTATA

+     MRE

| Site Name | Organism | Position | Strand | Matrix score. | sequence | function |
| --- | --- | --- | --- | --- | --- | --- |
| MRE | Petroselinum crispum | 776 | + | 7 | AACCTAA | MYB binding site involved in light responsiveness |

> 2018/04/13 10:10:12  
+ AGAAGGAAAG AAATAGGTGT AATGATAGTG GGGGGACAGA GTTTGCAACT ACCATTCCTT TATTTTTTCA   
  
  
+ TGGTGTTTTG GATTGAATGT CAAATTTAAG AATAAATTTT CTCAAATTTT CCTGATGATG ATGCATATTG   
  
  
+ TATCTGAAGT AAAGATTTTT TTTTTTTAAA ATATCCAAAG TAGTTAACAT TAAGTCATCT ACGGATCAGA   
  
  
+ TTTAATTCGG TCCATTCATA ATTTGTAAAA TTTTAGTTTA TTTAAATAAA ATTGTTGATA TTATTTTAAA   
  
  
+ AATAAAAATT ATTTAACACT TCAAACTATA TTTTATATAT TAAATCAAAG TTTAGTGTTT AAAAGTAATA   
  
  
+ AATCAAACTT TTAATTATTA ATTTGATTTA AATTAAATTT TGATTTAATA TTTTTTGTTA TTAAAAATAT   
  
  
+ TTTAAAAAAT AATTCAAATA AATTGTATAC TTTATTTTAA ATCAATTCTT TAATTTCAAA TAATTTTTGG   
  
  
+ ATTTATGAGT TATTTATTAT AAATTTTTAT AATAATTTTT CTAAAAACAT TTTAGTCAAA AAAACTTATA   
  
  
+ CTTTTTTATA ATGATAGTTT GAATATTTTT AAAAATTTTT AATATGATTT AATTTGATAT AAATTAATAA   
  
  
+ ATCAGATTTT AATTTGTTAC GTTTTAAACC TTAAGGTTAA ATTTGACAAA TCATATACTT AAAAGTTTTA   
  
  
+ ATTAATCTTT ACAATTAGTT TAATTAGATT TGGTACTATA AATAAAAATA CTCATAGTTT TATTTGAATT   
  
  
+ TATGAAACCT AAAATAAGTT TATATCTTTT TATTTTTTCT CAACAAATAT ATATCCAAAA TAGAAAAATA   
  
  
+ AATTTCAAAA TTTTCTTAAT ATACCATAAC CGTTTTATGT CATTAATAAA TAATGTCACA ATAAAAATAA   
  
  
+ TAAAATAAAA TCTCATTACC ACCTACAACT TTTAGCCTTT TACTATAGTA CAAAATTCTT TCATCTTTAA   
  
  
+ TAATAATTAC TTTCTTTCAT TCAGTGGTGT CATAAATTAA AGACCCTACA GTATACCATA TGCCATGAAA   
  
  
+ TTAATGAAAT CTAACACAAA TCATTATGAT TTTATCACTT ATAAAAAAAT TATATTTTTC AACCGCACCA   
  
  
+ TATAATATAA CATATATATT AGATTAATAT AGCAACCAAA ATGAATCATT TGGAGGGTAC ACGAGTCCTT   
  
  
+ AACATGGACC TGACTTCGTC GAATCATTCC CAATCGTTGT GACTAAAGAA ATATTAGAAT TGCGTAATTG   
  
  
+ CTTACACGTC CCCATATAAA CAAAACAACT GCCAATCTGT CATTACCTGA CGGTCCCATA TTAAACCCTC   
  
  
+ CTTAAGCACC CACTAATCAT ATCCACTTCT CTTTCACGTT TCTCGCCGGC CTCAGTCTGC CTTGCCGGTG   
  
  
+ ATCCTACGCC TCCATGATCA TCGCTACCAT TGTCGTCGTC GCCGTTTCAG TACGAAGCCG ACGGTCTTGA   
  
  
+ TTAACTTACC ACCTGATCCA GCACCATAT  

- TCTTCCTTTC TTTATCCACA TTACTATCAC CCCCCTGTCT CAAACGTTGA TGGTAAGGAA ATAAAAAAGT   
  
  
- ACCACAAAAC CTAACTTACA GTTTAAATTC TTATTTAAAA GAGTTTAAAA GGACTACTAC TACGTATAAC   
  
  
- ATAGACTTCA TTTCTAAAAA AAAAAAATTT TATAGGTTTC ATCAATTGTA ATTCAGTAGA TGCCTAGTCT   
  
  
- AAATTAAGCC AGGTAAGTAT TAAACATTTT AAAATCAAAT AAATTTATTT TAACAACTAT AATAAAATTT   
  
  
- TTATTTTTAA TAAATTGTGA AGTTTGATAT AAAATATATA ATTTAGTTTC AAATCACAAA TTTTCATTAT   
  
  
- TTAGTTTGAA AATTAATAAT TAAACTAAAT TTAATTTAAA ACTAAATTAT AAAAAACAAT AATTTTTATA   
  
  
- AAATTTTTTA TTAAGTTTAT TTAACATATG AAATAAAATT TAGTTAAGAA ATTAAAGTTT ATTAAAAACC   
  
  
- TAAATACTCA ATAAATAATA TTTAAAAATA TTATTAAAAA GATTTTTGTA AAATCAGTTT TTTTGAATAT   
  
  
- GAAAAAATAT TACTATCAAA CTTATAAAAA TTTTTAAAAA TTATACTAAA TTAAACTATA TTTAATTATT   
  
  
- TAGTCTAAAA TTAAACAATG CAAAATTTGG AATTCCAATT TAAACTGTTT AGTATATGAA TTTTCAAAAT   
  
  
- TAATTAGAAA TGTTAATCAA ATTAATCTAA ACCATGATAT TTATTTTTAT GAGTATCAAA ATAAACTTAA   
  
  
- ATACTTTGGA TTTTATTCAA ATATAGAAAA ATAAAAAAGA GTTGTTTATA TATAGGTTTT ATCTTTTTAT   
  
  
- TTAAAGTTTT AAAAGAATTA TATGGTATTG GCAAAATACA GTAATTATTT ATTACAGTGT TATTTTTATT   
  
  
- ATTTTATTTT AGAGTAATGG TGGATGTTGA AAATCGGAAA ATGATATCAT GTTTTAAGAA AGTAGAAATT   
  
  
- ATTATTAATG AAAGAAAGTA AGTCACCACA GTATTTAATT TCTGGGATGT CATATGGTAT ACGGTACTTT   
  
  
- AATTACTTTA GATTGTGTTT AGTAATACTA AAATAGTGAA TATTTTTTTA ATATAAAAAG TTGGCGTGGT   
  
  
- ATATTATATT GTATATATAA TCTAATTATA TCGTTGGTTT TACTTAGTAA ACCTCCCATG TGCTCAGGAA   
  
  
- TTGTACCTGG ACTGAAGCAG CTTAGTAAGG GTTAGCAACA CTGATTTCTT TATAATCTTA ACGCATTAAC   
  
  
- GAATGTGCAG GGGTATATTT GTTTTGTTGA CGGTTAGACA GTAATGGACT GCCAGGGTAT AATTTGGGAG   
  
  
- GAATTCGTGG GTGATTAGTA TAGGTGAAGA GAAAGTGCAA AGAGCGGCCG GAGTCAGACG GAACGGCCAC   
  
  
- TAGGATGCGG AGGTACTAGT AGCGATGGTA ACAGCAGCAG CGGCAAAGTC ATGCTTCGGC TGCCAGAACT   
  
  
- AATTGAATGG TGGACTAGGT CGTGGTATA

+     Skn-1\_motif

| Site Name | Organism | Position | Strand | Matrix score. | sequence | function |
| --- | --- | --- | --- | --- | --- | --- |
| Skn-1\_motif | Oryza sativa | 1009 | + | 5 | GTCAT | cis-acting regulatory element required for endosperm expression |
| Skn-1\_motif | Oryza sativa | 1299 | + | 5 | GTCAT | cis-acting regulatory element required for endosperm expression |
| Skn-1\_motif | Oryza sativa | 194 | + | 5 | GTCAT | cis-acting regulatory element required for endosperm expression |
| Skn-1\_motif | Oryza sativa | 879 | + | 5 | GTCAT | cis-acting regulatory element required for endosperm expression |

> 2018/04/13 10:10:12  
+ AGAAGGAAAG AAATAGGTGT AATGATAGTG GGGGGACAGA GTTTGCAACT ACCATTCCTT TATTTTTTCA   
  
  
+ TGGTGTTTTG GATTGAATGT CAAATTTAAG AATAAATTTT CTCAAATTTT CCTGATGATG ATGCATATTG   
  
  
+ TATCTGAAGT AAAGATTTTT TTTTTTTAAA ATATCCAAAG TAGTTAACAT TAAGTCATCT ACGGATCAGA   
  
  
+ TTTAATTCGG TCCATTCATA ATTTGTAAAA TTTTAGTTTA TTTAAATAAA ATTGTTGATA TTATTTTAAA   
  
  
+ AATAAAAATT ATTTAACACT TCAAACTATA TTTTATATAT TAAATCAAAG TTTAGTGTTT AAAAGTAATA   
  
  
+ AATCAAACTT TTAATTATTA ATTTGATTTA AATTAAATTT TGATTTAATA TTTTTTGTTA TTAAAAATAT   
  
  
+ TTTAAAAAAT AATTCAAATA AATTGTATAC TTTATTTTAA ATCAATTCTT TAATTTCAAA TAATTTTTGG   
  
  
+ ATTTATGAGT TATTTATTAT AAATTTTTAT AATAATTTTT CTAAAAACAT TTTAGTCAAA AAAACTTATA   
  
  
+ CTTTTTTATA ATGATAGTTT GAATATTTTT AAAAATTTTT AATATGATTT AATTTGATAT AAATTAATAA   
  
  
+ ATCAGATTTT AATTTGTTAC GTTTTAAACC TTAAGGTTAA ATTTGACAAA TCATATACTT AAAAGTTTTA   
  
  
+ ATTAATCTTT ACAATTAGTT TAATTAGATT TGGTACTATA AATAAAAATA CTCATAGTTT TATTTGAATT   
  
  
+ TATGAAACCT AAAATAAGTT TATATCTTTT TATTTTTTCT CAACAAATAT ATATCCAAAA TAGAAAAATA   
  
  
+ AATTTCAAAA TTTTCTTAAT ATACCATAAC CGTTTTATGT CATTAATAAA TAATGTCACA ATAAAAATAA   
  
  
+ TAAAATAAAA TCTCATTACC ACCTACAACT TTTAGCCTTT TACTATAGTA CAAAATTCTT TCATCTTTAA   
  
  
+ TAATAATTAC TTTCTTTCAT TCAGTGGTGT CATAAATTAA AGACCCTACA GTATACCATA TGCCATGAAA   
  
  
+ TTAATGAAAT CTAACACAAA TCATTATGAT TTTATCACTT ATAAAAAAAT TATATTTTTC AACCGCACCA   
  
  
+ TATAATATAA CATATATATT AGATTAATAT AGCAACCAAA ATGAATCATT TGGAGGGTAC ACGAGTCCTT   
  
  
+ AACATGGACC TGACTTCGTC GAATCATTCC CAATCGTTGT GACTAAAGAA ATATTAGAAT TGCGTAATTG   
  
  
+ CTTACACGTC CCCATATAAA CAAAACAACT GCCAATCTGT CATTACCTGA CGGTCCCATA TTAAACCCTC   
  
  
+ CTTAAGCACC CACTAATCAT ATCCACTTCT CTTTCACGTT TCTCGCCGGC CTCAGTCTGC CTTGCCGGTG   
  
  
+ ATCCTACGCC TCCATGATCA TCGCTACCAT TGTCGTCGTC GCCGTTTCAG TACGAAGCCG ACGGTCTTGA   
  
  
+ TTAACTTACC ACCTGATCCA GCACCATAT  

- TCTTCCTTTC TTTATCCACA TTACTATCAC CCCCCTGTCT CAAACGTTGA TGGTAAGGAA ATAAAAAAGT   
  
  
- ACCACAAAAC CTAACTTACA GTTTAAATTC TTATTTAAAA GAGTTTAAAA GGACTACTAC TACGTATAAC   
  
  
- ATAGACTTCA TTTCTAAAAA AAAAAAATTT TATAGGTTTC ATCAATTGTA ATTCAGTAGA TGCCTAGTCT   
  
  
- AAATTAAGCC AGGTAAGTAT TAAACATTTT AAAATCAAAT AAATTTATTT TAACAACTAT AATAAAATTT   
  
  
- TTATTTTTAA TAAATTGTGA AGTTTGATAT AAAATATATA ATTTAGTTTC AAATCACAAA TTTTCATTAT   
  
  
- TTAGTTTGAA AATTAATAAT TAAACTAAAT TTAATTTAAA ACTAAATTAT AAAAAACAAT AATTTTTATA   
  
  
- AAATTTTTTA TTAAGTTTAT TTAACATATG AAATAAAATT TAGTTAAGAA ATTAAAGTTT ATTAAAAACC   
  
  
- TAAATACTCA ATAAATAATA TTTAAAAATA TTATTAAAAA GATTTTTGTA AAATCAGTTT TTTTGAATAT   
  
  
- GAAAAAATAT TACTATCAAA CTTATAAAAA TTTTTAAAAA TTATACTAAA TTAAACTATA TTTAATTATT   
  
  
- TAGTCTAAAA TTAAACAATG CAAAATTTGG AATTCCAATT TAAACTGTTT AGTATATGAA TTTTCAAAAT   
  
  
- TAATTAGAAA TGTTAATCAA ATTAATCTAA ACCATGATAT TTATTTTTAT GAGTATCAAA ATAAACTTAA   
  
  
- ATACTTTGGA TTTTATTCAA ATATAGAAAA ATAAAAAAGA GTTGTTTATA TATAGGTTTT ATCTTTTTAT   
  
  
- TTAAAGTTTT AAAAGAATTA TATGGTATTG GCAAAATACA GTAATTATTT ATTACAGTGT TATTTTTATT   
  
  
- ATTTTATTTT AGAGTAATGG TGGATGTTGA AAATCGGAAA ATGATATCAT GTTTTAAGAA AGTAGAAATT   
  
  
- ATTATTAATG AAAGAAAGTA AGTCACCACA GTATTTAATT TCTGGGATGT CATATGGTAT ACGGTACTTT   
  
  
- AATTACTTTA GATTGTGTTT AGTAATACTA AAATAGTGAA TATTTTTTTA ATATAAAAAG TTGGCGTGGT   
  
  
- ATATTATATT GTATATATAA TCTAATTATA TCGTTGGTTT TACTTAGTAA ACCTCCCATG TGCTCAGGAA   
  
  
- TTGTACCTGG ACTGAAGCAG CTTAGTAAGG GTTAGCAACA CTGATTTCTT TATAATCTTA ACGCATTAAC   
  
  
- GAATGTGCAG GGGTATATTT GTTTTGTTGA CGGTTAGACA GTAATGGACT GCCAGGGTAT AATTTGGGAG   
  
  
- GAATTCGTGG GTGATTAGTA TAGGTGAAGA GAAAGTGCAA AGAGCGGCCG GAGTCAGACG GAACGGCCAC   
  
  
- TAGGATGCGG AGGTACTAGT AGCGATGGTA ACAGCAGCAG CGGCAAAGTC ATGCTTCGGC TGCCAGAACT   
  
  
- AATTGAATGG TGGACTAGGT CGTGGTATA

+     Sp1

| Site Name | Organism | Position | Strand | Matrix score. | sequence | function |
| --- | --- | --- | --- | --- | --- | --- |
| Sp1 | Zea mays | 30 | - | 5 | CC(G/A)CCC | light responsive element |

> 2018/04/13 10:10:12  
+ AGAAGGAAAG AAATAGGTGT AATGATAGTG GGGGGACAGA GTTTGCAACT ACCATTCCTT TATTTTTTCA   
  
  
+ TGGTGTTTTG GATTGAATGT CAAATTTAAG AATAAATTTT CTCAAATTTT CCTGATGATG ATGCATATTG   
  
  
+ TATCTGAAGT AAAGATTTTT TTTTTTTAAA ATATCCAAAG TAGTTAACAT TAAGTCATCT ACGGATCAGA   
  
  
+ TTTAATTCGG TCCATTCATA ATTTGTAAAA TTTTAGTTTA TTTAAATAAA ATTGTTGATA TTATTTTAAA   
  
  
+ AATAAAAATT ATTTAACACT TCAAACTATA TTTTATATAT TAAATCAAAG TTTAGTGTTT AAAAGTAATA   
  
  
+ AATCAAACTT TTAATTATTA ATTTGATTTA AATTAAATTT TGATTTAATA TTTTTTGTTA TTAAAAATAT   
  
  
+ TTTAAAAAAT AATTCAAATA AATTGTATAC TTTATTTTAA ATCAATTCTT TAATTTCAAA TAATTTTTGG   
  
  
+ ATTTATGAGT TATTTATTAT AAATTTTTAT AATAATTTTT CTAAAAACAT TTTAGTCAAA AAAACTTATA   
  
  
+ CTTTTTTATA ATGATAGTTT GAATATTTTT AAAAATTTTT AATATGATTT AATTTGATAT AAATTAATAA   
  
  
+ ATCAGATTTT AATTTGTTAC GTTTTAAACC TTAAGGTTAA ATTTGACAAA TCATATACTT AAAAGTTTTA   
  
  
+ ATTAATCTTT ACAATTAGTT TAATTAGATT TGGTACTATA AATAAAAATA CTCATAGTTT TATTTGAATT   
  
  
+ TATGAAACCT AAAATAAGTT TATATCTTTT TATTTTTTCT CAACAAATAT ATATCCAAAA TAGAAAAATA   
  
  
+ AATTTCAAAA TTTTCTTAAT ATACCATAAC CGTTTTATGT CATTAATAAA TAATGTCACA ATAAAAATAA   
  
  
+ TAAAATAAAA TCTCATTACC ACCTACAACT TTTAGCCTTT TACTATAGTA CAAAATTCTT TCATCTTTAA   
  
  
+ TAATAATTAC TTTCTTTCAT TCAGTGGTGT CATAAATTAA AGACCCTACA GTATACCATA TGCCATGAAA   
  
  
+ TTAATGAAAT CTAACACAAA TCATTATGAT TTTATCACTT ATAAAAAAAT TATATTTTTC AACCGCACCA   
  
  
+ TATAATATAA CATATATATT AGATTAATAT AGCAACCAAA ATGAATCATT TGGAGGGTAC ACGAGTCCTT   
  
  
+ AACATGGACC TGACTTCGTC GAATCATTCC CAATCGTTGT GACTAAAGAA ATATTAGAAT TGCGTAATTG   
  
  
+ CTTACACGTC CCCATATAAA CAAAACAACT GCCAATCTGT CATTACCTGA CGGTCCCATA TTAAACCCTC   
  
  
+ CTTAAGCACC CACTAATCAT ATCCACTTCT CTTTCACGTT TCTCGCCGGC CTCAGTCTGC CTTGCCGGTG   
  
  
+ ATCCTACGCC TCCATGATCA TCGCTACCAT TGTCGTCGTC GCCGTTTCAG TACGAAGCCG ACGGTCTTGA   
  
  
+ TTAACTTACC ACCTGATCCA GCACCATAT  

- TCTTCCTTTC TTTATCCACA TTACTATCAC CCCCCTGTCT CAAACGTTGA TGGTAAGGAA ATAAAAAAGT   
  
  
- ACCACAAAAC CTAACTTACA GTTTAAATTC TTATTTAAAA GAGTTTAAAA GGACTACTAC TACGTATAAC   
  
  
- ATAGACTTCA TTTCTAAAAA AAAAAAATTT TATAGGTTTC ATCAATTGTA ATTCAGTAGA TGCCTAGTCT   
  
  
- AAATTAAGCC AGGTAAGTAT TAAACATTTT AAAATCAAAT AAATTTATTT TAACAACTAT AATAAAATTT   
  
  
- TTATTTTTAA TAAATTGTGA AGTTTGATAT AAAATATATA ATTTAGTTTC AAATCACAAA TTTTCATTAT   
  
  
- TTAGTTTGAA AATTAATAAT TAAACTAAAT TTAATTTAAA ACTAAATTAT AAAAAACAAT AATTTTTATA   
  
  
- AAATTTTTTA TTAAGTTTAT TTAACATATG AAATAAAATT TAGTTAAGAA ATTAAAGTTT ATTAAAAACC   
  
  
- TAAATACTCA ATAAATAATA TTTAAAAATA TTATTAAAAA GATTTTTGTA AAATCAGTTT TTTTGAATAT   
  
  
- GAAAAAATAT TACTATCAAA CTTATAAAAA TTTTTAAAAA TTATACTAAA TTAAACTATA TTTAATTATT   
  
  
- TAGTCTAAAA TTAAACAATG CAAAATTTGG AATTCCAATT TAAACTGTTT AGTATATGAA TTTTCAAAAT   
  
  
- TAATTAGAAA TGTTAATCAA ATTAATCTAA ACCATGATAT TTATTTTTAT GAGTATCAAA ATAAACTTAA   
  
  
- ATACTTTGGA TTTTATTCAA ATATAGAAAA ATAAAAAAGA GTTGTTTATA TATAGGTTTT ATCTTTTTAT   
  
  
- TTAAAGTTTT AAAAGAATTA TATGGTATTG GCAAAATACA GTAATTATTT ATTACAGTGT TATTTTTATT   
  
  
- ATTTTATTTT AGAGTAATGG TGGATGTTGA AAATCGGAAA ATGATATCAT GTTTTAAGAA AGTAGAAATT   
  
  
- ATTATTAATG AAAGAAAGTA AGTCACCACA GTATTTAATT TCTGGGATGT CATATGGTAT ACGGTACTTT   
  
  
- AATTACTTTA GATTGTGTTT AGTAATACTA AAATAGTGAA TATTTTTTTA ATATAAAAAG TTGGCGTGGT   
  
  
- ATATTATATT GTATATATAA TCTAATTATA TCGTTGGTTT TACTTAGTAA ACCTCCCATG TGCTCAGGAA   
  
  
- TTGTACCTGG ACTGAAGCAG CTTAGTAAGG GTTAGCAACA CTGATTTCTT TATAATCTTA ACGCATTAAC   
  
  
- GAATGTGCAG GGGTATATTT GTTTTGTTGA CGGTTAGACA GTAATGGACT GCCAGGGTAT AATTTGGGAG   
  
  
- GAATTCGTGG GTGATTAGTA TAGGTGAAGA GAAAGTGCAA AGAGCGGCCG GAGTCAGACG GAACGGCCAC   
  
  
- TAGGATGCGG AGGTACTAGT AGCGATGGTA ACAGCAGCAG CGGCAAAGTC ATGCTTCGGC TGCCAGAACT   
  
  
- AATTGAATGG TGGACTAGGT CGTGGTATA

+     TATA-box

| Site Name | Organism | Position | Strand | Matrix score. | sequence | function |
| --- | --- | --- | --- | --- | --- | --- |
| TATA-box | Lycopersicon esculentum | 916 | - | 5 | TTTTA | core promoter element around -30 of transcription start |
| TATA-box | Brassica oleracea | 617 | + | 6 | ATATAA | core promoter element around -30 of transcription start |
| TATA-box | Glycine max | 1145 | + | 5 | TAATA | core promoter element around -30 of transcription start |
| TATA-box | Lycopersicon esculentum | 948 | + | 5 | TTTTA | core promoter element around -30 of transcription start |
| TATA-box | Arabidopsis thaliana | 1142 | - | 9 | taTATAAAtc | core promoter element around -30 of transcription start |
| TATA-box | Arabidopsis thaliana | 1133 | - | 4 | TATA | core promoter element around -30 of transcription start |
| TATA-box | Glycine max | 1137 | - | 5 | TAATA | core promoter element around -30 of transcription start |
| TATA-box | Zea mays | 586 | - | 8 | TTTAAAAA | core promoter element around -30 of transcription start |
| TATA-box | Arabidopsis thaliana | 1148 | - | 4 | TATA | core promoter element around -30 of transcription start |
| TATA-box | Arabidopsis thaliana | 820 | - | 4 | TATA | core promoter element around -30 of transcription start |
| TATA-box | Arabidopsis thaliana | 954 | - | 4 | TATA | core promoter element around -30 of transcription start |
| TATA-box | Lycopersicon esculentum | 587 | + | 5 | TTTTA | core promoter element around -30 of transcription start |
| TATA-box | Glycine max | 1123 | + | 5 | TAATA | core promoter element around -30 of transcription start |
| TATA-box | Arabidopsis thaliana | 684 | + | 4 | TATA | core promoter element around -30 of transcription start |
| TATA-box | Brassica oleracea | 1274 | + | 6 | ATATAA | core promoter element around -30 of transcription start |
| TATA-box | Glycine max | 1319 | - | 5 | TAATA | core promoter element around -30 of transcription start |
| TATA-box | Zea mays | 421 | + | 8 | TTTAAAAA | core promoter element around -30 of transcription start |
| TATA-box | Arabidopsis thaliana | 150 | + | 8 | TAAAGATT | core promoter element around -30 of transcription start |
| TATA-box | Arabidopsis thaliana | 1089 | - | 5 | TATAA | core promoter element around -30 of transcription start |
| TATA-box | Arabidopsis thaliana | 1135 | - | 4 | TATA | core promoter element around -30 of transcription start |
| TATA-box | Arabidopsis thaliana | 790 | - | 5 | TATAA | core promoter element around -30 of transcription start |
| TATA-box | Arabidopsis thaliana | 446 | + | 4 | TATA | core promoter element around -30 of transcription start |
| TATA-box | Helianthus annuus | 444 | - | 6 | TATACA | core promoter element around -30 of transcription start |
| TATA-box | Lycopersicon esculentum | 283 | - | 5 | TTTTA | core promoter element around -30 of transcription start |
| TATA-box | Arabidopsis thaliana | 902 | + | 9 | TAAAAATAA | core promoter element around -30 of transcription start |
| TATA-box | Brassica napus | 1132 | - | 6 | ATATAT | core promoter element around -30 of transcription start |
| TATA-box | Brassica napus | 1134 | - | 6 | ATATAT | core promoter element around -30 of transcription start |
| TATA-box | Arabidopsis thaliana | 1126 | - | 4 | TATA | core promoter element around -30 of transcription start |
| TATA-box | Arabidopsis thaliana | 1275 | + | 6 | TATAAA | core promoter element around -30 of transcription start |
| TATA-box | Arabidopsis thaliana | 860 | - | 4 | TATA | core promoter element around -30 of transcription start |
| TATA-box | Lycopersicon esculentum | 412 | - | 5 | TTTTA | core promoter element around -30 of transcription start |
| TATA-box | Arabidopsis thaliana | 312 | - | 6 | TATAAA | core promoter element around -30 of transcription start |
| TATA-box | Arabidopsis thaliana | 1088 | + | 9 | ccTATAAAaa | core promoter element around -30 of transcription start |
| TATA-box | Lycopersicon esculentum | 1080 | + | 5 | TTTTA | core promoter element around -30 of transcription start |
| TATA-box | Glycine max | 625 | + | 5 | TAATA | core promoter element around -30 of transcription start |
| TATA-box | Arabidopsis thaliana | 249 | + | 8 | TATTTAAA | core promoter element around -30 of transcription start |
| TATA-box | Lycopersicon esculentum | 241 | + | 5 | TTTTA | core promoter element around -30 of transcription start |
| TATA-box | Brassica oleracea | 1120 | + | 7 | ATATAAT | core promoter element around -30 of transcription start |
| TATA-box | Brassica napus | 819 | - | 6 | ATATAT | core promoter element around -30 of transcription start |
| TATA-box | Brassica napus | 1099 | + | 6 | ATTATA | core promoter element around -30 of transcription start |
| TATA-box | Glycine max | 505 | - | 5 | TAATA | core promoter element around -30 of transcription start |
| TATA-box | Lycopersicon esculentum | 597 | + | 5 | TTTTA | core promoter element around -30 of transcription start |
| TATA-box | Brassica napus | 506 | + | 6 | ATTATA | core promoter element around -30 of transcription start |
| TATA-box | Ac | 737 | + | 7 | TATAAAT | core promoter element around -30 of transcription start |
| TATA-box | Brassica oleracea | 1125 | + | 6 | ATATAA | core promoter element around -30 of transcription start |
| TATA-box | Arabidopsis thaliana | 316 | + | 4 | TATA | core promoter element around -30 of transcription start |
| TATA-box | Lycopersicon esculentum | 637 | + | 5 | TTTTA | core promoter element around -30 of transcription start |
| TATA-box | Glycine max | 409 | - | 5 | TAATA | core promoter element around -30 of transcription start |
| TATA-box | Glycine max | 396 | + | 5 | TAATA | core promoter element around -30 of transcription start |
| TATA-box | Arabidopsis thaliana | 789 | - | 6 | TATAAA | core promoter element around -30 of transcription start |
| TATA-box | Lycopersicon esculentum | 1092 | - | 5 | TTTTA | core promoter element around -30 of transcription start |
| TATA-box | Arabidopsis thaliana | 507 | - | 5 | TATAA | core promoter element around -30 of transcription start |
| TATA-box | Arabidopsis thaliana | 556 | - | 5 | TATAA | core promoter element around -30 of transcription start |
| TATA-box | Glycine max | 366 | - | 5 | TAATA | core promoter element around -30 of transcription start |
| TATA-box | Lycopersicon esculentum | 696 | + | 5 | TTTTA | core promoter element around -30 of transcription start |
| TATA-box | Arabidopsis thaliana | 566 | - | 5 | TATAA | core promoter element around -30 of transcription start |
| TATA-box | Pisum sativum | 1130 | - | 7 | TATATGT | core promoter element around -30 of transcription start |
| TATA-box | Arabidopsis thaliana | 704 | - | 8 | TAAAGATT | core promoter element around -30 of transcription start |
| TATA-box | Nicotiana tabacum | 735 | + | 9 | tcTATAAAta | core promoter element around -30 of transcription start |
| TATA-box | Arabidopsis thaliana | 1032 | - | 4 | TATA | core promoter element around -30 of transcription start |
| TATA-box | Glycine max | 1242 | - | 5 | TAATA | core promoter element around -30 of transcription start |
| TATA-box | Arabidopsis thaliana | 1100 | - | 5 | TATAA | core promoter element around -30 of transcription start |
| TATA-box | Glycine max | 981 | + | 5 | TAATA | core promoter element around -30 of transcription start |
| TATA-box | Arabidopsis thaliana | 1121 | - | 4 | TATA | core promoter element around -30 of transcription start |
| TATA-box | Oryza sativa | 959 | + | 7 | TACAAAA | core promoter element around -30 of transcription start |
| TATA-box | Glycine max | 908 | + | 5 | TAATA | core promoter element around -30 of transcription start |
| TATA-box | Arabidopsis thaliana | 818 | - | 4 | TATA | core promoter element around -30 of transcription start |
| TATA-box | Brassica napus | 817 | - | 6 | ATATAT | core promoter element around -30 of transcription start |
| TATA-box | Lycopersicon esculentum | 798 | + | 5 | TTTTA | core promoter element around -30 of transcription start |
| TATA-box | Lycopersicon esculentum | 780 | - | 5 | TTTTA | core promoter element around -30 of transcription start |
| TATA-box | Arabidopsis thaliana | 1090 | + | 6 | TATAAA | core promoter element around -30 of transcription start |
| TATA-box | Lycopersicon esculentum | 743 | - | 5 | TTTTA | core promoter element around -30 of transcription start |
| TATA-box | Lycopersicon esculentum | 340 | - | 5 | TTTTA | core promoter element around -30 of transcription start |
| TATA-box | Glycine max | 978 | + | 5 | TAATA | core promoter element around -30 of transcription start |
| TATA-box | Arabidopsis thaliana | 313 | - | 7 | TATATAA | core promoter element around -30 of transcription start |
| TATA-box | Lycopersicon esculentum | 423 | - | 5 | TTTTA | core promoter element around -30 of transcription start |
| TATA-box | Lycopersicon esculentum | 420 | + | 5 | TTTTA | core promoter element around -30 of transcription start |
| TATA-box | Lycopersicon esculentum | 911 | - | 5 | TTTTA | core promoter element around -30 of transcription start |
| TATA-box | Arabidopsis thaliana | 565 | - | 6 | TATAAA | core promoter element around -30 of transcription start |
| TATA-box | Arabidopsis thaliana | 1101 | - | 4 | TATA | core promoter element around -30 of transcription start |
| TATA-box | Pisum sativum | 310 | - | 8 | TATAAAAT | core promoter element around -30 of transcription start |
| TATA-box | Lycopersicon esculentum | 652 | + | 5 | TTTTA | core promoter element around -30 of transcription start |
| TATA-box | Lycopersicon esculentum | 873 | + | 5 | TTTTA | core promoter element around -30 of transcription start |
| TATA-box | Lycopersicon esculentum | 532 | - | 5 | TTTTA | core promoter element around -30 of transcription start |
| TATA-box | Glycine max | 520 | + | 5 | TAATA | core promoter element around -30 of transcription start |
| TATA-box | Arabidopsis thaliana | 314 | + | 4 | TATA | core promoter element around -30 of transcription start |
| TATA-box | Arabidopsis thaliana | 517 | - | 5 | TATAA | core promoter element around -30 of transcription start |
| TATA-box | Arabidopsis thaliana | 515 | - | 7 | TATAAAA | core promoter element around -30 of transcription start |
| TATA-box | Zea mays | 275 | + | 8 | TTTAAAAA | core promoter element around -30 of transcription start |
| TATA-box | Lycopersicon esculentum | 690 | - | 5 | TTTTA | core promoter element around -30 of transcription start |
| TATA-box | Glycine max | 884 | + | 5 | TAATA | core promoter element around -30 of transcription start |
| TATA-box | Lycopersicon esculentum | 167 | - | 5 | TTTTA | core promoter element around -30 of transcription start |
| TATA-box | Arabidopsis thaliana | 791 | - | 4 | TATA | core promoter element around -30 of transcription start |
| TATA-box | Arabidopsis thaliana | 567 | + | 4 | TATA | core promoter element around -30 of transcription start |
| TATA-box | Arabidopsis thaliana | 557 | + | 4 | TATA | core promoter element around -30 of transcription start |
| TATA-box | Arabidopsis thaliana | 564 | - | 7 | TATAAAA | core promoter element around -30 of transcription start |
| TATA-box | Lycopersicon esculentum | 257 | - | 5 | TTTTA | core promoter element around -30 of transcription start |
| TATA-box | Lycopersicon esculentum | 940 | + | 5 | TTTTA | core promoter element around -30 of transcription start |
| TATA-box | Lycopersicon esculentum | 758 | + | 5 | TTTTA | core promoter element around -30 of transcription start |
| TATA-box | Lycopersicon esculentum | 455 | + | 5 | TTTTA | core promoter element around -30 of transcription start |
| TATA-box | Ac | 508 | + | 7 | TATAAAT | core promoter element around -30 of transcription start |
| TATA-box | Glycine max | 857 | + | 5 | TAATA | core promoter element around -30 of transcription start |
| TATA-box | Zea mays | 163 | - | 8 | TTTAAAAA | core promoter element around -30 of transcription start |
| TATA-box | Glycine max | 318 | - | 5 | TAATA | core promoter element around -30 of transcription start |
| TATA-box | Ac | 618 | + | 7 | TATAAAT | core promoter element around -30 of transcription start |
| TATA-box | Lycopersicon esculentum | 590 | - | 5 | TTTTA | core promoter element around -30 of transcription start |
| TATA-box | Lycopersicon esculentum | 236 | - | 5 | TTTTA | core promoter element around -30 of transcription start |
| TATA-box | Arabidopsis thaliana | 307 | + | 4 | TATA | core promoter element around -30 of transcription start |
| TATA-box | Zea mays | 588 | + | 8 | TTTAAAAA | core promoter element around -30 of transcription start |
| TATA-box | Glycine max | 346 | + | 5 | TAATA | core promoter element around -30 of transcription start |
| TATA-box | Arabidopsis thaliana | 311 | - | 7 | TATAAAA | core promoter element around -30 of transcription start |
| TATA-box | Glycine max | 269 | - | 5 | TAATA | core promoter element around -30 of transcription start |
| TATA-box | Arabidopsis thaliana | 251 | - | 8 | TATTTAAA | core promoter element around -30 of transcription start |
| TATA-box | Arabidopsis thaliana | 277 | + | 9 | TAAAAATAA | core promoter element around -30 of transcription start |
| TATA-box | Glycine max | 600 | + | 5 | TAATA | core promoter element around -30 of transcription start |
| TATA-box | Lycopersicon esculentum | 164 | + | 5 | TTTTA | core promoter element around -30 of transcription start |
| TATA-box | Lycopersicon esculentum | 540 | + | 5 | TTTTA | core promoter element around -30 of transcription start |
| TATA-box | Arabidopsis thaliana | 516 | - | 6 | TATAAA | core promoter element around -30 of transcription start |
| TATA-box | Lycopersicon esculentum | 359 | + | 5 | TTTTA | core promoter element around -30 of transcription start |
| TATA-box | Lycopersicon esculentum | 274 | + | 5 | TTTTA | core promoter element around -30 of transcription start |
| TATA-box | Arabidopsis thaliana | 518 | + | 4 | TATA | core promoter element around -30 of transcription start |
| TATA-box | Brassica napus | 315 | + | 6 | ATATAT | core promoter element around -30 of transcription start |

> 2018/04/13 10:10:12  
+ AGAAGGAAAG AAATAGGTGT AATGATAGTG GGGGGACAGA GTTTGCAACT ACCATTCCTT TATTTTTTCA   
  
  
+ TGGTGTTTTG GATTGAATGT CAAATTTAAG AATAAATTTT CTCAAATTTT CCTGATGATG ATGCATATTG   
  
  
+ TATCTGAAGT AAAGATTTTT TTTTTTTAAA ATATCCAAAG TAGTTAACAT TAAGTCATCT ACGGATCAGA   
  
  
+ TTTAATTCGG TCCATTCATA ATTTGTAAAA TTTTAGTTTA TTTAAATAAA ATTGTTGATA TTATTTTAAA   
  
  
+ AATAAAAATT ATTTAACACT TCAAACTATA TTTTATATAT TAAATCAAAG TTTAGTGTTT AAAAGTAATA   
  
  
+ AATCAAACTT TTAATTATTA ATTTGATTTA AATTAAATTT TGATTTAATA TTTTTTGTTA TTAAAAATAT   
  
  
+ TTTAAAAAAT AATTCAAATA AATTGTATAC TTTATTTTAA ATCAATTCTT TAATTTCAAA TAATTTTTGG   
  
  
+ ATTTATGAGT TATTTATTAT AAATTTTTAT AATAATTTTT CTAAAAACAT TTTAGTCAAA AAAACTTATA   
  
  
+ CTTTTTTATA ATGATAGTTT GAATATTTTT AAAAATTTTT AATATGATTT AATTTGATAT AAATTAATAA   
  
  
+ ATCAGATTTT AATTTGTTAC GTTTTAAACC TTAAGGTTAA ATTTGACAAA TCATATACTT AAAAGTTTTA   
  
  
+ ATTAATCTTT ACAATTAGTT TAATTAGATT TGGTACTATA AATAAAAATA CTCATAGTTT TATTTGAATT   
  
  
+ TATGAAACCT AAAATAAGTT TATATCTTTT TATTTTTTCT CAACAAATAT ATATCCAAAA TAGAAAAATA   
  
  
+ AATTTCAAAA TTTTCTTAAT ATACCATAAC CGTTTTATGT CATTAATAAA TAATGTCACA ATAAAAATAA   
  
  
+ TAAAATAAAA TCTCATTACC ACCTACAACT TTTAGCCTTT TACTATAGTA CAAAATTCTT TCATCTTTAA   
  
  
+ TAATAATTAC TTTCTTTCAT TCAGTGGTGT CATAAATTAA AGACCCTACA GTATACCATA TGCCATGAAA   
  
  
+ TTAATGAAAT CTAACACAAA TCATTATGAT TTTATCACTT ATAAAAAAAT TATATTTTTC AACCGCACCA   
  
  
+ TATAATATAA CATATATATT AGATTAATAT AGCAACCAAA ATGAATCATT TGGAGGGTAC ACGAGTCCTT   
  
  
+ AACATGGACC TGACTTCGTC GAATCATTCC CAATCGTTGT GACTAAAGAA ATATTAGAAT TGCGTAATTG   
  
  
+ CTTACACGTC CCCATATAAA CAAAACAACT GCCAATCTGT CATTACCTGA CGGTCCCATA TTAAACCCTC   
  
  
+ CTTAAGCACC CACTAATCAT ATCCACTTCT CTTTCACGTT TCTCGCCGGC CTCAGTCTGC CTTGCCGGTG   
  
  
+ ATCCTACGCC TCCATGATCA TCGCTACCAT TGTCGTCGTC GCCGTTTCAG TACGAAGCCG ACGGTCTTGA   
  
  
+ TTAACTTACC ACCTGATCCA GCACCATAT  

- TCTTCCTTTC TTTATCCACA TTACTATCAC CCCCCTGTCT CAAACGTTGA TGGTAAGGAA ATAAAAAAGT   
  
  
- ACCACAAAAC CTAACTTACA GTTTAAATTC TTATTTAAAA GAGTTTAAAA GGACTACTAC TACGTATAAC   
  
  
- ATAGACTTCA TTTCTAAAAA AAAAAAATTT TATAGGTTTC ATCAATTGTA ATTCAGTAGA TGCCTAGTCT   
  
  
- AAATTAAGCC AGGTAAGTAT TAAACATTTT AAAATCAAAT AAATTTATTT TAACAACTAT AATAAAATTT   
  
  
- TTATTTTTAA TAAATTGTGA AGTTTGATAT AAAATATATA ATTTAGTTTC AAATCACAAA TTTTCATTAT   
  
  
- TTAGTTTGAA AATTAATAAT TAAACTAAAT TTAATTTAAA ACTAAATTAT AAAAAACAAT AATTTTTATA   
  
  
- AAATTTTTTA TTAAGTTTAT TTAACATATG AAATAAAATT TAGTTAAGAA ATTAAAGTTT ATTAAAAACC   
  
  
- TAAATACTCA ATAAATAATA TTTAAAAATA TTATTAAAAA GATTTTTGTA AAATCAGTTT TTTTGAATAT   
  
  
- GAAAAAATAT TACTATCAAA CTTATAAAAA TTTTTAAAAA TTATACTAAA TTAAACTATA TTTAATTATT   
  
  
- TAGTCTAAAA TTAAACAATG CAAAATTTGG AATTCCAATT TAAACTGTTT AGTATATGAA TTTTCAAAAT   
  
  
- TAATTAGAAA TGTTAATCAA ATTAATCTAA ACCATGATAT TTATTTTTAT GAGTATCAAA ATAAACTTAA   
  
  
- ATACTTTGGA TTTTATTCAA ATATAGAAAA ATAAAAAAGA GTTGTTTATA TATAGGTTTT ATCTTTTTAT   
  
  
- TTAAAGTTTT AAAAGAATTA TATGGTATTG GCAAAATACA GTAATTATTT ATTACAGTGT TATTTTTATT   
  
  
- ATTTTATTTT AGAGTAATGG TGGATGTTGA AAATCGGAAA ATGATATCAT GTTTTAAGAA AGTAGAAATT   
  
  
- ATTATTAATG AAAGAAAGTA AGTCACCACA GTATTTAATT TCTGGGATGT CATATGGTAT ACGGTACTTT   
  
  
- AATTACTTTA GATTGTGTTT AGTAATACTA AAATAGTGAA TATTTTTTTA ATATAAAAAG TTGGCGTGGT   
  
  
- ATATTATATT GTATATATAA TCTAATTATA TCGTTGGTTT TACTTAGTAA ACCTCCCATG TGCTCAGGAA   
  
  
- TTGTACCTGG ACTGAAGCAG CTTAGTAAGG GTTAGCAACA CTGATTTCTT TATAATCTTA ACGCATTAAC   
  
  
- GAATGTGCAG GGGTATATTT GTTTTGTTGA CGGTTAGACA GTAATGGACT GCCAGGGTAT AATTTGGGAG   
  
  
- GAATTCGTGG GTGATTAGTA TAGGTGAAGA GAAAGTGCAA AGAGCGGCCG GAGTCAGACG GAACGGCCAC   
  
  
- TAGGATGCGG AGGTACTAGT AGCGATGGTA ACAGCAGCAG CGGCAAAGTC ATGCTTCGGC TGCCAGAACT   
  
  
- AATTGAATGG TGGACTAGGT CGTGGTATA

+     TC-rich repeats

| Site Name | Organism | Position | Strand | Matrix score. | sequence | function |
| --- | --- | --- | --- | --- | --- | --- |
| TC-rich repeats | Nicotiana tabacum | 106 | + | 9 | ATTTTCTCCA | cis-acting element involved in defense and stress responsiveness |
| TC-rich repeats | Nicotiana tabacum | 850 | + | 9 | ATTTTCTTCA | cis-acting element involved in defense and stress responsiveness |

> 2018/04/13 10:10:12  
+ AGAAGGAAAG AAATAGGTGT AATGATAGTG GGGGGACAGA GTTTGCAACT ACCATTCCTT TATTTTTTCA   
  
  
+ TGGTGTTTTG GATTGAATGT CAAATTTAAG AATAAATTTT CTCAAATTTT CCTGATGATG ATGCATATTG   
  
  
+ TATCTGAAGT AAAGATTTTT TTTTTTTAAA ATATCCAAAG TAGTTAACAT TAAGTCATCT ACGGATCAGA   
  
  
+ TTTAATTCGG TCCATTCATA ATTTGTAAAA TTTTAGTTTA TTTAAATAAA ATTGTTGATA TTATTTTAAA   
  
  
+ AATAAAAATT ATTTAACACT TCAAACTATA TTTTATATAT TAAATCAAAG TTTAGTGTTT AAAAGTAATA   
  
  
+ AATCAAACTT TTAATTATTA ATTTGATTTA AATTAAATTT TGATTTAATA TTTTTTGTTA TTAAAAATAT   
  
  
+ TTTAAAAAAT AATTCAAATA AATTGTATAC TTTATTTTAA ATCAATTCTT TAATTTCAAA TAATTTTTGG   
  
  
+ ATTTATGAGT TATTTATTAT AAATTTTTAT AATAATTTTT CTAAAAACAT TTTAGTCAAA AAAACTTATA   
  
  
+ CTTTTTTATA ATGATAGTTT GAATATTTTT AAAAATTTTT AATATGATTT AATTTGATAT AAATTAATAA   
  
  
+ ATCAGATTTT AATTTGTTAC GTTTTAAACC TTAAGGTTAA ATTTGACAAA TCATATACTT AAAAGTTTTA   
  
  
+ ATTAATCTTT ACAATTAGTT TAATTAGATT TGGTACTATA AATAAAAATA CTCATAGTTT TATTTGAATT   
  
  
+ TATGAAACCT AAAATAAGTT TATATCTTTT TATTTTTTCT CAACAAATAT ATATCCAAAA TAGAAAAATA   
  
  
+ AATTTCAAAA TTTTCTTAAT ATACCATAAC CGTTTTATGT CATTAATAAA TAATGTCACA ATAAAAATAA   
  
  
+ TAAAATAAAA TCTCATTACC ACCTACAACT TTTAGCCTTT TACTATAGTA CAAAATTCTT TCATCTTTAA   
  
  
+ TAATAATTAC TTTCTTTCAT TCAGTGGTGT CATAAATTAA AGACCCTACA GTATACCATA TGCCATGAAA   
  
  
+ TTAATGAAAT CTAACACAAA TCATTATGAT TTTATCACTT ATAAAAAAAT TATATTTTTC AACCGCACCA   
  
  
+ TATAATATAA CATATATATT AGATTAATAT AGCAACCAAA ATGAATCATT TGGAGGGTAC ACGAGTCCTT   
  
  
+ AACATGGACC TGACTTCGTC GAATCATTCC CAATCGTTGT GACTAAAGAA ATATTAGAAT TGCGTAATTG   
  
  
+ CTTACACGTC CCCATATAAA CAAAACAACT GCCAATCTGT CATTACCTGA CGGTCCCATA TTAAACCCTC   
  
  
+ CTTAAGCACC CACTAATCAT ATCCACTTCT CTTTCACGTT TCTCGCCGGC CTCAGTCTGC CTTGCCGGTG   
  
  
+ ATCCTACGCC TCCATGATCA TCGCTACCAT TGTCGTCGTC GCCGTTTCAG TACGAAGCCG ACGGTCTTGA   
  
  
+ TTAACTTACC ACCTGATCCA GCACCATAT  

- TCTTCCTTTC TTTATCCACA TTACTATCAC CCCCCTGTCT CAAACGTTGA TGGTAAGGAA ATAAAAAAGT   
  
  
- ACCACAAAAC CTAACTTACA GTTTAAATTC TTATTTAAAA GAGTTTAAAA GGACTACTAC TACGTATAAC   
  
  
- ATAGACTTCA TTTCTAAAAA AAAAAAATTT TATAGGTTTC ATCAATTGTA ATTCAGTAGA TGCCTAGTCT   
  
  
- AAATTAAGCC AGGTAAGTAT TAAACATTTT AAAATCAAAT AAATTTATTT TAACAACTAT AATAAAATTT   
  
  
- TTATTTTTAA TAAATTGTGA AGTTTGATAT AAAATATATA ATTTAGTTTC AAATCACAAA TTTTCATTAT   
  
  
- TTAGTTTGAA AATTAATAAT TAAACTAAAT TTAATTTAAA ACTAAATTAT AAAAAACAAT AATTTTTATA   
  
  
- AAATTTTTTA TTAAGTTTAT TTAACATATG AAATAAAATT TAGTTAAGAA ATTAAAGTTT ATTAAAAACC   
  
  
- TAAATACTCA ATAAATAATA TTTAAAAATA TTATTAAAAA GATTTTTGTA AAATCAGTTT TTTTGAATAT   
  
  
- GAAAAAATAT TACTATCAAA CTTATAAAAA TTTTTAAAAA TTATACTAAA TTAAACTATA TTTAATTATT   
  
  
- TAGTCTAAAA TTAAACAATG CAAAATTTGG AATTCCAATT TAAACTGTTT AGTATATGAA TTTTCAAAAT   
  
  
- TAATTAGAAA TGTTAATCAA ATTAATCTAA ACCATGATAT TTATTTTTAT GAGTATCAAA ATAAACTTAA   
  
  
- ATACTTTGGA TTTTATTCAA ATATAGAAAA ATAAAAAAGA GTTGTTTATA TATAGGTTTT ATCTTTTTAT   
  
  
- TTAAAGTTTT AAAAGAATTA TATGGTATTG GCAAAATACA GTAATTATTT ATTACAGTGT TATTTTTATT   
  
  
- ATTTTATTTT AGAGTAATGG TGGATGTTGA AAATCGGAAA ATGATATCAT GTTTTAAGAA AGTAGAAATT   
  
  
- ATTATTAATG AAAGAAAGTA AGTCACCACA GTATTTAATT TCTGGGATGT CATATGGTAT ACGGTACTTT   
  
  
- AATTACTTTA GATTGTGTTT AGTAATACTA AAATAGTGAA TATTTTTTTA ATATAAAAAG TTGGCGTGGT   
  
  
- ATATTATATT GTATATATAA TCTAATTATA TCGTTGGTTT TACTTAGTAA ACCTCCCATG TGCTCAGGAA   
  
  
- TTGTACCTGG ACTGAAGCAG CTTAGTAAGG GTTAGCAACA CTGATTTCTT TATAATCTTA ACGCATTAAC   
  
  
- GAATGTGCAG GGGTATATTT GTTTTGTTGA CGGTTAGACA GTAATGGACT GCCAGGGTAT AATTTGGGAG   
  
  
- GAATTCGTGG GTGATTAGTA TAGGTGAAGA GAAAGTGCAA AGAGCGGCCG GAGTCAGACG GAACGGCCAC   
  
  
- TAGGATGCGG AGGTACTAGT AGCGATGGTA ACAGCAGCAG CGGCAAAGTC ATGCTTCGGC TGCCAGAACT   
  
  
- AATTGAATGG TGGACTAGGT CGTGGTATA

+     TGACG-motif

| Site Name | Organism | Position | Strand | Matrix score. | sequence | function |
| --- | --- | --- | --- | --- | --- | --- |
| TGACG-motif | Hordeum vulgare | 1308 | + | 5 | TGACG | cis-acting regulatory element involved in the MeJA-responsiveness |

> 2018/04/13 10:10:12  
+ AGAAGGAAAG AAATAGGTGT AATGATAGTG GGGGGACAGA GTTTGCAACT ACCATTCCTT TATTTTTTCA   
  
  
+ TGGTGTTTTG GATTGAATGT CAAATTTAAG AATAAATTTT CTCAAATTTT CCTGATGATG ATGCATATTG   
  
  
+ TATCTGAAGT AAAGATTTTT TTTTTTTAAA ATATCCAAAG TAGTTAACAT TAAGTCATCT ACGGATCAGA   
  
  
+ TTTAATTCGG TCCATTCATA ATTTGTAAAA TTTTAGTTTA TTTAAATAAA ATTGTTGATA TTATTTTAAA   
  
  
+ AATAAAAATT ATTTAACACT TCAAACTATA TTTTATATAT TAAATCAAAG TTTAGTGTTT AAAAGTAATA   
  
  
+ AATCAAACTT TTAATTATTA ATTTGATTTA AATTAAATTT TGATTTAATA TTTTTTGTTA TTAAAAATAT   
  
  
+ TTTAAAAAAT AATTCAAATA AATTGTATAC TTTATTTTAA ATCAATTCTT TAATTTCAAA TAATTTTTGG   
  
  
+ ATTTATGAGT TATTTATTAT AAATTTTTAT AATAATTTTT CTAAAAACAT TTTAGTCAAA AAAACTTATA   
  
  
+ CTTTTTTATA ATGATAGTTT GAATATTTTT AAAAATTTTT AATATGATTT AATTTGATAT AAATTAATAA   
  
  
+ ATCAGATTTT AATTTGTTAC GTTTTAAACC TTAAGGTTAA ATTTGACAAA TCATATACTT AAAAGTTTTA   
  
  
+ ATTAATCTTT ACAATTAGTT TAATTAGATT TGGTACTATA AATAAAAATA CTCATAGTTT TATTTGAATT   
  
  
+ TATGAAACCT AAAATAAGTT TATATCTTTT TATTTTTTCT CAACAAATAT ATATCCAAAA TAGAAAAATA   
  
  
+ AATTTCAAAA TTTTCTTAAT ATACCATAAC CGTTTTATGT CATTAATAAA TAATGTCACA ATAAAAATAA   
  
  
+ TAAAATAAAA TCTCATTACC ACCTACAACT TTTAGCCTTT TACTATAGTA CAAAATTCTT TCATCTTTAA   
  
  
+ TAATAATTAC TTTCTTTCAT TCAGTGGTGT CATAAATTAA AGACCCTACA GTATACCATA TGCCATGAAA   
  
  
+ TTAATGAAAT CTAACACAAA TCATTATGAT TTTATCACTT ATAAAAAAAT TATATTTTTC AACCGCACCA   
  
  
+ TATAATATAA CATATATATT AGATTAATAT AGCAACCAAA ATGAATCATT TGGAGGGTAC ACGAGTCCTT   
  
  
+ AACATGGACC TGACTTCGTC GAATCATTCC CAATCGTTGT GACTAAAGAA ATATTAGAAT TGCGTAATTG   
  
  
+ CTTACACGTC CCCATATAAA CAAAACAACT GCCAATCTGT CATTACCTGA CGGTCCCATA TTAAACCCTC   
  
  
+ CTTAAGCACC CACTAATCAT ATCCACTTCT CTTTCACGTT TCTCGCCGGC CTCAGTCTGC CTTGCCGGTG   
  
  
+ ATCCTACGCC TCCATGATCA TCGCTACCAT TGTCGTCGTC GCCGTTTCAG TACGAAGCCG ACGGTCTTGA   
  
  
+ TTAACTTACC ACCTGATCCA GCACCATAT  

- TCTTCCTTTC TTTATCCACA TTACTATCAC CCCCCTGTCT CAAACGTTGA TGGTAAGGAA ATAAAAAAGT   
  
  
- ACCACAAAAC CTAACTTACA GTTTAAATTC TTATTTAAAA GAGTTTAAAA GGACTACTAC TACGTATAAC   
  
  
- ATAGACTTCA TTTCTAAAAA AAAAAAATTT TATAGGTTTC ATCAATTGTA ATTCAGTAGA TGCCTAGTCT   
  
  
- AAATTAAGCC AGGTAAGTAT TAAACATTTT AAAATCAAAT AAATTTATTT TAACAACTAT AATAAAATTT   
  
  
- TTATTTTTAA TAAATTGTGA AGTTTGATAT AAAATATATA ATTTAGTTTC AAATCACAAA TTTTCATTAT   
  
  
- TTAGTTTGAA AATTAATAAT TAAACTAAAT TTAATTTAAA ACTAAATTAT AAAAAACAAT AATTTTTATA   
  
  
- AAATTTTTTA TTAAGTTTAT TTAACATATG AAATAAAATT TAGTTAAGAA ATTAAAGTTT ATTAAAAACC   
  
  
- TAAATACTCA ATAAATAATA TTTAAAAATA TTATTAAAAA GATTTTTGTA AAATCAGTTT TTTTGAATAT   
  
  
- GAAAAAATAT TACTATCAAA CTTATAAAAA TTTTTAAAAA TTATACTAAA TTAAACTATA TTTAATTATT   
  
  
- TAGTCTAAAA TTAAACAATG CAAAATTTGG AATTCCAATT TAAACTGTTT AGTATATGAA TTTTCAAAAT   
  
  
- TAATTAGAAA TGTTAATCAA ATTAATCTAA ACCATGATAT TTATTTTTAT GAGTATCAAA ATAAACTTAA   
  
  
- ATACTTTGGA TTTTATTCAA ATATAGAAAA ATAAAAAAGA GTTGTTTATA TATAGGTTTT ATCTTTTTAT   
  
  
- TTAAAGTTTT AAAAGAATTA TATGGTATTG GCAAAATACA GTAATTATTT ATTACAGTGT TATTTTTATT   
  
  
- ATTTTATTTT AGAGTAATGG TGGATGTTGA AAATCGGAAA ATGATATCAT GTTTTAAGAA AGTAGAAATT   
  
  
- ATTATTAATG AAAGAAAGTA AGTCACCACA GTATTTAATT TCTGGGATGT CATATGGTAT ACGGTACTTT   
  
  
- AATTACTTTA GATTGTGTTT AGTAATACTA AAATAGTGAA TATTTTTTTA ATATAAAAAG TTGGCGTGGT   
  
  
- ATATTATATT GTATATATAA TCTAATTATA TCGTTGGTTT TACTTAGTAA ACCTCCCATG TGCTCAGGAA   
  
  
- TTGTACCTGG ACTGAAGCAG CTTAGTAAGG GTTAGCAACA CTGATTTCTT TATAATCTTA ACGCATTAAC   
  
  
- GAATGTGCAG GGGTATATTT GTTTTGTTGA CGGTTAGACA GTAATGGACT GCCAGGGTAT AATTTGGGAG   
  
  
- GAATTCGTGG GTGATTAGTA TAGGTGAAGA GAAAGTGCAA AGAGCGGCCG GAGTCAGACG GAACGGCCAC   
  
  
- TAGGATGCGG AGGTACTAGT AGCGATGGTA ACAGCAGCAG CGGCAAAGTC ATGCTTCGGC TGCCAGAACT   
  
  
- AATTGAATGG TGGACTAGGT CGTGGTATA

+     Unnamed\_\_4

| Site Name | Organism | Position | Strand | Matrix score. | sequence | function |
| --- | --- | --- | --- | --- | --- | --- |
| Unnamed\_\_4 | Petroselinum hortense | 1328 | + | 4 | CTCC |  |
| Unnamed\_\_4 | Petroselinum hortense | 1172 | - | 4 | CTCC |  |
| Unnamed\_\_4 | Petroselinum hortense | 1410 | + | 4 | CTCC |  |

> 2018/04/13 10:10:12  
+ AGAAGGAAAG AAATAGGTGT AATGATAGTG GGGGGACAGA GTTTGCAACT ACCATTCCTT TATTTTTTCA   
  
  
+ TGGTGTTTTG GATTGAATGT CAAATTTAAG AATAAATTTT CTCAAATTTT CCTGATGATG ATGCATATTG   
  
  
+ TATCTGAAGT AAAGATTTTT TTTTTTTAAA ATATCCAAAG TAGTTAACAT TAAGTCATCT ACGGATCAGA   
  
  
+ TTTAATTCGG TCCATTCATA ATTTGTAAAA TTTTAGTTTA TTTAAATAAA ATTGTTGATA TTATTTTAAA   
  
  
+ AATAAAAATT ATTTAACACT TCAAACTATA TTTTATATAT TAAATCAAAG TTTAGTGTTT AAAAGTAATA   
  
  
+ AATCAAACTT TTAATTATTA ATTTGATTTA AATTAAATTT TGATTTAATA TTTTTTGTTA TTAAAAATAT   
  
  
+ TTTAAAAAAT AATTCAAATA AATTGTATAC TTTATTTTAA ATCAATTCTT TAATTTCAAA TAATTTTTGG   
  
  
+ ATTTATGAGT TATTTATTAT AAATTTTTAT AATAATTTTT CTAAAAACAT TTTAGTCAAA AAAACTTATA   
  
  
+ CTTTTTTATA ATGATAGTTT GAATATTTTT AAAAATTTTT AATATGATTT AATTTGATAT AAATTAATAA   
  
  
+ ATCAGATTTT AATTTGTTAC GTTTTAAACC TTAAGGTTAA ATTTGACAAA TCATATACTT AAAAGTTTTA   
  
  
+ ATTAATCTTT ACAATTAGTT TAATTAGATT TGGTACTATA AATAAAAATA CTCATAGTTT TATTTGAATT   
  
  
+ TATGAAACCT AAAATAAGTT TATATCTTTT TATTTTTTCT CAACAAATAT ATATCCAAAA TAGAAAAATA   
  
  
+ AATTTCAAAA TTTTCTTAAT ATACCATAAC CGTTTTATGT CATTAATAAA TAATGTCACA ATAAAAATAA   
  
  
+ TAAAATAAAA TCTCATTACC ACCTACAACT TTTAGCCTTT TACTATAGTA CAAAATTCTT TCATCTTTAA   
  
  
+ TAATAATTAC TTTCTTTCAT TCAGTGGTGT CATAAATTAA AGACCCTACA GTATACCATA TGCCATGAAA   
  
  
+ TTAATGAAAT CTAACACAAA TCATTATGAT TTTATCACTT ATAAAAAAAT TATATTTTTC AACCGCACCA   
  
  
+ TATAATATAA CATATATATT AGATTAATAT AGCAACCAAA ATGAATCATT TGGAGGGTAC ACGAGTCCTT   
  
  
+ AACATGGACC TGACTTCGTC GAATCATTCC CAATCGTTGT GACTAAAGAA ATATTAGAAT TGCGTAATTG   
  
  
+ CTTACACGTC CCCATATAAA CAAAACAACT GCCAATCTGT CATTACCTGA CGGTCCCATA TTAAACCCTC   
  
  
+ CTTAAGCACC CACTAATCAT ATCCACTTCT CTTTCACGTT TCTCGCCGGC CTCAGTCTGC CTTGCCGGTG   
  
  
+ ATCCTACGCC TCCATGATCA TCGCTACCAT TGTCGTCGTC GCCGTTTCAG TACGAAGCCG ACGGTCTTGA   
  
  
+ TTAACTTACC ACCTGATCCA GCACCATAT  

- TCTTCCTTTC TTTATCCACA TTACTATCAC CCCCCTGTCT CAAACGTTGA TGGTAAGGAA ATAAAAAAGT   
  
  
- ACCACAAAAC CTAACTTACA GTTTAAATTC TTATTTAAAA GAGTTTAAAA GGACTACTAC TACGTATAAC   
  
  
- ATAGACTTCA TTTCTAAAAA AAAAAAATTT TATAGGTTTC ATCAATTGTA ATTCAGTAGA TGCCTAGTCT   
  
  
- AAATTAAGCC AGGTAAGTAT TAAACATTTT AAAATCAAAT AAATTTATTT TAACAACTAT AATAAAATTT   
  
  
- TTATTTTTAA TAAATTGTGA AGTTTGATAT AAAATATATA ATTTAGTTTC AAATCACAAA TTTTCATTAT   
  
  
- TTAGTTTGAA AATTAATAAT TAAACTAAAT TTAATTTAAA ACTAAATTAT AAAAAACAAT AATTTTTATA   
  
  
- AAATTTTTTA TTAAGTTTAT TTAACATATG AAATAAAATT TAGTTAAGAA ATTAAAGTTT ATTAAAAACC   
  
  
- TAAATACTCA ATAAATAATA TTTAAAAATA TTATTAAAAA GATTTTTGTA AAATCAGTTT TTTTGAATAT   
  
  
- GAAAAAATAT TACTATCAAA CTTATAAAAA TTTTTAAAAA TTATACTAAA TTAAACTATA TTTAATTATT   
  
  
- TAGTCTAAAA TTAAACAATG CAAAATTTGG AATTCCAATT TAAACTGTTT AGTATATGAA TTTTCAAAAT   
  
  
- TAATTAGAAA TGTTAATCAA ATTAATCTAA ACCATGATAT TTATTTTTAT GAGTATCAAA ATAAACTTAA   
  
  
- ATACTTTGGA TTTTATTCAA ATATAGAAAA ATAAAAAAGA GTTGTTTATA TATAGGTTTT ATCTTTTTAT   
  
  
- TTAAAGTTTT AAAAGAATTA TATGGTATTG GCAAAATACA GTAATTATTT ATTACAGTGT TATTTTTATT   
  
  
- ATTTTATTTT AGAGTAATGG TGGATGTTGA AAATCGGAAA ATGATATCAT GTTTTAAGAA AGTAGAAATT   
  
  
- ATTATTAATG AAAGAAAGTA AGTCACCACA GTATTTAATT TCTGGGATGT CATATGGTAT ACGGTACTTT   
  
  
- AATTACTTTA GATTGTGTTT AGTAATACTA AAATAGTGAA TATTTTTTTA ATATAAAAAG TTGGCGTGGT   
  
  
- ATATTATATT GTATATATAA TCTAATTATA TCGTTGGTTT TACTTAGTAA ACCTCCCATG TGCTCAGGAA   
  
  
- TTGTACCTGG ACTGAAGCAG CTTAGTAAGG GTTAGCAACA CTGATTTCTT TATAATCTTA ACGCATTAAC   
  
  
- GAATGTGCAG GGGTATATTT GTTTTGTTGA CGGTTAGACA GTAATGGACT GCCAGGGTAT AATTTGGGAG   
  
  
- GAATTCGTGG GTGATTAGTA TAGGTGAAGA GAAAGTGCAA AGAGCGGCCG GAGTCAGACG GAACGGCCAC   
  
  
- TAGGATGCGG AGGTACTAGT AGCGATGGTA ACAGCAGCAG CGGCAAAGTC ATGCTTCGGC TGCCAGAACT   
  
  
- AATTGAATGG TGGACTAGGT CGTGGTATA

+     WUN-motif

| Site Name | Organism | Position | Strand | Matrix score. | sequence | function |
| --- | --- | --- | --- | --- | --- | --- |
| WUN-motif | Brassica oleracea | 1447 | + | 9 | TCATTACGAA | wound-responsive element |

> 2018/04/13 10:10:12  
+ AGAAGGAAAG AAATAGGTGT AATGATAGTG GGGGGACAGA GTTTGCAACT ACCATTCCTT TATTTTTTCA   
  
  
+ TGGTGTTTTG GATTGAATGT CAAATTTAAG AATAAATTTT CTCAAATTTT CCTGATGATG ATGCATATTG   
  
  
+ TATCTGAAGT AAAGATTTTT TTTTTTTAAA ATATCCAAAG TAGTTAACAT TAAGTCATCT ACGGATCAGA   
  
  
+ TTTAATTCGG TCCATTCATA ATTTGTAAAA TTTTAGTTTA TTTAAATAAA ATTGTTGATA TTATTTTAAA   
  
  
+ AATAAAAATT ATTTAACACT TCAAACTATA TTTTATATAT TAAATCAAAG TTTAGTGTTT AAAAGTAATA   
  
  
+ AATCAAACTT TTAATTATTA ATTTGATTTA AATTAAATTT TGATTTAATA TTTTTTGTTA TTAAAAATAT   
  
  
+ TTTAAAAAAT AATTCAAATA AATTGTATAC TTTATTTTAA ATCAATTCTT TAATTTCAAA TAATTTTTGG   
  
  
+ ATTTATGAGT TATTTATTAT AAATTTTTAT AATAATTTTT CTAAAAACAT TTTAGTCAAA AAAACTTATA   
  
  
+ CTTTTTTATA ATGATAGTTT GAATATTTTT AAAAATTTTT AATATGATTT AATTTGATAT AAATTAATAA   
  
  
+ ATCAGATTTT AATTTGTTAC GTTTTAAACC TTAAGGTTAA ATTTGACAAA TCATATACTT AAAAGTTTTA   
  
  
+ ATTAATCTTT ACAATTAGTT TAATTAGATT TGGTACTATA AATAAAAATA CTCATAGTTT TATTTGAATT   
  
  
+ TATGAAACCT AAAATAAGTT TATATCTTTT TATTTTTTCT CAACAAATAT ATATCCAAAA TAGAAAAATA   
  
  
+ AATTTCAAAA TTTTCTTAAT ATACCATAAC CGTTTTATGT CATTAATAAA TAATGTCACA ATAAAAATAA   
  
  
+ TAAAATAAAA TCTCATTACC ACCTACAACT TTTAGCCTTT TACTATAGTA CAAAATTCTT TCATCTTTAA   
  
  
+ TAATAATTAC TTTCTTTCAT TCAGTGGTGT CATAAATTAA AGACCCTACA GTATACCATA TGCCATGAAA   
  
  
+ TTAATGAAAT CTAACACAAA TCATTATGAT TTTATCACTT ATAAAAAAAT TATATTTTTC AACCGCACCA   
  
  
+ TATAATATAA CATATATATT AGATTAATAT AGCAACCAAA ATGAATCATT TGGAGGGTAC ACGAGTCCTT   
  
  
+ AACATGGACC TGACTTCGTC GAATCATTCC CAATCGTTGT GACTAAAGAA ATATTAGAAT TGCGTAATTG   
  
  
+ CTTACACGTC CCCATATAAA CAAAACAACT GCCAATCTGT CATTACCTGA CGGTCCCATA TTAAACCCTC   
  
  
+ CTTAAGCACC CACTAATCAT ATCCACTTCT CTTTCACGTT TCTCGCCGGC CTCAGTCTGC CTTGCCGGTG   
  
  
+ ATCCTACGCC TCCATGATCA TCGCTACCAT TGTCGTCGTC GCCGTTTCAG TACGAAGCCG ACGGTCTTGA   
  
  
+ TTAACTTACC ACCTGATCCA GCACCATAT  

- TCTTCCTTTC TTTATCCACA TTACTATCAC CCCCCTGTCT CAAACGTTGA TGGTAAGGAA ATAAAAAAGT   
  
  
- ACCACAAAAC CTAACTTACA GTTTAAATTC TTATTTAAAA GAGTTTAAAA GGACTACTAC TACGTATAAC   
  
  
- ATAGACTTCA TTTCTAAAAA AAAAAAATTT TATAGGTTTC ATCAATTGTA ATTCAGTAGA TGCCTAGTCT   
  
  
- AAATTAAGCC AGGTAAGTAT TAAACATTTT AAAATCAAAT AAATTTATTT TAACAACTAT AATAAAATTT   
  
  
- TTATTTTTAA TAAATTGTGA AGTTTGATAT AAAATATATA ATTTAGTTTC AAATCACAAA TTTTCATTAT   
  
  
- TTAGTTTGAA AATTAATAAT TAAACTAAAT TTAATTTAAA ACTAAATTAT AAAAAACAAT AATTTTTATA   
  
  
- AAATTTTTTA TTAAGTTTAT TTAACATATG AAATAAAATT TAGTTAAGAA ATTAAAGTTT ATTAAAAACC   
  
  
- TAAATACTCA ATAAATAATA TTTAAAAATA TTATTAAAAA GATTTTTGTA AAATCAGTTT TTTTGAATAT   
  
  
- GAAAAAATAT TACTATCAAA CTTATAAAAA TTTTTAAAAA TTATACTAAA TTAAACTATA TTTAATTATT   
  
  
- TAGTCTAAAA TTAAACAATG CAAAATTTGG AATTCCAATT TAAACTGTTT AGTATATGAA TTTTCAAAAT   
  
  
- TAATTAGAAA TGTTAATCAA ATTAATCTAA ACCATGATAT TTATTTTTAT GAGTATCAAA ATAAACTTAA   
  
  
- ATACTTTGGA TTTTATTCAA ATATAGAAAA ATAAAAAAGA GTTGTTTATA TATAGGTTTT ATCTTTTTAT   
  
  
- TTAAAGTTTT AAAAGAATTA TATGGTATTG GCAAAATACA GTAATTATTT ATTACAGTGT TATTTTTATT   
  
  
- ATTTTATTTT AGAGTAATGG TGGATGTTGA AAATCGGAAA ATGATATCAT GTTTTAAGAA AGTAGAAATT   
  
  
- ATTATTAATG AAAGAAAGTA AGTCACCACA GTATTTAATT TCTGGGATGT CATATGGTAT ACGGTACTTT   
  
  
- AATTACTTTA GATTGTGTTT AGTAATACTA AAATAGTGAA TATTTTTTTA ATATAAAAAG TTGGCGTGGT   
  
  
- ATATTATATT GTATATATAA TCTAATTATA TCGTTGGTTT TACTTAGTAA ACCTCCCATG TGCTCAGGAA   
  
  
- TTGTACCTGG ACTGAAGCAG CTTAGTAAGG GTTAGCAACA CTGATTTCTT TATAATCTTA ACGCATTAAC   
  
  
- GAATGTGCAG GGGTATATTT GTTTTGTTGA CGGTTAGACA GTAATGGACT GCCAGGGTAT AATTTGGGAG   
  
  
- GAATTCGTGG GTGATTAGTA TAGGTGAAGA GAAAGTGCAA AGAGCGGCCG GAGTCAGACG GAACGGCCAC   
  
  
- TAGGATGCGG AGGTACTAGT AGCGATGGTA ACAGCAGCAG CGGCAAAGTC ATGCTTCGGC TGCCAGAACT   
  
  
- AATTGAATGG TGGACTAGGT CGTGGTATA

+     as-2-box

| Site Name | Organism | Position | Strand | Matrix score. | sequence | function |
| --- | --- | --- | --- | --- | --- | --- |
| as-2-box | Nicotiana tabacum | 124 | + | 9 | GATAatGATG | involved in shoot-specific expression and light responsiveness |

> 2018/04/13 10:10:12  
+ AGAAGGAAAG AAATAGGTGT AATGATAGTG GGGGGACAGA GTTTGCAACT ACCATTCCTT TATTTTTTCA   
  
  
+ TGGTGTTTTG GATTGAATGT CAAATTTAAG AATAAATTTT CTCAAATTTT CCTGATGATG ATGCATATTG   
  
  
+ TATCTGAAGT AAAGATTTTT TTTTTTTAAA ATATCCAAAG TAGTTAACAT TAAGTCATCT ACGGATCAGA   
  
  
+ TTTAATTCGG TCCATTCATA ATTTGTAAAA TTTTAGTTTA TTTAAATAAA ATTGTTGATA TTATTTTAAA   
  
  
+ AATAAAAATT ATTTAACACT TCAAACTATA TTTTATATAT TAAATCAAAG TTTAGTGTTT AAAAGTAATA   
  
  
+ AATCAAACTT TTAATTATTA ATTTGATTTA AATTAAATTT TGATTTAATA TTTTTTGTTA TTAAAAATAT   
  
  
+ TTTAAAAAAT AATTCAAATA AATTGTATAC TTTATTTTAA ATCAATTCTT TAATTTCAAA TAATTTTTGG   
  
  
+ ATTTATGAGT TATTTATTAT AAATTTTTAT AATAATTTTT CTAAAAACAT TTTAGTCAAA AAAACTTATA   
  
  
+ CTTTTTTATA ATGATAGTTT GAATATTTTT AAAAATTTTT AATATGATTT AATTTGATAT AAATTAATAA   
  
  
+ ATCAGATTTT AATTTGTTAC GTTTTAAACC TTAAGGTTAA ATTTGACAAA TCATATACTT AAAAGTTTTA   
  
  
+ ATTAATCTTT ACAATTAGTT TAATTAGATT TGGTACTATA AATAAAAATA CTCATAGTTT TATTTGAATT   
  
  
+ TATGAAACCT AAAATAAGTT TATATCTTTT TATTTTTTCT CAACAAATAT ATATCCAAAA TAGAAAAATA   
  
  
+ AATTTCAAAA TTTTCTTAAT ATACCATAAC CGTTTTATGT CATTAATAAA TAATGTCACA ATAAAAATAA   
  
  
+ TAAAATAAAA TCTCATTACC ACCTACAACT TTTAGCCTTT TACTATAGTA CAAAATTCTT TCATCTTTAA   
  
  
+ TAATAATTAC TTTCTTTCAT TCAGTGGTGT CATAAATTAA AGACCCTACA GTATACCATA TGCCATGAAA   
  
  
+ TTAATGAAAT CTAACACAAA TCATTATGAT TTTATCACTT ATAAAAAAAT TATATTTTTC AACCGCACCA   
  
  
+ TATAATATAA CATATATATT AGATTAATAT AGCAACCAAA ATGAATCATT TGGAGGGTAC ACGAGTCCTT   
  
  
+ AACATGGACC TGACTTCGTC GAATCATTCC CAATCGTTGT GACTAAAGAA ATATTAGAAT TGCGTAATTG   
  
  
+ CTTACACGTC CCCATATAAA CAAAACAACT GCCAATCTGT CATTACCTGA CGGTCCCATA TTAAACCCTC   
  
  
+ CTTAAGCACC CACTAATCAT ATCCACTTCT CTTTCACGTT TCTCGCCGGC CTCAGTCTGC CTTGCCGGTG   
  
  
+ ATCCTACGCC TCCATGATCA TCGCTACCAT TGTCGTCGTC GCCGTTTCAG TACGAAGCCG ACGGTCTTGA   
  
  
+ TTAACTTACC ACCTGATCCA GCACCATAT  

- TCTTCCTTTC TTTATCCACA TTACTATCAC CCCCCTGTCT CAAACGTTGA TGGTAAGGAA ATAAAAAAGT   
  
  
- ACCACAAAAC CTAACTTACA GTTTAAATTC TTATTTAAAA GAGTTTAAAA GGACTACTAC TACGTATAAC   
  
  
- ATAGACTTCA TTTCTAAAAA AAAAAAATTT TATAGGTTTC ATCAATTGTA ATTCAGTAGA TGCCTAGTCT   
  
  
- AAATTAAGCC AGGTAAGTAT TAAACATTTT AAAATCAAAT AAATTTATTT TAACAACTAT AATAAAATTT   
  
  
- TTATTTTTAA TAAATTGTGA AGTTTGATAT AAAATATATA ATTTAGTTTC AAATCACAAA TTTTCATTAT   
  
  
- TTAGTTTGAA AATTAATAAT TAAACTAAAT TTAATTTAAA ACTAAATTAT AAAAAACAAT AATTTTTATA   
  
  
- AAATTTTTTA TTAAGTTTAT TTAACATATG AAATAAAATT TAGTTAAGAA ATTAAAGTTT ATTAAAAACC   
  
  
- TAAATACTCA ATAAATAATA TTTAAAAATA TTATTAAAAA GATTTTTGTA AAATCAGTTT TTTTGAATAT   
  
  
- GAAAAAATAT TACTATCAAA CTTATAAAAA TTTTTAAAAA TTATACTAAA TTAAACTATA TTTAATTATT   
  
  
- TAGTCTAAAA TTAAACAATG CAAAATTTGG AATTCCAATT TAAACTGTTT AGTATATGAA TTTTCAAAAT   
  
  
- TAATTAGAAA TGTTAATCAA ATTAATCTAA ACCATGATAT TTATTTTTAT GAGTATCAAA ATAAACTTAA   
  
  
- ATACTTTGGA TTTTATTCAA ATATAGAAAA ATAAAAAAGA GTTGTTTATA TATAGGTTTT ATCTTTTTAT   
  
  
- TTAAAGTTTT AAAAGAATTA TATGGTATTG GCAAAATACA GTAATTATTT ATTACAGTGT TATTTTTATT   
  
  
- ATTTTATTTT AGAGTAATGG TGGATGTTGA AAATCGGAAA ATGATATCAT GTTTTAAGAA AGTAGAAATT   
  
  
- ATTATTAATG AAAGAAAGTA AGTCACCACA GTATTTAATT TCTGGGATGT CATATGGTAT ACGGTACTTT   
  
  
- AATTACTTTA GATTGTGTTT AGTAATACTA AAATAGTGAA TATTTTTTTA ATATAAAAAG TTGGCGTGGT   
  
  
- ATATTATATT GTATATATAA TCTAATTATA TCGTTGGTTT TACTTAGTAA ACCTCCCATG TGCTCAGGAA   
  
  
- TTGTACCTGG ACTGAAGCAG CTTAGTAAGG GTTAGCAACA CTGATTTCTT TATAATCTTA ACGCATTAAC   
  
  
- GAATGTGCAG GGGTATATTT GTTTTGTTGA CGGTTAGACA GTAATGGACT GCCAGGGTAT AATTTGGGAG   
  
  
- GAATTCGTGG GTGATTAGTA TAGGTGAAGA GAAAGTGCAA AGAGCGGCCG GAGTCAGACG GAACGGCCAC   
  
  
- TAGGATGCGG AGGTACTAGT AGCGATGGTA ACAGCAGCAG CGGCAAAGTC ATGCTTCGGC TGCCAGAACT   
  
  
- AATTGAATGG TGGACTAGGT CGTGGTATA
