# Supplementary material for: Genome-wide analysis of the WRKY gene family in drumstick (Moringa oleifera Lam.)
Source: PeerJ. 2019 Jun 10;7:e7063. doi: 10.7717/peerj.7063 (PMC6563795; doi:10.7717/peerj.7063)
Supplement: Supplemental Information 1 [file peerj-07-7063-s003.gz › MoWRKY36_plantcare.html]

Content-Type: text/html; charset=ISO-8859-1


CallMat\_Firefox


Webmaster Firefox specific output  
To save the result:
click on the frame with the right mouse button and save the source code as a text file with extension .html  
REFERENCE:PlantCARE: a database of plant cis-acting regulatory elements and a portal to tools for in silico analysis of promoter sequences.  
Lescot, M., Déhais, P., Moreau, Y., De Moor, B., Rouzé ,P.,and Rombauts, S.  
Nucleic Acids Res., Database issue(2002), 30(1):325-327.   


---

> 2018/04/13 10:10:12  
+ AAGAAACTAC CCGCCGAATG TCGCTAGGTA GCTCGCTAGT TAATGTGGCG GCCGGCGTGG CTTTAAGACG   
  
  
+ GCGAGTTTCA GAGGCCCCCT CACGTCCTTA CACTTAAGTG CCGCAAGAAA TAGTAGGTAC CGACTTCTCT   
  
  
+ TCTTCTTTGA GAGAGAGAGA GAGAGAGAGA GAGAGAGAGA GAGTCTCCTC ATCATCAACA CATCGGAAAA   
  
  
+ GTTCTAAAGT TGTTGATTCC TATAAAGAAA TACCATACAT TCTGAAACGA ATCTCCCTCT ACGTTCCTAT   
  
  
+ TATTTTCCGC TCTCTCTCTC TCTCTCTTTC TCCTCCGCTT CCGCTTCTGG TCGGCAAAAG AAGCCCCCTC   
  
  
+ TCTATACTGT AGTACCAACT ACTACCGTGT GCACCGCTGT AGACCTTCAC TTTCTCCCCT ACTCTCACCC   
  
  
+ TGGATGCCCT TCCTAGCGGC AACTCATGGA AGTTCTCCAG CTGCCACTCT CCACTGCTTC TCACTATCCC   
  
  
+ ACGCCCGCCA AGTGCACCCC GCAGCAGAAG TCCAGTTGAT TCAACTGCCG TGCCGACATA GACTGCCACC   
  
  
+ TGTTCTTTTT ATTCTCCGCG CACACTCTGG TCTGCTCGTC TCATGGATTA GGGTGACTGG TAGGTTGGGT   
  
  
+ TGGGTCGGGA AGTGCAGTCA ATAAAACCTA GACAGCATCA GTTGCTTGGA GTACCCTCTC ACTTCTGGCA   
  
  
+ GGACGAGGGT ACACACCAGG CGAGCTGAGC CAACGATAGA GATTAATGGT TAAAAATAAT TTTTATTAAT   
  
  
+ AAAATAATAT TGTTTTTAAT TCTAATAAAT AACTTCATCT ATAAAAATAG AATTATAAGT ACGAATTTAA   
  
  
+ TCTTTAGTAA TTTTAAAACT TTTAAAAAAT TTTCTGTTTC TTAACAGGTT GTTATTCTAA TTATAATAAA   
  
  
+ TTTTATAGAA CTAGAGAAGA ATTCTATGAC TGTATATTAT TAATTAATAA TTTTTAAAAT AAAAAATAAT   
  
  
+ TATATTATTT AATGTTTTTT TCTATAAAAT AAAAATTTTA TGATATTTTT ATTTAAAATT TTTTTACAAA   
  
  
+ TAAGATACTA TCAATACATA GATCAACTTC ACCTCTTTAC ATTTCGACTT TAAGAACTGG AGAGGTTAAC   
  
  
+ TAGTTATAAC AGATATGAAA CTAAAACTTA TCTACTGGCT AACGTTCTTA CCGGGATTAC GTAATTTTTA   
  
  
+ GGTAAAATAC TTATATATAT ATATATATAT TATTCGATTA TGGAGTATAA TATTATTGGG TAATACATAT   
  
  
+ TTGTGTATCA GAATACCTGA ACTGAGATAA AAAAATCGAT CTAGGTGTAT TTAATTTATC TTTGTAATAA   
  
  
+ TAGTAATAAT AATAAATTTT ATTCTTTTAT ACTTTCTTTT ATTGTTATAA GAAACTTTAA AAATGATCGA   
  
  
+ ACCGATTCAT GTATTCATAT TTGCGTTACT ACTTGGTAAC AACTTGTATT TATATATTAC TTGTGTTATT   
  
  
+ TTTTTAATTA TTATTATTTA TAATCTCGT  

- TTCTTTGATG GGCGGCTTAC AGCGATCCAT CGAGCGATCA ATTACACCGC CGGCCGCACC GAAATTCTGC   
  
  
- CGCTCAAAGT CTCCGGGGGA GTGCAGGAAT GTGAATTCAC GGCGTTCTTT ATCATCCATG GCTGAAGAGA   
  
  
- AGAAGAAACT CTCTCTCTCT CTCTCTCTCT CTCTCTCTCT CTCAGAGGAG TAGTAGTTGT GTAGCCTTTT   
  
  
- CAAGATTTCA ACAACTAAGG ATATTTCTTT ATGGTATGTA AGACTTTGCT TAGAGGGAGA TGCAAGGATA   
  
  
- ATAAAAGGCG AGAGAGAGAG AGAGAGAAAG AGGAGGCGAA GGCGAAGACC AGCCGTTTTC TTCGGGGGAG   
  
  
- AGATATGACA TCATGGTTGA TGATGGCACA CGTGGCGACA TCTGGAAGTG AAAGAGGGGA TGAGAGTGGG   
  
  
- ACCTACGGGA AGGATCGCCG TTGAGTACCT TCAAGAGGTC GACGGTGAGA GGTGACGAAG AGTGATAGGG   
  
  
- TGCGGGCGGT TCACGTGGGG CGTCGTCTTC AGGTCAACTA AGTTGACGGC ACGGCTGTAT CTGACGGTGG   
  
  
- ACAAGAAAAA TAAGAGGCGC GTGTGAGACC AGACGAGCAG AGTACCTAAT CCCACTGACC ATCCAACCCA   
  
  
- ACCCAGCCCT TCACGTCAGT TATTTTGGAT CTGTCGTAGT CAACGAACCT CATGGGAGAG TGAAGACCGT   
  
  
- CCTGCTCCCA TGTGTGGTCC GCTCGACTCG GTTGCTATCT CTAATTACCA ATTTTTATTA AAAATAATTA   
  
  
- TTTTATTATA ACAAAAATTA AGATTATTTA TTGAAGTAGA TATTTTTATC TTAATATTCA TGCTTAAATT   
  
  
- AGAAATCATT AAAATTTTGA AAATTTTTTA AAAGACAAAG AATTGTCCAA CAATAAGATT AATATTATTT   
  
  
- AAAATATCTT GATCTCTTCT TAAGATACTG ACATATAATA ATTAATTATT AAAAATTTTA TTTTTTATTA   
  
  
- ATATAATAAA TTACAAAAAA AGATATTTTA TTTTTAAAAT ACTATAAAAA TAAATTTTAA AAAAATGTTT   
  
  
- ATTCTATGAT AGTTATGTAT CTAGTTGAAG TGGAGAAATG TAAAGCTGAA ATTCTTGACC TCTCCAATTG   
  
  
- ATCAATATTG TCTATACTTT GATTTTGAAT AGATGACCGA TTGCAAGAAT GGCCCTAATG CATTAAAAAT   
  
  
- CCATTTTATG AATATATATA TATATATATA ATAAGCTAAT ACCTCATATT ATAATAACCC ATTATGTATA   
  
  
- AACACATAGT CTTATGGACT TGACTCTATT TTTTTAGCTA GATCCACATA AATTAAATAG AAACATTATT   
  
  
- ATCATTATTA TTATTTAAAA TAAGAAAATA TGAAAGAAAA TAACAATATT CTTTGAAATT TTTACTAGCT   
  
  
- TGGCTAAGTA CATAAGTATA AACGCAATGA TGAACCATTG TTGAACATAA ATATATAATG AACACAATAA   
  
  
- AAAAATTAAT AATAATAAAT ATTAGAGCA

  
  
Motifs Found  

+     5UTR Py-rich stretch

| Site Name | Organism | Position | Strand | Matrix score. | sequence | function |
| --- | --- | --- | --- | --- | --- | --- |
| 5UTR Py-rich stretch | Lycopersicon esculentum | 153 | - | 13 | TTTCTCTCTCTCTC | cis-acting element conferring high transcription levels |
| 5UTR Py-rich stretch | Lycopersicon esculentum | 291 | + | 13 | TTTCTCTCTCTCTC | cis-acting element conferring high transcription levels |
| 5UTR Py-rich stretch | Lycopersicon esculentum | 167 | - | 13 | TTTCTCTCTCTCTC | cis-acting element conferring high transcription levels |
| 5UTR Py-rich stretch | Lycopersicon esculentum | 169 | - | 13 | TTTCTCTCTCTCTC | cis-acting element conferring high transcription levels |
| 5UTR Py-rich stretch | Lycopersicon esculentum | 165 | - | 13 | TTTCTCTCTCTCTC | cis-acting element conferring high transcription levels |
| 5UTR Py-rich stretch | Lycopersicon esculentum | 923 | - | 9 | TTTCTTCTCT | cis-acting element conferring high transcription levels |
| 5UTR Py-rich stretch | Lycopersicon esculentum | 293 | + | 13 | TTTCTCTCTCTCTC | cis-acting element conferring high transcription levels |
| 5UTR Py-rich stretch | Lycopersicon esculentum | 151 | - | 13 | TTTCTCTCTCTCTC | cis-acting element conferring high transcription levels |
| 5UTR Py-rich stretch | Lycopersicon esculentum | 163 | - | 13 | TTTCTCTCTCTCTC | cis-acting element conferring high transcription levels |
| 5UTR Py-rich stretch | Lycopersicon esculentum | 155 | - | 13 | TTTCTCTCTCTCTC | cis-acting element conferring high transcription levels |
| 5UTR Py-rich stretch | Lycopersicon esculentum | 149 | - | 13 | TTTCTCTCTCTCTC | cis-acting element conferring high transcription levels |
| 5UTR Py-rich stretch | Lycopersicon esculentum | 157 | - | 13 | TTTCTCTCTCTCTC | cis-acting element conferring high transcription levels |
| 5UTR Py-rich stretch | Lycopersicon esculentum | 161 | - | 13 | TTTCTCTCTCTCTC | cis-acting element conferring high transcription levels |
| 5UTR Py-rich stretch | Lycopersicon esculentum | 159 | - | 13 | TTTCTCTCTCTCTC | cis-acting element conferring high transcription levels |

> 2018/04/13 10:10:12  
+ AAGAAACTAC CCGCCGAATG TCGCTAGGTA GCTCGCTAGT TAATGTGGCG GCCGGCGTGG CTTTAAGACG   
  
  
+ GCGAGTTTCA GAGGCCCCCT CACGTCCTTA CACTTAAGTG CCGCAAGAAA TAGTAGGTAC CGACTTCTCT   
  
  
+ TCTTCTTTGA GAGAGAGAGA GAGAGAGAGA GAGAGAGAGA GAGTCTCCTC ATCATCAACA CATCGGAAAA   
  
  
+ GTTCTAAAGT TGTTGATTCC TATAAAGAAA TACCATACAT TCTGAAACGA ATCTCCCTCT ACGTTCCTAT   
  
  
+ TATTTTCCGC TCTCTCTCTC TCTCTCTTTC TCCTCCGCTT CCGCTTCTGG TCGGCAAAAG AAGCCCCCTC   
  
  
+ TCTATACTGT AGTACCAACT ACTACCGTGT GCACCGCTGT AGACCTTCAC TTTCTCCCCT ACTCTCACCC   
  
  
+ TGGATGCCCT TCCTAGCGGC AACTCATGGA AGTTCTCCAG CTGCCACTCT CCACTGCTTC TCACTATCCC   
  
  
+ ACGCCCGCCA AGTGCACCCC GCAGCAGAAG TCCAGTTGAT TCAACTGCCG TGCCGACATA GACTGCCACC   
  
  
+ TGTTCTTTTT ATTCTCCGCG CACACTCTGG TCTGCTCGTC TCATGGATTA GGGTGACTGG TAGGTTGGGT   
  
  
+ TGGGTCGGGA AGTGCAGTCA ATAAAACCTA GACAGCATCA GTTGCTTGGA GTACCCTCTC ACTTCTGGCA   
  
  
+ GGACGAGGGT ACACACCAGG CGAGCTGAGC CAACGATAGA GATTAATGGT TAAAAATAAT TTTTATTAAT   
  
  
+ AAAATAATAT TGTTTTTAAT TCTAATAAAT AACTTCATCT ATAAAAATAG AATTATAAGT ACGAATTTAA   
  
  
+ TCTTTAGTAA TTTTAAAACT TTTAAAAAAT TTTCTGTTTC TTAACAGGTT GTTATTCTAA TTATAATAAA   
  
  
+ TTTTATAGAA CTAGAGAAGA ATTCTATGAC TGTATATTAT TAATTAATAA TTTTTAAAAT AAAAAATAAT   
  
  
+ TATATTATTT AATGTTTTTT TCTATAAAAT AAAAATTTTA TGATATTTTT ATTTAAAATT TTTTTACAAA   
  
  
+ TAAGATACTA TCAATACATA GATCAACTTC ACCTCTTTAC ATTTCGACTT TAAGAACTGG AGAGGTTAAC   
  
  
+ TAGTTATAAC AGATATGAAA CTAAAACTTA TCTACTGGCT AACGTTCTTA CCGGGATTAC GTAATTTTTA   
  
  
+ GGTAAAATAC TTATATATAT ATATATATAT TATTCGATTA TGGAGTATAA TATTATTGGG TAATACATAT   
  
  
+ TTGTGTATCA GAATACCTGA ACTGAGATAA AAAAATCGAT CTAGGTGTAT TTAATTTATC TTTGTAATAA   
  
  
+ TAGTAATAAT AATAAATTTT ATTCTTTTAT ACTTTCTTTT ATTGTTATAA GAAACTTTAA AAATGATCGA   
  
  
+ ACCGATTCAT GTATTCATAT TTGCGTTACT ACTTGGTAAC AACTTGTATT TATATATTAC TTGTGTTATT   
  
  
+ TTTTTAATTA TTATTATTTA TAATCTCGT  

- TTCTTTGATG GGCGGCTTAC AGCGATCCAT CGAGCGATCA ATTACACCGC CGGCCGCACC GAAATTCTGC   
  
  
- CGCTCAAAGT CTCCGGGGGA GTGCAGGAAT GTGAATTCAC GGCGTTCTTT ATCATCCATG GCTGAAGAGA   
  
  
- AGAAGAAACT CTCTCTCTCT CTCTCTCTCT CTCTCTCTCT CTCAGAGGAG TAGTAGTTGT GTAGCCTTTT   
  
  
- CAAGATTTCA ACAACTAAGG ATATTTCTTT ATGGTATGTA AGACTTTGCT TAGAGGGAGA TGCAAGGATA   
  
  
- ATAAAAGGCG AGAGAGAGAG AGAGAGAAAG AGGAGGCGAA GGCGAAGACC AGCCGTTTTC TTCGGGGGAG   
  
  
- AGATATGACA TCATGGTTGA TGATGGCACA CGTGGCGACA TCTGGAAGTG AAAGAGGGGA TGAGAGTGGG   
  
  
- ACCTACGGGA AGGATCGCCG TTGAGTACCT TCAAGAGGTC GACGGTGAGA GGTGACGAAG AGTGATAGGG   
  
  
- TGCGGGCGGT TCACGTGGGG CGTCGTCTTC AGGTCAACTA AGTTGACGGC ACGGCTGTAT CTGACGGTGG   
  
  
- ACAAGAAAAA TAAGAGGCGC GTGTGAGACC AGACGAGCAG AGTACCTAAT CCCACTGACC ATCCAACCCA   
  
  
- ACCCAGCCCT TCACGTCAGT TATTTTGGAT CTGTCGTAGT CAACGAACCT CATGGGAGAG TGAAGACCGT   
  
  
- CCTGCTCCCA TGTGTGGTCC GCTCGACTCG GTTGCTATCT CTAATTACCA ATTTTTATTA AAAATAATTA   
  
  
- TTTTATTATA ACAAAAATTA AGATTATTTA TTGAAGTAGA TATTTTTATC TTAATATTCA TGCTTAAATT   
  
  
- AGAAATCATT AAAATTTTGA AAATTTTTTA AAAGACAAAG AATTGTCCAA CAATAAGATT AATATTATTT   
  
  
- AAAATATCTT GATCTCTTCT TAAGATACTG ACATATAATA ATTAATTATT AAAAATTTTA TTTTTTATTA   
  
  
- ATATAATAAA TTACAAAAAA AGATATTTTA TTTTTAAAAT ACTATAAAAA TAAATTTTAA AAAAATGTTT   
  
  
- ATTCTATGAT AGTTATGTAT CTAGTTGAAG TGGAGAAATG TAAAGCTGAA ATTCTTGACC TCTCCAATTG   
  
  
- ATCAATATTG TCTATACTTT GATTTTGAAT AGATGACCGA TTGCAAGAAT GGCCCTAATG CATTAAAAAT   
  
  
- CCATTTTATG AATATATATA TATATATATA ATAAGCTAAT ACCTCATATT ATAATAACCC ATTATGTATA   
  
  
- AACACATAGT CTTATGGACT TGACTCTATT TTTTTAGCTA GATCCACATA AATTAAATAG AAACATTATT   
  
  
- ATCATTATTA TTATTTAAAA TAAGAAAATA TGAAAGAAAA TAACAATATT CTTTGAAATT TTTACTAGCT   
  
  
- TGGCTAAGTA CATAAGTATA AACGCAATGA TGAACCATTG TTGAACATAA ATATATAATG AACACAATAA   
  
  
- AAAAATTAAT AATAATAAAT ATTAGAGCA

+     AAGAA-motif

| Site Name | Organism | Position | Strand | Matrix score. | sequence | function |
| --- | --- | --- | --- | --- | --- | --- |
| AAGAA-motif | Avena sativa | 1165 | - | 8 | gGTAAGAA |  |

> 2018/04/13 10:10:12  
+ AAGAAACTAC CCGCCGAATG TCGCTAGGTA GCTCGCTAGT TAATGTGGCG GCCGGCGTGG CTTTAAGACG   
  
  
+ GCGAGTTTCA GAGGCCCCCT CACGTCCTTA CACTTAAGTG CCGCAAGAAA TAGTAGGTAC CGACTTCTCT   
  
  
+ TCTTCTTTGA GAGAGAGAGA GAGAGAGAGA GAGAGAGAGA GAGTCTCCTC ATCATCAACA CATCGGAAAA   
  
  
+ GTTCTAAAGT TGTTGATTCC TATAAAGAAA TACCATACAT TCTGAAACGA ATCTCCCTCT ACGTTCCTAT   
  
  
+ TATTTTCCGC TCTCTCTCTC TCTCTCTTTC TCCTCCGCTT CCGCTTCTGG TCGGCAAAAG AAGCCCCCTC   
  
  
+ TCTATACTGT AGTACCAACT ACTACCGTGT GCACCGCTGT AGACCTTCAC TTTCTCCCCT ACTCTCACCC   
  
  
+ TGGATGCCCT TCCTAGCGGC AACTCATGGA AGTTCTCCAG CTGCCACTCT CCACTGCTTC TCACTATCCC   
  
  
+ ACGCCCGCCA AGTGCACCCC GCAGCAGAAG TCCAGTTGAT TCAACTGCCG TGCCGACATA GACTGCCACC   
  
  
+ TGTTCTTTTT ATTCTCCGCG CACACTCTGG TCTGCTCGTC TCATGGATTA GGGTGACTGG TAGGTTGGGT   
  
  
+ TGGGTCGGGA AGTGCAGTCA ATAAAACCTA GACAGCATCA GTTGCTTGGA GTACCCTCTC ACTTCTGGCA   
  
  
+ GGACGAGGGT ACACACCAGG CGAGCTGAGC CAACGATAGA GATTAATGGT TAAAAATAAT TTTTATTAAT   
  
  
+ AAAATAATAT TGTTTTTAAT TCTAATAAAT AACTTCATCT ATAAAAATAG AATTATAAGT ACGAATTTAA   
  
  
+ TCTTTAGTAA TTTTAAAACT TTTAAAAAAT TTTCTGTTTC TTAACAGGTT GTTATTCTAA TTATAATAAA   
  
  
+ TTTTATAGAA CTAGAGAAGA ATTCTATGAC TGTATATTAT TAATTAATAA TTTTTAAAAT AAAAAATAAT   
  
  
+ TATATTATTT AATGTTTTTT TCTATAAAAT AAAAATTTTA TGATATTTTT ATTTAAAATT TTTTTACAAA   
  
  
+ TAAGATACTA TCAATACATA GATCAACTTC ACCTCTTTAC ATTTCGACTT TAAGAACTGG AGAGGTTAAC   
  
  
+ TAGTTATAAC AGATATGAAA CTAAAACTTA TCTACTGGCT AACGTTCTTA CCGGGATTAC GTAATTTTTA   
  
  
+ GGTAAAATAC TTATATATAT ATATATATAT TATTCGATTA TGGAGTATAA TATTATTGGG TAATACATAT   
  
  
+ TTGTGTATCA GAATACCTGA ACTGAGATAA AAAAATCGAT CTAGGTGTAT TTAATTTATC TTTGTAATAA   
  
  
+ TAGTAATAAT AATAAATTTT ATTCTTTTAT ACTTTCTTTT ATTGTTATAA GAAACTTTAA AAATGATCGA   
  
  
+ ACCGATTCAT GTATTCATAT TTGCGTTACT ACTTGGTAAC AACTTGTATT TATATATTAC TTGTGTTATT   
  
  
+ TTTTTAATTA TTATTATTTA TAATCTCGT  

- TTCTTTGATG GGCGGCTTAC AGCGATCCAT CGAGCGATCA ATTACACCGC CGGCCGCACC GAAATTCTGC   
  
  
- CGCTCAAAGT CTCCGGGGGA GTGCAGGAAT GTGAATTCAC GGCGTTCTTT ATCATCCATG GCTGAAGAGA   
  
  
- AGAAGAAACT CTCTCTCTCT CTCTCTCTCT CTCTCTCTCT CTCAGAGGAG TAGTAGTTGT GTAGCCTTTT   
  
  
- CAAGATTTCA ACAACTAAGG ATATTTCTTT ATGGTATGTA AGACTTTGCT TAGAGGGAGA TGCAAGGATA   
  
  
- ATAAAAGGCG AGAGAGAGAG AGAGAGAAAG AGGAGGCGAA GGCGAAGACC AGCCGTTTTC TTCGGGGGAG   
  
  
- AGATATGACA TCATGGTTGA TGATGGCACA CGTGGCGACA TCTGGAAGTG AAAGAGGGGA TGAGAGTGGG   
  
  
- ACCTACGGGA AGGATCGCCG TTGAGTACCT TCAAGAGGTC GACGGTGAGA GGTGACGAAG AGTGATAGGG   
  
  
- TGCGGGCGGT TCACGTGGGG CGTCGTCTTC AGGTCAACTA AGTTGACGGC ACGGCTGTAT CTGACGGTGG   
  
  
- ACAAGAAAAA TAAGAGGCGC GTGTGAGACC AGACGAGCAG AGTACCTAAT CCCACTGACC ATCCAACCCA   
  
  
- ACCCAGCCCT TCACGTCAGT TATTTTGGAT CTGTCGTAGT CAACGAACCT CATGGGAGAG TGAAGACCGT   
  
  
- CCTGCTCCCA TGTGTGGTCC GCTCGACTCG GTTGCTATCT CTAATTACCA ATTTTTATTA AAAATAATTA   
  
  
- TTTTATTATA ACAAAAATTA AGATTATTTA TTGAAGTAGA TATTTTTATC TTAATATTCA TGCTTAAATT   
  
  
- AGAAATCATT AAAATTTTGA AAATTTTTTA AAAGACAAAG AATTGTCCAA CAATAAGATT AATATTATTT   
  
  
- AAAATATCTT GATCTCTTCT TAAGATACTG ACATATAATA ATTAATTATT AAAAATTTTA TTTTTTATTA   
  
  
- ATATAATAAA TTACAAAAAA AGATATTTTA TTTTTAAAAT ACTATAAAAA TAAATTTTAA AAAAATGTTT   
  
  
- ATTCTATGAT AGTTATGTAT CTAGTTGAAG TGGAGAAATG TAAAGCTGAA ATTCTTGACC TCTCCAATTG   
  
  
- ATCAATATTG TCTATACTTT GATTTTGAAT AGATGACCGA TTGCAAGAAT GGCCCTAATG CATTAAAAAT   
  
  
- CCATTTTATG AATATATATA TATATATATA ATAAGCTAAT ACCTCATATT ATAATAACCC ATTATGTATA   
  
  
- AACACATAGT CTTATGGACT TGACTCTATT TTTTTAGCTA GATCCACATA AATTAAATAG AAACATTATT   
  
  
- ATCATTATTA TTATTTAAAA TAAGAAAATA TGAAAGAAAA TAACAATATT CTTTGAAATT TTTACTAGCT   
  
  
- TGGCTAAGTA CATAAGTATA AACGCAATGA TGAACCATTG TTGAACATAA ATATATAATG AACACAATAA   
  
  
- AAAAATTAAT AATAATAAAT ATTAGAGCA

+     AC-I

| Site Name | Organism | Position | Strand | Matrix score. | sequence | function |
| --- | --- | --- | --- | --- | --- | --- |
| AC-I | Phaseolus vulgaris | 619 | - | 9 | CCCACCTACC |  |

> 2018/04/13 10:10:12  
+ AAGAAACTAC CCGCCGAATG TCGCTAGGTA GCTCGCTAGT TAATGTGGCG GCCGGCGTGG CTTTAAGACG   
  
  
+ GCGAGTTTCA GAGGCCCCCT CACGTCCTTA CACTTAAGTG CCGCAAGAAA TAGTAGGTAC CGACTTCTCT   
  
  
+ TCTTCTTTGA GAGAGAGAGA GAGAGAGAGA GAGAGAGAGA GAGTCTCCTC ATCATCAACA CATCGGAAAA   
  
  
+ GTTCTAAAGT TGTTGATTCC TATAAAGAAA TACCATACAT TCTGAAACGA ATCTCCCTCT ACGTTCCTAT   
  
  
+ TATTTTCCGC TCTCTCTCTC TCTCTCTTTC TCCTCCGCTT CCGCTTCTGG TCGGCAAAAG AAGCCCCCTC   
  
  
+ TCTATACTGT AGTACCAACT ACTACCGTGT GCACCGCTGT AGACCTTCAC TTTCTCCCCT ACTCTCACCC   
  
  
+ TGGATGCCCT TCCTAGCGGC AACTCATGGA AGTTCTCCAG CTGCCACTCT CCACTGCTTC TCACTATCCC   
  
  
+ ACGCCCGCCA AGTGCACCCC GCAGCAGAAG TCCAGTTGAT TCAACTGCCG TGCCGACATA GACTGCCACC   
  
  
+ TGTTCTTTTT ATTCTCCGCG CACACTCTGG TCTGCTCGTC TCATGGATTA GGGTGACTGG TAGGTTGGGT   
  
  
+ TGGGTCGGGA AGTGCAGTCA ATAAAACCTA GACAGCATCA GTTGCTTGGA GTACCCTCTC ACTTCTGGCA   
  
  
+ GGACGAGGGT ACACACCAGG CGAGCTGAGC CAACGATAGA GATTAATGGT TAAAAATAAT TTTTATTAAT   
  
  
+ AAAATAATAT TGTTTTTAAT TCTAATAAAT AACTTCATCT ATAAAAATAG AATTATAAGT ACGAATTTAA   
  
  
+ TCTTTAGTAA TTTTAAAACT TTTAAAAAAT TTTCTGTTTC TTAACAGGTT GTTATTCTAA TTATAATAAA   
  
  
+ TTTTATAGAA CTAGAGAAGA ATTCTATGAC TGTATATTAT TAATTAATAA TTTTTAAAAT AAAAAATAAT   
  
  
+ TATATTATTT AATGTTTTTT TCTATAAAAT AAAAATTTTA TGATATTTTT ATTTAAAATT TTTTTACAAA   
  
  
+ TAAGATACTA TCAATACATA GATCAACTTC ACCTCTTTAC ATTTCGACTT TAAGAACTGG AGAGGTTAAC   
  
  
+ TAGTTATAAC AGATATGAAA CTAAAACTTA TCTACTGGCT AACGTTCTTA CCGGGATTAC GTAATTTTTA   
  
  
+ GGTAAAATAC TTATATATAT ATATATATAT TATTCGATTA TGGAGTATAA TATTATTGGG TAATACATAT   
  
  
+ TTGTGTATCA GAATACCTGA ACTGAGATAA AAAAATCGAT CTAGGTGTAT TTAATTTATC TTTGTAATAA   
  
  
+ TAGTAATAAT AATAAATTTT ATTCTTTTAT ACTTTCTTTT ATTGTTATAA GAAACTTTAA AAATGATCGA   
  
  
+ ACCGATTCAT GTATTCATAT TTGCGTTACT ACTTGGTAAC AACTTGTATT TATATATTAC TTGTGTTATT   
  
  
+ TTTTTAATTA TTATTATTTA TAATCTCGT  

- TTCTTTGATG GGCGGCTTAC AGCGATCCAT CGAGCGATCA ATTACACCGC CGGCCGCACC GAAATTCTGC   
  
  
- CGCTCAAAGT CTCCGGGGGA GTGCAGGAAT GTGAATTCAC GGCGTTCTTT ATCATCCATG GCTGAAGAGA   
  
  
- AGAAGAAACT CTCTCTCTCT CTCTCTCTCT CTCTCTCTCT CTCAGAGGAG TAGTAGTTGT GTAGCCTTTT   
  
  
- CAAGATTTCA ACAACTAAGG ATATTTCTTT ATGGTATGTA AGACTTTGCT TAGAGGGAGA TGCAAGGATA   
  
  
- ATAAAAGGCG AGAGAGAGAG AGAGAGAAAG AGGAGGCGAA GGCGAAGACC AGCCGTTTTC TTCGGGGGAG   
  
  
- AGATATGACA TCATGGTTGA TGATGGCACA CGTGGCGACA TCTGGAAGTG AAAGAGGGGA TGAGAGTGGG   
  
  
- ACCTACGGGA AGGATCGCCG TTGAGTACCT TCAAGAGGTC GACGGTGAGA GGTGACGAAG AGTGATAGGG   
  
  
- TGCGGGCGGT TCACGTGGGG CGTCGTCTTC AGGTCAACTA AGTTGACGGC ACGGCTGTAT CTGACGGTGG   
  
  
- ACAAGAAAAA TAAGAGGCGC GTGTGAGACC AGACGAGCAG AGTACCTAAT CCCACTGACC ATCCAACCCA   
  
  
- ACCCAGCCCT TCACGTCAGT TATTTTGGAT CTGTCGTAGT CAACGAACCT CATGGGAGAG TGAAGACCGT   
  
  
- CCTGCTCCCA TGTGTGGTCC GCTCGACTCG GTTGCTATCT CTAATTACCA ATTTTTATTA AAAATAATTA   
  
  
- TTTTATTATA ACAAAAATTA AGATTATTTA TTGAAGTAGA TATTTTTATC TTAATATTCA TGCTTAAATT   
  
  
- AGAAATCATT AAAATTTTGA AAATTTTTTA AAAGACAAAG AATTGTCCAA CAATAAGATT AATATTATTT   
  
  
- AAAATATCTT GATCTCTTCT TAAGATACTG ACATATAATA ATTAATTATT AAAAATTTTA TTTTTTATTA   
  
  
- ATATAATAAA TTACAAAAAA AGATATTTTA TTTTTAAAAT ACTATAAAAA TAAATTTTAA AAAAATGTTT   
  
  
- ATTCTATGAT AGTTATGTAT CTAGTTGAAG TGGAGAAATG TAAAGCTGAA ATTCTTGACC TCTCCAATTG   
  
  
- ATCAATATTG TCTATACTTT GATTTTGAAT AGATGACCGA TTGCAAGAAT GGCCCTAATG CATTAAAAAT   
  
  
- CCATTTTATG AATATATATA TATATATATA ATAAGCTAAT ACCTCATATT ATAATAACCC ATTATGTATA   
  
  
- AACACATAGT CTTATGGACT TGACTCTATT TTTTTAGCTA GATCCACATA AATTAAATAG AAACATTATT   
  
  
- ATCATTATTA TTATTTAAAA TAAGAAAATA TGAAAGAAAA TAACAATATT CTTTGAAATT TTTACTAGCT   
  
  
- TGGCTAAGTA CATAAGTATA AACGCAATGA TGAACCATTG TTGAACATAA ATATATAATG AACACAATAA   
  
  
- AAAAATTAAT AATAATAAAT ATTAGAGCA

+     ACE

| Site Name | Organism | Position | Strand | Matrix score. | sequence | function |
| --- | --- | --- | --- | --- | --- | --- |
| ACE | Petroselinum crispum | 855 | + | 9 | AAAACGTTTA | cis-acting element involved in light responsiveness |

> 2018/04/13 10:10:12  
+ AAGAAACTAC CCGCCGAATG TCGCTAGGTA GCTCGCTAGT TAATGTGGCG GCCGGCGTGG CTTTAAGACG   
  
  
+ GCGAGTTTCA GAGGCCCCCT CACGTCCTTA CACTTAAGTG CCGCAAGAAA TAGTAGGTAC CGACTTCTCT   
  
  
+ TCTTCTTTGA GAGAGAGAGA GAGAGAGAGA GAGAGAGAGA GAGTCTCCTC ATCATCAACA CATCGGAAAA   
  
  
+ GTTCTAAAGT TGTTGATTCC TATAAAGAAA TACCATACAT TCTGAAACGA ATCTCCCTCT ACGTTCCTAT   
  
  
+ TATTTTCCGC TCTCTCTCTC TCTCTCTTTC TCCTCCGCTT CCGCTTCTGG TCGGCAAAAG AAGCCCCCTC   
  
  
+ TCTATACTGT AGTACCAACT ACTACCGTGT GCACCGCTGT AGACCTTCAC TTTCTCCCCT ACTCTCACCC   
  
  
+ TGGATGCCCT TCCTAGCGGC AACTCATGGA AGTTCTCCAG CTGCCACTCT CCACTGCTTC TCACTATCCC   
  
  
+ ACGCCCGCCA AGTGCACCCC GCAGCAGAAG TCCAGTTGAT TCAACTGCCG TGCCGACATA GACTGCCACC   
  
  
+ TGTTCTTTTT ATTCTCCGCG CACACTCTGG TCTGCTCGTC TCATGGATTA GGGTGACTGG TAGGTTGGGT   
  
  
+ TGGGTCGGGA AGTGCAGTCA ATAAAACCTA GACAGCATCA GTTGCTTGGA GTACCCTCTC ACTTCTGGCA   
  
  
+ GGACGAGGGT ACACACCAGG CGAGCTGAGC CAACGATAGA GATTAATGGT TAAAAATAAT TTTTATTAAT   
  
  
+ AAAATAATAT TGTTTTTAAT TCTAATAAAT AACTTCATCT ATAAAAATAG AATTATAAGT ACGAATTTAA   
  
  
+ TCTTTAGTAA TTTTAAAACT TTTAAAAAAT TTTCTGTTTC TTAACAGGTT GTTATTCTAA TTATAATAAA   
  
  
+ TTTTATAGAA CTAGAGAAGA ATTCTATGAC TGTATATTAT TAATTAATAA TTTTTAAAAT AAAAAATAAT   
  
  
+ TATATTATTT AATGTTTTTT TCTATAAAAT AAAAATTTTA TGATATTTTT ATTTAAAATT TTTTTACAAA   
  
  
+ TAAGATACTA TCAATACATA GATCAACTTC ACCTCTTTAC ATTTCGACTT TAAGAACTGG AGAGGTTAAC   
  
  
+ TAGTTATAAC AGATATGAAA CTAAAACTTA TCTACTGGCT AACGTTCTTA CCGGGATTAC GTAATTTTTA   
  
  
+ GGTAAAATAC TTATATATAT ATATATATAT TATTCGATTA TGGAGTATAA TATTATTGGG TAATACATAT   
  
  
+ TTGTGTATCA GAATACCTGA ACTGAGATAA AAAAATCGAT CTAGGTGTAT TTAATTTATC TTTGTAATAA   
  
  
+ TAGTAATAAT AATAAATTTT ATTCTTTTAT ACTTTCTTTT ATTGTTATAA GAAACTTTAA AAATGATCGA   
  
  
+ ACCGATTCAT GTATTCATAT TTGCGTTACT ACTTGGTAAC AACTTGTATT TATATATTAC TTGTGTTATT   
  
  
+ TTTTTAATTA TTATTATTTA TAATCTCGT  

- TTCTTTGATG GGCGGCTTAC AGCGATCCAT CGAGCGATCA ATTACACCGC CGGCCGCACC GAAATTCTGC   
  
  
- CGCTCAAAGT CTCCGGGGGA GTGCAGGAAT GTGAATTCAC GGCGTTCTTT ATCATCCATG GCTGAAGAGA   
  
  
- AGAAGAAACT CTCTCTCTCT CTCTCTCTCT CTCTCTCTCT CTCAGAGGAG TAGTAGTTGT GTAGCCTTTT   
  
  
- CAAGATTTCA ACAACTAAGG ATATTTCTTT ATGGTATGTA AGACTTTGCT TAGAGGGAGA TGCAAGGATA   
  
  
- ATAAAAGGCG AGAGAGAGAG AGAGAGAAAG AGGAGGCGAA GGCGAAGACC AGCCGTTTTC TTCGGGGGAG   
  
  
- AGATATGACA TCATGGTTGA TGATGGCACA CGTGGCGACA TCTGGAAGTG AAAGAGGGGA TGAGAGTGGG   
  
  
- ACCTACGGGA AGGATCGCCG TTGAGTACCT TCAAGAGGTC GACGGTGAGA GGTGACGAAG AGTGATAGGG   
  
  
- TGCGGGCGGT TCACGTGGGG CGTCGTCTTC AGGTCAACTA AGTTGACGGC ACGGCTGTAT CTGACGGTGG   
  
  
- ACAAGAAAAA TAAGAGGCGC GTGTGAGACC AGACGAGCAG AGTACCTAAT CCCACTGACC ATCCAACCCA   
  
  
- ACCCAGCCCT TCACGTCAGT TATTTTGGAT CTGTCGTAGT CAACGAACCT CATGGGAGAG TGAAGACCGT   
  
  
- CCTGCTCCCA TGTGTGGTCC GCTCGACTCG GTTGCTATCT CTAATTACCA ATTTTTATTA AAAATAATTA   
  
  
- TTTTATTATA ACAAAAATTA AGATTATTTA TTGAAGTAGA TATTTTTATC TTAATATTCA TGCTTAAATT   
  
  
- AGAAATCATT AAAATTTTGA AAATTTTTTA AAAGACAAAG AATTGTCCAA CAATAAGATT AATATTATTT   
  
  
- AAAATATCTT GATCTCTTCT TAAGATACTG ACATATAATA ATTAATTATT AAAAATTTTA TTTTTTATTA   
  
  
- ATATAATAAA TTACAAAAAA AGATATTTTA TTTTTAAAAT ACTATAAAAA TAAATTTTAA AAAAATGTTT   
  
  
- ATTCTATGAT AGTTATGTAT CTAGTTGAAG TGGAGAAATG TAAAGCTGAA ATTCTTGACC TCTCCAATTG   
  
  
- ATCAATATTG TCTATACTTT GATTTTGAAT AGATGACCGA TTGCAAGAAT GGCCCTAATG CATTAAAAAT   
  
  
- CCATTTTATG AATATATATA TATATATATA ATAAGCTAAT ACCTCATATT ATAATAACCC ATTATGTATA   
  
  
- AACACATAGT CTTATGGACT TGACTCTATT TTTTTAGCTA GATCCACATA AATTAAATAG AAACATTATT   
  
  
- ATCATTATTA TTATTTAAAA TAAGAAAATA TGAAAGAAAA TAACAATATT CTTTGAAATT TTTACTAGCT   
  
  
- TGGCTAAGTA CATAAGTATA AACGCAATGA TGAACCATTG TTGAACATAA ATATATAATG AACACAATAA   
  
  
- AAAAATTAAT AATAATAAAT ATTAGAGCA

+     AE-box

| Site Name | Organism | Position | Strand | Matrix score. | sequence | function |
| --- | --- | --- | --- | --- | --- | --- |
| AE-box | Arabidopsis thaliana | 1380 | + | 8 | AGAAACTT | part of a module for light response |

> 2018/04/13 10:10:12  
+ AAGAAACTAC CCGCCGAATG TCGCTAGGTA GCTCGCTAGT TAATGTGGCG GCCGGCGTGG CTTTAAGACG   
  
  
+ GCGAGTTTCA GAGGCCCCCT CACGTCCTTA CACTTAAGTG CCGCAAGAAA TAGTAGGTAC CGACTTCTCT   
  
  
+ TCTTCTTTGA GAGAGAGAGA GAGAGAGAGA GAGAGAGAGA GAGTCTCCTC ATCATCAACA CATCGGAAAA   
  
  
+ GTTCTAAAGT TGTTGATTCC TATAAAGAAA TACCATACAT TCTGAAACGA ATCTCCCTCT ACGTTCCTAT   
  
  
+ TATTTTCCGC TCTCTCTCTC TCTCTCTTTC TCCTCCGCTT CCGCTTCTGG TCGGCAAAAG AAGCCCCCTC   
  
  
+ TCTATACTGT AGTACCAACT ACTACCGTGT GCACCGCTGT AGACCTTCAC TTTCTCCCCT ACTCTCACCC   
  
  
+ TGGATGCCCT TCCTAGCGGC AACTCATGGA AGTTCTCCAG CTGCCACTCT CCACTGCTTC TCACTATCCC   
  
  
+ ACGCCCGCCA AGTGCACCCC GCAGCAGAAG TCCAGTTGAT TCAACTGCCG TGCCGACATA GACTGCCACC   
  
  
+ TGTTCTTTTT ATTCTCCGCG CACACTCTGG TCTGCTCGTC TCATGGATTA GGGTGACTGG TAGGTTGGGT   
  
  
+ TGGGTCGGGA AGTGCAGTCA ATAAAACCTA GACAGCATCA GTTGCTTGGA GTACCCTCTC ACTTCTGGCA   
  
  
+ GGACGAGGGT ACACACCAGG CGAGCTGAGC CAACGATAGA GATTAATGGT TAAAAATAAT TTTTATTAAT   
  
  
+ AAAATAATAT TGTTTTTAAT TCTAATAAAT AACTTCATCT ATAAAAATAG AATTATAAGT ACGAATTTAA   
  
  
+ TCTTTAGTAA TTTTAAAACT TTTAAAAAAT TTTCTGTTTC TTAACAGGTT GTTATTCTAA TTATAATAAA   
  
  
+ TTTTATAGAA CTAGAGAAGA ATTCTATGAC TGTATATTAT TAATTAATAA TTTTTAAAAT AAAAAATAAT   
  
  
+ TATATTATTT AATGTTTTTT TCTATAAAAT AAAAATTTTA TGATATTTTT ATTTAAAATT TTTTTACAAA   
  
  
+ TAAGATACTA TCAATACATA GATCAACTTC ACCTCTTTAC ATTTCGACTT TAAGAACTGG AGAGGTTAAC   
  
  
+ TAGTTATAAC AGATATGAAA CTAAAACTTA TCTACTGGCT AACGTTCTTA CCGGGATTAC GTAATTTTTA   
  
  
+ GGTAAAATAC TTATATATAT ATATATATAT TATTCGATTA TGGAGTATAA TATTATTGGG TAATACATAT   
  
  
+ TTGTGTATCA GAATACCTGA ACTGAGATAA AAAAATCGAT CTAGGTGTAT TTAATTTATC TTTGTAATAA   
  
  
+ TAGTAATAAT AATAAATTTT ATTCTTTTAT ACTTTCTTTT ATTGTTATAA GAAACTTTAA AAATGATCGA   
  
  
+ ACCGATTCAT GTATTCATAT TTGCGTTACT ACTTGGTAAC AACTTGTATT TATATATTAC TTGTGTTATT   
  
  
+ TTTTTAATTA TTATTATTTA TAATCTCGT  

- TTCTTTGATG GGCGGCTTAC AGCGATCCAT CGAGCGATCA ATTACACCGC CGGCCGCACC GAAATTCTGC   
  
  
- CGCTCAAAGT CTCCGGGGGA GTGCAGGAAT GTGAATTCAC GGCGTTCTTT ATCATCCATG GCTGAAGAGA   
  
  
- AGAAGAAACT CTCTCTCTCT CTCTCTCTCT CTCTCTCTCT CTCAGAGGAG TAGTAGTTGT GTAGCCTTTT   
  
  
- CAAGATTTCA ACAACTAAGG ATATTTCTTT ATGGTATGTA AGACTTTGCT TAGAGGGAGA TGCAAGGATA   
  
  
- ATAAAAGGCG AGAGAGAGAG AGAGAGAAAG AGGAGGCGAA GGCGAAGACC AGCCGTTTTC TTCGGGGGAG   
  
  
- AGATATGACA TCATGGTTGA TGATGGCACA CGTGGCGACA TCTGGAAGTG AAAGAGGGGA TGAGAGTGGG   
  
  
- ACCTACGGGA AGGATCGCCG TTGAGTACCT TCAAGAGGTC GACGGTGAGA GGTGACGAAG AGTGATAGGG   
  
  
- TGCGGGCGGT TCACGTGGGG CGTCGTCTTC AGGTCAACTA AGTTGACGGC ACGGCTGTAT CTGACGGTGG   
  
  
- ACAAGAAAAA TAAGAGGCGC GTGTGAGACC AGACGAGCAG AGTACCTAAT CCCACTGACC ATCCAACCCA   
  
  
- ACCCAGCCCT TCACGTCAGT TATTTTGGAT CTGTCGTAGT CAACGAACCT CATGGGAGAG TGAAGACCGT   
  
  
- CCTGCTCCCA TGTGTGGTCC GCTCGACTCG GTTGCTATCT CTAATTACCA ATTTTTATTA AAAATAATTA   
  
  
- TTTTATTATA ACAAAAATTA AGATTATTTA TTGAAGTAGA TATTTTTATC TTAATATTCA TGCTTAAATT   
  
  
- AGAAATCATT AAAATTTTGA AAATTTTTTA AAAGACAAAG AATTGTCCAA CAATAAGATT AATATTATTT   
  
  
- AAAATATCTT GATCTCTTCT TAAGATACTG ACATATAATA ATTAATTATT AAAAATTTTA TTTTTTATTA   
  
  
- ATATAATAAA TTACAAAAAA AGATATTTTA TTTTTAAAAT ACTATAAAAA TAAATTTTAA AAAAATGTTT   
  
  
- ATTCTATGAT AGTTATGTAT CTAGTTGAAG TGGAGAAATG TAAAGCTGAA ATTCTTGACC TCTCCAATTG   
  
  
- ATCAATATTG TCTATACTTT GATTTTGAAT AGATGACCGA TTGCAAGAAT GGCCCTAATG CATTAAAAAT   
  
  
- CCATTTTATG AATATATATA TATATATATA ATAAGCTAAT ACCTCATATT ATAATAACCC ATTATGTATA   
  
  
- AACACATAGT CTTATGGACT TGACTCTATT TTTTTAGCTA GATCCACATA AATTAAATAG AAACATTATT   
  
  
- ATCATTATTA TTATTTAAAA TAAGAAAATA TGAAAGAAAA TAACAATATT CTTTGAAATT TTTACTAGCT   
  
  
- TGGCTAAGTA CATAAGTATA AACGCAATGA TGAACCATTG TTGAACATAA ATATATAATG AACACAATAA   
  
  
- AAAAATTAAT AATAATAAAT ATTAGAGCA

+     AT-rich element

| Site Name | Organism | Position | Strand | Matrix score. | sequence | function |
| --- | --- | --- | --- | --- | --- | --- |
| AT-rich element | Glycine max | 223 | - | 10 | ATAGAAATCAA | binding site of AT-rich DNA binding protein (ATBP-1) |

> 2018/04/13 10:10:12  
+ AAGAAACTAC CCGCCGAATG TCGCTAGGTA GCTCGCTAGT TAATGTGGCG GCCGGCGTGG CTTTAAGACG   
  
  
+ GCGAGTTTCA GAGGCCCCCT CACGTCCTTA CACTTAAGTG CCGCAAGAAA TAGTAGGTAC CGACTTCTCT   
  
  
+ TCTTCTTTGA GAGAGAGAGA GAGAGAGAGA GAGAGAGAGA GAGTCTCCTC ATCATCAACA CATCGGAAAA   
  
  
+ GTTCTAAAGT TGTTGATTCC TATAAAGAAA TACCATACAT TCTGAAACGA ATCTCCCTCT ACGTTCCTAT   
  
  
+ TATTTTCCGC TCTCTCTCTC TCTCTCTTTC TCCTCCGCTT CCGCTTCTGG TCGGCAAAAG AAGCCCCCTC   
  
  
+ TCTATACTGT AGTACCAACT ACTACCGTGT GCACCGCTGT AGACCTTCAC TTTCTCCCCT ACTCTCACCC   
  
  
+ TGGATGCCCT TCCTAGCGGC AACTCATGGA AGTTCTCCAG CTGCCACTCT CCACTGCTTC TCACTATCCC   
  
  
+ ACGCCCGCCA AGTGCACCCC GCAGCAGAAG TCCAGTTGAT TCAACTGCCG TGCCGACATA GACTGCCACC   
  
  
+ TGTTCTTTTT ATTCTCCGCG CACACTCTGG TCTGCTCGTC TCATGGATTA GGGTGACTGG TAGGTTGGGT   
  
  
+ TGGGTCGGGA AGTGCAGTCA ATAAAACCTA GACAGCATCA GTTGCTTGGA GTACCCTCTC ACTTCTGGCA   
  
  
+ GGACGAGGGT ACACACCAGG CGAGCTGAGC CAACGATAGA GATTAATGGT TAAAAATAAT TTTTATTAAT   
  
  
+ AAAATAATAT TGTTTTTAAT TCTAATAAAT AACTTCATCT ATAAAAATAG AATTATAAGT ACGAATTTAA   
  
  
+ TCTTTAGTAA TTTTAAAACT TTTAAAAAAT TTTCTGTTTC TTAACAGGTT GTTATTCTAA TTATAATAAA   
  
  
+ TTTTATAGAA CTAGAGAAGA ATTCTATGAC TGTATATTAT TAATTAATAA TTTTTAAAAT AAAAAATAAT   
  
  
+ TATATTATTT AATGTTTTTT TCTATAAAAT AAAAATTTTA TGATATTTTT ATTTAAAATT TTTTTACAAA   
  
  
+ TAAGATACTA TCAATACATA GATCAACTTC ACCTCTTTAC ATTTCGACTT TAAGAACTGG AGAGGTTAAC   
  
  
+ TAGTTATAAC AGATATGAAA CTAAAACTTA TCTACTGGCT AACGTTCTTA CCGGGATTAC GTAATTTTTA   
  
  
+ GGTAAAATAC TTATATATAT ATATATATAT TATTCGATTA TGGAGTATAA TATTATTGGG TAATACATAT   
  
  
+ TTGTGTATCA GAATACCTGA ACTGAGATAA AAAAATCGAT CTAGGTGTAT TTAATTTATC TTTGTAATAA   
  
  
+ TAGTAATAAT AATAAATTTT ATTCTTTTAT ACTTTCTTTT ATTGTTATAA GAAACTTTAA AAATGATCGA   
  
  
+ ACCGATTCAT GTATTCATAT TTGCGTTACT ACTTGGTAAC AACTTGTATT TATATATTAC TTGTGTTATT   
  
  
+ TTTTTAATTA TTATTATTTA TAATCTCGT  

- TTCTTTGATG GGCGGCTTAC AGCGATCCAT CGAGCGATCA ATTACACCGC CGGCCGCACC GAAATTCTGC   
  
  
- CGCTCAAAGT CTCCGGGGGA GTGCAGGAAT GTGAATTCAC GGCGTTCTTT ATCATCCATG GCTGAAGAGA   
  
  
- AGAAGAAACT CTCTCTCTCT CTCTCTCTCT CTCTCTCTCT CTCAGAGGAG TAGTAGTTGT GTAGCCTTTT   
  
  
- CAAGATTTCA ACAACTAAGG ATATTTCTTT ATGGTATGTA AGACTTTGCT TAGAGGGAGA TGCAAGGATA   
  
  
- ATAAAAGGCG AGAGAGAGAG AGAGAGAAAG AGGAGGCGAA GGCGAAGACC AGCCGTTTTC TTCGGGGGAG   
  
  
- AGATATGACA TCATGGTTGA TGATGGCACA CGTGGCGACA TCTGGAAGTG AAAGAGGGGA TGAGAGTGGG   
  
  
- ACCTACGGGA AGGATCGCCG TTGAGTACCT TCAAGAGGTC GACGGTGAGA GGTGACGAAG AGTGATAGGG   
  
  
- TGCGGGCGGT TCACGTGGGG CGTCGTCTTC AGGTCAACTA AGTTGACGGC ACGGCTGTAT CTGACGGTGG   
  
  
- ACAAGAAAAA TAAGAGGCGC GTGTGAGACC AGACGAGCAG AGTACCTAAT CCCACTGACC ATCCAACCCA   
  
  
- ACCCAGCCCT TCACGTCAGT TATTTTGGAT CTGTCGTAGT CAACGAACCT CATGGGAGAG TGAAGACCGT   
  
  
- CCTGCTCCCA TGTGTGGTCC GCTCGACTCG GTTGCTATCT CTAATTACCA ATTTTTATTA AAAATAATTA   
  
  
- TTTTATTATA ACAAAAATTA AGATTATTTA TTGAAGTAGA TATTTTTATC TTAATATTCA TGCTTAAATT   
  
  
- AGAAATCATT AAAATTTTGA AAATTTTTTA AAAGACAAAG AATTGTCCAA CAATAAGATT AATATTATTT   
  
  
- AAAATATCTT GATCTCTTCT TAAGATACTG ACATATAATA ATTAATTATT AAAAATTTTA TTTTTTATTA   
  
  
- ATATAATAAA TTACAAAAAA AGATATTTTA TTTTTAAAAT ACTATAAAAA TAAATTTTAA AAAAATGTTT   
  
  
- ATTCTATGAT AGTTATGTAT CTAGTTGAAG TGGAGAAATG TAAAGCTGAA ATTCTTGACC TCTCCAATTG   
  
  
- ATCAATATTG TCTATACTTT GATTTTGAAT AGATGACCGA TTGCAAGAAT GGCCCTAATG CATTAAAAAT   
  
  
- CCATTTTATG AATATATATA TATATATATA ATAAGCTAAT ACCTCATATT ATAATAACCC ATTATGTATA   
  
  
- AACACATAGT CTTATGGACT TGACTCTATT TTTTTAGCTA GATCCACATA AATTAAATAG AAACATTATT   
  
  
- ATCATTATTA TTATTTAAAA TAAGAAAATA TGAAAGAAAA TAACAATATT CTTTGAAATT TTTACTAGCT   
  
  
- TGGCTAAGTA CATAAGTATA AACGCAATGA TGAACCATTG TTGAACATAA ATATATAATG AACACAATAA   
  
  
- AAAAATTAAT AATAATAAAT ATTAGAGCA

+     AT-rich sequence

| Site Name | Organism | Position | Strand | Matrix score. | sequence | function |
| --- | --- | --- | --- | --- | --- | --- |
| AT-rich sequence | Pisum sativum | 1193 | + | 9 | TAAAATACT | element for maximal elicitor-mediated activation (2copies) |

> 2018/04/13 10:10:12  
+ AAGAAACTAC CCGCCGAATG TCGCTAGGTA GCTCGCTAGT TAATGTGGCG GCCGGCGTGG CTTTAAGACG   
  
  
+ GCGAGTTTCA GAGGCCCCCT CACGTCCTTA CACTTAAGTG CCGCAAGAAA TAGTAGGTAC CGACTTCTCT   
  
  
+ TCTTCTTTGA GAGAGAGAGA GAGAGAGAGA GAGAGAGAGA GAGTCTCCTC ATCATCAACA CATCGGAAAA   
  
  
+ GTTCTAAAGT TGTTGATTCC TATAAAGAAA TACCATACAT TCTGAAACGA ATCTCCCTCT ACGTTCCTAT   
  
  
+ TATTTTCCGC TCTCTCTCTC TCTCTCTTTC TCCTCCGCTT CCGCTTCTGG TCGGCAAAAG AAGCCCCCTC   
  
  
+ TCTATACTGT AGTACCAACT ACTACCGTGT GCACCGCTGT AGACCTTCAC TTTCTCCCCT ACTCTCACCC   
  
  
+ TGGATGCCCT TCCTAGCGGC AACTCATGGA AGTTCTCCAG CTGCCACTCT CCACTGCTTC TCACTATCCC   
  
  
+ ACGCCCGCCA AGTGCACCCC GCAGCAGAAG TCCAGTTGAT TCAACTGCCG TGCCGACATA GACTGCCACC   
  
  
+ TGTTCTTTTT ATTCTCCGCG CACACTCTGG TCTGCTCGTC TCATGGATTA GGGTGACTGG TAGGTTGGGT   
  
  
+ TGGGTCGGGA AGTGCAGTCA ATAAAACCTA GACAGCATCA GTTGCTTGGA GTACCCTCTC ACTTCTGGCA   
  
  
+ GGACGAGGGT ACACACCAGG CGAGCTGAGC CAACGATAGA GATTAATGGT TAAAAATAAT TTTTATTAAT   
  
  
+ AAAATAATAT TGTTTTTAAT TCTAATAAAT AACTTCATCT ATAAAAATAG AATTATAAGT ACGAATTTAA   
  
  
+ TCTTTAGTAA TTTTAAAACT TTTAAAAAAT TTTCTGTTTC TTAACAGGTT GTTATTCTAA TTATAATAAA   
  
  
+ TTTTATAGAA CTAGAGAAGA ATTCTATGAC TGTATATTAT TAATTAATAA TTTTTAAAAT AAAAAATAAT   
  
  
+ TATATTATTT AATGTTTTTT TCTATAAAAT AAAAATTTTA TGATATTTTT ATTTAAAATT TTTTTACAAA   
  
  
+ TAAGATACTA TCAATACATA GATCAACTTC ACCTCTTTAC ATTTCGACTT TAAGAACTGG AGAGGTTAAC   
  
  
+ TAGTTATAAC AGATATGAAA CTAAAACTTA TCTACTGGCT AACGTTCTTA CCGGGATTAC GTAATTTTTA   
  
  
+ GGTAAAATAC TTATATATAT ATATATATAT TATTCGATTA TGGAGTATAA TATTATTGGG TAATACATAT   
  
  
+ TTGTGTATCA GAATACCTGA ACTGAGATAA AAAAATCGAT CTAGGTGTAT TTAATTTATC TTTGTAATAA   
  
  
+ TAGTAATAAT AATAAATTTT ATTCTTTTAT ACTTTCTTTT ATTGTTATAA GAAACTTTAA AAATGATCGA   
  
  
+ ACCGATTCAT GTATTCATAT TTGCGTTACT ACTTGGTAAC AACTTGTATT TATATATTAC TTGTGTTATT   
  
  
+ TTTTTAATTA TTATTATTTA TAATCTCGT  

- TTCTTTGATG GGCGGCTTAC AGCGATCCAT CGAGCGATCA ATTACACCGC CGGCCGCACC GAAATTCTGC   
  
  
- CGCTCAAAGT CTCCGGGGGA GTGCAGGAAT GTGAATTCAC GGCGTTCTTT ATCATCCATG GCTGAAGAGA   
  
  
- AGAAGAAACT CTCTCTCTCT CTCTCTCTCT CTCTCTCTCT CTCAGAGGAG TAGTAGTTGT GTAGCCTTTT   
  
  
- CAAGATTTCA ACAACTAAGG ATATTTCTTT ATGGTATGTA AGACTTTGCT TAGAGGGAGA TGCAAGGATA   
  
  
- ATAAAAGGCG AGAGAGAGAG AGAGAGAAAG AGGAGGCGAA GGCGAAGACC AGCCGTTTTC TTCGGGGGAG   
  
  
- AGATATGACA TCATGGTTGA TGATGGCACA CGTGGCGACA TCTGGAAGTG AAAGAGGGGA TGAGAGTGGG   
  
  
- ACCTACGGGA AGGATCGCCG TTGAGTACCT TCAAGAGGTC GACGGTGAGA GGTGACGAAG AGTGATAGGG   
  
  
- TGCGGGCGGT TCACGTGGGG CGTCGTCTTC AGGTCAACTA AGTTGACGGC ACGGCTGTAT CTGACGGTGG   
  
  
- ACAAGAAAAA TAAGAGGCGC GTGTGAGACC AGACGAGCAG AGTACCTAAT CCCACTGACC ATCCAACCCA   
  
  
- ACCCAGCCCT TCACGTCAGT TATTTTGGAT CTGTCGTAGT CAACGAACCT CATGGGAGAG TGAAGACCGT   
  
  
- CCTGCTCCCA TGTGTGGTCC GCTCGACTCG GTTGCTATCT CTAATTACCA ATTTTTATTA AAAATAATTA   
  
  
- TTTTATTATA ACAAAAATTA AGATTATTTA TTGAAGTAGA TATTTTTATC TTAATATTCA TGCTTAAATT   
  
  
- AGAAATCATT AAAATTTTGA AAATTTTTTA AAAGACAAAG AATTGTCCAA CAATAAGATT AATATTATTT   
  
  
- AAAATATCTT GATCTCTTCT TAAGATACTG ACATATAATA ATTAATTATT AAAAATTTTA TTTTTTATTA   
  
  
- ATATAATAAA TTACAAAAAA AGATATTTTA TTTTTAAAAT ACTATAAAAA TAAATTTTAA AAAAATGTTT   
  
  
- ATTCTATGAT AGTTATGTAT CTAGTTGAAG TGGAGAAATG TAAAGCTGAA ATTCTTGACC TCTCCAATTG   
  
  
- ATCAATATTG TCTATACTTT GATTTTGAAT AGATGACCGA TTGCAAGAAT GGCCCTAATG CATTAAAAAT   
  
  
- CCATTTTATG AATATATATA TATATATATA ATAAGCTAAT ACCTCATATT ATAATAACCC ATTATGTATA   
  
  
- AACACATAGT CTTATGGACT TGACTCTATT TTTTTAGCTA GATCCACATA AATTAAATAG AAACATTATT   
  
  
- ATCATTATTA TTATTTAAAA TAAGAAAATA TGAAAGAAAA TAACAATATT CTTTGAAATT TTTACTAGCT   
  
  
- TGGCTAAGTA CATAAGTATA AACGCAATGA TGAACCATTG TTGAACATAA ATATATAATG AACACAATAA   
  
  
- AAAAATTAAT AATAATAAAT ATTAGAGCA

+     AT1-motif

| Site Name | Organism | Position | Strand | Matrix score. | sequence | function |
| --- | --- | --- | --- | --- | --- | --- |
| AT1-motif | Solanum tuberosum | 968 | - | 14 | AATTATTTTTTATT | part of a light responsive module |

> 2018/04/13 10:10:12  
+ AAGAAACTAC CCGCCGAATG TCGCTAGGTA GCTCGCTAGT TAATGTGGCG GCCGGCGTGG CTTTAAGACG   
  
  
+ GCGAGTTTCA GAGGCCCCCT CACGTCCTTA CACTTAAGTG CCGCAAGAAA TAGTAGGTAC CGACTTCTCT   
  
  
+ TCTTCTTTGA GAGAGAGAGA GAGAGAGAGA GAGAGAGAGA GAGTCTCCTC ATCATCAACA CATCGGAAAA   
  
  
+ GTTCTAAAGT TGTTGATTCC TATAAAGAAA TACCATACAT TCTGAAACGA ATCTCCCTCT ACGTTCCTAT   
  
  
+ TATTTTCCGC TCTCTCTCTC TCTCTCTTTC TCCTCCGCTT CCGCTTCTGG TCGGCAAAAG AAGCCCCCTC   
  
  
+ TCTATACTGT AGTACCAACT ACTACCGTGT GCACCGCTGT AGACCTTCAC TTTCTCCCCT ACTCTCACCC   
  
  
+ TGGATGCCCT TCCTAGCGGC AACTCATGGA AGTTCTCCAG CTGCCACTCT CCACTGCTTC TCACTATCCC   
  
  
+ ACGCCCGCCA AGTGCACCCC GCAGCAGAAG TCCAGTTGAT TCAACTGCCG TGCCGACATA GACTGCCACC   
  
  
+ TGTTCTTTTT ATTCTCCGCG CACACTCTGG TCTGCTCGTC TCATGGATTA GGGTGACTGG TAGGTTGGGT   
  
  
+ TGGGTCGGGA AGTGCAGTCA ATAAAACCTA GACAGCATCA GTTGCTTGGA GTACCCTCTC ACTTCTGGCA   
  
  
+ GGACGAGGGT ACACACCAGG CGAGCTGAGC CAACGATAGA GATTAATGGT TAAAAATAAT TTTTATTAAT   
  
  
+ AAAATAATAT TGTTTTTAAT TCTAATAAAT AACTTCATCT ATAAAAATAG AATTATAAGT ACGAATTTAA   
  
  
+ TCTTTAGTAA TTTTAAAACT TTTAAAAAAT TTTCTGTTTC TTAACAGGTT GTTATTCTAA TTATAATAAA   
  
  
+ TTTTATAGAA CTAGAGAAGA ATTCTATGAC TGTATATTAT TAATTAATAA TTTTTAAAAT AAAAAATAAT   
  
  
+ TATATTATTT AATGTTTTTT TCTATAAAAT AAAAATTTTA TGATATTTTT ATTTAAAATT TTTTTACAAA   
  
  
+ TAAGATACTA TCAATACATA GATCAACTTC ACCTCTTTAC ATTTCGACTT TAAGAACTGG AGAGGTTAAC   
  
  
+ TAGTTATAAC AGATATGAAA CTAAAACTTA TCTACTGGCT AACGTTCTTA CCGGGATTAC GTAATTTTTA   
  
  
+ GGTAAAATAC TTATATATAT ATATATATAT TATTCGATTA TGGAGTATAA TATTATTGGG TAATACATAT   
  
  
+ TTGTGTATCA GAATACCTGA ACTGAGATAA AAAAATCGAT CTAGGTGTAT TTAATTTATC TTTGTAATAA   
  
  
+ TAGTAATAAT AATAAATTTT ATTCTTTTAT ACTTTCTTTT ATTGTTATAA GAAACTTTAA AAATGATCGA   
  
  
+ ACCGATTCAT GTATTCATAT TTGCGTTACT ACTTGGTAAC AACTTGTATT TATATATTAC TTGTGTTATT   
  
  
+ TTTTTAATTA TTATTATTTA TAATCTCGT  

- TTCTTTGATG GGCGGCTTAC AGCGATCCAT CGAGCGATCA ATTACACCGC CGGCCGCACC GAAATTCTGC   
  
  
- CGCTCAAAGT CTCCGGGGGA GTGCAGGAAT GTGAATTCAC GGCGTTCTTT ATCATCCATG GCTGAAGAGA   
  
  
- AGAAGAAACT CTCTCTCTCT CTCTCTCTCT CTCTCTCTCT CTCAGAGGAG TAGTAGTTGT GTAGCCTTTT   
  
  
- CAAGATTTCA ACAACTAAGG ATATTTCTTT ATGGTATGTA AGACTTTGCT TAGAGGGAGA TGCAAGGATA   
  
  
- ATAAAAGGCG AGAGAGAGAG AGAGAGAAAG AGGAGGCGAA GGCGAAGACC AGCCGTTTTC TTCGGGGGAG   
  
  
- AGATATGACA TCATGGTTGA TGATGGCACA CGTGGCGACA TCTGGAAGTG AAAGAGGGGA TGAGAGTGGG   
  
  
- ACCTACGGGA AGGATCGCCG TTGAGTACCT TCAAGAGGTC GACGGTGAGA GGTGACGAAG AGTGATAGGG   
  
  
- TGCGGGCGGT TCACGTGGGG CGTCGTCTTC AGGTCAACTA AGTTGACGGC ACGGCTGTAT CTGACGGTGG   
  
  
- ACAAGAAAAA TAAGAGGCGC GTGTGAGACC AGACGAGCAG AGTACCTAAT CCCACTGACC ATCCAACCCA   
  
  
- ACCCAGCCCT TCACGTCAGT TATTTTGGAT CTGTCGTAGT CAACGAACCT CATGGGAGAG TGAAGACCGT   
  
  
- CCTGCTCCCA TGTGTGGTCC GCTCGACTCG GTTGCTATCT CTAATTACCA ATTTTTATTA AAAATAATTA   
  
  
- TTTTATTATA ACAAAAATTA AGATTATTTA TTGAAGTAGA TATTTTTATC TTAATATTCA TGCTTAAATT   
  
  
- AGAAATCATT AAAATTTTGA AAATTTTTTA AAAGACAAAG AATTGTCCAA CAATAAGATT AATATTATTT   
  
  
- AAAATATCTT GATCTCTTCT TAAGATACTG ACATATAATA ATTAATTATT AAAAATTTTA TTTTTTATTA   
  
  
- ATATAATAAA TTACAAAAAA AGATATTTTA TTTTTAAAAT ACTATAAAAA TAAATTTTAA AAAAATGTTT   
  
  
- ATTCTATGAT AGTTATGTAT CTAGTTGAAG TGGAGAAATG TAAAGCTGAA ATTCTTGACC TCTCCAATTG   
  
  
- ATCAATATTG TCTATACTTT GATTTTGAAT AGATGACCGA TTGCAAGAAT GGCCCTAATG CATTAAAAAT   
  
  
- CCATTTTATG AATATATATA TATATATATA ATAAGCTAAT ACCTCATATT ATAATAACCC ATTATGTATA   
  
  
- AACACATAGT CTTATGGACT TGACTCTATT TTTTTAGCTA GATCCACATA AATTAAATAG AAACATTATT   
  
  
- ATCATTATTA TTATTTAAAA TAAGAAAATA TGAAAGAAAA TAACAATATT CTTTGAAATT TTTACTAGCT   
  
  
- TGGCTAAGTA CATAAGTATA AACGCAATGA TGAACCATTG TTGAACATAA ATATATAATG AACACAATAA   
  
  
- AAAAATTAAT AATAATAAAT ATTAGAGCA

+     ATCT-motif

| Site Name | Organism | Position | Strand | Matrix score. | sequence | function |
| --- | --- | --- | --- | --- | --- | --- |
| ATCT-motif | Arabidopsis thaliana | 834 | + | 9 | AATCTAATCT | part of a conserved DNA module involved in light responsiveness |

> 2018/04/13 10:10:12  
+ AAGAAACTAC CCGCCGAATG TCGCTAGGTA GCTCGCTAGT TAATGTGGCG GCCGGCGTGG CTTTAAGACG   
  
  
+ GCGAGTTTCA GAGGCCCCCT CACGTCCTTA CACTTAAGTG CCGCAAGAAA TAGTAGGTAC CGACTTCTCT   
  
  
+ TCTTCTTTGA GAGAGAGAGA GAGAGAGAGA GAGAGAGAGA GAGTCTCCTC ATCATCAACA CATCGGAAAA   
  
  
+ GTTCTAAAGT TGTTGATTCC TATAAAGAAA TACCATACAT TCTGAAACGA ATCTCCCTCT ACGTTCCTAT   
  
  
+ TATTTTCCGC TCTCTCTCTC TCTCTCTTTC TCCTCCGCTT CCGCTTCTGG TCGGCAAAAG AAGCCCCCTC   
  
  
+ TCTATACTGT AGTACCAACT ACTACCGTGT GCACCGCTGT AGACCTTCAC TTTCTCCCCT ACTCTCACCC   
  
  
+ TGGATGCCCT TCCTAGCGGC AACTCATGGA AGTTCTCCAG CTGCCACTCT CCACTGCTTC TCACTATCCC   
  
  
+ ACGCCCGCCA AGTGCACCCC GCAGCAGAAG TCCAGTTGAT TCAACTGCCG TGCCGACATA GACTGCCACC   
  
  
+ TGTTCTTTTT ATTCTCCGCG CACACTCTGG TCTGCTCGTC TCATGGATTA GGGTGACTGG TAGGTTGGGT   
  
  
+ TGGGTCGGGA AGTGCAGTCA ATAAAACCTA GACAGCATCA GTTGCTTGGA GTACCCTCTC ACTTCTGGCA   
  
  
+ GGACGAGGGT ACACACCAGG CGAGCTGAGC CAACGATAGA GATTAATGGT TAAAAATAAT TTTTATTAAT   
  
  
+ AAAATAATAT TGTTTTTAAT TCTAATAAAT AACTTCATCT ATAAAAATAG AATTATAAGT ACGAATTTAA   
  
  
+ TCTTTAGTAA TTTTAAAACT TTTAAAAAAT TTTCTGTTTC TTAACAGGTT GTTATTCTAA TTATAATAAA   
  
  
+ TTTTATAGAA CTAGAGAAGA ATTCTATGAC TGTATATTAT TAATTAATAA TTTTTAAAAT AAAAAATAAT   
  
  
+ TATATTATTT AATGTTTTTT TCTATAAAAT AAAAATTTTA TGATATTTTT ATTTAAAATT TTTTTACAAA   
  
  
+ TAAGATACTA TCAATACATA GATCAACTTC ACCTCTTTAC ATTTCGACTT TAAGAACTGG AGAGGTTAAC   
  
  
+ TAGTTATAAC AGATATGAAA CTAAAACTTA TCTACTGGCT AACGTTCTTA CCGGGATTAC GTAATTTTTA   
  
  
+ GGTAAAATAC TTATATATAT ATATATATAT TATTCGATTA TGGAGTATAA TATTATTGGG TAATACATAT   
  
  
+ TTGTGTATCA GAATACCTGA ACTGAGATAA AAAAATCGAT CTAGGTGTAT TTAATTTATC TTTGTAATAA   
  
  
+ TAGTAATAAT AATAAATTTT ATTCTTTTAT ACTTTCTTTT ATTGTTATAA GAAACTTTAA AAATGATCGA   
  
  
+ ACCGATTCAT GTATTCATAT TTGCGTTACT ACTTGGTAAC AACTTGTATT TATATATTAC TTGTGTTATT   
  
  
+ TTTTTAATTA TTATTATTTA TAATCTCGT  

- TTCTTTGATG GGCGGCTTAC AGCGATCCAT CGAGCGATCA ATTACACCGC CGGCCGCACC GAAATTCTGC   
  
  
- CGCTCAAAGT CTCCGGGGGA GTGCAGGAAT GTGAATTCAC GGCGTTCTTT ATCATCCATG GCTGAAGAGA   
  
  
- AGAAGAAACT CTCTCTCTCT CTCTCTCTCT CTCTCTCTCT CTCAGAGGAG TAGTAGTTGT GTAGCCTTTT   
  
  
- CAAGATTTCA ACAACTAAGG ATATTTCTTT ATGGTATGTA AGACTTTGCT TAGAGGGAGA TGCAAGGATA   
  
  
- ATAAAAGGCG AGAGAGAGAG AGAGAGAAAG AGGAGGCGAA GGCGAAGACC AGCCGTTTTC TTCGGGGGAG   
  
  
- AGATATGACA TCATGGTTGA TGATGGCACA CGTGGCGACA TCTGGAAGTG AAAGAGGGGA TGAGAGTGGG   
  
  
- ACCTACGGGA AGGATCGCCG TTGAGTACCT TCAAGAGGTC GACGGTGAGA GGTGACGAAG AGTGATAGGG   
  
  
- TGCGGGCGGT TCACGTGGGG CGTCGTCTTC AGGTCAACTA AGTTGACGGC ACGGCTGTAT CTGACGGTGG   
  
  
- ACAAGAAAAA TAAGAGGCGC GTGTGAGACC AGACGAGCAG AGTACCTAAT CCCACTGACC ATCCAACCCA   
  
  
- ACCCAGCCCT TCACGTCAGT TATTTTGGAT CTGTCGTAGT CAACGAACCT CATGGGAGAG TGAAGACCGT   
  
  
- CCTGCTCCCA TGTGTGGTCC GCTCGACTCG GTTGCTATCT CTAATTACCA ATTTTTATTA AAAATAATTA   
  
  
- TTTTATTATA ACAAAAATTA AGATTATTTA TTGAAGTAGA TATTTTTATC TTAATATTCA TGCTTAAATT   
  
  
- AGAAATCATT AAAATTTTGA AAATTTTTTA AAAGACAAAG AATTGTCCAA CAATAAGATT AATATTATTT   
  
  
- AAAATATCTT GATCTCTTCT TAAGATACTG ACATATAATA ATTAATTATT AAAAATTTTA TTTTTTATTA   
  
  
- ATATAATAAA TTACAAAAAA AGATATTTTA TTTTTAAAAT ACTATAAAAA TAAATTTTAA AAAAATGTTT   
  
  
- ATTCTATGAT AGTTATGTAT CTAGTTGAAG TGGAGAAATG TAAAGCTGAA ATTCTTGACC TCTCCAATTG   
  
  
- ATCAATATTG TCTATACTTT GATTTTGAAT AGATGACCGA TTGCAAGAAT GGCCCTAATG CATTAAAAAT   
  
  
- CCATTTTATG AATATATATA TATATATATA ATAAGCTAAT ACCTCATATT ATAATAACCC ATTATGTATA   
  
  
- AACACATAGT CTTATGGACT TGACTCTATT TTTTTAGCTA GATCCACATA AATTAAATAG AAACATTATT   
  
  
- ATCATTATTA TTATTTAAAA TAAGAAAATA TGAAAGAAAA TAACAATATT CTTTGAAATT TTTACTAGCT   
  
  
- TGGCTAAGTA CATAAGTATA AACGCAATGA TGAACCATTG TTGAACATAA ATATATAATG AACACAATAA   
  
  
- AAAAATTAAT AATAATAAAT ATTAGAGCA

+     Box 4

| Site Name | Organism | Position | Strand | Matrix score. | sequence | function |
| --- | --- | --- | --- | --- | --- | --- |
| Box 4 | Petroselinum crispum | 765 | - | 6 | ATTAAT | part of a conserved DNA module involved in light responsiveness |
| Box 4 | Petroselinum crispum | 953 | - | 6 | ATTAAT | part of a conserved DNA module involved in light responsiveness |
| Box 4 | Petroselinum crispum | 742 | + | 6 | ATTAAT | part of a conserved DNA module involved in light responsiveness |
| Box 4 | Petroselinum crispum | 949 | - | 6 | ATTAAT | part of a conserved DNA module involved in light responsiveness |

> 2018/04/13 10:10:12  
+ AAGAAACTAC CCGCCGAATG TCGCTAGGTA GCTCGCTAGT TAATGTGGCG GCCGGCGTGG CTTTAAGACG   
  
  
+ GCGAGTTTCA GAGGCCCCCT CACGTCCTTA CACTTAAGTG CCGCAAGAAA TAGTAGGTAC CGACTTCTCT   
  
  
+ TCTTCTTTGA GAGAGAGAGA GAGAGAGAGA GAGAGAGAGA GAGTCTCCTC ATCATCAACA CATCGGAAAA   
  
  
+ GTTCTAAAGT TGTTGATTCC TATAAAGAAA TACCATACAT TCTGAAACGA ATCTCCCTCT ACGTTCCTAT   
  
  
+ TATTTTCCGC TCTCTCTCTC TCTCTCTTTC TCCTCCGCTT CCGCTTCTGG TCGGCAAAAG AAGCCCCCTC   
  
  
+ TCTATACTGT AGTACCAACT ACTACCGTGT GCACCGCTGT AGACCTTCAC TTTCTCCCCT ACTCTCACCC   
  
  
+ TGGATGCCCT TCCTAGCGGC AACTCATGGA AGTTCTCCAG CTGCCACTCT CCACTGCTTC TCACTATCCC   
  
  
+ ACGCCCGCCA AGTGCACCCC GCAGCAGAAG TCCAGTTGAT TCAACTGCCG TGCCGACATA GACTGCCACC   
  
  
+ TGTTCTTTTT ATTCTCCGCG CACACTCTGG TCTGCTCGTC TCATGGATTA GGGTGACTGG TAGGTTGGGT   
  
  
+ TGGGTCGGGA AGTGCAGTCA ATAAAACCTA GACAGCATCA GTTGCTTGGA GTACCCTCTC ACTTCTGGCA   
  
  
+ GGACGAGGGT ACACACCAGG CGAGCTGAGC CAACGATAGA GATTAATGGT TAAAAATAAT TTTTATTAAT   
  
  
+ AAAATAATAT TGTTTTTAAT TCTAATAAAT AACTTCATCT ATAAAAATAG AATTATAAGT ACGAATTTAA   
  
  
+ TCTTTAGTAA TTTTAAAACT TTTAAAAAAT TTTCTGTTTC TTAACAGGTT GTTATTCTAA TTATAATAAA   
  
  
+ TTTTATAGAA CTAGAGAAGA ATTCTATGAC TGTATATTAT TAATTAATAA TTTTTAAAAT AAAAAATAAT   
  
  
+ TATATTATTT AATGTTTTTT TCTATAAAAT AAAAATTTTA TGATATTTTT ATTTAAAATT TTTTTACAAA   
  
  
+ TAAGATACTA TCAATACATA GATCAACTTC ACCTCTTTAC ATTTCGACTT TAAGAACTGG AGAGGTTAAC   
  
  
+ TAGTTATAAC AGATATGAAA CTAAAACTTA TCTACTGGCT AACGTTCTTA CCGGGATTAC GTAATTTTTA   
  
  
+ GGTAAAATAC TTATATATAT ATATATATAT TATTCGATTA TGGAGTATAA TATTATTGGG TAATACATAT   
  
  
+ TTGTGTATCA GAATACCTGA ACTGAGATAA AAAAATCGAT CTAGGTGTAT TTAATTTATC TTTGTAATAA   
  
  
+ TAGTAATAAT AATAAATTTT ATTCTTTTAT ACTTTCTTTT ATTGTTATAA GAAACTTTAA AAATGATCGA   
  
  
+ ACCGATTCAT GTATTCATAT TTGCGTTACT ACTTGGTAAC AACTTGTATT TATATATTAC TTGTGTTATT   
  
  
+ TTTTTAATTA TTATTATTTA TAATCTCGT  

- TTCTTTGATG GGCGGCTTAC AGCGATCCAT CGAGCGATCA ATTACACCGC CGGCCGCACC GAAATTCTGC   
  
  
- CGCTCAAAGT CTCCGGGGGA GTGCAGGAAT GTGAATTCAC GGCGTTCTTT ATCATCCATG GCTGAAGAGA   
  
  
- AGAAGAAACT CTCTCTCTCT CTCTCTCTCT CTCTCTCTCT CTCAGAGGAG TAGTAGTTGT GTAGCCTTTT   
  
  
- CAAGATTTCA ACAACTAAGG ATATTTCTTT ATGGTATGTA AGACTTTGCT TAGAGGGAGA TGCAAGGATA   
  
  
- ATAAAAGGCG AGAGAGAGAG AGAGAGAAAG AGGAGGCGAA GGCGAAGACC AGCCGTTTTC TTCGGGGGAG   
  
  
- AGATATGACA TCATGGTTGA TGATGGCACA CGTGGCGACA TCTGGAAGTG AAAGAGGGGA TGAGAGTGGG   
  
  
- ACCTACGGGA AGGATCGCCG TTGAGTACCT TCAAGAGGTC GACGGTGAGA GGTGACGAAG AGTGATAGGG   
  
  
- TGCGGGCGGT TCACGTGGGG CGTCGTCTTC AGGTCAACTA AGTTGACGGC ACGGCTGTAT CTGACGGTGG   
  
  
- ACAAGAAAAA TAAGAGGCGC GTGTGAGACC AGACGAGCAG AGTACCTAAT CCCACTGACC ATCCAACCCA   
  
  
- ACCCAGCCCT TCACGTCAGT TATTTTGGAT CTGTCGTAGT CAACGAACCT CATGGGAGAG TGAAGACCGT   
  
  
- CCTGCTCCCA TGTGTGGTCC GCTCGACTCG GTTGCTATCT CTAATTACCA ATTTTTATTA AAAATAATTA   
  
  
- TTTTATTATA ACAAAAATTA AGATTATTTA TTGAAGTAGA TATTTTTATC TTAATATTCA TGCTTAAATT   
  
  
- AGAAATCATT AAAATTTTGA AAATTTTTTA AAAGACAAAG AATTGTCCAA CAATAAGATT AATATTATTT   
  
  
- AAAATATCTT GATCTCTTCT TAAGATACTG ACATATAATA ATTAATTATT AAAAATTTTA TTTTTTATTA   
  
  
- ATATAATAAA TTACAAAAAA AGATATTTTA TTTTTAAAAT ACTATAAAAA TAAATTTTAA AAAAATGTTT   
  
  
- ATTCTATGAT AGTTATGTAT CTAGTTGAAG TGGAGAAATG TAAAGCTGAA ATTCTTGACC TCTCCAATTG   
  
  
- ATCAATATTG TCTATACTTT GATTTTGAAT AGATGACCGA TTGCAAGAAT GGCCCTAATG CATTAAAAAT   
  
  
- CCATTTTATG AATATATATA TATATATATA ATAAGCTAAT ACCTCATATT ATAATAACCC ATTATGTATA   
  
  
- AACACATAGT CTTATGGACT TGACTCTATT TTTTTAGCTA GATCCACATA AATTAAATAG AAACATTATT   
  
  
- ATCATTATTA TTATTTAAAA TAAGAAAATA TGAAAGAAAA TAACAATATT CTTTGAAATT TTTACTAGCT   
  
  
- TGGCTAAGTA CATAAGTATA AACGCAATGA TGAACCATTG TTGAACATAA ATATATAATG AACACAATAA   
  
  
- AAAAATTAAT AATAATAAAT ATTAGAGCA

+     CAAT-box

| Site Name | Organism | Position | Strand | Matrix score. | sequence | function |
| --- | --- | --- | --- | --- | --- | --- |
| CAAT-box | Hordeum vulgare | 1371 | - | 4 | CAAT | common cis-acting element in promoter and enhancer regions |
| CAAT-box | Hordeum vulgare | 649 | + | 4 | CAAT | common cis-acting element in promoter and enhancer regions |
| CAAT-box | Brassica rapa | 1047 | + | 5 | CAAAT | common cis-acting element in promoter and enhancer regions |
| CAAT-box | Hordeum vulgare | 1062 | + | 4 | CAAT | common cis-acting element in promoter and enhancer regions |
| CAAT-box | Hordeum vulgare | 779 | - | 4 | CAAT | common cis-acting element in promoter and enhancer regions |
| CAAT-box | Brassica rapa | 1419 | - | 5 | CAAAT | common cis-acting element in promoter and enhancer regions |
| CAAT-box | Brassica rapa | 1259 | - | 5 | CAAAT | common cis-acting element in promoter and enhancer regions |
| CAAT-box | Arabidopsis thaliana | 1245 | - | 5 | CCAAT | common cis-acting element in promoter and enhancer regions |

> 2018/04/13 10:10:12  
+ AAGAAACTAC CCGCCGAATG TCGCTAGGTA GCTCGCTAGT TAATGTGGCG GCCGGCGTGG CTTTAAGACG   
  
  
+ GCGAGTTTCA GAGGCCCCCT CACGTCCTTA CACTTAAGTG CCGCAAGAAA TAGTAGGTAC CGACTTCTCT   
  
  
+ TCTTCTTTGA GAGAGAGAGA GAGAGAGAGA GAGAGAGAGA GAGTCTCCTC ATCATCAACA CATCGGAAAA   
  
  
+ GTTCTAAAGT TGTTGATTCC TATAAAGAAA TACCATACAT TCTGAAACGA ATCTCCCTCT ACGTTCCTAT   
  
  
+ TATTTTCCGC TCTCTCTCTC TCTCTCTTTC TCCTCCGCTT CCGCTTCTGG TCGGCAAAAG AAGCCCCCTC   
  
  
+ TCTATACTGT AGTACCAACT ACTACCGTGT GCACCGCTGT AGACCTTCAC TTTCTCCCCT ACTCTCACCC   
  
  
+ TGGATGCCCT TCCTAGCGGC AACTCATGGA AGTTCTCCAG CTGCCACTCT CCACTGCTTC TCACTATCCC   
  
  
+ ACGCCCGCCA AGTGCACCCC GCAGCAGAAG TCCAGTTGAT TCAACTGCCG TGCCGACATA GACTGCCACC   
  
  
+ TGTTCTTTTT ATTCTCCGCG CACACTCTGG TCTGCTCGTC TCATGGATTA GGGTGACTGG TAGGTTGGGT   
  
  
+ TGGGTCGGGA AGTGCAGTCA ATAAAACCTA GACAGCATCA GTTGCTTGGA GTACCCTCTC ACTTCTGGCA   
  
  
+ GGACGAGGGT ACACACCAGG CGAGCTGAGC CAACGATAGA GATTAATGGT TAAAAATAAT TTTTATTAAT   
  
  
+ AAAATAATAT TGTTTTTAAT TCTAATAAAT AACTTCATCT ATAAAAATAG AATTATAAGT ACGAATTTAA   
  
  
+ TCTTTAGTAA TTTTAAAACT TTTAAAAAAT TTTCTGTTTC TTAACAGGTT GTTATTCTAA TTATAATAAA   
  
  
+ TTTTATAGAA CTAGAGAAGA ATTCTATGAC TGTATATTAT TAATTAATAA TTTTTAAAAT AAAAAATAAT   
  
  
+ TATATTATTT AATGTTTTTT TCTATAAAAT AAAAATTTTA TGATATTTTT ATTTAAAATT TTTTTACAAA   
  
  
+ TAAGATACTA TCAATACATA GATCAACTTC ACCTCTTTAC ATTTCGACTT TAAGAACTGG AGAGGTTAAC   
  
  
+ TAGTTATAAC AGATATGAAA CTAAAACTTA TCTACTGGCT AACGTTCTTA CCGGGATTAC GTAATTTTTA   
  
  
+ GGTAAAATAC TTATATATAT ATATATATAT TATTCGATTA TGGAGTATAA TATTATTGGG TAATACATAT   
  
  
+ TTGTGTATCA GAATACCTGA ACTGAGATAA AAAAATCGAT CTAGGTGTAT TTAATTTATC TTTGTAATAA   
  
  
+ TAGTAATAAT AATAAATTTT ATTCTTTTAT ACTTTCTTTT ATTGTTATAA GAAACTTTAA AAATGATCGA   
  
  
+ ACCGATTCAT GTATTCATAT TTGCGTTACT ACTTGGTAAC AACTTGTATT TATATATTAC TTGTGTTATT   
  
  
+ TTTTTAATTA TTATTATTTA TAATCTCGT  

- TTCTTTGATG GGCGGCTTAC AGCGATCCAT CGAGCGATCA ATTACACCGC CGGCCGCACC GAAATTCTGC   
  
  
- CGCTCAAAGT CTCCGGGGGA GTGCAGGAAT GTGAATTCAC GGCGTTCTTT ATCATCCATG GCTGAAGAGA   
  
  
- AGAAGAAACT CTCTCTCTCT CTCTCTCTCT CTCTCTCTCT CTCAGAGGAG TAGTAGTTGT GTAGCCTTTT   
  
  
- CAAGATTTCA ACAACTAAGG ATATTTCTTT ATGGTATGTA AGACTTTGCT TAGAGGGAGA TGCAAGGATA   
  
  
- ATAAAAGGCG AGAGAGAGAG AGAGAGAAAG AGGAGGCGAA GGCGAAGACC AGCCGTTTTC TTCGGGGGAG   
  
  
- AGATATGACA TCATGGTTGA TGATGGCACA CGTGGCGACA TCTGGAAGTG AAAGAGGGGA TGAGAGTGGG   
  
  
- ACCTACGGGA AGGATCGCCG TTGAGTACCT TCAAGAGGTC GACGGTGAGA GGTGACGAAG AGTGATAGGG   
  
  
- TGCGGGCGGT TCACGTGGGG CGTCGTCTTC AGGTCAACTA AGTTGACGGC ACGGCTGTAT CTGACGGTGG   
  
  
- ACAAGAAAAA TAAGAGGCGC GTGTGAGACC AGACGAGCAG AGTACCTAAT CCCACTGACC ATCCAACCCA   
  
  
- ACCCAGCCCT TCACGTCAGT TATTTTGGAT CTGTCGTAGT CAACGAACCT CATGGGAGAG TGAAGACCGT   
  
  
- CCTGCTCCCA TGTGTGGTCC GCTCGACTCG GTTGCTATCT CTAATTACCA ATTTTTATTA AAAATAATTA   
  
  
- TTTTATTATA ACAAAAATTA AGATTATTTA TTGAAGTAGA TATTTTTATC TTAATATTCA TGCTTAAATT   
  
  
- AGAAATCATT AAAATTTTGA AAATTTTTTA AAAGACAAAG AATTGTCCAA CAATAAGATT AATATTATTT   
  
  
- AAAATATCTT GATCTCTTCT TAAGATACTG ACATATAATA ATTAATTATT AAAAATTTTA TTTTTTATTA   
  
  
- ATATAATAAA TTACAAAAAA AGATATTTTA TTTTTAAAAT ACTATAAAAA TAAATTTTAA AAAAATGTTT   
  
  
- ATTCTATGAT AGTTATGTAT CTAGTTGAAG TGGAGAAATG TAAAGCTGAA ATTCTTGACC TCTCCAATTG   
  
  
- ATCAATATTG TCTATACTTT GATTTTGAAT AGATGACCGA TTGCAAGAAT GGCCCTAATG CATTAAAAAT   
  
  
- CCATTTTATG AATATATATA TATATATATA ATAAGCTAAT ACCTCATATT ATAATAACCC ATTATGTATA   
  
  
- AACACATAGT CTTATGGACT TGACTCTATT TTTTTAGCTA GATCCACATA AATTAAATAG AAACATTATT   
  
  
- ATCATTATTA TTATTTAAAA TAAGAAAATA TGAAAGAAAA TAACAATATT CTTTGAAATT TTTACTAGCT   
  
  
- TGGCTAAGTA CATAAGTATA AACGCAATGA TGAACCATTG TTGAACATAA ATATATAATG AACACAATAA   
  
  
- AAAAATTAAT AATAATAAAT ATTAGAGCA

+     CAT-box

| Site Name | Organism | Position | Strand | Matrix score. | sequence | function |
| --- | --- | --- | --- | --- | --- | --- |
| CAT-box | Arabidopsis thaliana | 463 | + | 6 | GCCACT | cis-acting regulatory element related to meristem expression |

> 2018/04/13 10:10:12  
+ AAGAAACTAC CCGCCGAATG TCGCTAGGTA GCTCGCTAGT TAATGTGGCG GCCGGCGTGG CTTTAAGACG   
  
  
+ GCGAGTTTCA GAGGCCCCCT CACGTCCTTA CACTTAAGTG CCGCAAGAAA TAGTAGGTAC CGACTTCTCT   
  
  
+ TCTTCTTTGA GAGAGAGAGA GAGAGAGAGA GAGAGAGAGA GAGTCTCCTC ATCATCAACA CATCGGAAAA   
  
  
+ GTTCTAAAGT TGTTGATTCC TATAAAGAAA TACCATACAT TCTGAAACGA ATCTCCCTCT ACGTTCCTAT   
  
  
+ TATTTTCCGC TCTCTCTCTC TCTCTCTTTC TCCTCCGCTT CCGCTTCTGG TCGGCAAAAG AAGCCCCCTC   
  
  
+ TCTATACTGT AGTACCAACT ACTACCGTGT GCACCGCTGT AGACCTTCAC TTTCTCCCCT ACTCTCACCC   
  
  
+ TGGATGCCCT TCCTAGCGGC AACTCATGGA AGTTCTCCAG CTGCCACTCT CCACTGCTTC TCACTATCCC   
  
  
+ ACGCCCGCCA AGTGCACCCC GCAGCAGAAG TCCAGTTGAT TCAACTGCCG TGCCGACATA GACTGCCACC   
  
  
+ TGTTCTTTTT ATTCTCCGCG CACACTCTGG TCTGCTCGTC TCATGGATTA GGGTGACTGG TAGGTTGGGT   
  
  
+ TGGGTCGGGA AGTGCAGTCA ATAAAACCTA GACAGCATCA GTTGCTTGGA GTACCCTCTC ACTTCTGGCA   
  
  
+ GGACGAGGGT ACACACCAGG CGAGCTGAGC CAACGATAGA GATTAATGGT TAAAAATAAT TTTTATTAAT   
  
  
+ AAAATAATAT TGTTTTTAAT TCTAATAAAT AACTTCATCT ATAAAAATAG AATTATAAGT ACGAATTTAA   
  
  
+ TCTTTAGTAA TTTTAAAACT TTTAAAAAAT TTTCTGTTTC TTAACAGGTT GTTATTCTAA TTATAATAAA   
  
  
+ TTTTATAGAA CTAGAGAAGA ATTCTATGAC TGTATATTAT TAATTAATAA TTTTTAAAAT AAAAAATAAT   
  
  
+ TATATTATTT AATGTTTTTT TCTATAAAAT AAAAATTTTA TGATATTTTT ATTTAAAATT TTTTTACAAA   
  
  
+ TAAGATACTA TCAATACATA GATCAACTTC ACCTCTTTAC ATTTCGACTT TAAGAACTGG AGAGGTTAAC   
  
  
+ TAGTTATAAC AGATATGAAA CTAAAACTTA TCTACTGGCT AACGTTCTTA CCGGGATTAC GTAATTTTTA   
  
  
+ GGTAAAATAC TTATATATAT ATATATATAT TATTCGATTA TGGAGTATAA TATTATTGGG TAATACATAT   
  
  
+ TTGTGTATCA GAATACCTGA ACTGAGATAA AAAAATCGAT CTAGGTGTAT TTAATTTATC TTTGTAATAA   
  
  
+ TAGTAATAAT AATAAATTTT ATTCTTTTAT ACTTTCTTTT ATTGTTATAA GAAACTTTAA AAATGATCGA   
  
  
+ ACCGATTCAT GTATTCATAT TTGCGTTACT ACTTGGTAAC AACTTGTATT TATATATTAC TTGTGTTATT   
  
  
+ TTTTTAATTA TTATTATTTA TAATCTCGT  

- TTCTTTGATG GGCGGCTTAC AGCGATCCAT CGAGCGATCA ATTACACCGC CGGCCGCACC GAAATTCTGC   
  
  
- CGCTCAAAGT CTCCGGGGGA GTGCAGGAAT GTGAATTCAC GGCGTTCTTT ATCATCCATG GCTGAAGAGA   
  
  
- AGAAGAAACT CTCTCTCTCT CTCTCTCTCT CTCTCTCTCT CTCAGAGGAG TAGTAGTTGT GTAGCCTTTT   
  
  
- CAAGATTTCA ACAACTAAGG ATATTTCTTT ATGGTATGTA AGACTTTGCT TAGAGGGAGA TGCAAGGATA   
  
  
- ATAAAAGGCG AGAGAGAGAG AGAGAGAAAG AGGAGGCGAA GGCGAAGACC AGCCGTTTTC TTCGGGGGAG   
  
  
- AGATATGACA TCATGGTTGA TGATGGCACA CGTGGCGACA TCTGGAAGTG AAAGAGGGGA TGAGAGTGGG   
  
  
- ACCTACGGGA AGGATCGCCG TTGAGTACCT TCAAGAGGTC GACGGTGAGA GGTGACGAAG AGTGATAGGG   
  
  
- TGCGGGCGGT TCACGTGGGG CGTCGTCTTC AGGTCAACTA AGTTGACGGC ACGGCTGTAT CTGACGGTGG   
  
  
- ACAAGAAAAA TAAGAGGCGC GTGTGAGACC AGACGAGCAG AGTACCTAAT CCCACTGACC ATCCAACCCA   
  
  
- ACCCAGCCCT TCACGTCAGT TATTTTGGAT CTGTCGTAGT CAACGAACCT CATGGGAGAG TGAAGACCGT   
  
  
- CCTGCTCCCA TGTGTGGTCC GCTCGACTCG GTTGCTATCT CTAATTACCA ATTTTTATTA AAAATAATTA   
  
  
- TTTTATTATA ACAAAAATTA AGATTATTTA TTGAAGTAGA TATTTTTATC TTAATATTCA TGCTTAAATT   
  
  
- AGAAATCATT AAAATTTTGA AAATTTTTTA AAAGACAAAG AATTGTCCAA CAATAAGATT AATATTATTT   
  
  
- AAAATATCTT GATCTCTTCT TAAGATACTG ACATATAATA ATTAATTATT AAAAATTTTA TTTTTTATTA   
  
  
- ATATAATAAA TTACAAAAAA AGATATTTTA TTTTTAAAAT ACTATAAAAA TAAATTTTAA AAAAATGTTT   
  
  
- ATTCTATGAT AGTTATGTAT CTAGTTGAAG TGGAGAAATG TAAAGCTGAA ATTCTTGACC TCTCCAATTG   
  
  
- ATCAATATTG TCTATACTTT GATTTTGAAT AGATGACCGA TTGCAAGAAT GGCCCTAATG CATTAAAAAT   
  
  
- CCATTTTATG AATATATATA TATATATATA ATAAGCTAAT ACCTCATATT ATAATAACCC ATTATGTATA   
  
  
- AACACATAGT CTTATGGACT TGACTCTATT TTTTTAGCTA GATCCACATA AATTAAATAG AAACATTATT   
  
  
- ATCATTATTA TTATTTAAAA TAAGAAAATA TGAAAGAAAA TAACAATATT CTTTGAAATT TTTACTAGCT   
  
  
- TGGCTAAGTA CATAAGTATA AACGCAATGA TGAACCATTG TTGAACATAA ATATATAATG AACACAATAA   
  
  
- AAAAATTAAT AATAATAAAT ATTAGAGCA

+     G-box

| Site Name | Organism | Position | Strand | Matrix score. | sequence | function |
| --- | --- | --- | --- | --- | --- | --- |
| G-box | Zea mays | 91 | + | 6 | CACGTC | cis-acting regulatory element involved in light responsiveness |

> 2018/04/13 10:10:12  
+ AAGAAACTAC CCGCCGAATG TCGCTAGGTA GCTCGCTAGT TAATGTGGCG GCCGGCGTGG CTTTAAGACG   
  
  
+ GCGAGTTTCA GAGGCCCCCT CACGTCCTTA CACTTAAGTG CCGCAAGAAA TAGTAGGTAC CGACTTCTCT   
  
  
+ TCTTCTTTGA GAGAGAGAGA GAGAGAGAGA GAGAGAGAGA GAGTCTCCTC ATCATCAACA CATCGGAAAA   
  
  
+ GTTCTAAAGT TGTTGATTCC TATAAAGAAA TACCATACAT TCTGAAACGA ATCTCCCTCT ACGTTCCTAT   
  
  
+ TATTTTCCGC TCTCTCTCTC TCTCTCTTTC TCCTCCGCTT CCGCTTCTGG TCGGCAAAAG AAGCCCCCTC   
  
  
+ TCTATACTGT AGTACCAACT ACTACCGTGT GCACCGCTGT AGACCTTCAC TTTCTCCCCT ACTCTCACCC   
  
  
+ TGGATGCCCT TCCTAGCGGC AACTCATGGA AGTTCTCCAG CTGCCACTCT CCACTGCTTC TCACTATCCC   
  
  
+ ACGCCCGCCA AGTGCACCCC GCAGCAGAAG TCCAGTTGAT TCAACTGCCG TGCCGACATA GACTGCCACC   
  
  
+ TGTTCTTTTT ATTCTCCGCG CACACTCTGG TCTGCTCGTC TCATGGATTA GGGTGACTGG TAGGTTGGGT   
  
  
+ TGGGTCGGGA AGTGCAGTCA ATAAAACCTA GACAGCATCA GTTGCTTGGA GTACCCTCTC ACTTCTGGCA   
  
  
+ GGACGAGGGT ACACACCAGG CGAGCTGAGC CAACGATAGA GATTAATGGT TAAAAATAAT TTTTATTAAT   
  
  
+ AAAATAATAT TGTTTTTAAT TCTAATAAAT AACTTCATCT ATAAAAATAG AATTATAAGT ACGAATTTAA   
  
  
+ TCTTTAGTAA TTTTAAAACT TTTAAAAAAT TTTCTGTTTC TTAACAGGTT GTTATTCTAA TTATAATAAA   
  
  
+ TTTTATAGAA CTAGAGAAGA ATTCTATGAC TGTATATTAT TAATTAATAA TTTTTAAAAT AAAAAATAAT   
  
  
+ TATATTATTT AATGTTTTTT TCTATAAAAT AAAAATTTTA TGATATTTTT ATTTAAAATT TTTTTACAAA   
  
  
+ TAAGATACTA TCAATACATA GATCAACTTC ACCTCTTTAC ATTTCGACTT TAAGAACTGG AGAGGTTAAC   
  
  
+ TAGTTATAAC AGATATGAAA CTAAAACTTA TCTACTGGCT AACGTTCTTA CCGGGATTAC GTAATTTTTA   
  
  
+ GGTAAAATAC TTATATATAT ATATATATAT TATTCGATTA TGGAGTATAA TATTATTGGG TAATACATAT   
  
  
+ TTGTGTATCA GAATACCTGA ACTGAGATAA AAAAATCGAT CTAGGTGTAT TTAATTTATC TTTGTAATAA   
  
  
+ TAGTAATAAT AATAAATTTT ATTCTTTTAT ACTTTCTTTT ATTGTTATAA GAAACTTTAA AAATGATCGA   
  
  
+ ACCGATTCAT GTATTCATAT TTGCGTTACT ACTTGGTAAC AACTTGTATT TATATATTAC TTGTGTTATT   
  
  
+ TTTTTAATTA TTATTATTTA TAATCTCGT  

- TTCTTTGATG GGCGGCTTAC AGCGATCCAT CGAGCGATCA ATTACACCGC CGGCCGCACC GAAATTCTGC   
  
  
- CGCTCAAAGT CTCCGGGGGA GTGCAGGAAT GTGAATTCAC GGCGTTCTTT ATCATCCATG GCTGAAGAGA   
  
  
- AGAAGAAACT CTCTCTCTCT CTCTCTCTCT CTCTCTCTCT CTCAGAGGAG TAGTAGTTGT GTAGCCTTTT   
  
  
- CAAGATTTCA ACAACTAAGG ATATTTCTTT ATGGTATGTA AGACTTTGCT TAGAGGGAGA TGCAAGGATA   
  
  
- ATAAAAGGCG AGAGAGAGAG AGAGAGAAAG AGGAGGCGAA GGCGAAGACC AGCCGTTTTC TTCGGGGGAG   
  
  
- AGATATGACA TCATGGTTGA TGATGGCACA CGTGGCGACA TCTGGAAGTG AAAGAGGGGA TGAGAGTGGG   
  
  
- ACCTACGGGA AGGATCGCCG TTGAGTACCT TCAAGAGGTC GACGGTGAGA GGTGACGAAG AGTGATAGGG   
  
  
- TGCGGGCGGT TCACGTGGGG CGTCGTCTTC AGGTCAACTA AGTTGACGGC ACGGCTGTAT CTGACGGTGG   
  
  
- ACAAGAAAAA TAAGAGGCGC GTGTGAGACC AGACGAGCAG AGTACCTAAT CCCACTGACC ATCCAACCCA   
  
  
- ACCCAGCCCT TCACGTCAGT TATTTTGGAT CTGTCGTAGT CAACGAACCT CATGGGAGAG TGAAGACCGT   
  
  
- CCTGCTCCCA TGTGTGGTCC GCTCGACTCG GTTGCTATCT CTAATTACCA ATTTTTATTA AAAATAATTA   
  
  
- TTTTATTATA ACAAAAATTA AGATTATTTA TTGAAGTAGA TATTTTTATC TTAATATTCA TGCTTAAATT   
  
  
- AGAAATCATT AAAATTTTGA AAATTTTTTA AAAGACAAAG AATTGTCCAA CAATAAGATT AATATTATTT   
  
  
- AAAATATCTT GATCTCTTCT TAAGATACTG ACATATAATA ATTAATTATT AAAAATTTTA TTTTTTATTA   
  
  
- ATATAATAAA TTACAAAAAA AGATATTTTA TTTTTAAAAT ACTATAAAAA TAAATTTTAA AAAAATGTTT   
  
  
- ATTCTATGAT AGTTATGTAT CTAGTTGAAG TGGAGAAATG TAAAGCTGAA ATTCTTGACC TCTCCAATTG   
  
  
- ATCAATATTG TCTATACTTT GATTTTGAAT AGATGACCGA TTGCAAGAAT GGCCCTAATG CATTAAAAAT   
  
  
- CCATTTTATG AATATATATA TATATATATA ATAAGCTAAT ACCTCATATT ATAATAACCC ATTATGTATA   
  
  
- AACACATAGT CTTATGGACT TGACTCTATT TTTTTAGCTA GATCCACATA AATTAAATAG AAACATTATT   
  
  
- ATCATTATTA TTATTTAAAA TAAGAAAATA TGAAAGAAAA TAACAATATT CTTTGAAATT TTTACTAGCT   
  
  
- TGGCTAAGTA CATAAGTATA AACGCAATGA TGAACCATTG TTGAACATAA ATATATAATG AACACAATAA   
  
  
- AAAAATTAAT AATAATAAAT ATTAGAGCA

+     GAG-motif

| Site Name | Organism | Position | Strand | Matrix score. | sequence | function |
| --- | --- | --- | --- | --- | --- | --- |
| GAG-motif | Arabidopsis thaliana | 178 | + | 7 | AGAGAGT | part of a light responsive element |

> 2018/04/13 10:10:12  
+ AAGAAACTAC CCGCCGAATG TCGCTAGGTA GCTCGCTAGT TAATGTGGCG GCCGGCGTGG CTTTAAGACG   
  
  
+ GCGAGTTTCA GAGGCCCCCT CACGTCCTTA CACTTAAGTG CCGCAAGAAA TAGTAGGTAC CGACTTCTCT   
  
  
+ TCTTCTTTGA GAGAGAGAGA GAGAGAGAGA GAGAGAGAGA GAGTCTCCTC ATCATCAACA CATCGGAAAA   
  
  
+ GTTCTAAAGT TGTTGATTCC TATAAAGAAA TACCATACAT TCTGAAACGA ATCTCCCTCT ACGTTCCTAT   
  
  
+ TATTTTCCGC TCTCTCTCTC TCTCTCTTTC TCCTCCGCTT CCGCTTCTGG TCGGCAAAAG AAGCCCCCTC   
  
  
+ TCTATACTGT AGTACCAACT ACTACCGTGT GCACCGCTGT AGACCTTCAC TTTCTCCCCT ACTCTCACCC   
  
  
+ TGGATGCCCT TCCTAGCGGC AACTCATGGA AGTTCTCCAG CTGCCACTCT CCACTGCTTC TCACTATCCC   
  
  
+ ACGCCCGCCA AGTGCACCCC GCAGCAGAAG TCCAGTTGAT TCAACTGCCG TGCCGACATA GACTGCCACC   
  
  
+ TGTTCTTTTT ATTCTCCGCG CACACTCTGG TCTGCTCGTC TCATGGATTA GGGTGACTGG TAGGTTGGGT   
  
  
+ TGGGTCGGGA AGTGCAGTCA ATAAAACCTA GACAGCATCA GTTGCTTGGA GTACCCTCTC ACTTCTGGCA   
  
  
+ GGACGAGGGT ACACACCAGG CGAGCTGAGC CAACGATAGA GATTAATGGT TAAAAATAAT TTTTATTAAT   
  
  
+ AAAATAATAT TGTTTTTAAT TCTAATAAAT AACTTCATCT ATAAAAATAG AATTATAAGT ACGAATTTAA   
  
  
+ TCTTTAGTAA TTTTAAAACT TTTAAAAAAT TTTCTGTTTC TTAACAGGTT GTTATTCTAA TTATAATAAA   
  
  
+ TTTTATAGAA CTAGAGAAGA ATTCTATGAC TGTATATTAT TAATTAATAA TTTTTAAAAT AAAAAATAAT   
  
  
+ TATATTATTT AATGTTTTTT TCTATAAAAT AAAAATTTTA TGATATTTTT ATTTAAAATT TTTTTACAAA   
  
  
+ TAAGATACTA TCAATACATA GATCAACTTC ACCTCTTTAC ATTTCGACTT TAAGAACTGG AGAGGTTAAC   
  
  
+ TAGTTATAAC AGATATGAAA CTAAAACTTA TCTACTGGCT AACGTTCTTA CCGGGATTAC GTAATTTTTA   
  
  
+ GGTAAAATAC TTATATATAT ATATATATAT TATTCGATTA TGGAGTATAA TATTATTGGG TAATACATAT   
  
  
+ TTGTGTATCA GAATACCTGA ACTGAGATAA AAAAATCGAT CTAGGTGTAT TTAATTTATC TTTGTAATAA   
  
  
+ TAGTAATAAT AATAAATTTT ATTCTTTTAT ACTTTCTTTT ATTGTTATAA GAAACTTTAA AAATGATCGA   
  
  
+ ACCGATTCAT GTATTCATAT TTGCGTTACT ACTTGGTAAC AACTTGTATT TATATATTAC TTGTGTTATT   
  
  
+ TTTTTAATTA TTATTATTTA TAATCTCGT  

- TTCTTTGATG GGCGGCTTAC AGCGATCCAT CGAGCGATCA ATTACACCGC CGGCCGCACC GAAATTCTGC   
  
  
- CGCTCAAAGT CTCCGGGGGA GTGCAGGAAT GTGAATTCAC GGCGTTCTTT ATCATCCATG GCTGAAGAGA   
  
  
- AGAAGAAACT CTCTCTCTCT CTCTCTCTCT CTCTCTCTCT CTCAGAGGAG TAGTAGTTGT GTAGCCTTTT   
  
  
- CAAGATTTCA ACAACTAAGG ATATTTCTTT ATGGTATGTA AGACTTTGCT TAGAGGGAGA TGCAAGGATA   
  
  
- ATAAAAGGCG AGAGAGAGAG AGAGAGAAAG AGGAGGCGAA GGCGAAGACC AGCCGTTTTC TTCGGGGGAG   
  
  
- AGATATGACA TCATGGTTGA TGATGGCACA CGTGGCGACA TCTGGAAGTG AAAGAGGGGA TGAGAGTGGG   
  
  
- ACCTACGGGA AGGATCGCCG TTGAGTACCT TCAAGAGGTC GACGGTGAGA GGTGACGAAG AGTGATAGGG   
  
  
- TGCGGGCGGT TCACGTGGGG CGTCGTCTTC AGGTCAACTA AGTTGACGGC ACGGCTGTAT CTGACGGTGG   
  
  
- ACAAGAAAAA TAAGAGGCGC GTGTGAGACC AGACGAGCAG AGTACCTAAT CCCACTGACC ATCCAACCCA   
  
  
- ACCCAGCCCT TCACGTCAGT TATTTTGGAT CTGTCGTAGT CAACGAACCT CATGGGAGAG TGAAGACCGT   
  
  
- CCTGCTCCCA TGTGTGGTCC GCTCGACTCG GTTGCTATCT CTAATTACCA ATTTTTATTA AAAATAATTA   
  
  
- TTTTATTATA ACAAAAATTA AGATTATTTA TTGAAGTAGA TATTTTTATC TTAATATTCA TGCTTAAATT   
  
  
- AGAAATCATT AAAATTTTGA AAATTTTTTA AAAGACAAAG AATTGTCCAA CAATAAGATT AATATTATTT   
  
  
- AAAATATCTT GATCTCTTCT TAAGATACTG ACATATAATA ATTAATTATT AAAAATTTTA TTTTTTATTA   
  
  
- ATATAATAAA TTACAAAAAA AGATATTTTA TTTTTAAAAT ACTATAAAAA TAAATTTTAA AAAAATGTTT   
  
  
- ATTCTATGAT AGTTATGTAT CTAGTTGAAG TGGAGAAATG TAAAGCTGAA ATTCTTGACC TCTCCAATTG   
  
  
- ATCAATATTG TCTATACTTT GATTTTGAAT AGATGACCGA TTGCAAGAAT GGCCCTAATG CATTAAAAAT   
  
  
- CCATTTTATG AATATATATA TATATATATA ATAAGCTAAT ACCTCATATT ATAATAACCC ATTATGTATA   
  
  
- AACACATAGT CTTATGGACT TGACTCTATT TTTTTAGCTA GATCCACATA AATTAAATAG AAACATTATT   
  
  
- ATCATTATTA TTATTTAAAA TAAGAAAATA TGAAAGAAAA TAACAATATT CTTTGAAATT TTTACTAGCT   
  
  
- TGGCTAAGTA CATAAGTATA AACGCAATGA TGAACCATTG TTGAACATAA ATATATAATG AACACAATAA   
  
  
- AAAAATTAAT AATAATAAAT ATTAGAGCA

+     GARE-motif

| Site Name | Organism | Position | Strand | Matrix score. | sequence | function |
| --- | --- | --- | --- | --- | --- | --- |
| GARE-motif | Brassica oleracea | 873 | - | 7 | AAACAGA | gibberellin-responsive element |

> 2018/04/13 10:10:12  
+ AAGAAACTAC CCGCCGAATG TCGCTAGGTA GCTCGCTAGT TAATGTGGCG GCCGGCGTGG CTTTAAGACG   
  
  
+ GCGAGTTTCA GAGGCCCCCT CACGTCCTTA CACTTAAGTG CCGCAAGAAA TAGTAGGTAC CGACTTCTCT   
  
  
+ TCTTCTTTGA GAGAGAGAGA GAGAGAGAGA GAGAGAGAGA GAGTCTCCTC ATCATCAACA CATCGGAAAA   
  
  
+ GTTCTAAAGT TGTTGATTCC TATAAAGAAA TACCATACAT TCTGAAACGA ATCTCCCTCT ACGTTCCTAT   
  
  
+ TATTTTCCGC TCTCTCTCTC TCTCTCTTTC TCCTCCGCTT CCGCTTCTGG TCGGCAAAAG AAGCCCCCTC   
  
  
+ TCTATACTGT AGTACCAACT ACTACCGTGT GCACCGCTGT AGACCTTCAC TTTCTCCCCT ACTCTCACCC   
  
  
+ TGGATGCCCT TCCTAGCGGC AACTCATGGA AGTTCTCCAG CTGCCACTCT CCACTGCTTC TCACTATCCC   
  
  
+ ACGCCCGCCA AGTGCACCCC GCAGCAGAAG TCCAGTTGAT TCAACTGCCG TGCCGACATA GACTGCCACC   
  
  
+ TGTTCTTTTT ATTCTCCGCG CACACTCTGG TCTGCTCGTC TCATGGATTA GGGTGACTGG TAGGTTGGGT   
  
  
+ TGGGTCGGGA AGTGCAGTCA ATAAAACCTA GACAGCATCA GTTGCTTGGA GTACCCTCTC ACTTCTGGCA   
  
  
+ GGACGAGGGT ACACACCAGG CGAGCTGAGC CAACGATAGA GATTAATGGT TAAAAATAAT TTTTATTAAT   
  
  
+ AAAATAATAT TGTTTTTAAT TCTAATAAAT AACTTCATCT ATAAAAATAG AATTATAAGT ACGAATTTAA   
  
  
+ TCTTTAGTAA TTTTAAAACT TTTAAAAAAT TTTCTGTTTC TTAACAGGTT GTTATTCTAA TTATAATAAA   
  
  
+ TTTTATAGAA CTAGAGAAGA ATTCTATGAC TGTATATTAT TAATTAATAA TTTTTAAAAT AAAAAATAAT   
  
  
+ TATATTATTT AATGTTTTTT TCTATAAAAT AAAAATTTTA TGATATTTTT ATTTAAAATT TTTTTACAAA   
  
  
+ TAAGATACTA TCAATACATA GATCAACTTC ACCTCTTTAC ATTTCGACTT TAAGAACTGG AGAGGTTAAC   
  
  
+ TAGTTATAAC AGATATGAAA CTAAAACTTA TCTACTGGCT AACGTTCTTA CCGGGATTAC GTAATTTTTA   
  
  
+ GGTAAAATAC TTATATATAT ATATATATAT TATTCGATTA TGGAGTATAA TATTATTGGG TAATACATAT   
  
  
+ TTGTGTATCA GAATACCTGA ACTGAGATAA AAAAATCGAT CTAGGTGTAT TTAATTTATC TTTGTAATAA   
  
  
+ TAGTAATAAT AATAAATTTT ATTCTTTTAT ACTTTCTTTT ATTGTTATAA GAAACTTTAA AAATGATCGA   
  
  
+ ACCGATTCAT GTATTCATAT TTGCGTTACT ACTTGGTAAC AACTTGTATT TATATATTAC TTGTGTTATT   
  
  
+ TTTTTAATTA TTATTATTTA TAATCTCGT  

- TTCTTTGATG GGCGGCTTAC AGCGATCCAT CGAGCGATCA ATTACACCGC CGGCCGCACC GAAATTCTGC   
  
  
- CGCTCAAAGT CTCCGGGGGA GTGCAGGAAT GTGAATTCAC GGCGTTCTTT ATCATCCATG GCTGAAGAGA   
  
  
- AGAAGAAACT CTCTCTCTCT CTCTCTCTCT CTCTCTCTCT CTCAGAGGAG TAGTAGTTGT GTAGCCTTTT   
  
  
- CAAGATTTCA ACAACTAAGG ATATTTCTTT ATGGTATGTA AGACTTTGCT TAGAGGGAGA TGCAAGGATA   
  
  
- ATAAAAGGCG AGAGAGAGAG AGAGAGAAAG AGGAGGCGAA GGCGAAGACC AGCCGTTTTC TTCGGGGGAG   
  
  
- AGATATGACA TCATGGTTGA TGATGGCACA CGTGGCGACA TCTGGAAGTG AAAGAGGGGA TGAGAGTGGG   
  
  
- ACCTACGGGA AGGATCGCCG TTGAGTACCT TCAAGAGGTC GACGGTGAGA GGTGACGAAG AGTGATAGGG   
  
  
- TGCGGGCGGT TCACGTGGGG CGTCGTCTTC AGGTCAACTA AGTTGACGGC ACGGCTGTAT CTGACGGTGG   
  
  
- ACAAGAAAAA TAAGAGGCGC GTGTGAGACC AGACGAGCAG AGTACCTAAT CCCACTGACC ATCCAACCCA   
  
  
- ACCCAGCCCT TCACGTCAGT TATTTTGGAT CTGTCGTAGT CAACGAACCT CATGGGAGAG TGAAGACCGT   
  
  
- CCTGCTCCCA TGTGTGGTCC GCTCGACTCG GTTGCTATCT CTAATTACCA ATTTTTATTA AAAATAATTA   
  
  
- TTTTATTATA ACAAAAATTA AGATTATTTA TTGAAGTAGA TATTTTTATC TTAATATTCA TGCTTAAATT   
  
  
- AGAAATCATT AAAATTTTGA AAATTTTTTA AAAGACAAAG AATTGTCCAA CAATAAGATT AATATTATTT   
  
  
- AAAATATCTT GATCTCTTCT TAAGATACTG ACATATAATA ATTAATTATT AAAAATTTTA TTTTTTATTA   
  
  
- ATATAATAAA TTACAAAAAA AGATATTTTA TTTTTAAAAT ACTATAAAAA TAAATTTTAA AAAAATGTTT   
  
  
- ATTCTATGAT AGTTATGTAT CTAGTTGAAG TGGAGAAATG TAAAGCTGAA ATTCTTGACC TCTCCAATTG   
  
  
- ATCAATATTG TCTATACTTT GATTTTGAAT AGATGACCGA TTGCAAGAAT GGCCCTAATG CATTAAAAAT   
  
  
- CCATTTTATG AATATATATA TATATATATA ATAAGCTAAT ACCTCATATT ATAATAACCC ATTATGTATA   
  
  
- AACACATAGT CTTATGGACT TGACTCTATT TTTTTAGCTA GATCCACATA AATTAAATAG AAACATTATT   
  
  
- ATCATTATTA TTATTTAAAA TAAGAAAATA TGAAAGAAAA TAACAATATT CTTTGAAATT TTTACTAGCT   
  
  
- TGGCTAAGTA CATAAGTATA AACGCAATGA TGAACCATTG TTGAACATAA ATATATAATG AACACAATAA   
  
  
- AAAAATTAAT AATAATAAAT ATTAGAGCA

+     GT1-motif

| Site Name | Organism | Position | Strand | Matrix score. | sequence | function |
| --- | --- | --- | --- | --- | --- | --- |
| GT1-motif | Arabidopsis thaliana | 1114 | + | 6 | GGTTAA | light responsive element |
| GT1-motif | Arabidopsis thaliana | 748 | + | 6 | GGTTAA | light responsive element |

> 2018/04/13 10:10:12  
+ AAGAAACTAC CCGCCGAATG TCGCTAGGTA GCTCGCTAGT TAATGTGGCG GCCGGCGTGG CTTTAAGACG   
  
  
+ GCGAGTTTCA GAGGCCCCCT CACGTCCTTA CACTTAAGTG CCGCAAGAAA TAGTAGGTAC CGACTTCTCT   
  
  
+ TCTTCTTTGA GAGAGAGAGA GAGAGAGAGA GAGAGAGAGA GAGTCTCCTC ATCATCAACA CATCGGAAAA   
  
  
+ GTTCTAAAGT TGTTGATTCC TATAAAGAAA TACCATACAT TCTGAAACGA ATCTCCCTCT ACGTTCCTAT   
  
  
+ TATTTTCCGC TCTCTCTCTC TCTCTCTTTC TCCTCCGCTT CCGCTTCTGG TCGGCAAAAG AAGCCCCCTC   
  
  
+ TCTATACTGT AGTACCAACT ACTACCGTGT GCACCGCTGT AGACCTTCAC TTTCTCCCCT ACTCTCACCC   
  
  
+ TGGATGCCCT TCCTAGCGGC AACTCATGGA AGTTCTCCAG CTGCCACTCT CCACTGCTTC TCACTATCCC   
  
  
+ ACGCCCGCCA AGTGCACCCC GCAGCAGAAG TCCAGTTGAT TCAACTGCCG TGCCGACATA GACTGCCACC   
  
  
+ TGTTCTTTTT ATTCTCCGCG CACACTCTGG TCTGCTCGTC TCATGGATTA GGGTGACTGG TAGGTTGGGT   
  
  
+ TGGGTCGGGA AGTGCAGTCA ATAAAACCTA GACAGCATCA GTTGCTTGGA GTACCCTCTC ACTTCTGGCA   
  
  
+ GGACGAGGGT ACACACCAGG CGAGCTGAGC CAACGATAGA GATTAATGGT TAAAAATAAT TTTTATTAAT   
  
  
+ AAAATAATAT TGTTTTTAAT TCTAATAAAT AACTTCATCT ATAAAAATAG AATTATAAGT ACGAATTTAA   
  
  
+ TCTTTAGTAA TTTTAAAACT TTTAAAAAAT TTTCTGTTTC TTAACAGGTT GTTATTCTAA TTATAATAAA   
  
  
+ TTTTATAGAA CTAGAGAAGA ATTCTATGAC TGTATATTAT TAATTAATAA TTTTTAAAAT AAAAAATAAT   
  
  
+ TATATTATTT AATGTTTTTT TCTATAAAAT AAAAATTTTA TGATATTTTT ATTTAAAATT TTTTTACAAA   
  
  
+ TAAGATACTA TCAATACATA GATCAACTTC ACCTCTTTAC ATTTCGACTT TAAGAACTGG AGAGGTTAAC   
  
  
+ TAGTTATAAC AGATATGAAA CTAAAACTTA TCTACTGGCT AACGTTCTTA CCGGGATTAC GTAATTTTTA   
  
  
+ GGTAAAATAC TTATATATAT ATATATATAT TATTCGATTA TGGAGTATAA TATTATTGGG TAATACATAT   
  
  
+ TTGTGTATCA GAATACCTGA ACTGAGATAA AAAAATCGAT CTAGGTGTAT TTAATTTATC TTTGTAATAA   
  
  
+ TAGTAATAAT AATAAATTTT ATTCTTTTAT ACTTTCTTTT ATTGTTATAA GAAACTTTAA AAATGATCGA   
  
  
+ ACCGATTCAT GTATTCATAT TTGCGTTACT ACTTGGTAAC AACTTGTATT TATATATTAC TTGTGTTATT   
  
  
+ TTTTTAATTA TTATTATTTA TAATCTCGT  

- TTCTTTGATG GGCGGCTTAC AGCGATCCAT CGAGCGATCA ATTACACCGC CGGCCGCACC GAAATTCTGC   
  
  
- CGCTCAAAGT CTCCGGGGGA GTGCAGGAAT GTGAATTCAC GGCGTTCTTT ATCATCCATG GCTGAAGAGA   
  
  
- AGAAGAAACT CTCTCTCTCT CTCTCTCTCT CTCTCTCTCT CTCAGAGGAG TAGTAGTTGT GTAGCCTTTT   
  
  
- CAAGATTTCA ACAACTAAGG ATATTTCTTT ATGGTATGTA AGACTTTGCT TAGAGGGAGA TGCAAGGATA   
  
  
- ATAAAAGGCG AGAGAGAGAG AGAGAGAAAG AGGAGGCGAA GGCGAAGACC AGCCGTTTTC TTCGGGGGAG   
  
  
- AGATATGACA TCATGGTTGA TGATGGCACA CGTGGCGACA TCTGGAAGTG AAAGAGGGGA TGAGAGTGGG   
  
  
- ACCTACGGGA AGGATCGCCG TTGAGTACCT TCAAGAGGTC GACGGTGAGA GGTGACGAAG AGTGATAGGG   
  
  
- TGCGGGCGGT TCACGTGGGG CGTCGTCTTC AGGTCAACTA AGTTGACGGC ACGGCTGTAT CTGACGGTGG   
  
  
- ACAAGAAAAA TAAGAGGCGC GTGTGAGACC AGACGAGCAG AGTACCTAAT CCCACTGACC ATCCAACCCA   
  
  
- ACCCAGCCCT TCACGTCAGT TATTTTGGAT CTGTCGTAGT CAACGAACCT CATGGGAGAG TGAAGACCGT   
  
  
- CCTGCTCCCA TGTGTGGTCC GCTCGACTCG GTTGCTATCT CTAATTACCA ATTTTTATTA AAAATAATTA   
  
  
- TTTTATTATA ACAAAAATTA AGATTATTTA TTGAAGTAGA TATTTTTATC TTAATATTCA TGCTTAAATT   
  
  
- AGAAATCATT AAAATTTTGA AAATTTTTTA AAAGACAAAG AATTGTCCAA CAATAAGATT AATATTATTT   
  
  
- AAAATATCTT GATCTCTTCT TAAGATACTG ACATATAATA ATTAATTATT AAAAATTTTA TTTTTTATTA   
  
  
- ATATAATAAA TTACAAAAAA AGATATTTTA TTTTTAAAAT ACTATAAAAA TAAATTTTAA AAAAATGTTT   
  
  
- ATTCTATGAT AGTTATGTAT CTAGTTGAAG TGGAGAAATG TAAAGCTGAA ATTCTTGACC TCTCCAATTG   
  
  
- ATCAATATTG TCTATACTTT GATTTTGAAT AGATGACCGA TTGCAAGAAT GGCCCTAATG CATTAAAAAT   
  
  
- CCATTTTATG AATATATATA TATATATATA ATAAGCTAAT ACCTCATATT ATAATAACCC ATTATGTATA   
  
  
- AACACATAGT CTTATGGACT TGACTCTATT TTTTTAGCTA GATCCACATA AATTAAATAG AAACATTATT   
  
  
- ATCATTATTA TTATTTAAAA TAAGAAAATA TGAAAGAAAA TAACAATATT CTTTGAAATT TTTACTAGCT   
  
  
- TGGCTAAGTA CATAAGTATA AACGCAATGA TGAACCATTG TTGAACATAA ATATATAATG AACACAATAA   
  
  
- AAAAATTAAT AATAATAAAT ATTAGAGCA

+     HSE

| Site Name | Organism | Position | Strand | Matrix score. | sequence | function |
| --- | --- | --- | --- | --- | --- | --- |
| HSE | Brassica oleracea | 1035 | - | 9 | AAAAAATTTC | cis-acting element involved in heat stress responsiveness |
| HSE | Brassica oleracea | 864 | + | 9 | AAAAAATTTC | cis-acting element involved in heat stress responsiveness |
| HSE | Brassica oleracea | 865 | + | 9 | AAAAAATTTC | cis-acting element involved in heat stress responsiveness |

> 2018/04/13 10:10:12  
+ AAGAAACTAC CCGCCGAATG TCGCTAGGTA GCTCGCTAGT TAATGTGGCG GCCGGCGTGG CTTTAAGACG   
  
  
+ GCGAGTTTCA GAGGCCCCCT CACGTCCTTA CACTTAAGTG CCGCAAGAAA TAGTAGGTAC CGACTTCTCT   
  
  
+ TCTTCTTTGA GAGAGAGAGA GAGAGAGAGA GAGAGAGAGA GAGTCTCCTC ATCATCAACA CATCGGAAAA   
  
  
+ GTTCTAAAGT TGTTGATTCC TATAAAGAAA TACCATACAT TCTGAAACGA ATCTCCCTCT ACGTTCCTAT   
  
  
+ TATTTTCCGC TCTCTCTCTC TCTCTCTTTC TCCTCCGCTT CCGCTTCTGG TCGGCAAAAG AAGCCCCCTC   
  
  
+ TCTATACTGT AGTACCAACT ACTACCGTGT GCACCGCTGT AGACCTTCAC TTTCTCCCCT ACTCTCACCC   
  
  
+ TGGATGCCCT TCCTAGCGGC AACTCATGGA AGTTCTCCAG CTGCCACTCT CCACTGCTTC TCACTATCCC   
  
  
+ ACGCCCGCCA AGTGCACCCC GCAGCAGAAG TCCAGTTGAT TCAACTGCCG TGCCGACATA GACTGCCACC   
  
  
+ TGTTCTTTTT ATTCTCCGCG CACACTCTGG TCTGCTCGTC TCATGGATTA GGGTGACTGG TAGGTTGGGT   
  
  
+ TGGGTCGGGA AGTGCAGTCA ATAAAACCTA GACAGCATCA GTTGCTTGGA GTACCCTCTC ACTTCTGGCA   
  
  
+ GGACGAGGGT ACACACCAGG CGAGCTGAGC CAACGATAGA GATTAATGGT TAAAAATAAT TTTTATTAAT   
  
  
+ AAAATAATAT TGTTTTTAAT TCTAATAAAT AACTTCATCT ATAAAAATAG AATTATAAGT ACGAATTTAA   
  
  
+ TCTTTAGTAA TTTTAAAACT TTTAAAAAAT TTTCTGTTTC TTAACAGGTT GTTATTCTAA TTATAATAAA   
  
  
+ TTTTATAGAA CTAGAGAAGA ATTCTATGAC TGTATATTAT TAATTAATAA TTTTTAAAAT AAAAAATAAT   
  
  
+ TATATTATTT AATGTTTTTT TCTATAAAAT AAAAATTTTA TGATATTTTT ATTTAAAATT TTTTTACAAA   
  
  
+ TAAGATACTA TCAATACATA GATCAACTTC ACCTCTTTAC ATTTCGACTT TAAGAACTGG AGAGGTTAAC   
  
  
+ TAGTTATAAC AGATATGAAA CTAAAACTTA TCTACTGGCT AACGTTCTTA CCGGGATTAC GTAATTTTTA   
  
  
+ GGTAAAATAC TTATATATAT ATATATATAT TATTCGATTA TGGAGTATAA TATTATTGGG TAATACATAT   
  
  
+ TTGTGTATCA GAATACCTGA ACTGAGATAA AAAAATCGAT CTAGGTGTAT TTAATTTATC TTTGTAATAA   
  
  
+ TAGTAATAAT AATAAATTTT ATTCTTTTAT ACTTTCTTTT ATTGTTATAA GAAACTTTAA AAATGATCGA   
  
  
+ ACCGATTCAT GTATTCATAT TTGCGTTACT ACTTGGTAAC AACTTGTATT TATATATTAC TTGTGTTATT   
  
  
+ TTTTTAATTA TTATTATTTA TAATCTCGT  

- TTCTTTGATG GGCGGCTTAC AGCGATCCAT CGAGCGATCA ATTACACCGC CGGCCGCACC GAAATTCTGC   
  
  
- CGCTCAAAGT CTCCGGGGGA GTGCAGGAAT GTGAATTCAC GGCGTTCTTT ATCATCCATG GCTGAAGAGA   
  
  
- AGAAGAAACT CTCTCTCTCT CTCTCTCTCT CTCTCTCTCT CTCAGAGGAG TAGTAGTTGT GTAGCCTTTT   
  
  
- CAAGATTTCA ACAACTAAGG ATATTTCTTT ATGGTATGTA AGACTTTGCT TAGAGGGAGA TGCAAGGATA   
  
  
- ATAAAAGGCG AGAGAGAGAG AGAGAGAAAG AGGAGGCGAA GGCGAAGACC AGCCGTTTTC TTCGGGGGAG   
  
  
- AGATATGACA TCATGGTTGA TGATGGCACA CGTGGCGACA TCTGGAAGTG AAAGAGGGGA TGAGAGTGGG   
  
  
- ACCTACGGGA AGGATCGCCG TTGAGTACCT TCAAGAGGTC GACGGTGAGA GGTGACGAAG AGTGATAGGG   
  
  
- TGCGGGCGGT TCACGTGGGG CGTCGTCTTC AGGTCAACTA AGTTGACGGC ACGGCTGTAT CTGACGGTGG   
  
  
- ACAAGAAAAA TAAGAGGCGC GTGTGAGACC AGACGAGCAG AGTACCTAAT CCCACTGACC ATCCAACCCA   
  
  
- ACCCAGCCCT TCACGTCAGT TATTTTGGAT CTGTCGTAGT CAACGAACCT CATGGGAGAG TGAAGACCGT   
  
  
- CCTGCTCCCA TGTGTGGTCC GCTCGACTCG GTTGCTATCT CTAATTACCA ATTTTTATTA AAAATAATTA   
  
  
- TTTTATTATA ACAAAAATTA AGATTATTTA TTGAAGTAGA TATTTTTATC TTAATATTCA TGCTTAAATT   
  
  
- AGAAATCATT AAAATTTTGA AAATTTTTTA AAAGACAAAG AATTGTCCAA CAATAAGATT AATATTATTT   
  
  
- AAAATATCTT GATCTCTTCT TAAGATACTG ACATATAATA ATTAATTATT AAAAATTTTA TTTTTTATTA   
  
  
- ATATAATAAA TTACAAAAAA AGATATTTTA TTTTTAAAAT ACTATAAAAA TAAATTTTAA AAAAATGTTT   
  
  
- ATTCTATGAT AGTTATGTAT CTAGTTGAAG TGGAGAAATG TAAAGCTGAA ATTCTTGACC TCTCCAATTG   
  
  
- ATCAATATTG TCTATACTTT GATTTTGAAT AGATGACCGA TTGCAAGAAT GGCCCTAATG CATTAAAAAT   
  
  
- CCATTTTATG AATATATATA TATATATATA ATAAGCTAAT ACCTCATATT ATAATAACCC ATTATGTATA   
  
  
- AACACATAGT CTTATGGACT TGACTCTATT TTTTTAGCTA GATCCACATA AATTAAATAG AAACATTATT   
  
  
- ATCATTATTA TTATTTAAAA TAAGAAAATA TGAAAGAAAA TAACAATATT CTTTGAAATT TTTACTAGCT   
  
  
- TGGCTAAGTA CATAAGTATA AACGCAATGA TGAACCATTG TTGAACATAA ATATATAATG AACACAATAA   
  
  
- AAAAATTAAT AATAATAAAT ATTAGAGCA

+     MBS

| Site Name | Organism | Position | Strand | Matrix score. | sequence | function |
| --- | --- | --- | --- | --- | --- | --- |
| MBS | Arabidopsis thaliana | 523 | - | 6 | CAACTG | MYB binding site involved in drought-inducibility |
| MBS | Arabidopsis thaliana | 532 | + | 6 | CAACTG | MYB binding site involved in drought-inducibility |
| MBS | Arabidopsis thaliana | 669 | - | 6 | CAACTG | MYB binding site involved in drought-inducibility |

> 2018/04/13 10:10:12  
+ AAGAAACTAC CCGCCGAATG TCGCTAGGTA GCTCGCTAGT TAATGTGGCG GCCGGCGTGG CTTTAAGACG   
  
  
+ GCGAGTTTCA GAGGCCCCCT CACGTCCTTA CACTTAAGTG CCGCAAGAAA TAGTAGGTAC CGACTTCTCT   
  
  
+ TCTTCTTTGA GAGAGAGAGA GAGAGAGAGA GAGAGAGAGA GAGTCTCCTC ATCATCAACA CATCGGAAAA   
  
  
+ GTTCTAAAGT TGTTGATTCC TATAAAGAAA TACCATACAT TCTGAAACGA ATCTCCCTCT ACGTTCCTAT   
  
  
+ TATTTTCCGC TCTCTCTCTC TCTCTCTTTC TCCTCCGCTT CCGCTTCTGG TCGGCAAAAG AAGCCCCCTC   
  
  
+ TCTATACTGT AGTACCAACT ACTACCGTGT GCACCGCTGT AGACCTTCAC TTTCTCCCCT ACTCTCACCC   
  
  
+ TGGATGCCCT TCCTAGCGGC AACTCATGGA AGTTCTCCAG CTGCCACTCT CCACTGCTTC TCACTATCCC   
  
  
+ ACGCCCGCCA AGTGCACCCC GCAGCAGAAG TCCAGTTGAT TCAACTGCCG TGCCGACATA GACTGCCACC   
  
  
+ TGTTCTTTTT ATTCTCCGCG CACACTCTGG TCTGCTCGTC TCATGGATTA GGGTGACTGG TAGGTTGGGT   
  
  
+ TGGGTCGGGA AGTGCAGTCA ATAAAACCTA GACAGCATCA GTTGCTTGGA GTACCCTCTC ACTTCTGGCA   
  
  
+ GGACGAGGGT ACACACCAGG CGAGCTGAGC CAACGATAGA GATTAATGGT TAAAAATAAT TTTTATTAAT   
  
  
+ AAAATAATAT TGTTTTTAAT TCTAATAAAT AACTTCATCT ATAAAAATAG AATTATAAGT ACGAATTTAA   
  
  
+ TCTTTAGTAA TTTTAAAACT TTTAAAAAAT TTTCTGTTTC TTAACAGGTT GTTATTCTAA TTATAATAAA   
  
  
+ TTTTATAGAA CTAGAGAAGA ATTCTATGAC TGTATATTAT TAATTAATAA TTTTTAAAAT AAAAAATAAT   
  
  
+ TATATTATTT AATGTTTTTT TCTATAAAAT AAAAATTTTA TGATATTTTT ATTTAAAATT TTTTTACAAA   
  
  
+ TAAGATACTA TCAATACATA GATCAACTTC ACCTCTTTAC ATTTCGACTT TAAGAACTGG AGAGGTTAAC   
  
  
+ TAGTTATAAC AGATATGAAA CTAAAACTTA TCTACTGGCT AACGTTCTTA CCGGGATTAC GTAATTTTTA   
  
  
+ GGTAAAATAC TTATATATAT ATATATATAT TATTCGATTA TGGAGTATAA TATTATTGGG TAATACATAT   
  
  
+ TTGTGTATCA GAATACCTGA ACTGAGATAA AAAAATCGAT CTAGGTGTAT TTAATTTATC TTTGTAATAA   
  
  
+ TAGTAATAAT AATAAATTTT ATTCTTTTAT ACTTTCTTTT ATTGTTATAA GAAACTTTAA AAATGATCGA   
  
  
+ ACCGATTCAT GTATTCATAT TTGCGTTACT ACTTGGTAAC AACTTGTATT TATATATTAC TTGTGTTATT   
  
  
+ TTTTTAATTA TTATTATTTA TAATCTCGT  

- TTCTTTGATG GGCGGCTTAC AGCGATCCAT CGAGCGATCA ATTACACCGC CGGCCGCACC GAAATTCTGC   
  
  
- CGCTCAAAGT CTCCGGGGGA GTGCAGGAAT GTGAATTCAC GGCGTTCTTT ATCATCCATG GCTGAAGAGA   
  
  
- AGAAGAAACT CTCTCTCTCT CTCTCTCTCT CTCTCTCTCT CTCAGAGGAG TAGTAGTTGT GTAGCCTTTT   
  
  
- CAAGATTTCA ACAACTAAGG ATATTTCTTT ATGGTATGTA AGACTTTGCT TAGAGGGAGA TGCAAGGATA   
  
  
- ATAAAAGGCG AGAGAGAGAG AGAGAGAAAG AGGAGGCGAA GGCGAAGACC AGCCGTTTTC TTCGGGGGAG   
  
  
- AGATATGACA TCATGGTTGA TGATGGCACA CGTGGCGACA TCTGGAAGTG AAAGAGGGGA TGAGAGTGGG   
  
  
- ACCTACGGGA AGGATCGCCG TTGAGTACCT TCAAGAGGTC GACGGTGAGA GGTGACGAAG AGTGATAGGG   
  
  
- TGCGGGCGGT TCACGTGGGG CGTCGTCTTC AGGTCAACTA AGTTGACGGC ACGGCTGTAT CTGACGGTGG   
  
  
- ACAAGAAAAA TAAGAGGCGC GTGTGAGACC AGACGAGCAG AGTACCTAAT CCCACTGACC ATCCAACCCA   
  
  
- ACCCAGCCCT TCACGTCAGT TATTTTGGAT CTGTCGTAGT CAACGAACCT CATGGGAGAG TGAAGACCGT   
  
  
- CCTGCTCCCA TGTGTGGTCC GCTCGACTCG GTTGCTATCT CTAATTACCA ATTTTTATTA AAAATAATTA   
  
  
- TTTTATTATA ACAAAAATTA AGATTATTTA TTGAAGTAGA TATTTTTATC TTAATATTCA TGCTTAAATT   
  
  
- AGAAATCATT AAAATTTTGA AAATTTTTTA AAAGACAAAG AATTGTCCAA CAATAAGATT AATATTATTT   
  
  
- AAAATATCTT GATCTCTTCT TAAGATACTG ACATATAATA ATTAATTATT AAAAATTTTA TTTTTTATTA   
  
  
- ATATAATAAA TTACAAAAAA AGATATTTTA TTTTTAAAAT ACTATAAAAA TAAATTTTAA AAAAATGTTT   
  
  
- ATTCTATGAT AGTTATGTAT CTAGTTGAAG TGGAGAAATG TAAAGCTGAA ATTCTTGACC TCTCCAATTG   
  
  
- ATCAATATTG TCTATACTTT GATTTTGAAT AGATGACCGA TTGCAAGAAT GGCCCTAATG CATTAAAAAT   
  
  
- CCATTTTATG AATATATATA TATATATATA ATAAGCTAAT ACCTCATATT ATAATAACCC ATTATGTATA   
  
  
- AACACATAGT CTTATGGACT TGACTCTATT TTTTTAGCTA GATCCACATA AATTAAATAG AAACATTATT   
  
  
- ATCATTATTA TTATTTAAAA TAAGAAAATA TGAAAGAAAA TAACAATATT CTTTGAAATT TTTACTAGCT   
  
  
- TGGCTAAGTA CATAAGTATA AACGCAATGA TGAACCATTG TTGAACATAA ATATATAATG AACACAATAA   
  
  
- AAAAATTAAT AATAATAAAT ATTAGAGCA

+     O2-site

| Site Name | Organism | Position | Strand | Matrix score. | sequence | function |
| --- | --- | --- | --- | --- | --- | --- |
| O2-site | Zea mays | 187 | - | 9 | GATGATGTGG | cis-acting regulatory element involved in zein metabolism regulation |

> 2018/04/13 10:10:12  
+ AAGAAACTAC CCGCCGAATG TCGCTAGGTA GCTCGCTAGT TAATGTGGCG GCCGGCGTGG CTTTAAGACG   
  
  
+ GCGAGTTTCA GAGGCCCCCT CACGTCCTTA CACTTAAGTG CCGCAAGAAA TAGTAGGTAC CGACTTCTCT   
  
  
+ TCTTCTTTGA GAGAGAGAGA GAGAGAGAGA GAGAGAGAGA GAGTCTCCTC ATCATCAACA CATCGGAAAA   
  
  
+ GTTCTAAAGT TGTTGATTCC TATAAAGAAA TACCATACAT TCTGAAACGA ATCTCCCTCT ACGTTCCTAT   
  
  
+ TATTTTCCGC TCTCTCTCTC TCTCTCTTTC TCCTCCGCTT CCGCTTCTGG TCGGCAAAAG AAGCCCCCTC   
  
  
+ TCTATACTGT AGTACCAACT ACTACCGTGT GCACCGCTGT AGACCTTCAC TTTCTCCCCT ACTCTCACCC   
  
  
+ TGGATGCCCT TCCTAGCGGC AACTCATGGA AGTTCTCCAG CTGCCACTCT CCACTGCTTC TCACTATCCC   
  
  
+ ACGCCCGCCA AGTGCACCCC GCAGCAGAAG TCCAGTTGAT TCAACTGCCG TGCCGACATA GACTGCCACC   
  
  
+ TGTTCTTTTT ATTCTCCGCG CACACTCTGG TCTGCTCGTC TCATGGATTA GGGTGACTGG TAGGTTGGGT   
  
  
+ TGGGTCGGGA AGTGCAGTCA ATAAAACCTA GACAGCATCA GTTGCTTGGA GTACCCTCTC ACTTCTGGCA   
  
  
+ GGACGAGGGT ACACACCAGG CGAGCTGAGC CAACGATAGA GATTAATGGT TAAAAATAAT TTTTATTAAT   
  
  
+ AAAATAATAT TGTTTTTAAT TCTAATAAAT AACTTCATCT ATAAAAATAG AATTATAAGT ACGAATTTAA   
  
  
+ TCTTTAGTAA TTTTAAAACT TTTAAAAAAT TTTCTGTTTC TTAACAGGTT GTTATTCTAA TTATAATAAA   
  
  
+ TTTTATAGAA CTAGAGAAGA ATTCTATGAC TGTATATTAT TAATTAATAA TTTTTAAAAT AAAAAATAAT   
  
  
+ TATATTATTT AATGTTTTTT TCTATAAAAT AAAAATTTTA TGATATTTTT ATTTAAAATT TTTTTACAAA   
  
  
+ TAAGATACTA TCAATACATA GATCAACTTC ACCTCTTTAC ATTTCGACTT TAAGAACTGG AGAGGTTAAC   
  
  
+ TAGTTATAAC AGATATGAAA CTAAAACTTA TCTACTGGCT AACGTTCTTA CCGGGATTAC GTAATTTTTA   
  
  
+ GGTAAAATAC TTATATATAT ATATATATAT TATTCGATTA TGGAGTATAA TATTATTGGG TAATACATAT   
  
  
+ TTGTGTATCA GAATACCTGA ACTGAGATAA AAAAATCGAT CTAGGTGTAT TTAATTTATC TTTGTAATAA   
  
  
+ TAGTAATAAT AATAAATTTT ATTCTTTTAT ACTTTCTTTT ATTGTTATAA GAAACTTTAA AAATGATCGA   
  
  
+ ACCGATTCAT GTATTCATAT TTGCGTTACT ACTTGGTAAC AACTTGTATT TATATATTAC TTGTGTTATT   
  
  
+ TTTTTAATTA TTATTATTTA TAATCTCGT  

- TTCTTTGATG GGCGGCTTAC AGCGATCCAT CGAGCGATCA ATTACACCGC CGGCCGCACC GAAATTCTGC   
  
  
- CGCTCAAAGT CTCCGGGGGA GTGCAGGAAT GTGAATTCAC GGCGTTCTTT ATCATCCATG GCTGAAGAGA   
  
  
- AGAAGAAACT CTCTCTCTCT CTCTCTCTCT CTCTCTCTCT CTCAGAGGAG TAGTAGTTGT GTAGCCTTTT   
  
  
- CAAGATTTCA ACAACTAAGG ATATTTCTTT ATGGTATGTA AGACTTTGCT TAGAGGGAGA TGCAAGGATA   
  
  
- ATAAAAGGCG AGAGAGAGAG AGAGAGAAAG AGGAGGCGAA GGCGAAGACC AGCCGTTTTC TTCGGGGGAG   
  
  
- AGATATGACA TCATGGTTGA TGATGGCACA CGTGGCGACA TCTGGAAGTG AAAGAGGGGA TGAGAGTGGG   
  
  
- ACCTACGGGA AGGATCGCCG TTGAGTACCT TCAAGAGGTC GACGGTGAGA GGTGACGAAG AGTGATAGGG   
  
  
- TGCGGGCGGT TCACGTGGGG CGTCGTCTTC AGGTCAACTA AGTTGACGGC ACGGCTGTAT CTGACGGTGG   
  
  
- ACAAGAAAAA TAAGAGGCGC GTGTGAGACC AGACGAGCAG AGTACCTAAT CCCACTGACC ATCCAACCCA   
  
  
- ACCCAGCCCT TCACGTCAGT TATTTTGGAT CTGTCGTAGT CAACGAACCT CATGGGAGAG TGAAGACCGT   
  
  
- CCTGCTCCCA TGTGTGGTCC GCTCGACTCG GTTGCTATCT CTAATTACCA ATTTTTATTA AAAATAATTA   
  
  
- TTTTATTATA ACAAAAATTA AGATTATTTA TTGAAGTAGA TATTTTTATC TTAATATTCA TGCTTAAATT   
  
  
- AGAAATCATT AAAATTTTGA AAATTTTTTA AAAGACAAAG AATTGTCCAA CAATAAGATT AATATTATTT   
  
  
- AAAATATCTT GATCTCTTCT TAAGATACTG ACATATAATA ATTAATTATT AAAAATTTTA TTTTTTATTA   
  
  
- ATATAATAAA TTACAAAAAA AGATATTTTA TTTTTAAAAT ACTATAAAAA TAAATTTTAA AAAAATGTTT   
  
  
- ATTCTATGAT AGTTATGTAT CTAGTTGAAG TGGAGAAATG TAAAGCTGAA ATTCTTGACC TCTCCAATTG   
  
  
- ATCAATATTG TCTATACTTT GATTTTGAAT AGATGACCGA TTGCAAGAAT GGCCCTAATG CATTAAAAAT   
  
  
- CCATTTTATG AATATATATA TATATATATA ATAAGCTAAT ACCTCATATT ATAATAACCC ATTATGTATA   
  
  
- AACACATAGT CTTATGGACT TGACTCTATT TTTTTAGCTA GATCCACATA AATTAAATAG AAACATTATT   
  
  
- ATCATTATTA TTATTTAAAA TAAGAAAATA TGAAAGAAAA TAACAATATT CTTTGAAATT TTTACTAGCT   
  
  
- TGGCTAAGTA CATAAGTATA AACGCAATGA TGAACCATTG TTGAACATAA ATATATAATG AACACAATAA   
  
  
- AAAAATTAAT AATAATAAAT ATTAGAGCA

+     Skn-1\_motif

| Site Name | Organism | Position | Strand | Matrix score. | sequence | function |
| --- | --- | --- | --- | --- | --- | --- |
| Skn-1\_motif | Oryza sativa | 936 | - | 5 | GTCAT | cis-acting regulatory element required for endosperm expression |

> 2018/04/13 10:10:12  
+ AAGAAACTAC CCGCCGAATG TCGCTAGGTA GCTCGCTAGT TAATGTGGCG GCCGGCGTGG CTTTAAGACG   
  
  
+ GCGAGTTTCA GAGGCCCCCT CACGTCCTTA CACTTAAGTG CCGCAAGAAA TAGTAGGTAC CGACTTCTCT   
  
  
+ TCTTCTTTGA GAGAGAGAGA GAGAGAGAGA GAGAGAGAGA GAGTCTCCTC ATCATCAACA CATCGGAAAA   
  
  
+ GTTCTAAAGT TGTTGATTCC TATAAAGAAA TACCATACAT TCTGAAACGA ATCTCCCTCT ACGTTCCTAT   
  
  
+ TATTTTCCGC TCTCTCTCTC TCTCTCTTTC TCCTCCGCTT CCGCTTCTGG TCGGCAAAAG AAGCCCCCTC   
  
  
+ TCTATACTGT AGTACCAACT ACTACCGTGT GCACCGCTGT AGACCTTCAC TTTCTCCCCT ACTCTCACCC   
  
  
+ TGGATGCCCT TCCTAGCGGC AACTCATGGA AGTTCTCCAG CTGCCACTCT CCACTGCTTC TCACTATCCC   
  
  
+ ACGCCCGCCA AGTGCACCCC GCAGCAGAAG TCCAGTTGAT TCAACTGCCG TGCCGACATA GACTGCCACC   
  
  
+ TGTTCTTTTT ATTCTCCGCG CACACTCTGG TCTGCTCGTC TCATGGATTA GGGTGACTGG TAGGTTGGGT   
  
  
+ TGGGTCGGGA AGTGCAGTCA ATAAAACCTA GACAGCATCA GTTGCTTGGA GTACCCTCTC ACTTCTGGCA   
  
  
+ GGACGAGGGT ACACACCAGG CGAGCTGAGC CAACGATAGA GATTAATGGT TAAAAATAAT TTTTATTAAT   
  
  
+ AAAATAATAT TGTTTTTAAT TCTAATAAAT AACTTCATCT ATAAAAATAG AATTATAAGT ACGAATTTAA   
  
  
+ TCTTTAGTAA TTTTAAAACT TTTAAAAAAT TTTCTGTTTC TTAACAGGTT GTTATTCTAA TTATAATAAA   
  
  
+ TTTTATAGAA CTAGAGAAGA ATTCTATGAC TGTATATTAT TAATTAATAA TTTTTAAAAT AAAAAATAAT   
  
  
+ TATATTATTT AATGTTTTTT TCTATAAAAT AAAAATTTTA TGATATTTTT ATTTAAAATT TTTTTACAAA   
  
  
+ TAAGATACTA TCAATACATA GATCAACTTC ACCTCTTTAC ATTTCGACTT TAAGAACTGG AGAGGTTAAC   
  
  
+ TAGTTATAAC AGATATGAAA CTAAAACTTA TCTACTGGCT AACGTTCTTA CCGGGATTAC GTAATTTTTA   
  
  
+ GGTAAAATAC TTATATATAT ATATATATAT TATTCGATTA TGGAGTATAA TATTATTGGG TAATACATAT   
  
  
+ TTGTGTATCA GAATACCTGA ACTGAGATAA AAAAATCGAT CTAGGTGTAT TTAATTTATC TTTGTAATAA   
  
  
+ TAGTAATAAT AATAAATTTT ATTCTTTTAT ACTTTCTTTT ATTGTTATAA GAAACTTTAA AAATGATCGA   
  
  
+ ACCGATTCAT GTATTCATAT TTGCGTTACT ACTTGGTAAC AACTTGTATT TATATATTAC TTGTGTTATT   
  
  
+ TTTTTAATTA TTATTATTTA TAATCTCGT  

- TTCTTTGATG GGCGGCTTAC AGCGATCCAT CGAGCGATCA ATTACACCGC CGGCCGCACC GAAATTCTGC   
  
  
- CGCTCAAAGT CTCCGGGGGA GTGCAGGAAT GTGAATTCAC GGCGTTCTTT ATCATCCATG GCTGAAGAGA   
  
  
- AGAAGAAACT CTCTCTCTCT CTCTCTCTCT CTCTCTCTCT CTCAGAGGAG TAGTAGTTGT GTAGCCTTTT   
  
  
- CAAGATTTCA ACAACTAAGG ATATTTCTTT ATGGTATGTA AGACTTTGCT TAGAGGGAGA TGCAAGGATA   
  
  
- ATAAAAGGCG AGAGAGAGAG AGAGAGAAAG AGGAGGCGAA GGCGAAGACC AGCCGTTTTC TTCGGGGGAG   
  
  
- AGATATGACA TCATGGTTGA TGATGGCACA CGTGGCGACA TCTGGAAGTG AAAGAGGGGA TGAGAGTGGG   
  
  
- ACCTACGGGA AGGATCGCCG TTGAGTACCT TCAAGAGGTC GACGGTGAGA GGTGACGAAG AGTGATAGGG   
  
  
- TGCGGGCGGT TCACGTGGGG CGTCGTCTTC AGGTCAACTA AGTTGACGGC ACGGCTGTAT CTGACGGTGG   
  
  
- ACAAGAAAAA TAAGAGGCGC GTGTGAGACC AGACGAGCAG AGTACCTAAT CCCACTGACC ATCCAACCCA   
  
  
- ACCCAGCCCT TCACGTCAGT TATTTTGGAT CTGTCGTAGT CAACGAACCT CATGGGAGAG TGAAGACCGT   
  
  
- CCTGCTCCCA TGTGTGGTCC GCTCGACTCG GTTGCTATCT CTAATTACCA ATTTTTATTA AAAATAATTA   
  
  
- TTTTATTATA ACAAAAATTA AGATTATTTA TTGAAGTAGA TATTTTTATC TTAATATTCA TGCTTAAATT   
  
  
- AGAAATCATT AAAATTTTGA AAATTTTTTA AAAGACAAAG AATTGTCCAA CAATAAGATT AATATTATTT   
  
  
- AAAATATCTT GATCTCTTCT TAAGATACTG ACATATAATA ATTAATTATT AAAAATTTTA TTTTTTATTA   
  
  
- ATATAATAAA TTACAAAAAA AGATATTTTA TTTTTAAAAT ACTATAAAAA TAAATTTTAA AAAAATGTTT   
  
  
- ATTCTATGAT AGTTATGTAT CTAGTTGAAG TGGAGAAATG TAAAGCTGAA ATTCTTGACC TCTCCAATTG   
  
  
- ATCAATATTG TCTATACTTT GATTTTGAAT AGATGACCGA TTGCAAGAAT GGCCCTAATG CATTAAAAAT   
  
  
- CCATTTTATG AATATATATA TATATATATA ATAAGCTAAT ACCTCATATT ATAATAACCC ATTATGTATA   
  
  
- AACACATAGT CTTATGGACT TGACTCTATT TTTTTAGCTA GATCCACATA AATTAAATAG AAACATTATT   
  
  
- ATCATTATTA TTATTTAAAA TAAGAAAATA TGAAAGAAAA TAACAATATT CTTTGAAATT TTTACTAGCT   
  
  
- TGGCTAAGTA CATAAGTATA AACGCAATGA TGAACCATTG TTGAACATAA ATATATAATG AACACAATAA   
  
  
- AAAAATTAAT AATAATAAAT ATTAGAGCA

+     TA-rich region

| Site Name | Organism | Position | Strand | Matrix score. | sequence | function |
| --- | --- | --- | --- | --- | --- | --- |
| TA-rich region | Nicotiana tabacum | 1198 | - | 20 | TATATATATATATATATATATA | enhancer |

> 2018/04/13 10:10:12  
+ AAGAAACTAC CCGCCGAATG TCGCTAGGTA GCTCGCTAGT TAATGTGGCG GCCGGCGTGG CTTTAAGACG   
  
  
+ GCGAGTTTCA GAGGCCCCCT CACGTCCTTA CACTTAAGTG CCGCAAGAAA TAGTAGGTAC CGACTTCTCT   
  
  
+ TCTTCTTTGA GAGAGAGAGA GAGAGAGAGA GAGAGAGAGA GAGTCTCCTC ATCATCAACA CATCGGAAAA   
  
  
+ GTTCTAAAGT TGTTGATTCC TATAAAGAAA TACCATACAT TCTGAAACGA ATCTCCCTCT ACGTTCCTAT   
  
  
+ TATTTTCCGC TCTCTCTCTC TCTCTCTTTC TCCTCCGCTT CCGCTTCTGG TCGGCAAAAG AAGCCCCCTC   
  
  
+ TCTATACTGT AGTACCAACT ACTACCGTGT GCACCGCTGT AGACCTTCAC TTTCTCCCCT ACTCTCACCC   
  
  
+ TGGATGCCCT TCCTAGCGGC AACTCATGGA AGTTCTCCAG CTGCCACTCT CCACTGCTTC TCACTATCCC   
  
  
+ ACGCCCGCCA AGTGCACCCC GCAGCAGAAG TCCAGTTGAT TCAACTGCCG TGCCGACATA GACTGCCACC   
  
  
+ TGTTCTTTTT ATTCTCCGCG CACACTCTGG TCTGCTCGTC TCATGGATTA GGGTGACTGG TAGGTTGGGT   
  
  
+ TGGGTCGGGA AGTGCAGTCA ATAAAACCTA GACAGCATCA GTTGCTTGGA GTACCCTCTC ACTTCTGGCA   
  
  
+ GGACGAGGGT ACACACCAGG CGAGCTGAGC CAACGATAGA GATTAATGGT TAAAAATAAT TTTTATTAAT   
  
  
+ AAAATAATAT TGTTTTTAAT TCTAATAAAT AACTTCATCT ATAAAAATAG AATTATAAGT ACGAATTTAA   
  
  
+ TCTTTAGTAA TTTTAAAACT TTTAAAAAAT TTTCTGTTTC TTAACAGGTT GTTATTCTAA TTATAATAAA   
  
  
+ TTTTATAGAA CTAGAGAAGA ATTCTATGAC TGTATATTAT TAATTAATAA TTTTTAAAAT AAAAAATAAT   
  
  
+ TATATTATTT AATGTTTTTT TCTATAAAAT AAAAATTTTA TGATATTTTT ATTTAAAATT TTTTTACAAA   
  
  
+ TAAGATACTA TCAATACATA GATCAACTTC ACCTCTTTAC ATTTCGACTT TAAGAACTGG AGAGGTTAAC   
  
  
+ TAGTTATAAC AGATATGAAA CTAAAACTTA TCTACTGGCT AACGTTCTTA CCGGGATTAC GTAATTTTTA   
  
  
+ GGTAAAATAC TTATATATAT ATATATATAT TATTCGATTA TGGAGTATAA TATTATTGGG TAATACATAT   
  
  
+ TTGTGTATCA GAATACCTGA ACTGAGATAA AAAAATCGAT CTAGGTGTAT TTAATTTATC TTTGTAATAA   
  
  
+ TAGTAATAAT AATAAATTTT ATTCTTTTAT ACTTTCTTTT ATTGTTATAA GAAACTTTAA AAATGATCGA   
  
  
+ ACCGATTCAT GTATTCATAT TTGCGTTACT ACTTGGTAAC AACTTGTATT TATATATTAC TTGTGTTATT   
  
  
+ TTTTTAATTA TTATTATTTA TAATCTCGT  

- TTCTTTGATG GGCGGCTTAC AGCGATCCAT CGAGCGATCA ATTACACCGC CGGCCGCACC GAAATTCTGC   
  
  
- CGCTCAAAGT CTCCGGGGGA GTGCAGGAAT GTGAATTCAC GGCGTTCTTT ATCATCCATG GCTGAAGAGA   
  
  
- AGAAGAAACT CTCTCTCTCT CTCTCTCTCT CTCTCTCTCT CTCAGAGGAG TAGTAGTTGT GTAGCCTTTT   
  
  
- CAAGATTTCA ACAACTAAGG ATATTTCTTT ATGGTATGTA AGACTTTGCT TAGAGGGAGA TGCAAGGATA   
  
  
- ATAAAAGGCG AGAGAGAGAG AGAGAGAAAG AGGAGGCGAA GGCGAAGACC AGCCGTTTTC TTCGGGGGAG   
  
  
- AGATATGACA TCATGGTTGA TGATGGCACA CGTGGCGACA TCTGGAAGTG AAAGAGGGGA TGAGAGTGGG   
  
  
- ACCTACGGGA AGGATCGCCG TTGAGTACCT TCAAGAGGTC GACGGTGAGA GGTGACGAAG AGTGATAGGG   
  
  
- TGCGGGCGGT TCACGTGGGG CGTCGTCTTC AGGTCAACTA AGTTGACGGC ACGGCTGTAT CTGACGGTGG   
  
  
- ACAAGAAAAA TAAGAGGCGC GTGTGAGACC AGACGAGCAG AGTACCTAAT CCCACTGACC ATCCAACCCA   
  
  
- ACCCAGCCCT TCACGTCAGT TATTTTGGAT CTGTCGTAGT CAACGAACCT CATGGGAGAG TGAAGACCGT   
  
  
- CCTGCTCCCA TGTGTGGTCC GCTCGACTCG GTTGCTATCT CTAATTACCA ATTTTTATTA AAAATAATTA   
  
  
- TTTTATTATA ACAAAAATTA AGATTATTTA TTGAAGTAGA TATTTTTATC TTAATATTCA TGCTTAAATT   
  
  
- AGAAATCATT AAAATTTTGA AAATTTTTTA AAAGACAAAG AATTGTCCAA CAATAAGATT AATATTATTT   
  
  
- AAAATATCTT GATCTCTTCT TAAGATACTG ACATATAATA ATTAATTATT AAAAATTTTA TTTTTTATTA   
  
  
- ATATAATAAA TTACAAAAAA AGATATTTTA TTTTTAAAAT ACTATAAAAA TAAATTTTAA AAAAATGTTT   
  
  
- ATTCTATGAT AGTTATGTAT CTAGTTGAAG TGGAGAAATG TAAAGCTGAA ATTCTTGACC TCTCCAATTG   
  
  
- ATCAATATTG TCTATACTTT GATTTTGAAT AGATGACCGA TTGCAAGAAT GGCCCTAATG CATTAAAAAT   
  
  
- CCATTTTATG AATATATATA TATATATATA ATAAGCTAAT ACCTCATATT ATAATAACCC ATTATGTATA   
  
  
- AACACATAGT CTTATGGACT TGACTCTATT TTTTTAGCTA GATCCACATA AATTAAATAG AAACATTATT   
  
  
- ATCATTATTA TTATTTAAAA TAAGAAAATA TGAAAGAAAA TAACAATATT CTTTGAAATT TTTACTAGCT   
  
  
- TGGCTAAGTA CATAAGTATA AACGCAATGA TGAACCATTG TTGAACATAA ATATATAATG AACACAATAA   
  
  
- AAAAATTAAT AATAATAAAT ATTAGAGCA

+     TATA-box

| Site Name | Organism | Position | Strand | Matrix score. | sequence | function |
| --- | --- | --- | --- | --- | --- | --- |
| TATA-box | Daucus carota | 1485 | - | 8 | TATAAATA | core promoter element around -30 of transcription start |
| TATA-box | Glycine max | 1479 | - | 5 | TAATA | core promoter element around -30 of transcription start |
| TATA-box | Arabidopsis thaliana | 901 | - | 5 | TATAA | core promoter element around -30 of transcription start |
| TATA-box | Glycine max | 1340 | + | 5 | TAATA | core promoter element around -30 of transcription start |
| TATA-box | Glycine max | 1241 | - | 5 | TAATA | core promoter element around -30 of transcription start |
| TATA-box | Brassica napus | 1205 | - | 6 | ATATAT | core promoter element around -30 of transcription start |
| TATA-box | Brassica napus | 900 | + | 6 | ATTATA | core promoter element around -30 of transcription start |
| TATA-box | Glycine max | 948 | - | 5 | TAATA | core promoter element around -30 of transcription start |
| TATA-box | Glycine max | 1337 | + | 5 | TAATA | core promoter element around -30 of transcription start |
| TATA-box | Ac | 1486 | - | 7 | TATAAAT | core promoter element around -30 of transcription start |
| TATA-box | Arabidopsis thaliana | 1376 | - | 4 | TATA | core promoter element around -30 of transcription start |
| TATA-box | Arabidopsis thaliana | 1357 | - | 5 | TATAA | core promoter element around -30 of transcription start |
| TATA-box | Glycine max | 1238 | + | 5 | TAATA | core promoter element around -30 of transcription start |
| TATA-box | Zea mays | 861 | + | 8 | TTTAAAAA | core promoter element around -30 of transcription start |
| TATA-box | Glycine max | 793 | + | 5 | TAATA | core promoter element around -30 of transcription start |
| TATA-box | Glycine max | 767 | + | 5 | TAATA | core promoter element around -30 of transcription start |
| TATA-box | Brassica napus | 1452 | - | 6 | ATATAT | core promoter element around -30 of transcription start |
| TATA-box | Arabidopsis thaliana | 1488 | - | 5 | TATAA | core promoter element around -30 of transcription start |
| TATA-box | Arabidopsis thaliana | 1236 | - | 4 | TATA | core promoter element around -30 of transcription start |
| TATA-box | Lycopersicon esculentum | 770 | - | 5 | TTTTA | core promoter element around -30 of transcription start |
| TATA-box | Arabidopsis thaliana | 353 | + | 4 | TATA | core promoter element around -30 of transcription start |
| TATA-box | Lycopersicon esculentum | 962 | + | 5 | TTTTA | core promoter element around -30 of transcription start |
| TATA-box | Glycine max | 278 | - | 5 | TAATA | core promoter element around -30 of transcription start |
| TATA-box | Arabidopsis thaliana | 1487 | - | 6 | TATAAA | core promoter element around -30 of transcription start |
| TATA-box | Brassica napus | 1203 | - | 6 | ATATAT | core promoter element around -30 of transcription start |
| TATA-box | Lycopersicon esculentum | 970 | - | 5 | TTTTA | core promoter element around -30 of transcription start |
| TATA-box | Arabidopsis thaliana | 1030 | + | 8 | TATTTAAA | core promoter element around -30 of transcription start |
| TATA-box | Glycine max | 955 | + | 5 | TAATA | core promoter element around -30 of transcription start |
| TATA-box | Arabidopsis thaliana | 943 | - | 4 | TATA | core promoter element around -30 of transcription start |
| TATA-box | Arabidopsis thaliana | 911 | - | 7 | TATAAAA | core promoter element around -30 of transcription start |
| TATA-box | Arabidopsis thaliana | 1202 | - | 8 | TATATATA | core promoter element around -30 of transcription start |
| TATA-box | Lycopersicon esculentum | 851 | + | 5 | TTTTA | core promoter element around -30 of transcription start |
| TATA-box | Lycopersicon esculentum | 1027 | + | 5 | TTTTA | core promoter element around -30 of transcription start |
| TATA-box | Glycine max | 983 | - | 5 | TAATA | core promoter element around -30 of transcription start |
| TATA-box | Glycine max | 1325 | + | 5 | TAATA | core promoter element around -30 of transcription start |
| TATA-box | Lycopersicon esculentum | 1288 | - | 5 | TTTTA | core promoter element around -30 of transcription start |
| TATA-box | Helianthus annuus | 941 | - | 6 | TATACA | core promoter element around -30 of transcription start |
| TATA-box | Glycine max | 1328 | + | 5 | TAATA | core promoter element around -30 of transcription start |
| TATA-box | Lycopersicon esculentum | 1388 | - | 5 | TTTTA | core promoter element around -30 of transcription start |
| TATA-box | Lycopersicon esculentum | 784 | + | 5 | TTTTA | core promoter element around -30 of transcription start |
| TATA-box | Glycine max | 1218 | - | 5 | TAATA | core promoter element around -30 of transcription start |
| TATA-box | Arabidopsis thaliana | 1216 | - | 4 | TATA | core promoter element around -30 of transcription start |
| TATA-box | Lycopersicon esculentum | 761 | + | 5 | TTTTA | core promoter element around -30 of transcription start |
| TATA-box | Brassica napus | 1207 | - | 6 | ATATAT | core promoter element around -30 of transcription start |
| TATA-box | Lycopersicon esculentum | 965 | - | 5 | TTTTA | core promoter element around -30 of transcription start |
| TATA-box | Arabidopsis thaliana | 751 | + | 9 | TAAAAATAA | core promoter element around -30 of transcription start |
| TATA-box | Glycine max | 1482 | - | 5 | TAATA | core promoter element around -30 of transcription start |
| TATA-box | Zea mays | 961 | - | 8 | TTTAAAAA | core promoter element around -30 of transcription start |
| TATA-box | Arabidopsis thaliana | 1210 | - | 8 | TATATATA | core promoter element around -30 of transcription start |
| TATA-box | Arabidopsis thaliana | 810 | + | 6 | TATAAA | core promoter element around -30 of transcription start |
| TATA-box | Lycopersicon esculentum | 1367 | + | 5 | TTTTA | core promoter element around -30 of transcription start |
| TATA-box | Arabidopsis thaliana | 1453 | - | 4 | TATA | core promoter element around -30 of transcription start |
| TATA-box | Arabidopsis thaliana | 1358 | - | 4 | TATA | core promoter element around -30 of transcription start |
| TATA-box | Glycine max | 945 | - | 5 | TAATA | core promoter element around -30 of transcription start |
| TATA-box | Lycopersicon esculentum | 652 | - | 5 | TTTTA | core promoter element around -30 of transcription start |
| TATA-box | Lycopersicon esculentum | 1034 | - | 5 | TTTTA | core promoter element around -30 of transcription start |
| TATA-box | Brassica napus | 1209 | - | 6 | ATATAT | core promoter element around -30 of transcription start |
| TATA-box | Arabidopsis thaliana | 1204 | - | 8 | TATATATA | core promoter element around -30 of transcription start |
| TATA-box | Lycopersicon esculentum | 863 | - | 5 | TTTTA | core promoter element around -30 of transcription start |
| TATA-box | Arabidopsis thaliana | 914 | - | 4 | TATA | core promoter element around -30 of transcription start |
| TATA-box | Arabidopsis thaliana | 912 | - | 6 | TATAAA | core promoter element around -30 of transcription start |
| TATA-box | Arabidopsis thaliana | 913 | - | 5 | TATAA | core promoter element around -30 of transcription start |
| TATA-box | Arabidopsis thaliana | 839 | - | 8 | TAAAGATT | core promoter element around -30 of transcription start |
| TATA-box | Arabidopsis thaliana | 229 | + | 9 | ccTATAAAaa | core promoter element around -30 of transcription start |
| TATA-box | Arabidopsis thaliana | 1206 | - | 8 | TATATATA | core promoter element around -30 of transcription start |
| TATA-box | Arabidopsis thaliana | 1063 | - | 9 | tcTATATAtt | core promoter element around -30 of transcription start |
| TATA-box | Lycopersicon esculentum | 567 | + | 5 | TTTTA | core promoter element around -30 of transcription start |
| TATA-box | Pisum sativum | 910 | - | 8 | TATAAAAT | core promoter element around -30 of transcription start |
| TATA-box | Glycine max | 904 | + | 5 | TAATA | core promoter element around -30 of transcription start |
| TATA-box | Arabidopsis thaliana | 902 | - | 4 | TATA | core promoter element around -30 of transcription start |
| TATA-box | Arabidopsis thaliana | 1355 | - | 7 | TATAAAA | core promoter element around -30 of transcription start |
| TATA-box | Lycopersicon esculentum | 1347 | + | 5 | TTTTA | core promoter element around -30 of transcription start |
| TATA-box | Glycine max | 764 | - | 5 | TAATA | core promoter element around -30 of transcription start |
| TATA-box | Arabidopsis thaliana | 231 | + | 6 | TATAAA | core promoter element around -30 of transcription start |
| TATA-box | Glycine max | 1251 | + | 5 | TAATA | core promoter element around -30 of transcription start |
| TATA-box | Brassica napus | 1211 | - | 6 | ATATAT | core promoter element around -30 of transcription start |
| TATA-box | Arabidopsis thaliana | 1003 | + | 6 | TATAAA | core promoter element around -30 of transcription start |
| TATA-box | Lycopersicon esculentum | 860 | + | 5 | TTTTA | core promoter element around -30 of transcription start |
| TATA-box | Zea mays | 1386 | + | 8 | TTTAAAAA | core promoter element around -30 of transcription start |
| TATA-box | Lycopersicon esculentum | 1016 | + | 5 | TTTTA | core promoter element around -30 of transcription start |
| TATA-box | Lycopersicon esculentum | 1042 | + | 5 | TTTTA | core promoter element around -30 of transcription start |
| TATA-box | Arabidopsis thaliana | 1356 | - | 6 | TATAAA | core promoter element around -30 of transcription start |
| TATA-box | Arabidopsis thaliana | 1201 | - | 7 | TATATAA | core promoter element around -30 of transcription start |
| TATA-box | Glycine max | 775 | + | 5 | TAATA | core promoter element around -30 of transcription start |
| TATA-box | Brassica napus | 822 | + | 6 | ATTATA | core promoter element around -30 of transcription start |
| TATA-box | Arabidopsis thaliana | 808 | + | 9 | ccTATAAAaa | core promoter element around -30 of transcription start |
| TATA-box | Arabidopsis thaliana | 981 | - | 4 | TATA | core promoter element around -30 of transcription start |
| TATA-box | Arabidopsis thaliana | 1125 | - | 4 | TATA | core promoter element around -30 of transcription start |
| TATA-box | Arabidopsis thaliana | 824 | - | 4 | TATA | core promoter element around -30 of transcription start |
| TATA-box | Brassica napus | 979 | + | 6 | ATTATA | core promoter element around -30 of transcription start |
| TATA-box | Arabidopsis thaliana | 1489 | - | 4 | TATA | core promoter element around -30 of transcription start |
| TATA-box | Arabidopsis thaliana | 1214 | - | 4 | TATA | core promoter element around -30 of transcription start |
| TATA-box | Lycopersicon esculentum | 854 | - | 5 | TTTTA | core promoter element around -30 of transcription start |
| TATA-box | Lycopersicon esculentum | 1005 | - | 5 | TTTTA | core promoter element around -30 of transcription start |
| TATA-box | Glycine max | 1334 | + | 5 | TAATA | core promoter element around -30 of transcription start |
| TATA-box | Lycopersicon esculentum | 1142 | - | 5 | TTTTA | core promoter element around -30 of transcription start |
| TATA-box | Arabidopsis thaliana | 980 | - | 5 | TATAA | core promoter element around -30 of transcription start |
| TATA-box | Arabidopsis thaliana | 1375 | - | 5 | TATAA | core promoter element around -30 of transcription start |
| TATA-box | Lycopersicon esculentum | 812 | - | 5 | TTTTA | core promoter element around -30 of transcription start |
| TATA-box | Arabidopsis thaliana | 1208 | - | 8 | TATATATA | core promoter element around -30 of transcription start |
| TATA-box | Arabidopsis thaliana | 823 | - | 5 | TATAA | core promoter element around -30 of transcription start |
| TATA-box | Lycopersicon esculentum | 1010 | - | 5 | TTTTA | core promoter element around -30 of transcription start |
| TATA-box | Glycine max | 1455 | - | 5 | TAATA | core promoter element around -30 of transcription start |
| TATA-box | Daucus carota | 1183 | - | 9 | ccTATAAATT | core promoter element around -30 of transcription start |
| TATA-box | Lycopersicon esculentum | 1472 | + | 5 | TTTTA | core promoter element around -30 of transcription start |
| TATA-box | Brassica napus | 1215 | - | 6 | ATATAT | core promoter element around -30 of transcription start |
| TATA-box | Arabidopsis thaliana | 1451 | - | 4 | TATA | core promoter element around -30 of transcription start |
| TATA-box | Arabidopsis thaliana | 1124 | - | 5 | TATAA | core promoter element around -30 of transcription start |
| TATA-box | Arabidopsis thaliana | 1450 | - | 7 | TATATAA | core promoter element around -30 of transcription start |
| TATA-box | Arabidopsis thaliana | 1212 | - | 8 | TATATATA | core promoter element around -30 of transcription start |
| TATA-box | Brassica napus | 1213 | - | 6 | ATATAT | core promoter element around -30 of transcription start |
| TATA-box | Arabidopsis thaliana | 1449 | - | 6 | TATAAA | core promoter element around -30 of transcription start |
| TATA-box | Ac | 1448 | - | 7 | TATAAAT | core promoter element around -30 of transcription start |
| TATA-box | Lycopersicon esculentum | 1193 | - | 5 | TTTTA | core promoter element around -30 of transcription start |
| TATA-box | Arabidopsis thaliana | 1447 | - | 9 | taTATAAAtc | core promoter element around -30 of transcription start |
| TATA-box | Lycopersicon esculentum | 1186 | + | 5 | TTTTA | core promoter element around -30 of transcription start |

> 2018/04/13 10:10:12  
+ AAGAAACTAC CCGCCGAATG TCGCTAGGTA GCTCGCTAGT TAATGTGGCG GCCGGCGTGG CTTTAAGACG   
  
  
+ GCGAGTTTCA GAGGCCCCCT CACGTCCTTA CACTTAAGTG CCGCAAGAAA TAGTAGGTAC CGACTTCTCT   
  
  
+ TCTTCTTTGA GAGAGAGAGA GAGAGAGAGA GAGAGAGAGA GAGTCTCCTC ATCATCAACA CATCGGAAAA   
  
  
+ GTTCTAAAGT TGTTGATTCC TATAAAGAAA TACCATACAT TCTGAAACGA ATCTCCCTCT ACGTTCCTAT   
  
  
+ TATTTTCCGC TCTCTCTCTC TCTCTCTTTC TCCTCCGCTT CCGCTTCTGG TCGGCAAAAG AAGCCCCCTC   
  
  
+ TCTATACTGT AGTACCAACT ACTACCGTGT GCACCGCTGT AGACCTTCAC TTTCTCCCCT ACTCTCACCC   
  
  
+ TGGATGCCCT TCCTAGCGGC AACTCATGGA AGTTCTCCAG CTGCCACTCT CCACTGCTTC TCACTATCCC   
  
  
+ ACGCCCGCCA AGTGCACCCC GCAGCAGAAG TCCAGTTGAT TCAACTGCCG TGCCGACATA GACTGCCACC   
  
  
+ TGTTCTTTTT ATTCTCCGCG CACACTCTGG TCTGCTCGTC TCATGGATTA GGGTGACTGG TAGGTTGGGT   
  
  
+ TGGGTCGGGA AGTGCAGTCA ATAAAACCTA GACAGCATCA GTTGCTTGGA GTACCCTCTC ACTTCTGGCA   
  
  
+ GGACGAGGGT ACACACCAGG CGAGCTGAGC CAACGATAGA GATTAATGGT TAAAAATAAT TTTTATTAAT   
  
  
+ AAAATAATAT TGTTTTTAAT TCTAATAAAT AACTTCATCT ATAAAAATAG AATTATAAGT ACGAATTTAA   
  
  
+ TCTTTAGTAA TTTTAAAACT TTTAAAAAAT TTTCTGTTTC TTAACAGGTT GTTATTCTAA TTATAATAAA   
  
  
+ TTTTATAGAA CTAGAGAAGA ATTCTATGAC TGTATATTAT TAATTAATAA TTTTTAAAAT AAAAAATAAT   
  
  
+ TATATTATTT AATGTTTTTT TCTATAAAAT AAAAATTTTA TGATATTTTT ATTTAAAATT TTTTTACAAA   
  
  
+ TAAGATACTA TCAATACATA GATCAACTTC ACCTCTTTAC ATTTCGACTT TAAGAACTGG AGAGGTTAAC   
  
  
+ TAGTTATAAC AGATATGAAA CTAAAACTTA TCTACTGGCT AACGTTCTTA CCGGGATTAC GTAATTTTTA   
  
  
+ GGTAAAATAC TTATATATAT ATATATATAT TATTCGATTA TGGAGTATAA TATTATTGGG TAATACATAT   
  
  
+ TTGTGTATCA GAATACCTGA ACTGAGATAA AAAAATCGAT CTAGGTGTAT TTAATTTATC TTTGTAATAA   
  
  
+ TAGTAATAAT AATAAATTTT ATTCTTTTAT ACTTTCTTTT ATTGTTATAA GAAACTTTAA AAATGATCGA   
  
  
+ ACCGATTCAT GTATTCATAT TTGCGTTACT ACTTGGTAAC AACTTGTATT TATATATTAC TTGTGTTATT   
  
  
+ TTTTTAATTA TTATTATTTA TAATCTCGT  

- TTCTTTGATG GGCGGCTTAC AGCGATCCAT CGAGCGATCA ATTACACCGC CGGCCGCACC GAAATTCTGC   
  
  
- CGCTCAAAGT CTCCGGGGGA GTGCAGGAAT GTGAATTCAC GGCGTTCTTT ATCATCCATG GCTGAAGAGA   
  
  
- AGAAGAAACT CTCTCTCTCT CTCTCTCTCT CTCTCTCTCT CTCAGAGGAG TAGTAGTTGT GTAGCCTTTT   
  
  
- CAAGATTTCA ACAACTAAGG ATATTTCTTT ATGGTATGTA AGACTTTGCT TAGAGGGAGA TGCAAGGATA   
  
  
- ATAAAAGGCG AGAGAGAGAG AGAGAGAAAG AGGAGGCGAA GGCGAAGACC AGCCGTTTTC TTCGGGGGAG   
  
  
- AGATATGACA TCATGGTTGA TGATGGCACA CGTGGCGACA TCTGGAAGTG AAAGAGGGGA TGAGAGTGGG   
  
  
- ACCTACGGGA AGGATCGCCG TTGAGTACCT TCAAGAGGTC GACGGTGAGA GGTGACGAAG AGTGATAGGG   
  
  
- TGCGGGCGGT TCACGTGGGG CGTCGTCTTC AGGTCAACTA AGTTGACGGC ACGGCTGTAT CTGACGGTGG   
  
  
- ACAAGAAAAA TAAGAGGCGC GTGTGAGACC AGACGAGCAG AGTACCTAAT CCCACTGACC ATCCAACCCA   
  
  
- ACCCAGCCCT TCACGTCAGT TATTTTGGAT CTGTCGTAGT CAACGAACCT CATGGGAGAG TGAAGACCGT   
  
  
- CCTGCTCCCA TGTGTGGTCC GCTCGACTCG GTTGCTATCT CTAATTACCA ATTTTTATTA AAAATAATTA   
  
  
- TTTTATTATA ACAAAAATTA AGATTATTTA TTGAAGTAGA TATTTTTATC TTAATATTCA TGCTTAAATT   
  
  
- AGAAATCATT AAAATTTTGA AAATTTTTTA AAAGACAAAG AATTGTCCAA CAATAAGATT AATATTATTT   
  
  
- AAAATATCTT GATCTCTTCT TAAGATACTG ACATATAATA ATTAATTATT AAAAATTTTA TTTTTTATTA   
  
  
- ATATAATAAA TTACAAAAAA AGATATTTTA TTTTTAAAAT ACTATAAAAA TAAATTTTAA AAAAATGTTT   
  
  
- ATTCTATGAT AGTTATGTAT CTAGTTGAAG TGGAGAAATG TAAAGCTGAA ATTCTTGACC TCTCCAATTG   
  
  
- ATCAATATTG TCTATACTTT GATTTTGAAT AGATGACCGA TTGCAAGAAT GGCCCTAATG CATTAAAAAT   
  
  
- CCATTTTATG AATATATATA TATATATATA ATAAGCTAAT ACCTCATATT ATAATAACCC ATTATGTATA   
  
  
- AACACATAGT CTTATGGACT TGACTCTATT TTTTTAGCTA GATCCACATA AATTAAATAG AAACATTATT   
  
  
- ATCATTATTA TTATTTAAAA TAAGAAAATA TGAAAGAAAA TAACAATATT CTTTGAAATT TTTACTAGCT   
  
  
- TGGCTAAGTA CATAAGTATA AACGCAATGA TGAACCATTG TTGAACATAA ATATATAATG AACACAATAA   
  
  
- AAAAATTAAT AATAATAAAT ATTAGAGCA

+     TATC-box

| Site Name | Organism | Position | Strand | Matrix score. | sequence | function |
| --- | --- | --- | --- | --- | --- | --- |
| TATC-box | Oryza sativa | 485 | + | 7 | TATCCCA | cis-acting element involved in gibberellin-responsiveness |

> 2018/04/13 10:10:12  
+ AAGAAACTAC CCGCCGAATG TCGCTAGGTA GCTCGCTAGT TAATGTGGCG GCCGGCGTGG CTTTAAGACG   
  
  
+ GCGAGTTTCA GAGGCCCCCT CACGTCCTTA CACTTAAGTG CCGCAAGAAA TAGTAGGTAC CGACTTCTCT   
  
  
+ TCTTCTTTGA GAGAGAGAGA GAGAGAGAGA GAGAGAGAGA GAGTCTCCTC ATCATCAACA CATCGGAAAA   
  
  
+ GTTCTAAAGT TGTTGATTCC TATAAAGAAA TACCATACAT TCTGAAACGA ATCTCCCTCT ACGTTCCTAT   
  
  
+ TATTTTCCGC TCTCTCTCTC TCTCTCTTTC TCCTCCGCTT CCGCTTCTGG TCGGCAAAAG AAGCCCCCTC   
  
  
+ TCTATACTGT AGTACCAACT ACTACCGTGT GCACCGCTGT AGACCTTCAC TTTCTCCCCT ACTCTCACCC   
  
  
+ TGGATGCCCT TCCTAGCGGC AACTCATGGA AGTTCTCCAG CTGCCACTCT CCACTGCTTC TCACTATCCC   
  
  
+ ACGCCCGCCA AGTGCACCCC GCAGCAGAAG TCCAGTTGAT TCAACTGCCG TGCCGACATA GACTGCCACC   
  
  
+ TGTTCTTTTT ATTCTCCGCG CACACTCTGG TCTGCTCGTC TCATGGATTA GGGTGACTGG TAGGTTGGGT   
  
  
+ TGGGTCGGGA AGTGCAGTCA ATAAAACCTA GACAGCATCA GTTGCTTGGA GTACCCTCTC ACTTCTGGCA   
  
  
+ GGACGAGGGT ACACACCAGG CGAGCTGAGC CAACGATAGA GATTAATGGT TAAAAATAAT TTTTATTAAT   
  
  
+ AAAATAATAT TGTTTTTAAT TCTAATAAAT AACTTCATCT ATAAAAATAG AATTATAAGT ACGAATTTAA   
  
  
+ TCTTTAGTAA TTTTAAAACT TTTAAAAAAT TTTCTGTTTC TTAACAGGTT GTTATTCTAA TTATAATAAA   
  
  
+ TTTTATAGAA CTAGAGAAGA ATTCTATGAC TGTATATTAT TAATTAATAA TTTTTAAAAT AAAAAATAAT   
  
  
+ TATATTATTT AATGTTTTTT TCTATAAAAT AAAAATTTTA TGATATTTTT ATTTAAAATT TTTTTACAAA   
  
  
+ TAAGATACTA TCAATACATA GATCAACTTC ACCTCTTTAC ATTTCGACTT TAAGAACTGG AGAGGTTAAC   
  
  
+ TAGTTATAAC AGATATGAAA CTAAAACTTA TCTACTGGCT AACGTTCTTA CCGGGATTAC GTAATTTTTA   
  
  
+ GGTAAAATAC TTATATATAT ATATATATAT TATTCGATTA TGGAGTATAA TATTATTGGG TAATACATAT   
  
  
+ TTGTGTATCA GAATACCTGA ACTGAGATAA AAAAATCGAT CTAGGTGTAT TTAATTTATC TTTGTAATAA   
  
  
+ TAGTAATAAT AATAAATTTT ATTCTTTTAT ACTTTCTTTT ATTGTTATAA GAAACTTTAA AAATGATCGA   
  
  
+ ACCGATTCAT GTATTCATAT TTGCGTTACT ACTTGGTAAC AACTTGTATT TATATATTAC TTGTGTTATT   
  
  
+ TTTTTAATTA TTATTATTTA TAATCTCGT  

- TTCTTTGATG GGCGGCTTAC AGCGATCCAT CGAGCGATCA ATTACACCGC CGGCCGCACC GAAATTCTGC   
  
  
- CGCTCAAAGT CTCCGGGGGA GTGCAGGAAT GTGAATTCAC GGCGTTCTTT ATCATCCATG GCTGAAGAGA   
  
  
- AGAAGAAACT CTCTCTCTCT CTCTCTCTCT CTCTCTCTCT CTCAGAGGAG TAGTAGTTGT GTAGCCTTTT   
  
  
- CAAGATTTCA ACAACTAAGG ATATTTCTTT ATGGTATGTA AGACTTTGCT TAGAGGGAGA TGCAAGGATA   
  
  
- ATAAAAGGCG AGAGAGAGAG AGAGAGAAAG AGGAGGCGAA GGCGAAGACC AGCCGTTTTC TTCGGGGGAG   
  
  
- AGATATGACA TCATGGTTGA TGATGGCACA CGTGGCGACA TCTGGAAGTG AAAGAGGGGA TGAGAGTGGG   
  
  
- ACCTACGGGA AGGATCGCCG TTGAGTACCT TCAAGAGGTC GACGGTGAGA GGTGACGAAG AGTGATAGGG   
  
  
- TGCGGGCGGT TCACGTGGGG CGTCGTCTTC AGGTCAACTA AGTTGACGGC ACGGCTGTAT CTGACGGTGG   
  
  
- ACAAGAAAAA TAAGAGGCGC GTGTGAGACC AGACGAGCAG AGTACCTAAT CCCACTGACC ATCCAACCCA   
  
  
- ACCCAGCCCT TCACGTCAGT TATTTTGGAT CTGTCGTAGT CAACGAACCT CATGGGAGAG TGAAGACCGT   
  
  
- CCTGCTCCCA TGTGTGGTCC GCTCGACTCG GTTGCTATCT CTAATTACCA ATTTTTATTA AAAATAATTA   
  
  
- TTTTATTATA ACAAAAATTA AGATTATTTA TTGAAGTAGA TATTTTTATC TTAATATTCA TGCTTAAATT   
  
  
- AGAAATCATT AAAATTTTGA AAATTTTTTA AAAGACAAAG AATTGTCCAA CAATAAGATT AATATTATTT   
  
  
- AAAATATCTT GATCTCTTCT TAAGATACTG ACATATAATA ATTAATTATT AAAAATTTTA TTTTTTATTA   
  
  
- ATATAATAAA TTACAAAAAA AGATATTTTA TTTTTAAAAT ACTATAAAAA TAAATTTTAA AAAAATGTTT   
  
  
- ATTCTATGAT AGTTATGTAT CTAGTTGAAG TGGAGAAATG TAAAGCTGAA ATTCTTGACC TCTCCAATTG   
  
  
- ATCAATATTG TCTATACTTT GATTTTGAAT AGATGACCGA TTGCAAGAAT GGCCCTAATG CATTAAAAAT   
  
  
- CCATTTTATG AATATATATA TATATATATA ATAAGCTAAT ACCTCATATT ATAATAACCC ATTATGTATA   
  
  
- AACACATAGT CTTATGGACT TGACTCTATT TTTTTAGCTA GATCCACATA AATTAAATAG AAACATTATT   
  
  
- ATCATTATTA TTATTTAAAA TAAGAAAATA TGAAAGAAAA TAACAATATT CTTTGAAATT TTTACTAGCT   
  
  
- TGGCTAAGTA CATAAGTATA AACGCAATGA TGAACCATTG TTGAACATAA ATATATAATG AACACAATAA   
  
  
- AAAAATTAAT AATAATAAAT ATTAGAGCA

+     TCA-element

| Site Name | Organism | Position | Strand | Matrix score. | sequence | function |
| --- | --- | --- | --- | --- | --- | --- |
| TCA-element | Brassica oleracea | 924 | + | 9 | GAGAAGAATA | cis-acting element involved in salicylic acid responsiveness |

> 2018/04/13 10:10:12  
+ AAGAAACTAC CCGCCGAATG TCGCTAGGTA GCTCGCTAGT TAATGTGGCG GCCGGCGTGG CTTTAAGACG   
  
  
+ GCGAGTTTCA GAGGCCCCCT CACGTCCTTA CACTTAAGTG CCGCAAGAAA TAGTAGGTAC CGACTTCTCT   
  
  
+ TCTTCTTTGA GAGAGAGAGA GAGAGAGAGA GAGAGAGAGA GAGTCTCCTC ATCATCAACA CATCGGAAAA   
  
  
+ GTTCTAAAGT TGTTGATTCC TATAAAGAAA TACCATACAT TCTGAAACGA ATCTCCCTCT ACGTTCCTAT   
  
  
+ TATTTTCCGC TCTCTCTCTC TCTCTCTTTC TCCTCCGCTT CCGCTTCTGG TCGGCAAAAG AAGCCCCCTC   
  
  
+ TCTATACTGT AGTACCAACT ACTACCGTGT GCACCGCTGT AGACCTTCAC TTTCTCCCCT ACTCTCACCC   
  
  
+ TGGATGCCCT TCCTAGCGGC AACTCATGGA AGTTCTCCAG CTGCCACTCT CCACTGCTTC TCACTATCCC   
  
  
+ ACGCCCGCCA AGTGCACCCC GCAGCAGAAG TCCAGTTGAT TCAACTGCCG TGCCGACATA GACTGCCACC   
  
  
+ TGTTCTTTTT ATTCTCCGCG CACACTCTGG TCTGCTCGTC TCATGGATTA GGGTGACTGG TAGGTTGGGT   
  
  
+ TGGGTCGGGA AGTGCAGTCA ATAAAACCTA GACAGCATCA GTTGCTTGGA GTACCCTCTC ACTTCTGGCA   
  
  
+ GGACGAGGGT ACACACCAGG CGAGCTGAGC CAACGATAGA GATTAATGGT TAAAAATAAT TTTTATTAAT   
  
  
+ AAAATAATAT TGTTTTTAAT TCTAATAAAT AACTTCATCT ATAAAAATAG AATTATAAGT ACGAATTTAA   
  
  
+ TCTTTAGTAA TTTTAAAACT TTTAAAAAAT TTTCTGTTTC TTAACAGGTT GTTATTCTAA TTATAATAAA   
  
  
+ TTTTATAGAA CTAGAGAAGA ATTCTATGAC TGTATATTAT TAATTAATAA TTTTTAAAAT AAAAAATAAT   
  
  
+ TATATTATTT AATGTTTTTT TCTATAAAAT AAAAATTTTA TGATATTTTT ATTTAAAATT TTTTTACAAA   
  
  
+ TAAGATACTA TCAATACATA GATCAACTTC ACCTCTTTAC ATTTCGACTT TAAGAACTGG AGAGGTTAAC   
  
  
+ TAGTTATAAC AGATATGAAA CTAAAACTTA TCTACTGGCT AACGTTCTTA CCGGGATTAC GTAATTTTTA   
  
  
+ GGTAAAATAC TTATATATAT ATATATATAT TATTCGATTA TGGAGTATAA TATTATTGGG TAATACATAT   
  
  
+ TTGTGTATCA GAATACCTGA ACTGAGATAA AAAAATCGAT CTAGGTGTAT TTAATTTATC TTTGTAATAA   
  
  
+ TAGTAATAAT AATAAATTTT ATTCTTTTAT ACTTTCTTTT ATTGTTATAA GAAACTTTAA AAATGATCGA   
  
  
+ ACCGATTCAT GTATTCATAT TTGCGTTACT ACTTGGTAAC AACTTGTATT TATATATTAC TTGTGTTATT   
  
  
+ TTTTTAATTA TTATTATTTA TAATCTCGT  

- TTCTTTGATG GGCGGCTTAC AGCGATCCAT CGAGCGATCA ATTACACCGC CGGCCGCACC GAAATTCTGC   
  
  
- CGCTCAAAGT CTCCGGGGGA GTGCAGGAAT GTGAATTCAC GGCGTTCTTT ATCATCCATG GCTGAAGAGA   
  
  
- AGAAGAAACT CTCTCTCTCT CTCTCTCTCT CTCTCTCTCT CTCAGAGGAG TAGTAGTTGT GTAGCCTTTT   
  
  
- CAAGATTTCA ACAACTAAGG ATATTTCTTT ATGGTATGTA AGACTTTGCT TAGAGGGAGA TGCAAGGATA   
  
  
- ATAAAAGGCG AGAGAGAGAG AGAGAGAAAG AGGAGGCGAA GGCGAAGACC AGCCGTTTTC TTCGGGGGAG   
  
  
- AGATATGACA TCATGGTTGA TGATGGCACA CGTGGCGACA TCTGGAAGTG AAAGAGGGGA TGAGAGTGGG   
  
  
- ACCTACGGGA AGGATCGCCG TTGAGTACCT TCAAGAGGTC GACGGTGAGA GGTGACGAAG AGTGATAGGG   
  
  
- TGCGGGCGGT TCACGTGGGG CGTCGTCTTC AGGTCAACTA AGTTGACGGC ACGGCTGTAT CTGACGGTGG   
  
  
- ACAAGAAAAA TAAGAGGCGC GTGTGAGACC AGACGAGCAG AGTACCTAAT CCCACTGACC ATCCAACCCA   
  
  
- ACCCAGCCCT TCACGTCAGT TATTTTGGAT CTGTCGTAGT CAACGAACCT CATGGGAGAG TGAAGACCGT   
  
  
- CCTGCTCCCA TGTGTGGTCC GCTCGACTCG GTTGCTATCT CTAATTACCA ATTTTTATTA AAAATAATTA   
  
  
- TTTTATTATA ACAAAAATTA AGATTATTTA TTGAAGTAGA TATTTTTATC TTAATATTCA TGCTTAAATT   
  
  
- AGAAATCATT AAAATTTTGA AAATTTTTTA AAAGACAAAG AATTGTCCAA CAATAAGATT AATATTATTT   
  
  
- AAAATATCTT GATCTCTTCT TAAGATACTG ACATATAATA ATTAATTATT AAAAATTTTA TTTTTTATTA   
  
  
- ATATAATAAA TTACAAAAAA AGATATTTTA TTTTTAAAAT ACTATAAAAA TAAATTTTAA AAAAATGTTT   
  
  
- ATTCTATGAT AGTTATGTAT CTAGTTGAAG TGGAGAAATG TAAAGCTGAA ATTCTTGACC TCTCCAATTG   
  
  
- ATCAATATTG TCTATACTTT GATTTTGAAT AGATGACCGA TTGCAAGAAT GGCCCTAATG CATTAAAAAT   
  
  
- CCATTTTATG AATATATATA TATATATATA ATAAGCTAAT ACCTCATATT ATAATAACCC ATTATGTATA   
  
  
- AACACATAGT CTTATGGACT TGACTCTATT TTTTTAGCTA GATCCACATA AATTAAATAG AAACATTATT   
  
  
- ATCATTATTA TTATTTAAAA TAAGAAAATA TGAAAGAAAA TAACAATATT CTTTGAAATT TTTACTAGCT   
  
  
- TGGCTAAGTA CATAAGTATA AACGCAATGA TGAACCATTG TTGAACATAA ATATATAATG AACACAATAA   
  
  
- AAAAATTAAT AATAATAAAT ATTAGAGCA

+     TCCC-motif

| Site Name | Organism | Position | Strand | Matrix score. | sequence | function |
| --- | --- | --- | --- | --- | --- | --- |
| TCCC-motif | Spinacia oleracea | 262 | + | 7 | TCTCCCT | part of a light responsive element |

> 2018/04/13 10:10:12  
+ AAGAAACTAC CCGCCGAATG TCGCTAGGTA GCTCGCTAGT TAATGTGGCG GCCGGCGTGG CTTTAAGACG   
  
  
+ GCGAGTTTCA GAGGCCCCCT CACGTCCTTA CACTTAAGTG CCGCAAGAAA TAGTAGGTAC CGACTTCTCT   
  
  
+ TCTTCTTTGA GAGAGAGAGA GAGAGAGAGA GAGAGAGAGA GAGTCTCCTC ATCATCAACA CATCGGAAAA   
  
  
+ GTTCTAAAGT TGTTGATTCC TATAAAGAAA TACCATACAT TCTGAAACGA ATCTCCCTCT ACGTTCCTAT   
  
  
+ TATTTTCCGC TCTCTCTCTC TCTCTCTTTC TCCTCCGCTT CCGCTTCTGG TCGGCAAAAG AAGCCCCCTC   
  
  
+ TCTATACTGT AGTACCAACT ACTACCGTGT GCACCGCTGT AGACCTTCAC TTTCTCCCCT ACTCTCACCC   
  
  
+ TGGATGCCCT TCCTAGCGGC AACTCATGGA AGTTCTCCAG CTGCCACTCT CCACTGCTTC TCACTATCCC   
  
  
+ ACGCCCGCCA AGTGCACCCC GCAGCAGAAG TCCAGTTGAT TCAACTGCCG TGCCGACATA GACTGCCACC   
  
  
+ TGTTCTTTTT ATTCTCCGCG CACACTCTGG TCTGCTCGTC TCATGGATTA GGGTGACTGG TAGGTTGGGT   
  
  
+ TGGGTCGGGA AGTGCAGTCA ATAAAACCTA GACAGCATCA GTTGCTTGGA GTACCCTCTC ACTTCTGGCA   
  
  
+ GGACGAGGGT ACACACCAGG CGAGCTGAGC CAACGATAGA GATTAATGGT TAAAAATAAT TTTTATTAAT   
  
  
+ AAAATAATAT TGTTTTTAAT TCTAATAAAT AACTTCATCT ATAAAAATAG AATTATAAGT ACGAATTTAA   
  
  
+ TCTTTAGTAA TTTTAAAACT TTTAAAAAAT TTTCTGTTTC TTAACAGGTT GTTATTCTAA TTATAATAAA   
  
  
+ TTTTATAGAA CTAGAGAAGA ATTCTATGAC TGTATATTAT TAATTAATAA TTTTTAAAAT AAAAAATAAT   
  
  
+ TATATTATTT AATGTTTTTT TCTATAAAAT AAAAATTTTA TGATATTTTT ATTTAAAATT TTTTTACAAA   
  
  
+ TAAGATACTA TCAATACATA GATCAACTTC ACCTCTTTAC ATTTCGACTT TAAGAACTGG AGAGGTTAAC   
  
  
+ TAGTTATAAC AGATATGAAA CTAAAACTTA TCTACTGGCT AACGTTCTTA CCGGGATTAC GTAATTTTTA   
  
  
+ GGTAAAATAC TTATATATAT ATATATATAT TATTCGATTA TGGAGTATAA TATTATTGGG TAATACATAT   
  
  
+ TTGTGTATCA GAATACCTGA ACTGAGATAA AAAAATCGAT CTAGGTGTAT TTAATTTATC TTTGTAATAA   
  
  
+ TAGTAATAAT AATAAATTTT ATTCTTTTAT ACTTTCTTTT ATTGTTATAA GAAACTTTAA AAATGATCGA   
  
  
+ ACCGATTCAT GTATTCATAT TTGCGTTACT ACTTGGTAAC AACTTGTATT TATATATTAC TTGTGTTATT   
  
  
+ TTTTTAATTA TTATTATTTA TAATCTCGT  

- TTCTTTGATG GGCGGCTTAC AGCGATCCAT CGAGCGATCA ATTACACCGC CGGCCGCACC GAAATTCTGC   
  
  
- CGCTCAAAGT CTCCGGGGGA GTGCAGGAAT GTGAATTCAC GGCGTTCTTT ATCATCCATG GCTGAAGAGA   
  
  
- AGAAGAAACT CTCTCTCTCT CTCTCTCTCT CTCTCTCTCT CTCAGAGGAG TAGTAGTTGT GTAGCCTTTT   
  
  
- CAAGATTTCA ACAACTAAGG ATATTTCTTT ATGGTATGTA AGACTTTGCT TAGAGGGAGA TGCAAGGATA   
  
  
- ATAAAAGGCG AGAGAGAGAG AGAGAGAAAG AGGAGGCGAA GGCGAAGACC AGCCGTTTTC TTCGGGGGAG   
  
  
- AGATATGACA TCATGGTTGA TGATGGCACA CGTGGCGACA TCTGGAAGTG AAAGAGGGGA TGAGAGTGGG   
  
  
- ACCTACGGGA AGGATCGCCG TTGAGTACCT TCAAGAGGTC GACGGTGAGA GGTGACGAAG AGTGATAGGG   
  
  
- TGCGGGCGGT TCACGTGGGG CGTCGTCTTC AGGTCAACTA AGTTGACGGC ACGGCTGTAT CTGACGGTGG   
  
  
- ACAAGAAAAA TAAGAGGCGC GTGTGAGACC AGACGAGCAG AGTACCTAAT CCCACTGACC ATCCAACCCA   
  
  
- ACCCAGCCCT TCACGTCAGT TATTTTGGAT CTGTCGTAGT CAACGAACCT CATGGGAGAG TGAAGACCGT   
  
  
- CCTGCTCCCA TGTGTGGTCC GCTCGACTCG GTTGCTATCT CTAATTACCA ATTTTTATTA AAAATAATTA   
  
  
- TTTTATTATA ACAAAAATTA AGATTATTTA TTGAAGTAGA TATTTTTATC TTAATATTCA TGCTTAAATT   
  
  
- AGAAATCATT AAAATTTTGA AAATTTTTTA AAAGACAAAG AATTGTCCAA CAATAAGATT AATATTATTT   
  
  
- AAAATATCTT GATCTCTTCT TAAGATACTG ACATATAATA ATTAATTATT AAAAATTTTA TTTTTTATTA   
  
  
- ATATAATAAA TTACAAAAAA AGATATTTTA TTTTTAAAAT ACTATAAAAA TAAATTTTAA AAAAATGTTT   
  
  
- ATTCTATGAT AGTTATGTAT CTAGTTGAAG TGGAGAAATG TAAAGCTGAA ATTCTTGACC TCTCCAATTG   
  
  
- ATCAATATTG TCTATACTTT GATTTTGAAT AGATGACCGA TTGCAAGAAT GGCCCTAATG CATTAAAAAT   
  
  
- CCATTTTATG AATATATATA TATATATATA ATAAGCTAAT ACCTCATATT ATAATAACCC ATTATGTATA   
  
  
- AACACATAGT CTTATGGACT TGACTCTATT TTTTTAGCTA GATCCACATA AATTAAATAG AAACATTATT   
  
  
- ATCATTATTA TTATTTAAAA TAAGAAAATA TGAAAGAAAA TAACAATATT CTTTGAAATT TTTACTAGCT   
  
  
- TGGCTAAGTA CATAAGTATA AACGCAATGA TGAACCATTG TTGAACATAA ATATATAATG AACACAATAA   
  
  
- AAAAATTAAT AATAATAAAT ATTAGAGCA

+     TCT-motif

| Site Name | Organism | Position | Strand | Matrix score. | sequence | function |
| --- | --- | --- | --- | --- | --- | --- |
| TCT-motif | Arabidopsis thaliana | 1166 | + | 6 | TCTTAC | part of a light responsive element |

> 2018/04/13 10:10:12  
+ AAGAAACTAC CCGCCGAATG TCGCTAGGTA GCTCGCTAGT TAATGTGGCG GCCGGCGTGG CTTTAAGACG   
  
  
+ GCGAGTTTCA GAGGCCCCCT CACGTCCTTA CACTTAAGTG CCGCAAGAAA TAGTAGGTAC CGACTTCTCT   
  
  
+ TCTTCTTTGA GAGAGAGAGA GAGAGAGAGA GAGAGAGAGA GAGTCTCCTC ATCATCAACA CATCGGAAAA   
  
  
+ GTTCTAAAGT TGTTGATTCC TATAAAGAAA TACCATACAT TCTGAAACGA ATCTCCCTCT ACGTTCCTAT   
  
  
+ TATTTTCCGC TCTCTCTCTC TCTCTCTTTC TCCTCCGCTT CCGCTTCTGG TCGGCAAAAG AAGCCCCCTC   
  
  
+ TCTATACTGT AGTACCAACT ACTACCGTGT GCACCGCTGT AGACCTTCAC TTTCTCCCCT ACTCTCACCC   
  
  
+ TGGATGCCCT TCCTAGCGGC AACTCATGGA AGTTCTCCAG CTGCCACTCT CCACTGCTTC TCACTATCCC   
  
  
+ ACGCCCGCCA AGTGCACCCC GCAGCAGAAG TCCAGTTGAT TCAACTGCCG TGCCGACATA GACTGCCACC   
  
  
+ TGTTCTTTTT ATTCTCCGCG CACACTCTGG TCTGCTCGTC TCATGGATTA GGGTGACTGG TAGGTTGGGT   
  
  
+ TGGGTCGGGA AGTGCAGTCA ATAAAACCTA GACAGCATCA GTTGCTTGGA GTACCCTCTC ACTTCTGGCA   
  
  
+ GGACGAGGGT ACACACCAGG CGAGCTGAGC CAACGATAGA GATTAATGGT TAAAAATAAT TTTTATTAAT   
  
  
+ AAAATAATAT TGTTTTTAAT TCTAATAAAT AACTTCATCT ATAAAAATAG AATTATAAGT ACGAATTTAA   
  
  
+ TCTTTAGTAA TTTTAAAACT TTTAAAAAAT TTTCTGTTTC TTAACAGGTT GTTATTCTAA TTATAATAAA   
  
  
+ TTTTATAGAA CTAGAGAAGA ATTCTATGAC TGTATATTAT TAATTAATAA TTTTTAAAAT AAAAAATAAT   
  
  
+ TATATTATTT AATGTTTTTT TCTATAAAAT AAAAATTTTA TGATATTTTT ATTTAAAATT TTTTTACAAA   
  
  
+ TAAGATACTA TCAATACATA GATCAACTTC ACCTCTTTAC ATTTCGACTT TAAGAACTGG AGAGGTTAAC   
  
  
+ TAGTTATAAC AGATATGAAA CTAAAACTTA TCTACTGGCT AACGTTCTTA CCGGGATTAC GTAATTTTTA   
  
  
+ GGTAAAATAC TTATATATAT ATATATATAT TATTCGATTA TGGAGTATAA TATTATTGGG TAATACATAT   
  
  
+ TTGTGTATCA GAATACCTGA ACTGAGATAA AAAAATCGAT CTAGGTGTAT TTAATTTATC TTTGTAATAA   
  
  
+ TAGTAATAAT AATAAATTTT ATTCTTTTAT ACTTTCTTTT ATTGTTATAA GAAACTTTAA AAATGATCGA   
  
  
+ ACCGATTCAT GTATTCATAT TTGCGTTACT ACTTGGTAAC AACTTGTATT TATATATTAC TTGTGTTATT   
  
  
+ TTTTTAATTA TTATTATTTA TAATCTCGT  

- TTCTTTGATG GGCGGCTTAC AGCGATCCAT CGAGCGATCA ATTACACCGC CGGCCGCACC GAAATTCTGC   
  
  
- CGCTCAAAGT CTCCGGGGGA GTGCAGGAAT GTGAATTCAC GGCGTTCTTT ATCATCCATG GCTGAAGAGA   
  
  
- AGAAGAAACT CTCTCTCTCT CTCTCTCTCT CTCTCTCTCT CTCAGAGGAG TAGTAGTTGT GTAGCCTTTT   
  
  
- CAAGATTTCA ACAACTAAGG ATATTTCTTT ATGGTATGTA AGACTTTGCT TAGAGGGAGA TGCAAGGATA   
  
  
- ATAAAAGGCG AGAGAGAGAG AGAGAGAAAG AGGAGGCGAA GGCGAAGACC AGCCGTTTTC TTCGGGGGAG   
  
  
- AGATATGACA TCATGGTTGA TGATGGCACA CGTGGCGACA TCTGGAAGTG AAAGAGGGGA TGAGAGTGGG   
  
  
- ACCTACGGGA AGGATCGCCG TTGAGTACCT TCAAGAGGTC GACGGTGAGA GGTGACGAAG AGTGATAGGG   
  
  
- TGCGGGCGGT TCACGTGGGG CGTCGTCTTC AGGTCAACTA AGTTGACGGC ACGGCTGTAT CTGACGGTGG   
  
  
- ACAAGAAAAA TAAGAGGCGC GTGTGAGACC AGACGAGCAG AGTACCTAAT CCCACTGACC ATCCAACCCA   
  
  
- ACCCAGCCCT TCACGTCAGT TATTTTGGAT CTGTCGTAGT CAACGAACCT CATGGGAGAG TGAAGACCGT   
  
  
- CCTGCTCCCA TGTGTGGTCC GCTCGACTCG GTTGCTATCT CTAATTACCA ATTTTTATTA AAAATAATTA   
  
  
- TTTTATTATA ACAAAAATTA AGATTATTTA TTGAAGTAGA TATTTTTATC TTAATATTCA TGCTTAAATT   
  
  
- AGAAATCATT AAAATTTTGA AAATTTTTTA AAAGACAAAG AATTGTCCAA CAATAAGATT AATATTATTT   
  
  
- AAAATATCTT GATCTCTTCT TAAGATACTG ACATATAATA ATTAATTATT AAAAATTTTA TTTTTTATTA   
  
  
- ATATAATAAA TTACAAAAAA AGATATTTTA TTTTTAAAAT ACTATAAAAA TAAATTTTAA AAAAATGTTT   
  
  
- ATTCTATGAT AGTTATGTAT CTAGTTGAAG TGGAGAAATG TAAAGCTGAA ATTCTTGACC TCTCCAATTG   
  
  
- ATCAATATTG TCTATACTTT GATTTTGAAT AGATGACCGA TTGCAAGAAT GGCCCTAATG CATTAAAAAT   
  
  
- CCATTTTATG AATATATATA TATATATATA ATAAGCTAAT ACCTCATATT ATAATAACCC ATTATGTATA   
  
  
- AACACATAGT CTTATGGACT TGACTCTATT TTTTTAGCTA GATCCACATA AATTAAATAG AAACATTATT   
  
  
- ATCATTATTA TTATTTAAAA TAAGAAAATA TGAAAGAAAA TAACAATATT CTTTGAAATT TTTACTAGCT   
  
  
- TGGCTAAGTA CATAAGTATA AACGCAATGA TGAACCATTG TTGAACATAA ATATATAATG AACACAATAA   
  
  
- AAAAATTAAT AATAATAAAT ATTAGAGCA

+     Unnamed\_\_1

| Site Name | Organism | Position | Strand | Matrix score. | sequence | function |
| --- | --- | --- | --- | --- | --- | --- |
| Unnamed\_\_1 | Zea mays | 489 | - | 5 | CGTGG |  |
| Unnamed\_\_1 | Zea mays | 56 | + | 5 | CGTGG |  |

> 2018/04/13 10:10:12  
+ AAGAAACTAC CCGCCGAATG TCGCTAGGTA GCTCGCTAGT TAATGTGGCG GCCGGCGTGG CTTTAAGACG   
  
  
+ GCGAGTTTCA GAGGCCCCCT CACGTCCTTA CACTTAAGTG CCGCAAGAAA TAGTAGGTAC CGACTTCTCT   
  
  
+ TCTTCTTTGA GAGAGAGAGA GAGAGAGAGA GAGAGAGAGA GAGTCTCCTC ATCATCAACA CATCGGAAAA   
  
  
+ GTTCTAAAGT TGTTGATTCC TATAAAGAAA TACCATACAT TCTGAAACGA ATCTCCCTCT ACGTTCCTAT   
  
  
+ TATTTTCCGC TCTCTCTCTC TCTCTCTTTC TCCTCCGCTT CCGCTTCTGG TCGGCAAAAG AAGCCCCCTC   
  
  
+ TCTATACTGT AGTACCAACT ACTACCGTGT GCACCGCTGT AGACCTTCAC TTTCTCCCCT ACTCTCACCC   
  
  
+ TGGATGCCCT TCCTAGCGGC AACTCATGGA AGTTCTCCAG CTGCCACTCT CCACTGCTTC TCACTATCCC   
  
  
+ ACGCCCGCCA AGTGCACCCC GCAGCAGAAG TCCAGTTGAT TCAACTGCCG TGCCGACATA GACTGCCACC   
  
  
+ TGTTCTTTTT ATTCTCCGCG CACACTCTGG TCTGCTCGTC TCATGGATTA GGGTGACTGG TAGGTTGGGT   
  
  
+ TGGGTCGGGA AGTGCAGTCA ATAAAACCTA GACAGCATCA GTTGCTTGGA GTACCCTCTC ACTTCTGGCA   
  
  
+ GGACGAGGGT ACACACCAGG CGAGCTGAGC CAACGATAGA GATTAATGGT TAAAAATAAT TTTTATTAAT   
  
  
+ AAAATAATAT TGTTTTTAAT TCTAATAAAT AACTTCATCT ATAAAAATAG AATTATAAGT ACGAATTTAA   
  
  
+ TCTTTAGTAA TTTTAAAACT TTTAAAAAAT TTTCTGTTTC TTAACAGGTT GTTATTCTAA TTATAATAAA   
  
  
+ TTTTATAGAA CTAGAGAAGA ATTCTATGAC TGTATATTAT TAATTAATAA TTTTTAAAAT AAAAAATAAT   
  
  
+ TATATTATTT AATGTTTTTT TCTATAAAAT AAAAATTTTA TGATATTTTT ATTTAAAATT TTTTTACAAA   
  
  
+ TAAGATACTA TCAATACATA GATCAACTTC ACCTCTTTAC ATTTCGACTT TAAGAACTGG AGAGGTTAAC   
  
  
+ TAGTTATAAC AGATATGAAA CTAAAACTTA TCTACTGGCT AACGTTCTTA CCGGGATTAC GTAATTTTTA   
  
  
+ GGTAAAATAC TTATATATAT ATATATATAT TATTCGATTA TGGAGTATAA TATTATTGGG TAATACATAT   
  
  
+ TTGTGTATCA GAATACCTGA ACTGAGATAA AAAAATCGAT CTAGGTGTAT TTAATTTATC TTTGTAATAA   
  
  
+ TAGTAATAAT AATAAATTTT ATTCTTTTAT ACTTTCTTTT ATTGTTATAA GAAACTTTAA AAATGATCGA   
  
  
+ ACCGATTCAT GTATTCATAT TTGCGTTACT ACTTGGTAAC AACTTGTATT TATATATTAC TTGTGTTATT   
  
  
+ TTTTTAATTA TTATTATTTA TAATCTCGT  

- TTCTTTGATG GGCGGCTTAC AGCGATCCAT CGAGCGATCA ATTACACCGC CGGCCGCACC GAAATTCTGC   
  
  
- CGCTCAAAGT CTCCGGGGGA GTGCAGGAAT GTGAATTCAC GGCGTTCTTT ATCATCCATG GCTGAAGAGA   
  
  
- AGAAGAAACT CTCTCTCTCT CTCTCTCTCT CTCTCTCTCT CTCAGAGGAG TAGTAGTTGT GTAGCCTTTT   
  
  
- CAAGATTTCA ACAACTAAGG ATATTTCTTT ATGGTATGTA AGACTTTGCT TAGAGGGAGA TGCAAGGATA   
  
  
- ATAAAAGGCG AGAGAGAGAG AGAGAGAAAG AGGAGGCGAA GGCGAAGACC AGCCGTTTTC TTCGGGGGAG   
  
  
- AGATATGACA TCATGGTTGA TGATGGCACA CGTGGCGACA TCTGGAAGTG AAAGAGGGGA TGAGAGTGGG   
  
  
- ACCTACGGGA AGGATCGCCG TTGAGTACCT TCAAGAGGTC GACGGTGAGA GGTGACGAAG AGTGATAGGG   
  
  
- TGCGGGCGGT TCACGTGGGG CGTCGTCTTC AGGTCAACTA AGTTGACGGC ACGGCTGTAT CTGACGGTGG   
  
  
- ACAAGAAAAA TAAGAGGCGC GTGTGAGACC AGACGAGCAG AGTACCTAAT CCCACTGACC ATCCAACCCA   
  
  
- ACCCAGCCCT TCACGTCAGT TATTTTGGAT CTGTCGTAGT CAACGAACCT CATGGGAGAG TGAAGACCGT   
  
  
- CCTGCTCCCA TGTGTGGTCC GCTCGACTCG GTTGCTATCT CTAATTACCA ATTTTTATTA AAAATAATTA   
  
  
- TTTTATTATA ACAAAAATTA AGATTATTTA TTGAAGTAGA TATTTTTATC TTAATATTCA TGCTTAAATT   
  
  
- AGAAATCATT AAAATTTTGA AAATTTTTTA AAAGACAAAG AATTGTCCAA CAATAAGATT AATATTATTT   
  
  
- AAAATATCTT GATCTCTTCT TAAGATACTG ACATATAATA ATTAATTATT AAAAATTTTA TTTTTTATTA   
  
  
- ATATAATAAA TTACAAAAAA AGATATTTTA TTTTTAAAAT ACTATAAAAA TAAATTTTAA AAAAATGTTT   
  
  
- ATTCTATGAT AGTTATGTAT CTAGTTGAAG TGGAGAAATG TAAAGCTGAA ATTCTTGACC TCTCCAATTG   
  
  
- ATCAATATTG TCTATACTTT GATTTTGAAT AGATGACCGA TTGCAAGAAT GGCCCTAATG CATTAAAAAT   
  
  
- CCATTTTATG AATATATATA TATATATATA ATAAGCTAAT ACCTCATATT ATAATAACCC ATTATGTATA   
  
  
- AACACATAGT CTTATGGACT TGACTCTATT TTTTTAGCTA GATCCACATA AATTAAATAG AAACATTATT   
  
  
- ATCATTATTA TTATTTAAAA TAAGAAAATA TGAAAGAAAA TAACAATATT CTTTGAAATT TTTACTAGCT   
  
  
- TGGCTAAGTA CATAAGTATA AACGCAATGA TGAACCATTG TTGAACATAA ATATATAATG AACACAATAA   
  
  
- AAAAATTAAT AATAATAAAT ATTAGAGCA

+     Unnamed\_\_2

| Site Name | Organism | Position | Strand | Matrix score. | sequence | function |
| --- | --- | --- | --- | --- | --- | --- |
| Unnamed\_\_2 | Petroselinum hortense | 622 | - | 9 | AACCTAACCT |  |

> 2018/04/13 10:10:12  
+ AAGAAACTAC CCGCCGAATG TCGCTAGGTA GCTCGCTAGT TAATGTGGCG GCCGGCGTGG CTTTAAGACG   
  
  
+ GCGAGTTTCA GAGGCCCCCT CACGTCCTTA CACTTAAGTG CCGCAAGAAA TAGTAGGTAC CGACTTCTCT   
  
  
+ TCTTCTTTGA GAGAGAGAGA GAGAGAGAGA GAGAGAGAGA GAGTCTCCTC ATCATCAACA CATCGGAAAA   
  
  
+ GTTCTAAAGT TGTTGATTCC TATAAAGAAA TACCATACAT TCTGAAACGA ATCTCCCTCT ACGTTCCTAT   
  
  
+ TATTTTCCGC TCTCTCTCTC TCTCTCTTTC TCCTCCGCTT CCGCTTCTGG TCGGCAAAAG AAGCCCCCTC   
  
  
+ TCTATACTGT AGTACCAACT ACTACCGTGT GCACCGCTGT AGACCTTCAC TTTCTCCCCT ACTCTCACCC   
  
  
+ TGGATGCCCT TCCTAGCGGC AACTCATGGA AGTTCTCCAG CTGCCACTCT CCACTGCTTC TCACTATCCC   
  
  
+ ACGCCCGCCA AGTGCACCCC GCAGCAGAAG TCCAGTTGAT TCAACTGCCG TGCCGACATA GACTGCCACC   
  
  
+ TGTTCTTTTT ATTCTCCGCG CACACTCTGG TCTGCTCGTC TCATGGATTA GGGTGACTGG TAGGTTGGGT   
  
  
+ TGGGTCGGGA AGTGCAGTCA ATAAAACCTA GACAGCATCA GTTGCTTGGA GTACCCTCTC ACTTCTGGCA   
  
  
+ GGACGAGGGT ACACACCAGG CGAGCTGAGC CAACGATAGA GATTAATGGT TAAAAATAAT TTTTATTAAT   
  
  
+ AAAATAATAT TGTTTTTAAT TCTAATAAAT AACTTCATCT ATAAAAATAG AATTATAAGT ACGAATTTAA   
  
  
+ TCTTTAGTAA TTTTAAAACT TTTAAAAAAT TTTCTGTTTC TTAACAGGTT GTTATTCTAA TTATAATAAA   
  
  
+ TTTTATAGAA CTAGAGAAGA ATTCTATGAC TGTATATTAT TAATTAATAA TTTTTAAAAT AAAAAATAAT   
  
  
+ TATATTATTT AATGTTTTTT TCTATAAAAT AAAAATTTTA TGATATTTTT ATTTAAAATT TTTTTACAAA   
  
  
+ TAAGATACTA TCAATACATA GATCAACTTC ACCTCTTTAC ATTTCGACTT TAAGAACTGG AGAGGTTAAC   
  
  
+ TAGTTATAAC AGATATGAAA CTAAAACTTA TCTACTGGCT AACGTTCTTA CCGGGATTAC GTAATTTTTA   
  
  
+ GGTAAAATAC TTATATATAT ATATATATAT TATTCGATTA TGGAGTATAA TATTATTGGG TAATACATAT   
  
  
+ TTGTGTATCA GAATACCTGA ACTGAGATAA AAAAATCGAT CTAGGTGTAT TTAATTTATC TTTGTAATAA   
  
  
+ TAGTAATAAT AATAAATTTT ATTCTTTTAT ACTTTCTTTT ATTGTTATAA GAAACTTTAA AAATGATCGA   
  
  
+ ACCGATTCAT GTATTCATAT TTGCGTTACT ACTTGGTAAC AACTTGTATT TATATATTAC TTGTGTTATT   
  
  
+ TTTTTAATTA TTATTATTTA TAATCTCGT  

- TTCTTTGATG GGCGGCTTAC AGCGATCCAT CGAGCGATCA ATTACACCGC CGGCCGCACC GAAATTCTGC   
  
  
- CGCTCAAAGT CTCCGGGGGA GTGCAGGAAT GTGAATTCAC GGCGTTCTTT ATCATCCATG GCTGAAGAGA   
  
  
- AGAAGAAACT CTCTCTCTCT CTCTCTCTCT CTCTCTCTCT CTCAGAGGAG TAGTAGTTGT GTAGCCTTTT   
  
  
- CAAGATTTCA ACAACTAAGG ATATTTCTTT ATGGTATGTA AGACTTTGCT TAGAGGGAGA TGCAAGGATA   
  
  
- ATAAAAGGCG AGAGAGAGAG AGAGAGAAAG AGGAGGCGAA GGCGAAGACC AGCCGTTTTC TTCGGGGGAG   
  
  
- AGATATGACA TCATGGTTGA TGATGGCACA CGTGGCGACA TCTGGAAGTG AAAGAGGGGA TGAGAGTGGG   
  
  
- ACCTACGGGA AGGATCGCCG TTGAGTACCT TCAAGAGGTC GACGGTGAGA GGTGACGAAG AGTGATAGGG   
  
  
- TGCGGGCGGT TCACGTGGGG CGTCGTCTTC AGGTCAACTA AGTTGACGGC ACGGCTGTAT CTGACGGTGG   
  
  
- ACAAGAAAAA TAAGAGGCGC GTGTGAGACC AGACGAGCAG AGTACCTAAT CCCACTGACC ATCCAACCCA   
  
  
- ACCCAGCCCT TCACGTCAGT TATTTTGGAT CTGTCGTAGT CAACGAACCT CATGGGAGAG TGAAGACCGT   
  
  
- CCTGCTCCCA TGTGTGGTCC GCTCGACTCG GTTGCTATCT CTAATTACCA ATTTTTATTA AAAATAATTA   
  
  
- TTTTATTATA ACAAAAATTA AGATTATTTA TTGAAGTAGA TATTTTTATC TTAATATTCA TGCTTAAATT   
  
  
- AGAAATCATT AAAATTTTGA AAATTTTTTA AAAGACAAAG AATTGTCCAA CAATAAGATT AATATTATTT   
  
  
- AAAATATCTT GATCTCTTCT TAAGATACTG ACATATAATA ATTAATTATT AAAAATTTTA TTTTTTATTA   
  
  
- ATATAATAAA TTACAAAAAA AGATATTTTA TTTTTAAAAT ACTATAAAAA TAAATTTTAA AAAAATGTTT   
  
  
- ATTCTATGAT AGTTATGTAT CTAGTTGAAG TGGAGAAATG TAAAGCTGAA ATTCTTGACC TCTCCAATTG   
  
  
- ATCAATATTG TCTATACTTT GATTTTGAAT AGATGACCGA TTGCAAGAAT GGCCCTAATG CATTAAAAAT   
  
  
- CCATTTTATG AATATATATA TATATATATA ATAAGCTAAT ACCTCATATT ATAATAACCC ATTATGTATA   
  
  
- AACACATAGT CTTATGGACT TGACTCTATT TTTTTAGCTA GATCCACATA AATTAAATAG AAACATTATT   
  
  
- ATCATTATTA TTATTTAAAA TAAGAAAATA TGAAAGAAAA TAACAATATT CTTTGAAATT TTTACTAGCT   
  
  
- TGGCTAAGTA CATAAGTATA AACGCAATGA TGAACCATTG TTGAACATAA ATATATAATG AACACAATAA   
  
  
- AAAAATTAAT AATAATAAAT ATTAGAGCA

+     Unnamed\_\_3

| Site Name | Organism | Position | Strand | Matrix score. | sequence | function |
| --- | --- | --- | --- | --- | --- | --- |
| Unnamed\_\_3 | Zea mays | 56 | + | 5 | CGTGG |  |
| Unnamed\_\_3 | Zea mays | 489 | - | 5 | CGTGG |  |

> 2018/04/13 10:10:12  
+ AAGAAACTAC CCGCCGAATG TCGCTAGGTA GCTCGCTAGT TAATGTGGCG GCCGGCGTGG CTTTAAGACG   
  
  
+ GCGAGTTTCA GAGGCCCCCT CACGTCCTTA CACTTAAGTG CCGCAAGAAA TAGTAGGTAC CGACTTCTCT   
  
  
+ TCTTCTTTGA GAGAGAGAGA GAGAGAGAGA GAGAGAGAGA GAGTCTCCTC ATCATCAACA CATCGGAAAA   
  
  
+ GTTCTAAAGT TGTTGATTCC TATAAAGAAA TACCATACAT TCTGAAACGA ATCTCCCTCT ACGTTCCTAT   
  
  
+ TATTTTCCGC TCTCTCTCTC TCTCTCTTTC TCCTCCGCTT CCGCTTCTGG TCGGCAAAAG AAGCCCCCTC   
  
  
+ TCTATACTGT AGTACCAACT ACTACCGTGT GCACCGCTGT AGACCTTCAC TTTCTCCCCT ACTCTCACCC   
  
  
+ TGGATGCCCT TCCTAGCGGC AACTCATGGA AGTTCTCCAG CTGCCACTCT CCACTGCTTC TCACTATCCC   
  
  
+ ACGCCCGCCA AGTGCACCCC GCAGCAGAAG TCCAGTTGAT TCAACTGCCG TGCCGACATA GACTGCCACC   
  
  
+ TGTTCTTTTT ATTCTCCGCG CACACTCTGG TCTGCTCGTC TCATGGATTA GGGTGACTGG TAGGTTGGGT   
  
  
+ TGGGTCGGGA AGTGCAGTCA ATAAAACCTA GACAGCATCA GTTGCTTGGA GTACCCTCTC ACTTCTGGCA   
  
  
+ GGACGAGGGT ACACACCAGG CGAGCTGAGC CAACGATAGA GATTAATGGT TAAAAATAAT TTTTATTAAT   
  
  
+ AAAATAATAT TGTTTTTAAT TCTAATAAAT AACTTCATCT ATAAAAATAG AATTATAAGT ACGAATTTAA   
  
  
+ TCTTTAGTAA TTTTAAAACT TTTAAAAAAT TTTCTGTTTC TTAACAGGTT GTTATTCTAA TTATAATAAA   
  
  
+ TTTTATAGAA CTAGAGAAGA ATTCTATGAC TGTATATTAT TAATTAATAA TTTTTAAAAT AAAAAATAAT   
  
  
+ TATATTATTT AATGTTTTTT TCTATAAAAT AAAAATTTTA TGATATTTTT ATTTAAAATT TTTTTACAAA   
  
  
+ TAAGATACTA TCAATACATA GATCAACTTC ACCTCTTTAC ATTTCGACTT TAAGAACTGG AGAGGTTAAC   
  
  
+ TAGTTATAAC AGATATGAAA CTAAAACTTA TCTACTGGCT AACGTTCTTA CCGGGATTAC GTAATTTTTA   
  
  
+ GGTAAAATAC TTATATATAT ATATATATAT TATTCGATTA TGGAGTATAA TATTATTGGG TAATACATAT   
  
  
+ TTGTGTATCA GAATACCTGA ACTGAGATAA AAAAATCGAT CTAGGTGTAT TTAATTTATC TTTGTAATAA   
  
  
+ TAGTAATAAT AATAAATTTT ATTCTTTTAT ACTTTCTTTT ATTGTTATAA GAAACTTTAA AAATGATCGA   
  
  
+ ACCGATTCAT GTATTCATAT TTGCGTTACT ACTTGGTAAC AACTTGTATT TATATATTAC TTGTGTTATT   
  
  
+ TTTTTAATTA TTATTATTTA TAATCTCGT  

- TTCTTTGATG GGCGGCTTAC AGCGATCCAT CGAGCGATCA ATTACACCGC CGGCCGCACC GAAATTCTGC   
  
  
- CGCTCAAAGT CTCCGGGGGA GTGCAGGAAT GTGAATTCAC GGCGTTCTTT ATCATCCATG GCTGAAGAGA   
  
  
- AGAAGAAACT CTCTCTCTCT CTCTCTCTCT CTCTCTCTCT CTCAGAGGAG TAGTAGTTGT GTAGCCTTTT   
  
  
- CAAGATTTCA ACAACTAAGG ATATTTCTTT ATGGTATGTA AGACTTTGCT TAGAGGGAGA TGCAAGGATA   
  
  
- ATAAAAGGCG AGAGAGAGAG AGAGAGAAAG AGGAGGCGAA GGCGAAGACC AGCCGTTTTC TTCGGGGGAG   
  
  
- AGATATGACA TCATGGTTGA TGATGGCACA CGTGGCGACA TCTGGAAGTG AAAGAGGGGA TGAGAGTGGG   
  
  
- ACCTACGGGA AGGATCGCCG TTGAGTACCT TCAAGAGGTC GACGGTGAGA GGTGACGAAG AGTGATAGGG   
  
  
- TGCGGGCGGT TCACGTGGGG CGTCGTCTTC AGGTCAACTA AGTTGACGGC ACGGCTGTAT CTGACGGTGG   
  
  
- ACAAGAAAAA TAAGAGGCGC GTGTGAGACC AGACGAGCAG AGTACCTAAT CCCACTGACC ATCCAACCCA   
  
  
- ACCCAGCCCT TCACGTCAGT TATTTTGGAT CTGTCGTAGT CAACGAACCT CATGGGAGAG TGAAGACCGT   
  
  
- CCTGCTCCCA TGTGTGGTCC GCTCGACTCG GTTGCTATCT CTAATTACCA ATTTTTATTA AAAATAATTA   
  
  
- TTTTATTATA ACAAAAATTA AGATTATTTA TTGAAGTAGA TATTTTTATC TTAATATTCA TGCTTAAATT   
  
  
- AGAAATCATT AAAATTTTGA AAATTTTTTA AAAGACAAAG AATTGTCCAA CAATAAGATT AATATTATTT   
  
  
- AAAATATCTT GATCTCTTCT TAAGATACTG ACATATAATA ATTAATTATT AAAAATTTTA TTTTTTATTA   
  
  
- ATATAATAAA TTACAAAAAA AGATATTTTA TTTTTAAAAT ACTATAAAAA TAAATTTTAA AAAAATGTTT   
  
  
- ATTCTATGAT AGTTATGTAT CTAGTTGAAG TGGAGAAATG TAAAGCTGAA ATTCTTGACC TCTCCAATTG   
  
  
- ATCAATATTG TCTATACTTT GATTTTGAAT AGATGACCGA TTGCAAGAAT GGCCCTAATG CATTAAAAAT   
  
  
- CCATTTTATG AATATATATA TATATATATA ATAAGCTAAT ACCTCATATT ATAATAACCC ATTATGTATA   
  
  
- AACACATAGT CTTATGGACT TGACTCTATT TTTTTAGCTA GATCCACATA AATTAAATAG AAACATTATT   
  
  
- ATCATTATTA TTATTTAAAA TAAGAAAATA TGAAAGAAAA TAACAATATT CTTTGAAATT TTTACTAGCT   
  
  
- TGGCTAAGTA CATAAGTATA AACGCAATGA TGAACCATTG TTGAACATAA ATATATAATG AACACAATAA   
  
  
- AAAAATTAAT AATAATAAAT ATTAGAGCA

+     Unnamed\_\_4

| Site Name | Organism | Position | Strand | Matrix score. | sequence | function |
| --- | --- | --- | --- | --- | --- | --- |
| Unnamed\_\_4 | Petroselinum hortense | 455 | + | 4 | CTCC |  |
| Unnamed\_\_4 | Petroselinum hortense | 1109 | - | 4 | CTCC |  |
| Unnamed\_\_4 | Petroselinum hortense | 404 | + | 4 | CTCC |  |
| Unnamed\_\_4 | Petroselinum hortense | 313 | + | 4 | CTCC |  |
| Unnamed\_\_4 | Petroselinum hortense | 263 | + | 4 | CTCC |  |
| Unnamed\_\_4 | Petroselinum hortense | 1232 | - | 4 | CTCC |  |
| Unnamed\_\_4 | Petroselinum hortense | 185 | + | 4 | CTCC |  |
| Unnamed\_\_4 | Petroselinum hortense | 574 | + | 4 | CTCC |  |
| Unnamed\_\_4 | Petroselinum hortense | 310 | + | 4 | CTCC |  |
| Unnamed\_\_4 | Petroselinum hortense | 469 | + | 4 | CTCC |  |
| Unnamed\_\_4 | Petroselinum hortense | 678 | - | 4 | CTCC |  |

> 2018/04/13 10:10:12  
+ AAGAAACTAC CCGCCGAATG TCGCTAGGTA GCTCGCTAGT TAATGTGGCG GCCGGCGTGG CTTTAAGACG   
  
  
+ GCGAGTTTCA GAGGCCCCCT CACGTCCTTA CACTTAAGTG CCGCAAGAAA TAGTAGGTAC CGACTTCTCT   
  
  
+ TCTTCTTTGA GAGAGAGAGA GAGAGAGAGA GAGAGAGAGA GAGTCTCCTC ATCATCAACA CATCGGAAAA   
  
  
+ GTTCTAAAGT TGTTGATTCC TATAAAGAAA TACCATACAT TCTGAAACGA ATCTCCCTCT ACGTTCCTAT   
  
  
+ TATTTTCCGC TCTCTCTCTC TCTCTCTTTC TCCTCCGCTT CCGCTTCTGG TCGGCAAAAG AAGCCCCCTC   
  
  
+ TCTATACTGT AGTACCAACT ACTACCGTGT GCACCGCTGT AGACCTTCAC TTTCTCCCCT ACTCTCACCC   
  
  
+ TGGATGCCCT TCCTAGCGGC AACTCATGGA AGTTCTCCAG CTGCCACTCT CCACTGCTTC TCACTATCCC   
  
  
+ ACGCCCGCCA AGTGCACCCC GCAGCAGAAG TCCAGTTGAT TCAACTGCCG TGCCGACATA GACTGCCACC   
  
  
+ TGTTCTTTTT ATTCTCCGCG CACACTCTGG TCTGCTCGTC TCATGGATTA GGGTGACTGG TAGGTTGGGT   
  
  
+ TGGGTCGGGA AGTGCAGTCA ATAAAACCTA GACAGCATCA GTTGCTTGGA GTACCCTCTC ACTTCTGGCA   
  
  
+ GGACGAGGGT ACACACCAGG CGAGCTGAGC CAACGATAGA GATTAATGGT TAAAAATAAT TTTTATTAAT   
  
  
+ AAAATAATAT TGTTTTTAAT TCTAATAAAT AACTTCATCT ATAAAAATAG AATTATAAGT ACGAATTTAA   
  
  
+ TCTTTAGTAA TTTTAAAACT TTTAAAAAAT TTTCTGTTTC TTAACAGGTT GTTATTCTAA TTATAATAAA   
  
  
+ TTTTATAGAA CTAGAGAAGA ATTCTATGAC TGTATATTAT TAATTAATAA TTTTTAAAAT AAAAAATAAT   
  
  
+ TATATTATTT AATGTTTTTT TCTATAAAAT AAAAATTTTA TGATATTTTT ATTTAAAATT TTTTTACAAA   
  
  
+ TAAGATACTA TCAATACATA GATCAACTTC ACCTCTTTAC ATTTCGACTT TAAGAACTGG AGAGGTTAAC   
  
  
+ TAGTTATAAC AGATATGAAA CTAAAACTTA TCTACTGGCT AACGTTCTTA CCGGGATTAC GTAATTTTTA   
  
  
+ GGTAAAATAC TTATATATAT ATATATATAT TATTCGATTA TGGAGTATAA TATTATTGGG TAATACATAT   
  
  
+ TTGTGTATCA GAATACCTGA ACTGAGATAA AAAAATCGAT CTAGGTGTAT TTAATTTATC TTTGTAATAA   
  
  
+ TAGTAATAAT AATAAATTTT ATTCTTTTAT ACTTTCTTTT ATTGTTATAA GAAACTTTAA AAATGATCGA   
  
  
+ ACCGATTCAT GTATTCATAT TTGCGTTACT ACTTGGTAAC AACTTGTATT TATATATTAC TTGTGTTATT   
  
  
+ TTTTTAATTA TTATTATTTA TAATCTCGT  

- TTCTTTGATG GGCGGCTTAC AGCGATCCAT CGAGCGATCA ATTACACCGC CGGCCGCACC GAAATTCTGC   
  
  
- CGCTCAAAGT CTCCGGGGGA GTGCAGGAAT GTGAATTCAC GGCGTTCTTT ATCATCCATG GCTGAAGAGA   
  
  
- AGAAGAAACT CTCTCTCTCT CTCTCTCTCT CTCTCTCTCT CTCAGAGGAG TAGTAGTTGT GTAGCCTTTT   
  
  
- CAAGATTTCA ACAACTAAGG ATATTTCTTT ATGGTATGTA AGACTTTGCT TAGAGGGAGA TGCAAGGATA   
  
  
- ATAAAAGGCG AGAGAGAGAG AGAGAGAAAG AGGAGGCGAA GGCGAAGACC AGCCGTTTTC TTCGGGGGAG   
  
  
- AGATATGACA TCATGGTTGA TGATGGCACA CGTGGCGACA TCTGGAAGTG AAAGAGGGGA TGAGAGTGGG   
  
  
- ACCTACGGGA AGGATCGCCG TTGAGTACCT TCAAGAGGTC GACGGTGAGA GGTGACGAAG AGTGATAGGG   
  
  
- TGCGGGCGGT TCACGTGGGG CGTCGTCTTC AGGTCAACTA AGTTGACGGC ACGGCTGTAT CTGACGGTGG   
  
  
- ACAAGAAAAA TAAGAGGCGC GTGTGAGACC AGACGAGCAG AGTACCTAAT CCCACTGACC ATCCAACCCA   
  
  
- ACCCAGCCCT TCACGTCAGT TATTTTGGAT CTGTCGTAGT CAACGAACCT CATGGGAGAG TGAAGACCGT   
  
  
- CCTGCTCCCA TGTGTGGTCC GCTCGACTCG GTTGCTATCT CTAATTACCA ATTTTTATTA AAAATAATTA   
  
  
- TTTTATTATA ACAAAAATTA AGATTATTTA TTGAAGTAGA TATTTTTATC TTAATATTCA TGCTTAAATT   
  
  
- AGAAATCATT AAAATTTTGA AAATTTTTTA AAAGACAAAG AATTGTCCAA CAATAAGATT AATATTATTT   
  
  
- AAAATATCTT GATCTCTTCT TAAGATACTG ACATATAATA ATTAATTATT AAAAATTTTA TTTTTTATTA   
  
  
- ATATAATAAA TTACAAAAAA AGATATTTTA TTTTTAAAAT ACTATAAAAA TAAATTTTAA AAAAATGTTT   
  
  
- ATTCTATGAT AGTTATGTAT CTAGTTGAAG TGGAGAAATG TAAAGCTGAA ATTCTTGACC TCTCCAATTG   
  
  
- ATCAATATTG TCTATACTTT GATTTTGAAT AGATGACCGA TTGCAAGAAT GGCCCTAATG CATTAAAAAT   
  
  
- CCATTTTATG AATATATATA TATATATATA ATAAGCTAAT ACCTCATATT ATAATAACCC ATTATGTATA   
  
  
- AACACATAGT CTTATGGACT TGACTCTATT TTTTTAGCTA GATCCACATA AATTAAATAG AAACATTATT   
  
  
- ATCATTATTA TTATTTAAAA TAAGAAAATA TGAAAGAAAA TAACAATATT CTTTGAAATT TTTACTAGCT   
  
  
- TGGCTAAGTA CATAAGTATA AACGCAATGA TGAACCATTG TTGAACATAA ATATATAATG AACACAATAA   
  
  
- AAAAATTAAT AATAATAAAT ATTAGAGCA
