# Supplementary material for: Genome-wide analysis of the WRKY gene family in drumstick (Moringa oleifera Lam.)
Source: PeerJ. 2019 Jun 10;7:e7063. doi: 10.7717/peerj.7063 (PMC6563795; doi:10.7717/peerj.7063)
Supplement: Supplemental Information 1 [file peerj-07-7063-s003.gz › MoWRKY37_plantcare.html]

Content-Type: text/html; charset=ISO-8859-1


CallMat\_Firefox


Webmaster Firefox specific output  
To save the result:
click on the frame with the right mouse button and save the source code as a text file with extension .html  
REFERENCE:PlantCARE: a database of plant cis-acting regulatory elements and a portal to tools for in silico analysis of promoter sequences.  
Lescot, M., Déhais, P., Moreau, Y., De Moor, B., Rouzé ,P.,and Rombauts, S.  
Nucleic Acids Res., Database issue(2002), 30(1):325-327.   


---

> 2018/04/13 10:10:12  
+ CTAATTTCTT TTTGAAGAGT CGCGCAGAGA GATATGTGCT TCTAGGCCTA TGGACGGCTA TTTACTGACC   
  
  
+ AAAACCGGCA TCTACTACTA CTGCTGCTGA TGACGTCGTC TATGTCTCTC ACCGAACTAA CGACCTTCCT   
  
  
+ TCATTTCTCC CTCTCTCTCT CTCTCTCTCT TCTCTTCTTT ACCTCCTCTT CCACCACTTC CTTTCTTTCC   
  
  
+ CACCAGGAAT GTCTTTTCCC GGCAGTACAC TCTGTCTTCC TCACAAACCA TTATATATAA ACATCAATCG   
  
  
+ TTTCCTGCAC GTTCGTTTAC ACTAAAGTTC GGTAGTGATA AAATAATATA GTAGTTGAAC TTTTGCTTGG   
  
  
+ GAGGTAAAAT GAAAAGAAAA AACAAATAAC TTTAGTAATT CGGGTGACTA ATGTCTCAGA CGTAATTAAT   
  
  
+ AAAAATTAAA ACCATCTTTT GCCCCGACAC AGTACAACCA ACCAAAAAGA CCAAACTTGT TTTGTACACA   
  
  
+ ACAAAACAGG ACAACGAAGT AACAAGAAAT TCTCGTCAAC AACAACTTTA GTCTAATGGC CATCCTCCCA   
  
  
+ TCCGGCTGAC TAACCAAAGT ATGGGTCTTC AACGGGAAAA TTTTGGTCTA CTCCTTTCAG GACAAATATA   
  
  
+ AGCGAATATA ATAAGAGTAC GTTTTAATCC CAAAGGTAAA AGAAAAAATA AAAGGGATCA CCTATATCAA   
  
  
+ CTCATTGTGT CACTAGGTGG GTTGAGTCGA CGGAGGGAAA TAGGCTGGGT TTTAGTACCC CTAGAGAAGA   
  
  
+ GTTTTCCCTC TCGCTCTCTC TCTCTTTAAA ATTTAAAGAA TTTTTTAAAA TACAATTAAA GTAGACCCAA   
  
  
+ AATAATTTTA AAATGAAAAA TTAATTGGGT TTTTTAATAG TTTTATAATA AAATCAGAAA TAATTTTTAT   
  
  
+ CTCGTGTATA GTTATCTCAT AGAATTAAGA TTTTTTATTA CAAAACTTAT CCTTTAAATT TACCTTACTT   
  
  
+ AACCAGTTTC AACATTAAAT CCCGGTTTTT TAATACCTAT TTTTTCCTTT ATTTCCCTAA GTTATTAAGT   
  
  
+ GTACATTATT TACGAACATA CCTTTCGTAA CTCGTACATA CCGACGGACG ATGCCACCAA CGAGACAATT   
  
  
+ ATCACGGGGT TCTTCTTCCC GGCGTGGATA TCCGATACCA GTCGGCTCCT TTAACGTTTA ACCGTGCTTT   
  
  
+ GAGAACGCGT GGAACTCAAA ATGAGAAATT GGACGTGTGA TTGGGAGACA CACCGCGGGT GTTATGGGGG   
  
  
+ TTCAGTGGGT CCTAGGGTGT GGGTCTCCCG TCATGTTTGC GGCGTGGCCC CCTCCTAGGG GCATTTGCTT   
  
  
+ TAACACTGTG CACTCCGTAT ACCTACATTA TAAAATTAAT ACTTTTAATT TTCCATAGTT AATACTTTTT   
  
  
+ AATTGCAGTG TTTCTAATGT CATAATTTTG ATATAATATA TAGCTTGACT TCGAATTTAA TTATTAATAA   
  
  
+ TTTCCTAGTG CCGTTATAAA AAGGAATAT  

- GATTAAAGAA AAACTTCTCA GCGCGTCTCT CTATACACGA AGATCCGGAT ACCTGCCGAT AAATGACTGG   
  
  
- TTTTGGCCGT AGATGATGAT GACGACGACT ACTGCAGCAG ATACAGAGAG TGGCTTGATT GCTGGAAGGA   
  
  
- AGTAAAGAGG GAGAGAGAGA GAGAGAGAGA AGAGAAGAAA TGGAGGAGAA GGTGGTGAAG GAAAGAAAGG   
  
  
- GTGGTCCTTA CAGAAAAGGG CCGTCATGTG AGACAGAAGG AGTGTTTGGT AATATATATT TGTAGTTAGC   
  
  
- AAAGGACGTG CAAGCAAATG TGATTTCAAG CCATCACTAT TTTATTATAT CATCAACTTG AAAACGAACC   
  
  
- CTCCATTTTA CTTTTCTTTT TTGTTTATTG AAATCATTAA GCCCACTGAT TACAGAGTCT GCATTAATTA   
  
  
- TTTTTAATTT TGGTAGAAAA CGGGGCTGTG TCATGTTGGT TGGTTTTTCT GGTTTGAACA AAACATGTGT   
  
  
- TGTTTTGTCC TGTTGCTTCA TTGTTCTTTA AGAGCAGTTG TTGTTGAAAT CAGATTACCG GTAGGAGGGT   
  
  
- AGGCCGACTG ATTGGTTTCA TACCCAGAAG TTGCCCTTTT AAAACCAGAT GAGGAAAGTC CTGTTTATAT   
  
  
- TCGCTTATAT TATTCTCATG CAAAATTAGG GTTTCCATTT TCTTTTTTAT TTTCCCTAGT GGATATAGTT   
  
  
- GAGTAACACA GTGATCCACC CAACTCAGCT GCCTCCCTTT ATCCGACCCA AAATCATGGG GATCTCTTCT   
  
  
- CAAAAGGGAG AGCGAGAGAG AGAGAAATTT TAAATTTCTT AAAAAATTTT ATGTTAATTT CATCTGGGTT   
  
  
- TTATTAAAAT TTTACTTTTT AATTAACCCA AAAAATTATC AAAATATTAT TTTAGTCTTT ATTAAAAATA   
  
  
- GAGCACATAT CAATAGAGTA TCTTAATTCT AAAAAATAAT GTTTTGAATA GGAAATTTAA ATGGAATGAA   
  
  
- TTGGTCAAAG TTGTAATTTA GGGCCAAAAA ATTATGGATA AAAAAGGAAA TAAAGGGATT CAATAATTCA   
  
  
- CATGTAATAA ATGCTTGTAT GGAAAGCATT GAGCATGTAT GGCTGCCTGC TACGGTGGTT GCTCTGTTAA   
  
  
- TAGTGCCCCA AGAAGAAGGG CCGCACCTAT AGGCTATGGT CAGCCGAGGA AATTGCAAAT TGGCACGAAA   
  
  
- CTCTTGCGCA CCTTGAGTTT TACTCTTTAA CCTGCACACT AACCCTCTGT GTGGCGCCCA CAATACCCCC   
  
  
- AAGTCACCCA GGATCCCACA CCCAGAGGGC AGTACAAACG CCGCACCGGG GGAGGATCCC CGTAAACGAA   
  
  
- ATTGTGACAC GTGAGGCATA TGGATGTAAT ATTTTAATTA TGAAAATTAA AAGGTATCAA TTATGAAAAA   
  
  
- TTAACGTCAC AAAGATTACA GTATTAAAAC TATATTATAT ATCGAACTGA AGCTTAAATT AATAATTATT   
  
  
- AAAGGATCAC GGCAATATTT TTCCTTATA

  
  
Motifs Found  

+     5UTR Py-rich stretch

| Site Name | Organism | Position | Strand | Matrix score. | sequence | function |
| --- | --- | --- | --- | --- | --- | --- |
| 5UTR Py-rich stretch | Lycopersicon esculentum | 156 | + | 13 | TTTCTCTCTCTCTC | cis-acting element conferring high transcription levels |
| 5UTR Py-rich stretch | Lycopersicon esculentum | 166 | + | 9 | TTTCTTCTCT | cis-acting element conferring high transcription levels |
| 5UTR Py-rich stretch | Lycopersicon esculentum | 144 | + | 13 | TTTCTCTCTCTCTC | cis-acting element conferring high transcription levels |
| 5UTR Py-rich stretch | Lycopersicon esculentum | 152 | + | 13 | TTTCTCTCTCTCTC | cis-acting element conferring high transcription levels |
| 5UTR Py-rich stretch | Lycopersicon esculentum | 154 | + | 13 | TTTCTCTCTCTCTC | cis-acting element conferring high transcription levels |

> 2018/04/13 10:10:12  
+ CTAATTTCTT TTTGAAGAGT CGCGCAGAGA GATATGTGCT TCTAGGCCTA TGGACGGCTA TTTACTGACC   
  
  
+ AAAACCGGCA TCTACTACTA CTGCTGCTGA TGACGTCGTC TATGTCTCTC ACCGAACTAA CGACCTTCCT   
  
  
+ TCATTTCTCC CTCTCTCTCT CTCTCTCTCT TCTCTTCTTT ACCTCCTCTT CCACCACTTC CTTTCTTTCC   
  
  
+ CACCAGGAAT GTCTTTTCCC GGCAGTACAC TCTGTCTTCC TCACAAACCA TTATATATAA ACATCAATCG   
  
  
+ TTTCCTGCAC GTTCGTTTAC ACTAAAGTTC GGTAGTGATA AAATAATATA GTAGTTGAAC TTTTGCTTGG   
  
  
+ GAGGTAAAAT GAAAAGAAAA AACAAATAAC TTTAGTAATT CGGGTGACTA ATGTCTCAGA CGTAATTAAT   
  
  
+ AAAAATTAAA ACCATCTTTT GCCCCGACAC AGTACAACCA ACCAAAAAGA CCAAACTTGT TTTGTACACA   
  
  
+ ACAAAACAGG ACAACGAAGT AACAAGAAAT TCTCGTCAAC AACAACTTTA GTCTAATGGC CATCCTCCCA   
  
  
+ TCCGGCTGAC TAACCAAAGT ATGGGTCTTC AACGGGAAAA TTTTGGTCTA CTCCTTTCAG GACAAATATA   
  
  
+ AGCGAATATA ATAAGAGTAC GTTTTAATCC CAAAGGTAAA AGAAAAAATA AAAGGGATCA CCTATATCAA   
  
  
+ CTCATTGTGT CACTAGGTGG GTTGAGTCGA CGGAGGGAAA TAGGCTGGGT TTTAGTACCC CTAGAGAAGA   
  
  
+ GTTTTCCCTC TCGCTCTCTC TCTCTTTAAA ATTTAAAGAA TTTTTTAAAA TACAATTAAA GTAGACCCAA   
  
  
+ AATAATTTTA AAATGAAAAA TTAATTGGGT TTTTTAATAG TTTTATAATA AAATCAGAAA TAATTTTTAT   
  
  
+ CTCGTGTATA GTTATCTCAT AGAATTAAGA TTTTTTATTA CAAAACTTAT CCTTTAAATT TACCTTACTT   
  
  
+ AACCAGTTTC AACATTAAAT CCCGGTTTTT TAATACCTAT TTTTTCCTTT ATTTCCCTAA GTTATTAAGT   
  
  
+ GTACATTATT TACGAACATA CCTTTCGTAA CTCGTACATA CCGACGGACG ATGCCACCAA CGAGACAATT   
  
  
+ ATCACGGGGT TCTTCTTCCC GGCGTGGATA TCCGATACCA GTCGGCTCCT TTAACGTTTA ACCGTGCTTT   
  
  
+ GAGAACGCGT GGAACTCAAA ATGAGAAATT GGACGTGTGA TTGGGAGACA CACCGCGGGT GTTATGGGGG   
  
  
+ TTCAGTGGGT CCTAGGGTGT GGGTCTCCCG TCATGTTTGC GGCGTGGCCC CCTCCTAGGG GCATTTGCTT   
  
  
+ TAACACTGTG CACTCCGTAT ACCTACATTA TAAAATTAAT ACTTTTAATT TTCCATAGTT AATACTTTTT   
  
  
+ AATTGCAGTG TTTCTAATGT CATAATTTTG ATATAATATA TAGCTTGACT TCGAATTTAA TTATTAATAA   
  
  
+ TTTCCTAGTG CCGTTATAAA AAGGAATAT  

- GATTAAAGAA AAACTTCTCA GCGCGTCTCT CTATACACGA AGATCCGGAT ACCTGCCGAT AAATGACTGG   
  
  
- TTTTGGCCGT AGATGATGAT GACGACGACT ACTGCAGCAG ATACAGAGAG TGGCTTGATT GCTGGAAGGA   
  
  
- AGTAAAGAGG GAGAGAGAGA GAGAGAGAGA AGAGAAGAAA TGGAGGAGAA GGTGGTGAAG GAAAGAAAGG   
  
  
- GTGGTCCTTA CAGAAAAGGG CCGTCATGTG AGACAGAAGG AGTGTTTGGT AATATATATT TGTAGTTAGC   
  
  
- AAAGGACGTG CAAGCAAATG TGATTTCAAG CCATCACTAT TTTATTATAT CATCAACTTG AAAACGAACC   
  
  
- CTCCATTTTA CTTTTCTTTT TTGTTTATTG AAATCATTAA GCCCACTGAT TACAGAGTCT GCATTAATTA   
  
  
- TTTTTAATTT TGGTAGAAAA CGGGGCTGTG TCATGTTGGT TGGTTTTTCT GGTTTGAACA AAACATGTGT   
  
  
- TGTTTTGTCC TGTTGCTTCA TTGTTCTTTA AGAGCAGTTG TTGTTGAAAT CAGATTACCG GTAGGAGGGT   
  
  
- AGGCCGACTG ATTGGTTTCA TACCCAGAAG TTGCCCTTTT AAAACCAGAT GAGGAAAGTC CTGTTTATAT   
  
  
- TCGCTTATAT TATTCTCATG CAAAATTAGG GTTTCCATTT TCTTTTTTAT TTTCCCTAGT GGATATAGTT   
  
  
- GAGTAACACA GTGATCCACC CAACTCAGCT GCCTCCCTTT ATCCGACCCA AAATCATGGG GATCTCTTCT   
  
  
- CAAAAGGGAG AGCGAGAGAG AGAGAAATTT TAAATTTCTT AAAAAATTTT ATGTTAATTT CATCTGGGTT   
  
  
- TTATTAAAAT TTTACTTTTT AATTAACCCA AAAAATTATC AAAATATTAT TTTAGTCTTT ATTAAAAATA   
  
  
- GAGCACATAT CAATAGAGTA TCTTAATTCT AAAAAATAAT GTTTTGAATA GGAAATTTAA ATGGAATGAA   
  
  
- TTGGTCAAAG TTGTAATTTA GGGCCAAAAA ATTATGGATA AAAAAGGAAA TAAAGGGATT CAATAATTCA   
  
  
- CATGTAATAA ATGCTTGTAT GGAAAGCATT GAGCATGTAT GGCTGCCTGC TACGGTGGTT GCTCTGTTAA   
  
  
- TAGTGCCCCA AGAAGAAGGG CCGCACCTAT AGGCTATGGT CAGCCGAGGA AATTGCAAAT TGGCACGAAA   
  
  
- CTCTTGCGCA CCTTGAGTTT TACTCTTTAA CCTGCACACT AACCCTCTGT GTGGCGCCCA CAATACCCCC   
  
  
- AAGTCACCCA GGATCCCACA CCCAGAGGGC AGTACAAACG CCGCACCGGG GGAGGATCCC CGTAAACGAA   
  
  
- ATTGTGACAC GTGAGGCATA TGGATGTAAT ATTTTAATTA TGAAAATTAA AAGGTATCAA TTATGAAAAA   
  
  
- TTAACGTCAC AAAGATTACA GTATTAAAAC TATATTATAT ATCGAACTGA AGCTTAAATT AATAATTATT   
  
  
- AAAGGATCAC GGCAATATTT TTCCTTATA

+     A-box

| Site Name | Organism | Position | Strand | Matrix score. | sequence | function |
| --- | --- | --- | --- | --- | --- | --- |
| A-box | Petroselinum crispum | 52 | - | 6 | CCGTCC | cis-acting regulatory element |

> 2018/04/13 10:10:12  
+ CTAATTTCTT TTTGAAGAGT CGCGCAGAGA GATATGTGCT TCTAGGCCTA TGGACGGCTA TTTACTGACC   
  
  
+ AAAACCGGCA TCTACTACTA CTGCTGCTGA TGACGTCGTC TATGTCTCTC ACCGAACTAA CGACCTTCCT   
  
  
+ TCATTTCTCC CTCTCTCTCT CTCTCTCTCT TCTCTTCTTT ACCTCCTCTT CCACCACTTC CTTTCTTTCC   
  
  
+ CACCAGGAAT GTCTTTTCCC GGCAGTACAC TCTGTCTTCC TCACAAACCA TTATATATAA ACATCAATCG   
  
  
+ TTTCCTGCAC GTTCGTTTAC ACTAAAGTTC GGTAGTGATA AAATAATATA GTAGTTGAAC TTTTGCTTGG   
  
  
+ GAGGTAAAAT GAAAAGAAAA AACAAATAAC TTTAGTAATT CGGGTGACTA ATGTCTCAGA CGTAATTAAT   
  
  
+ AAAAATTAAA ACCATCTTTT GCCCCGACAC AGTACAACCA ACCAAAAAGA CCAAACTTGT TTTGTACACA   
  
  
+ ACAAAACAGG ACAACGAAGT AACAAGAAAT TCTCGTCAAC AACAACTTTA GTCTAATGGC CATCCTCCCA   
  
  
+ TCCGGCTGAC TAACCAAAGT ATGGGTCTTC AACGGGAAAA TTTTGGTCTA CTCCTTTCAG GACAAATATA   
  
  
+ AGCGAATATA ATAAGAGTAC GTTTTAATCC CAAAGGTAAA AGAAAAAATA AAAGGGATCA CCTATATCAA   
  
  
+ CTCATTGTGT CACTAGGTGG GTTGAGTCGA CGGAGGGAAA TAGGCTGGGT TTTAGTACCC CTAGAGAAGA   
  
  
+ GTTTTCCCTC TCGCTCTCTC TCTCTTTAAA ATTTAAAGAA TTTTTTAAAA TACAATTAAA GTAGACCCAA   
  
  
+ AATAATTTTA AAATGAAAAA TTAATTGGGT TTTTTAATAG TTTTATAATA AAATCAGAAA TAATTTTTAT   
  
  
+ CTCGTGTATA GTTATCTCAT AGAATTAAGA TTTTTTATTA CAAAACTTAT CCTTTAAATT TACCTTACTT   
  
  
+ AACCAGTTTC AACATTAAAT CCCGGTTTTT TAATACCTAT TTTTTCCTTT ATTTCCCTAA GTTATTAAGT   
  
  
+ GTACATTATT TACGAACATA CCTTTCGTAA CTCGTACATA CCGACGGACG ATGCCACCAA CGAGACAATT   
  
  
+ ATCACGGGGT TCTTCTTCCC GGCGTGGATA TCCGATACCA GTCGGCTCCT TTAACGTTTA ACCGTGCTTT   
  
  
+ GAGAACGCGT GGAACTCAAA ATGAGAAATT GGACGTGTGA TTGGGAGACA CACCGCGGGT GTTATGGGGG   
  
  
+ TTCAGTGGGT CCTAGGGTGT GGGTCTCCCG TCATGTTTGC GGCGTGGCCC CCTCCTAGGG GCATTTGCTT   
  
  
+ TAACACTGTG CACTCCGTAT ACCTACATTA TAAAATTAAT ACTTTTAATT TTCCATAGTT AATACTTTTT   
  
  
+ AATTGCAGTG TTTCTAATGT CATAATTTTG ATATAATATA TAGCTTGACT TCGAATTTAA TTATTAATAA   
  
  
+ TTTCCTAGTG CCGTTATAAA AAGGAATAT  

- GATTAAAGAA AAACTTCTCA GCGCGTCTCT CTATACACGA AGATCCGGAT ACCTGCCGAT AAATGACTGG   
  
  
- TTTTGGCCGT AGATGATGAT GACGACGACT ACTGCAGCAG ATACAGAGAG TGGCTTGATT GCTGGAAGGA   
  
  
- AGTAAAGAGG GAGAGAGAGA GAGAGAGAGA AGAGAAGAAA TGGAGGAGAA GGTGGTGAAG GAAAGAAAGG   
  
  
- GTGGTCCTTA CAGAAAAGGG CCGTCATGTG AGACAGAAGG AGTGTTTGGT AATATATATT TGTAGTTAGC   
  
  
- AAAGGACGTG CAAGCAAATG TGATTTCAAG CCATCACTAT TTTATTATAT CATCAACTTG AAAACGAACC   
  
  
- CTCCATTTTA CTTTTCTTTT TTGTTTATTG AAATCATTAA GCCCACTGAT TACAGAGTCT GCATTAATTA   
  
  
- TTTTTAATTT TGGTAGAAAA CGGGGCTGTG TCATGTTGGT TGGTTTTTCT GGTTTGAACA AAACATGTGT   
  
  
- TGTTTTGTCC TGTTGCTTCA TTGTTCTTTA AGAGCAGTTG TTGTTGAAAT CAGATTACCG GTAGGAGGGT   
  
  
- AGGCCGACTG ATTGGTTTCA TACCCAGAAG TTGCCCTTTT AAAACCAGAT GAGGAAAGTC CTGTTTATAT   
  
  
- TCGCTTATAT TATTCTCATG CAAAATTAGG GTTTCCATTT TCTTTTTTAT TTTCCCTAGT GGATATAGTT   
  
  
- GAGTAACACA GTGATCCACC CAACTCAGCT GCCTCCCTTT ATCCGACCCA AAATCATGGG GATCTCTTCT   
  
  
- CAAAAGGGAG AGCGAGAGAG AGAGAAATTT TAAATTTCTT AAAAAATTTT ATGTTAATTT CATCTGGGTT   
  
  
- TTATTAAAAT TTTACTTTTT AATTAACCCA AAAAATTATC AAAATATTAT TTTAGTCTTT ATTAAAAATA   
  
  
- GAGCACATAT CAATAGAGTA TCTTAATTCT AAAAAATAAT GTTTTGAATA GGAAATTTAA ATGGAATGAA   
  
  
- TTGGTCAAAG TTGTAATTTA GGGCCAAAAA ATTATGGATA AAAAAGGAAA TAAAGGGATT CAATAATTCA   
  
  
- CATGTAATAA ATGCTTGTAT GGAAAGCATT GAGCATGTAT GGCTGCCTGC TACGGTGGTT GCTCTGTTAA   
  
  
- TAGTGCCCCA AGAAGAAGGG CCGCACCTAT AGGCTATGGT CAGCCGAGGA AATTGCAAAT TGGCACGAAA   
  
  
- CTCTTGCGCA CCTTGAGTTT TACTCTTTAA CCTGCACACT AACCCTCTGT GTGGCGCCCA CAATACCCCC   
  
  
- AAGTCACCCA GGATCCCACA CCCAGAGGGC AGTACAAACG CCGCACCGGG GGAGGATCCC CGTAAACGAA   
  
  
- ATTGTGACAC GTGAGGCATA TGGATGTAAT ATTTTAATTA TGAAAATTAA AAGGTATCAA TTATGAAAAA   
  
  
- TTAACGTCAC AAAGATTACA GTATTAAAAC TATATTATAT ATCGAACTGA AGCTTAAATT AATAATTATT   
  
  
- AAAGGATCAC GGCAATATTT TTCCTTATA

+     AAGAA-motif

| Site Name | Organism | Position | Strand | Matrix score. | sequence | function |
| --- | --- | --- | --- | --- | --- | --- |
| AAGAA-motif | Avena sativa | 202 | - | 9 | gGTAAAGAAA |  |
| AAGAA-motif | Avena sativa | 203 | - | 7 | GAAAGAA |  |
| AAGAA-motif | Avena sativa | 174 | - | 9 | gGTAAAGAAA |  |

> 2018/04/13 10:10:12  
+ CTAATTTCTT TTTGAAGAGT CGCGCAGAGA GATATGTGCT TCTAGGCCTA TGGACGGCTA TTTACTGACC   
  
  
+ AAAACCGGCA TCTACTACTA CTGCTGCTGA TGACGTCGTC TATGTCTCTC ACCGAACTAA CGACCTTCCT   
  
  
+ TCATTTCTCC CTCTCTCTCT CTCTCTCTCT TCTCTTCTTT ACCTCCTCTT CCACCACTTC CTTTCTTTCC   
  
  
+ CACCAGGAAT GTCTTTTCCC GGCAGTACAC TCTGTCTTCC TCACAAACCA TTATATATAA ACATCAATCG   
  
  
+ TTTCCTGCAC GTTCGTTTAC ACTAAAGTTC GGTAGTGATA AAATAATATA GTAGTTGAAC TTTTGCTTGG   
  
  
+ GAGGTAAAAT GAAAAGAAAA AACAAATAAC TTTAGTAATT CGGGTGACTA ATGTCTCAGA CGTAATTAAT   
  
  
+ AAAAATTAAA ACCATCTTTT GCCCCGACAC AGTACAACCA ACCAAAAAGA CCAAACTTGT TTTGTACACA   
  
  
+ ACAAAACAGG ACAACGAAGT AACAAGAAAT TCTCGTCAAC AACAACTTTA GTCTAATGGC CATCCTCCCA   
  
  
+ TCCGGCTGAC TAACCAAAGT ATGGGTCTTC AACGGGAAAA TTTTGGTCTA CTCCTTTCAG GACAAATATA   
  
  
+ AGCGAATATA ATAAGAGTAC GTTTTAATCC CAAAGGTAAA AGAAAAAATA AAAGGGATCA CCTATATCAA   
  
  
+ CTCATTGTGT CACTAGGTGG GTTGAGTCGA CGGAGGGAAA TAGGCTGGGT TTTAGTACCC CTAGAGAAGA   
  
  
+ GTTTTCCCTC TCGCTCTCTC TCTCTTTAAA ATTTAAAGAA TTTTTTAAAA TACAATTAAA GTAGACCCAA   
  
  
+ AATAATTTTA AAATGAAAAA TTAATTGGGT TTTTTAATAG TTTTATAATA AAATCAGAAA TAATTTTTAT   
  
  
+ CTCGTGTATA GTTATCTCAT AGAATTAAGA TTTTTTATTA CAAAACTTAT CCTTTAAATT TACCTTACTT   
  
  
+ AACCAGTTTC AACATTAAAT CCCGGTTTTT TAATACCTAT TTTTTCCTTT ATTTCCCTAA GTTATTAAGT   
  
  
+ GTACATTATT TACGAACATA CCTTTCGTAA CTCGTACATA CCGACGGACG ATGCCACCAA CGAGACAATT   
  
  
+ ATCACGGGGT TCTTCTTCCC GGCGTGGATA TCCGATACCA GTCGGCTCCT TTAACGTTTA ACCGTGCTTT   
  
  
+ GAGAACGCGT GGAACTCAAA ATGAGAAATT GGACGTGTGA TTGGGAGACA CACCGCGGGT GTTATGGGGG   
  
  
+ TTCAGTGGGT CCTAGGGTGT GGGTCTCCCG TCATGTTTGC GGCGTGGCCC CCTCCTAGGG GCATTTGCTT   
  
  
+ TAACACTGTG CACTCCGTAT ACCTACATTA TAAAATTAAT ACTTTTAATT TTCCATAGTT AATACTTTTT   
  
  
+ AATTGCAGTG TTTCTAATGT CATAATTTTG ATATAATATA TAGCTTGACT TCGAATTTAA TTATTAATAA   
  
  
+ TTTCCTAGTG CCGTTATAAA AAGGAATAT  

- GATTAAAGAA AAACTTCTCA GCGCGTCTCT CTATACACGA AGATCCGGAT ACCTGCCGAT AAATGACTGG   
  
  
- TTTTGGCCGT AGATGATGAT GACGACGACT ACTGCAGCAG ATACAGAGAG TGGCTTGATT GCTGGAAGGA   
  
  
- AGTAAAGAGG GAGAGAGAGA GAGAGAGAGA AGAGAAGAAA TGGAGGAGAA GGTGGTGAAG GAAAGAAAGG   
  
  
- GTGGTCCTTA CAGAAAAGGG CCGTCATGTG AGACAGAAGG AGTGTTTGGT AATATATATT TGTAGTTAGC   
  
  
- AAAGGACGTG CAAGCAAATG TGATTTCAAG CCATCACTAT TTTATTATAT CATCAACTTG AAAACGAACC   
  
  
- CTCCATTTTA CTTTTCTTTT TTGTTTATTG AAATCATTAA GCCCACTGAT TACAGAGTCT GCATTAATTA   
  
  
- TTTTTAATTT TGGTAGAAAA CGGGGCTGTG TCATGTTGGT TGGTTTTTCT GGTTTGAACA AAACATGTGT   
  
  
- TGTTTTGTCC TGTTGCTTCA TTGTTCTTTA AGAGCAGTTG TTGTTGAAAT CAGATTACCG GTAGGAGGGT   
  
  
- AGGCCGACTG ATTGGTTTCA TACCCAGAAG TTGCCCTTTT AAAACCAGAT GAGGAAAGTC CTGTTTATAT   
  
  
- TCGCTTATAT TATTCTCATG CAAAATTAGG GTTTCCATTT TCTTTTTTAT TTTCCCTAGT GGATATAGTT   
  
  
- GAGTAACACA GTGATCCACC CAACTCAGCT GCCTCCCTTT ATCCGACCCA AAATCATGGG GATCTCTTCT   
  
  
- CAAAAGGGAG AGCGAGAGAG AGAGAAATTT TAAATTTCTT AAAAAATTTT ATGTTAATTT CATCTGGGTT   
  
  
- TTATTAAAAT TTTACTTTTT AATTAACCCA AAAAATTATC AAAATATTAT TTTAGTCTTT ATTAAAAATA   
  
  
- GAGCACATAT CAATAGAGTA TCTTAATTCT AAAAAATAAT GTTTTGAATA GGAAATTTAA ATGGAATGAA   
  
  
- TTGGTCAAAG TTGTAATTTA GGGCCAAAAA ATTATGGATA AAAAAGGAAA TAAAGGGATT CAATAATTCA   
  
  
- CATGTAATAA ATGCTTGTAT GGAAAGCATT GAGCATGTAT GGCTGCCTGC TACGGTGGTT GCTCTGTTAA   
  
  
- TAGTGCCCCA AGAAGAAGGG CCGCACCTAT AGGCTATGGT CAGCCGAGGA AATTGCAAAT TGGCACGAAA   
  
  
- CTCTTGCGCA CCTTGAGTTT TACTCTTTAA CCTGCACACT AACCCTCTGT GTGGCGCCCA CAATACCCCC   
  
  
- AAGTCACCCA GGATCCCACA CCCAGAGGGC AGTACAAACG CCGCACCGGG GGAGGATCCC CGTAAACGAA   
  
  
- ATTGTGACAC GTGAGGCATA TGGATGTAAT ATTTTAATTA TGAAAATTAA AAGGTATCAA TTATGAAAAA   
  
  
- TTAACGTCAC AAAGATTACA GTATTAAAAC TATATTATAT ATCGAACTGA AGCTTAAATT AATAATTATT   
  
  
- AAAGGATCAC GGCAATATTT TTCCTTATA

+     ABRE

| Site Name | Organism | Position | Strand | Matrix score. | sequence | function |
| --- | --- | --- | --- | --- | --- | --- |
| ABRE | Hordeum vulgare | 286 | - | 9 | CGTACGTGCA | cis-acting element involved in the abscisic acid responsiveness |
| ABRE | Oryza sativa | 1299 | + | 9 | GCCGCGTGGC | cis-acting element involved in the abscisic acid responsiveness |

> 2018/04/13 10:10:12  
+ CTAATTTCTT TTTGAAGAGT CGCGCAGAGA GATATGTGCT TCTAGGCCTA TGGACGGCTA TTTACTGACC   
  
  
+ AAAACCGGCA TCTACTACTA CTGCTGCTGA TGACGTCGTC TATGTCTCTC ACCGAACTAA CGACCTTCCT   
  
  
+ TCATTTCTCC CTCTCTCTCT CTCTCTCTCT TCTCTTCTTT ACCTCCTCTT CCACCACTTC CTTTCTTTCC   
  
  
+ CACCAGGAAT GTCTTTTCCC GGCAGTACAC TCTGTCTTCC TCACAAACCA TTATATATAA ACATCAATCG   
  
  
+ TTTCCTGCAC GTTCGTTTAC ACTAAAGTTC GGTAGTGATA AAATAATATA GTAGTTGAAC TTTTGCTTGG   
  
  
+ GAGGTAAAAT GAAAAGAAAA AACAAATAAC TTTAGTAATT CGGGTGACTA ATGTCTCAGA CGTAATTAAT   
  
  
+ AAAAATTAAA ACCATCTTTT GCCCCGACAC AGTACAACCA ACCAAAAAGA CCAAACTTGT TTTGTACACA   
  
  
+ ACAAAACAGG ACAACGAAGT AACAAGAAAT TCTCGTCAAC AACAACTTTA GTCTAATGGC CATCCTCCCA   
  
  
+ TCCGGCTGAC TAACCAAAGT ATGGGTCTTC AACGGGAAAA TTTTGGTCTA CTCCTTTCAG GACAAATATA   
  
  
+ AGCGAATATA ATAAGAGTAC GTTTTAATCC CAAAGGTAAA AGAAAAAATA AAAGGGATCA CCTATATCAA   
  
  
+ CTCATTGTGT CACTAGGTGG GTTGAGTCGA CGGAGGGAAA TAGGCTGGGT TTTAGTACCC CTAGAGAAGA   
  
  
+ GTTTTCCCTC TCGCTCTCTC TCTCTTTAAA ATTTAAAGAA TTTTTTAAAA TACAATTAAA GTAGACCCAA   
  
  
+ AATAATTTTA AAATGAAAAA TTAATTGGGT TTTTTAATAG TTTTATAATA AAATCAGAAA TAATTTTTAT   
  
  
+ CTCGTGTATA GTTATCTCAT AGAATTAAGA TTTTTTATTA CAAAACTTAT CCTTTAAATT TACCTTACTT   
  
  
+ AACCAGTTTC AACATTAAAT CCCGGTTTTT TAATACCTAT TTTTTCCTTT ATTTCCCTAA GTTATTAAGT   
  
  
+ GTACATTATT TACGAACATA CCTTTCGTAA CTCGTACATA CCGACGGACG ATGCCACCAA CGAGACAATT   
  
  
+ ATCACGGGGT TCTTCTTCCC GGCGTGGATA TCCGATACCA GTCGGCTCCT TTAACGTTTA ACCGTGCTTT   
  
  
+ GAGAACGCGT GGAACTCAAA ATGAGAAATT GGACGTGTGA TTGGGAGACA CACCGCGGGT GTTATGGGGG   
  
  
+ TTCAGTGGGT CCTAGGGTGT GGGTCTCCCG TCATGTTTGC GGCGTGGCCC CCTCCTAGGG GCATTTGCTT   
  
  
+ TAACACTGTG CACTCCGTAT ACCTACATTA TAAAATTAAT ACTTTTAATT TTCCATAGTT AATACTTTTT   
  
  
+ AATTGCAGTG TTTCTAATGT CATAATTTTG ATATAATATA TAGCTTGACT TCGAATTTAA TTATTAATAA   
  
  
+ TTTCCTAGTG CCGTTATAAA AAGGAATAT  

- GATTAAAGAA AAACTTCTCA GCGCGTCTCT CTATACACGA AGATCCGGAT ACCTGCCGAT AAATGACTGG   
  
  
- TTTTGGCCGT AGATGATGAT GACGACGACT ACTGCAGCAG ATACAGAGAG TGGCTTGATT GCTGGAAGGA   
  
  
- AGTAAAGAGG GAGAGAGAGA GAGAGAGAGA AGAGAAGAAA TGGAGGAGAA GGTGGTGAAG GAAAGAAAGG   
  
  
- GTGGTCCTTA CAGAAAAGGG CCGTCATGTG AGACAGAAGG AGTGTTTGGT AATATATATT TGTAGTTAGC   
  
  
- AAAGGACGTG CAAGCAAATG TGATTTCAAG CCATCACTAT TTTATTATAT CATCAACTTG AAAACGAACC   
  
  
- CTCCATTTTA CTTTTCTTTT TTGTTTATTG AAATCATTAA GCCCACTGAT TACAGAGTCT GCATTAATTA   
  
  
- TTTTTAATTT TGGTAGAAAA CGGGGCTGTG TCATGTTGGT TGGTTTTTCT GGTTTGAACA AAACATGTGT   
  
  
- TGTTTTGTCC TGTTGCTTCA TTGTTCTTTA AGAGCAGTTG TTGTTGAAAT CAGATTACCG GTAGGAGGGT   
  
  
- AGGCCGACTG ATTGGTTTCA TACCCAGAAG TTGCCCTTTT AAAACCAGAT GAGGAAAGTC CTGTTTATAT   
  
  
- TCGCTTATAT TATTCTCATG CAAAATTAGG GTTTCCATTT TCTTTTTTAT TTTCCCTAGT GGATATAGTT   
  
  
- GAGTAACACA GTGATCCACC CAACTCAGCT GCCTCCCTTT ATCCGACCCA AAATCATGGG GATCTCTTCT   
  
  
- CAAAAGGGAG AGCGAGAGAG AGAGAAATTT TAAATTTCTT AAAAAATTTT ATGTTAATTT CATCTGGGTT   
  
  
- TTATTAAAAT TTTACTTTTT AATTAACCCA AAAAATTATC AAAATATTAT TTTAGTCTTT ATTAAAAATA   
  
  
- GAGCACATAT CAATAGAGTA TCTTAATTCT AAAAAATAAT GTTTTGAATA GGAAATTTAA ATGGAATGAA   
  
  
- TTGGTCAAAG TTGTAATTTA GGGCCAAAAA ATTATGGATA AAAAAGGAAA TAAAGGGATT CAATAATTCA   
  
  
- CATGTAATAA ATGCTTGTAT GGAAAGCATT GAGCATGTAT GGCTGCCTGC TACGGTGGTT GCTCTGTTAA   
  
  
- TAGTGCCCCA AGAAGAAGGG CCGCACCTAT AGGCTATGGT CAGCCGAGGA AATTGCAAAT TGGCACGAAA   
  
  
- CTCTTGCGCA CCTTGAGTTT TACTCTTTAA CCTGCACACT AACCCTCTGT GTGGCGCCCA CAATACCCCC   
  
  
- AAGTCACCCA GGATCCCACA CCCAGAGGGC AGTACAAACG CCGCACCGGG GGAGGATCCC CGTAAACGAA   
  
  
- ATTGTGACAC GTGAGGCATA TGGATGTAAT ATTTTAATTA TGAAAATTAA AAGGTATCAA TTATGAAAAA   
  
  
- TTAACGTCAC AAAGATTACA GTATTAAAAC TATATTATAT ATCGAACTGA AGCTTAAATT AATAATTATT   
  
  
- AAAGGATCAC GGCAATATTT TTCCTTATA

+     ARE

| Site Name | Organism | Position | Strand | Matrix score. | sequence | function |
| --- | --- | --- | --- | --- | --- | --- |
| ARE | Zea mays | 255 | - | 6 | TGGTTT | cis-acting regulatory element essential for the anaerobic induction |
| ARE | Zea mays | 429 | - | 6 | TGGTTT | cis-acting regulatory element essential for the anaerobic induction |

> 2018/04/13 10:10:12  
+ CTAATTTCTT TTTGAAGAGT CGCGCAGAGA GATATGTGCT TCTAGGCCTA TGGACGGCTA TTTACTGACC   
  
  
+ AAAACCGGCA TCTACTACTA CTGCTGCTGA TGACGTCGTC TATGTCTCTC ACCGAACTAA CGACCTTCCT   
  
  
+ TCATTTCTCC CTCTCTCTCT CTCTCTCTCT TCTCTTCTTT ACCTCCTCTT CCACCACTTC CTTTCTTTCC   
  
  
+ CACCAGGAAT GTCTTTTCCC GGCAGTACAC TCTGTCTTCC TCACAAACCA TTATATATAA ACATCAATCG   
  
  
+ TTTCCTGCAC GTTCGTTTAC ACTAAAGTTC GGTAGTGATA AAATAATATA GTAGTTGAAC TTTTGCTTGG   
  
  
+ GAGGTAAAAT GAAAAGAAAA AACAAATAAC TTTAGTAATT CGGGTGACTA ATGTCTCAGA CGTAATTAAT   
  
  
+ AAAAATTAAA ACCATCTTTT GCCCCGACAC AGTACAACCA ACCAAAAAGA CCAAACTTGT TTTGTACACA   
  
  
+ ACAAAACAGG ACAACGAAGT AACAAGAAAT TCTCGTCAAC AACAACTTTA GTCTAATGGC CATCCTCCCA   
  
  
+ TCCGGCTGAC TAACCAAAGT ATGGGTCTTC AACGGGAAAA TTTTGGTCTA CTCCTTTCAG GACAAATATA   
  
  
+ AGCGAATATA ATAAGAGTAC GTTTTAATCC CAAAGGTAAA AGAAAAAATA AAAGGGATCA CCTATATCAA   
  
  
+ CTCATTGTGT CACTAGGTGG GTTGAGTCGA CGGAGGGAAA TAGGCTGGGT TTTAGTACCC CTAGAGAAGA   
  
  
+ GTTTTCCCTC TCGCTCTCTC TCTCTTTAAA ATTTAAAGAA TTTTTTAAAA TACAATTAAA GTAGACCCAA   
  
  
+ AATAATTTTA AAATGAAAAA TTAATTGGGT TTTTTAATAG TTTTATAATA AAATCAGAAA TAATTTTTAT   
  
  
+ CTCGTGTATA GTTATCTCAT AGAATTAAGA TTTTTTATTA CAAAACTTAT CCTTTAAATT TACCTTACTT   
  
  
+ AACCAGTTTC AACATTAAAT CCCGGTTTTT TAATACCTAT TTTTTCCTTT ATTTCCCTAA GTTATTAAGT   
  
  
+ GTACATTATT TACGAACATA CCTTTCGTAA CTCGTACATA CCGACGGACG ATGCCACCAA CGAGACAATT   
  
  
+ ATCACGGGGT TCTTCTTCCC GGCGTGGATA TCCGATACCA GTCGGCTCCT TTAACGTTTA ACCGTGCTTT   
  
  
+ GAGAACGCGT GGAACTCAAA ATGAGAAATT GGACGTGTGA TTGGGAGACA CACCGCGGGT GTTATGGGGG   
  
  
+ TTCAGTGGGT CCTAGGGTGT GGGTCTCCCG TCATGTTTGC GGCGTGGCCC CCTCCTAGGG GCATTTGCTT   
  
  
+ TAACACTGTG CACTCCGTAT ACCTACATTA TAAAATTAAT ACTTTTAATT TTCCATAGTT AATACTTTTT   
  
  
+ AATTGCAGTG TTTCTAATGT CATAATTTTG ATATAATATA TAGCTTGACT TCGAATTTAA TTATTAATAA   
  
  
+ TTTCCTAGTG CCGTTATAAA AAGGAATAT  

- GATTAAAGAA AAACTTCTCA GCGCGTCTCT CTATACACGA AGATCCGGAT ACCTGCCGAT AAATGACTGG   
  
  
- TTTTGGCCGT AGATGATGAT GACGACGACT ACTGCAGCAG ATACAGAGAG TGGCTTGATT GCTGGAAGGA   
  
  
- AGTAAAGAGG GAGAGAGAGA GAGAGAGAGA AGAGAAGAAA TGGAGGAGAA GGTGGTGAAG GAAAGAAAGG   
  
  
- GTGGTCCTTA CAGAAAAGGG CCGTCATGTG AGACAGAAGG AGTGTTTGGT AATATATATT TGTAGTTAGC   
  
  
- AAAGGACGTG CAAGCAAATG TGATTTCAAG CCATCACTAT TTTATTATAT CATCAACTTG AAAACGAACC   
  
  
- CTCCATTTTA CTTTTCTTTT TTGTTTATTG AAATCATTAA GCCCACTGAT TACAGAGTCT GCATTAATTA   
  
  
- TTTTTAATTT TGGTAGAAAA CGGGGCTGTG TCATGTTGGT TGGTTTTTCT GGTTTGAACA AAACATGTGT   
  
  
- TGTTTTGTCC TGTTGCTTCA TTGTTCTTTA AGAGCAGTTG TTGTTGAAAT CAGATTACCG GTAGGAGGGT   
  
  
- AGGCCGACTG ATTGGTTTCA TACCCAGAAG TTGCCCTTTT AAAACCAGAT GAGGAAAGTC CTGTTTATAT   
  
  
- TCGCTTATAT TATTCTCATG CAAAATTAGG GTTTCCATTT TCTTTTTTAT TTTCCCTAGT GGATATAGTT   
  
  
- GAGTAACACA GTGATCCACC CAACTCAGCT GCCTCCCTTT ATCCGACCCA AAATCATGGG GATCTCTTCT   
  
  
- CAAAAGGGAG AGCGAGAGAG AGAGAAATTT TAAATTTCTT AAAAAATTTT ATGTTAATTT CATCTGGGTT   
  
  
- TTATTAAAAT TTTACTTTTT AATTAACCCA AAAAATTATC AAAATATTAT TTTAGTCTTT ATTAAAAATA   
  
  
- GAGCACATAT CAATAGAGTA TCTTAATTCT AAAAAATAAT GTTTTGAATA GGAAATTTAA ATGGAATGAA   
  
  
- TTGGTCAAAG TTGTAATTTA GGGCCAAAAA ATTATGGATA AAAAAGGAAA TAAAGGGATT CAATAATTCA   
  
  
- CATGTAATAA ATGCTTGTAT GGAAAGCATT GAGCATGTAT GGCTGCCTGC TACGGTGGTT GCTCTGTTAA   
  
  
- TAGTGCCCCA AGAAGAAGGG CCGCACCTAT AGGCTATGGT CAGCCGAGGA AATTGCAAAT TGGCACGAAA   
  
  
- CTCTTGCGCA CCTTGAGTTT TACTCTTTAA CCTGCACACT AACCCTCTGT GTGGCGCCCA CAATACCCCC   
  
  
- AAGTCACCCA GGATCCCACA CCCAGAGGGC AGTACAAACG CCGCACCGGG GGAGGATCCC CGTAAACGAA   
  
  
- ATTGTGACAC GTGAGGCATA TGGATGTAAT ATTTTAATTA TGAAAATTAA AAGGTATCAA TTATGAAAAA   
  
  
- TTAACGTCAC AAAGATTACA GTATTAAAAC TATATTATAT ATCGAACTGA AGCTTAAATT AATAATTATT   
  
  
- AAAGGATCAC GGCAATATTT TTCCTTATA

+     AT1-motif

| Site Name | Organism | Position | Strand | Matrix score. | sequence | function |
| --- | --- | --- | --- | --- | --- | --- |
| AT1-motif | Solanum tuberosum | 1359 | - | 11 | ATTAATTTTACA | part of a light responsive module |
| AT1-motif | Solanum tuberosum | 854 | - | 11 | ATTAATTTTACA | part of a light responsive module |

> 2018/04/13 10:10:12  
+ CTAATTTCTT TTTGAAGAGT CGCGCAGAGA GATATGTGCT TCTAGGCCTA TGGACGGCTA TTTACTGACC   
  
  
+ AAAACCGGCA TCTACTACTA CTGCTGCTGA TGACGTCGTC TATGTCTCTC ACCGAACTAA CGACCTTCCT   
  
  
+ TCATTTCTCC CTCTCTCTCT CTCTCTCTCT TCTCTTCTTT ACCTCCTCTT CCACCACTTC CTTTCTTTCC   
  
  
+ CACCAGGAAT GTCTTTTCCC GGCAGTACAC TCTGTCTTCC TCACAAACCA TTATATATAA ACATCAATCG   
  
  
+ TTTCCTGCAC GTTCGTTTAC ACTAAAGTTC GGTAGTGATA AAATAATATA GTAGTTGAAC TTTTGCTTGG   
  
  
+ GAGGTAAAAT GAAAAGAAAA AACAAATAAC TTTAGTAATT CGGGTGACTA ATGTCTCAGA CGTAATTAAT   
  
  
+ AAAAATTAAA ACCATCTTTT GCCCCGACAC AGTACAACCA ACCAAAAAGA CCAAACTTGT TTTGTACACA   
  
  
+ ACAAAACAGG ACAACGAAGT AACAAGAAAT TCTCGTCAAC AACAACTTTA GTCTAATGGC CATCCTCCCA   
  
  
+ TCCGGCTGAC TAACCAAAGT ATGGGTCTTC AACGGGAAAA TTTTGGTCTA CTCCTTTCAG GACAAATATA   
  
  
+ AGCGAATATA ATAAGAGTAC GTTTTAATCC CAAAGGTAAA AGAAAAAATA AAAGGGATCA CCTATATCAA   
  
  
+ CTCATTGTGT CACTAGGTGG GTTGAGTCGA CGGAGGGAAA TAGGCTGGGT TTTAGTACCC CTAGAGAAGA   
  
  
+ GTTTTCCCTC TCGCTCTCTC TCTCTTTAAA ATTTAAAGAA TTTTTTAAAA TACAATTAAA GTAGACCCAA   
  
  
+ AATAATTTTA AAATGAAAAA TTAATTGGGT TTTTTAATAG TTTTATAATA AAATCAGAAA TAATTTTTAT   
  
  
+ CTCGTGTATA GTTATCTCAT AGAATTAAGA TTTTTTATTA CAAAACTTAT CCTTTAAATT TACCTTACTT   
  
  
+ AACCAGTTTC AACATTAAAT CCCGGTTTTT TAATACCTAT TTTTTCCTTT ATTTCCCTAA GTTATTAAGT   
  
  
+ GTACATTATT TACGAACATA CCTTTCGTAA CTCGTACATA CCGACGGACG ATGCCACCAA CGAGACAATT   
  
  
+ ATCACGGGGT TCTTCTTCCC GGCGTGGATA TCCGATACCA GTCGGCTCCT TTAACGTTTA ACCGTGCTTT   
  
  
+ GAGAACGCGT GGAACTCAAA ATGAGAAATT GGACGTGTGA TTGGGAGACA CACCGCGGGT GTTATGGGGG   
  
  
+ TTCAGTGGGT CCTAGGGTGT GGGTCTCCCG TCATGTTTGC GGCGTGGCCC CCTCCTAGGG GCATTTGCTT   
  
  
+ TAACACTGTG CACTCCGTAT ACCTACATTA TAAAATTAAT ACTTTTAATT TTCCATAGTT AATACTTTTT   
  
  
+ AATTGCAGTG TTTCTAATGT CATAATTTTG ATATAATATA TAGCTTGACT TCGAATTTAA TTATTAATAA   
  
  
+ TTTCCTAGTG CCGTTATAAA AAGGAATAT  

- GATTAAAGAA AAACTTCTCA GCGCGTCTCT CTATACACGA AGATCCGGAT ACCTGCCGAT AAATGACTGG   
  
  
- TTTTGGCCGT AGATGATGAT GACGACGACT ACTGCAGCAG ATACAGAGAG TGGCTTGATT GCTGGAAGGA   
  
  
- AGTAAAGAGG GAGAGAGAGA GAGAGAGAGA AGAGAAGAAA TGGAGGAGAA GGTGGTGAAG GAAAGAAAGG   
  
  
- GTGGTCCTTA CAGAAAAGGG CCGTCATGTG AGACAGAAGG AGTGTTTGGT AATATATATT TGTAGTTAGC   
  
  
- AAAGGACGTG CAAGCAAATG TGATTTCAAG CCATCACTAT TTTATTATAT CATCAACTTG AAAACGAACC   
  
  
- CTCCATTTTA CTTTTCTTTT TTGTTTATTG AAATCATTAA GCCCACTGAT TACAGAGTCT GCATTAATTA   
  
  
- TTTTTAATTT TGGTAGAAAA CGGGGCTGTG TCATGTTGGT TGGTTTTTCT GGTTTGAACA AAACATGTGT   
  
  
- TGTTTTGTCC TGTTGCTTCA TTGTTCTTTA AGAGCAGTTG TTGTTGAAAT CAGATTACCG GTAGGAGGGT   
  
  
- AGGCCGACTG ATTGGTTTCA TACCCAGAAG TTGCCCTTTT AAAACCAGAT GAGGAAAGTC CTGTTTATAT   
  
  
- TCGCTTATAT TATTCTCATG CAAAATTAGG GTTTCCATTT TCTTTTTTAT TTTCCCTAGT GGATATAGTT   
  
  
- GAGTAACACA GTGATCCACC CAACTCAGCT GCCTCCCTTT ATCCGACCCA AAATCATGGG GATCTCTTCT   
  
  
- CAAAAGGGAG AGCGAGAGAG AGAGAAATTT TAAATTTCTT AAAAAATTTT ATGTTAATTT CATCTGGGTT   
  
  
- TTATTAAAAT TTTACTTTTT AATTAACCCA AAAAATTATC AAAATATTAT TTTAGTCTTT ATTAAAAATA   
  
  
- GAGCACATAT CAATAGAGTA TCTTAATTCT AAAAAATAAT GTTTTGAATA GGAAATTTAA ATGGAATGAA   
  
  
- TTGGTCAAAG TTGTAATTTA GGGCCAAAAA ATTATGGATA AAAAAGGAAA TAAAGGGATT CAATAATTCA   
  
  
- CATGTAATAA ATGCTTGTAT GGAAAGCATT GAGCATGTAT GGCTGCCTGC TACGGTGGTT GCTCTGTTAA   
  
  
- TAGTGCCCCA AGAAGAAGGG CCGCACCTAT AGGCTATGGT CAGCCGAGGA AATTGCAAAT TGGCACGAAA   
  
  
- CTCTTGCGCA CCTTGAGTTT TACTCTTTAA CCTGCACACT AACCCTCTGT GTGGCGCCCA CAATACCCCC   
  
  
- AAGTCACCCA GGATCCCACA CCCAGAGGGC AGTACAAACG CCGCACCGGG GGAGGATCCC CGTAAACGAA   
  
  
- ATTGTGACAC GTGAGGCATA TGGATGTAAT ATTTTAATTA TGAAAATTAA AAGGTATCAA TTATGAAAAA   
  
  
- TTAACGTCAC AAAGATTACA GTATTAAAAC TATATTATAT ATCGAACTGA AGCTTAAATT AATAATTATT   
  
  
- AAAGGATCAC GGCAATATTT TTCCTTATA

+     Box 4

| Site Name | Organism | Position | Strand | Matrix score. | sequence | function |
| --- | --- | --- | --- | --- | --- | --- |
| Box 4 | Petroselinum crispum | 415 | + | 6 | ATTAAT | part of a conserved DNA module involved in light responsiveness |
| Box 4 | Petroselinum crispum | 860 | - | 6 | ATTAAT | part of a conserved DNA module involved in light responsiveness |
| Box 4 | Petroselinum crispum | 1365 | - | 6 | ATTAAT | part of a conserved DNA module involved in light responsiveness |
| Box 4 | Petroselinum crispum | 1463 | - | 6 | ATTAAT | part of a conserved DNA module involved in light responsiveness |

> 2018/04/13 10:10:12  
+ CTAATTTCTT TTTGAAGAGT CGCGCAGAGA GATATGTGCT TCTAGGCCTA TGGACGGCTA TTTACTGACC   
  
  
+ AAAACCGGCA TCTACTACTA CTGCTGCTGA TGACGTCGTC TATGTCTCTC ACCGAACTAA CGACCTTCCT   
  
  
+ TCATTTCTCC CTCTCTCTCT CTCTCTCTCT TCTCTTCTTT ACCTCCTCTT CCACCACTTC CTTTCTTTCC   
  
  
+ CACCAGGAAT GTCTTTTCCC GGCAGTACAC TCTGTCTTCC TCACAAACCA TTATATATAA ACATCAATCG   
  
  
+ TTTCCTGCAC GTTCGTTTAC ACTAAAGTTC GGTAGTGATA AAATAATATA GTAGTTGAAC TTTTGCTTGG   
  
  
+ GAGGTAAAAT GAAAAGAAAA AACAAATAAC TTTAGTAATT CGGGTGACTA ATGTCTCAGA CGTAATTAAT   
  
  
+ AAAAATTAAA ACCATCTTTT GCCCCGACAC AGTACAACCA ACCAAAAAGA CCAAACTTGT TTTGTACACA   
  
  
+ ACAAAACAGG ACAACGAAGT AACAAGAAAT TCTCGTCAAC AACAACTTTA GTCTAATGGC CATCCTCCCA   
  
  
+ TCCGGCTGAC TAACCAAAGT ATGGGTCTTC AACGGGAAAA TTTTGGTCTA CTCCTTTCAG GACAAATATA   
  
  
+ AGCGAATATA ATAAGAGTAC GTTTTAATCC CAAAGGTAAA AGAAAAAATA AAAGGGATCA CCTATATCAA   
  
  
+ CTCATTGTGT CACTAGGTGG GTTGAGTCGA CGGAGGGAAA TAGGCTGGGT TTTAGTACCC CTAGAGAAGA   
  
  
+ GTTTTCCCTC TCGCTCTCTC TCTCTTTAAA ATTTAAAGAA TTTTTTAAAA TACAATTAAA GTAGACCCAA   
  
  
+ AATAATTTTA AAATGAAAAA TTAATTGGGT TTTTTAATAG TTTTATAATA AAATCAGAAA TAATTTTTAT   
  
  
+ CTCGTGTATA GTTATCTCAT AGAATTAAGA TTTTTTATTA CAAAACTTAT CCTTTAAATT TACCTTACTT   
  
  
+ AACCAGTTTC AACATTAAAT CCCGGTTTTT TAATACCTAT TTTTTCCTTT ATTTCCCTAA GTTATTAAGT   
  
  
+ GTACATTATT TACGAACATA CCTTTCGTAA CTCGTACATA CCGACGGACG ATGCCACCAA CGAGACAATT   
  
  
+ ATCACGGGGT TCTTCTTCCC GGCGTGGATA TCCGATACCA GTCGGCTCCT TTAACGTTTA ACCGTGCTTT   
  
  
+ GAGAACGCGT GGAACTCAAA ATGAGAAATT GGACGTGTGA TTGGGAGACA CACCGCGGGT GTTATGGGGG   
  
  
+ TTCAGTGGGT CCTAGGGTGT GGGTCTCCCG TCATGTTTGC GGCGTGGCCC CCTCCTAGGG GCATTTGCTT   
  
  
+ TAACACTGTG CACTCCGTAT ACCTACATTA TAAAATTAAT ACTTTTAATT TTCCATAGTT AATACTTTTT   
  
  
+ AATTGCAGTG TTTCTAATGT CATAATTTTG ATATAATATA TAGCTTGACT TCGAATTTAA TTATTAATAA   
  
  
+ TTTCCTAGTG CCGTTATAAA AAGGAATAT  

- GATTAAAGAA AAACTTCTCA GCGCGTCTCT CTATACACGA AGATCCGGAT ACCTGCCGAT AAATGACTGG   
  
  
- TTTTGGCCGT AGATGATGAT GACGACGACT ACTGCAGCAG ATACAGAGAG TGGCTTGATT GCTGGAAGGA   
  
  
- AGTAAAGAGG GAGAGAGAGA GAGAGAGAGA AGAGAAGAAA TGGAGGAGAA GGTGGTGAAG GAAAGAAAGG   
  
  
- GTGGTCCTTA CAGAAAAGGG CCGTCATGTG AGACAGAAGG AGTGTTTGGT AATATATATT TGTAGTTAGC   
  
  
- AAAGGACGTG CAAGCAAATG TGATTTCAAG CCATCACTAT TTTATTATAT CATCAACTTG AAAACGAACC   
  
  
- CTCCATTTTA CTTTTCTTTT TTGTTTATTG AAATCATTAA GCCCACTGAT TACAGAGTCT GCATTAATTA   
  
  
- TTTTTAATTT TGGTAGAAAA CGGGGCTGTG TCATGTTGGT TGGTTTTTCT GGTTTGAACA AAACATGTGT   
  
  
- TGTTTTGTCC TGTTGCTTCA TTGTTCTTTA AGAGCAGTTG TTGTTGAAAT CAGATTACCG GTAGGAGGGT   
  
  
- AGGCCGACTG ATTGGTTTCA TACCCAGAAG TTGCCCTTTT AAAACCAGAT GAGGAAAGTC CTGTTTATAT   
  
  
- TCGCTTATAT TATTCTCATG CAAAATTAGG GTTTCCATTT TCTTTTTTAT TTTCCCTAGT GGATATAGTT   
  
  
- GAGTAACACA GTGATCCACC CAACTCAGCT GCCTCCCTTT ATCCGACCCA AAATCATGGG GATCTCTTCT   
  
  
- CAAAAGGGAG AGCGAGAGAG AGAGAAATTT TAAATTTCTT AAAAAATTTT ATGTTAATTT CATCTGGGTT   
  
  
- TTATTAAAAT TTTACTTTTT AATTAACCCA AAAAATTATC AAAATATTAT TTTAGTCTTT ATTAAAAATA   
  
  
- GAGCACATAT CAATAGAGTA TCTTAATTCT AAAAAATAAT GTTTTGAATA GGAAATTTAA ATGGAATGAA   
  
  
- TTGGTCAAAG TTGTAATTTA GGGCCAAAAA ATTATGGATA AAAAAGGAAA TAAAGGGATT CAATAATTCA   
  
  
- CATGTAATAA ATGCTTGTAT GGAAAGCATT GAGCATGTAT GGCTGCCTGC TACGGTGGTT GCTCTGTTAA   
  
  
- TAGTGCCCCA AGAAGAAGGG CCGCACCTAT AGGCTATGGT CAGCCGAGGA AATTGCAAAT TGGCACGAAA   
  
  
- CTCTTGCGCA CCTTGAGTTT TACTCTTTAA CCTGCACACT AACCCTCTGT GTGGCGCCCA CAATACCCCC   
  
  
- AAGTCACCCA GGATCCCACA CCCAGAGGGC AGTACAAACG CCGCACCGGG GGAGGATCCC CGTAAACGAA   
  
  
- ATTGTGACAC GTGAGGCATA TGGATGTAAT ATTTTAATTA TGAAAATTAA AAGGTATCAA TTATGAAAAA   
  
  
- TTAACGTCAC AAAGATTACA GTATTAAAAC TATATTATAT ATCGAACTGA AGCTTAAATT AATAATTATT   
  
  
- AAAGGATCAC GGCAATATTT TTCCTTATA

+     Box III

| Site Name | Organism | Position | Strand | Matrix score. | sequence | function |
| --- | --- | --- | --- | --- | --- | --- |
| Box III | Pisum sativum | 294 | + | 9 | CATTTACACT | protein binding site |

> 2018/04/13 10:10:12  
+ CTAATTTCTT TTTGAAGAGT CGCGCAGAGA GATATGTGCT TCTAGGCCTA TGGACGGCTA TTTACTGACC   
  
  
+ AAAACCGGCA TCTACTACTA CTGCTGCTGA TGACGTCGTC TATGTCTCTC ACCGAACTAA CGACCTTCCT   
  
  
+ TCATTTCTCC CTCTCTCTCT CTCTCTCTCT TCTCTTCTTT ACCTCCTCTT CCACCACTTC CTTTCTTTCC   
  
  
+ CACCAGGAAT GTCTTTTCCC GGCAGTACAC TCTGTCTTCC TCACAAACCA TTATATATAA ACATCAATCG   
  
  
+ TTTCCTGCAC GTTCGTTTAC ACTAAAGTTC GGTAGTGATA AAATAATATA GTAGTTGAAC TTTTGCTTGG   
  
  
+ GAGGTAAAAT GAAAAGAAAA AACAAATAAC TTTAGTAATT CGGGTGACTA ATGTCTCAGA CGTAATTAAT   
  
  
+ AAAAATTAAA ACCATCTTTT GCCCCGACAC AGTACAACCA ACCAAAAAGA CCAAACTTGT TTTGTACACA   
  
  
+ ACAAAACAGG ACAACGAAGT AACAAGAAAT TCTCGTCAAC AACAACTTTA GTCTAATGGC CATCCTCCCA   
  
  
+ TCCGGCTGAC TAACCAAAGT ATGGGTCTTC AACGGGAAAA TTTTGGTCTA CTCCTTTCAG GACAAATATA   
  
  
+ AGCGAATATA ATAAGAGTAC GTTTTAATCC CAAAGGTAAA AGAAAAAATA AAAGGGATCA CCTATATCAA   
  
  
+ CTCATTGTGT CACTAGGTGG GTTGAGTCGA CGGAGGGAAA TAGGCTGGGT TTTAGTACCC CTAGAGAAGA   
  
  
+ GTTTTCCCTC TCGCTCTCTC TCTCTTTAAA ATTTAAAGAA TTTTTTAAAA TACAATTAAA GTAGACCCAA   
  
  
+ AATAATTTTA AAATGAAAAA TTAATTGGGT TTTTTAATAG TTTTATAATA AAATCAGAAA TAATTTTTAT   
  
  
+ CTCGTGTATA GTTATCTCAT AGAATTAAGA TTTTTTATTA CAAAACTTAT CCTTTAAATT TACCTTACTT   
  
  
+ AACCAGTTTC AACATTAAAT CCCGGTTTTT TAATACCTAT TTTTTCCTTT ATTTCCCTAA GTTATTAAGT   
  
  
+ GTACATTATT TACGAACATA CCTTTCGTAA CTCGTACATA CCGACGGACG ATGCCACCAA CGAGACAATT   
  
  
+ ATCACGGGGT TCTTCTTCCC GGCGTGGATA TCCGATACCA GTCGGCTCCT TTAACGTTTA ACCGTGCTTT   
  
  
+ GAGAACGCGT GGAACTCAAA ATGAGAAATT GGACGTGTGA TTGGGAGACA CACCGCGGGT GTTATGGGGG   
  
  
+ TTCAGTGGGT CCTAGGGTGT GGGTCTCCCG TCATGTTTGC GGCGTGGCCC CCTCCTAGGG GCATTTGCTT   
  
  
+ TAACACTGTG CACTCCGTAT ACCTACATTA TAAAATTAAT ACTTTTAATT TTCCATAGTT AATACTTTTT   
  
  
+ AATTGCAGTG TTTCTAATGT CATAATTTTG ATATAATATA TAGCTTGACT TCGAATTTAA TTATTAATAA   
  
  
+ TTTCCTAGTG CCGTTATAAA AAGGAATAT  

- GATTAAAGAA AAACTTCTCA GCGCGTCTCT CTATACACGA AGATCCGGAT ACCTGCCGAT AAATGACTGG   
  
  
- TTTTGGCCGT AGATGATGAT GACGACGACT ACTGCAGCAG ATACAGAGAG TGGCTTGATT GCTGGAAGGA   
  
  
- AGTAAAGAGG GAGAGAGAGA GAGAGAGAGA AGAGAAGAAA TGGAGGAGAA GGTGGTGAAG GAAAGAAAGG   
  
  
- GTGGTCCTTA CAGAAAAGGG CCGTCATGTG AGACAGAAGG AGTGTTTGGT AATATATATT TGTAGTTAGC   
  
  
- AAAGGACGTG CAAGCAAATG TGATTTCAAG CCATCACTAT TTTATTATAT CATCAACTTG AAAACGAACC   
  
  
- CTCCATTTTA CTTTTCTTTT TTGTTTATTG AAATCATTAA GCCCACTGAT TACAGAGTCT GCATTAATTA   
  
  
- TTTTTAATTT TGGTAGAAAA CGGGGCTGTG TCATGTTGGT TGGTTTTTCT GGTTTGAACA AAACATGTGT   
  
  
- TGTTTTGTCC TGTTGCTTCA TTGTTCTTTA AGAGCAGTTG TTGTTGAAAT CAGATTACCG GTAGGAGGGT   
  
  
- AGGCCGACTG ATTGGTTTCA TACCCAGAAG TTGCCCTTTT AAAACCAGAT GAGGAAAGTC CTGTTTATAT   
  
  
- TCGCTTATAT TATTCTCATG CAAAATTAGG GTTTCCATTT TCTTTTTTAT TTTCCCTAGT GGATATAGTT   
  
  
- GAGTAACACA GTGATCCACC CAACTCAGCT GCCTCCCTTT ATCCGACCCA AAATCATGGG GATCTCTTCT   
  
  
- CAAAAGGGAG AGCGAGAGAG AGAGAAATTT TAAATTTCTT AAAAAATTTT ATGTTAATTT CATCTGGGTT   
  
  
- TTATTAAAAT TTTACTTTTT AATTAACCCA AAAAATTATC AAAATATTAT TTTAGTCTTT ATTAAAAATA   
  
  
- GAGCACATAT CAATAGAGTA TCTTAATTCT AAAAAATAAT GTTTTGAATA GGAAATTTAA ATGGAATGAA   
  
  
- TTGGTCAAAG TTGTAATTTA GGGCCAAAAA ATTATGGATA AAAAAGGAAA TAAAGGGATT CAATAATTCA   
  
  
- CATGTAATAA ATGCTTGTAT GGAAAGCATT GAGCATGTAT GGCTGCCTGC TACGGTGGTT GCTCTGTTAA   
  
  
- TAGTGCCCCA AGAAGAAGGG CCGCACCTAT AGGCTATGGT CAGCCGAGGA AATTGCAAAT TGGCACGAAA   
  
  
- CTCTTGCGCA CCTTGAGTTT TACTCTTTAA CCTGCACACT AACCCTCTGT GTGGCGCCCA CAATACCCCC   
  
  
- AAGTCACCCA GGATCCCACA CCCAGAGGGC AGTACAAACG CCGCACCGGG GGAGGATCCC CGTAAACGAA   
  
  
- ATTGTGACAC GTGAGGCATA TGGATGTAAT ATTTTAATTA TGAAAATTAA AAGGTATCAA TTATGAAAAA   
  
  
- TTAACGTCAC AAAGATTACA GTATTAAAAC TATATTATAT ATCGAACTGA AGCTTAAATT AATAATTATT   
  
  
- AAAGGATCAC GGCAATATTT TTCCTTATA

+     CAAT-box

| Site Name | Organism | Position | Strand | Matrix score. | sequence | function |
| --- | --- | --- | --- | --- | --- | --- |
| CAAT-box | Arabidopsis thaliana | 1230 | - | 5 | CCAAT | common cis-acting element in promoter and enhancer regions |
| CAAT-box | Arabidopsis thaliana | 1218 | - | 5 | CCAAT | common cis-acting element in promoter and enhancer regions |
| CAAT-box | Brassica rapa | 623 | + | 5 | CAAAT | common cis-acting element in promoter and enhancer regions |
| CAAT-box | Brassica rapa | 373 | + | 5 | CAAAT | common cis-acting element in promoter and enhancer regions |
| CAAT-box | Glycine max | 1217 | - | 5 | CAATT | common cis-acting element in promoter and enhancer regions |
| CAAT-box | Glycine max | 1401 | - | 5 | CAATT | common cis-acting element in promoter and enhancer regions |
| CAAT-box | Hordeum vulgare | 704 | - | 4 | CAAT | common cis-acting element in promoter and enhancer regions |
| CAAT-box | Brassica rapa | 1323 | - | 5 | CAAAT | common cis-acting element in promoter and enhancer regions |
| CAAT-box | Hordeum vulgare | 275 | + | 4 | CAAT | common cis-acting element in promoter and enhancer regions |
| CAAT-box | Arabidopsis thaliana | 864 | - | 5 | CCAAT | common cis-acting element in promoter and enhancer regions |
| CAAT-box | Hordeum vulgare | 1402 | - | 4 | CAAT | common cis-acting element in promoter and enhancer regions |
| CAAT-box | Glycine max | 823 | + | 5 | CAATT | common cis-acting element in promoter and enhancer regions |
| CAAT-box | Glycine max | 863 | - | 5 | CAATT | common cis-acting element in promoter and enhancer regions |
| CAAT-box | Glycine max | 1116 | + | 5 | CAATT | common cis-acting element in promoter and enhancer regions |

> 2018/04/13 10:10:12  
+ CTAATTTCTT TTTGAAGAGT CGCGCAGAGA GATATGTGCT TCTAGGCCTA TGGACGGCTA TTTACTGACC   
  
  
+ AAAACCGGCA TCTACTACTA CTGCTGCTGA TGACGTCGTC TATGTCTCTC ACCGAACTAA CGACCTTCCT   
  
  
+ TCATTTCTCC CTCTCTCTCT CTCTCTCTCT TCTCTTCTTT ACCTCCTCTT CCACCACTTC CTTTCTTTCC   
  
  
+ CACCAGGAAT GTCTTTTCCC GGCAGTACAC TCTGTCTTCC TCACAAACCA TTATATATAA ACATCAATCG   
  
  
+ TTTCCTGCAC GTTCGTTTAC ACTAAAGTTC GGTAGTGATA AAATAATATA GTAGTTGAAC TTTTGCTTGG   
  
  
+ GAGGTAAAAT GAAAAGAAAA AACAAATAAC TTTAGTAATT CGGGTGACTA ATGTCTCAGA CGTAATTAAT   
  
  
+ AAAAATTAAA ACCATCTTTT GCCCCGACAC AGTACAACCA ACCAAAAAGA CCAAACTTGT TTTGTACACA   
  
  
+ ACAAAACAGG ACAACGAAGT AACAAGAAAT TCTCGTCAAC AACAACTTTA GTCTAATGGC CATCCTCCCA   
  
  
+ TCCGGCTGAC TAACCAAAGT ATGGGTCTTC AACGGGAAAA TTTTGGTCTA CTCCTTTCAG GACAAATATA   
  
  
+ AGCGAATATA ATAAGAGTAC GTTTTAATCC CAAAGGTAAA AGAAAAAATA AAAGGGATCA CCTATATCAA   
  
  
+ CTCATTGTGT CACTAGGTGG GTTGAGTCGA CGGAGGGAAA TAGGCTGGGT TTTAGTACCC CTAGAGAAGA   
  
  
+ GTTTTCCCTC TCGCTCTCTC TCTCTTTAAA ATTTAAAGAA TTTTTTAAAA TACAATTAAA GTAGACCCAA   
  
  
+ AATAATTTTA AAATGAAAAA TTAATTGGGT TTTTTAATAG TTTTATAATA AAATCAGAAA TAATTTTTAT   
  
  
+ CTCGTGTATA GTTATCTCAT AGAATTAAGA TTTTTTATTA CAAAACTTAT CCTTTAAATT TACCTTACTT   
  
  
+ AACCAGTTTC AACATTAAAT CCCGGTTTTT TAATACCTAT TTTTTCCTTT ATTTCCCTAA GTTATTAAGT   
  
  
+ GTACATTATT TACGAACATA CCTTTCGTAA CTCGTACATA CCGACGGACG ATGCCACCAA CGAGACAATT   
  
  
+ ATCACGGGGT TCTTCTTCCC GGCGTGGATA TCCGATACCA GTCGGCTCCT TTAACGTTTA ACCGTGCTTT   
  
  
+ GAGAACGCGT GGAACTCAAA ATGAGAAATT GGACGTGTGA TTGGGAGACA CACCGCGGGT GTTATGGGGG   
  
  
+ TTCAGTGGGT CCTAGGGTGT GGGTCTCCCG TCATGTTTGC GGCGTGGCCC CCTCCTAGGG GCATTTGCTT   
  
  
+ TAACACTGTG CACTCCGTAT ACCTACATTA TAAAATTAAT ACTTTTAATT TTCCATAGTT AATACTTTTT   
  
  
+ AATTGCAGTG TTTCTAATGT CATAATTTTG ATATAATATA TAGCTTGACT TCGAATTTAA TTATTAATAA   
  
  
+ TTTCCTAGTG CCGTTATAAA AAGGAATAT  

- GATTAAAGAA AAACTTCTCA GCGCGTCTCT CTATACACGA AGATCCGGAT ACCTGCCGAT AAATGACTGG   
  
  
- TTTTGGCCGT AGATGATGAT GACGACGACT ACTGCAGCAG ATACAGAGAG TGGCTTGATT GCTGGAAGGA   
  
  
- AGTAAAGAGG GAGAGAGAGA GAGAGAGAGA AGAGAAGAAA TGGAGGAGAA GGTGGTGAAG GAAAGAAAGG   
  
  
- GTGGTCCTTA CAGAAAAGGG CCGTCATGTG AGACAGAAGG AGTGTTTGGT AATATATATT TGTAGTTAGC   
  
  
- AAAGGACGTG CAAGCAAATG TGATTTCAAG CCATCACTAT TTTATTATAT CATCAACTTG AAAACGAACC   
  
  
- CTCCATTTTA CTTTTCTTTT TTGTTTATTG AAATCATTAA GCCCACTGAT TACAGAGTCT GCATTAATTA   
  
  
- TTTTTAATTT TGGTAGAAAA CGGGGCTGTG TCATGTTGGT TGGTTTTTCT GGTTTGAACA AAACATGTGT   
  
  
- TGTTTTGTCC TGTTGCTTCA TTGTTCTTTA AGAGCAGTTG TTGTTGAAAT CAGATTACCG GTAGGAGGGT   
  
  
- AGGCCGACTG ATTGGTTTCA TACCCAGAAG TTGCCCTTTT AAAACCAGAT GAGGAAAGTC CTGTTTATAT   
  
  
- TCGCTTATAT TATTCTCATG CAAAATTAGG GTTTCCATTT TCTTTTTTAT TTTCCCTAGT GGATATAGTT   
  
  
- GAGTAACACA GTGATCCACC CAACTCAGCT GCCTCCCTTT ATCCGACCCA AAATCATGGG GATCTCTTCT   
  
  
- CAAAAGGGAG AGCGAGAGAG AGAGAAATTT TAAATTTCTT AAAAAATTTT ATGTTAATTT CATCTGGGTT   
  
  
- TTATTAAAAT TTTACTTTTT AATTAACCCA AAAAATTATC AAAATATTAT TTTAGTCTTT ATTAAAAATA   
  
  
- GAGCACATAT CAATAGAGTA TCTTAATTCT AAAAAATAAT GTTTTGAATA GGAAATTTAA ATGGAATGAA   
  
  
- TTGGTCAAAG TTGTAATTTA GGGCCAAAAA ATTATGGATA AAAAAGGAAA TAAAGGGATT CAATAATTCA   
  
  
- CATGTAATAA ATGCTTGTAT GGAAAGCATT GAGCATGTAT GGCTGCCTGC TACGGTGGTT GCTCTGTTAA   
  
  
- TAGTGCCCCA AGAAGAAGGG CCGCACCTAT AGGCTATGGT CAGCCGAGGA AATTGCAAAT TGGCACGAAA   
  
  
- CTCTTGCGCA CCTTGAGTTT TACTCTTTAA CCTGCACACT AACCCTCTGT GTGGCGCCCA CAATACCCCC   
  
  
- AAGTCACCCA GGATCCCACA CCCAGAGGGC AGTACAAACG CCGCACCGGG GGAGGATCCC CGTAAACGAA   
  
  
- ATTGTGACAC GTGAGGCATA TGGATGTAAT ATTTTAATTA TGAAAATTAA AAGGTATCAA TTATGAAAAA   
  
  
- TTAACGTCAC AAAGATTACA GTATTAAAAC TATATTATAT ATCGAACTGA AGCTTAAATT AATAATTATT   
  
  
- AAAGGATCAC GGCAATATTT TTCCTTATA

+     CCAAT-box

| Site Name | Organism | Position | Strand | Matrix score. | sequence | function |
| --- | --- | --- | --- | --- | --- | --- |
| CCAAT-box | Hordeum vulgare | 590 | + | 6 | CAACGG | MYBHv1 binding site |

> 2018/04/13 10:10:12  
+ CTAATTTCTT TTTGAAGAGT CGCGCAGAGA GATATGTGCT TCTAGGCCTA TGGACGGCTA TTTACTGACC   
  
  
+ AAAACCGGCA TCTACTACTA CTGCTGCTGA TGACGTCGTC TATGTCTCTC ACCGAACTAA CGACCTTCCT   
  
  
+ TCATTTCTCC CTCTCTCTCT CTCTCTCTCT TCTCTTCTTT ACCTCCTCTT CCACCACTTC CTTTCTTTCC   
  
  
+ CACCAGGAAT GTCTTTTCCC GGCAGTACAC TCTGTCTTCC TCACAAACCA TTATATATAA ACATCAATCG   
  
  
+ TTTCCTGCAC GTTCGTTTAC ACTAAAGTTC GGTAGTGATA AAATAATATA GTAGTTGAAC TTTTGCTTGG   
  
  
+ GAGGTAAAAT GAAAAGAAAA AACAAATAAC TTTAGTAATT CGGGTGACTA ATGTCTCAGA CGTAATTAAT   
  
  
+ AAAAATTAAA ACCATCTTTT GCCCCGACAC AGTACAACCA ACCAAAAAGA CCAAACTTGT TTTGTACACA   
  
  
+ ACAAAACAGG ACAACGAAGT AACAAGAAAT TCTCGTCAAC AACAACTTTA GTCTAATGGC CATCCTCCCA   
  
  
+ TCCGGCTGAC TAACCAAAGT ATGGGTCTTC AACGGGAAAA TTTTGGTCTA CTCCTTTCAG GACAAATATA   
  
  
+ AGCGAATATA ATAAGAGTAC GTTTTAATCC CAAAGGTAAA AGAAAAAATA AAAGGGATCA CCTATATCAA   
  
  
+ CTCATTGTGT CACTAGGTGG GTTGAGTCGA CGGAGGGAAA TAGGCTGGGT TTTAGTACCC CTAGAGAAGA   
  
  
+ GTTTTCCCTC TCGCTCTCTC TCTCTTTAAA ATTTAAAGAA TTTTTTAAAA TACAATTAAA GTAGACCCAA   
  
  
+ AATAATTTTA AAATGAAAAA TTAATTGGGT TTTTTAATAG TTTTATAATA AAATCAGAAA TAATTTTTAT   
  
  
+ CTCGTGTATA GTTATCTCAT AGAATTAAGA TTTTTTATTA CAAAACTTAT CCTTTAAATT TACCTTACTT   
  
  
+ AACCAGTTTC AACATTAAAT CCCGGTTTTT TAATACCTAT TTTTTCCTTT ATTTCCCTAA GTTATTAAGT   
  
  
+ GTACATTATT TACGAACATA CCTTTCGTAA CTCGTACATA CCGACGGACG ATGCCACCAA CGAGACAATT   
  
  
+ ATCACGGGGT TCTTCTTCCC GGCGTGGATA TCCGATACCA GTCGGCTCCT TTAACGTTTA ACCGTGCTTT   
  
  
+ GAGAACGCGT GGAACTCAAA ATGAGAAATT GGACGTGTGA TTGGGAGACA CACCGCGGGT GTTATGGGGG   
  
  
+ TTCAGTGGGT CCTAGGGTGT GGGTCTCCCG TCATGTTTGC GGCGTGGCCC CCTCCTAGGG GCATTTGCTT   
  
  
+ TAACACTGTG CACTCCGTAT ACCTACATTA TAAAATTAAT ACTTTTAATT TTCCATAGTT AATACTTTTT   
  
  
+ AATTGCAGTG TTTCTAATGT CATAATTTTG ATATAATATA TAGCTTGACT TCGAATTTAA TTATTAATAA   
  
  
+ TTTCCTAGTG CCGTTATAAA AAGGAATAT  

- GATTAAAGAA AAACTTCTCA GCGCGTCTCT CTATACACGA AGATCCGGAT ACCTGCCGAT AAATGACTGG   
  
  
- TTTTGGCCGT AGATGATGAT GACGACGACT ACTGCAGCAG ATACAGAGAG TGGCTTGATT GCTGGAAGGA   
  
  
- AGTAAAGAGG GAGAGAGAGA GAGAGAGAGA AGAGAAGAAA TGGAGGAGAA GGTGGTGAAG GAAAGAAAGG   
  
  
- GTGGTCCTTA CAGAAAAGGG CCGTCATGTG AGACAGAAGG AGTGTTTGGT AATATATATT TGTAGTTAGC   
  
  
- AAAGGACGTG CAAGCAAATG TGATTTCAAG CCATCACTAT TTTATTATAT CATCAACTTG AAAACGAACC   
  
  
- CTCCATTTTA CTTTTCTTTT TTGTTTATTG AAATCATTAA GCCCACTGAT TACAGAGTCT GCATTAATTA   
  
  
- TTTTTAATTT TGGTAGAAAA CGGGGCTGTG TCATGTTGGT TGGTTTTTCT GGTTTGAACA AAACATGTGT   
  
  
- TGTTTTGTCC TGTTGCTTCA TTGTTCTTTA AGAGCAGTTG TTGTTGAAAT CAGATTACCG GTAGGAGGGT   
  
  
- AGGCCGACTG ATTGGTTTCA TACCCAGAAG TTGCCCTTTT AAAACCAGAT GAGGAAAGTC CTGTTTATAT   
  
  
- TCGCTTATAT TATTCTCATG CAAAATTAGG GTTTCCATTT TCTTTTTTAT TTTCCCTAGT GGATATAGTT   
  
  
- GAGTAACACA GTGATCCACC CAACTCAGCT GCCTCCCTTT ATCCGACCCA AAATCATGGG GATCTCTTCT   
  
  
- CAAAAGGGAG AGCGAGAGAG AGAGAAATTT TAAATTTCTT AAAAAATTTT ATGTTAATTT CATCTGGGTT   
  
  
- TTATTAAAAT TTTACTTTTT AATTAACCCA AAAAATTATC AAAATATTAT TTTAGTCTTT ATTAAAAATA   
  
  
- GAGCACATAT CAATAGAGTA TCTTAATTCT AAAAAATAAT GTTTTGAATA GGAAATTTAA ATGGAATGAA   
  
  
- TTGGTCAAAG TTGTAATTTA GGGCCAAAAA ATTATGGATA AAAAAGGAAA TAAAGGGATT CAATAATTCA   
  
  
- CATGTAATAA ATGCTTGTAT GGAAAGCATT GAGCATGTAT GGCTGCCTGC TACGGTGGTT GCTCTGTTAA   
  
  
- TAGTGCCCCA AGAAGAAGGG CCGCACCTAT AGGCTATGGT CAGCCGAGGA AATTGCAAAT TGGCACGAAA   
  
  
- CTCTTGCGCA CCTTGAGTTT TACTCTTTAA CCTGCACACT AACCCTCTGT GTGGCGCCCA CAATACCCCC   
  
  
- AAGTCACCCA GGATCCCACA CCCAGAGGGC AGTACAAACG CCGCACCGGG GGAGGATCCC CGTAAACGAA   
  
  
- ATTGTGACAC GTGAGGCATA TGGATGTAAT ATTTTAATTA TGAAAATTAA AAGGTATCAA TTATGAAAAA   
  
  
- TTAACGTCAC AAAGATTACA GTATTAAAAC TATATTATAT ATCGAACTGA AGCTTAAATT AATAATTATT   
  
  
- AAAGGATCAC GGCAATATTT TTCCTTATA

+     CCGTCC-box

| Site Name | Organism | Position | Strand | Matrix score. | sequence | function |
| --- | --- | --- | --- | --- | --- | --- |
| CCGTCC-box | Arabidopsis thaliana | 52 | - | 6 | CCGTCC | cis-acting regulatory element related to meristem specific activation |

> 2018/04/13 10:10:12  
+ CTAATTTCTT TTTGAAGAGT CGCGCAGAGA GATATGTGCT TCTAGGCCTA TGGACGGCTA TTTACTGACC   
  
  
+ AAAACCGGCA TCTACTACTA CTGCTGCTGA TGACGTCGTC TATGTCTCTC ACCGAACTAA CGACCTTCCT   
  
  
+ TCATTTCTCC CTCTCTCTCT CTCTCTCTCT TCTCTTCTTT ACCTCCTCTT CCACCACTTC CTTTCTTTCC   
  
  
+ CACCAGGAAT GTCTTTTCCC GGCAGTACAC TCTGTCTTCC TCACAAACCA TTATATATAA ACATCAATCG   
  
  
+ TTTCCTGCAC GTTCGTTTAC ACTAAAGTTC GGTAGTGATA AAATAATATA GTAGTTGAAC TTTTGCTTGG   
  
  
+ GAGGTAAAAT GAAAAGAAAA AACAAATAAC TTTAGTAATT CGGGTGACTA ATGTCTCAGA CGTAATTAAT   
  
  
+ AAAAATTAAA ACCATCTTTT GCCCCGACAC AGTACAACCA ACCAAAAAGA CCAAACTTGT TTTGTACACA   
  
  
+ ACAAAACAGG ACAACGAAGT AACAAGAAAT TCTCGTCAAC AACAACTTTA GTCTAATGGC CATCCTCCCA   
  
  
+ TCCGGCTGAC TAACCAAAGT ATGGGTCTTC AACGGGAAAA TTTTGGTCTA CTCCTTTCAG GACAAATATA   
  
  
+ AGCGAATATA ATAAGAGTAC GTTTTAATCC CAAAGGTAAA AGAAAAAATA AAAGGGATCA CCTATATCAA   
  
  
+ CTCATTGTGT CACTAGGTGG GTTGAGTCGA CGGAGGGAAA TAGGCTGGGT TTTAGTACCC CTAGAGAAGA   
  
  
+ GTTTTCCCTC TCGCTCTCTC TCTCTTTAAA ATTTAAAGAA TTTTTTAAAA TACAATTAAA GTAGACCCAA   
  
  
+ AATAATTTTA AAATGAAAAA TTAATTGGGT TTTTTAATAG TTTTATAATA AAATCAGAAA TAATTTTTAT   
  
  
+ CTCGTGTATA GTTATCTCAT AGAATTAAGA TTTTTTATTA CAAAACTTAT CCTTTAAATT TACCTTACTT   
  
  
+ AACCAGTTTC AACATTAAAT CCCGGTTTTT TAATACCTAT TTTTTCCTTT ATTTCCCTAA GTTATTAAGT   
  
  
+ GTACATTATT TACGAACATA CCTTTCGTAA CTCGTACATA CCGACGGACG ATGCCACCAA CGAGACAATT   
  
  
+ ATCACGGGGT TCTTCTTCCC GGCGTGGATA TCCGATACCA GTCGGCTCCT TTAACGTTTA ACCGTGCTTT   
  
  
+ GAGAACGCGT GGAACTCAAA ATGAGAAATT GGACGTGTGA TTGGGAGACA CACCGCGGGT GTTATGGGGG   
  
  
+ TTCAGTGGGT CCTAGGGTGT GGGTCTCCCG TCATGTTTGC GGCGTGGCCC CCTCCTAGGG GCATTTGCTT   
  
  
+ TAACACTGTG CACTCCGTAT ACCTACATTA TAAAATTAAT ACTTTTAATT TTCCATAGTT AATACTTTTT   
  
  
+ AATTGCAGTG TTTCTAATGT CATAATTTTG ATATAATATA TAGCTTGACT TCGAATTTAA TTATTAATAA   
  
  
+ TTTCCTAGTG CCGTTATAAA AAGGAATAT  

- GATTAAAGAA AAACTTCTCA GCGCGTCTCT CTATACACGA AGATCCGGAT ACCTGCCGAT AAATGACTGG   
  
  
- TTTTGGCCGT AGATGATGAT GACGACGACT ACTGCAGCAG ATACAGAGAG TGGCTTGATT GCTGGAAGGA   
  
  
- AGTAAAGAGG GAGAGAGAGA GAGAGAGAGA AGAGAAGAAA TGGAGGAGAA GGTGGTGAAG GAAAGAAAGG   
  
  
- GTGGTCCTTA CAGAAAAGGG CCGTCATGTG AGACAGAAGG AGTGTTTGGT AATATATATT TGTAGTTAGC   
  
  
- AAAGGACGTG CAAGCAAATG TGATTTCAAG CCATCACTAT TTTATTATAT CATCAACTTG AAAACGAACC   
  
  
- CTCCATTTTA CTTTTCTTTT TTGTTTATTG AAATCATTAA GCCCACTGAT TACAGAGTCT GCATTAATTA   
  
  
- TTTTTAATTT TGGTAGAAAA CGGGGCTGTG TCATGTTGGT TGGTTTTTCT GGTTTGAACA AAACATGTGT   
  
  
- TGTTTTGTCC TGTTGCTTCA TTGTTCTTTA AGAGCAGTTG TTGTTGAAAT CAGATTACCG GTAGGAGGGT   
  
  
- AGGCCGACTG ATTGGTTTCA TACCCAGAAG TTGCCCTTTT AAAACCAGAT GAGGAAAGTC CTGTTTATAT   
  
  
- TCGCTTATAT TATTCTCATG CAAAATTAGG GTTTCCATTT TCTTTTTTAT TTTCCCTAGT GGATATAGTT   
  
  
- GAGTAACACA GTGATCCACC CAACTCAGCT GCCTCCCTTT ATCCGACCCA AAATCATGGG GATCTCTTCT   
  
  
- CAAAAGGGAG AGCGAGAGAG AGAGAAATTT TAAATTTCTT AAAAAATTTT ATGTTAATTT CATCTGGGTT   
  
  
- TTATTAAAAT TTTACTTTTT AATTAACCCA AAAAATTATC AAAATATTAT TTTAGTCTTT ATTAAAAATA   
  
  
- GAGCACATAT CAATAGAGTA TCTTAATTCT AAAAAATAAT GTTTTGAATA GGAAATTTAA ATGGAATGAA   
  
  
- TTGGTCAAAG TTGTAATTTA GGGCCAAAAA ATTATGGATA AAAAAGGAAA TAAAGGGATT CAATAATTCA   
  
  
- CATGTAATAA ATGCTTGTAT GGAAAGCATT GAGCATGTAT GGCTGCCTGC TACGGTGGTT GCTCTGTTAA   
  
  
- TAGTGCCCCA AGAAGAAGGG CCGCACCTAT AGGCTATGGT CAGCCGAGGA AATTGCAAAT TGGCACGAAA   
  
  
- CTCTTGCGCA CCTTGAGTTT TACTCTTTAA CCTGCACACT AACCCTCTGT GTGGCGCCCA CAATACCCCC   
  
  
- AAGTCACCCA GGATCCCACA CCCAGAGGGC AGTACAAACG CCGCACCGGG GGAGGATCCC CGTAAACGAA   
  
  
- ATTGTGACAC GTGAGGCATA TGGATGTAAT ATTTTAATTA TGAAAATTAA AAGGTATCAA TTATGAAAAA   
  
  
- TTAACGTCAC AAAGATTACA GTATTAAAAC TATATTATAT ATCGAACTGA AGCTTAAATT AATAATTATT   
  
  
- AAAGGATCAC GGCAATATTT TTCCTTATA

+     CGTCA-motif

| Site Name | Organism | Position | Strand | Matrix score. | sequence | function |
| --- | --- | --- | --- | --- | --- | --- |
| CGTCA-motif | Hordeum vulgare | 1289 | + | 5 | CGTCA | cis-acting regulatory element involved in the MeJA-responsiveness |
| CGTCA-motif | Hordeum vulgare | 524 | + | 5 | CGTCA | cis-acting regulatory element involved in the MeJA-responsiveness |
| CGTCA-motif | Hordeum vulgare | 101 | - | 5 | CGTCA | cis-acting regulatory element involved in the MeJA-responsiveness |

> 2018/04/13 10:10:12  
+ CTAATTTCTT TTTGAAGAGT CGCGCAGAGA GATATGTGCT TCTAGGCCTA TGGACGGCTA TTTACTGACC   
  
  
+ AAAACCGGCA TCTACTACTA CTGCTGCTGA TGACGTCGTC TATGTCTCTC ACCGAACTAA CGACCTTCCT   
  
  
+ TCATTTCTCC CTCTCTCTCT CTCTCTCTCT TCTCTTCTTT ACCTCCTCTT CCACCACTTC CTTTCTTTCC   
  
  
+ CACCAGGAAT GTCTTTTCCC GGCAGTACAC TCTGTCTTCC TCACAAACCA TTATATATAA ACATCAATCG   
  
  
+ TTTCCTGCAC GTTCGTTTAC ACTAAAGTTC GGTAGTGATA AAATAATATA GTAGTTGAAC TTTTGCTTGG   
  
  
+ GAGGTAAAAT GAAAAGAAAA AACAAATAAC TTTAGTAATT CGGGTGACTA ATGTCTCAGA CGTAATTAAT   
  
  
+ AAAAATTAAA ACCATCTTTT GCCCCGACAC AGTACAACCA ACCAAAAAGA CCAAACTTGT TTTGTACACA   
  
  
+ ACAAAACAGG ACAACGAAGT AACAAGAAAT TCTCGTCAAC AACAACTTTA GTCTAATGGC CATCCTCCCA   
  
  
+ TCCGGCTGAC TAACCAAAGT ATGGGTCTTC AACGGGAAAA TTTTGGTCTA CTCCTTTCAG GACAAATATA   
  
  
+ AGCGAATATA ATAAGAGTAC GTTTTAATCC CAAAGGTAAA AGAAAAAATA AAAGGGATCA CCTATATCAA   
  
  
+ CTCATTGTGT CACTAGGTGG GTTGAGTCGA CGGAGGGAAA TAGGCTGGGT TTTAGTACCC CTAGAGAAGA   
  
  
+ GTTTTCCCTC TCGCTCTCTC TCTCTTTAAA ATTTAAAGAA TTTTTTAAAA TACAATTAAA GTAGACCCAA   
  
  
+ AATAATTTTA AAATGAAAAA TTAATTGGGT TTTTTAATAG TTTTATAATA AAATCAGAAA TAATTTTTAT   
  
  
+ CTCGTGTATA GTTATCTCAT AGAATTAAGA TTTTTTATTA CAAAACTTAT CCTTTAAATT TACCTTACTT   
  
  
+ AACCAGTTTC AACATTAAAT CCCGGTTTTT TAATACCTAT TTTTTCCTTT ATTTCCCTAA GTTATTAAGT   
  
  
+ GTACATTATT TACGAACATA CCTTTCGTAA CTCGTACATA CCGACGGACG ATGCCACCAA CGAGACAATT   
  
  
+ ATCACGGGGT TCTTCTTCCC GGCGTGGATA TCCGATACCA GTCGGCTCCT TTAACGTTTA ACCGTGCTTT   
  
  
+ GAGAACGCGT GGAACTCAAA ATGAGAAATT GGACGTGTGA TTGGGAGACA CACCGCGGGT GTTATGGGGG   
  
  
+ TTCAGTGGGT CCTAGGGTGT GGGTCTCCCG TCATGTTTGC GGCGTGGCCC CCTCCTAGGG GCATTTGCTT   
  
  
+ TAACACTGTG CACTCCGTAT ACCTACATTA TAAAATTAAT ACTTTTAATT TTCCATAGTT AATACTTTTT   
  
  
+ AATTGCAGTG TTTCTAATGT CATAATTTTG ATATAATATA TAGCTTGACT TCGAATTTAA TTATTAATAA   
  
  
+ TTTCCTAGTG CCGTTATAAA AAGGAATAT  

- GATTAAAGAA AAACTTCTCA GCGCGTCTCT CTATACACGA AGATCCGGAT ACCTGCCGAT AAATGACTGG   
  
  
- TTTTGGCCGT AGATGATGAT GACGACGACT ACTGCAGCAG ATACAGAGAG TGGCTTGATT GCTGGAAGGA   
  
  
- AGTAAAGAGG GAGAGAGAGA GAGAGAGAGA AGAGAAGAAA TGGAGGAGAA GGTGGTGAAG GAAAGAAAGG   
  
  
- GTGGTCCTTA CAGAAAAGGG CCGTCATGTG AGACAGAAGG AGTGTTTGGT AATATATATT TGTAGTTAGC   
  
  
- AAAGGACGTG CAAGCAAATG TGATTTCAAG CCATCACTAT TTTATTATAT CATCAACTTG AAAACGAACC   
  
  
- CTCCATTTTA CTTTTCTTTT TTGTTTATTG AAATCATTAA GCCCACTGAT TACAGAGTCT GCATTAATTA   
  
  
- TTTTTAATTT TGGTAGAAAA CGGGGCTGTG TCATGTTGGT TGGTTTTTCT GGTTTGAACA AAACATGTGT   
  
  
- TGTTTTGTCC TGTTGCTTCA TTGTTCTTTA AGAGCAGTTG TTGTTGAAAT CAGATTACCG GTAGGAGGGT   
  
  
- AGGCCGACTG ATTGGTTTCA TACCCAGAAG TTGCCCTTTT AAAACCAGAT GAGGAAAGTC CTGTTTATAT   
  
  
- TCGCTTATAT TATTCTCATG CAAAATTAGG GTTTCCATTT TCTTTTTTAT TTTCCCTAGT GGATATAGTT   
  
  
- GAGTAACACA GTGATCCACC CAACTCAGCT GCCTCCCTTT ATCCGACCCA AAATCATGGG GATCTCTTCT   
  
  
- CAAAAGGGAG AGCGAGAGAG AGAGAAATTT TAAATTTCTT AAAAAATTTT ATGTTAATTT CATCTGGGTT   
  
  
- TTATTAAAAT TTTACTTTTT AATTAACCCA AAAAATTATC AAAATATTAT TTTAGTCTTT ATTAAAAATA   
  
  
- GAGCACATAT CAATAGAGTA TCTTAATTCT AAAAAATAAT GTTTTGAATA GGAAATTTAA ATGGAATGAA   
  
  
- TTGGTCAAAG TTGTAATTTA GGGCCAAAAA ATTATGGATA AAAAAGGAAA TAAAGGGATT CAATAATTCA   
  
  
- CATGTAATAA ATGCTTGTAT GGAAAGCATT GAGCATGTAT GGCTGCCTGC TACGGTGGTT GCTCTGTTAA   
  
  
- TAGTGCCCCA AGAAGAAGGG CCGCACCTAT AGGCTATGGT CAGCCGAGGA AATTGCAAAT TGGCACGAAA   
  
  
- CTCTTGCGCA CCTTGAGTTT TACTCTTTAA CCTGCACACT AACCCTCTGT GTGGCGCCCA CAATACCCCC   
  
  
- AAGTCACCCA GGATCCCACA CCCAGAGGGC AGTACAAACG CCGCACCGGG GGAGGATCCC CGTAAACGAA   
  
  
- ATTGTGACAC GTGAGGCATA TGGATGTAAT ATTTTAATTA TGAAAATTAA AAGGTATCAA TTATGAAAAA   
  
  
- TTAACGTCAC AAAGATTACA GTATTAAAAC TATATTATAT ATCGAACTGA AGCTTAAATT AATAATTATT   
  
  
- AAAGGATCAC GGCAATATTT TTCCTTATA

+     G-Box

| Site Name | Organism | Position | Strand | Matrix score. | sequence | function |
| --- | --- | --- | --- | --- | --- | --- |
| G-Box | Pisum sativum | 288 | + | 6 | CACGTT | cis-acting regulatory element involved in light responsiveness |

> 2018/04/13 10:10:12  
+ CTAATTTCTT TTTGAAGAGT CGCGCAGAGA GATATGTGCT TCTAGGCCTA TGGACGGCTA TTTACTGACC   
  
  
+ AAAACCGGCA TCTACTACTA CTGCTGCTGA TGACGTCGTC TATGTCTCTC ACCGAACTAA CGACCTTCCT   
  
  
+ TCATTTCTCC CTCTCTCTCT CTCTCTCTCT TCTCTTCTTT ACCTCCTCTT CCACCACTTC CTTTCTTTCC   
  
  
+ CACCAGGAAT GTCTTTTCCC GGCAGTACAC TCTGTCTTCC TCACAAACCA TTATATATAA ACATCAATCG   
  
  
+ TTTCCTGCAC GTTCGTTTAC ACTAAAGTTC GGTAGTGATA AAATAATATA GTAGTTGAAC TTTTGCTTGG   
  
  
+ GAGGTAAAAT GAAAAGAAAA AACAAATAAC TTTAGTAATT CGGGTGACTA ATGTCTCAGA CGTAATTAAT   
  
  
+ AAAAATTAAA ACCATCTTTT GCCCCGACAC AGTACAACCA ACCAAAAAGA CCAAACTTGT TTTGTACACA   
  
  
+ ACAAAACAGG ACAACGAAGT AACAAGAAAT TCTCGTCAAC AACAACTTTA GTCTAATGGC CATCCTCCCA   
  
  
+ TCCGGCTGAC TAACCAAAGT ATGGGTCTTC AACGGGAAAA TTTTGGTCTA CTCCTTTCAG GACAAATATA   
  
  
+ AGCGAATATA ATAAGAGTAC GTTTTAATCC CAAAGGTAAA AGAAAAAATA AAAGGGATCA CCTATATCAA   
  
  
+ CTCATTGTGT CACTAGGTGG GTTGAGTCGA CGGAGGGAAA TAGGCTGGGT TTTAGTACCC CTAGAGAAGA   
  
  
+ GTTTTCCCTC TCGCTCTCTC TCTCTTTAAA ATTTAAAGAA TTTTTTAAAA TACAATTAAA GTAGACCCAA   
  
  
+ AATAATTTTA AAATGAAAAA TTAATTGGGT TTTTTAATAG TTTTATAATA AAATCAGAAA TAATTTTTAT   
  
  
+ CTCGTGTATA GTTATCTCAT AGAATTAAGA TTTTTTATTA CAAAACTTAT CCTTTAAATT TACCTTACTT   
  
  
+ AACCAGTTTC AACATTAAAT CCCGGTTTTT TAATACCTAT TTTTTCCTTT ATTTCCCTAA GTTATTAAGT   
  
  
+ GTACATTATT TACGAACATA CCTTTCGTAA CTCGTACATA CCGACGGACG ATGCCACCAA CGAGACAATT   
  
  
+ ATCACGGGGT TCTTCTTCCC GGCGTGGATA TCCGATACCA GTCGGCTCCT TTAACGTTTA ACCGTGCTTT   
  
  
+ GAGAACGCGT GGAACTCAAA ATGAGAAATT GGACGTGTGA TTGGGAGACA CACCGCGGGT GTTATGGGGG   
  
  
+ TTCAGTGGGT CCTAGGGTGT GGGTCTCCCG TCATGTTTGC GGCGTGGCCC CCTCCTAGGG GCATTTGCTT   
  
  
+ TAACACTGTG CACTCCGTAT ACCTACATTA TAAAATTAAT ACTTTTAATT TTCCATAGTT AATACTTTTT   
  
  
+ AATTGCAGTG TTTCTAATGT CATAATTTTG ATATAATATA TAGCTTGACT TCGAATTTAA TTATTAATAA   
  
  
+ TTTCCTAGTG CCGTTATAAA AAGGAATAT  

- GATTAAAGAA AAACTTCTCA GCGCGTCTCT CTATACACGA AGATCCGGAT ACCTGCCGAT AAATGACTGG   
  
  
- TTTTGGCCGT AGATGATGAT GACGACGACT ACTGCAGCAG ATACAGAGAG TGGCTTGATT GCTGGAAGGA   
  
  
- AGTAAAGAGG GAGAGAGAGA GAGAGAGAGA AGAGAAGAAA TGGAGGAGAA GGTGGTGAAG GAAAGAAAGG   
  
  
- GTGGTCCTTA CAGAAAAGGG CCGTCATGTG AGACAGAAGG AGTGTTTGGT AATATATATT TGTAGTTAGC   
  
  
- AAAGGACGTG CAAGCAAATG TGATTTCAAG CCATCACTAT TTTATTATAT CATCAACTTG AAAACGAACC   
  
  
- CTCCATTTTA CTTTTCTTTT TTGTTTATTG AAATCATTAA GCCCACTGAT TACAGAGTCT GCATTAATTA   
  
  
- TTTTTAATTT TGGTAGAAAA CGGGGCTGTG TCATGTTGGT TGGTTTTTCT GGTTTGAACA AAACATGTGT   
  
  
- TGTTTTGTCC TGTTGCTTCA TTGTTCTTTA AGAGCAGTTG TTGTTGAAAT CAGATTACCG GTAGGAGGGT   
  
  
- AGGCCGACTG ATTGGTTTCA TACCCAGAAG TTGCCCTTTT AAAACCAGAT GAGGAAAGTC CTGTTTATAT   
  
  
- TCGCTTATAT TATTCTCATG CAAAATTAGG GTTTCCATTT TCTTTTTTAT TTTCCCTAGT GGATATAGTT   
  
  
- GAGTAACACA GTGATCCACC CAACTCAGCT GCCTCCCTTT ATCCGACCCA AAATCATGGG GATCTCTTCT   
  
  
- CAAAAGGGAG AGCGAGAGAG AGAGAAATTT TAAATTTCTT AAAAAATTTT ATGTTAATTT CATCTGGGTT   
  
  
- TTATTAAAAT TTTACTTTTT AATTAACCCA AAAAATTATC AAAATATTAT TTTAGTCTTT ATTAAAAATA   
  
  
- GAGCACATAT CAATAGAGTA TCTTAATTCT AAAAAATAAT GTTTTGAATA GGAAATTTAA ATGGAATGAA   
  
  
- TTGGTCAAAG TTGTAATTTA GGGCCAAAAA ATTATGGATA AAAAAGGAAA TAAAGGGATT CAATAATTCA   
  
  
- CATGTAATAA ATGCTTGTAT GGAAAGCATT GAGCATGTAT GGCTGCCTGC TACGGTGGTT GCTCTGTTAA   
  
  
- TAGTGCCCCA AGAAGAAGGG CCGCACCTAT AGGCTATGGT CAGCCGAGGA AATTGCAAAT TGGCACGAAA   
  
  
- CTCTTGCGCA CCTTGAGTTT TACTCTTTAA CCTGCACACT AACCCTCTGT GTGGCGCCCA CAATACCCCC   
  
  
- AAGTCACCCA GGATCCCACA CCCAGAGGGC AGTACAAACG CCGCACCGGG GGAGGATCCC CGTAAACGAA   
  
  
- ATTGTGACAC GTGAGGCATA TGGATGTAAT ATTTTAATTA TGAAAATTAA AAGGTATCAA TTATGAAAAA   
  
  
- TTAACGTCAC AAAGATTACA GTATTAAAAC TATATTATAT ATCGAACTGA AGCTTAAATT AATAATTATT   
  
  
- AAAGGATCAC GGCAATATTT TTCCTTATA

+     G-box

| Site Name | Organism | Position | Strand | Matrix score. | sequence | function |
| --- | --- | --- | --- | --- | --- | --- |
| G-box | Zea mays | 288 | + | 6 | CACGTT | cis-acting regulatory element involved in light responsiveness |
| G-box | Zea mays | 1222 | - | 6 | CACGTC | cis-acting regulatory element involved in light responsiveness |

> 2018/04/13 10:10:12  
+ CTAATTTCTT TTTGAAGAGT CGCGCAGAGA GATATGTGCT TCTAGGCCTA TGGACGGCTA TTTACTGACC   
  
  
+ AAAACCGGCA TCTACTACTA CTGCTGCTGA TGACGTCGTC TATGTCTCTC ACCGAACTAA CGACCTTCCT   
  
  
+ TCATTTCTCC CTCTCTCTCT CTCTCTCTCT TCTCTTCTTT ACCTCCTCTT CCACCACTTC CTTTCTTTCC   
  
  
+ CACCAGGAAT GTCTTTTCCC GGCAGTACAC TCTGTCTTCC TCACAAACCA TTATATATAA ACATCAATCG   
  
  
+ TTTCCTGCAC GTTCGTTTAC ACTAAAGTTC GGTAGTGATA AAATAATATA GTAGTTGAAC TTTTGCTTGG   
  
  
+ GAGGTAAAAT GAAAAGAAAA AACAAATAAC TTTAGTAATT CGGGTGACTA ATGTCTCAGA CGTAATTAAT   
  
  
+ AAAAATTAAA ACCATCTTTT GCCCCGACAC AGTACAACCA ACCAAAAAGA CCAAACTTGT TTTGTACACA   
  
  
+ ACAAAACAGG ACAACGAAGT AACAAGAAAT TCTCGTCAAC AACAACTTTA GTCTAATGGC CATCCTCCCA   
  
  
+ TCCGGCTGAC TAACCAAAGT ATGGGTCTTC AACGGGAAAA TTTTGGTCTA CTCCTTTCAG GACAAATATA   
  
  
+ AGCGAATATA ATAAGAGTAC GTTTTAATCC CAAAGGTAAA AGAAAAAATA AAAGGGATCA CCTATATCAA   
  
  
+ CTCATTGTGT CACTAGGTGG GTTGAGTCGA CGGAGGGAAA TAGGCTGGGT TTTAGTACCC CTAGAGAAGA   
  
  
+ GTTTTCCCTC TCGCTCTCTC TCTCTTTAAA ATTTAAAGAA TTTTTTAAAA TACAATTAAA GTAGACCCAA   
  
  
+ AATAATTTTA AAATGAAAAA TTAATTGGGT TTTTTAATAG TTTTATAATA AAATCAGAAA TAATTTTTAT   
  
  
+ CTCGTGTATA GTTATCTCAT AGAATTAAGA TTTTTTATTA CAAAACTTAT CCTTTAAATT TACCTTACTT   
  
  
+ AACCAGTTTC AACATTAAAT CCCGGTTTTT TAATACCTAT TTTTTCCTTT ATTTCCCTAA GTTATTAAGT   
  
  
+ GTACATTATT TACGAACATA CCTTTCGTAA CTCGTACATA CCGACGGACG ATGCCACCAA CGAGACAATT   
  
  
+ ATCACGGGGT TCTTCTTCCC GGCGTGGATA TCCGATACCA GTCGGCTCCT TTAACGTTTA ACCGTGCTTT   
  
  
+ GAGAACGCGT GGAACTCAAA ATGAGAAATT GGACGTGTGA TTGGGAGACA CACCGCGGGT GTTATGGGGG   
  
  
+ TTCAGTGGGT CCTAGGGTGT GGGTCTCCCG TCATGTTTGC GGCGTGGCCC CCTCCTAGGG GCATTTGCTT   
  
  
+ TAACACTGTG CACTCCGTAT ACCTACATTA TAAAATTAAT ACTTTTAATT TTCCATAGTT AATACTTTTT   
  
  
+ AATTGCAGTG TTTCTAATGT CATAATTTTG ATATAATATA TAGCTTGACT TCGAATTTAA TTATTAATAA   
  
  
+ TTTCCTAGTG CCGTTATAAA AAGGAATAT  

- GATTAAAGAA AAACTTCTCA GCGCGTCTCT CTATACACGA AGATCCGGAT ACCTGCCGAT AAATGACTGG   
  
  
- TTTTGGCCGT AGATGATGAT GACGACGACT ACTGCAGCAG ATACAGAGAG TGGCTTGATT GCTGGAAGGA   
  
  
- AGTAAAGAGG GAGAGAGAGA GAGAGAGAGA AGAGAAGAAA TGGAGGAGAA GGTGGTGAAG GAAAGAAAGG   
  
  
- GTGGTCCTTA CAGAAAAGGG CCGTCATGTG AGACAGAAGG AGTGTTTGGT AATATATATT TGTAGTTAGC   
  
  
- AAAGGACGTG CAAGCAAATG TGATTTCAAG CCATCACTAT TTTATTATAT CATCAACTTG AAAACGAACC   
  
  
- CTCCATTTTA CTTTTCTTTT TTGTTTATTG AAATCATTAA GCCCACTGAT TACAGAGTCT GCATTAATTA   
  
  
- TTTTTAATTT TGGTAGAAAA CGGGGCTGTG TCATGTTGGT TGGTTTTTCT GGTTTGAACA AAACATGTGT   
  
  
- TGTTTTGTCC TGTTGCTTCA TTGTTCTTTA AGAGCAGTTG TTGTTGAAAT CAGATTACCG GTAGGAGGGT   
  
  
- AGGCCGACTG ATTGGTTTCA TACCCAGAAG TTGCCCTTTT AAAACCAGAT GAGGAAAGTC CTGTTTATAT   
  
  
- TCGCTTATAT TATTCTCATG CAAAATTAGG GTTTCCATTT TCTTTTTTAT TTTCCCTAGT GGATATAGTT   
  
  
- GAGTAACACA GTGATCCACC CAACTCAGCT GCCTCCCTTT ATCCGACCCA AAATCATGGG GATCTCTTCT   
  
  
- CAAAAGGGAG AGCGAGAGAG AGAGAAATTT TAAATTTCTT AAAAAATTTT ATGTTAATTT CATCTGGGTT   
  
  
- TTATTAAAAT TTTACTTTTT AATTAACCCA AAAAATTATC AAAATATTAT TTTAGTCTTT ATTAAAAATA   
  
  
- GAGCACATAT CAATAGAGTA TCTTAATTCT AAAAAATAAT GTTTTGAATA GGAAATTTAA ATGGAATGAA   
  
  
- TTGGTCAAAG TTGTAATTTA GGGCCAAAAA ATTATGGATA AAAAAGGAAA TAAAGGGATT CAATAATTCA   
  
  
- CATGTAATAA ATGCTTGTAT GGAAAGCATT GAGCATGTAT GGCTGCCTGC TACGGTGGTT GCTCTGTTAA   
  
  
- TAGTGCCCCA AGAAGAAGGG CCGCACCTAT AGGCTATGGT CAGCCGAGGA AATTGCAAAT TGGCACGAAA   
  
  
- CTCTTGCGCA CCTTGAGTTT TACTCTTTAA CCTGCACACT AACCCTCTGT GTGGCGCCCA CAATACCCCC   
  
  
- AAGTCACCCA GGATCCCACA CCCAGAGGGC AGTACAAACG CCGCACCGGG GGAGGATCCC CGTAAACGAA   
  
  
- ATTGTGACAC GTGAGGCATA TGGATGTAAT ATTTTAATTA TGAAAATTAA AAGGTATCAA TTATGAAAAA   
  
  
- TTAACGTCAC AAAGATTACA GTATTAAAAC TATATTATAT ATCGAACTGA AGCTTAAATT AATAATTATT   
  
  
- AAAGGATCAC GGCAATATTT TTCCTTATA

+     GATA-motif

| Site Name | Organism | Position | Strand | Matrix score. | sequence | function |
| --- | --- | --- | --- | --- | --- | --- |
| GATA-motif | Solanum tuberosum | 955 | - | 9 | AAGGATAAGG | part of a light responsive element |

> 2018/04/13 10:10:12  
+ CTAATTTCTT TTTGAAGAGT CGCGCAGAGA GATATGTGCT TCTAGGCCTA TGGACGGCTA TTTACTGACC   
  
  
+ AAAACCGGCA TCTACTACTA CTGCTGCTGA TGACGTCGTC TATGTCTCTC ACCGAACTAA CGACCTTCCT   
  
  
+ TCATTTCTCC CTCTCTCTCT CTCTCTCTCT TCTCTTCTTT ACCTCCTCTT CCACCACTTC CTTTCTTTCC   
  
  
+ CACCAGGAAT GTCTTTTCCC GGCAGTACAC TCTGTCTTCC TCACAAACCA TTATATATAA ACATCAATCG   
  
  
+ TTTCCTGCAC GTTCGTTTAC ACTAAAGTTC GGTAGTGATA AAATAATATA GTAGTTGAAC TTTTGCTTGG   
  
  
+ GAGGTAAAAT GAAAAGAAAA AACAAATAAC TTTAGTAATT CGGGTGACTA ATGTCTCAGA CGTAATTAAT   
  
  
+ AAAAATTAAA ACCATCTTTT GCCCCGACAC AGTACAACCA ACCAAAAAGA CCAAACTTGT TTTGTACACA   
  
  
+ ACAAAACAGG ACAACGAAGT AACAAGAAAT TCTCGTCAAC AACAACTTTA GTCTAATGGC CATCCTCCCA   
  
  
+ TCCGGCTGAC TAACCAAAGT ATGGGTCTTC AACGGGAAAA TTTTGGTCTA CTCCTTTCAG GACAAATATA   
  
  
+ AGCGAATATA ATAAGAGTAC GTTTTAATCC CAAAGGTAAA AGAAAAAATA AAAGGGATCA CCTATATCAA   
  
  
+ CTCATTGTGT CACTAGGTGG GTTGAGTCGA CGGAGGGAAA TAGGCTGGGT TTTAGTACCC CTAGAGAAGA   
  
  
+ GTTTTCCCTC TCGCTCTCTC TCTCTTTAAA ATTTAAAGAA TTTTTTAAAA TACAATTAAA GTAGACCCAA   
  
  
+ AATAATTTTA AAATGAAAAA TTAATTGGGT TTTTTAATAG TTTTATAATA AAATCAGAAA TAATTTTTAT   
  
  
+ CTCGTGTATA GTTATCTCAT AGAATTAAGA TTTTTTATTA CAAAACTTAT CCTTTAAATT TACCTTACTT   
  
  
+ AACCAGTTTC AACATTAAAT CCCGGTTTTT TAATACCTAT TTTTTCCTTT ATTTCCCTAA GTTATTAAGT   
  
  
+ GTACATTATT TACGAACATA CCTTTCGTAA CTCGTACATA CCGACGGACG ATGCCACCAA CGAGACAATT   
  
  
+ ATCACGGGGT TCTTCTTCCC GGCGTGGATA TCCGATACCA GTCGGCTCCT TTAACGTTTA ACCGTGCTTT   
  
  
+ GAGAACGCGT GGAACTCAAA ATGAGAAATT GGACGTGTGA TTGGGAGACA CACCGCGGGT GTTATGGGGG   
  
  
+ TTCAGTGGGT CCTAGGGTGT GGGTCTCCCG TCATGTTTGC GGCGTGGCCC CCTCCTAGGG GCATTTGCTT   
  
  
+ TAACACTGTG CACTCCGTAT ACCTACATTA TAAAATTAAT ACTTTTAATT TTCCATAGTT AATACTTTTT   
  
  
+ AATTGCAGTG TTTCTAATGT CATAATTTTG ATATAATATA TAGCTTGACT TCGAATTTAA TTATTAATAA   
  
  
+ TTTCCTAGTG CCGTTATAAA AAGGAATAT  

- GATTAAAGAA AAACTTCTCA GCGCGTCTCT CTATACACGA AGATCCGGAT ACCTGCCGAT AAATGACTGG   
  
  
- TTTTGGCCGT AGATGATGAT GACGACGACT ACTGCAGCAG ATACAGAGAG TGGCTTGATT GCTGGAAGGA   
  
  
- AGTAAAGAGG GAGAGAGAGA GAGAGAGAGA AGAGAAGAAA TGGAGGAGAA GGTGGTGAAG GAAAGAAAGG   
  
  
- GTGGTCCTTA CAGAAAAGGG CCGTCATGTG AGACAGAAGG AGTGTTTGGT AATATATATT TGTAGTTAGC   
  
  
- AAAGGACGTG CAAGCAAATG TGATTTCAAG CCATCACTAT TTTATTATAT CATCAACTTG AAAACGAACC   
  
  
- CTCCATTTTA CTTTTCTTTT TTGTTTATTG AAATCATTAA GCCCACTGAT TACAGAGTCT GCATTAATTA   
  
  
- TTTTTAATTT TGGTAGAAAA CGGGGCTGTG TCATGTTGGT TGGTTTTTCT GGTTTGAACA AAACATGTGT   
  
  
- TGTTTTGTCC TGTTGCTTCA TTGTTCTTTA AGAGCAGTTG TTGTTGAAAT CAGATTACCG GTAGGAGGGT   
  
  
- AGGCCGACTG ATTGGTTTCA TACCCAGAAG TTGCCCTTTT AAAACCAGAT GAGGAAAGTC CTGTTTATAT   
  
  
- TCGCTTATAT TATTCTCATG CAAAATTAGG GTTTCCATTT TCTTTTTTAT TTTCCCTAGT GGATATAGTT   
  
  
- GAGTAACACA GTGATCCACC CAACTCAGCT GCCTCCCTTT ATCCGACCCA AAATCATGGG GATCTCTTCT   
  
  
- CAAAAGGGAG AGCGAGAGAG AGAGAAATTT TAAATTTCTT AAAAAATTTT ATGTTAATTT CATCTGGGTT   
  
  
- TTATTAAAAT TTTACTTTTT AATTAACCCA AAAAATTATC AAAATATTAT TTTAGTCTTT ATTAAAAATA   
  
  
- GAGCACATAT CAATAGAGTA TCTTAATTCT AAAAAATAAT GTTTTGAATA GGAAATTTAA ATGGAATGAA   
  
  
- TTGGTCAAAG TTGTAATTTA GGGCCAAAAA ATTATGGATA AAAAAGGAAA TAAAGGGATT CAATAATTCA   
  
  
- CATGTAATAA ATGCTTGTAT GGAAAGCATT GAGCATGTAT GGCTGCCTGC TACGGTGGTT GCTCTGTTAA   
  
  
- TAGTGCCCCA AGAAGAAGGG CCGCACCTAT AGGCTATGGT CAGCCGAGGA AATTGCAAAT TGGCACGAAA   
  
  
- CTCTTGCGCA CCTTGAGTTT TACTCTTTAA CCTGCACACT AACCCTCTGT GTGGCGCCCA CAATACCCCC   
  
  
- AAGTCACCCA GGATCCCACA CCCAGAGGGC AGTACAAACG CCGCACCGGG GGAGGATCCC CGTAAACGAA   
  
  
- ATTGTGACAC GTGAGGCATA TGGATGTAAT ATTTTAATTA TGAAAATTAA AAGGTATCAA TTATGAAAAA   
  
  
- TTAACGTCAC AAAGATTACA GTATTAAAAC TATATTATAT ATCGAACTGA AGCTTAAATT AATAATTATT   
  
  
- AAAGGATCAC GGCAATATTT TTCCTTATA

+     GCN4\_motif

| Site Name | Organism | Position | Strand | Matrix score. | sequence | function |
| --- | --- | --- | --- | --- | --- | --- |
| GCN4\_motif | Oryza sativa | 706 | + | 7 | TGTGTCA | cis-regulatory element involved in endosperm expression |

> 2018/04/13 10:10:12  
+ CTAATTTCTT TTTGAAGAGT CGCGCAGAGA GATATGTGCT TCTAGGCCTA TGGACGGCTA TTTACTGACC   
  
  
+ AAAACCGGCA TCTACTACTA CTGCTGCTGA TGACGTCGTC TATGTCTCTC ACCGAACTAA CGACCTTCCT   
  
  
+ TCATTTCTCC CTCTCTCTCT CTCTCTCTCT TCTCTTCTTT ACCTCCTCTT CCACCACTTC CTTTCTTTCC   
  
  
+ CACCAGGAAT GTCTTTTCCC GGCAGTACAC TCTGTCTTCC TCACAAACCA TTATATATAA ACATCAATCG   
  
  
+ TTTCCTGCAC GTTCGTTTAC ACTAAAGTTC GGTAGTGATA AAATAATATA GTAGTTGAAC TTTTGCTTGG   
  
  
+ GAGGTAAAAT GAAAAGAAAA AACAAATAAC TTTAGTAATT CGGGTGACTA ATGTCTCAGA CGTAATTAAT   
  
  
+ AAAAATTAAA ACCATCTTTT GCCCCGACAC AGTACAACCA ACCAAAAAGA CCAAACTTGT TTTGTACACA   
  
  
+ ACAAAACAGG ACAACGAAGT AACAAGAAAT TCTCGTCAAC AACAACTTTA GTCTAATGGC CATCCTCCCA   
  
  
+ TCCGGCTGAC TAACCAAAGT ATGGGTCTTC AACGGGAAAA TTTTGGTCTA CTCCTTTCAG GACAAATATA   
  
  
+ AGCGAATATA ATAAGAGTAC GTTTTAATCC CAAAGGTAAA AGAAAAAATA AAAGGGATCA CCTATATCAA   
  
  
+ CTCATTGTGT CACTAGGTGG GTTGAGTCGA CGGAGGGAAA TAGGCTGGGT TTTAGTACCC CTAGAGAAGA   
  
  
+ GTTTTCCCTC TCGCTCTCTC TCTCTTTAAA ATTTAAAGAA TTTTTTAAAA TACAATTAAA GTAGACCCAA   
  
  
+ AATAATTTTA AAATGAAAAA TTAATTGGGT TTTTTAATAG TTTTATAATA AAATCAGAAA TAATTTTTAT   
  
  
+ CTCGTGTATA GTTATCTCAT AGAATTAAGA TTTTTTATTA CAAAACTTAT CCTTTAAATT TACCTTACTT   
  
  
+ AACCAGTTTC AACATTAAAT CCCGGTTTTT TAATACCTAT TTTTTCCTTT ATTTCCCTAA GTTATTAAGT   
  
  
+ GTACATTATT TACGAACATA CCTTTCGTAA CTCGTACATA CCGACGGACG ATGCCACCAA CGAGACAATT   
  
  
+ ATCACGGGGT TCTTCTTCCC GGCGTGGATA TCCGATACCA GTCGGCTCCT TTAACGTTTA ACCGTGCTTT   
  
  
+ GAGAACGCGT GGAACTCAAA ATGAGAAATT GGACGTGTGA TTGGGAGACA CACCGCGGGT GTTATGGGGG   
  
  
+ TTCAGTGGGT CCTAGGGTGT GGGTCTCCCG TCATGTTTGC GGCGTGGCCC CCTCCTAGGG GCATTTGCTT   
  
  
+ TAACACTGTG CACTCCGTAT ACCTACATTA TAAAATTAAT ACTTTTAATT TTCCATAGTT AATACTTTTT   
  
  
+ AATTGCAGTG TTTCTAATGT CATAATTTTG ATATAATATA TAGCTTGACT TCGAATTTAA TTATTAATAA   
  
  
+ TTTCCTAGTG CCGTTATAAA AAGGAATAT  

- GATTAAAGAA AAACTTCTCA GCGCGTCTCT CTATACACGA AGATCCGGAT ACCTGCCGAT AAATGACTGG   
  
  
- TTTTGGCCGT AGATGATGAT GACGACGACT ACTGCAGCAG ATACAGAGAG TGGCTTGATT GCTGGAAGGA   
  
  
- AGTAAAGAGG GAGAGAGAGA GAGAGAGAGA AGAGAAGAAA TGGAGGAGAA GGTGGTGAAG GAAAGAAAGG   
  
  
- GTGGTCCTTA CAGAAAAGGG CCGTCATGTG AGACAGAAGG AGTGTTTGGT AATATATATT TGTAGTTAGC   
  
  
- AAAGGACGTG CAAGCAAATG TGATTTCAAG CCATCACTAT TTTATTATAT CATCAACTTG AAAACGAACC   
  
  
- CTCCATTTTA CTTTTCTTTT TTGTTTATTG AAATCATTAA GCCCACTGAT TACAGAGTCT GCATTAATTA   
  
  
- TTTTTAATTT TGGTAGAAAA CGGGGCTGTG TCATGTTGGT TGGTTTTTCT GGTTTGAACA AAACATGTGT   
  
  
- TGTTTTGTCC TGTTGCTTCA TTGTTCTTTA AGAGCAGTTG TTGTTGAAAT CAGATTACCG GTAGGAGGGT   
  
  
- AGGCCGACTG ATTGGTTTCA TACCCAGAAG TTGCCCTTTT AAAACCAGAT GAGGAAAGTC CTGTTTATAT   
  
  
- TCGCTTATAT TATTCTCATG CAAAATTAGG GTTTCCATTT TCTTTTTTAT TTTCCCTAGT GGATATAGTT   
  
  
- GAGTAACACA GTGATCCACC CAACTCAGCT GCCTCCCTTT ATCCGACCCA AAATCATGGG GATCTCTTCT   
  
  
- CAAAAGGGAG AGCGAGAGAG AGAGAAATTT TAAATTTCTT AAAAAATTTT ATGTTAATTT CATCTGGGTT   
  
  
- TTATTAAAAT TTTACTTTTT AATTAACCCA AAAAATTATC AAAATATTAT TTTAGTCTTT ATTAAAAATA   
  
  
- GAGCACATAT CAATAGAGTA TCTTAATTCT AAAAAATAAT GTTTTGAATA GGAAATTTAA ATGGAATGAA   
  
  
- TTGGTCAAAG TTGTAATTTA GGGCCAAAAA ATTATGGATA AAAAAGGAAA TAAAGGGATT CAATAATTCA   
  
  
- CATGTAATAA ATGCTTGTAT GGAAAGCATT GAGCATGTAT GGCTGCCTGC TACGGTGGTT GCTCTGTTAA   
  
  
- TAGTGCCCCA AGAAGAAGGG CCGCACCTAT AGGCTATGGT CAGCCGAGGA AATTGCAAAT TGGCACGAAA   
  
  
- CTCTTGCGCA CCTTGAGTTT TACTCTTTAA CCTGCACACT AACCCTCTGT GTGGCGCCCA CAATACCCCC   
  
  
- AAGTCACCCA GGATCCCACA CCCAGAGGGC AGTACAAACG CCGCACCGGG GGAGGATCCC CGTAAACGAA   
  
  
- ATTGTGACAC GTGAGGCATA TGGATGTAAT ATTTTAATTA TGAAAATTAA AAGGTATCAA TTATGAAAAA   
  
  
- TTAACGTCAC AAAGATTACA GTATTAAAAC TATATTATAT ATCGAACTGA AGCTTAAATT AATAATTATT   
  
  
- AAAGGATCAC GGCAATATTT TTCCTTATA

+     GT1-motif

| Site Name | Organism | Position | Strand | Matrix score. | sequence | function |
| --- | --- | --- | --- | --- | --- | --- |
| GT1-motif | Arabidopsis thaliana | 979 | - | 6 | GGTTAA | light responsive element |
| GT1-motif | Arabidopsis thaliana | 1178 | - | 6 | GGTTAA | light responsive element |

> 2018/04/13 10:10:12  
+ CTAATTTCTT TTTGAAGAGT CGCGCAGAGA GATATGTGCT TCTAGGCCTA TGGACGGCTA TTTACTGACC   
  
  
+ AAAACCGGCA TCTACTACTA CTGCTGCTGA TGACGTCGTC TATGTCTCTC ACCGAACTAA CGACCTTCCT   
  
  
+ TCATTTCTCC CTCTCTCTCT CTCTCTCTCT TCTCTTCTTT ACCTCCTCTT CCACCACTTC CTTTCTTTCC   
  
  
+ CACCAGGAAT GTCTTTTCCC GGCAGTACAC TCTGTCTTCC TCACAAACCA TTATATATAA ACATCAATCG   
  
  
+ TTTCCTGCAC GTTCGTTTAC ACTAAAGTTC GGTAGTGATA AAATAATATA GTAGTTGAAC TTTTGCTTGG   
  
  
+ GAGGTAAAAT GAAAAGAAAA AACAAATAAC TTTAGTAATT CGGGTGACTA ATGTCTCAGA CGTAATTAAT   
  
  
+ AAAAATTAAA ACCATCTTTT GCCCCGACAC AGTACAACCA ACCAAAAAGA CCAAACTTGT TTTGTACACA   
  
  
+ ACAAAACAGG ACAACGAAGT AACAAGAAAT TCTCGTCAAC AACAACTTTA GTCTAATGGC CATCCTCCCA   
  
  
+ TCCGGCTGAC TAACCAAAGT ATGGGTCTTC AACGGGAAAA TTTTGGTCTA CTCCTTTCAG GACAAATATA   
  
  
+ AGCGAATATA ATAAGAGTAC GTTTTAATCC CAAAGGTAAA AGAAAAAATA AAAGGGATCA CCTATATCAA   
  
  
+ CTCATTGTGT CACTAGGTGG GTTGAGTCGA CGGAGGGAAA TAGGCTGGGT TTTAGTACCC CTAGAGAAGA   
  
  
+ GTTTTCCCTC TCGCTCTCTC TCTCTTTAAA ATTTAAAGAA TTTTTTAAAA TACAATTAAA GTAGACCCAA   
  
  
+ AATAATTTTA AAATGAAAAA TTAATTGGGT TTTTTAATAG TTTTATAATA AAATCAGAAA TAATTTTTAT   
  
  
+ CTCGTGTATA GTTATCTCAT AGAATTAAGA TTTTTTATTA CAAAACTTAT CCTTTAAATT TACCTTACTT   
  
  
+ AACCAGTTTC AACATTAAAT CCCGGTTTTT TAATACCTAT TTTTTCCTTT ATTTCCCTAA GTTATTAAGT   
  
  
+ GTACATTATT TACGAACATA CCTTTCGTAA CTCGTACATA CCGACGGACG ATGCCACCAA CGAGACAATT   
  
  
+ ATCACGGGGT TCTTCTTCCC GGCGTGGATA TCCGATACCA GTCGGCTCCT TTAACGTTTA ACCGTGCTTT   
  
  
+ GAGAACGCGT GGAACTCAAA ATGAGAAATT GGACGTGTGA TTGGGAGACA CACCGCGGGT GTTATGGGGG   
  
  
+ TTCAGTGGGT CCTAGGGTGT GGGTCTCCCG TCATGTTTGC GGCGTGGCCC CCTCCTAGGG GCATTTGCTT   
  
  
+ TAACACTGTG CACTCCGTAT ACCTACATTA TAAAATTAAT ACTTTTAATT TTCCATAGTT AATACTTTTT   
  
  
+ AATTGCAGTG TTTCTAATGT CATAATTTTG ATATAATATA TAGCTTGACT TCGAATTTAA TTATTAATAA   
  
  
+ TTTCCTAGTG CCGTTATAAA AAGGAATAT  

- GATTAAAGAA AAACTTCTCA GCGCGTCTCT CTATACACGA AGATCCGGAT ACCTGCCGAT AAATGACTGG   
  
  
- TTTTGGCCGT AGATGATGAT GACGACGACT ACTGCAGCAG ATACAGAGAG TGGCTTGATT GCTGGAAGGA   
  
  
- AGTAAAGAGG GAGAGAGAGA GAGAGAGAGA AGAGAAGAAA TGGAGGAGAA GGTGGTGAAG GAAAGAAAGG   
  
  
- GTGGTCCTTA CAGAAAAGGG CCGTCATGTG AGACAGAAGG AGTGTTTGGT AATATATATT TGTAGTTAGC   
  
  
- AAAGGACGTG CAAGCAAATG TGATTTCAAG CCATCACTAT TTTATTATAT CATCAACTTG AAAACGAACC   
  
  
- CTCCATTTTA CTTTTCTTTT TTGTTTATTG AAATCATTAA GCCCACTGAT TACAGAGTCT GCATTAATTA   
  
  
- TTTTTAATTT TGGTAGAAAA CGGGGCTGTG TCATGTTGGT TGGTTTTTCT GGTTTGAACA AAACATGTGT   
  
  
- TGTTTTGTCC TGTTGCTTCA TTGTTCTTTA AGAGCAGTTG TTGTTGAAAT CAGATTACCG GTAGGAGGGT   
  
  
- AGGCCGACTG ATTGGTTTCA TACCCAGAAG TTGCCCTTTT AAAACCAGAT GAGGAAAGTC CTGTTTATAT   
  
  
- TCGCTTATAT TATTCTCATG CAAAATTAGG GTTTCCATTT TCTTTTTTAT TTTCCCTAGT GGATATAGTT   
  
  
- GAGTAACACA GTGATCCACC CAACTCAGCT GCCTCCCTTT ATCCGACCCA AAATCATGGG GATCTCTTCT   
  
  
- CAAAAGGGAG AGCGAGAGAG AGAGAAATTT TAAATTTCTT AAAAAATTTT ATGTTAATTT CATCTGGGTT   
  
  
- TTATTAAAAT TTTACTTTTT AATTAACCCA AAAAATTATC AAAATATTAT TTTAGTCTTT ATTAAAAATA   
  
  
- GAGCACATAT CAATAGAGTA TCTTAATTCT AAAAAATAAT GTTTTGAATA GGAAATTTAA ATGGAATGAA   
  
  
- TTGGTCAAAG TTGTAATTTA GGGCCAAAAA ATTATGGATA AAAAAGGAAA TAAAGGGATT CAATAATTCA   
  
  
- CATGTAATAA ATGCTTGTAT GGAAAGCATT GAGCATGTAT GGCTGCCTGC TACGGTGGTT GCTCTGTTAA   
  
  
- TAGTGCCCCA AGAAGAAGGG CCGCACCTAT AGGCTATGGT CAGCCGAGGA AATTGCAAAT TGGCACGAAA   
  
  
- CTCTTGCGCA CCTTGAGTTT TACTCTTTAA CCTGCACACT AACCCTCTGT GTGGCGCCCA CAATACCCCC   
  
  
- AAGTCACCCA GGATCCCACA CCCAGAGGGC AGTACAAACG CCGCACCGGG GGAGGATCCC CGTAAACGAA   
  
  
- ATTGTGACAC GTGAGGCATA TGGATGTAAT ATTTTAATTA TGAAAATTAA AAGGTATCAA TTATGAAAAA   
  
  
- TTAACGTCAC AAAGATTACA GTATTAAAAC TATATTATAT ATCGAACTGA AGCTTAAATT AATAATTATT   
  
  
- AAAGGATCAC GGCAATATTT TTCCTTATA

+     O2-site

| Site Name | Organism | Position | Strand | Matrix score. | sequence | function |
| --- | --- | --- | --- | --- | --- | --- |
| O2-site | Zea mays | 521 | - | 9 | GTTGACGTGA | cis-acting regulatory element involved in zein metabolism regulation |

> 2018/04/13 10:10:12  
+ CTAATTTCTT TTTGAAGAGT CGCGCAGAGA GATATGTGCT TCTAGGCCTA TGGACGGCTA TTTACTGACC   
  
  
+ AAAACCGGCA TCTACTACTA CTGCTGCTGA TGACGTCGTC TATGTCTCTC ACCGAACTAA CGACCTTCCT   
  
  
+ TCATTTCTCC CTCTCTCTCT CTCTCTCTCT TCTCTTCTTT ACCTCCTCTT CCACCACTTC CTTTCTTTCC   
  
  
+ CACCAGGAAT GTCTTTTCCC GGCAGTACAC TCTGTCTTCC TCACAAACCA TTATATATAA ACATCAATCG   
  
  
+ TTTCCTGCAC GTTCGTTTAC ACTAAAGTTC GGTAGTGATA AAATAATATA GTAGTTGAAC TTTTGCTTGG   
  
  
+ GAGGTAAAAT GAAAAGAAAA AACAAATAAC TTTAGTAATT CGGGTGACTA ATGTCTCAGA CGTAATTAAT   
  
  
+ AAAAATTAAA ACCATCTTTT GCCCCGACAC AGTACAACCA ACCAAAAAGA CCAAACTTGT TTTGTACACA   
  
  
+ ACAAAACAGG ACAACGAAGT AACAAGAAAT TCTCGTCAAC AACAACTTTA GTCTAATGGC CATCCTCCCA   
  
  
+ TCCGGCTGAC TAACCAAAGT ATGGGTCTTC AACGGGAAAA TTTTGGTCTA CTCCTTTCAG GACAAATATA   
  
  
+ AGCGAATATA ATAAGAGTAC GTTTTAATCC CAAAGGTAAA AGAAAAAATA AAAGGGATCA CCTATATCAA   
  
  
+ CTCATTGTGT CACTAGGTGG GTTGAGTCGA CGGAGGGAAA TAGGCTGGGT TTTAGTACCC CTAGAGAAGA   
  
  
+ GTTTTCCCTC TCGCTCTCTC TCTCTTTAAA ATTTAAAGAA TTTTTTAAAA TACAATTAAA GTAGACCCAA   
  
  
+ AATAATTTTA AAATGAAAAA TTAATTGGGT TTTTTAATAG TTTTATAATA AAATCAGAAA TAATTTTTAT   
  
  
+ CTCGTGTATA GTTATCTCAT AGAATTAAGA TTTTTTATTA CAAAACTTAT CCTTTAAATT TACCTTACTT   
  
  
+ AACCAGTTTC AACATTAAAT CCCGGTTTTT TAATACCTAT TTTTTCCTTT ATTTCCCTAA GTTATTAAGT   
  
  
+ GTACATTATT TACGAACATA CCTTTCGTAA CTCGTACATA CCGACGGACG ATGCCACCAA CGAGACAATT   
  
  
+ ATCACGGGGT TCTTCTTCCC GGCGTGGATA TCCGATACCA GTCGGCTCCT TTAACGTTTA ACCGTGCTTT   
  
  
+ GAGAACGCGT GGAACTCAAA ATGAGAAATT GGACGTGTGA TTGGGAGACA CACCGCGGGT GTTATGGGGG   
  
  
+ TTCAGTGGGT CCTAGGGTGT GGGTCTCCCG TCATGTTTGC GGCGTGGCCC CCTCCTAGGG GCATTTGCTT   
  
  
+ TAACACTGTG CACTCCGTAT ACCTACATTA TAAAATTAAT ACTTTTAATT TTCCATAGTT AATACTTTTT   
  
  
+ AATTGCAGTG TTTCTAATGT CATAATTTTG ATATAATATA TAGCTTGACT TCGAATTTAA TTATTAATAA   
  
  
+ TTTCCTAGTG CCGTTATAAA AAGGAATAT  

- GATTAAAGAA AAACTTCTCA GCGCGTCTCT CTATACACGA AGATCCGGAT ACCTGCCGAT AAATGACTGG   
  
  
- TTTTGGCCGT AGATGATGAT GACGACGACT ACTGCAGCAG ATACAGAGAG TGGCTTGATT GCTGGAAGGA   
  
  
- AGTAAAGAGG GAGAGAGAGA GAGAGAGAGA AGAGAAGAAA TGGAGGAGAA GGTGGTGAAG GAAAGAAAGG   
  
  
- GTGGTCCTTA CAGAAAAGGG CCGTCATGTG AGACAGAAGG AGTGTTTGGT AATATATATT TGTAGTTAGC   
  
  
- AAAGGACGTG CAAGCAAATG TGATTTCAAG CCATCACTAT TTTATTATAT CATCAACTTG AAAACGAACC   
  
  
- CTCCATTTTA CTTTTCTTTT TTGTTTATTG AAATCATTAA GCCCACTGAT TACAGAGTCT GCATTAATTA   
  
  
- TTTTTAATTT TGGTAGAAAA CGGGGCTGTG TCATGTTGGT TGGTTTTTCT GGTTTGAACA AAACATGTGT   
  
  
- TGTTTTGTCC TGTTGCTTCA TTGTTCTTTA AGAGCAGTTG TTGTTGAAAT CAGATTACCG GTAGGAGGGT   
  
  
- AGGCCGACTG ATTGGTTTCA TACCCAGAAG TTGCCCTTTT AAAACCAGAT GAGGAAAGTC CTGTTTATAT   
  
  
- TCGCTTATAT TATTCTCATG CAAAATTAGG GTTTCCATTT TCTTTTTTAT TTTCCCTAGT GGATATAGTT   
  
  
- GAGTAACACA GTGATCCACC CAACTCAGCT GCCTCCCTTT ATCCGACCCA AAATCATGGG GATCTCTTCT   
  
  
- CAAAAGGGAG AGCGAGAGAG AGAGAAATTT TAAATTTCTT AAAAAATTTT ATGTTAATTT CATCTGGGTT   
  
  
- TTATTAAAAT TTTACTTTTT AATTAACCCA AAAAATTATC AAAATATTAT TTTAGTCTTT ATTAAAAATA   
  
  
- GAGCACATAT CAATAGAGTA TCTTAATTCT AAAAAATAAT GTTTTGAATA GGAAATTTAA ATGGAATGAA   
  
  
- TTGGTCAAAG TTGTAATTTA GGGCCAAAAA ATTATGGATA AAAAAGGAAA TAAAGGGATT CAATAATTCA   
  
  
- CATGTAATAA ATGCTTGTAT GGAAAGCATT GAGCATGTAT GGCTGCCTGC TACGGTGGTT GCTCTGTTAA   
  
  
- TAGTGCCCCA AGAAGAAGGG CCGCACCTAT AGGCTATGGT CAGCCGAGGA AATTGCAAAT TGGCACGAAA   
  
  
- CTCTTGCGCA CCTTGAGTTT TACTCTTTAA CCTGCACACT AACCCTCTGT GTGGCGCCCA CAATACCCCC   
  
  
- AAGTCACCCA GGATCCCACA CCCAGAGGGC AGTACAAACG CCGCACCGGG GGAGGATCCC CGTAAACGAA   
  
  
- ATTGTGACAC GTGAGGCATA TGGATGTAAT ATTTTAATTA TGAAAATTAA AAGGTATCAA TTATGAAAAA   
  
  
- TTAACGTCAC AAAGATTACA GTATTAAAAC TATATTATAT ATCGAACTGA AGCTTAAATT AATAATTATT   
  
  
- AAAGGATCAC GGCAATATTT TTCCTTATA

+     P-box

| Site Name | Organism | Position | Strand | Matrix score. | sequence | function |
| --- | --- | --- | --- | --- | --- | --- |
| P-box | Pisum sativum | 469 | + | 11 | GACCAAACTCGT | gibberellin-responsive element |

> 2018/04/13 10:10:12  
+ CTAATTTCTT TTTGAAGAGT CGCGCAGAGA GATATGTGCT TCTAGGCCTA TGGACGGCTA TTTACTGACC   
  
  
+ AAAACCGGCA TCTACTACTA CTGCTGCTGA TGACGTCGTC TATGTCTCTC ACCGAACTAA CGACCTTCCT   
  
  
+ TCATTTCTCC CTCTCTCTCT CTCTCTCTCT TCTCTTCTTT ACCTCCTCTT CCACCACTTC CTTTCTTTCC   
  
  
+ CACCAGGAAT GTCTTTTCCC GGCAGTACAC TCTGTCTTCC TCACAAACCA TTATATATAA ACATCAATCG   
  
  
+ TTTCCTGCAC GTTCGTTTAC ACTAAAGTTC GGTAGTGATA AAATAATATA GTAGTTGAAC TTTTGCTTGG   
  
  
+ GAGGTAAAAT GAAAAGAAAA AACAAATAAC TTTAGTAATT CGGGTGACTA ATGTCTCAGA CGTAATTAAT   
  
  
+ AAAAATTAAA ACCATCTTTT GCCCCGACAC AGTACAACCA ACCAAAAAGA CCAAACTTGT TTTGTACACA   
  
  
+ ACAAAACAGG ACAACGAAGT AACAAGAAAT TCTCGTCAAC AACAACTTTA GTCTAATGGC CATCCTCCCA   
  
  
+ TCCGGCTGAC TAACCAAAGT ATGGGTCTTC AACGGGAAAA TTTTGGTCTA CTCCTTTCAG GACAAATATA   
  
  
+ AGCGAATATA ATAAGAGTAC GTTTTAATCC CAAAGGTAAA AGAAAAAATA AAAGGGATCA CCTATATCAA   
  
  
+ CTCATTGTGT CACTAGGTGG GTTGAGTCGA CGGAGGGAAA TAGGCTGGGT TTTAGTACCC CTAGAGAAGA   
  
  
+ GTTTTCCCTC TCGCTCTCTC TCTCTTTAAA ATTTAAAGAA TTTTTTAAAA TACAATTAAA GTAGACCCAA   
  
  
+ AATAATTTTA AAATGAAAAA TTAATTGGGT TTTTTAATAG TTTTATAATA AAATCAGAAA TAATTTTTAT   
  
  
+ CTCGTGTATA GTTATCTCAT AGAATTAAGA TTTTTTATTA CAAAACTTAT CCTTTAAATT TACCTTACTT   
  
  
+ AACCAGTTTC AACATTAAAT CCCGGTTTTT TAATACCTAT TTTTTCCTTT ATTTCCCTAA GTTATTAAGT   
  
  
+ GTACATTATT TACGAACATA CCTTTCGTAA CTCGTACATA CCGACGGACG ATGCCACCAA CGAGACAATT   
  
  
+ ATCACGGGGT TCTTCTTCCC GGCGTGGATA TCCGATACCA GTCGGCTCCT TTAACGTTTA ACCGTGCTTT   
  
  
+ GAGAACGCGT GGAACTCAAA ATGAGAAATT GGACGTGTGA TTGGGAGACA CACCGCGGGT GTTATGGGGG   
  
  
+ TTCAGTGGGT CCTAGGGTGT GGGTCTCCCG TCATGTTTGC GGCGTGGCCC CCTCCTAGGG GCATTTGCTT   
  
  
+ TAACACTGTG CACTCCGTAT ACCTACATTA TAAAATTAAT ACTTTTAATT TTCCATAGTT AATACTTTTT   
  
  
+ AATTGCAGTG TTTCTAATGT CATAATTTTG ATATAATATA TAGCTTGACT TCGAATTTAA TTATTAATAA   
  
  
+ TTTCCTAGTG CCGTTATAAA AAGGAATAT  

- GATTAAAGAA AAACTTCTCA GCGCGTCTCT CTATACACGA AGATCCGGAT ACCTGCCGAT AAATGACTGG   
  
  
- TTTTGGCCGT AGATGATGAT GACGACGACT ACTGCAGCAG ATACAGAGAG TGGCTTGATT GCTGGAAGGA   
  
  
- AGTAAAGAGG GAGAGAGAGA GAGAGAGAGA AGAGAAGAAA TGGAGGAGAA GGTGGTGAAG GAAAGAAAGG   
  
  
- GTGGTCCTTA CAGAAAAGGG CCGTCATGTG AGACAGAAGG AGTGTTTGGT AATATATATT TGTAGTTAGC   
  
  
- AAAGGACGTG CAAGCAAATG TGATTTCAAG CCATCACTAT TTTATTATAT CATCAACTTG AAAACGAACC   
  
  
- CTCCATTTTA CTTTTCTTTT TTGTTTATTG AAATCATTAA GCCCACTGAT TACAGAGTCT GCATTAATTA   
  
  
- TTTTTAATTT TGGTAGAAAA CGGGGCTGTG TCATGTTGGT TGGTTTTTCT GGTTTGAACA AAACATGTGT   
  
  
- TGTTTTGTCC TGTTGCTTCA TTGTTCTTTA AGAGCAGTTG TTGTTGAAAT CAGATTACCG GTAGGAGGGT   
  
  
- AGGCCGACTG ATTGGTTTCA TACCCAGAAG TTGCCCTTTT AAAACCAGAT GAGGAAAGTC CTGTTTATAT   
  
  
- TCGCTTATAT TATTCTCATG CAAAATTAGG GTTTCCATTT TCTTTTTTAT TTTCCCTAGT GGATATAGTT   
  
  
- GAGTAACACA GTGATCCACC CAACTCAGCT GCCTCCCTTT ATCCGACCCA AAATCATGGG GATCTCTTCT   
  
  
- CAAAAGGGAG AGCGAGAGAG AGAGAAATTT TAAATTTCTT AAAAAATTTT ATGTTAATTT CATCTGGGTT   
  
  
- TTATTAAAAT TTTACTTTTT AATTAACCCA AAAAATTATC AAAATATTAT TTTAGTCTTT ATTAAAAATA   
  
  
- GAGCACATAT CAATAGAGTA TCTTAATTCT AAAAAATAAT GTTTTGAATA GGAAATTTAA ATGGAATGAA   
  
  
- TTGGTCAAAG TTGTAATTTA GGGCCAAAAA ATTATGGATA AAAAAGGAAA TAAAGGGATT CAATAATTCA   
  
  
- CATGTAATAA ATGCTTGTAT GGAAAGCATT GAGCATGTAT GGCTGCCTGC TACGGTGGTT GCTCTGTTAA   
  
  
- TAGTGCCCCA AGAAGAAGGG CCGCACCTAT AGGCTATGGT CAGCCGAGGA AATTGCAAAT TGGCACGAAA   
  
  
- CTCTTGCGCA CCTTGAGTTT TACTCTTTAA CCTGCACACT AACCCTCTGT GTGGCGCCCA CAATACCCCC   
  
  
- AAGTCACCCA GGATCCCACA CCCAGAGGGC AGTACAAACG CCGCACCGGG GGAGGATCCC CGTAAACGAA   
  
  
- ATTGTGACAC GTGAGGCATA TGGATGTAAT ATTTTAATTA TGAAAATTAA AAGGTATCAA TTATGAAAAA   
  
  
- TTAACGTCAC AAAGATTACA GTATTAAAAC TATATTATAT ATCGAACTGA AGCTTAAATT AATAATTATT   
  
  
- AAAGGATCAC GGCAATATTT TTCCTTATA

+     Skn-1\_motif

| Site Name | Organism | Position | Strand | Matrix score. | sequence | function |
| --- | --- | --- | --- | --- | --- | --- |
| Skn-1\_motif | Oryza sativa | 100 | - | 5 | GTCAT | cis-acting regulatory element required for endosperm expression |
| Skn-1\_motif | Oryza sativa | 1290 | + | 5 | GTCAT | cis-acting regulatory element required for endosperm expression |
| Skn-1\_motif | Oryza sativa | 1419 | + | 5 | GTCAT | cis-acting regulatory element required for endosperm expression |

> 2018/04/13 10:10:12  
+ CTAATTTCTT TTTGAAGAGT CGCGCAGAGA GATATGTGCT TCTAGGCCTA TGGACGGCTA TTTACTGACC   
  
  
+ AAAACCGGCA TCTACTACTA CTGCTGCTGA TGACGTCGTC TATGTCTCTC ACCGAACTAA CGACCTTCCT   
  
  
+ TCATTTCTCC CTCTCTCTCT CTCTCTCTCT TCTCTTCTTT ACCTCCTCTT CCACCACTTC CTTTCTTTCC   
  
  
+ CACCAGGAAT GTCTTTTCCC GGCAGTACAC TCTGTCTTCC TCACAAACCA TTATATATAA ACATCAATCG   
  
  
+ TTTCCTGCAC GTTCGTTTAC ACTAAAGTTC GGTAGTGATA AAATAATATA GTAGTTGAAC TTTTGCTTGG   
  
  
+ GAGGTAAAAT GAAAAGAAAA AACAAATAAC TTTAGTAATT CGGGTGACTA ATGTCTCAGA CGTAATTAAT   
  
  
+ AAAAATTAAA ACCATCTTTT GCCCCGACAC AGTACAACCA ACCAAAAAGA CCAAACTTGT TTTGTACACA   
  
  
+ ACAAAACAGG ACAACGAAGT AACAAGAAAT TCTCGTCAAC AACAACTTTA GTCTAATGGC CATCCTCCCA   
  
  
+ TCCGGCTGAC TAACCAAAGT ATGGGTCTTC AACGGGAAAA TTTTGGTCTA CTCCTTTCAG GACAAATATA   
  
  
+ AGCGAATATA ATAAGAGTAC GTTTTAATCC CAAAGGTAAA AGAAAAAATA AAAGGGATCA CCTATATCAA   
  
  
+ CTCATTGTGT CACTAGGTGG GTTGAGTCGA CGGAGGGAAA TAGGCTGGGT TTTAGTACCC CTAGAGAAGA   
  
  
+ GTTTTCCCTC TCGCTCTCTC TCTCTTTAAA ATTTAAAGAA TTTTTTAAAA TACAATTAAA GTAGACCCAA   
  
  
+ AATAATTTTA AAATGAAAAA TTAATTGGGT TTTTTAATAG TTTTATAATA AAATCAGAAA TAATTTTTAT   
  
  
+ CTCGTGTATA GTTATCTCAT AGAATTAAGA TTTTTTATTA CAAAACTTAT CCTTTAAATT TACCTTACTT   
  
  
+ AACCAGTTTC AACATTAAAT CCCGGTTTTT TAATACCTAT TTTTTCCTTT ATTTCCCTAA GTTATTAAGT   
  
  
+ GTACATTATT TACGAACATA CCTTTCGTAA CTCGTACATA CCGACGGACG ATGCCACCAA CGAGACAATT   
  
  
+ ATCACGGGGT TCTTCTTCCC GGCGTGGATA TCCGATACCA GTCGGCTCCT TTAACGTTTA ACCGTGCTTT   
  
  
+ GAGAACGCGT GGAACTCAAA ATGAGAAATT GGACGTGTGA TTGGGAGACA CACCGCGGGT GTTATGGGGG   
  
  
+ TTCAGTGGGT CCTAGGGTGT GGGTCTCCCG TCATGTTTGC GGCGTGGCCC CCTCCTAGGG GCATTTGCTT   
  
  
+ TAACACTGTG CACTCCGTAT ACCTACATTA TAAAATTAAT ACTTTTAATT TTCCATAGTT AATACTTTTT   
  
  
+ AATTGCAGTG TTTCTAATGT CATAATTTTG ATATAATATA TAGCTTGACT TCGAATTTAA TTATTAATAA   
  
  
+ TTTCCTAGTG CCGTTATAAA AAGGAATAT  

- GATTAAAGAA AAACTTCTCA GCGCGTCTCT CTATACACGA AGATCCGGAT ACCTGCCGAT AAATGACTGG   
  
  
- TTTTGGCCGT AGATGATGAT GACGACGACT ACTGCAGCAG ATACAGAGAG TGGCTTGATT GCTGGAAGGA   
  
  
- AGTAAAGAGG GAGAGAGAGA GAGAGAGAGA AGAGAAGAAA TGGAGGAGAA GGTGGTGAAG GAAAGAAAGG   
  
  
- GTGGTCCTTA CAGAAAAGGG CCGTCATGTG AGACAGAAGG AGTGTTTGGT AATATATATT TGTAGTTAGC   
  
  
- AAAGGACGTG CAAGCAAATG TGATTTCAAG CCATCACTAT TTTATTATAT CATCAACTTG AAAACGAACC   
  
  
- CTCCATTTTA CTTTTCTTTT TTGTTTATTG AAATCATTAA GCCCACTGAT TACAGAGTCT GCATTAATTA   
  
  
- TTTTTAATTT TGGTAGAAAA CGGGGCTGTG TCATGTTGGT TGGTTTTTCT GGTTTGAACA AAACATGTGT   
  
  
- TGTTTTGTCC TGTTGCTTCA TTGTTCTTTA AGAGCAGTTG TTGTTGAAAT CAGATTACCG GTAGGAGGGT   
  
  
- AGGCCGACTG ATTGGTTTCA TACCCAGAAG TTGCCCTTTT AAAACCAGAT GAGGAAAGTC CTGTTTATAT   
  
  
- TCGCTTATAT TATTCTCATG CAAAATTAGG GTTTCCATTT TCTTTTTTAT TTTCCCTAGT GGATATAGTT   
  
  
- GAGTAACACA GTGATCCACC CAACTCAGCT GCCTCCCTTT ATCCGACCCA AAATCATGGG GATCTCTTCT   
  
  
- CAAAAGGGAG AGCGAGAGAG AGAGAAATTT TAAATTTCTT AAAAAATTTT ATGTTAATTT CATCTGGGTT   
  
  
- TTATTAAAAT TTTACTTTTT AATTAACCCA AAAAATTATC AAAATATTAT TTTAGTCTTT ATTAAAAATA   
  
  
- GAGCACATAT CAATAGAGTA TCTTAATTCT AAAAAATAAT GTTTTGAATA GGAAATTTAA ATGGAATGAA   
  
  
- TTGGTCAAAG TTGTAATTTA GGGCCAAAAA ATTATGGATA AAAAAGGAAA TAAAGGGATT CAATAATTCA   
  
  
- CATGTAATAA ATGCTTGTAT GGAAAGCATT GAGCATGTAT GGCTGCCTGC TACGGTGGTT GCTCTGTTAA   
  
  
- TAGTGCCCCA AGAAGAAGGG CCGCACCTAT AGGCTATGGT CAGCCGAGGA AATTGCAAAT TGGCACGAAA   
  
  
- CTCTTGCGCA CCTTGAGTTT TACTCTTTAA CCTGCACACT AACCCTCTGT GTGGCGCCCA CAATACCCCC   
  
  
- AAGTCACCCA GGATCCCACA CCCAGAGGGC AGTACAAACG CCGCACCGGG GGAGGATCCC CGTAAACGAA   
  
  
- ATTGTGACAC GTGAGGCATA TGGATGTAAT ATTTTAATTA TGAAAATTAA AAGGTATCAA TTATGAAAAA   
  
  
- TTAACGTCAC AAAGATTACA GTATTAAAAC TATATTATAT ATCGAACTGA AGCTTAAATT AATAATTATT   
  
  
- AAAGGATCAC GGCAATATTT TTCCTTATA

+     Sp1

| Site Name | Organism | Position | Strand | Matrix score. | sequence | function |
| --- | --- | --- | --- | --- | --- | --- |
| Sp1 | Zea mays | 349 | - | 5 | CC(G/A)CCC | light responsive element |
| Sp1 | Zea mays | 554 | + | 5 | CC(G/A)CCC | light responsive element |

> 2018/04/13 10:10:12  
+ CTAATTTCTT TTTGAAGAGT CGCGCAGAGA GATATGTGCT TCTAGGCCTA TGGACGGCTA TTTACTGACC   
  
  
+ AAAACCGGCA TCTACTACTA CTGCTGCTGA TGACGTCGTC TATGTCTCTC ACCGAACTAA CGACCTTCCT   
  
  
+ TCATTTCTCC CTCTCTCTCT CTCTCTCTCT TCTCTTCTTT ACCTCCTCTT CCACCACTTC CTTTCTTTCC   
  
  
+ CACCAGGAAT GTCTTTTCCC GGCAGTACAC TCTGTCTTCC TCACAAACCA TTATATATAA ACATCAATCG   
  
  
+ TTTCCTGCAC GTTCGTTTAC ACTAAAGTTC GGTAGTGATA AAATAATATA GTAGTTGAAC TTTTGCTTGG   
  
  
+ GAGGTAAAAT GAAAAGAAAA AACAAATAAC TTTAGTAATT CGGGTGACTA ATGTCTCAGA CGTAATTAAT   
  
  
+ AAAAATTAAA ACCATCTTTT GCCCCGACAC AGTACAACCA ACCAAAAAGA CCAAACTTGT TTTGTACACA   
  
  
+ ACAAAACAGG ACAACGAAGT AACAAGAAAT TCTCGTCAAC AACAACTTTA GTCTAATGGC CATCCTCCCA   
  
  
+ TCCGGCTGAC TAACCAAAGT ATGGGTCTTC AACGGGAAAA TTTTGGTCTA CTCCTTTCAG GACAAATATA   
  
  
+ AGCGAATATA ATAAGAGTAC GTTTTAATCC CAAAGGTAAA AGAAAAAATA AAAGGGATCA CCTATATCAA   
  
  
+ CTCATTGTGT CACTAGGTGG GTTGAGTCGA CGGAGGGAAA TAGGCTGGGT TTTAGTACCC CTAGAGAAGA   
  
  
+ GTTTTCCCTC TCGCTCTCTC TCTCTTTAAA ATTTAAAGAA TTTTTTAAAA TACAATTAAA GTAGACCCAA   
  
  
+ AATAATTTTA AAATGAAAAA TTAATTGGGT TTTTTAATAG TTTTATAATA AAATCAGAAA TAATTTTTAT   
  
  
+ CTCGTGTATA GTTATCTCAT AGAATTAAGA TTTTTTATTA CAAAACTTAT CCTTTAAATT TACCTTACTT   
  
  
+ AACCAGTTTC AACATTAAAT CCCGGTTTTT TAATACCTAT TTTTTCCTTT ATTTCCCTAA GTTATTAAGT   
  
  
+ GTACATTATT TACGAACATA CCTTTCGTAA CTCGTACATA CCGACGGACG ATGCCACCAA CGAGACAATT   
  
  
+ ATCACGGGGT TCTTCTTCCC GGCGTGGATA TCCGATACCA GTCGGCTCCT TTAACGTTTA ACCGTGCTTT   
  
  
+ GAGAACGCGT GGAACTCAAA ATGAGAAATT GGACGTGTGA TTGGGAGACA CACCGCGGGT GTTATGGGGG   
  
  
+ TTCAGTGGGT CCTAGGGTGT GGGTCTCCCG TCATGTTTGC GGCGTGGCCC CCTCCTAGGG GCATTTGCTT   
  
  
+ TAACACTGTG CACTCCGTAT ACCTACATTA TAAAATTAAT ACTTTTAATT TTCCATAGTT AATACTTTTT   
  
  
+ AATTGCAGTG TTTCTAATGT CATAATTTTG ATATAATATA TAGCTTGACT TCGAATTTAA TTATTAATAA   
  
  
+ TTTCCTAGTG CCGTTATAAA AAGGAATAT  

- GATTAAAGAA AAACTTCTCA GCGCGTCTCT CTATACACGA AGATCCGGAT ACCTGCCGAT AAATGACTGG   
  
  
- TTTTGGCCGT AGATGATGAT GACGACGACT ACTGCAGCAG ATACAGAGAG TGGCTTGATT GCTGGAAGGA   
  
  
- AGTAAAGAGG GAGAGAGAGA GAGAGAGAGA AGAGAAGAAA TGGAGGAGAA GGTGGTGAAG GAAAGAAAGG   
  
  
- GTGGTCCTTA CAGAAAAGGG CCGTCATGTG AGACAGAAGG AGTGTTTGGT AATATATATT TGTAGTTAGC   
  
  
- AAAGGACGTG CAAGCAAATG TGATTTCAAG CCATCACTAT TTTATTATAT CATCAACTTG AAAACGAACC   
  
  
- CTCCATTTTA CTTTTCTTTT TTGTTTATTG AAATCATTAA GCCCACTGAT TACAGAGTCT GCATTAATTA   
  
  
- TTTTTAATTT TGGTAGAAAA CGGGGCTGTG TCATGTTGGT TGGTTTTTCT GGTTTGAACA AAACATGTGT   
  
  
- TGTTTTGTCC TGTTGCTTCA TTGTTCTTTA AGAGCAGTTG TTGTTGAAAT CAGATTACCG GTAGGAGGGT   
  
  
- AGGCCGACTG ATTGGTTTCA TACCCAGAAG TTGCCCTTTT AAAACCAGAT GAGGAAAGTC CTGTTTATAT   
  
  
- TCGCTTATAT TATTCTCATG CAAAATTAGG GTTTCCATTT TCTTTTTTAT TTTCCCTAGT GGATATAGTT   
  
  
- GAGTAACACA GTGATCCACC CAACTCAGCT GCCTCCCTTT ATCCGACCCA AAATCATGGG GATCTCTTCT   
  
  
- CAAAAGGGAG AGCGAGAGAG AGAGAAATTT TAAATTTCTT AAAAAATTTT ATGTTAATTT CATCTGGGTT   
  
  
- TTATTAAAAT TTTACTTTTT AATTAACCCA AAAAATTATC AAAATATTAT TTTAGTCTTT ATTAAAAATA   
  
  
- GAGCACATAT CAATAGAGTA TCTTAATTCT AAAAAATAAT GTTTTGAATA GGAAATTTAA ATGGAATGAA   
  
  
- TTGGTCAAAG TTGTAATTTA GGGCCAAAAA ATTATGGATA AAAAAGGAAA TAAAGGGATT CAATAATTCA   
  
  
- CATGTAATAA ATGCTTGTAT GGAAAGCATT GAGCATGTAT GGCTGCCTGC TACGGTGGTT GCTCTGTTAA   
  
  
- TAGTGCCCCA AGAAGAAGGG CCGCACCTAT AGGCTATGGT CAGCCGAGGA AATTGCAAAT TGGCACGAAA   
  
  
- CTCTTGCGCA CCTTGAGTTT TACTCTTTAA CCTGCACACT AACCCTCTGT GTGGCGCCCA CAATACCCCC   
  
  
- AAGTCACCCA GGATCCCACA CCCAGAGGGC AGTACAAACG CCGCACCGGG GGAGGATCCC CGTAAACGAA   
  
  
- ATTGTGACAC GTGAGGCATA TGGATGTAAT ATTTTAATTA TGAAAATTAA AAGGTATCAA TTATGAAAAA   
  
  
- TTAACGTCAC AAAGATTACA GTATTAAAAC TATATTATAT ATCGAACTGA AGCTTAAATT AATAATTATT   
  
  
- AAAGGATCAC GGCAATATTT TTCCTTATA

+     TATA-box

| Site Name | Organism | Position | Strand | Matrix score. | sequence | function |
| --- | --- | --- | --- | --- | --- | --- |
| TATA-box | Arabidopsis thaliana | 1484 | - | 5 | TATAA | core promoter element around -30 of transcription start |
| TATA-box | Brassica napus | 1436 | - | 6 | ATATAT | core promoter element around -30 of transcription start |
| TATA-box | Arabidopsis thaliana | 1359 | + | 6 | TATAAA | core promoter element around -30 of transcription start |
| TATA-box | Arabidopsis thaliana | 1358 | - | 5 | TATAA | core promoter element around -30 of transcription start |
| TATA-box | Brassica napus | 1357 | + | 6 | ATTATA | core promoter element around -30 of transcription start |
| TATA-box | Arabidopsis thaliana | 882 | - | 6 | TATAAA | core promoter element around -30 of transcription start |
| TATA-box | Arabidopsis thaliana | 1348 | - | 4 | TATA | core promoter element around -30 of transcription start |
| TATA-box | Zea mays | 812 | - | 8 | TTTAAAAA | core promoter element around -30 of transcription start |
| TATA-box | Arabidopsis thaliana | 1432 | - | 4 | TATA | core promoter element around -30 of transcription start |
| TATA-box | Brassica oleracea | 1431 | + | 7 | ATATAAT | core promoter element around -30 of transcription start |
| TATA-box | Glycine max | 1434 | + | 5 | TAATA | core promoter element around -30 of transcription start |
| TATA-box | Daucus carota | 961 | + | 9 | ccTATAAATT | core promoter element around -30 of transcription start |
| TATA-box | Arabidopsis thaliana | 627 | + | 4 | TATA | core promoter element around -30 of transcription start |
| TATA-box | Oryza sativa | 949 | + | 7 | TACAAAA | core promoter element around -30 of transcription start |
| TATA-box | Glycine max | 946 | - | 5 | TAATA | core promoter element around -30 of transcription start |
| TATA-box | Lycopersicon esculentum | 420 | - | 5 | TTTTA | core promoter element around -30 of transcription start |
| TATA-box | Lycopersicon esculentum | 889 | - | 5 | TTTTA | core promoter element around -30 of transcription start |
| TATA-box | Arabidopsis thaliana | 327 | + | 4 | TATA | core promoter element around -30 of transcription start |
| TATA-box | Arabidopsis thaliana | 1437 | - | 4 | TATA | core promoter element around -30 of transcription start |
| TATA-box | Brassica napus | 263 | + | 6 | ATATAT | core promoter element around -30 of transcription start |
| TATA-box | Lycopersicon esculentum | 319 | - | 5 | TTTTA | core promoter element around -30 of transcription start |
| TATA-box | Arabidopsis thaliana | 266 | + | 6 | TATAAA | core promoter element around -30 of transcription start |
| TATA-box | Arabidopsis thaliana | 693 | + | 4 | TATA | core promoter element around -30 of transcription start |
| TATA-box | Glycine max | 1043 | - | 5 | TAATA | core promoter element around -30 of transcription start |
| TATA-box | Arabidopsis thaliana | 264 | + | 4 | TATA | core promoter element around -30 of transcription start |
| TATA-box | Arabidopsis thaliana | 1435 | - | 9 | tcTATATAtt | core promoter element around -30 of transcription start |
| TATA-box | Brassica oleracea | 265 | + | 6 | ATATAA | core promoter element around -30 of transcription start |
| TATA-box | Arabidopsis thaliana | 262 | + | 8 | TATATATA | core promoter element around -30 of transcription start |
| TATA-box | Lycopersicon esculentum | 905 | + | 5 | TTTTA | core promoter element around -30 of transcription start |
| TATA-box | Lycopersicon esculentum | 1008 | + | 5 | TTTTA | core promoter element around -30 of transcription start |
| TATA-box | Lycopersicon esculentum | 1397 | + | 5 | TTTTA | core promoter element around -30 of transcription start |
| TATA-box | Arabidopsis thaliana | 917 | - | 4 | TATA | core promoter element around -30 of transcription start |
| TATA-box | Lycopersicon esculentum | 813 | + | 5 | TTTTA | core promoter element around -30 of transcription start |
| TATA-box | Lycopersicon esculentum | 1487 | - | 5 | TTTTA | core promoter element around -30 of transcription start |
| TATA-box | Arabidopsis thaliana | 881 | - | 7 | TATAAAA | core promoter element around -30 of transcription start |
| TATA-box | Lycopersicon esculentum | 846 | + | 5 | TTTTA | core promoter element around -30 of transcription start |
| TATA-box | Lycopersicon esculentum | 797 | - | 5 | TTTTA | core promoter element around -30 of transcription start |
| TATA-box | Lycopersicon esculentum | 1361 | - | 5 | TTTTA | core promoter element around -30 of transcription start |
| TATA-box | Glycine max | 1011 | + | 5 | TAATA | core promoter element around -30 of transcription start |
| TATA-box | Lycopersicon esculentum | 849 | - | 5 | TTTTA | core promoter element around -30 of transcription start |
| TATA-box | Lycopersicon esculentum | 943 | + | 5 | TTTTA | core promoter element around -30 of transcription start |
| TATA-box | Arabidopsis thaliana | 884 | - | 4 | TATA | core promoter element around -30 of transcription start |
| TATA-box | Arabidopsis thaliana | 258 | - | 9 | taTATAAAgg | core promoter element around -30 of transcription start |
| TATA-box | Glycine max | 417 | + | 5 | TAATA | core promoter element around -30 of transcription start |
| TATA-box | Glycine max | 1465 | + | 5 | TAATA | core promoter element around -30 of transcription start |
| TATA-box | Lycopersicon esculentum | 750 | + | 5 | TTTTA | core promoter element around -30 of transcription start |
| TATA-box | Helianthus annuus | 915 | - | 6 | TATACA | core promoter element around -30 of transcription start |
| TATA-box | Lycopersicon esculentum | 872 | + | 5 | TTTTA | core promoter element around -30 of transcription start |
| TATA-box | Glycine max | 1390 | + | 5 | TAATA | core promoter element around -30 of transcription start |
| TATA-box | Arabidopsis thaliana | 883 | - | 5 | TATAA | core promoter element around -30 of transcription start |
| TATA-box | Arabidopsis thaliana | 1439 | - | 4 | TATA | core promoter element around -30 of transcription start |
| TATA-box | Glycine max | 875 | + | 5 | TAATA | core promoter element around -30 of transcription start |
| TATA-box | Glycine max | 324 | + | 5 | TAATA | core promoter element around -30 of transcription start |
| TATA-box | Glycine max | 886 | + | 5 | TAATA | core promoter element around -30 of transcription start |
| TATA-box | Glycine max | 639 | + | 5 | TAATA | core promoter element around -30 of transcription start |
| TATA-box | Oryza sativa | 480 | - | 7 | TACAAAA | core promoter element around -30 of transcription start |
| TATA-box | Glycine max | 1367 | + | 5 | TAATA | core promoter element around -30 of transcription start |
| TATA-box | Lycopersicon esculentum | 816 | - | 5 | TTTTA | core promoter element around -30 of transcription start |
| TATA-box | Lycopersicon esculentum | 1373 | + | 5 | TTTTA | core promoter element around -30 of transcription start |
| TATA-box | Brassica napus | 260 | + | 6 | ATTATA | core promoter element around -30 of transcription start |
| TATA-box | Arabidopsis thaliana | 261 | - | 7 | TATATAA | core promoter element around -30 of transcription start |
| TATA-box | Arabidopsis thaliana | 1485 | + | 6 | TATAAA | core promoter element around -30 of transcription start |
| TATA-box | Lycopersicon esculentum | 427 | - | 5 | TTTTA | core promoter element around -30 of transcription start |
| TATA-box | Lycopersicon esculentum | 355 | - | 5 | TTTTA | core promoter element around -30 of transcription start |
| TATA-box | Glycine max | 1462 | - | 5 | TAATA | core promoter element around -30 of transcription start |
| TATA-box | Brassica oleracea | 626 | + | 6 | ATATAA | core promoter element around -30 of transcription start |
| TATA-box | Lycopersicon esculentum | 667 | - | 5 | TTTTA | core promoter element around -30 of transcription start |
| TATA-box | Brassica oleracea | 636 | + | 7 | ATATAAT | core promoter element around -30 of transcription start |
| TATA-box | Lycopersicon esculentum | 679 | - | 5 | TTTTA | core promoter element around -30 of transcription start |
| TATA-box | Arabidopsis thaliana | 637 | + | 4 | TATA | core promoter element around -30 of transcription start |
| TATA-box | Lycopersicon esculentum | 652 | + | 5 | TTTTA | core promoter element around -30 of transcription start |

> 2018/04/13 10:10:12  
+ CTAATTTCTT TTTGAAGAGT CGCGCAGAGA GATATGTGCT TCTAGGCCTA TGGACGGCTA TTTACTGACC   
  
  
+ AAAACCGGCA TCTACTACTA CTGCTGCTGA TGACGTCGTC TATGTCTCTC ACCGAACTAA CGACCTTCCT   
  
  
+ TCATTTCTCC CTCTCTCTCT CTCTCTCTCT TCTCTTCTTT ACCTCCTCTT CCACCACTTC CTTTCTTTCC   
  
  
+ CACCAGGAAT GTCTTTTCCC GGCAGTACAC TCTGTCTTCC TCACAAACCA TTATATATAA ACATCAATCG   
  
  
+ TTTCCTGCAC GTTCGTTTAC ACTAAAGTTC GGTAGTGATA AAATAATATA GTAGTTGAAC TTTTGCTTGG   
  
  
+ GAGGTAAAAT GAAAAGAAAA AACAAATAAC TTTAGTAATT CGGGTGACTA ATGTCTCAGA CGTAATTAAT   
  
  
+ AAAAATTAAA ACCATCTTTT GCCCCGACAC AGTACAACCA ACCAAAAAGA CCAAACTTGT TTTGTACACA   
  
  
+ ACAAAACAGG ACAACGAAGT AACAAGAAAT TCTCGTCAAC AACAACTTTA GTCTAATGGC CATCCTCCCA   
  
  
+ TCCGGCTGAC TAACCAAAGT ATGGGTCTTC AACGGGAAAA TTTTGGTCTA CTCCTTTCAG GACAAATATA   
  
  
+ AGCGAATATA ATAAGAGTAC GTTTTAATCC CAAAGGTAAA AGAAAAAATA AAAGGGATCA CCTATATCAA   
  
  
+ CTCATTGTGT CACTAGGTGG GTTGAGTCGA CGGAGGGAAA TAGGCTGGGT TTTAGTACCC CTAGAGAAGA   
  
  
+ GTTTTCCCTC TCGCTCTCTC TCTCTTTAAA ATTTAAAGAA TTTTTTAAAA TACAATTAAA GTAGACCCAA   
  
  
+ AATAATTTTA AAATGAAAAA TTAATTGGGT TTTTTAATAG TTTTATAATA AAATCAGAAA TAATTTTTAT   
  
  
+ CTCGTGTATA GTTATCTCAT AGAATTAAGA TTTTTTATTA CAAAACTTAT CCTTTAAATT TACCTTACTT   
  
  
+ AACCAGTTTC AACATTAAAT CCCGGTTTTT TAATACCTAT TTTTTCCTTT ATTTCCCTAA GTTATTAAGT   
  
  
+ GTACATTATT TACGAACATA CCTTTCGTAA CTCGTACATA CCGACGGACG ATGCCACCAA CGAGACAATT   
  
  
+ ATCACGGGGT TCTTCTTCCC GGCGTGGATA TCCGATACCA GTCGGCTCCT TTAACGTTTA ACCGTGCTTT   
  
  
+ GAGAACGCGT GGAACTCAAA ATGAGAAATT GGACGTGTGA TTGGGAGACA CACCGCGGGT GTTATGGGGG   
  
  
+ TTCAGTGGGT CCTAGGGTGT GGGTCTCCCG TCATGTTTGC GGCGTGGCCC CCTCCTAGGG GCATTTGCTT   
  
  
+ TAACACTGTG CACTCCGTAT ACCTACATTA TAAAATTAAT ACTTTTAATT TTCCATAGTT AATACTTTTT   
  
  
+ AATTGCAGTG TTTCTAATGT CATAATTTTG ATATAATATA TAGCTTGACT TCGAATTTAA TTATTAATAA   
  
  
+ TTTCCTAGTG CCGTTATAAA AAGGAATAT  

- GATTAAAGAA AAACTTCTCA GCGCGTCTCT CTATACACGA AGATCCGGAT ACCTGCCGAT AAATGACTGG   
  
  
- TTTTGGCCGT AGATGATGAT GACGACGACT ACTGCAGCAG ATACAGAGAG TGGCTTGATT GCTGGAAGGA   
  
  
- AGTAAAGAGG GAGAGAGAGA GAGAGAGAGA AGAGAAGAAA TGGAGGAGAA GGTGGTGAAG GAAAGAAAGG   
  
  
- GTGGTCCTTA CAGAAAAGGG CCGTCATGTG AGACAGAAGG AGTGTTTGGT AATATATATT TGTAGTTAGC   
  
  
- AAAGGACGTG CAAGCAAATG TGATTTCAAG CCATCACTAT TTTATTATAT CATCAACTTG AAAACGAACC   
  
  
- CTCCATTTTA CTTTTCTTTT TTGTTTATTG AAATCATTAA GCCCACTGAT TACAGAGTCT GCATTAATTA   
  
  
- TTTTTAATTT TGGTAGAAAA CGGGGCTGTG TCATGTTGGT TGGTTTTTCT GGTTTGAACA AAACATGTGT   
  
  
- TGTTTTGTCC TGTTGCTTCA TTGTTCTTTA AGAGCAGTTG TTGTTGAAAT CAGATTACCG GTAGGAGGGT   
  
  
- AGGCCGACTG ATTGGTTTCA TACCCAGAAG TTGCCCTTTT AAAACCAGAT GAGGAAAGTC CTGTTTATAT   
  
  
- TCGCTTATAT TATTCTCATG CAAAATTAGG GTTTCCATTT TCTTTTTTAT TTTCCCTAGT GGATATAGTT   
  
  
- GAGTAACACA GTGATCCACC CAACTCAGCT GCCTCCCTTT ATCCGACCCA AAATCATGGG GATCTCTTCT   
  
  
- CAAAAGGGAG AGCGAGAGAG AGAGAAATTT TAAATTTCTT AAAAAATTTT ATGTTAATTT CATCTGGGTT   
  
  
- TTATTAAAAT TTTACTTTTT AATTAACCCA AAAAATTATC AAAATATTAT TTTAGTCTTT ATTAAAAATA   
  
  
- GAGCACATAT CAATAGAGTA TCTTAATTCT AAAAAATAAT GTTTTGAATA GGAAATTTAA ATGGAATGAA   
  
  
- TTGGTCAAAG TTGTAATTTA GGGCCAAAAA ATTATGGATA AAAAAGGAAA TAAAGGGATT CAATAATTCA   
  
  
- CATGTAATAA ATGCTTGTAT GGAAAGCATT GAGCATGTAT GGCTGCCTGC TACGGTGGTT GCTCTGTTAA   
  
  
- TAGTGCCCCA AGAAGAAGGG CCGCACCTAT AGGCTATGGT CAGCCGAGGA AATTGCAAAT TGGCACGAAA   
  
  
- CTCTTGCGCA CCTTGAGTTT TACTCTTTAA CCTGCACACT AACCCTCTGT GTGGCGCCCA CAATACCCCC   
  
  
- AAGTCACCCA GGATCCCACA CCCAGAGGGC AGTACAAACG CCGCACCGGG GGAGGATCCC CGTAAACGAA   
  
  
- ATTGTGACAC GTGAGGCATA TGGATGTAAT ATTTTAATTA TGAAAATTAA AAGGTATCAA TTATGAAAAA   
  
  
- TTAACGTCAC AAAGATTACA GTATTAAAAC TATATTATAT ATCGAACTGA AGCTTAAATT AATAATTATT   
  
  
- AAAGGATCAC GGCAATATTT TTCCTTATA

+     TCA-element

| Site Name | Organism | Position | Strand | Matrix score. | sequence | function |
| --- | --- | --- | --- | --- | --- | --- |
| TCA-element | Nicotiana tabacum | 432 | + | 9 | CCATCTTTTT | cis-acting element involved in salicylic acid responsiveness |

> 2018/04/13 10:10:12  
+ CTAATTTCTT TTTGAAGAGT CGCGCAGAGA GATATGTGCT TCTAGGCCTA TGGACGGCTA TTTACTGACC   
  
  
+ AAAACCGGCA TCTACTACTA CTGCTGCTGA TGACGTCGTC TATGTCTCTC ACCGAACTAA CGACCTTCCT   
  
  
+ TCATTTCTCC CTCTCTCTCT CTCTCTCTCT TCTCTTCTTT ACCTCCTCTT CCACCACTTC CTTTCTTTCC   
  
  
+ CACCAGGAAT GTCTTTTCCC GGCAGTACAC TCTGTCTTCC TCACAAACCA TTATATATAA ACATCAATCG   
  
  
+ TTTCCTGCAC GTTCGTTTAC ACTAAAGTTC GGTAGTGATA AAATAATATA GTAGTTGAAC TTTTGCTTGG   
  
  
+ GAGGTAAAAT GAAAAGAAAA AACAAATAAC TTTAGTAATT CGGGTGACTA ATGTCTCAGA CGTAATTAAT   
  
  
+ AAAAATTAAA ACCATCTTTT GCCCCGACAC AGTACAACCA ACCAAAAAGA CCAAACTTGT TTTGTACACA   
  
  
+ ACAAAACAGG ACAACGAAGT AACAAGAAAT TCTCGTCAAC AACAACTTTA GTCTAATGGC CATCCTCCCA   
  
  
+ TCCGGCTGAC TAACCAAAGT ATGGGTCTTC AACGGGAAAA TTTTGGTCTA CTCCTTTCAG GACAAATATA   
  
  
+ AGCGAATATA ATAAGAGTAC GTTTTAATCC CAAAGGTAAA AGAAAAAATA AAAGGGATCA CCTATATCAA   
  
  
+ CTCATTGTGT CACTAGGTGG GTTGAGTCGA CGGAGGGAAA TAGGCTGGGT TTTAGTACCC CTAGAGAAGA   
  
  
+ GTTTTCCCTC TCGCTCTCTC TCTCTTTAAA ATTTAAAGAA TTTTTTAAAA TACAATTAAA GTAGACCCAA   
  
  
+ AATAATTTTA AAATGAAAAA TTAATTGGGT TTTTTAATAG TTTTATAATA AAATCAGAAA TAATTTTTAT   
  
  
+ CTCGTGTATA GTTATCTCAT AGAATTAAGA TTTTTTATTA CAAAACTTAT CCTTTAAATT TACCTTACTT   
  
  
+ AACCAGTTTC AACATTAAAT CCCGGTTTTT TAATACCTAT TTTTTCCTTT ATTTCCCTAA GTTATTAAGT   
  
  
+ GTACATTATT TACGAACATA CCTTTCGTAA CTCGTACATA CCGACGGACG ATGCCACCAA CGAGACAATT   
  
  
+ ATCACGGGGT TCTTCTTCCC GGCGTGGATA TCCGATACCA GTCGGCTCCT TTAACGTTTA ACCGTGCTTT   
  
  
+ GAGAACGCGT GGAACTCAAA ATGAGAAATT GGACGTGTGA TTGGGAGACA CACCGCGGGT GTTATGGGGG   
  
  
+ TTCAGTGGGT CCTAGGGTGT GGGTCTCCCG TCATGTTTGC GGCGTGGCCC CCTCCTAGGG GCATTTGCTT   
  
  
+ TAACACTGTG CACTCCGTAT ACCTACATTA TAAAATTAAT ACTTTTAATT TTCCATAGTT AATACTTTTT   
  
  
+ AATTGCAGTG TTTCTAATGT CATAATTTTG ATATAATATA TAGCTTGACT TCGAATTTAA TTATTAATAA   
  
  
+ TTTCCTAGTG CCGTTATAAA AAGGAATAT  

- GATTAAAGAA AAACTTCTCA GCGCGTCTCT CTATACACGA AGATCCGGAT ACCTGCCGAT AAATGACTGG   
  
  
- TTTTGGCCGT AGATGATGAT GACGACGACT ACTGCAGCAG ATACAGAGAG TGGCTTGATT GCTGGAAGGA   
  
  
- AGTAAAGAGG GAGAGAGAGA GAGAGAGAGA AGAGAAGAAA TGGAGGAGAA GGTGGTGAAG GAAAGAAAGG   
  
  
- GTGGTCCTTA CAGAAAAGGG CCGTCATGTG AGACAGAAGG AGTGTTTGGT AATATATATT TGTAGTTAGC   
  
  
- AAAGGACGTG CAAGCAAATG TGATTTCAAG CCATCACTAT TTTATTATAT CATCAACTTG AAAACGAACC   
  
  
- CTCCATTTTA CTTTTCTTTT TTGTTTATTG AAATCATTAA GCCCACTGAT TACAGAGTCT GCATTAATTA   
  
  
- TTTTTAATTT TGGTAGAAAA CGGGGCTGTG TCATGTTGGT TGGTTTTTCT GGTTTGAACA AAACATGTGT   
  
  
- TGTTTTGTCC TGTTGCTTCA TTGTTCTTTA AGAGCAGTTG TTGTTGAAAT CAGATTACCG GTAGGAGGGT   
  
  
- AGGCCGACTG ATTGGTTTCA TACCCAGAAG TTGCCCTTTT AAAACCAGAT GAGGAAAGTC CTGTTTATAT   
  
  
- TCGCTTATAT TATTCTCATG CAAAATTAGG GTTTCCATTT TCTTTTTTAT TTTCCCTAGT GGATATAGTT   
  
  
- GAGTAACACA GTGATCCACC CAACTCAGCT GCCTCCCTTT ATCCGACCCA AAATCATGGG GATCTCTTCT   
  
  
- CAAAAGGGAG AGCGAGAGAG AGAGAAATTT TAAATTTCTT AAAAAATTTT ATGTTAATTT CATCTGGGTT   
  
  
- TTATTAAAAT TTTACTTTTT AATTAACCCA AAAAATTATC AAAATATTAT TTTAGTCTTT ATTAAAAATA   
  
  
- GAGCACATAT CAATAGAGTA TCTTAATTCT AAAAAATAAT GTTTTGAATA GGAAATTTAA ATGGAATGAA   
  
  
- TTGGTCAAAG TTGTAATTTA GGGCCAAAAA ATTATGGATA AAAAAGGAAA TAAAGGGATT CAATAATTCA   
  
  
- CATGTAATAA ATGCTTGTAT GGAAAGCATT GAGCATGTAT GGCTGCCTGC TACGGTGGTT GCTCTGTTAA   
  
  
- TAGTGCCCCA AGAAGAAGGG CCGCACCTAT AGGCTATGGT CAGCCGAGGA AATTGCAAAT TGGCACGAAA   
  
  
- CTCTTGCGCA CCTTGAGTTT TACTCTTTAA CCTGCACACT AACCCTCTGT GTGGCGCCCA CAATACCCCC   
  
  
- AAGTCACCCA GGATCCCACA CCCAGAGGGC AGTACAAACG CCGCACCGGG GGAGGATCCC CGTAAACGAA   
  
  
- ATTGTGACAC GTGAGGCATA TGGATGTAAT ATTTTAATTA TGAAAATTAA AAGGTATCAA TTATGAAAAA   
  
  
- TTAACGTCAC AAAGATTACA GTATTAAAAC TATATTATAT ATCGAACTGA AGCTTAAATT AATAATTATT   
  
  
- AAAGGATCAC GGCAATATTT TTCCTTATA

+     TCCC-motif

| Site Name | Organism | Position | Strand | Matrix score. | sequence | function |
| --- | --- | --- | --- | --- | --- | --- |
| TCCC-motif | Spinacia oleracea | 146 | + | 7 | TCTCCCT | part of a light responsive element |

> 2018/04/13 10:10:12  
+ CTAATTTCTT TTTGAAGAGT CGCGCAGAGA GATATGTGCT TCTAGGCCTA TGGACGGCTA TTTACTGACC   
  
  
+ AAAACCGGCA TCTACTACTA CTGCTGCTGA TGACGTCGTC TATGTCTCTC ACCGAACTAA CGACCTTCCT   
  
  
+ TCATTTCTCC CTCTCTCTCT CTCTCTCTCT TCTCTTCTTT ACCTCCTCTT CCACCACTTC CTTTCTTTCC   
  
  
+ CACCAGGAAT GTCTTTTCCC GGCAGTACAC TCTGTCTTCC TCACAAACCA TTATATATAA ACATCAATCG   
  
  
+ TTTCCTGCAC GTTCGTTTAC ACTAAAGTTC GGTAGTGATA AAATAATATA GTAGTTGAAC TTTTGCTTGG   
  
  
+ GAGGTAAAAT GAAAAGAAAA AACAAATAAC TTTAGTAATT CGGGTGACTA ATGTCTCAGA CGTAATTAAT   
  
  
+ AAAAATTAAA ACCATCTTTT GCCCCGACAC AGTACAACCA ACCAAAAAGA CCAAACTTGT TTTGTACACA   
  
  
+ ACAAAACAGG ACAACGAAGT AACAAGAAAT TCTCGTCAAC AACAACTTTA GTCTAATGGC CATCCTCCCA   
  
  
+ TCCGGCTGAC TAACCAAAGT ATGGGTCTTC AACGGGAAAA TTTTGGTCTA CTCCTTTCAG GACAAATATA   
  
  
+ AGCGAATATA ATAAGAGTAC GTTTTAATCC CAAAGGTAAA AGAAAAAATA AAAGGGATCA CCTATATCAA   
  
  
+ CTCATTGTGT CACTAGGTGG GTTGAGTCGA CGGAGGGAAA TAGGCTGGGT TTTAGTACCC CTAGAGAAGA   
  
  
+ GTTTTCCCTC TCGCTCTCTC TCTCTTTAAA ATTTAAAGAA TTTTTTAAAA TACAATTAAA GTAGACCCAA   
  
  
+ AATAATTTTA AAATGAAAAA TTAATTGGGT TTTTTAATAG TTTTATAATA AAATCAGAAA TAATTTTTAT   
  
  
+ CTCGTGTATA GTTATCTCAT AGAATTAAGA TTTTTTATTA CAAAACTTAT CCTTTAAATT TACCTTACTT   
  
  
+ AACCAGTTTC AACATTAAAT CCCGGTTTTT TAATACCTAT TTTTTCCTTT ATTTCCCTAA GTTATTAAGT   
  
  
+ GTACATTATT TACGAACATA CCTTTCGTAA CTCGTACATA CCGACGGACG ATGCCACCAA CGAGACAATT   
  
  
+ ATCACGGGGT TCTTCTTCCC GGCGTGGATA TCCGATACCA GTCGGCTCCT TTAACGTTTA ACCGTGCTTT   
  
  
+ GAGAACGCGT GGAACTCAAA ATGAGAAATT GGACGTGTGA TTGGGAGACA CACCGCGGGT GTTATGGGGG   
  
  
+ TTCAGTGGGT CCTAGGGTGT GGGTCTCCCG TCATGTTTGC GGCGTGGCCC CCTCCTAGGG GCATTTGCTT   
  
  
+ TAACACTGTG CACTCCGTAT ACCTACATTA TAAAATTAAT ACTTTTAATT TTCCATAGTT AATACTTTTT   
  
  
+ AATTGCAGTG TTTCTAATGT CATAATTTTG ATATAATATA TAGCTTGACT TCGAATTTAA TTATTAATAA   
  
  
+ TTTCCTAGTG CCGTTATAAA AAGGAATAT  

- GATTAAAGAA AAACTTCTCA GCGCGTCTCT CTATACACGA AGATCCGGAT ACCTGCCGAT AAATGACTGG   
  
  
- TTTTGGCCGT AGATGATGAT GACGACGACT ACTGCAGCAG ATACAGAGAG TGGCTTGATT GCTGGAAGGA   
  
  
- AGTAAAGAGG GAGAGAGAGA GAGAGAGAGA AGAGAAGAAA TGGAGGAGAA GGTGGTGAAG GAAAGAAAGG   
  
  
- GTGGTCCTTA CAGAAAAGGG CCGTCATGTG AGACAGAAGG AGTGTTTGGT AATATATATT TGTAGTTAGC   
  
  
- AAAGGACGTG CAAGCAAATG TGATTTCAAG CCATCACTAT TTTATTATAT CATCAACTTG AAAACGAACC   
  
  
- CTCCATTTTA CTTTTCTTTT TTGTTTATTG AAATCATTAA GCCCACTGAT TACAGAGTCT GCATTAATTA   
  
  
- TTTTTAATTT TGGTAGAAAA CGGGGCTGTG TCATGTTGGT TGGTTTTTCT GGTTTGAACA AAACATGTGT   
  
  
- TGTTTTGTCC TGTTGCTTCA TTGTTCTTTA AGAGCAGTTG TTGTTGAAAT CAGATTACCG GTAGGAGGGT   
  
  
- AGGCCGACTG ATTGGTTTCA TACCCAGAAG TTGCCCTTTT AAAACCAGAT GAGGAAAGTC CTGTTTATAT   
  
  
- TCGCTTATAT TATTCTCATG CAAAATTAGG GTTTCCATTT TCTTTTTTAT TTTCCCTAGT GGATATAGTT   
  
  
- GAGTAACACA GTGATCCACC CAACTCAGCT GCCTCCCTTT ATCCGACCCA AAATCATGGG GATCTCTTCT   
  
  
- CAAAAGGGAG AGCGAGAGAG AGAGAAATTT TAAATTTCTT AAAAAATTTT ATGTTAATTT CATCTGGGTT   
  
  
- TTATTAAAAT TTTACTTTTT AATTAACCCA AAAAATTATC AAAATATTAT TTTAGTCTTT ATTAAAAATA   
  
  
- GAGCACATAT CAATAGAGTA TCTTAATTCT AAAAAATAAT GTTTTGAATA GGAAATTTAA ATGGAATGAA   
  
  
- TTGGTCAAAG TTGTAATTTA GGGCCAAAAA ATTATGGATA AAAAAGGAAA TAAAGGGATT CAATAATTCA   
  
  
- CATGTAATAA ATGCTTGTAT GGAAAGCATT GAGCATGTAT GGCTGCCTGC TACGGTGGTT GCTCTGTTAA   
  
  
- TAGTGCCCCA AGAAGAAGGG CCGCACCTAT AGGCTATGGT CAGCCGAGGA AATTGCAAAT TGGCACGAAA   
  
  
- CTCTTGCGCA CCTTGAGTTT TACTCTTTAA CCTGCACACT AACCCTCTGT GTGGCGCCCA CAATACCCCC   
  
  
- AAGTCACCCA GGATCCCACA CCCAGAGGGC AGTACAAACG CCGCACCGGG GGAGGATCCC CGTAAACGAA   
  
  
- ATTGTGACAC GTGAGGCATA TGGATGTAAT ATTTTAATTA TGAAAATTAA AAGGTATCAA TTATGAAAAA   
  
  
- TTAACGTCAC AAAGATTACA GTATTAAAAC TATATTATAT ATCGAACTGA AGCTTAAATT AATAATTATT   
  
  
- AAAGGATCAC GGCAATATTT TTCCTTATA

+     TGA-element

| Site Name | Organism | Position | Strand | Matrix score. | sequence | function |
| --- | --- | --- | --- | --- | --- | --- |
| TGA-element | Brassica oleracea | 129 | + | 6 | AACGAC | auxin-responsive element |

> 2018/04/13 10:10:12  
+ CTAATTTCTT TTTGAAGAGT CGCGCAGAGA GATATGTGCT TCTAGGCCTA TGGACGGCTA TTTACTGACC   
  
  
+ AAAACCGGCA TCTACTACTA CTGCTGCTGA TGACGTCGTC TATGTCTCTC ACCGAACTAA CGACCTTCCT   
  
  
+ TCATTTCTCC CTCTCTCTCT CTCTCTCTCT TCTCTTCTTT ACCTCCTCTT CCACCACTTC CTTTCTTTCC   
  
  
+ CACCAGGAAT GTCTTTTCCC GGCAGTACAC TCTGTCTTCC TCACAAACCA TTATATATAA ACATCAATCG   
  
  
+ TTTCCTGCAC GTTCGTTTAC ACTAAAGTTC GGTAGTGATA AAATAATATA GTAGTTGAAC TTTTGCTTGG   
  
  
+ GAGGTAAAAT GAAAAGAAAA AACAAATAAC TTTAGTAATT CGGGTGACTA ATGTCTCAGA CGTAATTAAT   
  
  
+ AAAAATTAAA ACCATCTTTT GCCCCGACAC AGTACAACCA ACCAAAAAGA CCAAACTTGT TTTGTACACA   
  
  
+ ACAAAACAGG ACAACGAAGT AACAAGAAAT TCTCGTCAAC AACAACTTTA GTCTAATGGC CATCCTCCCA   
  
  
+ TCCGGCTGAC TAACCAAAGT ATGGGTCTTC AACGGGAAAA TTTTGGTCTA CTCCTTTCAG GACAAATATA   
  
  
+ AGCGAATATA ATAAGAGTAC GTTTTAATCC CAAAGGTAAA AGAAAAAATA AAAGGGATCA CCTATATCAA   
  
  
+ CTCATTGTGT CACTAGGTGG GTTGAGTCGA CGGAGGGAAA TAGGCTGGGT TTTAGTACCC CTAGAGAAGA   
  
  
+ GTTTTCCCTC TCGCTCTCTC TCTCTTTAAA ATTTAAAGAA TTTTTTAAAA TACAATTAAA GTAGACCCAA   
  
  
+ AATAATTTTA AAATGAAAAA TTAATTGGGT TTTTTAATAG TTTTATAATA AAATCAGAAA TAATTTTTAT   
  
  
+ CTCGTGTATA GTTATCTCAT AGAATTAAGA TTTTTTATTA CAAAACTTAT CCTTTAAATT TACCTTACTT   
  
  
+ AACCAGTTTC AACATTAAAT CCCGGTTTTT TAATACCTAT TTTTTCCTTT ATTTCCCTAA GTTATTAAGT   
  
  
+ GTACATTATT TACGAACATA CCTTTCGTAA CTCGTACATA CCGACGGACG ATGCCACCAA CGAGACAATT   
  
  
+ ATCACGGGGT TCTTCTTCCC GGCGTGGATA TCCGATACCA GTCGGCTCCT TTAACGTTTA ACCGTGCTTT   
  
  
+ GAGAACGCGT GGAACTCAAA ATGAGAAATT GGACGTGTGA TTGGGAGACA CACCGCGGGT GTTATGGGGG   
  
  
+ TTCAGTGGGT CCTAGGGTGT GGGTCTCCCG TCATGTTTGC GGCGTGGCCC CCTCCTAGGG GCATTTGCTT   
  
  
+ TAACACTGTG CACTCCGTAT ACCTACATTA TAAAATTAAT ACTTTTAATT TTCCATAGTT AATACTTTTT   
  
  
+ AATTGCAGTG TTTCTAATGT CATAATTTTG ATATAATATA TAGCTTGACT TCGAATTTAA TTATTAATAA   
  
  
+ TTTCCTAGTG CCGTTATAAA AAGGAATAT  

- GATTAAAGAA AAACTTCTCA GCGCGTCTCT CTATACACGA AGATCCGGAT ACCTGCCGAT AAATGACTGG   
  
  
- TTTTGGCCGT AGATGATGAT GACGACGACT ACTGCAGCAG ATACAGAGAG TGGCTTGATT GCTGGAAGGA   
  
  
- AGTAAAGAGG GAGAGAGAGA GAGAGAGAGA AGAGAAGAAA TGGAGGAGAA GGTGGTGAAG GAAAGAAAGG   
  
  
- GTGGTCCTTA CAGAAAAGGG CCGTCATGTG AGACAGAAGG AGTGTTTGGT AATATATATT TGTAGTTAGC   
  
  
- AAAGGACGTG CAAGCAAATG TGATTTCAAG CCATCACTAT TTTATTATAT CATCAACTTG AAAACGAACC   
  
  
- CTCCATTTTA CTTTTCTTTT TTGTTTATTG AAATCATTAA GCCCACTGAT TACAGAGTCT GCATTAATTA   
  
  
- TTTTTAATTT TGGTAGAAAA CGGGGCTGTG TCATGTTGGT TGGTTTTTCT GGTTTGAACA AAACATGTGT   
  
  
- TGTTTTGTCC TGTTGCTTCA TTGTTCTTTA AGAGCAGTTG TTGTTGAAAT CAGATTACCG GTAGGAGGGT   
  
  
- AGGCCGACTG ATTGGTTTCA TACCCAGAAG TTGCCCTTTT AAAACCAGAT GAGGAAAGTC CTGTTTATAT   
  
  
- TCGCTTATAT TATTCTCATG CAAAATTAGG GTTTCCATTT TCTTTTTTAT TTTCCCTAGT GGATATAGTT   
  
  
- GAGTAACACA GTGATCCACC CAACTCAGCT GCCTCCCTTT ATCCGACCCA AAATCATGGG GATCTCTTCT   
  
  
- CAAAAGGGAG AGCGAGAGAG AGAGAAATTT TAAATTTCTT AAAAAATTTT ATGTTAATTT CATCTGGGTT   
  
  
- TTATTAAAAT TTTACTTTTT AATTAACCCA AAAAATTATC AAAATATTAT TTTAGTCTTT ATTAAAAATA   
  
  
- GAGCACATAT CAATAGAGTA TCTTAATTCT AAAAAATAAT GTTTTGAATA GGAAATTTAA ATGGAATGAA   
  
  
- TTGGTCAAAG TTGTAATTTA GGGCCAAAAA ATTATGGATA AAAAAGGAAA TAAAGGGATT CAATAATTCA   
  
  
- CATGTAATAA ATGCTTGTAT GGAAAGCATT GAGCATGTAT GGCTGCCTGC TACGGTGGTT GCTCTGTTAA   
  
  
- TAGTGCCCCA AGAAGAAGGG CCGCACCTAT AGGCTATGGT CAGCCGAGGA AATTGCAAAT TGGCACGAAA   
  
  
- CTCTTGCGCA CCTTGAGTTT TACTCTTTAA CCTGCACACT AACCCTCTGT GTGGCGCCCA CAATACCCCC   
  
  
- AAGTCACCCA GGATCCCACA CCCAGAGGGC AGTACAAACG CCGCACCGGG GGAGGATCCC CGTAAACGAA   
  
  
- ATTGTGACAC GTGAGGCATA TGGATGTAAT ATTTTAATTA TGAAAATTAA AAGGTATCAA TTATGAAAAA   
  
  
- TTAACGTCAC AAAGATTACA GTATTAAAAC TATATTATAT ATCGAACTGA AGCTTAAATT AATAATTATT   
  
  
- AAAGGATCAC GGCAATATTT TTCCTTATA

+     TGACG-motif

| Site Name | Organism | Position | Strand | Matrix score. | sequence | function |
| --- | --- | --- | --- | --- | --- | --- |
| TGACG-motif | Hordeum vulgare | 101 | + | 5 | TGACG | cis-acting regulatory element involved in the MeJA-responsiveness |
| TGACG-motif | Hordeum vulgare | 1289 | - | 5 | TGACG | cis-acting regulatory element involved in the MeJA-responsiveness |
| TGACG-motif | Hordeum vulgare | 524 | - | 5 | TGACG | cis-acting regulatory element involved in the MeJA-responsiveness |

> 2018/04/13 10:10:12  
+ CTAATTTCTT TTTGAAGAGT CGCGCAGAGA GATATGTGCT TCTAGGCCTA TGGACGGCTA TTTACTGACC   
  
  
+ AAAACCGGCA TCTACTACTA CTGCTGCTGA TGACGTCGTC TATGTCTCTC ACCGAACTAA CGACCTTCCT   
  
  
+ TCATTTCTCC CTCTCTCTCT CTCTCTCTCT TCTCTTCTTT ACCTCCTCTT CCACCACTTC CTTTCTTTCC   
  
  
+ CACCAGGAAT GTCTTTTCCC GGCAGTACAC TCTGTCTTCC TCACAAACCA TTATATATAA ACATCAATCG   
  
  
+ TTTCCTGCAC GTTCGTTTAC ACTAAAGTTC GGTAGTGATA AAATAATATA GTAGTTGAAC TTTTGCTTGG   
  
  
+ GAGGTAAAAT GAAAAGAAAA AACAAATAAC TTTAGTAATT CGGGTGACTA ATGTCTCAGA CGTAATTAAT   
  
  
+ AAAAATTAAA ACCATCTTTT GCCCCGACAC AGTACAACCA ACCAAAAAGA CCAAACTTGT TTTGTACACA   
  
  
+ ACAAAACAGG ACAACGAAGT AACAAGAAAT TCTCGTCAAC AACAACTTTA GTCTAATGGC CATCCTCCCA   
  
  
+ TCCGGCTGAC TAACCAAAGT ATGGGTCTTC AACGGGAAAA TTTTGGTCTA CTCCTTTCAG GACAAATATA   
  
  
+ AGCGAATATA ATAAGAGTAC GTTTTAATCC CAAAGGTAAA AGAAAAAATA AAAGGGATCA CCTATATCAA   
  
  
+ CTCATTGTGT CACTAGGTGG GTTGAGTCGA CGGAGGGAAA TAGGCTGGGT TTTAGTACCC CTAGAGAAGA   
  
  
+ GTTTTCCCTC TCGCTCTCTC TCTCTTTAAA ATTTAAAGAA TTTTTTAAAA TACAATTAAA GTAGACCCAA   
  
  
+ AATAATTTTA AAATGAAAAA TTAATTGGGT TTTTTAATAG TTTTATAATA AAATCAGAAA TAATTTTTAT   
  
  
+ CTCGTGTATA GTTATCTCAT AGAATTAAGA TTTTTTATTA CAAAACTTAT CCTTTAAATT TACCTTACTT   
  
  
+ AACCAGTTTC AACATTAAAT CCCGGTTTTT TAATACCTAT TTTTTCCTTT ATTTCCCTAA GTTATTAAGT   
  
  
+ GTACATTATT TACGAACATA CCTTTCGTAA CTCGTACATA CCGACGGACG ATGCCACCAA CGAGACAATT   
  
  
+ ATCACGGGGT TCTTCTTCCC GGCGTGGATA TCCGATACCA GTCGGCTCCT TTAACGTTTA ACCGTGCTTT   
  
  
+ GAGAACGCGT GGAACTCAAA ATGAGAAATT GGACGTGTGA TTGGGAGACA CACCGCGGGT GTTATGGGGG   
  
  
+ TTCAGTGGGT CCTAGGGTGT GGGTCTCCCG TCATGTTTGC GGCGTGGCCC CCTCCTAGGG GCATTTGCTT   
  
  
+ TAACACTGTG CACTCCGTAT ACCTACATTA TAAAATTAAT ACTTTTAATT TTCCATAGTT AATACTTTTT   
  
  
+ AATTGCAGTG TTTCTAATGT CATAATTTTG ATATAATATA TAGCTTGACT TCGAATTTAA TTATTAATAA   
  
  
+ TTTCCTAGTG CCGTTATAAA AAGGAATAT  

- GATTAAAGAA AAACTTCTCA GCGCGTCTCT CTATACACGA AGATCCGGAT ACCTGCCGAT AAATGACTGG   
  
  
- TTTTGGCCGT AGATGATGAT GACGACGACT ACTGCAGCAG ATACAGAGAG TGGCTTGATT GCTGGAAGGA   
  
  
- AGTAAAGAGG GAGAGAGAGA GAGAGAGAGA AGAGAAGAAA TGGAGGAGAA GGTGGTGAAG GAAAGAAAGG   
  
  
- GTGGTCCTTA CAGAAAAGGG CCGTCATGTG AGACAGAAGG AGTGTTTGGT AATATATATT TGTAGTTAGC   
  
  
- AAAGGACGTG CAAGCAAATG TGATTTCAAG CCATCACTAT TTTATTATAT CATCAACTTG AAAACGAACC   
  
  
- CTCCATTTTA CTTTTCTTTT TTGTTTATTG AAATCATTAA GCCCACTGAT TACAGAGTCT GCATTAATTA   
  
  
- TTTTTAATTT TGGTAGAAAA CGGGGCTGTG TCATGTTGGT TGGTTTTTCT GGTTTGAACA AAACATGTGT   
  
  
- TGTTTTGTCC TGTTGCTTCA TTGTTCTTTA AGAGCAGTTG TTGTTGAAAT CAGATTACCG GTAGGAGGGT   
  
  
- AGGCCGACTG ATTGGTTTCA TACCCAGAAG TTGCCCTTTT AAAACCAGAT GAGGAAAGTC CTGTTTATAT   
  
  
- TCGCTTATAT TATTCTCATG CAAAATTAGG GTTTCCATTT TCTTTTTTAT TTTCCCTAGT GGATATAGTT   
  
  
- GAGTAACACA GTGATCCACC CAACTCAGCT GCCTCCCTTT ATCCGACCCA AAATCATGGG GATCTCTTCT   
  
  
- CAAAAGGGAG AGCGAGAGAG AGAGAAATTT TAAATTTCTT AAAAAATTTT ATGTTAATTT CATCTGGGTT   
  
  
- TTATTAAAAT TTTACTTTTT AATTAACCCA AAAAATTATC AAAATATTAT TTTAGTCTTT ATTAAAAATA   
  
  
- GAGCACATAT CAATAGAGTA TCTTAATTCT AAAAAATAAT GTTTTGAATA GGAAATTTAA ATGGAATGAA   
  
  
- TTGGTCAAAG TTGTAATTTA GGGCCAAAAA ATTATGGATA AAAAAGGAAA TAAAGGGATT CAATAATTCA   
  
  
- CATGTAATAA ATGCTTGTAT GGAAAGCATT GAGCATGTAT GGCTGCCTGC TACGGTGGTT GCTCTGTTAA   
  
  
- TAGTGCCCCA AGAAGAAGGG CCGCACCTAT AGGCTATGGT CAGCCGAGGA AATTGCAAAT TGGCACGAAA   
  
  
- CTCTTGCGCA CCTTGAGTTT TACTCTTTAA CCTGCACACT AACCCTCTGT GTGGCGCCCA CAATACCCCC   
  
  
- AAGTCACCCA GGATCCCACA CCCAGAGGGC AGTACAAACG CCGCACCGGG GGAGGATCCC CGTAAACGAA   
  
  
- ATTGTGACAC GTGAGGCATA TGGATGTAAT ATTTTAATTA TGAAAATTAA AAGGTATCAA TTATGAAAAA   
  
  
- TTAACGTCAC AAAGATTACA GTATTAAAAC TATATTATAT ATCGAACTGA AGCTTAAATT AATAATTATT   
  
  
- AAAGGATCAC GGCAATATTT TTCCTTATA

+     Unnamed\_\_1

| Site Name | Organism | Position | Strand | Matrix score. | sequence | function |
| --- | --- | --- | --- | --- | --- | --- |
| Unnamed\_\_1 | Glycine max | 1453 | + | 11 | GAATTTAATTAA | 60K protein binding site |
| Unnamed\_\_1 | Zea mays | 1143 | + | 5 | CGTGG |  |
| Unnamed\_\_1 | Zea mays | 1198 | + | 5 | CGTGG |  |
| Unnamed\_\_1 | Zea mays | 1303 | + | 5 | CGTGG |  |

> 2018/04/13 10:10:12  
+ CTAATTTCTT TTTGAAGAGT CGCGCAGAGA GATATGTGCT TCTAGGCCTA TGGACGGCTA TTTACTGACC   
  
  
+ AAAACCGGCA TCTACTACTA CTGCTGCTGA TGACGTCGTC TATGTCTCTC ACCGAACTAA CGACCTTCCT   
  
  
+ TCATTTCTCC CTCTCTCTCT CTCTCTCTCT TCTCTTCTTT ACCTCCTCTT CCACCACTTC CTTTCTTTCC   
  
  
+ CACCAGGAAT GTCTTTTCCC GGCAGTACAC TCTGTCTTCC TCACAAACCA TTATATATAA ACATCAATCG   
  
  
+ TTTCCTGCAC GTTCGTTTAC ACTAAAGTTC GGTAGTGATA AAATAATATA GTAGTTGAAC TTTTGCTTGG   
  
  
+ GAGGTAAAAT GAAAAGAAAA AACAAATAAC TTTAGTAATT CGGGTGACTA ATGTCTCAGA CGTAATTAAT   
  
  
+ AAAAATTAAA ACCATCTTTT GCCCCGACAC AGTACAACCA ACCAAAAAGA CCAAACTTGT TTTGTACACA   
  
  
+ ACAAAACAGG ACAACGAAGT AACAAGAAAT TCTCGTCAAC AACAACTTTA GTCTAATGGC CATCCTCCCA   
  
  
+ TCCGGCTGAC TAACCAAAGT ATGGGTCTTC AACGGGAAAA TTTTGGTCTA CTCCTTTCAG GACAAATATA   
  
  
+ AGCGAATATA ATAAGAGTAC GTTTTAATCC CAAAGGTAAA AGAAAAAATA AAAGGGATCA CCTATATCAA   
  
  
+ CTCATTGTGT CACTAGGTGG GTTGAGTCGA CGGAGGGAAA TAGGCTGGGT TTTAGTACCC CTAGAGAAGA   
  
  
+ GTTTTCCCTC TCGCTCTCTC TCTCTTTAAA ATTTAAAGAA TTTTTTAAAA TACAATTAAA GTAGACCCAA   
  
  
+ AATAATTTTA AAATGAAAAA TTAATTGGGT TTTTTAATAG TTTTATAATA AAATCAGAAA TAATTTTTAT   
  
  
+ CTCGTGTATA GTTATCTCAT AGAATTAAGA TTTTTTATTA CAAAACTTAT CCTTTAAATT TACCTTACTT   
  
  
+ AACCAGTTTC AACATTAAAT CCCGGTTTTT TAATACCTAT TTTTTCCTTT ATTTCCCTAA GTTATTAAGT   
  
  
+ GTACATTATT TACGAACATA CCTTTCGTAA CTCGTACATA CCGACGGACG ATGCCACCAA CGAGACAATT   
  
  
+ ATCACGGGGT TCTTCTTCCC GGCGTGGATA TCCGATACCA GTCGGCTCCT TTAACGTTTA ACCGTGCTTT   
  
  
+ GAGAACGCGT GGAACTCAAA ATGAGAAATT GGACGTGTGA TTGGGAGACA CACCGCGGGT GTTATGGGGG   
  
  
+ TTCAGTGGGT CCTAGGGTGT GGGTCTCCCG TCATGTTTGC GGCGTGGCCC CCTCCTAGGG GCATTTGCTT   
  
  
+ TAACACTGTG CACTCCGTAT ACCTACATTA TAAAATTAAT ACTTTTAATT TTCCATAGTT AATACTTTTT   
  
  
+ AATTGCAGTG TTTCTAATGT CATAATTTTG ATATAATATA TAGCTTGACT TCGAATTTAA TTATTAATAA   
  
  
+ TTTCCTAGTG CCGTTATAAA AAGGAATAT  

- GATTAAAGAA AAACTTCTCA GCGCGTCTCT CTATACACGA AGATCCGGAT ACCTGCCGAT AAATGACTGG   
  
  
- TTTTGGCCGT AGATGATGAT GACGACGACT ACTGCAGCAG ATACAGAGAG TGGCTTGATT GCTGGAAGGA   
  
  
- AGTAAAGAGG GAGAGAGAGA GAGAGAGAGA AGAGAAGAAA TGGAGGAGAA GGTGGTGAAG GAAAGAAAGG   
  
  
- GTGGTCCTTA CAGAAAAGGG CCGTCATGTG AGACAGAAGG AGTGTTTGGT AATATATATT TGTAGTTAGC   
  
  
- AAAGGACGTG CAAGCAAATG TGATTTCAAG CCATCACTAT TTTATTATAT CATCAACTTG AAAACGAACC   
  
  
- CTCCATTTTA CTTTTCTTTT TTGTTTATTG AAATCATTAA GCCCACTGAT TACAGAGTCT GCATTAATTA   
  
  
- TTTTTAATTT TGGTAGAAAA CGGGGCTGTG TCATGTTGGT TGGTTTTTCT GGTTTGAACA AAACATGTGT   
  
  
- TGTTTTGTCC TGTTGCTTCA TTGTTCTTTA AGAGCAGTTG TTGTTGAAAT CAGATTACCG GTAGGAGGGT   
  
  
- AGGCCGACTG ATTGGTTTCA TACCCAGAAG TTGCCCTTTT AAAACCAGAT GAGGAAAGTC CTGTTTATAT   
  
  
- TCGCTTATAT TATTCTCATG CAAAATTAGG GTTTCCATTT TCTTTTTTAT TTTCCCTAGT GGATATAGTT   
  
  
- GAGTAACACA GTGATCCACC CAACTCAGCT GCCTCCCTTT ATCCGACCCA AAATCATGGG GATCTCTTCT   
  
  
- CAAAAGGGAG AGCGAGAGAG AGAGAAATTT TAAATTTCTT AAAAAATTTT ATGTTAATTT CATCTGGGTT   
  
  
- TTATTAAAAT TTTACTTTTT AATTAACCCA AAAAATTATC AAAATATTAT TTTAGTCTTT ATTAAAAATA   
  
  
- GAGCACATAT CAATAGAGTA TCTTAATTCT AAAAAATAAT GTTTTGAATA GGAAATTTAA ATGGAATGAA   
  
  
- TTGGTCAAAG TTGTAATTTA GGGCCAAAAA ATTATGGATA AAAAAGGAAA TAAAGGGATT CAATAATTCA   
  
  
- CATGTAATAA ATGCTTGTAT GGAAAGCATT GAGCATGTAT GGCTGCCTGC TACGGTGGTT GCTCTGTTAA   
  
  
- TAGTGCCCCA AGAAGAAGGG CCGCACCTAT AGGCTATGGT CAGCCGAGGA AATTGCAAAT TGGCACGAAA   
  
  
- CTCTTGCGCA CCTTGAGTTT TACTCTTTAA CCTGCACACT AACCCTCTGT GTGGCGCCCA CAATACCCCC   
  
  
- AAGTCACCCA GGATCCCACA CCCAGAGGGC AGTACAAACG CCGCACCGGG GGAGGATCCC CGTAAACGAA   
  
  
- ATTGTGACAC GTGAGGCATA TGGATGTAAT ATTTTAATTA TGAAAATTAA AAGGTATCAA TTATGAAAAA   
  
  
- TTAACGTCAC AAAGATTACA GTATTAAAAC TATATTATAT ATCGAACTGA AGCTTAAATT AATAATTATT   
  
  
- AAAGGATCAC GGCAATATTT TTCCTTATA

+     Unnamed\_\_3

| Site Name | Organism | Position | Strand | Matrix score. | sequence | function |
| --- | --- | --- | --- | --- | --- | --- |
| Unnamed\_\_3 | Zea mays | 1143 | + | 5 | CGTGG |  |
| Unnamed\_\_3 | Zea mays | 1303 | + | 5 | CGTGG |  |
| Unnamed\_\_3 | Zea mays | 1198 | + | 5 | CGTGG |  |

> 2018/04/13 10:10:12  
+ CTAATTTCTT TTTGAAGAGT CGCGCAGAGA GATATGTGCT TCTAGGCCTA TGGACGGCTA TTTACTGACC   
  
  
+ AAAACCGGCA TCTACTACTA CTGCTGCTGA TGACGTCGTC TATGTCTCTC ACCGAACTAA CGACCTTCCT   
  
  
+ TCATTTCTCC CTCTCTCTCT CTCTCTCTCT TCTCTTCTTT ACCTCCTCTT CCACCACTTC CTTTCTTTCC   
  
  
+ CACCAGGAAT GTCTTTTCCC GGCAGTACAC TCTGTCTTCC TCACAAACCA TTATATATAA ACATCAATCG   
  
  
+ TTTCCTGCAC GTTCGTTTAC ACTAAAGTTC GGTAGTGATA AAATAATATA GTAGTTGAAC TTTTGCTTGG   
  
  
+ GAGGTAAAAT GAAAAGAAAA AACAAATAAC TTTAGTAATT CGGGTGACTA ATGTCTCAGA CGTAATTAAT   
  
  
+ AAAAATTAAA ACCATCTTTT GCCCCGACAC AGTACAACCA ACCAAAAAGA CCAAACTTGT TTTGTACACA   
  
  
+ ACAAAACAGG ACAACGAAGT AACAAGAAAT TCTCGTCAAC AACAACTTTA GTCTAATGGC CATCCTCCCA   
  
  
+ TCCGGCTGAC TAACCAAAGT ATGGGTCTTC AACGGGAAAA TTTTGGTCTA CTCCTTTCAG GACAAATATA   
  
  
+ AGCGAATATA ATAAGAGTAC GTTTTAATCC CAAAGGTAAA AGAAAAAATA AAAGGGATCA CCTATATCAA   
  
  
+ CTCATTGTGT CACTAGGTGG GTTGAGTCGA CGGAGGGAAA TAGGCTGGGT TTTAGTACCC CTAGAGAAGA   
  
  
+ GTTTTCCCTC TCGCTCTCTC TCTCTTTAAA ATTTAAAGAA TTTTTTAAAA TACAATTAAA GTAGACCCAA   
  
  
+ AATAATTTTA AAATGAAAAA TTAATTGGGT TTTTTAATAG TTTTATAATA AAATCAGAAA TAATTTTTAT   
  
  
+ CTCGTGTATA GTTATCTCAT AGAATTAAGA TTTTTTATTA CAAAACTTAT CCTTTAAATT TACCTTACTT   
  
  
+ AACCAGTTTC AACATTAAAT CCCGGTTTTT TAATACCTAT TTTTTCCTTT ATTTCCCTAA GTTATTAAGT   
  
  
+ GTACATTATT TACGAACATA CCTTTCGTAA CTCGTACATA CCGACGGACG ATGCCACCAA CGAGACAATT   
  
  
+ ATCACGGGGT TCTTCTTCCC GGCGTGGATA TCCGATACCA GTCGGCTCCT TTAACGTTTA ACCGTGCTTT   
  
  
+ GAGAACGCGT GGAACTCAAA ATGAGAAATT GGACGTGTGA TTGGGAGACA CACCGCGGGT GTTATGGGGG   
  
  
+ TTCAGTGGGT CCTAGGGTGT GGGTCTCCCG TCATGTTTGC GGCGTGGCCC CCTCCTAGGG GCATTTGCTT   
  
  
+ TAACACTGTG CACTCCGTAT ACCTACATTA TAAAATTAAT ACTTTTAATT TTCCATAGTT AATACTTTTT   
  
  
+ AATTGCAGTG TTTCTAATGT CATAATTTTG ATATAATATA TAGCTTGACT TCGAATTTAA TTATTAATAA   
  
  
+ TTTCCTAGTG CCGTTATAAA AAGGAATAT  

- GATTAAAGAA AAACTTCTCA GCGCGTCTCT CTATACACGA AGATCCGGAT ACCTGCCGAT AAATGACTGG   
  
  
- TTTTGGCCGT AGATGATGAT GACGACGACT ACTGCAGCAG ATACAGAGAG TGGCTTGATT GCTGGAAGGA   
  
  
- AGTAAAGAGG GAGAGAGAGA GAGAGAGAGA AGAGAAGAAA TGGAGGAGAA GGTGGTGAAG GAAAGAAAGG   
  
  
- GTGGTCCTTA CAGAAAAGGG CCGTCATGTG AGACAGAAGG AGTGTTTGGT AATATATATT TGTAGTTAGC   
  
  
- AAAGGACGTG CAAGCAAATG TGATTTCAAG CCATCACTAT TTTATTATAT CATCAACTTG AAAACGAACC   
  
  
- CTCCATTTTA CTTTTCTTTT TTGTTTATTG AAATCATTAA GCCCACTGAT TACAGAGTCT GCATTAATTA   
  
  
- TTTTTAATTT TGGTAGAAAA CGGGGCTGTG TCATGTTGGT TGGTTTTTCT GGTTTGAACA AAACATGTGT   
  
  
- TGTTTTGTCC TGTTGCTTCA TTGTTCTTTA AGAGCAGTTG TTGTTGAAAT CAGATTACCG GTAGGAGGGT   
  
  
- AGGCCGACTG ATTGGTTTCA TACCCAGAAG TTGCCCTTTT AAAACCAGAT GAGGAAAGTC CTGTTTATAT   
  
  
- TCGCTTATAT TATTCTCATG CAAAATTAGG GTTTCCATTT TCTTTTTTAT TTTCCCTAGT GGATATAGTT   
  
  
- GAGTAACACA GTGATCCACC CAACTCAGCT GCCTCCCTTT ATCCGACCCA AAATCATGGG GATCTCTTCT   
  
  
- CAAAAGGGAG AGCGAGAGAG AGAGAAATTT TAAATTTCTT AAAAAATTTT ATGTTAATTT CATCTGGGTT   
  
  
- TTATTAAAAT TTTACTTTTT AATTAACCCA AAAAATTATC AAAATATTAT TTTAGTCTTT ATTAAAAATA   
  
  
- GAGCACATAT CAATAGAGTA TCTTAATTCT AAAAAATAAT GTTTTGAATA GGAAATTTAA ATGGAATGAA   
  
  
- TTGGTCAAAG TTGTAATTTA GGGCCAAAAA ATTATGGATA AAAAAGGAAA TAAAGGGATT CAATAATTCA   
  
  
- CATGTAATAA ATGCTTGTAT GGAAAGCATT GAGCATGTAT GGCTGCCTGC TACGGTGGTT GCTCTGTTAA   
  
  
- TAGTGCCCCA AGAAGAAGGG CCGCACCTAT AGGCTATGGT CAGCCGAGGA AATTGCAAAT TGGCACGAAA   
  
  
- CTCTTGCGCA CCTTGAGTTT TACTCTTTAA CCTGCACACT AACCCTCTGT GTGGCGCCCA CAATACCCCC   
  
  
- AAGTCACCCA GGATCCCACA CCCAGAGGGC AGTACAAACG CCGCACCGGG GGAGGATCCC CGTAAACGAA   
  
  
- ATTGTGACAC GTGAGGCATA TGGATGTAAT ATTTTAATTA TGAAAATTAA AAGGTATCAA TTATGAAAAA   
  
  
- TTAACGTCAC AAAGATTACA GTATTAAAAC TATATTATAT ATCGAACTGA AGCTTAAATT AATAATTATT   
  
  
- AAAGGATCAC GGCAATATTT TTCCTTATA

+     Unnamed\_\_4

| Site Name | Organism | Position | Strand | Matrix score. | sequence | function |
| --- | --- | --- | --- | --- | --- | --- |
| Unnamed\_\_4 | Petroselinum hortense | 1285 | + | 4 | CTCC |  |
| Unnamed\_\_4 | Petroselinum hortense | 350 | - | 4 | CTCC |  |
| Unnamed\_\_4 | Petroselinum hortense | 183 | + | 4 | CTCC |  |
| Unnamed\_\_4 | Petroselinum hortense | 1343 | + | 4 | CTCC |  |
| Unnamed\_\_4 | Petroselinum hortense | 611 | + | 4 | CTCC |  |
| Unnamed\_\_4 | Petroselinum hortense | 1234 | - | 4 | CTCC |  |
| Unnamed\_\_4 | Petroselinum hortense | 1166 | + | 4 | CTCC |  |
| Unnamed\_\_4 | Petroselinum hortense | 147 | + | 4 | CTCC |  |
| Unnamed\_\_4 | Petroselinum hortense | 732 | - | 4 | CTCC |  |
| Unnamed\_\_4 | Petroselinum hortense | 1312 | + | 4 | CTCC |  |
| Unnamed\_\_4 | Petroselinum hortense | 555 | + | 4 | CTCC |  |

> 2018/04/13 10:10:12  
+ CTAATTTCTT TTTGAAGAGT CGCGCAGAGA GATATGTGCT TCTAGGCCTA TGGACGGCTA TTTACTGACC   
  
  
+ AAAACCGGCA TCTACTACTA CTGCTGCTGA TGACGTCGTC TATGTCTCTC ACCGAACTAA CGACCTTCCT   
  
  
+ TCATTTCTCC CTCTCTCTCT CTCTCTCTCT TCTCTTCTTT ACCTCCTCTT CCACCACTTC CTTTCTTTCC   
  
  
+ CACCAGGAAT GTCTTTTCCC GGCAGTACAC TCTGTCTTCC TCACAAACCA TTATATATAA ACATCAATCG   
  
  
+ TTTCCTGCAC GTTCGTTTAC ACTAAAGTTC GGTAGTGATA AAATAATATA GTAGTTGAAC TTTTGCTTGG   
  
  
+ GAGGTAAAAT GAAAAGAAAA AACAAATAAC TTTAGTAATT CGGGTGACTA ATGTCTCAGA CGTAATTAAT   
  
  
+ AAAAATTAAA ACCATCTTTT GCCCCGACAC AGTACAACCA ACCAAAAAGA CCAAACTTGT TTTGTACACA   
  
  
+ ACAAAACAGG ACAACGAAGT AACAAGAAAT TCTCGTCAAC AACAACTTTA GTCTAATGGC CATCCTCCCA   
  
  
+ TCCGGCTGAC TAACCAAAGT ATGGGTCTTC AACGGGAAAA TTTTGGTCTA CTCCTTTCAG GACAAATATA   
  
  
+ AGCGAATATA ATAAGAGTAC GTTTTAATCC CAAAGGTAAA AGAAAAAATA AAAGGGATCA CCTATATCAA   
  
  
+ CTCATTGTGT CACTAGGTGG GTTGAGTCGA CGGAGGGAAA TAGGCTGGGT TTTAGTACCC CTAGAGAAGA   
  
  
+ GTTTTCCCTC TCGCTCTCTC TCTCTTTAAA ATTTAAAGAA TTTTTTAAAA TACAATTAAA GTAGACCCAA   
  
  
+ AATAATTTTA AAATGAAAAA TTAATTGGGT TTTTTAATAG TTTTATAATA AAATCAGAAA TAATTTTTAT   
  
  
+ CTCGTGTATA GTTATCTCAT AGAATTAAGA TTTTTTATTA CAAAACTTAT CCTTTAAATT TACCTTACTT   
  
  
+ AACCAGTTTC AACATTAAAT CCCGGTTTTT TAATACCTAT TTTTTCCTTT ATTTCCCTAA GTTATTAAGT   
  
  
+ GTACATTATT TACGAACATA CCTTTCGTAA CTCGTACATA CCGACGGACG ATGCCACCAA CGAGACAATT   
  
  
+ ATCACGGGGT TCTTCTTCCC GGCGTGGATA TCCGATACCA GTCGGCTCCT TTAACGTTTA ACCGTGCTTT   
  
  
+ GAGAACGCGT GGAACTCAAA ATGAGAAATT GGACGTGTGA TTGGGAGACA CACCGCGGGT GTTATGGGGG   
  
  
+ TTCAGTGGGT CCTAGGGTGT GGGTCTCCCG TCATGTTTGC GGCGTGGCCC CCTCCTAGGG GCATTTGCTT   
  
  
+ TAACACTGTG CACTCCGTAT ACCTACATTA TAAAATTAAT ACTTTTAATT TTCCATAGTT AATACTTTTT   
  
  
+ AATTGCAGTG TTTCTAATGT CATAATTTTG ATATAATATA TAGCTTGACT TCGAATTTAA TTATTAATAA   
  
  
+ TTTCCTAGTG CCGTTATAAA AAGGAATAT  

- GATTAAAGAA AAACTTCTCA GCGCGTCTCT CTATACACGA AGATCCGGAT ACCTGCCGAT AAATGACTGG   
  
  
- TTTTGGCCGT AGATGATGAT GACGACGACT ACTGCAGCAG ATACAGAGAG TGGCTTGATT GCTGGAAGGA   
  
  
- AGTAAAGAGG GAGAGAGAGA GAGAGAGAGA AGAGAAGAAA TGGAGGAGAA GGTGGTGAAG GAAAGAAAGG   
  
  
- GTGGTCCTTA CAGAAAAGGG CCGTCATGTG AGACAGAAGG AGTGTTTGGT AATATATATT TGTAGTTAGC   
  
  
- AAAGGACGTG CAAGCAAATG TGATTTCAAG CCATCACTAT TTTATTATAT CATCAACTTG AAAACGAACC   
  
  
- CTCCATTTTA CTTTTCTTTT TTGTTTATTG AAATCATTAA GCCCACTGAT TACAGAGTCT GCATTAATTA   
  
  
- TTTTTAATTT TGGTAGAAAA CGGGGCTGTG TCATGTTGGT TGGTTTTTCT GGTTTGAACA AAACATGTGT   
  
  
- TGTTTTGTCC TGTTGCTTCA TTGTTCTTTA AGAGCAGTTG TTGTTGAAAT CAGATTACCG GTAGGAGGGT   
  
  
- AGGCCGACTG ATTGGTTTCA TACCCAGAAG TTGCCCTTTT AAAACCAGAT GAGGAAAGTC CTGTTTATAT   
  
  
- TCGCTTATAT TATTCTCATG CAAAATTAGG GTTTCCATTT TCTTTTTTAT TTTCCCTAGT GGATATAGTT   
  
  
- GAGTAACACA GTGATCCACC CAACTCAGCT GCCTCCCTTT ATCCGACCCA AAATCATGGG GATCTCTTCT   
  
  
- CAAAAGGGAG AGCGAGAGAG AGAGAAATTT TAAATTTCTT AAAAAATTTT ATGTTAATTT CATCTGGGTT   
  
  
- TTATTAAAAT TTTACTTTTT AATTAACCCA AAAAATTATC AAAATATTAT TTTAGTCTTT ATTAAAAATA   
  
  
- GAGCACATAT CAATAGAGTA TCTTAATTCT AAAAAATAAT GTTTTGAATA GGAAATTTAA ATGGAATGAA   
  
  
- TTGGTCAAAG TTGTAATTTA GGGCCAAAAA ATTATGGATA AAAAAGGAAA TAAAGGGATT CAATAATTCA   
  
  
- CATGTAATAA ATGCTTGTAT GGAAAGCATT GAGCATGTAT GGCTGCCTGC TACGGTGGTT GCTCTGTTAA   
  
  
- TAGTGCCCCA AGAAGAAGGG CCGCACCTAT AGGCTATGGT CAGCCGAGGA AATTGCAAAT TGGCACGAAA   
  
  
- CTCTTGCGCA CCTTGAGTTT TACTCTTTAA CCTGCACACT AACCCTCTGT GTGGCGCCCA CAATACCCCC   
  
  
- AAGTCACCCA GGATCCCACA CCCAGAGGGC AGTACAAACG CCGCACCGGG GGAGGATCCC CGTAAACGAA   
  
  
- ATTGTGACAC GTGAGGCATA TGGATGTAAT ATTTTAATTA TGAAAATTAA AAGGTATCAA TTATGAAAAA   
  
  
- TTAACGTCAC AAAGATTACA GTATTAAAAC TATATTATAT ATCGAACTGA AGCTTAAATT AATAATTATT   
  
  
- AAAGGATCAC GGCAATATTT TTCCTTATA

+     chs-CMA1a

| Site Name | Organism | Position | Strand | Matrix score. | sequence | function |
| --- | --- | --- | --- | --- | --- | --- |
| chs-CMA1a | Daucus carota | 975 | + | 8 | TTACTTAA | part of a light responsive element |

> 2018/04/13 10:10:12  
+ CTAATTTCTT TTTGAAGAGT CGCGCAGAGA GATATGTGCT TCTAGGCCTA TGGACGGCTA TTTACTGACC   
  
  
+ AAAACCGGCA TCTACTACTA CTGCTGCTGA TGACGTCGTC TATGTCTCTC ACCGAACTAA CGACCTTCCT   
  
  
+ TCATTTCTCC CTCTCTCTCT CTCTCTCTCT TCTCTTCTTT ACCTCCTCTT CCACCACTTC CTTTCTTTCC   
  
  
+ CACCAGGAAT GTCTTTTCCC GGCAGTACAC TCTGTCTTCC TCACAAACCA TTATATATAA ACATCAATCG   
  
  
+ TTTCCTGCAC GTTCGTTTAC ACTAAAGTTC GGTAGTGATA AAATAATATA GTAGTTGAAC TTTTGCTTGG   
  
  
+ GAGGTAAAAT GAAAAGAAAA AACAAATAAC TTTAGTAATT CGGGTGACTA ATGTCTCAGA CGTAATTAAT   
  
  
+ AAAAATTAAA ACCATCTTTT GCCCCGACAC AGTACAACCA ACCAAAAAGA CCAAACTTGT TTTGTACACA   
  
  
+ ACAAAACAGG ACAACGAAGT AACAAGAAAT TCTCGTCAAC AACAACTTTA GTCTAATGGC CATCCTCCCA   
  
  
+ TCCGGCTGAC TAACCAAAGT ATGGGTCTTC AACGGGAAAA TTTTGGTCTA CTCCTTTCAG GACAAATATA   
  
  
+ AGCGAATATA ATAAGAGTAC GTTTTAATCC CAAAGGTAAA AGAAAAAATA AAAGGGATCA CCTATATCAA   
  
  
+ CTCATTGTGT CACTAGGTGG GTTGAGTCGA CGGAGGGAAA TAGGCTGGGT TTTAGTACCC CTAGAGAAGA   
  
  
+ GTTTTCCCTC TCGCTCTCTC TCTCTTTAAA ATTTAAAGAA TTTTTTAAAA TACAATTAAA GTAGACCCAA   
  
  
+ AATAATTTTA AAATGAAAAA TTAATTGGGT TTTTTAATAG TTTTATAATA AAATCAGAAA TAATTTTTAT   
  
  
+ CTCGTGTATA GTTATCTCAT AGAATTAAGA TTTTTTATTA CAAAACTTAT CCTTTAAATT TACCTTACTT   
  
  
+ AACCAGTTTC AACATTAAAT CCCGGTTTTT TAATACCTAT TTTTTCCTTT ATTTCCCTAA GTTATTAAGT   
  
  
+ GTACATTATT TACGAACATA CCTTTCGTAA CTCGTACATA CCGACGGACG ATGCCACCAA CGAGACAATT   
  
  
+ ATCACGGGGT TCTTCTTCCC GGCGTGGATA TCCGATACCA GTCGGCTCCT TTAACGTTTA ACCGTGCTTT   
  
  
+ GAGAACGCGT GGAACTCAAA ATGAGAAATT GGACGTGTGA TTGGGAGACA CACCGCGGGT GTTATGGGGG   
  
  
+ TTCAGTGGGT CCTAGGGTGT GGGTCTCCCG TCATGTTTGC GGCGTGGCCC CCTCCTAGGG GCATTTGCTT   
  
  
+ TAACACTGTG CACTCCGTAT ACCTACATTA TAAAATTAAT ACTTTTAATT TTCCATAGTT AATACTTTTT   
  
  
+ AATTGCAGTG TTTCTAATGT CATAATTTTG ATATAATATA TAGCTTGACT TCGAATTTAA TTATTAATAA   
  
  
+ TTTCCTAGTG CCGTTATAAA AAGGAATAT  

- GATTAAAGAA AAACTTCTCA GCGCGTCTCT CTATACACGA AGATCCGGAT ACCTGCCGAT AAATGACTGG   
  
  
- TTTTGGCCGT AGATGATGAT GACGACGACT ACTGCAGCAG ATACAGAGAG TGGCTTGATT GCTGGAAGGA   
  
  
- AGTAAAGAGG GAGAGAGAGA GAGAGAGAGA AGAGAAGAAA TGGAGGAGAA GGTGGTGAAG GAAAGAAAGG   
  
  
- GTGGTCCTTA CAGAAAAGGG CCGTCATGTG AGACAGAAGG AGTGTTTGGT AATATATATT TGTAGTTAGC   
  
  
- AAAGGACGTG CAAGCAAATG TGATTTCAAG CCATCACTAT TTTATTATAT CATCAACTTG AAAACGAACC   
  
  
- CTCCATTTTA CTTTTCTTTT TTGTTTATTG AAATCATTAA GCCCACTGAT TACAGAGTCT GCATTAATTA   
  
  
- TTTTTAATTT TGGTAGAAAA CGGGGCTGTG TCATGTTGGT TGGTTTTTCT GGTTTGAACA AAACATGTGT   
  
  
- TGTTTTGTCC TGTTGCTTCA TTGTTCTTTA AGAGCAGTTG TTGTTGAAAT CAGATTACCG GTAGGAGGGT   
  
  
- AGGCCGACTG ATTGGTTTCA TACCCAGAAG TTGCCCTTTT AAAACCAGAT GAGGAAAGTC CTGTTTATAT   
  
  
- TCGCTTATAT TATTCTCATG CAAAATTAGG GTTTCCATTT TCTTTTTTAT TTTCCCTAGT GGATATAGTT   
  
  
- GAGTAACACA GTGATCCACC CAACTCAGCT GCCTCCCTTT ATCCGACCCA AAATCATGGG GATCTCTTCT   
  
  
- CAAAAGGGAG AGCGAGAGAG AGAGAAATTT TAAATTTCTT AAAAAATTTT ATGTTAATTT CATCTGGGTT   
  
  
- TTATTAAAAT TTTACTTTTT AATTAACCCA AAAAATTATC AAAATATTAT TTTAGTCTTT ATTAAAAATA   
  
  
- GAGCACATAT CAATAGAGTA TCTTAATTCT AAAAAATAAT GTTTTGAATA GGAAATTTAA ATGGAATGAA   
  
  
- TTGGTCAAAG TTGTAATTTA GGGCCAAAAA ATTATGGATA AAAAAGGAAA TAAAGGGATT CAATAATTCA   
  
  
- CATGTAATAA ATGCTTGTAT GGAAAGCATT GAGCATGTAT GGCTGCCTGC TACGGTGGTT GCTCTGTTAA   
  
  
- TAGTGCCCCA AGAAGAAGGG CCGCACCTAT AGGCTATGGT CAGCCGAGGA AATTGCAAAT TGGCACGAAA   
  
  
- CTCTTGCGCA CCTTGAGTTT TACTCTTTAA CCTGCACACT AACCCTCTGT GTGGCGCCCA CAATACCCCC   
  
  
- AAGTCACCCA GGATCCCACA CCCAGAGGGC AGTACAAACG CCGCACCGGG GGAGGATCCC CGTAAACGAA   
  
  
- ATTGTGACAC GTGAGGCATA TGGATGTAAT ATTTTAATTA TGAAAATTAA AAGGTATCAA TTATGAAAAA   
  
  
- TTAACGTCAC AAAGATTACA GTATTAAAAC TATATTATAT ATCGAACTGA AGCTTAAATT AATAATTATT   
  
  
- AAAGGATCAC GGCAATATTT TTCCTTATA
