# Supplementary material for: Genome-wide analysis of the WRKY gene family in drumstick (Moringa oleifera Lam.)
Source: PeerJ. 2019 Jun 10;7:e7063. doi: 10.7717/peerj.7063 (PMC6563795; doi:10.7717/peerj.7063)
Supplement: Supplemental Information 1 [file peerj-07-7063-s003.gz › MoWRKY38_plantcare.html]

Content-Type: text/html; charset=ISO-8859-1


CallMat\_Firefox


Webmaster Firefox specific output  
To save the result:
click on the frame with the right mouse button and save the source code as a text file with extension .html  
REFERENCE:PlantCARE: a database of plant cis-acting regulatory elements and a portal to tools for in silico analysis of promoter sequences.  
Lescot, M., Déhais, P., Moreau, Y., De Moor, B., Rouzé ,P.,and Rombauts, S.  
Nucleic Acids Res., Database issue(2002), 30(1):325-327.   


---

> 2018/04/13 10:10:12  
+ TTTTTGTTAG GGTTTTTCGT TTCTTTCTTC TTTAAGTTCT TTACACAATA TTTCTTGTCG ACTTCGGACA   
  
  
+ TAGATGAGAG AGAGAGAGAG AGGGAGAGAG GAAGACGAAG AGGGTGATAC ACATACTTCA GACACTTCAG   
  
  
+ AAAGTGGCCA CGATATCCGA GCCGATAATT TTCCCAACCC GGCACCTGGT CTAAAACAAT AATCTTCTAC   
  
  
+ TGTGTGAGAA ACCTAAAGAG TGATAAAGGT TTAAAGGTTA GGCTCGAAGA ATGCAGTCGA CCACGCAGTC   
  
  
+ CAGCGGTCGA TCGTTGACCC AGGGTGAAGA CTGGTGGAGA CCGAACCAAT GGGCAGAAAA GGTGTTGGGC   
  
  
+ TTGAGCAATT GGGCACCGAC TAAACAGTAA AATAGACAAC TATATACACA CAACCGCCCA TACCTCGGCT   
  
  
+ GAAGCCCAAT CTGCATCTAT GCCTAGACAA ACTGCTTTCT CTTTTTTCAA TTTCTCCCTC TCCAAAGGAT   
  
  
+ TCGTAAAGCA GTTCTTTTCT CAAACTGAGC GAGCATTTAG TCTTCTTTCT TTACTTAGGT GGCGGGGCTT   
  
  
+ GCCACGCCGC GCAAGGTGCA CTGCTTTTTC CTTTATGTTT ATTGGGTTTT CTCCTTCTTT TGACTTCCTC   
  
  
+ TCTCTTCCTC TCTCTCTCTC TCTCTCTCCC CTCGGCTCAG TTGCTTAAAC TGCCGGGCCG AGAGACTCCT   
  
  
+ ATACTGGCCT CTGGCCCACC CCGTCACCGG ACCAGATACG CACCTGGGGT GCATCGTACT GTACTCTTTC   
  
  
+ GTGCAAAGTC TAGGCGGGGG CCCTTGGAAC AACTGACAAA TGGACACCCT GGCTCAGCGA ACGAAACTGA   
  
  
+ AAGACAATGT ACAACGAAAT GAGTAAACAG GGAGAGAAAG AGAAGTAGAG GATCAGTAGA AAAGGAAAAA   
  
  
+ AATAATTAAA GATTTTATTT CGAAATTGTA TGTACAAAAA GGAACACGTA CATAAAAGAG TTCATTTATC   
  
  
+ TTTTTATTAA TAAATAATGG TTTGAATAAA GGATATAATA ATTTTAAAAA CGTACAAATT TTATTCAAAT   
  
  
+ AATAAAATCA ATTTTTTAAT TAGTTTTTTT AATTAATAAA AAAAATACAT TAAATACTTC CCGTGTATCA   
  
  
+ TAAAATCCAC TTATAGTACT GTGTGCACAA TGAAACCTAA TCCTAAAAGA CAATCTAAAA ATAGAGGCGA   
  
  
+ TTTGAAAAAT TACTTTTCTA TTTTATCATA AAAATATTAA ATAATATAAT TTAATTATTT ATTTAATATT   
  
  
+ AATAAATAAA ATAATTTAAT ATTGTACAGT AATTGAAAAT TTTTTGAGTC GTGTTAAATC TACTCATTTA   
  
  
+ ATTTTTATTA CTATACATCA ATATTCATAG TTTTTATTTT TATAAACAAA TAAATTTTTA AAATTTTCGA   
  
  
+ TTTTGTATAA AATATTTGGG TAATTATGTT TTAATACAGT GATTAATTGT ATTTTGAGAT CAAAATAGGT   
  
  
+ TTTAACTACA GCAAAATGAT TATTGGATT  

- AAAAACAATC CCAAAAAGCA AAGAAAGAAG AAATTCAAGA AATGTGTTAT AAAGAACAGC TGAAGCCTGT   
  
  
- ATCTACTCTC TCTCTCTCTC TCCCTCTCTC CTTCTGCTTC TCCCACTATG TGTATGAAGT CTGTGAAGTC   
  
  
- TTTCACCGGT GCTATAGGCT CGGCTATTAA AAGGGTTGGG CCGTGGACCA GATTTTGTTA TTAGAAGATG   
  
  
- ACACACTCTT TGGATTTCTC ACTATTTCCA AATTTCCAAT CCGAGCTTCT TACGTCAGCT GGTGCGTCAG   
  
  
- GTCGCCAGCT AGCAACTGGG TCCCACTTCT GACCACCTCT GGCTTGGTTA CCCGTCTTTT CCACAACCCG   
  
  
- AACTCGTTAA CCCGTGGCTG ATTTGTCATT TTATCTGTTG ATATATGTGT GTTGGCGGGT ATGGAGCCGA   
  
  
- CTTCGGGTTA GACGTAGATA CGGATCTGTT TGACGAAAGA GAAAAAAGTT AAAGAGGGAG AGGTTTCCTA   
  
  
- AGCATTTCGT CAAGAAAAGA GTTTGACTCG CTCGTAAATC AGAAGAAAGA AATGAATCCA CCGCCCCGAA   
  
  
- CGGTGCGGCG CGTTCCACGT GACGAAAAAG GAAATACAAA TAACCCAAAA GAGGAAGAAA ACTGAAGGAG   
  
  
- AGAGAAGGAG AGAGAGAGAG AGAGAGAGGG GAGCCGAGTC AACGAATTTG ACGGCCCGGC TCTCTGAGGA   
  
  
- TATGACCGGA GACCGGGTGG GGCAGTGGCC TGGTCTATGC GTGGACCCCA CGTAGCATGA CATGAGAAAG   
  
  
- CACGTTTCAG ATCCGCCCCC GGGAACCTTG TTGACTGTTT ACCTGTGGGA CCGAGTCGCT TGCTTTGACT   
  
  
- TTCTGTTACA TGTTGCTTTA CTCATTTGTC CCTCTCTTTC TCTTCATCTC CTAGTCATCT TTTCCTTTTT   
  
  
- TTATTAATTT CTAAAATAAA GCTTTAACAT ACATGTTTTT CCTTGTGCAT GTATTTTCTC AAGTAAATAG   
  
  
- AAAAATAATT ATTTATTACC AAACTTATTT CCTATATTAT TAAAATTTTT GCATGTTTAA AATAAGTTTA   
  
  
- TTATTTTAGT TAAAAAATTA ATCAAAAAAA TTAATTATTT TTTTTATGTA ATTTATGAAG GGCACATAGT   
  
  
- ATTTTAGGTG AATATCATGA CACACGTGTT ACTTTGGATT AGGATTTTCT GTTAGATTTT TATCTCCGCT   
  
  
- AAACTTTTTA ATGAAAAGAT AAAATAGTAT TTTTATAATT TATTATATTA AATTAATAAA TAAATTATAA   
  
  
- TTATTTATTT TATTAAATTA TAACATGTCA TTAACTTTTA AAAAACTCAG CACAATTTAG ATGAGTAAAT   
  
  
- TAAAAATAAT GATATGTAGT TATAAGTATC AAAAATAAAA ATATTTGTTT ATTTAAAAAT TTTAAAAGCT   
  
  
- AAAACATATT TTATAAACCC ATTAATACAA AATTATGTCA CTAATTAACA TAAAACTCTA GTTTTATCCA   
  
  
- AAATTGATGT CGTTTTACTA ATAACCTAA

  
  
Motifs Found  

+     5UTR Py-rich stretch

| Site Name | Organism | Position | Strand | Matrix score. | sequence | function |
| --- | --- | --- | --- | --- | --- | --- |
| 5UTR Py-rich stretch | Lycopersicon esculentum | 643 | + | 13 | TTTCTCTCTCTCTC | cis-acting element conferring high transcription levels |
| 5UTR Py-rich stretch | Lycopersicon esculentum | 639 | + | 13 | TTTCTCTCTCTCTC | cis-acting element conferring high transcription levels |
| 5UTR Py-rich stretch | Lycopersicon esculentum | 641 | + | 13 | TTTCTCTCTCTCTC | cis-acting element conferring high transcription levels |
| 5UTR Py-rich stretch | Lycopersicon esculentum | 645 | + | 13 | TTTCTCTCTCTCTC | cis-acting element conferring high transcription levels |
| 5UTR Py-rich stretch | Lycopersicon esculentum | 635 | + | 13 | TTTCTCTCTCTCTC | cis-acting element conferring high transcription levels |
| 5UTR Py-rich stretch | Lycopersicon esculentum | 24 | + | 9 | TTTCTTCTCT | cis-acting element conferring high transcription levels |
| 5UTR Py-rich stretch | Lycopersicon esculentum | 76 | - | 13 | TTTCTCTCTCTCTC | cis-acting element conferring high transcription levels |
| 5UTR Py-rich stretch | Lycopersicon esculentum | 78 | - | 13 | TTTCTCTCTCTCTC | cis-acting element conferring high transcription levels |

> 2018/04/13 10:10:12  
+ TTTTTGTTAG GGTTTTTCGT TTCTTTCTTC TTTAAGTTCT TTACACAATA TTTCTTGTCG ACTTCGGACA   
  
  
+ TAGATGAGAG AGAGAGAGAG AGGGAGAGAG GAAGACGAAG AGGGTGATAC ACATACTTCA GACACTTCAG   
  
  
+ AAAGTGGCCA CGATATCCGA GCCGATAATT TTCCCAACCC GGCACCTGGT CTAAAACAAT AATCTTCTAC   
  
  
+ TGTGTGAGAA ACCTAAAGAG TGATAAAGGT TTAAAGGTTA GGCTCGAAGA ATGCAGTCGA CCACGCAGTC   
  
  
+ CAGCGGTCGA TCGTTGACCC AGGGTGAAGA CTGGTGGAGA CCGAACCAAT GGGCAGAAAA GGTGTTGGGC   
  
  
+ TTGAGCAATT GGGCACCGAC TAAACAGTAA AATAGACAAC TATATACACA CAACCGCCCA TACCTCGGCT   
  
  
+ GAAGCCCAAT CTGCATCTAT GCCTAGACAA ACTGCTTTCT CTTTTTTCAA TTTCTCCCTC TCCAAAGGAT   
  
  
+ TCGTAAAGCA GTTCTTTTCT CAAACTGAGC GAGCATTTAG TCTTCTTTCT TTACTTAGGT GGCGGGGCTT   
  
  
+ GCCACGCCGC GCAAGGTGCA CTGCTTTTTC CTTTATGTTT ATTGGGTTTT CTCCTTCTTT TGACTTCCTC   
  
  
+ TCTCTTCCTC TCTCTCTCTC TCTCTCTCCC CTCGGCTCAG TTGCTTAAAC TGCCGGGCCG AGAGACTCCT   
  
  
+ ATACTGGCCT CTGGCCCACC CCGTCACCGG ACCAGATACG CACCTGGGGT GCATCGTACT GTACTCTTTC   
  
  
+ GTGCAAAGTC TAGGCGGGGG CCCTTGGAAC AACTGACAAA TGGACACCCT GGCTCAGCGA ACGAAACTGA   
  
  
+ AAGACAATGT ACAACGAAAT GAGTAAACAG GGAGAGAAAG AGAAGTAGAG GATCAGTAGA AAAGGAAAAA   
  
  
+ AATAATTAAA GATTTTATTT CGAAATTGTA TGTACAAAAA GGAACACGTA CATAAAAGAG TTCATTTATC   
  
  
+ TTTTTATTAA TAAATAATGG TTTGAATAAA GGATATAATA ATTTTAAAAA CGTACAAATT TTATTCAAAT   
  
  
+ AATAAAATCA ATTTTTTAAT TAGTTTTTTT AATTAATAAA AAAAATACAT TAAATACTTC CCGTGTATCA   
  
  
+ TAAAATCCAC TTATAGTACT GTGTGCACAA TGAAACCTAA TCCTAAAAGA CAATCTAAAA ATAGAGGCGA   
  
  
+ TTTGAAAAAT TACTTTTCTA TTTTATCATA AAAATATTAA ATAATATAAT TTAATTATTT ATTTAATATT   
  
  
+ AATAAATAAA ATAATTTAAT ATTGTACAGT AATTGAAAAT TTTTTGAGTC GTGTTAAATC TACTCATTTA   
  
  
+ ATTTTTATTA CTATACATCA ATATTCATAG TTTTTATTTT TATAAACAAA TAAATTTTTA AAATTTTCGA   
  
  
+ TTTTGTATAA AATATTTGGG TAATTATGTT TTAATACAGT GATTAATTGT ATTTTGAGAT CAAAATAGGT   
  
  
+ TTTAACTACA GCAAAATGAT TATTGGATT  

- AAAAACAATC CCAAAAAGCA AAGAAAGAAG AAATTCAAGA AATGTGTTAT AAAGAACAGC TGAAGCCTGT   
  
  
- ATCTACTCTC TCTCTCTCTC TCCCTCTCTC CTTCTGCTTC TCCCACTATG TGTATGAAGT CTGTGAAGTC   
  
  
- TTTCACCGGT GCTATAGGCT CGGCTATTAA AAGGGTTGGG CCGTGGACCA GATTTTGTTA TTAGAAGATG   
  
  
- ACACACTCTT TGGATTTCTC ACTATTTCCA AATTTCCAAT CCGAGCTTCT TACGTCAGCT GGTGCGTCAG   
  
  
- GTCGCCAGCT AGCAACTGGG TCCCACTTCT GACCACCTCT GGCTTGGTTA CCCGTCTTTT CCACAACCCG   
  
  
- AACTCGTTAA CCCGTGGCTG ATTTGTCATT TTATCTGTTG ATATATGTGT GTTGGCGGGT ATGGAGCCGA   
  
  
- CTTCGGGTTA GACGTAGATA CGGATCTGTT TGACGAAAGA GAAAAAAGTT AAAGAGGGAG AGGTTTCCTA   
  
  
- AGCATTTCGT CAAGAAAAGA GTTTGACTCG CTCGTAAATC AGAAGAAAGA AATGAATCCA CCGCCCCGAA   
  
  
- CGGTGCGGCG CGTTCCACGT GACGAAAAAG GAAATACAAA TAACCCAAAA GAGGAAGAAA ACTGAAGGAG   
  
  
- AGAGAAGGAG AGAGAGAGAG AGAGAGAGGG GAGCCGAGTC AACGAATTTG ACGGCCCGGC TCTCTGAGGA   
  
  
- TATGACCGGA GACCGGGTGG GGCAGTGGCC TGGTCTATGC GTGGACCCCA CGTAGCATGA CATGAGAAAG   
  
  
- CACGTTTCAG ATCCGCCCCC GGGAACCTTG TTGACTGTTT ACCTGTGGGA CCGAGTCGCT TGCTTTGACT   
  
  
- TTCTGTTACA TGTTGCTTTA CTCATTTGTC CCTCTCTTTC TCTTCATCTC CTAGTCATCT TTTCCTTTTT   
  
  
- TTATTAATTT CTAAAATAAA GCTTTAACAT ACATGTTTTT CCTTGTGCAT GTATTTTCTC AAGTAAATAG   
  
  
- AAAAATAATT ATTTATTACC AAACTTATTT CCTATATTAT TAAAATTTTT GCATGTTTAA AATAAGTTTA   
  
  
- TTATTTTAGT TAAAAAATTA ATCAAAAAAA TTAATTATTT TTTTTATGTA ATTTATGAAG GGCACATAGT   
  
  
- ATTTTAGGTG AATATCATGA CACACGTGTT ACTTTGGATT AGGATTTTCT GTTAGATTTT TATCTCCGCT   
  
  
- AAACTTTTTA ATGAAAAGAT AAAATAGTAT TTTTATAATT TATTATATTA AATTAATAAA TAAATTATAA   
  
  
- TTATTTATTT TATTAAATTA TAACATGTCA TTAACTTTTA AAAAACTCAG CACAATTTAG ATGAGTAAAT   
  
  
- TAAAAATAAT GATATGTAGT TATAAGTATC AAAAATAAAA ATATTTGTTT ATTTAAAAAT TTTAAAAGCT   
  
  
- AAAACATATT TTATAAACCC ATTAATACAA AATTATGTCA CTAATTAACA TAAAACTCTA GTTTTATCCA   
  
  
- AAATTGATGT CGTTTTACTA ATAACCTAA

+     AAGAA-motif

| Site Name | Organism | Position | Strand | Matrix score. | sequence | function |
| --- | --- | --- | --- | --- | --- | --- |
| AAGAA-motif | Avena sativa | 536 | - | 9 | gGTAAAGAAA |  |
| AAGAA-motif | Avena sativa | 533 | - | 7 | GAAAGAA |  |
| AAGAA-motif | Avena sativa | 21 | - | 7 | GAAAGAA |  |

> 2018/04/13 10:10:12  
+ TTTTTGTTAG GGTTTTTCGT TTCTTTCTTC TTTAAGTTCT TTACACAATA TTTCTTGTCG ACTTCGGACA   
  
  
+ TAGATGAGAG AGAGAGAGAG AGGGAGAGAG GAAGACGAAG AGGGTGATAC ACATACTTCA GACACTTCAG   
  
  
+ AAAGTGGCCA CGATATCCGA GCCGATAATT TTCCCAACCC GGCACCTGGT CTAAAACAAT AATCTTCTAC   
  
  
+ TGTGTGAGAA ACCTAAAGAG TGATAAAGGT TTAAAGGTTA GGCTCGAAGA ATGCAGTCGA CCACGCAGTC   
  
  
+ CAGCGGTCGA TCGTTGACCC AGGGTGAAGA CTGGTGGAGA CCGAACCAAT GGGCAGAAAA GGTGTTGGGC   
  
  
+ TTGAGCAATT GGGCACCGAC TAAACAGTAA AATAGACAAC TATATACACA CAACCGCCCA TACCTCGGCT   
  
  
+ GAAGCCCAAT CTGCATCTAT GCCTAGACAA ACTGCTTTCT CTTTTTTCAA TTTCTCCCTC TCCAAAGGAT   
  
  
+ TCGTAAAGCA GTTCTTTTCT CAAACTGAGC GAGCATTTAG TCTTCTTTCT TTACTTAGGT GGCGGGGCTT   
  
  
+ GCCACGCCGC GCAAGGTGCA CTGCTTTTTC CTTTATGTTT ATTGGGTTTT CTCCTTCTTT TGACTTCCTC   
  
  
+ TCTCTTCCTC TCTCTCTCTC TCTCTCTCCC CTCGGCTCAG TTGCTTAAAC TGCCGGGCCG AGAGACTCCT   
  
  
+ ATACTGGCCT CTGGCCCACC CCGTCACCGG ACCAGATACG CACCTGGGGT GCATCGTACT GTACTCTTTC   
  
  
+ GTGCAAAGTC TAGGCGGGGG CCCTTGGAAC AACTGACAAA TGGACACCCT GGCTCAGCGA ACGAAACTGA   
  
  
+ AAGACAATGT ACAACGAAAT GAGTAAACAG GGAGAGAAAG AGAAGTAGAG GATCAGTAGA AAAGGAAAAA   
  
  
+ AATAATTAAA GATTTTATTT CGAAATTGTA TGTACAAAAA GGAACACGTA CATAAAAGAG TTCATTTATC   
  
  
+ TTTTTATTAA TAAATAATGG TTTGAATAAA GGATATAATA ATTTTAAAAA CGTACAAATT TTATTCAAAT   
  
  
+ AATAAAATCA ATTTTTTAAT TAGTTTTTTT AATTAATAAA AAAAATACAT TAAATACTTC CCGTGTATCA   
  
  
+ TAAAATCCAC TTATAGTACT GTGTGCACAA TGAAACCTAA TCCTAAAAGA CAATCTAAAA ATAGAGGCGA   
  
  
+ TTTGAAAAAT TACTTTTCTA TTTTATCATA AAAATATTAA ATAATATAAT TTAATTATTT ATTTAATATT   
  
  
+ AATAAATAAA ATAATTTAAT ATTGTACAGT AATTGAAAAT TTTTTGAGTC GTGTTAAATC TACTCATTTA   
  
  
+ ATTTTTATTA CTATACATCA ATATTCATAG TTTTTATTTT TATAAACAAA TAAATTTTTA AAATTTTCGA   
  
  
+ TTTTGTATAA AATATTTGGG TAATTATGTT TTAATACAGT GATTAATTGT ATTTTGAGAT CAAAATAGGT   
  
  
+ TTTAACTACA GCAAAATGAT TATTGGATT  

- AAAAACAATC CCAAAAAGCA AAGAAAGAAG AAATTCAAGA AATGTGTTAT AAAGAACAGC TGAAGCCTGT   
  
  
- ATCTACTCTC TCTCTCTCTC TCCCTCTCTC CTTCTGCTTC TCCCACTATG TGTATGAAGT CTGTGAAGTC   
  
  
- TTTCACCGGT GCTATAGGCT CGGCTATTAA AAGGGTTGGG CCGTGGACCA GATTTTGTTA TTAGAAGATG   
  
  
- ACACACTCTT TGGATTTCTC ACTATTTCCA AATTTCCAAT CCGAGCTTCT TACGTCAGCT GGTGCGTCAG   
  
  
- GTCGCCAGCT AGCAACTGGG TCCCACTTCT GACCACCTCT GGCTTGGTTA CCCGTCTTTT CCACAACCCG   
  
  
- AACTCGTTAA CCCGTGGCTG ATTTGTCATT TTATCTGTTG ATATATGTGT GTTGGCGGGT ATGGAGCCGA   
  
  
- CTTCGGGTTA GACGTAGATA CGGATCTGTT TGACGAAAGA GAAAAAAGTT AAAGAGGGAG AGGTTTCCTA   
  
  
- AGCATTTCGT CAAGAAAAGA GTTTGACTCG CTCGTAAATC AGAAGAAAGA AATGAATCCA CCGCCCCGAA   
  
  
- CGGTGCGGCG CGTTCCACGT GACGAAAAAG GAAATACAAA TAACCCAAAA GAGGAAGAAA ACTGAAGGAG   
  
  
- AGAGAAGGAG AGAGAGAGAG AGAGAGAGGG GAGCCGAGTC AACGAATTTG ACGGCCCGGC TCTCTGAGGA   
  
  
- TATGACCGGA GACCGGGTGG GGCAGTGGCC TGGTCTATGC GTGGACCCCA CGTAGCATGA CATGAGAAAG   
  
  
- CACGTTTCAG ATCCGCCCCC GGGAACCTTG TTGACTGTTT ACCTGTGGGA CCGAGTCGCT TGCTTTGACT   
  
  
- TTCTGTTACA TGTTGCTTTA CTCATTTGTC CCTCTCTTTC TCTTCATCTC CTAGTCATCT TTTCCTTTTT   
  
  
- TTATTAATTT CTAAAATAAA GCTTTAACAT ACATGTTTTT CCTTGTGCAT GTATTTTCTC AAGTAAATAG   
  
  
- AAAAATAATT ATTTATTACC AAACTTATTT CCTATATTAT TAAAATTTTT GCATGTTTAA AATAAGTTTA   
  
  
- TTATTTTAGT TAAAAAATTA ATCAAAAAAA TTAATTATTT TTTTTATGTA ATTTATGAAG GGCACATAGT   
  
  
- ATTTTAGGTG AATATCATGA CACACGTGTT ACTTTGGATT AGGATTTTCT GTTAGATTTT TATCTCCGCT   
  
  
- AAACTTTTTA ATGAAAAGAT AAAATAGTAT TTTTATAATT TATTATATTA AATTAATAAA TAAATTATAA   
  
  
- TTATTTATTT TATTAAATTA TAACATGTCA TTAACTTTTA AAAAACTCAG CACAATTTAG ATGAGTAAAT   
  
  
- TAAAAATAAT GATATGTAGT TATAAGTATC AAAAATAAAA ATATTTGTTT ATTTAAAAAT TTTAAAAGCT   
  
  
- AAAACATATT TTATAAACCC ATTAATACAA AATTATGTCA CTAATTAACA TAAAACTCTA GTTTTATCCA   
  
  
- AAATTGATGT CGTTTTACTA ATAACCTAA

+     ABRE

| Site Name | Organism | Position | Strand | Matrix score. | sequence | function |
| --- | --- | --- | --- | --- | --- | --- |
| ABRE | Arabidopsis thaliana | 955 | - | 6 | TACGTG | cis-acting element involved in the abscisic acid responsiveness |
| ABRE | Oryza sativa | 561 | - | 9 | GCCGCGTGGC | cis-acting element involved in the abscisic acid responsiveness |

> 2018/04/13 10:10:12  
+ TTTTTGTTAG GGTTTTTCGT TTCTTTCTTC TTTAAGTTCT TTACACAATA TTTCTTGTCG ACTTCGGACA   
  
  
+ TAGATGAGAG AGAGAGAGAG AGGGAGAGAG GAAGACGAAG AGGGTGATAC ACATACTTCA GACACTTCAG   
  
  
+ AAAGTGGCCA CGATATCCGA GCCGATAATT TTCCCAACCC GGCACCTGGT CTAAAACAAT AATCTTCTAC   
  
  
+ TGTGTGAGAA ACCTAAAGAG TGATAAAGGT TTAAAGGTTA GGCTCGAAGA ATGCAGTCGA CCACGCAGTC   
  
  
+ CAGCGGTCGA TCGTTGACCC AGGGTGAAGA CTGGTGGAGA CCGAACCAAT GGGCAGAAAA GGTGTTGGGC   
  
  
+ TTGAGCAATT GGGCACCGAC TAAACAGTAA AATAGACAAC TATATACACA CAACCGCCCA TACCTCGGCT   
  
  
+ GAAGCCCAAT CTGCATCTAT GCCTAGACAA ACTGCTTTCT CTTTTTTCAA TTTCTCCCTC TCCAAAGGAT   
  
  
+ TCGTAAAGCA GTTCTTTTCT CAAACTGAGC GAGCATTTAG TCTTCTTTCT TTACTTAGGT GGCGGGGCTT   
  
  
+ GCCACGCCGC GCAAGGTGCA CTGCTTTTTC CTTTATGTTT ATTGGGTTTT CTCCTTCTTT TGACTTCCTC   
  
  
+ TCTCTTCCTC TCTCTCTCTC TCTCTCTCCC CTCGGCTCAG TTGCTTAAAC TGCCGGGCCG AGAGACTCCT   
  
  
+ ATACTGGCCT CTGGCCCACC CCGTCACCGG ACCAGATACG CACCTGGGGT GCATCGTACT GTACTCTTTC   
  
  
+ GTGCAAAGTC TAGGCGGGGG CCCTTGGAAC AACTGACAAA TGGACACCCT GGCTCAGCGA ACGAAACTGA   
  
  
+ AAGACAATGT ACAACGAAAT GAGTAAACAG GGAGAGAAAG AGAAGTAGAG GATCAGTAGA AAAGGAAAAA   
  
  
+ AATAATTAAA GATTTTATTT CGAAATTGTA TGTACAAAAA GGAACACGTA CATAAAAGAG TTCATTTATC   
  
  
+ TTTTTATTAA TAAATAATGG TTTGAATAAA GGATATAATA ATTTTAAAAA CGTACAAATT TTATTCAAAT   
  
  
+ AATAAAATCA ATTTTTTAAT TAGTTTTTTT AATTAATAAA AAAAATACAT TAAATACTTC CCGTGTATCA   
  
  
+ TAAAATCCAC TTATAGTACT GTGTGCACAA TGAAACCTAA TCCTAAAAGA CAATCTAAAA ATAGAGGCGA   
  
  
+ TTTGAAAAAT TACTTTTCTA TTTTATCATA AAAATATTAA ATAATATAAT TTAATTATTT ATTTAATATT   
  
  
+ AATAAATAAA ATAATTTAAT ATTGTACAGT AATTGAAAAT TTTTTGAGTC GTGTTAAATC TACTCATTTA   
  
  
+ ATTTTTATTA CTATACATCA ATATTCATAG TTTTTATTTT TATAAACAAA TAAATTTTTA AAATTTTCGA   
  
  
+ TTTTGTATAA AATATTTGGG TAATTATGTT TTAATACAGT GATTAATTGT ATTTTGAGAT CAAAATAGGT   
  
  
+ TTTAACTACA GCAAAATGAT TATTGGATT  

- AAAAACAATC CCAAAAAGCA AAGAAAGAAG AAATTCAAGA AATGTGTTAT AAAGAACAGC TGAAGCCTGT   
  
  
- ATCTACTCTC TCTCTCTCTC TCCCTCTCTC CTTCTGCTTC TCCCACTATG TGTATGAAGT CTGTGAAGTC   
  
  
- TTTCACCGGT GCTATAGGCT CGGCTATTAA AAGGGTTGGG CCGTGGACCA GATTTTGTTA TTAGAAGATG   
  
  
- ACACACTCTT TGGATTTCTC ACTATTTCCA AATTTCCAAT CCGAGCTTCT TACGTCAGCT GGTGCGTCAG   
  
  
- GTCGCCAGCT AGCAACTGGG TCCCACTTCT GACCACCTCT GGCTTGGTTA CCCGTCTTTT CCACAACCCG   
  
  
- AACTCGTTAA CCCGTGGCTG ATTTGTCATT TTATCTGTTG ATATATGTGT GTTGGCGGGT ATGGAGCCGA   
  
  
- CTTCGGGTTA GACGTAGATA CGGATCTGTT TGACGAAAGA GAAAAAAGTT AAAGAGGGAG AGGTTTCCTA   
  
  
- AGCATTTCGT CAAGAAAAGA GTTTGACTCG CTCGTAAATC AGAAGAAAGA AATGAATCCA CCGCCCCGAA   
  
  
- CGGTGCGGCG CGTTCCACGT GACGAAAAAG GAAATACAAA TAACCCAAAA GAGGAAGAAA ACTGAAGGAG   
  
  
- AGAGAAGGAG AGAGAGAGAG AGAGAGAGGG GAGCCGAGTC AACGAATTTG ACGGCCCGGC TCTCTGAGGA   
  
  
- TATGACCGGA GACCGGGTGG GGCAGTGGCC TGGTCTATGC GTGGACCCCA CGTAGCATGA CATGAGAAAG   
  
  
- CACGTTTCAG ATCCGCCCCC GGGAACCTTG TTGACTGTTT ACCTGTGGGA CCGAGTCGCT TGCTTTGACT   
  
  
- TTCTGTTACA TGTTGCTTTA CTCATTTGTC CCTCTCTTTC TCTTCATCTC CTAGTCATCT TTTCCTTTTT   
  
  
- TTATTAATTT CTAAAATAAA GCTTTAACAT ACATGTTTTT CCTTGTGCAT GTATTTTCTC AAGTAAATAG   
  
  
- AAAAATAATT ATTTATTACC AAACTTATTT CCTATATTAT TAAAATTTTT GCATGTTTAA AATAAGTTTA   
  
  
- TTATTTTAGT TAAAAAATTA ATCAAAAAAA TTAATTATTT TTTTTATGTA ATTTATGAAG GGCACATAGT   
  
  
- ATTTTAGGTG AATATCATGA CACACGTGTT ACTTTGGATT AGGATTTTCT GTTAGATTTT TATCTCCGCT   
  
  
- AAACTTTTTA ATGAAAAGAT AAAATAGTAT TTTTATAATT TATTATATTA AATTAATAAA TAAATTATAA   
  
  
- TTATTTATTT TATTAAATTA TAACATGTCA TTAACTTTTA AAAAACTCAG CACAATTTAG ATGAGTAAAT   
  
  
- TAAAAATAAT GATATGTAGT TATAAGTATC AAAAATAAAA ATATTTGTTT ATTTAAAAAT TTTAAAAGCT   
  
  
- AAAACATATT TTATAAACCC ATTAATACAA AATTATGTCA CTAATTAACA TAAAACTCTA GTTTTATCCA   
  
  
- AAATTGATGT CGTTTTACTA ATAACCTAA

+     ARE

| Site Name | Organism | Position | Strand | Matrix score. | sequence | function |
| --- | --- | --- | --- | --- | --- | --- |
| ARE | Zea mays | 998 | + | 6 | TGGTTT | cis-acting regulatory element essential for the anaerobic induction |

> 2018/04/13 10:10:12  
+ TTTTTGTTAG GGTTTTTCGT TTCTTTCTTC TTTAAGTTCT TTACACAATA TTTCTTGTCG ACTTCGGACA   
  
  
+ TAGATGAGAG AGAGAGAGAG AGGGAGAGAG GAAGACGAAG AGGGTGATAC ACATACTTCA GACACTTCAG   
  
  
+ AAAGTGGCCA CGATATCCGA GCCGATAATT TTCCCAACCC GGCACCTGGT CTAAAACAAT AATCTTCTAC   
  
  
+ TGTGTGAGAA ACCTAAAGAG TGATAAAGGT TTAAAGGTTA GGCTCGAAGA ATGCAGTCGA CCACGCAGTC   
  
  
+ CAGCGGTCGA TCGTTGACCC AGGGTGAAGA CTGGTGGAGA CCGAACCAAT GGGCAGAAAA GGTGTTGGGC   
  
  
+ TTGAGCAATT GGGCACCGAC TAAACAGTAA AATAGACAAC TATATACACA CAACCGCCCA TACCTCGGCT   
  
  
+ GAAGCCCAAT CTGCATCTAT GCCTAGACAA ACTGCTTTCT CTTTTTTCAA TTTCTCCCTC TCCAAAGGAT   
  
  
+ TCGTAAAGCA GTTCTTTTCT CAAACTGAGC GAGCATTTAG TCTTCTTTCT TTACTTAGGT GGCGGGGCTT   
  
  
+ GCCACGCCGC GCAAGGTGCA CTGCTTTTTC CTTTATGTTT ATTGGGTTTT CTCCTTCTTT TGACTTCCTC   
  
  
+ TCTCTTCCTC TCTCTCTCTC TCTCTCTCCC CTCGGCTCAG TTGCTTAAAC TGCCGGGCCG AGAGACTCCT   
  
  
+ ATACTGGCCT CTGGCCCACC CCGTCACCGG ACCAGATACG CACCTGGGGT GCATCGTACT GTACTCTTTC   
  
  
+ GTGCAAAGTC TAGGCGGGGG CCCTTGGAAC AACTGACAAA TGGACACCCT GGCTCAGCGA ACGAAACTGA   
  
  
+ AAGACAATGT ACAACGAAAT GAGTAAACAG GGAGAGAAAG AGAAGTAGAG GATCAGTAGA AAAGGAAAAA   
  
  
+ AATAATTAAA GATTTTATTT CGAAATTGTA TGTACAAAAA GGAACACGTA CATAAAAGAG TTCATTTATC   
  
  
+ TTTTTATTAA TAAATAATGG TTTGAATAAA GGATATAATA ATTTTAAAAA CGTACAAATT TTATTCAAAT   
  
  
+ AATAAAATCA ATTTTTTAAT TAGTTTTTTT AATTAATAAA AAAAATACAT TAAATACTTC CCGTGTATCA   
  
  
+ TAAAATCCAC TTATAGTACT GTGTGCACAA TGAAACCTAA TCCTAAAAGA CAATCTAAAA ATAGAGGCGA   
  
  
+ TTTGAAAAAT TACTTTTCTA TTTTATCATA AAAATATTAA ATAATATAAT TTAATTATTT ATTTAATATT   
  
  
+ AATAAATAAA ATAATTTAAT ATTGTACAGT AATTGAAAAT TTTTTGAGTC GTGTTAAATC TACTCATTTA   
  
  
+ ATTTTTATTA CTATACATCA ATATTCATAG TTTTTATTTT TATAAACAAA TAAATTTTTA AAATTTTCGA   
  
  
+ TTTTGTATAA AATATTTGGG TAATTATGTT TTAATACAGT GATTAATTGT ATTTTGAGAT CAAAATAGGT   
  
  
+ TTTAACTACA GCAAAATGAT TATTGGATT  

- AAAAACAATC CCAAAAAGCA AAGAAAGAAG AAATTCAAGA AATGTGTTAT AAAGAACAGC TGAAGCCTGT   
  
  
- ATCTACTCTC TCTCTCTCTC TCCCTCTCTC CTTCTGCTTC TCCCACTATG TGTATGAAGT CTGTGAAGTC   
  
  
- TTTCACCGGT GCTATAGGCT CGGCTATTAA AAGGGTTGGG CCGTGGACCA GATTTTGTTA TTAGAAGATG   
  
  
- ACACACTCTT TGGATTTCTC ACTATTTCCA AATTTCCAAT CCGAGCTTCT TACGTCAGCT GGTGCGTCAG   
  
  
- GTCGCCAGCT AGCAACTGGG TCCCACTTCT GACCACCTCT GGCTTGGTTA CCCGTCTTTT CCACAACCCG   
  
  
- AACTCGTTAA CCCGTGGCTG ATTTGTCATT TTATCTGTTG ATATATGTGT GTTGGCGGGT ATGGAGCCGA   
  
  
- CTTCGGGTTA GACGTAGATA CGGATCTGTT TGACGAAAGA GAAAAAAGTT AAAGAGGGAG AGGTTTCCTA   
  
  
- AGCATTTCGT CAAGAAAAGA GTTTGACTCG CTCGTAAATC AGAAGAAAGA AATGAATCCA CCGCCCCGAA   
  
  
- CGGTGCGGCG CGTTCCACGT GACGAAAAAG GAAATACAAA TAACCCAAAA GAGGAAGAAA ACTGAAGGAG   
  
  
- AGAGAAGGAG AGAGAGAGAG AGAGAGAGGG GAGCCGAGTC AACGAATTTG ACGGCCCGGC TCTCTGAGGA   
  
  
- TATGACCGGA GACCGGGTGG GGCAGTGGCC TGGTCTATGC GTGGACCCCA CGTAGCATGA CATGAGAAAG   
  
  
- CACGTTTCAG ATCCGCCCCC GGGAACCTTG TTGACTGTTT ACCTGTGGGA CCGAGTCGCT TGCTTTGACT   
  
  
- TTCTGTTACA TGTTGCTTTA CTCATTTGTC CCTCTCTTTC TCTTCATCTC CTAGTCATCT TTTCCTTTTT   
  
  
- TTATTAATTT CTAAAATAAA GCTTTAACAT ACATGTTTTT CCTTGTGCAT GTATTTTCTC AAGTAAATAG   
  
  
- AAAAATAATT ATTTATTACC AAACTTATTT CCTATATTAT TAAAATTTTT GCATGTTTAA AATAAGTTTA   
  
  
- TTATTTTAGT TAAAAAATTA ATCAAAAAAA TTAATTATTT TTTTTATGTA ATTTATGAAG GGCACATAGT   
  
  
- ATTTTAGGTG AATATCATGA CACACGTGTT ACTTTGGATT AGGATTTTCT GTTAGATTTT TATCTCCGCT   
  
  
- AAACTTTTTA ATGAAAAGAT AAAATAGTAT TTTTATAATT TATTATATTA AATTAATAAA TAAATTATAA   
  
  
- TTATTTATTT TATTAAATTA TAACATGTCA TTAACTTTTA AAAAACTCAG CACAATTTAG ATGAGTAAAT   
  
  
- TAAAAATAAT GATATGTAGT TATAAGTATC AAAAATAAAA ATATTTGTTT ATTTAAAAAT TTTAAAAGCT   
  
  
- AAAACATATT TTATAAACCC ATTAATACAA AATTATGTCA CTAATTAACA TAAAACTCTA GTTTTATCCA   
  
  
- AAATTGATGT CGTTTTACTA ATAACCTAA

+     ATCT-motif

| Site Name | Organism | Position | Strand | Matrix score. | sequence | function |
| --- | --- | --- | --- | --- | --- | --- |
| ATCT-motif | Pisum sativum | 1154 | + | 9 | AATCTAATCC | part of a conserved DNA module involved in light responsiveness |

> 2018/04/13 10:10:12  
+ TTTTTGTTAG GGTTTTTCGT TTCTTTCTTC TTTAAGTTCT TTACACAATA TTTCTTGTCG ACTTCGGACA   
  
  
+ TAGATGAGAG AGAGAGAGAG AGGGAGAGAG GAAGACGAAG AGGGTGATAC ACATACTTCA GACACTTCAG   
  
  
+ AAAGTGGCCA CGATATCCGA GCCGATAATT TTCCCAACCC GGCACCTGGT CTAAAACAAT AATCTTCTAC   
  
  
+ TGTGTGAGAA ACCTAAAGAG TGATAAAGGT TTAAAGGTTA GGCTCGAAGA ATGCAGTCGA CCACGCAGTC   
  
  
+ CAGCGGTCGA TCGTTGACCC AGGGTGAAGA CTGGTGGAGA CCGAACCAAT GGGCAGAAAA GGTGTTGGGC   
  
  
+ TTGAGCAATT GGGCACCGAC TAAACAGTAA AATAGACAAC TATATACACA CAACCGCCCA TACCTCGGCT   
  
  
+ GAAGCCCAAT CTGCATCTAT GCCTAGACAA ACTGCTTTCT CTTTTTTCAA TTTCTCCCTC TCCAAAGGAT   
  
  
+ TCGTAAAGCA GTTCTTTTCT CAAACTGAGC GAGCATTTAG TCTTCTTTCT TTACTTAGGT GGCGGGGCTT   
  
  
+ GCCACGCCGC GCAAGGTGCA CTGCTTTTTC CTTTATGTTT ATTGGGTTTT CTCCTTCTTT TGACTTCCTC   
  
  
+ TCTCTTCCTC TCTCTCTCTC TCTCTCTCCC CTCGGCTCAG TTGCTTAAAC TGCCGGGCCG AGAGACTCCT   
  
  
+ ATACTGGCCT CTGGCCCACC CCGTCACCGG ACCAGATACG CACCTGGGGT GCATCGTACT GTACTCTTTC   
  
  
+ GTGCAAAGTC TAGGCGGGGG CCCTTGGAAC AACTGACAAA TGGACACCCT GGCTCAGCGA ACGAAACTGA   
  
  
+ AAGACAATGT ACAACGAAAT GAGTAAACAG GGAGAGAAAG AGAAGTAGAG GATCAGTAGA AAAGGAAAAA   
  
  
+ AATAATTAAA GATTTTATTT CGAAATTGTA TGTACAAAAA GGAACACGTA CATAAAAGAG TTCATTTATC   
  
  
+ TTTTTATTAA TAAATAATGG TTTGAATAAA GGATATAATA ATTTTAAAAA CGTACAAATT TTATTCAAAT   
  
  
+ AATAAAATCA ATTTTTTAAT TAGTTTTTTT AATTAATAAA AAAAATACAT TAAATACTTC CCGTGTATCA   
  
  
+ TAAAATCCAC TTATAGTACT GTGTGCACAA TGAAACCTAA TCCTAAAAGA CAATCTAAAA ATAGAGGCGA   
  
  
+ TTTGAAAAAT TACTTTTCTA TTTTATCATA AAAATATTAA ATAATATAAT TTAATTATTT ATTTAATATT   
  
  
+ AATAAATAAA ATAATTTAAT ATTGTACAGT AATTGAAAAT TTTTTGAGTC GTGTTAAATC TACTCATTTA   
  
  
+ ATTTTTATTA CTATACATCA ATATTCATAG TTTTTATTTT TATAAACAAA TAAATTTTTA AAATTTTCGA   
  
  
+ TTTTGTATAA AATATTTGGG TAATTATGTT TTAATACAGT GATTAATTGT ATTTTGAGAT CAAAATAGGT   
  
  
+ TTTAACTACA GCAAAATGAT TATTGGATT  

- AAAAACAATC CCAAAAAGCA AAGAAAGAAG AAATTCAAGA AATGTGTTAT AAAGAACAGC TGAAGCCTGT   
  
  
- ATCTACTCTC TCTCTCTCTC TCCCTCTCTC CTTCTGCTTC TCCCACTATG TGTATGAAGT CTGTGAAGTC   
  
  
- TTTCACCGGT GCTATAGGCT CGGCTATTAA AAGGGTTGGG CCGTGGACCA GATTTTGTTA TTAGAAGATG   
  
  
- ACACACTCTT TGGATTTCTC ACTATTTCCA AATTTCCAAT CCGAGCTTCT TACGTCAGCT GGTGCGTCAG   
  
  
- GTCGCCAGCT AGCAACTGGG TCCCACTTCT GACCACCTCT GGCTTGGTTA CCCGTCTTTT CCACAACCCG   
  
  
- AACTCGTTAA CCCGTGGCTG ATTTGTCATT TTATCTGTTG ATATATGTGT GTTGGCGGGT ATGGAGCCGA   
  
  
- CTTCGGGTTA GACGTAGATA CGGATCTGTT TGACGAAAGA GAAAAAAGTT AAAGAGGGAG AGGTTTCCTA   
  
  
- AGCATTTCGT CAAGAAAAGA GTTTGACTCG CTCGTAAATC AGAAGAAAGA AATGAATCCA CCGCCCCGAA   
  
  
- CGGTGCGGCG CGTTCCACGT GACGAAAAAG GAAATACAAA TAACCCAAAA GAGGAAGAAA ACTGAAGGAG   
  
  
- AGAGAAGGAG AGAGAGAGAG AGAGAGAGGG GAGCCGAGTC AACGAATTTG ACGGCCCGGC TCTCTGAGGA   
  
  
- TATGACCGGA GACCGGGTGG GGCAGTGGCC TGGTCTATGC GTGGACCCCA CGTAGCATGA CATGAGAAAG   
  
  
- CACGTTTCAG ATCCGCCCCC GGGAACCTTG TTGACTGTTT ACCTGTGGGA CCGAGTCGCT TGCTTTGACT   
  
  
- TTCTGTTACA TGTTGCTTTA CTCATTTGTC CCTCTCTTTC TCTTCATCTC CTAGTCATCT TTTCCTTTTT   
  
  
- TTATTAATTT CTAAAATAAA GCTTTAACAT ACATGTTTTT CCTTGTGCAT GTATTTTCTC AAGTAAATAG   
  
  
- AAAAATAATT ATTTATTACC AAACTTATTT CCTATATTAT TAAAATTTTT GCATGTTTAA AATAAGTTTA   
  
  
- TTATTTTAGT TAAAAAATTA ATCAAAAAAA TTAATTATTT TTTTTATGTA ATTTATGAAG GGCACATAGT   
  
  
- ATTTTAGGTG AATATCATGA CACACGTGTT ACTTTGGATT AGGATTTTCT GTTAGATTTT TATCTCCGCT   
  
  
- AAACTTTTTA ATGAAAAGAT AAAATAGTAT TTTTATAATT TATTATATTA AATTAATAAA TAAATTATAA   
  
  
- TTATTTATTT TATTAAATTA TAACATGTCA TTAACTTTTA AAAAACTCAG CACAATTTAG ATGAGTAAAT   
  
  
- TAAAAATAAT GATATGTAGT TATAAGTATC AAAAATAAAA ATATTTGTTT ATTTAAAAAT TTTAAAAGCT   
  
  
- AAAACATATT TTATAAACCC ATTAATACAA AATTATGTCA CTAATTAACA TAAAACTCTA GTTTTATCCA   
  
  
- AAATTGATGT CGTTTTACTA ATAACCTAA

+     Box 4

| Site Name | Organism | Position | Strand | Matrix score. | sequence | function |
| --- | --- | --- | --- | --- | --- | --- |
| Box 4 | Petroselinum crispum | 986 | - | 6 | ATTAAT | part of a conserved DNA module involved in light responsiveness |
| Box 4 | Petroselinum crispum | 1258 | - | 6 | ATTAAT | part of a conserved DNA module involved in light responsiveness |
| Box 4 | Petroselinum crispum | 1082 | - | 6 | ATTAAT | part of a conserved DNA module involved in light responsiveness |
| Box 4 | Petroselinum crispum | 1442 | - | 6 | ATTAAT | part of a conserved DNA module involved in light responsiveness |

> 2018/04/13 10:10:12  
+ TTTTTGTTAG GGTTTTTCGT TTCTTTCTTC TTTAAGTTCT TTACACAATA TTTCTTGTCG ACTTCGGACA   
  
  
+ TAGATGAGAG AGAGAGAGAG AGGGAGAGAG GAAGACGAAG AGGGTGATAC ACATACTTCA GACACTTCAG   
  
  
+ AAAGTGGCCA CGATATCCGA GCCGATAATT TTCCCAACCC GGCACCTGGT CTAAAACAAT AATCTTCTAC   
  
  
+ TGTGTGAGAA ACCTAAAGAG TGATAAAGGT TTAAAGGTTA GGCTCGAAGA ATGCAGTCGA CCACGCAGTC   
  
  
+ CAGCGGTCGA TCGTTGACCC AGGGTGAAGA CTGGTGGAGA CCGAACCAAT GGGCAGAAAA GGTGTTGGGC   
  
  
+ TTGAGCAATT GGGCACCGAC TAAACAGTAA AATAGACAAC TATATACACA CAACCGCCCA TACCTCGGCT   
  
  
+ GAAGCCCAAT CTGCATCTAT GCCTAGACAA ACTGCTTTCT CTTTTTTCAA TTTCTCCCTC TCCAAAGGAT   
  
  
+ TCGTAAAGCA GTTCTTTTCT CAAACTGAGC GAGCATTTAG TCTTCTTTCT TTACTTAGGT GGCGGGGCTT   
  
  
+ GCCACGCCGC GCAAGGTGCA CTGCTTTTTC CTTTATGTTT ATTGGGTTTT CTCCTTCTTT TGACTTCCTC   
  
  
+ TCTCTTCCTC TCTCTCTCTC TCTCTCTCCC CTCGGCTCAG TTGCTTAAAC TGCCGGGCCG AGAGACTCCT   
  
  
+ ATACTGGCCT CTGGCCCACC CCGTCACCGG ACCAGATACG CACCTGGGGT GCATCGTACT GTACTCTTTC   
  
  
+ GTGCAAAGTC TAGGCGGGGG CCCTTGGAAC AACTGACAAA TGGACACCCT GGCTCAGCGA ACGAAACTGA   
  
  
+ AAGACAATGT ACAACGAAAT GAGTAAACAG GGAGAGAAAG AGAAGTAGAG GATCAGTAGA AAAGGAAAAA   
  
  
+ AATAATTAAA GATTTTATTT CGAAATTGTA TGTACAAAAA GGAACACGTA CATAAAAGAG TTCATTTATC   
  
  
+ TTTTTATTAA TAAATAATGG TTTGAATAAA GGATATAATA ATTTTAAAAA CGTACAAATT TTATTCAAAT   
  
  
+ AATAAAATCA ATTTTTTAAT TAGTTTTTTT AATTAATAAA AAAAATACAT TAAATACTTC CCGTGTATCA   
  
  
+ TAAAATCCAC TTATAGTACT GTGTGCACAA TGAAACCTAA TCCTAAAAGA CAATCTAAAA ATAGAGGCGA   
  
  
+ TTTGAAAAAT TACTTTTCTA TTTTATCATA AAAATATTAA ATAATATAAT TTAATTATTT ATTTAATATT   
  
  
+ AATAAATAAA ATAATTTAAT ATTGTACAGT AATTGAAAAT TTTTTGAGTC GTGTTAAATC TACTCATTTA   
  
  
+ ATTTTTATTA CTATACATCA ATATTCATAG TTTTTATTTT TATAAACAAA TAAATTTTTA AAATTTTCGA   
  
  
+ TTTTGTATAA AATATTTGGG TAATTATGTT TTAATACAGT GATTAATTGT ATTTTGAGAT CAAAATAGGT   
  
  
+ TTTAACTACA GCAAAATGAT TATTGGATT  

- AAAAACAATC CCAAAAAGCA AAGAAAGAAG AAATTCAAGA AATGTGTTAT AAAGAACAGC TGAAGCCTGT   
  
  
- ATCTACTCTC TCTCTCTCTC TCCCTCTCTC CTTCTGCTTC TCCCACTATG TGTATGAAGT CTGTGAAGTC   
  
  
- TTTCACCGGT GCTATAGGCT CGGCTATTAA AAGGGTTGGG CCGTGGACCA GATTTTGTTA TTAGAAGATG   
  
  
- ACACACTCTT TGGATTTCTC ACTATTTCCA AATTTCCAAT CCGAGCTTCT TACGTCAGCT GGTGCGTCAG   
  
  
- GTCGCCAGCT AGCAACTGGG TCCCACTTCT GACCACCTCT GGCTTGGTTA CCCGTCTTTT CCACAACCCG   
  
  
- AACTCGTTAA CCCGTGGCTG ATTTGTCATT TTATCTGTTG ATATATGTGT GTTGGCGGGT ATGGAGCCGA   
  
  
- CTTCGGGTTA GACGTAGATA CGGATCTGTT TGACGAAAGA GAAAAAAGTT AAAGAGGGAG AGGTTTCCTA   
  
  
- AGCATTTCGT CAAGAAAAGA GTTTGACTCG CTCGTAAATC AGAAGAAAGA AATGAATCCA CCGCCCCGAA   
  
  
- CGGTGCGGCG CGTTCCACGT GACGAAAAAG GAAATACAAA TAACCCAAAA GAGGAAGAAA ACTGAAGGAG   
  
  
- AGAGAAGGAG AGAGAGAGAG AGAGAGAGGG GAGCCGAGTC AACGAATTTG ACGGCCCGGC TCTCTGAGGA   
  
  
- TATGACCGGA GACCGGGTGG GGCAGTGGCC TGGTCTATGC GTGGACCCCA CGTAGCATGA CATGAGAAAG   
  
  
- CACGTTTCAG ATCCGCCCCC GGGAACCTTG TTGACTGTTT ACCTGTGGGA CCGAGTCGCT TGCTTTGACT   
  
  
- TTCTGTTACA TGTTGCTTTA CTCATTTGTC CCTCTCTTTC TCTTCATCTC CTAGTCATCT TTTCCTTTTT   
  
  
- TTATTAATTT CTAAAATAAA GCTTTAACAT ACATGTTTTT CCTTGTGCAT GTATTTTCTC AAGTAAATAG   
  
  
- AAAAATAATT ATTTATTACC AAACTTATTT CCTATATTAT TAAAATTTTT GCATGTTTAA AATAAGTTTA   
  
  
- TTATTTTAGT TAAAAAATTA ATCAAAAAAA TTAATTATTT TTTTTATGTA ATTTATGAAG GGCACATAGT   
  
  
- ATTTTAGGTG AATATCATGA CACACGTGTT ACTTTGGATT AGGATTTTCT GTTAGATTTT TATCTCCGCT   
  
  
- AAACTTTTTA ATGAAAAGAT AAAATAGTAT TTTTATAATT TATTATATTA AATTAATAAA TAAATTATAA   
  
  
- TTATTTATTT TATTAAATTA TAACATGTCA TTAACTTTTA AAAAACTCAG CACAATTTAG ATGAGTAAAT   
  
  
- TAAAAATAAT GATATGTAGT TATAAGTATC AAAAATAAAA ATATTTGTTT ATTTAAAAAT TTTAAAAGCT   
  
  
- AAAACATATT TTATAAACCC ATTAATACAA AATTATGTCA CTAATTAACA TAAAACTCTA GTTTTATCCA   
  
  
- AAATTGATGT CGTTTTACTA ATAACCTAA

+     Box I

| Site Name | Organism | Position | Strand | Matrix score. | sequence | function |
| --- | --- | --- | --- | --- | --- | --- |
| Box I | Pisum sativum | 1191 | - | 7 | TTTCAAA | light responsive element |

> 2018/04/13 10:10:12  
+ TTTTTGTTAG GGTTTTTCGT TTCTTTCTTC TTTAAGTTCT TTACACAATA TTTCTTGTCG ACTTCGGACA   
  
  
+ TAGATGAGAG AGAGAGAGAG AGGGAGAGAG GAAGACGAAG AGGGTGATAC ACATACTTCA GACACTTCAG   
  
  
+ AAAGTGGCCA CGATATCCGA GCCGATAATT TTCCCAACCC GGCACCTGGT CTAAAACAAT AATCTTCTAC   
  
  
+ TGTGTGAGAA ACCTAAAGAG TGATAAAGGT TTAAAGGTTA GGCTCGAAGA ATGCAGTCGA CCACGCAGTC   
  
  
+ CAGCGGTCGA TCGTTGACCC AGGGTGAAGA CTGGTGGAGA CCGAACCAAT GGGCAGAAAA GGTGTTGGGC   
  
  
+ TTGAGCAATT GGGCACCGAC TAAACAGTAA AATAGACAAC TATATACACA CAACCGCCCA TACCTCGGCT   
  
  
+ GAAGCCCAAT CTGCATCTAT GCCTAGACAA ACTGCTTTCT CTTTTTTCAA TTTCTCCCTC TCCAAAGGAT   
  
  
+ TCGTAAAGCA GTTCTTTTCT CAAACTGAGC GAGCATTTAG TCTTCTTTCT TTACTTAGGT GGCGGGGCTT   
  
  
+ GCCACGCCGC GCAAGGTGCA CTGCTTTTTC CTTTATGTTT ATTGGGTTTT CTCCTTCTTT TGACTTCCTC   
  
  
+ TCTCTTCCTC TCTCTCTCTC TCTCTCTCCC CTCGGCTCAG TTGCTTAAAC TGCCGGGCCG AGAGACTCCT   
  
  
+ ATACTGGCCT CTGGCCCACC CCGTCACCGG ACCAGATACG CACCTGGGGT GCATCGTACT GTACTCTTTC   
  
  
+ GTGCAAAGTC TAGGCGGGGG CCCTTGGAAC AACTGACAAA TGGACACCCT GGCTCAGCGA ACGAAACTGA   
  
  
+ AAGACAATGT ACAACGAAAT GAGTAAACAG GGAGAGAAAG AGAAGTAGAG GATCAGTAGA AAAGGAAAAA   
  
  
+ AATAATTAAA GATTTTATTT CGAAATTGTA TGTACAAAAA GGAACACGTA CATAAAAGAG TTCATTTATC   
  
  
+ TTTTTATTAA TAAATAATGG TTTGAATAAA GGATATAATA ATTTTAAAAA CGTACAAATT TTATTCAAAT   
  
  
+ AATAAAATCA ATTTTTTAAT TAGTTTTTTT AATTAATAAA AAAAATACAT TAAATACTTC CCGTGTATCA   
  
  
+ TAAAATCCAC TTATAGTACT GTGTGCACAA TGAAACCTAA TCCTAAAAGA CAATCTAAAA ATAGAGGCGA   
  
  
+ TTTGAAAAAT TACTTTTCTA TTTTATCATA AAAATATTAA ATAATATAAT TTAATTATTT ATTTAATATT   
  
  
+ AATAAATAAA ATAATTTAAT ATTGTACAGT AATTGAAAAT TTTTTGAGTC GTGTTAAATC TACTCATTTA   
  
  
+ ATTTTTATTA CTATACATCA ATATTCATAG TTTTTATTTT TATAAACAAA TAAATTTTTA AAATTTTCGA   
  
  
+ TTTTGTATAA AATATTTGGG TAATTATGTT TTAATACAGT GATTAATTGT ATTTTGAGAT CAAAATAGGT   
  
  
+ TTTAACTACA GCAAAATGAT TATTGGATT  

- AAAAACAATC CCAAAAAGCA AAGAAAGAAG AAATTCAAGA AATGTGTTAT AAAGAACAGC TGAAGCCTGT   
  
  
- ATCTACTCTC TCTCTCTCTC TCCCTCTCTC CTTCTGCTTC TCCCACTATG TGTATGAAGT CTGTGAAGTC   
  
  
- TTTCACCGGT GCTATAGGCT CGGCTATTAA AAGGGTTGGG CCGTGGACCA GATTTTGTTA TTAGAAGATG   
  
  
- ACACACTCTT TGGATTTCTC ACTATTTCCA AATTTCCAAT CCGAGCTTCT TACGTCAGCT GGTGCGTCAG   
  
  
- GTCGCCAGCT AGCAACTGGG TCCCACTTCT GACCACCTCT GGCTTGGTTA CCCGTCTTTT CCACAACCCG   
  
  
- AACTCGTTAA CCCGTGGCTG ATTTGTCATT TTATCTGTTG ATATATGTGT GTTGGCGGGT ATGGAGCCGA   
  
  
- CTTCGGGTTA GACGTAGATA CGGATCTGTT TGACGAAAGA GAAAAAAGTT AAAGAGGGAG AGGTTTCCTA   
  
  
- AGCATTTCGT CAAGAAAAGA GTTTGACTCG CTCGTAAATC AGAAGAAAGA AATGAATCCA CCGCCCCGAA   
  
  
- CGGTGCGGCG CGTTCCACGT GACGAAAAAG GAAATACAAA TAACCCAAAA GAGGAAGAAA ACTGAAGGAG   
  
  
- AGAGAAGGAG AGAGAGAGAG AGAGAGAGGG GAGCCGAGTC AACGAATTTG ACGGCCCGGC TCTCTGAGGA   
  
  
- TATGACCGGA GACCGGGTGG GGCAGTGGCC TGGTCTATGC GTGGACCCCA CGTAGCATGA CATGAGAAAG   
  
  
- CACGTTTCAG ATCCGCCCCC GGGAACCTTG TTGACTGTTT ACCTGTGGGA CCGAGTCGCT TGCTTTGACT   
  
  
- TTCTGTTACA TGTTGCTTTA CTCATTTGTC CCTCTCTTTC TCTTCATCTC CTAGTCATCT TTTCCTTTTT   
  
  
- TTATTAATTT CTAAAATAAA GCTTTAACAT ACATGTTTTT CCTTGTGCAT GTATTTTCTC AAGTAAATAG   
  
  
- AAAAATAATT ATTTATTACC AAACTTATTT CCTATATTAT TAAAATTTTT GCATGTTTAA AATAAGTTTA   
  
  
- TTATTTTAGT TAAAAAATTA ATCAAAAAAA TTAATTATTT TTTTTATGTA ATTTATGAAG GGCACATAGT   
  
  
- ATTTTAGGTG AATATCATGA CACACGTGTT ACTTTGGATT AGGATTTTCT GTTAGATTTT TATCTCCGCT   
  
  
- AAACTTTTTA ATGAAAAGAT AAAATAGTAT TTTTATAATT TATTATATTA AATTAATAAA TAAATTATAA   
  
  
- TTATTTATTT TATTAAATTA TAACATGTCA TTAACTTTTA AAAAACTCAG CACAATTTAG ATGAGTAAAT   
  
  
- TAAAAATAAT GATATGTAGT TATAAGTATC AAAAATAAAA ATATTTGTTT ATTTAAAAAT TTTAAAAGCT   
  
  
- AAAACATATT TTATAAACCC ATTAATACAA AATTATGTCA CTAATTAACA TAAAACTCTA GTTTTATCCA   
  
  
- AAATTGATGT CGTTTTACTA ATAACCTAA

+     Box-W1

| Site Name | Organism | Position | Strand | Matrix score. | sequence | function |
| --- | --- | --- | --- | --- | --- | --- |
| Box-W1 | Petroselinum crispum | 294 | + | 6 | TTGACC | fungal elicitor responsive element |

> 2018/04/13 10:10:12  
+ TTTTTGTTAG GGTTTTTCGT TTCTTTCTTC TTTAAGTTCT TTACACAATA TTTCTTGTCG ACTTCGGACA   
  
  
+ TAGATGAGAG AGAGAGAGAG AGGGAGAGAG GAAGACGAAG AGGGTGATAC ACATACTTCA GACACTTCAG   
  
  
+ AAAGTGGCCA CGATATCCGA GCCGATAATT TTCCCAACCC GGCACCTGGT CTAAAACAAT AATCTTCTAC   
  
  
+ TGTGTGAGAA ACCTAAAGAG TGATAAAGGT TTAAAGGTTA GGCTCGAAGA ATGCAGTCGA CCACGCAGTC   
  
  
+ CAGCGGTCGA TCGTTGACCC AGGGTGAAGA CTGGTGGAGA CCGAACCAAT GGGCAGAAAA GGTGTTGGGC   
  
  
+ TTGAGCAATT GGGCACCGAC TAAACAGTAA AATAGACAAC TATATACACA CAACCGCCCA TACCTCGGCT   
  
  
+ GAAGCCCAAT CTGCATCTAT GCCTAGACAA ACTGCTTTCT CTTTTTTCAA TTTCTCCCTC TCCAAAGGAT   
  
  
+ TCGTAAAGCA GTTCTTTTCT CAAACTGAGC GAGCATTTAG TCTTCTTTCT TTACTTAGGT GGCGGGGCTT   
  
  
+ GCCACGCCGC GCAAGGTGCA CTGCTTTTTC CTTTATGTTT ATTGGGTTTT CTCCTTCTTT TGACTTCCTC   
  
  
+ TCTCTTCCTC TCTCTCTCTC TCTCTCTCCC CTCGGCTCAG TTGCTTAAAC TGCCGGGCCG AGAGACTCCT   
  
  
+ ATACTGGCCT CTGGCCCACC CCGTCACCGG ACCAGATACG CACCTGGGGT GCATCGTACT GTACTCTTTC   
  
  
+ GTGCAAAGTC TAGGCGGGGG CCCTTGGAAC AACTGACAAA TGGACACCCT GGCTCAGCGA ACGAAACTGA   
  
  
+ AAGACAATGT ACAACGAAAT GAGTAAACAG GGAGAGAAAG AGAAGTAGAG GATCAGTAGA AAAGGAAAAA   
  
  
+ AATAATTAAA GATTTTATTT CGAAATTGTA TGTACAAAAA GGAACACGTA CATAAAAGAG TTCATTTATC   
  
  
+ TTTTTATTAA TAAATAATGG TTTGAATAAA GGATATAATA ATTTTAAAAA CGTACAAATT TTATTCAAAT   
  
  
+ AATAAAATCA ATTTTTTAAT TAGTTTTTTT AATTAATAAA AAAAATACAT TAAATACTTC CCGTGTATCA   
  
  
+ TAAAATCCAC TTATAGTACT GTGTGCACAA TGAAACCTAA TCCTAAAAGA CAATCTAAAA ATAGAGGCGA   
  
  
+ TTTGAAAAAT TACTTTTCTA TTTTATCATA AAAATATTAA ATAATATAAT TTAATTATTT ATTTAATATT   
  
  
+ AATAAATAAA ATAATTTAAT ATTGTACAGT AATTGAAAAT TTTTTGAGTC GTGTTAAATC TACTCATTTA   
  
  
+ ATTTTTATTA CTATACATCA ATATTCATAG TTTTTATTTT TATAAACAAA TAAATTTTTA AAATTTTCGA   
  
  
+ TTTTGTATAA AATATTTGGG TAATTATGTT TTAATACAGT GATTAATTGT ATTTTGAGAT CAAAATAGGT   
  
  
+ TTTAACTACA GCAAAATGAT TATTGGATT  

- AAAAACAATC CCAAAAAGCA AAGAAAGAAG AAATTCAAGA AATGTGTTAT AAAGAACAGC TGAAGCCTGT   
  
  
- ATCTACTCTC TCTCTCTCTC TCCCTCTCTC CTTCTGCTTC TCCCACTATG TGTATGAAGT CTGTGAAGTC   
  
  
- TTTCACCGGT GCTATAGGCT CGGCTATTAA AAGGGTTGGG CCGTGGACCA GATTTTGTTA TTAGAAGATG   
  
  
- ACACACTCTT TGGATTTCTC ACTATTTCCA AATTTCCAAT CCGAGCTTCT TACGTCAGCT GGTGCGTCAG   
  
  
- GTCGCCAGCT AGCAACTGGG TCCCACTTCT GACCACCTCT GGCTTGGTTA CCCGTCTTTT CCACAACCCG   
  
  
- AACTCGTTAA CCCGTGGCTG ATTTGTCATT TTATCTGTTG ATATATGTGT GTTGGCGGGT ATGGAGCCGA   
  
  
- CTTCGGGTTA GACGTAGATA CGGATCTGTT TGACGAAAGA GAAAAAAGTT AAAGAGGGAG AGGTTTCCTA   
  
  
- AGCATTTCGT CAAGAAAAGA GTTTGACTCG CTCGTAAATC AGAAGAAAGA AATGAATCCA CCGCCCCGAA   
  
  
- CGGTGCGGCG CGTTCCACGT GACGAAAAAG GAAATACAAA TAACCCAAAA GAGGAAGAAA ACTGAAGGAG   
  
  
- AGAGAAGGAG AGAGAGAGAG AGAGAGAGGG GAGCCGAGTC AACGAATTTG ACGGCCCGGC TCTCTGAGGA   
  
  
- TATGACCGGA GACCGGGTGG GGCAGTGGCC TGGTCTATGC GTGGACCCCA CGTAGCATGA CATGAGAAAG   
  
  
- CACGTTTCAG ATCCGCCCCC GGGAACCTTG TTGACTGTTT ACCTGTGGGA CCGAGTCGCT TGCTTTGACT   
  
  
- TTCTGTTACA TGTTGCTTTA CTCATTTGTC CCTCTCTTTC TCTTCATCTC CTAGTCATCT TTTCCTTTTT   
  
  
- TTATTAATTT CTAAAATAAA GCTTTAACAT ACATGTTTTT CCTTGTGCAT GTATTTTCTC AAGTAAATAG   
  
  
- AAAAATAATT ATTTATTACC AAACTTATTT CCTATATTAT TAAAATTTTT GCATGTTTAA AATAAGTTTA   
  
  
- TTATTTTAGT TAAAAAATTA ATCAAAAAAA TTAATTATTT TTTTTATGTA ATTTATGAAG GGCACATAGT   
  
  
- ATTTTAGGTG AATATCATGA CACACGTGTT ACTTTGGATT AGGATTTTCT GTTAGATTTT TATCTCCGCT   
  
  
- AAACTTTTTA ATGAAAAGAT AAAATAGTAT TTTTATAATT TATTATATTA AATTAATAAA TAAATTATAA   
  
  
- TTATTTATTT TATTAAATTA TAACATGTCA TTAACTTTTA AAAAACTCAG CACAATTTAG ATGAGTAAAT   
  
  
- TAAAAATAAT GATATGTAGT TATAAGTATC AAAAATAAAA ATATTTGTTT ATTTAAAAAT TTTAAAAGCT   
  
  
- AAAACATATT TTATAAACCC ATTAATACAA AATTATGTCA CTAATTAACA TAAAACTCTA GTTTTATCCA   
  
  
- AAATTGATGT CGTTTTACTA ATAACCTAA

+     CAAT-box

| Site Name | Organism | Position | Strand | Matrix score. | sequence | function |
| --- | --- | --- | --- | --- | --- | --- |
| CAAT-box | Brassica rapa | 1190 | - | 5 | CAAAT | common cis-acting element in promoter and enhancer regions |
| CAAT-box | Glycine max | 356 | + | 5 | CAATT | common cis-acting element in promoter and enhancer regions |
| CAAT-box | Brassica rapa | 1035 | + | 5 | CAAAT | common cis-acting element in promoter and enhancer regions |
| CAAT-box | Hordeum vulgare | 935 | - | 4 | CAAT | common cis-acting element in promoter and enhancer regions |
| CAAT-box | Hordeum vulgare | 1446 | - | 4 | CAAT | common cis-acting element in promoter and enhancer regions |
| CAAT-box | Arabidopsis thaliana | 358 | - | 5 | CCAAT | common cis-acting element in promoter and enhancer regions |
| CAAT-box | Arabidopsis thaliana | 426 | + | 5 | CCAAT | common cis-acting element in promoter and enhancer regions |
| CAAT-box | Arabidopsis thaliana | 326 | + | 5 | CCAAT | common cis-acting element in promoter and enhancer regions |
| CAAT-box | Brassica rapa | 1046 | + | 5 | CAAAT | common cis-acting element in promoter and enhancer regions |
| CAAT-box | Hordeum vulgare | 327 | + | 4 | CAAT | common cis-acting element in promoter and enhancer regions |
| CAAT-box | Arabidopsis thaliana | 1492 | - | 5 | CCAAT | common cis-acting element in promoter and enhancer regions |
| CAAT-box | Hordeum vulgare | 1148 | + | 4 | CAAT | common cis-acting element in promoter and enhancer regions |
| CAAT-box | Hordeum vulgare | 1349 | + | 4 | CAAT | common cis-acting element in promoter and enhancer regions |
| CAAT-box | Glycine max | 357 | - | 5 | CAATT | common cis-acting element in promoter and enhancer regions |
| CAAT-box | Hordeum vulgare | 197 | + | 4 | CAAT | common cis-acting element in promoter and enhancer regions |
| CAAT-box | Hordeum vulgare | 46 | + | 4 | CAAT | common cis-acting element in promoter and enhancer regions |
| CAAT-box | Hordeum vulgare | 1281 | - | 4 | CAAT | common cis-acting element in promoter and enhancer regions |
| CAAT-box | Brassica rapa | 1377 | + | 5 | CAAAT | common cis-acting element in promoter and enhancer regions |
| CAAT-box | Hordeum vulgare | 845 | + | 4 | CAAT | common cis-acting element in promoter and enhancer regions |
| CAAT-box | Hordeum vulgare | 427 | + | 4 | CAAT | common cis-acting element in promoter and enhancer regions |
| CAAT-box | Glycine max | 1059 | + | 5 | CAATT | common cis-acting element in promoter and enhancer regions |
| CAAT-box | Glycine max | 1291 | - | 5 | CAATT | common cis-acting element in promoter and enhancer regions |
| CAAT-box | Glycine max | 934 | - | 5 | CAATT | common cis-acting element in promoter and enhancer regions |
| CAAT-box | Glycine max | 468 | + | 5 | CAATT | common cis-acting element in promoter and enhancer regions |
| CAAT-box | Brassica rapa | 807 | + | 5 | CAAAT | common cis-acting element in promoter and enhancer regions |
| CAAT-box | Arabidopsis thaliana | 601 | - | 5 | CCAAT | common cis-acting element in promoter and enhancer regions |
| CAAT-box | Hordeum vulgare | 1171 | + | 4 | CAAT | common cis-acting element in promoter and enhancer regions |
| CAAT-box | Glycine max | 1445 | - | 5 | CAATT | common cis-acting element in promoter and enhancer regions |
| CAAT-box | Brassica rapa | 1414 | - | 5 | CAAAT | common cis-acting element in promoter and enhancer regions |
| CAAT-box | Hordeum vulgare | 1292 | - | 4 | CAAT | common cis-acting element in promoter and enhancer regions |

> 2018/04/13 10:10:12  
+ TTTTTGTTAG GGTTTTTCGT TTCTTTCTTC TTTAAGTTCT TTACACAATA TTTCTTGTCG ACTTCGGACA   
  
  
+ TAGATGAGAG AGAGAGAGAG AGGGAGAGAG GAAGACGAAG AGGGTGATAC ACATACTTCA GACACTTCAG   
  
  
+ AAAGTGGCCA CGATATCCGA GCCGATAATT TTCCCAACCC GGCACCTGGT CTAAAACAAT AATCTTCTAC   
  
  
+ TGTGTGAGAA ACCTAAAGAG TGATAAAGGT TTAAAGGTTA GGCTCGAAGA ATGCAGTCGA CCACGCAGTC   
  
  
+ CAGCGGTCGA TCGTTGACCC AGGGTGAAGA CTGGTGGAGA CCGAACCAAT GGGCAGAAAA GGTGTTGGGC   
  
  
+ TTGAGCAATT GGGCACCGAC TAAACAGTAA AATAGACAAC TATATACACA CAACCGCCCA TACCTCGGCT   
  
  
+ GAAGCCCAAT CTGCATCTAT GCCTAGACAA ACTGCTTTCT CTTTTTTCAA TTTCTCCCTC TCCAAAGGAT   
  
  
+ TCGTAAAGCA GTTCTTTTCT CAAACTGAGC GAGCATTTAG TCTTCTTTCT TTACTTAGGT GGCGGGGCTT   
  
  
+ GCCACGCCGC GCAAGGTGCA CTGCTTTTTC CTTTATGTTT ATTGGGTTTT CTCCTTCTTT TGACTTCCTC   
  
  
+ TCTCTTCCTC TCTCTCTCTC TCTCTCTCCC CTCGGCTCAG TTGCTTAAAC TGCCGGGCCG AGAGACTCCT   
  
  
+ ATACTGGCCT CTGGCCCACC CCGTCACCGG ACCAGATACG CACCTGGGGT GCATCGTACT GTACTCTTTC   
  
  
+ GTGCAAAGTC TAGGCGGGGG CCCTTGGAAC AACTGACAAA TGGACACCCT GGCTCAGCGA ACGAAACTGA   
  
  
+ AAGACAATGT ACAACGAAAT GAGTAAACAG GGAGAGAAAG AGAAGTAGAG GATCAGTAGA AAAGGAAAAA   
  
  
+ AATAATTAAA GATTTTATTT CGAAATTGTA TGTACAAAAA GGAACACGTA CATAAAAGAG TTCATTTATC   
  
  
+ TTTTTATTAA TAAATAATGG TTTGAATAAA GGATATAATA ATTTTAAAAA CGTACAAATT TTATTCAAAT   
  
  
+ AATAAAATCA ATTTTTTAAT TAGTTTTTTT AATTAATAAA AAAAATACAT TAAATACTTC CCGTGTATCA   
  
  
+ TAAAATCCAC TTATAGTACT GTGTGCACAA TGAAACCTAA TCCTAAAAGA CAATCTAAAA ATAGAGGCGA   
  
  
+ TTTGAAAAAT TACTTTTCTA TTTTATCATA AAAATATTAA ATAATATAAT TTAATTATTT ATTTAATATT   
  
  
+ AATAAATAAA ATAATTTAAT ATTGTACAGT AATTGAAAAT TTTTTGAGTC GTGTTAAATC TACTCATTTA   
  
  
+ ATTTTTATTA CTATACATCA ATATTCATAG TTTTTATTTT TATAAACAAA TAAATTTTTA AAATTTTCGA   
  
  
+ TTTTGTATAA AATATTTGGG TAATTATGTT TTAATACAGT GATTAATTGT ATTTTGAGAT CAAAATAGGT   
  
  
+ TTTAACTACA GCAAAATGAT TATTGGATT  

- AAAAACAATC CCAAAAAGCA AAGAAAGAAG AAATTCAAGA AATGTGTTAT AAAGAACAGC TGAAGCCTGT   
  
  
- ATCTACTCTC TCTCTCTCTC TCCCTCTCTC CTTCTGCTTC TCCCACTATG TGTATGAAGT CTGTGAAGTC   
  
  
- TTTCACCGGT GCTATAGGCT CGGCTATTAA AAGGGTTGGG CCGTGGACCA GATTTTGTTA TTAGAAGATG   
  
  
- ACACACTCTT TGGATTTCTC ACTATTTCCA AATTTCCAAT CCGAGCTTCT TACGTCAGCT GGTGCGTCAG   
  
  
- GTCGCCAGCT AGCAACTGGG TCCCACTTCT GACCACCTCT GGCTTGGTTA CCCGTCTTTT CCACAACCCG   
  
  
- AACTCGTTAA CCCGTGGCTG ATTTGTCATT TTATCTGTTG ATATATGTGT GTTGGCGGGT ATGGAGCCGA   
  
  
- CTTCGGGTTA GACGTAGATA CGGATCTGTT TGACGAAAGA GAAAAAAGTT AAAGAGGGAG AGGTTTCCTA   
  
  
- AGCATTTCGT CAAGAAAAGA GTTTGACTCG CTCGTAAATC AGAAGAAAGA AATGAATCCA CCGCCCCGAA   
  
  
- CGGTGCGGCG CGTTCCACGT GACGAAAAAG GAAATACAAA TAACCCAAAA GAGGAAGAAA ACTGAAGGAG   
  
  
- AGAGAAGGAG AGAGAGAGAG AGAGAGAGGG GAGCCGAGTC AACGAATTTG ACGGCCCGGC TCTCTGAGGA   
  
  
- TATGACCGGA GACCGGGTGG GGCAGTGGCC TGGTCTATGC GTGGACCCCA CGTAGCATGA CATGAGAAAG   
  
  
- CACGTTTCAG ATCCGCCCCC GGGAACCTTG TTGACTGTTT ACCTGTGGGA CCGAGTCGCT TGCTTTGACT   
  
  
- TTCTGTTACA TGTTGCTTTA CTCATTTGTC CCTCTCTTTC TCTTCATCTC CTAGTCATCT TTTCCTTTTT   
  
  
- TTATTAATTT CTAAAATAAA GCTTTAACAT ACATGTTTTT CCTTGTGCAT GTATTTTCTC AAGTAAATAG   
  
  
- AAAAATAATT ATTTATTACC AAACTTATTT CCTATATTAT TAAAATTTTT GCATGTTTAA AATAAGTTTA   
  
  
- TTATTTTAGT TAAAAAATTA ATCAAAAAAA TTAATTATTT TTTTTATGTA ATTTATGAAG GGCACATAGT   
  
  
- ATTTTAGGTG AATATCATGA CACACGTGTT ACTTTGGATT AGGATTTTCT GTTAGATTTT TATCTCCGCT   
  
  
- AAACTTTTTA ATGAAAAGAT AAAATAGTAT TTTTATAATT TATTATATTA AATTAATAAA TAAATTATAA   
  
  
- TTATTTATTT TATTAAATTA TAACATGTCA TTAACTTTTA AAAAACTCAG CACAATTTAG ATGAGTAAAT   
  
  
- TAAAAATAAT GATATGTAGT TATAAGTATC AAAAATAAAA ATATTTGTTT ATTTAAAAAT TTTAAAAGCT   
  
  
- AAAACATATT TTATAAACCC ATTAATACAA AATTATGTCA CTAATTAACA TAAAACTCTA GTTTTATCCA   
  
  
- AAATTGATGT CGTTTTACTA ATAACCTAA

+     CAT-box

| Site Name | Organism | Position | Strand | Matrix score. | sequence | function |
| --- | --- | --- | --- | --- | --- | --- |
| CAT-box | Arabidopsis thaliana | 143 | - | 6 | GCCACT | cis-acting regulatory element related to meristem expression |

> 2018/04/13 10:10:12  
+ TTTTTGTTAG GGTTTTTCGT TTCTTTCTTC TTTAAGTTCT TTACACAATA TTTCTTGTCG ACTTCGGACA   
  
  
+ TAGATGAGAG AGAGAGAGAG AGGGAGAGAG GAAGACGAAG AGGGTGATAC ACATACTTCA GACACTTCAG   
  
  
+ AAAGTGGCCA CGATATCCGA GCCGATAATT TTCCCAACCC GGCACCTGGT CTAAAACAAT AATCTTCTAC   
  
  
+ TGTGTGAGAA ACCTAAAGAG TGATAAAGGT TTAAAGGTTA GGCTCGAAGA ATGCAGTCGA CCACGCAGTC   
  
  
+ CAGCGGTCGA TCGTTGACCC AGGGTGAAGA CTGGTGGAGA CCGAACCAAT GGGCAGAAAA GGTGTTGGGC   
  
  
+ TTGAGCAATT GGGCACCGAC TAAACAGTAA AATAGACAAC TATATACACA CAACCGCCCA TACCTCGGCT   
  
  
+ GAAGCCCAAT CTGCATCTAT GCCTAGACAA ACTGCTTTCT CTTTTTTCAA TTTCTCCCTC TCCAAAGGAT   
  
  
+ TCGTAAAGCA GTTCTTTTCT CAAACTGAGC GAGCATTTAG TCTTCTTTCT TTACTTAGGT GGCGGGGCTT   
  
  
+ GCCACGCCGC GCAAGGTGCA CTGCTTTTTC CTTTATGTTT ATTGGGTTTT CTCCTTCTTT TGACTTCCTC   
  
  
+ TCTCTTCCTC TCTCTCTCTC TCTCTCTCCC CTCGGCTCAG TTGCTTAAAC TGCCGGGCCG AGAGACTCCT   
  
  
+ ATACTGGCCT CTGGCCCACC CCGTCACCGG ACCAGATACG CACCTGGGGT GCATCGTACT GTACTCTTTC   
  
  
+ GTGCAAAGTC TAGGCGGGGG CCCTTGGAAC AACTGACAAA TGGACACCCT GGCTCAGCGA ACGAAACTGA   
  
  
+ AAGACAATGT ACAACGAAAT GAGTAAACAG GGAGAGAAAG AGAAGTAGAG GATCAGTAGA AAAGGAAAAA   
  
  
+ AATAATTAAA GATTTTATTT CGAAATTGTA TGTACAAAAA GGAACACGTA CATAAAAGAG TTCATTTATC   
  
  
+ TTTTTATTAA TAAATAATGG TTTGAATAAA GGATATAATA ATTTTAAAAA CGTACAAATT TTATTCAAAT   
  
  
+ AATAAAATCA ATTTTTTAAT TAGTTTTTTT AATTAATAAA AAAAATACAT TAAATACTTC CCGTGTATCA   
  
  
+ TAAAATCCAC TTATAGTACT GTGTGCACAA TGAAACCTAA TCCTAAAAGA CAATCTAAAA ATAGAGGCGA   
  
  
+ TTTGAAAAAT TACTTTTCTA TTTTATCATA AAAATATTAA ATAATATAAT TTAATTATTT ATTTAATATT   
  
  
+ AATAAATAAA ATAATTTAAT ATTGTACAGT AATTGAAAAT TTTTTGAGTC GTGTTAAATC TACTCATTTA   
  
  
+ ATTTTTATTA CTATACATCA ATATTCATAG TTTTTATTTT TATAAACAAA TAAATTTTTA AAATTTTCGA   
  
  
+ TTTTGTATAA AATATTTGGG TAATTATGTT TTAATACAGT GATTAATTGT ATTTTGAGAT CAAAATAGGT   
  
  
+ TTTAACTACA GCAAAATGAT TATTGGATT  

- AAAAACAATC CCAAAAAGCA AAGAAAGAAG AAATTCAAGA AATGTGTTAT AAAGAACAGC TGAAGCCTGT   
  
  
- ATCTACTCTC TCTCTCTCTC TCCCTCTCTC CTTCTGCTTC TCCCACTATG TGTATGAAGT CTGTGAAGTC   
  
  
- TTTCACCGGT GCTATAGGCT CGGCTATTAA AAGGGTTGGG CCGTGGACCA GATTTTGTTA TTAGAAGATG   
  
  
- ACACACTCTT TGGATTTCTC ACTATTTCCA AATTTCCAAT CCGAGCTTCT TACGTCAGCT GGTGCGTCAG   
  
  
- GTCGCCAGCT AGCAACTGGG TCCCACTTCT GACCACCTCT GGCTTGGTTA CCCGTCTTTT CCACAACCCG   
  
  
- AACTCGTTAA CCCGTGGCTG ATTTGTCATT TTATCTGTTG ATATATGTGT GTTGGCGGGT ATGGAGCCGA   
  
  
- CTTCGGGTTA GACGTAGATA CGGATCTGTT TGACGAAAGA GAAAAAAGTT AAAGAGGGAG AGGTTTCCTA   
  
  
- AGCATTTCGT CAAGAAAAGA GTTTGACTCG CTCGTAAATC AGAAGAAAGA AATGAATCCA CCGCCCCGAA   
  
  
- CGGTGCGGCG CGTTCCACGT GACGAAAAAG GAAATACAAA TAACCCAAAA GAGGAAGAAA ACTGAAGGAG   
  
  
- AGAGAAGGAG AGAGAGAGAG AGAGAGAGGG GAGCCGAGTC AACGAATTTG ACGGCCCGGC TCTCTGAGGA   
  
  
- TATGACCGGA GACCGGGTGG GGCAGTGGCC TGGTCTATGC GTGGACCCCA CGTAGCATGA CATGAGAAAG   
  
  
- CACGTTTCAG ATCCGCCCCC GGGAACCTTG TTGACTGTTT ACCTGTGGGA CCGAGTCGCT TGCTTTGACT   
  
  
- TTCTGTTACA TGTTGCTTTA CTCATTTGTC CCTCTCTTTC TCTTCATCTC CTAGTCATCT TTTCCTTTTT   
  
  
- TTATTAATTT CTAAAATAAA GCTTTAACAT ACATGTTTTT CCTTGTGCAT GTATTTTCTC AAGTAAATAG   
  
  
- AAAAATAATT ATTTATTACC AAACTTATTT CCTATATTAT TAAAATTTTT GCATGTTTAA AATAAGTTTA   
  
  
- TTATTTTAGT TAAAAAATTA ATCAAAAAAA TTAATTATTT TTTTTATGTA ATTTATGAAG GGCACATAGT   
  
  
- ATTTTAGGTG AATATCATGA CACACGTGTT ACTTTGGATT AGGATTTTCT GTTAGATTTT TATCTCCGCT   
  
  
- AAACTTTTTA ATGAAAAGAT AAAATAGTAT TTTTATAATT TATTATATTA AATTAATAAA TAAATTATAA   
  
  
- TTATTTATTT TATTAAATTA TAACATGTCA TTAACTTTTA AAAAACTCAG CACAATTTAG ATGAGTAAAT   
  
  
- TAAAAATAAT GATATGTAGT TATAAGTATC AAAAATAAAA ATATTTGTTT ATTTAAAAAT TTTAAAAGCT   
  
  
- AAAACATATT TTATAAACCC ATTAATACAA AATTATGTCA CTAATTAACA TAAAACTCTA GTTTTATCCA   
  
  
- AAATTGATGT CGTTTTACTA ATAACCTAA

+     CATT-motif

| Site Name | Organism | Position | Strand | Matrix score. | sequence | function |
| --- | --- | --- | --- | --- | --- | --- |
| CATT-motif | Zea mays | 259 | - | 6 | GCATTC | part of a light responsive element |

> 2018/04/13 10:10:12  
+ TTTTTGTTAG GGTTTTTCGT TTCTTTCTTC TTTAAGTTCT TTACACAATA TTTCTTGTCG ACTTCGGACA   
  
  
+ TAGATGAGAG AGAGAGAGAG AGGGAGAGAG GAAGACGAAG AGGGTGATAC ACATACTTCA GACACTTCAG   
  
  
+ AAAGTGGCCA CGATATCCGA GCCGATAATT TTCCCAACCC GGCACCTGGT CTAAAACAAT AATCTTCTAC   
  
  
+ TGTGTGAGAA ACCTAAAGAG TGATAAAGGT TTAAAGGTTA GGCTCGAAGA ATGCAGTCGA CCACGCAGTC   
  
  
+ CAGCGGTCGA TCGTTGACCC AGGGTGAAGA CTGGTGGAGA CCGAACCAAT GGGCAGAAAA GGTGTTGGGC   
  
  
+ TTGAGCAATT GGGCACCGAC TAAACAGTAA AATAGACAAC TATATACACA CAACCGCCCA TACCTCGGCT   
  
  
+ GAAGCCCAAT CTGCATCTAT GCCTAGACAA ACTGCTTTCT CTTTTTTCAA TTTCTCCCTC TCCAAAGGAT   
  
  
+ TCGTAAAGCA GTTCTTTTCT CAAACTGAGC GAGCATTTAG TCTTCTTTCT TTACTTAGGT GGCGGGGCTT   
  
  
+ GCCACGCCGC GCAAGGTGCA CTGCTTTTTC CTTTATGTTT ATTGGGTTTT CTCCTTCTTT TGACTTCCTC   
  
  
+ TCTCTTCCTC TCTCTCTCTC TCTCTCTCCC CTCGGCTCAG TTGCTTAAAC TGCCGGGCCG AGAGACTCCT   
  
  
+ ATACTGGCCT CTGGCCCACC CCGTCACCGG ACCAGATACG CACCTGGGGT GCATCGTACT GTACTCTTTC   
  
  
+ GTGCAAAGTC TAGGCGGGGG CCCTTGGAAC AACTGACAAA TGGACACCCT GGCTCAGCGA ACGAAACTGA   
  
  
+ AAGACAATGT ACAACGAAAT GAGTAAACAG GGAGAGAAAG AGAAGTAGAG GATCAGTAGA AAAGGAAAAA   
  
  
+ AATAATTAAA GATTTTATTT CGAAATTGTA TGTACAAAAA GGAACACGTA CATAAAAGAG TTCATTTATC   
  
  
+ TTTTTATTAA TAAATAATGG TTTGAATAAA GGATATAATA ATTTTAAAAA CGTACAAATT TTATTCAAAT   
  
  
+ AATAAAATCA ATTTTTTAAT TAGTTTTTTT AATTAATAAA AAAAATACAT TAAATACTTC CCGTGTATCA   
  
  
+ TAAAATCCAC TTATAGTACT GTGTGCACAA TGAAACCTAA TCCTAAAAGA CAATCTAAAA ATAGAGGCGA   
  
  
+ TTTGAAAAAT TACTTTTCTA TTTTATCATA AAAATATTAA ATAATATAAT TTAATTATTT ATTTAATATT   
  
  
+ AATAAATAAA ATAATTTAAT ATTGTACAGT AATTGAAAAT TTTTTGAGTC GTGTTAAATC TACTCATTTA   
  
  
+ ATTTTTATTA CTATACATCA ATATTCATAG TTTTTATTTT TATAAACAAA TAAATTTTTA AAATTTTCGA   
  
  
+ TTTTGTATAA AATATTTGGG TAATTATGTT TTAATACAGT GATTAATTGT ATTTTGAGAT CAAAATAGGT   
  
  
+ TTTAACTACA GCAAAATGAT TATTGGATT  

- AAAAACAATC CCAAAAAGCA AAGAAAGAAG AAATTCAAGA AATGTGTTAT AAAGAACAGC TGAAGCCTGT   
  
  
- ATCTACTCTC TCTCTCTCTC TCCCTCTCTC CTTCTGCTTC TCCCACTATG TGTATGAAGT CTGTGAAGTC   
  
  
- TTTCACCGGT GCTATAGGCT CGGCTATTAA AAGGGTTGGG CCGTGGACCA GATTTTGTTA TTAGAAGATG   
  
  
- ACACACTCTT TGGATTTCTC ACTATTTCCA AATTTCCAAT CCGAGCTTCT TACGTCAGCT GGTGCGTCAG   
  
  
- GTCGCCAGCT AGCAACTGGG TCCCACTTCT GACCACCTCT GGCTTGGTTA CCCGTCTTTT CCACAACCCG   
  
  
- AACTCGTTAA CCCGTGGCTG ATTTGTCATT TTATCTGTTG ATATATGTGT GTTGGCGGGT ATGGAGCCGA   
  
  
- CTTCGGGTTA GACGTAGATA CGGATCTGTT TGACGAAAGA GAAAAAAGTT AAAGAGGGAG AGGTTTCCTA   
  
  
- AGCATTTCGT CAAGAAAAGA GTTTGACTCG CTCGTAAATC AGAAGAAAGA AATGAATCCA CCGCCCCGAA   
  
  
- CGGTGCGGCG CGTTCCACGT GACGAAAAAG GAAATACAAA TAACCCAAAA GAGGAAGAAA ACTGAAGGAG   
  
  
- AGAGAAGGAG AGAGAGAGAG AGAGAGAGGG GAGCCGAGTC AACGAATTTG ACGGCCCGGC TCTCTGAGGA   
  
  
- TATGACCGGA GACCGGGTGG GGCAGTGGCC TGGTCTATGC GTGGACCCCA CGTAGCATGA CATGAGAAAG   
  
  
- CACGTTTCAG ATCCGCCCCC GGGAACCTTG TTGACTGTTT ACCTGTGGGA CCGAGTCGCT TGCTTTGACT   
  
  
- TTCTGTTACA TGTTGCTTTA CTCATTTGTC CCTCTCTTTC TCTTCATCTC CTAGTCATCT TTTCCTTTTT   
  
  
- TTATTAATTT CTAAAATAAA GCTTTAACAT ACATGTTTTT CCTTGTGCAT GTATTTTCTC AAGTAAATAG   
  
  
- AAAAATAATT ATTTATTACC AAACTTATTT CCTATATTAT TAAAATTTTT GCATGTTTAA AATAAGTTTA   
  
  
- TTATTTTAGT TAAAAAATTA ATCAAAAAAA TTAATTATTT TTTTTATGTA ATTTATGAAG GGCACATAGT   
  
  
- ATTTTAGGTG AATATCATGA CACACGTGTT ACTTTGGATT AGGATTTTCT GTTAGATTTT TATCTCCGCT   
  
  
- AAACTTTTTA ATGAAAAGAT AAAATAGTAT TTTTATAATT TATTATATTA AATTAATAAA TAAATTATAA   
  
  
- TTATTTATTT TATTAAATTA TAACATGTCA TTAACTTTTA AAAAACTCAG CACAATTTAG ATGAGTAAAT   
  
  
- TAAAAATAAT GATATGTAGT TATAAGTATC AAAAATAAAA ATATTTGTTT ATTTAAAAAT TTTAAAAGCT   
  
  
- AAAACATATT TTATAAACCC ATTAATACAA AATTATGTCA CTAATTAACA TAAAACTCTA GTTTTATCCA   
  
  
- AAATTGATGT CGTTTTACTA ATAACCTAA

+     CGTCA-motif

| Site Name | Organism | Position | Strand | Matrix score. | sequence | function |
| --- | --- | --- | --- | --- | --- | --- |
| CGTCA-motif | Hordeum vulgare | 722 | + | 5 | CGTCA | cis-acting regulatory element involved in the MeJA-responsiveness |

> 2018/04/13 10:10:12  
+ TTTTTGTTAG GGTTTTTCGT TTCTTTCTTC TTTAAGTTCT TTACACAATA TTTCTTGTCG ACTTCGGACA   
  
  
+ TAGATGAGAG AGAGAGAGAG AGGGAGAGAG GAAGACGAAG AGGGTGATAC ACATACTTCA GACACTTCAG   
  
  
+ AAAGTGGCCA CGATATCCGA GCCGATAATT TTCCCAACCC GGCACCTGGT CTAAAACAAT AATCTTCTAC   
  
  
+ TGTGTGAGAA ACCTAAAGAG TGATAAAGGT TTAAAGGTTA GGCTCGAAGA ATGCAGTCGA CCACGCAGTC   
  
  
+ CAGCGGTCGA TCGTTGACCC AGGGTGAAGA CTGGTGGAGA CCGAACCAAT GGGCAGAAAA GGTGTTGGGC   
  
  
+ TTGAGCAATT GGGCACCGAC TAAACAGTAA AATAGACAAC TATATACACA CAACCGCCCA TACCTCGGCT   
  
  
+ GAAGCCCAAT CTGCATCTAT GCCTAGACAA ACTGCTTTCT CTTTTTTCAA TTTCTCCCTC TCCAAAGGAT   
  
  
+ TCGTAAAGCA GTTCTTTTCT CAAACTGAGC GAGCATTTAG TCTTCTTTCT TTACTTAGGT GGCGGGGCTT   
  
  
+ GCCACGCCGC GCAAGGTGCA CTGCTTTTTC CTTTATGTTT ATTGGGTTTT CTCCTTCTTT TGACTTCCTC   
  
  
+ TCTCTTCCTC TCTCTCTCTC TCTCTCTCCC CTCGGCTCAG TTGCTTAAAC TGCCGGGCCG AGAGACTCCT   
  
  
+ ATACTGGCCT CTGGCCCACC CCGTCACCGG ACCAGATACG CACCTGGGGT GCATCGTACT GTACTCTTTC   
  
  
+ GTGCAAAGTC TAGGCGGGGG CCCTTGGAAC AACTGACAAA TGGACACCCT GGCTCAGCGA ACGAAACTGA   
  
  
+ AAGACAATGT ACAACGAAAT GAGTAAACAG GGAGAGAAAG AGAAGTAGAG GATCAGTAGA AAAGGAAAAA   
  
  
+ AATAATTAAA GATTTTATTT CGAAATTGTA TGTACAAAAA GGAACACGTA CATAAAAGAG TTCATTTATC   
  
  
+ TTTTTATTAA TAAATAATGG TTTGAATAAA GGATATAATA ATTTTAAAAA CGTACAAATT TTATTCAAAT   
  
  
+ AATAAAATCA ATTTTTTAAT TAGTTTTTTT AATTAATAAA AAAAATACAT TAAATACTTC CCGTGTATCA   
  
  
+ TAAAATCCAC TTATAGTACT GTGTGCACAA TGAAACCTAA TCCTAAAAGA CAATCTAAAA ATAGAGGCGA   
  
  
+ TTTGAAAAAT TACTTTTCTA TTTTATCATA AAAATATTAA ATAATATAAT TTAATTATTT ATTTAATATT   
  
  
+ AATAAATAAA ATAATTTAAT ATTGTACAGT AATTGAAAAT TTTTTGAGTC GTGTTAAATC TACTCATTTA   
  
  
+ ATTTTTATTA CTATACATCA ATATTCATAG TTTTTATTTT TATAAACAAA TAAATTTTTA AAATTTTCGA   
  
  
+ TTTTGTATAA AATATTTGGG TAATTATGTT TTAATACAGT GATTAATTGT ATTTTGAGAT CAAAATAGGT   
  
  
+ TTTAACTACA GCAAAATGAT TATTGGATT  

- AAAAACAATC CCAAAAAGCA AAGAAAGAAG AAATTCAAGA AATGTGTTAT AAAGAACAGC TGAAGCCTGT   
  
  
- ATCTACTCTC TCTCTCTCTC TCCCTCTCTC CTTCTGCTTC TCCCACTATG TGTATGAAGT CTGTGAAGTC   
  
  
- TTTCACCGGT GCTATAGGCT CGGCTATTAA AAGGGTTGGG CCGTGGACCA GATTTTGTTA TTAGAAGATG   
  
  
- ACACACTCTT TGGATTTCTC ACTATTTCCA AATTTCCAAT CCGAGCTTCT TACGTCAGCT GGTGCGTCAG   
  
  
- GTCGCCAGCT AGCAACTGGG TCCCACTTCT GACCACCTCT GGCTTGGTTA CCCGTCTTTT CCACAACCCG   
  
  
- AACTCGTTAA CCCGTGGCTG ATTTGTCATT TTATCTGTTG ATATATGTGT GTTGGCGGGT ATGGAGCCGA   
  
  
- CTTCGGGTTA GACGTAGATA CGGATCTGTT TGACGAAAGA GAAAAAAGTT AAAGAGGGAG AGGTTTCCTA   
  
  
- AGCATTTCGT CAAGAAAAGA GTTTGACTCG CTCGTAAATC AGAAGAAAGA AATGAATCCA CCGCCCCGAA   
  
  
- CGGTGCGGCG CGTTCCACGT GACGAAAAAG GAAATACAAA TAACCCAAAA GAGGAAGAAA ACTGAAGGAG   
  
  
- AGAGAAGGAG AGAGAGAGAG AGAGAGAGGG GAGCCGAGTC AACGAATTTG ACGGCCCGGC TCTCTGAGGA   
  
  
- TATGACCGGA GACCGGGTGG GGCAGTGGCC TGGTCTATGC GTGGACCCCA CGTAGCATGA CATGAGAAAG   
  
  
- CACGTTTCAG ATCCGCCCCC GGGAACCTTG TTGACTGTTT ACCTGTGGGA CCGAGTCGCT TGCTTTGACT   
  
  
- TTCTGTTACA TGTTGCTTTA CTCATTTGTC CCTCTCTTTC TCTTCATCTC CTAGTCATCT TTTCCTTTTT   
  
  
- TTATTAATTT CTAAAATAAA GCTTTAACAT ACATGTTTTT CCTTGTGCAT GTATTTTCTC AAGTAAATAG   
  
  
- AAAAATAATT ATTTATTACC AAACTTATTT CCTATATTAT TAAAATTTTT GCATGTTTAA AATAAGTTTA   
  
  
- TTATTTTAGT TAAAAAATTA ATCAAAAAAA TTAATTATTT TTTTTATGTA ATTTATGAAG GGCACATAGT   
  
  
- ATTTTAGGTG AATATCATGA CACACGTGTT ACTTTGGATT AGGATTTTCT GTTAGATTTT TATCTCCGCT   
  
  
- AAACTTTTTA ATGAAAAGAT AAAATAGTAT TTTTATAATT TATTATATTA AATTAATAAA TAAATTATAA   
  
  
- TTATTTATTT TATTAAATTA TAACATGTCA TTAACTTTTA AAAAACTCAG CACAATTTAG ATGAGTAAAT   
  
  
- TAAAAATAAT GATATGTAGT TATAAGTATC AAAAATAAAA ATATTTGTTT ATTTAAAAAT TTTAAAAGCT   
  
  
- AAAACATATT TTATAAACCC ATTAATACAA AATTATGTCA CTAATTAACA TAAAACTCTA GTTTTATCCA   
  
  
- AAATTGATGT CGTTTTACTA ATAACCTAA

+     G-Box

| Site Name | Organism | Position | Strand | Matrix score. | sequence | function |
| --- | --- | --- | --- | --- | --- | --- |
| G-Box | Antirrhinum majus | 955 | + | 6 | CACGTA | cis-acting regulatory element involved in light responsiveness |

> 2018/04/13 10:10:12  
+ TTTTTGTTAG GGTTTTTCGT TTCTTTCTTC TTTAAGTTCT TTACACAATA TTTCTTGTCG ACTTCGGACA   
  
  
+ TAGATGAGAG AGAGAGAGAG AGGGAGAGAG GAAGACGAAG AGGGTGATAC ACATACTTCA GACACTTCAG   
  
  
+ AAAGTGGCCA CGATATCCGA GCCGATAATT TTCCCAACCC GGCACCTGGT CTAAAACAAT AATCTTCTAC   
  
  
+ TGTGTGAGAA ACCTAAAGAG TGATAAAGGT TTAAAGGTTA GGCTCGAAGA ATGCAGTCGA CCACGCAGTC   
  
  
+ CAGCGGTCGA TCGTTGACCC AGGGTGAAGA CTGGTGGAGA CCGAACCAAT GGGCAGAAAA GGTGTTGGGC   
  
  
+ TTGAGCAATT GGGCACCGAC TAAACAGTAA AATAGACAAC TATATACACA CAACCGCCCA TACCTCGGCT   
  
  
+ GAAGCCCAAT CTGCATCTAT GCCTAGACAA ACTGCTTTCT CTTTTTTCAA TTTCTCCCTC TCCAAAGGAT   
  
  
+ TCGTAAAGCA GTTCTTTTCT CAAACTGAGC GAGCATTTAG TCTTCTTTCT TTACTTAGGT GGCGGGGCTT   
  
  
+ GCCACGCCGC GCAAGGTGCA CTGCTTTTTC CTTTATGTTT ATTGGGTTTT CTCCTTCTTT TGACTTCCTC   
  
  
+ TCTCTTCCTC TCTCTCTCTC TCTCTCTCCC CTCGGCTCAG TTGCTTAAAC TGCCGGGCCG AGAGACTCCT   
  
  
+ ATACTGGCCT CTGGCCCACC CCGTCACCGG ACCAGATACG CACCTGGGGT GCATCGTACT GTACTCTTTC   
  
  
+ GTGCAAAGTC TAGGCGGGGG CCCTTGGAAC AACTGACAAA TGGACACCCT GGCTCAGCGA ACGAAACTGA   
  
  
+ AAGACAATGT ACAACGAAAT GAGTAAACAG GGAGAGAAAG AGAAGTAGAG GATCAGTAGA AAAGGAAAAA   
  
  
+ AATAATTAAA GATTTTATTT CGAAATTGTA TGTACAAAAA GGAACACGTA CATAAAAGAG TTCATTTATC   
  
  
+ TTTTTATTAA TAAATAATGG TTTGAATAAA GGATATAATA ATTTTAAAAA CGTACAAATT TTATTCAAAT   
  
  
+ AATAAAATCA ATTTTTTAAT TAGTTTTTTT AATTAATAAA AAAAATACAT TAAATACTTC CCGTGTATCA   
  
  
+ TAAAATCCAC TTATAGTACT GTGTGCACAA TGAAACCTAA TCCTAAAAGA CAATCTAAAA ATAGAGGCGA   
  
  
+ TTTGAAAAAT TACTTTTCTA TTTTATCATA AAAATATTAA ATAATATAAT TTAATTATTT ATTTAATATT   
  
  
+ AATAAATAAA ATAATTTAAT ATTGTACAGT AATTGAAAAT TTTTTGAGTC GTGTTAAATC TACTCATTTA   
  
  
+ ATTTTTATTA CTATACATCA ATATTCATAG TTTTTATTTT TATAAACAAA TAAATTTTTA AAATTTTCGA   
  
  
+ TTTTGTATAA AATATTTGGG TAATTATGTT TTAATACAGT GATTAATTGT ATTTTGAGAT CAAAATAGGT   
  
  
+ TTTAACTACA GCAAAATGAT TATTGGATT  

- AAAAACAATC CCAAAAAGCA AAGAAAGAAG AAATTCAAGA AATGTGTTAT AAAGAACAGC TGAAGCCTGT   
  
  
- ATCTACTCTC TCTCTCTCTC TCCCTCTCTC CTTCTGCTTC TCCCACTATG TGTATGAAGT CTGTGAAGTC   
  
  
- TTTCACCGGT GCTATAGGCT CGGCTATTAA AAGGGTTGGG CCGTGGACCA GATTTTGTTA TTAGAAGATG   
  
  
- ACACACTCTT TGGATTTCTC ACTATTTCCA AATTTCCAAT CCGAGCTTCT TACGTCAGCT GGTGCGTCAG   
  
  
- GTCGCCAGCT AGCAACTGGG TCCCACTTCT GACCACCTCT GGCTTGGTTA CCCGTCTTTT CCACAACCCG   
  
  
- AACTCGTTAA CCCGTGGCTG ATTTGTCATT TTATCTGTTG ATATATGTGT GTTGGCGGGT ATGGAGCCGA   
  
  
- CTTCGGGTTA GACGTAGATA CGGATCTGTT TGACGAAAGA GAAAAAAGTT AAAGAGGGAG AGGTTTCCTA   
  
  
- AGCATTTCGT CAAGAAAAGA GTTTGACTCG CTCGTAAATC AGAAGAAAGA AATGAATCCA CCGCCCCGAA   
  
  
- CGGTGCGGCG CGTTCCACGT GACGAAAAAG GAAATACAAA TAACCCAAAA GAGGAAGAAA ACTGAAGGAG   
  
  
- AGAGAAGGAG AGAGAGAGAG AGAGAGAGGG GAGCCGAGTC AACGAATTTG ACGGCCCGGC TCTCTGAGGA   
  
  
- TATGACCGGA GACCGGGTGG GGCAGTGGCC TGGTCTATGC GTGGACCCCA CGTAGCATGA CATGAGAAAG   
  
  
- CACGTTTCAG ATCCGCCCCC GGGAACCTTG TTGACTGTTT ACCTGTGGGA CCGAGTCGCT TGCTTTGACT   
  
  
- TTCTGTTACA TGTTGCTTTA CTCATTTGTC CCTCTCTTTC TCTTCATCTC CTAGTCATCT TTTCCTTTTT   
  
  
- TTATTAATTT CTAAAATAAA GCTTTAACAT ACATGTTTTT CCTTGTGCAT GTATTTTCTC AAGTAAATAG   
  
  
- AAAAATAATT ATTTATTACC AAACTTATTT CCTATATTAT TAAAATTTTT GCATGTTTAA AATAAGTTTA   
  
  
- TTATTTTAGT TAAAAAATTA ATCAAAAAAA TTAATTATTT TTTTTATGTA ATTTATGAAG GGCACATAGT   
  
  
- ATTTTAGGTG AATATCATGA CACACGTGTT ACTTTGGATT AGGATTTTCT GTTAGATTTT TATCTCCGCT   
  
  
- AAACTTTTTA ATGAAAAGAT AAAATAGTAT TTTTATAATT TATTATATTA AATTAATAAA TAAATTATAA   
  
  
- TTATTTATTT TATTAAATTA TAACATGTCA TTAACTTTTA AAAAACTCAG CACAATTTAG ATGAGTAAAT   
  
  
- TAAAAATAAT GATATGTAGT TATAAGTATC AAAAATAAAA ATATTTGTTT ATTTAAAAAT TTTAAAAGCT   
  
  
- AAAACATATT TTATAAACCC ATTAATACAA AATTATGTCA CTAATTAACA TAAAACTCTA GTTTTATCCA   
  
  
- AAATTGATGT CGTTTTACTA ATAACCTAA

+     G-box

| Site Name | Organism | Position | Strand | Matrix score. | sequence | function |
| --- | --- | --- | --- | --- | --- | --- |
| G-box | Daucus carota | 955 | - | 6 | TACGTG | cis-acting regulatory element involved in light responsiveness |
| G-box | Zea mays | 1308 | - | 6 | CACGAC | cis-acting regulatory element involved in light responsiveness |

> 2018/04/13 10:10:12  
+ TTTTTGTTAG GGTTTTTCGT TTCTTTCTTC TTTAAGTTCT TTACACAATA TTTCTTGTCG ACTTCGGACA   
  
  
+ TAGATGAGAG AGAGAGAGAG AGGGAGAGAG GAAGACGAAG AGGGTGATAC ACATACTTCA GACACTTCAG   
  
  
+ AAAGTGGCCA CGATATCCGA GCCGATAATT TTCCCAACCC GGCACCTGGT CTAAAACAAT AATCTTCTAC   
  
  
+ TGTGTGAGAA ACCTAAAGAG TGATAAAGGT TTAAAGGTTA GGCTCGAAGA ATGCAGTCGA CCACGCAGTC   
  
  
+ CAGCGGTCGA TCGTTGACCC AGGGTGAAGA CTGGTGGAGA CCGAACCAAT GGGCAGAAAA GGTGTTGGGC   
  
  
+ TTGAGCAATT GGGCACCGAC TAAACAGTAA AATAGACAAC TATATACACA CAACCGCCCA TACCTCGGCT   
  
  
+ GAAGCCCAAT CTGCATCTAT GCCTAGACAA ACTGCTTTCT CTTTTTTCAA TTTCTCCCTC TCCAAAGGAT   
  
  
+ TCGTAAAGCA GTTCTTTTCT CAAACTGAGC GAGCATTTAG TCTTCTTTCT TTACTTAGGT GGCGGGGCTT   
  
  
+ GCCACGCCGC GCAAGGTGCA CTGCTTTTTC CTTTATGTTT ATTGGGTTTT CTCCTTCTTT TGACTTCCTC   
  
  
+ TCTCTTCCTC TCTCTCTCTC TCTCTCTCCC CTCGGCTCAG TTGCTTAAAC TGCCGGGCCG AGAGACTCCT   
  
  
+ ATACTGGCCT CTGGCCCACC CCGTCACCGG ACCAGATACG CACCTGGGGT GCATCGTACT GTACTCTTTC   
  
  
+ GTGCAAAGTC TAGGCGGGGG CCCTTGGAAC AACTGACAAA TGGACACCCT GGCTCAGCGA ACGAAACTGA   
  
  
+ AAGACAATGT ACAACGAAAT GAGTAAACAG GGAGAGAAAG AGAAGTAGAG GATCAGTAGA AAAGGAAAAA   
  
  
+ AATAATTAAA GATTTTATTT CGAAATTGTA TGTACAAAAA GGAACACGTA CATAAAAGAG TTCATTTATC   
  
  
+ TTTTTATTAA TAAATAATGG TTTGAATAAA GGATATAATA ATTTTAAAAA CGTACAAATT TTATTCAAAT   
  
  
+ AATAAAATCA ATTTTTTAAT TAGTTTTTTT AATTAATAAA AAAAATACAT TAAATACTTC CCGTGTATCA   
  
  
+ TAAAATCCAC TTATAGTACT GTGTGCACAA TGAAACCTAA TCCTAAAAGA CAATCTAAAA ATAGAGGCGA   
  
  
+ TTTGAAAAAT TACTTTTCTA TTTTATCATA AAAATATTAA ATAATATAAT TTAATTATTT ATTTAATATT   
  
  
+ AATAAATAAA ATAATTTAAT ATTGTACAGT AATTGAAAAT TTTTTGAGTC GTGTTAAATC TACTCATTTA   
  
  
+ ATTTTTATTA CTATACATCA ATATTCATAG TTTTTATTTT TATAAACAAA TAAATTTTTA AAATTTTCGA   
  
  
+ TTTTGTATAA AATATTTGGG TAATTATGTT TTAATACAGT GATTAATTGT ATTTTGAGAT CAAAATAGGT   
  
  
+ TTTAACTACA GCAAAATGAT TATTGGATT  

- AAAAACAATC CCAAAAAGCA AAGAAAGAAG AAATTCAAGA AATGTGTTAT AAAGAACAGC TGAAGCCTGT   
  
  
- ATCTACTCTC TCTCTCTCTC TCCCTCTCTC CTTCTGCTTC TCCCACTATG TGTATGAAGT CTGTGAAGTC   
  
  
- TTTCACCGGT GCTATAGGCT CGGCTATTAA AAGGGTTGGG CCGTGGACCA GATTTTGTTA TTAGAAGATG   
  
  
- ACACACTCTT TGGATTTCTC ACTATTTCCA AATTTCCAAT CCGAGCTTCT TACGTCAGCT GGTGCGTCAG   
  
  
- GTCGCCAGCT AGCAACTGGG TCCCACTTCT GACCACCTCT GGCTTGGTTA CCCGTCTTTT CCACAACCCG   
  
  
- AACTCGTTAA CCCGTGGCTG ATTTGTCATT TTATCTGTTG ATATATGTGT GTTGGCGGGT ATGGAGCCGA   
  
  
- CTTCGGGTTA GACGTAGATA CGGATCTGTT TGACGAAAGA GAAAAAAGTT AAAGAGGGAG AGGTTTCCTA   
  
  
- AGCATTTCGT CAAGAAAAGA GTTTGACTCG CTCGTAAATC AGAAGAAAGA AATGAATCCA CCGCCCCGAA   
  
  
- CGGTGCGGCG CGTTCCACGT GACGAAAAAG GAAATACAAA TAACCCAAAA GAGGAAGAAA ACTGAAGGAG   
  
  
- AGAGAAGGAG AGAGAGAGAG AGAGAGAGGG GAGCCGAGTC AACGAATTTG ACGGCCCGGC TCTCTGAGGA   
  
  
- TATGACCGGA GACCGGGTGG GGCAGTGGCC TGGTCTATGC GTGGACCCCA CGTAGCATGA CATGAGAAAG   
  
  
- CACGTTTCAG ATCCGCCCCC GGGAACCTTG TTGACTGTTT ACCTGTGGGA CCGAGTCGCT TGCTTTGACT   
  
  
- TTCTGTTACA TGTTGCTTTA CTCATTTGTC CCTCTCTTTC TCTTCATCTC CTAGTCATCT TTTCCTTTTT   
  
  
- TTATTAATTT CTAAAATAAA GCTTTAACAT ACATGTTTTT CCTTGTGCAT GTATTTTCTC AAGTAAATAG   
  
  
- AAAAATAATT ATTTATTACC AAACTTATTT CCTATATTAT TAAAATTTTT GCATGTTTAA AATAAGTTTA   
  
  
- TTATTTTAGT TAAAAAATTA ATCAAAAAAA TTAATTATTT TTTTTATGTA ATTTATGAAG GGCACATAGT   
  
  
- ATTTTAGGTG AATATCATGA CACACGTGTT ACTTTGGATT AGGATTTTCT GTTAGATTTT TATCTCCGCT   
  
  
- AAACTTTTTA ATGAAAAGAT AAAATAGTAT TTTTATAATT TATTATATTA AATTAATAAA TAAATTATAA   
  
  
- TTATTTATTT TATTAAATTA TAACATGTCA TTAACTTTTA AAAAACTCAG CACAATTTAG ATGAGTAAAT   
  
  
- TAAAAATAAT GATATGTAGT TATAAGTATC AAAAATAAAA ATATTTGTTT ATTTAAAAAT TTTAAAAGCT   
  
  
- AAAACATATT TTATAAACCC ATTAATACAA AATTATGTCA CTAATTAACA TAAAACTCTA GTTTTATCCA   
  
  
- AAATTGATGT CGTTTTACTA ATAACCTAA

+     GC-motif

| Site Name | Organism | Position | Strand | Matrix score. | sequence | function |
| --- | --- | --- | --- | --- | --- | --- |
| GC-motif | Oryza sativa | 565 | + | 9 | CGCCGCGCA | ? |
| GC-motif | Zea mays | 785 | - | 6 | CCCCCG | enhancer-like element involved in anoxic specific inducibility |

> 2018/04/13 10:10:12  
+ TTTTTGTTAG GGTTTTTCGT TTCTTTCTTC TTTAAGTTCT TTACACAATA TTTCTTGTCG ACTTCGGACA   
  
  
+ TAGATGAGAG AGAGAGAGAG AGGGAGAGAG GAAGACGAAG AGGGTGATAC ACATACTTCA GACACTTCAG   
  
  
+ AAAGTGGCCA CGATATCCGA GCCGATAATT TTCCCAACCC GGCACCTGGT CTAAAACAAT AATCTTCTAC   
  
  
+ TGTGTGAGAA ACCTAAAGAG TGATAAAGGT TTAAAGGTTA GGCTCGAAGA ATGCAGTCGA CCACGCAGTC   
  
  
+ CAGCGGTCGA TCGTTGACCC AGGGTGAAGA CTGGTGGAGA CCGAACCAAT GGGCAGAAAA GGTGTTGGGC   
  
  
+ TTGAGCAATT GGGCACCGAC TAAACAGTAA AATAGACAAC TATATACACA CAACCGCCCA TACCTCGGCT   
  
  
+ GAAGCCCAAT CTGCATCTAT GCCTAGACAA ACTGCTTTCT CTTTTTTCAA TTTCTCCCTC TCCAAAGGAT   
  
  
+ TCGTAAAGCA GTTCTTTTCT CAAACTGAGC GAGCATTTAG TCTTCTTTCT TTACTTAGGT GGCGGGGCTT   
  
  
+ GCCACGCCGC GCAAGGTGCA CTGCTTTTTC CTTTATGTTT ATTGGGTTTT CTCCTTCTTT TGACTTCCTC   
  
  
+ TCTCTTCCTC TCTCTCTCTC TCTCTCTCCC CTCGGCTCAG TTGCTTAAAC TGCCGGGCCG AGAGACTCCT   
  
  
+ ATACTGGCCT CTGGCCCACC CCGTCACCGG ACCAGATACG CACCTGGGGT GCATCGTACT GTACTCTTTC   
  
  
+ GTGCAAAGTC TAGGCGGGGG CCCTTGGAAC AACTGACAAA TGGACACCCT GGCTCAGCGA ACGAAACTGA   
  
  
+ AAGACAATGT ACAACGAAAT GAGTAAACAG GGAGAGAAAG AGAAGTAGAG GATCAGTAGA AAAGGAAAAA   
  
  
+ AATAATTAAA GATTTTATTT CGAAATTGTA TGTACAAAAA GGAACACGTA CATAAAAGAG TTCATTTATC   
  
  
+ TTTTTATTAA TAAATAATGG TTTGAATAAA GGATATAATA ATTTTAAAAA CGTACAAATT TTATTCAAAT   
  
  
+ AATAAAATCA ATTTTTTAAT TAGTTTTTTT AATTAATAAA AAAAATACAT TAAATACTTC CCGTGTATCA   
  
  
+ TAAAATCCAC TTATAGTACT GTGTGCACAA TGAAACCTAA TCCTAAAAGA CAATCTAAAA ATAGAGGCGA   
  
  
+ TTTGAAAAAT TACTTTTCTA TTTTATCATA AAAATATTAA ATAATATAAT TTAATTATTT ATTTAATATT   
  
  
+ AATAAATAAA ATAATTTAAT ATTGTACAGT AATTGAAAAT TTTTTGAGTC GTGTTAAATC TACTCATTTA   
  
  
+ ATTTTTATTA CTATACATCA ATATTCATAG TTTTTATTTT TATAAACAAA TAAATTTTTA AAATTTTCGA   
  
  
+ TTTTGTATAA AATATTTGGG TAATTATGTT TTAATACAGT GATTAATTGT ATTTTGAGAT CAAAATAGGT   
  
  
+ TTTAACTACA GCAAAATGAT TATTGGATT  

- AAAAACAATC CCAAAAAGCA AAGAAAGAAG AAATTCAAGA AATGTGTTAT AAAGAACAGC TGAAGCCTGT   
  
  
- ATCTACTCTC TCTCTCTCTC TCCCTCTCTC CTTCTGCTTC TCCCACTATG TGTATGAAGT CTGTGAAGTC   
  
  
- TTTCACCGGT GCTATAGGCT CGGCTATTAA AAGGGTTGGG CCGTGGACCA GATTTTGTTA TTAGAAGATG   
  
  
- ACACACTCTT TGGATTTCTC ACTATTTCCA AATTTCCAAT CCGAGCTTCT TACGTCAGCT GGTGCGTCAG   
  
  
- GTCGCCAGCT AGCAACTGGG TCCCACTTCT GACCACCTCT GGCTTGGTTA CCCGTCTTTT CCACAACCCG   
  
  
- AACTCGTTAA CCCGTGGCTG ATTTGTCATT TTATCTGTTG ATATATGTGT GTTGGCGGGT ATGGAGCCGA   
  
  
- CTTCGGGTTA GACGTAGATA CGGATCTGTT TGACGAAAGA GAAAAAAGTT AAAGAGGGAG AGGTTTCCTA   
  
  
- AGCATTTCGT CAAGAAAAGA GTTTGACTCG CTCGTAAATC AGAAGAAAGA AATGAATCCA CCGCCCCGAA   
  
  
- CGGTGCGGCG CGTTCCACGT GACGAAAAAG GAAATACAAA TAACCCAAAA GAGGAAGAAA ACTGAAGGAG   
  
  
- AGAGAAGGAG AGAGAGAGAG AGAGAGAGGG GAGCCGAGTC AACGAATTTG ACGGCCCGGC TCTCTGAGGA   
  
  
- TATGACCGGA GACCGGGTGG GGCAGTGGCC TGGTCTATGC GTGGACCCCA CGTAGCATGA CATGAGAAAG   
  
  
- CACGTTTCAG ATCCGCCCCC GGGAACCTTG TTGACTGTTT ACCTGTGGGA CCGAGTCGCT TGCTTTGACT   
  
  
- TTCTGTTACA TGTTGCTTTA CTCATTTGTC CCTCTCTTTC TCTTCATCTC CTAGTCATCT TTTCCTTTTT   
  
  
- TTATTAATTT CTAAAATAAA GCTTTAACAT ACATGTTTTT CCTTGTGCAT GTATTTTCTC AAGTAAATAG   
  
  
- AAAAATAATT ATTTATTACC AAACTTATTT CCTATATTAT TAAAATTTTT GCATGTTTAA AATAAGTTTA   
  
  
- TTATTTTAGT TAAAAAATTA ATCAAAAAAA TTAATTATTT TTTTTATGTA ATTTATGAAG GGCACATAGT   
  
  
- ATTTTAGGTG AATATCATGA CACACGTGTT ACTTTGGATT AGGATTTTCT GTTAGATTTT TATCTCCGCT   
  
  
- AAACTTTTTA ATGAAAAGAT AAAATAGTAT TTTTATAATT TATTATATTA AATTAATAAA TAAATTATAA   
  
  
- TTATTTATTT TATTAAATTA TAACATGTCA TTAACTTTTA AAAAACTCAG CACAATTTAG ATGAGTAAAT   
  
  
- TAAAAATAAT GATATGTAGT TATAAGTATC AAAAATAAAA ATATTTGTTT ATTTAAAAAT TTTAAAAGCT   
  
  
- AAAACATATT TTATAAACCC ATTAATACAA AATTATGTCA CTAATTAACA TAAAACTCTA GTTTTATCCA   
  
  
- AAATTGATGT CGTTTTACTA ATAACCTAA

+     HSE

| Site Name | Organism | Position | Strand | Matrix score. | sequence | function |
| --- | --- | --- | --- | --- | --- | --- |
| HSE | Brassica oleracea | 1296 | - | 9 | AAAAAATTTC | cis-acting element involved in heat stress responsiveness |
| HSE | Brassica oleracea | 1295 | - | 9 | AAAAAATTTC | cis-acting element involved in heat stress responsiveness |

> 2018/04/13 10:10:12  
+ TTTTTGTTAG GGTTTTTCGT TTCTTTCTTC TTTAAGTTCT TTACACAATA TTTCTTGTCG ACTTCGGACA   
  
  
+ TAGATGAGAG AGAGAGAGAG AGGGAGAGAG GAAGACGAAG AGGGTGATAC ACATACTTCA GACACTTCAG   
  
  
+ AAAGTGGCCA CGATATCCGA GCCGATAATT TTCCCAACCC GGCACCTGGT CTAAAACAAT AATCTTCTAC   
  
  
+ TGTGTGAGAA ACCTAAAGAG TGATAAAGGT TTAAAGGTTA GGCTCGAAGA ATGCAGTCGA CCACGCAGTC   
  
  
+ CAGCGGTCGA TCGTTGACCC AGGGTGAAGA CTGGTGGAGA CCGAACCAAT GGGCAGAAAA GGTGTTGGGC   
  
  
+ TTGAGCAATT GGGCACCGAC TAAACAGTAA AATAGACAAC TATATACACA CAACCGCCCA TACCTCGGCT   
  
  
+ GAAGCCCAAT CTGCATCTAT GCCTAGACAA ACTGCTTTCT CTTTTTTCAA TTTCTCCCTC TCCAAAGGAT   
  
  
+ TCGTAAAGCA GTTCTTTTCT CAAACTGAGC GAGCATTTAG TCTTCTTTCT TTACTTAGGT GGCGGGGCTT   
  
  
+ GCCACGCCGC GCAAGGTGCA CTGCTTTTTC CTTTATGTTT ATTGGGTTTT CTCCTTCTTT TGACTTCCTC   
  
  
+ TCTCTTCCTC TCTCTCTCTC TCTCTCTCCC CTCGGCTCAG TTGCTTAAAC TGCCGGGCCG AGAGACTCCT   
  
  
+ ATACTGGCCT CTGGCCCACC CCGTCACCGG ACCAGATACG CACCTGGGGT GCATCGTACT GTACTCTTTC   
  
  
+ GTGCAAAGTC TAGGCGGGGG CCCTTGGAAC AACTGACAAA TGGACACCCT GGCTCAGCGA ACGAAACTGA   
  
  
+ AAGACAATGT ACAACGAAAT GAGTAAACAG GGAGAGAAAG AGAAGTAGAG GATCAGTAGA AAAGGAAAAA   
  
  
+ AATAATTAAA GATTTTATTT CGAAATTGTA TGTACAAAAA GGAACACGTA CATAAAAGAG TTCATTTATC   
  
  
+ TTTTTATTAA TAAATAATGG TTTGAATAAA GGATATAATA ATTTTAAAAA CGTACAAATT TTATTCAAAT   
  
  
+ AATAAAATCA ATTTTTTAAT TAGTTTTTTT AATTAATAAA AAAAATACAT TAAATACTTC CCGTGTATCA   
  
  
+ TAAAATCCAC TTATAGTACT GTGTGCACAA TGAAACCTAA TCCTAAAAGA CAATCTAAAA ATAGAGGCGA   
  
  
+ TTTGAAAAAT TACTTTTCTA TTTTATCATA AAAATATTAA ATAATATAAT TTAATTATTT ATTTAATATT   
  
  
+ AATAAATAAA ATAATTTAAT ATTGTACAGT AATTGAAAAT TTTTTGAGTC GTGTTAAATC TACTCATTTA   
  
  
+ ATTTTTATTA CTATACATCA ATATTCATAG TTTTTATTTT TATAAACAAA TAAATTTTTA AAATTTTCGA   
  
  
+ TTTTGTATAA AATATTTGGG TAATTATGTT TTAATACAGT GATTAATTGT ATTTTGAGAT CAAAATAGGT   
  
  
+ TTTAACTACA GCAAAATGAT TATTGGATT  

- AAAAACAATC CCAAAAAGCA AAGAAAGAAG AAATTCAAGA AATGTGTTAT AAAGAACAGC TGAAGCCTGT   
  
  
- ATCTACTCTC TCTCTCTCTC TCCCTCTCTC CTTCTGCTTC TCCCACTATG TGTATGAAGT CTGTGAAGTC   
  
  
- TTTCACCGGT GCTATAGGCT CGGCTATTAA AAGGGTTGGG CCGTGGACCA GATTTTGTTA TTAGAAGATG   
  
  
- ACACACTCTT TGGATTTCTC ACTATTTCCA AATTTCCAAT CCGAGCTTCT TACGTCAGCT GGTGCGTCAG   
  
  
- GTCGCCAGCT AGCAACTGGG TCCCACTTCT GACCACCTCT GGCTTGGTTA CCCGTCTTTT CCACAACCCG   
  
  
- AACTCGTTAA CCCGTGGCTG ATTTGTCATT TTATCTGTTG ATATATGTGT GTTGGCGGGT ATGGAGCCGA   
  
  
- CTTCGGGTTA GACGTAGATA CGGATCTGTT TGACGAAAGA GAAAAAAGTT AAAGAGGGAG AGGTTTCCTA   
  
  
- AGCATTTCGT CAAGAAAAGA GTTTGACTCG CTCGTAAATC AGAAGAAAGA AATGAATCCA CCGCCCCGAA   
  
  
- CGGTGCGGCG CGTTCCACGT GACGAAAAAG GAAATACAAA TAACCCAAAA GAGGAAGAAA ACTGAAGGAG   
  
  
- AGAGAAGGAG AGAGAGAGAG AGAGAGAGGG GAGCCGAGTC AACGAATTTG ACGGCCCGGC TCTCTGAGGA   
  
  
- TATGACCGGA GACCGGGTGG GGCAGTGGCC TGGTCTATGC GTGGACCCCA CGTAGCATGA CATGAGAAAG   
  
  
- CACGTTTCAG ATCCGCCCCC GGGAACCTTG TTGACTGTTT ACCTGTGGGA CCGAGTCGCT TGCTTTGACT   
  
  
- TTCTGTTACA TGTTGCTTTA CTCATTTGTC CCTCTCTTTC TCTTCATCTC CTAGTCATCT TTTCCTTTTT   
  
  
- TTATTAATTT CTAAAATAAA GCTTTAACAT ACATGTTTTT CCTTGTGCAT GTATTTTCTC AAGTAAATAG   
  
  
- AAAAATAATT ATTTATTACC AAACTTATTT CCTATATTAT TAAAATTTTT GCATGTTTAA AATAAGTTTA   
  
  
- TTATTTTAGT TAAAAAATTA ATCAAAAAAA TTAATTATTT TTTTTATGTA ATTTATGAAG GGCACATAGT   
  
  
- ATTTTAGGTG AATATCATGA CACACGTGTT ACTTTGGATT AGGATTTTCT GTTAGATTTT TATCTCCGCT   
  
  
- AAACTTTTTA ATGAAAAGAT AAAATAGTAT TTTTATAATT TATTATATTA AATTAATAAA TAAATTATAA   
  
  
- TTATTTATTT TATTAAATTA TAACATGTCA TTAACTTTTA AAAAACTCAG CACAATTTAG ATGAGTAAAT   
  
  
- TAAAAATAAT GATATGTAGT TATAAGTATC AAAAATAAAA ATATTTGTTT ATTTAAAAAT TTTAAAAGCT   
  
  
- AAAACATATT TTATAAACCC ATTAATACAA AATTATGTCA CTAATTAACA TAAAACTCTA GTTTTATCCA   
  
  
- AAATTGATGT CGTTTTACTA ATAACCTAA

+     LAMP-element

| Site Name | Organism | Position | Strand | Matrix score. | sequence | function |
| --- | --- | --- | --- | --- | --- | --- |
| LAMP-element | Pisum sativum | 231 | - | 8 | CTTTATCA | part of a light responsive element |

> 2018/04/13 10:10:12  
+ TTTTTGTTAG GGTTTTTCGT TTCTTTCTTC TTTAAGTTCT TTACACAATA TTTCTTGTCG ACTTCGGACA   
  
  
+ TAGATGAGAG AGAGAGAGAG AGGGAGAGAG GAAGACGAAG AGGGTGATAC ACATACTTCA GACACTTCAG   
  
  
+ AAAGTGGCCA CGATATCCGA GCCGATAATT TTCCCAACCC GGCACCTGGT CTAAAACAAT AATCTTCTAC   
  
  
+ TGTGTGAGAA ACCTAAAGAG TGATAAAGGT TTAAAGGTTA GGCTCGAAGA ATGCAGTCGA CCACGCAGTC   
  
  
+ CAGCGGTCGA TCGTTGACCC AGGGTGAAGA CTGGTGGAGA CCGAACCAAT GGGCAGAAAA GGTGTTGGGC   
  
  
+ TTGAGCAATT GGGCACCGAC TAAACAGTAA AATAGACAAC TATATACACA CAACCGCCCA TACCTCGGCT   
  
  
+ GAAGCCCAAT CTGCATCTAT GCCTAGACAA ACTGCTTTCT CTTTTTTCAA TTTCTCCCTC TCCAAAGGAT   
  
  
+ TCGTAAAGCA GTTCTTTTCT CAAACTGAGC GAGCATTTAG TCTTCTTTCT TTACTTAGGT GGCGGGGCTT   
  
  
+ GCCACGCCGC GCAAGGTGCA CTGCTTTTTC CTTTATGTTT ATTGGGTTTT CTCCTTCTTT TGACTTCCTC   
  
  
+ TCTCTTCCTC TCTCTCTCTC TCTCTCTCCC CTCGGCTCAG TTGCTTAAAC TGCCGGGCCG AGAGACTCCT   
  
  
+ ATACTGGCCT CTGGCCCACC CCGTCACCGG ACCAGATACG CACCTGGGGT GCATCGTACT GTACTCTTTC   
  
  
+ GTGCAAAGTC TAGGCGGGGG CCCTTGGAAC AACTGACAAA TGGACACCCT GGCTCAGCGA ACGAAACTGA   
  
  
+ AAGACAATGT ACAACGAAAT GAGTAAACAG GGAGAGAAAG AGAAGTAGAG GATCAGTAGA AAAGGAAAAA   
  
  
+ AATAATTAAA GATTTTATTT CGAAATTGTA TGTACAAAAA GGAACACGTA CATAAAAGAG TTCATTTATC   
  
  
+ TTTTTATTAA TAAATAATGG TTTGAATAAA GGATATAATA ATTTTAAAAA CGTACAAATT TTATTCAAAT   
  
  
+ AATAAAATCA ATTTTTTAAT TAGTTTTTTT AATTAATAAA AAAAATACAT TAAATACTTC CCGTGTATCA   
  
  
+ TAAAATCCAC TTATAGTACT GTGTGCACAA TGAAACCTAA TCCTAAAAGA CAATCTAAAA ATAGAGGCGA   
  
  
+ TTTGAAAAAT TACTTTTCTA TTTTATCATA AAAATATTAA ATAATATAAT TTAATTATTT ATTTAATATT   
  
  
+ AATAAATAAA ATAATTTAAT ATTGTACAGT AATTGAAAAT TTTTTGAGTC GTGTTAAATC TACTCATTTA   
  
  
+ ATTTTTATTA CTATACATCA ATATTCATAG TTTTTATTTT TATAAACAAA TAAATTTTTA AAATTTTCGA   
  
  
+ TTTTGTATAA AATATTTGGG TAATTATGTT TTAATACAGT GATTAATTGT ATTTTGAGAT CAAAATAGGT   
  
  
+ TTTAACTACA GCAAAATGAT TATTGGATT  

- AAAAACAATC CCAAAAAGCA AAGAAAGAAG AAATTCAAGA AATGTGTTAT AAAGAACAGC TGAAGCCTGT   
  
  
- ATCTACTCTC TCTCTCTCTC TCCCTCTCTC CTTCTGCTTC TCCCACTATG TGTATGAAGT CTGTGAAGTC   
  
  
- TTTCACCGGT GCTATAGGCT CGGCTATTAA AAGGGTTGGG CCGTGGACCA GATTTTGTTA TTAGAAGATG   
  
  
- ACACACTCTT TGGATTTCTC ACTATTTCCA AATTTCCAAT CCGAGCTTCT TACGTCAGCT GGTGCGTCAG   
  
  
- GTCGCCAGCT AGCAACTGGG TCCCACTTCT GACCACCTCT GGCTTGGTTA CCCGTCTTTT CCACAACCCG   
  
  
- AACTCGTTAA CCCGTGGCTG ATTTGTCATT TTATCTGTTG ATATATGTGT GTTGGCGGGT ATGGAGCCGA   
  
  
- CTTCGGGTTA GACGTAGATA CGGATCTGTT TGACGAAAGA GAAAAAAGTT AAAGAGGGAG AGGTTTCCTA   
  
  
- AGCATTTCGT CAAGAAAAGA GTTTGACTCG CTCGTAAATC AGAAGAAAGA AATGAATCCA CCGCCCCGAA   
  
  
- CGGTGCGGCG CGTTCCACGT GACGAAAAAG GAAATACAAA TAACCCAAAA GAGGAAGAAA ACTGAAGGAG   
  
  
- AGAGAAGGAG AGAGAGAGAG AGAGAGAGGG GAGCCGAGTC AACGAATTTG ACGGCCCGGC TCTCTGAGGA   
  
  
- TATGACCGGA GACCGGGTGG GGCAGTGGCC TGGTCTATGC GTGGACCCCA CGTAGCATGA CATGAGAAAG   
  
  
- CACGTTTCAG ATCCGCCCCC GGGAACCTTG TTGACTGTTT ACCTGTGGGA CCGAGTCGCT TGCTTTGACT   
  
  
- TTCTGTTACA TGTTGCTTTA CTCATTTGTC CCTCTCTTTC TCTTCATCTC CTAGTCATCT TTTCCTTTTT   
  
  
- TTATTAATTT CTAAAATAAA GCTTTAACAT ACATGTTTTT CCTTGTGCAT GTATTTTCTC AAGTAAATAG   
  
  
- AAAAATAATT ATTTATTACC AAACTTATTT CCTATATTAT TAAAATTTTT GCATGTTTAA AATAAGTTTA   
  
  
- TTATTTTAGT TAAAAAATTA ATCAAAAAAA TTAATTATTT TTTTTATGTA ATTTATGAAG GGCACATAGT   
  
  
- ATTTTAGGTG AATATCATGA CACACGTGTT ACTTTGGATT AGGATTTTCT GTTAGATTTT TATCTCCGCT   
  
  
- AAACTTTTTA ATGAAAAGAT AAAATAGTAT TTTTATAATT TATTATATTA AATTAATAAA TAAATTATAA   
  
  
- TTATTTATTT TATTAAATTA TAACATGTCA TTAACTTTTA AAAAACTCAG CACAATTTAG ATGAGTAAAT   
  
  
- TAAAAATAAT GATATGTAGT TATAAGTATC AAAAATAAAA ATATTTGTTT ATTTAAAAAT TTTAAAAGCT   
  
  
- AAAACATATT TTATAAACCC ATTAATACAA AATTATGTCA CTAATTAACA TAAAACTCTA GTTTTATCCA   
  
  
- AAATTGATGT CGTTTTACTA ATAACCTAA

+     MBS

| Site Name | Organism | Position | Strand | Matrix score. | sequence | function |
| --- | --- | --- | --- | --- | --- | --- |
| MBS | Arabidopsis thaliana | 668 | - | 6 | CAACTG | MYB binding site involved in drought-inducibility |
| MBS | Arabidopsis thaliana | 800 | + | 6 | CAACTG | MYB binding site involved in drought-inducibility |

> 2018/04/13 10:10:12  
+ TTTTTGTTAG GGTTTTTCGT TTCTTTCTTC TTTAAGTTCT TTACACAATA TTTCTTGTCG ACTTCGGACA   
  
  
+ TAGATGAGAG AGAGAGAGAG AGGGAGAGAG GAAGACGAAG AGGGTGATAC ACATACTTCA GACACTTCAG   
  
  
+ AAAGTGGCCA CGATATCCGA GCCGATAATT TTCCCAACCC GGCACCTGGT CTAAAACAAT AATCTTCTAC   
  
  
+ TGTGTGAGAA ACCTAAAGAG TGATAAAGGT TTAAAGGTTA GGCTCGAAGA ATGCAGTCGA CCACGCAGTC   
  
  
+ CAGCGGTCGA TCGTTGACCC AGGGTGAAGA CTGGTGGAGA CCGAACCAAT GGGCAGAAAA GGTGTTGGGC   
  
  
+ TTGAGCAATT GGGCACCGAC TAAACAGTAA AATAGACAAC TATATACACA CAACCGCCCA TACCTCGGCT   
  
  
+ GAAGCCCAAT CTGCATCTAT GCCTAGACAA ACTGCTTTCT CTTTTTTCAA TTTCTCCCTC TCCAAAGGAT   
  
  
+ TCGTAAAGCA GTTCTTTTCT CAAACTGAGC GAGCATTTAG TCTTCTTTCT TTACTTAGGT GGCGGGGCTT   
  
  
+ GCCACGCCGC GCAAGGTGCA CTGCTTTTTC CTTTATGTTT ATTGGGTTTT CTCCTTCTTT TGACTTCCTC   
  
  
+ TCTCTTCCTC TCTCTCTCTC TCTCTCTCCC CTCGGCTCAG TTGCTTAAAC TGCCGGGCCG AGAGACTCCT   
  
  
+ ATACTGGCCT CTGGCCCACC CCGTCACCGG ACCAGATACG CACCTGGGGT GCATCGTACT GTACTCTTTC   
  
  
+ GTGCAAAGTC TAGGCGGGGG CCCTTGGAAC AACTGACAAA TGGACACCCT GGCTCAGCGA ACGAAACTGA   
  
  
+ AAGACAATGT ACAACGAAAT GAGTAAACAG GGAGAGAAAG AGAAGTAGAG GATCAGTAGA AAAGGAAAAA   
  
  
+ AATAATTAAA GATTTTATTT CGAAATTGTA TGTACAAAAA GGAACACGTA CATAAAAGAG TTCATTTATC   
  
  
+ TTTTTATTAA TAAATAATGG TTTGAATAAA GGATATAATA ATTTTAAAAA CGTACAAATT TTATTCAAAT   
  
  
+ AATAAAATCA ATTTTTTAAT TAGTTTTTTT AATTAATAAA AAAAATACAT TAAATACTTC CCGTGTATCA   
  
  
+ TAAAATCCAC TTATAGTACT GTGTGCACAA TGAAACCTAA TCCTAAAAGA CAATCTAAAA ATAGAGGCGA   
  
  
+ TTTGAAAAAT TACTTTTCTA TTTTATCATA AAAATATTAA ATAATATAAT TTAATTATTT ATTTAATATT   
  
  
+ AATAAATAAA ATAATTTAAT ATTGTACAGT AATTGAAAAT TTTTTGAGTC GTGTTAAATC TACTCATTTA   
  
  
+ ATTTTTATTA CTATACATCA ATATTCATAG TTTTTATTTT TATAAACAAA TAAATTTTTA AAATTTTCGA   
  
  
+ TTTTGTATAA AATATTTGGG TAATTATGTT TTAATACAGT GATTAATTGT ATTTTGAGAT CAAAATAGGT   
  
  
+ TTTAACTACA GCAAAATGAT TATTGGATT  

- AAAAACAATC CCAAAAAGCA AAGAAAGAAG AAATTCAAGA AATGTGTTAT AAAGAACAGC TGAAGCCTGT   
  
  
- ATCTACTCTC TCTCTCTCTC TCCCTCTCTC CTTCTGCTTC TCCCACTATG TGTATGAAGT CTGTGAAGTC   
  
  
- TTTCACCGGT GCTATAGGCT CGGCTATTAA AAGGGTTGGG CCGTGGACCA GATTTTGTTA TTAGAAGATG   
  
  
- ACACACTCTT TGGATTTCTC ACTATTTCCA AATTTCCAAT CCGAGCTTCT TACGTCAGCT GGTGCGTCAG   
  
  
- GTCGCCAGCT AGCAACTGGG TCCCACTTCT GACCACCTCT GGCTTGGTTA CCCGTCTTTT CCACAACCCG   
  
  
- AACTCGTTAA CCCGTGGCTG ATTTGTCATT TTATCTGTTG ATATATGTGT GTTGGCGGGT ATGGAGCCGA   
  
  
- CTTCGGGTTA GACGTAGATA CGGATCTGTT TGACGAAAGA GAAAAAAGTT AAAGAGGGAG AGGTTTCCTA   
  
  
- AGCATTTCGT CAAGAAAAGA GTTTGACTCG CTCGTAAATC AGAAGAAAGA AATGAATCCA CCGCCCCGAA   
  
  
- CGGTGCGGCG CGTTCCACGT GACGAAAAAG GAAATACAAA TAACCCAAAA GAGGAAGAAA ACTGAAGGAG   
  
  
- AGAGAAGGAG AGAGAGAGAG AGAGAGAGGG GAGCCGAGTC AACGAATTTG ACGGCCCGGC TCTCTGAGGA   
  
  
- TATGACCGGA GACCGGGTGG GGCAGTGGCC TGGTCTATGC GTGGACCCCA CGTAGCATGA CATGAGAAAG   
  
  
- CACGTTTCAG ATCCGCCCCC GGGAACCTTG TTGACTGTTT ACCTGTGGGA CCGAGTCGCT TGCTTTGACT   
  
  
- TTCTGTTACA TGTTGCTTTA CTCATTTGTC CCTCTCTTTC TCTTCATCTC CTAGTCATCT TTTCCTTTTT   
  
  
- TTATTAATTT CTAAAATAAA GCTTTAACAT ACATGTTTTT CCTTGTGCAT GTATTTTCTC AAGTAAATAG   
  
  
- AAAAATAATT ATTTATTACC AAACTTATTT CCTATATTAT TAAAATTTTT GCATGTTTAA AATAAGTTTA   
  
  
- TTATTTTAGT TAAAAAATTA ATCAAAAAAA TTAATTATTT TTTTTATGTA ATTTATGAAG GGCACATAGT   
  
  
- ATTTTAGGTG AATATCATGA CACACGTGTT ACTTTGGATT AGGATTTTCT GTTAGATTTT TATCTCCGCT   
  
  
- AAACTTTTTA ATGAAAAGAT AAAATAGTAT TTTTATAATT TATTATATTA AATTAATAAA TAAATTATAA   
  
  
- TTATTTATTT TATTAAATTA TAACATGTCA TTAACTTTTA AAAAACTCAG CACAATTTAG ATGAGTAAAT   
  
  
- TAAAAATAAT GATATGTAGT TATAAGTATC AAAAATAAAA ATATTTGTTT ATTTAAAAAT TTTAAAAGCT   
  
  
- AAAACATATT TTATAAACCC ATTAATACAA AATTATGTCA CTAATTAACA TAAAACTCTA GTTTTATCCA   
  
  
- AAATTGATGT CGTTTTACTA ATAACCTAA

+     MNF1

| Site Name | Organism | Position | Strand | Matrix score. | sequence | function |
| --- | --- | --- | --- | --- | --- | --- |
| MNF1 | Zea mays | 359 | - | 7 | GTGCCC(A/T)(A/T) | light responsive element |

> 2018/04/13 10:10:12  
+ TTTTTGTTAG GGTTTTTCGT TTCTTTCTTC TTTAAGTTCT TTACACAATA TTTCTTGTCG ACTTCGGACA   
  
  
+ TAGATGAGAG AGAGAGAGAG AGGGAGAGAG GAAGACGAAG AGGGTGATAC ACATACTTCA GACACTTCAG   
  
  
+ AAAGTGGCCA CGATATCCGA GCCGATAATT TTCCCAACCC GGCACCTGGT CTAAAACAAT AATCTTCTAC   
  
  
+ TGTGTGAGAA ACCTAAAGAG TGATAAAGGT TTAAAGGTTA GGCTCGAAGA ATGCAGTCGA CCACGCAGTC   
  
  
+ CAGCGGTCGA TCGTTGACCC AGGGTGAAGA CTGGTGGAGA CCGAACCAAT GGGCAGAAAA GGTGTTGGGC   
  
  
+ TTGAGCAATT GGGCACCGAC TAAACAGTAA AATAGACAAC TATATACACA CAACCGCCCA TACCTCGGCT   
  
  
+ GAAGCCCAAT CTGCATCTAT GCCTAGACAA ACTGCTTTCT CTTTTTTCAA TTTCTCCCTC TCCAAAGGAT   
  
  
+ TCGTAAAGCA GTTCTTTTCT CAAACTGAGC GAGCATTTAG TCTTCTTTCT TTACTTAGGT GGCGGGGCTT   
  
  
+ GCCACGCCGC GCAAGGTGCA CTGCTTTTTC CTTTATGTTT ATTGGGTTTT CTCCTTCTTT TGACTTCCTC   
  
  
+ TCTCTTCCTC TCTCTCTCTC TCTCTCTCCC CTCGGCTCAG TTGCTTAAAC TGCCGGGCCG AGAGACTCCT   
  
  
+ ATACTGGCCT CTGGCCCACC CCGTCACCGG ACCAGATACG CACCTGGGGT GCATCGTACT GTACTCTTTC   
  
  
+ GTGCAAAGTC TAGGCGGGGG CCCTTGGAAC AACTGACAAA TGGACACCCT GGCTCAGCGA ACGAAACTGA   
  
  
+ AAGACAATGT ACAACGAAAT GAGTAAACAG GGAGAGAAAG AGAAGTAGAG GATCAGTAGA AAAGGAAAAA   
  
  
+ AATAATTAAA GATTTTATTT CGAAATTGTA TGTACAAAAA GGAACACGTA CATAAAAGAG TTCATTTATC   
  
  
+ TTTTTATTAA TAAATAATGG TTTGAATAAA GGATATAATA ATTTTAAAAA CGTACAAATT TTATTCAAAT   
  
  
+ AATAAAATCA ATTTTTTAAT TAGTTTTTTT AATTAATAAA AAAAATACAT TAAATACTTC CCGTGTATCA   
  
  
+ TAAAATCCAC TTATAGTACT GTGTGCACAA TGAAACCTAA TCCTAAAAGA CAATCTAAAA ATAGAGGCGA   
  
  
+ TTTGAAAAAT TACTTTTCTA TTTTATCATA AAAATATTAA ATAATATAAT TTAATTATTT ATTTAATATT   
  
  
+ AATAAATAAA ATAATTTAAT ATTGTACAGT AATTGAAAAT TTTTTGAGTC GTGTTAAATC TACTCATTTA   
  
  
+ ATTTTTATTA CTATACATCA ATATTCATAG TTTTTATTTT TATAAACAAA TAAATTTTTA AAATTTTCGA   
  
  
+ TTTTGTATAA AATATTTGGG TAATTATGTT TTAATACAGT GATTAATTGT ATTTTGAGAT CAAAATAGGT   
  
  
+ TTTAACTACA GCAAAATGAT TATTGGATT  

- AAAAACAATC CCAAAAAGCA AAGAAAGAAG AAATTCAAGA AATGTGTTAT AAAGAACAGC TGAAGCCTGT   
  
  
- ATCTACTCTC TCTCTCTCTC TCCCTCTCTC CTTCTGCTTC TCCCACTATG TGTATGAAGT CTGTGAAGTC   
  
  
- TTTCACCGGT GCTATAGGCT CGGCTATTAA AAGGGTTGGG CCGTGGACCA GATTTTGTTA TTAGAAGATG   
  
  
- ACACACTCTT TGGATTTCTC ACTATTTCCA AATTTCCAAT CCGAGCTTCT TACGTCAGCT GGTGCGTCAG   
  
  
- GTCGCCAGCT AGCAACTGGG TCCCACTTCT GACCACCTCT GGCTTGGTTA CCCGTCTTTT CCACAACCCG   
  
  
- AACTCGTTAA CCCGTGGCTG ATTTGTCATT TTATCTGTTG ATATATGTGT GTTGGCGGGT ATGGAGCCGA   
  
  
- CTTCGGGTTA GACGTAGATA CGGATCTGTT TGACGAAAGA GAAAAAAGTT AAAGAGGGAG AGGTTTCCTA   
  
  
- AGCATTTCGT CAAGAAAAGA GTTTGACTCG CTCGTAAATC AGAAGAAAGA AATGAATCCA CCGCCCCGAA   
  
  
- CGGTGCGGCG CGTTCCACGT GACGAAAAAG GAAATACAAA TAACCCAAAA GAGGAAGAAA ACTGAAGGAG   
  
  
- AGAGAAGGAG AGAGAGAGAG AGAGAGAGGG GAGCCGAGTC AACGAATTTG ACGGCCCGGC TCTCTGAGGA   
  
  
- TATGACCGGA GACCGGGTGG GGCAGTGGCC TGGTCTATGC GTGGACCCCA CGTAGCATGA CATGAGAAAG   
  
  
- CACGTTTCAG ATCCGCCCCC GGGAACCTTG TTGACTGTTT ACCTGTGGGA CCGAGTCGCT TGCTTTGACT   
  
  
- TTCTGTTACA TGTTGCTTTA CTCATTTGTC CCTCTCTTTC TCTTCATCTC CTAGTCATCT TTTCCTTTTT   
  
  
- TTATTAATTT CTAAAATAAA GCTTTAACAT ACATGTTTTT CCTTGTGCAT GTATTTTCTC AAGTAAATAG   
  
  
- AAAAATAATT ATTTATTACC AAACTTATTT CCTATATTAT TAAAATTTTT GCATGTTTAA AATAAGTTTA   
  
  
- TTATTTTAGT TAAAAAATTA ATCAAAAAAA TTAATTATTT TTTTTATGTA ATTTATGAAG GGCACATAGT   
  
  
- ATTTTAGGTG AATATCATGA CACACGTGTT ACTTTGGATT AGGATTTTCT GTTAGATTTT TATCTCCGCT   
  
  
- AAACTTTTTA ATGAAAAGAT AAAATAGTAT TTTTATAATT TATTATATTA AATTAATAAA TAAATTATAA   
  
  
- TTATTTATTT TATTAAATTA TAACATGTCA TTAACTTTTA AAAAACTCAG CACAATTTAG ATGAGTAAAT   
  
  
- TAAAAATAAT GATATGTAGT TATAAGTATC AAAAATAAAA ATATTTGTTT ATTTAAAAAT TTTAAAAGCT   
  
  
- AAAACATATT TTATAAACCC ATTAATACAA AATTATGTCA CTAATTAACA TAAAACTCTA GTTTTATCCA   
  
  
- AAATTGATGT CGTTTTACTA ATAACCTAA

+     MRE

| Site Name | Organism | Position | Strand | Matrix score. | sequence | function |
| --- | --- | --- | --- | --- | --- | --- |
| MRE | Petroselinum crispum | 1154 | + | 7 | AACCTAA | MYB binding site involved in light responsiveness |
| MRE | Petroselinum crispum | 220 | + | 7 | AACCTAA | MYB binding site involved in light responsiveness |

> 2018/04/13 10:10:12  
+ TTTTTGTTAG GGTTTTTCGT TTCTTTCTTC TTTAAGTTCT TTACACAATA TTTCTTGTCG ACTTCGGACA   
  
  
+ TAGATGAGAG AGAGAGAGAG AGGGAGAGAG GAAGACGAAG AGGGTGATAC ACATACTTCA GACACTTCAG   
  
  
+ AAAGTGGCCA CGATATCCGA GCCGATAATT TTCCCAACCC GGCACCTGGT CTAAAACAAT AATCTTCTAC   
  
  
+ TGTGTGAGAA ACCTAAAGAG TGATAAAGGT TTAAAGGTTA GGCTCGAAGA ATGCAGTCGA CCACGCAGTC   
  
  
+ CAGCGGTCGA TCGTTGACCC AGGGTGAAGA CTGGTGGAGA CCGAACCAAT GGGCAGAAAA GGTGTTGGGC   
  
  
+ TTGAGCAATT GGGCACCGAC TAAACAGTAA AATAGACAAC TATATACACA CAACCGCCCA TACCTCGGCT   
  
  
+ GAAGCCCAAT CTGCATCTAT GCCTAGACAA ACTGCTTTCT CTTTTTTCAA TTTCTCCCTC TCCAAAGGAT   
  
  
+ TCGTAAAGCA GTTCTTTTCT CAAACTGAGC GAGCATTTAG TCTTCTTTCT TTACTTAGGT GGCGGGGCTT   
  
  
+ GCCACGCCGC GCAAGGTGCA CTGCTTTTTC CTTTATGTTT ATTGGGTTTT CTCCTTCTTT TGACTTCCTC   
  
  
+ TCTCTTCCTC TCTCTCTCTC TCTCTCTCCC CTCGGCTCAG TTGCTTAAAC TGCCGGGCCG AGAGACTCCT   
  
  
+ ATACTGGCCT CTGGCCCACC CCGTCACCGG ACCAGATACG CACCTGGGGT GCATCGTACT GTACTCTTTC   
  
  
+ GTGCAAAGTC TAGGCGGGGG CCCTTGGAAC AACTGACAAA TGGACACCCT GGCTCAGCGA ACGAAACTGA   
  
  
+ AAGACAATGT ACAACGAAAT GAGTAAACAG GGAGAGAAAG AGAAGTAGAG GATCAGTAGA AAAGGAAAAA   
  
  
+ AATAATTAAA GATTTTATTT CGAAATTGTA TGTACAAAAA GGAACACGTA CATAAAAGAG TTCATTTATC   
  
  
+ TTTTTATTAA TAAATAATGG TTTGAATAAA GGATATAATA ATTTTAAAAA CGTACAAATT TTATTCAAAT   
  
  
+ AATAAAATCA ATTTTTTAAT TAGTTTTTTT AATTAATAAA AAAAATACAT TAAATACTTC CCGTGTATCA   
  
  
+ TAAAATCCAC TTATAGTACT GTGTGCACAA TGAAACCTAA TCCTAAAAGA CAATCTAAAA ATAGAGGCGA   
  
  
+ TTTGAAAAAT TACTTTTCTA TTTTATCATA AAAATATTAA ATAATATAAT TTAATTATTT ATTTAATATT   
  
  
+ AATAAATAAA ATAATTTAAT ATTGTACAGT AATTGAAAAT TTTTTGAGTC GTGTTAAATC TACTCATTTA   
  
  
+ ATTTTTATTA CTATACATCA ATATTCATAG TTTTTATTTT TATAAACAAA TAAATTTTTA AAATTTTCGA   
  
  
+ TTTTGTATAA AATATTTGGG TAATTATGTT TTAATACAGT GATTAATTGT ATTTTGAGAT CAAAATAGGT   
  
  
+ TTTAACTACA GCAAAATGAT TATTGGATT  

- AAAAACAATC CCAAAAAGCA AAGAAAGAAG AAATTCAAGA AATGTGTTAT AAAGAACAGC TGAAGCCTGT   
  
  
- ATCTACTCTC TCTCTCTCTC TCCCTCTCTC CTTCTGCTTC TCCCACTATG TGTATGAAGT CTGTGAAGTC   
  
  
- TTTCACCGGT GCTATAGGCT CGGCTATTAA AAGGGTTGGG CCGTGGACCA GATTTTGTTA TTAGAAGATG   
  
  
- ACACACTCTT TGGATTTCTC ACTATTTCCA AATTTCCAAT CCGAGCTTCT TACGTCAGCT GGTGCGTCAG   
  
  
- GTCGCCAGCT AGCAACTGGG TCCCACTTCT GACCACCTCT GGCTTGGTTA CCCGTCTTTT CCACAACCCG   
  
  
- AACTCGTTAA CCCGTGGCTG ATTTGTCATT TTATCTGTTG ATATATGTGT GTTGGCGGGT ATGGAGCCGA   
  
  
- CTTCGGGTTA GACGTAGATA CGGATCTGTT TGACGAAAGA GAAAAAAGTT AAAGAGGGAG AGGTTTCCTA   
  
  
- AGCATTTCGT CAAGAAAAGA GTTTGACTCG CTCGTAAATC AGAAGAAAGA AATGAATCCA CCGCCCCGAA   
  
  
- CGGTGCGGCG CGTTCCACGT GACGAAAAAG GAAATACAAA TAACCCAAAA GAGGAAGAAA ACTGAAGGAG   
  
  
- AGAGAAGGAG AGAGAGAGAG AGAGAGAGGG GAGCCGAGTC AACGAATTTG ACGGCCCGGC TCTCTGAGGA   
  
  
- TATGACCGGA GACCGGGTGG GGCAGTGGCC TGGTCTATGC GTGGACCCCA CGTAGCATGA CATGAGAAAG   
  
  
- CACGTTTCAG ATCCGCCCCC GGGAACCTTG TTGACTGTTT ACCTGTGGGA CCGAGTCGCT TGCTTTGACT   
  
  
- TTCTGTTACA TGTTGCTTTA CTCATTTGTC CCTCTCTTTC TCTTCATCTC CTAGTCATCT TTTCCTTTTT   
  
  
- TTATTAATTT CTAAAATAAA GCTTTAACAT ACATGTTTTT CCTTGTGCAT GTATTTTCTC AAGTAAATAG   
  
  
- AAAAATAATT ATTTATTACC AAACTTATTT CCTATATTAT TAAAATTTTT GCATGTTTAA AATAAGTTTA   
  
  
- TTATTTTAGT TAAAAAATTA ATCAAAAAAA TTAATTATTT TTTTTATGTA ATTTATGAAG GGCACATAGT   
  
  
- ATTTTAGGTG AATATCATGA CACACGTGTT ACTTTGGATT AGGATTTTCT GTTAGATTTT TATCTCCGCT   
  
  
- AAACTTTTTA ATGAAAAGAT AAAATAGTAT TTTTATAATT TATTATATTA AATTAATAAA TAAATTATAA   
  
  
- TTATTTATTT TATTAAATTA TAACATGTCA TTAACTTTTA AAAAACTCAG CACAATTTAG ATGAGTAAAT   
  
  
- TAAAAATAAT GATATGTAGT TATAAGTATC AAAAATAAAA ATATTTGTTT ATTTAAAAAT TTTAAAAGCT   
  
  
- AAAACATATT TTATAAACCC ATTAATACAA AATTATGTCA CTAATTAACA TAAAACTCTA GTTTTATCCA   
  
  
- AAATTGATGT CGTTTTACTA ATAACCTAA

+     Sp1

| Site Name | Organism | Position | Strand | Matrix score. | sequence | function |
| --- | --- | --- | --- | --- | --- | --- |
| Sp1 | Oryza sativa | 404 | - | 6 | GGGCGG | light responsive element |
| Sp1 | Zea mays | 716 | + | 5.5 | CC(G/A)CCC | light responsive element |

> 2018/04/13 10:10:12  
+ TTTTTGTTAG GGTTTTTCGT TTCTTTCTTC TTTAAGTTCT TTACACAATA TTTCTTGTCG ACTTCGGACA   
  
  
+ TAGATGAGAG AGAGAGAGAG AGGGAGAGAG GAAGACGAAG AGGGTGATAC ACATACTTCA GACACTTCAG   
  
  
+ AAAGTGGCCA CGATATCCGA GCCGATAATT TTCCCAACCC GGCACCTGGT CTAAAACAAT AATCTTCTAC   
  
  
+ TGTGTGAGAA ACCTAAAGAG TGATAAAGGT TTAAAGGTTA GGCTCGAAGA ATGCAGTCGA CCACGCAGTC   
  
  
+ CAGCGGTCGA TCGTTGACCC AGGGTGAAGA CTGGTGGAGA CCGAACCAAT GGGCAGAAAA GGTGTTGGGC   
  
  
+ TTGAGCAATT GGGCACCGAC TAAACAGTAA AATAGACAAC TATATACACA CAACCGCCCA TACCTCGGCT   
  
  
+ GAAGCCCAAT CTGCATCTAT GCCTAGACAA ACTGCTTTCT CTTTTTTCAA TTTCTCCCTC TCCAAAGGAT   
  
  
+ TCGTAAAGCA GTTCTTTTCT CAAACTGAGC GAGCATTTAG TCTTCTTTCT TTACTTAGGT GGCGGGGCTT   
  
  
+ GCCACGCCGC GCAAGGTGCA CTGCTTTTTC CTTTATGTTT ATTGGGTTTT CTCCTTCTTT TGACTTCCTC   
  
  
+ TCTCTTCCTC TCTCTCTCTC TCTCTCTCCC CTCGGCTCAG TTGCTTAAAC TGCCGGGCCG AGAGACTCCT   
  
  
+ ATACTGGCCT CTGGCCCACC CCGTCACCGG ACCAGATACG CACCTGGGGT GCATCGTACT GTACTCTTTC   
  
  
+ GTGCAAAGTC TAGGCGGGGG CCCTTGGAAC AACTGACAAA TGGACACCCT GGCTCAGCGA ACGAAACTGA   
  
  
+ AAGACAATGT ACAACGAAAT GAGTAAACAG GGAGAGAAAG AGAAGTAGAG GATCAGTAGA AAAGGAAAAA   
  
  
+ AATAATTAAA GATTTTATTT CGAAATTGTA TGTACAAAAA GGAACACGTA CATAAAAGAG TTCATTTATC   
  
  
+ TTTTTATTAA TAAATAATGG TTTGAATAAA GGATATAATA ATTTTAAAAA CGTACAAATT TTATTCAAAT   
  
  
+ AATAAAATCA ATTTTTTAAT TAGTTTTTTT AATTAATAAA AAAAATACAT TAAATACTTC CCGTGTATCA   
  
  
+ TAAAATCCAC TTATAGTACT GTGTGCACAA TGAAACCTAA TCCTAAAAGA CAATCTAAAA ATAGAGGCGA   
  
  
+ TTTGAAAAAT TACTTTTCTA TTTTATCATA AAAATATTAA ATAATATAAT TTAATTATTT ATTTAATATT   
  
  
+ AATAAATAAA ATAATTTAAT ATTGTACAGT AATTGAAAAT TTTTTGAGTC GTGTTAAATC TACTCATTTA   
  
  
+ ATTTTTATTA CTATACATCA ATATTCATAG TTTTTATTTT TATAAACAAA TAAATTTTTA AAATTTTCGA   
  
  
+ TTTTGTATAA AATATTTGGG TAATTATGTT TTAATACAGT GATTAATTGT ATTTTGAGAT CAAAATAGGT   
  
  
+ TTTAACTACA GCAAAATGAT TATTGGATT  

- AAAAACAATC CCAAAAAGCA AAGAAAGAAG AAATTCAAGA AATGTGTTAT AAAGAACAGC TGAAGCCTGT   
  
  
- ATCTACTCTC TCTCTCTCTC TCCCTCTCTC CTTCTGCTTC TCCCACTATG TGTATGAAGT CTGTGAAGTC   
  
  
- TTTCACCGGT GCTATAGGCT CGGCTATTAA AAGGGTTGGG CCGTGGACCA GATTTTGTTA TTAGAAGATG   
  
  
- ACACACTCTT TGGATTTCTC ACTATTTCCA AATTTCCAAT CCGAGCTTCT TACGTCAGCT GGTGCGTCAG   
  
  
- GTCGCCAGCT AGCAACTGGG TCCCACTTCT GACCACCTCT GGCTTGGTTA CCCGTCTTTT CCACAACCCG   
  
  
- AACTCGTTAA CCCGTGGCTG ATTTGTCATT TTATCTGTTG ATATATGTGT GTTGGCGGGT ATGGAGCCGA   
  
  
- CTTCGGGTTA GACGTAGATA CGGATCTGTT TGACGAAAGA GAAAAAAGTT AAAGAGGGAG AGGTTTCCTA   
  
  
- AGCATTTCGT CAAGAAAAGA GTTTGACTCG CTCGTAAATC AGAAGAAAGA AATGAATCCA CCGCCCCGAA   
  
  
- CGGTGCGGCG CGTTCCACGT GACGAAAAAG GAAATACAAA TAACCCAAAA GAGGAAGAAA ACTGAAGGAG   
  
  
- AGAGAAGGAG AGAGAGAGAG AGAGAGAGGG GAGCCGAGTC AACGAATTTG ACGGCCCGGC TCTCTGAGGA   
  
  
- TATGACCGGA GACCGGGTGG GGCAGTGGCC TGGTCTATGC GTGGACCCCA CGTAGCATGA CATGAGAAAG   
  
  
- CACGTTTCAG ATCCGCCCCC GGGAACCTTG TTGACTGTTT ACCTGTGGGA CCGAGTCGCT TGCTTTGACT   
  
  
- TTCTGTTACA TGTTGCTTTA CTCATTTGTC CCTCTCTTTC TCTTCATCTC CTAGTCATCT TTTCCTTTTT   
  
  
- TTATTAATTT CTAAAATAAA GCTTTAACAT ACATGTTTTT CCTTGTGCAT GTATTTTCTC AAGTAAATAG   
  
  
- AAAAATAATT ATTTATTACC AAACTTATTT CCTATATTAT TAAAATTTTT GCATGTTTAA AATAAGTTTA   
  
  
- TTATTTTAGT TAAAAAATTA ATCAAAAAAA TTAATTATTT TTTTTATGTA ATTTATGAAG GGCACATAGT   
  
  
- ATTTTAGGTG AATATCATGA CACACGTGTT ACTTTGGATT AGGATTTTCT GTTAGATTTT TATCTCCGCT   
  
  
- AAACTTTTTA ATGAAAAGAT AAAATAGTAT TTTTATAATT TATTATATTA AATTAATAAA TAAATTATAA   
  
  
- TTATTTATTT TATTAAATTA TAACATGTCA TTAACTTTTA AAAAACTCAG CACAATTTAG ATGAGTAAAT   
  
  
- TAAAAATAAT GATATGTAGT TATAAGTATC AAAAATAAAA ATATTTGTTT ATTTAAAAAT TTTAAAAGCT   
  
  
- AAAACATATT TTATAAACCC ATTAATACAA AATTATGTCA CTAATTAACA TAAAACTCTA GTTTTATCCA   
  
  
- AAATTGATGT CGTTTTACTA ATAACCTAA

+     TATA-box

| Site Name | Organism | Position | Strand | Matrix score. | sequence | function |
| --- | --- | --- | --- | --- | --- | --- |
| TATA-box | Helianthus annuus | 1404 | - | 6 | TATACA | core promoter element around -30 of transcription start |
| TATA-box | Arabidopsis thaliana | 1371 | + | 6 | TATAAA | core promoter element around -30 of transcription start |
| TATA-box | Lycopersicon esculentum | 1386 | + | 5 | TTTTA | core promoter element around -30 of transcription start |
| TATA-box | Arabidopsis thaliana | 1369 | - | 6 | TATAAA | core promoter element around -30 of transcription start |
| TATA-box | Arabidopsis thaliana | 1235 | - | 4 | TATA | core promoter element around -30 of transcription start |
| TATA-box | Nicotiana tabacum | 1174 | + | 9 | tcTATAAAta | core promoter element around -30 of transcription start |
| TATA-box | Brassica oleracea | 1234 | + | 7 | ATATAAT | core promoter element around -30 of transcription start |
| TATA-box | Lycopersicon esculentum | 1176 | - | 5 | TTTTA | core promoter element around -30 of transcription start |
| TATA-box | Arabidopsis thaliana | 1132 | - | 4 | TATA | core promoter element around -30 of transcription start |
| TATA-box | Lycopersicon esculentum | 1121 | - | 5 | TTTTA | core promoter element around -30 of transcription start |
| TATA-box | Glycine max | 1084 | + | 5 | TAATA | core promoter element around -30 of transcription start |
| TATA-box | Lycopersicon esculentum | 1053 | - | 5 | TTTTA | core promoter element around -30 of transcription start |
| TATA-box | Arabidopsis thaliana | 1406 | + | 6 | TATAAA | core promoter element around -30 of transcription start |
| TATA-box | Lycopersicon esculentum | 1470 | + | 5 | TTTTA | core promoter element around -30 of transcription start |
| TATA-box | Lycopersicon esculentum | 1164 | - | 5 | TTTTA | core promoter element around -30 of transcription start |
| TATA-box | Glycine max | 1225 | - | 5 | TAATA | core promoter element around -30 of transcription start |
| TATA-box | Lycopersicon esculentum | 1211 | + | 5 | TTTTA | core promoter element around -30 of transcription start |
| TATA-box | Lycopersicon esculentum | 1219 | - | 5 | TTTTA | core promoter element around -30 of transcription start |
| TATA-box | Lycopersicon esculentum | 1025 | - | 5 | TTTTA | core promoter element around -30 of transcription start |
| TATA-box | Lycopersicon esculentum | 1408 | - | 5 | TTTTA | core promoter element around -30 of transcription start |
| TATA-box | Oryza sativa | 1401 | - | 7 | TACAAAA | core promoter element around -30 of transcription start |
| TATA-box | Lycopersicon esculentum | 1077 | + | 5 | TTTTA | core promoter element around -30 of transcription start |
| TATA-box | Lycopersicon esculentum | 1087 | - | 5 | TTTTA | core promoter element around -30 of transcription start |
| TATA-box | Lycopersicon esculentum | 1022 | + | 5 | TTTTA | core promoter element around -30 of transcription start |
| TATA-box | Glycine max | 1432 | + | 5 | TAATA | core promoter element around -30 of transcription start |
| TATA-box | Arabidopsis thaliana | 1363 | - | 11 | TATAAATATAAA | core promoter element around -30 of transcription start |
| TATA-box | Zea mays | 1023 | + | 8 | TTTAAAAA | core promoter element around -30 of transcription start |
| TATA-box | Arabidopsis thaliana | 1014 | - | 4 | TATA | core promoter element around -30 of transcription start |
| TATA-box | Glycine max | 1260 | + | 5 | TAATA | core promoter element around -30 of transcription start |
| TATA-box | Lycopersicon esculentum | 982 | + | 5 | TTTTA | core promoter element around -30 of transcription start |
| TATA-box | Arabidopsis thaliana | 1368 | - | 7 | TATAAAA | core promoter element around -30 of transcription start |
| TATA-box | Arabidopsis thaliana | 1364 | - | 9 | TAAAAATAA | core promoter element around -30 of transcription start |
| TATA-box | Arabidopsis thaliana | 1131 | - | 5 | TATAA | core promoter element around -30 of transcription start |
| TATA-box | Glycine max | 985 | - | 5 | TAATA | core promoter element around -30 of transcription start |
| TATA-box | Lycopersicon esculentum | 1064 | + | 5 | TTTTA | core promoter element around -30 of transcription start |
| TATA-box | Arabidopsis thaliana | 393 | + | 4 | TATA | core promoter element around -30 of transcription start |
| TATA-box | Glycine max | 1257 | - | 5 | TAATA | core promoter element around -30 of transcription start |
| TATA-box | Glycine max | 1254 | + | 5 | TAATA | core promoter element around -30 of transcription start |
| TATA-box | Glycine max | 1050 | + | 5 | TAATA | core promoter element around -30 of transcription start |
| TATA-box | Glycine max | 1232 | + | 5 | TAATA | core promoter element around -30 of transcription start |
| TATA-box | Arabidopsis thaliana | 391 | + | 4 | TATA | core promoter element around -30 of transcription start |
| TATA-box | Lycopersicon esculentum | 378 | - | 5 | TTTTA | core promoter element around -30 of transcription start |
| TATA-box | Lycopersicon esculentum | 1389 | - | 5 | TTTTA | core promoter element around -30 of transcription start |
| TATA-box | Glycine max | 1016 | + | 5 | TAATA | core promoter element around -30 of transcription start |
| TATA-box | Lycopersicon esculentum | 1333 | + | 5 | TTTTA | core promoter element around -30 of transcription start |
| TATA-box | Lycopersicon esculentum | 1429 | + | 5 | TTTTA | core promoter element around -30 of transcription start |
| TATA-box | Lycopersicon esculentum | 1039 | + | 5 | TTTTA | core promoter element around -30 of transcription start |
| TATA-box | Lycopersicon esculentum | 192 | - | 5 | TTTTA | core promoter element around -30 of transcription start |
| TATA-box | Arabidopsis thaliana | 917 | + | 8 | TAAAGATT | core promoter element around -30 of transcription start |
| TATA-box | Arabidopsis thaliana | 700 | + | 4 | TATA | core promoter element around -30 of transcription start |
| TATA-box | Brassica oleracea | 1013 | + | 7 | ATATAAT | core promoter element around -30 of transcription start |
| TATA-box | Zea mays | 1385 | - | 8 | TTTAAAAA | core promoter element around -30 of transcription start |
| TATA-box | Arabidopsis thaliana | 1342 | - | 4 | TATA | core promoter element around -30 of transcription start |
| TATA-box | Lycopersicon esculentum | 1267 | - | 5 | TTTTA | core promoter element around -30 of transcription start |
| TATA-box | Oryza sativa | 943 | + | 7 | TACAAAA | core promoter element around -30 of transcription start |
| TATA-box | Lycopersicon esculentum | 923 | + | 5 | TTTTA | core promoter element around -30 of transcription start |
| TATA-box | Lycopersicon esculentum | 963 | - | 5 | TTTTA | core promoter element around -30 of transcription start |
| TATA-box | Oryza sativa | 959 | + | 8 | TACATAAA | core promoter element around -30 of transcription start |
| TATA-box | Arabidopsis thaliana | 1370 | - | 5 | TATAA | core promoter element around -30 of transcription start |
| TATA-box | Glycine max | 1336 | - | 5 | TAATA | core promoter element around -30 of transcription start |
| TATA-box | Glycine max | 988 | + | 5 | TAATA | core promoter element around -30 of transcription start |
| TATA-box | Glycine max | 1277 | + | 5 | TAATA | core promoter element around -30 of transcription start |
| TATA-box | Lycopersicon esculentum | 1362 | + | 5 | TTTTA | core promoter element around -30 of transcription start |

> 2018/04/13 10:10:12  
+ TTTTTGTTAG GGTTTTTCGT TTCTTTCTTC TTTAAGTTCT TTACACAATA TTTCTTGTCG ACTTCGGACA   
  
  
+ TAGATGAGAG AGAGAGAGAG AGGGAGAGAG GAAGACGAAG AGGGTGATAC ACATACTTCA GACACTTCAG   
  
  
+ AAAGTGGCCA CGATATCCGA GCCGATAATT TTCCCAACCC GGCACCTGGT CTAAAACAAT AATCTTCTAC   
  
  
+ TGTGTGAGAA ACCTAAAGAG TGATAAAGGT TTAAAGGTTA GGCTCGAAGA ATGCAGTCGA CCACGCAGTC   
  
  
+ CAGCGGTCGA TCGTTGACCC AGGGTGAAGA CTGGTGGAGA CCGAACCAAT GGGCAGAAAA GGTGTTGGGC   
  
  
+ TTGAGCAATT GGGCACCGAC TAAACAGTAA AATAGACAAC TATATACACA CAACCGCCCA TACCTCGGCT   
  
  
+ GAAGCCCAAT CTGCATCTAT GCCTAGACAA ACTGCTTTCT CTTTTTTCAA TTTCTCCCTC TCCAAAGGAT   
  
  
+ TCGTAAAGCA GTTCTTTTCT CAAACTGAGC GAGCATTTAG TCTTCTTTCT TTACTTAGGT GGCGGGGCTT   
  
  
+ GCCACGCCGC GCAAGGTGCA CTGCTTTTTC CTTTATGTTT ATTGGGTTTT CTCCTTCTTT TGACTTCCTC   
  
  
+ TCTCTTCCTC TCTCTCTCTC TCTCTCTCCC CTCGGCTCAG TTGCTTAAAC TGCCGGGCCG AGAGACTCCT   
  
  
+ ATACTGGCCT CTGGCCCACC CCGTCACCGG ACCAGATACG CACCTGGGGT GCATCGTACT GTACTCTTTC   
  
  
+ GTGCAAAGTC TAGGCGGGGG CCCTTGGAAC AACTGACAAA TGGACACCCT GGCTCAGCGA ACGAAACTGA   
  
  
+ AAGACAATGT ACAACGAAAT GAGTAAACAG GGAGAGAAAG AGAAGTAGAG GATCAGTAGA AAAGGAAAAA   
  
  
+ AATAATTAAA GATTTTATTT CGAAATTGTA TGTACAAAAA GGAACACGTA CATAAAAGAG TTCATTTATC   
  
  
+ TTTTTATTAA TAAATAATGG TTTGAATAAA GGATATAATA ATTTTAAAAA CGTACAAATT TTATTCAAAT   
  
  
+ AATAAAATCA ATTTTTTAAT TAGTTTTTTT AATTAATAAA AAAAATACAT TAAATACTTC CCGTGTATCA   
  
  
+ TAAAATCCAC TTATAGTACT GTGTGCACAA TGAAACCTAA TCCTAAAAGA CAATCTAAAA ATAGAGGCGA   
  
  
+ TTTGAAAAAT TACTTTTCTA TTTTATCATA AAAATATTAA ATAATATAAT TTAATTATTT ATTTAATATT   
  
  
+ AATAAATAAA ATAATTTAAT ATTGTACAGT AATTGAAAAT TTTTTGAGTC GTGTTAAATC TACTCATTTA   
  
  
+ ATTTTTATTA CTATACATCA ATATTCATAG TTTTTATTTT TATAAACAAA TAAATTTTTA AAATTTTCGA   
  
  
+ TTTTGTATAA AATATTTGGG TAATTATGTT TTAATACAGT GATTAATTGT ATTTTGAGAT CAAAATAGGT   
  
  
+ TTTAACTACA GCAAAATGAT TATTGGATT  

- AAAAACAATC CCAAAAAGCA AAGAAAGAAG AAATTCAAGA AATGTGTTAT AAAGAACAGC TGAAGCCTGT   
  
  
- ATCTACTCTC TCTCTCTCTC TCCCTCTCTC CTTCTGCTTC TCCCACTATG TGTATGAAGT CTGTGAAGTC   
  
  
- TTTCACCGGT GCTATAGGCT CGGCTATTAA AAGGGTTGGG CCGTGGACCA GATTTTGTTA TTAGAAGATG   
  
  
- ACACACTCTT TGGATTTCTC ACTATTTCCA AATTTCCAAT CCGAGCTTCT TACGTCAGCT GGTGCGTCAG   
  
  
- GTCGCCAGCT AGCAACTGGG TCCCACTTCT GACCACCTCT GGCTTGGTTA CCCGTCTTTT CCACAACCCG   
  
  
- AACTCGTTAA CCCGTGGCTG ATTTGTCATT TTATCTGTTG ATATATGTGT GTTGGCGGGT ATGGAGCCGA   
  
  
- CTTCGGGTTA GACGTAGATA CGGATCTGTT TGACGAAAGA GAAAAAAGTT AAAGAGGGAG AGGTTTCCTA   
  
  
- AGCATTTCGT CAAGAAAAGA GTTTGACTCG CTCGTAAATC AGAAGAAAGA AATGAATCCA CCGCCCCGAA   
  
  
- CGGTGCGGCG CGTTCCACGT GACGAAAAAG GAAATACAAA TAACCCAAAA GAGGAAGAAA ACTGAAGGAG   
  
  
- AGAGAAGGAG AGAGAGAGAG AGAGAGAGGG GAGCCGAGTC AACGAATTTG ACGGCCCGGC TCTCTGAGGA   
  
  
- TATGACCGGA GACCGGGTGG GGCAGTGGCC TGGTCTATGC GTGGACCCCA CGTAGCATGA CATGAGAAAG   
  
  
- CACGTTTCAG ATCCGCCCCC GGGAACCTTG TTGACTGTTT ACCTGTGGGA CCGAGTCGCT TGCTTTGACT   
  
  
- TTCTGTTACA TGTTGCTTTA CTCATTTGTC CCTCTCTTTC TCTTCATCTC CTAGTCATCT TTTCCTTTTT   
  
  
- TTATTAATTT CTAAAATAAA GCTTTAACAT ACATGTTTTT CCTTGTGCAT GTATTTTCTC AAGTAAATAG   
  
  
- AAAAATAATT ATTTATTACC AAACTTATTT CCTATATTAT TAAAATTTTT GCATGTTTAA AATAAGTTTA   
  
  
- TTATTTTAGT TAAAAAATTA ATCAAAAAAA TTAATTATTT TTTTTATGTA ATTTATGAAG GGCACATAGT   
  
  
- ATTTTAGGTG AATATCATGA CACACGTGTT ACTTTGGATT AGGATTTTCT GTTAGATTTT TATCTCCGCT   
  
  
- AAACTTTTTA ATGAAAAGAT AAAATAGTAT TTTTATAATT TATTATATTA AATTAATAAA TAAATTATAA   
  
  
- TTATTTATTT TATTAAATTA TAACATGTCA TTAACTTTTA AAAAACTCAG CACAATTTAG ATGAGTAAAT   
  
  
- TAAAAATAAT GATATGTAGT TATAAGTATC AAAAATAAAA ATATTTGTTT ATTTAAAAAT TTTAAAAGCT   
  
  
- AAAACATATT TTATAAACCC ATTAATACAA AATTATGTCA CTAATTAACA TAAAACTCTA GTTTTATCCA   
  
  
- AAATTGATGT CGTTTTACTA ATAACCTAA

+     TC-rich repeats

| Site Name | Organism | Position | Strand | Matrix score. | sequence | function |
| --- | --- | --- | --- | --- | --- | --- |
| TC-rich repeats | Nicotiana tabacum | 1038 | + | 9 | ATTTTCTTCA | cis-acting element involved in defense and stress responsiveness |

> 2018/04/13 10:10:12  
+ TTTTTGTTAG GGTTTTTCGT TTCTTTCTTC TTTAAGTTCT TTACACAATA TTTCTTGTCG ACTTCGGACA   
  
  
+ TAGATGAGAG AGAGAGAGAG AGGGAGAGAG GAAGACGAAG AGGGTGATAC ACATACTTCA GACACTTCAG   
  
  
+ AAAGTGGCCA CGATATCCGA GCCGATAATT TTCCCAACCC GGCACCTGGT CTAAAACAAT AATCTTCTAC   
  
  
+ TGTGTGAGAA ACCTAAAGAG TGATAAAGGT TTAAAGGTTA GGCTCGAAGA ATGCAGTCGA CCACGCAGTC   
  
  
+ CAGCGGTCGA TCGTTGACCC AGGGTGAAGA CTGGTGGAGA CCGAACCAAT GGGCAGAAAA GGTGTTGGGC   
  
  
+ TTGAGCAATT GGGCACCGAC TAAACAGTAA AATAGACAAC TATATACACA CAACCGCCCA TACCTCGGCT   
  
  
+ GAAGCCCAAT CTGCATCTAT GCCTAGACAA ACTGCTTTCT CTTTTTTCAA TTTCTCCCTC TCCAAAGGAT   
  
  
+ TCGTAAAGCA GTTCTTTTCT CAAACTGAGC GAGCATTTAG TCTTCTTTCT TTACTTAGGT GGCGGGGCTT   
  
  
+ GCCACGCCGC GCAAGGTGCA CTGCTTTTTC CTTTATGTTT ATTGGGTTTT CTCCTTCTTT TGACTTCCTC   
  
  
+ TCTCTTCCTC TCTCTCTCTC TCTCTCTCCC CTCGGCTCAG TTGCTTAAAC TGCCGGGCCG AGAGACTCCT   
  
  
+ ATACTGGCCT CTGGCCCACC CCGTCACCGG ACCAGATACG CACCTGGGGT GCATCGTACT GTACTCTTTC   
  
  
+ GTGCAAAGTC TAGGCGGGGG CCCTTGGAAC AACTGACAAA TGGACACCCT GGCTCAGCGA ACGAAACTGA   
  
  
+ AAGACAATGT ACAACGAAAT GAGTAAACAG GGAGAGAAAG AGAAGTAGAG GATCAGTAGA AAAGGAAAAA   
  
  
+ AATAATTAAA GATTTTATTT CGAAATTGTA TGTACAAAAA GGAACACGTA CATAAAAGAG TTCATTTATC   
  
  
+ TTTTTATTAA TAAATAATGG TTTGAATAAA GGATATAATA ATTTTAAAAA CGTACAAATT TTATTCAAAT   
  
  
+ AATAAAATCA ATTTTTTAAT TAGTTTTTTT AATTAATAAA AAAAATACAT TAAATACTTC CCGTGTATCA   
  
  
+ TAAAATCCAC TTATAGTACT GTGTGCACAA TGAAACCTAA TCCTAAAAGA CAATCTAAAA ATAGAGGCGA   
  
  
+ TTTGAAAAAT TACTTTTCTA TTTTATCATA AAAATATTAA ATAATATAAT TTAATTATTT ATTTAATATT   
  
  
+ AATAAATAAA ATAATTTAAT ATTGTACAGT AATTGAAAAT TTTTTGAGTC GTGTTAAATC TACTCATTTA   
  
  
+ ATTTTTATTA CTATACATCA ATATTCATAG TTTTTATTTT TATAAACAAA TAAATTTTTA AAATTTTCGA   
  
  
+ TTTTGTATAA AATATTTGGG TAATTATGTT TTAATACAGT GATTAATTGT ATTTTGAGAT CAAAATAGGT   
  
  
+ TTTAACTACA GCAAAATGAT TATTGGATT  

- AAAAACAATC CCAAAAAGCA AAGAAAGAAG AAATTCAAGA AATGTGTTAT AAAGAACAGC TGAAGCCTGT   
  
  
- ATCTACTCTC TCTCTCTCTC TCCCTCTCTC CTTCTGCTTC TCCCACTATG TGTATGAAGT CTGTGAAGTC   
  
  
- TTTCACCGGT GCTATAGGCT CGGCTATTAA AAGGGTTGGG CCGTGGACCA GATTTTGTTA TTAGAAGATG   
  
  
- ACACACTCTT TGGATTTCTC ACTATTTCCA AATTTCCAAT CCGAGCTTCT TACGTCAGCT GGTGCGTCAG   
  
  
- GTCGCCAGCT AGCAACTGGG TCCCACTTCT GACCACCTCT GGCTTGGTTA CCCGTCTTTT CCACAACCCG   
  
  
- AACTCGTTAA CCCGTGGCTG ATTTGTCATT TTATCTGTTG ATATATGTGT GTTGGCGGGT ATGGAGCCGA   
  
  
- CTTCGGGTTA GACGTAGATA CGGATCTGTT TGACGAAAGA GAAAAAAGTT AAAGAGGGAG AGGTTTCCTA   
  
  
- AGCATTTCGT CAAGAAAAGA GTTTGACTCG CTCGTAAATC AGAAGAAAGA AATGAATCCA CCGCCCCGAA   
  
  
- CGGTGCGGCG CGTTCCACGT GACGAAAAAG GAAATACAAA TAACCCAAAA GAGGAAGAAA ACTGAAGGAG   
  
  
- AGAGAAGGAG AGAGAGAGAG AGAGAGAGGG GAGCCGAGTC AACGAATTTG ACGGCCCGGC TCTCTGAGGA   
  
  
- TATGACCGGA GACCGGGTGG GGCAGTGGCC TGGTCTATGC GTGGACCCCA CGTAGCATGA CATGAGAAAG   
  
  
- CACGTTTCAG ATCCGCCCCC GGGAACCTTG TTGACTGTTT ACCTGTGGGA CCGAGTCGCT TGCTTTGACT   
  
  
- TTCTGTTACA TGTTGCTTTA CTCATTTGTC CCTCTCTTTC TCTTCATCTC CTAGTCATCT TTTCCTTTTT   
  
  
- TTATTAATTT CTAAAATAAA GCTTTAACAT ACATGTTTTT CCTTGTGCAT GTATTTTCTC AAGTAAATAG   
  
  
- AAAAATAATT ATTTATTACC AAACTTATTT CCTATATTAT TAAAATTTTT GCATGTTTAA AATAAGTTTA   
  
  
- TTATTTTAGT TAAAAAATTA ATCAAAAAAA TTAATTATTT TTTTTATGTA ATTTATGAAG GGCACATAGT   
  
  
- ATTTTAGGTG AATATCATGA CACACGTGTT ACTTTGGATT AGGATTTTCT GTTAGATTTT TATCTCCGCT   
  
  
- AAACTTTTTA ATGAAAAGAT AAAATAGTAT TTTTATAATT TATTATATTA AATTAATAAA TAAATTATAA   
  
  
- TTATTTATTT TATTAAATTA TAACATGTCA TTAACTTTTA AAAAACTCAG CACAATTTAG ATGAGTAAAT   
  
  
- TAAAAATAAT GATATGTAGT TATAAGTATC AAAAATAAAA ATATTTGTTT ATTTAAAAAT TTTAAAAGCT   
  
  
- AAAACATATT TTATAAACCC ATTAATACAA AATTATGTCA CTAATTAACA TAAAACTCTA GTTTTATCCA   
  
  
- AAATTGATGT CGTTTTACTA ATAACCTAA

+     TCA-element

| Site Name | Organism | Position | Strand | Matrix score. | sequence | function |
| --- | --- | --- | --- | --- | --- | --- |
| TCA-element | Brassica oleracea | 334 | + | 9 | CAGAAAAGGA | cis-acting element involved in salicylic acid responsiveness |
| TCA-element | Brassica oleracea | 897 | + | 9 | CAGAAAAGGA | cis-acting element involved in salicylic acid responsiveness |

> 2018/04/13 10:10:12  
+ TTTTTGTTAG GGTTTTTCGT TTCTTTCTTC TTTAAGTTCT TTACACAATA TTTCTTGTCG ACTTCGGACA   
  
  
+ TAGATGAGAG AGAGAGAGAG AGGGAGAGAG GAAGACGAAG AGGGTGATAC ACATACTTCA GACACTTCAG   
  
  
+ AAAGTGGCCA CGATATCCGA GCCGATAATT TTCCCAACCC GGCACCTGGT CTAAAACAAT AATCTTCTAC   
  
  
+ TGTGTGAGAA ACCTAAAGAG TGATAAAGGT TTAAAGGTTA GGCTCGAAGA ATGCAGTCGA CCACGCAGTC   
  
  
+ CAGCGGTCGA TCGTTGACCC AGGGTGAAGA CTGGTGGAGA CCGAACCAAT GGGCAGAAAA GGTGTTGGGC   
  
  
+ TTGAGCAATT GGGCACCGAC TAAACAGTAA AATAGACAAC TATATACACA CAACCGCCCA TACCTCGGCT   
  
  
+ GAAGCCCAAT CTGCATCTAT GCCTAGACAA ACTGCTTTCT CTTTTTTCAA TTTCTCCCTC TCCAAAGGAT   
  
  
+ TCGTAAAGCA GTTCTTTTCT CAAACTGAGC GAGCATTTAG TCTTCTTTCT TTACTTAGGT GGCGGGGCTT   
  
  
+ GCCACGCCGC GCAAGGTGCA CTGCTTTTTC CTTTATGTTT ATTGGGTTTT CTCCTTCTTT TGACTTCCTC   
  
  
+ TCTCTTCCTC TCTCTCTCTC TCTCTCTCCC CTCGGCTCAG TTGCTTAAAC TGCCGGGCCG AGAGACTCCT   
  
  
+ ATACTGGCCT CTGGCCCACC CCGTCACCGG ACCAGATACG CACCTGGGGT GCATCGTACT GTACTCTTTC   
  
  
+ GTGCAAAGTC TAGGCGGGGG CCCTTGGAAC AACTGACAAA TGGACACCCT GGCTCAGCGA ACGAAACTGA   
  
  
+ AAGACAATGT ACAACGAAAT GAGTAAACAG GGAGAGAAAG AGAAGTAGAG GATCAGTAGA AAAGGAAAAA   
  
  
+ AATAATTAAA GATTTTATTT CGAAATTGTA TGTACAAAAA GGAACACGTA CATAAAAGAG TTCATTTATC   
  
  
+ TTTTTATTAA TAAATAATGG TTTGAATAAA GGATATAATA ATTTTAAAAA CGTACAAATT TTATTCAAAT   
  
  
+ AATAAAATCA ATTTTTTAAT TAGTTTTTTT AATTAATAAA AAAAATACAT TAAATACTTC CCGTGTATCA   
  
  
+ TAAAATCCAC TTATAGTACT GTGTGCACAA TGAAACCTAA TCCTAAAAGA CAATCTAAAA ATAGAGGCGA   
  
  
+ TTTGAAAAAT TACTTTTCTA TTTTATCATA AAAATATTAA ATAATATAAT TTAATTATTT ATTTAATATT   
  
  
+ AATAAATAAA ATAATTTAAT ATTGTACAGT AATTGAAAAT TTTTTGAGTC GTGTTAAATC TACTCATTTA   
  
  
+ ATTTTTATTA CTATACATCA ATATTCATAG TTTTTATTTT TATAAACAAA TAAATTTTTA AAATTTTCGA   
  
  
+ TTTTGTATAA AATATTTGGG TAATTATGTT TTAATACAGT GATTAATTGT ATTTTGAGAT CAAAATAGGT   
  
  
+ TTTAACTACA GCAAAATGAT TATTGGATT  

- AAAAACAATC CCAAAAAGCA AAGAAAGAAG AAATTCAAGA AATGTGTTAT AAAGAACAGC TGAAGCCTGT   
  
  
- ATCTACTCTC TCTCTCTCTC TCCCTCTCTC CTTCTGCTTC TCCCACTATG TGTATGAAGT CTGTGAAGTC   
  
  
- TTTCACCGGT GCTATAGGCT CGGCTATTAA AAGGGTTGGG CCGTGGACCA GATTTTGTTA TTAGAAGATG   
  
  
- ACACACTCTT TGGATTTCTC ACTATTTCCA AATTTCCAAT CCGAGCTTCT TACGTCAGCT GGTGCGTCAG   
  
  
- GTCGCCAGCT AGCAACTGGG TCCCACTTCT GACCACCTCT GGCTTGGTTA CCCGTCTTTT CCACAACCCG   
  
  
- AACTCGTTAA CCCGTGGCTG ATTTGTCATT TTATCTGTTG ATATATGTGT GTTGGCGGGT ATGGAGCCGA   
  
  
- CTTCGGGTTA GACGTAGATA CGGATCTGTT TGACGAAAGA GAAAAAAGTT AAAGAGGGAG AGGTTTCCTA   
  
  
- AGCATTTCGT CAAGAAAAGA GTTTGACTCG CTCGTAAATC AGAAGAAAGA AATGAATCCA CCGCCCCGAA   
  
  
- CGGTGCGGCG CGTTCCACGT GACGAAAAAG GAAATACAAA TAACCCAAAA GAGGAAGAAA ACTGAAGGAG   
  
  
- AGAGAAGGAG AGAGAGAGAG AGAGAGAGGG GAGCCGAGTC AACGAATTTG ACGGCCCGGC TCTCTGAGGA   
  
  
- TATGACCGGA GACCGGGTGG GGCAGTGGCC TGGTCTATGC GTGGACCCCA CGTAGCATGA CATGAGAAAG   
  
  
- CACGTTTCAG ATCCGCCCCC GGGAACCTTG TTGACTGTTT ACCTGTGGGA CCGAGTCGCT TGCTTTGACT   
  
  
- TTCTGTTACA TGTTGCTTTA CTCATTTGTC CCTCTCTTTC TCTTCATCTC CTAGTCATCT TTTCCTTTTT   
  
  
- TTATTAATTT CTAAAATAAA GCTTTAACAT ACATGTTTTT CCTTGTGCAT GTATTTTCTC AAGTAAATAG   
  
  
- AAAAATAATT ATTTATTACC AAACTTATTT CCTATATTAT TAAAATTTTT GCATGTTTAA AATAAGTTTA   
  
  
- TTATTTTAGT TAAAAAATTA ATCAAAAAAA TTAATTATTT TTTTTATGTA ATTTATGAAG GGCACATAGT   
  
  
- ATTTTAGGTG AATATCATGA CACACGTGTT ACTTTGGATT AGGATTTTCT GTTAGATTTT TATCTCCGCT   
  
  
- AAACTTTTTA ATGAAAAGAT AAAATAGTAT TTTTATAATT TATTATATTA AATTAATAAA TAAATTATAA   
  
  
- TTATTTATTT TATTAAATTA TAACATGTCA TTAACTTTTA AAAAACTCAG CACAATTTAG ATGAGTAAAT   
  
  
- TAAAAATAAT GATATGTAGT TATAAGTATC AAAAATAAAA ATATTTGTTT ATTTAAAAAT TTTAAAAGCT   
  
  
- AAAACATATT TTATAAACCC ATTAATACAA AATTATGTCA CTAATTAACA TAAAACTCTA GTTTTATCCA   
  
  
- AAATTGATGT CGTTTTACTA ATAACCTAA

+     TCCC-motif

| Site Name | Organism | Position | Strand | Matrix score. | sequence | function |
| --- | --- | --- | --- | --- | --- | --- |
| TCCC-motif | Spinacia oleracea | 869 | - | 7 | TCTCCCT | part of a light responsive element |
| TCCC-motif | Spinacia oleracea | 91 | - | 7 | TCTCCCT | part of a light responsive element |
| TCCC-motif | Spinacia oleracea | 473 | + | 7 | TCTCCCT | part of a light responsive element |

> 2018/04/13 10:10:12  
+ TTTTTGTTAG GGTTTTTCGT TTCTTTCTTC TTTAAGTTCT TTACACAATA TTTCTTGTCG ACTTCGGACA   
  
  
+ TAGATGAGAG AGAGAGAGAG AGGGAGAGAG GAAGACGAAG AGGGTGATAC ACATACTTCA GACACTTCAG   
  
  
+ AAAGTGGCCA CGATATCCGA GCCGATAATT TTCCCAACCC GGCACCTGGT CTAAAACAAT AATCTTCTAC   
  
  
+ TGTGTGAGAA ACCTAAAGAG TGATAAAGGT TTAAAGGTTA GGCTCGAAGA ATGCAGTCGA CCACGCAGTC   
  
  
+ CAGCGGTCGA TCGTTGACCC AGGGTGAAGA CTGGTGGAGA CCGAACCAAT GGGCAGAAAA GGTGTTGGGC   
  
  
+ TTGAGCAATT GGGCACCGAC TAAACAGTAA AATAGACAAC TATATACACA CAACCGCCCA TACCTCGGCT   
  
  
+ GAAGCCCAAT CTGCATCTAT GCCTAGACAA ACTGCTTTCT CTTTTTTCAA TTTCTCCCTC TCCAAAGGAT   
  
  
+ TCGTAAAGCA GTTCTTTTCT CAAACTGAGC GAGCATTTAG TCTTCTTTCT TTACTTAGGT GGCGGGGCTT   
  
  
+ GCCACGCCGC GCAAGGTGCA CTGCTTTTTC CTTTATGTTT ATTGGGTTTT CTCCTTCTTT TGACTTCCTC   
  
  
+ TCTCTTCCTC TCTCTCTCTC TCTCTCTCCC CTCGGCTCAG TTGCTTAAAC TGCCGGGCCG AGAGACTCCT   
  
  
+ ATACTGGCCT CTGGCCCACC CCGTCACCGG ACCAGATACG CACCTGGGGT GCATCGTACT GTACTCTTTC   
  
  
+ GTGCAAAGTC TAGGCGGGGG CCCTTGGAAC AACTGACAAA TGGACACCCT GGCTCAGCGA ACGAAACTGA   
  
  
+ AAGACAATGT ACAACGAAAT GAGTAAACAG GGAGAGAAAG AGAAGTAGAG GATCAGTAGA AAAGGAAAAA   
  
  
+ AATAATTAAA GATTTTATTT CGAAATTGTA TGTACAAAAA GGAACACGTA CATAAAAGAG TTCATTTATC   
  
  
+ TTTTTATTAA TAAATAATGG TTTGAATAAA GGATATAATA ATTTTAAAAA CGTACAAATT TTATTCAAAT   
  
  
+ AATAAAATCA ATTTTTTAAT TAGTTTTTTT AATTAATAAA AAAAATACAT TAAATACTTC CCGTGTATCA   
  
  
+ TAAAATCCAC TTATAGTACT GTGTGCACAA TGAAACCTAA TCCTAAAAGA CAATCTAAAA ATAGAGGCGA   
  
  
+ TTTGAAAAAT TACTTTTCTA TTTTATCATA AAAATATTAA ATAATATAAT TTAATTATTT ATTTAATATT   
  
  
+ AATAAATAAA ATAATTTAAT ATTGTACAGT AATTGAAAAT TTTTTGAGTC GTGTTAAATC TACTCATTTA   
  
  
+ ATTTTTATTA CTATACATCA ATATTCATAG TTTTTATTTT TATAAACAAA TAAATTTTTA AAATTTTCGA   
  
  
+ TTTTGTATAA AATATTTGGG TAATTATGTT TTAATACAGT GATTAATTGT ATTTTGAGAT CAAAATAGGT   
  
  
+ TTTAACTACA GCAAAATGAT TATTGGATT  

- AAAAACAATC CCAAAAAGCA AAGAAAGAAG AAATTCAAGA AATGTGTTAT AAAGAACAGC TGAAGCCTGT   
  
  
- ATCTACTCTC TCTCTCTCTC TCCCTCTCTC CTTCTGCTTC TCCCACTATG TGTATGAAGT CTGTGAAGTC   
  
  
- TTTCACCGGT GCTATAGGCT CGGCTATTAA AAGGGTTGGG CCGTGGACCA GATTTTGTTA TTAGAAGATG   
  
  
- ACACACTCTT TGGATTTCTC ACTATTTCCA AATTTCCAAT CCGAGCTTCT TACGTCAGCT GGTGCGTCAG   
  
  
- GTCGCCAGCT AGCAACTGGG TCCCACTTCT GACCACCTCT GGCTTGGTTA CCCGTCTTTT CCACAACCCG   
  
  
- AACTCGTTAA CCCGTGGCTG ATTTGTCATT TTATCTGTTG ATATATGTGT GTTGGCGGGT ATGGAGCCGA   
  
  
- CTTCGGGTTA GACGTAGATA CGGATCTGTT TGACGAAAGA GAAAAAAGTT AAAGAGGGAG AGGTTTCCTA   
  
  
- AGCATTTCGT CAAGAAAAGA GTTTGACTCG CTCGTAAATC AGAAGAAAGA AATGAATCCA CCGCCCCGAA   
  
  
- CGGTGCGGCG CGTTCCACGT GACGAAAAAG GAAATACAAA TAACCCAAAA GAGGAAGAAA ACTGAAGGAG   
  
  
- AGAGAAGGAG AGAGAGAGAG AGAGAGAGGG GAGCCGAGTC AACGAATTTG ACGGCCCGGC TCTCTGAGGA   
  
  
- TATGACCGGA GACCGGGTGG GGCAGTGGCC TGGTCTATGC GTGGACCCCA CGTAGCATGA CATGAGAAAG   
  
  
- CACGTTTCAG ATCCGCCCCC GGGAACCTTG TTGACTGTTT ACCTGTGGGA CCGAGTCGCT TGCTTTGACT   
  
  
- TTCTGTTACA TGTTGCTTTA CTCATTTGTC CCTCTCTTTC TCTTCATCTC CTAGTCATCT TTTCCTTTTT   
  
  
- TTATTAATTT CTAAAATAAA GCTTTAACAT ACATGTTTTT CCTTGTGCAT GTATTTTCTC AAGTAAATAG   
  
  
- AAAAATAATT ATTTATTACC AAACTTATTT CCTATATTAT TAAAATTTTT GCATGTTTAA AATAAGTTTA   
  
  
- TTATTTTAGT TAAAAAATTA ATCAAAAAAA TTAATTATTT TTTTTATGTA ATTTATGAAG GGCACATAGT   
  
  
- ATTTTAGGTG AATATCATGA CACACGTGTT ACTTTGGATT AGGATTTTCT GTTAGATTTT TATCTCCGCT   
  
  
- AAACTTTTTA ATGAAAAGAT AAAATAGTAT TTTTATAATT TATTATATTA AATTAATAAA TAAATTATAA   
  
  
- TTATTTATTT TATTAAATTA TAACATGTCA TTAACTTTTA AAAAACTCAG CACAATTTAG ATGAGTAAAT   
  
  
- TAAAAATAAT GATATGTAGT TATAAGTATC AAAAATAAAA ATATTTGTTT ATTTAAAAAT TTTAAAAGCT   
  
  
- AAAACATATT TTATAAACCC ATTAATACAA AATTATGTCA CTAATTAACA TAAAACTCTA GTTTTATCCA   
  
  
- AAATTGATGT CGTTTTACTA ATAACCTAA

+     TGACG-motif

| Site Name | Organism | Position | Strand | Matrix score. | sequence | function |
| --- | --- | --- | --- | --- | --- | --- |
| TGACG-motif | Hordeum vulgare | 722 | - | 5 | TGACG | cis-acting regulatory element involved in the MeJA-responsiveness |

> 2018/04/13 10:10:12  
+ TTTTTGTTAG GGTTTTTCGT TTCTTTCTTC TTTAAGTTCT TTACACAATA TTTCTTGTCG ACTTCGGACA   
  
  
+ TAGATGAGAG AGAGAGAGAG AGGGAGAGAG GAAGACGAAG AGGGTGATAC ACATACTTCA GACACTTCAG   
  
  
+ AAAGTGGCCA CGATATCCGA GCCGATAATT TTCCCAACCC GGCACCTGGT CTAAAACAAT AATCTTCTAC   
  
  
+ TGTGTGAGAA ACCTAAAGAG TGATAAAGGT TTAAAGGTTA GGCTCGAAGA ATGCAGTCGA CCACGCAGTC   
  
  
+ CAGCGGTCGA TCGTTGACCC AGGGTGAAGA CTGGTGGAGA CCGAACCAAT GGGCAGAAAA GGTGTTGGGC   
  
  
+ TTGAGCAATT GGGCACCGAC TAAACAGTAA AATAGACAAC TATATACACA CAACCGCCCA TACCTCGGCT   
  
  
+ GAAGCCCAAT CTGCATCTAT GCCTAGACAA ACTGCTTTCT CTTTTTTCAA TTTCTCCCTC TCCAAAGGAT   
  
  
+ TCGTAAAGCA GTTCTTTTCT CAAACTGAGC GAGCATTTAG TCTTCTTTCT TTACTTAGGT GGCGGGGCTT   
  
  
+ GCCACGCCGC GCAAGGTGCA CTGCTTTTTC CTTTATGTTT ATTGGGTTTT CTCCTTCTTT TGACTTCCTC   
  
  
+ TCTCTTCCTC TCTCTCTCTC TCTCTCTCCC CTCGGCTCAG TTGCTTAAAC TGCCGGGCCG AGAGACTCCT   
  
  
+ ATACTGGCCT CTGGCCCACC CCGTCACCGG ACCAGATACG CACCTGGGGT GCATCGTACT GTACTCTTTC   
  
  
+ GTGCAAAGTC TAGGCGGGGG CCCTTGGAAC AACTGACAAA TGGACACCCT GGCTCAGCGA ACGAAACTGA   
  
  
+ AAGACAATGT ACAACGAAAT GAGTAAACAG GGAGAGAAAG AGAAGTAGAG GATCAGTAGA AAAGGAAAAA   
  
  
+ AATAATTAAA GATTTTATTT CGAAATTGTA TGTACAAAAA GGAACACGTA CATAAAAGAG TTCATTTATC   
  
  
+ TTTTTATTAA TAAATAATGG TTTGAATAAA GGATATAATA ATTTTAAAAA CGTACAAATT TTATTCAAAT   
  
  
+ AATAAAATCA ATTTTTTAAT TAGTTTTTTT AATTAATAAA AAAAATACAT TAAATACTTC CCGTGTATCA   
  
  
+ TAAAATCCAC TTATAGTACT GTGTGCACAA TGAAACCTAA TCCTAAAAGA CAATCTAAAA ATAGAGGCGA   
  
  
+ TTTGAAAAAT TACTTTTCTA TTTTATCATA AAAATATTAA ATAATATAAT TTAATTATTT ATTTAATATT   
  
  
+ AATAAATAAA ATAATTTAAT ATTGTACAGT AATTGAAAAT TTTTTGAGTC GTGTTAAATC TACTCATTTA   
  
  
+ ATTTTTATTA CTATACATCA ATATTCATAG TTTTTATTTT TATAAACAAA TAAATTTTTA AAATTTTCGA   
  
  
+ TTTTGTATAA AATATTTGGG TAATTATGTT TTAATACAGT GATTAATTGT ATTTTGAGAT CAAAATAGGT   
  
  
+ TTTAACTACA GCAAAATGAT TATTGGATT  

- AAAAACAATC CCAAAAAGCA AAGAAAGAAG AAATTCAAGA AATGTGTTAT AAAGAACAGC TGAAGCCTGT   
  
  
- ATCTACTCTC TCTCTCTCTC TCCCTCTCTC CTTCTGCTTC TCCCACTATG TGTATGAAGT CTGTGAAGTC   
  
  
- TTTCACCGGT GCTATAGGCT CGGCTATTAA AAGGGTTGGG CCGTGGACCA GATTTTGTTA TTAGAAGATG   
  
  
- ACACACTCTT TGGATTTCTC ACTATTTCCA AATTTCCAAT CCGAGCTTCT TACGTCAGCT GGTGCGTCAG   
  
  
- GTCGCCAGCT AGCAACTGGG TCCCACTTCT GACCACCTCT GGCTTGGTTA CCCGTCTTTT CCACAACCCG   
  
  
- AACTCGTTAA CCCGTGGCTG ATTTGTCATT TTATCTGTTG ATATATGTGT GTTGGCGGGT ATGGAGCCGA   
  
  
- CTTCGGGTTA GACGTAGATA CGGATCTGTT TGACGAAAGA GAAAAAAGTT AAAGAGGGAG AGGTTTCCTA   
  
  
- AGCATTTCGT CAAGAAAAGA GTTTGACTCG CTCGTAAATC AGAAGAAAGA AATGAATCCA CCGCCCCGAA   
  
  
- CGGTGCGGCG CGTTCCACGT GACGAAAAAG GAAATACAAA TAACCCAAAA GAGGAAGAAA ACTGAAGGAG   
  
  
- AGAGAAGGAG AGAGAGAGAG AGAGAGAGGG GAGCCGAGTC AACGAATTTG ACGGCCCGGC TCTCTGAGGA   
  
  
- TATGACCGGA GACCGGGTGG GGCAGTGGCC TGGTCTATGC GTGGACCCCA CGTAGCATGA CATGAGAAAG   
  
  
- CACGTTTCAG ATCCGCCCCC GGGAACCTTG TTGACTGTTT ACCTGTGGGA CCGAGTCGCT TGCTTTGACT   
  
  
- TTCTGTTACA TGTTGCTTTA CTCATTTGTC CCTCTCTTTC TCTTCATCTC CTAGTCATCT TTTCCTTTTT   
  
  
- TTATTAATTT CTAAAATAAA GCTTTAACAT ACATGTTTTT CCTTGTGCAT GTATTTTCTC AAGTAAATAG   
  
  
- AAAAATAATT ATTTATTACC AAACTTATTT CCTATATTAT TAAAATTTTT GCATGTTTAA AATAAGTTTA   
  
  
- TTATTTTAGT TAAAAAATTA ATCAAAAAAA TTAATTATTT TTTTTATGTA ATTTATGAAG GGCACATAGT   
  
  
- ATTTTAGGTG AATATCATGA CACACGTGTT ACTTTGGATT AGGATTTTCT GTTAGATTTT TATCTCCGCT   
  
  
- AAACTTTTTA ATGAAAAGAT AAAATAGTAT TTTTATAATT TATTATATTA AATTAATAAA TAAATTATAA   
  
  
- TTATTTATTT TATTAAATTA TAACATGTCA TTAACTTTTA AAAAACTCAG CACAATTTAG ATGAGTAAAT   
  
  
- TAAAAATAAT GATATGTAGT TATAAGTATC AAAAATAAAA ATATTTGTTT ATTTAAAAAT TTTAAAAGCT   
  
  
- AAAACATATT TTATAAACCC ATTAATACAA AATTATGTCA CTAATTAACA TAAAACTCTA GTTTTATCCA   
  
  
- AAATTGATGT CGTTTTACTA ATAACCTAA

+     Unnamed\_\_1

| Site Name | Organism | Position | Strand | Matrix score. | sequence | function |
| --- | --- | --- | --- | --- | --- | --- |
| Unnamed\_\_1 | Zea mays | 148 | - | 5 | CGTGG |  |
| Unnamed\_\_1 | Zea mays | 562 | - | 5 | CGTGG |  |
| Unnamed\_\_1 | Zea mays | 271 | - | 5 | CGTGG |  |

> 2018/04/13 10:10:12  
+ TTTTTGTTAG GGTTTTTCGT TTCTTTCTTC TTTAAGTTCT TTACACAATA TTTCTTGTCG ACTTCGGACA   
  
  
+ TAGATGAGAG AGAGAGAGAG AGGGAGAGAG GAAGACGAAG AGGGTGATAC ACATACTTCA GACACTTCAG   
  
  
+ AAAGTGGCCA CGATATCCGA GCCGATAATT TTCCCAACCC GGCACCTGGT CTAAAACAAT AATCTTCTAC   
  
  
+ TGTGTGAGAA ACCTAAAGAG TGATAAAGGT TTAAAGGTTA GGCTCGAAGA ATGCAGTCGA CCACGCAGTC   
  
  
+ CAGCGGTCGA TCGTTGACCC AGGGTGAAGA CTGGTGGAGA CCGAACCAAT GGGCAGAAAA GGTGTTGGGC   
  
  
+ TTGAGCAATT GGGCACCGAC TAAACAGTAA AATAGACAAC TATATACACA CAACCGCCCA TACCTCGGCT   
  
  
+ GAAGCCCAAT CTGCATCTAT GCCTAGACAA ACTGCTTTCT CTTTTTTCAA TTTCTCCCTC TCCAAAGGAT   
  
  
+ TCGTAAAGCA GTTCTTTTCT CAAACTGAGC GAGCATTTAG TCTTCTTTCT TTACTTAGGT GGCGGGGCTT   
  
  
+ GCCACGCCGC GCAAGGTGCA CTGCTTTTTC CTTTATGTTT ATTGGGTTTT CTCCTTCTTT TGACTTCCTC   
  
  
+ TCTCTTCCTC TCTCTCTCTC TCTCTCTCCC CTCGGCTCAG TTGCTTAAAC TGCCGGGCCG AGAGACTCCT   
  
  
+ ATACTGGCCT CTGGCCCACC CCGTCACCGG ACCAGATACG CACCTGGGGT GCATCGTACT GTACTCTTTC   
  
  
+ GTGCAAAGTC TAGGCGGGGG CCCTTGGAAC AACTGACAAA TGGACACCCT GGCTCAGCGA ACGAAACTGA   
  
  
+ AAGACAATGT ACAACGAAAT GAGTAAACAG GGAGAGAAAG AGAAGTAGAG GATCAGTAGA AAAGGAAAAA   
  
  
+ AATAATTAAA GATTTTATTT CGAAATTGTA TGTACAAAAA GGAACACGTA CATAAAAGAG TTCATTTATC   
  
  
+ TTTTTATTAA TAAATAATGG TTTGAATAAA GGATATAATA ATTTTAAAAA CGTACAAATT TTATTCAAAT   
  
  
+ AATAAAATCA ATTTTTTAAT TAGTTTTTTT AATTAATAAA AAAAATACAT TAAATACTTC CCGTGTATCA   
  
  
+ TAAAATCCAC TTATAGTACT GTGTGCACAA TGAAACCTAA TCCTAAAAGA CAATCTAAAA ATAGAGGCGA   
  
  
+ TTTGAAAAAT TACTTTTCTA TTTTATCATA AAAATATTAA ATAATATAAT TTAATTATTT ATTTAATATT   
  
  
+ AATAAATAAA ATAATTTAAT ATTGTACAGT AATTGAAAAT TTTTTGAGTC GTGTTAAATC TACTCATTTA   
  
  
+ ATTTTTATTA CTATACATCA ATATTCATAG TTTTTATTTT TATAAACAAA TAAATTTTTA AAATTTTCGA   
  
  
+ TTTTGTATAA AATATTTGGG TAATTATGTT TTAATACAGT GATTAATTGT ATTTTGAGAT CAAAATAGGT   
  
  
+ TTTAACTACA GCAAAATGAT TATTGGATT  

- AAAAACAATC CCAAAAAGCA AAGAAAGAAG AAATTCAAGA AATGTGTTAT AAAGAACAGC TGAAGCCTGT   
  
  
- ATCTACTCTC TCTCTCTCTC TCCCTCTCTC CTTCTGCTTC TCCCACTATG TGTATGAAGT CTGTGAAGTC   
  
  
- TTTCACCGGT GCTATAGGCT CGGCTATTAA AAGGGTTGGG CCGTGGACCA GATTTTGTTA TTAGAAGATG   
  
  
- ACACACTCTT TGGATTTCTC ACTATTTCCA AATTTCCAAT CCGAGCTTCT TACGTCAGCT GGTGCGTCAG   
  
  
- GTCGCCAGCT AGCAACTGGG TCCCACTTCT GACCACCTCT GGCTTGGTTA CCCGTCTTTT CCACAACCCG   
  
  
- AACTCGTTAA CCCGTGGCTG ATTTGTCATT TTATCTGTTG ATATATGTGT GTTGGCGGGT ATGGAGCCGA   
  
  
- CTTCGGGTTA GACGTAGATA CGGATCTGTT TGACGAAAGA GAAAAAAGTT AAAGAGGGAG AGGTTTCCTA   
  
  
- AGCATTTCGT CAAGAAAAGA GTTTGACTCG CTCGTAAATC AGAAGAAAGA AATGAATCCA CCGCCCCGAA   
  
  
- CGGTGCGGCG CGTTCCACGT GACGAAAAAG GAAATACAAA TAACCCAAAA GAGGAAGAAA ACTGAAGGAG   
  
  
- AGAGAAGGAG AGAGAGAGAG AGAGAGAGGG GAGCCGAGTC AACGAATTTG ACGGCCCGGC TCTCTGAGGA   
  
  
- TATGACCGGA GACCGGGTGG GGCAGTGGCC TGGTCTATGC GTGGACCCCA CGTAGCATGA CATGAGAAAG   
  
  
- CACGTTTCAG ATCCGCCCCC GGGAACCTTG TTGACTGTTT ACCTGTGGGA CCGAGTCGCT TGCTTTGACT   
  
  
- TTCTGTTACA TGTTGCTTTA CTCATTTGTC CCTCTCTTTC TCTTCATCTC CTAGTCATCT TTTCCTTTTT   
  
  
- TTATTAATTT CTAAAATAAA GCTTTAACAT ACATGTTTTT CCTTGTGCAT GTATTTTCTC AAGTAAATAG   
  
  
- AAAAATAATT ATTTATTACC AAACTTATTT CCTATATTAT TAAAATTTTT GCATGTTTAA AATAAGTTTA   
  
  
- TTATTTTAGT TAAAAAATTA ATCAAAAAAA TTAATTATTT TTTTTATGTA ATTTATGAAG GGCACATAGT   
  
  
- ATTTTAGGTG AATATCATGA CACACGTGTT ACTTTGGATT AGGATTTTCT GTTAGATTTT TATCTCCGCT   
  
  
- AAACTTTTTA ATGAAAAGAT AAAATAGTAT TTTTATAATT TATTATATTA AATTAATAAA TAAATTATAA   
  
  
- TTATTTATTT TATTAAATTA TAACATGTCA TTAACTTTTA AAAAACTCAG CACAATTTAG ATGAGTAAAT   
  
  
- TAAAAATAAT GATATGTAGT TATAAGTATC AAAAATAAAA ATATTTGTTT ATTTAAAAAT TTTAAAAGCT   
  
  
- AAAACATATT TTATAAACCC ATTAATACAA AATTATGTCA CTAATTAACA TAAAACTCTA GTTTTATCCA   
  
  
- AAATTGATGT CGTTTTACTA ATAACCTAA

+     Unnamed\_\_2

| Site Name | Organism | Position | Strand | Matrix score. | sequence | function |
| --- | --- | --- | --- | --- | --- | --- |
| Unnamed\_\_2 | Petroselinum hortense | 245 | - | 9 | AACCTAACCT |  |
| Unnamed\_\_2 | Triticum aestivum | 719 | + | 10 | CCACGTCACCG | single-strand DNA-binding proteins site (ssDBP-1 and -2) |

> 2018/04/13 10:10:12  
+ TTTTTGTTAG GGTTTTTCGT TTCTTTCTTC TTTAAGTTCT TTACACAATA TTTCTTGTCG ACTTCGGACA   
  
  
+ TAGATGAGAG AGAGAGAGAG AGGGAGAGAG GAAGACGAAG AGGGTGATAC ACATACTTCA GACACTTCAG   
  
  
+ AAAGTGGCCA CGATATCCGA GCCGATAATT TTCCCAACCC GGCACCTGGT CTAAAACAAT AATCTTCTAC   
  
  
+ TGTGTGAGAA ACCTAAAGAG TGATAAAGGT TTAAAGGTTA GGCTCGAAGA ATGCAGTCGA CCACGCAGTC   
  
  
+ CAGCGGTCGA TCGTTGACCC AGGGTGAAGA CTGGTGGAGA CCGAACCAAT GGGCAGAAAA GGTGTTGGGC   
  
  
+ TTGAGCAATT GGGCACCGAC TAAACAGTAA AATAGACAAC TATATACACA CAACCGCCCA TACCTCGGCT   
  
  
+ GAAGCCCAAT CTGCATCTAT GCCTAGACAA ACTGCTTTCT CTTTTTTCAA TTTCTCCCTC TCCAAAGGAT   
  
  
+ TCGTAAAGCA GTTCTTTTCT CAAACTGAGC GAGCATTTAG TCTTCTTTCT TTACTTAGGT GGCGGGGCTT   
  
  
+ GCCACGCCGC GCAAGGTGCA CTGCTTTTTC CTTTATGTTT ATTGGGTTTT CTCCTTCTTT TGACTTCCTC   
  
  
+ TCTCTTCCTC TCTCTCTCTC TCTCTCTCCC CTCGGCTCAG TTGCTTAAAC TGCCGGGCCG AGAGACTCCT   
  
  
+ ATACTGGCCT CTGGCCCACC CCGTCACCGG ACCAGATACG CACCTGGGGT GCATCGTACT GTACTCTTTC   
  
  
+ GTGCAAAGTC TAGGCGGGGG CCCTTGGAAC AACTGACAAA TGGACACCCT GGCTCAGCGA ACGAAACTGA   
  
  
+ AAGACAATGT ACAACGAAAT GAGTAAACAG GGAGAGAAAG AGAAGTAGAG GATCAGTAGA AAAGGAAAAA   
  
  
+ AATAATTAAA GATTTTATTT CGAAATTGTA TGTACAAAAA GGAACACGTA CATAAAAGAG TTCATTTATC   
  
  
+ TTTTTATTAA TAAATAATGG TTTGAATAAA GGATATAATA ATTTTAAAAA CGTACAAATT TTATTCAAAT   
  
  
+ AATAAAATCA ATTTTTTAAT TAGTTTTTTT AATTAATAAA AAAAATACAT TAAATACTTC CCGTGTATCA   
  
  
+ TAAAATCCAC TTATAGTACT GTGTGCACAA TGAAACCTAA TCCTAAAAGA CAATCTAAAA ATAGAGGCGA   
  
  
+ TTTGAAAAAT TACTTTTCTA TTTTATCATA AAAATATTAA ATAATATAAT TTAATTATTT ATTTAATATT   
  
  
+ AATAAATAAA ATAATTTAAT ATTGTACAGT AATTGAAAAT TTTTTGAGTC GTGTTAAATC TACTCATTTA   
  
  
+ ATTTTTATTA CTATACATCA ATATTCATAG TTTTTATTTT TATAAACAAA TAAATTTTTA AAATTTTCGA   
  
  
+ TTTTGTATAA AATATTTGGG TAATTATGTT TTAATACAGT GATTAATTGT ATTTTGAGAT CAAAATAGGT   
  
  
+ TTTAACTACA GCAAAATGAT TATTGGATT  

- AAAAACAATC CCAAAAAGCA AAGAAAGAAG AAATTCAAGA AATGTGTTAT AAAGAACAGC TGAAGCCTGT   
  
  
- ATCTACTCTC TCTCTCTCTC TCCCTCTCTC CTTCTGCTTC TCCCACTATG TGTATGAAGT CTGTGAAGTC   
  
  
- TTTCACCGGT GCTATAGGCT CGGCTATTAA AAGGGTTGGG CCGTGGACCA GATTTTGTTA TTAGAAGATG   
  
  
- ACACACTCTT TGGATTTCTC ACTATTTCCA AATTTCCAAT CCGAGCTTCT TACGTCAGCT GGTGCGTCAG   
  
  
- GTCGCCAGCT AGCAACTGGG TCCCACTTCT GACCACCTCT GGCTTGGTTA CCCGTCTTTT CCACAACCCG   
  
  
- AACTCGTTAA CCCGTGGCTG ATTTGTCATT TTATCTGTTG ATATATGTGT GTTGGCGGGT ATGGAGCCGA   
  
  
- CTTCGGGTTA GACGTAGATA CGGATCTGTT TGACGAAAGA GAAAAAAGTT AAAGAGGGAG AGGTTTCCTA   
  
  
- AGCATTTCGT CAAGAAAAGA GTTTGACTCG CTCGTAAATC AGAAGAAAGA AATGAATCCA CCGCCCCGAA   
  
  
- CGGTGCGGCG CGTTCCACGT GACGAAAAAG GAAATACAAA TAACCCAAAA GAGGAAGAAA ACTGAAGGAG   
  
  
- AGAGAAGGAG AGAGAGAGAG AGAGAGAGGG GAGCCGAGTC AACGAATTTG ACGGCCCGGC TCTCTGAGGA   
  
  
- TATGACCGGA GACCGGGTGG GGCAGTGGCC TGGTCTATGC GTGGACCCCA CGTAGCATGA CATGAGAAAG   
  
  
- CACGTTTCAG ATCCGCCCCC GGGAACCTTG TTGACTGTTT ACCTGTGGGA CCGAGTCGCT TGCTTTGACT   
  
  
- TTCTGTTACA TGTTGCTTTA CTCATTTGTC CCTCTCTTTC TCTTCATCTC CTAGTCATCT TTTCCTTTTT   
  
  
- TTATTAATTT CTAAAATAAA GCTTTAACAT ACATGTTTTT CCTTGTGCAT GTATTTTCTC AAGTAAATAG   
  
  
- AAAAATAATT ATTTATTACC AAACTTATTT CCTATATTAT TAAAATTTTT GCATGTTTAA AATAAGTTTA   
  
  
- TTATTTTAGT TAAAAAATTA ATCAAAAAAA TTAATTATTT TTTTTATGTA ATTTATGAAG GGCACATAGT   
  
  
- ATTTTAGGTG AATATCATGA CACACGTGTT ACTTTGGATT AGGATTTTCT GTTAGATTTT TATCTCCGCT   
  
  
- AAACTTTTTA ATGAAAAGAT AAAATAGTAT TTTTATAATT TATTATATTA AATTAATAAA TAAATTATAA   
  
  
- TTATTTATTT TATTAAATTA TAACATGTCA TTAACTTTTA AAAAACTCAG CACAATTTAG ATGAGTAAAT   
  
  
- TAAAAATAAT GATATGTAGT TATAAGTATC AAAAATAAAA ATATTTGTTT ATTTAAAAAT TTTAAAAGCT   
  
  
- AAAACATATT TTATAAACCC ATTAATACAA AATTATGTCA CTAATTAACA TAAAACTCTA GTTTTATCCA   
  
  
- AAATTGATGT CGTTTTACTA ATAACCTAA

+     Unnamed\_\_3

| Site Name | Organism | Position | Strand | Matrix score. | sequence | function |
| --- | --- | --- | --- | --- | --- | --- |
| Unnamed\_\_3 | Zea mays | 148 | - | 5 | CGTGG |  |
| Unnamed\_\_3 | Zea mays | 271 | - | 5 | CGTGG |  |
| Unnamed\_\_3 | Zea mays | 562 | - | 5 | CGTGG |  |

> 2018/04/13 10:10:12  
+ TTTTTGTTAG GGTTTTTCGT TTCTTTCTTC TTTAAGTTCT TTACACAATA TTTCTTGTCG ACTTCGGACA   
  
  
+ TAGATGAGAG AGAGAGAGAG AGGGAGAGAG GAAGACGAAG AGGGTGATAC ACATACTTCA GACACTTCAG   
  
  
+ AAAGTGGCCA CGATATCCGA GCCGATAATT TTCCCAACCC GGCACCTGGT CTAAAACAAT AATCTTCTAC   
  
  
+ TGTGTGAGAA ACCTAAAGAG TGATAAAGGT TTAAAGGTTA GGCTCGAAGA ATGCAGTCGA CCACGCAGTC   
  
  
+ CAGCGGTCGA TCGTTGACCC AGGGTGAAGA CTGGTGGAGA CCGAACCAAT GGGCAGAAAA GGTGTTGGGC   
  
  
+ TTGAGCAATT GGGCACCGAC TAAACAGTAA AATAGACAAC TATATACACA CAACCGCCCA TACCTCGGCT   
  
  
+ GAAGCCCAAT CTGCATCTAT GCCTAGACAA ACTGCTTTCT CTTTTTTCAA TTTCTCCCTC TCCAAAGGAT   
  
  
+ TCGTAAAGCA GTTCTTTTCT CAAACTGAGC GAGCATTTAG TCTTCTTTCT TTACTTAGGT GGCGGGGCTT   
  
  
+ GCCACGCCGC GCAAGGTGCA CTGCTTTTTC CTTTATGTTT ATTGGGTTTT CTCCTTCTTT TGACTTCCTC   
  
  
+ TCTCTTCCTC TCTCTCTCTC TCTCTCTCCC CTCGGCTCAG TTGCTTAAAC TGCCGGGCCG AGAGACTCCT   
  
  
+ ATACTGGCCT CTGGCCCACC CCGTCACCGG ACCAGATACG CACCTGGGGT GCATCGTACT GTACTCTTTC   
  
  
+ GTGCAAAGTC TAGGCGGGGG CCCTTGGAAC AACTGACAAA TGGACACCCT GGCTCAGCGA ACGAAACTGA   
  
  
+ AAGACAATGT ACAACGAAAT GAGTAAACAG GGAGAGAAAG AGAAGTAGAG GATCAGTAGA AAAGGAAAAA   
  
  
+ AATAATTAAA GATTTTATTT CGAAATTGTA TGTACAAAAA GGAACACGTA CATAAAAGAG TTCATTTATC   
  
  
+ TTTTTATTAA TAAATAATGG TTTGAATAAA GGATATAATA ATTTTAAAAA CGTACAAATT TTATTCAAAT   
  
  
+ AATAAAATCA ATTTTTTAAT TAGTTTTTTT AATTAATAAA AAAAATACAT TAAATACTTC CCGTGTATCA   
  
  
+ TAAAATCCAC TTATAGTACT GTGTGCACAA TGAAACCTAA TCCTAAAAGA CAATCTAAAA ATAGAGGCGA   
  
  
+ TTTGAAAAAT TACTTTTCTA TTTTATCATA AAAATATTAA ATAATATAAT TTAATTATTT ATTTAATATT   
  
  
+ AATAAATAAA ATAATTTAAT ATTGTACAGT AATTGAAAAT TTTTTGAGTC GTGTTAAATC TACTCATTTA   
  
  
+ ATTTTTATTA CTATACATCA ATATTCATAG TTTTTATTTT TATAAACAAA TAAATTTTTA AAATTTTCGA   
  
  
+ TTTTGTATAA AATATTTGGG TAATTATGTT TTAATACAGT GATTAATTGT ATTTTGAGAT CAAAATAGGT   
  
  
+ TTTAACTACA GCAAAATGAT TATTGGATT  

- AAAAACAATC CCAAAAAGCA AAGAAAGAAG AAATTCAAGA AATGTGTTAT AAAGAACAGC TGAAGCCTGT   
  
  
- ATCTACTCTC TCTCTCTCTC TCCCTCTCTC CTTCTGCTTC TCCCACTATG TGTATGAAGT CTGTGAAGTC   
  
  
- TTTCACCGGT GCTATAGGCT CGGCTATTAA AAGGGTTGGG CCGTGGACCA GATTTTGTTA TTAGAAGATG   
  
  
- ACACACTCTT TGGATTTCTC ACTATTTCCA AATTTCCAAT CCGAGCTTCT TACGTCAGCT GGTGCGTCAG   
  
  
- GTCGCCAGCT AGCAACTGGG TCCCACTTCT GACCACCTCT GGCTTGGTTA CCCGTCTTTT CCACAACCCG   
  
  
- AACTCGTTAA CCCGTGGCTG ATTTGTCATT TTATCTGTTG ATATATGTGT GTTGGCGGGT ATGGAGCCGA   
  
  
- CTTCGGGTTA GACGTAGATA CGGATCTGTT TGACGAAAGA GAAAAAAGTT AAAGAGGGAG AGGTTTCCTA   
  
  
- AGCATTTCGT CAAGAAAAGA GTTTGACTCG CTCGTAAATC AGAAGAAAGA AATGAATCCA CCGCCCCGAA   
  
  
- CGGTGCGGCG CGTTCCACGT GACGAAAAAG GAAATACAAA TAACCCAAAA GAGGAAGAAA ACTGAAGGAG   
  
  
- AGAGAAGGAG AGAGAGAGAG AGAGAGAGGG GAGCCGAGTC AACGAATTTG ACGGCCCGGC TCTCTGAGGA   
  
  
- TATGACCGGA GACCGGGTGG GGCAGTGGCC TGGTCTATGC GTGGACCCCA CGTAGCATGA CATGAGAAAG   
  
  
- CACGTTTCAG ATCCGCCCCC GGGAACCTTG TTGACTGTTT ACCTGTGGGA CCGAGTCGCT TGCTTTGACT   
  
  
- TTCTGTTACA TGTTGCTTTA CTCATTTGTC CCTCTCTTTC TCTTCATCTC CTAGTCATCT TTTCCTTTTT   
  
  
- TTATTAATTT CTAAAATAAA GCTTTAACAT ACATGTTTTT CCTTGTGCAT GTATTTTCTC AAGTAAATAG   
  
  
- AAAAATAATT ATTTATTACC AAACTTATTT CCTATATTAT TAAAATTTTT GCATGTTTAA AATAAGTTTA   
  
  
- TTATTTTAGT TAAAAAATTA ATCAAAAAAA TTAATTATTT TTTTTATGTA ATTTATGAAG GGCACATAGT   
  
  
- ATTTTAGGTG AATATCATGA CACACGTGTT ACTTTGGATT AGGATTTTCT GTTAGATTTT TATCTCCGCT   
  
  
- AAACTTTTTA ATGAAAAGAT AAAATAGTAT TTTTATAATT TATTATATTA AATTAATAAA TAAATTATAA   
  
  
- TTATTTATTT TATTAAATTA TAACATGTCA TTAACTTTTA AAAAACTCAG CACAATTTAG ATGAGTAAAT   
  
  
- TAAAAATAAT GATATGTAGT TATAAGTATC AAAAATAAAA ATATTTGTTT ATTTAAAAAT TTTAAAAGCT   
  
  
- AAAACATATT TTATAAACCC ATTAATACAA AATTATGTCA CTAATTAACA TAAAACTCTA GTTTTATCCA   
  
  
- AAATTGATGT CGTTTTACTA ATAACCTAA

+     Unnamed\_\_4

| Site Name | Organism | Position | Strand | Matrix score. | sequence | function |
| --- | --- | --- | --- | --- | --- | --- |
| Unnamed\_\_4 | Petroselinum hortense | 696 | + | 4 | CTCC |  |
| Unnamed\_\_4 | Petroselinum hortense | 656 | + | 4 | CTCC |  |
| Unnamed\_\_4 | Petroselinum hortense | 611 | + | 4 | CTCC |  |
| Unnamed\_\_4 | Petroselinum hortense | 93 | - | 4 | CTCC |  |
| Unnamed\_\_4 | Petroselinum hortense | 474 | + | 4 | CTCC |  |
| Unnamed\_\_4 | Petroselinum hortense | 316 | - | 4 | CTCC |  |
| Unnamed\_\_4 | Petroselinum hortense | 871 | - | 4 | CTCC |  |
| Unnamed\_\_4 | Petroselinum hortense | 480 | + | 4 | CTCC |  |

> 2018/04/13 10:10:12  
+ TTTTTGTTAG GGTTTTTCGT TTCTTTCTTC TTTAAGTTCT TTACACAATA TTTCTTGTCG ACTTCGGACA   
  
  
+ TAGATGAGAG AGAGAGAGAG AGGGAGAGAG GAAGACGAAG AGGGTGATAC ACATACTTCA GACACTTCAG   
  
  
+ AAAGTGGCCA CGATATCCGA GCCGATAATT TTCCCAACCC GGCACCTGGT CTAAAACAAT AATCTTCTAC   
  
  
+ TGTGTGAGAA ACCTAAAGAG TGATAAAGGT TTAAAGGTTA GGCTCGAAGA ATGCAGTCGA CCACGCAGTC   
  
  
+ CAGCGGTCGA TCGTTGACCC AGGGTGAAGA CTGGTGGAGA CCGAACCAAT GGGCAGAAAA GGTGTTGGGC   
  
  
+ TTGAGCAATT GGGCACCGAC TAAACAGTAA AATAGACAAC TATATACACA CAACCGCCCA TACCTCGGCT   
  
  
+ GAAGCCCAAT CTGCATCTAT GCCTAGACAA ACTGCTTTCT CTTTTTTCAA TTTCTCCCTC TCCAAAGGAT   
  
  
+ TCGTAAAGCA GTTCTTTTCT CAAACTGAGC GAGCATTTAG TCTTCTTTCT TTACTTAGGT GGCGGGGCTT   
  
  
+ GCCACGCCGC GCAAGGTGCA CTGCTTTTTC CTTTATGTTT ATTGGGTTTT CTCCTTCTTT TGACTTCCTC   
  
  
+ TCTCTTCCTC TCTCTCTCTC TCTCTCTCCC CTCGGCTCAG TTGCTTAAAC TGCCGGGCCG AGAGACTCCT   
  
  
+ ATACTGGCCT CTGGCCCACC CCGTCACCGG ACCAGATACG CACCTGGGGT GCATCGTACT GTACTCTTTC   
  
  
+ GTGCAAAGTC TAGGCGGGGG CCCTTGGAAC AACTGACAAA TGGACACCCT GGCTCAGCGA ACGAAACTGA   
  
  
+ AAGACAATGT ACAACGAAAT GAGTAAACAG GGAGAGAAAG AGAAGTAGAG GATCAGTAGA AAAGGAAAAA   
  
  
+ AATAATTAAA GATTTTATTT CGAAATTGTA TGTACAAAAA GGAACACGTA CATAAAAGAG TTCATTTATC   
  
  
+ TTTTTATTAA TAAATAATGG TTTGAATAAA GGATATAATA ATTTTAAAAA CGTACAAATT TTATTCAAAT   
  
  
+ AATAAAATCA ATTTTTTAAT TAGTTTTTTT AATTAATAAA AAAAATACAT TAAATACTTC CCGTGTATCA   
  
  
+ TAAAATCCAC TTATAGTACT GTGTGCACAA TGAAACCTAA TCCTAAAAGA CAATCTAAAA ATAGAGGCGA   
  
  
+ TTTGAAAAAT TACTTTTCTA TTTTATCATA AAAATATTAA ATAATATAAT TTAATTATTT ATTTAATATT   
  
  
+ AATAAATAAA ATAATTTAAT ATTGTACAGT AATTGAAAAT TTTTTGAGTC GTGTTAAATC TACTCATTTA   
  
  
+ ATTTTTATTA CTATACATCA ATATTCATAG TTTTTATTTT TATAAACAAA TAAATTTTTA AAATTTTCGA   
  
  
+ TTTTGTATAA AATATTTGGG TAATTATGTT TTAATACAGT GATTAATTGT ATTTTGAGAT CAAAATAGGT   
  
  
+ TTTAACTACA GCAAAATGAT TATTGGATT  

- AAAAACAATC CCAAAAAGCA AAGAAAGAAG AAATTCAAGA AATGTGTTAT AAAGAACAGC TGAAGCCTGT   
  
  
- ATCTACTCTC TCTCTCTCTC TCCCTCTCTC CTTCTGCTTC TCCCACTATG TGTATGAAGT CTGTGAAGTC   
  
  
- TTTCACCGGT GCTATAGGCT CGGCTATTAA AAGGGTTGGG CCGTGGACCA GATTTTGTTA TTAGAAGATG   
  
  
- ACACACTCTT TGGATTTCTC ACTATTTCCA AATTTCCAAT CCGAGCTTCT TACGTCAGCT GGTGCGTCAG   
  
  
- GTCGCCAGCT AGCAACTGGG TCCCACTTCT GACCACCTCT GGCTTGGTTA CCCGTCTTTT CCACAACCCG   
  
  
- AACTCGTTAA CCCGTGGCTG ATTTGTCATT TTATCTGTTG ATATATGTGT GTTGGCGGGT ATGGAGCCGA   
  
  
- CTTCGGGTTA GACGTAGATA CGGATCTGTT TGACGAAAGA GAAAAAAGTT AAAGAGGGAG AGGTTTCCTA   
  
  
- AGCATTTCGT CAAGAAAAGA GTTTGACTCG CTCGTAAATC AGAAGAAAGA AATGAATCCA CCGCCCCGAA   
  
  
- CGGTGCGGCG CGTTCCACGT GACGAAAAAG GAAATACAAA TAACCCAAAA GAGGAAGAAA ACTGAAGGAG   
  
  
- AGAGAAGGAG AGAGAGAGAG AGAGAGAGGG GAGCCGAGTC AACGAATTTG ACGGCCCGGC TCTCTGAGGA   
  
  
- TATGACCGGA GACCGGGTGG GGCAGTGGCC TGGTCTATGC GTGGACCCCA CGTAGCATGA CATGAGAAAG   
  
  
- CACGTTTCAG ATCCGCCCCC GGGAACCTTG TTGACTGTTT ACCTGTGGGA CCGAGTCGCT TGCTTTGACT   
  
  
- TTCTGTTACA TGTTGCTTTA CTCATTTGTC CCTCTCTTTC TCTTCATCTC CTAGTCATCT TTTCCTTTTT   
  
  
- TTATTAATTT CTAAAATAAA GCTTTAACAT ACATGTTTTT CCTTGTGCAT GTATTTTCTC AAGTAAATAG   
  
  
- AAAAATAATT ATTTATTACC AAACTTATTT CCTATATTAT TAAAATTTTT GCATGTTTAA AATAAGTTTA   
  
  
- TTATTTTAGT TAAAAAATTA ATCAAAAAAA TTAATTATTT TTTTTATGTA ATTTATGAAG GGCACATAGT   
  
  
- ATTTTAGGTG AATATCATGA CACACGTGTT ACTTTGGATT AGGATTTTCT GTTAGATTTT TATCTCCGCT   
  
  
- AAACTTTTTA ATGAAAAGAT AAAATAGTAT TTTTATAATT TATTATATTA AATTAATAAA TAAATTATAA   
  
  
- TTATTTATTT TATTAAATTA TAACATGTCA TTAACTTTTA AAAAACTCAG CACAATTTAG ATGAGTAAAT   
  
  
- TAAAAATAAT GATATGTAGT TATAAGTATC AAAAATAAAA ATATTTGTTT ATTTAAAAAT TTTAAAAGCT   
  
  
- AAAACATATT TTATAAACCC ATTAATACAA AATTATGTCA CTAATTAACA TAAAACTCTA GTTTTATCCA   
  
  
- AAATTGATGT CGTTTTACTA ATAACCTAA

+     W box

| Site Name | Organism | Position | Strand | Matrix score. | sequence | function |
| --- | --- | --- | --- | --- | --- | --- |
| W box | Arabidopsis thaliana | 294 | + | 6 | TTGACC |  |

> 2018/04/13 10:10:12  
+ TTTTTGTTAG GGTTTTTCGT TTCTTTCTTC TTTAAGTTCT TTACACAATA TTTCTTGTCG ACTTCGGACA   
  
  
+ TAGATGAGAG AGAGAGAGAG AGGGAGAGAG GAAGACGAAG AGGGTGATAC ACATACTTCA GACACTTCAG   
  
  
+ AAAGTGGCCA CGATATCCGA GCCGATAATT TTCCCAACCC GGCACCTGGT CTAAAACAAT AATCTTCTAC   
  
  
+ TGTGTGAGAA ACCTAAAGAG TGATAAAGGT TTAAAGGTTA GGCTCGAAGA ATGCAGTCGA CCACGCAGTC   
  
  
+ CAGCGGTCGA TCGTTGACCC AGGGTGAAGA CTGGTGGAGA CCGAACCAAT GGGCAGAAAA GGTGTTGGGC   
  
  
+ TTGAGCAATT GGGCACCGAC TAAACAGTAA AATAGACAAC TATATACACA CAACCGCCCA TACCTCGGCT   
  
  
+ GAAGCCCAAT CTGCATCTAT GCCTAGACAA ACTGCTTTCT CTTTTTTCAA TTTCTCCCTC TCCAAAGGAT   
  
  
+ TCGTAAAGCA GTTCTTTTCT CAAACTGAGC GAGCATTTAG TCTTCTTTCT TTACTTAGGT GGCGGGGCTT   
  
  
+ GCCACGCCGC GCAAGGTGCA CTGCTTTTTC CTTTATGTTT ATTGGGTTTT CTCCTTCTTT TGACTTCCTC   
  
  
+ TCTCTTCCTC TCTCTCTCTC TCTCTCTCCC CTCGGCTCAG TTGCTTAAAC TGCCGGGCCG AGAGACTCCT   
  
  
+ ATACTGGCCT CTGGCCCACC CCGTCACCGG ACCAGATACG CACCTGGGGT GCATCGTACT GTACTCTTTC   
  
  
+ GTGCAAAGTC TAGGCGGGGG CCCTTGGAAC AACTGACAAA TGGACACCCT GGCTCAGCGA ACGAAACTGA   
  
  
+ AAGACAATGT ACAACGAAAT GAGTAAACAG GGAGAGAAAG AGAAGTAGAG GATCAGTAGA AAAGGAAAAA   
  
  
+ AATAATTAAA GATTTTATTT CGAAATTGTA TGTACAAAAA GGAACACGTA CATAAAAGAG TTCATTTATC   
  
  
+ TTTTTATTAA TAAATAATGG TTTGAATAAA GGATATAATA ATTTTAAAAA CGTACAAATT TTATTCAAAT   
  
  
+ AATAAAATCA ATTTTTTAAT TAGTTTTTTT AATTAATAAA AAAAATACAT TAAATACTTC CCGTGTATCA   
  
  
+ TAAAATCCAC TTATAGTACT GTGTGCACAA TGAAACCTAA TCCTAAAAGA CAATCTAAAA ATAGAGGCGA   
  
  
+ TTTGAAAAAT TACTTTTCTA TTTTATCATA AAAATATTAA ATAATATAAT TTAATTATTT ATTTAATATT   
  
  
+ AATAAATAAA ATAATTTAAT ATTGTACAGT AATTGAAAAT TTTTTGAGTC GTGTTAAATC TACTCATTTA   
  
  
+ ATTTTTATTA CTATACATCA ATATTCATAG TTTTTATTTT TATAAACAAA TAAATTTTTA AAATTTTCGA   
  
  
+ TTTTGTATAA AATATTTGGG TAATTATGTT TTAATACAGT GATTAATTGT ATTTTGAGAT CAAAATAGGT   
  
  
+ TTTAACTACA GCAAAATGAT TATTGGATT  

- AAAAACAATC CCAAAAAGCA AAGAAAGAAG AAATTCAAGA AATGTGTTAT AAAGAACAGC TGAAGCCTGT   
  
  
- ATCTACTCTC TCTCTCTCTC TCCCTCTCTC CTTCTGCTTC TCCCACTATG TGTATGAAGT CTGTGAAGTC   
  
  
- TTTCACCGGT GCTATAGGCT CGGCTATTAA AAGGGTTGGG CCGTGGACCA GATTTTGTTA TTAGAAGATG   
  
  
- ACACACTCTT TGGATTTCTC ACTATTTCCA AATTTCCAAT CCGAGCTTCT TACGTCAGCT GGTGCGTCAG   
  
  
- GTCGCCAGCT AGCAACTGGG TCCCACTTCT GACCACCTCT GGCTTGGTTA CCCGTCTTTT CCACAACCCG   
  
  
- AACTCGTTAA CCCGTGGCTG ATTTGTCATT TTATCTGTTG ATATATGTGT GTTGGCGGGT ATGGAGCCGA   
  
  
- CTTCGGGTTA GACGTAGATA CGGATCTGTT TGACGAAAGA GAAAAAAGTT AAAGAGGGAG AGGTTTCCTA   
  
  
- AGCATTTCGT CAAGAAAAGA GTTTGACTCG CTCGTAAATC AGAAGAAAGA AATGAATCCA CCGCCCCGAA   
  
  
- CGGTGCGGCG CGTTCCACGT GACGAAAAAG GAAATACAAA TAACCCAAAA GAGGAAGAAA ACTGAAGGAG   
  
  
- AGAGAAGGAG AGAGAGAGAG AGAGAGAGGG GAGCCGAGTC AACGAATTTG ACGGCCCGGC TCTCTGAGGA   
  
  
- TATGACCGGA GACCGGGTGG GGCAGTGGCC TGGTCTATGC GTGGACCCCA CGTAGCATGA CATGAGAAAG   
  
  
- CACGTTTCAG ATCCGCCCCC GGGAACCTTG TTGACTGTTT ACCTGTGGGA CCGAGTCGCT TGCTTTGACT   
  
  
- TTCTGTTACA TGTTGCTTTA CTCATTTGTC CCTCTCTTTC TCTTCATCTC CTAGTCATCT TTTCCTTTTT   
  
  
- TTATTAATTT CTAAAATAAA GCTTTAACAT ACATGTTTTT CCTTGTGCAT GTATTTTCTC AAGTAAATAG   
  
  
- AAAAATAATT ATTTATTACC AAACTTATTT CCTATATTAT TAAAATTTTT GCATGTTTAA AATAAGTTTA   
  
  
- TTATTTTAGT TAAAAAATTA ATCAAAAAAA TTAATTATTT TTTTTATGTA ATTTATGAAG GGCACATAGT   
  
  
- ATTTTAGGTG AATATCATGA CACACGTGTT ACTTTGGATT AGGATTTTCT GTTAGATTTT TATCTCCGCT   
  
  
- AAACTTTTTA ATGAAAAGAT AAAATAGTAT TTTTATAATT TATTATATTA AATTAATAAA TAAATTATAA   
  
  
- TTATTTATTT TATTAAATTA TAACATGTCA TTAACTTTTA AAAAACTCAG CACAATTTAG ATGAGTAAAT   
  
  
- TAAAAATAAT GATATGTAGT TATAAGTATC AAAAATAAAA ATATTTGTTT ATTTAAAAAT TTTAAAAGCT   
  
  
- AAAACATATT TTATAAACCC ATTAATACAA AATTATGTCA CTAATTAACA TAAAACTCTA GTTTTATCCA   
  
  
- AAATTGATGT CGTTTTACTA ATAACCTAA
