# Supplementary material for: Genome-wide analysis of the WRKY gene family in drumstick (Moringa oleifera Lam.)
Source: PeerJ. 2019 Jun 10;7:e7063. doi: 10.7717/peerj.7063 (PMC6563795; doi:10.7717/peerj.7063)
Supplement: Supplemental Information 1 [file peerj-07-7063-s003.gz › MoWRKY39_plantcare.html]

Content-Type: text/html; charset=ISO-8859-1


CallMat\_Firefox


Webmaster Firefox specific output  
To save the result:
click on the frame with the right mouse button and save the source code as a text file with extension .html  
REFERENCE:PlantCARE: a database of plant cis-acting regulatory elements and a portal to tools for in silico analysis of promoter sequences.  
Lescot, M., Déhais, P., Moreau, Y., De Moor, B., Rouzé ,P.,and Rombauts, S.  
Nucleic Acids Res., Database issue(2002), 30(1):325-327.   


---

> 2018/04/13 10:10:12  
+ CAATGAAAAG AAAGCTGCAT AGACATGTTG ACCCCTTCAT CCACTCTTTG TCTAGTTGCT TCTCTTCTAT   
  
  
+ GTATTACCAT TGACTGTTGA GTTTTCGAAG GATTAAAAGA AAAGTTTGTT CACAAAAAAT TTGATGCCCT   
  
  
+ AATTAAAACC CTTGAGGAAA TAAAAATCAT AATCTGGGCA ACCGGCTTCC TAAGTATATA TAGTATATAT   
  
  
+ TTCTAGGATT GGATGGCAGC TCGGCTTAAT TAATTATCTT TTAGTTATCT TAGAGCAATA AATTTAAGTC   
  
  
+ CGTTTTATAT GGTCGATTTC TATATATTGT GAATCATTGA CTGTTGAATT GAAATATTTT GTCATAATAT   
  
  
+ TTGTACATAT AACTTGGGGC AGCCAACGGA GCAATGGGAT CGGGGAGTCT AATATTTTAT CTACTAGAAG   
  
  
+ GTAAAAGCTT TTGAGTAACA AAGATGACTA CTATATAATA GCAAGCTTGA TAGTTACATA ACTAATAATA   
  
  
+ TCGGGTTTGA GATATGGTAA TCTATTGCTA TTTCATAAAA TAAGAATCGT ATTTACACCA CGATCCGTTT   
  
  
+ AATAATCCAC AAGTCTTTGT GTATATAGTT TTATATTAAC TTAATTCGAC TTAGTGAACA GATCAAAATC   
  
  
+ GATTATTTTT ATAGCTCGTG GAACTCGGAT CAGAAGAGAT GTATCCACCT GTGCTATTTC AAGAAGGGAA   
  
  
+ ATTTTGTCAT TAATATTAAG TGGATAATAA TGGACAAACT AACCGGGTAC TTAGACATCA ATCCATTAAC   
  
  
+ ATTGAATTAG TCCTTTTGTA CATATTATGG CGTTACATGT CCTGTATGAA GAAAAGAAAA CATCACCGTT   
  
  
+ TTAATTTATT ATTCAAATAT ACTTGTAATA CACAGAGATA TCAGAGATGA TAATCATAAT CATGATTAAC   
  
  
+ TAATCAGATC TGATGAAGCG CTTGGTACCT AACTAGTTGT GAGCTATACA ATAATATCAA CTAGGGTTTT   
  
  
+ CATACCGAGC AAGTGAAGGT CAATGGCGAG GTCAGTGGTG TGAGTGATCG TCACTCCGTG TTATACAAGC   
  
  
+ GTATTGACCA GGAGCTTAAT TATTGACACA GAAACAACAA AGCCTGCACC ATTGACACAG AGAAAAACCG   
  
  
+ ACCGGCCACC GGGGAGGCTA AAAGGTCACC AGTACTGGAT CTTTGACCCT TAGCCATGTG ACGTCGTCTC   
  
  
+ TATATGAAGC TTCAACGATG GTTACCATAG ATTATACAAT AGGGTCGTCG CCGCAAATTT AGCCTTTCTC   
  
  
+ CTTCTTTCTC TCTCTCTGCA AACTGATCAT ATCTCATCTG TTTCTTAACT GTGAAAGGTT GAAGTTGAAT   
  
  
+ CTTTCCATAT TGTTTACTCT TTTAAGGTAA TAAAATACTA AAAGGAAAAT GTGGTTGGGG TGTTGACATT   
  
  
+ GTCGGCCTGT GTATGAAGGT ACTATTATAA GAAGAGAAGT GCGAGAGAGA CATAACGGAT ACAGAAGCTA   
  
  
+ AAAGAGCAAA GAAGAATACC TTCTACAGC  

- GTTACTTTTC TTTCGACGTA TCTGTACAAC TGGGGAAGTA GGTGAGAAAC AGATCAACGA AGAGAAGATA   
  
  
- CATAATGGTA ACTGACAACT CAAAAGCTTC CTAATTTTCT TTTCAAACAA GTGTTTTTTA AACTACGGGA   
  
  
- TTAATTTTGG GAACTCCTTT ATTTTTAGTA TTAGACCCGT TGGCCGAAGG ATTCATATAT ATCATATATA   
  
  
- AAGATCCTAA CCTACCGTCG AGCCGAATTA ATTAATAGAA AATCAATAGA ATCTCGTTAT TTAAATTCAG   
  
  
- GCAAAATATA CCAGCTAAAG ATATATAACA CTTAGTAACT GACAACTTAA CTTTATAAAA CAGTATTATA   
  
  
- AACATGTATA TTGAACCCCG TCGGTTGCCT CGTTACCCTA GCCCCTCAGA TTATAAAATA GATGATCTTC   
  
  
- CATTTTCGAA AACTCATTGT TTCTACTGAT GATATATTAT CGTTCGAACT ATCAATGTAT TGATTATTAT   
  
  
- AGCCCAAACT CTATACCATT AGATAACGAT AAAGTATTTT ATTCTTAGCA TAAATGTGGT GCTAGGCAAA   
  
  
- TTATTAGGTG TTCAGAAACA CATATATCAA AATATAATTG AATTAAGCTG AATCACTTGT CTAGTTTTAG   
  
  
- CTAATAAAAA TATCGAGCAC CTTGAGCCTA GTCTTCTCTA CATAGGTGGA CACGATAAAG TTCTTCCCTT   
  
  
- TAAAACAGTA ATTATAATTC ACCTATTATT ACCTGTTTGA TTGGCCCATG AATCTGTAGT TAGGTAATTG   
  
  
- TAACTTAATC AGGAAAACAT GTATAATACC GCAATGTACA GGACATACTT CTTTTCTTTT GTAGTGGCAA   
  
  
- AATTAAATAA TAAGTTTATA TGAACATTAT GTGTCTCTAT AGTCTCTACT ATTAGTATTA GTACTAATTG   
  
  
- ATTAGTCTAG ACTACTTCGC GAACCATGGA TTGATCAACA CTCGATATGT TATTATAGTT GATCCCAAAA   
  
  
- GTATGGCTCG TTCACTTCCA GTTACCGCTC CAGTCACCAC ACTCACTAGC AGTGAGGCAC AATATGTTCG   
  
  
- CATAACTGGT CCTCGAATTA ATAACTGTGT CTTTGTTGTT TCGGACGTGG TAACTGTGTC TCTTTTTGGC   
  
  
- TGGCCGGTGG CCCCTCCGAT TTTCCAGTGG TCATGACCTA GAAACTGGGA ATCGGTACAC TGCAGCAGAG   
  
  
- ATATACTTCG AAGTTGCTAC CAATGGTATC TAATATGTTA TCCCAGCAGC GGCGTTTAAA TCGGAAAGAG   
  
  
- GAAGAAAGAG AGAGAGACGT TTGACTAGTA TAGAGTAGAC AAAGAATTGA CACTTTCCAA CTTCAACTTA   
  
  
- GAAAGGTATA ACAAATGAGA AAATTCCATT ATTTTATGAT TTTCCTTTTA CACCAACCCC ACAACTGTAA   
  
  
- CAGCCGGACA CATACTTCCA TGATAATATT CTTCTCTTCA CGCTCTCTCT GTATTGCCTA TGTCTTCGAT   
  
  
- TTTCTCGTTT CTTCTTATGG AAGATGTCG

  
  
Motifs Found  

+     5UTR Py-rich stretch

| Site Name | Organism | Position | Strand | Matrix score. | sequence | function |
| --- | --- | --- | --- | --- | --- | --- |
| 5UTR Py-rich stretch | Lycopersicon esculentum | 820 | - | 9 | TTTCTTCTCT | cis-acting element conferring high transcription levels |
| 5UTR Py-rich stretch | Lycopersicon esculentum | 1265 | + | 13 | TTTCTCTCTCTCTC | cis-acting element conferring high transcription levels |
| 5UTR Py-rich stretch | Lycopersicon esculentum | 56 | + | 9 | TTTCTTCTCT | cis-acting element conferring high transcription levels |

> 2018/04/13 10:10:12  
+ CAATGAAAAG AAAGCTGCAT AGACATGTTG ACCCCTTCAT CCACTCTTTG TCTAGTTGCT TCTCTTCTAT   
  
  
+ GTATTACCAT TGACTGTTGA GTTTTCGAAG GATTAAAAGA AAAGTTTGTT CACAAAAAAT TTGATGCCCT   
  
  
+ AATTAAAACC CTTGAGGAAA TAAAAATCAT AATCTGGGCA ACCGGCTTCC TAAGTATATA TAGTATATAT   
  
  
+ TTCTAGGATT GGATGGCAGC TCGGCTTAAT TAATTATCTT TTAGTTATCT TAGAGCAATA AATTTAAGTC   
  
  
+ CGTTTTATAT GGTCGATTTC TATATATTGT GAATCATTGA CTGTTGAATT GAAATATTTT GTCATAATAT   
  
  
+ TTGTACATAT AACTTGGGGC AGCCAACGGA GCAATGGGAT CGGGGAGTCT AATATTTTAT CTACTAGAAG   
  
  
+ GTAAAAGCTT TTGAGTAACA AAGATGACTA CTATATAATA GCAAGCTTGA TAGTTACATA ACTAATAATA   
  
  
+ TCGGGTTTGA GATATGGTAA TCTATTGCTA TTTCATAAAA TAAGAATCGT ATTTACACCA CGATCCGTTT   
  
  
+ AATAATCCAC AAGTCTTTGT GTATATAGTT TTATATTAAC TTAATTCGAC TTAGTGAACA GATCAAAATC   
  
  
+ GATTATTTTT ATAGCTCGTG GAACTCGGAT CAGAAGAGAT GTATCCACCT GTGCTATTTC AAGAAGGGAA   
  
  
+ ATTTTGTCAT TAATATTAAG TGGATAATAA TGGACAAACT AACCGGGTAC TTAGACATCA ATCCATTAAC   
  
  
+ ATTGAATTAG TCCTTTTGTA CATATTATGG CGTTACATGT CCTGTATGAA GAAAAGAAAA CATCACCGTT   
  
  
+ TTAATTTATT ATTCAAATAT ACTTGTAATA CACAGAGATA TCAGAGATGA TAATCATAAT CATGATTAAC   
  
  
+ TAATCAGATC TGATGAAGCG CTTGGTACCT AACTAGTTGT GAGCTATACA ATAATATCAA CTAGGGTTTT   
  
  
+ CATACCGAGC AAGTGAAGGT CAATGGCGAG GTCAGTGGTG TGAGTGATCG TCACTCCGTG TTATACAAGC   
  
  
+ GTATTGACCA GGAGCTTAAT TATTGACACA GAAACAACAA AGCCTGCACC ATTGACACAG AGAAAAACCG   
  
  
+ ACCGGCCACC GGGGAGGCTA AAAGGTCACC AGTACTGGAT CTTTGACCCT TAGCCATGTG ACGTCGTCTC   
  
  
+ TATATGAAGC TTCAACGATG GTTACCATAG ATTATACAAT AGGGTCGTCG CCGCAAATTT AGCCTTTCTC   
  
  
+ CTTCTTTCTC TCTCTCTGCA AACTGATCAT ATCTCATCTG TTTCTTAACT GTGAAAGGTT GAAGTTGAAT   
  
  
+ CTTTCCATAT TGTTTACTCT TTTAAGGTAA TAAAATACTA AAAGGAAAAT GTGGTTGGGG TGTTGACATT   
  
  
+ GTCGGCCTGT GTATGAAGGT ACTATTATAA GAAGAGAAGT GCGAGAGAGA CATAACGGAT ACAGAAGCTA   
  
  
+ AAAGAGCAAA GAAGAATACC TTCTACAGC  

- GTTACTTTTC TTTCGACGTA TCTGTACAAC TGGGGAAGTA GGTGAGAAAC AGATCAACGA AGAGAAGATA   
  
  
- CATAATGGTA ACTGACAACT CAAAAGCTTC CTAATTTTCT TTTCAAACAA GTGTTTTTTA AACTACGGGA   
  
  
- TTAATTTTGG GAACTCCTTT ATTTTTAGTA TTAGACCCGT TGGCCGAAGG ATTCATATAT ATCATATATA   
  
  
- AAGATCCTAA CCTACCGTCG AGCCGAATTA ATTAATAGAA AATCAATAGA ATCTCGTTAT TTAAATTCAG   
  
  
- GCAAAATATA CCAGCTAAAG ATATATAACA CTTAGTAACT GACAACTTAA CTTTATAAAA CAGTATTATA   
  
  
- AACATGTATA TTGAACCCCG TCGGTTGCCT CGTTACCCTA GCCCCTCAGA TTATAAAATA GATGATCTTC   
  
  
- CATTTTCGAA AACTCATTGT TTCTACTGAT GATATATTAT CGTTCGAACT ATCAATGTAT TGATTATTAT   
  
  
- AGCCCAAACT CTATACCATT AGATAACGAT AAAGTATTTT ATTCTTAGCA TAAATGTGGT GCTAGGCAAA   
  
  
- TTATTAGGTG TTCAGAAACA CATATATCAA AATATAATTG AATTAAGCTG AATCACTTGT CTAGTTTTAG   
  
  
- CTAATAAAAA TATCGAGCAC CTTGAGCCTA GTCTTCTCTA CATAGGTGGA CACGATAAAG TTCTTCCCTT   
  
  
- TAAAACAGTA ATTATAATTC ACCTATTATT ACCTGTTTGA TTGGCCCATG AATCTGTAGT TAGGTAATTG   
  
  
- TAACTTAATC AGGAAAACAT GTATAATACC GCAATGTACA GGACATACTT CTTTTCTTTT GTAGTGGCAA   
  
  
- AATTAAATAA TAAGTTTATA TGAACATTAT GTGTCTCTAT AGTCTCTACT ATTAGTATTA GTACTAATTG   
  
  
- ATTAGTCTAG ACTACTTCGC GAACCATGGA TTGATCAACA CTCGATATGT TATTATAGTT GATCCCAAAA   
  
  
- GTATGGCTCG TTCACTTCCA GTTACCGCTC CAGTCACCAC ACTCACTAGC AGTGAGGCAC AATATGTTCG   
  
  
- CATAACTGGT CCTCGAATTA ATAACTGTGT CTTTGTTGTT TCGGACGTGG TAACTGTGTC TCTTTTTGGC   
  
  
- TGGCCGGTGG CCCCTCCGAT TTTCCAGTGG TCATGACCTA GAAACTGGGA ATCGGTACAC TGCAGCAGAG   
  
  
- ATATACTTCG AAGTTGCTAC CAATGGTATC TAATATGTTA TCCCAGCAGC GGCGTTTAAA TCGGAAAGAG   
  
  
- GAAGAAAGAG AGAGAGACGT TTGACTAGTA TAGAGTAGAC AAAGAATTGA CACTTTCCAA CTTCAACTTA   
  
  
- GAAAGGTATA ACAAATGAGA AAATTCCATT ATTTTATGAT TTTCCTTTTA CACCAACCCC ACAACTGTAA   
  
  
- CAGCCGGACA CATACTTCCA TGATAATATT CTTCTCTTCA CGCTCTCTCT GTATTGCCTA TGTCTTCGAT   
  
  
- TTTCTCGTTT CTTCTTATGG AAGATGTCG

+     AAGAA-motif

| Site Name | Organism | Position | Strand | Matrix score. | sequence | function |
| --- | --- | --- | --- | --- | --- | --- |
| AAGAA-motif | Avena sativa | 1262 | - | 7 | GAAAGAA |  |

> 2018/04/13 10:10:12  
+ CAATGAAAAG AAAGCTGCAT AGACATGTTG ACCCCTTCAT CCACTCTTTG TCTAGTTGCT TCTCTTCTAT   
  
  
+ GTATTACCAT TGACTGTTGA GTTTTCGAAG GATTAAAAGA AAAGTTTGTT CACAAAAAAT TTGATGCCCT   
  
  
+ AATTAAAACC CTTGAGGAAA TAAAAATCAT AATCTGGGCA ACCGGCTTCC TAAGTATATA TAGTATATAT   
  
  
+ TTCTAGGATT GGATGGCAGC TCGGCTTAAT TAATTATCTT TTAGTTATCT TAGAGCAATA AATTTAAGTC   
  
  
+ CGTTTTATAT GGTCGATTTC TATATATTGT GAATCATTGA CTGTTGAATT GAAATATTTT GTCATAATAT   
  
  
+ TTGTACATAT AACTTGGGGC AGCCAACGGA GCAATGGGAT CGGGGAGTCT AATATTTTAT CTACTAGAAG   
  
  
+ GTAAAAGCTT TTGAGTAACA AAGATGACTA CTATATAATA GCAAGCTTGA TAGTTACATA ACTAATAATA   
  
  
+ TCGGGTTTGA GATATGGTAA TCTATTGCTA TTTCATAAAA TAAGAATCGT ATTTACACCA CGATCCGTTT   
  
  
+ AATAATCCAC AAGTCTTTGT GTATATAGTT TTATATTAAC TTAATTCGAC TTAGTGAACA GATCAAAATC   
  
  
+ GATTATTTTT ATAGCTCGTG GAACTCGGAT CAGAAGAGAT GTATCCACCT GTGCTATTTC AAGAAGGGAA   
  
  
+ ATTTTGTCAT TAATATTAAG TGGATAATAA TGGACAAACT AACCGGGTAC TTAGACATCA ATCCATTAAC   
  
  
+ ATTGAATTAG TCCTTTTGTA CATATTATGG CGTTACATGT CCTGTATGAA GAAAAGAAAA CATCACCGTT   
  
  
+ TTAATTTATT ATTCAAATAT ACTTGTAATA CACAGAGATA TCAGAGATGA TAATCATAAT CATGATTAAC   
  
  
+ TAATCAGATC TGATGAAGCG CTTGGTACCT AACTAGTTGT GAGCTATACA ATAATATCAA CTAGGGTTTT   
  
  
+ CATACCGAGC AAGTGAAGGT CAATGGCGAG GTCAGTGGTG TGAGTGATCG TCACTCCGTG TTATACAAGC   
  
  
+ GTATTGACCA GGAGCTTAAT TATTGACACA GAAACAACAA AGCCTGCACC ATTGACACAG AGAAAAACCG   
  
  
+ ACCGGCCACC GGGGAGGCTA AAAGGTCACC AGTACTGGAT CTTTGACCCT TAGCCATGTG ACGTCGTCTC   
  
  
+ TATATGAAGC TTCAACGATG GTTACCATAG ATTATACAAT AGGGTCGTCG CCGCAAATTT AGCCTTTCTC   
  
  
+ CTTCTTTCTC TCTCTCTGCA AACTGATCAT ATCTCATCTG TTTCTTAACT GTGAAAGGTT GAAGTTGAAT   
  
  
+ CTTTCCATAT TGTTTACTCT TTTAAGGTAA TAAAATACTA AAAGGAAAAT GTGGTTGGGG TGTTGACATT   
  
  
+ GTCGGCCTGT GTATGAAGGT ACTATTATAA GAAGAGAAGT GCGAGAGAGA CATAACGGAT ACAGAAGCTA   
  
  
+ AAAGAGCAAA GAAGAATACC TTCTACAGC  

- GTTACTTTTC TTTCGACGTA TCTGTACAAC TGGGGAAGTA GGTGAGAAAC AGATCAACGA AGAGAAGATA   
  
  
- CATAATGGTA ACTGACAACT CAAAAGCTTC CTAATTTTCT TTTCAAACAA GTGTTTTTTA AACTACGGGA   
  
  
- TTAATTTTGG GAACTCCTTT ATTTTTAGTA TTAGACCCGT TGGCCGAAGG ATTCATATAT ATCATATATA   
  
  
- AAGATCCTAA CCTACCGTCG AGCCGAATTA ATTAATAGAA AATCAATAGA ATCTCGTTAT TTAAATTCAG   
  
  
- GCAAAATATA CCAGCTAAAG ATATATAACA CTTAGTAACT GACAACTTAA CTTTATAAAA CAGTATTATA   
  
  
- AACATGTATA TTGAACCCCG TCGGTTGCCT CGTTACCCTA GCCCCTCAGA TTATAAAATA GATGATCTTC   
  
  
- CATTTTCGAA AACTCATTGT TTCTACTGAT GATATATTAT CGTTCGAACT ATCAATGTAT TGATTATTAT   
  
  
- AGCCCAAACT CTATACCATT AGATAACGAT AAAGTATTTT ATTCTTAGCA TAAATGTGGT GCTAGGCAAA   
  
  
- TTATTAGGTG TTCAGAAACA CATATATCAA AATATAATTG AATTAAGCTG AATCACTTGT CTAGTTTTAG   
  
  
- CTAATAAAAA TATCGAGCAC CTTGAGCCTA GTCTTCTCTA CATAGGTGGA CACGATAAAG TTCTTCCCTT   
  
  
- TAAAACAGTA ATTATAATTC ACCTATTATT ACCTGTTTGA TTGGCCCATG AATCTGTAGT TAGGTAATTG   
  
  
- TAACTTAATC AGGAAAACAT GTATAATACC GCAATGTACA GGACATACTT CTTTTCTTTT GTAGTGGCAA   
  
  
- AATTAAATAA TAAGTTTATA TGAACATTAT GTGTCTCTAT AGTCTCTACT ATTAGTATTA GTACTAATTG   
  
  
- ATTAGTCTAG ACTACTTCGC GAACCATGGA TTGATCAACA CTCGATATGT TATTATAGTT GATCCCAAAA   
  
  
- GTATGGCTCG TTCACTTCCA GTTACCGCTC CAGTCACCAC ACTCACTAGC AGTGAGGCAC AATATGTTCG   
  
  
- CATAACTGGT CCTCGAATTA ATAACTGTGT CTTTGTTGTT TCGGACGTGG TAACTGTGTC TCTTTTTGGC   
  
  
- TGGCCGGTGG CCCCTCCGAT TTTCCAGTGG TCATGACCTA GAAACTGGGA ATCGGTACAC TGCAGCAGAG   
  
  
- ATATACTTCG AAGTTGCTAC CAATGGTATC TAATATGTTA TCCCAGCAGC GGCGTTTAAA TCGGAAAGAG   
  
  
- GAAGAAAGAG AGAGAGACGT TTGACTAGTA TAGAGTAGAC AAAGAATTGA CACTTTCCAA CTTCAACTTA   
  
  
- GAAAGGTATA ACAAATGAGA AAATTCCATT ATTTTATGAT TTTCCTTTTA CACCAACCCC ACAACTGTAA   
  
  
- CAGCCGGACA CATACTTCCA TGATAATATT CTTCTCTTCA CGCTCTCTCT GTATTGCCTA TGTCTTCGAT   
  
  
- TTTCTCGTTT CTTCTTATGG AAGATGTCG

+     AE-box

| Site Name | Organism | Position | Strand | Matrix score. | sequence | function |
| --- | --- | --- | --- | --- | --- | --- |
| AE-box | Arabidopsis thaliana | 1080 | + | 8 | AGAAACAA | part of a module for light response |

> 2018/04/13 10:10:12  
+ CAATGAAAAG AAAGCTGCAT AGACATGTTG ACCCCTTCAT CCACTCTTTG TCTAGTTGCT TCTCTTCTAT   
  
  
+ GTATTACCAT TGACTGTTGA GTTTTCGAAG GATTAAAAGA AAAGTTTGTT CACAAAAAAT TTGATGCCCT   
  
  
+ AATTAAAACC CTTGAGGAAA TAAAAATCAT AATCTGGGCA ACCGGCTTCC TAAGTATATA TAGTATATAT   
  
  
+ TTCTAGGATT GGATGGCAGC TCGGCTTAAT TAATTATCTT TTAGTTATCT TAGAGCAATA AATTTAAGTC   
  
  
+ CGTTTTATAT GGTCGATTTC TATATATTGT GAATCATTGA CTGTTGAATT GAAATATTTT GTCATAATAT   
  
  
+ TTGTACATAT AACTTGGGGC AGCCAACGGA GCAATGGGAT CGGGGAGTCT AATATTTTAT CTACTAGAAG   
  
  
+ GTAAAAGCTT TTGAGTAACA AAGATGACTA CTATATAATA GCAAGCTTGA TAGTTACATA ACTAATAATA   
  
  
+ TCGGGTTTGA GATATGGTAA TCTATTGCTA TTTCATAAAA TAAGAATCGT ATTTACACCA CGATCCGTTT   
  
  
+ AATAATCCAC AAGTCTTTGT GTATATAGTT TTATATTAAC TTAATTCGAC TTAGTGAACA GATCAAAATC   
  
  
+ GATTATTTTT ATAGCTCGTG GAACTCGGAT CAGAAGAGAT GTATCCACCT GTGCTATTTC AAGAAGGGAA   
  
  
+ ATTTTGTCAT TAATATTAAG TGGATAATAA TGGACAAACT AACCGGGTAC TTAGACATCA ATCCATTAAC   
  
  
+ ATTGAATTAG TCCTTTTGTA CATATTATGG CGTTACATGT CCTGTATGAA GAAAAGAAAA CATCACCGTT   
  
  
+ TTAATTTATT ATTCAAATAT ACTTGTAATA CACAGAGATA TCAGAGATGA TAATCATAAT CATGATTAAC   
  
  
+ TAATCAGATC TGATGAAGCG CTTGGTACCT AACTAGTTGT GAGCTATACA ATAATATCAA CTAGGGTTTT   
  
  
+ CATACCGAGC AAGTGAAGGT CAATGGCGAG GTCAGTGGTG TGAGTGATCG TCACTCCGTG TTATACAAGC   
  
  
+ GTATTGACCA GGAGCTTAAT TATTGACACA GAAACAACAA AGCCTGCACC ATTGACACAG AGAAAAACCG   
  
  
+ ACCGGCCACC GGGGAGGCTA AAAGGTCACC AGTACTGGAT CTTTGACCCT TAGCCATGTG ACGTCGTCTC   
  
  
+ TATATGAAGC TTCAACGATG GTTACCATAG ATTATACAAT AGGGTCGTCG CCGCAAATTT AGCCTTTCTC   
  
  
+ CTTCTTTCTC TCTCTCTGCA AACTGATCAT ATCTCATCTG TTTCTTAACT GTGAAAGGTT GAAGTTGAAT   
  
  
+ CTTTCCATAT TGTTTACTCT TTTAAGGTAA TAAAATACTA AAAGGAAAAT GTGGTTGGGG TGTTGACATT   
  
  
+ GTCGGCCTGT GTATGAAGGT ACTATTATAA GAAGAGAAGT GCGAGAGAGA CATAACGGAT ACAGAAGCTA   
  
  
+ AAAGAGCAAA GAAGAATACC TTCTACAGC  

- GTTACTTTTC TTTCGACGTA TCTGTACAAC TGGGGAAGTA GGTGAGAAAC AGATCAACGA AGAGAAGATA   
  
  
- CATAATGGTA ACTGACAACT CAAAAGCTTC CTAATTTTCT TTTCAAACAA GTGTTTTTTA AACTACGGGA   
  
  
- TTAATTTTGG GAACTCCTTT ATTTTTAGTA TTAGACCCGT TGGCCGAAGG ATTCATATAT ATCATATATA   
  
  
- AAGATCCTAA CCTACCGTCG AGCCGAATTA ATTAATAGAA AATCAATAGA ATCTCGTTAT TTAAATTCAG   
  
  
- GCAAAATATA CCAGCTAAAG ATATATAACA CTTAGTAACT GACAACTTAA CTTTATAAAA CAGTATTATA   
  
  
- AACATGTATA TTGAACCCCG TCGGTTGCCT CGTTACCCTA GCCCCTCAGA TTATAAAATA GATGATCTTC   
  
  
- CATTTTCGAA AACTCATTGT TTCTACTGAT GATATATTAT CGTTCGAACT ATCAATGTAT TGATTATTAT   
  
  
- AGCCCAAACT CTATACCATT AGATAACGAT AAAGTATTTT ATTCTTAGCA TAAATGTGGT GCTAGGCAAA   
  
  
- TTATTAGGTG TTCAGAAACA CATATATCAA AATATAATTG AATTAAGCTG AATCACTTGT CTAGTTTTAG   
  
  
- CTAATAAAAA TATCGAGCAC CTTGAGCCTA GTCTTCTCTA CATAGGTGGA CACGATAAAG TTCTTCCCTT   
  
  
- TAAAACAGTA ATTATAATTC ACCTATTATT ACCTGTTTGA TTGGCCCATG AATCTGTAGT TAGGTAATTG   
  
  
- TAACTTAATC AGGAAAACAT GTATAATACC GCAATGTACA GGACATACTT CTTTTCTTTT GTAGTGGCAA   
  
  
- AATTAAATAA TAAGTTTATA TGAACATTAT GTGTCTCTAT AGTCTCTACT ATTAGTATTA GTACTAATTG   
  
  
- ATTAGTCTAG ACTACTTCGC GAACCATGGA TTGATCAACA CTCGATATGT TATTATAGTT GATCCCAAAA   
  
  
- GTATGGCTCG TTCACTTCCA GTTACCGCTC CAGTCACCAC ACTCACTAGC AGTGAGGCAC AATATGTTCG   
  
  
- CATAACTGGT CCTCGAATTA ATAACTGTGT CTTTGTTGTT TCGGACGTGG TAACTGTGTC TCTTTTTGGC   
  
  
- TGGCCGGTGG CCCCTCCGAT TTTCCAGTGG TCATGACCTA GAAACTGGGA ATCGGTACAC TGCAGCAGAG   
  
  
- ATATACTTCG AAGTTGCTAC CAATGGTATC TAATATGTTA TCCCAGCAGC GGCGTTTAAA TCGGAAAGAG   
  
  
- GAAGAAAGAG AGAGAGACGT TTGACTAGTA TAGAGTAGAC AAAGAATTGA CACTTTCCAA CTTCAACTTA   
  
  
- GAAAGGTATA ACAAATGAGA AAATTCCATT ATTTTATGAT TTTCCTTTTA CACCAACCCC ACAACTGTAA   
  
  
- CAGCCGGACA CATACTTCCA TGATAATATT CTTCTCTTCA CGCTCTCTCT GTATTGCCTA TGTCTTCGAT   
  
  
- TTTCTCGTTT CTTCTTATGG AAGATGTCG

+     AT-rich element

| Site Name | Organism | Position | Strand | Matrix score. | sequence | function |
| --- | --- | --- | --- | --- | --- | --- |
| AT-rich element | Glycine max | 293 | - | 10 | ATAGAAATCAA | binding site of AT-rich DNA binding protein (ATBP-1) |

> 2018/04/13 10:10:12  
+ CAATGAAAAG AAAGCTGCAT AGACATGTTG ACCCCTTCAT CCACTCTTTG TCTAGTTGCT TCTCTTCTAT   
  
  
+ GTATTACCAT TGACTGTTGA GTTTTCGAAG GATTAAAAGA AAAGTTTGTT CACAAAAAAT TTGATGCCCT   
  
  
+ AATTAAAACC CTTGAGGAAA TAAAAATCAT AATCTGGGCA ACCGGCTTCC TAAGTATATA TAGTATATAT   
  
  
+ TTCTAGGATT GGATGGCAGC TCGGCTTAAT TAATTATCTT TTAGTTATCT TAGAGCAATA AATTTAAGTC   
  
  
+ CGTTTTATAT GGTCGATTTC TATATATTGT GAATCATTGA CTGTTGAATT GAAATATTTT GTCATAATAT   
  
  
+ TTGTACATAT AACTTGGGGC AGCCAACGGA GCAATGGGAT CGGGGAGTCT AATATTTTAT CTACTAGAAG   
  
  
+ GTAAAAGCTT TTGAGTAACA AAGATGACTA CTATATAATA GCAAGCTTGA TAGTTACATA ACTAATAATA   
  
  
+ TCGGGTTTGA GATATGGTAA TCTATTGCTA TTTCATAAAA TAAGAATCGT ATTTACACCA CGATCCGTTT   
  
  
+ AATAATCCAC AAGTCTTTGT GTATATAGTT TTATATTAAC TTAATTCGAC TTAGTGAACA GATCAAAATC   
  
  
+ GATTATTTTT ATAGCTCGTG GAACTCGGAT CAGAAGAGAT GTATCCACCT GTGCTATTTC AAGAAGGGAA   
  
  
+ ATTTTGTCAT TAATATTAAG TGGATAATAA TGGACAAACT AACCGGGTAC TTAGACATCA ATCCATTAAC   
  
  
+ ATTGAATTAG TCCTTTTGTA CATATTATGG CGTTACATGT CCTGTATGAA GAAAAGAAAA CATCACCGTT   
  
  
+ TTAATTTATT ATTCAAATAT ACTTGTAATA CACAGAGATA TCAGAGATGA TAATCATAAT CATGATTAAC   
  
  
+ TAATCAGATC TGATGAAGCG CTTGGTACCT AACTAGTTGT GAGCTATACA ATAATATCAA CTAGGGTTTT   
  
  
+ CATACCGAGC AAGTGAAGGT CAATGGCGAG GTCAGTGGTG TGAGTGATCG TCACTCCGTG TTATACAAGC   
  
  
+ GTATTGACCA GGAGCTTAAT TATTGACACA GAAACAACAA AGCCTGCACC ATTGACACAG AGAAAAACCG   
  
  
+ ACCGGCCACC GGGGAGGCTA AAAGGTCACC AGTACTGGAT CTTTGACCCT TAGCCATGTG ACGTCGTCTC   
  
  
+ TATATGAAGC TTCAACGATG GTTACCATAG ATTATACAAT AGGGTCGTCG CCGCAAATTT AGCCTTTCTC   
  
  
+ CTTCTTTCTC TCTCTCTGCA AACTGATCAT ATCTCATCTG TTTCTTAACT GTGAAAGGTT GAAGTTGAAT   
  
  
+ CTTTCCATAT TGTTTACTCT TTTAAGGTAA TAAAATACTA AAAGGAAAAT GTGGTTGGGG TGTTGACATT   
  
  
+ GTCGGCCTGT GTATGAAGGT ACTATTATAA GAAGAGAAGT GCGAGAGAGA CATAACGGAT ACAGAAGCTA   
  
  
+ AAAGAGCAAA GAAGAATACC TTCTACAGC  

- GTTACTTTTC TTTCGACGTA TCTGTACAAC TGGGGAAGTA GGTGAGAAAC AGATCAACGA AGAGAAGATA   
  
  
- CATAATGGTA ACTGACAACT CAAAAGCTTC CTAATTTTCT TTTCAAACAA GTGTTTTTTA AACTACGGGA   
  
  
- TTAATTTTGG GAACTCCTTT ATTTTTAGTA TTAGACCCGT TGGCCGAAGG ATTCATATAT ATCATATATA   
  
  
- AAGATCCTAA CCTACCGTCG AGCCGAATTA ATTAATAGAA AATCAATAGA ATCTCGTTAT TTAAATTCAG   
  
  
- GCAAAATATA CCAGCTAAAG ATATATAACA CTTAGTAACT GACAACTTAA CTTTATAAAA CAGTATTATA   
  
  
- AACATGTATA TTGAACCCCG TCGGTTGCCT CGTTACCCTA GCCCCTCAGA TTATAAAATA GATGATCTTC   
  
  
- CATTTTCGAA AACTCATTGT TTCTACTGAT GATATATTAT CGTTCGAACT ATCAATGTAT TGATTATTAT   
  
  
- AGCCCAAACT CTATACCATT AGATAACGAT AAAGTATTTT ATTCTTAGCA TAAATGTGGT GCTAGGCAAA   
  
  
- TTATTAGGTG TTCAGAAACA CATATATCAA AATATAATTG AATTAAGCTG AATCACTTGT CTAGTTTTAG   
  
  
- CTAATAAAAA TATCGAGCAC CTTGAGCCTA GTCTTCTCTA CATAGGTGGA CACGATAAAG TTCTTCCCTT   
  
  
- TAAAACAGTA ATTATAATTC ACCTATTATT ACCTGTTTGA TTGGCCCATG AATCTGTAGT TAGGTAATTG   
  
  
- TAACTTAATC AGGAAAACAT GTATAATACC GCAATGTACA GGACATACTT CTTTTCTTTT GTAGTGGCAA   
  
  
- AATTAAATAA TAAGTTTATA TGAACATTAT GTGTCTCTAT AGTCTCTACT ATTAGTATTA GTACTAATTG   
  
  
- ATTAGTCTAG ACTACTTCGC GAACCATGGA TTGATCAACA CTCGATATGT TATTATAGTT GATCCCAAAA   
  
  
- GTATGGCTCG TTCACTTCCA GTTACCGCTC CAGTCACCAC ACTCACTAGC AGTGAGGCAC AATATGTTCG   
  
  
- CATAACTGGT CCTCGAATTA ATAACTGTGT CTTTGTTGTT TCGGACGTGG TAACTGTGTC TCTTTTTGGC   
  
  
- TGGCCGGTGG CCCCTCCGAT TTTCCAGTGG TCATGACCTA GAAACTGGGA ATCGGTACAC TGCAGCAGAG   
  
  
- ATATACTTCG AAGTTGCTAC CAATGGTATC TAATATGTTA TCCCAGCAGC GGCGTTTAAA TCGGAAAGAG   
  
  
- GAAGAAAGAG AGAGAGACGT TTGACTAGTA TAGAGTAGAC AAAGAATTGA CACTTTCCAA CTTCAACTTA   
  
  
- GAAAGGTATA ACAAATGAGA AAATTCCATT ATTTTATGAT TTTCCTTTTA CACCAACCCC ACAACTGTAA   
  
  
- CAGCCGGACA CATACTTCCA TGATAATATT CTTCTCTTCA CGCTCTCTCT GTATTGCCTA TGTCTTCGAT   
  
  
- TTTCTCGTTT CTTCTTATGG AAGATGTCG

+     AT-rich sequence

| Site Name | Organism | Position | Strand | Matrix score. | sequence | function |
| --- | --- | --- | --- | --- | --- | --- |
| AT-rich sequence | Pisum sativum | 1361 | + | 9 | TAAAATACT | element for maximal elicitor-mediated activation (2copies) |

> 2018/04/13 10:10:12  
+ CAATGAAAAG AAAGCTGCAT AGACATGTTG ACCCCTTCAT CCACTCTTTG TCTAGTTGCT TCTCTTCTAT   
  
  
+ GTATTACCAT TGACTGTTGA GTTTTCGAAG GATTAAAAGA AAAGTTTGTT CACAAAAAAT TTGATGCCCT   
  
  
+ AATTAAAACC CTTGAGGAAA TAAAAATCAT AATCTGGGCA ACCGGCTTCC TAAGTATATA TAGTATATAT   
  
  
+ TTCTAGGATT GGATGGCAGC TCGGCTTAAT TAATTATCTT TTAGTTATCT TAGAGCAATA AATTTAAGTC   
  
  
+ CGTTTTATAT GGTCGATTTC TATATATTGT GAATCATTGA CTGTTGAATT GAAATATTTT GTCATAATAT   
  
  
+ TTGTACATAT AACTTGGGGC AGCCAACGGA GCAATGGGAT CGGGGAGTCT AATATTTTAT CTACTAGAAG   
  
  
+ GTAAAAGCTT TTGAGTAACA AAGATGACTA CTATATAATA GCAAGCTTGA TAGTTACATA ACTAATAATA   
  
  
+ TCGGGTTTGA GATATGGTAA TCTATTGCTA TTTCATAAAA TAAGAATCGT ATTTACACCA CGATCCGTTT   
  
  
+ AATAATCCAC AAGTCTTTGT GTATATAGTT TTATATTAAC TTAATTCGAC TTAGTGAACA GATCAAAATC   
  
  
+ GATTATTTTT ATAGCTCGTG GAACTCGGAT CAGAAGAGAT GTATCCACCT GTGCTATTTC AAGAAGGGAA   
  
  
+ ATTTTGTCAT TAATATTAAG TGGATAATAA TGGACAAACT AACCGGGTAC TTAGACATCA ATCCATTAAC   
  
  
+ ATTGAATTAG TCCTTTTGTA CATATTATGG CGTTACATGT CCTGTATGAA GAAAAGAAAA CATCACCGTT   
  
  
+ TTAATTTATT ATTCAAATAT ACTTGTAATA CACAGAGATA TCAGAGATGA TAATCATAAT CATGATTAAC   
  
  
+ TAATCAGATC TGATGAAGCG CTTGGTACCT AACTAGTTGT GAGCTATACA ATAATATCAA CTAGGGTTTT   
  
  
+ CATACCGAGC AAGTGAAGGT CAATGGCGAG GTCAGTGGTG TGAGTGATCG TCACTCCGTG TTATACAAGC   
  
  
+ GTATTGACCA GGAGCTTAAT TATTGACACA GAAACAACAA AGCCTGCACC ATTGACACAG AGAAAAACCG   
  
  
+ ACCGGCCACC GGGGAGGCTA AAAGGTCACC AGTACTGGAT CTTTGACCCT TAGCCATGTG ACGTCGTCTC   
  
  
+ TATATGAAGC TTCAACGATG GTTACCATAG ATTATACAAT AGGGTCGTCG CCGCAAATTT AGCCTTTCTC   
  
  
+ CTTCTTTCTC TCTCTCTGCA AACTGATCAT ATCTCATCTG TTTCTTAACT GTGAAAGGTT GAAGTTGAAT   
  
  
+ CTTTCCATAT TGTTTACTCT TTTAAGGTAA TAAAATACTA AAAGGAAAAT GTGGTTGGGG TGTTGACATT   
  
  
+ GTCGGCCTGT GTATGAAGGT ACTATTATAA GAAGAGAAGT GCGAGAGAGA CATAACGGAT ACAGAAGCTA   
  
  
+ AAAGAGCAAA GAAGAATACC TTCTACAGC  

- GTTACTTTTC TTTCGACGTA TCTGTACAAC TGGGGAAGTA GGTGAGAAAC AGATCAACGA AGAGAAGATA   
  
  
- CATAATGGTA ACTGACAACT CAAAAGCTTC CTAATTTTCT TTTCAAACAA GTGTTTTTTA AACTACGGGA   
  
  
- TTAATTTTGG GAACTCCTTT ATTTTTAGTA TTAGACCCGT TGGCCGAAGG ATTCATATAT ATCATATATA   
  
  
- AAGATCCTAA CCTACCGTCG AGCCGAATTA ATTAATAGAA AATCAATAGA ATCTCGTTAT TTAAATTCAG   
  
  
- GCAAAATATA CCAGCTAAAG ATATATAACA CTTAGTAACT GACAACTTAA CTTTATAAAA CAGTATTATA   
  
  
- AACATGTATA TTGAACCCCG TCGGTTGCCT CGTTACCCTA GCCCCTCAGA TTATAAAATA GATGATCTTC   
  
  
- CATTTTCGAA AACTCATTGT TTCTACTGAT GATATATTAT CGTTCGAACT ATCAATGTAT TGATTATTAT   
  
  
- AGCCCAAACT CTATACCATT AGATAACGAT AAAGTATTTT ATTCTTAGCA TAAATGTGGT GCTAGGCAAA   
  
  
- TTATTAGGTG TTCAGAAACA CATATATCAA AATATAATTG AATTAAGCTG AATCACTTGT CTAGTTTTAG   
  
  
- CTAATAAAAA TATCGAGCAC CTTGAGCCTA GTCTTCTCTA CATAGGTGGA CACGATAAAG TTCTTCCCTT   
  
  
- TAAAACAGTA ATTATAATTC ACCTATTATT ACCTGTTTGA TTGGCCCATG AATCTGTAGT TAGGTAATTG   
  
  
- TAACTTAATC AGGAAAACAT GTATAATACC GCAATGTACA GGACATACTT CTTTTCTTTT GTAGTGGCAA   
  
  
- AATTAAATAA TAAGTTTATA TGAACATTAT GTGTCTCTAT AGTCTCTACT ATTAGTATTA GTACTAATTG   
  
  
- ATTAGTCTAG ACTACTTCGC GAACCATGGA TTGATCAACA CTCGATATGT TATTATAGTT GATCCCAAAA   
  
  
- GTATGGCTCG TTCACTTCCA GTTACCGCTC CAGTCACCAC ACTCACTAGC AGTGAGGCAC AATATGTTCG   
  
  
- CATAACTGGT CCTCGAATTA ATAACTGTGT CTTTGTTGTT TCGGACGTGG TAACTGTGTC TCTTTTTGGC   
  
  
- TGGCCGGTGG CCCCTCCGAT TTTCCAGTGG TCATGACCTA GAAACTGGGA ATCGGTACAC TGCAGCAGAG   
  
  
- ATATACTTCG AAGTTGCTAC CAATGGTATC TAATATGTTA TCCCAGCAGC GGCGTTTAAA TCGGAAAGAG   
  
  
- GAAGAAAGAG AGAGAGACGT TTGACTAGTA TAGAGTAGAC AAAGAATTGA CACTTTCCAA CTTCAACTTA   
  
  
- GAAAGGTATA ACAAATGAGA AAATTCCATT ATTTTATGAT TTTCCTTTTA CACCAACCCC ACAACTGTAA   
  
  
- CAGCCGGACA CATACTTCCA TGATAATATT CTTCTCTTCA CGCTCTCTCT GTATTGCCTA TGTCTTCGAT   
  
  
- TTTCTCGTTT CTTCTTATGG AAGATGTCG

+     Box 4

| Site Name | Organism | Position | Strand | Matrix score. | sequence | function |
| --- | --- | --- | --- | --- | --- | --- |
| Box 4 | Petroselinum crispum | 239 | + | 6 | ATTAAT | part of a conserved DNA module involved in light responsiveness |
| Box 4 | Petroselinum crispum | 709 | + | 6 | ATTAAT | part of a conserved DNA module involved in light responsiveness |

> 2018/04/13 10:10:12  
+ CAATGAAAAG AAAGCTGCAT AGACATGTTG ACCCCTTCAT CCACTCTTTG TCTAGTTGCT TCTCTTCTAT   
  
  
+ GTATTACCAT TGACTGTTGA GTTTTCGAAG GATTAAAAGA AAAGTTTGTT CACAAAAAAT TTGATGCCCT   
  
  
+ AATTAAAACC CTTGAGGAAA TAAAAATCAT AATCTGGGCA ACCGGCTTCC TAAGTATATA TAGTATATAT   
  
  
+ TTCTAGGATT GGATGGCAGC TCGGCTTAAT TAATTATCTT TTAGTTATCT TAGAGCAATA AATTTAAGTC   
  
  
+ CGTTTTATAT GGTCGATTTC TATATATTGT GAATCATTGA CTGTTGAATT GAAATATTTT GTCATAATAT   
  
  
+ TTGTACATAT AACTTGGGGC AGCCAACGGA GCAATGGGAT CGGGGAGTCT AATATTTTAT CTACTAGAAG   
  
  
+ GTAAAAGCTT TTGAGTAACA AAGATGACTA CTATATAATA GCAAGCTTGA TAGTTACATA ACTAATAATA   
  
  
+ TCGGGTTTGA GATATGGTAA TCTATTGCTA TTTCATAAAA TAAGAATCGT ATTTACACCA CGATCCGTTT   
  
  
+ AATAATCCAC AAGTCTTTGT GTATATAGTT TTATATTAAC TTAATTCGAC TTAGTGAACA GATCAAAATC   
  
  
+ GATTATTTTT ATAGCTCGTG GAACTCGGAT CAGAAGAGAT GTATCCACCT GTGCTATTTC AAGAAGGGAA   
  
  
+ ATTTTGTCAT TAATATTAAG TGGATAATAA TGGACAAACT AACCGGGTAC TTAGACATCA ATCCATTAAC   
  
  
+ ATTGAATTAG TCCTTTTGTA CATATTATGG CGTTACATGT CCTGTATGAA GAAAAGAAAA CATCACCGTT   
  
  
+ TTAATTTATT ATTCAAATAT ACTTGTAATA CACAGAGATA TCAGAGATGA TAATCATAAT CATGATTAAC   
  
  
+ TAATCAGATC TGATGAAGCG CTTGGTACCT AACTAGTTGT GAGCTATACA ATAATATCAA CTAGGGTTTT   
  
  
+ CATACCGAGC AAGTGAAGGT CAATGGCGAG GTCAGTGGTG TGAGTGATCG TCACTCCGTG TTATACAAGC   
  
  
+ GTATTGACCA GGAGCTTAAT TATTGACACA GAAACAACAA AGCCTGCACC ATTGACACAG AGAAAAACCG   
  
  
+ ACCGGCCACC GGGGAGGCTA AAAGGTCACC AGTACTGGAT CTTTGACCCT TAGCCATGTG ACGTCGTCTC   
  
  
+ TATATGAAGC TTCAACGATG GTTACCATAG ATTATACAAT AGGGTCGTCG CCGCAAATTT AGCCTTTCTC   
  
  
+ CTTCTTTCTC TCTCTCTGCA AACTGATCAT ATCTCATCTG TTTCTTAACT GTGAAAGGTT GAAGTTGAAT   
  
  
+ CTTTCCATAT TGTTTACTCT TTTAAGGTAA TAAAATACTA AAAGGAAAAT GTGGTTGGGG TGTTGACATT   
  
  
+ GTCGGCCTGT GTATGAAGGT ACTATTATAA GAAGAGAAGT GCGAGAGAGA CATAACGGAT ACAGAAGCTA   
  
  
+ AAAGAGCAAA GAAGAATACC TTCTACAGC  

- GTTACTTTTC TTTCGACGTA TCTGTACAAC TGGGGAAGTA GGTGAGAAAC AGATCAACGA AGAGAAGATA   
  
  
- CATAATGGTA ACTGACAACT CAAAAGCTTC CTAATTTTCT TTTCAAACAA GTGTTTTTTA AACTACGGGA   
  
  
- TTAATTTTGG GAACTCCTTT ATTTTTAGTA TTAGACCCGT TGGCCGAAGG ATTCATATAT ATCATATATA   
  
  
- AAGATCCTAA CCTACCGTCG AGCCGAATTA ATTAATAGAA AATCAATAGA ATCTCGTTAT TTAAATTCAG   
  
  
- GCAAAATATA CCAGCTAAAG ATATATAACA CTTAGTAACT GACAACTTAA CTTTATAAAA CAGTATTATA   
  
  
- AACATGTATA TTGAACCCCG TCGGTTGCCT CGTTACCCTA GCCCCTCAGA TTATAAAATA GATGATCTTC   
  
  
- CATTTTCGAA AACTCATTGT TTCTACTGAT GATATATTAT CGTTCGAACT ATCAATGTAT TGATTATTAT   
  
  
- AGCCCAAACT CTATACCATT AGATAACGAT AAAGTATTTT ATTCTTAGCA TAAATGTGGT GCTAGGCAAA   
  
  
- TTATTAGGTG TTCAGAAACA CATATATCAA AATATAATTG AATTAAGCTG AATCACTTGT CTAGTTTTAG   
  
  
- CTAATAAAAA TATCGAGCAC CTTGAGCCTA GTCTTCTCTA CATAGGTGGA CACGATAAAG TTCTTCCCTT   
  
  
- TAAAACAGTA ATTATAATTC ACCTATTATT ACCTGTTTGA TTGGCCCATG AATCTGTAGT TAGGTAATTG   
  
  
- TAACTTAATC AGGAAAACAT GTATAATACC GCAATGTACA GGACATACTT CTTTTCTTTT GTAGTGGCAA   
  
  
- AATTAAATAA TAAGTTTATA TGAACATTAT GTGTCTCTAT AGTCTCTACT ATTAGTATTA GTACTAATTG   
  
  
- ATTAGTCTAG ACTACTTCGC GAACCATGGA TTGATCAACA CTCGATATGT TATTATAGTT GATCCCAAAA   
  
  
- GTATGGCTCG TTCACTTCCA GTTACCGCTC CAGTCACCAC ACTCACTAGC AGTGAGGCAC AATATGTTCG   
  
  
- CATAACTGGT CCTCGAATTA ATAACTGTGT CTTTGTTGTT TCGGACGTGG TAACTGTGTC TCTTTTTGGC   
  
  
- TGGCCGGTGG CCCCTCCGAT TTTCCAGTGG TCATGACCTA GAAACTGGGA ATCGGTACAC TGCAGCAGAG   
  
  
- ATATACTTCG AAGTTGCTAC CAATGGTATC TAATATGTTA TCCCAGCAGC GGCGTTTAAA TCGGAAAGAG   
  
  
- GAAGAAAGAG AGAGAGACGT TTGACTAGTA TAGAGTAGAC AAAGAATTGA CACTTTCCAA CTTCAACTTA   
  
  
- GAAAGGTATA ACAAATGAGA AAATTCCATT ATTTTATGAT TTTCCTTTTA CACCAACCCC ACAACTGTAA   
  
  
- CAGCCGGACA CATACTTCCA TGATAATATT CTTCTCTTCA CGCTCTCTCT GTATTGCCTA TGTCTTCGAT   
  
  
- TTTCTCGTTT CTTCTTATGG AAGATGTCG

+     Box-W1

| Site Name | Organism | Position | Strand | Matrix score. | sequence | function |
| --- | --- | --- | --- | --- | --- | --- |
| Box-W1 | Petroselinum crispum | 1054 | + | 6 | TTGACC | fungal elicitor responsive element |
| Box-W1 | Petroselinum crispum | 998 | - | 6 | TTGACC | fungal elicitor responsive element |
| Box-W1 | Petroselinum crispum | 28 | + | 6 | TTGACC | fungal elicitor responsive element |
| Box-W1 | Petroselinum crispum | 1163 | + | 6 | TTGACC | fungal elicitor responsive element |

> 2018/04/13 10:10:12  
+ CAATGAAAAG AAAGCTGCAT AGACATGTTG ACCCCTTCAT CCACTCTTTG TCTAGTTGCT TCTCTTCTAT   
  
  
+ GTATTACCAT TGACTGTTGA GTTTTCGAAG GATTAAAAGA AAAGTTTGTT CACAAAAAAT TTGATGCCCT   
  
  
+ AATTAAAACC CTTGAGGAAA TAAAAATCAT AATCTGGGCA ACCGGCTTCC TAAGTATATA TAGTATATAT   
  
  
+ TTCTAGGATT GGATGGCAGC TCGGCTTAAT TAATTATCTT TTAGTTATCT TAGAGCAATA AATTTAAGTC   
  
  
+ CGTTTTATAT GGTCGATTTC TATATATTGT GAATCATTGA CTGTTGAATT GAAATATTTT GTCATAATAT   
  
  
+ TTGTACATAT AACTTGGGGC AGCCAACGGA GCAATGGGAT CGGGGAGTCT AATATTTTAT CTACTAGAAG   
  
  
+ GTAAAAGCTT TTGAGTAACA AAGATGACTA CTATATAATA GCAAGCTTGA TAGTTACATA ACTAATAATA   
  
  
+ TCGGGTTTGA GATATGGTAA TCTATTGCTA TTTCATAAAA TAAGAATCGT ATTTACACCA CGATCCGTTT   
  
  
+ AATAATCCAC AAGTCTTTGT GTATATAGTT TTATATTAAC TTAATTCGAC TTAGTGAACA GATCAAAATC   
  
  
+ GATTATTTTT ATAGCTCGTG GAACTCGGAT CAGAAGAGAT GTATCCACCT GTGCTATTTC AAGAAGGGAA   
  
  
+ ATTTTGTCAT TAATATTAAG TGGATAATAA TGGACAAACT AACCGGGTAC TTAGACATCA ATCCATTAAC   
  
  
+ ATTGAATTAG TCCTTTTGTA CATATTATGG CGTTACATGT CCTGTATGAA GAAAAGAAAA CATCACCGTT   
  
  
+ TTAATTTATT ATTCAAATAT ACTTGTAATA CACAGAGATA TCAGAGATGA TAATCATAAT CATGATTAAC   
  
  
+ TAATCAGATC TGATGAAGCG CTTGGTACCT AACTAGTTGT GAGCTATACA ATAATATCAA CTAGGGTTTT   
  
  
+ CATACCGAGC AAGTGAAGGT CAATGGCGAG GTCAGTGGTG TGAGTGATCG TCACTCCGTG TTATACAAGC   
  
  
+ GTATTGACCA GGAGCTTAAT TATTGACACA GAAACAACAA AGCCTGCACC ATTGACACAG AGAAAAACCG   
  
  
+ ACCGGCCACC GGGGAGGCTA AAAGGTCACC AGTACTGGAT CTTTGACCCT TAGCCATGTG ACGTCGTCTC   
  
  
+ TATATGAAGC TTCAACGATG GTTACCATAG ATTATACAAT AGGGTCGTCG CCGCAAATTT AGCCTTTCTC   
  
  
+ CTTCTTTCTC TCTCTCTGCA AACTGATCAT ATCTCATCTG TTTCTTAACT GTGAAAGGTT GAAGTTGAAT   
  
  
+ CTTTCCATAT TGTTTACTCT TTTAAGGTAA TAAAATACTA AAAGGAAAAT GTGGTTGGGG TGTTGACATT   
  
  
+ GTCGGCCTGT GTATGAAGGT ACTATTATAA GAAGAGAAGT GCGAGAGAGA CATAACGGAT ACAGAAGCTA   
  
  
+ AAAGAGCAAA GAAGAATACC TTCTACAGC  

- GTTACTTTTC TTTCGACGTA TCTGTACAAC TGGGGAAGTA GGTGAGAAAC AGATCAACGA AGAGAAGATA   
  
  
- CATAATGGTA ACTGACAACT CAAAAGCTTC CTAATTTTCT TTTCAAACAA GTGTTTTTTA AACTACGGGA   
  
  
- TTAATTTTGG GAACTCCTTT ATTTTTAGTA TTAGACCCGT TGGCCGAAGG ATTCATATAT ATCATATATA   
  
  
- AAGATCCTAA CCTACCGTCG AGCCGAATTA ATTAATAGAA AATCAATAGA ATCTCGTTAT TTAAATTCAG   
  
  
- GCAAAATATA CCAGCTAAAG ATATATAACA CTTAGTAACT GACAACTTAA CTTTATAAAA CAGTATTATA   
  
  
- AACATGTATA TTGAACCCCG TCGGTTGCCT CGTTACCCTA GCCCCTCAGA TTATAAAATA GATGATCTTC   
  
  
- CATTTTCGAA AACTCATTGT TTCTACTGAT GATATATTAT CGTTCGAACT ATCAATGTAT TGATTATTAT   
  
  
- AGCCCAAACT CTATACCATT AGATAACGAT AAAGTATTTT ATTCTTAGCA TAAATGTGGT GCTAGGCAAA   
  
  
- TTATTAGGTG TTCAGAAACA CATATATCAA AATATAATTG AATTAAGCTG AATCACTTGT CTAGTTTTAG   
  
  
- CTAATAAAAA TATCGAGCAC CTTGAGCCTA GTCTTCTCTA CATAGGTGGA CACGATAAAG TTCTTCCCTT   
  
  
- TAAAACAGTA ATTATAATTC ACCTATTATT ACCTGTTTGA TTGGCCCATG AATCTGTAGT TAGGTAATTG   
  
  
- TAACTTAATC AGGAAAACAT GTATAATACC GCAATGTACA GGACATACTT CTTTTCTTTT GTAGTGGCAA   
  
  
- AATTAAATAA TAAGTTTATA TGAACATTAT GTGTCTCTAT AGTCTCTACT ATTAGTATTA GTACTAATTG   
  
  
- ATTAGTCTAG ACTACTTCGC GAACCATGGA TTGATCAACA CTCGATATGT TATTATAGTT GATCCCAAAA   
  
  
- GTATGGCTCG TTCACTTCCA GTTACCGCTC CAGTCACCAC ACTCACTAGC AGTGAGGCAC AATATGTTCG   
  
  
- CATAACTGGT CCTCGAATTA ATAACTGTGT CTTTGTTGTT TCGGACGTGG TAACTGTGTC TCTTTTTGGC   
  
  
- TGGCCGGTGG CCCCTCCGAT TTTCCAGTGG TCATGACCTA GAAACTGGGA ATCGGTACAC TGCAGCAGAG   
  
  
- ATATACTTCG AAGTTGCTAC CAATGGTATC TAATATGTTA TCCCAGCAGC GGCGTTTAAA TCGGAAAGAG   
  
  
- GAAGAAAGAG AGAGAGACGT TTGACTAGTA TAGAGTAGAC AAAGAATTGA CACTTTCCAA CTTCAACTTA   
  
  
- GAAAGGTATA ACAAATGAGA AAATTCCATT ATTTTATGAT TTTCCTTTTA CACCAACCCC ACAACTGTAA   
  
  
- CAGCCGGACA CATACTTCCA TGATAATATT CTTCTCTTCA CGCTCTCTCT GTATTGCCTA TGTCTTCGAT   
  
  
- TTTCTCGTTT CTTCTTATGG AAGATGTCG

+     CAAT-box

| Site Name | Organism | Position | Strand | Matrix score. | sequence | function |
| --- | --- | --- | --- | --- | --- | --- |
| CAAT-box | Hordeum vulgare | 266 | + | 4 | CAAT | common cis-acting element in promoter and enhancer regions |
| CAAT-box | Hordeum vulgare | 306 | - | 4 | CAAT | common cis-acting element in promoter and enhancer regions |
| CAAT-box | Hordeum vulgare | 1072 | - | 4 | CAAT | common cis-acting element in promoter and enhancer regions |
| CAAT-box | Hordeum vulgare | 316 | - | 4 | CAAT | common cis-acting element in promoter and enhancer regions |
| CAAT-box | Hordeum vulgare | 1053 | - | 4 | CAAT | common cis-acting element in promoter and enhancer regions |
| CAAT-box | Hordeum vulgare | 1101 | - | 4 | CAAT | common cis-acting element in promoter and enhancer regions |
| CAAT-box | Hordeum vulgare | 328 | - | 4 | CAAT | common cis-acting element in promoter and enhancer regions |
| CAAT-box | Hordeum vulgare | 1227 | + | 4 | CAAT | common cis-acting element in promoter and enhancer regions |
| CAAT-box | Hordeum vulgare | 759 | + | 4 | CAAT | common cis-acting element in promoter and enhancer regions |
| CAAT-box | Hordeum vulgare | 514 | - | 4 | CAAT | common cis-acting element in promoter and enhancer regions |
| CAAT-box | Hordeum vulgare | 771 | - | 4 | CAAT | common cis-acting element in promoter and enhancer regions |
| CAAT-box | Hordeum vulgare | 959 | + | 4 | CAAT | common cis-acting element in promoter and enhancer regions |
| CAAT-box | Brassica rapa | 854 | + | 5 | CAAAT | common cis-acting element in promoter and enhancer regions |
| CAAT-box | Brassica rapa | 1244 | + | 5 | CAAAT | common cis-acting element in promoter and enhancer regions |
| CAAT-box | Hordeum vulgare | 1001 | + | 4 | CAAT | common cis-acting element in promoter and enhancer regions |
| CAAT-box | Hordeum vulgare | 382 | + | 4 | CAAT | common cis-acting element in promoter and enhancer regions |
| CAAT-box | Hordeum vulgare | 1339 | - | 4 | CAAT | common cis-acting element in promoter and enhancer regions |
| CAAT-box | Hordeum vulgare | 79 | - | 4 | CAAT | common cis-acting element in promoter and enhancer regions |
| CAAT-box | Hordeum vulgare | 1 | + | 4 | CAAT | common cis-acting element in promoter and enhancer regions |
| CAAT-box | Hordeum vulgare | 1398 | - | 4 | CAAT | common cis-acting element in promoter and enhancer regions |
| CAAT-box | Glycine max | 327 | - | 5 | CAATT | common cis-acting element in promoter and enhancer regions |
| CAAT-box | Brassica rapa | 349 | - | 5 | CAAAT | common cis-acting element in promoter and enhancer regions |
| CAAT-box | Brassica rapa | 129 | - | 5 | CAAAT | common cis-acting element in promoter and enhancer regions |
| CAAT-box | Arabidopsis thaliana | 218 | - | 5 | CCAAT | common cis-acting element in promoter and enhancer regions |

> 2018/04/13 10:10:12  
+ CAATGAAAAG AAAGCTGCAT AGACATGTTG ACCCCTTCAT CCACTCTTTG TCTAGTTGCT TCTCTTCTAT   
  
  
+ GTATTACCAT TGACTGTTGA GTTTTCGAAG GATTAAAAGA AAAGTTTGTT CACAAAAAAT TTGATGCCCT   
  
  
+ AATTAAAACC CTTGAGGAAA TAAAAATCAT AATCTGGGCA ACCGGCTTCC TAAGTATATA TAGTATATAT   
  
  
+ TTCTAGGATT GGATGGCAGC TCGGCTTAAT TAATTATCTT TTAGTTATCT TAGAGCAATA AATTTAAGTC   
  
  
+ CGTTTTATAT GGTCGATTTC TATATATTGT GAATCATTGA CTGTTGAATT GAAATATTTT GTCATAATAT   
  
  
+ TTGTACATAT AACTTGGGGC AGCCAACGGA GCAATGGGAT CGGGGAGTCT AATATTTTAT CTACTAGAAG   
  
  
+ GTAAAAGCTT TTGAGTAACA AAGATGACTA CTATATAATA GCAAGCTTGA TAGTTACATA ACTAATAATA   
  
  
+ TCGGGTTTGA GATATGGTAA TCTATTGCTA TTTCATAAAA TAAGAATCGT ATTTACACCA CGATCCGTTT   
  
  
+ AATAATCCAC AAGTCTTTGT GTATATAGTT TTATATTAAC TTAATTCGAC TTAGTGAACA GATCAAAATC   
  
  
+ GATTATTTTT ATAGCTCGTG GAACTCGGAT CAGAAGAGAT GTATCCACCT GTGCTATTTC AAGAAGGGAA   
  
  
+ ATTTTGTCAT TAATATTAAG TGGATAATAA TGGACAAACT AACCGGGTAC TTAGACATCA ATCCATTAAC   
  
  
+ ATTGAATTAG TCCTTTTGTA CATATTATGG CGTTACATGT CCTGTATGAA GAAAAGAAAA CATCACCGTT   
  
  
+ TTAATTTATT ATTCAAATAT ACTTGTAATA CACAGAGATA TCAGAGATGA TAATCATAAT CATGATTAAC   
  
  
+ TAATCAGATC TGATGAAGCG CTTGGTACCT AACTAGTTGT GAGCTATACA ATAATATCAA CTAGGGTTTT   
  
  
+ CATACCGAGC AAGTGAAGGT CAATGGCGAG GTCAGTGGTG TGAGTGATCG TCACTCCGTG TTATACAAGC   
  
  
+ GTATTGACCA GGAGCTTAAT TATTGACACA GAAACAACAA AGCCTGCACC ATTGACACAG AGAAAAACCG   
  
  
+ ACCGGCCACC GGGGAGGCTA AAAGGTCACC AGTACTGGAT CTTTGACCCT TAGCCATGTG ACGTCGTCTC   
  
  
+ TATATGAAGC TTCAACGATG GTTACCATAG ATTATACAAT AGGGTCGTCG CCGCAAATTT AGCCTTTCTC   
  
  
+ CTTCTTTCTC TCTCTCTGCA AACTGATCAT ATCTCATCTG TTTCTTAACT GTGAAAGGTT GAAGTTGAAT   
  
  
+ CTTTCCATAT TGTTTACTCT TTTAAGGTAA TAAAATACTA AAAGGAAAAT GTGGTTGGGG TGTTGACATT   
  
  
+ GTCGGCCTGT GTATGAAGGT ACTATTATAA GAAGAGAAGT GCGAGAGAGA CATAACGGAT ACAGAAGCTA   
  
  
+ AAAGAGCAAA GAAGAATACC TTCTACAGC  

- GTTACTTTTC TTTCGACGTA TCTGTACAAC TGGGGAAGTA GGTGAGAAAC AGATCAACGA AGAGAAGATA   
  
  
- CATAATGGTA ACTGACAACT CAAAAGCTTC CTAATTTTCT TTTCAAACAA GTGTTTTTTA AACTACGGGA   
  
  
- TTAATTTTGG GAACTCCTTT ATTTTTAGTA TTAGACCCGT TGGCCGAAGG ATTCATATAT ATCATATATA   
  
  
- AAGATCCTAA CCTACCGTCG AGCCGAATTA ATTAATAGAA AATCAATAGA ATCTCGTTAT TTAAATTCAG   
  
  
- GCAAAATATA CCAGCTAAAG ATATATAACA CTTAGTAACT GACAACTTAA CTTTATAAAA CAGTATTATA   
  
  
- AACATGTATA TTGAACCCCG TCGGTTGCCT CGTTACCCTA GCCCCTCAGA TTATAAAATA GATGATCTTC   
  
  
- CATTTTCGAA AACTCATTGT TTCTACTGAT GATATATTAT CGTTCGAACT ATCAATGTAT TGATTATTAT   
  
  
- AGCCCAAACT CTATACCATT AGATAACGAT AAAGTATTTT ATTCTTAGCA TAAATGTGGT GCTAGGCAAA   
  
  
- TTATTAGGTG TTCAGAAACA CATATATCAA AATATAATTG AATTAAGCTG AATCACTTGT CTAGTTTTAG   
  
  
- CTAATAAAAA TATCGAGCAC CTTGAGCCTA GTCTTCTCTA CATAGGTGGA CACGATAAAG TTCTTCCCTT   
  
  
- TAAAACAGTA ATTATAATTC ACCTATTATT ACCTGTTTGA TTGGCCCATG AATCTGTAGT TAGGTAATTG   
  
  
- TAACTTAATC AGGAAAACAT GTATAATACC GCAATGTACA GGACATACTT CTTTTCTTTT GTAGTGGCAA   
  
  
- AATTAAATAA TAAGTTTATA TGAACATTAT GTGTCTCTAT AGTCTCTACT ATTAGTATTA GTACTAATTG   
  
  
- ATTAGTCTAG ACTACTTCGC GAACCATGGA TTGATCAACA CTCGATATGT TATTATAGTT GATCCCAAAA   
  
  
- GTATGGCTCG TTCACTTCCA GTTACCGCTC CAGTCACCAC ACTCACTAGC AGTGAGGCAC AATATGTTCG   
  
  
- CATAACTGGT CCTCGAATTA ATAACTGTGT CTTTGTTGTT TCGGACGTGG TAACTGTGTC TCTTTTTGGC   
  
  
- TGGCCGGTGG CCCCTCCGAT TTTCCAGTGG TCATGACCTA GAAACTGGGA ATCGGTACAC TGCAGCAGAG   
  
  
- ATATACTTCG AAGTTGCTAC CAATGGTATC TAATATGTTA TCCCAGCAGC GGCGTTTAAA TCGGAAAGAG   
  
  
- GAAGAAAGAG AGAGAGACGT TTGACTAGTA TAGAGTAGAC AAAGAATTGA CACTTTCCAA CTTCAACTTA   
  
  
- GAAAGGTATA ACAAATGAGA AAATTCCATT ATTTTATGAT TTTCCTTTTA CACCAACCCC ACAACTGTAA   
  
  
- CAGCCGGACA CATACTTCCA TGATAATATT CTTCTCTTCA CGCTCTCTCT GTATTGCCTA TGTCTTCGAT   
  
  
- TTTCTCGTTT CTTCTTATGG AAGATGTCG

+     CCAAT-box

| Site Name | Organism | Position | Strand | Matrix score. | sequence | function |
| --- | --- | --- | --- | --- | --- | --- |
| CCAAT-box | Hordeum vulgare | 374 | + | 6 | CAACGG | MYBHv1 binding site |

> 2018/04/13 10:10:12  
+ CAATGAAAAG AAAGCTGCAT AGACATGTTG ACCCCTTCAT CCACTCTTTG TCTAGTTGCT TCTCTTCTAT   
  
  
+ GTATTACCAT TGACTGTTGA GTTTTCGAAG GATTAAAAGA AAAGTTTGTT CACAAAAAAT TTGATGCCCT   
  
  
+ AATTAAAACC CTTGAGGAAA TAAAAATCAT AATCTGGGCA ACCGGCTTCC TAAGTATATA TAGTATATAT   
  
  
+ TTCTAGGATT GGATGGCAGC TCGGCTTAAT TAATTATCTT TTAGTTATCT TAGAGCAATA AATTTAAGTC   
  
  
+ CGTTTTATAT GGTCGATTTC TATATATTGT GAATCATTGA CTGTTGAATT GAAATATTTT GTCATAATAT   
  
  
+ TTGTACATAT AACTTGGGGC AGCCAACGGA GCAATGGGAT CGGGGAGTCT AATATTTTAT CTACTAGAAG   
  
  
+ GTAAAAGCTT TTGAGTAACA AAGATGACTA CTATATAATA GCAAGCTTGA TAGTTACATA ACTAATAATA   
  
  
+ TCGGGTTTGA GATATGGTAA TCTATTGCTA TTTCATAAAA TAAGAATCGT ATTTACACCA CGATCCGTTT   
  
  
+ AATAATCCAC AAGTCTTTGT GTATATAGTT TTATATTAAC TTAATTCGAC TTAGTGAACA GATCAAAATC   
  
  
+ GATTATTTTT ATAGCTCGTG GAACTCGGAT CAGAAGAGAT GTATCCACCT GTGCTATTTC AAGAAGGGAA   
  
  
+ ATTTTGTCAT TAATATTAAG TGGATAATAA TGGACAAACT AACCGGGTAC TTAGACATCA ATCCATTAAC   
  
  
+ ATTGAATTAG TCCTTTTGTA CATATTATGG CGTTACATGT CCTGTATGAA GAAAAGAAAA CATCACCGTT   
  
  
+ TTAATTTATT ATTCAAATAT ACTTGTAATA CACAGAGATA TCAGAGATGA TAATCATAAT CATGATTAAC   
  
  
+ TAATCAGATC TGATGAAGCG CTTGGTACCT AACTAGTTGT GAGCTATACA ATAATATCAA CTAGGGTTTT   
  
  
+ CATACCGAGC AAGTGAAGGT CAATGGCGAG GTCAGTGGTG TGAGTGATCG TCACTCCGTG TTATACAAGC   
  
  
+ GTATTGACCA GGAGCTTAAT TATTGACACA GAAACAACAA AGCCTGCACC ATTGACACAG AGAAAAACCG   
  
  
+ ACCGGCCACC GGGGAGGCTA AAAGGTCACC AGTACTGGAT CTTTGACCCT TAGCCATGTG ACGTCGTCTC   
  
  
+ TATATGAAGC TTCAACGATG GTTACCATAG ATTATACAAT AGGGTCGTCG CCGCAAATTT AGCCTTTCTC   
  
  
+ CTTCTTTCTC TCTCTCTGCA AACTGATCAT ATCTCATCTG TTTCTTAACT GTGAAAGGTT GAAGTTGAAT   
  
  
+ CTTTCCATAT TGTTTACTCT TTTAAGGTAA TAAAATACTA AAAGGAAAAT GTGGTTGGGG TGTTGACATT   
  
  
+ GTCGGCCTGT GTATGAAGGT ACTATTATAA GAAGAGAAGT GCGAGAGAGA CATAACGGAT ACAGAAGCTA   
  
  
+ AAAGAGCAAA GAAGAATACC TTCTACAGC  

- GTTACTTTTC TTTCGACGTA TCTGTACAAC TGGGGAAGTA GGTGAGAAAC AGATCAACGA AGAGAAGATA   
  
  
- CATAATGGTA ACTGACAACT CAAAAGCTTC CTAATTTTCT TTTCAAACAA GTGTTTTTTA AACTACGGGA   
  
  
- TTAATTTTGG GAACTCCTTT ATTTTTAGTA TTAGACCCGT TGGCCGAAGG ATTCATATAT ATCATATATA   
  
  
- AAGATCCTAA CCTACCGTCG AGCCGAATTA ATTAATAGAA AATCAATAGA ATCTCGTTAT TTAAATTCAG   
  
  
- GCAAAATATA CCAGCTAAAG ATATATAACA CTTAGTAACT GACAACTTAA CTTTATAAAA CAGTATTATA   
  
  
- AACATGTATA TTGAACCCCG TCGGTTGCCT CGTTACCCTA GCCCCTCAGA TTATAAAATA GATGATCTTC   
  
  
- CATTTTCGAA AACTCATTGT TTCTACTGAT GATATATTAT CGTTCGAACT ATCAATGTAT TGATTATTAT   
  
  
- AGCCCAAACT CTATACCATT AGATAACGAT AAAGTATTTT ATTCTTAGCA TAAATGTGGT GCTAGGCAAA   
  
  
- TTATTAGGTG TTCAGAAACA CATATATCAA AATATAATTG AATTAAGCTG AATCACTTGT CTAGTTTTAG   
  
  
- CTAATAAAAA TATCGAGCAC CTTGAGCCTA GTCTTCTCTA CATAGGTGGA CACGATAAAG TTCTTCCCTT   
  
  
- TAAAACAGTA ATTATAATTC ACCTATTATT ACCTGTTTGA TTGGCCCATG AATCTGTAGT TAGGTAATTG   
  
  
- TAACTTAATC AGGAAAACAT GTATAATACC GCAATGTACA GGACATACTT CTTTTCTTTT GTAGTGGCAA   
  
  
- AATTAAATAA TAAGTTTATA TGAACATTAT GTGTCTCTAT AGTCTCTACT ATTAGTATTA GTACTAATTG   
  
  
- ATTAGTCTAG ACTACTTCGC GAACCATGGA TTGATCAACA CTCGATATGT TATTATAGTT GATCCCAAAA   
  
  
- GTATGGCTCG TTCACTTCCA GTTACCGCTC CAGTCACCAC ACTCACTAGC AGTGAGGCAC AATATGTTCG   
  
  
- CATAACTGGT CCTCGAATTA ATAACTGTGT CTTTGTTGTT TCGGACGTGG TAACTGTGTC TCTTTTTGGC   
  
  
- TGGCCGGTGG CCCCTCCGAT TTTCCAGTGG TCATGACCTA GAAACTGGGA ATCGGTACAC TGCAGCAGAG   
  
  
- ATATACTTCG AAGTTGCTAC CAATGGTATC TAATATGTTA TCCCAGCAGC GGCGTTTAAA TCGGAAAGAG   
  
  
- GAAGAAAGAG AGAGAGACGT TTGACTAGTA TAGAGTAGAC AAAGAATTGA CACTTTCCAA CTTCAACTTA   
  
  
- GAAAGGTATA ACAAATGAGA AAATTCCATT ATTTTATGAT TTTCCTTTTA CACCAACCCC ACAACTGTAA   
  
  
- CAGCCGGACA CATACTTCCA TGATAATATT CTTCTCTTCA CGCTCTCTCT GTATTGCCTA TGTCTTCGAT   
  
  
- TTTCTCGTTT CTTCTTATGG AAGATGTCG

+     CGTCA-motif

| Site Name | Organism | Position | Strand | Matrix score. | sequence | function |
| --- | --- | --- | --- | --- | --- | --- |
| CGTCA-motif | Hordeum vulgare | 1179 | - | 5 | CGTCA | cis-acting regulatory element involved in the MeJA-responsiveness |
| CGTCA-motif | Hordeum vulgare | 1029 | + | 5 | CGTCA | cis-acting regulatory element involved in the MeJA-responsiveness |

> 2018/04/13 10:10:12  
+ CAATGAAAAG AAAGCTGCAT AGACATGTTG ACCCCTTCAT CCACTCTTTG TCTAGTTGCT TCTCTTCTAT   
  
  
+ GTATTACCAT TGACTGTTGA GTTTTCGAAG GATTAAAAGA AAAGTTTGTT CACAAAAAAT TTGATGCCCT   
  
  
+ AATTAAAACC CTTGAGGAAA TAAAAATCAT AATCTGGGCA ACCGGCTTCC TAAGTATATA TAGTATATAT   
  
  
+ TTCTAGGATT GGATGGCAGC TCGGCTTAAT TAATTATCTT TTAGTTATCT TAGAGCAATA AATTTAAGTC   
  
  
+ CGTTTTATAT GGTCGATTTC TATATATTGT GAATCATTGA CTGTTGAATT GAAATATTTT GTCATAATAT   
  
  
+ TTGTACATAT AACTTGGGGC AGCCAACGGA GCAATGGGAT CGGGGAGTCT AATATTTTAT CTACTAGAAG   
  
  
+ GTAAAAGCTT TTGAGTAACA AAGATGACTA CTATATAATA GCAAGCTTGA TAGTTACATA ACTAATAATA   
  
  
+ TCGGGTTTGA GATATGGTAA TCTATTGCTA TTTCATAAAA TAAGAATCGT ATTTACACCA CGATCCGTTT   
  
  
+ AATAATCCAC AAGTCTTTGT GTATATAGTT TTATATTAAC TTAATTCGAC TTAGTGAACA GATCAAAATC   
  
  
+ GATTATTTTT ATAGCTCGTG GAACTCGGAT CAGAAGAGAT GTATCCACCT GTGCTATTTC AAGAAGGGAA   
  
  
+ ATTTTGTCAT TAATATTAAG TGGATAATAA TGGACAAACT AACCGGGTAC TTAGACATCA ATCCATTAAC   
  
  
+ ATTGAATTAG TCCTTTTGTA CATATTATGG CGTTACATGT CCTGTATGAA GAAAAGAAAA CATCACCGTT   
  
  
+ TTAATTTATT ATTCAAATAT ACTTGTAATA CACAGAGATA TCAGAGATGA TAATCATAAT CATGATTAAC   
  
  
+ TAATCAGATC TGATGAAGCG CTTGGTACCT AACTAGTTGT GAGCTATACA ATAATATCAA CTAGGGTTTT   
  
  
+ CATACCGAGC AAGTGAAGGT CAATGGCGAG GTCAGTGGTG TGAGTGATCG TCACTCCGTG TTATACAAGC   
  
  
+ GTATTGACCA GGAGCTTAAT TATTGACACA GAAACAACAA AGCCTGCACC ATTGACACAG AGAAAAACCG   
  
  
+ ACCGGCCACC GGGGAGGCTA AAAGGTCACC AGTACTGGAT CTTTGACCCT TAGCCATGTG ACGTCGTCTC   
  
  
+ TATATGAAGC TTCAACGATG GTTACCATAG ATTATACAAT AGGGTCGTCG CCGCAAATTT AGCCTTTCTC   
  
  
+ CTTCTTTCTC TCTCTCTGCA AACTGATCAT ATCTCATCTG TTTCTTAACT GTGAAAGGTT GAAGTTGAAT   
  
  
+ CTTTCCATAT TGTTTACTCT TTTAAGGTAA TAAAATACTA AAAGGAAAAT GTGGTTGGGG TGTTGACATT   
  
  
+ GTCGGCCTGT GTATGAAGGT ACTATTATAA GAAGAGAAGT GCGAGAGAGA CATAACGGAT ACAGAAGCTA   
  
  
+ AAAGAGCAAA GAAGAATACC TTCTACAGC  

- GTTACTTTTC TTTCGACGTA TCTGTACAAC TGGGGAAGTA GGTGAGAAAC AGATCAACGA AGAGAAGATA   
  
  
- CATAATGGTA ACTGACAACT CAAAAGCTTC CTAATTTTCT TTTCAAACAA GTGTTTTTTA AACTACGGGA   
  
  
- TTAATTTTGG GAACTCCTTT ATTTTTAGTA TTAGACCCGT TGGCCGAAGG ATTCATATAT ATCATATATA   
  
  
- AAGATCCTAA CCTACCGTCG AGCCGAATTA ATTAATAGAA AATCAATAGA ATCTCGTTAT TTAAATTCAG   
  
  
- GCAAAATATA CCAGCTAAAG ATATATAACA CTTAGTAACT GACAACTTAA CTTTATAAAA CAGTATTATA   
  
  
- AACATGTATA TTGAACCCCG TCGGTTGCCT CGTTACCCTA GCCCCTCAGA TTATAAAATA GATGATCTTC   
  
  
- CATTTTCGAA AACTCATTGT TTCTACTGAT GATATATTAT CGTTCGAACT ATCAATGTAT TGATTATTAT   
  
  
- AGCCCAAACT CTATACCATT AGATAACGAT AAAGTATTTT ATTCTTAGCA TAAATGTGGT GCTAGGCAAA   
  
  
- TTATTAGGTG TTCAGAAACA CATATATCAA AATATAATTG AATTAAGCTG AATCACTTGT CTAGTTTTAG   
  
  
- CTAATAAAAA TATCGAGCAC CTTGAGCCTA GTCTTCTCTA CATAGGTGGA CACGATAAAG TTCTTCCCTT   
  
  
- TAAAACAGTA ATTATAATTC ACCTATTATT ACCTGTTTGA TTGGCCCATG AATCTGTAGT TAGGTAATTG   
  
  
- TAACTTAATC AGGAAAACAT GTATAATACC GCAATGTACA GGACATACTT CTTTTCTTTT GTAGTGGCAA   
  
  
- AATTAAATAA TAAGTTTATA TGAACATTAT GTGTCTCTAT AGTCTCTACT ATTAGTATTA GTACTAATTG   
  
  
- ATTAGTCTAG ACTACTTCGC GAACCATGGA TTGATCAACA CTCGATATGT TATTATAGTT GATCCCAAAA   
  
  
- GTATGGCTCG TTCACTTCCA GTTACCGCTC CAGTCACCAC ACTCACTAGC AGTGAGGCAC AATATGTTCG   
  
  
- CATAACTGGT CCTCGAATTA ATAACTGTGT CTTTGTTGTT TCGGACGTGG TAACTGTGTC TCTTTTTGGC   
  
  
- TGGCCGGTGG CCCCTCCGAT TTTCCAGTGG TCATGACCTA GAAACTGGGA ATCGGTACAC TGCAGCAGAG   
  
  
- ATATACTTCG AAGTTGCTAC CAATGGTATC TAATATGTTA TCCCAGCAGC GGCGTTTAAA TCGGAAAGAG   
  
  
- GAAGAAAGAG AGAGAGACGT TTGACTAGTA TAGAGTAGAC AAAGAATTGA CACTTTCCAA CTTCAACTTA   
  
  
- GAAAGGTATA ACAAATGAGA AAATTCCATT ATTTTATGAT TTTCCTTTTA CACCAACCCC ACAACTGTAA   
  
  
- CAGCCGGACA CATACTTCCA TGATAATATT CTTCTCTTCA CGCTCTCTCT GTATTGCCTA TGTCTTCGAT   
  
  
- TTTCTCGTTT CTTCTTATGG AAGATGTCG

+     G-box

| Site Name | Organism | Position | Strand | Matrix score. | sequence | function |
| --- | --- | --- | --- | --- | --- | --- |
| G-box | Solanum tuberosum | 1174 | - | 7 | CACATGG | cis-acting regulatory element involved in light responsiveness |
| G-box | Brassica oleracea | 1034 | - | 9 | TAACACGTAG | cis-acting regulatory element involved in light responsiveness |

> 2018/04/13 10:10:12  
+ CAATGAAAAG AAAGCTGCAT AGACATGTTG ACCCCTTCAT CCACTCTTTG TCTAGTTGCT TCTCTTCTAT   
  
  
+ GTATTACCAT TGACTGTTGA GTTTTCGAAG GATTAAAAGA AAAGTTTGTT CACAAAAAAT TTGATGCCCT   
  
  
+ AATTAAAACC CTTGAGGAAA TAAAAATCAT AATCTGGGCA ACCGGCTTCC TAAGTATATA TAGTATATAT   
  
  
+ TTCTAGGATT GGATGGCAGC TCGGCTTAAT TAATTATCTT TTAGTTATCT TAGAGCAATA AATTTAAGTC   
  
  
+ CGTTTTATAT GGTCGATTTC TATATATTGT GAATCATTGA CTGTTGAATT GAAATATTTT GTCATAATAT   
  
  
+ TTGTACATAT AACTTGGGGC AGCCAACGGA GCAATGGGAT CGGGGAGTCT AATATTTTAT CTACTAGAAG   
  
  
+ GTAAAAGCTT TTGAGTAACA AAGATGACTA CTATATAATA GCAAGCTTGA TAGTTACATA ACTAATAATA   
  
  
+ TCGGGTTTGA GATATGGTAA TCTATTGCTA TTTCATAAAA TAAGAATCGT ATTTACACCA CGATCCGTTT   
  
  
+ AATAATCCAC AAGTCTTTGT GTATATAGTT TTATATTAAC TTAATTCGAC TTAGTGAACA GATCAAAATC   
  
  
+ GATTATTTTT ATAGCTCGTG GAACTCGGAT CAGAAGAGAT GTATCCACCT GTGCTATTTC AAGAAGGGAA   
  
  
+ ATTTTGTCAT TAATATTAAG TGGATAATAA TGGACAAACT AACCGGGTAC TTAGACATCA ATCCATTAAC   
  
  
+ ATTGAATTAG TCCTTTTGTA CATATTATGG CGTTACATGT CCTGTATGAA GAAAAGAAAA CATCACCGTT   
  
  
+ TTAATTTATT ATTCAAATAT ACTTGTAATA CACAGAGATA TCAGAGATGA TAATCATAAT CATGATTAAC   
  
  
+ TAATCAGATC TGATGAAGCG CTTGGTACCT AACTAGTTGT GAGCTATACA ATAATATCAA CTAGGGTTTT   
  
  
+ CATACCGAGC AAGTGAAGGT CAATGGCGAG GTCAGTGGTG TGAGTGATCG TCACTCCGTG TTATACAAGC   
  
  
+ GTATTGACCA GGAGCTTAAT TATTGACACA GAAACAACAA AGCCTGCACC ATTGACACAG AGAAAAACCG   
  
  
+ ACCGGCCACC GGGGAGGCTA AAAGGTCACC AGTACTGGAT CTTTGACCCT TAGCCATGTG ACGTCGTCTC   
  
  
+ TATATGAAGC TTCAACGATG GTTACCATAG ATTATACAAT AGGGTCGTCG CCGCAAATTT AGCCTTTCTC   
  
  
+ CTTCTTTCTC TCTCTCTGCA AACTGATCAT ATCTCATCTG TTTCTTAACT GTGAAAGGTT GAAGTTGAAT   
  
  
+ CTTTCCATAT TGTTTACTCT TTTAAGGTAA TAAAATACTA AAAGGAAAAT GTGGTTGGGG TGTTGACATT   
  
  
+ GTCGGCCTGT GTATGAAGGT ACTATTATAA GAAGAGAAGT GCGAGAGAGA CATAACGGAT ACAGAAGCTA   
  
  
+ AAAGAGCAAA GAAGAATACC TTCTACAGC  

- GTTACTTTTC TTTCGACGTA TCTGTACAAC TGGGGAAGTA GGTGAGAAAC AGATCAACGA AGAGAAGATA   
  
  
- CATAATGGTA ACTGACAACT CAAAAGCTTC CTAATTTTCT TTTCAAACAA GTGTTTTTTA AACTACGGGA   
  
  
- TTAATTTTGG GAACTCCTTT ATTTTTAGTA TTAGACCCGT TGGCCGAAGG ATTCATATAT ATCATATATA   
  
  
- AAGATCCTAA CCTACCGTCG AGCCGAATTA ATTAATAGAA AATCAATAGA ATCTCGTTAT TTAAATTCAG   
  
  
- GCAAAATATA CCAGCTAAAG ATATATAACA CTTAGTAACT GACAACTTAA CTTTATAAAA CAGTATTATA   
  
  
- AACATGTATA TTGAACCCCG TCGGTTGCCT CGTTACCCTA GCCCCTCAGA TTATAAAATA GATGATCTTC   
  
  
- CATTTTCGAA AACTCATTGT TTCTACTGAT GATATATTAT CGTTCGAACT ATCAATGTAT TGATTATTAT   
  
  
- AGCCCAAACT CTATACCATT AGATAACGAT AAAGTATTTT ATTCTTAGCA TAAATGTGGT GCTAGGCAAA   
  
  
- TTATTAGGTG TTCAGAAACA CATATATCAA AATATAATTG AATTAAGCTG AATCACTTGT CTAGTTTTAG   
  
  
- CTAATAAAAA TATCGAGCAC CTTGAGCCTA GTCTTCTCTA CATAGGTGGA CACGATAAAG TTCTTCCCTT   
  
  
- TAAAACAGTA ATTATAATTC ACCTATTATT ACCTGTTTGA TTGGCCCATG AATCTGTAGT TAGGTAATTG   
  
  
- TAACTTAATC AGGAAAACAT GTATAATACC GCAATGTACA GGACATACTT CTTTTCTTTT GTAGTGGCAA   
  
  
- AATTAAATAA TAAGTTTATA TGAACATTAT GTGTCTCTAT AGTCTCTACT ATTAGTATTA GTACTAATTG   
  
  
- ATTAGTCTAG ACTACTTCGC GAACCATGGA TTGATCAACA CTCGATATGT TATTATAGTT GATCCCAAAA   
  
  
- GTATGGCTCG TTCACTTCCA GTTACCGCTC CAGTCACCAC ACTCACTAGC AGTGAGGCAC AATATGTTCG   
  
  
- CATAACTGGT CCTCGAATTA ATAACTGTGT CTTTGTTGTT TCGGACGTGG TAACTGTGTC TCTTTTTGGC   
  
  
- TGGCCGGTGG CCCCTCCGAT TTTCCAGTGG TCATGACCTA GAAACTGGGA ATCGGTACAC TGCAGCAGAG   
  
  
- ATATACTTCG AAGTTGCTAC CAATGGTATC TAATATGTTA TCCCAGCAGC GGCGTTTAAA TCGGAAAGAG   
  
  
- GAAGAAAGAG AGAGAGACGT TTGACTAGTA TAGAGTAGAC AAAGAATTGA CACTTTCCAA CTTCAACTTA   
  
  
- GAAAGGTATA ACAAATGAGA AAATTCCATT ATTTTATGAT TTTCCTTTTA CACCAACCCC ACAACTGTAA   
  
  
- CAGCCGGACA CATACTTCCA TGATAATATT CTTCTCTTCA CGCTCTCTCT GTATTGCCTA TGTCTTCGAT   
  
  
- TTTCTCGTTT CTTCTTATGG AAGATGTCG

+     GA-motif

| Site Name | Organism | Position | Strand | Matrix score. | sequence | function |
| --- | --- | --- | --- | --- | --- | --- |
| GA-motif | Helianthus annuus | 440 | + | 8 | AAAGATGA | part of a light responsive element |

> 2018/04/13 10:10:12  
+ CAATGAAAAG AAAGCTGCAT AGACATGTTG ACCCCTTCAT CCACTCTTTG TCTAGTTGCT TCTCTTCTAT   
  
  
+ GTATTACCAT TGACTGTTGA GTTTTCGAAG GATTAAAAGA AAAGTTTGTT CACAAAAAAT TTGATGCCCT   
  
  
+ AATTAAAACC CTTGAGGAAA TAAAAATCAT AATCTGGGCA ACCGGCTTCC TAAGTATATA TAGTATATAT   
  
  
+ TTCTAGGATT GGATGGCAGC TCGGCTTAAT TAATTATCTT TTAGTTATCT TAGAGCAATA AATTTAAGTC   
  
  
+ CGTTTTATAT GGTCGATTTC TATATATTGT GAATCATTGA CTGTTGAATT GAAATATTTT GTCATAATAT   
  
  
+ TTGTACATAT AACTTGGGGC AGCCAACGGA GCAATGGGAT CGGGGAGTCT AATATTTTAT CTACTAGAAG   
  
  
+ GTAAAAGCTT TTGAGTAACA AAGATGACTA CTATATAATA GCAAGCTTGA TAGTTACATA ACTAATAATA   
  
  
+ TCGGGTTTGA GATATGGTAA TCTATTGCTA TTTCATAAAA TAAGAATCGT ATTTACACCA CGATCCGTTT   
  
  
+ AATAATCCAC AAGTCTTTGT GTATATAGTT TTATATTAAC TTAATTCGAC TTAGTGAACA GATCAAAATC   
  
  
+ GATTATTTTT ATAGCTCGTG GAACTCGGAT CAGAAGAGAT GTATCCACCT GTGCTATTTC AAGAAGGGAA   
  
  
+ ATTTTGTCAT TAATATTAAG TGGATAATAA TGGACAAACT AACCGGGTAC TTAGACATCA ATCCATTAAC   
  
  
+ ATTGAATTAG TCCTTTTGTA CATATTATGG CGTTACATGT CCTGTATGAA GAAAAGAAAA CATCACCGTT   
  
  
+ TTAATTTATT ATTCAAATAT ACTTGTAATA CACAGAGATA TCAGAGATGA TAATCATAAT CATGATTAAC   
  
  
+ TAATCAGATC TGATGAAGCG CTTGGTACCT AACTAGTTGT GAGCTATACA ATAATATCAA CTAGGGTTTT   
  
  
+ CATACCGAGC AAGTGAAGGT CAATGGCGAG GTCAGTGGTG TGAGTGATCG TCACTCCGTG TTATACAAGC   
  
  
+ GTATTGACCA GGAGCTTAAT TATTGACACA GAAACAACAA AGCCTGCACC ATTGACACAG AGAAAAACCG   
  
  
+ ACCGGCCACC GGGGAGGCTA AAAGGTCACC AGTACTGGAT CTTTGACCCT TAGCCATGTG ACGTCGTCTC   
  
  
+ TATATGAAGC TTCAACGATG GTTACCATAG ATTATACAAT AGGGTCGTCG CCGCAAATTT AGCCTTTCTC   
  
  
+ CTTCTTTCTC TCTCTCTGCA AACTGATCAT ATCTCATCTG TTTCTTAACT GTGAAAGGTT GAAGTTGAAT   
  
  
+ CTTTCCATAT TGTTTACTCT TTTAAGGTAA TAAAATACTA AAAGGAAAAT GTGGTTGGGG TGTTGACATT   
  
  
+ GTCGGCCTGT GTATGAAGGT ACTATTATAA GAAGAGAAGT GCGAGAGAGA CATAACGGAT ACAGAAGCTA   
  
  
+ AAAGAGCAAA GAAGAATACC TTCTACAGC  

- GTTACTTTTC TTTCGACGTA TCTGTACAAC TGGGGAAGTA GGTGAGAAAC AGATCAACGA AGAGAAGATA   
  
  
- CATAATGGTA ACTGACAACT CAAAAGCTTC CTAATTTTCT TTTCAAACAA GTGTTTTTTA AACTACGGGA   
  
  
- TTAATTTTGG GAACTCCTTT ATTTTTAGTA TTAGACCCGT TGGCCGAAGG ATTCATATAT ATCATATATA   
  
  
- AAGATCCTAA CCTACCGTCG AGCCGAATTA ATTAATAGAA AATCAATAGA ATCTCGTTAT TTAAATTCAG   
  
  
- GCAAAATATA CCAGCTAAAG ATATATAACA CTTAGTAACT GACAACTTAA CTTTATAAAA CAGTATTATA   
  
  
- AACATGTATA TTGAACCCCG TCGGTTGCCT CGTTACCCTA GCCCCTCAGA TTATAAAATA GATGATCTTC   
  
  
- CATTTTCGAA AACTCATTGT TTCTACTGAT GATATATTAT CGTTCGAACT ATCAATGTAT TGATTATTAT   
  
  
- AGCCCAAACT CTATACCATT AGATAACGAT AAAGTATTTT ATTCTTAGCA TAAATGTGGT GCTAGGCAAA   
  
  
- TTATTAGGTG TTCAGAAACA CATATATCAA AATATAATTG AATTAAGCTG AATCACTTGT CTAGTTTTAG   
  
  
- CTAATAAAAA TATCGAGCAC CTTGAGCCTA GTCTTCTCTA CATAGGTGGA CACGATAAAG TTCTTCCCTT   
  
  
- TAAAACAGTA ATTATAATTC ACCTATTATT ACCTGTTTGA TTGGCCCATG AATCTGTAGT TAGGTAATTG   
  
  
- TAACTTAATC AGGAAAACAT GTATAATACC GCAATGTACA GGACATACTT CTTTTCTTTT GTAGTGGCAA   
  
  
- AATTAAATAA TAAGTTTATA TGAACATTAT GTGTCTCTAT AGTCTCTACT ATTAGTATTA GTACTAATTG   
  
  
- ATTAGTCTAG ACTACTTCGC GAACCATGGA TTGATCAACA CTCGATATGT TATTATAGTT GATCCCAAAA   
  
  
- GTATGGCTCG TTCACTTCCA GTTACCGCTC CAGTCACCAC ACTCACTAGC AGTGAGGCAC AATATGTTCG   
  
  
- CATAACTGGT CCTCGAATTA ATAACTGTGT CTTTGTTGTT TCGGACGTGG TAACTGTGTC TCTTTTTGGC   
  
  
- TGGCCGGTGG CCCCTCCGAT TTTCCAGTGG TCATGACCTA GAAACTGGGA ATCGGTACAC TGCAGCAGAG   
  
  
- ATATACTTCG AAGTTGCTAC CAATGGTATC TAATATGTTA TCCCAGCAGC GGCGTTTAAA TCGGAAAGAG   
  
  
- GAAGAAAGAG AGAGAGACGT TTGACTAGTA TAGAGTAGAC AAAGAATTGA CACTTTCCAA CTTCAACTTA   
  
  
- GAAAGGTATA ACAAATGAGA AAATTCCATT ATTTTATGAT TTTCCTTTTA CACCAACCCC ACAACTGTAA   
  
  
- CAGCCGGACA CATACTTCCA TGATAATATT CTTCTCTTCA CGCTCTCTCT GTATTGCCTA TGTCTTCGAT   
  
  
- TTTCTCGTTT CTTCTTATGG AAGATGTCG

+     GAG-motif

| Site Name | Organism | Position | Strand | Matrix score. | sequence | function |
| --- | --- | --- | --- | --- | --- | --- |
| GAG-motif | Spinacia oleracea | 883 | + | 7 | AGAGATG | part of a light responsive element |
| GAG-motif | Spinacia oleracea | 665 | + | 7 | AGAGATG | part of a light responsive element |

> 2018/04/13 10:10:12  
+ CAATGAAAAG AAAGCTGCAT AGACATGTTG ACCCCTTCAT CCACTCTTTG TCTAGTTGCT TCTCTTCTAT   
  
  
+ GTATTACCAT TGACTGTTGA GTTTTCGAAG GATTAAAAGA AAAGTTTGTT CACAAAAAAT TTGATGCCCT   
  
  
+ AATTAAAACC CTTGAGGAAA TAAAAATCAT AATCTGGGCA ACCGGCTTCC TAAGTATATA TAGTATATAT   
  
  
+ TTCTAGGATT GGATGGCAGC TCGGCTTAAT TAATTATCTT TTAGTTATCT TAGAGCAATA AATTTAAGTC   
  
  
+ CGTTTTATAT GGTCGATTTC TATATATTGT GAATCATTGA CTGTTGAATT GAAATATTTT GTCATAATAT   
  
  
+ TTGTACATAT AACTTGGGGC AGCCAACGGA GCAATGGGAT CGGGGAGTCT AATATTTTAT CTACTAGAAG   
  
  
+ GTAAAAGCTT TTGAGTAACA AAGATGACTA CTATATAATA GCAAGCTTGA TAGTTACATA ACTAATAATA   
  
  
+ TCGGGTTTGA GATATGGTAA TCTATTGCTA TTTCATAAAA TAAGAATCGT ATTTACACCA CGATCCGTTT   
  
  
+ AATAATCCAC AAGTCTTTGT GTATATAGTT TTATATTAAC TTAATTCGAC TTAGTGAACA GATCAAAATC   
  
  
+ GATTATTTTT ATAGCTCGTG GAACTCGGAT CAGAAGAGAT GTATCCACCT GTGCTATTTC AAGAAGGGAA   
  
  
+ ATTTTGTCAT TAATATTAAG TGGATAATAA TGGACAAACT AACCGGGTAC TTAGACATCA ATCCATTAAC   
  
  
+ ATTGAATTAG TCCTTTTGTA CATATTATGG CGTTACATGT CCTGTATGAA GAAAAGAAAA CATCACCGTT   
  
  
+ TTAATTTATT ATTCAAATAT ACTTGTAATA CACAGAGATA TCAGAGATGA TAATCATAAT CATGATTAAC   
  
  
+ TAATCAGATC TGATGAAGCG CTTGGTACCT AACTAGTTGT GAGCTATACA ATAATATCAA CTAGGGTTTT   
  
  
+ CATACCGAGC AAGTGAAGGT CAATGGCGAG GTCAGTGGTG TGAGTGATCG TCACTCCGTG TTATACAAGC   
  
  
+ GTATTGACCA GGAGCTTAAT TATTGACACA GAAACAACAA AGCCTGCACC ATTGACACAG AGAAAAACCG   
  
  
+ ACCGGCCACC GGGGAGGCTA AAAGGTCACC AGTACTGGAT CTTTGACCCT TAGCCATGTG ACGTCGTCTC   
  
  
+ TATATGAAGC TTCAACGATG GTTACCATAG ATTATACAAT AGGGTCGTCG CCGCAAATTT AGCCTTTCTC   
  
  
+ CTTCTTTCTC TCTCTCTGCA AACTGATCAT ATCTCATCTG TTTCTTAACT GTGAAAGGTT GAAGTTGAAT   
  
  
+ CTTTCCATAT TGTTTACTCT TTTAAGGTAA TAAAATACTA AAAGGAAAAT GTGGTTGGGG TGTTGACATT   
  
  
+ GTCGGCCTGT GTATGAAGGT ACTATTATAA GAAGAGAAGT GCGAGAGAGA CATAACGGAT ACAGAAGCTA   
  
  
+ AAAGAGCAAA GAAGAATACC TTCTACAGC  

- GTTACTTTTC TTTCGACGTA TCTGTACAAC TGGGGAAGTA GGTGAGAAAC AGATCAACGA AGAGAAGATA   
  
  
- CATAATGGTA ACTGACAACT CAAAAGCTTC CTAATTTTCT TTTCAAACAA GTGTTTTTTA AACTACGGGA   
  
  
- TTAATTTTGG GAACTCCTTT ATTTTTAGTA TTAGACCCGT TGGCCGAAGG ATTCATATAT ATCATATATA   
  
  
- AAGATCCTAA CCTACCGTCG AGCCGAATTA ATTAATAGAA AATCAATAGA ATCTCGTTAT TTAAATTCAG   
  
  
- GCAAAATATA CCAGCTAAAG ATATATAACA CTTAGTAACT GACAACTTAA CTTTATAAAA CAGTATTATA   
  
  
- AACATGTATA TTGAACCCCG TCGGTTGCCT CGTTACCCTA GCCCCTCAGA TTATAAAATA GATGATCTTC   
  
  
- CATTTTCGAA AACTCATTGT TTCTACTGAT GATATATTAT CGTTCGAACT ATCAATGTAT TGATTATTAT   
  
  
- AGCCCAAACT CTATACCATT AGATAACGAT AAAGTATTTT ATTCTTAGCA TAAATGTGGT GCTAGGCAAA   
  
  
- TTATTAGGTG TTCAGAAACA CATATATCAA AATATAATTG AATTAAGCTG AATCACTTGT CTAGTTTTAG   
  
  
- CTAATAAAAA TATCGAGCAC CTTGAGCCTA GTCTTCTCTA CATAGGTGGA CACGATAAAG TTCTTCCCTT   
  
  
- TAAAACAGTA ATTATAATTC ACCTATTATT ACCTGTTTGA TTGGCCCATG AATCTGTAGT TAGGTAATTG   
  
  
- TAACTTAATC AGGAAAACAT GTATAATACC GCAATGTACA GGACATACTT CTTTTCTTTT GTAGTGGCAA   
  
  
- AATTAAATAA TAAGTTTATA TGAACATTAT GTGTCTCTAT AGTCTCTACT ATTAGTATTA GTACTAATTG   
  
  
- ATTAGTCTAG ACTACTTCGC GAACCATGGA TTGATCAACA CTCGATATGT TATTATAGTT GATCCCAAAA   
  
  
- GTATGGCTCG TTCACTTCCA GTTACCGCTC CAGTCACCAC ACTCACTAGC AGTGAGGCAC AATATGTTCG   
  
  
- CATAACTGGT CCTCGAATTA ATAACTGTGT CTTTGTTGTT TCGGACGTGG TAACTGTGTC TCTTTTTGGC   
  
  
- TGGCCGGTGG CCCCTCCGAT TTTCCAGTGG TCATGACCTA GAAACTGGGA ATCGGTACAC TGCAGCAGAG   
  
  
- ATATACTTCG AAGTTGCTAC CAATGGTATC TAATATGTTA TCCCAGCAGC GGCGTTTAAA TCGGAAAGAG   
  
  
- GAAGAAAGAG AGAGAGACGT TTGACTAGTA TAGAGTAGAC AAAGAATTGA CACTTTCCAA CTTCAACTTA   
  
  
- GAAAGGTATA ACAAATGAGA AAATTCCATT ATTTTATGAT TTTCCTTTTA CACCAACCCC ACAACTGTAA   
  
  
- CAGCCGGACA CATACTTCCA TGATAATATT CTTCTCTTCA CGCTCTCTCT GTATTGCCTA TGTCTTCGAT   
  
  
- TTTCTCGTTT CTTCTTATGG AAGATGTCG

+     GARE-motif

| Site Name | Organism | Position | Strand | Matrix score. | sequence | function |
| --- | --- | --- | --- | --- | --- | --- |
| GARE-motif | Brassica oleracea | 1297 | - | 7 | AAACAGA | gibberellin-responsive element |

> 2018/04/13 10:10:12  
+ CAATGAAAAG AAAGCTGCAT AGACATGTTG ACCCCTTCAT CCACTCTTTG TCTAGTTGCT TCTCTTCTAT   
  
  
+ GTATTACCAT TGACTGTTGA GTTTTCGAAG GATTAAAAGA AAAGTTTGTT CACAAAAAAT TTGATGCCCT   
  
  
+ AATTAAAACC CTTGAGGAAA TAAAAATCAT AATCTGGGCA ACCGGCTTCC TAAGTATATA TAGTATATAT   
  
  
+ TTCTAGGATT GGATGGCAGC TCGGCTTAAT TAATTATCTT TTAGTTATCT TAGAGCAATA AATTTAAGTC   
  
  
+ CGTTTTATAT GGTCGATTTC TATATATTGT GAATCATTGA CTGTTGAATT GAAATATTTT GTCATAATAT   
  
  
+ TTGTACATAT AACTTGGGGC AGCCAACGGA GCAATGGGAT CGGGGAGTCT AATATTTTAT CTACTAGAAG   
  
  
+ GTAAAAGCTT TTGAGTAACA AAGATGACTA CTATATAATA GCAAGCTTGA TAGTTACATA ACTAATAATA   
  
  
+ TCGGGTTTGA GATATGGTAA TCTATTGCTA TTTCATAAAA TAAGAATCGT ATTTACACCA CGATCCGTTT   
  
  
+ AATAATCCAC AAGTCTTTGT GTATATAGTT TTATATTAAC TTAATTCGAC TTAGTGAACA GATCAAAATC   
  
  
+ GATTATTTTT ATAGCTCGTG GAACTCGGAT CAGAAGAGAT GTATCCACCT GTGCTATTTC AAGAAGGGAA   
  
  
+ ATTTTGTCAT TAATATTAAG TGGATAATAA TGGACAAACT AACCGGGTAC TTAGACATCA ATCCATTAAC   
  
  
+ ATTGAATTAG TCCTTTTGTA CATATTATGG CGTTACATGT CCTGTATGAA GAAAAGAAAA CATCACCGTT   
  
  
+ TTAATTTATT ATTCAAATAT ACTTGTAATA CACAGAGATA TCAGAGATGA TAATCATAAT CATGATTAAC   
  
  
+ TAATCAGATC TGATGAAGCG CTTGGTACCT AACTAGTTGT GAGCTATACA ATAATATCAA CTAGGGTTTT   
  
  
+ CATACCGAGC AAGTGAAGGT CAATGGCGAG GTCAGTGGTG TGAGTGATCG TCACTCCGTG TTATACAAGC   
  
  
+ GTATTGACCA GGAGCTTAAT TATTGACACA GAAACAACAA AGCCTGCACC ATTGACACAG AGAAAAACCG   
  
  
+ ACCGGCCACC GGGGAGGCTA AAAGGTCACC AGTACTGGAT CTTTGACCCT TAGCCATGTG ACGTCGTCTC   
  
  
+ TATATGAAGC TTCAACGATG GTTACCATAG ATTATACAAT AGGGTCGTCG CCGCAAATTT AGCCTTTCTC   
  
  
+ CTTCTTTCTC TCTCTCTGCA AACTGATCAT ATCTCATCTG TTTCTTAACT GTGAAAGGTT GAAGTTGAAT   
  
  
+ CTTTCCATAT TGTTTACTCT TTTAAGGTAA TAAAATACTA AAAGGAAAAT GTGGTTGGGG TGTTGACATT   
  
  
+ GTCGGCCTGT GTATGAAGGT ACTATTATAA GAAGAGAAGT GCGAGAGAGA CATAACGGAT ACAGAAGCTA   
  
  
+ AAAGAGCAAA GAAGAATACC TTCTACAGC  

- GTTACTTTTC TTTCGACGTA TCTGTACAAC TGGGGAAGTA GGTGAGAAAC AGATCAACGA AGAGAAGATA   
  
  
- CATAATGGTA ACTGACAACT CAAAAGCTTC CTAATTTTCT TTTCAAACAA GTGTTTTTTA AACTACGGGA   
  
  
- TTAATTTTGG GAACTCCTTT ATTTTTAGTA TTAGACCCGT TGGCCGAAGG ATTCATATAT ATCATATATA   
  
  
- AAGATCCTAA CCTACCGTCG AGCCGAATTA ATTAATAGAA AATCAATAGA ATCTCGTTAT TTAAATTCAG   
  
  
- GCAAAATATA CCAGCTAAAG ATATATAACA CTTAGTAACT GACAACTTAA CTTTATAAAA CAGTATTATA   
  
  
- AACATGTATA TTGAACCCCG TCGGTTGCCT CGTTACCCTA GCCCCTCAGA TTATAAAATA GATGATCTTC   
  
  
- CATTTTCGAA AACTCATTGT TTCTACTGAT GATATATTAT CGTTCGAACT ATCAATGTAT TGATTATTAT   
  
  
- AGCCCAAACT CTATACCATT AGATAACGAT AAAGTATTTT ATTCTTAGCA TAAATGTGGT GCTAGGCAAA   
  
  
- TTATTAGGTG TTCAGAAACA CATATATCAA AATATAATTG AATTAAGCTG AATCACTTGT CTAGTTTTAG   
  
  
- CTAATAAAAA TATCGAGCAC CTTGAGCCTA GTCTTCTCTA CATAGGTGGA CACGATAAAG TTCTTCCCTT   
  
  
- TAAAACAGTA ATTATAATTC ACCTATTATT ACCTGTTTGA TTGGCCCATG AATCTGTAGT TAGGTAATTG   
  
  
- TAACTTAATC AGGAAAACAT GTATAATACC GCAATGTACA GGACATACTT CTTTTCTTTT GTAGTGGCAA   
  
  
- AATTAAATAA TAAGTTTATA TGAACATTAT GTGTCTCTAT AGTCTCTACT ATTAGTATTA GTACTAATTG   
  
  
- ATTAGTCTAG ACTACTTCGC GAACCATGGA TTGATCAACA CTCGATATGT TATTATAGTT GATCCCAAAA   
  
  
- GTATGGCTCG TTCACTTCCA GTTACCGCTC CAGTCACCAC ACTCACTAGC AGTGAGGCAC AATATGTTCG   
  
  
- CATAACTGGT CCTCGAATTA ATAACTGTGT CTTTGTTGTT TCGGACGTGG TAACTGTGTC TCTTTTTGGC   
  
  
- TGGCCGGTGG CCCCTCCGAT TTTCCAGTGG TCATGACCTA GAAACTGGGA ATCGGTACAC TGCAGCAGAG   
  
  
- ATATACTTCG AAGTTGCTAC CAATGGTATC TAATATGTTA TCCCAGCAGC GGCGTTTAAA TCGGAAAGAG   
  
  
- GAAGAAAGAG AGAGAGACGT TTGACTAGTA TAGAGTAGAC AAAGAATTGA CACTTTCCAA CTTCAACTTA   
  
  
- GAAAGGTATA ACAAATGAGA AAATTCCATT ATTTTATGAT TTTCCTTTTA CACCAACCCC ACAACTGTAA   
  
  
- CAGCCGGACA CATACTTCCA TGATAATATT CTTCTCTTCA CGCTCTCTCT GTATTGCCTA TGTCTTCGAT   
  
  
- TTTCTCGTTT CTTCTTATGG AAGATGTCG

+     GCN4\_motif

| Site Name | Organism | Position | Strand | Matrix score. | sequence | function |
| --- | --- | --- | --- | --- | --- | --- |
| GCN4\_motif | Oryza sativa | 1103 | - | 7 | TGTGTCA | cis-regulatory element involved in endosperm expression |
| GCN4\_motif | Oryza sativa | 1074 | - | 7 | TGTGTCA | cis-regulatory element involved in endosperm expression |

> 2018/04/13 10:10:12  
+ CAATGAAAAG AAAGCTGCAT AGACATGTTG ACCCCTTCAT CCACTCTTTG TCTAGTTGCT TCTCTTCTAT   
  
  
+ GTATTACCAT TGACTGTTGA GTTTTCGAAG GATTAAAAGA AAAGTTTGTT CACAAAAAAT TTGATGCCCT   
  
  
+ AATTAAAACC CTTGAGGAAA TAAAAATCAT AATCTGGGCA ACCGGCTTCC TAAGTATATA TAGTATATAT   
  
  
+ TTCTAGGATT GGATGGCAGC TCGGCTTAAT TAATTATCTT TTAGTTATCT TAGAGCAATA AATTTAAGTC   
  
  
+ CGTTTTATAT GGTCGATTTC TATATATTGT GAATCATTGA CTGTTGAATT GAAATATTTT GTCATAATAT   
  
  
+ TTGTACATAT AACTTGGGGC AGCCAACGGA GCAATGGGAT CGGGGAGTCT AATATTTTAT CTACTAGAAG   
  
  
+ GTAAAAGCTT TTGAGTAACA AAGATGACTA CTATATAATA GCAAGCTTGA TAGTTACATA ACTAATAATA   
  
  
+ TCGGGTTTGA GATATGGTAA TCTATTGCTA TTTCATAAAA TAAGAATCGT ATTTACACCA CGATCCGTTT   
  
  
+ AATAATCCAC AAGTCTTTGT GTATATAGTT TTATATTAAC TTAATTCGAC TTAGTGAACA GATCAAAATC   
  
  
+ GATTATTTTT ATAGCTCGTG GAACTCGGAT CAGAAGAGAT GTATCCACCT GTGCTATTTC AAGAAGGGAA   
  
  
+ ATTTTGTCAT TAATATTAAG TGGATAATAA TGGACAAACT AACCGGGTAC TTAGACATCA ATCCATTAAC   
  
  
+ ATTGAATTAG TCCTTTTGTA CATATTATGG CGTTACATGT CCTGTATGAA GAAAAGAAAA CATCACCGTT   
  
  
+ TTAATTTATT ATTCAAATAT ACTTGTAATA CACAGAGATA TCAGAGATGA TAATCATAAT CATGATTAAC   
  
  
+ TAATCAGATC TGATGAAGCG CTTGGTACCT AACTAGTTGT GAGCTATACA ATAATATCAA CTAGGGTTTT   
  
  
+ CATACCGAGC AAGTGAAGGT CAATGGCGAG GTCAGTGGTG TGAGTGATCG TCACTCCGTG TTATACAAGC   
  
  
+ GTATTGACCA GGAGCTTAAT TATTGACACA GAAACAACAA AGCCTGCACC ATTGACACAG AGAAAAACCG   
  
  
+ ACCGGCCACC GGGGAGGCTA AAAGGTCACC AGTACTGGAT CTTTGACCCT TAGCCATGTG ACGTCGTCTC   
  
  
+ TATATGAAGC TTCAACGATG GTTACCATAG ATTATACAAT AGGGTCGTCG CCGCAAATTT AGCCTTTCTC   
  
  
+ CTTCTTTCTC TCTCTCTGCA AACTGATCAT ATCTCATCTG TTTCTTAACT GTGAAAGGTT GAAGTTGAAT   
  
  
+ CTTTCCATAT TGTTTACTCT TTTAAGGTAA TAAAATACTA AAAGGAAAAT GTGGTTGGGG TGTTGACATT   
  
  
+ GTCGGCCTGT GTATGAAGGT ACTATTATAA GAAGAGAAGT GCGAGAGAGA CATAACGGAT ACAGAAGCTA   
  
  
+ AAAGAGCAAA GAAGAATACC TTCTACAGC  

- GTTACTTTTC TTTCGACGTA TCTGTACAAC TGGGGAAGTA GGTGAGAAAC AGATCAACGA AGAGAAGATA   
  
  
- CATAATGGTA ACTGACAACT CAAAAGCTTC CTAATTTTCT TTTCAAACAA GTGTTTTTTA AACTACGGGA   
  
  
- TTAATTTTGG GAACTCCTTT ATTTTTAGTA TTAGACCCGT TGGCCGAAGG ATTCATATAT ATCATATATA   
  
  
- AAGATCCTAA CCTACCGTCG AGCCGAATTA ATTAATAGAA AATCAATAGA ATCTCGTTAT TTAAATTCAG   
  
  
- GCAAAATATA CCAGCTAAAG ATATATAACA CTTAGTAACT GACAACTTAA CTTTATAAAA CAGTATTATA   
  
  
- AACATGTATA TTGAACCCCG TCGGTTGCCT CGTTACCCTA GCCCCTCAGA TTATAAAATA GATGATCTTC   
  
  
- CATTTTCGAA AACTCATTGT TTCTACTGAT GATATATTAT CGTTCGAACT ATCAATGTAT TGATTATTAT   
  
  
- AGCCCAAACT CTATACCATT AGATAACGAT AAAGTATTTT ATTCTTAGCA TAAATGTGGT GCTAGGCAAA   
  
  
- TTATTAGGTG TTCAGAAACA CATATATCAA AATATAATTG AATTAAGCTG AATCACTTGT CTAGTTTTAG   
  
  
- CTAATAAAAA TATCGAGCAC CTTGAGCCTA GTCTTCTCTA CATAGGTGGA CACGATAAAG TTCTTCCCTT   
  
  
- TAAAACAGTA ATTATAATTC ACCTATTATT ACCTGTTTGA TTGGCCCATG AATCTGTAGT TAGGTAATTG   
  
  
- TAACTTAATC AGGAAAACAT GTATAATACC GCAATGTACA GGACATACTT CTTTTCTTTT GTAGTGGCAA   
  
  
- AATTAAATAA TAAGTTTATA TGAACATTAT GTGTCTCTAT AGTCTCTACT ATTAGTATTA GTACTAATTG   
  
  
- ATTAGTCTAG ACTACTTCGC GAACCATGGA TTGATCAACA CTCGATATGT TATTATAGTT GATCCCAAAA   
  
  
- GTATGGCTCG TTCACTTCCA GTTACCGCTC CAGTCACCAC ACTCACTAGC AGTGAGGCAC AATATGTTCG   
  
  
- CATAACTGGT CCTCGAATTA ATAACTGTGT CTTTGTTGTT TCGGACGTGG TAACTGTGTC TCTTTTTGGC   
  
  
- TGGCCGGTGG CCCCTCCGAT TTTCCAGTGG TCATGACCTA GAAACTGGGA ATCGGTACAC TGCAGCAGAG   
  
  
- ATATACTTCG AAGTTGCTAC CAATGGTATC TAATATGTTA TCCCAGCAGC GGCGTTTAAA TCGGAAAGAG   
  
  
- GAAGAAAGAG AGAGAGACGT TTGACTAGTA TAGAGTAGAC AAAGAATTGA CACTTTCCAA CTTCAACTTA   
  
  
- GAAAGGTATA ACAAATGAGA AAATTCCATT ATTTTATGAT TTTCCTTTTA CACCAACCCC ACAACTGTAA   
  
  
- CAGCCGGACA CATACTTCCA TGATAATATT CTTCTCTTCA CGCTCTCTCT GTATTGCCTA TGTCTTCGAT   
  
  
- TTTCTCGTTT CTTCTTATGG AAGATGTCG

+     GT1-motif

| Site Name | Organism | Position | Strand | Matrix score. | sequence | function |
| --- | --- | --- | --- | --- | --- | --- |
| GT1-motif | Solanum tuberosum | 564 | + | 8 | AATCCACA | light responsive element |

> 2018/04/13 10:10:12  
+ CAATGAAAAG AAAGCTGCAT AGACATGTTG ACCCCTTCAT CCACTCTTTG TCTAGTTGCT TCTCTTCTAT   
  
  
+ GTATTACCAT TGACTGTTGA GTTTTCGAAG GATTAAAAGA AAAGTTTGTT CACAAAAAAT TTGATGCCCT   
  
  
+ AATTAAAACC CTTGAGGAAA TAAAAATCAT AATCTGGGCA ACCGGCTTCC TAAGTATATA TAGTATATAT   
  
  
+ TTCTAGGATT GGATGGCAGC TCGGCTTAAT TAATTATCTT TTAGTTATCT TAGAGCAATA AATTTAAGTC   
  
  
+ CGTTTTATAT GGTCGATTTC TATATATTGT GAATCATTGA CTGTTGAATT GAAATATTTT GTCATAATAT   
  
  
+ TTGTACATAT AACTTGGGGC AGCCAACGGA GCAATGGGAT CGGGGAGTCT AATATTTTAT CTACTAGAAG   
  
  
+ GTAAAAGCTT TTGAGTAACA AAGATGACTA CTATATAATA GCAAGCTTGA TAGTTACATA ACTAATAATA   
  
  
+ TCGGGTTTGA GATATGGTAA TCTATTGCTA TTTCATAAAA TAAGAATCGT ATTTACACCA CGATCCGTTT   
  
  
+ AATAATCCAC AAGTCTTTGT GTATATAGTT TTATATTAAC TTAATTCGAC TTAGTGAACA GATCAAAATC   
  
  
+ GATTATTTTT ATAGCTCGTG GAACTCGGAT CAGAAGAGAT GTATCCACCT GTGCTATTTC AAGAAGGGAA   
  
  
+ ATTTTGTCAT TAATATTAAG TGGATAATAA TGGACAAACT AACCGGGTAC TTAGACATCA ATCCATTAAC   
  
  
+ ATTGAATTAG TCCTTTTGTA CATATTATGG CGTTACATGT CCTGTATGAA GAAAAGAAAA CATCACCGTT   
  
  
+ TTAATTTATT ATTCAAATAT ACTTGTAATA CACAGAGATA TCAGAGATGA TAATCATAAT CATGATTAAC   
  
  
+ TAATCAGATC TGATGAAGCG CTTGGTACCT AACTAGTTGT GAGCTATACA ATAATATCAA CTAGGGTTTT   
  
  
+ CATACCGAGC AAGTGAAGGT CAATGGCGAG GTCAGTGGTG TGAGTGATCG TCACTCCGTG TTATACAAGC   
  
  
+ GTATTGACCA GGAGCTTAAT TATTGACACA GAAACAACAA AGCCTGCACC ATTGACACAG AGAAAAACCG   
  
  
+ ACCGGCCACC GGGGAGGCTA AAAGGTCACC AGTACTGGAT CTTTGACCCT TAGCCATGTG ACGTCGTCTC   
  
  
+ TATATGAAGC TTCAACGATG GTTACCATAG ATTATACAAT AGGGTCGTCG CCGCAAATTT AGCCTTTCTC   
  
  
+ CTTCTTTCTC TCTCTCTGCA AACTGATCAT ATCTCATCTG TTTCTTAACT GTGAAAGGTT GAAGTTGAAT   
  
  
+ CTTTCCATAT TGTTTACTCT TTTAAGGTAA TAAAATACTA AAAGGAAAAT GTGGTTGGGG TGTTGACATT   
  
  
+ GTCGGCCTGT GTATGAAGGT ACTATTATAA GAAGAGAAGT GCGAGAGAGA CATAACGGAT ACAGAAGCTA   
  
  
+ AAAGAGCAAA GAAGAATACC TTCTACAGC  

- GTTACTTTTC TTTCGACGTA TCTGTACAAC TGGGGAAGTA GGTGAGAAAC AGATCAACGA AGAGAAGATA   
  
  
- CATAATGGTA ACTGACAACT CAAAAGCTTC CTAATTTTCT TTTCAAACAA GTGTTTTTTA AACTACGGGA   
  
  
- TTAATTTTGG GAACTCCTTT ATTTTTAGTA TTAGACCCGT TGGCCGAAGG ATTCATATAT ATCATATATA   
  
  
- AAGATCCTAA CCTACCGTCG AGCCGAATTA ATTAATAGAA AATCAATAGA ATCTCGTTAT TTAAATTCAG   
  
  
- GCAAAATATA CCAGCTAAAG ATATATAACA CTTAGTAACT GACAACTTAA CTTTATAAAA CAGTATTATA   
  
  
- AACATGTATA TTGAACCCCG TCGGTTGCCT CGTTACCCTA GCCCCTCAGA TTATAAAATA GATGATCTTC   
  
  
- CATTTTCGAA AACTCATTGT TTCTACTGAT GATATATTAT CGTTCGAACT ATCAATGTAT TGATTATTAT   
  
  
- AGCCCAAACT CTATACCATT AGATAACGAT AAAGTATTTT ATTCTTAGCA TAAATGTGGT GCTAGGCAAA   
  
  
- TTATTAGGTG TTCAGAAACA CATATATCAA AATATAATTG AATTAAGCTG AATCACTTGT CTAGTTTTAG   
  
  
- CTAATAAAAA TATCGAGCAC CTTGAGCCTA GTCTTCTCTA CATAGGTGGA CACGATAAAG TTCTTCCCTT   
  
  
- TAAAACAGTA ATTATAATTC ACCTATTATT ACCTGTTTGA TTGGCCCATG AATCTGTAGT TAGGTAATTG   
  
  
- TAACTTAATC AGGAAAACAT GTATAATACC GCAATGTACA GGACATACTT CTTTTCTTTT GTAGTGGCAA   
  
  
- AATTAAATAA TAAGTTTATA TGAACATTAT GTGTCTCTAT AGTCTCTACT ATTAGTATTA GTACTAATTG   
  
  
- ATTAGTCTAG ACTACTTCGC GAACCATGGA TTGATCAACA CTCGATATGT TATTATAGTT GATCCCAAAA   
  
  
- GTATGGCTCG TTCACTTCCA GTTACCGCTC CAGTCACCAC ACTCACTAGC AGTGAGGCAC AATATGTTCG   
  
  
- CATAACTGGT CCTCGAATTA ATAACTGTGT CTTTGTTGTT TCGGACGTGG TAACTGTGTC TCTTTTTGGC   
  
  
- TGGCCGGTGG CCCCTCCGAT TTTCCAGTGG TCATGACCTA GAAACTGGGA ATCGGTACAC TGCAGCAGAG   
  
  
- ATATACTTCG AAGTTGCTAC CAATGGTATC TAATATGTTA TCCCAGCAGC GGCGTTTAAA TCGGAAAGAG   
  
  
- GAAGAAAGAG AGAGAGACGT TTGACTAGTA TAGAGTAGAC AAAGAATTGA CACTTTCCAA CTTCAACTTA   
  
  
- GAAAGGTATA ACAAATGAGA AAATTCCATT ATTTTATGAT TTTCCTTTTA CACCAACCCC ACAACTGTAA   
  
  
- CAGCCGGACA CATACTTCCA TGATAATATT CTTCTCTTCA CGCTCTCTCT GTATTGCCTA TGTCTTCGAT   
  
  
- TTTCTCGTTT CTTCTTATGG AAGATGTCG

+     HSE

| Site Name | Organism | Position | Strand | Matrix score. | sequence | function |
| --- | --- | --- | --- | --- | --- | --- |
| HSE | Brassica oleracea | 698 | - | 9 | AAAAAATTTC | cis-acting element involved in heat stress responsiveness |
| HSE | Brassica oleracea | 331 | - | 9 | AAAAAATTTC | cis-acting element involved in heat stress responsiveness |
| HSE | Brassica oleracea | 124 | + | 9 | AAAAAATTTC | cis-acting element involved in heat stress responsiveness |

> 2018/04/13 10:10:12  
+ CAATGAAAAG AAAGCTGCAT AGACATGTTG ACCCCTTCAT CCACTCTTTG TCTAGTTGCT TCTCTTCTAT   
  
  
+ GTATTACCAT TGACTGTTGA GTTTTCGAAG GATTAAAAGA AAAGTTTGTT CACAAAAAAT TTGATGCCCT   
  
  
+ AATTAAAACC CTTGAGGAAA TAAAAATCAT AATCTGGGCA ACCGGCTTCC TAAGTATATA TAGTATATAT   
  
  
+ TTCTAGGATT GGATGGCAGC TCGGCTTAAT TAATTATCTT TTAGTTATCT TAGAGCAATA AATTTAAGTC   
  
  
+ CGTTTTATAT GGTCGATTTC TATATATTGT GAATCATTGA CTGTTGAATT GAAATATTTT GTCATAATAT   
  
  
+ TTGTACATAT AACTTGGGGC AGCCAACGGA GCAATGGGAT CGGGGAGTCT AATATTTTAT CTACTAGAAG   
  
  
+ GTAAAAGCTT TTGAGTAACA AAGATGACTA CTATATAATA GCAAGCTTGA TAGTTACATA ACTAATAATA   
  
  
+ TCGGGTTTGA GATATGGTAA TCTATTGCTA TTTCATAAAA TAAGAATCGT ATTTACACCA CGATCCGTTT   
  
  
+ AATAATCCAC AAGTCTTTGT GTATATAGTT TTATATTAAC TTAATTCGAC TTAGTGAACA GATCAAAATC   
  
  
+ GATTATTTTT ATAGCTCGTG GAACTCGGAT CAGAAGAGAT GTATCCACCT GTGCTATTTC AAGAAGGGAA   
  
  
+ ATTTTGTCAT TAATATTAAG TGGATAATAA TGGACAAACT AACCGGGTAC TTAGACATCA ATCCATTAAC   
  
  
+ ATTGAATTAG TCCTTTTGTA CATATTATGG CGTTACATGT CCTGTATGAA GAAAAGAAAA CATCACCGTT   
  
  
+ TTAATTTATT ATTCAAATAT ACTTGTAATA CACAGAGATA TCAGAGATGA TAATCATAAT CATGATTAAC   
  
  
+ TAATCAGATC TGATGAAGCG CTTGGTACCT AACTAGTTGT GAGCTATACA ATAATATCAA CTAGGGTTTT   
  
  
+ CATACCGAGC AAGTGAAGGT CAATGGCGAG GTCAGTGGTG TGAGTGATCG TCACTCCGTG TTATACAAGC   
  
  
+ GTATTGACCA GGAGCTTAAT TATTGACACA GAAACAACAA AGCCTGCACC ATTGACACAG AGAAAAACCG   
  
  
+ ACCGGCCACC GGGGAGGCTA AAAGGTCACC AGTACTGGAT CTTTGACCCT TAGCCATGTG ACGTCGTCTC   
  
  
+ TATATGAAGC TTCAACGATG GTTACCATAG ATTATACAAT AGGGTCGTCG CCGCAAATTT AGCCTTTCTC   
  
  
+ CTTCTTTCTC TCTCTCTGCA AACTGATCAT ATCTCATCTG TTTCTTAACT GTGAAAGGTT GAAGTTGAAT   
  
  
+ CTTTCCATAT TGTTTACTCT TTTAAGGTAA TAAAATACTA AAAGGAAAAT GTGGTTGGGG TGTTGACATT   
  
  
+ GTCGGCCTGT GTATGAAGGT ACTATTATAA GAAGAGAAGT GCGAGAGAGA CATAACGGAT ACAGAAGCTA   
  
  
+ AAAGAGCAAA GAAGAATACC TTCTACAGC  

- GTTACTTTTC TTTCGACGTA TCTGTACAAC TGGGGAAGTA GGTGAGAAAC AGATCAACGA AGAGAAGATA   
  
  
- CATAATGGTA ACTGACAACT CAAAAGCTTC CTAATTTTCT TTTCAAACAA GTGTTTTTTA AACTACGGGA   
  
  
- TTAATTTTGG GAACTCCTTT ATTTTTAGTA TTAGACCCGT TGGCCGAAGG ATTCATATAT ATCATATATA   
  
  
- AAGATCCTAA CCTACCGTCG AGCCGAATTA ATTAATAGAA AATCAATAGA ATCTCGTTAT TTAAATTCAG   
  
  
- GCAAAATATA CCAGCTAAAG ATATATAACA CTTAGTAACT GACAACTTAA CTTTATAAAA CAGTATTATA   
  
  
- AACATGTATA TTGAACCCCG TCGGTTGCCT CGTTACCCTA GCCCCTCAGA TTATAAAATA GATGATCTTC   
  
  
- CATTTTCGAA AACTCATTGT TTCTACTGAT GATATATTAT CGTTCGAACT ATCAATGTAT TGATTATTAT   
  
  
- AGCCCAAACT CTATACCATT AGATAACGAT AAAGTATTTT ATTCTTAGCA TAAATGTGGT GCTAGGCAAA   
  
  
- TTATTAGGTG TTCAGAAACA CATATATCAA AATATAATTG AATTAAGCTG AATCACTTGT CTAGTTTTAG   
  
  
- CTAATAAAAA TATCGAGCAC CTTGAGCCTA GTCTTCTCTA CATAGGTGGA CACGATAAAG TTCTTCCCTT   
  
  
- TAAAACAGTA ATTATAATTC ACCTATTATT ACCTGTTTGA TTGGCCCATG AATCTGTAGT TAGGTAATTG   
  
  
- TAACTTAATC AGGAAAACAT GTATAATACC GCAATGTACA GGACATACTT CTTTTCTTTT GTAGTGGCAA   
  
  
- AATTAAATAA TAAGTTTATA TGAACATTAT GTGTCTCTAT AGTCTCTACT ATTAGTATTA GTACTAATTG   
  
  
- ATTAGTCTAG ACTACTTCGC GAACCATGGA TTGATCAACA CTCGATATGT TATTATAGTT GATCCCAAAA   
  
  
- GTATGGCTCG TTCACTTCCA GTTACCGCTC CAGTCACCAC ACTCACTAGC AGTGAGGCAC AATATGTTCG   
  
  
- CATAACTGGT CCTCGAATTA ATAACTGTGT CTTTGTTGTT TCGGACGTGG TAACTGTGTC TCTTTTTGGC   
  
  
- TGGCCGGTGG CCCCTCCGAT TTTCCAGTGG TCATGACCTA GAAACTGGGA ATCGGTACAC TGCAGCAGAG   
  
  
- ATATACTTCG AAGTTGCTAC CAATGGTATC TAATATGTTA TCCCAGCAGC GGCGTTTAAA TCGGAAAGAG   
  
  
- GAAGAAAGAG AGAGAGACGT TTGACTAGTA TAGAGTAGAC AAAGAATTGA CACTTTCCAA CTTCAACTTA   
  
  
- GAAAGGTATA ACAAATGAGA AAATTCCATT ATTTTATGAT TTTCCTTTTA CACCAACCCC ACAACTGTAA   
  
  
- CAGCCGGACA CATACTTCCA TGATAATATT CTTCTCTTCA CGCTCTCTCT GTATTGCCTA TGTCTTCGAT   
  
  
- TTTCTCGTTT CTTCTTATGG AAGATGTCG

+     I-box

| Site Name | Organism | Position | Strand | Matrix score. | sequence | function |
| --- | --- | --- | --- | --- | --- | --- |
| I-box | Flaveria trinervia | 501 | + | 7 | GATATGG | part of a light responsive element |

> 2018/04/13 10:10:12  
+ CAATGAAAAG AAAGCTGCAT AGACATGTTG ACCCCTTCAT CCACTCTTTG TCTAGTTGCT TCTCTTCTAT   
  
  
+ GTATTACCAT TGACTGTTGA GTTTTCGAAG GATTAAAAGA AAAGTTTGTT CACAAAAAAT TTGATGCCCT   
  
  
+ AATTAAAACC CTTGAGGAAA TAAAAATCAT AATCTGGGCA ACCGGCTTCC TAAGTATATA TAGTATATAT   
  
  
+ TTCTAGGATT GGATGGCAGC TCGGCTTAAT TAATTATCTT TTAGTTATCT TAGAGCAATA AATTTAAGTC   
  
  
+ CGTTTTATAT GGTCGATTTC TATATATTGT GAATCATTGA CTGTTGAATT GAAATATTTT GTCATAATAT   
  
  
+ TTGTACATAT AACTTGGGGC AGCCAACGGA GCAATGGGAT CGGGGAGTCT AATATTTTAT CTACTAGAAG   
  
  
+ GTAAAAGCTT TTGAGTAACA AAGATGACTA CTATATAATA GCAAGCTTGA TAGTTACATA ACTAATAATA   
  
  
+ TCGGGTTTGA GATATGGTAA TCTATTGCTA TTTCATAAAA TAAGAATCGT ATTTACACCA CGATCCGTTT   
  
  
+ AATAATCCAC AAGTCTTTGT GTATATAGTT TTATATTAAC TTAATTCGAC TTAGTGAACA GATCAAAATC   
  
  
+ GATTATTTTT ATAGCTCGTG GAACTCGGAT CAGAAGAGAT GTATCCACCT GTGCTATTTC AAGAAGGGAA   
  
  
+ ATTTTGTCAT TAATATTAAG TGGATAATAA TGGACAAACT AACCGGGTAC TTAGACATCA ATCCATTAAC   
  
  
+ ATTGAATTAG TCCTTTTGTA CATATTATGG CGTTACATGT CCTGTATGAA GAAAAGAAAA CATCACCGTT   
  
  
+ TTAATTTATT ATTCAAATAT ACTTGTAATA CACAGAGATA TCAGAGATGA TAATCATAAT CATGATTAAC   
  
  
+ TAATCAGATC TGATGAAGCG CTTGGTACCT AACTAGTTGT GAGCTATACA ATAATATCAA CTAGGGTTTT   
  
  
+ CATACCGAGC AAGTGAAGGT CAATGGCGAG GTCAGTGGTG TGAGTGATCG TCACTCCGTG TTATACAAGC   
  
  
+ GTATTGACCA GGAGCTTAAT TATTGACACA GAAACAACAA AGCCTGCACC ATTGACACAG AGAAAAACCG   
  
  
+ ACCGGCCACC GGGGAGGCTA AAAGGTCACC AGTACTGGAT CTTTGACCCT TAGCCATGTG ACGTCGTCTC   
  
  
+ TATATGAAGC TTCAACGATG GTTACCATAG ATTATACAAT AGGGTCGTCG CCGCAAATTT AGCCTTTCTC   
  
  
+ CTTCTTTCTC TCTCTCTGCA AACTGATCAT ATCTCATCTG TTTCTTAACT GTGAAAGGTT GAAGTTGAAT   
  
  
+ CTTTCCATAT TGTTTACTCT TTTAAGGTAA TAAAATACTA AAAGGAAAAT GTGGTTGGGG TGTTGACATT   
  
  
+ GTCGGCCTGT GTATGAAGGT ACTATTATAA GAAGAGAAGT GCGAGAGAGA CATAACGGAT ACAGAAGCTA   
  
  
+ AAAGAGCAAA GAAGAATACC TTCTACAGC  

- GTTACTTTTC TTTCGACGTA TCTGTACAAC TGGGGAAGTA GGTGAGAAAC AGATCAACGA AGAGAAGATA   
  
  
- CATAATGGTA ACTGACAACT CAAAAGCTTC CTAATTTTCT TTTCAAACAA GTGTTTTTTA AACTACGGGA   
  
  
- TTAATTTTGG GAACTCCTTT ATTTTTAGTA TTAGACCCGT TGGCCGAAGG ATTCATATAT ATCATATATA   
  
  
- AAGATCCTAA CCTACCGTCG AGCCGAATTA ATTAATAGAA AATCAATAGA ATCTCGTTAT TTAAATTCAG   
  
  
- GCAAAATATA CCAGCTAAAG ATATATAACA CTTAGTAACT GACAACTTAA CTTTATAAAA CAGTATTATA   
  
  
- AACATGTATA TTGAACCCCG TCGGTTGCCT CGTTACCCTA GCCCCTCAGA TTATAAAATA GATGATCTTC   
  
  
- CATTTTCGAA AACTCATTGT TTCTACTGAT GATATATTAT CGTTCGAACT ATCAATGTAT TGATTATTAT   
  
  
- AGCCCAAACT CTATACCATT AGATAACGAT AAAGTATTTT ATTCTTAGCA TAAATGTGGT GCTAGGCAAA   
  
  
- TTATTAGGTG TTCAGAAACA CATATATCAA AATATAATTG AATTAAGCTG AATCACTTGT CTAGTTTTAG   
  
  
- CTAATAAAAA TATCGAGCAC CTTGAGCCTA GTCTTCTCTA CATAGGTGGA CACGATAAAG TTCTTCCCTT   
  
  
- TAAAACAGTA ATTATAATTC ACCTATTATT ACCTGTTTGA TTGGCCCATG AATCTGTAGT TAGGTAATTG   
  
  
- TAACTTAATC AGGAAAACAT GTATAATACC GCAATGTACA GGACATACTT CTTTTCTTTT GTAGTGGCAA   
  
  
- AATTAAATAA TAAGTTTATA TGAACATTAT GTGTCTCTAT AGTCTCTACT ATTAGTATTA GTACTAATTG   
  
  
- ATTAGTCTAG ACTACTTCGC GAACCATGGA TTGATCAACA CTCGATATGT TATTATAGTT GATCCCAAAA   
  
  
- GTATGGCTCG TTCACTTCCA GTTACCGCTC CAGTCACCAC ACTCACTAGC AGTGAGGCAC AATATGTTCG   
  
  
- CATAACTGGT CCTCGAATTA ATAACTGTGT CTTTGTTGTT TCGGACGTGG TAACTGTGTC TCTTTTTGGC   
  
  
- TGGCCGGTGG CCCCTCCGAT TTTCCAGTGG TCATGACCTA GAAACTGGGA ATCGGTACAC TGCAGCAGAG   
  
  
- ATATACTTCG AAGTTGCTAC CAATGGTATC TAATATGTTA TCCCAGCAGC GGCGTTTAAA TCGGAAAGAG   
  
  
- GAAGAAAGAG AGAGAGACGT TTGACTAGTA TAGAGTAGAC AAAGAATTGA CACTTTCCAA CTTCAACTTA   
  
  
- GAAAGGTATA ACAAATGAGA AAATTCCATT ATTTTATGAT TTTCCTTTTA CACCAACCCC ACAACTGTAA   
  
  
- CAGCCGGACA CATACTTCCA TGATAATATT CTTCTCTTCA CGCTCTCTCT GTATTGCCTA TGTCTTCGAT   
  
  
- TTTCTCGTTT CTTCTTATGG AAGATGTCG

+     MBS

| Site Name | Organism | Position | Strand | Matrix score. | sequence | function |
| --- | --- | --- | --- | --- | --- | --- |
| MBS | Arabidopsis thaliana | 1306 | + | 6 | TAACTG | MYB binding site involved in drought-inducibility |

> 2018/04/13 10:10:12  
+ CAATGAAAAG AAAGCTGCAT AGACATGTTG ACCCCTTCAT CCACTCTTTG TCTAGTTGCT TCTCTTCTAT   
  
  
+ GTATTACCAT TGACTGTTGA GTTTTCGAAG GATTAAAAGA AAAGTTTGTT CACAAAAAAT TTGATGCCCT   
  
  
+ AATTAAAACC CTTGAGGAAA TAAAAATCAT AATCTGGGCA ACCGGCTTCC TAAGTATATA TAGTATATAT   
  
  
+ TTCTAGGATT GGATGGCAGC TCGGCTTAAT TAATTATCTT TTAGTTATCT TAGAGCAATA AATTTAAGTC   
  
  
+ CGTTTTATAT GGTCGATTTC TATATATTGT GAATCATTGA CTGTTGAATT GAAATATTTT GTCATAATAT   
  
  
+ TTGTACATAT AACTTGGGGC AGCCAACGGA GCAATGGGAT CGGGGAGTCT AATATTTTAT CTACTAGAAG   
  
  
+ GTAAAAGCTT TTGAGTAACA AAGATGACTA CTATATAATA GCAAGCTTGA TAGTTACATA ACTAATAATA   
  
  
+ TCGGGTTTGA GATATGGTAA TCTATTGCTA TTTCATAAAA TAAGAATCGT ATTTACACCA CGATCCGTTT   
  
  
+ AATAATCCAC AAGTCTTTGT GTATATAGTT TTATATTAAC TTAATTCGAC TTAGTGAACA GATCAAAATC   
  
  
+ GATTATTTTT ATAGCTCGTG GAACTCGGAT CAGAAGAGAT GTATCCACCT GTGCTATTTC AAGAAGGGAA   
  
  
+ ATTTTGTCAT TAATATTAAG TGGATAATAA TGGACAAACT AACCGGGTAC TTAGACATCA ATCCATTAAC   
  
  
+ ATTGAATTAG TCCTTTTGTA CATATTATGG CGTTACATGT CCTGTATGAA GAAAAGAAAA CATCACCGTT   
  
  
+ TTAATTTATT ATTCAAATAT ACTTGTAATA CACAGAGATA TCAGAGATGA TAATCATAAT CATGATTAAC   
  
  
+ TAATCAGATC TGATGAAGCG CTTGGTACCT AACTAGTTGT GAGCTATACA ATAATATCAA CTAGGGTTTT   
  
  
+ CATACCGAGC AAGTGAAGGT CAATGGCGAG GTCAGTGGTG TGAGTGATCG TCACTCCGTG TTATACAAGC   
  
  
+ GTATTGACCA GGAGCTTAAT TATTGACACA GAAACAACAA AGCCTGCACC ATTGACACAG AGAAAAACCG   
  
  
+ ACCGGCCACC GGGGAGGCTA AAAGGTCACC AGTACTGGAT CTTTGACCCT TAGCCATGTG ACGTCGTCTC   
  
  
+ TATATGAAGC TTCAACGATG GTTACCATAG ATTATACAAT AGGGTCGTCG CCGCAAATTT AGCCTTTCTC   
  
  
+ CTTCTTTCTC TCTCTCTGCA AACTGATCAT ATCTCATCTG TTTCTTAACT GTGAAAGGTT GAAGTTGAAT   
  
  
+ CTTTCCATAT TGTTTACTCT TTTAAGGTAA TAAAATACTA AAAGGAAAAT GTGGTTGGGG TGTTGACATT   
  
  
+ GTCGGCCTGT GTATGAAGGT ACTATTATAA GAAGAGAAGT GCGAGAGAGA CATAACGGAT ACAGAAGCTA   
  
  
+ AAAGAGCAAA GAAGAATACC TTCTACAGC  

- GTTACTTTTC TTTCGACGTA TCTGTACAAC TGGGGAAGTA GGTGAGAAAC AGATCAACGA AGAGAAGATA   
  
  
- CATAATGGTA ACTGACAACT CAAAAGCTTC CTAATTTTCT TTTCAAACAA GTGTTTTTTA AACTACGGGA   
  
  
- TTAATTTTGG GAACTCCTTT ATTTTTAGTA TTAGACCCGT TGGCCGAAGG ATTCATATAT ATCATATATA   
  
  
- AAGATCCTAA CCTACCGTCG AGCCGAATTA ATTAATAGAA AATCAATAGA ATCTCGTTAT TTAAATTCAG   
  
  
- GCAAAATATA CCAGCTAAAG ATATATAACA CTTAGTAACT GACAACTTAA CTTTATAAAA CAGTATTATA   
  
  
- AACATGTATA TTGAACCCCG TCGGTTGCCT CGTTACCCTA GCCCCTCAGA TTATAAAATA GATGATCTTC   
  
  
- CATTTTCGAA AACTCATTGT TTCTACTGAT GATATATTAT CGTTCGAACT ATCAATGTAT TGATTATTAT   
  
  
- AGCCCAAACT CTATACCATT AGATAACGAT AAAGTATTTT ATTCTTAGCA TAAATGTGGT GCTAGGCAAA   
  
  
- TTATTAGGTG TTCAGAAACA CATATATCAA AATATAATTG AATTAAGCTG AATCACTTGT CTAGTTTTAG   
  
  
- CTAATAAAAA TATCGAGCAC CTTGAGCCTA GTCTTCTCTA CATAGGTGGA CACGATAAAG TTCTTCCCTT   
  
  
- TAAAACAGTA ATTATAATTC ACCTATTATT ACCTGTTTGA TTGGCCCATG AATCTGTAGT TAGGTAATTG   
  
  
- TAACTTAATC AGGAAAACAT GTATAATACC GCAATGTACA GGACATACTT CTTTTCTTTT GTAGTGGCAA   
  
  
- AATTAAATAA TAAGTTTATA TGAACATTAT GTGTCTCTAT AGTCTCTACT ATTAGTATTA GTACTAATTG   
  
  
- ATTAGTCTAG ACTACTTCGC GAACCATGGA TTGATCAACA CTCGATATGT TATTATAGTT GATCCCAAAA   
  
  
- GTATGGCTCG TTCACTTCCA GTTACCGCTC CAGTCACCAC ACTCACTAGC AGTGAGGCAC AATATGTTCG   
  
  
- CATAACTGGT CCTCGAATTA ATAACTGTGT CTTTGTTGTT TCGGACGTGG TAACTGTGTC TCTTTTTGGC   
  
  
- TGGCCGGTGG CCCCTCCGAT TTTCCAGTGG TCATGACCTA GAAACTGGGA ATCGGTACAC TGCAGCAGAG   
  
  
- ATATACTTCG AAGTTGCTAC CAATGGTATC TAATATGTTA TCCCAGCAGC GGCGTTTAAA TCGGAAAGAG   
  
  
- GAAGAAAGAG AGAGAGACGT TTGACTAGTA TAGAGTAGAC AAAGAATTGA CACTTTCCAA CTTCAACTTA   
  
  
- GAAAGGTATA ACAAATGAGA AAATTCCATT ATTTTATGAT TTTCCTTTTA CACCAACCCC ACAACTGTAA   
  
  
- CAGCCGGACA CATACTTCCA TGATAATATT CTTCTCTTCA CGCTCTCTCT GTATTGCCTA TGTCTTCGAT   
  
  
- TTTCTCGTTT CTTCTTATGG AAGATGTCG

+     P-box

| Site Name | Organism | Position | Strand | Matrix score. | sequence | function |
| --- | --- | --- | --- | --- | --- | --- |
| P-box | Oryza sativa | 782 | + | 7 | CCTTTTG | gibberellin-responsive element |

> 2018/04/13 10:10:12  
+ CAATGAAAAG AAAGCTGCAT AGACATGTTG ACCCCTTCAT CCACTCTTTG TCTAGTTGCT TCTCTTCTAT   
  
  
+ GTATTACCAT TGACTGTTGA GTTTTCGAAG GATTAAAAGA AAAGTTTGTT CACAAAAAAT TTGATGCCCT   
  
  
+ AATTAAAACC CTTGAGGAAA TAAAAATCAT AATCTGGGCA ACCGGCTTCC TAAGTATATA TAGTATATAT   
  
  
+ TTCTAGGATT GGATGGCAGC TCGGCTTAAT TAATTATCTT TTAGTTATCT TAGAGCAATA AATTTAAGTC   
  
  
+ CGTTTTATAT GGTCGATTTC TATATATTGT GAATCATTGA CTGTTGAATT GAAATATTTT GTCATAATAT   
  
  
+ TTGTACATAT AACTTGGGGC AGCCAACGGA GCAATGGGAT CGGGGAGTCT AATATTTTAT CTACTAGAAG   
  
  
+ GTAAAAGCTT TTGAGTAACA AAGATGACTA CTATATAATA GCAAGCTTGA TAGTTACATA ACTAATAATA   
  
  
+ TCGGGTTTGA GATATGGTAA TCTATTGCTA TTTCATAAAA TAAGAATCGT ATTTACACCA CGATCCGTTT   
  
  
+ AATAATCCAC AAGTCTTTGT GTATATAGTT TTATATTAAC TTAATTCGAC TTAGTGAACA GATCAAAATC   
  
  
+ GATTATTTTT ATAGCTCGTG GAACTCGGAT CAGAAGAGAT GTATCCACCT GTGCTATTTC AAGAAGGGAA   
  
  
+ ATTTTGTCAT TAATATTAAG TGGATAATAA TGGACAAACT AACCGGGTAC TTAGACATCA ATCCATTAAC   
  
  
+ ATTGAATTAG TCCTTTTGTA CATATTATGG CGTTACATGT CCTGTATGAA GAAAAGAAAA CATCACCGTT   
  
  
+ TTAATTTATT ATTCAAATAT ACTTGTAATA CACAGAGATA TCAGAGATGA TAATCATAAT CATGATTAAC   
  
  
+ TAATCAGATC TGATGAAGCG CTTGGTACCT AACTAGTTGT GAGCTATACA ATAATATCAA CTAGGGTTTT   
  
  
+ CATACCGAGC AAGTGAAGGT CAATGGCGAG GTCAGTGGTG TGAGTGATCG TCACTCCGTG TTATACAAGC   
  
  
+ GTATTGACCA GGAGCTTAAT TATTGACACA GAAACAACAA AGCCTGCACC ATTGACACAG AGAAAAACCG   
  
  
+ ACCGGCCACC GGGGAGGCTA AAAGGTCACC AGTACTGGAT CTTTGACCCT TAGCCATGTG ACGTCGTCTC   
  
  
+ TATATGAAGC TTCAACGATG GTTACCATAG ATTATACAAT AGGGTCGTCG CCGCAAATTT AGCCTTTCTC   
  
  
+ CTTCTTTCTC TCTCTCTGCA AACTGATCAT ATCTCATCTG TTTCTTAACT GTGAAAGGTT GAAGTTGAAT   
  
  
+ CTTTCCATAT TGTTTACTCT TTTAAGGTAA TAAAATACTA AAAGGAAAAT GTGGTTGGGG TGTTGACATT   
  
  
+ GTCGGCCTGT GTATGAAGGT ACTATTATAA GAAGAGAAGT GCGAGAGAGA CATAACGGAT ACAGAAGCTA   
  
  
+ AAAGAGCAAA GAAGAATACC TTCTACAGC  

- GTTACTTTTC TTTCGACGTA TCTGTACAAC TGGGGAAGTA GGTGAGAAAC AGATCAACGA AGAGAAGATA   
  
  
- CATAATGGTA ACTGACAACT CAAAAGCTTC CTAATTTTCT TTTCAAACAA GTGTTTTTTA AACTACGGGA   
  
  
- TTAATTTTGG GAACTCCTTT ATTTTTAGTA TTAGACCCGT TGGCCGAAGG ATTCATATAT ATCATATATA   
  
  
- AAGATCCTAA CCTACCGTCG AGCCGAATTA ATTAATAGAA AATCAATAGA ATCTCGTTAT TTAAATTCAG   
  
  
- GCAAAATATA CCAGCTAAAG ATATATAACA CTTAGTAACT GACAACTTAA CTTTATAAAA CAGTATTATA   
  
  
- AACATGTATA TTGAACCCCG TCGGTTGCCT CGTTACCCTA GCCCCTCAGA TTATAAAATA GATGATCTTC   
  
  
- CATTTTCGAA AACTCATTGT TTCTACTGAT GATATATTAT CGTTCGAACT ATCAATGTAT TGATTATTAT   
  
  
- AGCCCAAACT CTATACCATT AGATAACGAT AAAGTATTTT ATTCTTAGCA TAAATGTGGT GCTAGGCAAA   
  
  
- TTATTAGGTG TTCAGAAACA CATATATCAA AATATAATTG AATTAAGCTG AATCACTTGT CTAGTTTTAG   
  
  
- CTAATAAAAA TATCGAGCAC CTTGAGCCTA GTCTTCTCTA CATAGGTGGA CACGATAAAG TTCTTCCCTT   
  
  
- TAAAACAGTA ATTATAATTC ACCTATTATT ACCTGTTTGA TTGGCCCATG AATCTGTAGT TAGGTAATTG   
  
  
- TAACTTAATC AGGAAAACAT GTATAATACC GCAATGTACA GGACATACTT CTTTTCTTTT GTAGTGGCAA   
  
  
- AATTAAATAA TAAGTTTATA TGAACATTAT GTGTCTCTAT AGTCTCTACT ATTAGTATTA GTACTAATTG   
  
  
- ATTAGTCTAG ACTACTTCGC GAACCATGGA TTGATCAACA CTCGATATGT TATTATAGTT GATCCCAAAA   
  
  
- GTATGGCTCG TTCACTTCCA GTTACCGCTC CAGTCACCAC ACTCACTAGC AGTGAGGCAC AATATGTTCG   
  
  
- CATAACTGGT CCTCGAATTA ATAACTGTGT CTTTGTTGTT TCGGACGTGG TAACTGTGTC TCTTTTTGGC   
  
  
- TGGCCGGTGG CCCCTCCGAT TTTCCAGTGG TCATGACCTA GAAACTGGGA ATCGGTACAC TGCAGCAGAG   
  
  
- ATATACTTCG AAGTTGCTAC CAATGGTATC TAATATGTTA TCCCAGCAGC GGCGTTTAAA TCGGAAAGAG   
  
  
- GAAGAAAGAG AGAGAGACGT TTGACTAGTA TAGAGTAGAC AAAGAATTGA CACTTTCCAA CTTCAACTTA   
  
  
- GAAAGGTATA ACAAATGAGA AAATTCCATT ATTTTATGAT TTTCCTTTTA CACCAACCCC ACAACTGTAA   
  
  
- CAGCCGGACA CATACTTCCA TGATAATATT CTTCTCTTCA CGCTCTCTCT GTATTGCCTA TGTCTTCGAT   
  
  
- TTTCTCGTTT CTTCTTATGG AAGATGTCG

+     Skn-1\_motif

| Site Name | Organism | Position | Strand | Matrix score. | sequence | function |
| --- | --- | --- | --- | --- | --- | --- |
| Skn-1\_motif | Oryza sativa | 706 | + | 5 | GTCAT | cis-acting regulatory element required for endosperm expression |
| Skn-1\_motif | Oryza sativa | 444 | - | 5 | GTCAT | cis-acting regulatory element required for endosperm expression |
| Skn-1\_motif | Oryza sativa | 341 | + | 5 | GTCAT | cis-acting regulatory element required for endosperm expression |

> 2018/04/13 10:10:12  
+ CAATGAAAAG AAAGCTGCAT AGACATGTTG ACCCCTTCAT CCACTCTTTG TCTAGTTGCT TCTCTTCTAT   
  
  
+ GTATTACCAT TGACTGTTGA GTTTTCGAAG GATTAAAAGA AAAGTTTGTT CACAAAAAAT TTGATGCCCT   
  
  
+ AATTAAAACC CTTGAGGAAA TAAAAATCAT AATCTGGGCA ACCGGCTTCC TAAGTATATA TAGTATATAT   
  
  
+ TTCTAGGATT GGATGGCAGC TCGGCTTAAT TAATTATCTT TTAGTTATCT TAGAGCAATA AATTTAAGTC   
  
  
+ CGTTTTATAT GGTCGATTTC TATATATTGT GAATCATTGA CTGTTGAATT GAAATATTTT GTCATAATAT   
  
  
+ TTGTACATAT AACTTGGGGC AGCCAACGGA GCAATGGGAT CGGGGAGTCT AATATTTTAT CTACTAGAAG   
  
  
+ GTAAAAGCTT TTGAGTAACA AAGATGACTA CTATATAATA GCAAGCTTGA TAGTTACATA ACTAATAATA   
  
  
+ TCGGGTTTGA GATATGGTAA TCTATTGCTA TTTCATAAAA TAAGAATCGT ATTTACACCA CGATCCGTTT   
  
  
+ AATAATCCAC AAGTCTTTGT GTATATAGTT TTATATTAAC TTAATTCGAC TTAGTGAACA GATCAAAATC   
  
  
+ GATTATTTTT ATAGCTCGTG GAACTCGGAT CAGAAGAGAT GTATCCACCT GTGCTATTTC AAGAAGGGAA   
  
  
+ ATTTTGTCAT TAATATTAAG TGGATAATAA TGGACAAACT AACCGGGTAC TTAGACATCA ATCCATTAAC   
  
  
+ ATTGAATTAG TCCTTTTGTA CATATTATGG CGTTACATGT CCTGTATGAA GAAAAGAAAA CATCACCGTT   
  
  
+ TTAATTTATT ATTCAAATAT ACTTGTAATA CACAGAGATA TCAGAGATGA TAATCATAAT CATGATTAAC   
  
  
+ TAATCAGATC TGATGAAGCG CTTGGTACCT AACTAGTTGT GAGCTATACA ATAATATCAA CTAGGGTTTT   
  
  
+ CATACCGAGC AAGTGAAGGT CAATGGCGAG GTCAGTGGTG TGAGTGATCG TCACTCCGTG TTATACAAGC   
  
  
+ GTATTGACCA GGAGCTTAAT TATTGACACA GAAACAACAA AGCCTGCACC ATTGACACAG AGAAAAACCG   
  
  
+ ACCGGCCACC GGGGAGGCTA AAAGGTCACC AGTACTGGAT CTTTGACCCT TAGCCATGTG ACGTCGTCTC   
  
  
+ TATATGAAGC TTCAACGATG GTTACCATAG ATTATACAAT AGGGTCGTCG CCGCAAATTT AGCCTTTCTC   
  
  
+ CTTCTTTCTC TCTCTCTGCA AACTGATCAT ATCTCATCTG TTTCTTAACT GTGAAAGGTT GAAGTTGAAT   
  
  
+ CTTTCCATAT TGTTTACTCT TTTAAGGTAA TAAAATACTA AAAGGAAAAT GTGGTTGGGG TGTTGACATT   
  
  
+ GTCGGCCTGT GTATGAAGGT ACTATTATAA GAAGAGAAGT GCGAGAGAGA CATAACGGAT ACAGAAGCTA   
  
  
+ AAAGAGCAAA GAAGAATACC TTCTACAGC  

- GTTACTTTTC TTTCGACGTA TCTGTACAAC TGGGGAAGTA GGTGAGAAAC AGATCAACGA AGAGAAGATA   
  
  
- CATAATGGTA ACTGACAACT CAAAAGCTTC CTAATTTTCT TTTCAAACAA GTGTTTTTTA AACTACGGGA   
  
  
- TTAATTTTGG GAACTCCTTT ATTTTTAGTA TTAGACCCGT TGGCCGAAGG ATTCATATAT ATCATATATA   
  
  
- AAGATCCTAA CCTACCGTCG AGCCGAATTA ATTAATAGAA AATCAATAGA ATCTCGTTAT TTAAATTCAG   
  
  
- GCAAAATATA CCAGCTAAAG ATATATAACA CTTAGTAACT GACAACTTAA CTTTATAAAA CAGTATTATA   
  
  
- AACATGTATA TTGAACCCCG TCGGTTGCCT CGTTACCCTA GCCCCTCAGA TTATAAAATA GATGATCTTC   
  
  
- CATTTTCGAA AACTCATTGT TTCTACTGAT GATATATTAT CGTTCGAACT ATCAATGTAT TGATTATTAT   
  
  
- AGCCCAAACT CTATACCATT AGATAACGAT AAAGTATTTT ATTCTTAGCA TAAATGTGGT GCTAGGCAAA   
  
  
- TTATTAGGTG TTCAGAAACA CATATATCAA AATATAATTG AATTAAGCTG AATCACTTGT CTAGTTTTAG   
  
  
- CTAATAAAAA TATCGAGCAC CTTGAGCCTA GTCTTCTCTA CATAGGTGGA CACGATAAAG TTCTTCCCTT   
  
  
- TAAAACAGTA ATTATAATTC ACCTATTATT ACCTGTTTGA TTGGCCCATG AATCTGTAGT TAGGTAATTG   
  
  
- TAACTTAATC AGGAAAACAT GTATAATACC GCAATGTACA GGACATACTT CTTTTCTTTT GTAGTGGCAA   
  
  
- AATTAAATAA TAAGTTTATA TGAACATTAT GTGTCTCTAT AGTCTCTACT ATTAGTATTA GTACTAATTG   
  
  
- ATTAGTCTAG ACTACTTCGC GAACCATGGA TTGATCAACA CTCGATATGT TATTATAGTT GATCCCAAAA   
  
  
- GTATGGCTCG TTCACTTCCA GTTACCGCTC CAGTCACCAC ACTCACTAGC AGTGAGGCAC AATATGTTCG   
  
  
- CATAACTGGT CCTCGAATTA ATAACTGTGT CTTTGTTGTT TCGGACGTGG TAACTGTGTC TCTTTTTGGC   
  
  
- TGGCCGGTGG CCCCTCCGAT TTTCCAGTGG TCATGACCTA GAAACTGGGA ATCGGTACAC TGCAGCAGAG   
  
  
- ATATACTTCG AAGTTGCTAC CAATGGTATC TAATATGTTA TCCCAGCAGC GGCGTTTAAA TCGGAAAGAG   
  
  
- GAAGAAAGAG AGAGAGACGT TTGACTAGTA TAGAGTAGAC AAAGAATTGA CACTTTCCAA CTTCAACTTA   
  
  
- GAAAGGTATA ACAAATGAGA AAATTCCATT ATTTTATGAT TTTCCTTTTA CACCAACCCC ACAACTGTAA   
  
  
- CAGCCGGACA CATACTTCCA TGATAATATT CTTCTCTTCA CGCTCTCTCT GTATTGCCTA TGTCTTCGAT   
  
  
- TTTCTCGTTT CTTCTTATGG AAGATGTCG

+     Sp1

| Site Name | Organism | Position | Strand | Matrix score. | sequence | function |
| --- | --- | --- | --- | --- | --- | --- |
| Sp1 | Zea mays | 1132 | - | 5 | CC(G/A)CCC | light responsive element |

> 2018/04/13 10:10:12  
+ CAATGAAAAG AAAGCTGCAT AGACATGTTG ACCCCTTCAT CCACTCTTTG TCTAGTTGCT TCTCTTCTAT   
  
  
+ GTATTACCAT TGACTGTTGA GTTTTCGAAG GATTAAAAGA AAAGTTTGTT CACAAAAAAT TTGATGCCCT   
  
  
+ AATTAAAACC CTTGAGGAAA TAAAAATCAT AATCTGGGCA ACCGGCTTCC TAAGTATATA TAGTATATAT   
  
  
+ TTCTAGGATT GGATGGCAGC TCGGCTTAAT TAATTATCTT TTAGTTATCT TAGAGCAATA AATTTAAGTC   
  
  
+ CGTTTTATAT GGTCGATTTC TATATATTGT GAATCATTGA CTGTTGAATT GAAATATTTT GTCATAATAT   
  
  
+ TTGTACATAT AACTTGGGGC AGCCAACGGA GCAATGGGAT CGGGGAGTCT AATATTTTAT CTACTAGAAG   
  
  
+ GTAAAAGCTT TTGAGTAACA AAGATGACTA CTATATAATA GCAAGCTTGA TAGTTACATA ACTAATAATA   
  
  
+ TCGGGTTTGA GATATGGTAA TCTATTGCTA TTTCATAAAA TAAGAATCGT ATTTACACCA CGATCCGTTT   
  
  
+ AATAATCCAC AAGTCTTTGT GTATATAGTT TTATATTAAC TTAATTCGAC TTAGTGAACA GATCAAAATC   
  
  
+ GATTATTTTT ATAGCTCGTG GAACTCGGAT CAGAAGAGAT GTATCCACCT GTGCTATTTC AAGAAGGGAA   
  
  
+ ATTTTGTCAT TAATATTAAG TGGATAATAA TGGACAAACT AACCGGGTAC TTAGACATCA ATCCATTAAC   
  
  
+ ATTGAATTAG TCCTTTTGTA CATATTATGG CGTTACATGT CCTGTATGAA GAAAAGAAAA CATCACCGTT   
  
  
+ TTAATTTATT ATTCAAATAT ACTTGTAATA CACAGAGATA TCAGAGATGA TAATCATAAT CATGATTAAC   
  
  
+ TAATCAGATC TGATGAAGCG CTTGGTACCT AACTAGTTGT GAGCTATACA ATAATATCAA CTAGGGTTTT   
  
  
+ CATACCGAGC AAGTGAAGGT CAATGGCGAG GTCAGTGGTG TGAGTGATCG TCACTCCGTG TTATACAAGC   
  
  
+ GTATTGACCA GGAGCTTAAT TATTGACACA GAAACAACAA AGCCTGCACC ATTGACACAG AGAAAAACCG   
  
  
+ ACCGGCCACC GGGGAGGCTA AAAGGTCACC AGTACTGGAT CTTTGACCCT TAGCCATGTG ACGTCGTCTC   
  
  
+ TATATGAAGC TTCAACGATG GTTACCATAG ATTATACAAT AGGGTCGTCG CCGCAAATTT AGCCTTTCTC   
  
  
+ CTTCTTTCTC TCTCTCTGCA AACTGATCAT ATCTCATCTG TTTCTTAACT GTGAAAGGTT GAAGTTGAAT   
  
  
+ CTTTCCATAT TGTTTACTCT TTTAAGGTAA TAAAATACTA AAAGGAAAAT GTGGTTGGGG TGTTGACATT   
  
  
+ GTCGGCCTGT GTATGAAGGT ACTATTATAA GAAGAGAAGT GCGAGAGAGA CATAACGGAT ACAGAAGCTA   
  
  
+ AAAGAGCAAA GAAGAATACC TTCTACAGC  

- GTTACTTTTC TTTCGACGTA TCTGTACAAC TGGGGAAGTA GGTGAGAAAC AGATCAACGA AGAGAAGATA   
  
  
- CATAATGGTA ACTGACAACT CAAAAGCTTC CTAATTTTCT TTTCAAACAA GTGTTTTTTA AACTACGGGA   
  
  
- TTAATTTTGG GAACTCCTTT ATTTTTAGTA TTAGACCCGT TGGCCGAAGG ATTCATATAT ATCATATATA   
  
  
- AAGATCCTAA CCTACCGTCG AGCCGAATTA ATTAATAGAA AATCAATAGA ATCTCGTTAT TTAAATTCAG   
  
  
- GCAAAATATA CCAGCTAAAG ATATATAACA CTTAGTAACT GACAACTTAA CTTTATAAAA CAGTATTATA   
  
  
- AACATGTATA TTGAACCCCG TCGGTTGCCT CGTTACCCTA GCCCCTCAGA TTATAAAATA GATGATCTTC   
  
  
- CATTTTCGAA AACTCATTGT TTCTACTGAT GATATATTAT CGTTCGAACT ATCAATGTAT TGATTATTAT   
  
  
- AGCCCAAACT CTATACCATT AGATAACGAT AAAGTATTTT ATTCTTAGCA TAAATGTGGT GCTAGGCAAA   
  
  
- TTATTAGGTG TTCAGAAACA CATATATCAA AATATAATTG AATTAAGCTG AATCACTTGT CTAGTTTTAG   
  
  
- CTAATAAAAA TATCGAGCAC CTTGAGCCTA GTCTTCTCTA CATAGGTGGA CACGATAAAG TTCTTCCCTT   
  
  
- TAAAACAGTA ATTATAATTC ACCTATTATT ACCTGTTTGA TTGGCCCATG AATCTGTAGT TAGGTAATTG   
  
  
- TAACTTAATC AGGAAAACAT GTATAATACC GCAATGTACA GGACATACTT CTTTTCTTTT GTAGTGGCAA   
  
  
- AATTAAATAA TAAGTTTATA TGAACATTAT GTGTCTCTAT AGTCTCTACT ATTAGTATTA GTACTAATTG   
  
  
- ATTAGTCTAG ACTACTTCGC GAACCATGGA TTGATCAACA CTCGATATGT TATTATAGTT GATCCCAAAA   
  
  
- GTATGGCTCG TTCACTTCCA GTTACCGCTC CAGTCACCAC ACTCACTAGC AGTGAGGCAC AATATGTTCG   
  
  
- CATAACTGGT CCTCGAATTA ATAACTGTGT CTTTGTTGTT TCGGACGTGG TAACTGTGTC TCTTTTTGGC   
  
  
- TGGCCGGTGG CCCCTCCGAT TTTCCAGTGG TCATGACCTA GAAACTGGGA ATCGGTACAC TGCAGCAGAG   
  
  
- ATATACTTCG AAGTTGCTAC CAATGGTATC TAATATGTTA TCCCAGCAGC GGCGTTTAAA TCGGAAAGAG   
  
  
- GAAGAAAGAG AGAGAGACGT TTGACTAGTA TAGAGTAGAC AAAGAATTGA CACTTTCCAA CTTCAACTTA   
  
  
- GAAAGGTATA ACAAATGAGA AAATTCCATT ATTTTATGAT TTTCCTTTTA CACCAACCCC ACAACTGTAA   
  
  
- CAGCCGGACA CATACTTCCA TGATAATATT CTTCTCTTCA CGCTCTCTCT GTATTGCCTA TGTCTTCGAT   
  
  
- TTTCTCGTTT CTTCTTATGG AAGATGTCG

+     TATA-box

| Site Name | Organism | Position | Strand | Matrix score. | sequence | function |
| --- | --- | --- | --- | --- | --- | --- |
| TATA-box | Arabidopsis thaliana | 1426 | - | 4 | TATA | core promoter element around -30 of transcription start |
| TATA-box | Lycopersicon esculentum | 1469 | - | 5 | TTTTA | core promoter element around -30 of transcription start |
| TATA-box | Lycopersicon esculentum | 839 | + | 5 | TTTTA | core promoter element around -30 of transcription start |
| TATA-box | Lycopersicon esculentum | 1369 | - | 5 | TTTTA | core promoter element around -30 of transcription start |
| TATA-box | Lycopersicon esculentum | 526 | - | 5 | TTTTA | core promoter element around -30 of transcription start |
| TATA-box | Arabidopsis thaliana | 295 | - | 9 | taTATAAAtc | core promoter element around -30 of transcription start |
| TATA-box | Lycopersicon esculentum | 422 | - | 5 | TTTTA | core promoter element around -30 of transcription start |
| TATA-box | Arabidopsis thaliana | 195 | + | 8 | TATATATA | core promoter element around -30 of transcription start |
| TATA-box | Glycine max | 1423 | - | 5 | TAATA | core promoter element around -30 of transcription start |
| TATA-box | Arabidopsis thaliana | 591 | - | 5 | TATAA | core promoter element around -30 of transcription start |
| TATA-box | Arabidopsis thaliana | 584 | + | 4 | TATA | core promoter element around -30 of transcription start |
| TATA-box | Brassica napus | 1424 | + | 6 | ATTATA | core promoter element around -30 of transcription start |
| TATA-box | Lycopersicon esculentum | 1361 | - | 5 | TTTTA | core promoter element around -30 of transcription start |
| TATA-box | Arabidopsis thaliana | 1223 | - | 4 | TATA | core promoter element around -30 of transcription start |
| TATA-box | Lycopersicon esculentum | 405 | + | 5 | TTTTA | core promoter element around -30 of transcription start |
| TATA-box | Glycine max | 486 | + | 5 | TAATA | core promoter element around -30 of transcription start |
| TATA-box | Pisum sativum | 355 | - | 7 | TATATGT | core promoter element around -30 of transcription start |
| TATA-box | Glycine max | 400 | + | 5 | TAATA | core promoter element around -30 of transcription start |
| TATA-box | Arabidopsis thaliana | 285 | - | 5 | TATAA | core promoter element around -30 of transcription start |
| TATA-box | Arabidopsis thaliana | 582 | + | 4 | TATA | core promoter element around -30 of transcription start |
| TATA-box | Arabidopsis thaliana | 1041 | - | 5 | TATAA | core promoter element around -30 of transcription start |
| TATA-box | Arabidopsis thaliana | 955 | - | 4 | TATA | core promoter element around -30 of transcription start |
| TATA-box | Brassica oleracea | 453 | + | 7 | ATATAAT | core promoter element around -30 of transcription start |
| TATA-box | Arabidopsis thaliana | 639 | - | 5 | TATAA | core promoter element around -30 of transcription start |
| TATA-box | Arabidopsis thaliana | 638 | - | 6 | TATAAA | core promoter element around -30 of transcription start |
| TATA-box | Glycine max | 456 | + | 5 | TAATA | core promoter element around -30 of transcription start |
| TATA-box | Arabidopsis thaliana | 358 | + | 4 | TATA | core promoter element around -30 of transcription start |
| TATA-box | Arabidopsis thaliana | 1425 | - | 5 | TATAA | core promoter element around -30 of transcription start |
| TATA-box | Arabidopsis thaliana | 633 | - | 9 | TAAAAATAA | core promoter element around -30 of transcription start |
| TATA-box | Arabidopsis thaliana | 286 | + | 4 | TATA | core promoter element around -30 of transcription start |
| TATA-box | Glycine max | 1358 | + | 5 | TAATA | core promoter element around -30 of transcription start |
| TATA-box | Brassica oleracea | 357 | + | 6 | ATATAA | core promoter element around -30 of transcription start |
| TATA-box | Lycopersicon esculentum | 161 | - | 5 | TTTTA | core promoter element around -30 of transcription start |
| TATA-box | Arabidopsis thaliana | 284 | - | 6 | TATAAA | core promoter element around -30 of transcription start |
| TATA-box | Lycopersicon esculentum | 144 | - | 5 | TTTTA | core promoter element around -30 of transcription start |
| TATA-box | Lycopersicon esculentum | 104 | - | 5 | TTTTA | core promoter element around -30 of transcription start |
| TATA-box | Arabidopsis thaliana | 66 | + | 9 | tcTATATAtt | core promoter element around -30 of transcription start |
| TATA-box | Glycine max | 711 | + | 5 | TAATA | core promoter element around -30 of transcription start |
| TATA-box | Glycine max | 72 | - | 5 | TAATA | core promoter element around -30 of transcription start |
| TATA-box | Brassica napus | 1221 | + | 6 | ATTATA | core promoter element around -30 of transcription start |
| TATA-box | Glycine max | 594 | - | 5 | TAATA | core promoter element around -30 of transcription start |
| TATA-box | Glycine max | 793 | - | 5 | TAATA | core promoter element around -30 of transcription start |
| TATA-box | Arabidopsis thaliana | 452 | + | 4 | TATA | core promoter element around -30 of transcription start |
| TATA-box | Glycine max | 560 | + | 5 | TAATA | core promoter element around -30 of transcription start |
| TATA-box | Arabidopsis thaliana | 199 | + | 4 | TATA | core promoter element around -30 of transcription start |
| TATA-box | Arabidopsis thaliana | 204 | + | 4 | TATA | core promoter element around -30 of transcription start |
| TATA-box | Glycine max | 345 | + | 5 | TAATA | core promoter element around -30 of transcription start |
| TATA-box | Arabidopsis thaliana | 299 | + | 10 | tcTATATAtt | core promoter element around -30 of transcription start |
| TATA-box | Arabidopsis thaliana | 303 | + | 4 | TATA | core promoter element around -30 of transcription start |
| TATA-box | Arabidopsis thaliana | 197 | + | 4 | TATA | core promoter element around -30 of transcription start |
| TATA-box | Arabidopsis thaliana | 454 | + | 4 | TATA | core promoter element around -30 of transcription start |
| TATA-box | Lycopersicon esculentum | 249 | + | 5 | TTTTA | core promoter element around -30 of transcription start |
| TATA-box | Arabidopsis thaliana | 1191 | - | 4 | TATA | core promoter element around -30 of transcription start |
| TATA-box | Lycopersicon esculentum | 1139 | - | 5 | TTTTA | core promoter element around -30 of transcription start |
| TATA-box | Helianthus annuus | 580 | - | 6 | TATACA | core promoter element around -30 of transcription start |
| TATA-box | Lycopersicon esculentum | 1350 | + | 5 | TTTTA | core promoter element around -30 of transcription start |
| TATA-box | Glycine max | 962 | + | 5 | TAATA | core promoter element around -30 of transcription start |
| TATA-box | Brassica napus | 196 | + | 6 | ATATAT | core promoter element around -30 of transcription start |
| TATA-box | Brassica napus | 302 | + | 6 | ATATAT | core promoter element around -30 of transcription start |
| TATA-box | Glycine max | 866 | + | 5 | TAATA | core promoter element around -30 of transcription start |
| TATA-box | Brassica napus | 205 | + | 6 | ATATAT | core promoter element around -30 of transcription start |
| TATA-box | Glycine max | 847 | - | 5 | TAATA | core promoter element around -30 of transcription start |
| TATA-box | Glycine max | 483 | + | 5 | TAATA | core promoter element around -30 of transcription start |
| TATA-box | Oryza sativa | 784 | - | 7 | TACAAAA | core promoter element around -30 of transcription start |
| TATA-box | Arabidopsis thaliana | 858 | - | 4 | TATA | core promoter element around -30 of transcription start |
| TATA-box | Arabidopsis thaliana | 1042 | - | 4 | TATA | core promoter element around -30 of transcription start |
| TATA-box | Arabidopsis thaliana | 301 | + | 4 | TATA | core promoter element around -30 of transcription start |
| TATA-box | Arabidopsis thaliana | 283 | - | 7 | TATAAAA | core promoter element around -30 of transcription start |
| TATA-box | Arabidopsis thaliana | 206 | + | 4 | TATA | core promoter element around -30 of transcription start |
| TATA-box | Arabidopsis thaliana | 636 | - | 9 | ccTATAAAaa | core promoter element around -30 of transcription start |
| TATA-box | Arabidopsis thaliana | 1222 | - | 5 | TATAA | core promoter element around -30 of transcription start |
| TATA-box | Glycine max | 714 | - | 5 | TAATA | core promoter element around -30 of transcription start |
| TATA-box | Arabidopsis thaliana | 637 | - | 7 | TATAAAA | core promoter element around -30 of transcription start |
| TATA-box | Glycine max | 725 | + | 5 | TAATA | core promoter element around -30 of transcription start |
| TATA-box | Arabidopsis thaliana | 640 | + | 4 | TATA | core promoter element around -30 of transcription start |
| TATA-box | Arabidopsis thaliana | 589 | - | 7 | TATAAAA | core promoter element around -30 of transcription start |
| TATA-box | Arabidopsis thaliana | 590 | - | 6 | TATAAA | core promoter element around -30 of transcription start |
| TATA-box | Arabidopsis thaliana | 592 | + | 4 | TATA | core promoter element around -30 of transcription start |

> 2018/04/13 10:10:12  
+ CAATGAAAAG AAAGCTGCAT AGACATGTTG ACCCCTTCAT CCACTCTTTG TCTAGTTGCT TCTCTTCTAT   
  
  
+ GTATTACCAT TGACTGTTGA GTTTTCGAAG GATTAAAAGA AAAGTTTGTT CACAAAAAAT TTGATGCCCT   
  
  
+ AATTAAAACC CTTGAGGAAA TAAAAATCAT AATCTGGGCA ACCGGCTTCC TAAGTATATA TAGTATATAT   
  
  
+ TTCTAGGATT GGATGGCAGC TCGGCTTAAT TAATTATCTT TTAGTTATCT TAGAGCAATA AATTTAAGTC   
  
  
+ CGTTTTATAT GGTCGATTTC TATATATTGT GAATCATTGA CTGTTGAATT GAAATATTTT GTCATAATAT   
  
  
+ TTGTACATAT AACTTGGGGC AGCCAACGGA GCAATGGGAT CGGGGAGTCT AATATTTTAT CTACTAGAAG   
  
  
+ GTAAAAGCTT TTGAGTAACA AAGATGACTA CTATATAATA GCAAGCTTGA TAGTTACATA ACTAATAATA   
  
  
+ TCGGGTTTGA GATATGGTAA TCTATTGCTA TTTCATAAAA TAAGAATCGT ATTTACACCA CGATCCGTTT   
  
  
+ AATAATCCAC AAGTCTTTGT GTATATAGTT TTATATTAAC TTAATTCGAC TTAGTGAACA GATCAAAATC   
  
  
+ GATTATTTTT ATAGCTCGTG GAACTCGGAT CAGAAGAGAT GTATCCACCT GTGCTATTTC AAGAAGGGAA   
  
  
+ ATTTTGTCAT TAATATTAAG TGGATAATAA TGGACAAACT AACCGGGTAC TTAGACATCA ATCCATTAAC   
  
  
+ ATTGAATTAG TCCTTTTGTA CATATTATGG CGTTACATGT CCTGTATGAA GAAAAGAAAA CATCACCGTT   
  
  
+ TTAATTTATT ATTCAAATAT ACTTGTAATA CACAGAGATA TCAGAGATGA TAATCATAAT CATGATTAAC   
  
  
+ TAATCAGATC TGATGAAGCG CTTGGTACCT AACTAGTTGT GAGCTATACA ATAATATCAA CTAGGGTTTT   
  
  
+ CATACCGAGC AAGTGAAGGT CAATGGCGAG GTCAGTGGTG TGAGTGATCG TCACTCCGTG TTATACAAGC   
  
  
+ GTATTGACCA GGAGCTTAAT TATTGACACA GAAACAACAA AGCCTGCACC ATTGACACAG AGAAAAACCG   
  
  
+ ACCGGCCACC GGGGAGGCTA AAAGGTCACC AGTACTGGAT CTTTGACCCT TAGCCATGTG ACGTCGTCTC   
  
  
+ TATATGAAGC TTCAACGATG GTTACCATAG ATTATACAAT AGGGTCGTCG CCGCAAATTT AGCCTTTCTC   
  
  
+ CTTCTTTCTC TCTCTCTGCA AACTGATCAT ATCTCATCTG TTTCTTAACT GTGAAAGGTT GAAGTTGAAT   
  
  
+ CTTTCCATAT TGTTTACTCT TTTAAGGTAA TAAAATACTA AAAGGAAAAT GTGGTTGGGG TGTTGACATT   
  
  
+ GTCGGCCTGT GTATGAAGGT ACTATTATAA GAAGAGAAGT GCGAGAGAGA CATAACGGAT ACAGAAGCTA   
  
  
+ AAAGAGCAAA GAAGAATACC TTCTACAGC  

- GTTACTTTTC TTTCGACGTA TCTGTACAAC TGGGGAAGTA GGTGAGAAAC AGATCAACGA AGAGAAGATA   
  
  
- CATAATGGTA ACTGACAACT CAAAAGCTTC CTAATTTTCT TTTCAAACAA GTGTTTTTTA AACTACGGGA   
  
  
- TTAATTTTGG GAACTCCTTT ATTTTTAGTA TTAGACCCGT TGGCCGAAGG ATTCATATAT ATCATATATA   
  
  
- AAGATCCTAA CCTACCGTCG AGCCGAATTA ATTAATAGAA AATCAATAGA ATCTCGTTAT TTAAATTCAG   
  
  
- GCAAAATATA CCAGCTAAAG ATATATAACA CTTAGTAACT GACAACTTAA CTTTATAAAA CAGTATTATA   
  
  
- AACATGTATA TTGAACCCCG TCGGTTGCCT CGTTACCCTA GCCCCTCAGA TTATAAAATA GATGATCTTC   
  
  
- CATTTTCGAA AACTCATTGT TTCTACTGAT GATATATTAT CGTTCGAACT ATCAATGTAT TGATTATTAT   
  
  
- AGCCCAAACT CTATACCATT AGATAACGAT AAAGTATTTT ATTCTTAGCA TAAATGTGGT GCTAGGCAAA   
  
  
- TTATTAGGTG TTCAGAAACA CATATATCAA AATATAATTG AATTAAGCTG AATCACTTGT CTAGTTTTAG   
  
  
- CTAATAAAAA TATCGAGCAC CTTGAGCCTA GTCTTCTCTA CATAGGTGGA CACGATAAAG TTCTTCCCTT   
  
  
- TAAAACAGTA ATTATAATTC ACCTATTATT ACCTGTTTGA TTGGCCCATG AATCTGTAGT TAGGTAATTG   
  
  
- TAACTTAATC AGGAAAACAT GTATAATACC GCAATGTACA GGACATACTT CTTTTCTTTT GTAGTGGCAA   
  
  
- AATTAAATAA TAAGTTTATA TGAACATTAT GTGTCTCTAT AGTCTCTACT ATTAGTATTA GTACTAATTG   
  
  
- ATTAGTCTAG ACTACTTCGC GAACCATGGA TTGATCAACA CTCGATATGT TATTATAGTT GATCCCAAAA   
  
  
- GTATGGCTCG TTCACTTCCA GTTACCGCTC CAGTCACCAC ACTCACTAGC AGTGAGGCAC AATATGTTCG   
  
  
- CATAACTGGT CCTCGAATTA ATAACTGTGT CTTTGTTGTT TCGGACGTGG TAACTGTGTC TCTTTTTGGC   
  
  
- TGGCCGGTGG CCCCTCCGAT TTTCCAGTGG TCATGACCTA GAAACTGGGA ATCGGTACAC TGCAGCAGAG   
  
  
- ATATACTTCG AAGTTGCTAC CAATGGTATC TAATATGTTA TCCCAGCAGC GGCGTTTAAA TCGGAAAGAG   
  
  
- GAAGAAAGAG AGAGAGACGT TTGACTAGTA TAGAGTAGAC AAAGAATTGA CACTTTCCAA CTTCAACTTA   
  
  
- GAAAGGTATA ACAAATGAGA AAATTCCATT ATTTTATGAT TTTCCTTTTA CACCAACCCC ACAACTGTAA   
  
  
- CAGCCGGACA CATACTTCCA TGATAATATT CTTCTCTTCA CGCTCTCTCT GTATTGCCTA TGTCTTCGAT   
  
  
- TTTCTCGTTT CTTCTTATGG AAGATGTCG

+     TC-rich repeats

| Site Name | Organism | Position | Strand | Matrix score. | sequence | function |
| --- | --- | --- | --- | --- | --- | --- |
| TC-rich repeats | Nicotiana tabacum | 817 | - | 9 | ATTTTCTTCA | cis-acting element involved in defense and stress responsiveness |
| TC-rich repeats | Nicotiana tabacum | 976 | + | 9 | GTTTTCTTAC | cis-acting element involved in defense and stress responsiveness |

> 2018/04/13 10:10:12  
+ CAATGAAAAG AAAGCTGCAT AGACATGTTG ACCCCTTCAT CCACTCTTTG TCTAGTTGCT TCTCTTCTAT   
  
  
+ GTATTACCAT TGACTGTTGA GTTTTCGAAG GATTAAAAGA AAAGTTTGTT CACAAAAAAT TTGATGCCCT   
  
  
+ AATTAAAACC CTTGAGGAAA TAAAAATCAT AATCTGGGCA ACCGGCTTCC TAAGTATATA TAGTATATAT   
  
  
+ TTCTAGGATT GGATGGCAGC TCGGCTTAAT TAATTATCTT TTAGTTATCT TAGAGCAATA AATTTAAGTC   
  
  
+ CGTTTTATAT GGTCGATTTC TATATATTGT GAATCATTGA CTGTTGAATT GAAATATTTT GTCATAATAT   
  
  
+ TTGTACATAT AACTTGGGGC AGCCAACGGA GCAATGGGAT CGGGGAGTCT AATATTTTAT CTACTAGAAG   
  
  
+ GTAAAAGCTT TTGAGTAACA AAGATGACTA CTATATAATA GCAAGCTTGA TAGTTACATA ACTAATAATA   
  
  
+ TCGGGTTTGA GATATGGTAA TCTATTGCTA TTTCATAAAA TAAGAATCGT ATTTACACCA CGATCCGTTT   
  
  
+ AATAATCCAC AAGTCTTTGT GTATATAGTT TTATATTAAC TTAATTCGAC TTAGTGAACA GATCAAAATC   
  
  
+ GATTATTTTT ATAGCTCGTG GAACTCGGAT CAGAAGAGAT GTATCCACCT GTGCTATTTC AAGAAGGGAA   
  
  
+ ATTTTGTCAT TAATATTAAG TGGATAATAA TGGACAAACT AACCGGGTAC TTAGACATCA ATCCATTAAC   
  
  
+ ATTGAATTAG TCCTTTTGTA CATATTATGG CGTTACATGT CCTGTATGAA GAAAAGAAAA CATCACCGTT   
  
  
+ TTAATTTATT ATTCAAATAT ACTTGTAATA CACAGAGATA TCAGAGATGA TAATCATAAT CATGATTAAC   
  
  
+ TAATCAGATC TGATGAAGCG CTTGGTACCT AACTAGTTGT GAGCTATACA ATAATATCAA CTAGGGTTTT   
  
  
+ CATACCGAGC AAGTGAAGGT CAATGGCGAG GTCAGTGGTG TGAGTGATCG TCACTCCGTG TTATACAAGC   
  
  
+ GTATTGACCA GGAGCTTAAT TATTGACACA GAAACAACAA AGCCTGCACC ATTGACACAG AGAAAAACCG   
  
  
+ ACCGGCCACC GGGGAGGCTA AAAGGTCACC AGTACTGGAT CTTTGACCCT TAGCCATGTG ACGTCGTCTC   
  
  
+ TATATGAAGC TTCAACGATG GTTACCATAG ATTATACAAT AGGGTCGTCG CCGCAAATTT AGCCTTTCTC   
  
  
+ CTTCTTTCTC TCTCTCTGCA AACTGATCAT ATCTCATCTG TTTCTTAACT GTGAAAGGTT GAAGTTGAAT   
  
  
+ CTTTCCATAT TGTTTACTCT TTTAAGGTAA TAAAATACTA AAAGGAAAAT GTGGTTGGGG TGTTGACATT   
  
  
+ GTCGGCCTGT GTATGAAGGT ACTATTATAA GAAGAGAAGT GCGAGAGAGA CATAACGGAT ACAGAAGCTA   
  
  
+ AAAGAGCAAA GAAGAATACC TTCTACAGC  

- GTTACTTTTC TTTCGACGTA TCTGTACAAC TGGGGAAGTA GGTGAGAAAC AGATCAACGA AGAGAAGATA   
  
  
- CATAATGGTA ACTGACAACT CAAAAGCTTC CTAATTTTCT TTTCAAACAA GTGTTTTTTA AACTACGGGA   
  
  
- TTAATTTTGG GAACTCCTTT ATTTTTAGTA TTAGACCCGT TGGCCGAAGG ATTCATATAT ATCATATATA   
  
  
- AAGATCCTAA CCTACCGTCG AGCCGAATTA ATTAATAGAA AATCAATAGA ATCTCGTTAT TTAAATTCAG   
  
  
- GCAAAATATA CCAGCTAAAG ATATATAACA CTTAGTAACT GACAACTTAA CTTTATAAAA CAGTATTATA   
  
  
- AACATGTATA TTGAACCCCG TCGGTTGCCT CGTTACCCTA GCCCCTCAGA TTATAAAATA GATGATCTTC   
  
  
- CATTTTCGAA AACTCATTGT TTCTACTGAT GATATATTAT CGTTCGAACT ATCAATGTAT TGATTATTAT   
  
  
- AGCCCAAACT CTATACCATT AGATAACGAT AAAGTATTTT ATTCTTAGCA TAAATGTGGT GCTAGGCAAA   
  
  
- TTATTAGGTG TTCAGAAACA CATATATCAA AATATAATTG AATTAAGCTG AATCACTTGT CTAGTTTTAG   
  
  
- CTAATAAAAA TATCGAGCAC CTTGAGCCTA GTCTTCTCTA CATAGGTGGA CACGATAAAG TTCTTCCCTT   
  
  
- TAAAACAGTA ATTATAATTC ACCTATTATT ACCTGTTTGA TTGGCCCATG AATCTGTAGT TAGGTAATTG   
  
  
- TAACTTAATC AGGAAAACAT GTATAATACC GCAATGTACA GGACATACTT CTTTTCTTTT GTAGTGGCAA   
  
  
- AATTAAATAA TAAGTTTATA TGAACATTAT GTGTCTCTAT AGTCTCTACT ATTAGTATTA GTACTAATTG   
  
  
- ATTAGTCTAG ACTACTTCGC GAACCATGGA TTGATCAACA CTCGATATGT TATTATAGTT GATCCCAAAA   
  
  
- GTATGGCTCG TTCACTTCCA GTTACCGCTC CAGTCACCAC ACTCACTAGC AGTGAGGCAC AATATGTTCG   
  
  
- CATAACTGGT CCTCGAATTA ATAACTGTGT CTTTGTTGTT TCGGACGTGG TAACTGTGTC TCTTTTTGGC   
  
  
- TGGCCGGTGG CCCCTCCGAT TTTCCAGTGG TCATGACCTA GAAACTGGGA ATCGGTACAC TGCAGCAGAG   
  
  
- ATATACTTCG AAGTTGCTAC CAATGGTATC TAATATGTTA TCCCAGCAGC GGCGTTTAAA TCGGAAAGAG   
  
  
- GAAGAAAGAG AGAGAGACGT TTGACTAGTA TAGAGTAGAC AAAGAATTGA CACTTTCCAA CTTCAACTTA   
  
  
- GAAAGGTATA ACAAATGAGA AAATTCCATT ATTTTATGAT TTTCCTTTTA CACCAACCCC ACAACTGTAA   
  
  
- CAGCCGGACA CATACTTCCA TGATAATATT CTTCTCTTCA CGCTCTCTCT GTATTGCCTA TGTCTTCGAT   
  
  
- TTTCTCGTTT CTTCTTATGG AAGATGTCG

+     TCA-element

| Site Name | Organism | Position | Strand | Matrix score. | sequence | function |
| --- | --- | --- | --- | --- | --- | --- |
| TCA-element | Brassica oleracea | 660 | + | 9 | TCAGAAGAGG | cis-acting element involved in salicylic acid responsiveness |
| TCA-element | Brassica oleracea | 1479 | + | 9 | GAGAAGAATA | cis-acting element involved in salicylic acid responsiveness |

> 2018/04/13 10:10:12  
+ CAATGAAAAG AAAGCTGCAT AGACATGTTG ACCCCTTCAT CCACTCTTTG TCTAGTTGCT TCTCTTCTAT   
  
  
+ GTATTACCAT TGACTGTTGA GTTTTCGAAG GATTAAAAGA AAAGTTTGTT CACAAAAAAT TTGATGCCCT   
  
  
+ AATTAAAACC CTTGAGGAAA TAAAAATCAT AATCTGGGCA ACCGGCTTCC TAAGTATATA TAGTATATAT   
  
  
+ TTCTAGGATT GGATGGCAGC TCGGCTTAAT TAATTATCTT TTAGTTATCT TAGAGCAATA AATTTAAGTC   
  
  
+ CGTTTTATAT GGTCGATTTC TATATATTGT GAATCATTGA CTGTTGAATT GAAATATTTT GTCATAATAT   
  
  
+ TTGTACATAT AACTTGGGGC AGCCAACGGA GCAATGGGAT CGGGGAGTCT AATATTTTAT CTACTAGAAG   
  
  
+ GTAAAAGCTT TTGAGTAACA AAGATGACTA CTATATAATA GCAAGCTTGA TAGTTACATA ACTAATAATA   
  
  
+ TCGGGTTTGA GATATGGTAA TCTATTGCTA TTTCATAAAA TAAGAATCGT ATTTACACCA CGATCCGTTT   
  
  
+ AATAATCCAC AAGTCTTTGT GTATATAGTT TTATATTAAC TTAATTCGAC TTAGTGAACA GATCAAAATC   
  
  
+ GATTATTTTT ATAGCTCGTG GAACTCGGAT CAGAAGAGAT GTATCCACCT GTGCTATTTC AAGAAGGGAA   
  
  
+ ATTTTGTCAT TAATATTAAG TGGATAATAA TGGACAAACT AACCGGGTAC TTAGACATCA ATCCATTAAC   
  
  
+ ATTGAATTAG TCCTTTTGTA CATATTATGG CGTTACATGT CCTGTATGAA GAAAAGAAAA CATCACCGTT   
  
  
+ TTAATTTATT ATTCAAATAT ACTTGTAATA CACAGAGATA TCAGAGATGA TAATCATAAT CATGATTAAC   
  
  
+ TAATCAGATC TGATGAAGCG CTTGGTACCT AACTAGTTGT GAGCTATACA ATAATATCAA CTAGGGTTTT   
  
  
+ CATACCGAGC AAGTGAAGGT CAATGGCGAG GTCAGTGGTG TGAGTGATCG TCACTCCGTG TTATACAAGC   
  
  
+ GTATTGACCA GGAGCTTAAT TATTGACACA GAAACAACAA AGCCTGCACC ATTGACACAG AGAAAAACCG   
  
  
+ ACCGGCCACC GGGGAGGCTA AAAGGTCACC AGTACTGGAT CTTTGACCCT TAGCCATGTG ACGTCGTCTC   
  
  
+ TATATGAAGC TTCAACGATG GTTACCATAG ATTATACAAT AGGGTCGTCG CCGCAAATTT AGCCTTTCTC   
  
  
+ CTTCTTTCTC TCTCTCTGCA AACTGATCAT ATCTCATCTG TTTCTTAACT GTGAAAGGTT GAAGTTGAAT   
  
  
+ CTTTCCATAT TGTTTACTCT TTTAAGGTAA TAAAATACTA AAAGGAAAAT GTGGTTGGGG TGTTGACATT   
  
  
+ GTCGGCCTGT GTATGAAGGT ACTATTATAA GAAGAGAAGT GCGAGAGAGA CATAACGGAT ACAGAAGCTA   
  
  
+ AAAGAGCAAA GAAGAATACC TTCTACAGC  

- GTTACTTTTC TTTCGACGTA TCTGTACAAC TGGGGAAGTA GGTGAGAAAC AGATCAACGA AGAGAAGATA   
  
  
- CATAATGGTA ACTGACAACT CAAAAGCTTC CTAATTTTCT TTTCAAACAA GTGTTTTTTA AACTACGGGA   
  
  
- TTAATTTTGG GAACTCCTTT ATTTTTAGTA TTAGACCCGT TGGCCGAAGG ATTCATATAT ATCATATATA   
  
  
- AAGATCCTAA CCTACCGTCG AGCCGAATTA ATTAATAGAA AATCAATAGA ATCTCGTTAT TTAAATTCAG   
  
  
- GCAAAATATA CCAGCTAAAG ATATATAACA CTTAGTAACT GACAACTTAA CTTTATAAAA CAGTATTATA   
  
  
- AACATGTATA TTGAACCCCG TCGGTTGCCT CGTTACCCTA GCCCCTCAGA TTATAAAATA GATGATCTTC   
  
  
- CATTTTCGAA AACTCATTGT TTCTACTGAT GATATATTAT CGTTCGAACT ATCAATGTAT TGATTATTAT   
  
  
- AGCCCAAACT CTATACCATT AGATAACGAT AAAGTATTTT ATTCTTAGCA TAAATGTGGT GCTAGGCAAA   
  
  
- TTATTAGGTG TTCAGAAACA CATATATCAA AATATAATTG AATTAAGCTG AATCACTTGT CTAGTTTTAG   
  
  
- CTAATAAAAA TATCGAGCAC CTTGAGCCTA GTCTTCTCTA CATAGGTGGA CACGATAAAG TTCTTCCCTT   
  
  
- TAAAACAGTA ATTATAATTC ACCTATTATT ACCTGTTTGA TTGGCCCATG AATCTGTAGT TAGGTAATTG   
  
  
- TAACTTAATC AGGAAAACAT GTATAATACC GCAATGTACA GGACATACTT CTTTTCTTTT GTAGTGGCAA   
  
  
- AATTAAATAA TAAGTTTATA TGAACATTAT GTGTCTCTAT AGTCTCTACT ATTAGTATTA GTACTAATTG   
  
  
- ATTAGTCTAG ACTACTTCGC GAACCATGGA TTGATCAACA CTCGATATGT TATTATAGTT GATCCCAAAA   
  
  
- GTATGGCTCG TTCACTTCCA GTTACCGCTC CAGTCACCAC ACTCACTAGC AGTGAGGCAC AATATGTTCG   
  
  
- CATAACTGGT CCTCGAATTA ATAACTGTGT CTTTGTTGTT TCGGACGTGG TAACTGTGTC TCTTTTTGGC   
  
  
- TGGCCGGTGG CCCCTCCGAT TTTCCAGTGG TCATGACCTA GAAACTGGGA ATCGGTACAC TGCAGCAGAG   
  
  
- ATATACTTCG AAGTTGCTAC CAATGGTATC TAATATGTTA TCCCAGCAGC GGCGTTTAAA TCGGAAAGAG   
  
  
- GAAGAAAGAG AGAGAGACGT TTGACTAGTA TAGAGTAGAC AAAGAATTGA CACTTTCCAA CTTCAACTTA   
  
  
- GAAAGGTATA ACAAATGAGA AAATTCCATT ATTTTATGAT TTTCCTTTTA CACCAACCCC ACAACTGTAA   
  
  
- CAGCCGGACA CATACTTCCA TGATAATATT CTTCTCTTCA CGCTCTCTCT GTATTGCCTA TGTCTTCGAT   
  
  
- TTTCTCGTTT CTTCTTATGG AAGATGTCG

+     TCCACCT-motif

| Site Name | Organism | Position | Strand | Matrix score. | sequence | function |
| --- | --- | --- | --- | --- | --- | --- |
| TCCACCT-motif | Petroselinum hortense | 674 | + | 7 | TCCACCT |  |

> 2018/04/13 10:10:12  
+ CAATGAAAAG AAAGCTGCAT AGACATGTTG ACCCCTTCAT CCACTCTTTG TCTAGTTGCT TCTCTTCTAT   
  
  
+ GTATTACCAT TGACTGTTGA GTTTTCGAAG GATTAAAAGA AAAGTTTGTT CACAAAAAAT TTGATGCCCT   
  
  
+ AATTAAAACC CTTGAGGAAA TAAAAATCAT AATCTGGGCA ACCGGCTTCC TAAGTATATA TAGTATATAT   
  
  
+ TTCTAGGATT GGATGGCAGC TCGGCTTAAT TAATTATCTT TTAGTTATCT TAGAGCAATA AATTTAAGTC   
  
  
+ CGTTTTATAT GGTCGATTTC TATATATTGT GAATCATTGA CTGTTGAATT GAAATATTTT GTCATAATAT   
  
  
+ TTGTACATAT AACTTGGGGC AGCCAACGGA GCAATGGGAT CGGGGAGTCT AATATTTTAT CTACTAGAAG   
  
  
+ GTAAAAGCTT TTGAGTAACA AAGATGACTA CTATATAATA GCAAGCTTGA TAGTTACATA ACTAATAATA   
  
  
+ TCGGGTTTGA GATATGGTAA TCTATTGCTA TTTCATAAAA TAAGAATCGT ATTTACACCA CGATCCGTTT   
  
  
+ AATAATCCAC AAGTCTTTGT GTATATAGTT TTATATTAAC TTAATTCGAC TTAGTGAACA GATCAAAATC   
  
  
+ GATTATTTTT ATAGCTCGTG GAACTCGGAT CAGAAGAGAT GTATCCACCT GTGCTATTTC AAGAAGGGAA   
  
  
+ ATTTTGTCAT TAATATTAAG TGGATAATAA TGGACAAACT AACCGGGTAC TTAGACATCA ATCCATTAAC   
  
  
+ ATTGAATTAG TCCTTTTGTA CATATTATGG CGTTACATGT CCTGTATGAA GAAAAGAAAA CATCACCGTT   
  
  
+ TTAATTTATT ATTCAAATAT ACTTGTAATA CACAGAGATA TCAGAGATGA TAATCATAAT CATGATTAAC   
  
  
+ TAATCAGATC TGATGAAGCG CTTGGTACCT AACTAGTTGT GAGCTATACA ATAATATCAA CTAGGGTTTT   
  
  
+ CATACCGAGC AAGTGAAGGT CAATGGCGAG GTCAGTGGTG TGAGTGATCG TCACTCCGTG TTATACAAGC   
  
  
+ GTATTGACCA GGAGCTTAAT TATTGACACA GAAACAACAA AGCCTGCACC ATTGACACAG AGAAAAACCG   
  
  
+ ACCGGCCACC GGGGAGGCTA AAAGGTCACC AGTACTGGAT CTTTGACCCT TAGCCATGTG ACGTCGTCTC   
  
  
+ TATATGAAGC TTCAACGATG GTTACCATAG ATTATACAAT AGGGTCGTCG CCGCAAATTT AGCCTTTCTC   
  
  
+ CTTCTTTCTC TCTCTCTGCA AACTGATCAT ATCTCATCTG TTTCTTAACT GTGAAAGGTT GAAGTTGAAT   
  
  
+ CTTTCCATAT TGTTTACTCT TTTAAGGTAA TAAAATACTA AAAGGAAAAT GTGGTTGGGG TGTTGACATT   
  
  
+ GTCGGCCTGT GTATGAAGGT ACTATTATAA GAAGAGAAGT GCGAGAGAGA CATAACGGAT ACAGAAGCTA   
  
  
+ AAAGAGCAAA GAAGAATACC TTCTACAGC  

- GTTACTTTTC TTTCGACGTA TCTGTACAAC TGGGGAAGTA GGTGAGAAAC AGATCAACGA AGAGAAGATA   
  
  
- CATAATGGTA ACTGACAACT CAAAAGCTTC CTAATTTTCT TTTCAAACAA GTGTTTTTTA AACTACGGGA   
  
  
- TTAATTTTGG GAACTCCTTT ATTTTTAGTA TTAGACCCGT TGGCCGAAGG ATTCATATAT ATCATATATA   
  
  
- AAGATCCTAA CCTACCGTCG AGCCGAATTA ATTAATAGAA AATCAATAGA ATCTCGTTAT TTAAATTCAG   
  
  
- GCAAAATATA CCAGCTAAAG ATATATAACA CTTAGTAACT GACAACTTAA CTTTATAAAA CAGTATTATA   
  
  
- AACATGTATA TTGAACCCCG TCGGTTGCCT CGTTACCCTA GCCCCTCAGA TTATAAAATA GATGATCTTC   
  
  
- CATTTTCGAA AACTCATTGT TTCTACTGAT GATATATTAT CGTTCGAACT ATCAATGTAT TGATTATTAT   
  
  
- AGCCCAAACT CTATACCATT AGATAACGAT AAAGTATTTT ATTCTTAGCA TAAATGTGGT GCTAGGCAAA   
  
  
- TTATTAGGTG TTCAGAAACA CATATATCAA AATATAATTG AATTAAGCTG AATCACTTGT CTAGTTTTAG   
  
  
- CTAATAAAAA TATCGAGCAC CTTGAGCCTA GTCTTCTCTA CATAGGTGGA CACGATAAAG TTCTTCCCTT   
  
  
- TAAAACAGTA ATTATAATTC ACCTATTATT ACCTGTTTGA TTGGCCCATG AATCTGTAGT TAGGTAATTG   
  
  
- TAACTTAATC AGGAAAACAT GTATAATACC GCAATGTACA GGACATACTT CTTTTCTTTT GTAGTGGCAA   
  
  
- AATTAAATAA TAAGTTTATA TGAACATTAT GTGTCTCTAT AGTCTCTACT ATTAGTATTA GTACTAATTG   
  
  
- ATTAGTCTAG ACTACTTCGC GAACCATGGA TTGATCAACA CTCGATATGT TATTATAGTT GATCCCAAAA   
  
  
- GTATGGCTCG TTCACTTCCA GTTACCGCTC CAGTCACCAC ACTCACTAGC AGTGAGGCAC AATATGTTCG   
  
  
- CATAACTGGT CCTCGAATTA ATAACTGTGT CTTTGTTGTT TCGGACGTGG TAACTGTGTC TCTTTTTGGC   
  
  
- TGGCCGGTGG CCCCTCCGAT TTTCCAGTGG TCATGACCTA GAAACTGGGA ATCGGTACAC TGCAGCAGAG   
  
  
- ATATACTTCG AAGTTGCTAC CAATGGTATC TAATATGTTA TCCCAGCAGC GGCGTTTAAA TCGGAAAGAG   
  
  
- GAAGAAAGAG AGAGAGACGT TTGACTAGTA TAGAGTAGAC AAAGAATTGA CACTTTCCAA CTTCAACTTA   
  
  
- GAAAGGTATA ACAAATGAGA AAATTCCATT ATTTTATGAT TTTCCTTTTA CACCAACCCC ACAACTGTAA   
  
  
- CAGCCGGACA CATACTTCCA TGATAATATT CTTCTCTTCA CGCTCTCTCT GTATTGCCTA TGTCTTCGAT   
  
  
- TTTCTCGTTT CTTCTTATGG AAGATGTCG

+     TGACG-motif

| Site Name | Organism | Position | Strand | Matrix score. | sequence | function |
| --- | --- | --- | --- | --- | --- | --- |
| TGACG-motif | Hordeum vulgare | 1029 | - | 5 | TGACG | cis-acting regulatory element involved in the MeJA-responsiveness |
| TGACG-motif | Hordeum vulgare | 1179 | + | 5 | TGACG | cis-acting regulatory element involved in the MeJA-responsiveness |

> 2018/04/13 10:10:12  
+ CAATGAAAAG AAAGCTGCAT AGACATGTTG ACCCCTTCAT CCACTCTTTG TCTAGTTGCT TCTCTTCTAT   
  
  
+ GTATTACCAT TGACTGTTGA GTTTTCGAAG GATTAAAAGA AAAGTTTGTT CACAAAAAAT TTGATGCCCT   
  
  
+ AATTAAAACC CTTGAGGAAA TAAAAATCAT AATCTGGGCA ACCGGCTTCC TAAGTATATA TAGTATATAT   
  
  
+ TTCTAGGATT GGATGGCAGC TCGGCTTAAT TAATTATCTT TTAGTTATCT TAGAGCAATA AATTTAAGTC   
  
  
+ CGTTTTATAT GGTCGATTTC TATATATTGT GAATCATTGA CTGTTGAATT GAAATATTTT GTCATAATAT   
  
  
+ TTGTACATAT AACTTGGGGC AGCCAACGGA GCAATGGGAT CGGGGAGTCT AATATTTTAT CTACTAGAAG   
  
  
+ GTAAAAGCTT TTGAGTAACA AAGATGACTA CTATATAATA GCAAGCTTGA TAGTTACATA ACTAATAATA   
  
  
+ TCGGGTTTGA GATATGGTAA TCTATTGCTA TTTCATAAAA TAAGAATCGT ATTTACACCA CGATCCGTTT   
  
  
+ AATAATCCAC AAGTCTTTGT GTATATAGTT TTATATTAAC TTAATTCGAC TTAGTGAACA GATCAAAATC   
  
  
+ GATTATTTTT ATAGCTCGTG GAACTCGGAT CAGAAGAGAT GTATCCACCT GTGCTATTTC AAGAAGGGAA   
  
  
+ ATTTTGTCAT TAATATTAAG TGGATAATAA TGGACAAACT AACCGGGTAC TTAGACATCA ATCCATTAAC   
  
  
+ ATTGAATTAG TCCTTTTGTA CATATTATGG CGTTACATGT CCTGTATGAA GAAAAGAAAA CATCACCGTT   
  
  
+ TTAATTTATT ATTCAAATAT ACTTGTAATA CACAGAGATA TCAGAGATGA TAATCATAAT CATGATTAAC   
  
  
+ TAATCAGATC TGATGAAGCG CTTGGTACCT AACTAGTTGT GAGCTATACA ATAATATCAA CTAGGGTTTT   
  
  
+ CATACCGAGC AAGTGAAGGT CAATGGCGAG GTCAGTGGTG TGAGTGATCG TCACTCCGTG TTATACAAGC   
  
  
+ GTATTGACCA GGAGCTTAAT TATTGACACA GAAACAACAA AGCCTGCACC ATTGACACAG AGAAAAACCG   
  
  
+ ACCGGCCACC GGGGAGGCTA AAAGGTCACC AGTACTGGAT CTTTGACCCT TAGCCATGTG ACGTCGTCTC   
  
  
+ TATATGAAGC TTCAACGATG GTTACCATAG ATTATACAAT AGGGTCGTCG CCGCAAATTT AGCCTTTCTC   
  
  
+ CTTCTTTCTC TCTCTCTGCA AACTGATCAT ATCTCATCTG TTTCTTAACT GTGAAAGGTT GAAGTTGAAT   
  
  
+ CTTTCCATAT TGTTTACTCT TTTAAGGTAA TAAAATACTA AAAGGAAAAT GTGGTTGGGG TGTTGACATT   
  
  
+ GTCGGCCTGT GTATGAAGGT ACTATTATAA GAAGAGAAGT GCGAGAGAGA CATAACGGAT ACAGAAGCTA   
  
  
+ AAAGAGCAAA GAAGAATACC TTCTACAGC  

- GTTACTTTTC TTTCGACGTA TCTGTACAAC TGGGGAAGTA GGTGAGAAAC AGATCAACGA AGAGAAGATA   
  
  
- CATAATGGTA ACTGACAACT CAAAAGCTTC CTAATTTTCT TTTCAAACAA GTGTTTTTTA AACTACGGGA   
  
  
- TTAATTTTGG GAACTCCTTT ATTTTTAGTA TTAGACCCGT TGGCCGAAGG ATTCATATAT ATCATATATA   
  
  
- AAGATCCTAA CCTACCGTCG AGCCGAATTA ATTAATAGAA AATCAATAGA ATCTCGTTAT TTAAATTCAG   
  
  
- GCAAAATATA CCAGCTAAAG ATATATAACA CTTAGTAACT GACAACTTAA CTTTATAAAA CAGTATTATA   
  
  
- AACATGTATA TTGAACCCCG TCGGTTGCCT CGTTACCCTA GCCCCTCAGA TTATAAAATA GATGATCTTC   
  
  
- CATTTTCGAA AACTCATTGT TTCTACTGAT GATATATTAT CGTTCGAACT ATCAATGTAT TGATTATTAT   
  
  
- AGCCCAAACT CTATACCATT AGATAACGAT AAAGTATTTT ATTCTTAGCA TAAATGTGGT GCTAGGCAAA   
  
  
- TTATTAGGTG TTCAGAAACA CATATATCAA AATATAATTG AATTAAGCTG AATCACTTGT CTAGTTTTAG   
  
  
- CTAATAAAAA TATCGAGCAC CTTGAGCCTA GTCTTCTCTA CATAGGTGGA CACGATAAAG TTCTTCCCTT   
  
  
- TAAAACAGTA ATTATAATTC ACCTATTATT ACCTGTTTGA TTGGCCCATG AATCTGTAGT TAGGTAATTG   
  
  
- TAACTTAATC AGGAAAACAT GTATAATACC GCAATGTACA GGACATACTT CTTTTCTTTT GTAGTGGCAA   
  
  
- AATTAAATAA TAAGTTTATA TGAACATTAT GTGTCTCTAT AGTCTCTACT ATTAGTATTA GTACTAATTG   
  
  
- ATTAGTCTAG ACTACTTCGC GAACCATGGA TTGATCAACA CTCGATATGT TATTATAGTT GATCCCAAAA   
  
  
- GTATGGCTCG TTCACTTCCA GTTACCGCTC CAGTCACCAC ACTCACTAGC AGTGAGGCAC AATATGTTCG   
  
  
- CATAACTGGT CCTCGAATTA ATAACTGTGT CTTTGTTGTT TCGGACGTGG TAACTGTGTC TCTTTTTGGC   
  
  
- TGGCCGGTGG CCCCTCCGAT TTTCCAGTGG TCATGACCTA GAAACTGGGA ATCGGTACAC TGCAGCAGAG   
  
  
- ATATACTTCG AAGTTGCTAC CAATGGTATC TAATATGTTA TCCCAGCAGC GGCGTTTAAA TCGGAAAGAG   
  
  
- GAAGAAAGAG AGAGAGACGT TTGACTAGTA TAGAGTAGAC AAAGAATTGA CACTTTCCAA CTTCAACTTA   
  
  
- GAAAGGTATA ACAAATGAGA AAATTCCATT ATTTTATGAT TTTCCTTTTA CACCAACCCC ACAACTGTAA   
  
  
- CAGCCGGACA CATACTTCCA TGATAATATT CTTCTCTTCA CGCTCTCTCT GTATTGCCTA TGTCTTCGAT   
  
  
- TTTCTCGTTT CTTCTTATGG AAGATGTCG

+     Unnamed\_\_1

| Site Name | Organism | Position | Strand | Matrix score. | sequence | function |
| --- | --- | --- | --- | --- | --- | --- |
| Unnamed\_\_1 | Zea mays | 647 | + | 5 | CGTGG |  |
| Unnamed\_\_1 | Zea mays | 548 | - | 5 | CGTGG |  |

> 2018/04/13 10:10:12  
+ CAATGAAAAG AAAGCTGCAT AGACATGTTG ACCCCTTCAT CCACTCTTTG TCTAGTTGCT TCTCTTCTAT   
  
  
+ GTATTACCAT TGACTGTTGA GTTTTCGAAG GATTAAAAGA AAAGTTTGTT CACAAAAAAT TTGATGCCCT   
  
  
+ AATTAAAACC CTTGAGGAAA TAAAAATCAT AATCTGGGCA ACCGGCTTCC TAAGTATATA TAGTATATAT   
  
  
+ TTCTAGGATT GGATGGCAGC TCGGCTTAAT TAATTATCTT TTAGTTATCT TAGAGCAATA AATTTAAGTC   
  
  
+ CGTTTTATAT GGTCGATTTC TATATATTGT GAATCATTGA CTGTTGAATT GAAATATTTT GTCATAATAT   
  
  
+ TTGTACATAT AACTTGGGGC AGCCAACGGA GCAATGGGAT CGGGGAGTCT AATATTTTAT CTACTAGAAG   
  
  
+ GTAAAAGCTT TTGAGTAACA AAGATGACTA CTATATAATA GCAAGCTTGA TAGTTACATA ACTAATAATA   
  
  
+ TCGGGTTTGA GATATGGTAA TCTATTGCTA TTTCATAAAA TAAGAATCGT ATTTACACCA CGATCCGTTT   
  
  
+ AATAATCCAC AAGTCTTTGT GTATATAGTT TTATATTAAC TTAATTCGAC TTAGTGAACA GATCAAAATC   
  
  
+ GATTATTTTT ATAGCTCGTG GAACTCGGAT CAGAAGAGAT GTATCCACCT GTGCTATTTC AAGAAGGGAA   
  
  
+ ATTTTGTCAT TAATATTAAG TGGATAATAA TGGACAAACT AACCGGGTAC TTAGACATCA ATCCATTAAC   
  
  
+ ATTGAATTAG TCCTTTTGTA CATATTATGG CGTTACATGT CCTGTATGAA GAAAAGAAAA CATCACCGTT   
  
  
+ TTAATTTATT ATTCAAATAT ACTTGTAATA CACAGAGATA TCAGAGATGA TAATCATAAT CATGATTAAC   
  
  
+ TAATCAGATC TGATGAAGCG CTTGGTACCT AACTAGTTGT GAGCTATACA ATAATATCAA CTAGGGTTTT   
  
  
+ CATACCGAGC AAGTGAAGGT CAATGGCGAG GTCAGTGGTG TGAGTGATCG TCACTCCGTG TTATACAAGC   
  
  
+ GTATTGACCA GGAGCTTAAT TATTGACACA GAAACAACAA AGCCTGCACC ATTGACACAG AGAAAAACCG   
  
  
+ ACCGGCCACC GGGGAGGCTA AAAGGTCACC AGTACTGGAT CTTTGACCCT TAGCCATGTG ACGTCGTCTC   
  
  
+ TATATGAAGC TTCAACGATG GTTACCATAG ATTATACAAT AGGGTCGTCG CCGCAAATTT AGCCTTTCTC   
  
  
+ CTTCTTTCTC TCTCTCTGCA AACTGATCAT ATCTCATCTG TTTCTTAACT GTGAAAGGTT GAAGTTGAAT   
  
  
+ CTTTCCATAT TGTTTACTCT TTTAAGGTAA TAAAATACTA AAAGGAAAAT GTGGTTGGGG TGTTGACATT   
  
  
+ GTCGGCCTGT GTATGAAGGT ACTATTATAA GAAGAGAAGT GCGAGAGAGA CATAACGGAT ACAGAAGCTA   
  
  
+ AAAGAGCAAA GAAGAATACC TTCTACAGC  

- GTTACTTTTC TTTCGACGTA TCTGTACAAC TGGGGAAGTA GGTGAGAAAC AGATCAACGA AGAGAAGATA   
  
  
- CATAATGGTA ACTGACAACT CAAAAGCTTC CTAATTTTCT TTTCAAACAA GTGTTTTTTA AACTACGGGA   
  
  
- TTAATTTTGG GAACTCCTTT ATTTTTAGTA TTAGACCCGT TGGCCGAAGG ATTCATATAT ATCATATATA   
  
  
- AAGATCCTAA CCTACCGTCG AGCCGAATTA ATTAATAGAA AATCAATAGA ATCTCGTTAT TTAAATTCAG   
  
  
- GCAAAATATA CCAGCTAAAG ATATATAACA CTTAGTAACT GACAACTTAA CTTTATAAAA CAGTATTATA   
  
  
- AACATGTATA TTGAACCCCG TCGGTTGCCT CGTTACCCTA GCCCCTCAGA TTATAAAATA GATGATCTTC   
  
  
- CATTTTCGAA AACTCATTGT TTCTACTGAT GATATATTAT CGTTCGAACT ATCAATGTAT TGATTATTAT   
  
  
- AGCCCAAACT CTATACCATT AGATAACGAT AAAGTATTTT ATTCTTAGCA TAAATGTGGT GCTAGGCAAA   
  
  
- TTATTAGGTG TTCAGAAACA CATATATCAA AATATAATTG AATTAAGCTG AATCACTTGT CTAGTTTTAG   
  
  
- CTAATAAAAA TATCGAGCAC CTTGAGCCTA GTCTTCTCTA CATAGGTGGA CACGATAAAG TTCTTCCCTT   
  
  
- TAAAACAGTA ATTATAATTC ACCTATTATT ACCTGTTTGA TTGGCCCATG AATCTGTAGT TAGGTAATTG   
  
  
- TAACTTAATC AGGAAAACAT GTATAATACC GCAATGTACA GGACATACTT CTTTTCTTTT GTAGTGGCAA   
  
  
- AATTAAATAA TAAGTTTATA TGAACATTAT GTGTCTCTAT AGTCTCTACT ATTAGTATTA GTACTAATTG   
  
  
- ATTAGTCTAG ACTACTTCGC GAACCATGGA TTGATCAACA CTCGATATGT TATTATAGTT GATCCCAAAA   
  
  
- GTATGGCTCG TTCACTTCCA GTTACCGCTC CAGTCACCAC ACTCACTAGC AGTGAGGCAC AATATGTTCG   
  
  
- CATAACTGGT CCTCGAATTA ATAACTGTGT CTTTGTTGTT TCGGACGTGG TAACTGTGTC TCTTTTTGGC   
  
  
- TGGCCGGTGG CCCCTCCGAT TTTCCAGTGG TCATGACCTA GAAACTGGGA ATCGGTACAC TGCAGCAGAG   
  
  
- ATATACTTCG AAGTTGCTAC CAATGGTATC TAATATGTTA TCCCAGCAGC GGCGTTTAAA TCGGAAAGAG   
  
  
- GAAGAAAGAG AGAGAGACGT TTGACTAGTA TAGAGTAGAC AAAGAATTGA CACTTTCCAA CTTCAACTTA   
  
  
- GAAAGGTATA ACAAATGAGA AAATTCCATT ATTTTATGAT TTTCCTTTTA CACCAACCCC ACAACTGTAA   
  
  
- CAGCCGGACA CATACTTCCA TGATAATATT CTTCTCTTCA CGCTCTCTCT GTATTGCCTA TGTCTTCGAT   
  
  
- TTTCTCGTTT CTTCTTATGG AAGATGTCG

+     Unnamed\_\_13

| Site Name | Organism | Position | Strand | Matrix score. | sequence | function |
| --- | --- | --- | --- | --- | --- | --- |
| Unnamed\_\_13 | Zea mays | 858 | - | 9 | TCCAAGTATA |  |

> 2018/04/13 10:10:12  
+ CAATGAAAAG AAAGCTGCAT AGACATGTTG ACCCCTTCAT CCACTCTTTG TCTAGTTGCT TCTCTTCTAT   
  
  
+ GTATTACCAT TGACTGTTGA GTTTTCGAAG GATTAAAAGA AAAGTTTGTT CACAAAAAAT TTGATGCCCT   
  
  
+ AATTAAAACC CTTGAGGAAA TAAAAATCAT AATCTGGGCA ACCGGCTTCC TAAGTATATA TAGTATATAT   
  
  
+ TTCTAGGATT GGATGGCAGC TCGGCTTAAT TAATTATCTT TTAGTTATCT TAGAGCAATA AATTTAAGTC   
  
  
+ CGTTTTATAT GGTCGATTTC TATATATTGT GAATCATTGA CTGTTGAATT GAAATATTTT GTCATAATAT   
  
  
+ TTGTACATAT AACTTGGGGC AGCCAACGGA GCAATGGGAT CGGGGAGTCT AATATTTTAT CTACTAGAAG   
  
  
+ GTAAAAGCTT TTGAGTAACA AAGATGACTA CTATATAATA GCAAGCTTGA TAGTTACATA ACTAATAATA   
  
  
+ TCGGGTTTGA GATATGGTAA TCTATTGCTA TTTCATAAAA TAAGAATCGT ATTTACACCA CGATCCGTTT   
  
  
+ AATAATCCAC AAGTCTTTGT GTATATAGTT TTATATTAAC TTAATTCGAC TTAGTGAACA GATCAAAATC   
  
  
+ GATTATTTTT ATAGCTCGTG GAACTCGGAT CAGAAGAGAT GTATCCACCT GTGCTATTTC AAGAAGGGAA   
  
  
+ ATTTTGTCAT TAATATTAAG TGGATAATAA TGGACAAACT AACCGGGTAC TTAGACATCA ATCCATTAAC   
  
  
+ ATTGAATTAG TCCTTTTGTA CATATTATGG CGTTACATGT CCTGTATGAA GAAAAGAAAA CATCACCGTT   
  
  
+ TTAATTTATT ATTCAAATAT ACTTGTAATA CACAGAGATA TCAGAGATGA TAATCATAAT CATGATTAAC   
  
  
+ TAATCAGATC TGATGAAGCG CTTGGTACCT AACTAGTTGT GAGCTATACA ATAATATCAA CTAGGGTTTT   
  
  
+ CATACCGAGC AAGTGAAGGT CAATGGCGAG GTCAGTGGTG TGAGTGATCG TCACTCCGTG TTATACAAGC   
  
  
+ GTATTGACCA GGAGCTTAAT TATTGACACA GAAACAACAA AGCCTGCACC ATTGACACAG AGAAAAACCG   
  
  
+ ACCGGCCACC GGGGAGGCTA AAAGGTCACC AGTACTGGAT CTTTGACCCT TAGCCATGTG ACGTCGTCTC   
  
  
+ TATATGAAGC TTCAACGATG GTTACCATAG ATTATACAAT AGGGTCGTCG CCGCAAATTT AGCCTTTCTC   
  
  
+ CTTCTTTCTC TCTCTCTGCA AACTGATCAT ATCTCATCTG TTTCTTAACT GTGAAAGGTT GAAGTTGAAT   
  
  
+ CTTTCCATAT TGTTTACTCT TTTAAGGTAA TAAAATACTA AAAGGAAAAT GTGGTTGGGG TGTTGACATT   
  
  
+ GTCGGCCTGT GTATGAAGGT ACTATTATAA GAAGAGAAGT GCGAGAGAGA CATAACGGAT ACAGAAGCTA   
  
  
+ AAAGAGCAAA GAAGAATACC TTCTACAGC  

- GTTACTTTTC TTTCGACGTA TCTGTACAAC TGGGGAAGTA GGTGAGAAAC AGATCAACGA AGAGAAGATA   
  
  
- CATAATGGTA ACTGACAACT CAAAAGCTTC CTAATTTTCT TTTCAAACAA GTGTTTTTTA AACTACGGGA   
  
  
- TTAATTTTGG GAACTCCTTT ATTTTTAGTA TTAGACCCGT TGGCCGAAGG ATTCATATAT ATCATATATA   
  
  
- AAGATCCTAA CCTACCGTCG AGCCGAATTA ATTAATAGAA AATCAATAGA ATCTCGTTAT TTAAATTCAG   
  
  
- GCAAAATATA CCAGCTAAAG ATATATAACA CTTAGTAACT GACAACTTAA CTTTATAAAA CAGTATTATA   
  
  
- AACATGTATA TTGAACCCCG TCGGTTGCCT CGTTACCCTA GCCCCTCAGA TTATAAAATA GATGATCTTC   
  
  
- CATTTTCGAA AACTCATTGT TTCTACTGAT GATATATTAT CGTTCGAACT ATCAATGTAT TGATTATTAT   
  
  
- AGCCCAAACT CTATACCATT AGATAACGAT AAAGTATTTT ATTCTTAGCA TAAATGTGGT GCTAGGCAAA   
  
  
- TTATTAGGTG TTCAGAAACA CATATATCAA AATATAATTG AATTAAGCTG AATCACTTGT CTAGTTTTAG   
  
  
- CTAATAAAAA TATCGAGCAC CTTGAGCCTA GTCTTCTCTA CATAGGTGGA CACGATAAAG TTCTTCCCTT   
  
  
- TAAAACAGTA ATTATAATTC ACCTATTATT ACCTGTTTGA TTGGCCCATG AATCTGTAGT TAGGTAATTG   
  
  
- TAACTTAATC AGGAAAACAT GTATAATACC GCAATGTACA GGACATACTT CTTTTCTTTT GTAGTGGCAA   
  
  
- AATTAAATAA TAAGTTTATA TGAACATTAT GTGTCTCTAT AGTCTCTACT ATTAGTATTA GTACTAATTG   
  
  
- ATTAGTCTAG ACTACTTCGC GAACCATGGA TTGATCAACA CTCGATATGT TATTATAGTT GATCCCAAAA   
  
  
- GTATGGCTCG TTCACTTCCA GTTACCGCTC CAGTCACCAC ACTCACTAGC AGTGAGGCAC AATATGTTCG   
  
  
- CATAACTGGT CCTCGAATTA ATAACTGTGT CTTTGTTGTT TCGGACGTGG TAACTGTGTC TCTTTTTGGC   
  
  
- TGGCCGGTGG CCCCTCCGAT TTTCCAGTGG TCATGACCTA GAAACTGGGA ATCGGTACAC TGCAGCAGAG   
  
  
- ATATACTTCG AAGTTGCTAC CAATGGTATC TAATATGTTA TCCCAGCAGC GGCGTTTAAA TCGGAAAGAG   
  
  
- GAAGAAAGAG AGAGAGACGT TTGACTAGTA TAGAGTAGAC AAAGAATTGA CACTTTCCAA CTTCAACTTA   
  
  
- GAAAGGTATA ACAAATGAGA AAATTCCATT ATTTTATGAT TTTCCTTTTA CACCAACCCC ACAACTGTAA   
  
  
- CAGCCGGACA CATACTTCCA TGATAATATT CTTCTCTTCA CGCTCTCTCT GTATTGCCTA TGTCTTCGAT   
  
  
- TTTCTCGTTT CTTCTTATGG AAGATGTCG

+     Unnamed\_\_2

| Site Name | Organism | Position | Strand | Matrix score. | sequence | function |
| --- | --- | --- | --- | --- | --- | --- |
| Unnamed\_\_2 | Zea mays | 1129 | - | 6 | CCCCGG |  |

> 2018/04/13 10:10:12  
+ CAATGAAAAG AAAGCTGCAT AGACATGTTG ACCCCTTCAT CCACTCTTTG TCTAGTTGCT TCTCTTCTAT   
  
  
+ GTATTACCAT TGACTGTTGA GTTTTCGAAG GATTAAAAGA AAAGTTTGTT CACAAAAAAT TTGATGCCCT   
  
  
+ AATTAAAACC CTTGAGGAAA TAAAAATCAT AATCTGGGCA ACCGGCTTCC TAAGTATATA TAGTATATAT   
  
  
+ TTCTAGGATT GGATGGCAGC TCGGCTTAAT TAATTATCTT TTAGTTATCT TAGAGCAATA AATTTAAGTC   
  
  
+ CGTTTTATAT GGTCGATTTC TATATATTGT GAATCATTGA CTGTTGAATT GAAATATTTT GTCATAATAT   
  
  
+ TTGTACATAT AACTTGGGGC AGCCAACGGA GCAATGGGAT CGGGGAGTCT AATATTTTAT CTACTAGAAG   
  
  
+ GTAAAAGCTT TTGAGTAACA AAGATGACTA CTATATAATA GCAAGCTTGA TAGTTACATA ACTAATAATA   
  
  
+ TCGGGTTTGA GATATGGTAA TCTATTGCTA TTTCATAAAA TAAGAATCGT ATTTACACCA CGATCCGTTT   
  
  
+ AATAATCCAC AAGTCTTTGT GTATATAGTT TTATATTAAC TTAATTCGAC TTAGTGAACA GATCAAAATC   
  
  
+ GATTATTTTT ATAGCTCGTG GAACTCGGAT CAGAAGAGAT GTATCCACCT GTGCTATTTC AAGAAGGGAA   
  
  
+ ATTTTGTCAT TAATATTAAG TGGATAATAA TGGACAAACT AACCGGGTAC TTAGACATCA ATCCATTAAC   
  
  
+ ATTGAATTAG TCCTTTTGTA CATATTATGG CGTTACATGT CCTGTATGAA GAAAAGAAAA CATCACCGTT   
  
  
+ TTAATTTATT ATTCAAATAT ACTTGTAATA CACAGAGATA TCAGAGATGA TAATCATAAT CATGATTAAC   
  
  
+ TAATCAGATC TGATGAAGCG CTTGGTACCT AACTAGTTGT GAGCTATACA ATAATATCAA CTAGGGTTTT   
  
  
+ CATACCGAGC AAGTGAAGGT CAATGGCGAG GTCAGTGGTG TGAGTGATCG TCACTCCGTG TTATACAAGC   
  
  
+ GTATTGACCA GGAGCTTAAT TATTGACACA GAAACAACAA AGCCTGCACC ATTGACACAG AGAAAAACCG   
  
  
+ ACCGGCCACC GGGGAGGCTA AAAGGTCACC AGTACTGGAT CTTTGACCCT TAGCCATGTG ACGTCGTCTC   
  
  
+ TATATGAAGC TTCAACGATG GTTACCATAG ATTATACAAT AGGGTCGTCG CCGCAAATTT AGCCTTTCTC   
  
  
+ CTTCTTTCTC TCTCTCTGCA AACTGATCAT ATCTCATCTG TTTCTTAACT GTGAAAGGTT GAAGTTGAAT   
  
  
+ CTTTCCATAT TGTTTACTCT TTTAAGGTAA TAAAATACTA AAAGGAAAAT GTGGTTGGGG TGTTGACATT   
  
  
+ GTCGGCCTGT GTATGAAGGT ACTATTATAA GAAGAGAAGT GCGAGAGAGA CATAACGGAT ACAGAAGCTA   
  
  
+ AAAGAGCAAA GAAGAATACC TTCTACAGC  

- GTTACTTTTC TTTCGACGTA TCTGTACAAC TGGGGAAGTA GGTGAGAAAC AGATCAACGA AGAGAAGATA   
  
  
- CATAATGGTA ACTGACAACT CAAAAGCTTC CTAATTTTCT TTTCAAACAA GTGTTTTTTA AACTACGGGA   
  
  
- TTAATTTTGG GAACTCCTTT ATTTTTAGTA TTAGACCCGT TGGCCGAAGG ATTCATATAT ATCATATATA   
  
  
- AAGATCCTAA CCTACCGTCG AGCCGAATTA ATTAATAGAA AATCAATAGA ATCTCGTTAT TTAAATTCAG   
  
  
- GCAAAATATA CCAGCTAAAG ATATATAACA CTTAGTAACT GACAACTTAA CTTTATAAAA CAGTATTATA   
  
  
- AACATGTATA TTGAACCCCG TCGGTTGCCT CGTTACCCTA GCCCCTCAGA TTATAAAATA GATGATCTTC   
  
  
- CATTTTCGAA AACTCATTGT TTCTACTGAT GATATATTAT CGTTCGAACT ATCAATGTAT TGATTATTAT   
  
  
- AGCCCAAACT CTATACCATT AGATAACGAT AAAGTATTTT ATTCTTAGCA TAAATGTGGT GCTAGGCAAA   
  
  
- TTATTAGGTG TTCAGAAACA CATATATCAA AATATAATTG AATTAAGCTG AATCACTTGT CTAGTTTTAG   
  
  
- CTAATAAAAA TATCGAGCAC CTTGAGCCTA GTCTTCTCTA CATAGGTGGA CACGATAAAG TTCTTCCCTT   
  
  
- TAAAACAGTA ATTATAATTC ACCTATTATT ACCTGTTTGA TTGGCCCATG AATCTGTAGT TAGGTAATTG   
  
  
- TAACTTAATC AGGAAAACAT GTATAATACC GCAATGTACA GGACATACTT CTTTTCTTTT GTAGTGGCAA   
  
  
- AATTAAATAA TAAGTTTATA TGAACATTAT GTGTCTCTAT AGTCTCTACT ATTAGTATTA GTACTAATTG   
  
  
- ATTAGTCTAG ACTACTTCGC GAACCATGGA TTGATCAACA CTCGATATGT TATTATAGTT GATCCCAAAA   
  
  
- GTATGGCTCG TTCACTTCCA GTTACCGCTC CAGTCACCAC ACTCACTAGC AGTGAGGCAC AATATGTTCG   
  
  
- CATAACTGGT CCTCGAATTA ATAACTGTGT CTTTGTTGTT TCGGACGTGG TAACTGTGTC TCTTTTTGGC   
  
  
- TGGCCGGTGG CCCCTCCGAT TTTCCAGTGG TCATGACCTA GAAACTGGGA ATCGGTACAC TGCAGCAGAG   
  
  
- ATATACTTCG AAGTTGCTAC CAATGGTATC TAATATGTTA TCCCAGCAGC GGCGTTTAAA TCGGAAAGAG   
  
  
- GAAGAAAGAG AGAGAGACGT TTGACTAGTA TAGAGTAGAC AAAGAATTGA CACTTTCCAA CTTCAACTTA   
  
  
- GAAAGGTATA ACAAATGAGA AAATTCCATT ATTTTATGAT TTTCCTTTTA CACCAACCCC ACAACTGTAA   
  
  
- CAGCCGGACA CATACTTCCA TGATAATATT CTTCTCTTCA CGCTCTCTCT GTATTGCCTA TGTCTTCGAT   
  
  
- TTTCTCGTTT CTTCTTATGG AAGATGTCG

+     Unnamed\_\_3

| Site Name | Organism | Position | Strand | Matrix score. | sequence | function |
| --- | --- | --- | --- | --- | --- | --- |
| Unnamed\_\_3 | Zea mays | 647 | + | 5 | CGTGG |  |
| Unnamed\_\_3 | Zea mays | 548 | - | 5 | CGTGG |  |

> 2018/04/13 10:10:12  
+ CAATGAAAAG AAAGCTGCAT AGACATGTTG ACCCCTTCAT CCACTCTTTG TCTAGTTGCT TCTCTTCTAT   
  
  
+ GTATTACCAT TGACTGTTGA GTTTTCGAAG GATTAAAAGA AAAGTTTGTT CACAAAAAAT TTGATGCCCT   
  
  
+ AATTAAAACC CTTGAGGAAA TAAAAATCAT AATCTGGGCA ACCGGCTTCC TAAGTATATA TAGTATATAT   
  
  
+ TTCTAGGATT GGATGGCAGC TCGGCTTAAT TAATTATCTT TTAGTTATCT TAGAGCAATA AATTTAAGTC   
  
  
+ CGTTTTATAT GGTCGATTTC TATATATTGT GAATCATTGA CTGTTGAATT GAAATATTTT GTCATAATAT   
  
  
+ TTGTACATAT AACTTGGGGC AGCCAACGGA GCAATGGGAT CGGGGAGTCT AATATTTTAT CTACTAGAAG   
  
  
+ GTAAAAGCTT TTGAGTAACA AAGATGACTA CTATATAATA GCAAGCTTGA TAGTTACATA ACTAATAATA   
  
  
+ TCGGGTTTGA GATATGGTAA TCTATTGCTA TTTCATAAAA TAAGAATCGT ATTTACACCA CGATCCGTTT   
  
  
+ AATAATCCAC AAGTCTTTGT GTATATAGTT TTATATTAAC TTAATTCGAC TTAGTGAACA GATCAAAATC   
  
  
+ GATTATTTTT ATAGCTCGTG GAACTCGGAT CAGAAGAGAT GTATCCACCT GTGCTATTTC AAGAAGGGAA   
  
  
+ ATTTTGTCAT TAATATTAAG TGGATAATAA TGGACAAACT AACCGGGTAC TTAGACATCA ATCCATTAAC   
  
  
+ ATTGAATTAG TCCTTTTGTA CATATTATGG CGTTACATGT CCTGTATGAA GAAAAGAAAA CATCACCGTT   
  
  
+ TTAATTTATT ATTCAAATAT ACTTGTAATA CACAGAGATA TCAGAGATGA TAATCATAAT CATGATTAAC   
  
  
+ TAATCAGATC TGATGAAGCG CTTGGTACCT AACTAGTTGT GAGCTATACA ATAATATCAA CTAGGGTTTT   
  
  
+ CATACCGAGC AAGTGAAGGT CAATGGCGAG GTCAGTGGTG TGAGTGATCG TCACTCCGTG TTATACAAGC   
  
  
+ GTATTGACCA GGAGCTTAAT TATTGACACA GAAACAACAA AGCCTGCACC ATTGACACAG AGAAAAACCG   
  
  
+ ACCGGCCACC GGGGAGGCTA AAAGGTCACC AGTACTGGAT CTTTGACCCT TAGCCATGTG ACGTCGTCTC   
  
  
+ TATATGAAGC TTCAACGATG GTTACCATAG ATTATACAAT AGGGTCGTCG CCGCAAATTT AGCCTTTCTC   
  
  
+ CTTCTTTCTC TCTCTCTGCA AACTGATCAT ATCTCATCTG TTTCTTAACT GTGAAAGGTT GAAGTTGAAT   
  
  
+ CTTTCCATAT TGTTTACTCT TTTAAGGTAA TAAAATACTA AAAGGAAAAT GTGGTTGGGG TGTTGACATT   
  
  
+ GTCGGCCTGT GTATGAAGGT ACTATTATAA GAAGAGAAGT GCGAGAGAGA CATAACGGAT ACAGAAGCTA   
  
  
+ AAAGAGCAAA GAAGAATACC TTCTACAGC  

- GTTACTTTTC TTTCGACGTA TCTGTACAAC TGGGGAAGTA GGTGAGAAAC AGATCAACGA AGAGAAGATA   
  
  
- CATAATGGTA ACTGACAACT CAAAAGCTTC CTAATTTTCT TTTCAAACAA GTGTTTTTTA AACTACGGGA   
  
  
- TTAATTTTGG GAACTCCTTT ATTTTTAGTA TTAGACCCGT TGGCCGAAGG ATTCATATAT ATCATATATA   
  
  
- AAGATCCTAA CCTACCGTCG AGCCGAATTA ATTAATAGAA AATCAATAGA ATCTCGTTAT TTAAATTCAG   
  
  
- GCAAAATATA CCAGCTAAAG ATATATAACA CTTAGTAACT GACAACTTAA CTTTATAAAA CAGTATTATA   
  
  
- AACATGTATA TTGAACCCCG TCGGTTGCCT CGTTACCCTA GCCCCTCAGA TTATAAAATA GATGATCTTC   
  
  
- CATTTTCGAA AACTCATTGT TTCTACTGAT GATATATTAT CGTTCGAACT ATCAATGTAT TGATTATTAT   
  
  
- AGCCCAAACT CTATACCATT AGATAACGAT AAAGTATTTT ATTCTTAGCA TAAATGTGGT GCTAGGCAAA   
  
  
- TTATTAGGTG TTCAGAAACA CATATATCAA AATATAATTG AATTAAGCTG AATCACTTGT CTAGTTTTAG   
  
  
- CTAATAAAAA TATCGAGCAC CTTGAGCCTA GTCTTCTCTA CATAGGTGGA CACGATAAAG TTCTTCCCTT   
  
  
- TAAAACAGTA ATTATAATTC ACCTATTATT ACCTGTTTGA TTGGCCCATG AATCTGTAGT TAGGTAATTG   
  
  
- TAACTTAATC AGGAAAACAT GTATAATACC GCAATGTACA GGACATACTT CTTTTCTTTT GTAGTGGCAA   
  
  
- AATTAAATAA TAAGTTTATA TGAACATTAT GTGTCTCTAT AGTCTCTACT ATTAGTATTA GTACTAATTG   
  
  
- ATTAGTCTAG ACTACTTCGC GAACCATGGA TTGATCAACA CTCGATATGT TATTATAGTT GATCCCAAAA   
  
  
- GTATGGCTCG TTCACTTCCA GTTACCGCTC CAGTCACCAC ACTCACTAGC AGTGAGGCAC AATATGTTCG   
  
  
- CATAACTGGT CCTCGAATTA ATAACTGTGT CTTTGTTGTT TCGGACGTGG TAACTGTGTC TCTTTTTGGC   
  
  
- TGGCCGGTGG CCCCTCCGAT TTTCCAGTGG TCATGACCTA GAAACTGGGA ATCGGTACAC TGCAGCAGAG   
  
  
- ATATACTTCG AAGTTGCTAC CAATGGTATC TAATATGTTA TCCCAGCAGC GGCGTTTAAA TCGGAAAGAG   
  
  
- GAAGAAAGAG AGAGAGACGT TTGACTAGTA TAGAGTAGAC AAAGAATTGA CACTTTCCAA CTTCAACTTA   
  
  
- GAAAGGTATA ACAAATGAGA AAATTCCATT ATTTTATGAT TTTCCTTTTA CACCAACCCC ACAACTGTAA   
  
  
- CAGCCGGACA CATACTTCCA TGATAATATT CTTCTCTTCA CGCTCTCTCT GTATTGCCTA TGTCTTCGAT   
  
  
- TTTCTCGTTT CTTCTTATGG AAGATGTCG

+     Unnamed\_\_4

| Site Name | Organism | Position | Strand | Matrix score. | sequence | function |
| --- | --- | --- | --- | --- | --- | --- |
| Unnamed\_\_4 | Petroselinum hortense | 1133 | - | 4 | CTCC |  |
| Unnamed\_\_4 | Petroselinum hortense | 1258 | + | 4 | CTCC |  |
| Unnamed\_\_4 | Petroselinum hortense | 394 | - | 4 | CTCC |  |
| Unnamed\_\_4 | Petroselinum hortense | 378 | - | 4 | CTCC |  |
| Unnamed\_\_4 | Petroselinum hortense | 1034 | + | 4 | CTCC |  |
| Unnamed\_\_4 | Petroselinum hortense | 1061 | - | 4 | CTCC |  |

> 2018/04/13 10:10:12  
+ CAATGAAAAG AAAGCTGCAT AGACATGTTG ACCCCTTCAT CCACTCTTTG TCTAGTTGCT TCTCTTCTAT   
  
  
+ GTATTACCAT TGACTGTTGA GTTTTCGAAG GATTAAAAGA AAAGTTTGTT CACAAAAAAT TTGATGCCCT   
  
  
+ AATTAAAACC CTTGAGGAAA TAAAAATCAT AATCTGGGCA ACCGGCTTCC TAAGTATATA TAGTATATAT   
  
  
+ TTCTAGGATT GGATGGCAGC TCGGCTTAAT TAATTATCTT TTAGTTATCT TAGAGCAATA AATTTAAGTC   
  
  
+ CGTTTTATAT GGTCGATTTC TATATATTGT GAATCATTGA CTGTTGAATT GAAATATTTT GTCATAATAT   
  
  
+ TTGTACATAT AACTTGGGGC AGCCAACGGA GCAATGGGAT CGGGGAGTCT AATATTTTAT CTACTAGAAG   
  
  
+ GTAAAAGCTT TTGAGTAACA AAGATGACTA CTATATAATA GCAAGCTTGA TAGTTACATA ACTAATAATA   
  
  
+ TCGGGTTTGA GATATGGTAA TCTATTGCTA TTTCATAAAA TAAGAATCGT ATTTACACCA CGATCCGTTT   
  
  
+ AATAATCCAC AAGTCTTTGT GTATATAGTT TTATATTAAC TTAATTCGAC TTAGTGAACA GATCAAAATC   
  
  
+ GATTATTTTT ATAGCTCGTG GAACTCGGAT CAGAAGAGAT GTATCCACCT GTGCTATTTC AAGAAGGGAA   
  
  
+ ATTTTGTCAT TAATATTAAG TGGATAATAA TGGACAAACT AACCGGGTAC TTAGACATCA ATCCATTAAC   
  
  
+ ATTGAATTAG TCCTTTTGTA CATATTATGG CGTTACATGT CCTGTATGAA GAAAAGAAAA CATCACCGTT   
  
  
+ TTAATTTATT ATTCAAATAT ACTTGTAATA CACAGAGATA TCAGAGATGA TAATCATAAT CATGATTAAC   
  
  
+ TAATCAGATC TGATGAAGCG CTTGGTACCT AACTAGTTGT GAGCTATACA ATAATATCAA CTAGGGTTTT   
  
  
+ CATACCGAGC AAGTGAAGGT CAATGGCGAG GTCAGTGGTG TGAGTGATCG TCACTCCGTG TTATACAAGC   
  
  
+ GTATTGACCA GGAGCTTAAT TATTGACACA GAAACAACAA AGCCTGCACC ATTGACACAG AGAAAAACCG   
  
  
+ ACCGGCCACC GGGGAGGCTA AAAGGTCACC AGTACTGGAT CTTTGACCCT TAGCCATGTG ACGTCGTCTC   
  
  
+ TATATGAAGC TTCAACGATG GTTACCATAG ATTATACAAT AGGGTCGTCG CCGCAAATTT AGCCTTTCTC   
  
  
+ CTTCTTTCTC TCTCTCTGCA AACTGATCAT ATCTCATCTG TTTCTTAACT GTGAAAGGTT GAAGTTGAAT   
  
  
+ CTTTCCATAT TGTTTACTCT TTTAAGGTAA TAAAATACTA AAAGGAAAAT GTGGTTGGGG TGTTGACATT   
  
  
+ GTCGGCCTGT GTATGAAGGT ACTATTATAA GAAGAGAAGT GCGAGAGAGA CATAACGGAT ACAGAAGCTA   
  
  
+ AAAGAGCAAA GAAGAATACC TTCTACAGC  

- GTTACTTTTC TTTCGACGTA TCTGTACAAC TGGGGAAGTA GGTGAGAAAC AGATCAACGA AGAGAAGATA   
  
  
- CATAATGGTA ACTGACAACT CAAAAGCTTC CTAATTTTCT TTTCAAACAA GTGTTTTTTA AACTACGGGA   
  
  
- TTAATTTTGG GAACTCCTTT ATTTTTAGTA TTAGACCCGT TGGCCGAAGG ATTCATATAT ATCATATATA   
  
  
- AAGATCCTAA CCTACCGTCG AGCCGAATTA ATTAATAGAA AATCAATAGA ATCTCGTTAT TTAAATTCAG   
  
  
- GCAAAATATA CCAGCTAAAG ATATATAACA CTTAGTAACT GACAACTTAA CTTTATAAAA CAGTATTATA   
  
  
- AACATGTATA TTGAACCCCG TCGGTTGCCT CGTTACCCTA GCCCCTCAGA TTATAAAATA GATGATCTTC   
  
  
- CATTTTCGAA AACTCATTGT TTCTACTGAT GATATATTAT CGTTCGAACT ATCAATGTAT TGATTATTAT   
  
  
- AGCCCAAACT CTATACCATT AGATAACGAT AAAGTATTTT ATTCTTAGCA TAAATGTGGT GCTAGGCAAA   
  
  
- TTATTAGGTG TTCAGAAACA CATATATCAA AATATAATTG AATTAAGCTG AATCACTTGT CTAGTTTTAG   
  
  
- CTAATAAAAA TATCGAGCAC CTTGAGCCTA GTCTTCTCTA CATAGGTGGA CACGATAAAG TTCTTCCCTT   
  
  
- TAAAACAGTA ATTATAATTC ACCTATTATT ACCTGTTTGA TTGGCCCATG AATCTGTAGT TAGGTAATTG   
  
  
- TAACTTAATC AGGAAAACAT GTATAATACC GCAATGTACA GGACATACTT CTTTTCTTTT GTAGTGGCAA   
  
  
- AATTAAATAA TAAGTTTATA TGAACATTAT GTGTCTCTAT AGTCTCTACT ATTAGTATTA GTACTAATTG   
  
  
- ATTAGTCTAG ACTACTTCGC GAACCATGGA TTGATCAACA CTCGATATGT TATTATAGTT GATCCCAAAA   
  
  
- GTATGGCTCG TTCACTTCCA GTTACCGCTC CAGTCACCAC ACTCACTAGC AGTGAGGCAC AATATGTTCG   
  
  
- CATAACTGGT CCTCGAATTA ATAACTGTGT CTTTGTTGTT TCGGACGTGG TAACTGTGTC TCTTTTTGGC   
  
  
- TGGCCGGTGG CCCCTCCGAT TTTCCAGTGG TCATGACCTA GAAACTGGGA ATCGGTACAC TGCAGCAGAG   
  
  
- ATATACTTCG AAGTTGCTAC CAATGGTATC TAATATGTTA TCCCAGCAGC GGCGTTTAAA TCGGAAAGAG   
  
  
- GAAGAAAGAG AGAGAGACGT TTGACTAGTA TAGAGTAGAC AAAGAATTGA CACTTTCCAA CTTCAACTTA   
  
  
- GAAAGGTATA ACAAATGAGA AAATTCCATT ATTTTATGAT TTTCCTTTTA CACCAACCCC ACAACTGTAA   
  
  
- CAGCCGGACA CATACTTCCA TGATAATATT CTTCTCTTCA CGCTCTCTCT GTATTGCCTA TGTCTTCGAT   
  
  
- TTTCTCGTTT CTTCTTATGG AAGATGTCG

+     W box

| Site Name | Organism | Position | Strand | Matrix score. | sequence | function |
| --- | --- | --- | --- | --- | --- | --- |
| W box | Arabidopsis thaliana | 1163 | + | 6 | TTGACC |  |
| W box | Arabidopsis thaliana | 1054 | + | 6 | TTGACC |  |
| W box | Arabidopsis thaliana | 28 | + | 6 | TTGACC |  |
| W box | Arabidopsis thaliana | 998 | - | 6 | TTGACC |  |

> 2018/04/13 10:10:12  
+ CAATGAAAAG AAAGCTGCAT AGACATGTTG ACCCCTTCAT CCACTCTTTG TCTAGTTGCT TCTCTTCTAT   
  
  
+ GTATTACCAT TGACTGTTGA GTTTTCGAAG GATTAAAAGA AAAGTTTGTT CACAAAAAAT TTGATGCCCT   
  
  
+ AATTAAAACC CTTGAGGAAA TAAAAATCAT AATCTGGGCA ACCGGCTTCC TAAGTATATA TAGTATATAT   
  
  
+ TTCTAGGATT GGATGGCAGC TCGGCTTAAT TAATTATCTT TTAGTTATCT TAGAGCAATA AATTTAAGTC   
  
  
+ CGTTTTATAT GGTCGATTTC TATATATTGT GAATCATTGA CTGTTGAATT GAAATATTTT GTCATAATAT   
  
  
+ TTGTACATAT AACTTGGGGC AGCCAACGGA GCAATGGGAT CGGGGAGTCT AATATTTTAT CTACTAGAAG   
  
  
+ GTAAAAGCTT TTGAGTAACA AAGATGACTA CTATATAATA GCAAGCTTGA TAGTTACATA ACTAATAATA   
  
  
+ TCGGGTTTGA GATATGGTAA TCTATTGCTA TTTCATAAAA TAAGAATCGT ATTTACACCA CGATCCGTTT   
  
  
+ AATAATCCAC AAGTCTTTGT GTATATAGTT TTATATTAAC TTAATTCGAC TTAGTGAACA GATCAAAATC   
  
  
+ GATTATTTTT ATAGCTCGTG GAACTCGGAT CAGAAGAGAT GTATCCACCT GTGCTATTTC AAGAAGGGAA   
  
  
+ ATTTTGTCAT TAATATTAAG TGGATAATAA TGGACAAACT AACCGGGTAC TTAGACATCA ATCCATTAAC   
  
  
+ ATTGAATTAG TCCTTTTGTA CATATTATGG CGTTACATGT CCTGTATGAA GAAAAGAAAA CATCACCGTT   
  
  
+ TTAATTTATT ATTCAAATAT ACTTGTAATA CACAGAGATA TCAGAGATGA TAATCATAAT CATGATTAAC   
  
  
+ TAATCAGATC TGATGAAGCG CTTGGTACCT AACTAGTTGT GAGCTATACA ATAATATCAA CTAGGGTTTT   
  
  
+ CATACCGAGC AAGTGAAGGT CAATGGCGAG GTCAGTGGTG TGAGTGATCG TCACTCCGTG TTATACAAGC   
  
  
+ GTATTGACCA GGAGCTTAAT TATTGACACA GAAACAACAA AGCCTGCACC ATTGACACAG AGAAAAACCG   
  
  
+ ACCGGCCACC GGGGAGGCTA AAAGGTCACC AGTACTGGAT CTTTGACCCT TAGCCATGTG ACGTCGTCTC   
  
  
+ TATATGAAGC TTCAACGATG GTTACCATAG ATTATACAAT AGGGTCGTCG CCGCAAATTT AGCCTTTCTC   
  
  
+ CTTCTTTCTC TCTCTCTGCA AACTGATCAT ATCTCATCTG TTTCTTAACT GTGAAAGGTT GAAGTTGAAT   
  
  
+ CTTTCCATAT TGTTTACTCT TTTAAGGTAA TAAAATACTA AAAGGAAAAT GTGGTTGGGG TGTTGACATT   
  
  
+ GTCGGCCTGT GTATGAAGGT ACTATTATAA GAAGAGAAGT GCGAGAGAGA CATAACGGAT ACAGAAGCTA   
  
  
+ AAAGAGCAAA GAAGAATACC TTCTACAGC  

- GTTACTTTTC TTTCGACGTA TCTGTACAAC TGGGGAAGTA GGTGAGAAAC AGATCAACGA AGAGAAGATA   
  
  
- CATAATGGTA ACTGACAACT CAAAAGCTTC CTAATTTTCT TTTCAAACAA GTGTTTTTTA AACTACGGGA   
  
  
- TTAATTTTGG GAACTCCTTT ATTTTTAGTA TTAGACCCGT TGGCCGAAGG ATTCATATAT ATCATATATA   
  
  
- AAGATCCTAA CCTACCGTCG AGCCGAATTA ATTAATAGAA AATCAATAGA ATCTCGTTAT TTAAATTCAG   
  
  
- GCAAAATATA CCAGCTAAAG ATATATAACA CTTAGTAACT GACAACTTAA CTTTATAAAA CAGTATTATA   
  
  
- AACATGTATA TTGAACCCCG TCGGTTGCCT CGTTACCCTA GCCCCTCAGA TTATAAAATA GATGATCTTC   
  
  
- CATTTTCGAA AACTCATTGT TTCTACTGAT GATATATTAT CGTTCGAACT ATCAATGTAT TGATTATTAT   
  
  
- AGCCCAAACT CTATACCATT AGATAACGAT AAAGTATTTT ATTCTTAGCA TAAATGTGGT GCTAGGCAAA   
  
  
- TTATTAGGTG TTCAGAAACA CATATATCAA AATATAATTG AATTAAGCTG AATCACTTGT CTAGTTTTAG   
  
  
- CTAATAAAAA TATCGAGCAC CTTGAGCCTA GTCTTCTCTA CATAGGTGGA CACGATAAAG TTCTTCCCTT   
  
  
- TAAAACAGTA ATTATAATTC ACCTATTATT ACCTGTTTGA TTGGCCCATG AATCTGTAGT TAGGTAATTG   
  
  
- TAACTTAATC AGGAAAACAT GTATAATACC GCAATGTACA GGACATACTT CTTTTCTTTT GTAGTGGCAA   
  
  
- AATTAAATAA TAAGTTTATA TGAACATTAT GTGTCTCTAT AGTCTCTACT ATTAGTATTA GTACTAATTG   
  
  
- ATTAGTCTAG ACTACTTCGC GAACCATGGA TTGATCAACA CTCGATATGT TATTATAGTT GATCCCAAAA   
  
  
- GTATGGCTCG TTCACTTCCA GTTACCGCTC CAGTCACCAC ACTCACTAGC AGTGAGGCAC AATATGTTCG   
  
  
- CATAACTGGT CCTCGAATTA ATAACTGTGT CTTTGTTGTT TCGGACGTGG TAACTGTGTC TCTTTTTGGC   
  
  
- TGGCCGGTGG CCCCTCCGAT TTTCCAGTGG TCATGACCTA GAAACTGGGA ATCGGTACAC TGCAGCAGAG   
  
  
- ATATACTTCG AAGTTGCTAC CAATGGTATC TAATATGTTA TCCCAGCAGC GGCGTTTAAA TCGGAAAGAG   
  
  
- GAAGAAAGAG AGAGAGACGT TTGACTAGTA TAGAGTAGAC AAAGAATTGA CACTTTCCAA CTTCAACTTA   
  
  
- GAAAGGTATA ACAAATGAGA AAATTCCATT ATTTTATGAT TTTCCTTTTA CACCAACCCC ACAACTGTAA   
  
  
- CAGCCGGACA CATACTTCCA TGATAATATT CTTCTCTTCA CGCTCTCTCT GTATTGCCTA TGTCTTCGAT   
  
  
- TTTCTCGTTT CTTCTTATGG AAGATGTCG

+     as-2-box

| Site Name | Organism | Position | Strand | Matrix score. | sequence | function |
| --- | --- | --- | --- | --- | --- | --- |
| as-2-box | Nicotiana tabacum | 723 | + | 9 | GATAatGATG | involved in shoot-specific expression and light responsiveness |

> 2018/04/13 10:10:12  
+ CAATGAAAAG AAAGCTGCAT AGACATGTTG ACCCCTTCAT CCACTCTTTG TCTAGTTGCT TCTCTTCTAT   
  
  
+ GTATTACCAT TGACTGTTGA GTTTTCGAAG GATTAAAAGA AAAGTTTGTT CACAAAAAAT TTGATGCCCT   
  
  
+ AATTAAAACC CTTGAGGAAA TAAAAATCAT AATCTGGGCA ACCGGCTTCC TAAGTATATA TAGTATATAT   
  
  
+ TTCTAGGATT GGATGGCAGC TCGGCTTAAT TAATTATCTT TTAGTTATCT TAGAGCAATA AATTTAAGTC   
  
  
+ CGTTTTATAT GGTCGATTTC TATATATTGT GAATCATTGA CTGTTGAATT GAAATATTTT GTCATAATAT   
  
  
+ TTGTACATAT AACTTGGGGC AGCCAACGGA GCAATGGGAT CGGGGAGTCT AATATTTTAT CTACTAGAAG   
  
  
+ GTAAAAGCTT TTGAGTAACA AAGATGACTA CTATATAATA GCAAGCTTGA TAGTTACATA ACTAATAATA   
  
  
+ TCGGGTTTGA GATATGGTAA TCTATTGCTA TTTCATAAAA TAAGAATCGT ATTTACACCA CGATCCGTTT   
  
  
+ AATAATCCAC AAGTCTTTGT GTATATAGTT TTATATTAAC TTAATTCGAC TTAGTGAACA GATCAAAATC   
  
  
+ GATTATTTTT ATAGCTCGTG GAACTCGGAT CAGAAGAGAT GTATCCACCT GTGCTATTTC AAGAAGGGAA   
  
  
+ ATTTTGTCAT TAATATTAAG TGGATAATAA TGGACAAACT AACCGGGTAC TTAGACATCA ATCCATTAAC   
  
  
+ ATTGAATTAG TCCTTTTGTA CATATTATGG CGTTACATGT CCTGTATGAA GAAAAGAAAA CATCACCGTT   
  
  
+ TTAATTTATT ATTCAAATAT ACTTGTAATA CACAGAGATA TCAGAGATGA TAATCATAAT CATGATTAAC   
  
  
+ TAATCAGATC TGATGAAGCG CTTGGTACCT AACTAGTTGT GAGCTATACA ATAATATCAA CTAGGGTTTT   
  
  
+ CATACCGAGC AAGTGAAGGT CAATGGCGAG GTCAGTGGTG TGAGTGATCG TCACTCCGTG TTATACAAGC   
  
  
+ GTATTGACCA GGAGCTTAAT TATTGACACA GAAACAACAA AGCCTGCACC ATTGACACAG AGAAAAACCG   
  
  
+ ACCGGCCACC GGGGAGGCTA AAAGGTCACC AGTACTGGAT CTTTGACCCT TAGCCATGTG ACGTCGTCTC   
  
  
+ TATATGAAGC TTCAACGATG GTTACCATAG ATTATACAAT AGGGTCGTCG CCGCAAATTT AGCCTTTCTC   
  
  
+ CTTCTTTCTC TCTCTCTGCA AACTGATCAT ATCTCATCTG TTTCTTAACT GTGAAAGGTT GAAGTTGAAT   
  
  
+ CTTTCCATAT TGTTTACTCT TTTAAGGTAA TAAAATACTA AAAGGAAAAT GTGGTTGGGG TGTTGACATT   
  
  
+ GTCGGCCTGT GTATGAAGGT ACTATTATAA GAAGAGAAGT GCGAGAGAGA CATAACGGAT ACAGAAGCTA   
  
  
+ AAAGAGCAAA GAAGAATACC TTCTACAGC  

- GTTACTTTTC TTTCGACGTA TCTGTACAAC TGGGGAAGTA GGTGAGAAAC AGATCAACGA AGAGAAGATA   
  
  
- CATAATGGTA ACTGACAACT CAAAAGCTTC CTAATTTTCT TTTCAAACAA GTGTTTTTTA AACTACGGGA   
  
  
- TTAATTTTGG GAACTCCTTT ATTTTTAGTA TTAGACCCGT TGGCCGAAGG ATTCATATAT ATCATATATA   
  
  
- AAGATCCTAA CCTACCGTCG AGCCGAATTA ATTAATAGAA AATCAATAGA ATCTCGTTAT TTAAATTCAG   
  
  
- GCAAAATATA CCAGCTAAAG ATATATAACA CTTAGTAACT GACAACTTAA CTTTATAAAA CAGTATTATA   
  
  
- AACATGTATA TTGAACCCCG TCGGTTGCCT CGTTACCCTA GCCCCTCAGA TTATAAAATA GATGATCTTC   
  
  
- CATTTTCGAA AACTCATTGT TTCTACTGAT GATATATTAT CGTTCGAACT ATCAATGTAT TGATTATTAT   
  
  
- AGCCCAAACT CTATACCATT AGATAACGAT AAAGTATTTT ATTCTTAGCA TAAATGTGGT GCTAGGCAAA   
  
  
- TTATTAGGTG TTCAGAAACA CATATATCAA AATATAATTG AATTAAGCTG AATCACTTGT CTAGTTTTAG   
  
  
- CTAATAAAAA TATCGAGCAC CTTGAGCCTA GTCTTCTCTA CATAGGTGGA CACGATAAAG TTCTTCCCTT   
  
  
- TAAAACAGTA ATTATAATTC ACCTATTATT ACCTGTTTGA TTGGCCCATG AATCTGTAGT TAGGTAATTG   
  
  
- TAACTTAATC AGGAAAACAT GTATAATACC GCAATGTACA GGACATACTT CTTTTCTTTT GTAGTGGCAA   
  
  
- AATTAAATAA TAAGTTTATA TGAACATTAT GTGTCTCTAT AGTCTCTACT ATTAGTATTA GTACTAATTG   
  
  
- ATTAGTCTAG ACTACTTCGC GAACCATGGA TTGATCAACA CTCGATATGT TATTATAGTT GATCCCAAAA   
  
  
- GTATGGCTCG TTCACTTCCA GTTACCGCTC CAGTCACCAC ACTCACTAGC AGTGAGGCAC AATATGTTCG   
  
  
- CATAACTGGT CCTCGAATTA ATAACTGTGT CTTTGTTGTT TCGGACGTGG TAACTGTGTC TCTTTTTGGC   
  
  
- TGGCCGGTGG CCCCTCCGAT TTTCCAGTGG TCATGACCTA GAAACTGGGA ATCGGTACAC TGCAGCAGAG   
  
  
- ATATACTTCG AAGTTGCTAC CAATGGTATC TAATATGTTA TCCCAGCAGC GGCGTTTAAA TCGGAAAGAG   
  
  
- GAAGAAAGAG AGAGAGACGT TTGACTAGTA TAGAGTAGAC AAAGAATTGA CACTTTCCAA CTTCAACTTA   
  
  
- GAAAGGTATA ACAAATGAGA AAATTCCATT ATTTTATGAT TTTCCTTTTA CACCAACCCC ACAACTGTAA   
  
  
- CAGCCGGACA CATACTTCCA TGATAATATT CTTCTCTTCA CGCTCTCTCT GTATTGCCTA TGTCTTCGAT   
  
  
- TTTCTCGTTT CTTCTTATGG AAGATGTCG

+     circadian

| Site Name | Organism | Position | Strand | Matrix score. | sequence | function |
| --- | --- | --- | --- | --- | --- | --- |
| circadian | Lycopersicon esculentum | 959 | + | 6 | CAANNNNATC | cis-acting regulatory element involved in circadian control |
| circadian | Lycopersicon esculentum | 1279 | + | 6 | CAANNNNATC | cis-acting regulatory element involved in circadian control |
| circadian | Lycopersicon esculentum | 873 | + | 9 | CAAAGATATC | cis-acting regulatory element involved in circadian control |
| circadian | Lycopersicon esculentum | 382 | + | 6 | CAANNNNATC | cis-acting regulatory element involved in circadian control |

> 2018/04/13 10:10:12  
+ CAATGAAAAG AAAGCTGCAT AGACATGTTG ACCCCTTCAT CCACTCTTTG TCTAGTTGCT TCTCTTCTAT   
  
  
+ GTATTACCAT TGACTGTTGA GTTTTCGAAG GATTAAAAGA AAAGTTTGTT CACAAAAAAT TTGATGCCCT   
  
  
+ AATTAAAACC CTTGAGGAAA TAAAAATCAT AATCTGGGCA ACCGGCTTCC TAAGTATATA TAGTATATAT   
  
  
+ TTCTAGGATT GGATGGCAGC TCGGCTTAAT TAATTATCTT TTAGTTATCT TAGAGCAATA AATTTAAGTC   
  
  
+ CGTTTTATAT GGTCGATTTC TATATATTGT GAATCATTGA CTGTTGAATT GAAATATTTT GTCATAATAT   
  
  
+ TTGTACATAT AACTTGGGGC AGCCAACGGA GCAATGGGAT CGGGGAGTCT AATATTTTAT CTACTAGAAG   
  
  
+ GTAAAAGCTT TTGAGTAACA AAGATGACTA CTATATAATA GCAAGCTTGA TAGTTACATA ACTAATAATA   
  
  
+ TCGGGTTTGA GATATGGTAA TCTATTGCTA TTTCATAAAA TAAGAATCGT ATTTACACCA CGATCCGTTT   
  
  
+ AATAATCCAC AAGTCTTTGT GTATATAGTT TTATATTAAC TTAATTCGAC TTAGTGAACA GATCAAAATC   
  
  
+ GATTATTTTT ATAGCTCGTG GAACTCGGAT CAGAAGAGAT GTATCCACCT GTGCTATTTC AAGAAGGGAA   
  
  
+ ATTTTGTCAT TAATATTAAG TGGATAATAA TGGACAAACT AACCGGGTAC TTAGACATCA ATCCATTAAC   
  
  
+ ATTGAATTAG TCCTTTTGTA CATATTATGG CGTTACATGT CCTGTATGAA GAAAAGAAAA CATCACCGTT   
  
  
+ TTAATTTATT ATTCAAATAT ACTTGTAATA CACAGAGATA TCAGAGATGA TAATCATAAT CATGATTAAC   
  
  
+ TAATCAGATC TGATGAAGCG CTTGGTACCT AACTAGTTGT GAGCTATACA ATAATATCAA CTAGGGTTTT   
  
  
+ CATACCGAGC AAGTGAAGGT CAATGGCGAG GTCAGTGGTG TGAGTGATCG TCACTCCGTG TTATACAAGC   
  
  
+ GTATTGACCA GGAGCTTAAT TATTGACACA GAAACAACAA AGCCTGCACC ATTGACACAG AGAAAAACCG   
  
  
+ ACCGGCCACC GGGGAGGCTA AAAGGTCACC AGTACTGGAT CTTTGACCCT TAGCCATGTG ACGTCGTCTC   
  
  
+ TATATGAAGC TTCAACGATG GTTACCATAG ATTATACAAT AGGGTCGTCG CCGCAAATTT AGCCTTTCTC   
  
  
+ CTTCTTTCTC TCTCTCTGCA AACTGATCAT ATCTCATCTG TTTCTTAACT GTGAAAGGTT GAAGTTGAAT   
  
  
+ CTTTCCATAT TGTTTACTCT TTTAAGGTAA TAAAATACTA AAAGGAAAAT GTGGTTGGGG TGTTGACATT   
  
  
+ GTCGGCCTGT GTATGAAGGT ACTATTATAA GAAGAGAAGT GCGAGAGAGA CATAACGGAT ACAGAAGCTA   
  
  
+ AAAGAGCAAA GAAGAATACC TTCTACAGC  

- GTTACTTTTC TTTCGACGTA TCTGTACAAC TGGGGAAGTA GGTGAGAAAC AGATCAACGA AGAGAAGATA   
  
  
- CATAATGGTA ACTGACAACT CAAAAGCTTC CTAATTTTCT TTTCAAACAA GTGTTTTTTA AACTACGGGA   
  
  
- TTAATTTTGG GAACTCCTTT ATTTTTAGTA TTAGACCCGT TGGCCGAAGG ATTCATATAT ATCATATATA   
  
  
- AAGATCCTAA CCTACCGTCG AGCCGAATTA ATTAATAGAA AATCAATAGA ATCTCGTTAT TTAAATTCAG   
  
  
- GCAAAATATA CCAGCTAAAG ATATATAACA CTTAGTAACT GACAACTTAA CTTTATAAAA CAGTATTATA   
  
  
- AACATGTATA TTGAACCCCG TCGGTTGCCT CGTTACCCTA GCCCCTCAGA TTATAAAATA GATGATCTTC   
  
  
- CATTTTCGAA AACTCATTGT TTCTACTGAT GATATATTAT CGTTCGAACT ATCAATGTAT TGATTATTAT   
  
  
- AGCCCAAACT CTATACCATT AGATAACGAT AAAGTATTTT ATTCTTAGCA TAAATGTGGT GCTAGGCAAA   
  
  
- TTATTAGGTG TTCAGAAACA CATATATCAA AATATAATTG AATTAAGCTG AATCACTTGT CTAGTTTTAG   
  
  
- CTAATAAAAA TATCGAGCAC CTTGAGCCTA GTCTTCTCTA CATAGGTGGA CACGATAAAG TTCTTCCCTT   
  
  
- TAAAACAGTA ATTATAATTC ACCTATTATT ACCTGTTTGA TTGGCCCATG AATCTGTAGT TAGGTAATTG   
  
  
- TAACTTAATC AGGAAAACAT GTATAATACC GCAATGTACA GGACATACTT CTTTTCTTTT GTAGTGGCAA   
  
  
- AATTAAATAA TAAGTTTATA TGAACATTAT GTGTCTCTAT AGTCTCTACT ATTAGTATTA GTACTAATTG   
  
  
- ATTAGTCTAG ACTACTTCGC GAACCATGGA TTGATCAACA CTCGATATGT TATTATAGTT GATCCCAAAA   
  
  
- GTATGGCTCG TTCACTTCCA GTTACCGCTC CAGTCACCAC ACTCACTAGC AGTGAGGCAC AATATGTTCG   
  
  
- CATAACTGGT CCTCGAATTA ATAACTGTGT CTTTGTTGTT TCGGACGTGG TAACTGTGTC TCTTTTTGGC   
  
  
- TGGCCGGTGG CCCCTCCGAT TTTCCAGTGG TCATGACCTA GAAACTGGGA ATCGGTACAC TGCAGCAGAG   
  
  
- ATATACTTCG AAGTTGCTAC CAATGGTATC TAATATGTTA TCCCAGCAGC GGCGTTTAAA TCGGAAAGAG   
  
  
- GAAGAAAGAG AGAGAGACGT TTGACTAGTA TAGAGTAGAC AAAGAATTGA CACTTTCCAA CTTCAACTTA   
  
  
- GAAAGGTATA ACAAATGAGA AAATTCCATT ATTTTATGAT TTTCCTTTTA CACCAACCCC ACAACTGTAA   
  
  
- CAGCCGGACA CATACTTCCA TGATAATATT CTTCTCTTCA CGCTCTCTCT GTATTGCCTA TGTCTTCGAT   
  
  
- TTTCTCGTTT CTTCTTATGG AAGATGTCG
