# Supplementary material for: Genome-wide analysis of the WRKY gene family in drumstick (Moringa oleifera Lam.)
Source: PeerJ. 2019 Jun 10;7:e7063. doi: 10.7717/peerj.7063 (PMC6563795; doi:10.7717/peerj.7063)
Supplement: Supplemental Information 1 [file peerj-07-7063-s003.gz › MoWRKY3_plantcare.html]

Content-Type: text/html; charset=ISO-8859-1


CallMat\_Firefox


Webmaster Firefox specific output  
To save the result:
click on the frame with the right mouse button and save the source code as a text file with extension .html  
REFERENCE:PlantCARE: a database of plant cis-acting regulatory elements and a portal to tools for in silico analysis of promoter sequences.  
Lescot, M., Déhais, P., Moreau, Y., De Moor, B., Rouzé ,P.,and Rombauts, S.  
Nucleic Acids Res., Database issue(2002), 30(1):325-327.   


---

> 2018/04/13 10:10:12  
+ CTAAAGTGGA TCGCTCTGTG CAGACTCTAC CATTTGCCTC TTTTCGTCGA GAACCTCGAA AACAACAAGC   
  
  
+ CCCAACAAGC AGATGTTTTT GAAGGCGAAG CGGGGTCACT AGTCAGCTAA CCTTGACTTT GGGTCTACTG   
  
  
+ TCTCTTTTGT TGTCTTTTAC CCTGAATTGT ACCGTAGGTT TCAACCAAAA CAGCCTCTCT GGGACCATCT   
  
  
+ TGTTTATGTT AAGGGTCACA AACCGAAAAG TCTTTCTTTC TCTATCTGTA CCTTCCTACG GCGTTGATTA   
  
  
+ ACAGGCCATA ACACTTTTAC GACTGTGCAT TAGTGTCTCT TTACTCAACC CGGGGTGTTG TGACCAGGTC   
  
  
+ CTAAAAGAAA AGAAAAGACA TGCTTAAAGA ATTAAACTTT TACTTACCCT AAAATTAATT ACATCCTATC   
  
  
+ TGTTAATTCG ACAAGGCTGT AAATACCGTG CACTGCTTTA CATCCGGTGT ACACAAAAAG AACAACTTTT   
  
  
+ ACCAAATGGG TGCACAACAT ATAGAAAACC GTTGGACTTA AGGCCTAAAA CAAGGTGTTT AGGGGTTAAA   
  
  
+ AATATGTGAC GAACCAAGTT TAAAAACGTT CACGGAGACG AATGTTATGT TACTGAATTA ACCTACTGTA   
  
  
+ ATAGAAGTTT TGATAAAAGA ATTTTTTAAT TATTAAAGTG GTCATTAGAC ATCCCCAATC TAGCAGAAAA   
  
  
+ TGAACGAACA GAACCGAGAA TGGACGTTAA GCTGTATCGA CAGGATGAAT GATGAAACGT ACCGCTAGAT   
  
  
+ AGTGGTGACG GTAACGGAGT TAGGCTTGCG TTTATTTATA TACTAAAACA AAGAAACCGT CGTTTTTATT   
  
  
+ TACTTGCAAC TTTTAAGTTT TGTTTATAAA TCATTAACCC AGTCAGTTCA AACTAAATTA CAATTTAAAA   
  
  
+ GAATTTATTA ATTTTTTATT CATTTATATC TTTTTTTAAT ACTTCATTCG AATGTAAAAT TAAAATTAAA   
  
  
+ TAAATAATTT AATGAACATA TAAATTGAAA ATTTGAATTA AAGTTAATAA AATTATTAAT ATTTTTAATT   
  
  
+ TTTATAAAAT TGCCAAATCT ATAGAAATTT AGATATAAAC AATGCATCTT TATTTAACTC TAATTTGTTT   
  
  
+ ACAATAGGAT TAGAATATAA ATTCACGTAA CTACTCTAAC TCTATTCAAA TAAAATACAA AAAAATTAAG   
  
  
+ TACTCTCAGG GGATTTAGTT CTATGATCTA GAACTAAAGT CAACATAATT TTAAAATTAT TTTTTATATA   
  
  
+ TAATATTAAA TTATTTTATT TAACAATTTT TATAAAGATG TAAAAAAAGA GTGTAATTAT ATGATTTGAC   
  
  
+ GTTTTATAAT AAAAATAGAA GAATAATTTT TTTGAATTGT GTTTTATAAT AAAAATAGAA GAATAGTTTT   
  
  
+ TTTGAATTGT AACACTTTAA AAATAGACTT AAAGTATCTC GAATTAGTTT TAACTTCGGA AACTAAAACT   
  
  
+ ATTATAATAA TATAAAATTT TTTAAATTA  

- GATTTCACCT AGCGAGACAC GTCTGAGATG GTAAACGGAG AAAAGCAGCT CTTGGAGCTT TTGTTGTTCG   
  
  
- GGGTTGTTCG TCTACAAAAA CTTCCGCTTC GCCCCAGTGA TCAGTCGATT GGAACTGAAA CCCAGATGAC   
  
  
- AGAGAAAACA ACAGAAAATG GGACTTAACA TGGCATCCAA AGTTGGTTTT GTCGGAGAGA CCCTGGTAGA   
  
  
- ACAAATACAA TTCCCAGTGT TTGGCTTTTC AGAAAGAAAG AGATAGACAT GGAAGGATGC CGCAACTAAT   
  
  
- TGTCCGGTAT TGTGAAAATG CTGACACGTA ATCACAGAGA AATGAGTTGG GCCCCACAAC ACTGGTCCAG   
  
  
- GATTTTCTTT TCTTTTCTGT ACGAATTTCT TAATTTGAAA ATGAATGGGA TTTTAATTAA TGTAGGATAG   
  
  
- ACAATTAAGC TGTTCCGACA TTTATGGCAC GTGACGAAAT GTAGGCCACA TGTGTTTTTC TTGTTGAAAA   
  
  
- TGGTTTACCC ACGTGTTGTA TATCTTTTGG CAACCTGAAT TCCGGATTTT GTTCCACAAA TCCCCAATTT   
  
  
- TTATACACTG CTTGGTTCAA ATTTTTGCAA GTGCCTCTGC TTACAATACA ATGACTTAAT TGGATGACAT   
  
  
- TATCTTCAAA ACTATTTTCT TAAAAAATTA ATAATTTCAC CAGTAATCTG TAGGGGTTAG ATCGTCTTTT   
  
  
- ACTTGCTTGT CTTGGCTCTT ACCTGCAATT CGACATAGCT GTCCTACTTA CTACTTTGCA TGGCGATCTA   
  
  
- TCACCACTGC CATTGCCTCA ATCCGAACGC AAATAAATAT ATGATTTTGT TTCTTTGGCA GCAAAAATAA   
  
  
- ATGAACGTTG AAAATTCAAA ACAAATATTT AGTAATTGGG TCAGTCAAGT TTGATTTAAT GTTAAATTTT   
  
  
- CTTAAATAAT TAAAAAATAA GTAAATATAG AAAAAAATTA TGAAGTAAGC TTACATTTTA ATTTTAATTT   
  
  
- ATTTATTAAA TTACTTGTAT ATTTAACTTT TAAACTTAAT TTCAATTATT TTAATAATTA TAAAAATTAA   
  
  
- AAATATTTTA ACGGTTTAGA TATCTTTAAA TCTATATTTG TTACGTAGAA ATAAATTGAG ATTAAACAAA   
  
  
- TGTTATCCTA ATCTTATATT TAAGTGCATT GATGAGATTG AGATAAGTTT ATTTTATGTT TTTTTAATTC   
  
  
- ATGAGAGTCC CCTAAATCAA GATACTAGAT CTTGATTTCA GTTGTATTAA AATTTTAATA AAAAATATAT   
  
  
- ATTATAATTT AATAAAATAA ATTGTTAAAA ATATTTCTAC ATTTTTTTCT CACATTAATA TACTAAACTG   
  
  
- CAAAATATTA TTTTTATCTT CTTATTAAAA AAACTTAACA CAAAATATTA TTTTTATCTT CTTATCAAAA   
  
  
- AAACTTAACA TTGTGAAATT TTTATCTGAA TTTCATAGAG CTTAATCAAA ATTGAAGCCT TTGATTTTGA   
  
  
- TAATATTATT ATATTTTAAA AAATTTAAT

  
  
Motifs Found  

+     3-AF3 binding site

| Site Name | Organism | Position | Strand | Matrix score. | sequence | function |
| --- | --- | --- | --- | --- | --- | --- |
| 3-AF3 binding site | Pisum sativum | 764 | - | 10 | CACTATCTAAC | part of a conserved DNA module array (CMA3) |

> 2018/04/13 10:10:12  
+ CTAAAGTGGA TCGCTCTGTG CAGACTCTAC CATTTGCCTC TTTTCGTCGA GAACCTCGAA AACAACAAGC   
  
  
+ CCCAACAAGC AGATGTTTTT GAAGGCGAAG CGGGGTCACT AGTCAGCTAA CCTTGACTTT GGGTCTACTG   
  
  
+ TCTCTTTTGT TGTCTTTTAC CCTGAATTGT ACCGTAGGTT TCAACCAAAA CAGCCTCTCT GGGACCATCT   
  
  
+ TGTTTATGTT AAGGGTCACA AACCGAAAAG TCTTTCTTTC TCTATCTGTA CCTTCCTACG GCGTTGATTA   
  
  
+ ACAGGCCATA ACACTTTTAC GACTGTGCAT TAGTGTCTCT TTACTCAACC CGGGGTGTTG TGACCAGGTC   
  
  
+ CTAAAAGAAA AGAAAAGACA TGCTTAAAGA ATTAAACTTT TACTTACCCT AAAATTAATT ACATCCTATC   
  
  
+ TGTTAATTCG ACAAGGCTGT AAATACCGTG CACTGCTTTA CATCCGGTGT ACACAAAAAG AACAACTTTT   
  
  
+ ACCAAATGGG TGCACAACAT ATAGAAAACC GTTGGACTTA AGGCCTAAAA CAAGGTGTTT AGGGGTTAAA   
  
  
+ AATATGTGAC GAACCAAGTT TAAAAACGTT CACGGAGACG AATGTTATGT TACTGAATTA ACCTACTGTA   
  
  
+ ATAGAAGTTT TGATAAAAGA ATTTTTTAAT TATTAAAGTG GTCATTAGAC ATCCCCAATC TAGCAGAAAA   
  
  
+ TGAACGAACA GAACCGAGAA TGGACGTTAA GCTGTATCGA CAGGATGAAT GATGAAACGT ACCGCTAGAT   
  
  
+ AGTGGTGACG GTAACGGAGT TAGGCTTGCG TTTATTTATA TACTAAAACA AAGAAACCGT CGTTTTTATT   
  
  
+ TACTTGCAAC TTTTAAGTTT TGTTTATAAA TCATTAACCC AGTCAGTTCA AACTAAATTA CAATTTAAAA   
  
  
+ GAATTTATTA ATTTTTTATT CATTTATATC TTTTTTTAAT ACTTCATTCG AATGTAAAAT TAAAATTAAA   
  
  
+ TAAATAATTT AATGAACATA TAAATTGAAA ATTTGAATTA AAGTTAATAA AATTATTAAT ATTTTTAATT   
  
  
+ TTTATAAAAT TGCCAAATCT ATAGAAATTT AGATATAAAC AATGCATCTT TATTTAACTC TAATTTGTTT   
  
  
+ ACAATAGGAT TAGAATATAA ATTCACGTAA CTACTCTAAC TCTATTCAAA TAAAATACAA AAAAATTAAG   
  
  
+ TACTCTCAGG GGATTTAGTT CTATGATCTA GAACTAAAGT CAACATAATT TTAAAATTAT TTTTTATATA   
  
  
+ TAATATTAAA TTATTTTATT TAACAATTTT TATAAAGATG TAAAAAAAGA GTGTAATTAT ATGATTTGAC   
  
  
+ GTTTTATAAT AAAAATAGAA GAATAATTTT TTTGAATTGT GTTTTATAAT AAAAATAGAA GAATAGTTTT   
  
  
+ TTTGAATTGT AACACTTTAA AAATAGACTT AAAGTATCTC GAATTAGTTT TAACTTCGGA AACTAAAACT   
  
  
+ ATTATAATAA TATAAAATTT TTTAAATTA  

- GATTTCACCT AGCGAGACAC GTCTGAGATG GTAAACGGAG AAAAGCAGCT CTTGGAGCTT TTGTTGTTCG   
  
  
- GGGTTGTTCG TCTACAAAAA CTTCCGCTTC GCCCCAGTGA TCAGTCGATT GGAACTGAAA CCCAGATGAC   
  
  
- AGAGAAAACA ACAGAAAATG GGACTTAACA TGGCATCCAA AGTTGGTTTT GTCGGAGAGA CCCTGGTAGA   
  
  
- ACAAATACAA TTCCCAGTGT TTGGCTTTTC AGAAAGAAAG AGATAGACAT GGAAGGATGC CGCAACTAAT   
  
  
- TGTCCGGTAT TGTGAAAATG CTGACACGTA ATCACAGAGA AATGAGTTGG GCCCCACAAC ACTGGTCCAG   
  
  
- GATTTTCTTT TCTTTTCTGT ACGAATTTCT TAATTTGAAA ATGAATGGGA TTTTAATTAA TGTAGGATAG   
  
  
- ACAATTAAGC TGTTCCGACA TTTATGGCAC GTGACGAAAT GTAGGCCACA TGTGTTTTTC TTGTTGAAAA   
  
  
- TGGTTTACCC ACGTGTTGTA TATCTTTTGG CAACCTGAAT TCCGGATTTT GTTCCACAAA TCCCCAATTT   
  
  
- TTATACACTG CTTGGTTCAA ATTTTTGCAA GTGCCTCTGC TTACAATACA ATGACTTAAT TGGATGACAT   
  
  
- TATCTTCAAA ACTATTTTCT TAAAAAATTA ATAATTTCAC CAGTAATCTG TAGGGGTTAG ATCGTCTTTT   
  
  
- ACTTGCTTGT CTTGGCTCTT ACCTGCAATT CGACATAGCT GTCCTACTTA CTACTTTGCA TGGCGATCTA   
  
  
- TCACCACTGC CATTGCCTCA ATCCGAACGC AAATAAATAT ATGATTTTGT TTCTTTGGCA GCAAAAATAA   
  
  
- ATGAACGTTG AAAATTCAAA ACAAATATTT AGTAATTGGG TCAGTCAAGT TTGATTTAAT GTTAAATTTT   
  
  
- CTTAAATAAT TAAAAAATAA GTAAATATAG AAAAAAATTA TGAAGTAAGC TTACATTTTA ATTTTAATTT   
  
  
- ATTTATTAAA TTACTTGTAT ATTTAACTTT TAAACTTAAT TTCAATTATT TTAATAATTA TAAAAATTAA   
  
  
- AAATATTTTA ACGGTTTAGA TATCTTTAAA TCTATATTTG TTACGTAGAA ATAAATTGAG ATTAAACAAA   
  
  
- TGTTATCCTA ATCTTATATT TAAGTGCATT GATGAGATTG AGATAAGTTT ATTTTATGTT TTTTTAATTC   
  
  
- ATGAGAGTCC CCTAAATCAA GATACTAGAT CTTGATTTCA GTTGTATTAA AATTTTAATA AAAAATATAT   
  
  
- ATTATAATTT AATAAAATAA ATTGTTAAAA ATATTTCTAC ATTTTTTTCT CACATTAATA TACTAAACTG   
  
  
- CAAAATATTA TTTTTATCTT CTTATTAAAA AAACTTAACA CAAAATATTA TTTTTATCTT CTTATCAAAA   
  
  
- AAACTTAACA TTGTGAAATT TTTATCTGAA TTTCATAGAG CTTAATCAAA ATTGAAGCCT TTGATTTTGA   
  
  
- TAATATTATT ATATTTTAAA AAATTTAAT

+     5UTR Py-rich stretch

| Site Name | Organism | Position | Strand | Matrix score. | sequence | function |
| --- | --- | --- | --- | --- | --- | --- |
| 5UTR Py-rich stretch | Lycopersicon esculentum | 356 | - | 9 | TTTCTTCTCT | cis-acting element conferring high transcription levels |

> 2018/04/13 10:10:12  
+ CTAAAGTGGA TCGCTCTGTG CAGACTCTAC CATTTGCCTC TTTTCGTCGA GAACCTCGAA AACAACAAGC   
  
  
+ CCCAACAAGC AGATGTTTTT GAAGGCGAAG CGGGGTCACT AGTCAGCTAA CCTTGACTTT GGGTCTACTG   
  
  
+ TCTCTTTTGT TGTCTTTTAC CCTGAATTGT ACCGTAGGTT TCAACCAAAA CAGCCTCTCT GGGACCATCT   
  
  
+ TGTTTATGTT AAGGGTCACA AACCGAAAAG TCTTTCTTTC TCTATCTGTA CCTTCCTACG GCGTTGATTA   
  
  
+ ACAGGCCATA ACACTTTTAC GACTGTGCAT TAGTGTCTCT TTACTCAACC CGGGGTGTTG TGACCAGGTC   
  
  
+ CTAAAAGAAA AGAAAAGACA TGCTTAAAGA ATTAAACTTT TACTTACCCT AAAATTAATT ACATCCTATC   
  
  
+ TGTTAATTCG ACAAGGCTGT AAATACCGTG CACTGCTTTA CATCCGGTGT ACACAAAAAG AACAACTTTT   
  
  
+ ACCAAATGGG TGCACAACAT ATAGAAAACC GTTGGACTTA AGGCCTAAAA CAAGGTGTTT AGGGGTTAAA   
  
  
+ AATATGTGAC GAACCAAGTT TAAAAACGTT CACGGAGACG AATGTTATGT TACTGAATTA ACCTACTGTA   
  
  
+ ATAGAAGTTT TGATAAAAGA ATTTTTTAAT TATTAAAGTG GTCATTAGAC ATCCCCAATC TAGCAGAAAA   
  
  
+ TGAACGAACA GAACCGAGAA TGGACGTTAA GCTGTATCGA CAGGATGAAT GATGAAACGT ACCGCTAGAT   
  
  
+ AGTGGTGACG GTAACGGAGT TAGGCTTGCG TTTATTTATA TACTAAAACA AAGAAACCGT CGTTTTTATT   
  
  
+ TACTTGCAAC TTTTAAGTTT TGTTTATAAA TCATTAACCC AGTCAGTTCA AACTAAATTA CAATTTAAAA   
  
  
+ GAATTTATTA ATTTTTTATT CATTTATATC TTTTTTTAAT ACTTCATTCG AATGTAAAAT TAAAATTAAA   
  
  
+ TAAATAATTT AATGAACATA TAAATTGAAA ATTTGAATTA AAGTTAATAA AATTATTAAT ATTTTTAATT   
  
  
+ TTTATAAAAT TGCCAAATCT ATAGAAATTT AGATATAAAC AATGCATCTT TATTTAACTC TAATTTGTTT   
  
  
+ ACAATAGGAT TAGAATATAA ATTCACGTAA CTACTCTAAC TCTATTCAAA TAAAATACAA AAAAATTAAG   
  
  
+ TACTCTCAGG GGATTTAGTT CTATGATCTA GAACTAAAGT CAACATAATT TTAAAATTAT TTTTTATATA   
  
  
+ TAATATTAAA TTATTTTATT TAACAATTTT TATAAAGATG TAAAAAAAGA GTGTAATTAT ATGATTTGAC   
  
  
+ GTTTTATAAT AAAAATAGAA GAATAATTTT TTTGAATTGT GTTTTATAAT AAAAATAGAA GAATAGTTTT   
  
  
+ TTTGAATTGT AACACTTTAA AAATAGACTT AAAGTATCTC GAATTAGTTT TAACTTCGGA AACTAAAACT   
  
  
+ ATTATAATAA TATAAAATTT TTTAAATTA  

- GATTTCACCT AGCGAGACAC GTCTGAGATG GTAAACGGAG AAAAGCAGCT CTTGGAGCTT TTGTTGTTCG   
  
  
- GGGTTGTTCG TCTACAAAAA CTTCCGCTTC GCCCCAGTGA TCAGTCGATT GGAACTGAAA CCCAGATGAC   
  
  
- AGAGAAAACA ACAGAAAATG GGACTTAACA TGGCATCCAA AGTTGGTTTT GTCGGAGAGA CCCTGGTAGA   
  
  
- ACAAATACAA TTCCCAGTGT TTGGCTTTTC AGAAAGAAAG AGATAGACAT GGAAGGATGC CGCAACTAAT   
  
  
- TGTCCGGTAT TGTGAAAATG CTGACACGTA ATCACAGAGA AATGAGTTGG GCCCCACAAC ACTGGTCCAG   
  
  
- GATTTTCTTT TCTTTTCTGT ACGAATTTCT TAATTTGAAA ATGAATGGGA TTTTAATTAA TGTAGGATAG   
  
  
- ACAATTAAGC TGTTCCGACA TTTATGGCAC GTGACGAAAT GTAGGCCACA TGTGTTTTTC TTGTTGAAAA   
  
  
- TGGTTTACCC ACGTGTTGTA TATCTTTTGG CAACCTGAAT TCCGGATTTT GTTCCACAAA TCCCCAATTT   
  
  
- TTATACACTG CTTGGTTCAA ATTTTTGCAA GTGCCTCTGC TTACAATACA ATGACTTAAT TGGATGACAT   
  
  
- TATCTTCAAA ACTATTTTCT TAAAAAATTA ATAATTTCAC CAGTAATCTG TAGGGGTTAG ATCGTCTTTT   
  
  
- ACTTGCTTGT CTTGGCTCTT ACCTGCAATT CGACATAGCT GTCCTACTTA CTACTTTGCA TGGCGATCTA   
  
  
- TCACCACTGC CATTGCCTCA ATCCGAACGC AAATAAATAT ATGATTTTGT TTCTTTGGCA GCAAAAATAA   
  
  
- ATGAACGTTG AAAATTCAAA ACAAATATTT AGTAATTGGG TCAGTCAAGT TTGATTTAAT GTTAAATTTT   
  
  
- CTTAAATAAT TAAAAAATAA GTAAATATAG AAAAAAATTA TGAAGTAAGC TTACATTTTA ATTTTAATTT   
  
  
- ATTTATTAAA TTACTTGTAT ATTTAACTTT TAAACTTAAT TTCAATTATT TTAATAATTA TAAAAATTAA   
  
  
- AAATATTTTA ACGGTTTAGA TATCTTTAAA TCTATATTTG TTACGTAGAA ATAAATTGAG ATTAAACAAA   
  
  
- TGTTATCCTA ATCTTATATT TAAGTGCATT GATGAGATTG AGATAAGTTT ATTTTATGTT TTTTTAATTC   
  
  
- ATGAGAGTCC CCTAAATCAA GATACTAGAT CTTGATTTCA GTTGTATTAA AATTTTAATA AAAAATATAT   
  
  
- ATTATAATTT AATAAAATAA ATTGTTAAAA ATATTTCTAC ATTTTTTTCT CACATTAATA TACTAAACTG   
  
  
- CAAAATATTA TTTTTATCTT CTTATTAAAA AAACTTAACA CAAAATATTA TTTTTATCTT CTTATCAAAA   
  
  
- AAACTTAACA TTGTGAAATT TTTATCTGAA TTTCATAGAG CTTAATCAAA ATTGAAGCCT TTGATTTTGA   
  
  
- TAATATTATT ATATTTTAAA AAATTTAAT

+     AAGAA-motif

| Site Name | Organism | Position | Strand | Matrix score. | sequence | function |
| --- | --- | --- | --- | --- | --- | --- |
| AAGAA-motif | Avena sativa | 244 | - | 7 | GAAAGAA |  |

> 2018/04/13 10:10:12  
+ CTAAAGTGGA TCGCTCTGTG CAGACTCTAC CATTTGCCTC TTTTCGTCGA GAACCTCGAA AACAACAAGC   
  
  
+ CCCAACAAGC AGATGTTTTT GAAGGCGAAG CGGGGTCACT AGTCAGCTAA CCTTGACTTT GGGTCTACTG   
  
  
+ TCTCTTTTGT TGTCTTTTAC CCTGAATTGT ACCGTAGGTT TCAACCAAAA CAGCCTCTCT GGGACCATCT   
  
  
+ TGTTTATGTT AAGGGTCACA AACCGAAAAG TCTTTCTTTC TCTATCTGTA CCTTCCTACG GCGTTGATTA   
  
  
+ ACAGGCCATA ACACTTTTAC GACTGTGCAT TAGTGTCTCT TTACTCAACC CGGGGTGTTG TGACCAGGTC   
  
  
+ CTAAAAGAAA AGAAAAGACA TGCTTAAAGA ATTAAACTTT TACTTACCCT AAAATTAATT ACATCCTATC   
  
  
+ TGTTAATTCG ACAAGGCTGT AAATACCGTG CACTGCTTTA CATCCGGTGT ACACAAAAAG AACAACTTTT   
  
  
+ ACCAAATGGG TGCACAACAT ATAGAAAACC GTTGGACTTA AGGCCTAAAA CAAGGTGTTT AGGGGTTAAA   
  
  
+ AATATGTGAC GAACCAAGTT TAAAAACGTT CACGGAGACG AATGTTATGT TACTGAATTA ACCTACTGTA   
  
  
+ ATAGAAGTTT TGATAAAAGA ATTTTTTAAT TATTAAAGTG GTCATTAGAC ATCCCCAATC TAGCAGAAAA   
  
  
+ TGAACGAACA GAACCGAGAA TGGACGTTAA GCTGTATCGA CAGGATGAAT GATGAAACGT ACCGCTAGAT   
  
  
+ AGTGGTGACG GTAACGGAGT TAGGCTTGCG TTTATTTATA TACTAAAACA AAGAAACCGT CGTTTTTATT   
  
  
+ TACTTGCAAC TTTTAAGTTT TGTTTATAAA TCATTAACCC AGTCAGTTCA AACTAAATTA CAATTTAAAA   
  
  
+ GAATTTATTA ATTTTTTATT CATTTATATC TTTTTTTAAT ACTTCATTCG AATGTAAAAT TAAAATTAAA   
  
  
+ TAAATAATTT AATGAACATA TAAATTGAAA ATTTGAATTA AAGTTAATAA AATTATTAAT ATTTTTAATT   
  
  
+ TTTATAAAAT TGCCAAATCT ATAGAAATTT AGATATAAAC AATGCATCTT TATTTAACTC TAATTTGTTT   
  
  
+ ACAATAGGAT TAGAATATAA ATTCACGTAA CTACTCTAAC TCTATTCAAA TAAAATACAA AAAAATTAAG   
  
  
+ TACTCTCAGG GGATTTAGTT CTATGATCTA GAACTAAAGT CAACATAATT TTAAAATTAT TTTTTATATA   
  
  
+ TAATATTAAA TTATTTTATT TAACAATTTT TATAAAGATG TAAAAAAAGA GTGTAATTAT ATGATTTGAC   
  
  
+ GTTTTATAAT AAAAATAGAA GAATAATTTT TTTGAATTGT GTTTTATAAT AAAAATAGAA GAATAGTTTT   
  
  
+ TTTGAATTGT AACACTTTAA AAATAGACTT AAAGTATCTC GAATTAGTTT TAACTTCGGA AACTAAAACT   
  
  
+ ATTATAATAA TATAAAATTT TTTAAATTA  

- GATTTCACCT AGCGAGACAC GTCTGAGATG GTAAACGGAG AAAAGCAGCT CTTGGAGCTT TTGTTGTTCG   
  
  
- GGGTTGTTCG TCTACAAAAA CTTCCGCTTC GCCCCAGTGA TCAGTCGATT GGAACTGAAA CCCAGATGAC   
  
  
- AGAGAAAACA ACAGAAAATG GGACTTAACA TGGCATCCAA AGTTGGTTTT GTCGGAGAGA CCCTGGTAGA   
  
  
- ACAAATACAA TTCCCAGTGT TTGGCTTTTC AGAAAGAAAG AGATAGACAT GGAAGGATGC CGCAACTAAT   
  
  
- TGTCCGGTAT TGTGAAAATG CTGACACGTA ATCACAGAGA AATGAGTTGG GCCCCACAAC ACTGGTCCAG   
  
  
- GATTTTCTTT TCTTTTCTGT ACGAATTTCT TAATTTGAAA ATGAATGGGA TTTTAATTAA TGTAGGATAG   
  
  
- ACAATTAAGC TGTTCCGACA TTTATGGCAC GTGACGAAAT GTAGGCCACA TGTGTTTTTC TTGTTGAAAA   
  
  
- TGGTTTACCC ACGTGTTGTA TATCTTTTGG CAACCTGAAT TCCGGATTTT GTTCCACAAA TCCCCAATTT   
  
  
- TTATACACTG CTTGGTTCAA ATTTTTGCAA GTGCCTCTGC TTACAATACA ATGACTTAAT TGGATGACAT   
  
  
- TATCTTCAAA ACTATTTTCT TAAAAAATTA ATAATTTCAC CAGTAATCTG TAGGGGTTAG ATCGTCTTTT   
  
  
- ACTTGCTTGT CTTGGCTCTT ACCTGCAATT CGACATAGCT GTCCTACTTA CTACTTTGCA TGGCGATCTA   
  
  
- TCACCACTGC CATTGCCTCA ATCCGAACGC AAATAAATAT ATGATTTTGT TTCTTTGGCA GCAAAAATAA   
  
  
- ATGAACGTTG AAAATTCAAA ACAAATATTT AGTAATTGGG TCAGTCAAGT TTGATTTAAT GTTAAATTTT   
  
  
- CTTAAATAAT TAAAAAATAA GTAAATATAG AAAAAAATTA TGAAGTAAGC TTACATTTTA ATTTTAATTT   
  
  
- ATTTATTAAA TTACTTGTAT ATTTAACTTT TAAACTTAAT TTCAATTATT TTAATAATTA TAAAAATTAA   
  
  
- AAATATTTTA ACGGTTTAGA TATCTTTAAA TCTATATTTG TTACGTAGAA ATAAATTGAG ATTAAACAAA   
  
  
- TGTTATCCTA ATCTTATATT TAAGTGCATT GATGAGATTG AGATAAGTTT ATTTTATGTT TTTTTAATTC   
  
  
- ATGAGAGTCC CCTAAATCAA GATACTAGAT CTTGATTTCA GTTGTATTAA AATTTTAATA AAAAATATAT   
  
  
- ATTATAATTT AATAAAATAA ATTGTTAAAA ATATTTCTAC ATTTTTTTCT CACATTAATA TACTAAACTG   
  
  
- CAAAATATTA TTTTTATCTT CTTATTAAAA AAACTTAACA CAAAATATTA TTTTTATCTT CTTATCAAAA   
  
  
- AAACTTAACA TTGTGAAATT TTTATCTGAA TTTCATAGAG CTTAATCAAA ATTGAAGCCT TTGATTTTGA   
  
  
- TAATATTATT ATATTTTAAA AAATTTAAT

+     ABRE

| Site Name | Organism | Position | Strand | Matrix score. | sequence | function |
| --- | --- | --- | --- | --- | --- | --- |
| ABRE | Arabidopsis thaliana | 1144 | - | 6 | TACGTG | cis-acting element involved in the abscisic acid responsiveness |

> 2018/04/13 10:10:12  
+ CTAAAGTGGA TCGCTCTGTG CAGACTCTAC CATTTGCCTC TTTTCGTCGA GAACCTCGAA AACAACAAGC   
  
  
+ CCCAACAAGC AGATGTTTTT GAAGGCGAAG CGGGGTCACT AGTCAGCTAA CCTTGACTTT GGGTCTACTG   
  
  
+ TCTCTTTTGT TGTCTTTTAC CCTGAATTGT ACCGTAGGTT TCAACCAAAA CAGCCTCTCT GGGACCATCT   
  
  
+ TGTTTATGTT AAGGGTCACA AACCGAAAAG TCTTTCTTTC TCTATCTGTA CCTTCCTACG GCGTTGATTA   
  
  
+ ACAGGCCATA ACACTTTTAC GACTGTGCAT TAGTGTCTCT TTACTCAACC CGGGGTGTTG TGACCAGGTC   
  
  
+ CTAAAAGAAA AGAAAAGACA TGCTTAAAGA ATTAAACTTT TACTTACCCT AAAATTAATT ACATCCTATC   
  
  
+ TGTTAATTCG ACAAGGCTGT AAATACCGTG CACTGCTTTA CATCCGGTGT ACACAAAAAG AACAACTTTT   
  
  
+ ACCAAATGGG TGCACAACAT ATAGAAAACC GTTGGACTTA AGGCCTAAAA CAAGGTGTTT AGGGGTTAAA   
  
  
+ AATATGTGAC GAACCAAGTT TAAAAACGTT CACGGAGACG AATGTTATGT TACTGAATTA ACCTACTGTA   
  
  
+ ATAGAAGTTT TGATAAAAGA ATTTTTTAAT TATTAAAGTG GTCATTAGAC ATCCCCAATC TAGCAGAAAA   
  
  
+ TGAACGAACA GAACCGAGAA TGGACGTTAA GCTGTATCGA CAGGATGAAT GATGAAACGT ACCGCTAGAT   
  
  
+ AGTGGTGACG GTAACGGAGT TAGGCTTGCG TTTATTTATA TACTAAAACA AAGAAACCGT CGTTTTTATT   
  
  
+ TACTTGCAAC TTTTAAGTTT TGTTTATAAA TCATTAACCC AGTCAGTTCA AACTAAATTA CAATTTAAAA   
  
  
+ GAATTTATTA ATTTTTTATT CATTTATATC TTTTTTTAAT ACTTCATTCG AATGTAAAAT TAAAATTAAA   
  
  
+ TAAATAATTT AATGAACATA TAAATTGAAA ATTTGAATTA AAGTTAATAA AATTATTAAT ATTTTTAATT   
  
  
+ TTTATAAAAT TGCCAAATCT ATAGAAATTT AGATATAAAC AATGCATCTT TATTTAACTC TAATTTGTTT   
  
  
+ ACAATAGGAT TAGAATATAA ATTCACGTAA CTACTCTAAC TCTATTCAAA TAAAATACAA AAAAATTAAG   
  
  
+ TACTCTCAGG GGATTTAGTT CTATGATCTA GAACTAAAGT CAACATAATT TTAAAATTAT TTTTTATATA   
  
  
+ TAATATTAAA TTATTTTATT TAACAATTTT TATAAAGATG TAAAAAAAGA GTGTAATTAT ATGATTTGAC   
  
  
+ GTTTTATAAT AAAAATAGAA GAATAATTTT TTTGAATTGT GTTTTATAAT AAAAATAGAA GAATAGTTTT   
  
  
+ TTTGAATTGT AACACTTTAA AAATAGACTT AAAGTATCTC GAATTAGTTT TAACTTCGGA AACTAAAACT   
  
  
+ ATTATAATAA TATAAAATTT TTTAAATTA  

- GATTTCACCT AGCGAGACAC GTCTGAGATG GTAAACGGAG AAAAGCAGCT CTTGGAGCTT TTGTTGTTCG   
  
  
- GGGTTGTTCG TCTACAAAAA CTTCCGCTTC GCCCCAGTGA TCAGTCGATT GGAACTGAAA CCCAGATGAC   
  
  
- AGAGAAAACA ACAGAAAATG GGACTTAACA TGGCATCCAA AGTTGGTTTT GTCGGAGAGA CCCTGGTAGA   
  
  
- ACAAATACAA TTCCCAGTGT TTGGCTTTTC AGAAAGAAAG AGATAGACAT GGAAGGATGC CGCAACTAAT   
  
  
- TGTCCGGTAT TGTGAAAATG CTGACACGTA ATCACAGAGA AATGAGTTGG GCCCCACAAC ACTGGTCCAG   
  
  
- GATTTTCTTT TCTTTTCTGT ACGAATTTCT TAATTTGAAA ATGAATGGGA TTTTAATTAA TGTAGGATAG   
  
  
- ACAATTAAGC TGTTCCGACA TTTATGGCAC GTGACGAAAT GTAGGCCACA TGTGTTTTTC TTGTTGAAAA   
  
  
- TGGTTTACCC ACGTGTTGTA TATCTTTTGG CAACCTGAAT TCCGGATTTT GTTCCACAAA TCCCCAATTT   
  
  
- TTATACACTG CTTGGTTCAA ATTTTTGCAA GTGCCTCTGC TTACAATACA ATGACTTAAT TGGATGACAT   
  
  
- TATCTTCAAA ACTATTTTCT TAAAAAATTA ATAATTTCAC CAGTAATCTG TAGGGGTTAG ATCGTCTTTT   
  
  
- ACTTGCTTGT CTTGGCTCTT ACCTGCAATT CGACATAGCT GTCCTACTTA CTACTTTGCA TGGCGATCTA   
  
  
- TCACCACTGC CATTGCCTCA ATCCGAACGC AAATAAATAT ATGATTTTGT TTCTTTGGCA GCAAAAATAA   
  
  
- ATGAACGTTG AAAATTCAAA ACAAATATTT AGTAATTGGG TCAGTCAAGT TTGATTTAAT GTTAAATTTT   
  
  
- CTTAAATAAT TAAAAAATAA GTAAATATAG AAAAAAATTA TGAAGTAAGC TTACATTTTA ATTTTAATTT   
  
  
- ATTTATTAAA TTACTTGTAT ATTTAACTTT TAAACTTAAT TTCAATTATT TTAATAATTA TAAAAATTAA   
  
  
- AAATATTTTA ACGGTTTAGA TATCTTTAAA TCTATATTTG TTACGTAGAA ATAAATTGAG ATTAAACAAA   
  
  
- TGTTATCCTA ATCTTATATT TAAGTGCATT GATGAGATTG AGATAAGTTT ATTTTATGTT TTTTTAATTC   
  
  
- ATGAGAGTCC CCTAAATCAA GATACTAGAT CTTGATTTCA GTTGTATTAA AATTTTAATA AAAAATATAT   
  
  
- ATTATAATTT AATAAAATAA ATTGTTAAAA ATATTTCTAC ATTTTTTTCT CACATTAATA TACTAAACTG   
  
  
- CAAAATATTA TTTTTATCTT CTTATTAAAA AAACTTAACA CAAAATATTA TTTTTATCTT CTTATCAAAA   
  
  
- AAACTTAACA TTGTGAAATT TTTATCTGAA TTTCATAGAG CTTAATCAAA ATTGAAGCCT TTGATTTTGA   
  
  
- TAATATTATT ATATTTTAAA AAATTTAAT

+     ACE

| Site Name | Organism | Position | Strand | Matrix score. | sequence | function |
| --- | --- | --- | --- | --- | --- | --- |
| ACE | Petroselinum crispum | 583 | + | 9 | AAAACGTTTA | cis-acting element involved in light responsiveness |

> 2018/04/13 10:10:12  
+ CTAAAGTGGA TCGCTCTGTG CAGACTCTAC CATTTGCCTC TTTTCGTCGA GAACCTCGAA AACAACAAGC   
  
  
+ CCCAACAAGC AGATGTTTTT GAAGGCGAAG CGGGGTCACT AGTCAGCTAA CCTTGACTTT GGGTCTACTG   
  
  
+ TCTCTTTTGT TGTCTTTTAC CCTGAATTGT ACCGTAGGTT TCAACCAAAA CAGCCTCTCT GGGACCATCT   
  
  
+ TGTTTATGTT AAGGGTCACA AACCGAAAAG TCTTTCTTTC TCTATCTGTA CCTTCCTACG GCGTTGATTA   
  
  
+ ACAGGCCATA ACACTTTTAC GACTGTGCAT TAGTGTCTCT TTACTCAACC CGGGGTGTTG TGACCAGGTC   
  
  
+ CTAAAAGAAA AGAAAAGACA TGCTTAAAGA ATTAAACTTT TACTTACCCT AAAATTAATT ACATCCTATC   
  
  
+ TGTTAATTCG ACAAGGCTGT AAATACCGTG CACTGCTTTA CATCCGGTGT ACACAAAAAG AACAACTTTT   
  
  
+ ACCAAATGGG TGCACAACAT ATAGAAAACC GTTGGACTTA AGGCCTAAAA CAAGGTGTTT AGGGGTTAAA   
  
  
+ AATATGTGAC GAACCAAGTT TAAAAACGTT CACGGAGACG AATGTTATGT TACTGAATTA ACCTACTGTA   
  
  
+ ATAGAAGTTT TGATAAAAGA ATTTTTTAAT TATTAAAGTG GTCATTAGAC ATCCCCAATC TAGCAGAAAA   
  
  
+ TGAACGAACA GAACCGAGAA TGGACGTTAA GCTGTATCGA CAGGATGAAT GATGAAACGT ACCGCTAGAT   
  
  
+ AGTGGTGACG GTAACGGAGT TAGGCTTGCG TTTATTTATA TACTAAAACA AAGAAACCGT CGTTTTTATT   
  
  
+ TACTTGCAAC TTTTAAGTTT TGTTTATAAA TCATTAACCC AGTCAGTTCA AACTAAATTA CAATTTAAAA   
  
  
+ GAATTTATTA ATTTTTTATT CATTTATATC TTTTTTTAAT ACTTCATTCG AATGTAAAAT TAAAATTAAA   
  
  
+ TAAATAATTT AATGAACATA TAAATTGAAA ATTTGAATTA AAGTTAATAA AATTATTAAT ATTTTTAATT   
  
  
+ TTTATAAAAT TGCCAAATCT ATAGAAATTT AGATATAAAC AATGCATCTT TATTTAACTC TAATTTGTTT   
  
  
+ ACAATAGGAT TAGAATATAA ATTCACGTAA CTACTCTAAC TCTATTCAAA TAAAATACAA AAAAATTAAG   
  
  
+ TACTCTCAGG GGATTTAGTT CTATGATCTA GAACTAAAGT CAACATAATT TTAAAATTAT TTTTTATATA   
  
  
+ TAATATTAAA TTATTTTATT TAACAATTTT TATAAAGATG TAAAAAAAGA GTGTAATTAT ATGATTTGAC   
  
  
+ GTTTTATAAT AAAAATAGAA GAATAATTTT TTTGAATTGT GTTTTATAAT AAAAATAGAA GAATAGTTTT   
  
  
+ TTTGAATTGT AACACTTTAA AAATAGACTT AAAGTATCTC GAATTAGTTT TAACTTCGGA AACTAAAACT   
  
  
+ ATTATAATAA TATAAAATTT TTTAAATTA  

- GATTTCACCT AGCGAGACAC GTCTGAGATG GTAAACGGAG AAAAGCAGCT CTTGGAGCTT TTGTTGTTCG   
  
  
- GGGTTGTTCG TCTACAAAAA CTTCCGCTTC GCCCCAGTGA TCAGTCGATT GGAACTGAAA CCCAGATGAC   
  
  
- AGAGAAAACA ACAGAAAATG GGACTTAACA TGGCATCCAA AGTTGGTTTT GTCGGAGAGA CCCTGGTAGA   
  
  
- ACAAATACAA TTCCCAGTGT TTGGCTTTTC AGAAAGAAAG AGATAGACAT GGAAGGATGC CGCAACTAAT   
  
  
- TGTCCGGTAT TGTGAAAATG CTGACACGTA ATCACAGAGA AATGAGTTGG GCCCCACAAC ACTGGTCCAG   
  
  
- GATTTTCTTT TCTTTTCTGT ACGAATTTCT TAATTTGAAA ATGAATGGGA TTTTAATTAA TGTAGGATAG   
  
  
- ACAATTAAGC TGTTCCGACA TTTATGGCAC GTGACGAAAT GTAGGCCACA TGTGTTTTTC TTGTTGAAAA   
  
  
- TGGTTTACCC ACGTGTTGTA TATCTTTTGG CAACCTGAAT TCCGGATTTT GTTCCACAAA TCCCCAATTT   
  
  
- TTATACACTG CTTGGTTCAA ATTTTTGCAA GTGCCTCTGC TTACAATACA ATGACTTAAT TGGATGACAT   
  
  
- TATCTTCAAA ACTATTTTCT TAAAAAATTA ATAATTTCAC CAGTAATCTG TAGGGGTTAG ATCGTCTTTT   
  
  
- ACTTGCTTGT CTTGGCTCTT ACCTGCAATT CGACATAGCT GTCCTACTTA CTACTTTGCA TGGCGATCTA   
  
  
- TCACCACTGC CATTGCCTCA ATCCGAACGC AAATAAATAT ATGATTTTGT TTCTTTGGCA GCAAAAATAA   
  
  
- ATGAACGTTG AAAATTCAAA ACAAATATTT AGTAATTGGG TCAGTCAAGT TTGATTTAAT GTTAAATTTT   
  
  
- CTTAAATAAT TAAAAAATAA GTAAATATAG AAAAAAATTA TGAAGTAAGC TTACATTTTA ATTTTAATTT   
  
  
- ATTTATTAAA TTACTTGTAT ATTTAACTTT TAAACTTAAT TTCAATTATT TTAATAATTA TAAAAATTAA   
  
  
- AAATATTTTA ACGGTTTAGA TATCTTTAAA TCTATATTTG TTACGTAGAA ATAAATTGAG ATTAAACAAA   
  
  
- TGTTATCCTA ATCTTATATT TAAGTGCATT GATGAGATTG AGATAAGTTT ATTTTATGTT TTTTTAATTC   
  
  
- ATGAGAGTCC CCTAAATCAA GATACTAGAT CTTGATTTCA GTTGTATTAA AATTTTAATA AAAAATATAT   
  
  
- ATTATAATTT AATAAAATAA ATTGTTAAAA ATATTTCTAC ATTTTTTTCT CACATTAATA TACTAAACTG   
  
  
- CAAAATATTA TTTTTATCTT CTTATTAAAA AAACTTAACA CAAAATATTA TTTTTATCTT CTTATCAAAA   
  
  
- AAACTTAACA TTGTGAAATT TTTATCTGAA TTTCATAGAG CTTAATCAAA ATTGAAGCCT TTGATTTTGA   
  
  
- TAATATTATT ATATTTTAAA AAATTTAAT

+     AT1-motif

| Site Name | Organism | Position | Strand | Matrix score. | sequence | function |
| --- | --- | --- | --- | --- | --- | --- |
| AT1-motif | Solanum tuberosum | 963 | - | 11 | ATTAATTTTACA | part of a light responsive module |
| AT1-motif | Solanum tuberosum | 1245 | + | 13 | AATTATTTTTTATT | part of a light responsive module |

> 2018/04/13 10:10:12  
+ CTAAAGTGGA TCGCTCTGTG CAGACTCTAC CATTTGCCTC TTTTCGTCGA GAACCTCGAA AACAACAAGC   
  
  
+ CCCAACAAGC AGATGTTTTT GAAGGCGAAG CGGGGTCACT AGTCAGCTAA CCTTGACTTT GGGTCTACTG   
  
  
+ TCTCTTTTGT TGTCTTTTAC CCTGAATTGT ACCGTAGGTT TCAACCAAAA CAGCCTCTCT GGGACCATCT   
  
  
+ TGTTTATGTT AAGGGTCACA AACCGAAAAG TCTTTCTTTC TCTATCTGTA CCTTCCTACG GCGTTGATTA   
  
  
+ ACAGGCCATA ACACTTTTAC GACTGTGCAT TAGTGTCTCT TTACTCAACC CGGGGTGTTG TGACCAGGTC   
  
  
+ CTAAAAGAAA AGAAAAGACA TGCTTAAAGA ATTAAACTTT TACTTACCCT AAAATTAATT ACATCCTATC   
  
  
+ TGTTAATTCG ACAAGGCTGT AAATACCGTG CACTGCTTTA CATCCGGTGT ACACAAAAAG AACAACTTTT   
  
  
+ ACCAAATGGG TGCACAACAT ATAGAAAACC GTTGGACTTA AGGCCTAAAA CAAGGTGTTT AGGGGTTAAA   
  
  
+ AATATGTGAC GAACCAAGTT TAAAAACGTT CACGGAGACG AATGTTATGT TACTGAATTA ACCTACTGTA   
  
  
+ ATAGAAGTTT TGATAAAAGA ATTTTTTAAT TATTAAAGTG GTCATTAGAC ATCCCCAATC TAGCAGAAAA   
  
  
+ TGAACGAACA GAACCGAGAA TGGACGTTAA GCTGTATCGA CAGGATGAAT GATGAAACGT ACCGCTAGAT   
  
  
+ AGTGGTGACG GTAACGGAGT TAGGCTTGCG TTTATTTATA TACTAAAACA AAGAAACCGT CGTTTTTATT   
  
  
+ TACTTGCAAC TTTTAAGTTT TGTTTATAAA TCATTAACCC AGTCAGTTCA AACTAAATTA CAATTTAAAA   
  
  
+ GAATTTATTA ATTTTTTATT CATTTATATC TTTTTTTAAT ACTTCATTCG AATGTAAAAT TAAAATTAAA   
  
  
+ TAAATAATTT AATGAACATA TAAATTGAAA ATTTGAATTA AAGTTAATAA AATTATTAAT ATTTTTAATT   
  
  
+ TTTATAAAAT TGCCAAATCT ATAGAAATTT AGATATAAAC AATGCATCTT TATTTAACTC TAATTTGTTT   
  
  
+ ACAATAGGAT TAGAATATAA ATTCACGTAA CTACTCTAAC TCTATTCAAA TAAAATACAA AAAAATTAAG   
  
  
+ TACTCTCAGG GGATTTAGTT CTATGATCTA GAACTAAAGT CAACATAATT TTAAAATTAT TTTTTATATA   
  
  
+ TAATATTAAA TTATTTTATT TAACAATTTT TATAAAGATG TAAAAAAAGA GTGTAATTAT ATGATTTGAC   
  
  
+ GTTTTATAAT AAAAATAGAA GAATAATTTT TTTGAATTGT GTTTTATAAT AAAAATAGAA GAATAGTTTT   
  
  
+ TTTGAATTGT AACACTTTAA AAATAGACTT AAAGTATCTC GAATTAGTTT TAACTTCGGA AACTAAAACT   
  
  
+ ATTATAATAA TATAAAATTT TTTAAATTA  

- GATTTCACCT AGCGAGACAC GTCTGAGATG GTAAACGGAG AAAAGCAGCT CTTGGAGCTT TTGTTGTTCG   
  
  
- GGGTTGTTCG TCTACAAAAA CTTCCGCTTC GCCCCAGTGA TCAGTCGATT GGAACTGAAA CCCAGATGAC   
  
  
- AGAGAAAACA ACAGAAAATG GGACTTAACA TGGCATCCAA AGTTGGTTTT GTCGGAGAGA CCCTGGTAGA   
  
  
- ACAAATACAA TTCCCAGTGT TTGGCTTTTC AGAAAGAAAG AGATAGACAT GGAAGGATGC CGCAACTAAT   
  
  
- TGTCCGGTAT TGTGAAAATG CTGACACGTA ATCACAGAGA AATGAGTTGG GCCCCACAAC ACTGGTCCAG   
  
  
- GATTTTCTTT TCTTTTCTGT ACGAATTTCT TAATTTGAAA ATGAATGGGA TTTTAATTAA TGTAGGATAG   
  
  
- ACAATTAAGC TGTTCCGACA TTTATGGCAC GTGACGAAAT GTAGGCCACA TGTGTTTTTC TTGTTGAAAA   
  
  
- TGGTTTACCC ACGTGTTGTA TATCTTTTGG CAACCTGAAT TCCGGATTTT GTTCCACAAA TCCCCAATTT   
  
  
- TTATACACTG CTTGGTTCAA ATTTTTGCAA GTGCCTCTGC TTACAATACA ATGACTTAAT TGGATGACAT   
  
  
- TATCTTCAAA ACTATTTTCT TAAAAAATTA ATAATTTCAC CAGTAATCTG TAGGGGTTAG ATCGTCTTTT   
  
  
- ACTTGCTTGT CTTGGCTCTT ACCTGCAATT CGACATAGCT GTCCTACTTA CTACTTTGCA TGGCGATCTA   
  
  
- TCACCACTGC CATTGCCTCA ATCCGAACGC AAATAAATAT ATGATTTTGT TTCTTTGGCA GCAAAAATAA   
  
  
- ATGAACGTTG AAAATTCAAA ACAAATATTT AGTAATTGGG TCAGTCAAGT TTGATTTAAT GTTAAATTTT   
  
  
- CTTAAATAAT TAAAAAATAA GTAAATATAG AAAAAAATTA TGAAGTAAGC TTACATTTTA ATTTTAATTT   
  
  
- ATTTATTAAA TTACTTGTAT ATTTAACTTT TAAACTTAAT TTCAATTATT TTAATAATTA TAAAAATTAA   
  
  
- AAATATTTTA ACGGTTTAGA TATCTTTAAA TCTATATTTG TTACGTAGAA ATAAATTGAG ATTAAACAAA   
  
  
- TGTTATCCTA ATCTTATATT TAAGTGCATT GATGAGATTG AGATAAGTTT ATTTTATGTT TTTTTAATTC   
  
  
- ATGAGAGTCC CCTAAATCAA GATACTAGAT CTTGATTTCA GTTGTATTAA AATTTTAATA AAAAATATAT   
  
  
- ATTATAATTT AATAAAATAA ATTGTTAAAA ATATTTCTAC ATTTTTTTCT CACATTAATA TACTAAACTG   
  
  
- CAAAATATTA TTTTTATCTT CTTATTAAAA AAACTTAACA CAAAATATTA TTTTTATCTT CTTATCAAAA   
  
  
- AAACTTAACA TTGTGAAATT TTTATCTGAA TTTCATAGAG CTTAATCAAA ATTGAAGCCT TTGATTTTGA   
  
  
- TAATATTATT ATATTTTAAA AAATTTAAT

+     ATCT-motif

| Site Name | Organism | Position | Strand | Matrix score. | sequence | function |
| --- | --- | --- | --- | --- | --- | --- |
| ATCT-motif | Pisum sativum | 1127 | - | 9 | AATCTAATCC | part of a conserved DNA module involved in light responsiveness |

> 2018/04/13 10:10:12  
+ CTAAAGTGGA TCGCTCTGTG CAGACTCTAC CATTTGCCTC TTTTCGTCGA GAACCTCGAA AACAACAAGC   
  
  
+ CCCAACAAGC AGATGTTTTT GAAGGCGAAG CGGGGTCACT AGTCAGCTAA CCTTGACTTT GGGTCTACTG   
  
  
+ TCTCTTTTGT TGTCTTTTAC CCTGAATTGT ACCGTAGGTT TCAACCAAAA CAGCCTCTCT GGGACCATCT   
  
  
+ TGTTTATGTT AAGGGTCACA AACCGAAAAG TCTTTCTTTC TCTATCTGTA CCTTCCTACG GCGTTGATTA   
  
  
+ ACAGGCCATA ACACTTTTAC GACTGTGCAT TAGTGTCTCT TTACTCAACC CGGGGTGTTG TGACCAGGTC   
  
  
+ CTAAAAGAAA AGAAAAGACA TGCTTAAAGA ATTAAACTTT TACTTACCCT AAAATTAATT ACATCCTATC   
  
  
+ TGTTAATTCG ACAAGGCTGT AAATACCGTG CACTGCTTTA CATCCGGTGT ACACAAAAAG AACAACTTTT   
  
  
+ ACCAAATGGG TGCACAACAT ATAGAAAACC GTTGGACTTA AGGCCTAAAA CAAGGTGTTT AGGGGTTAAA   
  
  
+ AATATGTGAC GAACCAAGTT TAAAAACGTT CACGGAGACG AATGTTATGT TACTGAATTA ACCTACTGTA   
  
  
+ ATAGAAGTTT TGATAAAAGA ATTTTTTAAT TATTAAAGTG GTCATTAGAC ATCCCCAATC TAGCAGAAAA   
  
  
+ TGAACGAACA GAACCGAGAA TGGACGTTAA GCTGTATCGA CAGGATGAAT GATGAAACGT ACCGCTAGAT   
  
  
+ AGTGGTGACG GTAACGGAGT TAGGCTTGCG TTTATTTATA TACTAAAACA AAGAAACCGT CGTTTTTATT   
  
  
+ TACTTGCAAC TTTTAAGTTT TGTTTATAAA TCATTAACCC AGTCAGTTCA AACTAAATTA CAATTTAAAA   
  
  
+ GAATTTATTA ATTTTTTATT CATTTATATC TTTTTTTAAT ACTTCATTCG AATGTAAAAT TAAAATTAAA   
  
  
+ TAAATAATTT AATGAACATA TAAATTGAAA ATTTGAATTA AAGTTAATAA AATTATTAAT ATTTTTAATT   
  
  
+ TTTATAAAAT TGCCAAATCT ATAGAAATTT AGATATAAAC AATGCATCTT TATTTAACTC TAATTTGTTT   
  
  
+ ACAATAGGAT TAGAATATAA ATTCACGTAA CTACTCTAAC TCTATTCAAA TAAAATACAA AAAAATTAAG   
  
  
+ TACTCTCAGG GGATTTAGTT CTATGATCTA GAACTAAAGT CAACATAATT TTAAAATTAT TTTTTATATA   
  
  
+ TAATATTAAA TTATTTTATT TAACAATTTT TATAAAGATG TAAAAAAAGA GTGTAATTAT ATGATTTGAC   
  
  
+ GTTTTATAAT AAAAATAGAA GAATAATTTT TTTGAATTGT GTTTTATAAT AAAAATAGAA GAATAGTTTT   
  
  
+ TTTGAATTGT AACACTTTAA AAATAGACTT AAAGTATCTC GAATTAGTTT TAACTTCGGA AACTAAAACT   
  
  
+ ATTATAATAA TATAAAATTT TTTAAATTA  

- GATTTCACCT AGCGAGACAC GTCTGAGATG GTAAACGGAG AAAAGCAGCT CTTGGAGCTT TTGTTGTTCG   
  
  
- GGGTTGTTCG TCTACAAAAA CTTCCGCTTC GCCCCAGTGA TCAGTCGATT GGAACTGAAA CCCAGATGAC   
  
  
- AGAGAAAACA ACAGAAAATG GGACTTAACA TGGCATCCAA AGTTGGTTTT GTCGGAGAGA CCCTGGTAGA   
  
  
- ACAAATACAA TTCCCAGTGT TTGGCTTTTC AGAAAGAAAG AGATAGACAT GGAAGGATGC CGCAACTAAT   
  
  
- TGTCCGGTAT TGTGAAAATG CTGACACGTA ATCACAGAGA AATGAGTTGG GCCCCACAAC ACTGGTCCAG   
  
  
- GATTTTCTTT TCTTTTCTGT ACGAATTTCT TAATTTGAAA ATGAATGGGA TTTTAATTAA TGTAGGATAG   
  
  
- ACAATTAAGC TGTTCCGACA TTTATGGCAC GTGACGAAAT GTAGGCCACA TGTGTTTTTC TTGTTGAAAA   
  
  
- TGGTTTACCC ACGTGTTGTA TATCTTTTGG CAACCTGAAT TCCGGATTTT GTTCCACAAA TCCCCAATTT   
  
  
- TTATACACTG CTTGGTTCAA ATTTTTGCAA GTGCCTCTGC TTACAATACA ATGACTTAAT TGGATGACAT   
  
  
- TATCTTCAAA ACTATTTTCT TAAAAAATTA ATAATTTCAC CAGTAATCTG TAGGGGTTAG ATCGTCTTTT   
  
  
- ACTTGCTTGT CTTGGCTCTT ACCTGCAATT CGACATAGCT GTCCTACTTA CTACTTTGCA TGGCGATCTA   
  
  
- TCACCACTGC CATTGCCTCA ATCCGAACGC AAATAAATAT ATGATTTTGT TTCTTTGGCA GCAAAAATAA   
  
  
- ATGAACGTTG AAAATTCAAA ACAAATATTT AGTAATTGGG TCAGTCAAGT TTGATTTAAT GTTAAATTTT   
  
  
- CTTAAATAAT TAAAAAATAA GTAAATATAG AAAAAAATTA TGAAGTAAGC TTACATTTTA ATTTTAATTT   
  
  
- ATTTATTAAA TTACTTGTAT ATTTAACTTT TAAACTTAAT TTCAATTATT TTAATAATTA TAAAAATTAA   
  
  
- AAATATTTTA ACGGTTTAGA TATCTTTAAA TCTATATTTG TTACGTAGAA ATAAATTGAG ATTAAACAAA   
  
  
- TGTTATCCTA ATCTTATATT TAAGTGCATT GATGAGATTG AGATAAGTTT ATTTTATGTT TTTTTAATTC   
  
  
- ATGAGAGTCC CCTAAATCAA GATACTAGAT CTTGATTTCA GTTGTATTAA AATTTTAATA AAAAATATAT   
  
  
- ATTATAATTT AATAAAATAA ATTGTTAAAA ATATTTCTAC ATTTTTTTCT CACATTAATA TACTAAACTG   
  
  
- CAAAATATTA TTTTTATCTT CTTATTAAAA AAACTTAACA CAAAATATTA TTTTTATCTT CTTATCAAAA   
  
  
- AAACTTAACA TTGTGAAATT TTTATCTGAA TTTCATAGAG CTTAATCAAA ATTGAAGCCT TTGATTTTGA   
  
  
- TAATATTATT ATATTTTAAA AAATTTAAT

+     Box 4

| Site Name | Organism | Position | Strand | Matrix score. | sequence | function |
| --- | --- | --- | --- | --- | --- | --- |
| Box 4 | Petroselinum crispum | 1035 | - | 6 | ATTAAT | part of a conserved DNA module involved in light responsiveness |
| Box 4 | Petroselinum crispum | 917 | - | 6 | ATTAAT | part of a conserved DNA module involved in light responsiveness |
| Box 4 | Petroselinum crispum | 404 | + | 6 | ATTAAT | part of a conserved DNA module involved in light responsiveness |

> 2018/04/13 10:10:12  
+ CTAAAGTGGA TCGCTCTGTG CAGACTCTAC CATTTGCCTC TTTTCGTCGA GAACCTCGAA AACAACAAGC   
  
  
+ CCCAACAAGC AGATGTTTTT GAAGGCGAAG CGGGGTCACT AGTCAGCTAA CCTTGACTTT GGGTCTACTG   
  
  
+ TCTCTTTTGT TGTCTTTTAC CCTGAATTGT ACCGTAGGTT TCAACCAAAA CAGCCTCTCT GGGACCATCT   
  
  
+ TGTTTATGTT AAGGGTCACA AACCGAAAAG TCTTTCTTTC TCTATCTGTA CCTTCCTACG GCGTTGATTA   
  
  
+ ACAGGCCATA ACACTTTTAC GACTGTGCAT TAGTGTCTCT TTACTCAACC CGGGGTGTTG TGACCAGGTC   
  
  
+ CTAAAAGAAA AGAAAAGACA TGCTTAAAGA ATTAAACTTT TACTTACCCT AAAATTAATT ACATCCTATC   
  
  
+ TGTTAATTCG ACAAGGCTGT AAATACCGTG CACTGCTTTA CATCCGGTGT ACACAAAAAG AACAACTTTT   
  
  
+ ACCAAATGGG TGCACAACAT ATAGAAAACC GTTGGACTTA AGGCCTAAAA CAAGGTGTTT AGGGGTTAAA   
  
  
+ AATATGTGAC GAACCAAGTT TAAAAACGTT CACGGAGACG AATGTTATGT TACTGAATTA ACCTACTGTA   
  
  
+ ATAGAAGTTT TGATAAAAGA ATTTTTTAAT TATTAAAGTG GTCATTAGAC ATCCCCAATC TAGCAGAAAA   
  
  
+ TGAACGAACA GAACCGAGAA TGGACGTTAA GCTGTATCGA CAGGATGAAT GATGAAACGT ACCGCTAGAT   
  
  
+ AGTGGTGACG GTAACGGAGT TAGGCTTGCG TTTATTTATA TACTAAAACA AAGAAACCGT CGTTTTTATT   
  
  
+ TACTTGCAAC TTTTAAGTTT TGTTTATAAA TCATTAACCC AGTCAGTTCA AACTAAATTA CAATTTAAAA   
  
  
+ GAATTTATTA ATTTTTTATT CATTTATATC TTTTTTTAAT ACTTCATTCG AATGTAAAAT TAAAATTAAA   
  
  
+ TAAATAATTT AATGAACATA TAAATTGAAA ATTTGAATTA AAGTTAATAA AATTATTAAT ATTTTTAATT   
  
  
+ TTTATAAAAT TGCCAAATCT ATAGAAATTT AGATATAAAC AATGCATCTT TATTTAACTC TAATTTGTTT   
  
  
+ ACAATAGGAT TAGAATATAA ATTCACGTAA CTACTCTAAC TCTATTCAAA TAAAATACAA AAAAATTAAG   
  
  
+ TACTCTCAGG GGATTTAGTT CTATGATCTA GAACTAAAGT CAACATAATT TTAAAATTAT TTTTTATATA   
  
  
+ TAATATTAAA TTATTTTATT TAACAATTTT TATAAAGATG TAAAAAAAGA GTGTAATTAT ATGATTTGAC   
  
  
+ GTTTTATAAT AAAAATAGAA GAATAATTTT TTTGAATTGT GTTTTATAAT AAAAATAGAA GAATAGTTTT   
  
  
+ TTTGAATTGT AACACTTTAA AAATAGACTT AAAGTATCTC GAATTAGTTT TAACTTCGGA AACTAAAACT   
  
  
+ ATTATAATAA TATAAAATTT TTTAAATTA  

- GATTTCACCT AGCGAGACAC GTCTGAGATG GTAAACGGAG AAAAGCAGCT CTTGGAGCTT TTGTTGTTCG   
  
  
- GGGTTGTTCG TCTACAAAAA CTTCCGCTTC GCCCCAGTGA TCAGTCGATT GGAACTGAAA CCCAGATGAC   
  
  
- AGAGAAAACA ACAGAAAATG GGACTTAACA TGGCATCCAA AGTTGGTTTT GTCGGAGAGA CCCTGGTAGA   
  
  
- ACAAATACAA TTCCCAGTGT TTGGCTTTTC AGAAAGAAAG AGATAGACAT GGAAGGATGC CGCAACTAAT   
  
  
- TGTCCGGTAT TGTGAAAATG CTGACACGTA ATCACAGAGA AATGAGTTGG GCCCCACAAC ACTGGTCCAG   
  
  
- GATTTTCTTT TCTTTTCTGT ACGAATTTCT TAATTTGAAA ATGAATGGGA TTTTAATTAA TGTAGGATAG   
  
  
- ACAATTAAGC TGTTCCGACA TTTATGGCAC GTGACGAAAT GTAGGCCACA TGTGTTTTTC TTGTTGAAAA   
  
  
- TGGTTTACCC ACGTGTTGTA TATCTTTTGG CAACCTGAAT TCCGGATTTT GTTCCACAAA TCCCCAATTT   
  
  
- TTATACACTG CTTGGTTCAA ATTTTTGCAA GTGCCTCTGC TTACAATACA ATGACTTAAT TGGATGACAT   
  
  
- TATCTTCAAA ACTATTTTCT TAAAAAATTA ATAATTTCAC CAGTAATCTG TAGGGGTTAG ATCGTCTTTT   
  
  
- ACTTGCTTGT CTTGGCTCTT ACCTGCAATT CGACATAGCT GTCCTACTTA CTACTTTGCA TGGCGATCTA   
  
  
- TCACCACTGC CATTGCCTCA ATCCGAACGC AAATAAATAT ATGATTTTGT TTCTTTGGCA GCAAAAATAA   
  
  
- ATGAACGTTG AAAATTCAAA ACAAATATTT AGTAATTGGG TCAGTCAAGT TTGATTTAAT GTTAAATTTT   
  
  
- CTTAAATAAT TAAAAAATAA GTAAATATAG AAAAAAATTA TGAAGTAAGC TTACATTTTA ATTTTAATTT   
  
  
- ATTTATTAAA TTACTTGTAT ATTTAACTTT TAAACTTAAT TTCAATTATT TTAATAATTA TAAAAATTAA   
  
  
- AAATATTTTA ACGGTTTAGA TATCTTTAAA TCTATATTTG TTACGTAGAA ATAAATTGAG ATTAAACAAA   
  
  
- TGTTATCCTA ATCTTATATT TAAGTGCATT GATGAGATTG AGATAAGTTT ATTTTATGTT TTTTTAATTC   
  
  
- ATGAGAGTCC CCTAAATCAA GATACTAGAT CTTGATTTCA GTTGTATTAA AATTTTAATA AAAAATATAT   
  
  
- ATTATAATTT AATAAAATAA ATTGTTAAAA ATATTTCTAC ATTTTTTTCT CACATTAATA TACTAAACTG   
  
  
- CAAAATATTA TTTTTATCTT CTTATTAAAA AAACTTAACA CAAAATATTA TTTTTATCTT CTTATCAAAA   
  
  
- AAACTTAACA TTGTGAAATT TTTATCTGAA TTTCATAGAG CTTAATCAAA ATTGAAGCCT TTGATTTTGA   
  
  
- TAATATTATT ATATTTTAAA AAATTTAAT

+     CAAT-box

| Site Name | Organism | Position | Strand | Matrix score. | sequence | function |
| --- | --- | --- | --- | --- | --- | --- |
| CAAT-box | Hordeum vulgare | 1406 | - | 4 | CAAT | common cis-acting element in promoter and enhancer regions |
| CAAT-box | Brassica rapa | 1167 | + | 5 | CAAAT | common cis-acting element in promoter and enhancer regions |
| CAAT-box | Hordeum vulgare | 686 | + | 4 | CAAT | common cis-acting element in promoter and enhancer regions |
| CAAT-box | Arabidopsis thaliana | 685 | + | 5 | CCAAT | common cis-acting element in promoter and enhancer regions |
| CAAT-box | Hordeum vulgare | 166 | - | 4 | CAAT | common cis-acting element in promoter and enhancer regions |
| CAAT-box | Hordeum vulgare | 1004 | - | 4 | CAAT | common cis-acting element in promoter and enhancer regions |
| CAAT-box | Glycine max | 901 | + | 5 | CAATT | common cis-acting element in promoter and enhancer regions |
| CAAT-box | Glycine max | 165 | - | 5 | CAATT | common cis-acting element in promoter and enhancer regions |
| CAAT-box | Glycine max | 1365 | - | 5 | CAATT | common cis-acting element in promoter and enhancer regions |
| CAAT-box | Glycine max | 1405 | - | 5 | CAATT | common cis-acting element in promoter and enhancer regions |
| CAAT-box | Hordeum vulgare | 1366 | - | 4 | CAAT | common cis-acting element in promoter and enhancer regions |
| CAAT-box | Brassica rapa | 32 | - | 5 | CAAAT | common cis-acting element in promoter and enhancer regions |
| CAAT-box | Brassica rapa | 1324 | - | 5 | CAAAT | common cis-acting element in promoter and enhancer regions |
| CAAT-box | Brassica rapa | 1064 | + | 5 | CAAAT | common cis-acting element in promoter and enhancer regions |
| CAAT-box | Hordeum vulgare | 1122 | + | 4 | CAAT | common cis-acting element in promoter and enhancer regions |
| CAAT-box | Glycine max | 1284 | + | 5 | CAATT | common cis-acting element in promoter and enhancer regions |
| CAAT-box | Brassica rapa | 1113 | - | 5 | CAAAT | common cis-acting element in promoter and enhancer regions |
| CAAT-box | Glycine max | 1003 | - | 5 | CAATT | common cis-acting element in promoter and enhancer regions |
| CAAT-box | Arabidopsis thaliana | 1059 | - | 6 | gGCAAT | common cis-acting element in promoter and enhancer regions |
| CAAT-box | Hordeum vulgare | 1090 | + | 4 | CAAT | common cis-acting element in promoter and enhancer regions |
| CAAT-box | Brassica rapa | 493 | + | 5 | CAAAT | common cis-acting element in promoter and enhancer regions |
| CAAT-box | Brassica rapa | 1011 | - | 5 | CAAAT | common cis-acting element in promoter and enhancer regions |
| CAAT-box | Glycine max | 1058 | - | 5 | CAATT | common cis-acting element in promoter and enhancer regions |

> 2018/04/13 10:10:12  
+ CTAAAGTGGA TCGCTCTGTG CAGACTCTAC CATTTGCCTC TTTTCGTCGA GAACCTCGAA AACAACAAGC   
  
  
+ CCCAACAAGC AGATGTTTTT GAAGGCGAAG CGGGGTCACT AGTCAGCTAA CCTTGACTTT GGGTCTACTG   
  
  
+ TCTCTTTTGT TGTCTTTTAC CCTGAATTGT ACCGTAGGTT TCAACCAAAA CAGCCTCTCT GGGACCATCT   
  
  
+ TGTTTATGTT AAGGGTCACA AACCGAAAAG TCTTTCTTTC TCTATCTGTA CCTTCCTACG GCGTTGATTA   
  
  
+ ACAGGCCATA ACACTTTTAC GACTGTGCAT TAGTGTCTCT TTACTCAACC CGGGGTGTTG TGACCAGGTC   
  
  
+ CTAAAAGAAA AGAAAAGACA TGCTTAAAGA ATTAAACTTT TACTTACCCT AAAATTAATT ACATCCTATC   
  
  
+ TGTTAATTCG ACAAGGCTGT AAATACCGTG CACTGCTTTA CATCCGGTGT ACACAAAAAG AACAACTTTT   
  
  
+ ACCAAATGGG TGCACAACAT ATAGAAAACC GTTGGACTTA AGGCCTAAAA CAAGGTGTTT AGGGGTTAAA   
  
  
+ AATATGTGAC GAACCAAGTT TAAAAACGTT CACGGAGACG AATGTTATGT TACTGAATTA ACCTACTGTA   
  
  
+ ATAGAAGTTT TGATAAAAGA ATTTTTTAAT TATTAAAGTG GTCATTAGAC ATCCCCAATC TAGCAGAAAA   
  
  
+ TGAACGAACA GAACCGAGAA TGGACGTTAA GCTGTATCGA CAGGATGAAT GATGAAACGT ACCGCTAGAT   
  
  
+ AGTGGTGACG GTAACGGAGT TAGGCTTGCG TTTATTTATA TACTAAAACA AAGAAACCGT CGTTTTTATT   
  
  
+ TACTTGCAAC TTTTAAGTTT TGTTTATAAA TCATTAACCC AGTCAGTTCA AACTAAATTA CAATTTAAAA   
  
  
+ GAATTTATTA ATTTTTTATT CATTTATATC TTTTTTTAAT ACTTCATTCG AATGTAAAAT TAAAATTAAA   
  
  
+ TAAATAATTT AATGAACATA TAAATTGAAA ATTTGAATTA AAGTTAATAA AATTATTAAT ATTTTTAATT   
  
  
+ TTTATAAAAT TGCCAAATCT ATAGAAATTT AGATATAAAC AATGCATCTT TATTTAACTC TAATTTGTTT   
  
  
+ ACAATAGGAT TAGAATATAA ATTCACGTAA CTACTCTAAC TCTATTCAAA TAAAATACAA AAAAATTAAG   
  
  
+ TACTCTCAGG GGATTTAGTT CTATGATCTA GAACTAAAGT CAACATAATT TTAAAATTAT TTTTTATATA   
  
  
+ TAATATTAAA TTATTTTATT TAACAATTTT TATAAAGATG TAAAAAAAGA GTGTAATTAT ATGATTTGAC   
  
  
+ GTTTTATAAT AAAAATAGAA GAATAATTTT TTTGAATTGT GTTTTATAAT AAAAATAGAA GAATAGTTTT   
  
  
+ TTTGAATTGT AACACTTTAA AAATAGACTT AAAGTATCTC GAATTAGTTT TAACTTCGGA AACTAAAACT   
  
  
+ ATTATAATAA TATAAAATTT TTTAAATTA  

- GATTTCACCT AGCGAGACAC GTCTGAGATG GTAAACGGAG AAAAGCAGCT CTTGGAGCTT TTGTTGTTCG   
  
  
- GGGTTGTTCG TCTACAAAAA CTTCCGCTTC GCCCCAGTGA TCAGTCGATT GGAACTGAAA CCCAGATGAC   
  
  
- AGAGAAAACA ACAGAAAATG GGACTTAACA TGGCATCCAA AGTTGGTTTT GTCGGAGAGA CCCTGGTAGA   
  
  
- ACAAATACAA TTCCCAGTGT TTGGCTTTTC AGAAAGAAAG AGATAGACAT GGAAGGATGC CGCAACTAAT   
  
  
- TGTCCGGTAT TGTGAAAATG CTGACACGTA ATCACAGAGA AATGAGTTGG GCCCCACAAC ACTGGTCCAG   
  
  
- GATTTTCTTT TCTTTTCTGT ACGAATTTCT TAATTTGAAA ATGAATGGGA TTTTAATTAA TGTAGGATAG   
  
  
- ACAATTAAGC TGTTCCGACA TTTATGGCAC GTGACGAAAT GTAGGCCACA TGTGTTTTTC TTGTTGAAAA   
  
  
- TGGTTTACCC ACGTGTTGTA TATCTTTTGG CAACCTGAAT TCCGGATTTT GTTCCACAAA TCCCCAATTT   
  
  
- TTATACACTG CTTGGTTCAA ATTTTTGCAA GTGCCTCTGC TTACAATACA ATGACTTAAT TGGATGACAT   
  
  
- TATCTTCAAA ACTATTTTCT TAAAAAATTA ATAATTTCAC CAGTAATCTG TAGGGGTTAG ATCGTCTTTT   
  
  
- ACTTGCTTGT CTTGGCTCTT ACCTGCAATT CGACATAGCT GTCCTACTTA CTACTTTGCA TGGCGATCTA   
  
  
- TCACCACTGC CATTGCCTCA ATCCGAACGC AAATAAATAT ATGATTTTGT TTCTTTGGCA GCAAAAATAA   
  
  
- ATGAACGTTG AAAATTCAAA ACAAATATTT AGTAATTGGG TCAGTCAAGT TTGATTTAAT GTTAAATTTT   
  
  
- CTTAAATAAT TAAAAAATAA GTAAATATAG AAAAAAATTA TGAAGTAAGC TTACATTTTA ATTTTAATTT   
  
  
- ATTTATTAAA TTACTTGTAT ATTTAACTTT TAAACTTAAT TTCAATTATT TTAATAATTA TAAAAATTAA   
  
  
- AAATATTTTA ACGGTTTAGA TATCTTTAAA TCTATATTTG TTACGTAGAA ATAAATTGAG ATTAAACAAA   
  
  
- TGTTATCCTA ATCTTATATT TAAGTGCATT GATGAGATTG AGATAAGTTT ATTTTATGTT TTTTTAATTC   
  
  
- ATGAGAGTCC CCTAAATCAA GATACTAGAT CTTGATTTCA GTTGTATTAA AATTTTAATA AAAAATATAT   
  
  
- ATTATAATTT AATAAAATAA ATTGTTAAAA ATATTTCTAC ATTTTTTTCT CACATTAATA TACTAAACTG   
  
  
- CAAAATATTA TTTTTATCTT CTTATTAAAA AAACTTAACA CAAAATATTA TTTTTATCTT CTTATCAAAA   
  
  
- AAACTTAACA TTGTGAAATT TTTATCTGAA TTTCATAGAG CTTAATCAAA ATTGAAGCCT TTGATTTTGA   
  
  
- TAATATTATT ATATTTTAAA AAATTTAAT

+     CCAAT-box

| Site Name | Organism | Position | Strand | Matrix score. | sequence | function |
| --- | --- | --- | --- | --- | --- | --- |
| CCAAT-box | Hordeum vulgare | 519 | - | 6 | CAACGG | MYBHv1 binding site |

> 2018/04/13 10:10:12  
+ CTAAAGTGGA TCGCTCTGTG CAGACTCTAC CATTTGCCTC TTTTCGTCGA GAACCTCGAA AACAACAAGC   
  
  
+ CCCAACAAGC AGATGTTTTT GAAGGCGAAG CGGGGTCACT AGTCAGCTAA CCTTGACTTT GGGTCTACTG   
  
  
+ TCTCTTTTGT TGTCTTTTAC CCTGAATTGT ACCGTAGGTT TCAACCAAAA CAGCCTCTCT GGGACCATCT   
  
  
+ TGTTTATGTT AAGGGTCACA AACCGAAAAG TCTTTCTTTC TCTATCTGTA CCTTCCTACG GCGTTGATTA   
  
  
+ ACAGGCCATA ACACTTTTAC GACTGTGCAT TAGTGTCTCT TTACTCAACC CGGGGTGTTG TGACCAGGTC   
  
  
+ CTAAAAGAAA AGAAAAGACA TGCTTAAAGA ATTAAACTTT TACTTACCCT AAAATTAATT ACATCCTATC   
  
  
+ TGTTAATTCG ACAAGGCTGT AAATACCGTG CACTGCTTTA CATCCGGTGT ACACAAAAAG AACAACTTTT   
  
  
+ ACCAAATGGG TGCACAACAT ATAGAAAACC GTTGGACTTA AGGCCTAAAA CAAGGTGTTT AGGGGTTAAA   
  
  
+ AATATGTGAC GAACCAAGTT TAAAAACGTT CACGGAGACG AATGTTATGT TACTGAATTA ACCTACTGTA   
  
  
+ ATAGAAGTTT TGATAAAAGA ATTTTTTAAT TATTAAAGTG GTCATTAGAC ATCCCCAATC TAGCAGAAAA   
  
  
+ TGAACGAACA GAACCGAGAA TGGACGTTAA GCTGTATCGA CAGGATGAAT GATGAAACGT ACCGCTAGAT   
  
  
+ AGTGGTGACG GTAACGGAGT TAGGCTTGCG TTTATTTATA TACTAAAACA AAGAAACCGT CGTTTTTATT   
  
  
+ TACTTGCAAC TTTTAAGTTT TGTTTATAAA TCATTAACCC AGTCAGTTCA AACTAAATTA CAATTTAAAA   
  
  
+ GAATTTATTA ATTTTTTATT CATTTATATC TTTTTTTAAT ACTTCATTCG AATGTAAAAT TAAAATTAAA   
  
  
+ TAAATAATTT AATGAACATA TAAATTGAAA ATTTGAATTA AAGTTAATAA AATTATTAAT ATTTTTAATT   
  
  
+ TTTATAAAAT TGCCAAATCT ATAGAAATTT AGATATAAAC AATGCATCTT TATTTAACTC TAATTTGTTT   
  
  
+ ACAATAGGAT TAGAATATAA ATTCACGTAA CTACTCTAAC TCTATTCAAA TAAAATACAA AAAAATTAAG   
  
  
+ TACTCTCAGG GGATTTAGTT CTATGATCTA GAACTAAAGT CAACATAATT TTAAAATTAT TTTTTATATA   
  
  
+ TAATATTAAA TTATTTTATT TAACAATTTT TATAAAGATG TAAAAAAAGA GTGTAATTAT ATGATTTGAC   
  
  
+ GTTTTATAAT AAAAATAGAA GAATAATTTT TTTGAATTGT GTTTTATAAT AAAAATAGAA GAATAGTTTT   
  
  
+ TTTGAATTGT AACACTTTAA AAATAGACTT AAAGTATCTC GAATTAGTTT TAACTTCGGA AACTAAAACT   
  
  
+ ATTATAATAA TATAAAATTT TTTAAATTA  

- GATTTCACCT AGCGAGACAC GTCTGAGATG GTAAACGGAG AAAAGCAGCT CTTGGAGCTT TTGTTGTTCG   
  
  
- GGGTTGTTCG TCTACAAAAA CTTCCGCTTC GCCCCAGTGA TCAGTCGATT GGAACTGAAA CCCAGATGAC   
  
  
- AGAGAAAACA ACAGAAAATG GGACTTAACA TGGCATCCAA AGTTGGTTTT GTCGGAGAGA CCCTGGTAGA   
  
  
- ACAAATACAA TTCCCAGTGT TTGGCTTTTC AGAAAGAAAG AGATAGACAT GGAAGGATGC CGCAACTAAT   
  
  
- TGTCCGGTAT TGTGAAAATG CTGACACGTA ATCACAGAGA AATGAGTTGG GCCCCACAAC ACTGGTCCAG   
  
  
- GATTTTCTTT TCTTTTCTGT ACGAATTTCT TAATTTGAAA ATGAATGGGA TTTTAATTAA TGTAGGATAG   
  
  
- ACAATTAAGC TGTTCCGACA TTTATGGCAC GTGACGAAAT GTAGGCCACA TGTGTTTTTC TTGTTGAAAA   
  
  
- TGGTTTACCC ACGTGTTGTA TATCTTTTGG CAACCTGAAT TCCGGATTTT GTTCCACAAA TCCCCAATTT   
  
  
- TTATACACTG CTTGGTTCAA ATTTTTGCAA GTGCCTCTGC TTACAATACA ATGACTTAAT TGGATGACAT   
  
  
- TATCTTCAAA ACTATTTTCT TAAAAAATTA ATAATTTCAC CAGTAATCTG TAGGGGTTAG ATCGTCTTTT   
  
  
- ACTTGCTTGT CTTGGCTCTT ACCTGCAATT CGACATAGCT GTCCTACTTA CTACTTTGCA TGGCGATCTA   
  
  
- TCACCACTGC CATTGCCTCA ATCCGAACGC AAATAAATAT ATGATTTTGT TTCTTTGGCA GCAAAAATAA   
  
  
- ATGAACGTTG AAAATTCAAA ACAAATATTT AGTAATTGGG TCAGTCAAGT TTGATTTAAT GTTAAATTTT   
  
  
- CTTAAATAAT TAAAAAATAA GTAAATATAG AAAAAAATTA TGAAGTAAGC TTACATTTTA ATTTTAATTT   
  
  
- ATTTATTAAA TTACTTGTAT ATTTAACTTT TAAACTTAAT TTCAATTATT TTAATAATTA TAAAAATTAA   
  
  
- AAATATTTTA ACGGTTTAGA TATCTTTAAA TCTATATTTG TTACGTAGAA ATAAATTGAG ATTAAACAAA   
  
  
- TGTTATCCTA ATCTTATATT TAAGTGCATT GATGAGATTG AGATAAGTTT ATTTTATGTT TTTTTAATTC   
  
  
- ATGAGAGTCC CCTAAATCAA GATACTAGAT CTTGATTTCA GTTGTATTAA AATTTTAATA AAAAATATAT   
  
  
- ATTATAATTT AATAAAATAA ATTGTTAAAA ATATTTCTAC ATTTTTTTCT CACATTAATA TACTAAACTG   
  
  
- CAAAATATTA TTTTTATCTT CTTATTAAAA AAACTTAACA CAAAATATTA TTTTTATCTT CTTATCAAAA   
  
  
- AAACTTAACA TTGTGAAATT TTTATCTGAA TTTCATAGAG CTTAATCAAA ATTGAAGCCT TTGATTTTGA   
  
  
- TAATATTATT ATATTTTAAA AAATTTAAT

+     CGTCA-motif

| Site Name | Organism | Position | Strand | Matrix score. | sequence | function |
| --- | --- | --- | --- | --- | --- | --- |
| CGTCA-motif | Hordeum vulgare | 567 | - | 5 | CGTCA | cis-acting regulatory element involved in the MeJA-responsiveness |
| CGTCA-motif | Hordeum vulgare | 776 | - | 5 | CGTCA | cis-acting regulatory element involved in the MeJA-responsiveness |
| CGTCA-motif | Hordeum vulgare | 1327 | - | 5 | CGTCA | cis-acting regulatory element involved in the MeJA-responsiveness |

> 2018/04/13 10:10:12  
+ CTAAAGTGGA TCGCTCTGTG CAGACTCTAC CATTTGCCTC TTTTCGTCGA GAACCTCGAA AACAACAAGC   
  
  
+ CCCAACAAGC AGATGTTTTT GAAGGCGAAG CGGGGTCACT AGTCAGCTAA CCTTGACTTT GGGTCTACTG   
  
  
+ TCTCTTTTGT TGTCTTTTAC CCTGAATTGT ACCGTAGGTT TCAACCAAAA CAGCCTCTCT GGGACCATCT   
  
  
+ TGTTTATGTT AAGGGTCACA AACCGAAAAG TCTTTCTTTC TCTATCTGTA CCTTCCTACG GCGTTGATTA   
  
  
+ ACAGGCCATA ACACTTTTAC GACTGTGCAT TAGTGTCTCT TTACTCAACC CGGGGTGTTG TGACCAGGTC   
  
  
+ CTAAAAGAAA AGAAAAGACA TGCTTAAAGA ATTAAACTTT TACTTACCCT AAAATTAATT ACATCCTATC   
  
  
+ TGTTAATTCG ACAAGGCTGT AAATACCGTG CACTGCTTTA CATCCGGTGT ACACAAAAAG AACAACTTTT   
  
  
+ ACCAAATGGG TGCACAACAT ATAGAAAACC GTTGGACTTA AGGCCTAAAA CAAGGTGTTT AGGGGTTAAA   
  
  
+ AATATGTGAC GAACCAAGTT TAAAAACGTT CACGGAGACG AATGTTATGT TACTGAATTA ACCTACTGTA   
  
  
+ ATAGAAGTTT TGATAAAAGA ATTTTTTAAT TATTAAAGTG GTCATTAGAC ATCCCCAATC TAGCAGAAAA   
  
  
+ TGAACGAACA GAACCGAGAA TGGACGTTAA GCTGTATCGA CAGGATGAAT GATGAAACGT ACCGCTAGAT   
  
  
+ AGTGGTGACG GTAACGGAGT TAGGCTTGCG TTTATTTATA TACTAAAACA AAGAAACCGT CGTTTTTATT   
  
  
+ TACTTGCAAC TTTTAAGTTT TGTTTATAAA TCATTAACCC AGTCAGTTCA AACTAAATTA CAATTTAAAA   
  
  
+ GAATTTATTA ATTTTTTATT CATTTATATC TTTTTTTAAT ACTTCATTCG AATGTAAAAT TAAAATTAAA   
  
  
+ TAAATAATTT AATGAACATA TAAATTGAAA ATTTGAATTA AAGTTAATAA AATTATTAAT ATTTTTAATT   
  
  
+ TTTATAAAAT TGCCAAATCT ATAGAAATTT AGATATAAAC AATGCATCTT TATTTAACTC TAATTTGTTT   
  
  
+ ACAATAGGAT TAGAATATAA ATTCACGTAA CTACTCTAAC TCTATTCAAA TAAAATACAA AAAAATTAAG   
  
  
+ TACTCTCAGG GGATTTAGTT CTATGATCTA GAACTAAAGT CAACATAATT TTAAAATTAT TTTTTATATA   
  
  
+ TAATATTAAA TTATTTTATT TAACAATTTT TATAAAGATG TAAAAAAAGA GTGTAATTAT ATGATTTGAC   
  
  
+ GTTTTATAAT AAAAATAGAA GAATAATTTT TTTGAATTGT GTTTTATAAT AAAAATAGAA GAATAGTTTT   
  
  
+ TTTGAATTGT AACACTTTAA AAATAGACTT AAAGTATCTC GAATTAGTTT TAACTTCGGA AACTAAAACT   
  
  
+ ATTATAATAA TATAAAATTT TTTAAATTA  

- GATTTCACCT AGCGAGACAC GTCTGAGATG GTAAACGGAG AAAAGCAGCT CTTGGAGCTT TTGTTGTTCG   
  
  
- GGGTTGTTCG TCTACAAAAA CTTCCGCTTC GCCCCAGTGA TCAGTCGATT GGAACTGAAA CCCAGATGAC   
  
  
- AGAGAAAACA ACAGAAAATG GGACTTAACA TGGCATCCAA AGTTGGTTTT GTCGGAGAGA CCCTGGTAGA   
  
  
- ACAAATACAA TTCCCAGTGT TTGGCTTTTC AGAAAGAAAG AGATAGACAT GGAAGGATGC CGCAACTAAT   
  
  
- TGTCCGGTAT TGTGAAAATG CTGACACGTA ATCACAGAGA AATGAGTTGG GCCCCACAAC ACTGGTCCAG   
  
  
- GATTTTCTTT TCTTTTCTGT ACGAATTTCT TAATTTGAAA ATGAATGGGA TTTTAATTAA TGTAGGATAG   
  
  
- ACAATTAAGC TGTTCCGACA TTTATGGCAC GTGACGAAAT GTAGGCCACA TGTGTTTTTC TTGTTGAAAA   
  
  
- TGGTTTACCC ACGTGTTGTA TATCTTTTGG CAACCTGAAT TCCGGATTTT GTTCCACAAA TCCCCAATTT   
  
  
- TTATACACTG CTTGGTTCAA ATTTTTGCAA GTGCCTCTGC TTACAATACA ATGACTTAAT TGGATGACAT   
  
  
- TATCTTCAAA ACTATTTTCT TAAAAAATTA ATAATTTCAC CAGTAATCTG TAGGGGTTAG ATCGTCTTTT   
  
  
- ACTTGCTTGT CTTGGCTCTT ACCTGCAATT CGACATAGCT GTCCTACTTA CTACTTTGCA TGGCGATCTA   
  
  
- TCACCACTGC CATTGCCTCA ATCCGAACGC AAATAAATAT ATGATTTTGT TTCTTTGGCA GCAAAAATAA   
  
  
- ATGAACGTTG AAAATTCAAA ACAAATATTT AGTAATTGGG TCAGTCAAGT TTGATTTAAT GTTAAATTTT   
  
  
- CTTAAATAAT TAAAAAATAA GTAAATATAG AAAAAAATTA TGAAGTAAGC TTACATTTTA ATTTTAATTT   
  
  
- ATTTATTAAA TTACTTGTAT ATTTAACTTT TAAACTTAAT TTCAATTATT TTAATAATTA TAAAAATTAA   
  
  
- AAATATTTTA ACGGTTTAGA TATCTTTAAA TCTATATTTG TTACGTAGAA ATAAATTGAG ATTAAACAAA   
  
  
- TGTTATCCTA ATCTTATATT TAAGTGCATT GATGAGATTG AGATAAGTTT ATTTTATGTT TTTTTAATTC   
  
  
- ATGAGAGTCC CCTAAATCAA GATACTAGAT CTTGATTTCA GTTGTATTAA AATTTTAATA AAAAATATAT   
  
  
- ATTATAATTT AATAAAATAA ATTGTTAAAA ATATTTCTAC ATTTTTTTCT CACATTAATA TACTAAACTG   
  
  
- CAAAATATTA TTTTTATCTT CTTATTAAAA AAACTTAACA CAAAATATTA TTTTTATCTT CTTATCAAAA   
  
  
- AAACTTAACA TTGTGAAATT TTTATCTGAA TTTCATAGAG CTTAATCAAA ATTGAAGCCT TTGATTTTGA   
  
  
- TAATATTATT ATATTTTAAA AAATTTAAT

+     CTAG-motif

| Site Name | Organism | Position | Strand | Matrix score. | sequence | function |
| --- | --- | --- | --- | --- | --- | --- |
| CTAG-motif | Avena sativa | 689 | + | 9 | ACTAGCAGAA |  |

> 2018/04/13 10:10:12  
+ CTAAAGTGGA TCGCTCTGTG CAGACTCTAC CATTTGCCTC TTTTCGTCGA GAACCTCGAA AACAACAAGC   
  
  
+ CCCAACAAGC AGATGTTTTT GAAGGCGAAG CGGGGTCACT AGTCAGCTAA CCTTGACTTT GGGTCTACTG   
  
  
+ TCTCTTTTGT TGTCTTTTAC CCTGAATTGT ACCGTAGGTT TCAACCAAAA CAGCCTCTCT GGGACCATCT   
  
  
+ TGTTTATGTT AAGGGTCACA AACCGAAAAG TCTTTCTTTC TCTATCTGTA CCTTCCTACG GCGTTGATTA   
  
  
+ ACAGGCCATA ACACTTTTAC GACTGTGCAT TAGTGTCTCT TTACTCAACC CGGGGTGTTG TGACCAGGTC   
  
  
+ CTAAAAGAAA AGAAAAGACA TGCTTAAAGA ATTAAACTTT TACTTACCCT AAAATTAATT ACATCCTATC   
  
  
+ TGTTAATTCG ACAAGGCTGT AAATACCGTG CACTGCTTTA CATCCGGTGT ACACAAAAAG AACAACTTTT   
  
  
+ ACCAAATGGG TGCACAACAT ATAGAAAACC GTTGGACTTA AGGCCTAAAA CAAGGTGTTT AGGGGTTAAA   
  
  
+ AATATGTGAC GAACCAAGTT TAAAAACGTT CACGGAGACG AATGTTATGT TACTGAATTA ACCTACTGTA   
  
  
+ ATAGAAGTTT TGATAAAAGA ATTTTTTAAT TATTAAAGTG GTCATTAGAC ATCCCCAATC TAGCAGAAAA   
  
  
+ TGAACGAACA GAACCGAGAA TGGACGTTAA GCTGTATCGA CAGGATGAAT GATGAAACGT ACCGCTAGAT   
  
  
+ AGTGGTGACG GTAACGGAGT TAGGCTTGCG TTTATTTATA TACTAAAACA AAGAAACCGT CGTTTTTATT   
  
  
+ TACTTGCAAC TTTTAAGTTT TGTTTATAAA TCATTAACCC AGTCAGTTCA AACTAAATTA CAATTTAAAA   
  
  
+ GAATTTATTA ATTTTTTATT CATTTATATC TTTTTTTAAT ACTTCATTCG AATGTAAAAT TAAAATTAAA   
  
  
+ TAAATAATTT AATGAACATA TAAATTGAAA ATTTGAATTA AAGTTAATAA AATTATTAAT ATTTTTAATT   
  
  
+ TTTATAAAAT TGCCAAATCT ATAGAAATTT AGATATAAAC AATGCATCTT TATTTAACTC TAATTTGTTT   
  
  
+ ACAATAGGAT TAGAATATAA ATTCACGTAA CTACTCTAAC TCTATTCAAA TAAAATACAA AAAAATTAAG   
  
  
+ TACTCTCAGG GGATTTAGTT CTATGATCTA GAACTAAAGT CAACATAATT TTAAAATTAT TTTTTATATA   
  
  
+ TAATATTAAA TTATTTTATT TAACAATTTT TATAAAGATG TAAAAAAAGA GTGTAATTAT ATGATTTGAC   
  
  
+ GTTTTATAAT AAAAATAGAA GAATAATTTT TTTGAATTGT GTTTTATAAT AAAAATAGAA GAATAGTTTT   
  
  
+ TTTGAATTGT AACACTTTAA AAATAGACTT AAAGTATCTC GAATTAGTTT TAACTTCGGA AACTAAAACT   
  
  
+ ATTATAATAA TATAAAATTT TTTAAATTA  

- GATTTCACCT AGCGAGACAC GTCTGAGATG GTAAACGGAG AAAAGCAGCT CTTGGAGCTT TTGTTGTTCG   
  
  
- GGGTTGTTCG TCTACAAAAA CTTCCGCTTC GCCCCAGTGA TCAGTCGATT GGAACTGAAA CCCAGATGAC   
  
  
- AGAGAAAACA ACAGAAAATG GGACTTAACA TGGCATCCAA AGTTGGTTTT GTCGGAGAGA CCCTGGTAGA   
  
  
- ACAAATACAA TTCCCAGTGT TTGGCTTTTC AGAAAGAAAG AGATAGACAT GGAAGGATGC CGCAACTAAT   
  
  
- TGTCCGGTAT TGTGAAAATG CTGACACGTA ATCACAGAGA AATGAGTTGG GCCCCACAAC ACTGGTCCAG   
  
  
- GATTTTCTTT TCTTTTCTGT ACGAATTTCT TAATTTGAAA ATGAATGGGA TTTTAATTAA TGTAGGATAG   
  
  
- ACAATTAAGC TGTTCCGACA TTTATGGCAC GTGACGAAAT GTAGGCCACA TGTGTTTTTC TTGTTGAAAA   
  
  
- TGGTTTACCC ACGTGTTGTA TATCTTTTGG CAACCTGAAT TCCGGATTTT GTTCCACAAA TCCCCAATTT   
  
  
- TTATACACTG CTTGGTTCAA ATTTTTGCAA GTGCCTCTGC TTACAATACA ATGACTTAAT TGGATGACAT   
  
  
- TATCTTCAAA ACTATTTTCT TAAAAAATTA ATAATTTCAC CAGTAATCTG TAGGGGTTAG ATCGTCTTTT   
  
  
- ACTTGCTTGT CTTGGCTCTT ACCTGCAATT CGACATAGCT GTCCTACTTA CTACTTTGCA TGGCGATCTA   
  
  
- TCACCACTGC CATTGCCTCA ATCCGAACGC AAATAAATAT ATGATTTTGT TTCTTTGGCA GCAAAAATAA   
  
  
- ATGAACGTTG AAAATTCAAA ACAAATATTT AGTAATTGGG TCAGTCAAGT TTGATTTAAT GTTAAATTTT   
  
  
- CTTAAATAAT TAAAAAATAA GTAAATATAG AAAAAAATTA TGAAGTAAGC TTACATTTTA ATTTTAATTT   
  
  
- ATTTATTAAA TTACTTGTAT ATTTAACTTT TAAACTTAAT TTCAATTATT TTAATAATTA TAAAAATTAA   
  
  
- AAATATTTTA ACGGTTTAGA TATCTTTAAA TCTATATTTG TTACGTAGAA ATAAATTGAG ATTAAACAAA   
  
  
- TGTTATCCTA ATCTTATATT TAAGTGCATT GATGAGATTG AGATAAGTTT ATTTTATGTT TTTTTAATTC   
  
  
- ATGAGAGTCC CCTAAATCAA GATACTAGAT CTTGATTTCA GTTGTATTAA AATTTTAATA AAAAATATAT   
  
  
- ATTATAATTT AATAAAATAA ATTGTTAAAA ATATTTCTAC ATTTTTTTCT CACATTAATA TACTAAACTG   
  
  
- CAAAATATTA TTTTTATCTT CTTATTAAAA AAACTTAACA CAAAATATTA TTTTTATCTT CTTATCAAAA   
  
  
- AAACTTAACA TTGTGAAATT TTTATCTGAA TTTCATAGAG CTTAATCAAA ATTGAAGCCT TTGATTTTGA   
  
  
- TAATATTATT ATATTTTAAA AAATTTAAT

+     G-Box

| Site Name | Organism | Position | Strand | Matrix score. | sequence | function |
| --- | --- | --- | --- | --- | --- | --- |
| G-Box | Antirrhinum majus | 1144 | + | 6 | CACGTA | cis-acting regulatory element involved in light responsiveness |

> 2018/04/13 10:10:12  
+ CTAAAGTGGA TCGCTCTGTG CAGACTCTAC CATTTGCCTC TTTTCGTCGA GAACCTCGAA AACAACAAGC   
  
  
+ CCCAACAAGC AGATGTTTTT GAAGGCGAAG CGGGGTCACT AGTCAGCTAA CCTTGACTTT GGGTCTACTG   
  
  
+ TCTCTTTTGT TGTCTTTTAC CCTGAATTGT ACCGTAGGTT TCAACCAAAA CAGCCTCTCT GGGACCATCT   
  
  
+ TGTTTATGTT AAGGGTCACA AACCGAAAAG TCTTTCTTTC TCTATCTGTA CCTTCCTACG GCGTTGATTA   
  
  
+ ACAGGCCATA ACACTTTTAC GACTGTGCAT TAGTGTCTCT TTACTCAACC CGGGGTGTTG TGACCAGGTC   
  
  
+ CTAAAAGAAA AGAAAAGACA TGCTTAAAGA ATTAAACTTT TACTTACCCT AAAATTAATT ACATCCTATC   
  
  
+ TGTTAATTCG ACAAGGCTGT AAATACCGTG CACTGCTTTA CATCCGGTGT ACACAAAAAG AACAACTTTT   
  
  
+ ACCAAATGGG TGCACAACAT ATAGAAAACC GTTGGACTTA AGGCCTAAAA CAAGGTGTTT AGGGGTTAAA   
  
  
+ AATATGTGAC GAACCAAGTT TAAAAACGTT CACGGAGACG AATGTTATGT TACTGAATTA ACCTACTGTA   
  
  
+ ATAGAAGTTT TGATAAAAGA ATTTTTTAAT TATTAAAGTG GTCATTAGAC ATCCCCAATC TAGCAGAAAA   
  
  
+ TGAACGAACA GAACCGAGAA TGGACGTTAA GCTGTATCGA CAGGATGAAT GATGAAACGT ACCGCTAGAT   
  
  
+ AGTGGTGACG GTAACGGAGT TAGGCTTGCG TTTATTTATA TACTAAAACA AAGAAACCGT CGTTTTTATT   
  
  
+ TACTTGCAAC TTTTAAGTTT TGTTTATAAA TCATTAACCC AGTCAGTTCA AACTAAATTA CAATTTAAAA   
  
  
+ GAATTTATTA ATTTTTTATT CATTTATATC TTTTTTTAAT ACTTCATTCG AATGTAAAAT TAAAATTAAA   
  
  
+ TAAATAATTT AATGAACATA TAAATTGAAA ATTTGAATTA AAGTTAATAA AATTATTAAT ATTTTTAATT   
  
  
+ TTTATAAAAT TGCCAAATCT ATAGAAATTT AGATATAAAC AATGCATCTT TATTTAACTC TAATTTGTTT   
  
  
+ ACAATAGGAT TAGAATATAA ATTCACGTAA CTACTCTAAC TCTATTCAAA TAAAATACAA AAAAATTAAG   
  
  
+ TACTCTCAGG GGATTTAGTT CTATGATCTA GAACTAAAGT CAACATAATT TTAAAATTAT TTTTTATATA   
  
  
+ TAATATTAAA TTATTTTATT TAACAATTTT TATAAAGATG TAAAAAAAGA GTGTAATTAT ATGATTTGAC   
  
  
+ GTTTTATAAT AAAAATAGAA GAATAATTTT TTTGAATTGT GTTTTATAAT AAAAATAGAA GAATAGTTTT   
  
  
+ TTTGAATTGT AACACTTTAA AAATAGACTT AAAGTATCTC GAATTAGTTT TAACTTCGGA AACTAAAACT   
  
  
+ ATTATAATAA TATAAAATTT TTTAAATTA  

- GATTTCACCT AGCGAGACAC GTCTGAGATG GTAAACGGAG AAAAGCAGCT CTTGGAGCTT TTGTTGTTCG   
  
  
- GGGTTGTTCG TCTACAAAAA CTTCCGCTTC GCCCCAGTGA TCAGTCGATT GGAACTGAAA CCCAGATGAC   
  
  
- AGAGAAAACA ACAGAAAATG GGACTTAACA TGGCATCCAA AGTTGGTTTT GTCGGAGAGA CCCTGGTAGA   
  
  
- ACAAATACAA TTCCCAGTGT TTGGCTTTTC AGAAAGAAAG AGATAGACAT GGAAGGATGC CGCAACTAAT   
  
  
- TGTCCGGTAT TGTGAAAATG CTGACACGTA ATCACAGAGA AATGAGTTGG GCCCCACAAC ACTGGTCCAG   
  
  
- GATTTTCTTT TCTTTTCTGT ACGAATTTCT TAATTTGAAA ATGAATGGGA TTTTAATTAA TGTAGGATAG   
  
  
- ACAATTAAGC TGTTCCGACA TTTATGGCAC GTGACGAAAT GTAGGCCACA TGTGTTTTTC TTGTTGAAAA   
  
  
- TGGTTTACCC ACGTGTTGTA TATCTTTTGG CAACCTGAAT TCCGGATTTT GTTCCACAAA TCCCCAATTT   
  
  
- TTATACACTG CTTGGTTCAA ATTTTTGCAA GTGCCTCTGC TTACAATACA ATGACTTAAT TGGATGACAT   
  
  
- TATCTTCAAA ACTATTTTCT TAAAAAATTA ATAATTTCAC CAGTAATCTG TAGGGGTTAG ATCGTCTTTT   
  
  
- ACTTGCTTGT CTTGGCTCTT ACCTGCAATT CGACATAGCT GTCCTACTTA CTACTTTGCA TGGCGATCTA   
  
  
- TCACCACTGC CATTGCCTCA ATCCGAACGC AAATAAATAT ATGATTTTGT TTCTTTGGCA GCAAAAATAA   
  
  
- ATGAACGTTG AAAATTCAAA ACAAATATTT AGTAATTGGG TCAGTCAAGT TTGATTTAAT GTTAAATTTT   
  
  
- CTTAAATAAT TAAAAAATAA GTAAATATAG AAAAAAATTA TGAAGTAAGC TTACATTTTA ATTTTAATTT   
  
  
- ATTTATTAAA TTACTTGTAT ATTTAACTTT TAAACTTAAT TTCAATTATT TTAATAATTA TAAAAATTAA   
  
  
- AAATATTTTA ACGGTTTAGA TATCTTTAAA TCTATATTTG TTACGTAGAA ATAAATTGAG ATTAAACAAA   
  
  
- TGTTATCCTA ATCTTATATT TAAGTGCATT GATGAGATTG AGATAAGTTT ATTTTATGTT TTTTTAATTC   
  
  
- ATGAGAGTCC CCTAAATCAA GATACTAGAT CTTGATTTCA GTTGTATTAA AATTTTAATA AAAAATATAT   
  
  
- ATTATAATTT AATAAAATAA ATTGTTAAAA ATATTTCTAC ATTTTTTTCT CACATTAATA TACTAAACTG   
  
  
- CAAAATATTA TTTTTATCTT CTTATTAAAA AAACTTAACA CAAAATATTA TTTTTATCTT CTTATCAAAA   
  
  
- AAACTTAACA TTGTGAAATT TTTATCTGAA TTTCATAGAG CTTAATCAAA ATTGAAGCCT TTGATTTTGA   
  
  
- TAATATTATT ATATTTTAAA AAATTTAAT

+     G-box

| Site Name | Organism | Position | Strand | Matrix score. | sequence | function |
| --- | --- | --- | --- | --- | --- | --- |
| G-box | Daucus carota | 1144 | - | 6 | TACGTG | cis-acting regulatory element involved in light responsiveness |

> 2018/04/13 10:10:12  
+ CTAAAGTGGA TCGCTCTGTG CAGACTCTAC CATTTGCCTC TTTTCGTCGA GAACCTCGAA AACAACAAGC   
  
  
+ CCCAACAAGC AGATGTTTTT GAAGGCGAAG CGGGGTCACT AGTCAGCTAA CCTTGACTTT GGGTCTACTG   
  
  
+ TCTCTTTTGT TGTCTTTTAC CCTGAATTGT ACCGTAGGTT TCAACCAAAA CAGCCTCTCT GGGACCATCT   
  
  
+ TGTTTATGTT AAGGGTCACA AACCGAAAAG TCTTTCTTTC TCTATCTGTA CCTTCCTACG GCGTTGATTA   
  
  
+ ACAGGCCATA ACACTTTTAC GACTGTGCAT TAGTGTCTCT TTACTCAACC CGGGGTGTTG TGACCAGGTC   
  
  
+ CTAAAAGAAA AGAAAAGACA TGCTTAAAGA ATTAAACTTT TACTTACCCT AAAATTAATT ACATCCTATC   
  
  
+ TGTTAATTCG ACAAGGCTGT AAATACCGTG CACTGCTTTA CATCCGGTGT ACACAAAAAG AACAACTTTT   
  
  
+ ACCAAATGGG TGCACAACAT ATAGAAAACC GTTGGACTTA AGGCCTAAAA CAAGGTGTTT AGGGGTTAAA   
  
  
+ AATATGTGAC GAACCAAGTT TAAAAACGTT CACGGAGACG AATGTTATGT TACTGAATTA ACCTACTGTA   
  
  
+ ATAGAAGTTT TGATAAAAGA ATTTTTTAAT TATTAAAGTG GTCATTAGAC ATCCCCAATC TAGCAGAAAA   
  
  
+ TGAACGAACA GAACCGAGAA TGGACGTTAA GCTGTATCGA CAGGATGAAT GATGAAACGT ACCGCTAGAT   
  
  
+ AGTGGTGACG GTAACGGAGT TAGGCTTGCG TTTATTTATA TACTAAAACA AAGAAACCGT CGTTTTTATT   
  
  
+ TACTTGCAAC TTTTAAGTTT TGTTTATAAA TCATTAACCC AGTCAGTTCA AACTAAATTA CAATTTAAAA   
  
  
+ GAATTTATTA ATTTTTTATT CATTTATATC TTTTTTTAAT ACTTCATTCG AATGTAAAAT TAAAATTAAA   
  
  
+ TAAATAATTT AATGAACATA TAAATTGAAA ATTTGAATTA AAGTTAATAA AATTATTAAT ATTTTTAATT   
  
  
+ TTTATAAAAT TGCCAAATCT ATAGAAATTT AGATATAAAC AATGCATCTT TATTTAACTC TAATTTGTTT   
  
  
+ ACAATAGGAT TAGAATATAA ATTCACGTAA CTACTCTAAC TCTATTCAAA TAAAATACAA AAAAATTAAG   
  
  
+ TACTCTCAGG GGATTTAGTT CTATGATCTA GAACTAAAGT CAACATAATT TTAAAATTAT TTTTTATATA   
  
  
+ TAATATTAAA TTATTTTATT TAACAATTTT TATAAAGATG TAAAAAAAGA GTGTAATTAT ATGATTTGAC   
  
  
+ GTTTTATAAT AAAAATAGAA GAATAATTTT TTTGAATTGT GTTTTATAAT AAAAATAGAA GAATAGTTTT   
  
  
+ TTTGAATTGT AACACTTTAA AAATAGACTT AAAGTATCTC GAATTAGTTT TAACTTCGGA AACTAAAACT   
  
  
+ ATTATAATAA TATAAAATTT TTTAAATTA  

- GATTTCACCT AGCGAGACAC GTCTGAGATG GTAAACGGAG AAAAGCAGCT CTTGGAGCTT TTGTTGTTCG   
  
  
- GGGTTGTTCG TCTACAAAAA CTTCCGCTTC GCCCCAGTGA TCAGTCGATT GGAACTGAAA CCCAGATGAC   
  
  
- AGAGAAAACA ACAGAAAATG GGACTTAACA TGGCATCCAA AGTTGGTTTT GTCGGAGAGA CCCTGGTAGA   
  
  
- ACAAATACAA TTCCCAGTGT TTGGCTTTTC AGAAAGAAAG AGATAGACAT GGAAGGATGC CGCAACTAAT   
  
  
- TGTCCGGTAT TGTGAAAATG CTGACACGTA ATCACAGAGA AATGAGTTGG GCCCCACAAC ACTGGTCCAG   
  
  
- GATTTTCTTT TCTTTTCTGT ACGAATTTCT TAATTTGAAA ATGAATGGGA TTTTAATTAA TGTAGGATAG   
  
  
- ACAATTAAGC TGTTCCGACA TTTATGGCAC GTGACGAAAT GTAGGCCACA TGTGTTTTTC TTGTTGAAAA   
  
  
- TGGTTTACCC ACGTGTTGTA TATCTTTTGG CAACCTGAAT TCCGGATTTT GTTCCACAAA TCCCCAATTT   
  
  
- TTATACACTG CTTGGTTCAA ATTTTTGCAA GTGCCTCTGC TTACAATACA ATGACTTAAT TGGATGACAT   
  
  
- TATCTTCAAA ACTATTTTCT TAAAAAATTA ATAATTTCAC CAGTAATCTG TAGGGGTTAG ATCGTCTTTT   
  
  
- ACTTGCTTGT CTTGGCTCTT ACCTGCAATT CGACATAGCT GTCCTACTTA CTACTTTGCA TGGCGATCTA   
  
  
- TCACCACTGC CATTGCCTCA ATCCGAACGC AAATAAATAT ATGATTTTGT TTCTTTGGCA GCAAAAATAA   
  
  
- ATGAACGTTG AAAATTCAAA ACAAATATTT AGTAATTGGG TCAGTCAAGT TTGATTTAAT GTTAAATTTT   
  
  
- CTTAAATAAT TAAAAAATAA GTAAATATAG AAAAAAATTA TGAAGTAAGC TTACATTTTA ATTTTAATTT   
  
  
- ATTTATTAAA TTACTTGTAT ATTTAACTTT TAAACTTAAT TTCAATTATT TTAATAATTA TAAAAATTAA   
  
  
- AAATATTTTA ACGGTTTAGA TATCTTTAAA TCTATATTTG TTACGTAGAA ATAAATTGAG ATTAAACAAA   
  
  
- TGTTATCCTA ATCTTATATT TAAGTGCATT GATGAGATTG AGATAAGTTT ATTTTATGTT TTTTTAATTC   
  
  
- ATGAGAGTCC CCTAAATCAA GATACTAGAT CTTGATTTCA GTTGTATTAA AATTTTAATA AAAAATATAT   
  
  
- ATTATAATTT AATAAAATAA ATTGTTAAAA ATATTTCTAC ATTTTTTTCT CACATTAATA TACTAAACTG   
  
  
- CAAAATATTA TTTTTATCTT CTTATTAAAA AAACTTAACA CAAAATATTA TTTTTATCTT CTTATCAAAA   
  
  
- AAACTTAACA TTGTGAAATT TTTATCTGAA TTTCATAGAG CTTAATCAAA ATTGAAGCCT TTGATTTTGA   
  
  
- TAATATTATT ATATTTTAAA AAATTTAAT

+     GATA-motif

| Site Name | Organism | Position | Strand | Matrix score. | sequence | function |
| --- | --- | --- | --- | --- | --- | --- |
| GATA-motif | Arabidopsis thaliana | 414 | - | 7 | GATAGGA | part of a light responsive element |

> 2018/04/13 10:10:12  
+ CTAAAGTGGA TCGCTCTGTG CAGACTCTAC CATTTGCCTC TTTTCGTCGA GAACCTCGAA AACAACAAGC   
  
  
+ CCCAACAAGC AGATGTTTTT GAAGGCGAAG CGGGGTCACT AGTCAGCTAA CCTTGACTTT GGGTCTACTG   
  
  
+ TCTCTTTTGT TGTCTTTTAC CCTGAATTGT ACCGTAGGTT TCAACCAAAA CAGCCTCTCT GGGACCATCT   
  
  
+ TGTTTATGTT AAGGGTCACA AACCGAAAAG TCTTTCTTTC TCTATCTGTA CCTTCCTACG GCGTTGATTA   
  
  
+ ACAGGCCATA ACACTTTTAC GACTGTGCAT TAGTGTCTCT TTACTCAACC CGGGGTGTTG TGACCAGGTC   
  
  
+ CTAAAAGAAA AGAAAAGACA TGCTTAAAGA ATTAAACTTT TACTTACCCT AAAATTAATT ACATCCTATC   
  
  
+ TGTTAATTCG ACAAGGCTGT AAATACCGTG CACTGCTTTA CATCCGGTGT ACACAAAAAG AACAACTTTT   
  
  
+ ACCAAATGGG TGCACAACAT ATAGAAAACC GTTGGACTTA AGGCCTAAAA CAAGGTGTTT AGGGGTTAAA   
  
  
+ AATATGTGAC GAACCAAGTT TAAAAACGTT CACGGAGACG AATGTTATGT TACTGAATTA ACCTACTGTA   
  
  
+ ATAGAAGTTT TGATAAAAGA ATTTTTTAAT TATTAAAGTG GTCATTAGAC ATCCCCAATC TAGCAGAAAA   
  
  
+ TGAACGAACA GAACCGAGAA TGGACGTTAA GCTGTATCGA CAGGATGAAT GATGAAACGT ACCGCTAGAT   
  
  
+ AGTGGTGACG GTAACGGAGT TAGGCTTGCG TTTATTTATA TACTAAAACA AAGAAACCGT CGTTTTTATT   
  
  
+ TACTTGCAAC TTTTAAGTTT TGTTTATAAA TCATTAACCC AGTCAGTTCA AACTAAATTA CAATTTAAAA   
  
  
+ GAATTTATTA ATTTTTTATT CATTTATATC TTTTTTTAAT ACTTCATTCG AATGTAAAAT TAAAATTAAA   
  
  
+ TAAATAATTT AATGAACATA TAAATTGAAA ATTTGAATTA AAGTTAATAA AATTATTAAT ATTTTTAATT   
  
  
+ TTTATAAAAT TGCCAAATCT ATAGAAATTT AGATATAAAC AATGCATCTT TATTTAACTC TAATTTGTTT   
  
  
+ ACAATAGGAT TAGAATATAA ATTCACGTAA CTACTCTAAC TCTATTCAAA TAAAATACAA AAAAATTAAG   
  
  
+ TACTCTCAGG GGATTTAGTT CTATGATCTA GAACTAAAGT CAACATAATT TTAAAATTAT TTTTTATATA   
  
  
+ TAATATTAAA TTATTTTATT TAACAATTTT TATAAAGATG TAAAAAAAGA GTGTAATTAT ATGATTTGAC   
  
  
+ GTTTTATAAT AAAAATAGAA GAATAATTTT TTTGAATTGT GTTTTATAAT AAAAATAGAA GAATAGTTTT   
  
  
+ TTTGAATTGT AACACTTTAA AAATAGACTT AAAGTATCTC GAATTAGTTT TAACTTCGGA AACTAAAACT   
  
  
+ ATTATAATAA TATAAAATTT TTTAAATTA  

- GATTTCACCT AGCGAGACAC GTCTGAGATG GTAAACGGAG AAAAGCAGCT CTTGGAGCTT TTGTTGTTCG   
  
  
- GGGTTGTTCG TCTACAAAAA CTTCCGCTTC GCCCCAGTGA TCAGTCGATT GGAACTGAAA CCCAGATGAC   
  
  
- AGAGAAAACA ACAGAAAATG GGACTTAACA TGGCATCCAA AGTTGGTTTT GTCGGAGAGA CCCTGGTAGA   
  
  
- ACAAATACAA TTCCCAGTGT TTGGCTTTTC AGAAAGAAAG AGATAGACAT GGAAGGATGC CGCAACTAAT   
  
  
- TGTCCGGTAT TGTGAAAATG CTGACACGTA ATCACAGAGA AATGAGTTGG GCCCCACAAC ACTGGTCCAG   
  
  
- GATTTTCTTT TCTTTTCTGT ACGAATTTCT TAATTTGAAA ATGAATGGGA TTTTAATTAA TGTAGGATAG   
  
  
- ACAATTAAGC TGTTCCGACA TTTATGGCAC GTGACGAAAT GTAGGCCACA TGTGTTTTTC TTGTTGAAAA   
  
  
- TGGTTTACCC ACGTGTTGTA TATCTTTTGG CAACCTGAAT TCCGGATTTT GTTCCACAAA TCCCCAATTT   
  
  
- TTATACACTG CTTGGTTCAA ATTTTTGCAA GTGCCTCTGC TTACAATACA ATGACTTAAT TGGATGACAT   
  
  
- TATCTTCAAA ACTATTTTCT TAAAAAATTA ATAATTTCAC CAGTAATCTG TAGGGGTTAG ATCGTCTTTT   
  
  
- ACTTGCTTGT CTTGGCTCTT ACCTGCAATT CGACATAGCT GTCCTACTTA CTACTTTGCA TGGCGATCTA   
  
  
- TCACCACTGC CATTGCCTCA ATCCGAACGC AAATAAATAT ATGATTTTGT TTCTTTGGCA GCAAAAATAA   
  
  
- ATGAACGTTG AAAATTCAAA ACAAATATTT AGTAATTGGG TCAGTCAAGT TTGATTTAAT GTTAAATTTT   
  
  
- CTTAAATAAT TAAAAAATAA GTAAATATAG AAAAAAATTA TGAAGTAAGC TTACATTTTA ATTTTAATTT   
  
  
- ATTTATTAAA TTACTTGTAT ATTTAACTTT TAAACTTAAT TTCAATTATT TTAATAATTA TAAAAATTAA   
  
  
- AAATATTTTA ACGGTTTAGA TATCTTTAAA TCTATATTTG TTACGTAGAA ATAAATTGAG ATTAAACAAA   
  
  
- TGTTATCCTA ATCTTATATT TAAGTGCATT GATGAGATTG AGATAAGTTT ATTTTATGTT TTTTTAATTC   
  
  
- ATGAGAGTCC CCTAAATCAA GATACTAGAT CTTGATTTCA GTTGTATTAA AATTTTAATA AAAAATATAT   
  
  
- ATTATAATTT AATAAAATAA ATTGTTAAAA ATATTTCTAC ATTTTTTTCT CACATTAATA TACTAAACTG   
  
  
- CAAAATATTA TTTTTATCTT CTTATTAAAA AAACTTAACA CAAAATATTA TTTTTATCTT CTTATCAAAA   
  
  
- AAACTTAACA TTGTGAAATT TTTATCTGAA TTTCATAGAG CTTAATCAAA ATTGAAGCCT TTGATTTTGA   
  
  
- TAATATTATT ATATTTTAAA AAATTTAAT

+     GT1-motif

| Site Name | Organism | Position | Strand | Matrix score. | sequence | function |
| --- | --- | --- | --- | --- | --- | --- |
| GT1-motif | Arabidopsis thaliana | 618 | - | 6 | GGTTAA | light responsive element |
| GT1-motif | Arabidopsis thaliana | 554 | + | 6 | GGTTAA | light responsive element |
| GT1-motif | Arabidopsis thaliana | 874 | - | 6 | GGTTAA | light responsive element |
| GT1-motif | Avena sativa | 617 | - | 7 | GGTTAAT | light responsive element |
| GT1-motif | Avena sativa | 873 | - | 7 | GGTTAAT | light responsive element |

> 2018/04/13 10:10:12  
+ CTAAAGTGGA TCGCTCTGTG CAGACTCTAC CATTTGCCTC TTTTCGTCGA GAACCTCGAA AACAACAAGC   
  
  
+ CCCAACAAGC AGATGTTTTT GAAGGCGAAG CGGGGTCACT AGTCAGCTAA CCTTGACTTT GGGTCTACTG   
  
  
+ TCTCTTTTGT TGTCTTTTAC CCTGAATTGT ACCGTAGGTT TCAACCAAAA CAGCCTCTCT GGGACCATCT   
  
  
+ TGTTTATGTT AAGGGTCACA AACCGAAAAG TCTTTCTTTC TCTATCTGTA CCTTCCTACG GCGTTGATTA   
  
  
+ ACAGGCCATA ACACTTTTAC GACTGTGCAT TAGTGTCTCT TTACTCAACC CGGGGTGTTG TGACCAGGTC   
  
  
+ CTAAAAGAAA AGAAAAGACA TGCTTAAAGA ATTAAACTTT TACTTACCCT AAAATTAATT ACATCCTATC   
  
  
+ TGTTAATTCG ACAAGGCTGT AAATACCGTG CACTGCTTTA CATCCGGTGT ACACAAAAAG AACAACTTTT   
  
  
+ ACCAAATGGG TGCACAACAT ATAGAAAACC GTTGGACTTA AGGCCTAAAA CAAGGTGTTT AGGGGTTAAA   
  
  
+ AATATGTGAC GAACCAAGTT TAAAAACGTT CACGGAGACG AATGTTATGT TACTGAATTA ACCTACTGTA   
  
  
+ ATAGAAGTTT TGATAAAAGA ATTTTTTAAT TATTAAAGTG GTCATTAGAC ATCCCCAATC TAGCAGAAAA   
  
  
+ TGAACGAACA GAACCGAGAA TGGACGTTAA GCTGTATCGA CAGGATGAAT GATGAAACGT ACCGCTAGAT   
  
  
+ AGTGGTGACG GTAACGGAGT TAGGCTTGCG TTTATTTATA TACTAAAACA AAGAAACCGT CGTTTTTATT   
  
  
+ TACTTGCAAC TTTTAAGTTT TGTTTATAAA TCATTAACCC AGTCAGTTCA AACTAAATTA CAATTTAAAA   
  
  
+ GAATTTATTA ATTTTTTATT CATTTATATC TTTTTTTAAT ACTTCATTCG AATGTAAAAT TAAAATTAAA   
  
  
+ TAAATAATTT AATGAACATA TAAATTGAAA ATTTGAATTA AAGTTAATAA AATTATTAAT ATTTTTAATT   
  
  
+ TTTATAAAAT TGCCAAATCT ATAGAAATTT AGATATAAAC AATGCATCTT TATTTAACTC TAATTTGTTT   
  
  
+ ACAATAGGAT TAGAATATAA ATTCACGTAA CTACTCTAAC TCTATTCAAA TAAAATACAA AAAAATTAAG   
  
  
+ TACTCTCAGG GGATTTAGTT CTATGATCTA GAACTAAAGT CAACATAATT TTAAAATTAT TTTTTATATA   
  
  
+ TAATATTAAA TTATTTTATT TAACAATTTT TATAAAGATG TAAAAAAAGA GTGTAATTAT ATGATTTGAC   
  
  
+ GTTTTATAAT AAAAATAGAA GAATAATTTT TTTGAATTGT GTTTTATAAT AAAAATAGAA GAATAGTTTT   
  
  
+ TTTGAATTGT AACACTTTAA AAATAGACTT AAAGTATCTC GAATTAGTTT TAACTTCGGA AACTAAAACT   
  
  
+ ATTATAATAA TATAAAATTT TTTAAATTA  

- GATTTCACCT AGCGAGACAC GTCTGAGATG GTAAACGGAG AAAAGCAGCT CTTGGAGCTT TTGTTGTTCG   
  
  
- GGGTTGTTCG TCTACAAAAA CTTCCGCTTC GCCCCAGTGA TCAGTCGATT GGAACTGAAA CCCAGATGAC   
  
  
- AGAGAAAACA ACAGAAAATG GGACTTAACA TGGCATCCAA AGTTGGTTTT GTCGGAGAGA CCCTGGTAGA   
  
  
- ACAAATACAA TTCCCAGTGT TTGGCTTTTC AGAAAGAAAG AGATAGACAT GGAAGGATGC CGCAACTAAT   
  
  
- TGTCCGGTAT TGTGAAAATG CTGACACGTA ATCACAGAGA AATGAGTTGG GCCCCACAAC ACTGGTCCAG   
  
  
- GATTTTCTTT TCTTTTCTGT ACGAATTTCT TAATTTGAAA ATGAATGGGA TTTTAATTAA TGTAGGATAG   
  
  
- ACAATTAAGC TGTTCCGACA TTTATGGCAC GTGACGAAAT GTAGGCCACA TGTGTTTTTC TTGTTGAAAA   
  
  
- TGGTTTACCC ACGTGTTGTA TATCTTTTGG CAACCTGAAT TCCGGATTTT GTTCCACAAA TCCCCAATTT   
  
  
- TTATACACTG CTTGGTTCAA ATTTTTGCAA GTGCCTCTGC TTACAATACA ATGACTTAAT TGGATGACAT   
  
  
- TATCTTCAAA ACTATTTTCT TAAAAAATTA ATAATTTCAC CAGTAATCTG TAGGGGTTAG ATCGTCTTTT   
  
  
- ACTTGCTTGT CTTGGCTCTT ACCTGCAATT CGACATAGCT GTCCTACTTA CTACTTTGCA TGGCGATCTA   
  
  
- TCACCACTGC CATTGCCTCA ATCCGAACGC AAATAAATAT ATGATTTTGT TTCTTTGGCA GCAAAAATAA   
  
  
- ATGAACGTTG AAAATTCAAA ACAAATATTT AGTAATTGGG TCAGTCAAGT TTGATTTAAT GTTAAATTTT   
  
  
- CTTAAATAAT TAAAAAATAA GTAAATATAG AAAAAAATTA TGAAGTAAGC TTACATTTTA ATTTTAATTT   
  
  
- ATTTATTAAA TTACTTGTAT ATTTAACTTT TAAACTTAAT TTCAATTATT TTAATAATTA TAAAAATTAA   
  
  
- AAATATTTTA ACGGTTTAGA TATCTTTAAA TCTATATTTG TTACGTAGAA ATAAATTGAG ATTAAACAAA   
  
  
- TGTTATCCTA ATCTTATATT TAAGTGCATT GATGAGATTG AGATAAGTTT ATTTTATGTT TTTTTAATTC   
  
  
- ATGAGAGTCC CCTAAATCAA GATACTAGAT CTTGATTTCA GTTGTATTAA AATTTTAATA AAAAATATAT   
  
  
- ATTATAATTT AATAAAATAA ATTGTTAAAA ATATTTCTAC ATTTTTTTCT CACATTAATA TACTAAACTG   
  
  
- CAAAATATTA TTTTTATCTT CTTATTAAAA AAACTTAACA CAAAATATTA TTTTTATCTT CTTATCAAAA   
  
  
- AAACTTAACA TTGTGAAATT TTTATCTGAA TTTCATAGAG CTTAATCAAA ATTGAAGCCT TTGATTTTGA   
  
  
- TAATATTATT ATATTTTAAA AAATTTAAT

+     HSE

| Site Name | Organism | Position | Strand | Matrix score. | sequence | function |
| --- | --- | --- | --- | --- | --- | --- |
| HSE | Brassica oleracea | 1484 | - | 9 | AAAAAATTTC | cis-acting element involved in heat stress responsiveness |

> 2018/04/13 10:10:12  
+ CTAAAGTGGA TCGCTCTGTG CAGACTCTAC CATTTGCCTC TTTTCGTCGA GAACCTCGAA AACAACAAGC   
  
  
+ CCCAACAAGC AGATGTTTTT GAAGGCGAAG CGGGGTCACT AGTCAGCTAA CCTTGACTTT GGGTCTACTG   
  
  
+ TCTCTTTTGT TGTCTTTTAC CCTGAATTGT ACCGTAGGTT TCAACCAAAA CAGCCTCTCT GGGACCATCT   
  
  
+ TGTTTATGTT AAGGGTCACA AACCGAAAAG TCTTTCTTTC TCTATCTGTA CCTTCCTACG GCGTTGATTA   
  
  
+ ACAGGCCATA ACACTTTTAC GACTGTGCAT TAGTGTCTCT TTACTCAACC CGGGGTGTTG TGACCAGGTC   
  
  
+ CTAAAAGAAA AGAAAAGACA TGCTTAAAGA ATTAAACTTT TACTTACCCT AAAATTAATT ACATCCTATC   
  
  
+ TGTTAATTCG ACAAGGCTGT AAATACCGTG CACTGCTTTA CATCCGGTGT ACACAAAAAG AACAACTTTT   
  
  
+ ACCAAATGGG TGCACAACAT ATAGAAAACC GTTGGACTTA AGGCCTAAAA CAAGGTGTTT AGGGGTTAAA   
  
  
+ AATATGTGAC GAACCAAGTT TAAAAACGTT CACGGAGACG AATGTTATGT TACTGAATTA ACCTACTGTA   
  
  
+ ATAGAAGTTT TGATAAAAGA ATTTTTTAAT TATTAAAGTG GTCATTAGAC ATCCCCAATC TAGCAGAAAA   
  
  
+ TGAACGAACA GAACCGAGAA TGGACGTTAA GCTGTATCGA CAGGATGAAT GATGAAACGT ACCGCTAGAT   
  
  
+ AGTGGTGACG GTAACGGAGT TAGGCTTGCG TTTATTTATA TACTAAAACA AAGAAACCGT CGTTTTTATT   
  
  
+ TACTTGCAAC TTTTAAGTTT TGTTTATAAA TCATTAACCC AGTCAGTTCA AACTAAATTA CAATTTAAAA   
  
  
+ GAATTTATTA ATTTTTTATT CATTTATATC TTTTTTTAAT ACTTCATTCG AATGTAAAAT TAAAATTAAA   
  
  
+ TAAATAATTT AATGAACATA TAAATTGAAA ATTTGAATTA AAGTTAATAA AATTATTAAT ATTTTTAATT   
  
  
+ TTTATAAAAT TGCCAAATCT ATAGAAATTT AGATATAAAC AATGCATCTT TATTTAACTC TAATTTGTTT   
  
  
+ ACAATAGGAT TAGAATATAA ATTCACGTAA CTACTCTAAC TCTATTCAAA TAAAATACAA AAAAATTAAG   
  
  
+ TACTCTCAGG GGATTTAGTT CTATGATCTA GAACTAAAGT CAACATAATT TTAAAATTAT TTTTTATATA   
  
  
+ TAATATTAAA TTATTTTATT TAACAATTTT TATAAAGATG TAAAAAAAGA GTGTAATTAT ATGATTTGAC   
  
  
+ GTTTTATAAT AAAAATAGAA GAATAATTTT TTTGAATTGT GTTTTATAAT AAAAATAGAA GAATAGTTTT   
  
  
+ TTTGAATTGT AACACTTTAA AAATAGACTT AAAGTATCTC GAATTAGTTT TAACTTCGGA AACTAAAACT   
  
  
+ ATTATAATAA TATAAAATTT TTTAAATTA  

- GATTTCACCT AGCGAGACAC GTCTGAGATG GTAAACGGAG AAAAGCAGCT CTTGGAGCTT TTGTTGTTCG   
  
  
- GGGTTGTTCG TCTACAAAAA CTTCCGCTTC GCCCCAGTGA TCAGTCGATT GGAACTGAAA CCCAGATGAC   
  
  
- AGAGAAAACA ACAGAAAATG GGACTTAACA TGGCATCCAA AGTTGGTTTT GTCGGAGAGA CCCTGGTAGA   
  
  
- ACAAATACAA TTCCCAGTGT TTGGCTTTTC AGAAAGAAAG AGATAGACAT GGAAGGATGC CGCAACTAAT   
  
  
- TGTCCGGTAT TGTGAAAATG CTGACACGTA ATCACAGAGA AATGAGTTGG GCCCCACAAC ACTGGTCCAG   
  
  
- GATTTTCTTT TCTTTTCTGT ACGAATTTCT TAATTTGAAA ATGAATGGGA TTTTAATTAA TGTAGGATAG   
  
  
- ACAATTAAGC TGTTCCGACA TTTATGGCAC GTGACGAAAT GTAGGCCACA TGTGTTTTTC TTGTTGAAAA   
  
  
- TGGTTTACCC ACGTGTTGTA TATCTTTTGG CAACCTGAAT TCCGGATTTT GTTCCACAAA TCCCCAATTT   
  
  
- TTATACACTG CTTGGTTCAA ATTTTTGCAA GTGCCTCTGC TTACAATACA ATGACTTAAT TGGATGACAT   
  
  
- TATCTTCAAA ACTATTTTCT TAAAAAATTA ATAATTTCAC CAGTAATCTG TAGGGGTTAG ATCGTCTTTT   
  
  
- ACTTGCTTGT CTTGGCTCTT ACCTGCAATT CGACATAGCT GTCCTACTTA CTACTTTGCA TGGCGATCTA   
  
  
- TCACCACTGC CATTGCCTCA ATCCGAACGC AAATAAATAT ATGATTTTGT TTCTTTGGCA GCAAAAATAA   
  
  
- ATGAACGTTG AAAATTCAAA ACAAATATTT AGTAATTGGG TCAGTCAAGT TTGATTTAAT GTTAAATTTT   
  
  
- CTTAAATAAT TAAAAAATAA GTAAATATAG AAAAAAATTA TGAAGTAAGC TTACATTTTA ATTTTAATTT   
  
  
- ATTTATTAAA TTACTTGTAT ATTTAACTTT TAAACTTAAT TTCAATTATT TTAATAATTA TAAAAATTAA   
  
  
- AAATATTTTA ACGGTTTAGA TATCTTTAAA TCTATATTTG TTACGTAGAA ATAAATTGAG ATTAAACAAA   
  
  
- TGTTATCCTA ATCTTATATT TAAGTGCATT GATGAGATTG AGATAAGTTT ATTTTATGTT TTTTTAATTC   
  
  
- ATGAGAGTCC CCTAAATCAA GATACTAGAT CTTGATTTCA GTTGTATTAA AATTTTAATA AAAAATATAT   
  
  
- ATTATAATTT AATAAAATAA ATTGTTAAAA ATATTTCTAC ATTTTTTTCT CACATTAATA TACTAAACTG   
  
  
- CAAAATATTA TTTTTATCTT CTTATTAAAA AAACTTAACA CAAAATATTA TTTTTATCTT CTTATCAAAA   
  
  
- AAACTTAACA TTGTGAAATT TTTATCTGAA TTTCATAGAG CTTAATCAAA ATTGAAGCCT TTGATTTTGA   
  
  
- TAATATTATT ATATTTTAAA AAATTTAAT

+     I-box

| Site Name | Organism | Position | Strand | Matrix score. | sequence | function |
| --- | --- | --- | --- | --- | --- | --- |
| I-box | Solanum tuberosum | 1212 | + | 10 | TATTATCTAGA | part of a light responsive element |

> 2018/04/13 10:10:12  
+ CTAAAGTGGA TCGCTCTGTG CAGACTCTAC CATTTGCCTC TTTTCGTCGA GAACCTCGAA AACAACAAGC   
  
  
+ CCCAACAAGC AGATGTTTTT GAAGGCGAAG CGGGGTCACT AGTCAGCTAA CCTTGACTTT GGGTCTACTG   
  
  
+ TCTCTTTTGT TGTCTTTTAC CCTGAATTGT ACCGTAGGTT TCAACCAAAA CAGCCTCTCT GGGACCATCT   
  
  
+ TGTTTATGTT AAGGGTCACA AACCGAAAAG TCTTTCTTTC TCTATCTGTA CCTTCCTACG GCGTTGATTA   
  
  
+ ACAGGCCATA ACACTTTTAC GACTGTGCAT TAGTGTCTCT TTACTCAACC CGGGGTGTTG TGACCAGGTC   
  
  
+ CTAAAAGAAA AGAAAAGACA TGCTTAAAGA ATTAAACTTT TACTTACCCT AAAATTAATT ACATCCTATC   
  
  
+ TGTTAATTCG ACAAGGCTGT AAATACCGTG CACTGCTTTA CATCCGGTGT ACACAAAAAG AACAACTTTT   
  
  
+ ACCAAATGGG TGCACAACAT ATAGAAAACC GTTGGACTTA AGGCCTAAAA CAAGGTGTTT AGGGGTTAAA   
  
  
+ AATATGTGAC GAACCAAGTT TAAAAACGTT CACGGAGACG AATGTTATGT TACTGAATTA ACCTACTGTA   
  
  
+ ATAGAAGTTT TGATAAAAGA ATTTTTTAAT TATTAAAGTG GTCATTAGAC ATCCCCAATC TAGCAGAAAA   
  
  
+ TGAACGAACA GAACCGAGAA TGGACGTTAA GCTGTATCGA CAGGATGAAT GATGAAACGT ACCGCTAGAT   
  
  
+ AGTGGTGACG GTAACGGAGT TAGGCTTGCG TTTATTTATA TACTAAAACA AAGAAACCGT CGTTTTTATT   
  
  
+ TACTTGCAAC TTTTAAGTTT TGTTTATAAA TCATTAACCC AGTCAGTTCA AACTAAATTA CAATTTAAAA   
  
  
+ GAATTTATTA ATTTTTTATT CATTTATATC TTTTTTTAAT ACTTCATTCG AATGTAAAAT TAAAATTAAA   
  
  
+ TAAATAATTT AATGAACATA TAAATTGAAA ATTTGAATTA AAGTTAATAA AATTATTAAT ATTTTTAATT   
  
  
+ TTTATAAAAT TGCCAAATCT ATAGAAATTT AGATATAAAC AATGCATCTT TATTTAACTC TAATTTGTTT   
  
  
+ ACAATAGGAT TAGAATATAA ATTCACGTAA CTACTCTAAC TCTATTCAAA TAAAATACAA AAAAATTAAG   
  
  
+ TACTCTCAGG GGATTTAGTT CTATGATCTA GAACTAAAGT CAACATAATT TTAAAATTAT TTTTTATATA   
  
  
+ TAATATTAAA TTATTTTATT TAACAATTTT TATAAAGATG TAAAAAAAGA GTGTAATTAT ATGATTTGAC   
  
  
+ GTTTTATAAT AAAAATAGAA GAATAATTTT TTTGAATTGT GTTTTATAAT AAAAATAGAA GAATAGTTTT   
  
  
+ TTTGAATTGT AACACTTTAA AAATAGACTT AAAGTATCTC GAATTAGTTT TAACTTCGGA AACTAAAACT   
  
  
+ ATTATAATAA TATAAAATTT TTTAAATTA  

- GATTTCACCT AGCGAGACAC GTCTGAGATG GTAAACGGAG AAAAGCAGCT CTTGGAGCTT TTGTTGTTCG   
  
  
- GGGTTGTTCG TCTACAAAAA CTTCCGCTTC GCCCCAGTGA TCAGTCGATT GGAACTGAAA CCCAGATGAC   
  
  
- AGAGAAAACA ACAGAAAATG GGACTTAACA TGGCATCCAA AGTTGGTTTT GTCGGAGAGA CCCTGGTAGA   
  
  
- ACAAATACAA TTCCCAGTGT TTGGCTTTTC AGAAAGAAAG AGATAGACAT GGAAGGATGC CGCAACTAAT   
  
  
- TGTCCGGTAT TGTGAAAATG CTGACACGTA ATCACAGAGA AATGAGTTGG GCCCCACAAC ACTGGTCCAG   
  
  
- GATTTTCTTT TCTTTTCTGT ACGAATTTCT TAATTTGAAA ATGAATGGGA TTTTAATTAA TGTAGGATAG   
  
  
- ACAATTAAGC TGTTCCGACA TTTATGGCAC GTGACGAAAT GTAGGCCACA TGTGTTTTTC TTGTTGAAAA   
  
  
- TGGTTTACCC ACGTGTTGTA TATCTTTTGG CAACCTGAAT TCCGGATTTT GTTCCACAAA TCCCCAATTT   
  
  
- TTATACACTG CTTGGTTCAA ATTTTTGCAA GTGCCTCTGC TTACAATACA ATGACTTAAT TGGATGACAT   
  
  
- TATCTTCAAA ACTATTTTCT TAAAAAATTA ATAATTTCAC CAGTAATCTG TAGGGGTTAG ATCGTCTTTT   
  
  
- ACTTGCTTGT CTTGGCTCTT ACCTGCAATT CGACATAGCT GTCCTACTTA CTACTTTGCA TGGCGATCTA   
  
  
- TCACCACTGC CATTGCCTCA ATCCGAACGC AAATAAATAT ATGATTTTGT TTCTTTGGCA GCAAAAATAA   
  
  
- ATGAACGTTG AAAATTCAAA ACAAATATTT AGTAATTGGG TCAGTCAAGT TTGATTTAAT GTTAAATTTT   
  
  
- CTTAAATAAT TAAAAAATAA GTAAATATAG AAAAAAATTA TGAAGTAAGC TTACATTTTA ATTTTAATTT   
  
  
- ATTTATTAAA TTACTTGTAT ATTTAACTTT TAAACTTAAT TTCAATTATT TTAATAATTA TAAAAATTAA   
  
  
- AAATATTTTA ACGGTTTAGA TATCTTTAAA TCTATATTTG TTACGTAGAA ATAAATTGAG ATTAAACAAA   
  
  
- TGTTATCCTA ATCTTATATT TAAGTGCATT GATGAGATTG AGATAAGTTT ATTTTATGTT TTTTTAATTC   
  
  
- ATGAGAGTCC CCTAAATCAA GATACTAGAT CTTGATTTCA GTTGTATTAA AATTTTAATA AAAAATATAT   
  
  
- ATTATAATTT AATAAAATAA ATTGTTAAAA ATATTTCTAC ATTTTTTTCT CACATTAATA TACTAAACTG   
  
  
- CAAAATATTA TTTTTATCTT CTTATTAAAA AAACTTAACA CAAAATATTA TTTTTATCTT CTTATCAAAA   
  
  
- AAACTTAACA TTGTGAAATT TTTATCTGAA TTTCATAGAG CTTAATCAAA ATTGAAGCCT TTGATTTTGA   
  
  
- TAATATTATT ATATTTTAAA AAATTTAAT

+     LTR

| Site Name | Organism | Position | Strand | Matrix score. | sequence | function |
| --- | --- | --- | --- | --- | --- | --- |
| LTR | Hordeum vulgare | 233 | + | 6 | CCGAAA | cis-acting element involved in low-temperature responsiveness |

> 2018/04/13 10:10:12  
+ CTAAAGTGGA TCGCTCTGTG CAGACTCTAC CATTTGCCTC TTTTCGTCGA GAACCTCGAA AACAACAAGC   
  
  
+ CCCAACAAGC AGATGTTTTT GAAGGCGAAG CGGGGTCACT AGTCAGCTAA CCTTGACTTT GGGTCTACTG   
  
  
+ TCTCTTTTGT TGTCTTTTAC CCTGAATTGT ACCGTAGGTT TCAACCAAAA CAGCCTCTCT GGGACCATCT   
  
  
+ TGTTTATGTT AAGGGTCACA AACCGAAAAG TCTTTCTTTC TCTATCTGTA CCTTCCTACG GCGTTGATTA   
  
  
+ ACAGGCCATA ACACTTTTAC GACTGTGCAT TAGTGTCTCT TTACTCAACC CGGGGTGTTG TGACCAGGTC   
  
  
+ CTAAAAGAAA AGAAAAGACA TGCTTAAAGA ATTAAACTTT TACTTACCCT AAAATTAATT ACATCCTATC   
  
  
+ TGTTAATTCG ACAAGGCTGT AAATACCGTG CACTGCTTTA CATCCGGTGT ACACAAAAAG AACAACTTTT   
  
  
+ ACCAAATGGG TGCACAACAT ATAGAAAACC GTTGGACTTA AGGCCTAAAA CAAGGTGTTT AGGGGTTAAA   
  
  
+ AATATGTGAC GAACCAAGTT TAAAAACGTT CACGGAGACG AATGTTATGT TACTGAATTA ACCTACTGTA   
  
  
+ ATAGAAGTTT TGATAAAAGA ATTTTTTAAT TATTAAAGTG GTCATTAGAC ATCCCCAATC TAGCAGAAAA   
  
  
+ TGAACGAACA GAACCGAGAA TGGACGTTAA GCTGTATCGA CAGGATGAAT GATGAAACGT ACCGCTAGAT   
  
  
+ AGTGGTGACG GTAACGGAGT TAGGCTTGCG TTTATTTATA TACTAAAACA AAGAAACCGT CGTTTTTATT   
  
  
+ TACTTGCAAC TTTTAAGTTT TGTTTATAAA TCATTAACCC AGTCAGTTCA AACTAAATTA CAATTTAAAA   
  
  
+ GAATTTATTA ATTTTTTATT CATTTATATC TTTTTTTAAT ACTTCATTCG AATGTAAAAT TAAAATTAAA   
  
  
+ TAAATAATTT AATGAACATA TAAATTGAAA ATTTGAATTA AAGTTAATAA AATTATTAAT ATTTTTAATT   
  
  
+ TTTATAAAAT TGCCAAATCT ATAGAAATTT AGATATAAAC AATGCATCTT TATTTAACTC TAATTTGTTT   
  
  
+ ACAATAGGAT TAGAATATAA ATTCACGTAA CTACTCTAAC TCTATTCAAA TAAAATACAA AAAAATTAAG   
  
  
+ TACTCTCAGG GGATTTAGTT CTATGATCTA GAACTAAAGT CAACATAATT TTAAAATTAT TTTTTATATA   
  
  
+ TAATATTAAA TTATTTTATT TAACAATTTT TATAAAGATG TAAAAAAAGA GTGTAATTAT ATGATTTGAC   
  
  
+ GTTTTATAAT AAAAATAGAA GAATAATTTT TTTGAATTGT GTTTTATAAT AAAAATAGAA GAATAGTTTT   
  
  
+ TTTGAATTGT AACACTTTAA AAATAGACTT AAAGTATCTC GAATTAGTTT TAACTTCGGA AACTAAAACT   
  
  
+ ATTATAATAA TATAAAATTT TTTAAATTA  

- GATTTCACCT AGCGAGACAC GTCTGAGATG GTAAACGGAG AAAAGCAGCT CTTGGAGCTT TTGTTGTTCG   
  
  
- GGGTTGTTCG TCTACAAAAA CTTCCGCTTC GCCCCAGTGA TCAGTCGATT GGAACTGAAA CCCAGATGAC   
  
  
- AGAGAAAACA ACAGAAAATG GGACTTAACA TGGCATCCAA AGTTGGTTTT GTCGGAGAGA CCCTGGTAGA   
  
  
- ACAAATACAA TTCCCAGTGT TTGGCTTTTC AGAAAGAAAG AGATAGACAT GGAAGGATGC CGCAACTAAT   
  
  
- TGTCCGGTAT TGTGAAAATG CTGACACGTA ATCACAGAGA AATGAGTTGG GCCCCACAAC ACTGGTCCAG   
  
  
- GATTTTCTTT TCTTTTCTGT ACGAATTTCT TAATTTGAAA ATGAATGGGA TTTTAATTAA TGTAGGATAG   
  
  
- ACAATTAAGC TGTTCCGACA TTTATGGCAC GTGACGAAAT GTAGGCCACA TGTGTTTTTC TTGTTGAAAA   
  
  
- TGGTTTACCC ACGTGTTGTA TATCTTTTGG CAACCTGAAT TCCGGATTTT GTTCCACAAA TCCCCAATTT   
  
  
- TTATACACTG CTTGGTTCAA ATTTTTGCAA GTGCCTCTGC TTACAATACA ATGACTTAAT TGGATGACAT   
  
  
- TATCTTCAAA ACTATTTTCT TAAAAAATTA ATAATTTCAC CAGTAATCTG TAGGGGTTAG ATCGTCTTTT   
  
  
- ACTTGCTTGT CTTGGCTCTT ACCTGCAATT CGACATAGCT GTCCTACTTA CTACTTTGCA TGGCGATCTA   
  
  
- TCACCACTGC CATTGCCTCA ATCCGAACGC AAATAAATAT ATGATTTTGT TTCTTTGGCA GCAAAAATAA   
  
  
- ATGAACGTTG AAAATTCAAA ACAAATATTT AGTAATTGGG TCAGTCAAGT TTGATTTAAT GTTAAATTTT   
  
  
- CTTAAATAAT TAAAAAATAA GTAAATATAG AAAAAAATTA TGAAGTAAGC TTACATTTTA ATTTTAATTT   
  
  
- ATTTATTAAA TTACTTGTAT ATTTAACTTT TAAACTTAAT TTCAATTATT TTAATAATTA TAAAAATTAA   
  
  
- AAATATTTTA ACGGTTTAGA TATCTTTAAA TCTATATTTG TTACGTAGAA ATAAATTGAG ATTAAACAAA   
  
  
- TGTTATCCTA ATCTTATATT TAAGTGCATT GATGAGATTG AGATAAGTTT ATTTTATGTT TTTTTAATTC   
  
  
- ATGAGAGTCC CCTAAATCAA GATACTAGAT CTTGATTTCA GTTGTATTAA AATTTTAATA AAAAATATAT   
  
  
- ATTATAATTT AATAAAATAA ATTGTTAAAA ATATTTCTAC ATTTTTTTCT CACATTAATA TACTAAACTG   
  
  
- CAAAATATTA TTTTTATCTT CTTATTAAAA AAACTTAACA CAAAATATTA TTTTTATCTT CTTATCAAAA   
  
  
- AAACTTAACA TTGTGAAATT TTTATCTGAA TTTCATAGAG CTTAATCAAA ATTGAAGCCT TTGATTTTGA   
  
  
- TAATATTATT ATATTTTAAA AAATTTAAT

+     MSA-like

| Site Name | Organism | Position | Strand | Matrix score. | sequence | function |
| --- | --- | --- | --- | --- | --- | --- |
| MSA-like | Catharanthus roseus | 518 | - | 9 | TCCAACGGT | cis-acting element involved in cell cycle regulation |

> 2018/04/13 10:10:12  
+ CTAAAGTGGA TCGCTCTGTG CAGACTCTAC CATTTGCCTC TTTTCGTCGA GAACCTCGAA AACAACAAGC   
  
  
+ CCCAACAAGC AGATGTTTTT GAAGGCGAAG CGGGGTCACT AGTCAGCTAA CCTTGACTTT GGGTCTACTG   
  
  
+ TCTCTTTTGT TGTCTTTTAC CCTGAATTGT ACCGTAGGTT TCAACCAAAA CAGCCTCTCT GGGACCATCT   
  
  
+ TGTTTATGTT AAGGGTCACA AACCGAAAAG TCTTTCTTTC TCTATCTGTA CCTTCCTACG GCGTTGATTA   
  
  
+ ACAGGCCATA ACACTTTTAC GACTGTGCAT TAGTGTCTCT TTACTCAACC CGGGGTGTTG TGACCAGGTC   
  
  
+ CTAAAAGAAA AGAAAAGACA TGCTTAAAGA ATTAAACTTT TACTTACCCT AAAATTAATT ACATCCTATC   
  
  
+ TGTTAATTCG ACAAGGCTGT AAATACCGTG CACTGCTTTA CATCCGGTGT ACACAAAAAG AACAACTTTT   
  
  
+ ACCAAATGGG TGCACAACAT ATAGAAAACC GTTGGACTTA AGGCCTAAAA CAAGGTGTTT AGGGGTTAAA   
  
  
+ AATATGTGAC GAACCAAGTT TAAAAACGTT CACGGAGACG AATGTTATGT TACTGAATTA ACCTACTGTA   
  
  
+ ATAGAAGTTT TGATAAAAGA ATTTTTTAAT TATTAAAGTG GTCATTAGAC ATCCCCAATC TAGCAGAAAA   
  
  
+ TGAACGAACA GAACCGAGAA TGGACGTTAA GCTGTATCGA CAGGATGAAT GATGAAACGT ACCGCTAGAT   
  
  
+ AGTGGTGACG GTAACGGAGT TAGGCTTGCG TTTATTTATA TACTAAAACA AAGAAACCGT CGTTTTTATT   
  
  
+ TACTTGCAAC TTTTAAGTTT TGTTTATAAA TCATTAACCC AGTCAGTTCA AACTAAATTA CAATTTAAAA   
  
  
+ GAATTTATTA ATTTTTTATT CATTTATATC TTTTTTTAAT ACTTCATTCG AATGTAAAAT TAAAATTAAA   
  
  
+ TAAATAATTT AATGAACATA TAAATTGAAA ATTTGAATTA AAGTTAATAA AATTATTAAT ATTTTTAATT   
  
  
+ TTTATAAAAT TGCCAAATCT ATAGAAATTT AGATATAAAC AATGCATCTT TATTTAACTC TAATTTGTTT   
  
  
+ ACAATAGGAT TAGAATATAA ATTCACGTAA CTACTCTAAC TCTATTCAAA TAAAATACAA AAAAATTAAG   
  
  
+ TACTCTCAGG GGATTTAGTT CTATGATCTA GAACTAAAGT CAACATAATT TTAAAATTAT TTTTTATATA   
  
  
+ TAATATTAAA TTATTTTATT TAACAATTTT TATAAAGATG TAAAAAAAGA GTGTAATTAT ATGATTTGAC   
  
  
+ GTTTTATAAT AAAAATAGAA GAATAATTTT TTTGAATTGT GTTTTATAAT AAAAATAGAA GAATAGTTTT   
  
  
+ TTTGAATTGT AACACTTTAA AAATAGACTT AAAGTATCTC GAATTAGTTT TAACTTCGGA AACTAAAACT   
  
  
+ ATTATAATAA TATAAAATTT TTTAAATTA  

- GATTTCACCT AGCGAGACAC GTCTGAGATG GTAAACGGAG AAAAGCAGCT CTTGGAGCTT TTGTTGTTCG   
  
  
- GGGTTGTTCG TCTACAAAAA CTTCCGCTTC GCCCCAGTGA TCAGTCGATT GGAACTGAAA CCCAGATGAC   
  
  
- AGAGAAAACA ACAGAAAATG GGACTTAACA TGGCATCCAA AGTTGGTTTT GTCGGAGAGA CCCTGGTAGA   
  
  
- ACAAATACAA TTCCCAGTGT TTGGCTTTTC AGAAAGAAAG AGATAGACAT GGAAGGATGC CGCAACTAAT   
  
  
- TGTCCGGTAT TGTGAAAATG CTGACACGTA ATCACAGAGA AATGAGTTGG GCCCCACAAC ACTGGTCCAG   
  
  
- GATTTTCTTT TCTTTTCTGT ACGAATTTCT TAATTTGAAA ATGAATGGGA TTTTAATTAA TGTAGGATAG   
  
  
- ACAATTAAGC TGTTCCGACA TTTATGGCAC GTGACGAAAT GTAGGCCACA TGTGTTTTTC TTGTTGAAAA   
  
  
- TGGTTTACCC ACGTGTTGTA TATCTTTTGG CAACCTGAAT TCCGGATTTT GTTCCACAAA TCCCCAATTT   
  
  
- TTATACACTG CTTGGTTCAA ATTTTTGCAA GTGCCTCTGC TTACAATACA ATGACTTAAT TGGATGACAT   
  
  
- TATCTTCAAA ACTATTTTCT TAAAAAATTA ATAATTTCAC CAGTAATCTG TAGGGGTTAG ATCGTCTTTT   
  
  
- ACTTGCTTGT CTTGGCTCTT ACCTGCAATT CGACATAGCT GTCCTACTTA CTACTTTGCA TGGCGATCTA   
  
  
- TCACCACTGC CATTGCCTCA ATCCGAACGC AAATAAATAT ATGATTTTGT TTCTTTGGCA GCAAAAATAA   
  
  
- ATGAACGTTG AAAATTCAAA ACAAATATTT AGTAATTGGG TCAGTCAAGT TTGATTTAAT GTTAAATTTT   
  
  
- CTTAAATAAT TAAAAAATAA GTAAATATAG AAAAAAATTA TGAAGTAAGC TTACATTTTA ATTTTAATTT   
  
  
- ATTTATTAAA TTACTTGTAT ATTTAACTTT TAAACTTAAT TTCAATTATT TTAATAATTA TAAAAATTAA   
  
  
- AAATATTTTA ACGGTTTAGA TATCTTTAAA TCTATATTTG TTACGTAGAA ATAAATTGAG ATTAAACAAA   
  
  
- TGTTATCCTA ATCTTATATT TAAGTGCATT GATGAGATTG AGATAAGTTT ATTTTATGTT TTTTTAATTC   
  
  
- ATGAGAGTCC CCTAAATCAA GATACTAGAT CTTGATTTCA GTTGTATTAA AATTTTAATA AAAAATATAT   
  
  
- ATTATAATTT AATAAAATAA ATTGTTAAAA ATATTTCTAC ATTTTTTTCT CACATTAATA TACTAAACTG   
  
  
- CAAAATATTA TTTTTATCTT CTTATTAAAA AAACTTAACA CAAAATATTA TTTTTATCTT CTTATCAAAA   
  
  
- AAACTTAACA TTGTGAAATT TTTATCTGAA TTTCATAGAG CTTAATCAAA ATTGAAGCCT TTGATTTTGA   
  
  
- TAATATTATT ATATTTTAAA AAATTTAAT

+     Nodule-site2

| Site Name | Organism | Position | Strand | Matrix score. | sequence | function |
| --- | --- | --- | --- | --- | --- | --- |
| Nodule-site2 | Glycine max | 978 | - | 15 | CTTAAATTATTTATTT | nodule specific factor binding site |

> 2018/04/13 10:10:12  
+ CTAAAGTGGA TCGCTCTGTG CAGACTCTAC CATTTGCCTC TTTTCGTCGA GAACCTCGAA AACAACAAGC   
  
  
+ CCCAACAAGC AGATGTTTTT GAAGGCGAAG CGGGGTCACT AGTCAGCTAA CCTTGACTTT GGGTCTACTG   
  
  
+ TCTCTTTTGT TGTCTTTTAC CCTGAATTGT ACCGTAGGTT TCAACCAAAA CAGCCTCTCT GGGACCATCT   
  
  
+ TGTTTATGTT AAGGGTCACA AACCGAAAAG TCTTTCTTTC TCTATCTGTA CCTTCCTACG GCGTTGATTA   
  
  
+ ACAGGCCATA ACACTTTTAC GACTGTGCAT TAGTGTCTCT TTACTCAACC CGGGGTGTTG TGACCAGGTC   
  
  
+ CTAAAAGAAA AGAAAAGACA TGCTTAAAGA ATTAAACTTT TACTTACCCT AAAATTAATT ACATCCTATC   
  
  
+ TGTTAATTCG ACAAGGCTGT AAATACCGTG CACTGCTTTA CATCCGGTGT ACACAAAAAG AACAACTTTT   
  
  
+ ACCAAATGGG TGCACAACAT ATAGAAAACC GTTGGACTTA AGGCCTAAAA CAAGGTGTTT AGGGGTTAAA   
  
  
+ AATATGTGAC GAACCAAGTT TAAAAACGTT CACGGAGACG AATGTTATGT TACTGAATTA ACCTACTGTA   
  
  
+ ATAGAAGTTT TGATAAAAGA ATTTTTTAAT TATTAAAGTG GTCATTAGAC ATCCCCAATC TAGCAGAAAA   
  
  
+ TGAACGAACA GAACCGAGAA TGGACGTTAA GCTGTATCGA CAGGATGAAT GATGAAACGT ACCGCTAGAT   
  
  
+ AGTGGTGACG GTAACGGAGT TAGGCTTGCG TTTATTTATA TACTAAAACA AAGAAACCGT CGTTTTTATT   
  
  
+ TACTTGCAAC TTTTAAGTTT TGTTTATAAA TCATTAACCC AGTCAGTTCA AACTAAATTA CAATTTAAAA   
  
  
+ GAATTTATTA ATTTTTTATT CATTTATATC TTTTTTTAAT ACTTCATTCG AATGTAAAAT TAAAATTAAA   
  
  
+ TAAATAATTT AATGAACATA TAAATTGAAA ATTTGAATTA AAGTTAATAA AATTATTAAT ATTTTTAATT   
  
  
+ TTTATAAAAT TGCCAAATCT ATAGAAATTT AGATATAAAC AATGCATCTT TATTTAACTC TAATTTGTTT   
  
  
+ ACAATAGGAT TAGAATATAA ATTCACGTAA CTACTCTAAC TCTATTCAAA TAAAATACAA AAAAATTAAG   
  
  
+ TACTCTCAGG GGATTTAGTT CTATGATCTA GAACTAAAGT CAACATAATT TTAAAATTAT TTTTTATATA   
  
  
+ TAATATTAAA TTATTTTATT TAACAATTTT TATAAAGATG TAAAAAAAGA GTGTAATTAT ATGATTTGAC   
  
  
+ GTTTTATAAT AAAAATAGAA GAATAATTTT TTTGAATTGT GTTTTATAAT AAAAATAGAA GAATAGTTTT   
  
  
+ TTTGAATTGT AACACTTTAA AAATAGACTT AAAGTATCTC GAATTAGTTT TAACTTCGGA AACTAAAACT   
  
  
+ ATTATAATAA TATAAAATTT TTTAAATTA  

- GATTTCACCT AGCGAGACAC GTCTGAGATG GTAAACGGAG AAAAGCAGCT CTTGGAGCTT TTGTTGTTCG   
  
  
- GGGTTGTTCG TCTACAAAAA CTTCCGCTTC GCCCCAGTGA TCAGTCGATT GGAACTGAAA CCCAGATGAC   
  
  
- AGAGAAAACA ACAGAAAATG GGACTTAACA TGGCATCCAA AGTTGGTTTT GTCGGAGAGA CCCTGGTAGA   
  
  
- ACAAATACAA TTCCCAGTGT TTGGCTTTTC AGAAAGAAAG AGATAGACAT GGAAGGATGC CGCAACTAAT   
  
  
- TGTCCGGTAT TGTGAAAATG CTGACACGTA ATCACAGAGA AATGAGTTGG GCCCCACAAC ACTGGTCCAG   
  
  
- GATTTTCTTT TCTTTTCTGT ACGAATTTCT TAATTTGAAA ATGAATGGGA TTTTAATTAA TGTAGGATAG   
  
  
- ACAATTAAGC TGTTCCGACA TTTATGGCAC GTGACGAAAT GTAGGCCACA TGTGTTTTTC TTGTTGAAAA   
  
  
- TGGTTTACCC ACGTGTTGTA TATCTTTTGG CAACCTGAAT TCCGGATTTT GTTCCACAAA TCCCCAATTT   
  
  
- TTATACACTG CTTGGTTCAA ATTTTTGCAA GTGCCTCTGC TTACAATACA ATGACTTAAT TGGATGACAT   
  
  
- TATCTTCAAA ACTATTTTCT TAAAAAATTA ATAATTTCAC CAGTAATCTG TAGGGGTTAG ATCGTCTTTT   
  
  
- ACTTGCTTGT CTTGGCTCTT ACCTGCAATT CGACATAGCT GTCCTACTTA CTACTTTGCA TGGCGATCTA   
  
  
- TCACCACTGC CATTGCCTCA ATCCGAACGC AAATAAATAT ATGATTTTGT TTCTTTGGCA GCAAAAATAA   
  
  
- ATGAACGTTG AAAATTCAAA ACAAATATTT AGTAATTGGG TCAGTCAAGT TTGATTTAAT GTTAAATTTT   
  
  
- CTTAAATAAT TAAAAAATAA GTAAATATAG AAAAAAATTA TGAAGTAAGC TTACATTTTA ATTTTAATTT   
  
  
- ATTTATTAAA TTACTTGTAT ATTTAACTTT TAAACTTAAT TTCAATTATT TTAATAATTA TAAAAATTAA   
  
  
- AAATATTTTA ACGGTTTAGA TATCTTTAAA TCTATATTTG TTACGTAGAA ATAAATTGAG ATTAAACAAA   
  
  
- TGTTATCCTA ATCTTATATT TAAGTGCATT GATGAGATTG AGATAAGTTT ATTTTATGTT TTTTTAATTC   
  
  
- ATGAGAGTCC CCTAAATCAA GATACTAGAT CTTGATTTCA GTTGTATTAA AATTTTAATA AAAAATATAT   
  
  
- ATTATAATTT AATAAAATAA ATTGTTAAAA ATATTTCTAC ATTTTTTTCT CACATTAATA TACTAAACTG   
  
  
- CAAAATATTA TTTTTATCTT CTTATTAAAA AAACTTAACA CAAAATATTA TTTTTATCTT CTTATCAAAA   
  
  
- AAACTTAACA TTGTGAAATT TTTATCTGAA TTTCATAGAG CTTAATCAAA ATTGAAGCCT TTGATTTTGA   
  
  
- TAATATTATT ATATTTTAAA AAATTTAAT

+     Skn-1\_motif

| Site Name | Organism | Position | Strand | Matrix score. | sequence | function |
| --- | --- | --- | --- | --- | --- | --- |
| Skn-1\_motif | Oryza sativa | 671 | + | 5 | GTCAT | cis-acting regulatory element required for endosperm expression |

> 2018/04/13 10:10:12  
+ CTAAAGTGGA TCGCTCTGTG CAGACTCTAC CATTTGCCTC TTTTCGTCGA GAACCTCGAA AACAACAAGC   
  
  
+ CCCAACAAGC AGATGTTTTT GAAGGCGAAG CGGGGTCACT AGTCAGCTAA CCTTGACTTT GGGTCTACTG   
  
  
+ TCTCTTTTGT TGTCTTTTAC CCTGAATTGT ACCGTAGGTT TCAACCAAAA CAGCCTCTCT GGGACCATCT   
  
  
+ TGTTTATGTT AAGGGTCACA AACCGAAAAG TCTTTCTTTC TCTATCTGTA CCTTCCTACG GCGTTGATTA   
  
  
+ ACAGGCCATA ACACTTTTAC GACTGTGCAT TAGTGTCTCT TTACTCAACC CGGGGTGTTG TGACCAGGTC   
  
  
+ CTAAAAGAAA AGAAAAGACA TGCTTAAAGA ATTAAACTTT TACTTACCCT AAAATTAATT ACATCCTATC   
  
  
+ TGTTAATTCG ACAAGGCTGT AAATACCGTG CACTGCTTTA CATCCGGTGT ACACAAAAAG AACAACTTTT   
  
  
+ ACCAAATGGG TGCACAACAT ATAGAAAACC GTTGGACTTA AGGCCTAAAA CAAGGTGTTT AGGGGTTAAA   
  
  
+ AATATGTGAC GAACCAAGTT TAAAAACGTT CACGGAGACG AATGTTATGT TACTGAATTA ACCTACTGTA   
  
  
+ ATAGAAGTTT TGATAAAAGA ATTTTTTAAT TATTAAAGTG GTCATTAGAC ATCCCCAATC TAGCAGAAAA   
  
  
+ TGAACGAACA GAACCGAGAA TGGACGTTAA GCTGTATCGA CAGGATGAAT GATGAAACGT ACCGCTAGAT   
  
  
+ AGTGGTGACG GTAACGGAGT TAGGCTTGCG TTTATTTATA TACTAAAACA AAGAAACCGT CGTTTTTATT   
  
  
+ TACTTGCAAC TTTTAAGTTT TGTTTATAAA TCATTAACCC AGTCAGTTCA AACTAAATTA CAATTTAAAA   
  
  
+ GAATTTATTA ATTTTTTATT CATTTATATC TTTTTTTAAT ACTTCATTCG AATGTAAAAT TAAAATTAAA   
  
  
+ TAAATAATTT AATGAACATA TAAATTGAAA ATTTGAATTA AAGTTAATAA AATTATTAAT ATTTTTAATT   
  
  
+ TTTATAAAAT TGCCAAATCT ATAGAAATTT AGATATAAAC AATGCATCTT TATTTAACTC TAATTTGTTT   
  
  
+ ACAATAGGAT TAGAATATAA ATTCACGTAA CTACTCTAAC TCTATTCAAA TAAAATACAA AAAAATTAAG   
  
  
+ TACTCTCAGG GGATTTAGTT CTATGATCTA GAACTAAAGT CAACATAATT TTAAAATTAT TTTTTATATA   
  
  
+ TAATATTAAA TTATTTTATT TAACAATTTT TATAAAGATG TAAAAAAAGA GTGTAATTAT ATGATTTGAC   
  
  
+ GTTTTATAAT AAAAATAGAA GAATAATTTT TTTGAATTGT GTTTTATAAT AAAAATAGAA GAATAGTTTT   
  
  
+ TTTGAATTGT AACACTTTAA AAATAGACTT AAAGTATCTC GAATTAGTTT TAACTTCGGA AACTAAAACT   
  
  
+ ATTATAATAA TATAAAATTT TTTAAATTA  

- GATTTCACCT AGCGAGACAC GTCTGAGATG GTAAACGGAG AAAAGCAGCT CTTGGAGCTT TTGTTGTTCG   
  
  
- GGGTTGTTCG TCTACAAAAA CTTCCGCTTC GCCCCAGTGA TCAGTCGATT GGAACTGAAA CCCAGATGAC   
  
  
- AGAGAAAACA ACAGAAAATG GGACTTAACA TGGCATCCAA AGTTGGTTTT GTCGGAGAGA CCCTGGTAGA   
  
  
- ACAAATACAA TTCCCAGTGT TTGGCTTTTC AGAAAGAAAG AGATAGACAT GGAAGGATGC CGCAACTAAT   
  
  
- TGTCCGGTAT TGTGAAAATG CTGACACGTA ATCACAGAGA AATGAGTTGG GCCCCACAAC ACTGGTCCAG   
  
  
- GATTTTCTTT TCTTTTCTGT ACGAATTTCT TAATTTGAAA ATGAATGGGA TTTTAATTAA TGTAGGATAG   
  
  
- ACAATTAAGC TGTTCCGACA TTTATGGCAC GTGACGAAAT GTAGGCCACA TGTGTTTTTC TTGTTGAAAA   
  
  
- TGGTTTACCC ACGTGTTGTA TATCTTTTGG CAACCTGAAT TCCGGATTTT GTTCCACAAA TCCCCAATTT   
  
  
- TTATACACTG CTTGGTTCAA ATTTTTGCAA GTGCCTCTGC TTACAATACA ATGACTTAAT TGGATGACAT   
  
  
- TATCTTCAAA ACTATTTTCT TAAAAAATTA ATAATTTCAC CAGTAATCTG TAGGGGTTAG ATCGTCTTTT   
  
  
- ACTTGCTTGT CTTGGCTCTT ACCTGCAATT CGACATAGCT GTCCTACTTA CTACTTTGCA TGGCGATCTA   
  
  
- TCACCACTGC CATTGCCTCA ATCCGAACGC AAATAAATAT ATGATTTTGT TTCTTTGGCA GCAAAAATAA   
  
  
- ATGAACGTTG AAAATTCAAA ACAAATATTT AGTAATTGGG TCAGTCAAGT TTGATTTAAT GTTAAATTTT   
  
  
- CTTAAATAAT TAAAAAATAA GTAAATATAG AAAAAAATTA TGAAGTAAGC TTACATTTTA ATTTTAATTT   
  
  
- ATTTATTAAA TTACTTGTAT ATTTAACTTT TAAACTTAAT TTCAATTATT TTAATAATTA TAAAAATTAA   
  
  
- AAATATTTTA ACGGTTTAGA TATCTTTAAA TCTATATTTG TTACGTAGAA ATAAATTGAG ATTAAACAAA   
  
  
- TGTTATCCTA ATCTTATATT TAAGTGCATT GATGAGATTG AGATAAGTTT ATTTTATGTT TTTTTAATTC   
  
  
- ATGAGAGTCC CCTAAATCAA GATACTAGAT CTTGATTTCA GTTGTATTAA AATTTTAATA AAAAATATAT   
  
  
- ATTATAATTT AATAAAATAA ATTGTTAAAA ATATTTCTAC ATTTTTTTCT CACATTAATA TACTAAACTG   
  
  
- CAAAATATTA TTTTTATCTT CTTATTAAAA AAACTTAACA CAAAATATTA TTTTTATCTT CTTATCAAAA   
  
  
- AAACTTAACA TTGTGAAATT TTTATCTGAA TTTCATAGAG CTTAATCAAA ATTGAAGCCT TTGATTTTGA   
  
  
- TAATATTATT ATATTTTAAA AAATTTAAT

+     TATA-box

| Site Name | Organism | Position | Strand | Matrix score. | sequence | function |
| --- | --- | --- | --- | --- | --- | --- |
| TATA-box | Brassica oleracea | 1480 | + | 6 | ATATAA | core promoter element around -30 of transcription start |
| TATA-box | Zea mays | 1416 | + | 8 | TTTAAAAA | core promoter element around -30 of transcription start |
| TATA-box | Glycine max | 1478 | + | 5 | TAATA | core promoter element around -30 of transcription start |
| TATA-box | Lycopersicon esculentum | 1490 | + | 5 | TTTTA | core promoter element around -30 of transcription start |
| TATA-box | Arabidopsis thaliana | 1317 | - | 5 | TATAA | core promoter element around -30 of transcription start |
| TATA-box | Arabidopsis thaliana | 1290 | - | 5 | TATAA | core promoter element around -30 of transcription start |
| TATA-box | Arabidopsis thaliana | 1070 | - | 4 | TATA | core promoter element around -30 of transcription start |
| TATA-box | Lycopersicon esculentum | 1043 | + | 5 | TTTTA | core promoter element around -30 of transcription start |
| TATA-box | Arabidopsis thaliana | 1053 | + | 6 | TATAAA | core promoter element around -30 of transcription start |
| TATA-box | Glycine max | 1037 | + | 5 | TAATA | core promoter element around -30 of transcription start |
| TATA-box | Glycine max | 916 | - | 5 | TAATA | core promoter element around -30 of transcription start |
| TATA-box | Pisum sativum | 996 | - | 7 | TATATGT | core promoter element around -30 of transcription start |
| TATA-box | Arabidopsis thaliana | 809 | - | 4 | TATA | core promoter element around -30 of transcription start |
| TATA-box | Arabidopsis thaliana | 1052 | - | 5 | TATAA | core promoter element around -30 of transcription start |
| TATA-box | Arabidopsis thaliana | 1051 | - | 6 | TATAAA | core promoter element around -30 of transcription start |
| TATA-box | Ac | 865 | + | 7 | TATAAAT | core promoter element around -30 of transcription start |
| TATA-box | Arabidopsis thaliana | 510 | + | 4 | TATA | core promoter element around -30 of transcription start |
| TATA-box | Arabidopsis thaliana | 506 | - | 9 | tcTATATAtt | core promoter element around -30 of transcription start |
| TATA-box | Lycopersicon esculentum | 1418 | - | 5 | TTTTA | core promoter element around -30 of transcription start |
| TATA-box | Lycopersicon esculentum | 1301 | - | 5 | TTTTA | core promoter element around -30 of transcription start |
| TATA-box | Arabidopsis thaliana | 1289 | - | 6 | TATAAA | core promoter element around -30 of transcription start |
| TATA-box | Arabidopsis thaliana | 1066 | - | 9 | tcTATATAtt | core promoter element around -30 of transcription start |
| TATA-box | Arabidopsis thaliana | 1288 | - | 7 | TATAAAA | core promoter element around -30 of transcription start |
| TATA-box | Lycopersicon esculentum | 1274 | + | 5 | TTTTA | core promoter element around -30 of transcription start |
| TATA-box | Ac | 804 | - | 7 | TATAAAT | core promoter element around -30 of transcription start |
| TATA-box | Glycine max | 1261 | + | 5 | TAATA | core promoter element around -30 of transcription start |
| TATA-box | Arabidopsis thaliana | 1259 | - | 4 | TATA | core promoter element around -30 of transcription start |
| TATA-box | Lycopersicon esculentum | 1380 | - | 5 | TTTTA | core promoter element around -30 of transcription start |
| TATA-box | Arabidopsis thaliana | 1375 | - | 4 | TATA | core promoter element around -30 of transcription start |
| TATA-box | Arabidopsis thaliana | 1372 | - | 7 | TATAAAA | core promoter element around -30 of transcription start |
| TATA-box | Arabidopsis thaliana | 1050 | - | 7 | TATAAAA | core promoter element around -30 of transcription start |
| TATA-box | Lycopersicon esculentum | 906 | - | 5 | TTTTA | core promoter element around -30 of transcription start |
| TATA-box | Pisum sativum | 507 | - | 7 | TATATGT | core promoter element around -30 of transcription start |
| TATA-box | Glycine max | 1475 | + | 5 | TAATA | core promoter element around -30 of transcription start |
| TATA-box | Arabidopsis thaliana | 1473 | - | 4 | TATA | core promoter element around -30 of transcription start |
| TATA-box | Glycine max | 1470 | - | 5 | TAATA | core promoter element around -30 of transcription start |
| TATA-box | Ac | 932 | - | 7 | TATAAAT | core promoter element around -30 of transcription start |
| TATA-box | Arabidopsis thaliana | 935 | - | 4 | TATA | core promoter element around -30 of transcription start |
| TATA-box | Glycine max | 1025 | + | 5 | TAATA | core promoter element around -30 of transcription start |
| TATA-box | Ac | 999 | + | 7 | TATAAAT | core promoter element around -30 of transcription start |
| TATA-box | Arabidopsis thaliana | 803 | - | 9 | taTATAAAtc | core promoter element around -30 of transcription start |
| TATA-box | Lycopersicon esculentum | 654 | + | 5 | TTTTA | core promoter element around -30 of transcription start |
| TATA-box | Arabidopsis thaliana | 1318 | - | 4 | TATA | core promoter element around -30 of transcription start |
| TATA-box | Glycine max | 1377 | + | 5 | TAATA | core promoter element around -30 of transcription start |
| TATA-box | Lycopersicon esculentum | 1055 | - | 5 | TTTTA | core promoter element around -30 of transcription start |
| TATA-box | Lycopersicon esculentum | 581 | - | 5 | TTTTA | core promoter element around -30 of transcription start |
| TATA-box | Daucus carota | 997 | + | 9 | ccTATAAATT | core promoter element around -30 of transcription start |
| TATA-box | Glycine max | 629 | + | 5 | TAATA | core promoter element around -30 of transcription start |
| TATA-box | Lycopersicon esculentum | 965 | - | 5 | TTTTA | core promoter element around -30 of transcription start |
| TATA-box | Glycine max | 947 | + | 5 | TAATA | core promoter element around -30 of transcription start |
| TATA-box | Glycine max | 1034 | - | 5 | TAATA | core promoter element around -30 of transcription start |
| TATA-box | Lycopersicon esculentum | 1464 | - | 5 | TTTTA | core promoter element around -30 of transcription start |
| TATA-box | Arabidopsis thaliana | 1332 | - | 7 | TATAAAA | core promoter element around -30 of transcription start |
| TATA-box | Brassica oleracea | 1258 | + | 7 | ATATAAT | core promoter element around -30 of transcription start |
| TATA-box | Brassica oleracea | 998 | + | 6 | ATATAA | core promoter element around -30 of transcription start |
| TATA-box | Arabidopsis thaliana | 863 | - | 6 | TATAAA | core promoter element around -30 of transcription start |
| TATA-box | Arabidopsis thaliana | 1481 | + | 6 | TATAAA | core promoter element around -30 of transcription start |
| TATA-box | Brassica napus | 1471 | + | 6 | ATTATA | core promoter element around -30 of transcription start |
| TATA-box | Arabidopsis thaliana | 1333 | - | 6 | TATAAA | core promoter element around -30 of transcription start |
| TATA-box | Lycopersicon esculentum | 155 | + | 5 | TTTTA | core promoter element around -30 of transcription start |
| TATA-box | Lycopersicon esculentum | 924 | + | 5 | TTTTA | core promoter element around -30 of transcription start |
| TATA-box | Glycine max | 1264 | - | 5 | TAATA | core promoter element around -30 of transcription start |
| TATA-box | Lycopersicon esculentum | 352 | - | 5 | TTTTA | core promoter element around -30 of transcription start |
| TATA-box | Brassica napus | 1316 | + | 6 | ATTATA | core promoter element around -30 of transcription start |
| TATA-box | Arabidopsis thaliana | 933 | - | 6 | TATAAA | core promoter element around -30 of transcription start |
| TATA-box | Arabidopsis thaliana | 934 | - | 5 | TATAA | core promoter element around -30 of transcription start |
| TATA-box | Ac | 1136 | + | 7 | TATAAAT | core promoter element around -30 of transcription start |
| TATA-box | Brassica oleracea | 1135 | + | 6 | ATATAA | core promoter element around -30 of transcription start |
| TATA-box | Arabidopsis thaliana | 864 | - | 5 | TATAA | core promoter element around -30 of transcription start |
| TATA-box | Lycopersicon esculentum | 971 | - | 5 | TTTTA | core promoter element around -30 of transcription start |
| TATA-box | Lycopersicon esculentum | 1239 | + | 5 | TTTTA | core promoter element around -30 of transcription start |
| TATA-box | Lycopersicon esculentum | 536 | - | 5 | TTTTA | core promoter element around -30 of transcription start |
| TATA-box | Arabidopsis thaliana | 805 | - | 6 | TATAAA | core promoter element around -30 of transcription start |
| TATA-box | Zea mays | 579 | + | 8 | TTTAAAAA | core promoter element around -30 of transcription start |
| TATA-box | Arabidopsis thaliana | 1374 | - | 5 | TATAA | core promoter element around -30 of transcription start |
| TATA-box | Brassica napus | 1256 | - | 6 | ATATAT | core promoter element around -30 of transcription start |
| TATA-box | Lycopersicon esculentum | 1028 | - | 5 | TTTTA | core promoter element around -30 of transcription start |
| TATA-box | Arabidopsis thaliana | 1255 | - | 8 | TATATATA | core promoter element around -30 of transcription start |
| TATA-box | Arabidopsis thaliana | 1254 | - | 7 | TATATAA | core promoter element around -30 of transcription start |
| TATA-box | Zea mays | 1489 | - | 8 | TTTAAAAA | core promoter element around -30 of transcription start |
| TATA-box | Lycopersicon esculentum | 944 | + | 5 | TTTTA | core promoter element around -30 of transcription start |
| TATA-box | Lycopersicon esculentum | 1448 | + | 5 | TTTTA | core promoter element around -30 of transcription start |
| TATA-box | Lycopersicon esculentum | 557 | - | 5 | TTTTA | core promoter element around -30 of transcription start |
| TATA-box | Lycopersicon esculentum | 644 | - | 5 | TTTTA | core promoter element around -30 of transcription start |
| TATA-box | Arabidopsis thaliana | 1291 | + | 6 | TATAAA | core promoter element around -30 of transcription start |
| TATA-box | Arabidopsis thaliana | 1253 | - | 6 | TATAAA | core promoter element around -30 of transcription start |
| TATA-box | Arabidopsis thaliana | 1257 | - | 4 | TATA | core promoter element around -30 of transcription start |
| TATA-box | Lycopersicon esculentum | 1483 | - | 5 | TTTTA | core promoter element around -30 of transcription start |
| TATA-box | Brassica oleracea | 1083 | + | 6 | ATATAA | core promoter element around -30 of transcription start |
| TATA-box | Lycopersicon esculentum | 1340 | - | 5 | TTTTA | core promoter element around -30 of transcription start |
| TATA-box | Lycopersicon esculentum | 400 | - | 5 | TTTTA | core promoter element around -30 of transcription start |
| TATA-box | Lycopersicon esculentum | 295 | + | 5 | TTTTA | core promoter element around -30 of transcription start |
| TATA-box | Oryza sativa | 1176 | + | 7 | TACAAAA | core promoter element around -30 of transcription start |
| TATA-box | Lycopersicon esculentum | 1171 | - | 5 | TTTTA | core promoter element around -30 of transcription start |
| TATA-box | Arabidopsis thaliana | 1472 | - | 5 | TATAA | core promoter element around -30 of transcription start |
| TATA-box | Lycopersicon esculentum | 1242 | - | 5 | TTTTA | core promoter element around -30 of transcription start |
| TATA-box | Lycopersicon esculentum | 851 | + | 5 | TTTTA | core promoter element around -30 of transcription start |
| TATA-box | Arabidopsis thaliana | 807 | - | 4 | TATA | core promoter element around -30 of transcription start |
| TATA-box | Arabidopsis thaliana | 1373 | - | 6 | TATAAA | core promoter element around -30 of transcription start |
| TATA-box | Arabidopsis thaliana | 1334 | - | 5 | TATAA | core promoter element around -30 of transcription start |
| TATA-box | Lycopersicon esculentum | 487 | + | 5 | TTTTA | core promoter element around -30 of transcription start |
| TATA-box | Lycopersicon esculentum | 388 | + | 5 | TTTTA | core promoter element around -30 of transcription start |
| TATA-box | Arabidopsis thaliana | 1252 | - | 7 | TATAAAA | core promoter element around -30 of transcription start |
| TATA-box | Arabidopsis thaliana | 1084 | + | 6 | TATAAA | core promoter element around -30 of transcription start |
| TATA-box | Lycopersicon esculentum | 814 | - | 5 | TTTTA | core promoter element around -30 of transcription start |
| TATA-box | Arabidopsis thaliana | 806 | - | 7 | TATATAA | core promoter element around -30 of transcription start |
| TATA-box | Lycopersicon esculentum | 834 | + | 5 | TTTTA | core promoter element around -30 of transcription start |
| TATA-box | Glycine max | 661 | - | 5 | TAATA | core promoter element around -30 of transcription start |
| TATA-box | Arabidopsis thaliana | 1335 | - | 4 | TATA | core promoter element around -30 of transcription start |
| TATA-box | Glycine max | 1337 | + | 5 | TAATA | core promoter element around -30 of transcription start |

> 2018/04/13 10:10:12  
+ CTAAAGTGGA TCGCTCTGTG CAGACTCTAC CATTTGCCTC TTTTCGTCGA GAACCTCGAA AACAACAAGC   
  
  
+ CCCAACAAGC AGATGTTTTT GAAGGCGAAG CGGGGTCACT AGTCAGCTAA CCTTGACTTT GGGTCTACTG   
  
  
+ TCTCTTTTGT TGTCTTTTAC CCTGAATTGT ACCGTAGGTT TCAACCAAAA CAGCCTCTCT GGGACCATCT   
  
  
+ TGTTTATGTT AAGGGTCACA AACCGAAAAG TCTTTCTTTC TCTATCTGTA CCTTCCTACG GCGTTGATTA   
  
  
+ ACAGGCCATA ACACTTTTAC GACTGTGCAT TAGTGTCTCT TTACTCAACC CGGGGTGTTG TGACCAGGTC   
  
  
+ CTAAAAGAAA AGAAAAGACA TGCTTAAAGA ATTAAACTTT TACTTACCCT AAAATTAATT ACATCCTATC   
  
  
+ TGTTAATTCG ACAAGGCTGT AAATACCGTG CACTGCTTTA CATCCGGTGT ACACAAAAAG AACAACTTTT   
  
  
+ ACCAAATGGG TGCACAACAT ATAGAAAACC GTTGGACTTA AGGCCTAAAA CAAGGTGTTT AGGGGTTAAA   
  
  
+ AATATGTGAC GAACCAAGTT TAAAAACGTT CACGGAGACG AATGTTATGT TACTGAATTA ACCTACTGTA   
  
  
+ ATAGAAGTTT TGATAAAAGA ATTTTTTAAT TATTAAAGTG GTCATTAGAC ATCCCCAATC TAGCAGAAAA   
  
  
+ TGAACGAACA GAACCGAGAA TGGACGTTAA GCTGTATCGA CAGGATGAAT GATGAAACGT ACCGCTAGAT   
  
  
+ AGTGGTGACG GTAACGGAGT TAGGCTTGCG TTTATTTATA TACTAAAACA AAGAAACCGT CGTTTTTATT   
  
  
+ TACTTGCAAC TTTTAAGTTT TGTTTATAAA TCATTAACCC AGTCAGTTCA AACTAAATTA CAATTTAAAA   
  
  
+ GAATTTATTA ATTTTTTATT CATTTATATC TTTTTTTAAT ACTTCATTCG AATGTAAAAT TAAAATTAAA   
  
  
+ TAAATAATTT AATGAACATA TAAATTGAAA ATTTGAATTA AAGTTAATAA AATTATTAAT ATTTTTAATT   
  
  
+ TTTATAAAAT TGCCAAATCT ATAGAAATTT AGATATAAAC AATGCATCTT TATTTAACTC TAATTTGTTT   
  
  
+ ACAATAGGAT TAGAATATAA ATTCACGTAA CTACTCTAAC TCTATTCAAA TAAAATACAA AAAAATTAAG   
  
  
+ TACTCTCAGG GGATTTAGTT CTATGATCTA GAACTAAAGT CAACATAATT TTAAAATTAT TTTTTATATA   
  
  
+ TAATATTAAA TTATTTTATT TAACAATTTT TATAAAGATG TAAAAAAAGA GTGTAATTAT ATGATTTGAC   
  
  
+ GTTTTATAAT AAAAATAGAA GAATAATTTT TTTGAATTGT GTTTTATAAT AAAAATAGAA GAATAGTTTT   
  
  
+ TTTGAATTGT AACACTTTAA AAATAGACTT AAAGTATCTC GAATTAGTTT TAACTTCGGA AACTAAAACT   
  
  
+ ATTATAATAA TATAAAATTT TTTAAATTA  

- GATTTCACCT AGCGAGACAC GTCTGAGATG GTAAACGGAG AAAAGCAGCT CTTGGAGCTT TTGTTGTTCG   
  
  
- GGGTTGTTCG TCTACAAAAA CTTCCGCTTC GCCCCAGTGA TCAGTCGATT GGAACTGAAA CCCAGATGAC   
  
  
- AGAGAAAACA ACAGAAAATG GGACTTAACA TGGCATCCAA AGTTGGTTTT GTCGGAGAGA CCCTGGTAGA   
  
  
- ACAAATACAA TTCCCAGTGT TTGGCTTTTC AGAAAGAAAG AGATAGACAT GGAAGGATGC CGCAACTAAT   
  
  
- TGTCCGGTAT TGTGAAAATG CTGACACGTA ATCACAGAGA AATGAGTTGG GCCCCACAAC ACTGGTCCAG   
  
  
- GATTTTCTTT TCTTTTCTGT ACGAATTTCT TAATTTGAAA ATGAATGGGA TTTTAATTAA TGTAGGATAG   
  
  
- ACAATTAAGC TGTTCCGACA TTTATGGCAC GTGACGAAAT GTAGGCCACA TGTGTTTTTC TTGTTGAAAA   
  
  
- TGGTTTACCC ACGTGTTGTA TATCTTTTGG CAACCTGAAT TCCGGATTTT GTTCCACAAA TCCCCAATTT   
  
  
- TTATACACTG CTTGGTTCAA ATTTTTGCAA GTGCCTCTGC TTACAATACA ATGACTTAAT TGGATGACAT   
  
  
- TATCTTCAAA ACTATTTTCT TAAAAAATTA ATAATTTCAC CAGTAATCTG TAGGGGTTAG ATCGTCTTTT   
  
  
- ACTTGCTTGT CTTGGCTCTT ACCTGCAATT CGACATAGCT GTCCTACTTA CTACTTTGCA TGGCGATCTA   
  
  
- TCACCACTGC CATTGCCTCA ATCCGAACGC AAATAAATAT ATGATTTTGT TTCTTTGGCA GCAAAAATAA   
  
  
- ATGAACGTTG AAAATTCAAA ACAAATATTT AGTAATTGGG TCAGTCAAGT TTGATTTAAT GTTAAATTTT   
  
  
- CTTAAATAAT TAAAAAATAA GTAAATATAG AAAAAAATTA TGAAGTAAGC TTACATTTTA ATTTTAATTT   
  
  
- ATTTATTAAA TTACTTGTAT ATTTAACTTT TAAACTTAAT TTCAATTATT TTAATAATTA TAAAAATTAA   
  
  
- AAATATTTTA ACGGTTTAGA TATCTTTAAA TCTATATTTG TTACGTAGAA ATAAATTGAG ATTAAACAAA   
  
  
- TGTTATCCTA ATCTTATATT TAAGTGCATT GATGAGATTG AGATAAGTTT ATTTTATGTT TTTTTAATTC   
  
  
- ATGAGAGTCC CCTAAATCAA GATACTAGAT CTTGATTTCA GTTGTATTAA AATTTTAATA AAAAATATAT   
  
  
- ATTATAATTT AATAAAATAA ATTGTTAAAA ATATTTCTAC ATTTTTTTCT CACATTAATA TACTAAACTG   
  
  
- CAAAATATTA TTTTTATCTT CTTATTAAAA AAACTTAACA CAAAATATTA TTTTTATCTT CTTATCAAAA   
  
  
- AAACTTAACA TTGTGAAATT TTTATCTGAA TTTCATAGAG CTTAATCAAA ATTGAAGCCT TTGATTTTGA   
  
  
- TAATATTATT ATATTTTAAA AAATTTAAT

+     TCA-element

| Site Name | Organism | Position | Strand | Matrix score. | sequence | function |
| --- | --- | --- | --- | --- | --- | --- |
| TCA-element | Brassica oleracea | 1386 | + | 9 | GAGAAGAATA | cis-acting element involved in salicylic acid responsiveness |
| TCA-element | Brassica oleracea | 1346 | + | 9 | GAGAAGAATA | cis-acting element involved in salicylic acid responsiveness |
| TCA-element | Nicotiana tabacum | 205 | + | 9 | CCATCTTTTT | cis-acting element involved in salicylic acid responsiveness |
| TCA-element | Brassica oleracea | 694 | + | 9 | CAGAAAAGGA | cis-acting element involved in salicylic acid responsiveness |

> 2018/04/13 10:10:12  
+ CTAAAGTGGA TCGCTCTGTG CAGACTCTAC CATTTGCCTC TTTTCGTCGA GAACCTCGAA AACAACAAGC   
  
  
+ CCCAACAAGC AGATGTTTTT GAAGGCGAAG CGGGGTCACT AGTCAGCTAA CCTTGACTTT GGGTCTACTG   
  
  
+ TCTCTTTTGT TGTCTTTTAC CCTGAATTGT ACCGTAGGTT TCAACCAAAA CAGCCTCTCT GGGACCATCT   
  
  
+ TGTTTATGTT AAGGGTCACA AACCGAAAAG TCTTTCTTTC TCTATCTGTA CCTTCCTACG GCGTTGATTA   
  
  
+ ACAGGCCATA ACACTTTTAC GACTGTGCAT TAGTGTCTCT TTACTCAACC CGGGGTGTTG TGACCAGGTC   
  
  
+ CTAAAAGAAA AGAAAAGACA TGCTTAAAGA ATTAAACTTT TACTTACCCT AAAATTAATT ACATCCTATC   
  
  
+ TGTTAATTCG ACAAGGCTGT AAATACCGTG CACTGCTTTA CATCCGGTGT ACACAAAAAG AACAACTTTT   
  
  
+ ACCAAATGGG TGCACAACAT ATAGAAAACC GTTGGACTTA AGGCCTAAAA CAAGGTGTTT AGGGGTTAAA   
  
  
+ AATATGTGAC GAACCAAGTT TAAAAACGTT CACGGAGACG AATGTTATGT TACTGAATTA ACCTACTGTA   
  
  
+ ATAGAAGTTT TGATAAAAGA ATTTTTTAAT TATTAAAGTG GTCATTAGAC ATCCCCAATC TAGCAGAAAA   
  
  
+ TGAACGAACA GAACCGAGAA TGGACGTTAA GCTGTATCGA CAGGATGAAT GATGAAACGT ACCGCTAGAT   
  
  
+ AGTGGTGACG GTAACGGAGT TAGGCTTGCG TTTATTTATA TACTAAAACA AAGAAACCGT CGTTTTTATT   
  
  
+ TACTTGCAAC TTTTAAGTTT TGTTTATAAA TCATTAACCC AGTCAGTTCA AACTAAATTA CAATTTAAAA   
  
  
+ GAATTTATTA ATTTTTTATT CATTTATATC TTTTTTTAAT ACTTCATTCG AATGTAAAAT TAAAATTAAA   
  
  
+ TAAATAATTT AATGAACATA TAAATTGAAA ATTTGAATTA AAGTTAATAA AATTATTAAT ATTTTTAATT   
  
  
+ TTTATAAAAT TGCCAAATCT ATAGAAATTT AGATATAAAC AATGCATCTT TATTTAACTC TAATTTGTTT   
  
  
+ ACAATAGGAT TAGAATATAA ATTCACGTAA CTACTCTAAC TCTATTCAAA TAAAATACAA AAAAATTAAG   
  
  
+ TACTCTCAGG GGATTTAGTT CTATGATCTA GAACTAAAGT CAACATAATT TTAAAATTAT TTTTTATATA   
  
  
+ TAATATTAAA TTATTTTATT TAACAATTTT TATAAAGATG TAAAAAAAGA GTGTAATTAT ATGATTTGAC   
  
  
+ GTTTTATAAT AAAAATAGAA GAATAATTTT TTTGAATTGT GTTTTATAAT AAAAATAGAA GAATAGTTTT   
  
  
+ TTTGAATTGT AACACTTTAA AAATAGACTT AAAGTATCTC GAATTAGTTT TAACTTCGGA AACTAAAACT   
  
  
+ ATTATAATAA TATAAAATTT TTTAAATTA  

- GATTTCACCT AGCGAGACAC GTCTGAGATG GTAAACGGAG AAAAGCAGCT CTTGGAGCTT TTGTTGTTCG   
  
  
- GGGTTGTTCG TCTACAAAAA CTTCCGCTTC GCCCCAGTGA TCAGTCGATT GGAACTGAAA CCCAGATGAC   
  
  
- AGAGAAAACA ACAGAAAATG GGACTTAACA TGGCATCCAA AGTTGGTTTT GTCGGAGAGA CCCTGGTAGA   
  
  
- ACAAATACAA TTCCCAGTGT TTGGCTTTTC AGAAAGAAAG AGATAGACAT GGAAGGATGC CGCAACTAAT   
  
  
- TGTCCGGTAT TGTGAAAATG CTGACACGTA ATCACAGAGA AATGAGTTGG GCCCCACAAC ACTGGTCCAG   
  
  
- GATTTTCTTT TCTTTTCTGT ACGAATTTCT TAATTTGAAA ATGAATGGGA TTTTAATTAA TGTAGGATAG   
  
  
- ACAATTAAGC TGTTCCGACA TTTATGGCAC GTGACGAAAT GTAGGCCACA TGTGTTTTTC TTGTTGAAAA   
  
  
- TGGTTTACCC ACGTGTTGTA TATCTTTTGG CAACCTGAAT TCCGGATTTT GTTCCACAAA TCCCCAATTT   
  
  
- TTATACACTG CTTGGTTCAA ATTTTTGCAA GTGCCTCTGC TTACAATACA ATGACTTAAT TGGATGACAT   
  
  
- TATCTTCAAA ACTATTTTCT TAAAAAATTA ATAATTTCAC CAGTAATCTG TAGGGGTTAG ATCGTCTTTT   
  
  
- ACTTGCTTGT CTTGGCTCTT ACCTGCAATT CGACATAGCT GTCCTACTTA CTACTTTGCA TGGCGATCTA   
  
  
- TCACCACTGC CATTGCCTCA ATCCGAACGC AAATAAATAT ATGATTTTGT TTCTTTGGCA GCAAAAATAA   
  
  
- ATGAACGTTG AAAATTCAAA ACAAATATTT AGTAATTGGG TCAGTCAAGT TTGATTTAAT GTTAAATTTT   
  
  
- CTTAAATAAT TAAAAAATAA GTAAATATAG AAAAAAATTA TGAAGTAAGC TTACATTTTA ATTTTAATTT   
  
  
- ATTTATTAAA TTACTTGTAT ATTTAACTTT TAAACTTAAT TTCAATTATT TTAATAATTA TAAAAATTAA   
  
  
- AAATATTTTA ACGGTTTAGA TATCTTTAAA TCTATATTTG TTACGTAGAA ATAAATTGAG ATTAAACAAA   
  
  
- TGTTATCCTA ATCTTATATT TAAGTGCATT GATGAGATTG AGATAAGTTT ATTTTATGTT TTTTTAATTC   
  
  
- ATGAGAGTCC CCTAAATCAA GATACTAGAT CTTGATTTCA GTTGTATTAA AATTTTAATA AAAAATATAT   
  
  
- ATTATAATTT AATAAAATAA ATTGTTAAAA ATATTTCTAC ATTTTTTTCT CACATTAATA TACTAAACTG   
  
  
- CAAAATATTA TTTTTATCTT CTTATTAAAA AAACTTAACA CAAAATATTA TTTTTATCTT CTTATCAAAA   
  
  
- AAACTTAACA TTGTGAAATT TTTATCTGAA TTTCATAGAG CTTAATCAAA ATTGAAGCCT TTGATTTTGA   
  
  
- TAATATTATT ATATTTTAAA AAATTTAAT

+     TGA-element

| Site Name | Organism | Position | Strand | Matrix score. | sequence | function |
| --- | --- | --- | --- | --- | --- | --- |
| TGA-element | Brassica oleracea | 829 | - | 6 | AACGAC | auxin-responsive element |

> 2018/04/13 10:10:12  
+ CTAAAGTGGA TCGCTCTGTG CAGACTCTAC CATTTGCCTC TTTTCGTCGA GAACCTCGAA AACAACAAGC   
  
  
+ CCCAACAAGC AGATGTTTTT GAAGGCGAAG CGGGGTCACT AGTCAGCTAA CCTTGACTTT GGGTCTACTG   
  
  
+ TCTCTTTTGT TGTCTTTTAC CCTGAATTGT ACCGTAGGTT TCAACCAAAA CAGCCTCTCT GGGACCATCT   
  
  
+ TGTTTATGTT AAGGGTCACA AACCGAAAAG TCTTTCTTTC TCTATCTGTA CCTTCCTACG GCGTTGATTA   
  
  
+ ACAGGCCATA ACACTTTTAC GACTGTGCAT TAGTGTCTCT TTACTCAACC CGGGGTGTTG TGACCAGGTC   
  
  
+ CTAAAAGAAA AGAAAAGACA TGCTTAAAGA ATTAAACTTT TACTTACCCT AAAATTAATT ACATCCTATC   
  
  
+ TGTTAATTCG ACAAGGCTGT AAATACCGTG CACTGCTTTA CATCCGGTGT ACACAAAAAG AACAACTTTT   
  
  
+ ACCAAATGGG TGCACAACAT ATAGAAAACC GTTGGACTTA AGGCCTAAAA CAAGGTGTTT AGGGGTTAAA   
  
  
+ AATATGTGAC GAACCAAGTT TAAAAACGTT CACGGAGACG AATGTTATGT TACTGAATTA ACCTACTGTA   
  
  
+ ATAGAAGTTT TGATAAAAGA ATTTTTTAAT TATTAAAGTG GTCATTAGAC ATCCCCAATC TAGCAGAAAA   
  
  
+ TGAACGAACA GAACCGAGAA TGGACGTTAA GCTGTATCGA CAGGATGAAT GATGAAACGT ACCGCTAGAT   
  
  
+ AGTGGTGACG GTAACGGAGT TAGGCTTGCG TTTATTTATA TACTAAAACA AAGAAACCGT CGTTTTTATT   
  
  
+ TACTTGCAAC TTTTAAGTTT TGTTTATAAA TCATTAACCC AGTCAGTTCA AACTAAATTA CAATTTAAAA   
  
  
+ GAATTTATTA ATTTTTTATT CATTTATATC TTTTTTTAAT ACTTCATTCG AATGTAAAAT TAAAATTAAA   
  
  
+ TAAATAATTT AATGAACATA TAAATTGAAA ATTTGAATTA AAGTTAATAA AATTATTAAT ATTTTTAATT   
  
  
+ TTTATAAAAT TGCCAAATCT ATAGAAATTT AGATATAAAC AATGCATCTT TATTTAACTC TAATTTGTTT   
  
  
+ ACAATAGGAT TAGAATATAA ATTCACGTAA CTACTCTAAC TCTATTCAAA TAAAATACAA AAAAATTAAG   
  
  
+ TACTCTCAGG GGATTTAGTT CTATGATCTA GAACTAAAGT CAACATAATT TTAAAATTAT TTTTTATATA   
  
  
+ TAATATTAAA TTATTTTATT TAACAATTTT TATAAAGATG TAAAAAAAGA GTGTAATTAT ATGATTTGAC   
  
  
+ GTTTTATAAT AAAAATAGAA GAATAATTTT TTTGAATTGT GTTTTATAAT AAAAATAGAA GAATAGTTTT   
  
  
+ TTTGAATTGT AACACTTTAA AAATAGACTT AAAGTATCTC GAATTAGTTT TAACTTCGGA AACTAAAACT   
  
  
+ ATTATAATAA TATAAAATTT TTTAAATTA  

- GATTTCACCT AGCGAGACAC GTCTGAGATG GTAAACGGAG AAAAGCAGCT CTTGGAGCTT TTGTTGTTCG   
  
  
- GGGTTGTTCG TCTACAAAAA CTTCCGCTTC GCCCCAGTGA TCAGTCGATT GGAACTGAAA CCCAGATGAC   
  
  
- AGAGAAAACA ACAGAAAATG GGACTTAACA TGGCATCCAA AGTTGGTTTT GTCGGAGAGA CCCTGGTAGA   
  
  
- ACAAATACAA TTCCCAGTGT TTGGCTTTTC AGAAAGAAAG AGATAGACAT GGAAGGATGC CGCAACTAAT   
  
  
- TGTCCGGTAT TGTGAAAATG CTGACACGTA ATCACAGAGA AATGAGTTGG GCCCCACAAC ACTGGTCCAG   
  
  
- GATTTTCTTT TCTTTTCTGT ACGAATTTCT TAATTTGAAA ATGAATGGGA TTTTAATTAA TGTAGGATAG   
  
  
- ACAATTAAGC TGTTCCGACA TTTATGGCAC GTGACGAAAT GTAGGCCACA TGTGTTTTTC TTGTTGAAAA   
  
  
- TGGTTTACCC ACGTGTTGTA TATCTTTTGG CAACCTGAAT TCCGGATTTT GTTCCACAAA TCCCCAATTT   
  
  
- TTATACACTG CTTGGTTCAA ATTTTTGCAA GTGCCTCTGC TTACAATACA ATGACTTAAT TGGATGACAT   
  
  
- TATCTTCAAA ACTATTTTCT TAAAAAATTA ATAATTTCAC CAGTAATCTG TAGGGGTTAG ATCGTCTTTT   
  
  
- ACTTGCTTGT CTTGGCTCTT ACCTGCAATT CGACATAGCT GTCCTACTTA CTACTTTGCA TGGCGATCTA   
  
  
- TCACCACTGC CATTGCCTCA ATCCGAACGC AAATAAATAT ATGATTTTGT TTCTTTGGCA GCAAAAATAA   
  
  
- ATGAACGTTG AAAATTCAAA ACAAATATTT AGTAATTGGG TCAGTCAAGT TTGATTTAAT GTTAAATTTT   
  
  
- CTTAAATAAT TAAAAAATAA GTAAATATAG AAAAAAATTA TGAAGTAAGC TTACATTTTA ATTTTAATTT   
  
  
- ATTTATTAAA TTACTTGTAT ATTTAACTTT TAAACTTAAT TTCAATTATT TTAATAATTA TAAAAATTAA   
  
  
- AAATATTTTA ACGGTTTAGA TATCTTTAAA TCTATATTTG TTACGTAGAA ATAAATTGAG ATTAAACAAA   
  
  
- TGTTATCCTA ATCTTATATT TAAGTGCATT GATGAGATTG AGATAAGTTT ATTTTATGTT TTTTTAATTC   
  
  
- ATGAGAGTCC CCTAAATCAA GATACTAGAT CTTGATTTCA GTTGTATTAA AATTTTAATA AAAAATATAT   
  
  
- ATTATAATTT AATAAAATAA ATTGTTAAAA ATATTTCTAC ATTTTTTTCT CACATTAATA TACTAAACTG   
  
  
- CAAAATATTA TTTTTATCTT CTTATTAAAA AAACTTAACA CAAAATATTA TTTTTATCTT CTTATCAAAA   
  
  
- AAACTTAACA TTGTGAAATT TTTATCTGAA TTTCATAGAG CTTAATCAAA ATTGAAGCCT TTGATTTTGA   
  
  
- TAATATTATT ATATTTTAAA AAATTTAAT

+     TGACG-motif

| Site Name | Organism | Position | Strand | Matrix score. | sequence | function |
| --- | --- | --- | --- | --- | --- | --- |
| TGACG-motif | Hordeum vulgare | 1327 | + | 5 | TGACG | cis-acting regulatory element involved in the MeJA-responsiveness |
| TGACG-motif | Hordeum vulgare | 776 | + | 5 | TGACG | cis-acting regulatory element involved in the MeJA-responsiveness |
| TGACG-motif | Hordeum vulgare | 567 | + | 5 | TGACG | cis-acting regulatory element involved in the MeJA-responsiveness |

> 2018/04/13 10:10:12  
+ CTAAAGTGGA TCGCTCTGTG CAGACTCTAC CATTTGCCTC TTTTCGTCGA GAACCTCGAA AACAACAAGC   
  
  
+ CCCAACAAGC AGATGTTTTT GAAGGCGAAG CGGGGTCACT AGTCAGCTAA CCTTGACTTT GGGTCTACTG   
  
  
+ TCTCTTTTGT TGTCTTTTAC CCTGAATTGT ACCGTAGGTT TCAACCAAAA CAGCCTCTCT GGGACCATCT   
  
  
+ TGTTTATGTT AAGGGTCACA AACCGAAAAG TCTTTCTTTC TCTATCTGTA CCTTCCTACG GCGTTGATTA   
  
  
+ ACAGGCCATA ACACTTTTAC GACTGTGCAT TAGTGTCTCT TTACTCAACC CGGGGTGTTG TGACCAGGTC   
  
  
+ CTAAAAGAAA AGAAAAGACA TGCTTAAAGA ATTAAACTTT TACTTACCCT AAAATTAATT ACATCCTATC   
  
  
+ TGTTAATTCG ACAAGGCTGT AAATACCGTG CACTGCTTTA CATCCGGTGT ACACAAAAAG AACAACTTTT   
  
  
+ ACCAAATGGG TGCACAACAT ATAGAAAACC GTTGGACTTA AGGCCTAAAA CAAGGTGTTT AGGGGTTAAA   
  
  
+ AATATGTGAC GAACCAAGTT TAAAAACGTT CACGGAGACG AATGTTATGT TACTGAATTA ACCTACTGTA   
  
  
+ ATAGAAGTTT TGATAAAAGA ATTTTTTAAT TATTAAAGTG GTCATTAGAC ATCCCCAATC TAGCAGAAAA   
  
  
+ TGAACGAACA GAACCGAGAA TGGACGTTAA GCTGTATCGA CAGGATGAAT GATGAAACGT ACCGCTAGAT   
  
  
+ AGTGGTGACG GTAACGGAGT TAGGCTTGCG TTTATTTATA TACTAAAACA AAGAAACCGT CGTTTTTATT   
  
  
+ TACTTGCAAC TTTTAAGTTT TGTTTATAAA TCATTAACCC AGTCAGTTCA AACTAAATTA CAATTTAAAA   
  
  
+ GAATTTATTA ATTTTTTATT CATTTATATC TTTTTTTAAT ACTTCATTCG AATGTAAAAT TAAAATTAAA   
  
  
+ TAAATAATTT AATGAACATA TAAATTGAAA ATTTGAATTA AAGTTAATAA AATTATTAAT ATTTTTAATT   
  
  
+ TTTATAAAAT TGCCAAATCT ATAGAAATTT AGATATAAAC AATGCATCTT TATTTAACTC TAATTTGTTT   
  
  
+ ACAATAGGAT TAGAATATAA ATTCACGTAA CTACTCTAAC TCTATTCAAA TAAAATACAA AAAAATTAAG   
  
  
+ TACTCTCAGG GGATTTAGTT CTATGATCTA GAACTAAAGT CAACATAATT TTAAAATTAT TTTTTATATA   
  
  
+ TAATATTAAA TTATTTTATT TAACAATTTT TATAAAGATG TAAAAAAAGA GTGTAATTAT ATGATTTGAC   
  
  
+ GTTTTATAAT AAAAATAGAA GAATAATTTT TTTGAATTGT GTTTTATAAT AAAAATAGAA GAATAGTTTT   
  
  
+ TTTGAATTGT AACACTTTAA AAATAGACTT AAAGTATCTC GAATTAGTTT TAACTTCGGA AACTAAAACT   
  
  
+ ATTATAATAA TATAAAATTT TTTAAATTA  

- GATTTCACCT AGCGAGACAC GTCTGAGATG GTAAACGGAG AAAAGCAGCT CTTGGAGCTT TTGTTGTTCG   
  
  
- GGGTTGTTCG TCTACAAAAA CTTCCGCTTC GCCCCAGTGA TCAGTCGATT GGAACTGAAA CCCAGATGAC   
  
  
- AGAGAAAACA ACAGAAAATG GGACTTAACA TGGCATCCAA AGTTGGTTTT GTCGGAGAGA CCCTGGTAGA   
  
  
- ACAAATACAA TTCCCAGTGT TTGGCTTTTC AGAAAGAAAG AGATAGACAT GGAAGGATGC CGCAACTAAT   
  
  
- TGTCCGGTAT TGTGAAAATG CTGACACGTA ATCACAGAGA AATGAGTTGG GCCCCACAAC ACTGGTCCAG   
  
  
- GATTTTCTTT TCTTTTCTGT ACGAATTTCT TAATTTGAAA ATGAATGGGA TTTTAATTAA TGTAGGATAG   
  
  
- ACAATTAAGC TGTTCCGACA TTTATGGCAC GTGACGAAAT GTAGGCCACA TGTGTTTTTC TTGTTGAAAA   
  
  
- TGGTTTACCC ACGTGTTGTA TATCTTTTGG CAACCTGAAT TCCGGATTTT GTTCCACAAA TCCCCAATTT   
  
  
- TTATACACTG CTTGGTTCAA ATTTTTGCAA GTGCCTCTGC TTACAATACA ATGACTTAAT TGGATGACAT   
  
  
- TATCTTCAAA ACTATTTTCT TAAAAAATTA ATAATTTCAC CAGTAATCTG TAGGGGTTAG ATCGTCTTTT   
  
  
- ACTTGCTTGT CTTGGCTCTT ACCTGCAATT CGACATAGCT GTCCTACTTA CTACTTTGCA TGGCGATCTA   
  
  
- TCACCACTGC CATTGCCTCA ATCCGAACGC AAATAAATAT ATGATTTTGT TTCTTTGGCA GCAAAAATAA   
  
  
- ATGAACGTTG AAAATTCAAA ACAAATATTT AGTAATTGGG TCAGTCAAGT TTGATTTAAT GTTAAATTTT   
  
  
- CTTAAATAAT TAAAAAATAA GTAAATATAG AAAAAAATTA TGAAGTAAGC TTACATTTTA ATTTTAATTT   
  
  
- ATTTATTAAA TTACTTGTAT ATTTAACTTT TAAACTTAAT TTCAATTATT TTAATAATTA TAAAAATTAA   
  
  
- AAATATTTTA ACGGTTTAGA TATCTTTAAA TCTATATTTG TTACGTAGAA ATAAATTGAG ATTAAACAAA   
  
  
- TGTTATCCTA ATCTTATATT TAAGTGCATT GATGAGATTG AGATAAGTTT ATTTTATGTT TTTTTAATTC   
  
  
- ATGAGAGTCC CCTAAATCAA GATACTAGAT CTTGATTTCA GTTGTATTAA AATTTTAATA AAAAATATAT   
  
  
- ATTATAATTT AATAAAATAA ATTGTTAAAA ATATTTCTAC ATTTTTTTCT CACATTAATA TACTAAACTG   
  
  
- CAAAATATTA TTTTTATCTT CTTATTAAAA AAACTTAACA CAAAATATTA TTTTTATCTT CTTATCAAAA   
  
  
- AAACTTAACA TTGTGAAATT TTTATCTGAA TTTCATAGAG CTTAATCAAA ATTGAAGCCT TTGATTTTGA   
  
  
- TAATATTATT ATATTTTAAA AAATTTAAT

+     Unnamed\_\_2

| Site Name | Organism | Position | Strand | Matrix score. | sequence | function |
| --- | --- | --- | --- | --- | --- | --- |
| Unnamed\_\_2 | Zea mays | 330 | - | 6 | CCCCGG |  |

> 2018/04/13 10:10:12  
+ CTAAAGTGGA TCGCTCTGTG CAGACTCTAC CATTTGCCTC TTTTCGTCGA GAACCTCGAA AACAACAAGC   
  
  
+ CCCAACAAGC AGATGTTTTT GAAGGCGAAG CGGGGTCACT AGTCAGCTAA CCTTGACTTT GGGTCTACTG   
  
  
+ TCTCTTTTGT TGTCTTTTAC CCTGAATTGT ACCGTAGGTT TCAACCAAAA CAGCCTCTCT GGGACCATCT   
  
  
+ TGTTTATGTT AAGGGTCACA AACCGAAAAG TCTTTCTTTC TCTATCTGTA CCTTCCTACG GCGTTGATTA   
  
  
+ ACAGGCCATA ACACTTTTAC GACTGTGCAT TAGTGTCTCT TTACTCAACC CGGGGTGTTG TGACCAGGTC   
  
  
+ CTAAAAGAAA AGAAAAGACA TGCTTAAAGA ATTAAACTTT TACTTACCCT AAAATTAATT ACATCCTATC   
  
  
+ TGTTAATTCG ACAAGGCTGT AAATACCGTG CACTGCTTTA CATCCGGTGT ACACAAAAAG AACAACTTTT   
  
  
+ ACCAAATGGG TGCACAACAT ATAGAAAACC GTTGGACTTA AGGCCTAAAA CAAGGTGTTT AGGGGTTAAA   
  
  
+ AATATGTGAC GAACCAAGTT TAAAAACGTT CACGGAGACG AATGTTATGT TACTGAATTA ACCTACTGTA   
  
  
+ ATAGAAGTTT TGATAAAAGA ATTTTTTAAT TATTAAAGTG GTCATTAGAC ATCCCCAATC TAGCAGAAAA   
  
  
+ TGAACGAACA GAACCGAGAA TGGACGTTAA GCTGTATCGA CAGGATGAAT GATGAAACGT ACCGCTAGAT   
  
  
+ AGTGGTGACG GTAACGGAGT TAGGCTTGCG TTTATTTATA TACTAAAACA AAGAAACCGT CGTTTTTATT   
  
  
+ TACTTGCAAC TTTTAAGTTT TGTTTATAAA TCATTAACCC AGTCAGTTCA AACTAAATTA CAATTTAAAA   
  
  
+ GAATTTATTA ATTTTTTATT CATTTATATC TTTTTTTAAT ACTTCATTCG AATGTAAAAT TAAAATTAAA   
  
  
+ TAAATAATTT AATGAACATA TAAATTGAAA ATTTGAATTA AAGTTAATAA AATTATTAAT ATTTTTAATT   
  
  
+ TTTATAAAAT TGCCAAATCT ATAGAAATTT AGATATAAAC AATGCATCTT TATTTAACTC TAATTTGTTT   
  
  
+ ACAATAGGAT TAGAATATAA ATTCACGTAA CTACTCTAAC TCTATTCAAA TAAAATACAA AAAAATTAAG   
  
  
+ TACTCTCAGG GGATTTAGTT CTATGATCTA GAACTAAAGT CAACATAATT TTAAAATTAT TTTTTATATA   
  
  
+ TAATATTAAA TTATTTTATT TAACAATTTT TATAAAGATG TAAAAAAAGA GTGTAATTAT ATGATTTGAC   
  
  
+ GTTTTATAAT AAAAATAGAA GAATAATTTT TTTGAATTGT GTTTTATAAT AAAAATAGAA GAATAGTTTT   
  
  
+ TTTGAATTGT AACACTTTAA AAATAGACTT AAAGTATCTC GAATTAGTTT TAACTTCGGA AACTAAAACT   
  
  
+ ATTATAATAA TATAAAATTT TTTAAATTA  

- GATTTCACCT AGCGAGACAC GTCTGAGATG GTAAACGGAG AAAAGCAGCT CTTGGAGCTT TTGTTGTTCG   
  
  
- GGGTTGTTCG TCTACAAAAA CTTCCGCTTC GCCCCAGTGA TCAGTCGATT GGAACTGAAA CCCAGATGAC   
  
  
- AGAGAAAACA ACAGAAAATG GGACTTAACA TGGCATCCAA AGTTGGTTTT GTCGGAGAGA CCCTGGTAGA   
  
  
- ACAAATACAA TTCCCAGTGT TTGGCTTTTC AGAAAGAAAG AGATAGACAT GGAAGGATGC CGCAACTAAT   
  
  
- TGTCCGGTAT TGTGAAAATG CTGACACGTA ATCACAGAGA AATGAGTTGG GCCCCACAAC ACTGGTCCAG   
  
  
- GATTTTCTTT TCTTTTCTGT ACGAATTTCT TAATTTGAAA ATGAATGGGA TTTTAATTAA TGTAGGATAG   
  
  
- ACAATTAAGC TGTTCCGACA TTTATGGCAC GTGACGAAAT GTAGGCCACA TGTGTTTTTC TTGTTGAAAA   
  
  
- TGGTTTACCC ACGTGTTGTA TATCTTTTGG CAACCTGAAT TCCGGATTTT GTTCCACAAA TCCCCAATTT   
  
  
- TTATACACTG CTTGGTTCAA ATTTTTGCAA GTGCCTCTGC TTACAATACA ATGACTTAAT TGGATGACAT   
  
  
- TATCTTCAAA ACTATTTTCT TAAAAAATTA ATAATTTCAC CAGTAATCTG TAGGGGTTAG ATCGTCTTTT   
  
  
- ACTTGCTTGT CTTGGCTCTT ACCTGCAATT CGACATAGCT GTCCTACTTA CTACTTTGCA TGGCGATCTA   
  
  
- TCACCACTGC CATTGCCTCA ATCCGAACGC AAATAAATAT ATGATTTTGT TTCTTTGGCA GCAAAAATAA   
  
  
- ATGAACGTTG AAAATTCAAA ACAAATATTT AGTAATTGGG TCAGTCAAGT TTGATTTAAT GTTAAATTTT   
  
  
- CTTAAATAAT TAAAAAATAA GTAAATATAG AAAAAAATTA TGAAGTAAGC TTACATTTTA ATTTTAATTT   
  
  
- ATTTATTAAA TTACTTGTAT ATTTAACTTT TAAACTTAAT TTCAATTATT TTAATAATTA TAAAAATTAA   
  
  
- AAATATTTTA ACGGTTTAGA TATCTTTAAA TCTATATTTG TTACGTAGAA ATAAATTGAG ATTAAACAAA   
  
  
- TGTTATCCTA ATCTTATATT TAAGTGCATT GATGAGATTG AGATAAGTTT ATTTTATGTT TTTTTAATTC   
  
  
- ATGAGAGTCC CCTAAATCAA GATACTAGAT CTTGATTTCA GTTGTATTAA AATTTTAATA AAAAATATAT   
  
  
- ATTATAATTT AATAAAATAA ATTGTTAAAA ATATTTCTAC ATTTTTTTCT CACATTAATA TACTAAACTG   
  
  
- CAAAATATTA TTTTTATCTT CTTATTAAAA AAACTTAACA CAAAATATTA TTTTTATCTT CTTATCAAAA   
  
  
- AAACTTAACA TTGTGAAATT TTTATCTGAA TTTCATAGAG CTTAATCAAA ATTGAAGCCT TTGATTTTGA   
  
  
- TAATATTATT ATATTTTAAA AAATTTAAT

+     Unnamed\_\_4

| Site Name | Organism | Position | Strand | Matrix score. | sequence | function |
| --- | --- | --- | --- | --- | --- | --- |
| Unnamed\_\_4 | Petroselinum hortense | 786 | - | 4 | CTCC |  |
| Unnamed\_\_4 | Petroselinum hortense | 594 | - | 4 | CTCC |  |

> 2018/04/13 10:10:12  
+ CTAAAGTGGA TCGCTCTGTG CAGACTCTAC CATTTGCCTC TTTTCGTCGA GAACCTCGAA AACAACAAGC   
  
  
+ CCCAACAAGC AGATGTTTTT GAAGGCGAAG CGGGGTCACT AGTCAGCTAA CCTTGACTTT GGGTCTACTG   
  
  
+ TCTCTTTTGT TGTCTTTTAC CCTGAATTGT ACCGTAGGTT TCAACCAAAA CAGCCTCTCT GGGACCATCT   
  
  
+ TGTTTATGTT AAGGGTCACA AACCGAAAAG TCTTTCTTTC TCTATCTGTA CCTTCCTACG GCGTTGATTA   
  
  
+ ACAGGCCATA ACACTTTTAC GACTGTGCAT TAGTGTCTCT TTACTCAACC CGGGGTGTTG TGACCAGGTC   
  
  
+ CTAAAAGAAA AGAAAAGACA TGCTTAAAGA ATTAAACTTT TACTTACCCT AAAATTAATT ACATCCTATC   
  
  
+ TGTTAATTCG ACAAGGCTGT AAATACCGTG CACTGCTTTA CATCCGGTGT ACACAAAAAG AACAACTTTT   
  
  
+ ACCAAATGGG TGCACAACAT ATAGAAAACC GTTGGACTTA AGGCCTAAAA CAAGGTGTTT AGGGGTTAAA   
  
  
+ AATATGTGAC GAACCAAGTT TAAAAACGTT CACGGAGACG AATGTTATGT TACTGAATTA ACCTACTGTA   
  
  
+ ATAGAAGTTT TGATAAAAGA ATTTTTTAAT TATTAAAGTG GTCATTAGAC ATCCCCAATC TAGCAGAAAA   
  
  
+ TGAACGAACA GAACCGAGAA TGGACGTTAA GCTGTATCGA CAGGATGAAT GATGAAACGT ACCGCTAGAT   
  
  
+ AGTGGTGACG GTAACGGAGT TAGGCTTGCG TTTATTTATA TACTAAAACA AAGAAACCGT CGTTTTTATT   
  
  
+ TACTTGCAAC TTTTAAGTTT TGTTTATAAA TCATTAACCC AGTCAGTTCA AACTAAATTA CAATTTAAAA   
  
  
+ GAATTTATTA ATTTTTTATT CATTTATATC TTTTTTTAAT ACTTCATTCG AATGTAAAAT TAAAATTAAA   
  
  
+ TAAATAATTT AATGAACATA TAAATTGAAA ATTTGAATTA AAGTTAATAA AATTATTAAT ATTTTTAATT   
  
  
+ TTTATAAAAT TGCCAAATCT ATAGAAATTT AGATATAAAC AATGCATCTT TATTTAACTC TAATTTGTTT   
  
  
+ ACAATAGGAT TAGAATATAA ATTCACGTAA CTACTCTAAC TCTATTCAAA TAAAATACAA AAAAATTAAG   
  
  
+ TACTCTCAGG GGATTTAGTT CTATGATCTA GAACTAAAGT CAACATAATT TTAAAATTAT TTTTTATATA   
  
  
+ TAATATTAAA TTATTTTATT TAACAATTTT TATAAAGATG TAAAAAAAGA GTGTAATTAT ATGATTTGAC   
  
  
+ GTTTTATAAT AAAAATAGAA GAATAATTTT TTTGAATTGT GTTTTATAAT AAAAATAGAA GAATAGTTTT   
  
  
+ TTTGAATTGT AACACTTTAA AAATAGACTT AAAGTATCTC GAATTAGTTT TAACTTCGGA AACTAAAACT   
  
  
+ ATTATAATAA TATAAAATTT TTTAAATTA  

- GATTTCACCT AGCGAGACAC GTCTGAGATG GTAAACGGAG AAAAGCAGCT CTTGGAGCTT TTGTTGTTCG   
  
  
- GGGTTGTTCG TCTACAAAAA CTTCCGCTTC GCCCCAGTGA TCAGTCGATT GGAACTGAAA CCCAGATGAC   
  
  
- AGAGAAAACA ACAGAAAATG GGACTTAACA TGGCATCCAA AGTTGGTTTT GTCGGAGAGA CCCTGGTAGA   
  
  
- ACAAATACAA TTCCCAGTGT TTGGCTTTTC AGAAAGAAAG AGATAGACAT GGAAGGATGC CGCAACTAAT   
  
  
- TGTCCGGTAT TGTGAAAATG CTGACACGTA ATCACAGAGA AATGAGTTGG GCCCCACAAC ACTGGTCCAG   
  
  
- GATTTTCTTT TCTTTTCTGT ACGAATTTCT TAATTTGAAA ATGAATGGGA TTTTAATTAA TGTAGGATAG   
  
  
- ACAATTAAGC TGTTCCGACA TTTATGGCAC GTGACGAAAT GTAGGCCACA TGTGTTTTTC TTGTTGAAAA   
  
  
- TGGTTTACCC ACGTGTTGTA TATCTTTTGG CAACCTGAAT TCCGGATTTT GTTCCACAAA TCCCCAATTT   
  
  
- TTATACACTG CTTGGTTCAA ATTTTTGCAA GTGCCTCTGC TTACAATACA ATGACTTAAT TGGATGACAT   
  
  
- TATCTTCAAA ACTATTTTCT TAAAAAATTA ATAATTTCAC CAGTAATCTG TAGGGGTTAG ATCGTCTTTT   
  
  
- ACTTGCTTGT CTTGGCTCTT ACCTGCAATT CGACATAGCT GTCCTACTTA CTACTTTGCA TGGCGATCTA   
  
  
- TCACCACTGC CATTGCCTCA ATCCGAACGC AAATAAATAT ATGATTTTGT TTCTTTGGCA GCAAAAATAA   
  
  
- ATGAACGTTG AAAATTCAAA ACAAATATTT AGTAATTGGG TCAGTCAAGT TTGATTTAAT GTTAAATTTT   
  
  
- CTTAAATAAT TAAAAAATAA GTAAATATAG AAAAAAATTA TGAAGTAAGC TTACATTTTA ATTTTAATTT   
  
  
- ATTTATTAAA TTACTTGTAT ATTTAACTTT TAAACTTAAT TTCAATTATT TTAATAATTA TAAAAATTAA   
  
  
- AAATATTTTA ACGGTTTAGA TATCTTTAAA TCTATATTTG TTACGTAGAA ATAAATTGAG ATTAAACAAA   
  
  
- TGTTATCCTA ATCTTATATT TAAGTGCATT GATGAGATTG AGATAAGTTT ATTTTATGTT TTTTTAATTC   
  
  
- ATGAGAGTCC CCTAAATCAA GATACTAGAT CTTGATTTCA GTTGTATTAA AATTTTAATA AAAAATATAT   
  
  
- ATTATAATTT AATAAAATAA ATTGTTAAAA ATATTTCTAC ATTTTTTTCT CACATTAATA TACTAAACTG   
  
  
- CAAAATATTA TTTTTATCTT CTTATTAAAA AAACTTAACA CAAAATATTA TTTTTATCTT CTTATCAAAA   
  
  
- AAACTTAACA TTGTGAAATT TTTATCTGAA TTTCATAGAG CTTAATCAAA ATTGAAGCCT TTGATTTTGA   
  
  
- TAATATTATT ATATTTTAAA AAATTTAAT

+     circadian

| Site Name | Organism | Position | Strand | Matrix score. | sequence | function |
| --- | --- | --- | --- | --- | --- | --- |
| circadian | Lycopersicon esculentum | 82 | - | 6 | CAANNNNATC | cis-acting regulatory element involved in circadian control |

> 2018/04/13 10:10:12  
+ CTAAAGTGGA TCGCTCTGTG CAGACTCTAC CATTTGCCTC TTTTCGTCGA GAACCTCGAA AACAACAAGC   
  
  
+ CCCAACAAGC AGATGTTTTT GAAGGCGAAG CGGGGTCACT AGTCAGCTAA CCTTGACTTT GGGTCTACTG   
  
  
+ TCTCTTTTGT TGTCTTTTAC CCTGAATTGT ACCGTAGGTT TCAACCAAAA CAGCCTCTCT GGGACCATCT   
  
  
+ TGTTTATGTT AAGGGTCACA AACCGAAAAG TCTTTCTTTC TCTATCTGTA CCTTCCTACG GCGTTGATTA   
  
  
+ ACAGGCCATA ACACTTTTAC GACTGTGCAT TAGTGTCTCT TTACTCAACC CGGGGTGTTG TGACCAGGTC   
  
  
+ CTAAAAGAAA AGAAAAGACA TGCTTAAAGA ATTAAACTTT TACTTACCCT AAAATTAATT ACATCCTATC   
  
  
+ TGTTAATTCG ACAAGGCTGT AAATACCGTG CACTGCTTTA CATCCGGTGT ACACAAAAAG AACAACTTTT   
  
  
+ ACCAAATGGG TGCACAACAT ATAGAAAACC GTTGGACTTA AGGCCTAAAA CAAGGTGTTT AGGGGTTAAA   
  
  
+ AATATGTGAC GAACCAAGTT TAAAAACGTT CACGGAGACG AATGTTATGT TACTGAATTA ACCTACTGTA   
  
  
+ ATAGAAGTTT TGATAAAAGA ATTTTTTAAT TATTAAAGTG GTCATTAGAC ATCCCCAATC TAGCAGAAAA   
  
  
+ TGAACGAACA GAACCGAGAA TGGACGTTAA GCTGTATCGA CAGGATGAAT GATGAAACGT ACCGCTAGAT   
  
  
+ AGTGGTGACG GTAACGGAGT TAGGCTTGCG TTTATTTATA TACTAAAACA AAGAAACCGT CGTTTTTATT   
  
  
+ TACTTGCAAC TTTTAAGTTT TGTTTATAAA TCATTAACCC AGTCAGTTCA AACTAAATTA CAATTTAAAA   
  
  
+ GAATTTATTA ATTTTTTATT CATTTATATC TTTTTTTAAT ACTTCATTCG AATGTAAAAT TAAAATTAAA   
  
  
+ TAAATAATTT AATGAACATA TAAATTGAAA ATTTGAATTA AAGTTAATAA AATTATTAAT ATTTTTAATT   
  
  
+ TTTATAAAAT TGCCAAATCT ATAGAAATTT AGATATAAAC AATGCATCTT TATTTAACTC TAATTTGTTT   
  
  
+ ACAATAGGAT TAGAATATAA ATTCACGTAA CTACTCTAAC TCTATTCAAA TAAAATACAA AAAAATTAAG   
  
  
+ TACTCTCAGG GGATTTAGTT CTATGATCTA GAACTAAAGT CAACATAATT TTAAAATTAT TTTTTATATA   
  
  
+ TAATATTAAA TTATTTTATT TAACAATTTT TATAAAGATG TAAAAAAAGA GTGTAATTAT ATGATTTGAC   
  
  
+ GTTTTATAAT AAAAATAGAA GAATAATTTT TTTGAATTGT GTTTTATAAT AAAAATAGAA GAATAGTTTT   
  
  
+ TTTGAATTGT AACACTTTAA AAATAGACTT AAAGTATCTC GAATTAGTTT TAACTTCGGA AACTAAAACT   
  
  
+ ATTATAATAA TATAAAATTT TTTAAATTA  

- GATTTCACCT AGCGAGACAC GTCTGAGATG GTAAACGGAG AAAAGCAGCT CTTGGAGCTT TTGTTGTTCG   
  
  
- GGGTTGTTCG TCTACAAAAA CTTCCGCTTC GCCCCAGTGA TCAGTCGATT GGAACTGAAA CCCAGATGAC   
  
  
- AGAGAAAACA ACAGAAAATG GGACTTAACA TGGCATCCAA AGTTGGTTTT GTCGGAGAGA CCCTGGTAGA   
  
  
- ACAAATACAA TTCCCAGTGT TTGGCTTTTC AGAAAGAAAG AGATAGACAT GGAAGGATGC CGCAACTAAT   
  
  
- TGTCCGGTAT TGTGAAAATG CTGACACGTA ATCACAGAGA AATGAGTTGG GCCCCACAAC ACTGGTCCAG   
  
  
- GATTTTCTTT TCTTTTCTGT ACGAATTTCT TAATTTGAAA ATGAATGGGA TTTTAATTAA TGTAGGATAG   
  
  
- ACAATTAAGC TGTTCCGACA TTTATGGCAC GTGACGAAAT GTAGGCCACA TGTGTTTTTC TTGTTGAAAA   
  
  
- TGGTTTACCC ACGTGTTGTA TATCTTTTGG CAACCTGAAT TCCGGATTTT GTTCCACAAA TCCCCAATTT   
  
  
- TTATACACTG CTTGGTTCAA ATTTTTGCAA GTGCCTCTGC TTACAATACA ATGACTTAAT TGGATGACAT   
  
  
- TATCTTCAAA ACTATTTTCT TAAAAAATTA ATAATTTCAC CAGTAATCTG TAGGGGTTAG ATCGTCTTTT   
  
  
- ACTTGCTTGT CTTGGCTCTT ACCTGCAATT CGACATAGCT GTCCTACTTA CTACTTTGCA TGGCGATCTA   
  
  
- TCACCACTGC CATTGCCTCA ATCCGAACGC AAATAAATAT ATGATTTTGT TTCTTTGGCA GCAAAAATAA   
  
  
- ATGAACGTTG AAAATTCAAA ACAAATATTT AGTAATTGGG TCAGTCAAGT TTGATTTAAT GTTAAATTTT   
  
  
- CTTAAATAAT TAAAAAATAA GTAAATATAG AAAAAAATTA TGAAGTAAGC TTACATTTTA ATTTTAATTT   
  
  
- ATTTATTAAA TTACTTGTAT ATTTAACTTT TAAACTTAAT TTCAATTATT TTAATAATTA TAAAAATTAA   
  
  
- AAATATTTTA ACGGTTTAGA TATCTTTAAA TCTATATTTG TTACGTAGAA ATAAATTGAG ATTAAACAAA   
  
  
- TGTTATCCTA ATCTTATATT TAAGTGCATT GATGAGATTG AGATAAGTTT ATTTTATGTT TTTTTAATTC   
  
  
- ATGAGAGTCC CCTAAATCAA GATACTAGAT CTTGATTTCA GTTGTATTAA AATTTTAATA AAAAATATAT   
  
  
- ATTATAATTT AATAAAATAA ATTGTTAAAA ATATTTCTAC ATTTTTTTCT CACATTAATA TACTAAACTG   
  
  
- CAAAATATTA TTTTTATCTT CTTATTAAAA AAACTTAACA CAAAATATTA TTTTTATCTT CTTATCAAAA   
  
  
- AAACTTAACA TTGTGAAATT TTTATCTGAA TTTCATAGAG CTTAATCAAA ATTGAAGCCT TTGATTTTGA   
  
  
- TAATATTATT ATATTTTAAA AAATTTAAT
