# Supplementary material for: Genome-wide analysis of the WRKY gene family in drumstick (Moringa oleifera Lam.)
Source: PeerJ. 2019 Jun 10;7:e7063. doi: 10.7717/peerj.7063 (PMC6563795; doi:10.7717/peerj.7063)
Supplement: Supplemental Information 1 [file peerj-07-7063-s003.gz › MoWRKY40_plantcare.html]

Content-Type: text/html; charset=ISO-8859-1


CallMat\_Firefox


Webmaster Firefox specific output  
To save the result:
click on the frame with the right mouse button and save the source code as a text file with extension .html  
REFERENCE:PlantCARE: a database of plant cis-acting regulatory elements and a portal to tools for in silico analysis of promoter sequences.  
Lescot, M., Déhais, P., Moreau, Y., De Moor, B., Rouzé ,P.,and Rombauts, S.  
Nucleic Acids Res., Database issue(2002), 30(1):325-327.   


---

> 2018/04/13 10:10:12  
+ ATAACCAAGA ACTTCTTTCG TTCGTTCGAA GGAAAGAAAC ACAAAGTTTC TTCAAAATCC CAAAATATAT   
  
  
+ AGTTGTCTCT CGATGGAGGT AGAGGTCTAA AACTGAAGTT TAATATATTA AGTTTTTGAT GACGGATAAT   
  
  
+ AGATAAAGAG AACGTACAGT GGATTAGAGC ATACAAGTCA GGTCGTTCGT TGCAATTAGA ACTTATAAAT   
  
  
+ AATTAAAATA TTACATTTAA TATATTTAAT TCGAATAAGG TTTTTAATAA AGTTGAACCC TCAAATAATT   
  
  
+ AATTAAATAT GTTATTACAA AAGACCTAGG CATGCACGCT GGTGACTTTT GCCACCAGTT TTGACTCCGC   
  
  
+ GGCAATAAAA CTGAAACACT GACTCACCCA GGGCATTAAC GATGGGCTGG GCCCCAGTTA ATACATGAAA   
  
  
+ ACGCATTATA AAGTTTAATA TGTCTCCGTG CAGTTAAGTT GCACATTTTG CTGCATTTGC GTCGAACTGA   
  
  
+ CAAGGATACA CTGGTACATT CTCAACTGAC ATTCGTCAAT GCCAGAAAAA AACTTTTTCT TTTATTCCCT   
  
  
+ TCGACCTCTT TCACCGCAAA GTAAATTTCA AGTCCTCGGT TTGGGAACCT TCTTTTTATC TTTTTTTATA   
  
  
+ TTTTTATTTA ATATCCGAAT TTCTTAGAAA TTTCTCCCAC ATATGTATAT AAAAAATTAT AAATAAATTA   
  
  
+ CAAAAAGTAC ACTGATTTTG TTTAGTTAAT ACTTCTATTT TTTTAATAAC AGTAATTAAT TAACTTTATT   
  
  
+ ACCACATTTC CATTACAAAA TTTTTAGCTT AATCCAACTA ACGAGGATAA CTAATTTAAT ATTTGATTTT   
  
  
+ AAAATTAATT AAATTAGATA AATTTTAGTT AAACTAAATA AATATAATAG TTTTTTTACA ACAAAAAATT   
  
  
+ TTTTTATATC ATTTAAAAAA TAAAATTTAT AAAAATTTTA TATTCTTTAA AATTTTTGTT TTTTAACCTA   
  
  
+ CGACGTAATC TCTCAACTTA ATTAACACTA CTCTTTTAAT ACTATAGTAC TTCTACCCAC TATAAAATAG   
  
  
+ GGTTTAACGA TTAGAATTAA TCATCCCATT TAGAACTGGT AAAGTTAAGA CTTCTCTGTC TTAATTAATT   
  
  
+ AAAACTCATA TATAATAGTT TTAAGATACG TGTTAGCTGT ATCATGGGTG CAGGATTCGA AGTAACTAAC   
  
  
+ GGGGAAGTTA AATAAAAACC GGAAGTGAAA GAAGGGTAAT TTTAGGTTAG TACTGTATAT ATATTTTTAA   
  
  
+ TAATTGTGTA TGGAATTGTA ATGGCATTAA AATAATATTT ATATATTATA TTTATAAGAT ACCAAATTGA   
  
  
+ ATATTTTAGA TTTTTTTTTA ATTTTTGAAA CTCTTTAAAT CAACCAATAG TTCATACGTA ACTGAATATT   
  
  
+ CGATCGAGGC TTCAAAGTTA AAGTAGTTTT TAATAACTAA AAAAATAAAT AATTGAAATT CTAAAACTTT   
  
  
+ TCTTATTTAG GGAGTAAATA ATTAATCTA  

- TATTGGTTCT TGAAGAAAGC AAGCAAGCTT CCTTTCTTTG TGTTTCAAAG AAGTTTTAGG GTTTTATATA   
  
  
- TCAACAGAGA GCTACCTCCA TCTCCAGATT TTGACTTCAA ATTATATAAT TCAAAAACTA CTGCCTATTA   
  
  
- TCTATTTCTC TTGCATGTCA CCTAATCTCG TATGTTCAGT CCAGCAAGCA ACGTTAATCT TGAATATTTA   
  
  
- TTAATTTTAT AATGTAAATT ATATAAATTA AGCTTATTCC AAAAATTATT TCAACTTGGG AGTTTATTAA   
  
  
- TTAATTTATA CAATAATGTT TTCTGGATCC GTACGTGCGA CCACTGAAAA CGGTGGTCAA AACTGAGGCG   
  
  
- CCGTTATTTT GACTTTGTGA CTGAGTGGGT CCCGTAATTG CTACCCGACC CGGGGTCAAT TATGTACTTT   
  
  
- TGCGTAATAT TTCAAATTAT ACAGAGGCAC GTCAATTCAA CGTGTAAAAC GACGTAAACG CAGCTTGACT   
  
  
- GTTCCTATGT GACCATGTAA GAGTTGACTG TAAGCAGTTA CGGTCTTTTT TTGAAAAAGA AAATAAGGGA   
  
  
- AGCTGGAGAA AGTGGCGTTT CATTTAAAGT TCAGGAGCCA AACCCTTGGA AGAAAAATAG AAAAAAATAT   
  
  
- AAAAATAAAT TATAGGCTTA AAGAATCTTT AAAGAGGGTG TATACATATA TTTTTTAATA TTTATTTAAT   
  
  
- GTTTTTCATG TGACTAAAAC AAATCAATTA TGAAGATAAA AAAATTATTG TCATTAATTA ATTGAAATAA   
  
  
- TGGTGTAAAG GTAATGTTTT AAAAATCGAA TTAGGTTGAT TGCTCCTATT GATTAAATTA TAAACTAAAA   
  
  
- TTTTAATTAA TTTAATCTAT TTAAAATCAA TTTGATTTAT TTATATTATC AAAAAAATGT TGTTTTTTAA   
  
  
- AAAAATATAG TAAATTTTTT ATTTTAAATA TTTTTAAAAT ATAAGAAATT TTAAAAACAA AAAATTGGAT   
  
  
- GCTGCATTAG AGAGTTGAAT TAATTGTGAT GAGAAAATTA TGATATCATG AAGATGGGTG ATATTTTATC   
  
  
- CCAAATTGCT AATCTTAATT AGTAGGGTAA ATCTTGACCA TTTCAATTCT GAAGAGACAG AATTAATTAA   
  
  
- TTTTGAGTAT ATATTATCAA AATTCTATGC ACAATCGACA TAGTACCCAC GTCCTAAGCT TCATTGATTG   
  
  
- CCCCTTCAAT TTATTTTTGG CCTTCACTTT CTTCCCATTA AAATCCAATC ATGACATATA TATAAAAATT   
  
  
- ATTAACACAT ACCTTAACAT TACCGTAATT TTATTATAAA TATATAATAT AAATATTCTA TGGTTTAACT   
  
  
- TATAAAATCT AAAAAAAAAT TAAAAACTTT GAGAAATTTA GTTGGTTATC AAGTATGCAT TGACTTATAA   
  
  
- GCTAGCTCCG AAGTTTCAAT TTCATCAAAA ATTATTGATT TTTTTATTTA TTAACTTTAA GATTTTGAAA   
  
  
- AGAATAAATC CCTCATTTAT TAATTAGAT

  
  
Motifs Found  

+     AAGAA-motif

| Site Name | Organism | Position | Strand | Matrix score. | sequence | function |
| --- | --- | --- | --- | --- | --- | --- |
| AAGAA-motif | Avena sativa | 32 | + | 7 | GAAAGAA |  |
| AAGAA-motif | Avena sativa | 1217 | + | 7 | GAAAGAA |  |
| AAGAA-motif | Avena sativa | 13 | - | 7 | GAAAGAA |  |

> 2018/04/13 10:10:12  
+ ATAACCAAGA ACTTCTTTCG TTCGTTCGAA GGAAAGAAAC ACAAAGTTTC TTCAAAATCC CAAAATATAT   
  
  
+ AGTTGTCTCT CGATGGAGGT AGAGGTCTAA AACTGAAGTT TAATATATTA AGTTTTTGAT GACGGATAAT   
  
  
+ AGATAAAGAG AACGTACAGT GGATTAGAGC ATACAAGTCA GGTCGTTCGT TGCAATTAGA ACTTATAAAT   
  
  
+ AATTAAAATA TTACATTTAA TATATTTAAT TCGAATAAGG TTTTTAATAA AGTTGAACCC TCAAATAATT   
  
  
+ AATTAAATAT GTTATTACAA AAGACCTAGG CATGCACGCT GGTGACTTTT GCCACCAGTT TTGACTCCGC   
  
  
+ GGCAATAAAA CTGAAACACT GACTCACCCA GGGCATTAAC GATGGGCTGG GCCCCAGTTA ATACATGAAA   
  
  
+ ACGCATTATA AAGTTTAATA TGTCTCCGTG CAGTTAAGTT GCACATTTTG CTGCATTTGC GTCGAACTGA   
  
  
+ CAAGGATACA CTGGTACATT CTCAACTGAC ATTCGTCAAT GCCAGAAAAA AACTTTTTCT TTTATTCCCT   
  
  
+ TCGACCTCTT TCACCGCAAA GTAAATTTCA AGTCCTCGGT TTGGGAACCT TCTTTTTATC TTTTTTTATA   
  
  
+ TTTTTATTTA ATATCCGAAT TTCTTAGAAA TTTCTCCCAC ATATGTATAT AAAAAATTAT AAATAAATTA   
  
  
+ CAAAAAGTAC ACTGATTTTG TTTAGTTAAT ACTTCTATTT TTTTAATAAC AGTAATTAAT TAACTTTATT   
  
  
+ ACCACATTTC CATTACAAAA TTTTTAGCTT AATCCAACTA ACGAGGATAA CTAATTTAAT ATTTGATTTT   
  
  
+ AAAATTAATT AAATTAGATA AATTTTAGTT AAACTAAATA AATATAATAG TTTTTTTACA ACAAAAAATT   
  
  
+ TTTTTATATC ATTTAAAAAA TAAAATTTAT AAAAATTTTA TATTCTTTAA AATTTTTGTT TTTTAACCTA   
  
  
+ CGACGTAATC TCTCAACTTA ATTAACACTA CTCTTTTAAT ACTATAGTAC TTCTACCCAC TATAAAATAG   
  
  
+ GGTTTAACGA TTAGAATTAA TCATCCCATT TAGAACTGGT AAAGTTAAGA CTTCTCTGTC TTAATTAATT   
  
  
+ AAAACTCATA TATAATAGTT TTAAGATACG TGTTAGCTGT ATCATGGGTG CAGGATTCGA AGTAACTAAC   
  
  
+ GGGGAAGTTA AATAAAAACC GGAAGTGAAA GAAGGGTAAT TTTAGGTTAG TACTGTATAT ATATTTTTAA   
  
  
+ TAATTGTGTA TGGAATTGTA ATGGCATTAA AATAATATTT ATATATTATA TTTATAAGAT ACCAAATTGA   
  
  
+ ATATTTTAGA TTTTTTTTTA ATTTTTGAAA CTCTTTAAAT CAACCAATAG TTCATACGTA ACTGAATATT   
  
  
+ CGATCGAGGC TTCAAAGTTA AAGTAGTTTT TAATAACTAA AAAAATAAAT AATTGAAATT CTAAAACTTT   
  
  
+ TCTTATTTAG GGAGTAAATA ATTAATCTA  

- TATTGGTTCT TGAAGAAAGC AAGCAAGCTT CCTTTCTTTG TGTTTCAAAG AAGTTTTAGG GTTTTATATA   
  
  
- TCAACAGAGA GCTACCTCCA TCTCCAGATT TTGACTTCAA ATTATATAAT TCAAAAACTA CTGCCTATTA   
  
  
- TCTATTTCTC TTGCATGTCA CCTAATCTCG TATGTTCAGT CCAGCAAGCA ACGTTAATCT TGAATATTTA   
  
  
- TTAATTTTAT AATGTAAATT ATATAAATTA AGCTTATTCC AAAAATTATT TCAACTTGGG AGTTTATTAA   
  
  
- TTAATTTATA CAATAATGTT TTCTGGATCC GTACGTGCGA CCACTGAAAA CGGTGGTCAA AACTGAGGCG   
  
  
- CCGTTATTTT GACTTTGTGA CTGAGTGGGT CCCGTAATTG CTACCCGACC CGGGGTCAAT TATGTACTTT   
  
  
- TGCGTAATAT TTCAAATTAT ACAGAGGCAC GTCAATTCAA CGTGTAAAAC GACGTAAACG CAGCTTGACT   
  
  
- GTTCCTATGT GACCATGTAA GAGTTGACTG TAAGCAGTTA CGGTCTTTTT TTGAAAAAGA AAATAAGGGA   
  
  
- AGCTGGAGAA AGTGGCGTTT CATTTAAAGT TCAGGAGCCA AACCCTTGGA AGAAAAATAG AAAAAAATAT   
  
  
- AAAAATAAAT TATAGGCTTA AAGAATCTTT AAAGAGGGTG TATACATATA TTTTTTAATA TTTATTTAAT   
  
  
- GTTTTTCATG TGACTAAAAC AAATCAATTA TGAAGATAAA AAAATTATTG TCATTAATTA ATTGAAATAA   
  
  
- TGGTGTAAAG GTAATGTTTT AAAAATCGAA TTAGGTTGAT TGCTCCTATT GATTAAATTA TAAACTAAAA   
  
  
- TTTTAATTAA TTTAATCTAT TTAAAATCAA TTTGATTTAT TTATATTATC AAAAAAATGT TGTTTTTTAA   
  
  
- AAAAATATAG TAAATTTTTT ATTTTAAATA TTTTTAAAAT ATAAGAAATT TTAAAAACAA AAAATTGGAT   
  
  
- GCTGCATTAG AGAGTTGAAT TAATTGTGAT GAGAAAATTA TGATATCATG AAGATGGGTG ATATTTTATC   
  
  
- CCAAATTGCT AATCTTAATT AGTAGGGTAA ATCTTGACCA TTTCAATTCT GAAGAGACAG AATTAATTAA   
  
  
- TTTTGAGTAT ATATTATCAA AATTCTATGC ACAATCGACA TAGTACCCAC GTCCTAAGCT TCATTGATTG   
  
  
- CCCCTTCAAT TTATTTTTGG CCTTCACTTT CTTCCCATTA AAATCCAATC ATGACATATA TATAAAAATT   
  
  
- ATTAACACAT ACCTTAACAT TACCGTAATT TTATTATAAA TATATAATAT AAATATTCTA TGGTTTAACT   
  
  
- TATAAAATCT AAAAAAAAAT TAAAAACTTT GAGAAATTTA GTTGGTTATC AAGTATGCAT TGACTTATAA   
  
  
- GCTAGCTCCG AAGTTTCAAT TTCATCAAAA ATTATTGATT TTTTTATTTA TTAACTTTAA GATTTTGAAA   
  
  
- AGAATAAATC CCTCATTTAT TAATTAGAT

+     ABRE

| Site Name | Organism | Position | Strand | Matrix score. | sequence | function |
| --- | --- | --- | --- | --- | --- | --- |
| ABRE | Arabidopsis thaliana | 1147 | + | 6 | TACGTG | cis-acting element involved in the abscisic acid responsiveness |

> 2018/04/13 10:10:12  
+ ATAACCAAGA ACTTCTTTCG TTCGTTCGAA GGAAAGAAAC ACAAAGTTTC TTCAAAATCC CAAAATATAT   
  
  
+ AGTTGTCTCT CGATGGAGGT AGAGGTCTAA AACTGAAGTT TAATATATTA AGTTTTTGAT GACGGATAAT   
  
  
+ AGATAAAGAG AACGTACAGT GGATTAGAGC ATACAAGTCA GGTCGTTCGT TGCAATTAGA ACTTATAAAT   
  
  
+ AATTAAAATA TTACATTTAA TATATTTAAT TCGAATAAGG TTTTTAATAA AGTTGAACCC TCAAATAATT   
  
  
+ AATTAAATAT GTTATTACAA AAGACCTAGG CATGCACGCT GGTGACTTTT GCCACCAGTT TTGACTCCGC   
  
  
+ GGCAATAAAA CTGAAACACT GACTCACCCA GGGCATTAAC GATGGGCTGG GCCCCAGTTA ATACATGAAA   
  
  
+ ACGCATTATA AAGTTTAATA TGTCTCCGTG CAGTTAAGTT GCACATTTTG CTGCATTTGC GTCGAACTGA   
  
  
+ CAAGGATACA CTGGTACATT CTCAACTGAC ATTCGTCAAT GCCAGAAAAA AACTTTTTCT TTTATTCCCT   
  
  
+ TCGACCTCTT TCACCGCAAA GTAAATTTCA AGTCCTCGGT TTGGGAACCT TCTTTTTATC TTTTTTTATA   
  
  
+ TTTTTATTTA ATATCCGAAT TTCTTAGAAA TTTCTCCCAC ATATGTATAT AAAAAATTAT AAATAAATTA   
  
  
+ CAAAAAGTAC ACTGATTTTG TTTAGTTAAT ACTTCTATTT TTTTAATAAC AGTAATTAAT TAACTTTATT   
  
  
+ ACCACATTTC CATTACAAAA TTTTTAGCTT AATCCAACTA ACGAGGATAA CTAATTTAAT ATTTGATTTT   
  
  
+ AAAATTAATT AAATTAGATA AATTTTAGTT AAACTAAATA AATATAATAG TTTTTTTACA ACAAAAAATT   
  
  
+ TTTTTATATC ATTTAAAAAA TAAAATTTAT AAAAATTTTA TATTCTTTAA AATTTTTGTT TTTTAACCTA   
  
  
+ CGACGTAATC TCTCAACTTA ATTAACACTA CTCTTTTAAT ACTATAGTAC TTCTACCCAC TATAAAATAG   
  
  
+ GGTTTAACGA TTAGAATTAA TCATCCCATT TAGAACTGGT AAAGTTAAGA CTTCTCTGTC TTAATTAATT   
  
  
+ AAAACTCATA TATAATAGTT TTAAGATACG TGTTAGCTGT ATCATGGGTG CAGGATTCGA AGTAACTAAC   
  
  
+ GGGGAAGTTA AATAAAAACC GGAAGTGAAA GAAGGGTAAT TTTAGGTTAG TACTGTATAT ATATTTTTAA   
  
  
+ TAATTGTGTA TGGAATTGTA ATGGCATTAA AATAATATTT ATATATTATA TTTATAAGAT ACCAAATTGA   
  
  
+ ATATTTTAGA TTTTTTTTTA ATTTTTGAAA CTCTTTAAAT CAACCAATAG TTCATACGTA ACTGAATATT   
  
  
+ CGATCGAGGC TTCAAAGTTA AAGTAGTTTT TAATAACTAA AAAAATAAAT AATTGAAATT CTAAAACTTT   
  
  
+ TCTTATTTAG GGAGTAAATA ATTAATCTA  

- TATTGGTTCT TGAAGAAAGC AAGCAAGCTT CCTTTCTTTG TGTTTCAAAG AAGTTTTAGG GTTTTATATA   
  
  
- TCAACAGAGA GCTACCTCCA TCTCCAGATT TTGACTTCAA ATTATATAAT TCAAAAACTA CTGCCTATTA   
  
  
- TCTATTTCTC TTGCATGTCA CCTAATCTCG TATGTTCAGT CCAGCAAGCA ACGTTAATCT TGAATATTTA   
  
  
- TTAATTTTAT AATGTAAATT ATATAAATTA AGCTTATTCC AAAAATTATT TCAACTTGGG AGTTTATTAA   
  
  
- TTAATTTATA CAATAATGTT TTCTGGATCC GTACGTGCGA CCACTGAAAA CGGTGGTCAA AACTGAGGCG   
  
  
- CCGTTATTTT GACTTTGTGA CTGAGTGGGT CCCGTAATTG CTACCCGACC CGGGGTCAAT TATGTACTTT   
  
  
- TGCGTAATAT TTCAAATTAT ACAGAGGCAC GTCAATTCAA CGTGTAAAAC GACGTAAACG CAGCTTGACT   
  
  
- GTTCCTATGT GACCATGTAA GAGTTGACTG TAAGCAGTTA CGGTCTTTTT TTGAAAAAGA AAATAAGGGA   
  
  
- AGCTGGAGAA AGTGGCGTTT CATTTAAAGT TCAGGAGCCA AACCCTTGGA AGAAAAATAG AAAAAAATAT   
  
  
- AAAAATAAAT TATAGGCTTA AAGAATCTTT AAAGAGGGTG TATACATATA TTTTTTAATA TTTATTTAAT   
  
  
- GTTTTTCATG TGACTAAAAC AAATCAATTA TGAAGATAAA AAAATTATTG TCATTAATTA ATTGAAATAA   
  
  
- TGGTGTAAAG GTAATGTTTT AAAAATCGAA TTAGGTTGAT TGCTCCTATT GATTAAATTA TAAACTAAAA   
  
  
- TTTTAATTAA TTTAATCTAT TTAAAATCAA TTTGATTTAT TTATATTATC AAAAAAATGT TGTTTTTTAA   
  
  
- AAAAATATAG TAAATTTTTT ATTTTAAATA TTTTTAAAAT ATAAGAAATT TTAAAAACAA AAAATTGGAT   
  
  
- GCTGCATTAG AGAGTTGAAT TAATTGTGAT GAGAAAATTA TGATATCATG AAGATGGGTG ATATTTTATC   
  
  
- CCAAATTGCT AATCTTAATT AGTAGGGTAA ATCTTGACCA TTTCAATTCT GAAGAGACAG AATTAATTAA   
  
  
- TTTTGAGTAT ATATTATCAA AATTCTATGC ACAATCGACA TAGTACCCAC GTCCTAAGCT TCATTGATTG   
  
  
- CCCCTTCAAT TTATTTTTGG CCTTCACTTT CTTCCCATTA AAATCCAATC ATGACATATA TATAAAAATT   
  
  
- ATTAACACAT ACCTTAACAT TACCGTAATT TTATTATAAA TATATAATAT AAATATTCTA TGGTTTAACT   
  
  
- TATAAAATCT AAAAAAAAAT TAAAAACTTT GAGAAATTTA GTTGGTTATC AAGTATGCAT TGACTTATAA   
  
  
- GCTAGCTCCG AAGTTTCAAT TTCATCAAAA ATTATTGATT TTTTTATTTA TTAACTTTAA GATTTTGAAA   
  
  
- AGAATAAATC CCTCATTTAT TAATTAGAT

+     AE-box

| Site Name | Organism | Position | Strand | Matrix score. | sequence | function |
| --- | --- | --- | --- | --- | --- | --- |
| AE-box | Arabidopsis thaliana | 44 | - | 8 | AGAAACTT | part of a module for light response |

> 2018/04/13 10:10:12  
+ ATAACCAAGA ACTTCTTTCG TTCGTTCGAA GGAAAGAAAC ACAAAGTTTC TTCAAAATCC CAAAATATAT   
  
  
+ AGTTGTCTCT CGATGGAGGT AGAGGTCTAA AACTGAAGTT TAATATATTA AGTTTTTGAT GACGGATAAT   
  
  
+ AGATAAAGAG AACGTACAGT GGATTAGAGC ATACAAGTCA GGTCGTTCGT TGCAATTAGA ACTTATAAAT   
  
  
+ AATTAAAATA TTACATTTAA TATATTTAAT TCGAATAAGG TTTTTAATAA AGTTGAACCC TCAAATAATT   
  
  
+ AATTAAATAT GTTATTACAA AAGACCTAGG CATGCACGCT GGTGACTTTT GCCACCAGTT TTGACTCCGC   
  
  
+ GGCAATAAAA CTGAAACACT GACTCACCCA GGGCATTAAC GATGGGCTGG GCCCCAGTTA ATACATGAAA   
  
  
+ ACGCATTATA AAGTTTAATA TGTCTCCGTG CAGTTAAGTT GCACATTTTG CTGCATTTGC GTCGAACTGA   
  
  
+ CAAGGATACA CTGGTACATT CTCAACTGAC ATTCGTCAAT GCCAGAAAAA AACTTTTTCT TTTATTCCCT   
  
  
+ TCGACCTCTT TCACCGCAAA GTAAATTTCA AGTCCTCGGT TTGGGAACCT TCTTTTTATC TTTTTTTATA   
  
  
+ TTTTTATTTA ATATCCGAAT TTCTTAGAAA TTTCTCCCAC ATATGTATAT AAAAAATTAT AAATAAATTA   
  
  
+ CAAAAAGTAC ACTGATTTTG TTTAGTTAAT ACTTCTATTT TTTTAATAAC AGTAATTAAT TAACTTTATT   
  
  
+ ACCACATTTC CATTACAAAA TTTTTAGCTT AATCCAACTA ACGAGGATAA CTAATTTAAT ATTTGATTTT   
  
  
+ AAAATTAATT AAATTAGATA AATTTTAGTT AAACTAAATA AATATAATAG TTTTTTTACA ACAAAAAATT   
  
  
+ TTTTTATATC ATTTAAAAAA TAAAATTTAT AAAAATTTTA TATTCTTTAA AATTTTTGTT TTTTAACCTA   
  
  
+ CGACGTAATC TCTCAACTTA ATTAACACTA CTCTTTTAAT ACTATAGTAC TTCTACCCAC TATAAAATAG   
  
  
+ GGTTTAACGA TTAGAATTAA TCATCCCATT TAGAACTGGT AAAGTTAAGA CTTCTCTGTC TTAATTAATT   
  
  
+ AAAACTCATA TATAATAGTT TTAAGATACG TGTTAGCTGT ATCATGGGTG CAGGATTCGA AGTAACTAAC   
  
  
+ GGGGAAGTTA AATAAAAACC GGAAGTGAAA GAAGGGTAAT TTTAGGTTAG TACTGTATAT ATATTTTTAA   
  
  
+ TAATTGTGTA TGGAATTGTA ATGGCATTAA AATAATATTT ATATATTATA TTTATAAGAT ACCAAATTGA   
  
  
+ ATATTTTAGA TTTTTTTTTA ATTTTTGAAA CTCTTTAAAT CAACCAATAG TTCATACGTA ACTGAATATT   
  
  
+ CGATCGAGGC TTCAAAGTTA AAGTAGTTTT TAATAACTAA AAAAATAAAT AATTGAAATT CTAAAACTTT   
  
  
+ TCTTATTTAG GGAGTAAATA ATTAATCTA  

- TATTGGTTCT TGAAGAAAGC AAGCAAGCTT CCTTTCTTTG TGTTTCAAAG AAGTTTTAGG GTTTTATATA   
  
  
- TCAACAGAGA GCTACCTCCA TCTCCAGATT TTGACTTCAA ATTATATAAT TCAAAAACTA CTGCCTATTA   
  
  
- TCTATTTCTC TTGCATGTCA CCTAATCTCG TATGTTCAGT CCAGCAAGCA ACGTTAATCT TGAATATTTA   
  
  
- TTAATTTTAT AATGTAAATT ATATAAATTA AGCTTATTCC AAAAATTATT TCAACTTGGG AGTTTATTAA   
  
  
- TTAATTTATA CAATAATGTT TTCTGGATCC GTACGTGCGA CCACTGAAAA CGGTGGTCAA AACTGAGGCG   
  
  
- CCGTTATTTT GACTTTGTGA CTGAGTGGGT CCCGTAATTG CTACCCGACC CGGGGTCAAT TATGTACTTT   
  
  
- TGCGTAATAT TTCAAATTAT ACAGAGGCAC GTCAATTCAA CGTGTAAAAC GACGTAAACG CAGCTTGACT   
  
  
- GTTCCTATGT GACCATGTAA GAGTTGACTG TAAGCAGTTA CGGTCTTTTT TTGAAAAAGA AAATAAGGGA   
  
  
- AGCTGGAGAA AGTGGCGTTT CATTTAAAGT TCAGGAGCCA AACCCTTGGA AGAAAAATAG AAAAAAATAT   
  
  
- AAAAATAAAT TATAGGCTTA AAGAATCTTT AAAGAGGGTG TATACATATA TTTTTTAATA TTTATTTAAT   
  
  
- GTTTTTCATG TGACTAAAAC AAATCAATTA TGAAGATAAA AAAATTATTG TCATTAATTA ATTGAAATAA   
  
  
- TGGTGTAAAG GTAATGTTTT AAAAATCGAA TTAGGTTGAT TGCTCCTATT GATTAAATTA TAAACTAAAA   
  
  
- TTTTAATTAA TTTAATCTAT TTAAAATCAA TTTGATTTAT TTATATTATC AAAAAAATGT TGTTTTTTAA   
  
  
- AAAAATATAG TAAATTTTTT ATTTTAAATA TTTTTAAAAT ATAAGAAATT TTAAAAACAA AAAATTGGAT   
  
  
- GCTGCATTAG AGAGTTGAAT TAATTGTGAT GAGAAAATTA TGATATCATG AAGATGGGTG ATATTTTATC   
  
  
- CCAAATTGCT AATCTTAATT AGTAGGGTAA ATCTTGACCA TTTCAATTCT GAAGAGACAG AATTAATTAA   
  
  
- TTTTGAGTAT ATATTATCAA AATTCTATGC ACAATCGACA TAGTACCCAC GTCCTAAGCT TCATTGATTG   
  
  
- CCCCTTCAAT TTATTTTTGG CCTTCACTTT CTTCCCATTA AAATCCAATC ATGACATATA TATAAAAATT   
  
  
- ATTAACACAT ACCTTAACAT TACCGTAATT TTATTATAAA TATATAATAT AAATATTCTA TGGTTTAACT   
  
  
- TATAAAATCT AAAAAAAAAT TAAAAACTTT GAGAAATTTA GTTGGTTATC AAGTATGCAT TGACTTATAA   
  
  
- GCTAGCTCCG AAGTTTCAAT TTCATCAAAA ATTATTGATT TTTTTATTTA TTAACTTTAA GATTTTGAAA   
  
  
- AGAATAAATC CCTCATTTAT TAATTAGAT

+     AT1-motif

| Site Name | Organism | Position | Strand | Matrix score. | sequence | function |
| --- | --- | --- | --- | --- | --- | --- |
| AT1-motif | Solanum tuberosum | 838 | - | 11 | ATTAATTTTACA | part of a light responsive module |

> 2018/04/13 10:10:12  
+ ATAACCAAGA ACTTCTTTCG TTCGTTCGAA GGAAAGAAAC ACAAAGTTTC TTCAAAATCC CAAAATATAT   
  
  
+ AGTTGTCTCT CGATGGAGGT AGAGGTCTAA AACTGAAGTT TAATATATTA AGTTTTTGAT GACGGATAAT   
  
  
+ AGATAAAGAG AACGTACAGT GGATTAGAGC ATACAAGTCA GGTCGTTCGT TGCAATTAGA ACTTATAAAT   
  
  
+ AATTAAAATA TTACATTTAA TATATTTAAT TCGAATAAGG TTTTTAATAA AGTTGAACCC TCAAATAATT   
  
  
+ AATTAAATAT GTTATTACAA AAGACCTAGG CATGCACGCT GGTGACTTTT GCCACCAGTT TTGACTCCGC   
  
  
+ GGCAATAAAA CTGAAACACT GACTCACCCA GGGCATTAAC GATGGGCTGG GCCCCAGTTA ATACATGAAA   
  
  
+ ACGCATTATA AAGTTTAATA TGTCTCCGTG CAGTTAAGTT GCACATTTTG CTGCATTTGC GTCGAACTGA   
  
  
+ CAAGGATACA CTGGTACATT CTCAACTGAC ATTCGTCAAT GCCAGAAAAA AACTTTTTCT TTTATTCCCT   
  
  
+ TCGACCTCTT TCACCGCAAA GTAAATTTCA AGTCCTCGGT TTGGGAACCT TCTTTTTATC TTTTTTTATA   
  
  
+ TTTTTATTTA ATATCCGAAT TTCTTAGAAA TTTCTCCCAC ATATGTATAT AAAAAATTAT AAATAAATTA   
  
  
+ CAAAAAGTAC ACTGATTTTG TTTAGTTAAT ACTTCTATTT TTTTAATAAC AGTAATTAAT TAACTTTATT   
  
  
+ ACCACATTTC CATTACAAAA TTTTTAGCTT AATCCAACTA ACGAGGATAA CTAATTTAAT ATTTGATTTT   
  
  
+ AAAATTAATT AAATTAGATA AATTTTAGTT AAACTAAATA AATATAATAG TTTTTTTACA ACAAAAAATT   
  
  
+ TTTTTATATC ATTTAAAAAA TAAAATTTAT AAAAATTTTA TATTCTTTAA AATTTTTGTT TTTTAACCTA   
  
  
+ CGACGTAATC TCTCAACTTA ATTAACACTA CTCTTTTAAT ACTATAGTAC TTCTACCCAC TATAAAATAG   
  
  
+ GGTTTAACGA TTAGAATTAA TCATCCCATT TAGAACTGGT AAAGTTAAGA CTTCTCTGTC TTAATTAATT   
  
  
+ AAAACTCATA TATAATAGTT TTAAGATACG TGTTAGCTGT ATCATGGGTG CAGGATTCGA AGTAACTAAC   
  
  
+ GGGGAAGTTA AATAAAAACC GGAAGTGAAA GAAGGGTAAT TTTAGGTTAG TACTGTATAT ATATTTTTAA   
  
  
+ TAATTGTGTA TGGAATTGTA ATGGCATTAA AATAATATTT ATATATTATA TTTATAAGAT ACCAAATTGA   
  
  
+ ATATTTTAGA TTTTTTTTTA ATTTTTGAAA CTCTTTAAAT CAACCAATAG TTCATACGTA ACTGAATATT   
  
  
+ CGATCGAGGC TTCAAAGTTA AAGTAGTTTT TAATAACTAA AAAAATAAAT AATTGAAATT CTAAAACTTT   
  
  
+ TCTTATTTAG GGAGTAAATA ATTAATCTA  

- TATTGGTTCT TGAAGAAAGC AAGCAAGCTT CCTTTCTTTG TGTTTCAAAG AAGTTTTAGG GTTTTATATA   
  
  
- TCAACAGAGA GCTACCTCCA TCTCCAGATT TTGACTTCAA ATTATATAAT TCAAAAACTA CTGCCTATTA   
  
  
- TCTATTTCTC TTGCATGTCA CCTAATCTCG TATGTTCAGT CCAGCAAGCA ACGTTAATCT TGAATATTTA   
  
  
- TTAATTTTAT AATGTAAATT ATATAAATTA AGCTTATTCC AAAAATTATT TCAACTTGGG AGTTTATTAA   
  
  
- TTAATTTATA CAATAATGTT TTCTGGATCC GTACGTGCGA CCACTGAAAA CGGTGGTCAA AACTGAGGCG   
  
  
- CCGTTATTTT GACTTTGTGA CTGAGTGGGT CCCGTAATTG CTACCCGACC CGGGGTCAAT TATGTACTTT   
  
  
- TGCGTAATAT TTCAAATTAT ACAGAGGCAC GTCAATTCAA CGTGTAAAAC GACGTAAACG CAGCTTGACT   
  
  
- GTTCCTATGT GACCATGTAA GAGTTGACTG TAAGCAGTTA CGGTCTTTTT TTGAAAAAGA AAATAAGGGA   
  
  
- AGCTGGAGAA AGTGGCGTTT CATTTAAAGT TCAGGAGCCA AACCCTTGGA AGAAAAATAG AAAAAAATAT   
  
  
- AAAAATAAAT TATAGGCTTA AAGAATCTTT AAAGAGGGTG TATACATATA TTTTTTAATA TTTATTTAAT   
  
  
- GTTTTTCATG TGACTAAAAC AAATCAATTA TGAAGATAAA AAAATTATTG TCATTAATTA ATTGAAATAA   
  
  
- TGGTGTAAAG GTAATGTTTT AAAAATCGAA TTAGGTTGAT TGCTCCTATT GATTAAATTA TAAACTAAAA   
  
  
- TTTTAATTAA TTTAATCTAT TTAAAATCAA TTTGATTTAT TTATATTATC AAAAAAATGT TGTTTTTTAA   
  
  
- AAAAATATAG TAAATTTTTT ATTTTAAATA TTTTTAAAAT ATAAGAAATT TTAAAAACAA AAAATTGGAT   
  
  
- GCTGCATTAG AGAGTTGAAT TAATTGTGAT GAGAAAATTA TGATATCATG AAGATGGGTG ATATTTTATC   
  
  
- CCAAATTGCT AATCTTAATT AGTAGGGTAA ATCTTGACCA TTTCAATTCT GAAGAGACAG AATTAATTAA   
  
  
- TTTTGAGTAT ATATTATCAA AATTCTATGC ACAATCGACA TAGTACCCAC GTCCTAAGCT TCATTGATTG   
  
  
- CCCCTTCAAT TTATTTTTGG CCTTCACTTT CTTCCCATTA AAATCCAATC ATGACATATA TATAAAAATT   
  
  
- ATTAACACAT ACCTTAACAT TACCGTAATT TTATTATAAA TATATAATAT AAATATTCTA TGGTTTAACT   
  
  
- TATAAAATCT AAAAAAAAAT TAAAAACTTT GAGAAATTTA GTTGGTTATC AAGTATGCAT TGACTTATAA   
  
  
- GCTAGCTCCG AAGTTTCAAT TTCATCAAAA ATTATTGATT TTTTTATTTA TTAACTTTAA GATTTTGAAA   
  
  
- AGAATAAATC CCTCATTTAT TAATTAGAT

+     Box 4

| Site Name | Organism | Position | Strand | Matrix score. | sequence | function |
| --- | --- | --- | --- | --- | --- | --- |
| Box 4 | Petroselinum crispum | 844 | - | 6 | ATTAAT | part of a conserved DNA module involved in light responsiveness |
| Box 4 | Petroselinum crispum | 1114 | - | 6 | ATTAAT | part of a conserved DNA module involved in light responsiveness |
| Box 4 | Petroselinum crispum | 278 | + | 6 | ATTAAT | part of a conserved DNA module involved in light responsiveness |
| Box 4 | Petroselinum crispum | 1491 | - | 6 | ATTAAT | part of a conserved DNA module involved in light responsiveness |
| Box 4 | Petroselinum crispum | 755 | - | 6 | ATTAAT | part of a conserved DNA module involved in light responsiveness |
| Box 4 | Petroselinum crispum | 1066 | - | 6 | ATTAAT | part of a conserved DNA module involved in light responsiveness |

> 2018/04/13 10:10:12  
+ ATAACCAAGA ACTTCTTTCG TTCGTTCGAA GGAAAGAAAC ACAAAGTTTC TTCAAAATCC CAAAATATAT   
  
  
+ AGTTGTCTCT CGATGGAGGT AGAGGTCTAA AACTGAAGTT TAATATATTA AGTTTTTGAT GACGGATAAT   
  
  
+ AGATAAAGAG AACGTACAGT GGATTAGAGC ATACAAGTCA GGTCGTTCGT TGCAATTAGA ACTTATAAAT   
  
  
+ AATTAAAATA TTACATTTAA TATATTTAAT TCGAATAAGG TTTTTAATAA AGTTGAACCC TCAAATAATT   
  
  
+ AATTAAATAT GTTATTACAA AAGACCTAGG CATGCACGCT GGTGACTTTT GCCACCAGTT TTGACTCCGC   
  
  
+ GGCAATAAAA CTGAAACACT GACTCACCCA GGGCATTAAC GATGGGCTGG GCCCCAGTTA ATACATGAAA   
  
  
+ ACGCATTATA AAGTTTAATA TGTCTCCGTG CAGTTAAGTT GCACATTTTG CTGCATTTGC GTCGAACTGA   
  
  
+ CAAGGATACA CTGGTACATT CTCAACTGAC ATTCGTCAAT GCCAGAAAAA AACTTTTTCT TTTATTCCCT   
  
  
+ TCGACCTCTT TCACCGCAAA GTAAATTTCA AGTCCTCGGT TTGGGAACCT TCTTTTTATC TTTTTTTATA   
  
  
+ TTTTTATTTA ATATCCGAAT TTCTTAGAAA TTTCTCCCAC ATATGTATAT AAAAAATTAT AAATAAATTA   
  
  
+ CAAAAAGTAC ACTGATTTTG TTTAGTTAAT ACTTCTATTT TTTTAATAAC AGTAATTAAT TAACTTTATT   
  
  
+ ACCACATTTC CATTACAAAA TTTTTAGCTT AATCCAACTA ACGAGGATAA CTAATTTAAT ATTTGATTTT   
  
  
+ AAAATTAATT AAATTAGATA AATTTTAGTT AAACTAAATA AATATAATAG TTTTTTTACA ACAAAAAATT   
  
  
+ TTTTTATATC ATTTAAAAAA TAAAATTTAT AAAAATTTTA TATTCTTTAA AATTTTTGTT TTTTAACCTA   
  
  
+ CGACGTAATC TCTCAACTTA ATTAACACTA CTCTTTTAAT ACTATAGTAC TTCTACCCAC TATAAAATAG   
  
  
+ GGTTTAACGA TTAGAATTAA TCATCCCATT TAGAACTGGT AAAGTTAAGA CTTCTCTGTC TTAATTAATT   
  
  
+ AAAACTCATA TATAATAGTT TTAAGATACG TGTTAGCTGT ATCATGGGTG CAGGATTCGA AGTAACTAAC   
  
  
+ GGGGAAGTTA AATAAAAACC GGAAGTGAAA GAAGGGTAAT TTTAGGTTAG TACTGTATAT ATATTTTTAA   
  
  
+ TAATTGTGTA TGGAATTGTA ATGGCATTAA AATAATATTT ATATATTATA TTTATAAGAT ACCAAATTGA   
  
  
+ ATATTTTAGA TTTTTTTTTA ATTTTTGAAA CTCTTTAAAT CAACCAATAG TTCATACGTA ACTGAATATT   
  
  
+ CGATCGAGGC TTCAAAGTTA AAGTAGTTTT TAATAACTAA AAAAATAAAT AATTGAAATT CTAAAACTTT   
  
  
+ TCTTATTTAG GGAGTAAATA ATTAATCTA  

- TATTGGTTCT TGAAGAAAGC AAGCAAGCTT CCTTTCTTTG TGTTTCAAAG AAGTTTTAGG GTTTTATATA   
  
  
- TCAACAGAGA GCTACCTCCA TCTCCAGATT TTGACTTCAA ATTATATAAT TCAAAAACTA CTGCCTATTA   
  
  
- TCTATTTCTC TTGCATGTCA CCTAATCTCG TATGTTCAGT CCAGCAAGCA ACGTTAATCT TGAATATTTA   
  
  
- TTAATTTTAT AATGTAAATT ATATAAATTA AGCTTATTCC AAAAATTATT TCAACTTGGG AGTTTATTAA   
  
  
- TTAATTTATA CAATAATGTT TTCTGGATCC GTACGTGCGA CCACTGAAAA CGGTGGTCAA AACTGAGGCG   
  
  
- CCGTTATTTT GACTTTGTGA CTGAGTGGGT CCCGTAATTG CTACCCGACC CGGGGTCAAT TATGTACTTT   
  
  
- TGCGTAATAT TTCAAATTAT ACAGAGGCAC GTCAATTCAA CGTGTAAAAC GACGTAAACG CAGCTTGACT   
  
  
- GTTCCTATGT GACCATGTAA GAGTTGACTG TAAGCAGTTA CGGTCTTTTT TTGAAAAAGA AAATAAGGGA   
  
  
- AGCTGGAGAA AGTGGCGTTT CATTTAAAGT TCAGGAGCCA AACCCTTGGA AGAAAAATAG AAAAAAATAT   
  
  
- AAAAATAAAT TATAGGCTTA AAGAATCTTT AAAGAGGGTG TATACATATA TTTTTTAATA TTTATTTAAT   
  
  
- GTTTTTCATG TGACTAAAAC AAATCAATTA TGAAGATAAA AAAATTATTG TCATTAATTA ATTGAAATAA   
  
  
- TGGTGTAAAG GTAATGTTTT AAAAATCGAA TTAGGTTGAT TGCTCCTATT GATTAAATTA TAAACTAAAA   
  
  
- TTTTAATTAA TTTAATCTAT TTAAAATCAA TTTGATTTAT TTATATTATC AAAAAAATGT TGTTTTTTAA   
  
  
- AAAAATATAG TAAATTTTTT ATTTTAAATA TTTTTAAAAT ATAAGAAATT TTAAAAACAA AAAATTGGAT   
  
  
- GCTGCATTAG AGAGTTGAAT TAATTGTGAT GAGAAAATTA TGATATCATG AAGATGGGTG ATATTTTATC   
  
  
- CCAAATTGCT AATCTTAATT AGTAGGGTAA ATCTTGACCA TTTCAATTCT GAAGAGACAG AATTAATTAA   
  
  
- TTTTGAGTAT ATATTATCAA AATTCTATGC ACAATCGACA TAGTACCCAC GTCCTAAGCT TCATTGATTG   
  
  
- CCCCTTCAAT TTATTTTTGG CCTTCACTTT CTTCCCATTA AAATCCAATC ATGACATATA TATAAAAATT   
  
  
- ATTAACACAT ACCTTAACAT TACCGTAATT TTATTATAAA TATATAATAT AAATATTCTA TGGTTTAACT   
  
  
- TATAAAATCT AAAAAAAAAT TAAAAACTTT GAGAAATTTA GTTGGTTATC AAGTATGCAT TGACTTATAA   
  
  
- GCTAGCTCCG AAGTTTCAAT TTCATCAAAA ATTATTGATT TTTTTATTTA TTAACTTTAA GATTTTGAAA   
  
  
- AGAATAAATC CCTCATTTAT TAATTAGAT

+     Box I

| Site Name | Organism | Position | Strand | Matrix score. | sequence | function |
| --- | --- | --- | --- | --- | --- | --- |
| Box I | Pisum sativum | 1354 | - | 7 | TTTCAAA | light responsive element |

> 2018/04/13 10:10:12  
+ ATAACCAAGA ACTTCTTTCG TTCGTTCGAA GGAAAGAAAC ACAAAGTTTC TTCAAAATCC CAAAATATAT   
  
  
+ AGTTGTCTCT CGATGGAGGT AGAGGTCTAA AACTGAAGTT TAATATATTA AGTTTTTGAT GACGGATAAT   
  
  
+ AGATAAAGAG AACGTACAGT GGATTAGAGC ATACAAGTCA GGTCGTTCGT TGCAATTAGA ACTTATAAAT   
  
  
+ AATTAAAATA TTACATTTAA TATATTTAAT TCGAATAAGG TTTTTAATAA AGTTGAACCC TCAAATAATT   
  
  
+ AATTAAATAT GTTATTACAA AAGACCTAGG CATGCACGCT GGTGACTTTT GCCACCAGTT TTGACTCCGC   
  
  
+ GGCAATAAAA CTGAAACACT GACTCACCCA GGGCATTAAC GATGGGCTGG GCCCCAGTTA ATACATGAAA   
  
  
+ ACGCATTATA AAGTTTAATA TGTCTCCGTG CAGTTAAGTT GCACATTTTG CTGCATTTGC GTCGAACTGA   
  
  
+ CAAGGATACA CTGGTACATT CTCAACTGAC ATTCGTCAAT GCCAGAAAAA AACTTTTTCT TTTATTCCCT   
  
  
+ TCGACCTCTT TCACCGCAAA GTAAATTTCA AGTCCTCGGT TTGGGAACCT TCTTTTTATC TTTTTTTATA   
  
  
+ TTTTTATTTA ATATCCGAAT TTCTTAGAAA TTTCTCCCAC ATATGTATAT AAAAAATTAT AAATAAATTA   
  
  
+ CAAAAAGTAC ACTGATTTTG TTTAGTTAAT ACTTCTATTT TTTTAATAAC AGTAATTAAT TAACTTTATT   
  
  
+ ACCACATTTC CATTACAAAA TTTTTAGCTT AATCCAACTA ACGAGGATAA CTAATTTAAT ATTTGATTTT   
  
  
+ AAAATTAATT AAATTAGATA AATTTTAGTT AAACTAAATA AATATAATAG TTTTTTTACA ACAAAAAATT   
  
  
+ TTTTTATATC ATTTAAAAAA TAAAATTTAT AAAAATTTTA TATTCTTTAA AATTTTTGTT TTTTAACCTA   
  
  
+ CGACGTAATC TCTCAACTTA ATTAACACTA CTCTTTTAAT ACTATAGTAC TTCTACCCAC TATAAAATAG   
  
  
+ GGTTTAACGA TTAGAATTAA TCATCCCATT TAGAACTGGT AAAGTTAAGA CTTCTCTGTC TTAATTAATT   
  
  
+ AAAACTCATA TATAATAGTT TTAAGATACG TGTTAGCTGT ATCATGGGTG CAGGATTCGA AGTAACTAAC   
  
  
+ GGGGAAGTTA AATAAAAACC GGAAGTGAAA GAAGGGTAAT TTTAGGTTAG TACTGTATAT ATATTTTTAA   
  
  
+ TAATTGTGTA TGGAATTGTA ATGGCATTAA AATAATATTT ATATATTATA TTTATAAGAT ACCAAATTGA   
  
  
+ ATATTTTAGA TTTTTTTTTA ATTTTTGAAA CTCTTTAAAT CAACCAATAG TTCATACGTA ACTGAATATT   
  
  
+ CGATCGAGGC TTCAAAGTTA AAGTAGTTTT TAATAACTAA AAAAATAAAT AATTGAAATT CTAAAACTTT   
  
  
+ TCTTATTTAG GGAGTAAATA ATTAATCTA  

- TATTGGTTCT TGAAGAAAGC AAGCAAGCTT CCTTTCTTTG TGTTTCAAAG AAGTTTTAGG GTTTTATATA   
  
  
- TCAACAGAGA GCTACCTCCA TCTCCAGATT TTGACTTCAA ATTATATAAT TCAAAAACTA CTGCCTATTA   
  
  
- TCTATTTCTC TTGCATGTCA CCTAATCTCG TATGTTCAGT CCAGCAAGCA ACGTTAATCT TGAATATTTA   
  
  
- TTAATTTTAT AATGTAAATT ATATAAATTA AGCTTATTCC AAAAATTATT TCAACTTGGG AGTTTATTAA   
  
  
- TTAATTTATA CAATAATGTT TTCTGGATCC GTACGTGCGA CCACTGAAAA CGGTGGTCAA AACTGAGGCG   
  
  
- CCGTTATTTT GACTTTGTGA CTGAGTGGGT CCCGTAATTG CTACCCGACC CGGGGTCAAT TATGTACTTT   
  
  
- TGCGTAATAT TTCAAATTAT ACAGAGGCAC GTCAATTCAA CGTGTAAAAC GACGTAAACG CAGCTTGACT   
  
  
- GTTCCTATGT GACCATGTAA GAGTTGACTG TAAGCAGTTA CGGTCTTTTT TTGAAAAAGA AAATAAGGGA   
  
  
- AGCTGGAGAA AGTGGCGTTT CATTTAAAGT TCAGGAGCCA AACCCTTGGA AGAAAAATAG AAAAAAATAT   
  
  
- AAAAATAAAT TATAGGCTTA AAGAATCTTT AAAGAGGGTG TATACATATA TTTTTTAATA TTTATTTAAT   
  
  
- GTTTTTCATG TGACTAAAAC AAATCAATTA TGAAGATAAA AAAATTATTG TCATTAATTA ATTGAAATAA   
  
  
- TGGTGTAAAG GTAATGTTTT AAAAATCGAA TTAGGTTGAT TGCTCCTATT GATTAAATTA TAAACTAAAA   
  
  
- TTTTAATTAA TTTAATCTAT TTAAAATCAA TTTGATTTAT TTATATTATC AAAAAAATGT TGTTTTTTAA   
  
  
- AAAAATATAG TAAATTTTTT ATTTTAAATA TTTTTAAAAT ATAAGAAATT TTAAAAACAA AAAATTGGAT   
  
  
- GCTGCATTAG AGAGTTGAAT TAATTGTGAT GAGAAAATTA TGATATCATG AAGATGGGTG ATATTTTATC   
  
  
- CCAAATTGCT AATCTTAATT AGTAGGGTAA ATCTTGACCA TTTCAATTCT GAAGAGACAG AATTAATTAA   
  
  
- TTTTGAGTAT ATATTATCAA AATTCTATGC ACAATCGACA TAGTACCCAC GTCCTAAGCT TCATTGATTG   
  
  
- CCCCTTCAAT TTATTTTTGG CCTTCACTTT CTTCCCATTA AAATCCAATC ATGACATATA TATAAAAATT   
  
  
- ATTAACACAT ACCTTAACAT TACCGTAATT TTATTATAAA TATATAATAT AAATATTCTA TGGTTTAACT   
  
  
- TATAAAATCT AAAAAAAAAT TAAAAACTTT GAGAAATTTA GTTGGTTATC AAGTATGCAT TGACTTATAA   
  
  
- GCTAGCTCCG AAGTTTCAAT TTCATCAAAA ATTATTGATT TTTTTATTTA TTAACTTTAA GATTTTGAAA   
  
  
- AGAATAAATC CCTCATTTAT TAATTAGAT

+     Box II

| Site Name | Organism | Position | Strand | Matrix score. | sequence | function |
| --- | --- | --- | --- | --- | --- | --- |
| Box II | Solanum tuberosum | 766 | - | 9 | TGGTAATAA | part of a light responsive element |

> 2018/04/13 10:10:12  
+ ATAACCAAGA ACTTCTTTCG TTCGTTCGAA GGAAAGAAAC ACAAAGTTTC TTCAAAATCC CAAAATATAT   
  
  
+ AGTTGTCTCT CGATGGAGGT AGAGGTCTAA AACTGAAGTT TAATATATTA AGTTTTTGAT GACGGATAAT   
  
  
+ AGATAAAGAG AACGTACAGT GGATTAGAGC ATACAAGTCA GGTCGTTCGT TGCAATTAGA ACTTATAAAT   
  
  
+ AATTAAAATA TTACATTTAA TATATTTAAT TCGAATAAGG TTTTTAATAA AGTTGAACCC TCAAATAATT   
  
  
+ AATTAAATAT GTTATTACAA AAGACCTAGG CATGCACGCT GGTGACTTTT GCCACCAGTT TTGACTCCGC   
  
  
+ GGCAATAAAA CTGAAACACT GACTCACCCA GGGCATTAAC GATGGGCTGG GCCCCAGTTA ATACATGAAA   
  
  
+ ACGCATTATA AAGTTTAATA TGTCTCCGTG CAGTTAAGTT GCACATTTTG CTGCATTTGC GTCGAACTGA   
  
  
+ CAAGGATACA CTGGTACATT CTCAACTGAC ATTCGTCAAT GCCAGAAAAA AACTTTTTCT TTTATTCCCT   
  
  
+ TCGACCTCTT TCACCGCAAA GTAAATTTCA AGTCCTCGGT TTGGGAACCT TCTTTTTATC TTTTTTTATA   
  
  
+ TTTTTATTTA ATATCCGAAT TTCTTAGAAA TTTCTCCCAC ATATGTATAT AAAAAATTAT AAATAAATTA   
  
  
+ CAAAAAGTAC ACTGATTTTG TTTAGTTAAT ACTTCTATTT TTTTAATAAC AGTAATTAAT TAACTTTATT   
  
  
+ ACCACATTTC CATTACAAAA TTTTTAGCTT AATCCAACTA ACGAGGATAA CTAATTTAAT ATTTGATTTT   
  
  
+ AAAATTAATT AAATTAGATA AATTTTAGTT AAACTAAATA AATATAATAG TTTTTTTACA ACAAAAAATT   
  
  
+ TTTTTATATC ATTTAAAAAA TAAAATTTAT AAAAATTTTA TATTCTTTAA AATTTTTGTT TTTTAACCTA   
  
  
+ CGACGTAATC TCTCAACTTA ATTAACACTA CTCTTTTAAT ACTATAGTAC TTCTACCCAC TATAAAATAG   
  
  
+ GGTTTAACGA TTAGAATTAA TCATCCCATT TAGAACTGGT AAAGTTAAGA CTTCTCTGTC TTAATTAATT   
  
  
+ AAAACTCATA TATAATAGTT TTAAGATACG TGTTAGCTGT ATCATGGGTG CAGGATTCGA AGTAACTAAC   
  
  
+ GGGGAAGTTA AATAAAAACC GGAAGTGAAA GAAGGGTAAT TTTAGGTTAG TACTGTATAT ATATTTTTAA   
  
  
+ TAATTGTGTA TGGAATTGTA ATGGCATTAA AATAATATTT ATATATTATA TTTATAAGAT ACCAAATTGA   
  
  
+ ATATTTTAGA TTTTTTTTTA ATTTTTGAAA CTCTTTAAAT CAACCAATAG TTCATACGTA ACTGAATATT   
  
  
+ CGATCGAGGC TTCAAAGTTA AAGTAGTTTT TAATAACTAA AAAAATAAAT AATTGAAATT CTAAAACTTT   
  
  
+ TCTTATTTAG GGAGTAAATA ATTAATCTA  

- TATTGGTTCT TGAAGAAAGC AAGCAAGCTT CCTTTCTTTG TGTTTCAAAG AAGTTTTAGG GTTTTATATA   
  
  
- TCAACAGAGA GCTACCTCCA TCTCCAGATT TTGACTTCAA ATTATATAAT TCAAAAACTA CTGCCTATTA   
  
  
- TCTATTTCTC TTGCATGTCA CCTAATCTCG TATGTTCAGT CCAGCAAGCA ACGTTAATCT TGAATATTTA   
  
  
- TTAATTTTAT AATGTAAATT ATATAAATTA AGCTTATTCC AAAAATTATT TCAACTTGGG AGTTTATTAA   
  
  
- TTAATTTATA CAATAATGTT TTCTGGATCC GTACGTGCGA CCACTGAAAA CGGTGGTCAA AACTGAGGCG   
  
  
- CCGTTATTTT GACTTTGTGA CTGAGTGGGT CCCGTAATTG CTACCCGACC CGGGGTCAAT TATGTACTTT   
  
  
- TGCGTAATAT TTCAAATTAT ACAGAGGCAC GTCAATTCAA CGTGTAAAAC GACGTAAACG CAGCTTGACT   
  
  
- GTTCCTATGT GACCATGTAA GAGTTGACTG TAAGCAGTTA CGGTCTTTTT TTGAAAAAGA AAATAAGGGA   
  
  
- AGCTGGAGAA AGTGGCGTTT CATTTAAAGT TCAGGAGCCA AACCCTTGGA AGAAAAATAG AAAAAAATAT   
  
  
- AAAAATAAAT TATAGGCTTA AAGAATCTTT AAAGAGGGTG TATACATATA TTTTTTAATA TTTATTTAAT   
  
  
- GTTTTTCATG TGACTAAAAC AAATCAATTA TGAAGATAAA AAAATTATTG TCATTAATTA ATTGAAATAA   
  
  
- TGGTGTAAAG GTAATGTTTT AAAAATCGAA TTAGGTTGAT TGCTCCTATT GATTAAATTA TAAACTAAAA   
  
  
- TTTTAATTAA TTTAATCTAT TTAAAATCAA TTTGATTTAT TTATATTATC AAAAAAATGT TGTTTTTTAA   
  
  
- AAAAATATAG TAAATTTTTT ATTTTAAATA TTTTTAAAAT ATAAGAAATT TTAAAAACAA AAAATTGGAT   
  
  
- GCTGCATTAG AGAGTTGAAT TAATTGTGAT GAGAAAATTA TGATATCATG AAGATGGGTG ATATTTTATC   
  
  
- CCAAATTGCT AATCTTAATT AGTAGGGTAA ATCTTGACCA TTTCAATTCT GAAGAGACAG AATTAATTAA   
  
  
- TTTTGAGTAT ATATTATCAA AATTCTATGC ACAATCGACA TAGTACCCAC GTCCTAAGCT TCATTGATTG   
  
  
- CCCCTTCAAT TTATTTTTGG CCTTCACTTT CTTCCCATTA AAATCCAATC ATGACATATA TATAAAAATT   
  
  
- ATTAACACAT ACCTTAACAT TACCGTAATT TTATTATAAA TATATAATAT AAATATTCTA TGGTTTAACT   
  
  
- TATAAAATCT AAAAAAAAAT TAAAAACTTT GAGAAATTTA GTTGGTTATC AAGTATGCAT TGACTTATAA   
  
  
- GCTAGCTCCG AAGTTTCAAT TTCATCAAAA ATTATTGATT TTTTTATTTA TTAACTTTAA GATTTTGAAA   
  
  
- AGAATAAATC CCTCATTTAT TAATTAGAT

+     CAAT-box

| Site Name | Organism | Position | Strand | Matrix score. | sequence | function |
| --- | --- | --- | --- | --- | --- | --- |
| CAAT-box | Hordeum vulgare | 1452 | - | 4 | CAAT | common cis-acting element in promoter and enhancer regions |
| CAAT-box | Glycine max | 1274 | - | 5 | CAATT | common cis-acting element in promoter and enhancer regions |
| CAAT-box | Hordeum vulgare | 353 | + | 4 | CAAT | common cis-acting element in promoter and enhancer regions |
| CAAT-box | Glycine max | 193 | + | 5 | CAATT | common cis-acting element in promoter and enhancer regions |
| CAAT-box | Hordeum vulgare | 1275 | - | 4 | CAAT | common cis-acting element in promoter and enhancer regions |
| CAAT-box | Hordeum vulgare | 1326 | - | 4 | CAAT | common cis-acting element in promoter and enhancer regions |
| CAAT-box | Hordeum vulgare | 1263 | - | 4 | CAAT | common cis-acting element in promoter and enhancer regions |
| CAAT-box | Glycine max | 1262 | - | 5 | CAATT | common cis-acting element in promoter and enhancer regions |
| CAAT-box | Brassica rapa | 831 | - | 5 | CAAAT | common cis-acting element in promoter and enhancer regions |
| CAAT-box | Arabidopsis thaliana | 1374 | + | 5 | CCAAT | common cis-acting element in promoter and enhancer regions |
| CAAT-box | Brassica rapa | 1323 | + | 5 | CAAAT | common cis-acting element in promoter and enhancer regions |
| CAAT-box | Hordeum vulgare | 1375 | + | 4 | CAAT | common cis-acting element in promoter and enhancer regions |
| CAAT-box | Brassica rapa | 475 | - | 5 | CAAAT | common cis-acting element in promoter and enhancer regions |
| CAAT-box | Arabidopsis thaliana | 351 | + | 6 | gGCAAT | common cis-acting element in promoter and enhancer regions |
| CAAT-box | Glycine max | 1325 | - | 5 | CAATT | common cis-acting element in promoter and enhancer regions |
| CAAT-box | Brassica rapa | 272 | + | 5 | CAAAT | common cis-acting element in promoter and enhancer regions |
| CAAT-box | Glycine max | 1451 | - | 5 | CAATT | common cis-acting element in promoter and enhancer regions |
| CAAT-box | Hordeum vulgare | 527 | + | 4 | CAAT | common cis-acting element in promoter and enhancer regions |

> 2018/04/13 10:10:12  
+ ATAACCAAGA ACTTCTTTCG TTCGTTCGAA GGAAAGAAAC ACAAAGTTTC TTCAAAATCC CAAAATATAT   
  
  
+ AGTTGTCTCT CGATGGAGGT AGAGGTCTAA AACTGAAGTT TAATATATTA AGTTTTTGAT GACGGATAAT   
  
  
+ AGATAAAGAG AACGTACAGT GGATTAGAGC ATACAAGTCA GGTCGTTCGT TGCAATTAGA ACTTATAAAT   
  
  
+ AATTAAAATA TTACATTTAA TATATTTAAT TCGAATAAGG TTTTTAATAA AGTTGAACCC TCAAATAATT   
  
  
+ AATTAAATAT GTTATTACAA AAGACCTAGG CATGCACGCT GGTGACTTTT GCCACCAGTT TTGACTCCGC   
  
  
+ GGCAATAAAA CTGAAACACT GACTCACCCA GGGCATTAAC GATGGGCTGG GCCCCAGTTA ATACATGAAA   
  
  
+ ACGCATTATA AAGTTTAATA TGTCTCCGTG CAGTTAAGTT GCACATTTTG CTGCATTTGC GTCGAACTGA   
  
  
+ CAAGGATACA CTGGTACATT CTCAACTGAC ATTCGTCAAT GCCAGAAAAA AACTTTTTCT TTTATTCCCT   
  
  
+ TCGACCTCTT TCACCGCAAA GTAAATTTCA AGTCCTCGGT TTGGGAACCT TCTTTTTATC TTTTTTTATA   
  
  
+ TTTTTATTTA ATATCCGAAT TTCTTAGAAA TTTCTCCCAC ATATGTATAT AAAAAATTAT AAATAAATTA   
  
  
+ CAAAAAGTAC ACTGATTTTG TTTAGTTAAT ACTTCTATTT TTTTAATAAC AGTAATTAAT TAACTTTATT   
  
  
+ ACCACATTTC CATTACAAAA TTTTTAGCTT AATCCAACTA ACGAGGATAA CTAATTTAAT ATTTGATTTT   
  
  
+ AAAATTAATT AAATTAGATA AATTTTAGTT AAACTAAATA AATATAATAG TTTTTTTACA ACAAAAAATT   
  
  
+ TTTTTATATC ATTTAAAAAA TAAAATTTAT AAAAATTTTA TATTCTTTAA AATTTTTGTT TTTTAACCTA   
  
  
+ CGACGTAATC TCTCAACTTA ATTAACACTA CTCTTTTAAT ACTATAGTAC TTCTACCCAC TATAAAATAG   
  
  
+ GGTTTAACGA TTAGAATTAA TCATCCCATT TAGAACTGGT AAAGTTAAGA CTTCTCTGTC TTAATTAATT   
  
  
+ AAAACTCATA TATAATAGTT TTAAGATACG TGTTAGCTGT ATCATGGGTG CAGGATTCGA AGTAACTAAC   
  
  
+ GGGGAAGTTA AATAAAAACC GGAAGTGAAA GAAGGGTAAT TTTAGGTTAG TACTGTATAT ATATTTTTAA   
  
  
+ TAATTGTGTA TGGAATTGTA ATGGCATTAA AATAATATTT ATATATTATA TTTATAAGAT ACCAAATTGA   
  
  
+ ATATTTTAGA TTTTTTTTTA ATTTTTGAAA CTCTTTAAAT CAACCAATAG TTCATACGTA ACTGAATATT   
  
  
+ CGATCGAGGC TTCAAAGTTA AAGTAGTTTT TAATAACTAA AAAAATAAAT AATTGAAATT CTAAAACTTT   
  
  
+ TCTTATTTAG GGAGTAAATA ATTAATCTA  

- TATTGGTTCT TGAAGAAAGC AAGCAAGCTT CCTTTCTTTG TGTTTCAAAG AAGTTTTAGG GTTTTATATA   
  
  
- TCAACAGAGA GCTACCTCCA TCTCCAGATT TTGACTTCAA ATTATATAAT TCAAAAACTA CTGCCTATTA   
  
  
- TCTATTTCTC TTGCATGTCA CCTAATCTCG TATGTTCAGT CCAGCAAGCA ACGTTAATCT TGAATATTTA   
  
  
- TTAATTTTAT AATGTAAATT ATATAAATTA AGCTTATTCC AAAAATTATT TCAACTTGGG AGTTTATTAA   
  
  
- TTAATTTATA CAATAATGTT TTCTGGATCC GTACGTGCGA CCACTGAAAA CGGTGGTCAA AACTGAGGCG   
  
  
- CCGTTATTTT GACTTTGTGA CTGAGTGGGT CCCGTAATTG CTACCCGACC CGGGGTCAAT TATGTACTTT   
  
  
- TGCGTAATAT TTCAAATTAT ACAGAGGCAC GTCAATTCAA CGTGTAAAAC GACGTAAACG CAGCTTGACT   
  
  
- GTTCCTATGT GACCATGTAA GAGTTGACTG TAAGCAGTTA CGGTCTTTTT TTGAAAAAGA AAATAAGGGA   
  
  
- AGCTGGAGAA AGTGGCGTTT CATTTAAAGT TCAGGAGCCA AACCCTTGGA AGAAAAATAG AAAAAAATAT   
  
  
- AAAAATAAAT TATAGGCTTA AAGAATCTTT AAAGAGGGTG TATACATATA TTTTTTAATA TTTATTTAAT   
  
  
- GTTTTTCATG TGACTAAAAC AAATCAATTA TGAAGATAAA AAAATTATTG TCATTAATTA ATTGAAATAA   
  
  
- TGGTGTAAAG GTAATGTTTT AAAAATCGAA TTAGGTTGAT TGCTCCTATT GATTAAATTA TAAACTAAAA   
  
  
- TTTTAATTAA TTTAATCTAT TTAAAATCAA TTTGATTTAT TTATATTATC AAAAAAATGT TGTTTTTTAA   
  
  
- AAAAATATAG TAAATTTTTT ATTTTAAATA TTTTTAAAAT ATAAGAAATT TTAAAAACAA AAAATTGGAT   
  
  
- GCTGCATTAG AGAGTTGAAT TAATTGTGAT GAGAAAATTA TGATATCATG AAGATGGGTG ATATTTTATC   
  
  
- CCAAATTGCT AATCTTAATT AGTAGGGTAA ATCTTGACCA TTTCAATTCT GAAGAGACAG AATTAATTAA   
  
  
- TTTTGAGTAT ATATTATCAA AATTCTATGC ACAATCGACA TAGTACCCAC GTCCTAAGCT TCATTGATTG   
  
  
- CCCCTTCAAT TTATTTTTGG CCTTCACTTT CTTCCCATTA AAATCCAATC ATGACATATA TATAAAAATT   
  
  
- ATTAACACAT ACCTTAACAT TACCGTAATT TTATTATAAA TATATAATAT AAATATTCTA TGGTTTAACT   
  
  
- TATAAAATCT AAAAAAAAAT TAAAAACTTT GAGAAATTTA GTTGGTTATC AAGTATGCAT TGACTTATAA   
  
  
- GCTAGCTCCG AAGTTTCAAT TTCATCAAAA ATTATTGATT TTTTTATTTA TTAACTTTAA GATTTTGAAA   
  
  
- AGAATAAATC CCTCATTTAT TAATTAGAT

+     CGTCA-motif

| Site Name | Organism | Position | Strand | Matrix score. | sequence | function |
| --- | --- | --- | --- | --- | --- | --- |
| CGTCA-motif | Hordeum vulgare | 130 | - | 5 | CGTCA | cis-acting regulatory element involved in the MeJA-responsiveness |
| CGTCA-motif | Hordeum vulgare | 524 | + | 5 | CGTCA | cis-acting regulatory element involved in the MeJA-responsiveness |

> 2018/04/13 10:10:12  
+ ATAACCAAGA ACTTCTTTCG TTCGTTCGAA GGAAAGAAAC ACAAAGTTTC TTCAAAATCC CAAAATATAT   
  
  
+ AGTTGTCTCT CGATGGAGGT AGAGGTCTAA AACTGAAGTT TAATATATTA AGTTTTTGAT GACGGATAAT   
  
  
+ AGATAAAGAG AACGTACAGT GGATTAGAGC ATACAAGTCA GGTCGTTCGT TGCAATTAGA ACTTATAAAT   
  
  
+ AATTAAAATA TTACATTTAA TATATTTAAT TCGAATAAGG TTTTTAATAA AGTTGAACCC TCAAATAATT   
  
  
+ AATTAAATAT GTTATTACAA AAGACCTAGG CATGCACGCT GGTGACTTTT GCCACCAGTT TTGACTCCGC   
  
  
+ GGCAATAAAA CTGAAACACT GACTCACCCA GGGCATTAAC GATGGGCTGG GCCCCAGTTA ATACATGAAA   
  
  
+ ACGCATTATA AAGTTTAATA TGTCTCCGTG CAGTTAAGTT GCACATTTTG CTGCATTTGC GTCGAACTGA   
  
  
+ CAAGGATACA CTGGTACATT CTCAACTGAC ATTCGTCAAT GCCAGAAAAA AACTTTTTCT TTTATTCCCT   
  
  
+ TCGACCTCTT TCACCGCAAA GTAAATTTCA AGTCCTCGGT TTGGGAACCT TCTTTTTATC TTTTTTTATA   
  
  
+ TTTTTATTTA ATATCCGAAT TTCTTAGAAA TTTCTCCCAC ATATGTATAT AAAAAATTAT AAATAAATTA   
  
  
+ CAAAAAGTAC ACTGATTTTG TTTAGTTAAT ACTTCTATTT TTTTAATAAC AGTAATTAAT TAACTTTATT   
  
  
+ ACCACATTTC CATTACAAAA TTTTTAGCTT AATCCAACTA ACGAGGATAA CTAATTTAAT ATTTGATTTT   
  
  
+ AAAATTAATT AAATTAGATA AATTTTAGTT AAACTAAATA AATATAATAG TTTTTTTACA ACAAAAAATT   
  
  
+ TTTTTATATC ATTTAAAAAA TAAAATTTAT AAAAATTTTA TATTCTTTAA AATTTTTGTT TTTTAACCTA   
  
  
+ CGACGTAATC TCTCAACTTA ATTAACACTA CTCTTTTAAT ACTATAGTAC TTCTACCCAC TATAAAATAG   
  
  
+ GGTTTAACGA TTAGAATTAA TCATCCCATT TAGAACTGGT AAAGTTAAGA CTTCTCTGTC TTAATTAATT   
  
  
+ AAAACTCATA TATAATAGTT TTAAGATACG TGTTAGCTGT ATCATGGGTG CAGGATTCGA AGTAACTAAC   
  
  
+ GGGGAAGTTA AATAAAAACC GGAAGTGAAA GAAGGGTAAT TTTAGGTTAG TACTGTATAT ATATTTTTAA   
  
  
+ TAATTGTGTA TGGAATTGTA ATGGCATTAA AATAATATTT ATATATTATA TTTATAAGAT ACCAAATTGA   
  
  
+ ATATTTTAGA TTTTTTTTTA ATTTTTGAAA CTCTTTAAAT CAACCAATAG TTCATACGTA ACTGAATATT   
  
  
+ CGATCGAGGC TTCAAAGTTA AAGTAGTTTT TAATAACTAA AAAAATAAAT AATTGAAATT CTAAAACTTT   
  
  
+ TCTTATTTAG GGAGTAAATA ATTAATCTA  

- TATTGGTTCT TGAAGAAAGC AAGCAAGCTT CCTTTCTTTG TGTTTCAAAG AAGTTTTAGG GTTTTATATA   
  
  
- TCAACAGAGA GCTACCTCCA TCTCCAGATT TTGACTTCAA ATTATATAAT TCAAAAACTA CTGCCTATTA   
  
  
- TCTATTTCTC TTGCATGTCA CCTAATCTCG TATGTTCAGT CCAGCAAGCA ACGTTAATCT TGAATATTTA   
  
  
- TTAATTTTAT AATGTAAATT ATATAAATTA AGCTTATTCC AAAAATTATT TCAACTTGGG AGTTTATTAA   
  
  
- TTAATTTATA CAATAATGTT TTCTGGATCC GTACGTGCGA CCACTGAAAA CGGTGGTCAA AACTGAGGCG   
  
  
- CCGTTATTTT GACTTTGTGA CTGAGTGGGT CCCGTAATTG CTACCCGACC CGGGGTCAAT TATGTACTTT   
  
  
- TGCGTAATAT TTCAAATTAT ACAGAGGCAC GTCAATTCAA CGTGTAAAAC GACGTAAACG CAGCTTGACT   
  
  
- GTTCCTATGT GACCATGTAA GAGTTGACTG TAAGCAGTTA CGGTCTTTTT TTGAAAAAGA AAATAAGGGA   
  
  
- AGCTGGAGAA AGTGGCGTTT CATTTAAAGT TCAGGAGCCA AACCCTTGGA AGAAAAATAG AAAAAAATAT   
  
  
- AAAAATAAAT TATAGGCTTA AAGAATCTTT AAAGAGGGTG TATACATATA TTTTTTAATA TTTATTTAAT   
  
  
- GTTTTTCATG TGACTAAAAC AAATCAATTA TGAAGATAAA AAAATTATTG TCATTAATTA ATTGAAATAA   
  
  
- TGGTGTAAAG GTAATGTTTT AAAAATCGAA TTAGGTTGAT TGCTCCTATT GATTAAATTA TAAACTAAAA   
  
  
- TTTTAATTAA TTTAATCTAT TTAAAATCAA TTTGATTTAT TTATATTATC AAAAAAATGT TGTTTTTTAA   
  
  
- AAAAATATAG TAAATTTTTT ATTTTAAATA TTTTTAAAAT ATAAGAAATT TTAAAAACAA AAAATTGGAT   
  
  
- GCTGCATTAG AGAGTTGAAT TAATTGTGAT GAGAAAATTA TGATATCATG AAGATGGGTG ATATTTTATC   
  
  
- CCAAATTGCT AATCTTAATT AGTAGGGTAA ATCTTGACCA TTTCAATTCT GAAGAGACAG AATTAATTAA   
  
  
- TTTTGAGTAT ATATTATCAA AATTCTATGC ACAATCGACA TAGTACCCAC GTCCTAAGCT TCATTGATTG   
  
  
- CCCCTTCAAT TTATTTTTGG CCTTCACTTT CTTCCCATTA AAATCCAATC ATGACATATA TATAAAAATT   
  
  
- ATTAACACAT ACCTTAACAT TACCGTAATT TTATTATAAA TATATAATAT AAATATTCTA TGGTTTAACT   
  
  
- TATAAAATCT AAAAAAAAAT TAAAAACTTT GAGAAATTTA GTTGGTTATC AAGTATGCAT TGACTTATAA   
  
  
- GCTAGCTCCG AAGTTTCAAT TTCATCAAAA ATTATTGATT TTTTTATTTA TTAACTTTAA GATTTTGAAA   
  
  
- AGAATAAATC CCTCATTTAT TAATTAGAT

+     EIRE

| Site Name | Organism | Position | Strand | Matrix score. | sequence | function |
| --- | --- | --- | --- | --- | --- | --- |
| EIRE | Nicotiana tabacum | 560 | + | 7 | TTCGACC | elicitor-responsive element |

> 2018/04/13 10:10:12  
+ ATAACCAAGA ACTTCTTTCG TTCGTTCGAA GGAAAGAAAC ACAAAGTTTC TTCAAAATCC CAAAATATAT   
  
  
+ AGTTGTCTCT CGATGGAGGT AGAGGTCTAA AACTGAAGTT TAATATATTA AGTTTTTGAT GACGGATAAT   
  
  
+ AGATAAAGAG AACGTACAGT GGATTAGAGC ATACAAGTCA GGTCGTTCGT TGCAATTAGA ACTTATAAAT   
  
  
+ AATTAAAATA TTACATTTAA TATATTTAAT TCGAATAAGG TTTTTAATAA AGTTGAACCC TCAAATAATT   
  
  
+ AATTAAATAT GTTATTACAA AAGACCTAGG CATGCACGCT GGTGACTTTT GCCACCAGTT TTGACTCCGC   
  
  
+ GGCAATAAAA CTGAAACACT GACTCACCCA GGGCATTAAC GATGGGCTGG GCCCCAGTTA ATACATGAAA   
  
  
+ ACGCATTATA AAGTTTAATA TGTCTCCGTG CAGTTAAGTT GCACATTTTG CTGCATTTGC GTCGAACTGA   
  
  
+ CAAGGATACA CTGGTACATT CTCAACTGAC ATTCGTCAAT GCCAGAAAAA AACTTTTTCT TTTATTCCCT   
  
  
+ TCGACCTCTT TCACCGCAAA GTAAATTTCA AGTCCTCGGT TTGGGAACCT TCTTTTTATC TTTTTTTATA   
  
  
+ TTTTTATTTA ATATCCGAAT TTCTTAGAAA TTTCTCCCAC ATATGTATAT AAAAAATTAT AAATAAATTA   
  
  
+ CAAAAAGTAC ACTGATTTTG TTTAGTTAAT ACTTCTATTT TTTTAATAAC AGTAATTAAT TAACTTTATT   
  
  
+ ACCACATTTC CATTACAAAA TTTTTAGCTT AATCCAACTA ACGAGGATAA CTAATTTAAT ATTTGATTTT   
  
  
+ AAAATTAATT AAATTAGATA AATTTTAGTT AAACTAAATA AATATAATAG TTTTTTTACA ACAAAAAATT   
  
  
+ TTTTTATATC ATTTAAAAAA TAAAATTTAT AAAAATTTTA TATTCTTTAA AATTTTTGTT TTTTAACCTA   
  
  
+ CGACGTAATC TCTCAACTTA ATTAACACTA CTCTTTTAAT ACTATAGTAC TTCTACCCAC TATAAAATAG   
  
  
+ GGTTTAACGA TTAGAATTAA TCATCCCATT TAGAACTGGT AAAGTTAAGA CTTCTCTGTC TTAATTAATT   
  
  
+ AAAACTCATA TATAATAGTT TTAAGATACG TGTTAGCTGT ATCATGGGTG CAGGATTCGA AGTAACTAAC   
  
  
+ GGGGAAGTTA AATAAAAACC GGAAGTGAAA GAAGGGTAAT TTTAGGTTAG TACTGTATAT ATATTTTTAA   
  
  
+ TAATTGTGTA TGGAATTGTA ATGGCATTAA AATAATATTT ATATATTATA TTTATAAGAT ACCAAATTGA   
  
  
+ ATATTTTAGA TTTTTTTTTA ATTTTTGAAA CTCTTTAAAT CAACCAATAG TTCATACGTA ACTGAATATT   
  
  
+ CGATCGAGGC TTCAAAGTTA AAGTAGTTTT TAATAACTAA AAAAATAAAT AATTGAAATT CTAAAACTTT   
  
  
+ TCTTATTTAG GGAGTAAATA ATTAATCTA  

- TATTGGTTCT TGAAGAAAGC AAGCAAGCTT CCTTTCTTTG TGTTTCAAAG AAGTTTTAGG GTTTTATATA   
  
  
- TCAACAGAGA GCTACCTCCA TCTCCAGATT TTGACTTCAA ATTATATAAT TCAAAAACTA CTGCCTATTA   
  
  
- TCTATTTCTC TTGCATGTCA CCTAATCTCG TATGTTCAGT CCAGCAAGCA ACGTTAATCT TGAATATTTA   
  
  
- TTAATTTTAT AATGTAAATT ATATAAATTA AGCTTATTCC AAAAATTATT TCAACTTGGG AGTTTATTAA   
  
  
- TTAATTTATA CAATAATGTT TTCTGGATCC GTACGTGCGA CCACTGAAAA CGGTGGTCAA AACTGAGGCG   
  
  
- CCGTTATTTT GACTTTGTGA CTGAGTGGGT CCCGTAATTG CTACCCGACC CGGGGTCAAT TATGTACTTT   
  
  
- TGCGTAATAT TTCAAATTAT ACAGAGGCAC GTCAATTCAA CGTGTAAAAC GACGTAAACG CAGCTTGACT   
  
  
- GTTCCTATGT GACCATGTAA GAGTTGACTG TAAGCAGTTA CGGTCTTTTT TTGAAAAAGA AAATAAGGGA   
  
  
- AGCTGGAGAA AGTGGCGTTT CATTTAAAGT TCAGGAGCCA AACCCTTGGA AGAAAAATAG AAAAAAATAT   
  
  
- AAAAATAAAT TATAGGCTTA AAGAATCTTT AAAGAGGGTG TATACATATA TTTTTTAATA TTTATTTAAT   
  
  
- GTTTTTCATG TGACTAAAAC AAATCAATTA TGAAGATAAA AAAATTATTG TCATTAATTA ATTGAAATAA   
  
  
- TGGTGTAAAG GTAATGTTTT AAAAATCGAA TTAGGTTGAT TGCTCCTATT GATTAAATTA TAAACTAAAA   
  
  
- TTTTAATTAA TTTAATCTAT TTAAAATCAA TTTGATTTAT TTATATTATC AAAAAAATGT TGTTTTTTAA   
  
  
- AAAAATATAG TAAATTTTTT ATTTTAAATA TTTTTAAAAT ATAAGAAATT TTAAAAACAA AAAATTGGAT   
  
  
- GCTGCATTAG AGAGTTGAAT TAATTGTGAT GAGAAAATTA TGATATCATG AAGATGGGTG ATATTTTATC   
  
  
- CCAAATTGCT AATCTTAATT AGTAGGGTAA ATCTTGACCA TTTCAATTCT GAAGAGACAG AATTAATTAA   
  
  
- TTTTGAGTAT ATATTATCAA AATTCTATGC ACAATCGACA TAGTACCCAC GTCCTAAGCT TCATTGATTG   
  
  
- CCCCTTCAAT TTATTTTTGG CCTTCACTTT CTTCCCATTA AAATCCAATC ATGACATATA TATAAAAATT   
  
  
- ATTAACACAT ACCTTAACAT TACCGTAATT TTATTATAAA TATATAATAT AAATATTCTA TGGTTTAACT   
  
  
- TATAAAATCT AAAAAAAAAT TAAAAACTTT GAGAAATTTA GTTGGTTATC AAGTATGCAT TGACTTATAA   
  
  
- GCTAGCTCCG AAGTTTCAAT TTCATCAAAA ATTATTGATT TTTTTATTTA TTAACTTTAA GATTTTGAAA   
  
  
- AGAATAAATC CCTCATTTAT TAATTAGAT

+     G-Box

| Site Name | Organism | Position | Strand | Matrix score. | sequence | function |
| --- | --- | --- | --- | --- | --- | --- |
| G-Box | Antirrhinum majus | 1147 | - | 6 | CACGTA | cis-acting regulatory element involved in light responsiveness |

> 2018/04/13 10:10:12  
+ ATAACCAAGA ACTTCTTTCG TTCGTTCGAA GGAAAGAAAC ACAAAGTTTC TTCAAAATCC CAAAATATAT   
  
  
+ AGTTGTCTCT CGATGGAGGT AGAGGTCTAA AACTGAAGTT TAATATATTA AGTTTTTGAT GACGGATAAT   
  
  
+ AGATAAAGAG AACGTACAGT GGATTAGAGC ATACAAGTCA GGTCGTTCGT TGCAATTAGA ACTTATAAAT   
  
  
+ AATTAAAATA TTACATTTAA TATATTTAAT TCGAATAAGG TTTTTAATAA AGTTGAACCC TCAAATAATT   
  
  
+ AATTAAATAT GTTATTACAA AAGACCTAGG CATGCACGCT GGTGACTTTT GCCACCAGTT TTGACTCCGC   
  
  
+ GGCAATAAAA CTGAAACACT GACTCACCCA GGGCATTAAC GATGGGCTGG GCCCCAGTTA ATACATGAAA   
  
  
+ ACGCATTATA AAGTTTAATA TGTCTCCGTG CAGTTAAGTT GCACATTTTG CTGCATTTGC GTCGAACTGA   
  
  
+ CAAGGATACA CTGGTACATT CTCAACTGAC ATTCGTCAAT GCCAGAAAAA AACTTTTTCT TTTATTCCCT   
  
  
+ TCGACCTCTT TCACCGCAAA GTAAATTTCA AGTCCTCGGT TTGGGAACCT TCTTTTTATC TTTTTTTATA   
  
  
+ TTTTTATTTA ATATCCGAAT TTCTTAGAAA TTTCTCCCAC ATATGTATAT AAAAAATTAT AAATAAATTA   
  
  
+ CAAAAAGTAC ACTGATTTTG TTTAGTTAAT ACTTCTATTT TTTTAATAAC AGTAATTAAT TAACTTTATT   
  
  
+ ACCACATTTC CATTACAAAA TTTTTAGCTT AATCCAACTA ACGAGGATAA CTAATTTAAT ATTTGATTTT   
  
  
+ AAAATTAATT AAATTAGATA AATTTTAGTT AAACTAAATA AATATAATAG TTTTTTTACA ACAAAAAATT   
  
  
+ TTTTTATATC ATTTAAAAAA TAAAATTTAT AAAAATTTTA TATTCTTTAA AATTTTTGTT TTTTAACCTA   
  
  
+ CGACGTAATC TCTCAACTTA ATTAACACTA CTCTTTTAAT ACTATAGTAC TTCTACCCAC TATAAAATAG   
  
  
+ GGTTTAACGA TTAGAATTAA TCATCCCATT TAGAACTGGT AAAGTTAAGA CTTCTCTGTC TTAATTAATT   
  
  
+ AAAACTCATA TATAATAGTT TTAAGATACG TGTTAGCTGT ATCATGGGTG CAGGATTCGA AGTAACTAAC   
  
  
+ GGGGAAGTTA AATAAAAACC GGAAGTGAAA GAAGGGTAAT TTTAGGTTAG TACTGTATAT ATATTTTTAA   
  
  
+ TAATTGTGTA TGGAATTGTA ATGGCATTAA AATAATATTT ATATATTATA TTTATAAGAT ACCAAATTGA   
  
  
+ ATATTTTAGA TTTTTTTTTA ATTTTTGAAA CTCTTTAAAT CAACCAATAG TTCATACGTA ACTGAATATT   
  
  
+ CGATCGAGGC TTCAAAGTTA AAGTAGTTTT TAATAACTAA AAAAATAAAT AATTGAAATT CTAAAACTTT   
  
  
+ TCTTATTTAG GGAGTAAATA ATTAATCTA  

- TATTGGTTCT TGAAGAAAGC AAGCAAGCTT CCTTTCTTTG TGTTTCAAAG AAGTTTTAGG GTTTTATATA   
  
  
- TCAACAGAGA GCTACCTCCA TCTCCAGATT TTGACTTCAA ATTATATAAT TCAAAAACTA CTGCCTATTA   
  
  
- TCTATTTCTC TTGCATGTCA CCTAATCTCG TATGTTCAGT CCAGCAAGCA ACGTTAATCT TGAATATTTA   
  
  
- TTAATTTTAT AATGTAAATT ATATAAATTA AGCTTATTCC AAAAATTATT TCAACTTGGG AGTTTATTAA   
  
  
- TTAATTTATA CAATAATGTT TTCTGGATCC GTACGTGCGA CCACTGAAAA CGGTGGTCAA AACTGAGGCG   
  
  
- CCGTTATTTT GACTTTGTGA CTGAGTGGGT CCCGTAATTG CTACCCGACC CGGGGTCAAT TATGTACTTT   
  
  
- TGCGTAATAT TTCAAATTAT ACAGAGGCAC GTCAATTCAA CGTGTAAAAC GACGTAAACG CAGCTTGACT   
  
  
- GTTCCTATGT GACCATGTAA GAGTTGACTG TAAGCAGTTA CGGTCTTTTT TTGAAAAAGA AAATAAGGGA   
  
  
- AGCTGGAGAA AGTGGCGTTT CATTTAAAGT TCAGGAGCCA AACCCTTGGA AGAAAAATAG AAAAAAATAT   
  
  
- AAAAATAAAT TATAGGCTTA AAGAATCTTT AAAGAGGGTG TATACATATA TTTTTTAATA TTTATTTAAT   
  
  
- GTTTTTCATG TGACTAAAAC AAATCAATTA TGAAGATAAA AAAATTATTG TCATTAATTA ATTGAAATAA   
  
  
- TGGTGTAAAG GTAATGTTTT AAAAATCGAA TTAGGTTGAT TGCTCCTATT GATTAAATTA TAAACTAAAA   
  
  
- TTTTAATTAA TTTAATCTAT TTAAAATCAA TTTGATTTAT TTATATTATC AAAAAAATGT TGTTTTTTAA   
  
  
- AAAAATATAG TAAATTTTTT ATTTTAAATA TTTTTAAAAT ATAAGAAATT TTAAAAACAA AAAATTGGAT   
  
  
- GCTGCATTAG AGAGTTGAAT TAATTGTGAT GAGAAAATTA TGATATCATG AAGATGGGTG ATATTTTATC   
  
  
- CCAAATTGCT AATCTTAATT AGTAGGGTAA ATCTTGACCA TTTCAATTCT GAAGAGACAG AATTAATTAA   
  
  
- TTTTGAGTAT ATATTATCAA AATTCTATGC ACAATCGACA TAGTACCCAC GTCCTAAGCT TCATTGATTG   
  
  
- CCCCTTCAAT TTATTTTTGG CCTTCACTTT CTTCCCATTA AAATCCAATC ATGACATATA TATAAAAATT   
  
  
- ATTAACACAT ACCTTAACAT TACCGTAATT TTATTATAAA TATATAATAT AAATATTCTA TGGTTTAACT   
  
  
- TATAAAATCT AAAAAAAAAT TAAAAACTTT GAGAAATTTA GTTGGTTATC AAGTATGCAT TGACTTATAA   
  
  
- GCTAGCTCCG AAGTTTCAAT TTCATCAAAA ATTATTGATT TTTTTATTTA TTAACTTTAA GATTTTGAAA   
  
  
- AGAATAAATC CCTCATTTAT TAATTAGAT

+     G-box

| Site Name | Organism | Position | Strand | Matrix score. | sequence | function |
| --- | --- | --- | --- | --- | --- | --- |
| G-box | Brassica oleracea | 1146 | - | 9 | TAACACGTAG | cis-acting regulatory element involved in light responsiveness |
| G-box | Daucus carota | 1147 | + | 6 | TACGTG | cis-acting regulatory element involved in light responsiveness |
| G-box | Zea mays | 771 | - | 9 | GACATGTGGT | cis-acting regulatory element involved in light responsiveness |

> 2018/04/13 10:10:12  
+ ATAACCAAGA ACTTCTTTCG TTCGTTCGAA GGAAAGAAAC ACAAAGTTTC TTCAAAATCC CAAAATATAT   
  
  
+ AGTTGTCTCT CGATGGAGGT AGAGGTCTAA AACTGAAGTT TAATATATTA AGTTTTTGAT GACGGATAAT   
  
  
+ AGATAAAGAG AACGTACAGT GGATTAGAGC ATACAAGTCA GGTCGTTCGT TGCAATTAGA ACTTATAAAT   
  
  
+ AATTAAAATA TTACATTTAA TATATTTAAT TCGAATAAGG TTTTTAATAA AGTTGAACCC TCAAATAATT   
  
  
+ AATTAAATAT GTTATTACAA AAGACCTAGG CATGCACGCT GGTGACTTTT GCCACCAGTT TTGACTCCGC   
  
  
+ GGCAATAAAA CTGAAACACT GACTCACCCA GGGCATTAAC GATGGGCTGG GCCCCAGTTA ATACATGAAA   
  
  
+ ACGCATTATA AAGTTTAATA TGTCTCCGTG CAGTTAAGTT GCACATTTTG CTGCATTTGC GTCGAACTGA   
  
  
+ CAAGGATACA CTGGTACATT CTCAACTGAC ATTCGTCAAT GCCAGAAAAA AACTTTTTCT TTTATTCCCT   
  
  
+ TCGACCTCTT TCACCGCAAA GTAAATTTCA AGTCCTCGGT TTGGGAACCT TCTTTTTATC TTTTTTTATA   
  
  
+ TTTTTATTTA ATATCCGAAT TTCTTAGAAA TTTCTCCCAC ATATGTATAT AAAAAATTAT AAATAAATTA   
  
  
+ CAAAAAGTAC ACTGATTTTG TTTAGTTAAT ACTTCTATTT TTTTAATAAC AGTAATTAAT TAACTTTATT   
  
  
+ ACCACATTTC CATTACAAAA TTTTTAGCTT AATCCAACTA ACGAGGATAA CTAATTTAAT ATTTGATTTT   
  
  
+ AAAATTAATT AAATTAGATA AATTTTAGTT AAACTAAATA AATATAATAG TTTTTTTACA ACAAAAAATT   
  
  
+ TTTTTATATC ATTTAAAAAA TAAAATTTAT AAAAATTTTA TATTCTTTAA AATTTTTGTT TTTTAACCTA   
  
  
+ CGACGTAATC TCTCAACTTA ATTAACACTA CTCTTTTAAT ACTATAGTAC TTCTACCCAC TATAAAATAG   
  
  
+ GGTTTAACGA TTAGAATTAA TCATCCCATT TAGAACTGGT AAAGTTAAGA CTTCTCTGTC TTAATTAATT   
  
  
+ AAAACTCATA TATAATAGTT TTAAGATACG TGTTAGCTGT ATCATGGGTG CAGGATTCGA AGTAACTAAC   
  
  
+ GGGGAAGTTA AATAAAAACC GGAAGTGAAA GAAGGGTAAT TTTAGGTTAG TACTGTATAT ATATTTTTAA   
  
  
+ TAATTGTGTA TGGAATTGTA ATGGCATTAA AATAATATTT ATATATTATA TTTATAAGAT ACCAAATTGA   
  
  
+ ATATTTTAGA TTTTTTTTTA ATTTTTGAAA CTCTTTAAAT CAACCAATAG TTCATACGTA ACTGAATATT   
  
  
+ CGATCGAGGC TTCAAAGTTA AAGTAGTTTT TAATAACTAA AAAAATAAAT AATTGAAATT CTAAAACTTT   
  
  
+ TCTTATTTAG GGAGTAAATA ATTAATCTA  

- TATTGGTTCT TGAAGAAAGC AAGCAAGCTT CCTTTCTTTG TGTTTCAAAG AAGTTTTAGG GTTTTATATA   
  
  
- TCAACAGAGA GCTACCTCCA TCTCCAGATT TTGACTTCAA ATTATATAAT TCAAAAACTA CTGCCTATTA   
  
  
- TCTATTTCTC TTGCATGTCA CCTAATCTCG TATGTTCAGT CCAGCAAGCA ACGTTAATCT TGAATATTTA   
  
  
- TTAATTTTAT AATGTAAATT ATATAAATTA AGCTTATTCC AAAAATTATT TCAACTTGGG AGTTTATTAA   
  
  
- TTAATTTATA CAATAATGTT TTCTGGATCC GTACGTGCGA CCACTGAAAA CGGTGGTCAA AACTGAGGCG   
  
  
- CCGTTATTTT GACTTTGTGA CTGAGTGGGT CCCGTAATTG CTACCCGACC CGGGGTCAAT TATGTACTTT   
  
  
- TGCGTAATAT TTCAAATTAT ACAGAGGCAC GTCAATTCAA CGTGTAAAAC GACGTAAACG CAGCTTGACT   
  
  
- GTTCCTATGT GACCATGTAA GAGTTGACTG TAAGCAGTTA CGGTCTTTTT TTGAAAAAGA AAATAAGGGA   
  
  
- AGCTGGAGAA AGTGGCGTTT CATTTAAAGT TCAGGAGCCA AACCCTTGGA AGAAAAATAG AAAAAAATAT   
  
  
- AAAAATAAAT TATAGGCTTA AAGAATCTTT AAAGAGGGTG TATACATATA TTTTTTAATA TTTATTTAAT   
  
  
- GTTTTTCATG TGACTAAAAC AAATCAATTA TGAAGATAAA AAAATTATTG TCATTAATTA ATTGAAATAA   
  
  
- TGGTGTAAAG GTAATGTTTT AAAAATCGAA TTAGGTTGAT TGCTCCTATT GATTAAATTA TAAACTAAAA   
  
  
- TTTTAATTAA TTTAATCTAT TTAAAATCAA TTTGATTTAT TTATATTATC AAAAAAATGT TGTTTTTTAA   
  
  
- AAAAATATAG TAAATTTTTT ATTTTAAATA TTTTTAAAAT ATAAGAAATT TTAAAAACAA AAAATTGGAT   
  
  
- GCTGCATTAG AGAGTTGAAT TAATTGTGAT GAGAAAATTA TGATATCATG AAGATGGGTG ATATTTTATC   
  
  
- CCAAATTGCT AATCTTAATT AGTAGGGTAA ATCTTGACCA TTTCAATTCT GAAGAGACAG AATTAATTAA   
  
  
- TTTTGAGTAT ATATTATCAA AATTCTATGC ACAATCGACA TAGTACCCAC GTCCTAAGCT TCATTGATTG   
  
  
- CCCCTTCAAT TTATTTTTGG CCTTCACTTT CTTCCCATTA AAATCCAATC ATGACATATA TATAAAAATT   
  
  
- ATTAACACAT ACCTTAACAT TACCGTAATT TTATTATAAA TATATAATAT AAATATTCTA TGGTTTAACT   
  
  
- TATAAAATCT AAAAAAAAAT TAAAAACTTT GAGAAATTTA GTTGGTTATC AAGTATGCAT TGACTTATAA   
  
  
- GCTAGCTCCG AAGTTTCAAT TTCATCAAAA ATTATTGATT TTTTTATTTA TTAACTTTAA GATTTTGAAA   
  
  
- AGAATAAATC CCTCATTTAT TAATTAGAT

+     GA-motif

| Site Name | Organism | Position | Strand | Matrix score. | sequence | function |
| --- | --- | --- | --- | --- | --- | --- |
| GA-motif | Arabidopsis thaliana | 139 | + | 8 | ATAGATAA | part of a light responsive element |

> 2018/04/13 10:10:12  
+ ATAACCAAGA ACTTCTTTCG TTCGTTCGAA GGAAAGAAAC ACAAAGTTTC TTCAAAATCC CAAAATATAT   
  
  
+ AGTTGTCTCT CGATGGAGGT AGAGGTCTAA AACTGAAGTT TAATATATTA AGTTTTTGAT GACGGATAAT   
  
  
+ AGATAAAGAG AACGTACAGT GGATTAGAGC ATACAAGTCA GGTCGTTCGT TGCAATTAGA ACTTATAAAT   
  
  
+ AATTAAAATA TTACATTTAA TATATTTAAT TCGAATAAGG TTTTTAATAA AGTTGAACCC TCAAATAATT   
  
  
+ AATTAAATAT GTTATTACAA AAGACCTAGG CATGCACGCT GGTGACTTTT GCCACCAGTT TTGACTCCGC   
  
  
+ GGCAATAAAA CTGAAACACT GACTCACCCA GGGCATTAAC GATGGGCTGG GCCCCAGTTA ATACATGAAA   
  
  
+ ACGCATTATA AAGTTTAATA TGTCTCCGTG CAGTTAAGTT GCACATTTTG CTGCATTTGC GTCGAACTGA   
  
  
+ CAAGGATACA CTGGTACATT CTCAACTGAC ATTCGTCAAT GCCAGAAAAA AACTTTTTCT TTTATTCCCT   
  
  
+ TCGACCTCTT TCACCGCAAA GTAAATTTCA AGTCCTCGGT TTGGGAACCT TCTTTTTATC TTTTTTTATA   
  
  
+ TTTTTATTTA ATATCCGAAT TTCTTAGAAA TTTCTCCCAC ATATGTATAT AAAAAATTAT AAATAAATTA   
  
  
+ CAAAAAGTAC ACTGATTTTG TTTAGTTAAT ACTTCTATTT TTTTAATAAC AGTAATTAAT TAACTTTATT   
  
  
+ ACCACATTTC CATTACAAAA TTTTTAGCTT AATCCAACTA ACGAGGATAA CTAATTTAAT ATTTGATTTT   
  
  
+ AAAATTAATT AAATTAGATA AATTTTAGTT AAACTAAATA AATATAATAG TTTTTTTACA ACAAAAAATT   
  
  
+ TTTTTATATC ATTTAAAAAA TAAAATTTAT AAAAATTTTA TATTCTTTAA AATTTTTGTT TTTTAACCTA   
  
  
+ CGACGTAATC TCTCAACTTA ATTAACACTA CTCTTTTAAT ACTATAGTAC TTCTACCCAC TATAAAATAG   
  
  
+ GGTTTAACGA TTAGAATTAA TCATCCCATT TAGAACTGGT AAAGTTAAGA CTTCTCTGTC TTAATTAATT   
  
  
+ AAAACTCATA TATAATAGTT TTAAGATACG TGTTAGCTGT ATCATGGGTG CAGGATTCGA AGTAACTAAC   
  
  
+ GGGGAAGTTA AATAAAAACC GGAAGTGAAA GAAGGGTAAT TTTAGGTTAG TACTGTATAT ATATTTTTAA   
  
  
+ TAATTGTGTA TGGAATTGTA ATGGCATTAA AATAATATTT ATATATTATA TTTATAAGAT ACCAAATTGA   
  
  
+ ATATTTTAGA TTTTTTTTTA ATTTTTGAAA CTCTTTAAAT CAACCAATAG TTCATACGTA ACTGAATATT   
  
  
+ CGATCGAGGC TTCAAAGTTA AAGTAGTTTT TAATAACTAA AAAAATAAAT AATTGAAATT CTAAAACTTT   
  
  
+ TCTTATTTAG GGAGTAAATA ATTAATCTA  

- TATTGGTTCT TGAAGAAAGC AAGCAAGCTT CCTTTCTTTG TGTTTCAAAG AAGTTTTAGG GTTTTATATA   
  
  
- TCAACAGAGA GCTACCTCCA TCTCCAGATT TTGACTTCAA ATTATATAAT TCAAAAACTA CTGCCTATTA   
  
  
- TCTATTTCTC TTGCATGTCA CCTAATCTCG TATGTTCAGT CCAGCAAGCA ACGTTAATCT TGAATATTTA   
  
  
- TTAATTTTAT AATGTAAATT ATATAAATTA AGCTTATTCC AAAAATTATT TCAACTTGGG AGTTTATTAA   
  
  
- TTAATTTATA CAATAATGTT TTCTGGATCC GTACGTGCGA CCACTGAAAA CGGTGGTCAA AACTGAGGCG   
  
  
- CCGTTATTTT GACTTTGTGA CTGAGTGGGT CCCGTAATTG CTACCCGACC CGGGGTCAAT TATGTACTTT   
  
  
- TGCGTAATAT TTCAAATTAT ACAGAGGCAC GTCAATTCAA CGTGTAAAAC GACGTAAACG CAGCTTGACT   
  
  
- GTTCCTATGT GACCATGTAA GAGTTGACTG TAAGCAGTTA CGGTCTTTTT TTGAAAAAGA AAATAAGGGA   
  
  
- AGCTGGAGAA AGTGGCGTTT CATTTAAAGT TCAGGAGCCA AACCCTTGGA AGAAAAATAG AAAAAAATAT   
  
  
- AAAAATAAAT TATAGGCTTA AAGAATCTTT AAAGAGGGTG TATACATATA TTTTTTAATA TTTATTTAAT   
  
  
- GTTTTTCATG TGACTAAAAC AAATCAATTA TGAAGATAAA AAAATTATTG TCATTAATTA ATTGAAATAA   
  
  
- TGGTGTAAAG GTAATGTTTT AAAAATCGAA TTAGGTTGAT TGCTCCTATT GATTAAATTA TAAACTAAAA   
  
  
- TTTTAATTAA TTTAATCTAT TTAAAATCAA TTTGATTTAT TTATATTATC AAAAAAATGT TGTTTTTTAA   
  
  
- AAAAATATAG TAAATTTTTT ATTTTAAATA TTTTTAAAAT ATAAGAAATT TTAAAAACAA AAAATTGGAT   
  
  
- GCTGCATTAG AGAGTTGAAT TAATTGTGAT GAGAAAATTA TGATATCATG AAGATGGGTG ATATTTTATC   
  
  
- CCAAATTGCT AATCTTAATT AGTAGGGTAA ATCTTGACCA TTTCAATTCT GAAGAGACAG AATTAATTAA   
  
  
- TTTTGAGTAT ATATTATCAA AATTCTATGC ACAATCGACA TAGTACCCAC GTCCTAAGCT TCATTGATTG   
  
  
- CCCCTTCAAT TTATTTTTGG CCTTCACTTT CTTCCCATTA AAATCCAATC ATGACATATA TATAAAAATT   
  
  
- ATTAACACAT ACCTTAACAT TACCGTAATT TTATTATAAA TATATAATAT AAATATTCTA TGGTTTAACT   
  
  
- TATAAAATCT AAAAAAAAAT TAAAAACTTT GAGAAATTTA GTTGGTTATC AAGTATGCAT TGACTTATAA   
  
  
- GCTAGCTCCG AAGTTTCAAT TTCATCAAAA ATTATTGATT TTTTTATTTA TTAACTTTAA GATTTTGAAA   
  
  
- AGAATAAATC CCTCATTTAT TAATTAGAT

+     GCN4\_motif

| Site Name | Organism | Position | Strand | Matrix score. | sequence | function |
| --- | --- | --- | --- | --- | --- | --- |
| GCN4\_motif | Oryza sativa | 370 | - | 7 | TGAGTCA | cis-regulatory element involved in endosperm expression |

> 2018/04/13 10:10:12  
+ ATAACCAAGA ACTTCTTTCG TTCGTTCGAA GGAAAGAAAC ACAAAGTTTC TTCAAAATCC CAAAATATAT   
  
  
+ AGTTGTCTCT CGATGGAGGT AGAGGTCTAA AACTGAAGTT TAATATATTA AGTTTTTGAT GACGGATAAT   
  
  
+ AGATAAAGAG AACGTACAGT GGATTAGAGC ATACAAGTCA GGTCGTTCGT TGCAATTAGA ACTTATAAAT   
  
  
+ AATTAAAATA TTACATTTAA TATATTTAAT TCGAATAAGG TTTTTAATAA AGTTGAACCC TCAAATAATT   
  
  
+ AATTAAATAT GTTATTACAA AAGACCTAGG CATGCACGCT GGTGACTTTT GCCACCAGTT TTGACTCCGC   
  
  
+ GGCAATAAAA CTGAAACACT GACTCACCCA GGGCATTAAC GATGGGCTGG GCCCCAGTTA ATACATGAAA   
  
  
+ ACGCATTATA AAGTTTAATA TGTCTCCGTG CAGTTAAGTT GCACATTTTG CTGCATTTGC GTCGAACTGA   
  
  
+ CAAGGATACA CTGGTACATT CTCAACTGAC ATTCGTCAAT GCCAGAAAAA AACTTTTTCT TTTATTCCCT   
  
  
+ TCGACCTCTT TCACCGCAAA GTAAATTTCA AGTCCTCGGT TTGGGAACCT TCTTTTTATC TTTTTTTATA   
  
  
+ TTTTTATTTA ATATCCGAAT TTCTTAGAAA TTTCTCCCAC ATATGTATAT AAAAAATTAT AAATAAATTA   
  
  
+ CAAAAAGTAC ACTGATTTTG TTTAGTTAAT ACTTCTATTT TTTTAATAAC AGTAATTAAT TAACTTTATT   
  
  
+ ACCACATTTC CATTACAAAA TTTTTAGCTT AATCCAACTA ACGAGGATAA CTAATTTAAT ATTTGATTTT   
  
  
+ AAAATTAATT AAATTAGATA AATTTTAGTT AAACTAAATA AATATAATAG TTTTTTTACA ACAAAAAATT   
  
  
+ TTTTTATATC ATTTAAAAAA TAAAATTTAT AAAAATTTTA TATTCTTTAA AATTTTTGTT TTTTAACCTA   
  
  
+ CGACGTAATC TCTCAACTTA ATTAACACTA CTCTTTTAAT ACTATAGTAC TTCTACCCAC TATAAAATAG   
  
  
+ GGTTTAACGA TTAGAATTAA TCATCCCATT TAGAACTGGT AAAGTTAAGA CTTCTCTGTC TTAATTAATT   
  
  
+ AAAACTCATA TATAATAGTT TTAAGATACG TGTTAGCTGT ATCATGGGTG CAGGATTCGA AGTAACTAAC   
  
  
+ GGGGAAGTTA AATAAAAACC GGAAGTGAAA GAAGGGTAAT TTTAGGTTAG TACTGTATAT ATATTTTTAA   
  
  
+ TAATTGTGTA TGGAATTGTA ATGGCATTAA AATAATATTT ATATATTATA TTTATAAGAT ACCAAATTGA   
  
  
+ ATATTTTAGA TTTTTTTTTA ATTTTTGAAA CTCTTTAAAT CAACCAATAG TTCATACGTA ACTGAATATT   
  
  
+ CGATCGAGGC TTCAAAGTTA AAGTAGTTTT TAATAACTAA AAAAATAAAT AATTGAAATT CTAAAACTTT   
  
  
+ TCTTATTTAG GGAGTAAATA ATTAATCTA  

- TATTGGTTCT TGAAGAAAGC AAGCAAGCTT CCTTTCTTTG TGTTTCAAAG AAGTTTTAGG GTTTTATATA   
  
  
- TCAACAGAGA GCTACCTCCA TCTCCAGATT TTGACTTCAA ATTATATAAT TCAAAAACTA CTGCCTATTA   
  
  
- TCTATTTCTC TTGCATGTCA CCTAATCTCG TATGTTCAGT CCAGCAAGCA ACGTTAATCT TGAATATTTA   
  
  
- TTAATTTTAT AATGTAAATT ATATAAATTA AGCTTATTCC AAAAATTATT TCAACTTGGG AGTTTATTAA   
  
  
- TTAATTTATA CAATAATGTT TTCTGGATCC GTACGTGCGA CCACTGAAAA CGGTGGTCAA AACTGAGGCG   
  
  
- CCGTTATTTT GACTTTGTGA CTGAGTGGGT CCCGTAATTG CTACCCGACC CGGGGTCAAT TATGTACTTT   
  
  
- TGCGTAATAT TTCAAATTAT ACAGAGGCAC GTCAATTCAA CGTGTAAAAC GACGTAAACG CAGCTTGACT   
  
  
- GTTCCTATGT GACCATGTAA GAGTTGACTG TAAGCAGTTA CGGTCTTTTT TTGAAAAAGA AAATAAGGGA   
  
  
- AGCTGGAGAA AGTGGCGTTT CATTTAAAGT TCAGGAGCCA AACCCTTGGA AGAAAAATAG AAAAAAATAT   
  
  
- AAAAATAAAT TATAGGCTTA AAGAATCTTT AAAGAGGGTG TATACATATA TTTTTTAATA TTTATTTAAT   
  
  
- GTTTTTCATG TGACTAAAAC AAATCAATTA TGAAGATAAA AAAATTATTG TCATTAATTA ATTGAAATAA   
  
  
- TGGTGTAAAG GTAATGTTTT AAAAATCGAA TTAGGTTGAT TGCTCCTATT GATTAAATTA TAAACTAAAA   
  
  
- TTTTAATTAA TTTAATCTAT TTAAAATCAA TTTGATTTAT TTATATTATC AAAAAAATGT TGTTTTTTAA   
  
  
- AAAAATATAG TAAATTTTTT ATTTTAAATA TTTTTAAAAT ATAAGAAATT TTAAAAACAA AAAATTGGAT   
  
  
- GCTGCATTAG AGAGTTGAAT TAATTGTGAT GAGAAAATTA TGATATCATG AAGATGGGTG ATATTTTATC   
  
  
- CCAAATTGCT AATCTTAATT AGTAGGGTAA ATCTTGACCA TTTCAATTCT GAAGAGACAG AATTAATTAA   
  
  
- TTTTGAGTAT ATATTATCAA AATTCTATGC ACAATCGACA TAGTACCCAC GTCCTAAGCT TCATTGATTG   
  
  
- CCCCTTCAAT TTATTTTTGG CCTTCACTTT CTTCCCATTA AAATCCAATC ATGACATATA TATAAAAATT   
  
  
- ATTAACACAT ACCTTAACAT TACCGTAATT TTATTATAAA TATATAATAT AAATATTCTA TGGTTTAACT   
  
  
- TATAAAATCT AAAAAAAAAT TAAAAACTTT GAGAAATTTA GTTGGTTATC AAGTATGCAT TGACTTATAA   
  
  
- GCTAGCTCCG AAGTTTCAAT TTCATCAAAA ATTATTGATT TTTTTATTTA TTAACTTTAA GATTTTGAAA   
  
  
- AGAATAAATC CCTCATTTAT TAATTAGAT

+     GT1-motif

| Site Name | Organism | Position | Strand | Matrix score. | sequence | function |
| --- | --- | --- | --- | --- | --- | --- |
| GT1-motif | Arabidopsis thaliana | 973 | - | 6 | GGTTAA | light responsive element |

> 2018/04/13 10:10:12  
+ ATAACCAAGA ACTTCTTTCG TTCGTTCGAA GGAAAGAAAC ACAAAGTTTC TTCAAAATCC CAAAATATAT   
  
  
+ AGTTGTCTCT CGATGGAGGT AGAGGTCTAA AACTGAAGTT TAATATATTA AGTTTTTGAT GACGGATAAT   
  
  
+ AGATAAAGAG AACGTACAGT GGATTAGAGC ATACAAGTCA GGTCGTTCGT TGCAATTAGA ACTTATAAAT   
  
  
+ AATTAAAATA TTACATTTAA TATATTTAAT TCGAATAAGG TTTTTAATAA AGTTGAACCC TCAAATAATT   
  
  
+ AATTAAATAT GTTATTACAA AAGACCTAGG CATGCACGCT GGTGACTTTT GCCACCAGTT TTGACTCCGC   
  
  
+ GGCAATAAAA CTGAAACACT GACTCACCCA GGGCATTAAC GATGGGCTGG GCCCCAGTTA ATACATGAAA   
  
  
+ ACGCATTATA AAGTTTAATA TGTCTCCGTG CAGTTAAGTT GCACATTTTG CTGCATTTGC GTCGAACTGA   
  
  
+ CAAGGATACA CTGGTACATT CTCAACTGAC ATTCGTCAAT GCCAGAAAAA AACTTTTTCT TTTATTCCCT   
  
  
+ TCGACCTCTT TCACCGCAAA GTAAATTTCA AGTCCTCGGT TTGGGAACCT TCTTTTTATC TTTTTTTATA   
  
  
+ TTTTTATTTA ATATCCGAAT TTCTTAGAAA TTTCTCCCAC ATATGTATAT AAAAAATTAT AAATAAATTA   
  
  
+ CAAAAAGTAC ACTGATTTTG TTTAGTTAAT ACTTCTATTT TTTTAATAAC AGTAATTAAT TAACTTTATT   
  
  
+ ACCACATTTC CATTACAAAA TTTTTAGCTT AATCCAACTA ACGAGGATAA CTAATTTAAT ATTTGATTTT   
  
  
+ AAAATTAATT AAATTAGATA AATTTTAGTT AAACTAAATA AATATAATAG TTTTTTTACA ACAAAAAATT   
  
  
+ TTTTTATATC ATTTAAAAAA TAAAATTTAT AAAAATTTTA TATTCTTTAA AATTTTTGTT TTTTAACCTA   
  
  
+ CGACGTAATC TCTCAACTTA ATTAACACTA CTCTTTTAAT ACTATAGTAC TTCTACCCAC TATAAAATAG   
  
  
+ GGTTTAACGA TTAGAATTAA TCATCCCATT TAGAACTGGT AAAGTTAAGA CTTCTCTGTC TTAATTAATT   
  
  
+ AAAACTCATA TATAATAGTT TTAAGATACG TGTTAGCTGT ATCATGGGTG CAGGATTCGA AGTAACTAAC   
  
  
+ GGGGAAGTTA AATAAAAACC GGAAGTGAAA GAAGGGTAAT TTTAGGTTAG TACTGTATAT ATATTTTTAA   
  
  
+ TAATTGTGTA TGGAATTGTA ATGGCATTAA AATAATATTT ATATATTATA TTTATAAGAT ACCAAATTGA   
  
  
+ ATATTTTAGA TTTTTTTTTA ATTTTTGAAA CTCTTTAAAT CAACCAATAG TTCATACGTA ACTGAATATT   
  
  
+ CGATCGAGGC TTCAAAGTTA AAGTAGTTTT TAATAACTAA AAAAATAAAT AATTGAAATT CTAAAACTTT   
  
  
+ TCTTATTTAG GGAGTAAATA ATTAATCTA  

- TATTGGTTCT TGAAGAAAGC AAGCAAGCTT CCTTTCTTTG TGTTTCAAAG AAGTTTTAGG GTTTTATATA   
  
  
- TCAACAGAGA GCTACCTCCA TCTCCAGATT TTGACTTCAA ATTATATAAT TCAAAAACTA CTGCCTATTA   
  
  
- TCTATTTCTC TTGCATGTCA CCTAATCTCG TATGTTCAGT CCAGCAAGCA ACGTTAATCT TGAATATTTA   
  
  
- TTAATTTTAT AATGTAAATT ATATAAATTA AGCTTATTCC AAAAATTATT TCAACTTGGG AGTTTATTAA   
  
  
- TTAATTTATA CAATAATGTT TTCTGGATCC GTACGTGCGA CCACTGAAAA CGGTGGTCAA AACTGAGGCG   
  
  
- CCGTTATTTT GACTTTGTGA CTGAGTGGGT CCCGTAATTG CTACCCGACC CGGGGTCAAT TATGTACTTT   
  
  
- TGCGTAATAT TTCAAATTAT ACAGAGGCAC GTCAATTCAA CGTGTAAAAC GACGTAAACG CAGCTTGACT   
  
  
- GTTCCTATGT GACCATGTAA GAGTTGACTG TAAGCAGTTA CGGTCTTTTT TTGAAAAAGA AAATAAGGGA   
  
  
- AGCTGGAGAA AGTGGCGTTT CATTTAAAGT TCAGGAGCCA AACCCTTGGA AGAAAAATAG AAAAAAATAT   
  
  
- AAAAATAAAT TATAGGCTTA AAGAATCTTT AAAGAGGGTG TATACATATA TTTTTTAATA TTTATTTAAT   
  
  
- GTTTTTCATG TGACTAAAAC AAATCAATTA TGAAGATAAA AAAATTATTG TCATTAATTA ATTGAAATAA   
  
  
- TGGTGTAAAG GTAATGTTTT AAAAATCGAA TTAGGTTGAT TGCTCCTATT GATTAAATTA TAAACTAAAA   
  
  
- TTTTAATTAA TTTAATCTAT TTAAAATCAA TTTGATTTAT TTATATTATC AAAAAAATGT TGTTTTTTAA   
  
  
- AAAAATATAG TAAATTTTTT ATTTTAAATA TTTTTAAAAT ATAAGAAATT TTAAAAACAA AAAATTGGAT   
  
  
- GCTGCATTAG AGAGTTGAAT TAATTGTGAT GAGAAAATTA TGATATCATG AAGATGGGTG ATATTTTATC   
  
  
- CCAAATTGCT AATCTTAATT AGTAGGGTAA ATCTTGACCA TTTCAATTCT GAAGAGACAG AATTAATTAA   
  
  
- TTTTGAGTAT ATATTATCAA AATTCTATGC ACAATCGACA TAGTACCCAC GTCCTAAGCT TCATTGATTG   
  
  
- CCCCTTCAAT TTATTTTTGG CCTTCACTTT CTTCCCATTA AAATCCAATC ATGACATATA TATAAAAATT   
  
  
- ATTAACACAT ACCTTAACAT TACCGTAATT TTATTATAAA TATATAATAT AAATATTCTA TGGTTTAACT   
  
  
- TATAAAATCT AAAAAAAAAT TAAAAACTTT GAGAAATTTA GTTGGTTATC AAGTATGCAT TGACTTATAA   
  
  
- GCTAGCTCCG AAGTTTCAAT TTCATCAAAA ATTATTGATT TTTTTATTTA TTAACTTTAA GATTTTGAAA   
  
  
- AGAATAAATC CCTCATTTAT TAATTAGAT

+     HSE

| Site Name | Organism | Position | Strand | Matrix score. | sequence | function |
| --- | --- | --- | --- | --- | --- | --- |
| HSE | Brassica oleracea | 903 | + | 9 | AAAAAATTTC | cis-acting element involved in heat stress responsiveness |
| HSE | Brassica oleracea | 905 | - | 9 | AAAAAATTTC | cis-acting element involved in heat stress responsiveness |

> 2018/04/13 10:10:12  
+ ATAACCAAGA ACTTCTTTCG TTCGTTCGAA GGAAAGAAAC ACAAAGTTTC TTCAAAATCC CAAAATATAT   
  
  
+ AGTTGTCTCT CGATGGAGGT AGAGGTCTAA AACTGAAGTT TAATATATTA AGTTTTTGAT GACGGATAAT   
  
  
+ AGATAAAGAG AACGTACAGT GGATTAGAGC ATACAAGTCA GGTCGTTCGT TGCAATTAGA ACTTATAAAT   
  
  
+ AATTAAAATA TTACATTTAA TATATTTAAT TCGAATAAGG TTTTTAATAA AGTTGAACCC TCAAATAATT   
  
  
+ AATTAAATAT GTTATTACAA AAGACCTAGG CATGCACGCT GGTGACTTTT GCCACCAGTT TTGACTCCGC   
  
  
+ GGCAATAAAA CTGAAACACT GACTCACCCA GGGCATTAAC GATGGGCTGG GCCCCAGTTA ATACATGAAA   
  
  
+ ACGCATTATA AAGTTTAATA TGTCTCCGTG CAGTTAAGTT GCACATTTTG CTGCATTTGC GTCGAACTGA   
  
  
+ CAAGGATACA CTGGTACATT CTCAACTGAC ATTCGTCAAT GCCAGAAAAA AACTTTTTCT TTTATTCCCT   
  
  
+ TCGACCTCTT TCACCGCAAA GTAAATTTCA AGTCCTCGGT TTGGGAACCT TCTTTTTATC TTTTTTTATA   
  
  
+ TTTTTATTTA ATATCCGAAT TTCTTAGAAA TTTCTCCCAC ATATGTATAT AAAAAATTAT AAATAAATTA   
  
  
+ CAAAAAGTAC ACTGATTTTG TTTAGTTAAT ACTTCTATTT TTTTAATAAC AGTAATTAAT TAACTTTATT   
  
  
+ ACCACATTTC CATTACAAAA TTTTTAGCTT AATCCAACTA ACGAGGATAA CTAATTTAAT ATTTGATTTT   
  
  
+ AAAATTAATT AAATTAGATA AATTTTAGTT AAACTAAATA AATATAATAG TTTTTTTACA ACAAAAAATT   
  
  
+ TTTTTATATC ATTTAAAAAA TAAAATTTAT AAAAATTTTA TATTCTTTAA AATTTTTGTT TTTTAACCTA   
  
  
+ CGACGTAATC TCTCAACTTA ATTAACACTA CTCTTTTAAT ACTATAGTAC TTCTACCCAC TATAAAATAG   
  
  
+ GGTTTAACGA TTAGAATTAA TCATCCCATT TAGAACTGGT AAAGTTAAGA CTTCTCTGTC TTAATTAATT   
  
  
+ AAAACTCATA TATAATAGTT TTAAGATACG TGTTAGCTGT ATCATGGGTG CAGGATTCGA AGTAACTAAC   
  
  
+ GGGGAAGTTA AATAAAAACC GGAAGTGAAA GAAGGGTAAT TTTAGGTTAG TACTGTATAT ATATTTTTAA   
  
  
+ TAATTGTGTA TGGAATTGTA ATGGCATTAA AATAATATTT ATATATTATA TTTATAAGAT ACCAAATTGA   
  
  
+ ATATTTTAGA TTTTTTTTTA ATTTTTGAAA CTCTTTAAAT CAACCAATAG TTCATACGTA ACTGAATATT   
  
  
+ CGATCGAGGC TTCAAAGTTA AAGTAGTTTT TAATAACTAA AAAAATAAAT AATTGAAATT CTAAAACTTT   
  
  
+ TCTTATTTAG GGAGTAAATA ATTAATCTA  

- TATTGGTTCT TGAAGAAAGC AAGCAAGCTT CCTTTCTTTG TGTTTCAAAG AAGTTTTAGG GTTTTATATA   
  
  
- TCAACAGAGA GCTACCTCCA TCTCCAGATT TTGACTTCAA ATTATATAAT TCAAAAACTA CTGCCTATTA   
  
  
- TCTATTTCTC TTGCATGTCA CCTAATCTCG TATGTTCAGT CCAGCAAGCA ACGTTAATCT TGAATATTTA   
  
  
- TTAATTTTAT AATGTAAATT ATATAAATTA AGCTTATTCC AAAAATTATT TCAACTTGGG AGTTTATTAA   
  
  
- TTAATTTATA CAATAATGTT TTCTGGATCC GTACGTGCGA CCACTGAAAA CGGTGGTCAA AACTGAGGCG   
  
  
- CCGTTATTTT GACTTTGTGA CTGAGTGGGT CCCGTAATTG CTACCCGACC CGGGGTCAAT TATGTACTTT   
  
  
- TGCGTAATAT TTCAAATTAT ACAGAGGCAC GTCAATTCAA CGTGTAAAAC GACGTAAACG CAGCTTGACT   
  
  
- GTTCCTATGT GACCATGTAA GAGTTGACTG TAAGCAGTTA CGGTCTTTTT TTGAAAAAGA AAATAAGGGA   
  
  
- AGCTGGAGAA AGTGGCGTTT CATTTAAAGT TCAGGAGCCA AACCCTTGGA AGAAAAATAG AAAAAAATAT   
  
  
- AAAAATAAAT TATAGGCTTA AAGAATCTTT AAAGAGGGTG TATACATATA TTTTTTAATA TTTATTTAAT   
  
  
- GTTTTTCATG TGACTAAAAC AAATCAATTA TGAAGATAAA AAAATTATTG TCATTAATTA ATTGAAATAA   
  
  
- TGGTGTAAAG GTAATGTTTT AAAAATCGAA TTAGGTTGAT TGCTCCTATT GATTAAATTA TAAACTAAAA   
  
  
- TTTTAATTAA TTTAATCTAT TTAAAATCAA TTTGATTTAT TTATATTATC AAAAAAATGT TGTTTTTTAA   
  
  
- AAAAATATAG TAAATTTTTT ATTTTAAATA TTTTTAAAAT ATAAGAAATT TTAAAAACAA AAAATTGGAT   
  
  
- GCTGCATTAG AGAGTTGAAT TAATTGTGAT GAGAAAATTA TGATATCATG AAGATGGGTG ATATTTTATC   
  
  
- CCAAATTGCT AATCTTAATT AGTAGGGTAA ATCTTGACCA TTTCAATTCT GAAGAGACAG AATTAATTAA   
  
  
- TTTTGAGTAT ATATTATCAA AATTCTATGC ACAATCGACA TAGTACCCAC GTCCTAAGCT TCATTGATTG   
  
  
- CCCCTTCAAT TTATTTTTGG CCTTCACTTT CTTCCCATTA AAATCCAATC ATGACATATA TATAAAAATT   
  
  
- ATTAACACAT ACCTTAACAT TACCGTAATT TTATTATAAA TATATAATAT AAATATTCTA TGGTTTAACT   
  
  
- TATAAAATCT AAAAAAAAAT TAAAAACTTT GAGAAATTTA GTTGGTTATC AAGTATGCAT TGACTTATAA   
  
  
- GCTAGCTCCG AAGTTTCAAT TTCATCAAAA ATTATTGATT TTTTTATTTA TTAACTTTAA GATTTTGAAA   
  
  
- AGAATAAATC CCTCATTTAT TAATTAGAT

+     MBS

| Site Name | Organism | Position | Strand | Matrix score. | sequence | function |
| --- | --- | --- | --- | --- | --- | --- |
| MBS | Arabidopsis thaliana | 513 | + | 6 | CAACTG | MYB binding site involved in drought-inducibility |
| MBS | Arabidopsis thaliana | 405 | - | 6 | TAACTG | MYB binding site involved in drought-inducibility |
| MBS | Arabidopsis thaliana | 451 | - | 6 | TAACTG | MYB binding site involved in drought-inducibility |
| MBS | Arabidopsis thaliana | 1389 | + | 6 | TAACTG | MYB binding site involved in drought-inducibility |

> 2018/04/13 10:10:12  
+ ATAACCAAGA ACTTCTTTCG TTCGTTCGAA GGAAAGAAAC ACAAAGTTTC TTCAAAATCC CAAAATATAT   
  
  
+ AGTTGTCTCT CGATGGAGGT AGAGGTCTAA AACTGAAGTT TAATATATTA AGTTTTTGAT GACGGATAAT   
  
  
+ AGATAAAGAG AACGTACAGT GGATTAGAGC ATACAAGTCA GGTCGTTCGT TGCAATTAGA ACTTATAAAT   
  
  
+ AATTAAAATA TTACATTTAA TATATTTAAT TCGAATAAGG TTTTTAATAA AGTTGAACCC TCAAATAATT   
  
  
+ AATTAAATAT GTTATTACAA AAGACCTAGG CATGCACGCT GGTGACTTTT GCCACCAGTT TTGACTCCGC   
  
  
+ GGCAATAAAA CTGAAACACT GACTCACCCA GGGCATTAAC GATGGGCTGG GCCCCAGTTA ATACATGAAA   
  
  
+ ACGCATTATA AAGTTTAATA TGTCTCCGTG CAGTTAAGTT GCACATTTTG CTGCATTTGC GTCGAACTGA   
  
  
+ CAAGGATACA CTGGTACATT CTCAACTGAC ATTCGTCAAT GCCAGAAAAA AACTTTTTCT TTTATTCCCT   
  
  
+ TCGACCTCTT TCACCGCAAA GTAAATTTCA AGTCCTCGGT TTGGGAACCT TCTTTTTATC TTTTTTTATA   
  
  
+ TTTTTATTTA ATATCCGAAT TTCTTAGAAA TTTCTCCCAC ATATGTATAT AAAAAATTAT AAATAAATTA   
  
  
+ CAAAAAGTAC ACTGATTTTG TTTAGTTAAT ACTTCTATTT TTTTAATAAC AGTAATTAAT TAACTTTATT   
  
  
+ ACCACATTTC CATTACAAAA TTTTTAGCTT AATCCAACTA ACGAGGATAA CTAATTTAAT ATTTGATTTT   
  
  
+ AAAATTAATT AAATTAGATA AATTTTAGTT AAACTAAATA AATATAATAG TTTTTTTACA ACAAAAAATT   
  
  
+ TTTTTATATC ATTTAAAAAA TAAAATTTAT AAAAATTTTA TATTCTTTAA AATTTTTGTT TTTTAACCTA   
  
  
+ CGACGTAATC TCTCAACTTA ATTAACACTA CTCTTTTAAT ACTATAGTAC TTCTACCCAC TATAAAATAG   
  
  
+ GGTTTAACGA TTAGAATTAA TCATCCCATT TAGAACTGGT AAAGTTAAGA CTTCTCTGTC TTAATTAATT   
  
  
+ AAAACTCATA TATAATAGTT TTAAGATACG TGTTAGCTGT ATCATGGGTG CAGGATTCGA AGTAACTAAC   
  
  
+ GGGGAAGTTA AATAAAAACC GGAAGTGAAA GAAGGGTAAT TTTAGGTTAG TACTGTATAT ATATTTTTAA   
  
  
+ TAATTGTGTA TGGAATTGTA ATGGCATTAA AATAATATTT ATATATTATA TTTATAAGAT ACCAAATTGA   
  
  
+ ATATTTTAGA TTTTTTTTTA ATTTTTGAAA CTCTTTAAAT CAACCAATAG TTCATACGTA ACTGAATATT   
  
  
+ CGATCGAGGC TTCAAAGTTA AAGTAGTTTT TAATAACTAA AAAAATAAAT AATTGAAATT CTAAAACTTT   
  
  
+ TCTTATTTAG GGAGTAAATA ATTAATCTA  

- TATTGGTTCT TGAAGAAAGC AAGCAAGCTT CCTTTCTTTG TGTTTCAAAG AAGTTTTAGG GTTTTATATA   
  
  
- TCAACAGAGA GCTACCTCCA TCTCCAGATT TTGACTTCAA ATTATATAAT TCAAAAACTA CTGCCTATTA   
  
  
- TCTATTTCTC TTGCATGTCA CCTAATCTCG TATGTTCAGT CCAGCAAGCA ACGTTAATCT TGAATATTTA   
  
  
- TTAATTTTAT AATGTAAATT ATATAAATTA AGCTTATTCC AAAAATTATT TCAACTTGGG AGTTTATTAA   
  
  
- TTAATTTATA CAATAATGTT TTCTGGATCC GTACGTGCGA CCACTGAAAA CGGTGGTCAA AACTGAGGCG   
  
  
- CCGTTATTTT GACTTTGTGA CTGAGTGGGT CCCGTAATTG CTACCCGACC CGGGGTCAAT TATGTACTTT   
  
  
- TGCGTAATAT TTCAAATTAT ACAGAGGCAC GTCAATTCAA CGTGTAAAAC GACGTAAACG CAGCTTGACT   
  
  
- GTTCCTATGT GACCATGTAA GAGTTGACTG TAAGCAGTTA CGGTCTTTTT TTGAAAAAGA AAATAAGGGA   
  
  
- AGCTGGAGAA AGTGGCGTTT CATTTAAAGT TCAGGAGCCA AACCCTTGGA AGAAAAATAG AAAAAAATAT   
  
  
- AAAAATAAAT TATAGGCTTA AAGAATCTTT AAAGAGGGTG TATACATATA TTTTTTAATA TTTATTTAAT   
  
  
- GTTTTTCATG TGACTAAAAC AAATCAATTA TGAAGATAAA AAAATTATTG TCATTAATTA ATTGAAATAA   
  
  
- TGGTGTAAAG GTAATGTTTT AAAAATCGAA TTAGGTTGAT TGCTCCTATT GATTAAATTA TAAACTAAAA   
  
  
- TTTTAATTAA TTTAATCTAT TTAAAATCAA TTTGATTTAT TTATATTATC AAAAAAATGT TGTTTTTTAA   
  
  
- AAAAATATAG TAAATTTTTT ATTTTAAATA TTTTTAAAAT ATAAGAAATT TTAAAAACAA AAAATTGGAT   
  
  
- GCTGCATTAG AGAGTTGAAT TAATTGTGAT GAGAAAATTA TGATATCATG AAGATGGGTG ATATTTTATC   
  
  
- CCAAATTGCT AATCTTAATT AGTAGGGTAA ATCTTGACCA TTTCAATTCT GAAGAGACAG AATTAATTAA   
  
  
- TTTTGAGTAT ATATTATCAA AATTCTATGC ACAATCGACA TAGTACCCAC GTCCTAAGCT TCATTGATTG   
  
  
- CCCCTTCAAT TTATTTTTGG CCTTCACTTT CTTCCCATTA AAATCCAATC ATGACATATA TATAAAAATT   
  
  
- ATTAACACAT ACCTTAACAT TACCGTAATT TTATTATAAA TATATAATAT AAATATTCTA TGGTTTAACT   
  
  
- TATAAAATCT AAAAAAAAAT TAAAAACTTT GAGAAATTTA GTTGGTTATC AAGTATGCAT TGACTTATAA   
  
  
- GCTAGCTCCG AAGTTTCAAT TTCATCAAAA ATTATTGATT TTTTTATTTA TTAACTTTAA GATTTTGAAA   
  
  
- AGAATAAATC CCTCATTTAT TAATTAGAT

+     MRE

| Site Name | Organism | Position | Strand | Matrix score. | sequence | function |
| --- | --- | --- | --- | --- | --- | --- |
| MRE | Petroselinum crispum | 1232 | - | 7 | AACCTAA | MYB binding site involved in light responsiveness |

> 2018/04/13 10:10:12  
+ ATAACCAAGA ACTTCTTTCG TTCGTTCGAA GGAAAGAAAC ACAAAGTTTC TTCAAAATCC CAAAATATAT   
  
  
+ AGTTGTCTCT CGATGGAGGT AGAGGTCTAA AACTGAAGTT TAATATATTA AGTTTTTGAT GACGGATAAT   
  
  
+ AGATAAAGAG AACGTACAGT GGATTAGAGC ATACAAGTCA GGTCGTTCGT TGCAATTAGA ACTTATAAAT   
  
  
+ AATTAAAATA TTACATTTAA TATATTTAAT TCGAATAAGG TTTTTAATAA AGTTGAACCC TCAAATAATT   
  
  
+ AATTAAATAT GTTATTACAA AAGACCTAGG CATGCACGCT GGTGACTTTT GCCACCAGTT TTGACTCCGC   
  
  
+ GGCAATAAAA CTGAAACACT GACTCACCCA GGGCATTAAC GATGGGCTGG GCCCCAGTTA ATACATGAAA   
  
  
+ ACGCATTATA AAGTTTAATA TGTCTCCGTG CAGTTAAGTT GCACATTTTG CTGCATTTGC GTCGAACTGA   
  
  
+ CAAGGATACA CTGGTACATT CTCAACTGAC ATTCGTCAAT GCCAGAAAAA AACTTTTTCT TTTATTCCCT   
  
  
+ TCGACCTCTT TCACCGCAAA GTAAATTTCA AGTCCTCGGT TTGGGAACCT TCTTTTTATC TTTTTTTATA   
  
  
+ TTTTTATTTA ATATCCGAAT TTCTTAGAAA TTTCTCCCAC ATATGTATAT AAAAAATTAT AAATAAATTA   
  
  
+ CAAAAAGTAC ACTGATTTTG TTTAGTTAAT ACTTCTATTT TTTTAATAAC AGTAATTAAT TAACTTTATT   
  
  
+ ACCACATTTC CATTACAAAA TTTTTAGCTT AATCCAACTA ACGAGGATAA CTAATTTAAT ATTTGATTTT   
  
  
+ AAAATTAATT AAATTAGATA AATTTTAGTT AAACTAAATA AATATAATAG TTTTTTTACA ACAAAAAATT   
  
  
+ TTTTTATATC ATTTAAAAAA TAAAATTTAT AAAAATTTTA TATTCTTTAA AATTTTTGTT TTTTAACCTA   
  
  
+ CGACGTAATC TCTCAACTTA ATTAACACTA CTCTTTTAAT ACTATAGTAC TTCTACCCAC TATAAAATAG   
  
  
+ GGTTTAACGA TTAGAATTAA TCATCCCATT TAGAACTGGT AAAGTTAAGA CTTCTCTGTC TTAATTAATT   
  
  
+ AAAACTCATA TATAATAGTT TTAAGATACG TGTTAGCTGT ATCATGGGTG CAGGATTCGA AGTAACTAAC   
  
  
+ GGGGAAGTTA AATAAAAACC GGAAGTGAAA GAAGGGTAAT TTTAGGTTAG TACTGTATAT ATATTTTTAA   
  
  
+ TAATTGTGTA TGGAATTGTA ATGGCATTAA AATAATATTT ATATATTATA TTTATAAGAT ACCAAATTGA   
  
  
+ ATATTTTAGA TTTTTTTTTA ATTTTTGAAA CTCTTTAAAT CAACCAATAG TTCATACGTA ACTGAATATT   
  
  
+ CGATCGAGGC TTCAAAGTTA AAGTAGTTTT TAATAACTAA AAAAATAAAT AATTGAAATT CTAAAACTTT   
  
  
+ TCTTATTTAG GGAGTAAATA ATTAATCTA  

- TATTGGTTCT TGAAGAAAGC AAGCAAGCTT CCTTTCTTTG TGTTTCAAAG AAGTTTTAGG GTTTTATATA   
  
  
- TCAACAGAGA GCTACCTCCA TCTCCAGATT TTGACTTCAA ATTATATAAT TCAAAAACTA CTGCCTATTA   
  
  
- TCTATTTCTC TTGCATGTCA CCTAATCTCG TATGTTCAGT CCAGCAAGCA ACGTTAATCT TGAATATTTA   
  
  
- TTAATTTTAT AATGTAAATT ATATAAATTA AGCTTATTCC AAAAATTATT TCAACTTGGG AGTTTATTAA   
  
  
- TTAATTTATA CAATAATGTT TTCTGGATCC GTACGTGCGA CCACTGAAAA CGGTGGTCAA AACTGAGGCG   
  
  
- CCGTTATTTT GACTTTGTGA CTGAGTGGGT CCCGTAATTG CTACCCGACC CGGGGTCAAT TATGTACTTT   
  
  
- TGCGTAATAT TTCAAATTAT ACAGAGGCAC GTCAATTCAA CGTGTAAAAC GACGTAAACG CAGCTTGACT   
  
  
- GTTCCTATGT GACCATGTAA GAGTTGACTG TAAGCAGTTA CGGTCTTTTT TTGAAAAAGA AAATAAGGGA   
  
  
- AGCTGGAGAA AGTGGCGTTT CATTTAAAGT TCAGGAGCCA AACCCTTGGA AGAAAAATAG AAAAAAATAT   
  
  
- AAAAATAAAT TATAGGCTTA AAGAATCTTT AAAGAGGGTG TATACATATA TTTTTTAATA TTTATTTAAT   
  
  
- GTTTTTCATG TGACTAAAAC AAATCAATTA TGAAGATAAA AAAATTATTG TCATTAATTA ATTGAAATAA   
  
  
- TGGTGTAAAG GTAATGTTTT AAAAATCGAA TTAGGTTGAT TGCTCCTATT GATTAAATTA TAAACTAAAA   
  
  
- TTTTAATTAA TTTAATCTAT TTAAAATCAA TTTGATTTAT TTATATTATC AAAAAAATGT TGTTTTTTAA   
  
  
- AAAAATATAG TAAATTTTTT ATTTTAAATA TTTTTAAAAT ATAAGAAATT TTAAAAACAA AAAATTGGAT   
  
  
- GCTGCATTAG AGAGTTGAAT TAATTGTGAT GAGAAAATTA TGATATCATG AAGATGGGTG ATATTTTATC   
  
  
- CCAAATTGCT AATCTTAATT AGTAGGGTAA ATCTTGACCA TTTCAATTCT GAAGAGACAG AATTAATTAA   
  
  
- TTTTGAGTAT ATATTATCAA AATTCTATGC ACAATCGACA TAGTACCCAC GTCCTAAGCT TCATTGATTG   
  
  
- CCCCTTCAAT TTATTTTTGG CCTTCACTTT CTTCCCATTA AAATCCAATC ATGACATATA TATAAAAATT   
  
  
- ATTAACACAT ACCTTAACAT TACCGTAATT TTATTATAAA TATATAATAT AAATATTCTA TGGTTTAACT   
  
  
- TATAAAATCT AAAAAAAAAT TAAAAACTTT GAGAAATTTA GTTGGTTATC AAGTATGCAT TGACTTATAA   
  
  
- GCTAGCTCCG AAGTTTCAAT TTCATCAAAA ATTATTGATT TTTTTATTTA TTAACTTTAA GATTTTGAAA   
  
  
- AGAATAAATC CCTCATTTAT TAATTAGAT

+     SARE

| Site Name | Organism | Position | Strand | Matrix score. | sequence | function |
| --- | --- | --- | --- | --- | --- | --- |
| SARE | Nicotiana tabacum | 560 | + | 11 | TTCGACCTCCTT | cis-acting element involved in salicylic acid responsiveness |

> 2018/04/13 10:10:12  
+ ATAACCAAGA ACTTCTTTCG TTCGTTCGAA GGAAAGAAAC ACAAAGTTTC TTCAAAATCC CAAAATATAT   
  
  
+ AGTTGTCTCT CGATGGAGGT AGAGGTCTAA AACTGAAGTT TAATATATTA AGTTTTTGAT GACGGATAAT   
  
  
+ AGATAAAGAG AACGTACAGT GGATTAGAGC ATACAAGTCA GGTCGTTCGT TGCAATTAGA ACTTATAAAT   
  
  
+ AATTAAAATA TTACATTTAA TATATTTAAT TCGAATAAGG TTTTTAATAA AGTTGAACCC TCAAATAATT   
  
  
+ AATTAAATAT GTTATTACAA AAGACCTAGG CATGCACGCT GGTGACTTTT GCCACCAGTT TTGACTCCGC   
  
  
+ GGCAATAAAA CTGAAACACT GACTCACCCA GGGCATTAAC GATGGGCTGG GCCCCAGTTA ATACATGAAA   
  
  
+ ACGCATTATA AAGTTTAATA TGTCTCCGTG CAGTTAAGTT GCACATTTTG CTGCATTTGC GTCGAACTGA   
  
  
+ CAAGGATACA CTGGTACATT CTCAACTGAC ATTCGTCAAT GCCAGAAAAA AACTTTTTCT TTTATTCCCT   
  
  
+ TCGACCTCTT TCACCGCAAA GTAAATTTCA AGTCCTCGGT TTGGGAACCT TCTTTTTATC TTTTTTTATA   
  
  
+ TTTTTATTTA ATATCCGAAT TTCTTAGAAA TTTCTCCCAC ATATGTATAT AAAAAATTAT AAATAAATTA   
  
  
+ CAAAAAGTAC ACTGATTTTG TTTAGTTAAT ACTTCTATTT TTTTAATAAC AGTAATTAAT TAACTTTATT   
  
  
+ ACCACATTTC CATTACAAAA TTTTTAGCTT AATCCAACTA ACGAGGATAA CTAATTTAAT ATTTGATTTT   
  
  
+ AAAATTAATT AAATTAGATA AATTTTAGTT AAACTAAATA AATATAATAG TTTTTTTACA ACAAAAAATT   
  
  
+ TTTTTATATC ATTTAAAAAA TAAAATTTAT AAAAATTTTA TATTCTTTAA AATTTTTGTT TTTTAACCTA   
  
  
+ CGACGTAATC TCTCAACTTA ATTAACACTA CTCTTTTAAT ACTATAGTAC TTCTACCCAC TATAAAATAG   
  
  
+ GGTTTAACGA TTAGAATTAA TCATCCCATT TAGAACTGGT AAAGTTAAGA CTTCTCTGTC TTAATTAATT   
  
  
+ AAAACTCATA TATAATAGTT TTAAGATACG TGTTAGCTGT ATCATGGGTG CAGGATTCGA AGTAACTAAC   
  
  
+ GGGGAAGTTA AATAAAAACC GGAAGTGAAA GAAGGGTAAT TTTAGGTTAG TACTGTATAT ATATTTTTAA   
  
  
+ TAATTGTGTA TGGAATTGTA ATGGCATTAA AATAATATTT ATATATTATA TTTATAAGAT ACCAAATTGA   
  
  
+ ATATTTTAGA TTTTTTTTTA ATTTTTGAAA CTCTTTAAAT CAACCAATAG TTCATACGTA ACTGAATATT   
  
  
+ CGATCGAGGC TTCAAAGTTA AAGTAGTTTT TAATAACTAA AAAAATAAAT AATTGAAATT CTAAAACTTT   
  
  
+ TCTTATTTAG GGAGTAAATA ATTAATCTA  

- TATTGGTTCT TGAAGAAAGC AAGCAAGCTT CCTTTCTTTG TGTTTCAAAG AAGTTTTAGG GTTTTATATA   
  
  
- TCAACAGAGA GCTACCTCCA TCTCCAGATT TTGACTTCAA ATTATATAAT TCAAAAACTA CTGCCTATTA   
  
  
- TCTATTTCTC TTGCATGTCA CCTAATCTCG TATGTTCAGT CCAGCAAGCA ACGTTAATCT TGAATATTTA   
  
  
- TTAATTTTAT AATGTAAATT ATATAAATTA AGCTTATTCC AAAAATTATT TCAACTTGGG AGTTTATTAA   
  
  
- TTAATTTATA CAATAATGTT TTCTGGATCC GTACGTGCGA CCACTGAAAA CGGTGGTCAA AACTGAGGCG   
  
  
- CCGTTATTTT GACTTTGTGA CTGAGTGGGT CCCGTAATTG CTACCCGACC CGGGGTCAAT TATGTACTTT   
  
  
- TGCGTAATAT TTCAAATTAT ACAGAGGCAC GTCAATTCAA CGTGTAAAAC GACGTAAACG CAGCTTGACT   
  
  
- GTTCCTATGT GACCATGTAA GAGTTGACTG TAAGCAGTTA CGGTCTTTTT TTGAAAAAGA AAATAAGGGA   
  
  
- AGCTGGAGAA AGTGGCGTTT CATTTAAAGT TCAGGAGCCA AACCCTTGGA AGAAAAATAG AAAAAAATAT   
  
  
- AAAAATAAAT TATAGGCTTA AAGAATCTTT AAAGAGGGTG TATACATATA TTTTTTAATA TTTATTTAAT   
  
  
- GTTTTTCATG TGACTAAAAC AAATCAATTA TGAAGATAAA AAAATTATTG TCATTAATTA ATTGAAATAA   
  
  
- TGGTGTAAAG GTAATGTTTT AAAAATCGAA TTAGGTTGAT TGCTCCTATT GATTAAATTA TAAACTAAAA   
  
  
- TTTTAATTAA TTTAATCTAT TTAAAATCAA TTTGATTTAT TTATATTATC AAAAAAATGT TGTTTTTTAA   
  
  
- AAAAATATAG TAAATTTTTT ATTTTAAATA TTTTTAAAAT ATAAGAAATT TTAAAAACAA AAAATTGGAT   
  
  
- GCTGCATTAG AGAGTTGAAT TAATTGTGAT GAGAAAATTA TGATATCATG AAGATGGGTG ATATTTTATC   
  
  
- CCAAATTGCT AATCTTAATT AGTAGGGTAA ATCTTGACCA TTTCAATTCT GAAGAGACAG AATTAATTAA   
  
  
- TTTTGAGTAT ATATTATCAA AATTCTATGC ACAATCGACA TAGTACCCAC GTCCTAAGCT TCATTGATTG   
  
  
- CCCCTTCAAT TTATTTTTGG CCTTCACTTT CTTCCCATTA AAATCCAATC ATGACATATA TATAAAAATT   
  
  
- ATTAACACAT ACCTTAACAT TACCGTAATT TTATTATAAA TATATAATAT AAATATTCTA TGGTTTAACT   
  
  
- TATAAAATCT AAAAAAAAAT TAAAAACTTT GAGAAATTTA GTTGGTTATC AAGTATGCAT TGACTTATAA   
  
  
- GCTAGCTCCG AAGTTTCAAT TTCATCAAAA ATTATTGATT TTTTTATTTA TTAACTTTAA GATTTTGAAA   
  
  
- AGAATAAATC CCTCATTTAT TAATTAGAT

+     Skn-1\_motif

| Site Name | Organism | Position | Strand | Matrix score. | sequence | function |
| --- | --- | --- | --- | --- | --- | --- |
| Skn-1\_motif | Oryza sativa | 129 | - | 5 | GTCAT | cis-acting regulatory element required for endosperm expression |

> 2018/04/13 10:10:12  
+ ATAACCAAGA ACTTCTTTCG TTCGTTCGAA GGAAAGAAAC ACAAAGTTTC TTCAAAATCC CAAAATATAT   
  
  
+ AGTTGTCTCT CGATGGAGGT AGAGGTCTAA AACTGAAGTT TAATATATTA AGTTTTTGAT GACGGATAAT   
  
  
+ AGATAAAGAG AACGTACAGT GGATTAGAGC ATACAAGTCA GGTCGTTCGT TGCAATTAGA ACTTATAAAT   
  
  
+ AATTAAAATA TTACATTTAA TATATTTAAT TCGAATAAGG TTTTTAATAA AGTTGAACCC TCAAATAATT   
  
  
+ AATTAAATAT GTTATTACAA AAGACCTAGG CATGCACGCT GGTGACTTTT GCCACCAGTT TTGACTCCGC   
  
  
+ GGCAATAAAA CTGAAACACT GACTCACCCA GGGCATTAAC GATGGGCTGG GCCCCAGTTA ATACATGAAA   
  
  
+ ACGCATTATA AAGTTTAATA TGTCTCCGTG CAGTTAAGTT GCACATTTTG CTGCATTTGC GTCGAACTGA   
  
  
+ CAAGGATACA CTGGTACATT CTCAACTGAC ATTCGTCAAT GCCAGAAAAA AACTTTTTCT TTTATTCCCT   
  
  
+ TCGACCTCTT TCACCGCAAA GTAAATTTCA AGTCCTCGGT TTGGGAACCT TCTTTTTATC TTTTTTTATA   
  
  
+ TTTTTATTTA ATATCCGAAT TTCTTAGAAA TTTCTCCCAC ATATGTATAT AAAAAATTAT AAATAAATTA   
  
  
+ CAAAAAGTAC ACTGATTTTG TTTAGTTAAT ACTTCTATTT TTTTAATAAC AGTAATTAAT TAACTTTATT   
  
  
+ ACCACATTTC CATTACAAAA TTTTTAGCTT AATCCAACTA ACGAGGATAA CTAATTTAAT ATTTGATTTT   
  
  
+ AAAATTAATT AAATTAGATA AATTTTAGTT AAACTAAATA AATATAATAG TTTTTTTACA ACAAAAAATT   
  
  
+ TTTTTATATC ATTTAAAAAA TAAAATTTAT AAAAATTTTA TATTCTTTAA AATTTTTGTT TTTTAACCTA   
  
  
+ CGACGTAATC TCTCAACTTA ATTAACACTA CTCTTTTAAT ACTATAGTAC TTCTACCCAC TATAAAATAG   
  
  
+ GGTTTAACGA TTAGAATTAA TCATCCCATT TAGAACTGGT AAAGTTAAGA CTTCTCTGTC TTAATTAATT   
  
  
+ AAAACTCATA TATAATAGTT TTAAGATACG TGTTAGCTGT ATCATGGGTG CAGGATTCGA AGTAACTAAC   
  
  
+ GGGGAAGTTA AATAAAAACC GGAAGTGAAA GAAGGGTAAT TTTAGGTTAG TACTGTATAT ATATTTTTAA   
  
  
+ TAATTGTGTA TGGAATTGTA ATGGCATTAA AATAATATTT ATATATTATA TTTATAAGAT ACCAAATTGA   
  
  
+ ATATTTTAGA TTTTTTTTTA ATTTTTGAAA CTCTTTAAAT CAACCAATAG TTCATACGTA ACTGAATATT   
  
  
+ CGATCGAGGC TTCAAAGTTA AAGTAGTTTT TAATAACTAA AAAAATAAAT AATTGAAATT CTAAAACTTT   
  
  
+ TCTTATTTAG GGAGTAAATA ATTAATCTA  

- TATTGGTTCT TGAAGAAAGC AAGCAAGCTT CCTTTCTTTG TGTTTCAAAG AAGTTTTAGG GTTTTATATA   
  
  
- TCAACAGAGA GCTACCTCCA TCTCCAGATT TTGACTTCAA ATTATATAAT TCAAAAACTA CTGCCTATTA   
  
  
- TCTATTTCTC TTGCATGTCA CCTAATCTCG TATGTTCAGT CCAGCAAGCA ACGTTAATCT TGAATATTTA   
  
  
- TTAATTTTAT AATGTAAATT ATATAAATTA AGCTTATTCC AAAAATTATT TCAACTTGGG AGTTTATTAA   
  
  
- TTAATTTATA CAATAATGTT TTCTGGATCC GTACGTGCGA CCACTGAAAA CGGTGGTCAA AACTGAGGCG   
  
  
- CCGTTATTTT GACTTTGTGA CTGAGTGGGT CCCGTAATTG CTACCCGACC CGGGGTCAAT TATGTACTTT   
  
  
- TGCGTAATAT TTCAAATTAT ACAGAGGCAC GTCAATTCAA CGTGTAAAAC GACGTAAACG CAGCTTGACT   
  
  
- GTTCCTATGT GACCATGTAA GAGTTGACTG TAAGCAGTTA CGGTCTTTTT TTGAAAAAGA AAATAAGGGA   
  
  
- AGCTGGAGAA AGTGGCGTTT CATTTAAAGT TCAGGAGCCA AACCCTTGGA AGAAAAATAG AAAAAAATAT   
  
  
- AAAAATAAAT TATAGGCTTA AAGAATCTTT AAAGAGGGTG TATACATATA TTTTTTAATA TTTATTTAAT   
  
  
- GTTTTTCATG TGACTAAAAC AAATCAATTA TGAAGATAAA AAAATTATTG TCATTAATTA ATTGAAATAA   
  
  
- TGGTGTAAAG GTAATGTTTT AAAAATCGAA TTAGGTTGAT TGCTCCTATT GATTAAATTA TAAACTAAAA   
  
  
- TTTTAATTAA TTTAATCTAT TTAAAATCAA TTTGATTTAT TTATATTATC AAAAAAATGT TGTTTTTTAA   
  
  
- AAAAATATAG TAAATTTTTT ATTTTAAATA TTTTTAAAAT ATAAGAAATT TTAAAAACAA AAAATTGGAT   
  
  
- GCTGCATTAG AGAGTTGAAT TAATTGTGAT GAGAAAATTA TGATATCATG AAGATGGGTG ATATTTTATC   
  
  
- CCAAATTGCT AATCTTAATT AGTAGGGTAA ATCTTGACCA TTTCAATTCT GAAGAGACAG AATTAATTAA   
  
  
- TTTTGAGTAT ATATTATCAA AATTCTATGC ACAATCGACA TAGTACCCAC GTCCTAAGCT TCATTGATTG   
  
  
- CCCCTTCAAT TTATTTTTGG CCTTCACTTT CTTCCCATTA AAATCCAATC ATGACATATA TATAAAAATT   
  
  
- ATTAACACAT ACCTTAACAT TACCGTAATT TTATTATAAA TATATAATAT AAATATTCTA TGGTTTAACT   
  
  
- TATAAAATCT AAAAAAAAAT TAAAAACTTT GAGAAATTTA GTTGGTTATC AAGTATGCAT TGACTTATAA   
  
  
- GCTAGCTCCG AAGTTTCAAT TTCATCAAAA ATTATTGATT TTTTTATTTA TTAACTTTAA GATTTTGAAA   
  
  
- AGAATAAATC CCTCATTTAT TAATTAGAT

+     TATA-box

| Site Name | Organism | Position | Strand | Matrix score. | sequence | function |
| --- | --- | --- | --- | --- | --- | --- |
| TATA-box | Lycopersicon esculentum | 1438 | - | 5 | TTTTA | core promoter element around -30 of transcription start |
| TATA-box | Glycine max | 1431 | + | 5 | TAATA | core promoter element around -30 of transcription start |
| TATA-box | Arabidopsis thaliana | 1023 | - | 4 | TATA | core promoter element around -30 of transcription start |
| TATA-box | Arabidopsis thaliana | 1296 | - | 9 | taTATAAAtc | core promoter element around -30 of transcription start |
| TATA-box | Lycopersicon esculentum | 1428 | + | 5 | TTTTA | core promoter element around -30 of transcription start |
| TATA-box | Arabidopsis thaliana | 676 | + | 4 | TATA | core promoter element around -30 of transcription start |
| TATA-box | Lycopersicon esculentum | 931 | - | 5 | TTTTA | core promoter element around -30 of transcription start |
| TATA-box | Arabidopsis thaliana | 626 | - | 5 | TATAA | core promoter element around -30 of transcription start |
| TATA-box | Arabidopsis thaliana | 625 | - | 11 | TATAAATATAAA | core promoter element around -30 of transcription start |
| TATA-box | Glycine max | 228 | + | 5 | TAATA | core promoter element around -30 of transcription start |
| TATA-box | Arabidopsis thaliana | 678 | + | 6 | TATAAA | core promoter element around -30 of transcription start |
| TATA-box | Avena sativa | 627 | + | 12 | TATATTTATATTT | core promoter element around -30 of transcription start |
| TATA-box | Arabidopsis thaliana | 914 | - | 5 | TATAA | core promoter element around -30 of transcription start |
| TATA-box | Arabidopsis thaliana | 427 | + | 6 | TATAAA | core promoter element around -30 of transcription start |
| TATA-box | Glycine max | 255 | + | 5 | TAATA | core promoter element around -30 of transcription start |
| TATA-box | Lycopersicon esculentum | 98 | - | 5 | TTTTA | core promoter element around -30 of transcription start |
| TATA-box | Lycopersicon esculentum | 1014 | + | 5 | TTTTA | core promoter element around -30 of transcription start |
| TATA-box | Arabidopsis thaliana | 949 | - | 4 | TATA | core promoter element around -30 of transcription start |
| TATA-box | Arabidopsis thaliana | 948 | - | 5 | TATAA | core promoter element around -30 of transcription start |
| TATA-box | Lycopersicon esculentum | 940 | - | 5 | TTTTA | core promoter element around -30 of transcription start |
| TATA-box | Arabidopsis thaliana | 687 | - | 5 | TATAA | core promoter element around -30 of transcription start |
| TATA-box | Arabidopsis thaliana | 915 | - | 4 | TATA | core promoter element around -30 of transcription start |
| TATA-box | Arabidopsis thaliana | 1299 | - | 7 | TATATAA | core promoter element around -30 of transcription start |
| TATA-box | Arabidopsis thaliana | 231 | + | 4 | TATA | core promoter element around -30 of transcription start |
| TATA-box | Glycine max | 885 | + | 5 | TAATA | core promoter element around -30 of transcription start |
| TATA-box | Brassica oleracea | 882 | + | 7 | ATATAAT | core promoter element around -30 of transcription start |
| TATA-box | Lycopersicon esculentum | 840 | - | 5 | TTTTA | core promoter element around -30 of transcription start |
| TATA-box | Lycopersicon esculentum | 1346 | + | 5 | TTTTA | core promoter element around -30 of transcription start |
| TATA-box | Arabidopsis thaliana | 1312 | - | 5 | TATAA | core promoter element around -30 of transcription start |
| TATA-box | Ac | 1310 | - | 7 | TATAAAT | core promoter element around -30 of transcription start |
| TATA-box | Lycopersicon esculentum | 894 | + | 5 | TTTTA | core promoter element around -30 of transcription start |
| TATA-box | Arabidopsis thaliana | 1307 | - | 4 | TATA | core promoter element around -30 of transcription start |
| TATA-box | Arabidopsis thaliana | 1248 | - | 4 | TATA | core promoter element around -30 of transcription start |
| TATA-box | Lycopersicon esculentum | 958 | - | 5 | TTTTA | core promoter element around -30 of transcription start |
| TATA-box | Arabidopsis thaliana | 913 | - | 6 | TATAAA | core promoter element around -30 of transcription start |
| TATA-box | Lycopersicon esculentum | 971 | + | 5 | TTTTA | core promoter element around -30 of transcription start |
| TATA-box | Arabidopsis thaliana | 426 | - | 5 | TATAA | core promoter element around -30 of transcription start |
| TATA-box | Arabidopsis thaliana | 624 | - | 7 | TATAAAA | core promoter element around -30 of transcription start |
| TATA-box | Lycopersicon esculentum | 356 | - | 5 | TTTTA | core promoter element around -30 of transcription start |
| TATA-box | Lycopersicon esculentum | 1334 | + | 5 | TTTTA | core promoter element around -30 of transcription start |
| TATA-box | Glycine max | 436 | + | 5 | TAATA | core promoter element around -30 of transcription start |
| TATA-box | Lycopersicon esculentum | 1288 | - | 5 | TTTTA | core promoter element around -30 of transcription start |
| TATA-box | Glycine max | 1258 | + | 5 | TAATA | core promoter element around -30 of transcription start |
| TATA-box | Glycine max | 639 | + | 5 | TAATA | core promoter element around -30 of transcription start |
| TATA-box | Arabidopsis thaliana | 1041 | + | 6 | TATAAA | core promoter element around -30 of transcription start |
| TATA-box | Brassica napus | 1247 | - | 6 | ATATAT | core promoter element around -30 of transcription start |
| TATA-box | Arabidopsis thaliana | 1306 | - | 5 | TATAA | core promoter element around -30 of transcription start |
| TATA-box | Arabidopsis thaliana | 1300 | - | 4 | TATA | core promoter element around -30 of transcription start |
| TATA-box | Lycopersicon esculentum | 550 | + | 5 | TTTTA | core promoter element around -30 of transcription start |
| TATA-box | Ac | 688 | + | 7 | TATAAAT | core promoter element around -30 of transcription start |
| TATA-box | Glycine max | 409 | + | 5 | TAATA | core promoter element around -30 of transcription start |
| TATA-box | Helianthus annuus | 1244 | - | 6 | TATACA | core promoter element around -30 of transcription start |
| TATA-box | Arabidopsis thaliana | 1131 | - | 4 | TATA | core promoter element around -30 of transcription start |
| TATA-box | Brassica oleracea | 1130 | + | 7 | ATATAAT | core promoter element around -30 of transcription start |
| TATA-box | Lycopersicon esculentum | 863 | + | 5 | TTTTA | core promoter element around -30 of transcription start |
| TATA-box | Arabidopsis thaliana | 912 | - | 7 | TATAAAA | core promoter element around -30 of transcription start |
| TATA-box | Lycopersicon esculentum | 837 | + | 5 | TTTTA | core promoter element around -30 of transcription start |
| TATA-box | Glycine max | 767 | - | 5 | TAATA | core promoter element around -30 of transcription start |
| TATA-box | Lycopersicon esculentum | 1462 | - | 5 | TTTTA | core promoter element around -30 of transcription start |
| TATA-box | Lycopersicon esculentum | 614 | + | 5 | TTTTA | core promoter element around -30 of transcription start |
| TATA-box | Arabidopsis thaliana | 938 | + | 6 | TATAAA | core promoter element around -30 of transcription start |
| TATA-box | Lycopersicon esculentum | 924 | - | 5 | TTTTA | core promoter element around -30 of transcription start |
| TATA-box | Lycopersicon esculentum | 741 | + | 5 | TTTTA | core promoter element around -30 of transcription start |
| TATA-box | Lycopersicon esculentum | 680 | - | 5 | TTTTA | core promoter element around -30 of transcription start |
| TATA-box | Arabidopsis thaliana | 1311 | - | 6 | TATAAA | core promoter element around -30 of transcription start |
| TATA-box | Glycine max | 827 | + | 5 | TAATA | core promoter element around -30 of transcription start |
| TATA-box | Brassica napus | 425 | + | 6 | ATTATA | core promoter element around -30 of transcription start |
| TATA-box | Arabidopsis thaliana | 947 | - | 6 | TATAAA | core promoter element around -30 of transcription start |
| TATA-box | Glycine max | 293 | - | 5 | TAATA | core promoter element around -30 of transcription start |
| TATA-box | Arabidopsis thaliana | 1305 | - | 11 | TATAAATATAAA | core promoter element around -30 of transcription start |
| TATA-box | Arabidopsis thaliana | 114 | + | 4 | TATA | core promoter element around -30 of transcription start |
| TATA-box | Glycine max | 1133 | + | 5 | TAATA | core promoter element around -30 of transcription start |
| TATA-box | Lycopersicon esculentum | 632 | + | 5 | TTTTA | core promoter element around -30 of transcription start |
| TATA-box | Lycopersicon esculentum | 792 | + | 5 | TTTTA | core promoter element around -30 of transcription start |
| TATA-box | Daucus carota | 1309 | - | 8 | TATAAATA | core promoter element around -30 of transcription start |
| TATA-box | Glycine max | 1017 | + | 5 | TAATA | core promoter element around -30 of transcription start |
| TATA-box | Glycine max | 116 | - | 5 | TAATA | core promoter element around -30 of transcription start |
| TATA-box | Glycine max | 137 | + | 5 | TAATA | core promoter element around -30 of transcription start |
| TATA-box | Brassica napus | 1249 | - | 6 | ATATAT | core promoter element around -30 of transcription start |
| TATA-box | Brassica oleracea | 677 | + | 6 | ATATAA | core promoter element around -30 of transcription start |
| TATA-box | Lycopersicon esculentum | 1255 | + | 5 | TTTTA | core promoter element around -30 of transcription start |
| TATA-box | Glycine max | 111 | + | 5 | TAATA | core promoter element around -30 of transcription start |
| TATA-box | Oryza sativa | 784 | + | 7 | TACAAAA | core promoter element around -30 of transcription start |
| TATA-box | Ac | 935 | - | 7 | TATAAAT | core promoter element around -30 of transcription start |
| TATA-box | Arabidopsis thaliana | 1129 | - | 4 | TATA | core promoter element around -30 of transcription start |
| TATA-box | Oryza sativa | 699 | + | 7 | TACAAAA | core promoter element around -30 of transcription start |
| TATA-box | Lycopersicon esculentum | 252 | + | 5 | TTTTA | core promoter element around -30 of transcription start |
| TATA-box | Brassica napus | 113 | + | 6 | ATATAT | core promoter element around -30 of transcription start |
| TATA-box | Arabidopsis thaliana | 1313 | - | 4 | TATA | core promoter element around -30 of transcription start |
| TATA-box | Lycopersicon esculentum | 1120 | - | 5 | TTTTA | core promoter element around -30 of transcription start |
| TATA-box | Oryza sativa | 296 | + | 7 | TACAAAA | core promoter element around -30 of transcription start |
| TATA-box | Lycopersicon esculentum | 214 | - | 5 | TTTTA | core promoter element around -30 of transcription start |
| TATA-box | Ac | 1297 | - | 7 | TATAAAT | core promoter element around -30 of transcription start |
| TATA-box | Arabidopsis thaliana | 883 | - | 4 | TATA | core promoter element around -30 of transcription start |
| TATA-box | Lycopersicon esculentum | 1043 | - | 5 | TTTTA | core promoter element around -30 of transcription start |
| TATA-box | Arabidopsis thaliana | 64 | - | 9 | tcTATATAtt | core promoter element around -30 of transcription start |
| TATA-box | Arabidopsis thaliana | 203 | - | 5 | TATAA | core promoter element around -30 of transcription start |
| TATA-box | Helianthus annuus | 674 | - | 6 | TATACA | core promoter element around -30 of transcription start |
| TATA-box | Zea mays | 922 | + | 8 | TTTAAAAA | core promoter element around -30 of transcription start |
| TATA-box | Glycine max | 219 | - | 5 | TAATA | core promoter element around -30 of transcription start |
| TATA-box | Arabidopsis thaliana | 1250 | - | 4 | TATA | core promoter element around -30 of transcription start |
| TATA-box | Brassica napus | 686 | + | 6 | ATTATA | core promoter element around -30 of transcription start |
| TATA-box | Pisum sativum | 945 | - | 8 | TATAAAAT | core promoter element around -30 of transcription start |
| TATA-box | Brassica napus | 1128 | - | 6 | ATATAT | core promoter element around -30 of transcription start |
| TATA-box | Glycine max | 744 | + | 5 | TAATA | core promoter element around -30 of transcription start |
| TATA-box | Glycine max | 1293 | + | 5 | TAATA | core promoter element around -30 of transcription start |
| TATA-box | Glycine max | 727 | + | 5 | TAATA | core promoter element around -30 of transcription start |
| TATA-box | Arabidopsis thaliana | 66 | + | 4 | TATA | core promoter element around -30 of transcription start |
| TATA-box | Arabidopsis thaliana | 936 | - | 6 | TATAAA | core promoter element around -30 of transcription start |
| TATA-box | Brassica napus | 65 | + | 6 | ATATAT | core promoter element around -30 of transcription start |
| TATA-box | Arabidopsis thaliana | 1302 | - | 4 | TATA | core promoter element around -30 of transcription start |
| TATA-box | Arabidopsis thaliana | 1298 | - | 6 | TATAAA | core promoter element around -30 of transcription start |
| TATA-box | Brassica napus | 230 | + | 6 | ATATAT | core promoter element around -30 of transcription start |
| TATA-box | Arabidopsis thaliana | 68 | + | 4 | TATA | core promoter element around -30 of transcription start |
| TATA-box | Lycopersicon esculentum | 1139 | + | 5 | TTTTA | core promoter element around -30 of transcription start |
| TATA-box | Ac | 204 | + | 7 | TATAAAT | core promoter element around -30 of transcription start |
| TATA-box | Arabidopsis thaliana | 946 | - | 7 | TATAAAA | core promoter element around -30 of transcription start |
| TATA-box | Brassica napus | 1301 | - | 6 | ATATAT | core promoter element around -30 of transcription start |
| TATA-box | Arabidopsis thaliana | 937 | - | 5 | TATAA | core promoter element around -30 of transcription start |
| TATA-box | Lycopersicon esculentum | 1203 | - | 5 | TTTTA | core promoter element around -30 of transcription start |
| TATA-box | Glycine max | 1304 | - | 5 | TAATA | core promoter element around -30 of transcription start |
| TATA-box | Antirrhinum majus | 934 | - | 8 | TATAAATT | core promoter element around -30 of transcription start |
| TATA-box | Arabidopsis thaliana | 1246 | - | 8 | TATATATA | core promoter element around -30 of transcription start |
| TATA-box | Lycopersicon esculentum | 1230 | + | 5 | TTTTA | core promoter element around -30 of transcription start |

> 2018/04/13 10:10:12  
+ ATAACCAAGA ACTTCTTTCG TTCGTTCGAA GGAAAGAAAC ACAAAGTTTC TTCAAAATCC CAAAATATAT   
  
  
+ AGTTGTCTCT CGATGGAGGT AGAGGTCTAA AACTGAAGTT TAATATATTA AGTTTTTGAT GACGGATAAT   
  
  
+ AGATAAAGAG AACGTACAGT GGATTAGAGC ATACAAGTCA GGTCGTTCGT TGCAATTAGA ACTTATAAAT   
  
  
+ AATTAAAATA TTACATTTAA TATATTTAAT TCGAATAAGG TTTTTAATAA AGTTGAACCC TCAAATAATT   
  
  
+ AATTAAATAT GTTATTACAA AAGACCTAGG CATGCACGCT GGTGACTTTT GCCACCAGTT TTGACTCCGC   
  
  
+ GGCAATAAAA CTGAAACACT GACTCACCCA GGGCATTAAC GATGGGCTGG GCCCCAGTTA ATACATGAAA   
  
  
+ ACGCATTATA AAGTTTAATA TGTCTCCGTG CAGTTAAGTT GCACATTTTG CTGCATTTGC GTCGAACTGA   
  
  
+ CAAGGATACA CTGGTACATT CTCAACTGAC ATTCGTCAAT GCCAGAAAAA AACTTTTTCT TTTATTCCCT   
  
  
+ TCGACCTCTT TCACCGCAAA GTAAATTTCA AGTCCTCGGT TTGGGAACCT TCTTTTTATC TTTTTTTATA   
  
  
+ TTTTTATTTA ATATCCGAAT TTCTTAGAAA TTTCTCCCAC ATATGTATAT AAAAAATTAT AAATAAATTA   
  
  
+ CAAAAAGTAC ACTGATTTTG TTTAGTTAAT ACTTCTATTT TTTTAATAAC AGTAATTAAT TAACTTTATT   
  
  
+ ACCACATTTC CATTACAAAA TTTTTAGCTT AATCCAACTA ACGAGGATAA CTAATTTAAT ATTTGATTTT   
  
  
+ AAAATTAATT AAATTAGATA AATTTTAGTT AAACTAAATA AATATAATAG TTTTTTTACA ACAAAAAATT   
  
  
+ TTTTTATATC ATTTAAAAAA TAAAATTTAT AAAAATTTTA TATTCTTTAA AATTTTTGTT TTTTAACCTA   
  
  
+ CGACGTAATC TCTCAACTTA ATTAACACTA CTCTTTTAAT ACTATAGTAC TTCTACCCAC TATAAAATAG   
  
  
+ GGTTTAACGA TTAGAATTAA TCATCCCATT TAGAACTGGT AAAGTTAAGA CTTCTCTGTC TTAATTAATT   
  
  
+ AAAACTCATA TATAATAGTT TTAAGATACG TGTTAGCTGT ATCATGGGTG CAGGATTCGA AGTAACTAAC   
  
  
+ GGGGAAGTTA AATAAAAACC GGAAGTGAAA GAAGGGTAAT TTTAGGTTAG TACTGTATAT ATATTTTTAA   
  
  
+ TAATTGTGTA TGGAATTGTA ATGGCATTAA AATAATATTT ATATATTATA TTTATAAGAT ACCAAATTGA   
  
  
+ ATATTTTAGA TTTTTTTTTA ATTTTTGAAA CTCTTTAAAT CAACCAATAG TTCATACGTA ACTGAATATT   
  
  
+ CGATCGAGGC TTCAAAGTTA AAGTAGTTTT TAATAACTAA AAAAATAAAT AATTGAAATT CTAAAACTTT   
  
  
+ TCTTATTTAG GGAGTAAATA ATTAATCTA  

- TATTGGTTCT TGAAGAAAGC AAGCAAGCTT CCTTTCTTTG TGTTTCAAAG AAGTTTTAGG GTTTTATATA   
  
  
- TCAACAGAGA GCTACCTCCA TCTCCAGATT TTGACTTCAA ATTATATAAT TCAAAAACTA CTGCCTATTA   
  
  
- TCTATTTCTC TTGCATGTCA CCTAATCTCG TATGTTCAGT CCAGCAAGCA ACGTTAATCT TGAATATTTA   
  
  
- TTAATTTTAT AATGTAAATT ATATAAATTA AGCTTATTCC AAAAATTATT TCAACTTGGG AGTTTATTAA   
  
  
- TTAATTTATA CAATAATGTT TTCTGGATCC GTACGTGCGA CCACTGAAAA CGGTGGTCAA AACTGAGGCG   
  
  
- CCGTTATTTT GACTTTGTGA CTGAGTGGGT CCCGTAATTG CTACCCGACC CGGGGTCAAT TATGTACTTT   
  
  
- TGCGTAATAT TTCAAATTAT ACAGAGGCAC GTCAATTCAA CGTGTAAAAC GACGTAAACG CAGCTTGACT   
  
  
- GTTCCTATGT GACCATGTAA GAGTTGACTG TAAGCAGTTA CGGTCTTTTT TTGAAAAAGA AAATAAGGGA   
  
  
- AGCTGGAGAA AGTGGCGTTT CATTTAAAGT TCAGGAGCCA AACCCTTGGA AGAAAAATAG AAAAAAATAT   
  
  
- AAAAATAAAT TATAGGCTTA AAGAATCTTT AAAGAGGGTG TATACATATA TTTTTTAATA TTTATTTAAT   
  
  
- GTTTTTCATG TGACTAAAAC AAATCAATTA TGAAGATAAA AAAATTATTG TCATTAATTA ATTGAAATAA   
  
  
- TGGTGTAAAG GTAATGTTTT AAAAATCGAA TTAGGTTGAT TGCTCCTATT GATTAAATTA TAAACTAAAA   
  
  
- TTTTAATTAA TTTAATCTAT TTAAAATCAA TTTGATTTAT TTATATTATC AAAAAAATGT TGTTTTTTAA   
  
  
- AAAAATATAG TAAATTTTTT ATTTTAAATA TTTTTAAAAT ATAAGAAATT TTAAAAACAA AAAATTGGAT   
  
  
- GCTGCATTAG AGAGTTGAAT TAATTGTGAT GAGAAAATTA TGATATCATG AAGATGGGTG ATATTTTATC   
  
  
- CCAAATTGCT AATCTTAATT AGTAGGGTAA ATCTTGACCA TTTCAATTCT GAAGAGACAG AATTAATTAA   
  
  
- TTTTGAGTAT ATATTATCAA AATTCTATGC ACAATCGACA TAGTACCCAC GTCCTAAGCT TCATTGATTG   
  
  
- CCCCTTCAAT TTATTTTTGG CCTTCACTTT CTTCCCATTA AAATCCAATC ATGACATATA TATAAAAATT   
  
  
- ATTAACACAT ACCTTAACAT TACCGTAATT TTATTATAAA TATATAATAT AAATATTCTA TGGTTTAACT   
  
  
- TATAAAATCT AAAAAAAAAT TAAAAACTTT GAGAAATTTA GTTGGTTATC AAGTATGCAT TGACTTATAA   
  
  
- GCTAGCTCCG AAGTTTCAAT TTCATCAAAA ATTATTGATT TTTTTATTTA TTAACTTTAA GATTTTGAAA   
  
  
- AGAATAAATC CCTCATTTAT TAATTAGAT

+     TC-rich repeats

| Site Name | Organism | Position | Strand | Matrix score. | sequence | function |
| --- | --- | --- | --- | --- | --- | --- |
| TC-rich repeats | Nicotiana tabacum | 45 | + | 9 | ATTTTCTTCA | cis-acting element involved in defense and stress responsiveness |
| TC-rich repeats | Nicotiana tabacum | 890 | + | 9 | GTTTTCTTAC | cis-acting element involved in defense and stress responsiveness |

> 2018/04/13 10:10:12  
+ ATAACCAAGA ACTTCTTTCG TTCGTTCGAA GGAAAGAAAC ACAAAGTTTC TTCAAAATCC CAAAATATAT   
  
  
+ AGTTGTCTCT CGATGGAGGT AGAGGTCTAA AACTGAAGTT TAATATATTA AGTTTTTGAT GACGGATAAT   
  
  
+ AGATAAAGAG AACGTACAGT GGATTAGAGC ATACAAGTCA GGTCGTTCGT TGCAATTAGA ACTTATAAAT   
  
  
+ AATTAAAATA TTACATTTAA TATATTTAAT TCGAATAAGG TTTTTAATAA AGTTGAACCC TCAAATAATT   
  
  
+ AATTAAATAT GTTATTACAA AAGACCTAGG CATGCACGCT GGTGACTTTT GCCACCAGTT TTGACTCCGC   
  
  
+ GGCAATAAAA CTGAAACACT GACTCACCCA GGGCATTAAC GATGGGCTGG GCCCCAGTTA ATACATGAAA   
  
  
+ ACGCATTATA AAGTTTAATA TGTCTCCGTG CAGTTAAGTT GCACATTTTG CTGCATTTGC GTCGAACTGA   
  
  
+ CAAGGATACA CTGGTACATT CTCAACTGAC ATTCGTCAAT GCCAGAAAAA AACTTTTTCT TTTATTCCCT   
  
  
+ TCGACCTCTT TCACCGCAAA GTAAATTTCA AGTCCTCGGT TTGGGAACCT TCTTTTTATC TTTTTTTATA   
  
  
+ TTTTTATTTA ATATCCGAAT TTCTTAGAAA TTTCTCCCAC ATATGTATAT AAAAAATTAT AAATAAATTA   
  
  
+ CAAAAAGTAC ACTGATTTTG TTTAGTTAAT ACTTCTATTT TTTTAATAAC AGTAATTAAT TAACTTTATT   
  
  
+ ACCACATTTC CATTACAAAA TTTTTAGCTT AATCCAACTA ACGAGGATAA CTAATTTAAT ATTTGATTTT   
  
  
+ AAAATTAATT AAATTAGATA AATTTTAGTT AAACTAAATA AATATAATAG TTTTTTTACA ACAAAAAATT   
  
  
+ TTTTTATATC ATTTAAAAAA TAAAATTTAT AAAAATTTTA TATTCTTTAA AATTTTTGTT TTTTAACCTA   
  
  
+ CGACGTAATC TCTCAACTTA ATTAACACTA CTCTTTTAAT ACTATAGTAC TTCTACCCAC TATAAAATAG   
  
  
+ GGTTTAACGA TTAGAATTAA TCATCCCATT TAGAACTGGT AAAGTTAAGA CTTCTCTGTC TTAATTAATT   
  
  
+ AAAACTCATA TATAATAGTT TTAAGATACG TGTTAGCTGT ATCATGGGTG CAGGATTCGA AGTAACTAAC   
  
  
+ GGGGAAGTTA AATAAAAACC GGAAGTGAAA GAAGGGTAAT TTTAGGTTAG TACTGTATAT ATATTTTTAA   
  
  
+ TAATTGTGTA TGGAATTGTA ATGGCATTAA AATAATATTT ATATATTATA TTTATAAGAT ACCAAATTGA   
  
  
+ ATATTTTAGA TTTTTTTTTA ATTTTTGAAA CTCTTTAAAT CAACCAATAG TTCATACGTA ACTGAATATT   
  
  
+ CGATCGAGGC TTCAAAGTTA AAGTAGTTTT TAATAACTAA AAAAATAAAT AATTGAAATT CTAAAACTTT   
  
  
+ TCTTATTTAG GGAGTAAATA ATTAATCTA  

- TATTGGTTCT TGAAGAAAGC AAGCAAGCTT CCTTTCTTTG TGTTTCAAAG AAGTTTTAGG GTTTTATATA   
  
  
- TCAACAGAGA GCTACCTCCA TCTCCAGATT TTGACTTCAA ATTATATAAT TCAAAAACTA CTGCCTATTA   
  
  
- TCTATTTCTC TTGCATGTCA CCTAATCTCG TATGTTCAGT CCAGCAAGCA ACGTTAATCT TGAATATTTA   
  
  
- TTAATTTTAT AATGTAAATT ATATAAATTA AGCTTATTCC AAAAATTATT TCAACTTGGG AGTTTATTAA   
  
  
- TTAATTTATA CAATAATGTT TTCTGGATCC GTACGTGCGA CCACTGAAAA CGGTGGTCAA AACTGAGGCG   
  
  
- CCGTTATTTT GACTTTGTGA CTGAGTGGGT CCCGTAATTG CTACCCGACC CGGGGTCAAT TATGTACTTT   
  
  
- TGCGTAATAT TTCAAATTAT ACAGAGGCAC GTCAATTCAA CGTGTAAAAC GACGTAAACG CAGCTTGACT   
  
  
- GTTCCTATGT GACCATGTAA GAGTTGACTG TAAGCAGTTA CGGTCTTTTT TTGAAAAAGA AAATAAGGGA   
  
  
- AGCTGGAGAA AGTGGCGTTT CATTTAAAGT TCAGGAGCCA AACCCTTGGA AGAAAAATAG AAAAAAATAT   
  
  
- AAAAATAAAT TATAGGCTTA AAGAATCTTT AAAGAGGGTG TATACATATA TTTTTTAATA TTTATTTAAT   
  
  
- GTTTTTCATG TGACTAAAAC AAATCAATTA TGAAGATAAA AAAATTATTG TCATTAATTA ATTGAAATAA   
  
  
- TGGTGTAAAG GTAATGTTTT AAAAATCGAA TTAGGTTGAT TGCTCCTATT GATTAAATTA TAAACTAAAA   
  
  
- TTTTAATTAA TTTAATCTAT TTAAAATCAA TTTGATTTAT TTATATTATC AAAAAAATGT TGTTTTTTAA   
  
  
- AAAAATATAG TAAATTTTTT ATTTTAAATA TTTTTAAAAT ATAAGAAATT TTAAAAACAA AAAATTGGAT   
  
  
- GCTGCATTAG AGAGTTGAAT TAATTGTGAT GAGAAAATTA TGATATCATG AAGATGGGTG ATATTTTATC   
  
  
- CCAAATTGCT AATCTTAATT AGTAGGGTAA ATCTTGACCA TTTCAATTCT GAAGAGACAG AATTAATTAA   
  
  
- TTTTGAGTAT ATATTATCAA AATTCTATGC ACAATCGACA TAGTACCCAC GTCCTAAGCT TCATTGATTG   
  
  
- CCCCTTCAAT TTATTTTTGG CCTTCACTTT CTTCCCATTA AAATCCAATC ATGACATATA TATAAAAATT   
  
  
- ATTAACACAT ACCTTAACAT TACCGTAATT TTATTATAAA TATATAATAT AAATATTCTA TGGTTTAACT   
  
  
- TATAAAATCT AAAAAAAAAT TAAAAACTTT GAGAAATTTA GTTGGTTATC AAGTATGCAT TGACTTATAA   
  
  
- GCTAGCTCCG AAGTTTCAAT TTCATCAAAA ATTATTGATT TTTTTATTTA TTAACTTTAA GATTTTGAAA   
  
  
- AGAATAAATC CCTCATTTAT TAATTAGAT

+     TCA-element

| Site Name | Organism | Position | Strand | Matrix score. | sequence | function |
| --- | --- | --- | --- | --- | --- | --- |
| TCA-element | Nicotiana tabacum | 608 | + | 9 | CCATCTTTTT | cis-acting element involved in salicylic acid responsiveness |

> 2018/04/13 10:10:12  
+ ATAACCAAGA ACTTCTTTCG TTCGTTCGAA GGAAAGAAAC ACAAAGTTTC TTCAAAATCC CAAAATATAT   
  
  
+ AGTTGTCTCT CGATGGAGGT AGAGGTCTAA AACTGAAGTT TAATATATTA AGTTTTTGAT GACGGATAAT   
  
  
+ AGATAAAGAG AACGTACAGT GGATTAGAGC ATACAAGTCA GGTCGTTCGT TGCAATTAGA ACTTATAAAT   
  
  
+ AATTAAAATA TTACATTTAA TATATTTAAT TCGAATAAGG TTTTTAATAA AGTTGAACCC TCAAATAATT   
  
  
+ AATTAAATAT GTTATTACAA AAGACCTAGG CATGCACGCT GGTGACTTTT GCCACCAGTT TTGACTCCGC   
  
  
+ GGCAATAAAA CTGAAACACT GACTCACCCA GGGCATTAAC GATGGGCTGG GCCCCAGTTA ATACATGAAA   
  
  
+ ACGCATTATA AAGTTTAATA TGTCTCCGTG CAGTTAAGTT GCACATTTTG CTGCATTTGC GTCGAACTGA   
  
  
+ CAAGGATACA CTGGTACATT CTCAACTGAC ATTCGTCAAT GCCAGAAAAA AACTTTTTCT TTTATTCCCT   
  
  
+ TCGACCTCTT TCACCGCAAA GTAAATTTCA AGTCCTCGGT TTGGGAACCT TCTTTTTATC TTTTTTTATA   
  
  
+ TTTTTATTTA ATATCCGAAT TTCTTAGAAA TTTCTCCCAC ATATGTATAT AAAAAATTAT AAATAAATTA   
  
  
+ CAAAAAGTAC ACTGATTTTG TTTAGTTAAT ACTTCTATTT TTTTAATAAC AGTAATTAAT TAACTTTATT   
  
  
+ ACCACATTTC CATTACAAAA TTTTTAGCTT AATCCAACTA ACGAGGATAA CTAATTTAAT ATTTGATTTT   
  
  
+ AAAATTAATT AAATTAGATA AATTTTAGTT AAACTAAATA AATATAATAG TTTTTTTACA ACAAAAAATT   
  
  
+ TTTTTATATC ATTTAAAAAA TAAAATTTAT AAAAATTTTA TATTCTTTAA AATTTTTGTT TTTTAACCTA   
  
  
+ CGACGTAATC TCTCAACTTA ATTAACACTA CTCTTTTAAT ACTATAGTAC TTCTACCCAC TATAAAATAG   
  
  
+ GGTTTAACGA TTAGAATTAA TCATCCCATT TAGAACTGGT AAAGTTAAGA CTTCTCTGTC TTAATTAATT   
  
  
+ AAAACTCATA TATAATAGTT TTAAGATACG TGTTAGCTGT ATCATGGGTG CAGGATTCGA AGTAACTAAC   
  
  
+ GGGGAAGTTA AATAAAAACC GGAAGTGAAA GAAGGGTAAT TTTAGGTTAG TACTGTATAT ATATTTTTAA   
  
  
+ TAATTGTGTA TGGAATTGTA ATGGCATTAA AATAATATTT ATATATTATA TTTATAAGAT ACCAAATTGA   
  
  
+ ATATTTTAGA TTTTTTTTTA ATTTTTGAAA CTCTTTAAAT CAACCAATAG TTCATACGTA ACTGAATATT   
  
  
+ CGATCGAGGC TTCAAAGTTA AAGTAGTTTT TAATAACTAA AAAAATAAAT AATTGAAATT CTAAAACTTT   
  
  
+ TCTTATTTAG GGAGTAAATA ATTAATCTA  

- TATTGGTTCT TGAAGAAAGC AAGCAAGCTT CCTTTCTTTG TGTTTCAAAG AAGTTTTAGG GTTTTATATA   
  
  
- TCAACAGAGA GCTACCTCCA TCTCCAGATT TTGACTTCAA ATTATATAAT TCAAAAACTA CTGCCTATTA   
  
  
- TCTATTTCTC TTGCATGTCA CCTAATCTCG TATGTTCAGT CCAGCAAGCA ACGTTAATCT TGAATATTTA   
  
  
- TTAATTTTAT AATGTAAATT ATATAAATTA AGCTTATTCC AAAAATTATT TCAACTTGGG AGTTTATTAA   
  
  
- TTAATTTATA CAATAATGTT TTCTGGATCC GTACGTGCGA CCACTGAAAA CGGTGGTCAA AACTGAGGCG   
  
  
- CCGTTATTTT GACTTTGTGA CTGAGTGGGT CCCGTAATTG CTACCCGACC CGGGGTCAAT TATGTACTTT   
  
  
- TGCGTAATAT TTCAAATTAT ACAGAGGCAC GTCAATTCAA CGTGTAAAAC GACGTAAACG CAGCTTGACT   
  
  
- GTTCCTATGT GACCATGTAA GAGTTGACTG TAAGCAGTTA CGGTCTTTTT TTGAAAAAGA AAATAAGGGA   
  
  
- AGCTGGAGAA AGTGGCGTTT CATTTAAAGT TCAGGAGCCA AACCCTTGGA AGAAAAATAG AAAAAAATAT   
  
  
- AAAAATAAAT TATAGGCTTA AAGAATCTTT AAAGAGGGTG TATACATATA TTTTTTAATA TTTATTTAAT   
  
  
- GTTTTTCATG TGACTAAAAC AAATCAATTA TGAAGATAAA AAAATTATTG TCATTAATTA ATTGAAATAA   
  
  
- TGGTGTAAAG GTAATGTTTT AAAAATCGAA TTAGGTTGAT TGCTCCTATT GATTAAATTA TAAACTAAAA   
  
  
- TTTTAATTAA TTTAATCTAT TTAAAATCAA TTTGATTTAT TTATATTATC AAAAAAATGT TGTTTTTTAA   
  
  
- AAAAATATAG TAAATTTTTT ATTTTAAATA TTTTTAAAAT ATAAGAAATT TTAAAAACAA AAAATTGGAT   
  
  
- GCTGCATTAG AGAGTTGAAT TAATTGTGAT GAGAAAATTA TGATATCATG AAGATGGGTG ATATTTTATC   
  
  
- CCAAATTGCT AATCTTAATT AGTAGGGTAA ATCTTGACCA TTTCAATTCT GAAGAGACAG AATTAATTAA   
  
  
- TTTTGAGTAT ATATTATCAA AATTCTATGC ACAATCGACA TAGTACCCAC GTCCTAAGCT TCATTGATTG   
  
  
- CCCCTTCAAT TTATTTTTGG CCTTCACTTT CTTCCCATTA AAATCCAATC ATGACATATA TATAAAAATT   
  
  
- ATTAACACAT ACCTTAACAT TACCGTAATT TTATTATAAA TATATAATAT AAATATTCTA TGGTTTAACT   
  
  
- TATAAAATCT AAAAAAAAAT TAAAAACTTT GAGAAATTTA GTTGGTTATC AAGTATGCAT TGACTTATAA   
  
  
- GCTAGCTCCG AAGTTTCAAT TTCATCAAAA ATTATTGATT TTTTTATTTA TTAACTTTAA GATTTTGAAA   
  
  
- AGAATAAATC CCTCATTTAT TAATTAGAT

+     TGA-element

| Site Name | Organism | Position | Strand | Matrix score. | sequence | function |
| --- | --- | --- | --- | --- | --- | --- |
| TGA-element | Brassica oleracea | 182 | - | 6 | AACGAC | auxin-responsive element |

> 2018/04/13 10:10:12  
+ ATAACCAAGA ACTTCTTTCG TTCGTTCGAA GGAAAGAAAC ACAAAGTTTC TTCAAAATCC CAAAATATAT   
  
  
+ AGTTGTCTCT CGATGGAGGT AGAGGTCTAA AACTGAAGTT TAATATATTA AGTTTTTGAT GACGGATAAT   
  
  
+ AGATAAAGAG AACGTACAGT GGATTAGAGC ATACAAGTCA GGTCGTTCGT TGCAATTAGA ACTTATAAAT   
  
  
+ AATTAAAATA TTACATTTAA TATATTTAAT TCGAATAAGG TTTTTAATAA AGTTGAACCC TCAAATAATT   
  
  
+ AATTAAATAT GTTATTACAA AAGACCTAGG CATGCACGCT GGTGACTTTT GCCACCAGTT TTGACTCCGC   
  
  
+ GGCAATAAAA CTGAAACACT GACTCACCCA GGGCATTAAC GATGGGCTGG GCCCCAGTTA ATACATGAAA   
  
  
+ ACGCATTATA AAGTTTAATA TGTCTCCGTG CAGTTAAGTT GCACATTTTG CTGCATTTGC GTCGAACTGA   
  
  
+ CAAGGATACA CTGGTACATT CTCAACTGAC ATTCGTCAAT GCCAGAAAAA AACTTTTTCT TTTATTCCCT   
  
  
+ TCGACCTCTT TCACCGCAAA GTAAATTTCA AGTCCTCGGT TTGGGAACCT TCTTTTTATC TTTTTTTATA   
  
  
+ TTTTTATTTA ATATCCGAAT TTCTTAGAAA TTTCTCCCAC ATATGTATAT AAAAAATTAT AAATAAATTA   
  
  
+ CAAAAAGTAC ACTGATTTTG TTTAGTTAAT ACTTCTATTT TTTTAATAAC AGTAATTAAT TAACTTTATT   
  
  
+ ACCACATTTC CATTACAAAA TTTTTAGCTT AATCCAACTA ACGAGGATAA CTAATTTAAT ATTTGATTTT   
  
  
+ AAAATTAATT AAATTAGATA AATTTTAGTT AAACTAAATA AATATAATAG TTTTTTTACA ACAAAAAATT   
  
  
+ TTTTTATATC ATTTAAAAAA TAAAATTTAT AAAAATTTTA TATTCTTTAA AATTTTTGTT TTTTAACCTA   
  
  
+ CGACGTAATC TCTCAACTTA ATTAACACTA CTCTTTTAAT ACTATAGTAC TTCTACCCAC TATAAAATAG   
  
  
+ GGTTTAACGA TTAGAATTAA TCATCCCATT TAGAACTGGT AAAGTTAAGA CTTCTCTGTC TTAATTAATT   
  
  
+ AAAACTCATA TATAATAGTT TTAAGATACG TGTTAGCTGT ATCATGGGTG CAGGATTCGA AGTAACTAAC   
  
  
+ GGGGAAGTTA AATAAAAACC GGAAGTGAAA GAAGGGTAAT TTTAGGTTAG TACTGTATAT ATATTTTTAA   
  
  
+ TAATTGTGTA TGGAATTGTA ATGGCATTAA AATAATATTT ATATATTATA TTTATAAGAT ACCAAATTGA   
  
  
+ ATATTTTAGA TTTTTTTTTA ATTTTTGAAA CTCTTTAAAT CAACCAATAG TTCATACGTA ACTGAATATT   
  
  
+ CGATCGAGGC TTCAAAGTTA AAGTAGTTTT TAATAACTAA AAAAATAAAT AATTGAAATT CTAAAACTTT   
  
  
+ TCTTATTTAG GGAGTAAATA ATTAATCTA  

- TATTGGTTCT TGAAGAAAGC AAGCAAGCTT CCTTTCTTTG TGTTTCAAAG AAGTTTTAGG GTTTTATATA   
  
  
- TCAACAGAGA GCTACCTCCA TCTCCAGATT TTGACTTCAA ATTATATAAT TCAAAAACTA CTGCCTATTA   
  
  
- TCTATTTCTC TTGCATGTCA CCTAATCTCG TATGTTCAGT CCAGCAAGCA ACGTTAATCT TGAATATTTA   
  
  
- TTAATTTTAT AATGTAAATT ATATAAATTA AGCTTATTCC AAAAATTATT TCAACTTGGG AGTTTATTAA   
  
  
- TTAATTTATA CAATAATGTT TTCTGGATCC GTACGTGCGA CCACTGAAAA CGGTGGTCAA AACTGAGGCG   
  
  
- CCGTTATTTT GACTTTGTGA CTGAGTGGGT CCCGTAATTG CTACCCGACC CGGGGTCAAT TATGTACTTT   
  
  
- TGCGTAATAT TTCAAATTAT ACAGAGGCAC GTCAATTCAA CGTGTAAAAC GACGTAAACG CAGCTTGACT   
  
  
- GTTCCTATGT GACCATGTAA GAGTTGACTG TAAGCAGTTA CGGTCTTTTT TTGAAAAAGA AAATAAGGGA   
  
  
- AGCTGGAGAA AGTGGCGTTT CATTTAAAGT TCAGGAGCCA AACCCTTGGA AGAAAAATAG AAAAAAATAT   
  
  
- AAAAATAAAT TATAGGCTTA AAGAATCTTT AAAGAGGGTG TATACATATA TTTTTTAATA TTTATTTAAT   
  
  
- GTTTTTCATG TGACTAAAAC AAATCAATTA TGAAGATAAA AAAATTATTG TCATTAATTA ATTGAAATAA   
  
  
- TGGTGTAAAG GTAATGTTTT AAAAATCGAA TTAGGTTGAT TGCTCCTATT GATTAAATTA TAAACTAAAA   
  
  
- TTTTAATTAA TTTAATCTAT TTAAAATCAA TTTGATTTAT TTATATTATC AAAAAAATGT TGTTTTTTAA   
  
  
- AAAAATATAG TAAATTTTTT ATTTTAAATA TTTTTAAAAT ATAAGAAATT TTAAAAACAA AAAATTGGAT   
  
  
- GCTGCATTAG AGAGTTGAAT TAATTGTGAT GAGAAAATTA TGATATCATG AAGATGGGTG ATATTTTATC   
  
  
- CCAAATTGCT AATCTTAATT AGTAGGGTAA ATCTTGACCA TTTCAATTCT GAAGAGACAG AATTAATTAA   
  
  
- TTTTGAGTAT ATATTATCAA AATTCTATGC ACAATCGACA TAGTACCCAC GTCCTAAGCT TCATTGATTG   
  
  
- CCCCTTCAAT TTATTTTTGG CCTTCACTTT CTTCCCATTA AAATCCAATC ATGACATATA TATAAAAATT   
  
  
- ATTAACACAT ACCTTAACAT TACCGTAATT TTATTATAAA TATATAATAT AAATATTCTA TGGTTTAACT   
  
  
- TATAAAATCT AAAAAAAAAT TAAAAACTTT GAGAAATTTA GTTGGTTATC AAGTATGCAT TGACTTATAA   
  
  
- GCTAGCTCCG AAGTTTCAAT TTCATCAAAA ATTATTGATT TTTTTATTTA TTAACTTTAA GATTTTGAAA   
  
  
- AGAATAAATC CCTCATTTAT TAATTAGAT

+     TGACG-motif

| Site Name | Organism | Position | Strand | Matrix score. | sequence | function |
| --- | --- | --- | --- | --- | --- | --- |
| TGACG-motif | Hordeum vulgare | 130 | + | 5 | TGACG | cis-acting regulatory element involved in the MeJA-responsiveness |
| TGACG-motif | Hordeum vulgare | 524 | - | 5 | TGACG | cis-acting regulatory element involved in the MeJA-responsiveness |

> 2018/04/13 10:10:12  
+ ATAACCAAGA ACTTCTTTCG TTCGTTCGAA GGAAAGAAAC ACAAAGTTTC TTCAAAATCC CAAAATATAT   
  
  
+ AGTTGTCTCT CGATGGAGGT AGAGGTCTAA AACTGAAGTT TAATATATTA AGTTTTTGAT GACGGATAAT   
  
  
+ AGATAAAGAG AACGTACAGT GGATTAGAGC ATACAAGTCA GGTCGTTCGT TGCAATTAGA ACTTATAAAT   
  
  
+ AATTAAAATA TTACATTTAA TATATTTAAT TCGAATAAGG TTTTTAATAA AGTTGAACCC TCAAATAATT   
  
  
+ AATTAAATAT GTTATTACAA AAGACCTAGG CATGCACGCT GGTGACTTTT GCCACCAGTT TTGACTCCGC   
  
  
+ GGCAATAAAA CTGAAACACT GACTCACCCA GGGCATTAAC GATGGGCTGG GCCCCAGTTA ATACATGAAA   
  
  
+ ACGCATTATA AAGTTTAATA TGTCTCCGTG CAGTTAAGTT GCACATTTTG CTGCATTTGC GTCGAACTGA   
  
  
+ CAAGGATACA CTGGTACATT CTCAACTGAC ATTCGTCAAT GCCAGAAAAA AACTTTTTCT TTTATTCCCT   
  
  
+ TCGACCTCTT TCACCGCAAA GTAAATTTCA AGTCCTCGGT TTGGGAACCT TCTTTTTATC TTTTTTTATA   
  
  
+ TTTTTATTTA ATATCCGAAT TTCTTAGAAA TTTCTCCCAC ATATGTATAT AAAAAATTAT AAATAAATTA   
  
  
+ CAAAAAGTAC ACTGATTTTG TTTAGTTAAT ACTTCTATTT TTTTAATAAC AGTAATTAAT TAACTTTATT   
  
  
+ ACCACATTTC CATTACAAAA TTTTTAGCTT AATCCAACTA ACGAGGATAA CTAATTTAAT ATTTGATTTT   
  
  
+ AAAATTAATT AAATTAGATA AATTTTAGTT AAACTAAATA AATATAATAG TTTTTTTACA ACAAAAAATT   
  
  
+ TTTTTATATC ATTTAAAAAA TAAAATTTAT AAAAATTTTA TATTCTTTAA AATTTTTGTT TTTTAACCTA   
  
  
+ CGACGTAATC TCTCAACTTA ATTAACACTA CTCTTTTAAT ACTATAGTAC TTCTACCCAC TATAAAATAG   
  
  
+ GGTTTAACGA TTAGAATTAA TCATCCCATT TAGAACTGGT AAAGTTAAGA CTTCTCTGTC TTAATTAATT   
  
  
+ AAAACTCATA TATAATAGTT TTAAGATACG TGTTAGCTGT ATCATGGGTG CAGGATTCGA AGTAACTAAC   
  
  
+ GGGGAAGTTA AATAAAAACC GGAAGTGAAA GAAGGGTAAT TTTAGGTTAG TACTGTATAT ATATTTTTAA   
  
  
+ TAATTGTGTA TGGAATTGTA ATGGCATTAA AATAATATTT ATATATTATA TTTATAAGAT ACCAAATTGA   
  
  
+ ATATTTTAGA TTTTTTTTTA ATTTTTGAAA CTCTTTAAAT CAACCAATAG TTCATACGTA ACTGAATATT   
  
  
+ CGATCGAGGC TTCAAAGTTA AAGTAGTTTT TAATAACTAA AAAAATAAAT AATTGAAATT CTAAAACTTT   
  
  
+ TCTTATTTAG GGAGTAAATA ATTAATCTA  

- TATTGGTTCT TGAAGAAAGC AAGCAAGCTT CCTTTCTTTG TGTTTCAAAG AAGTTTTAGG GTTTTATATA   
  
  
- TCAACAGAGA GCTACCTCCA TCTCCAGATT TTGACTTCAA ATTATATAAT TCAAAAACTA CTGCCTATTA   
  
  
- TCTATTTCTC TTGCATGTCA CCTAATCTCG TATGTTCAGT CCAGCAAGCA ACGTTAATCT TGAATATTTA   
  
  
- TTAATTTTAT AATGTAAATT ATATAAATTA AGCTTATTCC AAAAATTATT TCAACTTGGG AGTTTATTAA   
  
  
- TTAATTTATA CAATAATGTT TTCTGGATCC GTACGTGCGA CCACTGAAAA CGGTGGTCAA AACTGAGGCG   
  
  
- CCGTTATTTT GACTTTGTGA CTGAGTGGGT CCCGTAATTG CTACCCGACC CGGGGTCAAT TATGTACTTT   
  
  
- TGCGTAATAT TTCAAATTAT ACAGAGGCAC GTCAATTCAA CGTGTAAAAC GACGTAAACG CAGCTTGACT   
  
  
- GTTCCTATGT GACCATGTAA GAGTTGACTG TAAGCAGTTA CGGTCTTTTT TTGAAAAAGA AAATAAGGGA   
  
  
- AGCTGGAGAA AGTGGCGTTT CATTTAAAGT TCAGGAGCCA AACCCTTGGA AGAAAAATAG AAAAAAATAT   
  
  
- AAAAATAAAT TATAGGCTTA AAGAATCTTT AAAGAGGGTG TATACATATA TTTTTTAATA TTTATTTAAT   
  
  
- GTTTTTCATG TGACTAAAAC AAATCAATTA TGAAGATAAA AAAATTATTG TCATTAATTA ATTGAAATAA   
  
  
- TGGTGTAAAG GTAATGTTTT AAAAATCGAA TTAGGTTGAT TGCTCCTATT GATTAAATTA TAAACTAAAA   
  
  
- TTTTAATTAA TTTAATCTAT TTAAAATCAA TTTGATTTAT TTATATTATC AAAAAAATGT TGTTTTTTAA   
  
  
- AAAAATATAG TAAATTTTTT ATTTTAAATA TTTTTAAAAT ATAAGAAATT TTAAAAACAA AAAATTGGAT   
  
  
- GCTGCATTAG AGAGTTGAAT TAATTGTGAT GAGAAAATTA TGATATCATG AAGATGGGTG ATATTTTATC   
  
  
- CCAAATTGCT AATCTTAATT AGTAGGGTAA ATCTTGACCA TTTCAATTCT GAAGAGACAG AATTAATTAA   
  
  
- TTTTGAGTAT ATATTATCAA AATTCTATGC ACAATCGACA TAGTACCCAC GTCCTAAGCT TCATTGATTG   
  
  
- CCCCTTCAAT TTATTTTTGG CCTTCACTTT CTTCCCATTA AAATCCAATC ATGACATATA TATAAAAATT   
  
  
- ATTAACACAT ACCTTAACAT TACCGTAATT TTATTATAAA TATATAATAT AAATATTCTA TGGTTTAACT   
  
  
- TATAAAATCT AAAAAAAAAT TAAAAACTTT GAGAAATTTA GTTGGTTATC AAGTATGCAT TGACTTATAA   
  
  
- GCTAGCTCCG AAGTTTCAAT TTCATCAAAA ATTATTGATT TTTTTATTTA TTAACTTTAA GATTTTGAAA   
  
  
- AGAATAAATC CCTCATTTAT TAATTAGAT

+     Unnamed\_\_1

| Site Name | Organism | Position | Strand | Matrix score. | sequence | function |
| --- | --- | --- | --- | --- | --- | --- |
| Unnamed\_\_1 | Glycine max | 845 | - | 11 | GAATTTAATTAA | 60K protein binding site |

> 2018/04/13 10:10:12  
+ ATAACCAAGA ACTTCTTTCG TTCGTTCGAA GGAAAGAAAC ACAAAGTTTC TTCAAAATCC CAAAATATAT   
  
  
+ AGTTGTCTCT CGATGGAGGT AGAGGTCTAA AACTGAAGTT TAATATATTA AGTTTTTGAT GACGGATAAT   
  
  
+ AGATAAAGAG AACGTACAGT GGATTAGAGC ATACAAGTCA GGTCGTTCGT TGCAATTAGA ACTTATAAAT   
  
  
+ AATTAAAATA TTACATTTAA TATATTTAAT TCGAATAAGG TTTTTAATAA AGTTGAACCC TCAAATAATT   
  
  
+ AATTAAATAT GTTATTACAA AAGACCTAGG CATGCACGCT GGTGACTTTT GCCACCAGTT TTGACTCCGC   
  
  
+ GGCAATAAAA CTGAAACACT GACTCACCCA GGGCATTAAC GATGGGCTGG GCCCCAGTTA ATACATGAAA   
  
  
+ ACGCATTATA AAGTTTAATA TGTCTCCGTG CAGTTAAGTT GCACATTTTG CTGCATTTGC GTCGAACTGA   
  
  
+ CAAGGATACA CTGGTACATT CTCAACTGAC ATTCGTCAAT GCCAGAAAAA AACTTTTTCT TTTATTCCCT   
  
  
+ TCGACCTCTT TCACCGCAAA GTAAATTTCA AGTCCTCGGT TTGGGAACCT TCTTTTTATC TTTTTTTATA   
  
  
+ TTTTTATTTA ATATCCGAAT TTCTTAGAAA TTTCTCCCAC ATATGTATAT AAAAAATTAT AAATAAATTA   
  
  
+ CAAAAAGTAC ACTGATTTTG TTTAGTTAAT ACTTCTATTT TTTTAATAAC AGTAATTAAT TAACTTTATT   
  
  
+ ACCACATTTC CATTACAAAA TTTTTAGCTT AATCCAACTA ACGAGGATAA CTAATTTAAT ATTTGATTTT   
  
  
+ AAAATTAATT AAATTAGATA AATTTTAGTT AAACTAAATA AATATAATAG TTTTTTTACA ACAAAAAATT   
  
  
+ TTTTTATATC ATTTAAAAAA TAAAATTTAT AAAAATTTTA TATTCTTTAA AATTTTTGTT TTTTAACCTA   
  
  
+ CGACGTAATC TCTCAACTTA ATTAACACTA CTCTTTTAAT ACTATAGTAC TTCTACCCAC TATAAAATAG   
  
  
+ GGTTTAACGA TTAGAATTAA TCATCCCATT TAGAACTGGT AAAGTTAAGA CTTCTCTGTC TTAATTAATT   
  
  
+ AAAACTCATA TATAATAGTT TTAAGATACG TGTTAGCTGT ATCATGGGTG CAGGATTCGA AGTAACTAAC   
  
  
+ GGGGAAGTTA AATAAAAACC GGAAGTGAAA GAAGGGTAAT TTTAGGTTAG TACTGTATAT ATATTTTTAA   
  
  
+ TAATTGTGTA TGGAATTGTA ATGGCATTAA AATAATATTT ATATATTATA TTTATAAGAT ACCAAATTGA   
  
  
+ ATATTTTAGA TTTTTTTTTA ATTTTTGAAA CTCTTTAAAT CAACCAATAG TTCATACGTA ACTGAATATT   
  
  
+ CGATCGAGGC TTCAAAGTTA AAGTAGTTTT TAATAACTAA AAAAATAAAT AATTGAAATT CTAAAACTTT   
  
  
+ TCTTATTTAG GGAGTAAATA ATTAATCTA  

- TATTGGTTCT TGAAGAAAGC AAGCAAGCTT CCTTTCTTTG TGTTTCAAAG AAGTTTTAGG GTTTTATATA   
  
  
- TCAACAGAGA GCTACCTCCA TCTCCAGATT TTGACTTCAA ATTATATAAT TCAAAAACTA CTGCCTATTA   
  
  
- TCTATTTCTC TTGCATGTCA CCTAATCTCG TATGTTCAGT CCAGCAAGCA ACGTTAATCT TGAATATTTA   
  
  
- TTAATTTTAT AATGTAAATT ATATAAATTA AGCTTATTCC AAAAATTATT TCAACTTGGG AGTTTATTAA   
  
  
- TTAATTTATA CAATAATGTT TTCTGGATCC GTACGTGCGA CCACTGAAAA CGGTGGTCAA AACTGAGGCG   
  
  
- CCGTTATTTT GACTTTGTGA CTGAGTGGGT CCCGTAATTG CTACCCGACC CGGGGTCAAT TATGTACTTT   
  
  
- TGCGTAATAT TTCAAATTAT ACAGAGGCAC GTCAATTCAA CGTGTAAAAC GACGTAAACG CAGCTTGACT   
  
  
- GTTCCTATGT GACCATGTAA GAGTTGACTG TAAGCAGTTA CGGTCTTTTT TTGAAAAAGA AAATAAGGGA   
  
  
- AGCTGGAGAA AGTGGCGTTT CATTTAAAGT TCAGGAGCCA AACCCTTGGA AGAAAAATAG AAAAAAATAT   
  
  
- AAAAATAAAT TATAGGCTTA AAGAATCTTT AAAGAGGGTG TATACATATA TTTTTTAATA TTTATTTAAT   
  
  
- GTTTTTCATG TGACTAAAAC AAATCAATTA TGAAGATAAA AAAATTATTG TCATTAATTA ATTGAAATAA   
  
  
- TGGTGTAAAG GTAATGTTTT AAAAATCGAA TTAGGTTGAT TGCTCCTATT GATTAAATTA TAAACTAAAA   
  
  
- TTTTAATTAA TTTAATCTAT TTAAAATCAA TTTGATTTAT TTATATTATC AAAAAAATGT TGTTTTTTAA   
  
  
- AAAAATATAG TAAATTTTTT ATTTTAAATA TTTTTAAAAT ATAAGAAATT TTAAAAACAA AAAATTGGAT   
  
  
- GCTGCATTAG AGAGTTGAAT TAATTGTGAT GAGAAAATTA TGATATCATG AAGATGGGTG ATATTTTATC   
  
  
- CCAAATTGCT AATCTTAATT AGTAGGGTAA ATCTTGACCA TTTCAATTCT GAAGAGACAG AATTAATTAA   
  
  
- TTTTGAGTAT ATATTATCAA AATTCTATGC ACAATCGACA TAGTACCCAC GTCCTAAGCT TCATTGATTG   
  
  
- CCCCTTCAAT TTATTTTTGG CCTTCACTTT CTTCCCATTA AAATCCAATC ATGACATATA TATAAAAATT   
  
  
- ATTAACACAT ACCTTAACAT TACCGTAATT TTATTATAAA TATATAATAT AAATATTCTA TGGTTTAACT   
  
  
- TATAAAATCT AAAAAAAAAT TAAAAACTTT GAGAAATTTA GTTGGTTATC AAGTATGCAT TGACTTATAA   
  
  
- GCTAGCTCCG AAGTTTCAAT TTCATCAAAA ATTATTGATT TTTTTATTTA TTAACTTTAA GATTTTGAAA   
  
  
- AGAATAAATC CCTCATTTAT TAATTAGAT

+     Unnamed\_\_4

| Site Name | Organism | Position | Strand | Matrix score. | sequence | function |
| --- | --- | --- | --- | --- | --- | --- |
| Unnamed\_\_4 | Petroselinum hortense | 345 | + | 4 | CTCC |  |
| Unnamed\_\_4 | Petroselinum hortense | 1481 | - | 4 | CTCC |  |
| Unnamed\_\_4 | Petroselinum hortense | 444 | + | 4 | CTCC |  |
| Unnamed\_\_4 | Petroselinum hortense | 85 | - | 4 | CTCC |  |
| Unnamed\_\_4 | Petroselinum hortense | 664 | + | 4 | CTCC |  |

> 2018/04/13 10:10:12  
+ ATAACCAAGA ACTTCTTTCG TTCGTTCGAA GGAAAGAAAC ACAAAGTTTC TTCAAAATCC CAAAATATAT   
  
  
+ AGTTGTCTCT CGATGGAGGT AGAGGTCTAA AACTGAAGTT TAATATATTA AGTTTTTGAT GACGGATAAT   
  
  
+ AGATAAAGAG AACGTACAGT GGATTAGAGC ATACAAGTCA GGTCGTTCGT TGCAATTAGA ACTTATAAAT   
  
  
+ AATTAAAATA TTACATTTAA TATATTTAAT TCGAATAAGG TTTTTAATAA AGTTGAACCC TCAAATAATT   
  
  
+ AATTAAATAT GTTATTACAA AAGACCTAGG CATGCACGCT GGTGACTTTT GCCACCAGTT TTGACTCCGC   
  
  
+ GGCAATAAAA CTGAAACACT GACTCACCCA GGGCATTAAC GATGGGCTGG GCCCCAGTTA ATACATGAAA   
  
  
+ ACGCATTATA AAGTTTAATA TGTCTCCGTG CAGTTAAGTT GCACATTTTG CTGCATTTGC GTCGAACTGA   
  
  
+ CAAGGATACA CTGGTACATT CTCAACTGAC ATTCGTCAAT GCCAGAAAAA AACTTTTTCT TTTATTCCCT   
  
  
+ TCGACCTCTT TCACCGCAAA GTAAATTTCA AGTCCTCGGT TTGGGAACCT TCTTTTTATC TTTTTTTATA   
  
  
+ TTTTTATTTA ATATCCGAAT TTCTTAGAAA TTTCTCCCAC ATATGTATAT AAAAAATTAT AAATAAATTA   
  
  
+ CAAAAAGTAC ACTGATTTTG TTTAGTTAAT ACTTCTATTT TTTTAATAAC AGTAATTAAT TAACTTTATT   
  
  
+ ACCACATTTC CATTACAAAA TTTTTAGCTT AATCCAACTA ACGAGGATAA CTAATTTAAT ATTTGATTTT   
  
  
+ AAAATTAATT AAATTAGATA AATTTTAGTT AAACTAAATA AATATAATAG TTTTTTTACA ACAAAAAATT   
  
  
+ TTTTTATATC ATTTAAAAAA TAAAATTTAT AAAAATTTTA TATTCTTTAA AATTTTTGTT TTTTAACCTA   
  
  
+ CGACGTAATC TCTCAACTTA ATTAACACTA CTCTTTTAAT ACTATAGTAC TTCTACCCAC TATAAAATAG   
  
  
+ GGTTTAACGA TTAGAATTAA TCATCCCATT TAGAACTGGT AAAGTTAAGA CTTCTCTGTC TTAATTAATT   
  
  
+ AAAACTCATA TATAATAGTT TTAAGATACG TGTTAGCTGT ATCATGGGTG CAGGATTCGA AGTAACTAAC   
  
  
+ GGGGAAGTTA AATAAAAACC GGAAGTGAAA GAAGGGTAAT TTTAGGTTAG TACTGTATAT ATATTTTTAA   
  
  
+ TAATTGTGTA TGGAATTGTA ATGGCATTAA AATAATATTT ATATATTATA TTTATAAGAT ACCAAATTGA   
  
  
+ ATATTTTAGA TTTTTTTTTA ATTTTTGAAA CTCTTTAAAT CAACCAATAG TTCATACGTA ACTGAATATT   
  
  
+ CGATCGAGGC TTCAAAGTTA AAGTAGTTTT TAATAACTAA AAAAATAAAT AATTGAAATT CTAAAACTTT   
  
  
+ TCTTATTTAG GGAGTAAATA ATTAATCTA  

- TATTGGTTCT TGAAGAAAGC AAGCAAGCTT CCTTTCTTTG TGTTTCAAAG AAGTTTTAGG GTTTTATATA   
  
  
- TCAACAGAGA GCTACCTCCA TCTCCAGATT TTGACTTCAA ATTATATAAT TCAAAAACTA CTGCCTATTA   
  
  
- TCTATTTCTC TTGCATGTCA CCTAATCTCG TATGTTCAGT CCAGCAAGCA ACGTTAATCT TGAATATTTA   
  
  
- TTAATTTTAT AATGTAAATT ATATAAATTA AGCTTATTCC AAAAATTATT TCAACTTGGG AGTTTATTAA   
  
  
- TTAATTTATA CAATAATGTT TTCTGGATCC GTACGTGCGA CCACTGAAAA CGGTGGTCAA AACTGAGGCG   
  
  
- CCGTTATTTT GACTTTGTGA CTGAGTGGGT CCCGTAATTG CTACCCGACC CGGGGTCAAT TATGTACTTT   
  
  
- TGCGTAATAT TTCAAATTAT ACAGAGGCAC GTCAATTCAA CGTGTAAAAC GACGTAAACG CAGCTTGACT   
  
  
- GTTCCTATGT GACCATGTAA GAGTTGACTG TAAGCAGTTA CGGTCTTTTT TTGAAAAAGA AAATAAGGGA   
  
  
- AGCTGGAGAA AGTGGCGTTT CATTTAAAGT TCAGGAGCCA AACCCTTGGA AGAAAAATAG AAAAAAATAT   
  
  
- AAAAATAAAT TATAGGCTTA AAGAATCTTT AAAGAGGGTG TATACATATA TTTTTTAATA TTTATTTAAT   
  
  
- GTTTTTCATG TGACTAAAAC AAATCAATTA TGAAGATAAA AAAATTATTG TCATTAATTA ATTGAAATAA   
  
  
- TGGTGTAAAG GTAATGTTTT AAAAATCGAA TTAGGTTGAT TGCTCCTATT GATTAAATTA TAAACTAAAA   
  
  
- TTTTAATTAA TTTAATCTAT TTAAAATCAA TTTGATTTAT TTATATTATC AAAAAAATGT TGTTTTTTAA   
  
  
- AAAAATATAG TAAATTTTTT ATTTTAAATA TTTTTAAAAT ATAAGAAATT TTAAAAACAA AAAATTGGAT   
  
  
- GCTGCATTAG AGAGTTGAAT TAATTGTGAT GAGAAAATTA TGATATCATG AAGATGGGTG ATATTTTATC   
  
  
- CCAAATTGCT AATCTTAATT AGTAGGGTAA ATCTTGACCA TTTCAATTCT GAAGAGACAG AATTAATTAA   
  
  
- TTTTGAGTAT ATATTATCAA AATTCTATGC ACAATCGACA TAGTACCCAC GTCCTAAGCT TCATTGATTG   
  
  
- CCCCTTCAAT TTATTTTTGG CCTTCACTTT CTTCCCATTA AAATCCAATC ATGACATATA TATAAAAATT   
  
  
- ATTAACACAT ACCTTAACAT TACCGTAATT TTATTATAAA TATATAATAT AAATATTCTA TGGTTTAACT   
  
  
- TATAAAATCT AAAAAAAAAT TAAAAACTTT GAGAAATTTA GTTGGTTATC AAGTATGCAT TGACTTATAA   
  
  
- GCTAGCTCCG AAGTTTCAAT TTCATCAAAA ATTATTGATT TTTTTATTTA TTAACTTTAA GATTTTGAAA   
  
  
- AGAATAAATC CCTCATTTAT TAATTAGAT
