# Supplementary material for: Genome-wide analysis of the WRKY gene family in drumstick (Moringa oleifera Lam.)
Source: PeerJ. 2019 Jun 10;7:e7063. doi: 10.7717/peerj.7063 (PMC6563795; doi:10.7717/peerj.7063)
Supplement: Supplemental Information 1 [file peerj-07-7063-s003.gz › MoWRKY41_plantcare.html]

Content-Type: text/html; charset=ISO-8859-1


CallMat\_Firefox


Webmaster Firefox specific output  
To save the result:
click on the frame with the right mouse button and save the source code as a text file with extension .html  
REFERENCE:PlantCARE: a database of plant cis-acting regulatory elements and a portal to tools for in silico analysis of promoter sequences.  
Lescot, M., Déhais, P., Moreau, Y., De Moor, B., Rouzé ,P.,and Rombauts, S.  
Nucleic Acids Res., Database issue(2002), 30(1):325-327.   


---

> 2018/04/13 10:10:12  
+ TATTTTAGTG TTTGGTGCTT TCAAATTTCA TTTACCTAAT TATTTAAGTA CATGTTTGAT CATTTTGTTG   
  
  
+ TTTCCAATTT AATTTAATTC AATCATAATA AAAAAAATTT GCTAATTATT TTATCATGAA AAGATTTAAA   
  
  
+ ATTTTTTAAG TATTGAATAT ATATATATAT ATATATTAAA ATGTTATGTT ATTAAAAATT TCAAAAAGTT   
  
  
+ ATTAAAAATA ACTCATAAAT TTACAAATTA TTAAAATTAA AAAATTAAAA TTAAAATTTG TTTAAATTAT   
  
  
+ TTTAAAAATA TTTCAATACT AAAAATATTT TTAATTCTTC ATCACCCTTT GATTTGAAAT CTAATTAGAT   
  
  
+ CAAATTAAAA TATTACCCAT AAAATTAATC CAGCCGATTA CACCAAAGAG TATAGTTCAG ATTTTTTTTT   
  
  
+ TCTTTTTGAT TATGCTAACA TGTAAAATTT TGACTTTCTA TTTTAAAGCC AAAAAAATAC AATTATGAAA   
  
  
+ GATAAAAATC CATAATAAAT TAAAAAACTC AAAATAAACA ATATTTACGA TACAACAAGG ATCAAAACAA   
  
  
+ TATAATATCC AAAAATTAGA CGCCACCATT AATAATAGAT AATGATTTGC AAAAGAAAAT ATCATTTAGA   
  
  
+ GTACTCCTGT CAAAATTTTG TCTTTTCTTT TCAGATATGA AAGAATGAGA CCCGGTTCAA AGATTTACAC   
  
  
+ TCTTAGACTT GGTCAGTTCA AATAATCCTT GTAGTTTAGT TAAACCCATT AATCAATTGG GTAGATTAAT   
  
  
+ CATAATTAGT CCCAAACCAA CCAGAATCGT GAAGAATCGT AAGTACCTGA TTATGGTAAA ATAATGAGAC   
  
  
+ TATGGTCTTG TCTTCTCTTC TGCAAGGCCT AAAAGAACTG CGGAGCACTA ACGCGGAAGA TTGATTTTGA   
  
  
+ AATTTCTTGG TCCACTGCTC TCCTGTGCTG TTTCACCTCT CGACTTTTCT TAATCTCCTC AATCATGTGA   
  
  
+ TGTCTTCAAA GGACCAATCA GAGAAGAGAG ACCAAGAAAG AGACCCTTTT TGAATGAAGA ATTGGTCAGC   
  
  
+ CTTTGGGATT TTCCACTCTC CCACTACTCT CTTTCTTTCA GCTACTTCGT GTTTATGGGA CCGAAGACCA   
  
  
+ GTCTTTGCTG AAAAACTCGG TTTCCATCAG TCAAACTTCC ACAAAATTTC TATCCTCCCA GAAGGGTACC   
  
  
+ CCAAAGGGCT TCGCTTTATA AGCCAAAGCT TCACTTTCCT TCTTGACGCA AGACAGATGC TTTTACTCTT   
  
  
+ CCTGCTCCTT CACACAAAGA ACCTTCATTT TCTCTCTTTT CATATTATCA GTCATATACT TCTCATTCCA   
  
  
+ GTAAATTTCA TCATTGTAGC TTGTTACCCT TTAAGATTCC ATTGTCTAAA GGGGTATAAT CTTCGTTGTC   
  
  
+ TGCTTCGTGG TGGAAAGGTT TTGCTGCTTC AGACGGGAAG TAGCATGTGC TTTCAGACAA GCTGAGATAC   
  
  
+ TGTATTGCTT GTAGCTCTTG GTCGTTGAA  

- ATAAAATCAC AAACCACGAA AGTTTAAAGT AAATGGATTA ATAAATTCAT GTACAAACTA GTAAAACAAC   
  
  
- AAAGGTTAAA TTAAATTAAG TTAGTATTAT TTTTTTTAAA CGATTAATAA AATAGTACTT TTCTAAATTT   
  
  
- TAAAAAATTC ATAACTTATA TATATATATA TATATAATTT TACAATACAA TAATTTTTAA AGTTTTTCAA   
  
  
- TAATTTTTAT TGAGTATTTA AATGTTTAAT AATTTTAATT TTTTAATTTT AATTTTAAAC AAATTTAATA   
  
  
- AAATTTTTAT AAAGTTATGA TTTTTATAAA AATTAAGAAG TAGTGGGAAA CTAAACTTTA GATTAATCTA   
  
  
- GTTTAATTTT ATAATGGGTA TTTTAATTAG GTCGGCTAAT GTGGTTTCTC ATATCAAGTC TAAAAAAAAA   
  
  
- AGAAAAACTA ATACGATTGT ACATTTTAAA ACTGAAAGAT AAAATTTCGG TTTTTTTATG TTAATACTTT   
  
  
- CTATTTTTAG GTATTATTTA ATTTTTTGAG TTTTATTTGT TATAAATGCT ATGTTGTTCC TAGTTTTGTT   
  
  
- ATATTATAGG TTTTTAATCT GCGGTGGTAA TTATTATCTA TTACTAAACG TTTTCTTTTA TAGTAAATCT   
  
  
- CATGAGGACA GTTTTAAAAC AGAAAAGAAA AGTCTATACT TTCTTACTCT GGGCCAAGTT TCTAAATGTG   
  
  
- AGAATCTGAA CCAGTCAAGT TTATTAGGAA CATCAAATCA ATTTGGGTAA TTAGTTAACC CATCTAATTA   
  
  
- GTATTAATCA GGGTTTGGTT GGTCTTAGCA CTTCTTAGCA TTCATGGACT AATACCATTT TATTACTCTG   
  
  
- ATACCAGAAC AGAAGAGAAG ACGTTCCGGA TTTTCTTGAC GCCTCGTGAT TGCGCCTTCT AACTAAAACT   
  
  
- TTAAAGAACC AGGTGACGAG AGGACACGAC AAAGTGGAGA GCTGAAAAGA ATTAGAGGAG TTAGTACACT   
  
  
- ACAGAAGTTT CCTGGTTAGT CTCTTCTCTC TGGTTCTTTC TCTGGGAAAA ACTTACTTCT TAACCAGTCG   
  
  
- GAAACCCTAA AAGGTGAGAG GGTGATGAGA GAAAGAAAGT CGATGAAGCA CAAATACCCT GGCTTCTGGT   
  
  
- CAGAAACGAC TTTTTGAGCC AAAGGTAGTC AGTTTGAAGG TGTTTTAAAG ATAGGAGGGT CTTCCCATGG   
  
  
- GGTTTCCCGA AGCGAAATAT TCGGTTTCGA AGTGAAAGGA AGAACTGCGT TCTGTCTACG AAAATGAGAA   
  
  
- GGACGAGGAA GTGTGTTTCT TGGAAGTAAA AGAGAGAAAA GTATAATAGT CAGTATATGA AGAGTAAGGT   
  
  
- CATTTAAAGT AGTAACATCG AACAATGGGA AATTCTAAGG TAACAGATTT CCCCATATTA GAAGCAACAG   
  
  
- ACGAAGCACC ACCTTTCCAA AACGACGAAG TCTGCCCTTC ATCGTACACG AAAGTCTGTT CGACTCTATG   
  
  
- ACATAACGAA CATCGAGAAC CAGCAACTT

  
  
Motifs Found  

+     5UTR Py-rich stretch

| Site Name | Organism | Position | Strand | Matrix score. | sequence | function |
| --- | --- | --- | --- | --- | --- | --- |
| 5UTR Py-rich stretch | Lycopersicon esculentum | 849 | + | 9 | TTTCTTCTCT | cis-acting element conferring high transcription levels |
| 5UTR Py-rich stretch | Lycopersicon esculentum | 1000 | - | 9 | TTTCTTCTCT | cis-acting element conferring high transcription levels |

> 2018/04/13 10:10:12  
+ TATTTTAGTG TTTGGTGCTT TCAAATTTCA TTTACCTAAT TATTTAAGTA CATGTTTGAT CATTTTGTTG   
  
  
+ TTTCCAATTT AATTTAATTC AATCATAATA AAAAAAATTT GCTAATTATT TTATCATGAA AAGATTTAAA   
  
  
+ ATTTTTTAAG TATTGAATAT ATATATATAT ATATATTAAA ATGTTATGTT ATTAAAAATT TCAAAAAGTT   
  
  
+ ATTAAAAATA ACTCATAAAT TTACAAATTA TTAAAATTAA AAAATTAAAA TTAAAATTTG TTTAAATTAT   
  
  
+ TTTAAAAATA TTTCAATACT AAAAATATTT TTAATTCTTC ATCACCCTTT GATTTGAAAT CTAATTAGAT   
  
  
+ CAAATTAAAA TATTACCCAT AAAATTAATC CAGCCGATTA CACCAAAGAG TATAGTTCAG ATTTTTTTTT   
  
  
+ TCTTTTTGAT TATGCTAACA TGTAAAATTT TGACTTTCTA TTTTAAAGCC AAAAAAATAC AATTATGAAA   
  
  
+ GATAAAAATC CATAATAAAT TAAAAAACTC AAAATAAACA ATATTTACGA TACAACAAGG ATCAAAACAA   
  
  
+ TATAATATCC AAAAATTAGA CGCCACCATT AATAATAGAT AATGATTTGC AAAAGAAAAT ATCATTTAGA   
  
  
+ GTACTCCTGT CAAAATTTTG TCTTTTCTTT TCAGATATGA AAGAATGAGA CCCGGTTCAA AGATTTACAC   
  
  
+ TCTTAGACTT GGTCAGTTCA AATAATCCTT GTAGTTTAGT TAAACCCATT AATCAATTGG GTAGATTAAT   
  
  
+ CATAATTAGT CCCAAACCAA CCAGAATCGT GAAGAATCGT AAGTACCTGA TTATGGTAAA ATAATGAGAC   
  
  
+ TATGGTCTTG TCTTCTCTTC TGCAAGGCCT AAAAGAACTG CGGAGCACTA ACGCGGAAGA TTGATTTTGA   
  
  
+ AATTTCTTGG TCCACTGCTC TCCTGTGCTG TTTCACCTCT CGACTTTTCT TAATCTCCTC AATCATGTGA   
  
  
+ TGTCTTCAAA GGACCAATCA GAGAAGAGAG ACCAAGAAAG AGACCCTTTT TGAATGAAGA ATTGGTCAGC   
  
  
+ CTTTGGGATT TTCCACTCTC CCACTACTCT CTTTCTTTCA GCTACTTCGT GTTTATGGGA CCGAAGACCA   
  
  
+ GTCTTTGCTG AAAAACTCGG TTTCCATCAG TCAAACTTCC ACAAAATTTC TATCCTCCCA GAAGGGTACC   
  
  
+ CCAAAGGGCT TCGCTTTATA AGCCAAAGCT TCACTTTCCT TCTTGACGCA AGACAGATGC TTTTACTCTT   
  
  
+ CCTGCTCCTT CACACAAAGA ACCTTCATTT TCTCTCTTTT CATATTATCA GTCATATACT TCTCATTCCA   
  
  
+ GTAAATTTCA TCATTGTAGC TTGTTACCCT TTAAGATTCC ATTGTCTAAA GGGGTATAAT CTTCGTTGTC   
  
  
+ TGCTTCGTGG TGGAAAGGTT TTGCTGCTTC AGACGGGAAG TAGCATGTGC TTTCAGACAA GCTGAGATAC   
  
  
+ TGTATTGCTT GTAGCTCTTG GTCGTTGAA  

- ATAAAATCAC AAACCACGAA AGTTTAAAGT AAATGGATTA ATAAATTCAT GTACAAACTA GTAAAACAAC   
  
  
- AAAGGTTAAA TTAAATTAAG TTAGTATTAT TTTTTTTAAA CGATTAATAA AATAGTACTT TTCTAAATTT   
  
  
- TAAAAAATTC ATAACTTATA TATATATATA TATATAATTT TACAATACAA TAATTTTTAA AGTTTTTCAA   
  
  
- TAATTTTTAT TGAGTATTTA AATGTTTAAT AATTTTAATT TTTTAATTTT AATTTTAAAC AAATTTAATA   
  
  
- AAATTTTTAT AAAGTTATGA TTTTTATAAA AATTAAGAAG TAGTGGGAAA CTAAACTTTA GATTAATCTA   
  
  
- GTTTAATTTT ATAATGGGTA TTTTAATTAG GTCGGCTAAT GTGGTTTCTC ATATCAAGTC TAAAAAAAAA   
  
  
- AGAAAAACTA ATACGATTGT ACATTTTAAA ACTGAAAGAT AAAATTTCGG TTTTTTTATG TTAATACTTT   
  
  
- CTATTTTTAG GTATTATTTA ATTTTTTGAG TTTTATTTGT TATAAATGCT ATGTTGTTCC TAGTTTTGTT   
  
  
- ATATTATAGG TTTTTAATCT GCGGTGGTAA TTATTATCTA TTACTAAACG TTTTCTTTTA TAGTAAATCT   
  
  
- CATGAGGACA GTTTTAAAAC AGAAAAGAAA AGTCTATACT TTCTTACTCT GGGCCAAGTT TCTAAATGTG   
  
  
- AGAATCTGAA CCAGTCAAGT TTATTAGGAA CATCAAATCA ATTTGGGTAA TTAGTTAACC CATCTAATTA   
  
  
- GTATTAATCA GGGTTTGGTT GGTCTTAGCA CTTCTTAGCA TTCATGGACT AATACCATTT TATTACTCTG   
  
  
- ATACCAGAAC AGAAGAGAAG ACGTTCCGGA TTTTCTTGAC GCCTCGTGAT TGCGCCTTCT AACTAAAACT   
  
  
- TTAAAGAACC AGGTGACGAG AGGACACGAC AAAGTGGAGA GCTGAAAAGA ATTAGAGGAG TTAGTACACT   
  
  
- ACAGAAGTTT CCTGGTTAGT CTCTTCTCTC TGGTTCTTTC TCTGGGAAAA ACTTACTTCT TAACCAGTCG   
  
  
- GAAACCCTAA AAGGTGAGAG GGTGATGAGA GAAAGAAAGT CGATGAAGCA CAAATACCCT GGCTTCTGGT   
  
  
- CAGAAACGAC TTTTTGAGCC AAAGGTAGTC AGTTTGAAGG TGTTTTAAAG ATAGGAGGGT CTTCCCATGG   
  
  
- GGTTTCCCGA AGCGAAATAT TCGGTTTCGA AGTGAAAGGA AGAACTGCGT TCTGTCTACG AAAATGAGAA   
  
  
- GGACGAGGAA GTGTGTTTCT TGGAAGTAAA AGAGAGAAAA GTATAATAGT CAGTATATGA AGAGTAAGGT   
  
  
- CATTTAAAGT AGTAACATCG AACAATGGGA AATTCTAAGG TAACAGATTT CCCCATATTA GAAGCAACAG   
  
  
- ACGAAGCACC ACCTTTCCAA AACGACGAAG TCTGCCCTTC ATCGTACACG AAAGTCTGTT CGACTCTATG   
  
  
- ACATAACGAA CATCGAGAAC CAGCAACTT

+     AAGAA-motif

| Site Name | Organism | Position | Strand | Matrix score. | sequence | function |
| --- | --- | --- | --- | --- | --- | --- |
| AAGAA-motif | Avena sativa | 669 | + | 7 | GAAAGAA |  |
| AAGAA-motif | Avena sativa | 1083 | - | 7 | GAAAGAA |  |

> 2018/04/13 10:10:12  
+ TATTTTAGTG TTTGGTGCTT TCAAATTTCA TTTACCTAAT TATTTAAGTA CATGTTTGAT CATTTTGTTG   
  
  
+ TTTCCAATTT AATTTAATTC AATCATAATA AAAAAAATTT GCTAATTATT TTATCATGAA AAGATTTAAA   
  
  
+ ATTTTTTAAG TATTGAATAT ATATATATAT ATATATTAAA ATGTTATGTT ATTAAAAATT TCAAAAAGTT   
  
  
+ ATTAAAAATA ACTCATAAAT TTACAAATTA TTAAAATTAA AAAATTAAAA TTAAAATTTG TTTAAATTAT   
  
  
+ TTTAAAAATA TTTCAATACT AAAAATATTT TTAATTCTTC ATCACCCTTT GATTTGAAAT CTAATTAGAT   
  
  
+ CAAATTAAAA TATTACCCAT AAAATTAATC CAGCCGATTA CACCAAAGAG TATAGTTCAG ATTTTTTTTT   
  
  
+ TCTTTTTGAT TATGCTAACA TGTAAAATTT TGACTTTCTA TTTTAAAGCC AAAAAAATAC AATTATGAAA   
  
  
+ GATAAAAATC CATAATAAAT TAAAAAACTC AAAATAAACA ATATTTACGA TACAACAAGG ATCAAAACAA   
  
  
+ TATAATATCC AAAAATTAGA CGCCACCATT AATAATAGAT AATGATTTGC AAAAGAAAAT ATCATTTAGA   
  
  
+ GTACTCCTGT CAAAATTTTG TCTTTTCTTT TCAGATATGA AAGAATGAGA CCCGGTTCAA AGATTTACAC   
  
  
+ TCTTAGACTT GGTCAGTTCA AATAATCCTT GTAGTTTAGT TAAACCCATT AATCAATTGG GTAGATTAAT   
  
  
+ CATAATTAGT CCCAAACCAA CCAGAATCGT GAAGAATCGT AAGTACCTGA TTATGGTAAA ATAATGAGAC   
  
  
+ TATGGTCTTG TCTTCTCTTC TGCAAGGCCT AAAAGAACTG CGGAGCACTA ACGCGGAAGA TTGATTTTGA   
  
  
+ AATTTCTTGG TCCACTGCTC TCCTGTGCTG TTTCACCTCT CGACTTTTCT TAATCTCCTC AATCATGTGA   
  
  
+ TGTCTTCAAA GGACCAATCA GAGAAGAGAG ACCAAGAAAG AGACCCTTTT TGAATGAAGA ATTGGTCAGC   
  
  
+ CTTTGGGATT TTCCACTCTC CCACTACTCT CTTTCTTTCA GCTACTTCGT GTTTATGGGA CCGAAGACCA   
  
  
+ GTCTTTGCTG AAAAACTCGG TTTCCATCAG TCAAACTTCC ACAAAATTTC TATCCTCCCA GAAGGGTACC   
  
  
+ CCAAAGGGCT TCGCTTTATA AGCCAAAGCT TCACTTTCCT TCTTGACGCA AGACAGATGC TTTTACTCTT   
  
  
+ CCTGCTCCTT CACACAAAGA ACCTTCATTT TCTCTCTTTT CATATTATCA GTCATATACT TCTCATTCCA   
  
  
+ GTAAATTTCA TCATTGTAGC TTGTTACCCT TTAAGATTCC ATTGTCTAAA GGGGTATAAT CTTCGTTGTC   
  
  
+ TGCTTCGTGG TGGAAAGGTT TTGCTGCTTC AGACGGGAAG TAGCATGTGC TTTCAGACAA GCTGAGATAC   
  
  
+ TGTATTGCTT GTAGCTCTTG GTCGTTGAA  

- ATAAAATCAC AAACCACGAA AGTTTAAAGT AAATGGATTA ATAAATTCAT GTACAAACTA GTAAAACAAC   
  
  
- AAAGGTTAAA TTAAATTAAG TTAGTATTAT TTTTTTTAAA CGATTAATAA AATAGTACTT TTCTAAATTT   
  
  
- TAAAAAATTC ATAACTTATA TATATATATA TATATAATTT TACAATACAA TAATTTTTAA AGTTTTTCAA   
  
  
- TAATTTTTAT TGAGTATTTA AATGTTTAAT AATTTTAATT TTTTAATTTT AATTTTAAAC AAATTTAATA   
  
  
- AAATTTTTAT AAAGTTATGA TTTTTATAAA AATTAAGAAG TAGTGGGAAA CTAAACTTTA GATTAATCTA   
  
  
- GTTTAATTTT ATAATGGGTA TTTTAATTAG GTCGGCTAAT GTGGTTTCTC ATATCAAGTC TAAAAAAAAA   
  
  
- AGAAAAACTA ATACGATTGT ACATTTTAAA ACTGAAAGAT AAAATTTCGG TTTTTTTATG TTAATACTTT   
  
  
- CTATTTTTAG GTATTATTTA ATTTTTTGAG TTTTATTTGT TATAAATGCT ATGTTGTTCC TAGTTTTGTT   
  
  
- ATATTATAGG TTTTTAATCT GCGGTGGTAA TTATTATCTA TTACTAAACG TTTTCTTTTA TAGTAAATCT   
  
  
- CATGAGGACA GTTTTAAAAC AGAAAAGAAA AGTCTATACT TTCTTACTCT GGGCCAAGTT TCTAAATGTG   
  
  
- AGAATCTGAA CCAGTCAAGT TTATTAGGAA CATCAAATCA ATTTGGGTAA TTAGTTAACC CATCTAATTA   
  
  
- GTATTAATCA GGGTTTGGTT GGTCTTAGCA CTTCTTAGCA TTCATGGACT AATACCATTT TATTACTCTG   
  
  
- ATACCAGAAC AGAAGAGAAG ACGTTCCGGA TTTTCTTGAC GCCTCGTGAT TGCGCCTTCT AACTAAAACT   
  
  
- TTAAAGAACC AGGTGACGAG AGGACACGAC AAAGTGGAGA GCTGAAAAGA ATTAGAGGAG TTAGTACACT   
  
  
- ACAGAAGTTT CCTGGTTAGT CTCTTCTCTC TGGTTCTTTC TCTGGGAAAA ACTTACTTCT TAACCAGTCG   
  
  
- GAAACCCTAA AAGGTGAGAG GGTGATGAGA GAAAGAAAGT CGATGAAGCA CAAATACCCT GGCTTCTGGT   
  
  
- CAGAAACGAC TTTTTGAGCC AAAGGTAGTC AGTTTGAAGG TGTTTTAAAG ATAGGAGGGT CTTCCCATGG   
  
  
- GGTTTCCCGA AGCGAAATAT TCGGTTTCGA AGTGAAAGGA AGAACTGCGT TCTGTCTACG AAAATGAGAA   
  
  
- GGACGAGGAA GTGTGTTTCT TGGAAGTAAA AGAGAGAAAA GTATAATAGT CAGTATATGA AGAGTAAGGT   
  
  
- CATTTAAAGT AGTAACATCG AACAATGGGA AATTCTAAGG TAACAGATTT CCCCATATTA GAAGCAACAG   
  
  
- ACGAAGCACC ACCTTTCCAA AACGACGAAG TCTGCCCTTC ATCGTACACG AAAGTCTGTT CGACTCTATG   
  
  
- ACATAACGAA CATCGAGAAC CAGCAACTT

+     ARE

| Site Name | Organism | Position | Strand | Matrix score. | sequence | function |
| --- | --- | --- | --- | --- | --- | --- |
| ARE | Zea mays | 784 | - | 6 | TGGTTT | cis-acting regulatory element essential for the anaerobic induction |

> 2018/04/13 10:10:12  
+ TATTTTAGTG TTTGGTGCTT TCAAATTTCA TTTACCTAAT TATTTAAGTA CATGTTTGAT CATTTTGTTG   
  
  
+ TTTCCAATTT AATTTAATTC AATCATAATA AAAAAAATTT GCTAATTATT TTATCATGAA AAGATTTAAA   
  
  
+ ATTTTTTAAG TATTGAATAT ATATATATAT ATATATTAAA ATGTTATGTT ATTAAAAATT TCAAAAAGTT   
  
  
+ ATTAAAAATA ACTCATAAAT TTACAAATTA TTAAAATTAA AAAATTAAAA TTAAAATTTG TTTAAATTAT   
  
  
+ TTTAAAAATA TTTCAATACT AAAAATATTT TTAATTCTTC ATCACCCTTT GATTTGAAAT CTAATTAGAT   
  
  
+ CAAATTAAAA TATTACCCAT AAAATTAATC CAGCCGATTA CACCAAAGAG TATAGTTCAG ATTTTTTTTT   
  
  
+ TCTTTTTGAT TATGCTAACA TGTAAAATTT TGACTTTCTA TTTTAAAGCC AAAAAAATAC AATTATGAAA   
  
  
+ GATAAAAATC CATAATAAAT TAAAAAACTC AAAATAAACA ATATTTACGA TACAACAAGG ATCAAAACAA   
  
  
+ TATAATATCC AAAAATTAGA CGCCACCATT AATAATAGAT AATGATTTGC AAAAGAAAAT ATCATTTAGA   
  
  
+ GTACTCCTGT CAAAATTTTG TCTTTTCTTT TCAGATATGA AAGAATGAGA CCCGGTTCAA AGATTTACAC   
  
  
+ TCTTAGACTT GGTCAGTTCA AATAATCCTT GTAGTTTAGT TAAACCCATT AATCAATTGG GTAGATTAAT   
  
  
+ CATAATTAGT CCCAAACCAA CCAGAATCGT GAAGAATCGT AAGTACCTGA TTATGGTAAA ATAATGAGAC   
  
  
+ TATGGTCTTG TCTTCTCTTC TGCAAGGCCT AAAAGAACTG CGGAGCACTA ACGCGGAAGA TTGATTTTGA   
  
  
+ AATTTCTTGG TCCACTGCTC TCCTGTGCTG TTTCACCTCT CGACTTTTCT TAATCTCCTC AATCATGTGA   
  
  
+ TGTCTTCAAA GGACCAATCA GAGAAGAGAG ACCAAGAAAG AGACCCTTTT TGAATGAAGA ATTGGTCAGC   
  
  
+ CTTTGGGATT TTCCACTCTC CCACTACTCT CTTTCTTTCA GCTACTTCGT GTTTATGGGA CCGAAGACCA   
  
  
+ GTCTTTGCTG AAAAACTCGG TTTCCATCAG TCAAACTTCC ACAAAATTTC TATCCTCCCA GAAGGGTACC   
  
  
+ CCAAAGGGCT TCGCTTTATA AGCCAAAGCT TCACTTTCCT TCTTGACGCA AGACAGATGC TTTTACTCTT   
  
  
+ CCTGCTCCTT CACACAAAGA ACCTTCATTT TCTCTCTTTT CATATTATCA GTCATATACT TCTCATTCCA   
  
  
+ GTAAATTTCA TCATTGTAGC TTGTTACCCT TTAAGATTCC ATTGTCTAAA GGGGTATAAT CTTCGTTGTC   
  
  
+ TGCTTCGTGG TGGAAAGGTT TTGCTGCTTC AGACGGGAAG TAGCATGTGC TTTCAGACAA GCTGAGATAC   
  
  
+ TGTATTGCTT GTAGCTCTTG GTCGTTGAA  

- ATAAAATCAC AAACCACGAA AGTTTAAAGT AAATGGATTA ATAAATTCAT GTACAAACTA GTAAAACAAC   
  
  
- AAAGGTTAAA TTAAATTAAG TTAGTATTAT TTTTTTTAAA CGATTAATAA AATAGTACTT TTCTAAATTT   
  
  
- TAAAAAATTC ATAACTTATA TATATATATA TATATAATTT TACAATACAA TAATTTTTAA AGTTTTTCAA   
  
  
- TAATTTTTAT TGAGTATTTA AATGTTTAAT AATTTTAATT TTTTAATTTT AATTTTAAAC AAATTTAATA   
  
  
- AAATTTTTAT AAAGTTATGA TTTTTATAAA AATTAAGAAG TAGTGGGAAA CTAAACTTTA GATTAATCTA   
  
  
- GTTTAATTTT ATAATGGGTA TTTTAATTAG GTCGGCTAAT GTGGTTTCTC ATATCAAGTC TAAAAAAAAA   
  
  
- AGAAAAACTA ATACGATTGT ACATTTTAAA ACTGAAAGAT AAAATTTCGG TTTTTTTATG TTAATACTTT   
  
  
- CTATTTTTAG GTATTATTTA ATTTTTTGAG TTTTATTTGT TATAAATGCT ATGTTGTTCC TAGTTTTGTT   
  
  
- ATATTATAGG TTTTTAATCT GCGGTGGTAA TTATTATCTA TTACTAAACG TTTTCTTTTA TAGTAAATCT   
  
  
- CATGAGGACA GTTTTAAAAC AGAAAAGAAA AGTCTATACT TTCTTACTCT GGGCCAAGTT TCTAAATGTG   
  
  
- AGAATCTGAA CCAGTCAAGT TTATTAGGAA CATCAAATCA ATTTGGGTAA TTAGTTAACC CATCTAATTA   
  
  
- GTATTAATCA GGGTTTGGTT GGTCTTAGCA CTTCTTAGCA TTCATGGACT AATACCATTT TATTACTCTG   
  
  
- ATACCAGAAC AGAAGAGAAG ACGTTCCGGA TTTTCTTGAC GCCTCGTGAT TGCGCCTTCT AACTAAAACT   
  
  
- TTAAAGAACC AGGTGACGAG AGGACACGAC AAAGTGGAGA GCTGAAAAGA ATTAGAGGAG TTAGTACACT   
  
  
- ACAGAAGTTT CCTGGTTAGT CTCTTCTCTC TGGTTCTTTC TCTGGGAAAA ACTTACTTCT TAACCAGTCG   
  
  
- GAAACCCTAA AAGGTGAGAG GGTGATGAGA GAAAGAAAGT CGATGAAGCA CAAATACCCT GGCTTCTGGT   
  
  
- CAGAAACGAC TTTTTGAGCC AAAGGTAGTC AGTTTGAAGG TGTTTTAAAG ATAGGAGGGT CTTCCCATGG   
  
  
- GGTTTCCCGA AGCGAAATAT TCGGTTTCGA AGTGAAAGGA AGAACTGCGT TCTGTCTACG AAAATGAGAA   
  
  
- GGACGAGGAA GTGTGTTTCT TGGAAGTAAA AGAGAGAAAA GTATAATAGT CAGTATATGA AGAGTAAGGT   
  
  
- CATTTAAAGT AGTAACATCG AACAATGGGA AATTCTAAGG TAACAGATTT CCCCATATTA GAAGCAACAG   
  
  
- ACGAAGCACC ACCTTTCCAA AACGACGAAG TCTGCCCTTC ATCGTACACG AAAGTCTGTT CGACTCTATG   
  
  
- ACATAACGAA CATCGAGAAC CAGCAACTT

+     Box 4

| Site Name | Organism | Position | Strand | Matrix score. | sequence | function |
| --- | --- | --- | --- | --- | --- | --- |
| Box 4 | Petroselinum crispum | 374 | + | 6 | ATTAAT | part of a conserved DNA module involved in light responsiveness |
| Box 4 | Petroselinum crispum | 748 | + | 6 | ATTAAT | part of a conserved DNA module involved in light responsiveness |
| Box 4 | Petroselinum crispum | 588 | + | 6 | ATTAAT | part of a conserved DNA module involved in light responsiveness |
| Box 4 | Petroselinum crispum | 765 | - | 6 | ATTAAT | part of a conserved DNA module involved in light responsiveness |

> 2018/04/13 10:10:12  
+ TATTTTAGTG TTTGGTGCTT TCAAATTTCA TTTACCTAAT TATTTAAGTA CATGTTTGAT CATTTTGTTG   
  
  
+ TTTCCAATTT AATTTAATTC AATCATAATA AAAAAAATTT GCTAATTATT TTATCATGAA AAGATTTAAA   
  
  
+ ATTTTTTAAG TATTGAATAT ATATATATAT ATATATTAAA ATGTTATGTT ATTAAAAATT TCAAAAAGTT   
  
  
+ ATTAAAAATA ACTCATAAAT TTACAAATTA TTAAAATTAA AAAATTAAAA TTAAAATTTG TTTAAATTAT   
  
  
+ TTTAAAAATA TTTCAATACT AAAAATATTT TTAATTCTTC ATCACCCTTT GATTTGAAAT CTAATTAGAT   
  
  
+ CAAATTAAAA TATTACCCAT AAAATTAATC CAGCCGATTA CACCAAAGAG TATAGTTCAG ATTTTTTTTT   
  
  
+ TCTTTTTGAT TATGCTAACA TGTAAAATTT TGACTTTCTA TTTTAAAGCC AAAAAAATAC AATTATGAAA   
  
  
+ GATAAAAATC CATAATAAAT TAAAAAACTC AAAATAAACA ATATTTACGA TACAACAAGG ATCAAAACAA   
  
  
+ TATAATATCC AAAAATTAGA CGCCACCATT AATAATAGAT AATGATTTGC AAAAGAAAAT ATCATTTAGA   
  
  
+ GTACTCCTGT CAAAATTTTG TCTTTTCTTT TCAGATATGA AAGAATGAGA CCCGGTTCAA AGATTTACAC   
  
  
+ TCTTAGACTT GGTCAGTTCA AATAATCCTT GTAGTTTAGT TAAACCCATT AATCAATTGG GTAGATTAAT   
  
  
+ CATAATTAGT CCCAAACCAA CCAGAATCGT GAAGAATCGT AAGTACCTGA TTATGGTAAA ATAATGAGAC   
  
  
+ TATGGTCTTG TCTTCTCTTC TGCAAGGCCT AAAAGAACTG CGGAGCACTA ACGCGGAAGA TTGATTTTGA   
  
  
+ AATTTCTTGG TCCACTGCTC TCCTGTGCTG TTTCACCTCT CGACTTTTCT TAATCTCCTC AATCATGTGA   
  
  
+ TGTCTTCAAA GGACCAATCA GAGAAGAGAG ACCAAGAAAG AGACCCTTTT TGAATGAAGA ATTGGTCAGC   
  
  
+ CTTTGGGATT TTCCACTCTC CCACTACTCT CTTTCTTTCA GCTACTTCGT GTTTATGGGA CCGAAGACCA   
  
  
+ GTCTTTGCTG AAAAACTCGG TTTCCATCAG TCAAACTTCC ACAAAATTTC TATCCTCCCA GAAGGGTACC   
  
  
+ CCAAAGGGCT TCGCTTTATA AGCCAAAGCT TCACTTTCCT TCTTGACGCA AGACAGATGC TTTTACTCTT   
  
  
+ CCTGCTCCTT CACACAAAGA ACCTTCATTT TCTCTCTTTT CATATTATCA GTCATATACT TCTCATTCCA   
  
  
+ GTAAATTTCA TCATTGTAGC TTGTTACCCT TTAAGATTCC ATTGTCTAAA GGGGTATAAT CTTCGTTGTC   
  
  
+ TGCTTCGTGG TGGAAAGGTT TTGCTGCTTC AGACGGGAAG TAGCATGTGC TTTCAGACAA GCTGAGATAC   
  
  
+ TGTATTGCTT GTAGCTCTTG GTCGTTGAA  

- ATAAAATCAC AAACCACGAA AGTTTAAAGT AAATGGATTA ATAAATTCAT GTACAAACTA GTAAAACAAC   
  
  
- AAAGGTTAAA TTAAATTAAG TTAGTATTAT TTTTTTTAAA CGATTAATAA AATAGTACTT TTCTAAATTT   
  
  
- TAAAAAATTC ATAACTTATA TATATATATA TATATAATTT TACAATACAA TAATTTTTAA AGTTTTTCAA   
  
  
- TAATTTTTAT TGAGTATTTA AATGTTTAAT AATTTTAATT TTTTAATTTT AATTTTAAAC AAATTTAATA   
  
  
- AAATTTTTAT AAAGTTATGA TTTTTATAAA AATTAAGAAG TAGTGGGAAA CTAAACTTTA GATTAATCTA   
  
  
- GTTTAATTTT ATAATGGGTA TTTTAATTAG GTCGGCTAAT GTGGTTTCTC ATATCAAGTC TAAAAAAAAA   
  
  
- AGAAAAACTA ATACGATTGT ACATTTTAAA ACTGAAAGAT AAAATTTCGG TTTTTTTATG TTAATACTTT   
  
  
- CTATTTTTAG GTATTATTTA ATTTTTTGAG TTTTATTTGT TATAAATGCT ATGTTGTTCC TAGTTTTGTT   
  
  
- ATATTATAGG TTTTTAATCT GCGGTGGTAA TTATTATCTA TTACTAAACG TTTTCTTTTA TAGTAAATCT   
  
  
- CATGAGGACA GTTTTAAAAC AGAAAAGAAA AGTCTATACT TTCTTACTCT GGGCCAAGTT TCTAAATGTG   
  
  
- AGAATCTGAA CCAGTCAAGT TTATTAGGAA CATCAAATCA ATTTGGGTAA TTAGTTAACC CATCTAATTA   
  
  
- GTATTAATCA GGGTTTGGTT GGTCTTAGCA CTTCTTAGCA TTCATGGACT AATACCATTT TATTACTCTG   
  
  
- ATACCAGAAC AGAAGAGAAG ACGTTCCGGA TTTTCTTGAC GCCTCGTGAT TGCGCCTTCT AACTAAAACT   
  
  
- TTAAAGAACC AGGTGACGAG AGGACACGAC AAAGTGGAGA GCTGAAAAGA ATTAGAGGAG TTAGTACACT   
  
  
- ACAGAAGTTT CCTGGTTAGT CTCTTCTCTC TGGTTCTTTC TCTGGGAAAA ACTTACTTCT TAACCAGTCG   
  
  
- GAAACCCTAA AAGGTGAGAG GGTGATGAGA GAAAGAAAGT CGATGAAGCA CAAATACCCT GGCTTCTGGT   
  
  
- CAGAAACGAC TTTTTGAGCC AAAGGTAGTC AGTTTGAAGG TGTTTTAAAG ATAGGAGGGT CTTCCCATGG   
  
  
- GGTTTCCCGA AGCGAAATAT TCGGTTTCGA AGTGAAAGGA AGAACTGCGT TCTGTCTACG AAAATGAGAA   
  
  
- GGACGAGGAA GTGTGTTTCT TGGAAGTAAA AGAGAGAAAA GTATAATAGT CAGTATATGA AGAGTAAGGT   
  
  
- CATTTAAAGT AGTAACATCG AACAATGGGA AATTCTAAGG TAACAGATTT CCCCATATTA GAAGCAACAG   
  
  
- ACGAAGCACC ACCTTTCCAA AACGACGAAG TCTGCCCTTC ATCGTACACG AAAGTCTGTT CGACTCTATG   
  
  
- ACATAACGAA CATCGAGAAC CAGCAACTT

+     Box I

| Site Name | Organism | Position | Strand | Matrix score. | sequence | function |
| --- | --- | --- | --- | --- | --- | --- |
| Box I | Pisum sativum | 19 | + | 7 | TTTCAAA | light responsive element |
| Box I | Pisum sativum | 333 | - | 7 | TTTCAAA | light responsive element |
| Box I | Pisum sativum | 199 | + | 7 | TTTCAAA | light responsive element |
| Box I | Pisum sativum | 906 | - | 7 | TTTCAAA | light responsive element |

> 2018/04/13 10:10:12  
+ TATTTTAGTG TTTGGTGCTT TCAAATTTCA TTTACCTAAT TATTTAAGTA CATGTTTGAT CATTTTGTTG   
  
  
+ TTTCCAATTT AATTTAATTC AATCATAATA AAAAAAATTT GCTAATTATT TTATCATGAA AAGATTTAAA   
  
  
+ ATTTTTTAAG TATTGAATAT ATATATATAT ATATATTAAA ATGTTATGTT ATTAAAAATT TCAAAAAGTT   
  
  
+ ATTAAAAATA ACTCATAAAT TTACAAATTA TTAAAATTAA AAAATTAAAA TTAAAATTTG TTTAAATTAT   
  
  
+ TTTAAAAATA TTTCAATACT AAAAATATTT TTAATTCTTC ATCACCCTTT GATTTGAAAT CTAATTAGAT   
  
  
+ CAAATTAAAA TATTACCCAT AAAATTAATC CAGCCGATTA CACCAAAGAG TATAGTTCAG ATTTTTTTTT   
  
  
+ TCTTTTTGAT TATGCTAACA TGTAAAATTT TGACTTTCTA TTTTAAAGCC AAAAAAATAC AATTATGAAA   
  
  
+ GATAAAAATC CATAATAAAT TAAAAAACTC AAAATAAACA ATATTTACGA TACAACAAGG ATCAAAACAA   
  
  
+ TATAATATCC AAAAATTAGA CGCCACCATT AATAATAGAT AATGATTTGC AAAAGAAAAT ATCATTTAGA   
  
  
+ GTACTCCTGT CAAAATTTTG TCTTTTCTTT TCAGATATGA AAGAATGAGA CCCGGTTCAA AGATTTACAC   
  
  
+ TCTTAGACTT GGTCAGTTCA AATAATCCTT GTAGTTTAGT TAAACCCATT AATCAATTGG GTAGATTAAT   
  
  
+ CATAATTAGT CCCAAACCAA CCAGAATCGT GAAGAATCGT AAGTACCTGA TTATGGTAAA ATAATGAGAC   
  
  
+ TATGGTCTTG TCTTCTCTTC TGCAAGGCCT AAAAGAACTG CGGAGCACTA ACGCGGAAGA TTGATTTTGA   
  
  
+ AATTTCTTGG TCCACTGCTC TCCTGTGCTG TTTCACCTCT CGACTTTTCT TAATCTCCTC AATCATGTGA   
  
  
+ TGTCTTCAAA GGACCAATCA GAGAAGAGAG ACCAAGAAAG AGACCCTTTT TGAATGAAGA ATTGGTCAGC   
  
  
+ CTTTGGGATT TTCCACTCTC CCACTACTCT CTTTCTTTCA GCTACTTCGT GTTTATGGGA CCGAAGACCA   
  
  
+ GTCTTTGCTG AAAAACTCGG TTTCCATCAG TCAAACTTCC ACAAAATTTC TATCCTCCCA GAAGGGTACC   
  
  
+ CCAAAGGGCT TCGCTTTATA AGCCAAAGCT TCACTTTCCT TCTTGACGCA AGACAGATGC TTTTACTCTT   
  
  
+ CCTGCTCCTT CACACAAAGA ACCTTCATTT TCTCTCTTTT CATATTATCA GTCATATACT TCTCATTCCA   
  
  
+ GTAAATTTCA TCATTGTAGC TTGTTACCCT TTAAGATTCC ATTGTCTAAA GGGGTATAAT CTTCGTTGTC   
  
  
+ TGCTTCGTGG TGGAAAGGTT TTGCTGCTTC AGACGGGAAG TAGCATGTGC TTTCAGACAA GCTGAGATAC   
  
  
+ TGTATTGCTT GTAGCTCTTG GTCGTTGAA  

- ATAAAATCAC AAACCACGAA AGTTTAAAGT AAATGGATTA ATAAATTCAT GTACAAACTA GTAAAACAAC   
  
  
- AAAGGTTAAA TTAAATTAAG TTAGTATTAT TTTTTTTAAA CGATTAATAA AATAGTACTT TTCTAAATTT   
  
  
- TAAAAAATTC ATAACTTATA TATATATATA TATATAATTT TACAATACAA TAATTTTTAA AGTTTTTCAA   
  
  
- TAATTTTTAT TGAGTATTTA AATGTTTAAT AATTTTAATT TTTTAATTTT AATTTTAAAC AAATTTAATA   
  
  
- AAATTTTTAT AAAGTTATGA TTTTTATAAA AATTAAGAAG TAGTGGGAAA CTAAACTTTA GATTAATCTA   
  
  
- GTTTAATTTT ATAATGGGTA TTTTAATTAG GTCGGCTAAT GTGGTTTCTC ATATCAAGTC TAAAAAAAAA   
  
  
- AGAAAAACTA ATACGATTGT ACATTTTAAA ACTGAAAGAT AAAATTTCGG TTTTTTTATG TTAATACTTT   
  
  
- CTATTTTTAG GTATTATTTA ATTTTTTGAG TTTTATTTGT TATAAATGCT ATGTTGTTCC TAGTTTTGTT   
  
  
- ATATTATAGG TTTTTAATCT GCGGTGGTAA TTATTATCTA TTACTAAACG TTTTCTTTTA TAGTAAATCT   
  
  
- CATGAGGACA GTTTTAAAAC AGAAAAGAAA AGTCTATACT TTCTTACTCT GGGCCAAGTT TCTAAATGTG   
  
  
- AGAATCTGAA CCAGTCAAGT TTATTAGGAA CATCAAATCA ATTTGGGTAA TTAGTTAACC CATCTAATTA   
  
  
- GTATTAATCA GGGTTTGGTT GGTCTTAGCA CTTCTTAGCA TTCATGGACT AATACCATTT TATTACTCTG   
  
  
- ATACCAGAAC AGAAGAGAAG ACGTTCCGGA TTTTCTTGAC GCCTCGTGAT TGCGCCTTCT AACTAAAACT   
  
  
- TTAAAGAACC AGGTGACGAG AGGACACGAC AAAGTGGAGA GCTGAAAAGA ATTAGAGGAG TTAGTACACT   
  
  
- ACAGAAGTTT CCTGGTTAGT CTCTTCTCTC TGGTTCTTTC TCTGGGAAAA ACTTACTTCT TAACCAGTCG   
  
  
- GAAACCCTAA AAGGTGAGAG GGTGATGAGA GAAAGAAAGT CGATGAAGCA CAAATACCCT GGCTTCTGGT   
  
  
- CAGAAACGAC TTTTTGAGCC AAAGGTAGTC AGTTTGAAGG TGTTTTAAAG ATAGGAGGGT CTTCCCATGG   
  
  
- GGTTTCCCGA AGCGAAATAT TCGGTTTCGA AGTGAAAGGA AGAACTGCGT TCTGTCTACG AAAATGAGAA   
  
  
- GGACGAGGAA GTGTGTTTCT TGGAAGTAAA AGAGAGAAAA GTATAATAGT CAGTATATGA AGAGTAAGGT   
  
  
- CATTTAAAGT AGTAACATCG AACAATGGGA AATTCTAAGG TAACAGATTT CCCCATATTA GAAGCAACAG   
  
  
- ACGAAGCACC ACCTTTCCAA AACGACGAAG TCTGCCCTTC ATCGTACACG AAAGTCTGTT CGACTCTATG   
  
  
- ACATAACGAA CATCGAGAAC CAGCAACTT

+     Box II

| Site Name | Organism | Position | Strand | Matrix score. | sequence | function |
| --- | --- | --- | --- | --- | --- | --- |
| Box II | Nicotiana plumbaginifolia | 560 | - | 12 | GTGGATATTATAT | part of a conserved DNA module involved in light responsiveness |

> 2018/04/13 10:10:12  
+ TATTTTAGTG TTTGGTGCTT TCAAATTTCA TTTACCTAAT TATTTAAGTA CATGTTTGAT CATTTTGTTG   
  
  
+ TTTCCAATTT AATTTAATTC AATCATAATA AAAAAAATTT GCTAATTATT TTATCATGAA AAGATTTAAA   
  
  
+ ATTTTTTAAG TATTGAATAT ATATATATAT ATATATTAAA ATGTTATGTT ATTAAAAATT TCAAAAAGTT   
  
  
+ ATTAAAAATA ACTCATAAAT TTACAAATTA TTAAAATTAA AAAATTAAAA TTAAAATTTG TTTAAATTAT   
  
  
+ TTTAAAAATA TTTCAATACT AAAAATATTT TTAATTCTTC ATCACCCTTT GATTTGAAAT CTAATTAGAT   
  
  
+ CAAATTAAAA TATTACCCAT AAAATTAATC CAGCCGATTA CACCAAAGAG TATAGTTCAG ATTTTTTTTT   
  
  
+ TCTTTTTGAT TATGCTAACA TGTAAAATTT TGACTTTCTA TTTTAAAGCC AAAAAAATAC AATTATGAAA   
  
  
+ GATAAAAATC CATAATAAAT TAAAAAACTC AAAATAAACA ATATTTACGA TACAACAAGG ATCAAAACAA   
  
  
+ TATAATATCC AAAAATTAGA CGCCACCATT AATAATAGAT AATGATTTGC AAAAGAAAAT ATCATTTAGA   
  
  
+ GTACTCCTGT CAAAATTTTG TCTTTTCTTT TCAGATATGA AAGAATGAGA CCCGGTTCAA AGATTTACAC   
  
  
+ TCTTAGACTT GGTCAGTTCA AATAATCCTT GTAGTTTAGT TAAACCCATT AATCAATTGG GTAGATTAAT   
  
  
+ CATAATTAGT CCCAAACCAA CCAGAATCGT GAAGAATCGT AAGTACCTGA TTATGGTAAA ATAATGAGAC   
  
  
+ TATGGTCTTG TCTTCTCTTC TGCAAGGCCT AAAAGAACTG CGGAGCACTA ACGCGGAAGA TTGATTTTGA   
  
  
+ AATTTCTTGG TCCACTGCTC TCCTGTGCTG TTTCACCTCT CGACTTTTCT TAATCTCCTC AATCATGTGA   
  
  
+ TGTCTTCAAA GGACCAATCA GAGAAGAGAG ACCAAGAAAG AGACCCTTTT TGAATGAAGA ATTGGTCAGC   
  
  
+ CTTTGGGATT TTCCACTCTC CCACTACTCT CTTTCTTTCA GCTACTTCGT GTTTATGGGA CCGAAGACCA   
  
  
+ GTCTTTGCTG AAAAACTCGG TTTCCATCAG TCAAACTTCC ACAAAATTTC TATCCTCCCA GAAGGGTACC   
  
  
+ CCAAAGGGCT TCGCTTTATA AGCCAAAGCT TCACTTTCCT TCTTGACGCA AGACAGATGC TTTTACTCTT   
  
  
+ CCTGCTCCTT CACACAAAGA ACCTTCATTT TCTCTCTTTT CATATTATCA GTCATATACT TCTCATTCCA   
  
  
+ GTAAATTTCA TCATTGTAGC TTGTTACCCT TTAAGATTCC ATTGTCTAAA GGGGTATAAT CTTCGTTGTC   
  
  
+ TGCTTCGTGG TGGAAAGGTT TTGCTGCTTC AGACGGGAAG TAGCATGTGC TTTCAGACAA GCTGAGATAC   
  
  
+ TGTATTGCTT GTAGCTCTTG GTCGTTGAA  

- ATAAAATCAC AAACCACGAA AGTTTAAAGT AAATGGATTA ATAAATTCAT GTACAAACTA GTAAAACAAC   
  
  
- AAAGGTTAAA TTAAATTAAG TTAGTATTAT TTTTTTTAAA CGATTAATAA AATAGTACTT TTCTAAATTT   
  
  
- TAAAAAATTC ATAACTTATA TATATATATA TATATAATTT TACAATACAA TAATTTTTAA AGTTTTTCAA   
  
  
- TAATTTTTAT TGAGTATTTA AATGTTTAAT AATTTTAATT TTTTAATTTT AATTTTAAAC AAATTTAATA   
  
  
- AAATTTTTAT AAAGTTATGA TTTTTATAAA AATTAAGAAG TAGTGGGAAA CTAAACTTTA GATTAATCTA   
  
  
- GTTTAATTTT ATAATGGGTA TTTTAATTAG GTCGGCTAAT GTGGTTTCTC ATATCAAGTC TAAAAAAAAA   
  
  
- AGAAAAACTA ATACGATTGT ACATTTTAAA ACTGAAAGAT AAAATTTCGG TTTTTTTATG TTAATACTTT   
  
  
- CTATTTTTAG GTATTATTTA ATTTTTTGAG TTTTATTTGT TATAAATGCT ATGTTGTTCC TAGTTTTGTT   
  
  
- ATATTATAGG TTTTTAATCT GCGGTGGTAA TTATTATCTA TTACTAAACG TTTTCTTTTA TAGTAAATCT   
  
  
- CATGAGGACA GTTTTAAAAC AGAAAAGAAA AGTCTATACT TTCTTACTCT GGGCCAAGTT TCTAAATGTG   
  
  
- AGAATCTGAA CCAGTCAAGT TTATTAGGAA CATCAAATCA ATTTGGGTAA TTAGTTAACC CATCTAATTA   
  
  
- GTATTAATCA GGGTTTGGTT GGTCTTAGCA CTTCTTAGCA TTCATGGACT AATACCATTT TATTACTCTG   
  
  
- ATACCAGAAC AGAAGAGAAG ACGTTCCGGA TTTTCTTGAC GCCTCGTGAT TGCGCCTTCT AACTAAAACT   
  
  
- TTAAAGAACC AGGTGACGAG AGGACACGAC AAAGTGGAGA GCTGAAAAGA ATTAGAGGAG TTAGTACACT   
  
  
- ACAGAAGTTT CCTGGTTAGT CTCTTCTCTC TGGTTCTTTC TCTGGGAAAA ACTTACTTCT TAACCAGTCG   
  
  
- GAAACCCTAA AAGGTGAGAG GGTGATGAGA GAAAGAAAGT CGATGAAGCA CAAATACCCT GGCTTCTGGT   
  
  
- CAGAAACGAC TTTTTGAGCC AAAGGTAGTC AGTTTGAAGG TGTTTTAAAG ATAGGAGGGT CTTCCCATGG   
  
  
- GGTTTCCCGA AGCGAAATAT TCGGTTTCGA AGTGAAAGGA AGAACTGCGT TCTGTCTACG AAAATGAGAA   
  
  
- GGACGAGGAA GTGTGTTTCT TGGAAGTAAA AGAGAGAAAA GTATAATAGT CAGTATATGA AGAGTAAGGT   
  
  
- CATTTAAAGT AGTAACATCG AACAATGGGA AATTCTAAGG TAACAGATTT CCCCATATTA GAAGCAACAG   
  
  
- ACGAAGCACC ACCTTTCCAA AACGACGAAG TCTGCCCTTC ATCGTACACG AAAGTCTGTT CGACTCTATG   
  
  
- ACATAACGAA CATCGAGAAC CAGCAACTT

+     Box III

| Site Name | Organism | Position | Strand | Matrix score. | sequence | function |
| --- | --- | --- | --- | --- | --- | --- |
| Box III | Pisum sativum | 692 | + | 9 | CATTTACACT | protein binding site |

> 2018/04/13 10:10:12  
+ TATTTTAGTG TTTGGTGCTT TCAAATTTCA TTTACCTAAT TATTTAAGTA CATGTTTGAT CATTTTGTTG   
  
  
+ TTTCCAATTT AATTTAATTC AATCATAATA AAAAAAATTT GCTAATTATT TTATCATGAA AAGATTTAAA   
  
  
+ ATTTTTTAAG TATTGAATAT ATATATATAT ATATATTAAA ATGTTATGTT ATTAAAAATT TCAAAAAGTT   
  
  
+ ATTAAAAATA ACTCATAAAT TTACAAATTA TTAAAATTAA AAAATTAAAA TTAAAATTTG TTTAAATTAT   
  
  
+ TTTAAAAATA TTTCAATACT AAAAATATTT TTAATTCTTC ATCACCCTTT GATTTGAAAT CTAATTAGAT   
  
  
+ CAAATTAAAA TATTACCCAT AAAATTAATC CAGCCGATTA CACCAAAGAG TATAGTTCAG ATTTTTTTTT   
  
  
+ TCTTTTTGAT TATGCTAACA TGTAAAATTT TGACTTTCTA TTTTAAAGCC AAAAAAATAC AATTATGAAA   
  
  
+ GATAAAAATC CATAATAAAT TAAAAAACTC AAAATAAACA ATATTTACGA TACAACAAGG ATCAAAACAA   
  
  
+ TATAATATCC AAAAATTAGA CGCCACCATT AATAATAGAT AATGATTTGC AAAAGAAAAT ATCATTTAGA   
  
  
+ GTACTCCTGT CAAAATTTTG TCTTTTCTTT TCAGATATGA AAGAATGAGA CCCGGTTCAA AGATTTACAC   
  
  
+ TCTTAGACTT GGTCAGTTCA AATAATCCTT GTAGTTTAGT TAAACCCATT AATCAATTGG GTAGATTAAT   
  
  
+ CATAATTAGT CCCAAACCAA CCAGAATCGT GAAGAATCGT AAGTACCTGA TTATGGTAAA ATAATGAGAC   
  
  
+ TATGGTCTTG TCTTCTCTTC TGCAAGGCCT AAAAGAACTG CGGAGCACTA ACGCGGAAGA TTGATTTTGA   
  
  
+ AATTTCTTGG TCCACTGCTC TCCTGTGCTG TTTCACCTCT CGACTTTTCT TAATCTCCTC AATCATGTGA   
  
  
+ TGTCTTCAAA GGACCAATCA GAGAAGAGAG ACCAAGAAAG AGACCCTTTT TGAATGAAGA ATTGGTCAGC   
  
  
+ CTTTGGGATT TTCCACTCTC CCACTACTCT CTTTCTTTCA GCTACTTCGT GTTTATGGGA CCGAAGACCA   
  
  
+ GTCTTTGCTG AAAAACTCGG TTTCCATCAG TCAAACTTCC ACAAAATTTC TATCCTCCCA GAAGGGTACC   
  
  
+ CCAAAGGGCT TCGCTTTATA AGCCAAAGCT TCACTTTCCT TCTTGACGCA AGACAGATGC TTTTACTCTT   
  
  
+ CCTGCTCCTT CACACAAAGA ACCTTCATTT TCTCTCTTTT CATATTATCA GTCATATACT TCTCATTCCA   
  
  
+ GTAAATTTCA TCATTGTAGC TTGTTACCCT TTAAGATTCC ATTGTCTAAA GGGGTATAAT CTTCGTTGTC   
  
  
+ TGCTTCGTGG TGGAAAGGTT TTGCTGCTTC AGACGGGAAG TAGCATGTGC TTTCAGACAA GCTGAGATAC   
  
  
+ TGTATTGCTT GTAGCTCTTG GTCGTTGAA  

- ATAAAATCAC AAACCACGAA AGTTTAAAGT AAATGGATTA ATAAATTCAT GTACAAACTA GTAAAACAAC   
  
  
- AAAGGTTAAA TTAAATTAAG TTAGTATTAT TTTTTTTAAA CGATTAATAA AATAGTACTT TTCTAAATTT   
  
  
- TAAAAAATTC ATAACTTATA TATATATATA TATATAATTT TACAATACAA TAATTTTTAA AGTTTTTCAA   
  
  
- TAATTTTTAT TGAGTATTTA AATGTTTAAT AATTTTAATT TTTTAATTTT AATTTTAAAC AAATTTAATA   
  
  
- AAATTTTTAT AAAGTTATGA TTTTTATAAA AATTAAGAAG TAGTGGGAAA CTAAACTTTA GATTAATCTA   
  
  
- GTTTAATTTT ATAATGGGTA TTTTAATTAG GTCGGCTAAT GTGGTTTCTC ATATCAAGTC TAAAAAAAAA   
  
  
- AGAAAAACTA ATACGATTGT ACATTTTAAA ACTGAAAGAT AAAATTTCGG TTTTTTTATG TTAATACTTT   
  
  
- CTATTTTTAG GTATTATTTA ATTTTTTGAG TTTTATTTGT TATAAATGCT ATGTTGTTCC TAGTTTTGTT   
  
  
- ATATTATAGG TTTTTAATCT GCGGTGGTAA TTATTATCTA TTACTAAACG TTTTCTTTTA TAGTAAATCT   
  
  
- CATGAGGACA GTTTTAAAAC AGAAAAGAAA AGTCTATACT TTCTTACTCT GGGCCAAGTT TCTAAATGTG   
  
  
- AGAATCTGAA CCAGTCAAGT TTATTAGGAA CATCAAATCA ATTTGGGTAA TTAGTTAACC CATCTAATTA   
  
  
- GTATTAATCA GGGTTTGGTT GGTCTTAGCA CTTCTTAGCA TTCATGGACT AATACCATTT TATTACTCTG   
  
  
- ATACCAGAAC AGAAGAGAAG ACGTTCCGGA TTTTCTTGAC GCCTCGTGAT TGCGCCTTCT AACTAAAACT   
  
  
- TTAAAGAACC AGGTGACGAG AGGACACGAC AAAGTGGAGA GCTGAAAAGA ATTAGAGGAG TTAGTACACT   
  
  
- ACAGAAGTTT CCTGGTTAGT CTCTTCTCTC TGGTTCTTTC TCTGGGAAAA ACTTACTTCT TAACCAGTCG   
  
  
- GAAACCCTAA AAGGTGAGAG GGTGATGAGA GAAAGAAAGT CGATGAAGCA CAAATACCCT GGCTTCTGGT   
  
  
- CAGAAACGAC TTTTTGAGCC AAAGGTAGTC AGTTTGAAGG TGTTTTAAAG ATAGGAGGGT CTTCCCATGG   
  
  
- GGTTTCCCGA AGCGAAATAT TCGGTTTCGA AGTGAAAGGA AGAACTGCGT TCTGTCTACG AAAATGAGAA   
  
  
- GGACGAGGAA GTGTGTTTCT TGGAAGTAAA AGAGAGAAAA GTATAATAGT CAGTATATGA AGAGTAAGGT   
  
  
- CATTTAAAGT AGTAACATCG AACAATGGGA AATTCTAAGG TAACAGATTT CCCCATATTA GAAGCAACAG   
  
  
- ACGAAGCACC ACCTTTCCAA AACGACGAAG TCTGCCCTTC ATCGTACACG AAAGTCTGTT CGACTCTATG   
  
  
- ACATAACGAA CATCGAGAAC CAGCAACTT

+     CAAT-box

| Site Name | Organism | Position | Strand | Matrix score. | sequence | function |
| --- | --- | --- | --- | --- | --- | --- |
| CAAT-box | Hordeum vulgare | 900 | - | 4 | CAAT | common cis-acting element in promoter and enhancer regions |
| CAAT-box | Hordeum vulgare | 529 | + | 4 | CAAT | common cis-acting element in promoter and enhancer regions |
| CAAT-box | Arabidopsis thaliana | 756 | - | 5 | CCAAT | common cis-acting element in promoter and enhancer regions |
| CAAT-box | Arabidopsis thaliana | 994 | + | 5 | CCAAT | common cis-acting element in promoter and enhancer regions |
| CAAT-box | Brassica rapa | 605 | - | 5 | CAAAT | common cis-acting element in promoter and enhancer regions |
| CAAT-box | Brassica rapa | 351 | + | 5 | CAAAT | common cis-acting element in promoter and enhancer regions |
| CAAT-box | Hordeum vulgare | 558 | + | 4 | CAAT | common cis-acting element in promoter and enhancer regions |
| CAAT-box | Brassica rapa | 234 | + | 5 | CAAAT | common cis-acting element in promoter and enhancer regions |
| CAAT-box | Hordeum vulgare | 152 | - | 4 | CAAT | common cis-acting element in promoter and enhancer regions |
| CAAT-box | Hordeum vulgare | 90 | + | 4 | CAAT | common cis-acting element in promoter and enhancer regions |
| CAAT-box | Brassica rapa | 719 | + | 5 | CAAAT | common cis-acting element in promoter and enhancer regions |
| CAAT-box | Hordeum vulgare | 1474 | - | 4 | CAAT | common cis-acting element in promoter and enhancer regions |
| CAAT-box | Hordeum vulgare | 970 | + | 4 | CAAT | common cis-acting element in promoter and enhancer regions |
| CAAT-box | Hordeum vulgare | 1343 | - | 4 | CAAT | common cis-acting element in promoter and enhancer regions |
| CAAT-box | Hordeum vulgare | 1371 | - | 4 | CAAT | common cis-acting element in promoter and enhancer regions |
| CAAT-box | Glycine max | 1040 | - | 5 | CAATT | common cis-acting element in promoter and enhancer regions |
| CAAT-box | Glycine max | 480 | + | 5 | CAATT | common cis-acting element in promoter and enhancer regions |
| CAAT-box | Hordeum vulgare | 995 | + | 4 | CAAT | common cis-acting element in promoter and enhancer regions |
| CAAT-box | Arabidopsis thaliana | 1041 | - | 5 | CCAAT | common cis-acting element in promoter and enhancer regions |
| CAAT-box | Glycine max | 755 | - | 5 | CAATT | common cis-acting element in promoter and enhancer regions |
| CAAT-box | Hordeum vulgare | 294 | + | 4 | CAAT | common cis-acting element in promoter and enhancer regions |
| CAAT-box | Glycine max | 75 | + | 5 | CAATT | common cis-acting element in promoter and enhancer regions |
| CAAT-box | Glycine max | 754 | + | 5 | CAATT | common cis-acting element in promoter and enhancer regions |
| CAAT-box | Brassica rapa | 266 | - | 5 | CAAAT | common cis-acting element in promoter and enhancer regions |
| CAAT-box | Brassica rapa | 332 | - | 5 | CAAAT | common cis-acting element in promoter and enhancer regions |
| CAAT-box | Brassica rapa | 107 | - | 5 | CAAAT | common cis-acting element in promoter and enhancer regions |
| CAAT-box | Brassica rapa | 22 | + | 5 | CAAAT | common cis-acting element in promoter and enhancer regions |
| CAAT-box | Arabidopsis thaliana | 74 | + | 5 | CCAAT | common cis-acting element in promoter and enhancer regions |

> 2018/04/13 10:10:12  
+ TATTTTAGTG TTTGGTGCTT TCAAATTTCA TTTACCTAAT TATTTAAGTA CATGTTTGAT CATTTTGTTG   
  
  
+ TTTCCAATTT AATTTAATTC AATCATAATA AAAAAAATTT GCTAATTATT TTATCATGAA AAGATTTAAA   
  
  
+ ATTTTTTAAG TATTGAATAT ATATATATAT ATATATTAAA ATGTTATGTT ATTAAAAATT TCAAAAAGTT   
  
  
+ ATTAAAAATA ACTCATAAAT TTACAAATTA TTAAAATTAA AAAATTAAAA TTAAAATTTG TTTAAATTAT   
  
  
+ TTTAAAAATA TTTCAATACT AAAAATATTT TTAATTCTTC ATCACCCTTT GATTTGAAAT CTAATTAGAT   
  
  
+ CAAATTAAAA TATTACCCAT AAAATTAATC CAGCCGATTA CACCAAAGAG TATAGTTCAG ATTTTTTTTT   
  
  
+ TCTTTTTGAT TATGCTAACA TGTAAAATTT TGACTTTCTA TTTTAAAGCC AAAAAAATAC AATTATGAAA   
  
  
+ GATAAAAATC CATAATAAAT TAAAAAACTC AAAATAAACA ATATTTACGA TACAACAAGG ATCAAAACAA   
  
  
+ TATAATATCC AAAAATTAGA CGCCACCATT AATAATAGAT AATGATTTGC AAAAGAAAAT ATCATTTAGA   
  
  
+ GTACTCCTGT CAAAATTTTG TCTTTTCTTT TCAGATATGA AAGAATGAGA CCCGGTTCAA AGATTTACAC   
  
  
+ TCTTAGACTT GGTCAGTTCA AATAATCCTT GTAGTTTAGT TAAACCCATT AATCAATTGG GTAGATTAAT   
  
  
+ CATAATTAGT CCCAAACCAA CCAGAATCGT GAAGAATCGT AAGTACCTGA TTATGGTAAA ATAATGAGAC   
  
  
+ TATGGTCTTG TCTTCTCTTC TGCAAGGCCT AAAAGAACTG CGGAGCACTA ACGCGGAAGA TTGATTTTGA   
  
  
+ AATTTCTTGG TCCACTGCTC TCCTGTGCTG TTTCACCTCT CGACTTTTCT TAATCTCCTC AATCATGTGA   
  
  
+ TGTCTTCAAA GGACCAATCA GAGAAGAGAG ACCAAGAAAG AGACCCTTTT TGAATGAAGA ATTGGTCAGC   
  
  
+ CTTTGGGATT TTCCACTCTC CCACTACTCT CTTTCTTTCA GCTACTTCGT GTTTATGGGA CCGAAGACCA   
  
  
+ GTCTTTGCTG AAAAACTCGG TTTCCATCAG TCAAACTTCC ACAAAATTTC TATCCTCCCA GAAGGGTACC   
  
  
+ CCAAAGGGCT TCGCTTTATA AGCCAAAGCT TCACTTTCCT TCTTGACGCA AGACAGATGC TTTTACTCTT   
  
  
+ CCTGCTCCTT CACACAAAGA ACCTTCATTT TCTCTCTTTT CATATTATCA GTCATATACT TCTCATTCCA   
  
  
+ GTAAATTTCA TCATTGTAGC TTGTTACCCT TTAAGATTCC ATTGTCTAAA GGGGTATAAT CTTCGTTGTC   
  
  
+ TGCTTCGTGG TGGAAAGGTT TTGCTGCTTC AGACGGGAAG TAGCATGTGC TTTCAGACAA GCTGAGATAC   
  
  
+ TGTATTGCTT GTAGCTCTTG GTCGTTGAA  

- ATAAAATCAC AAACCACGAA AGTTTAAAGT AAATGGATTA ATAAATTCAT GTACAAACTA GTAAAACAAC   
  
  
- AAAGGTTAAA TTAAATTAAG TTAGTATTAT TTTTTTTAAA CGATTAATAA AATAGTACTT TTCTAAATTT   
  
  
- TAAAAAATTC ATAACTTATA TATATATATA TATATAATTT TACAATACAA TAATTTTTAA AGTTTTTCAA   
  
  
- TAATTTTTAT TGAGTATTTA AATGTTTAAT AATTTTAATT TTTTAATTTT AATTTTAAAC AAATTTAATA   
  
  
- AAATTTTTAT AAAGTTATGA TTTTTATAAA AATTAAGAAG TAGTGGGAAA CTAAACTTTA GATTAATCTA   
  
  
- GTTTAATTTT ATAATGGGTA TTTTAATTAG GTCGGCTAAT GTGGTTTCTC ATATCAAGTC TAAAAAAAAA   
  
  
- AGAAAAACTA ATACGATTGT ACATTTTAAA ACTGAAAGAT AAAATTTCGG TTTTTTTATG TTAATACTTT   
  
  
- CTATTTTTAG GTATTATTTA ATTTTTTGAG TTTTATTTGT TATAAATGCT ATGTTGTTCC TAGTTTTGTT   
  
  
- ATATTATAGG TTTTTAATCT GCGGTGGTAA TTATTATCTA TTACTAAACG TTTTCTTTTA TAGTAAATCT   
  
  
- CATGAGGACA GTTTTAAAAC AGAAAAGAAA AGTCTATACT TTCTTACTCT GGGCCAAGTT TCTAAATGTG   
  
  
- AGAATCTGAA CCAGTCAAGT TTATTAGGAA CATCAAATCA ATTTGGGTAA TTAGTTAACC CATCTAATTA   
  
  
- GTATTAATCA GGGTTTGGTT GGTCTTAGCA CTTCTTAGCA TTCATGGACT AATACCATTT TATTACTCTG   
  
  
- ATACCAGAAC AGAAGAGAAG ACGTTCCGGA TTTTCTTGAC GCCTCGTGAT TGCGCCTTCT AACTAAAACT   
  
  
- TTAAAGAACC AGGTGACGAG AGGACACGAC AAAGTGGAGA GCTGAAAAGA ATTAGAGGAG TTAGTACACT   
  
  
- ACAGAAGTTT CCTGGTTAGT CTCTTCTCTC TGGTTCTTTC TCTGGGAAAA ACTTACTTCT TAACCAGTCG   
  
  
- GAAACCCTAA AAGGTGAGAG GGTGATGAGA GAAAGAAAGT CGATGAAGCA CAAATACCCT GGCTTCTGGT   
  
  
- CAGAAACGAC TTTTTGAGCC AAAGGTAGTC AGTTTGAAGG TGTTTTAAAG ATAGGAGGGT CTTCCCATGG   
  
  
- GGTTTCCCGA AGCGAAATAT TCGGTTTCGA AGTGAAAGGA AGAACTGCGT TCTGTCTACG AAAATGAGAA   
  
  
- GGACGAGGAA GTGTGTTTCT TGGAAGTAAA AGAGAGAAAA GTATAATAGT CAGTATATGA AGAGTAAGGT   
  
  
- CATTTAAAGT AGTAACATCG AACAATGGGA AATTCTAAGG TAACAGATTT CCCCATATTA GAAGCAACAG   
  
  
- ACGAAGCACC ACCTTTCCAA AACGACGAAG TCTGCCCTTC ATCGTACACG AAAGTCTGTT CGACTCTATG   
  
  
- ACATAACGAA CATCGAGAAC CAGCAACTT

+     CGTCA-motif

| Site Name | Organism | Position | Strand | Matrix score. | sequence | function |
| --- | --- | --- | --- | --- | --- | --- |
| CGTCA-motif | Hordeum vulgare | 1234 | - | 5 | CGTCA | cis-acting regulatory element involved in the MeJA-responsiveness |

> 2018/04/13 10:10:12  
+ TATTTTAGTG TTTGGTGCTT TCAAATTTCA TTTACCTAAT TATTTAAGTA CATGTTTGAT CATTTTGTTG   
  
  
+ TTTCCAATTT AATTTAATTC AATCATAATA AAAAAAATTT GCTAATTATT TTATCATGAA AAGATTTAAA   
  
  
+ ATTTTTTAAG TATTGAATAT ATATATATAT ATATATTAAA ATGTTATGTT ATTAAAAATT TCAAAAAGTT   
  
  
+ ATTAAAAATA ACTCATAAAT TTACAAATTA TTAAAATTAA AAAATTAAAA TTAAAATTTG TTTAAATTAT   
  
  
+ TTTAAAAATA TTTCAATACT AAAAATATTT TTAATTCTTC ATCACCCTTT GATTTGAAAT CTAATTAGAT   
  
  
+ CAAATTAAAA TATTACCCAT AAAATTAATC CAGCCGATTA CACCAAAGAG TATAGTTCAG ATTTTTTTTT   
  
  
+ TCTTTTTGAT TATGCTAACA TGTAAAATTT TGACTTTCTA TTTTAAAGCC AAAAAAATAC AATTATGAAA   
  
  
+ GATAAAAATC CATAATAAAT TAAAAAACTC AAAATAAACA ATATTTACGA TACAACAAGG ATCAAAACAA   
  
  
+ TATAATATCC AAAAATTAGA CGCCACCATT AATAATAGAT AATGATTTGC AAAAGAAAAT ATCATTTAGA   
  
  
+ GTACTCCTGT CAAAATTTTG TCTTTTCTTT TCAGATATGA AAGAATGAGA CCCGGTTCAA AGATTTACAC   
  
  
+ TCTTAGACTT GGTCAGTTCA AATAATCCTT GTAGTTTAGT TAAACCCATT AATCAATTGG GTAGATTAAT   
  
  
+ CATAATTAGT CCCAAACCAA CCAGAATCGT GAAGAATCGT AAGTACCTGA TTATGGTAAA ATAATGAGAC   
  
  
+ TATGGTCTTG TCTTCTCTTC TGCAAGGCCT AAAAGAACTG CGGAGCACTA ACGCGGAAGA TTGATTTTGA   
  
  
+ AATTTCTTGG TCCACTGCTC TCCTGTGCTG TTTCACCTCT CGACTTTTCT TAATCTCCTC AATCATGTGA   
  
  
+ TGTCTTCAAA GGACCAATCA GAGAAGAGAG ACCAAGAAAG AGACCCTTTT TGAATGAAGA ATTGGTCAGC   
  
  
+ CTTTGGGATT TTCCACTCTC CCACTACTCT CTTTCTTTCA GCTACTTCGT GTTTATGGGA CCGAAGACCA   
  
  
+ GTCTTTGCTG AAAAACTCGG TTTCCATCAG TCAAACTTCC ACAAAATTTC TATCCTCCCA GAAGGGTACC   
  
  
+ CCAAAGGGCT TCGCTTTATA AGCCAAAGCT TCACTTTCCT TCTTGACGCA AGACAGATGC TTTTACTCTT   
  
  
+ CCTGCTCCTT CACACAAAGA ACCTTCATTT TCTCTCTTTT CATATTATCA GTCATATACT TCTCATTCCA   
  
  
+ GTAAATTTCA TCATTGTAGC TTGTTACCCT TTAAGATTCC ATTGTCTAAA GGGGTATAAT CTTCGTTGTC   
  
  
+ TGCTTCGTGG TGGAAAGGTT TTGCTGCTTC AGACGGGAAG TAGCATGTGC TTTCAGACAA GCTGAGATAC   
  
  
+ TGTATTGCTT GTAGCTCTTG GTCGTTGAA  

- ATAAAATCAC AAACCACGAA AGTTTAAAGT AAATGGATTA ATAAATTCAT GTACAAACTA GTAAAACAAC   
  
  
- AAAGGTTAAA TTAAATTAAG TTAGTATTAT TTTTTTTAAA CGATTAATAA AATAGTACTT TTCTAAATTT   
  
  
- TAAAAAATTC ATAACTTATA TATATATATA TATATAATTT TACAATACAA TAATTTTTAA AGTTTTTCAA   
  
  
- TAATTTTTAT TGAGTATTTA AATGTTTAAT AATTTTAATT TTTTAATTTT AATTTTAAAC AAATTTAATA   
  
  
- AAATTTTTAT AAAGTTATGA TTTTTATAAA AATTAAGAAG TAGTGGGAAA CTAAACTTTA GATTAATCTA   
  
  
- GTTTAATTTT ATAATGGGTA TTTTAATTAG GTCGGCTAAT GTGGTTTCTC ATATCAAGTC TAAAAAAAAA   
  
  
- AGAAAAACTA ATACGATTGT ACATTTTAAA ACTGAAAGAT AAAATTTCGG TTTTTTTATG TTAATACTTT   
  
  
- CTATTTTTAG GTATTATTTA ATTTTTTGAG TTTTATTTGT TATAAATGCT ATGTTGTTCC TAGTTTTGTT   
  
  
- ATATTATAGG TTTTTAATCT GCGGTGGTAA TTATTATCTA TTACTAAACG TTTTCTTTTA TAGTAAATCT   
  
  
- CATGAGGACA GTTTTAAAAC AGAAAAGAAA AGTCTATACT TTCTTACTCT GGGCCAAGTT TCTAAATGTG   
  
  
- AGAATCTGAA CCAGTCAAGT TTATTAGGAA CATCAAATCA ATTTGGGTAA TTAGTTAACC CATCTAATTA   
  
  
- GTATTAATCA GGGTTTGGTT GGTCTTAGCA CTTCTTAGCA TTCATGGACT AATACCATTT TATTACTCTG   
  
  
- ATACCAGAAC AGAAGAGAAG ACGTTCCGGA TTTTCTTGAC GCCTCGTGAT TGCGCCTTCT AACTAAAACT   
  
  
- TTAAAGAACC AGGTGACGAG AGGACACGAC AAAGTGGAGA GCTGAAAAGA ATTAGAGGAG TTAGTACACT   
  
  
- ACAGAAGTTT CCTGGTTAGT CTCTTCTCTC TGGTTCTTTC TCTGGGAAAA ACTTACTTCT TAACCAGTCG   
  
  
- GAAACCCTAA AAGGTGAGAG GGTGATGAGA GAAAGAAAGT CGATGAAGCA CAAATACCCT GGCTTCTGGT   
  
  
- CAGAAACGAC TTTTTGAGCC AAAGGTAGTC AGTTTGAAGG TGTTTTAAAG ATAGGAGGGT CTTCCCATGG   
  
  
- GGTTTCCCGA AGCGAAATAT TCGGTTTCGA AGTGAAAGGA AGAACTGCGT TCTGTCTACG AAAATGAGAA   
  
  
- GGACGAGGAA GTGTGTTTCT TGGAAGTAAA AGAGAGAAAA GTATAATAGT CAGTATATGA AGAGTAAGGT   
  
  
- CATTTAAAGT AGTAACATCG AACAATGGGA AATTCTAAGG TAACAGATTT CCCCATATTA GAAGCAACAG   
  
  
- ACGAAGCACC ACCTTTCCAA AACGACGAAG TCTGCCCTTC ATCGTACACG AAAGTCTGTT CGACTCTATG   
  
  
- ACATAACGAA CATCGAGAAC CAGCAACTT

+     ERE

| Site Name | Organism | Position | Strand | Matrix score. | sequence | function |
| --- | --- | --- | --- | --- | --- | --- |
| ERE | Dianthus caryophyllus | 198 | + | 8 | ATTTCAAA | ethylene-responsive element |
| ERE | Dianthus caryophyllus | 333 | - | 8 | ATTTCAAA | ethylene-responsive element |
| ERE | Dianthus caryophyllus | 906 | - | 8 | ATTTCAAA | ethylene-responsive element |

> 2018/04/13 10:10:12  
+ TATTTTAGTG TTTGGTGCTT TCAAATTTCA TTTACCTAAT TATTTAAGTA CATGTTTGAT CATTTTGTTG   
  
  
+ TTTCCAATTT AATTTAATTC AATCATAATA AAAAAAATTT GCTAATTATT TTATCATGAA AAGATTTAAA   
  
  
+ ATTTTTTAAG TATTGAATAT ATATATATAT ATATATTAAA ATGTTATGTT ATTAAAAATT TCAAAAAGTT   
  
  
+ ATTAAAAATA ACTCATAAAT TTACAAATTA TTAAAATTAA AAAATTAAAA TTAAAATTTG TTTAAATTAT   
  
  
+ TTTAAAAATA TTTCAATACT AAAAATATTT TTAATTCTTC ATCACCCTTT GATTTGAAAT CTAATTAGAT   
  
  
+ CAAATTAAAA TATTACCCAT AAAATTAATC CAGCCGATTA CACCAAAGAG TATAGTTCAG ATTTTTTTTT   
  
  
+ TCTTTTTGAT TATGCTAACA TGTAAAATTT TGACTTTCTA TTTTAAAGCC AAAAAAATAC AATTATGAAA   
  
  
+ GATAAAAATC CATAATAAAT TAAAAAACTC AAAATAAACA ATATTTACGA TACAACAAGG ATCAAAACAA   
  
  
+ TATAATATCC AAAAATTAGA CGCCACCATT AATAATAGAT AATGATTTGC AAAAGAAAAT ATCATTTAGA   
  
  
+ GTACTCCTGT CAAAATTTTG TCTTTTCTTT TCAGATATGA AAGAATGAGA CCCGGTTCAA AGATTTACAC   
  
  
+ TCTTAGACTT GGTCAGTTCA AATAATCCTT GTAGTTTAGT TAAACCCATT AATCAATTGG GTAGATTAAT   
  
  
+ CATAATTAGT CCCAAACCAA CCAGAATCGT GAAGAATCGT AAGTACCTGA TTATGGTAAA ATAATGAGAC   
  
  
+ TATGGTCTTG TCTTCTCTTC TGCAAGGCCT AAAAGAACTG CGGAGCACTA ACGCGGAAGA TTGATTTTGA   
  
  
+ AATTTCTTGG TCCACTGCTC TCCTGTGCTG TTTCACCTCT CGACTTTTCT TAATCTCCTC AATCATGTGA   
  
  
+ TGTCTTCAAA GGACCAATCA GAGAAGAGAG ACCAAGAAAG AGACCCTTTT TGAATGAAGA ATTGGTCAGC   
  
  
+ CTTTGGGATT TTCCACTCTC CCACTACTCT CTTTCTTTCA GCTACTTCGT GTTTATGGGA CCGAAGACCA   
  
  
+ GTCTTTGCTG AAAAACTCGG TTTCCATCAG TCAAACTTCC ACAAAATTTC TATCCTCCCA GAAGGGTACC   
  
  
+ CCAAAGGGCT TCGCTTTATA AGCCAAAGCT TCACTTTCCT TCTTGACGCA AGACAGATGC TTTTACTCTT   
  
  
+ CCTGCTCCTT CACACAAAGA ACCTTCATTT TCTCTCTTTT CATATTATCA GTCATATACT TCTCATTCCA   
  
  
+ GTAAATTTCA TCATTGTAGC TTGTTACCCT TTAAGATTCC ATTGTCTAAA GGGGTATAAT CTTCGTTGTC   
  
  
+ TGCTTCGTGG TGGAAAGGTT TTGCTGCTTC AGACGGGAAG TAGCATGTGC TTTCAGACAA GCTGAGATAC   
  
  
+ TGTATTGCTT GTAGCTCTTG GTCGTTGAA  

- ATAAAATCAC AAACCACGAA AGTTTAAAGT AAATGGATTA ATAAATTCAT GTACAAACTA GTAAAACAAC   
  
  
- AAAGGTTAAA TTAAATTAAG TTAGTATTAT TTTTTTTAAA CGATTAATAA AATAGTACTT TTCTAAATTT   
  
  
- TAAAAAATTC ATAACTTATA TATATATATA TATATAATTT TACAATACAA TAATTTTTAA AGTTTTTCAA   
  
  
- TAATTTTTAT TGAGTATTTA AATGTTTAAT AATTTTAATT TTTTAATTTT AATTTTAAAC AAATTTAATA   
  
  
- AAATTTTTAT AAAGTTATGA TTTTTATAAA AATTAAGAAG TAGTGGGAAA CTAAACTTTA GATTAATCTA   
  
  
- GTTTAATTTT ATAATGGGTA TTTTAATTAG GTCGGCTAAT GTGGTTTCTC ATATCAAGTC TAAAAAAAAA   
  
  
- AGAAAAACTA ATACGATTGT ACATTTTAAA ACTGAAAGAT AAAATTTCGG TTTTTTTATG TTAATACTTT   
  
  
- CTATTTTTAG GTATTATTTA ATTTTTTGAG TTTTATTTGT TATAAATGCT ATGTTGTTCC TAGTTTTGTT   
  
  
- ATATTATAGG TTTTTAATCT GCGGTGGTAA TTATTATCTA TTACTAAACG TTTTCTTTTA TAGTAAATCT   
  
  
- CATGAGGACA GTTTTAAAAC AGAAAAGAAA AGTCTATACT TTCTTACTCT GGGCCAAGTT TCTAAATGTG   
  
  
- AGAATCTGAA CCAGTCAAGT TTATTAGGAA CATCAAATCA ATTTGGGTAA TTAGTTAACC CATCTAATTA   
  
  
- GTATTAATCA GGGTTTGGTT GGTCTTAGCA CTTCTTAGCA TTCATGGACT AATACCATTT TATTACTCTG   
  
  
- ATACCAGAAC AGAAGAGAAG ACGTTCCGGA TTTTCTTGAC GCCTCGTGAT TGCGCCTTCT AACTAAAACT   
  
  
- TTAAAGAACC AGGTGACGAG AGGACACGAC AAAGTGGAGA GCTGAAAAGA ATTAGAGGAG TTAGTACACT   
  
  
- ACAGAAGTTT CCTGGTTAGT CTCTTCTCTC TGGTTCTTTC TCTGGGAAAA ACTTACTTCT TAACCAGTCG   
  
  
- GAAACCCTAA AAGGTGAGAG GGTGATGAGA GAAAGAAAGT CGATGAAGCA CAAATACCCT GGCTTCTGGT   
  
  
- CAGAAACGAC TTTTTGAGCC AAAGGTAGTC AGTTTGAAGG TGTTTTAAAG ATAGGAGGGT CTTCCCATGG   
  
  
- GGTTTCCCGA AGCGAAATAT TCGGTTTCGA AGTGAAAGGA AGAACTGCGT TCTGTCTACG AAAATGAGAA   
  
  
- GGACGAGGAA GTGTGTTTCT TGGAAGTAAA AGAGAGAAAA GTATAATAGT CAGTATATGA AGAGTAAGGT   
  
  
- CATTTAAAGT AGTAACATCG AACAATGGGA AATTCTAAGG TAACAGATTT CCCCATATTA GAAGCAACAG   
  
  
- ACGAAGCACC ACCTTTCCAA AACGACGAAG TCTGCCCTTC ATCGTACACG AAAGTCTGTT CGACTCTATG   
  
  
- ACATAACGAA CATCGAGAAC CAGCAACTT

+     GA-motif

| Site Name | Organism | Position | Strand | Matrix score. | sequence | function |
| --- | --- | --- | --- | --- | --- | --- |
| GA-motif | Arabidopsis thaliana | 595 | + | 8 | ATAGATAA | part of a light responsive element |

> 2018/04/13 10:10:12  
+ TATTTTAGTG TTTGGTGCTT TCAAATTTCA TTTACCTAAT TATTTAAGTA CATGTTTGAT CATTTTGTTG   
  
  
+ TTTCCAATTT AATTTAATTC AATCATAATA AAAAAAATTT GCTAATTATT TTATCATGAA AAGATTTAAA   
  
  
+ ATTTTTTAAG TATTGAATAT ATATATATAT ATATATTAAA ATGTTATGTT ATTAAAAATT TCAAAAAGTT   
  
  
+ ATTAAAAATA ACTCATAAAT TTACAAATTA TTAAAATTAA AAAATTAAAA TTAAAATTTG TTTAAATTAT   
  
  
+ TTTAAAAATA TTTCAATACT AAAAATATTT TTAATTCTTC ATCACCCTTT GATTTGAAAT CTAATTAGAT   
  
  
+ CAAATTAAAA TATTACCCAT AAAATTAATC CAGCCGATTA CACCAAAGAG TATAGTTCAG ATTTTTTTTT   
  
  
+ TCTTTTTGAT TATGCTAACA TGTAAAATTT TGACTTTCTA TTTTAAAGCC AAAAAAATAC AATTATGAAA   
  
  
+ GATAAAAATC CATAATAAAT TAAAAAACTC AAAATAAACA ATATTTACGA TACAACAAGG ATCAAAACAA   
  
  
+ TATAATATCC AAAAATTAGA CGCCACCATT AATAATAGAT AATGATTTGC AAAAGAAAAT ATCATTTAGA   
  
  
+ GTACTCCTGT CAAAATTTTG TCTTTTCTTT TCAGATATGA AAGAATGAGA CCCGGTTCAA AGATTTACAC   
  
  
+ TCTTAGACTT GGTCAGTTCA AATAATCCTT GTAGTTTAGT TAAACCCATT AATCAATTGG GTAGATTAAT   
  
  
+ CATAATTAGT CCCAAACCAA CCAGAATCGT GAAGAATCGT AAGTACCTGA TTATGGTAAA ATAATGAGAC   
  
  
+ TATGGTCTTG TCTTCTCTTC TGCAAGGCCT AAAAGAACTG CGGAGCACTA ACGCGGAAGA TTGATTTTGA   
  
  
+ AATTTCTTGG TCCACTGCTC TCCTGTGCTG TTTCACCTCT CGACTTTTCT TAATCTCCTC AATCATGTGA   
  
  
+ TGTCTTCAAA GGACCAATCA GAGAAGAGAG ACCAAGAAAG AGACCCTTTT TGAATGAAGA ATTGGTCAGC   
  
  
+ CTTTGGGATT TTCCACTCTC CCACTACTCT CTTTCTTTCA GCTACTTCGT GTTTATGGGA CCGAAGACCA   
  
  
+ GTCTTTGCTG AAAAACTCGG TTTCCATCAG TCAAACTTCC ACAAAATTTC TATCCTCCCA GAAGGGTACC   
  
  
+ CCAAAGGGCT TCGCTTTATA AGCCAAAGCT TCACTTTCCT TCTTGACGCA AGACAGATGC TTTTACTCTT   
  
  
+ CCTGCTCCTT CACACAAAGA ACCTTCATTT TCTCTCTTTT CATATTATCA GTCATATACT TCTCATTCCA   
  
  
+ GTAAATTTCA TCATTGTAGC TTGTTACCCT TTAAGATTCC ATTGTCTAAA GGGGTATAAT CTTCGTTGTC   
  
  
+ TGCTTCGTGG TGGAAAGGTT TTGCTGCTTC AGACGGGAAG TAGCATGTGC TTTCAGACAA GCTGAGATAC   
  
  
+ TGTATTGCTT GTAGCTCTTG GTCGTTGAA  

- ATAAAATCAC AAACCACGAA AGTTTAAAGT AAATGGATTA ATAAATTCAT GTACAAACTA GTAAAACAAC   
  
  
- AAAGGTTAAA TTAAATTAAG TTAGTATTAT TTTTTTTAAA CGATTAATAA AATAGTACTT TTCTAAATTT   
  
  
- TAAAAAATTC ATAACTTATA TATATATATA TATATAATTT TACAATACAA TAATTTTTAA AGTTTTTCAA   
  
  
- TAATTTTTAT TGAGTATTTA AATGTTTAAT AATTTTAATT TTTTAATTTT AATTTTAAAC AAATTTAATA   
  
  
- AAATTTTTAT AAAGTTATGA TTTTTATAAA AATTAAGAAG TAGTGGGAAA CTAAACTTTA GATTAATCTA   
  
  
- GTTTAATTTT ATAATGGGTA TTTTAATTAG GTCGGCTAAT GTGGTTTCTC ATATCAAGTC TAAAAAAAAA   
  
  
- AGAAAAACTA ATACGATTGT ACATTTTAAA ACTGAAAGAT AAAATTTCGG TTTTTTTATG TTAATACTTT   
  
  
- CTATTTTTAG GTATTATTTA ATTTTTTGAG TTTTATTTGT TATAAATGCT ATGTTGTTCC TAGTTTTGTT   
  
  
- ATATTATAGG TTTTTAATCT GCGGTGGTAA TTATTATCTA TTACTAAACG TTTTCTTTTA TAGTAAATCT   
  
  
- CATGAGGACA GTTTTAAAAC AGAAAAGAAA AGTCTATACT TTCTTACTCT GGGCCAAGTT TCTAAATGTG   
  
  
- AGAATCTGAA CCAGTCAAGT TTATTAGGAA CATCAAATCA ATTTGGGTAA TTAGTTAACC CATCTAATTA   
  
  
- GTATTAATCA GGGTTTGGTT GGTCTTAGCA CTTCTTAGCA TTCATGGACT AATACCATTT TATTACTCTG   
  
  
- ATACCAGAAC AGAAGAGAAG ACGTTCCGGA TTTTCTTGAC GCCTCGTGAT TGCGCCTTCT AACTAAAACT   
  
  
- TTAAAGAACC AGGTGACGAG AGGACACGAC AAAGTGGAGA GCTGAAAAGA ATTAGAGGAG TTAGTACACT   
  
  
- ACAGAAGTTT CCTGGTTAGT CTCTTCTCTC TGGTTCTTTC TCTGGGAAAA ACTTACTTCT TAACCAGTCG   
  
  
- GAAACCCTAA AAGGTGAGAG GGTGATGAGA GAAAGAAAGT CGATGAAGCA CAAATACCCT GGCTTCTGGT   
  
  
- CAGAAACGAC TTTTTGAGCC AAAGGTAGTC AGTTTGAAGG TGTTTTAAAG ATAGGAGGGT CTTCCCATGG   
  
  
- GGTTTCCCGA AGCGAAATAT TCGGTTTCGA AGTGAAAGGA AGAACTGCGT TCTGTCTACG AAAATGAGAA   
  
  
- GGACGAGGAA GTGTGTTTCT TGGAAGTAAA AGAGAGAAAA GTATAATAGT CAGTATATGA AGAGTAAGGT   
  
  
- CATTTAAAGT AGTAACATCG AACAATGGGA AATTCTAAGG TAACAGATTT CCCCATATTA GAAGCAACAG   
  
  
- ACGAAGCACC ACCTTTCCAA AACGACGAAG TCTGCCCTTC ATCGTACACG AAAGTCTGTT CGACTCTATG   
  
  
- ACATAACGAA CATCGAGAAC CAGCAACTT

+     GAG-motif

| Site Name | Organism | Position | Strand | Matrix score. | sequence | function |
| --- | --- | --- | --- | --- | --- | --- |
| GAG-motif | Arabidopsis thaliana | 1076 | - | 7 | AGAGAGT | part of a light responsive element |

> 2018/04/13 10:10:12  
+ TATTTTAGTG TTTGGTGCTT TCAAATTTCA TTTACCTAAT TATTTAAGTA CATGTTTGAT CATTTTGTTG   
  
  
+ TTTCCAATTT AATTTAATTC AATCATAATA AAAAAAATTT GCTAATTATT TTATCATGAA AAGATTTAAA   
  
  
+ ATTTTTTAAG TATTGAATAT ATATATATAT ATATATTAAA ATGTTATGTT ATTAAAAATT TCAAAAAGTT   
  
  
+ ATTAAAAATA ACTCATAAAT TTACAAATTA TTAAAATTAA AAAATTAAAA TTAAAATTTG TTTAAATTAT   
  
  
+ TTTAAAAATA TTTCAATACT AAAAATATTT TTAATTCTTC ATCACCCTTT GATTTGAAAT CTAATTAGAT   
  
  
+ CAAATTAAAA TATTACCCAT AAAATTAATC CAGCCGATTA CACCAAAGAG TATAGTTCAG ATTTTTTTTT   
  
  
+ TCTTTTTGAT TATGCTAACA TGTAAAATTT TGACTTTCTA TTTTAAAGCC AAAAAAATAC AATTATGAAA   
  
  
+ GATAAAAATC CATAATAAAT TAAAAAACTC AAAATAAACA ATATTTACGA TACAACAAGG ATCAAAACAA   
  
  
+ TATAATATCC AAAAATTAGA CGCCACCATT AATAATAGAT AATGATTTGC AAAAGAAAAT ATCATTTAGA   
  
  
+ GTACTCCTGT CAAAATTTTG TCTTTTCTTT TCAGATATGA AAGAATGAGA CCCGGTTCAA AGATTTACAC   
  
  
+ TCTTAGACTT GGTCAGTTCA AATAATCCTT GTAGTTTAGT TAAACCCATT AATCAATTGG GTAGATTAAT   
  
  
+ CATAATTAGT CCCAAACCAA CCAGAATCGT GAAGAATCGT AAGTACCTGA TTATGGTAAA ATAATGAGAC   
  
  
+ TATGGTCTTG TCTTCTCTTC TGCAAGGCCT AAAAGAACTG CGGAGCACTA ACGCGGAAGA TTGATTTTGA   
  
  
+ AATTTCTTGG TCCACTGCTC TCCTGTGCTG TTTCACCTCT CGACTTTTCT TAATCTCCTC AATCATGTGA   
  
  
+ TGTCTTCAAA GGACCAATCA GAGAAGAGAG ACCAAGAAAG AGACCCTTTT TGAATGAAGA ATTGGTCAGC   
  
  
+ CTTTGGGATT TTCCACTCTC CCACTACTCT CTTTCTTTCA GCTACTTCGT GTTTATGGGA CCGAAGACCA   
  
  
+ GTCTTTGCTG AAAAACTCGG TTTCCATCAG TCAAACTTCC ACAAAATTTC TATCCTCCCA GAAGGGTACC   
  
  
+ CCAAAGGGCT TCGCTTTATA AGCCAAAGCT TCACTTTCCT TCTTGACGCA AGACAGATGC TTTTACTCTT   
  
  
+ CCTGCTCCTT CACACAAAGA ACCTTCATTT TCTCTCTTTT CATATTATCA GTCATATACT TCTCATTCCA   
  
  
+ GTAAATTTCA TCATTGTAGC TTGTTACCCT TTAAGATTCC ATTGTCTAAA GGGGTATAAT CTTCGTTGTC   
  
  
+ TGCTTCGTGG TGGAAAGGTT TTGCTGCTTC AGACGGGAAG TAGCATGTGC TTTCAGACAA GCTGAGATAC   
  
  
+ TGTATTGCTT GTAGCTCTTG GTCGTTGAA  

- ATAAAATCAC AAACCACGAA AGTTTAAAGT AAATGGATTA ATAAATTCAT GTACAAACTA GTAAAACAAC   
  
  
- AAAGGTTAAA TTAAATTAAG TTAGTATTAT TTTTTTTAAA CGATTAATAA AATAGTACTT TTCTAAATTT   
  
  
- TAAAAAATTC ATAACTTATA TATATATATA TATATAATTT TACAATACAA TAATTTTTAA AGTTTTTCAA   
  
  
- TAATTTTTAT TGAGTATTTA AATGTTTAAT AATTTTAATT TTTTAATTTT AATTTTAAAC AAATTTAATA   
  
  
- AAATTTTTAT AAAGTTATGA TTTTTATAAA AATTAAGAAG TAGTGGGAAA CTAAACTTTA GATTAATCTA   
  
  
- GTTTAATTTT ATAATGGGTA TTTTAATTAG GTCGGCTAAT GTGGTTTCTC ATATCAAGTC TAAAAAAAAA   
  
  
- AGAAAAACTA ATACGATTGT ACATTTTAAA ACTGAAAGAT AAAATTTCGG TTTTTTTATG TTAATACTTT   
  
  
- CTATTTTTAG GTATTATTTA ATTTTTTGAG TTTTATTTGT TATAAATGCT ATGTTGTTCC TAGTTTTGTT   
  
  
- ATATTATAGG TTTTTAATCT GCGGTGGTAA TTATTATCTA TTACTAAACG TTTTCTTTTA TAGTAAATCT   
  
  
- CATGAGGACA GTTTTAAAAC AGAAAAGAAA AGTCTATACT TTCTTACTCT GGGCCAAGTT TCTAAATGTG   
  
  
- AGAATCTGAA CCAGTCAAGT TTATTAGGAA CATCAAATCA ATTTGGGTAA TTAGTTAACC CATCTAATTA   
  
  
- GTATTAATCA GGGTTTGGTT GGTCTTAGCA CTTCTTAGCA TTCATGGACT AATACCATTT TATTACTCTG   
  
  
- ATACCAGAAC AGAAGAGAAG ACGTTCCGGA TTTTCTTGAC GCCTCGTGAT TGCGCCTTCT AACTAAAACT   
  
  
- TTAAAGAACC AGGTGACGAG AGGACACGAC AAAGTGGAGA GCTGAAAAGA ATTAGAGGAG TTAGTACACT   
  
  
- ACAGAAGTTT CCTGGTTAGT CTCTTCTCTC TGGTTCTTTC TCTGGGAAAA ACTTACTTCT TAACCAGTCG   
  
  
- GAAACCCTAA AAGGTGAGAG GGTGATGAGA GAAAGAAAGT CGATGAAGCA CAAATACCCT GGCTTCTGGT   
  
  
- CAGAAACGAC TTTTTGAGCC AAAGGTAGTC AGTTTGAAGG TGTTTTAAAG ATAGGAGGGT CTTCCCATGG   
  
  
- GGTTTCCCGA AGCGAAATAT TCGGTTTCGA AGTGAAAGGA AGAACTGCGT TCTGTCTACG AAAATGAGAA   
  
  
- GGACGAGGAA GTGTGTTTCT TGGAAGTAAA AGAGAGAAAA GTATAATAGT CAGTATATGA AGAGTAAGGT   
  
  
- CATTTAAAGT AGTAACATCG AACAATGGGA AATTCTAAGG TAACAGATTT CCCCATATTA GAAGCAACAG   
  
  
- ACGAAGCACC ACCTTTCCAA AACGACGAAG TCTGCCCTTC ATCGTACACG AAAGTCTGTT CGACTCTATG   
  
  
- ACATAACGAA CATCGAGAAC CAGCAACTT

+     HSE

| Site Name | Organism | Position | Strand | Matrix score. | sequence | function |
| --- | --- | --- | --- | --- | --- | --- |
| HSE | Brassica oleracea | 102 | + | 9 | AAAAAATTTC | cis-acting element involved in heat stress responsiveness |
| HSE | Brassica oleracea | 138 | - | 9 | AAAAAATTTC | cis-acting element involved in heat stress responsiveness |
| HSE | Brassica oleracea | 909 | - | 9 | AAAAAATTTC | cis-acting element involved in heat stress responsiveness |
| HSE | Brassica oleracea | 951 | - | 9 | AGAAAATTCG | cis-acting element involved in heat stress responsiveness |
| HSE | Brassica oleracea | 285 | + | 9 | AAAAAATTTC | cis-acting element involved in heat stress responsiveness |
| HSE | Brassica oleracea | 193 | + | 9 | AAAAAATTTC | cis-acting element involved in heat stress responsiveness |
| HSE | Brassica oleracea | 1161 | + | 9 | AAAAAATTTC | cis-acting element involved in heat stress responsiveness |

> 2018/04/13 10:10:12  
+ TATTTTAGTG TTTGGTGCTT TCAAATTTCA TTTACCTAAT TATTTAAGTA CATGTTTGAT CATTTTGTTG   
  
  
+ TTTCCAATTT AATTTAATTC AATCATAATA AAAAAAATTT GCTAATTATT TTATCATGAA AAGATTTAAA   
  
  
+ ATTTTTTAAG TATTGAATAT ATATATATAT ATATATTAAA ATGTTATGTT ATTAAAAATT TCAAAAAGTT   
  
  
+ ATTAAAAATA ACTCATAAAT TTACAAATTA TTAAAATTAA AAAATTAAAA TTAAAATTTG TTTAAATTAT   
  
  
+ TTTAAAAATA TTTCAATACT AAAAATATTT TTAATTCTTC ATCACCCTTT GATTTGAAAT CTAATTAGAT   
  
  
+ CAAATTAAAA TATTACCCAT AAAATTAATC CAGCCGATTA CACCAAAGAG TATAGTTCAG ATTTTTTTTT   
  
  
+ TCTTTTTGAT TATGCTAACA TGTAAAATTT TGACTTTCTA TTTTAAAGCC AAAAAAATAC AATTATGAAA   
  
  
+ GATAAAAATC CATAATAAAT TAAAAAACTC AAAATAAACA ATATTTACGA TACAACAAGG ATCAAAACAA   
  
  
+ TATAATATCC AAAAATTAGA CGCCACCATT AATAATAGAT AATGATTTGC AAAAGAAAAT ATCATTTAGA   
  
  
+ GTACTCCTGT CAAAATTTTG TCTTTTCTTT TCAGATATGA AAGAATGAGA CCCGGTTCAA AGATTTACAC   
  
  
+ TCTTAGACTT GGTCAGTTCA AATAATCCTT GTAGTTTAGT TAAACCCATT AATCAATTGG GTAGATTAAT   
  
  
+ CATAATTAGT CCCAAACCAA CCAGAATCGT GAAGAATCGT AAGTACCTGA TTATGGTAAA ATAATGAGAC   
  
  
+ TATGGTCTTG TCTTCTCTTC TGCAAGGCCT AAAAGAACTG CGGAGCACTA ACGCGGAAGA TTGATTTTGA   
  
  
+ AATTTCTTGG TCCACTGCTC TCCTGTGCTG TTTCACCTCT CGACTTTTCT TAATCTCCTC AATCATGTGA   
  
  
+ TGTCTTCAAA GGACCAATCA GAGAAGAGAG ACCAAGAAAG AGACCCTTTT TGAATGAAGA ATTGGTCAGC   
  
  
+ CTTTGGGATT TTCCACTCTC CCACTACTCT CTTTCTTTCA GCTACTTCGT GTTTATGGGA CCGAAGACCA   
  
  
+ GTCTTTGCTG AAAAACTCGG TTTCCATCAG TCAAACTTCC ACAAAATTTC TATCCTCCCA GAAGGGTACC   
  
  
+ CCAAAGGGCT TCGCTTTATA AGCCAAAGCT TCACTTTCCT TCTTGACGCA AGACAGATGC TTTTACTCTT   
  
  
+ CCTGCTCCTT CACACAAAGA ACCTTCATTT TCTCTCTTTT CATATTATCA GTCATATACT TCTCATTCCA   
  
  
+ GTAAATTTCA TCATTGTAGC TTGTTACCCT TTAAGATTCC ATTGTCTAAA GGGGTATAAT CTTCGTTGTC   
  
  
+ TGCTTCGTGG TGGAAAGGTT TTGCTGCTTC AGACGGGAAG TAGCATGTGC TTTCAGACAA GCTGAGATAC   
  
  
+ TGTATTGCTT GTAGCTCTTG GTCGTTGAA  

- ATAAAATCAC AAACCACGAA AGTTTAAAGT AAATGGATTA ATAAATTCAT GTACAAACTA GTAAAACAAC   
  
  
- AAAGGTTAAA TTAAATTAAG TTAGTATTAT TTTTTTTAAA CGATTAATAA AATAGTACTT TTCTAAATTT   
  
  
- TAAAAAATTC ATAACTTATA TATATATATA TATATAATTT TACAATACAA TAATTTTTAA AGTTTTTCAA   
  
  
- TAATTTTTAT TGAGTATTTA AATGTTTAAT AATTTTAATT TTTTAATTTT AATTTTAAAC AAATTTAATA   
  
  
- AAATTTTTAT AAAGTTATGA TTTTTATAAA AATTAAGAAG TAGTGGGAAA CTAAACTTTA GATTAATCTA   
  
  
- GTTTAATTTT ATAATGGGTA TTTTAATTAG GTCGGCTAAT GTGGTTTCTC ATATCAAGTC TAAAAAAAAA   
  
  
- AGAAAAACTA ATACGATTGT ACATTTTAAA ACTGAAAGAT AAAATTTCGG TTTTTTTATG TTAATACTTT   
  
  
- CTATTTTTAG GTATTATTTA ATTTTTTGAG TTTTATTTGT TATAAATGCT ATGTTGTTCC TAGTTTTGTT   
  
  
- ATATTATAGG TTTTTAATCT GCGGTGGTAA TTATTATCTA TTACTAAACG TTTTCTTTTA TAGTAAATCT   
  
  
- CATGAGGACA GTTTTAAAAC AGAAAAGAAA AGTCTATACT TTCTTACTCT GGGCCAAGTT TCTAAATGTG   
  
  
- AGAATCTGAA CCAGTCAAGT TTATTAGGAA CATCAAATCA ATTTGGGTAA TTAGTTAACC CATCTAATTA   
  
  
- GTATTAATCA GGGTTTGGTT GGTCTTAGCA CTTCTTAGCA TTCATGGACT AATACCATTT TATTACTCTG   
  
  
- ATACCAGAAC AGAAGAGAAG ACGTTCCGGA TTTTCTTGAC GCCTCGTGAT TGCGCCTTCT AACTAAAACT   
  
  
- TTAAAGAACC AGGTGACGAG AGGACACGAC AAAGTGGAGA GCTGAAAAGA ATTAGAGGAG TTAGTACACT   
  
  
- ACAGAAGTTT CCTGGTTAGT CTCTTCTCTC TGGTTCTTTC TCTGGGAAAA ACTTACTTCT TAACCAGTCG   
  
  
- GAAACCCTAA AAGGTGAGAG GGTGATGAGA GAAAGAAAGT CGATGAAGCA CAAATACCCT GGCTTCTGGT   
  
  
- CAGAAACGAC TTTTTGAGCC AAAGGTAGTC AGTTTGAAGG TGTTTTAAAG ATAGGAGGGT CTTCCCATGG   
  
  
- GGTTTCCCGA AGCGAAATAT TCGGTTTCGA AGTGAAAGGA AGAACTGCGT TCTGTCTACG AAAATGAGAA   
  
  
- GGACGAGGAA GTGTGTTTCT TGGAAGTAAA AGAGAGAAAA GTATAATAGT CAGTATATGA AGAGTAAGGT   
  
  
- CATTTAAAGT AGTAACATCG AACAATGGGA AATTCTAAGG TAACAGATTT CCCCATATTA GAAGCAACAG   
  
  
- ACGAAGCACC ACCTTTCCAA AACGACGAAG TCTGCCCTTC ATCGTACACG AAAGTCTGTT CGACTCTATG   
  
  
- ACATAACGAA CATCGAGAAC CAGCAACTT

+     Skn-1\_motif

| Site Name | Organism | Position | Strand | Matrix score. | sequence | function |
| --- | --- | --- | --- | --- | --- | --- |
| Skn-1\_motif | Oryza sativa | 1311 | + | 5 | GTCAT | cis-acting regulatory element required for endosperm expression |

> 2018/04/13 10:10:12  
+ TATTTTAGTG TTTGGTGCTT TCAAATTTCA TTTACCTAAT TATTTAAGTA CATGTTTGAT CATTTTGTTG   
  
  
+ TTTCCAATTT AATTTAATTC AATCATAATA AAAAAAATTT GCTAATTATT TTATCATGAA AAGATTTAAA   
  
  
+ ATTTTTTAAG TATTGAATAT ATATATATAT ATATATTAAA ATGTTATGTT ATTAAAAATT TCAAAAAGTT   
  
  
+ ATTAAAAATA ACTCATAAAT TTACAAATTA TTAAAATTAA AAAATTAAAA TTAAAATTTG TTTAAATTAT   
  
  
+ TTTAAAAATA TTTCAATACT AAAAATATTT TTAATTCTTC ATCACCCTTT GATTTGAAAT CTAATTAGAT   
  
  
+ CAAATTAAAA TATTACCCAT AAAATTAATC CAGCCGATTA CACCAAAGAG TATAGTTCAG ATTTTTTTTT   
  
  
+ TCTTTTTGAT TATGCTAACA TGTAAAATTT TGACTTTCTA TTTTAAAGCC AAAAAAATAC AATTATGAAA   
  
  
+ GATAAAAATC CATAATAAAT TAAAAAACTC AAAATAAACA ATATTTACGA TACAACAAGG ATCAAAACAA   
  
  
+ TATAATATCC AAAAATTAGA CGCCACCATT AATAATAGAT AATGATTTGC AAAAGAAAAT ATCATTTAGA   
  
  
+ GTACTCCTGT CAAAATTTTG TCTTTTCTTT TCAGATATGA AAGAATGAGA CCCGGTTCAA AGATTTACAC   
  
  
+ TCTTAGACTT GGTCAGTTCA AATAATCCTT GTAGTTTAGT TAAACCCATT AATCAATTGG GTAGATTAAT   
  
  
+ CATAATTAGT CCCAAACCAA CCAGAATCGT GAAGAATCGT AAGTACCTGA TTATGGTAAA ATAATGAGAC   
  
  
+ TATGGTCTTG TCTTCTCTTC TGCAAGGCCT AAAAGAACTG CGGAGCACTA ACGCGGAAGA TTGATTTTGA   
  
  
+ AATTTCTTGG TCCACTGCTC TCCTGTGCTG TTTCACCTCT CGACTTTTCT TAATCTCCTC AATCATGTGA   
  
  
+ TGTCTTCAAA GGACCAATCA GAGAAGAGAG ACCAAGAAAG AGACCCTTTT TGAATGAAGA ATTGGTCAGC   
  
  
+ CTTTGGGATT TTCCACTCTC CCACTACTCT CTTTCTTTCA GCTACTTCGT GTTTATGGGA CCGAAGACCA   
  
  
+ GTCTTTGCTG AAAAACTCGG TTTCCATCAG TCAAACTTCC ACAAAATTTC TATCCTCCCA GAAGGGTACC   
  
  
+ CCAAAGGGCT TCGCTTTATA AGCCAAAGCT TCACTTTCCT TCTTGACGCA AGACAGATGC TTTTACTCTT   
  
  
+ CCTGCTCCTT CACACAAAGA ACCTTCATTT TCTCTCTTTT CATATTATCA GTCATATACT TCTCATTCCA   
  
  
+ GTAAATTTCA TCATTGTAGC TTGTTACCCT TTAAGATTCC ATTGTCTAAA GGGGTATAAT CTTCGTTGTC   
  
  
+ TGCTTCGTGG TGGAAAGGTT TTGCTGCTTC AGACGGGAAG TAGCATGTGC TTTCAGACAA GCTGAGATAC   
  
  
+ TGTATTGCTT GTAGCTCTTG GTCGTTGAA  

- ATAAAATCAC AAACCACGAA AGTTTAAAGT AAATGGATTA ATAAATTCAT GTACAAACTA GTAAAACAAC   
  
  
- AAAGGTTAAA TTAAATTAAG TTAGTATTAT TTTTTTTAAA CGATTAATAA AATAGTACTT TTCTAAATTT   
  
  
- TAAAAAATTC ATAACTTATA TATATATATA TATATAATTT TACAATACAA TAATTTTTAA AGTTTTTCAA   
  
  
- TAATTTTTAT TGAGTATTTA AATGTTTAAT AATTTTAATT TTTTAATTTT AATTTTAAAC AAATTTAATA   
  
  
- AAATTTTTAT AAAGTTATGA TTTTTATAAA AATTAAGAAG TAGTGGGAAA CTAAACTTTA GATTAATCTA   
  
  
- GTTTAATTTT ATAATGGGTA TTTTAATTAG GTCGGCTAAT GTGGTTTCTC ATATCAAGTC TAAAAAAAAA   
  
  
- AGAAAAACTA ATACGATTGT ACATTTTAAA ACTGAAAGAT AAAATTTCGG TTTTTTTATG TTAATACTTT   
  
  
- CTATTTTTAG GTATTATTTA ATTTTTTGAG TTTTATTTGT TATAAATGCT ATGTTGTTCC TAGTTTTGTT   
  
  
- ATATTATAGG TTTTTAATCT GCGGTGGTAA TTATTATCTA TTACTAAACG TTTTCTTTTA TAGTAAATCT   
  
  
- CATGAGGACA GTTTTAAAAC AGAAAAGAAA AGTCTATACT TTCTTACTCT GGGCCAAGTT TCTAAATGTG   
  
  
- AGAATCTGAA CCAGTCAAGT TTATTAGGAA CATCAAATCA ATTTGGGTAA TTAGTTAACC CATCTAATTA   
  
  
- GTATTAATCA GGGTTTGGTT GGTCTTAGCA CTTCTTAGCA TTCATGGACT AATACCATTT TATTACTCTG   
  
  
- ATACCAGAAC AGAAGAGAAG ACGTTCCGGA TTTTCTTGAC GCCTCGTGAT TGCGCCTTCT AACTAAAACT   
  
  
- TTAAAGAACC AGGTGACGAG AGGACACGAC AAAGTGGAGA GCTGAAAAGA ATTAGAGGAG TTAGTACACT   
  
  
- ACAGAAGTTT CCTGGTTAGT CTCTTCTCTC TGGTTCTTTC TCTGGGAAAA ACTTACTTCT TAACCAGTCG   
  
  
- GAAACCCTAA AAGGTGAGAG GGTGATGAGA GAAAGAAAGT CGATGAAGCA CAAATACCCT GGCTTCTGGT   
  
  
- CAGAAACGAC TTTTTGAGCC AAAGGTAGTC AGTTTGAAGG TGTTTTAAAG ATAGGAGGGT CTTCCCATGG   
  
  
- GGTTTCCCGA AGCGAAATAT TCGGTTTCGA AGTGAAAGGA AGAACTGCGT TCTGTCTACG AAAATGAGAA   
  
  
- GGACGAGGAA GTGTGTTTCT TGGAAGTAAA AGAGAGAAAA GTATAATAGT CAGTATATGA AGAGTAAGGT   
  
  
- CATTTAAAGT AGTAACATCG AACAATGGGA AATTCTAAGG TAACAGATTT CCCCATATTA GAAGCAACAG   
  
  
- ACGAAGCACC ACCTTTCCAA AACGACGAAG TCTGCCCTTC ATCGTACACG AAAGTCTGTT CGACTCTATG   
  
  
- ACATAACGAA CATCGAGAAC CAGCAACTT

+     Sp1

| Site Name | Organism | Position | Strand | Matrix score. | sequence | function |
| --- | --- | --- | --- | --- | --- | --- |
| Sp1 | Zea mays | 1174 | + | 5 | CC(G/A)CCC | light responsive element |

> 2018/04/13 10:10:12  
+ TATTTTAGTG TTTGGTGCTT TCAAATTTCA TTTACCTAAT TATTTAAGTA CATGTTTGAT CATTTTGTTG   
  
  
+ TTTCCAATTT AATTTAATTC AATCATAATA AAAAAAATTT GCTAATTATT TTATCATGAA AAGATTTAAA   
  
  
+ ATTTTTTAAG TATTGAATAT ATATATATAT ATATATTAAA ATGTTATGTT ATTAAAAATT TCAAAAAGTT   
  
  
+ ATTAAAAATA ACTCATAAAT TTACAAATTA TTAAAATTAA AAAATTAAAA TTAAAATTTG TTTAAATTAT   
  
  
+ TTTAAAAATA TTTCAATACT AAAAATATTT TTAATTCTTC ATCACCCTTT GATTTGAAAT CTAATTAGAT   
  
  
+ CAAATTAAAA TATTACCCAT AAAATTAATC CAGCCGATTA CACCAAAGAG TATAGTTCAG ATTTTTTTTT   
  
  
+ TCTTTTTGAT TATGCTAACA TGTAAAATTT TGACTTTCTA TTTTAAAGCC AAAAAAATAC AATTATGAAA   
  
  
+ GATAAAAATC CATAATAAAT TAAAAAACTC AAAATAAACA ATATTTACGA TACAACAAGG ATCAAAACAA   
  
  
+ TATAATATCC AAAAATTAGA CGCCACCATT AATAATAGAT AATGATTTGC AAAAGAAAAT ATCATTTAGA   
  
  
+ GTACTCCTGT CAAAATTTTG TCTTTTCTTT TCAGATATGA AAGAATGAGA CCCGGTTCAA AGATTTACAC   
  
  
+ TCTTAGACTT GGTCAGTTCA AATAATCCTT GTAGTTTAGT TAAACCCATT AATCAATTGG GTAGATTAAT   
  
  
+ CATAATTAGT CCCAAACCAA CCAGAATCGT GAAGAATCGT AAGTACCTGA TTATGGTAAA ATAATGAGAC   
  
  
+ TATGGTCTTG TCTTCTCTTC TGCAAGGCCT AAAAGAACTG CGGAGCACTA ACGCGGAAGA TTGATTTTGA   
  
  
+ AATTTCTTGG TCCACTGCTC TCCTGTGCTG TTTCACCTCT CGACTTTTCT TAATCTCCTC AATCATGTGA   
  
  
+ TGTCTTCAAA GGACCAATCA GAGAAGAGAG ACCAAGAAAG AGACCCTTTT TGAATGAAGA ATTGGTCAGC   
  
  
+ CTTTGGGATT TTCCACTCTC CCACTACTCT CTTTCTTTCA GCTACTTCGT GTTTATGGGA CCGAAGACCA   
  
  
+ GTCTTTGCTG AAAAACTCGG TTTCCATCAG TCAAACTTCC ACAAAATTTC TATCCTCCCA GAAGGGTACC   
  
  
+ CCAAAGGGCT TCGCTTTATA AGCCAAAGCT TCACTTTCCT TCTTGACGCA AGACAGATGC TTTTACTCTT   
  
  
+ CCTGCTCCTT CACACAAAGA ACCTTCATTT TCTCTCTTTT CATATTATCA GTCATATACT TCTCATTCCA   
  
  
+ GTAAATTTCA TCATTGTAGC TTGTTACCCT TTAAGATTCC ATTGTCTAAA GGGGTATAAT CTTCGTTGTC   
  
  
+ TGCTTCGTGG TGGAAAGGTT TTGCTGCTTC AGACGGGAAG TAGCATGTGC TTTCAGACAA GCTGAGATAC   
  
  
+ TGTATTGCTT GTAGCTCTTG GTCGTTGAA  

- ATAAAATCAC AAACCACGAA AGTTTAAAGT AAATGGATTA ATAAATTCAT GTACAAACTA GTAAAACAAC   
  
  
- AAAGGTTAAA TTAAATTAAG TTAGTATTAT TTTTTTTAAA CGATTAATAA AATAGTACTT TTCTAAATTT   
  
  
- TAAAAAATTC ATAACTTATA TATATATATA TATATAATTT TACAATACAA TAATTTTTAA AGTTTTTCAA   
  
  
- TAATTTTTAT TGAGTATTTA AATGTTTAAT AATTTTAATT TTTTAATTTT AATTTTAAAC AAATTTAATA   
  
  
- AAATTTTTAT AAAGTTATGA TTTTTATAAA AATTAAGAAG TAGTGGGAAA CTAAACTTTA GATTAATCTA   
  
  
- GTTTAATTTT ATAATGGGTA TTTTAATTAG GTCGGCTAAT GTGGTTTCTC ATATCAAGTC TAAAAAAAAA   
  
  
- AGAAAAACTA ATACGATTGT ACATTTTAAA ACTGAAAGAT AAAATTTCGG TTTTTTTATG TTAATACTTT   
  
  
- CTATTTTTAG GTATTATTTA ATTTTTTGAG TTTTATTTGT TATAAATGCT ATGTTGTTCC TAGTTTTGTT   
  
  
- ATATTATAGG TTTTTAATCT GCGGTGGTAA TTATTATCTA TTACTAAACG TTTTCTTTTA TAGTAAATCT   
  
  
- CATGAGGACA GTTTTAAAAC AGAAAAGAAA AGTCTATACT TTCTTACTCT GGGCCAAGTT TCTAAATGTG   
  
  
- AGAATCTGAA CCAGTCAAGT TTATTAGGAA CATCAAATCA ATTTGGGTAA TTAGTTAACC CATCTAATTA   
  
  
- GTATTAATCA GGGTTTGGTT GGTCTTAGCA CTTCTTAGCA TTCATGGACT AATACCATTT TATTACTCTG   
  
  
- ATACCAGAAC AGAAGAGAAG ACGTTCCGGA TTTTCTTGAC GCCTCGTGAT TGCGCCTTCT AACTAAAACT   
  
  
- TTAAAGAACC AGGTGACGAG AGGACACGAC AAAGTGGAGA GCTGAAAAGA ATTAGAGGAG TTAGTACACT   
  
  
- ACAGAAGTTT CCTGGTTAGT CTCTTCTCTC TGGTTCTTTC TCTGGGAAAA ACTTACTTCT TAACCAGTCG   
  
  
- GAAACCCTAA AAGGTGAGAG GGTGATGAGA GAAAGAAAGT CGATGAAGCA CAAATACCCT GGCTTCTGGT   
  
  
- CAGAAACGAC TTTTTGAGCC AAAGGTAGTC AGTTTGAAGG TGTTTTAAAG ATAGGAGGGT CTTCCCATGG   
  
  
- GGTTTCCCGA AGCGAAATAT TCGGTTTCGA AGTGAAAGGA AGAACTGCGT TCTGTCTACG AAAATGAGAA   
  
  
- GGACGAGGAA GTGTGTTTCT TGGAAGTAAA AGAGAGAAAA GTATAATAGT CAGTATATGA AGAGTAAGGT   
  
  
- CATTTAAAGT AGTAACATCG AACAATGGGA AATTCTAAGG TAACAGATTT CCCCATATTA GAAGCAACAG   
  
  
- ACGAAGCACC ACCTTTCCAA AACGACGAAG TCTGCCCTTC ATCGTACACG AAAGTCTGTT CGACTCTATG   
  
  
- ACATAACGAA CATCGAGAAC CAGCAACTT

+     TA-rich region

| Site Name | Organism | Position | Strand | Matrix score. | sequence | function |
| --- | --- | --- | --- | --- | --- | --- |
| TA-rich region | Nicotiana tabacum | 154 | + | 20 | TATATATATATATATATATATA | enhancer |
| TA-rich region | Nicotiana tabacum | 158 | + | 20 | TATATATATATATATATATATA | enhancer |
| TA-rich region | Nicotiana tabacum | 156 | + | 20 | TATATATATATATATATATATA | enhancer |

> 2018/04/13 10:10:12  
+ TATTTTAGTG TTTGGTGCTT TCAAATTTCA TTTACCTAAT TATTTAAGTA CATGTTTGAT CATTTTGTTG   
  
  
+ TTTCCAATTT AATTTAATTC AATCATAATA AAAAAAATTT GCTAATTATT TTATCATGAA AAGATTTAAA   
  
  
+ ATTTTTTAAG TATTGAATAT ATATATATAT ATATATTAAA ATGTTATGTT ATTAAAAATT TCAAAAAGTT   
  
  
+ ATTAAAAATA ACTCATAAAT TTACAAATTA TTAAAATTAA AAAATTAAAA TTAAAATTTG TTTAAATTAT   
  
  
+ TTTAAAAATA TTTCAATACT AAAAATATTT TTAATTCTTC ATCACCCTTT GATTTGAAAT CTAATTAGAT   
  
  
+ CAAATTAAAA TATTACCCAT AAAATTAATC CAGCCGATTA CACCAAAGAG TATAGTTCAG ATTTTTTTTT   
  
  
+ TCTTTTTGAT TATGCTAACA TGTAAAATTT TGACTTTCTA TTTTAAAGCC AAAAAAATAC AATTATGAAA   
  
  
+ GATAAAAATC CATAATAAAT TAAAAAACTC AAAATAAACA ATATTTACGA TACAACAAGG ATCAAAACAA   
  
  
+ TATAATATCC AAAAATTAGA CGCCACCATT AATAATAGAT AATGATTTGC AAAAGAAAAT ATCATTTAGA   
  
  
+ GTACTCCTGT CAAAATTTTG TCTTTTCTTT TCAGATATGA AAGAATGAGA CCCGGTTCAA AGATTTACAC   
  
  
+ TCTTAGACTT GGTCAGTTCA AATAATCCTT GTAGTTTAGT TAAACCCATT AATCAATTGG GTAGATTAAT   
  
  
+ CATAATTAGT CCCAAACCAA CCAGAATCGT GAAGAATCGT AAGTACCTGA TTATGGTAAA ATAATGAGAC   
  
  
+ TATGGTCTTG TCTTCTCTTC TGCAAGGCCT AAAAGAACTG CGGAGCACTA ACGCGGAAGA TTGATTTTGA   
  
  
+ AATTTCTTGG TCCACTGCTC TCCTGTGCTG TTTCACCTCT CGACTTTTCT TAATCTCCTC AATCATGTGA   
  
  
+ TGTCTTCAAA GGACCAATCA GAGAAGAGAG ACCAAGAAAG AGACCCTTTT TGAATGAAGA ATTGGTCAGC   
  
  
+ CTTTGGGATT TTCCACTCTC CCACTACTCT CTTTCTTTCA GCTACTTCGT GTTTATGGGA CCGAAGACCA   
  
  
+ GTCTTTGCTG AAAAACTCGG TTTCCATCAG TCAAACTTCC ACAAAATTTC TATCCTCCCA GAAGGGTACC   
  
  
+ CCAAAGGGCT TCGCTTTATA AGCCAAAGCT TCACTTTCCT TCTTGACGCA AGACAGATGC TTTTACTCTT   
  
  
+ CCTGCTCCTT CACACAAAGA ACCTTCATTT TCTCTCTTTT CATATTATCA GTCATATACT TCTCATTCCA   
  
  
+ GTAAATTTCA TCATTGTAGC TTGTTACCCT TTAAGATTCC ATTGTCTAAA GGGGTATAAT CTTCGTTGTC   
  
  
+ TGCTTCGTGG TGGAAAGGTT TTGCTGCTTC AGACGGGAAG TAGCATGTGC TTTCAGACAA GCTGAGATAC   
  
  
+ TGTATTGCTT GTAGCTCTTG GTCGTTGAA  

- ATAAAATCAC AAACCACGAA AGTTTAAAGT AAATGGATTA ATAAATTCAT GTACAAACTA GTAAAACAAC   
  
  
- AAAGGTTAAA TTAAATTAAG TTAGTATTAT TTTTTTTAAA CGATTAATAA AATAGTACTT TTCTAAATTT   
  
  
- TAAAAAATTC ATAACTTATA TATATATATA TATATAATTT TACAATACAA TAATTTTTAA AGTTTTTCAA   
  
  
- TAATTTTTAT TGAGTATTTA AATGTTTAAT AATTTTAATT TTTTAATTTT AATTTTAAAC AAATTTAATA   
  
  
- AAATTTTTAT AAAGTTATGA TTTTTATAAA AATTAAGAAG TAGTGGGAAA CTAAACTTTA GATTAATCTA   
  
  
- GTTTAATTTT ATAATGGGTA TTTTAATTAG GTCGGCTAAT GTGGTTTCTC ATATCAAGTC TAAAAAAAAA   
  
  
- AGAAAAACTA ATACGATTGT ACATTTTAAA ACTGAAAGAT AAAATTTCGG TTTTTTTATG TTAATACTTT   
  
  
- CTATTTTTAG GTATTATTTA ATTTTTTGAG TTTTATTTGT TATAAATGCT ATGTTGTTCC TAGTTTTGTT   
  
  
- ATATTATAGG TTTTTAATCT GCGGTGGTAA TTATTATCTA TTACTAAACG TTTTCTTTTA TAGTAAATCT   
  
  
- CATGAGGACA GTTTTAAAAC AGAAAAGAAA AGTCTATACT TTCTTACTCT GGGCCAAGTT TCTAAATGTG   
  
  
- AGAATCTGAA CCAGTCAAGT TTATTAGGAA CATCAAATCA ATTTGGGTAA TTAGTTAACC CATCTAATTA   
  
  
- GTATTAATCA GGGTTTGGTT GGTCTTAGCA CTTCTTAGCA TTCATGGACT AATACCATTT TATTACTCTG   
  
  
- ATACCAGAAC AGAAGAGAAG ACGTTCCGGA TTTTCTTGAC GCCTCGTGAT TGCGCCTTCT AACTAAAACT   
  
  
- TTAAAGAACC AGGTGACGAG AGGACACGAC AAAGTGGAGA GCTGAAAAGA ATTAGAGGAG TTAGTACACT   
  
  
- ACAGAAGTTT CCTGGTTAGT CTCTTCTCTC TGGTTCTTTC TCTGGGAAAA ACTTACTTCT TAACCAGTCG   
  
  
- GAAACCCTAA AAGGTGAGAG GGTGATGAGA GAAAGAAAGT CGATGAAGCA CAAATACCCT GGCTTCTGGT   
  
  
- CAGAAACGAC TTTTTGAGCC AAAGGTAGTC AGTTTGAAGG TGTTTTAAAG ATAGGAGGGT CTTCCCATGG   
  
  
- GGTTTCCCGA AGCGAAATAT TCGGTTTCGA AGTGAAAGGA AGAACTGCGT TCTGTCTACG AAAATGAGAA   
  
  
- GGACGAGGAA GTGTGTTTCT TGGAAGTAAA AGAGAGAAAA GTATAATAGT CAGTATATGA AGAGTAAGGT   
  
  
- CATTTAAAGT AGTAACATCG AACAATGGGA AATTCTAAGG TAACAGATTT CCCCATATTA GAAGCAACAG   
  
  
- ACGAAGCACC ACCTTTCCAA AACGACGAAG TCTGCCCTTC ATCGTACACG AAAGTCTGTT CGACTCTATG   
  
  
- ACATAACGAA CATCGAGAAC CAGCAACTT

+     TATA-box

| Site Name | Organism | Position | Strand | Matrix score. | sequence | function |
| --- | --- | --- | --- | --- | --- | --- |
| TATA-box | Arabidopsis thaliana | 1315 | - | 4 | TATA | core promoter element around -30 of transcription start |
| TATA-box | Lycopersicon esculentum | 309 | + | 5 | TTTTA | core promoter element around -30 of transcription start |
| TATA-box | Arabidopsis thaliana | 1385 | - | 4 | TATA | core promoter element around -30 of transcription start |
| TATA-box | Lycopersicon esculentum | 1251 | + | 5 | TTTTA | core promoter element around -30 of transcription start |
| TATA-box | Glycine max | 1303 | - | 5 | TAATA | core promoter element around -30 of transcription start |
| TATA-box | Arabidopsis thaliana | 1207 | - | 4 | TATA | core promoter element around -30 of transcription start |
| TATA-box | Arabidopsis thaliana | 1206 | - | 5 | TATAA | core promoter element around -30 of transcription start |
| TATA-box | Lycopersicon esculentum | 870 | - | 5 | TTTTA | core promoter element around -30 of transcription start |
| TATA-box | Glycine max | 563 | + | 5 | TAATA | core promoter element around -30 of transcription start |
| TATA-box | Lycopersicon esculentum | 3 | + | 5 | TTTTA | core promoter element around -30 of transcription start |
| TATA-box | Zea mays | 281 | + | 8 | TTTAAAAA | core promoter element around -30 of transcription start |
| TATA-box | Lycopersicon esculentum | 356 | - | 5 | TTTTA | core promoter element around -30 of transcription start |
| TATA-box | Arabidopsis thaliana | 156 | - | 9 | tcTATATAtt | core promoter element around -30 of transcription start |
| TATA-box | Lycopersicon esculentum | 443 | - | 5 | TTTTA | core promoter element around -30 of transcription start |
| TATA-box | Arabidopsis thaliana | 1205 | - | 6 | TATAAA | core promoter element around -30 of transcription start |
| TATA-box | Arabidopsis thaliana | 401 | + | 4 | TATA | core promoter element around -30 of transcription start |
| TATA-box | Arabidopsis thaliana | 213 | + | 9 | TAAAAATAA | core promoter element around -30 of transcription start |
| TATA-box | Arabidopsis thaliana | 158 | + | 8 | TATATATA | core promoter element around -30 of transcription start |
| TATA-box | Arabidopsis thaliana | 172 | + | 4 | TATA | core promoter element around -30 of transcription start |
| TATA-box | Brassica napus | 171 | + | 6 | ATATAT | core promoter element around -30 of transcription start |
| TATA-box | Glycine max | 593 | + | 5 | TAATA | core promoter element around -30 of transcription start |
| TATA-box | Glycine max | 590 | + | 5 | TAATA | core promoter element around -30 of transcription start |
| TATA-box | Lycopersicon esculentum | 827 | - | 5 | TTTTA | core promoter element around -30 of transcription start |
| TATA-box | Arabidopsis thaliana | 561 | + | 4 | TATA | core promoter element around -30 of transcription start |
| TATA-box | Lycopersicon esculentum | 256 | - | 5 | TTTTA | core promoter element around -30 of transcription start |
| TATA-box | Brassica oleracea | 560 | + | 7 | ATATAAT | core promoter element around -30 of transcription start |
| TATA-box | Glycine max | 361 | - | 5 | TAATA | core promoter element around -30 of transcription start |
| TATA-box | Lycopersicon esculentum | 262 | - | 5 | TTTTA | core promoter element around -30 of transcription start |
| TATA-box | Lycopersicon esculentum | 280 | + | 5 | TTTTA | core promoter element around -30 of transcription start |
| TATA-box | Lycopersicon esculentum | 242 | - | 5 | TTTTA | core promoter element around -30 of transcription start |
| TATA-box | Lycopersicon esculentum | 493 | - | 5 | TTTTA | core promoter element around -30 of transcription start |
| TATA-box | Lycopersicon esculentum | 370 | - | 5 | TTTTA | core promoter element around -30 of transcription start |
| TATA-box | Lycopersicon esculentum | 511 | - | 5 | TTTTA | core promoter element around -30 of transcription start |
| TATA-box | Lycopersicon esculentum | 283 | - | 5 | TTTTA | core promoter element around -30 of transcription start |
| TATA-box | Glycine max | 503 | + | 5 | TAATA | core promoter element around -30 of transcription start |
| TATA-box | Lycopersicon esculentum | 461 | + | 5 | TTTTA | core promoter element around -30 of transcription start |
| TATA-box | Lycopersicon esculentum | 99 | - | 5 | TTTTA | core promoter element around -30 of transcription start |
| TATA-box | Lycopersicon esculentum | 300 | - | 5 | TTTTA | core promoter element around -30 of transcription start |
| TATA-box | Arabidopsis thaliana | 168 | + | 8 | TATATATA | core promoter element around -30 of transcription start |
| TATA-box | Arabidopsis thaliana | 160 | + | 8 | TATATATA | core promoter element around -30 of transcription start |
| TATA-box | Lycopersicon esculentum | 137 | - | 5 | TTTTA | core promoter element around -30 of transcription start |
| TATA-box | Arabidopsis thaliana | 162 | + | 8 | TATATATA | core promoter element around -30 of transcription start |
| TATA-box | Arabidopsis thaliana | 164 | + | 8 | TATATATA | core promoter element around -30 of transcription start |
| TATA-box | Lycopersicon esculentum | 193 | - | 5 | TTTTA | core promoter element around -30 of transcription start |
| TATA-box | Lycopersicon esculentum | 119 | + | 5 | TTTTA | core promoter element around -30 of transcription start |
| TATA-box | Lycopersicon esculentum | 177 | - | 5 | TTTTA | core promoter element around -30 of transcription start |
| TATA-box | Brassica napus | 157 | + | 6 | ATATAT | core promoter element around -30 of transcription start |
| TATA-box | Glycine max | 96 | + | 5 | TAATA | core promoter element around -30 of transcription start |
| TATA-box | Brassica napus | 169 | + | 6 | ATATAT | core promoter element around -30 of transcription start |
| TATA-box | Arabidopsis thaliana | 170 | + | 4 | TATA | core promoter element around -30 of transcription start |
| TATA-box | Brassica napus | 159 | + | 6 | ATATAT | core promoter element around -30 of transcription start |
| TATA-box | Brassica napus | 163 | + | 6 | ATATAT | core promoter element around -30 of transcription start |
| TATA-box | Lycopersicon esculentum | 248 | - | 5 | TTTTA | core promoter element around -30 of transcription start |
| TATA-box | Glycine max | 174 | - | 5 | TAATA | core promoter element around -30 of transcription start |
| TATA-box | Lycopersicon esculentum | 144 | + | 5 | TTTTA | core promoter element around -30 of transcription start |
| TATA-box | Brassica napus | 165 | + | 6 | ATATAT | core promoter element around -30 of transcription start |
| TATA-box | Arabidopsis thaliana | 166 | + | 8 | TATATATA | core promoter element around -30 of transcription start |
| TATA-box | Glycine max | 190 | - | 5 | TAATA | core promoter element around -30 of transcription start |
| TATA-box | Brassica napus | 161 | + | 6 | ATATAT | core promoter element around -30 of transcription start |
| TATA-box | Brassica napus | 167 | + | 6 | ATATAT | core promoter element around -30 of transcription start |
| TATA-box | Glycine max | 210 | - | 5 | TAATA | core promoter element around -30 of transcription start |
| TATA-box | Glycine max | 239 | - | 5 | TAATA | core promoter element around -30 of transcription start |

> 2018/04/13 10:10:12  
+ TATTTTAGTG TTTGGTGCTT TCAAATTTCA TTTACCTAAT TATTTAAGTA CATGTTTGAT CATTTTGTTG   
  
  
+ TTTCCAATTT AATTTAATTC AATCATAATA AAAAAAATTT GCTAATTATT TTATCATGAA AAGATTTAAA   
  
  
+ ATTTTTTAAG TATTGAATAT ATATATATAT ATATATTAAA ATGTTATGTT ATTAAAAATT TCAAAAAGTT   
  
  
+ ATTAAAAATA ACTCATAAAT TTACAAATTA TTAAAATTAA AAAATTAAAA TTAAAATTTG TTTAAATTAT   
  
  
+ TTTAAAAATA TTTCAATACT AAAAATATTT TTAATTCTTC ATCACCCTTT GATTTGAAAT CTAATTAGAT   
  
  
+ CAAATTAAAA TATTACCCAT AAAATTAATC CAGCCGATTA CACCAAAGAG TATAGTTCAG ATTTTTTTTT   
  
  
+ TCTTTTTGAT TATGCTAACA TGTAAAATTT TGACTTTCTA TTTTAAAGCC AAAAAAATAC AATTATGAAA   
  
  
+ GATAAAAATC CATAATAAAT TAAAAAACTC AAAATAAACA ATATTTACGA TACAACAAGG ATCAAAACAA   
  
  
+ TATAATATCC AAAAATTAGA CGCCACCATT AATAATAGAT AATGATTTGC AAAAGAAAAT ATCATTTAGA   
  
  
+ GTACTCCTGT CAAAATTTTG TCTTTTCTTT TCAGATATGA AAGAATGAGA CCCGGTTCAA AGATTTACAC   
  
  
+ TCTTAGACTT GGTCAGTTCA AATAATCCTT GTAGTTTAGT TAAACCCATT AATCAATTGG GTAGATTAAT   
  
  
+ CATAATTAGT CCCAAACCAA CCAGAATCGT GAAGAATCGT AAGTACCTGA TTATGGTAAA ATAATGAGAC   
  
  
+ TATGGTCTTG TCTTCTCTTC TGCAAGGCCT AAAAGAACTG CGGAGCACTA ACGCGGAAGA TTGATTTTGA   
  
  
+ AATTTCTTGG TCCACTGCTC TCCTGTGCTG TTTCACCTCT CGACTTTTCT TAATCTCCTC AATCATGTGA   
  
  
+ TGTCTTCAAA GGACCAATCA GAGAAGAGAG ACCAAGAAAG AGACCCTTTT TGAATGAAGA ATTGGTCAGC   
  
  
+ CTTTGGGATT TTCCACTCTC CCACTACTCT CTTTCTTTCA GCTACTTCGT GTTTATGGGA CCGAAGACCA   
  
  
+ GTCTTTGCTG AAAAACTCGG TTTCCATCAG TCAAACTTCC ACAAAATTTC TATCCTCCCA GAAGGGTACC   
  
  
+ CCAAAGGGCT TCGCTTTATA AGCCAAAGCT TCACTTTCCT TCTTGACGCA AGACAGATGC TTTTACTCTT   
  
  
+ CCTGCTCCTT CACACAAAGA ACCTTCATTT TCTCTCTTTT CATATTATCA GTCATATACT TCTCATTCCA   
  
  
+ GTAAATTTCA TCATTGTAGC TTGTTACCCT TTAAGATTCC ATTGTCTAAA GGGGTATAAT CTTCGTTGTC   
  
  
+ TGCTTCGTGG TGGAAAGGTT TTGCTGCTTC AGACGGGAAG TAGCATGTGC TTTCAGACAA GCTGAGATAC   
  
  
+ TGTATTGCTT GTAGCTCTTG GTCGTTGAA  

- ATAAAATCAC AAACCACGAA AGTTTAAAGT AAATGGATTA ATAAATTCAT GTACAAACTA GTAAAACAAC   
  
  
- AAAGGTTAAA TTAAATTAAG TTAGTATTAT TTTTTTTAAA CGATTAATAA AATAGTACTT TTCTAAATTT   
  
  
- TAAAAAATTC ATAACTTATA TATATATATA TATATAATTT TACAATACAA TAATTTTTAA AGTTTTTCAA   
  
  
- TAATTTTTAT TGAGTATTTA AATGTTTAAT AATTTTAATT TTTTAATTTT AATTTTAAAC AAATTTAATA   
  
  
- AAATTTTTAT AAAGTTATGA TTTTTATAAA AATTAAGAAG TAGTGGGAAA CTAAACTTTA GATTAATCTA   
  
  
- GTTTAATTTT ATAATGGGTA TTTTAATTAG GTCGGCTAAT GTGGTTTCTC ATATCAAGTC TAAAAAAAAA   
  
  
- AGAAAAACTA ATACGATTGT ACATTTTAAA ACTGAAAGAT AAAATTTCGG TTTTTTTATG TTAATACTTT   
  
  
- CTATTTTTAG GTATTATTTA ATTTTTTGAG TTTTATTTGT TATAAATGCT ATGTTGTTCC TAGTTTTGTT   
  
  
- ATATTATAGG TTTTTAATCT GCGGTGGTAA TTATTATCTA TTACTAAACG TTTTCTTTTA TAGTAAATCT   
  
  
- CATGAGGACA GTTTTAAAAC AGAAAAGAAA AGTCTATACT TTCTTACTCT GGGCCAAGTT TCTAAATGTG   
  
  
- AGAATCTGAA CCAGTCAAGT TTATTAGGAA CATCAAATCA ATTTGGGTAA TTAGTTAACC CATCTAATTA   
  
  
- GTATTAATCA GGGTTTGGTT GGTCTTAGCA CTTCTTAGCA TTCATGGACT AATACCATTT TATTACTCTG   
  
  
- ATACCAGAAC AGAAGAGAAG ACGTTCCGGA TTTTCTTGAC GCCTCGTGAT TGCGCCTTCT AACTAAAACT   
  
  
- TTAAAGAACC AGGTGACGAG AGGACACGAC AAAGTGGAGA GCTGAAAAGA ATTAGAGGAG TTAGTACACT   
  
  
- ACAGAAGTTT CCTGGTTAGT CTCTTCTCTC TGGTTCTTTC TCTGGGAAAA ACTTACTTCT TAACCAGTCG   
  
  
- GAAACCCTAA AAGGTGAGAG GGTGATGAGA GAAAGAAAGT CGATGAAGCA CAAATACCCT GGCTTCTGGT   
  
  
- CAGAAACGAC TTTTTGAGCC AAAGGTAGTC AGTTTGAAGG TGTTTTAAAG ATAGGAGGGT CTTCCCATGG   
  
  
- GGTTTCCCGA AGCGAAATAT TCGGTTTCGA AGTGAAAGGA AGAACTGCGT TCTGTCTACG AAAATGAGAA   
  
  
- GGACGAGGAA GTGTGTTTCT TGGAAGTAAA AGAGAGAAAA GTATAATAGT CAGTATATGA AGAGTAAGGT   
  
  
- CATTTAAAGT AGTAACATCG AACAATGGGA AATTCTAAGG TAACAGATTT CCCCATATTA GAAGCAACAG   
  
  
- ACGAAGCACC ACCTTTCCAA AACGACGAAG TCTGCCCTTC ATCGTACACG AAAGTCTGTT CGACTCTATG   
  
  
- ACATAACGAA CATCGAGAAC CAGCAACTT

+     TCA-element

| Site Name | Organism | Position | Strand | Matrix score. | sequence | function |
| --- | --- | --- | --- | --- | --- | --- |
| TCA-element | Brassica oleracea | 1315 | - | 9 | GAGAAGAATA | cis-acting element involved in salicylic acid responsiveness |

> 2018/04/13 10:10:12  
+ TATTTTAGTG TTTGGTGCTT TCAAATTTCA TTTACCTAAT TATTTAAGTA CATGTTTGAT CATTTTGTTG   
  
  
+ TTTCCAATTT AATTTAATTC AATCATAATA AAAAAAATTT GCTAATTATT TTATCATGAA AAGATTTAAA   
  
  
+ ATTTTTTAAG TATTGAATAT ATATATATAT ATATATTAAA ATGTTATGTT ATTAAAAATT TCAAAAAGTT   
  
  
+ ATTAAAAATA ACTCATAAAT TTACAAATTA TTAAAATTAA AAAATTAAAA TTAAAATTTG TTTAAATTAT   
  
  
+ TTTAAAAATA TTTCAATACT AAAAATATTT TTAATTCTTC ATCACCCTTT GATTTGAAAT CTAATTAGAT   
  
  
+ CAAATTAAAA TATTACCCAT AAAATTAATC CAGCCGATTA CACCAAAGAG TATAGTTCAG ATTTTTTTTT   
  
  
+ TCTTTTTGAT TATGCTAACA TGTAAAATTT TGACTTTCTA TTTTAAAGCC AAAAAAATAC AATTATGAAA   
  
  
+ GATAAAAATC CATAATAAAT TAAAAAACTC AAAATAAACA ATATTTACGA TACAACAAGG ATCAAAACAA   
  
  
+ TATAATATCC AAAAATTAGA CGCCACCATT AATAATAGAT AATGATTTGC AAAAGAAAAT ATCATTTAGA   
  
  
+ GTACTCCTGT CAAAATTTTG TCTTTTCTTT TCAGATATGA AAGAATGAGA CCCGGTTCAA AGATTTACAC   
  
  
+ TCTTAGACTT GGTCAGTTCA AATAATCCTT GTAGTTTAGT TAAACCCATT AATCAATTGG GTAGATTAAT   
  
  
+ CATAATTAGT CCCAAACCAA CCAGAATCGT GAAGAATCGT AAGTACCTGA TTATGGTAAA ATAATGAGAC   
  
  
+ TATGGTCTTG TCTTCTCTTC TGCAAGGCCT AAAAGAACTG CGGAGCACTA ACGCGGAAGA TTGATTTTGA   
  
  
+ AATTTCTTGG TCCACTGCTC TCCTGTGCTG TTTCACCTCT CGACTTTTCT TAATCTCCTC AATCATGTGA   
  
  
+ TGTCTTCAAA GGACCAATCA GAGAAGAGAG ACCAAGAAAG AGACCCTTTT TGAATGAAGA ATTGGTCAGC   
  
  
+ CTTTGGGATT TTCCACTCTC CCACTACTCT CTTTCTTTCA GCTACTTCGT GTTTATGGGA CCGAAGACCA   
  
  
+ GTCTTTGCTG AAAAACTCGG TTTCCATCAG TCAAACTTCC ACAAAATTTC TATCCTCCCA GAAGGGTACC   
  
  
+ CCAAAGGGCT TCGCTTTATA AGCCAAAGCT TCACTTTCCT TCTTGACGCA AGACAGATGC TTTTACTCTT   
  
  
+ CCTGCTCCTT CACACAAAGA ACCTTCATTT TCTCTCTTTT CATATTATCA GTCATATACT TCTCATTCCA   
  
  
+ GTAAATTTCA TCATTGTAGC TTGTTACCCT TTAAGATTCC ATTGTCTAAA GGGGTATAAT CTTCGTTGTC   
  
  
+ TGCTTCGTGG TGGAAAGGTT TTGCTGCTTC AGACGGGAAG TAGCATGTGC TTTCAGACAA GCTGAGATAC   
  
  
+ TGTATTGCTT GTAGCTCTTG GTCGTTGAA  

- ATAAAATCAC AAACCACGAA AGTTTAAAGT AAATGGATTA ATAAATTCAT GTACAAACTA GTAAAACAAC   
  
  
- AAAGGTTAAA TTAAATTAAG TTAGTATTAT TTTTTTTAAA CGATTAATAA AATAGTACTT TTCTAAATTT   
  
  
- TAAAAAATTC ATAACTTATA TATATATATA TATATAATTT TACAATACAA TAATTTTTAA AGTTTTTCAA   
  
  
- TAATTTTTAT TGAGTATTTA AATGTTTAAT AATTTTAATT TTTTAATTTT AATTTTAAAC AAATTTAATA   
  
  
- AAATTTTTAT AAAGTTATGA TTTTTATAAA AATTAAGAAG TAGTGGGAAA CTAAACTTTA GATTAATCTA   
  
  
- GTTTAATTTT ATAATGGGTA TTTTAATTAG GTCGGCTAAT GTGGTTTCTC ATATCAAGTC TAAAAAAAAA   
  
  
- AGAAAAACTA ATACGATTGT ACATTTTAAA ACTGAAAGAT AAAATTTCGG TTTTTTTATG TTAATACTTT   
  
  
- CTATTTTTAG GTATTATTTA ATTTTTTGAG TTTTATTTGT TATAAATGCT ATGTTGTTCC TAGTTTTGTT   
  
  
- ATATTATAGG TTTTTAATCT GCGGTGGTAA TTATTATCTA TTACTAAACG TTTTCTTTTA TAGTAAATCT   
  
  
- CATGAGGACA GTTTTAAAAC AGAAAAGAAA AGTCTATACT TTCTTACTCT GGGCCAAGTT TCTAAATGTG   
  
  
- AGAATCTGAA CCAGTCAAGT TTATTAGGAA CATCAAATCA ATTTGGGTAA TTAGTTAACC CATCTAATTA   
  
  
- GTATTAATCA GGGTTTGGTT GGTCTTAGCA CTTCTTAGCA TTCATGGACT AATACCATTT TATTACTCTG   
  
  
- ATACCAGAAC AGAAGAGAAG ACGTTCCGGA TTTTCTTGAC GCCTCGTGAT TGCGCCTTCT AACTAAAACT   
  
  
- TTAAAGAACC AGGTGACGAG AGGACACGAC AAAGTGGAGA GCTGAAAAGA ATTAGAGGAG TTAGTACACT   
  
  
- ACAGAAGTTT CCTGGTTAGT CTCTTCTCTC TGGTTCTTTC TCTGGGAAAA ACTTACTTCT TAACCAGTCG   
  
  
- GAAACCCTAA AAGGTGAGAG GGTGATGAGA GAAAGAAAGT CGATGAAGCA CAAATACCCT GGCTTCTGGT   
  
  
- CAGAAACGAC TTTTTGAGCC AAAGGTAGTC AGTTTGAAGG TGTTTTAAAG ATAGGAGGGT CTTCCCATGG   
  
  
- GGTTTCCCGA AGCGAAATAT TCGGTTTCGA AGTGAAAGGA AGAACTGCGT TCTGTCTACG AAAATGAGAA   
  
  
- GGACGAGGAA GTGTGTTTCT TGGAAGTAAA AGAGAGAAAA GTATAATAGT CAGTATATGA AGAGTAAGGT   
  
  
- CATTTAAAGT AGTAACATCG AACAATGGGA AATTCTAAGG TAACAGATTT CCCCATATTA GAAGCAACAG   
  
  
- ACGAAGCACC ACCTTTCCAA AACGACGAAG TCTGCCCTTC ATCGTACACG AAAGTCTGTT CGACTCTATG   
  
  
- ACATAACGAA CATCGAGAAC CAGCAACTT

+     TGA-element

| Site Name | Organism | Position | Strand | Matrix score. | sequence | function |
| --- | --- | --- | --- | --- | --- | --- |
| TGA-element | Brassica oleracea | 1491 | - | 6 | AACGAC | auxin-responsive element |

> 2018/04/13 10:10:12  
+ TATTTTAGTG TTTGGTGCTT TCAAATTTCA TTTACCTAAT TATTTAAGTA CATGTTTGAT CATTTTGTTG   
  
  
+ TTTCCAATTT AATTTAATTC AATCATAATA AAAAAAATTT GCTAATTATT TTATCATGAA AAGATTTAAA   
  
  
+ ATTTTTTAAG TATTGAATAT ATATATATAT ATATATTAAA ATGTTATGTT ATTAAAAATT TCAAAAAGTT   
  
  
+ ATTAAAAATA ACTCATAAAT TTACAAATTA TTAAAATTAA AAAATTAAAA TTAAAATTTG TTTAAATTAT   
  
  
+ TTTAAAAATA TTTCAATACT AAAAATATTT TTAATTCTTC ATCACCCTTT GATTTGAAAT CTAATTAGAT   
  
  
+ CAAATTAAAA TATTACCCAT AAAATTAATC CAGCCGATTA CACCAAAGAG TATAGTTCAG ATTTTTTTTT   
  
  
+ TCTTTTTGAT TATGCTAACA TGTAAAATTT TGACTTTCTA TTTTAAAGCC AAAAAAATAC AATTATGAAA   
  
  
+ GATAAAAATC CATAATAAAT TAAAAAACTC AAAATAAACA ATATTTACGA TACAACAAGG ATCAAAACAA   
  
  
+ TATAATATCC AAAAATTAGA CGCCACCATT AATAATAGAT AATGATTTGC AAAAGAAAAT ATCATTTAGA   
  
  
+ GTACTCCTGT CAAAATTTTG TCTTTTCTTT TCAGATATGA AAGAATGAGA CCCGGTTCAA AGATTTACAC   
  
  
+ TCTTAGACTT GGTCAGTTCA AATAATCCTT GTAGTTTAGT TAAACCCATT AATCAATTGG GTAGATTAAT   
  
  
+ CATAATTAGT CCCAAACCAA CCAGAATCGT GAAGAATCGT AAGTACCTGA TTATGGTAAA ATAATGAGAC   
  
  
+ TATGGTCTTG TCTTCTCTTC TGCAAGGCCT AAAAGAACTG CGGAGCACTA ACGCGGAAGA TTGATTTTGA   
  
  
+ AATTTCTTGG TCCACTGCTC TCCTGTGCTG TTTCACCTCT CGACTTTTCT TAATCTCCTC AATCATGTGA   
  
  
+ TGTCTTCAAA GGACCAATCA GAGAAGAGAG ACCAAGAAAG AGACCCTTTT TGAATGAAGA ATTGGTCAGC   
  
  
+ CTTTGGGATT TTCCACTCTC CCACTACTCT CTTTCTTTCA GCTACTTCGT GTTTATGGGA CCGAAGACCA   
  
  
+ GTCTTTGCTG AAAAACTCGG TTTCCATCAG TCAAACTTCC ACAAAATTTC TATCCTCCCA GAAGGGTACC   
  
  
+ CCAAAGGGCT TCGCTTTATA AGCCAAAGCT TCACTTTCCT TCTTGACGCA AGACAGATGC TTTTACTCTT   
  
  
+ CCTGCTCCTT CACACAAAGA ACCTTCATTT TCTCTCTTTT CATATTATCA GTCATATACT TCTCATTCCA   
  
  
+ GTAAATTTCA TCATTGTAGC TTGTTACCCT TTAAGATTCC ATTGTCTAAA GGGGTATAAT CTTCGTTGTC   
  
  
+ TGCTTCGTGG TGGAAAGGTT TTGCTGCTTC AGACGGGAAG TAGCATGTGC TTTCAGACAA GCTGAGATAC   
  
  
+ TGTATTGCTT GTAGCTCTTG GTCGTTGAA  

- ATAAAATCAC AAACCACGAA AGTTTAAAGT AAATGGATTA ATAAATTCAT GTACAAACTA GTAAAACAAC   
  
  
- AAAGGTTAAA TTAAATTAAG TTAGTATTAT TTTTTTTAAA CGATTAATAA AATAGTACTT TTCTAAATTT   
  
  
- TAAAAAATTC ATAACTTATA TATATATATA TATATAATTT TACAATACAA TAATTTTTAA AGTTTTTCAA   
  
  
- TAATTTTTAT TGAGTATTTA AATGTTTAAT AATTTTAATT TTTTAATTTT AATTTTAAAC AAATTTAATA   
  
  
- AAATTTTTAT AAAGTTATGA TTTTTATAAA AATTAAGAAG TAGTGGGAAA CTAAACTTTA GATTAATCTA   
  
  
- GTTTAATTTT ATAATGGGTA TTTTAATTAG GTCGGCTAAT GTGGTTTCTC ATATCAAGTC TAAAAAAAAA   
  
  
- AGAAAAACTA ATACGATTGT ACATTTTAAA ACTGAAAGAT AAAATTTCGG TTTTTTTATG TTAATACTTT   
  
  
- CTATTTTTAG GTATTATTTA ATTTTTTGAG TTTTATTTGT TATAAATGCT ATGTTGTTCC TAGTTTTGTT   
  
  
- ATATTATAGG TTTTTAATCT GCGGTGGTAA TTATTATCTA TTACTAAACG TTTTCTTTTA TAGTAAATCT   
  
  
- CATGAGGACA GTTTTAAAAC AGAAAAGAAA AGTCTATACT TTCTTACTCT GGGCCAAGTT TCTAAATGTG   
  
  
- AGAATCTGAA CCAGTCAAGT TTATTAGGAA CATCAAATCA ATTTGGGTAA TTAGTTAACC CATCTAATTA   
  
  
- GTATTAATCA GGGTTTGGTT GGTCTTAGCA CTTCTTAGCA TTCATGGACT AATACCATTT TATTACTCTG   
  
  
- ATACCAGAAC AGAAGAGAAG ACGTTCCGGA TTTTCTTGAC GCCTCGTGAT TGCGCCTTCT AACTAAAACT   
  
  
- TTAAAGAACC AGGTGACGAG AGGACACGAC AAAGTGGAGA GCTGAAAAGA ATTAGAGGAG TTAGTACACT   
  
  
- ACAGAAGTTT CCTGGTTAGT CTCTTCTCTC TGGTTCTTTC TCTGGGAAAA ACTTACTTCT TAACCAGTCG   
  
  
- GAAACCCTAA AAGGTGAGAG GGTGATGAGA GAAAGAAAGT CGATGAAGCA CAAATACCCT GGCTTCTGGT   
  
  
- CAGAAACGAC TTTTTGAGCC AAAGGTAGTC AGTTTGAAGG TGTTTTAAAG ATAGGAGGGT CTTCCCATGG   
  
  
- GGTTTCCCGA AGCGAAATAT TCGGTTTCGA AGTGAAAGGA AGAACTGCGT TCTGTCTACG AAAATGAGAA   
  
  
- GGACGAGGAA GTGTGTTTCT TGGAAGTAAA AGAGAGAAAA GTATAATAGT CAGTATATGA AGAGTAAGGT   
  
  
- CATTTAAAGT AGTAACATCG AACAATGGGA AATTCTAAGG TAACAGATTT CCCCATATTA GAAGCAACAG   
  
  
- ACGAAGCACC ACCTTTCCAA AACGACGAAG TCTGCCCTTC ATCGTACACG AAAGTCTGTT CGACTCTATG   
  
  
- ACATAACGAA CATCGAGAAC CAGCAACTT

+     TGACG-motif

| Site Name | Organism | Position | Strand | Matrix score. | sequence | function |
| --- | --- | --- | --- | --- | --- | --- |
| TGACG-motif | Hordeum vulgare | 1234 | + | 5 | TGACG | cis-acting regulatory element involved in the MeJA-responsiveness |

> 2018/04/13 10:10:12  
+ TATTTTAGTG TTTGGTGCTT TCAAATTTCA TTTACCTAAT TATTTAAGTA CATGTTTGAT CATTTTGTTG   
  
  
+ TTTCCAATTT AATTTAATTC AATCATAATA AAAAAAATTT GCTAATTATT TTATCATGAA AAGATTTAAA   
  
  
+ ATTTTTTAAG TATTGAATAT ATATATATAT ATATATTAAA ATGTTATGTT ATTAAAAATT TCAAAAAGTT   
  
  
+ ATTAAAAATA ACTCATAAAT TTACAAATTA TTAAAATTAA AAAATTAAAA TTAAAATTTG TTTAAATTAT   
  
  
+ TTTAAAAATA TTTCAATACT AAAAATATTT TTAATTCTTC ATCACCCTTT GATTTGAAAT CTAATTAGAT   
  
  
+ CAAATTAAAA TATTACCCAT AAAATTAATC CAGCCGATTA CACCAAAGAG TATAGTTCAG ATTTTTTTTT   
  
  
+ TCTTTTTGAT TATGCTAACA TGTAAAATTT TGACTTTCTA TTTTAAAGCC AAAAAAATAC AATTATGAAA   
  
  
+ GATAAAAATC CATAATAAAT TAAAAAACTC AAAATAAACA ATATTTACGA TACAACAAGG ATCAAAACAA   
  
  
+ TATAATATCC AAAAATTAGA CGCCACCATT AATAATAGAT AATGATTTGC AAAAGAAAAT ATCATTTAGA   
  
  
+ GTACTCCTGT CAAAATTTTG TCTTTTCTTT TCAGATATGA AAGAATGAGA CCCGGTTCAA AGATTTACAC   
  
  
+ TCTTAGACTT GGTCAGTTCA AATAATCCTT GTAGTTTAGT TAAACCCATT AATCAATTGG GTAGATTAAT   
  
  
+ CATAATTAGT CCCAAACCAA CCAGAATCGT GAAGAATCGT AAGTACCTGA TTATGGTAAA ATAATGAGAC   
  
  
+ TATGGTCTTG TCTTCTCTTC TGCAAGGCCT AAAAGAACTG CGGAGCACTA ACGCGGAAGA TTGATTTTGA   
  
  
+ AATTTCTTGG TCCACTGCTC TCCTGTGCTG TTTCACCTCT CGACTTTTCT TAATCTCCTC AATCATGTGA   
  
  
+ TGTCTTCAAA GGACCAATCA GAGAAGAGAG ACCAAGAAAG AGACCCTTTT TGAATGAAGA ATTGGTCAGC   
  
  
+ CTTTGGGATT TTCCACTCTC CCACTACTCT CTTTCTTTCA GCTACTTCGT GTTTATGGGA CCGAAGACCA   
  
  
+ GTCTTTGCTG AAAAACTCGG TTTCCATCAG TCAAACTTCC ACAAAATTTC TATCCTCCCA GAAGGGTACC   
  
  
+ CCAAAGGGCT TCGCTTTATA AGCCAAAGCT TCACTTTCCT TCTTGACGCA AGACAGATGC TTTTACTCTT   
  
  
+ CCTGCTCCTT CACACAAAGA ACCTTCATTT TCTCTCTTTT CATATTATCA GTCATATACT TCTCATTCCA   
  
  
+ GTAAATTTCA TCATTGTAGC TTGTTACCCT TTAAGATTCC ATTGTCTAAA GGGGTATAAT CTTCGTTGTC   
  
  
+ TGCTTCGTGG TGGAAAGGTT TTGCTGCTTC AGACGGGAAG TAGCATGTGC TTTCAGACAA GCTGAGATAC   
  
  
+ TGTATTGCTT GTAGCTCTTG GTCGTTGAA  

- ATAAAATCAC AAACCACGAA AGTTTAAAGT AAATGGATTA ATAAATTCAT GTACAAACTA GTAAAACAAC   
  
  
- AAAGGTTAAA TTAAATTAAG TTAGTATTAT TTTTTTTAAA CGATTAATAA AATAGTACTT TTCTAAATTT   
  
  
- TAAAAAATTC ATAACTTATA TATATATATA TATATAATTT TACAATACAA TAATTTTTAA AGTTTTTCAA   
  
  
- TAATTTTTAT TGAGTATTTA AATGTTTAAT AATTTTAATT TTTTAATTTT AATTTTAAAC AAATTTAATA   
  
  
- AAATTTTTAT AAAGTTATGA TTTTTATAAA AATTAAGAAG TAGTGGGAAA CTAAACTTTA GATTAATCTA   
  
  
- GTTTAATTTT ATAATGGGTA TTTTAATTAG GTCGGCTAAT GTGGTTTCTC ATATCAAGTC TAAAAAAAAA   
  
  
- AGAAAAACTA ATACGATTGT ACATTTTAAA ACTGAAAGAT AAAATTTCGG TTTTTTTATG TTAATACTTT   
  
  
- CTATTTTTAG GTATTATTTA ATTTTTTGAG TTTTATTTGT TATAAATGCT ATGTTGTTCC TAGTTTTGTT   
  
  
- ATATTATAGG TTTTTAATCT GCGGTGGTAA TTATTATCTA TTACTAAACG TTTTCTTTTA TAGTAAATCT   
  
  
- CATGAGGACA GTTTTAAAAC AGAAAAGAAA AGTCTATACT TTCTTACTCT GGGCCAAGTT TCTAAATGTG   
  
  
- AGAATCTGAA CCAGTCAAGT TTATTAGGAA CATCAAATCA ATTTGGGTAA TTAGTTAACC CATCTAATTA   
  
  
- GTATTAATCA GGGTTTGGTT GGTCTTAGCA CTTCTTAGCA TTCATGGACT AATACCATTT TATTACTCTG   
  
  
- ATACCAGAAC AGAAGAGAAG ACGTTCCGGA TTTTCTTGAC GCCTCGTGAT TGCGCCTTCT AACTAAAACT   
  
  
- TTAAAGAACC AGGTGACGAG AGGACACGAC AAAGTGGAGA GCTGAAAAGA ATTAGAGGAG TTAGTACACT   
  
  
- ACAGAAGTTT CCTGGTTAGT CTCTTCTCTC TGGTTCTTTC TCTGGGAAAA ACTTACTTCT TAACCAGTCG   
  
  
- GAAACCCTAA AAGGTGAGAG GGTGATGAGA GAAAGAAAGT CGATGAAGCA CAAATACCCT GGCTTCTGGT   
  
  
- CAGAAACGAC TTTTTGAGCC AAAGGTAGTC AGTTTGAAGG TGTTTTAAAG ATAGGAGGGT CTTCCCATGG   
  
  
- GGTTTCCCGA AGCGAAATAT TCGGTTTCGA AGTGAAAGGA AGAACTGCGT TCTGTCTACG AAAATGAGAA   
  
  
- GGACGAGGAA GTGTGTTTCT TGGAAGTAAA AGAGAGAAAA GTATAATAGT CAGTATATGA AGAGTAAGGT   
  
  
- CATTTAAAGT AGTAACATCG AACAATGGGA AATTCTAAGG TAACAGATTT CCCCATATTA GAAGCAACAG   
  
  
- ACGAAGCACC ACCTTTCCAA AACGACGAAG TCTGCCCTTC ATCGTACACG AAAGTCTGTT CGACTCTATG   
  
  
- ACATAACGAA CATCGAGAAC CAGCAACTT

+     Unnamed\_\_1

| Site Name | Organism | Position | Strand | Matrix score. | sequence | function |
| --- | --- | --- | --- | --- | --- | --- |
| Unnamed\_\_1 | Glycine max | 79 | - | 11 | GAATTTAATTAA | 60K protein binding site |
| Unnamed\_\_1 | Zea mays | 1406 | + | 5 | CGTGG |  |

> 2018/04/13 10:10:12  
+ TATTTTAGTG TTTGGTGCTT TCAAATTTCA TTTACCTAAT TATTTAAGTA CATGTTTGAT CATTTTGTTG   
  
  
+ TTTCCAATTT AATTTAATTC AATCATAATA AAAAAAATTT GCTAATTATT TTATCATGAA AAGATTTAAA   
  
  
+ ATTTTTTAAG TATTGAATAT ATATATATAT ATATATTAAA ATGTTATGTT ATTAAAAATT TCAAAAAGTT   
  
  
+ ATTAAAAATA ACTCATAAAT TTACAAATTA TTAAAATTAA AAAATTAAAA TTAAAATTTG TTTAAATTAT   
  
  
+ TTTAAAAATA TTTCAATACT AAAAATATTT TTAATTCTTC ATCACCCTTT GATTTGAAAT CTAATTAGAT   
  
  
+ CAAATTAAAA TATTACCCAT AAAATTAATC CAGCCGATTA CACCAAAGAG TATAGTTCAG ATTTTTTTTT   
  
  
+ TCTTTTTGAT TATGCTAACA TGTAAAATTT TGACTTTCTA TTTTAAAGCC AAAAAAATAC AATTATGAAA   
  
  
+ GATAAAAATC CATAATAAAT TAAAAAACTC AAAATAAACA ATATTTACGA TACAACAAGG ATCAAAACAA   
  
  
+ TATAATATCC AAAAATTAGA CGCCACCATT AATAATAGAT AATGATTTGC AAAAGAAAAT ATCATTTAGA   
  
  
+ GTACTCCTGT CAAAATTTTG TCTTTTCTTT TCAGATATGA AAGAATGAGA CCCGGTTCAA AGATTTACAC   
  
  
+ TCTTAGACTT GGTCAGTTCA AATAATCCTT GTAGTTTAGT TAAACCCATT AATCAATTGG GTAGATTAAT   
  
  
+ CATAATTAGT CCCAAACCAA CCAGAATCGT GAAGAATCGT AAGTACCTGA TTATGGTAAA ATAATGAGAC   
  
  
+ TATGGTCTTG TCTTCTCTTC TGCAAGGCCT AAAAGAACTG CGGAGCACTA ACGCGGAAGA TTGATTTTGA   
  
  
+ AATTTCTTGG TCCACTGCTC TCCTGTGCTG TTTCACCTCT CGACTTTTCT TAATCTCCTC AATCATGTGA   
  
  
+ TGTCTTCAAA GGACCAATCA GAGAAGAGAG ACCAAGAAAG AGACCCTTTT TGAATGAAGA ATTGGTCAGC   
  
  
+ CTTTGGGATT TTCCACTCTC CCACTACTCT CTTTCTTTCA GCTACTTCGT GTTTATGGGA CCGAAGACCA   
  
  
+ GTCTTTGCTG AAAAACTCGG TTTCCATCAG TCAAACTTCC ACAAAATTTC TATCCTCCCA GAAGGGTACC   
  
  
+ CCAAAGGGCT TCGCTTTATA AGCCAAAGCT TCACTTTCCT TCTTGACGCA AGACAGATGC TTTTACTCTT   
  
  
+ CCTGCTCCTT CACACAAAGA ACCTTCATTT TCTCTCTTTT CATATTATCA GTCATATACT TCTCATTCCA   
  
  
+ GTAAATTTCA TCATTGTAGC TTGTTACCCT TTAAGATTCC ATTGTCTAAA GGGGTATAAT CTTCGTTGTC   
  
  
+ TGCTTCGTGG TGGAAAGGTT TTGCTGCTTC AGACGGGAAG TAGCATGTGC TTTCAGACAA GCTGAGATAC   
  
  
+ TGTATTGCTT GTAGCTCTTG GTCGTTGAA  

- ATAAAATCAC AAACCACGAA AGTTTAAAGT AAATGGATTA ATAAATTCAT GTACAAACTA GTAAAACAAC   
  
  
- AAAGGTTAAA TTAAATTAAG TTAGTATTAT TTTTTTTAAA CGATTAATAA AATAGTACTT TTCTAAATTT   
  
  
- TAAAAAATTC ATAACTTATA TATATATATA TATATAATTT TACAATACAA TAATTTTTAA AGTTTTTCAA   
  
  
- TAATTTTTAT TGAGTATTTA AATGTTTAAT AATTTTAATT TTTTAATTTT AATTTTAAAC AAATTTAATA   
  
  
- AAATTTTTAT AAAGTTATGA TTTTTATAAA AATTAAGAAG TAGTGGGAAA CTAAACTTTA GATTAATCTA   
  
  
- GTTTAATTTT ATAATGGGTA TTTTAATTAG GTCGGCTAAT GTGGTTTCTC ATATCAAGTC TAAAAAAAAA   
  
  
- AGAAAAACTA ATACGATTGT ACATTTTAAA ACTGAAAGAT AAAATTTCGG TTTTTTTATG TTAATACTTT   
  
  
- CTATTTTTAG GTATTATTTA ATTTTTTGAG TTTTATTTGT TATAAATGCT ATGTTGTTCC TAGTTTTGTT   
  
  
- ATATTATAGG TTTTTAATCT GCGGTGGTAA TTATTATCTA TTACTAAACG TTTTCTTTTA TAGTAAATCT   
  
  
- CATGAGGACA GTTTTAAAAC AGAAAAGAAA AGTCTATACT TTCTTACTCT GGGCCAAGTT TCTAAATGTG   
  
  
- AGAATCTGAA CCAGTCAAGT TTATTAGGAA CATCAAATCA ATTTGGGTAA TTAGTTAACC CATCTAATTA   
  
  
- GTATTAATCA GGGTTTGGTT GGTCTTAGCA CTTCTTAGCA TTCATGGACT AATACCATTT TATTACTCTG   
  
  
- ATACCAGAAC AGAAGAGAAG ACGTTCCGGA TTTTCTTGAC GCCTCGTGAT TGCGCCTTCT AACTAAAACT   
  
  
- TTAAAGAACC AGGTGACGAG AGGACACGAC AAAGTGGAGA GCTGAAAAGA ATTAGAGGAG TTAGTACACT   
  
  
- ACAGAAGTTT CCTGGTTAGT CTCTTCTCTC TGGTTCTTTC TCTGGGAAAA ACTTACTTCT TAACCAGTCG   
  
  
- GAAACCCTAA AAGGTGAGAG GGTGATGAGA GAAAGAAAGT CGATGAAGCA CAAATACCCT GGCTTCTGGT   
  
  
- CAGAAACGAC TTTTTGAGCC AAAGGTAGTC AGTTTGAAGG TGTTTTAAAG ATAGGAGGGT CTTCCCATGG   
  
  
- GGTTTCCCGA AGCGAAATAT TCGGTTTCGA AGTGAAAGGA AGAACTGCGT TCTGTCTACG AAAATGAGAA   
  
  
- GGACGAGGAA GTGTGTTTCT TGGAAGTAAA AGAGAGAAAA GTATAATAGT CAGTATATGA AGAGTAAGGT   
  
  
- CATTTAAAGT AGTAACATCG AACAATGGGA AATTCTAAGG TAACAGATTT CCCCATATTA GAAGCAACAG   
  
  
- ACGAAGCACC ACCTTTCCAA AACGACGAAG TCTGCCCTTC ATCGTACACG AAAGTCTGTT CGACTCTATG   
  
  
- ACATAACGAA CATCGAGAAC CAGCAACTT

+     Unnamed\_\_3

| Site Name | Organism | Position | Strand | Matrix score. | sequence | function |
| --- | --- | --- | --- | --- | --- | --- |
| Unnamed\_\_3 | Zea mays | 1406 | + | 5 | CGTGG |  |

> 2018/04/13 10:10:12  
+ TATTTTAGTG TTTGGTGCTT TCAAATTTCA TTTACCTAAT TATTTAAGTA CATGTTTGAT CATTTTGTTG   
  
  
+ TTTCCAATTT AATTTAATTC AATCATAATA AAAAAAATTT GCTAATTATT TTATCATGAA AAGATTTAAA   
  
  
+ ATTTTTTAAG TATTGAATAT ATATATATAT ATATATTAAA ATGTTATGTT ATTAAAAATT TCAAAAAGTT   
  
  
+ ATTAAAAATA ACTCATAAAT TTACAAATTA TTAAAATTAA AAAATTAAAA TTAAAATTTG TTTAAATTAT   
  
  
+ TTTAAAAATA TTTCAATACT AAAAATATTT TTAATTCTTC ATCACCCTTT GATTTGAAAT CTAATTAGAT   
  
  
+ CAAATTAAAA TATTACCCAT AAAATTAATC CAGCCGATTA CACCAAAGAG TATAGTTCAG ATTTTTTTTT   
  
  
+ TCTTTTTGAT TATGCTAACA TGTAAAATTT TGACTTTCTA TTTTAAAGCC AAAAAAATAC AATTATGAAA   
  
  
+ GATAAAAATC CATAATAAAT TAAAAAACTC AAAATAAACA ATATTTACGA TACAACAAGG ATCAAAACAA   
  
  
+ TATAATATCC AAAAATTAGA CGCCACCATT AATAATAGAT AATGATTTGC AAAAGAAAAT ATCATTTAGA   
  
  
+ GTACTCCTGT CAAAATTTTG TCTTTTCTTT TCAGATATGA AAGAATGAGA CCCGGTTCAA AGATTTACAC   
  
  
+ TCTTAGACTT GGTCAGTTCA AATAATCCTT GTAGTTTAGT TAAACCCATT AATCAATTGG GTAGATTAAT   
  
  
+ CATAATTAGT CCCAAACCAA CCAGAATCGT GAAGAATCGT AAGTACCTGA TTATGGTAAA ATAATGAGAC   
  
  
+ TATGGTCTTG TCTTCTCTTC TGCAAGGCCT AAAAGAACTG CGGAGCACTA ACGCGGAAGA TTGATTTTGA   
  
  
+ AATTTCTTGG TCCACTGCTC TCCTGTGCTG TTTCACCTCT CGACTTTTCT TAATCTCCTC AATCATGTGA   
  
  
+ TGTCTTCAAA GGACCAATCA GAGAAGAGAG ACCAAGAAAG AGACCCTTTT TGAATGAAGA ATTGGTCAGC   
  
  
+ CTTTGGGATT TTCCACTCTC CCACTACTCT CTTTCTTTCA GCTACTTCGT GTTTATGGGA CCGAAGACCA   
  
  
+ GTCTTTGCTG AAAAACTCGG TTTCCATCAG TCAAACTTCC ACAAAATTTC TATCCTCCCA GAAGGGTACC   
  
  
+ CCAAAGGGCT TCGCTTTATA AGCCAAAGCT TCACTTTCCT TCTTGACGCA AGACAGATGC TTTTACTCTT   
  
  
+ CCTGCTCCTT CACACAAAGA ACCTTCATTT TCTCTCTTTT CATATTATCA GTCATATACT TCTCATTCCA   
  
  
+ GTAAATTTCA TCATTGTAGC TTGTTACCCT TTAAGATTCC ATTGTCTAAA GGGGTATAAT CTTCGTTGTC   
  
  
+ TGCTTCGTGG TGGAAAGGTT TTGCTGCTTC AGACGGGAAG TAGCATGTGC TTTCAGACAA GCTGAGATAC   
  
  
+ TGTATTGCTT GTAGCTCTTG GTCGTTGAA  

- ATAAAATCAC AAACCACGAA AGTTTAAAGT AAATGGATTA ATAAATTCAT GTACAAACTA GTAAAACAAC   
  
  
- AAAGGTTAAA TTAAATTAAG TTAGTATTAT TTTTTTTAAA CGATTAATAA AATAGTACTT TTCTAAATTT   
  
  
- TAAAAAATTC ATAACTTATA TATATATATA TATATAATTT TACAATACAA TAATTTTTAA AGTTTTTCAA   
  
  
- TAATTTTTAT TGAGTATTTA AATGTTTAAT AATTTTAATT TTTTAATTTT AATTTTAAAC AAATTTAATA   
  
  
- AAATTTTTAT AAAGTTATGA TTTTTATAAA AATTAAGAAG TAGTGGGAAA CTAAACTTTA GATTAATCTA   
  
  
- GTTTAATTTT ATAATGGGTA TTTTAATTAG GTCGGCTAAT GTGGTTTCTC ATATCAAGTC TAAAAAAAAA   
  
  
- AGAAAAACTA ATACGATTGT ACATTTTAAA ACTGAAAGAT AAAATTTCGG TTTTTTTATG TTAATACTTT   
  
  
- CTATTTTTAG GTATTATTTA ATTTTTTGAG TTTTATTTGT TATAAATGCT ATGTTGTTCC TAGTTTTGTT   
  
  
- ATATTATAGG TTTTTAATCT GCGGTGGTAA TTATTATCTA TTACTAAACG TTTTCTTTTA TAGTAAATCT   
  
  
- CATGAGGACA GTTTTAAAAC AGAAAAGAAA AGTCTATACT TTCTTACTCT GGGCCAAGTT TCTAAATGTG   
  
  
- AGAATCTGAA CCAGTCAAGT TTATTAGGAA CATCAAATCA ATTTGGGTAA TTAGTTAACC CATCTAATTA   
  
  
- GTATTAATCA GGGTTTGGTT GGTCTTAGCA CTTCTTAGCA TTCATGGACT AATACCATTT TATTACTCTG   
  
  
- ATACCAGAAC AGAAGAGAAG ACGTTCCGGA TTTTCTTGAC GCCTCGTGAT TGCGCCTTCT AACTAAAACT   
  
  
- TTAAAGAACC AGGTGACGAG AGGACACGAC AAAGTGGAGA GCTGAAAAGA ATTAGAGGAG TTAGTACACT   
  
  
- ACAGAAGTTT CCTGGTTAGT CTCTTCTCTC TGGTTCTTTC TCTGGGAAAA ACTTACTTCT TAACCAGTCG   
  
  
- GAAACCCTAA AAGGTGAGAG GGTGATGAGA GAAAGAAAGT CGATGAAGCA CAAATACCCT GGCTTCTGGT   
  
  
- CAGAAACGAC TTTTTGAGCC AAAGGTAGTC AGTTTGAAGG TGTTTTAAAG ATAGGAGGGT CTTCCCATGG   
  
  
- GGTTTCCCGA AGCGAAATAT TCGGTTTCGA AGTGAAAGGA AGAACTGCGT TCTGTCTACG AAAATGAGAA   
  
  
- GGACGAGGAA GTGTGTTTCT TGGAAGTAAA AGAGAGAAAA GTATAATAGT CAGTATATGA AGAGTAAGGT   
  
  
- CATTTAAAGT AGTAACATCG AACAATGGGA AATTCTAAGG TAACAGATTT CCCCATATTA GAAGCAACAG   
  
  
- ACGAAGCACC ACCTTTCCAA AACGACGAAG TCTGCCCTTC ATCGTACACG AAAGTCTGTT CGACTCTATG   
  
  
- ACATAACGAA CATCGAGAAC CAGCAACTT

+     Unnamed\_\_4

| Site Name | Organism | Position | Strand | Matrix score. | sequence | function |
| --- | --- | --- | --- | --- | --- | --- |
| Unnamed\_\_4 | Petroselinum hortense | 930 | + | 4 | CTCC |  |
| Unnamed\_\_4 | Petroselinum hortense | 1068 | + | 4 | CTCC |  |
| Unnamed\_\_4 | Petroselinum hortense | 634 | + | 4 | CTCC |  |
| Unnamed\_\_4 | Petroselinum hortense | 882 | - | 4 | CTCC |  |
| Unnamed\_\_4 | Petroselinum hortense | 965 | + | 4 | CTCC |  |
| Unnamed\_\_4 | Petroselinum hortense | 1265 | + | 4 | CTCC |  |
| Unnamed\_\_4 | Petroselinum hortense | 1175 | + | 4 | CTCC |  |

> 2018/04/13 10:10:12  
+ TATTTTAGTG TTTGGTGCTT TCAAATTTCA TTTACCTAAT TATTTAAGTA CATGTTTGAT CATTTTGTTG   
  
  
+ TTTCCAATTT AATTTAATTC AATCATAATA AAAAAAATTT GCTAATTATT TTATCATGAA AAGATTTAAA   
  
  
+ ATTTTTTAAG TATTGAATAT ATATATATAT ATATATTAAA ATGTTATGTT ATTAAAAATT TCAAAAAGTT   
  
  
+ ATTAAAAATA ACTCATAAAT TTACAAATTA TTAAAATTAA AAAATTAAAA TTAAAATTTG TTTAAATTAT   
  
  
+ TTTAAAAATA TTTCAATACT AAAAATATTT TTAATTCTTC ATCACCCTTT GATTTGAAAT CTAATTAGAT   
  
  
+ CAAATTAAAA TATTACCCAT AAAATTAATC CAGCCGATTA CACCAAAGAG TATAGTTCAG ATTTTTTTTT   
  
  
+ TCTTTTTGAT TATGCTAACA TGTAAAATTT TGACTTTCTA TTTTAAAGCC AAAAAAATAC AATTATGAAA   
  
  
+ GATAAAAATC CATAATAAAT TAAAAAACTC AAAATAAACA ATATTTACGA TACAACAAGG ATCAAAACAA   
  
  
+ TATAATATCC AAAAATTAGA CGCCACCATT AATAATAGAT AATGATTTGC AAAAGAAAAT ATCATTTAGA   
  
  
+ GTACTCCTGT CAAAATTTTG TCTTTTCTTT TCAGATATGA AAGAATGAGA CCCGGTTCAA AGATTTACAC   
  
  
+ TCTTAGACTT GGTCAGTTCA AATAATCCTT GTAGTTTAGT TAAACCCATT AATCAATTGG GTAGATTAAT   
  
  
+ CATAATTAGT CCCAAACCAA CCAGAATCGT GAAGAATCGT AAGTACCTGA TTATGGTAAA ATAATGAGAC   
  
  
+ TATGGTCTTG TCTTCTCTTC TGCAAGGCCT AAAAGAACTG CGGAGCACTA ACGCGGAAGA TTGATTTTGA   
  
  
+ AATTTCTTGG TCCACTGCTC TCCTGTGCTG TTTCACCTCT CGACTTTTCT TAATCTCCTC AATCATGTGA   
  
  
+ TGTCTTCAAA GGACCAATCA GAGAAGAGAG ACCAAGAAAG AGACCCTTTT TGAATGAAGA ATTGGTCAGC   
  
  
+ CTTTGGGATT TTCCACTCTC CCACTACTCT CTTTCTTTCA GCTACTTCGT GTTTATGGGA CCGAAGACCA   
  
  
+ GTCTTTGCTG AAAAACTCGG TTTCCATCAG TCAAACTTCC ACAAAATTTC TATCCTCCCA GAAGGGTACC   
  
  
+ CCAAAGGGCT TCGCTTTATA AGCCAAAGCT TCACTTTCCT TCTTGACGCA AGACAGATGC TTTTACTCTT   
  
  
+ CCTGCTCCTT CACACAAAGA ACCTTCATTT TCTCTCTTTT CATATTATCA GTCATATACT TCTCATTCCA   
  
  
+ GTAAATTTCA TCATTGTAGC TTGTTACCCT TTAAGATTCC ATTGTCTAAA GGGGTATAAT CTTCGTTGTC   
  
  
+ TGCTTCGTGG TGGAAAGGTT TTGCTGCTTC AGACGGGAAG TAGCATGTGC TTTCAGACAA GCTGAGATAC   
  
  
+ TGTATTGCTT GTAGCTCTTG GTCGTTGAA  

- ATAAAATCAC AAACCACGAA AGTTTAAAGT AAATGGATTA ATAAATTCAT GTACAAACTA GTAAAACAAC   
  
  
- AAAGGTTAAA TTAAATTAAG TTAGTATTAT TTTTTTTAAA CGATTAATAA AATAGTACTT TTCTAAATTT   
  
  
- TAAAAAATTC ATAACTTATA TATATATATA TATATAATTT TACAATACAA TAATTTTTAA AGTTTTTCAA   
  
  
- TAATTTTTAT TGAGTATTTA AATGTTTAAT AATTTTAATT TTTTAATTTT AATTTTAAAC AAATTTAATA   
  
  
- AAATTTTTAT AAAGTTATGA TTTTTATAAA AATTAAGAAG TAGTGGGAAA CTAAACTTTA GATTAATCTA   
  
  
- GTTTAATTTT ATAATGGGTA TTTTAATTAG GTCGGCTAAT GTGGTTTCTC ATATCAAGTC TAAAAAAAAA   
  
  
- AGAAAAACTA ATACGATTGT ACATTTTAAA ACTGAAAGAT AAAATTTCGG TTTTTTTATG TTAATACTTT   
  
  
- CTATTTTTAG GTATTATTTA ATTTTTTGAG TTTTATTTGT TATAAATGCT ATGTTGTTCC TAGTTTTGTT   
  
  
- ATATTATAGG TTTTTAATCT GCGGTGGTAA TTATTATCTA TTACTAAACG TTTTCTTTTA TAGTAAATCT   
  
  
- CATGAGGACA GTTTTAAAAC AGAAAAGAAA AGTCTATACT TTCTTACTCT GGGCCAAGTT TCTAAATGTG   
  
  
- AGAATCTGAA CCAGTCAAGT TTATTAGGAA CATCAAATCA ATTTGGGTAA TTAGTTAACC CATCTAATTA   
  
  
- GTATTAATCA GGGTTTGGTT GGTCTTAGCA CTTCTTAGCA TTCATGGACT AATACCATTT TATTACTCTG   
  
  
- ATACCAGAAC AGAAGAGAAG ACGTTCCGGA TTTTCTTGAC GCCTCGTGAT TGCGCCTTCT AACTAAAACT   
  
  
- TTAAAGAACC AGGTGACGAG AGGACACGAC AAAGTGGAGA GCTGAAAAGA ATTAGAGGAG TTAGTACACT   
  
  
- ACAGAAGTTT CCTGGTTAGT CTCTTCTCTC TGGTTCTTTC TCTGGGAAAA ACTTACTTCT TAACCAGTCG   
  
  
- GAAACCCTAA AAGGTGAGAG GGTGATGAGA GAAAGAAAGT CGATGAAGCA CAAATACCCT GGCTTCTGGT   
  
  
- CAGAAACGAC TTTTTGAGCC AAAGGTAGTC AGTTTGAAGG TGTTTTAAAG ATAGGAGGGT CTTCCCATGG   
  
  
- GGTTTCCCGA AGCGAAATAT TCGGTTTCGA AGTGAAAGGA AGAACTGCGT TCTGTCTACG AAAATGAGAA   
  
  
- GGACGAGGAA GTGTGTTTCT TGGAAGTAAA AGAGAGAAAA GTATAATAGT CAGTATATGA AGAGTAAGGT   
  
  
- CATTTAAAGT AGTAACATCG AACAATGGGA AATTCTAAGG TAACAGATTT CCCCATATTA GAAGCAACAG   
  
  
- ACGAAGCACC ACCTTTCCAA AACGACGAAG TCTGCCCTTC ATCGTACACG AAAGTCTGTT CGACTCTATG   
  
  
- ACATAACGAA CATCGAGAAC CAGCAACTT

+     Y-box

| Site Name | Organism | Position | Strand | Matrix score. | sequence | function |
| --- | --- | --- | --- | --- | --- | --- |
| Y-box | Lemna gibba | 1263 | - | 11 | TGTGGAGGAGCA | ? |

> 2018/04/13 10:10:12  
+ TATTTTAGTG TTTGGTGCTT TCAAATTTCA TTTACCTAAT TATTTAAGTA CATGTTTGAT CATTTTGTTG   
  
  
+ TTTCCAATTT AATTTAATTC AATCATAATA AAAAAAATTT GCTAATTATT TTATCATGAA AAGATTTAAA   
  
  
+ ATTTTTTAAG TATTGAATAT ATATATATAT ATATATTAAA ATGTTATGTT ATTAAAAATT TCAAAAAGTT   
  
  
+ ATTAAAAATA ACTCATAAAT TTACAAATTA TTAAAATTAA AAAATTAAAA TTAAAATTTG TTTAAATTAT   
  
  
+ TTTAAAAATA TTTCAATACT AAAAATATTT TTAATTCTTC ATCACCCTTT GATTTGAAAT CTAATTAGAT   
  
  
+ CAAATTAAAA TATTACCCAT AAAATTAATC CAGCCGATTA CACCAAAGAG TATAGTTCAG ATTTTTTTTT   
  
  
+ TCTTTTTGAT TATGCTAACA TGTAAAATTT TGACTTTCTA TTTTAAAGCC AAAAAAATAC AATTATGAAA   
  
  
+ GATAAAAATC CATAATAAAT TAAAAAACTC AAAATAAACA ATATTTACGA TACAACAAGG ATCAAAACAA   
  
  
+ TATAATATCC AAAAATTAGA CGCCACCATT AATAATAGAT AATGATTTGC AAAAGAAAAT ATCATTTAGA   
  
  
+ GTACTCCTGT CAAAATTTTG TCTTTTCTTT TCAGATATGA AAGAATGAGA CCCGGTTCAA AGATTTACAC   
  
  
+ TCTTAGACTT GGTCAGTTCA AATAATCCTT GTAGTTTAGT TAAACCCATT AATCAATTGG GTAGATTAAT   
  
  
+ CATAATTAGT CCCAAACCAA CCAGAATCGT GAAGAATCGT AAGTACCTGA TTATGGTAAA ATAATGAGAC   
  
  
+ TATGGTCTTG TCTTCTCTTC TGCAAGGCCT AAAAGAACTG CGGAGCACTA ACGCGGAAGA TTGATTTTGA   
  
  
+ AATTTCTTGG TCCACTGCTC TCCTGTGCTG TTTCACCTCT CGACTTTTCT TAATCTCCTC AATCATGTGA   
  
  
+ TGTCTTCAAA GGACCAATCA GAGAAGAGAG ACCAAGAAAG AGACCCTTTT TGAATGAAGA ATTGGTCAGC   
  
  
+ CTTTGGGATT TTCCACTCTC CCACTACTCT CTTTCTTTCA GCTACTTCGT GTTTATGGGA CCGAAGACCA   
  
  
+ GTCTTTGCTG AAAAACTCGG TTTCCATCAG TCAAACTTCC ACAAAATTTC TATCCTCCCA GAAGGGTACC   
  
  
+ CCAAAGGGCT TCGCTTTATA AGCCAAAGCT TCACTTTCCT TCTTGACGCA AGACAGATGC TTTTACTCTT   
  
  
+ CCTGCTCCTT CACACAAAGA ACCTTCATTT TCTCTCTTTT CATATTATCA GTCATATACT TCTCATTCCA   
  
  
+ GTAAATTTCA TCATTGTAGC TTGTTACCCT TTAAGATTCC ATTGTCTAAA GGGGTATAAT CTTCGTTGTC   
  
  
+ TGCTTCGTGG TGGAAAGGTT TTGCTGCTTC AGACGGGAAG TAGCATGTGC TTTCAGACAA GCTGAGATAC   
  
  
+ TGTATTGCTT GTAGCTCTTG GTCGTTGAA  

- ATAAAATCAC AAACCACGAA AGTTTAAAGT AAATGGATTA ATAAATTCAT GTACAAACTA GTAAAACAAC   
  
  
- AAAGGTTAAA TTAAATTAAG TTAGTATTAT TTTTTTTAAA CGATTAATAA AATAGTACTT TTCTAAATTT   
  
  
- TAAAAAATTC ATAACTTATA TATATATATA TATATAATTT TACAATACAA TAATTTTTAA AGTTTTTCAA   
  
  
- TAATTTTTAT TGAGTATTTA AATGTTTAAT AATTTTAATT TTTTAATTTT AATTTTAAAC AAATTTAATA   
  
  
- AAATTTTTAT AAAGTTATGA TTTTTATAAA AATTAAGAAG TAGTGGGAAA CTAAACTTTA GATTAATCTA   
  
  
- GTTTAATTTT ATAATGGGTA TTTTAATTAG GTCGGCTAAT GTGGTTTCTC ATATCAAGTC TAAAAAAAAA   
  
  
- AGAAAAACTA ATACGATTGT ACATTTTAAA ACTGAAAGAT AAAATTTCGG TTTTTTTATG TTAATACTTT   
  
  
- CTATTTTTAG GTATTATTTA ATTTTTTGAG TTTTATTTGT TATAAATGCT ATGTTGTTCC TAGTTTTGTT   
  
  
- ATATTATAGG TTTTTAATCT GCGGTGGTAA TTATTATCTA TTACTAAACG TTTTCTTTTA TAGTAAATCT   
  
  
- CATGAGGACA GTTTTAAAAC AGAAAAGAAA AGTCTATACT TTCTTACTCT GGGCCAAGTT TCTAAATGTG   
  
  
- AGAATCTGAA CCAGTCAAGT TTATTAGGAA CATCAAATCA ATTTGGGTAA TTAGTTAACC CATCTAATTA   
  
  
- GTATTAATCA GGGTTTGGTT GGTCTTAGCA CTTCTTAGCA TTCATGGACT AATACCATTT TATTACTCTG   
  
  
- ATACCAGAAC AGAAGAGAAG ACGTTCCGGA TTTTCTTGAC GCCTCGTGAT TGCGCCTTCT AACTAAAACT   
  
  
- TTAAAGAACC AGGTGACGAG AGGACACGAC AAAGTGGAGA GCTGAAAAGA ATTAGAGGAG TTAGTACACT   
  
  
- ACAGAAGTTT CCTGGTTAGT CTCTTCTCTC TGGTTCTTTC TCTGGGAAAA ACTTACTTCT TAACCAGTCG   
  
  
- GAAACCCTAA AAGGTGAGAG GGTGATGAGA GAAAGAAAGT CGATGAAGCA CAAATACCCT GGCTTCTGGT   
  
  
- CAGAAACGAC TTTTTGAGCC AAAGGTAGTC AGTTTGAAGG TGTTTTAAAG ATAGGAGGGT CTTCCCATGG   
  
  
- GGTTTCCCGA AGCGAAATAT TCGGTTTCGA AGTGAAAGGA AGAACTGCGT TCTGTCTACG AAAATGAGAA   
  
  
- GGACGAGGAA GTGTGTTTCT TGGAAGTAAA AGAGAGAAAA GTATAATAGT CAGTATATGA AGAGTAAGGT   
  
  
- CATTTAAAGT AGTAACATCG AACAATGGGA AATTCTAAGG TAACAGATTT CCCCATATTA GAAGCAACAG   
  
  
- ACGAAGCACC ACCTTTCCAA AACGACGAAG TCTGCCCTTC ATCGTACACG AAAGTCTGTT CGACTCTATG   
  
  
- ACATAACGAA CATCGAGAAC CAGCAACTT

+     as-2-box

| Site Name | Organism | Position | Strand | Matrix score. | sequence | function |
| --- | --- | --- | --- | --- | --- | --- |
| as-2-box | Nicotiana tabacum | 598 | + | 9 | GATAatGATG | involved in shoot-specific expression and light responsiveness |

> 2018/04/13 10:10:12  
+ TATTTTAGTG TTTGGTGCTT TCAAATTTCA TTTACCTAAT TATTTAAGTA CATGTTTGAT CATTTTGTTG   
  
  
+ TTTCCAATTT AATTTAATTC AATCATAATA AAAAAAATTT GCTAATTATT TTATCATGAA AAGATTTAAA   
  
  
+ ATTTTTTAAG TATTGAATAT ATATATATAT ATATATTAAA ATGTTATGTT ATTAAAAATT TCAAAAAGTT   
  
  
+ ATTAAAAATA ACTCATAAAT TTACAAATTA TTAAAATTAA AAAATTAAAA TTAAAATTTG TTTAAATTAT   
  
  
+ TTTAAAAATA TTTCAATACT AAAAATATTT TTAATTCTTC ATCACCCTTT GATTTGAAAT CTAATTAGAT   
  
  
+ CAAATTAAAA TATTACCCAT AAAATTAATC CAGCCGATTA CACCAAAGAG TATAGTTCAG ATTTTTTTTT   
  
  
+ TCTTTTTGAT TATGCTAACA TGTAAAATTT TGACTTTCTA TTTTAAAGCC AAAAAAATAC AATTATGAAA   
  
  
+ GATAAAAATC CATAATAAAT TAAAAAACTC AAAATAAACA ATATTTACGA TACAACAAGG ATCAAAACAA   
  
  
+ TATAATATCC AAAAATTAGA CGCCACCATT AATAATAGAT AATGATTTGC AAAAGAAAAT ATCATTTAGA   
  
  
+ GTACTCCTGT CAAAATTTTG TCTTTTCTTT TCAGATATGA AAGAATGAGA CCCGGTTCAA AGATTTACAC   
  
  
+ TCTTAGACTT GGTCAGTTCA AATAATCCTT GTAGTTTAGT TAAACCCATT AATCAATTGG GTAGATTAAT   
  
  
+ CATAATTAGT CCCAAACCAA CCAGAATCGT GAAGAATCGT AAGTACCTGA TTATGGTAAA ATAATGAGAC   
  
  
+ TATGGTCTTG TCTTCTCTTC TGCAAGGCCT AAAAGAACTG CGGAGCACTA ACGCGGAAGA TTGATTTTGA   
  
  
+ AATTTCTTGG TCCACTGCTC TCCTGTGCTG TTTCACCTCT CGACTTTTCT TAATCTCCTC AATCATGTGA   
  
  
+ TGTCTTCAAA GGACCAATCA GAGAAGAGAG ACCAAGAAAG AGACCCTTTT TGAATGAAGA ATTGGTCAGC   
  
  
+ CTTTGGGATT TTCCACTCTC CCACTACTCT CTTTCTTTCA GCTACTTCGT GTTTATGGGA CCGAAGACCA   
  
  
+ GTCTTTGCTG AAAAACTCGG TTTCCATCAG TCAAACTTCC ACAAAATTTC TATCCTCCCA GAAGGGTACC   
  
  
+ CCAAAGGGCT TCGCTTTATA AGCCAAAGCT TCACTTTCCT TCTTGACGCA AGACAGATGC TTTTACTCTT   
  
  
+ CCTGCTCCTT CACACAAAGA ACCTTCATTT TCTCTCTTTT CATATTATCA GTCATATACT TCTCATTCCA   
  
  
+ GTAAATTTCA TCATTGTAGC TTGTTACCCT TTAAGATTCC ATTGTCTAAA GGGGTATAAT CTTCGTTGTC   
  
  
+ TGCTTCGTGG TGGAAAGGTT TTGCTGCTTC AGACGGGAAG TAGCATGTGC TTTCAGACAA GCTGAGATAC   
  
  
+ TGTATTGCTT GTAGCTCTTG GTCGTTGAA  

- ATAAAATCAC AAACCACGAA AGTTTAAAGT AAATGGATTA ATAAATTCAT GTACAAACTA GTAAAACAAC   
  
  
- AAAGGTTAAA TTAAATTAAG TTAGTATTAT TTTTTTTAAA CGATTAATAA AATAGTACTT TTCTAAATTT   
  
  
- TAAAAAATTC ATAACTTATA TATATATATA TATATAATTT TACAATACAA TAATTTTTAA AGTTTTTCAA   
  
  
- TAATTTTTAT TGAGTATTTA AATGTTTAAT AATTTTAATT TTTTAATTTT AATTTTAAAC AAATTTAATA   
  
  
- AAATTTTTAT AAAGTTATGA TTTTTATAAA AATTAAGAAG TAGTGGGAAA CTAAACTTTA GATTAATCTA   
  
  
- GTTTAATTTT ATAATGGGTA TTTTAATTAG GTCGGCTAAT GTGGTTTCTC ATATCAAGTC TAAAAAAAAA   
  
  
- AGAAAAACTA ATACGATTGT ACATTTTAAA ACTGAAAGAT AAAATTTCGG TTTTTTTATG TTAATACTTT   
  
  
- CTATTTTTAG GTATTATTTA ATTTTTTGAG TTTTATTTGT TATAAATGCT ATGTTGTTCC TAGTTTTGTT   
  
  
- ATATTATAGG TTTTTAATCT GCGGTGGTAA TTATTATCTA TTACTAAACG TTTTCTTTTA TAGTAAATCT   
  
  
- CATGAGGACA GTTTTAAAAC AGAAAAGAAA AGTCTATACT TTCTTACTCT GGGCCAAGTT TCTAAATGTG   
  
  
- AGAATCTGAA CCAGTCAAGT TTATTAGGAA CATCAAATCA ATTTGGGTAA TTAGTTAACC CATCTAATTA   
  
  
- GTATTAATCA GGGTTTGGTT GGTCTTAGCA CTTCTTAGCA TTCATGGACT AATACCATTT TATTACTCTG   
  
  
- ATACCAGAAC AGAAGAGAAG ACGTTCCGGA TTTTCTTGAC GCCTCGTGAT TGCGCCTTCT AACTAAAACT   
  
  
- TTAAAGAACC AGGTGACGAG AGGACACGAC AAAGTGGAGA GCTGAAAAGA ATTAGAGGAG TTAGTACACT   
  
  
- ACAGAAGTTT CCTGGTTAGT CTCTTCTCTC TGGTTCTTTC TCTGGGAAAA ACTTACTTCT TAACCAGTCG   
  
  
- GAAACCCTAA AAGGTGAGAG GGTGATGAGA GAAAGAAAGT CGATGAAGCA CAAATACCCT GGCTTCTGGT   
  
  
- CAGAAACGAC TTTTTGAGCC AAAGGTAGTC AGTTTGAAGG TGTTTTAAAG ATAGGAGGGT CTTCCCATGG   
  
  
- GGTTTCCCGA AGCGAAATAT TCGGTTTCGA AGTGAAAGGA AGAACTGCGT TCTGTCTACG AAAATGAGAA   
  
  
- GGACGAGGAA GTGTGTTTCT TGGAAGTAAA AGAGAGAAAA GTATAATAGT CAGTATATGA AGAGTAAGGT   
  
  
- CATTTAAAGT AGTAACATCG AACAATGGGA AATTCTAAGG TAACAGATTT CCCCATATTA GAAGCAACAG   
  
  
- ACGAAGCACC ACCTTTCCAA AACGACGAAG TCTGCCCTTC ATCGTACACG AAAGTCTGTT CGACTCTATG   
  
  
- ACATAACGAA CATCGAGAAC CAGCAACTT

+     circadian

| Site Name | Organism | Position | Strand | Matrix score. | sequence | function |
| --- | --- | --- | --- | --- | --- | --- |
| circadian | Lycopersicon esculentum | 58 | - | 6 | CAANNNNATC | cis-acting regulatory element involved in circadian control |
| circadian | Lycopersicon esculentum | 1365 | - | 6 | CAANNNNATC | cis-acting regulatory element involved in circadian control |

> 2018/04/13 10:10:12  
+ TATTTTAGTG TTTGGTGCTT TCAAATTTCA TTTACCTAAT TATTTAAGTA CATGTTTGAT CATTTTGTTG   
  
  
+ TTTCCAATTT AATTTAATTC AATCATAATA AAAAAAATTT GCTAATTATT TTATCATGAA AAGATTTAAA   
  
  
+ ATTTTTTAAG TATTGAATAT ATATATATAT ATATATTAAA ATGTTATGTT ATTAAAAATT TCAAAAAGTT   
  
  
+ ATTAAAAATA ACTCATAAAT TTACAAATTA TTAAAATTAA AAAATTAAAA TTAAAATTTG TTTAAATTAT   
  
  
+ TTTAAAAATA TTTCAATACT AAAAATATTT TTAATTCTTC ATCACCCTTT GATTTGAAAT CTAATTAGAT   
  
  
+ CAAATTAAAA TATTACCCAT AAAATTAATC CAGCCGATTA CACCAAAGAG TATAGTTCAG ATTTTTTTTT   
  
  
+ TCTTTTTGAT TATGCTAACA TGTAAAATTT TGACTTTCTA TTTTAAAGCC AAAAAAATAC AATTATGAAA   
  
  
+ GATAAAAATC CATAATAAAT TAAAAAACTC AAAATAAACA ATATTTACGA TACAACAAGG ATCAAAACAA   
  
  
+ TATAATATCC AAAAATTAGA CGCCACCATT AATAATAGAT AATGATTTGC AAAAGAAAAT ATCATTTAGA   
  
  
+ GTACTCCTGT CAAAATTTTG TCTTTTCTTT TCAGATATGA AAGAATGAGA CCCGGTTCAA AGATTTACAC   
  
  
+ TCTTAGACTT GGTCAGTTCA AATAATCCTT GTAGTTTAGT TAAACCCATT AATCAATTGG GTAGATTAAT   
  
  
+ CATAATTAGT CCCAAACCAA CCAGAATCGT GAAGAATCGT AAGTACCTGA TTATGGTAAA ATAATGAGAC   
  
  
+ TATGGTCTTG TCTTCTCTTC TGCAAGGCCT AAAAGAACTG CGGAGCACTA ACGCGGAAGA TTGATTTTGA   
  
  
+ AATTTCTTGG TCCACTGCTC TCCTGTGCTG TTTCACCTCT CGACTTTTCT TAATCTCCTC AATCATGTGA   
  
  
+ TGTCTTCAAA GGACCAATCA GAGAAGAGAG ACCAAGAAAG AGACCCTTTT TGAATGAAGA ATTGGTCAGC   
  
  
+ CTTTGGGATT TTCCACTCTC CCACTACTCT CTTTCTTTCA GCTACTTCGT GTTTATGGGA CCGAAGACCA   
  
  
+ GTCTTTGCTG AAAAACTCGG TTTCCATCAG TCAAACTTCC ACAAAATTTC TATCCTCCCA GAAGGGTACC   
  
  
+ CCAAAGGGCT TCGCTTTATA AGCCAAAGCT TCACTTTCCT TCTTGACGCA AGACAGATGC TTTTACTCTT   
  
  
+ CCTGCTCCTT CACACAAAGA ACCTTCATTT TCTCTCTTTT CATATTATCA GTCATATACT TCTCATTCCA   
  
  
+ GTAAATTTCA TCATTGTAGC TTGTTACCCT TTAAGATTCC ATTGTCTAAA GGGGTATAAT CTTCGTTGTC   
  
  
+ TGCTTCGTGG TGGAAAGGTT TTGCTGCTTC AGACGGGAAG TAGCATGTGC TTTCAGACAA GCTGAGATAC   
  
  
+ TGTATTGCTT GTAGCTCTTG GTCGTTGAA  

- ATAAAATCAC AAACCACGAA AGTTTAAAGT AAATGGATTA ATAAATTCAT GTACAAACTA GTAAAACAAC   
  
  
- AAAGGTTAAA TTAAATTAAG TTAGTATTAT TTTTTTTAAA CGATTAATAA AATAGTACTT TTCTAAATTT   
  
  
- TAAAAAATTC ATAACTTATA TATATATATA TATATAATTT TACAATACAA TAATTTTTAA AGTTTTTCAA   
  
  
- TAATTTTTAT TGAGTATTTA AATGTTTAAT AATTTTAATT TTTTAATTTT AATTTTAAAC AAATTTAATA   
  
  
- AAATTTTTAT AAAGTTATGA TTTTTATAAA AATTAAGAAG TAGTGGGAAA CTAAACTTTA GATTAATCTA   
  
  
- GTTTAATTTT ATAATGGGTA TTTTAATTAG GTCGGCTAAT GTGGTTTCTC ATATCAAGTC TAAAAAAAAA   
  
  
- AGAAAAACTA ATACGATTGT ACATTTTAAA ACTGAAAGAT AAAATTTCGG TTTTTTTATG TTAATACTTT   
  
  
- CTATTTTTAG GTATTATTTA ATTTTTTGAG TTTTATTTGT TATAAATGCT ATGTTGTTCC TAGTTTTGTT   
  
  
- ATATTATAGG TTTTTAATCT GCGGTGGTAA TTATTATCTA TTACTAAACG TTTTCTTTTA TAGTAAATCT   
  
  
- CATGAGGACA GTTTTAAAAC AGAAAAGAAA AGTCTATACT TTCTTACTCT GGGCCAAGTT TCTAAATGTG   
  
  
- AGAATCTGAA CCAGTCAAGT TTATTAGGAA CATCAAATCA ATTTGGGTAA TTAGTTAACC CATCTAATTA   
  
  
- GTATTAATCA GGGTTTGGTT GGTCTTAGCA CTTCTTAGCA TTCATGGACT AATACCATTT TATTACTCTG   
  
  
- ATACCAGAAC AGAAGAGAAG ACGTTCCGGA TTTTCTTGAC GCCTCGTGAT TGCGCCTTCT AACTAAAACT   
  
  
- TTAAAGAACC AGGTGACGAG AGGACACGAC AAAGTGGAGA GCTGAAAAGA ATTAGAGGAG TTAGTACACT   
  
  
- ACAGAAGTTT CCTGGTTAGT CTCTTCTCTC TGGTTCTTTC TCTGGGAAAA ACTTACTTCT TAACCAGTCG   
  
  
- GAAACCCTAA AAGGTGAGAG GGTGATGAGA GAAAGAAAGT CGATGAAGCA CAAATACCCT GGCTTCTGGT   
  
  
- CAGAAACGAC TTTTTGAGCC AAAGGTAGTC AGTTTGAAGG TGTTTTAAAG ATAGGAGGGT CTTCCCATGG   
  
  
- GGTTTCCCGA AGCGAAATAT TCGGTTTCGA AGTGAAAGGA AGAACTGCGT TCTGTCTACG AAAATGAGAA   
  
  
- GGACGAGGAA GTGTGTTTCT TGGAAGTAAA AGAGAGAAAA GTATAATAGT CAGTATATGA AGAGTAAGGT   
  
  
- CATTTAAAGT AGTAACATCG AACAATGGGA AATTCTAAGG TAACAGATTT CCCCATATTA GAAGCAACAG   
  
  
- ACGAAGCACC ACCTTTCCAA AACGACGAAG TCTGCCCTTC ATCGTACACG AAAGTCTGTT CGACTCTATG   
  
  
- ACATAACGAA CATCGAGAAC CAGCAACTT
