# Supplementary material for: Genome-wide analysis of the WRKY gene family in drumstick (Moringa oleifera Lam.)
Source: PeerJ. 2019 Jun 10;7:e7063. doi: 10.7717/peerj.7063 (PMC6563795; doi:10.7717/peerj.7063)
Supplement: Supplemental Information 1 [file peerj-07-7063-s003.gz › MoWRKY42_plantcare.html]

Content-Type: text/html; charset=ISO-8859-1


CallMat\_Firefox


Webmaster Firefox specific output  
To save the result:
click on the frame with the right mouse button and save the source code as a text file with extension .html  
REFERENCE:PlantCARE: a database of plant cis-acting regulatory elements and a portal to tools for in silico analysis of promoter sequences.  
Lescot, M., Déhais, P., Moreau, Y., De Moor, B., Rouzé ,P.,and Rombauts, S.  
Nucleic Acids Res., Database issue(2002), 30(1):325-327.   


---

> 2018/04/13 10:10:12  
+ ATTTATTTAA TATTTTACAA TTATTTTTAA TATTATTTTT TAAAAATAAT AAATAAAATT ATTTATGAAT   
  
  
+ TAATTTTCAT AAAAATTTAT TGACTTAATT CGTGTATAAT TTTTTTGAGA AAAAATAAAT TAATGTATTT   
  
  
+ AAAATATAAT AAAATAATTT GTTAGTATAA TGAAACTAAC TCTTACATAA TAAAATAATC TGTGAATATG   
  
  
+ ATCCAATTAT TAAAATTCAT GAATAACACA TTTTTTAAAT AGATGATATT ACCAATTGAA TTGACCTTGA   
  
  
+ CCATAATTTT ATGGGCCGGA TTAGTCCAAG CGGCTCCATT CCCGAGTTGC TTTCAGGACC TGCCACAGCT   
  
  
+ GGACAGGACA GATTGCAGAC CCAAAACGAT GGGGAAGTGG GGTTGTAGTA GTTACCTTAA TTACTCTCAA   
  
  
+ TGAGTTTCTG AAATCCAAGG AAGTACAAGT TGAATACAAT TTTACCTTAT CTGATAAGTA TGAAGGGGTA   
  
  
+ AGATAAGGAT GAAGGTACCT TAACTTCTCC TTCGCTTTTA TGTAAGTTTT CTGCATTTTC ATCTGCTTTT   
  
  
+ TGGCCAATGG ATTGGCTGGC AGTAGCCTGT GGTCCTTAGA GCTTGAGAGT CAGAGCGGCA TTGATGAGCT   
  
  
+ TCCCTTCTAT TTCATTTCAC CTTGATTAAC ACTCAACCTA TAGCTTCACT TTCATTTGGC GTACCACGTT   
  
  
+ ATTGTAATTT CGGAATTCAT TTTTTGGCCA AGTTCATGAA GTTTCCCTTA CTGGGTGGAG AGAGAGAGAG   
  
  
+ AGAGATTGCT CTGTTCACAG GTAGAGGAGA AGAGCTGGGC AATGGACACG TGCATCTGAA AGGGAACAGC   
  
  
+ AGAAGATACC CGAGAGACCC TGTGATTTCA CACGAGCCAT GGTGGTTCCA TTACACTTCC ACACCAAAAA   
  
  
+ AGAGAGTTAT TGACCTCTGC GATGACATGG TTTGCCCCAC GAAATCCCAT AAAATCCAAG TGCATTACAT   
  
  
+ CTCTTTTCTG GGGTTTCCGA GTTCTCGAAT GTGTCTATGT TCACAATTTG GCTCAAGAAA AGTAATACCT   
  
  
+ AATTGGCAAC AGGAGATCCA GAGTGTGTAT GCAAGCATGC ACATATGAAG GGCCCCATTC TTGCTTACGT   
  
  
+ AGCCAATCAA GGAGAGCTTG TGGGGCCTCA TGTTTCTCCA CTGAAATATC TTCAATTTAT GTTCAACTGT   
  
  
+ GTGGGTCTCT GCTTATTTTA TTTGGGAGTG GGGGTGGGAG TGGCAATGGG CTGGGGGTGG TGCTGAATGG   
  
  
+ CCTTAAAGTC ATTGTTGTGT TCATAGTTTA TATTAAATGA GTGAAAAAGA TAGGGAGAAA AAGCAATCTT   
  
  
+ TGAGTGTTTT TGTCATCTAT TGTCTTAACT TCCCCCTCCG CCTTTTCTCT CTCTTAATCA ATCAAAGCTC   
  
  
+ TTGCGCTCTC TTCTTTATGG GAAAAGTCTT CCATTTCCAA AACATCCATC TCTGTCTGTT TGAAAGCTTT   
  
  
+ TCGGGGTAAT ACAGTAAACA GGTTGCAGT  

- TAAATAAATT ATAAAATGTT AATAAAAATT ATAATAAAAA ATTTTTATTA TTTATTTTAA TAAATACTTA   
  
  
- ATTAAAAGTA TTTTTAAATA ACTGAATTAA GCACATATTA AAAAAACTCT TTTTTATTTA ATTACATAAA   
  
  
- TTTTATATTA TTTTATTAAA CAATCATATT ACTTTGATTG AGAATGTATT ATTTTATTAG ACACTTATAC   
  
  
- TAGGTTAATA ATTTTAAGTA CTTATTGTGT AAAAAATTTA TCTACTATAA TGGTTAACTT AACTGGAACT   
  
  
- GGTATTAAAA TACCCGGCCT AATCAGGTTC GCCGAGGTAA GGGCTCAACG AAAGTCCTGG ACGGTGTCGA   
  
  
- CCTGTCCTGT CTAACGTCTG GGTTTTGCTA CCCCTTCACC CCAACATCAT CAATGGAATT AATGAGAGTT   
  
  
- ACTCAAAGAC TTTAGGTTCC TTCATGTTCA ACTTATGTTA AAATGGAATA GACTATTCAT ACTTCCCCAT   
  
  
- TCTATTCCTA CTTCCATGGA ATTGAAGAGG AAGCGAAAAT ACATTCAAAA GACGTAAAAG TAGACGAAAA   
  
  
- ACCGGTTACC TAACCGACCG TCATCGGACA CCAGGAATCT CGAACTCTCA GTCTCGCCGT AACTACTCGA   
  
  
- AGGGAAGATA AAGTAAAGTG GAACTAATTG TGAGTTGGAT ATCGAAGTGA AAGTAAACCG CATGGTGCAA   
  
  
- TAACATTAAA GCCTTAAGTA AAAAACCGGT TCAAGTACTT CAAAGGGAAT GACCCACCTC TCTCTCTCTC   
  
  
- TCTCTAACGA GACAAGTGTC CATCTCCTCT TCTCGACCCG TTACCTGTGC ACGTAGACTT TCCCTTGTCG   
  
  
- TCTTCTATGG GCTCTCTGGG ACACTAAAGT GTGCTCGGTA CCACCAAGGT AATGTGAAGG TGTGGTTTTT   
  
  
- TCTCTCAATA ACTGGAGACG CTACTGTACC AAACGGGGTG CTTTAGGGTA TTTTAGGTTC ACGTAATGTA   
  
  
- GAGAAAAGAC CCCAAAGGCT CAAGAGCTTA CACAGATACA AGTGTTAAAC CGAGTTCTTT TCATTATGGA   
  
  
- TTAACCGTTG TCCTCTAGGT CTCACACATA CGTTCGTACG TGTATACTTC CCGGGGTAAG AACGAATGCA   
  
  
- TCGGTTAGTT CCTCTCGAAC ACCCCGGAGT ACAAAGAGGT GACTTTATAG AAGTTAAATA CAAGTTGACA   
  
  
- CACCCAGAGA CGAATAAAAT AAACCCTCAC CCCCACCCTC ACCGTTACCC GACCCCCACC ACGACTTACC   
  
  
- GGAATTTCAG TAACAACACA AGTATCAAAT ATAATTTACT CACTTTTTCT ATCCCTCTTT TTCGTTAGAA   
  
  
- ACTCACAAAA ACAGTAGATA ACAGAATTGA AGGGGGAGGC GGAAAAGAGA GAGAATTAGT TAGTTTCGAG   
  
  
- AACGCGAGAG AAGAAATACC CTTTTCAGAA GGTAAAGGTT TTGTAGGTAG AGACAGACAA ACTTTCGAAA   
  
  
- AGCCCCATTA TGTCATTTGT CCAACGTCA

  
  
Motifs Found  

+     5UTR Py-rich stretch

| Site Name | Organism | Position | Strand | Matrix score. | sequence | function |
| --- | --- | --- | --- | --- | --- | --- |
| 5UTR Py-rich stretch | Lycopersicon esculentum | 758 | - | 13 | TTTCTCTCTCTCTC | cis-acting element conferring high transcription levels |
| 5UTR Py-rich stretch | Lycopersicon esculentum | 760 | - | 13 | TTTCTCTCTCTCTC | cis-acting element conferring high transcription levels |
| 5UTR Py-rich stretch | Lycopersicon esculentum | 762 | - | 13 | TTTCTCTCTCTCTC | cis-acting element conferring high transcription levels |

> 2018/04/13 10:10:12  
+ ATTTATTTAA TATTTTACAA TTATTTTTAA TATTATTTTT TAAAAATAAT AAATAAAATT ATTTATGAAT   
  
  
+ TAATTTTCAT AAAAATTTAT TGACTTAATT CGTGTATAAT TTTTTTGAGA AAAAATAAAT TAATGTATTT   
  
  
+ AAAATATAAT AAAATAATTT GTTAGTATAA TGAAACTAAC TCTTACATAA TAAAATAATC TGTGAATATG   
  
  
+ ATCCAATTAT TAAAATTCAT GAATAACACA TTTTTTAAAT AGATGATATT ACCAATTGAA TTGACCTTGA   
  
  
+ CCATAATTTT ATGGGCCGGA TTAGTCCAAG CGGCTCCATT CCCGAGTTGC TTTCAGGACC TGCCACAGCT   
  
  
+ GGACAGGACA GATTGCAGAC CCAAAACGAT GGGGAAGTGG GGTTGTAGTA GTTACCTTAA TTACTCTCAA   
  
  
+ TGAGTTTCTG AAATCCAAGG AAGTACAAGT TGAATACAAT TTTACCTTAT CTGATAAGTA TGAAGGGGTA   
  
  
+ AGATAAGGAT GAAGGTACCT TAACTTCTCC TTCGCTTTTA TGTAAGTTTT CTGCATTTTC ATCTGCTTTT   
  
  
+ TGGCCAATGG ATTGGCTGGC AGTAGCCTGT GGTCCTTAGA GCTTGAGAGT CAGAGCGGCA TTGATGAGCT   
  
  
+ TCCCTTCTAT TTCATTTCAC CTTGATTAAC ACTCAACCTA TAGCTTCACT TTCATTTGGC GTACCACGTT   
  
  
+ ATTGTAATTT CGGAATTCAT TTTTTGGCCA AGTTCATGAA GTTTCCCTTA CTGGGTGGAG AGAGAGAGAG   
  
  
+ AGAGATTGCT CTGTTCACAG GTAGAGGAGA AGAGCTGGGC AATGGACACG TGCATCTGAA AGGGAACAGC   
  
  
+ AGAAGATACC CGAGAGACCC TGTGATTTCA CACGAGCCAT GGTGGTTCCA TTACACTTCC ACACCAAAAA   
  
  
+ AGAGAGTTAT TGACCTCTGC GATGACATGG TTTGCCCCAC GAAATCCCAT AAAATCCAAG TGCATTACAT   
  
  
+ CTCTTTTCTG GGGTTTCCGA GTTCTCGAAT GTGTCTATGT TCACAATTTG GCTCAAGAAA AGTAATACCT   
  
  
+ AATTGGCAAC AGGAGATCCA GAGTGTGTAT GCAAGCATGC ACATATGAAG GGCCCCATTC TTGCTTACGT   
  
  
+ AGCCAATCAA GGAGAGCTTG TGGGGCCTCA TGTTTCTCCA CTGAAATATC TTCAATTTAT GTTCAACTGT   
  
  
+ GTGGGTCTCT GCTTATTTTA TTTGGGAGTG GGGGTGGGAG TGGCAATGGG CTGGGGGTGG TGCTGAATGG   
  
  
+ CCTTAAAGTC ATTGTTGTGT TCATAGTTTA TATTAAATGA GTGAAAAAGA TAGGGAGAAA AAGCAATCTT   
  
  
+ TGAGTGTTTT TGTCATCTAT TGTCTTAACT TCCCCCTCCG CCTTTTCTCT CTCTTAATCA ATCAAAGCTC   
  
  
+ TTGCGCTCTC TTCTTTATGG GAAAAGTCTT CCATTTCCAA AACATCCATC TCTGTCTGTT TGAAAGCTTT   
  
  
+ TCGGGGTAAT ACAGTAAACA GGTTGCAGT  

- TAAATAAATT ATAAAATGTT AATAAAAATT ATAATAAAAA ATTTTTATTA TTTATTTTAA TAAATACTTA   
  
  
- ATTAAAAGTA TTTTTAAATA ACTGAATTAA GCACATATTA AAAAAACTCT TTTTTATTTA ATTACATAAA   
  
  
- TTTTATATTA TTTTATTAAA CAATCATATT ACTTTGATTG AGAATGTATT ATTTTATTAG ACACTTATAC   
  
  
- TAGGTTAATA ATTTTAAGTA CTTATTGTGT AAAAAATTTA TCTACTATAA TGGTTAACTT AACTGGAACT   
  
  
- GGTATTAAAA TACCCGGCCT AATCAGGTTC GCCGAGGTAA GGGCTCAACG AAAGTCCTGG ACGGTGTCGA   
  
  
- CCTGTCCTGT CTAACGTCTG GGTTTTGCTA CCCCTTCACC CCAACATCAT CAATGGAATT AATGAGAGTT   
  
  
- ACTCAAAGAC TTTAGGTTCC TTCATGTTCA ACTTATGTTA AAATGGAATA GACTATTCAT ACTTCCCCAT   
  
  
- TCTATTCCTA CTTCCATGGA ATTGAAGAGG AAGCGAAAAT ACATTCAAAA GACGTAAAAG TAGACGAAAA   
  
  
- ACCGGTTACC TAACCGACCG TCATCGGACA CCAGGAATCT CGAACTCTCA GTCTCGCCGT AACTACTCGA   
  
  
- AGGGAAGATA AAGTAAAGTG GAACTAATTG TGAGTTGGAT ATCGAAGTGA AAGTAAACCG CATGGTGCAA   
  
  
- TAACATTAAA GCCTTAAGTA AAAAACCGGT TCAAGTACTT CAAAGGGAAT GACCCACCTC TCTCTCTCTC   
  
  
- TCTCTAACGA GACAAGTGTC CATCTCCTCT TCTCGACCCG TTACCTGTGC ACGTAGACTT TCCCTTGTCG   
  
  
- TCTTCTATGG GCTCTCTGGG ACACTAAAGT GTGCTCGGTA CCACCAAGGT AATGTGAAGG TGTGGTTTTT   
  
  
- TCTCTCAATA ACTGGAGACG CTACTGTACC AAACGGGGTG CTTTAGGGTA TTTTAGGTTC ACGTAATGTA   
  
  
- GAGAAAAGAC CCCAAAGGCT CAAGAGCTTA CACAGATACA AGTGTTAAAC CGAGTTCTTT TCATTATGGA   
  
  
- TTAACCGTTG TCCTCTAGGT CTCACACATA CGTTCGTACG TGTATACTTC CCGGGGTAAG AACGAATGCA   
  
  
- TCGGTTAGTT CCTCTCGAAC ACCCCGGAGT ACAAAGAGGT GACTTTATAG AAGTTAAATA CAAGTTGACA   
  
  
- CACCCAGAGA CGAATAAAAT AAACCCTCAC CCCCACCCTC ACCGTTACCC GACCCCCACC ACGACTTACC   
  
  
- GGAATTTCAG TAACAACACA AGTATCAAAT ATAATTTACT CACTTTTTCT ATCCCTCTTT TTCGTTAGAA   
  
  
- ACTCACAAAA ACAGTAGATA ACAGAATTGA AGGGGGAGGC GGAAAAGAGA GAGAATTAGT TAGTTTCGAG   
  
  
- AACGCGAGAG AAGAAATACC CTTTTCAGAA GGTAAAGGTT TTGTAGGTAG AGACAGACAA ACTTTCGAAA   
  
  
- AGCCCCATTA TGTCATTTGT CCAACGTCA

+     ABRE

| Site Name | Organism | Position | Strand | Matrix score. | sequence | function |
| --- | --- | --- | --- | --- | --- | --- |
| ABRE | Arabidopsis thaliana | 817 | - | 6 | CACGTG | cis-acting element involved in the abscisic acid responsiveness |
| ABRE | Hordeum vulgare | 815 | - | 9 | CGCACGTGTC | cis-acting element involved in the abscisic acid responsiveness |

> 2018/04/13 10:10:12  
+ ATTTATTTAA TATTTTACAA TTATTTTTAA TATTATTTTT TAAAAATAAT AAATAAAATT ATTTATGAAT   
  
  
+ TAATTTTCAT AAAAATTTAT TGACTTAATT CGTGTATAAT TTTTTTGAGA AAAAATAAAT TAATGTATTT   
  
  
+ AAAATATAAT AAAATAATTT GTTAGTATAA TGAAACTAAC TCTTACATAA TAAAATAATC TGTGAATATG   
  
  
+ ATCCAATTAT TAAAATTCAT GAATAACACA TTTTTTAAAT AGATGATATT ACCAATTGAA TTGACCTTGA   
  
  
+ CCATAATTTT ATGGGCCGGA TTAGTCCAAG CGGCTCCATT CCCGAGTTGC TTTCAGGACC TGCCACAGCT   
  
  
+ GGACAGGACA GATTGCAGAC CCAAAACGAT GGGGAAGTGG GGTTGTAGTA GTTACCTTAA TTACTCTCAA   
  
  
+ TGAGTTTCTG AAATCCAAGG AAGTACAAGT TGAATACAAT TTTACCTTAT CTGATAAGTA TGAAGGGGTA   
  
  
+ AGATAAGGAT GAAGGTACCT TAACTTCTCC TTCGCTTTTA TGTAAGTTTT CTGCATTTTC ATCTGCTTTT   
  
  
+ TGGCCAATGG ATTGGCTGGC AGTAGCCTGT GGTCCTTAGA GCTTGAGAGT CAGAGCGGCA TTGATGAGCT   
  
  
+ TCCCTTCTAT TTCATTTCAC CTTGATTAAC ACTCAACCTA TAGCTTCACT TTCATTTGGC GTACCACGTT   
  
  
+ ATTGTAATTT CGGAATTCAT TTTTTGGCCA AGTTCATGAA GTTTCCCTTA CTGGGTGGAG AGAGAGAGAG   
  
  
+ AGAGATTGCT CTGTTCACAG GTAGAGGAGA AGAGCTGGGC AATGGACACG TGCATCTGAA AGGGAACAGC   
  
  
+ AGAAGATACC CGAGAGACCC TGTGATTTCA CACGAGCCAT GGTGGTTCCA TTACACTTCC ACACCAAAAA   
  
  
+ AGAGAGTTAT TGACCTCTGC GATGACATGG TTTGCCCCAC GAAATCCCAT AAAATCCAAG TGCATTACAT   
  
  
+ CTCTTTTCTG GGGTTTCCGA GTTCTCGAAT GTGTCTATGT TCACAATTTG GCTCAAGAAA AGTAATACCT   
  
  
+ AATTGGCAAC AGGAGATCCA GAGTGTGTAT GCAAGCATGC ACATATGAAG GGCCCCATTC TTGCTTACGT   
  
  
+ AGCCAATCAA GGAGAGCTTG TGGGGCCTCA TGTTTCTCCA CTGAAATATC TTCAATTTAT GTTCAACTGT   
  
  
+ GTGGGTCTCT GCTTATTTTA TTTGGGAGTG GGGGTGGGAG TGGCAATGGG CTGGGGGTGG TGCTGAATGG   
  
  
+ CCTTAAAGTC ATTGTTGTGT TCATAGTTTA TATTAAATGA GTGAAAAAGA TAGGGAGAAA AAGCAATCTT   
  
  
+ TGAGTGTTTT TGTCATCTAT TGTCTTAACT TCCCCCTCCG CCTTTTCTCT CTCTTAATCA ATCAAAGCTC   
  
  
+ TTGCGCTCTC TTCTTTATGG GAAAAGTCTT CCATTTCCAA AACATCCATC TCTGTCTGTT TGAAAGCTTT   
  
  
+ TCGGGGTAAT ACAGTAAACA GGTTGCAGT  

- TAAATAAATT ATAAAATGTT AATAAAAATT ATAATAAAAA ATTTTTATTA TTTATTTTAA TAAATACTTA   
  
  
- ATTAAAAGTA TTTTTAAATA ACTGAATTAA GCACATATTA AAAAAACTCT TTTTTATTTA ATTACATAAA   
  
  
- TTTTATATTA TTTTATTAAA CAATCATATT ACTTTGATTG AGAATGTATT ATTTTATTAG ACACTTATAC   
  
  
- TAGGTTAATA ATTTTAAGTA CTTATTGTGT AAAAAATTTA TCTACTATAA TGGTTAACTT AACTGGAACT   
  
  
- GGTATTAAAA TACCCGGCCT AATCAGGTTC GCCGAGGTAA GGGCTCAACG AAAGTCCTGG ACGGTGTCGA   
  
  
- CCTGTCCTGT CTAACGTCTG GGTTTTGCTA CCCCTTCACC CCAACATCAT CAATGGAATT AATGAGAGTT   
  
  
- ACTCAAAGAC TTTAGGTTCC TTCATGTTCA ACTTATGTTA AAATGGAATA GACTATTCAT ACTTCCCCAT   
  
  
- TCTATTCCTA CTTCCATGGA ATTGAAGAGG AAGCGAAAAT ACATTCAAAA GACGTAAAAG TAGACGAAAA   
  
  
- ACCGGTTACC TAACCGACCG TCATCGGACA CCAGGAATCT CGAACTCTCA GTCTCGCCGT AACTACTCGA   
  
  
- AGGGAAGATA AAGTAAAGTG GAACTAATTG TGAGTTGGAT ATCGAAGTGA AAGTAAACCG CATGGTGCAA   
  
  
- TAACATTAAA GCCTTAAGTA AAAAACCGGT TCAAGTACTT CAAAGGGAAT GACCCACCTC TCTCTCTCTC   
  
  
- TCTCTAACGA GACAAGTGTC CATCTCCTCT TCTCGACCCG TTACCTGTGC ACGTAGACTT TCCCTTGTCG   
  
  
- TCTTCTATGG GCTCTCTGGG ACACTAAAGT GTGCTCGGTA CCACCAAGGT AATGTGAAGG TGTGGTTTTT   
  
  
- TCTCTCAATA ACTGGAGACG CTACTGTACC AAACGGGGTG CTTTAGGGTA TTTTAGGTTC ACGTAATGTA   
  
  
- GAGAAAAGAC CCCAAAGGCT CAAGAGCTTA CACAGATACA AGTGTTAAAC CGAGTTCTTT TCATTATGGA   
  
  
- TTAACCGTTG TCCTCTAGGT CTCACACATA CGTTCGTACG TGTATACTTC CCGGGGTAAG AACGAATGCA   
  
  
- TCGGTTAGTT CCTCTCGAAC ACCCCGGAGT ACAAAGAGGT GACTTTATAG AAGTTAAATA CAAGTTGACA   
  
  
- CACCCAGAGA CGAATAAAAT AAACCCTCAC CCCCACCCTC ACCGTTACCC GACCCCCACC ACGACTTACC   
  
  
- GGAATTTCAG TAACAACACA AGTATCAAAT ATAATTTACT CACTTTTTCT ATCCCTCTTT TTCGTTAGAA   
  
  
- ACTCACAAAA ACAGTAGATA ACAGAATTGA AGGGGGAGGC GGAAAAGAGA GAGAATTAGT TAGTTTCGAG   
  
  
- AACGCGAGAG AAGAAATACC CTTTTCAGAA GGTAAAGGTT TTGTAGGTAG AGACAGACAA ACTTTCGAAA   
  
  
- AGCCCCATTA TGTCATTTGT CCAACGTCA

+     AE-box

| Site Name | Organism | Position | Strand | Matrix score. | sequence | function |
| --- | --- | --- | --- | --- | --- | --- |
| AE-box | Arabidopsis thaliana | 1150 | - | 8 | AGAAACAT | part of a module for light response |

> 2018/04/13 10:10:12  
+ ATTTATTTAA TATTTTACAA TTATTTTTAA TATTATTTTT TAAAAATAAT AAATAAAATT ATTTATGAAT   
  
  
+ TAATTTTCAT AAAAATTTAT TGACTTAATT CGTGTATAAT TTTTTTGAGA AAAAATAAAT TAATGTATTT   
  
  
+ AAAATATAAT AAAATAATTT GTTAGTATAA TGAAACTAAC TCTTACATAA TAAAATAATC TGTGAATATG   
  
  
+ ATCCAATTAT TAAAATTCAT GAATAACACA TTTTTTAAAT AGATGATATT ACCAATTGAA TTGACCTTGA   
  
  
+ CCATAATTTT ATGGGCCGGA TTAGTCCAAG CGGCTCCATT CCCGAGTTGC TTTCAGGACC TGCCACAGCT   
  
  
+ GGACAGGACA GATTGCAGAC CCAAAACGAT GGGGAAGTGG GGTTGTAGTA GTTACCTTAA TTACTCTCAA   
  
  
+ TGAGTTTCTG AAATCCAAGG AAGTACAAGT TGAATACAAT TTTACCTTAT CTGATAAGTA TGAAGGGGTA   
  
  
+ AGATAAGGAT GAAGGTACCT TAACTTCTCC TTCGCTTTTA TGTAAGTTTT CTGCATTTTC ATCTGCTTTT   
  
  
+ TGGCCAATGG ATTGGCTGGC AGTAGCCTGT GGTCCTTAGA GCTTGAGAGT CAGAGCGGCA TTGATGAGCT   
  
  
+ TCCCTTCTAT TTCATTTCAC CTTGATTAAC ACTCAACCTA TAGCTTCACT TTCATTTGGC GTACCACGTT   
  
  
+ ATTGTAATTT CGGAATTCAT TTTTTGGCCA AGTTCATGAA GTTTCCCTTA CTGGGTGGAG AGAGAGAGAG   
  
  
+ AGAGATTGCT CTGTTCACAG GTAGAGGAGA AGAGCTGGGC AATGGACACG TGCATCTGAA AGGGAACAGC   
  
  
+ AGAAGATACC CGAGAGACCC TGTGATTTCA CACGAGCCAT GGTGGTTCCA TTACACTTCC ACACCAAAAA   
  
  
+ AGAGAGTTAT TGACCTCTGC GATGACATGG TTTGCCCCAC GAAATCCCAT AAAATCCAAG TGCATTACAT   
  
  
+ CTCTTTTCTG GGGTTTCCGA GTTCTCGAAT GTGTCTATGT TCACAATTTG GCTCAAGAAA AGTAATACCT   
  
  
+ AATTGGCAAC AGGAGATCCA GAGTGTGTAT GCAAGCATGC ACATATGAAG GGCCCCATTC TTGCTTACGT   
  
  
+ AGCCAATCAA GGAGAGCTTG TGGGGCCTCA TGTTTCTCCA CTGAAATATC TTCAATTTAT GTTCAACTGT   
  
  
+ GTGGGTCTCT GCTTATTTTA TTTGGGAGTG GGGGTGGGAG TGGCAATGGG CTGGGGGTGG TGCTGAATGG   
  
  
+ CCTTAAAGTC ATTGTTGTGT TCATAGTTTA TATTAAATGA GTGAAAAAGA TAGGGAGAAA AAGCAATCTT   
  
  
+ TGAGTGTTTT TGTCATCTAT TGTCTTAACT TCCCCCTCCG CCTTTTCTCT CTCTTAATCA ATCAAAGCTC   
  
  
+ TTGCGCTCTC TTCTTTATGG GAAAAGTCTT CCATTTCCAA AACATCCATC TCTGTCTGTT TGAAAGCTTT   
  
  
+ TCGGGGTAAT ACAGTAAACA GGTTGCAGT  

- TAAATAAATT ATAAAATGTT AATAAAAATT ATAATAAAAA ATTTTTATTA TTTATTTTAA TAAATACTTA   
  
  
- ATTAAAAGTA TTTTTAAATA ACTGAATTAA GCACATATTA AAAAAACTCT TTTTTATTTA ATTACATAAA   
  
  
- TTTTATATTA TTTTATTAAA CAATCATATT ACTTTGATTG AGAATGTATT ATTTTATTAG ACACTTATAC   
  
  
- TAGGTTAATA ATTTTAAGTA CTTATTGTGT AAAAAATTTA TCTACTATAA TGGTTAACTT AACTGGAACT   
  
  
- GGTATTAAAA TACCCGGCCT AATCAGGTTC GCCGAGGTAA GGGCTCAACG AAAGTCCTGG ACGGTGTCGA   
  
  
- CCTGTCCTGT CTAACGTCTG GGTTTTGCTA CCCCTTCACC CCAACATCAT CAATGGAATT AATGAGAGTT   
  
  
- ACTCAAAGAC TTTAGGTTCC TTCATGTTCA ACTTATGTTA AAATGGAATA GACTATTCAT ACTTCCCCAT   
  
  
- TCTATTCCTA CTTCCATGGA ATTGAAGAGG AAGCGAAAAT ACATTCAAAA GACGTAAAAG TAGACGAAAA   
  
  
- ACCGGTTACC TAACCGACCG TCATCGGACA CCAGGAATCT CGAACTCTCA GTCTCGCCGT AACTACTCGA   
  
  
- AGGGAAGATA AAGTAAAGTG GAACTAATTG TGAGTTGGAT ATCGAAGTGA AAGTAAACCG CATGGTGCAA   
  
  
- TAACATTAAA GCCTTAAGTA AAAAACCGGT TCAAGTACTT CAAAGGGAAT GACCCACCTC TCTCTCTCTC   
  
  
- TCTCTAACGA GACAAGTGTC CATCTCCTCT TCTCGACCCG TTACCTGTGC ACGTAGACTT TCCCTTGTCG   
  
  
- TCTTCTATGG GCTCTCTGGG ACACTAAAGT GTGCTCGGTA CCACCAAGGT AATGTGAAGG TGTGGTTTTT   
  
  
- TCTCTCAATA ACTGGAGACG CTACTGTACC AAACGGGGTG CTTTAGGGTA TTTTAGGTTC ACGTAATGTA   
  
  
- GAGAAAAGAC CCCAAAGGCT CAAGAGCTTA CACAGATACA AGTGTTAAAC CGAGTTCTTT TCATTATGGA   
  
  
- TTAACCGTTG TCCTCTAGGT CTCACACATA CGTTCGTACG TGTATACTTC CCGGGGTAAG AACGAATGCA   
  
  
- TCGGTTAGTT CCTCTCGAAC ACCCCGGAGT ACAAAGAGGT GACTTTATAG AAGTTAAATA CAAGTTGACA   
  
  
- CACCCAGAGA CGAATAAAAT AAACCCTCAC CCCCACCCTC ACCGTTACCC GACCCCCACC ACGACTTACC   
  
  
- GGAATTTCAG TAACAACACA AGTATCAAAT ATAATTTACT CACTTTTTCT ATCCCTCTTT TTCGTTAGAA   
  
  
- ACTCACAAAA ACAGTAGATA ACAGAATTGA AGGGGGAGGC GGAAAAGAGA GAGAATTAGT TAGTTTCGAG   
  
  
- AACGCGAGAG AAGAAATACC CTTTTCAGAA GGTAAAGGTT TTGTAGGTAG AGACAGACAA ACTTTCGAAA   
  
  
- AGCCCCATTA TGTCATTTGT CCAACGTCA

+     ARE

| Site Name | Organism | Position | Strand | Matrix score. | sequence | function |
| --- | --- | --- | --- | --- | --- | --- |
| ARE | Zea mays | 938 | + | 6 | TGGTTT | cis-acting regulatory element essential for the anaerobic induction |

> 2018/04/13 10:10:12  
+ ATTTATTTAA TATTTTACAA TTATTTTTAA TATTATTTTT TAAAAATAAT AAATAAAATT ATTTATGAAT   
  
  
+ TAATTTTCAT AAAAATTTAT TGACTTAATT CGTGTATAAT TTTTTTGAGA AAAAATAAAT TAATGTATTT   
  
  
+ AAAATATAAT AAAATAATTT GTTAGTATAA TGAAACTAAC TCTTACATAA TAAAATAATC TGTGAATATG   
  
  
+ ATCCAATTAT TAAAATTCAT GAATAACACA TTTTTTAAAT AGATGATATT ACCAATTGAA TTGACCTTGA   
  
  
+ CCATAATTTT ATGGGCCGGA TTAGTCCAAG CGGCTCCATT CCCGAGTTGC TTTCAGGACC TGCCACAGCT   
  
  
+ GGACAGGACA GATTGCAGAC CCAAAACGAT GGGGAAGTGG GGTTGTAGTA GTTACCTTAA TTACTCTCAA   
  
  
+ TGAGTTTCTG AAATCCAAGG AAGTACAAGT TGAATACAAT TTTACCTTAT CTGATAAGTA TGAAGGGGTA   
  
  
+ AGATAAGGAT GAAGGTACCT TAACTTCTCC TTCGCTTTTA TGTAAGTTTT CTGCATTTTC ATCTGCTTTT   
  
  
+ TGGCCAATGG ATTGGCTGGC AGTAGCCTGT GGTCCTTAGA GCTTGAGAGT CAGAGCGGCA TTGATGAGCT   
  
  
+ TCCCTTCTAT TTCATTTCAC CTTGATTAAC ACTCAACCTA TAGCTTCACT TTCATTTGGC GTACCACGTT   
  
  
+ ATTGTAATTT CGGAATTCAT TTTTTGGCCA AGTTCATGAA GTTTCCCTTA CTGGGTGGAG AGAGAGAGAG   
  
  
+ AGAGATTGCT CTGTTCACAG GTAGAGGAGA AGAGCTGGGC AATGGACACG TGCATCTGAA AGGGAACAGC   
  
  
+ AGAAGATACC CGAGAGACCC TGTGATTTCA CACGAGCCAT GGTGGTTCCA TTACACTTCC ACACCAAAAA   
  
  
+ AGAGAGTTAT TGACCTCTGC GATGACATGG TTTGCCCCAC GAAATCCCAT AAAATCCAAG TGCATTACAT   
  
  
+ CTCTTTTCTG GGGTTTCCGA GTTCTCGAAT GTGTCTATGT TCACAATTTG GCTCAAGAAA AGTAATACCT   
  
  
+ AATTGGCAAC AGGAGATCCA GAGTGTGTAT GCAAGCATGC ACATATGAAG GGCCCCATTC TTGCTTACGT   
  
  
+ AGCCAATCAA GGAGAGCTTG TGGGGCCTCA TGTTTCTCCA CTGAAATATC TTCAATTTAT GTTCAACTGT   
  
  
+ GTGGGTCTCT GCTTATTTTA TTTGGGAGTG GGGGTGGGAG TGGCAATGGG CTGGGGGTGG TGCTGAATGG   
  
  
+ CCTTAAAGTC ATTGTTGTGT TCATAGTTTA TATTAAATGA GTGAAAAAGA TAGGGAGAAA AAGCAATCTT   
  
  
+ TGAGTGTTTT TGTCATCTAT TGTCTTAACT TCCCCCTCCG CCTTTTCTCT CTCTTAATCA ATCAAAGCTC   
  
  
+ TTGCGCTCTC TTCTTTATGG GAAAAGTCTT CCATTTCCAA AACATCCATC TCTGTCTGTT TGAAAGCTTT   
  
  
+ TCGGGGTAAT ACAGTAAACA GGTTGCAGT  

- TAAATAAATT ATAAAATGTT AATAAAAATT ATAATAAAAA ATTTTTATTA TTTATTTTAA TAAATACTTA   
  
  
- ATTAAAAGTA TTTTTAAATA ACTGAATTAA GCACATATTA AAAAAACTCT TTTTTATTTA ATTACATAAA   
  
  
- TTTTATATTA TTTTATTAAA CAATCATATT ACTTTGATTG AGAATGTATT ATTTTATTAG ACACTTATAC   
  
  
- TAGGTTAATA ATTTTAAGTA CTTATTGTGT AAAAAATTTA TCTACTATAA TGGTTAACTT AACTGGAACT   
  
  
- GGTATTAAAA TACCCGGCCT AATCAGGTTC GCCGAGGTAA GGGCTCAACG AAAGTCCTGG ACGGTGTCGA   
  
  
- CCTGTCCTGT CTAACGTCTG GGTTTTGCTA CCCCTTCACC CCAACATCAT CAATGGAATT AATGAGAGTT   
  
  
- ACTCAAAGAC TTTAGGTTCC TTCATGTTCA ACTTATGTTA AAATGGAATA GACTATTCAT ACTTCCCCAT   
  
  
- TCTATTCCTA CTTCCATGGA ATTGAAGAGG AAGCGAAAAT ACATTCAAAA GACGTAAAAG TAGACGAAAA   
  
  
- ACCGGTTACC TAACCGACCG TCATCGGACA CCAGGAATCT CGAACTCTCA GTCTCGCCGT AACTACTCGA   
  
  
- AGGGAAGATA AAGTAAAGTG GAACTAATTG TGAGTTGGAT ATCGAAGTGA AAGTAAACCG CATGGTGCAA   
  
  
- TAACATTAAA GCCTTAAGTA AAAAACCGGT TCAAGTACTT CAAAGGGAAT GACCCACCTC TCTCTCTCTC   
  
  
- TCTCTAACGA GACAAGTGTC CATCTCCTCT TCTCGACCCG TTACCTGTGC ACGTAGACTT TCCCTTGTCG   
  
  
- TCTTCTATGG GCTCTCTGGG ACACTAAAGT GTGCTCGGTA CCACCAAGGT AATGTGAAGG TGTGGTTTTT   
  
  
- TCTCTCAATA ACTGGAGACG CTACTGTACC AAACGGGGTG CTTTAGGGTA TTTTAGGTTC ACGTAATGTA   
  
  
- GAGAAAAGAC CCCAAAGGCT CAAGAGCTTA CACAGATACA AGTGTTAAAC CGAGTTCTTT TCATTATGGA   
  
  
- TTAACCGTTG TCCTCTAGGT CTCACACATA CGTTCGTACG TGTATACTTC CCGGGGTAAG AACGAATGCA   
  
  
- TCGGTTAGTT CCTCTCGAAC ACCCCGGAGT ACAAAGAGGT GACTTTATAG AAGTTAAATA CAAGTTGACA   
  
  
- CACCCAGAGA CGAATAAAAT AAACCCTCAC CCCCACCCTC ACCGTTACCC GACCCCCACC ACGACTTACC   
  
  
- GGAATTTCAG TAACAACACA AGTATCAAAT ATAATTTACT CACTTTTTCT ATCCCTCTTT TTCGTTAGAA   
  
  
- ACTCACAAAA ACAGTAGATA ACAGAATTGA AGGGGGAGGC GGAAAAGAGA GAGAATTAGT TAGTTTCGAG   
  
  
- AACGCGAGAG AAGAAATACC CTTTTCAGAA GGTAAAGGTT TTGTAGGTAG AGACAGACAA ACTTTCGAAA   
  
  
- AGCCCCATTA TGTCATTTGT CCAACGTCA

+     ATC-motif

| Site Name | Organism | Position | Strand | Matrix score. | sequence | function |
| --- | --- | --- | --- | --- | --- | --- |
| ATC-motif | Arabidopsis thaliana | 569 | - | 8 | GCCAATCC | part of a conserved DNA module involved in light responsiveness |

> 2018/04/13 10:10:12  
+ ATTTATTTAA TATTTTACAA TTATTTTTAA TATTATTTTT TAAAAATAAT AAATAAAATT ATTTATGAAT   
  
  
+ TAATTTTCAT AAAAATTTAT TGACTTAATT CGTGTATAAT TTTTTTGAGA AAAAATAAAT TAATGTATTT   
  
  
+ AAAATATAAT AAAATAATTT GTTAGTATAA TGAAACTAAC TCTTACATAA TAAAATAATC TGTGAATATG   
  
  
+ ATCCAATTAT TAAAATTCAT GAATAACACA TTTTTTAAAT AGATGATATT ACCAATTGAA TTGACCTTGA   
  
  
+ CCATAATTTT ATGGGCCGGA TTAGTCCAAG CGGCTCCATT CCCGAGTTGC TTTCAGGACC TGCCACAGCT   
  
  
+ GGACAGGACA GATTGCAGAC CCAAAACGAT GGGGAAGTGG GGTTGTAGTA GTTACCTTAA TTACTCTCAA   
  
  
+ TGAGTTTCTG AAATCCAAGG AAGTACAAGT TGAATACAAT TTTACCTTAT CTGATAAGTA TGAAGGGGTA   
  
  
+ AGATAAGGAT GAAGGTACCT TAACTTCTCC TTCGCTTTTA TGTAAGTTTT CTGCATTTTC ATCTGCTTTT   
  
  
+ TGGCCAATGG ATTGGCTGGC AGTAGCCTGT GGTCCTTAGA GCTTGAGAGT CAGAGCGGCA TTGATGAGCT   
  
  
+ TCCCTTCTAT TTCATTTCAC CTTGATTAAC ACTCAACCTA TAGCTTCACT TTCATTTGGC GTACCACGTT   
  
  
+ ATTGTAATTT CGGAATTCAT TTTTTGGCCA AGTTCATGAA GTTTCCCTTA CTGGGTGGAG AGAGAGAGAG   
  
  
+ AGAGATTGCT CTGTTCACAG GTAGAGGAGA AGAGCTGGGC AATGGACACG TGCATCTGAA AGGGAACAGC   
  
  
+ AGAAGATACC CGAGAGACCC TGTGATTTCA CACGAGCCAT GGTGGTTCCA TTACACTTCC ACACCAAAAA   
  
  
+ AGAGAGTTAT TGACCTCTGC GATGACATGG TTTGCCCCAC GAAATCCCAT AAAATCCAAG TGCATTACAT   
  
  
+ CTCTTTTCTG GGGTTTCCGA GTTCTCGAAT GTGTCTATGT TCACAATTTG GCTCAAGAAA AGTAATACCT   
  
  
+ AATTGGCAAC AGGAGATCCA GAGTGTGTAT GCAAGCATGC ACATATGAAG GGCCCCATTC TTGCTTACGT   
  
  
+ AGCCAATCAA GGAGAGCTTG TGGGGCCTCA TGTTTCTCCA CTGAAATATC TTCAATTTAT GTTCAACTGT   
  
  
+ GTGGGTCTCT GCTTATTTTA TTTGGGAGTG GGGGTGGGAG TGGCAATGGG CTGGGGGTGG TGCTGAATGG   
  
  
+ CCTTAAAGTC ATTGTTGTGT TCATAGTTTA TATTAAATGA GTGAAAAAGA TAGGGAGAAA AAGCAATCTT   
  
  
+ TGAGTGTTTT TGTCATCTAT TGTCTTAACT TCCCCCTCCG CCTTTTCTCT CTCTTAATCA ATCAAAGCTC   
  
  
+ TTGCGCTCTC TTCTTTATGG GAAAAGTCTT CCATTTCCAA AACATCCATC TCTGTCTGTT TGAAAGCTTT   
  
  
+ TCGGGGTAAT ACAGTAAACA GGTTGCAGT  

- TAAATAAATT ATAAAATGTT AATAAAAATT ATAATAAAAA ATTTTTATTA TTTATTTTAA TAAATACTTA   
  
  
- ATTAAAAGTA TTTTTAAATA ACTGAATTAA GCACATATTA AAAAAACTCT TTTTTATTTA ATTACATAAA   
  
  
- TTTTATATTA TTTTATTAAA CAATCATATT ACTTTGATTG AGAATGTATT ATTTTATTAG ACACTTATAC   
  
  
- TAGGTTAATA ATTTTAAGTA CTTATTGTGT AAAAAATTTA TCTACTATAA TGGTTAACTT AACTGGAACT   
  
  
- GGTATTAAAA TACCCGGCCT AATCAGGTTC GCCGAGGTAA GGGCTCAACG AAAGTCCTGG ACGGTGTCGA   
  
  
- CCTGTCCTGT CTAACGTCTG GGTTTTGCTA CCCCTTCACC CCAACATCAT CAATGGAATT AATGAGAGTT   
  
  
- ACTCAAAGAC TTTAGGTTCC TTCATGTTCA ACTTATGTTA AAATGGAATA GACTATTCAT ACTTCCCCAT   
  
  
- TCTATTCCTA CTTCCATGGA ATTGAAGAGG AAGCGAAAAT ACATTCAAAA GACGTAAAAG TAGACGAAAA   
  
  
- ACCGGTTACC TAACCGACCG TCATCGGACA CCAGGAATCT CGAACTCTCA GTCTCGCCGT AACTACTCGA   
  
  
- AGGGAAGATA AAGTAAAGTG GAACTAATTG TGAGTTGGAT ATCGAAGTGA AAGTAAACCG CATGGTGCAA   
  
  
- TAACATTAAA GCCTTAAGTA AAAAACCGGT TCAAGTACTT CAAAGGGAAT GACCCACCTC TCTCTCTCTC   
  
  
- TCTCTAACGA GACAAGTGTC CATCTCCTCT TCTCGACCCG TTACCTGTGC ACGTAGACTT TCCCTTGTCG   
  
  
- TCTTCTATGG GCTCTCTGGG ACACTAAAGT GTGCTCGGTA CCACCAAGGT AATGTGAAGG TGTGGTTTTT   
  
  
- TCTCTCAATA ACTGGAGACG CTACTGTACC AAACGGGGTG CTTTAGGGTA TTTTAGGTTC ACGTAATGTA   
  
  
- GAGAAAAGAC CCCAAAGGCT CAAGAGCTTA CACAGATACA AGTGTTAAAC CGAGTTCTTT TCATTATGGA   
  
  
- TTAACCGTTG TCCTCTAGGT CTCACACATA CGTTCGTACG TGTATACTTC CCGGGGTAAG AACGAATGCA   
  
  
- TCGGTTAGTT CCTCTCGAAC ACCCCGGAGT ACAAAGAGGT GACTTTATAG AAGTTAAATA CAAGTTGACA   
  
  
- CACCCAGAGA CGAATAAAAT AAACCCTCAC CCCCACCCTC ACCGTTACCC GACCCCCACC ACGACTTACC   
  
  
- GGAATTTCAG TAACAACACA AGTATCAAAT ATAATTTACT CACTTTTTCT ATCCCTCTTT TTCGTTAGAA   
  
  
- ACTCACAAAA ACAGTAGATA ACAGAATTGA AGGGGGAGGC GGAAAAGAGA GAGAATTAGT TAGTTTCGAG   
  
  
- AACGCGAGAG AAGAAATACC CTTTTCAGAA GGTAAAGGTT TTGTAGGTAG AGACAGACAA ACTTTCGAAA   
  
  
- AGCCCCATTA TGTCATTTGT CCAACGTCA

+     Box 4

| Site Name | Organism | Position | Strand | Matrix score. | sequence | function |
| --- | --- | --- | --- | --- | --- | --- |
| Box 4 | Petroselinum crispum | 129 | + | 6 | ATTAAT | part of a conserved DNA module involved in light responsiveness |
| Box 4 | Petroselinum crispum | 69 | + | 6 | ATTAAT | part of a conserved DNA module involved in light responsiveness |

> 2018/04/13 10:10:12  
+ ATTTATTTAA TATTTTACAA TTATTTTTAA TATTATTTTT TAAAAATAAT AAATAAAATT ATTTATGAAT   
  
  
+ TAATTTTCAT AAAAATTTAT TGACTTAATT CGTGTATAAT TTTTTTGAGA AAAAATAAAT TAATGTATTT   
  
  
+ AAAATATAAT AAAATAATTT GTTAGTATAA TGAAACTAAC TCTTACATAA TAAAATAATC TGTGAATATG   
  
  
+ ATCCAATTAT TAAAATTCAT GAATAACACA TTTTTTAAAT AGATGATATT ACCAATTGAA TTGACCTTGA   
  
  
+ CCATAATTTT ATGGGCCGGA TTAGTCCAAG CGGCTCCATT CCCGAGTTGC TTTCAGGACC TGCCACAGCT   
  
  
+ GGACAGGACA GATTGCAGAC CCAAAACGAT GGGGAAGTGG GGTTGTAGTA GTTACCTTAA TTACTCTCAA   
  
  
+ TGAGTTTCTG AAATCCAAGG AAGTACAAGT TGAATACAAT TTTACCTTAT CTGATAAGTA TGAAGGGGTA   
  
  
+ AGATAAGGAT GAAGGTACCT TAACTTCTCC TTCGCTTTTA TGTAAGTTTT CTGCATTTTC ATCTGCTTTT   
  
  
+ TGGCCAATGG ATTGGCTGGC AGTAGCCTGT GGTCCTTAGA GCTTGAGAGT CAGAGCGGCA TTGATGAGCT   
  
  
+ TCCCTTCTAT TTCATTTCAC CTTGATTAAC ACTCAACCTA TAGCTTCACT TTCATTTGGC GTACCACGTT   
  
  
+ ATTGTAATTT CGGAATTCAT TTTTTGGCCA AGTTCATGAA GTTTCCCTTA CTGGGTGGAG AGAGAGAGAG   
  
  
+ AGAGATTGCT CTGTTCACAG GTAGAGGAGA AGAGCTGGGC AATGGACACG TGCATCTGAA AGGGAACAGC   
  
  
+ AGAAGATACC CGAGAGACCC TGTGATTTCA CACGAGCCAT GGTGGTTCCA TTACACTTCC ACACCAAAAA   
  
  
+ AGAGAGTTAT TGACCTCTGC GATGACATGG TTTGCCCCAC GAAATCCCAT AAAATCCAAG TGCATTACAT   
  
  
+ CTCTTTTCTG GGGTTTCCGA GTTCTCGAAT GTGTCTATGT TCACAATTTG GCTCAAGAAA AGTAATACCT   
  
  
+ AATTGGCAAC AGGAGATCCA GAGTGTGTAT GCAAGCATGC ACATATGAAG GGCCCCATTC TTGCTTACGT   
  
  
+ AGCCAATCAA GGAGAGCTTG TGGGGCCTCA TGTTTCTCCA CTGAAATATC TTCAATTTAT GTTCAACTGT   
  
  
+ GTGGGTCTCT GCTTATTTTA TTTGGGAGTG GGGGTGGGAG TGGCAATGGG CTGGGGGTGG TGCTGAATGG   
  
  
+ CCTTAAAGTC ATTGTTGTGT TCATAGTTTA TATTAAATGA GTGAAAAAGA TAGGGAGAAA AAGCAATCTT   
  
  
+ TGAGTGTTTT TGTCATCTAT TGTCTTAACT TCCCCCTCCG CCTTTTCTCT CTCTTAATCA ATCAAAGCTC   
  
  
+ TTGCGCTCTC TTCTTTATGG GAAAAGTCTT CCATTTCCAA AACATCCATC TCTGTCTGTT TGAAAGCTTT   
  
  
+ TCGGGGTAAT ACAGTAAACA GGTTGCAGT  

- TAAATAAATT ATAAAATGTT AATAAAAATT ATAATAAAAA ATTTTTATTA TTTATTTTAA TAAATACTTA   
  
  
- ATTAAAAGTA TTTTTAAATA ACTGAATTAA GCACATATTA AAAAAACTCT TTTTTATTTA ATTACATAAA   
  
  
- TTTTATATTA TTTTATTAAA CAATCATATT ACTTTGATTG AGAATGTATT ATTTTATTAG ACACTTATAC   
  
  
- TAGGTTAATA ATTTTAAGTA CTTATTGTGT AAAAAATTTA TCTACTATAA TGGTTAACTT AACTGGAACT   
  
  
- GGTATTAAAA TACCCGGCCT AATCAGGTTC GCCGAGGTAA GGGCTCAACG AAAGTCCTGG ACGGTGTCGA   
  
  
- CCTGTCCTGT CTAACGTCTG GGTTTTGCTA CCCCTTCACC CCAACATCAT CAATGGAATT AATGAGAGTT   
  
  
- ACTCAAAGAC TTTAGGTTCC TTCATGTTCA ACTTATGTTA AAATGGAATA GACTATTCAT ACTTCCCCAT   
  
  
- TCTATTCCTA CTTCCATGGA ATTGAAGAGG AAGCGAAAAT ACATTCAAAA GACGTAAAAG TAGACGAAAA   
  
  
- ACCGGTTACC TAACCGACCG TCATCGGACA CCAGGAATCT CGAACTCTCA GTCTCGCCGT AACTACTCGA   
  
  
- AGGGAAGATA AAGTAAAGTG GAACTAATTG TGAGTTGGAT ATCGAAGTGA AAGTAAACCG CATGGTGCAA   
  
  
- TAACATTAAA GCCTTAAGTA AAAAACCGGT TCAAGTACTT CAAAGGGAAT GACCCACCTC TCTCTCTCTC   
  
  
- TCTCTAACGA GACAAGTGTC CATCTCCTCT TCTCGACCCG TTACCTGTGC ACGTAGACTT TCCCTTGTCG   
  
  
- TCTTCTATGG GCTCTCTGGG ACACTAAAGT GTGCTCGGTA CCACCAAGGT AATGTGAAGG TGTGGTTTTT   
  
  
- TCTCTCAATA ACTGGAGACG CTACTGTACC AAACGGGGTG CTTTAGGGTA TTTTAGGTTC ACGTAATGTA   
  
  
- GAGAAAAGAC CCCAAAGGCT CAAGAGCTTA CACAGATACA AGTGTTAAAC CGAGTTCTTT TCATTATGGA   
  
  
- TTAACCGTTG TCCTCTAGGT CTCACACATA CGTTCGTACG TGTATACTTC CCGGGGTAAG AACGAATGCA   
  
  
- TCGGTTAGTT CCTCTCGAAC ACCCCGGAGT ACAAAGAGGT GACTTTATAG AAGTTAAATA CAAGTTGACA   
  
  
- CACCCAGAGA CGAATAAAAT AAACCCTCAC CCCCACCCTC ACCGTTACCC GACCCCCACC ACGACTTACC   
  
  
- GGAATTTCAG TAACAACACA AGTATCAAAT ATAATTTACT CACTTTTTCT ATCCCTCTTT TTCGTTAGAA   
  
  
- ACTCACAAAA ACAGTAGATA ACAGAATTGA AGGGGGAGGC GGAAAAGAGA GAGAATTAGT TAGTTTCGAG   
  
  
- AACGCGAGAG AAGAAATACC CTTTTCAGAA GGTAAAGGTT TTGTAGGTAG AGACAGACAA ACTTTCGAAA   
  
  
- AGCCCCATTA TGTCATTTGT CCAACGTCA

+     Box I

| Site Name | Organism | Position | Strand | Matrix score. | sequence | function |
| --- | --- | --- | --- | --- | --- | --- |
| Box I | Pisum sativum | 1459 | - | 7 | TTTCAAA | light responsive element |

> 2018/04/13 10:10:12  
+ ATTTATTTAA TATTTTACAA TTATTTTTAA TATTATTTTT TAAAAATAAT AAATAAAATT ATTTATGAAT   
  
  
+ TAATTTTCAT AAAAATTTAT TGACTTAATT CGTGTATAAT TTTTTTGAGA AAAAATAAAT TAATGTATTT   
  
  
+ AAAATATAAT AAAATAATTT GTTAGTATAA TGAAACTAAC TCTTACATAA TAAAATAATC TGTGAATATG   
  
  
+ ATCCAATTAT TAAAATTCAT GAATAACACA TTTTTTAAAT AGATGATATT ACCAATTGAA TTGACCTTGA   
  
  
+ CCATAATTTT ATGGGCCGGA TTAGTCCAAG CGGCTCCATT CCCGAGTTGC TTTCAGGACC TGCCACAGCT   
  
  
+ GGACAGGACA GATTGCAGAC CCAAAACGAT GGGGAAGTGG GGTTGTAGTA GTTACCTTAA TTACTCTCAA   
  
  
+ TGAGTTTCTG AAATCCAAGG AAGTACAAGT TGAATACAAT TTTACCTTAT CTGATAAGTA TGAAGGGGTA   
  
  
+ AGATAAGGAT GAAGGTACCT TAACTTCTCC TTCGCTTTTA TGTAAGTTTT CTGCATTTTC ATCTGCTTTT   
  
  
+ TGGCCAATGG ATTGGCTGGC AGTAGCCTGT GGTCCTTAGA GCTTGAGAGT CAGAGCGGCA TTGATGAGCT   
  
  
+ TCCCTTCTAT TTCATTTCAC CTTGATTAAC ACTCAACCTA TAGCTTCACT TTCATTTGGC GTACCACGTT   
  
  
+ ATTGTAATTT CGGAATTCAT TTTTTGGCCA AGTTCATGAA GTTTCCCTTA CTGGGTGGAG AGAGAGAGAG   
  
  
+ AGAGATTGCT CTGTTCACAG GTAGAGGAGA AGAGCTGGGC AATGGACACG TGCATCTGAA AGGGAACAGC   
  
  
+ AGAAGATACC CGAGAGACCC TGTGATTTCA CACGAGCCAT GGTGGTTCCA TTACACTTCC ACACCAAAAA   
  
  
+ AGAGAGTTAT TGACCTCTGC GATGACATGG TTTGCCCCAC GAAATCCCAT AAAATCCAAG TGCATTACAT   
  
  
+ CTCTTTTCTG GGGTTTCCGA GTTCTCGAAT GTGTCTATGT TCACAATTTG GCTCAAGAAA AGTAATACCT   
  
  
+ AATTGGCAAC AGGAGATCCA GAGTGTGTAT GCAAGCATGC ACATATGAAG GGCCCCATTC TTGCTTACGT   
  
  
+ AGCCAATCAA GGAGAGCTTG TGGGGCCTCA TGTTTCTCCA CTGAAATATC TTCAATTTAT GTTCAACTGT   
  
  
+ GTGGGTCTCT GCTTATTTTA TTTGGGAGTG GGGGTGGGAG TGGCAATGGG CTGGGGGTGG TGCTGAATGG   
  
  
+ CCTTAAAGTC ATTGTTGTGT TCATAGTTTA TATTAAATGA GTGAAAAAGA TAGGGAGAAA AAGCAATCTT   
  
  
+ TGAGTGTTTT TGTCATCTAT TGTCTTAACT TCCCCCTCCG CCTTTTCTCT CTCTTAATCA ATCAAAGCTC   
  
  
+ TTGCGCTCTC TTCTTTATGG GAAAAGTCTT CCATTTCCAA AACATCCATC TCTGTCTGTT TGAAAGCTTT   
  
  
+ TCGGGGTAAT ACAGTAAACA GGTTGCAGT  

- TAAATAAATT ATAAAATGTT AATAAAAATT ATAATAAAAA ATTTTTATTA TTTATTTTAA TAAATACTTA   
  
  
- ATTAAAAGTA TTTTTAAATA ACTGAATTAA GCACATATTA AAAAAACTCT TTTTTATTTA ATTACATAAA   
  
  
- TTTTATATTA TTTTATTAAA CAATCATATT ACTTTGATTG AGAATGTATT ATTTTATTAG ACACTTATAC   
  
  
- TAGGTTAATA ATTTTAAGTA CTTATTGTGT AAAAAATTTA TCTACTATAA TGGTTAACTT AACTGGAACT   
  
  
- GGTATTAAAA TACCCGGCCT AATCAGGTTC GCCGAGGTAA GGGCTCAACG AAAGTCCTGG ACGGTGTCGA   
  
  
- CCTGTCCTGT CTAACGTCTG GGTTTTGCTA CCCCTTCACC CCAACATCAT CAATGGAATT AATGAGAGTT   
  
  
- ACTCAAAGAC TTTAGGTTCC TTCATGTTCA ACTTATGTTA AAATGGAATA GACTATTCAT ACTTCCCCAT   
  
  
- TCTATTCCTA CTTCCATGGA ATTGAAGAGG AAGCGAAAAT ACATTCAAAA GACGTAAAAG TAGACGAAAA   
  
  
- ACCGGTTACC TAACCGACCG TCATCGGACA CCAGGAATCT CGAACTCTCA GTCTCGCCGT AACTACTCGA   
  
  
- AGGGAAGATA AAGTAAAGTG GAACTAATTG TGAGTTGGAT ATCGAAGTGA AAGTAAACCG CATGGTGCAA   
  
  
- TAACATTAAA GCCTTAAGTA AAAAACCGGT TCAAGTACTT CAAAGGGAAT GACCCACCTC TCTCTCTCTC   
  
  
- TCTCTAACGA GACAAGTGTC CATCTCCTCT TCTCGACCCG TTACCTGTGC ACGTAGACTT TCCCTTGTCG   
  
  
- TCTTCTATGG GCTCTCTGGG ACACTAAAGT GTGCTCGGTA CCACCAAGGT AATGTGAAGG TGTGGTTTTT   
  
  
- TCTCTCAATA ACTGGAGACG CTACTGTACC AAACGGGGTG CTTTAGGGTA TTTTAGGTTC ACGTAATGTA   
  
  
- GAGAAAAGAC CCCAAAGGCT CAAGAGCTTA CACAGATACA AGTGTTAAAC CGAGTTCTTT TCATTATGGA   
  
  
- TTAACCGTTG TCCTCTAGGT CTCACACATA CGTTCGTACG TGTATACTTC CCGGGGTAAG AACGAATGCA   
  
  
- TCGGTTAGTT CCTCTCGAAC ACCCCGGAGT ACAAAGAGGT GACTTTATAG AAGTTAAATA CAAGTTGACA   
  
  
- CACCCAGAGA CGAATAAAAT AAACCCTCAC CCCCACCCTC ACCGTTACCC GACCCCCACC ACGACTTACC   
  
  
- GGAATTTCAG TAACAACACA AGTATCAAAT ATAATTTACT CACTTTTTCT ATCCCTCTTT TTCGTTAGAA   
  
  
- ACTCACAAAA ACAGTAGATA ACAGAATTGA AGGGGGAGGC GGAAAAGAGA GAGAATTAGT TAGTTTCGAG   
  
  
- AACGCGAGAG AAGAAATACC CTTTTCAGAA GGTAAAGGTT TTGTAGGTAG AGACAGACAA ACTTTCGAAA   
  
  
- AGCCCCATTA TGTCATTTGT CCAACGTCA

+     Box III

| Site Name | Organism | Position | Strand | Matrix score. | sequence | function |
| --- | --- | --- | --- | --- | --- | --- |
| Box III | Pisum sativum | 1300 | - | 11 | atCATTTTCACt | protein binding site |

> 2018/04/13 10:10:12  
+ ATTTATTTAA TATTTTACAA TTATTTTTAA TATTATTTTT TAAAAATAAT AAATAAAATT ATTTATGAAT   
  
  
+ TAATTTTCAT AAAAATTTAT TGACTTAATT CGTGTATAAT TTTTTTGAGA AAAAATAAAT TAATGTATTT   
  
  
+ AAAATATAAT AAAATAATTT GTTAGTATAA TGAAACTAAC TCTTACATAA TAAAATAATC TGTGAATATG   
  
  
+ ATCCAATTAT TAAAATTCAT GAATAACACA TTTTTTAAAT AGATGATATT ACCAATTGAA TTGACCTTGA   
  
  
+ CCATAATTTT ATGGGCCGGA TTAGTCCAAG CGGCTCCATT CCCGAGTTGC TTTCAGGACC TGCCACAGCT   
  
  
+ GGACAGGACA GATTGCAGAC CCAAAACGAT GGGGAAGTGG GGTTGTAGTA GTTACCTTAA TTACTCTCAA   
  
  
+ TGAGTTTCTG AAATCCAAGG AAGTACAAGT TGAATACAAT TTTACCTTAT CTGATAAGTA TGAAGGGGTA   
  
  
+ AGATAAGGAT GAAGGTACCT TAACTTCTCC TTCGCTTTTA TGTAAGTTTT CTGCATTTTC ATCTGCTTTT   
  
  
+ TGGCCAATGG ATTGGCTGGC AGTAGCCTGT GGTCCTTAGA GCTTGAGAGT CAGAGCGGCA TTGATGAGCT   
  
  
+ TCCCTTCTAT TTCATTTCAC CTTGATTAAC ACTCAACCTA TAGCTTCACT TTCATTTGGC GTACCACGTT   
  
  
+ ATTGTAATTT CGGAATTCAT TTTTTGGCCA AGTTCATGAA GTTTCCCTTA CTGGGTGGAG AGAGAGAGAG   
  
  
+ AGAGATTGCT CTGTTCACAG GTAGAGGAGA AGAGCTGGGC AATGGACACG TGCATCTGAA AGGGAACAGC   
  
  
+ AGAAGATACC CGAGAGACCC TGTGATTTCA CACGAGCCAT GGTGGTTCCA TTACACTTCC ACACCAAAAA   
  
  
+ AGAGAGTTAT TGACCTCTGC GATGACATGG TTTGCCCCAC GAAATCCCAT AAAATCCAAG TGCATTACAT   
  
  
+ CTCTTTTCTG GGGTTTCCGA GTTCTCGAAT GTGTCTATGT TCACAATTTG GCTCAAGAAA AGTAATACCT   
  
  
+ AATTGGCAAC AGGAGATCCA GAGTGTGTAT GCAAGCATGC ACATATGAAG GGCCCCATTC TTGCTTACGT   
  
  
+ AGCCAATCAA GGAGAGCTTG TGGGGCCTCA TGTTTCTCCA CTGAAATATC TTCAATTTAT GTTCAACTGT   
  
  
+ GTGGGTCTCT GCTTATTTTA TTTGGGAGTG GGGGTGGGAG TGGCAATGGG CTGGGGGTGG TGCTGAATGG   
  
  
+ CCTTAAAGTC ATTGTTGTGT TCATAGTTTA TATTAAATGA GTGAAAAAGA TAGGGAGAAA AAGCAATCTT   
  
  
+ TGAGTGTTTT TGTCATCTAT TGTCTTAACT TCCCCCTCCG CCTTTTCTCT CTCTTAATCA ATCAAAGCTC   
  
  
+ TTGCGCTCTC TTCTTTATGG GAAAAGTCTT CCATTTCCAA AACATCCATC TCTGTCTGTT TGAAAGCTTT   
  
  
+ TCGGGGTAAT ACAGTAAACA GGTTGCAGT  

- TAAATAAATT ATAAAATGTT AATAAAAATT ATAATAAAAA ATTTTTATTA TTTATTTTAA TAAATACTTA   
  
  
- ATTAAAAGTA TTTTTAAATA ACTGAATTAA GCACATATTA AAAAAACTCT TTTTTATTTA ATTACATAAA   
  
  
- TTTTATATTA TTTTATTAAA CAATCATATT ACTTTGATTG AGAATGTATT ATTTTATTAG ACACTTATAC   
  
  
- TAGGTTAATA ATTTTAAGTA CTTATTGTGT AAAAAATTTA TCTACTATAA TGGTTAACTT AACTGGAACT   
  
  
- GGTATTAAAA TACCCGGCCT AATCAGGTTC GCCGAGGTAA GGGCTCAACG AAAGTCCTGG ACGGTGTCGA   
  
  
- CCTGTCCTGT CTAACGTCTG GGTTTTGCTA CCCCTTCACC CCAACATCAT CAATGGAATT AATGAGAGTT   
  
  
- ACTCAAAGAC TTTAGGTTCC TTCATGTTCA ACTTATGTTA AAATGGAATA GACTATTCAT ACTTCCCCAT   
  
  
- TCTATTCCTA CTTCCATGGA ATTGAAGAGG AAGCGAAAAT ACATTCAAAA GACGTAAAAG TAGACGAAAA   
  
  
- ACCGGTTACC TAACCGACCG TCATCGGACA CCAGGAATCT CGAACTCTCA GTCTCGCCGT AACTACTCGA   
  
  
- AGGGAAGATA AAGTAAAGTG GAACTAATTG TGAGTTGGAT ATCGAAGTGA AAGTAAACCG CATGGTGCAA   
  
  
- TAACATTAAA GCCTTAAGTA AAAAACCGGT TCAAGTACTT CAAAGGGAAT GACCCACCTC TCTCTCTCTC   
  
  
- TCTCTAACGA GACAAGTGTC CATCTCCTCT TCTCGACCCG TTACCTGTGC ACGTAGACTT TCCCTTGTCG   
  
  
- TCTTCTATGG GCTCTCTGGG ACACTAAAGT GTGCTCGGTA CCACCAAGGT AATGTGAAGG TGTGGTTTTT   
  
  
- TCTCTCAATA ACTGGAGACG CTACTGTACC AAACGGGGTG CTTTAGGGTA TTTTAGGTTC ACGTAATGTA   
  
  
- GAGAAAAGAC CCCAAAGGCT CAAGAGCTTA CACAGATACA AGTGTTAAAC CGAGTTCTTT TCATTATGGA   
  
  
- TTAACCGTTG TCCTCTAGGT CTCACACATA CGTTCGTACG TGTATACTTC CCGGGGTAAG AACGAATGCA   
  
  
- TCGGTTAGTT CCTCTCGAAC ACCCCGGAGT ACAAAGAGGT GACTTTATAG AAGTTAAATA CAAGTTGACA   
  
  
- CACCCAGAGA CGAATAAAAT AAACCCTCAC CCCCACCCTC ACCGTTACCC GACCCCCACC ACGACTTACC   
  
  
- GGAATTTCAG TAACAACACA AGTATCAAAT ATAATTTACT CACTTTTTCT ATCCCTCTTT TTCGTTAGAA   
  
  
- ACTCACAAAA ACAGTAGATA ACAGAATTGA AGGGGGAGGC GGAAAAGAGA GAGAATTAGT TAGTTTCGAG   
  
  
- AACGCGAGAG AAGAAATACC CTTTTCAGAA GGTAAAGGTT TTGTAGGTAG AGACAGACAA ACTTTCGAAA   
  
  
- AGCCCCATTA TGTCATTTGT CCAACGTCA

+     Box-W1

| Site Name | Organism | Position | Strand | Matrix score. | sequence | function |
| --- | --- | --- | --- | --- | --- | --- |
| Box-W1 | Petroselinum crispum | 920 | + | 6 | TTGACC | fungal elicitor responsive element |
| Box-W1 | Petroselinum crispum | 277 | + | 6 | TTGACC | fungal elicitor responsive element |
| Box-W1 | Petroselinum crispum | 271 | + | 6 | TTGACC | fungal elicitor responsive element |

> 2018/04/13 10:10:12  
+ ATTTATTTAA TATTTTACAA TTATTTTTAA TATTATTTTT TAAAAATAAT AAATAAAATT ATTTATGAAT   
  
  
+ TAATTTTCAT AAAAATTTAT TGACTTAATT CGTGTATAAT TTTTTTGAGA AAAAATAAAT TAATGTATTT   
  
  
+ AAAATATAAT AAAATAATTT GTTAGTATAA TGAAACTAAC TCTTACATAA TAAAATAATC TGTGAATATG   
  
  
+ ATCCAATTAT TAAAATTCAT GAATAACACA TTTTTTAAAT AGATGATATT ACCAATTGAA TTGACCTTGA   
  
  
+ CCATAATTTT ATGGGCCGGA TTAGTCCAAG CGGCTCCATT CCCGAGTTGC TTTCAGGACC TGCCACAGCT   
  
  
+ GGACAGGACA GATTGCAGAC CCAAAACGAT GGGGAAGTGG GGTTGTAGTA GTTACCTTAA TTACTCTCAA   
  
  
+ TGAGTTTCTG AAATCCAAGG AAGTACAAGT TGAATACAAT TTTACCTTAT CTGATAAGTA TGAAGGGGTA   
  
  
+ AGATAAGGAT GAAGGTACCT TAACTTCTCC TTCGCTTTTA TGTAAGTTTT CTGCATTTTC ATCTGCTTTT   
  
  
+ TGGCCAATGG ATTGGCTGGC AGTAGCCTGT GGTCCTTAGA GCTTGAGAGT CAGAGCGGCA TTGATGAGCT   
  
  
+ TCCCTTCTAT TTCATTTCAC CTTGATTAAC ACTCAACCTA TAGCTTCACT TTCATTTGGC GTACCACGTT   
  
  
+ ATTGTAATTT CGGAATTCAT TTTTTGGCCA AGTTCATGAA GTTTCCCTTA CTGGGTGGAG AGAGAGAGAG   
  
  
+ AGAGATTGCT CTGTTCACAG GTAGAGGAGA AGAGCTGGGC AATGGACACG TGCATCTGAA AGGGAACAGC   
  
  
+ AGAAGATACC CGAGAGACCC TGTGATTTCA CACGAGCCAT GGTGGTTCCA TTACACTTCC ACACCAAAAA   
  
  
+ AGAGAGTTAT TGACCTCTGC GATGACATGG TTTGCCCCAC GAAATCCCAT AAAATCCAAG TGCATTACAT   
  
  
+ CTCTTTTCTG GGGTTTCCGA GTTCTCGAAT GTGTCTATGT TCACAATTTG GCTCAAGAAA AGTAATACCT   
  
  
+ AATTGGCAAC AGGAGATCCA GAGTGTGTAT GCAAGCATGC ACATATGAAG GGCCCCATTC TTGCTTACGT   
  
  
+ AGCCAATCAA GGAGAGCTTG TGGGGCCTCA TGTTTCTCCA CTGAAATATC TTCAATTTAT GTTCAACTGT   
  
  
+ GTGGGTCTCT GCTTATTTTA TTTGGGAGTG GGGGTGGGAG TGGCAATGGG CTGGGGGTGG TGCTGAATGG   
  
  
+ CCTTAAAGTC ATTGTTGTGT TCATAGTTTA TATTAAATGA GTGAAAAAGA TAGGGAGAAA AAGCAATCTT   
  
  
+ TGAGTGTTTT TGTCATCTAT TGTCTTAACT TCCCCCTCCG CCTTTTCTCT CTCTTAATCA ATCAAAGCTC   
  
  
+ TTGCGCTCTC TTCTTTATGG GAAAAGTCTT CCATTTCCAA AACATCCATC TCTGTCTGTT TGAAAGCTTT   
  
  
+ TCGGGGTAAT ACAGTAAACA GGTTGCAGT  

- TAAATAAATT ATAAAATGTT AATAAAAATT ATAATAAAAA ATTTTTATTA TTTATTTTAA TAAATACTTA   
  
  
- ATTAAAAGTA TTTTTAAATA ACTGAATTAA GCACATATTA AAAAAACTCT TTTTTATTTA ATTACATAAA   
  
  
- TTTTATATTA TTTTATTAAA CAATCATATT ACTTTGATTG AGAATGTATT ATTTTATTAG ACACTTATAC   
  
  
- TAGGTTAATA ATTTTAAGTA CTTATTGTGT AAAAAATTTA TCTACTATAA TGGTTAACTT AACTGGAACT   
  
  
- GGTATTAAAA TACCCGGCCT AATCAGGTTC GCCGAGGTAA GGGCTCAACG AAAGTCCTGG ACGGTGTCGA   
  
  
- CCTGTCCTGT CTAACGTCTG GGTTTTGCTA CCCCTTCACC CCAACATCAT CAATGGAATT AATGAGAGTT   
  
  
- ACTCAAAGAC TTTAGGTTCC TTCATGTTCA ACTTATGTTA AAATGGAATA GACTATTCAT ACTTCCCCAT   
  
  
- TCTATTCCTA CTTCCATGGA ATTGAAGAGG AAGCGAAAAT ACATTCAAAA GACGTAAAAG TAGACGAAAA   
  
  
- ACCGGTTACC TAACCGACCG TCATCGGACA CCAGGAATCT CGAACTCTCA GTCTCGCCGT AACTACTCGA   
  
  
- AGGGAAGATA AAGTAAAGTG GAACTAATTG TGAGTTGGAT ATCGAAGTGA AAGTAAACCG CATGGTGCAA   
  
  
- TAACATTAAA GCCTTAAGTA AAAAACCGGT TCAAGTACTT CAAAGGGAAT GACCCACCTC TCTCTCTCTC   
  
  
- TCTCTAACGA GACAAGTGTC CATCTCCTCT TCTCGACCCG TTACCTGTGC ACGTAGACTT TCCCTTGTCG   
  
  
- TCTTCTATGG GCTCTCTGGG ACACTAAAGT GTGCTCGGTA CCACCAAGGT AATGTGAAGG TGTGGTTTTT   
  
  
- TCTCTCAATA ACTGGAGACG CTACTGTACC AAACGGGGTG CTTTAGGGTA TTTTAGGTTC ACGTAATGTA   
  
  
- GAGAAAAGAC CCCAAAGGCT CAAGAGCTTA CACAGATACA AGTGTTAAAC CGAGTTCTTT TCATTATGGA   
  
  
- TTAACCGTTG TCCTCTAGGT CTCACACATA CGTTCGTACG TGTATACTTC CCGGGGTAAG AACGAATGCA   
  
  
- TCGGTTAGTT CCTCTCGAAC ACCCCGGAGT ACAAAGAGGT GACTTTATAG AAGTTAAATA CAAGTTGACA   
  
  
- CACCCAGAGA CGAATAAAAT AAACCCTCAC CCCCACCCTC ACCGTTACCC GACCCCCACC ACGACTTACC   
  
  
- GGAATTTCAG TAACAACACA AGTATCAAAT ATAATTTACT CACTTTTTCT ATCCCTCTTT TTCGTTAGAA   
  
  
- ACTCACAAAA ACAGTAGATA ACAGAATTGA AGGGGGAGGC GGAAAAGAGA GAGAATTAGT TAGTTTCGAG   
  
  
- AACGCGAGAG AAGAAATACC CTTTTCAGAA GGTAAAGGTT TTGTAGGTAG AGACAGACAA ACTTTCGAAA   
  
  
- AGCCCCATTA TGTCATTTGT CCAACGTCA

+     CAAT-box

| Site Name | Organism | Position | Strand | Matrix score. | sequence | function |
| --- | --- | --- | --- | --- | --- | --- |
| CAAT-box | Arabidopsis thaliana | 571 | - | 5 | CCAAT | common cis-acting element in promoter and enhancer regions |
| CAAT-box | Hordeum vulgare | 620 | - | 4 | CAAT | common cis-acting element in promoter and enhancer regions |
| CAAT-box | Arabidopsis thaliana | 564 | + | 5 | CCAAT | common cis-acting element in promoter and enhancer regions |
| CAAT-box | Hordeum vulgare | 1234 | + | 4 | CAAT | common cis-acting element in promoter and enhancer regions |
| CAAT-box | Arabidopsis thaliana | 1232 | + | 6 | gGCAAT | common cis-acting element in promoter and enhancer regions |
| CAAT-box | Brassica rapa | 1210 | - | 5 | CAAAT | common cis-acting element in promoter and enhancer regions |
| CAAT-box | Glycine max | 264 | - | 5 | CAATT | common cis-acting element in promoter and enhancer regions |
| CAAT-box | Arabidopsis thaliana | 213 | + | 5 | CCAAT | common cis-acting element in promoter and enhancer regions |
| CAAT-box | Glycine max | 1173 | + | 5 | CAATT | common cis-acting element in promoter and enhancer regions |
| CAAT-box | Hordeum vulgare | 1389 | + | 4 | CAAT | common cis-acting element in promoter and enhancer regions |
| CAAT-box | Hordeum vulgare | 89 | - | 4 | CAAT | common cis-acting element in promoter and enhancer regions |
| CAAT-box | Hordeum vulgare | 1324 | + | 4 | CAAT | common cis-acting element in promoter and enhancer regions |
| CAAT-box | Hordeum vulgare | 1349 | - | 4 | CAAT | common cis-acting element in promoter and enhancer regions |
| CAAT-box | Hordeum vulgare | 1271 | - | 4 | CAAT | common cis-acting element in promoter and enhancer regions |
| CAAT-box | Arabidopsis thaliana | 1123 | + | 5 | CCAAT | common cis-acting element in promoter and enhancer regions |
| CAAT-box | Hordeum vulgare | 265 | - | 4 | CAAT | common cis-acting element in promoter and enhancer regions |
| CAAT-box | Glycine max | 269 | - | 5 | CAATT | common cis-acting element in promoter and enhancer regions |
| CAAT-box | Arabidopsis thaliana | 1052 | - | 5 | CCAAT | common cis-acting element in promoter and enhancer regions |
| CAAT-box | Glycine max | 263 | + | 5 | CAATT | common cis-acting element in promoter and enhancer regions |
| CAAT-box | Glycine max | 1051 | - | 5 | CAATT | common cis-acting element in promoter and enhancer regions |
| CAAT-box | Hordeum vulgare | 701 | - | 4 | CAAT | common cis-acting element in promoter and enhancer regions |
| CAAT-box | Brassica rapa | 684 | - | 5 | CAAAT | common cis-acting element in promoter and enhancer regions |
| CAAT-box | Hordeum vulgare | 1124 | + | 4 | CAAT | common cis-acting element in promoter and enhancer regions |
| CAAT-box | Glycine max | 457 | + | 5 | CAATT | common cis-acting element in promoter and enhancer regions |
| CAAT-box | Hordeum vulgare | 362 | - | 4 | CAAT | common cis-acting element in promoter and enhancer regions |
| CAAT-box | Hordeum vulgare | 418 | + | 4 | CAAT | common cis-acting element in promoter and enhancer regions |
| CAAT-box | Hordeum vulgare | 270 | - | 4 | CAAT | common cis-acting element in promoter and enhancer regions |
| CAAT-box | Glycine max | 1024 | + | 5 | CAATT | common cis-acting element in promoter and enhancer regions |
| CAAT-box | Brassica rapa | 1026 | - | 5 | CAAAT | common cis-acting element in promoter and enhancer regions |
| CAAT-box | Hordeum vulgare | 919 | - | 4 | CAAT | common cis-acting element in promoter and enhancer regions |
| CAAT-box | Hordeum vulgare | 810 | + | 4 | CAAT | common cis-acting element in promoter and enhancer regions |
| CAAT-box | Arabidopsis thaliana | 808 | + | 6 | gGCAAT | common cis-acting element in promoter and enhancer regions |
| CAAT-box | Arabidopsis thaliana | 262 | + | 5 | CCAAT | common cis-acting element in promoter and enhancer regions |
| CAAT-box | Hordeum vulgare | 775 | - | 4 | CAAT | common cis-acting element in promoter and enhancer regions |
| CAAT-box | Glycine max | 18 | + | 5 | CAATT | common cis-acting element in promoter and enhancer regions |
| CAAT-box | Glycine max | 214 | + | 5 | CAATT | common cis-acting element in promoter and enhancer regions |
| CAAT-box | Brassica rapa | 157 | - | 5 | CAAAT | common cis-acting element in promoter and enhancer regions |
| CAAT-box | Hordeum vulgare | 565 | + | 4 | CAAT | common cis-acting element in promoter and enhancer regions |

> 2018/04/13 10:10:12  
+ ATTTATTTAA TATTTTACAA TTATTTTTAA TATTATTTTT TAAAAATAAT AAATAAAATT ATTTATGAAT   
  
  
+ TAATTTTCAT AAAAATTTAT TGACTTAATT CGTGTATAAT TTTTTTGAGA AAAAATAAAT TAATGTATTT   
  
  
+ AAAATATAAT AAAATAATTT GTTAGTATAA TGAAACTAAC TCTTACATAA TAAAATAATC TGTGAATATG   
  
  
+ ATCCAATTAT TAAAATTCAT GAATAACACA TTTTTTAAAT AGATGATATT ACCAATTGAA TTGACCTTGA   
  
  
+ CCATAATTTT ATGGGCCGGA TTAGTCCAAG CGGCTCCATT CCCGAGTTGC TTTCAGGACC TGCCACAGCT   
  
  
+ GGACAGGACA GATTGCAGAC CCAAAACGAT GGGGAAGTGG GGTTGTAGTA GTTACCTTAA TTACTCTCAA   
  
  
+ TGAGTTTCTG AAATCCAAGG AAGTACAAGT TGAATACAAT TTTACCTTAT CTGATAAGTA TGAAGGGGTA   
  
  
+ AGATAAGGAT GAAGGTACCT TAACTTCTCC TTCGCTTTTA TGTAAGTTTT CTGCATTTTC ATCTGCTTTT   
  
  
+ TGGCCAATGG ATTGGCTGGC AGTAGCCTGT GGTCCTTAGA GCTTGAGAGT CAGAGCGGCA TTGATGAGCT   
  
  
+ TCCCTTCTAT TTCATTTCAC CTTGATTAAC ACTCAACCTA TAGCTTCACT TTCATTTGGC GTACCACGTT   
  
  
+ ATTGTAATTT CGGAATTCAT TTTTTGGCCA AGTTCATGAA GTTTCCCTTA CTGGGTGGAG AGAGAGAGAG   
  
  
+ AGAGATTGCT CTGTTCACAG GTAGAGGAGA AGAGCTGGGC AATGGACACG TGCATCTGAA AGGGAACAGC   
  
  
+ AGAAGATACC CGAGAGACCC TGTGATTTCA CACGAGCCAT GGTGGTTCCA TTACACTTCC ACACCAAAAA   
  
  
+ AGAGAGTTAT TGACCTCTGC GATGACATGG TTTGCCCCAC GAAATCCCAT AAAATCCAAG TGCATTACAT   
  
  
+ CTCTTTTCTG GGGTTTCCGA GTTCTCGAAT GTGTCTATGT TCACAATTTG GCTCAAGAAA AGTAATACCT   
  
  
+ AATTGGCAAC AGGAGATCCA GAGTGTGTAT GCAAGCATGC ACATATGAAG GGCCCCATTC TTGCTTACGT   
  
  
+ AGCCAATCAA GGAGAGCTTG TGGGGCCTCA TGTTTCTCCA CTGAAATATC TTCAATTTAT GTTCAACTGT   
  
  
+ GTGGGTCTCT GCTTATTTTA TTTGGGAGTG GGGGTGGGAG TGGCAATGGG CTGGGGGTGG TGCTGAATGG   
  
  
+ CCTTAAAGTC ATTGTTGTGT TCATAGTTTA TATTAAATGA GTGAAAAAGA TAGGGAGAAA AAGCAATCTT   
  
  
+ TGAGTGTTTT TGTCATCTAT TGTCTTAACT TCCCCCTCCG CCTTTTCTCT CTCTTAATCA ATCAAAGCTC   
  
  
+ TTGCGCTCTC TTCTTTATGG GAAAAGTCTT CCATTTCCAA AACATCCATC TCTGTCTGTT TGAAAGCTTT   
  
  
+ TCGGGGTAAT ACAGTAAACA GGTTGCAGT  

- TAAATAAATT ATAAAATGTT AATAAAAATT ATAATAAAAA ATTTTTATTA TTTATTTTAA TAAATACTTA   
  
  
- ATTAAAAGTA TTTTTAAATA ACTGAATTAA GCACATATTA AAAAAACTCT TTTTTATTTA ATTACATAAA   
  
  
- TTTTATATTA TTTTATTAAA CAATCATATT ACTTTGATTG AGAATGTATT ATTTTATTAG ACACTTATAC   
  
  
- TAGGTTAATA ATTTTAAGTA CTTATTGTGT AAAAAATTTA TCTACTATAA TGGTTAACTT AACTGGAACT   
  
  
- GGTATTAAAA TACCCGGCCT AATCAGGTTC GCCGAGGTAA GGGCTCAACG AAAGTCCTGG ACGGTGTCGA   
  
  
- CCTGTCCTGT CTAACGTCTG GGTTTTGCTA CCCCTTCACC CCAACATCAT CAATGGAATT AATGAGAGTT   
  
  
- ACTCAAAGAC TTTAGGTTCC TTCATGTTCA ACTTATGTTA AAATGGAATA GACTATTCAT ACTTCCCCAT   
  
  
- TCTATTCCTA CTTCCATGGA ATTGAAGAGG AAGCGAAAAT ACATTCAAAA GACGTAAAAG TAGACGAAAA   
  
  
- ACCGGTTACC TAACCGACCG TCATCGGACA CCAGGAATCT CGAACTCTCA GTCTCGCCGT AACTACTCGA   
  
  
- AGGGAAGATA AAGTAAAGTG GAACTAATTG TGAGTTGGAT ATCGAAGTGA AAGTAAACCG CATGGTGCAA   
  
  
- TAACATTAAA GCCTTAAGTA AAAAACCGGT TCAAGTACTT CAAAGGGAAT GACCCACCTC TCTCTCTCTC   
  
  
- TCTCTAACGA GACAAGTGTC CATCTCCTCT TCTCGACCCG TTACCTGTGC ACGTAGACTT TCCCTTGTCG   
  
  
- TCTTCTATGG GCTCTCTGGG ACACTAAAGT GTGCTCGGTA CCACCAAGGT AATGTGAAGG TGTGGTTTTT   
  
  
- TCTCTCAATA ACTGGAGACG CTACTGTACC AAACGGGGTG CTTTAGGGTA TTTTAGGTTC ACGTAATGTA   
  
  
- GAGAAAAGAC CCCAAAGGCT CAAGAGCTTA CACAGATACA AGTGTTAAAC CGAGTTCTTT TCATTATGGA   
  
  
- TTAACCGTTG TCCTCTAGGT CTCACACATA CGTTCGTACG TGTATACTTC CCGGGGTAAG AACGAATGCA   
  
  
- TCGGTTAGTT CCTCTCGAAC ACCCCGGAGT ACAAAGAGGT GACTTTATAG AAGTTAAATA CAAGTTGACA   
  
  
- CACCCAGAGA CGAATAAAAT AAACCCTCAC CCCCACCCTC ACCGTTACCC GACCCCCACC ACGACTTACC   
  
  
- GGAATTTCAG TAACAACACA AGTATCAAAT ATAATTTACT CACTTTTTCT ATCCCTCTTT TTCGTTAGAA   
  
  
- ACTCACAAAA ACAGTAGATA ACAGAATTGA AGGGGGAGGC GGAAAAGAGA GAGAATTAGT TAGTTTCGAG   
  
  
- AACGCGAGAG AAGAAATACC CTTTTCAGAA GGTAAAGGTT TTGTAGGTAG AGACAGACAA ACTTTCGAAA   
  
  
- AGCCCCATTA TGTCATTTGT CCAACGTCA

+     CAT-box

| Site Name | Organism | Position | Strand | Matrix score. | sequence | function |
| --- | --- | --- | --- | --- | --- | --- |
| CAT-box | Arabidopsis thaliana | 1229 | - | 6 | GCCACT | cis-acting regulatory element related to meristem expression |

> 2018/04/13 10:10:12  
+ ATTTATTTAA TATTTTACAA TTATTTTTAA TATTATTTTT TAAAAATAAT AAATAAAATT ATTTATGAAT   
  
  
+ TAATTTTCAT AAAAATTTAT TGACTTAATT CGTGTATAAT TTTTTTGAGA AAAAATAAAT TAATGTATTT   
  
  
+ AAAATATAAT AAAATAATTT GTTAGTATAA TGAAACTAAC TCTTACATAA TAAAATAATC TGTGAATATG   
  
  
+ ATCCAATTAT TAAAATTCAT GAATAACACA TTTTTTAAAT AGATGATATT ACCAATTGAA TTGACCTTGA   
  
  
+ CCATAATTTT ATGGGCCGGA TTAGTCCAAG CGGCTCCATT CCCGAGTTGC TTTCAGGACC TGCCACAGCT   
  
  
+ GGACAGGACA GATTGCAGAC CCAAAACGAT GGGGAAGTGG GGTTGTAGTA GTTACCTTAA TTACTCTCAA   
  
  
+ TGAGTTTCTG AAATCCAAGG AAGTACAAGT TGAATACAAT TTTACCTTAT CTGATAAGTA TGAAGGGGTA   
  
  
+ AGATAAGGAT GAAGGTACCT TAACTTCTCC TTCGCTTTTA TGTAAGTTTT CTGCATTTTC ATCTGCTTTT   
  
  
+ TGGCCAATGG ATTGGCTGGC AGTAGCCTGT GGTCCTTAGA GCTTGAGAGT CAGAGCGGCA TTGATGAGCT   
  
  
+ TCCCTTCTAT TTCATTTCAC CTTGATTAAC ACTCAACCTA TAGCTTCACT TTCATTTGGC GTACCACGTT   
  
  
+ ATTGTAATTT CGGAATTCAT TTTTTGGCCA AGTTCATGAA GTTTCCCTTA CTGGGTGGAG AGAGAGAGAG   
  
  
+ AGAGATTGCT CTGTTCACAG GTAGAGGAGA AGAGCTGGGC AATGGACACG TGCATCTGAA AGGGAACAGC   
  
  
+ AGAAGATACC CGAGAGACCC TGTGATTTCA CACGAGCCAT GGTGGTTCCA TTACACTTCC ACACCAAAAA   
  
  
+ AGAGAGTTAT TGACCTCTGC GATGACATGG TTTGCCCCAC GAAATCCCAT AAAATCCAAG TGCATTACAT   
  
  
+ CTCTTTTCTG GGGTTTCCGA GTTCTCGAAT GTGTCTATGT TCACAATTTG GCTCAAGAAA AGTAATACCT   
  
  
+ AATTGGCAAC AGGAGATCCA GAGTGTGTAT GCAAGCATGC ACATATGAAG GGCCCCATTC TTGCTTACGT   
  
  
+ AGCCAATCAA GGAGAGCTTG TGGGGCCTCA TGTTTCTCCA CTGAAATATC TTCAATTTAT GTTCAACTGT   
  
  
+ GTGGGTCTCT GCTTATTTTA TTTGGGAGTG GGGGTGGGAG TGGCAATGGG CTGGGGGTGG TGCTGAATGG   
  
  
+ CCTTAAAGTC ATTGTTGTGT TCATAGTTTA TATTAAATGA GTGAAAAAGA TAGGGAGAAA AAGCAATCTT   
  
  
+ TGAGTGTTTT TGTCATCTAT TGTCTTAACT TCCCCCTCCG CCTTTTCTCT CTCTTAATCA ATCAAAGCTC   
  
  
+ TTGCGCTCTC TTCTTTATGG GAAAAGTCTT CCATTTCCAA AACATCCATC TCTGTCTGTT TGAAAGCTTT   
  
  
+ TCGGGGTAAT ACAGTAAACA GGTTGCAGT  

- TAAATAAATT ATAAAATGTT AATAAAAATT ATAATAAAAA ATTTTTATTA TTTATTTTAA TAAATACTTA   
  
  
- ATTAAAAGTA TTTTTAAATA ACTGAATTAA GCACATATTA AAAAAACTCT TTTTTATTTA ATTACATAAA   
  
  
- TTTTATATTA TTTTATTAAA CAATCATATT ACTTTGATTG AGAATGTATT ATTTTATTAG ACACTTATAC   
  
  
- TAGGTTAATA ATTTTAAGTA CTTATTGTGT AAAAAATTTA TCTACTATAA TGGTTAACTT AACTGGAACT   
  
  
- GGTATTAAAA TACCCGGCCT AATCAGGTTC GCCGAGGTAA GGGCTCAACG AAAGTCCTGG ACGGTGTCGA   
  
  
- CCTGTCCTGT CTAACGTCTG GGTTTTGCTA CCCCTTCACC CCAACATCAT CAATGGAATT AATGAGAGTT   
  
  
- ACTCAAAGAC TTTAGGTTCC TTCATGTTCA ACTTATGTTA AAATGGAATA GACTATTCAT ACTTCCCCAT   
  
  
- TCTATTCCTA CTTCCATGGA ATTGAAGAGG AAGCGAAAAT ACATTCAAAA GACGTAAAAG TAGACGAAAA   
  
  
- ACCGGTTACC TAACCGACCG TCATCGGACA CCAGGAATCT CGAACTCTCA GTCTCGCCGT AACTACTCGA   
  
  
- AGGGAAGATA AAGTAAAGTG GAACTAATTG TGAGTTGGAT ATCGAAGTGA AAGTAAACCG CATGGTGCAA   
  
  
- TAACATTAAA GCCTTAAGTA AAAAACCGGT TCAAGTACTT CAAAGGGAAT GACCCACCTC TCTCTCTCTC   
  
  
- TCTCTAACGA GACAAGTGTC CATCTCCTCT TCTCGACCCG TTACCTGTGC ACGTAGACTT TCCCTTGTCG   
  
  
- TCTTCTATGG GCTCTCTGGG ACACTAAAGT GTGCTCGGTA CCACCAAGGT AATGTGAAGG TGTGGTTTTT   
  
  
- TCTCTCAATA ACTGGAGACG CTACTGTACC AAACGGGGTG CTTTAGGGTA TTTTAGGTTC ACGTAATGTA   
  
  
- GAGAAAAGAC CCCAAAGGCT CAAGAGCTTA CACAGATACA AGTGTTAAAC CGAGTTCTTT TCATTATGGA   
  
  
- TTAACCGTTG TCCTCTAGGT CTCACACATA CGTTCGTACG TGTATACTTC CCGGGGTAAG AACGAATGCA   
  
  
- TCGGTTAGTT CCTCTCGAAC ACCCCGGAGT ACAAAGAGGT GACTTTATAG AAGTTAAATA CAAGTTGACA   
  
  
- CACCCAGAGA CGAATAAAAT AAACCCTCAC CCCCACCCTC ACCGTTACCC GACCCCCACC ACGACTTACC   
  
  
- GGAATTTCAG TAACAACACA AGTATCAAAT ATAATTTACT CACTTTTTCT ATCCCTCTTT TTCGTTAGAA   
  
  
- ACTCACAAAA ACAGTAGATA ACAGAATTGA AGGGGGAGGC GGAAAAGAGA GAGAATTAGT TAGTTTCGAG   
  
  
- AACGCGAGAG AAGAAATACC CTTTTCAGAA GGTAAAGGTT TTGTAGGTAG AGACAGACAA ACTTTCGAAA   
  
  
- AGCCCCATTA TGTCATTTGT CCAACGTCA

+     G-Box

| Site Name | Organism | Position | Strand | Matrix score. | sequence | function |
| --- | --- | --- | --- | --- | --- | --- |
| G-Box | Pisum sativum | 817 | - | 6 | CACGTG | cis-acting regulatory element involved in light responsiveness |
| G-Box | Pisum sativum | 695 | + | 6 | CACGTT | cis-acting regulatory element involved in light responsiveness |

> 2018/04/13 10:10:12  
+ ATTTATTTAA TATTTTACAA TTATTTTTAA TATTATTTTT TAAAAATAAT AAATAAAATT ATTTATGAAT   
  
  
+ TAATTTTCAT AAAAATTTAT TGACTTAATT CGTGTATAAT TTTTTTGAGA AAAAATAAAT TAATGTATTT   
  
  
+ AAAATATAAT AAAATAATTT GTTAGTATAA TGAAACTAAC TCTTACATAA TAAAATAATC TGTGAATATG   
  
  
+ ATCCAATTAT TAAAATTCAT GAATAACACA TTTTTTAAAT AGATGATATT ACCAATTGAA TTGACCTTGA   
  
  
+ CCATAATTTT ATGGGCCGGA TTAGTCCAAG CGGCTCCATT CCCGAGTTGC TTTCAGGACC TGCCACAGCT   
  
  
+ GGACAGGACA GATTGCAGAC CCAAAACGAT GGGGAAGTGG GGTTGTAGTA GTTACCTTAA TTACTCTCAA   
  
  
+ TGAGTTTCTG AAATCCAAGG AAGTACAAGT TGAATACAAT TTTACCTTAT CTGATAAGTA TGAAGGGGTA   
  
  
+ AGATAAGGAT GAAGGTACCT TAACTTCTCC TTCGCTTTTA TGTAAGTTTT CTGCATTTTC ATCTGCTTTT   
  
  
+ TGGCCAATGG ATTGGCTGGC AGTAGCCTGT GGTCCTTAGA GCTTGAGAGT CAGAGCGGCA TTGATGAGCT   
  
  
+ TCCCTTCTAT TTCATTTCAC CTTGATTAAC ACTCAACCTA TAGCTTCACT TTCATTTGGC GTACCACGTT   
  
  
+ ATTGTAATTT CGGAATTCAT TTTTTGGCCA AGTTCATGAA GTTTCCCTTA CTGGGTGGAG AGAGAGAGAG   
  
  
+ AGAGATTGCT CTGTTCACAG GTAGAGGAGA AGAGCTGGGC AATGGACACG TGCATCTGAA AGGGAACAGC   
  
  
+ AGAAGATACC CGAGAGACCC TGTGATTTCA CACGAGCCAT GGTGGTTCCA TTACACTTCC ACACCAAAAA   
  
  
+ AGAGAGTTAT TGACCTCTGC GATGACATGG TTTGCCCCAC GAAATCCCAT AAAATCCAAG TGCATTACAT   
  
  
+ CTCTTTTCTG GGGTTTCCGA GTTCTCGAAT GTGTCTATGT TCACAATTTG GCTCAAGAAA AGTAATACCT   
  
  
+ AATTGGCAAC AGGAGATCCA GAGTGTGTAT GCAAGCATGC ACATATGAAG GGCCCCATTC TTGCTTACGT   
  
  
+ AGCCAATCAA GGAGAGCTTG TGGGGCCTCA TGTTTCTCCA CTGAAATATC TTCAATTTAT GTTCAACTGT   
  
  
+ GTGGGTCTCT GCTTATTTTA TTTGGGAGTG GGGGTGGGAG TGGCAATGGG CTGGGGGTGG TGCTGAATGG   
  
  
+ CCTTAAAGTC ATTGTTGTGT TCATAGTTTA TATTAAATGA GTGAAAAAGA TAGGGAGAAA AAGCAATCTT   
  
  
+ TGAGTGTTTT TGTCATCTAT TGTCTTAACT TCCCCCTCCG CCTTTTCTCT CTCTTAATCA ATCAAAGCTC   
  
  
+ TTGCGCTCTC TTCTTTATGG GAAAAGTCTT CCATTTCCAA AACATCCATC TCTGTCTGTT TGAAAGCTTT   
  
  
+ TCGGGGTAAT ACAGTAAACA GGTTGCAGT  

- TAAATAAATT ATAAAATGTT AATAAAAATT ATAATAAAAA ATTTTTATTA TTTATTTTAA TAAATACTTA   
  
  
- ATTAAAAGTA TTTTTAAATA ACTGAATTAA GCACATATTA AAAAAACTCT TTTTTATTTA ATTACATAAA   
  
  
- TTTTATATTA TTTTATTAAA CAATCATATT ACTTTGATTG AGAATGTATT ATTTTATTAG ACACTTATAC   
  
  
- TAGGTTAATA ATTTTAAGTA CTTATTGTGT AAAAAATTTA TCTACTATAA TGGTTAACTT AACTGGAACT   
  
  
- GGTATTAAAA TACCCGGCCT AATCAGGTTC GCCGAGGTAA GGGCTCAACG AAAGTCCTGG ACGGTGTCGA   
  
  
- CCTGTCCTGT CTAACGTCTG GGTTTTGCTA CCCCTTCACC CCAACATCAT CAATGGAATT AATGAGAGTT   
  
  
- ACTCAAAGAC TTTAGGTTCC TTCATGTTCA ACTTATGTTA AAATGGAATA GACTATTCAT ACTTCCCCAT   
  
  
- TCTATTCCTA CTTCCATGGA ATTGAAGAGG AAGCGAAAAT ACATTCAAAA GACGTAAAAG TAGACGAAAA   
  
  
- ACCGGTTACC TAACCGACCG TCATCGGACA CCAGGAATCT CGAACTCTCA GTCTCGCCGT AACTACTCGA   
  
  
- AGGGAAGATA AAGTAAAGTG GAACTAATTG TGAGTTGGAT ATCGAAGTGA AAGTAAACCG CATGGTGCAA   
  
  
- TAACATTAAA GCCTTAAGTA AAAAACCGGT TCAAGTACTT CAAAGGGAAT GACCCACCTC TCTCTCTCTC   
  
  
- TCTCTAACGA GACAAGTGTC CATCTCCTCT TCTCGACCCG TTACCTGTGC ACGTAGACTT TCCCTTGTCG   
  
  
- TCTTCTATGG GCTCTCTGGG ACACTAAAGT GTGCTCGGTA CCACCAAGGT AATGTGAAGG TGTGGTTTTT   
  
  
- TCTCTCAATA ACTGGAGACG CTACTGTACC AAACGGGGTG CTTTAGGGTA TTTTAGGTTC ACGTAATGTA   
  
  
- GAGAAAAGAC CCCAAAGGCT CAAGAGCTTA CACAGATACA AGTGTTAAAC CGAGTTCTTT TCATTATGGA   
  
  
- TTAACCGTTG TCCTCTAGGT CTCACACATA CGTTCGTACG TGTATACTTC CCGGGGTAAG AACGAATGCA   
  
  
- TCGGTTAGTT CCTCTCGAAC ACCCCGGAGT ACAAAGAGGT GACTTTATAG AAGTTAAATA CAAGTTGACA   
  
  
- CACCCAGAGA CGAATAAAAT AAACCCTCAC CCCCACCCTC ACCGTTACCC GACCCCCACC ACGACTTACC   
  
  
- GGAATTTCAG TAACAACACA AGTATCAAAT ATAATTTACT CACTTTTTCT ATCCCTCTTT TTCGTTAGAA   
  
  
- ACTCACAAAA ACAGTAGATA ACAGAATTGA AGGGGGAGGC GGAAAAGAGA GAGAATTAGT TAGTTTCGAG   
  
  
- AACGCGAGAG AAGAAATACC CTTTTCAGAA GGTAAAGGTT TTGTAGGTAG AGACAGACAA ACTTTCGAAA   
  
  
- AGCCCCATTA TGTCATTTGT CCAACGTCA

+     G-box

| Site Name | Organism | Position | Strand | Matrix score. | sequence | function |
| --- | --- | --- | --- | --- | --- | --- |
| G-box | Arabidopsis thaliana | 817 | - | 6 | CACGTG | cis-acting regulatory element involved in light responsiveness |
| G-box | Zea mays | 695 | + | 6 | CACGTT | cis-acting regulatory element involved in light responsiveness |

> 2018/04/13 10:10:12  
+ ATTTATTTAA TATTTTACAA TTATTTTTAA TATTATTTTT TAAAAATAAT AAATAAAATT ATTTATGAAT   
  
  
+ TAATTTTCAT AAAAATTTAT TGACTTAATT CGTGTATAAT TTTTTTGAGA AAAAATAAAT TAATGTATTT   
  
  
+ AAAATATAAT AAAATAATTT GTTAGTATAA TGAAACTAAC TCTTACATAA TAAAATAATC TGTGAATATG   
  
  
+ ATCCAATTAT TAAAATTCAT GAATAACACA TTTTTTAAAT AGATGATATT ACCAATTGAA TTGACCTTGA   
  
  
+ CCATAATTTT ATGGGCCGGA TTAGTCCAAG CGGCTCCATT CCCGAGTTGC TTTCAGGACC TGCCACAGCT   
  
  
+ GGACAGGACA GATTGCAGAC CCAAAACGAT GGGGAAGTGG GGTTGTAGTA GTTACCTTAA TTACTCTCAA   
  
  
+ TGAGTTTCTG AAATCCAAGG AAGTACAAGT TGAATACAAT TTTACCTTAT CTGATAAGTA TGAAGGGGTA   
  
  
+ AGATAAGGAT GAAGGTACCT TAACTTCTCC TTCGCTTTTA TGTAAGTTTT CTGCATTTTC ATCTGCTTTT   
  
  
+ TGGCCAATGG ATTGGCTGGC AGTAGCCTGT GGTCCTTAGA GCTTGAGAGT CAGAGCGGCA TTGATGAGCT   
  
  
+ TCCCTTCTAT TTCATTTCAC CTTGATTAAC ACTCAACCTA TAGCTTCACT TTCATTTGGC GTACCACGTT   
  
  
+ ATTGTAATTT CGGAATTCAT TTTTTGGCCA AGTTCATGAA GTTTCCCTTA CTGGGTGGAG AGAGAGAGAG   
  
  
+ AGAGATTGCT CTGTTCACAG GTAGAGGAGA AGAGCTGGGC AATGGACACG TGCATCTGAA AGGGAACAGC   
  
  
+ AGAAGATACC CGAGAGACCC TGTGATTTCA CACGAGCCAT GGTGGTTCCA TTACACTTCC ACACCAAAAA   
  
  
+ AGAGAGTTAT TGACCTCTGC GATGACATGG TTTGCCCCAC GAAATCCCAT AAAATCCAAG TGCATTACAT   
  
  
+ CTCTTTTCTG GGGTTTCCGA GTTCTCGAAT GTGTCTATGT TCACAATTTG GCTCAAGAAA AGTAATACCT   
  
  
+ AATTGGCAAC AGGAGATCCA GAGTGTGTAT GCAAGCATGC ACATATGAAG GGCCCCATTC TTGCTTACGT   
  
  
+ AGCCAATCAA GGAGAGCTTG TGGGGCCTCA TGTTTCTCCA CTGAAATATC TTCAATTTAT GTTCAACTGT   
  
  
+ GTGGGTCTCT GCTTATTTTA TTTGGGAGTG GGGGTGGGAG TGGCAATGGG CTGGGGGTGG TGCTGAATGG   
  
  
+ CCTTAAAGTC ATTGTTGTGT TCATAGTTTA TATTAAATGA GTGAAAAAGA TAGGGAGAAA AAGCAATCTT   
  
  
+ TGAGTGTTTT TGTCATCTAT TGTCTTAACT TCCCCCTCCG CCTTTTCTCT CTCTTAATCA ATCAAAGCTC   
  
  
+ TTGCGCTCTC TTCTTTATGG GAAAAGTCTT CCATTTCCAA AACATCCATC TCTGTCTGTT TGAAAGCTTT   
  
  
+ TCGGGGTAAT ACAGTAAACA GGTTGCAGT  

- TAAATAAATT ATAAAATGTT AATAAAAATT ATAATAAAAA ATTTTTATTA TTTATTTTAA TAAATACTTA   
  
  
- ATTAAAAGTA TTTTTAAATA ACTGAATTAA GCACATATTA AAAAAACTCT TTTTTATTTA ATTACATAAA   
  
  
- TTTTATATTA TTTTATTAAA CAATCATATT ACTTTGATTG AGAATGTATT ATTTTATTAG ACACTTATAC   
  
  
- TAGGTTAATA ATTTTAAGTA CTTATTGTGT AAAAAATTTA TCTACTATAA TGGTTAACTT AACTGGAACT   
  
  
- GGTATTAAAA TACCCGGCCT AATCAGGTTC GCCGAGGTAA GGGCTCAACG AAAGTCCTGG ACGGTGTCGA   
  
  
- CCTGTCCTGT CTAACGTCTG GGTTTTGCTA CCCCTTCACC CCAACATCAT CAATGGAATT AATGAGAGTT   
  
  
- ACTCAAAGAC TTTAGGTTCC TTCATGTTCA ACTTATGTTA AAATGGAATA GACTATTCAT ACTTCCCCAT   
  
  
- TCTATTCCTA CTTCCATGGA ATTGAAGAGG AAGCGAAAAT ACATTCAAAA GACGTAAAAG TAGACGAAAA   
  
  
- ACCGGTTACC TAACCGACCG TCATCGGACA CCAGGAATCT CGAACTCTCA GTCTCGCCGT AACTACTCGA   
  
  
- AGGGAAGATA AAGTAAAGTG GAACTAATTG TGAGTTGGAT ATCGAAGTGA AAGTAAACCG CATGGTGCAA   
  
  
- TAACATTAAA GCCTTAAGTA AAAAACCGGT TCAAGTACTT CAAAGGGAAT GACCCACCTC TCTCTCTCTC   
  
  
- TCTCTAACGA GACAAGTGTC CATCTCCTCT TCTCGACCCG TTACCTGTGC ACGTAGACTT TCCCTTGTCG   
  
  
- TCTTCTATGG GCTCTCTGGG ACACTAAAGT GTGCTCGGTA CCACCAAGGT AATGTGAAGG TGTGGTTTTT   
  
  
- TCTCTCAATA ACTGGAGACG CTACTGTACC AAACGGGGTG CTTTAGGGTA TTTTAGGTTC ACGTAATGTA   
  
  
- GAGAAAAGAC CCCAAAGGCT CAAGAGCTTA CACAGATACA AGTGTTAAAC CGAGTTCTTT TCATTATGGA   
  
  
- TTAACCGTTG TCCTCTAGGT CTCACACATA CGTTCGTACG TGTATACTTC CCGGGGTAAG AACGAATGCA   
  
  
- TCGGTTAGTT CCTCTCGAAC ACCCCGGAGT ACAAAGAGGT GACTTTATAG AAGTTAAATA CAAGTTGACA   
  
  
- CACCCAGAGA CGAATAAAAT AAACCCTCAC CCCCACCCTC ACCGTTACCC GACCCCCACC ACGACTTACC   
  
  
- GGAATTTCAG TAACAACACA AGTATCAAAT ATAATTTACT CACTTTTTCT ATCCCTCTTT TTCGTTAGAA   
  
  
- ACTCACAAAA ACAGTAGATA ACAGAATTGA AGGGGGAGGC GGAAAAGAGA GAGAATTAGT TAGTTTCGAG   
  
  
- AACGCGAGAG AAGAAATACC CTTTTCAGAA GGTAAAGGTT TTGTAGGTAG AGACAGACAA ACTTTCGAAA   
  
  
- AGCCCCATTA TGTCATTTGT CCAACGTCA

+     GAG-motif

| Site Name | Organism | Position | Strand | Matrix score. | sequence | function |
| --- | --- | --- | --- | --- | --- | --- |
| GAG-motif | Spinacia oleracea | 978 | - | 7 | AGAGATG | part of a light responsive element |
| GAG-motif | Arabidopsis thaliana | 911 | + | 7 | AGAGAGT | part of a light responsive element |
| GAG-motif | Spinacia oleracea | 1447 | - | 7 | AGAGATG | part of a light responsive element |

> 2018/04/13 10:10:12  
+ ATTTATTTAA TATTTTACAA TTATTTTTAA TATTATTTTT TAAAAATAAT AAATAAAATT ATTTATGAAT   
  
  
+ TAATTTTCAT AAAAATTTAT TGACTTAATT CGTGTATAAT TTTTTTGAGA AAAAATAAAT TAATGTATTT   
  
  
+ AAAATATAAT AAAATAATTT GTTAGTATAA TGAAACTAAC TCTTACATAA TAAAATAATC TGTGAATATG   
  
  
+ ATCCAATTAT TAAAATTCAT GAATAACACA TTTTTTAAAT AGATGATATT ACCAATTGAA TTGACCTTGA   
  
  
+ CCATAATTTT ATGGGCCGGA TTAGTCCAAG CGGCTCCATT CCCGAGTTGC TTTCAGGACC TGCCACAGCT   
  
  
+ GGACAGGACA GATTGCAGAC CCAAAACGAT GGGGAAGTGG GGTTGTAGTA GTTACCTTAA TTACTCTCAA   
  
  
+ TGAGTTTCTG AAATCCAAGG AAGTACAAGT TGAATACAAT TTTACCTTAT CTGATAAGTA TGAAGGGGTA   
  
  
+ AGATAAGGAT GAAGGTACCT TAACTTCTCC TTCGCTTTTA TGTAAGTTTT CTGCATTTTC ATCTGCTTTT   
  
  
+ TGGCCAATGG ATTGGCTGGC AGTAGCCTGT GGTCCTTAGA GCTTGAGAGT CAGAGCGGCA TTGATGAGCT   
  
  
+ TCCCTTCTAT TTCATTTCAC CTTGATTAAC ACTCAACCTA TAGCTTCACT TTCATTTGGC GTACCACGTT   
  
  
+ ATTGTAATTT CGGAATTCAT TTTTTGGCCA AGTTCATGAA GTTTCCCTTA CTGGGTGGAG AGAGAGAGAG   
  
  
+ AGAGATTGCT CTGTTCACAG GTAGAGGAGA AGAGCTGGGC AATGGACACG TGCATCTGAA AGGGAACAGC   
  
  
+ AGAAGATACC CGAGAGACCC TGTGATTTCA CACGAGCCAT GGTGGTTCCA TTACACTTCC ACACCAAAAA   
  
  
+ AGAGAGTTAT TGACCTCTGC GATGACATGG TTTGCCCCAC GAAATCCCAT AAAATCCAAG TGCATTACAT   
  
  
+ CTCTTTTCTG GGGTTTCCGA GTTCTCGAAT GTGTCTATGT TCACAATTTG GCTCAAGAAA AGTAATACCT   
  
  
+ AATTGGCAAC AGGAGATCCA GAGTGTGTAT GCAAGCATGC ACATATGAAG GGCCCCATTC TTGCTTACGT   
  
  
+ AGCCAATCAA GGAGAGCTTG TGGGGCCTCA TGTTTCTCCA CTGAAATATC TTCAATTTAT GTTCAACTGT   
  
  
+ GTGGGTCTCT GCTTATTTTA TTTGGGAGTG GGGGTGGGAG TGGCAATGGG CTGGGGGTGG TGCTGAATGG   
  
  
+ CCTTAAAGTC ATTGTTGTGT TCATAGTTTA TATTAAATGA GTGAAAAAGA TAGGGAGAAA AAGCAATCTT   
  
  
+ TGAGTGTTTT TGTCATCTAT TGTCTTAACT TCCCCCTCCG CCTTTTCTCT CTCTTAATCA ATCAAAGCTC   
  
  
+ TTGCGCTCTC TTCTTTATGG GAAAAGTCTT CCATTTCCAA AACATCCATC TCTGTCTGTT TGAAAGCTTT   
  
  
+ TCGGGGTAAT ACAGTAAACA GGTTGCAGT  

- TAAATAAATT ATAAAATGTT AATAAAAATT ATAATAAAAA ATTTTTATTA TTTATTTTAA TAAATACTTA   
  
  
- ATTAAAAGTA TTTTTAAATA ACTGAATTAA GCACATATTA AAAAAACTCT TTTTTATTTA ATTACATAAA   
  
  
- TTTTATATTA TTTTATTAAA CAATCATATT ACTTTGATTG AGAATGTATT ATTTTATTAG ACACTTATAC   
  
  
- TAGGTTAATA ATTTTAAGTA CTTATTGTGT AAAAAATTTA TCTACTATAA TGGTTAACTT AACTGGAACT   
  
  
- GGTATTAAAA TACCCGGCCT AATCAGGTTC GCCGAGGTAA GGGCTCAACG AAAGTCCTGG ACGGTGTCGA   
  
  
- CCTGTCCTGT CTAACGTCTG GGTTTTGCTA CCCCTTCACC CCAACATCAT CAATGGAATT AATGAGAGTT   
  
  
- ACTCAAAGAC TTTAGGTTCC TTCATGTTCA ACTTATGTTA AAATGGAATA GACTATTCAT ACTTCCCCAT   
  
  
- TCTATTCCTA CTTCCATGGA ATTGAAGAGG AAGCGAAAAT ACATTCAAAA GACGTAAAAG TAGACGAAAA   
  
  
- ACCGGTTACC TAACCGACCG TCATCGGACA CCAGGAATCT CGAACTCTCA GTCTCGCCGT AACTACTCGA   
  
  
- AGGGAAGATA AAGTAAAGTG GAACTAATTG TGAGTTGGAT ATCGAAGTGA AAGTAAACCG CATGGTGCAA   
  
  
- TAACATTAAA GCCTTAAGTA AAAAACCGGT TCAAGTACTT CAAAGGGAAT GACCCACCTC TCTCTCTCTC   
  
  
- TCTCTAACGA GACAAGTGTC CATCTCCTCT TCTCGACCCG TTACCTGTGC ACGTAGACTT TCCCTTGTCG   
  
  
- TCTTCTATGG GCTCTCTGGG ACACTAAAGT GTGCTCGGTA CCACCAAGGT AATGTGAAGG TGTGGTTTTT   
  
  
- TCTCTCAATA ACTGGAGACG CTACTGTACC AAACGGGGTG CTTTAGGGTA TTTTAGGTTC ACGTAATGTA   
  
  
- GAGAAAAGAC CCCAAAGGCT CAAGAGCTTA CACAGATACA AGTGTTAAAC CGAGTTCTTT TCATTATGGA   
  
  
- TTAACCGTTG TCCTCTAGGT CTCACACATA CGTTCGTACG TGTATACTTC CCGGGGTAAG AACGAATGCA   
  
  
- TCGGTTAGTT CCTCTCGAAC ACCCCGGAGT ACAAAGAGGT GACTTTATAG AAGTTAAATA CAAGTTGACA   
  
  
- CACCCAGAGA CGAATAAAAT AAACCCTCAC CCCCACCCTC ACCGTTACCC GACCCCCACC ACGACTTACC   
  
  
- GGAATTTCAG TAACAACACA AGTATCAAAT ATAATTTACT CACTTTTTCT ATCCCTCTTT TTCGTTAGAA   
  
  
- ACTCACAAAA ACAGTAGATA ACAGAATTGA AGGGGGAGGC GGAAAAGAGA GAGAATTAGT TAGTTTCGAG   
  
  
- AACGCGAGAG AAGAAATACC CTTTTCAGAA GGTAAAGGTT TTGTAGGTAG AGACAGACAA ACTTTCGAAA   
  
  
- AGCCCCATTA TGTCATTTGT CCAACGTCA

+     GARE-motif

| Site Name | Organism | Position | Strand | Matrix score. | sequence | function |
| --- | --- | --- | --- | --- | --- | --- |
| GARE-motif | Brassica oleracea | 1455 | - | 7 | AAACAGA | gibberellin-responsive element |

> 2018/04/13 10:10:12  
+ ATTTATTTAA TATTTTACAA TTATTTTTAA TATTATTTTT TAAAAATAAT AAATAAAATT ATTTATGAAT   
  
  
+ TAATTTTCAT AAAAATTTAT TGACTTAATT CGTGTATAAT TTTTTTGAGA AAAAATAAAT TAATGTATTT   
  
  
+ AAAATATAAT AAAATAATTT GTTAGTATAA TGAAACTAAC TCTTACATAA TAAAATAATC TGTGAATATG   
  
  
+ ATCCAATTAT TAAAATTCAT GAATAACACA TTTTTTAAAT AGATGATATT ACCAATTGAA TTGACCTTGA   
  
  
+ CCATAATTTT ATGGGCCGGA TTAGTCCAAG CGGCTCCATT CCCGAGTTGC TTTCAGGACC TGCCACAGCT   
  
  
+ GGACAGGACA GATTGCAGAC CCAAAACGAT GGGGAAGTGG GGTTGTAGTA GTTACCTTAA TTACTCTCAA   
  
  
+ TGAGTTTCTG AAATCCAAGG AAGTACAAGT TGAATACAAT TTTACCTTAT CTGATAAGTA TGAAGGGGTA   
  
  
+ AGATAAGGAT GAAGGTACCT TAACTTCTCC TTCGCTTTTA TGTAAGTTTT CTGCATTTTC ATCTGCTTTT   
  
  
+ TGGCCAATGG ATTGGCTGGC AGTAGCCTGT GGTCCTTAGA GCTTGAGAGT CAGAGCGGCA TTGATGAGCT   
  
  
+ TCCCTTCTAT TTCATTTCAC CTTGATTAAC ACTCAACCTA TAGCTTCACT TTCATTTGGC GTACCACGTT   
  
  
+ ATTGTAATTT CGGAATTCAT TTTTTGGCCA AGTTCATGAA GTTTCCCTTA CTGGGTGGAG AGAGAGAGAG   
  
  
+ AGAGATTGCT CTGTTCACAG GTAGAGGAGA AGAGCTGGGC AATGGACACG TGCATCTGAA AGGGAACAGC   
  
  
+ AGAAGATACC CGAGAGACCC TGTGATTTCA CACGAGCCAT GGTGGTTCCA TTACACTTCC ACACCAAAAA   
  
  
+ AGAGAGTTAT TGACCTCTGC GATGACATGG TTTGCCCCAC GAAATCCCAT AAAATCCAAG TGCATTACAT   
  
  
+ CTCTTTTCTG GGGTTTCCGA GTTCTCGAAT GTGTCTATGT TCACAATTTG GCTCAAGAAA AGTAATACCT   
  
  
+ AATTGGCAAC AGGAGATCCA GAGTGTGTAT GCAAGCATGC ACATATGAAG GGCCCCATTC TTGCTTACGT   
  
  
+ AGCCAATCAA GGAGAGCTTG TGGGGCCTCA TGTTTCTCCA CTGAAATATC TTCAATTTAT GTTCAACTGT   
  
  
+ GTGGGTCTCT GCTTATTTTA TTTGGGAGTG GGGGTGGGAG TGGCAATGGG CTGGGGGTGG TGCTGAATGG   
  
  
+ CCTTAAAGTC ATTGTTGTGT TCATAGTTTA TATTAAATGA GTGAAAAAGA TAGGGAGAAA AAGCAATCTT   
  
  
+ TGAGTGTTTT TGTCATCTAT TGTCTTAACT TCCCCCTCCG CCTTTTCTCT CTCTTAATCA ATCAAAGCTC   
  
  
+ TTGCGCTCTC TTCTTTATGG GAAAAGTCTT CCATTTCCAA AACATCCATC TCTGTCTGTT TGAAAGCTTT   
  
  
+ TCGGGGTAAT ACAGTAAACA GGTTGCAGT  

- TAAATAAATT ATAAAATGTT AATAAAAATT ATAATAAAAA ATTTTTATTA TTTATTTTAA TAAATACTTA   
  
  
- ATTAAAAGTA TTTTTAAATA ACTGAATTAA GCACATATTA AAAAAACTCT TTTTTATTTA ATTACATAAA   
  
  
- TTTTATATTA TTTTATTAAA CAATCATATT ACTTTGATTG AGAATGTATT ATTTTATTAG ACACTTATAC   
  
  
- TAGGTTAATA ATTTTAAGTA CTTATTGTGT AAAAAATTTA TCTACTATAA TGGTTAACTT AACTGGAACT   
  
  
- GGTATTAAAA TACCCGGCCT AATCAGGTTC GCCGAGGTAA GGGCTCAACG AAAGTCCTGG ACGGTGTCGA   
  
  
- CCTGTCCTGT CTAACGTCTG GGTTTTGCTA CCCCTTCACC CCAACATCAT CAATGGAATT AATGAGAGTT   
  
  
- ACTCAAAGAC TTTAGGTTCC TTCATGTTCA ACTTATGTTA AAATGGAATA GACTATTCAT ACTTCCCCAT   
  
  
- TCTATTCCTA CTTCCATGGA ATTGAAGAGG AAGCGAAAAT ACATTCAAAA GACGTAAAAG TAGACGAAAA   
  
  
- ACCGGTTACC TAACCGACCG TCATCGGACA CCAGGAATCT CGAACTCTCA GTCTCGCCGT AACTACTCGA   
  
  
- AGGGAAGATA AAGTAAAGTG GAACTAATTG TGAGTTGGAT ATCGAAGTGA AAGTAAACCG CATGGTGCAA   
  
  
- TAACATTAAA GCCTTAAGTA AAAAACCGGT TCAAGTACTT CAAAGGGAAT GACCCACCTC TCTCTCTCTC   
  
  
- TCTCTAACGA GACAAGTGTC CATCTCCTCT TCTCGACCCG TTACCTGTGC ACGTAGACTT TCCCTTGTCG   
  
  
- TCTTCTATGG GCTCTCTGGG ACACTAAAGT GTGCTCGGTA CCACCAAGGT AATGTGAAGG TGTGGTTTTT   
  
  
- TCTCTCAATA ACTGGAGACG CTACTGTACC AAACGGGGTG CTTTAGGGTA TTTTAGGTTC ACGTAATGTA   
  
  
- GAGAAAAGAC CCCAAAGGCT CAAGAGCTTA CACAGATACA AGTGTTAAAC CGAGTTCTTT TCATTATGGA   
  
  
- TTAACCGTTG TCCTCTAGGT CTCACACATA CGTTCGTACG TGTATACTTC CCGGGGTAAG AACGAATGCA   
  
  
- TCGGTTAGTT CCTCTCGAAC ACCCCGGAGT ACAAAGAGGT GACTTTATAG AAGTTAAATA CAAGTTGACA   
  
  
- CACCCAGAGA CGAATAAAAT AAACCCTCAC CCCCACCCTC ACCGTTACCC GACCCCCACC ACGACTTACC   
  
  
- GGAATTTCAG TAACAACACA AGTATCAAAT ATAATTTACT CACTTTTTCT ATCCCTCTTT TTCGTTAGAA   
  
  
- ACTCACAAAA ACAGTAGATA ACAGAATTGA AGGGGGAGGC GGAAAAGAGA GAGAATTAGT TAGTTTCGAG   
  
  
- AACGCGAGAG AAGAAATACC CTTTTCAGAA GGTAAAGGTT TTGTAGGTAG AGACAGACAA ACTTTCGAAA   
  
  
- AGCCCCATTA TGTCATTTGT CCAACGTCA

+     GATA-motif

| Site Name | Organism | Position | Strand | Matrix score. | sequence | function |
| --- | --- | --- | --- | --- | --- | --- |
| GATA-motif | Pisum sativum | 1309 | + | 7 | GATAGGG | part of a light responsive element |

> 2018/04/13 10:10:12  
+ ATTTATTTAA TATTTTACAA TTATTTTTAA TATTATTTTT TAAAAATAAT AAATAAAATT ATTTATGAAT   
  
  
+ TAATTTTCAT AAAAATTTAT TGACTTAATT CGTGTATAAT TTTTTTGAGA AAAAATAAAT TAATGTATTT   
  
  
+ AAAATATAAT AAAATAATTT GTTAGTATAA TGAAACTAAC TCTTACATAA TAAAATAATC TGTGAATATG   
  
  
+ ATCCAATTAT TAAAATTCAT GAATAACACA TTTTTTAAAT AGATGATATT ACCAATTGAA TTGACCTTGA   
  
  
+ CCATAATTTT ATGGGCCGGA TTAGTCCAAG CGGCTCCATT CCCGAGTTGC TTTCAGGACC TGCCACAGCT   
  
  
+ GGACAGGACA GATTGCAGAC CCAAAACGAT GGGGAAGTGG GGTTGTAGTA GTTACCTTAA TTACTCTCAA   
  
  
+ TGAGTTTCTG AAATCCAAGG AAGTACAAGT TGAATACAAT TTTACCTTAT CTGATAAGTA TGAAGGGGTA   
  
  
+ AGATAAGGAT GAAGGTACCT TAACTTCTCC TTCGCTTTTA TGTAAGTTTT CTGCATTTTC ATCTGCTTTT   
  
  
+ TGGCCAATGG ATTGGCTGGC AGTAGCCTGT GGTCCTTAGA GCTTGAGAGT CAGAGCGGCA TTGATGAGCT   
  
  
+ TCCCTTCTAT TTCATTTCAC CTTGATTAAC ACTCAACCTA TAGCTTCACT TTCATTTGGC GTACCACGTT   
  
  
+ ATTGTAATTT CGGAATTCAT TTTTTGGCCA AGTTCATGAA GTTTCCCTTA CTGGGTGGAG AGAGAGAGAG   
  
  
+ AGAGATTGCT CTGTTCACAG GTAGAGGAGA AGAGCTGGGC AATGGACACG TGCATCTGAA AGGGAACAGC   
  
  
+ AGAAGATACC CGAGAGACCC TGTGATTTCA CACGAGCCAT GGTGGTTCCA TTACACTTCC ACACCAAAAA   
  
  
+ AGAGAGTTAT TGACCTCTGC GATGACATGG TTTGCCCCAC GAAATCCCAT AAAATCCAAG TGCATTACAT   
  
  
+ CTCTTTTCTG GGGTTTCCGA GTTCTCGAAT GTGTCTATGT TCACAATTTG GCTCAAGAAA AGTAATACCT   
  
  
+ AATTGGCAAC AGGAGATCCA GAGTGTGTAT GCAAGCATGC ACATATGAAG GGCCCCATTC TTGCTTACGT   
  
  
+ AGCCAATCAA GGAGAGCTTG TGGGGCCTCA TGTTTCTCCA CTGAAATATC TTCAATTTAT GTTCAACTGT   
  
  
+ GTGGGTCTCT GCTTATTTTA TTTGGGAGTG GGGGTGGGAG TGGCAATGGG CTGGGGGTGG TGCTGAATGG   
  
  
+ CCTTAAAGTC ATTGTTGTGT TCATAGTTTA TATTAAATGA GTGAAAAAGA TAGGGAGAAA AAGCAATCTT   
  
  
+ TGAGTGTTTT TGTCATCTAT TGTCTTAACT TCCCCCTCCG CCTTTTCTCT CTCTTAATCA ATCAAAGCTC   
  
  
+ TTGCGCTCTC TTCTTTATGG GAAAAGTCTT CCATTTCCAA AACATCCATC TCTGTCTGTT TGAAAGCTTT   
  
  
+ TCGGGGTAAT ACAGTAAACA GGTTGCAGT  

- TAAATAAATT ATAAAATGTT AATAAAAATT ATAATAAAAA ATTTTTATTA TTTATTTTAA TAAATACTTA   
  
  
- ATTAAAAGTA TTTTTAAATA ACTGAATTAA GCACATATTA AAAAAACTCT TTTTTATTTA ATTACATAAA   
  
  
- TTTTATATTA TTTTATTAAA CAATCATATT ACTTTGATTG AGAATGTATT ATTTTATTAG ACACTTATAC   
  
  
- TAGGTTAATA ATTTTAAGTA CTTATTGTGT AAAAAATTTA TCTACTATAA TGGTTAACTT AACTGGAACT   
  
  
- GGTATTAAAA TACCCGGCCT AATCAGGTTC GCCGAGGTAA GGGCTCAACG AAAGTCCTGG ACGGTGTCGA   
  
  
- CCTGTCCTGT CTAACGTCTG GGTTTTGCTA CCCCTTCACC CCAACATCAT CAATGGAATT AATGAGAGTT   
  
  
- ACTCAAAGAC TTTAGGTTCC TTCATGTTCA ACTTATGTTA AAATGGAATA GACTATTCAT ACTTCCCCAT   
  
  
- TCTATTCCTA CTTCCATGGA ATTGAAGAGG AAGCGAAAAT ACATTCAAAA GACGTAAAAG TAGACGAAAA   
  
  
- ACCGGTTACC TAACCGACCG TCATCGGACA CCAGGAATCT CGAACTCTCA GTCTCGCCGT AACTACTCGA   
  
  
- AGGGAAGATA AAGTAAAGTG GAACTAATTG TGAGTTGGAT ATCGAAGTGA AAGTAAACCG CATGGTGCAA   
  
  
- TAACATTAAA GCCTTAAGTA AAAAACCGGT TCAAGTACTT CAAAGGGAAT GACCCACCTC TCTCTCTCTC   
  
  
- TCTCTAACGA GACAAGTGTC CATCTCCTCT TCTCGACCCG TTACCTGTGC ACGTAGACTT TCCCTTGTCG   
  
  
- TCTTCTATGG GCTCTCTGGG ACACTAAAGT GTGCTCGGTA CCACCAAGGT AATGTGAAGG TGTGGTTTTT   
  
  
- TCTCTCAATA ACTGGAGACG CTACTGTACC AAACGGGGTG CTTTAGGGTA TTTTAGGTTC ACGTAATGTA   
  
  
- GAGAAAAGAC CCCAAAGGCT CAAGAGCTTA CACAGATACA AGTGTTAAAC CGAGTTCTTT TCATTATGGA   
  
  
- TTAACCGTTG TCCTCTAGGT CTCACACATA CGTTCGTACG TGTATACTTC CCGGGGTAAG AACGAATGCA   
  
  
- TCGGTTAGTT CCTCTCGAAC ACCCCGGAGT ACAAAGAGGT GACTTTATAG AAGTTAAATA CAAGTTGACA   
  
  
- CACCCAGAGA CGAATAAAAT AAACCCTCAC CCCCACCCTC ACCGTTACCC GACCCCCACC ACGACTTACC   
  
  
- GGAATTTCAG TAACAACACA AGTATCAAAT ATAATTTACT CACTTTTTCT ATCCCTCTTT TTCGTTAGAA   
  
  
- ACTCACAAAA ACAGTAGATA ACAGAATTGA AGGGGGAGGC GGAAAAGAGA GAGAATTAGT TAGTTTCGAG   
  
  
- AACGCGAGAG AAGAAATACC CTTTTCAGAA GGTAAAGGTT TTGTAGGTAG AGACAGACAA ACTTTCGAAA   
  
  
- AGCCCCATTA TGTCATTTGT CCAACGTCA

+     I-box

| Site Name | Organism | Position | Strand | Matrix score. | sequence | function |
| --- | --- | --- | --- | --- | --- | --- |
| I-box | Zea mays | 1309 | + | 7 | GATAGGG | part of a light responsive element |
| I-box | Triticum aestivum | 491 | + | 8 | AGATAAGG | part of a light responsive element |
| I-box | Triticum aestivum | 465 | - | 8 | AGATAAGG | part of a light responsive element |
| I-box | Gossypium hirsutum | 490 | + | 10 | AAGATAAGGCT | part of a light responsive element |

> 2018/04/13 10:10:12  
+ ATTTATTTAA TATTTTACAA TTATTTTTAA TATTATTTTT TAAAAATAAT AAATAAAATT ATTTATGAAT   
  
  
+ TAATTTTCAT AAAAATTTAT TGACTTAATT CGTGTATAAT TTTTTTGAGA AAAAATAAAT TAATGTATTT   
  
  
+ AAAATATAAT AAAATAATTT GTTAGTATAA TGAAACTAAC TCTTACATAA TAAAATAATC TGTGAATATG   
  
  
+ ATCCAATTAT TAAAATTCAT GAATAACACA TTTTTTAAAT AGATGATATT ACCAATTGAA TTGACCTTGA   
  
  
+ CCATAATTTT ATGGGCCGGA TTAGTCCAAG CGGCTCCATT CCCGAGTTGC TTTCAGGACC TGCCACAGCT   
  
  
+ GGACAGGACA GATTGCAGAC CCAAAACGAT GGGGAAGTGG GGTTGTAGTA GTTACCTTAA TTACTCTCAA   
  
  
+ TGAGTTTCTG AAATCCAAGG AAGTACAAGT TGAATACAAT TTTACCTTAT CTGATAAGTA TGAAGGGGTA   
  
  
+ AGATAAGGAT GAAGGTACCT TAACTTCTCC TTCGCTTTTA TGTAAGTTTT CTGCATTTTC ATCTGCTTTT   
  
  
+ TGGCCAATGG ATTGGCTGGC AGTAGCCTGT GGTCCTTAGA GCTTGAGAGT CAGAGCGGCA TTGATGAGCT   
  
  
+ TCCCTTCTAT TTCATTTCAC CTTGATTAAC ACTCAACCTA TAGCTTCACT TTCATTTGGC GTACCACGTT   
  
  
+ ATTGTAATTT CGGAATTCAT TTTTTGGCCA AGTTCATGAA GTTTCCCTTA CTGGGTGGAG AGAGAGAGAG   
  
  
+ AGAGATTGCT CTGTTCACAG GTAGAGGAGA AGAGCTGGGC AATGGACACG TGCATCTGAA AGGGAACAGC   
  
  
+ AGAAGATACC CGAGAGACCC TGTGATTTCA CACGAGCCAT GGTGGTTCCA TTACACTTCC ACACCAAAAA   
  
  
+ AGAGAGTTAT TGACCTCTGC GATGACATGG TTTGCCCCAC GAAATCCCAT AAAATCCAAG TGCATTACAT   
  
  
+ CTCTTTTCTG GGGTTTCCGA GTTCTCGAAT GTGTCTATGT TCACAATTTG GCTCAAGAAA AGTAATACCT   
  
  
+ AATTGGCAAC AGGAGATCCA GAGTGTGTAT GCAAGCATGC ACATATGAAG GGCCCCATTC TTGCTTACGT   
  
  
+ AGCCAATCAA GGAGAGCTTG TGGGGCCTCA TGTTTCTCCA CTGAAATATC TTCAATTTAT GTTCAACTGT   
  
  
+ GTGGGTCTCT GCTTATTTTA TTTGGGAGTG GGGGTGGGAG TGGCAATGGG CTGGGGGTGG TGCTGAATGG   
  
  
+ CCTTAAAGTC ATTGTTGTGT TCATAGTTTA TATTAAATGA GTGAAAAAGA TAGGGAGAAA AAGCAATCTT   
  
  
+ TGAGTGTTTT TGTCATCTAT TGTCTTAACT TCCCCCTCCG CCTTTTCTCT CTCTTAATCA ATCAAAGCTC   
  
  
+ TTGCGCTCTC TTCTTTATGG GAAAAGTCTT CCATTTCCAA AACATCCATC TCTGTCTGTT TGAAAGCTTT   
  
  
+ TCGGGGTAAT ACAGTAAACA GGTTGCAGT  

- TAAATAAATT ATAAAATGTT AATAAAAATT ATAATAAAAA ATTTTTATTA TTTATTTTAA TAAATACTTA   
  
  
- ATTAAAAGTA TTTTTAAATA ACTGAATTAA GCACATATTA AAAAAACTCT TTTTTATTTA ATTACATAAA   
  
  
- TTTTATATTA TTTTATTAAA CAATCATATT ACTTTGATTG AGAATGTATT ATTTTATTAG ACACTTATAC   
  
  
- TAGGTTAATA ATTTTAAGTA CTTATTGTGT AAAAAATTTA TCTACTATAA TGGTTAACTT AACTGGAACT   
  
  
- GGTATTAAAA TACCCGGCCT AATCAGGTTC GCCGAGGTAA GGGCTCAACG AAAGTCCTGG ACGGTGTCGA   
  
  
- CCTGTCCTGT CTAACGTCTG GGTTTTGCTA CCCCTTCACC CCAACATCAT CAATGGAATT AATGAGAGTT   
  
  
- ACTCAAAGAC TTTAGGTTCC TTCATGTTCA ACTTATGTTA AAATGGAATA GACTATTCAT ACTTCCCCAT   
  
  
- TCTATTCCTA CTTCCATGGA ATTGAAGAGG AAGCGAAAAT ACATTCAAAA GACGTAAAAG TAGACGAAAA   
  
  
- ACCGGTTACC TAACCGACCG TCATCGGACA CCAGGAATCT CGAACTCTCA GTCTCGCCGT AACTACTCGA   
  
  
- AGGGAAGATA AAGTAAAGTG GAACTAATTG TGAGTTGGAT ATCGAAGTGA AAGTAAACCG CATGGTGCAA   
  
  
- TAACATTAAA GCCTTAAGTA AAAAACCGGT TCAAGTACTT CAAAGGGAAT GACCCACCTC TCTCTCTCTC   
  
  
- TCTCTAACGA GACAAGTGTC CATCTCCTCT TCTCGACCCG TTACCTGTGC ACGTAGACTT TCCCTTGTCG   
  
  
- TCTTCTATGG GCTCTCTGGG ACACTAAAGT GTGCTCGGTA CCACCAAGGT AATGTGAAGG TGTGGTTTTT   
  
  
- TCTCTCAATA ACTGGAGACG CTACTGTACC AAACGGGGTG CTTTAGGGTA TTTTAGGTTC ACGTAATGTA   
  
  
- GAGAAAAGAC CCCAAAGGCT CAAGAGCTTA CACAGATACA AGTGTTAAAC CGAGTTCTTT TCATTATGGA   
  
  
- TTAACCGTTG TCCTCTAGGT CTCACACATA CGTTCGTACG TGTATACTTC CCGGGGTAAG AACGAATGCA   
  
  
- TCGGTTAGTT CCTCTCGAAC ACCCCGGAGT ACAAAGAGGT GACTTTATAG AAGTTAAATA CAAGTTGACA   
  
  
- CACCCAGAGA CGAATAAAAT AAACCCTCAC CCCCACCCTC ACCGTTACCC GACCCCCACC ACGACTTACC   
  
  
- GGAATTTCAG TAACAACACA AGTATCAAAT ATAATTTACT CACTTTTTCT ATCCCTCTTT TTCGTTAGAA   
  
  
- ACTCACAAAA ACAGTAGATA ACAGAATTGA AGGGGGAGGC GGAAAAGAGA GAGAATTAGT TAGTTTCGAG   
  
  
- AACGCGAGAG AAGAAATACC CTTTTCAGAA GGTAAAGGTT TTGTAGGTAG AGACAGACAA ACTTTCGAAA   
  
  
- AGCCCCATTA TGTCATTTGT CCAACGTCA

+     LTR

| Site Name | Organism | Position | Strand | Matrix score. | sequence | function |
| --- | --- | --- | --- | --- | --- | --- |
| LTR | Hordeum vulgare | 708 | - | 6 | CCGAAA | cis-acting element involved in low-temperature responsiveness |
| LTR | Hordeum vulgare | 1469 | - | 6 | CCGAAA | cis-acting element involved in low-temperature responsiveness |

> 2018/04/13 10:10:12  
+ ATTTATTTAA TATTTTACAA TTATTTTTAA TATTATTTTT TAAAAATAAT AAATAAAATT ATTTATGAAT   
  
  
+ TAATTTTCAT AAAAATTTAT TGACTTAATT CGTGTATAAT TTTTTTGAGA AAAAATAAAT TAATGTATTT   
  
  
+ AAAATATAAT AAAATAATTT GTTAGTATAA TGAAACTAAC TCTTACATAA TAAAATAATC TGTGAATATG   
  
  
+ ATCCAATTAT TAAAATTCAT GAATAACACA TTTTTTAAAT AGATGATATT ACCAATTGAA TTGACCTTGA   
  
  
+ CCATAATTTT ATGGGCCGGA TTAGTCCAAG CGGCTCCATT CCCGAGTTGC TTTCAGGACC TGCCACAGCT   
  
  
+ GGACAGGACA GATTGCAGAC CCAAAACGAT GGGGAAGTGG GGTTGTAGTA GTTACCTTAA TTACTCTCAA   
  
  
+ TGAGTTTCTG AAATCCAAGG AAGTACAAGT TGAATACAAT TTTACCTTAT CTGATAAGTA TGAAGGGGTA   
  
  
+ AGATAAGGAT GAAGGTACCT TAACTTCTCC TTCGCTTTTA TGTAAGTTTT CTGCATTTTC ATCTGCTTTT   
  
  
+ TGGCCAATGG ATTGGCTGGC AGTAGCCTGT GGTCCTTAGA GCTTGAGAGT CAGAGCGGCA TTGATGAGCT   
  
  
+ TCCCTTCTAT TTCATTTCAC CTTGATTAAC ACTCAACCTA TAGCTTCACT TTCATTTGGC GTACCACGTT   
  
  
+ ATTGTAATTT CGGAATTCAT TTTTTGGCCA AGTTCATGAA GTTTCCCTTA CTGGGTGGAG AGAGAGAGAG   
  
  
+ AGAGATTGCT CTGTTCACAG GTAGAGGAGA AGAGCTGGGC AATGGACACG TGCATCTGAA AGGGAACAGC   
  
  
+ AGAAGATACC CGAGAGACCC TGTGATTTCA CACGAGCCAT GGTGGTTCCA TTACACTTCC ACACCAAAAA   
  
  
+ AGAGAGTTAT TGACCTCTGC GATGACATGG TTTGCCCCAC GAAATCCCAT AAAATCCAAG TGCATTACAT   
  
  
+ CTCTTTTCTG GGGTTTCCGA GTTCTCGAAT GTGTCTATGT TCACAATTTG GCTCAAGAAA AGTAATACCT   
  
  
+ AATTGGCAAC AGGAGATCCA GAGTGTGTAT GCAAGCATGC ACATATGAAG GGCCCCATTC TTGCTTACGT   
  
  
+ AGCCAATCAA GGAGAGCTTG TGGGGCCTCA TGTTTCTCCA CTGAAATATC TTCAATTTAT GTTCAACTGT   
  
  
+ GTGGGTCTCT GCTTATTTTA TTTGGGAGTG GGGGTGGGAG TGGCAATGGG CTGGGGGTGG TGCTGAATGG   
  
  
+ CCTTAAAGTC ATTGTTGTGT TCATAGTTTA TATTAAATGA GTGAAAAAGA TAGGGAGAAA AAGCAATCTT   
  
  
+ TGAGTGTTTT TGTCATCTAT TGTCTTAACT TCCCCCTCCG CCTTTTCTCT CTCTTAATCA ATCAAAGCTC   
  
  
+ TTGCGCTCTC TTCTTTATGG GAAAAGTCTT CCATTTCCAA AACATCCATC TCTGTCTGTT TGAAAGCTTT   
  
  
+ TCGGGGTAAT ACAGTAAACA GGTTGCAGT  

- TAAATAAATT ATAAAATGTT AATAAAAATT ATAATAAAAA ATTTTTATTA TTTATTTTAA TAAATACTTA   
  
  
- ATTAAAAGTA TTTTTAAATA ACTGAATTAA GCACATATTA AAAAAACTCT TTTTTATTTA ATTACATAAA   
  
  
- TTTTATATTA TTTTATTAAA CAATCATATT ACTTTGATTG AGAATGTATT ATTTTATTAG ACACTTATAC   
  
  
- TAGGTTAATA ATTTTAAGTA CTTATTGTGT AAAAAATTTA TCTACTATAA TGGTTAACTT AACTGGAACT   
  
  
- GGTATTAAAA TACCCGGCCT AATCAGGTTC GCCGAGGTAA GGGCTCAACG AAAGTCCTGG ACGGTGTCGA   
  
  
- CCTGTCCTGT CTAACGTCTG GGTTTTGCTA CCCCTTCACC CCAACATCAT CAATGGAATT AATGAGAGTT   
  
  
- ACTCAAAGAC TTTAGGTTCC TTCATGTTCA ACTTATGTTA AAATGGAATA GACTATTCAT ACTTCCCCAT   
  
  
- TCTATTCCTA CTTCCATGGA ATTGAAGAGG AAGCGAAAAT ACATTCAAAA GACGTAAAAG TAGACGAAAA   
  
  
- ACCGGTTACC TAACCGACCG TCATCGGACA CCAGGAATCT CGAACTCTCA GTCTCGCCGT AACTACTCGA   
  
  
- AGGGAAGATA AAGTAAAGTG GAACTAATTG TGAGTTGGAT ATCGAAGTGA AAGTAAACCG CATGGTGCAA   
  
  
- TAACATTAAA GCCTTAAGTA AAAAACCGGT TCAAGTACTT CAAAGGGAAT GACCCACCTC TCTCTCTCTC   
  
  
- TCTCTAACGA GACAAGTGTC CATCTCCTCT TCTCGACCCG TTACCTGTGC ACGTAGACTT TCCCTTGTCG   
  
  
- TCTTCTATGG GCTCTCTGGG ACACTAAAGT GTGCTCGGTA CCACCAAGGT AATGTGAAGG TGTGGTTTTT   
  
  
- TCTCTCAATA ACTGGAGACG CTACTGTACC AAACGGGGTG CTTTAGGGTA TTTTAGGTTC ACGTAATGTA   
  
  
- GAGAAAAGAC CCCAAAGGCT CAAGAGCTTA CACAGATACA AGTGTTAAAC CGAGTTCTTT TCATTATGGA   
  
  
- TTAACCGTTG TCCTCTAGGT CTCACACATA CGTTCGTACG TGTATACTTC CCGGGGTAAG AACGAATGCA   
  
  
- TCGGTTAGTT CCTCTCGAAC ACCCCGGAGT ACAAAGAGGT GACTTTATAG AAGTTAAATA CAAGTTGACA   
  
  
- CACCCAGAGA CGAATAAAAT AAACCCTCAC CCCCACCCTC ACCGTTACCC GACCCCCACC ACGACTTACC   
  
  
- GGAATTTCAG TAACAACACA AGTATCAAAT ATAATTTACT CACTTTTTCT ATCCCTCTTT TTCGTTAGAA   
  
  
- ACTCACAAAA ACAGTAGATA ACAGAATTGA AGGGGGAGGC GGAAAAGAGA GAGAATTAGT TAGTTTCGAG   
  
  
- AACGCGAGAG AAGAAATACC CTTTTCAGAA GGTAAAGGTT TTGTAGGTAG AGACAGACAA ACTTTCGAAA   
  
  
- AGCCCCATTA TGTCATTTGT CCAACGTCA

+     MBS

| Site Name | Organism | Position | Strand | Matrix score. | sequence | function |
| --- | --- | --- | --- | --- | --- | --- |
| MBS | Arabidopsis thaliana | 1184 | + | 6 | CAACTG | MYB binding site involved in drought-inducibility |

> 2018/04/13 10:10:12  
+ ATTTATTTAA TATTTTACAA TTATTTTTAA TATTATTTTT TAAAAATAAT AAATAAAATT ATTTATGAAT   
  
  
+ TAATTTTCAT AAAAATTTAT TGACTTAATT CGTGTATAAT TTTTTTGAGA AAAAATAAAT TAATGTATTT   
  
  
+ AAAATATAAT AAAATAATTT GTTAGTATAA TGAAACTAAC TCTTACATAA TAAAATAATC TGTGAATATG   
  
  
+ ATCCAATTAT TAAAATTCAT GAATAACACA TTTTTTAAAT AGATGATATT ACCAATTGAA TTGACCTTGA   
  
  
+ CCATAATTTT ATGGGCCGGA TTAGTCCAAG CGGCTCCATT CCCGAGTTGC TTTCAGGACC TGCCACAGCT   
  
  
+ GGACAGGACA GATTGCAGAC CCAAAACGAT GGGGAAGTGG GGTTGTAGTA GTTACCTTAA TTACTCTCAA   
  
  
+ TGAGTTTCTG AAATCCAAGG AAGTACAAGT TGAATACAAT TTTACCTTAT CTGATAAGTA TGAAGGGGTA   
  
  
+ AGATAAGGAT GAAGGTACCT TAACTTCTCC TTCGCTTTTA TGTAAGTTTT CTGCATTTTC ATCTGCTTTT   
  
  
+ TGGCCAATGG ATTGGCTGGC AGTAGCCTGT GGTCCTTAGA GCTTGAGAGT CAGAGCGGCA TTGATGAGCT   
  
  
+ TCCCTTCTAT TTCATTTCAC CTTGATTAAC ACTCAACCTA TAGCTTCACT TTCATTTGGC GTACCACGTT   
  
  
+ ATTGTAATTT CGGAATTCAT TTTTTGGCCA AGTTCATGAA GTTTCCCTTA CTGGGTGGAG AGAGAGAGAG   
  
  
+ AGAGATTGCT CTGTTCACAG GTAGAGGAGA AGAGCTGGGC AATGGACACG TGCATCTGAA AGGGAACAGC   
  
  
+ AGAAGATACC CGAGAGACCC TGTGATTTCA CACGAGCCAT GGTGGTTCCA TTACACTTCC ACACCAAAAA   
  
  
+ AGAGAGTTAT TGACCTCTGC GATGACATGG TTTGCCCCAC GAAATCCCAT AAAATCCAAG TGCATTACAT   
  
  
+ CTCTTTTCTG GGGTTTCCGA GTTCTCGAAT GTGTCTATGT TCACAATTTG GCTCAAGAAA AGTAATACCT   
  
  
+ AATTGGCAAC AGGAGATCCA GAGTGTGTAT GCAAGCATGC ACATATGAAG GGCCCCATTC TTGCTTACGT   
  
  
+ AGCCAATCAA GGAGAGCTTG TGGGGCCTCA TGTTTCTCCA CTGAAATATC TTCAATTTAT GTTCAACTGT   
  
  
+ GTGGGTCTCT GCTTATTTTA TTTGGGAGTG GGGGTGGGAG TGGCAATGGG CTGGGGGTGG TGCTGAATGG   
  
  
+ CCTTAAAGTC ATTGTTGTGT TCATAGTTTA TATTAAATGA GTGAAAAAGA TAGGGAGAAA AAGCAATCTT   
  
  
+ TGAGTGTTTT TGTCATCTAT TGTCTTAACT TCCCCCTCCG CCTTTTCTCT CTCTTAATCA ATCAAAGCTC   
  
  
+ TTGCGCTCTC TTCTTTATGG GAAAAGTCTT CCATTTCCAA AACATCCATC TCTGTCTGTT TGAAAGCTTT   
  
  
+ TCGGGGTAAT ACAGTAAACA GGTTGCAGT  

- TAAATAAATT ATAAAATGTT AATAAAAATT ATAATAAAAA ATTTTTATTA TTTATTTTAA TAAATACTTA   
  
  
- ATTAAAAGTA TTTTTAAATA ACTGAATTAA GCACATATTA AAAAAACTCT TTTTTATTTA ATTACATAAA   
  
  
- TTTTATATTA TTTTATTAAA CAATCATATT ACTTTGATTG AGAATGTATT ATTTTATTAG ACACTTATAC   
  
  
- TAGGTTAATA ATTTTAAGTA CTTATTGTGT AAAAAATTTA TCTACTATAA TGGTTAACTT AACTGGAACT   
  
  
- GGTATTAAAA TACCCGGCCT AATCAGGTTC GCCGAGGTAA GGGCTCAACG AAAGTCCTGG ACGGTGTCGA   
  
  
- CCTGTCCTGT CTAACGTCTG GGTTTTGCTA CCCCTTCACC CCAACATCAT CAATGGAATT AATGAGAGTT   
  
  
- ACTCAAAGAC TTTAGGTTCC TTCATGTTCA ACTTATGTTA AAATGGAATA GACTATTCAT ACTTCCCCAT   
  
  
- TCTATTCCTA CTTCCATGGA ATTGAAGAGG AAGCGAAAAT ACATTCAAAA GACGTAAAAG TAGACGAAAA   
  
  
- ACCGGTTACC TAACCGACCG TCATCGGACA CCAGGAATCT CGAACTCTCA GTCTCGCCGT AACTACTCGA   
  
  
- AGGGAAGATA AAGTAAAGTG GAACTAATTG TGAGTTGGAT ATCGAAGTGA AAGTAAACCG CATGGTGCAA   
  
  
- TAACATTAAA GCCTTAAGTA AAAAACCGGT TCAAGTACTT CAAAGGGAAT GACCCACCTC TCTCTCTCTC   
  
  
- TCTCTAACGA GACAAGTGTC CATCTCCTCT TCTCGACCCG TTACCTGTGC ACGTAGACTT TCCCTTGTCG   
  
  
- TCTTCTATGG GCTCTCTGGG ACACTAAAGT GTGCTCGGTA CCACCAAGGT AATGTGAAGG TGTGGTTTTT   
  
  
- TCTCTCAATA ACTGGAGACG CTACTGTACC AAACGGGGTG CTTTAGGGTA TTTTAGGTTC ACGTAATGTA   
  
  
- GAGAAAAGAC CCCAAAGGCT CAAGAGCTTA CACAGATACA AGTGTTAAAC CGAGTTCTTT TCATTATGGA   
  
  
- TTAACCGTTG TCCTCTAGGT CTCACACATA CGTTCGTACG TGTATACTTC CCGGGGTAAG AACGAATGCA   
  
  
- TCGGTTAGTT CCTCTCGAAC ACCCCGGAGT ACAAAGAGGT GACTTTATAG AAGTTAAATA CAAGTTGACA   
  
  
- CACCCAGAGA CGAATAAAAT AAACCCTCAC CCCCACCCTC ACCGTTACCC GACCCCCACC ACGACTTACC   
  
  
- GGAATTTCAG TAACAACACA AGTATCAAAT ATAATTTACT CACTTTTTCT ATCCCTCTTT TTCGTTAGAA   
  
  
- ACTCACAAAA ACAGTAGATA ACAGAATTGA AGGGGGAGGC GGAAAAGAGA GAGAATTAGT TAGTTTCGAG   
  
  
- AACGCGAGAG AAGAAATACC CTTTTCAGAA GGTAAAGGTT TTGTAGGTAG AGACAGACAA ACTTTCGAAA   
  
  
- AGCCCCATTA TGTCATTTGT CCAACGTCA

+     O2-site

| Site Name | Organism | Position | Strand | Matrix score. | sequence | function |
| --- | --- | --- | --- | --- | --- | --- |
| O2-site | Zea mays | 931 | + | 10 | GATGACATGG | cis-acting regulatory element involved in zein metabolism regulation |

> 2018/04/13 10:10:12  
+ ATTTATTTAA TATTTTACAA TTATTTTTAA TATTATTTTT TAAAAATAAT AAATAAAATT ATTTATGAAT   
  
  
+ TAATTTTCAT AAAAATTTAT TGACTTAATT CGTGTATAAT TTTTTTGAGA AAAAATAAAT TAATGTATTT   
  
  
+ AAAATATAAT AAAATAATTT GTTAGTATAA TGAAACTAAC TCTTACATAA TAAAATAATC TGTGAATATG   
  
  
+ ATCCAATTAT TAAAATTCAT GAATAACACA TTTTTTAAAT AGATGATATT ACCAATTGAA TTGACCTTGA   
  
  
+ CCATAATTTT ATGGGCCGGA TTAGTCCAAG CGGCTCCATT CCCGAGTTGC TTTCAGGACC TGCCACAGCT   
  
  
+ GGACAGGACA GATTGCAGAC CCAAAACGAT GGGGAAGTGG GGTTGTAGTA GTTACCTTAA TTACTCTCAA   
  
  
+ TGAGTTTCTG AAATCCAAGG AAGTACAAGT TGAATACAAT TTTACCTTAT CTGATAAGTA TGAAGGGGTA   
  
  
+ AGATAAGGAT GAAGGTACCT TAACTTCTCC TTCGCTTTTA TGTAAGTTTT CTGCATTTTC ATCTGCTTTT   
  
  
+ TGGCCAATGG ATTGGCTGGC AGTAGCCTGT GGTCCTTAGA GCTTGAGAGT CAGAGCGGCA TTGATGAGCT   
  
  
+ TCCCTTCTAT TTCATTTCAC CTTGATTAAC ACTCAACCTA TAGCTTCACT TTCATTTGGC GTACCACGTT   
  
  
+ ATTGTAATTT CGGAATTCAT TTTTTGGCCA AGTTCATGAA GTTTCCCTTA CTGGGTGGAG AGAGAGAGAG   
  
  
+ AGAGATTGCT CTGTTCACAG GTAGAGGAGA AGAGCTGGGC AATGGACACG TGCATCTGAA AGGGAACAGC   
  
  
+ AGAAGATACC CGAGAGACCC TGTGATTTCA CACGAGCCAT GGTGGTTCCA TTACACTTCC ACACCAAAAA   
  
  
+ AGAGAGTTAT TGACCTCTGC GATGACATGG TTTGCCCCAC GAAATCCCAT AAAATCCAAG TGCATTACAT   
  
  
+ CTCTTTTCTG GGGTTTCCGA GTTCTCGAAT GTGTCTATGT TCACAATTTG GCTCAAGAAA AGTAATACCT   
  
  
+ AATTGGCAAC AGGAGATCCA GAGTGTGTAT GCAAGCATGC ACATATGAAG GGCCCCATTC TTGCTTACGT   
  
  
+ AGCCAATCAA GGAGAGCTTG TGGGGCCTCA TGTTTCTCCA CTGAAATATC TTCAATTTAT GTTCAACTGT   
  
  
+ GTGGGTCTCT GCTTATTTTA TTTGGGAGTG GGGGTGGGAG TGGCAATGGG CTGGGGGTGG TGCTGAATGG   
  
  
+ CCTTAAAGTC ATTGTTGTGT TCATAGTTTA TATTAAATGA GTGAAAAAGA TAGGGAGAAA AAGCAATCTT   
  
  
+ TGAGTGTTTT TGTCATCTAT TGTCTTAACT TCCCCCTCCG CCTTTTCTCT CTCTTAATCA ATCAAAGCTC   
  
  
+ TTGCGCTCTC TTCTTTATGG GAAAAGTCTT CCATTTCCAA AACATCCATC TCTGTCTGTT TGAAAGCTTT   
  
  
+ TCGGGGTAAT ACAGTAAACA GGTTGCAGT  

- TAAATAAATT ATAAAATGTT AATAAAAATT ATAATAAAAA ATTTTTATTA TTTATTTTAA TAAATACTTA   
  
  
- ATTAAAAGTA TTTTTAAATA ACTGAATTAA GCACATATTA AAAAAACTCT TTTTTATTTA ATTACATAAA   
  
  
- TTTTATATTA TTTTATTAAA CAATCATATT ACTTTGATTG AGAATGTATT ATTTTATTAG ACACTTATAC   
  
  
- TAGGTTAATA ATTTTAAGTA CTTATTGTGT AAAAAATTTA TCTACTATAA TGGTTAACTT AACTGGAACT   
  
  
- GGTATTAAAA TACCCGGCCT AATCAGGTTC GCCGAGGTAA GGGCTCAACG AAAGTCCTGG ACGGTGTCGA   
  
  
- CCTGTCCTGT CTAACGTCTG GGTTTTGCTA CCCCTTCACC CCAACATCAT CAATGGAATT AATGAGAGTT   
  
  
- ACTCAAAGAC TTTAGGTTCC TTCATGTTCA ACTTATGTTA AAATGGAATA GACTATTCAT ACTTCCCCAT   
  
  
- TCTATTCCTA CTTCCATGGA ATTGAAGAGG AAGCGAAAAT ACATTCAAAA GACGTAAAAG TAGACGAAAA   
  
  
- ACCGGTTACC TAACCGACCG TCATCGGACA CCAGGAATCT CGAACTCTCA GTCTCGCCGT AACTACTCGA   
  
  
- AGGGAAGATA AAGTAAAGTG GAACTAATTG TGAGTTGGAT ATCGAAGTGA AAGTAAACCG CATGGTGCAA   
  
  
- TAACATTAAA GCCTTAAGTA AAAAACCGGT TCAAGTACTT CAAAGGGAAT GACCCACCTC TCTCTCTCTC   
  
  
- TCTCTAACGA GACAAGTGTC CATCTCCTCT TCTCGACCCG TTACCTGTGC ACGTAGACTT TCCCTTGTCG   
  
  
- TCTTCTATGG GCTCTCTGGG ACACTAAAGT GTGCTCGGTA CCACCAAGGT AATGTGAAGG TGTGGTTTTT   
  
  
- TCTCTCAATA ACTGGAGACG CTACTGTACC AAACGGGGTG CTTTAGGGTA TTTTAGGTTC ACGTAATGTA   
  
  
- GAGAAAAGAC CCCAAAGGCT CAAGAGCTTA CACAGATACA AGTGTTAAAC CGAGTTCTTT TCATTATGGA   
  
  
- TTAACCGTTG TCCTCTAGGT CTCACACATA CGTTCGTACG TGTATACTTC CCGGGGTAAG AACGAATGCA   
  
  
- TCGGTTAGTT CCTCTCGAAC ACCCCGGAGT ACAAAGAGGT GACTTTATAG AAGTTAAATA CAAGTTGACA   
  
  
- CACCCAGAGA CGAATAAAAT AAACCCTCAC CCCCACCCTC ACCGTTACCC GACCCCCACC ACGACTTACC   
  
  
- GGAATTTCAG TAACAACACA AGTATCAAAT ATAATTTACT CACTTTTTCT ATCCCTCTTT TTCGTTAGAA   
  
  
- ACTCACAAAA ACAGTAGATA ACAGAATTGA AGGGGGAGGC GGAAAAGAGA GAGAATTAGT TAGTTTCGAG   
  
  
- AACGCGAGAG AAGAAATACC CTTTTCAGAA GGTAAAGGTT TTGTAGGTAG AGACAGACAA ACTTTCGAAA   
  
  
- AGCCCCATTA TGTCATTTGT CCAACGTCA

+     Skn-1\_motif

| Site Name | Organism | Position | Strand | Matrix score. | sequence | function |
| --- | --- | --- | --- | --- | --- | --- |
| Skn-1\_motif | Oryza sativa | 1268 | + | 5 | GTCAT | cis-acting regulatory element required for endosperm expression |
| Skn-1\_motif | Oryza sativa | 932 | - | 5 | GTCAT | cis-acting regulatory element required for endosperm expression |
| Skn-1\_motif | Oryza sativa | 1342 | + | 5 | GTCAT | cis-acting regulatory element required for endosperm expression |

> 2018/04/13 10:10:12  
+ ATTTATTTAA TATTTTACAA TTATTTTTAA TATTATTTTT TAAAAATAAT AAATAAAATT ATTTATGAAT   
  
  
+ TAATTTTCAT AAAAATTTAT TGACTTAATT CGTGTATAAT TTTTTTGAGA AAAAATAAAT TAATGTATTT   
  
  
+ AAAATATAAT AAAATAATTT GTTAGTATAA TGAAACTAAC TCTTACATAA TAAAATAATC TGTGAATATG   
  
  
+ ATCCAATTAT TAAAATTCAT GAATAACACA TTTTTTAAAT AGATGATATT ACCAATTGAA TTGACCTTGA   
  
  
+ CCATAATTTT ATGGGCCGGA TTAGTCCAAG CGGCTCCATT CCCGAGTTGC TTTCAGGACC TGCCACAGCT   
  
  
+ GGACAGGACA GATTGCAGAC CCAAAACGAT GGGGAAGTGG GGTTGTAGTA GTTACCTTAA TTACTCTCAA   
  
  
+ TGAGTTTCTG AAATCCAAGG AAGTACAAGT TGAATACAAT TTTACCTTAT CTGATAAGTA TGAAGGGGTA   
  
  
+ AGATAAGGAT GAAGGTACCT TAACTTCTCC TTCGCTTTTA TGTAAGTTTT CTGCATTTTC ATCTGCTTTT   
  
  
+ TGGCCAATGG ATTGGCTGGC AGTAGCCTGT GGTCCTTAGA GCTTGAGAGT CAGAGCGGCA TTGATGAGCT   
  
  
+ TCCCTTCTAT TTCATTTCAC CTTGATTAAC ACTCAACCTA TAGCTTCACT TTCATTTGGC GTACCACGTT   
  
  
+ ATTGTAATTT CGGAATTCAT TTTTTGGCCA AGTTCATGAA GTTTCCCTTA CTGGGTGGAG AGAGAGAGAG   
  
  
+ AGAGATTGCT CTGTTCACAG GTAGAGGAGA AGAGCTGGGC AATGGACACG TGCATCTGAA AGGGAACAGC   
  
  
+ AGAAGATACC CGAGAGACCC TGTGATTTCA CACGAGCCAT GGTGGTTCCA TTACACTTCC ACACCAAAAA   
  
  
+ AGAGAGTTAT TGACCTCTGC GATGACATGG TTTGCCCCAC GAAATCCCAT AAAATCCAAG TGCATTACAT   
  
  
+ CTCTTTTCTG GGGTTTCCGA GTTCTCGAAT GTGTCTATGT TCACAATTTG GCTCAAGAAA AGTAATACCT   
  
  
+ AATTGGCAAC AGGAGATCCA GAGTGTGTAT GCAAGCATGC ACATATGAAG GGCCCCATTC TTGCTTACGT   
  
  
+ AGCCAATCAA GGAGAGCTTG TGGGGCCTCA TGTTTCTCCA CTGAAATATC TTCAATTTAT GTTCAACTGT   
  
  
+ GTGGGTCTCT GCTTATTTTA TTTGGGAGTG GGGGTGGGAG TGGCAATGGG CTGGGGGTGG TGCTGAATGG   
  
  
+ CCTTAAAGTC ATTGTTGTGT TCATAGTTTA TATTAAATGA GTGAAAAAGA TAGGGAGAAA AAGCAATCTT   
  
  
+ TGAGTGTTTT TGTCATCTAT TGTCTTAACT TCCCCCTCCG CCTTTTCTCT CTCTTAATCA ATCAAAGCTC   
  
  
+ TTGCGCTCTC TTCTTTATGG GAAAAGTCTT CCATTTCCAA AACATCCATC TCTGTCTGTT TGAAAGCTTT   
  
  
+ TCGGGGTAAT ACAGTAAACA GGTTGCAGT  

- TAAATAAATT ATAAAATGTT AATAAAAATT ATAATAAAAA ATTTTTATTA TTTATTTTAA TAAATACTTA   
  
  
- ATTAAAAGTA TTTTTAAATA ACTGAATTAA GCACATATTA AAAAAACTCT TTTTTATTTA ATTACATAAA   
  
  
- TTTTATATTA TTTTATTAAA CAATCATATT ACTTTGATTG AGAATGTATT ATTTTATTAG ACACTTATAC   
  
  
- TAGGTTAATA ATTTTAAGTA CTTATTGTGT AAAAAATTTA TCTACTATAA TGGTTAACTT AACTGGAACT   
  
  
- GGTATTAAAA TACCCGGCCT AATCAGGTTC GCCGAGGTAA GGGCTCAACG AAAGTCCTGG ACGGTGTCGA   
  
  
- CCTGTCCTGT CTAACGTCTG GGTTTTGCTA CCCCTTCACC CCAACATCAT CAATGGAATT AATGAGAGTT   
  
  
- ACTCAAAGAC TTTAGGTTCC TTCATGTTCA ACTTATGTTA AAATGGAATA GACTATTCAT ACTTCCCCAT   
  
  
- TCTATTCCTA CTTCCATGGA ATTGAAGAGG AAGCGAAAAT ACATTCAAAA GACGTAAAAG TAGACGAAAA   
  
  
- ACCGGTTACC TAACCGACCG TCATCGGACA CCAGGAATCT CGAACTCTCA GTCTCGCCGT AACTACTCGA   
  
  
- AGGGAAGATA AAGTAAAGTG GAACTAATTG TGAGTTGGAT ATCGAAGTGA AAGTAAACCG CATGGTGCAA   
  
  
- TAACATTAAA GCCTTAAGTA AAAAACCGGT TCAAGTACTT CAAAGGGAAT GACCCACCTC TCTCTCTCTC   
  
  
- TCTCTAACGA GACAAGTGTC CATCTCCTCT TCTCGACCCG TTACCTGTGC ACGTAGACTT TCCCTTGTCG   
  
  
- TCTTCTATGG GCTCTCTGGG ACACTAAAGT GTGCTCGGTA CCACCAAGGT AATGTGAAGG TGTGGTTTTT   
  
  
- TCTCTCAATA ACTGGAGACG CTACTGTACC AAACGGGGTG CTTTAGGGTA TTTTAGGTTC ACGTAATGTA   
  
  
- GAGAAAAGAC CCCAAAGGCT CAAGAGCTTA CACAGATACA AGTGTTAAAC CGAGTTCTTT TCATTATGGA   
  
  
- TTAACCGTTG TCCTCTAGGT CTCACACATA CGTTCGTACG TGTATACTTC CCGGGGTAAG AACGAATGCA   
  
  
- TCGGTTAGTT CCTCTCGAAC ACCCCGGAGT ACAAAGAGGT GACTTTATAG AAGTTAAATA CAAGTTGACA   
  
  
- CACCCAGAGA CGAATAAAAT AAACCCTCAC CCCCACCCTC ACCGTTACCC GACCCCCACC ACGACTTACC   
  
  
- GGAATTTCAG TAACAACACA AGTATCAAAT ATAATTTACT CACTTTTTCT ATCCCTCTTT TTCGTTAGAA   
  
  
- ACTCACAAAA ACAGTAGATA ACAGAATTGA AGGGGGAGGC GGAAAAGAGA GAGAATTAGT TAGTTTCGAG   
  
  
- AACGCGAGAG AAGAAATACC CTTTTCAGAA GGTAAAGGTT TTGTAGGTAG AGACAGACAA ACTTTCGAAA   
  
  
- AGCCCCATTA TGTCATTTGT CCAACGTCA

+     Sp1

| Site Name | Organism | Position | Strand | Matrix score. | sequence | function |
| --- | --- | --- | --- | --- | --- | --- |
| Sp1 | Zea mays | 753 | - | 5.5 | CC(G/A)CCC | light responsive element |
| Sp1 | Zea mays | 1245 | - | 5.5 | CC(G/A)CCC | light responsive element |
| Sp1 | Zea mays | 1222 | - | 5.5 | CC(G/A)CCC | light responsive element |

> 2018/04/13 10:10:12  
+ ATTTATTTAA TATTTTACAA TTATTTTTAA TATTATTTTT TAAAAATAAT AAATAAAATT ATTTATGAAT   
  
  
+ TAATTTTCAT AAAAATTTAT TGACTTAATT CGTGTATAAT TTTTTTGAGA AAAAATAAAT TAATGTATTT   
  
  
+ AAAATATAAT AAAATAATTT GTTAGTATAA TGAAACTAAC TCTTACATAA TAAAATAATC TGTGAATATG   
  
  
+ ATCCAATTAT TAAAATTCAT GAATAACACA TTTTTTAAAT AGATGATATT ACCAATTGAA TTGACCTTGA   
  
  
+ CCATAATTTT ATGGGCCGGA TTAGTCCAAG CGGCTCCATT CCCGAGTTGC TTTCAGGACC TGCCACAGCT   
  
  
+ GGACAGGACA GATTGCAGAC CCAAAACGAT GGGGAAGTGG GGTTGTAGTA GTTACCTTAA TTACTCTCAA   
  
  
+ TGAGTTTCTG AAATCCAAGG AAGTACAAGT TGAATACAAT TTTACCTTAT CTGATAAGTA TGAAGGGGTA   
  
  
+ AGATAAGGAT GAAGGTACCT TAACTTCTCC TTCGCTTTTA TGTAAGTTTT CTGCATTTTC ATCTGCTTTT   
  
  
+ TGGCCAATGG ATTGGCTGGC AGTAGCCTGT GGTCCTTAGA GCTTGAGAGT CAGAGCGGCA TTGATGAGCT   
  
  
+ TCCCTTCTAT TTCATTTCAC CTTGATTAAC ACTCAACCTA TAGCTTCACT TTCATTTGGC GTACCACGTT   
  
  
+ ATTGTAATTT CGGAATTCAT TTTTTGGCCA AGTTCATGAA GTTTCCCTTA CTGGGTGGAG AGAGAGAGAG   
  
  
+ AGAGATTGCT CTGTTCACAG GTAGAGGAGA AGAGCTGGGC AATGGACACG TGCATCTGAA AGGGAACAGC   
  
  
+ AGAAGATACC CGAGAGACCC TGTGATTTCA CACGAGCCAT GGTGGTTCCA TTACACTTCC ACACCAAAAA   
  
  
+ AGAGAGTTAT TGACCTCTGC GATGACATGG TTTGCCCCAC GAAATCCCAT AAAATCCAAG TGCATTACAT   
  
  
+ CTCTTTTCTG GGGTTTCCGA GTTCTCGAAT GTGTCTATGT TCACAATTTG GCTCAAGAAA AGTAATACCT   
  
  
+ AATTGGCAAC AGGAGATCCA GAGTGTGTAT GCAAGCATGC ACATATGAAG GGCCCCATTC TTGCTTACGT   
  
  
+ AGCCAATCAA GGAGAGCTTG TGGGGCCTCA TGTTTCTCCA CTGAAATATC TTCAATTTAT GTTCAACTGT   
  
  
+ GTGGGTCTCT GCTTATTTTA TTTGGGAGTG GGGGTGGGAG TGGCAATGGG CTGGGGGTGG TGCTGAATGG   
  
  
+ CCTTAAAGTC ATTGTTGTGT TCATAGTTTA TATTAAATGA GTGAAAAAGA TAGGGAGAAA AAGCAATCTT   
  
  
+ TGAGTGTTTT TGTCATCTAT TGTCTTAACT TCCCCCTCCG CCTTTTCTCT CTCTTAATCA ATCAAAGCTC   
  
  
+ TTGCGCTCTC TTCTTTATGG GAAAAGTCTT CCATTTCCAA AACATCCATC TCTGTCTGTT TGAAAGCTTT   
  
  
+ TCGGGGTAAT ACAGTAAACA GGTTGCAGT  

- TAAATAAATT ATAAAATGTT AATAAAAATT ATAATAAAAA ATTTTTATTA TTTATTTTAA TAAATACTTA   
  
  
- ATTAAAAGTA TTTTTAAATA ACTGAATTAA GCACATATTA AAAAAACTCT TTTTTATTTA ATTACATAAA   
  
  
- TTTTATATTA TTTTATTAAA CAATCATATT ACTTTGATTG AGAATGTATT ATTTTATTAG ACACTTATAC   
  
  
- TAGGTTAATA ATTTTAAGTA CTTATTGTGT AAAAAATTTA TCTACTATAA TGGTTAACTT AACTGGAACT   
  
  
- GGTATTAAAA TACCCGGCCT AATCAGGTTC GCCGAGGTAA GGGCTCAACG AAAGTCCTGG ACGGTGTCGA   
  
  
- CCTGTCCTGT CTAACGTCTG GGTTTTGCTA CCCCTTCACC CCAACATCAT CAATGGAATT AATGAGAGTT   
  
  
- ACTCAAAGAC TTTAGGTTCC TTCATGTTCA ACTTATGTTA AAATGGAATA GACTATTCAT ACTTCCCCAT   
  
  
- TCTATTCCTA CTTCCATGGA ATTGAAGAGG AAGCGAAAAT ACATTCAAAA GACGTAAAAG TAGACGAAAA   
  
  
- ACCGGTTACC TAACCGACCG TCATCGGACA CCAGGAATCT CGAACTCTCA GTCTCGCCGT AACTACTCGA   
  
  
- AGGGAAGATA AAGTAAAGTG GAACTAATTG TGAGTTGGAT ATCGAAGTGA AAGTAAACCG CATGGTGCAA   
  
  
- TAACATTAAA GCCTTAAGTA AAAAACCGGT TCAAGTACTT CAAAGGGAAT GACCCACCTC TCTCTCTCTC   
  
  
- TCTCTAACGA GACAAGTGTC CATCTCCTCT TCTCGACCCG TTACCTGTGC ACGTAGACTT TCCCTTGTCG   
  
  
- TCTTCTATGG GCTCTCTGGG ACACTAAAGT GTGCTCGGTA CCACCAAGGT AATGTGAAGG TGTGGTTTTT   
  
  
- TCTCTCAATA ACTGGAGACG CTACTGTACC AAACGGGGTG CTTTAGGGTA TTTTAGGTTC ACGTAATGTA   
  
  
- GAGAAAAGAC CCCAAAGGCT CAAGAGCTTA CACAGATACA AGTGTTAAAC CGAGTTCTTT TCATTATGGA   
  
  
- TTAACCGTTG TCCTCTAGGT CTCACACATA CGTTCGTACG TGTATACTTC CCGGGGTAAG AACGAATGCA   
  
  
- TCGGTTAGTT CCTCTCGAAC ACCCCGGAGT ACAAAGAGGT GACTTTATAG AAGTTAAATA CAAGTTGACA   
  
  
- CACCCAGAGA CGAATAAAAT AAACCCTCAC CCCCACCCTC ACCGTTACCC GACCCCCACC ACGACTTACC   
  
  
- GGAATTTCAG TAACAACACA AGTATCAAAT ATAATTTACT CACTTTTTCT ATCCCTCTTT TTCGTTAGAA   
  
  
- ACTCACAAAA ACAGTAGATA ACAGAATTGA AGGGGGAGGC GGAAAAGAGA GAGAATTAGT TAGTTTCGAG   
  
  
- AACGCGAGAG AAGAAATACC CTTTTCAGAA GGTAAAGGTT TTGTAGGTAG AGACAGACAA ACTTTCGAAA   
  
  
- AGCCCCATTA TGTCATTTGT CCAACGTCA

+     TATA-box

| Site Name | Organism | Position | Strand | Matrix score. | sequence | function |
| --- | --- | --- | --- | --- | --- | --- |
| TATA-box | Arabidopsis thaliana | 1288 | - | 5 | TATAA | core promoter element around -30 of transcription start |
| TATA-box | Arabidopsis thaliana | 1287 | - | 6 | TATAAA | core promoter element around -30 of transcription start |
| TATA-box | Lycopersicon esculentum | 1206 | + | 5 | TTTTA | core promoter element around -30 of transcription start |
| TATA-box | Lycopersicon esculentum | 460 | + | 5 | TTTTA | core promoter element around -30 of transcription start |
| TATA-box | Oryza sativa | 527 | - | 8 | TACATAAA | core promoter element around -30 of transcription start |
| TATA-box | Glycine max | 257 | - | 5 | TAATA | core promoter element around -30 of transcription start |
| TATA-box | Arabidopsis thaliana | 166 | + | 4 | TATA | core promoter element around -30 of transcription start |
| TATA-box | Glycine max | 147 | + | 5 | TAATA | core promoter element around -30 of transcription start |
| TATA-box | Arabidopsis thaliana | 1289 | - | 4 | TATA | core promoter element around -30 of transcription start |
| TATA-box | Brassica oleracea | 144 | + | 7 | ATATAAT | core promoter element around -30 of transcription start |
| TATA-box | Arabidopsis thaliana | 145 | + | 4 | TATA | core promoter element around -30 of transcription start |
| TATA-box | Lycopersicon esculentum | 54 | - | 5 | TTTTA | core promoter element around -30 of transcription start |
| TATA-box | Lycopersicon esculentum | 140 | - | 5 | TTTTA | core promoter element around -30 of transcription start |
| TATA-box | Arabidopsis thaliana | 136 | + | 8 | TATTTAAA | core promoter element around -30 of transcription start |
| TATA-box | Zea mays | 39 | + | 8 | TTTAAAAA | core promoter element around -30 of transcription start |
| TATA-box | Lycopersicon esculentum | 150 | - | 5 | TTTTA | core promoter element around -30 of transcription start |
| TATA-box | Lycopersicon esculentum | 287 | + | 5 | TTTTA | core promoter element around -30 of transcription start |
| TATA-box | Glycine max | 31 | - | 5 | TAATA | core promoter element around -30 of transcription start |
| TATA-box | Lycopersicon esculentum | 960 | - | 5 | TTTTA | core promoter element around -30 of transcription start |
| TATA-box | Arabidopsis thaliana | 244 | - | 8 | TATTTAAA | core promoter element around -30 of transcription start |
| TATA-box | Arabidopsis thaliana | 669 | + | 4 | TATA | core promoter element around -30 of transcription start |
| TATA-box | Lycopersicon esculentum | 80 | - | 5 | TTTTA | core promoter element around -30 of transcription start |
| TATA-box | Lycopersicon esculentum | 38 | + | 5 | TTTTA | core promoter element around -30 of transcription start |
| TATA-box | Zea mays | 37 | - | 8 | TTTAAAAA | core promoter element around -30 of transcription start |
| TATA-box | Lycopersicon esculentum | 526 | + | 5 | TTTTA | core promoter element around -30 of transcription start |
| TATA-box | Lycopersicon esculentum | 25 | + | 5 | TTTTA | core promoter element around -30 of transcription start |
| TATA-box | Glycine max | 218 | - | 5 | TAATA | core promoter element around -30 of transcription start |
| TATA-box | Glycine max | 1477 | + | 5 | TAATA | core promoter element around -30 of transcription start |
| TATA-box | Zea mays | 242 | - | 8 | TTTAAAAA | core promoter element around -30 of transcription start |
| TATA-box | Helianthus annuus | 103 | - | 6 | TATACA | core promoter element around -30 of transcription start |
| TATA-box | Arabidopsis thaliana | 105 | + | 4 | TATA | core promoter element around -30 of transcription start |
| TATA-box | Glycine max | 1043 | + | 5 | TAATA | core promoter element around -30 of transcription start |
| TATA-box | Glycine max | 1291 | - | 5 | TAATA | core promoter element around -30 of transcription start |
| TATA-box | Lycopersicon esculentum | 191 | - | 5 | TTTTA | core promoter element around -30 of transcription start |
| TATA-box | Glycine max | 47 | + | 5 | TAATA | core promoter element around -30 of transcription start |
| TATA-box | Glycine max | 28 | + | 5 | TAATA | core promoter element around -30 of transcription start |
| TATA-box | Glycine max | 8 | + | 5 | TAATA | core promoter element around -30 of transcription start |
| TATA-box | Lycopersicon esculentum | 13 | + | 5 | TTTTA | core promoter element around -30 of transcription start |
| TATA-box | Glycine max | 188 | + | 5 | TAATA | core promoter element around -30 of transcription start |
| TATA-box | Arabidopsis thaliana | 41 | + | 9 | TAAAAATAA | core promoter element around -30 of transcription start |
| TATA-box | Lycopersicon esculentum | 243 | + | 5 | TTTTA | core promoter element around -30 of transcription start |
| TATA-box | Lycopersicon esculentum | 221 | - | 5 | TTTTA | core promoter element around -30 of transcription start |
| TATA-box | Arabidopsis thaliana | 21 | - | 9 | TAAAAATAA | core promoter element around -30 of transcription start |

> 2018/04/13 10:10:12  
+ ATTTATTTAA TATTTTACAA TTATTTTTAA TATTATTTTT TAAAAATAAT AAATAAAATT ATTTATGAAT   
  
  
+ TAATTTTCAT AAAAATTTAT TGACTTAATT CGTGTATAAT TTTTTTGAGA AAAAATAAAT TAATGTATTT   
  
  
+ AAAATATAAT AAAATAATTT GTTAGTATAA TGAAACTAAC TCTTACATAA TAAAATAATC TGTGAATATG   
  
  
+ ATCCAATTAT TAAAATTCAT GAATAACACA TTTTTTAAAT AGATGATATT ACCAATTGAA TTGACCTTGA   
  
  
+ CCATAATTTT ATGGGCCGGA TTAGTCCAAG CGGCTCCATT CCCGAGTTGC TTTCAGGACC TGCCACAGCT   
  
  
+ GGACAGGACA GATTGCAGAC CCAAAACGAT GGGGAAGTGG GGTTGTAGTA GTTACCTTAA TTACTCTCAA   
  
  
+ TGAGTTTCTG AAATCCAAGG AAGTACAAGT TGAATACAAT TTTACCTTAT CTGATAAGTA TGAAGGGGTA   
  
  
+ AGATAAGGAT GAAGGTACCT TAACTTCTCC TTCGCTTTTA TGTAAGTTTT CTGCATTTTC ATCTGCTTTT   
  
  
+ TGGCCAATGG ATTGGCTGGC AGTAGCCTGT GGTCCTTAGA GCTTGAGAGT CAGAGCGGCA TTGATGAGCT   
  
  
+ TCCCTTCTAT TTCATTTCAC CTTGATTAAC ACTCAACCTA TAGCTTCACT TTCATTTGGC GTACCACGTT   
  
  
+ ATTGTAATTT CGGAATTCAT TTTTTGGCCA AGTTCATGAA GTTTCCCTTA CTGGGTGGAG AGAGAGAGAG   
  
  
+ AGAGATTGCT CTGTTCACAG GTAGAGGAGA AGAGCTGGGC AATGGACACG TGCATCTGAA AGGGAACAGC   
  
  
+ AGAAGATACC CGAGAGACCC TGTGATTTCA CACGAGCCAT GGTGGTTCCA TTACACTTCC ACACCAAAAA   
  
  
+ AGAGAGTTAT TGACCTCTGC GATGACATGG TTTGCCCCAC GAAATCCCAT AAAATCCAAG TGCATTACAT   
  
  
+ CTCTTTTCTG GGGTTTCCGA GTTCTCGAAT GTGTCTATGT TCACAATTTG GCTCAAGAAA AGTAATACCT   
  
  
+ AATTGGCAAC AGGAGATCCA GAGTGTGTAT GCAAGCATGC ACATATGAAG GGCCCCATTC TTGCTTACGT   
  
  
+ AGCCAATCAA GGAGAGCTTG TGGGGCCTCA TGTTTCTCCA CTGAAATATC TTCAATTTAT GTTCAACTGT   
  
  
+ GTGGGTCTCT GCTTATTTTA TTTGGGAGTG GGGGTGGGAG TGGCAATGGG CTGGGGGTGG TGCTGAATGG   
  
  
+ CCTTAAAGTC ATTGTTGTGT TCATAGTTTA TATTAAATGA GTGAAAAAGA TAGGGAGAAA AAGCAATCTT   
  
  
+ TGAGTGTTTT TGTCATCTAT TGTCTTAACT TCCCCCTCCG CCTTTTCTCT CTCTTAATCA ATCAAAGCTC   
  
  
+ TTGCGCTCTC TTCTTTATGG GAAAAGTCTT CCATTTCCAA AACATCCATC TCTGTCTGTT TGAAAGCTTT   
  
  
+ TCGGGGTAAT ACAGTAAACA GGTTGCAGT  

- TAAATAAATT ATAAAATGTT AATAAAAATT ATAATAAAAA ATTTTTATTA TTTATTTTAA TAAATACTTA   
  
  
- ATTAAAAGTA TTTTTAAATA ACTGAATTAA GCACATATTA AAAAAACTCT TTTTTATTTA ATTACATAAA   
  
  
- TTTTATATTA TTTTATTAAA CAATCATATT ACTTTGATTG AGAATGTATT ATTTTATTAG ACACTTATAC   
  
  
- TAGGTTAATA ATTTTAAGTA CTTATTGTGT AAAAAATTTA TCTACTATAA TGGTTAACTT AACTGGAACT   
  
  
- GGTATTAAAA TACCCGGCCT AATCAGGTTC GCCGAGGTAA GGGCTCAACG AAAGTCCTGG ACGGTGTCGA   
  
  
- CCTGTCCTGT CTAACGTCTG GGTTTTGCTA CCCCTTCACC CCAACATCAT CAATGGAATT AATGAGAGTT   
  
  
- ACTCAAAGAC TTTAGGTTCC TTCATGTTCA ACTTATGTTA AAATGGAATA GACTATTCAT ACTTCCCCAT   
  
  
- TCTATTCCTA CTTCCATGGA ATTGAAGAGG AAGCGAAAAT ACATTCAAAA GACGTAAAAG TAGACGAAAA   
  
  
- ACCGGTTACC TAACCGACCG TCATCGGACA CCAGGAATCT CGAACTCTCA GTCTCGCCGT AACTACTCGA   
  
  
- AGGGAAGATA AAGTAAAGTG GAACTAATTG TGAGTTGGAT ATCGAAGTGA AAGTAAACCG CATGGTGCAA   
  
  
- TAACATTAAA GCCTTAAGTA AAAAACCGGT TCAAGTACTT CAAAGGGAAT GACCCACCTC TCTCTCTCTC   
  
  
- TCTCTAACGA GACAAGTGTC CATCTCCTCT TCTCGACCCG TTACCTGTGC ACGTAGACTT TCCCTTGTCG   
  
  
- TCTTCTATGG GCTCTCTGGG ACACTAAAGT GTGCTCGGTA CCACCAAGGT AATGTGAAGG TGTGGTTTTT   
  
  
- TCTCTCAATA ACTGGAGACG CTACTGTACC AAACGGGGTG CTTTAGGGTA TTTTAGGTTC ACGTAATGTA   
  
  
- GAGAAAAGAC CCCAAAGGCT CAAGAGCTTA CACAGATACA AGTGTTAAAC CGAGTTCTTT TCATTATGGA   
  
  
- TTAACCGTTG TCCTCTAGGT CTCACACATA CGTTCGTACG TGTATACTTC CCGGGGTAAG AACGAATGCA   
  
  
- TCGGTTAGTT CCTCTCGAAC ACCCCGGAGT ACAAAGAGGT GACTTTATAG AAGTTAAATA CAAGTTGACA   
  
  
- CACCCAGAGA CGAATAAAAT AAACCCTCAC CCCCACCCTC ACCGTTACCC GACCCCCACC ACGACTTACC   
  
  
- GGAATTTCAG TAACAACACA AGTATCAAAT ATAATTTACT CACTTTTTCT ATCCCTCTTT TTCGTTAGAA   
  
  
- ACTCACAAAA ACAGTAGATA ACAGAATTGA AGGGGGAGGC GGAAAAGAGA GAGAATTAGT TAGTTTCGAG   
  
  
- AACGCGAGAG AAGAAATACC CTTTTCAGAA GGTAAAGGTT TTGTAGGTAG AGACAGACAA ACTTTCGAAA   
  
  
- AGCCCCATTA TGTCATTTGT CCAACGTCA

+     TCA-element

| Site Name | Organism | Position | Strand | Matrix score. | sequence | function |
| --- | --- | --- | --- | --- | --- | --- |
| TCA-element | Nicotiana tabacum | 373 | - | 9 | CCATCTTTTT | cis-acting element involved in salicylic acid responsiveness |
| TCA-element | Nicotiana tabacum | 1304 | - | 9 | CCATCTTTTT | cis-acting element involved in salicylic acid responsiveness |

> 2018/04/13 10:10:12  
+ ATTTATTTAA TATTTTACAA TTATTTTTAA TATTATTTTT TAAAAATAAT AAATAAAATT ATTTATGAAT   
  
  
+ TAATTTTCAT AAAAATTTAT TGACTTAATT CGTGTATAAT TTTTTTGAGA AAAAATAAAT TAATGTATTT   
  
  
+ AAAATATAAT AAAATAATTT GTTAGTATAA TGAAACTAAC TCTTACATAA TAAAATAATC TGTGAATATG   
  
  
+ ATCCAATTAT TAAAATTCAT GAATAACACA TTTTTTAAAT AGATGATATT ACCAATTGAA TTGACCTTGA   
  
  
+ CCATAATTTT ATGGGCCGGA TTAGTCCAAG CGGCTCCATT CCCGAGTTGC TTTCAGGACC TGCCACAGCT   
  
  
+ GGACAGGACA GATTGCAGAC CCAAAACGAT GGGGAAGTGG GGTTGTAGTA GTTACCTTAA TTACTCTCAA   
  
  
+ TGAGTTTCTG AAATCCAAGG AAGTACAAGT TGAATACAAT TTTACCTTAT CTGATAAGTA TGAAGGGGTA   
  
  
+ AGATAAGGAT GAAGGTACCT TAACTTCTCC TTCGCTTTTA TGTAAGTTTT CTGCATTTTC ATCTGCTTTT   
  
  
+ TGGCCAATGG ATTGGCTGGC AGTAGCCTGT GGTCCTTAGA GCTTGAGAGT CAGAGCGGCA TTGATGAGCT   
  
  
+ TCCCTTCTAT TTCATTTCAC CTTGATTAAC ACTCAACCTA TAGCTTCACT TTCATTTGGC GTACCACGTT   
  
  
+ ATTGTAATTT CGGAATTCAT TTTTTGGCCA AGTTCATGAA GTTTCCCTTA CTGGGTGGAG AGAGAGAGAG   
  
  
+ AGAGATTGCT CTGTTCACAG GTAGAGGAGA AGAGCTGGGC AATGGACACG TGCATCTGAA AGGGAACAGC   
  
  
+ AGAAGATACC CGAGAGACCC TGTGATTTCA CACGAGCCAT GGTGGTTCCA TTACACTTCC ACACCAAAAA   
  
  
+ AGAGAGTTAT TGACCTCTGC GATGACATGG TTTGCCCCAC GAAATCCCAT AAAATCCAAG TGCATTACAT   
  
  
+ CTCTTTTCTG GGGTTTCCGA GTTCTCGAAT GTGTCTATGT TCACAATTTG GCTCAAGAAA AGTAATACCT   
  
  
+ AATTGGCAAC AGGAGATCCA GAGTGTGTAT GCAAGCATGC ACATATGAAG GGCCCCATTC TTGCTTACGT   
  
  
+ AGCCAATCAA GGAGAGCTTG TGGGGCCTCA TGTTTCTCCA CTGAAATATC TTCAATTTAT GTTCAACTGT   
  
  
+ GTGGGTCTCT GCTTATTTTA TTTGGGAGTG GGGGTGGGAG TGGCAATGGG CTGGGGGTGG TGCTGAATGG   
  
  
+ CCTTAAAGTC ATTGTTGTGT TCATAGTTTA TATTAAATGA GTGAAAAAGA TAGGGAGAAA AAGCAATCTT   
  
  
+ TGAGTGTTTT TGTCATCTAT TGTCTTAACT TCCCCCTCCG CCTTTTCTCT CTCTTAATCA ATCAAAGCTC   
  
  
+ TTGCGCTCTC TTCTTTATGG GAAAAGTCTT CCATTTCCAA AACATCCATC TCTGTCTGTT TGAAAGCTTT   
  
  
+ TCGGGGTAAT ACAGTAAACA GGTTGCAGT  

- TAAATAAATT ATAAAATGTT AATAAAAATT ATAATAAAAA ATTTTTATTA TTTATTTTAA TAAATACTTA   
  
  
- ATTAAAAGTA TTTTTAAATA ACTGAATTAA GCACATATTA AAAAAACTCT TTTTTATTTA ATTACATAAA   
  
  
- TTTTATATTA TTTTATTAAA CAATCATATT ACTTTGATTG AGAATGTATT ATTTTATTAG ACACTTATAC   
  
  
- TAGGTTAATA ATTTTAAGTA CTTATTGTGT AAAAAATTTA TCTACTATAA TGGTTAACTT AACTGGAACT   
  
  
- GGTATTAAAA TACCCGGCCT AATCAGGTTC GCCGAGGTAA GGGCTCAACG AAAGTCCTGG ACGGTGTCGA   
  
  
- CCTGTCCTGT CTAACGTCTG GGTTTTGCTA CCCCTTCACC CCAACATCAT CAATGGAATT AATGAGAGTT   
  
  
- ACTCAAAGAC TTTAGGTTCC TTCATGTTCA ACTTATGTTA AAATGGAATA GACTATTCAT ACTTCCCCAT   
  
  
- TCTATTCCTA CTTCCATGGA ATTGAAGAGG AAGCGAAAAT ACATTCAAAA GACGTAAAAG TAGACGAAAA   
  
  
- ACCGGTTACC TAACCGACCG TCATCGGACA CCAGGAATCT CGAACTCTCA GTCTCGCCGT AACTACTCGA   
  
  
- AGGGAAGATA AAGTAAAGTG GAACTAATTG TGAGTTGGAT ATCGAAGTGA AAGTAAACCG CATGGTGCAA   
  
  
- TAACATTAAA GCCTTAAGTA AAAAACCGGT TCAAGTACTT CAAAGGGAAT GACCCACCTC TCTCTCTCTC   
  
  
- TCTCTAACGA GACAAGTGTC CATCTCCTCT TCTCGACCCG TTACCTGTGC ACGTAGACTT TCCCTTGTCG   
  
  
- TCTTCTATGG GCTCTCTGGG ACACTAAAGT GTGCTCGGTA CCACCAAGGT AATGTGAAGG TGTGGTTTTT   
  
  
- TCTCTCAATA ACTGGAGACG CTACTGTACC AAACGGGGTG CTTTAGGGTA TTTTAGGTTC ACGTAATGTA   
  
  
- GAGAAAAGAC CCCAAAGGCT CAAGAGCTTA CACAGATACA AGTGTTAAAC CGAGTTCTTT TCATTATGGA   
  
  
- TTAACCGTTG TCCTCTAGGT CTCACACATA CGTTCGTACG TGTATACTTC CCGGGGTAAG AACGAATGCA   
  
  
- TCGGTTAGTT CCTCTCGAAC ACCCCGGAGT ACAAAGAGGT GACTTTATAG AAGTTAAATA CAAGTTGACA   
  
  
- CACCCAGAGA CGAATAAAAT AAACCCTCAC CCCCACCCTC ACCGTTACCC GACCCCCACC ACGACTTACC   
  
  
- GGAATTTCAG TAACAACACA AGTATCAAAT ATAATTTACT CACTTTTTCT ATCCCTCTTT TTCGTTAGAA   
  
  
- ACTCACAAAA ACAGTAGATA ACAGAATTGA AGGGGGAGGC GGAAAAGAGA GAGAATTAGT TAGTTTCGAG   
  
  
- AACGCGAGAG AAGAAATACC CTTTTCAGAA GGTAAAGGTT TTGTAGGTAG AGACAGACAA ACTTTCGAAA   
  
  
- AGCCCCATTA TGTCATTTGT CCAACGTCA

+     TCCC-motif

| Site Name | Organism | Position | Strand | Matrix score. | sequence | function |
| --- | --- | --- | --- | --- | --- | --- |
| TCCC-motif | Spinacia oleracea | 1312 | - | 7 | TCTCCCT | part of a light responsive element |

> 2018/04/13 10:10:12  
+ ATTTATTTAA TATTTTACAA TTATTTTTAA TATTATTTTT TAAAAATAAT AAATAAAATT ATTTATGAAT   
  
  
+ TAATTTTCAT AAAAATTTAT TGACTTAATT CGTGTATAAT TTTTTTGAGA AAAAATAAAT TAATGTATTT   
  
  
+ AAAATATAAT AAAATAATTT GTTAGTATAA TGAAACTAAC TCTTACATAA TAAAATAATC TGTGAATATG   
  
  
+ ATCCAATTAT TAAAATTCAT GAATAACACA TTTTTTAAAT AGATGATATT ACCAATTGAA TTGACCTTGA   
  
  
+ CCATAATTTT ATGGGCCGGA TTAGTCCAAG CGGCTCCATT CCCGAGTTGC TTTCAGGACC TGCCACAGCT   
  
  
+ GGACAGGACA GATTGCAGAC CCAAAACGAT GGGGAAGTGG GGTTGTAGTA GTTACCTTAA TTACTCTCAA   
  
  
+ TGAGTTTCTG AAATCCAAGG AAGTACAAGT TGAATACAAT TTTACCTTAT CTGATAAGTA TGAAGGGGTA   
  
  
+ AGATAAGGAT GAAGGTACCT TAACTTCTCC TTCGCTTTTA TGTAAGTTTT CTGCATTTTC ATCTGCTTTT   
  
  
+ TGGCCAATGG ATTGGCTGGC AGTAGCCTGT GGTCCTTAGA GCTTGAGAGT CAGAGCGGCA TTGATGAGCT   
  
  
+ TCCCTTCTAT TTCATTTCAC CTTGATTAAC ACTCAACCTA TAGCTTCACT TTCATTTGGC GTACCACGTT   
  
  
+ ATTGTAATTT CGGAATTCAT TTTTTGGCCA AGTTCATGAA GTTTCCCTTA CTGGGTGGAG AGAGAGAGAG   
  
  
+ AGAGATTGCT CTGTTCACAG GTAGAGGAGA AGAGCTGGGC AATGGACACG TGCATCTGAA AGGGAACAGC   
  
  
+ AGAAGATACC CGAGAGACCC TGTGATTTCA CACGAGCCAT GGTGGTTCCA TTACACTTCC ACACCAAAAA   
  
  
+ AGAGAGTTAT TGACCTCTGC GATGACATGG TTTGCCCCAC GAAATCCCAT AAAATCCAAG TGCATTACAT   
  
  
+ CTCTTTTCTG GGGTTTCCGA GTTCTCGAAT GTGTCTATGT TCACAATTTG GCTCAAGAAA AGTAATACCT   
  
  
+ AATTGGCAAC AGGAGATCCA GAGTGTGTAT GCAAGCATGC ACATATGAAG GGCCCCATTC TTGCTTACGT   
  
  
+ AGCCAATCAA GGAGAGCTTG TGGGGCCTCA TGTTTCTCCA CTGAAATATC TTCAATTTAT GTTCAACTGT   
  
  
+ GTGGGTCTCT GCTTATTTTA TTTGGGAGTG GGGGTGGGAG TGGCAATGGG CTGGGGGTGG TGCTGAATGG   
  
  
+ CCTTAAAGTC ATTGTTGTGT TCATAGTTTA TATTAAATGA GTGAAAAAGA TAGGGAGAAA AAGCAATCTT   
  
  
+ TGAGTGTTTT TGTCATCTAT TGTCTTAACT TCCCCCTCCG CCTTTTCTCT CTCTTAATCA ATCAAAGCTC   
  
  
+ TTGCGCTCTC TTCTTTATGG GAAAAGTCTT CCATTTCCAA AACATCCATC TCTGTCTGTT TGAAAGCTTT   
  
  
+ TCGGGGTAAT ACAGTAAACA GGTTGCAGT  

- TAAATAAATT ATAAAATGTT AATAAAAATT ATAATAAAAA ATTTTTATTA TTTATTTTAA TAAATACTTA   
  
  
- ATTAAAAGTA TTTTTAAATA ACTGAATTAA GCACATATTA AAAAAACTCT TTTTTATTTA ATTACATAAA   
  
  
- TTTTATATTA TTTTATTAAA CAATCATATT ACTTTGATTG AGAATGTATT ATTTTATTAG ACACTTATAC   
  
  
- TAGGTTAATA ATTTTAAGTA CTTATTGTGT AAAAAATTTA TCTACTATAA TGGTTAACTT AACTGGAACT   
  
  
- GGTATTAAAA TACCCGGCCT AATCAGGTTC GCCGAGGTAA GGGCTCAACG AAAGTCCTGG ACGGTGTCGA   
  
  
- CCTGTCCTGT CTAACGTCTG GGTTTTGCTA CCCCTTCACC CCAACATCAT CAATGGAATT AATGAGAGTT   
  
  
- ACTCAAAGAC TTTAGGTTCC TTCATGTTCA ACTTATGTTA AAATGGAATA GACTATTCAT ACTTCCCCAT   
  
  
- TCTATTCCTA CTTCCATGGA ATTGAAGAGG AAGCGAAAAT ACATTCAAAA GACGTAAAAG TAGACGAAAA   
  
  
- ACCGGTTACC TAACCGACCG TCATCGGACA CCAGGAATCT CGAACTCTCA GTCTCGCCGT AACTACTCGA   
  
  
- AGGGAAGATA AAGTAAAGTG GAACTAATTG TGAGTTGGAT ATCGAAGTGA AAGTAAACCG CATGGTGCAA   
  
  
- TAACATTAAA GCCTTAAGTA AAAAACCGGT TCAAGTACTT CAAAGGGAAT GACCCACCTC TCTCTCTCTC   
  
  
- TCTCTAACGA GACAAGTGTC CATCTCCTCT TCTCGACCCG TTACCTGTGC ACGTAGACTT TCCCTTGTCG   
  
  
- TCTTCTATGG GCTCTCTGGG ACACTAAAGT GTGCTCGGTA CCACCAAGGT AATGTGAAGG TGTGGTTTTT   
  
  
- TCTCTCAATA ACTGGAGACG CTACTGTACC AAACGGGGTG CTTTAGGGTA TTTTAGGTTC ACGTAATGTA   
  
  
- GAGAAAAGAC CCCAAAGGCT CAAGAGCTTA CACAGATACA AGTGTTAAAC CGAGTTCTTT TCATTATGGA   
  
  
- TTAACCGTTG TCCTCTAGGT CTCACACATA CGTTCGTACG TGTATACTTC CCGGGGTAAG AACGAATGCA   
  
  
- TCGGTTAGTT CCTCTCGAAC ACCCCGGAGT ACAAAGAGGT GACTTTATAG AAGTTAAATA CAAGTTGACA   
  
  
- CACCCAGAGA CGAATAAAAT AAACCCTCAC CCCCACCCTC ACCGTTACCC GACCCCCACC ACGACTTACC   
  
  
- GGAATTTCAG TAACAACACA AGTATCAAAT ATAATTTACT CACTTTTTCT ATCCCTCTTT TTCGTTAGAA   
  
  
- ACTCACAAAA ACAGTAGATA ACAGAATTGA AGGGGGAGGC GGAAAAGAGA GAGAATTAGT TAGTTTCGAG   
  
  
- AACGCGAGAG AAGAAATACC CTTTTCAGAA GGTAAAGGTT TTGTAGGTAG AGACAGACAA ACTTTCGAAA   
  
  
- AGCCCCATTA TGTCATTTGT CCAACGTCA

+     TCT-motif

| Site Name | Organism | Position | Strand | Matrix score. | sequence | function |
| --- | --- | --- | --- | --- | --- | --- |
| TCT-motif | Arabidopsis thaliana | 488 | - | 6 | TCTTAC | part of a light responsive element |
| TCT-motif | Arabidopsis thaliana | 181 | + | 6 | TCTTAC | part of a light responsive element |

> 2018/04/13 10:10:12  
+ ATTTATTTAA TATTTTACAA TTATTTTTAA TATTATTTTT TAAAAATAAT AAATAAAATT ATTTATGAAT   
  
  
+ TAATTTTCAT AAAAATTTAT TGACTTAATT CGTGTATAAT TTTTTTGAGA AAAAATAAAT TAATGTATTT   
  
  
+ AAAATATAAT AAAATAATTT GTTAGTATAA TGAAACTAAC TCTTACATAA TAAAATAATC TGTGAATATG   
  
  
+ ATCCAATTAT TAAAATTCAT GAATAACACA TTTTTTAAAT AGATGATATT ACCAATTGAA TTGACCTTGA   
  
  
+ CCATAATTTT ATGGGCCGGA TTAGTCCAAG CGGCTCCATT CCCGAGTTGC TTTCAGGACC TGCCACAGCT   
  
  
+ GGACAGGACA GATTGCAGAC CCAAAACGAT GGGGAAGTGG GGTTGTAGTA GTTACCTTAA TTACTCTCAA   
  
  
+ TGAGTTTCTG AAATCCAAGG AAGTACAAGT TGAATACAAT TTTACCTTAT CTGATAAGTA TGAAGGGGTA   
  
  
+ AGATAAGGAT GAAGGTACCT TAACTTCTCC TTCGCTTTTA TGTAAGTTTT CTGCATTTTC ATCTGCTTTT   
  
  
+ TGGCCAATGG ATTGGCTGGC AGTAGCCTGT GGTCCTTAGA GCTTGAGAGT CAGAGCGGCA TTGATGAGCT   
  
  
+ TCCCTTCTAT TTCATTTCAC CTTGATTAAC ACTCAACCTA TAGCTTCACT TTCATTTGGC GTACCACGTT   
  
  
+ ATTGTAATTT CGGAATTCAT TTTTTGGCCA AGTTCATGAA GTTTCCCTTA CTGGGTGGAG AGAGAGAGAG   
  
  
+ AGAGATTGCT CTGTTCACAG GTAGAGGAGA AGAGCTGGGC AATGGACACG TGCATCTGAA AGGGAACAGC   
  
  
+ AGAAGATACC CGAGAGACCC TGTGATTTCA CACGAGCCAT GGTGGTTCCA TTACACTTCC ACACCAAAAA   
  
  
+ AGAGAGTTAT TGACCTCTGC GATGACATGG TTTGCCCCAC GAAATCCCAT AAAATCCAAG TGCATTACAT   
  
  
+ CTCTTTTCTG GGGTTTCCGA GTTCTCGAAT GTGTCTATGT TCACAATTTG GCTCAAGAAA AGTAATACCT   
  
  
+ AATTGGCAAC AGGAGATCCA GAGTGTGTAT GCAAGCATGC ACATATGAAG GGCCCCATTC TTGCTTACGT   
  
  
+ AGCCAATCAA GGAGAGCTTG TGGGGCCTCA TGTTTCTCCA CTGAAATATC TTCAATTTAT GTTCAACTGT   
  
  
+ GTGGGTCTCT GCTTATTTTA TTTGGGAGTG GGGGTGGGAG TGGCAATGGG CTGGGGGTGG TGCTGAATGG   
  
  
+ CCTTAAAGTC ATTGTTGTGT TCATAGTTTA TATTAAATGA GTGAAAAAGA TAGGGAGAAA AAGCAATCTT   
  
  
+ TGAGTGTTTT TGTCATCTAT TGTCTTAACT TCCCCCTCCG CCTTTTCTCT CTCTTAATCA ATCAAAGCTC   
  
  
+ TTGCGCTCTC TTCTTTATGG GAAAAGTCTT CCATTTCCAA AACATCCATC TCTGTCTGTT TGAAAGCTTT   
  
  
+ TCGGGGTAAT ACAGTAAACA GGTTGCAGT  

- TAAATAAATT ATAAAATGTT AATAAAAATT ATAATAAAAA ATTTTTATTA TTTATTTTAA TAAATACTTA   
  
  
- ATTAAAAGTA TTTTTAAATA ACTGAATTAA GCACATATTA AAAAAACTCT TTTTTATTTA ATTACATAAA   
  
  
- TTTTATATTA TTTTATTAAA CAATCATATT ACTTTGATTG AGAATGTATT ATTTTATTAG ACACTTATAC   
  
  
- TAGGTTAATA ATTTTAAGTA CTTATTGTGT AAAAAATTTA TCTACTATAA TGGTTAACTT AACTGGAACT   
  
  
- GGTATTAAAA TACCCGGCCT AATCAGGTTC GCCGAGGTAA GGGCTCAACG AAAGTCCTGG ACGGTGTCGA   
  
  
- CCTGTCCTGT CTAACGTCTG GGTTTTGCTA CCCCTTCACC CCAACATCAT CAATGGAATT AATGAGAGTT   
  
  
- ACTCAAAGAC TTTAGGTTCC TTCATGTTCA ACTTATGTTA AAATGGAATA GACTATTCAT ACTTCCCCAT   
  
  
- TCTATTCCTA CTTCCATGGA ATTGAAGAGG AAGCGAAAAT ACATTCAAAA GACGTAAAAG TAGACGAAAA   
  
  
- ACCGGTTACC TAACCGACCG TCATCGGACA CCAGGAATCT CGAACTCTCA GTCTCGCCGT AACTACTCGA   
  
  
- AGGGAAGATA AAGTAAAGTG GAACTAATTG TGAGTTGGAT ATCGAAGTGA AAGTAAACCG CATGGTGCAA   
  
  
- TAACATTAAA GCCTTAAGTA AAAAACCGGT TCAAGTACTT CAAAGGGAAT GACCCACCTC TCTCTCTCTC   
  
  
- TCTCTAACGA GACAAGTGTC CATCTCCTCT TCTCGACCCG TTACCTGTGC ACGTAGACTT TCCCTTGTCG   
  
  
- TCTTCTATGG GCTCTCTGGG ACACTAAAGT GTGCTCGGTA CCACCAAGGT AATGTGAAGG TGTGGTTTTT   
  
  
- TCTCTCAATA ACTGGAGACG CTACTGTACC AAACGGGGTG CTTTAGGGTA TTTTAGGTTC ACGTAATGTA   
  
  
- GAGAAAAGAC CCCAAAGGCT CAAGAGCTTA CACAGATACA AGTGTTAAAC CGAGTTCTTT TCATTATGGA   
  
  
- TTAACCGTTG TCCTCTAGGT CTCACACATA CGTTCGTACG TGTATACTTC CCGGGGTAAG AACGAATGCA   
  
  
- TCGGTTAGTT CCTCTCGAAC ACCCCGGAGT ACAAAGAGGT GACTTTATAG AAGTTAAATA CAAGTTGACA   
  
  
- CACCCAGAGA CGAATAAAAT AAACCCTCAC CCCCACCCTC ACCGTTACCC GACCCCCACC ACGACTTACC   
  
  
- GGAATTTCAG TAACAACACA AGTATCAAAT ATAATTTACT CACTTTTTCT ATCCCTCTTT TTCGTTAGAA   
  
  
- ACTCACAAAA ACAGTAGATA ACAGAATTGA AGGGGGAGGC GGAAAAGAGA GAGAATTAGT TAGTTTCGAG   
  
  
- AACGCGAGAG AAGAAATACC CTTTTCAGAA GGTAAAGGTT TTGTAGGTAG AGACAGACAA ACTTTCGAAA   
  
  
- AGCCCCATTA TGTCATTTGT CCAACGTCA

+     Unnamed\_\_1

| Site Name | Organism | Position | Strand | Matrix score. | sequence | function |
| --- | --- | --- | --- | --- | --- | --- |
| Unnamed\_\_1 | Zea mays | 694 | - | 5 | CGTGG |  |
| Unnamed\_\_1 | Zea mays | 947 | - | 5 | CGTGG |  |

> 2018/04/13 10:10:12  
+ ATTTATTTAA TATTTTACAA TTATTTTTAA TATTATTTTT TAAAAATAAT AAATAAAATT ATTTATGAAT   
  
  
+ TAATTTTCAT AAAAATTTAT TGACTTAATT CGTGTATAAT TTTTTTGAGA AAAAATAAAT TAATGTATTT   
  
  
+ AAAATATAAT AAAATAATTT GTTAGTATAA TGAAACTAAC TCTTACATAA TAAAATAATC TGTGAATATG   
  
  
+ ATCCAATTAT TAAAATTCAT GAATAACACA TTTTTTAAAT AGATGATATT ACCAATTGAA TTGACCTTGA   
  
  
+ CCATAATTTT ATGGGCCGGA TTAGTCCAAG CGGCTCCATT CCCGAGTTGC TTTCAGGACC TGCCACAGCT   
  
  
+ GGACAGGACA GATTGCAGAC CCAAAACGAT GGGGAAGTGG GGTTGTAGTA GTTACCTTAA TTACTCTCAA   
  
  
+ TGAGTTTCTG AAATCCAAGG AAGTACAAGT TGAATACAAT TTTACCTTAT CTGATAAGTA TGAAGGGGTA   
  
  
+ AGATAAGGAT GAAGGTACCT TAACTTCTCC TTCGCTTTTA TGTAAGTTTT CTGCATTTTC ATCTGCTTTT   
  
  
+ TGGCCAATGG ATTGGCTGGC AGTAGCCTGT GGTCCTTAGA GCTTGAGAGT CAGAGCGGCA TTGATGAGCT   
  
  
+ TCCCTTCTAT TTCATTTCAC CTTGATTAAC ACTCAACCTA TAGCTTCACT TTCATTTGGC GTACCACGTT   
  
  
+ ATTGTAATTT CGGAATTCAT TTTTTGGCCA AGTTCATGAA GTTTCCCTTA CTGGGTGGAG AGAGAGAGAG   
  
  
+ AGAGATTGCT CTGTTCACAG GTAGAGGAGA AGAGCTGGGC AATGGACACG TGCATCTGAA AGGGAACAGC   
  
  
+ AGAAGATACC CGAGAGACCC TGTGATTTCA CACGAGCCAT GGTGGTTCCA TTACACTTCC ACACCAAAAA   
  
  
+ AGAGAGTTAT TGACCTCTGC GATGACATGG TTTGCCCCAC GAAATCCCAT AAAATCCAAG TGCATTACAT   
  
  
+ CTCTTTTCTG GGGTTTCCGA GTTCTCGAAT GTGTCTATGT TCACAATTTG GCTCAAGAAA AGTAATACCT   
  
  
+ AATTGGCAAC AGGAGATCCA GAGTGTGTAT GCAAGCATGC ACATATGAAG GGCCCCATTC TTGCTTACGT   
  
  
+ AGCCAATCAA GGAGAGCTTG TGGGGCCTCA TGTTTCTCCA CTGAAATATC TTCAATTTAT GTTCAACTGT   
  
  
+ GTGGGTCTCT GCTTATTTTA TTTGGGAGTG GGGGTGGGAG TGGCAATGGG CTGGGGGTGG TGCTGAATGG   
  
  
+ CCTTAAAGTC ATTGTTGTGT TCATAGTTTA TATTAAATGA GTGAAAAAGA TAGGGAGAAA AAGCAATCTT   
  
  
+ TGAGTGTTTT TGTCATCTAT TGTCTTAACT TCCCCCTCCG CCTTTTCTCT CTCTTAATCA ATCAAAGCTC   
  
  
+ TTGCGCTCTC TTCTTTATGG GAAAAGTCTT CCATTTCCAA AACATCCATC TCTGTCTGTT TGAAAGCTTT   
  
  
+ TCGGGGTAAT ACAGTAAACA GGTTGCAGT  

- TAAATAAATT ATAAAATGTT AATAAAAATT ATAATAAAAA ATTTTTATTA TTTATTTTAA TAAATACTTA   
  
  
- ATTAAAAGTA TTTTTAAATA ACTGAATTAA GCACATATTA AAAAAACTCT TTTTTATTTA ATTACATAAA   
  
  
- TTTTATATTA TTTTATTAAA CAATCATATT ACTTTGATTG AGAATGTATT ATTTTATTAG ACACTTATAC   
  
  
- TAGGTTAATA ATTTTAAGTA CTTATTGTGT AAAAAATTTA TCTACTATAA TGGTTAACTT AACTGGAACT   
  
  
- GGTATTAAAA TACCCGGCCT AATCAGGTTC GCCGAGGTAA GGGCTCAACG AAAGTCCTGG ACGGTGTCGA   
  
  
- CCTGTCCTGT CTAACGTCTG GGTTTTGCTA CCCCTTCACC CCAACATCAT CAATGGAATT AATGAGAGTT   
  
  
- ACTCAAAGAC TTTAGGTTCC TTCATGTTCA ACTTATGTTA AAATGGAATA GACTATTCAT ACTTCCCCAT   
  
  
- TCTATTCCTA CTTCCATGGA ATTGAAGAGG AAGCGAAAAT ACATTCAAAA GACGTAAAAG TAGACGAAAA   
  
  
- ACCGGTTACC TAACCGACCG TCATCGGACA CCAGGAATCT CGAACTCTCA GTCTCGCCGT AACTACTCGA   
  
  
- AGGGAAGATA AAGTAAAGTG GAACTAATTG TGAGTTGGAT ATCGAAGTGA AAGTAAACCG CATGGTGCAA   
  
  
- TAACATTAAA GCCTTAAGTA AAAAACCGGT TCAAGTACTT CAAAGGGAAT GACCCACCTC TCTCTCTCTC   
  
  
- TCTCTAACGA GACAAGTGTC CATCTCCTCT TCTCGACCCG TTACCTGTGC ACGTAGACTT TCCCTTGTCG   
  
  
- TCTTCTATGG GCTCTCTGGG ACACTAAAGT GTGCTCGGTA CCACCAAGGT AATGTGAAGG TGTGGTTTTT   
  
  
- TCTCTCAATA ACTGGAGACG CTACTGTACC AAACGGGGTG CTTTAGGGTA TTTTAGGTTC ACGTAATGTA   
  
  
- GAGAAAAGAC CCCAAAGGCT CAAGAGCTTA CACAGATACA AGTGTTAAAC CGAGTTCTTT TCATTATGGA   
  
  
- TTAACCGTTG TCCTCTAGGT CTCACACATA CGTTCGTACG TGTATACTTC CCGGGGTAAG AACGAATGCA   
  
  
- TCGGTTAGTT CCTCTCGAAC ACCCCGGAGT ACAAAGAGGT GACTTTATAG AAGTTAAATA CAAGTTGACA   
  
  
- CACCCAGAGA CGAATAAAAT AAACCCTCAC CCCCACCCTC ACCGTTACCC GACCCCCACC ACGACTTACC   
  
  
- GGAATTTCAG TAACAACACA AGTATCAAAT ATAATTTACT CACTTTTTCT ATCCCTCTTT TTCGTTAGAA   
  
  
- ACTCACAAAA ACAGTAGATA ACAGAATTGA AGGGGGAGGC GGAAAAGAGA GAGAATTAGT TAGTTTCGAG   
  
  
- AACGCGAGAG AAGAAATACC CTTTTCAGAA GGTAAAGGTT TTGTAGGTAG AGACAGACAA ACTTTCGAAA   
  
  
- AGCCCCATTA TGTCATTTGT CCAACGTCA

+     Unnamed\_\_3

| Site Name | Organism | Position | Strand | Matrix score. | sequence | function |
| --- | --- | --- | --- | --- | --- | --- |
| Unnamed\_\_3 | Zea mays | 694 | - | 5 | CGTGG |  |
| Unnamed\_\_3 | Zea mays | 947 | - | 5 | CGTGG |  |

> 2018/04/13 10:10:12  
+ ATTTATTTAA TATTTTACAA TTATTTTTAA TATTATTTTT TAAAAATAAT AAATAAAATT ATTTATGAAT   
  
  
+ TAATTTTCAT AAAAATTTAT TGACTTAATT CGTGTATAAT TTTTTTGAGA AAAAATAAAT TAATGTATTT   
  
  
+ AAAATATAAT AAAATAATTT GTTAGTATAA TGAAACTAAC TCTTACATAA TAAAATAATC TGTGAATATG   
  
  
+ ATCCAATTAT TAAAATTCAT GAATAACACA TTTTTTAAAT AGATGATATT ACCAATTGAA TTGACCTTGA   
  
  
+ CCATAATTTT ATGGGCCGGA TTAGTCCAAG CGGCTCCATT CCCGAGTTGC TTTCAGGACC TGCCACAGCT   
  
  
+ GGACAGGACA GATTGCAGAC CCAAAACGAT GGGGAAGTGG GGTTGTAGTA GTTACCTTAA TTACTCTCAA   
  
  
+ TGAGTTTCTG AAATCCAAGG AAGTACAAGT TGAATACAAT TTTACCTTAT CTGATAAGTA TGAAGGGGTA   
  
  
+ AGATAAGGAT GAAGGTACCT TAACTTCTCC TTCGCTTTTA TGTAAGTTTT CTGCATTTTC ATCTGCTTTT   
  
  
+ TGGCCAATGG ATTGGCTGGC AGTAGCCTGT GGTCCTTAGA GCTTGAGAGT CAGAGCGGCA TTGATGAGCT   
  
  
+ TCCCTTCTAT TTCATTTCAC CTTGATTAAC ACTCAACCTA TAGCTTCACT TTCATTTGGC GTACCACGTT   
  
  
+ ATTGTAATTT CGGAATTCAT TTTTTGGCCA AGTTCATGAA GTTTCCCTTA CTGGGTGGAG AGAGAGAGAG   
  
  
+ AGAGATTGCT CTGTTCACAG GTAGAGGAGA AGAGCTGGGC AATGGACACG TGCATCTGAA AGGGAACAGC   
  
  
+ AGAAGATACC CGAGAGACCC TGTGATTTCA CACGAGCCAT GGTGGTTCCA TTACACTTCC ACACCAAAAA   
  
  
+ AGAGAGTTAT TGACCTCTGC GATGACATGG TTTGCCCCAC GAAATCCCAT AAAATCCAAG TGCATTACAT   
  
  
+ CTCTTTTCTG GGGTTTCCGA GTTCTCGAAT GTGTCTATGT TCACAATTTG GCTCAAGAAA AGTAATACCT   
  
  
+ AATTGGCAAC AGGAGATCCA GAGTGTGTAT GCAAGCATGC ACATATGAAG GGCCCCATTC TTGCTTACGT   
  
  
+ AGCCAATCAA GGAGAGCTTG TGGGGCCTCA TGTTTCTCCA CTGAAATATC TTCAATTTAT GTTCAACTGT   
  
  
+ GTGGGTCTCT GCTTATTTTA TTTGGGAGTG GGGGTGGGAG TGGCAATGGG CTGGGGGTGG TGCTGAATGG   
  
  
+ CCTTAAAGTC ATTGTTGTGT TCATAGTTTA TATTAAATGA GTGAAAAAGA TAGGGAGAAA AAGCAATCTT   
  
  
+ TGAGTGTTTT TGTCATCTAT TGTCTTAACT TCCCCCTCCG CCTTTTCTCT CTCTTAATCA ATCAAAGCTC   
  
  
+ TTGCGCTCTC TTCTTTATGG GAAAAGTCTT CCATTTCCAA AACATCCATC TCTGTCTGTT TGAAAGCTTT   
  
  
+ TCGGGGTAAT ACAGTAAACA GGTTGCAGT  

- TAAATAAATT ATAAAATGTT AATAAAAATT ATAATAAAAA ATTTTTATTA TTTATTTTAA TAAATACTTA   
  
  
- ATTAAAAGTA TTTTTAAATA ACTGAATTAA GCACATATTA AAAAAACTCT TTTTTATTTA ATTACATAAA   
  
  
- TTTTATATTA TTTTATTAAA CAATCATATT ACTTTGATTG AGAATGTATT ATTTTATTAG ACACTTATAC   
  
  
- TAGGTTAATA ATTTTAAGTA CTTATTGTGT AAAAAATTTA TCTACTATAA TGGTTAACTT AACTGGAACT   
  
  
- GGTATTAAAA TACCCGGCCT AATCAGGTTC GCCGAGGTAA GGGCTCAACG AAAGTCCTGG ACGGTGTCGA   
  
  
- CCTGTCCTGT CTAACGTCTG GGTTTTGCTA CCCCTTCACC CCAACATCAT CAATGGAATT AATGAGAGTT   
  
  
- ACTCAAAGAC TTTAGGTTCC TTCATGTTCA ACTTATGTTA AAATGGAATA GACTATTCAT ACTTCCCCAT   
  
  
- TCTATTCCTA CTTCCATGGA ATTGAAGAGG AAGCGAAAAT ACATTCAAAA GACGTAAAAG TAGACGAAAA   
  
  
- ACCGGTTACC TAACCGACCG TCATCGGACA CCAGGAATCT CGAACTCTCA GTCTCGCCGT AACTACTCGA   
  
  
- AGGGAAGATA AAGTAAAGTG GAACTAATTG TGAGTTGGAT ATCGAAGTGA AAGTAAACCG CATGGTGCAA   
  
  
- TAACATTAAA GCCTTAAGTA AAAAACCGGT TCAAGTACTT CAAAGGGAAT GACCCACCTC TCTCTCTCTC   
  
  
- TCTCTAACGA GACAAGTGTC CATCTCCTCT TCTCGACCCG TTACCTGTGC ACGTAGACTT TCCCTTGTCG   
  
  
- TCTTCTATGG GCTCTCTGGG ACACTAAAGT GTGCTCGGTA CCACCAAGGT AATGTGAAGG TGTGGTTTTT   
  
  
- TCTCTCAATA ACTGGAGACG CTACTGTACC AAACGGGGTG CTTTAGGGTA TTTTAGGTTC ACGTAATGTA   
  
  
- GAGAAAAGAC CCCAAAGGCT CAAGAGCTTA CACAGATACA AGTGTTAAAC CGAGTTCTTT TCATTATGGA   
  
  
- TTAACCGTTG TCCTCTAGGT CTCACACATA CGTTCGTACG TGTATACTTC CCGGGGTAAG AACGAATGCA   
  
  
- TCGGTTAGTT CCTCTCGAAC ACCCCGGAGT ACAAAGAGGT GACTTTATAG AAGTTAAATA CAAGTTGACA   
  
  
- CACCCAGAGA CGAATAAAAT AAACCCTCAC CCCCACCCTC ACCGTTACCC GACCCCCACC ACGACTTACC   
  
  
- GGAATTTCAG TAACAACACA AGTATCAAAT ATAATTTACT CACTTTTTCT ATCCCTCTTT TTCGTTAGAA   
  
  
- ACTCACAAAA ACAGTAGATA ACAGAATTGA AGGGGGAGGC GGAAAAGAGA GAGAATTAGT TAGTTTCGAG   
  
  
- AACGCGAGAG AAGAAATACC CTTTTCAGAA GGTAAAGGTT TTGTAGGTAG AGACAGACAA ACTTTCGAAA   
  
  
- AGCCCCATTA TGTCATTTGT CCAACGTCA

+     Unnamed\_\_4

| Site Name | Organism | Position | Strand | Matrix score. | sequence | function |
| --- | --- | --- | --- | --- | --- | --- |
| Unnamed\_\_4 | Petroselinum hortense | 1366 | + | 4 | CTCC |  |
| Unnamed\_\_4 | Petroselinum hortense | 1227 | - | 4 | CTCC |  |
| Unnamed\_\_4 | Petroselinum hortense | 1156 | + | 4 | CTCC |  |
| Unnamed\_\_4 | Petroselinum hortense | 1215 | - | 4 | CTCC |  |
| Unnamed\_\_4 | Petroselinum hortense | 1314 | - | 4 | CTCC |  |
| Unnamed\_\_4 | Petroselinum hortense | 757 | - | 4 | CTCC |  |
| Unnamed\_\_4 | Petroselinum hortense | 796 | - | 4 | CTCC |  |
| Unnamed\_\_4 | Petroselinum hortense | 517 | + | 4 | CTCC |  |
| Unnamed\_\_4 | Petroselinum hortense | 1131 | - | 4 | CTCC |  |
| Unnamed\_\_4 | Petroselinum hortense | 1062 | - | 4 | CTCC |  |
| Unnamed\_\_4 | Petroselinum hortense | 314 | + | 4 | CTCC |  |

> 2018/04/13 10:10:12  
+ ATTTATTTAA TATTTTACAA TTATTTTTAA TATTATTTTT TAAAAATAAT AAATAAAATT ATTTATGAAT   
  
  
+ TAATTTTCAT AAAAATTTAT TGACTTAATT CGTGTATAAT TTTTTTGAGA AAAAATAAAT TAATGTATTT   
  
  
+ AAAATATAAT AAAATAATTT GTTAGTATAA TGAAACTAAC TCTTACATAA TAAAATAATC TGTGAATATG   
  
  
+ ATCCAATTAT TAAAATTCAT GAATAACACA TTTTTTAAAT AGATGATATT ACCAATTGAA TTGACCTTGA   
  
  
+ CCATAATTTT ATGGGCCGGA TTAGTCCAAG CGGCTCCATT CCCGAGTTGC TTTCAGGACC TGCCACAGCT   
  
  
+ GGACAGGACA GATTGCAGAC CCAAAACGAT GGGGAAGTGG GGTTGTAGTA GTTACCTTAA TTACTCTCAA   
  
  
+ TGAGTTTCTG AAATCCAAGG AAGTACAAGT TGAATACAAT TTTACCTTAT CTGATAAGTA TGAAGGGGTA   
  
  
+ AGATAAGGAT GAAGGTACCT TAACTTCTCC TTCGCTTTTA TGTAAGTTTT CTGCATTTTC ATCTGCTTTT   
  
  
+ TGGCCAATGG ATTGGCTGGC AGTAGCCTGT GGTCCTTAGA GCTTGAGAGT CAGAGCGGCA TTGATGAGCT   
  
  
+ TCCCTTCTAT TTCATTTCAC CTTGATTAAC ACTCAACCTA TAGCTTCACT TTCATTTGGC GTACCACGTT   
  
  
+ ATTGTAATTT CGGAATTCAT TTTTTGGCCA AGTTCATGAA GTTTCCCTTA CTGGGTGGAG AGAGAGAGAG   
  
  
+ AGAGATTGCT CTGTTCACAG GTAGAGGAGA AGAGCTGGGC AATGGACACG TGCATCTGAA AGGGAACAGC   
  
  
+ AGAAGATACC CGAGAGACCC TGTGATTTCA CACGAGCCAT GGTGGTTCCA TTACACTTCC ACACCAAAAA   
  
  
+ AGAGAGTTAT TGACCTCTGC GATGACATGG TTTGCCCCAC GAAATCCCAT AAAATCCAAG TGCATTACAT   
  
  
+ CTCTTTTCTG GGGTTTCCGA GTTCTCGAAT GTGTCTATGT TCACAATTTG GCTCAAGAAA AGTAATACCT   
  
  
+ AATTGGCAAC AGGAGATCCA GAGTGTGTAT GCAAGCATGC ACATATGAAG GGCCCCATTC TTGCTTACGT   
  
  
+ AGCCAATCAA GGAGAGCTTG TGGGGCCTCA TGTTTCTCCA CTGAAATATC TTCAATTTAT GTTCAACTGT   
  
  
+ GTGGGTCTCT GCTTATTTTA TTTGGGAGTG GGGGTGGGAG TGGCAATGGG CTGGGGGTGG TGCTGAATGG   
  
  
+ CCTTAAAGTC ATTGTTGTGT TCATAGTTTA TATTAAATGA GTGAAAAAGA TAGGGAGAAA AAGCAATCTT   
  
  
+ TGAGTGTTTT TGTCATCTAT TGTCTTAACT TCCCCCTCCG CCTTTTCTCT CTCTTAATCA ATCAAAGCTC   
  
  
+ TTGCGCTCTC TTCTTTATGG GAAAAGTCTT CCATTTCCAA AACATCCATC TCTGTCTGTT TGAAAGCTTT   
  
  
+ TCGGGGTAAT ACAGTAAACA GGTTGCAGT  

- TAAATAAATT ATAAAATGTT AATAAAAATT ATAATAAAAA ATTTTTATTA TTTATTTTAA TAAATACTTA   
  
  
- ATTAAAAGTA TTTTTAAATA ACTGAATTAA GCACATATTA AAAAAACTCT TTTTTATTTA ATTACATAAA   
  
  
- TTTTATATTA TTTTATTAAA CAATCATATT ACTTTGATTG AGAATGTATT ATTTTATTAG ACACTTATAC   
  
  
- TAGGTTAATA ATTTTAAGTA CTTATTGTGT AAAAAATTTA TCTACTATAA TGGTTAACTT AACTGGAACT   
  
  
- GGTATTAAAA TACCCGGCCT AATCAGGTTC GCCGAGGTAA GGGCTCAACG AAAGTCCTGG ACGGTGTCGA   
  
  
- CCTGTCCTGT CTAACGTCTG GGTTTTGCTA CCCCTTCACC CCAACATCAT CAATGGAATT AATGAGAGTT   
  
  
- ACTCAAAGAC TTTAGGTTCC TTCATGTTCA ACTTATGTTA AAATGGAATA GACTATTCAT ACTTCCCCAT   
  
  
- TCTATTCCTA CTTCCATGGA ATTGAAGAGG AAGCGAAAAT ACATTCAAAA GACGTAAAAG TAGACGAAAA   
  
  
- ACCGGTTACC TAACCGACCG TCATCGGACA CCAGGAATCT CGAACTCTCA GTCTCGCCGT AACTACTCGA   
  
  
- AGGGAAGATA AAGTAAAGTG GAACTAATTG TGAGTTGGAT ATCGAAGTGA AAGTAAACCG CATGGTGCAA   
  
  
- TAACATTAAA GCCTTAAGTA AAAAACCGGT TCAAGTACTT CAAAGGGAAT GACCCACCTC TCTCTCTCTC   
  
  
- TCTCTAACGA GACAAGTGTC CATCTCCTCT TCTCGACCCG TTACCTGTGC ACGTAGACTT TCCCTTGTCG   
  
  
- TCTTCTATGG GCTCTCTGGG ACACTAAAGT GTGCTCGGTA CCACCAAGGT AATGTGAAGG TGTGGTTTTT   
  
  
- TCTCTCAATA ACTGGAGACG CTACTGTACC AAACGGGGTG CTTTAGGGTA TTTTAGGTTC ACGTAATGTA   
  
  
- GAGAAAAGAC CCCAAAGGCT CAAGAGCTTA CACAGATACA AGTGTTAAAC CGAGTTCTTT TCATTATGGA   
  
  
- TTAACCGTTG TCCTCTAGGT CTCACACATA CGTTCGTACG TGTATACTTC CCGGGGTAAG AACGAATGCA   
  
  
- TCGGTTAGTT CCTCTCGAAC ACCCCGGAGT ACAAAGAGGT GACTTTATAG AAGTTAAATA CAAGTTGACA   
  
  
- CACCCAGAGA CGAATAAAAT AAACCCTCAC CCCCACCCTC ACCGTTACCC GACCCCCACC ACGACTTACC   
  
  
- GGAATTTCAG TAACAACACA AGTATCAAAT ATAATTTACT CACTTTTTCT ATCCCTCTTT TTCGTTAGAA   
  
  
- ACTCACAAAA ACAGTAGATA ACAGAATTGA AGGGGGAGGC GGAAAAGAGA GAGAATTAGT TAGTTTCGAG   
  
  
- AACGCGAGAG AAGAAATACC CTTTTCAGAA GGTAAAGGTT TTGTAGGTAG AGACAGACAA ACTTTCGAAA   
  
  
- AGCCCCATTA TGTCATTTGT CCAACGTCA

+     W box

| Site Name | Organism | Position | Strand | Matrix score. | sequence | function |
| --- | --- | --- | --- | --- | --- | --- |
| W box | Arabidopsis thaliana | 920 | + | 6 | TTGACC |  |
| W box | Arabidopsis thaliana | 277 | + | 6 | TTGACC |  |
| W box | Arabidopsis thaliana | 271 | + | 6 | TTGACC |  |

> 2018/04/13 10:10:12  
+ ATTTATTTAA TATTTTACAA TTATTTTTAA TATTATTTTT TAAAAATAAT AAATAAAATT ATTTATGAAT   
  
  
+ TAATTTTCAT AAAAATTTAT TGACTTAATT CGTGTATAAT TTTTTTGAGA AAAAATAAAT TAATGTATTT   
  
  
+ AAAATATAAT AAAATAATTT GTTAGTATAA TGAAACTAAC TCTTACATAA TAAAATAATC TGTGAATATG   
  
  
+ ATCCAATTAT TAAAATTCAT GAATAACACA TTTTTTAAAT AGATGATATT ACCAATTGAA TTGACCTTGA   
  
  
+ CCATAATTTT ATGGGCCGGA TTAGTCCAAG CGGCTCCATT CCCGAGTTGC TTTCAGGACC TGCCACAGCT   
  
  
+ GGACAGGACA GATTGCAGAC CCAAAACGAT GGGGAAGTGG GGTTGTAGTA GTTACCTTAA TTACTCTCAA   
  
  
+ TGAGTTTCTG AAATCCAAGG AAGTACAAGT TGAATACAAT TTTACCTTAT CTGATAAGTA TGAAGGGGTA   
  
  
+ AGATAAGGAT GAAGGTACCT TAACTTCTCC TTCGCTTTTA TGTAAGTTTT CTGCATTTTC ATCTGCTTTT   
  
  
+ TGGCCAATGG ATTGGCTGGC AGTAGCCTGT GGTCCTTAGA GCTTGAGAGT CAGAGCGGCA TTGATGAGCT   
  
  
+ TCCCTTCTAT TTCATTTCAC CTTGATTAAC ACTCAACCTA TAGCTTCACT TTCATTTGGC GTACCACGTT   
  
  
+ ATTGTAATTT CGGAATTCAT TTTTTGGCCA AGTTCATGAA GTTTCCCTTA CTGGGTGGAG AGAGAGAGAG   
  
  
+ AGAGATTGCT CTGTTCACAG GTAGAGGAGA AGAGCTGGGC AATGGACACG TGCATCTGAA AGGGAACAGC   
  
  
+ AGAAGATACC CGAGAGACCC TGTGATTTCA CACGAGCCAT GGTGGTTCCA TTACACTTCC ACACCAAAAA   
  
  
+ AGAGAGTTAT TGACCTCTGC GATGACATGG TTTGCCCCAC GAAATCCCAT AAAATCCAAG TGCATTACAT   
  
  
+ CTCTTTTCTG GGGTTTCCGA GTTCTCGAAT GTGTCTATGT TCACAATTTG GCTCAAGAAA AGTAATACCT   
  
  
+ AATTGGCAAC AGGAGATCCA GAGTGTGTAT GCAAGCATGC ACATATGAAG GGCCCCATTC TTGCTTACGT   
  
  
+ AGCCAATCAA GGAGAGCTTG TGGGGCCTCA TGTTTCTCCA CTGAAATATC TTCAATTTAT GTTCAACTGT   
  
  
+ GTGGGTCTCT GCTTATTTTA TTTGGGAGTG GGGGTGGGAG TGGCAATGGG CTGGGGGTGG TGCTGAATGG   
  
  
+ CCTTAAAGTC ATTGTTGTGT TCATAGTTTA TATTAAATGA GTGAAAAAGA TAGGGAGAAA AAGCAATCTT   
  
  
+ TGAGTGTTTT TGTCATCTAT TGTCTTAACT TCCCCCTCCG CCTTTTCTCT CTCTTAATCA ATCAAAGCTC   
  
  
+ TTGCGCTCTC TTCTTTATGG GAAAAGTCTT CCATTTCCAA AACATCCATC TCTGTCTGTT TGAAAGCTTT   
  
  
+ TCGGGGTAAT ACAGTAAACA GGTTGCAGT  

- TAAATAAATT ATAAAATGTT AATAAAAATT ATAATAAAAA ATTTTTATTA TTTATTTTAA TAAATACTTA   
  
  
- ATTAAAAGTA TTTTTAAATA ACTGAATTAA GCACATATTA AAAAAACTCT TTTTTATTTA ATTACATAAA   
  
  
- TTTTATATTA TTTTATTAAA CAATCATATT ACTTTGATTG AGAATGTATT ATTTTATTAG ACACTTATAC   
  
  
- TAGGTTAATA ATTTTAAGTA CTTATTGTGT AAAAAATTTA TCTACTATAA TGGTTAACTT AACTGGAACT   
  
  
- GGTATTAAAA TACCCGGCCT AATCAGGTTC GCCGAGGTAA GGGCTCAACG AAAGTCCTGG ACGGTGTCGA   
  
  
- CCTGTCCTGT CTAACGTCTG GGTTTTGCTA CCCCTTCACC CCAACATCAT CAATGGAATT AATGAGAGTT   
  
  
- ACTCAAAGAC TTTAGGTTCC TTCATGTTCA ACTTATGTTA AAATGGAATA GACTATTCAT ACTTCCCCAT   
  
  
- TCTATTCCTA CTTCCATGGA ATTGAAGAGG AAGCGAAAAT ACATTCAAAA GACGTAAAAG TAGACGAAAA   
  
  
- ACCGGTTACC TAACCGACCG TCATCGGACA CCAGGAATCT CGAACTCTCA GTCTCGCCGT AACTACTCGA   
  
  
- AGGGAAGATA AAGTAAAGTG GAACTAATTG TGAGTTGGAT ATCGAAGTGA AAGTAAACCG CATGGTGCAA   
  
  
- TAACATTAAA GCCTTAAGTA AAAAACCGGT TCAAGTACTT CAAAGGGAAT GACCCACCTC TCTCTCTCTC   
  
  
- TCTCTAACGA GACAAGTGTC CATCTCCTCT TCTCGACCCG TTACCTGTGC ACGTAGACTT TCCCTTGTCG   
  
  
- TCTTCTATGG GCTCTCTGGG ACACTAAAGT GTGCTCGGTA CCACCAAGGT AATGTGAAGG TGTGGTTTTT   
  
  
- TCTCTCAATA ACTGGAGACG CTACTGTACC AAACGGGGTG CTTTAGGGTA TTTTAGGTTC ACGTAATGTA   
  
  
- GAGAAAAGAC CCCAAAGGCT CAAGAGCTTA CACAGATACA AGTGTTAAAC CGAGTTCTTT TCATTATGGA   
  
  
- TTAACCGTTG TCCTCTAGGT CTCACACATA CGTTCGTACG TGTATACTTC CCGGGGTAAG AACGAATGCA   
  
  
- TCGGTTAGTT CCTCTCGAAC ACCCCGGAGT ACAAAGAGGT GACTTTATAG AAGTTAAATA CAAGTTGACA   
  
  
- CACCCAGAGA CGAATAAAAT AAACCCTCAC CCCCACCCTC ACCGTTACCC GACCCCCACC ACGACTTACC   
  
  
- GGAATTTCAG TAACAACACA AGTATCAAAT ATAATTTACT CACTTTTTCT ATCCCTCTTT TTCGTTAGAA   
  
  
- ACTCACAAAA ACAGTAGATA ACAGAATTGA AGGGGGAGGC GGAAAAGAGA GAGAATTAGT TAGTTTCGAG   
  
  
- AACGCGAGAG AAGAAATACC CTTTTCAGAA GGTAAAGGTT TTGTAGGTAG AGACAGACAA ACTTTCGAAA   
  
  
- AGCCCCATTA TGTCATTTGT CCAACGTCA

+     as-2-box

| Site Name | Organism | Position | Strand | Matrix score. | sequence | function |
| --- | --- | --- | --- | --- | --- | --- |
| as-2-box | Nicotiana tabacum | 492 | + | 9 | GATAatGATG | involved in shoot-specific expression and light responsiveness |

> 2018/04/13 10:10:12  
+ ATTTATTTAA TATTTTACAA TTATTTTTAA TATTATTTTT TAAAAATAAT AAATAAAATT ATTTATGAAT   
  
  
+ TAATTTTCAT AAAAATTTAT TGACTTAATT CGTGTATAAT TTTTTTGAGA AAAAATAAAT TAATGTATTT   
  
  
+ AAAATATAAT AAAATAATTT GTTAGTATAA TGAAACTAAC TCTTACATAA TAAAATAATC TGTGAATATG   
  
  
+ ATCCAATTAT TAAAATTCAT GAATAACACA TTTTTTAAAT AGATGATATT ACCAATTGAA TTGACCTTGA   
  
  
+ CCATAATTTT ATGGGCCGGA TTAGTCCAAG CGGCTCCATT CCCGAGTTGC TTTCAGGACC TGCCACAGCT   
  
  
+ GGACAGGACA GATTGCAGAC CCAAAACGAT GGGGAAGTGG GGTTGTAGTA GTTACCTTAA TTACTCTCAA   
  
  
+ TGAGTTTCTG AAATCCAAGG AAGTACAAGT TGAATACAAT TTTACCTTAT CTGATAAGTA TGAAGGGGTA   
  
  
+ AGATAAGGAT GAAGGTACCT TAACTTCTCC TTCGCTTTTA TGTAAGTTTT CTGCATTTTC ATCTGCTTTT   
  
  
+ TGGCCAATGG ATTGGCTGGC AGTAGCCTGT GGTCCTTAGA GCTTGAGAGT CAGAGCGGCA TTGATGAGCT   
  
  
+ TCCCTTCTAT TTCATTTCAC CTTGATTAAC ACTCAACCTA TAGCTTCACT TTCATTTGGC GTACCACGTT   
  
  
+ ATTGTAATTT CGGAATTCAT TTTTTGGCCA AGTTCATGAA GTTTCCCTTA CTGGGTGGAG AGAGAGAGAG   
  
  
+ AGAGATTGCT CTGTTCACAG GTAGAGGAGA AGAGCTGGGC AATGGACACG TGCATCTGAA AGGGAACAGC   
  
  
+ AGAAGATACC CGAGAGACCC TGTGATTTCA CACGAGCCAT GGTGGTTCCA TTACACTTCC ACACCAAAAA   
  
  
+ AGAGAGTTAT TGACCTCTGC GATGACATGG TTTGCCCCAC GAAATCCCAT AAAATCCAAG TGCATTACAT   
  
  
+ CTCTTTTCTG GGGTTTCCGA GTTCTCGAAT GTGTCTATGT TCACAATTTG GCTCAAGAAA AGTAATACCT   
  
  
+ AATTGGCAAC AGGAGATCCA GAGTGTGTAT GCAAGCATGC ACATATGAAG GGCCCCATTC TTGCTTACGT   
  
  
+ AGCCAATCAA GGAGAGCTTG TGGGGCCTCA TGTTTCTCCA CTGAAATATC TTCAATTTAT GTTCAACTGT   
  
  
+ GTGGGTCTCT GCTTATTTTA TTTGGGAGTG GGGGTGGGAG TGGCAATGGG CTGGGGGTGG TGCTGAATGG   
  
  
+ CCTTAAAGTC ATTGTTGTGT TCATAGTTTA TATTAAATGA GTGAAAAAGA TAGGGAGAAA AAGCAATCTT   
  
  
+ TGAGTGTTTT TGTCATCTAT TGTCTTAACT TCCCCCTCCG CCTTTTCTCT CTCTTAATCA ATCAAAGCTC   
  
  
+ TTGCGCTCTC TTCTTTATGG GAAAAGTCTT CCATTTCCAA AACATCCATC TCTGTCTGTT TGAAAGCTTT   
  
  
+ TCGGGGTAAT ACAGTAAACA GGTTGCAGT  

- TAAATAAATT ATAAAATGTT AATAAAAATT ATAATAAAAA ATTTTTATTA TTTATTTTAA TAAATACTTA   
  
  
- ATTAAAAGTA TTTTTAAATA ACTGAATTAA GCACATATTA AAAAAACTCT TTTTTATTTA ATTACATAAA   
  
  
- TTTTATATTA TTTTATTAAA CAATCATATT ACTTTGATTG AGAATGTATT ATTTTATTAG ACACTTATAC   
  
  
- TAGGTTAATA ATTTTAAGTA CTTATTGTGT AAAAAATTTA TCTACTATAA TGGTTAACTT AACTGGAACT   
  
  
- GGTATTAAAA TACCCGGCCT AATCAGGTTC GCCGAGGTAA GGGCTCAACG AAAGTCCTGG ACGGTGTCGA   
  
  
- CCTGTCCTGT CTAACGTCTG GGTTTTGCTA CCCCTTCACC CCAACATCAT CAATGGAATT AATGAGAGTT   
  
  
- ACTCAAAGAC TTTAGGTTCC TTCATGTTCA ACTTATGTTA AAATGGAATA GACTATTCAT ACTTCCCCAT   
  
  
- TCTATTCCTA CTTCCATGGA ATTGAAGAGG AAGCGAAAAT ACATTCAAAA GACGTAAAAG TAGACGAAAA   
  
  
- ACCGGTTACC TAACCGACCG TCATCGGACA CCAGGAATCT CGAACTCTCA GTCTCGCCGT AACTACTCGA   
  
  
- AGGGAAGATA AAGTAAAGTG GAACTAATTG TGAGTTGGAT ATCGAAGTGA AAGTAAACCG CATGGTGCAA   
  
  
- TAACATTAAA GCCTTAAGTA AAAAACCGGT TCAAGTACTT CAAAGGGAAT GACCCACCTC TCTCTCTCTC   
  
  
- TCTCTAACGA GACAAGTGTC CATCTCCTCT TCTCGACCCG TTACCTGTGC ACGTAGACTT TCCCTTGTCG   
  
  
- TCTTCTATGG GCTCTCTGGG ACACTAAAGT GTGCTCGGTA CCACCAAGGT AATGTGAAGG TGTGGTTTTT   
  
  
- TCTCTCAATA ACTGGAGACG CTACTGTACC AAACGGGGTG CTTTAGGGTA TTTTAGGTTC ACGTAATGTA   
  
  
- GAGAAAAGAC CCCAAAGGCT CAAGAGCTTA CACAGATACA AGTGTTAAAC CGAGTTCTTT TCATTATGGA   
  
  
- TTAACCGTTG TCCTCTAGGT CTCACACATA CGTTCGTACG TGTATACTTC CCGGGGTAAG AACGAATGCA   
  
  
- TCGGTTAGTT CCTCTCGAAC ACCCCGGAGT ACAAAGAGGT GACTTTATAG AAGTTAAATA CAAGTTGACA   
  
  
- CACCCAGAGA CGAATAAAAT AAACCCTCAC CCCCACCCTC ACCGTTACCC GACCCCCACC ACGACTTACC   
  
  
- GGAATTTCAG TAACAACACA AGTATCAAAT ATAATTTACT CACTTTTTCT ATCCCTCTTT TTCGTTAGAA   
  
  
- ACTCACAAAA ACAGTAGATA ACAGAATTGA AGGGGGAGGC GGAAAAGAGA GAGAATTAGT TAGTTTCGAG   
  
  
- AACGCGAGAG AAGAAATACC CTTTTCAGAA GGTAAAGGTT TTGTAGGTAG AGACAGACAA ACTTTCGAAA   
  
  
- AGCCCCATTA TGTCATTTGT CCAACGTCA
