# Supplementary material for: Genome-wide analysis of the WRKY gene family in drumstick (Moringa oleifera Lam.)
Source: PeerJ. 2019 Jun 10;7:e7063. doi: 10.7717/peerj.7063 (PMC6563795; doi:10.7717/peerj.7063)
Supplement: Supplemental Information 1 [file peerj-07-7063-s003.gz › MoWRKY43_plantcare.html]

Content-Type: text/html; charset=ISO-8859-1


CallMat\_Firefox


Webmaster Firefox specific output  
To save the result:
click on the frame with the right mouse button and save the source code as a text file with extension .html  
REFERENCE:PlantCARE: a database of plant cis-acting regulatory elements and a portal to tools for in silico analysis of promoter sequences.  
Lescot, M., Déhais, P., Moreau, Y., De Moor, B., Rouzé ,P.,and Rombauts, S.  
Nucleic Acids Res., Database issue(2002), 30(1):325-327.   


---

> 2018/04/13 10:10:12  
+ GGTGTTGTTT GTACAATCAT ATGCGTGTCA TTGCGTACAA AACAAAAAAT ATGCTAGAGC ATCCATGAAA   
  
  
+ GCAATAATGA ATGGCAAAAC TACATAAAAG AAAAACAAAA CGCAGAAGCG GACCAACAAA ATTTAGAAGA   
  
  
+ AGTGAACGTG AACTATGGCG AAACAAAAAA AAAAAAAAAA CTTACAAAAA TGGAGAAAAG GGACAACCTT   
  
  
+ CTTCAAAAAA AAAAAAAAAA AAAAACTTAC AAAAATGGAG AAAAGGGACA ACCTTCTTCA AGCAGACGCT   
  
  
+ GCATAAGATA ACTTCAATCT CTCTCCAAGA GAGAGCGCCC TGAACACAGT TACTGTGCGC TTGAAAGCTC   
  
  
+ TCTAGCGGTC TATTAATGCT CACTAAAAGG ACTTTTTCTA TTGGTCATGA GGTTCATGCT TCCTACAAAA   
  
  
+ TTTCTTCCTA CCTAAATTAT TCCGCAGCTG CAGTTCAATT CATTAAAAAA GGTCTCGGTA GCATGGCTAT   
  
  
+ ACGTGTGGTC ACACTGGTCA GCTCTTTTTC CAACCCATGT GGGGGATAAT TGGAGCCCTC AATTGGGTCA   
  
  
+ TCGTTGAAAC TCAGAAGAAA CCAAAGTAAT AGCATCTGCT TCCCTGAAGT CATATCTTGT ACCCATCTAT   
  
  
+ GGGCACTCCA TGAAGATGCT GATCTGTGTC ATGCCATTCT TAAATTCATC AAAGGTTAAC CTAATTATTA   
  
  
+ CTTTGCAGAG TTTTCTTTTT CTTTCTATGC CCAAAAGTTT CCTACTTTAG CACTATAACC CCATCAATTC   
  
  
+ CACTGCTTCA TTAAGTTCAT TACATGGGCT TATCATTTAA GAAAACAAAG CAAAGTTTGT TGCCTAACTG   
  
  
+ ATTAGGAATT CCCTGTAATA CTACTTACCC TAATACTTCA ATATTATTCA AGCCAAAGAC CAGCAGTTTC   
  
  
+ TCCAAGTATT TATTTGCCTA TAAGGTTAGT TATCCATTCA CATACGAAAG AGATGAAAGC AAAAGGCAGG   
  
  
+ AAATACGGTG GTAACATTTT CCTTCATGCA AAAGTTTTAC TCGTACAGTT GGCTTTTAGG CTACAAGGTG   
  
  
+ TCCACTCCTA AGAAAAAGTG TTTAATTAAT GAATTAATCA TTAAAAAGAA AAACCAGTAT ATCCAAGTCA   
  
  
+ AAAATGCAAT AACAAACGGC CACCTTCACC GTTAGGGAAC CCGTCAAGTC AACAAAGGCA ACGATGATCC   
  
  
+ ACGCACCACA TCTAACGGAG AAATCAACGG AGTTTGTTAC ACAGAGGAAA CACCTGAGTC AATTCCAGTC   
  
  
+ AAAGACATAT ATACCCCCAA CACGACAGAC ATACAACCCC TCTCTCTTTC CTTTAAAGCA AACCCTTTCC   
  
  
+ ACTCTCTCTC TCTCTCTCTC CATCTTAATC CATTCCGATA CTTTTAAAAA ATCGGAAAAA TTTCAACGGT   
  
  
+ ACATATCGCT TCCGCCGCAA AATATCGGAC TTTTTTTAAG TGTCACCACA CTGTTGTTAC TTTACTCTCT   
  
  
+ GTTTCTGTCT CTCCTTTGGA AAAATCGGT  

- CCACAACAAA CATGTTAGTA TACGCACAGT AACGCATGTT TTGTTTTTTA TACGATCTCG TAGGTACTTT   
  
  
- CGTTATTACT TACCGTTTTG ATGTATTTTC TTTTTGTTTT GCGTCTTCGC CTGGTTGTTT TAAATCTTCT   
  
  
- TCACTTGCAC TTGATACCGC TTTGTTTTTT TTTTTTTTTT GAATGTTTTT ACCTCTTTTC CCTGTTGGAA   
  
  
- GAAGTTTTTT TTTTTTTTTT TTTTTGAATG TTTTTACCTC TTTTCCCTGT TGGAAGAAGT TCGTCTGCGA   
  
  
- CGTATTCTAT TGAAGTTAGA GAGAGGTTCT CTCTCGCGGG ACTTGTGTCA ATGACACGCG AACTTTCGAG   
  
  
- AGATCGCCAG ATAATTACGA GTGATTTTCC TGAAAAAGAT AACCAGTACT CCAAGTACGA AGGATGTTTT   
  
  
- AAAGAAGGAT GGATTTAATA AGGCGTCGAC GTCAAGTTAA GTAATTTTTT CCAGAGCCAT CGTACCGATA   
  
  
- TGCACACCAG TGTGACCAGT CGAGAAAAAG GTTGGGTACA CCCCCTATTA ACCTCGGGAG TTAACCCAGT   
  
  
- AGCAACTTTG AGTCTTCTTT GGTTTCATTA TCGTAGACGA AGGGACTTCA GTATAGAACA TGGGTAGATA   
  
  
- CCCGTGAGGT ACTTCTACGA CTAGACACAG TACGGTAAGA ATTTAAGTAG TTTCCAATTG GATTAATAAT   
  
  
- GAAACGTCTC AAAAGAAAAA GAAAGATACG GGTTTTCAAA GGATGAAATC GTGATATTGG GGTAGTTAAG   
  
  
- GTGACGAAGT AATTCAAGTA ATGTACCCGA ATAGTAAATT CTTTTGTTTC GTTTCAAACA ACGGATTGAC   
  
  
- TAATCCTTAA GGGACATTAT GATGAATGGG ATTATGAAGT TATAATAAGT TCGGTTTCTG GTCGTCAAAG   
  
  
- AGGTTCATAA ATAAACGGAT ATTCCAATCA ATAGGTAAGT GTATGCTTTC TCTACTTTCG TTTTCCGTCC   
  
  
- TTTATGCCAC CATTGTAAAA GGAAGTACGT TTTCAAAATG AGCATGTCAA CCGAAAATCC GATGTTCCAC   
  
  
- AGGTGAGGAT TCTTTTTCAC AAATTAATTA CTTAATTAGT AATTTTTCTT TTTGGTCATA TAGGTTCAGT   
  
  
- TTTTACGTTA TTGTTTGCCG GTGGAAGTGG CAATCCCTTG GGCAGTTCAG TTGTTTCCGT TGCTACTAGG   
  
  
- TGCGTGGTGT AGATTGCCTC TTTAGTTGCC TCAAACAATG TGTCTCCTTT GTGGACTCAG TTAAGGTCAG   
  
  
- TTTCTGTATA TATGGGGGTT GTGCTGTCTG TATGTTGGGG AGAGAGAAAG GAAATTTCGT TTGGGAAAGG   
  
  
- TGAGAGAGAG AGAGAGAGAG GTAGAATTAG GTAAGGCTAT GAAAATTTTT TAGCCTTTTT AAAGTTGCCA   
  
  
- TGTATAGCGA AGGCGGCGTT TTATAGCCTG AAAAAAATTC ACAGTGGTGT GACAACAATG AAATGAGAGA   
  
  
- CAAAGACAGA GAGGAAACCT TTTTAGCCA

  
  
Motifs Found  

+     5UTR Py-rich stretch

| Site Name | Organism | Position | Strand | Matrix score. | sequence | function |
| --- | --- | --- | --- | --- | --- | --- |
| 5UTR Py-rich stretch | Lycopersicon esculentum | 1335 | + | 13 | TTTCTCTCTCTCTC | cis-acting element conferring high transcription levels |
| 5UTR Py-rich stretch | Lycopersicon esculentum | 1337 | + | 13 | TTTCTCTCTCTCTC | cis-acting element conferring high transcription levels |
| 5UTR Py-rich stretch | Lycopersicon esculentum | 1333 | + | 13 | TTTCTCTCTCTCTC | cis-acting element conferring high transcription levels |
| 5UTR Py-rich stretch | Lycopersicon esculentum | 959 | - | 9 | TTTCTTCTCT | cis-acting element conferring high transcription levels |

> 2018/04/13 10:10:12  
+ GGTGTTGTTT GTACAATCAT ATGCGTGTCA TTGCGTACAA AACAAAAAAT ATGCTAGAGC ATCCATGAAA   
  
  
+ GCAATAATGA ATGGCAAAAC TACATAAAAG AAAAACAAAA CGCAGAAGCG GACCAACAAA ATTTAGAAGA   
  
  
+ AGTGAACGTG AACTATGGCG AAACAAAAAA AAAAAAAAAA CTTACAAAAA TGGAGAAAAG GGACAACCTT   
  
  
+ CTTCAAAAAA AAAAAAAAAA AAAAACTTAC AAAAATGGAG AAAAGGGACA ACCTTCTTCA AGCAGACGCT   
  
  
+ GCATAAGATA ACTTCAATCT CTCTCCAAGA GAGAGCGCCC TGAACACAGT TACTGTGCGC TTGAAAGCTC   
  
  
+ TCTAGCGGTC TATTAATGCT CACTAAAAGG ACTTTTTCTA TTGGTCATGA GGTTCATGCT TCCTACAAAA   
  
  
+ TTTCTTCCTA CCTAAATTAT TCCGCAGCTG CAGTTCAATT CATTAAAAAA GGTCTCGGTA GCATGGCTAT   
  
  
+ ACGTGTGGTC ACACTGGTCA GCTCTTTTTC CAACCCATGT GGGGGATAAT TGGAGCCCTC AATTGGGTCA   
  
  
+ TCGTTGAAAC TCAGAAGAAA CCAAAGTAAT AGCATCTGCT TCCCTGAAGT CATATCTTGT ACCCATCTAT   
  
  
+ GGGCACTCCA TGAAGATGCT GATCTGTGTC ATGCCATTCT TAAATTCATC AAAGGTTAAC CTAATTATTA   
  
  
+ CTTTGCAGAG TTTTCTTTTT CTTTCTATGC CCAAAAGTTT CCTACTTTAG CACTATAACC CCATCAATTC   
  
  
+ CACTGCTTCA TTAAGTTCAT TACATGGGCT TATCATTTAA GAAAACAAAG CAAAGTTTGT TGCCTAACTG   
  
  
+ ATTAGGAATT CCCTGTAATA CTACTTACCC TAATACTTCA ATATTATTCA AGCCAAAGAC CAGCAGTTTC   
  
  
+ TCCAAGTATT TATTTGCCTA TAAGGTTAGT TATCCATTCA CATACGAAAG AGATGAAAGC AAAAGGCAGG   
  
  
+ AAATACGGTG GTAACATTTT CCTTCATGCA AAAGTTTTAC TCGTACAGTT GGCTTTTAGG CTACAAGGTG   
  
  
+ TCCACTCCTA AGAAAAAGTG TTTAATTAAT GAATTAATCA TTAAAAAGAA AAACCAGTAT ATCCAAGTCA   
  
  
+ AAAATGCAAT AACAAACGGC CACCTTCACC GTTAGGGAAC CCGTCAAGTC AACAAAGGCA ACGATGATCC   
  
  
+ ACGCACCACA TCTAACGGAG AAATCAACGG AGTTTGTTAC ACAGAGGAAA CACCTGAGTC AATTCCAGTC   
  
  
+ AAAGACATAT ATACCCCCAA CACGACAGAC ATACAACCCC TCTCTCTTTC CTTTAAAGCA AACCCTTTCC   
  
  
+ ACTCTCTCTC TCTCTCTCTC CATCTTAATC CATTCCGATA CTTTTAAAAA ATCGGAAAAA TTTCAACGGT   
  
  
+ ACATATCGCT TCCGCCGCAA AATATCGGAC TTTTTTTAAG TGTCACCACA CTGTTGTTAC TTTACTCTCT   
  
  
+ GTTTCTGTCT CTCCTTTGGA AAAATCGGT  

- CCACAACAAA CATGTTAGTA TACGCACAGT AACGCATGTT TTGTTTTTTA TACGATCTCG TAGGTACTTT   
  
  
- CGTTATTACT TACCGTTTTG ATGTATTTTC TTTTTGTTTT GCGTCTTCGC CTGGTTGTTT TAAATCTTCT   
  
  
- TCACTTGCAC TTGATACCGC TTTGTTTTTT TTTTTTTTTT GAATGTTTTT ACCTCTTTTC CCTGTTGGAA   
  
  
- GAAGTTTTTT TTTTTTTTTT TTTTTGAATG TTTTTACCTC TTTTCCCTGT TGGAAGAAGT TCGTCTGCGA   
  
  
- CGTATTCTAT TGAAGTTAGA GAGAGGTTCT CTCTCGCGGG ACTTGTGTCA ATGACACGCG AACTTTCGAG   
  
  
- AGATCGCCAG ATAATTACGA GTGATTTTCC TGAAAAAGAT AACCAGTACT CCAAGTACGA AGGATGTTTT   
  
  
- AAAGAAGGAT GGATTTAATA AGGCGTCGAC GTCAAGTTAA GTAATTTTTT CCAGAGCCAT CGTACCGATA   
  
  
- TGCACACCAG TGTGACCAGT CGAGAAAAAG GTTGGGTACA CCCCCTATTA ACCTCGGGAG TTAACCCAGT   
  
  
- AGCAACTTTG AGTCTTCTTT GGTTTCATTA TCGTAGACGA AGGGACTTCA GTATAGAACA TGGGTAGATA   
  
  
- CCCGTGAGGT ACTTCTACGA CTAGACACAG TACGGTAAGA ATTTAAGTAG TTTCCAATTG GATTAATAAT   
  
  
- GAAACGTCTC AAAAGAAAAA GAAAGATACG GGTTTTCAAA GGATGAAATC GTGATATTGG GGTAGTTAAG   
  
  
- GTGACGAAGT AATTCAAGTA ATGTACCCGA ATAGTAAATT CTTTTGTTTC GTTTCAAACA ACGGATTGAC   
  
  
- TAATCCTTAA GGGACATTAT GATGAATGGG ATTATGAAGT TATAATAAGT TCGGTTTCTG GTCGTCAAAG   
  
  
- AGGTTCATAA ATAAACGGAT ATTCCAATCA ATAGGTAAGT GTATGCTTTC TCTACTTTCG TTTTCCGTCC   
  
  
- TTTATGCCAC CATTGTAAAA GGAAGTACGT TTTCAAAATG AGCATGTCAA CCGAAAATCC GATGTTCCAC   
  
  
- AGGTGAGGAT TCTTTTTCAC AAATTAATTA CTTAATTAGT AATTTTTCTT TTTGGTCATA TAGGTTCAGT   
  
  
- TTTTACGTTA TTGTTTGCCG GTGGAAGTGG CAATCCCTTG GGCAGTTCAG TTGTTTCCGT TGCTACTAGG   
  
  
- TGCGTGGTGT AGATTGCCTC TTTAGTTGCC TCAAACAATG TGTCTCCTTT GTGGACTCAG TTAAGGTCAG   
  
  
- TTTCTGTATA TATGGGGGTT GTGCTGTCTG TATGTTGGGG AGAGAGAAAG GAAATTTCGT TTGGGAAAGG   
  
  
- TGAGAGAGAG AGAGAGAGAG GTAGAATTAG GTAAGGCTAT GAAAATTTTT TAGCCTTTTT AAAGTTGCCA   
  
  
- TGTATAGCGA AGGCGGCGTT TTATAGCCTG AAAAAAATTC ACAGTGGTGT GACAACAATG AAATGAGAGA   
  
  
- CAAAGACAGA GAGGAAACCT TTTTAGCCA

+     AACA\_motif

| Site Name | Organism | Position | Strand | Matrix score. | sequence | function |
| --- | --- | --- | --- | --- | --- | --- |
| AACA\_motif | Oryza sativa | 1218 | - | 11 | TAACAAACTCCA | involved in endosperm-specific negative expression |

> 2018/04/13 10:10:12  
+ GGTGTTGTTT GTACAATCAT ATGCGTGTCA TTGCGTACAA AACAAAAAAT ATGCTAGAGC ATCCATGAAA   
  
  
+ GCAATAATGA ATGGCAAAAC TACATAAAAG AAAAACAAAA CGCAGAAGCG GACCAACAAA ATTTAGAAGA   
  
  
+ AGTGAACGTG AACTATGGCG AAACAAAAAA AAAAAAAAAA CTTACAAAAA TGGAGAAAAG GGACAACCTT   
  
  
+ CTTCAAAAAA AAAAAAAAAA AAAAACTTAC AAAAATGGAG AAAAGGGACA ACCTTCTTCA AGCAGACGCT   
  
  
+ GCATAAGATA ACTTCAATCT CTCTCCAAGA GAGAGCGCCC TGAACACAGT TACTGTGCGC TTGAAAGCTC   
  
  
+ TCTAGCGGTC TATTAATGCT CACTAAAAGG ACTTTTTCTA TTGGTCATGA GGTTCATGCT TCCTACAAAA   
  
  
+ TTTCTTCCTA CCTAAATTAT TCCGCAGCTG CAGTTCAATT CATTAAAAAA GGTCTCGGTA GCATGGCTAT   
  
  
+ ACGTGTGGTC ACACTGGTCA GCTCTTTTTC CAACCCATGT GGGGGATAAT TGGAGCCCTC AATTGGGTCA   
  
  
+ TCGTTGAAAC TCAGAAGAAA CCAAAGTAAT AGCATCTGCT TCCCTGAAGT CATATCTTGT ACCCATCTAT   
  
  
+ GGGCACTCCA TGAAGATGCT GATCTGTGTC ATGCCATTCT TAAATTCATC AAAGGTTAAC CTAATTATTA   
  
  
+ CTTTGCAGAG TTTTCTTTTT CTTTCTATGC CCAAAAGTTT CCTACTTTAG CACTATAACC CCATCAATTC   
  
  
+ CACTGCTTCA TTAAGTTCAT TACATGGGCT TATCATTTAA GAAAACAAAG CAAAGTTTGT TGCCTAACTG   
  
  
+ ATTAGGAATT CCCTGTAATA CTACTTACCC TAATACTTCA ATATTATTCA AGCCAAAGAC CAGCAGTTTC   
  
  
+ TCCAAGTATT TATTTGCCTA TAAGGTTAGT TATCCATTCA CATACGAAAG AGATGAAAGC AAAAGGCAGG   
  
  
+ AAATACGGTG GTAACATTTT CCTTCATGCA AAAGTTTTAC TCGTACAGTT GGCTTTTAGG CTACAAGGTG   
  
  
+ TCCACTCCTA AGAAAAAGTG TTTAATTAAT GAATTAATCA TTAAAAAGAA AAACCAGTAT ATCCAAGTCA   
  
  
+ AAAATGCAAT AACAAACGGC CACCTTCACC GTTAGGGAAC CCGTCAAGTC AACAAAGGCA ACGATGATCC   
  
  
+ ACGCACCACA TCTAACGGAG AAATCAACGG AGTTTGTTAC ACAGAGGAAA CACCTGAGTC AATTCCAGTC   
  
  
+ AAAGACATAT ATACCCCCAA CACGACAGAC ATACAACCCC TCTCTCTTTC CTTTAAAGCA AACCCTTTCC   
  
  
+ ACTCTCTCTC TCTCTCTCTC CATCTTAATC CATTCCGATA CTTTTAAAAA ATCGGAAAAA TTTCAACGGT   
  
  
+ ACATATCGCT TCCGCCGCAA AATATCGGAC TTTTTTTAAG TGTCACCACA CTGTTGTTAC TTTACTCTCT   
  
  
+ GTTTCTGTCT CTCCTTTGGA AAAATCGGT  

- CCACAACAAA CATGTTAGTA TACGCACAGT AACGCATGTT TTGTTTTTTA TACGATCTCG TAGGTACTTT   
  
  
- CGTTATTACT TACCGTTTTG ATGTATTTTC TTTTTGTTTT GCGTCTTCGC CTGGTTGTTT TAAATCTTCT   
  
  
- TCACTTGCAC TTGATACCGC TTTGTTTTTT TTTTTTTTTT GAATGTTTTT ACCTCTTTTC CCTGTTGGAA   
  
  
- GAAGTTTTTT TTTTTTTTTT TTTTTGAATG TTTTTACCTC TTTTCCCTGT TGGAAGAAGT TCGTCTGCGA   
  
  
- CGTATTCTAT TGAAGTTAGA GAGAGGTTCT CTCTCGCGGG ACTTGTGTCA ATGACACGCG AACTTTCGAG   
  
  
- AGATCGCCAG ATAATTACGA GTGATTTTCC TGAAAAAGAT AACCAGTACT CCAAGTACGA AGGATGTTTT   
  
  
- AAAGAAGGAT GGATTTAATA AGGCGTCGAC GTCAAGTTAA GTAATTTTTT CCAGAGCCAT CGTACCGATA   
  
  
- TGCACACCAG TGTGACCAGT CGAGAAAAAG GTTGGGTACA CCCCCTATTA ACCTCGGGAG TTAACCCAGT   
  
  
- AGCAACTTTG AGTCTTCTTT GGTTTCATTA TCGTAGACGA AGGGACTTCA GTATAGAACA TGGGTAGATA   
  
  
- CCCGTGAGGT ACTTCTACGA CTAGACACAG TACGGTAAGA ATTTAAGTAG TTTCCAATTG GATTAATAAT   
  
  
- GAAACGTCTC AAAAGAAAAA GAAAGATACG GGTTTTCAAA GGATGAAATC GTGATATTGG GGTAGTTAAG   
  
  
- GTGACGAAGT AATTCAAGTA ATGTACCCGA ATAGTAAATT CTTTTGTTTC GTTTCAAACA ACGGATTGAC   
  
  
- TAATCCTTAA GGGACATTAT GATGAATGGG ATTATGAAGT TATAATAAGT TCGGTTTCTG GTCGTCAAAG   
  
  
- AGGTTCATAA ATAAACGGAT ATTCCAATCA ATAGGTAAGT GTATGCTTTC TCTACTTTCG TTTTCCGTCC   
  
  
- TTTATGCCAC CATTGTAAAA GGAAGTACGT TTTCAAAATG AGCATGTCAA CCGAAAATCC GATGTTCCAC   
  
  
- AGGTGAGGAT TCTTTTTCAC AAATTAATTA CTTAATTAGT AATTTTTCTT TTTGGTCATA TAGGTTCAGT   
  
  
- TTTTACGTTA TTGTTTGCCG GTGGAAGTGG CAATCCCTTG GGCAGTTCAG TTGTTTCCGT TGCTACTAGG   
  
  
- TGCGTGGTGT AGATTGCCTC TTTAGTTGCC TCAAACAATG TGTCTCCTTT GTGGACTCAG TTAAGGTCAG   
  
  
- TTTCTGTATA TATGGGGGTT GTGCTGTCTG TATGTTGGGG AGAGAGAAAG GAAATTTCGT TTGGGAAAGG   
  
  
- TGAGAGAGAG AGAGAGAGAG GTAGAATTAG GTAAGGCTAT GAAAATTTTT TAGCCTTTTT AAAGTTGCCA   
  
  
- TGTATAGCGA AGGCGGCGTT TTATAGCCTG AAAAAAATTC ACAGTGGTGT GACAACAATG AAATGAGAGA   
  
  
- CAAAGACAGA GAGGAAACCT TTTTAGCCA

+     AAGAA-motif

| Site Name | Organism | Position | Strand | Matrix score. | sequence | function |
| --- | --- | --- | --- | --- | --- | --- |
| AAGAA-motif | Avena sativa | 719 | - | 7 | GAAAGAA |  |

> 2018/04/13 10:10:12  
+ GGTGTTGTTT GTACAATCAT ATGCGTGTCA TTGCGTACAA AACAAAAAAT ATGCTAGAGC ATCCATGAAA   
  
  
+ GCAATAATGA ATGGCAAAAC TACATAAAAG AAAAACAAAA CGCAGAAGCG GACCAACAAA ATTTAGAAGA   
  
  
+ AGTGAACGTG AACTATGGCG AAACAAAAAA AAAAAAAAAA CTTACAAAAA TGGAGAAAAG GGACAACCTT   
  
  
+ CTTCAAAAAA AAAAAAAAAA AAAAACTTAC AAAAATGGAG AAAAGGGACA ACCTTCTTCA AGCAGACGCT   
  
  
+ GCATAAGATA ACTTCAATCT CTCTCCAAGA GAGAGCGCCC TGAACACAGT TACTGTGCGC TTGAAAGCTC   
  
  
+ TCTAGCGGTC TATTAATGCT CACTAAAAGG ACTTTTTCTA TTGGTCATGA GGTTCATGCT TCCTACAAAA   
  
  
+ TTTCTTCCTA CCTAAATTAT TCCGCAGCTG CAGTTCAATT CATTAAAAAA GGTCTCGGTA GCATGGCTAT   
  
  
+ ACGTGTGGTC ACACTGGTCA GCTCTTTTTC CAACCCATGT GGGGGATAAT TGGAGCCCTC AATTGGGTCA   
  
  
+ TCGTTGAAAC TCAGAAGAAA CCAAAGTAAT AGCATCTGCT TCCCTGAAGT CATATCTTGT ACCCATCTAT   
  
  
+ GGGCACTCCA TGAAGATGCT GATCTGTGTC ATGCCATTCT TAAATTCATC AAAGGTTAAC CTAATTATTA   
  
  
+ CTTTGCAGAG TTTTCTTTTT CTTTCTATGC CCAAAAGTTT CCTACTTTAG CACTATAACC CCATCAATTC   
  
  
+ CACTGCTTCA TTAAGTTCAT TACATGGGCT TATCATTTAA GAAAACAAAG CAAAGTTTGT TGCCTAACTG   
  
  
+ ATTAGGAATT CCCTGTAATA CTACTTACCC TAATACTTCA ATATTATTCA AGCCAAAGAC CAGCAGTTTC   
  
  
+ TCCAAGTATT TATTTGCCTA TAAGGTTAGT TATCCATTCA CATACGAAAG AGATGAAAGC AAAAGGCAGG   
  
  
+ AAATACGGTG GTAACATTTT CCTTCATGCA AAAGTTTTAC TCGTACAGTT GGCTTTTAGG CTACAAGGTG   
  
  
+ TCCACTCCTA AGAAAAAGTG TTTAATTAAT GAATTAATCA TTAAAAAGAA AAACCAGTAT ATCCAAGTCA   
  
  
+ AAAATGCAAT AACAAACGGC CACCTTCACC GTTAGGGAAC CCGTCAAGTC AACAAAGGCA ACGATGATCC   
  
  
+ ACGCACCACA TCTAACGGAG AAATCAACGG AGTTTGTTAC ACAGAGGAAA CACCTGAGTC AATTCCAGTC   
  
  
+ AAAGACATAT ATACCCCCAA CACGACAGAC ATACAACCCC TCTCTCTTTC CTTTAAAGCA AACCCTTTCC   
  
  
+ ACTCTCTCTC TCTCTCTCTC CATCTTAATC CATTCCGATA CTTTTAAAAA ATCGGAAAAA TTTCAACGGT   
  
  
+ ACATATCGCT TCCGCCGCAA AATATCGGAC TTTTTTTAAG TGTCACCACA CTGTTGTTAC TTTACTCTCT   
  
  
+ GTTTCTGTCT CTCCTTTGGA AAAATCGGT  

- CCACAACAAA CATGTTAGTA TACGCACAGT AACGCATGTT TTGTTTTTTA TACGATCTCG TAGGTACTTT   
  
  
- CGTTATTACT TACCGTTTTG ATGTATTTTC TTTTTGTTTT GCGTCTTCGC CTGGTTGTTT TAAATCTTCT   
  
  
- TCACTTGCAC TTGATACCGC TTTGTTTTTT TTTTTTTTTT GAATGTTTTT ACCTCTTTTC CCTGTTGGAA   
  
  
- GAAGTTTTTT TTTTTTTTTT TTTTTGAATG TTTTTACCTC TTTTCCCTGT TGGAAGAAGT TCGTCTGCGA   
  
  
- CGTATTCTAT TGAAGTTAGA GAGAGGTTCT CTCTCGCGGG ACTTGTGTCA ATGACACGCG AACTTTCGAG   
  
  
- AGATCGCCAG ATAATTACGA GTGATTTTCC TGAAAAAGAT AACCAGTACT CCAAGTACGA AGGATGTTTT   
  
  
- AAAGAAGGAT GGATTTAATA AGGCGTCGAC GTCAAGTTAA GTAATTTTTT CCAGAGCCAT CGTACCGATA   
  
  
- TGCACACCAG TGTGACCAGT CGAGAAAAAG GTTGGGTACA CCCCCTATTA ACCTCGGGAG TTAACCCAGT   
  
  
- AGCAACTTTG AGTCTTCTTT GGTTTCATTA TCGTAGACGA AGGGACTTCA GTATAGAACA TGGGTAGATA   
  
  
- CCCGTGAGGT ACTTCTACGA CTAGACACAG TACGGTAAGA ATTTAAGTAG TTTCCAATTG GATTAATAAT   
  
  
- GAAACGTCTC AAAAGAAAAA GAAAGATACG GGTTTTCAAA GGATGAAATC GTGATATTGG GGTAGTTAAG   
  
  
- GTGACGAAGT AATTCAAGTA ATGTACCCGA ATAGTAAATT CTTTTGTTTC GTTTCAAACA ACGGATTGAC   
  
  
- TAATCCTTAA GGGACATTAT GATGAATGGG ATTATGAAGT TATAATAAGT TCGGTTTCTG GTCGTCAAAG   
  
  
- AGGTTCATAA ATAAACGGAT ATTCCAATCA ATAGGTAAGT GTATGCTTTC TCTACTTTCG TTTTCCGTCC   
  
  
- TTTATGCCAC CATTGTAAAA GGAAGTACGT TTTCAAAATG AGCATGTCAA CCGAAAATCC GATGTTCCAC   
  
  
- AGGTGAGGAT TCTTTTTCAC AAATTAATTA CTTAATTAGT AATTTTTCTT TTTGGTCATA TAGGTTCAGT   
  
  
- TTTTACGTTA TTGTTTGCCG GTGGAAGTGG CAATCCCTTG GGCAGTTCAG TTGTTTCCGT TGCTACTAGG   
  
  
- TGCGTGGTGT AGATTGCCTC TTTAGTTGCC TCAAACAATG TGTCTCCTTT GTGGACTCAG TTAAGGTCAG   
  
  
- TTTCTGTATA TATGGGGGTT GTGCTGTCTG TATGTTGGGG AGAGAGAAAG GAAATTTCGT TTGGGAAAGG   
  
  
- TGAGAGAGAG AGAGAGAGAG GTAGAATTAG GTAAGGCTAT GAAAATTTTT TAGCCTTTTT AAAGTTGCCA   
  
  
- TGTATAGCGA AGGCGGCGTT TTATAGCCTG AAAAAAATTC ACAGTGGTGT GACAACAATG AAATGAGAGA   
  
  
- CAAAGACAGA GAGGAAACCT TTTTAGCCA

+     ABRE

| Site Name | Organism | Position | Strand | Matrix score. | sequence | function |
| --- | --- | --- | --- | --- | --- | --- |
| ABRE | Arabidopsis thaliana | 490 | + | 6 | TACGTG | cis-acting element involved in the abscisic acid responsiveness |

> 2018/04/13 10:10:12  
+ GGTGTTGTTT GTACAATCAT ATGCGTGTCA TTGCGTACAA AACAAAAAAT ATGCTAGAGC ATCCATGAAA   
  
  
+ GCAATAATGA ATGGCAAAAC TACATAAAAG AAAAACAAAA CGCAGAAGCG GACCAACAAA ATTTAGAAGA   
  
  
+ AGTGAACGTG AACTATGGCG AAACAAAAAA AAAAAAAAAA CTTACAAAAA TGGAGAAAAG GGACAACCTT   
  
  
+ CTTCAAAAAA AAAAAAAAAA AAAAACTTAC AAAAATGGAG AAAAGGGACA ACCTTCTTCA AGCAGACGCT   
  
  
+ GCATAAGATA ACTTCAATCT CTCTCCAAGA GAGAGCGCCC TGAACACAGT TACTGTGCGC TTGAAAGCTC   
  
  
+ TCTAGCGGTC TATTAATGCT CACTAAAAGG ACTTTTTCTA TTGGTCATGA GGTTCATGCT TCCTACAAAA   
  
  
+ TTTCTTCCTA CCTAAATTAT TCCGCAGCTG CAGTTCAATT CATTAAAAAA GGTCTCGGTA GCATGGCTAT   
  
  
+ ACGTGTGGTC ACACTGGTCA GCTCTTTTTC CAACCCATGT GGGGGATAAT TGGAGCCCTC AATTGGGTCA   
  
  
+ TCGTTGAAAC TCAGAAGAAA CCAAAGTAAT AGCATCTGCT TCCCTGAAGT CATATCTTGT ACCCATCTAT   
  
  
+ GGGCACTCCA TGAAGATGCT GATCTGTGTC ATGCCATTCT TAAATTCATC AAAGGTTAAC CTAATTATTA   
  
  
+ CTTTGCAGAG TTTTCTTTTT CTTTCTATGC CCAAAAGTTT CCTACTTTAG CACTATAACC CCATCAATTC   
  
  
+ CACTGCTTCA TTAAGTTCAT TACATGGGCT TATCATTTAA GAAAACAAAG CAAAGTTTGT TGCCTAACTG   
  
  
+ ATTAGGAATT CCCTGTAATA CTACTTACCC TAATACTTCA ATATTATTCA AGCCAAAGAC CAGCAGTTTC   
  
  
+ TCCAAGTATT TATTTGCCTA TAAGGTTAGT TATCCATTCA CATACGAAAG AGATGAAAGC AAAAGGCAGG   
  
  
+ AAATACGGTG GTAACATTTT CCTTCATGCA AAAGTTTTAC TCGTACAGTT GGCTTTTAGG CTACAAGGTG   
  
  
+ TCCACTCCTA AGAAAAAGTG TTTAATTAAT GAATTAATCA TTAAAAAGAA AAACCAGTAT ATCCAAGTCA   
  
  
+ AAAATGCAAT AACAAACGGC CACCTTCACC GTTAGGGAAC CCGTCAAGTC AACAAAGGCA ACGATGATCC   
  
  
+ ACGCACCACA TCTAACGGAG AAATCAACGG AGTTTGTTAC ACAGAGGAAA CACCTGAGTC AATTCCAGTC   
  
  
+ AAAGACATAT ATACCCCCAA CACGACAGAC ATACAACCCC TCTCTCTTTC CTTTAAAGCA AACCCTTTCC   
  
  
+ ACTCTCTCTC TCTCTCTCTC CATCTTAATC CATTCCGATA CTTTTAAAAA ATCGGAAAAA TTTCAACGGT   
  
  
+ ACATATCGCT TCCGCCGCAA AATATCGGAC TTTTTTTAAG TGTCACCACA CTGTTGTTAC TTTACTCTCT   
  
  
+ GTTTCTGTCT CTCCTTTGGA AAAATCGGT  

- CCACAACAAA CATGTTAGTA TACGCACAGT AACGCATGTT TTGTTTTTTA TACGATCTCG TAGGTACTTT   
  
  
- CGTTATTACT TACCGTTTTG ATGTATTTTC TTTTTGTTTT GCGTCTTCGC CTGGTTGTTT TAAATCTTCT   
  
  
- TCACTTGCAC TTGATACCGC TTTGTTTTTT TTTTTTTTTT GAATGTTTTT ACCTCTTTTC CCTGTTGGAA   
  
  
- GAAGTTTTTT TTTTTTTTTT TTTTTGAATG TTTTTACCTC TTTTCCCTGT TGGAAGAAGT TCGTCTGCGA   
  
  
- CGTATTCTAT TGAAGTTAGA GAGAGGTTCT CTCTCGCGGG ACTTGTGTCA ATGACACGCG AACTTTCGAG   
  
  
- AGATCGCCAG ATAATTACGA GTGATTTTCC TGAAAAAGAT AACCAGTACT CCAAGTACGA AGGATGTTTT   
  
  
- AAAGAAGGAT GGATTTAATA AGGCGTCGAC GTCAAGTTAA GTAATTTTTT CCAGAGCCAT CGTACCGATA   
  
  
- TGCACACCAG TGTGACCAGT CGAGAAAAAG GTTGGGTACA CCCCCTATTA ACCTCGGGAG TTAACCCAGT   
  
  
- AGCAACTTTG AGTCTTCTTT GGTTTCATTA TCGTAGACGA AGGGACTTCA GTATAGAACA TGGGTAGATA   
  
  
- CCCGTGAGGT ACTTCTACGA CTAGACACAG TACGGTAAGA ATTTAAGTAG TTTCCAATTG GATTAATAAT   
  
  
- GAAACGTCTC AAAAGAAAAA GAAAGATACG GGTTTTCAAA GGATGAAATC GTGATATTGG GGTAGTTAAG   
  
  
- GTGACGAAGT AATTCAAGTA ATGTACCCGA ATAGTAAATT CTTTTGTTTC GTTTCAAACA ACGGATTGAC   
  
  
- TAATCCTTAA GGGACATTAT GATGAATGGG ATTATGAAGT TATAATAAGT TCGGTTTCTG GTCGTCAAAG   
  
  
- AGGTTCATAA ATAAACGGAT ATTCCAATCA ATAGGTAAGT GTATGCTTTC TCTACTTTCG TTTTCCGTCC   
  
  
- TTTATGCCAC CATTGTAAAA GGAAGTACGT TTTCAAAATG AGCATGTCAA CCGAAAATCC GATGTTCCAC   
  
  
- AGGTGAGGAT TCTTTTTCAC AAATTAATTA CTTAATTAGT AATTTTTCTT TTTGGTCATA TAGGTTCAGT   
  
  
- TTTTACGTTA TTGTTTGCCG GTGGAAGTGG CAATCCCTTG GGCAGTTCAG TTGTTTCCGT TGCTACTAGG   
  
  
- TGCGTGGTGT AGATTGCCTC TTTAGTTGCC TCAAACAATG TGTCTCCTTT GTGGACTCAG TTAAGGTCAG   
  
  
- TTTCTGTATA TATGGGGGTT GTGCTGTCTG TATGTTGGGG AGAGAGAAAG GAAATTTCGT TTGGGAAAGG   
  
  
- TGAGAGAGAG AGAGAGAGAG GTAGAATTAG GTAAGGCTAT GAAAATTTTT TAGCCTTTTT AAAGTTGCCA   
  
  
- TGTATAGCGA AGGCGGCGTT TTATAGCCTG AAAAAAATTC ACAGTGGTGT GACAACAATG AAATGAGAGA   
  
  
- CAAAGACAGA GAGGAAACCT TTTTAGCCA

+     ARE

| Site Name | Organism | Position | Strand | Matrix score. | sequence | function |
| --- | --- | --- | --- | --- | --- | --- |
| ARE | Zea mays | 1101 | - | 6 | TGGTTT | cis-acting regulatory element essential for the anaerobic induction |
| ARE | Zea mays | 578 | - | 6 | TGGTTT | cis-acting regulatory element essential for the anaerobic induction |

> 2018/04/13 10:10:12  
+ GGTGTTGTTT GTACAATCAT ATGCGTGTCA TTGCGTACAA AACAAAAAAT ATGCTAGAGC ATCCATGAAA   
  
  
+ GCAATAATGA ATGGCAAAAC TACATAAAAG AAAAACAAAA CGCAGAAGCG GACCAACAAA ATTTAGAAGA   
  
  
+ AGTGAACGTG AACTATGGCG AAACAAAAAA AAAAAAAAAA CTTACAAAAA TGGAGAAAAG GGACAACCTT   
  
  
+ CTTCAAAAAA AAAAAAAAAA AAAAACTTAC AAAAATGGAG AAAAGGGACA ACCTTCTTCA AGCAGACGCT   
  
  
+ GCATAAGATA ACTTCAATCT CTCTCCAAGA GAGAGCGCCC TGAACACAGT TACTGTGCGC TTGAAAGCTC   
  
  
+ TCTAGCGGTC TATTAATGCT CACTAAAAGG ACTTTTTCTA TTGGTCATGA GGTTCATGCT TCCTACAAAA   
  
  
+ TTTCTTCCTA CCTAAATTAT TCCGCAGCTG CAGTTCAATT CATTAAAAAA GGTCTCGGTA GCATGGCTAT   
  
  
+ ACGTGTGGTC ACACTGGTCA GCTCTTTTTC CAACCCATGT GGGGGATAAT TGGAGCCCTC AATTGGGTCA   
  
  
+ TCGTTGAAAC TCAGAAGAAA CCAAAGTAAT AGCATCTGCT TCCCTGAAGT CATATCTTGT ACCCATCTAT   
  
  
+ GGGCACTCCA TGAAGATGCT GATCTGTGTC ATGCCATTCT TAAATTCATC AAAGGTTAAC CTAATTATTA   
  
  
+ CTTTGCAGAG TTTTCTTTTT CTTTCTATGC CCAAAAGTTT CCTACTTTAG CACTATAACC CCATCAATTC   
  
  
+ CACTGCTTCA TTAAGTTCAT TACATGGGCT TATCATTTAA GAAAACAAAG CAAAGTTTGT TGCCTAACTG   
  
  
+ ATTAGGAATT CCCTGTAATA CTACTTACCC TAATACTTCA ATATTATTCA AGCCAAAGAC CAGCAGTTTC   
  
  
+ TCCAAGTATT TATTTGCCTA TAAGGTTAGT TATCCATTCA CATACGAAAG AGATGAAAGC AAAAGGCAGG   
  
  
+ AAATACGGTG GTAACATTTT CCTTCATGCA AAAGTTTTAC TCGTACAGTT GGCTTTTAGG CTACAAGGTG   
  
  
+ TCCACTCCTA AGAAAAAGTG TTTAATTAAT GAATTAATCA TTAAAAAGAA AAACCAGTAT ATCCAAGTCA   
  
  
+ AAAATGCAAT AACAAACGGC CACCTTCACC GTTAGGGAAC CCGTCAAGTC AACAAAGGCA ACGATGATCC   
  
  
+ ACGCACCACA TCTAACGGAG AAATCAACGG AGTTTGTTAC ACAGAGGAAA CACCTGAGTC AATTCCAGTC   
  
  
+ AAAGACATAT ATACCCCCAA CACGACAGAC ATACAACCCC TCTCTCTTTC CTTTAAAGCA AACCCTTTCC   
  
  
+ ACTCTCTCTC TCTCTCTCTC CATCTTAATC CATTCCGATA CTTTTAAAAA ATCGGAAAAA TTTCAACGGT   
  
  
+ ACATATCGCT TCCGCCGCAA AATATCGGAC TTTTTTTAAG TGTCACCACA CTGTTGTTAC TTTACTCTCT   
  
  
+ GTTTCTGTCT CTCCTTTGGA AAAATCGGT  

- CCACAACAAA CATGTTAGTA TACGCACAGT AACGCATGTT TTGTTTTTTA TACGATCTCG TAGGTACTTT   
  
  
- CGTTATTACT TACCGTTTTG ATGTATTTTC TTTTTGTTTT GCGTCTTCGC CTGGTTGTTT TAAATCTTCT   
  
  
- TCACTTGCAC TTGATACCGC TTTGTTTTTT TTTTTTTTTT GAATGTTTTT ACCTCTTTTC CCTGTTGGAA   
  
  
- GAAGTTTTTT TTTTTTTTTT TTTTTGAATG TTTTTACCTC TTTTCCCTGT TGGAAGAAGT TCGTCTGCGA   
  
  
- CGTATTCTAT TGAAGTTAGA GAGAGGTTCT CTCTCGCGGG ACTTGTGTCA ATGACACGCG AACTTTCGAG   
  
  
- AGATCGCCAG ATAATTACGA GTGATTTTCC TGAAAAAGAT AACCAGTACT CCAAGTACGA AGGATGTTTT   
  
  
- AAAGAAGGAT GGATTTAATA AGGCGTCGAC GTCAAGTTAA GTAATTTTTT CCAGAGCCAT CGTACCGATA   
  
  
- TGCACACCAG TGTGACCAGT CGAGAAAAAG GTTGGGTACA CCCCCTATTA ACCTCGGGAG TTAACCCAGT   
  
  
- AGCAACTTTG AGTCTTCTTT GGTTTCATTA TCGTAGACGA AGGGACTTCA GTATAGAACA TGGGTAGATA   
  
  
- CCCGTGAGGT ACTTCTACGA CTAGACACAG TACGGTAAGA ATTTAAGTAG TTTCCAATTG GATTAATAAT   
  
  
- GAAACGTCTC AAAAGAAAAA GAAAGATACG GGTTTTCAAA GGATGAAATC GTGATATTGG GGTAGTTAAG   
  
  
- GTGACGAAGT AATTCAAGTA ATGTACCCGA ATAGTAAATT CTTTTGTTTC GTTTCAAACA ACGGATTGAC   
  
  
- TAATCCTTAA GGGACATTAT GATGAATGGG ATTATGAAGT TATAATAAGT TCGGTTTCTG GTCGTCAAAG   
  
  
- AGGTTCATAA ATAAACGGAT ATTCCAATCA ATAGGTAAGT GTATGCTTTC TCTACTTTCG TTTTCCGTCC   
  
  
- TTTATGCCAC CATTGTAAAA GGAAGTACGT TTTCAAAATG AGCATGTCAA CCGAAAATCC GATGTTCCAC   
  
  
- AGGTGAGGAT TCTTTTTCAC AAATTAATTA CTTAATTAGT AATTTTTCTT TTTGGTCATA TAGGTTCAGT   
  
  
- TTTTACGTTA TTGTTTGCCG GTGGAAGTGG CAATCCCTTG GGCAGTTCAG TTGTTTCCGT TGCTACTAGG   
  
  
- TGCGTGGTGT AGATTGCCTC TTTAGTTGCC TCAAACAATG TGTCTCCTTT GTGGACTCAG TTAAGGTCAG   
  
  
- TTTCTGTATA TATGGGGGTT GTGCTGTCTG TATGTTGGGG AGAGAGAAAG GAAATTTCGT TTGGGAAAGG   
  
  
- TGAGAGAGAG AGAGAGAGAG GTAGAATTAG GTAAGGCTAT GAAAATTTTT TAGCCTTTTT AAAGTTGCCA   
  
  
- TGTATAGCGA AGGCGGCGTT TTATAGCCTG AAAAAAATTC ACAGTGGTGT GACAACAATG AAATGAGAGA   
  
  
- CAAAGACAGA GAGGAAACCT TTTTAGCCA

+     Box 4

| Site Name | Organism | Position | Strand | Matrix score. | sequence | function |
| --- | --- | --- | --- | --- | --- | --- |
| Box 4 | Petroselinum crispum | 1075 | - | 6 | ATTAAT | part of a conserved DNA module involved in light responsiveness |
| Box 4 | Petroselinum crispum | 362 | + | 6 | ATTAAT | part of a conserved DNA module involved in light responsiveness |
| Box 4 | Petroselinum crispum | 1083 | - | 6 | ATTAAT | part of a conserved DNA module involved in light responsiveness |

> 2018/04/13 10:10:12  
+ GGTGTTGTTT GTACAATCAT ATGCGTGTCA TTGCGTACAA AACAAAAAAT ATGCTAGAGC ATCCATGAAA   
  
  
+ GCAATAATGA ATGGCAAAAC TACATAAAAG AAAAACAAAA CGCAGAAGCG GACCAACAAA ATTTAGAAGA   
  
  
+ AGTGAACGTG AACTATGGCG AAACAAAAAA AAAAAAAAAA CTTACAAAAA TGGAGAAAAG GGACAACCTT   
  
  
+ CTTCAAAAAA AAAAAAAAAA AAAAACTTAC AAAAATGGAG AAAAGGGACA ACCTTCTTCA AGCAGACGCT   
  
  
+ GCATAAGATA ACTTCAATCT CTCTCCAAGA GAGAGCGCCC TGAACACAGT TACTGTGCGC TTGAAAGCTC   
  
  
+ TCTAGCGGTC TATTAATGCT CACTAAAAGG ACTTTTTCTA TTGGTCATGA GGTTCATGCT TCCTACAAAA   
  
  
+ TTTCTTCCTA CCTAAATTAT TCCGCAGCTG CAGTTCAATT CATTAAAAAA GGTCTCGGTA GCATGGCTAT   
  
  
+ ACGTGTGGTC ACACTGGTCA GCTCTTTTTC CAACCCATGT GGGGGATAAT TGGAGCCCTC AATTGGGTCA   
  
  
+ TCGTTGAAAC TCAGAAGAAA CCAAAGTAAT AGCATCTGCT TCCCTGAAGT CATATCTTGT ACCCATCTAT   
  
  
+ GGGCACTCCA TGAAGATGCT GATCTGTGTC ATGCCATTCT TAAATTCATC AAAGGTTAAC CTAATTATTA   
  
  
+ CTTTGCAGAG TTTTCTTTTT CTTTCTATGC CCAAAAGTTT CCTACTTTAG CACTATAACC CCATCAATTC   
  
  
+ CACTGCTTCA TTAAGTTCAT TACATGGGCT TATCATTTAA GAAAACAAAG CAAAGTTTGT TGCCTAACTG   
  
  
+ ATTAGGAATT CCCTGTAATA CTACTTACCC TAATACTTCA ATATTATTCA AGCCAAAGAC CAGCAGTTTC   
  
  
+ TCCAAGTATT TATTTGCCTA TAAGGTTAGT TATCCATTCA CATACGAAAG AGATGAAAGC AAAAGGCAGG   
  
  
+ AAATACGGTG GTAACATTTT CCTTCATGCA AAAGTTTTAC TCGTACAGTT GGCTTTTAGG CTACAAGGTG   
  
  
+ TCCACTCCTA AGAAAAAGTG TTTAATTAAT GAATTAATCA TTAAAAAGAA AAACCAGTAT ATCCAAGTCA   
  
  
+ AAAATGCAAT AACAAACGGC CACCTTCACC GTTAGGGAAC CCGTCAAGTC AACAAAGGCA ACGATGATCC   
  
  
+ ACGCACCACA TCTAACGGAG AAATCAACGG AGTTTGTTAC ACAGAGGAAA CACCTGAGTC AATTCCAGTC   
  
  
+ AAAGACATAT ATACCCCCAA CACGACAGAC ATACAACCCC TCTCTCTTTC CTTTAAAGCA AACCCTTTCC   
  
  
+ ACTCTCTCTC TCTCTCTCTC CATCTTAATC CATTCCGATA CTTTTAAAAA ATCGGAAAAA TTTCAACGGT   
  
  
+ ACATATCGCT TCCGCCGCAA AATATCGGAC TTTTTTTAAG TGTCACCACA CTGTTGTTAC TTTACTCTCT   
  
  
+ GTTTCTGTCT CTCCTTTGGA AAAATCGGT  

- CCACAACAAA CATGTTAGTA TACGCACAGT AACGCATGTT TTGTTTTTTA TACGATCTCG TAGGTACTTT   
  
  
- CGTTATTACT TACCGTTTTG ATGTATTTTC TTTTTGTTTT GCGTCTTCGC CTGGTTGTTT TAAATCTTCT   
  
  
- TCACTTGCAC TTGATACCGC TTTGTTTTTT TTTTTTTTTT GAATGTTTTT ACCTCTTTTC CCTGTTGGAA   
  
  
- GAAGTTTTTT TTTTTTTTTT TTTTTGAATG TTTTTACCTC TTTTCCCTGT TGGAAGAAGT TCGTCTGCGA   
  
  
- CGTATTCTAT TGAAGTTAGA GAGAGGTTCT CTCTCGCGGG ACTTGTGTCA ATGACACGCG AACTTTCGAG   
  
  
- AGATCGCCAG ATAATTACGA GTGATTTTCC TGAAAAAGAT AACCAGTACT CCAAGTACGA AGGATGTTTT   
  
  
- AAAGAAGGAT GGATTTAATA AGGCGTCGAC GTCAAGTTAA GTAATTTTTT CCAGAGCCAT CGTACCGATA   
  
  
- TGCACACCAG TGTGACCAGT CGAGAAAAAG GTTGGGTACA CCCCCTATTA ACCTCGGGAG TTAACCCAGT   
  
  
- AGCAACTTTG AGTCTTCTTT GGTTTCATTA TCGTAGACGA AGGGACTTCA GTATAGAACA TGGGTAGATA   
  
  
- CCCGTGAGGT ACTTCTACGA CTAGACACAG TACGGTAAGA ATTTAAGTAG TTTCCAATTG GATTAATAAT   
  
  
- GAAACGTCTC AAAAGAAAAA GAAAGATACG GGTTTTCAAA GGATGAAATC GTGATATTGG GGTAGTTAAG   
  
  
- GTGACGAAGT AATTCAAGTA ATGTACCCGA ATAGTAAATT CTTTTGTTTC GTTTCAAACA ACGGATTGAC   
  
  
- TAATCCTTAA GGGACATTAT GATGAATGGG ATTATGAAGT TATAATAAGT TCGGTTTCTG GTCGTCAAAG   
  
  
- AGGTTCATAA ATAAACGGAT ATTCCAATCA ATAGGTAAGT GTATGCTTTC TCTACTTTCG TTTTCCGTCC   
  
  
- TTTATGCCAC CATTGTAAAA GGAAGTACGT TTTCAAAATG AGCATGTCAA CCGAAAATCC GATGTTCCAC   
  
  
- AGGTGAGGAT TCTTTTTCAC AAATTAATTA CTTAATTAGT AATTTTTCTT TTTGGTCATA TAGGTTCAGT   
  
  
- TTTTACGTTA TTGTTTGCCG GTGGAAGTGG CAATCCCTTG GGCAGTTCAG TTGTTTCCGT TGCTACTAGG   
  
  
- TGCGTGGTGT AGATTGCCTC TTTAGTTGCC TCAAACAATG TGTCTCCTTT GTGGACTCAG TTAAGGTCAG   
  
  
- TTTCTGTATA TATGGGGGTT GTGCTGTCTG TATGTTGGGG AGAGAGAAAG GAAATTTCGT TTGGGAAAGG   
  
  
- TGAGAGAGAG AGAGAGAGAG GTAGAATTAG GTAAGGCTAT GAAAATTTTT TAGCCTTTTT AAAGTTGCCA   
  
  
- TGTATAGCGA AGGCGGCGTT TTATAGCCTG AAAAAAATTC ACAGTGGTGT GACAACAATG AAATGAGAGA   
  
  
- CAAAGACAGA GAGGAAACCT TTTTAGCCA

+     CAAT-box

| Site Name | Organism | Position | Strand | Matrix score. | sequence | function |
| --- | --- | --- | --- | --- | --- | --- |
| CAAT-box | Brassica rapa | 922 | - | 5 | CAAAT | common cis-acting element in promoter and enhancer regions |
| CAAT-box | Glycine max | 765 | + | 5 | CAATT | common cis-acting element in promoter and enhancer regions |
| CAAT-box | Glycine max | 551 | - | 5 | CAATT | common cis-acting element in promoter and enhancer regions |
| CAAT-box | Hordeum vulgare | 1127 | + | 4 | CAAT | common cis-acting element in promoter and enhancer regions |
| CAAT-box | Arabidopsis thaliana | 539 | - | 5 | CCAAT | common cis-acting element in promoter and enhancer regions |
| CAAT-box | Glycine max | 538 | - | 5 | CAATT | common cis-acting element in promoter and enhancer regions |
| CAAT-box | Glycine max | 550 | + | 5 | CAATT | common cis-acting element in promoter and enhancer regions |
| CAAT-box | Glycine max | 456 | + | 5 | CAATT | common cis-acting element in promoter and enhancer regions |
| CAAT-box | Arabidopsis thaliana | 390 | - | 5 | CCAAT | common cis-acting element in promoter and enhancer regions |
| CAAT-box | Hordeum vulgare | 72 | + | 4 | CAAT | common cis-acting element in promoter and enhancer regions |
| CAAT-box | Hordeum vulgare | 295 | + | 4 | CAAT | common cis-acting element in promoter and enhancer regions |
| CAAT-box | Hordeum vulgare | 14 | + | 4 | CAAT | common cis-acting element in promoter and enhancer regions |
| CAAT-box | Hordeum vulgare | 879 | + | 4 | CAAT | common cis-acting element in promoter and enhancer regions |
| CAAT-box | Arabidopsis thaliana | 552 | - | 5 | CCAAT | common cis-acting element in promoter and enhancer regions |
| CAAT-box | Glycine max | 1250 | + | 5 | CAATT | common cis-acting element in promoter and enhancer regions |
| CAAT-box | Hordeum vulgare | 30 | - | 4 | CAAT | common cis-acting element in promoter and enhancer regions |

> 2018/04/13 10:10:12  
+ GGTGTTGTTT GTACAATCAT ATGCGTGTCA TTGCGTACAA AACAAAAAAT ATGCTAGAGC ATCCATGAAA   
  
  
+ GCAATAATGA ATGGCAAAAC TACATAAAAG AAAAACAAAA CGCAGAAGCG GACCAACAAA ATTTAGAAGA   
  
  
+ AGTGAACGTG AACTATGGCG AAACAAAAAA AAAAAAAAAA CTTACAAAAA TGGAGAAAAG GGACAACCTT   
  
  
+ CTTCAAAAAA AAAAAAAAAA AAAAACTTAC AAAAATGGAG AAAAGGGACA ACCTTCTTCA AGCAGACGCT   
  
  
+ GCATAAGATA ACTTCAATCT CTCTCCAAGA GAGAGCGCCC TGAACACAGT TACTGTGCGC TTGAAAGCTC   
  
  
+ TCTAGCGGTC TATTAATGCT CACTAAAAGG ACTTTTTCTA TTGGTCATGA GGTTCATGCT TCCTACAAAA   
  
  
+ TTTCTTCCTA CCTAAATTAT TCCGCAGCTG CAGTTCAATT CATTAAAAAA GGTCTCGGTA GCATGGCTAT   
  
  
+ ACGTGTGGTC ACACTGGTCA GCTCTTTTTC CAACCCATGT GGGGGATAAT TGGAGCCCTC AATTGGGTCA   
  
  
+ TCGTTGAAAC TCAGAAGAAA CCAAAGTAAT AGCATCTGCT TCCCTGAAGT CATATCTTGT ACCCATCTAT   
  
  
+ GGGCACTCCA TGAAGATGCT GATCTGTGTC ATGCCATTCT TAAATTCATC AAAGGTTAAC CTAATTATTA   
  
  
+ CTTTGCAGAG TTTTCTTTTT CTTTCTATGC CCAAAAGTTT CCTACTTTAG CACTATAACC CCATCAATTC   
  
  
+ CACTGCTTCA TTAAGTTCAT TACATGGGCT TATCATTTAA GAAAACAAAG CAAAGTTTGT TGCCTAACTG   
  
  
+ ATTAGGAATT CCCTGTAATA CTACTTACCC TAATACTTCA ATATTATTCA AGCCAAAGAC CAGCAGTTTC   
  
  
+ TCCAAGTATT TATTTGCCTA TAAGGTTAGT TATCCATTCA CATACGAAAG AGATGAAAGC AAAAGGCAGG   
  
  
+ AAATACGGTG GTAACATTTT CCTTCATGCA AAAGTTTTAC TCGTACAGTT GGCTTTTAGG CTACAAGGTG   
  
  
+ TCCACTCCTA AGAAAAAGTG TTTAATTAAT GAATTAATCA TTAAAAAGAA AAACCAGTAT ATCCAAGTCA   
  
  
+ AAAATGCAAT AACAAACGGC CACCTTCACC GTTAGGGAAC CCGTCAAGTC AACAAAGGCA ACGATGATCC   
  
  
+ ACGCACCACA TCTAACGGAG AAATCAACGG AGTTTGTTAC ACAGAGGAAA CACCTGAGTC AATTCCAGTC   
  
  
+ AAAGACATAT ATACCCCCAA CACGACAGAC ATACAACCCC TCTCTCTTTC CTTTAAAGCA AACCCTTTCC   
  
  
+ ACTCTCTCTC TCTCTCTCTC CATCTTAATC CATTCCGATA CTTTTAAAAA ATCGGAAAAA TTTCAACGGT   
  
  
+ ACATATCGCT TCCGCCGCAA AATATCGGAC TTTTTTTAAG TGTCACCACA CTGTTGTTAC TTTACTCTCT   
  
  
+ GTTTCTGTCT CTCCTTTGGA AAAATCGGT  

- CCACAACAAA CATGTTAGTA TACGCACAGT AACGCATGTT TTGTTTTTTA TACGATCTCG TAGGTACTTT   
  
  
- CGTTATTACT TACCGTTTTG ATGTATTTTC TTTTTGTTTT GCGTCTTCGC CTGGTTGTTT TAAATCTTCT   
  
  
- TCACTTGCAC TTGATACCGC TTTGTTTTTT TTTTTTTTTT GAATGTTTTT ACCTCTTTTC CCTGTTGGAA   
  
  
- GAAGTTTTTT TTTTTTTTTT TTTTTGAATG TTTTTACCTC TTTTCCCTGT TGGAAGAAGT TCGTCTGCGA   
  
  
- CGTATTCTAT TGAAGTTAGA GAGAGGTTCT CTCTCGCGGG ACTTGTGTCA ATGACACGCG AACTTTCGAG   
  
  
- AGATCGCCAG ATAATTACGA GTGATTTTCC TGAAAAAGAT AACCAGTACT CCAAGTACGA AGGATGTTTT   
  
  
- AAAGAAGGAT GGATTTAATA AGGCGTCGAC GTCAAGTTAA GTAATTTTTT CCAGAGCCAT CGTACCGATA   
  
  
- TGCACACCAG TGTGACCAGT CGAGAAAAAG GTTGGGTACA CCCCCTATTA ACCTCGGGAG TTAACCCAGT   
  
  
- AGCAACTTTG AGTCTTCTTT GGTTTCATTA TCGTAGACGA AGGGACTTCA GTATAGAACA TGGGTAGATA   
  
  
- CCCGTGAGGT ACTTCTACGA CTAGACACAG TACGGTAAGA ATTTAAGTAG TTTCCAATTG GATTAATAAT   
  
  
- GAAACGTCTC AAAAGAAAAA GAAAGATACG GGTTTTCAAA GGATGAAATC GTGATATTGG GGTAGTTAAG   
  
  
- GTGACGAAGT AATTCAAGTA ATGTACCCGA ATAGTAAATT CTTTTGTTTC GTTTCAAACA ACGGATTGAC   
  
  
- TAATCCTTAA GGGACATTAT GATGAATGGG ATTATGAAGT TATAATAAGT TCGGTTTCTG GTCGTCAAAG   
  
  
- AGGTTCATAA ATAAACGGAT ATTCCAATCA ATAGGTAAGT GTATGCTTTC TCTACTTTCG TTTTCCGTCC   
  
  
- TTTATGCCAC CATTGTAAAA GGAAGTACGT TTTCAAAATG AGCATGTCAA CCGAAAATCC GATGTTCCAC   
  
  
- AGGTGAGGAT TCTTTTTCAC AAATTAATTA CTTAATTAGT AATTTTTCTT TTTGGTCATA TAGGTTCAGT   
  
  
- TTTTACGTTA TTGTTTGCCG GTGGAAGTGG CAATCCCTTG GGCAGTTCAG TTGTTTCCGT TGCTACTAGG   
  
  
- TGCGTGGTGT AGATTGCCTC TTTAGTTGCC TCAAACAATG TGTCTCCTTT GTGGACTCAG TTAAGGTCAG   
  
  
- TTTCTGTATA TATGGGGGTT GTGCTGTCTG TATGTTGGGG AGAGAGAAAG GAAATTTCGT TTGGGAAAGG   
  
  
- TGAGAGAGAG AGAGAGAGAG GTAGAATTAG GTAAGGCTAT GAAAATTTTT TAGCCTTTTT AAAGTTGCCA   
  
  
- TGTATAGCGA AGGCGGCGTT TTATAGCCTG AAAAAAATTC ACAGTGGTGT GACAACAATG AAATGAGAGA   
  
  
- CAAAGACAGA GAGGAAACCT TTTTAGCCA

+     CCAAT-box

| Site Name | Organism | Position | Strand | Matrix score. | sequence | function |
| --- | --- | --- | --- | --- | --- | --- |
| CCAAT-box | Hordeum vulgare | 1394 | + | 6 | CAACGG | MYBHv1 binding site |
| CCAAT-box | Hordeum vulgare | 1215 | + | 6 | CAACGG | MYBHv1 binding site |

> 2018/04/13 10:10:12  
+ GGTGTTGTTT GTACAATCAT ATGCGTGTCA TTGCGTACAA AACAAAAAAT ATGCTAGAGC ATCCATGAAA   
  
  
+ GCAATAATGA ATGGCAAAAC TACATAAAAG AAAAACAAAA CGCAGAAGCG GACCAACAAA ATTTAGAAGA   
  
  
+ AGTGAACGTG AACTATGGCG AAACAAAAAA AAAAAAAAAA CTTACAAAAA TGGAGAAAAG GGACAACCTT   
  
  
+ CTTCAAAAAA AAAAAAAAAA AAAAACTTAC AAAAATGGAG AAAAGGGACA ACCTTCTTCA AGCAGACGCT   
  
  
+ GCATAAGATA ACTTCAATCT CTCTCCAAGA GAGAGCGCCC TGAACACAGT TACTGTGCGC TTGAAAGCTC   
  
  
+ TCTAGCGGTC TATTAATGCT CACTAAAAGG ACTTTTTCTA TTGGTCATGA GGTTCATGCT TCCTACAAAA   
  
  
+ TTTCTTCCTA CCTAAATTAT TCCGCAGCTG CAGTTCAATT CATTAAAAAA GGTCTCGGTA GCATGGCTAT   
  
  
+ ACGTGTGGTC ACACTGGTCA GCTCTTTTTC CAACCCATGT GGGGGATAAT TGGAGCCCTC AATTGGGTCA   
  
  
+ TCGTTGAAAC TCAGAAGAAA CCAAAGTAAT AGCATCTGCT TCCCTGAAGT CATATCTTGT ACCCATCTAT   
  
  
+ GGGCACTCCA TGAAGATGCT GATCTGTGTC ATGCCATTCT TAAATTCATC AAAGGTTAAC CTAATTATTA   
  
  
+ CTTTGCAGAG TTTTCTTTTT CTTTCTATGC CCAAAAGTTT CCTACTTTAG CACTATAACC CCATCAATTC   
  
  
+ CACTGCTTCA TTAAGTTCAT TACATGGGCT TATCATTTAA GAAAACAAAG CAAAGTTTGT TGCCTAACTG   
  
  
+ ATTAGGAATT CCCTGTAATA CTACTTACCC TAATACTTCA ATATTATTCA AGCCAAAGAC CAGCAGTTTC   
  
  
+ TCCAAGTATT TATTTGCCTA TAAGGTTAGT TATCCATTCA CATACGAAAG AGATGAAAGC AAAAGGCAGG   
  
  
+ AAATACGGTG GTAACATTTT CCTTCATGCA AAAGTTTTAC TCGTACAGTT GGCTTTTAGG CTACAAGGTG   
  
  
+ TCCACTCCTA AGAAAAAGTG TTTAATTAAT GAATTAATCA TTAAAAAGAA AAACCAGTAT ATCCAAGTCA   
  
  
+ AAAATGCAAT AACAAACGGC CACCTTCACC GTTAGGGAAC CCGTCAAGTC AACAAAGGCA ACGATGATCC   
  
  
+ ACGCACCACA TCTAACGGAG AAATCAACGG AGTTTGTTAC ACAGAGGAAA CACCTGAGTC AATTCCAGTC   
  
  
+ AAAGACATAT ATACCCCCAA CACGACAGAC ATACAACCCC TCTCTCTTTC CTTTAAAGCA AACCCTTTCC   
  
  
+ ACTCTCTCTC TCTCTCTCTC CATCTTAATC CATTCCGATA CTTTTAAAAA ATCGGAAAAA TTTCAACGGT   
  
  
+ ACATATCGCT TCCGCCGCAA AATATCGGAC TTTTTTTAAG TGTCACCACA CTGTTGTTAC TTTACTCTCT   
  
  
+ GTTTCTGTCT CTCCTTTGGA AAAATCGGT  

- CCACAACAAA CATGTTAGTA TACGCACAGT AACGCATGTT TTGTTTTTTA TACGATCTCG TAGGTACTTT   
  
  
- CGTTATTACT TACCGTTTTG ATGTATTTTC TTTTTGTTTT GCGTCTTCGC CTGGTTGTTT TAAATCTTCT   
  
  
- TCACTTGCAC TTGATACCGC TTTGTTTTTT TTTTTTTTTT GAATGTTTTT ACCTCTTTTC CCTGTTGGAA   
  
  
- GAAGTTTTTT TTTTTTTTTT TTTTTGAATG TTTTTACCTC TTTTCCCTGT TGGAAGAAGT TCGTCTGCGA   
  
  
- CGTATTCTAT TGAAGTTAGA GAGAGGTTCT CTCTCGCGGG ACTTGTGTCA ATGACACGCG AACTTTCGAG   
  
  
- AGATCGCCAG ATAATTACGA GTGATTTTCC TGAAAAAGAT AACCAGTACT CCAAGTACGA AGGATGTTTT   
  
  
- AAAGAAGGAT GGATTTAATA AGGCGTCGAC GTCAAGTTAA GTAATTTTTT CCAGAGCCAT CGTACCGATA   
  
  
- TGCACACCAG TGTGACCAGT CGAGAAAAAG GTTGGGTACA CCCCCTATTA ACCTCGGGAG TTAACCCAGT   
  
  
- AGCAACTTTG AGTCTTCTTT GGTTTCATTA TCGTAGACGA AGGGACTTCA GTATAGAACA TGGGTAGATA   
  
  
- CCCGTGAGGT ACTTCTACGA CTAGACACAG TACGGTAAGA ATTTAAGTAG TTTCCAATTG GATTAATAAT   
  
  
- GAAACGTCTC AAAAGAAAAA GAAAGATACG GGTTTTCAAA GGATGAAATC GTGATATTGG GGTAGTTAAG   
  
  
- GTGACGAAGT AATTCAAGTA ATGTACCCGA ATAGTAAATT CTTTTGTTTC GTTTCAAACA ACGGATTGAC   
  
  
- TAATCCTTAA GGGACATTAT GATGAATGGG ATTATGAAGT TATAATAAGT TCGGTTTCTG GTCGTCAAAG   
  
  
- AGGTTCATAA ATAAACGGAT ATTCCAATCA ATAGGTAAGT GTATGCTTTC TCTACTTTCG TTTTCCGTCC   
  
  
- TTTATGCCAC CATTGTAAAA GGAAGTACGT TTTCAAAATG AGCATGTCAA CCGAAAATCC GATGTTCCAC   
  
  
- AGGTGAGGAT TCTTTTTCAC AAATTAATTA CTTAATTAGT AATTTTTCTT TTTGGTCATA TAGGTTCAGT   
  
  
- TTTTACGTTA TTGTTTGCCG GTGGAAGTGG CAATCCCTTG GGCAGTTCAG TTGTTTCCGT TGCTACTAGG   
  
  
- TGCGTGGTGT AGATTGCCTC TTTAGTTGCC TCAAACAATG TGTCTCCTTT GTGGACTCAG TTAAGGTCAG   
  
  
- TTTCTGTATA TATGGGGGTT GTGCTGTCTG TATGTTGGGG AGAGAGAAAG GAAATTTCGT TTGGGAAAGG   
  
  
- TGAGAGAGAG AGAGAGAGAG GTAGAATTAG GTAAGGCTAT GAAAATTTTT TAGCCTTTTT AAAGTTGCCA   
  
  
- TGTATAGCGA AGGCGGCGTT TTATAGCCTG AAAAAAATTC ACAGTGGTGT GACAACAATG AAATGAGAGA   
  
  
- CAAAGACAGA GAGGAAACCT TTTTAGCCA

+     CGTCA-motif

| Site Name | Organism | Position | Strand | Matrix score. | sequence | function |
| --- | --- | --- | --- | --- | --- | --- |
| CGTCA-motif | Hordeum vulgare | 1162 | + | 5 | CGTCA | cis-acting regulatory element involved in the MeJA-responsiveness |

> 2018/04/13 10:10:12  
+ GGTGTTGTTT GTACAATCAT ATGCGTGTCA TTGCGTACAA AACAAAAAAT ATGCTAGAGC ATCCATGAAA   
  
  
+ GCAATAATGA ATGGCAAAAC TACATAAAAG AAAAACAAAA CGCAGAAGCG GACCAACAAA ATTTAGAAGA   
  
  
+ AGTGAACGTG AACTATGGCG AAACAAAAAA AAAAAAAAAA CTTACAAAAA TGGAGAAAAG GGACAACCTT   
  
  
+ CTTCAAAAAA AAAAAAAAAA AAAAACTTAC AAAAATGGAG AAAAGGGACA ACCTTCTTCA AGCAGACGCT   
  
  
+ GCATAAGATA ACTTCAATCT CTCTCCAAGA GAGAGCGCCC TGAACACAGT TACTGTGCGC TTGAAAGCTC   
  
  
+ TCTAGCGGTC TATTAATGCT CACTAAAAGG ACTTTTTCTA TTGGTCATGA GGTTCATGCT TCCTACAAAA   
  
  
+ TTTCTTCCTA CCTAAATTAT TCCGCAGCTG CAGTTCAATT CATTAAAAAA GGTCTCGGTA GCATGGCTAT   
  
  
+ ACGTGTGGTC ACACTGGTCA GCTCTTTTTC CAACCCATGT GGGGGATAAT TGGAGCCCTC AATTGGGTCA   
  
  
+ TCGTTGAAAC TCAGAAGAAA CCAAAGTAAT AGCATCTGCT TCCCTGAAGT CATATCTTGT ACCCATCTAT   
  
  
+ GGGCACTCCA TGAAGATGCT GATCTGTGTC ATGCCATTCT TAAATTCATC AAAGGTTAAC CTAATTATTA   
  
  
+ CTTTGCAGAG TTTTCTTTTT CTTTCTATGC CCAAAAGTTT CCTACTTTAG CACTATAACC CCATCAATTC   
  
  
+ CACTGCTTCA TTAAGTTCAT TACATGGGCT TATCATTTAA GAAAACAAAG CAAAGTTTGT TGCCTAACTG   
  
  
+ ATTAGGAATT CCCTGTAATA CTACTTACCC TAATACTTCA ATATTATTCA AGCCAAAGAC CAGCAGTTTC   
  
  
+ TCCAAGTATT TATTTGCCTA TAAGGTTAGT TATCCATTCA CATACGAAAG AGATGAAAGC AAAAGGCAGG   
  
  
+ AAATACGGTG GTAACATTTT CCTTCATGCA AAAGTTTTAC TCGTACAGTT GGCTTTTAGG CTACAAGGTG   
  
  
+ TCCACTCCTA AGAAAAAGTG TTTAATTAAT GAATTAATCA TTAAAAAGAA AAACCAGTAT ATCCAAGTCA   
  
  
+ AAAATGCAAT AACAAACGGC CACCTTCACC GTTAGGGAAC CCGTCAAGTC AACAAAGGCA ACGATGATCC   
  
  
+ ACGCACCACA TCTAACGGAG AAATCAACGG AGTTTGTTAC ACAGAGGAAA CACCTGAGTC AATTCCAGTC   
  
  
+ AAAGACATAT ATACCCCCAA CACGACAGAC ATACAACCCC TCTCTCTTTC CTTTAAAGCA AACCCTTTCC   
  
  
+ ACTCTCTCTC TCTCTCTCTC CATCTTAATC CATTCCGATA CTTTTAAAAA ATCGGAAAAA TTTCAACGGT   
  
  
+ ACATATCGCT TCCGCCGCAA AATATCGGAC TTTTTTTAAG TGTCACCACA CTGTTGTTAC TTTACTCTCT   
  
  
+ GTTTCTGTCT CTCCTTTGGA AAAATCGGT  

- CCACAACAAA CATGTTAGTA TACGCACAGT AACGCATGTT TTGTTTTTTA TACGATCTCG TAGGTACTTT   
  
  
- CGTTATTACT TACCGTTTTG ATGTATTTTC TTTTTGTTTT GCGTCTTCGC CTGGTTGTTT TAAATCTTCT   
  
  
- TCACTTGCAC TTGATACCGC TTTGTTTTTT TTTTTTTTTT GAATGTTTTT ACCTCTTTTC CCTGTTGGAA   
  
  
- GAAGTTTTTT TTTTTTTTTT TTTTTGAATG TTTTTACCTC TTTTCCCTGT TGGAAGAAGT TCGTCTGCGA   
  
  
- CGTATTCTAT TGAAGTTAGA GAGAGGTTCT CTCTCGCGGG ACTTGTGTCA ATGACACGCG AACTTTCGAG   
  
  
- AGATCGCCAG ATAATTACGA GTGATTTTCC TGAAAAAGAT AACCAGTACT CCAAGTACGA AGGATGTTTT   
  
  
- AAAGAAGGAT GGATTTAATA AGGCGTCGAC GTCAAGTTAA GTAATTTTTT CCAGAGCCAT CGTACCGATA   
  
  
- TGCACACCAG TGTGACCAGT CGAGAAAAAG GTTGGGTACA CCCCCTATTA ACCTCGGGAG TTAACCCAGT   
  
  
- AGCAACTTTG AGTCTTCTTT GGTTTCATTA TCGTAGACGA AGGGACTTCA GTATAGAACA TGGGTAGATA   
  
  
- CCCGTGAGGT ACTTCTACGA CTAGACACAG TACGGTAAGA ATTTAAGTAG TTTCCAATTG GATTAATAAT   
  
  
- GAAACGTCTC AAAAGAAAAA GAAAGATACG GGTTTTCAAA GGATGAAATC GTGATATTGG GGTAGTTAAG   
  
  
- GTGACGAAGT AATTCAAGTA ATGTACCCGA ATAGTAAATT CTTTTGTTTC GTTTCAAACA ACGGATTGAC   
  
  
- TAATCCTTAA GGGACATTAT GATGAATGGG ATTATGAAGT TATAATAAGT TCGGTTTCTG GTCGTCAAAG   
  
  
- AGGTTCATAA ATAAACGGAT ATTCCAATCA ATAGGTAAGT GTATGCTTTC TCTACTTTCG TTTTCCGTCC   
  
  
- TTTATGCCAC CATTGTAAAA GGAAGTACGT TTTCAAAATG AGCATGTCAA CCGAAAATCC GATGTTCCAC   
  
  
- AGGTGAGGAT TCTTTTTCAC AAATTAATTA CTTAATTAGT AATTTTTCTT TTTGGTCATA TAGGTTCAGT   
  
  
- TTTTACGTTA TTGTTTGCCG GTGGAAGTGG CAATCCCTTG GGCAGTTCAG TTGTTTCCGT TGCTACTAGG   
  
  
- TGCGTGGTGT AGATTGCCTC TTTAGTTGCC TCAAACAATG TGTCTCCTTT GTGGACTCAG TTAAGGTCAG   
  
  
- TTTCTGTATA TATGGGGGTT GTGCTGTCTG TATGTTGGGG AGAGAGAAAG GAAATTTCGT TTGGGAAAGG   
  
  
- TGAGAGAGAG AGAGAGAGAG GTAGAATTAG GTAAGGCTAT GAAAATTTTT TAGCCTTTTT AAAGTTGCCA   
  
  
- TGTATAGCGA AGGCGGCGTT TTATAGCCTG AAAAAAATTC ACAGTGGTGT GACAACAATG AAATGAGAGA   
  
  
- CAAAGACAGA GAGGAAACCT TTTTAGCCA

+     G-Box

| Site Name | Organism | Position | Strand | Matrix score. | sequence | function |
| --- | --- | --- | --- | --- | --- | --- |
| G-Box | Antirrhinum majus | 490 | - | 6 | CACGTA | cis-acting regulatory element involved in light responsiveness |
| G-Box | Pisum sativum | 145 | - | 6 | CACGTT | cis-acting regulatory element involved in light responsiveness |

> 2018/04/13 10:10:12  
+ GGTGTTGTTT GTACAATCAT ATGCGTGTCA TTGCGTACAA AACAAAAAAT ATGCTAGAGC ATCCATGAAA   
  
  
+ GCAATAATGA ATGGCAAAAC TACATAAAAG AAAAACAAAA CGCAGAAGCG GACCAACAAA ATTTAGAAGA   
  
  
+ AGTGAACGTG AACTATGGCG AAACAAAAAA AAAAAAAAAA CTTACAAAAA TGGAGAAAAG GGACAACCTT   
  
  
+ CTTCAAAAAA AAAAAAAAAA AAAAACTTAC AAAAATGGAG AAAAGGGACA ACCTTCTTCA AGCAGACGCT   
  
  
+ GCATAAGATA ACTTCAATCT CTCTCCAAGA GAGAGCGCCC TGAACACAGT TACTGTGCGC TTGAAAGCTC   
  
  
+ TCTAGCGGTC TATTAATGCT CACTAAAAGG ACTTTTTCTA TTGGTCATGA GGTTCATGCT TCCTACAAAA   
  
  
+ TTTCTTCCTA CCTAAATTAT TCCGCAGCTG CAGTTCAATT CATTAAAAAA GGTCTCGGTA GCATGGCTAT   
  
  
+ ACGTGTGGTC ACACTGGTCA GCTCTTTTTC CAACCCATGT GGGGGATAAT TGGAGCCCTC AATTGGGTCA   
  
  
+ TCGTTGAAAC TCAGAAGAAA CCAAAGTAAT AGCATCTGCT TCCCTGAAGT CATATCTTGT ACCCATCTAT   
  
  
+ GGGCACTCCA TGAAGATGCT GATCTGTGTC ATGCCATTCT TAAATTCATC AAAGGTTAAC CTAATTATTA   
  
  
+ CTTTGCAGAG TTTTCTTTTT CTTTCTATGC CCAAAAGTTT CCTACTTTAG CACTATAACC CCATCAATTC   
  
  
+ CACTGCTTCA TTAAGTTCAT TACATGGGCT TATCATTTAA GAAAACAAAG CAAAGTTTGT TGCCTAACTG   
  
  
+ ATTAGGAATT CCCTGTAATA CTACTTACCC TAATACTTCA ATATTATTCA AGCCAAAGAC CAGCAGTTTC   
  
  
+ TCCAAGTATT TATTTGCCTA TAAGGTTAGT TATCCATTCA CATACGAAAG AGATGAAAGC AAAAGGCAGG   
  
  
+ AAATACGGTG GTAACATTTT CCTTCATGCA AAAGTTTTAC TCGTACAGTT GGCTTTTAGG CTACAAGGTG   
  
  
+ TCCACTCCTA AGAAAAAGTG TTTAATTAAT GAATTAATCA TTAAAAAGAA AAACCAGTAT ATCCAAGTCA   
  
  
+ AAAATGCAAT AACAAACGGC CACCTTCACC GTTAGGGAAC CCGTCAAGTC AACAAAGGCA ACGATGATCC   
  
  
+ ACGCACCACA TCTAACGGAG AAATCAACGG AGTTTGTTAC ACAGAGGAAA CACCTGAGTC AATTCCAGTC   
  
  
+ AAAGACATAT ATACCCCCAA CACGACAGAC ATACAACCCC TCTCTCTTTC CTTTAAAGCA AACCCTTTCC   
  
  
+ ACTCTCTCTC TCTCTCTCTC CATCTTAATC CATTCCGATA CTTTTAAAAA ATCGGAAAAA TTTCAACGGT   
  
  
+ ACATATCGCT TCCGCCGCAA AATATCGGAC TTTTTTTAAG TGTCACCACA CTGTTGTTAC TTTACTCTCT   
  
  
+ GTTTCTGTCT CTCCTTTGGA AAAATCGGT  

- CCACAACAAA CATGTTAGTA TACGCACAGT AACGCATGTT TTGTTTTTTA TACGATCTCG TAGGTACTTT   
  
  
- CGTTATTACT TACCGTTTTG ATGTATTTTC TTTTTGTTTT GCGTCTTCGC CTGGTTGTTT TAAATCTTCT   
  
  
- TCACTTGCAC TTGATACCGC TTTGTTTTTT TTTTTTTTTT GAATGTTTTT ACCTCTTTTC CCTGTTGGAA   
  
  
- GAAGTTTTTT TTTTTTTTTT TTTTTGAATG TTTTTACCTC TTTTCCCTGT TGGAAGAAGT TCGTCTGCGA   
  
  
- CGTATTCTAT TGAAGTTAGA GAGAGGTTCT CTCTCGCGGG ACTTGTGTCA ATGACACGCG AACTTTCGAG   
  
  
- AGATCGCCAG ATAATTACGA GTGATTTTCC TGAAAAAGAT AACCAGTACT CCAAGTACGA AGGATGTTTT   
  
  
- AAAGAAGGAT GGATTTAATA AGGCGTCGAC GTCAAGTTAA GTAATTTTTT CCAGAGCCAT CGTACCGATA   
  
  
- TGCACACCAG TGTGACCAGT CGAGAAAAAG GTTGGGTACA CCCCCTATTA ACCTCGGGAG TTAACCCAGT   
  
  
- AGCAACTTTG AGTCTTCTTT GGTTTCATTA TCGTAGACGA AGGGACTTCA GTATAGAACA TGGGTAGATA   
  
  
- CCCGTGAGGT ACTTCTACGA CTAGACACAG TACGGTAAGA ATTTAAGTAG TTTCCAATTG GATTAATAAT   
  
  
- GAAACGTCTC AAAAGAAAAA GAAAGATACG GGTTTTCAAA GGATGAAATC GTGATATTGG GGTAGTTAAG   
  
  
- GTGACGAAGT AATTCAAGTA ATGTACCCGA ATAGTAAATT CTTTTGTTTC GTTTCAAACA ACGGATTGAC   
  
  
- TAATCCTTAA GGGACATTAT GATGAATGGG ATTATGAAGT TATAATAAGT TCGGTTTCTG GTCGTCAAAG   
  
  
- AGGTTCATAA ATAAACGGAT ATTCCAATCA ATAGGTAAGT GTATGCTTTC TCTACTTTCG TTTTCCGTCC   
  
  
- TTTATGCCAC CATTGTAAAA GGAAGTACGT TTTCAAAATG AGCATGTCAA CCGAAAATCC GATGTTCCAC   
  
  
- AGGTGAGGAT TCTTTTTCAC AAATTAATTA CTTAATTAGT AATTTTTCTT TTTGGTCATA TAGGTTCAGT   
  
  
- TTTTACGTTA TTGTTTGCCG GTGGAAGTGG CAATCCCTTG GGCAGTTCAG TTGTTTCCGT TGCTACTAGG   
  
  
- TGCGTGGTGT AGATTGCCTC TTTAGTTGCC TCAAACAATG TGTCTCCTTT GTGGACTCAG TTAAGGTCAG   
  
  
- TTTCTGTATA TATGGGGGTT GTGCTGTCTG TATGTTGGGG AGAGAGAAAG GAAATTTCGT TTGGGAAAGG   
  
  
- TGAGAGAGAG AGAGAGAGAG GTAGAATTAG GTAAGGCTAT GAAAATTTTT TAGCCTTTTT AAAGTTGCCA   
  
  
- TGTATAGCGA AGGCGGCGTT TTATAGCCTG AAAAAAATTC ACAGTGGTGT GACAACAATG AAATGAGAGA   
  
  
- CAAAGACAGA GAGGAAACCT TTTTAGCCA

+     G-box

| Site Name | Organism | Position | Strand | Matrix score. | sequence | function |
| --- | --- | --- | --- | --- | --- | --- |
| G-box | Zea mays | 1281 | + | 6 | CACGAC | cis-acting regulatory element involved in light responsiveness |
| G-box | Daucus carota | 490 | + | 6 | TACGTG | cis-acting regulatory element involved in light responsiveness |
| G-box | Solanum tuberosum | 525 | - | 7 | CACATGG | cis-acting regulatory element involved in light responsiveness |
| G-box | Zea mays | 145 | - | 6 | CACGTT | cis-acting regulatory element involved in light responsiveness |

> 2018/04/13 10:10:12  
+ GGTGTTGTTT GTACAATCAT ATGCGTGTCA TTGCGTACAA AACAAAAAAT ATGCTAGAGC ATCCATGAAA   
  
  
+ GCAATAATGA ATGGCAAAAC TACATAAAAG AAAAACAAAA CGCAGAAGCG GACCAACAAA ATTTAGAAGA   
  
  
+ AGTGAACGTG AACTATGGCG AAACAAAAAA AAAAAAAAAA CTTACAAAAA TGGAGAAAAG GGACAACCTT   
  
  
+ CTTCAAAAAA AAAAAAAAAA AAAAACTTAC AAAAATGGAG AAAAGGGACA ACCTTCTTCA AGCAGACGCT   
  
  
+ GCATAAGATA ACTTCAATCT CTCTCCAAGA GAGAGCGCCC TGAACACAGT TACTGTGCGC TTGAAAGCTC   
  
  
+ TCTAGCGGTC TATTAATGCT CACTAAAAGG ACTTTTTCTA TTGGTCATGA GGTTCATGCT TCCTACAAAA   
  
  
+ TTTCTTCCTA CCTAAATTAT TCCGCAGCTG CAGTTCAATT CATTAAAAAA GGTCTCGGTA GCATGGCTAT   
  
  
+ ACGTGTGGTC ACACTGGTCA GCTCTTTTTC CAACCCATGT GGGGGATAAT TGGAGCCCTC AATTGGGTCA   
  
  
+ TCGTTGAAAC TCAGAAGAAA CCAAAGTAAT AGCATCTGCT TCCCTGAAGT CATATCTTGT ACCCATCTAT   
  
  
+ GGGCACTCCA TGAAGATGCT GATCTGTGTC ATGCCATTCT TAAATTCATC AAAGGTTAAC CTAATTATTA   
  
  
+ CTTTGCAGAG TTTTCTTTTT CTTTCTATGC CCAAAAGTTT CCTACTTTAG CACTATAACC CCATCAATTC   
  
  
+ CACTGCTTCA TTAAGTTCAT TACATGGGCT TATCATTTAA GAAAACAAAG CAAAGTTTGT TGCCTAACTG   
  
  
+ ATTAGGAATT CCCTGTAATA CTACTTACCC TAATACTTCA ATATTATTCA AGCCAAAGAC CAGCAGTTTC   
  
  
+ TCCAAGTATT TATTTGCCTA TAAGGTTAGT TATCCATTCA CATACGAAAG AGATGAAAGC AAAAGGCAGG   
  
  
+ AAATACGGTG GTAACATTTT CCTTCATGCA AAAGTTTTAC TCGTACAGTT GGCTTTTAGG CTACAAGGTG   
  
  
+ TCCACTCCTA AGAAAAAGTG TTTAATTAAT GAATTAATCA TTAAAAAGAA AAACCAGTAT ATCCAAGTCA   
  
  
+ AAAATGCAAT AACAAACGGC CACCTTCACC GTTAGGGAAC CCGTCAAGTC AACAAAGGCA ACGATGATCC   
  
  
+ ACGCACCACA TCTAACGGAG AAATCAACGG AGTTTGTTAC ACAGAGGAAA CACCTGAGTC AATTCCAGTC   
  
  
+ AAAGACATAT ATACCCCCAA CACGACAGAC ATACAACCCC TCTCTCTTTC CTTTAAAGCA AACCCTTTCC   
  
  
+ ACTCTCTCTC TCTCTCTCTC CATCTTAATC CATTCCGATA CTTTTAAAAA ATCGGAAAAA TTTCAACGGT   
  
  
+ ACATATCGCT TCCGCCGCAA AATATCGGAC TTTTTTTAAG TGTCACCACA CTGTTGTTAC TTTACTCTCT   
  
  
+ GTTTCTGTCT CTCCTTTGGA AAAATCGGT  

- CCACAACAAA CATGTTAGTA TACGCACAGT AACGCATGTT TTGTTTTTTA TACGATCTCG TAGGTACTTT   
  
  
- CGTTATTACT TACCGTTTTG ATGTATTTTC TTTTTGTTTT GCGTCTTCGC CTGGTTGTTT TAAATCTTCT   
  
  
- TCACTTGCAC TTGATACCGC TTTGTTTTTT TTTTTTTTTT GAATGTTTTT ACCTCTTTTC CCTGTTGGAA   
  
  
- GAAGTTTTTT TTTTTTTTTT TTTTTGAATG TTTTTACCTC TTTTCCCTGT TGGAAGAAGT TCGTCTGCGA   
  
  
- CGTATTCTAT TGAAGTTAGA GAGAGGTTCT CTCTCGCGGG ACTTGTGTCA ATGACACGCG AACTTTCGAG   
  
  
- AGATCGCCAG ATAATTACGA GTGATTTTCC TGAAAAAGAT AACCAGTACT CCAAGTACGA AGGATGTTTT   
  
  
- AAAGAAGGAT GGATTTAATA AGGCGTCGAC GTCAAGTTAA GTAATTTTTT CCAGAGCCAT CGTACCGATA   
  
  
- TGCACACCAG TGTGACCAGT CGAGAAAAAG GTTGGGTACA CCCCCTATTA ACCTCGGGAG TTAACCCAGT   
  
  
- AGCAACTTTG AGTCTTCTTT GGTTTCATTA TCGTAGACGA AGGGACTTCA GTATAGAACA TGGGTAGATA   
  
  
- CCCGTGAGGT ACTTCTACGA CTAGACACAG TACGGTAAGA ATTTAAGTAG TTTCCAATTG GATTAATAAT   
  
  
- GAAACGTCTC AAAAGAAAAA GAAAGATACG GGTTTTCAAA GGATGAAATC GTGATATTGG GGTAGTTAAG   
  
  
- GTGACGAAGT AATTCAAGTA ATGTACCCGA ATAGTAAATT CTTTTGTTTC GTTTCAAACA ACGGATTGAC   
  
  
- TAATCCTTAA GGGACATTAT GATGAATGGG ATTATGAAGT TATAATAAGT TCGGTTTCTG GTCGTCAAAG   
  
  
- AGGTTCATAA ATAAACGGAT ATTCCAATCA ATAGGTAAGT GTATGCTTTC TCTACTTTCG TTTTCCGTCC   
  
  
- TTTATGCCAC CATTGTAAAA GGAAGTACGT TTTCAAAATG AGCATGTCAA CCGAAAATCC GATGTTCCAC   
  
  
- AGGTGAGGAT TCTTTTTCAC AAATTAATTA CTTAATTAGT AATTTTTCTT TTTGGTCATA TAGGTTCAGT   
  
  
- TTTTACGTTA TTGTTTGCCG GTGGAAGTGG CAATCCCTTG GGCAGTTCAG TTGTTTCCGT TGCTACTAGG   
  
  
- TGCGTGGTGT AGATTGCCTC TTTAGTTGCC TCAAACAATG TGTCTCCTTT GTGGACTCAG TTAAGGTCAG   
  
  
- TTTCTGTATA TATGGGGGTT GTGCTGTCTG TATGTTGGGG AGAGAGAAAG GAAATTTCGT TTGGGAAAGG   
  
  
- TGAGAGAGAG AGAGAGAGAG GTAGAATTAG GTAAGGCTAT GAAAATTTTT TAGCCTTTTT AAAGTTGCCA   
  
  
- TGTATAGCGA AGGCGGCGTT TTATAGCCTG AAAAAAATTC ACAGTGGTGT GACAACAATG AAATGAGAGA   
  
  
- CAAAGACAGA GAGGAAACCT TTTTAGCCA

+     GAG-motif

| Site Name | Organism | Position | Strand | Matrix score. | sequence | function |
| --- | --- | --- | --- | --- | --- | --- |
| GAG-motif | Arabidopsis thaliana | 1464 | - | 7 | AGAGAGT | part of a light responsive element |
| GAG-motif | Arabidopsis thaliana | 1331 | - | 7 | AGAGAGT | part of a light responsive element |
| GAG-motif | Spinacia oleracea | 959 | + | 7 | AGAGATG | part of a light responsive element |

> 2018/04/13 10:10:12  
+ GGTGTTGTTT GTACAATCAT ATGCGTGTCA TTGCGTACAA AACAAAAAAT ATGCTAGAGC ATCCATGAAA   
  
  
+ GCAATAATGA ATGGCAAAAC TACATAAAAG AAAAACAAAA CGCAGAAGCG GACCAACAAA ATTTAGAAGA   
  
  
+ AGTGAACGTG AACTATGGCG AAACAAAAAA AAAAAAAAAA CTTACAAAAA TGGAGAAAAG GGACAACCTT   
  
  
+ CTTCAAAAAA AAAAAAAAAA AAAAACTTAC AAAAATGGAG AAAAGGGACA ACCTTCTTCA AGCAGACGCT   
  
  
+ GCATAAGATA ACTTCAATCT CTCTCCAAGA GAGAGCGCCC TGAACACAGT TACTGTGCGC TTGAAAGCTC   
  
  
+ TCTAGCGGTC TATTAATGCT CACTAAAAGG ACTTTTTCTA TTGGTCATGA GGTTCATGCT TCCTACAAAA   
  
  
+ TTTCTTCCTA CCTAAATTAT TCCGCAGCTG CAGTTCAATT CATTAAAAAA GGTCTCGGTA GCATGGCTAT   
  
  
+ ACGTGTGGTC ACACTGGTCA GCTCTTTTTC CAACCCATGT GGGGGATAAT TGGAGCCCTC AATTGGGTCA   
  
  
+ TCGTTGAAAC TCAGAAGAAA CCAAAGTAAT AGCATCTGCT TCCCTGAAGT CATATCTTGT ACCCATCTAT   
  
  
+ GGGCACTCCA TGAAGATGCT GATCTGTGTC ATGCCATTCT TAAATTCATC AAAGGTTAAC CTAATTATTA   
  
  
+ CTTTGCAGAG TTTTCTTTTT CTTTCTATGC CCAAAAGTTT CCTACTTTAG CACTATAACC CCATCAATTC   
  
  
+ CACTGCTTCA TTAAGTTCAT TACATGGGCT TATCATTTAA GAAAACAAAG CAAAGTTTGT TGCCTAACTG   
  
  
+ ATTAGGAATT CCCTGTAATA CTACTTACCC TAATACTTCA ATATTATTCA AGCCAAAGAC CAGCAGTTTC   
  
  
+ TCCAAGTATT TATTTGCCTA TAAGGTTAGT TATCCATTCA CATACGAAAG AGATGAAAGC AAAAGGCAGG   
  
  
+ AAATACGGTG GTAACATTTT CCTTCATGCA AAAGTTTTAC TCGTACAGTT GGCTTTTAGG CTACAAGGTG   
  
  
+ TCCACTCCTA AGAAAAAGTG TTTAATTAAT GAATTAATCA TTAAAAAGAA AAACCAGTAT ATCCAAGTCA   
  
  
+ AAAATGCAAT AACAAACGGC CACCTTCACC GTTAGGGAAC CCGTCAAGTC AACAAAGGCA ACGATGATCC   
  
  
+ ACGCACCACA TCTAACGGAG AAATCAACGG AGTTTGTTAC ACAGAGGAAA CACCTGAGTC AATTCCAGTC   
  
  
+ AAAGACATAT ATACCCCCAA CACGACAGAC ATACAACCCC TCTCTCTTTC CTTTAAAGCA AACCCTTTCC   
  
  
+ ACTCTCTCTC TCTCTCTCTC CATCTTAATC CATTCCGATA CTTTTAAAAA ATCGGAAAAA TTTCAACGGT   
  
  
+ ACATATCGCT TCCGCCGCAA AATATCGGAC TTTTTTTAAG TGTCACCACA CTGTTGTTAC TTTACTCTCT   
  
  
+ GTTTCTGTCT CTCCTTTGGA AAAATCGGT  

- CCACAACAAA CATGTTAGTA TACGCACAGT AACGCATGTT TTGTTTTTTA TACGATCTCG TAGGTACTTT   
  
  
- CGTTATTACT TACCGTTTTG ATGTATTTTC TTTTTGTTTT GCGTCTTCGC CTGGTTGTTT TAAATCTTCT   
  
  
- TCACTTGCAC TTGATACCGC TTTGTTTTTT TTTTTTTTTT GAATGTTTTT ACCTCTTTTC CCTGTTGGAA   
  
  
- GAAGTTTTTT TTTTTTTTTT TTTTTGAATG TTTTTACCTC TTTTCCCTGT TGGAAGAAGT TCGTCTGCGA   
  
  
- CGTATTCTAT TGAAGTTAGA GAGAGGTTCT CTCTCGCGGG ACTTGTGTCA ATGACACGCG AACTTTCGAG   
  
  
- AGATCGCCAG ATAATTACGA GTGATTTTCC TGAAAAAGAT AACCAGTACT CCAAGTACGA AGGATGTTTT   
  
  
- AAAGAAGGAT GGATTTAATA AGGCGTCGAC GTCAAGTTAA GTAATTTTTT CCAGAGCCAT CGTACCGATA   
  
  
- TGCACACCAG TGTGACCAGT CGAGAAAAAG GTTGGGTACA CCCCCTATTA ACCTCGGGAG TTAACCCAGT   
  
  
- AGCAACTTTG AGTCTTCTTT GGTTTCATTA TCGTAGACGA AGGGACTTCA GTATAGAACA TGGGTAGATA   
  
  
- CCCGTGAGGT ACTTCTACGA CTAGACACAG TACGGTAAGA ATTTAAGTAG TTTCCAATTG GATTAATAAT   
  
  
- GAAACGTCTC AAAAGAAAAA GAAAGATACG GGTTTTCAAA GGATGAAATC GTGATATTGG GGTAGTTAAG   
  
  
- GTGACGAAGT AATTCAAGTA ATGTACCCGA ATAGTAAATT CTTTTGTTTC GTTTCAAACA ACGGATTGAC   
  
  
- TAATCCTTAA GGGACATTAT GATGAATGGG ATTATGAAGT TATAATAAGT TCGGTTTCTG GTCGTCAAAG   
  
  
- AGGTTCATAA ATAAACGGAT ATTCCAATCA ATAGGTAAGT GTATGCTTTC TCTACTTTCG TTTTCCGTCC   
  
  
- TTTATGCCAC CATTGTAAAA GGAAGTACGT TTTCAAAATG AGCATGTCAA CCGAAAATCC GATGTTCCAC   
  
  
- AGGTGAGGAT TCTTTTTCAC AAATTAATTA CTTAATTAGT AATTTTTCTT TTTGGTCATA TAGGTTCAGT   
  
  
- TTTTACGTTA TTGTTTGCCG GTGGAAGTGG CAATCCCTTG GGCAGTTCAG TTGTTTCCGT TGCTACTAGG   
  
  
- TGCGTGGTGT AGATTGCCTC TTTAGTTGCC TCAAACAATG TGTCTCCTTT GTGGACTCAG TTAAGGTCAG   
  
  
- TTTCTGTATA TATGGGGGTT GTGCTGTCTG TATGTTGGGG AGAGAGAAAG GAAATTTCGT TTGGGAAAGG   
  
  
- TGAGAGAGAG AGAGAGAGAG GTAGAATTAG GTAAGGCTAT GAAAATTTTT TAGCCTTTTT AAAGTTGCCA   
  
  
- TGTATAGCGA AGGCGGCGTT TTATAGCCTG AAAAAAATTC ACAGTGGTGT GACAACAATG AAATGAGAGA   
  
  
- CAAAGACAGA GAGGAAACCT TTTTAGCCA

+     GARE-motif

| Site Name | Organism | Position | Strand | Matrix score. | sequence | function |
| --- | --- | --- | --- | --- | --- | --- |
| GARE-motif | Brassica oleracea | 1468 | - | 7 | AAACAGA | gibberellin-responsive element |

> 2018/04/13 10:10:12  
+ GGTGTTGTTT GTACAATCAT ATGCGTGTCA TTGCGTACAA AACAAAAAAT ATGCTAGAGC ATCCATGAAA   
  
  
+ GCAATAATGA ATGGCAAAAC TACATAAAAG AAAAACAAAA CGCAGAAGCG GACCAACAAA ATTTAGAAGA   
  
  
+ AGTGAACGTG AACTATGGCG AAACAAAAAA AAAAAAAAAA CTTACAAAAA TGGAGAAAAG GGACAACCTT   
  
  
+ CTTCAAAAAA AAAAAAAAAA AAAAACTTAC AAAAATGGAG AAAAGGGACA ACCTTCTTCA AGCAGACGCT   
  
  
+ GCATAAGATA ACTTCAATCT CTCTCCAAGA GAGAGCGCCC TGAACACAGT TACTGTGCGC TTGAAAGCTC   
  
  
+ TCTAGCGGTC TATTAATGCT CACTAAAAGG ACTTTTTCTA TTGGTCATGA GGTTCATGCT TCCTACAAAA   
  
  
+ TTTCTTCCTA CCTAAATTAT TCCGCAGCTG CAGTTCAATT CATTAAAAAA GGTCTCGGTA GCATGGCTAT   
  
  
+ ACGTGTGGTC ACACTGGTCA GCTCTTTTTC CAACCCATGT GGGGGATAAT TGGAGCCCTC AATTGGGTCA   
  
  
+ TCGTTGAAAC TCAGAAGAAA CCAAAGTAAT AGCATCTGCT TCCCTGAAGT CATATCTTGT ACCCATCTAT   
  
  
+ GGGCACTCCA TGAAGATGCT GATCTGTGTC ATGCCATTCT TAAATTCATC AAAGGTTAAC CTAATTATTA   
  
  
+ CTTTGCAGAG TTTTCTTTTT CTTTCTATGC CCAAAAGTTT CCTACTTTAG CACTATAACC CCATCAATTC   
  
  
+ CACTGCTTCA TTAAGTTCAT TACATGGGCT TATCATTTAA GAAAACAAAG CAAAGTTTGT TGCCTAACTG   
  
  
+ ATTAGGAATT CCCTGTAATA CTACTTACCC TAATACTTCA ATATTATTCA AGCCAAAGAC CAGCAGTTTC   
  
  
+ TCCAAGTATT TATTTGCCTA TAAGGTTAGT TATCCATTCA CATACGAAAG AGATGAAAGC AAAAGGCAGG   
  
  
+ AAATACGGTG GTAACATTTT CCTTCATGCA AAAGTTTTAC TCGTACAGTT GGCTTTTAGG CTACAAGGTG   
  
  
+ TCCACTCCTA AGAAAAAGTG TTTAATTAAT GAATTAATCA TTAAAAAGAA AAACCAGTAT ATCCAAGTCA   
  
  
+ AAAATGCAAT AACAAACGGC CACCTTCACC GTTAGGGAAC CCGTCAAGTC AACAAAGGCA ACGATGATCC   
  
  
+ ACGCACCACA TCTAACGGAG AAATCAACGG AGTTTGTTAC ACAGAGGAAA CACCTGAGTC AATTCCAGTC   
  
  
+ AAAGACATAT ATACCCCCAA CACGACAGAC ATACAACCCC TCTCTCTTTC CTTTAAAGCA AACCCTTTCC   
  
  
+ ACTCTCTCTC TCTCTCTCTC CATCTTAATC CATTCCGATA CTTTTAAAAA ATCGGAAAAA TTTCAACGGT   
  
  
+ ACATATCGCT TCCGCCGCAA AATATCGGAC TTTTTTTAAG TGTCACCACA CTGTTGTTAC TTTACTCTCT   
  
  
+ GTTTCTGTCT CTCCTTTGGA AAAATCGGT  

- CCACAACAAA CATGTTAGTA TACGCACAGT AACGCATGTT TTGTTTTTTA TACGATCTCG TAGGTACTTT   
  
  
- CGTTATTACT TACCGTTTTG ATGTATTTTC TTTTTGTTTT GCGTCTTCGC CTGGTTGTTT TAAATCTTCT   
  
  
- TCACTTGCAC TTGATACCGC TTTGTTTTTT TTTTTTTTTT GAATGTTTTT ACCTCTTTTC CCTGTTGGAA   
  
  
- GAAGTTTTTT TTTTTTTTTT TTTTTGAATG TTTTTACCTC TTTTCCCTGT TGGAAGAAGT TCGTCTGCGA   
  
  
- CGTATTCTAT TGAAGTTAGA GAGAGGTTCT CTCTCGCGGG ACTTGTGTCA ATGACACGCG AACTTTCGAG   
  
  
- AGATCGCCAG ATAATTACGA GTGATTTTCC TGAAAAAGAT AACCAGTACT CCAAGTACGA AGGATGTTTT   
  
  
- AAAGAAGGAT GGATTTAATA AGGCGTCGAC GTCAAGTTAA GTAATTTTTT CCAGAGCCAT CGTACCGATA   
  
  
- TGCACACCAG TGTGACCAGT CGAGAAAAAG GTTGGGTACA CCCCCTATTA ACCTCGGGAG TTAACCCAGT   
  
  
- AGCAACTTTG AGTCTTCTTT GGTTTCATTA TCGTAGACGA AGGGACTTCA GTATAGAACA TGGGTAGATA   
  
  
- CCCGTGAGGT ACTTCTACGA CTAGACACAG TACGGTAAGA ATTTAAGTAG TTTCCAATTG GATTAATAAT   
  
  
- GAAACGTCTC AAAAGAAAAA GAAAGATACG GGTTTTCAAA GGATGAAATC GTGATATTGG GGTAGTTAAG   
  
  
- GTGACGAAGT AATTCAAGTA ATGTACCCGA ATAGTAAATT CTTTTGTTTC GTTTCAAACA ACGGATTGAC   
  
  
- TAATCCTTAA GGGACATTAT GATGAATGGG ATTATGAAGT TATAATAAGT TCGGTTTCTG GTCGTCAAAG   
  
  
- AGGTTCATAA ATAAACGGAT ATTCCAATCA ATAGGTAAGT GTATGCTTTC TCTACTTTCG TTTTCCGTCC   
  
  
- TTTATGCCAC CATTGTAAAA GGAAGTACGT TTTCAAAATG AGCATGTCAA CCGAAAATCC GATGTTCCAC   
  
  
- AGGTGAGGAT TCTTTTTCAC AAATTAATTA CTTAATTAGT AATTTTTCTT TTTGGTCATA TAGGTTCAGT   
  
  
- TTTTACGTTA TTGTTTGCCG GTGGAAGTGG CAATCCCTTG GGCAGTTCAG TTGTTTCCGT TGCTACTAGG   
  
  
- TGCGTGGTGT AGATTGCCTC TTTAGTTGCC TCAAACAATG TGTCTCCTTT GTGGACTCAG TTAAGGTCAG   
  
  
- TTTCTGTATA TATGGGGGTT GTGCTGTCTG TATGTTGGGG AGAGAGAAAG GAAATTTCGT TTGGGAAAGG   
  
  
- TGAGAGAGAG AGAGAGAGAG GTAGAATTAG GTAAGGCTAT GAAAATTTTT TAGCCTTTTT AAAGTTGCCA   
  
  
- TGTATAGCGA AGGCGGCGTT TTATAGCCTG AAAAAAATTC ACAGTGGTGT GACAACAATG AAATGAGAGA   
  
  
- CAAAGACAGA GAGGAAACCT TTTTAGCCA

+     GCN4\_motif

| Site Name | Organism | Position | Strand | Matrix score. | sequence | function |
| --- | --- | --- | --- | --- | --- | --- |
| GCN4\_motif | Oryza sativa | 889 | + | 7 | CAAGCCA | cis-regulatory element involved in endosperm expression |
| GCN4\_motif | Oryza sativa | 655 | + | 7 | TGTGTCA | cis-regulatory element involved in endosperm expression |
| GCN4\_motif | Oryza sativa | 1245 | + | 7 | TGAGTCA | cis-regulatory element involved in endosperm expression |

> 2018/04/13 10:10:12  
+ GGTGTTGTTT GTACAATCAT ATGCGTGTCA TTGCGTACAA AACAAAAAAT ATGCTAGAGC ATCCATGAAA   
  
  
+ GCAATAATGA ATGGCAAAAC TACATAAAAG AAAAACAAAA CGCAGAAGCG GACCAACAAA ATTTAGAAGA   
  
  
+ AGTGAACGTG AACTATGGCG AAACAAAAAA AAAAAAAAAA CTTACAAAAA TGGAGAAAAG GGACAACCTT   
  
  
+ CTTCAAAAAA AAAAAAAAAA AAAAACTTAC AAAAATGGAG AAAAGGGACA ACCTTCTTCA AGCAGACGCT   
  
  
+ GCATAAGATA ACTTCAATCT CTCTCCAAGA GAGAGCGCCC TGAACACAGT TACTGTGCGC TTGAAAGCTC   
  
  
+ TCTAGCGGTC TATTAATGCT CACTAAAAGG ACTTTTTCTA TTGGTCATGA GGTTCATGCT TCCTACAAAA   
  
  
+ TTTCTTCCTA CCTAAATTAT TCCGCAGCTG CAGTTCAATT CATTAAAAAA GGTCTCGGTA GCATGGCTAT   
  
  
+ ACGTGTGGTC ACACTGGTCA GCTCTTTTTC CAACCCATGT GGGGGATAAT TGGAGCCCTC AATTGGGTCA   
  
  
+ TCGTTGAAAC TCAGAAGAAA CCAAAGTAAT AGCATCTGCT TCCCTGAAGT CATATCTTGT ACCCATCTAT   
  
  
+ GGGCACTCCA TGAAGATGCT GATCTGTGTC ATGCCATTCT TAAATTCATC AAAGGTTAAC CTAATTATTA   
  
  
+ CTTTGCAGAG TTTTCTTTTT CTTTCTATGC CCAAAAGTTT CCTACTTTAG CACTATAACC CCATCAATTC   
  
  
+ CACTGCTTCA TTAAGTTCAT TACATGGGCT TATCATTTAA GAAAACAAAG CAAAGTTTGT TGCCTAACTG   
  
  
+ ATTAGGAATT CCCTGTAATA CTACTTACCC TAATACTTCA ATATTATTCA AGCCAAAGAC CAGCAGTTTC   
  
  
+ TCCAAGTATT TATTTGCCTA TAAGGTTAGT TATCCATTCA CATACGAAAG AGATGAAAGC AAAAGGCAGG   
  
  
+ AAATACGGTG GTAACATTTT CCTTCATGCA AAAGTTTTAC TCGTACAGTT GGCTTTTAGG CTACAAGGTG   
  
  
+ TCCACTCCTA AGAAAAAGTG TTTAATTAAT GAATTAATCA TTAAAAAGAA AAACCAGTAT ATCCAAGTCA   
  
  
+ AAAATGCAAT AACAAACGGC CACCTTCACC GTTAGGGAAC CCGTCAAGTC AACAAAGGCA ACGATGATCC   
  
  
+ ACGCACCACA TCTAACGGAG AAATCAACGG AGTTTGTTAC ACAGAGGAAA CACCTGAGTC AATTCCAGTC   
  
  
+ AAAGACATAT ATACCCCCAA CACGACAGAC ATACAACCCC TCTCTCTTTC CTTTAAAGCA AACCCTTTCC   
  
  
+ ACTCTCTCTC TCTCTCTCTC CATCTTAATC CATTCCGATA CTTTTAAAAA ATCGGAAAAA TTTCAACGGT   
  
  
+ ACATATCGCT TCCGCCGCAA AATATCGGAC TTTTTTTAAG TGTCACCACA CTGTTGTTAC TTTACTCTCT   
  
  
+ GTTTCTGTCT CTCCTTTGGA AAAATCGGT  

- CCACAACAAA CATGTTAGTA TACGCACAGT AACGCATGTT TTGTTTTTTA TACGATCTCG TAGGTACTTT   
  
  
- CGTTATTACT TACCGTTTTG ATGTATTTTC TTTTTGTTTT GCGTCTTCGC CTGGTTGTTT TAAATCTTCT   
  
  
- TCACTTGCAC TTGATACCGC TTTGTTTTTT TTTTTTTTTT GAATGTTTTT ACCTCTTTTC CCTGTTGGAA   
  
  
- GAAGTTTTTT TTTTTTTTTT TTTTTGAATG TTTTTACCTC TTTTCCCTGT TGGAAGAAGT TCGTCTGCGA   
  
  
- CGTATTCTAT TGAAGTTAGA GAGAGGTTCT CTCTCGCGGG ACTTGTGTCA ATGACACGCG AACTTTCGAG   
  
  
- AGATCGCCAG ATAATTACGA GTGATTTTCC TGAAAAAGAT AACCAGTACT CCAAGTACGA AGGATGTTTT   
  
  
- AAAGAAGGAT GGATTTAATA AGGCGTCGAC GTCAAGTTAA GTAATTTTTT CCAGAGCCAT CGTACCGATA   
  
  
- TGCACACCAG TGTGACCAGT CGAGAAAAAG GTTGGGTACA CCCCCTATTA ACCTCGGGAG TTAACCCAGT   
  
  
- AGCAACTTTG AGTCTTCTTT GGTTTCATTA TCGTAGACGA AGGGACTTCA GTATAGAACA TGGGTAGATA   
  
  
- CCCGTGAGGT ACTTCTACGA CTAGACACAG TACGGTAAGA ATTTAAGTAG TTTCCAATTG GATTAATAAT   
  
  
- GAAACGTCTC AAAAGAAAAA GAAAGATACG GGTTTTCAAA GGATGAAATC GTGATATTGG GGTAGTTAAG   
  
  
- GTGACGAAGT AATTCAAGTA ATGTACCCGA ATAGTAAATT CTTTTGTTTC GTTTCAAACA ACGGATTGAC   
  
  
- TAATCCTTAA GGGACATTAT GATGAATGGG ATTATGAAGT TATAATAAGT TCGGTTTCTG GTCGTCAAAG   
  
  
- AGGTTCATAA ATAAACGGAT ATTCCAATCA ATAGGTAAGT GTATGCTTTC TCTACTTTCG TTTTCCGTCC   
  
  
- TTTATGCCAC CATTGTAAAA GGAAGTACGT TTTCAAAATG AGCATGTCAA CCGAAAATCC GATGTTCCAC   
  
  
- AGGTGAGGAT TCTTTTTCAC AAATTAATTA CTTAATTAGT AATTTTTCTT TTTGGTCATA TAGGTTCAGT   
  
  
- TTTTACGTTA TTGTTTGCCG GTGGAAGTGG CAATCCCTTG GGCAGTTCAG TTGTTTCCGT TGCTACTAGG   
  
  
- TGCGTGGTGT AGATTGCCTC TTTAGTTGCC TCAAACAATG TGTCTCCTTT GTGGACTCAG TTAAGGTCAG   
  
  
- TTTCTGTATA TATGGGGGTT GTGCTGTCTG TATGTTGGGG AGAGAGAAAG GAAATTTCGT TTGGGAAAGG   
  
  
- TGAGAGAGAG AGAGAGAGAG GTAGAATTAG GTAAGGCTAT GAAAATTTTT TAGCCTTTTT AAAGTTGCCA   
  
  
- TGTATAGCGA AGGCGGCGTT TTATAGCCTG AAAAAAATTC ACAGTGGTGT GACAACAATG AAATGAGAGA   
  
  
- CAAAGACAGA GAGGAAACCT TTTTAGCCA

+     GT1-motif

| Site Name | Organism | Position | Strand | Matrix score. | sequence | function |
| --- | --- | --- | --- | --- | --- | --- |
| GT1-motif | Arabidopsis thaliana | 686 | - | 6 | GGTTAA | light responsive element |
| GT1-motif | Arabidopsis thaliana | 684 | + | 6 | GGTTAA | light responsive element |

> 2018/04/13 10:10:12  
+ GGTGTTGTTT GTACAATCAT ATGCGTGTCA TTGCGTACAA AACAAAAAAT ATGCTAGAGC ATCCATGAAA   
  
  
+ GCAATAATGA ATGGCAAAAC TACATAAAAG AAAAACAAAA CGCAGAAGCG GACCAACAAA ATTTAGAAGA   
  
  
+ AGTGAACGTG AACTATGGCG AAACAAAAAA AAAAAAAAAA CTTACAAAAA TGGAGAAAAG GGACAACCTT   
  
  
+ CTTCAAAAAA AAAAAAAAAA AAAAACTTAC AAAAATGGAG AAAAGGGACA ACCTTCTTCA AGCAGACGCT   
  
  
+ GCATAAGATA ACTTCAATCT CTCTCCAAGA GAGAGCGCCC TGAACACAGT TACTGTGCGC TTGAAAGCTC   
  
  
+ TCTAGCGGTC TATTAATGCT CACTAAAAGG ACTTTTTCTA TTGGTCATGA GGTTCATGCT TCCTACAAAA   
  
  
+ TTTCTTCCTA CCTAAATTAT TCCGCAGCTG CAGTTCAATT CATTAAAAAA GGTCTCGGTA GCATGGCTAT   
  
  
+ ACGTGTGGTC ACACTGGTCA GCTCTTTTTC CAACCCATGT GGGGGATAAT TGGAGCCCTC AATTGGGTCA   
  
  
+ TCGTTGAAAC TCAGAAGAAA CCAAAGTAAT AGCATCTGCT TCCCTGAAGT CATATCTTGT ACCCATCTAT   
  
  
+ GGGCACTCCA TGAAGATGCT GATCTGTGTC ATGCCATTCT TAAATTCATC AAAGGTTAAC CTAATTATTA   
  
  
+ CTTTGCAGAG TTTTCTTTTT CTTTCTATGC CCAAAAGTTT CCTACTTTAG CACTATAACC CCATCAATTC   
  
  
+ CACTGCTTCA TTAAGTTCAT TACATGGGCT TATCATTTAA GAAAACAAAG CAAAGTTTGT TGCCTAACTG   
  
  
+ ATTAGGAATT CCCTGTAATA CTACTTACCC TAATACTTCA ATATTATTCA AGCCAAAGAC CAGCAGTTTC   
  
  
+ TCCAAGTATT TATTTGCCTA TAAGGTTAGT TATCCATTCA CATACGAAAG AGATGAAAGC AAAAGGCAGG   
  
  
+ AAATACGGTG GTAACATTTT CCTTCATGCA AAAGTTTTAC TCGTACAGTT GGCTTTTAGG CTACAAGGTG   
  
  
+ TCCACTCCTA AGAAAAAGTG TTTAATTAAT GAATTAATCA TTAAAAAGAA AAACCAGTAT ATCCAAGTCA   
  
  
+ AAAATGCAAT AACAAACGGC CACCTTCACC GTTAGGGAAC CCGTCAAGTC AACAAAGGCA ACGATGATCC   
  
  
+ ACGCACCACA TCTAACGGAG AAATCAACGG AGTTTGTTAC ACAGAGGAAA CACCTGAGTC AATTCCAGTC   
  
  
+ AAAGACATAT ATACCCCCAA CACGACAGAC ATACAACCCC TCTCTCTTTC CTTTAAAGCA AACCCTTTCC   
  
  
+ ACTCTCTCTC TCTCTCTCTC CATCTTAATC CATTCCGATA CTTTTAAAAA ATCGGAAAAA TTTCAACGGT   
  
  
+ ACATATCGCT TCCGCCGCAA AATATCGGAC TTTTTTTAAG TGTCACCACA CTGTTGTTAC TTTACTCTCT   
  
  
+ GTTTCTGTCT CTCCTTTGGA AAAATCGGT  

- CCACAACAAA CATGTTAGTA TACGCACAGT AACGCATGTT TTGTTTTTTA TACGATCTCG TAGGTACTTT   
  
  
- CGTTATTACT TACCGTTTTG ATGTATTTTC TTTTTGTTTT GCGTCTTCGC CTGGTTGTTT TAAATCTTCT   
  
  
- TCACTTGCAC TTGATACCGC TTTGTTTTTT TTTTTTTTTT GAATGTTTTT ACCTCTTTTC CCTGTTGGAA   
  
  
- GAAGTTTTTT TTTTTTTTTT TTTTTGAATG TTTTTACCTC TTTTCCCTGT TGGAAGAAGT TCGTCTGCGA   
  
  
- CGTATTCTAT TGAAGTTAGA GAGAGGTTCT CTCTCGCGGG ACTTGTGTCA ATGACACGCG AACTTTCGAG   
  
  
- AGATCGCCAG ATAATTACGA GTGATTTTCC TGAAAAAGAT AACCAGTACT CCAAGTACGA AGGATGTTTT   
  
  
- AAAGAAGGAT GGATTTAATA AGGCGTCGAC GTCAAGTTAA GTAATTTTTT CCAGAGCCAT CGTACCGATA   
  
  
- TGCACACCAG TGTGACCAGT CGAGAAAAAG GTTGGGTACA CCCCCTATTA ACCTCGGGAG TTAACCCAGT   
  
  
- AGCAACTTTG AGTCTTCTTT GGTTTCATTA TCGTAGACGA AGGGACTTCA GTATAGAACA TGGGTAGATA   
  
  
- CCCGTGAGGT ACTTCTACGA CTAGACACAG TACGGTAAGA ATTTAAGTAG TTTCCAATTG GATTAATAAT   
  
  
- GAAACGTCTC AAAAGAAAAA GAAAGATACG GGTTTTCAAA GGATGAAATC GTGATATTGG GGTAGTTAAG   
  
  
- GTGACGAAGT AATTCAAGTA ATGTACCCGA ATAGTAAATT CTTTTGTTTC GTTTCAAACA ACGGATTGAC   
  
  
- TAATCCTTAA GGGACATTAT GATGAATGGG ATTATGAAGT TATAATAAGT TCGGTTTCTG GTCGTCAAAG   
  
  
- AGGTTCATAA ATAAACGGAT ATTCCAATCA ATAGGTAAGT GTATGCTTTC TCTACTTTCG TTTTCCGTCC   
  
  
- TTTATGCCAC CATTGTAAAA GGAAGTACGT TTTCAAAATG AGCATGTCAA CCGAAAATCC GATGTTCCAC   
  
  
- AGGTGAGGAT TCTTTTTCAC AAATTAATTA CTTAATTAGT AATTTTTCTT TTTGGTCATA TAGGTTCAGT   
  
  
- TTTTACGTTA TTGTTTGCCG GTGGAAGTGG CAATCCCTTG GGCAGTTCAG TTGTTTCCGT TGCTACTAGG   
  
  
- TGCGTGGTGT AGATTGCCTC TTTAGTTGCC TCAAACAATG TGTCTCCTTT GTGGACTCAG TTAAGGTCAG   
  
  
- TTTCTGTATA TATGGGGGTT GTGCTGTCTG TATGTTGGGG AGAGAGAAAG GAAATTTCGT TTGGGAAAGG   
  
  
- TGAGAGAGAG AGAGAGAGAG GTAGAATTAG GTAAGGCTAT GAAAATTTTT TAGCCTTTTT AAAGTTGCCA   
  
  
- TGTATAGCGA AGGCGGCGTT TTATAGCCTG AAAAAAATTC ACAGTGGTGT GACAACAATG AAATGAGAGA   
  
  
- CAAAGACAGA GAGGAAACCT TTTTAGCCA

+     Gap-box

| Site Name | Organism | Position | Strand | Matrix score. | sequence | function |
| --- | --- | --- | --- | --- | --- | --- |
| Gap-box | Arabidopsis thaliana | 243 | + | 9 | AAATGGAGA | part of a light responsive element |
| Gap-box | Arabidopsis thaliana | 188 | + | 9 | AAATGGAGA | part of a light responsive element |

> 2018/04/13 10:10:12  
+ GGTGTTGTTT GTACAATCAT ATGCGTGTCA TTGCGTACAA AACAAAAAAT ATGCTAGAGC ATCCATGAAA   
  
  
+ GCAATAATGA ATGGCAAAAC TACATAAAAG AAAAACAAAA CGCAGAAGCG GACCAACAAA ATTTAGAAGA   
  
  
+ AGTGAACGTG AACTATGGCG AAACAAAAAA AAAAAAAAAA CTTACAAAAA TGGAGAAAAG GGACAACCTT   
  
  
+ CTTCAAAAAA AAAAAAAAAA AAAAACTTAC AAAAATGGAG AAAAGGGACA ACCTTCTTCA AGCAGACGCT   
  
  
+ GCATAAGATA ACTTCAATCT CTCTCCAAGA GAGAGCGCCC TGAACACAGT TACTGTGCGC TTGAAAGCTC   
  
  
+ TCTAGCGGTC TATTAATGCT CACTAAAAGG ACTTTTTCTA TTGGTCATGA GGTTCATGCT TCCTACAAAA   
  
  
+ TTTCTTCCTA CCTAAATTAT TCCGCAGCTG CAGTTCAATT CATTAAAAAA GGTCTCGGTA GCATGGCTAT   
  
  
+ ACGTGTGGTC ACACTGGTCA GCTCTTTTTC CAACCCATGT GGGGGATAAT TGGAGCCCTC AATTGGGTCA   
  
  
+ TCGTTGAAAC TCAGAAGAAA CCAAAGTAAT AGCATCTGCT TCCCTGAAGT CATATCTTGT ACCCATCTAT   
  
  
+ GGGCACTCCA TGAAGATGCT GATCTGTGTC ATGCCATTCT TAAATTCATC AAAGGTTAAC CTAATTATTA   
  
  
+ CTTTGCAGAG TTTTCTTTTT CTTTCTATGC CCAAAAGTTT CCTACTTTAG CACTATAACC CCATCAATTC   
  
  
+ CACTGCTTCA TTAAGTTCAT TACATGGGCT TATCATTTAA GAAAACAAAG CAAAGTTTGT TGCCTAACTG   
  
  
+ ATTAGGAATT CCCTGTAATA CTACTTACCC TAATACTTCA ATATTATTCA AGCCAAAGAC CAGCAGTTTC   
  
  
+ TCCAAGTATT TATTTGCCTA TAAGGTTAGT TATCCATTCA CATACGAAAG AGATGAAAGC AAAAGGCAGG   
  
  
+ AAATACGGTG GTAACATTTT CCTTCATGCA AAAGTTTTAC TCGTACAGTT GGCTTTTAGG CTACAAGGTG   
  
  
+ TCCACTCCTA AGAAAAAGTG TTTAATTAAT GAATTAATCA TTAAAAAGAA AAACCAGTAT ATCCAAGTCA   
  
  
+ AAAATGCAAT AACAAACGGC CACCTTCACC GTTAGGGAAC CCGTCAAGTC AACAAAGGCA ACGATGATCC   
  
  
+ ACGCACCACA TCTAACGGAG AAATCAACGG AGTTTGTTAC ACAGAGGAAA CACCTGAGTC AATTCCAGTC   
  
  
+ AAAGACATAT ATACCCCCAA CACGACAGAC ATACAACCCC TCTCTCTTTC CTTTAAAGCA AACCCTTTCC   
  
  
+ ACTCTCTCTC TCTCTCTCTC CATCTTAATC CATTCCGATA CTTTTAAAAA ATCGGAAAAA TTTCAACGGT   
  
  
+ ACATATCGCT TCCGCCGCAA AATATCGGAC TTTTTTTAAG TGTCACCACA CTGTTGTTAC TTTACTCTCT   
  
  
+ GTTTCTGTCT CTCCTTTGGA AAAATCGGT  

- CCACAACAAA CATGTTAGTA TACGCACAGT AACGCATGTT TTGTTTTTTA TACGATCTCG TAGGTACTTT   
  
  
- CGTTATTACT TACCGTTTTG ATGTATTTTC TTTTTGTTTT GCGTCTTCGC CTGGTTGTTT TAAATCTTCT   
  
  
- TCACTTGCAC TTGATACCGC TTTGTTTTTT TTTTTTTTTT GAATGTTTTT ACCTCTTTTC CCTGTTGGAA   
  
  
- GAAGTTTTTT TTTTTTTTTT TTTTTGAATG TTTTTACCTC TTTTCCCTGT TGGAAGAAGT TCGTCTGCGA   
  
  
- CGTATTCTAT TGAAGTTAGA GAGAGGTTCT CTCTCGCGGG ACTTGTGTCA ATGACACGCG AACTTTCGAG   
  
  
- AGATCGCCAG ATAATTACGA GTGATTTTCC TGAAAAAGAT AACCAGTACT CCAAGTACGA AGGATGTTTT   
  
  
- AAAGAAGGAT GGATTTAATA AGGCGTCGAC GTCAAGTTAA GTAATTTTTT CCAGAGCCAT CGTACCGATA   
  
  
- TGCACACCAG TGTGACCAGT CGAGAAAAAG GTTGGGTACA CCCCCTATTA ACCTCGGGAG TTAACCCAGT   
  
  
- AGCAACTTTG AGTCTTCTTT GGTTTCATTA TCGTAGACGA AGGGACTTCA GTATAGAACA TGGGTAGATA   
  
  
- CCCGTGAGGT ACTTCTACGA CTAGACACAG TACGGTAAGA ATTTAAGTAG TTTCCAATTG GATTAATAAT   
  
  
- GAAACGTCTC AAAAGAAAAA GAAAGATACG GGTTTTCAAA GGATGAAATC GTGATATTGG GGTAGTTAAG   
  
  
- GTGACGAAGT AATTCAAGTA ATGTACCCGA ATAGTAAATT CTTTTGTTTC GTTTCAAACA ACGGATTGAC   
  
  
- TAATCCTTAA GGGACATTAT GATGAATGGG ATTATGAAGT TATAATAAGT TCGGTTTCTG GTCGTCAAAG   
  
  
- AGGTTCATAA ATAAACGGAT ATTCCAATCA ATAGGTAAGT GTATGCTTTC TCTACTTTCG TTTTCCGTCC   
  
  
- TTTATGCCAC CATTGTAAAA GGAAGTACGT TTTCAAAATG AGCATGTCAA CCGAAAATCC GATGTTCCAC   
  
  
- AGGTGAGGAT TCTTTTTCAC AAATTAATTA CTTAATTAGT AATTTTTCTT TTTGGTCATA TAGGTTCAGT   
  
  
- TTTTACGTTA TTGTTTGCCG GTGGAAGTGG CAATCCCTTG GGCAGTTCAG TTGTTTCCGT TGCTACTAGG   
  
  
- TGCGTGGTGT AGATTGCCTC TTTAGTTGCC TCAAACAATG TGTCTCCTTT GTGGACTCAG TTAAGGTCAG   
  
  
- TTTCTGTATA TATGGGGGTT GTGCTGTCTG TATGTTGGGG AGAGAGAAAG GAAATTTCGT TTGGGAAAGG   
  
  
- TGAGAGAGAG AGAGAGAGAG GTAGAATTAG GTAAGGCTAT GAAAATTTTT TAGCCTTTTT AAAGTTGCCA   
  
  
- TGTATAGCGA AGGCGGCGTT TTATAGCCTG AAAAAAATTC ACAGTGGTGT GACAACAATG AAATGAGAGA   
  
  
- CAAAGACAGA GAGGAAACCT TTTTAGCCA

+     HSE

| Site Name | Organism | Position | Strand | Matrix score. | sequence | function |
| --- | --- | --- | --- | --- | --- | --- |
| HSE | Brassica oleracea | 1385 | + | 9 | AAAAAATTTC | cis-acting element involved in heat stress responsiveness |
| HSE | Brassica oleracea | 415 | + | 9 | AAAAAATTTC | cis-acting element involved in heat stress responsiveness |

> 2018/04/13 10:10:12  
+ GGTGTTGTTT GTACAATCAT ATGCGTGTCA TTGCGTACAA AACAAAAAAT ATGCTAGAGC ATCCATGAAA   
  
  
+ GCAATAATGA ATGGCAAAAC TACATAAAAG AAAAACAAAA CGCAGAAGCG GACCAACAAA ATTTAGAAGA   
  
  
+ AGTGAACGTG AACTATGGCG AAACAAAAAA AAAAAAAAAA CTTACAAAAA TGGAGAAAAG GGACAACCTT   
  
  
+ CTTCAAAAAA AAAAAAAAAA AAAAACTTAC AAAAATGGAG AAAAGGGACA ACCTTCTTCA AGCAGACGCT   
  
  
+ GCATAAGATA ACTTCAATCT CTCTCCAAGA GAGAGCGCCC TGAACACAGT TACTGTGCGC TTGAAAGCTC   
  
  
+ TCTAGCGGTC TATTAATGCT CACTAAAAGG ACTTTTTCTA TTGGTCATGA GGTTCATGCT TCCTACAAAA   
  
  
+ TTTCTTCCTA CCTAAATTAT TCCGCAGCTG CAGTTCAATT CATTAAAAAA GGTCTCGGTA GCATGGCTAT   
  
  
+ ACGTGTGGTC ACACTGGTCA GCTCTTTTTC CAACCCATGT GGGGGATAAT TGGAGCCCTC AATTGGGTCA   
  
  
+ TCGTTGAAAC TCAGAAGAAA CCAAAGTAAT AGCATCTGCT TCCCTGAAGT CATATCTTGT ACCCATCTAT   
  
  
+ GGGCACTCCA TGAAGATGCT GATCTGTGTC ATGCCATTCT TAAATTCATC AAAGGTTAAC CTAATTATTA   
  
  
+ CTTTGCAGAG TTTTCTTTTT CTTTCTATGC CCAAAAGTTT CCTACTTTAG CACTATAACC CCATCAATTC   
  
  
+ CACTGCTTCA TTAAGTTCAT TACATGGGCT TATCATTTAA GAAAACAAAG CAAAGTTTGT TGCCTAACTG   
  
  
+ ATTAGGAATT CCCTGTAATA CTACTTACCC TAATACTTCA ATATTATTCA AGCCAAAGAC CAGCAGTTTC   
  
  
+ TCCAAGTATT TATTTGCCTA TAAGGTTAGT TATCCATTCA CATACGAAAG AGATGAAAGC AAAAGGCAGG   
  
  
+ AAATACGGTG GTAACATTTT CCTTCATGCA AAAGTTTTAC TCGTACAGTT GGCTTTTAGG CTACAAGGTG   
  
  
+ TCCACTCCTA AGAAAAAGTG TTTAATTAAT GAATTAATCA TTAAAAAGAA AAACCAGTAT ATCCAAGTCA   
  
  
+ AAAATGCAAT AACAAACGGC CACCTTCACC GTTAGGGAAC CCGTCAAGTC AACAAAGGCA ACGATGATCC   
  
  
+ ACGCACCACA TCTAACGGAG AAATCAACGG AGTTTGTTAC ACAGAGGAAA CACCTGAGTC AATTCCAGTC   
  
  
+ AAAGACATAT ATACCCCCAA CACGACAGAC ATACAACCCC TCTCTCTTTC CTTTAAAGCA AACCCTTTCC   
  
  
+ ACTCTCTCTC TCTCTCTCTC CATCTTAATC CATTCCGATA CTTTTAAAAA ATCGGAAAAA TTTCAACGGT   
  
  
+ ACATATCGCT TCCGCCGCAA AATATCGGAC TTTTTTTAAG TGTCACCACA CTGTTGTTAC TTTACTCTCT   
  
  
+ GTTTCTGTCT CTCCTTTGGA AAAATCGGT  

- CCACAACAAA CATGTTAGTA TACGCACAGT AACGCATGTT TTGTTTTTTA TACGATCTCG TAGGTACTTT   
  
  
- CGTTATTACT TACCGTTTTG ATGTATTTTC TTTTTGTTTT GCGTCTTCGC CTGGTTGTTT TAAATCTTCT   
  
  
- TCACTTGCAC TTGATACCGC TTTGTTTTTT TTTTTTTTTT GAATGTTTTT ACCTCTTTTC CCTGTTGGAA   
  
  
- GAAGTTTTTT TTTTTTTTTT TTTTTGAATG TTTTTACCTC TTTTCCCTGT TGGAAGAAGT TCGTCTGCGA   
  
  
- CGTATTCTAT TGAAGTTAGA GAGAGGTTCT CTCTCGCGGG ACTTGTGTCA ATGACACGCG AACTTTCGAG   
  
  
- AGATCGCCAG ATAATTACGA GTGATTTTCC TGAAAAAGAT AACCAGTACT CCAAGTACGA AGGATGTTTT   
  
  
- AAAGAAGGAT GGATTTAATA AGGCGTCGAC GTCAAGTTAA GTAATTTTTT CCAGAGCCAT CGTACCGATA   
  
  
- TGCACACCAG TGTGACCAGT CGAGAAAAAG GTTGGGTACA CCCCCTATTA ACCTCGGGAG TTAACCCAGT   
  
  
- AGCAACTTTG AGTCTTCTTT GGTTTCATTA TCGTAGACGA AGGGACTTCA GTATAGAACA TGGGTAGATA   
  
  
- CCCGTGAGGT ACTTCTACGA CTAGACACAG TACGGTAAGA ATTTAAGTAG TTTCCAATTG GATTAATAAT   
  
  
- GAAACGTCTC AAAAGAAAAA GAAAGATACG GGTTTTCAAA GGATGAAATC GTGATATTGG GGTAGTTAAG   
  
  
- GTGACGAAGT AATTCAAGTA ATGTACCCGA ATAGTAAATT CTTTTGTTTC GTTTCAAACA ACGGATTGAC   
  
  
- TAATCCTTAA GGGACATTAT GATGAATGGG ATTATGAAGT TATAATAAGT TCGGTTTCTG GTCGTCAAAG   
  
  
- AGGTTCATAA ATAAACGGAT ATTCCAATCA ATAGGTAAGT GTATGCTTTC TCTACTTTCG TTTTCCGTCC   
  
  
- TTTATGCCAC CATTGTAAAA GGAAGTACGT TTTCAAAATG AGCATGTCAA CCGAAAATCC GATGTTCCAC   
  
  
- AGGTGAGGAT TCTTTTTCAC AAATTAATTA CTTAATTAGT AATTTTTCTT TTTGGTCATA TAGGTTCAGT   
  
  
- TTTTACGTTA TTGTTTGCCG GTGGAAGTGG CAATCCCTTG GGCAGTTCAG TTGTTTCCGT TGCTACTAGG   
  
  
- TGCGTGGTGT AGATTGCCTC TTTAGTTGCC TCAAACAATG TGTCTCCTTT GTGGACTCAG TTAAGGTCAG   
  
  
- TTTCTGTATA TATGGGGGTT GTGCTGTCTG TATGTTGGGG AGAGAGAAAG GAAATTTCGT TTGGGAAAGG   
  
  
- TGAGAGAGAG AGAGAGAGAG GTAGAATTAG GTAAGGCTAT GAAAATTTTT TAGCCTTTTT AAAGTTGCCA   
  
  
- TGTATAGCGA AGGCGGCGTT TTATAGCCTG AAAAAAATTC ACAGTGGTGT GACAACAATG AAATGAGAGA   
  
  
- CAAAGACAGA GAGGAAACCT TTTTAGCCA

+     MBS

| Site Name | Organism | Position | Strand | Matrix score. | sequence | function |
| --- | --- | --- | --- | --- | --- | --- |
| MBS | Arabidopsis thaliana | 1026 | - | 6 | CAACTG | MYB binding site involved in drought-inducibility |
| MBS | Arabidopsis thaliana | 835 | + | 6 | TAACTG | MYB binding site involved in drought-inducibility |
| MBS | Arabidopsis thaliana | 327 | - | 6 | TAACTG | MYB binding site involved in drought-inducibility |

> 2018/04/13 10:10:12  
+ GGTGTTGTTT GTACAATCAT ATGCGTGTCA TTGCGTACAA AACAAAAAAT ATGCTAGAGC ATCCATGAAA   
  
  
+ GCAATAATGA ATGGCAAAAC TACATAAAAG AAAAACAAAA CGCAGAAGCG GACCAACAAA ATTTAGAAGA   
  
  
+ AGTGAACGTG AACTATGGCG AAACAAAAAA AAAAAAAAAA CTTACAAAAA TGGAGAAAAG GGACAACCTT   
  
  
+ CTTCAAAAAA AAAAAAAAAA AAAAACTTAC AAAAATGGAG AAAAGGGACA ACCTTCTTCA AGCAGACGCT   
  
  
+ GCATAAGATA ACTTCAATCT CTCTCCAAGA GAGAGCGCCC TGAACACAGT TACTGTGCGC TTGAAAGCTC   
  
  
+ TCTAGCGGTC TATTAATGCT CACTAAAAGG ACTTTTTCTA TTGGTCATGA GGTTCATGCT TCCTACAAAA   
  
  
+ TTTCTTCCTA CCTAAATTAT TCCGCAGCTG CAGTTCAATT CATTAAAAAA GGTCTCGGTA GCATGGCTAT   
  
  
+ ACGTGTGGTC ACACTGGTCA GCTCTTTTTC CAACCCATGT GGGGGATAAT TGGAGCCCTC AATTGGGTCA   
  
  
+ TCGTTGAAAC TCAGAAGAAA CCAAAGTAAT AGCATCTGCT TCCCTGAAGT CATATCTTGT ACCCATCTAT   
  
  
+ GGGCACTCCA TGAAGATGCT GATCTGTGTC ATGCCATTCT TAAATTCATC AAAGGTTAAC CTAATTATTA   
  
  
+ CTTTGCAGAG TTTTCTTTTT CTTTCTATGC CCAAAAGTTT CCTACTTTAG CACTATAACC CCATCAATTC   
  
  
+ CACTGCTTCA TTAAGTTCAT TACATGGGCT TATCATTTAA GAAAACAAAG CAAAGTTTGT TGCCTAACTG   
  
  
+ ATTAGGAATT CCCTGTAATA CTACTTACCC TAATACTTCA ATATTATTCA AGCCAAAGAC CAGCAGTTTC   
  
  
+ TCCAAGTATT TATTTGCCTA TAAGGTTAGT TATCCATTCA CATACGAAAG AGATGAAAGC AAAAGGCAGG   
  
  
+ AAATACGGTG GTAACATTTT CCTTCATGCA AAAGTTTTAC TCGTACAGTT GGCTTTTAGG CTACAAGGTG   
  
  
+ TCCACTCCTA AGAAAAAGTG TTTAATTAAT GAATTAATCA TTAAAAAGAA AAACCAGTAT ATCCAAGTCA   
  
  
+ AAAATGCAAT AACAAACGGC CACCTTCACC GTTAGGGAAC CCGTCAAGTC AACAAAGGCA ACGATGATCC   
  
  
+ ACGCACCACA TCTAACGGAG AAATCAACGG AGTTTGTTAC ACAGAGGAAA CACCTGAGTC AATTCCAGTC   
  
  
+ AAAGACATAT ATACCCCCAA CACGACAGAC ATACAACCCC TCTCTCTTTC CTTTAAAGCA AACCCTTTCC   
  
  
+ ACTCTCTCTC TCTCTCTCTC CATCTTAATC CATTCCGATA CTTTTAAAAA ATCGGAAAAA TTTCAACGGT   
  
  
+ ACATATCGCT TCCGCCGCAA AATATCGGAC TTTTTTTAAG TGTCACCACA CTGTTGTTAC TTTACTCTCT   
  
  
+ GTTTCTGTCT CTCCTTTGGA AAAATCGGT  

- CCACAACAAA CATGTTAGTA TACGCACAGT AACGCATGTT TTGTTTTTTA TACGATCTCG TAGGTACTTT   
  
  
- CGTTATTACT TACCGTTTTG ATGTATTTTC TTTTTGTTTT GCGTCTTCGC CTGGTTGTTT TAAATCTTCT   
  
  
- TCACTTGCAC TTGATACCGC TTTGTTTTTT TTTTTTTTTT GAATGTTTTT ACCTCTTTTC CCTGTTGGAA   
  
  
- GAAGTTTTTT TTTTTTTTTT TTTTTGAATG TTTTTACCTC TTTTCCCTGT TGGAAGAAGT TCGTCTGCGA   
  
  
- CGTATTCTAT TGAAGTTAGA GAGAGGTTCT CTCTCGCGGG ACTTGTGTCA ATGACACGCG AACTTTCGAG   
  
  
- AGATCGCCAG ATAATTACGA GTGATTTTCC TGAAAAAGAT AACCAGTACT CCAAGTACGA AGGATGTTTT   
  
  
- AAAGAAGGAT GGATTTAATA AGGCGTCGAC GTCAAGTTAA GTAATTTTTT CCAGAGCCAT CGTACCGATA   
  
  
- TGCACACCAG TGTGACCAGT CGAGAAAAAG GTTGGGTACA CCCCCTATTA ACCTCGGGAG TTAACCCAGT   
  
  
- AGCAACTTTG AGTCTTCTTT GGTTTCATTA TCGTAGACGA AGGGACTTCA GTATAGAACA TGGGTAGATA   
  
  
- CCCGTGAGGT ACTTCTACGA CTAGACACAG TACGGTAAGA ATTTAAGTAG TTTCCAATTG GATTAATAAT   
  
  
- GAAACGTCTC AAAAGAAAAA GAAAGATACG GGTTTTCAAA GGATGAAATC GTGATATTGG GGTAGTTAAG   
  
  
- GTGACGAAGT AATTCAAGTA ATGTACCCGA ATAGTAAATT CTTTTGTTTC GTTTCAAACA ACGGATTGAC   
  
  
- TAATCCTTAA GGGACATTAT GATGAATGGG ATTATGAAGT TATAATAAGT TCGGTTTCTG GTCGTCAAAG   
  
  
- AGGTTCATAA ATAAACGGAT ATTCCAATCA ATAGGTAAGT GTATGCTTTC TCTACTTTCG TTTTCCGTCC   
  
  
- TTTATGCCAC CATTGTAAAA GGAAGTACGT TTTCAAAATG AGCATGTCAA CCGAAAATCC GATGTTCCAC   
  
  
- AGGTGAGGAT TCTTTTTCAC AAATTAATTA CTTAATTAGT AATTTTTCTT TTTGGTCATA TAGGTTCAGT   
  
  
- TTTTACGTTA TTGTTTGCCG GTGGAAGTGG CAATCCCTTG GGCAGTTCAG TTGTTTCCGT TGCTACTAGG   
  
  
- TGCGTGGTGT AGATTGCCTC TTTAGTTGCC TCAAACAATG TGTCTCCTTT GTGGACTCAG TTAAGGTCAG   
  
  
- TTTCTGTATA TATGGGGGTT GTGCTGTCTG TATGTTGGGG AGAGAGAAAG GAAATTTCGT TTGGGAAAGG   
  
  
- TGAGAGAGAG AGAGAGAGAG GTAGAATTAG GTAAGGCTAT GAAAATTTTT TAGCCTTTTT AAAGTTGCCA   
  
  
- TGTATAGCGA AGGCGGCGTT TTATAGCCTG AAAAAAATTC ACAGTGGTGT GACAACAATG AAATGAGAGA   
  
  
- CAAAGACAGA GAGGAAACCT TTTTAGCCA

+     MNF1

| Site Name | Organism | Position | Strand | Matrix score. | sequence | function |
| --- | --- | --- | --- | --- | --- | --- |
| MNF1 | Zea mays | 629 | - | 7 | GTGCCC(A/T)(A/T) | light responsive element |

> 2018/04/13 10:10:12  
+ GGTGTTGTTT GTACAATCAT ATGCGTGTCA TTGCGTACAA AACAAAAAAT ATGCTAGAGC ATCCATGAAA   
  
  
+ GCAATAATGA ATGGCAAAAC TACATAAAAG AAAAACAAAA CGCAGAAGCG GACCAACAAA ATTTAGAAGA   
  
  
+ AGTGAACGTG AACTATGGCG AAACAAAAAA AAAAAAAAAA CTTACAAAAA TGGAGAAAAG GGACAACCTT   
  
  
+ CTTCAAAAAA AAAAAAAAAA AAAAACTTAC AAAAATGGAG AAAAGGGACA ACCTTCTTCA AGCAGACGCT   
  
  
+ GCATAAGATA ACTTCAATCT CTCTCCAAGA GAGAGCGCCC TGAACACAGT TACTGTGCGC TTGAAAGCTC   
  
  
+ TCTAGCGGTC TATTAATGCT CACTAAAAGG ACTTTTTCTA TTGGTCATGA GGTTCATGCT TCCTACAAAA   
  
  
+ TTTCTTCCTA CCTAAATTAT TCCGCAGCTG CAGTTCAATT CATTAAAAAA GGTCTCGGTA GCATGGCTAT   
  
  
+ ACGTGTGGTC ACACTGGTCA GCTCTTTTTC CAACCCATGT GGGGGATAAT TGGAGCCCTC AATTGGGTCA   
  
  
+ TCGTTGAAAC TCAGAAGAAA CCAAAGTAAT AGCATCTGCT TCCCTGAAGT CATATCTTGT ACCCATCTAT   
  
  
+ GGGCACTCCA TGAAGATGCT GATCTGTGTC ATGCCATTCT TAAATTCATC AAAGGTTAAC CTAATTATTA   
  
  
+ CTTTGCAGAG TTTTCTTTTT CTTTCTATGC CCAAAAGTTT CCTACTTTAG CACTATAACC CCATCAATTC   
  
  
+ CACTGCTTCA TTAAGTTCAT TACATGGGCT TATCATTTAA GAAAACAAAG CAAAGTTTGT TGCCTAACTG   
  
  
+ ATTAGGAATT CCCTGTAATA CTACTTACCC TAATACTTCA ATATTATTCA AGCCAAAGAC CAGCAGTTTC   
  
  
+ TCCAAGTATT TATTTGCCTA TAAGGTTAGT TATCCATTCA CATACGAAAG AGATGAAAGC AAAAGGCAGG   
  
  
+ AAATACGGTG GTAACATTTT CCTTCATGCA AAAGTTTTAC TCGTACAGTT GGCTTTTAGG CTACAAGGTG   
  
  
+ TCCACTCCTA AGAAAAAGTG TTTAATTAAT GAATTAATCA TTAAAAAGAA AAACCAGTAT ATCCAAGTCA   
  
  
+ AAAATGCAAT AACAAACGGC CACCTTCACC GTTAGGGAAC CCGTCAAGTC AACAAAGGCA ACGATGATCC   
  
  
+ ACGCACCACA TCTAACGGAG AAATCAACGG AGTTTGTTAC ACAGAGGAAA CACCTGAGTC AATTCCAGTC   
  
  
+ AAAGACATAT ATACCCCCAA CACGACAGAC ATACAACCCC TCTCTCTTTC CTTTAAAGCA AACCCTTTCC   
  
  
+ ACTCTCTCTC TCTCTCTCTC CATCTTAATC CATTCCGATA CTTTTAAAAA ATCGGAAAAA TTTCAACGGT   
  
  
+ ACATATCGCT TCCGCCGCAA AATATCGGAC TTTTTTTAAG TGTCACCACA CTGTTGTTAC TTTACTCTCT   
  
  
+ GTTTCTGTCT CTCCTTTGGA AAAATCGGT  

- CCACAACAAA CATGTTAGTA TACGCACAGT AACGCATGTT TTGTTTTTTA TACGATCTCG TAGGTACTTT   
  
  
- CGTTATTACT TACCGTTTTG ATGTATTTTC TTTTTGTTTT GCGTCTTCGC CTGGTTGTTT TAAATCTTCT   
  
  
- TCACTTGCAC TTGATACCGC TTTGTTTTTT TTTTTTTTTT GAATGTTTTT ACCTCTTTTC CCTGTTGGAA   
  
  
- GAAGTTTTTT TTTTTTTTTT TTTTTGAATG TTTTTACCTC TTTTCCCTGT TGGAAGAAGT TCGTCTGCGA   
  
  
- CGTATTCTAT TGAAGTTAGA GAGAGGTTCT CTCTCGCGGG ACTTGTGTCA ATGACACGCG AACTTTCGAG   
  
  
- AGATCGCCAG ATAATTACGA GTGATTTTCC TGAAAAAGAT AACCAGTACT CCAAGTACGA AGGATGTTTT   
  
  
- AAAGAAGGAT GGATTTAATA AGGCGTCGAC GTCAAGTTAA GTAATTTTTT CCAGAGCCAT CGTACCGATA   
  
  
- TGCACACCAG TGTGACCAGT CGAGAAAAAG GTTGGGTACA CCCCCTATTA ACCTCGGGAG TTAACCCAGT   
  
  
- AGCAACTTTG AGTCTTCTTT GGTTTCATTA TCGTAGACGA AGGGACTTCA GTATAGAACA TGGGTAGATA   
  
  
- CCCGTGAGGT ACTTCTACGA CTAGACACAG TACGGTAAGA ATTTAAGTAG TTTCCAATTG GATTAATAAT   
  
  
- GAAACGTCTC AAAAGAAAAA GAAAGATACG GGTTTTCAAA GGATGAAATC GTGATATTGG GGTAGTTAAG   
  
  
- GTGACGAAGT AATTCAAGTA ATGTACCCGA ATAGTAAATT CTTTTGTTTC GTTTCAAACA ACGGATTGAC   
  
  
- TAATCCTTAA GGGACATTAT GATGAATGGG ATTATGAAGT TATAATAAGT TCGGTTTCTG GTCGTCAAAG   
  
  
- AGGTTCATAA ATAAACGGAT ATTCCAATCA ATAGGTAAGT GTATGCTTTC TCTACTTTCG TTTTCCGTCC   
  
  
- TTTATGCCAC CATTGTAAAA GGAAGTACGT TTTCAAAATG AGCATGTCAA CCGAAAATCC GATGTTCCAC   
  
  
- AGGTGAGGAT TCTTTTTCAC AAATTAATTA CTTAATTAGT AATTTTTCTT TTTGGTCATA TAGGTTCAGT   
  
  
- TTTTACGTTA TTGTTTGCCG GTGGAAGTGG CAATCCCTTG GGCAGTTCAG TTGTTTCCGT TGCTACTAGG   
  
  
- TGCGTGGTGT AGATTGCCTC TTTAGTTGCC TCAAACAATG TGTCTCCTTT GTGGACTCAG TTAAGGTCAG   
  
  
- TTTCTGTATA TATGGGGGTT GTGCTGTCTG TATGTTGGGG AGAGAGAAAG GAAATTTCGT TTGGGAAAGG   
  
  
- TGAGAGAGAG AGAGAGAGAG GTAGAATTAG GTAAGGCTAT GAAAATTTTT TAGCCTTTTT AAAGTTGCCA   
  
  
- TGTATAGCGA AGGCGGCGTT TTATAGCCTG AAAAAAATTC ACAGTGGTGT GACAACAATG AAATGAGAGA   
  
  
- CAAAGACAGA GAGGAAACCT TTTTAGCCA

+     MRE

| Site Name | Organism | Position | Strand | Matrix score. | sequence | function |
| --- | --- | --- | --- | --- | --- | --- |
| MRE | Petroselinum crispum | 688 | + | 7 | AACCTAA | MYB binding site involved in light responsiveness |

> 2018/04/13 10:10:12  
+ GGTGTTGTTT GTACAATCAT ATGCGTGTCA TTGCGTACAA AACAAAAAAT ATGCTAGAGC ATCCATGAAA   
  
  
+ GCAATAATGA ATGGCAAAAC TACATAAAAG AAAAACAAAA CGCAGAAGCG GACCAACAAA ATTTAGAAGA   
  
  
+ AGTGAACGTG AACTATGGCG AAACAAAAAA AAAAAAAAAA CTTACAAAAA TGGAGAAAAG GGACAACCTT   
  
  
+ CTTCAAAAAA AAAAAAAAAA AAAAACTTAC AAAAATGGAG AAAAGGGACA ACCTTCTTCA AGCAGACGCT   
  
  
+ GCATAAGATA ACTTCAATCT CTCTCCAAGA GAGAGCGCCC TGAACACAGT TACTGTGCGC TTGAAAGCTC   
  
  
+ TCTAGCGGTC TATTAATGCT CACTAAAAGG ACTTTTTCTA TTGGTCATGA GGTTCATGCT TCCTACAAAA   
  
  
+ TTTCTTCCTA CCTAAATTAT TCCGCAGCTG CAGTTCAATT CATTAAAAAA GGTCTCGGTA GCATGGCTAT   
  
  
+ ACGTGTGGTC ACACTGGTCA GCTCTTTTTC CAACCCATGT GGGGGATAAT TGGAGCCCTC AATTGGGTCA   
  
  
+ TCGTTGAAAC TCAGAAGAAA CCAAAGTAAT AGCATCTGCT TCCCTGAAGT CATATCTTGT ACCCATCTAT   
  
  
+ GGGCACTCCA TGAAGATGCT GATCTGTGTC ATGCCATTCT TAAATTCATC AAAGGTTAAC CTAATTATTA   
  
  
+ CTTTGCAGAG TTTTCTTTTT CTTTCTATGC CCAAAAGTTT CCTACTTTAG CACTATAACC CCATCAATTC   
  
  
+ CACTGCTTCA TTAAGTTCAT TACATGGGCT TATCATTTAA GAAAACAAAG CAAAGTTTGT TGCCTAACTG   
  
  
+ ATTAGGAATT CCCTGTAATA CTACTTACCC TAATACTTCA ATATTATTCA AGCCAAAGAC CAGCAGTTTC   
  
  
+ TCCAAGTATT TATTTGCCTA TAAGGTTAGT TATCCATTCA CATACGAAAG AGATGAAAGC AAAAGGCAGG   
  
  
+ AAATACGGTG GTAACATTTT CCTTCATGCA AAAGTTTTAC TCGTACAGTT GGCTTTTAGG CTACAAGGTG   
  
  
+ TCCACTCCTA AGAAAAAGTG TTTAATTAAT GAATTAATCA TTAAAAAGAA AAACCAGTAT ATCCAAGTCA   
  
  
+ AAAATGCAAT AACAAACGGC CACCTTCACC GTTAGGGAAC CCGTCAAGTC AACAAAGGCA ACGATGATCC   
  
  
+ ACGCACCACA TCTAACGGAG AAATCAACGG AGTTTGTTAC ACAGAGGAAA CACCTGAGTC AATTCCAGTC   
  
  
+ AAAGACATAT ATACCCCCAA CACGACAGAC ATACAACCCC TCTCTCTTTC CTTTAAAGCA AACCCTTTCC   
  
  
+ ACTCTCTCTC TCTCTCTCTC CATCTTAATC CATTCCGATA CTTTTAAAAA ATCGGAAAAA TTTCAACGGT   
  
  
+ ACATATCGCT TCCGCCGCAA AATATCGGAC TTTTTTTAAG TGTCACCACA CTGTTGTTAC TTTACTCTCT   
  
  
+ GTTTCTGTCT CTCCTTTGGA AAAATCGGT  

- CCACAACAAA CATGTTAGTA TACGCACAGT AACGCATGTT TTGTTTTTTA TACGATCTCG TAGGTACTTT   
  
  
- CGTTATTACT TACCGTTTTG ATGTATTTTC TTTTTGTTTT GCGTCTTCGC CTGGTTGTTT TAAATCTTCT   
  
  
- TCACTTGCAC TTGATACCGC TTTGTTTTTT TTTTTTTTTT GAATGTTTTT ACCTCTTTTC CCTGTTGGAA   
  
  
- GAAGTTTTTT TTTTTTTTTT TTTTTGAATG TTTTTACCTC TTTTCCCTGT TGGAAGAAGT TCGTCTGCGA   
  
  
- CGTATTCTAT TGAAGTTAGA GAGAGGTTCT CTCTCGCGGG ACTTGTGTCA ATGACACGCG AACTTTCGAG   
  
  
- AGATCGCCAG ATAATTACGA GTGATTTTCC TGAAAAAGAT AACCAGTACT CCAAGTACGA AGGATGTTTT   
  
  
- AAAGAAGGAT GGATTTAATA AGGCGTCGAC GTCAAGTTAA GTAATTTTTT CCAGAGCCAT CGTACCGATA   
  
  
- TGCACACCAG TGTGACCAGT CGAGAAAAAG GTTGGGTACA CCCCCTATTA ACCTCGGGAG TTAACCCAGT   
  
  
- AGCAACTTTG AGTCTTCTTT GGTTTCATTA TCGTAGACGA AGGGACTTCA GTATAGAACA TGGGTAGATA   
  
  
- CCCGTGAGGT ACTTCTACGA CTAGACACAG TACGGTAAGA ATTTAAGTAG TTTCCAATTG GATTAATAAT   
  
  
- GAAACGTCTC AAAAGAAAAA GAAAGATACG GGTTTTCAAA GGATGAAATC GTGATATTGG GGTAGTTAAG   
  
  
- GTGACGAAGT AATTCAAGTA ATGTACCCGA ATAGTAAATT CTTTTGTTTC GTTTCAAACA ACGGATTGAC   
  
  
- TAATCCTTAA GGGACATTAT GATGAATGGG ATTATGAAGT TATAATAAGT TCGGTTTCTG GTCGTCAAAG   
  
  
- AGGTTCATAA ATAAACGGAT ATTCCAATCA ATAGGTAAGT GTATGCTTTC TCTACTTTCG TTTTCCGTCC   
  
  
- TTTATGCCAC CATTGTAAAA GGAAGTACGT TTTCAAAATG AGCATGTCAA CCGAAAATCC GATGTTCCAC   
  
  
- AGGTGAGGAT TCTTTTTCAC AAATTAATTA CTTAATTAGT AATTTTTCTT TTTGGTCATA TAGGTTCAGT   
  
  
- TTTTACGTTA TTGTTTGCCG GTGGAAGTGG CAATCCCTTG GGCAGTTCAG TTGTTTCCGT TGCTACTAGG   
  
  
- TGCGTGGTGT AGATTGCCTC TTTAGTTGCC TCAAACAATG TGTCTCCTTT GTGGACTCAG TTAAGGTCAG   
  
  
- TTTCTGTATA TATGGGGGTT GTGCTGTCTG TATGTTGGGG AGAGAGAAAG GAAATTTCGT TTGGGAAAGG   
  
  
- TGAGAGAGAG AGAGAGAGAG GTAGAATTAG GTAAGGCTAT GAAAATTTTT TAGCCTTTTT AAAGTTGCCA   
  
  
- TGTATAGCGA AGGCGGCGTT TTATAGCCTG AAAAAAATTC ACAGTGGTGT GACAACAATG AAATGAGAGA   
  
  
- CAAAGACAGA GAGGAAACCT TTTTAGCCA

+     MSA-like

| Site Name | Organism | Position | Strand | Matrix score. | sequence | function |
| --- | --- | --- | --- | --- | --- | --- |
| MSA-like | Catharanthus roseus | 1146 | - | 8.5 | (T/C)C(T/C)AACGG(T/C)(T/C)A | cis-acting element involved in cell cycle regulation |

> 2018/04/13 10:10:12  
+ GGTGTTGTTT GTACAATCAT ATGCGTGTCA TTGCGTACAA AACAAAAAAT ATGCTAGAGC ATCCATGAAA   
  
  
+ GCAATAATGA ATGGCAAAAC TACATAAAAG AAAAACAAAA CGCAGAAGCG GACCAACAAA ATTTAGAAGA   
  
  
+ AGTGAACGTG AACTATGGCG AAACAAAAAA AAAAAAAAAA CTTACAAAAA TGGAGAAAAG GGACAACCTT   
  
  
+ CTTCAAAAAA AAAAAAAAAA AAAAACTTAC AAAAATGGAG AAAAGGGACA ACCTTCTTCA AGCAGACGCT   
  
  
+ GCATAAGATA ACTTCAATCT CTCTCCAAGA GAGAGCGCCC TGAACACAGT TACTGTGCGC TTGAAAGCTC   
  
  
+ TCTAGCGGTC TATTAATGCT CACTAAAAGG ACTTTTTCTA TTGGTCATGA GGTTCATGCT TCCTACAAAA   
  
  
+ TTTCTTCCTA CCTAAATTAT TCCGCAGCTG CAGTTCAATT CATTAAAAAA GGTCTCGGTA GCATGGCTAT   
  
  
+ ACGTGTGGTC ACACTGGTCA GCTCTTTTTC CAACCCATGT GGGGGATAAT TGGAGCCCTC AATTGGGTCA   
  
  
+ TCGTTGAAAC TCAGAAGAAA CCAAAGTAAT AGCATCTGCT TCCCTGAAGT CATATCTTGT ACCCATCTAT   
  
  
+ GGGCACTCCA TGAAGATGCT GATCTGTGTC ATGCCATTCT TAAATTCATC AAAGGTTAAC CTAATTATTA   
  
  
+ CTTTGCAGAG TTTTCTTTTT CTTTCTATGC CCAAAAGTTT CCTACTTTAG CACTATAACC CCATCAATTC   
  
  
+ CACTGCTTCA TTAAGTTCAT TACATGGGCT TATCATTTAA GAAAACAAAG CAAAGTTTGT TGCCTAACTG   
  
  
+ ATTAGGAATT CCCTGTAATA CTACTTACCC TAATACTTCA ATATTATTCA AGCCAAAGAC CAGCAGTTTC   
  
  
+ TCCAAGTATT TATTTGCCTA TAAGGTTAGT TATCCATTCA CATACGAAAG AGATGAAAGC AAAAGGCAGG   
  
  
+ AAATACGGTG GTAACATTTT CCTTCATGCA AAAGTTTTAC TCGTACAGTT GGCTTTTAGG CTACAAGGTG   
  
  
+ TCCACTCCTA AGAAAAAGTG TTTAATTAAT GAATTAATCA TTAAAAAGAA AAACCAGTAT ATCCAAGTCA   
  
  
+ AAAATGCAAT AACAAACGGC CACCTTCACC GTTAGGGAAC CCGTCAAGTC AACAAAGGCA ACGATGATCC   
  
  
+ ACGCACCACA TCTAACGGAG AAATCAACGG AGTTTGTTAC ACAGAGGAAA CACCTGAGTC AATTCCAGTC   
  
  
+ AAAGACATAT ATACCCCCAA CACGACAGAC ATACAACCCC TCTCTCTTTC CTTTAAAGCA AACCCTTTCC   
  
  
+ ACTCTCTCTC TCTCTCTCTC CATCTTAATC CATTCCGATA CTTTTAAAAA ATCGGAAAAA TTTCAACGGT   
  
  
+ ACATATCGCT TCCGCCGCAA AATATCGGAC TTTTTTTAAG TGTCACCACA CTGTTGTTAC TTTACTCTCT   
  
  
+ GTTTCTGTCT CTCCTTTGGA AAAATCGGT  

- CCACAACAAA CATGTTAGTA TACGCACAGT AACGCATGTT TTGTTTTTTA TACGATCTCG TAGGTACTTT   
  
  
- CGTTATTACT TACCGTTTTG ATGTATTTTC TTTTTGTTTT GCGTCTTCGC CTGGTTGTTT TAAATCTTCT   
  
  
- TCACTTGCAC TTGATACCGC TTTGTTTTTT TTTTTTTTTT GAATGTTTTT ACCTCTTTTC CCTGTTGGAA   
  
  
- GAAGTTTTTT TTTTTTTTTT TTTTTGAATG TTTTTACCTC TTTTCCCTGT TGGAAGAAGT TCGTCTGCGA   
  
  
- CGTATTCTAT TGAAGTTAGA GAGAGGTTCT CTCTCGCGGG ACTTGTGTCA ATGACACGCG AACTTTCGAG   
  
  
- AGATCGCCAG ATAATTACGA GTGATTTTCC TGAAAAAGAT AACCAGTACT CCAAGTACGA AGGATGTTTT   
  
  
- AAAGAAGGAT GGATTTAATA AGGCGTCGAC GTCAAGTTAA GTAATTTTTT CCAGAGCCAT CGTACCGATA   
  
  
- TGCACACCAG TGTGACCAGT CGAGAAAAAG GTTGGGTACA CCCCCTATTA ACCTCGGGAG TTAACCCAGT   
  
  
- AGCAACTTTG AGTCTTCTTT GGTTTCATTA TCGTAGACGA AGGGACTTCA GTATAGAACA TGGGTAGATA   
  
  
- CCCGTGAGGT ACTTCTACGA CTAGACACAG TACGGTAAGA ATTTAAGTAG TTTCCAATTG GATTAATAAT   
  
  
- GAAACGTCTC AAAAGAAAAA GAAAGATACG GGTTTTCAAA GGATGAAATC GTGATATTGG GGTAGTTAAG   
  
  
- GTGACGAAGT AATTCAAGTA ATGTACCCGA ATAGTAAATT CTTTTGTTTC GTTTCAAACA ACGGATTGAC   
  
  
- TAATCCTTAA GGGACATTAT GATGAATGGG ATTATGAAGT TATAATAAGT TCGGTTTCTG GTCGTCAAAG   
  
  
- AGGTTCATAA ATAAACGGAT ATTCCAATCA ATAGGTAAGT GTATGCTTTC TCTACTTTCG TTTTCCGTCC   
  
  
- TTTATGCCAC CATTGTAAAA GGAAGTACGT TTTCAAAATG AGCATGTCAA CCGAAAATCC GATGTTCCAC   
  
  
- AGGTGAGGAT TCTTTTTCAC AAATTAATTA CTTAATTAGT AATTTTTCTT TTTGGTCATA TAGGTTCAGT   
  
  
- TTTTACGTTA TTGTTTGCCG GTGGAAGTGG CAATCCCTTG GGCAGTTCAG TTGTTTCCGT TGCTACTAGG   
  
  
- TGCGTGGTGT AGATTGCCTC TTTAGTTGCC TCAAACAATG TGTCTCCTTT GTGGACTCAG TTAAGGTCAG   
  
  
- TTTCTGTATA TATGGGGGTT GTGCTGTCTG TATGTTGGGG AGAGAGAAAG GAAATTTCGT TTGGGAAAGG   
  
  
- TGAGAGAGAG AGAGAGAGAG GTAGAATTAG GTAAGGCTAT GAAAATTTTT TAGCCTTTTT AAAGTTGCCA   
  
  
- TGTATAGCGA AGGCGGCGTT TTATAGCCTG AAAAAAATTC ACAGTGGTGT GACAACAATG AAATGAGAGA   
  
  
- CAAAGACAGA GAGGAAACCT TTTTAGCCA

+     O2-site

| Site Name | Organism | Position | Strand | Matrix score. | sequence | function |
| --- | --- | --- | --- | --- | --- | --- |
| O2-site | Zea mays | 1164 | - | 9 | GTTGACGTGA | cis-acting regulatory element involved in zein metabolism regulation |

> 2018/04/13 10:10:12  
+ GGTGTTGTTT GTACAATCAT ATGCGTGTCA TTGCGTACAA AACAAAAAAT ATGCTAGAGC ATCCATGAAA   
  
  
+ GCAATAATGA ATGGCAAAAC TACATAAAAG AAAAACAAAA CGCAGAAGCG GACCAACAAA ATTTAGAAGA   
  
  
+ AGTGAACGTG AACTATGGCG AAACAAAAAA AAAAAAAAAA CTTACAAAAA TGGAGAAAAG GGACAACCTT   
  
  
+ CTTCAAAAAA AAAAAAAAAA AAAAACTTAC AAAAATGGAG AAAAGGGACA ACCTTCTTCA AGCAGACGCT   
  
  
+ GCATAAGATA ACTTCAATCT CTCTCCAAGA GAGAGCGCCC TGAACACAGT TACTGTGCGC TTGAAAGCTC   
  
  
+ TCTAGCGGTC TATTAATGCT CACTAAAAGG ACTTTTTCTA TTGGTCATGA GGTTCATGCT TCCTACAAAA   
  
  
+ TTTCTTCCTA CCTAAATTAT TCCGCAGCTG CAGTTCAATT CATTAAAAAA GGTCTCGGTA GCATGGCTAT   
  
  
+ ACGTGTGGTC ACACTGGTCA GCTCTTTTTC CAACCCATGT GGGGGATAAT TGGAGCCCTC AATTGGGTCA   
  
  
+ TCGTTGAAAC TCAGAAGAAA CCAAAGTAAT AGCATCTGCT TCCCTGAAGT CATATCTTGT ACCCATCTAT   
  
  
+ GGGCACTCCA TGAAGATGCT GATCTGTGTC ATGCCATTCT TAAATTCATC AAAGGTTAAC CTAATTATTA   
  
  
+ CTTTGCAGAG TTTTCTTTTT CTTTCTATGC CCAAAAGTTT CCTACTTTAG CACTATAACC CCATCAATTC   
  
  
+ CACTGCTTCA TTAAGTTCAT TACATGGGCT TATCATTTAA GAAAACAAAG CAAAGTTTGT TGCCTAACTG   
  
  
+ ATTAGGAATT CCCTGTAATA CTACTTACCC TAATACTTCA ATATTATTCA AGCCAAAGAC CAGCAGTTTC   
  
  
+ TCCAAGTATT TATTTGCCTA TAAGGTTAGT TATCCATTCA CATACGAAAG AGATGAAAGC AAAAGGCAGG   
  
  
+ AAATACGGTG GTAACATTTT CCTTCATGCA AAAGTTTTAC TCGTACAGTT GGCTTTTAGG CTACAAGGTG   
  
  
+ TCCACTCCTA AGAAAAAGTG TTTAATTAAT GAATTAATCA TTAAAAAGAA AAACCAGTAT ATCCAAGTCA   
  
  
+ AAAATGCAAT AACAAACGGC CACCTTCACC GTTAGGGAAC CCGTCAAGTC AACAAAGGCA ACGATGATCC   
  
  
+ ACGCACCACA TCTAACGGAG AAATCAACGG AGTTTGTTAC ACAGAGGAAA CACCTGAGTC AATTCCAGTC   
  
  
+ AAAGACATAT ATACCCCCAA CACGACAGAC ATACAACCCC TCTCTCTTTC CTTTAAAGCA AACCCTTTCC   
  
  
+ ACTCTCTCTC TCTCTCTCTC CATCTTAATC CATTCCGATA CTTTTAAAAA ATCGGAAAAA TTTCAACGGT   
  
  
+ ACATATCGCT TCCGCCGCAA AATATCGGAC TTTTTTTAAG TGTCACCACA CTGTTGTTAC TTTACTCTCT   
  
  
+ GTTTCTGTCT CTCCTTTGGA AAAATCGGT  

- CCACAACAAA CATGTTAGTA TACGCACAGT AACGCATGTT TTGTTTTTTA TACGATCTCG TAGGTACTTT   
  
  
- CGTTATTACT TACCGTTTTG ATGTATTTTC TTTTTGTTTT GCGTCTTCGC CTGGTTGTTT TAAATCTTCT   
  
  
- TCACTTGCAC TTGATACCGC TTTGTTTTTT TTTTTTTTTT GAATGTTTTT ACCTCTTTTC CCTGTTGGAA   
  
  
- GAAGTTTTTT TTTTTTTTTT TTTTTGAATG TTTTTACCTC TTTTCCCTGT TGGAAGAAGT TCGTCTGCGA   
  
  
- CGTATTCTAT TGAAGTTAGA GAGAGGTTCT CTCTCGCGGG ACTTGTGTCA ATGACACGCG AACTTTCGAG   
  
  
- AGATCGCCAG ATAATTACGA GTGATTTTCC TGAAAAAGAT AACCAGTACT CCAAGTACGA AGGATGTTTT   
  
  
- AAAGAAGGAT GGATTTAATA AGGCGTCGAC GTCAAGTTAA GTAATTTTTT CCAGAGCCAT CGTACCGATA   
  
  
- TGCACACCAG TGTGACCAGT CGAGAAAAAG GTTGGGTACA CCCCCTATTA ACCTCGGGAG TTAACCCAGT   
  
  
- AGCAACTTTG AGTCTTCTTT GGTTTCATTA TCGTAGACGA AGGGACTTCA GTATAGAACA TGGGTAGATA   
  
  
- CCCGTGAGGT ACTTCTACGA CTAGACACAG TACGGTAAGA ATTTAAGTAG TTTCCAATTG GATTAATAAT   
  
  
- GAAACGTCTC AAAAGAAAAA GAAAGATACG GGTTTTCAAA GGATGAAATC GTGATATTGG GGTAGTTAAG   
  
  
- GTGACGAAGT AATTCAAGTA ATGTACCCGA ATAGTAAATT CTTTTGTTTC GTTTCAAACA ACGGATTGAC   
  
  
- TAATCCTTAA GGGACATTAT GATGAATGGG ATTATGAAGT TATAATAAGT TCGGTTTCTG GTCGTCAAAG   
  
  
- AGGTTCATAA ATAAACGGAT ATTCCAATCA ATAGGTAAGT GTATGCTTTC TCTACTTTCG TTTTCCGTCC   
  
  
- TTTATGCCAC CATTGTAAAA GGAAGTACGT TTTCAAAATG AGCATGTCAA CCGAAAATCC GATGTTCCAC   
  
  
- AGGTGAGGAT TCTTTTTCAC AAATTAATTA CTTAATTAGT AATTTTTCTT TTTGGTCATA TAGGTTCAGT   
  
  
- TTTTACGTTA TTGTTTGCCG GTGGAAGTGG CAATCCCTTG GGCAGTTCAG TTGTTTCCGT TGCTACTAGG   
  
  
- TGCGTGGTGT AGATTGCCTC TTTAGTTGCC TCAAACAATG TGTCTCCTTT GTGGACTCAG TTAAGGTCAG   
  
  
- TTTCTGTATA TATGGGGGTT GTGCTGTCTG TATGTTGGGG AGAGAGAAAG GAAATTTCGT TTGGGAAAGG   
  
  
- TGAGAGAGAG AGAGAGAGAG GTAGAATTAG GTAAGGCTAT GAAAATTTTT TAGCCTTTTT AAAGTTGCCA   
  
  
- TGTATAGCGA AGGCGGCGTT TTATAGCCTG AAAAAAATTC ACAGTGGTGT GACAACAATG AAATGAGAGA   
  
  
- CAAAGACAGA GAGGAAACCT TTTTAGCCA

+     P-box

| Site Name | Organism | Position | Strand | Matrix score. | sequence | function |
| --- | --- | --- | --- | --- | --- | --- |
| P-box | Oryza sativa | 970 | - | 7 | CCTTTTG | gibberellin-responsive element |

> 2018/04/13 10:10:12  
+ GGTGTTGTTT GTACAATCAT ATGCGTGTCA TTGCGTACAA AACAAAAAAT ATGCTAGAGC ATCCATGAAA   
  
  
+ GCAATAATGA ATGGCAAAAC TACATAAAAG AAAAACAAAA CGCAGAAGCG GACCAACAAA ATTTAGAAGA   
  
  
+ AGTGAACGTG AACTATGGCG AAACAAAAAA AAAAAAAAAA CTTACAAAAA TGGAGAAAAG GGACAACCTT   
  
  
+ CTTCAAAAAA AAAAAAAAAA AAAAACTTAC AAAAATGGAG AAAAGGGACA ACCTTCTTCA AGCAGACGCT   
  
  
+ GCATAAGATA ACTTCAATCT CTCTCCAAGA GAGAGCGCCC TGAACACAGT TACTGTGCGC TTGAAAGCTC   
  
  
+ TCTAGCGGTC TATTAATGCT CACTAAAAGG ACTTTTTCTA TTGGTCATGA GGTTCATGCT TCCTACAAAA   
  
  
+ TTTCTTCCTA CCTAAATTAT TCCGCAGCTG CAGTTCAATT CATTAAAAAA GGTCTCGGTA GCATGGCTAT   
  
  
+ ACGTGTGGTC ACACTGGTCA GCTCTTTTTC CAACCCATGT GGGGGATAAT TGGAGCCCTC AATTGGGTCA   
  
  
+ TCGTTGAAAC TCAGAAGAAA CCAAAGTAAT AGCATCTGCT TCCCTGAAGT CATATCTTGT ACCCATCTAT   
  
  
+ GGGCACTCCA TGAAGATGCT GATCTGTGTC ATGCCATTCT TAAATTCATC AAAGGTTAAC CTAATTATTA   
  
  
+ CTTTGCAGAG TTTTCTTTTT CTTTCTATGC CCAAAAGTTT CCTACTTTAG CACTATAACC CCATCAATTC   
  
  
+ CACTGCTTCA TTAAGTTCAT TACATGGGCT TATCATTTAA GAAAACAAAG CAAAGTTTGT TGCCTAACTG   
  
  
+ ATTAGGAATT CCCTGTAATA CTACTTACCC TAATACTTCA ATATTATTCA AGCCAAAGAC CAGCAGTTTC   
  
  
+ TCCAAGTATT TATTTGCCTA TAAGGTTAGT TATCCATTCA CATACGAAAG AGATGAAAGC AAAAGGCAGG   
  
  
+ AAATACGGTG GTAACATTTT CCTTCATGCA AAAGTTTTAC TCGTACAGTT GGCTTTTAGG CTACAAGGTG   
  
  
+ TCCACTCCTA AGAAAAAGTG TTTAATTAAT GAATTAATCA TTAAAAAGAA AAACCAGTAT ATCCAAGTCA   
  
  
+ AAAATGCAAT AACAAACGGC CACCTTCACC GTTAGGGAAC CCGTCAAGTC AACAAAGGCA ACGATGATCC   
  
  
+ ACGCACCACA TCTAACGGAG AAATCAACGG AGTTTGTTAC ACAGAGGAAA CACCTGAGTC AATTCCAGTC   
  
  
+ AAAGACATAT ATACCCCCAA CACGACAGAC ATACAACCCC TCTCTCTTTC CTTTAAAGCA AACCCTTTCC   
  
  
+ ACTCTCTCTC TCTCTCTCTC CATCTTAATC CATTCCGATA CTTTTAAAAA ATCGGAAAAA TTTCAACGGT   
  
  
+ ACATATCGCT TCCGCCGCAA AATATCGGAC TTTTTTTAAG TGTCACCACA CTGTTGTTAC TTTACTCTCT   
  
  
+ GTTTCTGTCT CTCCTTTGGA AAAATCGGT  

- CCACAACAAA CATGTTAGTA TACGCACAGT AACGCATGTT TTGTTTTTTA TACGATCTCG TAGGTACTTT   
  
  
- CGTTATTACT TACCGTTTTG ATGTATTTTC TTTTTGTTTT GCGTCTTCGC CTGGTTGTTT TAAATCTTCT   
  
  
- TCACTTGCAC TTGATACCGC TTTGTTTTTT TTTTTTTTTT GAATGTTTTT ACCTCTTTTC CCTGTTGGAA   
  
  
- GAAGTTTTTT TTTTTTTTTT TTTTTGAATG TTTTTACCTC TTTTCCCTGT TGGAAGAAGT TCGTCTGCGA   
  
  
- CGTATTCTAT TGAAGTTAGA GAGAGGTTCT CTCTCGCGGG ACTTGTGTCA ATGACACGCG AACTTTCGAG   
  
  
- AGATCGCCAG ATAATTACGA GTGATTTTCC TGAAAAAGAT AACCAGTACT CCAAGTACGA AGGATGTTTT   
  
  
- AAAGAAGGAT GGATTTAATA AGGCGTCGAC GTCAAGTTAA GTAATTTTTT CCAGAGCCAT CGTACCGATA   
  
  
- TGCACACCAG TGTGACCAGT CGAGAAAAAG GTTGGGTACA CCCCCTATTA ACCTCGGGAG TTAACCCAGT   
  
  
- AGCAACTTTG AGTCTTCTTT GGTTTCATTA TCGTAGACGA AGGGACTTCA GTATAGAACA TGGGTAGATA   
  
  
- CCCGTGAGGT ACTTCTACGA CTAGACACAG TACGGTAAGA ATTTAAGTAG TTTCCAATTG GATTAATAAT   
  
  
- GAAACGTCTC AAAAGAAAAA GAAAGATACG GGTTTTCAAA GGATGAAATC GTGATATTGG GGTAGTTAAG   
  
  
- GTGACGAAGT AATTCAAGTA ATGTACCCGA ATAGTAAATT CTTTTGTTTC GTTTCAAACA ACGGATTGAC   
  
  
- TAATCCTTAA GGGACATTAT GATGAATGGG ATTATGAAGT TATAATAAGT TCGGTTTCTG GTCGTCAAAG   
  
  
- AGGTTCATAA ATAAACGGAT ATTCCAATCA ATAGGTAAGT GTATGCTTTC TCTACTTTCG TTTTCCGTCC   
  
  
- TTTATGCCAC CATTGTAAAA GGAAGTACGT TTTCAAAATG AGCATGTCAA CCGAAAATCC GATGTTCCAC   
  
  
- AGGTGAGGAT TCTTTTTCAC AAATTAATTA CTTAATTAGT AATTTTTCTT TTTGGTCATA TAGGTTCAGT   
  
  
- TTTTACGTTA TTGTTTGCCG GTGGAAGTGG CAATCCCTTG GGCAGTTCAG TTGTTTCCGT TGCTACTAGG   
  
  
- TGCGTGGTGT AGATTGCCTC TTTAGTTGCC TCAAACAATG TGTCTCCTTT GTGGACTCAG TTAAGGTCAG   
  
  
- TTTCTGTATA TATGGGGGTT GTGCTGTCTG TATGTTGGGG AGAGAGAAAG GAAATTTCGT TTGGGAAAGG   
  
  
- TGAGAGAGAG AGAGAGAGAG GTAGAATTAG GTAAGGCTAT GAAAATTTTT TAGCCTTTTT AAAGTTGCCA   
  
  
- TGTATAGCGA AGGCGGCGTT TTATAGCCTG AAAAAAATTC ACAGTGGTGT GACAACAATG AAATGAGAGA   
  
  
- CAAAGACAGA GAGGAAACCT TTTTAGCCA

+     Skn-1\_motif

| Site Name | Organism | Position | Strand | Matrix score. | sequence | function |
| --- | --- | --- | --- | --- | --- | --- |
| Skn-1\_motif | Oryza sativa | 658 | + | 5 | GTCAT | cis-acting regulatory element required for endosperm expression |
| Skn-1\_motif | Oryza sativa | 27 | + | 5 | GTCAT | cis-acting regulatory element required for endosperm expression |
| Skn-1\_motif | Oryza sativa | 609 | + | 5 | GTCAT | cis-acting regulatory element required for endosperm expression |
| Skn-1\_motif | Oryza sativa | 557 | + | 5 | GTCAT | cis-acting regulatory element required for endosperm expression |
| Skn-1\_motif | Oryza sativa | 394 | + | 5 | GTCAT | cis-acting regulatory element required for endosperm expression |

> 2018/04/13 10:10:12  
+ GGTGTTGTTT GTACAATCAT ATGCGTGTCA TTGCGTACAA AACAAAAAAT ATGCTAGAGC ATCCATGAAA   
  
  
+ GCAATAATGA ATGGCAAAAC TACATAAAAG AAAAACAAAA CGCAGAAGCG GACCAACAAA ATTTAGAAGA   
  
  
+ AGTGAACGTG AACTATGGCG AAACAAAAAA AAAAAAAAAA CTTACAAAAA TGGAGAAAAG GGACAACCTT   
  
  
+ CTTCAAAAAA AAAAAAAAAA AAAAACTTAC AAAAATGGAG AAAAGGGACA ACCTTCTTCA AGCAGACGCT   
  
  
+ GCATAAGATA ACTTCAATCT CTCTCCAAGA GAGAGCGCCC TGAACACAGT TACTGTGCGC TTGAAAGCTC   
  
  
+ TCTAGCGGTC TATTAATGCT CACTAAAAGG ACTTTTTCTA TTGGTCATGA GGTTCATGCT TCCTACAAAA   
  
  
+ TTTCTTCCTA CCTAAATTAT TCCGCAGCTG CAGTTCAATT CATTAAAAAA GGTCTCGGTA GCATGGCTAT   
  
  
+ ACGTGTGGTC ACACTGGTCA GCTCTTTTTC CAACCCATGT GGGGGATAAT TGGAGCCCTC AATTGGGTCA   
  
  
+ TCGTTGAAAC TCAGAAGAAA CCAAAGTAAT AGCATCTGCT TCCCTGAAGT CATATCTTGT ACCCATCTAT   
  
  
+ GGGCACTCCA TGAAGATGCT GATCTGTGTC ATGCCATTCT TAAATTCATC AAAGGTTAAC CTAATTATTA   
  
  
+ CTTTGCAGAG TTTTCTTTTT CTTTCTATGC CCAAAAGTTT CCTACTTTAG CACTATAACC CCATCAATTC   
  
  
+ CACTGCTTCA TTAAGTTCAT TACATGGGCT TATCATTTAA GAAAACAAAG CAAAGTTTGT TGCCTAACTG   
  
  
+ ATTAGGAATT CCCTGTAATA CTACTTACCC TAATACTTCA ATATTATTCA AGCCAAAGAC CAGCAGTTTC   
  
  
+ TCCAAGTATT TATTTGCCTA TAAGGTTAGT TATCCATTCA CATACGAAAG AGATGAAAGC AAAAGGCAGG   
  
  
+ AAATACGGTG GTAACATTTT CCTTCATGCA AAAGTTTTAC TCGTACAGTT GGCTTTTAGG CTACAAGGTG   
  
  
+ TCCACTCCTA AGAAAAAGTG TTTAATTAAT GAATTAATCA TTAAAAAGAA AAACCAGTAT ATCCAAGTCA   
  
  
+ AAAATGCAAT AACAAACGGC CACCTTCACC GTTAGGGAAC CCGTCAAGTC AACAAAGGCA ACGATGATCC   
  
  
+ ACGCACCACA TCTAACGGAG AAATCAACGG AGTTTGTTAC ACAGAGGAAA CACCTGAGTC AATTCCAGTC   
  
  
+ AAAGACATAT ATACCCCCAA CACGACAGAC ATACAACCCC TCTCTCTTTC CTTTAAAGCA AACCCTTTCC   
  
  
+ ACTCTCTCTC TCTCTCTCTC CATCTTAATC CATTCCGATA CTTTTAAAAA ATCGGAAAAA TTTCAACGGT   
  
  
+ ACATATCGCT TCCGCCGCAA AATATCGGAC TTTTTTTAAG TGTCACCACA CTGTTGTTAC TTTACTCTCT   
  
  
+ GTTTCTGTCT CTCCTTTGGA AAAATCGGT  

- CCACAACAAA CATGTTAGTA TACGCACAGT AACGCATGTT TTGTTTTTTA TACGATCTCG TAGGTACTTT   
  
  
- CGTTATTACT TACCGTTTTG ATGTATTTTC TTTTTGTTTT GCGTCTTCGC CTGGTTGTTT TAAATCTTCT   
  
  
- TCACTTGCAC TTGATACCGC TTTGTTTTTT TTTTTTTTTT GAATGTTTTT ACCTCTTTTC CCTGTTGGAA   
  
  
- GAAGTTTTTT TTTTTTTTTT TTTTTGAATG TTTTTACCTC TTTTCCCTGT TGGAAGAAGT TCGTCTGCGA   
  
  
- CGTATTCTAT TGAAGTTAGA GAGAGGTTCT CTCTCGCGGG ACTTGTGTCA ATGACACGCG AACTTTCGAG   
  
  
- AGATCGCCAG ATAATTACGA GTGATTTTCC TGAAAAAGAT AACCAGTACT CCAAGTACGA AGGATGTTTT   
  
  
- AAAGAAGGAT GGATTTAATA AGGCGTCGAC GTCAAGTTAA GTAATTTTTT CCAGAGCCAT CGTACCGATA   
  
  
- TGCACACCAG TGTGACCAGT CGAGAAAAAG GTTGGGTACA CCCCCTATTA ACCTCGGGAG TTAACCCAGT   
  
  
- AGCAACTTTG AGTCTTCTTT GGTTTCATTA TCGTAGACGA AGGGACTTCA GTATAGAACA TGGGTAGATA   
  
  
- CCCGTGAGGT ACTTCTACGA CTAGACACAG TACGGTAAGA ATTTAAGTAG TTTCCAATTG GATTAATAAT   
  
  
- GAAACGTCTC AAAAGAAAAA GAAAGATACG GGTTTTCAAA GGATGAAATC GTGATATTGG GGTAGTTAAG   
  
  
- GTGACGAAGT AATTCAAGTA ATGTACCCGA ATAGTAAATT CTTTTGTTTC GTTTCAAACA ACGGATTGAC   
  
  
- TAATCCTTAA GGGACATTAT GATGAATGGG ATTATGAAGT TATAATAAGT TCGGTTTCTG GTCGTCAAAG   
  
  
- AGGTTCATAA ATAAACGGAT ATTCCAATCA ATAGGTAAGT GTATGCTTTC TCTACTTTCG TTTTCCGTCC   
  
  
- TTTATGCCAC CATTGTAAAA GGAAGTACGT TTTCAAAATG AGCATGTCAA CCGAAAATCC GATGTTCCAC   
  
  
- AGGTGAGGAT TCTTTTTCAC AAATTAATTA CTTAATTAGT AATTTTTCTT TTTGGTCATA TAGGTTCAGT   
  
  
- TTTTACGTTA TTGTTTGCCG GTGGAAGTGG CAATCCCTTG GGCAGTTCAG TTGTTTCCGT TGCTACTAGG   
  
  
- TGCGTGGTGT AGATTGCCTC TTTAGTTGCC TCAAACAATG TGTCTCCTTT GTGGACTCAG TTAAGGTCAG   
  
  
- TTTCTGTATA TATGGGGGTT GTGCTGTCTG TATGTTGGGG AGAGAGAAAG GAAATTTCGT TTGGGAAAGG   
  
  
- TGAGAGAGAG AGAGAGAGAG GTAGAATTAG GTAAGGCTAT GAAAATTTTT TAGCCTTTTT AAAGTTGCCA   
  
  
- TGTATAGCGA AGGCGGCGTT TTATAGCCTG AAAAAAATTC ACAGTGGTGT GACAACAATG AAATGAGAGA   
  
  
- CAAAGACAGA GAGGAAACCT TTTTAGCCA

+     TATA-box

| Site Name | Organism | Position | Strand | Matrix score. | sequence | function |
| --- | --- | --- | --- | --- | --- | --- |
| TATA-box | Lycopersicon esculentum | 1092 | - | 5 | TTTTA | core promoter element around -30 of transcription start |
| TATA-box | Lycopersicon esculentum | 1434 | + | 5 | TTTTA | core promoter element around -30 of transcription start |
| TATA-box | Lycopersicon esculentum | 1375 | - | 5 | TTTTA | core promoter element around -30 of transcription start |
| TATA-box | Glycine max | 587 | + | 5 | TAATA | core promoter element around -30 of transcription start |
| TATA-box | Lycopersicon esculentum | 464 | - | 5 | TTTTA | core promoter element around -30 of transcription start |
| TATA-box | Lycopersicon esculentum | 1372 | + | 5 | TTTTA | core promoter element around -30 of transcription start |
| TATA-box | Lycopersicon esculentum | 374 | - | 5 | TTTTA | core promoter element around -30 of transcription start |
| TATA-box | Glycine max | 871 | + | 5 | TAATA | core promoter element around -30 of transcription start |
| TATA-box | Arabidopsis thaliana | 488 | + | 4 | TATA | core promoter element around -30 of transcription start |
| TATA-box | Oryza sativa | 183 | + | 7 | TACAAAA | core promoter element around -30 of transcription start |
| TATA-box | Arabidopsis thaliana | 754 | - | 4 | TATA | core promoter element around -30 of transcription start |
| TATA-box | Lycopersicon esculentum | 1015 | + | 5 | TTTTA | core promoter element around -30 of transcription start |
| TATA-box | Lycopersicon esculentum | 95 | - | 5 | TTTTA | core promoter element around -30 of transcription start |
| TATA-box | Lycopersicon esculentum | 1034 | + | 5 | TTTTA | core promoter element around -30 of transcription start |
| TATA-box | Glycine max | 361 | - | 5 | TAATA | core promoter element around -30 of transcription start |
| TATA-box | Arabidopsis thaliana | 1270 | - | 4 | TATA | core promoter element around -30 of transcription start |
| TATA-box | Oryza sativa | 36 | + | 7 | TACAAAA | core promoter element around -30 of transcription start |
| TATA-box | Brassica napus | 1267 | - | 6 | ATATAT | core promoter element around -30 of transcription start |
| TATA-box | Glycine max | 696 | - | 5 | TAATA | core promoter element around -30 of transcription start |
| TATA-box | Zea mays | 1373 | + | 8 | TTTAAAAA | core promoter element around -30 of transcription start |
| TATA-box | Glycine max | 882 | - | 5 | TAATA | core promoter element around -30 of transcription start |
| TATA-box | Arabidopsis thaliana | 1108 | - | 4 | TATA | core promoter element around -30 of transcription start |
| TATA-box | Arabidopsis thaliana | 929 | - | 4 | TATA | core promoter element around -30 of transcription start |
| TATA-box | Arabidopsis thaliana | 1268 | - | 4 | TATA | core promoter element around -30 of transcription start |
| TATA-box | Oryza sativa | 238 | + | 7 | TACAAAA | core promoter element around -30 of transcription start |
| TATA-box | Pisum sativum | 1265 | - | 7 | TATATGT | core promoter element around -30 of transcription start |
| TATA-box | Oryza sativa | 91 | + | 8 | TACATAAA | core promoter element around -30 of transcription start |
| TATA-box | Glycine max | 856 | + | 5 | TAATA | core promoter element around -30 of transcription start |
| TATA-box | Oryza sativa | 414 | + | 7 | TACAAAA | core promoter element around -30 of transcription start |

> 2018/04/13 10:10:12  
+ GGTGTTGTTT GTACAATCAT ATGCGTGTCA TTGCGTACAA AACAAAAAAT ATGCTAGAGC ATCCATGAAA   
  
  
+ GCAATAATGA ATGGCAAAAC TACATAAAAG AAAAACAAAA CGCAGAAGCG GACCAACAAA ATTTAGAAGA   
  
  
+ AGTGAACGTG AACTATGGCG AAACAAAAAA AAAAAAAAAA CTTACAAAAA TGGAGAAAAG GGACAACCTT   
  
  
+ CTTCAAAAAA AAAAAAAAAA AAAAACTTAC AAAAATGGAG AAAAGGGACA ACCTTCTTCA AGCAGACGCT   
  
  
+ GCATAAGATA ACTTCAATCT CTCTCCAAGA GAGAGCGCCC TGAACACAGT TACTGTGCGC TTGAAAGCTC   
  
  
+ TCTAGCGGTC TATTAATGCT CACTAAAAGG ACTTTTTCTA TTGGTCATGA GGTTCATGCT TCCTACAAAA   
  
  
+ TTTCTTCCTA CCTAAATTAT TCCGCAGCTG CAGTTCAATT CATTAAAAAA GGTCTCGGTA GCATGGCTAT   
  
  
+ ACGTGTGGTC ACACTGGTCA GCTCTTTTTC CAACCCATGT GGGGGATAAT TGGAGCCCTC AATTGGGTCA   
  
  
+ TCGTTGAAAC TCAGAAGAAA CCAAAGTAAT AGCATCTGCT TCCCTGAAGT CATATCTTGT ACCCATCTAT   
  
  
+ GGGCACTCCA TGAAGATGCT GATCTGTGTC ATGCCATTCT TAAATTCATC AAAGGTTAAC CTAATTATTA   
  
  
+ CTTTGCAGAG TTTTCTTTTT CTTTCTATGC CCAAAAGTTT CCTACTTTAG CACTATAACC CCATCAATTC   
  
  
+ CACTGCTTCA TTAAGTTCAT TACATGGGCT TATCATTTAA GAAAACAAAG CAAAGTTTGT TGCCTAACTG   
  
  
+ ATTAGGAATT CCCTGTAATA CTACTTACCC TAATACTTCA ATATTATTCA AGCCAAAGAC CAGCAGTTTC   
  
  
+ TCCAAGTATT TATTTGCCTA TAAGGTTAGT TATCCATTCA CATACGAAAG AGATGAAAGC AAAAGGCAGG   
  
  
+ AAATACGGTG GTAACATTTT CCTTCATGCA AAAGTTTTAC TCGTACAGTT GGCTTTTAGG CTACAAGGTG   
  
  
+ TCCACTCCTA AGAAAAAGTG TTTAATTAAT GAATTAATCA TTAAAAAGAA AAACCAGTAT ATCCAAGTCA   
  
  
+ AAAATGCAAT AACAAACGGC CACCTTCACC GTTAGGGAAC CCGTCAAGTC AACAAAGGCA ACGATGATCC   
  
  
+ ACGCACCACA TCTAACGGAG AAATCAACGG AGTTTGTTAC ACAGAGGAAA CACCTGAGTC AATTCCAGTC   
  
  
+ AAAGACATAT ATACCCCCAA CACGACAGAC ATACAACCCC TCTCTCTTTC CTTTAAAGCA AACCCTTTCC   
  
  
+ ACTCTCTCTC TCTCTCTCTC CATCTTAATC CATTCCGATA CTTTTAAAAA ATCGGAAAAA TTTCAACGGT   
  
  
+ ACATATCGCT TCCGCCGCAA AATATCGGAC TTTTTTTAAG TGTCACCACA CTGTTGTTAC TTTACTCTCT   
  
  
+ GTTTCTGTCT CTCCTTTGGA AAAATCGGT  

- CCACAACAAA CATGTTAGTA TACGCACAGT AACGCATGTT TTGTTTTTTA TACGATCTCG TAGGTACTTT   
  
  
- CGTTATTACT TACCGTTTTG ATGTATTTTC TTTTTGTTTT GCGTCTTCGC CTGGTTGTTT TAAATCTTCT   
  
  
- TCACTTGCAC TTGATACCGC TTTGTTTTTT TTTTTTTTTT GAATGTTTTT ACCTCTTTTC CCTGTTGGAA   
  
  
- GAAGTTTTTT TTTTTTTTTT TTTTTGAATG TTTTTACCTC TTTTCCCTGT TGGAAGAAGT TCGTCTGCGA   
  
  
- CGTATTCTAT TGAAGTTAGA GAGAGGTTCT CTCTCGCGGG ACTTGTGTCA ATGACACGCG AACTTTCGAG   
  
  
- AGATCGCCAG ATAATTACGA GTGATTTTCC TGAAAAAGAT AACCAGTACT CCAAGTACGA AGGATGTTTT   
  
  
- AAAGAAGGAT GGATTTAATA AGGCGTCGAC GTCAAGTTAA GTAATTTTTT CCAGAGCCAT CGTACCGATA   
  
  
- TGCACACCAG TGTGACCAGT CGAGAAAAAG GTTGGGTACA CCCCCTATTA ACCTCGGGAG TTAACCCAGT   
  
  
- AGCAACTTTG AGTCTTCTTT GGTTTCATTA TCGTAGACGA AGGGACTTCA GTATAGAACA TGGGTAGATA   
  
  
- CCCGTGAGGT ACTTCTACGA CTAGACACAG TACGGTAAGA ATTTAAGTAG TTTCCAATTG GATTAATAAT   
  
  
- GAAACGTCTC AAAAGAAAAA GAAAGATACG GGTTTTCAAA GGATGAAATC GTGATATTGG GGTAGTTAAG   
  
  
- GTGACGAAGT AATTCAAGTA ATGTACCCGA ATAGTAAATT CTTTTGTTTC GTTTCAAACA ACGGATTGAC   
  
  
- TAATCCTTAA GGGACATTAT GATGAATGGG ATTATGAAGT TATAATAAGT TCGGTTTCTG GTCGTCAAAG   
  
  
- AGGTTCATAA ATAAACGGAT ATTCCAATCA ATAGGTAAGT GTATGCTTTC TCTACTTTCG TTTTCCGTCC   
  
  
- TTTATGCCAC CATTGTAAAA GGAAGTACGT TTTCAAAATG AGCATGTCAA CCGAAAATCC GATGTTCCAC   
  
  
- AGGTGAGGAT TCTTTTTCAC AAATTAATTA CTTAATTAGT AATTTTTCTT TTTGGTCATA TAGGTTCAGT   
  
  
- TTTTACGTTA TTGTTTGCCG GTGGAAGTGG CAATCCCTTG GGCAGTTCAG TTGTTTCCGT TGCTACTAGG   
  
  
- TGCGTGGTGT AGATTGCCTC TTTAGTTGCC TCAAACAATG TGTCTCCTTT GTGGACTCAG TTAAGGTCAG   
  
  
- TTTCTGTATA TATGGGGGTT GTGCTGTCTG TATGTTGGGG AGAGAGAAAG GAAATTTCGT TTGGGAAAGG   
  
  
- TGAGAGAGAG AGAGAGAGAG GTAGAATTAG GTAAGGCTAT GAAAATTTTT TAGCCTTTTT AAAGTTGCCA   
  
  
- TGTATAGCGA AGGCGGCGTT TTATAGCCTG AAAAAAATTC ACAGTGGTGT GACAACAATG AAATGAGAGA   
  
  
- CAAAGACAGA GAGGAAACCT TTTTAGCCA

+     TATCCAT/C-motif

| Site Name | Organism | Position | Strand | Matrix score. | sequence | function |
| --- | --- | --- | --- | --- | --- | --- |
| TATCCAT/C-motif | Oryza sativa | 941 | + | 7 | TATCCAT |  |

> 2018/04/13 10:10:12  
+ GGTGTTGTTT GTACAATCAT ATGCGTGTCA TTGCGTACAA AACAAAAAAT ATGCTAGAGC ATCCATGAAA   
  
  
+ GCAATAATGA ATGGCAAAAC TACATAAAAG AAAAACAAAA CGCAGAAGCG GACCAACAAA ATTTAGAAGA   
  
  
+ AGTGAACGTG AACTATGGCG AAACAAAAAA AAAAAAAAAA CTTACAAAAA TGGAGAAAAG GGACAACCTT   
  
  
+ CTTCAAAAAA AAAAAAAAAA AAAAACTTAC AAAAATGGAG AAAAGGGACA ACCTTCTTCA AGCAGACGCT   
  
  
+ GCATAAGATA ACTTCAATCT CTCTCCAAGA GAGAGCGCCC TGAACACAGT TACTGTGCGC TTGAAAGCTC   
  
  
+ TCTAGCGGTC TATTAATGCT CACTAAAAGG ACTTTTTCTA TTGGTCATGA GGTTCATGCT TCCTACAAAA   
  
  
+ TTTCTTCCTA CCTAAATTAT TCCGCAGCTG CAGTTCAATT CATTAAAAAA GGTCTCGGTA GCATGGCTAT   
  
  
+ ACGTGTGGTC ACACTGGTCA GCTCTTTTTC CAACCCATGT GGGGGATAAT TGGAGCCCTC AATTGGGTCA   
  
  
+ TCGTTGAAAC TCAGAAGAAA CCAAAGTAAT AGCATCTGCT TCCCTGAAGT CATATCTTGT ACCCATCTAT   
  
  
+ GGGCACTCCA TGAAGATGCT GATCTGTGTC ATGCCATTCT TAAATTCATC AAAGGTTAAC CTAATTATTA   
  
  
+ CTTTGCAGAG TTTTCTTTTT CTTTCTATGC CCAAAAGTTT CCTACTTTAG CACTATAACC CCATCAATTC   
  
  
+ CACTGCTTCA TTAAGTTCAT TACATGGGCT TATCATTTAA GAAAACAAAG CAAAGTTTGT TGCCTAACTG   
  
  
+ ATTAGGAATT CCCTGTAATA CTACTTACCC TAATACTTCA ATATTATTCA AGCCAAAGAC CAGCAGTTTC   
  
  
+ TCCAAGTATT TATTTGCCTA TAAGGTTAGT TATCCATTCA CATACGAAAG AGATGAAAGC AAAAGGCAGG   
  
  
+ AAATACGGTG GTAACATTTT CCTTCATGCA AAAGTTTTAC TCGTACAGTT GGCTTTTAGG CTACAAGGTG   
  
  
+ TCCACTCCTA AGAAAAAGTG TTTAATTAAT GAATTAATCA TTAAAAAGAA AAACCAGTAT ATCCAAGTCA   
  
  
+ AAAATGCAAT AACAAACGGC CACCTTCACC GTTAGGGAAC CCGTCAAGTC AACAAAGGCA ACGATGATCC   
  
  
+ ACGCACCACA TCTAACGGAG AAATCAACGG AGTTTGTTAC ACAGAGGAAA CACCTGAGTC AATTCCAGTC   
  
  
+ AAAGACATAT ATACCCCCAA CACGACAGAC ATACAACCCC TCTCTCTTTC CTTTAAAGCA AACCCTTTCC   
  
  
+ ACTCTCTCTC TCTCTCTCTC CATCTTAATC CATTCCGATA CTTTTAAAAA ATCGGAAAAA TTTCAACGGT   
  
  
+ ACATATCGCT TCCGCCGCAA AATATCGGAC TTTTTTTAAG TGTCACCACA CTGTTGTTAC TTTACTCTCT   
  
  
+ GTTTCTGTCT CTCCTTTGGA AAAATCGGT  

- CCACAACAAA CATGTTAGTA TACGCACAGT AACGCATGTT TTGTTTTTTA TACGATCTCG TAGGTACTTT   
  
  
- CGTTATTACT TACCGTTTTG ATGTATTTTC TTTTTGTTTT GCGTCTTCGC CTGGTTGTTT TAAATCTTCT   
  
  
- TCACTTGCAC TTGATACCGC TTTGTTTTTT TTTTTTTTTT GAATGTTTTT ACCTCTTTTC CCTGTTGGAA   
  
  
- GAAGTTTTTT TTTTTTTTTT TTTTTGAATG TTTTTACCTC TTTTCCCTGT TGGAAGAAGT TCGTCTGCGA   
  
  
- CGTATTCTAT TGAAGTTAGA GAGAGGTTCT CTCTCGCGGG ACTTGTGTCA ATGACACGCG AACTTTCGAG   
  
  
- AGATCGCCAG ATAATTACGA GTGATTTTCC TGAAAAAGAT AACCAGTACT CCAAGTACGA AGGATGTTTT   
  
  
- AAAGAAGGAT GGATTTAATA AGGCGTCGAC GTCAAGTTAA GTAATTTTTT CCAGAGCCAT CGTACCGATA   
  
  
- TGCACACCAG TGTGACCAGT CGAGAAAAAG GTTGGGTACA CCCCCTATTA ACCTCGGGAG TTAACCCAGT   
  
  
- AGCAACTTTG AGTCTTCTTT GGTTTCATTA TCGTAGACGA AGGGACTTCA GTATAGAACA TGGGTAGATA   
  
  
- CCCGTGAGGT ACTTCTACGA CTAGACACAG TACGGTAAGA ATTTAAGTAG TTTCCAATTG GATTAATAAT   
  
  
- GAAACGTCTC AAAAGAAAAA GAAAGATACG GGTTTTCAAA GGATGAAATC GTGATATTGG GGTAGTTAAG   
  
  
- GTGACGAAGT AATTCAAGTA ATGTACCCGA ATAGTAAATT CTTTTGTTTC GTTTCAAACA ACGGATTGAC   
  
  
- TAATCCTTAA GGGACATTAT GATGAATGGG ATTATGAAGT TATAATAAGT TCGGTTTCTG GTCGTCAAAG   
  
  
- AGGTTCATAA ATAAACGGAT ATTCCAATCA ATAGGTAAGT GTATGCTTTC TCTACTTTCG TTTTCCGTCC   
  
  
- TTTATGCCAC CATTGTAAAA GGAAGTACGT TTTCAAAATG AGCATGTCAA CCGAAAATCC GATGTTCCAC   
  
  
- AGGTGAGGAT TCTTTTTCAC AAATTAATTA CTTAATTAGT AATTTTTCTT TTTGGTCATA TAGGTTCAGT   
  
  
- TTTTACGTTA TTGTTTGCCG GTGGAAGTGG CAATCCCTTG GGCAGTTCAG TTGTTTCCGT TGCTACTAGG   
  
  
- TGCGTGGTGT AGATTGCCTC TTTAGTTGCC TCAAACAATG TGTCTCCTTT GTGGACTCAG TTAAGGTCAG   
  
  
- TTTCTGTATA TATGGGGGTT GTGCTGTCTG TATGTTGGGG AGAGAGAAAG GAAATTTCGT TTGGGAAAGG   
  
  
- TGAGAGAGAG AGAGAGAGAG GTAGAATTAG GTAAGGCTAT GAAAATTTTT TAGCCTTTTT AAAGTTGCCA   
  
  
- TGTATAGCGA AGGCGGCGTT TTATAGCCTG AAAAAAATTC ACAGTGGTGT GACAACAATG AAATGAGAGA   
  
  
- CAAAGACAGA GAGGAAACCT TTTTAGCCA

+     TC-rich repeats

| Site Name | Organism | Position | Strand | Matrix score. | sequence | function |
| --- | --- | --- | --- | --- | --- | --- |
| TC-rich repeats | Nicotiana tabacum | 905 | + | 9 | ATTTTCTCCA | cis-acting element involved in defense and stress responsiveness |
| TC-rich repeats | Nicotiana tabacum | 246 | - | 9 | ATTTTCTCCA | cis-acting element involved in defense and stress responsiveness |
| TC-rich repeats | Nicotiana tabacum | 191 | - | 9 | ATTTTCTCCA | cis-acting element involved in defense and stress responsiveness |
| TC-rich repeats | Nicotiana tabacum | 807 | - | 9 | GTTTTCTTAC | cis-acting element involved in defense and stress responsiveness |

> 2018/04/13 10:10:12  
+ GGTGTTGTTT GTACAATCAT ATGCGTGTCA TTGCGTACAA AACAAAAAAT ATGCTAGAGC ATCCATGAAA   
  
  
+ GCAATAATGA ATGGCAAAAC TACATAAAAG AAAAACAAAA CGCAGAAGCG GACCAACAAA ATTTAGAAGA   
  
  
+ AGTGAACGTG AACTATGGCG AAACAAAAAA AAAAAAAAAA CTTACAAAAA TGGAGAAAAG GGACAACCTT   
  
  
+ CTTCAAAAAA AAAAAAAAAA AAAAACTTAC AAAAATGGAG AAAAGGGACA ACCTTCTTCA AGCAGACGCT   
  
  
+ GCATAAGATA ACTTCAATCT CTCTCCAAGA GAGAGCGCCC TGAACACAGT TACTGTGCGC TTGAAAGCTC   
  
  
+ TCTAGCGGTC TATTAATGCT CACTAAAAGG ACTTTTTCTA TTGGTCATGA GGTTCATGCT TCCTACAAAA   
  
  
+ TTTCTTCCTA CCTAAATTAT TCCGCAGCTG CAGTTCAATT CATTAAAAAA GGTCTCGGTA GCATGGCTAT   
  
  
+ ACGTGTGGTC ACACTGGTCA GCTCTTTTTC CAACCCATGT GGGGGATAAT TGGAGCCCTC AATTGGGTCA   
  
  
+ TCGTTGAAAC TCAGAAGAAA CCAAAGTAAT AGCATCTGCT TCCCTGAAGT CATATCTTGT ACCCATCTAT   
  
  
+ GGGCACTCCA TGAAGATGCT GATCTGTGTC ATGCCATTCT TAAATTCATC AAAGGTTAAC CTAATTATTA   
  
  
+ CTTTGCAGAG TTTTCTTTTT CTTTCTATGC CCAAAAGTTT CCTACTTTAG CACTATAACC CCATCAATTC   
  
  
+ CACTGCTTCA TTAAGTTCAT TACATGGGCT TATCATTTAA GAAAACAAAG CAAAGTTTGT TGCCTAACTG   
  
  
+ ATTAGGAATT CCCTGTAATA CTACTTACCC TAATACTTCA ATATTATTCA AGCCAAAGAC CAGCAGTTTC   
  
  
+ TCCAAGTATT TATTTGCCTA TAAGGTTAGT TATCCATTCA CATACGAAAG AGATGAAAGC AAAAGGCAGG   
  
  
+ AAATACGGTG GTAACATTTT CCTTCATGCA AAAGTTTTAC TCGTACAGTT GGCTTTTAGG CTACAAGGTG   
  
  
+ TCCACTCCTA AGAAAAAGTG TTTAATTAAT GAATTAATCA TTAAAAAGAA AAACCAGTAT ATCCAAGTCA   
  
  
+ AAAATGCAAT AACAAACGGC CACCTTCACC GTTAGGGAAC CCGTCAAGTC AACAAAGGCA ACGATGATCC   
  
  
+ ACGCACCACA TCTAACGGAG AAATCAACGG AGTTTGTTAC ACAGAGGAAA CACCTGAGTC AATTCCAGTC   
  
  
+ AAAGACATAT ATACCCCCAA CACGACAGAC ATACAACCCC TCTCTCTTTC CTTTAAAGCA AACCCTTTCC   
  
  
+ ACTCTCTCTC TCTCTCTCTC CATCTTAATC CATTCCGATA CTTTTAAAAA ATCGGAAAAA TTTCAACGGT   
  
  
+ ACATATCGCT TCCGCCGCAA AATATCGGAC TTTTTTTAAG TGTCACCACA CTGTTGTTAC TTTACTCTCT   
  
  
+ GTTTCTGTCT CTCCTTTGGA AAAATCGGT  

- CCACAACAAA CATGTTAGTA TACGCACAGT AACGCATGTT TTGTTTTTTA TACGATCTCG TAGGTACTTT   
  
  
- CGTTATTACT TACCGTTTTG ATGTATTTTC TTTTTGTTTT GCGTCTTCGC CTGGTTGTTT TAAATCTTCT   
  
  
- TCACTTGCAC TTGATACCGC TTTGTTTTTT TTTTTTTTTT GAATGTTTTT ACCTCTTTTC CCTGTTGGAA   
  
  
- GAAGTTTTTT TTTTTTTTTT TTTTTGAATG TTTTTACCTC TTTTCCCTGT TGGAAGAAGT TCGTCTGCGA   
  
  
- CGTATTCTAT TGAAGTTAGA GAGAGGTTCT CTCTCGCGGG ACTTGTGTCA ATGACACGCG AACTTTCGAG   
  
  
- AGATCGCCAG ATAATTACGA GTGATTTTCC TGAAAAAGAT AACCAGTACT CCAAGTACGA AGGATGTTTT   
  
  
- AAAGAAGGAT GGATTTAATA AGGCGTCGAC GTCAAGTTAA GTAATTTTTT CCAGAGCCAT CGTACCGATA   
  
  
- TGCACACCAG TGTGACCAGT CGAGAAAAAG GTTGGGTACA CCCCCTATTA ACCTCGGGAG TTAACCCAGT   
  
  
- AGCAACTTTG AGTCTTCTTT GGTTTCATTA TCGTAGACGA AGGGACTTCA GTATAGAACA TGGGTAGATA   
  
  
- CCCGTGAGGT ACTTCTACGA CTAGACACAG TACGGTAAGA ATTTAAGTAG TTTCCAATTG GATTAATAAT   
  
  
- GAAACGTCTC AAAAGAAAAA GAAAGATACG GGTTTTCAAA GGATGAAATC GTGATATTGG GGTAGTTAAG   
  
  
- GTGACGAAGT AATTCAAGTA ATGTACCCGA ATAGTAAATT CTTTTGTTTC GTTTCAAACA ACGGATTGAC   
  
  
- TAATCCTTAA GGGACATTAT GATGAATGGG ATTATGAAGT TATAATAAGT TCGGTTTCTG GTCGTCAAAG   
  
  
- AGGTTCATAA ATAAACGGAT ATTCCAATCA ATAGGTAAGT GTATGCTTTC TCTACTTTCG TTTTCCGTCC   
  
  
- TTTATGCCAC CATTGTAAAA GGAAGTACGT TTTCAAAATG AGCATGTCAA CCGAAAATCC GATGTTCCAC   
  
  
- AGGTGAGGAT TCTTTTTCAC AAATTAATTA CTTAATTAGT AATTTTTCTT TTTGGTCATA TAGGTTCAGT   
  
  
- TTTTACGTTA TTGTTTGCCG GTGGAAGTGG CAATCCCTTG GGCAGTTCAG TTGTTTCCGT TGCTACTAGG   
  
  
- TGCGTGGTGT AGATTGCCTC TTTAGTTGCC TCAAACAATG TGTCTCCTTT GTGGACTCAG TTAAGGTCAG   
  
  
- TTTCTGTATA TATGGGGGTT GTGCTGTCTG TATGTTGGGG AGAGAGAAAG GAAATTTCGT TTGGGAAAGG   
  
  
- TGAGAGAGAG AGAGAGAGAG GTAGAATTAG GTAAGGCTAT GAAAATTTTT TAGCCTTTTT AAAGTTGCCA   
  
  
- TGTATAGCGA AGGCGGCGTT TTATAGCCTG AAAAAAATTC ACAGTGGTGT GACAACAATG AAATGAGAGA   
  
  
- CAAAGACAGA GAGGAAACCT TTTTAGCCA

+     TGACG-motif

| Site Name | Organism | Position | Strand | Matrix score. | sequence | function |
| --- | --- | --- | --- | --- | --- | --- |
| TGACG-motif | Hordeum vulgare | 1162 | - | 5 | TGACG | cis-acting regulatory element involved in the MeJA-responsiveness |

> 2018/04/13 10:10:12  
+ GGTGTTGTTT GTACAATCAT ATGCGTGTCA TTGCGTACAA AACAAAAAAT ATGCTAGAGC ATCCATGAAA   
  
  
+ GCAATAATGA ATGGCAAAAC TACATAAAAG AAAAACAAAA CGCAGAAGCG GACCAACAAA ATTTAGAAGA   
  
  
+ AGTGAACGTG AACTATGGCG AAACAAAAAA AAAAAAAAAA CTTACAAAAA TGGAGAAAAG GGACAACCTT   
  
  
+ CTTCAAAAAA AAAAAAAAAA AAAAACTTAC AAAAATGGAG AAAAGGGACA ACCTTCTTCA AGCAGACGCT   
  
  
+ GCATAAGATA ACTTCAATCT CTCTCCAAGA GAGAGCGCCC TGAACACAGT TACTGTGCGC TTGAAAGCTC   
  
  
+ TCTAGCGGTC TATTAATGCT CACTAAAAGG ACTTTTTCTA TTGGTCATGA GGTTCATGCT TCCTACAAAA   
  
  
+ TTTCTTCCTA CCTAAATTAT TCCGCAGCTG CAGTTCAATT CATTAAAAAA GGTCTCGGTA GCATGGCTAT   
  
  
+ ACGTGTGGTC ACACTGGTCA GCTCTTTTTC CAACCCATGT GGGGGATAAT TGGAGCCCTC AATTGGGTCA   
  
  
+ TCGTTGAAAC TCAGAAGAAA CCAAAGTAAT AGCATCTGCT TCCCTGAAGT CATATCTTGT ACCCATCTAT   
  
  
+ GGGCACTCCA TGAAGATGCT GATCTGTGTC ATGCCATTCT TAAATTCATC AAAGGTTAAC CTAATTATTA   
  
  
+ CTTTGCAGAG TTTTCTTTTT CTTTCTATGC CCAAAAGTTT CCTACTTTAG CACTATAACC CCATCAATTC   
  
  
+ CACTGCTTCA TTAAGTTCAT TACATGGGCT TATCATTTAA GAAAACAAAG CAAAGTTTGT TGCCTAACTG   
  
  
+ ATTAGGAATT CCCTGTAATA CTACTTACCC TAATACTTCA ATATTATTCA AGCCAAAGAC CAGCAGTTTC   
  
  
+ TCCAAGTATT TATTTGCCTA TAAGGTTAGT TATCCATTCA CATACGAAAG AGATGAAAGC AAAAGGCAGG   
  
  
+ AAATACGGTG GTAACATTTT CCTTCATGCA AAAGTTTTAC TCGTACAGTT GGCTTTTAGG CTACAAGGTG   
  
  
+ TCCACTCCTA AGAAAAAGTG TTTAATTAAT GAATTAATCA TTAAAAAGAA AAACCAGTAT ATCCAAGTCA   
  
  
+ AAAATGCAAT AACAAACGGC CACCTTCACC GTTAGGGAAC CCGTCAAGTC AACAAAGGCA ACGATGATCC   
  
  
+ ACGCACCACA TCTAACGGAG AAATCAACGG AGTTTGTTAC ACAGAGGAAA CACCTGAGTC AATTCCAGTC   
  
  
+ AAAGACATAT ATACCCCCAA CACGACAGAC ATACAACCCC TCTCTCTTTC CTTTAAAGCA AACCCTTTCC   
  
  
+ ACTCTCTCTC TCTCTCTCTC CATCTTAATC CATTCCGATA CTTTTAAAAA ATCGGAAAAA TTTCAACGGT   
  
  
+ ACATATCGCT TCCGCCGCAA AATATCGGAC TTTTTTTAAG TGTCACCACA CTGTTGTTAC TTTACTCTCT   
  
  
+ GTTTCTGTCT CTCCTTTGGA AAAATCGGT  

- CCACAACAAA CATGTTAGTA TACGCACAGT AACGCATGTT TTGTTTTTTA TACGATCTCG TAGGTACTTT   
  
  
- CGTTATTACT TACCGTTTTG ATGTATTTTC TTTTTGTTTT GCGTCTTCGC CTGGTTGTTT TAAATCTTCT   
  
  
- TCACTTGCAC TTGATACCGC TTTGTTTTTT TTTTTTTTTT GAATGTTTTT ACCTCTTTTC CCTGTTGGAA   
  
  
- GAAGTTTTTT TTTTTTTTTT TTTTTGAATG TTTTTACCTC TTTTCCCTGT TGGAAGAAGT TCGTCTGCGA   
  
  
- CGTATTCTAT TGAAGTTAGA GAGAGGTTCT CTCTCGCGGG ACTTGTGTCA ATGACACGCG AACTTTCGAG   
  
  
- AGATCGCCAG ATAATTACGA GTGATTTTCC TGAAAAAGAT AACCAGTACT CCAAGTACGA AGGATGTTTT   
  
  
- AAAGAAGGAT GGATTTAATA AGGCGTCGAC GTCAAGTTAA GTAATTTTTT CCAGAGCCAT CGTACCGATA   
  
  
- TGCACACCAG TGTGACCAGT CGAGAAAAAG GTTGGGTACA CCCCCTATTA ACCTCGGGAG TTAACCCAGT   
  
  
- AGCAACTTTG AGTCTTCTTT GGTTTCATTA TCGTAGACGA AGGGACTTCA GTATAGAACA TGGGTAGATA   
  
  
- CCCGTGAGGT ACTTCTACGA CTAGACACAG TACGGTAAGA ATTTAAGTAG TTTCCAATTG GATTAATAAT   
  
  
- GAAACGTCTC AAAAGAAAAA GAAAGATACG GGTTTTCAAA GGATGAAATC GTGATATTGG GGTAGTTAAG   
  
  
- GTGACGAAGT AATTCAAGTA ATGTACCCGA ATAGTAAATT CTTTTGTTTC GTTTCAAACA ACGGATTGAC   
  
  
- TAATCCTTAA GGGACATTAT GATGAATGGG ATTATGAAGT TATAATAAGT TCGGTTTCTG GTCGTCAAAG   
  
  
- AGGTTCATAA ATAAACGGAT ATTCCAATCA ATAGGTAAGT GTATGCTTTC TCTACTTTCG TTTTCCGTCC   
  
  
- TTTATGCCAC CATTGTAAAA GGAAGTACGT TTTCAAAATG AGCATGTCAA CCGAAAATCC GATGTTCCAC   
  
  
- AGGTGAGGAT TCTTTTTCAC AAATTAATTA CTTAATTAGT AATTTTTCTT TTTGGTCATA TAGGTTCAGT   
  
  
- TTTTACGTTA TTGTTTGCCG GTGGAAGTGG CAATCCCTTG GGCAGTTCAG TTGTTTCCGT TGCTACTAGG   
  
  
- TGCGTGGTGT AGATTGCCTC TTTAGTTGCC TCAAACAATG TGTCTCCTTT GTGGACTCAG TTAAGGTCAG   
  
  
- TTTCTGTATA TATGGGGGTT GTGCTGTCTG TATGTTGGGG AGAGAGAAAG GAAATTTCGT TTGGGAAAGG   
  
  
- TGAGAGAGAG AGAGAGAGAG GTAGAATTAG GTAAGGCTAT GAAAATTTTT TAGCCTTTTT AAAGTTGCCA   
  
  
- TGTATAGCGA AGGCGGCGTT TTATAGCCTG AAAAAAATTC ACAGTGGTGT GACAACAATG AAATGAGAGA   
  
  
- CAAAGACAGA GAGGAAACCT TTTTAGCCA

+     Unnamed\_\_1

| Site Name | Organism | Position | Strand | Matrix score. | sequence | function |
| --- | --- | --- | --- | --- | --- | --- |
| Unnamed\_\_1 | Zea mays | 1189 | - | 5 | CGTGG |  |

> 2018/04/13 10:10:12  
+ GGTGTTGTTT GTACAATCAT ATGCGTGTCA TTGCGTACAA AACAAAAAAT ATGCTAGAGC ATCCATGAAA   
  
  
+ GCAATAATGA ATGGCAAAAC TACATAAAAG AAAAACAAAA CGCAGAAGCG GACCAACAAA ATTTAGAAGA   
  
  
+ AGTGAACGTG AACTATGGCG AAACAAAAAA AAAAAAAAAA CTTACAAAAA TGGAGAAAAG GGACAACCTT   
  
  
+ CTTCAAAAAA AAAAAAAAAA AAAAACTTAC AAAAATGGAG AAAAGGGACA ACCTTCTTCA AGCAGACGCT   
  
  
+ GCATAAGATA ACTTCAATCT CTCTCCAAGA GAGAGCGCCC TGAACACAGT TACTGTGCGC TTGAAAGCTC   
  
  
+ TCTAGCGGTC TATTAATGCT CACTAAAAGG ACTTTTTCTA TTGGTCATGA GGTTCATGCT TCCTACAAAA   
  
  
+ TTTCTTCCTA CCTAAATTAT TCCGCAGCTG CAGTTCAATT CATTAAAAAA GGTCTCGGTA GCATGGCTAT   
  
  
+ ACGTGTGGTC ACACTGGTCA GCTCTTTTTC CAACCCATGT GGGGGATAAT TGGAGCCCTC AATTGGGTCA   
  
  
+ TCGTTGAAAC TCAGAAGAAA CCAAAGTAAT AGCATCTGCT TCCCTGAAGT CATATCTTGT ACCCATCTAT   
  
  
+ GGGCACTCCA TGAAGATGCT GATCTGTGTC ATGCCATTCT TAAATTCATC AAAGGTTAAC CTAATTATTA   
  
  
+ CTTTGCAGAG TTTTCTTTTT CTTTCTATGC CCAAAAGTTT CCTACTTTAG CACTATAACC CCATCAATTC   
  
  
+ CACTGCTTCA TTAAGTTCAT TACATGGGCT TATCATTTAA GAAAACAAAG CAAAGTTTGT TGCCTAACTG   
  
  
+ ATTAGGAATT CCCTGTAATA CTACTTACCC TAATACTTCA ATATTATTCA AGCCAAAGAC CAGCAGTTTC   
  
  
+ TCCAAGTATT TATTTGCCTA TAAGGTTAGT TATCCATTCA CATACGAAAG AGATGAAAGC AAAAGGCAGG   
  
  
+ AAATACGGTG GTAACATTTT CCTTCATGCA AAAGTTTTAC TCGTACAGTT GGCTTTTAGG CTACAAGGTG   
  
  
+ TCCACTCCTA AGAAAAAGTG TTTAATTAAT GAATTAATCA TTAAAAAGAA AAACCAGTAT ATCCAAGTCA   
  
  
+ AAAATGCAAT AACAAACGGC CACCTTCACC GTTAGGGAAC CCGTCAAGTC AACAAAGGCA ACGATGATCC   
  
  
+ ACGCACCACA TCTAACGGAG AAATCAACGG AGTTTGTTAC ACAGAGGAAA CACCTGAGTC AATTCCAGTC   
  
  
+ AAAGACATAT ATACCCCCAA CACGACAGAC ATACAACCCC TCTCTCTTTC CTTTAAAGCA AACCCTTTCC   
  
  
+ ACTCTCTCTC TCTCTCTCTC CATCTTAATC CATTCCGATA CTTTTAAAAA ATCGGAAAAA TTTCAACGGT   
  
  
+ ACATATCGCT TCCGCCGCAA AATATCGGAC TTTTTTTAAG TGTCACCACA CTGTTGTTAC TTTACTCTCT   
  
  
+ GTTTCTGTCT CTCCTTTGGA AAAATCGGT  

- CCACAACAAA CATGTTAGTA TACGCACAGT AACGCATGTT TTGTTTTTTA TACGATCTCG TAGGTACTTT   
  
  
- CGTTATTACT TACCGTTTTG ATGTATTTTC TTTTTGTTTT GCGTCTTCGC CTGGTTGTTT TAAATCTTCT   
  
  
- TCACTTGCAC TTGATACCGC TTTGTTTTTT TTTTTTTTTT GAATGTTTTT ACCTCTTTTC CCTGTTGGAA   
  
  
- GAAGTTTTTT TTTTTTTTTT TTTTTGAATG TTTTTACCTC TTTTCCCTGT TGGAAGAAGT TCGTCTGCGA   
  
  
- CGTATTCTAT TGAAGTTAGA GAGAGGTTCT CTCTCGCGGG ACTTGTGTCA ATGACACGCG AACTTTCGAG   
  
  
- AGATCGCCAG ATAATTACGA GTGATTTTCC TGAAAAAGAT AACCAGTACT CCAAGTACGA AGGATGTTTT   
  
  
- AAAGAAGGAT GGATTTAATA AGGCGTCGAC GTCAAGTTAA GTAATTTTTT CCAGAGCCAT CGTACCGATA   
  
  
- TGCACACCAG TGTGACCAGT CGAGAAAAAG GTTGGGTACA CCCCCTATTA ACCTCGGGAG TTAACCCAGT   
  
  
- AGCAACTTTG AGTCTTCTTT GGTTTCATTA TCGTAGACGA AGGGACTTCA GTATAGAACA TGGGTAGATA   
  
  
- CCCGTGAGGT ACTTCTACGA CTAGACACAG TACGGTAAGA ATTTAAGTAG TTTCCAATTG GATTAATAAT   
  
  
- GAAACGTCTC AAAAGAAAAA GAAAGATACG GGTTTTCAAA GGATGAAATC GTGATATTGG GGTAGTTAAG   
  
  
- GTGACGAAGT AATTCAAGTA ATGTACCCGA ATAGTAAATT CTTTTGTTTC GTTTCAAACA ACGGATTGAC   
  
  
- TAATCCTTAA GGGACATTAT GATGAATGGG ATTATGAAGT TATAATAAGT TCGGTTTCTG GTCGTCAAAG   
  
  
- AGGTTCATAA ATAAACGGAT ATTCCAATCA ATAGGTAAGT GTATGCTTTC TCTACTTTCG TTTTCCGTCC   
  
  
- TTTATGCCAC CATTGTAAAA GGAAGTACGT TTTCAAAATG AGCATGTCAA CCGAAAATCC GATGTTCCAC   
  
  
- AGGTGAGGAT TCTTTTTCAC AAATTAATTA CTTAATTAGT AATTTTTCTT TTTGGTCATA TAGGTTCAGT   
  
  
- TTTTACGTTA TTGTTTGCCG GTGGAAGTGG CAATCCCTTG GGCAGTTCAG TTGTTTCCGT TGCTACTAGG   
  
  
- TGCGTGGTGT AGATTGCCTC TTTAGTTGCC TCAAACAATG TGTCTCCTTT GTGGACTCAG TTAAGGTCAG   
  
  
- TTTCTGTATA TATGGGGGTT GTGCTGTCTG TATGTTGGGG AGAGAGAAAG GAAATTTCGT TTGGGAAAGG   
  
  
- TGAGAGAGAG AGAGAGAGAG GTAGAATTAG GTAAGGCTAT GAAAATTTTT TAGCCTTTTT AAAGTTGCCA   
  
  
- TGTATAGCGA AGGCGGCGTT TTATAGCCTG AAAAAAATTC ACAGTGGTGT GACAACAATG AAATGAGAGA   
  
  
- CAAAGACAGA GAGGAAACCT TTTTAGCCA

+     Unnamed\_\_13

| Site Name | Organism | Position | Strand | Matrix score. | sequence | function |
| --- | --- | --- | --- | --- | --- | --- |
| Unnamed\_\_13 | Zea mays | 911 | + | 9 | TCCAAGTATA |  |

> 2018/04/13 10:10:12  
+ GGTGTTGTTT GTACAATCAT ATGCGTGTCA TTGCGTACAA AACAAAAAAT ATGCTAGAGC ATCCATGAAA   
  
  
+ GCAATAATGA ATGGCAAAAC TACATAAAAG AAAAACAAAA CGCAGAAGCG GACCAACAAA ATTTAGAAGA   
  
  
+ AGTGAACGTG AACTATGGCG AAACAAAAAA AAAAAAAAAA CTTACAAAAA TGGAGAAAAG GGACAACCTT   
  
  
+ CTTCAAAAAA AAAAAAAAAA AAAAACTTAC AAAAATGGAG AAAAGGGACA ACCTTCTTCA AGCAGACGCT   
  
  
+ GCATAAGATA ACTTCAATCT CTCTCCAAGA GAGAGCGCCC TGAACACAGT TACTGTGCGC TTGAAAGCTC   
  
  
+ TCTAGCGGTC TATTAATGCT CACTAAAAGG ACTTTTTCTA TTGGTCATGA GGTTCATGCT TCCTACAAAA   
  
  
+ TTTCTTCCTA CCTAAATTAT TCCGCAGCTG CAGTTCAATT CATTAAAAAA GGTCTCGGTA GCATGGCTAT   
  
  
+ ACGTGTGGTC ACACTGGTCA GCTCTTTTTC CAACCCATGT GGGGGATAAT TGGAGCCCTC AATTGGGTCA   
  
  
+ TCGTTGAAAC TCAGAAGAAA CCAAAGTAAT AGCATCTGCT TCCCTGAAGT CATATCTTGT ACCCATCTAT   
  
  
+ GGGCACTCCA TGAAGATGCT GATCTGTGTC ATGCCATTCT TAAATTCATC AAAGGTTAAC CTAATTATTA   
  
  
+ CTTTGCAGAG TTTTCTTTTT CTTTCTATGC CCAAAAGTTT CCTACTTTAG CACTATAACC CCATCAATTC   
  
  
+ CACTGCTTCA TTAAGTTCAT TACATGGGCT TATCATTTAA GAAAACAAAG CAAAGTTTGT TGCCTAACTG   
  
  
+ ATTAGGAATT CCCTGTAATA CTACTTACCC TAATACTTCA ATATTATTCA AGCCAAAGAC CAGCAGTTTC   
  
  
+ TCCAAGTATT TATTTGCCTA TAAGGTTAGT TATCCATTCA CATACGAAAG AGATGAAAGC AAAAGGCAGG   
  
  
+ AAATACGGTG GTAACATTTT CCTTCATGCA AAAGTTTTAC TCGTACAGTT GGCTTTTAGG CTACAAGGTG   
  
  
+ TCCACTCCTA AGAAAAAGTG TTTAATTAAT GAATTAATCA TTAAAAAGAA AAACCAGTAT ATCCAAGTCA   
  
  
+ AAAATGCAAT AACAAACGGC CACCTTCACC GTTAGGGAAC CCGTCAAGTC AACAAAGGCA ACGATGATCC   
  
  
+ ACGCACCACA TCTAACGGAG AAATCAACGG AGTTTGTTAC ACAGAGGAAA CACCTGAGTC AATTCCAGTC   
  
  
+ AAAGACATAT ATACCCCCAA CACGACAGAC ATACAACCCC TCTCTCTTTC CTTTAAAGCA AACCCTTTCC   
  
  
+ ACTCTCTCTC TCTCTCTCTC CATCTTAATC CATTCCGATA CTTTTAAAAA ATCGGAAAAA TTTCAACGGT   
  
  
+ ACATATCGCT TCCGCCGCAA AATATCGGAC TTTTTTTAAG TGTCACCACA CTGTTGTTAC TTTACTCTCT   
  
  
+ GTTTCTGTCT CTCCTTTGGA AAAATCGGT  

- CCACAACAAA CATGTTAGTA TACGCACAGT AACGCATGTT TTGTTTTTTA TACGATCTCG TAGGTACTTT   
  
  
- CGTTATTACT TACCGTTTTG ATGTATTTTC TTTTTGTTTT GCGTCTTCGC CTGGTTGTTT TAAATCTTCT   
  
  
- TCACTTGCAC TTGATACCGC TTTGTTTTTT TTTTTTTTTT GAATGTTTTT ACCTCTTTTC CCTGTTGGAA   
  
  
- GAAGTTTTTT TTTTTTTTTT TTTTTGAATG TTTTTACCTC TTTTCCCTGT TGGAAGAAGT TCGTCTGCGA   
  
  
- CGTATTCTAT TGAAGTTAGA GAGAGGTTCT CTCTCGCGGG ACTTGTGTCA ATGACACGCG AACTTTCGAG   
  
  
- AGATCGCCAG ATAATTACGA GTGATTTTCC TGAAAAAGAT AACCAGTACT CCAAGTACGA AGGATGTTTT   
  
  
- AAAGAAGGAT GGATTTAATA AGGCGTCGAC GTCAAGTTAA GTAATTTTTT CCAGAGCCAT CGTACCGATA   
  
  
- TGCACACCAG TGTGACCAGT CGAGAAAAAG GTTGGGTACA CCCCCTATTA ACCTCGGGAG TTAACCCAGT   
  
  
- AGCAACTTTG AGTCTTCTTT GGTTTCATTA TCGTAGACGA AGGGACTTCA GTATAGAACA TGGGTAGATA   
  
  
- CCCGTGAGGT ACTTCTACGA CTAGACACAG TACGGTAAGA ATTTAAGTAG TTTCCAATTG GATTAATAAT   
  
  
- GAAACGTCTC AAAAGAAAAA GAAAGATACG GGTTTTCAAA GGATGAAATC GTGATATTGG GGTAGTTAAG   
  
  
- GTGACGAAGT AATTCAAGTA ATGTACCCGA ATAGTAAATT CTTTTGTTTC GTTTCAAACA ACGGATTGAC   
  
  
- TAATCCTTAA GGGACATTAT GATGAATGGG ATTATGAAGT TATAATAAGT TCGGTTTCTG GTCGTCAAAG   
  
  
- AGGTTCATAA ATAAACGGAT ATTCCAATCA ATAGGTAAGT GTATGCTTTC TCTACTTTCG TTTTCCGTCC   
  
  
- TTTATGCCAC CATTGTAAAA GGAAGTACGT TTTCAAAATG AGCATGTCAA CCGAAAATCC GATGTTCCAC   
  
  
- AGGTGAGGAT TCTTTTTCAC AAATTAATTA CTTAATTAGT AATTTTTCTT TTTGGTCATA TAGGTTCAGT   
  
  
- TTTTACGTTA TTGTTTGCCG GTGGAAGTGG CAATCCCTTG GGCAGTTCAG TTGTTTCCGT TGCTACTAGG   
  
  
- TGCGTGGTGT AGATTGCCTC TTTAGTTGCC TCAAACAATG TGTCTCCTTT GTGGACTCAG TTAAGGTCAG   
  
  
- TTTCTGTATA TATGGGGGTT GTGCTGTCTG TATGTTGGGG AGAGAGAAAG GAAATTTCGT TTGGGAAAGG   
  
  
- TGAGAGAGAG AGAGAGAGAG GTAGAATTAG GTAAGGCTAT GAAAATTTTT TAGCCTTTTT AAAGTTGCCA   
  
  
- TGTATAGCGA AGGCGGCGTT TTATAGCCTG AAAAAAATTC ACAGTGGTGT GACAACAATG AAATGAGAGA   
  
  
- CAAAGACAGA GAGGAAACCT TTTTAGCCA

+     Unnamed\_\_3

| Site Name | Organism | Position | Strand | Matrix score. | sequence | function |
| --- | --- | --- | --- | --- | --- | --- |
| Unnamed\_\_3 | Zea mays | 1189 | - | 5 | CGTGG |  |

> 2018/04/13 10:10:12  
+ GGTGTTGTTT GTACAATCAT ATGCGTGTCA TTGCGTACAA AACAAAAAAT ATGCTAGAGC ATCCATGAAA   
  
  
+ GCAATAATGA ATGGCAAAAC TACATAAAAG AAAAACAAAA CGCAGAAGCG GACCAACAAA ATTTAGAAGA   
  
  
+ AGTGAACGTG AACTATGGCG AAACAAAAAA AAAAAAAAAA CTTACAAAAA TGGAGAAAAG GGACAACCTT   
  
  
+ CTTCAAAAAA AAAAAAAAAA AAAAACTTAC AAAAATGGAG AAAAGGGACA ACCTTCTTCA AGCAGACGCT   
  
  
+ GCATAAGATA ACTTCAATCT CTCTCCAAGA GAGAGCGCCC TGAACACAGT TACTGTGCGC TTGAAAGCTC   
  
  
+ TCTAGCGGTC TATTAATGCT CACTAAAAGG ACTTTTTCTA TTGGTCATGA GGTTCATGCT TCCTACAAAA   
  
  
+ TTTCTTCCTA CCTAAATTAT TCCGCAGCTG CAGTTCAATT CATTAAAAAA GGTCTCGGTA GCATGGCTAT   
  
  
+ ACGTGTGGTC ACACTGGTCA GCTCTTTTTC CAACCCATGT GGGGGATAAT TGGAGCCCTC AATTGGGTCA   
  
  
+ TCGTTGAAAC TCAGAAGAAA CCAAAGTAAT AGCATCTGCT TCCCTGAAGT CATATCTTGT ACCCATCTAT   
  
  
+ GGGCACTCCA TGAAGATGCT GATCTGTGTC ATGCCATTCT TAAATTCATC AAAGGTTAAC CTAATTATTA   
  
  
+ CTTTGCAGAG TTTTCTTTTT CTTTCTATGC CCAAAAGTTT CCTACTTTAG CACTATAACC CCATCAATTC   
  
  
+ CACTGCTTCA TTAAGTTCAT TACATGGGCT TATCATTTAA GAAAACAAAG CAAAGTTTGT TGCCTAACTG   
  
  
+ ATTAGGAATT CCCTGTAATA CTACTTACCC TAATACTTCA ATATTATTCA AGCCAAAGAC CAGCAGTTTC   
  
  
+ TCCAAGTATT TATTTGCCTA TAAGGTTAGT TATCCATTCA CATACGAAAG AGATGAAAGC AAAAGGCAGG   
  
  
+ AAATACGGTG GTAACATTTT CCTTCATGCA AAAGTTTTAC TCGTACAGTT GGCTTTTAGG CTACAAGGTG   
  
  
+ TCCACTCCTA AGAAAAAGTG TTTAATTAAT GAATTAATCA TTAAAAAGAA AAACCAGTAT ATCCAAGTCA   
  
  
+ AAAATGCAAT AACAAACGGC CACCTTCACC GTTAGGGAAC CCGTCAAGTC AACAAAGGCA ACGATGATCC   
  
  
+ ACGCACCACA TCTAACGGAG AAATCAACGG AGTTTGTTAC ACAGAGGAAA CACCTGAGTC AATTCCAGTC   
  
  
+ AAAGACATAT ATACCCCCAA CACGACAGAC ATACAACCCC TCTCTCTTTC CTTTAAAGCA AACCCTTTCC   
  
  
+ ACTCTCTCTC TCTCTCTCTC CATCTTAATC CATTCCGATA CTTTTAAAAA ATCGGAAAAA TTTCAACGGT   
  
  
+ ACATATCGCT TCCGCCGCAA AATATCGGAC TTTTTTTAAG TGTCACCACA CTGTTGTTAC TTTACTCTCT   
  
  
+ GTTTCTGTCT CTCCTTTGGA AAAATCGGT  

- CCACAACAAA CATGTTAGTA TACGCACAGT AACGCATGTT TTGTTTTTTA TACGATCTCG TAGGTACTTT   
  
  
- CGTTATTACT TACCGTTTTG ATGTATTTTC TTTTTGTTTT GCGTCTTCGC CTGGTTGTTT TAAATCTTCT   
  
  
- TCACTTGCAC TTGATACCGC TTTGTTTTTT TTTTTTTTTT GAATGTTTTT ACCTCTTTTC CCTGTTGGAA   
  
  
- GAAGTTTTTT TTTTTTTTTT TTTTTGAATG TTTTTACCTC TTTTCCCTGT TGGAAGAAGT TCGTCTGCGA   
  
  
- CGTATTCTAT TGAAGTTAGA GAGAGGTTCT CTCTCGCGGG ACTTGTGTCA ATGACACGCG AACTTTCGAG   
  
  
- AGATCGCCAG ATAATTACGA GTGATTTTCC TGAAAAAGAT AACCAGTACT CCAAGTACGA AGGATGTTTT   
  
  
- AAAGAAGGAT GGATTTAATA AGGCGTCGAC GTCAAGTTAA GTAATTTTTT CCAGAGCCAT CGTACCGATA   
  
  
- TGCACACCAG TGTGACCAGT CGAGAAAAAG GTTGGGTACA CCCCCTATTA ACCTCGGGAG TTAACCCAGT   
  
  
- AGCAACTTTG AGTCTTCTTT GGTTTCATTA TCGTAGACGA AGGGACTTCA GTATAGAACA TGGGTAGATA   
  
  
- CCCGTGAGGT ACTTCTACGA CTAGACACAG TACGGTAAGA ATTTAAGTAG TTTCCAATTG GATTAATAAT   
  
  
- GAAACGTCTC AAAAGAAAAA GAAAGATACG GGTTTTCAAA GGATGAAATC GTGATATTGG GGTAGTTAAG   
  
  
- GTGACGAAGT AATTCAAGTA ATGTACCCGA ATAGTAAATT CTTTTGTTTC GTTTCAAACA ACGGATTGAC   
  
  
- TAATCCTTAA GGGACATTAT GATGAATGGG ATTATGAAGT TATAATAAGT TCGGTTTCTG GTCGTCAAAG   
  
  
- AGGTTCATAA ATAAACGGAT ATTCCAATCA ATAGGTAAGT GTATGCTTTC TCTACTTTCG TTTTCCGTCC   
  
  
- TTTATGCCAC CATTGTAAAA GGAAGTACGT TTTCAAAATG AGCATGTCAA CCGAAAATCC GATGTTCCAC   
  
  
- AGGTGAGGAT TCTTTTTCAC AAATTAATTA CTTAATTAGT AATTTTTCTT TTTGGTCATA TAGGTTCAGT   
  
  
- TTTTACGTTA TTGTTTGCCG GTGGAAGTGG CAATCCCTTG GGCAGTTCAG TTGTTTCCGT TGCTACTAGG   
  
  
- TGCGTGGTGT AGATTGCCTC TTTAGTTGCC TCAAACAATG TGTCTCCTTT GTGGACTCAG TTAAGGTCAG   
  
  
- TTTCTGTATA TATGGGGGTT GTGCTGTCTG TATGTTGGGG AGAGAGAAAG GAAATTTCGT TTGGGAAAGG   
  
  
- TGAGAGAGAG AGAGAGAGAG GTAGAATTAG GTAAGGCTAT GAAAATTTTT TAGCCTTTTT AAAGTTGCCA   
  
  
- TGTATAGCGA AGGCGGCGTT TTATAGCCTG AAAAAAATTC ACAGTGGTGT GACAACAATG AAATGAGAGA   
  
  
- CAAAGACAGA GAGGAAACCT TTTTAGCCA

+     Unnamed\_\_4

| Site Name | Organism | Position | Strand | Matrix score. | sequence | function |
| --- | --- | --- | --- | --- | --- | --- |
| Unnamed\_\_4 | Petroselinum hortense | 192 | - | 4 | CTCC |  |
| Unnamed\_\_4 | Petroselinum hortense | 1481 | + | 4 | CTCC |  |
| Unnamed\_\_4 | Petroselinum hortense | 1219 | - | 4 | CTCC |  |
| Unnamed\_\_4 | Petroselinum hortense | 247 | - | 4 | CTCC |  |
| Unnamed\_\_4 | Petroselinum hortense | 910 | + | 4 | CTCC |  |
| Unnamed\_\_4 | Petroselinum hortense | 542 | - | 4 | CTCC |  |
| Unnamed\_\_4 | Petroselinum hortense | 303 | + | 4 | CTCC |  |
| Unnamed\_\_4 | Petroselinum hortense | 1055 | + | 4 | CTCC |  |
| Unnamed\_\_4 | Petroselinum hortense | 636 | + | 4 | CTCC |  |
| Unnamed\_\_4 | Petroselinum hortense | 1348 | + | 4 | CTCC |  |
| Unnamed\_\_4 | Petroselinum hortense | 1207 | - | 4 | CTCC |  |

> 2018/04/13 10:10:12  
+ GGTGTTGTTT GTACAATCAT ATGCGTGTCA TTGCGTACAA AACAAAAAAT ATGCTAGAGC ATCCATGAAA   
  
  
+ GCAATAATGA ATGGCAAAAC TACATAAAAG AAAAACAAAA CGCAGAAGCG GACCAACAAA ATTTAGAAGA   
  
  
+ AGTGAACGTG AACTATGGCG AAACAAAAAA AAAAAAAAAA CTTACAAAAA TGGAGAAAAG GGACAACCTT   
  
  
+ CTTCAAAAAA AAAAAAAAAA AAAAACTTAC AAAAATGGAG AAAAGGGACA ACCTTCTTCA AGCAGACGCT   
  
  
+ GCATAAGATA ACTTCAATCT CTCTCCAAGA GAGAGCGCCC TGAACACAGT TACTGTGCGC TTGAAAGCTC   
  
  
+ TCTAGCGGTC TATTAATGCT CACTAAAAGG ACTTTTTCTA TTGGTCATGA GGTTCATGCT TCCTACAAAA   
  
  
+ TTTCTTCCTA CCTAAATTAT TCCGCAGCTG CAGTTCAATT CATTAAAAAA GGTCTCGGTA GCATGGCTAT   
  
  
+ ACGTGTGGTC ACACTGGTCA GCTCTTTTTC CAACCCATGT GGGGGATAAT TGGAGCCCTC AATTGGGTCA   
  
  
+ TCGTTGAAAC TCAGAAGAAA CCAAAGTAAT AGCATCTGCT TCCCTGAAGT CATATCTTGT ACCCATCTAT   
  
  
+ GGGCACTCCA TGAAGATGCT GATCTGTGTC ATGCCATTCT TAAATTCATC AAAGGTTAAC CTAATTATTA   
  
  
+ CTTTGCAGAG TTTTCTTTTT CTTTCTATGC CCAAAAGTTT CCTACTTTAG CACTATAACC CCATCAATTC   
  
  
+ CACTGCTTCA TTAAGTTCAT TACATGGGCT TATCATTTAA GAAAACAAAG CAAAGTTTGT TGCCTAACTG   
  
  
+ ATTAGGAATT CCCTGTAATA CTACTTACCC TAATACTTCA ATATTATTCA AGCCAAAGAC CAGCAGTTTC   
  
  
+ TCCAAGTATT TATTTGCCTA TAAGGTTAGT TATCCATTCA CATACGAAAG AGATGAAAGC AAAAGGCAGG   
  
  
+ AAATACGGTG GTAACATTTT CCTTCATGCA AAAGTTTTAC TCGTACAGTT GGCTTTTAGG CTACAAGGTG   
  
  
+ TCCACTCCTA AGAAAAAGTG TTTAATTAAT GAATTAATCA TTAAAAAGAA AAACCAGTAT ATCCAAGTCA   
  
  
+ AAAATGCAAT AACAAACGGC CACCTTCACC GTTAGGGAAC CCGTCAAGTC AACAAAGGCA ACGATGATCC   
  
  
+ ACGCACCACA TCTAACGGAG AAATCAACGG AGTTTGTTAC ACAGAGGAAA CACCTGAGTC AATTCCAGTC   
  
  
+ AAAGACATAT ATACCCCCAA CACGACAGAC ATACAACCCC TCTCTCTTTC CTTTAAAGCA AACCCTTTCC   
  
  
+ ACTCTCTCTC TCTCTCTCTC CATCTTAATC CATTCCGATA CTTTTAAAAA ATCGGAAAAA TTTCAACGGT   
  
  
+ ACATATCGCT TCCGCCGCAA AATATCGGAC TTTTTTTAAG TGTCACCACA CTGTTGTTAC TTTACTCTCT   
  
  
+ GTTTCTGTCT CTCCTTTGGA AAAATCGGT  

- CCACAACAAA CATGTTAGTA TACGCACAGT AACGCATGTT TTGTTTTTTA TACGATCTCG TAGGTACTTT   
  
  
- CGTTATTACT TACCGTTTTG ATGTATTTTC TTTTTGTTTT GCGTCTTCGC CTGGTTGTTT TAAATCTTCT   
  
  
- TCACTTGCAC TTGATACCGC TTTGTTTTTT TTTTTTTTTT GAATGTTTTT ACCTCTTTTC CCTGTTGGAA   
  
  
- GAAGTTTTTT TTTTTTTTTT TTTTTGAATG TTTTTACCTC TTTTCCCTGT TGGAAGAAGT TCGTCTGCGA   
  
  
- CGTATTCTAT TGAAGTTAGA GAGAGGTTCT CTCTCGCGGG ACTTGTGTCA ATGACACGCG AACTTTCGAG   
  
  
- AGATCGCCAG ATAATTACGA GTGATTTTCC TGAAAAAGAT AACCAGTACT CCAAGTACGA AGGATGTTTT   
  
  
- AAAGAAGGAT GGATTTAATA AGGCGTCGAC GTCAAGTTAA GTAATTTTTT CCAGAGCCAT CGTACCGATA   
  
  
- TGCACACCAG TGTGACCAGT CGAGAAAAAG GTTGGGTACA CCCCCTATTA ACCTCGGGAG TTAACCCAGT   
  
  
- AGCAACTTTG AGTCTTCTTT GGTTTCATTA TCGTAGACGA AGGGACTTCA GTATAGAACA TGGGTAGATA   
  
  
- CCCGTGAGGT ACTTCTACGA CTAGACACAG TACGGTAAGA ATTTAAGTAG TTTCCAATTG GATTAATAAT   
  
  
- GAAACGTCTC AAAAGAAAAA GAAAGATACG GGTTTTCAAA GGATGAAATC GTGATATTGG GGTAGTTAAG   
  
  
- GTGACGAAGT AATTCAAGTA ATGTACCCGA ATAGTAAATT CTTTTGTTTC GTTTCAAACA ACGGATTGAC   
  
  
- TAATCCTTAA GGGACATTAT GATGAATGGG ATTATGAAGT TATAATAAGT TCGGTTTCTG GTCGTCAAAG   
  
  
- AGGTTCATAA ATAAACGGAT ATTCCAATCA ATAGGTAAGT GTATGCTTTC TCTACTTTCG TTTTCCGTCC   
  
  
- TTTATGCCAC CATTGTAAAA GGAAGTACGT TTTCAAAATG AGCATGTCAA CCGAAAATCC GATGTTCCAC   
  
  
- AGGTGAGGAT TCTTTTTCAC AAATTAATTA CTTAATTAGT AATTTTTCTT TTTGGTCATA TAGGTTCAGT   
  
  
- TTTTACGTTA TTGTTTGCCG GTGGAAGTGG CAATCCCTTG GGCAGTTCAG TTGTTTCCGT TGCTACTAGG   
  
  
- TGCGTGGTGT AGATTGCCTC TTTAGTTGCC TCAAACAATG TGTCTCCTTT GTGGACTCAG TTAAGGTCAG   
  
  
- TTTCTGTATA TATGGGGGTT GTGCTGTCTG TATGTTGGGG AGAGAGAAAG GAAATTTCGT TTGGGAAAGG   
  
  
- TGAGAGAGAG AGAGAGAGAG GTAGAATTAG GTAAGGCTAT GAAAATTTTT TAGCCTTTTT AAAGTTGCCA   
  
  
- TGTATAGCGA AGGCGGCGTT TTATAGCCTG AAAAAAATTC ACAGTGGTGT GACAACAATG AAATGAGAGA   
  
  
- CAAAGACAGA GAGGAAACCT TTTTAGCCA

+     Unnamed\_\_8

| Site Name | Organism | Position | Strand | Matrix score. | sequence | function |
| --- | --- | --- | --- | --- | --- | --- |
| Unnamed\_\_8 | Glycine max cv. Provar | 184 | - | 9 | CATTTTTGT |  |
| Unnamed\_\_8 | Glycine max cv. Provar | 239 | - | 9 | CATTTTTGT |  |

> 2018/04/13 10:10:12  
+ GGTGTTGTTT GTACAATCAT ATGCGTGTCA TTGCGTACAA AACAAAAAAT ATGCTAGAGC ATCCATGAAA   
  
  
+ GCAATAATGA ATGGCAAAAC TACATAAAAG AAAAACAAAA CGCAGAAGCG GACCAACAAA ATTTAGAAGA   
  
  
+ AGTGAACGTG AACTATGGCG AAACAAAAAA AAAAAAAAAA CTTACAAAAA TGGAGAAAAG GGACAACCTT   
  
  
+ CTTCAAAAAA AAAAAAAAAA AAAAACTTAC AAAAATGGAG AAAAGGGACA ACCTTCTTCA AGCAGACGCT   
  
  
+ GCATAAGATA ACTTCAATCT CTCTCCAAGA GAGAGCGCCC TGAACACAGT TACTGTGCGC TTGAAAGCTC   
  
  
+ TCTAGCGGTC TATTAATGCT CACTAAAAGG ACTTTTTCTA TTGGTCATGA GGTTCATGCT TCCTACAAAA   
  
  
+ TTTCTTCCTA CCTAAATTAT TCCGCAGCTG CAGTTCAATT CATTAAAAAA GGTCTCGGTA GCATGGCTAT   
  
  
+ ACGTGTGGTC ACACTGGTCA GCTCTTTTTC CAACCCATGT GGGGGATAAT TGGAGCCCTC AATTGGGTCA   
  
  
+ TCGTTGAAAC TCAGAAGAAA CCAAAGTAAT AGCATCTGCT TCCCTGAAGT CATATCTTGT ACCCATCTAT   
  
  
+ GGGCACTCCA TGAAGATGCT GATCTGTGTC ATGCCATTCT TAAATTCATC AAAGGTTAAC CTAATTATTA   
  
  
+ CTTTGCAGAG TTTTCTTTTT CTTTCTATGC CCAAAAGTTT CCTACTTTAG CACTATAACC CCATCAATTC   
  
  
+ CACTGCTTCA TTAAGTTCAT TACATGGGCT TATCATTTAA GAAAACAAAG CAAAGTTTGT TGCCTAACTG   
  
  
+ ATTAGGAATT CCCTGTAATA CTACTTACCC TAATACTTCA ATATTATTCA AGCCAAAGAC CAGCAGTTTC   
  
  
+ TCCAAGTATT TATTTGCCTA TAAGGTTAGT TATCCATTCA CATACGAAAG AGATGAAAGC AAAAGGCAGG   
  
  
+ AAATACGGTG GTAACATTTT CCTTCATGCA AAAGTTTTAC TCGTACAGTT GGCTTTTAGG CTACAAGGTG   
  
  
+ TCCACTCCTA AGAAAAAGTG TTTAATTAAT GAATTAATCA TTAAAAAGAA AAACCAGTAT ATCCAAGTCA   
  
  
+ AAAATGCAAT AACAAACGGC CACCTTCACC GTTAGGGAAC CCGTCAAGTC AACAAAGGCA ACGATGATCC   
  
  
+ ACGCACCACA TCTAACGGAG AAATCAACGG AGTTTGTTAC ACAGAGGAAA CACCTGAGTC AATTCCAGTC   
  
  
+ AAAGACATAT ATACCCCCAA CACGACAGAC ATACAACCCC TCTCTCTTTC CTTTAAAGCA AACCCTTTCC   
  
  
+ ACTCTCTCTC TCTCTCTCTC CATCTTAATC CATTCCGATA CTTTTAAAAA ATCGGAAAAA TTTCAACGGT   
  
  
+ ACATATCGCT TCCGCCGCAA AATATCGGAC TTTTTTTAAG TGTCACCACA CTGTTGTTAC TTTACTCTCT   
  
  
+ GTTTCTGTCT CTCCTTTGGA AAAATCGGT  

- CCACAACAAA CATGTTAGTA TACGCACAGT AACGCATGTT TTGTTTTTTA TACGATCTCG TAGGTACTTT   
  
  
- CGTTATTACT TACCGTTTTG ATGTATTTTC TTTTTGTTTT GCGTCTTCGC CTGGTTGTTT TAAATCTTCT   
  
  
- TCACTTGCAC TTGATACCGC TTTGTTTTTT TTTTTTTTTT GAATGTTTTT ACCTCTTTTC CCTGTTGGAA   
  
  
- GAAGTTTTTT TTTTTTTTTT TTTTTGAATG TTTTTACCTC TTTTCCCTGT TGGAAGAAGT TCGTCTGCGA   
  
  
- CGTATTCTAT TGAAGTTAGA GAGAGGTTCT CTCTCGCGGG ACTTGTGTCA ATGACACGCG AACTTTCGAG   
  
  
- AGATCGCCAG ATAATTACGA GTGATTTTCC TGAAAAAGAT AACCAGTACT CCAAGTACGA AGGATGTTTT   
  
  
- AAAGAAGGAT GGATTTAATA AGGCGTCGAC GTCAAGTTAA GTAATTTTTT CCAGAGCCAT CGTACCGATA   
  
  
- TGCACACCAG TGTGACCAGT CGAGAAAAAG GTTGGGTACA CCCCCTATTA ACCTCGGGAG TTAACCCAGT   
  
  
- AGCAACTTTG AGTCTTCTTT GGTTTCATTA TCGTAGACGA AGGGACTTCA GTATAGAACA TGGGTAGATA   
  
  
- CCCGTGAGGT ACTTCTACGA CTAGACACAG TACGGTAAGA ATTTAAGTAG TTTCCAATTG GATTAATAAT   
  
  
- GAAACGTCTC AAAAGAAAAA GAAAGATACG GGTTTTCAAA GGATGAAATC GTGATATTGG GGTAGTTAAG   
  
  
- GTGACGAAGT AATTCAAGTA ATGTACCCGA ATAGTAAATT CTTTTGTTTC GTTTCAAACA ACGGATTGAC   
  
  
- TAATCCTTAA GGGACATTAT GATGAATGGG ATTATGAAGT TATAATAAGT TCGGTTTCTG GTCGTCAAAG   
  
  
- AGGTTCATAA ATAAACGGAT ATTCCAATCA ATAGGTAAGT GTATGCTTTC TCTACTTTCG TTTTCCGTCC   
  
  
- TTTATGCCAC CATTGTAAAA GGAAGTACGT TTTCAAAATG AGCATGTCAA CCGAAAATCC GATGTTCCAC   
  
  
- AGGTGAGGAT TCTTTTTCAC AAATTAATTA CTTAATTAGT AATTTTTCTT TTTGGTCATA TAGGTTCAGT   
  
  
- TTTTACGTTA TTGTTTGCCG GTGGAAGTGG CAATCCCTTG GGCAGTTCAG TTGTTTCCGT TGCTACTAGG   
  
  
- TGCGTGGTGT AGATTGCCTC TTTAGTTGCC TCAAACAATG TGTCTCCTTT GTGGACTCAG TTAAGGTCAG   
  
  
- TTTCTGTATA TATGGGGGTT GTGCTGTCTG TATGTTGGGG AGAGAGAAAG GAAATTTCGT TTGGGAAAGG   
  
  
- TGAGAGAGAG AGAGAGAGAG GTAGAATTAG GTAAGGCTAT GAAAATTTTT TAGCCTTTTT AAAGTTGCCA   
  
  
- TGTATAGCGA AGGCGGCGTT TTATAGCCTG AAAAAAATTC ACAGTGGTGT GACAACAATG AAATGAGAGA   
  
  
- CAAAGACAGA GAGGAAACCT TTTTAGCCA

+     box E

| Site Name | Organism | Position | Strand | Matrix score. | sequence | function |
| --- | --- | --- | --- | --- | --- | --- |
| box E | Petroselinum crispum | 1159 | + | 9 | ACCCATCAAG |  |

> 2018/04/13 10:10:12  
+ GGTGTTGTTT GTACAATCAT ATGCGTGTCA TTGCGTACAA AACAAAAAAT ATGCTAGAGC ATCCATGAAA   
  
  
+ GCAATAATGA ATGGCAAAAC TACATAAAAG AAAAACAAAA CGCAGAAGCG GACCAACAAA ATTTAGAAGA   
  
  
+ AGTGAACGTG AACTATGGCG AAACAAAAAA AAAAAAAAAA CTTACAAAAA TGGAGAAAAG GGACAACCTT   
  
  
+ CTTCAAAAAA AAAAAAAAAA AAAAACTTAC AAAAATGGAG AAAAGGGACA ACCTTCTTCA AGCAGACGCT   
  
  
+ GCATAAGATA ACTTCAATCT CTCTCCAAGA GAGAGCGCCC TGAACACAGT TACTGTGCGC TTGAAAGCTC   
  
  
+ TCTAGCGGTC TATTAATGCT CACTAAAAGG ACTTTTTCTA TTGGTCATGA GGTTCATGCT TCCTACAAAA   
  
  
+ TTTCTTCCTA CCTAAATTAT TCCGCAGCTG CAGTTCAATT CATTAAAAAA GGTCTCGGTA GCATGGCTAT   
  
  
+ ACGTGTGGTC ACACTGGTCA GCTCTTTTTC CAACCCATGT GGGGGATAAT TGGAGCCCTC AATTGGGTCA   
  
  
+ TCGTTGAAAC TCAGAAGAAA CCAAAGTAAT AGCATCTGCT TCCCTGAAGT CATATCTTGT ACCCATCTAT   
  
  
+ GGGCACTCCA TGAAGATGCT GATCTGTGTC ATGCCATTCT TAAATTCATC AAAGGTTAAC CTAATTATTA   
  
  
+ CTTTGCAGAG TTTTCTTTTT CTTTCTATGC CCAAAAGTTT CCTACTTTAG CACTATAACC CCATCAATTC   
  
  
+ CACTGCTTCA TTAAGTTCAT TACATGGGCT TATCATTTAA GAAAACAAAG CAAAGTTTGT TGCCTAACTG   
  
  
+ ATTAGGAATT CCCTGTAATA CTACTTACCC TAATACTTCA ATATTATTCA AGCCAAAGAC CAGCAGTTTC   
  
  
+ TCCAAGTATT TATTTGCCTA TAAGGTTAGT TATCCATTCA CATACGAAAG AGATGAAAGC AAAAGGCAGG   
  
  
+ AAATACGGTG GTAACATTTT CCTTCATGCA AAAGTTTTAC TCGTACAGTT GGCTTTTAGG CTACAAGGTG   
  
  
+ TCCACTCCTA AGAAAAAGTG TTTAATTAAT GAATTAATCA TTAAAAAGAA AAACCAGTAT ATCCAAGTCA   
  
  
+ AAAATGCAAT AACAAACGGC CACCTTCACC GTTAGGGAAC CCGTCAAGTC AACAAAGGCA ACGATGATCC   
  
  
+ ACGCACCACA TCTAACGGAG AAATCAACGG AGTTTGTTAC ACAGAGGAAA CACCTGAGTC AATTCCAGTC   
  
  
+ AAAGACATAT ATACCCCCAA CACGACAGAC ATACAACCCC TCTCTCTTTC CTTTAAAGCA AACCCTTTCC   
  
  
+ ACTCTCTCTC TCTCTCTCTC CATCTTAATC CATTCCGATA CTTTTAAAAA ATCGGAAAAA TTTCAACGGT   
  
  
+ ACATATCGCT TCCGCCGCAA AATATCGGAC TTTTTTTAAG TGTCACCACA CTGTTGTTAC TTTACTCTCT   
  
  
+ GTTTCTGTCT CTCCTTTGGA AAAATCGGT  

- CCACAACAAA CATGTTAGTA TACGCACAGT AACGCATGTT TTGTTTTTTA TACGATCTCG TAGGTACTTT   
  
  
- CGTTATTACT TACCGTTTTG ATGTATTTTC TTTTTGTTTT GCGTCTTCGC CTGGTTGTTT TAAATCTTCT   
  
  
- TCACTTGCAC TTGATACCGC TTTGTTTTTT TTTTTTTTTT GAATGTTTTT ACCTCTTTTC CCTGTTGGAA   
  
  
- GAAGTTTTTT TTTTTTTTTT TTTTTGAATG TTTTTACCTC TTTTCCCTGT TGGAAGAAGT TCGTCTGCGA   
  
  
- CGTATTCTAT TGAAGTTAGA GAGAGGTTCT CTCTCGCGGG ACTTGTGTCA ATGACACGCG AACTTTCGAG   
  
  
- AGATCGCCAG ATAATTACGA GTGATTTTCC TGAAAAAGAT AACCAGTACT CCAAGTACGA AGGATGTTTT   
  
  
- AAAGAAGGAT GGATTTAATA AGGCGTCGAC GTCAAGTTAA GTAATTTTTT CCAGAGCCAT CGTACCGATA   
  
  
- TGCACACCAG TGTGACCAGT CGAGAAAAAG GTTGGGTACA CCCCCTATTA ACCTCGGGAG TTAACCCAGT   
  
  
- AGCAACTTTG AGTCTTCTTT GGTTTCATTA TCGTAGACGA AGGGACTTCA GTATAGAACA TGGGTAGATA   
  
  
- CCCGTGAGGT ACTTCTACGA CTAGACACAG TACGGTAAGA ATTTAAGTAG TTTCCAATTG GATTAATAAT   
  
  
- GAAACGTCTC AAAAGAAAAA GAAAGATACG GGTTTTCAAA GGATGAAATC GTGATATTGG GGTAGTTAAG   
  
  
- GTGACGAAGT AATTCAAGTA ATGTACCCGA ATAGTAAATT CTTTTGTTTC GTTTCAAACA ACGGATTGAC   
  
  
- TAATCCTTAA GGGACATTAT GATGAATGGG ATTATGAAGT TATAATAAGT TCGGTTTCTG GTCGTCAAAG   
  
  
- AGGTTCATAA ATAAACGGAT ATTCCAATCA ATAGGTAAGT GTATGCTTTC TCTACTTTCG TTTTCCGTCC   
  
  
- TTTATGCCAC CATTGTAAAA GGAAGTACGT TTTCAAAATG AGCATGTCAA CCGAAAATCC GATGTTCCAC   
  
  
- AGGTGAGGAT TCTTTTTCAC AAATTAATTA CTTAATTAGT AATTTTTCTT TTTGGTCATA TAGGTTCAGT   
  
  
- TTTTACGTTA TTGTTTGCCG GTGGAAGTGG CAATCCCTTG GGCAGTTCAG TTGTTTCCGT TGCTACTAGG   
  
  
- TGCGTGGTGT AGATTGCCTC TTTAGTTGCC TCAAACAATG TGTCTCCTTT GTGGACTCAG TTAAGGTCAG   
  
  
- TTTCTGTATA TATGGGGGTT GTGCTGTCTG TATGTTGGGG AGAGAGAAAG GAAATTTCGT TTGGGAAAGG   
  
  
- TGAGAGAGAG AGAGAGAGAG GTAGAATTAG GTAAGGCTAT GAAAATTTTT TAGCCTTTTT AAAGTTGCCA   
  
  
- TGTATAGCGA AGGCGGCGTT TTATAGCCTG AAAAAAATTC ACAGTGGTGT GACAACAATG AAATGAGAGA   
  
  
- CAAAGACAGA GAGGAAACCT TTTTAGCCA
