# Supplementary material for: Genome-wide analysis of the WRKY gene family in drumstick (Moringa oleifera Lam.)
Source: PeerJ. 2019 Jun 10;7:e7063. doi: 10.7717/peerj.7063 (PMC6563795; doi:10.7717/peerj.7063)
Supplement: Supplemental Information 1 [file peerj-07-7063-s003.gz › MoWRKY44_plantcare.html]

Content-Type: text/html; charset=ISO-8859-1


CallMat\_Firefox


Webmaster Firefox specific output  
To save the result:
click on the frame with the right mouse button and save the source code as a text file with extension .html  
REFERENCE:PlantCARE: a database of plant cis-acting regulatory elements and a portal to tools for in silico analysis of promoter sequences.  
Lescot, M., Déhais, P., Moreau, Y., De Moor, B., Rouzé ,P.,and Rombauts, S.  
Nucleic Acids Res., Database issue(2002), 30(1):325-327.   


---

> 2018/04/13 10:10:12  
+ ATCCCAATTG GTCAATTCTT TCCGACTTTT CGGAAGTGTA ACTGTTCCTT AAATAAATTC TTCTCTCAAC   
  
  
+ GGTTTATCAG AGTACGTACG CTTCTCGTTA TGACATTCTA AAAATTCTGT TTTGAAACCC AATTTAAGTA   
  
  
+ TCGCTAGCAA CTTCGGTTGA TGTGAAGTGG ATGAAAGACA AGTTGGGGGG AACACTTTTG TTTCTGCATG   
  
  
+ CACGTACGGG TTTTTAGGGT AGCAGTAGTA GCTTCGTACG TTCTTTTGAG TTATAGAGAG AGAGAGAGAG   
  
  
+ AGAGTCTATT TGTGTGTTCT ACTTCGTCTC TCTCTCTCTG TCTCTCTCTC TCTCTCTCAT TCAAATTAGG   
  
  
+ TATTACTTCA TTTTCATACA ACTATACTGT CTACTAACCG AGACTTTACC GAATTTCATG TCCCAAATCT   
  
  
+ ATAACAATTT GCCGATTAGG TCATAGACTC CAGACCAACA ACTCCATCAA ACCTTATTAA GACCTGACTA   
  
  
+ TCTGACTAAA GTCCGCTAGT TATGTTGGGA AAGGAATCCC GTGTACGTAT CAAGATACAT CAAAGGTATA   
  
  
+ CATCTAGTTC AACGATTATA AAGTACATAT GAACAGCACA CTTTACTTTA CTTTACTAGT TATACTATTC   
  
  
+ AATAATATTA TTTCGACACC TTCTCGGTCT CAAGATCTAC TAGTAAAGAA TGCCTTCTAC GAACAAAACA   
  
  
+ TTCAAGCAAT TAAGATCTCA ACTTCCATTA ATTGTGGTAG AAATAGAAGA ACAAACCTCC AAATGATCAT   
  
  
+ TGTACGTTAA CAATAATATG AAACCGTTTG TTCGTTACGT TTTTATTCTT TAATAACGGG TACGTGTACT   
  
  
+ CTCAAAACCC GATCGACCGA TCCGGTTTAC TCTGTACTTC TAATTCAACT TTATATATAT ATATACGGTG   
  
  
+ TACCATACGG TTCTTTTAAT ATTGTACGTA CAATAAAAAT CTTGGCCGAG TACAACTGAT CAATTAATTT   
  
  
+ TTTTTAATAT ATCCAAAAAG TTATAAAGTA TTATAATTAC ATTTTTATCT TATATTCTTT TATTTAAATA   
  
  
+ ATTAAAATAA TTTAATAGTA CATAATATTA AAATAAATTT TGAGTTTATT TGTTTTTTTA GAGAACTCTG   
  
  
+ ACTAAGAAAA AATAGTCTTT TTTTTTACTA ATATTAATTT GATTGAGAAA ACTGGAATCT CGTTACGATA   
  
  
+ ACTCGGGTTT AGGTATAAAT AAACTAGTAC AGTGGAACAA CAACTAATAC AAAGATTAGT TACCCCGAGG   
  
  
+ TTATATACTT TTTTACCGTC TATCACGGTT AAAAGATACC AGTAAATATA CTGACGTTTA ATACTTGTGT   
  
  
+ CGTCTTTGTA GTCGTTTATT TCGTTGATCT TAAGTATTAG GTATAGAATA GGTATTTGGA TGTACTACCA   
  
  
+ TTCACTTCTT CGATCTATAT TTCTAACGTA CGTATACGCA TGTTCCTTCT TAATTTCCTG TTTCTTTGTT   
  
  
+ GGTGGGTAGA ACGAGGGGTA CGAGTAGAA  

- TAGGGTTAAC CAGTTAAGAA AGGCTGAAAA GCCTTCACAT TGACAAGGAA TTTATTTAAG AAGAGAGTTG   
  
  
- CCAAATAGTC TCATGCATGC GAAGAGCAAT ACTGTAAGAT TTTTAAGACA AAACTTTGGG TTAAATTCAT   
  
  
- AGCGATCGTT GAAGCCAACT ACACTTCACC TACTTTCTGT TCAACCCCCC TTGTGAAAAC AAAGACGTAC   
  
  
- GTGCATGCCC AAAAATCCCA TCGTCATCAT CGAAGCATGC AAGAAAACTC AATATCTCTC TCTCTCTCTC   
  
  
- TCTCAGATAA ACACACAAGA TGAAGCAGAG AGAGAGAGAC AGAGAGAGAG AGAGAGAGTA AGTTTAATCC   
  
  
- ATAATGAAGT AAAAGTATGT TGATATGACA GATGATTGGC TCTGAAATGG CTTAAAGTAC AGGGTTTAGA   
  
  
- TATTGTTAAA CGGCTAATCC AGTATCTGAG GTCTGGTTGT TGAGGTAGTT TGGAATAATT CTGGACTGAT   
  
  
- AGACTGATTT CAGGCGATCA ATACAACCCT TTCCTTAGGG CACATGCATA GTTCTATGTA GTTTCCATAT   
  
  
- GTAGATCAAG TTGCTAATAT TTCATGTATA CTTGTCGTGT GAAATGAAAT GAAATGATCA ATATGATAAG   
  
  
- TTATTATAAT AAAGCTGTGG AAGAGCCAGA GTTCTAGATG ATCATTTCTT ACGGAAGATG CTTGTTTTGT   
  
  
- AAGTTCGTTA ATTCTAGAGT TGAAGGTAAT TAACACCATC TTTATCTTCT TGTTTGGAGG TTTACTAGTA   
  
  
- ACATGCAATT GTTATTATAC TTTGGCAAAC AAGCAATGCA AAAATAAGAA ATTATTGCCC ATGCACATGA   
  
  
- GAGTTTTGGG CTAGCTGGCT AGGCCAAATG AGACATGAAG ATTAAGTTGA AATATATATA TATATGCCAC   
  
  
- ATGGTATGCC AAGAAAATTA TAACATGCAT GTTATTTTTA GAACCGGCTC ATGTTGACTA GTTAATTAAA   
  
  
- AAAAATTATA TAGGTTTTTC AATATTTCAT AATATTAATG TAAAAATAGA ATATAAGAAA ATAAATTTAT   
  
  
- TAATTTTATT AAATTATCAT GTATTATAAT TTTATTTAAA ACTCAAATAA ACAAAAAAAT CTCTTGAGAC   
  
  
- TGATTCTTTT TTATCAGAAA AAAAAATGAT TATAATTAAA CTAACTCTTT TGACCTTAGA GCAATGCTAT   
  
  
- TGAGCCCAAA TCCATATTTA TTTGATCATG TCACCTTGTT GTTGATTATG TTTCTAATCA ATGGGGCTCC   
  
  
- AATATATGAA AAAATGGCAG ATAGTGCCAA TTTTCTATGG TCATTTATAT GACTGCAAAT TATGAACACA   
  
  
- GCAGAAACAT CAGCAAATAA AGCAACTAGA ATTCATAATC CATATCTTAT CCATAAACCT ACATGATGGT   
  
  
- AAGTGAAGAA GCTAGATATA AAGATTGCAT GCATATGCGT ACAAGGAAGA ATTAAAGGAC AAAGAAACAA   
  
  
- CCACCCATCT TGCTCCCCAT GCTCATCTT

  
  
Motifs Found  

+     5UTR Py-rich stretch

| Site Name | Organism | Position | Strand | Matrix score. | sequence | function |
| --- | --- | --- | --- | --- | --- | --- |
| 5UTR Py-rich stretch | Lycopersicon esculentum | 323 | + | 13 | TTTCTCTCTCTCTC | cis-acting element conferring high transcription levels |
| 5UTR Py-rich stretch | Lycopersicon esculentum | 266 | - | 13 | TTTCTCTCTCTCTC | cis-acting element conferring high transcription levels |
| 5UTR Py-rich stretch | Lycopersicon esculentum | 57 | + | 9 | TTTCTTCTCT | cis-acting element conferring high transcription levels |
| 5UTR Py-rich stretch | Lycopersicon esculentum | 321 | + | 13 | TTTCTCTCTCTCTC | cis-acting element conferring high transcription levels |
| 5UTR Py-rich stretch | Lycopersicon esculentum | 268 | - | 13 | TTTCTCTCTCTCTC | cis-acting element conferring high transcription levels |
| 5UTR Py-rich stretch | Lycopersicon esculentum | 319 | + | 13 | TTTCTCTCTCTCTC | cis-acting element conferring high transcription levels |
| 5UTR Py-rich stretch | Lycopersicon esculentum | 270 | - | 13 | TTTCTCTCTCTCTC | cis-acting element conferring high transcription levels |
| 5UTR Py-rich stretch | Lycopersicon esculentum | 325 | + | 13 | TTTCTCTCTCTCTC | cis-acting element conferring high transcription levels |

> 2018/04/13 10:10:12  
+ ATCCCAATTG GTCAATTCTT TCCGACTTTT CGGAAGTGTA ACTGTTCCTT AAATAAATTC TTCTCTCAAC   
  
  
+ GGTTTATCAG AGTACGTACG CTTCTCGTTA TGACATTCTA AAAATTCTGT TTTGAAACCC AATTTAAGTA   
  
  
+ TCGCTAGCAA CTTCGGTTGA TGTGAAGTGG ATGAAAGACA AGTTGGGGGG AACACTTTTG TTTCTGCATG   
  
  
+ CACGTACGGG TTTTTAGGGT AGCAGTAGTA GCTTCGTACG TTCTTTTGAG TTATAGAGAG AGAGAGAGAG   
  
  
+ AGAGTCTATT TGTGTGTTCT ACTTCGTCTC TCTCTCTCTG TCTCTCTCTC TCTCTCTCAT TCAAATTAGG   
  
  
+ TATTACTTCA TTTTCATACA ACTATACTGT CTACTAACCG AGACTTTACC GAATTTCATG TCCCAAATCT   
  
  
+ ATAACAATTT GCCGATTAGG TCATAGACTC CAGACCAACA ACTCCATCAA ACCTTATTAA GACCTGACTA   
  
  
+ TCTGACTAAA GTCCGCTAGT TATGTTGGGA AAGGAATCCC GTGTACGTAT CAAGATACAT CAAAGGTATA   
  
  
+ CATCTAGTTC AACGATTATA AAGTACATAT GAACAGCACA CTTTACTTTA CTTTACTAGT TATACTATTC   
  
  
+ AATAATATTA TTTCGACACC TTCTCGGTCT CAAGATCTAC TAGTAAAGAA TGCCTTCTAC GAACAAAACA   
  
  
+ TTCAAGCAAT TAAGATCTCA ACTTCCATTA ATTGTGGTAG AAATAGAAGA ACAAACCTCC AAATGATCAT   
  
  
+ TGTACGTTAA CAATAATATG AAACCGTTTG TTCGTTACGT TTTTATTCTT TAATAACGGG TACGTGTACT   
  
  
+ CTCAAAACCC GATCGACCGA TCCGGTTTAC TCTGTACTTC TAATTCAACT TTATATATAT ATATACGGTG   
  
  
+ TACCATACGG TTCTTTTAAT ATTGTACGTA CAATAAAAAT CTTGGCCGAG TACAACTGAT CAATTAATTT   
  
  
+ TTTTTAATAT ATCCAAAAAG TTATAAAGTA TTATAATTAC ATTTTTATCT TATATTCTTT TATTTAAATA   
  
  
+ ATTAAAATAA TTTAATAGTA CATAATATTA AAATAAATTT TGAGTTTATT TGTTTTTTTA GAGAACTCTG   
  
  
+ ACTAAGAAAA AATAGTCTTT TTTTTTACTA ATATTAATTT GATTGAGAAA ACTGGAATCT CGTTACGATA   
  
  
+ ACTCGGGTTT AGGTATAAAT AAACTAGTAC AGTGGAACAA CAACTAATAC AAAGATTAGT TACCCCGAGG   
  
  
+ TTATATACTT TTTTACCGTC TATCACGGTT AAAAGATACC AGTAAATATA CTGACGTTTA ATACTTGTGT   
  
  
+ CGTCTTTGTA GTCGTTTATT TCGTTGATCT TAAGTATTAG GTATAGAATA GGTATTTGGA TGTACTACCA   
  
  
+ TTCACTTCTT CGATCTATAT TTCTAACGTA CGTATACGCA TGTTCCTTCT TAATTTCCTG TTTCTTTGTT   
  
  
+ GGTGGGTAGA ACGAGGGGTA CGAGTAGAA  

- TAGGGTTAAC CAGTTAAGAA AGGCTGAAAA GCCTTCACAT TGACAAGGAA TTTATTTAAG AAGAGAGTTG   
  
  
- CCAAATAGTC TCATGCATGC GAAGAGCAAT ACTGTAAGAT TTTTAAGACA AAACTTTGGG TTAAATTCAT   
  
  
- AGCGATCGTT GAAGCCAACT ACACTTCACC TACTTTCTGT TCAACCCCCC TTGTGAAAAC AAAGACGTAC   
  
  
- GTGCATGCCC AAAAATCCCA TCGTCATCAT CGAAGCATGC AAGAAAACTC AATATCTCTC TCTCTCTCTC   
  
  
- TCTCAGATAA ACACACAAGA TGAAGCAGAG AGAGAGAGAC AGAGAGAGAG AGAGAGAGTA AGTTTAATCC   
  
  
- ATAATGAAGT AAAAGTATGT TGATATGACA GATGATTGGC TCTGAAATGG CTTAAAGTAC AGGGTTTAGA   
  
  
- TATTGTTAAA CGGCTAATCC AGTATCTGAG GTCTGGTTGT TGAGGTAGTT TGGAATAATT CTGGACTGAT   
  
  
- AGACTGATTT CAGGCGATCA ATACAACCCT TTCCTTAGGG CACATGCATA GTTCTATGTA GTTTCCATAT   
  
  
- GTAGATCAAG TTGCTAATAT TTCATGTATA CTTGTCGTGT GAAATGAAAT GAAATGATCA ATATGATAAG   
  
  
- TTATTATAAT AAAGCTGTGG AAGAGCCAGA GTTCTAGATG ATCATTTCTT ACGGAAGATG CTTGTTTTGT   
  
  
- AAGTTCGTTA ATTCTAGAGT TGAAGGTAAT TAACACCATC TTTATCTTCT TGTTTGGAGG TTTACTAGTA   
  
  
- ACATGCAATT GTTATTATAC TTTGGCAAAC AAGCAATGCA AAAATAAGAA ATTATTGCCC ATGCACATGA   
  
  
- GAGTTTTGGG CTAGCTGGCT AGGCCAAATG AGACATGAAG ATTAAGTTGA AATATATATA TATATGCCAC   
  
  
- ATGGTATGCC AAGAAAATTA TAACATGCAT GTTATTTTTA GAACCGGCTC ATGTTGACTA GTTAATTAAA   
  
  
- AAAAATTATA TAGGTTTTTC AATATTTCAT AATATTAATG TAAAAATAGA ATATAAGAAA ATAAATTTAT   
  
  
- TAATTTTATT AAATTATCAT GTATTATAAT TTTATTTAAA ACTCAAATAA ACAAAAAAAT CTCTTGAGAC   
  
  
- TGATTCTTTT TTATCAGAAA AAAAAATGAT TATAATTAAA CTAACTCTTT TGACCTTAGA GCAATGCTAT   
  
  
- TGAGCCCAAA TCCATATTTA TTTGATCATG TCACCTTGTT GTTGATTATG TTTCTAATCA ATGGGGCTCC   
  
  
- AATATATGAA AAAATGGCAG ATAGTGCCAA TTTTCTATGG TCATTTATAT GACTGCAAAT TATGAACACA   
  
  
- GCAGAAACAT CAGCAAATAA AGCAACTAGA ATTCATAATC CATATCTTAT CCATAAACCT ACATGATGGT   
  
  
- AAGTGAAGAA GCTAGATATA AAGATTGCAT GCATATGCGT ACAAGGAAGA ATTAAAGGAC AAAGAAACAA   
  
  
- CCACCCATCT TGCTCCCCAT GCTCATCTT

+     AAGAA-motif

| Site Name | Organism | Position | Strand | Matrix score. | sequence | function |
| --- | --- | --- | --- | --- | --- | --- |
| AAGAA-motif | Avena sativa | 16 | - | 7 | GAAAGAA |  |

> 2018/04/13 10:10:12  
+ ATCCCAATTG GTCAATTCTT TCCGACTTTT CGGAAGTGTA ACTGTTCCTT AAATAAATTC TTCTCTCAAC   
  
  
+ GGTTTATCAG AGTACGTACG CTTCTCGTTA TGACATTCTA AAAATTCTGT TTTGAAACCC AATTTAAGTA   
  
  
+ TCGCTAGCAA CTTCGGTTGA TGTGAAGTGG ATGAAAGACA AGTTGGGGGG AACACTTTTG TTTCTGCATG   
  
  
+ CACGTACGGG TTTTTAGGGT AGCAGTAGTA GCTTCGTACG TTCTTTTGAG TTATAGAGAG AGAGAGAGAG   
  
  
+ AGAGTCTATT TGTGTGTTCT ACTTCGTCTC TCTCTCTCTG TCTCTCTCTC TCTCTCTCAT TCAAATTAGG   
  
  
+ TATTACTTCA TTTTCATACA ACTATACTGT CTACTAACCG AGACTTTACC GAATTTCATG TCCCAAATCT   
  
  
+ ATAACAATTT GCCGATTAGG TCATAGACTC CAGACCAACA ACTCCATCAA ACCTTATTAA GACCTGACTA   
  
  
+ TCTGACTAAA GTCCGCTAGT TATGTTGGGA AAGGAATCCC GTGTACGTAT CAAGATACAT CAAAGGTATA   
  
  
+ CATCTAGTTC AACGATTATA AAGTACATAT GAACAGCACA CTTTACTTTA CTTTACTAGT TATACTATTC   
  
  
+ AATAATATTA TTTCGACACC TTCTCGGTCT CAAGATCTAC TAGTAAAGAA TGCCTTCTAC GAACAAAACA   
  
  
+ TTCAAGCAAT TAAGATCTCA ACTTCCATTA ATTGTGGTAG AAATAGAAGA ACAAACCTCC AAATGATCAT   
  
  
+ TGTACGTTAA CAATAATATG AAACCGTTTG TTCGTTACGT TTTTATTCTT TAATAACGGG TACGTGTACT   
  
  
+ CTCAAAACCC GATCGACCGA TCCGGTTTAC TCTGTACTTC TAATTCAACT TTATATATAT ATATACGGTG   
  
  
+ TACCATACGG TTCTTTTAAT ATTGTACGTA CAATAAAAAT CTTGGCCGAG TACAACTGAT CAATTAATTT   
  
  
+ TTTTTAATAT ATCCAAAAAG TTATAAAGTA TTATAATTAC ATTTTTATCT TATATTCTTT TATTTAAATA   
  
  
+ ATTAAAATAA TTTAATAGTA CATAATATTA AAATAAATTT TGAGTTTATT TGTTTTTTTA GAGAACTCTG   
  
  
+ ACTAAGAAAA AATAGTCTTT TTTTTTACTA ATATTAATTT GATTGAGAAA ACTGGAATCT CGTTACGATA   
  
  
+ ACTCGGGTTT AGGTATAAAT AAACTAGTAC AGTGGAACAA CAACTAATAC AAAGATTAGT TACCCCGAGG   
  
  
+ TTATATACTT TTTTACCGTC TATCACGGTT AAAAGATACC AGTAAATATA CTGACGTTTA ATACTTGTGT   
  
  
+ CGTCTTTGTA GTCGTTTATT TCGTTGATCT TAAGTATTAG GTATAGAATA GGTATTTGGA TGTACTACCA   
  
  
+ TTCACTTCTT CGATCTATAT TTCTAACGTA CGTATACGCA TGTTCCTTCT TAATTTCCTG TTTCTTTGTT   
  
  
+ GGTGGGTAGA ACGAGGGGTA CGAGTAGAA  

- TAGGGTTAAC CAGTTAAGAA AGGCTGAAAA GCCTTCACAT TGACAAGGAA TTTATTTAAG AAGAGAGTTG   
  
  
- CCAAATAGTC TCATGCATGC GAAGAGCAAT ACTGTAAGAT TTTTAAGACA AAACTTTGGG TTAAATTCAT   
  
  
- AGCGATCGTT GAAGCCAACT ACACTTCACC TACTTTCTGT TCAACCCCCC TTGTGAAAAC AAAGACGTAC   
  
  
- GTGCATGCCC AAAAATCCCA TCGTCATCAT CGAAGCATGC AAGAAAACTC AATATCTCTC TCTCTCTCTC   
  
  
- TCTCAGATAA ACACACAAGA TGAAGCAGAG AGAGAGAGAC AGAGAGAGAG AGAGAGAGTA AGTTTAATCC   
  
  
- ATAATGAAGT AAAAGTATGT TGATATGACA GATGATTGGC TCTGAAATGG CTTAAAGTAC AGGGTTTAGA   
  
  
- TATTGTTAAA CGGCTAATCC AGTATCTGAG GTCTGGTTGT TGAGGTAGTT TGGAATAATT CTGGACTGAT   
  
  
- AGACTGATTT CAGGCGATCA ATACAACCCT TTCCTTAGGG CACATGCATA GTTCTATGTA GTTTCCATAT   
  
  
- GTAGATCAAG TTGCTAATAT TTCATGTATA CTTGTCGTGT GAAATGAAAT GAAATGATCA ATATGATAAG   
  
  
- TTATTATAAT AAAGCTGTGG AAGAGCCAGA GTTCTAGATG ATCATTTCTT ACGGAAGATG CTTGTTTTGT   
  
  
- AAGTTCGTTA ATTCTAGAGT TGAAGGTAAT TAACACCATC TTTATCTTCT TGTTTGGAGG TTTACTAGTA   
  
  
- ACATGCAATT GTTATTATAC TTTGGCAAAC AAGCAATGCA AAAATAAGAA ATTATTGCCC ATGCACATGA   
  
  
- GAGTTTTGGG CTAGCTGGCT AGGCCAAATG AGACATGAAG ATTAAGTTGA AATATATATA TATATGCCAC   
  
  
- ATGGTATGCC AAGAAAATTA TAACATGCAT GTTATTTTTA GAACCGGCTC ATGTTGACTA GTTAATTAAA   
  
  
- AAAAATTATA TAGGTTTTTC AATATTTCAT AATATTAATG TAAAAATAGA ATATAAGAAA ATAAATTTAT   
  
  
- TAATTTTATT AAATTATCAT GTATTATAAT TTTATTTAAA ACTCAAATAA ACAAAAAAAT CTCTTGAGAC   
  
  
- TGATTCTTTT TTATCAGAAA AAAAAATGAT TATAATTAAA CTAACTCTTT TGACCTTAGA GCAATGCTAT   
  
  
- TGAGCCCAAA TCCATATTTA TTTGATCATG TCACCTTGTT GTTGATTATG TTTCTAATCA ATGGGGCTCC   
  
  
- AATATATGAA AAAATGGCAG ATAGTGCCAA TTTTCTATGG TCATTTATAT GACTGCAAAT TATGAACACA   
  
  
- GCAGAAACAT CAGCAAATAA AGCAACTAGA ATTCATAATC CATATCTTAT CCATAAACCT ACATGATGGT   
  
  
- AAGTGAAGAA GCTAGATATA AAGATTGCAT GCATATGCGT ACAAGGAAGA ATTAAAGGAC AAAGAAACAA   
  
  
- CCACCCATCT TGCTCCCCAT GCTCATCTT

+     ABRE

| Site Name | Organism | Position | Strand | Matrix score. | sequence | function |
| --- | --- | --- | --- | --- | --- | --- |
| ABRE | Arabidopsis thaliana | 831 | + | 6 | TACGTG | cis-acting element involved in the abscisic acid responsiveness |
| ABRE | Arabidopsis thaliana | 211 | - | 6 | TACGTG | cis-acting element involved in the abscisic acid responsiveness |
| ABRE | Hordeum vulgare | 209 | - | 10 | CGTACGTGCA | cis-acting element involved in the abscisic acid responsiveness |

> 2018/04/13 10:10:12  
+ ATCCCAATTG GTCAATTCTT TCCGACTTTT CGGAAGTGTA ACTGTTCCTT AAATAAATTC TTCTCTCAAC   
  
  
+ GGTTTATCAG AGTACGTACG CTTCTCGTTA TGACATTCTA AAAATTCTGT TTTGAAACCC AATTTAAGTA   
  
  
+ TCGCTAGCAA CTTCGGTTGA TGTGAAGTGG ATGAAAGACA AGTTGGGGGG AACACTTTTG TTTCTGCATG   
  
  
+ CACGTACGGG TTTTTAGGGT AGCAGTAGTA GCTTCGTACG TTCTTTTGAG TTATAGAGAG AGAGAGAGAG   
  
  
+ AGAGTCTATT TGTGTGTTCT ACTTCGTCTC TCTCTCTCTG TCTCTCTCTC TCTCTCTCAT TCAAATTAGG   
  
  
+ TATTACTTCA TTTTCATACA ACTATACTGT CTACTAACCG AGACTTTACC GAATTTCATG TCCCAAATCT   
  
  
+ ATAACAATTT GCCGATTAGG TCATAGACTC CAGACCAACA ACTCCATCAA ACCTTATTAA GACCTGACTA   
  
  
+ TCTGACTAAA GTCCGCTAGT TATGTTGGGA AAGGAATCCC GTGTACGTAT CAAGATACAT CAAAGGTATA   
  
  
+ CATCTAGTTC AACGATTATA AAGTACATAT GAACAGCACA CTTTACTTTA CTTTACTAGT TATACTATTC   
  
  
+ AATAATATTA TTTCGACACC TTCTCGGTCT CAAGATCTAC TAGTAAAGAA TGCCTTCTAC GAACAAAACA   
  
  
+ TTCAAGCAAT TAAGATCTCA ACTTCCATTA ATTGTGGTAG AAATAGAAGA ACAAACCTCC AAATGATCAT   
  
  
+ TGTACGTTAA CAATAATATG AAACCGTTTG TTCGTTACGT TTTTATTCTT TAATAACGGG TACGTGTACT   
  
  
+ CTCAAAACCC GATCGACCGA TCCGGTTTAC TCTGTACTTC TAATTCAACT TTATATATAT ATATACGGTG   
  
  
+ TACCATACGG TTCTTTTAAT ATTGTACGTA CAATAAAAAT CTTGGCCGAG TACAACTGAT CAATTAATTT   
  
  
+ TTTTTAATAT ATCCAAAAAG TTATAAAGTA TTATAATTAC ATTTTTATCT TATATTCTTT TATTTAAATA   
  
  
+ ATTAAAATAA TTTAATAGTA CATAATATTA AAATAAATTT TGAGTTTATT TGTTTTTTTA GAGAACTCTG   
  
  
+ ACTAAGAAAA AATAGTCTTT TTTTTTACTA ATATTAATTT GATTGAGAAA ACTGGAATCT CGTTACGATA   
  
  
+ ACTCGGGTTT AGGTATAAAT AAACTAGTAC AGTGGAACAA CAACTAATAC AAAGATTAGT TACCCCGAGG   
  
  
+ TTATATACTT TTTTACCGTC TATCACGGTT AAAAGATACC AGTAAATATA CTGACGTTTA ATACTTGTGT   
  
  
+ CGTCTTTGTA GTCGTTTATT TCGTTGATCT TAAGTATTAG GTATAGAATA GGTATTTGGA TGTACTACCA   
  
  
+ TTCACTTCTT CGATCTATAT TTCTAACGTA CGTATACGCA TGTTCCTTCT TAATTTCCTG TTTCTTTGTT   
  
  
+ GGTGGGTAGA ACGAGGGGTA CGAGTAGAA  

- TAGGGTTAAC CAGTTAAGAA AGGCTGAAAA GCCTTCACAT TGACAAGGAA TTTATTTAAG AAGAGAGTTG   
  
  
- CCAAATAGTC TCATGCATGC GAAGAGCAAT ACTGTAAGAT TTTTAAGACA AAACTTTGGG TTAAATTCAT   
  
  
- AGCGATCGTT GAAGCCAACT ACACTTCACC TACTTTCTGT TCAACCCCCC TTGTGAAAAC AAAGACGTAC   
  
  
- GTGCATGCCC AAAAATCCCA TCGTCATCAT CGAAGCATGC AAGAAAACTC AATATCTCTC TCTCTCTCTC   
  
  
- TCTCAGATAA ACACACAAGA TGAAGCAGAG AGAGAGAGAC AGAGAGAGAG AGAGAGAGTA AGTTTAATCC   
  
  
- ATAATGAAGT AAAAGTATGT TGATATGACA GATGATTGGC TCTGAAATGG CTTAAAGTAC AGGGTTTAGA   
  
  
- TATTGTTAAA CGGCTAATCC AGTATCTGAG GTCTGGTTGT TGAGGTAGTT TGGAATAATT CTGGACTGAT   
  
  
- AGACTGATTT CAGGCGATCA ATACAACCCT TTCCTTAGGG CACATGCATA GTTCTATGTA GTTTCCATAT   
  
  
- GTAGATCAAG TTGCTAATAT TTCATGTATA CTTGTCGTGT GAAATGAAAT GAAATGATCA ATATGATAAG   
  
  
- TTATTATAAT AAAGCTGTGG AAGAGCCAGA GTTCTAGATG ATCATTTCTT ACGGAAGATG CTTGTTTTGT   
  
  
- AAGTTCGTTA ATTCTAGAGT TGAAGGTAAT TAACACCATC TTTATCTTCT TGTTTGGAGG TTTACTAGTA   
  
  
- ACATGCAATT GTTATTATAC TTTGGCAAAC AAGCAATGCA AAAATAAGAA ATTATTGCCC ATGCACATGA   
  
  
- GAGTTTTGGG CTAGCTGGCT AGGCCAAATG AGACATGAAG ATTAAGTTGA AATATATATA TATATGCCAC   
  
  
- ATGGTATGCC AAGAAAATTA TAACATGCAT GTTATTTTTA GAACCGGCTC ATGTTGACTA GTTAATTAAA   
  
  
- AAAAATTATA TAGGTTTTTC AATATTTCAT AATATTAATG TAAAAATAGA ATATAAGAAA ATAAATTTAT   
  
  
- TAATTTTATT AAATTATCAT GTATTATAAT TTTATTTAAA ACTCAAATAA ACAAAAAAAT CTCTTGAGAC   
  
  
- TGATTCTTTT TTATCAGAAA AAAAAATGAT TATAATTAAA CTAACTCTTT TGACCTTAGA GCAATGCTAT   
  
  
- TGAGCCCAAA TCCATATTTA TTTGATCATG TCACCTTGTT GTTGATTATG TTTCTAATCA ATGGGGCTCC   
  
  
- AATATATGAA AAAATGGCAG ATAGTGCCAA TTTTCTATGG TCATTTATAT GACTGCAAAT TATGAACACA   
  
  
- GCAGAAACAT CAGCAAATAA AGCAACTAGA ATTCATAATC CATATCTTAT CCATAAACCT ACATGATGGT   
  
  
- AAGTGAAGAA GCTAGATATA AAGATTGCAT GCATATGCGT ACAAGGAAGA ATTAAAGGAC AAAGAAACAA   
  
  
- CCACCCATCT TGCTCCCCAT GCTCATCTT

+     AC-II

| Site Name | Organism | Position | Strand | Matrix score. | sequence | function |
| --- | --- | --- | --- | --- | --- | --- |
| AC-II | Phaseolus vulgaris | 1468 | - | 9.5 | (C/T)T(T/C)(C/T)(A/C)(A/C)C(A/C)A(A/C)C(C/A)(C/A)C |  |

> 2018/04/13 10:10:12  
+ ATCCCAATTG GTCAATTCTT TCCGACTTTT CGGAAGTGTA ACTGTTCCTT AAATAAATTC TTCTCTCAAC   
  
  
+ GGTTTATCAG AGTACGTACG CTTCTCGTTA TGACATTCTA AAAATTCTGT TTTGAAACCC AATTTAAGTA   
  
  
+ TCGCTAGCAA CTTCGGTTGA TGTGAAGTGG ATGAAAGACA AGTTGGGGGG AACACTTTTG TTTCTGCATG   
  
  
+ CACGTACGGG TTTTTAGGGT AGCAGTAGTA GCTTCGTACG TTCTTTTGAG TTATAGAGAG AGAGAGAGAG   
  
  
+ AGAGTCTATT TGTGTGTTCT ACTTCGTCTC TCTCTCTCTG TCTCTCTCTC TCTCTCTCAT TCAAATTAGG   
  
  
+ TATTACTTCA TTTTCATACA ACTATACTGT CTACTAACCG AGACTTTACC GAATTTCATG TCCCAAATCT   
  
  
+ ATAACAATTT GCCGATTAGG TCATAGACTC CAGACCAACA ACTCCATCAA ACCTTATTAA GACCTGACTA   
  
  
+ TCTGACTAAA GTCCGCTAGT TATGTTGGGA AAGGAATCCC GTGTACGTAT CAAGATACAT CAAAGGTATA   
  
  
+ CATCTAGTTC AACGATTATA AAGTACATAT GAACAGCACA CTTTACTTTA CTTTACTAGT TATACTATTC   
  
  
+ AATAATATTA TTTCGACACC TTCTCGGTCT CAAGATCTAC TAGTAAAGAA TGCCTTCTAC GAACAAAACA   
  
  
+ TTCAAGCAAT TAAGATCTCA ACTTCCATTA ATTGTGGTAG AAATAGAAGA ACAAACCTCC AAATGATCAT   
  
  
+ TGTACGTTAA CAATAATATG AAACCGTTTG TTCGTTACGT TTTTATTCTT TAATAACGGG TACGTGTACT   
  
  
+ CTCAAAACCC GATCGACCGA TCCGGTTTAC TCTGTACTTC TAATTCAACT TTATATATAT ATATACGGTG   
  
  
+ TACCATACGG TTCTTTTAAT ATTGTACGTA CAATAAAAAT CTTGGCCGAG TACAACTGAT CAATTAATTT   
  
  
+ TTTTTAATAT ATCCAAAAAG TTATAAAGTA TTATAATTAC ATTTTTATCT TATATTCTTT TATTTAAATA   
  
  
+ ATTAAAATAA TTTAATAGTA CATAATATTA AAATAAATTT TGAGTTTATT TGTTTTTTTA GAGAACTCTG   
  
  
+ ACTAAGAAAA AATAGTCTTT TTTTTTACTA ATATTAATTT GATTGAGAAA ACTGGAATCT CGTTACGATA   
  
  
+ ACTCGGGTTT AGGTATAAAT AAACTAGTAC AGTGGAACAA CAACTAATAC AAAGATTAGT TACCCCGAGG   
  
  
+ TTATATACTT TTTTACCGTC TATCACGGTT AAAAGATACC AGTAAATATA CTGACGTTTA ATACTTGTGT   
  
  
+ CGTCTTTGTA GTCGTTTATT TCGTTGATCT TAAGTATTAG GTATAGAATA GGTATTTGGA TGTACTACCA   
  
  
+ TTCACTTCTT CGATCTATAT TTCTAACGTA CGTATACGCA TGTTCCTTCT TAATTTCCTG TTTCTTTGTT   
  
  
+ GGTGGGTAGA ACGAGGGGTA CGAGTAGAA  

- TAGGGTTAAC CAGTTAAGAA AGGCTGAAAA GCCTTCACAT TGACAAGGAA TTTATTTAAG AAGAGAGTTG   
  
  
- CCAAATAGTC TCATGCATGC GAAGAGCAAT ACTGTAAGAT TTTTAAGACA AAACTTTGGG TTAAATTCAT   
  
  
- AGCGATCGTT GAAGCCAACT ACACTTCACC TACTTTCTGT TCAACCCCCC TTGTGAAAAC AAAGACGTAC   
  
  
- GTGCATGCCC AAAAATCCCA TCGTCATCAT CGAAGCATGC AAGAAAACTC AATATCTCTC TCTCTCTCTC   
  
  
- TCTCAGATAA ACACACAAGA TGAAGCAGAG AGAGAGAGAC AGAGAGAGAG AGAGAGAGTA AGTTTAATCC   
  
  
- ATAATGAAGT AAAAGTATGT TGATATGACA GATGATTGGC TCTGAAATGG CTTAAAGTAC AGGGTTTAGA   
  
  
- TATTGTTAAA CGGCTAATCC AGTATCTGAG GTCTGGTTGT TGAGGTAGTT TGGAATAATT CTGGACTGAT   
  
  
- AGACTGATTT CAGGCGATCA ATACAACCCT TTCCTTAGGG CACATGCATA GTTCTATGTA GTTTCCATAT   
  
  
- GTAGATCAAG TTGCTAATAT TTCATGTATA CTTGTCGTGT GAAATGAAAT GAAATGATCA ATATGATAAG   
  
  
- TTATTATAAT AAAGCTGTGG AAGAGCCAGA GTTCTAGATG ATCATTTCTT ACGGAAGATG CTTGTTTTGT   
  
  
- AAGTTCGTTA ATTCTAGAGT TGAAGGTAAT TAACACCATC TTTATCTTCT TGTTTGGAGG TTTACTAGTA   
  
  
- ACATGCAATT GTTATTATAC TTTGGCAAAC AAGCAATGCA AAAATAAGAA ATTATTGCCC ATGCACATGA   
  
  
- GAGTTTTGGG CTAGCTGGCT AGGCCAAATG AGACATGAAG ATTAAGTTGA AATATATATA TATATGCCAC   
  
  
- ATGGTATGCC AAGAAAATTA TAACATGCAT GTTATTTTTA GAACCGGCTC ATGTTGACTA GTTAATTAAA   
  
  
- AAAAATTATA TAGGTTTTTC AATATTTCAT AATATTAATG TAAAAATAGA ATATAAGAAA ATAAATTTAT   
  
  
- TAATTTTATT AAATTATCAT GTATTATAAT TTTATTTAAA ACTCAAATAA ACAAAAAAAT CTCTTGAGAC   
  
  
- TGATTCTTTT TTATCAGAAA AAAAAATGAT TATAATTAAA CTAACTCTTT TGACCTTAGA GCAATGCTAT   
  
  
- TGAGCCCAAA TCCATATTTA TTTGATCATG TCACCTTGTT GTTGATTATG TTTCTAATCA ATGGGGCTCC   
  
  
- AATATATGAA AAAATGGCAG ATAGTGCCAA TTTTCTATGG TCATTTATAT GACTGCAAAT TATGAACACA   
  
  
- GCAGAAACAT CAGCAAATAA AGCAACTAGA ATTCATAATC CATATCTTAT CCATAAACCT ACATGATGGT   
  
  
- AAGTGAAGAA GCTAGATATA AAGATTGCAT GCATATGCGT ACAAGGAAGA ATTAAAGGAC AAAGAAACAA   
  
  
- CCACCCATCT TGCTCCCCAT GCTCATCTTT

+     AE-box

| Site Name | Organism | Position | Strand | Matrix score. | sequence | function |
| --- | --- | --- | --- | --- | --- | --- |
| AE-box | Arabidopsis thaliana | 198 | - | 8 | AGAAACAA | part of a module for light response |

> 2018/04/13 10:10:12  
+ ATCCCAATTG GTCAATTCTT TCCGACTTTT CGGAAGTGTA ACTGTTCCTT AAATAAATTC TTCTCTCAAC   
  
  
+ GGTTTATCAG AGTACGTACG CTTCTCGTTA TGACATTCTA AAAATTCTGT TTTGAAACCC AATTTAAGTA   
  
  
+ TCGCTAGCAA CTTCGGTTGA TGTGAAGTGG ATGAAAGACA AGTTGGGGGG AACACTTTTG TTTCTGCATG   
  
  
+ CACGTACGGG TTTTTAGGGT AGCAGTAGTA GCTTCGTACG TTCTTTTGAG TTATAGAGAG AGAGAGAGAG   
  
  
+ AGAGTCTATT TGTGTGTTCT ACTTCGTCTC TCTCTCTCTG TCTCTCTCTC TCTCTCTCAT TCAAATTAGG   
  
  
+ TATTACTTCA TTTTCATACA ACTATACTGT CTACTAACCG AGACTTTACC GAATTTCATG TCCCAAATCT   
  
  
+ ATAACAATTT GCCGATTAGG TCATAGACTC CAGACCAACA ACTCCATCAA ACCTTATTAA GACCTGACTA   
  
  
+ TCTGACTAAA GTCCGCTAGT TATGTTGGGA AAGGAATCCC GTGTACGTAT CAAGATACAT CAAAGGTATA   
  
  
+ CATCTAGTTC AACGATTATA AAGTACATAT GAACAGCACA CTTTACTTTA CTTTACTAGT TATACTATTC   
  
  
+ AATAATATTA TTTCGACACC TTCTCGGTCT CAAGATCTAC TAGTAAAGAA TGCCTTCTAC GAACAAAACA   
  
  
+ TTCAAGCAAT TAAGATCTCA ACTTCCATTA ATTGTGGTAG AAATAGAAGA ACAAACCTCC AAATGATCAT   
  
  
+ TGTACGTTAA CAATAATATG AAACCGTTTG TTCGTTACGT TTTTATTCTT TAATAACGGG TACGTGTACT   
  
  
+ CTCAAAACCC GATCGACCGA TCCGGTTTAC TCTGTACTTC TAATTCAACT TTATATATAT ATATACGGTG   
  
  
+ TACCATACGG TTCTTTTAAT ATTGTACGTA CAATAAAAAT CTTGGCCGAG TACAACTGAT CAATTAATTT   
  
  
+ TTTTTAATAT ATCCAAAAAG TTATAAAGTA TTATAATTAC ATTTTTATCT TATATTCTTT TATTTAAATA   
  
  
+ ATTAAAATAA TTTAATAGTA CATAATATTA AAATAAATTT TGAGTTTATT TGTTTTTTTA GAGAACTCTG   
  
  
+ ACTAAGAAAA AATAGTCTTT TTTTTTACTA ATATTAATTT GATTGAGAAA ACTGGAATCT CGTTACGATA   
  
  
+ ACTCGGGTTT AGGTATAAAT AAACTAGTAC AGTGGAACAA CAACTAATAC AAAGATTAGT TACCCCGAGG   
  
  
+ TTATATACTT TTTTACCGTC TATCACGGTT AAAAGATACC AGTAAATATA CTGACGTTTA ATACTTGTGT   
  
  
+ CGTCTTTGTA GTCGTTTATT TCGTTGATCT TAAGTATTAG GTATAGAATA GGTATTTGGA TGTACTACCA   
  
  
+ TTCACTTCTT CGATCTATAT TTCTAACGTA CGTATACGCA TGTTCCTTCT TAATTTCCTG TTTCTTTGTT   
  
  
+ GGTGGGTAGA ACGAGGGGTA CGAGTAGAA  

- TAGGGTTAAC CAGTTAAGAA AGGCTGAAAA GCCTTCACAT TGACAAGGAA TTTATTTAAG AAGAGAGTTG   
  
  
- CCAAATAGTC TCATGCATGC GAAGAGCAAT ACTGTAAGAT TTTTAAGACA AAACTTTGGG TTAAATTCAT   
  
  
- AGCGATCGTT GAAGCCAACT ACACTTCACC TACTTTCTGT TCAACCCCCC TTGTGAAAAC AAAGACGTAC   
  
  
- GTGCATGCCC AAAAATCCCA TCGTCATCAT CGAAGCATGC AAGAAAACTC AATATCTCTC TCTCTCTCTC   
  
  
- TCTCAGATAA ACACACAAGA TGAAGCAGAG AGAGAGAGAC AGAGAGAGAG AGAGAGAGTA AGTTTAATCC   
  
  
- ATAATGAAGT AAAAGTATGT TGATATGACA GATGATTGGC TCTGAAATGG CTTAAAGTAC AGGGTTTAGA   
  
  
- TATTGTTAAA CGGCTAATCC AGTATCTGAG GTCTGGTTGT TGAGGTAGTT TGGAATAATT CTGGACTGAT   
  
  
- AGACTGATTT CAGGCGATCA ATACAACCCT TTCCTTAGGG CACATGCATA GTTCTATGTA GTTTCCATAT   
  
  
- GTAGATCAAG TTGCTAATAT TTCATGTATA CTTGTCGTGT GAAATGAAAT GAAATGATCA ATATGATAAG   
  
  
- TTATTATAAT AAAGCTGTGG AAGAGCCAGA GTTCTAGATG ATCATTTCTT ACGGAAGATG CTTGTTTTGT   
  
  
- AAGTTCGTTA ATTCTAGAGT TGAAGGTAAT TAACACCATC TTTATCTTCT TGTTTGGAGG TTTACTAGTA   
  
  
- ACATGCAATT GTTATTATAC TTTGGCAAAC AAGCAATGCA AAAATAAGAA ATTATTGCCC ATGCACATGA   
  
  
- GAGTTTTGGG CTAGCTGGCT AGGCCAAATG AGACATGAAG ATTAAGTTGA AATATATATA TATATGCCAC   
  
  
- ATGGTATGCC AAGAAAATTA TAACATGCAT GTTATTTTTA GAACCGGCTC ATGTTGACTA GTTAATTAAA   
  
  
- AAAAATTATA TAGGTTTTTC AATATTTCAT AATATTAATG TAAAAATAGA ATATAAGAAA ATAAATTTAT   
  
  
- TAATTTTATT AAATTATCAT GTATTATAAT TTTATTTAAA ACTCAAATAA ACAAAAAAAT CTCTTGAGAC   
  
  
- TGATTCTTTT TTATCAGAAA AAAAAATGAT TATAATTAAA CTAACTCTTT TGACCTTAGA GCAATGCTAT   
  
  
- TGAGCCCAAA TCCATATTTA TTTGATCATG TCACCTTGTT GTTGATTATG TTTCTAATCA ATGGGGCTCC   
  
  
- AATATATGAA AAAATGGCAG ATAGTGCCAA TTTTCTATGG TCATTTATAT GACTGCAAAT TATGAACACA   
  
  
- GCAGAAACAT CAGCAAATAA AGCAACTAGA ATTCATAATC CATATCTTAT CCATAAACCT ACATGATGGT   
  
  
- AAGTGAAGAA GCTAGATATA AAGATTGCAT GCATATGCGT ACAAGGAAGA ATTAAAGGAC AAAGAAACAA   
  
  
- CCACCCATCT TGCTCCCCAT GCTCATCTT

+     Box 4

| Site Name | Organism | Position | Strand | Matrix score. | sequence | function |
| --- | --- | --- | --- | --- | --- | --- |
| Box 4 | Petroselinum crispum | 973 | - | 6 | ATTAAT | part of a conserved DNA module involved in light responsiveness |
| Box 4 | Petroselinum crispum | 1153 | - | 6 | ATTAAT | part of a conserved DNA module involved in light responsiveness |
| Box 4 | Petroselinum crispum | 727 | + | 6 | ATTAAT | part of a conserved DNA module involved in light responsiveness |

> 2018/04/13 10:10:12  
+ ATCCCAATTG GTCAATTCTT TCCGACTTTT CGGAAGTGTA ACTGTTCCTT AAATAAATTC TTCTCTCAAC   
  
  
+ GGTTTATCAG AGTACGTACG CTTCTCGTTA TGACATTCTA AAAATTCTGT TTTGAAACCC AATTTAAGTA   
  
  
+ TCGCTAGCAA CTTCGGTTGA TGTGAAGTGG ATGAAAGACA AGTTGGGGGG AACACTTTTG TTTCTGCATG   
  
  
+ CACGTACGGG TTTTTAGGGT AGCAGTAGTA GCTTCGTACG TTCTTTTGAG TTATAGAGAG AGAGAGAGAG   
  
  
+ AGAGTCTATT TGTGTGTTCT ACTTCGTCTC TCTCTCTCTG TCTCTCTCTC TCTCTCTCAT TCAAATTAGG   
  
  
+ TATTACTTCA TTTTCATACA ACTATACTGT CTACTAACCG AGACTTTACC GAATTTCATG TCCCAAATCT   
  
  
+ ATAACAATTT GCCGATTAGG TCATAGACTC CAGACCAACA ACTCCATCAA ACCTTATTAA GACCTGACTA   
  
  
+ TCTGACTAAA GTCCGCTAGT TATGTTGGGA AAGGAATCCC GTGTACGTAT CAAGATACAT CAAAGGTATA   
  
  
+ CATCTAGTTC AACGATTATA AAGTACATAT GAACAGCACA CTTTACTTTA CTTTACTAGT TATACTATTC   
  
  
+ AATAATATTA TTTCGACACC TTCTCGGTCT CAAGATCTAC TAGTAAAGAA TGCCTTCTAC GAACAAAACA   
  
  
+ TTCAAGCAAT TAAGATCTCA ACTTCCATTA ATTGTGGTAG AAATAGAAGA ACAAACCTCC AAATGATCAT   
  
  
+ TGTACGTTAA CAATAATATG AAACCGTTTG TTCGTTACGT TTTTATTCTT TAATAACGGG TACGTGTACT   
  
  
+ CTCAAAACCC GATCGACCGA TCCGGTTTAC TCTGTACTTC TAATTCAACT TTATATATAT ATATACGGTG   
  
  
+ TACCATACGG TTCTTTTAAT ATTGTACGTA CAATAAAAAT CTTGGCCGAG TACAACTGAT CAATTAATTT   
  
  
+ TTTTTAATAT ATCCAAAAAG TTATAAAGTA TTATAATTAC ATTTTTATCT TATATTCTTT TATTTAAATA   
  
  
+ ATTAAAATAA TTTAATAGTA CATAATATTA AAATAAATTT TGAGTTTATT TGTTTTTTTA GAGAACTCTG   
  
  
+ ACTAAGAAAA AATAGTCTTT TTTTTTACTA ATATTAATTT GATTGAGAAA ACTGGAATCT CGTTACGATA   
  
  
+ ACTCGGGTTT AGGTATAAAT AAACTAGTAC AGTGGAACAA CAACTAATAC AAAGATTAGT TACCCCGAGG   
  
  
+ TTATATACTT TTTTACCGTC TATCACGGTT AAAAGATACC AGTAAATATA CTGACGTTTA ATACTTGTGT   
  
  
+ CGTCTTTGTA GTCGTTTATT TCGTTGATCT TAAGTATTAG GTATAGAATA GGTATTTGGA TGTACTACCA   
  
  
+ TTCACTTCTT CGATCTATAT TTCTAACGTA CGTATACGCA TGTTCCTTCT TAATTTCCTG TTTCTTTGTT   
  
  
+ GGTGGGTAGA ACGAGGGGTA CGAGTAGAA  

- TAGGGTTAAC CAGTTAAGAA AGGCTGAAAA GCCTTCACAT TGACAAGGAA TTTATTTAAG AAGAGAGTTG   
  
  
- CCAAATAGTC TCATGCATGC GAAGAGCAAT ACTGTAAGAT TTTTAAGACA AAACTTTGGG TTAAATTCAT   
  
  
- AGCGATCGTT GAAGCCAACT ACACTTCACC TACTTTCTGT TCAACCCCCC TTGTGAAAAC AAAGACGTAC   
  
  
- GTGCATGCCC AAAAATCCCA TCGTCATCAT CGAAGCATGC AAGAAAACTC AATATCTCTC TCTCTCTCTC   
  
  
- TCTCAGATAA ACACACAAGA TGAAGCAGAG AGAGAGAGAC AGAGAGAGAG AGAGAGAGTA AGTTTAATCC   
  
  
- ATAATGAAGT AAAAGTATGT TGATATGACA GATGATTGGC TCTGAAATGG CTTAAAGTAC AGGGTTTAGA   
  
  
- TATTGTTAAA CGGCTAATCC AGTATCTGAG GTCTGGTTGT TGAGGTAGTT TGGAATAATT CTGGACTGAT   
  
  
- AGACTGATTT CAGGCGATCA ATACAACCCT TTCCTTAGGG CACATGCATA GTTCTATGTA GTTTCCATAT   
  
  
- GTAGATCAAG TTGCTAATAT TTCATGTATA CTTGTCGTGT GAAATGAAAT GAAATGATCA ATATGATAAG   
  
  
- TTATTATAAT AAAGCTGTGG AAGAGCCAGA GTTCTAGATG ATCATTTCTT ACGGAAGATG CTTGTTTTGT   
  
  
- AAGTTCGTTA ATTCTAGAGT TGAAGGTAAT TAACACCATC TTTATCTTCT TGTTTGGAGG TTTACTAGTA   
  
  
- ACATGCAATT GTTATTATAC TTTGGCAAAC AAGCAATGCA AAAATAAGAA ATTATTGCCC ATGCACATGA   
  
  
- GAGTTTTGGG CTAGCTGGCT AGGCCAAATG AGACATGAAG ATTAAGTTGA AATATATATA TATATGCCAC   
  
  
- ATGGTATGCC AAGAAAATTA TAACATGCAT GTTATTTTTA GAACCGGCTC ATGTTGACTA GTTAATTAAA   
  
  
- AAAAATTATA TAGGTTTTTC AATATTTCAT AATATTAATG TAAAAATAGA ATATAAGAAA ATAAATTTAT   
  
  
- TAATTTTATT AAATTATCAT GTATTATAAT TTTATTTAAA ACTCAAATAA ACAAAAAAAT CTCTTGAGAC   
  
  
- TGATTCTTTT TTATCAGAAA AAAAAATGAT TATAATTAAA CTAACTCTTT TGACCTTAGA GCAATGCTAT   
  
  
- TGAGCCCAAA TCCATATTTA TTTGATCATG TCACCTTGTT GTTGATTATG TTTCTAATCA ATGGGGCTCC   
  
  
- AATATATGAA AAAATGGCAG ATAGTGCCAA TTTTCTATGG TCATTTATAT GACTGCAAAT TATGAACACA   
  
  
- GCAGAAACAT CAGCAAATAA AGCAACTAGA ATTCATAATC CATATCTTAT CCATAAACCT ACATGATGGT   
  
  
- AAGTGAAGAA GCTAGATATA AAGATTGCAT GCATATGCGT ACAAGGAAGA ATTAAAGGAC AAAGAAACAA   
  
  
- CCACCCATCT TGCTCCCCAT GCTCATCTT

+     Box I

| Site Name | Organism | Position | Strand | Matrix score. | sequence | function |
| --- | --- | --- | --- | --- | --- | --- |
| Box I | Pisum sativum | 121 | - | 7 | TTTCAAA | light responsive element |

> 2018/04/13 10:10:12  
+ ATCCCAATTG GTCAATTCTT TCCGACTTTT CGGAAGTGTA ACTGTTCCTT AAATAAATTC TTCTCTCAAC   
  
  
+ GGTTTATCAG AGTACGTACG CTTCTCGTTA TGACATTCTA AAAATTCTGT TTTGAAACCC AATTTAAGTA   
  
  
+ TCGCTAGCAA CTTCGGTTGA TGTGAAGTGG ATGAAAGACA AGTTGGGGGG AACACTTTTG TTTCTGCATG   
  
  
+ CACGTACGGG TTTTTAGGGT AGCAGTAGTA GCTTCGTACG TTCTTTTGAG TTATAGAGAG AGAGAGAGAG   
  
  
+ AGAGTCTATT TGTGTGTTCT ACTTCGTCTC TCTCTCTCTG TCTCTCTCTC TCTCTCTCAT TCAAATTAGG   
  
  
+ TATTACTTCA TTTTCATACA ACTATACTGT CTACTAACCG AGACTTTACC GAATTTCATG TCCCAAATCT   
  
  
+ ATAACAATTT GCCGATTAGG TCATAGACTC CAGACCAACA ACTCCATCAA ACCTTATTAA GACCTGACTA   
  
  
+ TCTGACTAAA GTCCGCTAGT TATGTTGGGA AAGGAATCCC GTGTACGTAT CAAGATACAT CAAAGGTATA   
  
  
+ CATCTAGTTC AACGATTATA AAGTACATAT GAACAGCACA CTTTACTTTA CTTTACTAGT TATACTATTC   
  
  
+ AATAATATTA TTTCGACACC TTCTCGGTCT CAAGATCTAC TAGTAAAGAA TGCCTTCTAC GAACAAAACA   
  
  
+ TTCAAGCAAT TAAGATCTCA ACTTCCATTA ATTGTGGTAG AAATAGAAGA ACAAACCTCC AAATGATCAT   
  
  
+ TGTACGTTAA CAATAATATG AAACCGTTTG TTCGTTACGT TTTTATTCTT TAATAACGGG TACGTGTACT   
  
  
+ CTCAAAACCC GATCGACCGA TCCGGTTTAC TCTGTACTTC TAATTCAACT TTATATATAT ATATACGGTG   
  
  
+ TACCATACGG TTCTTTTAAT ATTGTACGTA CAATAAAAAT CTTGGCCGAG TACAACTGAT CAATTAATTT   
  
  
+ TTTTTAATAT ATCCAAAAAG TTATAAAGTA TTATAATTAC ATTTTTATCT TATATTCTTT TATTTAAATA   
  
  
+ ATTAAAATAA TTTAATAGTA CATAATATTA AAATAAATTT TGAGTTTATT TGTTTTTTTA GAGAACTCTG   
  
  
+ ACTAAGAAAA AATAGTCTTT TTTTTTACTA ATATTAATTT GATTGAGAAA ACTGGAATCT CGTTACGATA   
  
  
+ ACTCGGGTTT AGGTATAAAT AAACTAGTAC AGTGGAACAA CAACTAATAC AAAGATTAGT TACCCCGAGG   
  
  
+ TTATATACTT TTTTACCGTC TATCACGGTT AAAAGATACC AGTAAATATA CTGACGTTTA ATACTTGTGT   
  
  
+ CGTCTTTGTA GTCGTTTATT TCGTTGATCT TAAGTATTAG GTATAGAATA GGTATTTGGA TGTACTACCA   
  
  
+ TTCACTTCTT CGATCTATAT TTCTAACGTA CGTATACGCA TGTTCCTTCT TAATTTCCTG TTTCTTTGTT   
  
  
+ GGTGGGTAGA ACGAGGGGTA CGAGTAGAA  

- TAGGGTTAAC CAGTTAAGAA AGGCTGAAAA GCCTTCACAT TGACAAGGAA TTTATTTAAG AAGAGAGTTG   
  
  
- CCAAATAGTC TCATGCATGC GAAGAGCAAT ACTGTAAGAT TTTTAAGACA AAACTTTGGG TTAAATTCAT   
  
  
- AGCGATCGTT GAAGCCAACT ACACTTCACC TACTTTCTGT TCAACCCCCC TTGTGAAAAC AAAGACGTAC   
  
  
- GTGCATGCCC AAAAATCCCA TCGTCATCAT CGAAGCATGC AAGAAAACTC AATATCTCTC TCTCTCTCTC   
  
  
- TCTCAGATAA ACACACAAGA TGAAGCAGAG AGAGAGAGAC AGAGAGAGAG AGAGAGAGTA AGTTTAATCC   
  
  
- ATAATGAAGT AAAAGTATGT TGATATGACA GATGATTGGC TCTGAAATGG CTTAAAGTAC AGGGTTTAGA   
  
  
- TATTGTTAAA CGGCTAATCC AGTATCTGAG GTCTGGTTGT TGAGGTAGTT TGGAATAATT CTGGACTGAT   
  
  
- AGACTGATTT CAGGCGATCA ATACAACCCT TTCCTTAGGG CACATGCATA GTTCTATGTA GTTTCCATAT   
  
  
- GTAGATCAAG TTGCTAATAT TTCATGTATA CTTGTCGTGT GAAATGAAAT GAAATGATCA ATATGATAAG   
  
  
- TTATTATAAT AAAGCTGTGG AAGAGCCAGA GTTCTAGATG ATCATTTCTT ACGGAAGATG CTTGTTTTGT   
  
  
- AAGTTCGTTA ATTCTAGAGT TGAAGGTAAT TAACACCATC TTTATCTTCT TGTTTGGAGG TTTACTAGTA   
  
  
- ACATGCAATT GTTATTATAC TTTGGCAAAC AAGCAATGCA AAAATAAGAA ATTATTGCCC ATGCACATGA   
  
  
- GAGTTTTGGG CTAGCTGGCT AGGCCAAATG AGACATGAAG ATTAAGTTGA AATATATATA TATATGCCAC   
  
  
- ATGGTATGCC AAGAAAATTA TAACATGCAT GTTATTTTTA GAACCGGCTC ATGTTGACTA GTTAATTAAA   
  
  
- AAAAATTATA TAGGTTTTTC AATATTTCAT AATATTAATG TAAAAATAGA ATATAAGAAA ATAAATTTAT   
  
  
- TAATTTTATT AAATTATCAT GTATTATAAT TTTATTTAAA ACTCAAATAA ACAAAAAAAT CTCTTGAGAC   
  
  
- TGATTCTTTT TTATCAGAAA AAAAAATGAT TATAATTAAA CTAACTCTTT TGACCTTAGA GCAATGCTAT   
  
  
- TGAGCCCAAA TCCATATTTA TTTGATCATG TCACCTTGTT GTTGATTATG TTTCTAATCA ATGGGGCTCC   
  
  
- AATATATGAA AAAATGGCAG ATAGTGCCAA TTTTCTATGG TCATTTATAT GACTGCAAAT TATGAACACA   
  
  
- GCAGAAACAT CAGCAAATAA AGCAACTAGA ATTCATAATC CATATCTTAT CCATAAACCT ACATGATGGT   
  
  
- AAGTGAAGAA GCTAGATATA AAGATTGCAT GCATATGCGT ACAAGGAAGA ATTAAAGGAC AAAGAAACAA   
  
  
- CCACCCATCT TGCTCCCCAT GCTCATCTT

+     Box III

| Site Name | Organism | Position | Strand | Matrix score. | sequence | function |
| --- | --- | --- | --- | --- | --- | --- |
| Box III | Pisum sativum | 35 | - | 9 | CATTTACACT | protein binding site |

> 2018/04/13 10:10:12  
+ ATCCCAATTG GTCAATTCTT TCCGACTTTT CGGAAGTGTA ACTGTTCCTT AAATAAATTC TTCTCTCAAC   
  
  
+ GGTTTATCAG AGTACGTACG CTTCTCGTTA TGACATTCTA AAAATTCTGT TTTGAAACCC AATTTAAGTA   
  
  
+ TCGCTAGCAA CTTCGGTTGA TGTGAAGTGG ATGAAAGACA AGTTGGGGGG AACACTTTTG TTTCTGCATG   
  
  
+ CACGTACGGG TTTTTAGGGT AGCAGTAGTA GCTTCGTACG TTCTTTTGAG TTATAGAGAG AGAGAGAGAG   
  
  
+ AGAGTCTATT TGTGTGTTCT ACTTCGTCTC TCTCTCTCTG TCTCTCTCTC TCTCTCTCAT TCAAATTAGG   
  
  
+ TATTACTTCA TTTTCATACA ACTATACTGT CTACTAACCG AGACTTTACC GAATTTCATG TCCCAAATCT   
  
  
+ ATAACAATTT GCCGATTAGG TCATAGACTC CAGACCAACA ACTCCATCAA ACCTTATTAA GACCTGACTA   
  
  
+ TCTGACTAAA GTCCGCTAGT TATGTTGGGA AAGGAATCCC GTGTACGTAT CAAGATACAT CAAAGGTATA   
  
  
+ CATCTAGTTC AACGATTATA AAGTACATAT GAACAGCACA CTTTACTTTA CTTTACTAGT TATACTATTC   
  
  
+ AATAATATTA TTTCGACACC TTCTCGGTCT CAAGATCTAC TAGTAAAGAA TGCCTTCTAC GAACAAAACA   
  
  
+ TTCAAGCAAT TAAGATCTCA ACTTCCATTA ATTGTGGTAG AAATAGAAGA ACAAACCTCC AAATGATCAT   
  
  
+ TGTACGTTAA CAATAATATG AAACCGTTTG TTCGTTACGT TTTTATTCTT TAATAACGGG TACGTGTACT   
  
  
+ CTCAAAACCC GATCGACCGA TCCGGTTTAC TCTGTACTTC TAATTCAACT TTATATATAT ATATACGGTG   
  
  
+ TACCATACGG TTCTTTTAAT ATTGTACGTA CAATAAAAAT CTTGGCCGAG TACAACTGAT CAATTAATTT   
  
  
+ TTTTTAATAT ATCCAAAAAG TTATAAAGTA TTATAATTAC ATTTTTATCT TATATTCTTT TATTTAAATA   
  
  
+ ATTAAAATAA TTTAATAGTA CATAATATTA AAATAAATTT TGAGTTTATT TGTTTTTTTA GAGAACTCTG   
  
  
+ ACTAAGAAAA AATAGTCTTT TTTTTTACTA ATATTAATTT GATTGAGAAA ACTGGAATCT CGTTACGATA   
  
  
+ ACTCGGGTTT AGGTATAAAT AAACTAGTAC AGTGGAACAA CAACTAATAC AAAGATTAGT TACCCCGAGG   
  
  
+ TTATATACTT TTTTACCGTC TATCACGGTT AAAAGATACC AGTAAATATA CTGACGTTTA ATACTTGTGT   
  
  
+ CGTCTTTGTA GTCGTTTATT TCGTTGATCT TAAGTATTAG GTATAGAATA GGTATTTGGA TGTACTACCA   
  
  
+ TTCACTTCTT CGATCTATAT TTCTAACGTA CGTATACGCA TGTTCCTTCT TAATTTCCTG TTTCTTTGTT   
  
  
+ GGTGGGTAGA ACGAGGGGTA CGAGTAGAA  

- TAGGGTTAAC CAGTTAAGAA AGGCTGAAAA GCCTTCACAT TGACAAGGAA TTTATTTAAG AAGAGAGTTG   
  
  
- CCAAATAGTC TCATGCATGC GAAGAGCAAT ACTGTAAGAT TTTTAAGACA AAACTTTGGG TTAAATTCAT   
  
  
- AGCGATCGTT GAAGCCAACT ACACTTCACC TACTTTCTGT TCAACCCCCC TTGTGAAAAC AAAGACGTAC   
  
  
- GTGCATGCCC AAAAATCCCA TCGTCATCAT CGAAGCATGC AAGAAAACTC AATATCTCTC TCTCTCTCTC   
  
  
- TCTCAGATAA ACACACAAGA TGAAGCAGAG AGAGAGAGAC AGAGAGAGAG AGAGAGAGTA AGTTTAATCC   
  
  
- ATAATGAAGT AAAAGTATGT TGATATGACA GATGATTGGC TCTGAAATGG CTTAAAGTAC AGGGTTTAGA   
  
  
- TATTGTTAAA CGGCTAATCC AGTATCTGAG GTCTGGTTGT TGAGGTAGTT TGGAATAATT CTGGACTGAT   
  
  
- AGACTGATTT CAGGCGATCA ATACAACCCT TTCCTTAGGG CACATGCATA GTTCTATGTA GTTTCCATAT   
  
  
- GTAGATCAAG TTGCTAATAT TTCATGTATA CTTGTCGTGT GAAATGAAAT GAAATGATCA ATATGATAAG   
  
  
- TTATTATAAT AAAGCTGTGG AAGAGCCAGA GTTCTAGATG ATCATTTCTT ACGGAAGATG CTTGTTTTGT   
  
  
- AAGTTCGTTA ATTCTAGAGT TGAAGGTAAT TAACACCATC TTTATCTTCT TGTTTGGAGG TTTACTAGTA   
  
  
- ACATGCAATT GTTATTATAC TTTGGCAAAC AAGCAATGCA AAAATAAGAA ATTATTGCCC ATGCACATGA   
  
  
- GAGTTTTGGG CTAGCTGGCT AGGCCAAATG AGACATGAAG ATTAAGTTGA AATATATATA TATATGCCAC   
  
  
- ATGGTATGCC AAGAAAATTA TAACATGCAT GTTATTTTTA GAACCGGCTC ATGTTGACTA GTTAATTAAA   
  
  
- AAAAATTATA TAGGTTTTTC AATATTTCAT AATATTAATG TAAAAATAGA ATATAAGAAA ATAAATTTAT   
  
  
- TAATTTTATT AAATTATCAT GTATTATAAT TTTATTTAAA ACTCAAATAA ACAAAAAAAT CTCTTGAGAC   
  
  
- TGATTCTTTT TTATCAGAAA AAAAAATGAT TATAATTAAA CTAACTCTTT TGACCTTAGA GCAATGCTAT   
  
  
- TGAGCCCAAA TCCATATTTA TTTGATCATG TCACCTTGTT GTTGATTATG TTTCTAATCA ATGGGGCTCC   
  
  
- AATATATGAA AAAATGGCAG ATAGTGCCAA TTTTCTATGG TCATTTATAT GACTGCAAAT TATGAACACA   
  
  
- GCAGAAACAT CAGCAAATAA AGCAACTAGA ATTCATAATC CATATCTTAT CCATAAACCT ACATGATGGT   
  
  
- AAGTGAAGAA GCTAGATATA AAGATTGCAT GCATATGCGT ACAAGGAAGA ATTAAAGGAC AAAGAAACAA   
  
  
- CCACCCATCT TGCTCCCCAT GCTCATCTT

+     Box-W1

| Site Name | Organism | Position | Strand | Matrix score. | sequence | function |
| --- | --- | --- | --- | --- | --- | --- |
| Box-W1 | Petroselinum crispum | 10 | - | 6 | TTGACC | fungal elicitor responsive element |

> 2018/04/13 10:10:12  
+ ATCCCAATTG GTCAATTCTT TCCGACTTTT CGGAAGTGTA ACTGTTCCTT AAATAAATTC TTCTCTCAAC   
  
  
+ GGTTTATCAG AGTACGTACG CTTCTCGTTA TGACATTCTA AAAATTCTGT TTTGAAACCC AATTTAAGTA   
  
  
+ TCGCTAGCAA CTTCGGTTGA TGTGAAGTGG ATGAAAGACA AGTTGGGGGG AACACTTTTG TTTCTGCATG   
  
  
+ CACGTACGGG TTTTTAGGGT AGCAGTAGTA GCTTCGTACG TTCTTTTGAG TTATAGAGAG AGAGAGAGAG   
  
  
+ AGAGTCTATT TGTGTGTTCT ACTTCGTCTC TCTCTCTCTG TCTCTCTCTC TCTCTCTCAT TCAAATTAGG   
  
  
+ TATTACTTCA TTTTCATACA ACTATACTGT CTACTAACCG AGACTTTACC GAATTTCATG TCCCAAATCT   
  
  
+ ATAACAATTT GCCGATTAGG TCATAGACTC CAGACCAACA ACTCCATCAA ACCTTATTAA GACCTGACTA   
  
  
+ TCTGACTAAA GTCCGCTAGT TATGTTGGGA AAGGAATCCC GTGTACGTAT CAAGATACAT CAAAGGTATA   
  
  
+ CATCTAGTTC AACGATTATA AAGTACATAT GAACAGCACA CTTTACTTTA CTTTACTAGT TATACTATTC   
  
  
+ AATAATATTA TTTCGACACC TTCTCGGTCT CAAGATCTAC TAGTAAAGAA TGCCTTCTAC GAACAAAACA   
  
  
+ TTCAAGCAAT TAAGATCTCA ACTTCCATTA ATTGTGGTAG AAATAGAAGA ACAAACCTCC AAATGATCAT   
  
  
+ TGTACGTTAA CAATAATATG AAACCGTTTG TTCGTTACGT TTTTATTCTT TAATAACGGG TACGTGTACT   
  
  
+ CTCAAAACCC GATCGACCGA TCCGGTTTAC TCTGTACTTC TAATTCAACT TTATATATAT ATATACGGTG   
  
  
+ TACCATACGG TTCTTTTAAT ATTGTACGTA CAATAAAAAT CTTGGCCGAG TACAACTGAT CAATTAATTT   
  
  
+ TTTTTAATAT ATCCAAAAAG TTATAAAGTA TTATAATTAC ATTTTTATCT TATATTCTTT TATTTAAATA   
  
  
+ ATTAAAATAA TTTAATAGTA CATAATATTA AAATAAATTT TGAGTTTATT TGTTTTTTTA GAGAACTCTG   
  
  
+ ACTAAGAAAA AATAGTCTTT TTTTTTACTA ATATTAATTT GATTGAGAAA ACTGGAATCT CGTTACGATA   
  
  
+ ACTCGGGTTT AGGTATAAAT AAACTAGTAC AGTGGAACAA CAACTAATAC AAAGATTAGT TACCCCGAGG   
  
  
+ TTATATACTT TTTTACCGTC TATCACGGTT AAAAGATACC AGTAAATATA CTGACGTTTA ATACTTGTGT   
  
  
+ CGTCTTTGTA GTCGTTTATT TCGTTGATCT TAAGTATTAG GTATAGAATA GGTATTTGGA TGTACTACCA   
  
  
+ TTCACTTCTT CGATCTATAT TTCTAACGTA CGTATACGCA TGTTCCTTCT TAATTTCCTG TTTCTTTGTT   
  
  
+ GGTGGGTAGA ACGAGGGGTA CGAGTAGAA  

- TAGGGTTAAC CAGTTAAGAA AGGCTGAAAA GCCTTCACAT TGACAAGGAA TTTATTTAAG AAGAGAGTTG   
  
  
- CCAAATAGTC TCATGCATGC GAAGAGCAAT ACTGTAAGAT TTTTAAGACA AAACTTTGGG TTAAATTCAT   
  
  
- AGCGATCGTT GAAGCCAACT ACACTTCACC TACTTTCTGT TCAACCCCCC TTGTGAAAAC AAAGACGTAC   
  
  
- GTGCATGCCC AAAAATCCCA TCGTCATCAT CGAAGCATGC AAGAAAACTC AATATCTCTC TCTCTCTCTC   
  
  
- TCTCAGATAA ACACACAAGA TGAAGCAGAG AGAGAGAGAC AGAGAGAGAG AGAGAGAGTA AGTTTAATCC   
  
  
- ATAATGAAGT AAAAGTATGT TGATATGACA GATGATTGGC TCTGAAATGG CTTAAAGTAC AGGGTTTAGA   
  
  
- TATTGTTAAA CGGCTAATCC AGTATCTGAG GTCTGGTTGT TGAGGTAGTT TGGAATAATT CTGGACTGAT   
  
  
- AGACTGATTT CAGGCGATCA ATACAACCCT TTCCTTAGGG CACATGCATA GTTCTATGTA GTTTCCATAT   
  
  
- GTAGATCAAG TTGCTAATAT TTCATGTATA CTTGTCGTGT GAAATGAAAT GAAATGATCA ATATGATAAG   
  
  
- TTATTATAAT AAAGCTGTGG AAGAGCCAGA GTTCTAGATG ATCATTTCTT ACGGAAGATG CTTGTTTTGT   
  
  
- AAGTTCGTTA ATTCTAGAGT TGAAGGTAAT TAACACCATC TTTATCTTCT TGTTTGGAGG TTTACTAGTA   
  
  
- ACATGCAATT GTTATTATAC TTTGGCAAAC AAGCAATGCA AAAATAAGAA ATTATTGCCC ATGCACATGA   
  
  
- GAGTTTTGGG CTAGCTGGCT AGGCCAAATG AGACATGAAG ATTAAGTTGA AATATATATA TATATGCCAC   
  
  
- ATGGTATGCC AAGAAAATTA TAACATGCAT GTTATTTTTA GAACCGGCTC ATGTTGACTA GTTAATTAAA   
  
  
- AAAAATTATA TAGGTTTTTC AATATTTCAT AATATTAATG TAAAAATAGA ATATAAGAAA ATAAATTTAT   
  
  
- TAATTTTATT AAATTATCAT GTATTATAAT TTTATTTAAA ACTCAAATAA ACAAAAAAAT CTCTTGAGAC   
  
  
- TGATTCTTTT TTATCAGAAA AAAAAATGAT TATAATTAAA CTAACTCTTT TGACCTTAGA GCAATGCTAT   
  
  
- TGAGCCCAAA TCCATATTTA TTTGATCATG TCACCTTGTT GTTGATTATG TTTCTAATCA ATGGGGCTCC   
  
  
- AATATATGAA AAAATGGCAG ATAGTGCCAA TTTTCTATGG TCATTTATAT GACTGCAAAT TATGAACACA   
  
  
- GCAGAAACAT CAGCAAATAA AGCAACTAGA ATTCATAATC CATATCTTAT CCATAAACCT ACATGATGGT   
  
  
- AAGTGAAGAA GCTAGATATA AAGATTGCAT GCATATGCGT ACAAGGAAGA ATTAAAGGAC AAAGAAACAA   
  
  
- CCACCCATCT TGCTCCCCAT GCTCATCTT

+     CAAT-box

| Site Name | Organism | Position | Strand | Matrix score. | sequence | function |
| --- | --- | --- | --- | --- | --- | --- |
| CAAT-box | Brassica rapa | 1384 | - | 5 | CAAAT | common cis-acting element in promoter and enhancer regions |
| CAAT-box | Hordeum vulgare | 1162 | - | 4 | CAAT | common cis-acting element in promoter and enhancer regions |
| CAAT-box | Brassica rapa | 1098 | - | 5 | CAAAT | common cis-acting element in promoter and enhancer regions |
| CAAT-box | Brassica rapa | 414 | + | 5 | CAAAT | common cis-acting element in promoter and enhancer regions |
| CAAT-box | Glycine max | 425 | + | 5 | CAATT | common cis-acting element in promoter and enhancer regions |
| CAAT-box | Glycine max | 971 | + | 5 | CAATT | common cis-acting element in promoter and enhancer regions |
| CAAT-box | Brassica rapa | 1157 | - | 5 | CAAAT | common cis-acting element in promoter and enhancer regions |
| CAAT-box | Glycine max | 5 | + | 5 | CAATT | common cis-acting element in promoter and enhancer regions |
| CAAT-box | Arabidopsis thaliana | 4 | + | 5 | CCAAT | common cis-acting element in promoter and enhancer regions |
| CAAT-box | Glycine max | 707 | + | 5 | CAATT | common cis-acting element in promoter and enhancer regions |
| CAAT-box | Glycine max | 730 | - | 5 | CAATT | common cis-acting element in promoter and enhancer regions |
| CAAT-box | Brassica rapa | 427 | - | 5 | CAAAT | common cis-acting element in promoter and enhancer regions |
| CAAT-box | Glycine max | 6 | - | 5 | CAATT | common cis-acting element in promoter and enhancer regions |
| CAAT-box | Glycine max | 130 | + | 5 | CAATT | common cis-acting element in promoter and enhancer regions |
| CAAT-box | Hordeum vulgare | 630 | + | 4 | CAAT | common cis-acting element in promoter and enhancer regions |
| CAAT-box | Arabidopsis thaliana | 129 | + | 5 | CCAAT | common cis-acting element in promoter and enhancer regions |
| CAAT-box | Brassica rapa | 760 | + | 5 | CAAAT | common cis-acting element in promoter and enhancer regions |
| CAAT-box | Hordeum vulgare | 731 | - | 4 | CAAT | common cis-acting element in promoter and enhancer regions |
| CAAT-box | Brassica rapa | 342 | + | 5 | CAAAT | common cis-acting element in promoter and enhancer regions |
| CAAT-box | Arabidopsis thaliana | 7 | - | 5 | CCAAT | common cis-acting element in promoter and enhancer regions |
| CAAT-box | Hordeum vulgare | 769 | - | 4 | CAAT | common cis-acting element in promoter and enhancer regions |
| CAAT-box | Brassica rapa | 288 | - | 5 | CAAAT | common cis-acting element in promoter and enhancer regions |
| CAAT-box | Hordeum vulgare | 781 | + | 4 | CAAT | common cis-acting element in promoter and enhancer regions |
| CAAT-box | Hordeum vulgare | 931 | - | 4 | CAAT | common cis-acting element in promoter and enhancer regions |
| CAAT-box | Glycine max | 13 | + | 5 | CAATT | common cis-acting element in promoter and enhancer regions |
| CAAT-box | Hordeum vulgare | 941 | + | 4 | CAAT | common cis-acting element in promoter and enhancer regions |
| CAAT-box | Arabidopsis thaliana | 128 | + | 8 | CCCAATTT | common cis-acting element in promoter and enhancer regions |

> 2018/04/13 10:10:12  
+ ATCCCAATTG GTCAATTCTT TCCGACTTTT CGGAAGTGTA ACTGTTCCTT AAATAAATTC TTCTCTCAAC   
  
  
+ GGTTTATCAG AGTACGTACG CTTCTCGTTA TGACATTCTA AAAATTCTGT TTTGAAACCC AATTTAAGTA   
  
  
+ TCGCTAGCAA CTTCGGTTGA TGTGAAGTGG ATGAAAGACA AGTTGGGGGG AACACTTTTG TTTCTGCATG   
  
  
+ CACGTACGGG TTTTTAGGGT AGCAGTAGTA GCTTCGTACG TTCTTTTGAG TTATAGAGAG AGAGAGAGAG   
  
  
+ AGAGTCTATT TGTGTGTTCT ACTTCGTCTC TCTCTCTCTG TCTCTCTCTC TCTCTCTCAT TCAAATTAGG   
  
  
+ TATTACTTCA TTTTCATACA ACTATACTGT CTACTAACCG AGACTTTACC GAATTTCATG TCCCAAATCT   
  
  
+ ATAACAATTT GCCGATTAGG TCATAGACTC CAGACCAACA ACTCCATCAA ACCTTATTAA GACCTGACTA   
  
  
+ TCTGACTAAA GTCCGCTAGT TATGTTGGGA AAGGAATCCC GTGTACGTAT CAAGATACAT CAAAGGTATA   
  
  
+ CATCTAGTTC AACGATTATA AAGTACATAT GAACAGCACA CTTTACTTTA CTTTACTAGT TATACTATTC   
  
  
+ AATAATATTA TTTCGACACC TTCTCGGTCT CAAGATCTAC TAGTAAAGAA TGCCTTCTAC GAACAAAACA   
  
  
+ TTCAAGCAAT TAAGATCTCA ACTTCCATTA ATTGTGGTAG AAATAGAAGA ACAAACCTCC AAATGATCAT   
  
  
+ TGTACGTTAA CAATAATATG AAACCGTTTG TTCGTTACGT TTTTATTCTT TAATAACGGG TACGTGTACT   
  
  
+ CTCAAAACCC GATCGACCGA TCCGGTTTAC TCTGTACTTC TAATTCAACT TTATATATAT ATATACGGTG   
  
  
+ TACCATACGG TTCTTTTAAT ATTGTACGTA CAATAAAAAT CTTGGCCGAG TACAACTGAT CAATTAATTT   
  
  
+ TTTTTAATAT ATCCAAAAAG TTATAAAGTA TTATAATTAC ATTTTTATCT TATATTCTTT TATTTAAATA   
  
  
+ ATTAAAATAA TTTAATAGTA CATAATATTA AAATAAATTT TGAGTTTATT TGTTTTTTTA GAGAACTCTG   
  
  
+ ACTAAGAAAA AATAGTCTTT TTTTTTACTA ATATTAATTT GATTGAGAAA ACTGGAATCT CGTTACGATA   
  
  
+ ACTCGGGTTT AGGTATAAAT AAACTAGTAC AGTGGAACAA CAACTAATAC AAAGATTAGT TACCCCGAGG   
  
  
+ TTATATACTT TTTTACCGTC TATCACGGTT AAAAGATACC AGTAAATATA CTGACGTTTA ATACTTGTGT   
  
  
+ CGTCTTTGTA GTCGTTTATT TCGTTGATCT TAAGTATTAG GTATAGAATA GGTATTTGGA TGTACTACCA   
  
  
+ TTCACTTCTT CGATCTATAT TTCTAACGTA CGTATACGCA TGTTCCTTCT TAATTTCCTG TTTCTTTGTT   
  
  
+ GGTGGGTAGA ACGAGGGGTA CGAGTAGAA  

- TAGGGTTAAC CAGTTAAGAA AGGCTGAAAA GCCTTCACAT TGACAAGGAA TTTATTTAAG AAGAGAGTTG   
  
  
- CCAAATAGTC TCATGCATGC GAAGAGCAAT ACTGTAAGAT TTTTAAGACA AAACTTTGGG TTAAATTCAT   
  
  
- AGCGATCGTT GAAGCCAACT ACACTTCACC TACTTTCTGT TCAACCCCCC TTGTGAAAAC AAAGACGTAC   
  
  
- GTGCATGCCC AAAAATCCCA TCGTCATCAT CGAAGCATGC AAGAAAACTC AATATCTCTC TCTCTCTCTC   
  
  
- TCTCAGATAA ACACACAAGA TGAAGCAGAG AGAGAGAGAC AGAGAGAGAG AGAGAGAGTA AGTTTAATCC   
  
  
- ATAATGAAGT AAAAGTATGT TGATATGACA GATGATTGGC TCTGAAATGG CTTAAAGTAC AGGGTTTAGA   
  
  
- TATTGTTAAA CGGCTAATCC AGTATCTGAG GTCTGGTTGT TGAGGTAGTT TGGAATAATT CTGGACTGAT   
  
  
- AGACTGATTT CAGGCGATCA ATACAACCCT TTCCTTAGGG CACATGCATA GTTCTATGTA GTTTCCATAT   
  
  
- GTAGATCAAG TTGCTAATAT TTCATGTATA CTTGTCGTGT GAAATGAAAT GAAATGATCA ATATGATAAG   
  
  
- TTATTATAAT AAAGCTGTGG AAGAGCCAGA GTTCTAGATG ATCATTTCTT ACGGAAGATG CTTGTTTTGT   
  
  
- AAGTTCGTTA ATTCTAGAGT TGAAGGTAAT TAACACCATC TTTATCTTCT TGTTTGGAGG TTTACTAGTA   
  
  
- ACATGCAATT GTTATTATAC TTTGGCAAAC AAGCAATGCA AAAATAAGAA ATTATTGCCC ATGCACATGA   
  
  
- GAGTTTTGGG CTAGCTGGCT AGGCCAAATG AGACATGAAG ATTAAGTTGA AATATATATA TATATGCCAC   
  
  
- ATGGTATGCC AAGAAAATTA TAACATGCAT GTTATTTTTA GAACCGGCTC ATGTTGACTA GTTAATTAAA   
  
  
- AAAAATTATA TAGGTTTTTC AATATTTCAT AATATTAATG TAAAAATAGA ATATAAGAAA ATAAATTTAT   
  
  
- TAATTTTATT AAATTATCAT GTATTATAAT TTTATTTAAA ACTCAAATAA ACAAAAAAAT CTCTTGAGAC   
  
  
- TGATTCTTTT TTATCAGAAA AAAAAATGAT TATAATTAAA CTAACTCTTT TGACCTTAGA GCAATGCTAT   
  
  
- TGAGCCCAAA TCCATATTTA TTTGATCATG TCACCTTGTT GTTGATTATG TTTCTAATCA ATGGGGCTCC   
  
  
- AATATATGAA AAAATGGCAG ATAGTGCCAA TTTTCTATGG TCATTTATAT GACTGCAAAT TATGAACACA   
  
  
- GCAGAAACAT CAGCAAATAA AGCAACTAGA ATTCATAATC CATATCTTAT CCATAAACCT ACATGATGGT   
  
  
- AAGTGAAGAA GCTAGATATA AAGATTGCAT GCATATGCGT ACAAGGAAGA ATTAAAGGAC AAAGAAACAA   
  
  
- CCACCCATCT TGCTCCCCAT GCTCATCTT

+     CATT-motif

| Site Name | Organism | Position | Strand | Matrix score. | sequence | function |
| --- | --- | --- | --- | --- | --- | --- |
| CATT-motif | Zea mays | 678 | - | 6 | GCATTC | part of a light responsive element |

> 2018/04/13 10:10:12  
+ ATCCCAATTG GTCAATTCTT TCCGACTTTT CGGAAGTGTA ACTGTTCCTT AAATAAATTC TTCTCTCAAC   
  
  
+ GGTTTATCAG AGTACGTACG CTTCTCGTTA TGACATTCTA AAAATTCTGT TTTGAAACCC AATTTAAGTA   
  
  
+ TCGCTAGCAA CTTCGGTTGA TGTGAAGTGG ATGAAAGACA AGTTGGGGGG AACACTTTTG TTTCTGCATG   
  
  
+ CACGTACGGG TTTTTAGGGT AGCAGTAGTA GCTTCGTACG TTCTTTTGAG TTATAGAGAG AGAGAGAGAG   
  
  
+ AGAGTCTATT TGTGTGTTCT ACTTCGTCTC TCTCTCTCTG TCTCTCTCTC TCTCTCTCAT TCAAATTAGG   
  
  
+ TATTACTTCA TTTTCATACA ACTATACTGT CTACTAACCG AGACTTTACC GAATTTCATG TCCCAAATCT   
  
  
+ ATAACAATTT GCCGATTAGG TCATAGACTC CAGACCAACA ACTCCATCAA ACCTTATTAA GACCTGACTA   
  
  
+ TCTGACTAAA GTCCGCTAGT TATGTTGGGA AAGGAATCCC GTGTACGTAT CAAGATACAT CAAAGGTATA   
  
  
+ CATCTAGTTC AACGATTATA AAGTACATAT GAACAGCACA CTTTACTTTA CTTTACTAGT TATACTATTC   
  
  
+ AATAATATTA TTTCGACACC TTCTCGGTCT CAAGATCTAC TAGTAAAGAA TGCCTTCTAC GAACAAAACA   
  
  
+ TTCAAGCAAT TAAGATCTCA ACTTCCATTA ATTGTGGTAG AAATAGAAGA ACAAACCTCC AAATGATCAT   
  
  
+ TGTACGTTAA CAATAATATG AAACCGTTTG TTCGTTACGT TTTTATTCTT TAATAACGGG TACGTGTACT   
  
  
+ CTCAAAACCC GATCGACCGA TCCGGTTTAC TCTGTACTTC TAATTCAACT TTATATATAT ATATACGGTG   
  
  
+ TACCATACGG TTCTTTTAAT ATTGTACGTA CAATAAAAAT CTTGGCCGAG TACAACTGAT CAATTAATTT   
  
  
+ TTTTTAATAT ATCCAAAAAG TTATAAAGTA TTATAATTAC ATTTTTATCT TATATTCTTT TATTTAAATA   
  
  
+ ATTAAAATAA TTTAATAGTA CATAATATTA AAATAAATTT TGAGTTTATT TGTTTTTTTA GAGAACTCTG   
  
  
+ ACTAAGAAAA AATAGTCTTT TTTTTTACTA ATATTAATTT GATTGAGAAA ACTGGAATCT CGTTACGATA   
  
  
+ ACTCGGGTTT AGGTATAAAT AAACTAGTAC AGTGGAACAA CAACTAATAC AAAGATTAGT TACCCCGAGG   
  
  
+ TTATATACTT TTTTACCGTC TATCACGGTT AAAAGATACC AGTAAATATA CTGACGTTTA ATACTTGTGT   
  
  
+ CGTCTTTGTA GTCGTTTATT TCGTTGATCT TAAGTATTAG GTATAGAATA GGTATTTGGA TGTACTACCA   
  
  
+ TTCACTTCTT CGATCTATAT TTCTAACGTA CGTATACGCA TGTTCCTTCT TAATTTCCTG TTTCTTTGTT   
  
  
+ GGTGGGTAGA ACGAGGGGTA CGAGTAGAA  

- TAGGGTTAAC CAGTTAAGAA AGGCTGAAAA GCCTTCACAT TGACAAGGAA TTTATTTAAG AAGAGAGTTG   
  
  
- CCAAATAGTC TCATGCATGC GAAGAGCAAT ACTGTAAGAT TTTTAAGACA AAACTTTGGG TTAAATTCAT   
  
  
- AGCGATCGTT GAAGCCAACT ACACTTCACC TACTTTCTGT TCAACCCCCC TTGTGAAAAC AAAGACGTAC   
  
  
- GTGCATGCCC AAAAATCCCA TCGTCATCAT CGAAGCATGC AAGAAAACTC AATATCTCTC TCTCTCTCTC   
  
  
- TCTCAGATAA ACACACAAGA TGAAGCAGAG AGAGAGAGAC AGAGAGAGAG AGAGAGAGTA AGTTTAATCC   
  
  
- ATAATGAAGT AAAAGTATGT TGATATGACA GATGATTGGC TCTGAAATGG CTTAAAGTAC AGGGTTTAGA   
  
  
- TATTGTTAAA CGGCTAATCC AGTATCTGAG GTCTGGTTGT TGAGGTAGTT TGGAATAATT CTGGACTGAT   
  
  
- AGACTGATTT CAGGCGATCA ATACAACCCT TTCCTTAGGG CACATGCATA GTTCTATGTA GTTTCCATAT   
  
  
- GTAGATCAAG TTGCTAATAT TTCATGTATA CTTGTCGTGT GAAATGAAAT GAAATGATCA ATATGATAAG   
  
  
- TTATTATAAT AAAGCTGTGG AAGAGCCAGA GTTCTAGATG ATCATTTCTT ACGGAAGATG CTTGTTTTGT   
  
  
- AAGTTCGTTA ATTCTAGAGT TGAAGGTAAT TAACACCATC TTTATCTTCT TGTTTGGAGG TTTACTAGTA   
  
  
- ACATGCAATT GTTATTATAC TTTGGCAAAC AAGCAATGCA AAAATAAGAA ATTATTGCCC ATGCACATGA   
  
  
- GAGTTTTGGG CTAGCTGGCT AGGCCAAATG AGACATGAAG ATTAAGTTGA AATATATATA TATATGCCAC   
  
  
- ATGGTATGCC AAGAAAATTA TAACATGCAT GTTATTTTTA GAACCGGCTC ATGTTGACTA GTTAATTAAA   
  
  
- AAAAATTATA TAGGTTTTTC AATATTTCAT AATATTAATG TAAAAATAGA ATATAAGAAA ATAAATTTAT   
  
  
- TAATTTTATT AAATTATCAT GTATTATAAT TTTATTTAAA ACTCAAATAA ACAAAAAAAT CTCTTGAGAC   
  
  
- TGATTCTTTT TTATCAGAAA AAAAAATGAT TATAATTAAA CTAACTCTTT TGACCTTAGA GCAATGCTAT   
  
  
- TGAGCCCAAA TCCATATTTA TTTGATCATG TCACCTTGTT GTTGATTATG TTTCTAATCA ATGGGGCTCC   
  
  
- AATATATGAA AAAATGGCAG ATAGTGCCAA TTTTCTATGG TCATTTATAT GACTGCAAAT TATGAACACA   
  
  
- GCAGAAACAT CAGCAAATAA AGCAACTAGA ATTCATAATC CATATCTTAT CCATAAACCT ACATGATGGT   
  
  
- AAGTGAAGAA GCTAGATATA AAGATTGCAT GCATATGCGT ACAAGGAAGA ATTAAAGGAC AAAGAAACAA   
  
  
- CCACCCATCT TGCTCCCCAT GCTCATCTT

+     CCAAT-box

| Site Name | Organism | Position | Strand | Matrix score. | sequence | function |
| --- | --- | --- | --- | --- | --- | --- |
| CCAAT-box | Hordeum vulgare | 67 | + | 6 | CAACGG | MYBHv1 binding site |

> 2018/04/13 10:10:12  
+ ATCCCAATTG GTCAATTCTT TCCGACTTTT CGGAAGTGTA ACTGTTCCTT AAATAAATTC TTCTCTCAAC   
  
  
+ GGTTTATCAG AGTACGTACG CTTCTCGTTA TGACATTCTA AAAATTCTGT TTTGAAACCC AATTTAAGTA   
  
  
+ TCGCTAGCAA CTTCGGTTGA TGTGAAGTGG ATGAAAGACA AGTTGGGGGG AACACTTTTG TTTCTGCATG   
  
  
+ CACGTACGGG TTTTTAGGGT AGCAGTAGTA GCTTCGTACG TTCTTTTGAG TTATAGAGAG AGAGAGAGAG   
  
  
+ AGAGTCTATT TGTGTGTTCT ACTTCGTCTC TCTCTCTCTG TCTCTCTCTC TCTCTCTCAT TCAAATTAGG   
  
  
+ TATTACTTCA TTTTCATACA ACTATACTGT CTACTAACCG AGACTTTACC GAATTTCATG TCCCAAATCT   
  
  
+ ATAACAATTT GCCGATTAGG TCATAGACTC CAGACCAACA ACTCCATCAA ACCTTATTAA GACCTGACTA   
  
  
+ TCTGACTAAA GTCCGCTAGT TATGTTGGGA AAGGAATCCC GTGTACGTAT CAAGATACAT CAAAGGTATA   
  
  
+ CATCTAGTTC AACGATTATA AAGTACATAT GAACAGCACA CTTTACTTTA CTTTACTAGT TATACTATTC   
  
  
+ AATAATATTA TTTCGACACC TTCTCGGTCT CAAGATCTAC TAGTAAAGAA TGCCTTCTAC GAACAAAACA   
  
  
+ TTCAAGCAAT TAAGATCTCA ACTTCCATTA ATTGTGGTAG AAATAGAAGA ACAAACCTCC AAATGATCAT   
  
  
+ TGTACGTTAA CAATAATATG AAACCGTTTG TTCGTTACGT TTTTATTCTT TAATAACGGG TACGTGTACT   
  
  
+ CTCAAAACCC GATCGACCGA TCCGGTTTAC TCTGTACTTC TAATTCAACT TTATATATAT ATATACGGTG   
  
  
+ TACCATACGG TTCTTTTAAT ATTGTACGTA CAATAAAAAT CTTGGCCGAG TACAACTGAT CAATTAATTT   
  
  
+ TTTTTAATAT ATCCAAAAAG TTATAAAGTA TTATAATTAC ATTTTTATCT TATATTCTTT TATTTAAATA   
  
  
+ ATTAAAATAA TTTAATAGTA CATAATATTA AAATAAATTT TGAGTTTATT TGTTTTTTTA GAGAACTCTG   
  
  
+ ACTAAGAAAA AATAGTCTTT TTTTTTACTA ATATTAATTT GATTGAGAAA ACTGGAATCT CGTTACGATA   
  
  
+ ACTCGGGTTT AGGTATAAAT AAACTAGTAC AGTGGAACAA CAACTAATAC AAAGATTAGT TACCCCGAGG   
  
  
+ TTATATACTT TTTTACCGTC TATCACGGTT AAAAGATACC AGTAAATATA CTGACGTTTA ATACTTGTGT   
  
  
+ CGTCTTTGTA GTCGTTTATT TCGTTGATCT TAAGTATTAG GTATAGAATA GGTATTTGGA TGTACTACCA   
  
  
+ TTCACTTCTT CGATCTATAT TTCTAACGTA CGTATACGCA TGTTCCTTCT TAATTTCCTG TTTCTTTGTT   
  
  
+ GGTGGGTAGA ACGAGGGGTA CGAGTAGAA  

- TAGGGTTAAC CAGTTAAGAA AGGCTGAAAA GCCTTCACAT TGACAAGGAA TTTATTTAAG AAGAGAGTTG   
  
  
- CCAAATAGTC TCATGCATGC GAAGAGCAAT ACTGTAAGAT TTTTAAGACA AAACTTTGGG TTAAATTCAT   
  
  
- AGCGATCGTT GAAGCCAACT ACACTTCACC TACTTTCTGT TCAACCCCCC TTGTGAAAAC AAAGACGTAC   
  
  
- GTGCATGCCC AAAAATCCCA TCGTCATCAT CGAAGCATGC AAGAAAACTC AATATCTCTC TCTCTCTCTC   
  
  
- TCTCAGATAA ACACACAAGA TGAAGCAGAG AGAGAGAGAC AGAGAGAGAG AGAGAGAGTA AGTTTAATCC   
  
  
- ATAATGAAGT AAAAGTATGT TGATATGACA GATGATTGGC TCTGAAATGG CTTAAAGTAC AGGGTTTAGA   
  
  
- TATTGTTAAA CGGCTAATCC AGTATCTGAG GTCTGGTTGT TGAGGTAGTT TGGAATAATT CTGGACTGAT   
  
  
- AGACTGATTT CAGGCGATCA ATACAACCCT TTCCTTAGGG CACATGCATA GTTCTATGTA GTTTCCATAT   
  
  
- GTAGATCAAG TTGCTAATAT TTCATGTATA CTTGTCGTGT GAAATGAAAT GAAATGATCA ATATGATAAG   
  
  
- TTATTATAAT AAAGCTGTGG AAGAGCCAGA GTTCTAGATG ATCATTTCTT ACGGAAGATG CTTGTTTTGT   
  
  
- AAGTTCGTTA ATTCTAGAGT TGAAGGTAAT TAACACCATC TTTATCTTCT TGTTTGGAGG TTTACTAGTA   
  
  
- ACATGCAATT GTTATTATAC TTTGGCAAAC AAGCAATGCA AAAATAAGAA ATTATTGCCC ATGCACATGA   
  
  
- GAGTTTTGGG CTAGCTGGCT AGGCCAAATG AGACATGAAG ATTAAGTTGA AATATATATA TATATGCCAC   
  
  
- ATGGTATGCC AAGAAAATTA TAACATGCAT GTTATTTTTA GAACCGGCTC ATGTTGACTA GTTAATTAAA   
  
  
- AAAAATTATA TAGGTTTTTC AATATTTCAT AATATTAATG TAAAAATAGA ATATAAGAAA ATAAATTTAT   
  
  
- TAATTTTATT AAATTATCAT GTATTATAAT TTTATTTAAA ACTCAAATAA ACAAAAAAAT CTCTTGAGAC   
  
  
- TGATTCTTTT TTATCAGAAA AAAAAATGAT TATAATTAAA CTAACTCTTT TGACCTTAGA GCAATGCTAT   
  
  
- TGAGCCCAAA TCCATATTTA TTTGATCATG TCACCTTGTT GTTGATTATG TTTCTAATCA ATGGGGCTCC   
  
  
- AATATATGAA AAAATGGCAG ATAGTGCCAA TTTTCTATGG TCATTTATAT GACTGCAAAT TATGAACACA   
  
  
- GCAGAAACAT CAGCAAATAA AGCAACTAGA ATTCATAATC CATATCTTAT CCATAAACCT ACATGATGGT   
  
  
- AAGTGAAGAA GCTAGATATA AAGATTGCAT GCATATGCGT ACAAGGAAGA ATTAAAGGAC AAAGAAACAA   
  
  
- CCACCCATCT TGCTCCCCAT GCTCATCTT

+     CGTCA-motif

| Site Name | Organism | Position | Strand | Matrix score. | sequence | function |
| --- | --- | --- | --- | --- | --- | --- |
| CGTCA-motif | Hordeum vulgare | 1312 | - | 5 | CGTCA | cis-acting regulatory element involved in the MeJA-responsiveness |

> 2018/04/13 10:10:12  
+ ATCCCAATTG GTCAATTCTT TCCGACTTTT CGGAAGTGTA ACTGTTCCTT AAATAAATTC TTCTCTCAAC   
  
  
+ GGTTTATCAG AGTACGTACG CTTCTCGTTA TGACATTCTA AAAATTCTGT TTTGAAACCC AATTTAAGTA   
  
  
+ TCGCTAGCAA CTTCGGTTGA TGTGAAGTGG ATGAAAGACA AGTTGGGGGG AACACTTTTG TTTCTGCATG   
  
  
+ CACGTACGGG TTTTTAGGGT AGCAGTAGTA GCTTCGTACG TTCTTTTGAG TTATAGAGAG AGAGAGAGAG   
  
  
+ AGAGTCTATT TGTGTGTTCT ACTTCGTCTC TCTCTCTCTG TCTCTCTCTC TCTCTCTCAT TCAAATTAGG   
  
  
+ TATTACTTCA TTTTCATACA ACTATACTGT CTACTAACCG AGACTTTACC GAATTTCATG TCCCAAATCT   
  
  
+ ATAACAATTT GCCGATTAGG TCATAGACTC CAGACCAACA ACTCCATCAA ACCTTATTAA GACCTGACTA   
  
  
+ TCTGACTAAA GTCCGCTAGT TATGTTGGGA AAGGAATCCC GTGTACGTAT CAAGATACAT CAAAGGTATA   
  
  
+ CATCTAGTTC AACGATTATA AAGTACATAT GAACAGCACA CTTTACTTTA CTTTACTAGT TATACTATTC   
  
  
+ AATAATATTA TTTCGACACC TTCTCGGTCT CAAGATCTAC TAGTAAAGAA TGCCTTCTAC GAACAAAACA   
  
  
+ TTCAAGCAAT TAAGATCTCA ACTTCCATTA ATTGTGGTAG AAATAGAAGA ACAAACCTCC AAATGATCAT   
  
  
+ TGTACGTTAA CAATAATATG AAACCGTTTG TTCGTTACGT TTTTATTCTT TAATAACGGG TACGTGTACT   
  
  
+ CTCAAAACCC GATCGACCGA TCCGGTTTAC TCTGTACTTC TAATTCAACT TTATATATAT ATATACGGTG   
  
  
+ TACCATACGG TTCTTTTAAT ATTGTACGTA CAATAAAAAT CTTGGCCGAG TACAACTGAT CAATTAATTT   
  
  
+ TTTTTAATAT ATCCAAAAAG TTATAAAGTA TTATAATTAC ATTTTTATCT TATATTCTTT TATTTAAATA   
  
  
+ ATTAAAATAA TTTAATAGTA CATAATATTA AAATAAATTT TGAGTTTATT TGTTTTTTTA GAGAACTCTG   
  
  
+ ACTAAGAAAA AATAGTCTTT TTTTTTACTA ATATTAATTT GATTGAGAAA ACTGGAATCT CGTTACGATA   
  
  
+ ACTCGGGTTT AGGTATAAAT AAACTAGTAC AGTGGAACAA CAACTAATAC AAAGATTAGT TACCCCGAGG   
  
  
+ TTATATACTT TTTTACCGTC TATCACGGTT AAAAGATACC AGTAAATATA CTGACGTTTA ATACTTGTGT   
  
  
+ CGTCTTTGTA GTCGTTTATT TCGTTGATCT TAAGTATTAG GTATAGAATA GGTATTTGGA TGTACTACCA   
  
  
+ TTCACTTCTT CGATCTATAT TTCTAACGTA CGTATACGCA TGTTCCTTCT TAATTTCCTG TTTCTTTGTT   
  
  
+ GGTGGGTAGA ACGAGGGGTA CGAGTAGAA  

- TAGGGTTAAC CAGTTAAGAA AGGCTGAAAA GCCTTCACAT TGACAAGGAA TTTATTTAAG AAGAGAGTTG   
  
  
- CCAAATAGTC TCATGCATGC GAAGAGCAAT ACTGTAAGAT TTTTAAGACA AAACTTTGGG TTAAATTCAT   
  
  
- AGCGATCGTT GAAGCCAACT ACACTTCACC TACTTTCTGT TCAACCCCCC TTGTGAAAAC AAAGACGTAC   
  
  
- GTGCATGCCC AAAAATCCCA TCGTCATCAT CGAAGCATGC AAGAAAACTC AATATCTCTC TCTCTCTCTC   
  
  
- TCTCAGATAA ACACACAAGA TGAAGCAGAG AGAGAGAGAC AGAGAGAGAG AGAGAGAGTA AGTTTAATCC   
  
  
- ATAATGAAGT AAAAGTATGT TGATATGACA GATGATTGGC TCTGAAATGG CTTAAAGTAC AGGGTTTAGA   
  
  
- TATTGTTAAA CGGCTAATCC AGTATCTGAG GTCTGGTTGT TGAGGTAGTT TGGAATAATT CTGGACTGAT   
  
  
- AGACTGATTT CAGGCGATCA ATACAACCCT TTCCTTAGGG CACATGCATA GTTCTATGTA GTTTCCATAT   
  
  
- GTAGATCAAG TTGCTAATAT TTCATGTATA CTTGTCGTGT GAAATGAAAT GAAATGATCA ATATGATAAG   
  
  
- TTATTATAAT AAAGCTGTGG AAGAGCCAGA GTTCTAGATG ATCATTTCTT ACGGAAGATG CTTGTTTTGT   
  
  
- AAGTTCGTTA ATTCTAGAGT TGAAGGTAAT TAACACCATC TTTATCTTCT TGTTTGGAGG TTTACTAGTA   
  
  
- ACATGCAATT GTTATTATAC TTTGGCAAAC AAGCAATGCA AAAATAAGAA ATTATTGCCC ATGCACATGA   
  
  
- GAGTTTTGGG CTAGCTGGCT AGGCCAAATG AGACATGAAG ATTAAGTTGA AATATATATA TATATGCCAC   
  
  
- ATGGTATGCC AAGAAAATTA TAACATGCAT GTTATTTTTA GAACCGGCTC ATGTTGACTA GTTAATTAAA   
  
  
- AAAAATTATA TAGGTTTTTC AATATTTCAT AATATTAATG TAAAAATAGA ATATAAGAAA ATAAATTTAT   
  
  
- TAATTTTATT AAATTATCAT GTATTATAAT TTTATTTAAA ACTCAAATAA ACAAAAAAAT CTCTTGAGAC   
  
  
- TGATTCTTTT TTATCAGAAA AAAAAATGAT TATAATTAAA CTAACTCTTT TGACCTTAGA GCAATGCTAT   
  
  
- TGAGCCCAAA TCCATATTTA TTTGATCATG TCACCTTGTT GTTGATTATG TTTCTAATCA ATGGGGCTCC   
  
  
- AATATATGAA AAAATGGCAG ATAGTGCCAA TTTTCTATGG TCATTTATAT GACTGCAAAT TATGAACACA   
  
  
- GCAGAAACAT CAGCAAATAA AGCAACTAGA ATTCATAATC CATATCTTAT CCATAAACCT ACATGATGGT   
  
  
- AAGTGAAGAA GCTAGATATA AAGATTGCAT GCATATGCGT ACAAGGAAGA ATTAAAGGAC AAAGAAACAA   
  
  
- CCACCCATCT TGCTCCCCAT GCTCATCTT

+     G-Box

| Site Name | Organism | Position | Strand | Matrix score. | sequence | function |
| --- | --- | --- | --- | --- | --- | --- |
| G-Box | Antirrhinum majus | 211 | + | 6 | CACGTA | cis-acting regulatory element involved in light responsiveness |
| G-Box | Antirrhinum majus | 831 | - | 6 | CACGTA | cis-acting regulatory element involved in light responsiveness |

> 2018/04/13 10:10:12  
+ ATCCCAATTG GTCAATTCTT TCCGACTTTT CGGAAGTGTA ACTGTTCCTT AAATAAATTC TTCTCTCAAC   
  
  
+ GGTTTATCAG AGTACGTACG CTTCTCGTTA TGACATTCTA AAAATTCTGT TTTGAAACCC AATTTAAGTA   
  
  
+ TCGCTAGCAA CTTCGGTTGA TGTGAAGTGG ATGAAAGACA AGTTGGGGGG AACACTTTTG TTTCTGCATG   
  
  
+ CACGTACGGG TTTTTAGGGT AGCAGTAGTA GCTTCGTACG TTCTTTTGAG TTATAGAGAG AGAGAGAGAG   
  
  
+ AGAGTCTATT TGTGTGTTCT ACTTCGTCTC TCTCTCTCTG TCTCTCTCTC TCTCTCTCAT TCAAATTAGG   
  
  
+ TATTACTTCA TTTTCATACA ACTATACTGT CTACTAACCG AGACTTTACC GAATTTCATG TCCCAAATCT   
  
  
+ ATAACAATTT GCCGATTAGG TCATAGACTC CAGACCAACA ACTCCATCAA ACCTTATTAA GACCTGACTA   
  
  
+ TCTGACTAAA GTCCGCTAGT TATGTTGGGA AAGGAATCCC GTGTACGTAT CAAGATACAT CAAAGGTATA   
  
  
+ CATCTAGTTC AACGATTATA AAGTACATAT GAACAGCACA CTTTACTTTA CTTTACTAGT TATACTATTC   
  
  
+ AATAATATTA TTTCGACACC TTCTCGGTCT CAAGATCTAC TAGTAAAGAA TGCCTTCTAC GAACAAAACA   
  
  
+ TTCAAGCAAT TAAGATCTCA ACTTCCATTA ATTGTGGTAG AAATAGAAGA ACAAACCTCC AAATGATCAT   
  
  
+ TGTACGTTAA CAATAATATG AAACCGTTTG TTCGTTACGT TTTTATTCTT TAATAACGGG TACGTGTACT   
  
  
+ CTCAAAACCC GATCGACCGA TCCGGTTTAC TCTGTACTTC TAATTCAACT TTATATATAT ATATACGGTG   
  
  
+ TACCATACGG TTCTTTTAAT ATTGTACGTA CAATAAAAAT CTTGGCCGAG TACAACTGAT CAATTAATTT   
  
  
+ TTTTTAATAT ATCCAAAAAG TTATAAAGTA TTATAATTAC ATTTTTATCT TATATTCTTT TATTTAAATA   
  
  
+ ATTAAAATAA TTTAATAGTA CATAATATTA AAATAAATTT TGAGTTTATT TGTTTTTTTA GAGAACTCTG   
  
  
+ ACTAAGAAAA AATAGTCTTT TTTTTTACTA ATATTAATTT GATTGAGAAA ACTGGAATCT CGTTACGATA   
  
  
+ ACTCGGGTTT AGGTATAAAT AAACTAGTAC AGTGGAACAA CAACTAATAC AAAGATTAGT TACCCCGAGG   
  
  
+ TTATATACTT TTTTACCGTC TATCACGGTT AAAAGATACC AGTAAATATA CTGACGTTTA ATACTTGTGT   
  
  
+ CGTCTTTGTA GTCGTTTATT TCGTTGATCT TAAGTATTAG GTATAGAATA GGTATTTGGA TGTACTACCA   
  
  
+ TTCACTTCTT CGATCTATAT TTCTAACGTA CGTATACGCA TGTTCCTTCT TAATTTCCTG TTTCTTTGTT   
  
  
+ GGTGGGTAGA ACGAGGGGTA CGAGTAGAA  

- TAGGGTTAAC CAGTTAAGAA AGGCTGAAAA GCCTTCACAT TGACAAGGAA TTTATTTAAG AAGAGAGTTG   
  
  
- CCAAATAGTC TCATGCATGC GAAGAGCAAT ACTGTAAGAT TTTTAAGACA AAACTTTGGG TTAAATTCAT   
  
  
- AGCGATCGTT GAAGCCAACT ACACTTCACC TACTTTCTGT TCAACCCCCC TTGTGAAAAC AAAGACGTAC   
  
  
- GTGCATGCCC AAAAATCCCA TCGTCATCAT CGAAGCATGC AAGAAAACTC AATATCTCTC TCTCTCTCTC   
  
  
- TCTCAGATAA ACACACAAGA TGAAGCAGAG AGAGAGAGAC AGAGAGAGAG AGAGAGAGTA AGTTTAATCC   
  
  
- ATAATGAAGT AAAAGTATGT TGATATGACA GATGATTGGC TCTGAAATGG CTTAAAGTAC AGGGTTTAGA   
  
  
- TATTGTTAAA CGGCTAATCC AGTATCTGAG GTCTGGTTGT TGAGGTAGTT TGGAATAATT CTGGACTGAT   
  
  
- AGACTGATTT CAGGCGATCA ATACAACCCT TTCCTTAGGG CACATGCATA GTTCTATGTA GTTTCCATAT   
  
  
- GTAGATCAAG TTGCTAATAT TTCATGTATA CTTGTCGTGT GAAATGAAAT GAAATGATCA ATATGATAAG   
  
  
- TTATTATAAT AAAGCTGTGG AAGAGCCAGA GTTCTAGATG ATCATTTCTT ACGGAAGATG CTTGTTTTGT   
  
  
- AAGTTCGTTA ATTCTAGAGT TGAAGGTAAT TAACACCATC TTTATCTTCT TGTTTGGAGG TTTACTAGTA   
  
  
- ACATGCAATT GTTATTATAC TTTGGCAAAC AAGCAATGCA AAAATAAGAA ATTATTGCCC ATGCACATGA   
  
  
- GAGTTTTGGG CTAGCTGGCT AGGCCAAATG AGACATGAAG ATTAAGTTGA AATATATATA TATATGCCAC   
  
  
- ATGGTATGCC AAGAAAATTA TAACATGCAT GTTATTTTTA GAACCGGCTC ATGTTGACTA GTTAATTAAA   
  
  
- AAAAATTATA TAGGTTTTTC AATATTTCAT AATATTAATG TAAAAATAGA ATATAAGAAA ATAAATTTAT   
  
  
- TAATTTTATT AAATTATCAT GTATTATAAT TTTATTTAAA ACTCAAATAA ACAAAAAAAT CTCTTGAGAC   
  
  
- TGATTCTTTT TTATCAGAAA AAAAAATGAT TATAATTAAA CTAACTCTTT TGACCTTAGA GCAATGCTAT   
  
  
- TGAGCCCAAA TCCATATTTA TTTGATCATG TCACCTTGTT GTTGATTATG TTTCTAATCA ATGGGGCTCC   
  
  
- AATATATGAA AAAATGGCAG ATAGTGCCAA TTTTCTATGG TCATTTATAT GACTGCAAAT TATGAACACA   
  
  
- GCAGAAACAT CAGCAAATAA AGCAACTAGA ATTCATAATC CATATCTTAT CCATAAACCT ACATGATGGT   
  
  
- AAGTGAAGAA GCTAGATATA AAGATTGCAT GCATATGCGT ACAAGGAAGA ATTAAAGGAC AAAGAAACAA   
  
  
- CCACCCATCT TGCTCCCCAT GCTCATCTT

+     G-box

| Site Name | Organism | Position | Strand | Matrix score. | sequence | function |
| --- | --- | --- | --- | --- | --- | --- |
| G-box | Daucus carota | 831 | + | 6 | TACGTG | cis-acting regulatory element involved in light responsiveness |
| G-box | Oryza sativa | 830 | + | 7 | GTACGTG | cis-acting regulatory element involved in light responsiveness |
| G-box | Daucus carota | 211 | - | 6 | TACGTG | cis-acting regulatory element involved in light responsiveness |

> 2018/04/13 10:10:12  
+ ATCCCAATTG GTCAATTCTT TCCGACTTTT CGGAAGTGTA ACTGTTCCTT AAATAAATTC TTCTCTCAAC   
  
  
+ GGTTTATCAG AGTACGTACG CTTCTCGTTA TGACATTCTA AAAATTCTGT TTTGAAACCC AATTTAAGTA   
  
  
+ TCGCTAGCAA CTTCGGTTGA TGTGAAGTGG ATGAAAGACA AGTTGGGGGG AACACTTTTG TTTCTGCATG   
  
  
+ CACGTACGGG TTTTTAGGGT AGCAGTAGTA GCTTCGTACG TTCTTTTGAG TTATAGAGAG AGAGAGAGAG   
  
  
+ AGAGTCTATT TGTGTGTTCT ACTTCGTCTC TCTCTCTCTG TCTCTCTCTC TCTCTCTCAT TCAAATTAGG   
  
  
+ TATTACTTCA TTTTCATACA ACTATACTGT CTACTAACCG AGACTTTACC GAATTTCATG TCCCAAATCT   
  
  
+ ATAACAATTT GCCGATTAGG TCATAGACTC CAGACCAACA ACTCCATCAA ACCTTATTAA GACCTGACTA   
  
  
+ TCTGACTAAA GTCCGCTAGT TATGTTGGGA AAGGAATCCC GTGTACGTAT CAAGATACAT CAAAGGTATA   
  
  
+ CATCTAGTTC AACGATTATA AAGTACATAT GAACAGCACA CTTTACTTTA CTTTACTAGT TATACTATTC   
  
  
+ AATAATATTA TTTCGACACC TTCTCGGTCT CAAGATCTAC TAGTAAAGAA TGCCTTCTAC GAACAAAACA   
  
  
+ TTCAAGCAAT TAAGATCTCA ACTTCCATTA ATTGTGGTAG AAATAGAAGA ACAAACCTCC AAATGATCAT   
  
  
+ TGTACGTTAA CAATAATATG AAACCGTTTG TTCGTTACGT TTTTATTCTT TAATAACGGG TACGTGTACT   
  
  
+ CTCAAAACCC GATCGACCGA TCCGGTTTAC TCTGTACTTC TAATTCAACT TTATATATAT ATATACGGTG   
  
  
+ TACCATACGG TTCTTTTAAT ATTGTACGTA CAATAAAAAT CTTGGCCGAG TACAACTGAT CAATTAATTT   
  
  
+ TTTTTAATAT ATCCAAAAAG TTATAAAGTA TTATAATTAC ATTTTTATCT TATATTCTTT TATTTAAATA   
  
  
+ ATTAAAATAA TTTAATAGTA CATAATATTA AAATAAATTT TGAGTTTATT TGTTTTTTTA GAGAACTCTG   
  
  
+ ACTAAGAAAA AATAGTCTTT TTTTTTACTA ATATTAATTT GATTGAGAAA ACTGGAATCT CGTTACGATA   
  
  
+ ACTCGGGTTT AGGTATAAAT AAACTAGTAC AGTGGAACAA CAACTAATAC AAAGATTAGT TACCCCGAGG   
  
  
+ TTATATACTT TTTTACCGTC TATCACGGTT AAAAGATACC AGTAAATATA CTGACGTTTA ATACTTGTGT   
  
  
+ CGTCTTTGTA GTCGTTTATT TCGTTGATCT TAAGTATTAG GTATAGAATA GGTATTTGGA TGTACTACCA   
  
  
+ TTCACTTCTT CGATCTATAT TTCTAACGTA CGTATACGCA TGTTCCTTCT TAATTTCCTG TTTCTTTGTT   
  
  
+ GGTGGGTAGA ACGAGGGGTA CGAGTAGAA  

- TAGGGTTAAC CAGTTAAGAA AGGCTGAAAA GCCTTCACAT TGACAAGGAA TTTATTTAAG AAGAGAGTTG   
  
  
- CCAAATAGTC TCATGCATGC GAAGAGCAAT ACTGTAAGAT TTTTAAGACA AAACTTTGGG TTAAATTCAT   
  
  
- AGCGATCGTT GAAGCCAACT ACACTTCACC TACTTTCTGT TCAACCCCCC TTGTGAAAAC AAAGACGTAC   
  
  
- GTGCATGCCC AAAAATCCCA TCGTCATCAT CGAAGCATGC AAGAAAACTC AATATCTCTC TCTCTCTCTC   
  
  
- TCTCAGATAA ACACACAAGA TGAAGCAGAG AGAGAGAGAC AGAGAGAGAG AGAGAGAGTA AGTTTAATCC   
  
  
- ATAATGAAGT AAAAGTATGT TGATATGACA GATGATTGGC TCTGAAATGG CTTAAAGTAC AGGGTTTAGA   
  
  
- TATTGTTAAA CGGCTAATCC AGTATCTGAG GTCTGGTTGT TGAGGTAGTT TGGAATAATT CTGGACTGAT   
  
  
- AGACTGATTT CAGGCGATCA ATACAACCCT TTCCTTAGGG CACATGCATA GTTCTATGTA GTTTCCATAT   
  
  
- GTAGATCAAG TTGCTAATAT TTCATGTATA CTTGTCGTGT GAAATGAAAT GAAATGATCA ATATGATAAG   
  
  
- TTATTATAAT AAAGCTGTGG AAGAGCCAGA GTTCTAGATG ATCATTTCTT ACGGAAGATG CTTGTTTTGT   
  
  
- AAGTTCGTTA ATTCTAGAGT TGAAGGTAAT TAACACCATC TTTATCTTCT TGTTTGGAGG TTTACTAGTA   
  
  
- ACATGCAATT GTTATTATAC TTTGGCAAAC AAGCAATGCA AAAATAAGAA ATTATTGCCC ATGCACATGA   
  
  
- GAGTTTTGGG CTAGCTGGCT AGGCCAAATG AGACATGAAG ATTAAGTTGA AATATATATA TATATGCCAC   
  
  
- ATGGTATGCC AAGAAAATTA TAACATGCAT GTTATTTTTA GAACCGGCTC ATGTTGACTA GTTAATTAAA   
  
  
- AAAAATTATA TAGGTTTTTC AATATTTCAT AATATTAATG TAAAAATAGA ATATAAGAAA ATAAATTTAT   
  
  
- TAATTTTATT AAATTATCAT GTATTATAAT TTTATTTAAA ACTCAAATAA ACAAAAAAAT CTCTTGAGAC   
  
  
- TGATTCTTTT TTATCAGAAA AAAAAATGAT TATAATTAAA CTAACTCTTT TGACCTTAGA GCAATGCTAT   
  
  
- TGAGCCCAAA TCCATATTTA TTTGATCATG TCACCTTGTT GTTGATTATG TTTCTAATCA ATGGGGCTCC   
  
  
- AATATATGAA AAAATGGCAG ATAGTGCCAA TTTTCTATGG TCATTTATAT GACTGCAAAT TATGAACACA   
  
  
- GCAGAAACAT CAGCAAATAA AGCAACTAGA ATTCATAATC CATATCTTAT CCATAAACCT ACATGATGGT   
  
  
- AAGTGAAGAA GCTAGATATA AAGATTGCAT GCATATGCGT ACAAGGAAGA ATTAAAGGAC AAAGAAACAA   
  
  
- CCACCCATCT TGCTCCCCAT GCTCATCTT

+     GAG-motif

| Site Name | Organism | Position | Strand | Matrix score. | sequence | function |
| --- | --- | --- | --- | --- | --- | --- |
| GAG-motif | Arabidopsis thaliana | 279 | + | 7 | AGAGAGT | part of a light responsive element |

> 2018/04/13 10:10:12  
+ ATCCCAATTG GTCAATTCTT TCCGACTTTT CGGAAGTGTA ACTGTTCCTT AAATAAATTC TTCTCTCAAC   
  
  
+ GGTTTATCAG AGTACGTACG CTTCTCGTTA TGACATTCTA AAAATTCTGT TTTGAAACCC AATTTAAGTA   
  
  
+ TCGCTAGCAA CTTCGGTTGA TGTGAAGTGG ATGAAAGACA AGTTGGGGGG AACACTTTTG TTTCTGCATG   
  
  
+ CACGTACGGG TTTTTAGGGT AGCAGTAGTA GCTTCGTACG TTCTTTTGAG TTATAGAGAG AGAGAGAGAG   
  
  
+ AGAGTCTATT TGTGTGTTCT ACTTCGTCTC TCTCTCTCTG TCTCTCTCTC TCTCTCTCAT TCAAATTAGG   
  
  
+ TATTACTTCA TTTTCATACA ACTATACTGT CTACTAACCG AGACTTTACC GAATTTCATG TCCCAAATCT   
  
  
+ ATAACAATTT GCCGATTAGG TCATAGACTC CAGACCAACA ACTCCATCAA ACCTTATTAA GACCTGACTA   
  
  
+ TCTGACTAAA GTCCGCTAGT TATGTTGGGA AAGGAATCCC GTGTACGTAT CAAGATACAT CAAAGGTATA   
  
  
+ CATCTAGTTC AACGATTATA AAGTACATAT GAACAGCACA CTTTACTTTA CTTTACTAGT TATACTATTC   
  
  
+ AATAATATTA TTTCGACACC TTCTCGGTCT CAAGATCTAC TAGTAAAGAA TGCCTTCTAC GAACAAAACA   
  
  
+ TTCAAGCAAT TAAGATCTCA ACTTCCATTA ATTGTGGTAG AAATAGAAGA ACAAACCTCC AAATGATCAT   
  
  
+ TGTACGTTAA CAATAATATG AAACCGTTTG TTCGTTACGT TTTTATTCTT TAATAACGGG TACGTGTACT   
  
  
+ CTCAAAACCC GATCGACCGA TCCGGTTTAC TCTGTACTTC TAATTCAACT TTATATATAT ATATACGGTG   
  
  
+ TACCATACGG TTCTTTTAAT ATTGTACGTA CAATAAAAAT CTTGGCCGAG TACAACTGAT CAATTAATTT   
  
  
+ TTTTTAATAT ATCCAAAAAG TTATAAAGTA TTATAATTAC ATTTTTATCT TATATTCTTT TATTTAAATA   
  
  
+ ATTAAAATAA TTTAATAGTA CATAATATTA AAATAAATTT TGAGTTTATT TGTTTTTTTA GAGAACTCTG   
  
  
+ ACTAAGAAAA AATAGTCTTT TTTTTTACTA ATATTAATTT GATTGAGAAA ACTGGAATCT CGTTACGATA   
  
  
+ ACTCGGGTTT AGGTATAAAT AAACTAGTAC AGTGGAACAA CAACTAATAC AAAGATTAGT TACCCCGAGG   
  
  
+ TTATATACTT TTTTACCGTC TATCACGGTT AAAAGATACC AGTAAATATA CTGACGTTTA ATACTTGTGT   
  
  
+ CGTCTTTGTA GTCGTTTATT TCGTTGATCT TAAGTATTAG GTATAGAATA GGTATTTGGA TGTACTACCA   
  
  
+ TTCACTTCTT CGATCTATAT TTCTAACGTA CGTATACGCA TGTTCCTTCT TAATTTCCTG TTTCTTTGTT   
  
  
+ GGTGGGTAGA ACGAGGGGTA CGAGTAGAA  

- TAGGGTTAAC CAGTTAAGAA AGGCTGAAAA GCCTTCACAT TGACAAGGAA TTTATTTAAG AAGAGAGTTG   
  
  
- CCAAATAGTC TCATGCATGC GAAGAGCAAT ACTGTAAGAT TTTTAAGACA AAACTTTGGG TTAAATTCAT   
  
  
- AGCGATCGTT GAAGCCAACT ACACTTCACC TACTTTCTGT TCAACCCCCC TTGTGAAAAC AAAGACGTAC   
  
  
- GTGCATGCCC AAAAATCCCA TCGTCATCAT CGAAGCATGC AAGAAAACTC AATATCTCTC TCTCTCTCTC   
  
  
- TCTCAGATAA ACACACAAGA TGAAGCAGAG AGAGAGAGAC AGAGAGAGAG AGAGAGAGTA AGTTTAATCC   
  
  
- ATAATGAAGT AAAAGTATGT TGATATGACA GATGATTGGC TCTGAAATGG CTTAAAGTAC AGGGTTTAGA   
  
  
- TATTGTTAAA CGGCTAATCC AGTATCTGAG GTCTGGTTGT TGAGGTAGTT TGGAATAATT CTGGACTGAT   
  
  
- AGACTGATTT CAGGCGATCA ATACAACCCT TTCCTTAGGG CACATGCATA GTTCTATGTA GTTTCCATAT   
  
  
- GTAGATCAAG TTGCTAATAT TTCATGTATA CTTGTCGTGT GAAATGAAAT GAAATGATCA ATATGATAAG   
  
  
- TTATTATAAT AAAGCTGTGG AAGAGCCAGA GTTCTAGATG ATCATTTCTT ACGGAAGATG CTTGTTTTGT   
  
  
- AAGTTCGTTA ATTCTAGAGT TGAAGGTAAT TAACACCATC TTTATCTTCT TGTTTGGAGG TTTACTAGTA   
  
  
- ACATGCAATT GTTATTATAC TTTGGCAAAC AAGCAATGCA AAAATAAGAA ATTATTGCCC ATGCACATGA   
  
  
- GAGTTTTGGG CTAGCTGGCT AGGCCAAATG AGACATGAAG ATTAAGTTGA AATATATATA TATATGCCAC   
  
  
- ATGGTATGCC AAGAAAATTA TAACATGCAT GTTATTTTTA GAACCGGCTC ATGTTGACTA GTTAATTAAA   
  
  
- AAAAATTATA TAGGTTTTTC AATATTTCAT AATATTAATG TAAAAATAGA ATATAAGAAA ATAAATTTAT   
  
  
- TAATTTTATT AAATTATCAT GTATTATAAT TTTATTTAAA ACTCAAATAA ACAAAAAAAT CTCTTGAGAC   
  
  
- TGATTCTTTT TTATCAGAAA AAAAAATGAT TATAATTAAA CTAACTCTTT TGACCTTAGA GCAATGCTAT   
  
  
- TGAGCCCAAA TCCATATTTA TTTGATCATG TCACCTTGTT GTTGATTATG TTTCTAATCA ATGGGGCTCC   
  
  
- AATATATGAA AAAATGGCAG ATAGTGCCAA TTTTCTATGG TCATTTATAT GACTGCAAAT TATGAACACA   
  
  
- GCAGAAACAT CAGCAAATAA AGCAACTAGA ATTCATAATC CATATCTTAT CCATAAACCT ACATGATGGT   
  
  
- AAGTGAAGAA GCTAGATATA AAGATTGCAT GCATATGCGT ACAAGGAAGA ATTAAAGGAC AAAGAAACAA   
  
  
- CCACCCATCT TGCTCCCCAT GCTCATCTT

+     GARE-motif

| Site Name | Organism | Position | Strand | Matrix score. | sequence | function |
| --- | --- | --- | --- | --- | --- | --- |
| GARE-motif | Brassica oleracea | 116 | - | 7 | AAACAGA | gibberellin-responsive element |

> 2018/04/13 10:10:12  
+ ATCCCAATTG GTCAATTCTT TCCGACTTTT CGGAAGTGTA ACTGTTCCTT AAATAAATTC TTCTCTCAAC   
  
  
+ GGTTTATCAG AGTACGTACG CTTCTCGTTA TGACATTCTA AAAATTCTGT TTTGAAACCC AATTTAAGTA   
  
  
+ TCGCTAGCAA CTTCGGTTGA TGTGAAGTGG ATGAAAGACA AGTTGGGGGG AACACTTTTG TTTCTGCATG   
  
  
+ CACGTACGGG TTTTTAGGGT AGCAGTAGTA GCTTCGTACG TTCTTTTGAG TTATAGAGAG AGAGAGAGAG   
  
  
+ AGAGTCTATT TGTGTGTTCT ACTTCGTCTC TCTCTCTCTG TCTCTCTCTC TCTCTCTCAT TCAAATTAGG   
  
  
+ TATTACTTCA TTTTCATACA ACTATACTGT CTACTAACCG AGACTTTACC GAATTTCATG TCCCAAATCT   
  
  
+ ATAACAATTT GCCGATTAGG TCATAGACTC CAGACCAACA ACTCCATCAA ACCTTATTAA GACCTGACTA   
  
  
+ TCTGACTAAA GTCCGCTAGT TATGTTGGGA AAGGAATCCC GTGTACGTAT CAAGATACAT CAAAGGTATA   
  
  
+ CATCTAGTTC AACGATTATA AAGTACATAT GAACAGCACA CTTTACTTTA CTTTACTAGT TATACTATTC   
  
  
+ AATAATATTA TTTCGACACC TTCTCGGTCT CAAGATCTAC TAGTAAAGAA TGCCTTCTAC GAACAAAACA   
  
  
+ TTCAAGCAAT TAAGATCTCA ACTTCCATTA ATTGTGGTAG AAATAGAAGA ACAAACCTCC AAATGATCAT   
  
  
+ TGTACGTTAA CAATAATATG AAACCGTTTG TTCGTTACGT TTTTATTCTT TAATAACGGG TACGTGTACT   
  
  
+ CTCAAAACCC GATCGACCGA TCCGGTTTAC TCTGTACTTC TAATTCAACT TTATATATAT ATATACGGTG   
  
  
+ TACCATACGG TTCTTTTAAT ATTGTACGTA CAATAAAAAT CTTGGCCGAG TACAACTGAT CAATTAATTT   
  
  
+ TTTTTAATAT ATCCAAAAAG TTATAAAGTA TTATAATTAC ATTTTTATCT TATATTCTTT TATTTAAATA   
  
  
+ ATTAAAATAA TTTAATAGTA CATAATATTA AAATAAATTT TGAGTTTATT TGTTTTTTTA GAGAACTCTG   
  
  
+ ACTAAGAAAA AATAGTCTTT TTTTTTACTA ATATTAATTT GATTGAGAAA ACTGGAATCT CGTTACGATA   
  
  
+ ACTCGGGTTT AGGTATAAAT AAACTAGTAC AGTGGAACAA CAACTAATAC AAAGATTAGT TACCCCGAGG   
  
  
+ TTATATACTT TTTTACCGTC TATCACGGTT AAAAGATACC AGTAAATATA CTGACGTTTA ATACTTGTGT   
  
  
+ CGTCTTTGTA GTCGTTTATT TCGTTGATCT TAAGTATTAG GTATAGAATA GGTATTTGGA TGTACTACCA   
  
  
+ TTCACTTCTT CGATCTATAT TTCTAACGTA CGTATACGCA TGTTCCTTCT TAATTTCCTG TTTCTTTGTT   
  
  
+ GGTGGGTAGA ACGAGGGGTA CGAGTAGAA  

- TAGGGTTAAC CAGTTAAGAA AGGCTGAAAA GCCTTCACAT TGACAAGGAA TTTATTTAAG AAGAGAGTTG   
  
  
- CCAAATAGTC TCATGCATGC GAAGAGCAAT ACTGTAAGAT TTTTAAGACA AAACTTTGGG TTAAATTCAT   
  
  
- AGCGATCGTT GAAGCCAACT ACACTTCACC TACTTTCTGT TCAACCCCCC TTGTGAAAAC AAAGACGTAC   
  
  
- GTGCATGCCC AAAAATCCCA TCGTCATCAT CGAAGCATGC AAGAAAACTC AATATCTCTC TCTCTCTCTC   
  
  
- TCTCAGATAA ACACACAAGA TGAAGCAGAG AGAGAGAGAC AGAGAGAGAG AGAGAGAGTA AGTTTAATCC   
  
  
- ATAATGAAGT AAAAGTATGT TGATATGACA GATGATTGGC TCTGAAATGG CTTAAAGTAC AGGGTTTAGA   
  
  
- TATTGTTAAA CGGCTAATCC AGTATCTGAG GTCTGGTTGT TGAGGTAGTT TGGAATAATT CTGGACTGAT   
  
  
- AGACTGATTT CAGGCGATCA ATACAACCCT TTCCTTAGGG CACATGCATA GTTCTATGTA GTTTCCATAT   
  
  
- GTAGATCAAG TTGCTAATAT TTCATGTATA CTTGTCGTGT GAAATGAAAT GAAATGATCA ATATGATAAG   
  
  
- TTATTATAAT AAAGCTGTGG AAGAGCCAGA GTTCTAGATG ATCATTTCTT ACGGAAGATG CTTGTTTTGT   
  
  
- AAGTTCGTTA ATTCTAGAGT TGAAGGTAAT TAACACCATC TTTATCTTCT TGTTTGGAGG TTTACTAGTA   
  
  
- ACATGCAATT GTTATTATAC TTTGGCAAAC AAGCAATGCA AAAATAAGAA ATTATTGCCC ATGCACATGA   
  
  
- GAGTTTTGGG CTAGCTGGCT AGGCCAAATG AGACATGAAG ATTAAGTTGA AATATATATA TATATGCCAC   
  
  
- ATGGTATGCC AAGAAAATTA TAACATGCAT GTTATTTTTA GAACCGGCTC ATGTTGACTA GTTAATTAAA   
  
  
- AAAAATTATA TAGGTTTTTC AATATTTCAT AATATTAATG TAAAAATAGA ATATAAGAAA ATAAATTTAT   
  
  
- TAATTTTATT AAATTATCAT GTATTATAAT TTTATTTAAA ACTCAAATAA ACAAAAAAAT CTCTTGAGAC   
  
  
- TGATTCTTTT TTATCAGAAA AAAAAATGAT TATAATTAAA CTAACTCTTT TGACCTTAGA GCAATGCTAT   
  
  
- TGAGCCCAAA TCCATATTTA TTTGATCATG TCACCTTGTT GTTGATTATG TTTCTAATCA ATGGGGCTCC   
  
  
- AATATATGAA AAAATGGCAG ATAGTGCCAA TTTTCTATGG TCATTTATAT GACTGCAAAT TATGAACACA   
  
  
- GCAGAAACAT CAGCAAATAA AGCAACTAGA ATTCATAATC CATATCTTAT CCATAAACCT ACATGATGGT   
  
  
- AAGTGAAGAA GCTAGATATA AAGATTGCAT GCATATGCGT ACAAGGAAGA ATTAAAGGAC AAAGAAACAA   
  
  
- CCACCCATCT TGCTCCCCAT GCTCATCTT

+     GT1-motif

| Site Name | Organism | Position | Strand | Matrix score. | sequence | function |
| --- | --- | --- | --- | --- | --- | --- |
| GT1-motif | Arabidopsis thaliana | 1287 | + | 6 | GGTTAA | light responsive element |

> 2018/04/13 10:10:12  
+ ATCCCAATTG GTCAATTCTT TCCGACTTTT CGGAAGTGTA ACTGTTCCTT AAATAAATTC TTCTCTCAAC   
  
  
+ GGTTTATCAG AGTACGTACG CTTCTCGTTA TGACATTCTA AAAATTCTGT TTTGAAACCC AATTTAAGTA   
  
  
+ TCGCTAGCAA CTTCGGTTGA TGTGAAGTGG ATGAAAGACA AGTTGGGGGG AACACTTTTG TTTCTGCATG   
  
  
+ CACGTACGGG TTTTTAGGGT AGCAGTAGTA GCTTCGTACG TTCTTTTGAG TTATAGAGAG AGAGAGAGAG   
  
  
+ AGAGTCTATT TGTGTGTTCT ACTTCGTCTC TCTCTCTCTG TCTCTCTCTC TCTCTCTCAT TCAAATTAGG   
  
  
+ TATTACTTCA TTTTCATACA ACTATACTGT CTACTAACCG AGACTTTACC GAATTTCATG TCCCAAATCT   
  
  
+ ATAACAATTT GCCGATTAGG TCATAGACTC CAGACCAACA ACTCCATCAA ACCTTATTAA GACCTGACTA   
  
  
+ TCTGACTAAA GTCCGCTAGT TATGTTGGGA AAGGAATCCC GTGTACGTAT CAAGATACAT CAAAGGTATA   
  
  
+ CATCTAGTTC AACGATTATA AAGTACATAT GAACAGCACA CTTTACTTTA CTTTACTAGT TATACTATTC   
  
  
+ AATAATATTA TTTCGACACC TTCTCGGTCT CAAGATCTAC TAGTAAAGAA TGCCTTCTAC GAACAAAACA   
  
  
+ TTCAAGCAAT TAAGATCTCA ACTTCCATTA ATTGTGGTAG AAATAGAAGA ACAAACCTCC AAATGATCAT   
  
  
+ TGTACGTTAA CAATAATATG AAACCGTTTG TTCGTTACGT TTTTATTCTT TAATAACGGG TACGTGTACT   
  
  
+ CTCAAAACCC GATCGACCGA TCCGGTTTAC TCTGTACTTC TAATTCAACT TTATATATAT ATATACGGTG   
  
  
+ TACCATACGG TTCTTTTAAT ATTGTACGTA CAATAAAAAT CTTGGCCGAG TACAACTGAT CAATTAATTT   
  
  
+ TTTTTAATAT ATCCAAAAAG TTATAAAGTA TTATAATTAC ATTTTTATCT TATATTCTTT TATTTAAATA   
  
  
+ ATTAAAATAA TTTAATAGTA CATAATATTA AAATAAATTT TGAGTTTATT TGTTTTTTTA GAGAACTCTG   
  
  
+ ACTAAGAAAA AATAGTCTTT TTTTTTACTA ATATTAATTT GATTGAGAAA ACTGGAATCT CGTTACGATA   
  
  
+ ACTCGGGTTT AGGTATAAAT AAACTAGTAC AGTGGAACAA CAACTAATAC AAAGATTAGT TACCCCGAGG   
  
  
+ TTATATACTT TTTTACCGTC TATCACGGTT AAAAGATACC AGTAAATATA CTGACGTTTA ATACTTGTGT   
  
  
+ CGTCTTTGTA GTCGTTTATT TCGTTGATCT TAAGTATTAG GTATAGAATA GGTATTTGGA TGTACTACCA   
  
  
+ TTCACTTCTT CGATCTATAT TTCTAACGTA CGTATACGCA TGTTCCTTCT TAATTTCCTG TTTCTTTGTT   
  
  
+ GGTGGGTAGA ACGAGGGGTA CGAGTAGAA  

- TAGGGTTAAC CAGTTAAGAA AGGCTGAAAA GCCTTCACAT TGACAAGGAA TTTATTTAAG AAGAGAGTTG   
  
  
- CCAAATAGTC TCATGCATGC GAAGAGCAAT ACTGTAAGAT TTTTAAGACA AAACTTTGGG TTAAATTCAT   
  
  
- AGCGATCGTT GAAGCCAACT ACACTTCACC TACTTTCTGT TCAACCCCCC TTGTGAAAAC AAAGACGTAC   
  
  
- GTGCATGCCC AAAAATCCCA TCGTCATCAT CGAAGCATGC AAGAAAACTC AATATCTCTC TCTCTCTCTC   
  
  
- TCTCAGATAA ACACACAAGA TGAAGCAGAG AGAGAGAGAC AGAGAGAGAG AGAGAGAGTA AGTTTAATCC   
  
  
- ATAATGAAGT AAAAGTATGT TGATATGACA GATGATTGGC TCTGAAATGG CTTAAAGTAC AGGGTTTAGA   
  
  
- TATTGTTAAA CGGCTAATCC AGTATCTGAG GTCTGGTTGT TGAGGTAGTT TGGAATAATT CTGGACTGAT   
  
  
- AGACTGATTT CAGGCGATCA ATACAACCCT TTCCTTAGGG CACATGCATA GTTCTATGTA GTTTCCATAT   
  
  
- GTAGATCAAG TTGCTAATAT TTCATGTATA CTTGTCGTGT GAAATGAAAT GAAATGATCA ATATGATAAG   
  
  
- TTATTATAAT AAAGCTGTGG AAGAGCCAGA GTTCTAGATG ATCATTTCTT ACGGAAGATG CTTGTTTTGT   
  
  
- AAGTTCGTTA ATTCTAGAGT TGAAGGTAAT TAACACCATC TTTATCTTCT TGTTTGGAGG TTTACTAGTA   
  
  
- ACATGCAATT GTTATTATAC TTTGGCAAAC AAGCAATGCA AAAATAAGAA ATTATTGCCC ATGCACATGA   
  
  
- GAGTTTTGGG CTAGCTGGCT AGGCCAAATG AGACATGAAG ATTAAGTTGA AATATATATA TATATGCCAC   
  
  
- ATGGTATGCC AAGAAAATTA TAACATGCAT GTTATTTTTA GAACCGGCTC ATGTTGACTA GTTAATTAAA   
  
  
- AAAAATTATA TAGGTTTTTC AATATTTCAT AATATTAATG TAAAAATAGA ATATAAGAAA ATAAATTTAT   
  
  
- TAATTTTATT AAATTATCAT GTATTATAAT TTTATTTAAA ACTCAAATAA ACAAAAAAAT CTCTTGAGAC   
  
  
- TGATTCTTTT TTATCAGAAA AAAAAATGAT TATAATTAAA CTAACTCTTT TGACCTTAGA GCAATGCTAT   
  
  
- TGAGCCCAAA TCCATATTTA TTTGATCATG TCACCTTGTT GTTGATTATG TTTCTAATCA ATGGGGCTCC   
  
  
- AATATATGAA AAAATGGCAG ATAGTGCCAA TTTTCTATGG TCATTTATAT GACTGCAAAT TATGAACACA   
  
  
- GCAGAAACAT CAGCAAATAA AGCAACTAGA ATTCATAATC CATATCTTAT CCATAAACCT ACATGATGGT   
  
  
- AAGTGAAGAA GCTAGATATA AAGATTGCAT GCATATGCGT ACAAGGAAGA ATTAAAGGAC AAAGAAACAA   
  
  
- CCACCCATCT TGCTCCCCAT GCTCATCTT

+     LTR

| Site Name | Organism | Position | Strand | Matrix score. | sequence | function |
| --- | --- | --- | --- | --- | --- | --- |
| LTR | Hordeum vulgare | 28 | - | 6 | CCGAAA | cis-acting element involved in low-temperature responsiveness |

> 2018/04/13 10:10:12  
+ ATCCCAATTG GTCAATTCTT TCCGACTTTT CGGAAGTGTA ACTGTTCCTT AAATAAATTC TTCTCTCAAC   
  
  
+ GGTTTATCAG AGTACGTACG CTTCTCGTTA TGACATTCTA AAAATTCTGT TTTGAAACCC AATTTAAGTA   
  
  
+ TCGCTAGCAA CTTCGGTTGA TGTGAAGTGG ATGAAAGACA AGTTGGGGGG AACACTTTTG TTTCTGCATG   
  
  
+ CACGTACGGG TTTTTAGGGT AGCAGTAGTA GCTTCGTACG TTCTTTTGAG TTATAGAGAG AGAGAGAGAG   
  
  
+ AGAGTCTATT TGTGTGTTCT ACTTCGTCTC TCTCTCTCTG TCTCTCTCTC TCTCTCTCAT TCAAATTAGG   
  
  
+ TATTACTTCA TTTTCATACA ACTATACTGT CTACTAACCG AGACTTTACC GAATTTCATG TCCCAAATCT   
  
  
+ ATAACAATTT GCCGATTAGG TCATAGACTC CAGACCAACA ACTCCATCAA ACCTTATTAA GACCTGACTA   
  
  
+ TCTGACTAAA GTCCGCTAGT TATGTTGGGA AAGGAATCCC GTGTACGTAT CAAGATACAT CAAAGGTATA   
  
  
+ CATCTAGTTC AACGATTATA AAGTACATAT GAACAGCACA CTTTACTTTA CTTTACTAGT TATACTATTC   
  
  
+ AATAATATTA TTTCGACACC TTCTCGGTCT CAAGATCTAC TAGTAAAGAA TGCCTTCTAC GAACAAAACA   
  
  
+ TTCAAGCAAT TAAGATCTCA ACTTCCATTA ATTGTGGTAG AAATAGAAGA ACAAACCTCC AAATGATCAT   
  
  
+ TGTACGTTAA CAATAATATG AAACCGTTTG TTCGTTACGT TTTTATTCTT TAATAACGGG TACGTGTACT   
  
  
+ CTCAAAACCC GATCGACCGA TCCGGTTTAC TCTGTACTTC TAATTCAACT TTATATATAT ATATACGGTG   
  
  
+ TACCATACGG TTCTTTTAAT ATTGTACGTA CAATAAAAAT CTTGGCCGAG TACAACTGAT CAATTAATTT   
  
  
+ TTTTTAATAT ATCCAAAAAG TTATAAAGTA TTATAATTAC ATTTTTATCT TATATTCTTT TATTTAAATA   
  
  
+ ATTAAAATAA TTTAATAGTA CATAATATTA AAATAAATTT TGAGTTTATT TGTTTTTTTA GAGAACTCTG   
  
  
+ ACTAAGAAAA AATAGTCTTT TTTTTTACTA ATATTAATTT GATTGAGAAA ACTGGAATCT CGTTACGATA   
  
  
+ ACTCGGGTTT AGGTATAAAT AAACTAGTAC AGTGGAACAA CAACTAATAC AAAGATTAGT TACCCCGAGG   
  
  
+ TTATATACTT TTTTACCGTC TATCACGGTT AAAAGATACC AGTAAATATA CTGACGTTTA ATACTTGTGT   
  
  
+ CGTCTTTGTA GTCGTTTATT TCGTTGATCT TAAGTATTAG GTATAGAATA GGTATTTGGA TGTACTACCA   
  
  
+ TTCACTTCTT CGATCTATAT TTCTAACGTA CGTATACGCA TGTTCCTTCT TAATTTCCTG TTTCTTTGTT   
  
  
+ GGTGGGTAGA ACGAGGGGTA CGAGTAGAA  

- TAGGGTTAAC CAGTTAAGAA AGGCTGAAAA GCCTTCACAT TGACAAGGAA TTTATTTAAG AAGAGAGTTG   
  
  
- CCAAATAGTC TCATGCATGC GAAGAGCAAT ACTGTAAGAT TTTTAAGACA AAACTTTGGG TTAAATTCAT   
  
  
- AGCGATCGTT GAAGCCAACT ACACTTCACC TACTTTCTGT TCAACCCCCC TTGTGAAAAC AAAGACGTAC   
  
  
- GTGCATGCCC AAAAATCCCA TCGTCATCAT CGAAGCATGC AAGAAAACTC AATATCTCTC TCTCTCTCTC   
  
  
- TCTCAGATAA ACACACAAGA TGAAGCAGAG AGAGAGAGAC AGAGAGAGAG AGAGAGAGTA AGTTTAATCC   
  
  
- ATAATGAAGT AAAAGTATGT TGATATGACA GATGATTGGC TCTGAAATGG CTTAAAGTAC AGGGTTTAGA   
  
  
- TATTGTTAAA CGGCTAATCC AGTATCTGAG GTCTGGTTGT TGAGGTAGTT TGGAATAATT CTGGACTGAT   
  
  
- AGACTGATTT CAGGCGATCA ATACAACCCT TTCCTTAGGG CACATGCATA GTTCTATGTA GTTTCCATAT   
  
  
- GTAGATCAAG TTGCTAATAT TTCATGTATA CTTGTCGTGT GAAATGAAAT GAAATGATCA ATATGATAAG   
  
  
- TTATTATAAT AAAGCTGTGG AAGAGCCAGA GTTCTAGATG ATCATTTCTT ACGGAAGATG CTTGTTTTGT   
  
  
- AAGTTCGTTA ATTCTAGAGT TGAAGGTAAT TAACACCATC TTTATCTTCT TGTTTGGAGG TTTACTAGTA   
  
  
- ACATGCAATT GTTATTATAC TTTGGCAAAC AAGCAATGCA AAAATAAGAA ATTATTGCCC ATGCACATGA   
  
  
- GAGTTTTGGG CTAGCTGGCT AGGCCAAATG AGACATGAAG ATTAAGTTGA AATATATATA TATATGCCAC   
  
  
- ATGGTATGCC AAGAAAATTA TAACATGCAT GTTATTTTTA GAACCGGCTC ATGTTGACTA GTTAATTAAA   
  
  
- AAAAATTATA TAGGTTTTTC AATATTTCAT AATATTAATG TAAAAATAGA ATATAAGAAA ATAAATTTAT   
  
  
- TAATTTTATT AAATTATCAT GTATTATAAT TTTATTTAAA ACTCAAATAA ACAAAAAAAT CTCTTGAGAC   
  
  
- TGATTCTTTT TTATCAGAAA AAAAAATGAT TATAATTAAA CTAACTCTTT TGACCTTAGA GCAATGCTAT   
  
  
- TGAGCCCAAA TCCATATTTA TTTGATCATG TCACCTTGTT GTTGATTATG TTTCTAATCA ATGGGGCTCC   
  
  
- AATATATGAA AAAATGGCAG ATAGTGCCAA TTTTCTATGG TCATTTATAT GACTGCAAAT TATGAACACA   
  
  
- GCAGAAACAT CAGCAAATAA AGCAACTAGA ATTCATAATC CATATCTTAT CCATAAACCT ACATGATGGT   
  
  
- AAGTGAAGAA GCTAGATATA AAGATTGCAT GCATATGCGT ACAAGGAAGA ATTAAAGGAC AAAGAAACAA   
  
  
- CCACCCATCT TGCTCCCCAT GCTCATCTT

+     MBS

| Site Name | Organism | Position | Strand | Matrix score. | sequence | function |
| --- | --- | --- | --- | --- | --- | --- |
| MBS | Arabidopsis thaliana | 39 | + | 6 | TAACTG | MYB binding site involved in drought-inducibility |
| MBS | Arabidopsis thaliana | 963 | + | 6 | CAACTG | MYB binding site involved in drought-inducibility |

> 2018/04/13 10:10:12  
+ ATCCCAATTG GTCAATTCTT TCCGACTTTT CGGAAGTGTA ACTGTTCCTT AAATAAATTC TTCTCTCAAC   
  
  
+ GGTTTATCAG AGTACGTACG CTTCTCGTTA TGACATTCTA AAAATTCTGT TTTGAAACCC AATTTAAGTA   
  
  
+ TCGCTAGCAA CTTCGGTTGA TGTGAAGTGG ATGAAAGACA AGTTGGGGGG AACACTTTTG TTTCTGCATG   
  
  
+ CACGTACGGG TTTTTAGGGT AGCAGTAGTA GCTTCGTACG TTCTTTTGAG TTATAGAGAG AGAGAGAGAG   
  
  
+ AGAGTCTATT TGTGTGTTCT ACTTCGTCTC TCTCTCTCTG TCTCTCTCTC TCTCTCTCAT TCAAATTAGG   
  
  
+ TATTACTTCA TTTTCATACA ACTATACTGT CTACTAACCG AGACTTTACC GAATTTCATG TCCCAAATCT   
  
  
+ ATAACAATTT GCCGATTAGG TCATAGACTC CAGACCAACA ACTCCATCAA ACCTTATTAA GACCTGACTA   
  
  
+ TCTGACTAAA GTCCGCTAGT TATGTTGGGA AAGGAATCCC GTGTACGTAT CAAGATACAT CAAAGGTATA   
  
  
+ CATCTAGTTC AACGATTATA AAGTACATAT GAACAGCACA CTTTACTTTA CTTTACTAGT TATACTATTC   
  
  
+ AATAATATTA TTTCGACACC TTCTCGGTCT CAAGATCTAC TAGTAAAGAA TGCCTTCTAC GAACAAAACA   
  
  
+ TTCAAGCAAT TAAGATCTCA ACTTCCATTA ATTGTGGTAG AAATAGAAGA ACAAACCTCC AAATGATCAT   
  
  
+ TGTACGTTAA CAATAATATG AAACCGTTTG TTCGTTACGT TTTTATTCTT TAATAACGGG TACGTGTACT   
  
  
+ CTCAAAACCC GATCGACCGA TCCGGTTTAC TCTGTACTTC TAATTCAACT TTATATATAT ATATACGGTG   
  
  
+ TACCATACGG TTCTTTTAAT ATTGTACGTA CAATAAAAAT CTTGGCCGAG TACAACTGAT CAATTAATTT   
  
  
+ TTTTTAATAT ATCCAAAAAG TTATAAAGTA TTATAATTAC ATTTTTATCT TATATTCTTT TATTTAAATA   
  
  
+ ATTAAAATAA TTTAATAGTA CATAATATTA AAATAAATTT TGAGTTTATT TGTTTTTTTA GAGAACTCTG   
  
  
+ ACTAAGAAAA AATAGTCTTT TTTTTTACTA ATATTAATTT GATTGAGAAA ACTGGAATCT CGTTACGATA   
  
  
+ ACTCGGGTTT AGGTATAAAT AAACTAGTAC AGTGGAACAA CAACTAATAC AAAGATTAGT TACCCCGAGG   
  
  
+ TTATATACTT TTTTACCGTC TATCACGGTT AAAAGATACC AGTAAATATA CTGACGTTTA ATACTTGTGT   
  
  
+ CGTCTTTGTA GTCGTTTATT TCGTTGATCT TAAGTATTAG GTATAGAATA GGTATTTGGA TGTACTACCA   
  
  
+ TTCACTTCTT CGATCTATAT TTCTAACGTA CGTATACGCA TGTTCCTTCT TAATTTCCTG TTTCTTTGTT   
  
  
+ GGTGGGTAGA ACGAGGGGTA CGAGTAGAA  

- TAGGGTTAAC CAGTTAAGAA AGGCTGAAAA GCCTTCACAT TGACAAGGAA TTTATTTAAG AAGAGAGTTG   
  
  
- CCAAATAGTC TCATGCATGC GAAGAGCAAT ACTGTAAGAT TTTTAAGACA AAACTTTGGG TTAAATTCAT   
  
  
- AGCGATCGTT GAAGCCAACT ACACTTCACC TACTTTCTGT TCAACCCCCC TTGTGAAAAC AAAGACGTAC   
  
  
- GTGCATGCCC AAAAATCCCA TCGTCATCAT CGAAGCATGC AAGAAAACTC AATATCTCTC TCTCTCTCTC   
  
  
- TCTCAGATAA ACACACAAGA TGAAGCAGAG AGAGAGAGAC AGAGAGAGAG AGAGAGAGTA AGTTTAATCC   
  
  
- ATAATGAAGT AAAAGTATGT TGATATGACA GATGATTGGC TCTGAAATGG CTTAAAGTAC AGGGTTTAGA   
  
  
- TATTGTTAAA CGGCTAATCC AGTATCTGAG GTCTGGTTGT TGAGGTAGTT TGGAATAATT CTGGACTGAT   
  
  
- AGACTGATTT CAGGCGATCA ATACAACCCT TTCCTTAGGG CACATGCATA GTTCTATGTA GTTTCCATAT   
  
  
- GTAGATCAAG TTGCTAATAT TTCATGTATA CTTGTCGTGT GAAATGAAAT GAAATGATCA ATATGATAAG   
  
  
- TTATTATAAT AAAGCTGTGG AAGAGCCAGA GTTCTAGATG ATCATTTCTT ACGGAAGATG CTTGTTTTGT   
  
  
- AAGTTCGTTA ATTCTAGAGT TGAAGGTAAT TAACACCATC TTTATCTTCT TGTTTGGAGG TTTACTAGTA   
  
  
- ACATGCAATT GTTATTATAC TTTGGCAAAC AAGCAATGCA AAAATAAGAA ATTATTGCCC ATGCACATGA   
  
  
- GAGTTTTGGG CTAGCTGGCT AGGCCAAATG AGACATGAAG ATTAAGTTGA AATATATATA TATATGCCAC   
  
  
- ATGGTATGCC AAGAAAATTA TAACATGCAT GTTATTTTTA GAACCGGCTC ATGTTGACTA GTTAATTAAA   
  
  
- AAAAATTATA TAGGTTTTTC AATATTTCAT AATATTAATG TAAAAATAGA ATATAAGAAA ATAAATTTAT   
  
  
- TAATTTTATT AAATTATCAT GTATTATAAT TTTATTTAAA ACTCAAATAA ACAAAAAAAT CTCTTGAGAC   
  
  
- TGATTCTTTT TTATCAGAAA AAAAAATGAT TATAATTAAA CTAACTCTTT TGACCTTAGA GCAATGCTAT   
  
  
- TGAGCCCAAA TCCATATTTA TTTGATCATG TCACCTTGTT GTTGATTATG TTTCTAATCA ATGGGGCTCC   
  
  
- AATATATGAA AAAATGGCAG ATAGTGCCAA TTTTCTATGG TCATTTATAT GACTGCAAAT TATGAACACA   
  
  
- GCAGAAACAT CAGCAAATAA AGCAACTAGA ATTCATAATC CATATCTTAT CCATAAACCT ACATGATGGT   
  
  
- AAGTGAAGAA GCTAGATATA AAGATTGCAT GCATATGCGT ACAAGGAAGA ATTAAAGGAC AAAGAAACAA   
  
  
- CCACCCATCT TGCTCCCCAT GCTCATCTT

+     O2-site

| Site Name | Organism | Position | Strand | Matrix score. | sequence | function |
| --- | --- | --- | --- | --- | --- | --- |
| O2-site | Zea mays | 156 | + | 9 | GTTGACGTGA | cis-acting regulatory element involved in zein metabolism regulation |

> 2018/04/13 10:10:12  
+ ATCCCAATTG GTCAATTCTT TCCGACTTTT CGGAAGTGTA ACTGTTCCTT AAATAAATTC TTCTCTCAAC   
  
  
+ GGTTTATCAG AGTACGTACG CTTCTCGTTA TGACATTCTA AAAATTCTGT TTTGAAACCC AATTTAAGTA   
  
  
+ TCGCTAGCAA CTTCGGTTGA TGTGAAGTGG ATGAAAGACA AGTTGGGGGG AACACTTTTG TTTCTGCATG   
  
  
+ CACGTACGGG TTTTTAGGGT AGCAGTAGTA GCTTCGTACG TTCTTTTGAG TTATAGAGAG AGAGAGAGAG   
  
  
+ AGAGTCTATT TGTGTGTTCT ACTTCGTCTC TCTCTCTCTG TCTCTCTCTC TCTCTCTCAT TCAAATTAGG   
  
  
+ TATTACTTCA TTTTCATACA ACTATACTGT CTACTAACCG AGACTTTACC GAATTTCATG TCCCAAATCT   
  
  
+ ATAACAATTT GCCGATTAGG TCATAGACTC CAGACCAACA ACTCCATCAA ACCTTATTAA GACCTGACTA   
  
  
+ TCTGACTAAA GTCCGCTAGT TATGTTGGGA AAGGAATCCC GTGTACGTAT CAAGATACAT CAAAGGTATA   
  
  
+ CATCTAGTTC AACGATTATA AAGTACATAT GAACAGCACA CTTTACTTTA CTTTACTAGT TATACTATTC   
  
  
+ AATAATATTA TTTCGACACC TTCTCGGTCT CAAGATCTAC TAGTAAAGAA TGCCTTCTAC GAACAAAACA   
  
  
+ TTCAAGCAAT TAAGATCTCA ACTTCCATTA ATTGTGGTAG AAATAGAAGA ACAAACCTCC AAATGATCAT   
  
  
+ TGTACGTTAA CAATAATATG AAACCGTTTG TTCGTTACGT TTTTATTCTT TAATAACGGG TACGTGTACT   
  
  
+ CTCAAAACCC GATCGACCGA TCCGGTTTAC TCTGTACTTC TAATTCAACT TTATATATAT ATATACGGTG   
  
  
+ TACCATACGG TTCTTTTAAT ATTGTACGTA CAATAAAAAT CTTGGCCGAG TACAACTGAT CAATTAATTT   
  
  
+ TTTTTAATAT ATCCAAAAAG TTATAAAGTA TTATAATTAC ATTTTTATCT TATATTCTTT TATTTAAATA   
  
  
+ ATTAAAATAA TTTAATAGTA CATAATATTA AAATAAATTT TGAGTTTATT TGTTTTTTTA GAGAACTCTG   
  
  
+ ACTAAGAAAA AATAGTCTTT TTTTTTACTA ATATTAATTT GATTGAGAAA ACTGGAATCT CGTTACGATA   
  
  
+ ACTCGGGTTT AGGTATAAAT AAACTAGTAC AGTGGAACAA CAACTAATAC AAAGATTAGT TACCCCGAGG   
  
  
+ TTATATACTT TTTTACCGTC TATCACGGTT AAAAGATACC AGTAAATATA CTGACGTTTA ATACTTGTGT   
  
  
+ CGTCTTTGTA GTCGTTTATT TCGTTGATCT TAAGTATTAG GTATAGAATA GGTATTTGGA TGTACTACCA   
  
  
+ TTCACTTCTT CGATCTATAT TTCTAACGTA CGTATACGCA TGTTCCTTCT TAATTTCCTG TTTCTTTGTT   
  
  
+ GGTGGGTAGA ACGAGGGGTA CGAGTAGAA  

- TAGGGTTAAC CAGTTAAGAA AGGCTGAAAA GCCTTCACAT TGACAAGGAA TTTATTTAAG AAGAGAGTTG   
  
  
- CCAAATAGTC TCATGCATGC GAAGAGCAAT ACTGTAAGAT TTTTAAGACA AAACTTTGGG TTAAATTCAT   
  
  
- AGCGATCGTT GAAGCCAACT ACACTTCACC TACTTTCTGT TCAACCCCCC TTGTGAAAAC AAAGACGTAC   
  
  
- GTGCATGCCC AAAAATCCCA TCGTCATCAT CGAAGCATGC AAGAAAACTC AATATCTCTC TCTCTCTCTC   
  
  
- TCTCAGATAA ACACACAAGA TGAAGCAGAG AGAGAGAGAC AGAGAGAGAG AGAGAGAGTA AGTTTAATCC   
  
  
- ATAATGAAGT AAAAGTATGT TGATATGACA GATGATTGGC TCTGAAATGG CTTAAAGTAC AGGGTTTAGA   
  
  
- TATTGTTAAA CGGCTAATCC AGTATCTGAG GTCTGGTTGT TGAGGTAGTT TGGAATAATT CTGGACTGAT   
  
  
- AGACTGATTT CAGGCGATCA ATACAACCCT TTCCTTAGGG CACATGCATA GTTCTATGTA GTTTCCATAT   
  
  
- GTAGATCAAG TTGCTAATAT TTCATGTATA CTTGTCGTGT GAAATGAAAT GAAATGATCA ATATGATAAG   
  
  
- TTATTATAAT AAAGCTGTGG AAGAGCCAGA GTTCTAGATG ATCATTTCTT ACGGAAGATG CTTGTTTTGT   
  
  
- AAGTTCGTTA ATTCTAGAGT TGAAGGTAAT TAACACCATC TTTATCTTCT TGTTTGGAGG TTTACTAGTA   
  
  
- ACATGCAATT GTTATTATAC TTTGGCAAAC AAGCAATGCA AAAATAAGAA ATTATTGCCC ATGCACATGA   
  
  
- GAGTTTTGGG CTAGCTGGCT AGGCCAAATG AGACATGAAG ATTAAGTTGA AATATATATA TATATGCCAC   
  
  
- ATGGTATGCC AAGAAAATTA TAACATGCAT GTTATTTTTA GAACCGGCTC ATGTTGACTA GTTAATTAAA   
  
  
- AAAAATTATA TAGGTTTTTC AATATTTCAT AATATTAATG TAAAAATAGA ATATAAGAAA ATAAATTTAT   
  
  
- TAATTTTATT AAATTATCAT GTATTATAAT TTTATTTAAA ACTCAAATAA ACAAAAAAAT CTCTTGAGAC   
  
  
- TGATTCTTTT TTATCAGAAA AAAAAATGAT TATAATTAAA CTAACTCTTT TGACCTTAGA GCAATGCTAT   
  
  
- TGAGCCCAAA TCCATATTTA TTTGATCATG TCACCTTGTT GTTGATTATG TTTCTAATCA ATGGGGCTCC   
  
  
- AATATATGAA AAAATGGCAG ATAGTGCCAA TTTTCTATGG TCATTTATAT GACTGCAAAT TATGAACACA   
  
  
- GCAGAAACAT CAGCAAATAA AGCAACTAGA ATTCATAATC CATATCTTAT CCATAAACCT ACATGATGGT   
  
  
- AAGTGAAGAA GCTAGATATA AAGATTGCAT GCATATGCGT ACAAGGAAGA ATTAAAGGAC AAAGAAACAA   
  
  
- CCACCCATCT TGCTCCCCAT GCTCATCTT

+     Skn-1\_motif

| Site Name | Organism | Position | Strand | Matrix score. | sequence | function |
| --- | --- | --- | --- | --- | --- | --- |
| Skn-1\_motif | Oryza sativa | 440 | + | 5 | GTCAT | cis-acting regulatory element required for endosperm expression |
| Skn-1\_motif | Oryza sativa | 100 | - | 5 | GTCAT | cis-acting regulatory element required for endosperm expression |

> 2018/04/13 10:10:12  
+ ATCCCAATTG GTCAATTCTT TCCGACTTTT CGGAAGTGTA ACTGTTCCTT AAATAAATTC TTCTCTCAAC   
  
  
+ GGTTTATCAG AGTACGTACG CTTCTCGTTA TGACATTCTA AAAATTCTGT TTTGAAACCC AATTTAAGTA   
  
  
+ TCGCTAGCAA CTTCGGTTGA TGTGAAGTGG ATGAAAGACA AGTTGGGGGG AACACTTTTG TTTCTGCATG   
  
  
+ CACGTACGGG TTTTTAGGGT AGCAGTAGTA GCTTCGTACG TTCTTTTGAG TTATAGAGAG AGAGAGAGAG   
  
  
+ AGAGTCTATT TGTGTGTTCT ACTTCGTCTC TCTCTCTCTG TCTCTCTCTC TCTCTCTCAT TCAAATTAGG   
  
  
+ TATTACTTCA TTTTCATACA ACTATACTGT CTACTAACCG AGACTTTACC GAATTTCATG TCCCAAATCT   
  
  
+ ATAACAATTT GCCGATTAGG TCATAGACTC CAGACCAACA ACTCCATCAA ACCTTATTAA GACCTGACTA   
  
  
+ TCTGACTAAA GTCCGCTAGT TATGTTGGGA AAGGAATCCC GTGTACGTAT CAAGATACAT CAAAGGTATA   
  
  
+ CATCTAGTTC AACGATTATA AAGTACATAT GAACAGCACA CTTTACTTTA CTTTACTAGT TATACTATTC   
  
  
+ AATAATATTA TTTCGACACC TTCTCGGTCT CAAGATCTAC TAGTAAAGAA TGCCTTCTAC GAACAAAACA   
  
  
+ TTCAAGCAAT TAAGATCTCA ACTTCCATTA ATTGTGGTAG AAATAGAAGA ACAAACCTCC AAATGATCAT   
  
  
+ TGTACGTTAA CAATAATATG AAACCGTTTG TTCGTTACGT TTTTATTCTT TAATAACGGG TACGTGTACT   
  
  
+ CTCAAAACCC GATCGACCGA TCCGGTTTAC TCTGTACTTC TAATTCAACT TTATATATAT ATATACGGTG   
  
  
+ TACCATACGG TTCTTTTAAT ATTGTACGTA CAATAAAAAT CTTGGCCGAG TACAACTGAT CAATTAATTT   
  
  
+ TTTTTAATAT ATCCAAAAAG TTATAAAGTA TTATAATTAC ATTTTTATCT TATATTCTTT TATTTAAATA   
  
  
+ ATTAAAATAA TTTAATAGTA CATAATATTA AAATAAATTT TGAGTTTATT TGTTTTTTTA GAGAACTCTG   
  
  
+ ACTAAGAAAA AATAGTCTTT TTTTTTACTA ATATTAATTT GATTGAGAAA ACTGGAATCT CGTTACGATA   
  
  
+ ACTCGGGTTT AGGTATAAAT AAACTAGTAC AGTGGAACAA CAACTAATAC AAAGATTAGT TACCCCGAGG   
  
  
+ TTATATACTT TTTTACCGTC TATCACGGTT AAAAGATACC AGTAAATATA CTGACGTTTA ATACTTGTGT   
  
  
+ CGTCTTTGTA GTCGTTTATT TCGTTGATCT TAAGTATTAG GTATAGAATA GGTATTTGGA TGTACTACCA   
  
  
+ TTCACTTCTT CGATCTATAT TTCTAACGTA CGTATACGCA TGTTCCTTCT TAATTTCCTG TTTCTTTGTT   
  
  
+ GGTGGGTAGA ACGAGGGGTA CGAGTAGAA  

- TAGGGTTAAC CAGTTAAGAA AGGCTGAAAA GCCTTCACAT TGACAAGGAA TTTATTTAAG AAGAGAGTTG   
  
  
- CCAAATAGTC TCATGCATGC GAAGAGCAAT ACTGTAAGAT TTTTAAGACA AAACTTTGGG TTAAATTCAT   
  
  
- AGCGATCGTT GAAGCCAACT ACACTTCACC TACTTTCTGT TCAACCCCCC TTGTGAAAAC AAAGACGTAC   
  
  
- GTGCATGCCC AAAAATCCCA TCGTCATCAT CGAAGCATGC AAGAAAACTC AATATCTCTC TCTCTCTCTC   
  
  
- TCTCAGATAA ACACACAAGA TGAAGCAGAG AGAGAGAGAC AGAGAGAGAG AGAGAGAGTA AGTTTAATCC   
  
  
- ATAATGAAGT AAAAGTATGT TGATATGACA GATGATTGGC TCTGAAATGG CTTAAAGTAC AGGGTTTAGA   
  
  
- TATTGTTAAA CGGCTAATCC AGTATCTGAG GTCTGGTTGT TGAGGTAGTT TGGAATAATT CTGGACTGAT   
  
  
- AGACTGATTT CAGGCGATCA ATACAACCCT TTCCTTAGGG CACATGCATA GTTCTATGTA GTTTCCATAT   
  
  
- GTAGATCAAG TTGCTAATAT TTCATGTATA CTTGTCGTGT GAAATGAAAT GAAATGATCA ATATGATAAG   
  
  
- TTATTATAAT AAAGCTGTGG AAGAGCCAGA GTTCTAGATG ATCATTTCTT ACGGAAGATG CTTGTTTTGT   
  
  
- AAGTTCGTTA ATTCTAGAGT TGAAGGTAAT TAACACCATC TTTATCTTCT TGTTTGGAGG TTTACTAGTA   
  
  
- ACATGCAATT GTTATTATAC TTTGGCAAAC AAGCAATGCA AAAATAAGAA ATTATTGCCC ATGCACATGA   
  
  
- GAGTTTTGGG CTAGCTGGCT AGGCCAAATG AGACATGAAG ATTAAGTTGA AATATATATA TATATGCCAC   
  
  
- ATGGTATGCC AAGAAAATTA TAACATGCAT GTTATTTTTA GAACCGGCTC ATGTTGACTA GTTAATTAAA   
  
  
- AAAAATTATA TAGGTTTTTC AATATTTCAT AATATTAATG TAAAAATAGA ATATAAGAAA ATAAATTTAT   
  
  
- TAATTTTATT AAATTATCAT GTATTATAAT TTTATTTAAA ACTCAAATAA ACAAAAAAAT CTCTTGAGAC   
  
  
- TGATTCTTTT TTATCAGAAA AAAAAATGAT TATAATTAAA CTAACTCTTT TGACCTTAGA GCAATGCTAT   
  
  
- TGAGCCCAAA TCCATATTTA TTTGATCATG TCACCTTGTT GTTGATTATG TTTCTAATCA ATGGGGCTCC   
  
  
- AATATATGAA AAAATGGCAG ATAGTGCCAA TTTTCTATGG TCATTTATAT GACTGCAAAT TATGAACACA   
  
  
- GCAGAAACAT CAGCAAATAA AGCAACTAGA ATTCATAATC CATATCTTAT CCATAAACCT ACATGATGGT   
  
  
- AAGTGAAGAA GCTAGATATA AAGATTGCAT GCATATGCGT ACAAGGAAGA ATTAAAGGAC AAAGAAACAA   
  
  
- CCACCCATCT TGCTCCCCAT GCTCATCTT

+     Sp1

| Site Name | Organism | Position | Strand | Matrix score. | sequence | function |
| --- | --- | --- | --- | --- | --- | --- |
| Sp1 | Zea mays | 185 | - | 5 | CC(G/A)CCC | light responsive element |

> 2018/04/13 10:10:12  
+ ATCCCAATTG GTCAATTCTT TCCGACTTTT CGGAAGTGTA ACTGTTCCTT AAATAAATTC TTCTCTCAAC   
  
  
+ GGTTTATCAG AGTACGTACG CTTCTCGTTA TGACATTCTA AAAATTCTGT TTTGAAACCC AATTTAAGTA   
  
  
+ TCGCTAGCAA CTTCGGTTGA TGTGAAGTGG ATGAAAGACA AGTTGGGGGG AACACTTTTG TTTCTGCATG   
  
  
+ CACGTACGGG TTTTTAGGGT AGCAGTAGTA GCTTCGTACG TTCTTTTGAG TTATAGAGAG AGAGAGAGAG   
  
  
+ AGAGTCTATT TGTGTGTTCT ACTTCGTCTC TCTCTCTCTG TCTCTCTCTC TCTCTCTCAT TCAAATTAGG   
  
  
+ TATTACTTCA TTTTCATACA ACTATACTGT CTACTAACCG AGACTTTACC GAATTTCATG TCCCAAATCT   
  
  
+ ATAACAATTT GCCGATTAGG TCATAGACTC CAGACCAACA ACTCCATCAA ACCTTATTAA GACCTGACTA   
  
  
+ TCTGACTAAA GTCCGCTAGT TATGTTGGGA AAGGAATCCC GTGTACGTAT CAAGATACAT CAAAGGTATA   
  
  
+ CATCTAGTTC AACGATTATA AAGTACATAT GAACAGCACA CTTTACTTTA CTTTACTAGT TATACTATTC   
  
  
+ AATAATATTA TTTCGACACC TTCTCGGTCT CAAGATCTAC TAGTAAAGAA TGCCTTCTAC GAACAAAACA   
  
  
+ TTCAAGCAAT TAAGATCTCA ACTTCCATTA ATTGTGGTAG AAATAGAAGA ACAAACCTCC AAATGATCAT   
  
  
+ TGTACGTTAA CAATAATATG AAACCGTTTG TTCGTTACGT TTTTATTCTT TAATAACGGG TACGTGTACT   
  
  
+ CTCAAAACCC GATCGACCGA TCCGGTTTAC TCTGTACTTC TAATTCAACT TTATATATAT ATATACGGTG   
  
  
+ TACCATACGG TTCTTTTAAT ATTGTACGTA CAATAAAAAT CTTGGCCGAG TACAACTGAT CAATTAATTT   
  
  
+ TTTTTAATAT ATCCAAAAAG TTATAAAGTA TTATAATTAC ATTTTTATCT TATATTCTTT TATTTAAATA   
  
  
+ ATTAAAATAA TTTAATAGTA CATAATATTA AAATAAATTT TGAGTTTATT TGTTTTTTTA GAGAACTCTG   
  
  
+ ACTAAGAAAA AATAGTCTTT TTTTTTACTA ATATTAATTT GATTGAGAAA ACTGGAATCT CGTTACGATA   
  
  
+ ACTCGGGTTT AGGTATAAAT AAACTAGTAC AGTGGAACAA CAACTAATAC AAAGATTAGT TACCCCGAGG   
  
  
+ TTATATACTT TTTTACCGTC TATCACGGTT AAAAGATACC AGTAAATATA CTGACGTTTA ATACTTGTGT   
  
  
+ CGTCTTTGTA GTCGTTTATT TCGTTGATCT TAAGTATTAG GTATAGAATA GGTATTTGGA TGTACTACCA   
  
  
+ TTCACTTCTT CGATCTATAT TTCTAACGTA CGTATACGCA TGTTCCTTCT TAATTTCCTG TTTCTTTGTT   
  
  
+ GGTGGGTAGA ACGAGGGGTA CGAGTAGAA  

- TAGGGTTAAC CAGTTAAGAA AGGCTGAAAA GCCTTCACAT TGACAAGGAA TTTATTTAAG AAGAGAGTTG   
  
  
- CCAAATAGTC TCATGCATGC GAAGAGCAAT ACTGTAAGAT TTTTAAGACA AAACTTTGGG TTAAATTCAT   
  
  
- AGCGATCGTT GAAGCCAACT ACACTTCACC TACTTTCTGT TCAACCCCCC TTGTGAAAAC AAAGACGTAC   
  
  
- GTGCATGCCC AAAAATCCCA TCGTCATCAT CGAAGCATGC AAGAAAACTC AATATCTCTC TCTCTCTCTC   
  
  
- TCTCAGATAA ACACACAAGA TGAAGCAGAG AGAGAGAGAC AGAGAGAGAG AGAGAGAGTA AGTTTAATCC   
  
  
- ATAATGAAGT AAAAGTATGT TGATATGACA GATGATTGGC TCTGAAATGG CTTAAAGTAC AGGGTTTAGA   
  
  
- TATTGTTAAA CGGCTAATCC AGTATCTGAG GTCTGGTTGT TGAGGTAGTT TGGAATAATT CTGGACTGAT   
  
  
- AGACTGATTT CAGGCGATCA ATACAACCCT TTCCTTAGGG CACATGCATA GTTCTATGTA GTTTCCATAT   
  
  
- GTAGATCAAG TTGCTAATAT TTCATGTATA CTTGTCGTGT GAAATGAAAT GAAATGATCA ATATGATAAG   
  
  
- TTATTATAAT AAAGCTGTGG AAGAGCCAGA GTTCTAGATG ATCATTTCTT ACGGAAGATG CTTGTTTTGT   
  
  
- AAGTTCGTTA ATTCTAGAGT TGAAGGTAAT TAACACCATC TTTATCTTCT TGTTTGGAGG TTTACTAGTA   
  
  
- ACATGCAATT GTTATTATAC TTTGGCAAAC AAGCAATGCA AAAATAAGAA ATTATTGCCC ATGCACATGA   
  
  
- GAGTTTTGGG CTAGCTGGCT AGGCCAAATG AGACATGAAG ATTAAGTTGA AATATATATA TATATGCCAC   
  
  
- ATGGTATGCC AAGAAAATTA TAACATGCAT GTTATTTTTA GAACCGGCTC ATGTTGACTA GTTAATTAAA   
  
  
- AAAAATTATA TAGGTTTTTC AATATTTCAT AATATTAATG TAAAAATAGA ATATAAGAAA ATAAATTTAT   
  
  
- TAATTTTATT AAATTATCAT GTATTATAAT TTTATTTAAA ACTCAAATAA ACAAAAAAAT CTCTTGAGAC   
  
  
- TGATTCTTTT TTATCAGAAA AAAAAATGAT TATAATTAAA CTAACTCTTT TGACCTTAGA GCAATGCTAT   
  
  
- TGAGCCCAAA TCCATATTTA TTTGATCATG TCACCTTGTT GTTGATTATG TTTCTAATCA ATGGGGCTCC   
  
  
- AATATATGAA AAAATGGCAG ATAGTGCCAA TTTTCTATGG TCATTTATAT GACTGCAAAT TATGAACACA   
  
  
- GCAGAAACAT CAGCAAATAA AGCAACTAGA ATTCATAATC CATATCTTAT CCATAAACCT ACATGATGGT   
  
  
- AAGTGAAGAA GCTAGATATA AAGATTGCAT GCATATGCGT ACAAGGAAGA ATTAAAGGAC AAAGAAACAA   
  
  
- CCACCCATCT TGCTCCCCAT GCTCATCTT

+     TATA-box

| Site Name | Organism | Position | Strand | Matrix score. | sequence | function |
| --- | --- | --- | --- | --- | --- | --- |
| TATA-box | Arabidopsis thaliana | 1264 | - | 4 | TATA | core promoter element around -30 of transcription start |
| TATA-box | Lycopersicon esculentum | 1023 | + | 5 | TTTTA | core promoter element around -30 of transcription start |
| TATA-box | Arabidopsis thaliana | 898 | - | 8 | TATATATA | core promoter element around -30 of transcription start |
| TATA-box | Glycine max | 1149 | + | 5 | TAATA | core promoter element around -30 of transcription start |
| TATA-box | Arabidopsis thaliana | 1372 | - | 4 | TATA | core promoter element around -30 of transcription start |
| TATA-box | Arabidopsis thaliana | 1011 | - | 5 | TATAA | core promoter element around -30 of transcription start |
| TATA-box | Arabidopsis thaliana | 892 | - | 8 | TATATATA | core promoter element around -30 of transcription start |
| TATA-box | Brassica napus | 897 | - | 6 | ATATAT | core promoter element around -30 of transcription start |
| TATA-box | Arabidopsis thaliana | 896 | - | 8 | TATATATA | core promoter element around -30 of transcription start |
| TATA-box | Arabidopsis thaliana | 888 | - | 9 | taTATAAAgg | core promoter element around -30 of transcription start |
| TATA-box | Arabidopsis thaliana | 1012 | - | 4 | TATA | core promoter element around -30 of transcription start |
| TATA-box | Arabidopsis thaliana | 1043 | - | 8 | TATTTAAA | core promoter element around -30 of transcription start |
| TATA-box | Lycopersicon esculentum | 944 | - | 5 | TTTTA | core promoter element around -30 of transcription start |
| TATA-box | Lycopersicon esculentum | 811 | + | 5 | TTTTA | core promoter element around -30 of transcription start |
| TATA-box | Arabidopsis thaliana | 891 | - | 7 | TATATAA | core promoter element around -30 of transcription start |
| TATA-box | Glycine max | 1009 | - | 5 | TAATA | core promoter element around -30 of transcription start |
| TATA-box | Arabidopsis thaliana | 988 | - | 4 | TATA | core promoter element around -30 of transcription start |
| TATA-box | Glycine max | 1152 | - | 5 | TAATA | core promoter element around -30 of transcription start |
| TATA-box | Lycopersicon esculentum | 1038 | + | 5 | TTTTA | core promoter element around -30 of transcription start |
| TATA-box | Arabidopsis thaliana | 1031 | - | 4 | TATA | core promoter element around -30 of transcription start |
| TATA-box | Glycine max | 1076 | - | 5 | TAATA | core promoter element around -30 of transcription start |
| TATA-box | Glycine max | 1073 | + | 5 | TAATA | core promoter element around -30 of transcription start |
| TATA-box | Brassica napus | 895 | - | 6 | ATATAT | core promoter element around -30 of transcription start |
| TATA-box | Arabidopsis thaliana | 1416 | - | 4 | TATA | core promoter element around -30 of transcription start |
| TATA-box | Arabidopsis thaliana | 890 | - | 11 | TATAAATATAAA | core promoter element around -30 of transcription start |
| TATA-box | Glycine max | 784 | + | 5 | TAATA | core promoter element around -30 of transcription start |
| TATA-box | Arabidopsis thaliana | 1030 | - | 5 | TATAA | core promoter element around -30 of transcription start |
| TATA-box | Brassica napus | 987 | - | 6 | ATATAT | core promoter element around -30 of transcription start |
| TATA-box | Lycopersicon esculentum | 982 | + | 5 | TTTTA | core promoter element around -30 of transcription start |
| TATA-box | Arabidopsis thaliana | 1002 | + | 6 | TATAAA | core promoter element around -30 of transcription start |
| TATA-box | Arabidopsis thaliana | 1307 | - | 4 | TATA | core promoter element around -30 of transcription start |
| TATA-box | Lycopersicon esculentum | 1053 | - | 5 | TTTTA | core promoter element around -30 of transcription start |
| TATA-box | Lycopersicon esculentum | 1290 | - | 5 | TTTTA | core promoter element around -30 of transcription start |
| TATA-box | Brassica napus | 1010 | + | 6 | ATTATA | core promoter element around -30 of transcription start |
| TATA-box | Glycine max | 1365 | - | 5 | TAATA | core promoter element around -30 of transcription start |
| TATA-box | Glycine max | 985 | + | 5 | TAATA | core promoter element around -30 of transcription start |
| TATA-box | Lycopersicon esculentum | 889 | - | 9 | taTATAAAg | core promoter element around -30 of transcription start |
| TATA-box | Arabidopsis thaliana | 902 | - | 4 | TATA | core promoter element around -30 of transcription start |
| TATA-box | Arabidopsis thaliana | 1041 | + | 8 | TATTTAAA | core promoter element around -30 of transcription start |
| TATA-box | Arabidopsis thaliana | 894 | - | 8 | TATATATA | core promoter element around -30 of transcription start |
| TATA-box | Arabidopsis thaliana | 1262 | - | 4 | TATA | core promoter element around -30 of transcription start |
| TATA-box | Arabidopsis thaliana | 900 | - | 4 | TATA | core promoter element around -30 of transcription start |
| TATA-box | Brassica napus | 899 | - | 6 | ATATAT | core promoter element around -30 of transcription start |
| TATA-box | Glycine max | 821 | + | 5 | TAATA | core promoter element around -30 of transcription start |
| TATA-box | Arabidopsis thaliana | 577 | + | 6 | TATAAA | core promoter element around -30 of transcription start |
| TATA-box | Glycine max | 351 | - | 5 | TAATA | core promoter element around -30 of transcription start |
| TATA-box | Glycine max | 1063 | + | 5 | TAATA | core promoter element around -30 of transcription start |
| TATA-box | Brassica napus | 893 | - | 6 | ATATAT | core promoter element around -30 of transcription start |
| TATA-box | Lycopersicon esculentum | 1106 | + | 5 | TTTTA | core promoter element around -30 of transcription start |
| TATA-box | Lycopersicon esculentum | 1079 | - | 5 | TTTTA | core promoter element around -30 of transcription start |
| TATA-box | Arabidopsis thaliana | 620 | - | 5 | TATAA | core promoter element around -30 of transcription start |
| TATA-box | Arabidopsis thaliana | 621 | + | 4 | TATA | core promoter element around -30 of transcription start |
| TATA-box | Glycine max | 633 | + | 5 | TAATA | core promoter element around -30 of transcription start |
| TATA-box | Brassica napus | 575 | + | 6 | ATTATA | core promoter element around -30 of transcription start |
| TATA-box | Arabidopsis thaliana | 557 | + | 4 | TATA | core promoter element around -30 of transcription start |
| TATA-box | Arabidopsis thaliana | 1001 | - | 5 | TATAA | core promoter element around -30 of transcription start |
| TATA-box | Ac | 1204 | + | 7 | TATAAAT | core promoter element around -30 of transcription start |
| TATA-box | Arabidopsis thaliana | 1261 | - | 7 | TATATAA | core promoter element around -30 of transcription start |
| TATA-box | Glycine max | 927 | + | 5 | TAATA | core promoter element around -30 of transcription start |
| TATA-box | Arabidopsis thaliana | 576 | - | 5 | TATAA | core promoter element around -30 of transcription start |
| TATA-box | Lycopersicon esculentum | 1143 | + | 5 | TTTTA | core promoter element around -30 of transcription start |
| TATA-box | Lycopersicon esculentum | 924 | + | 5 | TTTTA | core promoter element around -30 of transcription start |
| TATA-box | Glycine max | 1235 | + | 5 | TAATA | core promoter element around -30 of transcription start |
| TATA-box | Arabidopsis thaliana | 373 | + | 4 | TATA | core promoter element around -30 of transcription start |
| TATA-box | Lycopersicon esculentum | 1271 | + | 5 | TTTTA | core promoter element around -30 of transcription start |
| TATA-box | Arabidopsis thaliana | 1433 | - | 4 | TATA | core promoter element around -30 of transcription start |
| TATA-box | Arabidopsis thaliana | 420 | + | 4 | TATA | core promoter element around -30 of transcription start |
| TATA-box | Glycine max | 636 | - | 5 | TAATA | core promoter element around -30 of transcription start |
| TATA-box | Glycine max | 1319 | + | 5 | TAATA | core promoter element around -30 of transcription start |
| TATA-box | Glycine max | 475 | - | 5 | TAATA | core promoter element around -30 of transcription start |
| TATA-box | Lycopersicon esculentum | 109 | - | 5 | TTTTA | core promoter element around -30 of transcription start |
| TATA-box | Lycopersicon esculentum | 222 | + | 5 | TTTTA | core promoter element around -30 of transcription start |
| TATA-box | Arabidopsis thaliana | 261 | - | 5 | TATAA | core promoter element around -30 of transcription start |
| TATA-box | Arabidopsis thaliana | 262 | + | 4 | TATA | core promoter element around -30 of transcription start |

> 2018/04/13 10:10:12  
+ ATCCCAATTG GTCAATTCTT TCCGACTTTT CGGAAGTGTA ACTGTTCCTT AAATAAATTC TTCTCTCAAC   
  
  
+ GGTTTATCAG AGTACGTACG CTTCTCGTTA TGACATTCTA AAAATTCTGT TTTGAAACCC AATTTAAGTA   
  
  
+ TCGCTAGCAA CTTCGGTTGA TGTGAAGTGG ATGAAAGACA AGTTGGGGGG AACACTTTTG TTTCTGCATG   
  
  
+ CACGTACGGG TTTTTAGGGT AGCAGTAGTA GCTTCGTACG TTCTTTTGAG TTATAGAGAG AGAGAGAGAG   
  
  
+ AGAGTCTATT TGTGTGTTCT ACTTCGTCTC TCTCTCTCTG TCTCTCTCTC TCTCTCTCAT TCAAATTAGG   
  
  
+ TATTACTTCA TTTTCATACA ACTATACTGT CTACTAACCG AGACTTTACC GAATTTCATG TCCCAAATCT   
  
  
+ ATAACAATTT GCCGATTAGG TCATAGACTC CAGACCAACA ACTCCATCAA ACCTTATTAA GACCTGACTA   
  
  
+ TCTGACTAAA GTCCGCTAGT TATGTTGGGA AAGGAATCCC GTGTACGTAT CAAGATACAT CAAAGGTATA   
  
  
+ CATCTAGTTC AACGATTATA AAGTACATAT GAACAGCACA CTTTACTTTA CTTTACTAGT TATACTATTC   
  
  
+ AATAATATTA TTTCGACACC TTCTCGGTCT CAAGATCTAC TAGTAAAGAA TGCCTTCTAC GAACAAAACA   
  
  
+ TTCAAGCAAT TAAGATCTCA ACTTCCATTA ATTGTGGTAG AAATAGAAGA ACAAACCTCC AAATGATCAT   
  
  
+ TGTACGTTAA CAATAATATG AAACCGTTTG TTCGTTACGT TTTTATTCTT TAATAACGGG TACGTGTACT   
  
  
+ CTCAAAACCC GATCGACCGA TCCGGTTTAC TCTGTACTTC TAATTCAACT TTATATATAT ATATACGGTG   
  
  
+ TACCATACGG TTCTTTTAAT ATTGTACGTA CAATAAAAAT CTTGGCCGAG TACAACTGAT CAATTAATTT   
  
  
+ TTTTTAATAT ATCCAAAAAG TTATAAAGTA TTATAATTAC ATTTTTATCT TATATTCTTT TATTTAAATA   
  
  
+ ATTAAAATAA TTTAATAGTA CATAATATTA AAATAAATTT TGAGTTTATT TGTTTTTTTA GAGAACTCTG   
  
  
+ ACTAAGAAAA AATAGTCTTT TTTTTTACTA ATATTAATTT GATTGAGAAA ACTGGAATCT CGTTACGATA   
  
  
+ ACTCGGGTTT AGGTATAAAT AAACTAGTAC AGTGGAACAA CAACTAATAC AAAGATTAGT TACCCCGAGG   
  
  
+ TTATATACTT TTTTACCGTC TATCACGGTT AAAAGATACC AGTAAATATA CTGACGTTTA ATACTTGTGT   
  
  
+ CGTCTTTGTA GTCGTTTATT TCGTTGATCT TAAGTATTAG GTATAGAATA GGTATTTGGA TGTACTACCA   
  
  
+ TTCACTTCTT CGATCTATAT TTCTAACGTA CGTATACGCA TGTTCCTTCT TAATTTCCTG TTTCTTTGTT   
  
  
+ GGTGGGTAGA ACGAGGGGTA CGAGTAGAA  

- TAGGGTTAAC CAGTTAAGAA AGGCTGAAAA GCCTTCACAT TGACAAGGAA TTTATTTAAG AAGAGAGTTG   
  
  
- CCAAATAGTC TCATGCATGC GAAGAGCAAT ACTGTAAGAT TTTTAAGACA AAACTTTGGG TTAAATTCAT   
  
  
- AGCGATCGTT GAAGCCAACT ACACTTCACC TACTTTCTGT TCAACCCCCC TTGTGAAAAC AAAGACGTAC   
  
  
- GTGCATGCCC AAAAATCCCA TCGTCATCAT CGAAGCATGC AAGAAAACTC AATATCTCTC TCTCTCTCTC   
  
  
- TCTCAGATAA ACACACAAGA TGAAGCAGAG AGAGAGAGAC AGAGAGAGAG AGAGAGAGTA AGTTTAATCC   
  
  
- ATAATGAAGT AAAAGTATGT TGATATGACA GATGATTGGC TCTGAAATGG CTTAAAGTAC AGGGTTTAGA   
  
  
- TATTGTTAAA CGGCTAATCC AGTATCTGAG GTCTGGTTGT TGAGGTAGTT TGGAATAATT CTGGACTGAT   
  
  
- AGACTGATTT CAGGCGATCA ATACAACCCT TTCCTTAGGG CACATGCATA GTTCTATGTA GTTTCCATAT   
  
  
- GTAGATCAAG TTGCTAATAT TTCATGTATA CTTGTCGTGT GAAATGAAAT GAAATGATCA ATATGATAAG   
  
  
- TTATTATAAT AAAGCTGTGG AAGAGCCAGA GTTCTAGATG ATCATTTCTT ACGGAAGATG CTTGTTTTGT   
  
  
- AAGTTCGTTA ATTCTAGAGT TGAAGGTAAT TAACACCATC TTTATCTTCT TGTTTGGAGG TTTACTAGTA   
  
  
- ACATGCAATT GTTATTATAC TTTGGCAAAC AAGCAATGCA AAAATAAGAA ATTATTGCCC ATGCACATGA   
  
  
- GAGTTTTGGG CTAGCTGGCT AGGCCAAATG AGACATGAAG ATTAAGTTGA AATATATATA TATATGCCAC   
  
  
- ATGGTATGCC AAGAAAATTA TAACATGCAT GTTATTTTTA GAACCGGCTC ATGTTGACTA GTTAATTAAA   
  
  
- AAAAATTATA TAGGTTTTTC AATATTTCAT AATATTAATG TAAAAATAGA ATATAAGAAA ATAAATTTAT   
  
  
- TAATTTTATT AAATTATCAT GTATTATAAT TTTATTTAAA ACTCAAATAA ACAAAAAAAT CTCTTGAGAC   
  
  
- TGATTCTTTT TTATCAGAAA AAAAAATGAT TATAATTAAA CTAACTCTTT TGACCTTAGA GCAATGCTAT   
  
  
- TGAGCCCAAA TCCATATTTA TTTGATCATG TCACCTTGTT GTTGATTATG TTTCTAATCA ATGGGGCTCC   
  
  
- AATATATGAA AAAATGGCAG ATAGTGCCAA TTTTCTATGG TCATTTATAT GACTGCAAAT TATGAACACA   
  
  
- GCAGAAACAT CAGCAAATAA AGCAACTAGA ATTCATAATC CATATCTTAT CCATAAACCT ACATGATGGT   
  
  
- AAGTGAAGAA GCTAGATATA AAGATTGCAT GCATATGCGT ACAAGGAAGA ATTAAAGGAC AAAGAAACAA   
  
  
- CCACCCATCT TGCTCCCCAT GCTCATCTT

+     TCA-element

| Site Name | Organism | Position | Strand | Matrix score. | sequence | function |
| --- | --- | --- | --- | --- | --- | --- |
| TCA-element | Brassica oleracea | 56 | - | 9 | GAGAAGAATA | cis-acting element involved in salicylic acid responsiveness |

> 2018/04/13 10:10:12  
+ ATCCCAATTG GTCAATTCTT TCCGACTTTT CGGAAGTGTA ACTGTTCCTT AAATAAATTC TTCTCTCAAC   
  
  
+ GGTTTATCAG AGTACGTACG CTTCTCGTTA TGACATTCTA AAAATTCTGT TTTGAAACCC AATTTAAGTA   
  
  
+ TCGCTAGCAA CTTCGGTTGA TGTGAAGTGG ATGAAAGACA AGTTGGGGGG AACACTTTTG TTTCTGCATG   
  
  
+ CACGTACGGG TTTTTAGGGT AGCAGTAGTA GCTTCGTACG TTCTTTTGAG TTATAGAGAG AGAGAGAGAG   
  
  
+ AGAGTCTATT TGTGTGTTCT ACTTCGTCTC TCTCTCTCTG TCTCTCTCTC TCTCTCTCAT TCAAATTAGG   
  
  
+ TATTACTTCA TTTTCATACA ACTATACTGT CTACTAACCG AGACTTTACC GAATTTCATG TCCCAAATCT   
  
  
+ ATAACAATTT GCCGATTAGG TCATAGACTC CAGACCAACA ACTCCATCAA ACCTTATTAA GACCTGACTA   
  
  
+ TCTGACTAAA GTCCGCTAGT TATGTTGGGA AAGGAATCCC GTGTACGTAT CAAGATACAT CAAAGGTATA   
  
  
+ CATCTAGTTC AACGATTATA AAGTACATAT GAACAGCACA CTTTACTTTA CTTTACTAGT TATACTATTC   
  
  
+ AATAATATTA TTTCGACACC TTCTCGGTCT CAAGATCTAC TAGTAAAGAA TGCCTTCTAC GAACAAAACA   
  
  
+ TTCAAGCAAT TAAGATCTCA ACTTCCATTA ATTGTGGTAG AAATAGAAGA ACAAACCTCC AAATGATCAT   
  
  
+ TGTACGTTAA CAATAATATG AAACCGTTTG TTCGTTACGT TTTTATTCTT TAATAACGGG TACGTGTACT   
  
  
+ CTCAAAACCC GATCGACCGA TCCGGTTTAC TCTGTACTTC TAATTCAACT TTATATATAT ATATACGGTG   
  
  
+ TACCATACGG TTCTTTTAAT ATTGTACGTA CAATAAAAAT CTTGGCCGAG TACAACTGAT CAATTAATTT   
  
  
+ TTTTTAATAT ATCCAAAAAG TTATAAAGTA TTATAATTAC ATTTTTATCT TATATTCTTT TATTTAAATA   
  
  
+ ATTAAAATAA TTTAATAGTA CATAATATTA AAATAAATTT TGAGTTTATT TGTTTTTTTA GAGAACTCTG   
  
  
+ ACTAAGAAAA AATAGTCTTT TTTTTTACTA ATATTAATTT GATTGAGAAA ACTGGAATCT CGTTACGATA   
  
  
+ ACTCGGGTTT AGGTATAAAT AAACTAGTAC AGTGGAACAA CAACTAATAC AAAGATTAGT TACCCCGAGG   
  
  
+ TTATATACTT TTTTACCGTC TATCACGGTT AAAAGATACC AGTAAATATA CTGACGTTTA ATACTTGTGT   
  
  
+ CGTCTTTGTA GTCGTTTATT TCGTTGATCT TAAGTATTAG GTATAGAATA GGTATTTGGA TGTACTACCA   
  
  
+ TTCACTTCTT CGATCTATAT TTCTAACGTA CGTATACGCA TGTTCCTTCT TAATTTCCTG TTTCTTTGTT   
  
  
+ GGTGGGTAGA ACGAGGGGTA CGAGTAGAA  

- TAGGGTTAAC CAGTTAAGAA AGGCTGAAAA GCCTTCACAT TGACAAGGAA TTTATTTAAG AAGAGAGTTG   
  
  
- CCAAATAGTC TCATGCATGC GAAGAGCAAT ACTGTAAGAT TTTTAAGACA AAACTTTGGG TTAAATTCAT   
  
  
- AGCGATCGTT GAAGCCAACT ACACTTCACC TACTTTCTGT TCAACCCCCC TTGTGAAAAC AAAGACGTAC   
  
  
- GTGCATGCCC AAAAATCCCA TCGTCATCAT CGAAGCATGC AAGAAAACTC AATATCTCTC TCTCTCTCTC   
  
  
- TCTCAGATAA ACACACAAGA TGAAGCAGAG AGAGAGAGAC AGAGAGAGAG AGAGAGAGTA AGTTTAATCC   
  
  
- ATAATGAAGT AAAAGTATGT TGATATGACA GATGATTGGC TCTGAAATGG CTTAAAGTAC AGGGTTTAGA   
  
  
- TATTGTTAAA CGGCTAATCC AGTATCTGAG GTCTGGTTGT TGAGGTAGTT TGGAATAATT CTGGACTGAT   
  
  
- AGACTGATTT CAGGCGATCA ATACAACCCT TTCCTTAGGG CACATGCATA GTTCTATGTA GTTTCCATAT   
  
  
- GTAGATCAAG TTGCTAATAT TTCATGTATA CTTGTCGTGT GAAATGAAAT GAAATGATCA ATATGATAAG   
  
  
- TTATTATAAT AAAGCTGTGG AAGAGCCAGA GTTCTAGATG ATCATTTCTT ACGGAAGATG CTTGTTTTGT   
  
  
- AAGTTCGTTA ATTCTAGAGT TGAAGGTAAT TAACACCATC TTTATCTTCT TGTTTGGAGG TTTACTAGTA   
  
  
- ACATGCAATT GTTATTATAC TTTGGCAAAC AAGCAATGCA AAAATAAGAA ATTATTGCCC ATGCACATGA   
  
  
- GAGTTTTGGG CTAGCTGGCT AGGCCAAATG AGACATGAAG ATTAAGTTGA AATATATATA TATATGCCAC   
  
  
- ATGGTATGCC AAGAAAATTA TAACATGCAT GTTATTTTTA GAACCGGCTC ATGTTGACTA GTTAATTAAA   
  
  
- AAAAATTATA TAGGTTTTTC AATATTTCAT AATATTAATG TAAAAATAGA ATATAAGAAA ATAAATTTAT   
  
  
- TAATTTTATT AAATTATCAT GTATTATAAT TTTATTTAAA ACTCAAATAA ACAAAAAAAT CTCTTGAGAC   
  
  
- TGATTCTTTT TTATCAGAAA AAAAAATGAT TATAATTAAA CTAACTCTTT TGACCTTAGA GCAATGCTAT   
  
  
- TGAGCCCAAA TCCATATTTA TTTGATCATG TCACCTTGTT GTTGATTATG TTTCTAATCA ATGGGGCTCC   
  
  
- AATATATGAA AAAATGGCAG ATAGTGCCAA TTTTCTATGG TCATTTATAT GACTGCAAAT TATGAACACA   
  
  
- GCAGAAACAT CAGCAAATAA AGCAACTAGA ATTCATAATC CATATCTTAT CCATAAACCT ACATGATGGT   
  
  
- AAGTGAAGAA GCTAGATATA AAGATTGCAT GCATATGCGT ACAAGGAAGA ATTAAAGGAC AAAGAAACAA   
  
  
- CCACCCATCT TGCTCCCCAT GCTCATCTT

+     TGA-element

| Site Name | Organism | Position | Strand | Matrix score. | sequence | function |
| --- | --- | --- | --- | --- | --- | --- |
| TGA-element | Brassica oleracea | 1341 | - | 6 | AACGAC | auxin-responsive element |

> 2018/04/13 10:10:12  
+ ATCCCAATTG GTCAATTCTT TCCGACTTTT CGGAAGTGTA ACTGTTCCTT AAATAAATTC TTCTCTCAAC   
  
  
+ GGTTTATCAG AGTACGTACG CTTCTCGTTA TGACATTCTA AAAATTCTGT TTTGAAACCC AATTTAAGTA   
  
  
+ TCGCTAGCAA CTTCGGTTGA TGTGAAGTGG ATGAAAGACA AGTTGGGGGG AACACTTTTG TTTCTGCATG   
  
  
+ CACGTACGGG TTTTTAGGGT AGCAGTAGTA GCTTCGTACG TTCTTTTGAG TTATAGAGAG AGAGAGAGAG   
  
  
+ AGAGTCTATT TGTGTGTTCT ACTTCGTCTC TCTCTCTCTG TCTCTCTCTC TCTCTCTCAT TCAAATTAGG   
  
  
+ TATTACTTCA TTTTCATACA ACTATACTGT CTACTAACCG AGACTTTACC GAATTTCATG TCCCAAATCT   
  
  
+ ATAACAATTT GCCGATTAGG TCATAGACTC CAGACCAACA ACTCCATCAA ACCTTATTAA GACCTGACTA   
  
  
+ TCTGACTAAA GTCCGCTAGT TATGTTGGGA AAGGAATCCC GTGTACGTAT CAAGATACAT CAAAGGTATA   
  
  
+ CATCTAGTTC AACGATTATA AAGTACATAT GAACAGCACA CTTTACTTTA CTTTACTAGT TATACTATTC   
  
  
+ AATAATATTA TTTCGACACC TTCTCGGTCT CAAGATCTAC TAGTAAAGAA TGCCTTCTAC GAACAAAACA   
  
  
+ TTCAAGCAAT TAAGATCTCA ACTTCCATTA ATTGTGGTAG AAATAGAAGA ACAAACCTCC AAATGATCAT   
  
  
+ TGTACGTTAA CAATAATATG AAACCGTTTG TTCGTTACGT TTTTATTCTT TAATAACGGG TACGTGTACT   
  
  
+ CTCAAAACCC GATCGACCGA TCCGGTTTAC TCTGTACTTC TAATTCAACT TTATATATAT ATATACGGTG   
  
  
+ TACCATACGG TTCTTTTAAT ATTGTACGTA CAATAAAAAT CTTGGCCGAG TACAACTGAT CAATTAATTT   
  
  
+ TTTTTAATAT ATCCAAAAAG TTATAAAGTA TTATAATTAC ATTTTTATCT TATATTCTTT TATTTAAATA   
  
  
+ ATTAAAATAA TTTAATAGTA CATAATATTA AAATAAATTT TGAGTTTATT TGTTTTTTTA GAGAACTCTG   
  
  
+ ACTAAGAAAA AATAGTCTTT TTTTTTACTA ATATTAATTT GATTGAGAAA ACTGGAATCT CGTTACGATA   
  
  
+ ACTCGGGTTT AGGTATAAAT AAACTAGTAC AGTGGAACAA CAACTAATAC AAAGATTAGT TACCCCGAGG   
  
  
+ TTATATACTT TTTTACCGTC TATCACGGTT AAAAGATACC AGTAAATATA CTGACGTTTA ATACTTGTGT   
  
  
+ CGTCTTTGTA GTCGTTTATT TCGTTGATCT TAAGTATTAG GTATAGAATA GGTATTTGGA TGTACTACCA   
  
  
+ TTCACTTCTT CGATCTATAT TTCTAACGTA CGTATACGCA TGTTCCTTCT TAATTTCCTG TTTCTTTGTT   
  
  
+ GGTGGGTAGA ACGAGGGGTA CGAGTAGAA  

- TAGGGTTAAC CAGTTAAGAA AGGCTGAAAA GCCTTCACAT TGACAAGGAA TTTATTTAAG AAGAGAGTTG   
  
  
- CCAAATAGTC TCATGCATGC GAAGAGCAAT ACTGTAAGAT TTTTAAGACA AAACTTTGGG TTAAATTCAT   
  
  
- AGCGATCGTT GAAGCCAACT ACACTTCACC TACTTTCTGT TCAACCCCCC TTGTGAAAAC AAAGACGTAC   
  
  
- GTGCATGCCC AAAAATCCCA TCGTCATCAT CGAAGCATGC AAGAAAACTC AATATCTCTC TCTCTCTCTC   
  
  
- TCTCAGATAA ACACACAAGA TGAAGCAGAG AGAGAGAGAC AGAGAGAGAG AGAGAGAGTA AGTTTAATCC   
  
  
- ATAATGAAGT AAAAGTATGT TGATATGACA GATGATTGGC TCTGAAATGG CTTAAAGTAC AGGGTTTAGA   
  
  
- TATTGTTAAA CGGCTAATCC AGTATCTGAG GTCTGGTTGT TGAGGTAGTT TGGAATAATT CTGGACTGAT   
  
  
- AGACTGATTT CAGGCGATCA ATACAACCCT TTCCTTAGGG CACATGCATA GTTCTATGTA GTTTCCATAT   
  
  
- GTAGATCAAG TTGCTAATAT TTCATGTATA CTTGTCGTGT GAAATGAAAT GAAATGATCA ATATGATAAG   
  
  
- TTATTATAAT AAAGCTGTGG AAGAGCCAGA GTTCTAGATG ATCATTTCTT ACGGAAGATG CTTGTTTTGT   
  
  
- AAGTTCGTTA ATTCTAGAGT TGAAGGTAAT TAACACCATC TTTATCTTCT TGTTTGGAGG TTTACTAGTA   
  
  
- ACATGCAATT GTTATTATAC TTTGGCAAAC AAGCAATGCA AAAATAAGAA ATTATTGCCC ATGCACATGA   
  
  
- GAGTTTTGGG CTAGCTGGCT AGGCCAAATG AGACATGAAG ATTAAGTTGA AATATATATA TATATGCCAC   
  
  
- ATGGTATGCC AAGAAAATTA TAACATGCAT GTTATTTTTA GAACCGGCTC ATGTTGACTA GTTAATTAAA   
  
  
- AAAAATTATA TAGGTTTTTC AATATTTCAT AATATTAATG TAAAAATAGA ATATAAGAAA ATAAATTTAT   
  
  
- TAATTTTATT AAATTATCAT GTATTATAAT TTTATTTAAA ACTCAAATAA ACAAAAAAAT CTCTTGAGAC   
  
  
- TGATTCTTTT TTATCAGAAA AAAAAATGAT TATAATTAAA CTAACTCTTT TGACCTTAGA GCAATGCTAT   
  
  
- TGAGCCCAAA TCCATATTTA TTTGATCATG TCACCTTGTT GTTGATTATG TTTCTAATCA ATGGGGCTCC   
  
  
- AATATATGAA AAAATGGCAG ATAGTGCCAA TTTTCTATGG TCATTTATAT GACTGCAAAT TATGAACACA   
  
  
- GCAGAAACAT CAGCAAATAA AGCAACTAGA ATTCATAATC CATATCTTAT CCATAAACCT ACATGATGGT   
  
  
- AAGTGAAGAA GCTAGATATA AAGATTGCAT GCATATGCGT ACAAGGAAGA ATTAAAGGAC AAAGAAACAA   
  
  
- CCACCCATCT TGCTCCCCAT GCTCATCTT

+     TGACG-motif

| Site Name | Organism | Position | Strand | Matrix score. | sequence | function |
| --- | --- | --- | --- | --- | --- | --- |
| TGACG-motif | Hordeum vulgare | 1312 | + | 5 | TGACG | cis-acting regulatory element involved in the MeJA-responsiveness |

> 2018/04/13 10:10:12  
+ ATCCCAATTG GTCAATTCTT TCCGACTTTT CGGAAGTGTA ACTGTTCCTT AAATAAATTC TTCTCTCAAC   
  
  
+ GGTTTATCAG AGTACGTACG CTTCTCGTTA TGACATTCTA AAAATTCTGT TTTGAAACCC AATTTAAGTA   
  
  
+ TCGCTAGCAA CTTCGGTTGA TGTGAAGTGG ATGAAAGACA AGTTGGGGGG AACACTTTTG TTTCTGCATG   
  
  
+ CACGTACGGG TTTTTAGGGT AGCAGTAGTA GCTTCGTACG TTCTTTTGAG TTATAGAGAG AGAGAGAGAG   
  
  
+ AGAGTCTATT TGTGTGTTCT ACTTCGTCTC TCTCTCTCTG TCTCTCTCTC TCTCTCTCAT TCAAATTAGG   
  
  
+ TATTACTTCA TTTTCATACA ACTATACTGT CTACTAACCG AGACTTTACC GAATTTCATG TCCCAAATCT   
  
  
+ ATAACAATTT GCCGATTAGG TCATAGACTC CAGACCAACA ACTCCATCAA ACCTTATTAA GACCTGACTA   
  
  
+ TCTGACTAAA GTCCGCTAGT TATGTTGGGA AAGGAATCCC GTGTACGTAT CAAGATACAT CAAAGGTATA   
  
  
+ CATCTAGTTC AACGATTATA AAGTACATAT GAACAGCACA CTTTACTTTA CTTTACTAGT TATACTATTC   
  
  
+ AATAATATTA TTTCGACACC TTCTCGGTCT CAAGATCTAC TAGTAAAGAA TGCCTTCTAC GAACAAAACA   
  
  
+ TTCAAGCAAT TAAGATCTCA ACTTCCATTA ATTGTGGTAG AAATAGAAGA ACAAACCTCC AAATGATCAT   
  
  
+ TGTACGTTAA CAATAATATG AAACCGTTTG TTCGTTACGT TTTTATTCTT TAATAACGGG TACGTGTACT   
  
  
+ CTCAAAACCC GATCGACCGA TCCGGTTTAC TCTGTACTTC TAATTCAACT TTATATATAT ATATACGGTG   
  
  
+ TACCATACGG TTCTTTTAAT ATTGTACGTA CAATAAAAAT CTTGGCCGAG TACAACTGAT CAATTAATTT   
  
  
+ TTTTTAATAT ATCCAAAAAG TTATAAAGTA TTATAATTAC ATTTTTATCT TATATTCTTT TATTTAAATA   
  
  
+ ATTAAAATAA TTTAATAGTA CATAATATTA AAATAAATTT TGAGTTTATT TGTTTTTTTA GAGAACTCTG   
  
  
+ ACTAAGAAAA AATAGTCTTT TTTTTTACTA ATATTAATTT GATTGAGAAA ACTGGAATCT CGTTACGATA   
  
  
+ ACTCGGGTTT AGGTATAAAT AAACTAGTAC AGTGGAACAA CAACTAATAC AAAGATTAGT TACCCCGAGG   
  
  
+ TTATATACTT TTTTACCGTC TATCACGGTT AAAAGATACC AGTAAATATA CTGACGTTTA ATACTTGTGT   
  
  
+ CGTCTTTGTA GTCGTTTATT TCGTTGATCT TAAGTATTAG GTATAGAATA GGTATTTGGA TGTACTACCA   
  
  
+ TTCACTTCTT CGATCTATAT TTCTAACGTA CGTATACGCA TGTTCCTTCT TAATTTCCTG TTTCTTTGTT   
  
  
+ GGTGGGTAGA ACGAGGGGTA CGAGTAGAA  

- TAGGGTTAAC CAGTTAAGAA AGGCTGAAAA GCCTTCACAT TGACAAGGAA TTTATTTAAG AAGAGAGTTG   
  
  
- CCAAATAGTC TCATGCATGC GAAGAGCAAT ACTGTAAGAT TTTTAAGACA AAACTTTGGG TTAAATTCAT   
  
  
- AGCGATCGTT GAAGCCAACT ACACTTCACC TACTTTCTGT TCAACCCCCC TTGTGAAAAC AAAGACGTAC   
  
  
- GTGCATGCCC AAAAATCCCA TCGTCATCAT CGAAGCATGC AAGAAAACTC AATATCTCTC TCTCTCTCTC   
  
  
- TCTCAGATAA ACACACAAGA TGAAGCAGAG AGAGAGAGAC AGAGAGAGAG AGAGAGAGTA AGTTTAATCC   
  
  
- ATAATGAAGT AAAAGTATGT TGATATGACA GATGATTGGC TCTGAAATGG CTTAAAGTAC AGGGTTTAGA   
  
  
- TATTGTTAAA CGGCTAATCC AGTATCTGAG GTCTGGTTGT TGAGGTAGTT TGGAATAATT CTGGACTGAT   
  
  
- AGACTGATTT CAGGCGATCA ATACAACCCT TTCCTTAGGG CACATGCATA GTTCTATGTA GTTTCCATAT   
  
  
- GTAGATCAAG TTGCTAATAT TTCATGTATA CTTGTCGTGT GAAATGAAAT GAAATGATCA ATATGATAAG   
  
  
- TTATTATAAT AAAGCTGTGG AAGAGCCAGA GTTCTAGATG ATCATTTCTT ACGGAAGATG CTTGTTTTGT   
  
  
- AAGTTCGTTA ATTCTAGAGT TGAAGGTAAT TAACACCATC TTTATCTTCT TGTTTGGAGG TTTACTAGTA   
  
  
- ACATGCAATT GTTATTATAC TTTGGCAAAC AAGCAATGCA AAAATAAGAA ATTATTGCCC ATGCACATGA   
  
  
- GAGTTTTGGG CTAGCTGGCT AGGCCAAATG AGACATGAAG ATTAAGTTGA AATATATATA TATATGCCAC   
  
  
- ATGGTATGCC AAGAAAATTA TAACATGCAT GTTATTTTTA GAACCGGCTC ATGTTGACTA GTTAATTAAA   
  
  
- AAAAATTATA TAGGTTTTTC AATATTTCAT AATATTAATG TAAAAATAGA ATATAAGAAA ATAAATTTAT   
  
  
- TAATTTTATT AAATTATCAT GTATTATAAT TTTATTTAAA ACTCAAATAA ACAAAAAAAT CTCTTGAGAC   
  
  
- TGATTCTTTT TTATCAGAAA AAAAAATGAT TATAATTAAA CTAACTCTTT TGACCTTAGA GCAATGCTAT   
  
  
- TGAGCCCAAA TCCATATTTA TTTGATCATG TCACCTTGTT GTTGATTATG TTTCTAATCA ATGGGGCTCC   
  
  
- AATATATGAA AAAATGGCAG ATAGTGCCAA TTTTCTATGG TCATTTATAT GACTGCAAAT TATGAACACA   
  
  
- GCAGAAACAT CAGCAAATAA AGCAACTAGA ATTCATAATC CATATCTTAT CCATAAACCT ACATGATGGT   
  
  
- AAGTGAAGAA GCTAGATATA AAGATTGCAT GCATATGCGT ACAAGGAAGA ATTAAAGGAC AAAGAAACAA   
  
  
- CCACCCATCT TGCTCCCCAT GCTCATCTT

+     Unnamed\_\_1

| Site Name | Organism | Position | Strand | Matrix score. | sequence | function |
| --- | --- | --- | --- | --- | --- | --- |
| Unnamed\_\_1 | Glycine max | 49 | - | 11 | GAATTTAATTAA | 60K protein binding site |

> 2018/04/13 10:10:12  
+ ATCCCAATTG GTCAATTCTT TCCGACTTTT CGGAAGTGTA ACTGTTCCTT AAATAAATTC TTCTCTCAAC   
  
  
+ GGTTTATCAG AGTACGTACG CTTCTCGTTA TGACATTCTA AAAATTCTGT TTTGAAACCC AATTTAAGTA   
  
  
+ TCGCTAGCAA CTTCGGTTGA TGTGAAGTGG ATGAAAGACA AGTTGGGGGG AACACTTTTG TTTCTGCATG   
  
  
+ CACGTACGGG TTTTTAGGGT AGCAGTAGTA GCTTCGTACG TTCTTTTGAG TTATAGAGAG AGAGAGAGAG   
  
  
+ AGAGTCTATT TGTGTGTTCT ACTTCGTCTC TCTCTCTCTG TCTCTCTCTC TCTCTCTCAT TCAAATTAGG   
  
  
+ TATTACTTCA TTTTCATACA ACTATACTGT CTACTAACCG AGACTTTACC GAATTTCATG TCCCAAATCT   
  
  
+ ATAACAATTT GCCGATTAGG TCATAGACTC CAGACCAACA ACTCCATCAA ACCTTATTAA GACCTGACTA   
  
  
+ TCTGACTAAA GTCCGCTAGT TATGTTGGGA AAGGAATCCC GTGTACGTAT CAAGATACAT CAAAGGTATA   
  
  
+ CATCTAGTTC AACGATTATA AAGTACATAT GAACAGCACA CTTTACTTTA CTTTACTAGT TATACTATTC   
  
  
+ AATAATATTA TTTCGACACC TTCTCGGTCT CAAGATCTAC TAGTAAAGAA TGCCTTCTAC GAACAAAACA   
  
  
+ TTCAAGCAAT TAAGATCTCA ACTTCCATTA ATTGTGGTAG AAATAGAAGA ACAAACCTCC AAATGATCAT   
  
  
+ TGTACGTTAA CAATAATATG AAACCGTTTG TTCGTTACGT TTTTATTCTT TAATAACGGG TACGTGTACT   
  
  
+ CTCAAAACCC GATCGACCGA TCCGGTTTAC TCTGTACTTC TAATTCAACT TTATATATAT ATATACGGTG   
  
  
+ TACCATACGG TTCTTTTAAT ATTGTACGTA CAATAAAAAT CTTGGCCGAG TACAACTGAT CAATTAATTT   
  
  
+ TTTTTAATAT ATCCAAAAAG TTATAAAGTA TTATAATTAC ATTTTTATCT TATATTCTTT TATTTAAATA   
  
  
+ ATTAAAATAA TTTAATAGTA CATAATATTA AAATAAATTT TGAGTTTATT TGTTTTTTTA GAGAACTCTG   
  
  
+ ACTAAGAAAA AATAGTCTTT TTTTTTACTA ATATTAATTT GATTGAGAAA ACTGGAATCT CGTTACGATA   
  
  
+ ACTCGGGTTT AGGTATAAAT AAACTAGTAC AGTGGAACAA CAACTAATAC AAAGATTAGT TACCCCGAGG   
  
  
+ TTATATACTT TTTTACCGTC TATCACGGTT AAAAGATACC AGTAAATATA CTGACGTTTA ATACTTGTGT   
  
  
+ CGTCTTTGTA GTCGTTTATT TCGTTGATCT TAAGTATTAG GTATAGAATA GGTATTTGGA TGTACTACCA   
  
  
+ TTCACTTCTT CGATCTATAT TTCTAACGTA CGTATACGCA TGTTCCTTCT TAATTTCCTG TTTCTTTGTT   
  
  
+ GGTGGGTAGA ACGAGGGGTA CGAGTAGAA  

- TAGGGTTAAC CAGTTAAGAA AGGCTGAAAA GCCTTCACAT TGACAAGGAA TTTATTTAAG AAGAGAGTTG   
  
  
- CCAAATAGTC TCATGCATGC GAAGAGCAAT ACTGTAAGAT TTTTAAGACA AAACTTTGGG TTAAATTCAT   
  
  
- AGCGATCGTT GAAGCCAACT ACACTTCACC TACTTTCTGT TCAACCCCCC TTGTGAAAAC AAAGACGTAC   
  
  
- GTGCATGCCC AAAAATCCCA TCGTCATCAT CGAAGCATGC AAGAAAACTC AATATCTCTC TCTCTCTCTC   
  
  
- TCTCAGATAA ACACACAAGA TGAAGCAGAG AGAGAGAGAC AGAGAGAGAG AGAGAGAGTA AGTTTAATCC   
  
  
- ATAATGAAGT AAAAGTATGT TGATATGACA GATGATTGGC TCTGAAATGG CTTAAAGTAC AGGGTTTAGA   
  
  
- TATTGTTAAA CGGCTAATCC AGTATCTGAG GTCTGGTTGT TGAGGTAGTT TGGAATAATT CTGGACTGAT   
  
  
- AGACTGATTT CAGGCGATCA ATACAACCCT TTCCTTAGGG CACATGCATA GTTCTATGTA GTTTCCATAT   
  
  
- GTAGATCAAG TTGCTAATAT TTCATGTATA CTTGTCGTGT GAAATGAAAT GAAATGATCA ATATGATAAG   
  
  
- TTATTATAAT AAAGCTGTGG AAGAGCCAGA GTTCTAGATG ATCATTTCTT ACGGAAGATG CTTGTTTTGT   
  
  
- AAGTTCGTTA ATTCTAGAGT TGAAGGTAAT TAACACCATC TTTATCTTCT TGTTTGGAGG TTTACTAGTA   
  
  
- ACATGCAATT GTTATTATAC TTTGGCAAAC AAGCAATGCA AAAATAAGAA ATTATTGCCC ATGCACATGA   
  
  
- GAGTTTTGGG CTAGCTGGCT AGGCCAAATG AGACATGAAG ATTAAGTTGA AATATATATA TATATGCCAC   
  
  
- ATGGTATGCC AAGAAAATTA TAACATGCAT GTTATTTTTA GAACCGGCTC ATGTTGACTA GTTAATTAAA   
  
  
- AAAAATTATA TAGGTTTTTC AATATTTCAT AATATTAATG TAAAAATAGA ATATAAGAAA ATAAATTTAT   
  
  
- TAATTTTATT AAATTATCAT GTATTATAAT TTTATTTAAA ACTCAAATAA ACAAAAAAAT CTCTTGAGAC   
  
  
- TGATTCTTTT TTATCAGAAA AAAAAATGAT TATAATTAAA CTAACTCTTT TGACCTTAGA GCAATGCTAT   
  
  
- TGAGCCCAAA TCCATATTTA TTTGATCATG TCACCTTGTT GTTGATTATG TTTCTAATCA ATGGGGCTCC   
  
  
- AATATATGAA AAAATGGCAG ATAGTGCCAA TTTTCTATGG TCATTTATAT GACTGCAAAT TATGAACACA   
  
  
- GCAGAAACAT CAGCAAATAA AGCAACTAGA ATTCATAATC CATATCTTAT CCATAAACCT ACATGATGGT   
  
  
- AAGTGAAGAA GCTAGATATA AAGATTGCAT GCATATGCGT ACAAGGAAGA ATTAAAGGAC AAAGAAACAA   
  
  
- CCACCCATCT TGCTCCCCAT GCTCATCTT

+     Unnamed\_\_4

| Site Name | Organism | Position | Strand | Matrix score. | sequence | function |
| --- | --- | --- | --- | --- | --- | --- |
| Unnamed\_\_4 | Petroselinum hortense | 462 | + | 4 | CTCC |  |
| Unnamed\_\_4 | Petroselinum hortense | 757 | + | 4 | CTCC |  |
| Unnamed\_\_4 | Petroselinum hortense | 448 | + | 4 | CTCC |  |

> 2018/04/13 10:10:12  
+ ATCCCAATTG GTCAATTCTT TCCGACTTTT CGGAAGTGTA ACTGTTCCTT AAATAAATTC TTCTCTCAAC   
  
  
+ GGTTTATCAG AGTACGTACG CTTCTCGTTA TGACATTCTA AAAATTCTGT TTTGAAACCC AATTTAAGTA   
  
  
+ TCGCTAGCAA CTTCGGTTGA TGTGAAGTGG ATGAAAGACA AGTTGGGGGG AACACTTTTG TTTCTGCATG   
  
  
+ CACGTACGGG TTTTTAGGGT AGCAGTAGTA GCTTCGTACG TTCTTTTGAG TTATAGAGAG AGAGAGAGAG   
  
  
+ AGAGTCTATT TGTGTGTTCT ACTTCGTCTC TCTCTCTCTG TCTCTCTCTC TCTCTCTCAT TCAAATTAGG   
  
  
+ TATTACTTCA TTTTCATACA ACTATACTGT CTACTAACCG AGACTTTACC GAATTTCATG TCCCAAATCT   
  
  
+ ATAACAATTT GCCGATTAGG TCATAGACTC CAGACCAACA ACTCCATCAA ACCTTATTAA GACCTGACTA   
  
  
+ TCTGACTAAA GTCCGCTAGT TATGTTGGGA AAGGAATCCC GTGTACGTAT CAAGATACAT CAAAGGTATA   
  
  
+ CATCTAGTTC AACGATTATA AAGTACATAT GAACAGCACA CTTTACTTTA CTTTACTAGT TATACTATTC   
  
  
+ AATAATATTA TTTCGACACC TTCTCGGTCT CAAGATCTAC TAGTAAAGAA TGCCTTCTAC GAACAAAACA   
  
  
+ TTCAAGCAAT TAAGATCTCA ACTTCCATTA ATTGTGGTAG AAATAGAAGA ACAAACCTCC AAATGATCAT   
  
  
+ TGTACGTTAA CAATAATATG AAACCGTTTG TTCGTTACGT TTTTATTCTT TAATAACGGG TACGTGTACT   
  
  
+ CTCAAAACCC GATCGACCGA TCCGGTTTAC TCTGTACTTC TAATTCAACT TTATATATAT ATATACGGTG   
  
  
+ TACCATACGG TTCTTTTAAT ATTGTACGTA CAATAAAAAT CTTGGCCGAG TACAACTGAT CAATTAATTT   
  
  
+ TTTTTAATAT ATCCAAAAAG TTATAAAGTA TTATAATTAC ATTTTTATCT TATATTCTTT TATTTAAATA   
  
  
+ ATTAAAATAA TTTAATAGTA CATAATATTA AAATAAATTT TGAGTTTATT TGTTTTTTTA GAGAACTCTG   
  
  
+ ACTAAGAAAA AATAGTCTTT TTTTTTACTA ATATTAATTT GATTGAGAAA ACTGGAATCT CGTTACGATA   
  
  
+ ACTCGGGTTT AGGTATAAAT AAACTAGTAC AGTGGAACAA CAACTAATAC AAAGATTAGT TACCCCGAGG   
  
  
+ TTATATACTT TTTTACCGTC TATCACGGTT AAAAGATACC AGTAAATATA CTGACGTTTA ATACTTGTGT   
  
  
+ CGTCTTTGTA GTCGTTTATT TCGTTGATCT TAAGTATTAG GTATAGAATA GGTATTTGGA TGTACTACCA   
  
  
+ TTCACTTCTT CGATCTATAT TTCTAACGTA CGTATACGCA TGTTCCTTCT TAATTTCCTG TTTCTTTGTT   
  
  
+ GGTGGGTAGA ACGAGGGGTA CGAGTAGAA  

- TAGGGTTAAC CAGTTAAGAA AGGCTGAAAA GCCTTCACAT TGACAAGGAA TTTATTTAAG AAGAGAGTTG   
  
  
- CCAAATAGTC TCATGCATGC GAAGAGCAAT ACTGTAAGAT TTTTAAGACA AAACTTTGGG TTAAATTCAT   
  
  
- AGCGATCGTT GAAGCCAACT ACACTTCACC TACTTTCTGT TCAACCCCCC TTGTGAAAAC AAAGACGTAC   
  
  
- GTGCATGCCC AAAAATCCCA TCGTCATCAT CGAAGCATGC AAGAAAACTC AATATCTCTC TCTCTCTCTC   
  
  
- TCTCAGATAA ACACACAAGA TGAAGCAGAG AGAGAGAGAC AGAGAGAGAG AGAGAGAGTA AGTTTAATCC   
  
  
- ATAATGAAGT AAAAGTATGT TGATATGACA GATGATTGGC TCTGAAATGG CTTAAAGTAC AGGGTTTAGA   
  
  
- TATTGTTAAA CGGCTAATCC AGTATCTGAG GTCTGGTTGT TGAGGTAGTT TGGAATAATT CTGGACTGAT   
  
  
- AGACTGATTT CAGGCGATCA ATACAACCCT TTCCTTAGGG CACATGCATA GTTCTATGTA GTTTCCATAT   
  
  
- GTAGATCAAG TTGCTAATAT TTCATGTATA CTTGTCGTGT GAAATGAAAT GAAATGATCA ATATGATAAG   
  
  
- TTATTATAAT AAAGCTGTGG AAGAGCCAGA GTTCTAGATG ATCATTTCTT ACGGAAGATG CTTGTTTTGT   
  
  
- AAGTTCGTTA ATTCTAGAGT TGAAGGTAAT TAACACCATC TTTATCTTCT TGTTTGGAGG TTTACTAGTA   
  
  
- ACATGCAATT GTTATTATAC TTTGGCAAAC AAGCAATGCA AAAATAAGAA ATTATTGCCC ATGCACATGA   
  
  
- GAGTTTTGGG CTAGCTGGCT AGGCCAAATG AGACATGAAG ATTAAGTTGA AATATATATA TATATGCCAC   
  
  
- ATGGTATGCC AAGAAAATTA TAACATGCAT GTTATTTTTA GAACCGGCTC ATGTTGACTA GTTAATTAAA   
  
  
- AAAAATTATA TAGGTTTTTC AATATTTCAT AATATTAATG TAAAAATAGA ATATAAGAAA ATAAATTTAT   
  
  
- TAATTTTATT AAATTATCAT GTATTATAAT TTTATTTAAA ACTCAAATAA ACAAAAAAAT CTCTTGAGAC   
  
  
- TGATTCTTTT TTATCAGAAA AAAAAATGAT TATAATTAAA CTAACTCTTT TGACCTTAGA GCAATGCTAT   
  
  
- TGAGCCCAAA TCCATATTTA TTTGATCATG TCACCTTGTT GTTGATTATG TTTCTAATCA ATGGGGCTCC   
  
  
- AATATATGAA AAAATGGCAG ATAGTGCCAA TTTTCTATGG TCATTTATAT GACTGCAAAT TATGAACACA   
  
  
- GCAGAAACAT CAGCAAATAA AGCAACTAGA ATTCATAATC CATATCTTAT CCATAAACCT ACATGATGGT   
  
  
- AAGTGAAGAA GCTAGATATA AAGATTGCAT GCATATGCGT ACAAGGAAGA ATTAAAGGAC AAAGAAACAA   
  
  
- CCACCCATCT TGCTCCCCAT GCTCATCTT

+     W box

| Site Name | Organism | Position | Strand | Matrix score. | sequence | function |
| --- | --- | --- | --- | --- | --- | --- |
| W box | Arabidopsis thaliana | 10 | - | 6 | TTGACC |  |

> 2018/04/13 10:10:12  
+ ATCCCAATTG GTCAATTCTT TCCGACTTTT CGGAAGTGTA ACTGTTCCTT AAATAAATTC TTCTCTCAAC   
  
  
+ GGTTTATCAG AGTACGTACG CTTCTCGTTA TGACATTCTA AAAATTCTGT TTTGAAACCC AATTTAAGTA   
  
  
+ TCGCTAGCAA CTTCGGTTGA TGTGAAGTGG ATGAAAGACA AGTTGGGGGG AACACTTTTG TTTCTGCATG   
  
  
+ CACGTACGGG TTTTTAGGGT AGCAGTAGTA GCTTCGTACG TTCTTTTGAG TTATAGAGAG AGAGAGAGAG   
  
  
+ AGAGTCTATT TGTGTGTTCT ACTTCGTCTC TCTCTCTCTG TCTCTCTCTC TCTCTCTCAT TCAAATTAGG   
  
  
+ TATTACTTCA TTTTCATACA ACTATACTGT CTACTAACCG AGACTTTACC GAATTTCATG TCCCAAATCT   
  
  
+ ATAACAATTT GCCGATTAGG TCATAGACTC CAGACCAACA ACTCCATCAA ACCTTATTAA GACCTGACTA   
  
  
+ TCTGACTAAA GTCCGCTAGT TATGTTGGGA AAGGAATCCC GTGTACGTAT CAAGATACAT CAAAGGTATA   
  
  
+ CATCTAGTTC AACGATTATA AAGTACATAT GAACAGCACA CTTTACTTTA CTTTACTAGT TATACTATTC   
  
  
+ AATAATATTA TTTCGACACC TTCTCGGTCT CAAGATCTAC TAGTAAAGAA TGCCTTCTAC GAACAAAACA   
  
  
+ TTCAAGCAAT TAAGATCTCA ACTTCCATTA ATTGTGGTAG AAATAGAAGA ACAAACCTCC AAATGATCAT   
  
  
+ TGTACGTTAA CAATAATATG AAACCGTTTG TTCGTTACGT TTTTATTCTT TAATAACGGG TACGTGTACT   
  
  
+ CTCAAAACCC GATCGACCGA TCCGGTTTAC TCTGTACTTC TAATTCAACT TTATATATAT ATATACGGTG   
  
  
+ TACCATACGG TTCTTTTAAT ATTGTACGTA CAATAAAAAT CTTGGCCGAG TACAACTGAT CAATTAATTT   
  
  
+ TTTTTAATAT ATCCAAAAAG TTATAAAGTA TTATAATTAC ATTTTTATCT TATATTCTTT TATTTAAATA   
  
  
+ ATTAAAATAA TTTAATAGTA CATAATATTA AAATAAATTT TGAGTTTATT TGTTTTTTTA GAGAACTCTG   
  
  
+ ACTAAGAAAA AATAGTCTTT TTTTTTACTA ATATTAATTT GATTGAGAAA ACTGGAATCT CGTTACGATA   
  
  
+ ACTCGGGTTT AGGTATAAAT AAACTAGTAC AGTGGAACAA CAACTAATAC AAAGATTAGT TACCCCGAGG   
  
  
+ TTATATACTT TTTTACCGTC TATCACGGTT AAAAGATACC AGTAAATATA CTGACGTTTA ATACTTGTGT   
  
  
+ CGTCTTTGTA GTCGTTTATT TCGTTGATCT TAAGTATTAG GTATAGAATA GGTATTTGGA TGTACTACCA   
  
  
+ TTCACTTCTT CGATCTATAT TTCTAACGTA CGTATACGCA TGTTCCTTCT TAATTTCCTG TTTCTTTGTT   
  
  
+ GGTGGGTAGA ACGAGGGGTA CGAGTAGAA  

- TAGGGTTAAC CAGTTAAGAA AGGCTGAAAA GCCTTCACAT TGACAAGGAA TTTATTTAAG AAGAGAGTTG   
  
  
- CCAAATAGTC TCATGCATGC GAAGAGCAAT ACTGTAAGAT TTTTAAGACA AAACTTTGGG TTAAATTCAT   
  
  
- AGCGATCGTT GAAGCCAACT ACACTTCACC TACTTTCTGT TCAACCCCCC TTGTGAAAAC AAAGACGTAC   
  
  
- GTGCATGCCC AAAAATCCCA TCGTCATCAT CGAAGCATGC AAGAAAACTC AATATCTCTC TCTCTCTCTC   
  
  
- TCTCAGATAA ACACACAAGA TGAAGCAGAG AGAGAGAGAC AGAGAGAGAG AGAGAGAGTA AGTTTAATCC   
  
  
- ATAATGAAGT AAAAGTATGT TGATATGACA GATGATTGGC TCTGAAATGG CTTAAAGTAC AGGGTTTAGA   
  
  
- TATTGTTAAA CGGCTAATCC AGTATCTGAG GTCTGGTTGT TGAGGTAGTT TGGAATAATT CTGGACTGAT   
  
  
- AGACTGATTT CAGGCGATCA ATACAACCCT TTCCTTAGGG CACATGCATA GTTCTATGTA GTTTCCATAT   
  
  
- GTAGATCAAG TTGCTAATAT TTCATGTATA CTTGTCGTGT GAAATGAAAT GAAATGATCA ATATGATAAG   
  
  
- TTATTATAAT AAAGCTGTGG AAGAGCCAGA GTTCTAGATG ATCATTTCTT ACGGAAGATG CTTGTTTTGT   
  
  
- AAGTTCGTTA ATTCTAGAGT TGAAGGTAAT TAACACCATC TTTATCTTCT TGTTTGGAGG TTTACTAGTA   
  
  
- ACATGCAATT GTTATTATAC TTTGGCAAAC AAGCAATGCA AAAATAAGAA ATTATTGCCC ATGCACATGA   
  
  
- GAGTTTTGGG CTAGCTGGCT AGGCCAAATG AGACATGAAG ATTAAGTTGA AATATATATA TATATGCCAC   
  
  
- ATGGTATGCC AAGAAAATTA TAACATGCAT GTTATTTTTA GAACCGGCTC ATGTTGACTA GTTAATTAAA   
  
  
- AAAAATTATA TAGGTTTTTC AATATTTCAT AATATTAATG TAAAAATAGA ATATAAGAAA ATAAATTTAT   
  
  
- TAATTTTATT AAATTATCAT GTATTATAAT TTTATTTAAA ACTCAAATAA ACAAAAAAAT CTCTTGAGAC   
  
  
- TGATTCTTTT TTATCAGAAA AAAAAATGAT TATAATTAAA CTAACTCTTT TGACCTTAGA GCAATGCTAT   
  
  
- TGAGCCCAAA TCCATATTTA TTTGATCATG TCACCTTGTT GTTGATTATG TTTCTAATCA ATGGGGCTCC   
  
  
- AATATATGAA AAAATGGCAG ATAGTGCCAA TTTTCTATGG TCATTTATAT GACTGCAAAT TATGAACACA   
  
  
- GCAGAAACAT CAGCAAATAA AGCAACTAGA ATTCATAATC CATATCTTAT CCATAAACCT ACATGATGGT   
  
  
- AAGTGAAGAA GCTAGATATA AAGATTGCAT GCATATGCGT ACAAGGAAGA ATTAAAGGAC AAAGAAACAA   
  
  
- CCACCCATCT TGCTCCCCAT GCTCATCTT

+     circadian

| Site Name | Organism | Position | Strand | Matrix score. | sequence | function |
| --- | --- | --- | --- | --- | --- | --- |
| circadian | Lycopersicon esculentum | 459 | + | 6 | CAANNNNATC | cis-acting regulatory element involved in circadian control |

> 2018/04/13 10:10:12  
+ ATCCCAATTG GTCAATTCTT TCCGACTTTT CGGAAGTGTA ACTGTTCCTT AAATAAATTC TTCTCTCAAC   
  
  
+ GGTTTATCAG AGTACGTACG CTTCTCGTTA TGACATTCTA AAAATTCTGT TTTGAAACCC AATTTAAGTA   
  
  
+ TCGCTAGCAA CTTCGGTTGA TGTGAAGTGG ATGAAAGACA AGTTGGGGGG AACACTTTTG TTTCTGCATG   
  
  
+ CACGTACGGG TTTTTAGGGT AGCAGTAGTA GCTTCGTACG TTCTTTTGAG TTATAGAGAG AGAGAGAGAG   
  
  
+ AGAGTCTATT TGTGTGTTCT ACTTCGTCTC TCTCTCTCTG TCTCTCTCTC TCTCTCTCAT TCAAATTAGG   
  
  
+ TATTACTTCA TTTTCATACA ACTATACTGT CTACTAACCG AGACTTTACC GAATTTCATG TCCCAAATCT   
  
  
+ ATAACAATTT GCCGATTAGG TCATAGACTC CAGACCAACA ACTCCATCAA ACCTTATTAA GACCTGACTA   
  
  
+ TCTGACTAAA GTCCGCTAGT TATGTTGGGA AAGGAATCCC GTGTACGTAT CAAGATACAT CAAAGGTATA   
  
  
+ CATCTAGTTC AACGATTATA AAGTACATAT GAACAGCACA CTTTACTTTA CTTTACTAGT TATACTATTC   
  
  
+ AATAATATTA TTTCGACACC TTCTCGGTCT CAAGATCTAC TAGTAAAGAA TGCCTTCTAC GAACAAAACA   
  
  
+ TTCAAGCAAT TAAGATCTCA ACTTCCATTA ATTGTGGTAG AAATAGAAGA ACAAACCTCC AAATGATCAT   
  
  
+ TGTACGTTAA CAATAATATG AAACCGTTTG TTCGTTACGT TTTTATTCTT TAATAACGGG TACGTGTACT   
  
  
+ CTCAAAACCC GATCGACCGA TCCGGTTTAC TCTGTACTTC TAATTCAACT TTATATATAT ATATACGGTG   
  
  
+ TACCATACGG TTCTTTTAAT ATTGTACGTA CAATAAAAAT CTTGGCCGAG TACAACTGAT CAATTAATTT   
  
  
+ TTTTTAATAT ATCCAAAAAG TTATAAAGTA TTATAATTAC ATTTTTATCT TATATTCTTT TATTTAAATA   
  
  
+ ATTAAAATAA TTTAATAGTA CATAATATTA AAATAAATTT TGAGTTTATT TGTTTTTTTA GAGAACTCTG   
  
  
+ ACTAAGAAAA AATAGTCTTT TTTTTTACTA ATATTAATTT GATTGAGAAA ACTGGAATCT CGTTACGATA   
  
  
+ ACTCGGGTTT AGGTATAAAT AAACTAGTAC AGTGGAACAA CAACTAATAC AAAGATTAGT TACCCCGAGG   
  
  
+ TTATATACTT TTTTACCGTC TATCACGGTT AAAAGATACC AGTAAATATA CTGACGTTTA ATACTTGTGT   
  
  
+ CGTCTTTGTA GTCGTTTATT TCGTTGATCT TAAGTATTAG GTATAGAATA GGTATTTGGA TGTACTACCA   
  
  
+ TTCACTTCTT CGATCTATAT TTCTAACGTA CGTATACGCA TGTTCCTTCT TAATTTCCTG TTTCTTTGTT   
  
  
+ GGTGGGTAGA ACGAGGGGTA CGAGTAGAA  

- TAGGGTTAAC CAGTTAAGAA AGGCTGAAAA GCCTTCACAT TGACAAGGAA TTTATTTAAG AAGAGAGTTG   
  
  
- CCAAATAGTC TCATGCATGC GAAGAGCAAT ACTGTAAGAT TTTTAAGACA AAACTTTGGG TTAAATTCAT   
  
  
- AGCGATCGTT GAAGCCAACT ACACTTCACC TACTTTCTGT TCAACCCCCC TTGTGAAAAC AAAGACGTAC   
  
  
- GTGCATGCCC AAAAATCCCA TCGTCATCAT CGAAGCATGC AAGAAAACTC AATATCTCTC TCTCTCTCTC   
  
  
- TCTCAGATAA ACACACAAGA TGAAGCAGAG AGAGAGAGAC AGAGAGAGAG AGAGAGAGTA AGTTTAATCC   
  
  
- ATAATGAAGT AAAAGTATGT TGATATGACA GATGATTGGC TCTGAAATGG CTTAAAGTAC AGGGTTTAGA   
  
  
- TATTGTTAAA CGGCTAATCC AGTATCTGAG GTCTGGTTGT TGAGGTAGTT TGGAATAATT CTGGACTGAT   
  
  
- AGACTGATTT CAGGCGATCA ATACAACCCT TTCCTTAGGG CACATGCATA GTTCTATGTA GTTTCCATAT   
  
  
- GTAGATCAAG TTGCTAATAT TTCATGTATA CTTGTCGTGT GAAATGAAAT GAAATGATCA ATATGATAAG   
  
  
- TTATTATAAT AAAGCTGTGG AAGAGCCAGA GTTCTAGATG ATCATTTCTT ACGGAAGATG CTTGTTTTGT   
  
  
- AAGTTCGTTA ATTCTAGAGT TGAAGGTAAT TAACACCATC TTTATCTTCT TGTTTGGAGG TTTACTAGTA   
  
  
- ACATGCAATT GTTATTATAC TTTGGCAAAC AAGCAATGCA AAAATAAGAA ATTATTGCCC ATGCACATGA   
  
  
- GAGTTTTGGG CTAGCTGGCT AGGCCAAATG AGACATGAAG ATTAAGTTGA AATATATATA TATATGCCAC   
  
  
- ATGGTATGCC AAGAAAATTA TAACATGCAT GTTATTTTTA GAACCGGCTC ATGTTGACTA GTTAATTAAA   
  
  
- AAAAATTATA TAGGTTTTTC AATATTTCAT AATATTAATG TAAAAATAGA ATATAAGAAA ATAAATTTAT   
  
  
- TAATTTTATT AAATTATCAT GTATTATAAT TTTATTTAAA ACTCAAATAA ACAAAAAAAT CTCTTGAGAC   
  
  
- TGATTCTTTT TTATCAGAAA AAAAAATGAT TATAATTAAA CTAACTCTTT TGACCTTAGA GCAATGCTAT   
  
  
- TGAGCCCAAA TCCATATTTA TTTGATCATG TCACCTTGTT GTTGATTATG TTTCTAATCA ATGGGGCTCC   
  
  
- AATATATGAA AAAATGGCAG ATAGTGCCAA TTTTCTATGG TCATTTATAT GACTGCAAAT TATGAACACA   
  
  
- GCAGAAACAT CAGCAAATAA AGCAACTAGA ATTCATAATC CATATCTTAT CCATAAACCT ACATGATGGT   
  
  
- AAGTGAAGAA GCTAGATATA AAGATTGCAT GCATATGCGT ACAAGGAAGA ATTAAAGGAC AAAGAAACAA   
  
  
- CCACCCATCT TGCTCCCCAT GCTCATCTT
