# Supplementary material for: Genome-wide analysis of the WRKY gene family in drumstick (Moringa oleifera Lam.)
Source: PeerJ. 2019 Jun 10;7:e7063. doi: 10.7717/peerj.7063 (PMC6563795; doi:10.7717/peerj.7063)
Supplement: Supplemental Information 1 [file peerj-07-7063-s003.gz › MoWRKY45_plantcare.html]

Content-Type: text/html; charset=ISO-8859-1


CallMat\_Firefox


Webmaster Firefox specific output  
To save the result:
click on the frame with the right mouse button and save the source code as a text file with extension .html  
REFERENCE:PlantCARE: a database of plant cis-acting regulatory elements and a portal to tools for in silico analysis of promoter sequences.  
Lescot, M., Déhais, P., Moreau, Y., De Moor, B., Rouzé ,P.,and Rombauts, S.  
Nucleic Acids Res., Database issue(2002), 30(1):325-327.   


---

> 2018/04/13 10:10:12  
+ TCGAGAAGAA GCAAGAGAAC TTACCTGGTA GAGTTGTGAA ACAAGAGAAG AAAATATCTC TCCGACGTTT   
  
  
+ ACAAACTTAG GAAATTATAT GTGTTGCAGT TGTCGACGGA TAGGTTAACG TGATTTTTTG TCACGGGGTT   
  
  
+ GGTATATAAT TAATATGCTA GGGATTGGTA TTAATTAATG CGAATTAATT CATGCTTATT AAGTTGAAGA   
  
  
+ AGGTAGTAAA TCTAATATGT TTTACAGACG TAACTGTTGT TCTCTCTCGA AAGTCGGCAC TCCGGGCCTC   
  
  
+ GGTTGGTAGG TCCATATGAG TACGGGATAT AGGTACTTCA AAGCATGCCA TGTATTTCAG TGTACCTGTG   
  
  
+ TTGGCAAAGC AACCAGTTTA ATTGTCAAAA TATGTATCAA GGCAGAATTA CAGAATTTAT GTCCGTTATA   
  
  
+ CTGTTTTCAA AGAAGACTTT CCAGAGGAAA CAGCAGACCA AGGACGTAAT CCCAAAATGG CACCTCTCTC   
  
  
+ TGTACCCTAA TAGTTCTTAA CATTGTAGAT TTTTTTAATG GATTGTAGAT TTTTATGCTC AGCCAGGTTG   
  
  
+ GGCACAACAG TAAAAAGTGG TATATAATAT TAATACAGAG AAATAAAAAC CATTAGAATT ACAACGTACA   
  
  
+ TTCCGATCCG TACAGAGAAC TCGGCTTATA TATCTGTCAG TTCCCAGTTT ATATTATAAC CATTCATCCC   
  
  
+ ATACGCATAT GTAGAATTAG AACTTTTTAG AAAGATACAA AGCGGCCACT GAATGAAAGG CACCCAAGAA   
  
  
+ TACGTTATCT TTTTAGTCTT TAAAAAAACA GACGAAAACC ACCTCATTTT ATAACGGTTT AAGTAATGCT   
  
  
+ TTGTTAATTA ATTTTTTTAC ACAAATTAAC TAAATAGTTG GAAATAATAT GTCTTCTAAA AGTACTACGA   
  
  
+ ATTCGTACTA CTACTAGCCT AACGCCTTTA CAGATTTAGT TATTAGGCGT TTTAACCTTG AACAAAACGT   
  
  
+ TATAATTTTA TATATAACCC CAATTAAAAC ACTAAGTTTT AATAATTTTC CTTCCGTCTC GTCTTATTTC   
  
  
+ CTTAATTTAT ATAGGTTTCA GCAAACTTAT TAATTTAAAA CCTCGGAAAA ATTTTCAGTT CTTTTATCTC   
  
  
+ TTCTGTACGA ACCTAACAAA TTATTCCAGC GAAAATTCAA AATTGAGAAA AGAACCTTTA ATCATACTTA   
  
  
+ AAAGTCTCCT TAATCTCACC GTTATATATT ATTTTATTTT ATTTATTTGT TATATGAAAA AATTATAATA   
  
  
+ TTAATATAAT ACTTTCGAAA TGAAAAATTA AACGTTTATA AAATAACTTT TTTATTATAA TAGTAAAAAA   
  
  
+ ATATTAGTTT CATAGTTAAC CCCTAACCTT GGGATTTGAA GACAGAACGA ATAATCACTC TACCTAGATA   
  
  
+ GTTTTAATGA AGTAAAGTTT TAAATTTTTA CTTTAATGCT CTTAACACTA AAAAATAAAA CTCTGATTTT   
  
  
+ TTAACAATCA GTCGTCCAAC TAGTGTAGC  

- AGCTCTTCTT CGTTCTCTTG AATGGACCAT CTCAACACTT TGTTCTCTTC TTTTATAGAG AGGCTGCAAA   
  
  
- TGTTTGAATC CTTTAATATA CACAACGTCA ACAGCTGCCT ATCCAATTGC ACTAAAAAAC AGTGCCCCAA   
  
  
- CCATATATTA ATTATACGAT CCCTAACCAT AATTAATTAC GCTTAATTAA GTACGAATAA TTCAACTTCT   
  
  
- TCCATCATTT AGATTATACA AAATGTCTGC ATTGACAACA AGAGAGAGCT TTCAGCCGTG AGGCCCGGAG   
  
  
- CCAACCATCC AGGTATACTC ATGCCCTATA TCCATGAAGT TTCGTACGGT ACATAAAGTC ACATGGACAC   
  
  
- AACCGTTTCG TTGGTCAAAT TAACAGTTTT ATACATAGTT CCGTCTTAAT GTCTTAAATA CAGGCAATAT   
  
  
- GACAAAAGTT TCTTCTGAAA GGTCTCCTTT GTCGTCTGGT TCCTGCATTA GGGTTTTACC GTGGAGAGAG   
  
  
- ACATGGGATT ATCAAGAATT GTAACATCTA AAAAAATTAC CTAACATCTA AAAATACGAG TCGGTCCAAC   
  
  
- CCGTGTTGTC ATTTTTCACC ATATATTATA ATTATGTCTC TTTATTTTTG GTAATCTTAA TGTTGCATGT   
  
  
- AAGGCTAGGC ATGTCTCTTG AGCCGAATAT ATAGACAGTC AAGGGTCAAA TATAATATTG GTAAGTAGGG   
  
  
- TATGCGTATA CATCTTAATC TTGAAAAATC TTTCTATGTT TCGCCGGTGA CTTACTTTCC GTGGGTTCTT   
  
  
- ATGCAATAGA AAAATCAGAA ATTTTTTTGT CTGCTTTTGG TGGAGTAAAA TATTGCCAAA TTCATTACGA   
  
  
- AACAATTAAT TAAAAAAATG TGTTTAATTG ATTTATCAAC CTTTATTATA CAGAAGATTT TCATGATGCT   
  
  
- TAAGCATGAT GATGATCGGA TTGCGGAAAT GTCTAAATCA ATAATCCGCA AAATTGGAAC TTGTTTTGCA   
  
  
- ATATTAAAAT ATATATTGGG GTTAATTTTG TGATTCAAAA TTATTAAAAG GAAGGCAGAG CAGAATAAAG   
  
  
- GAATTAAATA TATCCAAAGT CGTTTGAATA ATTAAATTTT GGAGCCTTTT TAAAAGTCAA GAAAATAGAG   
  
  
- AAGACATGCT TGGATTGTTT AATAAGGTCG CTTTTAAGTT TTAACTCTTT TCTTGGAAAT TAGTATGAAT   
  
  
- TTTCAGAGGA ATTAGAGTGG CAATATATAA TAAAATAAAA TAAATAAACA ATATACTTTT TTAATATTAT   
  
  
- AATTATATTA TGAAAGCTTT ACTTTTTAAT TTGCAAATAT TTTATTGAAA AAATAATATT ATCATTTTTT   
  
  
- TATAATCAAA GTATCAATTG GGGATTGGAA CCCTAAACTT CTGTCTTGCT TATTAGTGAG ATGGATCTAT   
  
  
- CAAAATTACT TCATTTCAAA ATTTAAAAAT GAAATTACGA GAATTGTGAT TTTTTATTTT GAGACTAAAA   
  
  
- AATTGTTAGT CAGCAGGTTG ATCACATCG

  
  
Motifs Found  

+     3-AF1 binding site

| Site Name | Organism | Position | Strand | Matrix score. | sequence | function |
| --- | --- | --- | --- | --- | --- | --- |
| 3-AF1 binding site | Solanum tuberosum | 52 | - | 10 | AAGAGATATTT | light responsive element |

> 2018/04/13 10:10:12  
+ TCGAGAAGAA GCAAGAGAAC TTACCTGGTA GAGTTGTGAA ACAAGAGAAG AAAATATCTC TCCGACGTTT   
  
  
+ ACAAACTTAG GAAATTATAT GTGTTGCAGT TGTCGACGGA TAGGTTAACG TGATTTTTTG TCACGGGGTT   
  
  
+ GGTATATAAT TAATATGCTA GGGATTGGTA TTAATTAATG CGAATTAATT CATGCTTATT AAGTTGAAGA   
  
  
+ AGGTAGTAAA TCTAATATGT TTTACAGACG TAACTGTTGT TCTCTCTCGA AAGTCGGCAC TCCGGGCCTC   
  
  
+ GGTTGGTAGG TCCATATGAG TACGGGATAT AGGTACTTCA AAGCATGCCA TGTATTTCAG TGTACCTGTG   
  
  
+ TTGGCAAAGC AACCAGTTTA ATTGTCAAAA TATGTATCAA GGCAGAATTA CAGAATTTAT GTCCGTTATA   
  
  
+ CTGTTTTCAA AGAAGACTTT CCAGAGGAAA CAGCAGACCA AGGACGTAAT CCCAAAATGG CACCTCTCTC   
  
  
+ TGTACCCTAA TAGTTCTTAA CATTGTAGAT TTTTTTAATG GATTGTAGAT TTTTATGCTC AGCCAGGTTG   
  
  
+ GGCACAACAG TAAAAAGTGG TATATAATAT TAATACAGAG AAATAAAAAC CATTAGAATT ACAACGTACA   
  
  
+ TTCCGATCCG TACAGAGAAC TCGGCTTATA TATCTGTCAG TTCCCAGTTT ATATTATAAC CATTCATCCC   
  
  
+ ATACGCATAT GTAGAATTAG AACTTTTTAG AAAGATACAA AGCGGCCACT GAATGAAAGG CACCCAAGAA   
  
  
+ TACGTTATCT TTTTAGTCTT TAAAAAAACA GACGAAAACC ACCTCATTTT ATAACGGTTT AAGTAATGCT   
  
  
+ TTGTTAATTA ATTTTTTTAC ACAAATTAAC TAAATAGTTG GAAATAATAT GTCTTCTAAA AGTACTACGA   
  
  
+ ATTCGTACTA CTACTAGCCT AACGCCTTTA CAGATTTAGT TATTAGGCGT TTTAACCTTG AACAAAACGT   
  
  
+ TATAATTTTA TATATAACCC CAATTAAAAC ACTAAGTTTT AATAATTTTC CTTCCGTCTC GTCTTATTTC   
  
  
+ CTTAATTTAT ATAGGTTTCA GCAAACTTAT TAATTTAAAA CCTCGGAAAA ATTTTCAGTT CTTTTATCTC   
  
  
+ TTCTGTACGA ACCTAACAAA TTATTCCAGC GAAAATTCAA AATTGAGAAA AGAACCTTTA ATCATACTTA   
  
  
+ AAAGTCTCCT TAATCTCACC GTTATATATT ATTTTATTTT ATTTATTTGT TATATGAAAA AATTATAATA   
  
  
+ TTAATATAAT ACTTTCGAAA TGAAAAATTA AACGTTTATA AAATAACTTT TTTATTATAA TAGTAAAAAA   
  
  
+ ATATTAGTTT CATAGTTAAC CCCTAACCTT GGGATTTGAA GACAGAACGA ATAATCACTC TACCTAGATA   
  
  
+ GTTTTAATGA AGTAAAGTTT TAAATTTTTA CTTTAATGCT CTTAACACTA AAAAATAAAA CTCTGATTTT   
  
  
+ TTAACAATCA GTCGTCCAAC TAGTGTAGC  

- AGCTCTTCTT CGTTCTCTTG AATGGACCAT CTCAACACTT TGTTCTCTTC TTTTATAGAG AGGCTGCAAA   
  
  
- TGTTTGAATC CTTTAATATA CACAACGTCA ACAGCTGCCT ATCCAATTGC ACTAAAAAAC AGTGCCCCAA   
  
  
- CCATATATTA ATTATACGAT CCCTAACCAT AATTAATTAC GCTTAATTAA GTACGAATAA TTCAACTTCT   
  
  
- TCCATCATTT AGATTATACA AAATGTCTGC ATTGACAACA AGAGAGAGCT TTCAGCCGTG AGGCCCGGAG   
  
  
- CCAACCATCC AGGTATACTC ATGCCCTATA TCCATGAAGT TTCGTACGGT ACATAAAGTC ACATGGACAC   
  
  
- AACCGTTTCG TTGGTCAAAT TAACAGTTTT ATACATAGTT CCGTCTTAAT GTCTTAAATA CAGGCAATAT   
  
  
- GACAAAAGTT TCTTCTGAAA GGTCTCCTTT GTCGTCTGGT TCCTGCATTA GGGTTTTACC GTGGAGAGAG   
  
  
- ACATGGGATT ATCAAGAATT GTAACATCTA AAAAAATTAC CTAACATCTA AAAATACGAG TCGGTCCAAC   
  
  
- CCGTGTTGTC ATTTTTCACC ATATATTATA ATTATGTCTC TTTATTTTTG GTAATCTTAA TGTTGCATGT   
  
  
- AAGGCTAGGC ATGTCTCTTG AGCCGAATAT ATAGACAGTC AAGGGTCAAA TATAATATTG GTAAGTAGGG   
  
  
- TATGCGTATA CATCTTAATC TTGAAAAATC TTTCTATGTT TCGCCGGTGA CTTACTTTCC GTGGGTTCTT   
  
  
- ATGCAATAGA AAAATCAGAA ATTTTTTTGT CTGCTTTTGG TGGAGTAAAA TATTGCCAAA TTCATTACGA   
  
  
- AACAATTAAT TAAAAAAATG TGTTTAATTG ATTTATCAAC CTTTATTATA CAGAAGATTT TCATGATGCT   
  
  
- TAAGCATGAT GATGATCGGA TTGCGGAAAT GTCTAAATCA ATAATCCGCA AAATTGGAAC TTGTTTTGCA   
  
  
- ATATTAAAAT ATATATTGGG GTTAATTTTG TGATTCAAAA TTATTAAAAG GAAGGCAGAG CAGAATAAAG   
  
  
- GAATTAAATA TATCCAAAGT CGTTTGAATA ATTAAATTTT GGAGCCTTTT TAAAAGTCAA GAAAATAGAG   
  
  
- AAGACATGCT TGGATTGTTT AATAAGGTCG CTTTTAAGTT TTAACTCTTT TCTTGGAAAT TAGTATGAAT   
  
  
- TTTCAGAGGA ATTAGAGTGG CAATATATAA TAAAATAAAA TAAATAAACA ATATACTTTT TTAATATTAT   
  
  
- AATTATATTA TGAAAGCTTT ACTTTTTAAT TTGCAAATAT TTTATTGAAA AAATAATATT ATCATTTTTT   
  
  
- TATAATCAAA GTATCAATTG GGGATTGGAA CCCTAAACTT CTGTCTTGCT TATTAGTGAG ATGGATCTAT   
  
  
- CAAAATTACT TCATTTCAAA ATTTAAAAAT GAAATTACGA GAATTGTGAT TTTTTATTTT GAGACTAAAA   
  
  
- AATTGTTAGT CAGCAGGTTG ATCACATCG

+     5UTR Py-rich stretch

| Site Name | Organism | Position | Strand | Matrix score. | sequence | function |
| --- | --- | --- | --- | --- | --- | --- |
| 5UTR Py-rich stretch | Lycopersicon esculentum | 44 | - | 10 | TTTCTTCTCT | cis-acting element conferring high transcription levels |

> 2018/04/13 10:10:12  
+ TCGAGAAGAA GCAAGAGAAC TTACCTGGTA GAGTTGTGAA ACAAGAGAAG AAAATATCTC TCCGACGTTT   
  
  
+ ACAAACTTAG GAAATTATAT GTGTTGCAGT TGTCGACGGA TAGGTTAACG TGATTTTTTG TCACGGGGTT   
  
  
+ GGTATATAAT TAATATGCTA GGGATTGGTA TTAATTAATG CGAATTAATT CATGCTTATT AAGTTGAAGA   
  
  
+ AGGTAGTAAA TCTAATATGT TTTACAGACG TAACTGTTGT TCTCTCTCGA AAGTCGGCAC TCCGGGCCTC   
  
  
+ GGTTGGTAGG TCCATATGAG TACGGGATAT AGGTACTTCA AAGCATGCCA TGTATTTCAG TGTACCTGTG   
  
  
+ TTGGCAAAGC AACCAGTTTA ATTGTCAAAA TATGTATCAA GGCAGAATTA CAGAATTTAT GTCCGTTATA   
  
  
+ CTGTTTTCAA AGAAGACTTT CCAGAGGAAA CAGCAGACCA AGGACGTAAT CCCAAAATGG CACCTCTCTC   
  
  
+ TGTACCCTAA TAGTTCTTAA CATTGTAGAT TTTTTTAATG GATTGTAGAT TTTTATGCTC AGCCAGGTTG   
  
  
+ GGCACAACAG TAAAAAGTGG TATATAATAT TAATACAGAG AAATAAAAAC CATTAGAATT ACAACGTACA   
  
  
+ TTCCGATCCG TACAGAGAAC TCGGCTTATA TATCTGTCAG TTCCCAGTTT ATATTATAAC CATTCATCCC   
  
  
+ ATACGCATAT GTAGAATTAG AACTTTTTAG AAAGATACAA AGCGGCCACT GAATGAAAGG CACCCAAGAA   
  
  
+ TACGTTATCT TTTTAGTCTT TAAAAAAACA GACGAAAACC ACCTCATTTT ATAACGGTTT AAGTAATGCT   
  
  
+ TTGTTAATTA ATTTTTTTAC ACAAATTAAC TAAATAGTTG GAAATAATAT GTCTTCTAAA AGTACTACGA   
  
  
+ ATTCGTACTA CTACTAGCCT AACGCCTTTA CAGATTTAGT TATTAGGCGT TTTAACCTTG AACAAAACGT   
  
  
+ TATAATTTTA TATATAACCC CAATTAAAAC ACTAAGTTTT AATAATTTTC CTTCCGTCTC GTCTTATTTC   
  
  
+ CTTAATTTAT ATAGGTTTCA GCAAACTTAT TAATTTAAAA CCTCGGAAAA ATTTTCAGTT CTTTTATCTC   
  
  
+ TTCTGTACGA ACCTAACAAA TTATTCCAGC GAAAATTCAA AATTGAGAAA AGAACCTTTA ATCATACTTA   
  
  
+ AAAGTCTCCT TAATCTCACC GTTATATATT ATTTTATTTT ATTTATTTGT TATATGAAAA AATTATAATA   
  
  
+ TTAATATAAT ACTTTCGAAA TGAAAAATTA AACGTTTATA AAATAACTTT TTTATTATAA TAGTAAAAAA   
  
  
+ ATATTAGTTT CATAGTTAAC CCCTAACCTT GGGATTTGAA GACAGAACGA ATAATCACTC TACCTAGATA   
  
  
+ GTTTTAATGA AGTAAAGTTT TAAATTTTTA CTTTAATGCT CTTAACACTA AAAAATAAAA CTCTGATTTT   
  
  
+ TTAACAATCA GTCGTCCAAC TAGTGTAGC  

- AGCTCTTCTT CGTTCTCTTG AATGGACCAT CTCAACACTT TGTTCTCTTC TTTTATAGAG AGGCTGCAAA   
  
  
- TGTTTGAATC CTTTAATATA CACAACGTCA ACAGCTGCCT ATCCAATTGC ACTAAAAAAC AGTGCCCCAA   
  
  
- CCATATATTA ATTATACGAT CCCTAACCAT AATTAATTAC GCTTAATTAA GTACGAATAA TTCAACTTCT   
  
  
- TCCATCATTT AGATTATACA AAATGTCTGC ATTGACAACA AGAGAGAGCT TTCAGCCGTG AGGCCCGGAG   
  
  
- CCAACCATCC AGGTATACTC ATGCCCTATA TCCATGAAGT TTCGTACGGT ACATAAAGTC ACATGGACAC   
  
  
- AACCGTTTCG TTGGTCAAAT TAACAGTTTT ATACATAGTT CCGTCTTAAT GTCTTAAATA CAGGCAATAT   
  
  
- GACAAAAGTT TCTTCTGAAA GGTCTCCTTT GTCGTCTGGT TCCTGCATTA GGGTTTTACC GTGGAGAGAG   
  
  
- ACATGGGATT ATCAAGAATT GTAACATCTA AAAAAATTAC CTAACATCTA AAAATACGAG TCGGTCCAAC   
  
  
- CCGTGTTGTC ATTTTTCACC ATATATTATA ATTATGTCTC TTTATTTTTG GTAATCTTAA TGTTGCATGT   
  
  
- AAGGCTAGGC ATGTCTCTTG AGCCGAATAT ATAGACAGTC AAGGGTCAAA TATAATATTG GTAAGTAGGG   
  
  
- TATGCGTATA CATCTTAATC TTGAAAAATC TTTCTATGTT TCGCCGGTGA CTTACTTTCC GTGGGTTCTT   
  
  
- ATGCAATAGA AAAATCAGAA ATTTTTTTGT CTGCTTTTGG TGGAGTAAAA TATTGCCAAA TTCATTACGA   
  
  
- AACAATTAAT TAAAAAAATG TGTTTAATTG ATTTATCAAC CTTTATTATA CAGAAGATTT TCATGATGCT   
  
  
- TAAGCATGAT GATGATCGGA TTGCGGAAAT GTCTAAATCA ATAATCCGCA AAATTGGAAC TTGTTTTGCA   
  
  
- ATATTAAAAT ATATATTGGG GTTAATTTTG TGATTCAAAA TTATTAAAAG GAAGGCAGAG CAGAATAAAG   
  
  
- GAATTAAATA TATCCAAAGT CGTTTGAATA ATTAAATTTT GGAGCCTTTT TAAAAGTCAA GAAAATAGAG   
  
  
- AAGACATGCT TGGATTGTTT AATAAGGTCG CTTTTAAGTT TTAACTCTTT TCTTGGAAAT TAGTATGAAT   
  
  
- TTTCAGAGGA ATTAGAGTGG CAATATATAA TAAAATAAAA TAAATAAACA ATATACTTTT TTAATATTAT   
  
  
- AATTATATTA TGAAAGCTTT ACTTTTTAAT TTGCAAATAT TTTATTGAAA AAATAATATT ATCATTTTTT   
  
  
- TATAATCAAA GTATCAATTG GGGATTGGAA CCCTAAACTT CTGTCTTGCT TATTAGTGAG ATGGATCTAT   
  
  
- CAAAATTACT TCATTTCAAA ATTTAAAAAT GAAATTACGA GAATTGTGAT TTTTTATTTT GAGACTAAAA   
  
  
- AATTGTTAGT CAGCAGGTTG ATCACATCG

+     ACE

| Site Name | Organism | Position | Strand | Matrix score. | sequence | function |
| --- | --- | --- | --- | --- | --- | --- |
| ACE | Petroselinum crispum | 769 | - | 9 | CTAACGTATT | cis-acting element involved in light responsiveness |
| ACE | Petroselinum crispum | 1289 | - | 9 | AAAACGTTTA | cis-acting element involved in light responsiveness |

> 2018/04/13 10:10:12  
+ TCGAGAAGAA GCAAGAGAAC TTACCTGGTA GAGTTGTGAA ACAAGAGAAG AAAATATCTC TCCGACGTTT   
  
  
+ ACAAACTTAG GAAATTATAT GTGTTGCAGT TGTCGACGGA TAGGTTAACG TGATTTTTTG TCACGGGGTT   
  
  
+ GGTATATAAT TAATATGCTA GGGATTGGTA TTAATTAATG CGAATTAATT CATGCTTATT AAGTTGAAGA   
  
  
+ AGGTAGTAAA TCTAATATGT TTTACAGACG TAACTGTTGT TCTCTCTCGA AAGTCGGCAC TCCGGGCCTC   
  
  
+ GGTTGGTAGG TCCATATGAG TACGGGATAT AGGTACTTCA AAGCATGCCA TGTATTTCAG TGTACCTGTG   
  
  
+ TTGGCAAAGC AACCAGTTTA ATTGTCAAAA TATGTATCAA GGCAGAATTA CAGAATTTAT GTCCGTTATA   
  
  
+ CTGTTTTCAA AGAAGACTTT CCAGAGGAAA CAGCAGACCA AGGACGTAAT CCCAAAATGG CACCTCTCTC   
  
  
+ TGTACCCTAA TAGTTCTTAA CATTGTAGAT TTTTTTAATG GATTGTAGAT TTTTATGCTC AGCCAGGTTG   
  
  
+ GGCACAACAG TAAAAAGTGG TATATAATAT TAATACAGAG AAATAAAAAC CATTAGAATT ACAACGTACA   
  
  
+ TTCCGATCCG TACAGAGAAC TCGGCTTATA TATCTGTCAG TTCCCAGTTT ATATTATAAC CATTCATCCC   
  
  
+ ATACGCATAT GTAGAATTAG AACTTTTTAG AAAGATACAA AGCGGCCACT GAATGAAAGG CACCCAAGAA   
  
  
+ TACGTTATCT TTTTAGTCTT TAAAAAAACA GACGAAAACC ACCTCATTTT ATAACGGTTT AAGTAATGCT   
  
  
+ TTGTTAATTA ATTTTTTTAC ACAAATTAAC TAAATAGTTG GAAATAATAT GTCTTCTAAA AGTACTACGA   
  
  
+ ATTCGTACTA CTACTAGCCT AACGCCTTTA CAGATTTAGT TATTAGGCGT TTTAACCTTG AACAAAACGT   
  
  
+ TATAATTTTA TATATAACCC CAATTAAAAC ACTAAGTTTT AATAATTTTC CTTCCGTCTC GTCTTATTTC   
  
  
+ CTTAATTTAT ATAGGTTTCA GCAAACTTAT TAATTTAAAA CCTCGGAAAA ATTTTCAGTT CTTTTATCTC   
  
  
+ TTCTGTACGA ACCTAACAAA TTATTCCAGC GAAAATTCAA AATTGAGAAA AGAACCTTTA ATCATACTTA   
  
  
+ AAAGTCTCCT TAATCTCACC GTTATATATT ATTTTATTTT ATTTATTTGT TATATGAAAA AATTATAATA   
  
  
+ TTAATATAAT ACTTTCGAAA TGAAAAATTA AACGTTTATA AAATAACTTT TTTATTATAA TAGTAAAAAA   
  
  
+ ATATTAGTTT CATAGTTAAC CCCTAACCTT GGGATTTGAA GACAGAACGA ATAATCACTC TACCTAGATA   
  
  
+ GTTTTAATGA AGTAAAGTTT TAAATTTTTA CTTTAATGCT CTTAACACTA AAAAATAAAA CTCTGATTTT   
  
  
+ TTAACAATCA GTCGTCCAAC TAGTGTAGC  

- AGCTCTTCTT CGTTCTCTTG AATGGACCAT CTCAACACTT TGTTCTCTTC TTTTATAGAG AGGCTGCAAA   
  
  
- TGTTTGAATC CTTTAATATA CACAACGTCA ACAGCTGCCT ATCCAATTGC ACTAAAAAAC AGTGCCCCAA   
  
  
- CCATATATTA ATTATACGAT CCCTAACCAT AATTAATTAC GCTTAATTAA GTACGAATAA TTCAACTTCT   
  
  
- TCCATCATTT AGATTATACA AAATGTCTGC ATTGACAACA AGAGAGAGCT TTCAGCCGTG AGGCCCGGAG   
  
  
- CCAACCATCC AGGTATACTC ATGCCCTATA TCCATGAAGT TTCGTACGGT ACATAAAGTC ACATGGACAC   
  
  
- AACCGTTTCG TTGGTCAAAT TAACAGTTTT ATACATAGTT CCGTCTTAAT GTCTTAAATA CAGGCAATAT   
  
  
- GACAAAAGTT TCTTCTGAAA GGTCTCCTTT GTCGTCTGGT TCCTGCATTA GGGTTTTACC GTGGAGAGAG   
  
  
- ACATGGGATT ATCAAGAATT GTAACATCTA AAAAAATTAC CTAACATCTA AAAATACGAG TCGGTCCAAC   
  
  
- CCGTGTTGTC ATTTTTCACC ATATATTATA ATTATGTCTC TTTATTTTTG GTAATCTTAA TGTTGCATGT   
  
  
- AAGGCTAGGC ATGTCTCTTG AGCCGAATAT ATAGACAGTC AAGGGTCAAA TATAATATTG GTAAGTAGGG   
  
  
- TATGCGTATA CATCTTAATC TTGAAAAATC TTTCTATGTT TCGCCGGTGA CTTACTTTCC GTGGGTTCTT   
  
  
- ATGCAATAGA AAAATCAGAA ATTTTTTTGT CTGCTTTTGG TGGAGTAAAA TATTGCCAAA TTCATTACGA   
  
  
- AACAATTAAT TAAAAAAATG TGTTTAATTG ATTTATCAAC CTTTATTATA CAGAAGATTT TCATGATGCT   
  
  
- TAAGCATGAT GATGATCGGA TTGCGGAAAT GTCTAAATCA ATAATCCGCA AAATTGGAAC TTGTTTTGCA   
  
  
- ATATTAAAAT ATATATTGGG GTTAATTTTG TGATTCAAAA TTATTAAAAG GAAGGCAGAG CAGAATAAAG   
  
  
- GAATTAAATA TATCCAAAGT CGTTTGAATA ATTAAATTTT GGAGCCTTTT TAAAAGTCAA GAAAATAGAG   
  
  
- AAGACATGCT TGGATTGTTT AATAAGGTCG CTTTTAAGTT TTAACTCTTT TCTTGGAAAT TAGTATGAAT   
  
  
- TTTCAGAGGA ATTAGAGTGG CAATATATAA TAAAATAAAA TAAATAAACA ATATACTTTT TTAATATTAT   
  
  
- AATTATATTA TGAAAGCTTT ACTTTTTAAT TTGCAAATAT TTTATTGAAA AAATAATATT ATCATTTTTT   
  
  
- TATAATCAAA GTATCAATTG GGGATTGGAA CCCTAAACTT CTGTCTTGCT TATTAGTGAG ATGGATCTAT   
  
  
- CAAAATTACT TCATTTCAAA ATTTAAAAAT GAAATTACGA GAATTGTGAT TTTTTATTTT GAGACTAAAA   
  
  
- AATTGTTAGT CAGCAGGTTG ATCACATCG

+     ARE

| Site Name | Organism | Position | Strand | Matrix score. | sequence | function |
| --- | --- | --- | --- | --- | --- | --- |
| ARE | Zea mays | 607 | - | 6 | TGGTTT | cis-acting regulatory element essential for the anaerobic induction |
| ARE | Zea mays | 806 | - | 6 | TGGTTT | cis-acting regulatory element essential for the anaerobic induction |

> 2018/04/13 10:10:12  
+ TCGAGAAGAA GCAAGAGAAC TTACCTGGTA GAGTTGTGAA ACAAGAGAAG AAAATATCTC TCCGACGTTT   
  
  
+ ACAAACTTAG GAAATTATAT GTGTTGCAGT TGTCGACGGA TAGGTTAACG TGATTTTTTG TCACGGGGTT   
  
  
+ GGTATATAAT TAATATGCTA GGGATTGGTA TTAATTAATG CGAATTAATT CATGCTTATT AAGTTGAAGA   
  
  
+ AGGTAGTAAA TCTAATATGT TTTACAGACG TAACTGTTGT TCTCTCTCGA AAGTCGGCAC TCCGGGCCTC   
  
  
+ GGTTGGTAGG TCCATATGAG TACGGGATAT AGGTACTTCA AAGCATGCCA TGTATTTCAG TGTACCTGTG   
  
  
+ TTGGCAAAGC AACCAGTTTA ATTGTCAAAA TATGTATCAA GGCAGAATTA CAGAATTTAT GTCCGTTATA   
  
  
+ CTGTTTTCAA AGAAGACTTT CCAGAGGAAA CAGCAGACCA AGGACGTAAT CCCAAAATGG CACCTCTCTC   
  
  
+ TGTACCCTAA TAGTTCTTAA CATTGTAGAT TTTTTTAATG GATTGTAGAT TTTTATGCTC AGCCAGGTTG   
  
  
+ GGCACAACAG TAAAAAGTGG TATATAATAT TAATACAGAG AAATAAAAAC CATTAGAATT ACAACGTACA   
  
  
+ TTCCGATCCG TACAGAGAAC TCGGCTTATA TATCTGTCAG TTCCCAGTTT ATATTATAAC CATTCATCCC   
  
  
+ ATACGCATAT GTAGAATTAG AACTTTTTAG AAAGATACAA AGCGGCCACT GAATGAAAGG CACCCAAGAA   
  
  
+ TACGTTATCT TTTTAGTCTT TAAAAAAACA GACGAAAACC ACCTCATTTT ATAACGGTTT AAGTAATGCT   
  
  
+ TTGTTAATTA ATTTTTTTAC ACAAATTAAC TAAATAGTTG GAAATAATAT GTCTTCTAAA AGTACTACGA   
  
  
+ ATTCGTACTA CTACTAGCCT AACGCCTTTA CAGATTTAGT TATTAGGCGT TTTAACCTTG AACAAAACGT   
  
  
+ TATAATTTTA TATATAACCC CAATTAAAAC ACTAAGTTTT AATAATTTTC CTTCCGTCTC GTCTTATTTC   
  
  
+ CTTAATTTAT ATAGGTTTCA GCAAACTTAT TAATTTAAAA CCTCGGAAAA ATTTTCAGTT CTTTTATCTC   
  
  
+ TTCTGTACGA ACCTAACAAA TTATTCCAGC GAAAATTCAA AATTGAGAAA AGAACCTTTA ATCATACTTA   
  
  
+ AAAGTCTCCT TAATCTCACC GTTATATATT ATTTTATTTT ATTTATTTGT TATATGAAAA AATTATAATA   
  
  
+ TTAATATAAT ACTTTCGAAA TGAAAAATTA AACGTTTATA AAATAACTTT TTTATTATAA TAGTAAAAAA   
  
  
+ ATATTAGTTT CATAGTTAAC CCCTAACCTT GGGATTTGAA GACAGAACGA ATAATCACTC TACCTAGATA   
  
  
+ GTTTTAATGA AGTAAAGTTT TAAATTTTTA CTTTAATGCT CTTAACACTA AAAAATAAAA CTCTGATTTT   
  
  
+ TTAACAATCA GTCGTCCAAC TAGTGTAGC  

- AGCTCTTCTT CGTTCTCTTG AATGGACCAT CTCAACACTT TGTTCTCTTC TTTTATAGAG AGGCTGCAAA   
  
  
- TGTTTGAATC CTTTAATATA CACAACGTCA ACAGCTGCCT ATCCAATTGC ACTAAAAAAC AGTGCCCCAA   
  
  
- CCATATATTA ATTATACGAT CCCTAACCAT AATTAATTAC GCTTAATTAA GTACGAATAA TTCAACTTCT   
  
  
- TCCATCATTT AGATTATACA AAATGTCTGC ATTGACAACA AGAGAGAGCT TTCAGCCGTG AGGCCCGGAG   
  
  
- CCAACCATCC AGGTATACTC ATGCCCTATA TCCATGAAGT TTCGTACGGT ACATAAAGTC ACATGGACAC   
  
  
- AACCGTTTCG TTGGTCAAAT TAACAGTTTT ATACATAGTT CCGTCTTAAT GTCTTAAATA CAGGCAATAT   
  
  
- GACAAAAGTT TCTTCTGAAA GGTCTCCTTT GTCGTCTGGT TCCTGCATTA GGGTTTTACC GTGGAGAGAG   
  
  
- ACATGGGATT ATCAAGAATT GTAACATCTA AAAAAATTAC CTAACATCTA AAAATACGAG TCGGTCCAAC   
  
  
- CCGTGTTGTC ATTTTTCACC ATATATTATA ATTATGTCTC TTTATTTTTG GTAATCTTAA TGTTGCATGT   
  
  
- AAGGCTAGGC ATGTCTCTTG AGCCGAATAT ATAGACAGTC AAGGGTCAAA TATAATATTG GTAAGTAGGG   
  
  
- TATGCGTATA CATCTTAATC TTGAAAAATC TTTCTATGTT TCGCCGGTGA CTTACTTTCC GTGGGTTCTT   
  
  
- ATGCAATAGA AAAATCAGAA ATTTTTTTGT CTGCTTTTGG TGGAGTAAAA TATTGCCAAA TTCATTACGA   
  
  
- AACAATTAAT TAAAAAAATG TGTTTAATTG ATTTATCAAC CTTTATTATA CAGAAGATTT TCATGATGCT   
  
  
- TAAGCATGAT GATGATCGGA TTGCGGAAAT GTCTAAATCA ATAATCCGCA AAATTGGAAC TTGTTTTGCA   
  
  
- ATATTAAAAT ATATATTGGG GTTAATTTTG TGATTCAAAA TTATTAAAAG GAAGGCAGAG CAGAATAAAG   
  
  
- GAATTAAATA TATCCAAAGT CGTTTGAATA ATTAAATTTT GGAGCCTTTT TAAAAGTCAA GAAAATAGAG   
  
  
- AAGACATGCT TGGATTGTTT AATAAGGTCG CTTTTAAGTT TTAACTCTTT TCTTGGAAAT TAGTATGAAT   
  
  
- TTTCAGAGGA ATTAGAGTGG CAATATATAA TAAAATAAAA TAAATAAACA ATATACTTTT TTAATATTAT   
  
  
- AATTATATTA TGAAAGCTTT ACTTTTTAAT TTGCAAATAT TTTATTGAAA AAATAATATT ATCATTTTTT   
  
  
- TATAATCAAA GTATCAATTG GGGATTGGAA CCCTAAACTT CTGTCTTGCT TATTAGTGAG ATGGATCTAT   
  
  
- CAAAATTACT TCATTTCAAA ATTTAAAAAT GAAATTACGA GAATTGTGAT TTTTTATTTT GAGACTAAAA   
  
  
- AATTGTTAGT CAGCAGGTTG ATCACATCG

+     ATCT-motif

| Site Name | Organism | Position | Strand | Matrix score. | sequence | function |
| --- | --- | --- | --- | --- | --- | --- |
| ATCT-motif | Arabidopsis thaliana | 219 | + | 9 | AATCTAATCT | part of a conserved DNA module involved in light responsiveness |

> 2018/04/13 10:10:12  
+ TCGAGAAGAA GCAAGAGAAC TTACCTGGTA GAGTTGTGAA ACAAGAGAAG AAAATATCTC TCCGACGTTT   
  
  
+ ACAAACTTAG GAAATTATAT GTGTTGCAGT TGTCGACGGA TAGGTTAACG TGATTTTTTG TCACGGGGTT   
  
  
+ GGTATATAAT TAATATGCTA GGGATTGGTA TTAATTAATG CGAATTAATT CATGCTTATT AAGTTGAAGA   
  
  
+ AGGTAGTAAA TCTAATATGT TTTACAGACG TAACTGTTGT TCTCTCTCGA AAGTCGGCAC TCCGGGCCTC   
  
  
+ GGTTGGTAGG TCCATATGAG TACGGGATAT AGGTACTTCA AAGCATGCCA TGTATTTCAG TGTACCTGTG   
  
  
+ TTGGCAAAGC AACCAGTTTA ATTGTCAAAA TATGTATCAA GGCAGAATTA CAGAATTTAT GTCCGTTATA   
  
  
+ CTGTTTTCAA AGAAGACTTT CCAGAGGAAA CAGCAGACCA AGGACGTAAT CCCAAAATGG CACCTCTCTC   
  
  
+ TGTACCCTAA TAGTTCTTAA CATTGTAGAT TTTTTTAATG GATTGTAGAT TTTTATGCTC AGCCAGGTTG   
  
  
+ GGCACAACAG TAAAAAGTGG TATATAATAT TAATACAGAG AAATAAAAAC CATTAGAATT ACAACGTACA   
  
  
+ TTCCGATCCG TACAGAGAAC TCGGCTTATA TATCTGTCAG TTCCCAGTTT ATATTATAAC CATTCATCCC   
  
  
+ ATACGCATAT GTAGAATTAG AACTTTTTAG AAAGATACAA AGCGGCCACT GAATGAAAGG CACCCAAGAA   
  
  
+ TACGTTATCT TTTTAGTCTT TAAAAAAACA GACGAAAACC ACCTCATTTT ATAACGGTTT AAGTAATGCT   
  
  
+ TTGTTAATTA ATTTTTTTAC ACAAATTAAC TAAATAGTTG GAAATAATAT GTCTTCTAAA AGTACTACGA   
  
  
+ ATTCGTACTA CTACTAGCCT AACGCCTTTA CAGATTTAGT TATTAGGCGT TTTAACCTTG AACAAAACGT   
  
  
+ TATAATTTTA TATATAACCC CAATTAAAAC ACTAAGTTTT AATAATTTTC CTTCCGTCTC GTCTTATTTC   
  
  
+ CTTAATTTAT ATAGGTTTCA GCAAACTTAT TAATTTAAAA CCTCGGAAAA ATTTTCAGTT CTTTTATCTC   
  
  
+ TTCTGTACGA ACCTAACAAA TTATTCCAGC GAAAATTCAA AATTGAGAAA AGAACCTTTA ATCATACTTA   
  
  
+ AAAGTCTCCT TAATCTCACC GTTATATATT ATTTTATTTT ATTTATTTGT TATATGAAAA AATTATAATA   
  
  
+ TTAATATAAT ACTTTCGAAA TGAAAAATTA AACGTTTATA AAATAACTTT TTTATTATAA TAGTAAAAAA   
  
  
+ ATATTAGTTT CATAGTTAAC CCCTAACCTT GGGATTTGAA GACAGAACGA ATAATCACTC TACCTAGATA   
  
  
+ GTTTTAATGA AGTAAAGTTT TAAATTTTTA CTTTAATGCT CTTAACACTA AAAAATAAAA CTCTGATTTT   
  
  
+ TTAACAATCA GTCGTCCAAC TAGTGTAGC  

- AGCTCTTCTT CGTTCTCTTG AATGGACCAT CTCAACACTT TGTTCTCTTC TTTTATAGAG AGGCTGCAAA   
  
  
- TGTTTGAATC CTTTAATATA CACAACGTCA ACAGCTGCCT ATCCAATTGC ACTAAAAAAC AGTGCCCCAA   
  
  
- CCATATATTA ATTATACGAT CCCTAACCAT AATTAATTAC GCTTAATTAA GTACGAATAA TTCAACTTCT   
  
  
- TCCATCATTT AGATTATACA AAATGTCTGC ATTGACAACA AGAGAGAGCT TTCAGCCGTG AGGCCCGGAG   
  
  
- CCAACCATCC AGGTATACTC ATGCCCTATA TCCATGAAGT TTCGTACGGT ACATAAAGTC ACATGGACAC   
  
  
- AACCGTTTCG TTGGTCAAAT TAACAGTTTT ATACATAGTT CCGTCTTAAT GTCTTAAATA CAGGCAATAT   
  
  
- GACAAAAGTT TCTTCTGAAA GGTCTCCTTT GTCGTCTGGT TCCTGCATTA GGGTTTTACC GTGGAGAGAG   
  
  
- ACATGGGATT ATCAAGAATT GTAACATCTA AAAAAATTAC CTAACATCTA AAAATACGAG TCGGTCCAAC   
  
  
- CCGTGTTGTC ATTTTTCACC ATATATTATA ATTATGTCTC TTTATTTTTG GTAATCTTAA TGTTGCATGT   
  
  
- AAGGCTAGGC ATGTCTCTTG AGCCGAATAT ATAGACAGTC AAGGGTCAAA TATAATATTG GTAAGTAGGG   
  
  
- TATGCGTATA CATCTTAATC TTGAAAAATC TTTCTATGTT TCGCCGGTGA CTTACTTTCC GTGGGTTCTT   
  
  
- ATGCAATAGA AAAATCAGAA ATTTTTTTGT CTGCTTTTGG TGGAGTAAAA TATTGCCAAA TTCATTACGA   
  
  
- AACAATTAAT TAAAAAAATG TGTTTAATTG ATTTATCAAC CTTTATTATA CAGAAGATTT TCATGATGCT   
  
  
- TAAGCATGAT GATGATCGGA TTGCGGAAAT GTCTAAATCA ATAATCCGCA AAATTGGAAC TTGTTTTGCA   
  
  
- ATATTAAAAT ATATATTGGG GTTAATTTTG TGATTCAAAA TTATTAAAAG GAAGGCAGAG CAGAATAAAG   
  
  
- GAATTAAATA TATCCAAAGT CGTTTGAATA ATTAAATTTT GGAGCCTTTT TAAAAGTCAA GAAAATAGAG   
  
  
- AAGACATGCT TGGATTGTTT AATAAGGTCG CTTTTAAGTT TTAACTCTTT TCTTGGAAAT TAGTATGAAT   
  
  
- TTTCAGAGGA ATTAGAGTGG CAATATATAA TAAAATAAAA TAAATAAACA ATATACTTTT TTAATATTAT   
  
  
- AATTATATTA TGAAAGCTTT ACTTTTTAAT TTGCAAATAT TTTATTGAAA AAATAATATT ATCATTTTTT   
  
  
- TATAATCAAA GTATCAATTG GGGATTGGAA CCCTAAACTT CTGTCTTGCT TATTAGTGAG ATGGATCTAT   
  
  
- CAAAATTACT TCATTTCAAA ATTTAAAAAT GAAATTACGA GAATTGTGAT TTTTTATTTT GAGACTAAAA   
  
  
- AATTGTTAGT CAGCAGGTTG ATCACATCG

+     AuxRR-core

| Site Name | Organism | Position | Strand | Matrix score. | sequence | function |
| --- | --- | --- | --- | --- | --- | --- |
| AuxRR-core | Nicotiana tabacum | 289 | + | 7 | GGTCCAT | cis-acting regulatory element involved in auxin responsiveness |

> 2018/04/13 10:10:12  
+ TCGAGAAGAA GCAAGAGAAC TTACCTGGTA GAGTTGTGAA ACAAGAGAAG AAAATATCTC TCCGACGTTT   
  
  
+ ACAAACTTAG GAAATTATAT GTGTTGCAGT TGTCGACGGA TAGGTTAACG TGATTTTTTG TCACGGGGTT   
  
  
+ GGTATATAAT TAATATGCTA GGGATTGGTA TTAATTAATG CGAATTAATT CATGCTTATT AAGTTGAAGA   
  
  
+ AGGTAGTAAA TCTAATATGT TTTACAGACG TAACTGTTGT TCTCTCTCGA AAGTCGGCAC TCCGGGCCTC   
  
  
+ GGTTGGTAGG TCCATATGAG TACGGGATAT AGGTACTTCA AAGCATGCCA TGTATTTCAG TGTACCTGTG   
  
  
+ TTGGCAAAGC AACCAGTTTA ATTGTCAAAA TATGTATCAA GGCAGAATTA CAGAATTTAT GTCCGTTATA   
  
  
+ CTGTTTTCAA AGAAGACTTT CCAGAGGAAA CAGCAGACCA AGGACGTAAT CCCAAAATGG CACCTCTCTC   
  
  
+ TGTACCCTAA TAGTTCTTAA CATTGTAGAT TTTTTTAATG GATTGTAGAT TTTTATGCTC AGCCAGGTTG   
  
  
+ GGCACAACAG TAAAAAGTGG TATATAATAT TAATACAGAG AAATAAAAAC CATTAGAATT ACAACGTACA   
  
  
+ TTCCGATCCG TACAGAGAAC TCGGCTTATA TATCTGTCAG TTCCCAGTTT ATATTATAAC CATTCATCCC   
  
  
+ ATACGCATAT GTAGAATTAG AACTTTTTAG AAAGATACAA AGCGGCCACT GAATGAAAGG CACCCAAGAA   
  
  
+ TACGTTATCT TTTTAGTCTT TAAAAAAACA GACGAAAACC ACCTCATTTT ATAACGGTTT AAGTAATGCT   
  
  
+ TTGTTAATTA ATTTTTTTAC ACAAATTAAC TAAATAGTTG GAAATAATAT GTCTTCTAAA AGTACTACGA   
  
  
+ ATTCGTACTA CTACTAGCCT AACGCCTTTA CAGATTTAGT TATTAGGCGT TTTAACCTTG AACAAAACGT   
  
  
+ TATAATTTTA TATATAACCC CAATTAAAAC ACTAAGTTTT AATAATTTTC CTTCCGTCTC GTCTTATTTC   
  
  
+ CTTAATTTAT ATAGGTTTCA GCAAACTTAT TAATTTAAAA CCTCGGAAAA ATTTTCAGTT CTTTTATCTC   
  
  
+ TTCTGTACGA ACCTAACAAA TTATTCCAGC GAAAATTCAA AATTGAGAAA AGAACCTTTA ATCATACTTA   
  
  
+ AAAGTCTCCT TAATCTCACC GTTATATATT ATTTTATTTT ATTTATTTGT TATATGAAAA AATTATAATA   
  
  
+ TTAATATAAT ACTTTCGAAA TGAAAAATTA AACGTTTATA AAATAACTTT TTTATTATAA TAGTAAAAAA   
  
  
+ ATATTAGTTT CATAGTTAAC CCCTAACCTT GGGATTTGAA GACAGAACGA ATAATCACTC TACCTAGATA   
  
  
+ GTTTTAATGA AGTAAAGTTT TAAATTTTTA CTTTAATGCT CTTAACACTA AAAAATAAAA CTCTGATTTT   
  
  
+ TTAACAATCA GTCGTCCAAC TAGTGTAGC  

- AGCTCTTCTT CGTTCTCTTG AATGGACCAT CTCAACACTT TGTTCTCTTC TTTTATAGAG AGGCTGCAAA   
  
  
- TGTTTGAATC CTTTAATATA CACAACGTCA ACAGCTGCCT ATCCAATTGC ACTAAAAAAC AGTGCCCCAA   
  
  
- CCATATATTA ATTATACGAT CCCTAACCAT AATTAATTAC GCTTAATTAA GTACGAATAA TTCAACTTCT   
  
  
- TCCATCATTT AGATTATACA AAATGTCTGC ATTGACAACA AGAGAGAGCT TTCAGCCGTG AGGCCCGGAG   
  
  
- CCAACCATCC AGGTATACTC ATGCCCTATA TCCATGAAGT TTCGTACGGT ACATAAAGTC ACATGGACAC   
  
  
- AACCGTTTCG TTGGTCAAAT TAACAGTTTT ATACATAGTT CCGTCTTAAT GTCTTAAATA CAGGCAATAT   
  
  
- GACAAAAGTT TCTTCTGAAA GGTCTCCTTT GTCGTCTGGT TCCTGCATTA GGGTTTTACC GTGGAGAGAG   
  
  
- ACATGGGATT ATCAAGAATT GTAACATCTA AAAAAATTAC CTAACATCTA AAAATACGAG TCGGTCCAAC   
  
  
- CCGTGTTGTC ATTTTTCACC ATATATTATA ATTATGTCTC TTTATTTTTG GTAATCTTAA TGTTGCATGT   
  
  
- AAGGCTAGGC ATGTCTCTTG AGCCGAATAT ATAGACAGTC AAGGGTCAAA TATAATATTG GTAAGTAGGG   
  
  
- TATGCGTATA CATCTTAATC TTGAAAAATC TTTCTATGTT TCGCCGGTGA CTTACTTTCC GTGGGTTCTT   
  
  
- ATGCAATAGA AAAATCAGAA ATTTTTTTGT CTGCTTTTGG TGGAGTAAAA TATTGCCAAA TTCATTACGA   
  
  
- AACAATTAAT TAAAAAAATG TGTTTAATTG ATTTATCAAC CTTTATTATA CAGAAGATTT TCATGATGCT   
  
  
- TAAGCATGAT GATGATCGGA TTGCGGAAAT GTCTAAATCA ATAATCCGCA AAATTGGAAC TTGTTTTGCA   
  
  
- ATATTAAAAT ATATATTGGG GTTAATTTTG TGATTCAAAA TTATTAAAAG GAAGGCAGAG CAGAATAAAG   
  
  
- GAATTAAATA TATCCAAAGT CGTTTGAATA ATTAAATTTT GGAGCCTTTT TAAAAGTCAA GAAAATAGAG   
  
  
- AAGACATGCT TGGATTGTTT AATAAGGTCG CTTTTAAGTT TTAACTCTTT TCTTGGAAAT TAGTATGAAT   
  
  
- TTTCAGAGGA ATTAGAGTGG CAATATATAA TAAAATAAAA TAAATAAACA ATATACTTTT TTAATATTAT   
  
  
- AATTATATTA TGAAAGCTTT ACTTTTTAAT TTGCAAATAT TTTATTGAAA AAATAATATT ATCATTTTTT   
  
  
- TATAATCAAA GTATCAATTG GGGATTGGAA CCCTAAACTT CTGTCTTGCT TATTAGTGAG ATGGATCTAT   
  
  
- CAAAATTACT TCATTTCAAA ATTTAAAAAT GAAATTACGA GAATTGTGAT TTTTTATTTT GAGACTAAAA   
  
  
- AATTGTTAGT CAGCAGGTTG ATCACATCG

+     Box 4

| Site Name | Organism | Position | Strand | Matrix score. | sequence | function |
| --- | --- | --- | --- | --- | --- | --- |
| Box 4 | Petroselinum crispum | 149 | + | 6 | ATTAAT | part of a conserved DNA module involved in light responsiveness |
| Box 4 | Petroselinum crispum | 847 | - | 6 | ATTAAT | part of a conserved DNA module involved in light responsiveness |
| Box 4 | Petroselinum crispum | 170 | + | 6 | ATTAAT | part of a conserved DNA module involved in light responsiveness |
| Box 4 | Petroselinum crispum | 589 | + | 6 | ATTAAT | part of a conserved DNA module involved in light responsiveness |
| Box 4 | Petroselinum crispum | 1079 | - | 6 | ATTAAT | part of a conserved DNA module involved in light responsiveness |
| Box 4 | Petroselinum crispum | 1260 | - | 6 | ATTAAT | part of a conserved DNA module involved in light responsiveness |
| Box 4 | Petroselinum crispum | 174 | + | 6 | ATTAAT | part of a conserved DNA module involved in light responsiveness |
| Box 4 | Petroselinum crispum | 184 | + | 6 | ATTAAT | part of a conserved DNA module involved in light responsiveness |

> 2018/04/13 10:10:12  
+ TCGAGAAGAA GCAAGAGAAC TTACCTGGTA GAGTTGTGAA ACAAGAGAAG AAAATATCTC TCCGACGTTT   
  
  
+ ACAAACTTAG GAAATTATAT GTGTTGCAGT TGTCGACGGA TAGGTTAACG TGATTTTTTG TCACGGGGTT   
  
  
+ GGTATATAAT TAATATGCTA GGGATTGGTA TTAATTAATG CGAATTAATT CATGCTTATT AAGTTGAAGA   
  
  
+ AGGTAGTAAA TCTAATATGT TTTACAGACG TAACTGTTGT TCTCTCTCGA AAGTCGGCAC TCCGGGCCTC   
  
  
+ GGTTGGTAGG TCCATATGAG TACGGGATAT AGGTACTTCA AAGCATGCCA TGTATTTCAG TGTACCTGTG   
  
  
+ TTGGCAAAGC AACCAGTTTA ATTGTCAAAA TATGTATCAA GGCAGAATTA CAGAATTTAT GTCCGTTATA   
  
  
+ CTGTTTTCAA AGAAGACTTT CCAGAGGAAA CAGCAGACCA AGGACGTAAT CCCAAAATGG CACCTCTCTC   
  
  
+ TGTACCCTAA TAGTTCTTAA CATTGTAGAT TTTTTTAATG GATTGTAGAT TTTTATGCTC AGCCAGGTTG   
  
  
+ GGCACAACAG TAAAAAGTGG TATATAATAT TAATACAGAG AAATAAAAAC CATTAGAATT ACAACGTACA   
  
  
+ TTCCGATCCG TACAGAGAAC TCGGCTTATA TATCTGTCAG TTCCCAGTTT ATATTATAAC CATTCATCCC   
  
  
+ ATACGCATAT GTAGAATTAG AACTTTTTAG AAAGATACAA AGCGGCCACT GAATGAAAGG CACCCAAGAA   
  
  
+ TACGTTATCT TTTTAGTCTT TAAAAAAACA GACGAAAACC ACCTCATTTT ATAACGGTTT AAGTAATGCT   
  
  
+ TTGTTAATTA ATTTTTTTAC ACAAATTAAC TAAATAGTTG GAAATAATAT GTCTTCTAAA AGTACTACGA   
  
  
+ ATTCGTACTA CTACTAGCCT AACGCCTTTA CAGATTTAGT TATTAGGCGT TTTAACCTTG AACAAAACGT   
  
  
+ TATAATTTTA TATATAACCC CAATTAAAAC ACTAAGTTTT AATAATTTTC CTTCCGTCTC GTCTTATTTC   
  
  
+ CTTAATTTAT ATAGGTTTCA GCAAACTTAT TAATTTAAAA CCTCGGAAAA ATTTTCAGTT CTTTTATCTC   
  
  
+ TTCTGTACGA ACCTAACAAA TTATTCCAGC GAAAATTCAA AATTGAGAAA AGAACCTTTA ATCATACTTA   
  
  
+ AAAGTCTCCT TAATCTCACC GTTATATATT ATTTTATTTT ATTTATTTGT TATATGAAAA AATTATAATA   
  
  
+ TTAATATAAT ACTTTCGAAA TGAAAAATTA AACGTTTATA AAATAACTTT TTTATTATAA TAGTAAAAAA   
  
  
+ ATATTAGTTT CATAGTTAAC CCCTAACCTT GGGATTTGAA GACAGAACGA ATAATCACTC TACCTAGATA   
  
  
+ GTTTTAATGA AGTAAAGTTT TAAATTTTTA CTTTAATGCT CTTAACACTA AAAAATAAAA CTCTGATTTT   
  
  
+ TTAACAATCA GTCGTCCAAC TAGTGTAGC  

- AGCTCTTCTT CGTTCTCTTG AATGGACCAT CTCAACACTT TGTTCTCTTC TTTTATAGAG AGGCTGCAAA   
  
  
- TGTTTGAATC CTTTAATATA CACAACGTCA ACAGCTGCCT ATCCAATTGC ACTAAAAAAC AGTGCCCCAA   
  
  
- CCATATATTA ATTATACGAT CCCTAACCAT AATTAATTAC GCTTAATTAA GTACGAATAA TTCAACTTCT   
  
  
- TCCATCATTT AGATTATACA AAATGTCTGC ATTGACAACA AGAGAGAGCT TTCAGCCGTG AGGCCCGGAG   
  
  
- CCAACCATCC AGGTATACTC ATGCCCTATA TCCATGAAGT TTCGTACGGT ACATAAAGTC ACATGGACAC   
  
  
- AACCGTTTCG TTGGTCAAAT TAACAGTTTT ATACATAGTT CCGTCTTAAT GTCTTAAATA CAGGCAATAT   
  
  
- GACAAAAGTT TCTTCTGAAA GGTCTCCTTT GTCGTCTGGT TCCTGCATTA GGGTTTTACC GTGGAGAGAG   
  
  
- ACATGGGATT ATCAAGAATT GTAACATCTA AAAAAATTAC CTAACATCTA AAAATACGAG TCGGTCCAAC   
  
  
- CCGTGTTGTC ATTTTTCACC ATATATTATA ATTATGTCTC TTTATTTTTG GTAATCTTAA TGTTGCATGT   
  
  
- AAGGCTAGGC ATGTCTCTTG AGCCGAATAT ATAGACAGTC AAGGGTCAAA TATAATATTG GTAAGTAGGG   
  
  
- TATGCGTATA CATCTTAATC TTGAAAAATC TTTCTATGTT TCGCCGGTGA CTTACTTTCC GTGGGTTCTT   
  
  
- ATGCAATAGA AAAATCAGAA ATTTTTTTGT CTGCTTTTGG TGGAGTAAAA TATTGCCAAA TTCATTACGA   
  
  
- AACAATTAAT TAAAAAAATG TGTTTAATTG ATTTATCAAC CTTTATTATA CAGAAGATTT TCATGATGCT   
  
  
- TAAGCATGAT GATGATCGGA TTGCGGAAAT GTCTAAATCA ATAATCCGCA AAATTGGAAC TTGTTTTGCA   
  
  
- ATATTAAAAT ATATATTGGG GTTAATTTTG TGATTCAAAA TTATTAAAAG GAAGGCAGAG CAGAATAAAG   
  
  
- GAATTAAATA TATCCAAAGT CGTTTGAATA ATTAAATTTT GGAGCCTTTT TAAAAGTCAA GAAAATAGAG   
  
  
- AAGACATGCT TGGATTGTTT AATAAGGTCG CTTTTAAGTT TTAACTCTTT TCTTGGAAAT TAGTATGAAT   
  
  
- TTTCAGAGGA ATTAGAGTGG CAATATATAA TAAAATAAAA TAAATAAACA ATATACTTTT TTAATATTAT   
  
  
- AATTATATTA TGAAAGCTTT ACTTTTTAAT TTGCAAATAT TTTATTGAAA AAATAATATT ATCATTTTTT   
  
  
- TATAATCAAA GTATCAATTG GGGATTGGAA CCCTAAACTT CTGTCTTGCT TATTAGTGAG ATGGATCTAT   
  
  
- CAAAATTACT TCATTTCAAA ATTTAAAAAT GAAATTACGA GAATTGTGAT TTTTTATTTT GAGACTAAAA   
  
  
- AATTGTTAGT CAGCAGGTTG ATCACATCG

+     Box I

| Site Name | Organism | Position | Strand | Matrix score. | sequence | function |
| --- | --- | --- | --- | --- | --- | --- |
| Box I | Pisum sativum | 425 | + | 7 | TTTCAAA | light responsive element |

> 2018/04/13 10:10:12  
+ TCGAGAAGAA GCAAGAGAAC TTACCTGGTA GAGTTGTGAA ACAAGAGAAG AAAATATCTC TCCGACGTTT   
  
  
+ ACAAACTTAG GAAATTATAT GTGTTGCAGT TGTCGACGGA TAGGTTAACG TGATTTTTTG TCACGGGGTT   
  
  
+ GGTATATAAT TAATATGCTA GGGATTGGTA TTAATTAATG CGAATTAATT CATGCTTATT AAGTTGAAGA   
  
  
+ AGGTAGTAAA TCTAATATGT TTTACAGACG TAACTGTTGT TCTCTCTCGA AAGTCGGCAC TCCGGGCCTC   
  
  
+ GGTTGGTAGG TCCATATGAG TACGGGATAT AGGTACTTCA AAGCATGCCA TGTATTTCAG TGTACCTGTG   
  
  
+ TTGGCAAAGC AACCAGTTTA ATTGTCAAAA TATGTATCAA GGCAGAATTA CAGAATTTAT GTCCGTTATA   
  
  
+ CTGTTTTCAA AGAAGACTTT CCAGAGGAAA CAGCAGACCA AGGACGTAAT CCCAAAATGG CACCTCTCTC   
  
  
+ TGTACCCTAA TAGTTCTTAA CATTGTAGAT TTTTTTAATG GATTGTAGAT TTTTATGCTC AGCCAGGTTG   
  
  
+ GGCACAACAG TAAAAAGTGG TATATAATAT TAATACAGAG AAATAAAAAC CATTAGAATT ACAACGTACA   
  
  
+ TTCCGATCCG TACAGAGAAC TCGGCTTATA TATCTGTCAG TTCCCAGTTT ATATTATAAC CATTCATCCC   
  
  
+ ATACGCATAT GTAGAATTAG AACTTTTTAG AAAGATACAA AGCGGCCACT GAATGAAAGG CACCCAAGAA   
  
  
+ TACGTTATCT TTTTAGTCTT TAAAAAAACA GACGAAAACC ACCTCATTTT ATAACGGTTT AAGTAATGCT   
  
  
+ TTGTTAATTA ATTTTTTTAC ACAAATTAAC TAAATAGTTG GAAATAATAT GTCTTCTAAA AGTACTACGA   
  
  
+ ATTCGTACTA CTACTAGCCT AACGCCTTTA CAGATTTAGT TATTAGGCGT TTTAACCTTG AACAAAACGT   
  
  
+ TATAATTTTA TATATAACCC CAATTAAAAC ACTAAGTTTT AATAATTTTC CTTCCGTCTC GTCTTATTTC   
  
  
+ CTTAATTTAT ATAGGTTTCA GCAAACTTAT TAATTTAAAA CCTCGGAAAA ATTTTCAGTT CTTTTATCTC   
  
  
+ TTCTGTACGA ACCTAACAAA TTATTCCAGC GAAAATTCAA AATTGAGAAA AGAACCTTTA ATCATACTTA   
  
  
+ AAAGTCTCCT TAATCTCACC GTTATATATT ATTTTATTTT ATTTATTTGT TATATGAAAA AATTATAATA   
  
  
+ TTAATATAAT ACTTTCGAAA TGAAAAATTA AACGTTTATA AAATAACTTT TTTATTATAA TAGTAAAAAA   
  
  
+ ATATTAGTTT CATAGTTAAC CCCTAACCTT GGGATTTGAA GACAGAACGA ATAATCACTC TACCTAGATA   
  
  
+ GTTTTAATGA AGTAAAGTTT TAAATTTTTA CTTTAATGCT CTTAACACTA AAAAATAAAA CTCTGATTTT   
  
  
+ TTAACAATCA GTCGTCCAAC TAGTGTAGC  

- AGCTCTTCTT CGTTCTCTTG AATGGACCAT CTCAACACTT TGTTCTCTTC TTTTATAGAG AGGCTGCAAA   
  
  
- TGTTTGAATC CTTTAATATA CACAACGTCA ACAGCTGCCT ATCCAATTGC ACTAAAAAAC AGTGCCCCAA   
  
  
- CCATATATTA ATTATACGAT CCCTAACCAT AATTAATTAC GCTTAATTAA GTACGAATAA TTCAACTTCT   
  
  
- TCCATCATTT AGATTATACA AAATGTCTGC ATTGACAACA AGAGAGAGCT TTCAGCCGTG AGGCCCGGAG   
  
  
- CCAACCATCC AGGTATACTC ATGCCCTATA TCCATGAAGT TTCGTACGGT ACATAAAGTC ACATGGACAC   
  
  
- AACCGTTTCG TTGGTCAAAT TAACAGTTTT ATACATAGTT CCGTCTTAAT GTCTTAAATA CAGGCAATAT   
  
  
- GACAAAAGTT TCTTCTGAAA GGTCTCCTTT GTCGTCTGGT TCCTGCATTA GGGTTTTACC GTGGAGAGAG   
  
  
- ACATGGGATT ATCAAGAATT GTAACATCTA AAAAAATTAC CTAACATCTA AAAATACGAG TCGGTCCAAC   
  
  
- CCGTGTTGTC ATTTTTCACC ATATATTATA ATTATGTCTC TTTATTTTTG GTAATCTTAA TGTTGCATGT   
  
  
- AAGGCTAGGC ATGTCTCTTG AGCCGAATAT ATAGACAGTC AAGGGTCAAA TATAATATTG GTAAGTAGGG   
  
  
- TATGCGTATA CATCTTAATC TTGAAAAATC TTTCTATGTT TCGCCGGTGA CTTACTTTCC GTGGGTTCTT   
  
  
- ATGCAATAGA AAAATCAGAA ATTTTTTTGT CTGCTTTTGG TGGAGTAAAA TATTGCCAAA TTCATTACGA   
  
  
- AACAATTAAT TAAAAAAATG TGTTTAATTG ATTTATCAAC CTTTATTATA CAGAAGATTT TCATGATGCT   
  
  
- TAAGCATGAT GATGATCGGA TTGCGGAAAT GTCTAAATCA ATAATCCGCA AAATTGGAAC TTGTTTTGCA   
  
  
- ATATTAAAAT ATATATTGGG GTTAATTTTG TGATTCAAAA TTATTAAAAG GAAGGCAGAG CAGAATAAAG   
  
  
- GAATTAAATA TATCCAAAGT CGTTTGAATA ATTAAATTTT GGAGCCTTTT TAAAAGTCAA GAAAATAGAG   
  
  
- AAGACATGCT TGGATTGTTT AATAAGGTCG CTTTTAAGTT TTAACTCTTT TCTTGGAAAT TAGTATGAAT   
  
  
- TTTCAGAGGA ATTAGAGTGG CAATATATAA TAAAATAAAA TAAATAAACA ATATACTTTT TTAATATTAT   
  
  
- AATTATATTA TGAAAGCTTT ACTTTTTAAT TTGCAAATAT TTTATTGAAA AAATAATATT ATCATTTTTT   
  
  
- TATAATCAAA GTATCAATTG GGGATTGGAA CCCTAAACTT CTGTCTTGCT TATTAGTGAG ATGGATCTAT   
  
  
- CAAAATTACT TCATTTCAAA ATTTAAAAAT GAAATTACGA GAATTGTGAT TTTTTATTTT GAGACTAAAA   
  
  
- AATTGTTAGT CAGCAGGTTG ATCACATCG

+     CAAT-box

| Site Name | Organism | Position | Strand | Matrix score. | sequence | function |
| --- | --- | --- | --- | --- | --- | --- |
| CAAT-box | Hordeum vulgare | 1162 | - | 4 | CAAT | common cis-acting element in promoter and enhancer regions |
| CAAT-box | Hordeum vulgare | 371 | - | 4 | CAAT | common cis-acting element in promoter and enhancer regions |
| CAAT-box | Arabidopsis thaliana | 164 | - | 5 | CCAAT | common cis-acting element in promoter and enhancer regions |
| CAAT-box | Hordeum vulgare | 532 | - | 4 | CAAT | common cis-acting element in promoter and enhancer regions |
| CAAT-box | Glycine max | 370 | - | 5 | CAATT | common cis-acting element in promoter and enhancer regions |
| CAAT-box | Brassica rapa | 1235 | - | 5 | CAAAT | common cis-acting element in promoter and enhancer regions |
| CAAT-box | Hordeum vulgare | 512 | - | 4 | CAAT | common cis-acting element in promoter and enhancer regions |
| CAAT-box | Arabidopsis thaliana | 1000 | + | 5 | CCAAT | common cis-acting element in promoter and enhancer regions |
| CAAT-box | Glycine max | 1001 | + | 5 | CAATT | common cis-acting element in promoter and enhancer regions |
| CAAT-box | Brassica rapa | 862 | + | 5 | CAAAT | common cis-acting element in promoter and enhancer regions |
| CAAT-box | Hordeum vulgare | 1475 | + | 4 | CAAT | common cis-acting element in promoter and enhancer regions |
| CAAT-box | Petunia hybrida | 350 | - | 7 | TGCCAAC | common cis-acting element in promoter and enhancer regions |
| CAAT-box | Brassica rapa | 1137 | + | 5 | CAAAT | common cis-acting element in promoter and enhancer regions |
| CAAT-box | Brassica rapa | 1364 | - | 5 | CAAAT | common cis-acting element in promoter and enhancer regions |
| CAAT-box | Glycine max | 1161 | - | 5 | CAATT | common cis-acting element in promoter and enhancer regions |

> 2018/04/13 10:10:12  
+ TCGAGAAGAA GCAAGAGAAC TTACCTGGTA GAGTTGTGAA ACAAGAGAAG AAAATATCTC TCCGACGTTT   
  
  
+ ACAAACTTAG GAAATTATAT GTGTTGCAGT TGTCGACGGA TAGGTTAACG TGATTTTTTG TCACGGGGTT   
  
  
+ GGTATATAAT TAATATGCTA GGGATTGGTA TTAATTAATG CGAATTAATT CATGCTTATT AAGTTGAAGA   
  
  
+ AGGTAGTAAA TCTAATATGT TTTACAGACG TAACTGTTGT TCTCTCTCGA AAGTCGGCAC TCCGGGCCTC   
  
  
+ GGTTGGTAGG TCCATATGAG TACGGGATAT AGGTACTTCA AAGCATGCCA TGTATTTCAG TGTACCTGTG   
  
  
+ TTGGCAAAGC AACCAGTTTA ATTGTCAAAA TATGTATCAA GGCAGAATTA CAGAATTTAT GTCCGTTATA   
  
  
+ CTGTTTTCAA AGAAGACTTT CCAGAGGAAA CAGCAGACCA AGGACGTAAT CCCAAAATGG CACCTCTCTC   
  
  
+ TGTACCCTAA TAGTTCTTAA CATTGTAGAT TTTTTTAATG GATTGTAGAT TTTTATGCTC AGCCAGGTTG   
  
  
+ GGCACAACAG TAAAAAGTGG TATATAATAT TAATACAGAG AAATAAAAAC CATTAGAATT ACAACGTACA   
  
  
+ TTCCGATCCG TACAGAGAAC TCGGCTTATA TATCTGTCAG TTCCCAGTTT ATATTATAAC CATTCATCCC   
  
  
+ ATACGCATAT GTAGAATTAG AACTTTTTAG AAAGATACAA AGCGGCCACT GAATGAAAGG CACCCAAGAA   
  
  
+ TACGTTATCT TTTTAGTCTT TAAAAAAACA GACGAAAACC ACCTCATTTT ATAACGGTTT AAGTAATGCT   
  
  
+ TTGTTAATTA ATTTTTTTAC ACAAATTAAC TAAATAGTTG GAAATAATAT GTCTTCTAAA AGTACTACGA   
  
  
+ ATTCGTACTA CTACTAGCCT AACGCCTTTA CAGATTTAGT TATTAGGCGT TTTAACCTTG AACAAAACGT   
  
  
+ TATAATTTTA TATATAACCC CAATTAAAAC ACTAAGTTTT AATAATTTTC CTTCCGTCTC GTCTTATTTC   
  
  
+ CTTAATTTAT ATAGGTTTCA GCAAACTTAT TAATTTAAAA CCTCGGAAAA ATTTTCAGTT CTTTTATCTC   
  
  
+ TTCTGTACGA ACCTAACAAA TTATTCCAGC GAAAATTCAA AATTGAGAAA AGAACCTTTA ATCATACTTA   
  
  
+ AAAGTCTCCT TAATCTCACC GTTATATATT ATTTTATTTT ATTTATTTGT TATATGAAAA AATTATAATA   
  
  
+ TTAATATAAT ACTTTCGAAA TGAAAAATTA AACGTTTATA AAATAACTTT TTTATTATAA TAGTAAAAAA   
  
  
+ ATATTAGTTT CATAGTTAAC CCCTAACCTT GGGATTTGAA GACAGAACGA ATAATCACTC TACCTAGATA   
  
  
+ GTTTTAATGA AGTAAAGTTT TAAATTTTTA CTTTAATGCT CTTAACACTA AAAAATAAAA CTCTGATTTT   
  
  
+ TTAACAATCA GTCGTCCAAC TAGTGTAGC  

- AGCTCTTCTT CGTTCTCTTG AATGGACCAT CTCAACACTT TGTTCTCTTC TTTTATAGAG AGGCTGCAAA   
  
  
- TGTTTGAATC CTTTAATATA CACAACGTCA ACAGCTGCCT ATCCAATTGC ACTAAAAAAC AGTGCCCCAA   
  
  
- CCATATATTA ATTATACGAT CCCTAACCAT AATTAATTAC GCTTAATTAA GTACGAATAA TTCAACTTCT   
  
  
- TCCATCATTT AGATTATACA AAATGTCTGC ATTGACAACA AGAGAGAGCT TTCAGCCGTG AGGCCCGGAG   
  
  
- CCAACCATCC AGGTATACTC ATGCCCTATA TCCATGAAGT TTCGTACGGT ACATAAAGTC ACATGGACAC   
  
  
- AACCGTTTCG TTGGTCAAAT TAACAGTTTT ATACATAGTT CCGTCTTAAT GTCTTAAATA CAGGCAATAT   
  
  
- GACAAAAGTT TCTTCTGAAA GGTCTCCTTT GTCGTCTGGT TCCTGCATTA GGGTTTTACC GTGGAGAGAG   
  
  
- ACATGGGATT ATCAAGAATT GTAACATCTA AAAAAATTAC CTAACATCTA AAAATACGAG TCGGTCCAAC   
  
  
- CCGTGTTGTC ATTTTTCACC ATATATTATA ATTATGTCTC TTTATTTTTG GTAATCTTAA TGTTGCATGT   
  
  
- AAGGCTAGGC ATGTCTCTTG AGCCGAATAT ATAGACAGTC AAGGGTCAAA TATAATATTG GTAAGTAGGG   
  
  
- TATGCGTATA CATCTTAATC TTGAAAAATC TTTCTATGTT TCGCCGGTGA CTTACTTTCC GTGGGTTCTT   
  
  
- ATGCAATAGA AAAATCAGAA ATTTTTTTGT CTGCTTTTGG TGGAGTAAAA TATTGCCAAA TTCATTACGA   
  
  
- AACAATTAAT TAAAAAAATG TGTTTAATTG ATTTATCAAC CTTTATTATA CAGAAGATTT TCATGATGCT   
  
  
- TAAGCATGAT GATGATCGGA TTGCGGAAAT GTCTAAATCA ATAATCCGCA AAATTGGAAC TTGTTTTGCA   
  
  
- ATATTAAAAT ATATATTGGG GTTAATTTTG TGATTCAAAA TTATTAAAAG GAAGGCAGAG CAGAATAAAG   
  
  
- GAATTAAATA TATCCAAAGT CGTTTGAATA ATTAAATTTT GGAGCCTTTT TAAAAGTCAA GAAAATAGAG   
  
  
- AAGACATGCT TGGATTGTTT AATAAGGTCG CTTTTAAGTT TTAACTCTTT TCTTGGAAAT TAGTATGAAT   
  
  
- TTTCAGAGGA ATTAGAGTGG CAATATATAA TAAAATAAAA TAAATAAACA ATATACTTTT TTAATATTAT   
  
  
- AATTATATTA TGAAAGCTTT ACTTTTTAAT TTGCAAATAT TTTATTGAAA AAATAATATT ATCATTTTTT   
  
  
- TATAATCAAA GTATCAATTG GGGATTGGAA CCCTAAACTT CTGTCTTGCT TATTAGTGAG ATGGATCTAT   
  
  
- CAAAATTACT TCATTTCAAA ATTTAAAAAT GAAATTACGA GAATTGTGAT TTTTTATTTT GAGACTAAAA   
  
  
- AATTGTTAGT CAGCAGGTTG ATCACATCG

+     CAT-box

| Site Name | Organism | Position | Strand | Matrix score. | sequence | function |
| --- | --- | --- | --- | --- | --- | --- |
| CAT-box | Arabidopsis thaliana | 745 | + | 6 | GCCACT | cis-acting regulatory element related to meristem expression |

> 2018/04/13 10:10:12  
+ TCGAGAAGAA GCAAGAGAAC TTACCTGGTA GAGTTGTGAA ACAAGAGAAG AAAATATCTC TCCGACGTTT   
  
  
+ ACAAACTTAG GAAATTATAT GTGTTGCAGT TGTCGACGGA TAGGTTAACG TGATTTTTTG TCACGGGGTT   
  
  
+ GGTATATAAT TAATATGCTA GGGATTGGTA TTAATTAATG CGAATTAATT CATGCTTATT AAGTTGAAGA   
  
  
+ AGGTAGTAAA TCTAATATGT TTTACAGACG TAACTGTTGT TCTCTCTCGA AAGTCGGCAC TCCGGGCCTC   
  
  
+ GGTTGGTAGG TCCATATGAG TACGGGATAT AGGTACTTCA AAGCATGCCA TGTATTTCAG TGTACCTGTG   
  
  
+ TTGGCAAAGC AACCAGTTTA ATTGTCAAAA TATGTATCAA GGCAGAATTA CAGAATTTAT GTCCGTTATA   
  
  
+ CTGTTTTCAA AGAAGACTTT CCAGAGGAAA CAGCAGACCA AGGACGTAAT CCCAAAATGG CACCTCTCTC   
  
  
+ TGTACCCTAA TAGTTCTTAA CATTGTAGAT TTTTTTAATG GATTGTAGAT TTTTATGCTC AGCCAGGTTG   
  
  
+ GGCACAACAG TAAAAAGTGG TATATAATAT TAATACAGAG AAATAAAAAC CATTAGAATT ACAACGTACA   
  
  
+ TTCCGATCCG TACAGAGAAC TCGGCTTATA TATCTGTCAG TTCCCAGTTT ATATTATAAC CATTCATCCC   
  
  
+ ATACGCATAT GTAGAATTAG AACTTTTTAG AAAGATACAA AGCGGCCACT GAATGAAAGG CACCCAAGAA   
  
  
+ TACGTTATCT TTTTAGTCTT TAAAAAAACA GACGAAAACC ACCTCATTTT ATAACGGTTT AAGTAATGCT   
  
  
+ TTGTTAATTA ATTTTTTTAC ACAAATTAAC TAAATAGTTG GAAATAATAT GTCTTCTAAA AGTACTACGA   
  
  
+ ATTCGTACTA CTACTAGCCT AACGCCTTTA CAGATTTAGT TATTAGGCGT TTTAACCTTG AACAAAACGT   
  
  
+ TATAATTTTA TATATAACCC CAATTAAAAC ACTAAGTTTT AATAATTTTC CTTCCGTCTC GTCTTATTTC   
  
  
+ CTTAATTTAT ATAGGTTTCA GCAAACTTAT TAATTTAAAA CCTCGGAAAA ATTTTCAGTT CTTTTATCTC   
  
  
+ TTCTGTACGA ACCTAACAAA TTATTCCAGC GAAAATTCAA AATTGAGAAA AGAACCTTTA ATCATACTTA   
  
  
+ AAAGTCTCCT TAATCTCACC GTTATATATT ATTTTATTTT ATTTATTTGT TATATGAAAA AATTATAATA   
  
  
+ TTAATATAAT ACTTTCGAAA TGAAAAATTA AACGTTTATA AAATAACTTT TTTATTATAA TAGTAAAAAA   
  
  
+ ATATTAGTTT CATAGTTAAC CCCTAACCTT GGGATTTGAA GACAGAACGA ATAATCACTC TACCTAGATA   
  
  
+ GTTTTAATGA AGTAAAGTTT TAAATTTTTA CTTTAATGCT CTTAACACTA AAAAATAAAA CTCTGATTTT   
  
  
+ TTAACAATCA GTCGTCCAAC TAGTGTAGC  

- AGCTCTTCTT CGTTCTCTTG AATGGACCAT CTCAACACTT TGTTCTCTTC TTTTATAGAG AGGCTGCAAA   
  
  
- TGTTTGAATC CTTTAATATA CACAACGTCA ACAGCTGCCT ATCCAATTGC ACTAAAAAAC AGTGCCCCAA   
  
  
- CCATATATTA ATTATACGAT CCCTAACCAT AATTAATTAC GCTTAATTAA GTACGAATAA TTCAACTTCT   
  
  
- TCCATCATTT AGATTATACA AAATGTCTGC ATTGACAACA AGAGAGAGCT TTCAGCCGTG AGGCCCGGAG   
  
  
- CCAACCATCC AGGTATACTC ATGCCCTATA TCCATGAAGT TTCGTACGGT ACATAAAGTC ACATGGACAC   
  
  
- AACCGTTTCG TTGGTCAAAT TAACAGTTTT ATACATAGTT CCGTCTTAAT GTCTTAAATA CAGGCAATAT   
  
  
- GACAAAAGTT TCTTCTGAAA GGTCTCCTTT GTCGTCTGGT TCCTGCATTA GGGTTTTACC GTGGAGAGAG   
  
  
- ACATGGGATT ATCAAGAATT GTAACATCTA AAAAAATTAC CTAACATCTA AAAATACGAG TCGGTCCAAC   
  
  
- CCGTGTTGTC ATTTTTCACC ATATATTATA ATTATGTCTC TTTATTTTTG GTAATCTTAA TGTTGCATGT   
  
  
- AAGGCTAGGC ATGTCTCTTG AGCCGAATAT ATAGACAGTC AAGGGTCAAA TATAATATTG GTAAGTAGGG   
  
  
- TATGCGTATA CATCTTAATC TTGAAAAATC TTTCTATGTT TCGCCGGTGA CTTACTTTCC GTGGGTTCTT   
  
  
- ATGCAATAGA AAAATCAGAA ATTTTTTTGT CTGCTTTTGG TGGAGTAAAA TATTGCCAAA TTCATTACGA   
  
  
- AACAATTAAT TAAAAAAATG TGTTTAATTG ATTTATCAAC CTTTATTATA CAGAAGATTT TCATGATGCT   
  
  
- TAAGCATGAT GATGATCGGA TTGCGGAAAT GTCTAAATCA ATAATCCGCA AAATTGGAAC TTGTTTTGCA   
  
  
- ATATTAAAAT ATATATTGGG GTTAATTTTG TGATTCAAAA TTATTAAAAG GAAGGCAGAG CAGAATAAAG   
  
  
- GAATTAAATA TATCCAAAGT CGTTTGAATA ATTAAATTTT GGAGCCTTTT TAAAAGTCAA GAAAATAGAG   
  
  
- AAGACATGCT TGGATTGTTT AATAAGGTCG CTTTTAAGTT TTAACTCTTT TCTTGGAAAT TAGTATGAAT   
  
  
- TTTCAGAGGA ATTAGAGTGG CAATATATAA TAAAATAAAA TAAATAAACA ATATACTTTT TTAATATTAT   
  
  
- AATTATATTA TGAAAGCTTT ACTTTTTAAT TTGCAAATAT TTTATTGAAA AAATAATATT ATCATTTTTT   
  
  
- TATAATCAAA GTATCAATTG GGGATTGGAA CCCTAAACTT CTGTCTTGCT TATTAGTGAG ATGGATCTAT   
  
  
- CAAAATTACT TCATTTCAAA ATTTAAAAAT GAAATTACGA GAATTGTGAT TTTTTATTTT GAGACTAAAA   
  
  
- AATTGTTAGT CAGCAGGTTG ATCACATCG

+     G-Box

| Site Name | Organism | Position | Strand | Matrix score. | sequence | function |
| --- | --- | --- | --- | --- | --- | --- |
| G-Box | Pisum sativum | 117 | - | 6 | CACGTT | cis-acting regulatory element involved in light responsiveness |

> 2018/04/13 10:10:12  
+ TCGAGAAGAA GCAAGAGAAC TTACCTGGTA GAGTTGTGAA ACAAGAGAAG AAAATATCTC TCCGACGTTT   
  
  
+ ACAAACTTAG GAAATTATAT GTGTTGCAGT TGTCGACGGA TAGGTTAACG TGATTTTTTG TCACGGGGTT   
  
  
+ GGTATATAAT TAATATGCTA GGGATTGGTA TTAATTAATG CGAATTAATT CATGCTTATT AAGTTGAAGA   
  
  
+ AGGTAGTAAA TCTAATATGT TTTACAGACG TAACTGTTGT TCTCTCTCGA AAGTCGGCAC TCCGGGCCTC   
  
  
+ GGTTGGTAGG TCCATATGAG TACGGGATAT AGGTACTTCA AAGCATGCCA TGTATTTCAG TGTACCTGTG   
  
  
+ TTGGCAAAGC AACCAGTTTA ATTGTCAAAA TATGTATCAA GGCAGAATTA CAGAATTTAT GTCCGTTATA   
  
  
+ CTGTTTTCAA AGAAGACTTT CCAGAGGAAA CAGCAGACCA AGGACGTAAT CCCAAAATGG CACCTCTCTC   
  
  
+ TGTACCCTAA TAGTTCTTAA CATTGTAGAT TTTTTTAATG GATTGTAGAT TTTTATGCTC AGCCAGGTTG   
  
  
+ GGCACAACAG TAAAAAGTGG TATATAATAT TAATACAGAG AAATAAAAAC CATTAGAATT ACAACGTACA   
  
  
+ TTCCGATCCG TACAGAGAAC TCGGCTTATA TATCTGTCAG TTCCCAGTTT ATATTATAAC CATTCATCCC   
  
  
+ ATACGCATAT GTAGAATTAG AACTTTTTAG AAAGATACAA AGCGGCCACT GAATGAAAGG CACCCAAGAA   
  
  
+ TACGTTATCT TTTTAGTCTT TAAAAAAACA GACGAAAACC ACCTCATTTT ATAACGGTTT AAGTAATGCT   
  
  
+ TTGTTAATTA ATTTTTTTAC ACAAATTAAC TAAATAGTTG GAAATAATAT GTCTTCTAAA AGTACTACGA   
  
  
+ ATTCGTACTA CTACTAGCCT AACGCCTTTA CAGATTTAGT TATTAGGCGT TTTAACCTTG AACAAAACGT   
  
  
+ TATAATTTTA TATATAACCC CAATTAAAAC ACTAAGTTTT AATAATTTTC CTTCCGTCTC GTCTTATTTC   
  
  
+ CTTAATTTAT ATAGGTTTCA GCAAACTTAT TAATTTAAAA CCTCGGAAAA ATTTTCAGTT CTTTTATCTC   
  
  
+ TTCTGTACGA ACCTAACAAA TTATTCCAGC GAAAATTCAA AATTGAGAAA AGAACCTTTA ATCATACTTA   
  
  
+ AAAGTCTCCT TAATCTCACC GTTATATATT ATTTTATTTT ATTTATTTGT TATATGAAAA AATTATAATA   
  
  
+ TTAATATAAT ACTTTCGAAA TGAAAAATTA AACGTTTATA AAATAACTTT TTTATTATAA TAGTAAAAAA   
  
  
+ ATATTAGTTT CATAGTTAAC CCCTAACCTT GGGATTTGAA GACAGAACGA ATAATCACTC TACCTAGATA   
  
  
+ GTTTTAATGA AGTAAAGTTT TAAATTTTTA CTTTAATGCT CTTAACACTA AAAAATAAAA CTCTGATTTT   
  
  
+ TTAACAATCA GTCGTCCAAC TAGTGTAGC  

- AGCTCTTCTT CGTTCTCTTG AATGGACCAT CTCAACACTT TGTTCTCTTC TTTTATAGAG AGGCTGCAAA   
  
  
- TGTTTGAATC CTTTAATATA CACAACGTCA ACAGCTGCCT ATCCAATTGC ACTAAAAAAC AGTGCCCCAA   
  
  
- CCATATATTA ATTATACGAT CCCTAACCAT AATTAATTAC GCTTAATTAA GTACGAATAA TTCAACTTCT   
  
  
- TCCATCATTT AGATTATACA AAATGTCTGC ATTGACAACA AGAGAGAGCT TTCAGCCGTG AGGCCCGGAG   
  
  
- CCAACCATCC AGGTATACTC ATGCCCTATA TCCATGAAGT TTCGTACGGT ACATAAAGTC ACATGGACAC   
  
  
- AACCGTTTCG TTGGTCAAAT TAACAGTTTT ATACATAGTT CCGTCTTAAT GTCTTAAATA CAGGCAATAT   
  
  
- GACAAAAGTT TCTTCTGAAA GGTCTCCTTT GTCGTCTGGT TCCTGCATTA GGGTTTTACC GTGGAGAGAG   
  
  
- ACATGGGATT ATCAAGAATT GTAACATCTA AAAAAATTAC CTAACATCTA AAAATACGAG TCGGTCCAAC   
  
  
- CCGTGTTGTC ATTTTTCACC ATATATTATA ATTATGTCTC TTTATTTTTG GTAATCTTAA TGTTGCATGT   
  
  
- AAGGCTAGGC ATGTCTCTTG AGCCGAATAT ATAGACAGTC AAGGGTCAAA TATAATATTG GTAAGTAGGG   
  
  
- TATGCGTATA CATCTTAATC TTGAAAAATC TTTCTATGTT TCGCCGGTGA CTTACTTTCC GTGGGTTCTT   
  
  
- ATGCAATAGA AAAATCAGAA ATTTTTTTGT CTGCTTTTGG TGGAGTAAAA TATTGCCAAA TTCATTACGA   
  
  
- AACAATTAAT TAAAAAAATG TGTTTAATTG ATTTATCAAC CTTTATTATA CAGAAGATTT TCATGATGCT   
  
  
- TAAGCATGAT GATGATCGGA TTGCGGAAAT GTCTAAATCA ATAATCCGCA AAATTGGAAC TTGTTTTGCA   
  
  
- ATATTAAAAT ATATATTGGG GTTAATTTTG TGATTCAAAA TTATTAAAAG GAAGGCAGAG CAGAATAAAG   
  
  
- GAATTAAATA TATCCAAAGT CGTTTGAATA ATTAAATTTT GGAGCCTTTT TAAAAGTCAA GAAAATAGAG   
  
  
- AAGACATGCT TGGATTGTTT AATAAGGTCG CTTTTAAGTT TTAACTCTTT TCTTGGAAAT TAGTATGAAT   
  
  
- TTTCAGAGGA ATTAGAGTGG CAATATATAA TAAAATAAAA TAAATAAACA ATATACTTTT TTAATATTAT   
  
  
- AATTATATTA TGAAAGCTTT ACTTTTTAAT TTGCAAATAT TTTATTGAAA AAATAATATT ATCATTTTTT   
  
  
- TATAATCAAA GTATCAATTG GGGATTGGAA CCCTAAACTT CTGTCTTGCT TATTAGTGAG ATGGATCTAT   
  
  
- CAAAATTACT TCATTTCAAA ATTTAAAAAT GAAATTACGA GAATTGTGAT TTTTTATTTT GAGACTAAAA   
  
  
- AATTGTTAGT CAGCAGGTTG ATCACATCG

+     G-box

| Site Name | Organism | Position | Strand | Matrix score. | sequence | function |
| --- | --- | --- | --- | --- | --- | --- |
| G-box | Zea mays | 117 | - | 6 | CACGTT | cis-acting regulatory element involved in light responsiveness |

> 2018/04/13 10:10:12  
+ TCGAGAAGAA GCAAGAGAAC TTACCTGGTA GAGTTGTGAA ACAAGAGAAG AAAATATCTC TCCGACGTTT   
  
  
+ ACAAACTTAG GAAATTATAT GTGTTGCAGT TGTCGACGGA TAGGTTAACG TGATTTTTTG TCACGGGGTT   
  
  
+ GGTATATAAT TAATATGCTA GGGATTGGTA TTAATTAATG CGAATTAATT CATGCTTATT AAGTTGAAGA   
  
  
+ AGGTAGTAAA TCTAATATGT TTTACAGACG TAACTGTTGT TCTCTCTCGA AAGTCGGCAC TCCGGGCCTC   
  
  
+ GGTTGGTAGG TCCATATGAG TACGGGATAT AGGTACTTCA AAGCATGCCA TGTATTTCAG TGTACCTGTG   
  
  
+ TTGGCAAAGC AACCAGTTTA ATTGTCAAAA TATGTATCAA GGCAGAATTA CAGAATTTAT GTCCGTTATA   
  
  
+ CTGTTTTCAA AGAAGACTTT CCAGAGGAAA CAGCAGACCA AGGACGTAAT CCCAAAATGG CACCTCTCTC   
  
  
+ TGTACCCTAA TAGTTCTTAA CATTGTAGAT TTTTTTAATG GATTGTAGAT TTTTATGCTC AGCCAGGTTG   
  
  
+ GGCACAACAG TAAAAAGTGG TATATAATAT TAATACAGAG AAATAAAAAC CATTAGAATT ACAACGTACA   
  
  
+ TTCCGATCCG TACAGAGAAC TCGGCTTATA TATCTGTCAG TTCCCAGTTT ATATTATAAC CATTCATCCC   
  
  
+ ATACGCATAT GTAGAATTAG AACTTTTTAG AAAGATACAA AGCGGCCACT GAATGAAAGG CACCCAAGAA   
  
  
+ TACGTTATCT TTTTAGTCTT TAAAAAAACA GACGAAAACC ACCTCATTTT ATAACGGTTT AAGTAATGCT   
  
  
+ TTGTTAATTA ATTTTTTTAC ACAAATTAAC TAAATAGTTG GAAATAATAT GTCTTCTAAA AGTACTACGA   
  
  
+ ATTCGTACTA CTACTAGCCT AACGCCTTTA CAGATTTAGT TATTAGGCGT TTTAACCTTG AACAAAACGT   
  
  
+ TATAATTTTA TATATAACCC CAATTAAAAC ACTAAGTTTT AATAATTTTC CTTCCGTCTC GTCTTATTTC   
  
  
+ CTTAATTTAT ATAGGTTTCA GCAAACTTAT TAATTTAAAA CCTCGGAAAA ATTTTCAGTT CTTTTATCTC   
  
  
+ TTCTGTACGA ACCTAACAAA TTATTCCAGC GAAAATTCAA AATTGAGAAA AGAACCTTTA ATCATACTTA   
  
  
+ AAAGTCTCCT TAATCTCACC GTTATATATT ATTTTATTTT ATTTATTTGT TATATGAAAA AATTATAATA   
  
  
+ TTAATATAAT ACTTTCGAAA TGAAAAATTA AACGTTTATA AAATAACTTT TTTATTATAA TAGTAAAAAA   
  
  
+ ATATTAGTTT CATAGTTAAC CCCTAACCTT GGGATTTGAA GACAGAACGA ATAATCACTC TACCTAGATA   
  
  
+ GTTTTAATGA AGTAAAGTTT TAAATTTTTA CTTTAATGCT CTTAACACTA AAAAATAAAA CTCTGATTTT   
  
  
+ TTAACAATCA GTCGTCCAAC TAGTGTAGC  

- AGCTCTTCTT CGTTCTCTTG AATGGACCAT CTCAACACTT TGTTCTCTTC TTTTATAGAG AGGCTGCAAA   
  
  
- TGTTTGAATC CTTTAATATA CACAACGTCA ACAGCTGCCT ATCCAATTGC ACTAAAAAAC AGTGCCCCAA   
  
  
- CCATATATTA ATTATACGAT CCCTAACCAT AATTAATTAC GCTTAATTAA GTACGAATAA TTCAACTTCT   
  
  
- TCCATCATTT AGATTATACA AAATGTCTGC ATTGACAACA AGAGAGAGCT TTCAGCCGTG AGGCCCGGAG   
  
  
- CCAACCATCC AGGTATACTC ATGCCCTATA TCCATGAAGT TTCGTACGGT ACATAAAGTC ACATGGACAC   
  
  
- AACCGTTTCG TTGGTCAAAT TAACAGTTTT ATACATAGTT CCGTCTTAAT GTCTTAAATA CAGGCAATAT   
  
  
- GACAAAAGTT TCTTCTGAAA GGTCTCCTTT GTCGTCTGGT TCCTGCATTA GGGTTTTACC GTGGAGAGAG   
  
  
- ACATGGGATT ATCAAGAATT GTAACATCTA AAAAAATTAC CTAACATCTA AAAATACGAG TCGGTCCAAC   
  
  
- CCGTGTTGTC ATTTTTCACC ATATATTATA ATTATGTCTC TTTATTTTTG GTAATCTTAA TGTTGCATGT   
  
  
- AAGGCTAGGC ATGTCTCTTG AGCCGAATAT ATAGACAGTC AAGGGTCAAA TATAATATTG GTAAGTAGGG   
  
  
- TATGCGTATA CATCTTAATC TTGAAAAATC TTTCTATGTT TCGCCGGTGA CTTACTTTCC GTGGGTTCTT   
  
  
- ATGCAATAGA AAAATCAGAA ATTTTTTTGT CTGCTTTTGG TGGAGTAAAA TATTGCCAAA TTCATTACGA   
  
  
- AACAATTAAT TAAAAAAATG TGTTTAATTG ATTTATCAAC CTTTATTATA CAGAAGATTT TCATGATGCT   
  
  
- TAAGCATGAT GATGATCGGA TTGCGGAAAT GTCTAAATCA ATAATCCGCA AAATTGGAAC TTGTTTTGCA   
  
  
- ATATTAAAAT ATATATTGGG GTTAATTTTG TGATTCAAAA TTATTAAAAG GAAGGCAGAG CAGAATAAAG   
  
  
- GAATTAAATA TATCCAAAGT CGTTTGAATA ATTAAATTTT GGAGCCTTTT TAAAAGTCAA GAAAATAGAG   
  
  
- AAGACATGCT TGGATTGTTT AATAAGGTCG CTTTTAAGTT TTAACTCTTT TCTTGGAAAT TAGTATGAAT   
  
  
- TTTCAGAGGA ATTAGAGTGG CAATATATAA TAAAATAAAA TAAATAAACA ATATACTTTT TTAATATTAT   
  
  
- AATTATATTA TGAAAGCTTT ACTTTTTAAT TTGCAAATAT TTTATTGAAA AAATAATATT ATCATTTTTT   
  
  
- TATAATCAAA GTATCAATTG GGGATTGGAA CCCTAAACTT CTGTCTTGCT TATTAGTGAG ATGGATCTAT   
  
  
- CAAAATTACT TCATTTCAAA ATTTAAAAAT GAAATTACGA GAATTGTGAT TTTTTATTTT GAGACTAAAA   
  
  
- AATTGTTAGT CAGCAGGTTG ATCACATCG

+     GARE-motif

| Site Name | Organism | Position | Strand | Matrix score. | sequence | function |
| --- | --- | --- | --- | --- | --- | --- |
| GARE-motif | Brassica oleracea | 796 | + | 7 | AAACAGA | gibberellin-responsive element |

> 2018/04/13 10:10:12  
+ TCGAGAAGAA GCAAGAGAAC TTACCTGGTA GAGTTGTGAA ACAAGAGAAG AAAATATCTC TCCGACGTTT   
  
  
+ ACAAACTTAG GAAATTATAT GTGTTGCAGT TGTCGACGGA TAGGTTAACG TGATTTTTTG TCACGGGGTT   
  
  
+ GGTATATAAT TAATATGCTA GGGATTGGTA TTAATTAATG CGAATTAATT CATGCTTATT AAGTTGAAGA   
  
  
+ AGGTAGTAAA TCTAATATGT TTTACAGACG TAACTGTTGT TCTCTCTCGA AAGTCGGCAC TCCGGGCCTC   
  
  
+ GGTTGGTAGG TCCATATGAG TACGGGATAT AGGTACTTCA AAGCATGCCA TGTATTTCAG TGTACCTGTG   
  
  
+ TTGGCAAAGC AACCAGTTTA ATTGTCAAAA TATGTATCAA GGCAGAATTA CAGAATTTAT GTCCGTTATA   
  
  
+ CTGTTTTCAA AGAAGACTTT CCAGAGGAAA CAGCAGACCA AGGACGTAAT CCCAAAATGG CACCTCTCTC   
  
  
+ TGTACCCTAA TAGTTCTTAA CATTGTAGAT TTTTTTAATG GATTGTAGAT TTTTATGCTC AGCCAGGTTG   
  
  
+ GGCACAACAG TAAAAAGTGG TATATAATAT TAATACAGAG AAATAAAAAC CATTAGAATT ACAACGTACA   
  
  
+ TTCCGATCCG TACAGAGAAC TCGGCTTATA TATCTGTCAG TTCCCAGTTT ATATTATAAC CATTCATCCC   
  
  
+ ATACGCATAT GTAGAATTAG AACTTTTTAG AAAGATACAA AGCGGCCACT GAATGAAAGG CACCCAAGAA   
  
  
+ TACGTTATCT TTTTAGTCTT TAAAAAAACA GACGAAAACC ACCTCATTTT ATAACGGTTT AAGTAATGCT   
  
  
+ TTGTTAATTA ATTTTTTTAC ACAAATTAAC TAAATAGTTG GAAATAATAT GTCTTCTAAA AGTACTACGA   
  
  
+ ATTCGTACTA CTACTAGCCT AACGCCTTTA CAGATTTAGT TATTAGGCGT TTTAACCTTG AACAAAACGT   
  
  
+ TATAATTTTA TATATAACCC CAATTAAAAC ACTAAGTTTT AATAATTTTC CTTCCGTCTC GTCTTATTTC   
  
  
+ CTTAATTTAT ATAGGTTTCA GCAAACTTAT TAATTTAAAA CCTCGGAAAA ATTTTCAGTT CTTTTATCTC   
  
  
+ TTCTGTACGA ACCTAACAAA TTATTCCAGC GAAAATTCAA AATTGAGAAA AGAACCTTTA ATCATACTTA   
  
  
+ AAAGTCTCCT TAATCTCACC GTTATATATT ATTTTATTTT ATTTATTTGT TATATGAAAA AATTATAATA   
  
  
+ TTAATATAAT ACTTTCGAAA TGAAAAATTA AACGTTTATA AAATAACTTT TTTATTATAA TAGTAAAAAA   
  
  
+ ATATTAGTTT CATAGTTAAC CCCTAACCTT GGGATTTGAA GACAGAACGA ATAATCACTC TACCTAGATA   
  
  
+ GTTTTAATGA AGTAAAGTTT TAAATTTTTA CTTTAATGCT CTTAACACTA AAAAATAAAA CTCTGATTTT   
  
  
+ TTAACAATCA GTCGTCCAAC TAGTGTAGC  

- AGCTCTTCTT CGTTCTCTTG AATGGACCAT CTCAACACTT TGTTCTCTTC TTTTATAGAG AGGCTGCAAA   
  
  
- TGTTTGAATC CTTTAATATA CACAACGTCA ACAGCTGCCT ATCCAATTGC ACTAAAAAAC AGTGCCCCAA   
  
  
- CCATATATTA ATTATACGAT CCCTAACCAT AATTAATTAC GCTTAATTAA GTACGAATAA TTCAACTTCT   
  
  
- TCCATCATTT AGATTATACA AAATGTCTGC ATTGACAACA AGAGAGAGCT TTCAGCCGTG AGGCCCGGAG   
  
  
- CCAACCATCC AGGTATACTC ATGCCCTATA TCCATGAAGT TTCGTACGGT ACATAAAGTC ACATGGACAC   
  
  
- AACCGTTTCG TTGGTCAAAT TAACAGTTTT ATACATAGTT CCGTCTTAAT GTCTTAAATA CAGGCAATAT   
  
  
- GACAAAAGTT TCTTCTGAAA GGTCTCCTTT GTCGTCTGGT TCCTGCATTA GGGTTTTACC GTGGAGAGAG   
  
  
- ACATGGGATT ATCAAGAATT GTAACATCTA AAAAAATTAC CTAACATCTA AAAATACGAG TCGGTCCAAC   
  
  
- CCGTGTTGTC ATTTTTCACC ATATATTATA ATTATGTCTC TTTATTTTTG GTAATCTTAA TGTTGCATGT   
  
  
- AAGGCTAGGC ATGTCTCTTG AGCCGAATAT ATAGACAGTC AAGGGTCAAA TATAATATTG GTAAGTAGGG   
  
  
- TATGCGTATA CATCTTAATC TTGAAAAATC TTTCTATGTT TCGCCGGTGA CTTACTTTCC GTGGGTTCTT   
  
  
- ATGCAATAGA AAAATCAGAA ATTTTTTTGT CTGCTTTTGG TGGAGTAAAA TATTGCCAAA TTCATTACGA   
  
  
- AACAATTAAT TAAAAAAATG TGTTTAATTG ATTTATCAAC CTTTATTATA CAGAAGATTT TCATGATGCT   
  
  
- TAAGCATGAT GATGATCGGA TTGCGGAAAT GTCTAAATCA ATAATCCGCA AAATTGGAAC TTGTTTTGCA   
  
  
- ATATTAAAAT ATATATTGGG GTTAATTTTG TGATTCAAAA TTATTAAAAG GAAGGCAGAG CAGAATAAAG   
  
  
- GAATTAAATA TATCCAAAGT CGTTTGAATA ATTAAATTTT GGAGCCTTTT TAAAAGTCAA GAAAATAGAG   
  
  
- AAGACATGCT TGGATTGTTT AATAAGGTCG CTTTTAAGTT TTAACTCTTT TCTTGGAAAT TAGTATGAAT   
  
  
- TTTCAGAGGA ATTAGAGTGG CAATATATAA TAAAATAAAA TAAATAAACA ATATACTTTT TTAATATTAT   
  
  
- AATTATATTA TGAAAGCTTT ACTTTTTAAT TTGCAAATAT TTTATTGAAA AAATAATATT ATCATTTTTT   
  
  
- TATAATCAAA GTATCAATTG GGGATTGGAA CCCTAAACTT CTGTCTTGCT TATTAGTGAG ATGGATCTAT   
  
  
- CAAAATTACT TCATTTCAAA ATTTAAAAAT GAAATTACGA GAATTGTGAT TTTTTATTTT GAGACTAAAA   
  
  
- AATTGTTAGT CAGCAGGTTG ATCACATCG

+     GT1-motif

| Site Name | Organism | Position | Strand | Matrix score. | sequence | function |
| --- | --- | --- | --- | --- | --- | --- |
| GT1-motif | Arabidopsis thaliana | 1346 | - | 6 | GGTTAA | light responsive element |
| GT1-motif | Arabidopsis thaliana | 962 | - | 6 | GGTTAA | light responsive element |
| GT1-motif | Arabidopsis thaliana | 113 | + | 6 | GGTTAA | light responsive element |

> 2018/04/13 10:10:12  
+ TCGAGAAGAA GCAAGAGAAC TTACCTGGTA GAGTTGTGAA ACAAGAGAAG AAAATATCTC TCCGACGTTT   
  
  
+ ACAAACTTAG GAAATTATAT GTGTTGCAGT TGTCGACGGA TAGGTTAACG TGATTTTTTG TCACGGGGTT   
  
  
+ GGTATATAAT TAATATGCTA GGGATTGGTA TTAATTAATG CGAATTAATT CATGCTTATT AAGTTGAAGA   
  
  
+ AGGTAGTAAA TCTAATATGT TTTACAGACG TAACTGTTGT TCTCTCTCGA AAGTCGGCAC TCCGGGCCTC   
  
  
+ GGTTGGTAGG TCCATATGAG TACGGGATAT AGGTACTTCA AAGCATGCCA TGTATTTCAG TGTACCTGTG   
  
  
+ TTGGCAAAGC AACCAGTTTA ATTGTCAAAA TATGTATCAA GGCAGAATTA CAGAATTTAT GTCCGTTATA   
  
  
+ CTGTTTTCAA AGAAGACTTT CCAGAGGAAA CAGCAGACCA AGGACGTAAT CCCAAAATGG CACCTCTCTC   
  
  
+ TGTACCCTAA TAGTTCTTAA CATTGTAGAT TTTTTTAATG GATTGTAGAT TTTTATGCTC AGCCAGGTTG   
  
  
+ GGCACAACAG TAAAAAGTGG TATATAATAT TAATACAGAG AAATAAAAAC CATTAGAATT ACAACGTACA   
  
  
+ TTCCGATCCG TACAGAGAAC TCGGCTTATA TATCTGTCAG TTCCCAGTTT ATATTATAAC CATTCATCCC   
  
  
+ ATACGCATAT GTAGAATTAG AACTTTTTAG AAAGATACAA AGCGGCCACT GAATGAAAGG CACCCAAGAA   
  
  
+ TACGTTATCT TTTTAGTCTT TAAAAAAACA GACGAAAACC ACCTCATTTT ATAACGGTTT AAGTAATGCT   
  
  
+ TTGTTAATTA ATTTTTTTAC ACAAATTAAC TAAATAGTTG GAAATAATAT GTCTTCTAAA AGTACTACGA   
  
  
+ ATTCGTACTA CTACTAGCCT AACGCCTTTA CAGATTTAGT TATTAGGCGT TTTAACCTTG AACAAAACGT   
  
  
+ TATAATTTTA TATATAACCC CAATTAAAAC ACTAAGTTTT AATAATTTTC CTTCCGTCTC GTCTTATTTC   
  
  
+ CTTAATTTAT ATAGGTTTCA GCAAACTTAT TAATTTAAAA CCTCGGAAAA ATTTTCAGTT CTTTTATCTC   
  
  
+ TTCTGTACGA ACCTAACAAA TTATTCCAGC GAAAATTCAA AATTGAGAAA AGAACCTTTA ATCATACTTA   
  
  
+ AAAGTCTCCT TAATCTCACC GTTATATATT ATTTTATTTT ATTTATTTGT TATATGAAAA AATTATAATA   
  
  
+ TTAATATAAT ACTTTCGAAA TGAAAAATTA AACGTTTATA AAATAACTTT TTTATTATAA TAGTAAAAAA   
  
  
+ ATATTAGTTT CATAGTTAAC CCCTAACCTT GGGATTTGAA GACAGAACGA ATAATCACTC TACCTAGATA   
  
  
+ GTTTTAATGA AGTAAAGTTT TAAATTTTTA CTTTAATGCT CTTAACACTA AAAAATAAAA CTCTGATTTT   
  
  
+ TTAACAATCA GTCGTCCAAC TAGTGTAGC  

- AGCTCTTCTT CGTTCTCTTG AATGGACCAT CTCAACACTT TGTTCTCTTC TTTTATAGAG AGGCTGCAAA   
  
  
- TGTTTGAATC CTTTAATATA CACAACGTCA ACAGCTGCCT ATCCAATTGC ACTAAAAAAC AGTGCCCCAA   
  
  
- CCATATATTA ATTATACGAT CCCTAACCAT AATTAATTAC GCTTAATTAA GTACGAATAA TTCAACTTCT   
  
  
- TCCATCATTT AGATTATACA AAATGTCTGC ATTGACAACA AGAGAGAGCT TTCAGCCGTG AGGCCCGGAG   
  
  
- CCAACCATCC AGGTATACTC ATGCCCTATA TCCATGAAGT TTCGTACGGT ACATAAAGTC ACATGGACAC   
  
  
- AACCGTTTCG TTGGTCAAAT TAACAGTTTT ATACATAGTT CCGTCTTAAT GTCTTAAATA CAGGCAATAT   
  
  
- GACAAAAGTT TCTTCTGAAA GGTCTCCTTT GTCGTCTGGT TCCTGCATTA GGGTTTTACC GTGGAGAGAG   
  
  
- ACATGGGATT ATCAAGAATT GTAACATCTA AAAAAATTAC CTAACATCTA AAAATACGAG TCGGTCCAAC   
  
  
- CCGTGTTGTC ATTTTTCACC ATATATTATA ATTATGTCTC TTTATTTTTG GTAATCTTAA TGTTGCATGT   
  
  
- AAGGCTAGGC ATGTCTCTTG AGCCGAATAT ATAGACAGTC AAGGGTCAAA TATAATATTG GTAAGTAGGG   
  
  
- TATGCGTATA CATCTTAATC TTGAAAAATC TTTCTATGTT TCGCCGGTGA CTTACTTTCC GTGGGTTCTT   
  
  
- ATGCAATAGA AAAATCAGAA ATTTTTTTGT CTGCTTTTGG TGGAGTAAAA TATTGCCAAA TTCATTACGA   
  
  
- AACAATTAAT TAAAAAAATG TGTTTAATTG ATTTATCAAC CTTTATTATA CAGAAGATTT TCATGATGCT   
  
  
- TAAGCATGAT GATGATCGGA TTGCGGAAAT GTCTAAATCA ATAATCCGCA AAATTGGAAC TTGTTTTGCA   
  
  
- ATATTAAAAT ATATATTGGG GTTAATTTTG TGATTCAAAA TTATTAAAAG GAAGGCAGAG CAGAATAAAG   
  
  
- GAATTAAATA TATCCAAAGT CGTTTGAATA ATTAAATTTT GGAGCCTTTT TAAAAGTCAA GAAAATAGAG   
  
  
- AAGACATGCT TGGATTGTTT AATAAGGTCG CTTTTAAGTT TTAACTCTTT TCTTGGAAAT TAGTATGAAT   
  
  
- TTTCAGAGGA ATTAGAGTGG CAATATATAA TAAAATAAAA TAAATAAACA ATATACTTTT TTAATATTAT   
  
  
- AATTATATTA TGAAAGCTTT ACTTTTTAAT TTGCAAATAT TTTATTGAAA AAATAATATT ATCATTTTTT   
  
  
- TATAATCAAA GTATCAATTG GGGATTGGAA CCCTAAACTT CTGTCTTGCT TATTAGTGAG ATGGATCTAT   
  
  
- CAAAATTACT TCATTTCAAA ATTTAAAAAT GAAATTACGA GAATTGTGAT TTTTTATTTT GAGACTAAAA   
  
  
- AATTGTTAGT CAGCAGGTTG ATCACATCG

+     HSE

| Site Name | Organism | Position | Strand | Matrix score. | sequence | function |
| --- | --- | --- | --- | --- | --- | --- |
| HSE | Brassica oleracea | 1097 | + | 9 | AAAAAATTTC | cis-acting element involved in heat stress responsiveness |

> 2018/04/13 10:10:12  
+ TCGAGAAGAA GCAAGAGAAC TTACCTGGTA GAGTTGTGAA ACAAGAGAAG AAAATATCTC TCCGACGTTT   
  
  
+ ACAAACTTAG GAAATTATAT GTGTTGCAGT TGTCGACGGA TAGGTTAACG TGATTTTTTG TCACGGGGTT   
  
  
+ GGTATATAAT TAATATGCTA GGGATTGGTA TTAATTAATG CGAATTAATT CATGCTTATT AAGTTGAAGA   
  
  
+ AGGTAGTAAA TCTAATATGT TTTACAGACG TAACTGTTGT TCTCTCTCGA AAGTCGGCAC TCCGGGCCTC   
  
  
+ GGTTGGTAGG TCCATATGAG TACGGGATAT AGGTACTTCA AAGCATGCCA TGTATTTCAG TGTACCTGTG   
  
  
+ TTGGCAAAGC AACCAGTTTA ATTGTCAAAA TATGTATCAA GGCAGAATTA CAGAATTTAT GTCCGTTATA   
  
  
+ CTGTTTTCAA AGAAGACTTT CCAGAGGAAA CAGCAGACCA AGGACGTAAT CCCAAAATGG CACCTCTCTC   
  
  
+ TGTACCCTAA TAGTTCTTAA CATTGTAGAT TTTTTTAATG GATTGTAGAT TTTTATGCTC AGCCAGGTTG   
  
  
+ GGCACAACAG TAAAAAGTGG TATATAATAT TAATACAGAG AAATAAAAAC CATTAGAATT ACAACGTACA   
  
  
+ TTCCGATCCG TACAGAGAAC TCGGCTTATA TATCTGTCAG TTCCCAGTTT ATATTATAAC CATTCATCCC   
  
  
+ ATACGCATAT GTAGAATTAG AACTTTTTAG AAAGATACAA AGCGGCCACT GAATGAAAGG CACCCAAGAA   
  
  
+ TACGTTATCT TTTTAGTCTT TAAAAAAACA GACGAAAACC ACCTCATTTT ATAACGGTTT AAGTAATGCT   
  
  
+ TTGTTAATTA ATTTTTTTAC ACAAATTAAC TAAATAGTTG GAAATAATAT GTCTTCTAAA AGTACTACGA   
  
  
+ ATTCGTACTA CTACTAGCCT AACGCCTTTA CAGATTTAGT TATTAGGCGT TTTAACCTTG AACAAAACGT   
  
  
+ TATAATTTTA TATATAACCC CAATTAAAAC ACTAAGTTTT AATAATTTTC CTTCCGTCTC GTCTTATTTC   
  
  
+ CTTAATTTAT ATAGGTTTCA GCAAACTTAT TAATTTAAAA CCTCGGAAAA ATTTTCAGTT CTTTTATCTC   
  
  
+ TTCTGTACGA ACCTAACAAA TTATTCCAGC GAAAATTCAA AATTGAGAAA AGAACCTTTA ATCATACTTA   
  
  
+ AAAGTCTCCT TAATCTCACC GTTATATATT ATTTTATTTT ATTTATTTGT TATATGAAAA AATTATAATA   
  
  
+ TTAATATAAT ACTTTCGAAA TGAAAAATTA AACGTTTATA AAATAACTTT TTTATTATAA TAGTAAAAAA   
  
  
+ ATATTAGTTT CATAGTTAAC CCCTAACCTT GGGATTTGAA GACAGAACGA ATAATCACTC TACCTAGATA   
  
  
+ GTTTTAATGA AGTAAAGTTT TAAATTTTTA CTTTAATGCT CTTAACACTA AAAAATAAAA CTCTGATTTT   
  
  
+ TTAACAATCA GTCGTCCAAC TAGTGTAGC  

- AGCTCTTCTT CGTTCTCTTG AATGGACCAT CTCAACACTT TGTTCTCTTC TTTTATAGAG AGGCTGCAAA   
  
  
- TGTTTGAATC CTTTAATATA CACAACGTCA ACAGCTGCCT ATCCAATTGC ACTAAAAAAC AGTGCCCCAA   
  
  
- CCATATATTA ATTATACGAT CCCTAACCAT AATTAATTAC GCTTAATTAA GTACGAATAA TTCAACTTCT   
  
  
- TCCATCATTT AGATTATACA AAATGTCTGC ATTGACAACA AGAGAGAGCT TTCAGCCGTG AGGCCCGGAG   
  
  
- CCAACCATCC AGGTATACTC ATGCCCTATA TCCATGAAGT TTCGTACGGT ACATAAAGTC ACATGGACAC   
  
  
- AACCGTTTCG TTGGTCAAAT TAACAGTTTT ATACATAGTT CCGTCTTAAT GTCTTAAATA CAGGCAATAT   
  
  
- GACAAAAGTT TCTTCTGAAA GGTCTCCTTT GTCGTCTGGT TCCTGCATTA GGGTTTTACC GTGGAGAGAG   
  
  
- ACATGGGATT ATCAAGAATT GTAACATCTA AAAAAATTAC CTAACATCTA AAAATACGAG TCGGTCCAAC   
  
  
- CCGTGTTGTC ATTTTTCACC ATATATTATA ATTATGTCTC TTTATTTTTG GTAATCTTAA TGTTGCATGT   
  
  
- AAGGCTAGGC ATGTCTCTTG AGCCGAATAT ATAGACAGTC AAGGGTCAAA TATAATATTG GTAAGTAGGG   
  
  
- TATGCGTATA CATCTTAATC TTGAAAAATC TTTCTATGTT TCGCCGGTGA CTTACTTTCC GTGGGTTCTT   
  
  
- ATGCAATAGA AAAATCAGAA ATTTTTTTGT CTGCTTTTGG TGGAGTAAAA TATTGCCAAA TTCATTACGA   
  
  
- AACAATTAAT TAAAAAAATG TGTTTAATTG ATTTATCAAC CTTTATTATA CAGAAGATTT TCATGATGCT   
  
  
- TAAGCATGAT GATGATCGGA TTGCGGAAAT GTCTAAATCA ATAATCCGCA AAATTGGAAC TTGTTTTGCA   
  
  
- ATATTAAAAT ATATATTGGG GTTAATTTTG TGATTCAAAA TTATTAAAAG GAAGGCAGAG CAGAATAAAG   
  
  
- GAATTAAATA TATCCAAAGT CGTTTGAATA ATTAAATTTT GGAGCCTTTT TAAAAGTCAA GAAAATAGAG   
  
  
- AAGACATGCT TGGATTGTTT AATAAGGTCG CTTTTAAGTT TTAACTCTTT TCTTGGAAAT TAGTATGAAT   
  
  
- TTTCAGAGGA ATTAGAGTGG CAATATATAA TAAAATAAAA TAAATAAACA ATATACTTTT TTAATATTAT   
  
  
- AATTATATTA TGAAAGCTTT ACTTTTTAAT TTGCAAATAT TTTATTGAAA AAATAATATT ATCATTTTTT   
  
  
- TATAATCAAA GTATCAATTG GGGATTGGAA CCCTAAACTT CTGTCTTGCT TATTAGTGAG ATGGATCTAT   
  
  
- CAAAATTACT TCATTTCAAA ATTTAAAAAT GAAATTACGA GAATTGTGAT TTTTTATTTT GAGACTAAAA   
  
  
- AATTGTTAGT CAGCAGGTTG ATCACATCG

+     MBS

| Site Name | Organism | Position | Strand | Matrix score. | sequence | function |
| --- | --- | --- | --- | --- | --- | --- |
| MBS | Arabidopsis thaliana | 97 | - | 6 | CAACTG | MYB binding site involved in drought-inducibility |
| MBS | Arabidopsis thaliana | 241 | + | 6 | TAACTG | MYB binding site involved in drought-inducibility |

> 2018/04/13 10:10:12  
+ TCGAGAAGAA GCAAGAGAAC TTACCTGGTA GAGTTGTGAA ACAAGAGAAG AAAATATCTC TCCGACGTTT   
  
  
+ ACAAACTTAG GAAATTATAT GTGTTGCAGT TGTCGACGGA TAGGTTAACG TGATTTTTTG TCACGGGGTT   
  
  
+ GGTATATAAT TAATATGCTA GGGATTGGTA TTAATTAATG CGAATTAATT CATGCTTATT AAGTTGAAGA   
  
  
+ AGGTAGTAAA TCTAATATGT TTTACAGACG TAACTGTTGT TCTCTCTCGA AAGTCGGCAC TCCGGGCCTC   
  
  
+ GGTTGGTAGG TCCATATGAG TACGGGATAT AGGTACTTCA AAGCATGCCA TGTATTTCAG TGTACCTGTG   
  
  
+ TTGGCAAAGC AACCAGTTTA ATTGTCAAAA TATGTATCAA GGCAGAATTA CAGAATTTAT GTCCGTTATA   
  
  
+ CTGTTTTCAA AGAAGACTTT CCAGAGGAAA CAGCAGACCA AGGACGTAAT CCCAAAATGG CACCTCTCTC   
  
  
+ TGTACCCTAA TAGTTCTTAA CATTGTAGAT TTTTTTAATG GATTGTAGAT TTTTATGCTC AGCCAGGTTG   
  
  
+ GGCACAACAG TAAAAAGTGG TATATAATAT TAATACAGAG AAATAAAAAC CATTAGAATT ACAACGTACA   
  
  
+ TTCCGATCCG TACAGAGAAC TCGGCTTATA TATCTGTCAG TTCCCAGTTT ATATTATAAC CATTCATCCC   
  
  
+ ATACGCATAT GTAGAATTAG AACTTTTTAG AAAGATACAA AGCGGCCACT GAATGAAAGG CACCCAAGAA   
  
  
+ TACGTTATCT TTTTAGTCTT TAAAAAAACA GACGAAAACC ACCTCATTTT ATAACGGTTT AAGTAATGCT   
  
  
+ TTGTTAATTA ATTTTTTTAC ACAAATTAAC TAAATAGTTG GAAATAATAT GTCTTCTAAA AGTACTACGA   
  
  
+ ATTCGTACTA CTACTAGCCT AACGCCTTTA CAGATTTAGT TATTAGGCGT TTTAACCTTG AACAAAACGT   
  
  
+ TATAATTTTA TATATAACCC CAATTAAAAC ACTAAGTTTT AATAATTTTC CTTCCGTCTC GTCTTATTTC   
  
  
+ CTTAATTTAT ATAGGTTTCA GCAAACTTAT TAATTTAAAA CCTCGGAAAA ATTTTCAGTT CTTTTATCTC   
  
  
+ TTCTGTACGA ACCTAACAAA TTATTCCAGC GAAAATTCAA AATTGAGAAA AGAACCTTTA ATCATACTTA   
  
  
+ AAAGTCTCCT TAATCTCACC GTTATATATT ATTTTATTTT ATTTATTTGT TATATGAAAA AATTATAATA   
  
  
+ TTAATATAAT ACTTTCGAAA TGAAAAATTA AACGTTTATA AAATAACTTT TTTATTATAA TAGTAAAAAA   
  
  
+ ATATTAGTTT CATAGTTAAC CCCTAACCTT GGGATTTGAA GACAGAACGA ATAATCACTC TACCTAGATA   
  
  
+ GTTTTAATGA AGTAAAGTTT TAAATTTTTA CTTTAATGCT CTTAACACTA AAAAATAAAA CTCTGATTTT   
  
  
+ TTAACAATCA GTCGTCCAAC TAGTGTAGC  

- AGCTCTTCTT CGTTCTCTTG AATGGACCAT CTCAACACTT TGTTCTCTTC TTTTATAGAG AGGCTGCAAA   
  
  
- TGTTTGAATC CTTTAATATA CACAACGTCA ACAGCTGCCT ATCCAATTGC ACTAAAAAAC AGTGCCCCAA   
  
  
- CCATATATTA ATTATACGAT CCCTAACCAT AATTAATTAC GCTTAATTAA GTACGAATAA TTCAACTTCT   
  
  
- TCCATCATTT AGATTATACA AAATGTCTGC ATTGACAACA AGAGAGAGCT TTCAGCCGTG AGGCCCGGAG   
  
  
- CCAACCATCC AGGTATACTC ATGCCCTATA TCCATGAAGT TTCGTACGGT ACATAAAGTC ACATGGACAC   
  
  
- AACCGTTTCG TTGGTCAAAT TAACAGTTTT ATACATAGTT CCGTCTTAAT GTCTTAAATA CAGGCAATAT   
  
  
- GACAAAAGTT TCTTCTGAAA GGTCTCCTTT GTCGTCTGGT TCCTGCATTA GGGTTTTACC GTGGAGAGAG   
  
  
- ACATGGGATT ATCAAGAATT GTAACATCTA AAAAAATTAC CTAACATCTA AAAATACGAG TCGGTCCAAC   
  
  
- CCGTGTTGTC ATTTTTCACC ATATATTATA ATTATGTCTC TTTATTTTTG GTAATCTTAA TGTTGCATGT   
  
  
- AAGGCTAGGC ATGTCTCTTG AGCCGAATAT ATAGACAGTC AAGGGTCAAA TATAATATTG GTAAGTAGGG   
  
  
- TATGCGTATA CATCTTAATC TTGAAAAATC TTTCTATGTT TCGCCGGTGA CTTACTTTCC GTGGGTTCTT   
  
  
- ATGCAATAGA AAAATCAGAA ATTTTTTTGT CTGCTTTTGG TGGAGTAAAA TATTGCCAAA TTCATTACGA   
  
  
- AACAATTAAT TAAAAAAATG TGTTTAATTG ATTTATCAAC CTTTATTATA CAGAAGATTT TCATGATGCT   
  
  
- TAAGCATGAT GATGATCGGA TTGCGGAAAT GTCTAAATCA ATAATCCGCA AAATTGGAAC TTGTTTTGCA   
  
  
- ATATTAAAAT ATATATTGGG GTTAATTTTG TGATTCAAAA TTATTAAAAG GAAGGCAGAG CAGAATAAAG   
  
  
- GAATTAAATA TATCCAAAGT CGTTTGAATA ATTAAATTTT GGAGCCTTTT TAAAAGTCAA GAAAATAGAG   
  
  
- AAGACATGCT TGGATTGTTT AATAAGGTCG CTTTTAAGTT TTAACTCTTT TCTTGGAAAT TAGTATGAAT   
  
  
- TTTCAGAGGA ATTAGAGTGG CAATATATAA TAAAATAAAA TAAATAAACA ATATACTTTT TTAATATTAT   
  
  
- AATTATATTA TGAAAGCTTT ACTTTTTAAT TTGCAAATAT TTTATTGAAA AAATAATATT ATCATTTTTT   
  
  
- TATAATCAAA GTATCAATTG GGGATTGGAA CCCTAAACTT CTGTCTTGCT TATTAGTGAG ATGGATCTAT   
  
  
- CAAAATTACT TCATTTCAAA ATTTAAAAAT GAAATTACGA GAATTGTGAT TTTTTATTTT GAGACTAAAA   
  
  
- AATTGTTAGT CAGCAGGTTG ATCACATCG

+     MNF1

| Site Name | Organism | Position | Strand | Matrix score. | sequence | function |
| --- | --- | --- | --- | --- | --- | --- |
| MNF1 | Zea mays | 558 | - | 7 | GTGCCC(A/T)(A/T) | light responsive element |

> 2018/04/13 10:10:12  
+ TCGAGAAGAA GCAAGAGAAC TTACCTGGTA GAGTTGTGAA ACAAGAGAAG AAAATATCTC TCCGACGTTT   
  
  
+ ACAAACTTAG GAAATTATAT GTGTTGCAGT TGTCGACGGA TAGGTTAACG TGATTTTTTG TCACGGGGTT   
  
  
+ GGTATATAAT TAATATGCTA GGGATTGGTA TTAATTAATG CGAATTAATT CATGCTTATT AAGTTGAAGA   
  
  
+ AGGTAGTAAA TCTAATATGT TTTACAGACG TAACTGTTGT TCTCTCTCGA AAGTCGGCAC TCCGGGCCTC   
  
  
+ GGTTGGTAGG TCCATATGAG TACGGGATAT AGGTACTTCA AAGCATGCCA TGTATTTCAG TGTACCTGTG   
  
  
+ TTGGCAAAGC AACCAGTTTA ATTGTCAAAA TATGTATCAA GGCAGAATTA CAGAATTTAT GTCCGTTATA   
  
  
+ CTGTTTTCAA AGAAGACTTT CCAGAGGAAA CAGCAGACCA AGGACGTAAT CCCAAAATGG CACCTCTCTC   
  
  
+ TGTACCCTAA TAGTTCTTAA CATTGTAGAT TTTTTTAATG GATTGTAGAT TTTTATGCTC AGCCAGGTTG   
  
  
+ GGCACAACAG TAAAAAGTGG TATATAATAT TAATACAGAG AAATAAAAAC CATTAGAATT ACAACGTACA   
  
  
+ TTCCGATCCG TACAGAGAAC TCGGCTTATA TATCTGTCAG TTCCCAGTTT ATATTATAAC CATTCATCCC   
  
  
+ ATACGCATAT GTAGAATTAG AACTTTTTAG AAAGATACAA AGCGGCCACT GAATGAAAGG CACCCAAGAA   
  
  
+ TACGTTATCT TTTTAGTCTT TAAAAAAACA GACGAAAACC ACCTCATTTT ATAACGGTTT AAGTAATGCT   
  
  
+ TTGTTAATTA ATTTTTTTAC ACAAATTAAC TAAATAGTTG GAAATAATAT GTCTTCTAAA AGTACTACGA   
  
  
+ ATTCGTACTA CTACTAGCCT AACGCCTTTA CAGATTTAGT TATTAGGCGT TTTAACCTTG AACAAAACGT   
  
  
+ TATAATTTTA TATATAACCC CAATTAAAAC ACTAAGTTTT AATAATTTTC CTTCCGTCTC GTCTTATTTC   
  
  
+ CTTAATTTAT ATAGGTTTCA GCAAACTTAT TAATTTAAAA CCTCGGAAAA ATTTTCAGTT CTTTTATCTC   
  
  
+ TTCTGTACGA ACCTAACAAA TTATTCCAGC GAAAATTCAA AATTGAGAAA AGAACCTTTA ATCATACTTA   
  
  
+ AAAGTCTCCT TAATCTCACC GTTATATATT ATTTTATTTT ATTTATTTGT TATATGAAAA AATTATAATA   
  
  
+ TTAATATAAT ACTTTCGAAA TGAAAAATTA AACGTTTATA AAATAACTTT TTTATTATAA TAGTAAAAAA   
  
  
+ ATATTAGTTT CATAGTTAAC CCCTAACCTT GGGATTTGAA GACAGAACGA ATAATCACTC TACCTAGATA   
  
  
+ GTTTTAATGA AGTAAAGTTT TAAATTTTTA CTTTAATGCT CTTAACACTA AAAAATAAAA CTCTGATTTT   
  
  
+ TTAACAATCA GTCGTCCAAC TAGTGTAGC  

- AGCTCTTCTT CGTTCTCTTG AATGGACCAT CTCAACACTT TGTTCTCTTC TTTTATAGAG AGGCTGCAAA   
  
  
- TGTTTGAATC CTTTAATATA CACAACGTCA ACAGCTGCCT ATCCAATTGC ACTAAAAAAC AGTGCCCCAA   
  
  
- CCATATATTA ATTATACGAT CCCTAACCAT AATTAATTAC GCTTAATTAA GTACGAATAA TTCAACTTCT   
  
  
- TCCATCATTT AGATTATACA AAATGTCTGC ATTGACAACA AGAGAGAGCT TTCAGCCGTG AGGCCCGGAG   
  
  
- CCAACCATCC AGGTATACTC ATGCCCTATA TCCATGAAGT TTCGTACGGT ACATAAAGTC ACATGGACAC   
  
  
- AACCGTTTCG TTGGTCAAAT TAACAGTTTT ATACATAGTT CCGTCTTAAT GTCTTAAATA CAGGCAATAT   
  
  
- GACAAAAGTT TCTTCTGAAA GGTCTCCTTT GTCGTCTGGT TCCTGCATTA GGGTTTTACC GTGGAGAGAG   
  
  
- ACATGGGATT ATCAAGAATT GTAACATCTA AAAAAATTAC CTAACATCTA AAAATACGAG TCGGTCCAAC   
  
  
- CCGTGTTGTC ATTTTTCACC ATATATTATA ATTATGTCTC TTTATTTTTG GTAATCTTAA TGTTGCATGT   
  
  
- AAGGCTAGGC ATGTCTCTTG AGCCGAATAT ATAGACAGTC AAGGGTCAAA TATAATATTG GTAAGTAGGG   
  
  
- TATGCGTATA CATCTTAATC TTGAAAAATC TTTCTATGTT TCGCCGGTGA CTTACTTTCC GTGGGTTCTT   
  
  
- ATGCAATAGA AAAATCAGAA ATTTTTTTGT CTGCTTTTGG TGGAGTAAAA TATTGCCAAA TTCATTACGA   
  
  
- AACAATTAAT TAAAAAAATG TGTTTAATTG ATTTATCAAC CTTTATTATA CAGAAGATTT TCATGATGCT   
  
  
- TAAGCATGAT GATGATCGGA TTGCGGAAAT GTCTAAATCA ATAATCCGCA AAATTGGAAC TTGTTTTGCA   
  
  
- ATATTAAAAT ATATATTGGG GTTAATTTTG TGATTCAAAA TTATTAAAAG GAAGGCAGAG CAGAATAAAG   
  
  
- GAATTAAATA TATCCAAAGT CGTTTGAATA ATTAAATTTT GGAGCCTTTT TAAAAGTCAA GAAAATAGAG   
  
  
- AAGACATGCT TGGATTGTTT AATAAGGTCG CTTTTAAGTT TTAACTCTTT TCTTGGAAAT TAGTATGAAT   
  
  
- TTTCAGAGGA ATTAGAGTGG CAATATATAA TAAAATAAAA TAAATAAACA ATATACTTTT TTAATATTAT   
  
  
- AATTATATTA TGAAAGCTTT ACTTTTTAAT TTGCAAATAT TTTATTGAAA AAATAATATT ATCATTTTTT   
  
  
- TATAATCAAA GTATCAATTG GGGATTGGAA CCCTAAACTT CTGTCTTGCT TATTAGTGAG ATGGATCTAT   
  
  
- CAAAATTACT TCATTTCAAA ATTTAAAAAT GAAATTACGA GAATTGTGAT TTTTTATTTT GAGACTAAAA   
  
  
- AATTGTTAGT CAGCAGGTTG ATCACATCG

+     MRE

| Site Name | Organism | Position | Strand | Matrix score. | sequence | function |
| --- | --- | --- | --- | --- | --- | --- |
| MRE | Petroselinum crispum | 1130 | + | 7 | AACCTAA | MYB binding site involved in light responsiveness |

> 2018/04/13 10:10:12  
+ TCGAGAAGAA GCAAGAGAAC TTACCTGGTA GAGTTGTGAA ACAAGAGAAG AAAATATCTC TCCGACGTTT   
  
  
+ ACAAACTTAG GAAATTATAT GTGTTGCAGT TGTCGACGGA TAGGTTAACG TGATTTTTTG TCACGGGGTT   
  
  
+ GGTATATAAT TAATATGCTA GGGATTGGTA TTAATTAATG CGAATTAATT CATGCTTATT AAGTTGAAGA   
  
  
+ AGGTAGTAAA TCTAATATGT TTTACAGACG TAACTGTTGT TCTCTCTCGA AAGTCGGCAC TCCGGGCCTC   
  
  
+ GGTTGGTAGG TCCATATGAG TACGGGATAT AGGTACTTCA AAGCATGCCA TGTATTTCAG TGTACCTGTG   
  
  
+ TTGGCAAAGC AACCAGTTTA ATTGTCAAAA TATGTATCAA GGCAGAATTA CAGAATTTAT GTCCGTTATA   
  
  
+ CTGTTTTCAA AGAAGACTTT CCAGAGGAAA CAGCAGACCA AGGACGTAAT CCCAAAATGG CACCTCTCTC   
  
  
+ TGTACCCTAA TAGTTCTTAA CATTGTAGAT TTTTTTAATG GATTGTAGAT TTTTATGCTC AGCCAGGTTG   
  
  
+ GGCACAACAG TAAAAAGTGG TATATAATAT TAATACAGAG AAATAAAAAC CATTAGAATT ACAACGTACA   
  
  
+ TTCCGATCCG TACAGAGAAC TCGGCTTATA TATCTGTCAG TTCCCAGTTT ATATTATAAC CATTCATCCC   
  
  
+ ATACGCATAT GTAGAATTAG AACTTTTTAG AAAGATACAA AGCGGCCACT GAATGAAAGG CACCCAAGAA   
  
  
+ TACGTTATCT TTTTAGTCTT TAAAAAAACA GACGAAAACC ACCTCATTTT ATAACGGTTT AAGTAATGCT   
  
  
+ TTGTTAATTA ATTTTTTTAC ACAAATTAAC TAAATAGTTG GAAATAATAT GTCTTCTAAA AGTACTACGA   
  
  
+ ATTCGTACTA CTACTAGCCT AACGCCTTTA CAGATTTAGT TATTAGGCGT TTTAACCTTG AACAAAACGT   
  
  
+ TATAATTTTA TATATAACCC CAATTAAAAC ACTAAGTTTT AATAATTTTC CTTCCGTCTC GTCTTATTTC   
  
  
+ CTTAATTTAT ATAGGTTTCA GCAAACTTAT TAATTTAAAA CCTCGGAAAA ATTTTCAGTT CTTTTATCTC   
  
  
+ TTCTGTACGA ACCTAACAAA TTATTCCAGC GAAAATTCAA AATTGAGAAA AGAACCTTTA ATCATACTTA   
  
  
+ AAAGTCTCCT TAATCTCACC GTTATATATT ATTTTATTTT ATTTATTTGT TATATGAAAA AATTATAATA   
  
  
+ TTAATATAAT ACTTTCGAAA TGAAAAATTA AACGTTTATA AAATAACTTT TTTATTATAA TAGTAAAAAA   
  
  
+ ATATTAGTTT CATAGTTAAC CCCTAACCTT GGGATTTGAA GACAGAACGA ATAATCACTC TACCTAGATA   
  
  
+ GTTTTAATGA AGTAAAGTTT TAAATTTTTA CTTTAATGCT CTTAACACTA AAAAATAAAA CTCTGATTTT   
  
  
+ TTAACAATCA GTCGTCCAAC TAGTGTAGC  

- AGCTCTTCTT CGTTCTCTTG AATGGACCAT CTCAACACTT TGTTCTCTTC TTTTATAGAG AGGCTGCAAA   
  
  
- TGTTTGAATC CTTTAATATA CACAACGTCA ACAGCTGCCT ATCCAATTGC ACTAAAAAAC AGTGCCCCAA   
  
  
- CCATATATTA ATTATACGAT CCCTAACCAT AATTAATTAC GCTTAATTAA GTACGAATAA TTCAACTTCT   
  
  
- TCCATCATTT AGATTATACA AAATGTCTGC ATTGACAACA AGAGAGAGCT TTCAGCCGTG AGGCCCGGAG   
  
  
- CCAACCATCC AGGTATACTC ATGCCCTATA TCCATGAAGT TTCGTACGGT ACATAAAGTC ACATGGACAC   
  
  
- AACCGTTTCG TTGGTCAAAT TAACAGTTTT ATACATAGTT CCGTCTTAAT GTCTTAAATA CAGGCAATAT   
  
  
- GACAAAAGTT TCTTCTGAAA GGTCTCCTTT GTCGTCTGGT TCCTGCATTA GGGTTTTACC GTGGAGAGAG   
  
  
- ACATGGGATT ATCAAGAATT GTAACATCTA AAAAAATTAC CTAACATCTA AAAATACGAG TCGGTCCAAC   
  
  
- CCGTGTTGTC ATTTTTCACC ATATATTATA ATTATGTCTC TTTATTTTTG GTAATCTTAA TGTTGCATGT   
  
  
- AAGGCTAGGC ATGTCTCTTG AGCCGAATAT ATAGACAGTC AAGGGTCAAA TATAATATTG GTAAGTAGGG   
  
  
- TATGCGTATA CATCTTAATC TTGAAAAATC TTTCTATGTT TCGCCGGTGA CTTACTTTCC GTGGGTTCTT   
  
  
- ATGCAATAGA AAAATCAGAA ATTTTTTTGT CTGCTTTTGG TGGAGTAAAA TATTGCCAAA TTCATTACGA   
  
  
- AACAATTAAT TAAAAAAATG TGTTTAATTG ATTTATCAAC CTTTATTATA CAGAAGATTT TCATGATGCT   
  
  
- TAAGCATGAT GATGATCGGA TTGCGGAAAT GTCTAAATCA ATAATCCGCA AAATTGGAAC TTGTTTTGCA   
  
  
- ATATTAAAAT ATATATTGGG GTTAATTTTG TGATTCAAAA TTATTAAAAG GAAGGCAGAG CAGAATAAAG   
  
  
- GAATTAAATA TATCCAAAGT CGTTTGAATA ATTAAATTTT GGAGCCTTTT TAAAAGTCAA GAAAATAGAG   
  
  
- AAGACATGCT TGGATTGTTT AATAAGGTCG CTTTTAAGTT TTAACTCTTT TCTTGGAAAT TAGTATGAAT   
  
  
- TTTCAGAGGA ATTAGAGTGG CAATATATAA TAAAATAAAA TAAATAAACA ATATACTTTT TTAATATTAT   
  
  
- AATTATATTA TGAAAGCTTT ACTTTTTAAT TTGCAAATAT TTTATTGAAA AAATAATATT ATCATTTTTT   
  
  
- TATAATCAAA GTATCAATTG GGGATTGGAA CCCTAAACTT CTGTCTTGCT TATTAGTGAG ATGGATCTAT   
  
  
- CAAAATTACT TCATTTCAAA ATTTAAAAAT GAAATTACGA GAATTGTGAT TTTTTATTTT GAGACTAAAA   
  
  
- AATTGTTAGT CAGCAGGTTG ATCACATCG

+     O2-site

| Site Name | Organism | Position | Strand | Matrix score. | sequence | function |
| --- | --- | --- | --- | --- | --- | --- |
| O2-site | Zea mays | 114 | + | 9 | GTTGACGTGA | cis-acting regulatory element involved in zein metabolism regulation |

> 2018/04/13 10:10:12  
+ TCGAGAAGAA GCAAGAGAAC TTACCTGGTA GAGTTGTGAA ACAAGAGAAG AAAATATCTC TCCGACGTTT   
  
  
+ ACAAACTTAG GAAATTATAT GTGTTGCAGT TGTCGACGGA TAGGTTAACG TGATTTTTTG TCACGGGGTT   
  
  
+ GGTATATAAT TAATATGCTA GGGATTGGTA TTAATTAATG CGAATTAATT CATGCTTATT AAGTTGAAGA   
  
  
+ AGGTAGTAAA TCTAATATGT TTTACAGACG TAACTGTTGT TCTCTCTCGA AAGTCGGCAC TCCGGGCCTC   
  
  
+ GGTTGGTAGG TCCATATGAG TACGGGATAT AGGTACTTCA AAGCATGCCA TGTATTTCAG TGTACCTGTG   
  
  
+ TTGGCAAAGC AACCAGTTTA ATTGTCAAAA TATGTATCAA GGCAGAATTA CAGAATTTAT GTCCGTTATA   
  
  
+ CTGTTTTCAA AGAAGACTTT CCAGAGGAAA CAGCAGACCA AGGACGTAAT CCCAAAATGG CACCTCTCTC   
  
  
+ TGTACCCTAA TAGTTCTTAA CATTGTAGAT TTTTTTAATG GATTGTAGAT TTTTATGCTC AGCCAGGTTG   
  
  
+ GGCACAACAG TAAAAAGTGG TATATAATAT TAATACAGAG AAATAAAAAC CATTAGAATT ACAACGTACA   
  
  
+ TTCCGATCCG TACAGAGAAC TCGGCTTATA TATCTGTCAG TTCCCAGTTT ATATTATAAC CATTCATCCC   
  
  
+ ATACGCATAT GTAGAATTAG AACTTTTTAG AAAGATACAA AGCGGCCACT GAATGAAAGG CACCCAAGAA   
  
  
+ TACGTTATCT TTTTAGTCTT TAAAAAAACA GACGAAAACC ACCTCATTTT ATAACGGTTT AAGTAATGCT   
  
  
+ TTGTTAATTA ATTTTTTTAC ACAAATTAAC TAAATAGTTG GAAATAATAT GTCTTCTAAA AGTACTACGA   
  
  
+ ATTCGTACTA CTACTAGCCT AACGCCTTTA CAGATTTAGT TATTAGGCGT TTTAACCTTG AACAAAACGT   
  
  
+ TATAATTTTA TATATAACCC CAATTAAAAC ACTAAGTTTT AATAATTTTC CTTCCGTCTC GTCTTATTTC   
  
  
+ CTTAATTTAT ATAGGTTTCA GCAAACTTAT TAATTTAAAA CCTCGGAAAA ATTTTCAGTT CTTTTATCTC   
  
  
+ TTCTGTACGA ACCTAACAAA TTATTCCAGC GAAAATTCAA AATTGAGAAA AGAACCTTTA ATCATACTTA   
  
  
+ AAAGTCTCCT TAATCTCACC GTTATATATT ATTTTATTTT ATTTATTTGT TATATGAAAA AATTATAATA   
  
  
+ TTAATATAAT ACTTTCGAAA TGAAAAATTA AACGTTTATA AAATAACTTT TTTATTATAA TAGTAAAAAA   
  
  
+ ATATTAGTTT CATAGTTAAC CCCTAACCTT GGGATTTGAA GACAGAACGA ATAATCACTC TACCTAGATA   
  
  
+ GTTTTAATGA AGTAAAGTTT TAAATTTTTA CTTTAATGCT CTTAACACTA AAAAATAAAA CTCTGATTTT   
  
  
+ TTAACAATCA GTCGTCCAAC TAGTGTAGC  

- AGCTCTTCTT CGTTCTCTTG AATGGACCAT CTCAACACTT TGTTCTCTTC TTTTATAGAG AGGCTGCAAA   
  
  
- TGTTTGAATC CTTTAATATA CACAACGTCA ACAGCTGCCT ATCCAATTGC ACTAAAAAAC AGTGCCCCAA   
  
  
- CCATATATTA ATTATACGAT CCCTAACCAT AATTAATTAC GCTTAATTAA GTACGAATAA TTCAACTTCT   
  
  
- TCCATCATTT AGATTATACA AAATGTCTGC ATTGACAACA AGAGAGAGCT TTCAGCCGTG AGGCCCGGAG   
  
  
- CCAACCATCC AGGTATACTC ATGCCCTATA TCCATGAAGT TTCGTACGGT ACATAAAGTC ACATGGACAC   
  
  
- AACCGTTTCG TTGGTCAAAT TAACAGTTTT ATACATAGTT CCGTCTTAAT GTCTTAAATA CAGGCAATAT   
  
  
- GACAAAAGTT TCTTCTGAAA GGTCTCCTTT GTCGTCTGGT TCCTGCATTA GGGTTTTACC GTGGAGAGAG   
  
  
- ACATGGGATT ATCAAGAATT GTAACATCTA AAAAAATTAC CTAACATCTA AAAATACGAG TCGGTCCAAC   
  
  
- CCGTGTTGTC ATTTTTCACC ATATATTATA ATTATGTCTC TTTATTTTTG GTAATCTTAA TGTTGCATGT   
  
  
- AAGGCTAGGC ATGTCTCTTG AGCCGAATAT ATAGACAGTC AAGGGTCAAA TATAATATTG GTAAGTAGGG   
  
  
- TATGCGTATA CATCTTAATC TTGAAAAATC TTTCTATGTT TCGCCGGTGA CTTACTTTCC GTGGGTTCTT   
  
  
- ATGCAATAGA AAAATCAGAA ATTTTTTTGT CTGCTTTTGG TGGAGTAAAA TATTGCCAAA TTCATTACGA   
  
  
- AACAATTAAT TAAAAAAATG TGTTTAATTG ATTTATCAAC CTTTATTATA CAGAAGATTT TCATGATGCT   
  
  
- TAAGCATGAT GATGATCGGA TTGCGGAAAT GTCTAAATCA ATAATCCGCA AAATTGGAAC TTGTTTTGCA   
  
  
- ATATTAAAAT ATATATTGGG GTTAATTTTG TGATTCAAAA TTATTAAAAG GAAGGCAGAG CAGAATAAAG   
  
  
- GAATTAAATA TATCCAAAGT CGTTTGAATA ATTAAATTTT GGAGCCTTTT TAAAAGTCAA GAAAATAGAG   
  
  
- AAGACATGCT TGGATTGTTT AATAAGGTCG CTTTTAAGTT TTAACTCTTT TCTTGGAAAT TAGTATGAAT   
  
  
- TTTCAGAGGA ATTAGAGTGG CAATATATAA TAAAATAAAA TAAATAAACA ATATACTTTT TTAATATTAT   
  
  
- AATTATATTA TGAAAGCTTT ACTTTTTAAT TTGCAAATAT TTTATTGAAA AAATAATATT ATCATTTTTT   
  
  
- TATAATCAAA GTATCAATTG GGGATTGGAA CCCTAAACTT CTGTCTTGCT TATTAGTGAG ATGGATCTAT   
  
  
- CAAAATTACT TCATTTCAAA ATTTAAAAAT GAAATTACGA GAATTGTGAT TTTTTATTTT GAGACTAAAA   
  
  
- AATTGTTAGT CAGCAGGTTG ATCACATCG

+     TATA-box

| Site Name | Organism | Position | Strand | Matrix score. | sequence | function |
| --- | --- | --- | --- | --- | --- | --- |
| TATA-box | Glycine max | 1332 | - | 5 | TAATA | core promoter element around -30 of transcription start |
| TATA-box | Arabidopsis thaliana | 988 | - | 7 | TATATAA | core promoter element around -30 of transcription start |
| TATA-box | Glycine max | 1078 | - | 5 | TAATA | core promoter element around -30 of transcription start |
| TATA-box | Brassica napus | 683 | + | 6 | ATTATA | core promoter element around -30 of transcription start |
| TATA-box | Glycine max | 1318 | + | 5 | TAATA | core promoter element around -30 of transcription start |
| TATA-box | Arabidopsis thaliana | 1058 | - | 4 | TATA | core promoter element around -30 of transcription start |
| TATA-box | Pisum sativum | 985 | - | 8 | TATAAAAT | core promoter element around -30 of transcription start |
| TATA-box | Glycine max | 1217 | - | 5 | TAATA | core promoter element around -30 of transcription start |
| TATA-box | Lycopersicon esculentum | 1469 | + | 5 | TTTTA | core promoter element around -30 of transcription start |
| TATA-box | Arabidopsis thaliana | 1265 | - | 4 | TATA | core promoter element around -30 of transcription start |
| TATA-box | Lycopersicon esculentum | 1227 | + | 5 | TTTTA | core promoter element around -30 of transcription start |
| TATA-box | Lycopersicon esculentum | 960 | + | 5 | TTTTA | core promoter element around -30 of transcription start |
| TATA-box | Arabidopsis thaliana | 417 | + | 4 | TATA | core promoter element around -30 of transcription start |
| TATA-box | Lycopersicon esculentum | 1299 | - | 5 | TTTTA | core promoter element around -30 of transcription start |
| TATA-box | Arabidopsis thaliana | 1297 | + | 6 | TATAAA | core promoter element around -30 of transcription start |
| TATA-box | Arabidopsis thaliana | 1056 | - | 6 | TATAAA | core promoter element around -30 of transcription start |
| TATA-box | Arabidopsis thaliana | 981 | - | 4 | TATA | core promoter element around -30 of transcription start |
| TATA-box | Arabidopsis thaliana | 1057 | - | 7 | TATATAA | core promoter element around -30 of transcription start |
| TATA-box | Lycopersicon esculentum | 1324 | - | 5 | TTTTA | core promoter element around -30 of transcription start |
| TATA-box | Glycine max | 197 | - | 5 | TAATA | core promoter element around -30 of transcription start |
| TATA-box | Glycine max | 223 | + | 5 | TAATA | core promoter element around -30 of transcription start |
| TATA-box | Arabidopsis thaliana | 308 | + | 4 | TATA | core promoter element around -30 of transcription start |
| TATA-box | Lycopersicon esculentum | 230 | + | 5 | TTTTA | core promoter element around -30 of transcription start |
| TATA-box | Lycopersicon esculentum | 1189 | - | 5 | TTTTA | core promoter element around -30 of transcription start |
| TATA-box | Lycopersicon esculentum | 604 | - | 5 | TTTTA | core promoter element around -30 of transcription start |
| TATA-box | Glycine max | 585 | + | 5 | TAATA | core promoter element around -30 of transcription start |
| TATA-box | Arabidopsis thaliana | 986 | - | 7 | TATAAAA | core promoter element around -30 of transcription start |
| TATA-box | Lycopersicon esculentum | 1449 | - | 5 | TTTTA | core promoter element around -30 of transcription start |
| TATA-box | Arabidopsis thaliana | 416 | - | 5 | TATAA | core promoter element around -30 of transcription start |
| TATA-box | Arabidopsis thaliana | 145 | + | 4 | TATA | core promoter element around -30 of transcription start |
| TATA-box | Arabidopsis thaliana | 143 | + | 4 | TATA | core promoter element around -30 of transcription start |
| TATA-box | Brassica napus | 1314 | + | 6 | ATTATA | core promoter element around -30 of transcription start |
| TATA-box | Arabidopsis thaliana | 1295 | - | 6 | TATAAA | core promoter element around -30 of transcription start |
| TATA-box | Glycine max | 1313 | - | 5 | TAATA | core promoter element around -30 of transcription start |
| TATA-box | Lycopersicon esculentum | 1418 | + | 5 | TTTTA | core promoter element around -30 of transcription start |
| TATA-box | Arabidopsis thaliana | 86 | + | 4 | TATA | core promoter element around -30 of transcription start |
| TATA-box | Arabidopsis thaliana | 987 | - | 6 | TATAAA | core promoter element around -30 of transcription start |
| TATA-box | Brassica napus | 1252 | + | 6 | ATTATA | core promoter element around -30 of transcription start |
| TATA-box | Arabidopsis thaliana | 1254 | - | 4 | TATA | core promoter element around -30 of transcription start |
| TATA-box | Glycine max | 588 | - | 5 | TAATA | core promoter element around -30 of transcription start |
| TATA-box | Arabidopsis thaliana | 583 | + | 4 | TATA | core promoter element around -30 of transcription start |
| TATA-box | Brassica oleracea | 582 | + | 7 | ATATAAT | core promoter element around -30 of transcription start |
| TATA-box | Lycopersicon esculentum | 571 | - | 5 | TTTTA | core promoter element around -30 of transcription start |
| TATA-box | Arabidopsis thaliana | 1060 | - | 4 | TATA | core promoter element around -30 of transcription start |
| TATA-box | Arabidopsis thaliana | 581 | + | 4 | TATA | core promoter element around -30 of transcription start |
| TATA-box | Pisum sativum | 816 | - | 8 | TATAAAAT | core promoter element around -30 of transcription start |
| TATA-box | Glycine max | 682 | - | 5 | TAATA | core promoter element around -30 of transcription start |
| TATA-box | Arabidopsis thaliana | 1213 | - | 4 | TATA | core promoter element around -30 of transcription start |
| TATA-box | Brassica napus | 1214 | - | 6 | ATATAT | core promoter element around -30 of transcription start |
| TATA-box | Arabidopsis thaliana | 1209 | - | 9 | taTATAAAgg | core promoter element around -30 of transcription start |
| TATA-box | Arabidopsis thaliana | 817 | - | 7 | TATAAAA | core promoter element around -30 of transcription start |
| TATA-box | Brassica oleracea | 1264 | + | 7 | ATATAAT | core promoter element around -30 of transcription start |
| TATA-box | Glycine max | 1262 | + | 5 | TAATA | core promoter element around -30 of transcription start |
| TATA-box | Lycopersicon esculentum | 1426 | + | 5 | TTTTA | core promoter element around -30 of transcription start |
| TATA-box | Arabidopsis thaliana | 820 | - | 4 | TATA | core promoter element around -30 of transcription start |
| TATA-box | Glycine max | 151 | + | 5 | TAATA | core promoter element around -30 of transcription start |
| TATA-box | Arabidopsis thaliana | 993 | - | 4 | TATA | core promoter element around -30 of transcription start |
| TATA-box | Glycine max | 169 | - | 5 | TAATA | core promoter element around -30 of transcription start |
| TATA-box | Glycine max | 1020 | + | 5 | TAATA | core promoter element around -30 of transcription start |
| TATA-box | Arabidopsis thaliana | 684 | - | 5 | TATAA | core promoter element around -30 of transcription start |
| TATA-box | Lycopersicon esculentum | 541 | + | 5 | TTTTA | core promoter element around -30 of transcription start |
| TATA-box | Arabidopsis thaliana | 1315 | - | 5 | TATAA | core promoter element around -30 of transcription start |
| TATA-box | Arabidopsis thaliana | 678 | - | 6 | TATAAA | core promoter element around -30 of transcription start |
| TATA-box | Arabidopsis thaliana | 685 | + | 4 | TATA | core promoter element around -30 of transcription start |
| TATA-box | Arabidopsis thaliana | 1240 | - | 5 | TATAA | core promoter element around -30 of transcription start |
| TATA-box | Brassica oleracea | 144 | + | 7 | ATATAAT | core promoter element around -30 of transcription start |
| TATA-box | Lycopersicon esculentum | 1310 | + | 5 | TTTTA | core promoter element around -30 of transcription start |
| TATA-box | Lycopersicon esculentum | 1112 | + | 5 | TTTTA | core promoter element around -30 of transcription start |
| TATA-box | Brassica napus | 84 | + | 6 | ATTATA | core promoter element around -30 of transcription start |
| TATA-box | Antirrhinum majus | 1054 | - | 8 | TATAAATT | core promoter element around -30 of transcription start |
| TATA-box | Lycopersicon esculentum | 791 | - | 5 | TTTTA | core promoter element around -30 of transcription start |
| TATA-box | Arabidopsis thaliana | 659 | + | 4 | TATA | core promoter element around -30 of transcription start |
| TATA-box | Lycopersicon esculentum | 897 | - | 5 | TTTTA | core promoter element around -30 of transcription start |
| TATA-box | Ac | 1055 | - | 7 | TATAAAT | core promoter element around -30 of transcription start |
| TATA-box | Lycopersicon esculentum | 1017 | + | 5 | TTTTA | core promoter element around -30 of transcription start |
| TATA-box | Arabidopsis thaliana | 991 | - | 4 | TATA | core promoter element around -30 of transcription start |
| TATA-box | Arabidopsis thaliana | 85 | - | 5 | TATAA | core promoter element around -30 of transcription start |
| TATA-box | Glycine max | 591 | + | 5 | TAATA | core promoter element around -30 of transcription start |
| TATA-box | Glycine max | 1256 | + | 5 | TAATA | core promoter element around -30 of transcription start |
| TATA-box | Arabidopsis thaliana | 1241 | - | 4 | TATA | core promoter element around -30 of transcription start |
| TATA-box | Lycopersicon esculentum | 781 | + | 5 | TTTTA | core promoter element around -30 of transcription start |
| TATA-box | Glycine max | 498 | + | 5 | TAATA | core promoter element around -30 of transcription start |
| TATA-box | Lycopersicon esculentum | 1402 | + | 5 | TTTTA | core promoter element around -30 of transcription start |
| TATA-box | Arabidopsis thaliana | 980 | - | 5 | TATAA | core promoter element around -30 of transcription start |
| TATA-box | Arabidopsis thaliana | 818 | - | 6 | TATAAA | core promoter element around -30 of transcription start |
| TATA-box | Lycopersicon esculentum | 1005 | - | 5 | TTTTA | core promoter element around -30 of transcription start |
| TATA-box | Arabidopsis thaliana | 656 | - | 7 | TATATAA | core promoter element around -30 of transcription start |
| TATA-box | Glycine max | 1267 | + | 5 | TAATA | core promoter element around -30 of transcription start |
| TATA-box | Arabidopsis thaliana | 989 | - | 8 | TATATATA | core promoter element around -30 of transcription start |
| TATA-box | Lycopersicon esculentum | 523 | + | 5 | TTTTA | core promoter element around -30 of transcription start |
| TATA-box | Brassica napus | 658 | + | 6 | ATATAT | core promoter element around -30 of transcription start |
| TATA-box | Brassica napus | 990 | - | 6 | ATATAT | core promoter element around -30 of transcription start |
| TATA-box | Arabidopsis thaliana | 679 | - | 5 | TATAA | core promoter element around -30 of transcription start |
| TATA-box | Lycopersicon esculentum | 1456 | - | 5 | TTTTA | core promoter element around -30 of transcription start |
| TATA-box | Arabidopsis thaliana | 680 | + | 4 | TATA | core promoter element around -30 of transcription start |
| TATA-box | Lycopersicon esculentum | 1086 | - | 5 | TTTTA | core promoter element around -30 of transcription start |
| TATA-box | Arabidopsis thaliana | 657 | + | 4 | TATA | core promoter element around -30 of transcription start |
| TATA-box | Arabidopsis thaliana | 1253 | - | 5 | TATAA | core promoter element around -30 of transcription start |
| TATA-box | Arabidopsis thaliana | 819 | - | 5 | TATAA | core promoter element around -30 of transcription start |
| TATA-box | Arabidopsis thaliana | 1215 | - | 4 | TATA | core promoter element around -30 of transcription start |
| TATA-box | Arabidopsis thaliana | 1316 | - | 4 | TATA | core promoter element around -30 of transcription start |
| TATA-box | Lycopersicon esculentum | 725 | + | 5 | TTTTA | core promoter element around -30 of transcription start |
| TATA-box | Glycine max | 885 | + | 5 | TAATA | core promoter element around -30 of transcription start |
| TATA-box | Brassica oleracea | 992 | + | 6 | ATATAA | core promoter element around -30 of transcription start |
| TATA-box | Zea mays | 789 | + | 8 | TTTAAAAA | core promoter element around -30 of transcription start |
| TATA-box | Arabidopsis thaliana | 1296 | - | 5 | TATAA | core promoter element around -30 of transcription start |
| TATA-box | Lycopersicon esculentum | 855 | + | 5 | TTTTA | core promoter element around -30 of transcription start |
| TATA-box | Arabidopsis thaliana | 1212 | - | 7 | TATATAA | core promoter element around -30 of transcription start |
| TATA-box | Lycopersicon esculentum | 1222 | + | 5 | TTTTA | core promoter element around -30 of transcription start |
| TATA-box | Glycine max | 951 | - | 5 | TAATA | core promoter element around -30 of transcription start |
| TATA-box | Glycine max | 1259 | - | 5 | TAATA | core promoter element around -30 of transcription start |

> 2018/04/13 10:10:12  
+ TCGAGAAGAA GCAAGAGAAC TTACCTGGTA GAGTTGTGAA ACAAGAGAAG AAAATATCTC TCCGACGTTT   
  
  
+ ACAAACTTAG GAAATTATAT GTGTTGCAGT TGTCGACGGA TAGGTTAACG TGATTTTTTG TCACGGGGTT   
  
  
+ GGTATATAAT TAATATGCTA GGGATTGGTA TTAATTAATG CGAATTAATT CATGCTTATT AAGTTGAAGA   
  
  
+ AGGTAGTAAA TCTAATATGT TTTACAGACG TAACTGTTGT TCTCTCTCGA AAGTCGGCAC TCCGGGCCTC   
  
  
+ GGTTGGTAGG TCCATATGAG TACGGGATAT AGGTACTTCA AAGCATGCCA TGTATTTCAG TGTACCTGTG   
  
  
+ TTGGCAAAGC AACCAGTTTA ATTGTCAAAA TATGTATCAA GGCAGAATTA CAGAATTTAT GTCCGTTATA   
  
  
+ CTGTTTTCAA AGAAGACTTT CCAGAGGAAA CAGCAGACCA AGGACGTAAT CCCAAAATGG CACCTCTCTC   
  
  
+ TGTACCCTAA TAGTTCTTAA CATTGTAGAT TTTTTTAATG GATTGTAGAT TTTTATGCTC AGCCAGGTTG   
  
  
+ GGCACAACAG TAAAAAGTGG TATATAATAT TAATACAGAG AAATAAAAAC CATTAGAATT ACAACGTACA   
  
  
+ TTCCGATCCG TACAGAGAAC TCGGCTTATA TATCTGTCAG TTCCCAGTTT ATATTATAAC CATTCATCCC   
  
  
+ ATACGCATAT GTAGAATTAG AACTTTTTAG AAAGATACAA AGCGGCCACT GAATGAAAGG CACCCAAGAA   
  
  
+ TACGTTATCT TTTTAGTCTT TAAAAAAACA GACGAAAACC ACCTCATTTT ATAACGGTTT AAGTAATGCT   
  
  
+ TTGTTAATTA ATTTTTTTAC ACAAATTAAC TAAATAGTTG GAAATAATAT GTCTTCTAAA AGTACTACGA   
  
  
+ ATTCGTACTA CTACTAGCCT AACGCCTTTA CAGATTTAGT TATTAGGCGT TTTAACCTTG AACAAAACGT   
  
  
+ TATAATTTTA TATATAACCC CAATTAAAAC ACTAAGTTTT AATAATTTTC CTTCCGTCTC GTCTTATTTC   
  
  
+ CTTAATTTAT ATAGGTTTCA GCAAACTTAT TAATTTAAAA CCTCGGAAAA ATTTTCAGTT CTTTTATCTC   
  
  
+ TTCTGTACGA ACCTAACAAA TTATTCCAGC GAAAATTCAA AATTGAGAAA AGAACCTTTA ATCATACTTA   
  
  
+ AAAGTCTCCT TAATCTCACC GTTATATATT ATTTTATTTT ATTTATTTGT TATATGAAAA AATTATAATA   
  
  
+ TTAATATAAT ACTTTCGAAA TGAAAAATTA AACGTTTATA AAATAACTTT TTTATTATAA TAGTAAAAAA   
  
  
+ ATATTAGTTT CATAGTTAAC CCCTAACCTT GGGATTTGAA GACAGAACGA ATAATCACTC TACCTAGATA   
  
  
+ GTTTTAATGA AGTAAAGTTT TAAATTTTTA CTTTAATGCT CTTAACACTA AAAAATAAAA CTCTGATTTT   
  
  
+ TTAACAATCA GTCGTCCAAC TAGTGTAGC  

- AGCTCTTCTT CGTTCTCTTG AATGGACCAT CTCAACACTT TGTTCTCTTC TTTTATAGAG AGGCTGCAAA   
  
  
- TGTTTGAATC CTTTAATATA CACAACGTCA ACAGCTGCCT ATCCAATTGC ACTAAAAAAC AGTGCCCCAA   
  
  
- CCATATATTA ATTATACGAT CCCTAACCAT AATTAATTAC GCTTAATTAA GTACGAATAA TTCAACTTCT   
  
  
- TCCATCATTT AGATTATACA AAATGTCTGC ATTGACAACA AGAGAGAGCT TTCAGCCGTG AGGCCCGGAG   
  
  
- CCAACCATCC AGGTATACTC ATGCCCTATA TCCATGAAGT TTCGTACGGT ACATAAAGTC ACATGGACAC   
  
  
- AACCGTTTCG TTGGTCAAAT TAACAGTTTT ATACATAGTT CCGTCTTAAT GTCTTAAATA CAGGCAATAT   
  
  
- GACAAAAGTT TCTTCTGAAA GGTCTCCTTT GTCGTCTGGT TCCTGCATTA GGGTTTTACC GTGGAGAGAG   
  
  
- ACATGGGATT ATCAAGAATT GTAACATCTA AAAAAATTAC CTAACATCTA AAAATACGAG TCGGTCCAAC   
  
  
- CCGTGTTGTC ATTTTTCACC ATATATTATA ATTATGTCTC TTTATTTTTG GTAATCTTAA TGTTGCATGT   
  
  
- AAGGCTAGGC ATGTCTCTTG AGCCGAATAT ATAGACAGTC AAGGGTCAAA TATAATATTG GTAAGTAGGG   
  
  
- TATGCGTATA CATCTTAATC TTGAAAAATC TTTCTATGTT TCGCCGGTGA CTTACTTTCC GTGGGTTCTT   
  
  
- ATGCAATAGA AAAATCAGAA ATTTTTTTGT CTGCTTTTGG TGGAGTAAAA TATTGCCAAA TTCATTACGA   
  
  
- AACAATTAAT TAAAAAAATG TGTTTAATTG ATTTATCAAC CTTTATTATA CAGAAGATTT TCATGATGCT   
  
  
- TAAGCATGAT GATGATCGGA TTGCGGAAAT GTCTAAATCA ATAATCCGCA AAATTGGAAC TTGTTTTGCA   
  
  
- ATATTAAAAT ATATATTGGG GTTAATTTTG TGATTCAAAA TTATTAAAAG GAAGGCAGAG CAGAATAAAG   
  
  
- GAATTAAATA TATCCAAAGT CGTTTGAATA ATTAAATTTT GGAGCCTTTT TAAAAGTCAA GAAAATAGAG   
  
  
- AAGACATGCT TGGATTGTTT AATAAGGTCG CTTTTAAGTT TTAACTCTTT TCTTGGAAAT TAGTATGAAT   
  
  
- TTTCAGAGGA ATTAGAGTGG CAATATATAA TAAAATAAAA TAAATAAACA ATATACTTTT TTAATATTAT   
  
  
- AATTATATTA TGAAAGCTTT ACTTTTTAAT TTGCAAATAT TTTATTGAAA AAATAATATT ATCATTTTTT   
  
  
- TATAATCAAA GTATCAATTG GGGATTGGAA CCCTAAACTT CTGTCTTGCT TATTAGTGAG ATGGATCTAT   
  
  
- CAAAATTACT TCATTTCAAA ATTTAAAAAT GAAATTACGA GAATTGTGAT TTTTTATTTT GAGACTAAAA   
  
  
- AATTGTTAGT CAGCAGGTTG ATCACATCG

+     TC-rich repeats

| Site Name | Organism | Position | Strand | Matrix score. | sequence | function |
| --- | --- | --- | --- | --- | --- | --- |
| TC-rich repeats | Nicotiana tabacum | 46 | - | 9 | ATTTTCTTCA | cis-acting element involved in defense and stress responsiveness |

> 2018/04/13 10:10:12  
+ TCGAGAAGAA GCAAGAGAAC TTACCTGGTA GAGTTGTGAA ACAAGAGAAG AAAATATCTC TCCGACGTTT   
  
  
+ ACAAACTTAG GAAATTATAT GTGTTGCAGT TGTCGACGGA TAGGTTAACG TGATTTTTTG TCACGGGGTT   
  
  
+ GGTATATAAT TAATATGCTA GGGATTGGTA TTAATTAATG CGAATTAATT CATGCTTATT AAGTTGAAGA   
  
  
+ AGGTAGTAAA TCTAATATGT TTTACAGACG TAACTGTTGT TCTCTCTCGA AAGTCGGCAC TCCGGGCCTC   
  
  
+ GGTTGGTAGG TCCATATGAG TACGGGATAT AGGTACTTCA AAGCATGCCA TGTATTTCAG TGTACCTGTG   
  
  
+ TTGGCAAAGC AACCAGTTTA ATTGTCAAAA TATGTATCAA GGCAGAATTA CAGAATTTAT GTCCGTTATA   
  
  
+ CTGTTTTCAA AGAAGACTTT CCAGAGGAAA CAGCAGACCA AGGACGTAAT CCCAAAATGG CACCTCTCTC   
  
  
+ TGTACCCTAA TAGTTCTTAA CATTGTAGAT TTTTTTAATG GATTGTAGAT TTTTATGCTC AGCCAGGTTG   
  
  
+ GGCACAACAG TAAAAAGTGG TATATAATAT TAATACAGAG AAATAAAAAC CATTAGAATT ACAACGTACA   
  
  
+ TTCCGATCCG TACAGAGAAC TCGGCTTATA TATCTGTCAG TTCCCAGTTT ATATTATAAC CATTCATCCC   
  
  
+ ATACGCATAT GTAGAATTAG AACTTTTTAG AAAGATACAA AGCGGCCACT GAATGAAAGG CACCCAAGAA   
  
  
+ TACGTTATCT TTTTAGTCTT TAAAAAAACA GACGAAAACC ACCTCATTTT ATAACGGTTT AAGTAATGCT   
  
  
+ TTGTTAATTA ATTTTTTTAC ACAAATTAAC TAAATAGTTG GAAATAATAT GTCTTCTAAA AGTACTACGA   
  
  
+ ATTCGTACTA CTACTAGCCT AACGCCTTTA CAGATTTAGT TATTAGGCGT TTTAACCTTG AACAAAACGT   
  
  
+ TATAATTTTA TATATAACCC CAATTAAAAC ACTAAGTTTT AATAATTTTC CTTCCGTCTC GTCTTATTTC   
  
  
+ CTTAATTTAT ATAGGTTTCA GCAAACTTAT TAATTTAAAA CCTCGGAAAA ATTTTCAGTT CTTTTATCTC   
  
  
+ TTCTGTACGA ACCTAACAAA TTATTCCAGC GAAAATTCAA AATTGAGAAA AGAACCTTTA ATCATACTTA   
  
  
+ AAAGTCTCCT TAATCTCACC GTTATATATT ATTTTATTTT ATTTATTTGT TATATGAAAA AATTATAATA   
  
  
+ TTAATATAAT ACTTTCGAAA TGAAAAATTA AACGTTTATA AAATAACTTT TTTATTATAA TAGTAAAAAA   
  
  
+ ATATTAGTTT CATAGTTAAC CCCTAACCTT GGGATTTGAA GACAGAACGA ATAATCACTC TACCTAGATA   
  
  
+ GTTTTAATGA AGTAAAGTTT TAAATTTTTA CTTTAATGCT CTTAACACTA AAAAATAAAA CTCTGATTTT   
  
  
+ TTAACAATCA GTCGTCCAAC TAGTGTAGC  

- AGCTCTTCTT CGTTCTCTTG AATGGACCAT CTCAACACTT TGTTCTCTTC TTTTATAGAG AGGCTGCAAA   
  
  
- TGTTTGAATC CTTTAATATA CACAACGTCA ACAGCTGCCT ATCCAATTGC ACTAAAAAAC AGTGCCCCAA   
  
  
- CCATATATTA ATTATACGAT CCCTAACCAT AATTAATTAC GCTTAATTAA GTACGAATAA TTCAACTTCT   
  
  
- TCCATCATTT AGATTATACA AAATGTCTGC ATTGACAACA AGAGAGAGCT TTCAGCCGTG AGGCCCGGAG   
  
  
- CCAACCATCC AGGTATACTC ATGCCCTATA TCCATGAAGT TTCGTACGGT ACATAAAGTC ACATGGACAC   
  
  
- AACCGTTTCG TTGGTCAAAT TAACAGTTTT ATACATAGTT CCGTCTTAAT GTCTTAAATA CAGGCAATAT   
  
  
- GACAAAAGTT TCTTCTGAAA GGTCTCCTTT GTCGTCTGGT TCCTGCATTA GGGTTTTACC GTGGAGAGAG   
  
  
- ACATGGGATT ATCAAGAATT GTAACATCTA AAAAAATTAC CTAACATCTA AAAATACGAG TCGGTCCAAC   
  
  
- CCGTGTTGTC ATTTTTCACC ATATATTATA ATTATGTCTC TTTATTTTTG GTAATCTTAA TGTTGCATGT   
  
  
- AAGGCTAGGC ATGTCTCTTG AGCCGAATAT ATAGACAGTC AAGGGTCAAA TATAATATTG GTAAGTAGGG   
  
  
- TATGCGTATA CATCTTAATC TTGAAAAATC TTTCTATGTT TCGCCGGTGA CTTACTTTCC GTGGGTTCTT   
  
  
- ATGCAATAGA AAAATCAGAA ATTTTTTTGT CTGCTTTTGG TGGAGTAAAA TATTGCCAAA TTCATTACGA   
  
  
- AACAATTAAT TAAAAAAATG TGTTTAATTG ATTTATCAAC CTTTATTATA CAGAAGATTT TCATGATGCT   
  
  
- TAAGCATGAT GATGATCGGA TTGCGGAAAT GTCTAAATCA ATAATCCGCA AAATTGGAAC TTGTTTTGCA   
  
  
- ATATTAAAAT ATATATTGGG GTTAATTTTG TGATTCAAAA TTATTAAAAG GAAGGCAGAG CAGAATAAAG   
  
  
- GAATTAAATA TATCCAAAGT CGTTTGAATA ATTAAATTTT GGAGCCTTTT TAAAAGTCAA GAAAATAGAG   
  
  
- AAGACATGCT TGGATTGTTT AATAAGGTCG CTTTTAAGTT TTAACTCTTT TCTTGGAAAT TAGTATGAAT   
  
  
- TTTCAGAGGA ATTAGAGTGG CAATATATAA TAAAATAAAA TAAATAAACA ATATACTTTT TTAATATTAT   
  
  
- AATTATATTA TGAAAGCTTT ACTTTTTAAT TTGCAAATAT TTTATTGAAA AAATAATATT ATCATTTTTT   
  
  
- TATAATCAAA GTATCAATTG GGGATTGGAA CCCTAAACTT CTGTCTTGCT TATTAGTGAG ATGGATCTAT   
  
  
- CAAAATTACT TCATTTCAAA ATTTAAAAAT GAAATTACGA GAATTGTGAT TTTTTATTTT GAGACTAAAA   
  
  
- AATTGTTAGT CAGCAGGTTG ATCACATCG

+     TCA-element

| Site Name | Organism | Position | Strand | Matrix score. | sequence | function |
| --- | --- | --- | --- | --- | --- | --- |
| TCA-element | Brassica oleracea | 45 | + | 9 | GAGAAGAATA | cis-acting element involved in salicylic acid responsiveness |

> 2018/04/13 10:10:12  
+ TCGAGAAGAA GCAAGAGAAC TTACCTGGTA GAGTTGTGAA ACAAGAGAAG AAAATATCTC TCCGACGTTT   
  
  
+ ACAAACTTAG GAAATTATAT GTGTTGCAGT TGTCGACGGA TAGGTTAACG TGATTTTTTG TCACGGGGTT   
  
  
+ GGTATATAAT TAATATGCTA GGGATTGGTA TTAATTAATG CGAATTAATT CATGCTTATT AAGTTGAAGA   
  
  
+ AGGTAGTAAA TCTAATATGT TTTACAGACG TAACTGTTGT TCTCTCTCGA AAGTCGGCAC TCCGGGCCTC   
  
  
+ GGTTGGTAGG TCCATATGAG TACGGGATAT AGGTACTTCA AAGCATGCCA TGTATTTCAG TGTACCTGTG   
  
  
+ TTGGCAAAGC AACCAGTTTA ATTGTCAAAA TATGTATCAA GGCAGAATTA CAGAATTTAT GTCCGTTATA   
  
  
+ CTGTTTTCAA AGAAGACTTT CCAGAGGAAA CAGCAGACCA AGGACGTAAT CCCAAAATGG CACCTCTCTC   
  
  
+ TGTACCCTAA TAGTTCTTAA CATTGTAGAT TTTTTTAATG GATTGTAGAT TTTTATGCTC AGCCAGGTTG   
  
  
+ GGCACAACAG TAAAAAGTGG TATATAATAT TAATACAGAG AAATAAAAAC CATTAGAATT ACAACGTACA   
  
  
+ TTCCGATCCG TACAGAGAAC TCGGCTTATA TATCTGTCAG TTCCCAGTTT ATATTATAAC CATTCATCCC   
  
  
+ ATACGCATAT GTAGAATTAG AACTTTTTAG AAAGATACAA AGCGGCCACT GAATGAAAGG CACCCAAGAA   
  
  
+ TACGTTATCT TTTTAGTCTT TAAAAAAACA GACGAAAACC ACCTCATTTT ATAACGGTTT AAGTAATGCT   
  
  
+ TTGTTAATTA ATTTTTTTAC ACAAATTAAC TAAATAGTTG GAAATAATAT GTCTTCTAAA AGTACTACGA   
  
  
+ ATTCGTACTA CTACTAGCCT AACGCCTTTA CAGATTTAGT TATTAGGCGT TTTAACCTTG AACAAAACGT   
  
  
+ TATAATTTTA TATATAACCC CAATTAAAAC ACTAAGTTTT AATAATTTTC CTTCCGTCTC GTCTTATTTC   
  
  
+ CTTAATTTAT ATAGGTTTCA GCAAACTTAT TAATTTAAAA CCTCGGAAAA ATTTTCAGTT CTTTTATCTC   
  
  
+ TTCTGTACGA ACCTAACAAA TTATTCCAGC GAAAATTCAA AATTGAGAAA AGAACCTTTA ATCATACTTA   
  
  
+ AAAGTCTCCT TAATCTCACC GTTATATATT ATTTTATTTT ATTTATTTGT TATATGAAAA AATTATAATA   
  
  
+ TTAATATAAT ACTTTCGAAA TGAAAAATTA AACGTTTATA AAATAACTTT TTTATTATAA TAGTAAAAAA   
  
  
+ ATATTAGTTT CATAGTTAAC CCCTAACCTT GGGATTTGAA GACAGAACGA ATAATCACTC TACCTAGATA   
  
  
+ GTTTTAATGA AGTAAAGTTT TAAATTTTTA CTTTAATGCT CTTAACACTA AAAAATAAAA CTCTGATTTT   
  
  
+ TTAACAATCA GTCGTCCAAC TAGTGTAGC  

- AGCTCTTCTT CGTTCTCTTG AATGGACCAT CTCAACACTT TGTTCTCTTC TTTTATAGAG AGGCTGCAAA   
  
  
- TGTTTGAATC CTTTAATATA CACAACGTCA ACAGCTGCCT ATCCAATTGC ACTAAAAAAC AGTGCCCCAA   
  
  
- CCATATATTA ATTATACGAT CCCTAACCAT AATTAATTAC GCTTAATTAA GTACGAATAA TTCAACTTCT   
  
  
- TCCATCATTT AGATTATACA AAATGTCTGC ATTGACAACA AGAGAGAGCT TTCAGCCGTG AGGCCCGGAG   
  
  
- CCAACCATCC AGGTATACTC ATGCCCTATA TCCATGAAGT TTCGTACGGT ACATAAAGTC ACATGGACAC   
  
  
- AACCGTTTCG TTGGTCAAAT TAACAGTTTT ATACATAGTT CCGTCTTAAT GTCTTAAATA CAGGCAATAT   
  
  
- GACAAAAGTT TCTTCTGAAA GGTCTCCTTT GTCGTCTGGT TCCTGCATTA GGGTTTTACC GTGGAGAGAG   
  
  
- ACATGGGATT ATCAAGAATT GTAACATCTA AAAAAATTAC CTAACATCTA AAAATACGAG TCGGTCCAAC   
  
  
- CCGTGTTGTC ATTTTTCACC ATATATTATA ATTATGTCTC TTTATTTTTG GTAATCTTAA TGTTGCATGT   
  
  
- AAGGCTAGGC ATGTCTCTTG AGCCGAATAT ATAGACAGTC AAGGGTCAAA TATAATATTG GTAAGTAGGG   
  
  
- TATGCGTATA CATCTTAATC TTGAAAAATC TTTCTATGTT TCGCCGGTGA CTTACTTTCC GTGGGTTCTT   
  
  
- ATGCAATAGA AAAATCAGAA ATTTTTTTGT CTGCTTTTGG TGGAGTAAAA TATTGCCAAA TTCATTACGA   
  
  
- AACAATTAAT TAAAAAAATG TGTTTAATTG ATTTATCAAC CTTTATTATA CAGAAGATTT TCATGATGCT   
  
  
- TAAGCATGAT GATGATCGGA TTGCGGAAAT GTCTAAATCA ATAATCCGCA AAATTGGAAC TTGTTTTGCA   
  
  
- ATATTAAAAT ATATATTGGG GTTAATTTTG TGATTCAAAA TTATTAAAAG GAAGGCAGAG CAGAATAAAG   
  
  
- GAATTAAATA TATCCAAAGT CGTTTGAATA ATTAAATTTT GGAGCCTTTT TAAAAGTCAA GAAAATAGAG   
  
  
- AAGACATGCT TGGATTGTTT AATAAGGTCG CTTTTAAGTT TTAACTCTTT TCTTGGAAAT TAGTATGAAT   
  
  
- TTTCAGAGGA ATTAGAGTGG CAATATATAA TAAAATAAAA TAAATAAACA ATATACTTTT TTAATATTAT   
  
  
- AATTATATTA TGAAAGCTTT ACTTTTTAAT TTGCAAATAT TTTATTGAAA AAATAATATT ATCATTTTTT   
  
  
- TATAATCAAA GTATCAATTG GGGATTGGAA CCCTAAACTT CTGTCTTGCT TATTAGTGAG ATGGATCTAT   
  
  
- CAAAATTACT TCATTTCAAA ATTTAAAAAT GAAATTACGA GAATTGTGAT TTTTTATTTT GAGACTAAAA   
  
  
- AATTGTTAGT CAGCAGGTTG ATCACATCG

+     Unnamed\_\_4

| Site Name | Organism | Position | Strand | Matrix score. | sequence | function |
| --- | --- | --- | --- | --- | --- | --- |
| Unnamed\_\_4 | Petroselinum hortense | 270 | + | 4 | CTCC |  |
| Unnamed\_\_4 | Petroselinum hortense | 1196 | + | 4 | CTCC |  |
| Unnamed\_\_4 | Petroselinum hortense | 60 | + | 4 | CTCC |  |

> 2018/04/13 10:10:12  
+ TCGAGAAGAA GCAAGAGAAC TTACCTGGTA GAGTTGTGAA ACAAGAGAAG AAAATATCTC TCCGACGTTT   
  
  
+ ACAAACTTAG GAAATTATAT GTGTTGCAGT TGTCGACGGA TAGGTTAACG TGATTTTTTG TCACGGGGTT   
  
  
+ GGTATATAAT TAATATGCTA GGGATTGGTA TTAATTAATG CGAATTAATT CATGCTTATT AAGTTGAAGA   
  
  
+ AGGTAGTAAA TCTAATATGT TTTACAGACG TAACTGTTGT TCTCTCTCGA AAGTCGGCAC TCCGGGCCTC   
  
  
+ GGTTGGTAGG TCCATATGAG TACGGGATAT AGGTACTTCA AAGCATGCCA TGTATTTCAG TGTACCTGTG   
  
  
+ TTGGCAAAGC AACCAGTTTA ATTGTCAAAA TATGTATCAA GGCAGAATTA CAGAATTTAT GTCCGTTATA   
  
  
+ CTGTTTTCAA AGAAGACTTT CCAGAGGAAA CAGCAGACCA AGGACGTAAT CCCAAAATGG CACCTCTCTC   
  
  
+ TGTACCCTAA TAGTTCTTAA CATTGTAGAT TTTTTTAATG GATTGTAGAT TTTTATGCTC AGCCAGGTTG   
  
  
+ GGCACAACAG TAAAAAGTGG TATATAATAT TAATACAGAG AAATAAAAAC CATTAGAATT ACAACGTACA   
  
  
+ TTCCGATCCG TACAGAGAAC TCGGCTTATA TATCTGTCAG TTCCCAGTTT ATATTATAAC CATTCATCCC   
  
  
+ ATACGCATAT GTAGAATTAG AACTTTTTAG AAAGATACAA AGCGGCCACT GAATGAAAGG CACCCAAGAA   
  
  
+ TACGTTATCT TTTTAGTCTT TAAAAAAACA GACGAAAACC ACCTCATTTT ATAACGGTTT AAGTAATGCT   
  
  
+ TTGTTAATTA ATTTTTTTAC ACAAATTAAC TAAATAGTTG GAAATAATAT GTCTTCTAAA AGTACTACGA   
  
  
+ ATTCGTACTA CTACTAGCCT AACGCCTTTA CAGATTTAGT TATTAGGCGT TTTAACCTTG AACAAAACGT   
  
  
+ TATAATTTTA TATATAACCC CAATTAAAAC ACTAAGTTTT AATAATTTTC CTTCCGTCTC GTCTTATTTC   
  
  
+ CTTAATTTAT ATAGGTTTCA GCAAACTTAT TAATTTAAAA CCTCGGAAAA ATTTTCAGTT CTTTTATCTC   
  
  
+ TTCTGTACGA ACCTAACAAA TTATTCCAGC GAAAATTCAA AATTGAGAAA AGAACCTTTA ATCATACTTA   
  
  
+ AAAGTCTCCT TAATCTCACC GTTATATATT ATTTTATTTT ATTTATTTGT TATATGAAAA AATTATAATA   
  
  
+ TTAATATAAT ACTTTCGAAA TGAAAAATTA AACGTTTATA AAATAACTTT TTTATTATAA TAGTAAAAAA   
  
  
+ ATATTAGTTT CATAGTTAAC CCCTAACCTT GGGATTTGAA GACAGAACGA ATAATCACTC TACCTAGATA   
  
  
+ GTTTTAATGA AGTAAAGTTT TAAATTTTTA CTTTAATGCT CTTAACACTA AAAAATAAAA CTCTGATTTT   
  
  
+ TTAACAATCA GTCGTCCAAC TAGTGTAGC  

- AGCTCTTCTT CGTTCTCTTG AATGGACCAT CTCAACACTT TGTTCTCTTC TTTTATAGAG AGGCTGCAAA   
  
  
- TGTTTGAATC CTTTAATATA CACAACGTCA ACAGCTGCCT ATCCAATTGC ACTAAAAAAC AGTGCCCCAA   
  
  
- CCATATATTA ATTATACGAT CCCTAACCAT AATTAATTAC GCTTAATTAA GTACGAATAA TTCAACTTCT   
  
  
- TCCATCATTT AGATTATACA AAATGTCTGC ATTGACAACA AGAGAGAGCT TTCAGCCGTG AGGCCCGGAG   
  
  
- CCAACCATCC AGGTATACTC ATGCCCTATA TCCATGAAGT TTCGTACGGT ACATAAAGTC ACATGGACAC   
  
  
- AACCGTTTCG TTGGTCAAAT TAACAGTTTT ATACATAGTT CCGTCTTAAT GTCTTAAATA CAGGCAATAT   
  
  
- GACAAAAGTT TCTTCTGAAA GGTCTCCTTT GTCGTCTGGT TCCTGCATTA GGGTTTTACC GTGGAGAGAG   
  
  
- ACATGGGATT ATCAAGAATT GTAACATCTA AAAAAATTAC CTAACATCTA AAAATACGAG TCGGTCCAAC   
  
  
- CCGTGTTGTC ATTTTTCACC ATATATTATA ATTATGTCTC TTTATTTTTG GTAATCTTAA TGTTGCATGT   
  
  
- AAGGCTAGGC ATGTCTCTTG AGCCGAATAT ATAGACAGTC AAGGGTCAAA TATAATATTG GTAAGTAGGG   
  
  
- TATGCGTATA CATCTTAATC TTGAAAAATC TTTCTATGTT TCGCCGGTGA CTTACTTTCC GTGGGTTCTT   
  
  
- ATGCAATAGA AAAATCAGAA ATTTTTTTGT CTGCTTTTGG TGGAGTAAAA TATTGCCAAA TTCATTACGA   
  
  
- AACAATTAAT TAAAAAAATG TGTTTAATTG ATTTATCAAC CTTTATTATA CAGAAGATTT TCATGATGCT   
  
  
- TAAGCATGAT GATGATCGGA TTGCGGAAAT GTCTAAATCA ATAATCCGCA AAATTGGAAC TTGTTTTGCA   
  
  
- ATATTAAAAT ATATATTGGG GTTAATTTTG TGATTCAAAA TTATTAAAAG GAAGGCAGAG CAGAATAAAG   
  
  
- GAATTAAATA TATCCAAAGT CGTTTGAATA ATTAAATTTT GGAGCCTTTT TAAAAGTCAA GAAAATAGAG   
  
  
- AAGACATGCT TGGATTGTTT AATAAGGTCG CTTTTAAGTT TTAACTCTTT TCTTGGAAAT TAGTATGAAT   
  
  
- TTTCAGAGGA ATTAGAGTGG CAATATATAA TAAAATAAAA TAAATAAACA ATATACTTTT TTAATATTAT   
  
  
- AATTATATTA TGAAAGCTTT ACTTTTTAAT TTGCAAATAT TTTATTGAAA AAATAATATT ATCATTTTTT   
  
  
- TATAATCAAA GTATCAATTG GGGATTGGAA CCCTAAACTT CTGTCTTGCT TATTAGTGAG ATGGATCTAT   
  
  
- CAAAATTACT TCATTTCAAA ATTTAAAAAT GAAATTACGA GAATTGTGAT TTTTTATTTT GAGACTAAAA   
  
  
- AATTGTTAGT CAGCAGGTTG ATCACATCG

+     WUN-motif

| Site Name | Organism | Position | Strand | Matrix score. | sequence | function |
| --- | --- | --- | --- | --- | --- | --- |
| WUN-motif | Brassica oleracea | 1274 | - | 9 | TCATTACGAA | wound-responsive element |

> 2018/04/13 10:10:12  
+ TCGAGAAGAA GCAAGAGAAC TTACCTGGTA GAGTTGTGAA ACAAGAGAAG AAAATATCTC TCCGACGTTT   
  
  
+ ACAAACTTAG GAAATTATAT GTGTTGCAGT TGTCGACGGA TAGGTTAACG TGATTTTTTG TCACGGGGTT   
  
  
+ GGTATATAAT TAATATGCTA GGGATTGGTA TTAATTAATG CGAATTAATT CATGCTTATT AAGTTGAAGA   
  
  
+ AGGTAGTAAA TCTAATATGT TTTACAGACG TAACTGTTGT TCTCTCTCGA AAGTCGGCAC TCCGGGCCTC   
  
  
+ GGTTGGTAGG TCCATATGAG TACGGGATAT AGGTACTTCA AAGCATGCCA TGTATTTCAG TGTACCTGTG   
  
  
+ TTGGCAAAGC AACCAGTTTA ATTGTCAAAA TATGTATCAA GGCAGAATTA CAGAATTTAT GTCCGTTATA   
  
  
+ CTGTTTTCAA AGAAGACTTT CCAGAGGAAA CAGCAGACCA AGGACGTAAT CCCAAAATGG CACCTCTCTC   
  
  
+ TGTACCCTAA TAGTTCTTAA CATTGTAGAT TTTTTTAATG GATTGTAGAT TTTTATGCTC AGCCAGGTTG   
  
  
+ GGCACAACAG TAAAAAGTGG TATATAATAT TAATACAGAG AAATAAAAAC CATTAGAATT ACAACGTACA   
  
  
+ TTCCGATCCG TACAGAGAAC TCGGCTTATA TATCTGTCAG TTCCCAGTTT ATATTATAAC CATTCATCCC   
  
  
+ ATACGCATAT GTAGAATTAG AACTTTTTAG AAAGATACAA AGCGGCCACT GAATGAAAGG CACCCAAGAA   
  
  
+ TACGTTATCT TTTTAGTCTT TAAAAAAACA GACGAAAACC ACCTCATTTT ATAACGGTTT AAGTAATGCT   
  
  
+ TTGTTAATTA ATTTTTTTAC ACAAATTAAC TAAATAGTTG GAAATAATAT GTCTTCTAAA AGTACTACGA   
  
  
+ ATTCGTACTA CTACTAGCCT AACGCCTTTA CAGATTTAGT TATTAGGCGT TTTAACCTTG AACAAAACGT   
  
  
+ TATAATTTTA TATATAACCC CAATTAAAAC ACTAAGTTTT AATAATTTTC CTTCCGTCTC GTCTTATTTC   
  
  
+ CTTAATTTAT ATAGGTTTCA GCAAACTTAT TAATTTAAAA CCTCGGAAAA ATTTTCAGTT CTTTTATCTC   
  
  
+ TTCTGTACGA ACCTAACAAA TTATTCCAGC GAAAATTCAA AATTGAGAAA AGAACCTTTA ATCATACTTA   
  
  
+ AAAGTCTCCT TAATCTCACC GTTATATATT ATTTTATTTT ATTTATTTGT TATATGAAAA AATTATAATA   
  
  
+ TTAATATAAT ACTTTCGAAA TGAAAAATTA AACGTTTATA AAATAACTTT TTTATTATAA TAGTAAAAAA   
  
  
+ ATATTAGTTT CATAGTTAAC CCCTAACCTT GGGATTTGAA GACAGAACGA ATAATCACTC TACCTAGATA   
  
  
+ GTTTTAATGA AGTAAAGTTT TAAATTTTTA CTTTAATGCT CTTAACACTA AAAAATAAAA CTCTGATTTT   
  
  
+ TTAACAATCA GTCGTCCAAC TAGTGTAGC  

- AGCTCTTCTT CGTTCTCTTG AATGGACCAT CTCAACACTT TGTTCTCTTC TTTTATAGAG AGGCTGCAAA   
  
  
- TGTTTGAATC CTTTAATATA CACAACGTCA ACAGCTGCCT ATCCAATTGC ACTAAAAAAC AGTGCCCCAA   
  
  
- CCATATATTA ATTATACGAT CCCTAACCAT AATTAATTAC GCTTAATTAA GTACGAATAA TTCAACTTCT   
  
  
- TCCATCATTT AGATTATACA AAATGTCTGC ATTGACAACA AGAGAGAGCT TTCAGCCGTG AGGCCCGGAG   
  
  
- CCAACCATCC AGGTATACTC ATGCCCTATA TCCATGAAGT TTCGTACGGT ACATAAAGTC ACATGGACAC   
  
  
- AACCGTTTCG TTGGTCAAAT TAACAGTTTT ATACATAGTT CCGTCTTAAT GTCTTAAATA CAGGCAATAT   
  
  
- GACAAAAGTT TCTTCTGAAA GGTCTCCTTT GTCGTCTGGT TCCTGCATTA GGGTTTTACC GTGGAGAGAG   
  
  
- ACATGGGATT ATCAAGAATT GTAACATCTA AAAAAATTAC CTAACATCTA AAAATACGAG TCGGTCCAAC   
  
  
- CCGTGTTGTC ATTTTTCACC ATATATTATA ATTATGTCTC TTTATTTTTG GTAATCTTAA TGTTGCATGT   
  
  
- AAGGCTAGGC ATGTCTCTTG AGCCGAATAT ATAGACAGTC AAGGGTCAAA TATAATATTG GTAAGTAGGG   
  
  
- TATGCGTATA CATCTTAATC TTGAAAAATC TTTCTATGTT TCGCCGGTGA CTTACTTTCC GTGGGTTCTT   
  
  
- ATGCAATAGA AAAATCAGAA ATTTTTTTGT CTGCTTTTGG TGGAGTAAAA TATTGCCAAA TTCATTACGA   
  
  
- AACAATTAAT TAAAAAAATG TGTTTAATTG ATTTATCAAC CTTTATTATA CAGAAGATTT TCATGATGCT   
  
  
- TAAGCATGAT GATGATCGGA TTGCGGAAAT GTCTAAATCA ATAATCCGCA AAATTGGAAC TTGTTTTGCA   
  
  
- ATATTAAAAT ATATATTGGG GTTAATTTTG TGATTCAAAA TTATTAAAAG GAAGGCAGAG CAGAATAAAG   
  
  
- GAATTAAATA TATCCAAAGT CGTTTGAATA ATTAAATTTT GGAGCCTTTT TAAAAGTCAA GAAAATAGAG   
  
  
- AAGACATGCT TGGATTGTTT AATAAGGTCG CTTTTAAGTT TTAACTCTTT TCTTGGAAAT TAGTATGAAT   
  
  
- TTTCAGAGGA ATTAGAGTGG CAATATATAA TAAAATAAAA TAAATAAACA ATATACTTTT TTAATATTAT   
  
  
- AATTATATTA TGAAAGCTTT ACTTTTTAAT TTGCAAATAT TTTATTGAAA AAATAATATT ATCATTTTTT   
  
  
- TATAATCAAA GTATCAATTG GGGATTGGAA CCCTAAACTT CTGTCTTGCT TATTAGTGAG ATGGATCTAT   
  
  
- CAAAATTACT TCATTTCAAA ATTTAAAAAT GAAATTACGA GAATTGTGAT TTTTTATTTT GAGACTAAAA   
  
  
- AATTGTTAGT CAGCAGGTTG ATCACATCG

+     chs-CMA1a

| Site Name | Organism | Position | Strand | Matrix score. | sequence | function |
| --- | --- | --- | --- | --- | --- | --- |
| chs-CMA1a | Daucus carota | 829 | - | 8 | TTACTTAA | part of a light responsive element |

> 2018/04/13 10:10:12  
+ TCGAGAAGAA GCAAGAGAAC TTACCTGGTA GAGTTGTGAA ACAAGAGAAG AAAATATCTC TCCGACGTTT   
  
  
+ ACAAACTTAG GAAATTATAT GTGTTGCAGT TGTCGACGGA TAGGTTAACG TGATTTTTTG TCACGGGGTT   
  
  
+ GGTATATAAT TAATATGCTA GGGATTGGTA TTAATTAATG CGAATTAATT CATGCTTATT AAGTTGAAGA   
  
  
+ AGGTAGTAAA TCTAATATGT TTTACAGACG TAACTGTTGT TCTCTCTCGA AAGTCGGCAC TCCGGGCCTC   
  
  
+ GGTTGGTAGG TCCATATGAG TACGGGATAT AGGTACTTCA AAGCATGCCA TGTATTTCAG TGTACCTGTG   
  
  
+ TTGGCAAAGC AACCAGTTTA ATTGTCAAAA TATGTATCAA GGCAGAATTA CAGAATTTAT GTCCGTTATA   
  
  
+ CTGTTTTCAA AGAAGACTTT CCAGAGGAAA CAGCAGACCA AGGACGTAAT CCCAAAATGG CACCTCTCTC   
  
  
+ TGTACCCTAA TAGTTCTTAA CATTGTAGAT TTTTTTAATG GATTGTAGAT TTTTATGCTC AGCCAGGTTG   
  
  
+ GGCACAACAG TAAAAAGTGG TATATAATAT TAATACAGAG AAATAAAAAC CATTAGAATT ACAACGTACA   
  
  
+ TTCCGATCCG TACAGAGAAC TCGGCTTATA TATCTGTCAG TTCCCAGTTT ATATTATAAC CATTCATCCC   
  
  
+ ATACGCATAT GTAGAATTAG AACTTTTTAG AAAGATACAA AGCGGCCACT GAATGAAAGG CACCCAAGAA   
  
  
+ TACGTTATCT TTTTAGTCTT TAAAAAAACA GACGAAAACC ACCTCATTTT ATAACGGTTT AAGTAATGCT   
  
  
+ TTGTTAATTA ATTTTTTTAC ACAAATTAAC TAAATAGTTG GAAATAATAT GTCTTCTAAA AGTACTACGA   
  
  
+ ATTCGTACTA CTACTAGCCT AACGCCTTTA CAGATTTAGT TATTAGGCGT TTTAACCTTG AACAAAACGT   
  
  
+ TATAATTTTA TATATAACCC CAATTAAAAC ACTAAGTTTT AATAATTTTC CTTCCGTCTC GTCTTATTTC   
  
  
+ CTTAATTTAT ATAGGTTTCA GCAAACTTAT TAATTTAAAA CCTCGGAAAA ATTTTCAGTT CTTTTATCTC   
  
  
+ TTCTGTACGA ACCTAACAAA TTATTCCAGC GAAAATTCAA AATTGAGAAA AGAACCTTTA ATCATACTTA   
  
  
+ AAAGTCTCCT TAATCTCACC GTTATATATT ATTTTATTTT ATTTATTTGT TATATGAAAA AATTATAATA   
  
  
+ TTAATATAAT ACTTTCGAAA TGAAAAATTA AACGTTTATA AAATAACTTT TTTATTATAA TAGTAAAAAA   
  
  
+ ATATTAGTTT CATAGTTAAC CCCTAACCTT GGGATTTGAA GACAGAACGA ATAATCACTC TACCTAGATA   
  
  
+ GTTTTAATGA AGTAAAGTTT TAAATTTTTA CTTTAATGCT CTTAACACTA AAAAATAAAA CTCTGATTTT   
  
  
+ TTAACAATCA GTCGTCCAAC TAGTGTAGC  

- AGCTCTTCTT CGTTCTCTTG AATGGACCAT CTCAACACTT TGTTCTCTTC TTTTATAGAG AGGCTGCAAA   
  
  
- TGTTTGAATC CTTTAATATA CACAACGTCA ACAGCTGCCT ATCCAATTGC ACTAAAAAAC AGTGCCCCAA   
  
  
- CCATATATTA ATTATACGAT CCCTAACCAT AATTAATTAC GCTTAATTAA GTACGAATAA TTCAACTTCT   
  
  
- TCCATCATTT AGATTATACA AAATGTCTGC ATTGACAACA AGAGAGAGCT TTCAGCCGTG AGGCCCGGAG   
  
  
- CCAACCATCC AGGTATACTC ATGCCCTATA TCCATGAAGT TTCGTACGGT ACATAAAGTC ACATGGACAC   
  
  
- AACCGTTTCG TTGGTCAAAT TAACAGTTTT ATACATAGTT CCGTCTTAAT GTCTTAAATA CAGGCAATAT   
  
  
- GACAAAAGTT TCTTCTGAAA GGTCTCCTTT GTCGTCTGGT TCCTGCATTA GGGTTTTACC GTGGAGAGAG   
  
  
- ACATGGGATT ATCAAGAATT GTAACATCTA AAAAAATTAC CTAACATCTA AAAATACGAG TCGGTCCAAC   
  
  
- CCGTGTTGTC ATTTTTCACC ATATATTATA ATTATGTCTC TTTATTTTTG GTAATCTTAA TGTTGCATGT   
  
  
- AAGGCTAGGC ATGTCTCTTG AGCCGAATAT ATAGACAGTC AAGGGTCAAA TATAATATTG GTAAGTAGGG   
  
  
- TATGCGTATA CATCTTAATC TTGAAAAATC TTTCTATGTT TCGCCGGTGA CTTACTTTCC GTGGGTTCTT   
  
  
- ATGCAATAGA AAAATCAGAA ATTTTTTTGT CTGCTTTTGG TGGAGTAAAA TATTGCCAAA TTCATTACGA   
  
  
- AACAATTAAT TAAAAAAATG TGTTTAATTG ATTTATCAAC CTTTATTATA CAGAAGATTT TCATGATGCT   
  
  
- TAAGCATGAT GATGATCGGA TTGCGGAAAT GTCTAAATCA ATAATCCGCA AAATTGGAAC TTGTTTTGCA   
  
  
- ATATTAAAAT ATATATTGGG GTTAATTTTG TGATTCAAAA TTATTAAAAG GAAGGCAGAG CAGAATAAAG   
  
  
- GAATTAAATA TATCCAAAGT CGTTTGAATA ATTAAATTTT GGAGCCTTTT TAAAAGTCAA GAAAATAGAG   
  
  
- AAGACATGCT TGGATTGTTT AATAAGGTCG CTTTTAAGTT TTAACTCTTT TCTTGGAAAT TAGTATGAAT   
  
  
- TTTCAGAGGA ATTAGAGTGG CAATATATAA TAAAATAAAA TAAATAAACA ATATACTTTT TTAATATTAT   
  
  
- AATTATATTA TGAAAGCTTT ACTTTTTAAT TTGCAAATAT TTTATTGAAA AAATAATATT ATCATTTTTT   
  
  
- TATAATCAAA GTATCAATTG GGGATTGGAA CCCTAAACTT CTGTCTTGCT TATTAGTGAG ATGGATCTAT   
  
  
- CAAAATTACT TCATTTCAAA ATTTAAAAAT GAAATTACGA GAATTGTGAT TTTTTATTTT GAGACTAAAA   
  
  
- AATTGTTAGT CAGCAGGTTG ATCACATCG
