# Supplementary material for: Genome-wide analysis of the WRKY gene family in drumstick (Moringa oleifera Lam.)
Source: PeerJ. 2019 Jun 10;7:e7063. doi: 10.7717/peerj.7063 (PMC6563795; doi:10.7717/peerj.7063)
Supplement: Supplemental Information 1 [file peerj-07-7063-s003.gz › MoWRKY46_plantcare.html]

Content-Type: text/html; charset=ISO-8859-1


CallMat\_Firefox


Webmaster Firefox specific output  
To save the result:
click on the frame with the right mouse button and save the source code as a text file with extension .html  
REFERENCE:PlantCARE: a database of plant cis-acting regulatory elements and a portal to tools for in silico analysis of promoter sequences.  
Lescot, M., Déhais, P., Moreau, Y., De Moor, B., Rouzé ,P.,and Rombauts, S.  
Nucleic Acids Res., Database issue(2002), 30(1):325-327.   


---

> 2018/04/13 10:10:12  
+ AAGTCGTCGG TTAGTTGGAA CTTTGAAAGT TGGTGGGTTA GAAGAGTGGA CGGAAAGGAC CCATGACAAA   
  
  
+ TGTCTACTCC GCTCCTTTCT TTCTGTCTCT TCCGTGCTTC CTGCCGCGAC TGAAAAACCT TCTTCTCTTT   
  
  
+ TTATAGCCGT TGGCTCTCTC TTCCTCTTTT CATATTTGAT TAGTCTGTAC CGTCCTCCTC TCTCTCTCTC   
  
  
+ TCCTTTTACC GTCACGTGAG TGACTGAACC ATTAACAATT AATAAATATG TAAGTTCTTT TCTAAACCTT   
  
  
+ TCTCCTCTTA TCGGTCTCTC GAAAAGGAAG TACCTTCAAC CCCCATTACC GGAAGCACTA AAGAGACTTT   
  
  
+ TACAGAGAAA ACGAAAAAGA AACTGAAACA CTTTTACAGT TTTCATCCCG GTTCGATTAC ATGGGGCATA   
  
  
+ ACAAGGAGAC GAACGTTTCA TCGGTTGTGC GAAATAAGAT GATGCGGAGA TGAGCTTTAT AATCACCCAC   
  
  
+ GAACACACAG ATCACGACAC TGAAAACACT TGGAGGCTTT ATAGTGAGTG CGCGAAGGGT GGCCGCACAT   
  
  
+ TGAAGGAGGC CGTCCGTCGT GAATGCGAGG GGACTTCTCG CACCCCTACA AGGAAAATCA TATAAGTTCA   
  
  
+ ACGAGACTGC GTTGGTGGCA CCTCCGCACC TGGACCTGCT TGATTCTATG AGCAAGTCAG TGGGCGCGCA   
  
  
+ CGCGGTAAGA CAGCGTGCAA CTCTAGAACC AGTCACGCTT AAGTTACATT TTCGACAGGA AACCGTGCGT   
  
  
+ GGTCCTCGTC CGAAGGTACC GACACGGCAA ACCACTGTCT GTTTAAGACT AGCATGCTAG TTTGGGTGCA   
  
  
+ CCGGGTTTTG AAAGACTAAG GGACCCGGGG TGTACTGGGC GGTACCTTCA CCCCTTCCCT TTTCCCCTTC   
  
  
+ GACAAGGAGA CAACTAACGG AGTCTAAACC ACTTCCTAGA ACCCGGCGAC TTGAACCCAA CCATAAAAAG   
  
  
+ TATATACTCT ACGGGAAAAG ATACACCCCT TAGCACCGTA AAAATGCCAC CGACTAAGCC ACCATGACAC   
  
  
+ GTCATTTTTC ATCTCATTTA GACGGAGACA AGTAGAATCA ACCGTAGAAA GAAAGGACTC AACCTTTCCA   
  
  
+ CTAGTCCATC CACGACCTGG TCCTTGGTTT ACAACGAGAG AAAAGATAAG TACCCTCTCT TGGTTGGCAG   
  
  
+ AGAGTCGAAC GGACTGCGCG TGCTTCTACG GTTTCAACAT AATAAGTAAA GTCACATCGG CATGCACATG   
  
  
+ GACCCGCACA CCTGCACTGC GAGTAAAGTA CTTACTGGGC TATAAAAAAA AAGTAAATAA AATAAATCTC   
  
  
+ ATTTATATTT TATTAAAATT TATTATAAAT AAAAAATAAA ATTTAAAATT TTATAATAAT TTAGTTAATA   
  
  
+ CGTCTTTATT ACAAAATTTA ATTAAATTAT TTTTAAATAA ACAATAATTA AATAAAATTT TTTCAATAAG   
  
  
+ TTCAATTCTA AACATATTAA TTAAAACTA  

- TTCAGCAGCC AATCAACCTT GAAACTTTCA ACCACCCAAT CTTCTCACCT GCCTTTCCTG GGTACTGTTT   
  
  
- ACAGATGAGG CGAGGAAAGA AAGACAGAGA AGGCACGAAG GACGGCGCTG ACTTTTTGGA AGAAGAGAAA   
  
  
- AATATCGGCA ACCGAGAGAG AAGGAGAAAA GTATAAACTA ATCAGACATG GCAGGAGGAG AGAGAGAGAG   
  
  
- AGGAAAATGG CAGTGCACTC ACTGACTTGG TAATTGTTAA TTATTTATAC ATTCAAGAAA AGATTTGGAA   
  
  
- AGAGGAGAAT AGCCAGAGAG CTTTTCCTTC ATGGAAGTTG GGGGTAATGG CCTTCGTGAT TTCTCTGAAA   
  
  
- ATGTCTCTTT TGCTTTTTCT TTGACTTTGT GAAAATGTCA AAAGTAGGGC CAAGCTAATG TACCCCGTAT   
  
  
- TGTTCCTCTG CTTGCAAAGT AGCCAACACG CTTTATTCTA CTACGCCTCT ACTCGAAATA TTAGTGGGTG   
  
  
- CTTGTGTGTC TAGTGCTGTG ACTTTTGTGA ACCTCCGAAA TATCACTCAC GCGCTTCCCA CCGGCGTGTA   
  
  
- ACTTCCTCCG GCAGGCAGCA CTTACGCTCC CCTGAAGAGC GTGGGGATGT TCCTTTTAGT ATATTCAAGT   
  
  
- TGCTCTGACG CAACCACCGT GGAGGCGTGG ACCTGGACGA ACTAAGATAC TCGTTCAGTC ACCCGCGCGT   
  
  
- GCGCCATTCT GTCGCACGTT GAGATCTTGG TCAGTGCGAA TTCAATGTAA AAGCTGTCCT TTGGCACGCA   
  
  
- CCAGGAGCAG GCTTCCATGG CTGTGCCGTT TGGTGACAGA CAAATTCTGA TCGTACGATC AAACCCACGT   
  
  
- GGCCCAAAAC TTTCTGATTC CCTGGGCCCC ACATGACCCG CCATGGAAGT GGGGAAGGGA AAAGGGGAAG   
  
  
- CTGTTCCTCT GTTGATTGCC TCAGATTTGG TGAAGGATCT TGGGCCGCTG AACTTGGGTT GGTATTTTTC   
  
  
- ATATATGAGA TGCCCTTTTC TATGTGGGGA ATCGTGGCAT TTTTACGGTG GCTGATTCGG TGGTACTGTG   
  
  
- CAGTAAAAAG TAGAGTAAAT CTGCCTCTGT TCATCTTAGT TGGCATCTTT CTTTCCTGAG TTGGAAAGGT   
  
  
- GATCAGGTAG GTGCTGGACC AGGAACCAAA TGTTGCTCTC TTTTCTATTC ATGGGAGAGA ACCAACCGTC   
  
  
- TCTCAGCTTG CCTGACGCGC ACGAAGATGC CAAAGTTGTA TTATTCATTT CAGTGTAGCC GTACGTGTAC   
  
  
- CTGGGCGTGT GGACGTGACG CTCATTTCAT GAATGACCCG ATATTTTTTT TTCATTTATT TTATTTAGAG   
  
  
- TAAATATAAA ATAATTTTAA ATAATATTTA TTTTTTATTT TAAATTTTAA AATATTATTA AATCAATTAT   
  
  
- GCAGAAATAA TGTTTTAAAT TAATTTAATA AAAATTTATT TGTTATTAAT TTATTTTAAA AAAGTTATTC   
  
  
- AAGTTAAGAT TTGTATAATT AATTTTGAT

  
  
Motifs Found  

+     5UTR Py-rich stretch

| Site Name | Organism | Position | Strand | Matrix score. | sequence | function |
| --- | --- | --- | --- | --- | --- | --- |
| 5UTR Py-rich stretch | Lycopersicon esculentum | 279 | + | 9 | TTTCTTCTCT | cis-acting element conferring high transcription levels |
| 5UTR Py-rich stretch | Lycopersicon esculentum | 199 | + | 13 | TTTCTCTCTCTCTC | cis-acting element conferring high transcription levels |
| 5UTR Py-rich stretch | Lycopersicon esculentum | 129 | + | 9 | TTTCTTCTCT | cis-acting element conferring high transcription levels |

> 2018/04/13 10:10:12  
+ AAGTCGTCGG TTAGTTGGAA CTTTGAAAGT TGGTGGGTTA GAAGAGTGGA CGGAAAGGAC CCATGACAAA   
  
  
+ TGTCTACTCC GCTCCTTTCT TTCTGTCTCT TCCGTGCTTC CTGCCGCGAC TGAAAAACCT TCTTCTCTTT   
  
  
+ TTATAGCCGT TGGCTCTCTC TTCCTCTTTT CATATTTGAT TAGTCTGTAC CGTCCTCCTC TCTCTCTCTC   
  
  
+ TCCTTTTACC GTCACGTGAG TGACTGAACC ATTAACAATT AATAAATATG TAAGTTCTTT TCTAAACCTT   
  
  
+ TCTCCTCTTA TCGGTCTCTC GAAAAGGAAG TACCTTCAAC CCCCATTACC GGAAGCACTA AAGAGACTTT   
  
  
+ TACAGAGAAA ACGAAAAAGA AACTGAAACA CTTTTACAGT TTTCATCCCG GTTCGATTAC ATGGGGCATA   
  
  
+ ACAAGGAGAC GAACGTTTCA TCGGTTGTGC GAAATAAGAT GATGCGGAGA TGAGCTTTAT AATCACCCAC   
  
  
+ GAACACACAG ATCACGACAC TGAAAACACT TGGAGGCTTT ATAGTGAGTG CGCGAAGGGT GGCCGCACAT   
  
  
+ TGAAGGAGGC CGTCCGTCGT GAATGCGAGG GGACTTCTCG CACCCCTACA AGGAAAATCA TATAAGTTCA   
  
  
+ ACGAGACTGC GTTGGTGGCA CCTCCGCACC TGGACCTGCT TGATTCTATG AGCAAGTCAG TGGGCGCGCA   
  
  
+ CGCGGTAAGA CAGCGTGCAA CTCTAGAACC AGTCACGCTT AAGTTACATT TTCGACAGGA AACCGTGCGT   
  
  
+ GGTCCTCGTC CGAAGGTACC GACACGGCAA ACCACTGTCT GTTTAAGACT AGCATGCTAG TTTGGGTGCA   
  
  
+ CCGGGTTTTG AAAGACTAAG GGACCCGGGG TGTACTGGGC GGTACCTTCA CCCCTTCCCT TTTCCCCTTC   
  
  
+ GACAAGGAGA CAACTAACGG AGTCTAAACC ACTTCCTAGA ACCCGGCGAC TTGAACCCAA CCATAAAAAG   
  
  
+ TATATACTCT ACGGGAAAAG ATACACCCCT TAGCACCGTA AAAATGCCAC CGACTAAGCC ACCATGACAC   
  
  
+ GTCATTTTTC ATCTCATTTA GACGGAGACA AGTAGAATCA ACCGTAGAAA GAAAGGACTC AACCTTTCCA   
  
  
+ CTAGTCCATC CACGACCTGG TCCTTGGTTT ACAACGAGAG AAAAGATAAG TACCCTCTCT TGGTTGGCAG   
  
  
+ AGAGTCGAAC GGACTGCGCG TGCTTCTACG GTTTCAACAT AATAAGTAAA GTCACATCGG CATGCACATG   
  
  
+ GACCCGCACA CCTGCACTGC GAGTAAAGTA CTTACTGGGC TATAAAAAAA AAGTAAATAA AATAAATCTC   
  
  
+ ATTTATATTT TATTAAAATT TATTATAAAT AAAAAATAAA ATTTAAAATT TTATAATAAT TTAGTTAATA   
  
  
+ CGTCTTTATT ACAAAATTTA ATTAAATTAT TTTTAAATAA ACAATAATTA AATAAAATTT TTTCAATAAG   
  
  
+ TTCAATTCTA AACATATTAA TTAAAACTA  

- TTCAGCAGCC AATCAACCTT GAAACTTTCA ACCACCCAAT CTTCTCACCT GCCTTTCCTG GGTACTGTTT   
  
  
- ACAGATGAGG CGAGGAAAGA AAGACAGAGA AGGCACGAAG GACGGCGCTG ACTTTTTGGA AGAAGAGAAA   
  
  
- AATATCGGCA ACCGAGAGAG AAGGAGAAAA GTATAAACTA ATCAGACATG GCAGGAGGAG AGAGAGAGAG   
  
  
- AGGAAAATGG CAGTGCACTC ACTGACTTGG TAATTGTTAA TTATTTATAC ATTCAAGAAA AGATTTGGAA   
  
  
- AGAGGAGAAT AGCCAGAGAG CTTTTCCTTC ATGGAAGTTG GGGGTAATGG CCTTCGTGAT TTCTCTGAAA   
  
  
- ATGTCTCTTT TGCTTTTTCT TTGACTTTGT GAAAATGTCA AAAGTAGGGC CAAGCTAATG TACCCCGTAT   
  
  
- TGTTCCTCTG CTTGCAAAGT AGCCAACACG CTTTATTCTA CTACGCCTCT ACTCGAAATA TTAGTGGGTG   
  
  
- CTTGTGTGTC TAGTGCTGTG ACTTTTGTGA ACCTCCGAAA TATCACTCAC GCGCTTCCCA CCGGCGTGTA   
  
  
- ACTTCCTCCG GCAGGCAGCA CTTACGCTCC CCTGAAGAGC GTGGGGATGT TCCTTTTAGT ATATTCAAGT   
  
  
- TGCTCTGACG CAACCACCGT GGAGGCGTGG ACCTGGACGA ACTAAGATAC TCGTTCAGTC ACCCGCGCGT   
  
  
- GCGCCATTCT GTCGCACGTT GAGATCTTGG TCAGTGCGAA TTCAATGTAA AAGCTGTCCT TTGGCACGCA   
  
  
- CCAGGAGCAG GCTTCCATGG CTGTGCCGTT TGGTGACAGA CAAATTCTGA TCGTACGATC AAACCCACGT   
  
  
- GGCCCAAAAC TTTCTGATTC CCTGGGCCCC ACATGACCCG CCATGGAAGT GGGGAAGGGA AAAGGGGAAG   
  
  
- CTGTTCCTCT GTTGATTGCC TCAGATTTGG TGAAGGATCT TGGGCCGCTG AACTTGGGTT GGTATTTTTC   
  
  
- ATATATGAGA TGCCCTTTTC TATGTGGGGA ATCGTGGCAT TTTTACGGTG GCTGATTCGG TGGTACTGTG   
  
  
- CAGTAAAAAG TAGAGTAAAT CTGCCTCTGT TCATCTTAGT TGGCATCTTT CTTTCCTGAG TTGGAAAGGT   
  
  
- GATCAGGTAG GTGCTGGACC AGGAACCAAA TGTTGCTCTC TTTTCTATTC ATGGGAGAGA ACCAACCGTC   
  
  
- TCTCAGCTTG CCTGACGCGC ACGAAGATGC CAAAGTTGTA TTATTCATTT CAGTGTAGCC GTACGTGTAC   
  
  
- CTGGGCGTGT GGACGTGACG CTCATTTCAT GAATGACCCG ATATTTTTTT TTCATTTATT TTATTTAGAG   
  
  
- TAAATATAAA ATAATTTTAA ATAATATTTA TTTTTTATTT TAAATTTTAA AATATTATTA AATCAATTAT   
  
  
- GCAGAAATAA TGTTTTAAAT TAATTTAATA AAAATTTATT TGTTATTAAT TTATTTTAAA AAAGTTATTC   
  
  
- AAGTTAAGAT TTGTATAATT AATTTTGAT

+     A-box

| Site Name | Organism | Position | Strand | Matrix score. | sequence | function |
| --- | --- | --- | --- | --- | --- | --- |
| A-box | Petroselinum crispum | 190 | + | 6 | CCGTCC | cis-acting regulatory element |
| A-box | Petroselinum crispum | 48 | - | 6 | CCGTCC | cis-acting regulatory element |
| A-box | Petroselinum crispum | 570 | + | 6 | CCGTCC | cis-acting regulatory element |

> 2018/04/13 10:10:12  
+ AAGTCGTCGG TTAGTTGGAA CTTTGAAAGT TGGTGGGTTA GAAGAGTGGA CGGAAAGGAC CCATGACAAA   
  
  
+ TGTCTACTCC GCTCCTTTCT TTCTGTCTCT TCCGTGCTTC CTGCCGCGAC TGAAAAACCT TCTTCTCTTT   
  
  
+ TTATAGCCGT TGGCTCTCTC TTCCTCTTTT CATATTTGAT TAGTCTGTAC CGTCCTCCTC TCTCTCTCTC   
  
  
+ TCCTTTTACC GTCACGTGAG TGACTGAACC ATTAACAATT AATAAATATG TAAGTTCTTT TCTAAACCTT   
  
  
+ TCTCCTCTTA TCGGTCTCTC GAAAAGGAAG TACCTTCAAC CCCCATTACC GGAAGCACTA AAGAGACTTT   
  
  
+ TACAGAGAAA ACGAAAAAGA AACTGAAACA CTTTTACAGT TTTCATCCCG GTTCGATTAC ATGGGGCATA   
  
  
+ ACAAGGAGAC GAACGTTTCA TCGGTTGTGC GAAATAAGAT GATGCGGAGA TGAGCTTTAT AATCACCCAC   
  
  
+ GAACACACAG ATCACGACAC TGAAAACACT TGGAGGCTTT ATAGTGAGTG CGCGAAGGGT GGCCGCACAT   
  
  
+ TGAAGGAGGC CGTCCGTCGT GAATGCGAGG GGACTTCTCG CACCCCTACA AGGAAAATCA TATAAGTTCA   
  
  
+ ACGAGACTGC GTTGGTGGCA CCTCCGCACC TGGACCTGCT TGATTCTATG AGCAAGTCAG TGGGCGCGCA   
  
  
+ CGCGGTAAGA CAGCGTGCAA CTCTAGAACC AGTCACGCTT AAGTTACATT TTCGACAGGA AACCGTGCGT   
  
  
+ GGTCCTCGTC CGAAGGTACC GACACGGCAA ACCACTGTCT GTTTAAGACT AGCATGCTAG TTTGGGTGCA   
  
  
+ CCGGGTTTTG AAAGACTAAG GGACCCGGGG TGTACTGGGC GGTACCTTCA CCCCTTCCCT TTTCCCCTTC   
  
  
+ GACAAGGAGA CAACTAACGG AGTCTAAACC ACTTCCTAGA ACCCGGCGAC TTGAACCCAA CCATAAAAAG   
  
  
+ TATATACTCT ACGGGAAAAG ATACACCCCT TAGCACCGTA AAAATGCCAC CGACTAAGCC ACCATGACAC   
  
  
+ GTCATTTTTC ATCTCATTTA GACGGAGACA AGTAGAATCA ACCGTAGAAA GAAAGGACTC AACCTTTCCA   
  
  
+ CTAGTCCATC CACGACCTGG TCCTTGGTTT ACAACGAGAG AAAAGATAAG TACCCTCTCT TGGTTGGCAG   
  
  
+ AGAGTCGAAC GGACTGCGCG TGCTTCTACG GTTTCAACAT AATAAGTAAA GTCACATCGG CATGCACATG   
  
  
+ GACCCGCACA CCTGCACTGC GAGTAAAGTA CTTACTGGGC TATAAAAAAA AAGTAAATAA AATAAATCTC   
  
  
+ ATTTATATTT TATTAAAATT TATTATAAAT AAAAAATAAA ATTTAAAATT TTATAATAAT TTAGTTAATA   
  
  
+ CGTCTTTATT ACAAAATTTA ATTAAATTAT TTTTAAATAA ACAATAATTA AATAAAATTT TTTCAATAAG   
  
  
+ TTCAATTCTA AACATATTAA TTAAAACTA  

- TTCAGCAGCC AATCAACCTT GAAACTTTCA ACCACCCAAT CTTCTCACCT GCCTTTCCTG GGTACTGTTT   
  
  
- ACAGATGAGG CGAGGAAAGA AAGACAGAGA AGGCACGAAG GACGGCGCTG ACTTTTTGGA AGAAGAGAAA   
  
  
- AATATCGGCA ACCGAGAGAG AAGGAGAAAA GTATAAACTA ATCAGACATG GCAGGAGGAG AGAGAGAGAG   
  
  
- AGGAAAATGG CAGTGCACTC ACTGACTTGG TAATTGTTAA TTATTTATAC ATTCAAGAAA AGATTTGGAA   
  
  
- AGAGGAGAAT AGCCAGAGAG CTTTTCCTTC ATGGAAGTTG GGGGTAATGG CCTTCGTGAT TTCTCTGAAA   
  
  
- ATGTCTCTTT TGCTTTTTCT TTGACTTTGT GAAAATGTCA AAAGTAGGGC CAAGCTAATG TACCCCGTAT   
  
  
- TGTTCCTCTG CTTGCAAAGT AGCCAACACG CTTTATTCTA CTACGCCTCT ACTCGAAATA TTAGTGGGTG   
  
  
- CTTGTGTGTC TAGTGCTGTG ACTTTTGTGA ACCTCCGAAA TATCACTCAC GCGCTTCCCA CCGGCGTGTA   
  
  
- ACTTCCTCCG GCAGGCAGCA CTTACGCTCC CCTGAAGAGC GTGGGGATGT TCCTTTTAGT ATATTCAAGT   
  
  
- TGCTCTGACG CAACCACCGT GGAGGCGTGG ACCTGGACGA ACTAAGATAC TCGTTCAGTC ACCCGCGCGT   
  
  
- GCGCCATTCT GTCGCACGTT GAGATCTTGG TCAGTGCGAA TTCAATGTAA AAGCTGTCCT TTGGCACGCA   
  
  
- CCAGGAGCAG GCTTCCATGG CTGTGCCGTT TGGTGACAGA CAAATTCTGA TCGTACGATC AAACCCACGT   
  
  
- GGCCCAAAAC TTTCTGATTC CCTGGGCCCC ACATGACCCG CCATGGAAGT GGGGAAGGGA AAAGGGGAAG   
  
  
- CTGTTCCTCT GTTGATTGCC TCAGATTTGG TGAAGGATCT TGGGCCGCTG AACTTGGGTT GGTATTTTTC   
  
  
- ATATATGAGA TGCCCTTTTC TATGTGGGGA ATCGTGGCAT TTTTACGGTG GCTGATTCGG TGGTACTGTG   
  
  
- CAGTAAAAAG TAGAGTAAAT CTGCCTCTGT TCATCTTAGT TGGCATCTTT CTTTCCTGAG TTGGAAAGGT   
  
  
- GATCAGGTAG GTGCTGGACC AGGAACCAAA TGTTGCTCTC TTTTCTATTC ATGGGAGAGA ACCAACCGTC   
  
  
- TCTCAGCTTG CCTGACGCGC ACGAAGATGC CAAAGTTGTA TTATTCATTT CAGTGTAGCC GTACGTGTAC   
  
  
- CTGGGCGTGT GGACGTGACG CTCATTTCAT GAATGACCCG ATATTTTTTT TTCATTTATT TTATTTAGAG   
  
  
- TAAATATAAA ATAATTTTAA ATAATATTTA TTTTTTATTT TAAATTTTAA AATATTATTA AATCAATTAT   
  
  
- GCAGAAATAA TGTTTTAAAT TAATTTAATA AAAATTTATT TGTTATTAAT TTATTTTAAA AAAGTTATTC   
  
  
- AAGTTAAGAT TTGTATAATT AATTTTGAT

+     AAGAA-motif

| Site Name | Organism | Position | Strand | Matrix score. | sequence | function |
| --- | --- | --- | --- | --- | --- | --- |
| AAGAA-motif | Avena sativa | 87 | - | 7 | GAAAGAA |  |
| AAGAA-motif | Avena sativa | 1097 | + | 7 | GAAAGAA |  |

> 2018/04/13 10:10:12  
+ AAGTCGTCGG TTAGTTGGAA CTTTGAAAGT TGGTGGGTTA GAAGAGTGGA CGGAAAGGAC CCATGACAAA   
  
  
+ TGTCTACTCC GCTCCTTTCT TTCTGTCTCT TCCGTGCTTC CTGCCGCGAC TGAAAAACCT TCTTCTCTTT   
  
  
+ TTATAGCCGT TGGCTCTCTC TTCCTCTTTT CATATTTGAT TAGTCTGTAC CGTCCTCCTC TCTCTCTCTC   
  
  
+ TCCTTTTACC GTCACGTGAG TGACTGAACC ATTAACAATT AATAAATATG TAAGTTCTTT TCTAAACCTT   
  
  
+ TCTCCTCTTA TCGGTCTCTC GAAAAGGAAG TACCTTCAAC CCCCATTACC GGAAGCACTA AAGAGACTTT   
  
  
+ TACAGAGAAA ACGAAAAAGA AACTGAAACA CTTTTACAGT TTTCATCCCG GTTCGATTAC ATGGGGCATA   
  
  
+ ACAAGGAGAC GAACGTTTCA TCGGTTGTGC GAAATAAGAT GATGCGGAGA TGAGCTTTAT AATCACCCAC   
  
  
+ GAACACACAG ATCACGACAC TGAAAACACT TGGAGGCTTT ATAGTGAGTG CGCGAAGGGT GGCCGCACAT   
  
  
+ TGAAGGAGGC CGTCCGTCGT GAATGCGAGG GGACTTCTCG CACCCCTACA AGGAAAATCA TATAAGTTCA   
  
  
+ ACGAGACTGC GTTGGTGGCA CCTCCGCACC TGGACCTGCT TGATTCTATG AGCAAGTCAG TGGGCGCGCA   
  
  
+ CGCGGTAAGA CAGCGTGCAA CTCTAGAACC AGTCACGCTT AAGTTACATT TTCGACAGGA AACCGTGCGT   
  
  
+ GGTCCTCGTC CGAAGGTACC GACACGGCAA ACCACTGTCT GTTTAAGACT AGCATGCTAG TTTGGGTGCA   
  
  
+ CCGGGTTTTG AAAGACTAAG GGACCCGGGG TGTACTGGGC GGTACCTTCA CCCCTTCCCT TTTCCCCTTC   
  
  
+ GACAAGGAGA CAACTAACGG AGTCTAAACC ACTTCCTAGA ACCCGGCGAC TTGAACCCAA CCATAAAAAG   
  
  
+ TATATACTCT ACGGGAAAAG ATACACCCCT TAGCACCGTA AAAATGCCAC CGACTAAGCC ACCATGACAC   
  
  
+ GTCATTTTTC ATCTCATTTA GACGGAGACA AGTAGAATCA ACCGTAGAAA GAAAGGACTC AACCTTTCCA   
  
  
+ CTAGTCCATC CACGACCTGG TCCTTGGTTT ACAACGAGAG AAAAGATAAG TACCCTCTCT TGGTTGGCAG   
  
  
+ AGAGTCGAAC GGACTGCGCG TGCTTCTACG GTTTCAACAT AATAAGTAAA GTCACATCGG CATGCACATG   
  
  
+ GACCCGCACA CCTGCACTGC GAGTAAAGTA CTTACTGGGC TATAAAAAAA AAGTAAATAA AATAAATCTC   
  
  
+ ATTTATATTT TATTAAAATT TATTATAAAT AAAAAATAAA ATTTAAAATT TTATAATAAT TTAGTTAATA   
  
  
+ CGTCTTTATT ACAAAATTTA ATTAAATTAT TTTTAAATAA ACAATAATTA AATAAAATTT TTTCAATAAG   
  
  
+ TTCAATTCTA AACATATTAA TTAAAACTA  

- TTCAGCAGCC AATCAACCTT GAAACTTTCA ACCACCCAAT CTTCTCACCT GCCTTTCCTG GGTACTGTTT   
  
  
- ACAGATGAGG CGAGGAAAGA AAGACAGAGA AGGCACGAAG GACGGCGCTG ACTTTTTGGA AGAAGAGAAA   
  
  
- AATATCGGCA ACCGAGAGAG AAGGAGAAAA GTATAAACTA ATCAGACATG GCAGGAGGAG AGAGAGAGAG   
  
  
- AGGAAAATGG CAGTGCACTC ACTGACTTGG TAATTGTTAA TTATTTATAC ATTCAAGAAA AGATTTGGAA   
  
  
- AGAGGAGAAT AGCCAGAGAG CTTTTCCTTC ATGGAAGTTG GGGGTAATGG CCTTCGTGAT TTCTCTGAAA   
  
  
- ATGTCTCTTT TGCTTTTTCT TTGACTTTGT GAAAATGTCA AAAGTAGGGC CAAGCTAATG TACCCCGTAT   
  
  
- TGTTCCTCTG CTTGCAAAGT AGCCAACACG CTTTATTCTA CTACGCCTCT ACTCGAAATA TTAGTGGGTG   
  
  
- CTTGTGTGTC TAGTGCTGTG ACTTTTGTGA ACCTCCGAAA TATCACTCAC GCGCTTCCCA CCGGCGTGTA   
  
  
- ACTTCCTCCG GCAGGCAGCA CTTACGCTCC CCTGAAGAGC GTGGGGATGT TCCTTTTAGT ATATTCAAGT   
  
  
- TGCTCTGACG CAACCACCGT GGAGGCGTGG ACCTGGACGA ACTAAGATAC TCGTTCAGTC ACCCGCGCGT   
  
  
- GCGCCATTCT GTCGCACGTT GAGATCTTGG TCAGTGCGAA TTCAATGTAA AAGCTGTCCT TTGGCACGCA   
  
  
- CCAGGAGCAG GCTTCCATGG CTGTGCCGTT TGGTGACAGA CAAATTCTGA TCGTACGATC AAACCCACGT   
  
  
- GGCCCAAAAC TTTCTGATTC CCTGGGCCCC ACATGACCCG CCATGGAAGT GGGGAAGGGA AAAGGGGAAG   
  
  
- CTGTTCCTCT GTTGATTGCC TCAGATTTGG TGAAGGATCT TGGGCCGCTG AACTTGGGTT GGTATTTTTC   
  
  
- ATATATGAGA TGCCCTTTTC TATGTGGGGA ATCGTGGCAT TTTTACGGTG GCTGATTCGG TGGTACTGTG   
  
  
- CAGTAAAAAG TAGAGTAAAT CTGCCTCTGT TCATCTTAGT TGGCATCTTT CTTTCCTGAG TTGGAAAGGT   
  
  
- GATCAGGTAG GTGCTGGACC AGGAACCAAA TGTTGCTCTC TTTTCTATTC ATGGGAGAGA ACCAACCGTC   
  
  
- TCTCAGCTTG CCTGACGCGC ACGAAGATGC CAAAGTTGTA TTATTCATTT CAGTGTAGCC GTACGTGTAC   
  
  
- CTGGGCGTGT GGACGTGACG CTCATTTCAT GAATGACCCG ATATTTTTTT TTCATTTATT TTATTTAGAG   
  
  
- TAAATATAAA ATAATTTTAA ATAATATTTA TTTTTTATTT TAAATTTTAA AATATTATTA AATCAATTAT   
  
  
- GCAGAAATAA TGTTTTAAAT TAATTTAATA AAAATTTATT TGTTATTAAT TTATTTTAAA AAAGTTATTC   
  
  
- AAGTTAAGAT TTGTATAATT AATTTTGAT

+     ABRE

| Site Name | Organism | Position | Strand | Matrix score. | sequence | function |
| --- | --- | --- | --- | --- | --- | --- |
| ABRE | Arabidopsis thaliana | 223 | + | 6 | CACGTG | cis-acting element involved in the abscisic acid responsiveness |

> 2018/04/13 10:10:12  
+ AAGTCGTCGG TTAGTTGGAA CTTTGAAAGT TGGTGGGTTA GAAGAGTGGA CGGAAAGGAC CCATGACAAA   
  
  
+ TGTCTACTCC GCTCCTTTCT TTCTGTCTCT TCCGTGCTTC CTGCCGCGAC TGAAAAACCT TCTTCTCTTT   
  
  
+ TTATAGCCGT TGGCTCTCTC TTCCTCTTTT CATATTTGAT TAGTCTGTAC CGTCCTCCTC TCTCTCTCTC   
  
  
+ TCCTTTTACC GTCACGTGAG TGACTGAACC ATTAACAATT AATAAATATG TAAGTTCTTT TCTAAACCTT   
  
  
+ TCTCCTCTTA TCGGTCTCTC GAAAAGGAAG TACCTTCAAC CCCCATTACC GGAAGCACTA AAGAGACTTT   
  
  
+ TACAGAGAAA ACGAAAAAGA AACTGAAACA CTTTTACAGT TTTCATCCCG GTTCGATTAC ATGGGGCATA   
  
  
+ ACAAGGAGAC GAACGTTTCA TCGGTTGTGC GAAATAAGAT GATGCGGAGA TGAGCTTTAT AATCACCCAC   
  
  
+ GAACACACAG ATCACGACAC TGAAAACACT TGGAGGCTTT ATAGTGAGTG CGCGAAGGGT GGCCGCACAT   
  
  
+ TGAAGGAGGC CGTCCGTCGT GAATGCGAGG GGACTTCTCG CACCCCTACA AGGAAAATCA TATAAGTTCA   
  
  
+ ACGAGACTGC GTTGGTGGCA CCTCCGCACC TGGACCTGCT TGATTCTATG AGCAAGTCAG TGGGCGCGCA   
  
  
+ CGCGGTAAGA CAGCGTGCAA CTCTAGAACC AGTCACGCTT AAGTTACATT TTCGACAGGA AACCGTGCGT   
  
  
+ GGTCCTCGTC CGAAGGTACC GACACGGCAA ACCACTGTCT GTTTAAGACT AGCATGCTAG TTTGGGTGCA   
  
  
+ CCGGGTTTTG AAAGACTAAG GGACCCGGGG TGTACTGGGC GGTACCTTCA CCCCTTCCCT TTTCCCCTTC   
  
  
+ GACAAGGAGA CAACTAACGG AGTCTAAACC ACTTCCTAGA ACCCGGCGAC TTGAACCCAA CCATAAAAAG   
  
  
+ TATATACTCT ACGGGAAAAG ATACACCCCT TAGCACCGTA AAAATGCCAC CGACTAAGCC ACCATGACAC   
  
  
+ GTCATTTTTC ATCTCATTTA GACGGAGACA AGTAGAATCA ACCGTAGAAA GAAAGGACTC AACCTTTCCA   
  
  
+ CTAGTCCATC CACGACCTGG TCCTTGGTTT ACAACGAGAG AAAAGATAAG TACCCTCTCT TGGTTGGCAG   
  
  
+ AGAGTCGAAC GGACTGCGCG TGCTTCTACG GTTTCAACAT AATAAGTAAA GTCACATCGG CATGCACATG   
  
  
+ GACCCGCACA CCTGCACTGC GAGTAAAGTA CTTACTGGGC TATAAAAAAA AAGTAAATAA AATAAATCTC   
  
  
+ ATTTATATTT TATTAAAATT TATTATAAAT AAAAAATAAA ATTTAAAATT TTATAATAAT TTAGTTAATA   
  
  
+ CGTCTTTATT ACAAAATTTA ATTAAATTAT TTTTAAATAA ACAATAATTA AATAAAATTT TTTCAATAAG   
  
  
+ TTCAATTCTA AACATATTAA TTAAAACTA  

- TTCAGCAGCC AATCAACCTT GAAACTTTCA ACCACCCAAT CTTCTCACCT GCCTTTCCTG GGTACTGTTT   
  
  
- ACAGATGAGG CGAGGAAAGA AAGACAGAGA AGGCACGAAG GACGGCGCTG ACTTTTTGGA AGAAGAGAAA   
  
  
- AATATCGGCA ACCGAGAGAG AAGGAGAAAA GTATAAACTA ATCAGACATG GCAGGAGGAG AGAGAGAGAG   
  
  
- AGGAAAATGG CAGTGCACTC ACTGACTTGG TAATTGTTAA TTATTTATAC ATTCAAGAAA AGATTTGGAA   
  
  
- AGAGGAGAAT AGCCAGAGAG CTTTTCCTTC ATGGAAGTTG GGGGTAATGG CCTTCGTGAT TTCTCTGAAA   
  
  
- ATGTCTCTTT TGCTTTTTCT TTGACTTTGT GAAAATGTCA AAAGTAGGGC CAAGCTAATG TACCCCGTAT   
  
  
- TGTTCCTCTG CTTGCAAAGT AGCCAACACG CTTTATTCTA CTACGCCTCT ACTCGAAATA TTAGTGGGTG   
  
  
- CTTGTGTGTC TAGTGCTGTG ACTTTTGTGA ACCTCCGAAA TATCACTCAC GCGCTTCCCA CCGGCGTGTA   
  
  
- ACTTCCTCCG GCAGGCAGCA CTTACGCTCC CCTGAAGAGC GTGGGGATGT TCCTTTTAGT ATATTCAAGT   
  
  
- TGCTCTGACG CAACCACCGT GGAGGCGTGG ACCTGGACGA ACTAAGATAC TCGTTCAGTC ACCCGCGCGT   
  
  
- GCGCCATTCT GTCGCACGTT GAGATCTTGG TCAGTGCGAA TTCAATGTAA AAGCTGTCCT TTGGCACGCA   
  
  
- CCAGGAGCAG GCTTCCATGG CTGTGCCGTT TGGTGACAGA CAAATTCTGA TCGTACGATC AAACCCACGT   
  
  
- GGCCCAAAAC TTTCTGATTC CCTGGGCCCC ACATGACCCG CCATGGAAGT GGGGAAGGGA AAAGGGGAAG   
  
  
- CTGTTCCTCT GTTGATTGCC TCAGATTTGG TGAAGGATCT TGGGCCGCTG AACTTGGGTT GGTATTTTTC   
  
  
- ATATATGAGA TGCCCTTTTC TATGTGGGGA ATCGTGGCAT TTTTACGGTG GCTGATTCGG TGGTACTGTG   
  
  
- CAGTAAAAAG TAGAGTAAAT CTGCCTCTGT TCATCTTAGT TGGCATCTTT CTTTCCTGAG TTGGAAAGGT   
  
  
- GATCAGGTAG GTGCTGGACC AGGAACCAAA TGTTGCTCTC TTTTCTATTC ATGGGAGAGA ACCAACCGTC   
  
  
- TCTCAGCTTG CCTGACGCGC ACGAAGATGC CAAAGTTGTA TTATTCATTT CAGTGTAGCC GTACGTGTAC   
  
  
- CTGGGCGTGT GGACGTGACG CTCATTTCAT GAATGACCCG ATATTTTTTT TTCATTTATT TTATTTAGAG   
  
  
- TAAATATAAA ATAATTTTAA ATAATATTTA TTTTTTATTT TAAATTTTAA AATATTATTA AATCAATTAT   
  
  
- GCAGAAATAA TGTTTTAAAT TAATTTAATA AAAATTTATT TGTTATTAAT TTATTTTAAA AAAGTTATTC   
  
  
- AAGTTAAGAT TTGTATAATT AATTTTGAT

+     AC-I

| Site Name | Organism | Position | Strand | Matrix score. | sequence | function |
| --- | --- | --- | --- | --- | --- | --- |
| AC-I | Phaseolus vulgaris | 32 | - | 8.5 | (T/C)C(T/C)(C/T)ACC(T/C)ACC |  |

> 2018/04/13 10:10:12  
+ AAGTCGTCGG TTAGTTGGAA CTTTGAAAGT TGGTGGGTTA GAAGAGTGGA CGGAAAGGAC CCATGACAAA   
  
  
+ TGTCTACTCC GCTCCTTTCT TTCTGTCTCT TCCGTGCTTC CTGCCGCGAC TGAAAAACCT TCTTCTCTTT   
  
  
+ TTATAGCCGT TGGCTCTCTC TTCCTCTTTT CATATTTGAT TAGTCTGTAC CGTCCTCCTC TCTCTCTCTC   
  
  
+ TCCTTTTACC GTCACGTGAG TGACTGAACC ATTAACAATT AATAAATATG TAAGTTCTTT TCTAAACCTT   
  
  
+ TCTCCTCTTA TCGGTCTCTC GAAAAGGAAG TACCTTCAAC CCCCATTACC GGAAGCACTA AAGAGACTTT   
  
  
+ TACAGAGAAA ACGAAAAAGA AACTGAAACA CTTTTACAGT TTTCATCCCG GTTCGATTAC ATGGGGCATA   
  
  
+ ACAAGGAGAC GAACGTTTCA TCGGTTGTGC GAAATAAGAT GATGCGGAGA TGAGCTTTAT AATCACCCAC   
  
  
+ GAACACACAG ATCACGACAC TGAAAACACT TGGAGGCTTT ATAGTGAGTG CGCGAAGGGT GGCCGCACAT   
  
  
+ TGAAGGAGGC CGTCCGTCGT GAATGCGAGG GGACTTCTCG CACCCCTACA AGGAAAATCA TATAAGTTCA   
  
  
+ ACGAGACTGC GTTGGTGGCA CCTCCGCACC TGGACCTGCT TGATTCTATG AGCAAGTCAG TGGGCGCGCA   
  
  
+ CGCGGTAAGA CAGCGTGCAA CTCTAGAACC AGTCACGCTT AAGTTACATT TTCGACAGGA AACCGTGCGT   
  
  
+ GGTCCTCGTC CGAAGGTACC GACACGGCAA ACCACTGTCT GTTTAAGACT AGCATGCTAG TTTGGGTGCA   
  
  
+ CCGGGTTTTG AAAGACTAAG GGACCCGGGG TGTACTGGGC GGTACCTTCA CCCCTTCCCT TTTCCCCTTC   
  
  
+ GACAAGGAGA CAACTAACGG AGTCTAAACC ACTTCCTAGA ACCCGGCGAC TTGAACCCAA CCATAAAAAG   
  
  
+ TATATACTCT ACGGGAAAAG ATACACCCCT TAGCACCGTA AAAATGCCAC CGACTAAGCC ACCATGACAC   
  
  
+ GTCATTTTTC ATCTCATTTA GACGGAGACA AGTAGAATCA ACCGTAGAAA GAAAGGACTC AACCTTTCCA   
  
  
+ CTAGTCCATC CACGACCTGG TCCTTGGTTT ACAACGAGAG AAAAGATAAG TACCCTCTCT TGGTTGGCAG   
  
  
+ AGAGTCGAAC GGACTGCGCG TGCTTCTACG GTTTCAACAT AATAAGTAAA GTCACATCGG CATGCACATG   
  
  
+ GACCCGCACA CCTGCACTGC GAGTAAAGTA CTTACTGGGC TATAAAAAAA AAGTAAATAA AATAAATCTC   
  
  
+ ATTTATATTT TATTAAAATT TATTATAAAT AAAAAATAAA ATTTAAAATT TTATAATAAT TTAGTTAATA   
  
  
+ CGTCTTTATT ACAAAATTTA ATTAAATTAT TTTTAAATAA ACAATAATTA AATAAAATTT TTTCAATAAG   
  
  
+ TTCAATTCTA AACATATTAA TTAAAACTA  

- TTCAGCAGCC AATCAACCTT GAAACTTTCA ACCACCCAAT CTTCTCACCT GCCTTTCCTG GGTACTGTTT   
  
  
- ACAGATGAGG CGAGGAAAGA AAGACAGAGA AGGCACGAAG GACGGCGCTG ACTTTTTGGA AGAAGAGAAA   
  
  
- AATATCGGCA ACCGAGAGAG AAGGAGAAAA GTATAAACTA ATCAGACATG GCAGGAGGAG AGAGAGAGAG   
  
  
- AGGAAAATGG CAGTGCACTC ACTGACTTGG TAATTGTTAA TTATTTATAC ATTCAAGAAA AGATTTGGAA   
  
  
- AGAGGAGAAT AGCCAGAGAG CTTTTCCTTC ATGGAAGTTG GGGGTAATGG CCTTCGTGAT TTCTCTGAAA   
  
  
- ATGTCTCTTT TGCTTTTTCT TTGACTTTGT GAAAATGTCA AAAGTAGGGC CAAGCTAATG TACCCCGTAT   
  
  
- TGTTCCTCTG CTTGCAAAGT AGCCAACACG CTTTATTCTA CTACGCCTCT ACTCGAAATA TTAGTGGGTG   
  
  
- CTTGTGTGTC TAGTGCTGTG ACTTTTGTGA ACCTCCGAAA TATCACTCAC GCGCTTCCCA CCGGCGTGTA   
  
  
- ACTTCCTCCG GCAGGCAGCA CTTACGCTCC CCTGAAGAGC GTGGGGATGT TCCTTTTAGT ATATTCAAGT   
  
  
- TGCTCTGACG CAACCACCGT GGAGGCGTGG ACCTGGACGA ACTAAGATAC TCGTTCAGTC ACCCGCGCGT   
  
  
- GCGCCATTCT GTCGCACGTT GAGATCTTGG TCAGTGCGAA TTCAATGTAA AAGCTGTCCT TTGGCACGCA   
  
  
- CCAGGAGCAG GCTTCCATGG CTGTGCCGTT TGGTGACAGA CAAATTCTGA TCGTACGATC AAACCCACGT   
  
  
- GGCCCAAAAC TTTCTGATTC CCTGGGCCCC ACATGACCCG CCATGGAAGT GGGGAAGGGA AAAGGGGAAG   
  
  
- CTGTTCCTCT GTTGATTGCC TCAGATTTGG TGAAGGATCT TGGGCCGCTG AACTTGGGTT GGTATTTTTC   
  
  
- ATATATGAGA TGCCCTTTTC TATGTGGGGA ATCGTGGCAT TTTTACGGTG GCTGATTCGG TGGTACTGTG   
  
  
- CAGTAAAAAG TAGAGTAAAT CTGCCTCTGT TCATCTTAGT TGGCATCTTT CTTTCCTGAG TTGGAAAGGT   
  
  
- GATCAGGTAG GTGCTGGACC AGGAACCAAA TGTTGCTCTC TTTTCTATTC ATGGGAGAGA ACCAACCGTC   
  
  
- TCTCAGCTTG CCTGACGCGC ACGAAGATGC CAAAGTTGTA TTATTCATTT CAGTGTAGCC GTACGTGTAC   
  
  
- CTGGGCGTGT GGACGTGACG CTCATTTCAT GAATGACCCG ATATTTTTTT TTCATTTATT TTATTTAGAG   
  
  
- TAAATATAAA ATAATTTTAA ATAATATTTA TTTTTTATTT TAAATTTTAA AATATTATTA AATCAATTAT   
  
  
- GCAGAAATAA TGTTTTAAAT TAATTTAATA AAAATTTATT TGTTATTAAT TTATTTTAAA AAAGTTATTC   
  
  
- AAGTTAAGAT TTGTATAATT AATTTTGAT

+     ACE

| Site Name | Organism | Position | Strand | Matrix score. | sequence | function |
| --- | --- | --- | --- | --- | --- | --- |
| ACE | Petroselinum crispum | 636 | + | 9 | ACTACGTTGG | cis-acting element involved in light responsiveness |

> 2018/04/13 10:10:12  
+ AAGTCGTCGG TTAGTTGGAA CTTTGAAAGT TGGTGGGTTA GAAGAGTGGA CGGAAAGGAC CCATGACAAA   
  
  
+ TGTCTACTCC GCTCCTTTCT TTCTGTCTCT TCCGTGCTTC CTGCCGCGAC TGAAAAACCT TCTTCTCTTT   
  
  
+ TTATAGCCGT TGGCTCTCTC TTCCTCTTTT CATATTTGAT TAGTCTGTAC CGTCCTCCTC TCTCTCTCTC   
  
  
+ TCCTTTTACC GTCACGTGAG TGACTGAACC ATTAACAATT AATAAATATG TAAGTTCTTT TCTAAACCTT   
  
  
+ TCTCCTCTTA TCGGTCTCTC GAAAAGGAAG TACCTTCAAC CCCCATTACC GGAAGCACTA AAGAGACTTT   
  
  
+ TACAGAGAAA ACGAAAAAGA AACTGAAACA CTTTTACAGT TTTCATCCCG GTTCGATTAC ATGGGGCATA   
  
  
+ ACAAGGAGAC GAACGTTTCA TCGGTTGTGC GAAATAAGAT GATGCGGAGA TGAGCTTTAT AATCACCCAC   
  
  
+ GAACACACAG ATCACGACAC TGAAAACACT TGGAGGCTTT ATAGTGAGTG CGCGAAGGGT GGCCGCACAT   
  
  
+ TGAAGGAGGC CGTCCGTCGT GAATGCGAGG GGACTTCTCG CACCCCTACA AGGAAAATCA TATAAGTTCA   
  
  
+ ACGAGACTGC GTTGGTGGCA CCTCCGCACC TGGACCTGCT TGATTCTATG AGCAAGTCAG TGGGCGCGCA   
  
  
+ CGCGGTAAGA CAGCGTGCAA CTCTAGAACC AGTCACGCTT AAGTTACATT TTCGACAGGA AACCGTGCGT   
  
  
+ GGTCCTCGTC CGAAGGTACC GACACGGCAA ACCACTGTCT GTTTAAGACT AGCATGCTAG TTTGGGTGCA   
  
  
+ CCGGGTTTTG AAAGACTAAG GGACCCGGGG TGTACTGGGC GGTACCTTCA CCCCTTCCCT TTTCCCCTTC   
  
  
+ GACAAGGAGA CAACTAACGG AGTCTAAACC ACTTCCTAGA ACCCGGCGAC TTGAACCCAA CCATAAAAAG   
  
  
+ TATATACTCT ACGGGAAAAG ATACACCCCT TAGCACCGTA AAAATGCCAC CGACTAAGCC ACCATGACAC   
  
  
+ GTCATTTTTC ATCTCATTTA GACGGAGACA AGTAGAATCA ACCGTAGAAA GAAAGGACTC AACCTTTCCA   
  
  
+ CTAGTCCATC CACGACCTGG TCCTTGGTTT ACAACGAGAG AAAAGATAAG TACCCTCTCT TGGTTGGCAG   
  
  
+ AGAGTCGAAC GGACTGCGCG TGCTTCTACG GTTTCAACAT AATAAGTAAA GTCACATCGG CATGCACATG   
  
  
+ GACCCGCACA CCTGCACTGC GAGTAAAGTA CTTACTGGGC TATAAAAAAA AAGTAAATAA AATAAATCTC   
  
  
+ ATTTATATTT TATTAAAATT TATTATAAAT AAAAAATAAA ATTTAAAATT TTATAATAAT TTAGTTAATA   
  
  
+ CGTCTTTATT ACAAAATTTA ATTAAATTAT TTTTAAATAA ACAATAATTA AATAAAATTT TTTCAATAAG   
  
  
+ TTCAATTCTA AACATATTAA TTAAAACTA  

- TTCAGCAGCC AATCAACCTT GAAACTTTCA ACCACCCAAT CTTCTCACCT GCCTTTCCTG GGTACTGTTT   
  
  
- ACAGATGAGG CGAGGAAAGA AAGACAGAGA AGGCACGAAG GACGGCGCTG ACTTTTTGGA AGAAGAGAAA   
  
  
- AATATCGGCA ACCGAGAGAG AAGGAGAAAA GTATAAACTA ATCAGACATG GCAGGAGGAG AGAGAGAGAG   
  
  
- AGGAAAATGG CAGTGCACTC ACTGACTTGG TAATTGTTAA TTATTTATAC ATTCAAGAAA AGATTTGGAA   
  
  
- AGAGGAGAAT AGCCAGAGAG CTTTTCCTTC ATGGAAGTTG GGGGTAATGG CCTTCGTGAT TTCTCTGAAA   
  
  
- ATGTCTCTTT TGCTTTTTCT TTGACTTTGT GAAAATGTCA AAAGTAGGGC CAAGCTAATG TACCCCGTAT   
  
  
- TGTTCCTCTG CTTGCAAAGT AGCCAACACG CTTTATTCTA CTACGCCTCT ACTCGAAATA TTAGTGGGTG   
  
  
- CTTGTGTGTC TAGTGCTGTG ACTTTTGTGA ACCTCCGAAA TATCACTCAC GCGCTTCCCA CCGGCGTGTA   
  
  
- ACTTCCTCCG GCAGGCAGCA CTTACGCTCC CCTGAAGAGC GTGGGGATGT TCCTTTTAGT ATATTCAAGT   
  
  
- TGCTCTGACG CAACCACCGT GGAGGCGTGG ACCTGGACGA ACTAAGATAC TCGTTCAGTC ACCCGCGCGT   
  
  
- GCGCCATTCT GTCGCACGTT GAGATCTTGG TCAGTGCGAA TTCAATGTAA AAGCTGTCCT TTGGCACGCA   
  
  
- CCAGGAGCAG GCTTCCATGG CTGTGCCGTT TGGTGACAGA CAAATTCTGA TCGTACGATC AAACCCACGT   
  
  
- GGCCCAAAAC TTTCTGATTC CCTGGGCCCC ACATGACCCG CCATGGAAGT GGGGAAGGGA AAAGGGGAAG   
  
  
- CTGTTCCTCT GTTGATTGCC TCAGATTTGG TGAAGGATCT TGGGCCGCTG AACTTGGGTT GGTATTTTTC   
  
  
- ATATATGAGA TGCCCTTTTC TATGTGGGGA ATCGTGGCAT TTTTACGGTG GCTGATTCGG TGGTACTGTG   
  
  
- CAGTAAAAAG TAGAGTAAAT CTGCCTCTGT TCATCTTAGT TGGCATCTTT CTTTCCTGAG TTGGAAAGGT   
  
  
- GATCAGGTAG GTGCTGGACC AGGAACCAAA TGTTGCTCTC TTTTCTATTC ATGGGAGAGA ACCAACCGTC   
  
  
- TCTCAGCTTG CCTGACGCGC ACGAAGATGC CAAAGTTGTA TTATTCATTT CAGTGTAGCC GTACGTGTAC   
  
  
- CTGGGCGTGT GGACGTGACG CTCATTTCAT GAATGACCCG ATATTTTTTT TTCATTTATT TTATTTAGAG   
  
  
- TAAATATAAA ATAATTTTAA ATAATATTTA TTTTTTATTT TAAATTTTAA AATATTATTA AATCAATTAT   
  
  
- GCAGAAATAA TGTTTTAAAT TAATTTAATA AAAATTTATT TGTTATTAAT TTATTTTAAA AAAGTTATTC   
  
  
- AAGTTAAGAT TTGTATAATT AATTTTGAT

+     ARE

| Site Name | Organism | Position | Strand | Matrix score. | sequence | function |
| --- | --- | --- | --- | --- | --- | --- |
| ARE | Zea mays | 1145 | + | 6 | TGGTTT | cis-acting regulatory element essential for the anaerobic induction |
| ARE | Zea mays | 936 | - | 6 | TGGTTT | cis-acting regulatory element essential for the anaerobic induction |
| ARE | Zea mays | 799 | - | 6 | TGGTTT | cis-acting regulatory element essential for the anaerobic induction |

> 2018/04/13 10:10:12  
+ AAGTCGTCGG TTAGTTGGAA CTTTGAAAGT TGGTGGGTTA GAAGAGTGGA CGGAAAGGAC CCATGACAAA   
  
  
+ TGTCTACTCC GCTCCTTTCT TTCTGTCTCT TCCGTGCTTC CTGCCGCGAC TGAAAAACCT TCTTCTCTTT   
  
  
+ TTATAGCCGT TGGCTCTCTC TTCCTCTTTT CATATTTGAT TAGTCTGTAC CGTCCTCCTC TCTCTCTCTC   
  
  
+ TCCTTTTACC GTCACGTGAG TGACTGAACC ATTAACAATT AATAAATATG TAAGTTCTTT TCTAAACCTT   
  
  
+ TCTCCTCTTA TCGGTCTCTC GAAAAGGAAG TACCTTCAAC CCCCATTACC GGAAGCACTA AAGAGACTTT   
  
  
+ TACAGAGAAA ACGAAAAAGA AACTGAAACA CTTTTACAGT TTTCATCCCG GTTCGATTAC ATGGGGCATA   
  
  
+ ACAAGGAGAC GAACGTTTCA TCGGTTGTGC GAAATAAGAT GATGCGGAGA TGAGCTTTAT AATCACCCAC   
  
  
+ GAACACACAG ATCACGACAC TGAAAACACT TGGAGGCTTT ATAGTGAGTG CGCGAAGGGT GGCCGCACAT   
  
  
+ TGAAGGAGGC CGTCCGTCGT GAATGCGAGG GGACTTCTCG CACCCCTACA AGGAAAATCA TATAAGTTCA   
  
  
+ ACGAGACTGC GTTGGTGGCA CCTCCGCACC TGGACCTGCT TGATTCTATG AGCAAGTCAG TGGGCGCGCA   
  
  
+ CGCGGTAAGA CAGCGTGCAA CTCTAGAACC AGTCACGCTT AAGTTACATT TTCGACAGGA AACCGTGCGT   
  
  
+ GGTCCTCGTC CGAAGGTACC GACACGGCAA ACCACTGTCT GTTTAAGACT AGCATGCTAG TTTGGGTGCA   
  
  
+ CCGGGTTTTG AAAGACTAAG GGACCCGGGG TGTACTGGGC GGTACCTTCA CCCCTTCCCT TTTCCCCTTC   
  
  
+ GACAAGGAGA CAACTAACGG AGTCTAAACC ACTTCCTAGA ACCCGGCGAC TTGAACCCAA CCATAAAAAG   
  
  
+ TATATACTCT ACGGGAAAAG ATACACCCCT TAGCACCGTA AAAATGCCAC CGACTAAGCC ACCATGACAC   
  
  
+ GTCATTTTTC ATCTCATTTA GACGGAGACA AGTAGAATCA ACCGTAGAAA GAAAGGACTC AACCTTTCCA   
  
  
+ CTAGTCCATC CACGACCTGG TCCTTGGTTT ACAACGAGAG AAAAGATAAG TACCCTCTCT TGGTTGGCAG   
  
  
+ AGAGTCGAAC GGACTGCGCG TGCTTCTACG GTTTCAACAT AATAAGTAAA GTCACATCGG CATGCACATG   
  
  
+ GACCCGCACA CCTGCACTGC GAGTAAAGTA CTTACTGGGC TATAAAAAAA AAGTAAATAA AATAAATCTC   
  
  
+ ATTTATATTT TATTAAAATT TATTATAAAT AAAAAATAAA ATTTAAAATT TTATAATAAT TTAGTTAATA   
  
  
+ CGTCTTTATT ACAAAATTTA ATTAAATTAT TTTTAAATAA ACAATAATTA AATAAAATTT TTTCAATAAG   
  
  
+ TTCAATTCTA AACATATTAA TTAAAACTA  

- TTCAGCAGCC AATCAACCTT GAAACTTTCA ACCACCCAAT CTTCTCACCT GCCTTTCCTG GGTACTGTTT   
  
  
- ACAGATGAGG CGAGGAAAGA AAGACAGAGA AGGCACGAAG GACGGCGCTG ACTTTTTGGA AGAAGAGAAA   
  
  
- AATATCGGCA ACCGAGAGAG AAGGAGAAAA GTATAAACTA ATCAGACATG GCAGGAGGAG AGAGAGAGAG   
  
  
- AGGAAAATGG CAGTGCACTC ACTGACTTGG TAATTGTTAA TTATTTATAC ATTCAAGAAA AGATTTGGAA   
  
  
- AGAGGAGAAT AGCCAGAGAG CTTTTCCTTC ATGGAAGTTG GGGGTAATGG CCTTCGTGAT TTCTCTGAAA   
  
  
- ATGTCTCTTT TGCTTTTTCT TTGACTTTGT GAAAATGTCA AAAGTAGGGC CAAGCTAATG TACCCCGTAT   
  
  
- TGTTCCTCTG CTTGCAAAGT AGCCAACACG CTTTATTCTA CTACGCCTCT ACTCGAAATA TTAGTGGGTG   
  
  
- CTTGTGTGTC TAGTGCTGTG ACTTTTGTGA ACCTCCGAAA TATCACTCAC GCGCTTCCCA CCGGCGTGTA   
  
  
- ACTTCCTCCG GCAGGCAGCA CTTACGCTCC CCTGAAGAGC GTGGGGATGT TCCTTTTAGT ATATTCAAGT   
  
  
- TGCTCTGACG CAACCACCGT GGAGGCGTGG ACCTGGACGA ACTAAGATAC TCGTTCAGTC ACCCGCGCGT   
  
  
- GCGCCATTCT GTCGCACGTT GAGATCTTGG TCAGTGCGAA TTCAATGTAA AAGCTGTCCT TTGGCACGCA   
  
  
- CCAGGAGCAG GCTTCCATGG CTGTGCCGTT TGGTGACAGA CAAATTCTGA TCGTACGATC AAACCCACGT   
  
  
- GGCCCAAAAC TTTCTGATTC CCTGGGCCCC ACATGACCCG CCATGGAAGT GGGGAAGGGA AAAGGGGAAG   
  
  
- CTGTTCCTCT GTTGATTGCC TCAGATTTGG TGAAGGATCT TGGGCCGCTG AACTTGGGTT GGTATTTTTC   
  
  
- ATATATGAGA TGCCCTTTTC TATGTGGGGA ATCGTGGCAT TTTTACGGTG GCTGATTCGG TGGTACTGTG   
  
  
- CAGTAAAAAG TAGAGTAAAT CTGCCTCTGT TCATCTTAGT TGGCATCTTT CTTTCCTGAG TTGGAAAGGT   
  
  
- GATCAGGTAG GTGCTGGACC AGGAACCAAA TGTTGCTCTC TTTTCTATTC ATGGGAGAGA ACCAACCGTC   
  
  
- TCTCAGCTTG CCTGACGCGC ACGAAGATGC CAAAGTTGTA TTATTCATTT CAGTGTAGCC GTACGTGTAC   
  
  
- CTGGGCGTGT GGACGTGACG CTCATTTCAT GAATGACCCG ATATTTTTTT TTCATTTATT TTATTTAGAG   
  
  
- TAAATATAAA ATAATTTTAA ATAATATTTA TTTTTTATTT TAAATTTTAA AATATTATTA AATCAATTAT   
  
  
- GCAGAAATAA TGTTTTAAAT TAATTTAATA AAAATTTATT TGTTATTAAT TTATTTTAAA AAAGTTATTC   
  
  
- AAGTTAAGAT TTGTATAATT AATTTTGAT

+     AT1-motif

| Site Name | Organism | Position | Strand | Matrix score. | sequence | function |
| --- | --- | --- | --- | --- | --- | --- |
| AT1-motif | Solanum tuberosum | 1436 | - | 13 | AATTATTTTTTATT | part of a light responsive module |

> 2018/04/13 10:10:12  
+ AAGTCGTCGG TTAGTTGGAA CTTTGAAAGT TGGTGGGTTA GAAGAGTGGA CGGAAAGGAC CCATGACAAA   
  
  
+ TGTCTACTCC GCTCCTTTCT TTCTGTCTCT TCCGTGCTTC CTGCCGCGAC TGAAAAACCT TCTTCTCTTT   
  
  
+ TTATAGCCGT TGGCTCTCTC TTCCTCTTTT CATATTTGAT TAGTCTGTAC CGTCCTCCTC TCTCTCTCTC   
  
  
+ TCCTTTTACC GTCACGTGAG TGACTGAACC ATTAACAATT AATAAATATG TAAGTTCTTT TCTAAACCTT   
  
  
+ TCTCCTCTTA TCGGTCTCTC GAAAAGGAAG TACCTTCAAC CCCCATTACC GGAAGCACTA AAGAGACTTT   
  
  
+ TACAGAGAAA ACGAAAAAGA AACTGAAACA CTTTTACAGT TTTCATCCCG GTTCGATTAC ATGGGGCATA   
  
  
+ ACAAGGAGAC GAACGTTTCA TCGGTTGTGC GAAATAAGAT GATGCGGAGA TGAGCTTTAT AATCACCCAC   
  
  
+ GAACACACAG ATCACGACAC TGAAAACACT TGGAGGCTTT ATAGTGAGTG CGCGAAGGGT GGCCGCACAT   
  
  
+ TGAAGGAGGC CGTCCGTCGT GAATGCGAGG GGACTTCTCG CACCCCTACA AGGAAAATCA TATAAGTTCA   
  
  
+ ACGAGACTGC GTTGGTGGCA CCTCCGCACC TGGACCTGCT TGATTCTATG AGCAAGTCAG TGGGCGCGCA   
  
  
+ CGCGGTAAGA CAGCGTGCAA CTCTAGAACC AGTCACGCTT AAGTTACATT TTCGACAGGA AACCGTGCGT   
  
  
+ GGTCCTCGTC CGAAGGTACC GACACGGCAA ACCACTGTCT GTTTAAGACT AGCATGCTAG TTTGGGTGCA   
  
  
+ CCGGGTTTTG AAAGACTAAG GGACCCGGGG TGTACTGGGC GGTACCTTCA CCCCTTCCCT TTTCCCCTTC   
  
  
+ GACAAGGAGA CAACTAACGG AGTCTAAACC ACTTCCTAGA ACCCGGCGAC TTGAACCCAA CCATAAAAAG   
  
  
+ TATATACTCT ACGGGAAAAG ATACACCCCT TAGCACCGTA AAAATGCCAC CGACTAAGCC ACCATGACAC   
  
  
+ GTCATTTTTC ATCTCATTTA GACGGAGACA AGTAGAATCA ACCGTAGAAA GAAAGGACTC AACCTTTCCA   
  
  
+ CTAGTCCATC CACGACCTGG TCCTTGGTTT ACAACGAGAG AAAAGATAAG TACCCTCTCT TGGTTGGCAG   
  
  
+ AGAGTCGAAC GGACTGCGCG TGCTTCTACG GTTTCAACAT AATAAGTAAA GTCACATCGG CATGCACATG   
  
  
+ GACCCGCACA CCTGCACTGC GAGTAAAGTA CTTACTGGGC TATAAAAAAA AAGTAAATAA AATAAATCTC   
  
  
+ ATTTATATTT TATTAAAATT TATTATAAAT AAAAAATAAA ATTTAAAATT TTATAATAAT TTAGTTAATA   
  
  
+ CGTCTTTATT ACAAAATTTA ATTAAATTAT TTTTAAATAA ACAATAATTA AATAAAATTT TTTCAATAAG   
  
  
+ TTCAATTCTA AACATATTAA TTAAAACTA  

- TTCAGCAGCC AATCAACCTT GAAACTTTCA ACCACCCAAT CTTCTCACCT GCCTTTCCTG GGTACTGTTT   
  
  
- ACAGATGAGG CGAGGAAAGA AAGACAGAGA AGGCACGAAG GACGGCGCTG ACTTTTTGGA AGAAGAGAAA   
  
  
- AATATCGGCA ACCGAGAGAG AAGGAGAAAA GTATAAACTA ATCAGACATG GCAGGAGGAG AGAGAGAGAG   
  
  
- AGGAAAATGG CAGTGCACTC ACTGACTTGG TAATTGTTAA TTATTTATAC ATTCAAGAAA AGATTTGGAA   
  
  
- AGAGGAGAAT AGCCAGAGAG CTTTTCCTTC ATGGAAGTTG GGGGTAATGG CCTTCGTGAT TTCTCTGAAA   
  
  
- ATGTCTCTTT TGCTTTTTCT TTGACTTTGT GAAAATGTCA AAAGTAGGGC CAAGCTAATG TACCCCGTAT   
  
  
- TGTTCCTCTG CTTGCAAAGT AGCCAACACG CTTTATTCTA CTACGCCTCT ACTCGAAATA TTAGTGGGTG   
  
  
- CTTGTGTGTC TAGTGCTGTG ACTTTTGTGA ACCTCCGAAA TATCACTCAC GCGCTTCCCA CCGGCGTGTA   
  
  
- ACTTCCTCCG GCAGGCAGCA CTTACGCTCC CCTGAAGAGC GTGGGGATGT TCCTTTTAGT ATATTCAAGT   
  
  
- TGCTCTGACG CAACCACCGT GGAGGCGTGG ACCTGGACGA ACTAAGATAC TCGTTCAGTC ACCCGCGCGT   
  
  
- GCGCCATTCT GTCGCACGTT GAGATCTTGG TCAGTGCGAA TTCAATGTAA AAGCTGTCCT TTGGCACGCA   
  
  
- CCAGGAGCAG GCTTCCATGG CTGTGCCGTT TGGTGACAGA CAAATTCTGA TCGTACGATC AAACCCACGT   
  
  
- GGCCCAAAAC TTTCTGATTC CCTGGGCCCC ACATGACCCG CCATGGAAGT GGGGAAGGGA AAAGGGGAAG   
  
  
- CTGTTCCTCT GTTGATTGCC TCAGATTTGG TGAAGGATCT TGGGCCGCTG AACTTGGGTT GGTATTTTTC   
  
  
- ATATATGAGA TGCCCTTTTC TATGTGGGGA ATCGTGGCAT TTTTACGGTG GCTGATTCGG TGGTACTGTG   
  
  
- CAGTAAAAAG TAGAGTAAAT CTGCCTCTGT TCATCTTAGT TGGCATCTTT CTTTCCTGAG TTGGAAAGGT   
  
  
- GATCAGGTAG GTGCTGGACC AGGAACCAAA TGTTGCTCTC TTTTCTATTC ATGGGAGAGA ACCAACCGTC   
  
  
- TCTCAGCTTG CCTGACGCGC ACGAAGATGC CAAAGTTGTA TTATTCATTT CAGTGTAGCC GTACGTGTAC   
  
  
- CTGGGCGTGT GGACGTGACG CTCATTTCAT GAATGACCCG ATATTTTTTT TTCATTTATT TTATTTAGAG   
  
  
- TAAATATAAA ATAATTTTAA ATAATATTTA TTTTTTATTT TAAATTTTAA AATATTATTA AATCAATTAT   
  
  
- GCAGAAATAA TGTTTTAAAT TAATTTAATA AAAATTTATT TGTTATTAAT TTATTTTAAA AAAGTTATTC   
  
  
- AAGTTAAGAT TTGTATAATT AATTTTGAT

+     AuxRR-core

| Site Name | Organism | Position | Strand | Matrix score. | sequence | function |
| --- | --- | --- | --- | --- | --- | --- |
| AuxRR-core | Nicotiana tabacum | 1258 | - | 7 | GGTCCAT | cis-acting regulatory element involved in auxin responsiveness |

> 2018/04/13 10:10:12  
+ AAGTCGTCGG TTAGTTGGAA CTTTGAAAGT TGGTGGGTTA GAAGAGTGGA CGGAAAGGAC CCATGACAAA   
  
  
+ TGTCTACTCC GCTCCTTTCT TTCTGTCTCT TCCGTGCTTC CTGCCGCGAC TGAAAAACCT TCTTCTCTTT   
  
  
+ TTATAGCCGT TGGCTCTCTC TTCCTCTTTT CATATTTGAT TAGTCTGTAC CGTCCTCCTC TCTCTCTCTC   
  
  
+ TCCTTTTACC GTCACGTGAG TGACTGAACC ATTAACAATT AATAAATATG TAAGTTCTTT TCTAAACCTT   
  
  
+ TCTCCTCTTA TCGGTCTCTC GAAAAGGAAG TACCTTCAAC CCCCATTACC GGAAGCACTA AAGAGACTTT   
  
  
+ TACAGAGAAA ACGAAAAAGA AACTGAAACA CTTTTACAGT TTTCATCCCG GTTCGATTAC ATGGGGCATA   
  
  
+ ACAAGGAGAC GAACGTTTCA TCGGTTGTGC GAAATAAGAT GATGCGGAGA TGAGCTTTAT AATCACCCAC   
  
  
+ GAACACACAG ATCACGACAC TGAAAACACT TGGAGGCTTT ATAGTGAGTG CGCGAAGGGT GGCCGCACAT   
  
  
+ TGAAGGAGGC CGTCCGTCGT GAATGCGAGG GGACTTCTCG CACCCCTACA AGGAAAATCA TATAAGTTCA   
  
  
+ ACGAGACTGC GTTGGTGGCA CCTCCGCACC TGGACCTGCT TGATTCTATG AGCAAGTCAG TGGGCGCGCA   
  
  
+ CGCGGTAAGA CAGCGTGCAA CTCTAGAACC AGTCACGCTT AAGTTACATT TTCGACAGGA AACCGTGCGT   
  
  
+ GGTCCTCGTC CGAAGGTACC GACACGGCAA ACCACTGTCT GTTTAAGACT AGCATGCTAG TTTGGGTGCA   
  
  
+ CCGGGTTTTG AAAGACTAAG GGACCCGGGG TGTACTGGGC GGTACCTTCA CCCCTTCCCT TTTCCCCTTC   
  
  
+ GACAAGGAGA CAACTAACGG AGTCTAAACC ACTTCCTAGA ACCCGGCGAC TTGAACCCAA CCATAAAAAG   
  
  
+ TATATACTCT ACGGGAAAAG ATACACCCCT TAGCACCGTA AAAATGCCAC CGACTAAGCC ACCATGACAC   
  
  
+ GTCATTTTTC ATCTCATTTA GACGGAGACA AGTAGAATCA ACCGTAGAAA GAAAGGACTC AACCTTTCCA   
  
  
+ CTAGTCCATC CACGACCTGG TCCTTGGTTT ACAACGAGAG AAAAGATAAG TACCCTCTCT TGGTTGGCAG   
  
  
+ AGAGTCGAAC GGACTGCGCG TGCTTCTACG GTTTCAACAT AATAAGTAAA GTCACATCGG CATGCACATG   
  
  
+ GACCCGCACA CCTGCACTGC GAGTAAAGTA CTTACTGGGC TATAAAAAAA AAGTAAATAA AATAAATCTC   
  
  
+ ATTTATATTT TATTAAAATT TATTATAAAT AAAAAATAAA ATTTAAAATT TTATAATAAT TTAGTTAATA   
  
  
+ CGTCTTTATT ACAAAATTTA ATTAAATTAT TTTTAAATAA ACAATAATTA AATAAAATTT TTTCAATAAG   
  
  
+ TTCAATTCTA AACATATTAA TTAAAACTA  

- TTCAGCAGCC AATCAACCTT GAAACTTTCA ACCACCCAAT CTTCTCACCT GCCTTTCCTG GGTACTGTTT   
  
  
- ACAGATGAGG CGAGGAAAGA AAGACAGAGA AGGCACGAAG GACGGCGCTG ACTTTTTGGA AGAAGAGAAA   
  
  
- AATATCGGCA ACCGAGAGAG AAGGAGAAAA GTATAAACTA ATCAGACATG GCAGGAGGAG AGAGAGAGAG   
  
  
- AGGAAAATGG CAGTGCACTC ACTGACTTGG TAATTGTTAA TTATTTATAC ATTCAAGAAA AGATTTGGAA   
  
  
- AGAGGAGAAT AGCCAGAGAG CTTTTCCTTC ATGGAAGTTG GGGGTAATGG CCTTCGTGAT TTCTCTGAAA   
  
  
- ATGTCTCTTT TGCTTTTTCT TTGACTTTGT GAAAATGTCA AAAGTAGGGC CAAGCTAATG TACCCCGTAT   
  
  
- TGTTCCTCTG CTTGCAAAGT AGCCAACACG CTTTATTCTA CTACGCCTCT ACTCGAAATA TTAGTGGGTG   
  
  
- CTTGTGTGTC TAGTGCTGTG ACTTTTGTGA ACCTCCGAAA TATCACTCAC GCGCTTCCCA CCGGCGTGTA   
  
  
- ACTTCCTCCG GCAGGCAGCA CTTACGCTCC CCTGAAGAGC GTGGGGATGT TCCTTTTAGT ATATTCAAGT   
  
  
- TGCTCTGACG CAACCACCGT GGAGGCGTGG ACCTGGACGA ACTAAGATAC TCGTTCAGTC ACCCGCGCGT   
  
  
- GCGCCATTCT GTCGCACGTT GAGATCTTGG TCAGTGCGAA TTCAATGTAA AAGCTGTCCT TTGGCACGCA   
  
  
- CCAGGAGCAG GCTTCCATGG CTGTGCCGTT TGGTGACAGA CAAATTCTGA TCGTACGATC AAACCCACGT   
  
  
- GGCCCAAAAC TTTCTGATTC CCTGGGCCCC ACATGACCCG CCATGGAAGT GGGGAAGGGA AAAGGGGAAG   
  
  
- CTGTTCCTCT GTTGATTGCC TCAGATTTGG TGAAGGATCT TGGGCCGCTG AACTTGGGTT GGTATTTTTC   
  
  
- ATATATGAGA TGCCCTTTTC TATGTGGGGA ATCGTGGCAT TTTTACGGTG GCTGATTCGG TGGTACTGTG   
  
  
- CAGTAAAAAG TAGAGTAAAT CTGCCTCTGT TCATCTTAGT TGGCATCTTT CTTTCCTGAG TTGGAAAGGT   
  
  
- GATCAGGTAG GTGCTGGACC AGGAACCAAA TGTTGCTCTC TTTTCTATTC ATGGGAGAGA ACCAACCGTC   
  
  
- TCTCAGCTTG CCTGACGCGC ACGAAGATGC CAAAGTTGTA TTATTCATTT CAGTGTAGCC GTACGTGTAC   
  
  
- CTGGGCGTGT GGACGTGACG CTCATTTCAT GAATGACCCG ATATTTTTTT TTCATTTATT TTATTTAGAG   
  
  
- TAAATATAAA ATAATTTTAA ATAATATTTA TTTTTTATTT TAAATTTTAA AATATTATTA AATCAATTAT   
  
  
- GCAGAAATAA TGTTTTAAAT TAATTTAATA AAAATTTATT TGTTATTAAT TTATTTTAAA AAAGTTATTC   
  
  
- AAGTTAAGAT TTGTATAATT AATTTTGAT

+     Box 4

| Site Name | Organism | Position | Strand | Matrix score. | sequence | function |
| --- | --- | --- | --- | --- | --- | --- |
| Box 4 | Petroselinum crispum | 1486 | - | 6 | ATTAAT | part of a conserved DNA module involved in light responsiveness |
| Box 4 | Petroselinum crispum | 248 | + | 6 | ATTAAT | part of a conserved DNA module involved in light responsiveness |

> 2018/04/13 10:10:12  
+ AAGTCGTCGG TTAGTTGGAA CTTTGAAAGT TGGTGGGTTA GAAGAGTGGA CGGAAAGGAC CCATGACAAA   
  
  
+ TGTCTACTCC GCTCCTTTCT TTCTGTCTCT TCCGTGCTTC CTGCCGCGAC TGAAAAACCT TCTTCTCTTT   
  
  
+ TTATAGCCGT TGGCTCTCTC TTCCTCTTTT CATATTTGAT TAGTCTGTAC CGTCCTCCTC TCTCTCTCTC   
  
  
+ TCCTTTTACC GTCACGTGAG TGACTGAACC ATTAACAATT AATAAATATG TAAGTTCTTT TCTAAACCTT   
  
  
+ TCTCCTCTTA TCGGTCTCTC GAAAAGGAAG TACCTTCAAC CCCCATTACC GGAAGCACTA AAGAGACTTT   
  
  
+ TACAGAGAAA ACGAAAAAGA AACTGAAACA CTTTTACAGT TTTCATCCCG GTTCGATTAC ATGGGGCATA   
  
  
+ ACAAGGAGAC GAACGTTTCA TCGGTTGTGC GAAATAAGAT GATGCGGAGA TGAGCTTTAT AATCACCCAC   
  
  
+ GAACACACAG ATCACGACAC TGAAAACACT TGGAGGCTTT ATAGTGAGTG CGCGAAGGGT GGCCGCACAT   
  
  
+ TGAAGGAGGC CGTCCGTCGT GAATGCGAGG GGACTTCTCG CACCCCTACA AGGAAAATCA TATAAGTTCA   
  
  
+ ACGAGACTGC GTTGGTGGCA CCTCCGCACC TGGACCTGCT TGATTCTATG AGCAAGTCAG TGGGCGCGCA   
  
  
+ CGCGGTAAGA CAGCGTGCAA CTCTAGAACC AGTCACGCTT AAGTTACATT TTCGACAGGA AACCGTGCGT   
  
  
+ GGTCCTCGTC CGAAGGTACC GACACGGCAA ACCACTGTCT GTTTAAGACT AGCATGCTAG TTTGGGTGCA   
  
  
+ CCGGGTTTTG AAAGACTAAG GGACCCGGGG TGTACTGGGC GGTACCTTCA CCCCTTCCCT TTTCCCCTTC   
  
  
+ GACAAGGAGA CAACTAACGG AGTCTAAACC ACTTCCTAGA ACCCGGCGAC TTGAACCCAA CCATAAAAAG   
  
  
+ TATATACTCT ACGGGAAAAG ATACACCCCT TAGCACCGTA AAAATGCCAC CGACTAAGCC ACCATGACAC   
  
  
+ GTCATTTTTC ATCTCATTTA GACGGAGACA AGTAGAATCA ACCGTAGAAA GAAAGGACTC AACCTTTCCA   
  
  
+ CTAGTCCATC CACGACCTGG TCCTTGGTTT ACAACGAGAG AAAAGATAAG TACCCTCTCT TGGTTGGCAG   
  
  
+ AGAGTCGAAC GGACTGCGCG TGCTTCTACG GTTTCAACAT AATAAGTAAA GTCACATCGG CATGCACATG   
  
  
+ GACCCGCACA CCTGCACTGC GAGTAAAGTA CTTACTGGGC TATAAAAAAA AAGTAAATAA AATAAATCTC   
  
  
+ ATTTATATTT TATTAAAATT TATTATAAAT AAAAAATAAA ATTTAAAATT TTATAATAAT TTAGTTAATA   
  
  
+ CGTCTTTATT ACAAAATTTA ATTAAATTAT TTTTAAATAA ACAATAATTA AATAAAATTT TTTCAATAAG   
  
  
+ TTCAATTCTA AACATATTAA TTAAAACTA  

- TTCAGCAGCC AATCAACCTT GAAACTTTCA ACCACCCAAT CTTCTCACCT GCCTTTCCTG GGTACTGTTT   
  
  
- ACAGATGAGG CGAGGAAAGA AAGACAGAGA AGGCACGAAG GACGGCGCTG ACTTTTTGGA AGAAGAGAAA   
  
  
- AATATCGGCA ACCGAGAGAG AAGGAGAAAA GTATAAACTA ATCAGACATG GCAGGAGGAG AGAGAGAGAG   
  
  
- AGGAAAATGG CAGTGCACTC ACTGACTTGG TAATTGTTAA TTATTTATAC ATTCAAGAAA AGATTTGGAA   
  
  
- AGAGGAGAAT AGCCAGAGAG CTTTTCCTTC ATGGAAGTTG GGGGTAATGG CCTTCGTGAT TTCTCTGAAA   
  
  
- ATGTCTCTTT TGCTTTTTCT TTGACTTTGT GAAAATGTCA AAAGTAGGGC CAAGCTAATG TACCCCGTAT   
  
  
- TGTTCCTCTG CTTGCAAAGT AGCCAACACG CTTTATTCTA CTACGCCTCT ACTCGAAATA TTAGTGGGTG   
  
  
- CTTGTGTGTC TAGTGCTGTG ACTTTTGTGA ACCTCCGAAA TATCACTCAC GCGCTTCCCA CCGGCGTGTA   
  
  
- ACTTCCTCCG GCAGGCAGCA CTTACGCTCC CCTGAAGAGC GTGGGGATGT TCCTTTTAGT ATATTCAAGT   
  
  
- TGCTCTGACG CAACCACCGT GGAGGCGTGG ACCTGGACGA ACTAAGATAC TCGTTCAGTC ACCCGCGCGT   
  
  
- GCGCCATTCT GTCGCACGTT GAGATCTTGG TCAGTGCGAA TTCAATGTAA AAGCTGTCCT TTGGCACGCA   
  
  
- CCAGGAGCAG GCTTCCATGG CTGTGCCGTT TGGTGACAGA CAAATTCTGA TCGTACGATC AAACCCACGT   
  
  
- GGCCCAAAAC TTTCTGATTC CCTGGGCCCC ACATGACCCG CCATGGAAGT GGGGAAGGGA AAAGGGGAAG   
  
  
- CTGTTCCTCT GTTGATTGCC TCAGATTTGG TGAAGGATCT TGGGCCGCTG AACTTGGGTT GGTATTTTTC   
  
  
- ATATATGAGA TGCCCTTTTC TATGTGGGGA ATCGTGGCAT TTTTACGGTG GCTGATTCGG TGGTACTGTG   
  
  
- CAGTAAAAAG TAGAGTAAAT CTGCCTCTGT TCATCTTAGT TGGCATCTTT CTTTCCTGAG TTGGAAAGGT   
  
  
- GATCAGGTAG GTGCTGGACC AGGAACCAAA TGTTGCTCTC TTTTCTATTC ATGGGAGAGA ACCAACCGTC   
  
  
- TCTCAGCTTG CCTGACGCGC ACGAAGATGC CAAAGTTGTA TTATTCATTT CAGTGTAGCC GTACGTGTAC   
  
  
- CTGGGCGTGT GGACGTGACG CTCATTTCAT GAATGACCCG ATATTTTTTT TTCATTTATT TTATTTAGAG   
  
  
- TAAATATAAA ATAATTTTAA ATAATATTTA TTTTTTATTT TAAATTTTAA AATATTATTA AATCAATTAT   
  
  
- GCAGAAATAA TGTTTTAAAT TAATTTAATA AAAATTTATT TGTTATTAAT TTATTTTAAA AAAGTTATTC   
  
  
- AAGTTAAGAT TTGTATAATT AATTTTGAT

+     Box I

| Site Name | Organism | Position | Strand | Matrix score. | sequence | function |
| --- | --- | --- | --- | --- | --- | --- |
| Box I | Pisum sativum | 847 | - | 7 | TTTCAAA | light responsive element |
| Box I | Pisum sativum | 22 | - | 7 | TTTCAAA | light responsive element |

> 2018/04/13 10:10:12  
+ AAGTCGTCGG TTAGTTGGAA CTTTGAAAGT TGGTGGGTTA GAAGAGTGGA CGGAAAGGAC CCATGACAAA   
  
  
+ TGTCTACTCC GCTCCTTTCT TTCTGTCTCT TCCGTGCTTC CTGCCGCGAC TGAAAAACCT TCTTCTCTTT   
  
  
+ TTATAGCCGT TGGCTCTCTC TTCCTCTTTT CATATTTGAT TAGTCTGTAC CGTCCTCCTC TCTCTCTCTC   
  
  
+ TCCTTTTACC GTCACGTGAG TGACTGAACC ATTAACAATT AATAAATATG TAAGTTCTTT TCTAAACCTT   
  
  
+ TCTCCTCTTA TCGGTCTCTC GAAAAGGAAG TACCTTCAAC CCCCATTACC GGAAGCACTA AAGAGACTTT   
  
  
+ TACAGAGAAA ACGAAAAAGA AACTGAAACA CTTTTACAGT TTTCATCCCG GTTCGATTAC ATGGGGCATA   
  
  
+ ACAAGGAGAC GAACGTTTCA TCGGTTGTGC GAAATAAGAT GATGCGGAGA TGAGCTTTAT AATCACCCAC   
  
  
+ GAACACACAG ATCACGACAC TGAAAACACT TGGAGGCTTT ATAGTGAGTG CGCGAAGGGT GGCCGCACAT   
  
  
+ TGAAGGAGGC CGTCCGTCGT GAATGCGAGG GGACTTCTCG CACCCCTACA AGGAAAATCA TATAAGTTCA   
  
  
+ ACGAGACTGC GTTGGTGGCA CCTCCGCACC TGGACCTGCT TGATTCTATG AGCAAGTCAG TGGGCGCGCA   
  
  
+ CGCGGTAAGA CAGCGTGCAA CTCTAGAACC AGTCACGCTT AAGTTACATT TTCGACAGGA AACCGTGCGT   
  
  
+ GGTCCTCGTC CGAAGGTACC GACACGGCAA ACCACTGTCT GTTTAAGACT AGCATGCTAG TTTGGGTGCA   
  
  
+ CCGGGTTTTG AAAGACTAAG GGACCCGGGG TGTACTGGGC GGTACCTTCA CCCCTTCCCT TTTCCCCTTC   
  
  
+ GACAAGGAGA CAACTAACGG AGTCTAAACC ACTTCCTAGA ACCCGGCGAC TTGAACCCAA CCATAAAAAG   
  
  
+ TATATACTCT ACGGGAAAAG ATACACCCCT TAGCACCGTA AAAATGCCAC CGACTAAGCC ACCATGACAC   
  
  
+ GTCATTTTTC ATCTCATTTA GACGGAGACA AGTAGAATCA ACCGTAGAAA GAAAGGACTC AACCTTTCCA   
  
  
+ CTAGTCCATC CACGACCTGG TCCTTGGTTT ACAACGAGAG AAAAGATAAG TACCCTCTCT TGGTTGGCAG   
  
  
+ AGAGTCGAAC GGACTGCGCG TGCTTCTACG GTTTCAACAT AATAAGTAAA GTCACATCGG CATGCACATG   
  
  
+ GACCCGCACA CCTGCACTGC GAGTAAAGTA CTTACTGGGC TATAAAAAAA AAGTAAATAA AATAAATCTC   
  
  
+ ATTTATATTT TATTAAAATT TATTATAAAT AAAAAATAAA ATTTAAAATT TTATAATAAT TTAGTTAATA   
  
  
+ CGTCTTTATT ACAAAATTTA ATTAAATTAT TTTTAAATAA ACAATAATTA AATAAAATTT TTTCAATAAG   
  
  
+ TTCAATTCTA AACATATTAA TTAAAACTA  

- TTCAGCAGCC AATCAACCTT GAAACTTTCA ACCACCCAAT CTTCTCACCT GCCTTTCCTG GGTACTGTTT   
  
  
- ACAGATGAGG CGAGGAAAGA AAGACAGAGA AGGCACGAAG GACGGCGCTG ACTTTTTGGA AGAAGAGAAA   
  
  
- AATATCGGCA ACCGAGAGAG AAGGAGAAAA GTATAAACTA ATCAGACATG GCAGGAGGAG AGAGAGAGAG   
  
  
- AGGAAAATGG CAGTGCACTC ACTGACTTGG TAATTGTTAA TTATTTATAC ATTCAAGAAA AGATTTGGAA   
  
  
- AGAGGAGAAT AGCCAGAGAG CTTTTCCTTC ATGGAAGTTG GGGGTAATGG CCTTCGTGAT TTCTCTGAAA   
  
  
- ATGTCTCTTT TGCTTTTTCT TTGACTTTGT GAAAATGTCA AAAGTAGGGC CAAGCTAATG TACCCCGTAT   
  
  
- TGTTCCTCTG CTTGCAAAGT AGCCAACACG CTTTATTCTA CTACGCCTCT ACTCGAAATA TTAGTGGGTG   
  
  
- CTTGTGTGTC TAGTGCTGTG ACTTTTGTGA ACCTCCGAAA TATCACTCAC GCGCTTCCCA CCGGCGTGTA   
  
  
- ACTTCCTCCG GCAGGCAGCA CTTACGCTCC CCTGAAGAGC GTGGGGATGT TCCTTTTAGT ATATTCAAGT   
  
  
- TGCTCTGACG CAACCACCGT GGAGGCGTGG ACCTGGACGA ACTAAGATAC TCGTTCAGTC ACCCGCGCGT   
  
  
- GCGCCATTCT GTCGCACGTT GAGATCTTGG TCAGTGCGAA TTCAATGTAA AAGCTGTCCT TTGGCACGCA   
  
  
- CCAGGAGCAG GCTTCCATGG CTGTGCCGTT TGGTGACAGA CAAATTCTGA TCGTACGATC AAACCCACGT   
  
  
- GGCCCAAAAC TTTCTGATTC CCTGGGCCCC ACATGACCCG CCATGGAAGT GGGGAAGGGA AAAGGGGAAG   
  
  
- CTGTTCCTCT GTTGATTGCC TCAGATTTGG TGAAGGATCT TGGGCCGCTG AACTTGGGTT GGTATTTTTC   
  
  
- ATATATGAGA TGCCCTTTTC TATGTGGGGA ATCGTGGCAT TTTTACGGTG GCTGATTCGG TGGTACTGTG   
  
  
- CAGTAAAAAG TAGAGTAAAT CTGCCTCTGT TCATCTTAGT TGGCATCTTT CTTTCCTGAG TTGGAAAGGT   
  
  
- GATCAGGTAG GTGCTGGACC AGGAACCAAA TGTTGCTCTC TTTTCTATTC ATGGGAGAGA ACCAACCGTC   
  
  
- TCTCAGCTTG CCTGACGCGC ACGAAGATGC CAAAGTTGTA TTATTCATTT CAGTGTAGCC GTACGTGTAC   
  
  
- CTGGGCGTGT GGACGTGACG CTCATTTCAT GAATGACCCG ATATTTTTTT TTCATTTATT TTATTTAGAG   
  
  
- TAAATATAAA ATAATTTTAA ATAATATTTA TTTTTTATTT TAAATTTTAA AATATTATTA AATCAATTAT   
  
  
- GCAGAAATAA TGTTTTAAAT TAATTTAATA AAAATTTATT TGTTATTAAT TTATTTTAAA AAAGTTATTC   
  
  
- AAGTTAAGAT TTGTATAATT AATTTTGAT

+     CAAT-box

| Site Name | Organism | Position | Strand | Matrix score. | sequence | function |
| --- | --- | --- | --- | --- | --- | --- |
| CAAT-box | Glycine max | 1473 | + | 5 | CAATT | common cis-acting element in promoter and enhancer regions |
| CAAT-box | Hordeum vulgare | 1442 | + | 4 | CAAT | common cis-acting element in promoter and enhancer regions |
| CAAT-box | Hordeum vulgare | 559 | - | 4 | CAAT | common cis-acting element in promoter and enhancer regions |
| CAAT-box | Hordeum vulgare | 1464 | + | 4 | CAAT | common cis-acting element in promoter and enhancer regions |
| CAAT-box | Petunia hybrida | 1183 | - | 7 | TGCCAAC | common cis-acting element in promoter and enhancer regions |
| CAAT-box | Brassica rapa | 174 | - | 5 | CAAAT | common cis-acting element in promoter and enhancer regions |
| CAAT-box | Brassica rapa | 67 | + | 5 | CAAAT | common cis-acting element in promoter and enhancer regions |
| CAAT-box | Glycine max | 246 | + | 5 | CAATT | common cis-acting element in promoter and enhancer regions |

> 2018/04/13 10:10:12  
+ AAGTCGTCGG TTAGTTGGAA CTTTGAAAGT TGGTGGGTTA GAAGAGTGGA CGGAAAGGAC CCATGACAAA   
  
  
+ TGTCTACTCC GCTCCTTTCT TTCTGTCTCT TCCGTGCTTC CTGCCGCGAC TGAAAAACCT TCTTCTCTTT   
  
  
+ TTATAGCCGT TGGCTCTCTC TTCCTCTTTT CATATTTGAT TAGTCTGTAC CGTCCTCCTC TCTCTCTCTC   
  
  
+ TCCTTTTACC GTCACGTGAG TGACTGAACC ATTAACAATT AATAAATATG TAAGTTCTTT TCTAAACCTT   
  
  
+ TCTCCTCTTA TCGGTCTCTC GAAAAGGAAG TACCTTCAAC CCCCATTACC GGAAGCACTA AAGAGACTTT   
  
  
+ TACAGAGAAA ACGAAAAAGA AACTGAAACA CTTTTACAGT TTTCATCCCG GTTCGATTAC ATGGGGCATA   
  
  
+ ACAAGGAGAC GAACGTTTCA TCGGTTGTGC GAAATAAGAT GATGCGGAGA TGAGCTTTAT AATCACCCAC   
  
  
+ GAACACACAG ATCACGACAC TGAAAACACT TGGAGGCTTT ATAGTGAGTG CGCGAAGGGT GGCCGCACAT   
  
  
+ TGAAGGAGGC CGTCCGTCGT GAATGCGAGG GGACTTCTCG CACCCCTACA AGGAAAATCA TATAAGTTCA   
  
  
+ ACGAGACTGC GTTGGTGGCA CCTCCGCACC TGGACCTGCT TGATTCTATG AGCAAGTCAG TGGGCGCGCA   
  
  
+ CGCGGTAAGA CAGCGTGCAA CTCTAGAACC AGTCACGCTT AAGTTACATT TTCGACAGGA AACCGTGCGT   
  
  
+ GGTCCTCGTC CGAAGGTACC GACACGGCAA ACCACTGTCT GTTTAAGACT AGCATGCTAG TTTGGGTGCA   
  
  
+ CCGGGTTTTG AAAGACTAAG GGACCCGGGG TGTACTGGGC GGTACCTTCA CCCCTTCCCT TTTCCCCTTC   
  
  
+ GACAAGGAGA CAACTAACGG AGTCTAAACC ACTTCCTAGA ACCCGGCGAC TTGAACCCAA CCATAAAAAG   
  
  
+ TATATACTCT ACGGGAAAAG ATACACCCCT TAGCACCGTA AAAATGCCAC CGACTAAGCC ACCATGACAC   
  
  
+ GTCATTTTTC ATCTCATTTA GACGGAGACA AGTAGAATCA ACCGTAGAAA GAAAGGACTC AACCTTTCCA   
  
  
+ CTAGTCCATC CACGACCTGG TCCTTGGTTT ACAACGAGAG AAAAGATAAG TACCCTCTCT TGGTTGGCAG   
  
  
+ AGAGTCGAAC GGACTGCGCG TGCTTCTACG GTTTCAACAT AATAAGTAAA GTCACATCGG CATGCACATG   
  
  
+ GACCCGCACA CCTGCACTGC GAGTAAAGTA CTTACTGGGC TATAAAAAAA AAGTAAATAA AATAAATCTC   
  
  
+ ATTTATATTT TATTAAAATT TATTATAAAT AAAAAATAAA ATTTAAAATT TTATAATAAT TTAGTTAATA   
  
  
+ CGTCTTTATT ACAAAATTTA ATTAAATTAT TTTTAAATAA ACAATAATTA AATAAAATTT TTTCAATAAG   
  
  
+ TTCAATTCTA AACATATTAA TTAAAACTA  

- TTCAGCAGCC AATCAACCTT GAAACTTTCA ACCACCCAAT CTTCTCACCT GCCTTTCCTG GGTACTGTTT   
  
  
- ACAGATGAGG CGAGGAAAGA AAGACAGAGA AGGCACGAAG GACGGCGCTG ACTTTTTGGA AGAAGAGAAA   
  
  
- AATATCGGCA ACCGAGAGAG AAGGAGAAAA GTATAAACTA ATCAGACATG GCAGGAGGAG AGAGAGAGAG   
  
  
- AGGAAAATGG CAGTGCACTC ACTGACTTGG TAATTGTTAA TTATTTATAC ATTCAAGAAA AGATTTGGAA   
  
  
- AGAGGAGAAT AGCCAGAGAG CTTTTCCTTC ATGGAAGTTG GGGGTAATGG CCTTCGTGAT TTCTCTGAAA   
  
  
- ATGTCTCTTT TGCTTTTTCT TTGACTTTGT GAAAATGTCA AAAGTAGGGC CAAGCTAATG TACCCCGTAT   
  
  
- TGTTCCTCTG CTTGCAAAGT AGCCAACACG CTTTATTCTA CTACGCCTCT ACTCGAAATA TTAGTGGGTG   
  
  
- CTTGTGTGTC TAGTGCTGTG ACTTTTGTGA ACCTCCGAAA TATCACTCAC GCGCTTCCCA CCGGCGTGTA   
  
  
- ACTTCCTCCG GCAGGCAGCA CTTACGCTCC CCTGAAGAGC GTGGGGATGT TCCTTTTAGT ATATTCAAGT   
  
  
- TGCTCTGACG CAACCACCGT GGAGGCGTGG ACCTGGACGA ACTAAGATAC TCGTTCAGTC ACCCGCGCGT   
  
  
- GCGCCATTCT GTCGCACGTT GAGATCTTGG TCAGTGCGAA TTCAATGTAA AAGCTGTCCT TTGGCACGCA   
  
  
- CCAGGAGCAG GCTTCCATGG CTGTGCCGTT TGGTGACAGA CAAATTCTGA TCGTACGATC AAACCCACGT   
  
  
- GGCCCAAAAC TTTCTGATTC CCTGGGCCCC ACATGACCCG CCATGGAAGT GGGGAAGGGA AAAGGGGAAG   
  
  
- CTGTTCCTCT GTTGATTGCC TCAGATTTGG TGAAGGATCT TGGGCCGCTG AACTTGGGTT GGTATTTTTC   
  
  
- ATATATGAGA TGCCCTTTTC TATGTGGGGA ATCGTGGCAT TTTTACGGTG GCTGATTCGG TGGTACTGTG   
  
  
- CAGTAAAAAG TAGAGTAAAT CTGCCTCTGT TCATCTTAGT TGGCATCTTT CTTTCCTGAG TTGGAAAGGT   
  
  
- GATCAGGTAG GTGCTGGACC AGGAACCAAA TGTTGCTCTC TTTTCTATTC ATGGGAGAGA ACCAACCGTC   
  
  
- TCTCAGCTTG CCTGACGCGC ACGAAGATGC CAAAGTTGTA TTATTCATTT CAGTGTAGCC GTACGTGTAC   
  
  
- CTGGGCGTGT GGACGTGACG CTCATTTCAT GAATGACCCG ATATTTTTTT TTCATTTATT TTATTTAGAG   
  
  
- TAAATATAAA ATAATTTTAA ATAATATTTA TTTTTTATTT TAAATTTTAA AATATTATTA AATCAATTAT   
  
  
- GCAGAAATAA TGTTTTAAAT TAATTTAATA AAAATTTATT TGTTATTAAT TTATTTTAAA AAAGTTATTC   
  
  
- AAGTTAAGAT TTGTATAATT AATTTTGAT

+     CATT-motif

| Site Name | Organism | Position | Strand | Matrix score. | sequence | function |
| --- | --- | --- | --- | --- | --- | --- |
| CATT-motif | Zea mays | 581 | - | 6 | GCATTC | part of a light responsive element |

> 2018/04/13 10:10:12  
+ AAGTCGTCGG TTAGTTGGAA CTTTGAAAGT TGGTGGGTTA GAAGAGTGGA CGGAAAGGAC CCATGACAAA   
  
  
+ TGTCTACTCC GCTCCTTTCT TTCTGTCTCT TCCGTGCTTC CTGCCGCGAC TGAAAAACCT TCTTCTCTTT   
  
  
+ TTATAGCCGT TGGCTCTCTC TTCCTCTTTT CATATTTGAT TAGTCTGTAC CGTCCTCCTC TCTCTCTCTC   
  
  
+ TCCTTTTACC GTCACGTGAG TGACTGAACC ATTAACAATT AATAAATATG TAAGTTCTTT TCTAAACCTT   
  
  
+ TCTCCTCTTA TCGGTCTCTC GAAAAGGAAG TACCTTCAAC CCCCATTACC GGAAGCACTA AAGAGACTTT   
  
  
+ TACAGAGAAA ACGAAAAAGA AACTGAAACA CTTTTACAGT TTTCATCCCG GTTCGATTAC ATGGGGCATA   
  
  
+ ACAAGGAGAC GAACGTTTCA TCGGTTGTGC GAAATAAGAT GATGCGGAGA TGAGCTTTAT AATCACCCAC   
  
  
+ GAACACACAG ATCACGACAC TGAAAACACT TGGAGGCTTT ATAGTGAGTG CGCGAAGGGT GGCCGCACAT   
  
  
+ TGAAGGAGGC CGTCCGTCGT GAATGCGAGG GGACTTCTCG CACCCCTACA AGGAAAATCA TATAAGTTCA   
  
  
+ ACGAGACTGC GTTGGTGGCA CCTCCGCACC TGGACCTGCT TGATTCTATG AGCAAGTCAG TGGGCGCGCA   
  
  
+ CGCGGTAAGA CAGCGTGCAA CTCTAGAACC AGTCACGCTT AAGTTACATT TTCGACAGGA AACCGTGCGT   
  
  
+ GGTCCTCGTC CGAAGGTACC GACACGGCAA ACCACTGTCT GTTTAAGACT AGCATGCTAG TTTGGGTGCA   
  
  
+ CCGGGTTTTG AAAGACTAAG GGACCCGGGG TGTACTGGGC GGTACCTTCA CCCCTTCCCT TTTCCCCTTC   
  
  
+ GACAAGGAGA CAACTAACGG AGTCTAAACC ACTTCCTAGA ACCCGGCGAC TTGAACCCAA CCATAAAAAG   
  
  
+ TATATACTCT ACGGGAAAAG ATACACCCCT TAGCACCGTA AAAATGCCAC CGACTAAGCC ACCATGACAC   
  
  
+ GTCATTTTTC ATCTCATTTA GACGGAGACA AGTAGAATCA ACCGTAGAAA GAAAGGACTC AACCTTTCCA   
  
  
+ CTAGTCCATC CACGACCTGG TCCTTGGTTT ACAACGAGAG AAAAGATAAG TACCCTCTCT TGGTTGGCAG   
  
  
+ AGAGTCGAAC GGACTGCGCG TGCTTCTACG GTTTCAACAT AATAAGTAAA GTCACATCGG CATGCACATG   
  
  
+ GACCCGCACA CCTGCACTGC GAGTAAAGTA CTTACTGGGC TATAAAAAAA AAGTAAATAA AATAAATCTC   
  
  
+ ATTTATATTT TATTAAAATT TATTATAAAT AAAAAATAAA ATTTAAAATT TTATAATAAT TTAGTTAATA   
  
  
+ CGTCTTTATT ACAAAATTTA ATTAAATTAT TTTTAAATAA ACAATAATTA AATAAAATTT TTTCAATAAG   
  
  
+ TTCAATTCTA AACATATTAA TTAAAACTA  

- TTCAGCAGCC AATCAACCTT GAAACTTTCA ACCACCCAAT CTTCTCACCT GCCTTTCCTG GGTACTGTTT   
  
  
- ACAGATGAGG CGAGGAAAGA AAGACAGAGA AGGCACGAAG GACGGCGCTG ACTTTTTGGA AGAAGAGAAA   
  
  
- AATATCGGCA ACCGAGAGAG AAGGAGAAAA GTATAAACTA ATCAGACATG GCAGGAGGAG AGAGAGAGAG   
  
  
- AGGAAAATGG CAGTGCACTC ACTGACTTGG TAATTGTTAA TTATTTATAC ATTCAAGAAA AGATTTGGAA   
  
  
- AGAGGAGAAT AGCCAGAGAG CTTTTCCTTC ATGGAAGTTG GGGGTAATGG CCTTCGTGAT TTCTCTGAAA   
  
  
- ATGTCTCTTT TGCTTTTTCT TTGACTTTGT GAAAATGTCA AAAGTAGGGC CAAGCTAATG TACCCCGTAT   
  
  
- TGTTCCTCTG CTTGCAAAGT AGCCAACACG CTTTATTCTA CTACGCCTCT ACTCGAAATA TTAGTGGGTG   
  
  
- CTTGTGTGTC TAGTGCTGTG ACTTTTGTGA ACCTCCGAAA TATCACTCAC GCGCTTCCCA CCGGCGTGTA   
  
  
- ACTTCCTCCG GCAGGCAGCA CTTACGCTCC CCTGAAGAGC GTGGGGATGT TCCTTTTAGT ATATTCAAGT   
  
  
- TGCTCTGACG CAACCACCGT GGAGGCGTGG ACCTGGACGA ACTAAGATAC TCGTTCAGTC ACCCGCGCGT   
  
  
- GCGCCATTCT GTCGCACGTT GAGATCTTGG TCAGTGCGAA TTCAATGTAA AAGCTGTCCT TTGGCACGCA   
  
  
- CCAGGAGCAG GCTTCCATGG CTGTGCCGTT TGGTGACAGA CAAATTCTGA TCGTACGATC AAACCCACGT   
  
  
- GGCCCAAAAC TTTCTGATTC CCTGGGCCCC ACATGACCCG CCATGGAAGT GGGGAAGGGA AAAGGGGAAG   
  
  
- CTGTTCCTCT GTTGATTGCC TCAGATTTGG TGAAGGATCT TGGGCCGCTG AACTTGGGTT GGTATTTTTC   
  
  
- ATATATGAGA TGCCCTTTTC TATGTGGGGA ATCGTGGCAT TTTTACGGTG GCTGATTCGG TGGTACTGTG   
  
  
- CAGTAAAAAG TAGAGTAAAT CTGCCTCTGT TCATCTTAGT TGGCATCTTT CTTTCCTGAG TTGGAAAGGT   
  
  
- GATCAGGTAG GTGCTGGACC AGGAACCAAA TGTTGCTCTC TTTTCTATTC ATGGGAGAGA ACCAACCGTC   
  
  
- TCTCAGCTTG CCTGACGCGC ACGAAGATGC CAAAGTTGTA TTATTCATTT CAGTGTAGCC GTACGTGTAC   
  
  
- CTGGGCGTGT GGACGTGACG CTCATTTCAT GAATGACCCG ATATTTTTTT TTCATTTATT TTATTTAGAG   
  
  
- TAAATATAAA ATAATTTTAA ATAATATTTA TTTTTTATTT TAAATTTTAA AATATTATTA AATCAATTAT   
  
  
- GCAGAAATAA TGTTTTAAAT TAATTTAATA AAAATTTATT TGTTATTAAT TTATTTTAAA AAAGTTATTC   
  
  
- AAGTTAAGAT TTGTATAATT AATTTTGAT

+     CCAAT-box

| Site Name | Organism | Position | Strand | Matrix score. | sequence | function |
| --- | --- | --- | --- | --- | --- | --- |
| CCAAT-box | Hordeum vulgare | 147 | - | 6 | CAACGG | MYBHv1 binding site |

> 2018/04/13 10:10:12  
+ AAGTCGTCGG TTAGTTGGAA CTTTGAAAGT TGGTGGGTTA GAAGAGTGGA CGGAAAGGAC CCATGACAAA   
  
  
+ TGTCTACTCC GCTCCTTTCT TTCTGTCTCT TCCGTGCTTC CTGCCGCGAC TGAAAAACCT TCTTCTCTTT   
  
  
+ TTATAGCCGT TGGCTCTCTC TTCCTCTTTT CATATTTGAT TAGTCTGTAC CGTCCTCCTC TCTCTCTCTC   
  
  
+ TCCTTTTACC GTCACGTGAG TGACTGAACC ATTAACAATT AATAAATATG TAAGTTCTTT TCTAAACCTT   
  
  
+ TCTCCTCTTA TCGGTCTCTC GAAAAGGAAG TACCTTCAAC CCCCATTACC GGAAGCACTA AAGAGACTTT   
  
  
+ TACAGAGAAA ACGAAAAAGA AACTGAAACA CTTTTACAGT TTTCATCCCG GTTCGATTAC ATGGGGCATA   
  
  
+ ACAAGGAGAC GAACGTTTCA TCGGTTGTGC GAAATAAGAT GATGCGGAGA TGAGCTTTAT AATCACCCAC   
  
  
+ GAACACACAG ATCACGACAC TGAAAACACT TGGAGGCTTT ATAGTGAGTG CGCGAAGGGT GGCCGCACAT   
  
  
+ TGAAGGAGGC CGTCCGTCGT GAATGCGAGG GGACTTCTCG CACCCCTACA AGGAAAATCA TATAAGTTCA   
  
  
+ ACGAGACTGC GTTGGTGGCA CCTCCGCACC TGGACCTGCT TGATTCTATG AGCAAGTCAG TGGGCGCGCA   
  
  
+ CGCGGTAAGA CAGCGTGCAA CTCTAGAACC AGTCACGCTT AAGTTACATT TTCGACAGGA AACCGTGCGT   
  
  
+ GGTCCTCGTC CGAAGGTACC GACACGGCAA ACCACTGTCT GTTTAAGACT AGCATGCTAG TTTGGGTGCA   
  
  
+ CCGGGTTTTG AAAGACTAAG GGACCCGGGG TGTACTGGGC GGTACCTTCA CCCCTTCCCT TTTCCCCTTC   
  
  
+ GACAAGGAGA CAACTAACGG AGTCTAAACC ACTTCCTAGA ACCCGGCGAC TTGAACCCAA CCATAAAAAG   
  
  
+ TATATACTCT ACGGGAAAAG ATACACCCCT TAGCACCGTA AAAATGCCAC CGACTAAGCC ACCATGACAC   
  
  
+ GTCATTTTTC ATCTCATTTA GACGGAGACA AGTAGAATCA ACCGTAGAAA GAAAGGACTC AACCTTTCCA   
  
  
+ CTAGTCCATC CACGACCTGG TCCTTGGTTT ACAACGAGAG AAAAGATAAG TACCCTCTCT TGGTTGGCAG   
  
  
+ AGAGTCGAAC GGACTGCGCG TGCTTCTACG GTTTCAACAT AATAAGTAAA GTCACATCGG CATGCACATG   
  
  
+ GACCCGCACA CCTGCACTGC GAGTAAAGTA CTTACTGGGC TATAAAAAAA AAGTAAATAA AATAAATCTC   
  
  
+ ATTTATATTT TATTAAAATT TATTATAAAT AAAAAATAAA ATTTAAAATT TTATAATAAT TTAGTTAATA   
  
  
+ CGTCTTTATT ACAAAATTTA ATTAAATTAT TTTTAAATAA ACAATAATTA AATAAAATTT TTTCAATAAG   
  
  
+ TTCAATTCTA AACATATTAA TTAAAACTA  

- TTCAGCAGCC AATCAACCTT GAAACTTTCA ACCACCCAAT CTTCTCACCT GCCTTTCCTG GGTACTGTTT   
  
  
- ACAGATGAGG CGAGGAAAGA AAGACAGAGA AGGCACGAAG GACGGCGCTG ACTTTTTGGA AGAAGAGAAA   
  
  
- AATATCGGCA ACCGAGAGAG AAGGAGAAAA GTATAAACTA ATCAGACATG GCAGGAGGAG AGAGAGAGAG   
  
  
- AGGAAAATGG CAGTGCACTC ACTGACTTGG TAATTGTTAA TTATTTATAC ATTCAAGAAA AGATTTGGAA   
  
  
- AGAGGAGAAT AGCCAGAGAG CTTTTCCTTC ATGGAAGTTG GGGGTAATGG CCTTCGTGAT TTCTCTGAAA   
  
  
- ATGTCTCTTT TGCTTTTTCT TTGACTTTGT GAAAATGTCA AAAGTAGGGC CAAGCTAATG TACCCCGTAT   
  
  
- TGTTCCTCTG CTTGCAAAGT AGCCAACACG CTTTATTCTA CTACGCCTCT ACTCGAAATA TTAGTGGGTG   
  
  
- CTTGTGTGTC TAGTGCTGTG ACTTTTGTGA ACCTCCGAAA TATCACTCAC GCGCTTCCCA CCGGCGTGTA   
  
  
- ACTTCCTCCG GCAGGCAGCA CTTACGCTCC CCTGAAGAGC GTGGGGATGT TCCTTTTAGT ATATTCAAGT   
  
  
- TGCTCTGACG CAACCACCGT GGAGGCGTGG ACCTGGACGA ACTAAGATAC TCGTTCAGTC ACCCGCGCGT   
  
  
- GCGCCATTCT GTCGCACGTT GAGATCTTGG TCAGTGCGAA TTCAATGTAA AAGCTGTCCT TTGGCACGCA   
  
  
- CCAGGAGCAG GCTTCCATGG CTGTGCCGTT TGGTGACAGA CAAATTCTGA TCGTACGATC AAACCCACGT   
  
  
- GGCCCAAAAC TTTCTGATTC CCTGGGCCCC ACATGACCCG CCATGGAAGT GGGGAAGGGA AAAGGGGAAG   
  
  
- CTGTTCCTCT GTTGATTGCC TCAGATTTGG TGAAGGATCT TGGGCCGCTG AACTTGGGTT GGTATTTTTC   
  
  
- ATATATGAGA TGCCCTTTTC TATGTGGGGA ATCGTGGCAT TTTTACGGTG GCTGATTCGG TGGTACTGTG   
  
  
- CAGTAAAAAG TAGAGTAAAT CTGCCTCTGT TCATCTTAGT TGGCATCTTT CTTTCCTGAG TTGGAAAGGT   
  
  
- GATCAGGTAG GTGCTGGACC AGGAACCAAA TGTTGCTCTC TTTTCTATTC ATGGGAGAGA ACCAACCGTC   
  
  
- TCTCAGCTTG CCTGACGCGC ACGAAGATGC CAAAGTTGTA TTATTCATTT CAGTGTAGCC GTACGTGTAC   
  
  
- CTGGGCGTGT GGACGTGACG CTCATTTCAT GAATGACCCG ATATTTTTTT TTCATTTATT TTATTTAGAG   
  
  
- TAAATATAAA ATAATTTTAA ATAATATTTA TTTTTTATTT TAAATTTTAA AATATTATTA AATCAATTAT   
  
  
- GCAGAAATAA TGTTTTAAAT TAATTTAATA AAAATTTATT TGTTATTAAT TTATTTTAAA AAAGTTATTC   
  
  
- AAGTTAAGAT TTGTATAATT AATTTTGAT

+     CCGTCC-box

| Site Name | Organism | Position | Strand | Matrix score. | sequence | function |
| --- | --- | --- | --- | --- | --- | --- |
| CCGTCC-box | Arabidopsis thaliana | 570 | + | 6 | CCGTCC | cis-acting regulatory element related to meristem specific activation |
| CCGTCC-box | Arabidopsis thaliana | 190 | + | 6 | CCGTCC | cis-acting regulatory element related to meristem specific activation |
| CCGTCC-box | Arabidopsis thaliana | 48 | - | 6 | CCGTCC | cis-acting regulatory element related to meristem specific activation |

> 2018/04/13 10:10:12  
+ AAGTCGTCGG TTAGTTGGAA CTTTGAAAGT TGGTGGGTTA GAAGAGTGGA CGGAAAGGAC CCATGACAAA   
  
  
+ TGTCTACTCC GCTCCTTTCT TTCTGTCTCT TCCGTGCTTC CTGCCGCGAC TGAAAAACCT TCTTCTCTTT   
  
  
+ TTATAGCCGT TGGCTCTCTC TTCCTCTTTT CATATTTGAT TAGTCTGTAC CGTCCTCCTC TCTCTCTCTC   
  
  
+ TCCTTTTACC GTCACGTGAG TGACTGAACC ATTAACAATT AATAAATATG TAAGTTCTTT TCTAAACCTT   
  
  
+ TCTCCTCTTA TCGGTCTCTC GAAAAGGAAG TACCTTCAAC CCCCATTACC GGAAGCACTA AAGAGACTTT   
  
  
+ TACAGAGAAA ACGAAAAAGA AACTGAAACA CTTTTACAGT TTTCATCCCG GTTCGATTAC ATGGGGCATA   
  
  
+ ACAAGGAGAC GAACGTTTCA TCGGTTGTGC GAAATAAGAT GATGCGGAGA TGAGCTTTAT AATCACCCAC   
  
  
+ GAACACACAG ATCACGACAC TGAAAACACT TGGAGGCTTT ATAGTGAGTG CGCGAAGGGT GGCCGCACAT   
  
  
+ TGAAGGAGGC CGTCCGTCGT GAATGCGAGG GGACTTCTCG CACCCCTACA AGGAAAATCA TATAAGTTCA   
  
  
+ ACGAGACTGC GTTGGTGGCA CCTCCGCACC TGGACCTGCT TGATTCTATG AGCAAGTCAG TGGGCGCGCA   
  
  
+ CGCGGTAAGA CAGCGTGCAA CTCTAGAACC AGTCACGCTT AAGTTACATT TTCGACAGGA AACCGTGCGT   
  
  
+ GGTCCTCGTC CGAAGGTACC GACACGGCAA ACCACTGTCT GTTTAAGACT AGCATGCTAG TTTGGGTGCA   
  
  
+ CCGGGTTTTG AAAGACTAAG GGACCCGGGG TGTACTGGGC GGTACCTTCA CCCCTTCCCT TTTCCCCTTC   
  
  
+ GACAAGGAGA CAACTAACGG AGTCTAAACC ACTTCCTAGA ACCCGGCGAC TTGAACCCAA CCATAAAAAG   
  
  
+ TATATACTCT ACGGGAAAAG ATACACCCCT TAGCACCGTA AAAATGCCAC CGACTAAGCC ACCATGACAC   
  
  
+ GTCATTTTTC ATCTCATTTA GACGGAGACA AGTAGAATCA ACCGTAGAAA GAAAGGACTC AACCTTTCCA   
  
  
+ CTAGTCCATC CACGACCTGG TCCTTGGTTT ACAACGAGAG AAAAGATAAG TACCCTCTCT TGGTTGGCAG   
  
  
+ AGAGTCGAAC GGACTGCGCG TGCTTCTACG GTTTCAACAT AATAAGTAAA GTCACATCGG CATGCACATG   
  
  
+ GACCCGCACA CCTGCACTGC GAGTAAAGTA CTTACTGGGC TATAAAAAAA AAGTAAATAA AATAAATCTC   
  
  
+ ATTTATATTT TATTAAAATT TATTATAAAT AAAAAATAAA ATTTAAAATT TTATAATAAT TTAGTTAATA   
  
  
+ CGTCTTTATT ACAAAATTTA ATTAAATTAT TTTTAAATAA ACAATAATTA AATAAAATTT TTTCAATAAG   
  
  
+ TTCAATTCTA AACATATTAA TTAAAACTA  

- TTCAGCAGCC AATCAACCTT GAAACTTTCA ACCACCCAAT CTTCTCACCT GCCTTTCCTG GGTACTGTTT   
  
  
- ACAGATGAGG CGAGGAAAGA AAGACAGAGA AGGCACGAAG GACGGCGCTG ACTTTTTGGA AGAAGAGAAA   
  
  
- AATATCGGCA ACCGAGAGAG AAGGAGAAAA GTATAAACTA ATCAGACATG GCAGGAGGAG AGAGAGAGAG   
  
  
- AGGAAAATGG CAGTGCACTC ACTGACTTGG TAATTGTTAA TTATTTATAC ATTCAAGAAA AGATTTGGAA   
  
  
- AGAGGAGAAT AGCCAGAGAG CTTTTCCTTC ATGGAAGTTG GGGGTAATGG CCTTCGTGAT TTCTCTGAAA   
  
  
- ATGTCTCTTT TGCTTTTTCT TTGACTTTGT GAAAATGTCA AAAGTAGGGC CAAGCTAATG TACCCCGTAT   
  
  
- TGTTCCTCTG CTTGCAAAGT AGCCAACACG CTTTATTCTA CTACGCCTCT ACTCGAAATA TTAGTGGGTG   
  
  
- CTTGTGTGTC TAGTGCTGTG ACTTTTGTGA ACCTCCGAAA TATCACTCAC GCGCTTCCCA CCGGCGTGTA   
  
  
- ACTTCCTCCG GCAGGCAGCA CTTACGCTCC CCTGAAGAGC GTGGGGATGT TCCTTTTAGT ATATTCAAGT   
  
  
- TGCTCTGACG CAACCACCGT GGAGGCGTGG ACCTGGACGA ACTAAGATAC TCGTTCAGTC ACCCGCGCGT   
  
  
- GCGCCATTCT GTCGCACGTT GAGATCTTGG TCAGTGCGAA TTCAATGTAA AAGCTGTCCT TTGGCACGCA   
  
  
- CCAGGAGCAG GCTTCCATGG CTGTGCCGTT TGGTGACAGA CAAATTCTGA TCGTACGATC AAACCCACGT   
  
  
- GGCCCAAAAC TTTCTGATTC CCTGGGCCCC ACATGACCCG CCATGGAAGT GGGGAAGGGA AAAGGGGAAG   
  
  
- CTGTTCCTCT GTTGATTGCC TCAGATTTGG TGAAGGATCT TGGGCCGCTG AACTTGGGTT GGTATTTTTC   
  
  
- ATATATGAGA TGCCCTTTTC TATGTGGGGA ATCGTGGCAT TTTTACGGTG GCTGATTCGG TGGTACTGTG   
  
  
- CAGTAAAAAG TAGAGTAAAT CTGCCTCTGT TCATCTTAGT TGGCATCTTT CTTTCCTGAG TTGGAAAGGT   
  
  
- GATCAGGTAG GTGCTGGACC AGGAACCAAA TGTTGCTCTC TTTTCTATTC ATGGGAGAGA ACCAACCGTC   
  
  
- TCTCAGCTTG CCTGACGCGC ACGAAGATGC CAAAGTTGTA TTATTCATTT CAGTGTAGCC GTACGTGTAC   
  
  
- CTGGGCGTGT GGACGTGACG CTCATTTCAT GAATGACCCG ATATTTTTTT TTCATTTATT TTATTTAGAG   
  
  
- TAAATATAAA ATAATTTTAA ATAATATTTA TTTTTTATTT TAAATTTTAA AATATTATTA AATCAATTAT   
  
  
- GCAGAAATAA TGTTTTAAAT TAATTTAATA AAAATTTATT TGTTATTAAT TTATTTTAAA AAAGTTATTC   
  
  
- AAGTTAAGAT TTGTATAATT AATTTTGAT

+     CGTCA-motif

| Site Name | Organism | Position | Strand | Matrix score. | sequence | function |
| --- | --- | --- | --- | --- | --- | --- |
| CGTCA-motif | Hordeum vulgare | 1050 | + | 5 | CGTCA | cis-acting regulatory element involved in the MeJA-responsiveness |
| CGTCA-motif | Hordeum vulgare | 220 | + | 5 | CGTCA | cis-acting regulatory element involved in the MeJA-responsiveness |

> 2018/04/13 10:10:12  
+ AAGTCGTCGG TTAGTTGGAA CTTTGAAAGT TGGTGGGTTA GAAGAGTGGA CGGAAAGGAC CCATGACAAA   
  
  
+ TGTCTACTCC GCTCCTTTCT TTCTGTCTCT TCCGTGCTTC CTGCCGCGAC TGAAAAACCT TCTTCTCTTT   
  
  
+ TTATAGCCGT TGGCTCTCTC TTCCTCTTTT CATATTTGAT TAGTCTGTAC CGTCCTCCTC TCTCTCTCTC   
  
  
+ TCCTTTTACC GTCACGTGAG TGACTGAACC ATTAACAATT AATAAATATG TAAGTTCTTT TCTAAACCTT   
  
  
+ TCTCCTCTTA TCGGTCTCTC GAAAAGGAAG TACCTTCAAC CCCCATTACC GGAAGCACTA AAGAGACTTT   
  
  
+ TACAGAGAAA ACGAAAAAGA AACTGAAACA CTTTTACAGT TTTCATCCCG GTTCGATTAC ATGGGGCATA   
  
  
+ ACAAGGAGAC GAACGTTTCA TCGGTTGTGC GAAATAAGAT GATGCGGAGA TGAGCTTTAT AATCACCCAC   
  
  
+ GAACACACAG ATCACGACAC TGAAAACACT TGGAGGCTTT ATAGTGAGTG CGCGAAGGGT GGCCGCACAT   
  
  
+ TGAAGGAGGC CGTCCGTCGT GAATGCGAGG GGACTTCTCG CACCCCTACA AGGAAAATCA TATAAGTTCA   
  
  
+ ACGAGACTGC GTTGGTGGCA CCTCCGCACC TGGACCTGCT TGATTCTATG AGCAAGTCAG TGGGCGCGCA   
  
  
+ CGCGGTAAGA CAGCGTGCAA CTCTAGAACC AGTCACGCTT AAGTTACATT TTCGACAGGA AACCGTGCGT   
  
  
+ GGTCCTCGTC CGAAGGTACC GACACGGCAA ACCACTGTCT GTTTAAGACT AGCATGCTAG TTTGGGTGCA   
  
  
+ CCGGGTTTTG AAAGACTAAG GGACCCGGGG TGTACTGGGC GGTACCTTCA CCCCTTCCCT TTTCCCCTTC   
  
  
+ GACAAGGAGA CAACTAACGG AGTCTAAACC ACTTCCTAGA ACCCGGCGAC TTGAACCCAA CCATAAAAAG   
  
  
+ TATATACTCT ACGGGAAAAG ATACACCCCT TAGCACCGTA AAAATGCCAC CGACTAAGCC ACCATGACAC   
  
  
+ GTCATTTTTC ATCTCATTTA GACGGAGACA AGTAGAATCA ACCGTAGAAA GAAAGGACTC AACCTTTCCA   
  
  
+ CTAGTCCATC CACGACCTGG TCCTTGGTTT ACAACGAGAG AAAAGATAAG TACCCTCTCT TGGTTGGCAG   
  
  
+ AGAGTCGAAC GGACTGCGCG TGCTTCTACG GTTTCAACAT AATAAGTAAA GTCACATCGG CATGCACATG   
  
  
+ GACCCGCACA CCTGCACTGC GAGTAAAGTA CTTACTGGGC TATAAAAAAA AAGTAAATAA AATAAATCTC   
  
  
+ ATTTATATTT TATTAAAATT TATTATAAAT AAAAAATAAA ATTTAAAATT TTATAATAAT TTAGTTAATA   
  
  
+ CGTCTTTATT ACAAAATTTA ATTAAATTAT TTTTAAATAA ACAATAATTA AATAAAATTT TTTCAATAAG   
  
  
+ TTCAATTCTA AACATATTAA TTAAAACTA  

- TTCAGCAGCC AATCAACCTT GAAACTTTCA ACCACCCAAT CTTCTCACCT GCCTTTCCTG GGTACTGTTT   
  
  
- ACAGATGAGG CGAGGAAAGA AAGACAGAGA AGGCACGAAG GACGGCGCTG ACTTTTTGGA AGAAGAGAAA   
  
  
- AATATCGGCA ACCGAGAGAG AAGGAGAAAA GTATAAACTA ATCAGACATG GCAGGAGGAG AGAGAGAGAG   
  
  
- AGGAAAATGG CAGTGCACTC ACTGACTTGG TAATTGTTAA TTATTTATAC ATTCAAGAAA AGATTTGGAA   
  
  
- AGAGGAGAAT AGCCAGAGAG CTTTTCCTTC ATGGAAGTTG GGGGTAATGG CCTTCGTGAT TTCTCTGAAA   
  
  
- ATGTCTCTTT TGCTTTTTCT TTGACTTTGT GAAAATGTCA AAAGTAGGGC CAAGCTAATG TACCCCGTAT   
  
  
- TGTTCCTCTG CTTGCAAAGT AGCCAACACG CTTTATTCTA CTACGCCTCT ACTCGAAATA TTAGTGGGTG   
  
  
- CTTGTGTGTC TAGTGCTGTG ACTTTTGTGA ACCTCCGAAA TATCACTCAC GCGCTTCCCA CCGGCGTGTA   
  
  
- ACTTCCTCCG GCAGGCAGCA CTTACGCTCC CCTGAAGAGC GTGGGGATGT TCCTTTTAGT ATATTCAAGT   
  
  
- TGCTCTGACG CAACCACCGT GGAGGCGTGG ACCTGGACGA ACTAAGATAC TCGTTCAGTC ACCCGCGCGT   
  
  
- GCGCCATTCT GTCGCACGTT GAGATCTTGG TCAGTGCGAA TTCAATGTAA AAGCTGTCCT TTGGCACGCA   
  
  
- CCAGGAGCAG GCTTCCATGG CTGTGCCGTT TGGTGACAGA CAAATTCTGA TCGTACGATC AAACCCACGT   
  
  
- GGCCCAAAAC TTTCTGATTC CCTGGGCCCC ACATGACCCG CCATGGAAGT GGGGAAGGGA AAAGGGGAAG   
  
  
- CTGTTCCTCT GTTGATTGCC TCAGATTTGG TGAAGGATCT TGGGCCGCTG AACTTGGGTT GGTATTTTTC   
  
  
- ATATATGAGA TGCCCTTTTC TATGTGGGGA ATCGTGGCAT TTTTACGGTG GCTGATTCGG TGGTACTGTG   
  
  
- CAGTAAAAAG TAGAGTAAAT CTGCCTCTGT TCATCTTAGT TGGCATCTTT CTTTCCTGAG TTGGAAAGGT   
  
  
- GATCAGGTAG GTGCTGGACC AGGAACCAAA TGTTGCTCTC TTTTCTATTC ATGGGAGAGA ACCAACCGTC   
  
  
- TCTCAGCTTG CCTGACGCGC ACGAAGATGC CAAAGTTGTA TTATTCATTT CAGTGTAGCC GTACGTGTAC   
  
  
- CTGGGCGTGT GGACGTGACG CTCATTTCAT GAATGACCCG ATATTTTTTT TTCATTTATT TTATTTAGAG   
  
  
- TAAATATAAA ATAATTTTAA ATAATATTTA TTTTTTATTT TAAATTTTAA AATATTATTA AATCAATTAT   
  
  
- GCAGAAATAA TGTTTTAAAT TAATTTAATA AAAATTTATT TGTTATTAAT TTATTTTAAA AAAGTTATTC   
  
  
- AAGTTAAGAT TTGTATAATT AATTTTGAT

+     G-Box

| Site Name | Organism | Position | Strand | Matrix score. | sequence | function |
| --- | --- | --- | --- | --- | --- | --- |
| G-Box | Pisum sativum | 223 | + | 6 | CACGTG | cis-acting regulatory element involved in light responsiveness |

> 2018/04/13 10:10:12  
+ AAGTCGTCGG TTAGTTGGAA CTTTGAAAGT TGGTGGGTTA GAAGAGTGGA CGGAAAGGAC CCATGACAAA   
  
  
+ TGTCTACTCC GCTCCTTTCT TTCTGTCTCT TCCGTGCTTC CTGCCGCGAC TGAAAAACCT TCTTCTCTTT   
  
  
+ TTATAGCCGT TGGCTCTCTC TTCCTCTTTT CATATTTGAT TAGTCTGTAC CGTCCTCCTC TCTCTCTCTC   
  
  
+ TCCTTTTACC GTCACGTGAG TGACTGAACC ATTAACAATT AATAAATATG TAAGTTCTTT TCTAAACCTT   
  
  
+ TCTCCTCTTA TCGGTCTCTC GAAAAGGAAG TACCTTCAAC CCCCATTACC GGAAGCACTA AAGAGACTTT   
  
  
+ TACAGAGAAA ACGAAAAAGA AACTGAAACA CTTTTACAGT TTTCATCCCG GTTCGATTAC ATGGGGCATA   
  
  
+ ACAAGGAGAC GAACGTTTCA TCGGTTGTGC GAAATAAGAT GATGCGGAGA TGAGCTTTAT AATCACCCAC   
  
  
+ GAACACACAG ATCACGACAC TGAAAACACT TGGAGGCTTT ATAGTGAGTG CGCGAAGGGT GGCCGCACAT   
  
  
+ TGAAGGAGGC CGTCCGTCGT GAATGCGAGG GGACTTCTCG CACCCCTACA AGGAAAATCA TATAAGTTCA   
  
  
+ ACGAGACTGC GTTGGTGGCA CCTCCGCACC TGGACCTGCT TGATTCTATG AGCAAGTCAG TGGGCGCGCA   
  
  
+ CGCGGTAAGA CAGCGTGCAA CTCTAGAACC AGTCACGCTT AAGTTACATT TTCGACAGGA AACCGTGCGT   
  
  
+ GGTCCTCGTC CGAAGGTACC GACACGGCAA ACCACTGTCT GTTTAAGACT AGCATGCTAG TTTGGGTGCA   
  
  
+ CCGGGTTTTG AAAGACTAAG GGACCCGGGG TGTACTGGGC GGTACCTTCA CCCCTTCCCT TTTCCCCTTC   
  
  
+ GACAAGGAGA CAACTAACGG AGTCTAAACC ACTTCCTAGA ACCCGGCGAC TTGAACCCAA CCATAAAAAG   
  
  
+ TATATACTCT ACGGGAAAAG ATACACCCCT TAGCACCGTA AAAATGCCAC CGACTAAGCC ACCATGACAC   
  
  
+ GTCATTTTTC ATCTCATTTA GACGGAGACA AGTAGAATCA ACCGTAGAAA GAAAGGACTC AACCTTTCCA   
  
  
+ CTAGTCCATC CACGACCTGG TCCTTGGTTT ACAACGAGAG AAAAGATAAG TACCCTCTCT TGGTTGGCAG   
  
  
+ AGAGTCGAAC GGACTGCGCG TGCTTCTACG GTTTCAACAT AATAAGTAAA GTCACATCGG CATGCACATG   
  
  
+ GACCCGCACA CCTGCACTGC GAGTAAAGTA CTTACTGGGC TATAAAAAAA AAGTAAATAA AATAAATCTC   
  
  
+ ATTTATATTT TATTAAAATT TATTATAAAT AAAAAATAAA ATTTAAAATT TTATAATAAT TTAGTTAATA   
  
  
+ CGTCTTTATT ACAAAATTTA ATTAAATTAT TTTTAAATAA ACAATAATTA AATAAAATTT TTTCAATAAG   
  
  
+ TTCAATTCTA AACATATTAA TTAAAACTA  

- TTCAGCAGCC AATCAACCTT GAAACTTTCA ACCACCCAAT CTTCTCACCT GCCTTTCCTG GGTACTGTTT   
  
  
- ACAGATGAGG CGAGGAAAGA AAGACAGAGA AGGCACGAAG GACGGCGCTG ACTTTTTGGA AGAAGAGAAA   
  
  
- AATATCGGCA ACCGAGAGAG AAGGAGAAAA GTATAAACTA ATCAGACATG GCAGGAGGAG AGAGAGAGAG   
  
  
- AGGAAAATGG CAGTGCACTC ACTGACTTGG TAATTGTTAA TTATTTATAC ATTCAAGAAA AGATTTGGAA   
  
  
- AGAGGAGAAT AGCCAGAGAG CTTTTCCTTC ATGGAAGTTG GGGGTAATGG CCTTCGTGAT TTCTCTGAAA   
  
  
- ATGTCTCTTT TGCTTTTTCT TTGACTTTGT GAAAATGTCA AAAGTAGGGC CAAGCTAATG TACCCCGTAT   
  
  
- TGTTCCTCTG CTTGCAAAGT AGCCAACACG CTTTATTCTA CTACGCCTCT ACTCGAAATA TTAGTGGGTG   
  
  
- CTTGTGTGTC TAGTGCTGTG ACTTTTGTGA ACCTCCGAAA TATCACTCAC GCGCTTCCCA CCGGCGTGTA   
  
  
- ACTTCCTCCG GCAGGCAGCA CTTACGCTCC CCTGAAGAGC GTGGGGATGT TCCTTTTAGT ATATTCAAGT   
  
  
- TGCTCTGACG CAACCACCGT GGAGGCGTGG ACCTGGACGA ACTAAGATAC TCGTTCAGTC ACCCGCGCGT   
  
  
- GCGCCATTCT GTCGCACGTT GAGATCTTGG TCAGTGCGAA TTCAATGTAA AAGCTGTCCT TTGGCACGCA   
  
  
- CCAGGAGCAG GCTTCCATGG CTGTGCCGTT TGGTGACAGA CAAATTCTGA TCGTACGATC AAACCCACGT   
  
  
- GGCCCAAAAC TTTCTGATTC CCTGGGCCCC ACATGACCCG CCATGGAAGT GGGGAAGGGA AAAGGGGAAG   
  
  
- CTGTTCCTCT GTTGATTGCC TCAGATTTGG TGAAGGATCT TGGGCCGCTG AACTTGGGTT GGTATTTTTC   
  
  
- ATATATGAGA TGCCCTTTTC TATGTGGGGA ATCGTGGCAT TTTTACGGTG GCTGATTCGG TGGTACTGTG   
  
  
- CAGTAAAAAG TAGAGTAAAT CTGCCTCTGT TCATCTTAGT TGGCATCTTT CTTTCCTGAG TTGGAAAGGT   
  
  
- GATCAGGTAG GTGCTGGACC AGGAACCAAA TGTTGCTCTC TTTTCTATTC ATGGGAGAGA ACCAACCGTC   
  
  
- TCTCAGCTTG CCTGACGCGC ACGAAGATGC CAAAGTTGTA TTATTCATTT CAGTGTAGCC GTACGTGTAC   
  
  
- CTGGGCGTGT GGACGTGACG CTCATTTCAT GAATGACCCG ATATTTTTTT TTCATTTATT TTATTTAGAG   
  
  
- TAAATATAAA ATAATTTTAA ATAATATTTA TTTTTTATTT TAAATTTTAA AATATTATTA AATCAATTAT   
  
  
- GCAGAAATAA TGTTTTAAAT TAATTTAATA AAAATTTATT TGTTATTAAT TTATTTTAAA AAAGTTATTC   
  
  
- AAGTTAAGAT TTGTATAATT AATTTTGAT

+     G-box

| Site Name | Organism | Position | Strand | Matrix score. | sequence | function |
| --- | --- | --- | --- | --- | --- | --- |
| G-box | Solanum tuberosum | 1255 | + | 7 | CACATGG | cis-acting regulatory element involved in light responsiveness |
| G-box | Zea mays | 1131 | + | 6 | CACGAC | cis-acting regulatory element involved in light responsiveness |
| G-box | Zea mays | 1048 | + | 6 | CACGTC | cis-acting regulatory element involved in light responsiveness |
| G-box | Zea mays | 576 | - | 6 | CACGAC | cis-acting regulatory element involved in light responsiveness |
| G-box | Zea mays | 503 | + | 6 | CACGAC | cis-acting regulatory element involved in light responsiveness |
| G-box | Arabidopsis thaliana | 223 | + | 6 | CACGTG | cis-acting regulatory element involved in light responsiveness |

> 2018/04/13 10:10:12  
+ AAGTCGTCGG TTAGTTGGAA CTTTGAAAGT TGGTGGGTTA GAAGAGTGGA CGGAAAGGAC CCATGACAAA   
  
  
+ TGTCTACTCC GCTCCTTTCT TTCTGTCTCT TCCGTGCTTC CTGCCGCGAC TGAAAAACCT TCTTCTCTTT   
  
  
+ TTATAGCCGT TGGCTCTCTC TTCCTCTTTT CATATTTGAT TAGTCTGTAC CGTCCTCCTC TCTCTCTCTC   
  
  
+ TCCTTTTACC GTCACGTGAG TGACTGAACC ATTAACAATT AATAAATATG TAAGTTCTTT TCTAAACCTT   
  
  
+ TCTCCTCTTA TCGGTCTCTC GAAAAGGAAG TACCTTCAAC CCCCATTACC GGAAGCACTA AAGAGACTTT   
  
  
+ TACAGAGAAA ACGAAAAAGA AACTGAAACA CTTTTACAGT TTTCATCCCG GTTCGATTAC ATGGGGCATA   
  
  
+ ACAAGGAGAC GAACGTTTCA TCGGTTGTGC GAAATAAGAT GATGCGGAGA TGAGCTTTAT AATCACCCAC   
  
  
+ GAACACACAG ATCACGACAC TGAAAACACT TGGAGGCTTT ATAGTGAGTG CGCGAAGGGT GGCCGCACAT   
  
  
+ TGAAGGAGGC CGTCCGTCGT GAATGCGAGG GGACTTCTCG CACCCCTACA AGGAAAATCA TATAAGTTCA   
  
  
+ ACGAGACTGC GTTGGTGGCA CCTCCGCACC TGGACCTGCT TGATTCTATG AGCAAGTCAG TGGGCGCGCA   
  
  
+ CGCGGTAAGA CAGCGTGCAA CTCTAGAACC AGTCACGCTT AAGTTACATT TTCGACAGGA AACCGTGCGT   
  
  
+ GGTCCTCGTC CGAAGGTACC GACACGGCAA ACCACTGTCT GTTTAAGACT AGCATGCTAG TTTGGGTGCA   
  
  
+ CCGGGTTTTG AAAGACTAAG GGACCCGGGG TGTACTGGGC GGTACCTTCA CCCCTTCCCT TTTCCCCTTC   
  
  
+ GACAAGGAGA CAACTAACGG AGTCTAAACC ACTTCCTAGA ACCCGGCGAC TTGAACCCAA CCATAAAAAG   
  
  
+ TATATACTCT ACGGGAAAAG ATACACCCCT TAGCACCGTA AAAATGCCAC CGACTAAGCC ACCATGACAC   
  
  
+ GTCATTTTTC ATCTCATTTA GACGGAGACA AGTAGAATCA ACCGTAGAAA GAAAGGACTC AACCTTTCCA   
  
  
+ CTAGTCCATC CACGACCTGG TCCTTGGTTT ACAACGAGAG AAAAGATAAG TACCCTCTCT TGGTTGGCAG   
  
  
+ AGAGTCGAAC GGACTGCGCG TGCTTCTACG GTTTCAACAT AATAAGTAAA GTCACATCGG CATGCACATG   
  
  
+ GACCCGCACA CCTGCACTGC GAGTAAAGTA CTTACTGGGC TATAAAAAAA AAGTAAATAA AATAAATCTC   
  
  
+ ATTTATATTT TATTAAAATT TATTATAAAT AAAAAATAAA ATTTAAAATT TTATAATAAT TTAGTTAATA   
  
  
+ CGTCTTTATT ACAAAATTTA ATTAAATTAT TTTTAAATAA ACAATAATTA AATAAAATTT TTTCAATAAG   
  
  
+ TTCAATTCTA AACATATTAA TTAAAACTA  

- TTCAGCAGCC AATCAACCTT GAAACTTTCA ACCACCCAAT CTTCTCACCT GCCTTTCCTG GGTACTGTTT   
  
  
- ACAGATGAGG CGAGGAAAGA AAGACAGAGA AGGCACGAAG GACGGCGCTG ACTTTTTGGA AGAAGAGAAA   
  
  
- AATATCGGCA ACCGAGAGAG AAGGAGAAAA GTATAAACTA ATCAGACATG GCAGGAGGAG AGAGAGAGAG   
  
  
- AGGAAAATGG CAGTGCACTC ACTGACTTGG TAATTGTTAA TTATTTATAC ATTCAAGAAA AGATTTGGAA   
  
  
- AGAGGAGAAT AGCCAGAGAG CTTTTCCTTC ATGGAAGTTG GGGGTAATGG CCTTCGTGAT TTCTCTGAAA   
  
  
- ATGTCTCTTT TGCTTTTTCT TTGACTTTGT GAAAATGTCA AAAGTAGGGC CAAGCTAATG TACCCCGTAT   
  
  
- TGTTCCTCTG CTTGCAAAGT AGCCAACACG CTTTATTCTA CTACGCCTCT ACTCGAAATA TTAGTGGGTG   
  
  
- CTTGTGTGTC TAGTGCTGTG ACTTTTGTGA ACCTCCGAAA TATCACTCAC GCGCTTCCCA CCGGCGTGTA   
  
  
- ACTTCCTCCG GCAGGCAGCA CTTACGCTCC CCTGAAGAGC GTGGGGATGT TCCTTTTAGT ATATTCAAGT   
  
  
- TGCTCTGACG CAACCACCGT GGAGGCGTGG ACCTGGACGA ACTAAGATAC TCGTTCAGTC ACCCGCGCGT   
  
  
- GCGCCATTCT GTCGCACGTT GAGATCTTGG TCAGTGCGAA TTCAATGTAA AAGCTGTCCT TTGGCACGCA   
  
  
- CCAGGAGCAG GCTTCCATGG CTGTGCCGTT TGGTGACAGA CAAATTCTGA TCGTACGATC AAACCCACGT   
  
  
- GGCCCAAAAC TTTCTGATTC CCTGGGCCCC ACATGACCCG CCATGGAAGT GGGGAAGGGA AAAGGGGAAG   
  
  
- CTGTTCCTCT GTTGATTGCC TCAGATTTGG TGAAGGATCT TGGGCCGCTG AACTTGGGTT GGTATTTTTC   
  
  
- ATATATGAGA TGCCCTTTTC TATGTGGGGA ATCGTGGCAT TTTTACGGTG GCTGATTCGG TGGTACTGTG   
  
  
- CAGTAAAAAG TAGAGTAAAT CTGCCTCTGT TCATCTTAGT TGGCATCTTT CTTTCCTGAG TTGGAAAGGT   
  
  
- GATCAGGTAG GTGCTGGACC AGGAACCAAA TGTTGCTCTC TTTTCTATTC ATGGGAGAGA ACCAACCGTC   
  
  
- TCTCAGCTTG CCTGACGCGC ACGAAGATGC CAAAGTTGTA TTATTCATTT CAGTGTAGCC GTACGTGTAC   
  
  
- CTGGGCGTGT GGACGTGACG CTCATTTCAT GAATGACCCG ATATTTTTTT TTCATTTATT TTATTTAGAG   
  
  
- TAAATATAAA ATAATTTTAA ATAATATTTA TTTTTTATTT TAAATTTTAA AATATTATTA AATCAATTAT   
  
  
- GCAGAAATAA TGTTTTAAAT TAATTTAATA AAAATTTATT TGTTATTAAT TTATTTTAAA AAAGTTATTC   
  
  
- AAGTTAAGAT TTGTATAATT AATTTTGAT

+     GAG-motif

| Site Name | Organism | Position | Strand | Matrix score. | sequence | function |
| --- | --- | --- | --- | --- | --- | --- |
| GAG-motif | Hordeum vulgare | 466 | + | 7 | GGAGATG | part of a light responsive element |
| GAG-motif | Arabidopsis thaliana | 1189 | + | 7 | AGAGAGT | part of a light responsive element |

> 2018/04/13 10:10:12  
+ AAGTCGTCGG TTAGTTGGAA CTTTGAAAGT TGGTGGGTTA GAAGAGTGGA CGGAAAGGAC CCATGACAAA   
  
  
+ TGTCTACTCC GCTCCTTTCT TTCTGTCTCT TCCGTGCTTC CTGCCGCGAC TGAAAAACCT TCTTCTCTTT   
  
  
+ TTATAGCCGT TGGCTCTCTC TTCCTCTTTT CATATTTGAT TAGTCTGTAC CGTCCTCCTC TCTCTCTCTC   
  
  
+ TCCTTTTACC GTCACGTGAG TGACTGAACC ATTAACAATT AATAAATATG TAAGTTCTTT TCTAAACCTT   
  
  
+ TCTCCTCTTA TCGGTCTCTC GAAAAGGAAG TACCTTCAAC CCCCATTACC GGAAGCACTA AAGAGACTTT   
  
  
+ TACAGAGAAA ACGAAAAAGA AACTGAAACA CTTTTACAGT TTTCATCCCG GTTCGATTAC ATGGGGCATA   
  
  
+ ACAAGGAGAC GAACGTTTCA TCGGTTGTGC GAAATAAGAT GATGCGGAGA TGAGCTTTAT AATCACCCAC   
  
  
+ GAACACACAG ATCACGACAC TGAAAACACT TGGAGGCTTT ATAGTGAGTG CGCGAAGGGT GGCCGCACAT   
  
  
+ TGAAGGAGGC CGTCCGTCGT GAATGCGAGG GGACTTCTCG CACCCCTACA AGGAAAATCA TATAAGTTCA   
  
  
+ ACGAGACTGC GTTGGTGGCA CCTCCGCACC TGGACCTGCT TGATTCTATG AGCAAGTCAG TGGGCGCGCA   
  
  
+ CGCGGTAAGA CAGCGTGCAA CTCTAGAACC AGTCACGCTT AAGTTACATT TTCGACAGGA AACCGTGCGT   
  
  
+ GGTCCTCGTC CGAAGGTACC GACACGGCAA ACCACTGTCT GTTTAAGACT AGCATGCTAG TTTGGGTGCA   
  
  
+ CCGGGTTTTG AAAGACTAAG GGACCCGGGG TGTACTGGGC GGTACCTTCA CCCCTTCCCT TTTCCCCTTC   
  
  
+ GACAAGGAGA CAACTAACGG AGTCTAAACC ACTTCCTAGA ACCCGGCGAC TTGAACCCAA CCATAAAAAG   
  
  
+ TATATACTCT ACGGGAAAAG ATACACCCCT TAGCACCGTA AAAATGCCAC CGACTAAGCC ACCATGACAC   
  
  
+ GTCATTTTTC ATCTCATTTA GACGGAGACA AGTAGAATCA ACCGTAGAAA GAAAGGACTC AACCTTTCCA   
  
  
+ CTAGTCCATC CACGACCTGG TCCTTGGTTT ACAACGAGAG AAAAGATAAG TACCCTCTCT TGGTTGGCAG   
  
  
+ AGAGTCGAAC GGACTGCGCG TGCTTCTACG GTTTCAACAT AATAAGTAAA GTCACATCGG CATGCACATG   
  
  
+ GACCCGCACA CCTGCACTGC GAGTAAAGTA CTTACTGGGC TATAAAAAAA AAGTAAATAA AATAAATCTC   
  
  
+ ATTTATATTT TATTAAAATT TATTATAAAT AAAAAATAAA ATTTAAAATT TTATAATAAT TTAGTTAATA   
  
  
+ CGTCTTTATT ACAAAATTTA ATTAAATTAT TTTTAAATAA ACAATAATTA AATAAAATTT TTTCAATAAG   
  
  
+ TTCAATTCTA AACATATTAA TTAAAACTA  

- TTCAGCAGCC AATCAACCTT GAAACTTTCA ACCACCCAAT CTTCTCACCT GCCTTTCCTG GGTACTGTTT   
  
  
- ACAGATGAGG CGAGGAAAGA AAGACAGAGA AGGCACGAAG GACGGCGCTG ACTTTTTGGA AGAAGAGAAA   
  
  
- AATATCGGCA ACCGAGAGAG AAGGAGAAAA GTATAAACTA ATCAGACATG GCAGGAGGAG AGAGAGAGAG   
  
  
- AGGAAAATGG CAGTGCACTC ACTGACTTGG TAATTGTTAA TTATTTATAC ATTCAAGAAA AGATTTGGAA   
  
  
- AGAGGAGAAT AGCCAGAGAG CTTTTCCTTC ATGGAAGTTG GGGGTAATGG CCTTCGTGAT TTCTCTGAAA   
  
  
- ATGTCTCTTT TGCTTTTTCT TTGACTTTGT GAAAATGTCA AAAGTAGGGC CAAGCTAATG TACCCCGTAT   
  
  
- TGTTCCTCTG CTTGCAAAGT AGCCAACACG CTTTATTCTA CTACGCCTCT ACTCGAAATA TTAGTGGGTG   
  
  
- CTTGTGTGTC TAGTGCTGTG ACTTTTGTGA ACCTCCGAAA TATCACTCAC GCGCTTCCCA CCGGCGTGTA   
  
  
- ACTTCCTCCG GCAGGCAGCA CTTACGCTCC CCTGAAGAGC GTGGGGATGT TCCTTTTAGT ATATTCAAGT   
  
  
- TGCTCTGACG CAACCACCGT GGAGGCGTGG ACCTGGACGA ACTAAGATAC TCGTTCAGTC ACCCGCGCGT   
  
  
- GCGCCATTCT GTCGCACGTT GAGATCTTGG TCAGTGCGAA TTCAATGTAA AAGCTGTCCT TTGGCACGCA   
  
  
- CCAGGAGCAG GCTTCCATGG CTGTGCCGTT TGGTGACAGA CAAATTCTGA TCGTACGATC AAACCCACGT   
  
  
- GGCCCAAAAC TTTCTGATTC CCTGGGCCCC ACATGACCCG CCATGGAAGT GGGGAAGGGA AAAGGGGAAG   
  
  
- CTGTTCCTCT GTTGATTGCC TCAGATTTGG TGAAGGATCT TGGGCCGCTG AACTTGGGTT GGTATTTTTC   
  
  
- ATATATGAGA TGCCCTTTTC TATGTGGGGA ATCGTGGCAT TTTTACGGTG GCTGATTCGG TGGTACTGTG   
  
  
- CAGTAAAAAG TAGAGTAAAT CTGCCTCTGT TCATCTTAGT TGGCATCTTT CTTTCCTGAG TTGGAAAGGT   
  
  
- GATCAGGTAG GTGCTGGACC AGGAACCAAA TGTTGCTCTC TTTTCTATTC ATGGGAGAGA ACCAACCGTC   
  
  
- TCTCAGCTTG CCTGACGCGC ACGAAGATGC CAAAGTTGTA TTATTCATTT CAGTGTAGCC GTACGTGTAC   
  
  
- CTGGGCGTGT GGACGTGACG CTCATTTCAT GAATGACCCG ATATTTTTTT TTCATTTATT TTATTTAGAG   
  
  
- TAAATATAAA ATAATTTTAA ATAATATTTA TTTTTTATTT TAAATTTTAA AATATTATTA AATCAATTAT   
  
  
- GCAGAAATAA TGTTTTAAAT TAATTTAATA AAAATTTATT TGTTATTAAT TTATTTTAAA AAAGTTATTC   
  
  
- AAGTTAAGAT TTGTATAATT AATTTTGAT

+     GARE-motif

| Site Name | Organism | Position | Strand | Matrix score. | sequence | function |
| --- | --- | --- | --- | --- | --- | --- |
| GARE-motif | Brassica oleracea | 808 | - | 7 | AAACAGA | gibberellin-responsive element |

> 2018/04/13 10:10:12  
+ AAGTCGTCGG TTAGTTGGAA CTTTGAAAGT TGGTGGGTTA GAAGAGTGGA CGGAAAGGAC CCATGACAAA   
  
  
+ TGTCTACTCC GCTCCTTTCT TTCTGTCTCT TCCGTGCTTC CTGCCGCGAC TGAAAAACCT TCTTCTCTTT   
  
  
+ TTATAGCCGT TGGCTCTCTC TTCCTCTTTT CATATTTGAT TAGTCTGTAC CGTCCTCCTC TCTCTCTCTC   
  
  
+ TCCTTTTACC GTCACGTGAG TGACTGAACC ATTAACAATT AATAAATATG TAAGTTCTTT TCTAAACCTT   
  
  
+ TCTCCTCTTA TCGGTCTCTC GAAAAGGAAG TACCTTCAAC CCCCATTACC GGAAGCACTA AAGAGACTTT   
  
  
+ TACAGAGAAA ACGAAAAAGA AACTGAAACA CTTTTACAGT TTTCATCCCG GTTCGATTAC ATGGGGCATA   
  
  
+ ACAAGGAGAC GAACGTTTCA TCGGTTGTGC GAAATAAGAT GATGCGGAGA TGAGCTTTAT AATCACCCAC   
  
  
+ GAACACACAG ATCACGACAC TGAAAACACT TGGAGGCTTT ATAGTGAGTG CGCGAAGGGT GGCCGCACAT   
  
  
+ TGAAGGAGGC CGTCCGTCGT GAATGCGAGG GGACTTCTCG CACCCCTACA AGGAAAATCA TATAAGTTCA   
  
  
+ ACGAGACTGC GTTGGTGGCA CCTCCGCACC TGGACCTGCT TGATTCTATG AGCAAGTCAG TGGGCGCGCA   
  
  
+ CGCGGTAAGA CAGCGTGCAA CTCTAGAACC AGTCACGCTT AAGTTACATT TTCGACAGGA AACCGTGCGT   
  
  
+ GGTCCTCGTC CGAAGGTACC GACACGGCAA ACCACTGTCT GTTTAAGACT AGCATGCTAG TTTGGGTGCA   
  
  
+ CCGGGTTTTG AAAGACTAAG GGACCCGGGG TGTACTGGGC GGTACCTTCA CCCCTTCCCT TTTCCCCTTC   
  
  
+ GACAAGGAGA CAACTAACGG AGTCTAAACC ACTTCCTAGA ACCCGGCGAC TTGAACCCAA CCATAAAAAG   
  
  
+ TATATACTCT ACGGGAAAAG ATACACCCCT TAGCACCGTA AAAATGCCAC CGACTAAGCC ACCATGACAC   
  
  
+ GTCATTTTTC ATCTCATTTA GACGGAGACA AGTAGAATCA ACCGTAGAAA GAAAGGACTC AACCTTTCCA   
  
  
+ CTAGTCCATC CACGACCTGG TCCTTGGTTT ACAACGAGAG AAAAGATAAG TACCCTCTCT TGGTTGGCAG   
  
  
+ AGAGTCGAAC GGACTGCGCG TGCTTCTACG GTTTCAACAT AATAAGTAAA GTCACATCGG CATGCACATG   
  
  
+ GACCCGCACA CCTGCACTGC GAGTAAAGTA CTTACTGGGC TATAAAAAAA AAGTAAATAA AATAAATCTC   
  
  
+ ATTTATATTT TATTAAAATT TATTATAAAT AAAAAATAAA ATTTAAAATT TTATAATAAT TTAGTTAATA   
  
  
+ CGTCTTTATT ACAAAATTTA ATTAAATTAT TTTTAAATAA ACAATAATTA AATAAAATTT TTTCAATAAG   
  
  
+ TTCAATTCTA AACATATTAA TTAAAACTA  

- TTCAGCAGCC AATCAACCTT GAAACTTTCA ACCACCCAAT CTTCTCACCT GCCTTTCCTG GGTACTGTTT   
  
  
- ACAGATGAGG CGAGGAAAGA AAGACAGAGA AGGCACGAAG GACGGCGCTG ACTTTTTGGA AGAAGAGAAA   
  
  
- AATATCGGCA ACCGAGAGAG AAGGAGAAAA GTATAAACTA ATCAGACATG GCAGGAGGAG AGAGAGAGAG   
  
  
- AGGAAAATGG CAGTGCACTC ACTGACTTGG TAATTGTTAA TTATTTATAC ATTCAAGAAA AGATTTGGAA   
  
  
- AGAGGAGAAT AGCCAGAGAG CTTTTCCTTC ATGGAAGTTG GGGGTAATGG CCTTCGTGAT TTCTCTGAAA   
  
  
- ATGTCTCTTT TGCTTTTTCT TTGACTTTGT GAAAATGTCA AAAGTAGGGC CAAGCTAATG TACCCCGTAT   
  
  
- TGTTCCTCTG CTTGCAAAGT AGCCAACACG CTTTATTCTA CTACGCCTCT ACTCGAAATA TTAGTGGGTG   
  
  
- CTTGTGTGTC TAGTGCTGTG ACTTTTGTGA ACCTCCGAAA TATCACTCAC GCGCTTCCCA CCGGCGTGTA   
  
  
- ACTTCCTCCG GCAGGCAGCA CTTACGCTCC CCTGAAGAGC GTGGGGATGT TCCTTTTAGT ATATTCAAGT   
  
  
- TGCTCTGACG CAACCACCGT GGAGGCGTGG ACCTGGACGA ACTAAGATAC TCGTTCAGTC ACCCGCGCGT   
  
  
- GCGCCATTCT GTCGCACGTT GAGATCTTGG TCAGTGCGAA TTCAATGTAA AAGCTGTCCT TTGGCACGCA   
  
  
- CCAGGAGCAG GCTTCCATGG CTGTGCCGTT TGGTGACAGA CAAATTCTGA TCGTACGATC AAACCCACGT   
  
  
- GGCCCAAAAC TTTCTGATTC CCTGGGCCCC ACATGACCCG CCATGGAAGT GGGGAAGGGA AAAGGGGAAG   
  
  
- CTGTTCCTCT GTTGATTGCC TCAGATTTGG TGAAGGATCT TGGGCCGCTG AACTTGGGTT GGTATTTTTC   
  
  
- ATATATGAGA TGCCCTTTTC TATGTGGGGA ATCGTGGCAT TTTTACGGTG GCTGATTCGG TGGTACTGTG   
  
  
- CAGTAAAAAG TAGAGTAAAT CTGCCTCTGT TCATCTTAGT TGGCATCTTT CTTTCCTGAG TTGGAAAGGT   
  
  
- GATCAGGTAG GTGCTGGACC AGGAACCAAA TGTTGCTCTC TTTTCTATTC ATGGGAGAGA ACCAACCGTC   
  
  
- TCTCAGCTTG CCTGACGCGC ACGAAGATGC CAAAGTTGTA TTATTCATTT CAGTGTAGCC GTACGTGTAC   
  
  
- CTGGGCGTGT GGACGTGACG CTCATTTCAT GAATGACCCG ATATTTTTTT TTCATTTATT TTATTTAGAG   
  
  
- TAAATATAAA ATAATTTTAA ATAATATTTA TTTTTTATTT TAAATTTTAA AATATTATTA AATCAATTAT   
  
  
- GCAGAAATAA TGTTTTAAAT TAATTTAATA AAAATTTATT TGTTATTAAT TTATTTTAAA AAAGTTATTC   
  
  
- AAGTTAAGAT TTGTATAATT AATTTTGAT

+     HSE

| Site Name | Organism | Position | Strand | Matrix score. | sequence | function |
| --- | --- | --- | --- | --- | --- | --- |
| HSE | Brassica oleracea | 1454 | - | 9 | AAAAAATTTC | cis-acting element involved in heat stress responsiveness |

> 2018/04/13 10:10:12  
+ AAGTCGTCGG TTAGTTGGAA CTTTGAAAGT TGGTGGGTTA GAAGAGTGGA CGGAAAGGAC CCATGACAAA   
  
  
+ TGTCTACTCC GCTCCTTTCT TTCTGTCTCT TCCGTGCTTC CTGCCGCGAC TGAAAAACCT TCTTCTCTTT   
  
  
+ TTATAGCCGT TGGCTCTCTC TTCCTCTTTT CATATTTGAT TAGTCTGTAC CGTCCTCCTC TCTCTCTCTC   
  
  
+ TCCTTTTACC GTCACGTGAG TGACTGAACC ATTAACAATT AATAAATATG TAAGTTCTTT TCTAAACCTT   
  
  
+ TCTCCTCTTA TCGGTCTCTC GAAAAGGAAG TACCTTCAAC CCCCATTACC GGAAGCACTA AAGAGACTTT   
  
  
+ TACAGAGAAA ACGAAAAAGA AACTGAAACA CTTTTACAGT TTTCATCCCG GTTCGATTAC ATGGGGCATA   
  
  
+ ACAAGGAGAC GAACGTTTCA TCGGTTGTGC GAAATAAGAT GATGCGGAGA TGAGCTTTAT AATCACCCAC   
  
  
+ GAACACACAG ATCACGACAC TGAAAACACT TGGAGGCTTT ATAGTGAGTG CGCGAAGGGT GGCCGCACAT   
  
  
+ TGAAGGAGGC CGTCCGTCGT GAATGCGAGG GGACTTCTCG CACCCCTACA AGGAAAATCA TATAAGTTCA   
  
  
+ ACGAGACTGC GTTGGTGGCA CCTCCGCACC TGGACCTGCT TGATTCTATG AGCAAGTCAG TGGGCGCGCA   
  
  
+ CGCGGTAAGA CAGCGTGCAA CTCTAGAACC AGTCACGCTT AAGTTACATT TTCGACAGGA AACCGTGCGT   
  
  
+ GGTCCTCGTC CGAAGGTACC GACACGGCAA ACCACTGTCT GTTTAAGACT AGCATGCTAG TTTGGGTGCA   
  
  
+ CCGGGTTTTG AAAGACTAAG GGACCCGGGG TGTACTGGGC GGTACCTTCA CCCCTTCCCT TTTCCCCTTC   
  
  
+ GACAAGGAGA CAACTAACGG AGTCTAAACC ACTTCCTAGA ACCCGGCGAC TTGAACCCAA CCATAAAAAG   
  
  
+ TATATACTCT ACGGGAAAAG ATACACCCCT TAGCACCGTA AAAATGCCAC CGACTAAGCC ACCATGACAC   
  
  
+ GTCATTTTTC ATCTCATTTA GACGGAGACA AGTAGAATCA ACCGTAGAAA GAAAGGACTC AACCTTTCCA   
  
  
+ CTAGTCCATC CACGACCTGG TCCTTGGTTT ACAACGAGAG AAAAGATAAG TACCCTCTCT TGGTTGGCAG   
  
  
+ AGAGTCGAAC GGACTGCGCG TGCTTCTACG GTTTCAACAT AATAAGTAAA GTCACATCGG CATGCACATG   
  
  
+ GACCCGCACA CCTGCACTGC GAGTAAAGTA CTTACTGGGC TATAAAAAAA AAGTAAATAA AATAAATCTC   
  
  
+ ATTTATATTT TATTAAAATT TATTATAAAT AAAAAATAAA ATTTAAAATT TTATAATAAT TTAGTTAATA   
  
  
+ CGTCTTTATT ACAAAATTTA ATTAAATTAT TTTTAAATAA ACAATAATTA AATAAAATTT TTTCAATAAG   
  
  
+ TTCAATTCTA AACATATTAA TTAAAACTA  

- TTCAGCAGCC AATCAACCTT GAAACTTTCA ACCACCCAAT CTTCTCACCT GCCTTTCCTG GGTACTGTTT   
  
  
- ACAGATGAGG CGAGGAAAGA AAGACAGAGA AGGCACGAAG GACGGCGCTG ACTTTTTGGA AGAAGAGAAA   
  
  
- AATATCGGCA ACCGAGAGAG AAGGAGAAAA GTATAAACTA ATCAGACATG GCAGGAGGAG AGAGAGAGAG   
  
  
- AGGAAAATGG CAGTGCACTC ACTGACTTGG TAATTGTTAA TTATTTATAC ATTCAAGAAA AGATTTGGAA   
  
  
- AGAGGAGAAT AGCCAGAGAG CTTTTCCTTC ATGGAAGTTG GGGGTAATGG CCTTCGTGAT TTCTCTGAAA   
  
  
- ATGTCTCTTT TGCTTTTTCT TTGACTTTGT GAAAATGTCA AAAGTAGGGC CAAGCTAATG TACCCCGTAT   
  
  
- TGTTCCTCTG CTTGCAAAGT AGCCAACACG CTTTATTCTA CTACGCCTCT ACTCGAAATA TTAGTGGGTG   
  
  
- CTTGTGTGTC TAGTGCTGTG ACTTTTGTGA ACCTCCGAAA TATCACTCAC GCGCTTCCCA CCGGCGTGTA   
  
  
- ACTTCCTCCG GCAGGCAGCA CTTACGCTCC CCTGAAGAGC GTGGGGATGT TCCTTTTAGT ATATTCAAGT   
  
  
- TGCTCTGACG CAACCACCGT GGAGGCGTGG ACCTGGACGA ACTAAGATAC TCGTTCAGTC ACCCGCGCGT   
  
  
- GCGCCATTCT GTCGCACGTT GAGATCTTGG TCAGTGCGAA TTCAATGTAA AAGCTGTCCT TTGGCACGCA   
  
  
- CCAGGAGCAG GCTTCCATGG CTGTGCCGTT TGGTGACAGA CAAATTCTGA TCGTACGATC AAACCCACGT   
  
  
- GGCCCAAAAC TTTCTGATTC CCTGGGCCCC ACATGACCCG CCATGGAAGT GGGGAAGGGA AAAGGGGAAG   
  
  
- CTGTTCCTCT GTTGATTGCC TCAGATTTGG TGAAGGATCT TGGGCCGCTG AACTTGGGTT GGTATTTTTC   
  
  
- ATATATGAGA TGCCCTTTTC TATGTGGGGA ATCGTGGCAT TTTTACGGTG GCTGATTCGG TGGTACTGTG   
  
  
- CAGTAAAAAG TAGAGTAAAT CTGCCTCTGT TCATCTTAGT TGGCATCTTT CTTTCCTGAG TTGGAAAGGT   
  
  
- GATCAGGTAG GTGCTGGACC AGGAACCAAA TGTTGCTCTC TTTTCTATTC ATGGGAGAGA ACCAACCGTC   
  
  
- TCTCAGCTTG CCTGACGCGC ACGAAGATGC CAAAGTTGTA TTATTCATTT CAGTGTAGCC GTACGTGTAC   
  
  
- CTGGGCGTGT GGACGTGACG CTCATTTCAT GAATGACCCG ATATTTTTTT TTCATTTATT TTATTTAGAG   
  
  
- TAAATATAAA ATAATTTTAA ATAATATTTA TTTTTTATTT TAAATTTTAA AATATTATTA AATCAATTAT   
  
  
- GCAGAAATAA TGTTTTAAAT TAATTTAATA AAAATTTATT TGTTATTAAT TTATTTTAAA AAAGTTATTC   
  
  
- AAGTTAAGAT TTGTATAATT AATTTTGAT

+     MSA-like

| Site Name | Organism | Position | Strand | Matrix score. | sequence | function |
| --- | --- | --- | --- | --- | --- | --- |
| MSA-like | Catharanthus roseus | 144 | - | 8.5 | (T/C)C(T/C)AACGG(T/C)(T/C)A | cis-acting element involved in cell cycle regulation |

> 2018/04/13 10:10:12  
+ AAGTCGTCGG TTAGTTGGAA CTTTGAAAGT TGGTGGGTTA GAAGAGTGGA CGGAAAGGAC CCATGACAAA   
  
  
+ TGTCTACTCC GCTCCTTTCT TTCTGTCTCT TCCGTGCTTC CTGCCGCGAC TGAAAAACCT TCTTCTCTTT   
  
  
+ TTATAGCCGT TGGCTCTCTC TTCCTCTTTT CATATTTGAT TAGTCTGTAC CGTCCTCCTC TCTCTCTCTC   
  
  
+ TCCTTTTACC GTCACGTGAG TGACTGAACC ATTAACAATT AATAAATATG TAAGTTCTTT TCTAAACCTT   
  
  
+ TCTCCTCTTA TCGGTCTCTC GAAAAGGAAG TACCTTCAAC CCCCATTACC GGAAGCACTA AAGAGACTTT   
  
  
+ TACAGAGAAA ACGAAAAAGA AACTGAAACA CTTTTACAGT TTTCATCCCG GTTCGATTAC ATGGGGCATA   
  
  
+ ACAAGGAGAC GAACGTTTCA TCGGTTGTGC GAAATAAGAT GATGCGGAGA TGAGCTTTAT AATCACCCAC   
  
  
+ GAACACACAG ATCACGACAC TGAAAACACT TGGAGGCTTT ATAGTGAGTG CGCGAAGGGT GGCCGCACAT   
  
  
+ TGAAGGAGGC CGTCCGTCGT GAATGCGAGG GGACTTCTCG CACCCCTACA AGGAAAATCA TATAAGTTCA   
  
  
+ ACGAGACTGC GTTGGTGGCA CCTCCGCACC TGGACCTGCT TGATTCTATG AGCAAGTCAG TGGGCGCGCA   
  
  
+ CGCGGTAAGA CAGCGTGCAA CTCTAGAACC AGTCACGCTT AAGTTACATT TTCGACAGGA AACCGTGCGT   
  
  
+ GGTCCTCGTC CGAAGGTACC GACACGGCAA ACCACTGTCT GTTTAAGACT AGCATGCTAG TTTGGGTGCA   
  
  
+ CCGGGTTTTG AAAGACTAAG GGACCCGGGG TGTACTGGGC GGTACCTTCA CCCCTTCCCT TTTCCCCTTC   
  
  
+ GACAAGGAGA CAACTAACGG AGTCTAAACC ACTTCCTAGA ACCCGGCGAC TTGAACCCAA CCATAAAAAG   
  
  
+ TATATACTCT ACGGGAAAAG ATACACCCCT TAGCACCGTA AAAATGCCAC CGACTAAGCC ACCATGACAC   
  
  
+ GTCATTTTTC ATCTCATTTA GACGGAGACA AGTAGAATCA ACCGTAGAAA GAAAGGACTC AACCTTTCCA   
  
  
+ CTAGTCCATC CACGACCTGG TCCTTGGTTT ACAACGAGAG AAAAGATAAG TACCCTCTCT TGGTTGGCAG   
  
  
+ AGAGTCGAAC GGACTGCGCG TGCTTCTACG GTTTCAACAT AATAAGTAAA GTCACATCGG CATGCACATG   
  
  
+ GACCCGCACA CCTGCACTGC GAGTAAAGTA CTTACTGGGC TATAAAAAAA AAGTAAATAA AATAAATCTC   
  
  
+ ATTTATATTT TATTAAAATT TATTATAAAT AAAAAATAAA ATTTAAAATT TTATAATAAT TTAGTTAATA   
  
  
+ CGTCTTTATT ACAAAATTTA ATTAAATTAT TTTTAAATAA ACAATAATTA AATAAAATTT TTTCAATAAG   
  
  
+ TTCAATTCTA AACATATTAA TTAAAACTA  

- TTCAGCAGCC AATCAACCTT GAAACTTTCA ACCACCCAAT CTTCTCACCT GCCTTTCCTG GGTACTGTTT   
  
  
- ACAGATGAGG CGAGGAAAGA AAGACAGAGA AGGCACGAAG GACGGCGCTG ACTTTTTGGA AGAAGAGAAA   
  
  
- AATATCGGCA ACCGAGAGAG AAGGAGAAAA GTATAAACTA ATCAGACATG GCAGGAGGAG AGAGAGAGAG   
  
  
- AGGAAAATGG CAGTGCACTC ACTGACTTGG TAATTGTTAA TTATTTATAC ATTCAAGAAA AGATTTGGAA   
  
  
- AGAGGAGAAT AGCCAGAGAG CTTTTCCTTC ATGGAAGTTG GGGGTAATGG CCTTCGTGAT TTCTCTGAAA   
  
  
- ATGTCTCTTT TGCTTTTTCT TTGACTTTGT GAAAATGTCA AAAGTAGGGC CAAGCTAATG TACCCCGTAT   
  
  
- TGTTCCTCTG CTTGCAAAGT AGCCAACACG CTTTATTCTA CTACGCCTCT ACTCGAAATA TTAGTGGGTG   
  
  
- CTTGTGTGTC TAGTGCTGTG ACTTTTGTGA ACCTCCGAAA TATCACTCAC GCGCTTCCCA CCGGCGTGTA   
  
  
- ACTTCCTCCG GCAGGCAGCA CTTACGCTCC CCTGAAGAGC GTGGGGATGT TCCTTTTAGT ATATTCAAGT   
  
  
- TGCTCTGACG CAACCACCGT GGAGGCGTGG ACCTGGACGA ACTAAGATAC TCGTTCAGTC ACCCGCGCGT   
  
  
- GCGCCATTCT GTCGCACGTT GAGATCTTGG TCAGTGCGAA TTCAATGTAA AAGCTGTCCT TTGGCACGCA   
  
  
- CCAGGAGCAG GCTTCCATGG CTGTGCCGTT TGGTGACAGA CAAATTCTGA TCGTACGATC AAACCCACGT   
  
  
- GGCCCAAAAC TTTCTGATTC CCTGGGCCCC ACATGACCCG CCATGGAAGT GGGGAAGGGA AAAGGGGAAG   
  
  
- CTGTTCCTCT GTTGATTGCC TCAGATTTGG TGAAGGATCT TGGGCCGCTG AACTTGGGTT GGTATTTTTC   
  
  
- ATATATGAGA TGCCCTTTTC TATGTGGGGA ATCGTGGCAT TTTTACGGTG GCTGATTCGG TGGTACTGTG   
  
  
- CAGTAAAAAG TAGAGTAAAT CTGCCTCTGT TCATCTTAGT TGGCATCTTT CTTTCCTGAG TTGGAAAGGT   
  
  
- GATCAGGTAG GTGCTGGACC AGGAACCAAA TGTTGCTCTC TTTTCTATTC ATGGGAGAGA ACCAACCGTC   
  
  
- TCTCAGCTTG CCTGACGCGC ACGAAGATGC CAAAGTTGTA TTATTCATTT CAGTGTAGCC GTACGTGTAC   
  
  
- CTGGGCGTGT GGACGTGACG CTCATTTCAT GAATGACCCG ATATTTTTTT TTCATTTATT TTATTTAGAG   
  
  
- TAAATATAAA ATAATTTTAA ATAATATTTA TTTTTTATTT TAAATTTTAA AATATTATTA AATCAATTAT   
  
  
- GCAGAAATAA TGTTTTAAAT TAATTTAATA AAAATTTATT TGTTATTAAT TTATTTTAAA AAAGTTATTC   
  
  
- AAGTTAAGAT TTGTATAATT AATTTTGAT

+     O2-site

| Site Name | Organism | Position | Strand | Matrix score. | sequence | function |
| --- | --- | --- | --- | --- | --- | --- |
| O2-site | Zea mays | 458 | + | 9 | GATGATGTGG | cis-acting regulatory element involved in zein metabolism regulation |
| O2-site | Zea mays | 405 | + | 9 | GATGACATGG | cis-acting regulatory element involved in zein metabolism regulation |

> 2018/04/13 10:10:12  
+ AAGTCGTCGG TTAGTTGGAA CTTTGAAAGT TGGTGGGTTA GAAGAGTGGA CGGAAAGGAC CCATGACAAA   
  
  
+ TGTCTACTCC GCTCCTTTCT TTCTGTCTCT TCCGTGCTTC CTGCCGCGAC TGAAAAACCT TCTTCTCTTT   
  
  
+ TTATAGCCGT TGGCTCTCTC TTCCTCTTTT CATATTTGAT TAGTCTGTAC CGTCCTCCTC TCTCTCTCTC   
  
  
+ TCCTTTTACC GTCACGTGAG TGACTGAACC ATTAACAATT AATAAATATG TAAGTTCTTT TCTAAACCTT   
  
  
+ TCTCCTCTTA TCGGTCTCTC GAAAAGGAAG TACCTTCAAC CCCCATTACC GGAAGCACTA AAGAGACTTT   
  
  
+ TACAGAGAAA ACGAAAAAGA AACTGAAACA CTTTTACAGT TTTCATCCCG GTTCGATTAC ATGGGGCATA   
  
  
+ ACAAGGAGAC GAACGTTTCA TCGGTTGTGC GAAATAAGAT GATGCGGAGA TGAGCTTTAT AATCACCCAC   
  
  
+ GAACACACAG ATCACGACAC TGAAAACACT TGGAGGCTTT ATAGTGAGTG CGCGAAGGGT GGCCGCACAT   
  
  
+ TGAAGGAGGC CGTCCGTCGT GAATGCGAGG GGACTTCTCG CACCCCTACA AGGAAAATCA TATAAGTTCA   
  
  
+ ACGAGACTGC GTTGGTGGCA CCTCCGCACC TGGACCTGCT TGATTCTATG AGCAAGTCAG TGGGCGCGCA   
  
  
+ CGCGGTAAGA CAGCGTGCAA CTCTAGAACC AGTCACGCTT AAGTTACATT TTCGACAGGA AACCGTGCGT   
  
  
+ GGTCCTCGTC CGAAGGTACC GACACGGCAA ACCACTGTCT GTTTAAGACT AGCATGCTAG TTTGGGTGCA   
  
  
+ CCGGGTTTTG AAAGACTAAG GGACCCGGGG TGTACTGGGC GGTACCTTCA CCCCTTCCCT TTTCCCCTTC   
  
  
+ GACAAGGAGA CAACTAACGG AGTCTAAACC ACTTCCTAGA ACCCGGCGAC TTGAACCCAA CCATAAAAAG   
  
  
+ TATATACTCT ACGGGAAAAG ATACACCCCT TAGCACCGTA AAAATGCCAC CGACTAAGCC ACCATGACAC   
  
  
+ GTCATTTTTC ATCTCATTTA GACGGAGACA AGTAGAATCA ACCGTAGAAA GAAAGGACTC AACCTTTCCA   
  
  
+ CTAGTCCATC CACGACCTGG TCCTTGGTTT ACAACGAGAG AAAAGATAAG TACCCTCTCT TGGTTGGCAG   
  
  
+ AGAGTCGAAC GGACTGCGCG TGCTTCTACG GTTTCAACAT AATAAGTAAA GTCACATCGG CATGCACATG   
  
  
+ GACCCGCACA CCTGCACTGC GAGTAAAGTA CTTACTGGGC TATAAAAAAA AAGTAAATAA AATAAATCTC   
  
  
+ ATTTATATTT TATTAAAATT TATTATAAAT AAAAAATAAA ATTTAAAATT TTATAATAAT TTAGTTAATA   
  
  
+ CGTCTTTATT ACAAAATTTA ATTAAATTAT TTTTAAATAA ACAATAATTA AATAAAATTT TTTCAATAAG   
  
  
+ TTCAATTCTA AACATATTAA TTAAAACTA  

- TTCAGCAGCC AATCAACCTT GAAACTTTCA ACCACCCAAT CTTCTCACCT GCCTTTCCTG GGTACTGTTT   
  
  
- ACAGATGAGG CGAGGAAAGA AAGACAGAGA AGGCACGAAG GACGGCGCTG ACTTTTTGGA AGAAGAGAAA   
  
  
- AATATCGGCA ACCGAGAGAG AAGGAGAAAA GTATAAACTA ATCAGACATG GCAGGAGGAG AGAGAGAGAG   
  
  
- AGGAAAATGG CAGTGCACTC ACTGACTTGG TAATTGTTAA TTATTTATAC ATTCAAGAAA AGATTTGGAA   
  
  
- AGAGGAGAAT AGCCAGAGAG CTTTTCCTTC ATGGAAGTTG GGGGTAATGG CCTTCGTGAT TTCTCTGAAA   
  
  
- ATGTCTCTTT TGCTTTTTCT TTGACTTTGT GAAAATGTCA AAAGTAGGGC CAAGCTAATG TACCCCGTAT   
  
  
- TGTTCCTCTG CTTGCAAAGT AGCCAACACG CTTTATTCTA CTACGCCTCT ACTCGAAATA TTAGTGGGTG   
  
  
- CTTGTGTGTC TAGTGCTGTG ACTTTTGTGA ACCTCCGAAA TATCACTCAC GCGCTTCCCA CCGGCGTGTA   
  
  
- ACTTCCTCCG GCAGGCAGCA CTTACGCTCC CCTGAAGAGC GTGGGGATGT TCCTTTTAGT ATATTCAAGT   
  
  
- TGCTCTGACG CAACCACCGT GGAGGCGTGG ACCTGGACGA ACTAAGATAC TCGTTCAGTC ACCCGCGCGT   
  
  
- GCGCCATTCT GTCGCACGTT GAGATCTTGG TCAGTGCGAA TTCAATGTAA AAGCTGTCCT TTGGCACGCA   
  
  
- CCAGGAGCAG GCTTCCATGG CTGTGCCGTT TGGTGACAGA CAAATTCTGA TCGTACGATC AAACCCACGT   
  
  
- GGCCCAAAAC TTTCTGATTC CCTGGGCCCC ACATGACCCG CCATGGAAGT GGGGAAGGGA AAAGGGGAAG   
  
  
- CTGTTCCTCT GTTGATTGCC TCAGATTTGG TGAAGGATCT TGGGCCGCTG AACTTGGGTT GGTATTTTTC   
  
  
- ATATATGAGA TGCCCTTTTC TATGTGGGGA ATCGTGGCAT TTTTACGGTG GCTGATTCGG TGGTACTGTG   
  
  
- CAGTAAAAAG TAGAGTAAAT CTGCCTCTGT TCATCTTAGT TGGCATCTTT CTTTCCTGAG TTGGAAAGGT   
  
  
- GATCAGGTAG GTGCTGGACC AGGAACCAAA TGTTGCTCTC TTTTCTATTC ATGGGAGAGA ACCAACCGTC   
  
  
- TCTCAGCTTG CCTGACGCGC ACGAAGATGC CAAAGTTGTA TTATTCATTT CAGTGTAGCC GTACGTGTAC   
  
  
- CTGGGCGTGT GGACGTGACG CTCATTTCAT GAATGACCCG ATATTTTTTT TTCATTTATT TTATTTAGAG   
  
  
- TAAATATAAA ATAATTTTAA ATAATATTTA TTTTTTATTT TAAATTTTAA AATATTATTA AATCAATTAT   
  
  
- GCAGAAATAA TGTTTTAAAT TAATTTAATA AAAATTTATT TGTTATTAAT TTATTTTAAA AAAGTTATTC   
  
  
- AAGTTAAGAT TTGTATAATT AATTTTGAT

+     Skn-1\_motif

| Site Name | Organism | Position | Strand | Matrix score. | sequence | function |
| --- | --- | --- | --- | --- | --- | --- |
| Skn-1\_motif | Oryza sativa | 1044 | - | 5 | GTCAT | cis-acting regulatory element required for endosperm expression |
| Skn-1\_motif | Oryza sativa | 63 | - | 5 | GTCAT | cis-acting regulatory element required for endosperm expression |
| Skn-1\_motif | Oryza sativa | 1051 | + | 5 | GTCAT | cis-acting regulatory element required for endosperm expression |

> 2018/04/13 10:10:12  
+ AAGTCGTCGG TTAGTTGGAA CTTTGAAAGT TGGTGGGTTA GAAGAGTGGA CGGAAAGGAC CCATGACAAA   
  
  
+ TGTCTACTCC GCTCCTTTCT TTCTGTCTCT TCCGTGCTTC CTGCCGCGAC TGAAAAACCT TCTTCTCTTT   
  
  
+ TTATAGCCGT TGGCTCTCTC TTCCTCTTTT CATATTTGAT TAGTCTGTAC CGTCCTCCTC TCTCTCTCTC   
  
  
+ TCCTTTTACC GTCACGTGAG TGACTGAACC ATTAACAATT AATAAATATG TAAGTTCTTT TCTAAACCTT   
  
  
+ TCTCCTCTTA TCGGTCTCTC GAAAAGGAAG TACCTTCAAC CCCCATTACC GGAAGCACTA AAGAGACTTT   
  
  
+ TACAGAGAAA ACGAAAAAGA AACTGAAACA CTTTTACAGT TTTCATCCCG GTTCGATTAC ATGGGGCATA   
  
  
+ ACAAGGAGAC GAACGTTTCA TCGGTTGTGC GAAATAAGAT GATGCGGAGA TGAGCTTTAT AATCACCCAC   
  
  
+ GAACACACAG ATCACGACAC TGAAAACACT TGGAGGCTTT ATAGTGAGTG CGCGAAGGGT GGCCGCACAT   
  
  
+ TGAAGGAGGC CGTCCGTCGT GAATGCGAGG GGACTTCTCG CACCCCTACA AGGAAAATCA TATAAGTTCA   
  
  
+ ACGAGACTGC GTTGGTGGCA CCTCCGCACC TGGACCTGCT TGATTCTATG AGCAAGTCAG TGGGCGCGCA   
  
  
+ CGCGGTAAGA CAGCGTGCAA CTCTAGAACC AGTCACGCTT AAGTTACATT TTCGACAGGA AACCGTGCGT   
  
  
+ GGTCCTCGTC CGAAGGTACC GACACGGCAA ACCACTGTCT GTTTAAGACT AGCATGCTAG TTTGGGTGCA   
  
  
+ CCGGGTTTTG AAAGACTAAG GGACCCGGGG TGTACTGGGC GGTACCTTCA CCCCTTCCCT TTTCCCCTTC   
  
  
+ GACAAGGAGA CAACTAACGG AGTCTAAACC ACTTCCTAGA ACCCGGCGAC TTGAACCCAA CCATAAAAAG   
  
  
+ TATATACTCT ACGGGAAAAG ATACACCCCT TAGCACCGTA AAAATGCCAC CGACTAAGCC ACCATGACAC   
  
  
+ GTCATTTTTC ATCTCATTTA GACGGAGACA AGTAGAATCA ACCGTAGAAA GAAAGGACTC AACCTTTCCA   
  
  
+ CTAGTCCATC CACGACCTGG TCCTTGGTTT ACAACGAGAG AAAAGATAAG TACCCTCTCT TGGTTGGCAG   
  
  
+ AGAGTCGAAC GGACTGCGCG TGCTTCTACG GTTTCAACAT AATAAGTAAA GTCACATCGG CATGCACATG   
  
  
+ GACCCGCACA CCTGCACTGC GAGTAAAGTA CTTACTGGGC TATAAAAAAA AAGTAAATAA AATAAATCTC   
  
  
+ ATTTATATTT TATTAAAATT TATTATAAAT AAAAAATAAA ATTTAAAATT TTATAATAAT TTAGTTAATA   
  
  
+ CGTCTTTATT ACAAAATTTA ATTAAATTAT TTTTAAATAA ACAATAATTA AATAAAATTT TTTCAATAAG   
  
  
+ TTCAATTCTA AACATATTAA TTAAAACTA  

- TTCAGCAGCC AATCAACCTT GAAACTTTCA ACCACCCAAT CTTCTCACCT GCCTTTCCTG GGTACTGTTT   
  
  
- ACAGATGAGG CGAGGAAAGA AAGACAGAGA AGGCACGAAG GACGGCGCTG ACTTTTTGGA AGAAGAGAAA   
  
  
- AATATCGGCA ACCGAGAGAG AAGGAGAAAA GTATAAACTA ATCAGACATG GCAGGAGGAG AGAGAGAGAG   
  
  
- AGGAAAATGG CAGTGCACTC ACTGACTTGG TAATTGTTAA TTATTTATAC ATTCAAGAAA AGATTTGGAA   
  
  
- AGAGGAGAAT AGCCAGAGAG CTTTTCCTTC ATGGAAGTTG GGGGTAATGG CCTTCGTGAT TTCTCTGAAA   
  
  
- ATGTCTCTTT TGCTTTTTCT TTGACTTTGT GAAAATGTCA AAAGTAGGGC CAAGCTAATG TACCCCGTAT   
  
  
- TGTTCCTCTG CTTGCAAAGT AGCCAACACG CTTTATTCTA CTACGCCTCT ACTCGAAATA TTAGTGGGTG   
  
  
- CTTGTGTGTC TAGTGCTGTG ACTTTTGTGA ACCTCCGAAA TATCACTCAC GCGCTTCCCA CCGGCGTGTA   
  
  
- ACTTCCTCCG GCAGGCAGCA CTTACGCTCC CCTGAAGAGC GTGGGGATGT TCCTTTTAGT ATATTCAAGT   
  
  
- TGCTCTGACG CAACCACCGT GGAGGCGTGG ACCTGGACGA ACTAAGATAC TCGTTCAGTC ACCCGCGCGT   
  
  
- GCGCCATTCT GTCGCACGTT GAGATCTTGG TCAGTGCGAA TTCAATGTAA AAGCTGTCCT TTGGCACGCA   
  
  
- CCAGGAGCAG GCTTCCATGG CTGTGCCGTT TGGTGACAGA CAAATTCTGA TCGTACGATC AAACCCACGT   
  
  
- GGCCCAAAAC TTTCTGATTC CCTGGGCCCC ACATGACCCG CCATGGAAGT GGGGAAGGGA AAAGGGGAAG   
  
  
- CTGTTCCTCT GTTGATTGCC TCAGATTTGG TGAAGGATCT TGGGCCGCTG AACTTGGGTT GGTATTTTTC   
  
  
- ATATATGAGA TGCCCTTTTC TATGTGGGGA ATCGTGGCAT TTTTACGGTG GCTGATTCGG TGGTACTGTG   
  
  
- CAGTAAAAAG TAGAGTAAAT CTGCCTCTGT TCATCTTAGT TGGCATCTTT CTTTCCTGAG TTGGAAAGGT   
  
  
- GATCAGGTAG GTGCTGGACC AGGAACCAAA TGTTGCTCTC TTTTCTATTC ATGGGAGAGA ACCAACCGTC   
  
  
- TCTCAGCTTG CCTGACGCGC ACGAAGATGC CAAAGTTGTA TTATTCATTT CAGTGTAGCC GTACGTGTAC   
  
  
- CTGGGCGTGT GGACGTGACG CTCATTTCAT GAATGACCCG ATATTTTTTT TTCATTTATT TTATTTAGAG   
  
  
- TAAATATAAA ATAATTTTAA ATAATATTTA TTTTTTATTT TAAATTTTAA AATATTATTA AATCAATTAT   
  
  
- GCAGAAATAA TGTTTTAAAT TAATTTAATA AAAATTTATT TGTTATTAAT TTATTTTAAA AAAGTTATTC   
  
  
- AAGTTAAGAT TTGTATAATT AATTTTGAT

+     Sp1

| Site Name | Organism | Position | Strand | Matrix score. | sequence | function |
| --- | --- | --- | --- | --- | --- | --- |
| Sp1 | Zea mays | 547 | - | 5.5 | CC(G/A)CCC | light responsive element |
| Sp1 | Oryza sativa | 877 | + | 6 | GGGCGG | light responsive element |

> 2018/04/13 10:10:12  
+ AAGTCGTCGG TTAGTTGGAA CTTTGAAAGT TGGTGGGTTA GAAGAGTGGA CGGAAAGGAC CCATGACAAA   
  
  
+ TGTCTACTCC GCTCCTTTCT TTCTGTCTCT TCCGTGCTTC CTGCCGCGAC TGAAAAACCT TCTTCTCTTT   
  
  
+ TTATAGCCGT TGGCTCTCTC TTCCTCTTTT CATATTTGAT TAGTCTGTAC CGTCCTCCTC TCTCTCTCTC   
  
  
+ TCCTTTTACC GTCACGTGAG TGACTGAACC ATTAACAATT AATAAATATG TAAGTTCTTT TCTAAACCTT   
  
  
+ TCTCCTCTTA TCGGTCTCTC GAAAAGGAAG TACCTTCAAC CCCCATTACC GGAAGCACTA AAGAGACTTT   
  
  
+ TACAGAGAAA ACGAAAAAGA AACTGAAACA CTTTTACAGT TTTCATCCCG GTTCGATTAC ATGGGGCATA   
  
  
+ ACAAGGAGAC GAACGTTTCA TCGGTTGTGC GAAATAAGAT GATGCGGAGA TGAGCTTTAT AATCACCCAC   
  
  
+ GAACACACAG ATCACGACAC TGAAAACACT TGGAGGCTTT ATAGTGAGTG CGCGAAGGGT GGCCGCACAT   
  
  
+ TGAAGGAGGC CGTCCGTCGT GAATGCGAGG GGACTTCTCG CACCCCTACA AGGAAAATCA TATAAGTTCA   
  
  
+ ACGAGACTGC GTTGGTGGCA CCTCCGCACC TGGACCTGCT TGATTCTATG AGCAAGTCAG TGGGCGCGCA   
  
  
+ CGCGGTAAGA CAGCGTGCAA CTCTAGAACC AGTCACGCTT AAGTTACATT TTCGACAGGA AACCGTGCGT   
  
  
+ GGTCCTCGTC CGAAGGTACC GACACGGCAA ACCACTGTCT GTTTAAGACT AGCATGCTAG TTTGGGTGCA   
  
  
+ CCGGGTTTTG AAAGACTAAG GGACCCGGGG TGTACTGGGC GGTACCTTCA CCCCTTCCCT TTTCCCCTTC   
  
  
+ GACAAGGAGA CAACTAACGG AGTCTAAACC ACTTCCTAGA ACCCGGCGAC TTGAACCCAA CCATAAAAAG   
  
  
+ TATATACTCT ACGGGAAAAG ATACACCCCT TAGCACCGTA AAAATGCCAC CGACTAAGCC ACCATGACAC   
  
  
+ GTCATTTTTC ATCTCATTTA GACGGAGACA AGTAGAATCA ACCGTAGAAA GAAAGGACTC AACCTTTCCA   
  
  
+ CTAGTCCATC CACGACCTGG TCCTTGGTTT ACAACGAGAG AAAAGATAAG TACCCTCTCT TGGTTGGCAG   
  
  
+ AGAGTCGAAC GGACTGCGCG TGCTTCTACG GTTTCAACAT AATAAGTAAA GTCACATCGG CATGCACATG   
  
  
+ GACCCGCACA CCTGCACTGC GAGTAAAGTA CTTACTGGGC TATAAAAAAA AAGTAAATAA AATAAATCTC   
  
  
+ ATTTATATTT TATTAAAATT TATTATAAAT AAAAAATAAA ATTTAAAATT TTATAATAAT TTAGTTAATA   
  
  
+ CGTCTTTATT ACAAAATTTA ATTAAATTAT TTTTAAATAA ACAATAATTA AATAAAATTT TTTCAATAAG   
  
  
+ TTCAATTCTA AACATATTAA TTAAAACTA  

- TTCAGCAGCC AATCAACCTT GAAACTTTCA ACCACCCAAT CTTCTCACCT GCCTTTCCTG GGTACTGTTT   
  
  
- ACAGATGAGG CGAGGAAAGA AAGACAGAGA AGGCACGAAG GACGGCGCTG ACTTTTTGGA AGAAGAGAAA   
  
  
- AATATCGGCA ACCGAGAGAG AAGGAGAAAA GTATAAACTA ATCAGACATG GCAGGAGGAG AGAGAGAGAG   
  
  
- AGGAAAATGG CAGTGCACTC ACTGACTTGG TAATTGTTAA TTATTTATAC ATTCAAGAAA AGATTTGGAA   
  
  
- AGAGGAGAAT AGCCAGAGAG CTTTTCCTTC ATGGAAGTTG GGGGTAATGG CCTTCGTGAT TTCTCTGAAA   
  
  
- ATGTCTCTTT TGCTTTTTCT TTGACTTTGT GAAAATGTCA AAAGTAGGGC CAAGCTAATG TACCCCGTAT   
  
  
- TGTTCCTCTG CTTGCAAAGT AGCCAACACG CTTTATTCTA CTACGCCTCT ACTCGAAATA TTAGTGGGTG   
  
  
- CTTGTGTGTC TAGTGCTGTG ACTTTTGTGA ACCTCCGAAA TATCACTCAC GCGCTTCCCA CCGGCGTGTA   
  
  
- ACTTCCTCCG GCAGGCAGCA CTTACGCTCC CCTGAAGAGC GTGGGGATGT TCCTTTTAGT ATATTCAAGT   
  
  
- TGCTCTGACG CAACCACCGT GGAGGCGTGG ACCTGGACGA ACTAAGATAC TCGTTCAGTC ACCCGCGCGT   
  
  
- GCGCCATTCT GTCGCACGTT GAGATCTTGG TCAGTGCGAA TTCAATGTAA AAGCTGTCCT TTGGCACGCA   
  
  
- CCAGGAGCAG GCTTCCATGG CTGTGCCGTT TGGTGACAGA CAAATTCTGA TCGTACGATC AAACCCACGT   
  
  
- GGCCCAAAAC TTTCTGATTC CCTGGGCCCC ACATGACCCG CCATGGAAGT GGGGAAGGGA AAAGGGGAAG   
  
  
- CTGTTCCTCT GTTGATTGCC TCAGATTTGG TGAAGGATCT TGGGCCGCTG AACTTGGGTT GGTATTTTTC   
  
  
- ATATATGAGA TGCCCTTTTC TATGTGGGGA ATCGTGGCAT TTTTACGGTG GCTGATTCGG TGGTACTGTG   
  
  
- CAGTAAAAAG TAGAGTAAAT CTGCCTCTGT TCATCTTAGT TGGCATCTTT CTTTCCTGAG TTGGAAAGGT   
  
  
- GATCAGGTAG GTGCTGGACC AGGAACCAAA TGTTGCTCTC TTTTCTATTC ATGGGAGAGA ACCAACCGTC   
  
  
- TCTCAGCTTG CCTGACGCGC ACGAAGATGC CAAAGTTGTA TTATTCATTT CAGTGTAGCC GTACGTGTAC   
  
  
- CTGGGCGTGT GGACGTGACG CTCATTTCAT GAATGACCCG ATATTTTTTT TTCATTTATT TTATTTAGAG   
  
  
- TAAATATAAA ATAATTTTAA ATAATATTTA TTTTTTATTT TAAATTTTAA AATATTATTA AATCAATTAT   
  
  
- GCAGAAATAA TGTTTTAAAT TAATTTAATA AAAATTTATT TGTTATTAAT TTATTTTAAA AAAGTTATTC   
  
  
- AAGTTAAGAT TTGTATAATT AATTTTGAT

+     TATA-box

| Site Name | Organism | Position | Strand | Matrix score. | sequence | function |
| --- | --- | --- | --- | --- | --- | --- |
| TATA-box | Oryza sativa | 1410 | + | 7 | TACAAAA | core promoter element around -30 of transcription start |
| TATA-box | Glycine max | 1407 | - | 5 | TAATA | core promoter element around -30 of transcription start |
| TATA-box | Glycine max | 1396 | + | 5 | TAATA | core promoter element around -30 of transcription start |
| TATA-box | Glycine max | 1384 | + | 5 | TAATA | core promoter element around -30 of transcription start |
| TATA-box | Glycine max | 1230 | + | 5 | TAATA | core promoter element around -30 of transcription start |
| TATA-box | Arabidopsis thaliana | 1381 | - | 5 | TATAA | core promoter element around -30 of transcription start |
| TATA-box | Arabidopsis thaliana | 1379 | - | 7 | TATAAAA | core promoter element around -30 of transcription start |
| TATA-box | Lycopersicon esculentum | 1492 | - | 5 | TTTTA | core promoter element around -30 of transcription start |
| TATA-box | Glycine max | 1485 | - | 5 | TAATA | core promoter element around -30 of transcription start |
| TATA-box | Lycopersicon esculentum | 1374 | - | 5 | TTTTA | core promoter element around -30 of transcription start |
| TATA-box | Lycopersicon esculentum | 1453 | - | 5 | TTTTA | core promoter element around -30 of transcription start |
| TATA-box | Arabidopsis thaliana | 1299 | + | 9 | ccTATAAAaa | core promoter element around -30 of transcription start |
| TATA-box | Pisum sativum | 1378 | - | 8 | TATAAAAT | core promoter element around -30 of transcription start |
| TATA-box | Lycopersicon esculentum | 1367 | - | 5 | TTTTA | core promoter element around -30 of transcription start |
| TATA-box | Lycopersicon esculentum | 1360 | - | 5 | TTTTA | core promoter element around -30 of transcription start |
| TATA-box | Arabidopsis thaliana | 1353 | - | 5 | TATAA | core promoter element around -30 of transcription start |
| TATA-box | Lycopersicon esculentum | 1019 | - | 5 | TTTTA | core promoter element around -30 of transcription start |
| TATA-box | Arabidopsis thaliana | 529 | - | 5 | TATAA | core promoter element around -30 of transcription start |
| TATA-box | Arabidopsis thaliana | 1427 | - | 9 | TAAAAATAA | core promoter element around -30 of transcription start |
| TATA-box | Arabidopsis thaliana | 477 | - | 5 | TATAA | core promoter element around -30 of transcription start |
| TATA-box | Lycopersicon esculentum | 1344 | - | 5 | TTTTA | core promoter element around -30 of transcription start |
| TATA-box | Brassica napus | 1352 | + | 6 | ATTATA | core promoter element around -30 of transcription start |
| TATA-box | Lycopersicon esculentum | 1431 | + | 5 | TTTTA | core promoter element around -30 of transcription start |
| TATA-box | Zea mays | 1430 | - | 8 | TTTAAAAA | core promoter element around -30 of transcription start |
| TATA-box | Glycine max | 1351 | - | 5 | TAATA | core promoter element around -30 of transcription start |
| TATA-box | Glycine max | 1341 | - | 5 | TAATA | core promoter element around -30 of transcription start |
| TATA-box | Lycopersicon esculentum | 1338 | + | 5 | TTTTA | core promoter element around -30 of transcription start |
| TATA-box | Arabidopsis thaliana | 1333 | - | 5 | TATAA | core promoter element around -30 of transcription start |
| TATA-box | Ac | 1331 | - | 7 | TATAAAT | core promoter element around -30 of transcription start |
| TATA-box | Lycopersicon esculentum | 382 | + | 5 | TTTTA | core promoter element around -30 of transcription start |
| TATA-box | Arabidopsis thaliana | 139 | - | 7 | TATAAAA | core promoter element around -30 of transcription start |
| TATA-box | Arabidopsis thaliana | 138 | - | 9 | ccTATAAAaa | core promoter element around -30 of transcription start |
| TATA-box | Lycopersicon esculentum | 348 | + | 5 | TTTTA | core promoter element around -30 of transcription start |
| TATA-box | Glycine max | 250 | + | 5 | TAATA | core promoter element around -30 of transcription start |
| TATA-box | Arabidopsis thaliana | 1380 | - | 6 | TATAAA | core promoter element around -30 of transcription start |
| TATA-box | Arabidopsis thaliana | 530 | + | 4 | TATA | core promoter element around -30 of transcription start |
| TATA-box | Lycopersicon esculentum | 1303 | - | 5 | TTTTA | core promoter element around -30 of transcription start |
| TATA-box | Lycopersicon esculentum | 974 | - | 5 | TTTTA | core promoter element around -30 of transcription start |
| TATA-box | Ac | 1354 | + | 7 | TATAAAT | core promoter element around -30 of transcription start |
| TATA-box | Arabidopsis thaliana | 528 | - | 6 | TATAAA | core promoter element around -30 of transcription start |
| TATA-box | Arabidopsis thaliana | 141 | - | 5 | TATAA | core promoter element around -30 of transcription start |
| TATA-box | Arabidopsis thaliana | 1382 | - | 4 | TATA | core promoter element around -30 of transcription start |
| TATA-box | Arabidopsis thaliana | 1332 | - | 6 | TATAAA | core promoter element around -30 of transcription start |
| TATA-box | Arabidopsis thaliana | 1334 | - | 4 | TATA | core promoter element around -30 of transcription start |
| TATA-box | Lycopersicon esculentum | 1318 | - | 5 | TTTTA | core promoter element around -30 of transcription start |
| TATA-box | Arabidopsis thaliana | 1432 | - | 8 | TATTTAAA | core promoter element around -30 of transcription start |
| TATA-box | Arabidopsis thaliana | 476 | - | 6 | TATAAA | core promoter element around -30 of transcription start |
| TATA-box | Lycopersicon esculentum | 214 | + | 5 | TTTTA | core promoter element around -30 of transcription start |
| TATA-box | Arabidopsis thaliana | 981 | - | 4 | TATA | core promoter element around -30 of transcription start |
| TATA-box | Arabidopsis thaliana | 478 | + | 4 | TATA | core promoter element around -30 of transcription start |
| TATA-box | Brassica oleracea | 620 | + | 6 | ATATAA | core promoter element around -30 of transcription start |
| TATA-box | Arabidopsis thaliana | 140 | - | 6 | TATAAA | core promoter element around -30 of transcription start |
| TATA-box | Arabidopsis thaliana | 1301 | + | 6 | TATAAA | core promoter element around -30 of transcription start |
| TATA-box | Arabidopsis thaliana | 142 | + | 4 | TATA | core promoter element around -30 of transcription start |
| TATA-box | Arabidopsis thaliana | 983 | - | 4 | TATA | core promoter element around -30 of transcription start |
| TATA-box | Arabidopsis thaliana | 621 | + | 4 | TATA | core promoter element around -30 of transcription start |

> 2018/04/13 10:10:12  
+ AAGTCGTCGG TTAGTTGGAA CTTTGAAAGT TGGTGGGTTA GAAGAGTGGA CGGAAAGGAC CCATGACAAA   
  
  
+ TGTCTACTCC GCTCCTTTCT TTCTGTCTCT TCCGTGCTTC CTGCCGCGAC TGAAAAACCT TCTTCTCTTT   
  
  
+ TTATAGCCGT TGGCTCTCTC TTCCTCTTTT CATATTTGAT TAGTCTGTAC CGTCCTCCTC TCTCTCTCTC   
  
  
+ TCCTTTTACC GTCACGTGAG TGACTGAACC ATTAACAATT AATAAATATG TAAGTTCTTT TCTAAACCTT   
  
  
+ TCTCCTCTTA TCGGTCTCTC GAAAAGGAAG TACCTTCAAC CCCCATTACC GGAAGCACTA AAGAGACTTT   
  
  
+ TACAGAGAAA ACGAAAAAGA AACTGAAACA CTTTTACAGT TTTCATCCCG GTTCGATTAC ATGGGGCATA   
  
  
+ ACAAGGAGAC GAACGTTTCA TCGGTTGTGC GAAATAAGAT GATGCGGAGA TGAGCTTTAT AATCACCCAC   
  
  
+ GAACACACAG ATCACGACAC TGAAAACACT TGGAGGCTTT ATAGTGAGTG CGCGAAGGGT GGCCGCACAT   
  
  
+ TGAAGGAGGC CGTCCGTCGT GAATGCGAGG GGACTTCTCG CACCCCTACA AGGAAAATCA TATAAGTTCA   
  
  
+ ACGAGACTGC GTTGGTGGCA CCTCCGCACC TGGACCTGCT TGATTCTATG AGCAAGTCAG TGGGCGCGCA   
  
  
+ CGCGGTAAGA CAGCGTGCAA CTCTAGAACC AGTCACGCTT AAGTTACATT TTCGACAGGA AACCGTGCGT   
  
  
+ GGTCCTCGTC CGAAGGTACC GACACGGCAA ACCACTGTCT GTTTAAGACT AGCATGCTAG TTTGGGTGCA   
  
  
+ CCGGGTTTTG AAAGACTAAG GGACCCGGGG TGTACTGGGC GGTACCTTCA CCCCTTCCCT TTTCCCCTTC   
  
  
+ GACAAGGAGA CAACTAACGG AGTCTAAACC ACTTCCTAGA ACCCGGCGAC TTGAACCCAA CCATAAAAAG   
  
  
+ TATATACTCT ACGGGAAAAG ATACACCCCT TAGCACCGTA AAAATGCCAC CGACTAAGCC ACCATGACAC   
  
  
+ GTCATTTTTC ATCTCATTTA GACGGAGACA AGTAGAATCA ACCGTAGAAA GAAAGGACTC AACCTTTCCA   
  
  
+ CTAGTCCATC CACGACCTGG TCCTTGGTTT ACAACGAGAG AAAAGATAAG TACCCTCTCT TGGTTGGCAG   
  
  
+ AGAGTCGAAC GGACTGCGCG TGCTTCTACG GTTTCAACAT AATAAGTAAA GTCACATCGG CATGCACATG   
  
  
+ GACCCGCACA CCTGCACTGC GAGTAAAGTA CTTACTGGGC TATAAAAAAA AAGTAAATAA AATAAATCTC   
  
  
+ ATTTATATTT TATTAAAATT TATTATAAAT AAAAAATAAA ATTTAAAATT TTATAATAAT TTAGTTAATA   
  
  
+ CGTCTTTATT ACAAAATTTA ATTAAATTAT TTTTAAATAA ACAATAATTA AATAAAATTT TTTCAATAAG   
  
  
+ TTCAATTCTA AACATATTAA TTAAAACTA  

- TTCAGCAGCC AATCAACCTT GAAACTTTCA ACCACCCAAT CTTCTCACCT GCCTTTCCTG GGTACTGTTT   
  
  
- ACAGATGAGG CGAGGAAAGA AAGACAGAGA AGGCACGAAG GACGGCGCTG ACTTTTTGGA AGAAGAGAAA   
  
  
- AATATCGGCA ACCGAGAGAG AAGGAGAAAA GTATAAACTA ATCAGACATG GCAGGAGGAG AGAGAGAGAG   
  
  
- AGGAAAATGG CAGTGCACTC ACTGACTTGG TAATTGTTAA TTATTTATAC ATTCAAGAAA AGATTTGGAA   
  
  
- AGAGGAGAAT AGCCAGAGAG CTTTTCCTTC ATGGAAGTTG GGGGTAATGG CCTTCGTGAT TTCTCTGAAA   
  
  
- ATGTCTCTTT TGCTTTTTCT TTGACTTTGT GAAAATGTCA AAAGTAGGGC CAAGCTAATG TACCCCGTAT   
  
  
- TGTTCCTCTG CTTGCAAAGT AGCCAACACG CTTTATTCTA CTACGCCTCT ACTCGAAATA TTAGTGGGTG   
  
  
- CTTGTGTGTC TAGTGCTGTG ACTTTTGTGA ACCTCCGAAA TATCACTCAC GCGCTTCCCA CCGGCGTGTA   
  
  
- ACTTCCTCCG GCAGGCAGCA CTTACGCTCC CCTGAAGAGC GTGGGGATGT TCCTTTTAGT ATATTCAAGT   
  
  
- TGCTCTGACG CAACCACCGT GGAGGCGTGG ACCTGGACGA ACTAAGATAC TCGTTCAGTC ACCCGCGCGT   
  
  
- GCGCCATTCT GTCGCACGTT GAGATCTTGG TCAGTGCGAA TTCAATGTAA AAGCTGTCCT TTGGCACGCA   
  
  
- CCAGGAGCAG GCTTCCATGG CTGTGCCGTT TGGTGACAGA CAAATTCTGA TCGTACGATC AAACCCACGT   
  
  
- GGCCCAAAAC TTTCTGATTC CCTGGGCCCC ACATGACCCG CCATGGAAGT GGGGAAGGGA AAAGGGGAAG   
  
  
- CTGTTCCTCT GTTGATTGCC TCAGATTTGG TGAAGGATCT TGGGCCGCTG AACTTGGGTT GGTATTTTTC   
  
  
- ATATATGAGA TGCCCTTTTC TATGTGGGGA ATCGTGGCAT TTTTACGGTG GCTGATTCGG TGGTACTGTG   
  
  
- CAGTAAAAAG TAGAGTAAAT CTGCCTCTGT TCATCTTAGT TGGCATCTTT CTTTCCTGAG TTGGAAAGGT   
  
  
- GATCAGGTAG GTGCTGGACC AGGAACCAAA TGTTGCTCTC TTTTCTATTC ATGGGAGAGA ACCAACCGTC   
  
  
- TCTCAGCTTG CCTGACGCGC ACGAAGATGC CAAAGTTGTA TTATTCATTT CAGTGTAGCC GTACGTGTAC   
  
  
- CTGGGCGTGT GGACGTGACG CTCATTTCAT GAATGACCCG ATATTTTTTT TTCATTTATT TTATTTAGAG   
  
  
- TAAATATAAA ATAATTTTAA ATAATATTTA TTTTTTATTT TAAATTTTAA AATATTATTA AATCAATTAT   
  
  
- GCAGAAATAA TGTTTTAAAT TAATTTAATA AAAATTTATT TGTTATTAAT TTATTTTAAA AAAGTTATTC   
  
  
- AAGTTAAGAT TTGTATAATT AATTTTGAT

+     TCT-motif

| Site Name | Organism | Position | Strand | Matrix score. | sequence | function |
| --- | --- | --- | --- | --- | --- | --- |
| TCT-motif | Arabidopsis thaliana | 705 | - | 6 | TCTTAC | part of a light responsive element |

> 2018/04/13 10:10:12  
+ AAGTCGTCGG TTAGTTGGAA CTTTGAAAGT TGGTGGGTTA GAAGAGTGGA CGGAAAGGAC CCATGACAAA   
  
  
+ TGTCTACTCC GCTCCTTTCT TTCTGTCTCT TCCGTGCTTC CTGCCGCGAC TGAAAAACCT TCTTCTCTTT   
  
  
+ TTATAGCCGT TGGCTCTCTC TTCCTCTTTT CATATTTGAT TAGTCTGTAC CGTCCTCCTC TCTCTCTCTC   
  
  
+ TCCTTTTACC GTCACGTGAG TGACTGAACC ATTAACAATT AATAAATATG TAAGTTCTTT TCTAAACCTT   
  
  
+ TCTCCTCTTA TCGGTCTCTC GAAAAGGAAG TACCTTCAAC CCCCATTACC GGAAGCACTA AAGAGACTTT   
  
  
+ TACAGAGAAA ACGAAAAAGA AACTGAAACA CTTTTACAGT TTTCATCCCG GTTCGATTAC ATGGGGCATA   
  
  
+ ACAAGGAGAC GAACGTTTCA TCGGTTGTGC GAAATAAGAT GATGCGGAGA TGAGCTTTAT AATCACCCAC   
  
  
+ GAACACACAG ATCACGACAC TGAAAACACT TGGAGGCTTT ATAGTGAGTG CGCGAAGGGT GGCCGCACAT   
  
  
+ TGAAGGAGGC CGTCCGTCGT GAATGCGAGG GGACTTCTCG CACCCCTACA AGGAAAATCA TATAAGTTCA   
  
  
+ ACGAGACTGC GTTGGTGGCA CCTCCGCACC TGGACCTGCT TGATTCTATG AGCAAGTCAG TGGGCGCGCA   
  
  
+ CGCGGTAAGA CAGCGTGCAA CTCTAGAACC AGTCACGCTT AAGTTACATT TTCGACAGGA AACCGTGCGT   
  
  
+ GGTCCTCGTC CGAAGGTACC GACACGGCAA ACCACTGTCT GTTTAAGACT AGCATGCTAG TTTGGGTGCA   
  
  
+ CCGGGTTTTG AAAGACTAAG GGACCCGGGG TGTACTGGGC GGTACCTTCA CCCCTTCCCT TTTCCCCTTC   
  
  
+ GACAAGGAGA CAACTAACGG AGTCTAAACC ACTTCCTAGA ACCCGGCGAC TTGAACCCAA CCATAAAAAG   
  
  
+ TATATACTCT ACGGGAAAAG ATACACCCCT TAGCACCGTA AAAATGCCAC CGACTAAGCC ACCATGACAC   
  
  
+ GTCATTTTTC ATCTCATTTA GACGGAGACA AGTAGAATCA ACCGTAGAAA GAAAGGACTC AACCTTTCCA   
  
  
+ CTAGTCCATC CACGACCTGG TCCTTGGTTT ACAACGAGAG AAAAGATAAG TACCCTCTCT TGGTTGGCAG   
  
  
+ AGAGTCGAAC GGACTGCGCG TGCTTCTACG GTTTCAACAT AATAAGTAAA GTCACATCGG CATGCACATG   
  
  
+ GACCCGCACA CCTGCACTGC GAGTAAAGTA CTTACTGGGC TATAAAAAAA AAGTAAATAA AATAAATCTC   
  
  
+ ATTTATATTT TATTAAAATT TATTATAAAT AAAAAATAAA ATTTAAAATT TTATAATAAT TTAGTTAATA   
  
  
+ CGTCTTTATT ACAAAATTTA ATTAAATTAT TTTTAAATAA ACAATAATTA AATAAAATTT TTTCAATAAG   
  
  
+ TTCAATTCTA AACATATTAA TTAAAACTA  

- TTCAGCAGCC AATCAACCTT GAAACTTTCA ACCACCCAAT CTTCTCACCT GCCTTTCCTG GGTACTGTTT   
  
  
- ACAGATGAGG CGAGGAAAGA AAGACAGAGA AGGCACGAAG GACGGCGCTG ACTTTTTGGA AGAAGAGAAA   
  
  
- AATATCGGCA ACCGAGAGAG AAGGAGAAAA GTATAAACTA ATCAGACATG GCAGGAGGAG AGAGAGAGAG   
  
  
- AGGAAAATGG CAGTGCACTC ACTGACTTGG TAATTGTTAA TTATTTATAC ATTCAAGAAA AGATTTGGAA   
  
  
- AGAGGAGAAT AGCCAGAGAG CTTTTCCTTC ATGGAAGTTG GGGGTAATGG CCTTCGTGAT TTCTCTGAAA   
  
  
- ATGTCTCTTT TGCTTTTTCT TTGACTTTGT GAAAATGTCA AAAGTAGGGC CAAGCTAATG TACCCCGTAT   
  
  
- TGTTCCTCTG CTTGCAAAGT AGCCAACACG CTTTATTCTA CTACGCCTCT ACTCGAAATA TTAGTGGGTG   
  
  
- CTTGTGTGTC TAGTGCTGTG ACTTTTGTGA ACCTCCGAAA TATCACTCAC GCGCTTCCCA CCGGCGTGTA   
  
  
- ACTTCCTCCG GCAGGCAGCA CTTACGCTCC CCTGAAGAGC GTGGGGATGT TCCTTTTAGT ATATTCAAGT   
  
  
- TGCTCTGACG CAACCACCGT GGAGGCGTGG ACCTGGACGA ACTAAGATAC TCGTTCAGTC ACCCGCGCGT   
  
  
- GCGCCATTCT GTCGCACGTT GAGATCTTGG TCAGTGCGAA TTCAATGTAA AAGCTGTCCT TTGGCACGCA   
  
  
- CCAGGAGCAG GCTTCCATGG CTGTGCCGTT TGGTGACAGA CAAATTCTGA TCGTACGATC AAACCCACGT   
  
  
- GGCCCAAAAC TTTCTGATTC CCTGGGCCCC ACATGACCCG CCATGGAAGT GGGGAAGGGA AAAGGGGAAG   
  
  
- CTGTTCCTCT GTTGATTGCC TCAGATTTGG TGAAGGATCT TGGGCCGCTG AACTTGGGTT GGTATTTTTC   
  
  
- ATATATGAGA TGCCCTTTTC TATGTGGGGA ATCGTGGCAT TTTTACGGTG GCTGATTCGG TGGTACTGTG   
  
  
- CAGTAAAAAG TAGAGTAAAT CTGCCTCTGT TCATCTTAGT TGGCATCTTT CTTTCCTGAG TTGGAAAGGT   
  
  
- GATCAGGTAG GTGCTGGACC AGGAACCAAA TGTTGCTCTC TTTTCTATTC ATGGGAGAGA ACCAACCGTC   
  
  
- TCTCAGCTTG CCTGACGCGC ACGAAGATGC CAAAGTTGTA TTATTCATTT CAGTGTAGCC GTACGTGTAC   
  
  
- CTGGGCGTGT GGACGTGACG CTCATTTCAT GAATGACCCG ATATTTTTTT TTCATTTATT TTATTTAGAG   
  
  
- TAAATATAAA ATAATTTTAA ATAATATTTA TTTTTTATTT TAAATTTTAA AATATTATTA AATCAATTAT   
  
  
- GCAGAAATAA TGTTTTAAAT TAATTTAATA AAAATTTATT TGTTATTAAT TTATTTTAAA AAAGTTATTC   
  
  
- AAGTTAAGAT TTGTATAATT AATTTTGAT

+     TGACG-motif

| Site Name | Organism | Position | Strand | Matrix score. | sequence | function |
| --- | --- | --- | --- | --- | --- | --- |
| TGACG-motif | Hordeum vulgare | 1050 | - | 5 | TGACG | cis-acting regulatory element involved in the MeJA-responsiveness |
| TGACG-motif | Hordeum vulgare | 220 | - | 5 | TGACG | cis-acting regulatory element involved in the MeJA-responsiveness |

> 2018/04/13 10:10:12  
+ AAGTCGTCGG TTAGTTGGAA CTTTGAAAGT TGGTGGGTTA GAAGAGTGGA CGGAAAGGAC CCATGACAAA   
  
  
+ TGTCTACTCC GCTCCTTTCT TTCTGTCTCT TCCGTGCTTC CTGCCGCGAC TGAAAAACCT TCTTCTCTTT   
  
  
+ TTATAGCCGT TGGCTCTCTC TTCCTCTTTT CATATTTGAT TAGTCTGTAC CGTCCTCCTC TCTCTCTCTC   
  
  
+ TCCTTTTACC GTCACGTGAG TGACTGAACC ATTAACAATT AATAAATATG TAAGTTCTTT TCTAAACCTT   
  
  
+ TCTCCTCTTA TCGGTCTCTC GAAAAGGAAG TACCTTCAAC CCCCATTACC GGAAGCACTA AAGAGACTTT   
  
  
+ TACAGAGAAA ACGAAAAAGA AACTGAAACA CTTTTACAGT TTTCATCCCG GTTCGATTAC ATGGGGCATA   
  
  
+ ACAAGGAGAC GAACGTTTCA TCGGTTGTGC GAAATAAGAT GATGCGGAGA TGAGCTTTAT AATCACCCAC   
  
  
+ GAACACACAG ATCACGACAC TGAAAACACT TGGAGGCTTT ATAGTGAGTG CGCGAAGGGT GGCCGCACAT   
  
  
+ TGAAGGAGGC CGTCCGTCGT GAATGCGAGG GGACTTCTCG CACCCCTACA AGGAAAATCA TATAAGTTCA   
  
  
+ ACGAGACTGC GTTGGTGGCA CCTCCGCACC TGGACCTGCT TGATTCTATG AGCAAGTCAG TGGGCGCGCA   
  
  
+ CGCGGTAAGA CAGCGTGCAA CTCTAGAACC AGTCACGCTT AAGTTACATT TTCGACAGGA AACCGTGCGT   
  
  
+ GGTCCTCGTC CGAAGGTACC GACACGGCAA ACCACTGTCT GTTTAAGACT AGCATGCTAG TTTGGGTGCA   
  
  
+ CCGGGTTTTG AAAGACTAAG GGACCCGGGG TGTACTGGGC GGTACCTTCA CCCCTTCCCT TTTCCCCTTC   
  
  
+ GACAAGGAGA CAACTAACGG AGTCTAAACC ACTTCCTAGA ACCCGGCGAC TTGAACCCAA CCATAAAAAG   
  
  
+ TATATACTCT ACGGGAAAAG ATACACCCCT TAGCACCGTA AAAATGCCAC CGACTAAGCC ACCATGACAC   
  
  
+ GTCATTTTTC ATCTCATTTA GACGGAGACA AGTAGAATCA ACCGTAGAAA GAAAGGACTC AACCTTTCCA   
  
  
+ CTAGTCCATC CACGACCTGG TCCTTGGTTT ACAACGAGAG AAAAGATAAG TACCCTCTCT TGGTTGGCAG   
  
  
+ AGAGTCGAAC GGACTGCGCG TGCTTCTACG GTTTCAACAT AATAAGTAAA GTCACATCGG CATGCACATG   
  
  
+ GACCCGCACA CCTGCACTGC GAGTAAAGTA CTTACTGGGC TATAAAAAAA AAGTAAATAA AATAAATCTC   
  
  
+ ATTTATATTT TATTAAAATT TATTATAAAT AAAAAATAAA ATTTAAAATT TTATAATAAT TTAGTTAATA   
  
  
+ CGTCTTTATT ACAAAATTTA ATTAAATTAT TTTTAAATAA ACAATAATTA AATAAAATTT TTTCAATAAG   
  
  
+ TTCAATTCTA AACATATTAA TTAAAACTA  

- TTCAGCAGCC AATCAACCTT GAAACTTTCA ACCACCCAAT CTTCTCACCT GCCTTTCCTG GGTACTGTTT   
  
  
- ACAGATGAGG CGAGGAAAGA AAGACAGAGA AGGCACGAAG GACGGCGCTG ACTTTTTGGA AGAAGAGAAA   
  
  
- AATATCGGCA ACCGAGAGAG AAGGAGAAAA GTATAAACTA ATCAGACATG GCAGGAGGAG AGAGAGAGAG   
  
  
- AGGAAAATGG CAGTGCACTC ACTGACTTGG TAATTGTTAA TTATTTATAC ATTCAAGAAA AGATTTGGAA   
  
  
- AGAGGAGAAT AGCCAGAGAG CTTTTCCTTC ATGGAAGTTG GGGGTAATGG CCTTCGTGAT TTCTCTGAAA   
  
  
- ATGTCTCTTT TGCTTTTTCT TTGACTTTGT GAAAATGTCA AAAGTAGGGC CAAGCTAATG TACCCCGTAT   
  
  
- TGTTCCTCTG CTTGCAAAGT AGCCAACACG CTTTATTCTA CTACGCCTCT ACTCGAAATA TTAGTGGGTG   
  
  
- CTTGTGTGTC TAGTGCTGTG ACTTTTGTGA ACCTCCGAAA TATCACTCAC GCGCTTCCCA CCGGCGTGTA   
  
  
- ACTTCCTCCG GCAGGCAGCA CTTACGCTCC CCTGAAGAGC GTGGGGATGT TCCTTTTAGT ATATTCAAGT   
  
  
- TGCTCTGACG CAACCACCGT GGAGGCGTGG ACCTGGACGA ACTAAGATAC TCGTTCAGTC ACCCGCGCGT   
  
  
- GCGCCATTCT GTCGCACGTT GAGATCTTGG TCAGTGCGAA TTCAATGTAA AAGCTGTCCT TTGGCACGCA   
  
  
- CCAGGAGCAG GCTTCCATGG CTGTGCCGTT TGGTGACAGA CAAATTCTGA TCGTACGATC AAACCCACGT   
  
  
- GGCCCAAAAC TTTCTGATTC CCTGGGCCCC ACATGACCCG CCATGGAAGT GGGGAAGGGA AAAGGGGAAG   
  
  
- CTGTTCCTCT GTTGATTGCC TCAGATTTGG TGAAGGATCT TGGGCCGCTG AACTTGGGTT GGTATTTTTC   
  
  
- ATATATGAGA TGCCCTTTTC TATGTGGGGA ATCGTGGCAT TTTTACGGTG GCTGATTCGG TGGTACTGTG   
  
  
- CAGTAAAAAG TAGAGTAAAT CTGCCTCTGT TCATCTTAGT TGGCATCTTT CTTTCCTGAG TTGGAAAGGT   
  
  
- GATCAGGTAG GTGCTGGACC AGGAACCAAA TGTTGCTCTC TTTTCTATTC ATGGGAGAGA ACCAACCGTC   
  
  
- TCTCAGCTTG CCTGACGCGC ACGAAGATGC CAAAGTTGTA TTATTCATTT CAGTGTAGCC GTACGTGTAC   
  
  
- CTGGGCGTGT GGACGTGACG CTCATTTCAT GAATGACCCG ATATTTTTTT TTCATTTATT TTATTTAGAG   
  
  
- TAAATATAAA ATAATTTTAA ATAATATTTA TTTTTTATTT TAAATTTTAA AATATTATTA AATCAATTAT   
  
  
- GCAGAAATAA TGTTTTAAAT TAATTTAATA AAAATTTATT TGTTATTAAT TTATTTTAAA AAAGTTATTC   
  
  
- AAGTTAAGAT TTGTATAATT AATTTTGAT

+     Unnamed\_\_1

| Site Name | Organism | Position | Strand | Matrix score. | sequence | function |
| --- | --- | --- | --- | --- | --- | --- |
| Unnamed\_\_1 | Glycine max | 1418 | - | 11 | GAATTTAATTAA | 60K protein binding site |
| Unnamed\_\_1 | Zea mays | 1130 | - | 5 | CGTGG |  |
| Unnamed\_\_1 | Glycine max | 1414 | + | 11 | GAATTTAATTAA | 60K protein binding site |
| Unnamed\_\_1 | Zea mays | 487 | - | 5 | CGTGG |  |
| Unnamed\_\_1 | Zea mays | 768 | + | 5 | CGTGG |  |

> 2018/04/13 10:10:12  
+ AAGTCGTCGG TTAGTTGGAA CTTTGAAAGT TGGTGGGTTA GAAGAGTGGA CGGAAAGGAC CCATGACAAA   
  
  
+ TGTCTACTCC GCTCCTTTCT TTCTGTCTCT TCCGTGCTTC CTGCCGCGAC TGAAAAACCT TCTTCTCTTT   
  
  
+ TTATAGCCGT TGGCTCTCTC TTCCTCTTTT CATATTTGAT TAGTCTGTAC CGTCCTCCTC TCTCTCTCTC   
  
  
+ TCCTTTTACC GTCACGTGAG TGACTGAACC ATTAACAATT AATAAATATG TAAGTTCTTT TCTAAACCTT   
  
  
+ TCTCCTCTTA TCGGTCTCTC GAAAAGGAAG TACCTTCAAC CCCCATTACC GGAAGCACTA AAGAGACTTT   
  
  
+ TACAGAGAAA ACGAAAAAGA AACTGAAACA CTTTTACAGT TTTCATCCCG GTTCGATTAC ATGGGGCATA   
  
  
+ ACAAGGAGAC GAACGTTTCA TCGGTTGTGC GAAATAAGAT GATGCGGAGA TGAGCTTTAT AATCACCCAC   
  
  
+ GAACACACAG ATCACGACAC TGAAAACACT TGGAGGCTTT ATAGTGAGTG CGCGAAGGGT GGCCGCACAT   
  
  
+ TGAAGGAGGC CGTCCGTCGT GAATGCGAGG GGACTTCTCG CACCCCTACA AGGAAAATCA TATAAGTTCA   
  
  
+ ACGAGACTGC GTTGGTGGCA CCTCCGCACC TGGACCTGCT TGATTCTATG AGCAAGTCAG TGGGCGCGCA   
  
  
+ CGCGGTAAGA CAGCGTGCAA CTCTAGAACC AGTCACGCTT AAGTTACATT TTCGACAGGA AACCGTGCGT   
  
  
+ GGTCCTCGTC CGAAGGTACC GACACGGCAA ACCACTGTCT GTTTAAGACT AGCATGCTAG TTTGGGTGCA   
  
  
+ CCGGGTTTTG AAAGACTAAG GGACCCGGGG TGTACTGGGC GGTACCTTCA CCCCTTCCCT TTTCCCCTTC   
  
  
+ GACAAGGAGA CAACTAACGG AGTCTAAACC ACTTCCTAGA ACCCGGCGAC TTGAACCCAA CCATAAAAAG   
  
  
+ TATATACTCT ACGGGAAAAG ATACACCCCT TAGCACCGTA AAAATGCCAC CGACTAAGCC ACCATGACAC   
  
  
+ GTCATTTTTC ATCTCATTTA GACGGAGACA AGTAGAATCA ACCGTAGAAA GAAAGGACTC AACCTTTCCA   
  
  
+ CTAGTCCATC CACGACCTGG TCCTTGGTTT ACAACGAGAG AAAAGATAAG TACCCTCTCT TGGTTGGCAG   
  
  
+ AGAGTCGAAC GGACTGCGCG TGCTTCTACG GTTTCAACAT AATAAGTAAA GTCACATCGG CATGCACATG   
  
  
+ GACCCGCACA CCTGCACTGC GAGTAAAGTA CTTACTGGGC TATAAAAAAA AAGTAAATAA AATAAATCTC   
  
  
+ ATTTATATTT TATTAAAATT TATTATAAAT AAAAAATAAA ATTTAAAATT TTATAATAAT TTAGTTAATA   
  
  
+ CGTCTTTATT ACAAAATTTA ATTAAATTAT TTTTAAATAA ACAATAATTA AATAAAATTT TTTCAATAAG   
  
  
+ TTCAATTCTA AACATATTAA TTAAAACTA  

- TTCAGCAGCC AATCAACCTT GAAACTTTCA ACCACCCAAT CTTCTCACCT GCCTTTCCTG GGTACTGTTT   
  
  
- ACAGATGAGG CGAGGAAAGA AAGACAGAGA AGGCACGAAG GACGGCGCTG ACTTTTTGGA AGAAGAGAAA   
  
  
- AATATCGGCA ACCGAGAGAG AAGGAGAAAA GTATAAACTA ATCAGACATG GCAGGAGGAG AGAGAGAGAG   
  
  
- AGGAAAATGG CAGTGCACTC ACTGACTTGG TAATTGTTAA TTATTTATAC ATTCAAGAAA AGATTTGGAA   
  
  
- AGAGGAGAAT AGCCAGAGAG CTTTTCCTTC ATGGAAGTTG GGGGTAATGG CCTTCGTGAT TTCTCTGAAA   
  
  
- ATGTCTCTTT TGCTTTTTCT TTGACTTTGT GAAAATGTCA AAAGTAGGGC CAAGCTAATG TACCCCGTAT   
  
  
- TGTTCCTCTG CTTGCAAAGT AGCCAACACG CTTTATTCTA CTACGCCTCT ACTCGAAATA TTAGTGGGTG   
  
  
- CTTGTGTGTC TAGTGCTGTG ACTTTTGTGA ACCTCCGAAA TATCACTCAC GCGCTTCCCA CCGGCGTGTA   
  
  
- ACTTCCTCCG GCAGGCAGCA CTTACGCTCC CCTGAAGAGC GTGGGGATGT TCCTTTTAGT ATATTCAAGT   
  
  
- TGCTCTGACG CAACCACCGT GGAGGCGTGG ACCTGGACGA ACTAAGATAC TCGTTCAGTC ACCCGCGCGT   
  
  
- GCGCCATTCT GTCGCACGTT GAGATCTTGG TCAGTGCGAA TTCAATGTAA AAGCTGTCCT TTGGCACGCA   
  
  
- CCAGGAGCAG GCTTCCATGG CTGTGCCGTT TGGTGACAGA CAAATTCTGA TCGTACGATC AAACCCACGT   
  
  
- GGCCCAAAAC TTTCTGATTC CCTGGGCCCC ACATGACCCG CCATGGAAGT GGGGAAGGGA AAAGGGGAAG   
  
  
- CTGTTCCTCT GTTGATTGCC TCAGATTTGG TGAAGGATCT TGGGCCGCTG AACTTGGGTT GGTATTTTTC   
  
  
- ATATATGAGA TGCCCTTTTC TATGTGGGGA ATCGTGGCAT TTTTACGGTG GCTGATTCGG TGGTACTGTG   
  
  
- CAGTAAAAAG TAGAGTAAAT CTGCCTCTGT TCATCTTAGT TGGCATCTTT CTTTCCTGAG TTGGAAAGGT   
  
  
- GATCAGGTAG GTGCTGGACC AGGAACCAAA TGTTGCTCTC TTTTCTATTC ATGGGAGAGA ACCAACCGTC   
  
  
- TCTCAGCTTG CCTGACGCGC ACGAAGATGC CAAAGTTGTA TTATTCATTT CAGTGTAGCC GTACGTGTAC   
  
  
- CTGGGCGTGT GGACGTGACG CTCATTTCAT GAATGACCCG ATATTTTTTT TTCATTTATT TTATTTAGAG   
  
  
- TAAATATAAA ATAATTTTAA ATAATATTTA TTTTTTATTT TAAATTTTAA AATATTATTA AATCAATTAT   
  
  
- GCAGAAATAA TGTTTTAAAT TAATTTAATA AAAATTTATT TGTTATTAAT TTATTTTAAA AAAGTTATTC   
  
  
- AAGTTAAGAT TTGTATAATT AATTTTGAT

+     Unnamed\_\_2

| Site Name | Organism | Position | Strand | Matrix score. | sequence | function |
| --- | --- | --- | --- | --- | --- | --- |
| Unnamed\_\_2 | Zea mays | 865 | - | 6 | CCCCGG |  |
| Unnamed\_\_2 | Glycine max | 1370 | - | 14 | ATTAAATTTTAAATT |  |

> 2018/04/13 10:10:12  
+ AAGTCGTCGG TTAGTTGGAA CTTTGAAAGT TGGTGGGTTA GAAGAGTGGA CGGAAAGGAC CCATGACAAA   
  
  
+ TGTCTACTCC GCTCCTTTCT TTCTGTCTCT TCCGTGCTTC CTGCCGCGAC TGAAAAACCT TCTTCTCTTT   
  
  
+ TTATAGCCGT TGGCTCTCTC TTCCTCTTTT CATATTTGAT TAGTCTGTAC CGTCCTCCTC TCTCTCTCTC   
  
  
+ TCCTTTTACC GTCACGTGAG TGACTGAACC ATTAACAATT AATAAATATG TAAGTTCTTT TCTAAACCTT   
  
  
+ TCTCCTCTTA TCGGTCTCTC GAAAAGGAAG TACCTTCAAC CCCCATTACC GGAAGCACTA AAGAGACTTT   
  
  
+ TACAGAGAAA ACGAAAAAGA AACTGAAACA CTTTTACAGT TTTCATCCCG GTTCGATTAC ATGGGGCATA   
  
  
+ ACAAGGAGAC GAACGTTTCA TCGGTTGTGC GAAATAAGAT GATGCGGAGA TGAGCTTTAT AATCACCCAC   
  
  
+ GAACACACAG ATCACGACAC TGAAAACACT TGGAGGCTTT ATAGTGAGTG CGCGAAGGGT GGCCGCACAT   
  
  
+ TGAAGGAGGC CGTCCGTCGT GAATGCGAGG GGACTTCTCG CACCCCTACA AGGAAAATCA TATAAGTTCA   
  
  
+ ACGAGACTGC GTTGGTGGCA CCTCCGCACC TGGACCTGCT TGATTCTATG AGCAAGTCAG TGGGCGCGCA   
  
  
+ CGCGGTAAGA CAGCGTGCAA CTCTAGAACC AGTCACGCTT AAGTTACATT TTCGACAGGA AACCGTGCGT   
  
  
+ GGTCCTCGTC CGAAGGTACC GACACGGCAA ACCACTGTCT GTTTAAGACT AGCATGCTAG TTTGGGTGCA   
  
  
+ CCGGGTTTTG AAAGACTAAG GGACCCGGGG TGTACTGGGC GGTACCTTCA CCCCTTCCCT TTTCCCCTTC   
  
  
+ GACAAGGAGA CAACTAACGG AGTCTAAACC ACTTCCTAGA ACCCGGCGAC TTGAACCCAA CCATAAAAAG   
  
  
+ TATATACTCT ACGGGAAAAG ATACACCCCT TAGCACCGTA AAAATGCCAC CGACTAAGCC ACCATGACAC   
  
  
+ GTCATTTTTC ATCTCATTTA GACGGAGACA AGTAGAATCA ACCGTAGAAA GAAAGGACTC AACCTTTCCA   
  
  
+ CTAGTCCATC CACGACCTGG TCCTTGGTTT ACAACGAGAG AAAAGATAAG TACCCTCTCT TGGTTGGCAG   
  
  
+ AGAGTCGAAC GGACTGCGCG TGCTTCTACG GTTTCAACAT AATAAGTAAA GTCACATCGG CATGCACATG   
  
  
+ GACCCGCACA CCTGCACTGC GAGTAAAGTA CTTACTGGGC TATAAAAAAA AAGTAAATAA AATAAATCTC   
  
  
+ ATTTATATTT TATTAAAATT TATTATAAAT AAAAAATAAA ATTTAAAATT TTATAATAAT TTAGTTAATA   
  
  
+ CGTCTTTATT ACAAAATTTA ATTAAATTAT TTTTAAATAA ACAATAATTA AATAAAATTT TTTCAATAAG   
  
  
+ TTCAATTCTA AACATATTAA TTAAAACTA  

- TTCAGCAGCC AATCAACCTT GAAACTTTCA ACCACCCAAT CTTCTCACCT GCCTTTCCTG GGTACTGTTT   
  
  
- ACAGATGAGG CGAGGAAAGA AAGACAGAGA AGGCACGAAG GACGGCGCTG ACTTTTTGGA AGAAGAGAAA   
  
  
- AATATCGGCA ACCGAGAGAG AAGGAGAAAA GTATAAACTA ATCAGACATG GCAGGAGGAG AGAGAGAGAG   
  
  
- AGGAAAATGG CAGTGCACTC ACTGACTTGG TAATTGTTAA TTATTTATAC ATTCAAGAAA AGATTTGGAA   
  
  
- AGAGGAGAAT AGCCAGAGAG CTTTTCCTTC ATGGAAGTTG GGGGTAATGG CCTTCGTGAT TTCTCTGAAA   
  
  
- ATGTCTCTTT TGCTTTTTCT TTGACTTTGT GAAAATGTCA AAAGTAGGGC CAAGCTAATG TACCCCGTAT   
  
  
- TGTTCCTCTG CTTGCAAAGT AGCCAACACG CTTTATTCTA CTACGCCTCT ACTCGAAATA TTAGTGGGTG   
  
  
- CTTGTGTGTC TAGTGCTGTG ACTTTTGTGA ACCTCCGAAA TATCACTCAC GCGCTTCCCA CCGGCGTGTA   
  
  
- ACTTCCTCCG GCAGGCAGCA CTTACGCTCC CCTGAAGAGC GTGGGGATGT TCCTTTTAGT ATATTCAAGT   
  
  
- TGCTCTGACG CAACCACCGT GGAGGCGTGG ACCTGGACGA ACTAAGATAC TCGTTCAGTC ACCCGCGCGT   
  
  
- GCGCCATTCT GTCGCACGTT GAGATCTTGG TCAGTGCGAA TTCAATGTAA AAGCTGTCCT TTGGCACGCA   
  
  
- CCAGGAGCAG GCTTCCATGG CTGTGCCGTT TGGTGACAGA CAAATTCTGA TCGTACGATC AAACCCACGT   
  
  
- GGCCCAAAAC TTTCTGATTC CCTGGGCCCC ACATGACCCG CCATGGAAGT GGGGAAGGGA AAAGGGGAAG   
  
  
- CTGTTCCTCT GTTGATTGCC TCAGATTTGG TGAAGGATCT TGGGCCGCTG AACTTGGGTT GGTATTTTTC   
  
  
- ATATATGAGA TGCCCTTTTC TATGTGGGGA ATCGTGGCAT TTTTACGGTG GCTGATTCGG TGGTACTGTG   
  
  
- CAGTAAAAAG TAGAGTAAAT CTGCCTCTGT TCATCTTAGT TGGCATCTTT CTTTCCTGAG TTGGAAAGGT   
  
  
- GATCAGGTAG GTGCTGGACC AGGAACCAAA TGTTGCTCTC TTTTCTATTC ATGGGAGAGA ACCAACCGTC   
  
  
- TCTCAGCTTG CCTGACGCGC ACGAAGATGC CAAAGTTGTA TTATTCATTT CAGTGTAGCC GTACGTGTAC   
  
  
- CTGGGCGTGT GGACGTGACG CTCATTTCAT GAATGACCCG ATATTTTTTT TTCATTTATT TTATTTAGAG   
  
  
- TAAATATAAA ATAATTTTAA ATAATATTTA TTTTTTATTT TAAATTTTAA AATATTATTA AATCAATTAT   
  
  
- GCAGAAATAA TGTTTTAAAT TAATTTAATA AAAATTTATT TGTTATTAAT TTATTTTAAA AAAGTTATTC   
  
  
- AAGTTAAGAT TTGTATAATT AATTTTGAT

+     Unnamed\_\_3

| Site Name | Organism | Position | Strand | Matrix score. | sequence | function |
| --- | --- | --- | --- | --- | --- | --- |
| Unnamed\_\_3 | Zea mays | 1130 | - | 5 | CGTGG |  |
| Unnamed\_\_3 | Zea mays | 487 | - | 5 | CGTGG |  |
| Unnamed\_\_3 | Zea mays | 768 | + | 5 | CGTGG |  |

> 2018/04/13 10:10:12  
+ AAGTCGTCGG TTAGTTGGAA CTTTGAAAGT TGGTGGGTTA GAAGAGTGGA CGGAAAGGAC CCATGACAAA   
  
  
+ TGTCTACTCC GCTCCTTTCT TTCTGTCTCT TCCGTGCTTC CTGCCGCGAC TGAAAAACCT TCTTCTCTTT   
  
  
+ TTATAGCCGT TGGCTCTCTC TTCCTCTTTT CATATTTGAT TAGTCTGTAC CGTCCTCCTC TCTCTCTCTC   
  
  
+ TCCTTTTACC GTCACGTGAG TGACTGAACC ATTAACAATT AATAAATATG TAAGTTCTTT TCTAAACCTT   
  
  
+ TCTCCTCTTA TCGGTCTCTC GAAAAGGAAG TACCTTCAAC CCCCATTACC GGAAGCACTA AAGAGACTTT   
  
  
+ TACAGAGAAA ACGAAAAAGA AACTGAAACA CTTTTACAGT TTTCATCCCG GTTCGATTAC ATGGGGCATA   
  
  
+ ACAAGGAGAC GAACGTTTCA TCGGTTGTGC GAAATAAGAT GATGCGGAGA TGAGCTTTAT AATCACCCAC   
  
  
+ GAACACACAG ATCACGACAC TGAAAACACT TGGAGGCTTT ATAGTGAGTG CGCGAAGGGT GGCCGCACAT   
  
  
+ TGAAGGAGGC CGTCCGTCGT GAATGCGAGG GGACTTCTCG CACCCCTACA AGGAAAATCA TATAAGTTCA   
  
  
+ ACGAGACTGC GTTGGTGGCA CCTCCGCACC TGGACCTGCT TGATTCTATG AGCAAGTCAG TGGGCGCGCA   
  
  
+ CGCGGTAAGA CAGCGTGCAA CTCTAGAACC AGTCACGCTT AAGTTACATT TTCGACAGGA AACCGTGCGT   
  
  
+ GGTCCTCGTC CGAAGGTACC GACACGGCAA ACCACTGTCT GTTTAAGACT AGCATGCTAG TTTGGGTGCA   
  
  
+ CCGGGTTTTG AAAGACTAAG GGACCCGGGG TGTACTGGGC GGTACCTTCA CCCCTTCCCT TTTCCCCTTC   
  
  
+ GACAAGGAGA CAACTAACGG AGTCTAAACC ACTTCCTAGA ACCCGGCGAC TTGAACCCAA CCATAAAAAG   
  
  
+ TATATACTCT ACGGGAAAAG ATACACCCCT TAGCACCGTA AAAATGCCAC CGACTAAGCC ACCATGACAC   
  
  
+ GTCATTTTTC ATCTCATTTA GACGGAGACA AGTAGAATCA ACCGTAGAAA GAAAGGACTC AACCTTTCCA   
  
  
+ CTAGTCCATC CACGACCTGG TCCTTGGTTT ACAACGAGAG AAAAGATAAG TACCCTCTCT TGGTTGGCAG   
  
  
+ AGAGTCGAAC GGACTGCGCG TGCTTCTACG GTTTCAACAT AATAAGTAAA GTCACATCGG CATGCACATG   
  
  
+ GACCCGCACA CCTGCACTGC GAGTAAAGTA CTTACTGGGC TATAAAAAAA AAGTAAATAA AATAAATCTC   
  
  
+ ATTTATATTT TATTAAAATT TATTATAAAT AAAAAATAAA ATTTAAAATT TTATAATAAT TTAGTTAATA   
  
  
+ CGTCTTTATT ACAAAATTTA ATTAAATTAT TTTTAAATAA ACAATAATTA AATAAAATTT TTTCAATAAG   
  
  
+ TTCAATTCTA AACATATTAA TTAAAACTA  

- TTCAGCAGCC AATCAACCTT GAAACTTTCA ACCACCCAAT CTTCTCACCT GCCTTTCCTG GGTACTGTTT   
  
  
- ACAGATGAGG CGAGGAAAGA AAGACAGAGA AGGCACGAAG GACGGCGCTG ACTTTTTGGA AGAAGAGAAA   
  
  
- AATATCGGCA ACCGAGAGAG AAGGAGAAAA GTATAAACTA ATCAGACATG GCAGGAGGAG AGAGAGAGAG   
  
  
- AGGAAAATGG CAGTGCACTC ACTGACTTGG TAATTGTTAA TTATTTATAC ATTCAAGAAA AGATTTGGAA   
  
  
- AGAGGAGAAT AGCCAGAGAG CTTTTCCTTC ATGGAAGTTG GGGGTAATGG CCTTCGTGAT TTCTCTGAAA   
  
  
- ATGTCTCTTT TGCTTTTTCT TTGACTTTGT GAAAATGTCA AAAGTAGGGC CAAGCTAATG TACCCCGTAT   
  
  
- TGTTCCTCTG CTTGCAAAGT AGCCAACACG CTTTATTCTA CTACGCCTCT ACTCGAAATA TTAGTGGGTG   
  
  
- CTTGTGTGTC TAGTGCTGTG ACTTTTGTGA ACCTCCGAAA TATCACTCAC GCGCTTCCCA CCGGCGTGTA   
  
  
- ACTTCCTCCG GCAGGCAGCA CTTACGCTCC CCTGAAGAGC GTGGGGATGT TCCTTTTAGT ATATTCAAGT   
  
  
- TGCTCTGACG CAACCACCGT GGAGGCGTGG ACCTGGACGA ACTAAGATAC TCGTTCAGTC ACCCGCGCGT   
  
  
- GCGCCATTCT GTCGCACGTT GAGATCTTGG TCAGTGCGAA TTCAATGTAA AAGCTGTCCT TTGGCACGCA   
  
  
- CCAGGAGCAG GCTTCCATGG CTGTGCCGTT TGGTGACAGA CAAATTCTGA TCGTACGATC AAACCCACGT   
  
  
- GGCCCAAAAC TTTCTGATTC CCTGGGCCCC ACATGACCCG CCATGGAAGT GGGGAAGGGA AAAGGGGAAG   
  
  
- CTGTTCCTCT GTTGATTGCC TCAGATTTGG TGAAGGATCT TGGGCCGCTG AACTTGGGTT GGTATTTTTC   
  
  
- ATATATGAGA TGCCCTTTTC TATGTGGGGA ATCGTGGCAT TTTTACGGTG GCTGATTCGG TGGTACTGTG   
  
  
- CAGTAAAAAG TAGAGTAAAT CTGCCTCTGT TCATCTTAGT TGGCATCTTT CTTTCCTGAG TTGGAAAGGT   
  
  
- GATCAGGTAG GTGCTGGACC AGGAACCAAA TGTTGCTCTC TTTTCTATTC ATGGGAGAGA ACCAACCGTC   
  
  
- TCTCAGCTTG CCTGACGCGC ACGAAGATGC CAAAGTTGTA TTATTCATTT CAGTGTAGCC GTACGTGTAC   
  
  
- CTGGGCGTGT GGACGTGACG CTCATTTCAT GAATGACCCG ATATTTTTTT TTCATTTATT TTATTTAGAG   
  
  
- TAAATATAAA ATAATTTTAA ATAATATTTA TTTTTTATTT TAAATTTTAA AATATTATTA AATCAATTAT   
  
  
- GCAGAAATAA TGTTTTAAAT TAATTTAATA AAAATTTATT TGTTATTAAT TTATTTTAAA AAAGTTATTC   
  
  
- AAGTTAAGAT TTGTATAATT AATTTTGAT

+     Unnamed\_\_4

| Site Name | Organism | Position | Strand | Matrix score. | sequence | function |
| --- | --- | --- | --- | --- | --- | --- |
| Unnamed\_\_4 | Petroselinum hortense | 929 | - | 4 | CTCC |  |
| Unnamed\_\_4 | Petroselinum hortense | 916 | - | 4 | CTCC |  |
| Unnamed\_\_4 | Petroselinum hortense | 425 | - | 4 | CTCC |  |
| Unnamed\_\_4 | Petroselinum hortense | 1074 | - | 4 | CTCC |  |
| Unnamed\_\_4 | Petroselinum hortense | 652 | + | 4 | CTCC |  |
| Unnamed\_\_4 | Petroselinum hortense | 565 | - | 4 | CTCC |  |
| Unnamed\_\_4 | Petroselinum hortense | 282 | + | 4 | CTCC |  |
| Unnamed\_\_4 | Petroselinum hortense | 466 | - | 4 | CTCC |  |
| Unnamed\_\_4 | Petroselinum hortense | 522 | - | 4 | CTCC |  |
| Unnamed\_\_4 | Petroselinum hortense | 210 | + | 4 | CTCC |  |
| Unnamed\_\_4 | Petroselinum hortense | 195 | + | 4 | CTCC |  |
| Unnamed\_\_4 | Petroselinum hortense | 82 | + | 4 | CTCC |  |
| Unnamed\_\_4 | Petroselinum hortense | 77 | + | 4 | CTCC |  |

> 2018/04/13 10:10:12  
+ AAGTCGTCGG TTAGTTGGAA CTTTGAAAGT TGGTGGGTTA GAAGAGTGGA CGGAAAGGAC CCATGACAAA   
  
  
+ TGTCTACTCC GCTCCTTTCT TTCTGTCTCT TCCGTGCTTC CTGCCGCGAC TGAAAAACCT TCTTCTCTTT   
  
  
+ TTATAGCCGT TGGCTCTCTC TTCCTCTTTT CATATTTGAT TAGTCTGTAC CGTCCTCCTC TCTCTCTCTC   
  
  
+ TCCTTTTACC GTCACGTGAG TGACTGAACC ATTAACAATT AATAAATATG TAAGTTCTTT TCTAAACCTT   
  
  
+ TCTCCTCTTA TCGGTCTCTC GAAAAGGAAG TACCTTCAAC CCCCATTACC GGAAGCACTA AAGAGACTTT   
  
  
+ TACAGAGAAA ACGAAAAAGA AACTGAAACA CTTTTACAGT TTTCATCCCG GTTCGATTAC ATGGGGCATA   
  
  
+ ACAAGGAGAC GAACGTTTCA TCGGTTGTGC GAAATAAGAT GATGCGGAGA TGAGCTTTAT AATCACCCAC   
  
  
+ GAACACACAG ATCACGACAC TGAAAACACT TGGAGGCTTT ATAGTGAGTG CGCGAAGGGT GGCCGCACAT   
  
  
+ TGAAGGAGGC CGTCCGTCGT GAATGCGAGG GGACTTCTCG CACCCCTACA AGGAAAATCA TATAAGTTCA   
  
  
+ ACGAGACTGC GTTGGTGGCA CCTCCGCACC TGGACCTGCT TGATTCTATG AGCAAGTCAG TGGGCGCGCA   
  
  
+ CGCGGTAAGA CAGCGTGCAA CTCTAGAACC AGTCACGCTT AAGTTACATT TTCGACAGGA AACCGTGCGT   
  
  
+ GGTCCTCGTC CGAAGGTACC GACACGGCAA ACCACTGTCT GTTTAAGACT AGCATGCTAG TTTGGGTGCA   
  
  
+ CCGGGTTTTG AAAGACTAAG GGACCCGGGG TGTACTGGGC GGTACCTTCA CCCCTTCCCT TTTCCCCTTC   
  
  
+ GACAAGGAGA CAACTAACGG AGTCTAAACC ACTTCCTAGA ACCCGGCGAC TTGAACCCAA CCATAAAAAG   
  
  
+ TATATACTCT ACGGGAAAAG ATACACCCCT TAGCACCGTA AAAATGCCAC CGACTAAGCC ACCATGACAC   
  
  
+ GTCATTTTTC ATCTCATTTA GACGGAGACA AGTAGAATCA ACCGTAGAAA GAAAGGACTC AACCTTTCCA   
  
  
+ CTAGTCCATC CACGACCTGG TCCTTGGTTT ACAACGAGAG AAAAGATAAG TACCCTCTCT TGGTTGGCAG   
  
  
+ AGAGTCGAAC GGACTGCGCG TGCTTCTACG GTTTCAACAT AATAAGTAAA GTCACATCGG CATGCACATG   
  
  
+ GACCCGCACA CCTGCACTGC GAGTAAAGTA CTTACTGGGC TATAAAAAAA AAGTAAATAA AATAAATCTC   
  
  
+ ATTTATATTT TATTAAAATT TATTATAAAT AAAAAATAAA ATTTAAAATT TTATAATAAT TTAGTTAATA   
  
  
+ CGTCTTTATT ACAAAATTTA ATTAAATTAT TTTTAAATAA ACAATAATTA AATAAAATTT TTTCAATAAG   
  
  
+ TTCAATTCTA AACATATTAA TTAAAACTA  

- TTCAGCAGCC AATCAACCTT GAAACTTTCA ACCACCCAAT CTTCTCACCT GCCTTTCCTG GGTACTGTTT   
  
  
- ACAGATGAGG CGAGGAAAGA AAGACAGAGA AGGCACGAAG GACGGCGCTG ACTTTTTGGA AGAAGAGAAA   
  
  
- AATATCGGCA ACCGAGAGAG AAGGAGAAAA GTATAAACTA ATCAGACATG GCAGGAGGAG AGAGAGAGAG   
  
  
- AGGAAAATGG CAGTGCACTC ACTGACTTGG TAATTGTTAA TTATTTATAC ATTCAAGAAA AGATTTGGAA   
  
  
- AGAGGAGAAT AGCCAGAGAG CTTTTCCTTC ATGGAAGTTG GGGGTAATGG CCTTCGTGAT TTCTCTGAAA   
  
  
- ATGTCTCTTT TGCTTTTTCT TTGACTTTGT GAAAATGTCA AAAGTAGGGC CAAGCTAATG TACCCCGTAT   
  
  
- TGTTCCTCTG CTTGCAAAGT AGCCAACACG CTTTATTCTA CTACGCCTCT ACTCGAAATA TTAGTGGGTG   
  
  
- CTTGTGTGTC TAGTGCTGTG ACTTTTGTGA ACCTCCGAAA TATCACTCAC GCGCTTCCCA CCGGCGTGTA   
  
  
- ACTTCCTCCG GCAGGCAGCA CTTACGCTCC CCTGAAGAGC GTGGGGATGT TCCTTTTAGT ATATTCAAGT   
  
  
- TGCTCTGACG CAACCACCGT GGAGGCGTGG ACCTGGACGA ACTAAGATAC TCGTTCAGTC ACCCGCGCGT   
  
  
- GCGCCATTCT GTCGCACGTT GAGATCTTGG TCAGTGCGAA TTCAATGTAA AAGCTGTCCT TTGGCACGCA   
  
  
- CCAGGAGCAG GCTTCCATGG CTGTGCCGTT TGGTGACAGA CAAATTCTGA TCGTACGATC AAACCCACGT   
  
  
- GGCCCAAAAC TTTCTGATTC CCTGGGCCCC ACATGACCCG CCATGGAAGT GGGGAAGGGA AAAGGGGAAG   
  
  
- CTGTTCCTCT GTTGATTGCC TCAGATTTGG TGAAGGATCT TGGGCCGCTG AACTTGGGTT GGTATTTTTC   
  
  
- ATATATGAGA TGCCCTTTTC TATGTGGGGA ATCGTGGCAT TTTTACGGTG GCTGATTCGG TGGTACTGTG   
  
  
- CAGTAAAAAG TAGAGTAAAT CTGCCTCTGT TCATCTTAGT TGGCATCTTT CTTTCCTGAG TTGGAAAGGT   
  
  
- GATCAGGTAG GTGCTGGACC AGGAACCAAA TGTTGCTCTC TTTTCTATTC ATGGGAGAGA ACCAACCGTC   
  
  
- TCTCAGCTTG CCTGACGCGC ACGAAGATGC CAAAGTTGTA TTATTCATTT CAGTGTAGCC GTACGTGTAC   
  
  
- CTGGGCGTGT GGACGTGACG CTCATTTCAT GAATGACCCG ATATTTTTTT TTCATTTATT TTATTTAGAG   
  
  
- TAAATATAAA ATAATTTTAA ATAATATTTA TTTTTTATTT TAAATTTTAA AATATTATTA AATCAATTAT   
  
  
- GCAGAAATAA TGTTTTAAAT TAATTTAATA AAAATTTATT TGTTATTAAT TTATTTTAAA AAAGTTATTC   
  
  
- AAGTTAAGAT TTGTATAATT AATTTTGAT

+     box S

| Site Name | Organism | Position | Strand | Matrix score. | sequence | function |
| --- | --- | --- | --- | --- | --- | --- |
| box S | Arabidopsis thaliana | 1037 | + | 7 | AGCCACC |  |

> 2018/04/13 10:10:12  
+ AAGTCGTCGG TTAGTTGGAA CTTTGAAAGT TGGTGGGTTA GAAGAGTGGA CGGAAAGGAC CCATGACAAA   
  
  
+ TGTCTACTCC GCTCCTTTCT TTCTGTCTCT TCCGTGCTTC CTGCCGCGAC TGAAAAACCT TCTTCTCTTT   
  
  
+ TTATAGCCGT TGGCTCTCTC TTCCTCTTTT CATATTTGAT TAGTCTGTAC CGTCCTCCTC TCTCTCTCTC   
  
  
+ TCCTTTTACC GTCACGTGAG TGACTGAACC ATTAACAATT AATAAATATG TAAGTTCTTT TCTAAACCTT   
  
  
+ TCTCCTCTTA TCGGTCTCTC GAAAAGGAAG TACCTTCAAC CCCCATTACC GGAAGCACTA AAGAGACTTT   
  
  
+ TACAGAGAAA ACGAAAAAGA AACTGAAACA CTTTTACAGT TTTCATCCCG GTTCGATTAC ATGGGGCATA   
  
  
+ ACAAGGAGAC GAACGTTTCA TCGGTTGTGC GAAATAAGAT GATGCGGAGA TGAGCTTTAT AATCACCCAC   
  
  
+ GAACACACAG ATCACGACAC TGAAAACACT TGGAGGCTTT ATAGTGAGTG CGCGAAGGGT GGCCGCACAT   
  
  
+ TGAAGGAGGC CGTCCGTCGT GAATGCGAGG GGACTTCTCG CACCCCTACA AGGAAAATCA TATAAGTTCA   
  
  
+ ACGAGACTGC GTTGGTGGCA CCTCCGCACC TGGACCTGCT TGATTCTATG AGCAAGTCAG TGGGCGCGCA   
  
  
+ CGCGGTAAGA CAGCGTGCAA CTCTAGAACC AGTCACGCTT AAGTTACATT TTCGACAGGA AACCGTGCGT   
  
  
+ GGTCCTCGTC CGAAGGTACC GACACGGCAA ACCACTGTCT GTTTAAGACT AGCATGCTAG TTTGGGTGCA   
  
  
+ CCGGGTTTTG AAAGACTAAG GGACCCGGGG TGTACTGGGC GGTACCTTCA CCCCTTCCCT TTTCCCCTTC   
  
  
+ GACAAGGAGA CAACTAACGG AGTCTAAACC ACTTCCTAGA ACCCGGCGAC TTGAACCCAA CCATAAAAAG   
  
  
+ TATATACTCT ACGGGAAAAG ATACACCCCT TAGCACCGTA AAAATGCCAC CGACTAAGCC ACCATGACAC   
  
  
+ GTCATTTTTC ATCTCATTTA GACGGAGACA AGTAGAATCA ACCGTAGAAA GAAAGGACTC AACCTTTCCA   
  
  
+ CTAGTCCATC CACGACCTGG TCCTTGGTTT ACAACGAGAG AAAAGATAAG TACCCTCTCT TGGTTGGCAG   
  
  
+ AGAGTCGAAC GGACTGCGCG TGCTTCTACG GTTTCAACAT AATAAGTAAA GTCACATCGG CATGCACATG   
  
  
+ GACCCGCACA CCTGCACTGC GAGTAAAGTA CTTACTGGGC TATAAAAAAA AAGTAAATAA AATAAATCTC   
  
  
+ ATTTATATTT TATTAAAATT TATTATAAAT AAAAAATAAA ATTTAAAATT TTATAATAAT TTAGTTAATA   
  
  
+ CGTCTTTATT ACAAAATTTA ATTAAATTAT TTTTAAATAA ACAATAATTA AATAAAATTT TTTCAATAAG   
  
  
+ TTCAATTCTA AACATATTAA TTAAAACTA  

- TTCAGCAGCC AATCAACCTT GAAACTTTCA ACCACCCAAT CTTCTCACCT GCCTTTCCTG GGTACTGTTT   
  
  
- ACAGATGAGG CGAGGAAAGA AAGACAGAGA AGGCACGAAG GACGGCGCTG ACTTTTTGGA AGAAGAGAAA   
  
  
- AATATCGGCA ACCGAGAGAG AAGGAGAAAA GTATAAACTA ATCAGACATG GCAGGAGGAG AGAGAGAGAG   
  
  
- AGGAAAATGG CAGTGCACTC ACTGACTTGG TAATTGTTAA TTATTTATAC ATTCAAGAAA AGATTTGGAA   
  
  
- AGAGGAGAAT AGCCAGAGAG CTTTTCCTTC ATGGAAGTTG GGGGTAATGG CCTTCGTGAT TTCTCTGAAA   
  
  
- ATGTCTCTTT TGCTTTTTCT TTGACTTTGT GAAAATGTCA AAAGTAGGGC CAAGCTAATG TACCCCGTAT   
  
  
- TGTTCCTCTG CTTGCAAAGT AGCCAACACG CTTTATTCTA CTACGCCTCT ACTCGAAATA TTAGTGGGTG   
  
  
- CTTGTGTGTC TAGTGCTGTG ACTTTTGTGA ACCTCCGAAA TATCACTCAC GCGCTTCCCA CCGGCGTGTA   
  
  
- ACTTCCTCCG GCAGGCAGCA CTTACGCTCC CCTGAAGAGC GTGGGGATGT TCCTTTTAGT ATATTCAAGT   
  
  
- TGCTCTGACG CAACCACCGT GGAGGCGTGG ACCTGGACGA ACTAAGATAC TCGTTCAGTC ACCCGCGCGT   
  
  
- GCGCCATTCT GTCGCACGTT GAGATCTTGG TCAGTGCGAA TTCAATGTAA AAGCTGTCCT TTGGCACGCA   
  
  
- CCAGGAGCAG GCTTCCATGG CTGTGCCGTT TGGTGACAGA CAAATTCTGA TCGTACGATC AAACCCACGT   
  
  
- GGCCCAAAAC TTTCTGATTC CCTGGGCCCC ACATGACCCG CCATGGAAGT GGGGAAGGGA AAAGGGGAAG   
  
  
- CTGTTCCTCT GTTGATTGCC TCAGATTTGG TGAAGGATCT TGGGCCGCTG AACTTGGGTT GGTATTTTTC   
  
  
- ATATATGAGA TGCCCTTTTC TATGTGGGGA ATCGTGGCAT TTTTACGGTG GCTGATTCGG TGGTACTGTG   
  
  
- CAGTAAAAAG TAGAGTAAAT CTGCCTCTGT TCATCTTAGT TGGCATCTTT CTTTCCTGAG TTGGAAAGGT   
  
  
- GATCAGGTAG GTGCTGGACC AGGAACCAAA TGTTGCTCTC TTTTCTATTC ATGGGAGAGA ACCAACCGTC   
  
  
- TCTCAGCTTG CCTGACGCGC ACGAAGATGC CAAAGTTGTA TTATTCATTT CAGTGTAGCC GTACGTGTAC   
  
  
- CTGGGCGTGT GGACGTGACG CTCATTTCAT GAATGACCCG ATATTTTTTT TTCATTTATT TTATTTAGAG   
  
  
- TAAATATAAA ATAATTTTAA ATAATATTTA TTTTTTATTT TAAATTTTAA AATATTATTA AATCAATTAT   
  
  
- GCAGAAATAA TGTTTTAAAT TAATTTAATA AAAATTTATT TGTTATTAAT TTATTTTAAA AAAGTTATTC   
  
  
- AAGTTAAGAT TTGTATAATT AATTTTGAT
