# Supplementary material for: Genome-wide analysis of the WRKY gene family in drumstick (Moringa oleifera Lam.)
Source: PeerJ. 2019 Jun 10;7:e7063. doi: 10.7717/peerj.7063 (PMC6563795; doi:10.7717/peerj.7063)
Supplement: Supplemental Information 1 [file peerj-07-7063-s003.gz › MoWRKY47_plantcare.html]

Content-Type: text/html; charset=ISO-8859-1


CallMat\_Firefox


Webmaster Firefox specific output  
To save the result:
click on the frame with the right mouse button and save the source code as a text file with extension .html  
REFERENCE:PlantCARE: a database of plant cis-acting regulatory elements and a portal to tools for in silico analysis of promoter sequences.  
Lescot, M., Déhais, P., Moreau, Y., De Moor, B., Rouzé ,P.,and Rombauts, S.  
Nucleic Acids Res., Database issue(2002), 30(1):325-327.   


---

> 2018/04/13 10:10:12  
+ CCGATGGGGT TTCTTCGACG TGCATGATCA CTACGTTCTT GTCCTTCACT TCGTCTCGAT GAGCGAAGTA   
  
  
+ CACCACGTGG TAGACGACCC ACAAGCCGCT GTTTGTTTGC ATCTTCTGTA CGTTTTAAGA ATAGGGAGAG   
  
  
+ AGAGCCTCAA CACTCGAACA CTAGACCCCA CCCAAATACG AAAGAAAAAA CAAAAAACCT AATTCCATTT   
  
  
+ GTTCTTAAAG AAAGCCCATA GCCCTAAACC AACGATCCTA CGTTCTTTCG TTTCTTTTGT CCATACAGTT   
  
  
+ CCAACTTTTA TGACTACAAG GAGAGTTTTT GTTTGTTTGT TCTTGCCAAA CACTTCTCTC TCTCTCTCTC   
  
  
+ TCTCTCTCTC TCTCCCTTCC TCTCAGCGAC TGAAAAGTGT GTTTTGTGGA GAAGAGACAT GGGTTGTACA   
  
  
+ GAACTCTCTC TCTCCTTTCT CTCTCTCCAA ATCATATTTT ATTCTACCAT TCCTATTATT AGTTTCCTTT   
  
  
+ TCTTCTTTCT TCTTCTTCTT CTTCTACCAG TATTTCTTAA TACCGGTCAC TCGACCCACT AAATAGTACA   
  
  
+ TCGTAGTTTC CCAGTTTAAC TACGTTGACA AAAAGAATAA TATTATATAA AAGCATTATA AAATGAGAGA   
  
  
+ GAGAGAGAGA GAGAGAGATA GACTCGAAGT AAAGAAGTTC TCTCTCAATT TTTTTTTTTT TTTTTTGCTG   
  
  
+ GAATCCCCTT CAGTAGGAAG TTTTTGTTCA CTACCCTTTC ATTTCTCCGC CGTTCATCGG CGTAAATAGC   
  
  
+ CTTTTTCAAA GTTGGTCCGC TTGTTGGGGG CTTTGCAAAG CTTCGCCCGT CGAAGGAACG AATACTCTAC   
  
  
+ ACTTGGGGAG TTACACTACG TCTATGGGTG AACCGAAAAA CGAGTAAATT GCTTGCTCCT GTAGCTATAA   
  
  
+ CGTATATGGG TGAACCGAAA AACGAGTAAA TTGCTTGCTC CTGTAGTTAT AACGTATATG GGTGAAGGGG   
  
  
+ TGAAAGTAGG GGTGAGTTCC CCTGTGTATG AGTATATGAA TAGTTTTATA CAAGGAAATT ACATGGCTAA   
  
  
+ GGTATGCCTT GATATTTTTT ATTTATATAC AAACATAATA GTACGTTATT AGTTTATTTT TTCTTTAATT   
  
  
+ TAACAATTGT ATCCATTTAC CGTATAAGTA GCCTTTTCTT ATATTTTTCT TTTTCTTTAT TTGTTCCTAA   
  
  
+ CTCGGAGTGG GTGTACACAT AATTAGTGGT ATGTTCTTAG GTTAAGGTGT TGGGGTATCT AATACTTCCT   
  
  
+ AAGCTGGGGA TTGGTAAACT TATAGTGTGG GTTACACTTC ATTGTTTCGT ATTGCTATTA CGGAACTTTC   
  
  
+ GTATTTCACT GATAACAGCC TTATAAGTCT AACACACCAT TAAGTCATCG AAACCCATGG GTCCATCGAT   
  
  
+ GTTCCTGATT CTTCTTTAAT TGAAACTTCA AAAATATATG TTAAGTACCA TGATGTGTGT AAGTATACCC   
  
  
+ TTCTTTGATA CTTTTGTTCC TTGTTCTTC  

- GGCTACCCCA AAGAAGCTGC ACGTACTAGT GATGCAAGAA CAGGAAGTGA AGCAGAGCTA CTCGCTTCAT   
  
  
- GTGGTGCACC ATCTGCTGGG TGTTCGGCGA CAAACAAACG TAGAAGACAT GCAAAATTCT TATCCCTCTC   
  
  
- TCTCGGAGTT GTGAGCTTGT GATCTGGGGT GGGTTTATGC TTTCTTTTTT GTTTTTTGGA TTAAGGTAAA   
  
  
- CAAGAATTTC TTTCGGGTAT CGGGATTTGG TTGCTAGGAT GCAAGAAAGC AAAGAAAACA GGTATGTCAA   
  
  
- GGTTGAAAAT ACTGATGTTC CTCTCAAAAA CAAACAAACA AGAACGGTTT GTGAAGAGAG AGAGAGAGAG   
  
  
- AGAGAGAGAG AGAGGGAAGG AGAGTCGCTG ACTTTTCACA CAAAACACCT CTTCTCTGTA CCCAACATGT   
  
  
- CTTGAGAGAG AGAGGAAAGA GAGAGAGGTT TAGTATAAAA TAAGATGGTA AGGATAATAA TCAAAGGAAA   
  
  
- AGAAGAAAGA AGAAGAAGAA GAAGATGGTC ATAAAGAATT ATGGCCAGTG AGCTGGGTGA TTTATCATGT   
  
  
- AGCATCAAAG GGTCAAATTG ATGCAACTGT TTTTCTTATT ATAATATATT TTCGTAATAT TTTACTCTCT   
  
  
- CTCTCTCTCT CTCTCTCTAT CTGAGCTTCA TTTCTTCAAG AGAGAGTTAA AAAAAAAAAA AAAAAACGAC   
  
  
- CTTAGGGGAA GTCATCCTTC AAAAACAAGT GATGGGAAAG TAAAGAGGCG GCAAGTAGCC GCATTTATCG   
  
  
- GAAAAAGTTT CAACCAGGCG AACAACCCCC GAAACGTTTC GAAGCGGGCA GCTTCCTTGC TTATGAGATG   
  
  
- TGAACCCCTC AATGTGATGC AGATACCCAC TTGGCTTTTT GCTCATTTAA CGAACGAGGA CATCGATATT   
  
  
- GCATATACCC ACTTGGCTTT TTGCTCATTT AACGAACGAG GACATCAATA TTGCATATAC CCACTTCCCC   
  
  
- ACTTTCATCC CCACTCAAGG GGACACATAC TCATATACTT ATCAAAATAT GTTCCTTTAA TGTACCGATT   
  
  
- CCATACGGAA CTATAAAAAA TAAATATATG TTTGTATTAT CATGCAATAA TCAAATAAAA AAGAAATTAA   
  
  
- ATTGTTAACA TAGGTAAATG GCATATTCAT CGGAAAAGAA TATAAAAAGA AAAAGAAATA AACAAGGATT   
  
  
- GAGCCTCACC CACATGTGTA TTAATCACCA TACAAGAATC CAATTCCACA ACCCCATAGA TTATGAAGGA   
  
  
- TTCGACCCCT AACCATTTGA ATATCACACC CAATGTGAAG TAACAAAGCA TAACGATAAT GCCTTGAAAG   
  
  
- CATAAAGTGA CTATTGTCGG AATATTCAGA TTGTGTGGTA ATTCAGTAGC TTTGGGTACC CAGGTAGCTA   
  
  
- CAAGGACTAA GAAGAAATTA ACTTTGAAGT TTTTATATAC AATTCATGGT ACTACACACA TTCATATGGG   
  
  
- AAGAAACTAT GAAAACAAGG AACAAGAAG

  
  
Motifs Found  

+     5UTR Py-rich stretch

| Site Name | Organism | Position | Strand | Matrix score. | sequence | function |
| --- | --- | --- | --- | --- | --- | --- |
| 5UTR Py-rich stretch | Lycopersicon esculentum | 637 | - | 13 | TTTCTCTCTCTCTC | cis-acting element conferring high transcription levels |
| 5UTR Py-rich stretch | Lycopersicon esculentum | 349 | + | 13 | TTTCTCTCTCTCTC | cis-acting element conferring high transcription levels |
| 5UTR Py-rich stretch | Lycopersicon esculentum | 635 | - | 13 | TTTCTCTCTCTCTC | cis-acting element conferring high transcription levels |
| 5UTR Py-rich stretch | Lycopersicon esculentum | 351 | + | 13 | TTTCTCTCTCTCTC | cis-acting element conferring high transcription levels |
| 5UTR Py-rich stretch | Lycopersicon esculentum | 633 | - | 13 | TTTCTCTCTCTCTC | cis-acting element conferring high transcription levels |
| 5UTR Py-rich stretch | Lycopersicon esculentum | 627 | - | 13 | TTTCTCTCTCTCTC | cis-acting element conferring high transcription levels |
| 5UTR Py-rich stretch | Lycopersicon esculentum | 333 | + | 13 | TTTCTCTCTCTCTC | cis-acting element conferring high transcription levels |
| 5UTR Py-rich stretch | Lycopersicon esculentum | 345 | + | 13 | TTTCTCTCTCTCTC | cis-acting element conferring high transcription levels |
| 5UTR Py-rich stretch | Lycopersicon esculentum | 629 | - | 13 | TTTCTCTCTCTCTC | cis-acting element conferring high transcription levels |
| 5UTR Py-rich stretch | Lycopersicon esculentum | 341 | + | 13 | TTTCTCTCTCTCTC | cis-acting element conferring high transcription levels |
| 5UTR Py-rich stretch | Lycopersicon esculentum | 631 | - | 13 | TTTCTCTCTCTCTC | cis-acting element conferring high transcription levels |
| 5UTR Py-rich stretch | Lycopersicon esculentum | 347 | + | 13 | TTTCTCTCTCTCTC | cis-acting element conferring high transcription levels |
| 5UTR Py-rich stretch | Lycopersicon esculentum | 489 | + | 9 | TTTCTTCTCT | cis-acting element conferring high transcription levels |
| 5UTR Py-rich stretch | Lycopersicon esculentum | 335 | + | 13 | TTTCTCTCTCTCTC | cis-acting element conferring high transcription levels |
| 5UTR Py-rich stretch | Lycopersicon esculentum | 625 | - | 13 | TTTCTCTCTCTCTC | cis-acting element conferring high transcription levels |
| 5UTR Py-rich stretch | Lycopersicon esculentum | 337 | + | 13 | TTTCTCTCTCTCTC | cis-acting element conferring high transcription levels |
| 5UTR Py-rich stretch | Lycopersicon esculentum | 339 | + | 13 | TTTCTCTCTCTCTC | cis-acting element conferring high transcription levels |
| 5UTR Py-rich stretch | Lycopersicon esculentum | 343 | + | 13 | TTTCTCTCTCTCTC | cis-acting element conferring high transcription levels |

> 2018/04/13 10:10:12  
+ CCGATGGGGT TTCTTCGACG TGCATGATCA CTACGTTCTT GTCCTTCACT TCGTCTCGAT GAGCGAAGTA   
  
  
+ CACCACGTGG TAGACGACCC ACAAGCCGCT GTTTGTTTGC ATCTTCTGTA CGTTTTAAGA ATAGGGAGAG   
  
  
+ AGAGCCTCAA CACTCGAACA CTAGACCCCA CCCAAATACG AAAGAAAAAA CAAAAAACCT AATTCCATTT   
  
  
+ GTTCTTAAAG AAAGCCCATA GCCCTAAACC AACGATCCTA CGTTCTTTCG TTTCTTTTGT CCATACAGTT   
  
  
+ CCAACTTTTA TGACTACAAG GAGAGTTTTT GTTTGTTTGT TCTTGCCAAA CACTTCTCTC TCTCTCTCTC   
  
  
+ TCTCTCTCTC TCTCCCTTCC TCTCAGCGAC TGAAAAGTGT GTTTTGTGGA GAAGAGACAT GGGTTGTACA   
  
  
+ GAACTCTCTC TCTCCTTTCT CTCTCTCCAA ATCATATTTT ATTCTACCAT TCCTATTATT AGTTTCCTTT   
  
  
+ TCTTCTTTCT TCTTCTTCTT CTTCTACCAG TATTTCTTAA TACCGGTCAC TCGACCCACT AAATAGTACA   
  
  
+ TCGTAGTTTC CCAGTTTAAC TACGTTGACA AAAAGAATAA TATTATATAA AAGCATTATA AAATGAGAGA   
  
  
+ GAGAGAGAGA GAGAGAGATA GACTCGAAGT AAAGAAGTTC TCTCTCAATT TTTTTTTTTT TTTTTTGCTG   
  
  
+ GAATCCCCTT CAGTAGGAAG TTTTTGTTCA CTACCCTTTC ATTTCTCCGC CGTTCATCGG CGTAAATAGC   
  
  
+ CTTTTTCAAA GTTGGTCCGC TTGTTGGGGG CTTTGCAAAG CTTCGCCCGT CGAAGGAACG AATACTCTAC   
  
  
+ ACTTGGGGAG TTACACTACG TCTATGGGTG AACCGAAAAA CGAGTAAATT GCTTGCTCCT GTAGCTATAA   
  
  
+ CGTATATGGG TGAACCGAAA AACGAGTAAA TTGCTTGCTC CTGTAGTTAT AACGTATATG GGTGAAGGGG   
  
  
+ TGAAAGTAGG GGTGAGTTCC CCTGTGTATG AGTATATGAA TAGTTTTATA CAAGGAAATT ACATGGCTAA   
  
  
+ GGTATGCCTT GATATTTTTT ATTTATATAC AAACATAATA GTACGTTATT AGTTTATTTT TTCTTTAATT   
  
  
+ TAACAATTGT ATCCATTTAC CGTATAAGTA GCCTTTTCTT ATATTTTTCT TTTTCTTTAT TTGTTCCTAA   
  
  
+ CTCGGAGTGG GTGTACACAT AATTAGTGGT ATGTTCTTAG GTTAAGGTGT TGGGGTATCT AATACTTCCT   
  
  
+ AAGCTGGGGA TTGGTAAACT TATAGTGTGG GTTACACTTC ATTGTTTCGT ATTGCTATTA CGGAACTTTC   
  
  
+ GTATTTCACT GATAACAGCC TTATAAGTCT AACACACCAT TAAGTCATCG AAACCCATGG GTCCATCGAT   
  
  
+ GTTCCTGATT CTTCTTTAAT TGAAACTTCA AAAATATATG TTAAGTACCA TGATGTGTGT AAGTATACCC   
  
  
+ TTCTTTGATA CTTTTGTTCC TTGTTCTTC  

- GGCTACCCCA AAGAAGCTGC ACGTACTAGT GATGCAAGAA CAGGAAGTGA AGCAGAGCTA CTCGCTTCAT   
  
  
- GTGGTGCACC ATCTGCTGGG TGTTCGGCGA CAAACAAACG TAGAAGACAT GCAAAATTCT TATCCCTCTC   
  
  
- TCTCGGAGTT GTGAGCTTGT GATCTGGGGT GGGTTTATGC TTTCTTTTTT GTTTTTTGGA TTAAGGTAAA   
  
  
- CAAGAATTTC TTTCGGGTAT CGGGATTTGG TTGCTAGGAT GCAAGAAAGC AAAGAAAACA GGTATGTCAA   
  
  
- GGTTGAAAAT ACTGATGTTC CTCTCAAAAA CAAACAAACA AGAACGGTTT GTGAAGAGAG AGAGAGAGAG   
  
  
- AGAGAGAGAG AGAGGGAAGG AGAGTCGCTG ACTTTTCACA CAAAACACCT CTTCTCTGTA CCCAACATGT   
  
  
- CTTGAGAGAG AGAGGAAAGA GAGAGAGGTT TAGTATAAAA TAAGATGGTA AGGATAATAA TCAAAGGAAA   
  
  
- AGAAGAAAGA AGAAGAAGAA GAAGATGGTC ATAAAGAATT ATGGCCAGTG AGCTGGGTGA TTTATCATGT   
  
  
- AGCATCAAAG GGTCAAATTG ATGCAACTGT TTTTCTTATT ATAATATATT TTCGTAATAT TTTACTCTCT   
  
  
- CTCTCTCTCT CTCTCTCTAT CTGAGCTTCA TTTCTTCAAG AGAGAGTTAA AAAAAAAAAA AAAAAACGAC   
  
  
- CTTAGGGGAA GTCATCCTTC AAAAACAAGT GATGGGAAAG TAAAGAGGCG GCAAGTAGCC GCATTTATCG   
  
  
- GAAAAAGTTT CAACCAGGCG AACAACCCCC GAAACGTTTC GAAGCGGGCA GCTTCCTTGC TTATGAGATG   
  
  
- TGAACCCCTC AATGTGATGC AGATACCCAC TTGGCTTTTT GCTCATTTAA CGAACGAGGA CATCGATATT   
  
  
- GCATATACCC ACTTGGCTTT TTGCTCATTT AACGAACGAG GACATCAATA TTGCATATAC CCACTTCCCC   
  
  
- ACTTTCATCC CCACTCAAGG GGACACATAC TCATATACTT ATCAAAATAT GTTCCTTTAA TGTACCGATT   
  
  
- CCATACGGAA CTATAAAAAA TAAATATATG TTTGTATTAT CATGCAATAA TCAAATAAAA AAGAAATTAA   
  
  
- ATTGTTAACA TAGGTAAATG GCATATTCAT CGGAAAAGAA TATAAAAAGA AAAAGAAATA AACAAGGATT   
  
  
- GAGCCTCACC CACATGTGTA TTAATCACCA TACAAGAATC CAATTCCACA ACCCCATAGA TTATGAAGGA   
  
  
- TTCGACCCCT AACCATTTGA ATATCACACC CAATGTGAAG TAACAAAGCA TAACGATAAT GCCTTGAAAG   
  
  
- CATAAAGTGA CTATTGTCGG AATATTCAGA TTGTGTGGTA ATTCAGTAGC TTTGGGTACC CAGGTAGCTA   
  
  
- CAAGGACTAA GAAGAAATTA ACTTTGAAGT TTTTATATAC AATTCATGGT ACTACACACA TTCATATGGG   
  
  
- AAGAAACTAT GAAAACAAGG AACAAGAAG

+     AAGAA-motif

| Site Name | Organism | Position | Strand | Matrix score. | sequence | function |
| --- | --- | --- | --- | --- | --- | --- |
| AAGAA-motif | Avena sativa | 253 | - | 7 | GAAAGAA |  |
| AAGAA-motif | Avena sativa | 493 | - | 7 | GAAAGAA |  |
| AAGAA-motif | Avena sativa | 180 | + | 7 | GAAAGAA |  |

> 2018/04/13 10:10:12  
+ CCGATGGGGT TTCTTCGACG TGCATGATCA CTACGTTCTT GTCCTTCACT TCGTCTCGAT GAGCGAAGTA   
  
  
+ CACCACGTGG TAGACGACCC ACAAGCCGCT GTTTGTTTGC ATCTTCTGTA CGTTTTAAGA ATAGGGAGAG   
  
  
+ AGAGCCTCAA CACTCGAACA CTAGACCCCA CCCAAATACG AAAGAAAAAA CAAAAAACCT AATTCCATTT   
  
  
+ GTTCTTAAAG AAAGCCCATA GCCCTAAACC AACGATCCTA CGTTCTTTCG TTTCTTTTGT CCATACAGTT   
  
  
+ CCAACTTTTA TGACTACAAG GAGAGTTTTT GTTTGTTTGT TCTTGCCAAA CACTTCTCTC TCTCTCTCTC   
  
  
+ TCTCTCTCTC TCTCCCTTCC TCTCAGCGAC TGAAAAGTGT GTTTTGTGGA GAAGAGACAT GGGTTGTACA   
  
  
+ GAACTCTCTC TCTCCTTTCT CTCTCTCCAA ATCATATTTT ATTCTACCAT TCCTATTATT AGTTTCCTTT   
  
  
+ TCTTCTTTCT TCTTCTTCTT CTTCTACCAG TATTTCTTAA TACCGGTCAC TCGACCCACT AAATAGTACA   
  
  
+ TCGTAGTTTC CCAGTTTAAC TACGTTGACA AAAAGAATAA TATTATATAA AAGCATTATA AAATGAGAGA   
  
  
+ GAGAGAGAGA GAGAGAGATA GACTCGAAGT AAAGAAGTTC TCTCTCAATT TTTTTTTTTT TTTTTTGCTG   
  
  
+ GAATCCCCTT CAGTAGGAAG TTTTTGTTCA CTACCCTTTC ATTTCTCCGC CGTTCATCGG CGTAAATAGC   
  
  
+ CTTTTTCAAA GTTGGTCCGC TTGTTGGGGG CTTTGCAAAG CTTCGCCCGT CGAAGGAACG AATACTCTAC   
  
  
+ ACTTGGGGAG TTACACTACG TCTATGGGTG AACCGAAAAA CGAGTAAATT GCTTGCTCCT GTAGCTATAA   
  
  
+ CGTATATGGG TGAACCGAAA AACGAGTAAA TTGCTTGCTC CTGTAGTTAT AACGTATATG GGTGAAGGGG   
  
  
+ TGAAAGTAGG GGTGAGTTCC CCTGTGTATG AGTATATGAA TAGTTTTATA CAAGGAAATT ACATGGCTAA   
  
  
+ GGTATGCCTT GATATTTTTT ATTTATATAC AAACATAATA GTACGTTATT AGTTTATTTT TTCTTTAATT   
  
  
+ TAACAATTGT ATCCATTTAC CGTATAAGTA GCCTTTTCTT ATATTTTTCT TTTTCTTTAT TTGTTCCTAA   
  
  
+ CTCGGAGTGG GTGTACACAT AATTAGTGGT ATGTTCTTAG GTTAAGGTGT TGGGGTATCT AATACTTCCT   
  
  
+ AAGCTGGGGA TTGGTAAACT TATAGTGTGG GTTACACTTC ATTGTTTCGT ATTGCTATTA CGGAACTTTC   
  
  
+ GTATTTCACT GATAACAGCC TTATAAGTCT AACACACCAT TAAGTCATCG AAACCCATGG GTCCATCGAT   
  
  
+ GTTCCTGATT CTTCTTTAAT TGAAACTTCA AAAATATATG TTAAGTACCA TGATGTGTGT AAGTATACCC   
  
  
+ TTCTTTGATA CTTTTGTTCC TTGTTCTTC  

- GGCTACCCCA AAGAAGCTGC ACGTACTAGT GATGCAAGAA CAGGAAGTGA AGCAGAGCTA CTCGCTTCAT   
  
  
- GTGGTGCACC ATCTGCTGGG TGTTCGGCGA CAAACAAACG TAGAAGACAT GCAAAATTCT TATCCCTCTC   
  
  
- TCTCGGAGTT GTGAGCTTGT GATCTGGGGT GGGTTTATGC TTTCTTTTTT GTTTTTTGGA TTAAGGTAAA   
  
  
- CAAGAATTTC TTTCGGGTAT CGGGATTTGG TTGCTAGGAT GCAAGAAAGC AAAGAAAACA GGTATGTCAA   
  
  
- GGTTGAAAAT ACTGATGTTC CTCTCAAAAA CAAACAAACA AGAACGGTTT GTGAAGAGAG AGAGAGAGAG   
  
  
- AGAGAGAGAG AGAGGGAAGG AGAGTCGCTG ACTTTTCACA CAAAACACCT CTTCTCTGTA CCCAACATGT   
  
  
- CTTGAGAGAG AGAGGAAAGA GAGAGAGGTT TAGTATAAAA TAAGATGGTA AGGATAATAA TCAAAGGAAA   
  
  
- AGAAGAAAGA AGAAGAAGAA GAAGATGGTC ATAAAGAATT ATGGCCAGTG AGCTGGGTGA TTTATCATGT   
  
  
- AGCATCAAAG GGTCAAATTG ATGCAACTGT TTTTCTTATT ATAATATATT TTCGTAATAT TTTACTCTCT   
  
  
- CTCTCTCTCT CTCTCTCTAT CTGAGCTTCA TTTCTTCAAG AGAGAGTTAA AAAAAAAAAA AAAAAACGAC   
  
  
- CTTAGGGGAA GTCATCCTTC AAAAACAAGT GATGGGAAAG TAAAGAGGCG GCAAGTAGCC GCATTTATCG   
  
  
- GAAAAAGTTT CAACCAGGCG AACAACCCCC GAAACGTTTC GAAGCGGGCA GCTTCCTTGC TTATGAGATG   
  
  
- TGAACCCCTC AATGTGATGC AGATACCCAC TTGGCTTTTT GCTCATTTAA CGAACGAGGA CATCGATATT   
  
  
- GCATATACCC ACTTGGCTTT TTGCTCATTT AACGAACGAG GACATCAATA TTGCATATAC CCACTTCCCC   
  
  
- ACTTTCATCC CCACTCAAGG GGACACATAC TCATATACTT ATCAAAATAT GTTCCTTTAA TGTACCGATT   
  
  
- CCATACGGAA CTATAAAAAA TAAATATATG TTTGTATTAT CATGCAATAA TCAAATAAAA AAGAAATTAA   
  
  
- ATTGTTAACA TAGGTAAATG GCATATTCAT CGGAAAAGAA TATAAAAAGA AAAAGAAATA AACAAGGATT   
  
  
- GAGCCTCACC CACATGTGTA TTAATCACCA TACAAGAATC CAATTCCACA ACCCCATAGA TTATGAAGGA   
  
  
- TTCGACCCCT AACCATTTGA ATATCACACC CAATGTGAAG TAACAAAGCA TAACGATAAT GCCTTGAAAG   
  
  
- CATAAAGTGA CTATTGTCGG AATATTCAGA TTGTGTGGTA ATTCAGTAGC TTTGGGTACC CAGGTAGCTA   
  
  
- CAAGGACTAA GAAGAAATTA ACTTTGAAGT TTTTATATAC AATTCATGGT ACTACACACA TTCATATGGG   
  
  
- AAGAAACTAT GAAAACAAGG AACAAGAAG

+     ABRE

| Site Name | Organism | Position | Strand | Matrix score. | sequence | function |
| --- | --- | --- | --- | --- | --- | --- |
| ABRE | Arabidopsis thaliana | 74 | + | 6 | CACGTG | cis-acting element involved in the abscisic acid responsiveness |

> 2018/04/13 10:10:12  
+ CCGATGGGGT TTCTTCGACG TGCATGATCA CTACGTTCTT GTCCTTCACT TCGTCTCGAT GAGCGAAGTA   
  
  
+ CACCACGTGG TAGACGACCC ACAAGCCGCT GTTTGTTTGC ATCTTCTGTA CGTTTTAAGA ATAGGGAGAG   
  
  
+ AGAGCCTCAA CACTCGAACA CTAGACCCCA CCCAAATACG AAAGAAAAAA CAAAAAACCT AATTCCATTT   
  
  
+ GTTCTTAAAG AAAGCCCATA GCCCTAAACC AACGATCCTA CGTTCTTTCG TTTCTTTTGT CCATACAGTT   
  
  
+ CCAACTTTTA TGACTACAAG GAGAGTTTTT GTTTGTTTGT TCTTGCCAAA CACTTCTCTC TCTCTCTCTC   
  
  
+ TCTCTCTCTC TCTCCCTTCC TCTCAGCGAC TGAAAAGTGT GTTTTGTGGA GAAGAGACAT GGGTTGTACA   
  
  
+ GAACTCTCTC TCTCCTTTCT CTCTCTCCAA ATCATATTTT ATTCTACCAT TCCTATTATT AGTTTCCTTT   
  
  
+ TCTTCTTTCT TCTTCTTCTT CTTCTACCAG TATTTCTTAA TACCGGTCAC TCGACCCACT AAATAGTACA   
  
  
+ TCGTAGTTTC CCAGTTTAAC TACGTTGACA AAAAGAATAA TATTATATAA AAGCATTATA AAATGAGAGA   
  
  
+ GAGAGAGAGA GAGAGAGATA GACTCGAAGT AAAGAAGTTC TCTCTCAATT TTTTTTTTTT TTTTTTGCTG   
  
  
+ GAATCCCCTT CAGTAGGAAG TTTTTGTTCA CTACCCTTTC ATTTCTCCGC CGTTCATCGG CGTAAATAGC   
  
  
+ CTTTTTCAAA GTTGGTCCGC TTGTTGGGGG CTTTGCAAAG CTTCGCCCGT CGAAGGAACG AATACTCTAC   
  
  
+ ACTTGGGGAG TTACACTACG TCTATGGGTG AACCGAAAAA CGAGTAAATT GCTTGCTCCT GTAGCTATAA   
  
  
+ CGTATATGGG TGAACCGAAA AACGAGTAAA TTGCTTGCTC CTGTAGTTAT AACGTATATG GGTGAAGGGG   
  
  
+ TGAAAGTAGG GGTGAGTTCC CCTGTGTATG AGTATATGAA TAGTTTTATA CAAGGAAATT ACATGGCTAA   
  
  
+ GGTATGCCTT GATATTTTTT ATTTATATAC AAACATAATA GTACGTTATT AGTTTATTTT TTCTTTAATT   
  
  
+ TAACAATTGT ATCCATTTAC CGTATAAGTA GCCTTTTCTT ATATTTTTCT TTTTCTTTAT TTGTTCCTAA   
  
  
+ CTCGGAGTGG GTGTACACAT AATTAGTGGT ATGTTCTTAG GTTAAGGTGT TGGGGTATCT AATACTTCCT   
  
  
+ AAGCTGGGGA TTGGTAAACT TATAGTGTGG GTTACACTTC ATTGTTTCGT ATTGCTATTA CGGAACTTTC   
  
  
+ GTATTTCACT GATAACAGCC TTATAAGTCT AACACACCAT TAAGTCATCG AAACCCATGG GTCCATCGAT   
  
  
+ GTTCCTGATT CTTCTTTAAT TGAAACTTCA AAAATATATG TTAAGTACCA TGATGTGTGT AAGTATACCC   
  
  
+ TTCTTTGATA CTTTTGTTCC TTGTTCTTC  

- GGCTACCCCA AAGAAGCTGC ACGTACTAGT GATGCAAGAA CAGGAAGTGA AGCAGAGCTA CTCGCTTCAT   
  
  
- GTGGTGCACC ATCTGCTGGG TGTTCGGCGA CAAACAAACG TAGAAGACAT GCAAAATTCT TATCCCTCTC   
  
  
- TCTCGGAGTT GTGAGCTTGT GATCTGGGGT GGGTTTATGC TTTCTTTTTT GTTTTTTGGA TTAAGGTAAA   
  
  
- CAAGAATTTC TTTCGGGTAT CGGGATTTGG TTGCTAGGAT GCAAGAAAGC AAAGAAAACA GGTATGTCAA   
  
  
- GGTTGAAAAT ACTGATGTTC CTCTCAAAAA CAAACAAACA AGAACGGTTT GTGAAGAGAG AGAGAGAGAG   
  
  
- AGAGAGAGAG AGAGGGAAGG AGAGTCGCTG ACTTTTCACA CAAAACACCT CTTCTCTGTA CCCAACATGT   
  
  
- CTTGAGAGAG AGAGGAAAGA GAGAGAGGTT TAGTATAAAA TAAGATGGTA AGGATAATAA TCAAAGGAAA   
  
  
- AGAAGAAAGA AGAAGAAGAA GAAGATGGTC ATAAAGAATT ATGGCCAGTG AGCTGGGTGA TTTATCATGT   
  
  
- AGCATCAAAG GGTCAAATTG ATGCAACTGT TTTTCTTATT ATAATATATT TTCGTAATAT TTTACTCTCT   
  
  
- CTCTCTCTCT CTCTCTCTAT CTGAGCTTCA TTTCTTCAAG AGAGAGTTAA AAAAAAAAAA AAAAAACGAC   
  
  
- CTTAGGGGAA GTCATCCTTC AAAAACAAGT GATGGGAAAG TAAAGAGGCG GCAAGTAGCC GCATTTATCG   
  
  
- GAAAAAGTTT CAACCAGGCG AACAACCCCC GAAACGTTTC GAAGCGGGCA GCTTCCTTGC TTATGAGATG   
  
  
- TGAACCCCTC AATGTGATGC AGATACCCAC TTGGCTTTTT GCTCATTTAA CGAACGAGGA CATCGATATT   
  
  
- GCATATACCC ACTTGGCTTT TTGCTCATTT AACGAACGAG GACATCAATA TTGCATATAC CCACTTCCCC   
  
  
- ACTTTCATCC CCACTCAAGG GGACACATAC TCATATACTT ATCAAAATAT GTTCCTTTAA TGTACCGATT   
  
  
- CCATACGGAA CTATAAAAAA TAAATATATG TTTGTATTAT CATGCAATAA TCAAATAAAA AAGAAATTAA   
  
  
- ATTGTTAACA TAGGTAAATG GCATATTCAT CGGAAAAGAA TATAAAAAGA AAAAGAAATA AACAAGGATT   
  
  
- GAGCCTCACC CACATGTGTA TTAATCACCA TACAAGAATC CAATTCCACA ACCCCATAGA TTATGAAGGA   
  
  
- TTCGACCCCT AACCATTTGA ATATCACACC CAATGTGAAG TAACAAAGCA TAACGATAAT GCCTTGAAAG   
  
  
- CATAAAGTGA CTATTGTCGG AATATTCAGA TTGTGTGGTA ATTCAGTAGC TTTGGGTACC CAGGTAGCTA   
  
  
- CAAGGACTAA GAAGAAATTA ACTTTGAAGT TTTTATATAC AATTCATGGT ACTACACACA TTCATATGGG   
  
  
- AAGAAACTAT GAAAACAAGG AACAAGAAG

+     ACE

| Site Name | Organism | Position | Strand | Matrix score. | sequence | function |
| --- | --- | --- | --- | --- | --- | --- |
| ACE | Petroselinum crispum | 579 | + | 9 | ACTACGTTGG | cis-acting element involved in light responsiveness |

> 2018/04/13 10:10:12  
+ CCGATGGGGT TTCTTCGACG TGCATGATCA CTACGTTCTT GTCCTTCACT TCGTCTCGAT GAGCGAAGTA   
  
  
+ CACCACGTGG TAGACGACCC ACAAGCCGCT GTTTGTTTGC ATCTTCTGTA CGTTTTAAGA ATAGGGAGAG   
  
  
+ AGAGCCTCAA CACTCGAACA CTAGACCCCA CCCAAATACG AAAGAAAAAA CAAAAAACCT AATTCCATTT   
  
  
+ GTTCTTAAAG AAAGCCCATA GCCCTAAACC AACGATCCTA CGTTCTTTCG TTTCTTTTGT CCATACAGTT   
  
  
+ CCAACTTTTA TGACTACAAG GAGAGTTTTT GTTTGTTTGT TCTTGCCAAA CACTTCTCTC TCTCTCTCTC   
  
  
+ TCTCTCTCTC TCTCCCTTCC TCTCAGCGAC TGAAAAGTGT GTTTTGTGGA GAAGAGACAT GGGTTGTACA   
  
  
+ GAACTCTCTC TCTCCTTTCT CTCTCTCCAA ATCATATTTT ATTCTACCAT TCCTATTATT AGTTTCCTTT   
  
  
+ TCTTCTTTCT TCTTCTTCTT CTTCTACCAG TATTTCTTAA TACCGGTCAC TCGACCCACT AAATAGTACA   
  
  
+ TCGTAGTTTC CCAGTTTAAC TACGTTGACA AAAAGAATAA TATTATATAA AAGCATTATA AAATGAGAGA   
  
  
+ GAGAGAGAGA GAGAGAGATA GACTCGAAGT AAAGAAGTTC TCTCTCAATT TTTTTTTTTT TTTTTTGCTG   
  
  
+ GAATCCCCTT CAGTAGGAAG TTTTTGTTCA CTACCCTTTC ATTTCTCCGC CGTTCATCGG CGTAAATAGC   
  
  
+ CTTTTTCAAA GTTGGTCCGC TTGTTGGGGG CTTTGCAAAG CTTCGCCCGT CGAAGGAACG AATACTCTAC   
  
  
+ ACTTGGGGAG TTACACTACG TCTATGGGTG AACCGAAAAA CGAGTAAATT GCTTGCTCCT GTAGCTATAA   
  
  
+ CGTATATGGG TGAACCGAAA AACGAGTAAA TTGCTTGCTC CTGTAGTTAT AACGTATATG GGTGAAGGGG   
  
  
+ TGAAAGTAGG GGTGAGTTCC CCTGTGTATG AGTATATGAA TAGTTTTATA CAAGGAAATT ACATGGCTAA   
  
  
+ GGTATGCCTT GATATTTTTT ATTTATATAC AAACATAATA GTACGTTATT AGTTTATTTT TTCTTTAATT   
  
  
+ TAACAATTGT ATCCATTTAC CGTATAAGTA GCCTTTTCTT ATATTTTTCT TTTTCTTTAT TTGTTCCTAA   
  
  
+ CTCGGAGTGG GTGTACACAT AATTAGTGGT ATGTTCTTAG GTTAAGGTGT TGGGGTATCT AATACTTCCT   
  
  
+ AAGCTGGGGA TTGGTAAACT TATAGTGTGG GTTACACTTC ATTGTTTCGT ATTGCTATTA CGGAACTTTC   
  
  
+ GTATTTCACT GATAACAGCC TTATAAGTCT AACACACCAT TAAGTCATCG AAACCCATGG GTCCATCGAT   
  
  
+ GTTCCTGATT CTTCTTTAAT TGAAACTTCA AAAATATATG TTAAGTACCA TGATGTGTGT AAGTATACCC   
  
  
+ TTCTTTGATA CTTTTGTTCC TTGTTCTTC  

- GGCTACCCCA AAGAAGCTGC ACGTACTAGT GATGCAAGAA CAGGAAGTGA AGCAGAGCTA CTCGCTTCAT   
  
  
- GTGGTGCACC ATCTGCTGGG TGTTCGGCGA CAAACAAACG TAGAAGACAT GCAAAATTCT TATCCCTCTC   
  
  
- TCTCGGAGTT GTGAGCTTGT GATCTGGGGT GGGTTTATGC TTTCTTTTTT GTTTTTTGGA TTAAGGTAAA   
  
  
- CAAGAATTTC TTTCGGGTAT CGGGATTTGG TTGCTAGGAT GCAAGAAAGC AAAGAAAACA GGTATGTCAA   
  
  
- GGTTGAAAAT ACTGATGTTC CTCTCAAAAA CAAACAAACA AGAACGGTTT GTGAAGAGAG AGAGAGAGAG   
  
  
- AGAGAGAGAG AGAGGGAAGG AGAGTCGCTG ACTTTTCACA CAAAACACCT CTTCTCTGTA CCCAACATGT   
  
  
- CTTGAGAGAG AGAGGAAAGA GAGAGAGGTT TAGTATAAAA TAAGATGGTA AGGATAATAA TCAAAGGAAA   
  
  
- AGAAGAAAGA AGAAGAAGAA GAAGATGGTC ATAAAGAATT ATGGCCAGTG AGCTGGGTGA TTTATCATGT   
  
  
- AGCATCAAAG GGTCAAATTG ATGCAACTGT TTTTCTTATT ATAATATATT TTCGTAATAT TTTACTCTCT   
  
  
- CTCTCTCTCT CTCTCTCTAT CTGAGCTTCA TTTCTTCAAG AGAGAGTTAA AAAAAAAAAA AAAAAACGAC   
  
  
- CTTAGGGGAA GTCATCCTTC AAAAACAAGT GATGGGAAAG TAAAGAGGCG GCAAGTAGCC GCATTTATCG   
  
  
- GAAAAAGTTT CAACCAGGCG AACAACCCCC GAAACGTTTC GAAGCGGGCA GCTTCCTTGC TTATGAGATG   
  
  
- TGAACCCCTC AATGTGATGC AGATACCCAC TTGGCTTTTT GCTCATTTAA CGAACGAGGA CATCGATATT   
  
  
- GCATATACCC ACTTGGCTTT TTGCTCATTT AACGAACGAG GACATCAATA TTGCATATAC CCACTTCCCC   
  
  
- ACTTTCATCC CCACTCAAGG GGACACATAC TCATATACTT ATCAAAATAT GTTCCTTTAA TGTACCGATT   
  
  
- CCATACGGAA CTATAAAAAA TAAATATATG TTTGTATTAT CATGCAATAA TCAAATAAAA AAGAAATTAA   
  
  
- ATTGTTAACA TAGGTAAATG GCATATTCAT CGGAAAAGAA TATAAAAAGA AAAAGAAATA AACAAGGATT   
  
  
- GAGCCTCACC CACATGTGTA TTAATCACCA TACAAGAATC CAATTCCACA ACCCCATAGA TTATGAAGGA   
  
  
- TTCGACCCCT AACCATTTGA ATATCACACC CAATGTGAAG TAACAAAGCA TAACGATAAT GCCTTGAAAG   
  
  
- CATAAAGTGA CTATTGTCGG AATATTCAGA TTGTGTGGTA ATTCAGTAGC TTTGGGTACC CAGGTAGCTA   
  
  
- CAAGGACTAA GAAGAAATTA ACTTTGAAGT TTTTATATAC AATTCATGGT ACTACACACA TTCATATGGG   
  
  
- AAGAAACTAT GAAAACAAGG AACAAGAAG

+     ARE

| Site Name | Organism | Position | Strand | Matrix score. | sequence | function |
| --- | --- | --- | --- | --- | --- | --- |
| ARE | Zea mays | 236 | - | 6 | TGGTTT | cis-acting regulatory element essential for the anaerobic induction |

> 2018/04/13 10:10:12  
+ CCGATGGGGT TTCTTCGACG TGCATGATCA CTACGTTCTT GTCCTTCACT TCGTCTCGAT GAGCGAAGTA   
  
  
+ CACCACGTGG TAGACGACCC ACAAGCCGCT GTTTGTTTGC ATCTTCTGTA CGTTTTAAGA ATAGGGAGAG   
  
  
+ AGAGCCTCAA CACTCGAACA CTAGACCCCA CCCAAATACG AAAGAAAAAA CAAAAAACCT AATTCCATTT   
  
  
+ GTTCTTAAAG AAAGCCCATA GCCCTAAACC AACGATCCTA CGTTCTTTCG TTTCTTTTGT CCATACAGTT   
  
  
+ CCAACTTTTA TGACTACAAG GAGAGTTTTT GTTTGTTTGT TCTTGCCAAA CACTTCTCTC TCTCTCTCTC   
  
  
+ TCTCTCTCTC TCTCCCTTCC TCTCAGCGAC TGAAAAGTGT GTTTTGTGGA GAAGAGACAT GGGTTGTACA   
  
  
+ GAACTCTCTC TCTCCTTTCT CTCTCTCCAA ATCATATTTT ATTCTACCAT TCCTATTATT AGTTTCCTTT   
  
  
+ TCTTCTTTCT TCTTCTTCTT CTTCTACCAG TATTTCTTAA TACCGGTCAC TCGACCCACT AAATAGTACA   
  
  
+ TCGTAGTTTC CCAGTTTAAC TACGTTGACA AAAAGAATAA TATTATATAA AAGCATTATA AAATGAGAGA   
  
  
+ GAGAGAGAGA GAGAGAGATA GACTCGAAGT AAAGAAGTTC TCTCTCAATT TTTTTTTTTT TTTTTTGCTG   
  
  
+ GAATCCCCTT CAGTAGGAAG TTTTTGTTCA CTACCCTTTC ATTTCTCCGC CGTTCATCGG CGTAAATAGC   
  
  
+ CTTTTTCAAA GTTGGTCCGC TTGTTGGGGG CTTTGCAAAG CTTCGCCCGT CGAAGGAACG AATACTCTAC   
  
  
+ ACTTGGGGAG TTACACTACG TCTATGGGTG AACCGAAAAA CGAGTAAATT GCTTGCTCCT GTAGCTATAA   
  
  
+ CGTATATGGG TGAACCGAAA AACGAGTAAA TTGCTTGCTC CTGTAGTTAT AACGTATATG GGTGAAGGGG   
  
  
+ TGAAAGTAGG GGTGAGTTCC CCTGTGTATG AGTATATGAA TAGTTTTATA CAAGGAAATT ACATGGCTAA   
  
  
+ GGTATGCCTT GATATTTTTT ATTTATATAC AAACATAATA GTACGTTATT AGTTTATTTT TTCTTTAATT   
  
  
+ TAACAATTGT ATCCATTTAC CGTATAAGTA GCCTTTTCTT ATATTTTTCT TTTTCTTTAT TTGTTCCTAA   
  
  
+ CTCGGAGTGG GTGTACACAT AATTAGTGGT ATGTTCTTAG GTTAAGGTGT TGGGGTATCT AATACTTCCT   
  
  
+ AAGCTGGGGA TTGGTAAACT TATAGTGTGG GTTACACTTC ATTGTTTCGT ATTGCTATTA CGGAACTTTC   
  
  
+ GTATTTCACT GATAACAGCC TTATAAGTCT AACACACCAT TAAGTCATCG AAACCCATGG GTCCATCGAT   
  
  
+ GTTCCTGATT CTTCTTTAAT TGAAACTTCA AAAATATATG TTAAGTACCA TGATGTGTGT AAGTATACCC   
  
  
+ TTCTTTGATA CTTTTGTTCC TTGTTCTTC  

- GGCTACCCCA AAGAAGCTGC ACGTACTAGT GATGCAAGAA CAGGAAGTGA AGCAGAGCTA CTCGCTTCAT   
  
  
- GTGGTGCACC ATCTGCTGGG TGTTCGGCGA CAAACAAACG TAGAAGACAT GCAAAATTCT TATCCCTCTC   
  
  
- TCTCGGAGTT GTGAGCTTGT GATCTGGGGT GGGTTTATGC TTTCTTTTTT GTTTTTTGGA TTAAGGTAAA   
  
  
- CAAGAATTTC TTTCGGGTAT CGGGATTTGG TTGCTAGGAT GCAAGAAAGC AAAGAAAACA GGTATGTCAA   
  
  
- GGTTGAAAAT ACTGATGTTC CTCTCAAAAA CAAACAAACA AGAACGGTTT GTGAAGAGAG AGAGAGAGAG   
  
  
- AGAGAGAGAG AGAGGGAAGG AGAGTCGCTG ACTTTTCACA CAAAACACCT CTTCTCTGTA CCCAACATGT   
  
  
- CTTGAGAGAG AGAGGAAAGA GAGAGAGGTT TAGTATAAAA TAAGATGGTA AGGATAATAA TCAAAGGAAA   
  
  
- AGAAGAAAGA AGAAGAAGAA GAAGATGGTC ATAAAGAATT ATGGCCAGTG AGCTGGGTGA TTTATCATGT   
  
  
- AGCATCAAAG GGTCAAATTG ATGCAACTGT TTTTCTTATT ATAATATATT TTCGTAATAT TTTACTCTCT   
  
  
- CTCTCTCTCT CTCTCTCTAT CTGAGCTTCA TTTCTTCAAG AGAGAGTTAA AAAAAAAAAA AAAAAACGAC   
  
  
- CTTAGGGGAA GTCATCCTTC AAAAACAAGT GATGGGAAAG TAAAGAGGCG GCAAGTAGCC GCATTTATCG   
  
  
- GAAAAAGTTT CAACCAGGCG AACAACCCCC GAAACGTTTC GAAGCGGGCA GCTTCCTTGC TTATGAGATG   
  
  
- TGAACCCCTC AATGTGATGC AGATACCCAC TTGGCTTTTT GCTCATTTAA CGAACGAGGA CATCGATATT   
  
  
- GCATATACCC ACTTGGCTTT TTGCTCATTT AACGAACGAG GACATCAATA TTGCATATAC CCACTTCCCC   
  
  
- ACTTTCATCC CCACTCAAGG GGACACATAC TCATATACTT ATCAAAATAT GTTCCTTTAA TGTACCGATT   
  
  
- CCATACGGAA CTATAAAAAA TAAATATATG TTTGTATTAT CATGCAATAA TCAAATAAAA AAGAAATTAA   
  
  
- ATTGTTAACA TAGGTAAATG GCATATTCAT CGGAAAAGAA TATAAAAAGA AAAAGAAATA AACAAGGATT   
  
  
- GAGCCTCACC CACATGTGTA TTAATCACCA TACAAGAATC CAATTCCACA ACCCCATAGA TTATGAAGGA   
  
  
- TTCGACCCCT AACCATTTGA ATATCACACC CAATGTGAAG TAACAAAGCA TAACGATAAT GCCTTGAAAG   
  
  
- CATAAAGTGA CTATTGTCGG AATATTCAGA TTGTGTGGTA ATTCAGTAGC TTTGGGTACC CAGGTAGCTA   
  
  
- CAAGGACTAA GAAGAAATTA ACTTTGAAGT TTTTATATAC AATTCATGGT ACTACACACA TTCATATGGG   
  
  
- AAGAAACTAT GAAAACAAGG AACAAGAAG

+     AuxRR-core

| Site Name | Organism | Position | Strand | Matrix score. | sequence | function |
| --- | --- | --- | --- | --- | --- | --- |
| AuxRR-core | Nicotiana tabacum | 1390 | + | 7 | GGTCCAT | cis-acting regulatory element involved in auxin responsiveness |

> 2018/04/13 10:10:12  
+ CCGATGGGGT TTCTTCGACG TGCATGATCA CTACGTTCTT GTCCTTCACT TCGTCTCGAT GAGCGAAGTA   
  
  
+ CACCACGTGG TAGACGACCC ACAAGCCGCT GTTTGTTTGC ATCTTCTGTA CGTTTTAAGA ATAGGGAGAG   
  
  
+ AGAGCCTCAA CACTCGAACA CTAGACCCCA CCCAAATACG AAAGAAAAAA CAAAAAACCT AATTCCATTT   
  
  
+ GTTCTTAAAG AAAGCCCATA GCCCTAAACC AACGATCCTA CGTTCTTTCG TTTCTTTTGT CCATACAGTT   
  
  
+ CCAACTTTTA TGACTACAAG GAGAGTTTTT GTTTGTTTGT TCTTGCCAAA CACTTCTCTC TCTCTCTCTC   
  
  
+ TCTCTCTCTC TCTCCCTTCC TCTCAGCGAC TGAAAAGTGT GTTTTGTGGA GAAGAGACAT GGGTTGTACA   
  
  
+ GAACTCTCTC TCTCCTTTCT CTCTCTCCAA ATCATATTTT ATTCTACCAT TCCTATTATT AGTTTCCTTT   
  
  
+ TCTTCTTTCT TCTTCTTCTT CTTCTACCAG TATTTCTTAA TACCGGTCAC TCGACCCACT AAATAGTACA   
  
  
+ TCGTAGTTTC CCAGTTTAAC TACGTTGACA AAAAGAATAA TATTATATAA AAGCATTATA AAATGAGAGA   
  
  
+ GAGAGAGAGA GAGAGAGATA GACTCGAAGT AAAGAAGTTC TCTCTCAATT TTTTTTTTTT TTTTTTGCTG   
  
  
+ GAATCCCCTT CAGTAGGAAG TTTTTGTTCA CTACCCTTTC ATTTCTCCGC CGTTCATCGG CGTAAATAGC   
  
  
+ CTTTTTCAAA GTTGGTCCGC TTGTTGGGGG CTTTGCAAAG CTTCGCCCGT CGAAGGAACG AATACTCTAC   
  
  
+ ACTTGGGGAG TTACACTACG TCTATGGGTG AACCGAAAAA CGAGTAAATT GCTTGCTCCT GTAGCTATAA   
  
  
+ CGTATATGGG TGAACCGAAA AACGAGTAAA TTGCTTGCTC CTGTAGTTAT AACGTATATG GGTGAAGGGG   
  
  
+ TGAAAGTAGG GGTGAGTTCC CCTGTGTATG AGTATATGAA TAGTTTTATA CAAGGAAATT ACATGGCTAA   
  
  
+ GGTATGCCTT GATATTTTTT ATTTATATAC AAACATAATA GTACGTTATT AGTTTATTTT TTCTTTAATT   
  
  
+ TAACAATTGT ATCCATTTAC CGTATAAGTA GCCTTTTCTT ATATTTTTCT TTTTCTTTAT TTGTTCCTAA   
  
  
+ CTCGGAGTGG GTGTACACAT AATTAGTGGT ATGTTCTTAG GTTAAGGTGT TGGGGTATCT AATACTTCCT   
  
  
+ AAGCTGGGGA TTGGTAAACT TATAGTGTGG GTTACACTTC ATTGTTTCGT ATTGCTATTA CGGAACTTTC   
  
  
+ GTATTTCACT GATAACAGCC TTATAAGTCT AACACACCAT TAAGTCATCG AAACCCATGG GTCCATCGAT   
  
  
+ GTTCCTGATT CTTCTTTAAT TGAAACTTCA AAAATATATG TTAAGTACCA TGATGTGTGT AAGTATACCC   
  
  
+ TTCTTTGATA CTTTTGTTCC TTGTTCTTC  

- GGCTACCCCA AAGAAGCTGC ACGTACTAGT GATGCAAGAA CAGGAAGTGA AGCAGAGCTA CTCGCTTCAT   
  
  
- GTGGTGCACC ATCTGCTGGG TGTTCGGCGA CAAACAAACG TAGAAGACAT GCAAAATTCT TATCCCTCTC   
  
  
- TCTCGGAGTT GTGAGCTTGT GATCTGGGGT GGGTTTATGC TTTCTTTTTT GTTTTTTGGA TTAAGGTAAA   
  
  
- CAAGAATTTC TTTCGGGTAT CGGGATTTGG TTGCTAGGAT GCAAGAAAGC AAAGAAAACA GGTATGTCAA   
  
  
- GGTTGAAAAT ACTGATGTTC CTCTCAAAAA CAAACAAACA AGAACGGTTT GTGAAGAGAG AGAGAGAGAG   
  
  
- AGAGAGAGAG AGAGGGAAGG AGAGTCGCTG ACTTTTCACA CAAAACACCT CTTCTCTGTA CCCAACATGT   
  
  
- CTTGAGAGAG AGAGGAAAGA GAGAGAGGTT TAGTATAAAA TAAGATGGTA AGGATAATAA TCAAAGGAAA   
  
  
- AGAAGAAAGA AGAAGAAGAA GAAGATGGTC ATAAAGAATT ATGGCCAGTG AGCTGGGTGA TTTATCATGT   
  
  
- AGCATCAAAG GGTCAAATTG ATGCAACTGT TTTTCTTATT ATAATATATT TTCGTAATAT TTTACTCTCT   
  
  
- CTCTCTCTCT CTCTCTCTAT CTGAGCTTCA TTTCTTCAAG AGAGAGTTAA AAAAAAAAAA AAAAAACGAC   
  
  
- CTTAGGGGAA GTCATCCTTC AAAAACAAGT GATGGGAAAG TAAAGAGGCG GCAAGTAGCC GCATTTATCG   
  
  
- GAAAAAGTTT CAACCAGGCG AACAACCCCC GAAACGTTTC GAAGCGGGCA GCTTCCTTGC TTATGAGATG   
  
  
- TGAACCCCTC AATGTGATGC AGATACCCAC TTGGCTTTTT GCTCATTTAA CGAACGAGGA CATCGATATT   
  
  
- GCATATACCC ACTTGGCTTT TTGCTCATTT AACGAACGAG GACATCAATA TTGCATATAC CCACTTCCCC   
  
  
- ACTTTCATCC CCACTCAAGG GGACACATAC TCATATACTT ATCAAAATAT GTTCCTTTAA TGTACCGATT   
  
  
- CCATACGGAA CTATAAAAAA TAAATATATG TTTGTATTAT CATGCAATAA TCAAATAAAA AAGAAATTAA   
  
  
- ATTGTTAACA TAGGTAAATG GCATATTCAT CGGAAAAGAA TATAAAAAGA AAAAGAAATA AACAAGGATT   
  
  
- GAGCCTCACC CACATGTGTA TTAATCACCA TACAAGAATC CAATTCCACA ACCCCATAGA TTATGAAGGA   
  
  
- TTCGACCCCT AACCATTTGA ATATCACACC CAATGTGAAG TAACAAAGCA TAACGATAAT GCCTTGAAAG   
  
  
- CATAAAGTGA CTATTGTCGG AATATTCAGA TTGTGTGGTA ATTCAGTAGC TTTGGGTACC CAGGTAGCTA   
  
  
- CAAGGACTAA GAAGAAATTA ACTTTGAAGT TTTTATATAC AATTCATGGT ACTACACACA TTCATATGGG   
  
  
- AAGAAACTAT GAAAACAAGG AACAAGAAG

+     Box I

| Site Name | Organism | Position | Strand | Matrix score. | sequence | function |
| --- | --- | --- | --- | --- | --- | --- |
| Box I | Pisum sativum | 774 | + | 7 | TTTCAAA | light responsive element |

> 2018/04/13 10:10:12  
+ CCGATGGGGT TTCTTCGACG TGCATGATCA CTACGTTCTT GTCCTTCACT TCGTCTCGAT GAGCGAAGTA   
  
  
+ CACCACGTGG TAGACGACCC ACAAGCCGCT GTTTGTTTGC ATCTTCTGTA CGTTTTAAGA ATAGGGAGAG   
  
  
+ AGAGCCTCAA CACTCGAACA CTAGACCCCA CCCAAATACG AAAGAAAAAA CAAAAAACCT AATTCCATTT   
  
  
+ GTTCTTAAAG AAAGCCCATA GCCCTAAACC AACGATCCTA CGTTCTTTCG TTTCTTTTGT CCATACAGTT   
  
  
+ CCAACTTTTA TGACTACAAG GAGAGTTTTT GTTTGTTTGT TCTTGCCAAA CACTTCTCTC TCTCTCTCTC   
  
  
+ TCTCTCTCTC TCTCCCTTCC TCTCAGCGAC TGAAAAGTGT GTTTTGTGGA GAAGAGACAT GGGTTGTACA   
  
  
+ GAACTCTCTC TCTCCTTTCT CTCTCTCCAA ATCATATTTT ATTCTACCAT TCCTATTATT AGTTTCCTTT   
  
  
+ TCTTCTTTCT TCTTCTTCTT CTTCTACCAG TATTTCTTAA TACCGGTCAC TCGACCCACT AAATAGTACA   
  
  
+ TCGTAGTTTC CCAGTTTAAC TACGTTGACA AAAAGAATAA TATTATATAA AAGCATTATA AAATGAGAGA   
  
  
+ GAGAGAGAGA GAGAGAGATA GACTCGAAGT AAAGAAGTTC TCTCTCAATT TTTTTTTTTT TTTTTTGCTG   
  
  
+ GAATCCCCTT CAGTAGGAAG TTTTTGTTCA CTACCCTTTC ATTTCTCCGC CGTTCATCGG CGTAAATAGC   
  
  
+ CTTTTTCAAA GTTGGTCCGC TTGTTGGGGG CTTTGCAAAG CTTCGCCCGT CGAAGGAACG AATACTCTAC   
  
  
+ ACTTGGGGAG TTACACTACG TCTATGGGTG AACCGAAAAA CGAGTAAATT GCTTGCTCCT GTAGCTATAA   
  
  
+ CGTATATGGG TGAACCGAAA AACGAGTAAA TTGCTTGCTC CTGTAGTTAT AACGTATATG GGTGAAGGGG   
  
  
+ TGAAAGTAGG GGTGAGTTCC CCTGTGTATG AGTATATGAA TAGTTTTATA CAAGGAAATT ACATGGCTAA   
  
  
+ GGTATGCCTT GATATTTTTT ATTTATATAC AAACATAATA GTACGTTATT AGTTTATTTT TTCTTTAATT   
  
  
+ TAACAATTGT ATCCATTTAC CGTATAAGTA GCCTTTTCTT ATATTTTTCT TTTTCTTTAT TTGTTCCTAA   
  
  
+ CTCGGAGTGG GTGTACACAT AATTAGTGGT ATGTTCTTAG GTTAAGGTGT TGGGGTATCT AATACTTCCT   
  
  
+ AAGCTGGGGA TTGGTAAACT TATAGTGTGG GTTACACTTC ATTGTTTCGT ATTGCTATTA CGGAACTTTC   
  
  
+ GTATTTCACT GATAACAGCC TTATAAGTCT AACACACCAT TAAGTCATCG AAACCCATGG GTCCATCGAT   
  
  
+ GTTCCTGATT CTTCTTTAAT TGAAACTTCA AAAATATATG TTAAGTACCA TGATGTGTGT AAGTATACCC   
  
  
+ TTCTTTGATA CTTTTGTTCC TTGTTCTTC  

- GGCTACCCCA AAGAAGCTGC ACGTACTAGT GATGCAAGAA CAGGAAGTGA AGCAGAGCTA CTCGCTTCAT   
  
  
- GTGGTGCACC ATCTGCTGGG TGTTCGGCGA CAAACAAACG TAGAAGACAT GCAAAATTCT TATCCCTCTC   
  
  
- TCTCGGAGTT GTGAGCTTGT GATCTGGGGT GGGTTTATGC TTTCTTTTTT GTTTTTTGGA TTAAGGTAAA   
  
  
- CAAGAATTTC TTTCGGGTAT CGGGATTTGG TTGCTAGGAT GCAAGAAAGC AAAGAAAACA GGTATGTCAA   
  
  
- GGTTGAAAAT ACTGATGTTC CTCTCAAAAA CAAACAAACA AGAACGGTTT GTGAAGAGAG AGAGAGAGAG   
  
  
- AGAGAGAGAG AGAGGGAAGG AGAGTCGCTG ACTTTTCACA CAAAACACCT CTTCTCTGTA CCCAACATGT   
  
  
- CTTGAGAGAG AGAGGAAAGA GAGAGAGGTT TAGTATAAAA TAAGATGGTA AGGATAATAA TCAAAGGAAA   
  
  
- AGAAGAAAGA AGAAGAAGAA GAAGATGGTC ATAAAGAATT ATGGCCAGTG AGCTGGGTGA TTTATCATGT   
  
  
- AGCATCAAAG GGTCAAATTG ATGCAACTGT TTTTCTTATT ATAATATATT TTCGTAATAT TTTACTCTCT   
  
  
- CTCTCTCTCT CTCTCTCTAT CTGAGCTTCA TTTCTTCAAG AGAGAGTTAA AAAAAAAAAA AAAAAACGAC   
  
  
- CTTAGGGGAA GTCATCCTTC AAAAACAAGT GATGGGAAAG TAAAGAGGCG GCAAGTAGCC GCATTTATCG   
  
  
- GAAAAAGTTT CAACCAGGCG AACAACCCCC GAAACGTTTC GAAGCGGGCA GCTTCCTTGC TTATGAGATG   
  
  
- TGAACCCCTC AATGTGATGC AGATACCCAC TTGGCTTTTT GCTCATTTAA CGAACGAGGA CATCGATATT   
  
  
- GCATATACCC ACTTGGCTTT TTGCTCATTT AACGAACGAG GACATCAATA TTGCATATAC CCACTTCCCC   
  
  
- ACTTTCATCC CCACTCAAGG GGACACATAC TCATATACTT ATCAAAATAT GTTCCTTTAA TGTACCGATT   
  
  
- CCATACGGAA CTATAAAAAA TAAATATATG TTTGTATTAT CATGCAATAA TCAAATAAAA AAGAAATTAA   
  
  
- ATTGTTAACA TAGGTAAATG GCATATTCAT CGGAAAAGAA TATAAAAAGA AAAAGAAATA AACAAGGATT   
  
  
- GAGCCTCACC CACATGTGTA TTAATCACCA TACAAGAATC CAATTCCACA ACCCCATAGA TTATGAAGGA   
  
  
- TTCGACCCCT AACCATTTGA ATATCACACC CAATGTGAAG TAACAAAGCA TAACGATAAT GCCTTGAAAG   
  
  
- CATAAAGTGA CTATTGTCGG AATATTCAGA TTGTGTGGTA ATTCAGTAGC TTTGGGTACC CAGGTAGCTA   
  
  
- CAAGGACTAA GAAGAAATTA ACTTTGAAGT TTTTATATAC AATTCATGGT ACTACACACA TTCATATGGG   
  
  
- AAGAAACTAT GAAAACAAGG AACAAGAAG

+     CAAT-box

| Site Name | Organism | Position | Strand | Matrix score. | sequence | function |
| --- | --- | --- | --- | --- | --- | --- |
| CAAT-box | Hordeum vulgare | 1311 | - | 4 | CAAT | common cis-acting element in promoter and enhancer regions |
| CAAT-box | Hordeum vulgare | 888 | - | 4 | CAAT | common cis-acting element in promoter and enhancer regions |
| CAAT-box | Hordeum vulgare | 1419 | - | 4 | CAAT | common cis-acting element in promoter and enhancer regions |
| CAAT-box | Glycine max | 1124 | + | 5 | CAATT | common cis-acting element in promoter and enhancer regions |
| CAAT-box | Glycine max | 887 | - | 5 | CAATT | common cis-acting element in promoter and enhancer regions |
| CAAT-box | Hordeum vulgare | 940 | - | 4 | CAAT | common cis-acting element in promoter and enhancer regions |
| CAAT-box | Glycine max | 1418 | - | 5 | CAATT | common cis-acting element in promoter and enhancer regions |
| CAAT-box | Hordeum vulgare | 1126 | - | 4 | CAAT | common cis-acting element in promoter and enhancer regions |
| CAAT-box | Brassica rapa | 173 | + | 5 | CAAAT | common cis-acting element in promoter and enhancer regions |
| CAAT-box | Brassica rapa | 207 | - | 5 | CAAAT | common cis-acting element in promoter and enhancer regions |
| CAAT-box | Hordeum vulgare | 1301 | - | 4 | CAAT | common cis-acting element in promoter and enhancer regions |
| CAAT-box | Glycine max | 939 | - | 5 | CAATT | common cis-acting element in promoter and enhancer regions |
| CAAT-box | Brassica rapa | 1179 | - | 5 | CAAAT | common cis-acting element in promoter and enhancer regions |
| CAAT-box | Arabidopsis thaliana | 1270 | - | 5 | CCAAT | common cis-acting element in promoter and enhancer regions |
| CAAT-box | Brassica rapa | 448 | + | 5 | CAAAT | common cis-acting element in promoter and enhancer regions |
| CAAT-box | Glycine max | 1125 | - | 5 | CAATT | common cis-acting element in promoter and enhancer regions |
| CAAT-box | Glycine max | 676 | + | 5 | CAATT | common cis-acting element in promoter and enhancer regions |

> 2018/04/13 10:10:12  
+ CCGATGGGGT TTCTTCGACG TGCATGATCA CTACGTTCTT GTCCTTCACT TCGTCTCGAT GAGCGAAGTA   
  
  
+ CACCACGTGG TAGACGACCC ACAAGCCGCT GTTTGTTTGC ATCTTCTGTA CGTTTTAAGA ATAGGGAGAG   
  
  
+ AGAGCCTCAA CACTCGAACA CTAGACCCCA CCCAAATACG AAAGAAAAAA CAAAAAACCT AATTCCATTT   
  
  
+ GTTCTTAAAG AAAGCCCATA GCCCTAAACC AACGATCCTA CGTTCTTTCG TTTCTTTTGT CCATACAGTT   
  
  
+ CCAACTTTTA TGACTACAAG GAGAGTTTTT GTTTGTTTGT TCTTGCCAAA CACTTCTCTC TCTCTCTCTC   
  
  
+ TCTCTCTCTC TCTCCCTTCC TCTCAGCGAC TGAAAAGTGT GTTTTGTGGA GAAGAGACAT GGGTTGTACA   
  
  
+ GAACTCTCTC TCTCCTTTCT CTCTCTCCAA ATCATATTTT ATTCTACCAT TCCTATTATT AGTTTCCTTT   
  
  
+ TCTTCTTTCT TCTTCTTCTT CTTCTACCAG TATTTCTTAA TACCGGTCAC TCGACCCACT AAATAGTACA   
  
  
+ TCGTAGTTTC CCAGTTTAAC TACGTTGACA AAAAGAATAA TATTATATAA AAGCATTATA AAATGAGAGA   
  
  
+ GAGAGAGAGA GAGAGAGATA GACTCGAAGT AAAGAAGTTC TCTCTCAATT TTTTTTTTTT TTTTTTGCTG   
  
  
+ GAATCCCCTT CAGTAGGAAG TTTTTGTTCA CTACCCTTTC ATTTCTCCGC CGTTCATCGG CGTAAATAGC   
  
  
+ CTTTTTCAAA GTTGGTCCGC TTGTTGGGGG CTTTGCAAAG CTTCGCCCGT CGAAGGAACG AATACTCTAC   
  
  
+ ACTTGGGGAG TTACACTACG TCTATGGGTG AACCGAAAAA CGAGTAAATT GCTTGCTCCT GTAGCTATAA   
  
  
+ CGTATATGGG TGAACCGAAA AACGAGTAAA TTGCTTGCTC CTGTAGTTAT AACGTATATG GGTGAAGGGG   
  
  
+ TGAAAGTAGG GGTGAGTTCC CCTGTGTATG AGTATATGAA TAGTTTTATA CAAGGAAATT ACATGGCTAA   
  
  
+ GGTATGCCTT GATATTTTTT ATTTATATAC AAACATAATA GTACGTTATT AGTTTATTTT TTCTTTAATT   
  
  
+ TAACAATTGT ATCCATTTAC CGTATAAGTA GCCTTTTCTT ATATTTTTCT TTTTCTTTAT TTGTTCCTAA   
  
  
+ CTCGGAGTGG GTGTACACAT AATTAGTGGT ATGTTCTTAG GTTAAGGTGT TGGGGTATCT AATACTTCCT   
  
  
+ AAGCTGGGGA TTGGTAAACT TATAGTGTGG GTTACACTTC ATTGTTTCGT ATTGCTATTA CGGAACTTTC   
  
  
+ GTATTTCACT GATAACAGCC TTATAAGTCT AACACACCAT TAAGTCATCG AAACCCATGG GTCCATCGAT   
  
  
+ GTTCCTGATT CTTCTTTAAT TGAAACTTCA AAAATATATG TTAAGTACCA TGATGTGTGT AAGTATACCC   
  
  
+ TTCTTTGATA CTTTTGTTCC TTGTTCTTC  

- GGCTACCCCA AAGAAGCTGC ACGTACTAGT GATGCAAGAA CAGGAAGTGA AGCAGAGCTA CTCGCTTCAT   
  
  
- GTGGTGCACC ATCTGCTGGG TGTTCGGCGA CAAACAAACG TAGAAGACAT GCAAAATTCT TATCCCTCTC   
  
  
- TCTCGGAGTT GTGAGCTTGT GATCTGGGGT GGGTTTATGC TTTCTTTTTT GTTTTTTGGA TTAAGGTAAA   
  
  
- CAAGAATTTC TTTCGGGTAT CGGGATTTGG TTGCTAGGAT GCAAGAAAGC AAAGAAAACA GGTATGTCAA   
  
  
- GGTTGAAAAT ACTGATGTTC CTCTCAAAAA CAAACAAACA AGAACGGTTT GTGAAGAGAG AGAGAGAGAG   
  
  
- AGAGAGAGAG AGAGGGAAGG AGAGTCGCTG ACTTTTCACA CAAAACACCT CTTCTCTGTA CCCAACATGT   
  
  
- CTTGAGAGAG AGAGGAAAGA GAGAGAGGTT TAGTATAAAA TAAGATGGTA AGGATAATAA TCAAAGGAAA   
  
  
- AGAAGAAAGA AGAAGAAGAA GAAGATGGTC ATAAAGAATT ATGGCCAGTG AGCTGGGTGA TTTATCATGT   
  
  
- AGCATCAAAG GGTCAAATTG ATGCAACTGT TTTTCTTATT ATAATATATT TTCGTAATAT TTTACTCTCT   
  
  
- CTCTCTCTCT CTCTCTCTAT CTGAGCTTCA TTTCTTCAAG AGAGAGTTAA AAAAAAAAAA AAAAAACGAC   
  
  
- CTTAGGGGAA GTCATCCTTC AAAAACAAGT GATGGGAAAG TAAAGAGGCG GCAAGTAGCC GCATTTATCG   
  
  
- GAAAAAGTTT CAACCAGGCG AACAACCCCC GAAACGTTTC GAAGCGGGCA GCTTCCTTGC TTATGAGATG   
  
  
- TGAACCCCTC AATGTGATGC AGATACCCAC TTGGCTTTTT GCTCATTTAA CGAACGAGGA CATCGATATT   
  
  
- GCATATACCC ACTTGGCTTT TTGCTCATTT AACGAACGAG GACATCAATA TTGCATATAC CCACTTCCCC   
  
  
- ACTTTCATCC CCACTCAAGG GGACACATAC TCATATACTT ATCAAAATAT GTTCCTTTAA TGTACCGATT   
  
  
- CCATACGGAA CTATAAAAAA TAAATATATG TTTGTATTAT CATGCAATAA TCAAATAAAA AAGAAATTAA   
  
  
- ATTGTTAACA TAGGTAAATG GCATATTCAT CGGAAAAGAA TATAAAAAGA AAAAGAAATA AACAAGGATT   
  
  
- GAGCCTCACC CACATGTGTA TTAATCACCA TACAAGAATC CAATTCCACA ACCCCATAGA TTATGAAGGA   
  
  
- TTCGACCCCT AACCATTTGA ATATCACACC CAATGTGAAG TAACAAAGCA TAACGATAAT GCCTTGAAAG   
  
  
- CATAAAGTGA CTATTGTCGG AATATTCAGA TTGTGTGGTA ATTCAGTAGC TTTGGGTACC CAGGTAGCTA   
  
  
- CAAGGACTAA GAAGAAATTA ACTTTGAAGT TTTTATATAC AATTCATGGT ACTACACACA TTCATATGGG   
  
  
- AAGAAACTAT GAAAACAAGG AACAAGAAG

+     G-Box

| Site Name | Organism | Position | Strand | Matrix score. | sequence | function |
| --- | --- | --- | --- | --- | --- | --- |
| G-Box | Pisum sativum | 74 | + | 6 | CACGTG | cis-acting regulatory element involved in light responsiveness |

> 2018/04/13 10:10:12  
+ CCGATGGGGT TTCTTCGACG TGCATGATCA CTACGTTCTT GTCCTTCACT TCGTCTCGAT GAGCGAAGTA   
  
  
+ CACCACGTGG TAGACGACCC ACAAGCCGCT GTTTGTTTGC ATCTTCTGTA CGTTTTAAGA ATAGGGAGAG   
  
  
+ AGAGCCTCAA CACTCGAACA CTAGACCCCA CCCAAATACG AAAGAAAAAA CAAAAAACCT AATTCCATTT   
  
  
+ GTTCTTAAAG AAAGCCCATA GCCCTAAACC AACGATCCTA CGTTCTTTCG TTTCTTTTGT CCATACAGTT   
  
  
+ CCAACTTTTA TGACTACAAG GAGAGTTTTT GTTTGTTTGT TCTTGCCAAA CACTTCTCTC TCTCTCTCTC   
  
  
+ TCTCTCTCTC TCTCCCTTCC TCTCAGCGAC TGAAAAGTGT GTTTTGTGGA GAAGAGACAT GGGTTGTACA   
  
  
+ GAACTCTCTC TCTCCTTTCT CTCTCTCCAA ATCATATTTT ATTCTACCAT TCCTATTATT AGTTTCCTTT   
  
  
+ TCTTCTTTCT TCTTCTTCTT CTTCTACCAG TATTTCTTAA TACCGGTCAC TCGACCCACT AAATAGTACA   
  
  
+ TCGTAGTTTC CCAGTTTAAC TACGTTGACA AAAAGAATAA TATTATATAA AAGCATTATA AAATGAGAGA   
  
  
+ GAGAGAGAGA GAGAGAGATA GACTCGAAGT AAAGAAGTTC TCTCTCAATT TTTTTTTTTT TTTTTTGCTG   
  
  
+ GAATCCCCTT CAGTAGGAAG TTTTTGTTCA CTACCCTTTC ATTTCTCCGC CGTTCATCGG CGTAAATAGC   
  
  
+ CTTTTTCAAA GTTGGTCCGC TTGTTGGGGG CTTTGCAAAG CTTCGCCCGT CGAAGGAACG AATACTCTAC   
  
  
+ ACTTGGGGAG TTACACTACG TCTATGGGTG AACCGAAAAA CGAGTAAATT GCTTGCTCCT GTAGCTATAA   
  
  
+ CGTATATGGG TGAACCGAAA AACGAGTAAA TTGCTTGCTC CTGTAGTTAT AACGTATATG GGTGAAGGGG   
  
  
+ TGAAAGTAGG GGTGAGTTCC CCTGTGTATG AGTATATGAA TAGTTTTATA CAAGGAAATT ACATGGCTAA   
  
  
+ GGTATGCCTT GATATTTTTT ATTTATATAC AAACATAATA GTACGTTATT AGTTTATTTT TTCTTTAATT   
  
  
+ TAACAATTGT ATCCATTTAC CGTATAAGTA GCCTTTTCTT ATATTTTTCT TTTTCTTTAT TTGTTCCTAA   
  
  
+ CTCGGAGTGG GTGTACACAT AATTAGTGGT ATGTTCTTAG GTTAAGGTGT TGGGGTATCT AATACTTCCT   
  
  
+ AAGCTGGGGA TTGGTAAACT TATAGTGTGG GTTACACTTC ATTGTTTCGT ATTGCTATTA CGGAACTTTC   
  
  
+ GTATTTCACT GATAACAGCC TTATAAGTCT AACACACCAT TAAGTCATCG AAACCCATGG GTCCATCGAT   
  
  
+ GTTCCTGATT CTTCTTTAAT TGAAACTTCA AAAATATATG TTAAGTACCA TGATGTGTGT AAGTATACCC   
  
  
+ TTCTTTGATA CTTTTGTTCC TTGTTCTTC  

- GGCTACCCCA AAGAAGCTGC ACGTACTAGT GATGCAAGAA CAGGAAGTGA AGCAGAGCTA CTCGCTTCAT   
  
  
- GTGGTGCACC ATCTGCTGGG TGTTCGGCGA CAAACAAACG TAGAAGACAT GCAAAATTCT TATCCCTCTC   
  
  
- TCTCGGAGTT GTGAGCTTGT GATCTGGGGT GGGTTTATGC TTTCTTTTTT GTTTTTTGGA TTAAGGTAAA   
  
  
- CAAGAATTTC TTTCGGGTAT CGGGATTTGG TTGCTAGGAT GCAAGAAAGC AAAGAAAACA GGTATGTCAA   
  
  
- GGTTGAAAAT ACTGATGTTC CTCTCAAAAA CAAACAAACA AGAACGGTTT GTGAAGAGAG AGAGAGAGAG   
  
  
- AGAGAGAGAG AGAGGGAAGG AGAGTCGCTG ACTTTTCACA CAAAACACCT CTTCTCTGTA CCCAACATGT   
  
  
- CTTGAGAGAG AGAGGAAAGA GAGAGAGGTT TAGTATAAAA TAAGATGGTA AGGATAATAA TCAAAGGAAA   
  
  
- AGAAGAAAGA AGAAGAAGAA GAAGATGGTC ATAAAGAATT ATGGCCAGTG AGCTGGGTGA TTTATCATGT   
  
  
- AGCATCAAAG GGTCAAATTG ATGCAACTGT TTTTCTTATT ATAATATATT TTCGTAATAT TTTACTCTCT   
  
  
- CTCTCTCTCT CTCTCTCTAT CTGAGCTTCA TTTCTTCAAG AGAGAGTTAA AAAAAAAAAA AAAAAACGAC   
  
  
- CTTAGGGGAA GTCATCCTTC AAAAACAAGT GATGGGAAAG TAAAGAGGCG GCAAGTAGCC GCATTTATCG   
  
  
- GAAAAAGTTT CAACCAGGCG AACAACCCCC GAAACGTTTC GAAGCGGGCA GCTTCCTTGC TTATGAGATG   
  
  
- TGAACCCCTC AATGTGATGC AGATACCCAC TTGGCTTTTT GCTCATTTAA CGAACGAGGA CATCGATATT   
  
  
- GCATATACCC ACTTGGCTTT TTGCTCATTT AACGAACGAG GACATCAATA TTGCATATAC CCACTTCCCC   
  
  
- ACTTTCATCC CCACTCAAGG GGACACATAC TCATATACTT ATCAAAATAT GTTCCTTTAA TGTACCGATT   
  
  
- CCATACGGAA CTATAAAAAA TAAATATATG TTTGTATTAT CATGCAATAA TCAAATAAAA AAGAAATTAA   
  
  
- ATTGTTAACA TAGGTAAATG GCATATTCAT CGGAAAAGAA TATAAAAAGA AAAAGAAATA AACAAGGATT   
  
  
- GAGCCTCACC CACATGTGTA TTAATCACCA TACAAGAATC CAATTCCACA ACCCCATAGA TTATGAAGGA   
  
  
- TTCGACCCCT AACCATTTGA ATATCACACC CAATGTGAAG TAACAAAGCA TAACGATAAT GCCTTGAAAG   
  
  
- CATAAAGTGA CTATTGTCGG AATATTCAGA TTGTGTGGTA ATTCAGTAGC TTTGGGTACC CAGGTAGCTA   
  
  
- CAAGGACTAA GAAGAAATTA ACTTTGAAGT TTTTATATAC AATTCATGGT ACTACACACA TTCATATGGG   
  
  
- AAGAAACTAT GAAAACAAGG AACAAGAAG

+     G-box

| Site Name | Organism | Position | Strand | Matrix score. | sequence | function |
| --- | --- | --- | --- | --- | --- | --- |
| G-box | Zea mays | 17 | - | 6 | CACGTC | cis-acting regulatory element involved in light responsiveness |
| G-box | Brassica napus | 73 | - | 7 | CACGTGG | cis-acting regulatory element involved in light responsiveness |
| G-box | Arabidopsis thaliana | 72 | + | 10 | GCCACGTGGTA | cis-acting regulatory element involved in light responsiveness |
| G-box | Arabidopsis thaliana | 74 | + | 6 | CACGTG | cis-acting regulatory element involved in light responsiveness |

> 2018/04/13 10:10:12  
+ CCGATGGGGT TTCTTCGACG TGCATGATCA CTACGTTCTT GTCCTTCACT TCGTCTCGAT GAGCGAAGTA   
  
  
+ CACCACGTGG TAGACGACCC ACAAGCCGCT GTTTGTTTGC ATCTTCTGTA CGTTTTAAGA ATAGGGAGAG   
  
  
+ AGAGCCTCAA CACTCGAACA CTAGACCCCA CCCAAATACG AAAGAAAAAA CAAAAAACCT AATTCCATTT   
  
  
+ GTTCTTAAAG AAAGCCCATA GCCCTAAACC AACGATCCTA CGTTCTTTCG TTTCTTTTGT CCATACAGTT   
  
  
+ CCAACTTTTA TGACTACAAG GAGAGTTTTT GTTTGTTTGT TCTTGCCAAA CACTTCTCTC TCTCTCTCTC   
  
  
+ TCTCTCTCTC TCTCCCTTCC TCTCAGCGAC TGAAAAGTGT GTTTTGTGGA GAAGAGACAT GGGTTGTACA   
  
  
+ GAACTCTCTC TCTCCTTTCT CTCTCTCCAA ATCATATTTT ATTCTACCAT TCCTATTATT AGTTTCCTTT   
  
  
+ TCTTCTTTCT TCTTCTTCTT CTTCTACCAG TATTTCTTAA TACCGGTCAC TCGACCCACT AAATAGTACA   
  
  
+ TCGTAGTTTC CCAGTTTAAC TACGTTGACA AAAAGAATAA TATTATATAA AAGCATTATA AAATGAGAGA   
  
  
+ GAGAGAGAGA GAGAGAGATA GACTCGAAGT AAAGAAGTTC TCTCTCAATT TTTTTTTTTT TTTTTTGCTG   
  
  
+ GAATCCCCTT CAGTAGGAAG TTTTTGTTCA CTACCCTTTC ATTTCTCCGC CGTTCATCGG CGTAAATAGC   
  
  
+ CTTTTTCAAA GTTGGTCCGC TTGTTGGGGG CTTTGCAAAG CTTCGCCCGT CGAAGGAACG AATACTCTAC   
  
  
+ ACTTGGGGAG TTACACTACG TCTATGGGTG AACCGAAAAA CGAGTAAATT GCTTGCTCCT GTAGCTATAA   
  
  
+ CGTATATGGG TGAACCGAAA AACGAGTAAA TTGCTTGCTC CTGTAGTTAT AACGTATATG GGTGAAGGGG   
  
  
+ TGAAAGTAGG GGTGAGTTCC CCTGTGTATG AGTATATGAA TAGTTTTATA CAAGGAAATT ACATGGCTAA   
  
  
+ GGTATGCCTT GATATTTTTT ATTTATATAC AAACATAATA GTACGTTATT AGTTTATTTT TTCTTTAATT   
  
  
+ TAACAATTGT ATCCATTTAC CGTATAAGTA GCCTTTTCTT ATATTTTTCT TTTTCTTTAT TTGTTCCTAA   
  
  
+ CTCGGAGTGG GTGTACACAT AATTAGTGGT ATGTTCTTAG GTTAAGGTGT TGGGGTATCT AATACTTCCT   
  
  
+ AAGCTGGGGA TTGGTAAACT TATAGTGTGG GTTACACTTC ATTGTTTCGT ATTGCTATTA CGGAACTTTC   
  
  
+ GTATTTCACT GATAACAGCC TTATAAGTCT AACACACCAT TAAGTCATCG AAACCCATGG GTCCATCGAT   
  
  
+ GTTCCTGATT CTTCTTTAAT TGAAACTTCA AAAATATATG TTAAGTACCA TGATGTGTGT AAGTATACCC   
  
  
+ TTCTTTGATA CTTTTGTTCC TTGTTCTTC  

- GGCTACCCCA AAGAAGCTGC ACGTACTAGT GATGCAAGAA CAGGAAGTGA AGCAGAGCTA CTCGCTTCAT   
  
  
- GTGGTGCACC ATCTGCTGGG TGTTCGGCGA CAAACAAACG TAGAAGACAT GCAAAATTCT TATCCCTCTC   
  
  
- TCTCGGAGTT GTGAGCTTGT GATCTGGGGT GGGTTTATGC TTTCTTTTTT GTTTTTTGGA TTAAGGTAAA   
  
  
- CAAGAATTTC TTTCGGGTAT CGGGATTTGG TTGCTAGGAT GCAAGAAAGC AAAGAAAACA GGTATGTCAA   
  
  
- GGTTGAAAAT ACTGATGTTC CTCTCAAAAA CAAACAAACA AGAACGGTTT GTGAAGAGAG AGAGAGAGAG   
  
  
- AGAGAGAGAG AGAGGGAAGG AGAGTCGCTG ACTTTTCACA CAAAACACCT CTTCTCTGTA CCCAACATGT   
  
  
- CTTGAGAGAG AGAGGAAAGA GAGAGAGGTT TAGTATAAAA TAAGATGGTA AGGATAATAA TCAAAGGAAA   
  
  
- AGAAGAAAGA AGAAGAAGAA GAAGATGGTC ATAAAGAATT ATGGCCAGTG AGCTGGGTGA TTTATCATGT   
  
  
- AGCATCAAAG GGTCAAATTG ATGCAACTGT TTTTCTTATT ATAATATATT TTCGTAATAT TTTACTCTCT   
  
  
- CTCTCTCTCT CTCTCTCTAT CTGAGCTTCA TTTCTTCAAG AGAGAGTTAA AAAAAAAAAA AAAAAACGAC   
  
  
- CTTAGGGGAA GTCATCCTTC AAAAACAAGT GATGGGAAAG TAAAGAGGCG GCAAGTAGCC GCATTTATCG   
  
  
- GAAAAAGTTT CAACCAGGCG AACAACCCCC GAAACGTTTC GAAGCGGGCA GCTTCCTTGC TTATGAGATG   
  
  
- TGAACCCCTC AATGTGATGC AGATACCCAC TTGGCTTTTT GCTCATTTAA CGAACGAGGA CATCGATATT   
  
  
- GCATATACCC ACTTGGCTTT TTGCTCATTT AACGAACGAG GACATCAATA TTGCATATAC CCACTTCCCC   
  
  
- ACTTTCATCC CCACTCAAGG GGACACATAC TCATATACTT ATCAAAATAT GTTCCTTTAA TGTACCGATT   
  
  
- CCATACGGAA CTATAAAAAA TAAATATATG TTTGTATTAT CATGCAATAA TCAAATAAAA AAGAAATTAA   
  
  
- ATTGTTAACA TAGGTAAATG GCATATTCAT CGGAAAAGAA TATAAAAAGA AAAAGAAATA AACAAGGATT   
  
  
- GAGCCTCACC CACATGTGTA TTAATCACCA TACAAGAATC CAATTCCACA ACCCCATAGA TTATGAAGGA   
  
  
- TTCGACCCCT AACCATTTGA ATATCACACC CAATGTGAAG TAACAAAGCA TAACGATAAT GCCTTGAAAG   
  
  
- CATAAAGTGA CTATTGTCGG AATATTCAGA TTGTGTGGTA ATTCAGTAGC TTTGGGTACC CAGGTAGCTA   
  
  
- CAAGGACTAA GAAGAAATTA ACTTTGAAGT TTTTATATAC AATTCATGGT ACTACACACA TTCATATGGG   
  
  
- AAGAAACTAT GAAAACAAGG AACAAGAAG

+     GAG-motif

| Site Name | Organism | Position | Strand | Matrix score. | sequence | function |
| --- | --- | --- | --- | --- | --- | --- |
| GAG-motif | Arabidopsis thaliana | 423 | - | 7 | AGAGAGT | part of a light responsive element |

> 2018/04/13 10:10:12  
+ CCGATGGGGT TTCTTCGACG TGCATGATCA CTACGTTCTT GTCCTTCACT TCGTCTCGAT GAGCGAAGTA   
  
  
+ CACCACGTGG TAGACGACCC ACAAGCCGCT GTTTGTTTGC ATCTTCTGTA CGTTTTAAGA ATAGGGAGAG   
  
  
+ AGAGCCTCAA CACTCGAACA CTAGACCCCA CCCAAATACG AAAGAAAAAA CAAAAAACCT AATTCCATTT   
  
  
+ GTTCTTAAAG AAAGCCCATA GCCCTAAACC AACGATCCTA CGTTCTTTCG TTTCTTTTGT CCATACAGTT   
  
  
+ CCAACTTTTA TGACTACAAG GAGAGTTTTT GTTTGTTTGT TCTTGCCAAA CACTTCTCTC TCTCTCTCTC   
  
  
+ TCTCTCTCTC TCTCCCTTCC TCTCAGCGAC TGAAAAGTGT GTTTTGTGGA GAAGAGACAT GGGTTGTACA   
  
  
+ GAACTCTCTC TCTCCTTTCT CTCTCTCCAA ATCATATTTT ATTCTACCAT TCCTATTATT AGTTTCCTTT   
  
  
+ TCTTCTTTCT TCTTCTTCTT CTTCTACCAG TATTTCTTAA TACCGGTCAC TCGACCCACT AAATAGTACA   
  
  
+ TCGTAGTTTC CCAGTTTAAC TACGTTGACA AAAAGAATAA TATTATATAA AAGCATTATA AAATGAGAGA   
  
  
+ GAGAGAGAGA GAGAGAGATA GACTCGAAGT AAAGAAGTTC TCTCTCAATT TTTTTTTTTT TTTTTTGCTG   
  
  
+ GAATCCCCTT CAGTAGGAAG TTTTTGTTCA CTACCCTTTC ATTTCTCCGC CGTTCATCGG CGTAAATAGC   
  
  
+ CTTTTTCAAA GTTGGTCCGC TTGTTGGGGG CTTTGCAAAG CTTCGCCCGT CGAAGGAACG AATACTCTAC   
  
  
+ ACTTGGGGAG TTACACTACG TCTATGGGTG AACCGAAAAA CGAGTAAATT GCTTGCTCCT GTAGCTATAA   
  
  
+ CGTATATGGG TGAACCGAAA AACGAGTAAA TTGCTTGCTC CTGTAGTTAT AACGTATATG GGTGAAGGGG   
  
  
+ TGAAAGTAGG GGTGAGTTCC CCTGTGTATG AGTATATGAA TAGTTTTATA CAAGGAAATT ACATGGCTAA   
  
  
+ GGTATGCCTT GATATTTTTT ATTTATATAC AAACATAATA GTACGTTATT AGTTTATTTT TTCTTTAATT   
  
  
+ TAACAATTGT ATCCATTTAC CGTATAAGTA GCCTTTTCTT ATATTTTTCT TTTTCTTTAT TTGTTCCTAA   
  
  
+ CTCGGAGTGG GTGTACACAT AATTAGTGGT ATGTTCTTAG GTTAAGGTGT TGGGGTATCT AATACTTCCT   
  
  
+ AAGCTGGGGA TTGGTAAACT TATAGTGTGG GTTACACTTC ATTGTTTCGT ATTGCTATTA CGGAACTTTC   
  
  
+ GTATTTCACT GATAACAGCC TTATAAGTCT AACACACCAT TAAGTCATCG AAACCCATGG GTCCATCGAT   
  
  
+ GTTCCTGATT CTTCTTTAAT TGAAACTTCA AAAATATATG TTAAGTACCA TGATGTGTGT AAGTATACCC   
  
  
+ TTCTTTGATA CTTTTGTTCC TTGTTCTTC  

- GGCTACCCCA AAGAAGCTGC ACGTACTAGT GATGCAAGAA CAGGAAGTGA AGCAGAGCTA CTCGCTTCAT   
  
  
- GTGGTGCACC ATCTGCTGGG TGTTCGGCGA CAAACAAACG TAGAAGACAT GCAAAATTCT TATCCCTCTC   
  
  
- TCTCGGAGTT GTGAGCTTGT GATCTGGGGT GGGTTTATGC TTTCTTTTTT GTTTTTTGGA TTAAGGTAAA   
  
  
- CAAGAATTTC TTTCGGGTAT CGGGATTTGG TTGCTAGGAT GCAAGAAAGC AAAGAAAACA GGTATGTCAA   
  
  
- GGTTGAAAAT ACTGATGTTC CTCTCAAAAA CAAACAAACA AGAACGGTTT GTGAAGAGAG AGAGAGAGAG   
  
  
- AGAGAGAGAG AGAGGGAAGG AGAGTCGCTG ACTTTTCACA CAAAACACCT CTTCTCTGTA CCCAACATGT   
  
  
- CTTGAGAGAG AGAGGAAAGA GAGAGAGGTT TAGTATAAAA TAAGATGGTA AGGATAATAA TCAAAGGAAA   
  
  
- AGAAGAAAGA AGAAGAAGAA GAAGATGGTC ATAAAGAATT ATGGCCAGTG AGCTGGGTGA TTTATCATGT   
  
  
- AGCATCAAAG GGTCAAATTG ATGCAACTGT TTTTCTTATT ATAATATATT TTCGTAATAT TTTACTCTCT   
  
  
- CTCTCTCTCT CTCTCTCTAT CTGAGCTTCA TTTCTTCAAG AGAGAGTTAA AAAAAAAAAA AAAAAACGAC   
  
  
- CTTAGGGGAA GTCATCCTTC AAAAACAAGT GATGGGAAAG TAAAGAGGCG GCAAGTAGCC GCATTTATCG   
  
  
- GAAAAAGTTT CAACCAGGCG AACAACCCCC GAAACGTTTC GAAGCGGGCA GCTTCCTTGC TTATGAGATG   
  
  
- TGAACCCCTC AATGTGATGC AGATACCCAC TTGGCTTTTT GCTCATTTAA CGAACGAGGA CATCGATATT   
  
  
- GCATATACCC ACTTGGCTTT TTGCTCATTT AACGAACGAG GACATCAATA TTGCATATAC CCACTTCCCC   
  
  
- ACTTTCATCC CCACTCAAGG GGACACATAC TCATATACTT ATCAAAATAT GTTCCTTTAA TGTACCGATT   
  
  
- CCATACGGAA CTATAAAAAA TAAATATATG TTTGTATTAT CATGCAATAA TCAAATAAAA AAGAAATTAA   
  
  
- ATTGTTAACA TAGGTAAATG GCATATTCAT CGGAAAAGAA TATAAAAAGA AAAAGAAATA AACAAGGATT   
  
  
- GAGCCTCACC CACATGTGTA TTAATCACCA TACAAGAATC CAATTCCACA ACCCCATAGA TTATGAAGGA   
  
  
- TTCGACCCCT AACCATTTGA ATATCACACC CAATGTGAAG TAACAAAGCA TAACGATAAT GCCTTGAAAG   
  
  
- CATAAAGTGA CTATTGTCGG AATATTCAGA TTGTGTGGTA ATTCAGTAGC TTTGGGTACC CAGGTAGCTA   
  
  
- CAAGGACTAA GAAGAAATTA ACTTTGAAGT TTTTATATAC AATTCATGGT ACTACACACA TTCATATGGG   
  
  
- AAGAAACTAT GAAAACAAGG AACAAGAAG

+     GT1-motif

| Site Name | Organism | Position | Strand | Matrix score. | sequence | function |
| --- | --- | --- | --- | --- | --- | --- |
| GT1-motif | Arabidopsis thaliana | 1230 | + | 6 | GGTTAA | light responsive element |

> 2018/04/13 10:10:12  
+ CCGATGGGGT TTCTTCGACG TGCATGATCA CTACGTTCTT GTCCTTCACT TCGTCTCGAT GAGCGAAGTA   
  
  
+ CACCACGTGG TAGACGACCC ACAAGCCGCT GTTTGTTTGC ATCTTCTGTA CGTTTTAAGA ATAGGGAGAG   
  
  
+ AGAGCCTCAA CACTCGAACA CTAGACCCCA CCCAAATACG AAAGAAAAAA CAAAAAACCT AATTCCATTT   
  
  
+ GTTCTTAAAG AAAGCCCATA GCCCTAAACC AACGATCCTA CGTTCTTTCG TTTCTTTTGT CCATACAGTT   
  
  
+ CCAACTTTTA TGACTACAAG GAGAGTTTTT GTTTGTTTGT TCTTGCCAAA CACTTCTCTC TCTCTCTCTC   
  
  
+ TCTCTCTCTC TCTCCCTTCC TCTCAGCGAC TGAAAAGTGT GTTTTGTGGA GAAGAGACAT GGGTTGTACA   
  
  
+ GAACTCTCTC TCTCCTTTCT CTCTCTCCAA ATCATATTTT ATTCTACCAT TCCTATTATT AGTTTCCTTT   
  
  
+ TCTTCTTTCT TCTTCTTCTT CTTCTACCAG TATTTCTTAA TACCGGTCAC TCGACCCACT AAATAGTACA   
  
  
+ TCGTAGTTTC CCAGTTTAAC TACGTTGACA AAAAGAATAA TATTATATAA AAGCATTATA AAATGAGAGA   
  
  
+ GAGAGAGAGA GAGAGAGATA GACTCGAAGT AAAGAAGTTC TCTCTCAATT TTTTTTTTTT TTTTTTGCTG   
  
  
+ GAATCCCCTT CAGTAGGAAG TTTTTGTTCA CTACCCTTTC ATTTCTCCGC CGTTCATCGG CGTAAATAGC   
  
  
+ CTTTTTCAAA GTTGGTCCGC TTGTTGGGGG CTTTGCAAAG CTTCGCCCGT CGAAGGAACG AATACTCTAC   
  
  
+ ACTTGGGGAG TTACACTACG TCTATGGGTG AACCGAAAAA CGAGTAAATT GCTTGCTCCT GTAGCTATAA   
  
  
+ CGTATATGGG TGAACCGAAA AACGAGTAAA TTGCTTGCTC CTGTAGTTAT AACGTATATG GGTGAAGGGG   
  
  
+ TGAAAGTAGG GGTGAGTTCC CCTGTGTATG AGTATATGAA TAGTTTTATA CAAGGAAATT ACATGGCTAA   
  
  
+ GGTATGCCTT GATATTTTTT ATTTATATAC AAACATAATA GTACGTTATT AGTTTATTTT TTCTTTAATT   
  
  
+ TAACAATTGT ATCCATTTAC CGTATAAGTA GCCTTTTCTT ATATTTTTCT TTTTCTTTAT TTGTTCCTAA   
  
  
+ CTCGGAGTGG GTGTACACAT AATTAGTGGT ATGTTCTTAG GTTAAGGTGT TGGGGTATCT AATACTTCCT   
  
  
+ AAGCTGGGGA TTGGTAAACT TATAGTGTGG GTTACACTTC ATTGTTTCGT ATTGCTATTA CGGAACTTTC   
  
  
+ GTATTTCACT GATAACAGCC TTATAAGTCT AACACACCAT TAAGTCATCG AAACCCATGG GTCCATCGAT   
  
  
+ GTTCCTGATT CTTCTTTAAT TGAAACTTCA AAAATATATG TTAAGTACCA TGATGTGTGT AAGTATACCC   
  
  
+ TTCTTTGATA CTTTTGTTCC TTGTTCTTC  

- GGCTACCCCA AAGAAGCTGC ACGTACTAGT GATGCAAGAA CAGGAAGTGA AGCAGAGCTA CTCGCTTCAT   
  
  
- GTGGTGCACC ATCTGCTGGG TGTTCGGCGA CAAACAAACG TAGAAGACAT GCAAAATTCT TATCCCTCTC   
  
  
- TCTCGGAGTT GTGAGCTTGT GATCTGGGGT GGGTTTATGC TTTCTTTTTT GTTTTTTGGA TTAAGGTAAA   
  
  
- CAAGAATTTC TTTCGGGTAT CGGGATTTGG TTGCTAGGAT GCAAGAAAGC AAAGAAAACA GGTATGTCAA   
  
  
- GGTTGAAAAT ACTGATGTTC CTCTCAAAAA CAAACAAACA AGAACGGTTT GTGAAGAGAG AGAGAGAGAG   
  
  
- AGAGAGAGAG AGAGGGAAGG AGAGTCGCTG ACTTTTCACA CAAAACACCT CTTCTCTGTA CCCAACATGT   
  
  
- CTTGAGAGAG AGAGGAAAGA GAGAGAGGTT TAGTATAAAA TAAGATGGTA AGGATAATAA TCAAAGGAAA   
  
  
- AGAAGAAAGA AGAAGAAGAA GAAGATGGTC ATAAAGAATT ATGGCCAGTG AGCTGGGTGA TTTATCATGT   
  
  
- AGCATCAAAG GGTCAAATTG ATGCAACTGT TTTTCTTATT ATAATATATT TTCGTAATAT TTTACTCTCT   
  
  
- CTCTCTCTCT CTCTCTCTAT CTGAGCTTCA TTTCTTCAAG AGAGAGTTAA AAAAAAAAAA AAAAAACGAC   
  
  
- CTTAGGGGAA GTCATCCTTC AAAAACAAGT GATGGGAAAG TAAAGAGGCG GCAAGTAGCC GCATTTATCG   
  
  
- GAAAAAGTTT CAACCAGGCG AACAACCCCC GAAACGTTTC GAAGCGGGCA GCTTCCTTGC TTATGAGATG   
  
  
- TGAACCCCTC AATGTGATGC AGATACCCAC TTGGCTTTTT GCTCATTTAA CGAACGAGGA CATCGATATT   
  
  
- GCATATACCC ACTTGGCTTT TTGCTCATTT AACGAACGAG GACATCAATA TTGCATATAC CCACTTCCCC   
  
  
- ACTTTCATCC CCACTCAAGG GGACACATAC TCATATACTT ATCAAAATAT GTTCCTTTAA TGTACCGATT   
  
  
- CCATACGGAA CTATAAAAAA TAAATATATG TTTGTATTAT CATGCAATAA TCAAATAAAA AAGAAATTAA   
  
  
- ATTGTTAACA TAGGTAAATG GCATATTCAT CGGAAAAGAA TATAAAAAGA AAAAGAAATA AACAAGGATT   
  
  
- GAGCCTCACC CACATGTGTA TTAATCACCA TACAAGAATC CAATTCCACA ACCCCATAGA TTATGAAGGA   
  
  
- TTCGACCCCT AACCATTTGA ATATCACACC CAATGTGAAG TAACAAAGCA TAACGATAAT GCCTTGAAAG   
  
  
- CATAAAGTGA CTATTGTCGG AATATTCAGA TTGTGTGGTA ATTCAGTAGC TTTGGGTACC CAGGTAGCTA   
  
  
- CAAGGACTAA GAAGAAATTA ACTTTGAAGT TTTTATATAC AATTCATGGT ACTACACACA TTCATATGGG   
  
  
- AAGAAACTAT GAAAACAAGG AACAAGAAG

+     HSE

| Site Name | Organism | Position | Strand | Matrix score. | sequence | function |
| --- | --- | --- | --- | --- | --- | --- |
| HSE | Brassica oleracea | 1061 | - | 9 | AAAAAATTTC | cis-acting element involved in heat stress responsiveness |

> 2018/04/13 10:10:12  
+ CCGATGGGGT TTCTTCGACG TGCATGATCA CTACGTTCTT GTCCTTCACT TCGTCTCGAT GAGCGAAGTA   
  
  
+ CACCACGTGG TAGACGACCC ACAAGCCGCT GTTTGTTTGC ATCTTCTGTA CGTTTTAAGA ATAGGGAGAG   
  
  
+ AGAGCCTCAA CACTCGAACA CTAGACCCCA CCCAAATACG AAAGAAAAAA CAAAAAACCT AATTCCATTT   
  
  
+ GTTCTTAAAG AAAGCCCATA GCCCTAAACC AACGATCCTA CGTTCTTTCG TTTCTTTTGT CCATACAGTT   
  
  
+ CCAACTTTTA TGACTACAAG GAGAGTTTTT GTTTGTTTGT TCTTGCCAAA CACTTCTCTC TCTCTCTCTC   
  
  
+ TCTCTCTCTC TCTCCCTTCC TCTCAGCGAC TGAAAAGTGT GTTTTGTGGA GAAGAGACAT GGGTTGTACA   
  
  
+ GAACTCTCTC TCTCCTTTCT CTCTCTCCAA ATCATATTTT ATTCTACCAT TCCTATTATT AGTTTCCTTT   
  
  
+ TCTTCTTTCT TCTTCTTCTT CTTCTACCAG TATTTCTTAA TACCGGTCAC TCGACCCACT AAATAGTACA   
  
  
+ TCGTAGTTTC CCAGTTTAAC TACGTTGACA AAAAGAATAA TATTATATAA AAGCATTATA AAATGAGAGA   
  
  
+ GAGAGAGAGA GAGAGAGATA GACTCGAAGT AAAGAAGTTC TCTCTCAATT TTTTTTTTTT TTTTTTGCTG   
  
  
+ GAATCCCCTT CAGTAGGAAG TTTTTGTTCA CTACCCTTTC ATTTCTCCGC CGTTCATCGG CGTAAATAGC   
  
  
+ CTTTTTCAAA GTTGGTCCGC TTGTTGGGGG CTTTGCAAAG CTTCGCCCGT CGAAGGAACG AATACTCTAC   
  
  
+ ACTTGGGGAG TTACACTACG TCTATGGGTG AACCGAAAAA CGAGTAAATT GCTTGCTCCT GTAGCTATAA   
  
  
+ CGTATATGGG TGAACCGAAA AACGAGTAAA TTGCTTGCTC CTGTAGTTAT AACGTATATG GGTGAAGGGG   
  
  
+ TGAAAGTAGG GGTGAGTTCC CCTGTGTATG AGTATATGAA TAGTTTTATA CAAGGAAATT ACATGGCTAA   
  
  
+ GGTATGCCTT GATATTTTTT ATTTATATAC AAACATAATA GTACGTTATT AGTTTATTTT TTCTTTAATT   
  
  
+ TAACAATTGT ATCCATTTAC CGTATAAGTA GCCTTTTCTT ATATTTTTCT TTTTCTTTAT TTGTTCCTAA   
  
  
+ CTCGGAGTGG GTGTACACAT AATTAGTGGT ATGTTCTTAG GTTAAGGTGT TGGGGTATCT AATACTTCCT   
  
  
+ AAGCTGGGGA TTGGTAAACT TATAGTGTGG GTTACACTTC ATTGTTTCGT ATTGCTATTA CGGAACTTTC   
  
  
+ GTATTTCACT GATAACAGCC TTATAAGTCT AACACACCAT TAAGTCATCG AAACCCATGG GTCCATCGAT   
  
  
+ GTTCCTGATT CTTCTTTAAT TGAAACTTCA AAAATATATG TTAAGTACCA TGATGTGTGT AAGTATACCC   
  
  
+ TTCTTTGATA CTTTTGTTCC TTGTTCTTC  

- GGCTACCCCA AAGAAGCTGC ACGTACTAGT GATGCAAGAA CAGGAAGTGA AGCAGAGCTA CTCGCTTCAT   
  
  
- GTGGTGCACC ATCTGCTGGG TGTTCGGCGA CAAACAAACG TAGAAGACAT GCAAAATTCT TATCCCTCTC   
  
  
- TCTCGGAGTT GTGAGCTTGT GATCTGGGGT GGGTTTATGC TTTCTTTTTT GTTTTTTGGA TTAAGGTAAA   
  
  
- CAAGAATTTC TTTCGGGTAT CGGGATTTGG TTGCTAGGAT GCAAGAAAGC AAAGAAAACA GGTATGTCAA   
  
  
- GGTTGAAAAT ACTGATGTTC CTCTCAAAAA CAAACAAACA AGAACGGTTT GTGAAGAGAG AGAGAGAGAG   
  
  
- AGAGAGAGAG AGAGGGAAGG AGAGTCGCTG ACTTTTCACA CAAAACACCT CTTCTCTGTA CCCAACATGT   
  
  
- CTTGAGAGAG AGAGGAAAGA GAGAGAGGTT TAGTATAAAA TAAGATGGTA AGGATAATAA TCAAAGGAAA   
  
  
- AGAAGAAAGA AGAAGAAGAA GAAGATGGTC ATAAAGAATT ATGGCCAGTG AGCTGGGTGA TTTATCATGT   
  
  
- AGCATCAAAG GGTCAAATTG ATGCAACTGT TTTTCTTATT ATAATATATT TTCGTAATAT TTTACTCTCT   
  
  
- CTCTCTCTCT CTCTCTCTAT CTGAGCTTCA TTTCTTCAAG AGAGAGTTAA AAAAAAAAAA AAAAAACGAC   
  
  
- CTTAGGGGAA GTCATCCTTC AAAAACAAGT GATGGGAAAG TAAAGAGGCG GCAAGTAGCC GCATTTATCG   
  
  
- GAAAAAGTTT CAACCAGGCG AACAACCCCC GAAACGTTTC GAAGCGGGCA GCTTCCTTGC TTATGAGATG   
  
  
- TGAACCCCTC AATGTGATGC AGATACCCAC TTGGCTTTTT GCTCATTTAA CGAACGAGGA CATCGATATT   
  
  
- GCATATACCC ACTTGGCTTT TTGCTCATTT AACGAACGAG GACATCAATA TTGCATATAC CCACTTCCCC   
  
  
- ACTTTCATCC CCACTCAAGG GGACACATAC TCATATACTT ATCAAAATAT GTTCCTTTAA TGTACCGATT   
  
  
- CCATACGGAA CTATAAAAAA TAAATATATG TTTGTATTAT CATGCAATAA TCAAATAAAA AAGAAATTAA   
  
  
- ATTGTTAACA TAGGTAAATG GCATATTCAT CGGAAAAGAA TATAAAAAGA AAAAGAAATA AACAAGGATT   
  
  
- GAGCCTCACC CACATGTGTA TTAATCACCA TACAAGAATC CAATTCCACA ACCCCATAGA TTATGAAGGA   
  
  
- TTCGACCCCT AACCATTTGA ATATCACACC CAATGTGAAG TAACAAAGCA TAACGATAAT GCCTTGAAAG   
  
  
- CATAAAGTGA CTATTGTCGG AATATTCAGA TTGTGTGGTA ATTCAGTAGC TTTGGGTACC CAGGTAGCTA   
  
  
- CAAGGACTAA GAAGAAATTA ACTTTGAAGT TTTTATATAC AATTCATGGT ACTACACACA TTCATATGGG   
  
  
- AAGAAACTAT GAAAACAAGG AACAAGAAG

+     I-box

| Site Name | Organism | Position | Strand | Matrix score. | sequence | function |
| --- | --- | --- | --- | --- | --- | --- |
| I-box | Zea mays | 1230 | + | 9 | gGATAAGGTG | part of a light responsive element |
| I-box | Gossypium hirsutum | 1150 | - | 10 | AAGATAAGGCT | part of a light responsive element |

> 2018/04/13 10:10:12  
+ CCGATGGGGT TTCTTCGACG TGCATGATCA CTACGTTCTT GTCCTTCACT TCGTCTCGAT GAGCGAAGTA   
  
  
+ CACCACGTGG TAGACGACCC ACAAGCCGCT GTTTGTTTGC ATCTTCTGTA CGTTTTAAGA ATAGGGAGAG   
  
  
+ AGAGCCTCAA CACTCGAACA CTAGACCCCA CCCAAATACG AAAGAAAAAA CAAAAAACCT AATTCCATTT   
  
  
+ GTTCTTAAAG AAAGCCCATA GCCCTAAACC AACGATCCTA CGTTCTTTCG TTTCTTTTGT CCATACAGTT   
  
  
+ CCAACTTTTA TGACTACAAG GAGAGTTTTT GTTTGTTTGT TCTTGCCAAA CACTTCTCTC TCTCTCTCTC   
  
  
+ TCTCTCTCTC TCTCCCTTCC TCTCAGCGAC TGAAAAGTGT GTTTTGTGGA GAAGAGACAT GGGTTGTACA   
  
  
+ GAACTCTCTC TCTCCTTTCT CTCTCTCCAA ATCATATTTT ATTCTACCAT TCCTATTATT AGTTTCCTTT   
  
  
+ TCTTCTTTCT TCTTCTTCTT CTTCTACCAG TATTTCTTAA TACCGGTCAC TCGACCCACT AAATAGTACA   
  
  
+ TCGTAGTTTC CCAGTTTAAC TACGTTGACA AAAAGAATAA TATTATATAA AAGCATTATA AAATGAGAGA   
  
  
+ GAGAGAGAGA GAGAGAGATA GACTCGAAGT AAAGAAGTTC TCTCTCAATT TTTTTTTTTT TTTTTTGCTG   
  
  
+ GAATCCCCTT CAGTAGGAAG TTTTTGTTCA CTACCCTTTC ATTTCTCCGC CGTTCATCGG CGTAAATAGC   
  
  
+ CTTTTTCAAA GTTGGTCCGC TTGTTGGGGG CTTTGCAAAG CTTCGCCCGT CGAAGGAACG AATACTCTAC   
  
  
+ ACTTGGGGAG TTACACTACG TCTATGGGTG AACCGAAAAA CGAGTAAATT GCTTGCTCCT GTAGCTATAA   
  
  
+ CGTATATGGG TGAACCGAAA AACGAGTAAA TTGCTTGCTC CTGTAGTTAT AACGTATATG GGTGAAGGGG   
  
  
+ TGAAAGTAGG GGTGAGTTCC CCTGTGTATG AGTATATGAA TAGTTTTATA CAAGGAAATT ACATGGCTAA   
  
  
+ GGTATGCCTT GATATTTTTT ATTTATATAC AAACATAATA GTACGTTATT AGTTTATTTT TTCTTTAATT   
  
  
+ TAACAATTGT ATCCATTTAC CGTATAAGTA GCCTTTTCTT ATATTTTTCT TTTTCTTTAT TTGTTCCTAA   
  
  
+ CTCGGAGTGG GTGTACACAT AATTAGTGGT ATGTTCTTAG GTTAAGGTGT TGGGGTATCT AATACTTCCT   
  
  
+ AAGCTGGGGA TTGGTAAACT TATAGTGTGG GTTACACTTC ATTGTTTCGT ATTGCTATTA CGGAACTTTC   
  
  
+ GTATTTCACT GATAACAGCC TTATAAGTCT AACACACCAT TAAGTCATCG AAACCCATGG GTCCATCGAT   
  
  
+ GTTCCTGATT CTTCTTTAAT TGAAACTTCA AAAATATATG TTAAGTACCA TGATGTGTGT AAGTATACCC   
  
  
+ TTCTTTGATA CTTTTGTTCC TTGTTCTTC  

- GGCTACCCCA AAGAAGCTGC ACGTACTAGT GATGCAAGAA CAGGAAGTGA AGCAGAGCTA CTCGCTTCAT   
  
  
- GTGGTGCACC ATCTGCTGGG TGTTCGGCGA CAAACAAACG TAGAAGACAT GCAAAATTCT TATCCCTCTC   
  
  
- TCTCGGAGTT GTGAGCTTGT GATCTGGGGT GGGTTTATGC TTTCTTTTTT GTTTTTTGGA TTAAGGTAAA   
  
  
- CAAGAATTTC TTTCGGGTAT CGGGATTTGG TTGCTAGGAT GCAAGAAAGC AAAGAAAACA GGTATGTCAA   
  
  
- GGTTGAAAAT ACTGATGTTC CTCTCAAAAA CAAACAAACA AGAACGGTTT GTGAAGAGAG AGAGAGAGAG   
  
  
- AGAGAGAGAG AGAGGGAAGG AGAGTCGCTG ACTTTTCACA CAAAACACCT CTTCTCTGTA CCCAACATGT   
  
  
- CTTGAGAGAG AGAGGAAAGA GAGAGAGGTT TAGTATAAAA TAAGATGGTA AGGATAATAA TCAAAGGAAA   
  
  
- AGAAGAAAGA AGAAGAAGAA GAAGATGGTC ATAAAGAATT ATGGCCAGTG AGCTGGGTGA TTTATCATGT   
  
  
- AGCATCAAAG GGTCAAATTG ATGCAACTGT TTTTCTTATT ATAATATATT TTCGTAATAT TTTACTCTCT   
  
  
- CTCTCTCTCT CTCTCTCTAT CTGAGCTTCA TTTCTTCAAG AGAGAGTTAA AAAAAAAAAA AAAAAACGAC   
  
  
- CTTAGGGGAA GTCATCCTTC AAAAACAAGT GATGGGAAAG TAAAGAGGCG GCAAGTAGCC GCATTTATCG   
  
  
- GAAAAAGTTT CAACCAGGCG AACAACCCCC GAAACGTTTC GAAGCGGGCA GCTTCCTTGC TTATGAGATG   
  
  
- TGAACCCCTC AATGTGATGC AGATACCCAC TTGGCTTTTT GCTCATTTAA CGAACGAGGA CATCGATATT   
  
  
- GCATATACCC ACTTGGCTTT TTGCTCATTT AACGAACGAG GACATCAATA TTGCATATAC CCACTTCCCC   
  
  
- ACTTTCATCC CCACTCAAGG GGACACATAC TCATATACTT ATCAAAATAT GTTCCTTTAA TGTACCGATT   
  
  
- CCATACGGAA CTATAAAAAA TAAATATATG TTTGTATTAT CATGCAATAA TCAAATAAAA AAGAAATTAA   
  
  
- ATTGTTAACA TAGGTAAATG GCATATTCAT CGGAAAAGAA TATAAAAAGA AAAAGAAATA AACAAGGATT   
  
  
- GAGCCTCACC CACATGTGTA TTAATCACCA TACAAGAATC CAATTCCACA ACCCCATAGA TTATGAAGGA   
  
  
- TTCGACCCCT AACCATTTGA ATATCACACC CAATGTGAAG TAACAAAGCA TAACGATAAT GCCTTGAAAG   
  
  
- CATAAAGTGA CTATTGTCGG AATATTCAGA TTGTGTGGTA ATTCAGTAGC TTTGGGTACC CAGGTAGCTA   
  
  
- CAAGGACTAA GAAGAAATTA ACTTTGAAGT TTTTATATAC AATTCATGGT ACTACACACA TTCATATGGG   
  
  
- AAGAAACTAT GAAAACAAGG AACAAGAAG

+     LTR

| Site Name | Organism | Position | Strand | Matrix score. | sequence | function |
| --- | --- | --- | --- | --- | --- | --- |
| LTR | Hordeum vulgare | 873 | + | 6 | CCGAAA | cis-acting element involved in low-temperature responsiveness |
| LTR | Hordeum vulgare | 925 | + | 6 | CCGAAA | cis-acting element involved in low-temperature responsiveness |

> 2018/04/13 10:10:12  
+ CCGATGGGGT TTCTTCGACG TGCATGATCA CTACGTTCTT GTCCTTCACT TCGTCTCGAT GAGCGAAGTA   
  
  
+ CACCACGTGG TAGACGACCC ACAAGCCGCT GTTTGTTTGC ATCTTCTGTA CGTTTTAAGA ATAGGGAGAG   
  
  
+ AGAGCCTCAA CACTCGAACA CTAGACCCCA CCCAAATACG AAAGAAAAAA CAAAAAACCT AATTCCATTT   
  
  
+ GTTCTTAAAG AAAGCCCATA GCCCTAAACC AACGATCCTA CGTTCTTTCG TTTCTTTTGT CCATACAGTT   
  
  
+ CCAACTTTTA TGACTACAAG GAGAGTTTTT GTTTGTTTGT TCTTGCCAAA CACTTCTCTC TCTCTCTCTC   
  
  
+ TCTCTCTCTC TCTCCCTTCC TCTCAGCGAC TGAAAAGTGT GTTTTGTGGA GAAGAGACAT GGGTTGTACA   
  
  
+ GAACTCTCTC TCTCCTTTCT CTCTCTCCAA ATCATATTTT ATTCTACCAT TCCTATTATT AGTTTCCTTT   
  
  
+ TCTTCTTTCT TCTTCTTCTT CTTCTACCAG TATTTCTTAA TACCGGTCAC TCGACCCACT AAATAGTACA   
  
  
+ TCGTAGTTTC CCAGTTTAAC TACGTTGACA AAAAGAATAA TATTATATAA AAGCATTATA AAATGAGAGA   
  
  
+ GAGAGAGAGA GAGAGAGATA GACTCGAAGT AAAGAAGTTC TCTCTCAATT TTTTTTTTTT TTTTTTGCTG   
  
  
+ GAATCCCCTT CAGTAGGAAG TTTTTGTTCA CTACCCTTTC ATTTCTCCGC CGTTCATCGG CGTAAATAGC   
  
  
+ CTTTTTCAAA GTTGGTCCGC TTGTTGGGGG CTTTGCAAAG CTTCGCCCGT CGAAGGAACG AATACTCTAC   
  
  
+ ACTTGGGGAG TTACACTACG TCTATGGGTG AACCGAAAAA CGAGTAAATT GCTTGCTCCT GTAGCTATAA   
  
  
+ CGTATATGGG TGAACCGAAA AACGAGTAAA TTGCTTGCTC CTGTAGTTAT AACGTATATG GGTGAAGGGG   
  
  
+ TGAAAGTAGG GGTGAGTTCC CCTGTGTATG AGTATATGAA TAGTTTTATA CAAGGAAATT ACATGGCTAA   
  
  
+ GGTATGCCTT GATATTTTTT ATTTATATAC AAACATAATA GTACGTTATT AGTTTATTTT TTCTTTAATT   
  
  
+ TAACAATTGT ATCCATTTAC CGTATAAGTA GCCTTTTCTT ATATTTTTCT TTTTCTTTAT TTGTTCCTAA   
  
  
+ CTCGGAGTGG GTGTACACAT AATTAGTGGT ATGTTCTTAG GTTAAGGTGT TGGGGTATCT AATACTTCCT   
  
  
+ AAGCTGGGGA TTGGTAAACT TATAGTGTGG GTTACACTTC ATTGTTTCGT ATTGCTATTA CGGAACTTTC   
  
  
+ GTATTTCACT GATAACAGCC TTATAAGTCT AACACACCAT TAAGTCATCG AAACCCATGG GTCCATCGAT   
  
  
+ GTTCCTGATT CTTCTTTAAT TGAAACTTCA AAAATATATG TTAAGTACCA TGATGTGTGT AAGTATACCC   
  
  
+ TTCTTTGATA CTTTTGTTCC TTGTTCTTC  

- GGCTACCCCA AAGAAGCTGC ACGTACTAGT GATGCAAGAA CAGGAAGTGA AGCAGAGCTA CTCGCTTCAT   
  
  
- GTGGTGCACC ATCTGCTGGG TGTTCGGCGA CAAACAAACG TAGAAGACAT GCAAAATTCT TATCCCTCTC   
  
  
- TCTCGGAGTT GTGAGCTTGT GATCTGGGGT GGGTTTATGC TTTCTTTTTT GTTTTTTGGA TTAAGGTAAA   
  
  
- CAAGAATTTC TTTCGGGTAT CGGGATTTGG TTGCTAGGAT GCAAGAAAGC AAAGAAAACA GGTATGTCAA   
  
  
- GGTTGAAAAT ACTGATGTTC CTCTCAAAAA CAAACAAACA AGAACGGTTT GTGAAGAGAG AGAGAGAGAG   
  
  
- AGAGAGAGAG AGAGGGAAGG AGAGTCGCTG ACTTTTCACA CAAAACACCT CTTCTCTGTA CCCAACATGT   
  
  
- CTTGAGAGAG AGAGGAAAGA GAGAGAGGTT TAGTATAAAA TAAGATGGTA AGGATAATAA TCAAAGGAAA   
  
  
- AGAAGAAAGA AGAAGAAGAA GAAGATGGTC ATAAAGAATT ATGGCCAGTG AGCTGGGTGA TTTATCATGT   
  
  
- AGCATCAAAG GGTCAAATTG ATGCAACTGT TTTTCTTATT ATAATATATT TTCGTAATAT TTTACTCTCT   
  
  
- CTCTCTCTCT CTCTCTCTAT CTGAGCTTCA TTTCTTCAAG AGAGAGTTAA AAAAAAAAAA AAAAAACGAC   
  
  
- CTTAGGGGAA GTCATCCTTC AAAAACAAGT GATGGGAAAG TAAAGAGGCG GCAAGTAGCC GCATTTATCG   
  
  
- GAAAAAGTTT CAACCAGGCG AACAACCCCC GAAACGTTTC GAAGCGGGCA GCTTCCTTGC TTATGAGATG   
  
  
- TGAACCCCTC AATGTGATGC AGATACCCAC TTGGCTTTTT GCTCATTTAA CGAACGAGGA CATCGATATT   
  
  
- GCATATACCC ACTTGGCTTT TTGCTCATTT AACGAACGAG GACATCAATA TTGCATATAC CCACTTCCCC   
  
  
- ACTTTCATCC CCACTCAAGG GGACACATAC TCATATACTT ATCAAAATAT GTTCCTTTAA TGTACCGATT   
  
  
- CCATACGGAA CTATAAAAAA TAAATATATG TTTGTATTAT CATGCAATAA TCAAATAAAA AAGAAATTAA   
  
  
- ATTGTTAACA TAGGTAAATG GCATATTCAT CGGAAAAGAA TATAAAAAGA AAAAGAAATA AACAAGGATT   
  
  
- GAGCCTCACC CACATGTGTA TTAATCACCA TACAAGAATC CAATTCCACA ACCCCATAGA TTATGAAGGA   
  
  
- TTCGACCCCT AACCATTTGA ATATCACACC CAATGTGAAG TAACAAAGCA TAACGATAAT GCCTTGAAAG   
  
  
- CATAAAGTGA CTATTGTCGG AATATTCAGA TTGTGTGGTA ATTCAGTAGC TTTGGGTACC CAGGTAGCTA   
  
  
- CAAGGACTAA GAAGAAATTA ACTTTGAAGT TTTTATATAC AATTCATGGT ACTACACACA TTCATATGGG   
  
  
- AAGAAACTAT GAAAACAAGG AACAAGAAG

+     MBS

| Site Name | Organism | Position | Strand | Matrix score. | sequence | function |
| --- | --- | --- | --- | --- | --- | --- |
| MBS | Zea mays | 534 | + | 6 | CGGTCA | MYB Binding Site |

> 2018/04/13 10:10:12  
+ CCGATGGGGT TTCTTCGACG TGCATGATCA CTACGTTCTT GTCCTTCACT TCGTCTCGAT GAGCGAAGTA   
  
  
+ CACCACGTGG TAGACGACCC ACAAGCCGCT GTTTGTTTGC ATCTTCTGTA CGTTTTAAGA ATAGGGAGAG   
  
  
+ AGAGCCTCAA CACTCGAACA CTAGACCCCA CCCAAATACG AAAGAAAAAA CAAAAAACCT AATTCCATTT   
  
  
+ GTTCTTAAAG AAAGCCCATA GCCCTAAACC AACGATCCTA CGTTCTTTCG TTTCTTTTGT CCATACAGTT   
  
  
+ CCAACTTTTA TGACTACAAG GAGAGTTTTT GTTTGTTTGT TCTTGCCAAA CACTTCTCTC TCTCTCTCTC   
  
  
+ TCTCTCTCTC TCTCCCTTCC TCTCAGCGAC TGAAAAGTGT GTTTTGTGGA GAAGAGACAT GGGTTGTACA   
  
  
+ GAACTCTCTC TCTCCTTTCT CTCTCTCCAA ATCATATTTT ATTCTACCAT TCCTATTATT AGTTTCCTTT   
  
  
+ TCTTCTTTCT TCTTCTTCTT CTTCTACCAG TATTTCTTAA TACCGGTCAC TCGACCCACT AAATAGTACA   
  
  
+ TCGTAGTTTC CCAGTTTAAC TACGTTGACA AAAAGAATAA TATTATATAA AAGCATTATA AAATGAGAGA   
  
  
+ GAGAGAGAGA GAGAGAGATA GACTCGAAGT AAAGAAGTTC TCTCTCAATT TTTTTTTTTT TTTTTTGCTG   
  
  
+ GAATCCCCTT CAGTAGGAAG TTTTTGTTCA CTACCCTTTC ATTTCTCCGC CGTTCATCGG CGTAAATAGC   
  
  
+ CTTTTTCAAA GTTGGTCCGC TTGTTGGGGG CTTTGCAAAG CTTCGCCCGT CGAAGGAACG AATACTCTAC   
  
  
+ ACTTGGGGAG TTACACTACG TCTATGGGTG AACCGAAAAA CGAGTAAATT GCTTGCTCCT GTAGCTATAA   
  
  
+ CGTATATGGG TGAACCGAAA AACGAGTAAA TTGCTTGCTC CTGTAGTTAT AACGTATATG GGTGAAGGGG   
  
  
+ TGAAAGTAGG GGTGAGTTCC CCTGTGTATG AGTATATGAA TAGTTTTATA CAAGGAAATT ACATGGCTAA   
  
  
+ GGTATGCCTT GATATTTTTT ATTTATATAC AAACATAATA GTACGTTATT AGTTTATTTT TTCTTTAATT   
  
  
+ TAACAATTGT ATCCATTTAC CGTATAAGTA GCCTTTTCTT ATATTTTTCT TTTTCTTTAT TTGTTCCTAA   
  
  
+ CTCGGAGTGG GTGTACACAT AATTAGTGGT ATGTTCTTAG GTTAAGGTGT TGGGGTATCT AATACTTCCT   
  
  
+ AAGCTGGGGA TTGGTAAACT TATAGTGTGG GTTACACTTC ATTGTTTCGT ATTGCTATTA CGGAACTTTC   
  
  
+ GTATTTCACT GATAACAGCC TTATAAGTCT AACACACCAT TAAGTCATCG AAACCCATGG GTCCATCGAT   
  
  
+ GTTCCTGATT CTTCTTTAAT TGAAACTTCA AAAATATATG TTAAGTACCA TGATGTGTGT AAGTATACCC   
  
  
+ TTCTTTGATA CTTTTGTTCC TTGTTCTTC  

- GGCTACCCCA AAGAAGCTGC ACGTACTAGT GATGCAAGAA CAGGAAGTGA AGCAGAGCTA CTCGCTTCAT   
  
  
- GTGGTGCACC ATCTGCTGGG TGTTCGGCGA CAAACAAACG TAGAAGACAT GCAAAATTCT TATCCCTCTC   
  
  
- TCTCGGAGTT GTGAGCTTGT GATCTGGGGT GGGTTTATGC TTTCTTTTTT GTTTTTTGGA TTAAGGTAAA   
  
  
- CAAGAATTTC TTTCGGGTAT CGGGATTTGG TTGCTAGGAT GCAAGAAAGC AAAGAAAACA GGTATGTCAA   
  
  
- GGTTGAAAAT ACTGATGTTC CTCTCAAAAA CAAACAAACA AGAACGGTTT GTGAAGAGAG AGAGAGAGAG   
  
  
- AGAGAGAGAG AGAGGGAAGG AGAGTCGCTG ACTTTTCACA CAAAACACCT CTTCTCTGTA CCCAACATGT   
  
  
- CTTGAGAGAG AGAGGAAAGA GAGAGAGGTT TAGTATAAAA TAAGATGGTA AGGATAATAA TCAAAGGAAA   
  
  
- AGAAGAAAGA AGAAGAAGAA GAAGATGGTC ATAAAGAATT ATGGCCAGTG AGCTGGGTGA TTTATCATGT   
  
  
- AGCATCAAAG GGTCAAATTG ATGCAACTGT TTTTCTTATT ATAATATATT TTCGTAATAT TTTACTCTCT   
  
  
- CTCTCTCTCT CTCTCTCTAT CTGAGCTTCA TTTCTTCAAG AGAGAGTTAA AAAAAAAAAA AAAAAACGAC   
  
  
- CTTAGGGGAA GTCATCCTTC AAAAACAAGT GATGGGAAAG TAAAGAGGCG GCAAGTAGCC GCATTTATCG   
  
  
- GAAAAAGTTT CAACCAGGCG AACAACCCCC GAAACGTTTC GAAGCGGGCA GCTTCCTTGC TTATGAGATG   
  
  
- TGAACCCCTC AATGTGATGC AGATACCCAC TTGGCTTTTT GCTCATTTAA CGAACGAGGA CATCGATATT   
  
  
- GCATATACCC ACTTGGCTTT TTGCTCATTT AACGAACGAG GACATCAATA TTGCATATAC CCACTTCCCC   
  
  
- ACTTTCATCC CCACTCAAGG GGACACATAC TCATATACTT ATCAAAATAT GTTCCTTTAA TGTACCGATT   
  
  
- CCATACGGAA CTATAAAAAA TAAATATATG TTTGTATTAT CATGCAATAA TCAAATAAAA AAGAAATTAA   
  
  
- ATTGTTAACA TAGGTAAATG GCATATTCAT CGGAAAAGAA TATAAAAAGA AAAAGAAATA AACAAGGATT   
  
  
- GAGCCTCACC CACATGTGTA TTAATCACCA TACAAGAATC CAATTCCACA ACCCCATAGA TTATGAAGGA   
  
  
- TTCGACCCCT AACCATTTGA ATATCACACC CAATGTGAAG TAACAAAGCA TAACGATAAT GCCTTGAAAG   
  
  
- CATAAAGTGA CTATTGTCGG AATATTCAGA TTGTGTGGTA ATTCAGTAGC TTTGGGTACC CAGGTAGCTA   
  
  
- CAAGGACTAA GAAGAAATTA ACTTTGAAGT TTTTATATAC AATTCATGGT ACTACACACA TTCATATGGG   
  
  
- AAGAAACTAT GAAAACAAGG AACAAGAAG

+     MRE

| Site Name | Organism | Position | Strand | Matrix score. | sequence | function |
| --- | --- | --- | --- | --- | --- | --- |
| MRE | Petroselinum crispum | 196 | + | 7 | AACCTAA | MYB binding site involved in light responsiveness |
| MRE | Petroselinum crispum | 1227 | - | 7 | AACCTAA | MYB binding site involved in light responsiveness |

> 2018/04/13 10:10:12  
+ CCGATGGGGT TTCTTCGACG TGCATGATCA CTACGTTCTT GTCCTTCACT TCGTCTCGAT GAGCGAAGTA   
  
  
+ CACCACGTGG TAGACGACCC ACAAGCCGCT GTTTGTTTGC ATCTTCTGTA CGTTTTAAGA ATAGGGAGAG   
  
  
+ AGAGCCTCAA CACTCGAACA CTAGACCCCA CCCAAATACG AAAGAAAAAA CAAAAAACCT AATTCCATTT   
  
  
+ GTTCTTAAAG AAAGCCCATA GCCCTAAACC AACGATCCTA CGTTCTTTCG TTTCTTTTGT CCATACAGTT   
  
  
+ CCAACTTTTA TGACTACAAG GAGAGTTTTT GTTTGTTTGT TCTTGCCAAA CACTTCTCTC TCTCTCTCTC   
  
  
+ TCTCTCTCTC TCTCCCTTCC TCTCAGCGAC TGAAAAGTGT GTTTTGTGGA GAAGAGACAT GGGTTGTACA   
  
  
+ GAACTCTCTC TCTCCTTTCT CTCTCTCCAA ATCATATTTT ATTCTACCAT TCCTATTATT AGTTTCCTTT   
  
  
+ TCTTCTTTCT TCTTCTTCTT CTTCTACCAG TATTTCTTAA TACCGGTCAC TCGACCCACT AAATAGTACA   
  
  
+ TCGTAGTTTC CCAGTTTAAC TACGTTGACA AAAAGAATAA TATTATATAA AAGCATTATA AAATGAGAGA   
  
  
+ GAGAGAGAGA GAGAGAGATA GACTCGAAGT AAAGAAGTTC TCTCTCAATT TTTTTTTTTT TTTTTTGCTG   
  
  
+ GAATCCCCTT CAGTAGGAAG TTTTTGTTCA CTACCCTTTC ATTTCTCCGC CGTTCATCGG CGTAAATAGC   
  
  
+ CTTTTTCAAA GTTGGTCCGC TTGTTGGGGG CTTTGCAAAG CTTCGCCCGT CGAAGGAACG AATACTCTAC   
  
  
+ ACTTGGGGAG TTACACTACG TCTATGGGTG AACCGAAAAA CGAGTAAATT GCTTGCTCCT GTAGCTATAA   
  
  
+ CGTATATGGG TGAACCGAAA AACGAGTAAA TTGCTTGCTC CTGTAGTTAT AACGTATATG GGTGAAGGGG   
  
  
+ TGAAAGTAGG GGTGAGTTCC CCTGTGTATG AGTATATGAA TAGTTTTATA CAAGGAAATT ACATGGCTAA   
  
  
+ GGTATGCCTT GATATTTTTT ATTTATATAC AAACATAATA GTACGTTATT AGTTTATTTT TTCTTTAATT   
  
  
+ TAACAATTGT ATCCATTTAC CGTATAAGTA GCCTTTTCTT ATATTTTTCT TTTTCTTTAT TTGTTCCTAA   
  
  
+ CTCGGAGTGG GTGTACACAT AATTAGTGGT ATGTTCTTAG GTTAAGGTGT TGGGGTATCT AATACTTCCT   
  
  
+ AAGCTGGGGA TTGGTAAACT TATAGTGTGG GTTACACTTC ATTGTTTCGT ATTGCTATTA CGGAACTTTC   
  
  
+ GTATTTCACT GATAACAGCC TTATAAGTCT AACACACCAT TAAGTCATCG AAACCCATGG GTCCATCGAT   
  
  
+ GTTCCTGATT CTTCTTTAAT TGAAACTTCA AAAATATATG TTAAGTACCA TGATGTGTGT AAGTATACCC   
  
  
+ TTCTTTGATA CTTTTGTTCC TTGTTCTTC  

- GGCTACCCCA AAGAAGCTGC ACGTACTAGT GATGCAAGAA CAGGAAGTGA AGCAGAGCTA CTCGCTTCAT   
  
  
- GTGGTGCACC ATCTGCTGGG TGTTCGGCGA CAAACAAACG TAGAAGACAT GCAAAATTCT TATCCCTCTC   
  
  
- TCTCGGAGTT GTGAGCTTGT GATCTGGGGT GGGTTTATGC TTTCTTTTTT GTTTTTTGGA TTAAGGTAAA   
  
  
- CAAGAATTTC TTTCGGGTAT CGGGATTTGG TTGCTAGGAT GCAAGAAAGC AAAGAAAACA GGTATGTCAA   
  
  
- GGTTGAAAAT ACTGATGTTC CTCTCAAAAA CAAACAAACA AGAACGGTTT GTGAAGAGAG AGAGAGAGAG   
  
  
- AGAGAGAGAG AGAGGGAAGG AGAGTCGCTG ACTTTTCACA CAAAACACCT CTTCTCTGTA CCCAACATGT   
  
  
- CTTGAGAGAG AGAGGAAAGA GAGAGAGGTT TAGTATAAAA TAAGATGGTA AGGATAATAA TCAAAGGAAA   
  
  
- AGAAGAAAGA AGAAGAAGAA GAAGATGGTC ATAAAGAATT ATGGCCAGTG AGCTGGGTGA TTTATCATGT   
  
  
- AGCATCAAAG GGTCAAATTG ATGCAACTGT TTTTCTTATT ATAATATATT TTCGTAATAT TTTACTCTCT   
  
  
- CTCTCTCTCT CTCTCTCTAT CTGAGCTTCA TTTCTTCAAG AGAGAGTTAA AAAAAAAAAA AAAAAACGAC   
  
  
- CTTAGGGGAA GTCATCCTTC AAAAACAAGT GATGGGAAAG TAAAGAGGCG GCAAGTAGCC GCATTTATCG   
  
  
- GAAAAAGTTT CAACCAGGCG AACAACCCCC GAAACGTTTC GAAGCGGGCA GCTTCCTTGC TTATGAGATG   
  
  
- TGAACCCCTC AATGTGATGC AGATACCCAC TTGGCTTTTT GCTCATTTAA CGAACGAGGA CATCGATATT   
  
  
- GCATATACCC ACTTGGCTTT TTGCTCATTT AACGAACGAG GACATCAATA TTGCATATAC CCACTTCCCC   
  
  
- ACTTTCATCC CCACTCAAGG GGACACATAC TCATATACTT ATCAAAATAT GTTCCTTTAA TGTACCGATT   
  
  
- CCATACGGAA CTATAAAAAA TAAATATATG TTTGTATTAT CATGCAATAA TCAAATAAAA AAGAAATTAA   
  
  
- ATTGTTAACA TAGGTAAATG GCATATTCAT CGGAAAAGAA TATAAAAAGA AAAAGAAATA AACAAGGATT   
  
  
- GAGCCTCACC CACATGTGTA TTAATCACCA TACAAGAATC CAATTCCACA ACCCCATAGA TTATGAAGGA   
  
  
- TTCGACCCCT AACCATTTGA ATATCACACC CAATGTGAAG TAACAAAGCA TAACGATAAT GCCTTGAAAG   
  
  
- CATAAAGTGA CTATTGTCGG AATATTCAGA TTGTGTGGTA ATTCAGTAGC TTTGGGTACC CAGGTAGCTA   
  
  
- CAAGGACTAA GAAGAAATTA ACTTTGAAGT TTTTATATAC AATTCATGGT ACTACACACA TTCATATGGG   
  
  
- AAGAAACTAT GAAAACAAGG AACAAGAAG

+     Skn-1\_motif

| Site Name | Organism | Position | Strand | Matrix score. | sequence | function |
| --- | --- | --- | --- | --- | --- | --- |
| Skn-1\_motif | Oryza sativa | 1374 | + | 5 | GTCAT | cis-acting regulatory element required for endosperm expression |
| Skn-1\_motif | Oryza sativa | 290 | - | 5 | GTCAT | cis-acting regulatory element required for endosperm expression |

> 2018/04/13 10:10:12  
+ CCGATGGGGT TTCTTCGACG TGCATGATCA CTACGTTCTT GTCCTTCACT TCGTCTCGAT GAGCGAAGTA   
  
  
+ CACCACGTGG TAGACGACCC ACAAGCCGCT GTTTGTTTGC ATCTTCTGTA CGTTTTAAGA ATAGGGAGAG   
  
  
+ AGAGCCTCAA CACTCGAACA CTAGACCCCA CCCAAATACG AAAGAAAAAA CAAAAAACCT AATTCCATTT   
  
  
+ GTTCTTAAAG AAAGCCCATA GCCCTAAACC AACGATCCTA CGTTCTTTCG TTTCTTTTGT CCATACAGTT   
  
  
+ CCAACTTTTA TGACTACAAG GAGAGTTTTT GTTTGTTTGT TCTTGCCAAA CACTTCTCTC TCTCTCTCTC   
  
  
+ TCTCTCTCTC TCTCCCTTCC TCTCAGCGAC TGAAAAGTGT GTTTTGTGGA GAAGAGACAT GGGTTGTACA   
  
  
+ GAACTCTCTC TCTCCTTTCT CTCTCTCCAA ATCATATTTT ATTCTACCAT TCCTATTATT AGTTTCCTTT   
  
  
+ TCTTCTTTCT TCTTCTTCTT CTTCTACCAG TATTTCTTAA TACCGGTCAC TCGACCCACT AAATAGTACA   
  
  
+ TCGTAGTTTC CCAGTTTAAC TACGTTGACA AAAAGAATAA TATTATATAA AAGCATTATA AAATGAGAGA   
  
  
+ GAGAGAGAGA GAGAGAGATA GACTCGAAGT AAAGAAGTTC TCTCTCAATT TTTTTTTTTT TTTTTTGCTG   
  
  
+ GAATCCCCTT CAGTAGGAAG TTTTTGTTCA CTACCCTTTC ATTTCTCCGC CGTTCATCGG CGTAAATAGC   
  
  
+ CTTTTTCAAA GTTGGTCCGC TTGTTGGGGG CTTTGCAAAG CTTCGCCCGT CGAAGGAACG AATACTCTAC   
  
  
+ ACTTGGGGAG TTACACTACG TCTATGGGTG AACCGAAAAA CGAGTAAATT GCTTGCTCCT GTAGCTATAA   
  
  
+ CGTATATGGG TGAACCGAAA AACGAGTAAA TTGCTTGCTC CTGTAGTTAT AACGTATATG GGTGAAGGGG   
  
  
+ TGAAAGTAGG GGTGAGTTCC CCTGTGTATG AGTATATGAA TAGTTTTATA CAAGGAAATT ACATGGCTAA   
  
  
+ GGTATGCCTT GATATTTTTT ATTTATATAC AAACATAATA GTACGTTATT AGTTTATTTT TTCTTTAATT   
  
  
+ TAACAATTGT ATCCATTTAC CGTATAAGTA GCCTTTTCTT ATATTTTTCT TTTTCTTTAT TTGTTCCTAA   
  
  
+ CTCGGAGTGG GTGTACACAT AATTAGTGGT ATGTTCTTAG GTTAAGGTGT TGGGGTATCT AATACTTCCT   
  
  
+ AAGCTGGGGA TTGGTAAACT TATAGTGTGG GTTACACTTC ATTGTTTCGT ATTGCTATTA CGGAACTTTC   
  
  
+ GTATTTCACT GATAACAGCC TTATAAGTCT AACACACCAT TAAGTCATCG AAACCCATGG GTCCATCGAT   
  
  
+ GTTCCTGATT CTTCTTTAAT TGAAACTTCA AAAATATATG TTAAGTACCA TGATGTGTGT AAGTATACCC   
  
  
+ TTCTTTGATA CTTTTGTTCC TTGTTCTTC  

- GGCTACCCCA AAGAAGCTGC ACGTACTAGT GATGCAAGAA CAGGAAGTGA AGCAGAGCTA CTCGCTTCAT   
  
  
- GTGGTGCACC ATCTGCTGGG TGTTCGGCGA CAAACAAACG TAGAAGACAT GCAAAATTCT TATCCCTCTC   
  
  
- TCTCGGAGTT GTGAGCTTGT GATCTGGGGT GGGTTTATGC TTTCTTTTTT GTTTTTTGGA TTAAGGTAAA   
  
  
- CAAGAATTTC TTTCGGGTAT CGGGATTTGG TTGCTAGGAT GCAAGAAAGC AAAGAAAACA GGTATGTCAA   
  
  
- GGTTGAAAAT ACTGATGTTC CTCTCAAAAA CAAACAAACA AGAACGGTTT GTGAAGAGAG AGAGAGAGAG   
  
  
- AGAGAGAGAG AGAGGGAAGG AGAGTCGCTG ACTTTTCACA CAAAACACCT CTTCTCTGTA CCCAACATGT   
  
  
- CTTGAGAGAG AGAGGAAAGA GAGAGAGGTT TAGTATAAAA TAAGATGGTA AGGATAATAA TCAAAGGAAA   
  
  
- AGAAGAAAGA AGAAGAAGAA GAAGATGGTC ATAAAGAATT ATGGCCAGTG AGCTGGGTGA TTTATCATGT   
  
  
- AGCATCAAAG GGTCAAATTG ATGCAACTGT TTTTCTTATT ATAATATATT TTCGTAATAT TTTACTCTCT   
  
  
- CTCTCTCTCT CTCTCTCTAT CTGAGCTTCA TTTCTTCAAG AGAGAGTTAA AAAAAAAAAA AAAAAACGAC   
  
  
- CTTAGGGGAA GTCATCCTTC AAAAACAAGT GATGGGAAAG TAAAGAGGCG GCAAGTAGCC GCATTTATCG   
  
  
- GAAAAAGTTT CAACCAGGCG AACAACCCCC GAAACGTTTC GAAGCGGGCA GCTTCCTTGC TTATGAGATG   
  
  
- TGAACCCCTC AATGTGATGC AGATACCCAC TTGGCTTTTT GCTCATTTAA CGAACGAGGA CATCGATATT   
  
  
- GCATATACCC ACTTGGCTTT TTGCTCATTT AACGAACGAG GACATCAATA TTGCATATAC CCACTTCCCC   
  
  
- ACTTTCATCC CCACTCAAGG GGACACATAC TCATATACTT ATCAAAATAT GTTCCTTTAA TGTACCGATT   
  
  
- CCATACGGAA CTATAAAAAA TAAATATATG TTTGTATTAT CATGCAATAA TCAAATAAAA AAGAAATTAA   
  
  
- ATTGTTAACA TAGGTAAATG GCATATTCAT CGGAAAAGAA TATAAAAAGA AAAAGAAATA AACAAGGATT   
  
  
- GAGCCTCACC CACATGTGTA TTAATCACCA TACAAGAATC CAATTCCACA ACCCCATAGA TTATGAAGGA   
  
  
- TTCGACCCCT AACCATTTGA ATATCACACC CAATGTGAAG TAACAAAGCA TAACGATAAT GCCTTGAAAG   
  
  
- CATAAAGTGA CTATTGTCGG AATATTCAGA TTGTGTGGTA ATTCAGTAGC TTTGGGTACC CAGGTAGCTA   
  
  
- CAAGGACTAA GAAGAAATTA ACTTTGAAGT TTTTATATAC AATTCATGGT ACTACACACA TTCATATGGG   
  
  
- AAGAAACTAT GAAAACAAGG AACAAGAAG

+     Sp1

| Site Name | Organism | Position | Strand | Matrix score. | sequence | function |
| --- | --- | --- | --- | --- | --- | --- |
| Sp1 | Zea mays | 168 | + | 5.5 | CC(G/A)CCC | light responsive element |

> 2018/04/13 10:10:12  
+ CCGATGGGGT TTCTTCGACG TGCATGATCA CTACGTTCTT GTCCTTCACT TCGTCTCGAT GAGCGAAGTA   
  
  
+ CACCACGTGG TAGACGACCC ACAAGCCGCT GTTTGTTTGC ATCTTCTGTA CGTTTTAAGA ATAGGGAGAG   
  
  
+ AGAGCCTCAA CACTCGAACA CTAGACCCCA CCCAAATACG AAAGAAAAAA CAAAAAACCT AATTCCATTT   
  
  
+ GTTCTTAAAG AAAGCCCATA GCCCTAAACC AACGATCCTA CGTTCTTTCG TTTCTTTTGT CCATACAGTT   
  
  
+ CCAACTTTTA TGACTACAAG GAGAGTTTTT GTTTGTTTGT TCTTGCCAAA CACTTCTCTC TCTCTCTCTC   
  
  
+ TCTCTCTCTC TCTCCCTTCC TCTCAGCGAC TGAAAAGTGT GTTTTGTGGA GAAGAGACAT GGGTTGTACA   
  
  
+ GAACTCTCTC TCTCCTTTCT CTCTCTCCAA ATCATATTTT ATTCTACCAT TCCTATTATT AGTTTCCTTT   
  
  
+ TCTTCTTTCT TCTTCTTCTT CTTCTACCAG TATTTCTTAA TACCGGTCAC TCGACCCACT AAATAGTACA   
  
  
+ TCGTAGTTTC CCAGTTTAAC TACGTTGACA AAAAGAATAA TATTATATAA AAGCATTATA AAATGAGAGA   
  
  
+ GAGAGAGAGA GAGAGAGATA GACTCGAAGT AAAGAAGTTC TCTCTCAATT TTTTTTTTTT TTTTTTGCTG   
  
  
+ GAATCCCCTT CAGTAGGAAG TTTTTGTTCA CTACCCTTTC ATTTCTCCGC CGTTCATCGG CGTAAATAGC   
  
  
+ CTTTTTCAAA GTTGGTCCGC TTGTTGGGGG CTTTGCAAAG CTTCGCCCGT CGAAGGAACG AATACTCTAC   
  
  
+ ACTTGGGGAG TTACACTACG TCTATGGGTG AACCGAAAAA CGAGTAAATT GCTTGCTCCT GTAGCTATAA   
  
  
+ CGTATATGGG TGAACCGAAA AACGAGTAAA TTGCTTGCTC CTGTAGTTAT AACGTATATG GGTGAAGGGG   
  
  
+ TGAAAGTAGG GGTGAGTTCC CCTGTGTATG AGTATATGAA TAGTTTTATA CAAGGAAATT ACATGGCTAA   
  
  
+ GGTATGCCTT GATATTTTTT ATTTATATAC AAACATAATA GTACGTTATT AGTTTATTTT TTCTTTAATT   
  
  
+ TAACAATTGT ATCCATTTAC CGTATAAGTA GCCTTTTCTT ATATTTTTCT TTTTCTTTAT TTGTTCCTAA   
  
  
+ CTCGGAGTGG GTGTACACAT AATTAGTGGT ATGTTCTTAG GTTAAGGTGT TGGGGTATCT AATACTTCCT   
  
  
+ AAGCTGGGGA TTGGTAAACT TATAGTGTGG GTTACACTTC ATTGTTTCGT ATTGCTATTA CGGAACTTTC   
  
  
+ GTATTTCACT GATAACAGCC TTATAAGTCT AACACACCAT TAAGTCATCG AAACCCATGG GTCCATCGAT   
  
  
+ GTTCCTGATT CTTCTTTAAT TGAAACTTCA AAAATATATG TTAAGTACCA TGATGTGTGT AAGTATACCC   
  
  
+ TTCTTTGATA CTTTTGTTCC TTGTTCTTC  

- GGCTACCCCA AAGAAGCTGC ACGTACTAGT GATGCAAGAA CAGGAAGTGA AGCAGAGCTA CTCGCTTCAT   
  
  
- GTGGTGCACC ATCTGCTGGG TGTTCGGCGA CAAACAAACG TAGAAGACAT GCAAAATTCT TATCCCTCTC   
  
  
- TCTCGGAGTT GTGAGCTTGT GATCTGGGGT GGGTTTATGC TTTCTTTTTT GTTTTTTGGA TTAAGGTAAA   
  
  
- CAAGAATTTC TTTCGGGTAT CGGGATTTGG TTGCTAGGAT GCAAGAAAGC AAAGAAAACA GGTATGTCAA   
  
  
- GGTTGAAAAT ACTGATGTTC CTCTCAAAAA CAAACAAACA AGAACGGTTT GTGAAGAGAG AGAGAGAGAG   
  
  
- AGAGAGAGAG AGAGGGAAGG AGAGTCGCTG ACTTTTCACA CAAAACACCT CTTCTCTGTA CCCAACATGT   
  
  
- CTTGAGAGAG AGAGGAAAGA GAGAGAGGTT TAGTATAAAA TAAGATGGTA AGGATAATAA TCAAAGGAAA   
  
  
- AGAAGAAAGA AGAAGAAGAA GAAGATGGTC ATAAAGAATT ATGGCCAGTG AGCTGGGTGA TTTATCATGT   
  
  
- AGCATCAAAG GGTCAAATTG ATGCAACTGT TTTTCTTATT ATAATATATT TTCGTAATAT TTTACTCTCT   
  
  
- CTCTCTCTCT CTCTCTCTAT CTGAGCTTCA TTTCTTCAAG AGAGAGTTAA AAAAAAAAAA AAAAAACGAC   
  
  
- CTTAGGGGAA GTCATCCTTC AAAAACAAGT GATGGGAAAG TAAAGAGGCG GCAAGTAGCC GCATTTATCG   
  
  
- GAAAAAGTTT CAACCAGGCG AACAACCCCC GAAACGTTTC GAAGCGGGCA GCTTCCTTGC TTATGAGATG   
  
  
- TGAACCCCTC AATGTGATGC AGATACCCAC TTGGCTTTTT GCTCATTTAA CGAACGAGGA CATCGATATT   
  
  
- GCATATACCC ACTTGGCTTT TTGCTCATTT AACGAACGAG GACATCAATA TTGCATATAC CCACTTCCCC   
  
  
- ACTTTCATCC CCACTCAAGG GGACACATAC TCATATACTT ATCAAAATAT GTTCCTTTAA TGTACCGATT   
  
  
- CCATACGGAA CTATAAAAAA TAAATATATG TTTGTATTAT CATGCAATAA TCAAATAAAA AAGAAATTAA   
  
  
- ATTGTTAACA TAGGTAAATG GCATATTCAT CGGAAAAGAA TATAAAAAGA AAAAGAAATA AACAAGGATT   
  
  
- GAGCCTCACC CACATGTGTA TTAATCACCA TACAAGAATC CAATTCCACA ACCCCATAGA TTATGAAGGA   
  
  
- TTCGACCCCT AACCATTTGA ATATCACACC CAATGTGAAG TAACAAAGCA TAACGATAAT GCCTTGAAAG   
  
  
- CATAAAGTGA CTATTGTCGG AATATTCAGA TTGTGTGGTA ATTCAGTAGC TTTGGGTACC CAGGTAGCTA   
  
  
- CAAGGACTAA GAAGAAATTA ACTTTGAAGT TTTTATATAC AATTCATGGT ACTACACACA TTCATATGGG   
  
  
- AAGAAACTAT GAAAACAAGG AACAAGAAG

+     TATA-box

| Site Name | Organism | Position | Strand | Matrix score. | sequence | function |
| --- | --- | --- | --- | --- | --- | --- |
| TATA-box | Arabidopsis thaliana | 1352 | - | 4 | TATA | core promoter element around -30 of transcription start |
| TATA-box | Arabidopsis thaliana | 1066 | - | 11 | TATAAATATAAA | core promoter element around -30 of transcription start |
| TATA-box | Arabidopsis thaliana | 1026 | - | 5 | TATAA | core promoter element around -30 of transcription start |
| TATA-box | Glycine max | 1086 | + | 5 | TAATA | core promoter element around -30 of transcription start |
| TATA-box | Arabidopsis thaliana | 913 | - | 4 | TATA | core promoter element around -30 of transcription start |
| TATA-box | Arabidopsis thaliana | 606 | + | 6 | TATAAA | core promoter element around -30 of transcription start |
| TATA-box | Arabidopsis thaliana | 1027 | - | 4 | TATA | core promoter element around -30 of transcription start |
| TATA-box | Lycopersicon esculentum | 123 | + | 5 | TTTTA | core promoter element around -30 of transcription start |
| TATA-box | Arabidopsis thaliana | 1464 | - | 4 | TATA | core promoter element around -30 of transcription start |
| TATA-box | Brassica napus | 1434 | - | 6 | ATATAT | core promoter element around -30 of transcription start |
| TATA-box | Arabidopsis thaliana | 1280 | - | 5 | TATAA | core promoter element around -30 of transcription start |
| TATA-box | Arabidopsis thaliana | 1076 | - | 4 | TATA | core promoter element around -30 of transcription start |
| TATA-box | Arabidopsis thaliana | 1024 | - | 7 | TATAAAA | core promoter element around -30 of transcription start |
| TATA-box | Glycine max | 477 | - | 5 | TAATA | core promoter element around -30 of transcription start |
| TATA-box | Ac | 1071 | - | 7 | TATAAAT | core promoter element around -30 of transcription start |
| TATA-box | Lycopersicon esculentum | 457 | + | 5 | TTTTA | core promoter element around -30 of transcription start |
| TATA-box | Glycine max | 1316 | - | 5 | TAATA | core promoter element around -30 of transcription start |
| TATA-box | Lycopersicon esculentum | 608 | - | 5 | TTTTA | core promoter element around -30 of transcription start |
| TATA-box | Brassica oleracea | 605 | + | 6 | ATATAA | core promoter element around -30 of transcription start |
| TATA-box | Arabidopsis thaliana | 1435 | - | 4 | TATA | core promoter element around -30 of transcription start |
| TATA-box | Lycopersicon esculentum | 286 | + | 5 | TTTTA | core promoter element around -30 of transcription start |
| TATA-box | Arabidopsis thaliana | 603 | - | 7 | TATATAA | core promoter element around -30 of transcription start |
| TATA-box | Lycopersicon esculentum | 1067 | + | 5 | TTTTA | core promoter element around -30 of transcription start |
| TATA-box | Arabidopsis thaliana | 616 | - | 5 | TATAA | core promoter element around -30 of transcription start |
| TATA-box | Glycine max | 474 | - | 5 | TAATA | core promoter element around -30 of transcription start |
| TATA-box | Arabidopsis thaliana | 1073 | - | 7 | TATATAA | core promoter element around -30 of transcription start |
| TATA-box | Arabidopsis thaliana | 957 | - | 5 | TATAA | core promoter element around -30 of transcription start |
| TATA-box | Glycine max | 528 | + | 5 | TAATA | core promoter element around -30 of transcription start |
| TATA-box | Arabidopsis thaliana | 906 | - | 4 | TATA | core promoter element around -30 of transcription start |
| TATA-box | Arabidopsis thaliana | 1072 | - | 6 | TATAAA | core promoter element around -30 of transcription start |
| TATA-box | Brassica napus | 602 | + | 6 | ATTATA | core promoter element around -30 of transcription start |
| TATA-box | Arabidopsis thaliana | 1159 | - | 5 | TATAA | core promoter element around -30 of transcription start |
| TATA-box | Arabidopsis thaliana | 958 | - | 4 | TATA | core promoter element around -30 of transcription start |
| TATA-box | Oryza sativa | 1156 | - | 8 | TATAAGAA | core promoter element around -30 of transcription start |
| TATA-box | Glycine max | 598 | + | 5 | TAATA | core promoter element around -30 of transcription start |
| TATA-box | Arabidopsis thaliana | 965 | - | 4 | TATA | core promoter element around -30 of transcription start |
| TATA-box | Arabidopsis thaliana | 604 | + | 4 | TATA | core promoter element around -30 of transcription start |
| TATA-box | Arabidopsis thaliana | 1143 | - | 4 | TATA | core promoter element around -30 of transcription start |
| TATA-box | Arabidopsis thaliana | 1351 | - | 5 | TATAA | core promoter element around -30 of transcription start |
| TATA-box | Arabidopsis thaliana | 617 | + | 6 | TATAAA | core promoter element around -30 of transcription start |
| TATA-box | Glycine max | 601 | - | 5 | TAATA | core promoter element around -30 of transcription start |
| TATA-box | Arabidopsis thaliana | 1025 | - | 6 | TATAAA | core promoter element around -30 of transcription start |
| TATA-box | Arabidopsis thaliana | 1013 | - | 4 | TATA | core promoter element around -30 of transcription start |
| TATA-box | Arabidopsis thaliana | 1070 | - | 9 | taTATAAAtc | core promoter element around -30 of transcription start |
| TATA-box | Glycine max | 1097 | - | 5 | TAATA | core promoter element around -30 of transcription start |
| TATA-box | Lycopersicon esculentum | 619 | - | 5 | TTTTA | core promoter element around -30 of transcription start |
| TATA-box | Brassica napus | 615 | + | 6 | ATTATA | core promoter element around -30 of transcription start |
| TATA-box | Arabidopsis thaliana | 1281 | - | 4 | TATA | core promoter element around -30 of transcription start |
| TATA-box | Arabidopsis thaliana | 1074 | - | 4 | TATA | core promoter element around -30 of transcription start |
| TATA-box | Arabidopsis thaliana | 1160 | - | 4 | TATA | core promoter element around -30 of transcription start |
| TATA-box | Glycine max | 1250 | + | 5 | TAATA | core promoter element around -30 of transcription start |

> 2018/04/13 10:10:12  
+ CCGATGGGGT TTCTTCGACG TGCATGATCA CTACGTTCTT GTCCTTCACT TCGTCTCGAT GAGCGAAGTA   
  
  
+ CACCACGTGG TAGACGACCC ACAAGCCGCT GTTTGTTTGC ATCTTCTGTA CGTTTTAAGA ATAGGGAGAG   
  
  
+ AGAGCCTCAA CACTCGAACA CTAGACCCCA CCCAAATACG AAAGAAAAAA CAAAAAACCT AATTCCATTT   
  
  
+ GTTCTTAAAG AAAGCCCATA GCCCTAAACC AACGATCCTA CGTTCTTTCG TTTCTTTTGT CCATACAGTT   
  
  
+ CCAACTTTTA TGACTACAAG GAGAGTTTTT GTTTGTTTGT TCTTGCCAAA CACTTCTCTC TCTCTCTCTC   
  
  
+ TCTCTCTCTC TCTCCCTTCC TCTCAGCGAC TGAAAAGTGT GTTTTGTGGA GAAGAGACAT GGGTTGTACA   
  
  
+ GAACTCTCTC TCTCCTTTCT CTCTCTCCAA ATCATATTTT ATTCTACCAT TCCTATTATT AGTTTCCTTT   
  
  
+ TCTTCTTTCT TCTTCTTCTT CTTCTACCAG TATTTCTTAA TACCGGTCAC TCGACCCACT AAATAGTACA   
  
  
+ TCGTAGTTTC CCAGTTTAAC TACGTTGACA AAAAGAATAA TATTATATAA AAGCATTATA AAATGAGAGA   
  
  
+ GAGAGAGAGA GAGAGAGATA GACTCGAAGT AAAGAAGTTC TCTCTCAATT TTTTTTTTTT TTTTTTGCTG   
  
  
+ GAATCCCCTT CAGTAGGAAG TTTTTGTTCA CTACCCTTTC ATTTCTCCGC CGTTCATCGG CGTAAATAGC   
  
  
+ CTTTTTCAAA GTTGGTCCGC TTGTTGGGGG CTTTGCAAAG CTTCGCCCGT CGAAGGAACG AATACTCTAC   
  
  
+ ACTTGGGGAG TTACACTACG TCTATGGGTG AACCGAAAAA CGAGTAAATT GCTTGCTCCT GTAGCTATAA   
  
  
+ CGTATATGGG TGAACCGAAA AACGAGTAAA TTGCTTGCTC CTGTAGTTAT AACGTATATG GGTGAAGGGG   
  
  
+ TGAAAGTAGG GGTGAGTTCC CCTGTGTATG AGTATATGAA TAGTTTTATA CAAGGAAATT ACATGGCTAA   
  
  
+ GGTATGCCTT GATATTTTTT ATTTATATAC AAACATAATA GTACGTTATT AGTTTATTTT TTCTTTAATT   
  
  
+ TAACAATTGT ATCCATTTAC CGTATAAGTA GCCTTTTCTT ATATTTTTCT TTTTCTTTAT TTGTTCCTAA   
  
  
+ CTCGGAGTGG GTGTACACAT AATTAGTGGT ATGTTCTTAG GTTAAGGTGT TGGGGTATCT AATACTTCCT   
  
  
+ AAGCTGGGGA TTGGTAAACT TATAGTGTGG GTTACACTTC ATTGTTTCGT ATTGCTATTA CGGAACTTTC   
  
  
+ GTATTTCACT GATAACAGCC TTATAAGTCT AACACACCAT TAAGTCATCG AAACCCATGG GTCCATCGAT   
  
  
+ GTTCCTGATT CTTCTTTAAT TGAAACTTCA AAAATATATG TTAAGTACCA TGATGTGTGT AAGTATACCC   
  
  
+ TTCTTTGATA CTTTTGTTCC TTGTTCTTC  

- GGCTACCCCA AAGAAGCTGC ACGTACTAGT GATGCAAGAA CAGGAAGTGA AGCAGAGCTA CTCGCTTCAT   
  
  
- GTGGTGCACC ATCTGCTGGG TGTTCGGCGA CAAACAAACG TAGAAGACAT GCAAAATTCT TATCCCTCTC   
  
  
- TCTCGGAGTT GTGAGCTTGT GATCTGGGGT GGGTTTATGC TTTCTTTTTT GTTTTTTGGA TTAAGGTAAA   
  
  
- CAAGAATTTC TTTCGGGTAT CGGGATTTGG TTGCTAGGAT GCAAGAAAGC AAAGAAAACA GGTATGTCAA   
  
  
- GGTTGAAAAT ACTGATGTTC CTCTCAAAAA CAAACAAACA AGAACGGTTT GTGAAGAGAG AGAGAGAGAG   
  
  
- AGAGAGAGAG AGAGGGAAGG AGAGTCGCTG ACTTTTCACA CAAAACACCT CTTCTCTGTA CCCAACATGT   
  
  
- CTTGAGAGAG AGAGGAAAGA GAGAGAGGTT TAGTATAAAA TAAGATGGTA AGGATAATAA TCAAAGGAAA   
  
  
- AGAAGAAAGA AGAAGAAGAA GAAGATGGTC ATAAAGAATT ATGGCCAGTG AGCTGGGTGA TTTATCATGT   
  
  
- AGCATCAAAG GGTCAAATTG ATGCAACTGT TTTTCTTATT ATAATATATT TTCGTAATAT TTTACTCTCT   
  
  
- CTCTCTCTCT CTCTCTCTAT CTGAGCTTCA TTTCTTCAAG AGAGAGTTAA AAAAAAAAAA AAAAAACGAC   
  
  
- CTTAGGGGAA GTCATCCTTC AAAAACAAGT GATGGGAAAG TAAAGAGGCG GCAAGTAGCC GCATTTATCG   
  
  
- GAAAAAGTTT CAACCAGGCG AACAACCCCC GAAACGTTTC GAAGCGGGCA GCTTCCTTGC TTATGAGATG   
  
  
- TGAACCCCTC AATGTGATGC AGATACCCAC TTGGCTTTTT GCTCATTTAA CGAACGAGGA CATCGATATT   
  
  
- GCATATACCC ACTTGGCTTT TTGCTCATTT AACGAACGAG GACATCAATA TTGCATATAC CCACTTCCCC   
  
  
- ACTTTCATCC CCACTCAAGG GGACACATAC TCATATACTT ATCAAAATAT GTTCCTTTAA TGTACCGATT   
  
  
- CCATACGGAA CTATAAAAAA TAAATATATG TTTGTATTAT CATGCAATAA TCAAATAAAA AAGAAATTAA   
  
  
- ATTGTTAACA TAGGTAAATG GCATATTCAT CGGAAAAGAA TATAAAAAGA AAAAGAAATA AACAAGGATT   
  
  
- GAGCCTCACC CACATGTGTA TTAATCACCA TACAAGAATC CAATTCCACA ACCCCATAGA TTATGAAGGA   
  
  
- TTCGACCCCT AACCATTTGA ATATCACACC CAATGTGAAG TAACAAAGCA TAACGATAAT GCCTTGAAAG   
  
  
- CATAAAGTGA CTATTGTCGG AATATTCAGA TTGTGTGGTA ATTCAGTAGC TTTGGGTACC CAGGTAGCTA   
  
  
- CAAGGACTAA GAAGAAATTA ACTTTGAAGT TTTTATATAC AATTCATGGT ACTACACACA TTCATATGGG   
  
  
- AAGAAACTAT GAAAACAAGG AACAAGAAG

+     TATCCAT/C-motif

| Site Name | Organism | Position | Strand | Matrix score. | sequence | function |
| --- | --- | --- | --- | --- | --- | --- |
| TATCCAT/C-motif | Oryza sativa | 1130 | + | 7 | TATCCAT |  |

> 2018/04/13 10:10:12  
+ CCGATGGGGT TTCTTCGACG TGCATGATCA CTACGTTCTT GTCCTTCACT TCGTCTCGAT GAGCGAAGTA   
  
  
+ CACCACGTGG TAGACGACCC ACAAGCCGCT GTTTGTTTGC ATCTTCTGTA CGTTTTAAGA ATAGGGAGAG   
  
  
+ AGAGCCTCAA CACTCGAACA CTAGACCCCA CCCAAATACG AAAGAAAAAA CAAAAAACCT AATTCCATTT   
  
  
+ GTTCTTAAAG AAAGCCCATA GCCCTAAACC AACGATCCTA CGTTCTTTCG TTTCTTTTGT CCATACAGTT   
  
  
+ CCAACTTTTA TGACTACAAG GAGAGTTTTT GTTTGTTTGT TCTTGCCAAA CACTTCTCTC TCTCTCTCTC   
  
  
+ TCTCTCTCTC TCTCCCTTCC TCTCAGCGAC TGAAAAGTGT GTTTTGTGGA GAAGAGACAT GGGTTGTACA   
  
  
+ GAACTCTCTC TCTCCTTTCT CTCTCTCCAA ATCATATTTT ATTCTACCAT TCCTATTATT AGTTTCCTTT   
  
  
+ TCTTCTTTCT TCTTCTTCTT CTTCTACCAG TATTTCTTAA TACCGGTCAC TCGACCCACT AAATAGTACA   
  
  
+ TCGTAGTTTC CCAGTTTAAC TACGTTGACA AAAAGAATAA TATTATATAA AAGCATTATA AAATGAGAGA   
  
  
+ GAGAGAGAGA GAGAGAGATA GACTCGAAGT AAAGAAGTTC TCTCTCAATT TTTTTTTTTT TTTTTTGCTG   
  
  
+ GAATCCCCTT CAGTAGGAAG TTTTTGTTCA CTACCCTTTC ATTTCTCCGC CGTTCATCGG CGTAAATAGC   
  
  
+ CTTTTTCAAA GTTGGTCCGC TTGTTGGGGG CTTTGCAAAG CTTCGCCCGT CGAAGGAACG AATACTCTAC   
  
  
+ ACTTGGGGAG TTACACTACG TCTATGGGTG AACCGAAAAA CGAGTAAATT GCTTGCTCCT GTAGCTATAA   
  
  
+ CGTATATGGG TGAACCGAAA AACGAGTAAA TTGCTTGCTC CTGTAGTTAT AACGTATATG GGTGAAGGGG   
  
  
+ TGAAAGTAGG GGTGAGTTCC CCTGTGTATG AGTATATGAA TAGTTTTATA CAAGGAAATT ACATGGCTAA   
  
  
+ GGTATGCCTT GATATTTTTT ATTTATATAC AAACATAATA GTACGTTATT AGTTTATTTT TTCTTTAATT   
  
  
+ TAACAATTGT ATCCATTTAC CGTATAAGTA GCCTTTTCTT ATATTTTTCT TTTTCTTTAT TTGTTCCTAA   
  
  
+ CTCGGAGTGG GTGTACACAT AATTAGTGGT ATGTTCTTAG GTTAAGGTGT TGGGGTATCT AATACTTCCT   
  
  
+ AAGCTGGGGA TTGGTAAACT TATAGTGTGG GTTACACTTC ATTGTTTCGT ATTGCTATTA CGGAACTTTC   
  
  
+ GTATTTCACT GATAACAGCC TTATAAGTCT AACACACCAT TAAGTCATCG AAACCCATGG GTCCATCGAT   
  
  
+ GTTCCTGATT CTTCTTTAAT TGAAACTTCA AAAATATATG TTAAGTACCA TGATGTGTGT AAGTATACCC   
  
  
+ TTCTTTGATA CTTTTGTTCC TTGTTCTTC  

- GGCTACCCCA AAGAAGCTGC ACGTACTAGT GATGCAAGAA CAGGAAGTGA AGCAGAGCTA CTCGCTTCAT   
  
  
- GTGGTGCACC ATCTGCTGGG TGTTCGGCGA CAAACAAACG TAGAAGACAT GCAAAATTCT TATCCCTCTC   
  
  
- TCTCGGAGTT GTGAGCTTGT GATCTGGGGT GGGTTTATGC TTTCTTTTTT GTTTTTTGGA TTAAGGTAAA   
  
  
- CAAGAATTTC TTTCGGGTAT CGGGATTTGG TTGCTAGGAT GCAAGAAAGC AAAGAAAACA GGTATGTCAA   
  
  
- GGTTGAAAAT ACTGATGTTC CTCTCAAAAA CAAACAAACA AGAACGGTTT GTGAAGAGAG AGAGAGAGAG   
  
  
- AGAGAGAGAG AGAGGGAAGG AGAGTCGCTG ACTTTTCACA CAAAACACCT CTTCTCTGTA CCCAACATGT   
  
  
- CTTGAGAGAG AGAGGAAAGA GAGAGAGGTT TAGTATAAAA TAAGATGGTA AGGATAATAA TCAAAGGAAA   
  
  
- AGAAGAAAGA AGAAGAAGAA GAAGATGGTC ATAAAGAATT ATGGCCAGTG AGCTGGGTGA TTTATCATGT   
  
  
- AGCATCAAAG GGTCAAATTG ATGCAACTGT TTTTCTTATT ATAATATATT TTCGTAATAT TTTACTCTCT   
  
  
- CTCTCTCTCT CTCTCTCTAT CTGAGCTTCA TTTCTTCAAG AGAGAGTTAA AAAAAAAAAA AAAAAACGAC   
  
  
- CTTAGGGGAA GTCATCCTTC AAAAACAAGT GATGGGAAAG TAAAGAGGCG GCAAGTAGCC GCATTTATCG   
  
  
- GAAAAAGTTT CAACCAGGCG AACAACCCCC GAAACGTTTC GAAGCGGGCA GCTTCCTTGC TTATGAGATG   
  
  
- TGAACCCCTC AATGTGATGC AGATACCCAC TTGGCTTTTT GCTCATTTAA CGAACGAGGA CATCGATATT   
  
  
- GCATATACCC ACTTGGCTTT TTGCTCATTT AACGAACGAG GACATCAATA TTGCATATAC CCACTTCCCC   
  
  
- ACTTTCATCC CCACTCAAGG GGACACATAC TCATATACTT ATCAAAATAT GTTCCTTTAA TGTACCGATT   
  
  
- CCATACGGAA CTATAAAAAA TAAATATATG TTTGTATTAT CATGCAATAA TCAAATAAAA AAGAAATTAA   
  
  
- ATTGTTAACA TAGGTAAATG GCATATTCAT CGGAAAAGAA TATAAAAAGA AAAAGAAATA AACAAGGATT   
  
  
- GAGCCTCACC CACATGTGTA TTAATCACCA TACAAGAATC CAATTCCACA ACCCCATAGA TTATGAAGGA   
  
  
- TTCGACCCCT AACCATTTGA ATATCACACC CAATGTGAAG TAACAAAGCA TAACGATAAT GCCTTGAAAG   
  
  
- CATAAAGTGA CTATTGTCGG AATATTCAGA TTGTGTGGTA ATTCAGTAGC TTTGGGTACC CAGGTAGCTA   
  
  
- CAAGGACTAA GAAGAAATTA ACTTTGAAGT TTTTATATAC AATTCATGGT ACTACACACA TTCATATGGG   
  
  
- AAGAAACTAT GAAAACAAGG AACAAGAAG

+     TCA-element

| Site Name | Organism | Position | Strand | Matrix score. | sequence | function |
| --- | --- | --- | --- | --- | --- | --- |
| TCA-element | Brassica oleracea | 485 | - | 9 | CAGAAAAGGA | cis-acting element involved in salicylic acid responsiveness |

> 2018/04/13 10:10:12  
+ CCGATGGGGT TTCTTCGACG TGCATGATCA CTACGTTCTT GTCCTTCACT TCGTCTCGAT GAGCGAAGTA   
  
  
+ CACCACGTGG TAGACGACCC ACAAGCCGCT GTTTGTTTGC ATCTTCTGTA CGTTTTAAGA ATAGGGAGAG   
  
  
+ AGAGCCTCAA CACTCGAACA CTAGACCCCA CCCAAATACG AAAGAAAAAA CAAAAAACCT AATTCCATTT   
  
  
+ GTTCTTAAAG AAAGCCCATA GCCCTAAACC AACGATCCTA CGTTCTTTCG TTTCTTTTGT CCATACAGTT   
  
  
+ CCAACTTTTA TGACTACAAG GAGAGTTTTT GTTTGTTTGT TCTTGCCAAA CACTTCTCTC TCTCTCTCTC   
  
  
+ TCTCTCTCTC TCTCCCTTCC TCTCAGCGAC TGAAAAGTGT GTTTTGTGGA GAAGAGACAT GGGTTGTACA   
  
  
+ GAACTCTCTC TCTCCTTTCT CTCTCTCCAA ATCATATTTT ATTCTACCAT TCCTATTATT AGTTTCCTTT   
  
  
+ TCTTCTTTCT TCTTCTTCTT CTTCTACCAG TATTTCTTAA TACCGGTCAC TCGACCCACT AAATAGTACA   
  
  
+ TCGTAGTTTC CCAGTTTAAC TACGTTGACA AAAAGAATAA TATTATATAA AAGCATTATA AAATGAGAGA   
  
  
+ GAGAGAGAGA GAGAGAGATA GACTCGAAGT AAAGAAGTTC TCTCTCAATT TTTTTTTTTT TTTTTTGCTG   
  
  
+ GAATCCCCTT CAGTAGGAAG TTTTTGTTCA CTACCCTTTC ATTTCTCCGC CGTTCATCGG CGTAAATAGC   
  
  
+ CTTTTTCAAA GTTGGTCCGC TTGTTGGGGG CTTTGCAAAG CTTCGCCCGT CGAAGGAACG AATACTCTAC   
  
  
+ ACTTGGGGAG TTACACTACG TCTATGGGTG AACCGAAAAA CGAGTAAATT GCTTGCTCCT GTAGCTATAA   
  
  
+ CGTATATGGG TGAACCGAAA AACGAGTAAA TTGCTTGCTC CTGTAGTTAT AACGTATATG GGTGAAGGGG   
  
  
+ TGAAAGTAGG GGTGAGTTCC CCTGTGTATG AGTATATGAA TAGTTTTATA CAAGGAAATT ACATGGCTAA   
  
  
+ GGTATGCCTT GATATTTTTT ATTTATATAC AAACATAATA GTACGTTATT AGTTTATTTT TTCTTTAATT   
  
  
+ TAACAATTGT ATCCATTTAC CGTATAAGTA GCCTTTTCTT ATATTTTTCT TTTTCTTTAT TTGTTCCTAA   
  
  
+ CTCGGAGTGG GTGTACACAT AATTAGTGGT ATGTTCTTAG GTTAAGGTGT TGGGGTATCT AATACTTCCT   
  
  
+ AAGCTGGGGA TTGGTAAACT TATAGTGTGG GTTACACTTC ATTGTTTCGT ATTGCTATTA CGGAACTTTC   
  
  
+ GTATTTCACT GATAACAGCC TTATAAGTCT AACACACCAT TAAGTCATCG AAACCCATGG GTCCATCGAT   
  
  
+ GTTCCTGATT CTTCTTTAAT TGAAACTTCA AAAATATATG TTAAGTACCA TGATGTGTGT AAGTATACCC   
  
  
+ TTCTTTGATA CTTTTGTTCC TTGTTCTTC  

- GGCTACCCCA AAGAAGCTGC ACGTACTAGT GATGCAAGAA CAGGAAGTGA AGCAGAGCTA CTCGCTTCAT   
  
  
- GTGGTGCACC ATCTGCTGGG TGTTCGGCGA CAAACAAACG TAGAAGACAT GCAAAATTCT TATCCCTCTC   
  
  
- TCTCGGAGTT GTGAGCTTGT GATCTGGGGT GGGTTTATGC TTTCTTTTTT GTTTTTTGGA TTAAGGTAAA   
  
  
- CAAGAATTTC TTTCGGGTAT CGGGATTTGG TTGCTAGGAT GCAAGAAAGC AAAGAAAACA GGTATGTCAA   
  
  
- GGTTGAAAAT ACTGATGTTC CTCTCAAAAA CAAACAAACA AGAACGGTTT GTGAAGAGAG AGAGAGAGAG   
  
  
- AGAGAGAGAG AGAGGGAAGG AGAGTCGCTG ACTTTTCACA CAAAACACCT CTTCTCTGTA CCCAACATGT   
  
  
- CTTGAGAGAG AGAGGAAAGA GAGAGAGGTT TAGTATAAAA TAAGATGGTA AGGATAATAA TCAAAGGAAA   
  
  
- AGAAGAAAGA AGAAGAAGAA GAAGATGGTC ATAAAGAATT ATGGCCAGTG AGCTGGGTGA TTTATCATGT   
  
  
- AGCATCAAAG GGTCAAATTG ATGCAACTGT TTTTCTTATT ATAATATATT TTCGTAATAT TTTACTCTCT   
  
  
- CTCTCTCTCT CTCTCTCTAT CTGAGCTTCA TTTCTTCAAG AGAGAGTTAA AAAAAAAAAA AAAAAACGAC   
  
  
- CTTAGGGGAA GTCATCCTTC AAAAACAAGT GATGGGAAAG TAAAGAGGCG GCAAGTAGCC GCATTTATCG   
  
  
- GAAAAAGTTT CAACCAGGCG AACAACCCCC GAAACGTTTC GAAGCGGGCA GCTTCCTTGC TTATGAGATG   
  
  
- TGAACCCCTC AATGTGATGC AGATACCCAC TTGGCTTTTT GCTCATTTAA CGAACGAGGA CATCGATATT   
  
  
- GCATATACCC ACTTGGCTTT TTGCTCATTT AACGAACGAG GACATCAATA TTGCATATAC CCACTTCCCC   
  
  
- ACTTTCATCC CCACTCAAGG GGACACATAC TCATATACTT ATCAAAATAT GTTCCTTTAA TGTACCGATT   
  
  
- CCATACGGAA CTATAAAAAA TAAATATATG TTTGTATTAT CATGCAATAA TCAAATAAAA AAGAAATTAA   
  
  
- ATTGTTAACA TAGGTAAATG GCATATTCAT CGGAAAAGAA TATAAAAAGA AAAAGAAATA AACAAGGATT   
  
  
- GAGCCTCACC CACATGTGTA TTAATCACCA TACAAGAATC CAATTCCACA ACCCCATAGA TTATGAAGGA   
  
  
- TTCGACCCCT AACCATTTGA ATATCACACC CAATGTGAAG TAACAAAGCA TAACGATAAT GCCTTGAAAG   
  
  
- CATAAAGTGA CTATTGTCGG AATATTCAGA TTGTGTGGTA ATTCAGTAGC TTTGGGTACC CAGGTAGCTA   
  
  
- CAAGGACTAA GAAGAAATTA ACTTTGAAGT TTTTATATAC AATTCATGGT ACTACACACA TTCATATGGG   
  
  
- AAGAAACTAT GAAAACAAGG AACAAGAAG

+     TCCC-motif

| Site Name | Organism | Position | Strand | Matrix score. | sequence | function |
| --- | --- | --- | --- | --- | --- | --- |
| TCCC-motif | Spinacia oleracea | 361 | + | 7 | TCTCCCT | part of a light responsive element |
| TCCC-motif | Spinacia oleracea | 133 | - | 7 | TCTCCCT | part of a light responsive element |

> 2018/04/13 10:10:12  
+ CCGATGGGGT TTCTTCGACG TGCATGATCA CTACGTTCTT GTCCTTCACT TCGTCTCGAT GAGCGAAGTA   
  
  
+ CACCACGTGG TAGACGACCC ACAAGCCGCT GTTTGTTTGC ATCTTCTGTA CGTTTTAAGA ATAGGGAGAG   
  
  
+ AGAGCCTCAA CACTCGAACA CTAGACCCCA CCCAAATACG AAAGAAAAAA CAAAAAACCT AATTCCATTT   
  
  
+ GTTCTTAAAG AAAGCCCATA GCCCTAAACC AACGATCCTA CGTTCTTTCG TTTCTTTTGT CCATACAGTT   
  
  
+ CCAACTTTTA TGACTACAAG GAGAGTTTTT GTTTGTTTGT TCTTGCCAAA CACTTCTCTC TCTCTCTCTC   
  
  
+ TCTCTCTCTC TCTCCCTTCC TCTCAGCGAC TGAAAAGTGT GTTTTGTGGA GAAGAGACAT GGGTTGTACA   
  
  
+ GAACTCTCTC TCTCCTTTCT CTCTCTCCAA ATCATATTTT ATTCTACCAT TCCTATTATT AGTTTCCTTT   
  
  
+ TCTTCTTTCT TCTTCTTCTT CTTCTACCAG TATTTCTTAA TACCGGTCAC TCGACCCACT AAATAGTACA   
  
  
+ TCGTAGTTTC CCAGTTTAAC TACGTTGACA AAAAGAATAA TATTATATAA AAGCATTATA AAATGAGAGA   
  
  
+ GAGAGAGAGA GAGAGAGATA GACTCGAAGT AAAGAAGTTC TCTCTCAATT TTTTTTTTTT TTTTTTGCTG   
  
  
+ GAATCCCCTT CAGTAGGAAG TTTTTGTTCA CTACCCTTTC ATTTCTCCGC CGTTCATCGG CGTAAATAGC   
  
  
+ CTTTTTCAAA GTTGGTCCGC TTGTTGGGGG CTTTGCAAAG CTTCGCCCGT CGAAGGAACG AATACTCTAC   
  
  
+ ACTTGGGGAG TTACACTACG TCTATGGGTG AACCGAAAAA CGAGTAAATT GCTTGCTCCT GTAGCTATAA   
  
  
+ CGTATATGGG TGAACCGAAA AACGAGTAAA TTGCTTGCTC CTGTAGTTAT AACGTATATG GGTGAAGGGG   
  
  
+ TGAAAGTAGG GGTGAGTTCC CCTGTGTATG AGTATATGAA TAGTTTTATA CAAGGAAATT ACATGGCTAA   
  
  
+ GGTATGCCTT GATATTTTTT ATTTATATAC AAACATAATA GTACGTTATT AGTTTATTTT TTCTTTAATT   
  
  
+ TAACAATTGT ATCCATTTAC CGTATAAGTA GCCTTTTCTT ATATTTTTCT TTTTCTTTAT TTGTTCCTAA   
  
  
+ CTCGGAGTGG GTGTACACAT AATTAGTGGT ATGTTCTTAG GTTAAGGTGT TGGGGTATCT AATACTTCCT   
  
  
+ AAGCTGGGGA TTGGTAAACT TATAGTGTGG GTTACACTTC ATTGTTTCGT ATTGCTATTA CGGAACTTTC   
  
  
+ GTATTTCACT GATAACAGCC TTATAAGTCT AACACACCAT TAAGTCATCG AAACCCATGG GTCCATCGAT   
  
  
+ GTTCCTGATT CTTCTTTAAT TGAAACTTCA AAAATATATG TTAAGTACCA TGATGTGTGT AAGTATACCC   
  
  
+ TTCTTTGATA CTTTTGTTCC TTGTTCTTC  

- GGCTACCCCA AAGAAGCTGC ACGTACTAGT GATGCAAGAA CAGGAAGTGA AGCAGAGCTA CTCGCTTCAT   
  
  
- GTGGTGCACC ATCTGCTGGG TGTTCGGCGA CAAACAAACG TAGAAGACAT GCAAAATTCT TATCCCTCTC   
  
  
- TCTCGGAGTT GTGAGCTTGT GATCTGGGGT GGGTTTATGC TTTCTTTTTT GTTTTTTGGA TTAAGGTAAA   
  
  
- CAAGAATTTC TTTCGGGTAT CGGGATTTGG TTGCTAGGAT GCAAGAAAGC AAAGAAAACA GGTATGTCAA   
  
  
- GGTTGAAAAT ACTGATGTTC CTCTCAAAAA CAAACAAACA AGAACGGTTT GTGAAGAGAG AGAGAGAGAG   
  
  
- AGAGAGAGAG AGAGGGAAGG AGAGTCGCTG ACTTTTCACA CAAAACACCT CTTCTCTGTA CCCAACATGT   
  
  
- CTTGAGAGAG AGAGGAAAGA GAGAGAGGTT TAGTATAAAA TAAGATGGTA AGGATAATAA TCAAAGGAAA   
  
  
- AGAAGAAAGA AGAAGAAGAA GAAGATGGTC ATAAAGAATT ATGGCCAGTG AGCTGGGTGA TTTATCATGT   
  
  
- AGCATCAAAG GGTCAAATTG ATGCAACTGT TTTTCTTATT ATAATATATT TTCGTAATAT TTTACTCTCT   
  
  
- CTCTCTCTCT CTCTCTCTAT CTGAGCTTCA TTTCTTCAAG AGAGAGTTAA AAAAAAAAAA AAAAAACGAC   
  
  
- CTTAGGGGAA GTCATCCTTC AAAAACAAGT GATGGGAAAG TAAAGAGGCG GCAAGTAGCC GCATTTATCG   
  
  
- GAAAAAGTTT CAACCAGGCG AACAACCCCC GAAACGTTTC GAAGCGGGCA GCTTCCTTGC TTATGAGATG   
  
  
- TGAACCCCTC AATGTGATGC AGATACCCAC TTGGCTTTTT GCTCATTTAA CGAACGAGGA CATCGATATT   
  
  
- GCATATACCC ACTTGGCTTT TTGCTCATTT AACGAACGAG GACATCAATA TTGCATATAC CCACTTCCCC   
  
  
- ACTTTCATCC CCACTCAAGG GGACACATAC TCATATACTT ATCAAAATAT GTTCCTTTAA TGTACCGATT   
  
  
- CCATACGGAA CTATAAAAAA TAAATATATG TTTGTATTAT CATGCAATAA TCAAATAAAA AAGAAATTAA   
  
  
- ATTGTTAACA TAGGTAAATG GCATATTCAT CGGAAAAGAA TATAAAAAGA AAAAGAAATA AACAAGGATT   
  
  
- GAGCCTCACC CACATGTGTA TTAATCACCA TACAAGAATC CAATTCCACA ACCCCATAGA TTATGAAGGA   
  
  
- TTCGACCCCT AACCATTTGA ATATCACACC CAATGTGAAG TAACAAAGCA TAACGATAAT GCCTTGAAAG   
  
  
- CATAAAGTGA CTATTGTCGG AATATTCAGA TTGTGTGGTA ATTCAGTAGC TTTGGGTACC CAGGTAGCTA   
  
  
- CAAGGACTAA GAAGAAATTA ACTTTGAAGT TTTTATATAC AATTCATGGT ACTACACACA TTCATATGGG   
  
  
- AAGAAACTAT GAAAACAAGG AACAAGAAG

+     Unnamed\_\_1

| Site Name | Organism | Position | Strand | Matrix score. | sequence | function |
| --- | --- | --- | --- | --- | --- | --- |
| Unnamed\_\_1 | Zea mays | 73 | - | 5 | CGTGG |  |
| Unnamed\_\_1 | Zea mays | 76 | + | 5 | CGTGG |  |

> 2018/04/13 10:10:12  
+ CCGATGGGGT TTCTTCGACG TGCATGATCA CTACGTTCTT GTCCTTCACT TCGTCTCGAT GAGCGAAGTA   
  
  
+ CACCACGTGG TAGACGACCC ACAAGCCGCT GTTTGTTTGC ATCTTCTGTA CGTTTTAAGA ATAGGGAGAG   
  
  
+ AGAGCCTCAA CACTCGAACA CTAGACCCCA CCCAAATACG AAAGAAAAAA CAAAAAACCT AATTCCATTT   
  
  
+ GTTCTTAAAG AAAGCCCATA GCCCTAAACC AACGATCCTA CGTTCTTTCG TTTCTTTTGT CCATACAGTT   
  
  
+ CCAACTTTTA TGACTACAAG GAGAGTTTTT GTTTGTTTGT TCTTGCCAAA CACTTCTCTC TCTCTCTCTC   
  
  
+ TCTCTCTCTC TCTCCCTTCC TCTCAGCGAC TGAAAAGTGT GTTTTGTGGA GAAGAGACAT GGGTTGTACA   
  
  
+ GAACTCTCTC TCTCCTTTCT CTCTCTCCAA ATCATATTTT ATTCTACCAT TCCTATTATT AGTTTCCTTT   
  
  
+ TCTTCTTTCT TCTTCTTCTT CTTCTACCAG TATTTCTTAA TACCGGTCAC TCGACCCACT AAATAGTACA   
  
  
+ TCGTAGTTTC CCAGTTTAAC TACGTTGACA AAAAGAATAA TATTATATAA AAGCATTATA AAATGAGAGA   
  
  
+ GAGAGAGAGA GAGAGAGATA GACTCGAAGT AAAGAAGTTC TCTCTCAATT TTTTTTTTTT TTTTTTGCTG   
  
  
+ GAATCCCCTT CAGTAGGAAG TTTTTGTTCA CTACCCTTTC ATTTCTCCGC CGTTCATCGG CGTAAATAGC   
  
  
+ CTTTTTCAAA GTTGGTCCGC TTGTTGGGGG CTTTGCAAAG CTTCGCCCGT CGAAGGAACG AATACTCTAC   
  
  
+ ACTTGGGGAG TTACACTACG TCTATGGGTG AACCGAAAAA CGAGTAAATT GCTTGCTCCT GTAGCTATAA   
  
  
+ CGTATATGGG TGAACCGAAA AACGAGTAAA TTGCTTGCTC CTGTAGTTAT AACGTATATG GGTGAAGGGG   
  
  
+ TGAAAGTAGG GGTGAGTTCC CCTGTGTATG AGTATATGAA TAGTTTTATA CAAGGAAATT ACATGGCTAA   
  
  
+ GGTATGCCTT GATATTTTTT ATTTATATAC AAACATAATA GTACGTTATT AGTTTATTTT TTCTTTAATT   
  
  
+ TAACAATTGT ATCCATTTAC CGTATAAGTA GCCTTTTCTT ATATTTTTCT TTTTCTTTAT TTGTTCCTAA   
  
  
+ CTCGGAGTGG GTGTACACAT AATTAGTGGT ATGTTCTTAG GTTAAGGTGT TGGGGTATCT AATACTTCCT   
  
  
+ AAGCTGGGGA TTGGTAAACT TATAGTGTGG GTTACACTTC ATTGTTTCGT ATTGCTATTA CGGAACTTTC   
  
  
+ GTATTTCACT GATAACAGCC TTATAAGTCT AACACACCAT TAAGTCATCG AAACCCATGG GTCCATCGAT   
  
  
+ GTTCCTGATT CTTCTTTAAT TGAAACTTCA AAAATATATG TTAAGTACCA TGATGTGTGT AAGTATACCC   
  
  
+ TTCTTTGATA CTTTTGTTCC TTGTTCTTC  

- GGCTACCCCA AAGAAGCTGC ACGTACTAGT GATGCAAGAA CAGGAAGTGA AGCAGAGCTA CTCGCTTCAT   
  
  
- GTGGTGCACC ATCTGCTGGG TGTTCGGCGA CAAACAAACG TAGAAGACAT GCAAAATTCT TATCCCTCTC   
  
  
- TCTCGGAGTT GTGAGCTTGT GATCTGGGGT GGGTTTATGC TTTCTTTTTT GTTTTTTGGA TTAAGGTAAA   
  
  
- CAAGAATTTC TTTCGGGTAT CGGGATTTGG TTGCTAGGAT GCAAGAAAGC AAAGAAAACA GGTATGTCAA   
  
  
- GGTTGAAAAT ACTGATGTTC CTCTCAAAAA CAAACAAACA AGAACGGTTT GTGAAGAGAG AGAGAGAGAG   
  
  
- AGAGAGAGAG AGAGGGAAGG AGAGTCGCTG ACTTTTCACA CAAAACACCT CTTCTCTGTA CCCAACATGT   
  
  
- CTTGAGAGAG AGAGGAAAGA GAGAGAGGTT TAGTATAAAA TAAGATGGTA AGGATAATAA TCAAAGGAAA   
  
  
- AGAAGAAAGA AGAAGAAGAA GAAGATGGTC ATAAAGAATT ATGGCCAGTG AGCTGGGTGA TTTATCATGT   
  
  
- AGCATCAAAG GGTCAAATTG ATGCAACTGT TTTTCTTATT ATAATATATT TTCGTAATAT TTTACTCTCT   
  
  
- CTCTCTCTCT CTCTCTCTAT CTGAGCTTCA TTTCTTCAAG AGAGAGTTAA AAAAAAAAAA AAAAAACGAC   
  
  
- CTTAGGGGAA GTCATCCTTC AAAAACAAGT GATGGGAAAG TAAAGAGGCG GCAAGTAGCC GCATTTATCG   
  
  
- GAAAAAGTTT CAACCAGGCG AACAACCCCC GAAACGTTTC GAAGCGGGCA GCTTCCTTGC TTATGAGATG   
  
  
- TGAACCCCTC AATGTGATGC AGATACCCAC TTGGCTTTTT GCTCATTTAA CGAACGAGGA CATCGATATT   
  
  
- GCATATACCC ACTTGGCTTT TTGCTCATTT AACGAACGAG GACATCAATA TTGCATATAC CCACTTCCCC   
  
  
- ACTTTCATCC CCACTCAAGG GGACACATAC TCATATACTT ATCAAAATAT GTTCCTTTAA TGTACCGATT   
  
  
- CCATACGGAA CTATAAAAAA TAAATATATG TTTGTATTAT CATGCAATAA TCAAATAAAA AAGAAATTAA   
  
  
- ATTGTTAACA TAGGTAAATG GCATATTCAT CGGAAAAGAA TATAAAAAGA AAAAGAAATA AACAAGGATT   
  
  
- GAGCCTCACC CACATGTGTA TTAATCACCA TACAAGAATC CAATTCCACA ACCCCATAGA TTATGAAGGA   
  
  
- TTCGACCCCT AACCATTTGA ATATCACACC CAATGTGAAG TAACAAAGCA TAACGATAAT GCCTTGAAAG   
  
  
- CATAAAGTGA CTATTGTCGG AATATTCAGA TTGTGTGGTA ATTCAGTAGC TTTGGGTACC CAGGTAGCTA   
  
  
- CAAGGACTAA GAAGAAATTA ACTTTGAAGT TTTTATATAC AATTCATGGT ACTACACACA TTCATATGGG   
  
  
- AAGAAACTAT GAAAACAAGG AACAAGAAG

+     Unnamed\_\_3

| Site Name | Organism | Position | Strand | Matrix score. | sequence | function |
| --- | --- | --- | --- | --- | --- | --- |
| Unnamed\_\_3 | Zea mays | 73 | - | 5 | CGTGG |  |
| Unnamed\_\_3 | Zea mays | 76 | + | 5 | CGTGG |  |

> 2018/04/13 10:10:12  
+ CCGATGGGGT TTCTTCGACG TGCATGATCA CTACGTTCTT GTCCTTCACT TCGTCTCGAT GAGCGAAGTA   
  
  
+ CACCACGTGG TAGACGACCC ACAAGCCGCT GTTTGTTTGC ATCTTCTGTA CGTTTTAAGA ATAGGGAGAG   
  
  
+ AGAGCCTCAA CACTCGAACA CTAGACCCCA CCCAAATACG AAAGAAAAAA CAAAAAACCT AATTCCATTT   
  
  
+ GTTCTTAAAG AAAGCCCATA GCCCTAAACC AACGATCCTA CGTTCTTTCG TTTCTTTTGT CCATACAGTT   
  
  
+ CCAACTTTTA TGACTACAAG GAGAGTTTTT GTTTGTTTGT TCTTGCCAAA CACTTCTCTC TCTCTCTCTC   
  
  
+ TCTCTCTCTC TCTCCCTTCC TCTCAGCGAC TGAAAAGTGT GTTTTGTGGA GAAGAGACAT GGGTTGTACA   
  
  
+ GAACTCTCTC TCTCCTTTCT CTCTCTCCAA ATCATATTTT ATTCTACCAT TCCTATTATT AGTTTCCTTT   
  
  
+ TCTTCTTTCT TCTTCTTCTT CTTCTACCAG TATTTCTTAA TACCGGTCAC TCGACCCACT AAATAGTACA   
  
  
+ TCGTAGTTTC CCAGTTTAAC TACGTTGACA AAAAGAATAA TATTATATAA AAGCATTATA AAATGAGAGA   
  
  
+ GAGAGAGAGA GAGAGAGATA GACTCGAAGT AAAGAAGTTC TCTCTCAATT TTTTTTTTTT TTTTTTGCTG   
  
  
+ GAATCCCCTT CAGTAGGAAG TTTTTGTTCA CTACCCTTTC ATTTCTCCGC CGTTCATCGG CGTAAATAGC   
  
  
+ CTTTTTCAAA GTTGGTCCGC TTGTTGGGGG CTTTGCAAAG CTTCGCCCGT CGAAGGAACG AATACTCTAC   
  
  
+ ACTTGGGGAG TTACACTACG TCTATGGGTG AACCGAAAAA CGAGTAAATT GCTTGCTCCT GTAGCTATAA   
  
  
+ CGTATATGGG TGAACCGAAA AACGAGTAAA TTGCTTGCTC CTGTAGTTAT AACGTATATG GGTGAAGGGG   
  
  
+ TGAAAGTAGG GGTGAGTTCC CCTGTGTATG AGTATATGAA TAGTTTTATA CAAGGAAATT ACATGGCTAA   
  
  
+ GGTATGCCTT GATATTTTTT ATTTATATAC AAACATAATA GTACGTTATT AGTTTATTTT TTCTTTAATT   
  
  
+ TAACAATTGT ATCCATTTAC CGTATAAGTA GCCTTTTCTT ATATTTTTCT TTTTCTTTAT TTGTTCCTAA   
  
  
+ CTCGGAGTGG GTGTACACAT AATTAGTGGT ATGTTCTTAG GTTAAGGTGT TGGGGTATCT AATACTTCCT   
  
  
+ AAGCTGGGGA TTGGTAAACT TATAGTGTGG GTTACACTTC ATTGTTTCGT ATTGCTATTA CGGAACTTTC   
  
  
+ GTATTTCACT GATAACAGCC TTATAAGTCT AACACACCAT TAAGTCATCG AAACCCATGG GTCCATCGAT   
  
  
+ GTTCCTGATT CTTCTTTAAT TGAAACTTCA AAAATATATG TTAAGTACCA TGATGTGTGT AAGTATACCC   
  
  
+ TTCTTTGATA CTTTTGTTCC TTGTTCTTC  

- GGCTACCCCA AAGAAGCTGC ACGTACTAGT GATGCAAGAA CAGGAAGTGA AGCAGAGCTA CTCGCTTCAT   
  
  
- GTGGTGCACC ATCTGCTGGG TGTTCGGCGA CAAACAAACG TAGAAGACAT GCAAAATTCT TATCCCTCTC   
  
  
- TCTCGGAGTT GTGAGCTTGT GATCTGGGGT GGGTTTATGC TTTCTTTTTT GTTTTTTGGA TTAAGGTAAA   
  
  
- CAAGAATTTC TTTCGGGTAT CGGGATTTGG TTGCTAGGAT GCAAGAAAGC AAAGAAAACA GGTATGTCAA   
  
  
- GGTTGAAAAT ACTGATGTTC CTCTCAAAAA CAAACAAACA AGAACGGTTT GTGAAGAGAG AGAGAGAGAG   
  
  
- AGAGAGAGAG AGAGGGAAGG AGAGTCGCTG ACTTTTCACA CAAAACACCT CTTCTCTGTA CCCAACATGT   
  
  
- CTTGAGAGAG AGAGGAAAGA GAGAGAGGTT TAGTATAAAA TAAGATGGTA AGGATAATAA TCAAAGGAAA   
  
  
- AGAAGAAAGA AGAAGAAGAA GAAGATGGTC ATAAAGAATT ATGGCCAGTG AGCTGGGTGA TTTATCATGT   
  
  
- AGCATCAAAG GGTCAAATTG ATGCAACTGT TTTTCTTATT ATAATATATT TTCGTAATAT TTTACTCTCT   
  
  
- CTCTCTCTCT CTCTCTCTAT CTGAGCTTCA TTTCTTCAAG AGAGAGTTAA AAAAAAAAAA AAAAAACGAC   
  
  
- CTTAGGGGAA GTCATCCTTC AAAAACAAGT GATGGGAAAG TAAAGAGGCG GCAAGTAGCC GCATTTATCG   
  
  
- GAAAAAGTTT CAACCAGGCG AACAACCCCC GAAACGTTTC GAAGCGGGCA GCTTCCTTGC TTATGAGATG   
  
  
- TGAACCCCTC AATGTGATGC AGATACCCAC TTGGCTTTTT GCTCATTTAA CGAACGAGGA CATCGATATT   
  
  
- GCATATACCC ACTTGGCTTT TTGCTCATTT AACGAACGAG GACATCAATA TTGCATATAC CCACTTCCCC   
  
  
- ACTTTCATCC CCACTCAAGG GGACACATAC TCATATACTT ATCAAAATAT GTTCCTTTAA TGTACCGATT   
  
  
- CCATACGGAA CTATAAAAAA TAAATATATG TTTGTATTAT CATGCAATAA TCAAATAAAA AAGAAATTAA   
  
  
- ATTGTTAACA TAGGTAAATG GCATATTCAT CGGAAAAGAA TATAAAAAGA AAAAGAAATA AACAAGGATT   
  
  
- GAGCCTCACC CACATGTGTA TTAATCACCA TACAAGAATC CAATTCCACA ACCCCATAGA TTATGAAGGA   
  
  
- TTCGACCCCT AACCATTTGA ATATCACACC CAATGTGAAG TAACAAAGCA TAACGATAAT GCCTTGAAAG   
  
  
- CATAAAGTGA CTATTGTCGG AATATTCAGA TTGTGTGGTA ATTCAGTAGC TTTGGGTACC CAGGTAGCTA   
  
  
- CAAGGACTAA GAAGAAATTA ACTTTGAAGT TTTTATATAC AATTCATGGT ACTACACACA TTCATATGGG   
  
  
- AAGAAACTAT GAAAACAAGG AACAAGAAG

+     Unnamed\_\_4

| Site Name | Organism | Position | Strand | Matrix score. | sequence | function |
| --- | --- | --- | --- | --- | --- | --- |
| Unnamed\_\_4 | Petroselinum hortense | 362 | + | 4 | CTCC |  |
| Unnamed\_\_4 | Petroselinum hortense | 445 | + | 4 | CTCC |  |
| Unnamed\_\_4 | Petroselinum hortense | 432 | + | 4 | CTCC |  |
| Unnamed\_\_4 | Petroselinum hortense | 1194 | - | 4 | CTCC |  |
| Unnamed\_\_4 | Petroselinum hortense | 398 | - | 4 | CTCC |  |
| Unnamed\_\_4 | Petroselinum hortense | 745 | + | 4 | CTCC |  |
| Unnamed\_\_4 | Petroselinum hortense | 135 | - | 4 | CTCC |  |
| Unnamed\_\_4 | Petroselinum hortense | 896 | + | 4 | CTCC |  |
| Unnamed\_\_4 | Petroselinum hortense | 300 | - | 4 | CTCC |  |
| Unnamed\_\_4 | Petroselinum hortense | 847 | - | 4 | CTCC |  |
| Unnamed\_\_4 | Petroselinum hortense | 948 | + | 4 | CTCC |  |

> 2018/04/13 10:10:12  
+ CCGATGGGGT TTCTTCGACG TGCATGATCA CTACGTTCTT GTCCTTCACT TCGTCTCGAT GAGCGAAGTA   
  
  
+ CACCACGTGG TAGACGACCC ACAAGCCGCT GTTTGTTTGC ATCTTCTGTA CGTTTTAAGA ATAGGGAGAG   
  
  
+ AGAGCCTCAA CACTCGAACA CTAGACCCCA CCCAAATACG AAAGAAAAAA CAAAAAACCT AATTCCATTT   
  
  
+ GTTCTTAAAG AAAGCCCATA GCCCTAAACC AACGATCCTA CGTTCTTTCG TTTCTTTTGT CCATACAGTT   
  
  
+ CCAACTTTTA TGACTACAAG GAGAGTTTTT GTTTGTTTGT TCTTGCCAAA CACTTCTCTC TCTCTCTCTC   
  
  
+ TCTCTCTCTC TCTCCCTTCC TCTCAGCGAC TGAAAAGTGT GTTTTGTGGA GAAGAGACAT GGGTTGTACA   
  
  
+ GAACTCTCTC TCTCCTTTCT CTCTCTCCAA ATCATATTTT ATTCTACCAT TCCTATTATT AGTTTCCTTT   
  
  
+ TCTTCTTTCT TCTTCTTCTT CTTCTACCAG TATTTCTTAA TACCGGTCAC TCGACCCACT AAATAGTACA   
  
  
+ TCGTAGTTTC CCAGTTTAAC TACGTTGACA AAAAGAATAA TATTATATAA AAGCATTATA AAATGAGAGA   
  
  
+ GAGAGAGAGA GAGAGAGATA GACTCGAAGT AAAGAAGTTC TCTCTCAATT TTTTTTTTTT TTTTTTGCTG   
  
  
+ GAATCCCCTT CAGTAGGAAG TTTTTGTTCA CTACCCTTTC ATTTCTCCGC CGTTCATCGG CGTAAATAGC   
  
  
+ CTTTTTCAAA GTTGGTCCGC TTGTTGGGGG CTTTGCAAAG CTTCGCCCGT CGAAGGAACG AATACTCTAC   
  
  
+ ACTTGGGGAG TTACACTACG TCTATGGGTG AACCGAAAAA CGAGTAAATT GCTTGCTCCT GTAGCTATAA   
  
  
+ CGTATATGGG TGAACCGAAA AACGAGTAAA TTGCTTGCTC CTGTAGTTAT AACGTATATG GGTGAAGGGG   
  
  
+ TGAAAGTAGG GGTGAGTTCC CCTGTGTATG AGTATATGAA TAGTTTTATA CAAGGAAATT ACATGGCTAA   
  
  
+ GGTATGCCTT GATATTTTTT ATTTATATAC AAACATAATA GTACGTTATT AGTTTATTTT TTCTTTAATT   
  
  
+ TAACAATTGT ATCCATTTAC CGTATAAGTA GCCTTTTCTT ATATTTTTCT TTTTCTTTAT TTGTTCCTAA   
  
  
+ CTCGGAGTGG GTGTACACAT AATTAGTGGT ATGTTCTTAG GTTAAGGTGT TGGGGTATCT AATACTTCCT   
  
  
+ AAGCTGGGGA TTGGTAAACT TATAGTGTGG GTTACACTTC ATTGTTTCGT ATTGCTATTA CGGAACTTTC   
  
  
+ GTATTTCACT GATAACAGCC TTATAAGTCT AACACACCAT TAAGTCATCG AAACCCATGG GTCCATCGAT   
  
  
+ GTTCCTGATT CTTCTTTAAT TGAAACTTCA AAAATATATG TTAAGTACCA TGATGTGTGT AAGTATACCC   
  
  
+ TTCTTTGATA CTTTTGTTCC TTGTTCTTC  

- GGCTACCCCA AAGAAGCTGC ACGTACTAGT GATGCAAGAA CAGGAAGTGA AGCAGAGCTA CTCGCTTCAT   
  
  
- GTGGTGCACC ATCTGCTGGG TGTTCGGCGA CAAACAAACG TAGAAGACAT GCAAAATTCT TATCCCTCTC   
  
  
- TCTCGGAGTT GTGAGCTTGT GATCTGGGGT GGGTTTATGC TTTCTTTTTT GTTTTTTGGA TTAAGGTAAA   
  
  
- CAAGAATTTC TTTCGGGTAT CGGGATTTGG TTGCTAGGAT GCAAGAAAGC AAAGAAAACA GGTATGTCAA   
  
  
- GGTTGAAAAT ACTGATGTTC CTCTCAAAAA CAAACAAACA AGAACGGTTT GTGAAGAGAG AGAGAGAGAG   
  
  
- AGAGAGAGAG AGAGGGAAGG AGAGTCGCTG ACTTTTCACA CAAAACACCT CTTCTCTGTA CCCAACATGT   
  
  
- CTTGAGAGAG AGAGGAAAGA GAGAGAGGTT TAGTATAAAA TAAGATGGTA AGGATAATAA TCAAAGGAAA   
  
  
- AGAAGAAAGA AGAAGAAGAA GAAGATGGTC ATAAAGAATT ATGGCCAGTG AGCTGGGTGA TTTATCATGT   
  
  
- AGCATCAAAG GGTCAAATTG ATGCAACTGT TTTTCTTATT ATAATATATT TTCGTAATAT TTTACTCTCT   
  
  
- CTCTCTCTCT CTCTCTCTAT CTGAGCTTCA TTTCTTCAAG AGAGAGTTAA AAAAAAAAAA AAAAAACGAC   
  
  
- CTTAGGGGAA GTCATCCTTC AAAAACAAGT GATGGGAAAG TAAAGAGGCG GCAAGTAGCC GCATTTATCG   
  
  
- GAAAAAGTTT CAACCAGGCG AACAACCCCC GAAACGTTTC GAAGCGGGCA GCTTCCTTGC TTATGAGATG   
  
  
- TGAACCCCTC AATGTGATGC AGATACCCAC TTGGCTTTTT GCTCATTTAA CGAACGAGGA CATCGATATT   
  
  
- GCATATACCC ACTTGGCTTT TTGCTCATTT AACGAACGAG GACATCAATA TTGCATATAC CCACTTCCCC   
  
  
- ACTTTCATCC CCACTCAAGG GGACACATAC TCATATACTT ATCAAAATAT GTTCCTTTAA TGTACCGATT   
  
  
- CCATACGGAA CTATAAAAAA TAAATATATG TTTGTATTAT CATGCAATAA TCAAATAAAA AAGAAATTAA   
  
  
- ATTGTTAACA TAGGTAAATG GCATATTCAT CGGAAAAGAA TATAAAAAGA AAAAGAAATA AACAAGGATT   
  
  
- GAGCCTCACC CACATGTGTA TTAATCACCA TACAAGAATC CAATTCCACA ACCCCATAGA TTATGAAGGA   
  
  
- TTCGACCCCT AACCATTTGA ATATCACACC CAATGTGAAG TAACAAAGCA TAACGATAAT GCCTTGAAAG   
  
  
- CATAAAGTGA CTATTGTCGG AATATTCAGA TTGTGTGGTA ATTCAGTAGC TTTGGGTACC CAGGTAGCTA   
  
  
- CAAGGACTAA GAAGAAATTA ACTTTGAAGT TTTTATATAC AATTCATGGT ACTACACACA TTCATATGGG   
  
  
- AAGAAACTAT GAAAACAAGG AACAAGAAG

+     circadian

| Site Name | Organism | Position | Strand | Matrix score. | sequence | function |
| --- | --- | --- | --- | --- | --- | --- |
| circadian | Lycopersicon esculentum | 1477 | - | 6 | CAANNNNATC | cis-acting regulatory element involved in circadian control |
| circadian | Lycopersicon esculentum | 1124 | + | 6 | CAANNNNATC | cis-acting regulatory element involved in circadian control |

> 2018/04/13 10:10:12  
+ CCGATGGGGT TTCTTCGACG TGCATGATCA CTACGTTCTT GTCCTTCACT TCGTCTCGAT GAGCGAAGTA   
  
  
+ CACCACGTGG TAGACGACCC ACAAGCCGCT GTTTGTTTGC ATCTTCTGTA CGTTTTAAGA ATAGGGAGAG   
  
  
+ AGAGCCTCAA CACTCGAACA CTAGACCCCA CCCAAATACG AAAGAAAAAA CAAAAAACCT AATTCCATTT   
  
  
+ GTTCTTAAAG AAAGCCCATA GCCCTAAACC AACGATCCTA CGTTCTTTCG TTTCTTTTGT CCATACAGTT   
  
  
+ CCAACTTTTA TGACTACAAG GAGAGTTTTT GTTTGTTTGT TCTTGCCAAA CACTTCTCTC TCTCTCTCTC   
  
  
+ TCTCTCTCTC TCTCCCTTCC TCTCAGCGAC TGAAAAGTGT GTTTTGTGGA GAAGAGACAT GGGTTGTACA   
  
  
+ GAACTCTCTC TCTCCTTTCT CTCTCTCCAA ATCATATTTT ATTCTACCAT TCCTATTATT AGTTTCCTTT   
  
  
+ TCTTCTTTCT TCTTCTTCTT CTTCTACCAG TATTTCTTAA TACCGGTCAC TCGACCCACT AAATAGTACA   
  
  
+ TCGTAGTTTC CCAGTTTAAC TACGTTGACA AAAAGAATAA TATTATATAA AAGCATTATA AAATGAGAGA   
  
  
+ GAGAGAGAGA GAGAGAGATA GACTCGAAGT AAAGAAGTTC TCTCTCAATT TTTTTTTTTT TTTTTTGCTG   
  
  
+ GAATCCCCTT CAGTAGGAAG TTTTTGTTCA CTACCCTTTC ATTTCTCCGC CGTTCATCGG CGTAAATAGC   
  
  
+ CTTTTTCAAA GTTGGTCCGC TTGTTGGGGG CTTTGCAAAG CTTCGCCCGT CGAAGGAACG AATACTCTAC   
  
  
+ ACTTGGGGAG TTACACTACG TCTATGGGTG AACCGAAAAA CGAGTAAATT GCTTGCTCCT GTAGCTATAA   
  
  
+ CGTATATGGG TGAACCGAAA AACGAGTAAA TTGCTTGCTC CTGTAGTTAT AACGTATATG GGTGAAGGGG   
  
  
+ TGAAAGTAGG GGTGAGTTCC CCTGTGTATG AGTATATGAA TAGTTTTATA CAAGGAAATT ACATGGCTAA   
  
  
+ GGTATGCCTT GATATTTTTT ATTTATATAC AAACATAATA GTACGTTATT AGTTTATTTT TTCTTTAATT   
  
  
+ TAACAATTGT ATCCATTTAC CGTATAAGTA GCCTTTTCTT ATATTTTTCT TTTTCTTTAT TTGTTCCTAA   
  
  
+ CTCGGAGTGG GTGTACACAT AATTAGTGGT ATGTTCTTAG GTTAAGGTGT TGGGGTATCT AATACTTCCT   
  
  
+ AAGCTGGGGA TTGGTAAACT TATAGTGTGG GTTACACTTC ATTGTTTCGT ATTGCTATTA CGGAACTTTC   
  
  
+ GTATTTCACT GATAACAGCC TTATAAGTCT AACACACCAT TAAGTCATCG AAACCCATGG GTCCATCGAT   
  
  
+ GTTCCTGATT CTTCTTTAAT TGAAACTTCA AAAATATATG TTAAGTACCA TGATGTGTGT AAGTATACCC   
  
  
+ TTCTTTGATA CTTTTGTTCC TTGTTCTTC  

- GGCTACCCCA AAGAAGCTGC ACGTACTAGT GATGCAAGAA CAGGAAGTGA AGCAGAGCTA CTCGCTTCAT   
  
  
- GTGGTGCACC ATCTGCTGGG TGTTCGGCGA CAAACAAACG TAGAAGACAT GCAAAATTCT TATCCCTCTC   
  
  
- TCTCGGAGTT GTGAGCTTGT GATCTGGGGT GGGTTTATGC TTTCTTTTTT GTTTTTTGGA TTAAGGTAAA   
  
  
- CAAGAATTTC TTTCGGGTAT CGGGATTTGG TTGCTAGGAT GCAAGAAAGC AAAGAAAACA GGTATGTCAA   
  
  
- GGTTGAAAAT ACTGATGTTC CTCTCAAAAA CAAACAAACA AGAACGGTTT GTGAAGAGAG AGAGAGAGAG   
  
  
- AGAGAGAGAG AGAGGGAAGG AGAGTCGCTG ACTTTTCACA CAAAACACCT CTTCTCTGTA CCCAACATGT   
  
  
- CTTGAGAGAG AGAGGAAAGA GAGAGAGGTT TAGTATAAAA TAAGATGGTA AGGATAATAA TCAAAGGAAA   
  
  
- AGAAGAAAGA AGAAGAAGAA GAAGATGGTC ATAAAGAATT ATGGCCAGTG AGCTGGGTGA TTTATCATGT   
  
  
- AGCATCAAAG GGTCAAATTG ATGCAACTGT TTTTCTTATT ATAATATATT TTCGTAATAT TTTACTCTCT   
  
  
- CTCTCTCTCT CTCTCTCTAT CTGAGCTTCA TTTCTTCAAG AGAGAGTTAA AAAAAAAAAA AAAAAACGAC   
  
  
- CTTAGGGGAA GTCATCCTTC AAAAACAAGT GATGGGAAAG TAAAGAGGCG GCAAGTAGCC GCATTTATCG   
  
  
- GAAAAAGTTT CAACCAGGCG AACAACCCCC GAAACGTTTC GAAGCGGGCA GCTTCCTTGC TTATGAGATG   
  
  
- TGAACCCCTC AATGTGATGC AGATACCCAC TTGGCTTTTT GCTCATTTAA CGAACGAGGA CATCGATATT   
  
  
- GCATATACCC ACTTGGCTTT TTGCTCATTT AACGAACGAG GACATCAATA TTGCATATAC CCACTTCCCC   
  
  
- ACTTTCATCC CCACTCAAGG GGACACATAC TCATATACTT ATCAAAATAT GTTCCTTTAA TGTACCGATT   
  
  
- CCATACGGAA CTATAAAAAA TAAATATATG TTTGTATTAT CATGCAATAA TCAAATAAAA AAGAAATTAA   
  
  
- ATTGTTAACA TAGGTAAATG GCATATTCAT CGGAAAAGAA TATAAAAAGA AAAAGAAATA AACAAGGATT   
  
  
- GAGCCTCACC CACATGTGTA TTAATCACCA TACAAGAATC CAATTCCACA ACCCCATAGA TTATGAAGGA   
  
  
- TTCGACCCCT AACCATTTGA ATATCACACC CAATGTGAAG TAACAAAGCA TAACGATAAT GCCTTGAAAG   
  
  
- CATAAAGTGA CTATTGTCGG AATATTCAGA TTGTGTGGTA ATTCAGTAGC TTTGGGTACC CAGGTAGCTA   
  
  
- CAAGGACTAA GAAGAAATTA ACTTTGAAGT TTTTATATAC AATTCATGGT ACTACACACA TTCATATGGG   
  
  
- AAGAAACTAT GAAAACAAGG AACAAGAAG
