# Supplementary material for: Genome-wide analysis of the WRKY gene family in drumstick (Moringa oleifera Lam.)
Source: PeerJ. 2019 Jun 10;7:e7063. doi: 10.7717/peerj.7063 (PMC6563795; doi:10.7717/peerj.7063)
Supplement: Supplemental Information 1 [file peerj-07-7063-s003.gz › MoWRKY48_plantcare.html]

Content-Type: text/html; charset=ISO-8859-1


CallMat\_Firefox


Webmaster Firefox specific output  
To save the result:
click on the frame with the right mouse button and save the source code as a text file with extension .html  
REFERENCE:PlantCARE: a database of plant cis-acting regulatory elements and a portal to tools for in silico analysis of promoter sequences.  
Lescot, M., Déhais, P., Moreau, Y., De Moor, B., Rouzé ,P.,and Rombauts, S.  
Nucleic Acids Res., Database issue(2002), 30(1):325-327.   


---

> 2018/04/13 10:10:12  
+ CTGGGAAAAA CATAGAGAGC GAGATGCCGG CAAAGTCGCT GTTCCGAGAC ACTTAGGCAT TGTCTGGAGG   
  
  
+ CGTCCCTGAC AATCTCTTCT CTGTCTCTCT CTTTCTCTCT TCTGTTTTCT TACGCGACTG AAAACTCATT   
  
  
+ TCTCTCTCTT TTACCATCCC CGATCATTAC TCACCCCAAT GGTTATATAT ATCTCTCTCT CTCTCTGCCG   
  
  
+ TGTCATGTGT ACGTGGACAT ACATCTCTCT CTCTCGGCTT CACCAATAAA TCATAAGTGT TCAGATCTAT   
  
  
+ CTCTCTCTTT GATCTCTTCA ACCGTAGAGT TACGGGTAAC AACAAGAAAG AGAGAGACGA TAGGCTGTTA   
  
  
+ ACTTTTGGTC TTTGCCAAAG ACCAAAACCC CAACTGAAGA GCGTGTAGAG CAACCCAAGC GGGAGAGGTT   
  
  
+ AAGAAAAAAG TGTGTTTTTC AGGTGCGCGA GAGAAAAAGA GAGAGAGAGA GCACCCGCGA GCACTTAGTG   
  
  
+ GCTGTTTTCT CTCCTTAATT TTCTATCGAA GACGTTTCTC CTAATTTGCT TTCACCTGCG GTCAATTGCT   
  
  
+ CCCGCGAGGA TAGTTAAAGT TGACACAAAA ATGCTAAAAT GGGTATTGTT TTCTTCTTTC CTAAATTTAG   
  
  
+ TTAAATCAAC AACCAACGAA GGCATCAATT AATACACGGA GAAAAATACA GCGGCCCAAA CAAGCAGGGC   
  
  
+ ACGTACTGTG CACGGCTCAG TGACCGTCAA GCTAATTTGG TGTGGCCAAG GATGAAAGTG AGGGACACAA   
  
  
+ ACGAAGAAGG TGAGGTAATA AGGAAAGAAG AATGCCTTTA ATGGAAACCT GTATCGTTTT TATCGTGTCT   
  
  
+ TACTGCCTTT TTAGATTGTC AATGCTGCAC AGTGGGAATT TTCCGCTCAA CCTATAACCG TATGAGCTTA   
  
  
+ CCTCAAAGTA CAACTGGTGT GTAGTTCTAG CTGCCGACGT CGACCACCAA GCACACACCG TGATTGCACA   
  
  
+ TTAACTGGTC TCTCCTCCGC CTCTCGCACC GCCACGTCCT CTATTCCTCC ACTCCACCGG TCTACTGTCG   
  
  
+ CTCGGCTAAA ACACCCCAGG TGCAAGAAAA CGGTTTTCCC TACGGCAAAC TGTCGTAGAG CACCGAGCAC   
  
  
+ TAGAGTTACG GTTGGTCGAC ACGAGGGAAG AGCGTAGCAT ACTAAGTCCT AGGTTGCCGG CTTTTGCCAA   
  
  
+ GTGGAATAGT AGGTCAGTCG AATTACTAGT TTATAAAAGT TGAGAGGTGC CGACTCTACG CGACTGTCTC   
  
  
+ AGAGACATAT ATATTCGCTC TCATGCTTTT GGTAACTCCT AAATCTTCAT CCTCACCCGT AGTGCTCTGT   
  
  
+ ACCCTAACGT TCGCACACCC CAGTGTGAAA ATAAAATTAA ACAGACAAAT CCTGATTATT CAACCTTCAC   
  
  
+ CGCTCAGAAC ACAACCTGGG TTAAAGTCCG GGGGGTAGTA CCAAAGAGGG CGGTGTATTA ATGTCACAGA   
  
  
+ CTGGCTTAAT GTAATAACAA TAAGAACAA  

- GACCCTTTTT GTATCTCTCG CTCTACGGCC GTTTCAGCGA CAAGGCTCTG TGAATCCGTA ACAGACCTCC   
  
  
- GCAGGGACTG TTAGAGAAGA GACAGAGAGA GAAAGAGAGA AGACAAAAGA ATGCGCTGAC TTTTGAGTAA   
  
  
- AGAGAGAGAA AATGGTAGGG GCTAGTAATG AGTGGGGTTA CCAATATATA TAGAGAGAGA GAGAGACGGC   
  
  
- ACAGTACACA TGCACCTGTA TGTAGAGAGA GAGAGCCGAA GTGGTTATTT AGTATTCACA AGTCTAGATA   
  
  
- GAGAGAGAAA CTAGAGAAGT TGGCATCTCA ATGCCCATTG TTGTTCTTTC TCTCTCTGCT ATCCGACAAT   
  
  
- TGAAAACCAG AAACGGTTTC TGGTTTTGGG GTTGACTTCT CGCACATCTC GTTGGGTTCG CCCTCTCCAA   
  
  
- TTCTTTTTTC ACACAAAAAG TCCACGCGCT CTCTTTTTCT CTCTCTCTCT CGTGGGCGCT CGTGAATCAC   
  
  
- CGACAAAAGA GAGGAATTAA AAGATAGCTT CTGCAAAGAG GATTAAACGA AAGTGGACGC CAGTTAACGA   
  
  
- GGGCGCTCCT ATCAATTTCA ACTGTGTTTT TACGATTTTA CCCATAACAA AAGAAGAAAG GATTTAAATC   
  
  
- AATTTAGTTG TTGGTTGCTT CCGTAGTTAA TTATGTGCCT CTTTTTATGT CGCCGGGTTT GTTCGTCCCG   
  
  
- TGCATGACAC GTGCCGAGTC ACTGGCAGTT CGATTAAACC ACACCGGTTC CTACTTTCAC TCCCTGTGTT   
  
  
- TGCTTCTTCC ACTCCATTAT TCCTTTCTTC TTACGGAAAT TACCTTTGGA CATAGCAAAA ATAGCACAGA   
  
  
- ATGACGGAAA AATCTAACAG TTACGACGTG TCACCCTTAA AAGGCGAGTT GGATATTGGC ATACTCGAAT   
  
  
- GGAGTTTCAT GTTGACCACA CATCAAGATC GACGGCTGCA GCTGGTGGTT CGTGTGTGGC ACTAACGTGT   
  
  
- AATTGACCAG AGAGGAGGCG GAGAGCGTGG CGGTGCAGGA GATAAGGAGG TGAGGTGGCC AGATGACAGC   
  
  
- GAGCCGATTT TGTGGGGTCC ACGTTCTTTT GCCAAAAGGG ATGCCGTTTG ACAGCATCTC GTGGCTCGTG   
  
  
- ATCTCAATGC CAACCAGCTG TGCTCCCTTC TCGCATCGTA TGATTCAGGA TCCAACGGCC GAAAACGGTT   
  
  
- CACCTTATCA TCCAGTCAGC TTAATGATCA AATATTTTCA ACTCTCCACG GCTGAGATGC GCTGACAGAG   
  
  
- TCTCTGTATA TATAAGCGAG AGTACGAAAA CCATTGAGGA TTTAGAAGTA GGAGTGGGCA TCACGAGACA   
  
  
- TGGGATTGCA AGCGTGTGGG GTCACACTTT TATTTTAATT TGTCTGTTTA GGACTAATAA GTTGGAAGTG   
  
  
- GCGAGTCTTG TGTTGGACCC AATTTCAGGC CCCCCATCAT GGTTTCTCCC GCCACATAAT TACAGTGTCT   
  
  
- GACCGAATTA CATTATTGTT ATTCTTGTT

  
  
Motifs Found  

+     5UTR Py-rich stretch

| Site Name | Organism | Position | Strand | Matrix score. | sequence | function |
| --- | --- | --- | --- | --- | --- | --- |
| 5UTR Py-rich stretch | Lycopersicon esculentum | 610 | + | 9 | TTTCTTCTCT | cis-acting element conferring high transcription levels |
| 5UTR Py-rich stretch | Lycopersicon esculentum | 83 | + | 9 | TTTCTTCTCT | cis-acting element conferring high transcription levels |
| 5UTR Py-rich stretch | Lycopersicon esculentum | 190 | + | 13 | TTTCTCTCTCTCTC | cis-acting element conferring high transcription levels |
| 5UTR Py-rich stretch | Lycopersicon esculentum | 192 | + | 13 | TTTCTCTCTCTCTC | cis-acting element conferring high transcription levels |

> 2018/04/13 10:10:12  
+ CTGGGAAAAA CATAGAGAGC GAGATGCCGG CAAAGTCGCT GTTCCGAGAC ACTTAGGCAT TGTCTGGAGG   
  
  
+ CGTCCCTGAC AATCTCTTCT CTGTCTCTCT CTTTCTCTCT TCTGTTTTCT TACGCGACTG AAAACTCATT   
  
  
+ TCTCTCTCTT TTACCATCCC CGATCATTAC TCACCCCAAT GGTTATATAT ATCTCTCTCT CTCTCTGCCG   
  
  
+ TGTCATGTGT ACGTGGACAT ACATCTCTCT CTCTCGGCTT CACCAATAAA TCATAAGTGT TCAGATCTAT   
  
  
+ CTCTCTCTTT GATCTCTTCA ACCGTAGAGT TACGGGTAAC AACAAGAAAG AGAGAGACGA TAGGCTGTTA   
  
  
+ ACTTTTGGTC TTTGCCAAAG ACCAAAACCC CAACTGAAGA GCGTGTAGAG CAACCCAAGC GGGAGAGGTT   
  
  
+ AAGAAAAAAG TGTGTTTTTC AGGTGCGCGA GAGAAAAAGA GAGAGAGAGA GCACCCGCGA GCACTTAGTG   
  
  
+ GCTGTTTTCT CTCCTTAATT TTCTATCGAA GACGTTTCTC CTAATTTGCT TTCACCTGCG GTCAATTGCT   
  
  
+ CCCGCGAGGA TAGTTAAAGT TGACACAAAA ATGCTAAAAT GGGTATTGTT TTCTTCTTTC CTAAATTTAG   
  
  
+ TTAAATCAAC AACCAACGAA GGCATCAATT AATACACGGA GAAAAATACA GCGGCCCAAA CAAGCAGGGC   
  
  
+ ACGTACTGTG CACGGCTCAG TGACCGTCAA GCTAATTTGG TGTGGCCAAG GATGAAAGTG AGGGACACAA   
  
  
+ ACGAAGAAGG TGAGGTAATA AGGAAAGAAG AATGCCTTTA ATGGAAACCT GTATCGTTTT TATCGTGTCT   
  
  
+ TACTGCCTTT TTAGATTGTC AATGCTGCAC AGTGGGAATT TTCCGCTCAA CCTATAACCG TATGAGCTTA   
  
  
+ CCTCAAAGTA CAACTGGTGT GTAGTTCTAG CTGCCGACGT CGACCACCAA GCACACACCG TGATTGCACA   
  
  
+ TTAACTGGTC TCTCCTCCGC CTCTCGCACC GCCACGTCCT CTATTCCTCC ACTCCACCGG TCTACTGTCG   
  
  
+ CTCGGCTAAA ACACCCCAGG TGCAAGAAAA CGGTTTTCCC TACGGCAAAC TGTCGTAGAG CACCGAGCAC   
  
  
+ TAGAGTTACG GTTGGTCGAC ACGAGGGAAG AGCGTAGCAT ACTAAGTCCT AGGTTGCCGG CTTTTGCCAA   
  
  
+ GTGGAATAGT AGGTCAGTCG AATTACTAGT TTATAAAAGT TGAGAGGTGC CGACTCTACG CGACTGTCTC   
  
  
+ AGAGACATAT ATATTCGCTC TCATGCTTTT GGTAACTCCT AAATCTTCAT CCTCACCCGT AGTGCTCTGT   
  
  
+ ACCCTAACGT TCGCACACCC CAGTGTGAAA ATAAAATTAA ACAGACAAAT CCTGATTATT CAACCTTCAC   
  
  
+ CGCTCAGAAC ACAACCTGGG TTAAAGTCCG GGGGGTAGTA CCAAAGAGGG CGGTGTATTA ATGTCACAGA   
  
  
+ CTGGCTTAAT GTAATAACAA TAAGAACAA  

- GACCCTTTTT GTATCTCTCG CTCTACGGCC GTTTCAGCGA CAAGGCTCTG TGAATCCGTA ACAGACCTCC   
  
  
- GCAGGGACTG TTAGAGAAGA GACAGAGAGA GAAAGAGAGA AGACAAAAGA ATGCGCTGAC TTTTGAGTAA   
  
  
- AGAGAGAGAA AATGGTAGGG GCTAGTAATG AGTGGGGTTA CCAATATATA TAGAGAGAGA GAGAGACGGC   
  
  
- ACAGTACACA TGCACCTGTA TGTAGAGAGA GAGAGCCGAA GTGGTTATTT AGTATTCACA AGTCTAGATA   
  
  
- GAGAGAGAAA CTAGAGAAGT TGGCATCTCA ATGCCCATTG TTGTTCTTTC TCTCTCTGCT ATCCGACAAT   
  
  
- TGAAAACCAG AAACGGTTTC TGGTTTTGGG GTTGACTTCT CGCACATCTC GTTGGGTTCG CCCTCTCCAA   
  
  
- TTCTTTTTTC ACACAAAAAG TCCACGCGCT CTCTTTTTCT CTCTCTCTCT CGTGGGCGCT CGTGAATCAC   
  
  
- CGACAAAAGA GAGGAATTAA AAGATAGCTT CTGCAAAGAG GATTAAACGA AAGTGGACGC CAGTTAACGA   
  
  
- GGGCGCTCCT ATCAATTTCA ACTGTGTTTT TACGATTTTA CCCATAACAA AAGAAGAAAG GATTTAAATC   
  
  
- AATTTAGTTG TTGGTTGCTT CCGTAGTTAA TTATGTGCCT CTTTTTATGT CGCCGGGTTT GTTCGTCCCG   
  
  
- TGCATGACAC GTGCCGAGTC ACTGGCAGTT CGATTAAACC ACACCGGTTC CTACTTTCAC TCCCTGTGTT   
  
  
- TGCTTCTTCC ACTCCATTAT TCCTTTCTTC TTACGGAAAT TACCTTTGGA CATAGCAAAA ATAGCACAGA   
  
  
- ATGACGGAAA AATCTAACAG TTACGACGTG TCACCCTTAA AAGGCGAGTT GGATATTGGC ATACTCGAAT   
  
  
- GGAGTTTCAT GTTGACCACA CATCAAGATC GACGGCTGCA GCTGGTGGTT CGTGTGTGGC ACTAACGTGT   
  
  
- AATTGACCAG AGAGGAGGCG GAGAGCGTGG CGGTGCAGGA GATAAGGAGG TGAGGTGGCC AGATGACAGC   
  
  
- GAGCCGATTT TGTGGGGTCC ACGTTCTTTT GCCAAAAGGG ATGCCGTTTG ACAGCATCTC GTGGCTCGTG   
  
  
- ATCTCAATGC CAACCAGCTG TGCTCCCTTC TCGCATCGTA TGATTCAGGA TCCAACGGCC GAAAACGGTT   
  
  
- CACCTTATCA TCCAGTCAGC TTAATGATCA AATATTTTCA ACTCTCCACG GCTGAGATGC GCTGACAGAG   
  
  
- TCTCTGTATA TATAAGCGAG AGTACGAAAA CCATTGAGGA TTTAGAAGTA GGAGTGGGCA TCACGAGACA   
  
  
- TGGGATTGCA AGCGTGTGGG GTCACACTTT TATTTTAATT TGTCTGTTTA GGACTAATAA GTTGGAAGTG   
  
  
- GCGAGTCTTG TGTTGGACCC AATTTCAGGC CCCCCATCAT GGTTTCTCCC GCCACATAAT TACAGTGTCT   
  
  
- GACCGAATTA CATTATTGTT ATTCTTGTT

+     AAGAA-motif

| Site Name | Organism | Position | Strand | Matrix score. | sequence | function |
| --- | --- | --- | --- | --- | --- | --- |
| AAGAA-motif | Avena sativa | 417 | + | 9 | gGTAAAGAAA |  |
| AAGAA-motif | Avena sativa | 793 | + | 7 | GAAAGAA |  |
| AAGAA-motif | Avena sativa | 614 | - | 7 | GAAAGAA |  |

> 2018/04/13 10:10:12  
+ CTGGGAAAAA CATAGAGAGC GAGATGCCGG CAAAGTCGCT GTTCCGAGAC ACTTAGGCAT TGTCTGGAGG   
  
  
+ CGTCCCTGAC AATCTCTTCT CTGTCTCTCT CTTTCTCTCT TCTGTTTTCT TACGCGACTG AAAACTCATT   
  
  
+ TCTCTCTCTT TTACCATCCC CGATCATTAC TCACCCCAAT GGTTATATAT ATCTCTCTCT CTCTCTGCCG   
  
  
+ TGTCATGTGT ACGTGGACAT ACATCTCTCT CTCTCGGCTT CACCAATAAA TCATAAGTGT TCAGATCTAT   
  
  
+ CTCTCTCTTT GATCTCTTCA ACCGTAGAGT TACGGGTAAC AACAAGAAAG AGAGAGACGA TAGGCTGTTA   
  
  
+ ACTTTTGGTC TTTGCCAAAG ACCAAAACCC CAACTGAAGA GCGTGTAGAG CAACCCAAGC GGGAGAGGTT   
  
  
+ AAGAAAAAAG TGTGTTTTTC AGGTGCGCGA GAGAAAAAGA GAGAGAGAGA GCACCCGCGA GCACTTAGTG   
  
  
+ GCTGTTTTCT CTCCTTAATT TTCTATCGAA GACGTTTCTC CTAATTTGCT TTCACCTGCG GTCAATTGCT   
  
  
+ CCCGCGAGGA TAGTTAAAGT TGACACAAAA ATGCTAAAAT GGGTATTGTT TTCTTCTTTC CTAAATTTAG   
  
  
+ TTAAATCAAC AACCAACGAA GGCATCAATT AATACACGGA GAAAAATACA GCGGCCCAAA CAAGCAGGGC   
  
  
+ ACGTACTGTG CACGGCTCAG TGACCGTCAA GCTAATTTGG TGTGGCCAAG GATGAAAGTG AGGGACACAA   
  
  
+ ACGAAGAAGG TGAGGTAATA AGGAAAGAAG AATGCCTTTA ATGGAAACCT GTATCGTTTT TATCGTGTCT   
  
  
+ TACTGCCTTT TTAGATTGTC AATGCTGCAC AGTGGGAATT TTCCGCTCAA CCTATAACCG TATGAGCTTA   
  
  
+ CCTCAAAGTA CAACTGGTGT GTAGTTCTAG CTGCCGACGT CGACCACCAA GCACACACCG TGATTGCACA   
  
  
+ TTAACTGGTC TCTCCTCCGC CTCTCGCACC GCCACGTCCT CTATTCCTCC ACTCCACCGG TCTACTGTCG   
  
  
+ CTCGGCTAAA ACACCCCAGG TGCAAGAAAA CGGTTTTCCC TACGGCAAAC TGTCGTAGAG CACCGAGCAC   
  
  
+ TAGAGTTACG GTTGGTCGAC ACGAGGGAAG AGCGTAGCAT ACTAAGTCCT AGGTTGCCGG CTTTTGCCAA   
  
  
+ GTGGAATAGT AGGTCAGTCG AATTACTAGT TTATAAAAGT TGAGAGGTGC CGACTCTACG CGACTGTCTC   
  
  
+ AGAGACATAT ATATTCGCTC TCATGCTTTT GGTAACTCCT AAATCTTCAT CCTCACCCGT AGTGCTCTGT   
  
  
+ ACCCTAACGT TCGCACACCC CAGTGTGAAA ATAAAATTAA ACAGACAAAT CCTGATTATT CAACCTTCAC   
  
  
+ CGCTCAGAAC ACAACCTGGG TTAAAGTCCG GGGGGTAGTA CCAAAGAGGG CGGTGTATTA ATGTCACAGA   
  
  
+ CTGGCTTAAT GTAATAACAA TAAGAACAA  

- GACCCTTTTT GTATCTCTCG CTCTACGGCC GTTTCAGCGA CAAGGCTCTG TGAATCCGTA ACAGACCTCC   
  
  
- GCAGGGACTG TTAGAGAAGA GACAGAGAGA GAAAGAGAGA AGACAAAAGA ATGCGCTGAC TTTTGAGTAA   
  
  
- AGAGAGAGAA AATGGTAGGG GCTAGTAATG AGTGGGGTTA CCAATATATA TAGAGAGAGA GAGAGACGGC   
  
  
- ACAGTACACA TGCACCTGTA TGTAGAGAGA GAGAGCCGAA GTGGTTATTT AGTATTCACA AGTCTAGATA   
  
  
- GAGAGAGAAA CTAGAGAAGT TGGCATCTCA ATGCCCATTG TTGTTCTTTC TCTCTCTGCT ATCCGACAAT   
  
  
- TGAAAACCAG AAACGGTTTC TGGTTTTGGG GTTGACTTCT CGCACATCTC GTTGGGTTCG CCCTCTCCAA   
  
  
- TTCTTTTTTC ACACAAAAAG TCCACGCGCT CTCTTTTTCT CTCTCTCTCT CGTGGGCGCT CGTGAATCAC   
  
  
- CGACAAAAGA GAGGAATTAA AAGATAGCTT CTGCAAAGAG GATTAAACGA AAGTGGACGC CAGTTAACGA   
  
  
- GGGCGCTCCT ATCAATTTCA ACTGTGTTTT TACGATTTTA CCCATAACAA AAGAAGAAAG GATTTAAATC   
  
  
- AATTTAGTTG TTGGTTGCTT CCGTAGTTAA TTATGTGCCT CTTTTTATGT CGCCGGGTTT GTTCGTCCCG   
  
  
- TGCATGACAC GTGCCGAGTC ACTGGCAGTT CGATTAAACC ACACCGGTTC CTACTTTCAC TCCCTGTGTT   
  
  
- TGCTTCTTCC ACTCCATTAT TCCTTTCTTC TTACGGAAAT TACCTTTGGA CATAGCAAAA ATAGCACAGA   
  
  
- ATGACGGAAA AATCTAACAG TTACGACGTG TCACCCTTAA AAGGCGAGTT GGATATTGGC ATACTCGAAT   
  
  
- GGAGTTTCAT GTTGACCACA CATCAAGATC GACGGCTGCA GCTGGTGGTT CGTGTGTGGC ACTAACGTGT   
  
  
- AATTGACCAG AGAGGAGGCG GAGAGCGTGG CGGTGCAGGA GATAAGGAGG TGAGGTGGCC AGATGACAGC   
  
  
- GAGCCGATTT TGTGGGGTCC ACGTTCTTTT GCCAAAAGGG ATGCCGTTTG ACAGCATCTC GTGGCTCGTG   
  
  
- ATCTCAATGC CAACCAGCTG TGCTCCCTTC TCGCATCGTA TGATTCAGGA TCCAACGGCC GAAAACGGTT   
  
  
- CACCTTATCA TCCAGTCAGC TTAATGATCA AATATTTTCA ACTCTCCACG GCTGAGATGC GCTGACAGAG   
  
  
- TCTCTGTATA TATAAGCGAG AGTACGAAAA CCATTGAGGA TTTAGAAGTA GGAGTGGGCA TCACGAGACA   
  
  
- TGGGATTGCA AGCGTGTGGG GTCACACTTT TATTTTAATT TGTCTGTTTA GGACTAATAA GTTGGAAGTG   
  
  
- GCGAGTCTTG TGTTGGACCC AATTTCAGGC CCCCCATCAT GGTTTCTCCC GCCACATAAT TACAGTGTCT   
  
  
- GACCGAATTA CATTATTGTT ATTCTTGTT

+     ABRE

| Site Name | Organism | Position | Strand | Matrix score. | sequence | function |
| --- | --- | --- | --- | --- | --- | --- |
| ABRE | Arabidopsis thaliana | 700 | - | 6 | TACGTG | cis-acting element involved in the abscisic acid responsiveness |
| ABRE | Oryza sativa | 698 | - | 9 | AGTACGTGGC | cis-acting element involved in the abscisic acid responsiveness |
| ABRE | Arabidopsis thaliana | 1011 | - | 7 | ACGTGGC | cis-acting element involved in the abscisic acid responsiveness |
| ABRE | Hordeum vulgare | 218 | - | 9 | GCCACGTACA | cis-acting element involved in the abscisic acid responsiveness |
| ABRE | Arabidopsis thaliana | 220 | + | 6 | TACGTG | cis-acting element involved in the abscisic acid responsiveness |

> 2018/04/13 10:10:12  
+ CTGGGAAAAA CATAGAGAGC GAGATGCCGG CAAAGTCGCT GTTCCGAGAC ACTTAGGCAT TGTCTGGAGG   
  
  
+ CGTCCCTGAC AATCTCTTCT CTGTCTCTCT CTTTCTCTCT TCTGTTTTCT TACGCGACTG AAAACTCATT   
  
  
+ TCTCTCTCTT TTACCATCCC CGATCATTAC TCACCCCAAT GGTTATATAT ATCTCTCTCT CTCTCTGCCG   
  
  
+ TGTCATGTGT ACGTGGACAT ACATCTCTCT CTCTCGGCTT CACCAATAAA TCATAAGTGT TCAGATCTAT   
  
  
+ CTCTCTCTTT GATCTCTTCA ACCGTAGAGT TACGGGTAAC AACAAGAAAG AGAGAGACGA TAGGCTGTTA   
  
  
+ ACTTTTGGTC TTTGCCAAAG ACCAAAACCC CAACTGAAGA GCGTGTAGAG CAACCCAAGC GGGAGAGGTT   
  
  
+ AAGAAAAAAG TGTGTTTTTC AGGTGCGCGA GAGAAAAAGA GAGAGAGAGA GCACCCGCGA GCACTTAGTG   
  
  
+ GCTGTTTTCT CTCCTTAATT TTCTATCGAA GACGTTTCTC CTAATTTGCT TTCACCTGCG GTCAATTGCT   
  
  
+ CCCGCGAGGA TAGTTAAAGT TGACACAAAA ATGCTAAAAT GGGTATTGTT TTCTTCTTTC CTAAATTTAG   
  
  
+ TTAAATCAAC AACCAACGAA GGCATCAATT AATACACGGA GAAAAATACA GCGGCCCAAA CAAGCAGGGC   
  
  
+ ACGTACTGTG CACGGCTCAG TGACCGTCAA GCTAATTTGG TGTGGCCAAG GATGAAAGTG AGGGACACAA   
  
  
+ ACGAAGAAGG TGAGGTAATA AGGAAAGAAG AATGCCTTTA ATGGAAACCT GTATCGTTTT TATCGTGTCT   
  
  
+ TACTGCCTTT TTAGATTGTC AATGCTGCAC AGTGGGAATT TTCCGCTCAA CCTATAACCG TATGAGCTTA   
  
  
+ CCTCAAAGTA CAACTGGTGT GTAGTTCTAG CTGCCGACGT CGACCACCAA GCACACACCG TGATTGCACA   
  
  
+ TTAACTGGTC TCTCCTCCGC CTCTCGCACC GCCACGTCCT CTATTCCTCC ACTCCACCGG TCTACTGTCG   
  
  
+ CTCGGCTAAA ACACCCCAGG TGCAAGAAAA CGGTTTTCCC TACGGCAAAC TGTCGTAGAG CACCGAGCAC   
  
  
+ TAGAGTTACG GTTGGTCGAC ACGAGGGAAG AGCGTAGCAT ACTAAGTCCT AGGTTGCCGG CTTTTGCCAA   
  
  
+ GTGGAATAGT AGGTCAGTCG AATTACTAGT TTATAAAAGT TGAGAGGTGC CGACTCTACG CGACTGTCTC   
  
  
+ AGAGACATAT ATATTCGCTC TCATGCTTTT GGTAACTCCT AAATCTTCAT CCTCACCCGT AGTGCTCTGT   
  
  
+ ACCCTAACGT TCGCACACCC CAGTGTGAAA ATAAAATTAA ACAGACAAAT CCTGATTATT CAACCTTCAC   
  
  
+ CGCTCAGAAC ACAACCTGGG TTAAAGTCCG GGGGGTAGTA CCAAAGAGGG CGGTGTATTA ATGTCACAGA   
  
  
+ CTGGCTTAAT GTAATAACAA TAAGAACAA  

- GACCCTTTTT GTATCTCTCG CTCTACGGCC GTTTCAGCGA CAAGGCTCTG TGAATCCGTA ACAGACCTCC   
  
  
- GCAGGGACTG TTAGAGAAGA GACAGAGAGA GAAAGAGAGA AGACAAAAGA ATGCGCTGAC TTTTGAGTAA   
  
  
- AGAGAGAGAA AATGGTAGGG GCTAGTAATG AGTGGGGTTA CCAATATATA TAGAGAGAGA GAGAGACGGC   
  
  
- ACAGTACACA TGCACCTGTA TGTAGAGAGA GAGAGCCGAA GTGGTTATTT AGTATTCACA AGTCTAGATA   
  
  
- GAGAGAGAAA CTAGAGAAGT TGGCATCTCA ATGCCCATTG TTGTTCTTTC TCTCTCTGCT ATCCGACAAT   
  
  
- TGAAAACCAG AAACGGTTTC TGGTTTTGGG GTTGACTTCT CGCACATCTC GTTGGGTTCG CCCTCTCCAA   
  
  
- TTCTTTTTTC ACACAAAAAG TCCACGCGCT CTCTTTTTCT CTCTCTCTCT CGTGGGCGCT CGTGAATCAC   
  
  
- CGACAAAAGA GAGGAATTAA AAGATAGCTT CTGCAAAGAG GATTAAACGA AAGTGGACGC CAGTTAACGA   
  
  
- GGGCGCTCCT ATCAATTTCA ACTGTGTTTT TACGATTTTA CCCATAACAA AAGAAGAAAG GATTTAAATC   
  
  
- AATTTAGTTG TTGGTTGCTT CCGTAGTTAA TTATGTGCCT CTTTTTATGT CGCCGGGTTT GTTCGTCCCG   
  
  
- TGCATGACAC GTGCCGAGTC ACTGGCAGTT CGATTAAACC ACACCGGTTC CTACTTTCAC TCCCTGTGTT   
  
  
- TGCTTCTTCC ACTCCATTAT TCCTTTCTTC TTACGGAAAT TACCTTTGGA CATAGCAAAA ATAGCACAGA   
  
  
- ATGACGGAAA AATCTAACAG TTACGACGTG TCACCCTTAA AAGGCGAGTT GGATATTGGC ATACTCGAAT   
  
  
- GGAGTTTCAT GTTGACCACA CATCAAGATC GACGGCTGCA GCTGGTGGTT CGTGTGTGGC ACTAACGTGT   
  
  
- AATTGACCAG AGAGGAGGCG GAGAGCGTGG CGGTGCAGGA GATAAGGAGG TGAGGTGGCC AGATGACAGC   
  
  
- GAGCCGATTT TGTGGGGTCC ACGTTCTTTT GCCAAAAGGG ATGCCGTTTG ACAGCATCTC GTGGCTCGTG   
  
  
- ATCTCAATGC CAACCAGCTG TGCTCCCTTC TCGCATCGTA TGATTCAGGA TCCAACGGCC GAAAACGGTT   
  
  
- CACCTTATCA TCCAGTCAGC TTAATGATCA AATATTTTCA ACTCTCCACG GCTGAGATGC GCTGACAGAG   
  
  
- TCTCTGTATA TATAAGCGAG AGTACGAAAA CCATTGAGGA TTTAGAAGTA GGAGTGGGCA TCACGAGACA   
  
  
- TGGGATTGCA AGCGTGTGGG GTCACACTTT TATTTTAATT TGTCTGTTTA GGACTAATAA GTTGGAAGTG   
  
  
- GCGAGTCTTG TGTTGGACCC AATTTCAGGC CCCCCATCAT GGTTTCTCCC GCCACATAAT TACAGTGTCT   
  
  
- GACCGAATTA CATTATTGTT ATTCTTGTT

+     ACE

| Site Name | Organism | Position | Strand | Matrix score. | sequence | function |
| --- | --- | --- | --- | --- | --- | --- |
| ACE | Petroselinum hortense | 221 | + | 7 | ACGTGGA | cis-acting element involved in light responsiveness |

> 2018/04/13 10:10:12  
+ CTGGGAAAAA CATAGAGAGC GAGATGCCGG CAAAGTCGCT GTTCCGAGAC ACTTAGGCAT TGTCTGGAGG   
  
  
+ CGTCCCTGAC AATCTCTTCT CTGTCTCTCT CTTTCTCTCT TCTGTTTTCT TACGCGACTG AAAACTCATT   
  
  
+ TCTCTCTCTT TTACCATCCC CGATCATTAC TCACCCCAAT GGTTATATAT ATCTCTCTCT CTCTCTGCCG   
  
  
+ TGTCATGTGT ACGTGGACAT ACATCTCTCT CTCTCGGCTT CACCAATAAA TCATAAGTGT TCAGATCTAT   
  
  
+ CTCTCTCTTT GATCTCTTCA ACCGTAGAGT TACGGGTAAC AACAAGAAAG AGAGAGACGA TAGGCTGTTA   
  
  
+ ACTTTTGGTC TTTGCCAAAG ACCAAAACCC CAACTGAAGA GCGTGTAGAG CAACCCAAGC GGGAGAGGTT   
  
  
+ AAGAAAAAAG TGTGTTTTTC AGGTGCGCGA GAGAAAAAGA GAGAGAGAGA GCACCCGCGA GCACTTAGTG   
  
  
+ GCTGTTTTCT CTCCTTAATT TTCTATCGAA GACGTTTCTC CTAATTTGCT TTCACCTGCG GTCAATTGCT   
  
  
+ CCCGCGAGGA TAGTTAAAGT TGACACAAAA ATGCTAAAAT GGGTATTGTT TTCTTCTTTC CTAAATTTAG   
  
  
+ TTAAATCAAC AACCAACGAA GGCATCAATT AATACACGGA GAAAAATACA GCGGCCCAAA CAAGCAGGGC   
  
  
+ ACGTACTGTG CACGGCTCAG TGACCGTCAA GCTAATTTGG TGTGGCCAAG GATGAAAGTG AGGGACACAA   
  
  
+ ACGAAGAAGG TGAGGTAATA AGGAAAGAAG AATGCCTTTA ATGGAAACCT GTATCGTTTT TATCGTGTCT   
  
  
+ TACTGCCTTT TTAGATTGTC AATGCTGCAC AGTGGGAATT TTCCGCTCAA CCTATAACCG TATGAGCTTA   
  
  
+ CCTCAAAGTA CAACTGGTGT GTAGTTCTAG CTGCCGACGT CGACCACCAA GCACACACCG TGATTGCACA   
  
  
+ TTAACTGGTC TCTCCTCCGC CTCTCGCACC GCCACGTCCT CTATTCCTCC ACTCCACCGG TCTACTGTCG   
  
  
+ CTCGGCTAAA ACACCCCAGG TGCAAGAAAA CGGTTTTCCC TACGGCAAAC TGTCGTAGAG CACCGAGCAC   
  
  
+ TAGAGTTACG GTTGGTCGAC ACGAGGGAAG AGCGTAGCAT ACTAAGTCCT AGGTTGCCGG CTTTTGCCAA   
  
  
+ GTGGAATAGT AGGTCAGTCG AATTACTAGT TTATAAAAGT TGAGAGGTGC CGACTCTACG CGACTGTCTC   
  
  
+ AGAGACATAT ATATTCGCTC TCATGCTTTT GGTAACTCCT AAATCTTCAT CCTCACCCGT AGTGCTCTGT   
  
  
+ ACCCTAACGT TCGCACACCC CAGTGTGAAA ATAAAATTAA ACAGACAAAT CCTGATTATT CAACCTTCAC   
  
  
+ CGCTCAGAAC ACAACCTGGG TTAAAGTCCG GGGGGTAGTA CCAAAGAGGG CGGTGTATTA ATGTCACAGA   
  
  
+ CTGGCTTAAT GTAATAACAA TAAGAACAA  

- GACCCTTTTT GTATCTCTCG CTCTACGGCC GTTTCAGCGA CAAGGCTCTG TGAATCCGTA ACAGACCTCC   
  
  
- GCAGGGACTG TTAGAGAAGA GACAGAGAGA GAAAGAGAGA AGACAAAAGA ATGCGCTGAC TTTTGAGTAA   
  
  
- AGAGAGAGAA AATGGTAGGG GCTAGTAATG AGTGGGGTTA CCAATATATA TAGAGAGAGA GAGAGACGGC   
  
  
- ACAGTACACA TGCACCTGTA TGTAGAGAGA GAGAGCCGAA GTGGTTATTT AGTATTCACA AGTCTAGATA   
  
  
- GAGAGAGAAA CTAGAGAAGT TGGCATCTCA ATGCCCATTG TTGTTCTTTC TCTCTCTGCT ATCCGACAAT   
  
  
- TGAAAACCAG AAACGGTTTC TGGTTTTGGG GTTGACTTCT CGCACATCTC GTTGGGTTCG CCCTCTCCAA   
  
  
- TTCTTTTTTC ACACAAAAAG TCCACGCGCT CTCTTTTTCT CTCTCTCTCT CGTGGGCGCT CGTGAATCAC   
  
  
- CGACAAAAGA GAGGAATTAA AAGATAGCTT CTGCAAAGAG GATTAAACGA AAGTGGACGC CAGTTAACGA   
  
  
- GGGCGCTCCT ATCAATTTCA ACTGTGTTTT TACGATTTTA CCCATAACAA AAGAAGAAAG GATTTAAATC   
  
  
- AATTTAGTTG TTGGTTGCTT CCGTAGTTAA TTATGTGCCT CTTTTTATGT CGCCGGGTTT GTTCGTCCCG   
  
  
- TGCATGACAC GTGCCGAGTC ACTGGCAGTT CGATTAAACC ACACCGGTTC CTACTTTCAC TCCCTGTGTT   
  
  
- TGCTTCTTCC ACTCCATTAT TCCTTTCTTC TTACGGAAAT TACCTTTGGA CATAGCAAAA ATAGCACAGA   
  
  
- ATGACGGAAA AATCTAACAG TTACGACGTG TCACCCTTAA AAGGCGAGTT GGATATTGGC ATACTCGAAT   
  
  
- GGAGTTTCAT GTTGACCACA CATCAAGATC GACGGCTGCA GCTGGTGGTT CGTGTGTGGC ACTAACGTGT   
  
  
- AATTGACCAG AGAGGAGGCG GAGAGCGTGG CGGTGCAGGA GATAAGGAGG TGAGGTGGCC AGATGACAGC   
  
  
- GAGCCGATTT TGTGGGGTCC ACGTTCTTTT GCCAAAAGGG ATGCCGTTTG ACAGCATCTC GTGGCTCGTG   
  
  
- ATCTCAATGC CAACCAGCTG TGCTCCCTTC TCGCATCGTA TGATTCAGGA TCCAACGGCC GAAAACGGTT   
  
  
- CACCTTATCA TCCAGTCAGC TTAATGATCA AATATTTTCA ACTCTCCACG GCTGAGATGC GCTGACAGAG   
  
  
- TCTCTGTATA TATAAGCGAG AGTACGAAAA CCATTGAGGA TTTAGAAGTA GGAGTGGGCA TCACGAGACA   
  
  
- TGGGATTGCA AGCGTGTGGG GTCACACTTT TATTTTAATT TGTCTGTTTA GGACTAATAA GTTGGAAGTG   
  
  
- GCGAGTCTTG TGTTGGACCC AATTTCAGGC CCCCCATCAT GGTTTCTCCC GCCACATAAT TACAGTGTCT   
  
  
- GACCGAATTA CATTATTGTT ATTCTTGTT

+     Box 4

| Site Name | Organism | Position | Strand | Matrix score. | sequence | function |
| --- | --- | --- | --- | --- | --- | --- |
| Box 4 | Petroselinum crispum | 658 | + | 6 | ATTAAT | part of a conserved DNA module involved in light responsiveness |
| Box 4 | Petroselinum crispum | 1457 | - | 6 | ATTAAT | part of a conserved DNA module involved in light responsiveness |

> 2018/04/13 10:10:12  
+ CTGGGAAAAA CATAGAGAGC GAGATGCCGG CAAAGTCGCT GTTCCGAGAC ACTTAGGCAT TGTCTGGAGG   
  
  
+ CGTCCCTGAC AATCTCTTCT CTGTCTCTCT CTTTCTCTCT TCTGTTTTCT TACGCGACTG AAAACTCATT   
  
  
+ TCTCTCTCTT TTACCATCCC CGATCATTAC TCACCCCAAT GGTTATATAT ATCTCTCTCT CTCTCTGCCG   
  
  
+ TGTCATGTGT ACGTGGACAT ACATCTCTCT CTCTCGGCTT CACCAATAAA TCATAAGTGT TCAGATCTAT   
  
  
+ CTCTCTCTTT GATCTCTTCA ACCGTAGAGT TACGGGTAAC AACAAGAAAG AGAGAGACGA TAGGCTGTTA   
  
  
+ ACTTTTGGTC TTTGCCAAAG ACCAAAACCC CAACTGAAGA GCGTGTAGAG CAACCCAAGC GGGAGAGGTT   
  
  
+ AAGAAAAAAG TGTGTTTTTC AGGTGCGCGA GAGAAAAAGA GAGAGAGAGA GCACCCGCGA GCACTTAGTG   
  
  
+ GCTGTTTTCT CTCCTTAATT TTCTATCGAA GACGTTTCTC CTAATTTGCT TTCACCTGCG GTCAATTGCT   
  
  
+ CCCGCGAGGA TAGTTAAAGT TGACACAAAA ATGCTAAAAT GGGTATTGTT TTCTTCTTTC CTAAATTTAG   
  
  
+ TTAAATCAAC AACCAACGAA GGCATCAATT AATACACGGA GAAAAATACA GCGGCCCAAA CAAGCAGGGC   
  
  
+ ACGTACTGTG CACGGCTCAG TGACCGTCAA GCTAATTTGG TGTGGCCAAG GATGAAAGTG AGGGACACAA   
  
  
+ ACGAAGAAGG TGAGGTAATA AGGAAAGAAG AATGCCTTTA ATGGAAACCT GTATCGTTTT TATCGTGTCT   
  
  
+ TACTGCCTTT TTAGATTGTC AATGCTGCAC AGTGGGAATT TTCCGCTCAA CCTATAACCG TATGAGCTTA   
  
  
+ CCTCAAAGTA CAACTGGTGT GTAGTTCTAG CTGCCGACGT CGACCACCAA GCACACACCG TGATTGCACA   
  
  
+ TTAACTGGTC TCTCCTCCGC CTCTCGCACC GCCACGTCCT CTATTCCTCC ACTCCACCGG TCTACTGTCG   
  
  
+ CTCGGCTAAA ACACCCCAGG TGCAAGAAAA CGGTTTTCCC TACGGCAAAC TGTCGTAGAG CACCGAGCAC   
  
  
+ TAGAGTTACG GTTGGTCGAC ACGAGGGAAG AGCGTAGCAT ACTAAGTCCT AGGTTGCCGG CTTTTGCCAA   
  
  
+ GTGGAATAGT AGGTCAGTCG AATTACTAGT TTATAAAAGT TGAGAGGTGC CGACTCTACG CGACTGTCTC   
  
  
+ AGAGACATAT ATATTCGCTC TCATGCTTTT GGTAACTCCT AAATCTTCAT CCTCACCCGT AGTGCTCTGT   
  
  
+ ACCCTAACGT TCGCACACCC CAGTGTGAAA ATAAAATTAA ACAGACAAAT CCTGATTATT CAACCTTCAC   
  
  
+ CGCTCAGAAC ACAACCTGGG TTAAAGTCCG GGGGGTAGTA CCAAAGAGGG CGGTGTATTA ATGTCACAGA   
  
  
+ CTGGCTTAAT GTAATAACAA TAAGAACAA  

- GACCCTTTTT GTATCTCTCG CTCTACGGCC GTTTCAGCGA CAAGGCTCTG TGAATCCGTA ACAGACCTCC   
  
  
- GCAGGGACTG TTAGAGAAGA GACAGAGAGA GAAAGAGAGA AGACAAAAGA ATGCGCTGAC TTTTGAGTAA   
  
  
- AGAGAGAGAA AATGGTAGGG GCTAGTAATG AGTGGGGTTA CCAATATATA TAGAGAGAGA GAGAGACGGC   
  
  
- ACAGTACACA TGCACCTGTA TGTAGAGAGA GAGAGCCGAA GTGGTTATTT AGTATTCACA AGTCTAGATA   
  
  
- GAGAGAGAAA CTAGAGAAGT TGGCATCTCA ATGCCCATTG TTGTTCTTTC TCTCTCTGCT ATCCGACAAT   
  
  
- TGAAAACCAG AAACGGTTTC TGGTTTTGGG GTTGACTTCT CGCACATCTC GTTGGGTTCG CCCTCTCCAA   
  
  
- TTCTTTTTTC ACACAAAAAG TCCACGCGCT CTCTTTTTCT CTCTCTCTCT CGTGGGCGCT CGTGAATCAC   
  
  
- CGACAAAAGA GAGGAATTAA AAGATAGCTT CTGCAAAGAG GATTAAACGA AAGTGGACGC CAGTTAACGA   
  
  
- GGGCGCTCCT ATCAATTTCA ACTGTGTTTT TACGATTTTA CCCATAACAA AAGAAGAAAG GATTTAAATC   
  
  
- AATTTAGTTG TTGGTTGCTT CCGTAGTTAA TTATGTGCCT CTTTTTATGT CGCCGGGTTT GTTCGTCCCG   
  
  
- TGCATGACAC GTGCCGAGTC ACTGGCAGTT CGATTAAACC ACACCGGTTC CTACTTTCAC TCCCTGTGTT   
  
  
- TGCTTCTTCC ACTCCATTAT TCCTTTCTTC TTACGGAAAT TACCTTTGGA CATAGCAAAA ATAGCACAGA   
  
  
- ATGACGGAAA AATCTAACAG TTACGACGTG TCACCCTTAA AAGGCGAGTT GGATATTGGC ATACTCGAAT   
  
  
- GGAGTTTCAT GTTGACCACA CATCAAGATC GACGGCTGCA GCTGGTGGTT CGTGTGTGGC ACTAACGTGT   
  
  
- AATTGACCAG AGAGGAGGCG GAGAGCGTGG CGGTGCAGGA GATAAGGAGG TGAGGTGGCC AGATGACAGC   
  
  
- GAGCCGATTT TGTGGGGTCC ACGTTCTTTT GCCAAAAGGG ATGCCGTTTG ACAGCATCTC GTGGCTCGTG   
  
  
- ATCTCAATGC CAACCAGCTG TGCTCCCTTC TCGCATCGTA TGATTCAGGA TCCAACGGCC GAAAACGGTT   
  
  
- CACCTTATCA TCCAGTCAGC TTAATGATCA AATATTTTCA ACTCTCCACG GCTGAGATGC GCTGACAGAG   
  
  
- TCTCTGTATA TATAAGCGAG AGTACGAAAA CCATTGAGGA TTTAGAAGTA GGAGTGGGCA TCACGAGACA   
  
  
- TGGGATTGCA AGCGTGTGGG GTCACACTTT TATTTTAATT TGTCTGTTTA GGACTAATAA GTTGGAAGTG   
  
  
- GCGAGTCTTG TGTTGGACCC AATTTCAGGC CCCCCATCAT GGTTTCTCCC GCCACATAAT TACAGTGTCT   
  
  
- GACCGAATTA CATTATTGTT ATTCTTGTT

+     Box II

| Site Name | Organism | Position | Strand | Matrix score. | sequence | function |
| --- | --- | --- | --- | --- | --- | --- |
| Box II | Pisum sativum | 780 | + | 11 | GTGAGGTAATAT | part of a light responsive element |

> 2018/04/13 10:10:12  
+ CTGGGAAAAA CATAGAGAGC GAGATGCCGG CAAAGTCGCT GTTCCGAGAC ACTTAGGCAT TGTCTGGAGG   
  
  
+ CGTCCCTGAC AATCTCTTCT CTGTCTCTCT CTTTCTCTCT TCTGTTTTCT TACGCGACTG AAAACTCATT   
  
  
+ TCTCTCTCTT TTACCATCCC CGATCATTAC TCACCCCAAT GGTTATATAT ATCTCTCTCT CTCTCTGCCG   
  
  
+ TGTCATGTGT ACGTGGACAT ACATCTCTCT CTCTCGGCTT CACCAATAAA TCATAAGTGT TCAGATCTAT   
  
  
+ CTCTCTCTTT GATCTCTTCA ACCGTAGAGT TACGGGTAAC AACAAGAAAG AGAGAGACGA TAGGCTGTTA   
  
  
+ ACTTTTGGTC TTTGCCAAAG ACCAAAACCC CAACTGAAGA GCGTGTAGAG CAACCCAAGC GGGAGAGGTT   
  
  
+ AAGAAAAAAG TGTGTTTTTC AGGTGCGCGA GAGAAAAAGA GAGAGAGAGA GCACCCGCGA GCACTTAGTG   
  
  
+ GCTGTTTTCT CTCCTTAATT TTCTATCGAA GACGTTTCTC CTAATTTGCT TTCACCTGCG GTCAATTGCT   
  
  
+ CCCGCGAGGA TAGTTAAAGT TGACACAAAA ATGCTAAAAT GGGTATTGTT TTCTTCTTTC CTAAATTTAG   
  
  
+ TTAAATCAAC AACCAACGAA GGCATCAATT AATACACGGA GAAAAATACA GCGGCCCAAA CAAGCAGGGC   
  
  
+ ACGTACTGTG CACGGCTCAG TGACCGTCAA GCTAATTTGG TGTGGCCAAG GATGAAAGTG AGGGACACAA   
  
  
+ ACGAAGAAGG TGAGGTAATA AGGAAAGAAG AATGCCTTTA ATGGAAACCT GTATCGTTTT TATCGTGTCT   
  
  
+ TACTGCCTTT TTAGATTGTC AATGCTGCAC AGTGGGAATT TTCCGCTCAA CCTATAACCG TATGAGCTTA   
  
  
+ CCTCAAAGTA CAACTGGTGT GTAGTTCTAG CTGCCGACGT CGACCACCAA GCACACACCG TGATTGCACA   
  
  
+ TTAACTGGTC TCTCCTCCGC CTCTCGCACC GCCACGTCCT CTATTCCTCC ACTCCACCGG TCTACTGTCG   
  
  
+ CTCGGCTAAA ACACCCCAGG TGCAAGAAAA CGGTTTTCCC TACGGCAAAC TGTCGTAGAG CACCGAGCAC   
  
  
+ TAGAGTTACG GTTGGTCGAC ACGAGGGAAG AGCGTAGCAT ACTAAGTCCT AGGTTGCCGG CTTTTGCCAA   
  
  
+ GTGGAATAGT AGGTCAGTCG AATTACTAGT TTATAAAAGT TGAGAGGTGC CGACTCTACG CGACTGTCTC   
  
  
+ AGAGACATAT ATATTCGCTC TCATGCTTTT GGTAACTCCT AAATCTTCAT CCTCACCCGT AGTGCTCTGT   
  
  
+ ACCCTAACGT TCGCACACCC CAGTGTGAAA ATAAAATTAA ACAGACAAAT CCTGATTATT CAACCTTCAC   
  
  
+ CGCTCAGAAC ACAACCTGGG TTAAAGTCCG GGGGGTAGTA CCAAAGAGGG CGGTGTATTA ATGTCACAGA   
  
  
+ CTGGCTTAAT GTAATAACAA TAAGAACAA  

- GACCCTTTTT GTATCTCTCG CTCTACGGCC GTTTCAGCGA CAAGGCTCTG TGAATCCGTA ACAGACCTCC   
  
  
- GCAGGGACTG TTAGAGAAGA GACAGAGAGA GAAAGAGAGA AGACAAAAGA ATGCGCTGAC TTTTGAGTAA   
  
  
- AGAGAGAGAA AATGGTAGGG GCTAGTAATG AGTGGGGTTA CCAATATATA TAGAGAGAGA GAGAGACGGC   
  
  
- ACAGTACACA TGCACCTGTA TGTAGAGAGA GAGAGCCGAA GTGGTTATTT AGTATTCACA AGTCTAGATA   
  
  
- GAGAGAGAAA CTAGAGAAGT TGGCATCTCA ATGCCCATTG TTGTTCTTTC TCTCTCTGCT ATCCGACAAT   
  
  
- TGAAAACCAG AAACGGTTTC TGGTTTTGGG GTTGACTTCT CGCACATCTC GTTGGGTTCG CCCTCTCCAA   
  
  
- TTCTTTTTTC ACACAAAAAG TCCACGCGCT CTCTTTTTCT CTCTCTCTCT CGTGGGCGCT CGTGAATCAC   
  
  
- CGACAAAAGA GAGGAATTAA AAGATAGCTT CTGCAAAGAG GATTAAACGA AAGTGGACGC CAGTTAACGA   
  
  
- GGGCGCTCCT ATCAATTTCA ACTGTGTTTT TACGATTTTA CCCATAACAA AAGAAGAAAG GATTTAAATC   
  
  
- AATTTAGTTG TTGGTTGCTT CCGTAGTTAA TTATGTGCCT CTTTTTATGT CGCCGGGTTT GTTCGTCCCG   
  
  
- TGCATGACAC GTGCCGAGTC ACTGGCAGTT CGATTAAACC ACACCGGTTC CTACTTTCAC TCCCTGTGTT   
  
  
- TGCTTCTTCC ACTCCATTAT TCCTTTCTTC TTACGGAAAT TACCTTTGGA CATAGCAAAA ATAGCACAGA   
  
  
- ATGACGGAAA AATCTAACAG TTACGACGTG TCACCCTTAA AAGGCGAGTT GGATATTGGC ATACTCGAAT   
  
  
- GGAGTTTCAT GTTGACCACA CATCAAGATC GACGGCTGCA GCTGGTGGTT CGTGTGTGGC ACTAACGTGT   
  
  
- AATTGACCAG AGAGGAGGCG GAGAGCGTGG CGGTGCAGGA GATAAGGAGG TGAGGTGGCC AGATGACAGC   
  
  
- GAGCCGATTT TGTGGGGTCC ACGTTCTTTT GCCAAAAGGG ATGCCGTTTG ACAGCATCTC GTGGCTCGTG   
  
  
- ATCTCAATGC CAACCAGCTG TGCTCCCTTC TCGCATCGTA TGATTCAGGA TCCAACGGCC GAAAACGGTT   
  
  
- CACCTTATCA TCCAGTCAGC TTAATGATCA AATATTTTCA ACTCTCCACG GCTGAGATGC GCTGACAGAG   
  
  
- TCTCTGTATA TATAAGCGAG AGTACGAAAA CCATTGAGGA TTTAGAAGTA GGAGTGGGCA TCACGAGACA   
  
  
- TGGGATTGCA AGCGTGTGGG GTCACACTTT TATTTTAATT TGTCTGTTTA GGACTAATAA GTTGGAAGTG   
  
  
- GCGAGTCTTG TGTTGGACCC AATTTCAGGC CCCCCATCAT GGTTTCTCCC GCCACATAAT TACAGTGTCT   
  
  
- GACCGAATTA CATTATTGTT ATTCTTGTT

+     Box-W1

| Site Name | Organism | Position | Strand | Matrix score. | sequence | function |
| --- | --- | --- | --- | --- | --- | --- |
| Box-W1 | Petroselinum crispum | 550 | - | 6 | TTGACC | fungal elicitor responsive element |

> 2018/04/13 10:10:12  
+ CTGGGAAAAA CATAGAGAGC GAGATGCCGG CAAAGTCGCT GTTCCGAGAC ACTTAGGCAT TGTCTGGAGG   
  
  
+ CGTCCCTGAC AATCTCTTCT CTGTCTCTCT CTTTCTCTCT TCTGTTTTCT TACGCGACTG AAAACTCATT   
  
  
+ TCTCTCTCTT TTACCATCCC CGATCATTAC TCACCCCAAT GGTTATATAT ATCTCTCTCT CTCTCTGCCG   
  
  
+ TGTCATGTGT ACGTGGACAT ACATCTCTCT CTCTCGGCTT CACCAATAAA TCATAAGTGT TCAGATCTAT   
  
  
+ CTCTCTCTTT GATCTCTTCA ACCGTAGAGT TACGGGTAAC AACAAGAAAG AGAGAGACGA TAGGCTGTTA   
  
  
+ ACTTTTGGTC TTTGCCAAAG ACCAAAACCC CAACTGAAGA GCGTGTAGAG CAACCCAAGC GGGAGAGGTT   
  
  
+ AAGAAAAAAG TGTGTTTTTC AGGTGCGCGA GAGAAAAAGA GAGAGAGAGA GCACCCGCGA GCACTTAGTG   
  
  
+ GCTGTTTTCT CTCCTTAATT TTCTATCGAA GACGTTTCTC CTAATTTGCT TTCACCTGCG GTCAATTGCT   
  
  
+ CCCGCGAGGA TAGTTAAAGT TGACACAAAA ATGCTAAAAT GGGTATTGTT TTCTTCTTTC CTAAATTTAG   
  
  
+ TTAAATCAAC AACCAACGAA GGCATCAATT AATACACGGA GAAAAATACA GCGGCCCAAA CAAGCAGGGC   
  
  
+ ACGTACTGTG CACGGCTCAG TGACCGTCAA GCTAATTTGG TGTGGCCAAG GATGAAAGTG AGGGACACAA   
  
  
+ ACGAAGAAGG TGAGGTAATA AGGAAAGAAG AATGCCTTTA ATGGAAACCT GTATCGTTTT TATCGTGTCT   
  
  
+ TACTGCCTTT TTAGATTGTC AATGCTGCAC AGTGGGAATT TTCCGCTCAA CCTATAACCG TATGAGCTTA   
  
  
+ CCTCAAAGTA CAACTGGTGT GTAGTTCTAG CTGCCGACGT CGACCACCAA GCACACACCG TGATTGCACA   
  
  
+ TTAACTGGTC TCTCCTCCGC CTCTCGCACC GCCACGTCCT CTATTCCTCC ACTCCACCGG TCTACTGTCG   
  
  
+ CTCGGCTAAA ACACCCCAGG TGCAAGAAAA CGGTTTTCCC TACGGCAAAC TGTCGTAGAG CACCGAGCAC   
  
  
+ TAGAGTTACG GTTGGTCGAC ACGAGGGAAG AGCGTAGCAT ACTAAGTCCT AGGTTGCCGG CTTTTGCCAA   
  
  
+ GTGGAATAGT AGGTCAGTCG AATTACTAGT TTATAAAAGT TGAGAGGTGC CGACTCTACG CGACTGTCTC   
  
  
+ AGAGACATAT ATATTCGCTC TCATGCTTTT GGTAACTCCT AAATCTTCAT CCTCACCCGT AGTGCTCTGT   
  
  
+ ACCCTAACGT TCGCACACCC CAGTGTGAAA ATAAAATTAA ACAGACAAAT CCTGATTATT CAACCTTCAC   
  
  
+ CGCTCAGAAC ACAACCTGGG TTAAAGTCCG GGGGGTAGTA CCAAAGAGGG CGGTGTATTA ATGTCACAGA   
  
  
+ CTGGCTTAAT GTAATAACAA TAAGAACAA  

- GACCCTTTTT GTATCTCTCG CTCTACGGCC GTTTCAGCGA CAAGGCTCTG TGAATCCGTA ACAGACCTCC   
  
  
- GCAGGGACTG TTAGAGAAGA GACAGAGAGA GAAAGAGAGA AGACAAAAGA ATGCGCTGAC TTTTGAGTAA   
  
  
- AGAGAGAGAA AATGGTAGGG GCTAGTAATG AGTGGGGTTA CCAATATATA TAGAGAGAGA GAGAGACGGC   
  
  
- ACAGTACACA TGCACCTGTA TGTAGAGAGA GAGAGCCGAA GTGGTTATTT AGTATTCACA AGTCTAGATA   
  
  
- GAGAGAGAAA CTAGAGAAGT TGGCATCTCA ATGCCCATTG TTGTTCTTTC TCTCTCTGCT ATCCGACAAT   
  
  
- TGAAAACCAG AAACGGTTTC TGGTTTTGGG GTTGACTTCT CGCACATCTC GTTGGGTTCG CCCTCTCCAA   
  
  
- TTCTTTTTTC ACACAAAAAG TCCACGCGCT CTCTTTTTCT CTCTCTCTCT CGTGGGCGCT CGTGAATCAC   
  
  
- CGACAAAAGA GAGGAATTAA AAGATAGCTT CTGCAAAGAG GATTAAACGA AAGTGGACGC CAGTTAACGA   
  
  
- GGGCGCTCCT ATCAATTTCA ACTGTGTTTT TACGATTTTA CCCATAACAA AAGAAGAAAG GATTTAAATC   
  
  
- AATTTAGTTG TTGGTTGCTT CCGTAGTTAA TTATGTGCCT CTTTTTATGT CGCCGGGTTT GTTCGTCCCG   
  
  
- TGCATGACAC GTGCCGAGTC ACTGGCAGTT CGATTAAACC ACACCGGTTC CTACTTTCAC TCCCTGTGTT   
  
  
- TGCTTCTTCC ACTCCATTAT TCCTTTCTTC TTACGGAAAT TACCTTTGGA CATAGCAAAA ATAGCACAGA   
  
  
- ATGACGGAAA AATCTAACAG TTACGACGTG TCACCCTTAA AAGGCGAGTT GGATATTGGC ATACTCGAAT   
  
  
- GGAGTTTCAT GTTGACCACA CATCAAGATC GACGGCTGCA GCTGGTGGTT CGTGTGTGGC ACTAACGTGT   
  
  
- AATTGACCAG AGAGGAGGCG GAGAGCGTGG CGGTGCAGGA GATAAGGAGG TGAGGTGGCC AGATGACAGC   
  
  
- GAGCCGATTT TGTGGGGTCC ACGTTCTTTT GCCAAAAGGG ATGCCGTTTG ACAGCATCTC GTGGCTCGTG   
  
  
- ATCTCAATGC CAACCAGCTG TGCTCCCTTC TCGCATCGTA TGATTCAGGA TCCAACGGCC GAAAACGGTT   
  
  
- CACCTTATCA TCCAGTCAGC TTAATGATCA AATATTTTCA ACTCTCCACG GCTGAGATGC GCTGACAGAG   
  
  
- TCTCTGTATA TATAAGCGAG AGTACGAAAA CCATTGAGGA TTTAGAAGTA GGAGTGGGCA TCACGAGACA   
  
  
- TGGGATTGCA AGCGTGTGGG GTCACACTTT TATTTTAATT TGTCTGTTTA GGACTAATAA GTTGGAAGTG   
  
  
- GCGAGTCTTG TGTTGGACCC AATTTCAGGC CCCCCATCAT GGTTTCTCCC GCCACATAAT TACAGTGTCT   
  
  
- GACCGAATTA CATTATTGTT ATTCTTGTT

+     CAAT-box

| Site Name | Organism | Position | Strand | Matrix score. | sequence | function |
| --- | --- | --- | --- | --- | --- | --- |
| CAAT-box | Glycine max | 656 | + | 5 | CAATT | common cis-acting element in promoter and enhancer regions |
| CAAT-box | Hordeum vulgare | 605 | - | 4 | CAAT | common cis-acting element in promoter and enhancer regions |
| CAAT-box | Glycine max | 554 | - | 5 | CAATT | common cis-acting element in promoter and enhancer regions |
| CAAT-box | Hordeum vulgare | 254 | + | 4 | CAAT | common cis-acting element in promoter and enhancer regions |
| CAAT-box | Hordeum vulgare | 80 | + | 4 | CAAT | common cis-acting element in promoter and enhancer regions |
| CAAT-box | Arabidopsis thaliana | 176 | + | 5 | CCAAT | common cis-acting element in promoter and enhancer regions |
| CAAT-box | Hordeum vulgare | 59 | - | 4 | CAAT | common cis-acting element in promoter and enhancer regions |
| CAAT-box | Hordeum vulgare | 860 | + | 4 | CAAT | common cis-acting element in promoter and enhancer regions |
| CAAT-box | Arabidopsis thaliana | 253 | + | 5 | CCAAT | common cis-acting element in promoter and enhancer regions |
| CAAT-box | Brassica rapa | 735 | - | 5 | CAAAT | common cis-acting element in promoter and enhancer regions |
| CAAT-box | Hordeum vulgare | 855 | - | 4 | CAAT | common cis-acting element in promoter and enhancer regions |
| CAAT-box | Hordeum vulgare | 555 | - | 4 | CAAT | common cis-acting element in promoter and enhancer regions |
| CAAT-box | Hordeum vulgare | 177 | + | 4 | CAAT | common cis-acting element in promoter and enhancer regions |
| CAAT-box | Brassica rapa | 1376 | + | 5 | CAAAT | common cis-acting element in promoter and enhancer regions |
| CAAT-box | Hordeum vulgare | 1488 | + | 4 | CAAT | common cis-acting element in promoter and enhancer regions |
| CAAT-box | Brassica rapa | 534 | - | 5 | CAAAT | common cis-acting element in promoter and enhancer regions |
| CAAT-box | Glycine max | 553 | + | 5 | CAATT | common cis-acting element in promoter and enhancer regions |
| CAAT-box | Hordeum vulgare | 973 | - | 4 | CAAT | common cis-acting element in promoter and enhancer regions |

> 2018/04/13 10:10:12  
+ CTGGGAAAAA CATAGAGAGC GAGATGCCGG CAAAGTCGCT GTTCCGAGAC ACTTAGGCAT TGTCTGGAGG   
  
  
+ CGTCCCTGAC AATCTCTTCT CTGTCTCTCT CTTTCTCTCT TCTGTTTTCT TACGCGACTG AAAACTCATT   
  
  
+ TCTCTCTCTT TTACCATCCC CGATCATTAC TCACCCCAAT GGTTATATAT ATCTCTCTCT CTCTCTGCCG   
  
  
+ TGTCATGTGT ACGTGGACAT ACATCTCTCT CTCTCGGCTT CACCAATAAA TCATAAGTGT TCAGATCTAT   
  
  
+ CTCTCTCTTT GATCTCTTCA ACCGTAGAGT TACGGGTAAC AACAAGAAAG AGAGAGACGA TAGGCTGTTA   
  
  
+ ACTTTTGGTC TTTGCCAAAG ACCAAAACCC CAACTGAAGA GCGTGTAGAG CAACCCAAGC GGGAGAGGTT   
  
  
+ AAGAAAAAAG TGTGTTTTTC AGGTGCGCGA GAGAAAAAGA GAGAGAGAGA GCACCCGCGA GCACTTAGTG   
  
  
+ GCTGTTTTCT CTCCTTAATT TTCTATCGAA GACGTTTCTC CTAATTTGCT TTCACCTGCG GTCAATTGCT   
  
  
+ CCCGCGAGGA TAGTTAAAGT TGACACAAAA ATGCTAAAAT GGGTATTGTT TTCTTCTTTC CTAAATTTAG   
  
  
+ TTAAATCAAC AACCAACGAA GGCATCAATT AATACACGGA GAAAAATACA GCGGCCCAAA CAAGCAGGGC   
  
  
+ ACGTACTGTG CACGGCTCAG TGACCGTCAA GCTAATTTGG TGTGGCCAAG GATGAAAGTG AGGGACACAA   
  
  
+ ACGAAGAAGG TGAGGTAATA AGGAAAGAAG AATGCCTTTA ATGGAAACCT GTATCGTTTT TATCGTGTCT   
  
  
+ TACTGCCTTT TTAGATTGTC AATGCTGCAC AGTGGGAATT TTCCGCTCAA CCTATAACCG TATGAGCTTA   
  
  
+ CCTCAAAGTA CAACTGGTGT GTAGTTCTAG CTGCCGACGT CGACCACCAA GCACACACCG TGATTGCACA   
  
  
+ TTAACTGGTC TCTCCTCCGC CTCTCGCACC GCCACGTCCT CTATTCCTCC ACTCCACCGG TCTACTGTCG   
  
  
+ CTCGGCTAAA ACACCCCAGG TGCAAGAAAA CGGTTTTCCC TACGGCAAAC TGTCGTAGAG CACCGAGCAC   
  
  
+ TAGAGTTACG GTTGGTCGAC ACGAGGGAAG AGCGTAGCAT ACTAAGTCCT AGGTTGCCGG CTTTTGCCAA   
  
  
+ GTGGAATAGT AGGTCAGTCG AATTACTAGT TTATAAAAGT TGAGAGGTGC CGACTCTACG CGACTGTCTC   
  
  
+ AGAGACATAT ATATTCGCTC TCATGCTTTT GGTAACTCCT AAATCTTCAT CCTCACCCGT AGTGCTCTGT   
  
  
+ ACCCTAACGT TCGCACACCC CAGTGTGAAA ATAAAATTAA ACAGACAAAT CCTGATTATT CAACCTTCAC   
  
  
+ CGCTCAGAAC ACAACCTGGG TTAAAGTCCG GGGGGTAGTA CCAAAGAGGG CGGTGTATTA ATGTCACAGA   
  
  
+ CTGGCTTAAT GTAATAACAA TAAGAACAA  

- GACCCTTTTT GTATCTCTCG CTCTACGGCC GTTTCAGCGA CAAGGCTCTG TGAATCCGTA ACAGACCTCC   
  
  
- GCAGGGACTG TTAGAGAAGA GACAGAGAGA GAAAGAGAGA AGACAAAAGA ATGCGCTGAC TTTTGAGTAA   
  
  
- AGAGAGAGAA AATGGTAGGG GCTAGTAATG AGTGGGGTTA CCAATATATA TAGAGAGAGA GAGAGACGGC   
  
  
- ACAGTACACA TGCACCTGTA TGTAGAGAGA GAGAGCCGAA GTGGTTATTT AGTATTCACA AGTCTAGATA   
  
  
- GAGAGAGAAA CTAGAGAAGT TGGCATCTCA ATGCCCATTG TTGTTCTTTC TCTCTCTGCT ATCCGACAAT   
  
  
- TGAAAACCAG AAACGGTTTC TGGTTTTGGG GTTGACTTCT CGCACATCTC GTTGGGTTCG CCCTCTCCAA   
  
  
- TTCTTTTTTC ACACAAAAAG TCCACGCGCT CTCTTTTTCT CTCTCTCTCT CGTGGGCGCT CGTGAATCAC   
  
  
- CGACAAAAGA GAGGAATTAA AAGATAGCTT CTGCAAAGAG GATTAAACGA AAGTGGACGC CAGTTAACGA   
  
  
- GGGCGCTCCT ATCAATTTCA ACTGTGTTTT TACGATTTTA CCCATAACAA AAGAAGAAAG GATTTAAATC   
  
  
- AATTTAGTTG TTGGTTGCTT CCGTAGTTAA TTATGTGCCT CTTTTTATGT CGCCGGGTTT GTTCGTCCCG   
  
  
- TGCATGACAC GTGCCGAGTC ACTGGCAGTT CGATTAAACC ACACCGGTTC CTACTTTCAC TCCCTGTGTT   
  
  
- TGCTTCTTCC ACTCCATTAT TCCTTTCTTC TTACGGAAAT TACCTTTGGA CATAGCAAAA ATAGCACAGA   
  
  
- ATGACGGAAA AATCTAACAG TTACGACGTG TCACCCTTAA AAGGCGAGTT GGATATTGGC ATACTCGAAT   
  
  
- GGAGTTTCAT GTTGACCACA CATCAAGATC GACGGCTGCA GCTGGTGGTT CGTGTGTGGC ACTAACGTGT   
  
  
- AATTGACCAG AGAGGAGGCG GAGAGCGTGG CGGTGCAGGA GATAAGGAGG TGAGGTGGCC AGATGACAGC   
  
  
- GAGCCGATTT TGTGGGGTCC ACGTTCTTTT GCCAAAAGGG ATGCCGTTTG ACAGCATCTC GTGGCTCGTG   
  
  
- ATCTCAATGC CAACCAGCTG TGCTCCCTTC TCGCATCGTA TGATTCAGGA TCCAACGGCC GAAAACGGTT   
  
  
- CACCTTATCA TCCAGTCAGC TTAATGATCA AATATTTTCA ACTCTCCACG GCTGAGATGC GCTGACAGAG   
  
  
- TCTCTGTATA TATAAGCGAG AGTACGAAAA CCATTGAGGA TTTAGAAGTA GGAGTGGGCA TCACGAGACA   
  
  
- TGGGATTGCA AGCGTGTGGG GTCACACTTT TATTTTAATT TGTCTGTTTA GGACTAATAA GTTGGAAGTG   
  
  
- GCGAGTCTTG TGTTGGACCC AATTTCAGGC CCCCCATCAT GGTTTCTCCC GCCACATAAT TACAGTGTCT   
  
  
- GACCGAATTA CATTATTGTT ATTCTTGTT

+     CAT-box

| Site Name | Organism | Position | Strand | Matrix score. | sequence | function |
| --- | --- | --- | --- | --- | --- | --- |
| CAT-box | Arabidopsis thaliana | 487 | - | 6 | GCCACT | cis-acting regulatory element related to meristem expression |

> 2018/04/13 10:10:12  
+ CTGGGAAAAA CATAGAGAGC GAGATGCCGG CAAAGTCGCT GTTCCGAGAC ACTTAGGCAT TGTCTGGAGG   
  
  
+ CGTCCCTGAC AATCTCTTCT CTGTCTCTCT CTTTCTCTCT TCTGTTTTCT TACGCGACTG AAAACTCATT   
  
  
+ TCTCTCTCTT TTACCATCCC CGATCATTAC TCACCCCAAT GGTTATATAT ATCTCTCTCT CTCTCTGCCG   
  
  
+ TGTCATGTGT ACGTGGACAT ACATCTCTCT CTCTCGGCTT CACCAATAAA TCATAAGTGT TCAGATCTAT   
  
  
+ CTCTCTCTTT GATCTCTTCA ACCGTAGAGT TACGGGTAAC AACAAGAAAG AGAGAGACGA TAGGCTGTTA   
  
  
+ ACTTTTGGTC TTTGCCAAAG ACCAAAACCC CAACTGAAGA GCGTGTAGAG CAACCCAAGC GGGAGAGGTT   
  
  
+ AAGAAAAAAG TGTGTTTTTC AGGTGCGCGA GAGAAAAAGA GAGAGAGAGA GCACCCGCGA GCACTTAGTG   
  
  
+ GCTGTTTTCT CTCCTTAATT TTCTATCGAA GACGTTTCTC CTAATTTGCT TTCACCTGCG GTCAATTGCT   
  
  
+ CCCGCGAGGA TAGTTAAAGT TGACACAAAA ATGCTAAAAT GGGTATTGTT TTCTTCTTTC CTAAATTTAG   
  
  
+ TTAAATCAAC AACCAACGAA GGCATCAATT AATACACGGA GAAAAATACA GCGGCCCAAA CAAGCAGGGC   
  
  
+ ACGTACTGTG CACGGCTCAG TGACCGTCAA GCTAATTTGG TGTGGCCAAG GATGAAAGTG AGGGACACAA   
  
  
+ ACGAAGAAGG TGAGGTAATA AGGAAAGAAG AATGCCTTTA ATGGAAACCT GTATCGTTTT TATCGTGTCT   
  
  
+ TACTGCCTTT TTAGATTGTC AATGCTGCAC AGTGGGAATT TTCCGCTCAA CCTATAACCG TATGAGCTTA   
  
  
+ CCTCAAAGTA CAACTGGTGT GTAGTTCTAG CTGCCGACGT CGACCACCAA GCACACACCG TGATTGCACA   
  
  
+ TTAACTGGTC TCTCCTCCGC CTCTCGCACC GCCACGTCCT CTATTCCTCC ACTCCACCGG TCTACTGTCG   
  
  
+ CTCGGCTAAA ACACCCCAGG TGCAAGAAAA CGGTTTTCCC TACGGCAAAC TGTCGTAGAG CACCGAGCAC   
  
  
+ TAGAGTTACG GTTGGTCGAC ACGAGGGAAG AGCGTAGCAT ACTAAGTCCT AGGTTGCCGG CTTTTGCCAA   
  
  
+ GTGGAATAGT AGGTCAGTCG AATTACTAGT TTATAAAAGT TGAGAGGTGC CGACTCTACG CGACTGTCTC   
  
  
+ AGAGACATAT ATATTCGCTC TCATGCTTTT GGTAACTCCT AAATCTTCAT CCTCACCCGT AGTGCTCTGT   
  
  
+ ACCCTAACGT TCGCACACCC CAGTGTGAAA ATAAAATTAA ACAGACAAAT CCTGATTATT CAACCTTCAC   
  
  
+ CGCTCAGAAC ACAACCTGGG TTAAAGTCCG GGGGGTAGTA CCAAAGAGGG CGGTGTATTA ATGTCACAGA   
  
  
+ CTGGCTTAAT GTAATAACAA TAAGAACAA  

- GACCCTTTTT GTATCTCTCG CTCTACGGCC GTTTCAGCGA CAAGGCTCTG TGAATCCGTA ACAGACCTCC   
  
  
- GCAGGGACTG TTAGAGAAGA GACAGAGAGA GAAAGAGAGA AGACAAAAGA ATGCGCTGAC TTTTGAGTAA   
  
  
- AGAGAGAGAA AATGGTAGGG GCTAGTAATG AGTGGGGTTA CCAATATATA TAGAGAGAGA GAGAGACGGC   
  
  
- ACAGTACACA TGCACCTGTA TGTAGAGAGA GAGAGCCGAA GTGGTTATTT AGTATTCACA AGTCTAGATA   
  
  
- GAGAGAGAAA CTAGAGAAGT TGGCATCTCA ATGCCCATTG TTGTTCTTTC TCTCTCTGCT ATCCGACAAT   
  
  
- TGAAAACCAG AAACGGTTTC TGGTTTTGGG GTTGACTTCT CGCACATCTC GTTGGGTTCG CCCTCTCCAA   
  
  
- TTCTTTTTTC ACACAAAAAG TCCACGCGCT CTCTTTTTCT CTCTCTCTCT CGTGGGCGCT CGTGAATCAC   
  
  
- CGACAAAAGA GAGGAATTAA AAGATAGCTT CTGCAAAGAG GATTAAACGA AAGTGGACGC CAGTTAACGA   
  
  
- GGGCGCTCCT ATCAATTTCA ACTGTGTTTT TACGATTTTA CCCATAACAA AAGAAGAAAG GATTTAAATC   
  
  
- AATTTAGTTG TTGGTTGCTT CCGTAGTTAA TTATGTGCCT CTTTTTATGT CGCCGGGTTT GTTCGTCCCG   
  
  
- TGCATGACAC GTGCCGAGTC ACTGGCAGTT CGATTAAACC ACACCGGTTC CTACTTTCAC TCCCTGTGTT   
  
  
- TGCTTCTTCC ACTCCATTAT TCCTTTCTTC TTACGGAAAT TACCTTTGGA CATAGCAAAA ATAGCACAGA   
  
  
- ATGACGGAAA AATCTAACAG TTACGACGTG TCACCCTTAA AAGGCGAGTT GGATATTGGC ATACTCGAAT   
  
  
- GGAGTTTCAT GTTGACCACA CATCAAGATC GACGGCTGCA GCTGGTGGTT CGTGTGTGGC ACTAACGTGT   
  
  
- AATTGACCAG AGAGGAGGCG GAGAGCGTGG CGGTGCAGGA GATAAGGAGG TGAGGTGGCC AGATGACAGC   
  
  
- GAGCCGATTT TGTGGGGTCC ACGTTCTTTT GCCAAAAGGG ATGCCGTTTG ACAGCATCTC GTGGCTCGTG   
  
  
- ATCTCAATGC CAACCAGCTG TGCTCCCTTC TCGCATCGTA TGATTCAGGA TCCAACGGCC GAAAACGGTT   
  
  
- CACCTTATCA TCCAGTCAGC TTAATGATCA AATATTTTCA ACTCTCCACG GCTGAGATGC GCTGACAGAG   
  
  
- TCTCTGTATA TATAAGCGAG AGTACGAAAA CCATTGAGGA TTTAGAAGTA GGAGTGGGCA TCACGAGACA   
  
  
- TGGGATTGCA AGCGTGTGGG GTCACACTTT TATTTTAATT TGTCTGTTTA GGACTAATAA GTTGGAAGTG   
  
  
- GCGAGTCTTG TGTTGGACCC AATTTCAGGC CCCCCATCAT GGTTTCTCCC GCCACATAAT TACAGTGTCT   
  
  
- GACCGAATTA CATTATTGTT ATTCTTGTT

+     CATT-motif

| Site Name | Organism | Position | Strand | Matrix score. | sequence | function |
| --- | --- | --- | --- | --- | --- | --- |
| CATT-motif | Zea mays | 800 | - | 6 | GCATTC | part of a light responsive element |

> 2018/04/13 10:10:12  
+ CTGGGAAAAA CATAGAGAGC GAGATGCCGG CAAAGTCGCT GTTCCGAGAC ACTTAGGCAT TGTCTGGAGG   
  
  
+ CGTCCCTGAC AATCTCTTCT CTGTCTCTCT CTTTCTCTCT TCTGTTTTCT TACGCGACTG AAAACTCATT   
  
  
+ TCTCTCTCTT TTACCATCCC CGATCATTAC TCACCCCAAT GGTTATATAT ATCTCTCTCT CTCTCTGCCG   
  
  
+ TGTCATGTGT ACGTGGACAT ACATCTCTCT CTCTCGGCTT CACCAATAAA TCATAAGTGT TCAGATCTAT   
  
  
+ CTCTCTCTTT GATCTCTTCA ACCGTAGAGT TACGGGTAAC AACAAGAAAG AGAGAGACGA TAGGCTGTTA   
  
  
+ ACTTTTGGTC TTTGCCAAAG ACCAAAACCC CAACTGAAGA GCGTGTAGAG CAACCCAAGC GGGAGAGGTT   
  
  
+ AAGAAAAAAG TGTGTTTTTC AGGTGCGCGA GAGAAAAAGA GAGAGAGAGA GCACCCGCGA GCACTTAGTG   
  
  
+ GCTGTTTTCT CTCCTTAATT TTCTATCGAA GACGTTTCTC CTAATTTGCT TTCACCTGCG GTCAATTGCT   
  
  
+ CCCGCGAGGA TAGTTAAAGT TGACACAAAA ATGCTAAAAT GGGTATTGTT TTCTTCTTTC CTAAATTTAG   
  
  
+ TTAAATCAAC AACCAACGAA GGCATCAATT AATACACGGA GAAAAATACA GCGGCCCAAA CAAGCAGGGC   
  
  
+ ACGTACTGTG CACGGCTCAG TGACCGTCAA GCTAATTTGG TGTGGCCAAG GATGAAAGTG AGGGACACAA   
  
  
+ ACGAAGAAGG TGAGGTAATA AGGAAAGAAG AATGCCTTTA ATGGAAACCT GTATCGTTTT TATCGTGTCT   
  
  
+ TACTGCCTTT TTAGATTGTC AATGCTGCAC AGTGGGAATT TTCCGCTCAA CCTATAACCG TATGAGCTTA   
  
  
+ CCTCAAAGTA CAACTGGTGT GTAGTTCTAG CTGCCGACGT CGACCACCAA GCACACACCG TGATTGCACA   
  
  
+ TTAACTGGTC TCTCCTCCGC CTCTCGCACC GCCACGTCCT CTATTCCTCC ACTCCACCGG TCTACTGTCG   
  
  
+ CTCGGCTAAA ACACCCCAGG TGCAAGAAAA CGGTTTTCCC TACGGCAAAC TGTCGTAGAG CACCGAGCAC   
  
  
+ TAGAGTTACG GTTGGTCGAC ACGAGGGAAG AGCGTAGCAT ACTAAGTCCT AGGTTGCCGG CTTTTGCCAA   
  
  
+ GTGGAATAGT AGGTCAGTCG AATTACTAGT TTATAAAAGT TGAGAGGTGC CGACTCTACG CGACTGTCTC   
  
  
+ AGAGACATAT ATATTCGCTC TCATGCTTTT GGTAACTCCT AAATCTTCAT CCTCACCCGT AGTGCTCTGT   
  
  
+ ACCCTAACGT TCGCACACCC CAGTGTGAAA ATAAAATTAA ACAGACAAAT CCTGATTATT CAACCTTCAC   
  
  
+ CGCTCAGAAC ACAACCTGGG TTAAAGTCCG GGGGGTAGTA CCAAAGAGGG CGGTGTATTA ATGTCACAGA   
  
  
+ CTGGCTTAAT GTAATAACAA TAAGAACAA  

- GACCCTTTTT GTATCTCTCG CTCTACGGCC GTTTCAGCGA CAAGGCTCTG TGAATCCGTA ACAGACCTCC   
  
  
- GCAGGGACTG TTAGAGAAGA GACAGAGAGA GAAAGAGAGA AGACAAAAGA ATGCGCTGAC TTTTGAGTAA   
  
  
- AGAGAGAGAA AATGGTAGGG GCTAGTAATG AGTGGGGTTA CCAATATATA TAGAGAGAGA GAGAGACGGC   
  
  
- ACAGTACACA TGCACCTGTA TGTAGAGAGA GAGAGCCGAA GTGGTTATTT AGTATTCACA AGTCTAGATA   
  
  
- GAGAGAGAAA CTAGAGAAGT TGGCATCTCA ATGCCCATTG TTGTTCTTTC TCTCTCTGCT ATCCGACAAT   
  
  
- TGAAAACCAG AAACGGTTTC TGGTTTTGGG GTTGACTTCT CGCACATCTC GTTGGGTTCG CCCTCTCCAA   
  
  
- TTCTTTTTTC ACACAAAAAG TCCACGCGCT CTCTTTTTCT CTCTCTCTCT CGTGGGCGCT CGTGAATCAC   
  
  
- CGACAAAAGA GAGGAATTAA AAGATAGCTT CTGCAAAGAG GATTAAACGA AAGTGGACGC CAGTTAACGA   
  
  
- GGGCGCTCCT ATCAATTTCA ACTGTGTTTT TACGATTTTA CCCATAACAA AAGAAGAAAG GATTTAAATC   
  
  
- AATTTAGTTG TTGGTTGCTT CCGTAGTTAA TTATGTGCCT CTTTTTATGT CGCCGGGTTT GTTCGTCCCG   
  
  
- TGCATGACAC GTGCCGAGTC ACTGGCAGTT CGATTAAACC ACACCGGTTC CTACTTTCAC TCCCTGTGTT   
  
  
- TGCTTCTTCC ACTCCATTAT TCCTTTCTTC TTACGGAAAT TACCTTTGGA CATAGCAAAA ATAGCACAGA   
  
  
- ATGACGGAAA AATCTAACAG TTACGACGTG TCACCCTTAA AAGGCGAGTT GGATATTGGC ATACTCGAAT   
  
  
- GGAGTTTCAT GTTGACCACA CATCAAGATC GACGGCTGCA GCTGGTGGTT CGTGTGTGGC ACTAACGTGT   
  
  
- AATTGACCAG AGAGGAGGCG GAGAGCGTGG CGGTGCAGGA GATAAGGAGG TGAGGTGGCC AGATGACAGC   
  
  
- GAGCCGATTT TGTGGGGTCC ACGTTCTTTT GCCAAAAGGG ATGCCGTTTG ACAGCATCTC GTGGCTCGTG   
  
  
- ATCTCAATGC CAACCAGCTG TGCTCCCTTC TCGCATCGTA TGATTCAGGA TCCAACGGCC GAAAACGGTT   
  
  
- CACCTTATCA TCCAGTCAGC TTAATGATCA AATATTTTCA ACTCTCCACG GCTGAGATGC GCTGACAGAG   
  
  
- TCTCTGTATA TATAAGCGAG AGTACGAAAA CCATTGAGGA TTTAGAAGTA GGAGTGGGCA TCACGAGACA   
  
  
- TGGGATTGCA AGCGTGTGGG GTCACACTTT TATTTTAATT TGTCTGTTTA GGACTAATAA GTTGGAAGTG   
  
  
- GCGAGTCTTG TGTTGGACCC AATTTCAGGC CCCCCATCAT GGTTTCTCCC GCCACATAAT TACAGTGTCT   
  
  
- GACCGAATTA CATTATTGTT ATTCTTGTT

+     CGTCA-motif

| Site Name | Organism | Position | Strand | Matrix score. | sequence | function |
| --- | --- | --- | --- | --- | --- | --- |
| CGTCA-motif | Hordeum vulgare | 725 | + | 5 | CGTCA | cis-acting regulatory element involved in the MeJA-responsiveness |

> 2018/04/13 10:10:12  
+ CTGGGAAAAA CATAGAGAGC GAGATGCCGG CAAAGTCGCT GTTCCGAGAC ACTTAGGCAT TGTCTGGAGG   
  
  
+ CGTCCCTGAC AATCTCTTCT CTGTCTCTCT CTTTCTCTCT TCTGTTTTCT TACGCGACTG AAAACTCATT   
  
  
+ TCTCTCTCTT TTACCATCCC CGATCATTAC TCACCCCAAT GGTTATATAT ATCTCTCTCT CTCTCTGCCG   
  
  
+ TGTCATGTGT ACGTGGACAT ACATCTCTCT CTCTCGGCTT CACCAATAAA TCATAAGTGT TCAGATCTAT   
  
  
+ CTCTCTCTTT GATCTCTTCA ACCGTAGAGT TACGGGTAAC AACAAGAAAG AGAGAGACGA TAGGCTGTTA   
  
  
+ ACTTTTGGTC TTTGCCAAAG ACCAAAACCC CAACTGAAGA GCGTGTAGAG CAACCCAAGC GGGAGAGGTT   
  
  
+ AAGAAAAAAG TGTGTTTTTC AGGTGCGCGA GAGAAAAAGA GAGAGAGAGA GCACCCGCGA GCACTTAGTG   
  
  
+ GCTGTTTTCT CTCCTTAATT TTCTATCGAA GACGTTTCTC CTAATTTGCT TTCACCTGCG GTCAATTGCT   
  
  
+ CCCGCGAGGA TAGTTAAAGT TGACACAAAA ATGCTAAAAT GGGTATTGTT TTCTTCTTTC CTAAATTTAG   
  
  
+ TTAAATCAAC AACCAACGAA GGCATCAATT AATACACGGA GAAAAATACA GCGGCCCAAA CAAGCAGGGC   
  
  
+ ACGTACTGTG CACGGCTCAG TGACCGTCAA GCTAATTTGG TGTGGCCAAG GATGAAAGTG AGGGACACAA   
  
  
+ ACGAAGAAGG TGAGGTAATA AGGAAAGAAG AATGCCTTTA ATGGAAACCT GTATCGTTTT TATCGTGTCT   
  
  
+ TACTGCCTTT TTAGATTGTC AATGCTGCAC AGTGGGAATT TTCCGCTCAA CCTATAACCG TATGAGCTTA   
  
  
+ CCTCAAAGTA CAACTGGTGT GTAGTTCTAG CTGCCGACGT CGACCACCAA GCACACACCG TGATTGCACA   
  
  
+ TTAACTGGTC TCTCCTCCGC CTCTCGCACC GCCACGTCCT CTATTCCTCC ACTCCACCGG TCTACTGTCG   
  
  
+ CTCGGCTAAA ACACCCCAGG TGCAAGAAAA CGGTTTTCCC TACGGCAAAC TGTCGTAGAG CACCGAGCAC   
  
  
+ TAGAGTTACG GTTGGTCGAC ACGAGGGAAG AGCGTAGCAT ACTAAGTCCT AGGTTGCCGG CTTTTGCCAA   
  
  
+ GTGGAATAGT AGGTCAGTCG AATTACTAGT TTATAAAAGT TGAGAGGTGC CGACTCTACG CGACTGTCTC   
  
  
+ AGAGACATAT ATATTCGCTC TCATGCTTTT GGTAACTCCT AAATCTTCAT CCTCACCCGT AGTGCTCTGT   
  
  
+ ACCCTAACGT TCGCACACCC CAGTGTGAAA ATAAAATTAA ACAGACAAAT CCTGATTATT CAACCTTCAC   
  
  
+ CGCTCAGAAC ACAACCTGGG TTAAAGTCCG GGGGGTAGTA CCAAAGAGGG CGGTGTATTA ATGTCACAGA   
  
  
+ CTGGCTTAAT GTAATAACAA TAAGAACAA  

- GACCCTTTTT GTATCTCTCG CTCTACGGCC GTTTCAGCGA CAAGGCTCTG TGAATCCGTA ACAGACCTCC   
  
  
- GCAGGGACTG TTAGAGAAGA GACAGAGAGA GAAAGAGAGA AGACAAAAGA ATGCGCTGAC TTTTGAGTAA   
  
  
- AGAGAGAGAA AATGGTAGGG GCTAGTAATG AGTGGGGTTA CCAATATATA TAGAGAGAGA GAGAGACGGC   
  
  
- ACAGTACACA TGCACCTGTA TGTAGAGAGA GAGAGCCGAA GTGGTTATTT AGTATTCACA AGTCTAGATA   
  
  
- GAGAGAGAAA CTAGAGAAGT TGGCATCTCA ATGCCCATTG TTGTTCTTTC TCTCTCTGCT ATCCGACAAT   
  
  
- TGAAAACCAG AAACGGTTTC TGGTTTTGGG GTTGACTTCT CGCACATCTC GTTGGGTTCG CCCTCTCCAA   
  
  
- TTCTTTTTTC ACACAAAAAG TCCACGCGCT CTCTTTTTCT CTCTCTCTCT CGTGGGCGCT CGTGAATCAC   
  
  
- CGACAAAAGA GAGGAATTAA AAGATAGCTT CTGCAAAGAG GATTAAACGA AAGTGGACGC CAGTTAACGA   
  
  
- GGGCGCTCCT ATCAATTTCA ACTGTGTTTT TACGATTTTA CCCATAACAA AAGAAGAAAG GATTTAAATC   
  
  
- AATTTAGTTG TTGGTTGCTT CCGTAGTTAA TTATGTGCCT CTTTTTATGT CGCCGGGTTT GTTCGTCCCG   
  
  
- TGCATGACAC GTGCCGAGTC ACTGGCAGTT CGATTAAACC ACACCGGTTC CTACTTTCAC TCCCTGTGTT   
  
  
- TGCTTCTTCC ACTCCATTAT TCCTTTCTTC TTACGGAAAT TACCTTTGGA CATAGCAAAA ATAGCACAGA   
  
  
- ATGACGGAAA AATCTAACAG TTACGACGTG TCACCCTTAA AAGGCGAGTT GGATATTGGC ATACTCGAAT   
  
  
- GGAGTTTCAT GTTGACCACA CATCAAGATC GACGGCTGCA GCTGGTGGTT CGTGTGTGGC ACTAACGTGT   
  
  
- AATTGACCAG AGAGGAGGCG GAGAGCGTGG CGGTGCAGGA GATAAGGAGG TGAGGTGGCC AGATGACAGC   
  
  
- GAGCCGATTT TGTGGGGTCC ACGTTCTTTT GCCAAAAGGG ATGCCGTTTG ACAGCATCTC GTGGCTCGTG   
  
  
- ATCTCAATGC CAACCAGCTG TGCTCCCTTC TCGCATCGTA TGATTCAGGA TCCAACGGCC GAAAACGGTT   
  
  
- CACCTTATCA TCCAGTCAGC TTAATGATCA AATATTTTCA ACTCTCCACG GCTGAGATGC GCTGACAGAG   
  
  
- TCTCTGTATA TATAAGCGAG AGTACGAAAA CCATTGAGGA TTTAGAAGTA GGAGTGGGCA TCACGAGACA   
  
  
- TGGGATTGCA AGCGTGTGGG GTCACACTTT TATTTTAATT TGTCTGTTTA GGACTAATAA GTTGGAAGTG   
  
  
- GCGAGTCTTG TGTTGGACCC AATTTCAGGC CCCCCATCAT GGTTTCTCCC GCCACATAAT TACAGTGTCT   
  
  
- GACCGAATTA CATTATTGTT ATTCTTGTT

+     G-Box

| Site Name | Organism | Position | Strand | Matrix score. | sequence | function |
| --- | --- | --- | --- | --- | --- | --- |
| G-Box | Antirrhinum majus | 700 | + | 6 | CACGTA | cis-acting regulatory element involved in light responsiveness |
| G-Box | Antirrhinum majus | 220 | - | 6 | CACGTA | cis-acting regulatory element involved in light responsiveness |
| G-Box | Triticum aestivum | 1185 | - | 10 | TCCACATGGCA | cis-acting regulatory element involved in light responsiveness |

> 2018/04/13 10:10:12  
+ CTGGGAAAAA CATAGAGAGC GAGATGCCGG CAAAGTCGCT GTTCCGAGAC ACTTAGGCAT TGTCTGGAGG   
  
  
+ CGTCCCTGAC AATCTCTTCT CTGTCTCTCT CTTTCTCTCT TCTGTTTTCT TACGCGACTG AAAACTCATT   
  
  
+ TCTCTCTCTT TTACCATCCC CGATCATTAC TCACCCCAAT GGTTATATAT ATCTCTCTCT CTCTCTGCCG   
  
  
+ TGTCATGTGT ACGTGGACAT ACATCTCTCT CTCTCGGCTT CACCAATAAA TCATAAGTGT TCAGATCTAT   
  
  
+ CTCTCTCTTT GATCTCTTCA ACCGTAGAGT TACGGGTAAC AACAAGAAAG AGAGAGACGA TAGGCTGTTA   
  
  
+ ACTTTTGGTC TTTGCCAAAG ACCAAAACCC CAACTGAAGA GCGTGTAGAG CAACCCAAGC GGGAGAGGTT   
  
  
+ AAGAAAAAAG TGTGTTTTTC AGGTGCGCGA GAGAAAAAGA GAGAGAGAGA GCACCCGCGA GCACTTAGTG   
  
  
+ GCTGTTTTCT CTCCTTAATT TTCTATCGAA GACGTTTCTC CTAATTTGCT TTCACCTGCG GTCAATTGCT   
  
  
+ CCCGCGAGGA TAGTTAAAGT TGACACAAAA ATGCTAAAAT GGGTATTGTT TTCTTCTTTC CTAAATTTAG   
  
  
+ TTAAATCAAC AACCAACGAA GGCATCAATT AATACACGGA GAAAAATACA GCGGCCCAAA CAAGCAGGGC   
  
  
+ ACGTACTGTG CACGGCTCAG TGACCGTCAA GCTAATTTGG TGTGGCCAAG GATGAAAGTG AGGGACACAA   
  
  
+ ACGAAGAAGG TGAGGTAATA AGGAAAGAAG AATGCCTTTA ATGGAAACCT GTATCGTTTT TATCGTGTCT   
  
  
+ TACTGCCTTT TTAGATTGTC AATGCTGCAC AGTGGGAATT TTCCGCTCAA CCTATAACCG TATGAGCTTA   
  
  
+ CCTCAAAGTA CAACTGGTGT GTAGTTCTAG CTGCCGACGT CGACCACCAA GCACACACCG TGATTGCACA   
  
  
+ TTAACTGGTC TCTCCTCCGC CTCTCGCACC GCCACGTCCT CTATTCCTCC ACTCCACCGG TCTACTGTCG   
  
  
+ CTCGGCTAAA ACACCCCAGG TGCAAGAAAA CGGTTTTCCC TACGGCAAAC TGTCGTAGAG CACCGAGCAC   
  
  
+ TAGAGTTACG GTTGGTCGAC ACGAGGGAAG AGCGTAGCAT ACTAAGTCCT AGGTTGCCGG CTTTTGCCAA   
  
  
+ GTGGAATAGT AGGTCAGTCG AATTACTAGT TTATAAAAGT TGAGAGGTGC CGACTCTACG CGACTGTCTC   
  
  
+ AGAGACATAT ATATTCGCTC TCATGCTTTT GGTAACTCCT AAATCTTCAT CCTCACCCGT AGTGCTCTGT   
  
  
+ ACCCTAACGT TCGCACACCC CAGTGTGAAA ATAAAATTAA ACAGACAAAT CCTGATTATT CAACCTTCAC   
  
  
+ CGCTCAGAAC ACAACCTGGG TTAAAGTCCG GGGGGTAGTA CCAAAGAGGG CGGTGTATTA ATGTCACAGA   
  
  
+ CTGGCTTAAT GTAATAACAA TAAGAACAA  

- GACCCTTTTT GTATCTCTCG CTCTACGGCC GTTTCAGCGA CAAGGCTCTG TGAATCCGTA ACAGACCTCC   
  
  
- GCAGGGACTG TTAGAGAAGA GACAGAGAGA GAAAGAGAGA AGACAAAAGA ATGCGCTGAC TTTTGAGTAA   
  
  
- AGAGAGAGAA AATGGTAGGG GCTAGTAATG AGTGGGGTTA CCAATATATA TAGAGAGAGA GAGAGACGGC   
  
  
- ACAGTACACA TGCACCTGTA TGTAGAGAGA GAGAGCCGAA GTGGTTATTT AGTATTCACA AGTCTAGATA   
  
  
- GAGAGAGAAA CTAGAGAAGT TGGCATCTCA ATGCCCATTG TTGTTCTTTC TCTCTCTGCT ATCCGACAAT   
  
  
- TGAAAACCAG AAACGGTTTC TGGTTTTGGG GTTGACTTCT CGCACATCTC GTTGGGTTCG CCCTCTCCAA   
  
  
- TTCTTTTTTC ACACAAAAAG TCCACGCGCT CTCTTTTTCT CTCTCTCTCT CGTGGGCGCT CGTGAATCAC   
  
  
- CGACAAAAGA GAGGAATTAA AAGATAGCTT CTGCAAAGAG GATTAAACGA AAGTGGACGC CAGTTAACGA   
  
  
- GGGCGCTCCT ATCAATTTCA ACTGTGTTTT TACGATTTTA CCCATAACAA AAGAAGAAAG GATTTAAATC   
  
  
- AATTTAGTTG TTGGTTGCTT CCGTAGTTAA TTATGTGCCT CTTTTTATGT CGCCGGGTTT GTTCGTCCCG   
  
  
- TGCATGACAC GTGCCGAGTC ACTGGCAGTT CGATTAAACC ACACCGGTTC CTACTTTCAC TCCCTGTGTT   
  
  
- TGCTTCTTCC ACTCCATTAT TCCTTTCTTC TTACGGAAAT TACCTTTGGA CATAGCAAAA ATAGCACAGA   
  
  
- ATGACGGAAA AATCTAACAG TTACGACGTG TCACCCTTAA AAGGCGAGTT GGATATTGGC ATACTCGAAT   
  
  
- GGAGTTTCAT GTTGACCACA CATCAAGATC GACGGCTGCA GCTGGTGGTT CGTGTGTGGC ACTAACGTGT   
  
  
- AATTGACCAG AGAGGAGGCG GAGAGCGTGG CGGTGCAGGA GATAAGGAGG TGAGGTGGCC AGATGACAGC   
  
  
- GAGCCGATTT TGTGGGGTCC ACGTTCTTTT GCCAAAAGGG ATGCCGTTTG ACAGCATCTC GTGGCTCGTG   
  
  
- ATCTCAATGC CAACCAGCTG TGCTCCCTTC TCGCATCGTA TGATTCAGGA TCCAACGGCC GAAAACGGTT   
  
  
- CACCTTATCA TCCAGTCAGC TTAATGATCA AATATTTTCA ACTCTCCACG GCTGAGATGC GCTGACAGAG   
  
  
- TCTCTGTATA TATAAGCGAG AGTACGAAAA CCATTGAGGA TTTAGAAGTA GGAGTGGGCA TCACGAGACA   
  
  
- TGGGATTGCA AGCGTGTGGG GTCACACTTT TATTTTAATT TGTCTGTTTA GGACTAATAA GTTGGAAGTG   
  
  
- GCGAGTCTTG TGTTGGACCC AATTTCAGGC CCCCCATCAT GGTTTCTCCC GCCACATAAT TACAGTGTCT   
  
  
- GACCGAATTA CATTATTGTT ATTCTTGTT

+     G-box

| Site Name | Organism | Position | Strand | Matrix score. | sequence | function |
| --- | --- | --- | --- | --- | --- | --- |
| G-box | Arabidopsis thaliana | 1186 | + | 9 | GCCACGTGGA | cis-acting regulatory element involved in light responsiveness |
| G-box | Gossypium hirsutum | 1185 | - | 11 | TTCCACGTGGCA | cis-acting regulatory element involved in light responsiveness |
| G-box | Oryza sativa | 219 | + | 7 | GTACGTG | cis-acting regulatory element involved in light responsiveness |
| G-box | Daucus carota | 700 | - | 6 | TACGTG | cis-acting regulatory element involved in light responsiveness |
| G-box | Daucus carota | 220 | + | 6 | TACGTG | cis-acting regulatory element involved in light responsiveness |
| G-box | Zea mays | 1013 | + | 6 | CACGTC | cis-acting regulatory element involved in light responsiveness |

> 2018/04/13 10:10:12  
+ CTGGGAAAAA CATAGAGAGC GAGATGCCGG CAAAGTCGCT GTTCCGAGAC ACTTAGGCAT TGTCTGGAGG   
  
  
+ CGTCCCTGAC AATCTCTTCT CTGTCTCTCT CTTTCTCTCT TCTGTTTTCT TACGCGACTG AAAACTCATT   
  
  
+ TCTCTCTCTT TTACCATCCC CGATCATTAC TCACCCCAAT GGTTATATAT ATCTCTCTCT CTCTCTGCCG   
  
  
+ TGTCATGTGT ACGTGGACAT ACATCTCTCT CTCTCGGCTT CACCAATAAA TCATAAGTGT TCAGATCTAT   
  
  
+ CTCTCTCTTT GATCTCTTCA ACCGTAGAGT TACGGGTAAC AACAAGAAAG AGAGAGACGA TAGGCTGTTA   
  
  
+ ACTTTTGGTC TTTGCCAAAG ACCAAAACCC CAACTGAAGA GCGTGTAGAG CAACCCAAGC GGGAGAGGTT   
  
  
+ AAGAAAAAAG TGTGTTTTTC AGGTGCGCGA GAGAAAAAGA GAGAGAGAGA GCACCCGCGA GCACTTAGTG   
  
  
+ GCTGTTTTCT CTCCTTAATT TTCTATCGAA GACGTTTCTC CTAATTTGCT TTCACCTGCG GTCAATTGCT   
  
  
+ CCCGCGAGGA TAGTTAAAGT TGACACAAAA ATGCTAAAAT GGGTATTGTT TTCTTCTTTC CTAAATTTAG   
  
  
+ TTAAATCAAC AACCAACGAA GGCATCAATT AATACACGGA GAAAAATACA GCGGCCCAAA CAAGCAGGGC   
  
  
+ ACGTACTGTG CACGGCTCAG TGACCGTCAA GCTAATTTGG TGTGGCCAAG GATGAAAGTG AGGGACACAA   
  
  
+ ACGAAGAAGG TGAGGTAATA AGGAAAGAAG AATGCCTTTA ATGGAAACCT GTATCGTTTT TATCGTGTCT   
  
  
+ TACTGCCTTT TTAGATTGTC AATGCTGCAC AGTGGGAATT TTCCGCTCAA CCTATAACCG TATGAGCTTA   
  
  
+ CCTCAAAGTA CAACTGGTGT GTAGTTCTAG CTGCCGACGT CGACCACCAA GCACACACCG TGATTGCACA   
  
  
+ TTAACTGGTC TCTCCTCCGC CTCTCGCACC GCCACGTCCT CTATTCCTCC ACTCCACCGG TCTACTGTCG   
  
  
+ CTCGGCTAAA ACACCCCAGG TGCAAGAAAA CGGTTTTCCC TACGGCAAAC TGTCGTAGAG CACCGAGCAC   
  
  
+ TAGAGTTACG GTTGGTCGAC ACGAGGGAAG AGCGTAGCAT ACTAAGTCCT AGGTTGCCGG CTTTTGCCAA   
  
  
+ GTGGAATAGT AGGTCAGTCG AATTACTAGT TTATAAAAGT TGAGAGGTGC CGACTCTACG CGACTGTCTC   
  
  
+ AGAGACATAT ATATTCGCTC TCATGCTTTT GGTAACTCCT AAATCTTCAT CCTCACCCGT AGTGCTCTGT   
  
  
+ ACCCTAACGT TCGCACACCC CAGTGTGAAA ATAAAATTAA ACAGACAAAT CCTGATTATT CAACCTTCAC   
  
  
+ CGCTCAGAAC ACAACCTGGG TTAAAGTCCG GGGGGTAGTA CCAAAGAGGG CGGTGTATTA ATGTCACAGA   
  
  
+ CTGGCTTAAT GTAATAACAA TAAGAACAA  

- GACCCTTTTT GTATCTCTCG CTCTACGGCC GTTTCAGCGA CAAGGCTCTG TGAATCCGTA ACAGACCTCC   
  
  
- GCAGGGACTG TTAGAGAAGA GACAGAGAGA GAAAGAGAGA AGACAAAAGA ATGCGCTGAC TTTTGAGTAA   
  
  
- AGAGAGAGAA AATGGTAGGG GCTAGTAATG AGTGGGGTTA CCAATATATA TAGAGAGAGA GAGAGACGGC   
  
  
- ACAGTACACA TGCACCTGTA TGTAGAGAGA GAGAGCCGAA GTGGTTATTT AGTATTCACA AGTCTAGATA   
  
  
- GAGAGAGAAA CTAGAGAAGT TGGCATCTCA ATGCCCATTG TTGTTCTTTC TCTCTCTGCT ATCCGACAAT   
  
  
- TGAAAACCAG AAACGGTTTC TGGTTTTGGG GTTGACTTCT CGCACATCTC GTTGGGTTCG CCCTCTCCAA   
  
  
- TTCTTTTTTC ACACAAAAAG TCCACGCGCT CTCTTTTTCT CTCTCTCTCT CGTGGGCGCT CGTGAATCAC   
  
  
- CGACAAAAGA GAGGAATTAA AAGATAGCTT CTGCAAAGAG GATTAAACGA AAGTGGACGC CAGTTAACGA   
  
  
- GGGCGCTCCT ATCAATTTCA ACTGTGTTTT TACGATTTTA CCCATAACAA AAGAAGAAAG GATTTAAATC   
  
  
- AATTTAGTTG TTGGTTGCTT CCGTAGTTAA TTATGTGCCT CTTTTTATGT CGCCGGGTTT GTTCGTCCCG   
  
  
- TGCATGACAC GTGCCGAGTC ACTGGCAGTT CGATTAAACC ACACCGGTTC CTACTTTCAC TCCCTGTGTT   
  
  
- TGCTTCTTCC ACTCCATTAT TCCTTTCTTC TTACGGAAAT TACCTTTGGA CATAGCAAAA ATAGCACAGA   
  
  
- ATGACGGAAA AATCTAACAG TTACGACGTG TCACCCTTAA AAGGCGAGTT GGATATTGGC ATACTCGAAT   
  
  
- GGAGTTTCAT GTTGACCACA CATCAAGATC GACGGCTGCA GCTGGTGGTT CGTGTGTGGC ACTAACGTGT   
  
  
- AATTGACCAG AGAGGAGGCG GAGAGCGTGG CGGTGCAGGA GATAAGGAGG TGAGGTGGCC AGATGACAGC   
  
  
- GAGCCGATTT TGTGGGGTCC ACGTTCTTTT GCCAAAAGGG ATGCCGTTTG ACAGCATCTC GTGGCTCGTG   
  
  
- ATCTCAATGC CAACCAGCTG TGCTCCCTTC TCGCATCGTA TGATTCAGGA TCCAACGGCC GAAAACGGTT   
  
  
- CACCTTATCA TCCAGTCAGC TTAATGATCA AATATTTTCA ACTCTCCACG GCTGAGATGC GCTGACAGAG   
  
  
- TCTCTGTATA TATAAGCGAG AGTACGAAAA CCATTGAGGA TTTAGAAGTA GGAGTGGGCA TCACGAGACA   
  
  
- TGGGATTGCA AGCGTGTGGG GTCACACTTT TATTTTAATT TGTCTGTTTA GGACTAATAA GTTGGAAGTG   
  
  
- GCGAGTCTTG TGTTGGACCC AATTTCAGGC CCCCCATCAT GGTTTCTCCC GCCACATAAT TACAGTGTCT   
  
  
- GACCGAATTA CATTATTGTT ATTCTTGTT

+     GAG-motif

| Site Name | Organism | Position | Strand | Matrix score. | sequence | function |
| --- | --- | --- | --- | --- | --- | --- |
| GAG-motif | Spinacia oleracea | 232 | - | 7 | AGAGATG | part of a light responsive element |

> 2018/04/13 10:10:12  
+ CTGGGAAAAA CATAGAGAGC GAGATGCCGG CAAAGTCGCT GTTCCGAGAC ACTTAGGCAT TGTCTGGAGG   
  
  
+ CGTCCCTGAC AATCTCTTCT CTGTCTCTCT CTTTCTCTCT TCTGTTTTCT TACGCGACTG AAAACTCATT   
  
  
+ TCTCTCTCTT TTACCATCCC CGATCATTAC TCACCCCAAT GGTTATATAT ATCTCTCTCT CTCTCTGCCG   
  
  
+ TGTCATGTGT ACGTGGACAT ACATCTCTCT CTCTCGGCTT CACCAATAAA TCATAAGTGT TCAGATCTAT   
  
  
+ CTCTCTCTTT GATCTCTTCA ACCGTAGAGT TACGGGTAAC AACAAGAAAG AGAGAGACGA TAGGCTGTTA   
  
  
+ ACTTTTGGTC TTTGCCAAAG ACCAAAACCC CAACTGAAGA GCGTGTAGAG CAACCCAAGC GGGAGAGGTT   
  
  
+ AAGAAAAAAG TGTGTTTTTC AGGTGCGCGA GAGAAAAAGA GAGAGAGAGA GCACCCGCGA GCACTTAGTG   
  
  
+ GCTGTTTTCT CTCCTTAATT TTCTATCGAA GACGTTTCTC CTAATTTGCT TTCACCTGCG GTCAATTGCT   
  
  
+ CCCGCGAGGA TAGTTAAAGT TGACACAAAA ATGCTAAAAT GGGTATTGTT TTCTTCTTTC CTAAATTTAG   
  
  
+ TTAAATCAAC AACCAACGAA GGCATCAATT AATACACGGA GAAAAATACA GCGGCCCAAA CAAGCAGGGC   
  
  
+ ACGTACTGTG CACGGCTCAG TGACCGTCAA GCTAATTTGG TGTGGCCAAG GATGAAAGTG AGGGACACAA   
  
  
+ ACGAAGAAGG TGAGGTAATA AGGAAAGAAG AATGCCTTTA ATGGAAACCT GTATCGTTTT TATCGTGTCT   
  
  
+ TACTGCCTTT TTAGATTGTC AATGCTGCAC AGTGGGAATT TTCCGCTCAA CCTATAACCG TATGAGCTTA   
  
  
+ CCTCAAAGTA CAACTGGTGT GTAGTTCTAG CTGCCGACGT CGACCACCAA GCACACACCG TGATTGCACA   
  
  
+ TTAACTGGTC TCTCCTCCGC CTCTCGCACC GCCACGTCCT CTATTCCTCC ACTCCACCGG TCTACTGTCG   
  
  
+ CTCGGCTAAA ACACCCCAGG TGCAAGAAAA CGGTTTTCCC TACGGCAAAC TGTCGTAGAG CACCGAGCAC   
  
  
+ TAGAGTTACG GTTGGTCGAC ACGAGGGAAG AGCGTAGCAT ACTAAGTCCT AGGTTGCCGG CTTTTGCCAA   
  
  
+ GTGGAATAGT AGGTCAGTCG AATTACTAGT TTATAAAAGT TGAGAGGTGC CGACTCTACG CGACTGTCTC   
  
  
+ AGAGACATAT ATATTCGCTC TCATGCTTTT GGTAACTCCT AAATCTTCAT CCTCACCCGT AGTGCTCTGT   
  
  
+ ACCCTAACGT TCGCACACCC CAGTGTGAAA ATAAAATTAA ACAGACAAAT CCTGATTATT CAACCTTCAC   
  
  
+ CGCTCAGAAC ACAACCTGGG TTAAAGTCCG GGGGGTAGTA CCAAAGAGGG CGGTGTATTA ATGTCACAGA   
  
  
+ CTGGCTTAAT GTAATAACAA TAAGAACAA  

- GACCCTTTTT GTATCTCTCG CTCTACGGCC GTTTCAGCGA CAAGGCTCTG TGAATCCGTA ACAGACCTCC   
  
  
- GCAGGGACTG TTAGAGAAGA GACAGAGAGA GAAAGAGAGA AGACAAAAGA ATGCGCTGAC TTTTGAGTAA   
  
  
- AGAGAGAGAA AATGGTAGGG GCTAGTAATG AGTGGGGTTA CCAATATATA TAGAGAGAGA GAGAGACGGC   
  
  
- ACAGTACACA TGCACCTGTA TGTAGAGAGA GAGAGCCGAA GTGGTTATTT AGTATTCACA AGTCTAGATA   
  
  
- GAGAGAGAAA CTAGAGAAGT TGGCATCTCA ATGCCCATTG TTGTTCTTTC TCTCTCTGCT ATCCGACAAT   
  
  
- TGAAAACCAG AAACGGTTTC TGGTTTTGGG GTTGACTTCT CGCACATCTC GTTGGGTTCG CCCTCTCCAA   
  
  
- TTCTTTTTTC ACACAAAAAG TCCACGCGCT CTCTTTTTCT CTCTCTCTCT CGTGGGCGCT CGTGAATCAC   
  
  
- CGACAAAAGA GAGGAATTAA AAGATAGCTT CTGCAAAGAG GATTAAACGA AAGTGGACGC CAGTTAACGA   
  
  
- GGGCGCTCCT ATCAATTTCA ACTGTGTTTT TACGATTTTA CCCATAACAA AAGAAGAAAG GATTTAAATC   
  
  
- AATTTAGTTG TTGGTTGCTT CCGTAGTTAA TTATGTGCCT CTTTTTATGT CGCCGGGTTT GTTCGTCCCG   
  
  
- TGCATGACAC GTGCCGAGTC ACTGGCAGTT CGATTAAACC ACACCGGTTC CTACTTTCAC TCCCTGTGTT   
  
  
- TGCTTCTTCC ACTCCATTAT TCCTTTCTTC TTACGGAAAT TACCTTTGGA CATAGCAAAA ATAGCACAGA   
  
  
- ATGACGGAAA AATCTAACAG TTACGACGTG TCACCCTTAA AAGGCGAGTT GGATATTGGC ATACTCGAAT   
  
  
- GGAGTTTCAT GTTGACCACA CATCAAGATC GACGGCTGCA GCTGGTGGTT CGTGTGTGGC ACTAACGTGT   
  
  
- AATTGACCAG AGAGGAGGCG GAGAGCGTGG CGGTGCAGGA GATAAGGAGG TGAGGTGGCC AGATGACAGC   
  
  
- GAGCCGATTT TGTGGGGTCC ACGTTCTTTT GCCAAAAGGG ATGCCGTTTG ACAGCATCTC GTGGCTCGTG   
  
  
- ATCTCAATGC CAACCAGCTG TGCTCCCTTC TCGCATCGTA TGATTCAGGA TCCAACGGCC GAAAACGGTT   
  
  
- CACCTTATCA TCCAGTCAGC TTAATGATCA AATATTTTCA ACTCTCCACG GCTGAGATGC GCTGACAGAG   
  
  
- TCTCTGTATA TATAAGCGAG AGTACGAAAA CCATTGAGGA TTTAGAAGTA GGAGTGGGCA TCACGAGACA   
  
  
- TGGGATTGCA AGCGTGTGGG GTCACACTTT TATTTTAATT TGTCTGTTTA GGACTAATAA GTTGGAAGTG   
  
  
- GCGAGTCTTG TGTTGGACCC AATTTCAGGC CCCCCATCAT GGTTTCTCCC GCCACATAAT TACAGTGTCT   
  
  
- GACCGAATTA CATTATTGTT ATTCTTGTT

+     GARE-motif

| Site Name | Organism | Position | Strand | Matrix score. | sequence | function |
| --- | --- | --- | --- | --- | --- | --- |
| GARE-motif | Brassica oleracea | 111 | - | 7 | AAACAGA | gibberellin-responsive element |
| GARE-motif | Brassica oleracea | 1369 | + | 7 | AAACAGA | gibberellin-responsive element |

> 2018/04/13 10:10:12  
+ CTGGGAAAAA CATAGAGAGC GAGATGCCGG CAAAGTCGCT GTTCCGAGAC ACTTAGGCAT TGTCTGGAGG   
  
  
+ CGTCCCTGAC AATCTCTTCT CTGTCTCTCT CTTTCTCTCT TCTGTTTTCT TACGCGACTG AAAACTCATT   
  
  
+ TCTCTCTCTT TTACCATCCC CGATCATTAC TCACCCCAAT GGTTATATAT ATCTCTCTCT CTCTCTGCCG   
  
  
+ TGTCATGTGT ACGTGGACAT ACATCTCTCT CTCTCGGCTT CACCAATAAA TCATAAGTGT TCAGATCTAT   
  
  
+ CTCTCTCTTT GATCTCTTCA ACCGTAGAGT TACGGGTAAC AACAAGAAAG AGAGAGACGA TAGGCTGTTA   
  
  
+ ACTTTTGGTC TTTGCCAAAG ACCAAAACCC CAACTGAAGA GCGTGTAGAG CAACCCAAGC GGGAGAGGTT   
  
  
+ AAGAAAAAAG TGTGTTTTTC AGGTGCGCGA GAGAAAAAGA GAGAGAGAGA GCACCCGCGA GCACTTAGTG   
  
  
+ GCTGTTTTCT CTCCTTAATT TTCTATCGAA GACGTTTCTC CTAATTTGCT TTCACCTGCG GTCAATTGCT   
  
  
+ CCCGCGAGGA TAGTTAAAGT TGACACAAAA ATGCTAAAAT GGGTATTGTT TTCTTCTTTC CTAAATTTAG   
  
  
+ TTAAATCAAC AACCAACGAA GGCATCAATT AATACACGGA GAAAAATACA GCGGCCCAAA CAAGCAGGGC   
  
  
+ ACGTACTGTG CACGGCTCAG TGACCGTCAA GCTAATTTGG TGTGGCCAAG GATGAAAGTG AGGGACACAA   
  
  
+ ACGAAGAAGG TGAGGTAATA AGGAAAGAAG AATGCCTTTA ATGGAAACCT GTATCGTTTT TATCGTGTCT   
  
  
+ TACTGCCTTT TTAGATTGTC AATGCTGCAC AGTGGGAATT TTCCGCTCAA CCTATAACCG TATGAGCTTA   
  
  
+ CCTCAAAGTA CAACTGGTGT GTAGTTCTAG CTGCCGACGT CGACCACCAA GCACACACCG TGATTGCACA   
  
  
+ TTAACTGGTC TCTCCTCCGC CTCTCGCACC GCCACGTCCT CTATTCCTCC ACTCCACCGG TCTACTGTCG   
  
  
+ CTCGGCTAAA ACACCCCAGG TGCAAGAAAA CGGTTTTCCC TACGGCAAAC TGTCGTAGAG CACCGAGCAC   
  
  
+ TAGAGTTACG GTTGGTCGAC ACGAGGGAAG AGCGTAGCAT ACTAAGTCCT AGGTTGCCGG CTTTTGCCAA   
  
  
+ GTGGAATAGT AGGTCAGTCG AATTACTAGT TTATAAAAGT TGAGAGGTGC CGACTCTACG CGACTGTCTC   
  
  
+ AGAGACATAT ATATTCGCTC TCATGCTTTT GGTAACTCCT AAATCTTCAT CCTCACCCGT AGTGCTCTGT   
  
  
+ ACCCTAACGT TCGCACACCC CAGTGTGAAA ATAAAATTAA ACAGACAAAT CCTGATTATT CAACCTTCAC   
  
  
+ CGCTCAGAAC ACAACCTGGG TTAAAGTCCG GGGGGTAGTA CCAAAGAGGG CGGTGTATTA ATGTCACAGA   
  
  
+ CTGGCTTAAT GTAATAACAA TAAGAACAA  

- GACCCTTTTT GTATCTCTCG CTCTACGGCC GTTTCAGCGA CAAGGCTCTG TGAATCCGTA ACAGACCTCC   
  
  
- GCAGGGACTG TTAGAGAAGA GACAGAGAGA GAAAGAGAGA AGACAAAAGA ATGCGCTGAC TTTTGAGTAA   
  
  
- AGAGAGAGAA AATGGTAGGG GCTAGTAATG AGTGGGGTTA CCAATATATA TAGAGAGAGA GAGAGACGGC   
  
  
- ACAGTACACA TGCACCTGTA TGTAGAGAGA GAGAGCCGAA GTGGTTATTT AGTATTCACA AGTCTAGATA   
  
  
- GAGAGAGAAA CTAGAGAAGT TGGCATCTCA ATGCCCATTG TTGTTCTTTC TCTCTCTGCT ATCCGACAAT   
  
  
- TGAAAACCAG AAACGGTTTC TGGTTTTGGG GTTGACTTCT CGCACATCTC GTTGGGTTCG CCCTCTCCAA   
  
  
- TTCTTTTTTC ACACAAAAAG TCCACGCGCT CTCTTTTTCT CTCTCTCTCT CGTGGGCGCT CGTGAATCAC   
  
  
- CGACAAAAGA GAGGAATTAA AAGATAGCTT CTGCAAAGAG GATTAAACGA AAGTGGACGC CAGTTAACGA   
  
  
- GGGCGCTCCT ATCAATTTCA ACTGTGTTTT TACGATTTTA CCCATAACAA AAGAAGAAAG GATTTAAATC   
  
  
- AATTTAGTTG TTGGTTGCTT CCGTAGTTAA TTATGTGCCT CTTTTTATGT CGCCGGGTTT GTTCGTCCCG   
  
  
- TGCATGACAC GTGCCGAGTC ACTGGCAGTT CGATTAAACC ACACCGGTTC CTACTTTCAC TCCCTGTGTT   
  
  
- TGCTTCTTCC ACTCCATTAT TCCTTTCTTC TTACGGAAAT TACCTTTGGA CATAGCAAAA ATAGCACAGA   
  
  
- ATGACGGAAA AATCTAACAG TTACGACGTG TCACCCTTAA AAGGCGAGTT GGATATTGGC ATACTCGAAT   
  
  
- GGAGTTTCAT GTTGACCACA CATCAAGATC GACGGCTGCA GCTGGTGGTT CGTGTGTGGC ACTAACGTGT   
  
  
- AATTGACCAG AGAGGAGGCG GAGAGCGTGG CGGTGCAGGA GATAAGGAGG TGAGGTGGCC AGATGACAGC   
  
  
- GAGCCGATTT TGTGGGGTCC ACGTTCTTTT GCCAAAAGGG ATGCCGTTTG ACAGCATCTC GTGGCTCGTG   
  
  
- ATCTCAATGC CAACCAGCTG TGCTCCCTTC TCGCATCGTA TGATTCAGGA TCCAACGGCC GAAAACGGTT   
  
  
- CACCTTATCA TCCAGTCAGC TTAATGATCA AATATTTTCA ACTCTCCACG GCTGAGATGC GCTGACAGAG   
  
  
- TCTCTGTATA TATAAGCGAG AGTACGAAAA CCATTGAGGA TTTAGAAGTA GGAGTGGGCA TCACGAGACA   
  
  
- TGGGATTGCA AGCGTGTGGG GTCACACTTT TATTTTAATT TGTCTGTTTA GGACTAATAA GTTGGAAGTG   
  
  
- GCGAGTCTTG TGTTGGACCC AATTTCAGGC CCCCCATCAT GGTTTCTCCC GCCACATAAT TACAGTGTCT   
  
  
- GACCGAATTA CATTATTGTT ATTCTTGTT

+     GC-motif

| Site Name | Organism | Position | Strand | Matrix score. | sequence | function |
| --- | --- | --- | --- | --- | --- | --- |
| GC-motif | Zea mays | 1429 | - | 6 | CCCCCG | enhancer-like element involved in anoxic specific inducibility |

> 2018/04/13 10:10:12  
+ CTGGGAAAAA CATAGAGAGC GAGATGCCGG CAAAGTCGCT GTTCCGAGAC ACTTAGGCAT TGTCTGGAGG   
  
  
+ CGTCCCTGAC AATCTCTTCT CTGTCTCTCT CTTTCTCTCT TCTGTTTTCT TACGCGACTG AAAACTCATT   
  
  
+ TCTCTCTCTT TTACCATCCC CGATCATTAC TCACCCCAAT GGTTATATAT ATCTCTCTCT CTCTCTGCCG   
  
  
+ TGTCATGTGT ACGTGGACAT ACATCTCTCT CTCTCGGCTT CACCAATAAA TCATAAGTGT TCAGATCTAT   
  
  
+ CTCTCTCTTT GATCTCTTCA ACCGTAGAGT TACGGGTAAC AACAAGAAAG AGAGAGACGA TAGGCTGTTA   
  
  
+ ACTTTTGGTC TTTGCCAAAG ACCAAAACCC CAACTGAAGA GCGTGTAGAG CAACCCAAGC GGGAGAGGTT   
  
  
+ AAGAAAAAAG TGTGTTTTTC AGGTGCGCGA GAGAAAAAGA GAGAGAGAGA GCACCCGCGA GCACTTAGTG   
  
  
+ GCTGTTTTCT CTCCTTAATT TTCTATCGAA GACGTTTCTC CTAATTTGCT TTCACCTGCG GTCAATTGCT   
  
  
+ CCCGCGAGGA TAGTTAAAGT TGACACAAAA ATGCTAAAAT GGGTATTGTT TTCTTCTTTC CTAAATTTAG   
  
  
+ TTAAATCAAC AACCAACGAA GGCATCAATT AATACACGGA GAAAAATACA GCGGCCCAAA CAAGCAGGGC   
  
  
+ ACGTACTGTG CACGGCTCAG TGACCGTCAA GCTAATTTGG TGTGGCCAAG GATGAAAGTG AGGGACACAA   
  
  
+ ACGAAGAAGG TGAGGTAATA AGGAAAGAAG AATGCCTTTA ATGGAAACCT GTATCGTTTT TATCGTGTCT   
  
  
+ TACTGCCTTT TTAGATTGTC AATGCTGCAC AGTGGGAATT TTCCGCTCAA CCTATAACCG TATGAGCTTA   
  
  
+ CCTCAAAGTA CAACTGGTGT GTAGTTCTAG CTGCCGACGT CGACCACCAA GCACACACCG TGATTGCACA   
  
  
+ TTAACTGGTC TCTCCTCCGC CTCTCGCACC GCCACGTCCT CTATTCCTCC ACTCCACCGG TCTACTGTCG   
  
  
+ CTCGGCTAAA ACACCCCAGG TGCAAGAAAA CGGTTTTCCC TACGGCAAAC TGTCGTAGAG CACCGAGCAC   
  
  
+ TAGAGTTACG GTTGGTCGAC ACGAGGGAAG AGCGTAGCAT ACTAAGTCCT AGGTTGCCGG CTTTTGCCAA   
  
  
+ GTGGAATAGT AGGTCAGTCG AATTACTAGT TTATAAAAGT TGAGAGGTGC CGACTCTACG CGACTGTCTC   
  
  
+ AGAGACATAT ATATTCGCTC TCATGCTTTT GGTAACTCCT AAATCTTCAT CCTCACCCGT AGTGCTCTGT   
  
  
+ ACCCTAACGT TCGCACACCC CAGTGTGAAA ATAAAATTAA ACAGACAAAT CCTGATTATT CAACCTTCAC   
  
  
+ CGCTCAGAAC ACAACCTGGG TTAAAGTCCG GGGGGTAGTA CCAAAGAGGG CGGTGTATTA ATGTCACAGA   
  
  
+ CTGGCTTAAT GTAATAACAA TAAGAACAA  

- GACCCTTTTT GTATCTCTCG CTCTACGGCC GTTTCAGCGA CAAGGCTCTG TGAATCCGTA ACAGACCTCC   
  
  
- GCAGGGACTG TTAGAGAAGA GACAGAGAGA GAAAGAGAGA AGACAAAAGA ATGCGCTGAC TTTTGAGTAA   
  
  
- AGAGAGAGAA AATGGTAGGG GCTAGTAATG AGTGGGGTTA CCAATATATA TAGAGAGAGA GAGAGACGGC   
  
  
- ACAGTACACA TGCACCTGTA TGTAGAGAGA GAGAGCCGAA GTGGTTATTT AGTATTCACA AGTCTAGATA   
  
  
- GAGAGAGAAA CTAGAGAAGT TGGCATCTCA ATGCCCATTG TTGTTCTTTC TCTCTCTGCT ATCCGACAAT   
  
  
- TGAAAACCAG AAACGGTTTC TGGTTTTGGG GTTGACTTCT CGCACATCTC GTTGGGTTCG CCCTCTCCAA   
  
  
- TTCTTTTTTC ACACAAAAAG TCCACGCGCT CTCTTTTTCT CTCTCTCTCT CGTGGGCGCT CGTGAATCAC   
  
  
- CGACAAAAGA GAGGAATTAA AAGATAGCTT CTGCAAAGAG GATTAAACGA AAGTGGACGC CAGTTAACGA   
  
  
- GGGCGCTCCT ATCAATTTCA ACTGTGTTTT TACGATTTTA CCCATAACAA AAGAAGAAAG GATTTAAATC   
  
  
- AATTTAGTTG TTGGTTGCTT CCGTAGTTAA TTATGTGCCT CTTTTTATGT CGCCGGGTTT GTTCGTCCCG   
  
  
- TGCATGACAC GTGCCGAGTC ACTGGCAGTT CGATTAAACC ACACCGGTTC CTACTTTCAC TCCCTGTGTT   
  
  
- TGCTTCTTCC ACTCCATTAT TCCTTTCTTC TTACGGAAAT TACCTTTGGA CATAGCAAAA ATAGCACAGA   
  
  
- ATGACGGAAA AATCTAACAG TTACGACGTG TCACCCTTAA AAGGCGAGTT GGATATTGGC ATACTCGAAT   
  
  
- GGAGTTTCAT GTTGACCACA CATCAAGATC GACGGCTGCA GCTGGTGGTT CGTGTGTGGC ACTAACGTGT   
  
  
- AATTGACCAG AGAGGAGGCG GAGAGCGTGG CGGTGCAGGA GATAAGGAGG TGAGGTGGCC AGATGACAGC   
  
  
- GAGCCGATTT TGTGGGGTCC ACGTTCTTTT GCCAAAAGGG ATGCCGTTTG ACAGCATCTC GTGGCTCGTG   
  
  
- ATCTCAATGC CAACCAGCTG TGCTCCCTTC TCGCATCGTA TGATTCAGGA TCCAACGGCC GAAAACGGTT   
  
  
- CACCTTATCA TCCAGTCAGC TTAATGATCA AATATTTTCA ACTCTCCACG GCTGAGATGC GCTGACAGAG   
  
  
- TCTCTGTATA TATAAGCGAG AGTACGAAAA CCATTGAGGA TTTAGAAGTA GGAGTGGGCA TCACGAGACA   
  
  
- TGGGATTGCA AGCGTGTGGG GTCACACTTT TATTTTAATT TGTCTGTTTA GGACTAATAA GTTGGAAGTG   
  
  
- GCGAGTCTTG TGTTGGACCC AATTTCAGGC CCCCCATCAT GGTTTCTCCC GCCACATAAT TACAGTGTCT   
  
  
- GACCGAATTA CATTATTGTT ATTCTTGTT

+     GC-repeat

| Site Name | Organism | Position | Strand | Matrix score. | sequence | function |
| --- | --- | --- | --- | --- | --- | --- |
| GC-repeat | Oryza sativa | 1110 | + | 9 | GCACCGAGC | ? |

> 2018/04/13 10:10:12  
+ CTGGGAAAAA CATAGAGAGC GAGATGCCGG CAAAGTCGCT GTTCCGAGAC ACTTAGGCAT TGTCTGGAGG   
  
  
+ CGTCCCTGAC AATCTCTTCT CTGTCTCTCT CTTTCTCTCT TCTGTTTTCT TACGCGACTG AAAACTCATT   
  
  
+ TCTCTCTCTT TTACCATCCC CGATCATTAC TCACCCCAAT GGTTATATAT ATCTCTCTCT CTCTCTGCCG   
  
  
+ TGTCATGTGT ACGTGGACAT ACATCTCTCT CTCTCGGCTT CACCAATAAA TCATAAGTGT TCAGATCTAT   
  
  
+ CTCTCTCTTT GATCTCTTCA ACCGTAGAGT TACGGGTAAC AACAAGAAAG AGAGAGACGA TAGGCTGTTA   
  
  
+ ACTTTTGGTC TTTGCCAAAG ACCAAAACCC CAACTGAAGA GCGTGTAGAG CAACCCAAGC GGGAGAGGTT   
  
  
+ AAGAAAAAAG TGTGTTTTTC AGGTGCGCGA GAGAAAAAGA GAGAGAGAGA GCACCCGCGA GCACTTAGTG   
  
  
+ GCTGTTTTCT CTCCTTAATT TTCTATCGAA GACGTTTCTC CTAATTTGCT TTCACCTGCG GTCAATTGCT   
  
  
+ CCCGCGAGGA TAGTTAAAGT TGACACAAAA ATGCTAAAAT GGGTATTGTT TTCTTCTTTC CTAAATTTAG   
  
  
+ TTAAATCAAC AACCAACGAA GGCATCAATT AATACACGGA GAAAAATACA GCGGCCCAAA CAAGCAGGGC   
  
  
+ ACGTACTGTG CACGGCTCAG TGACCGTCAA GCTAATTTGG TGTGGCCAAG GATGAAAGTG AGGGACACAA   
  
  
+ ACGAAGAAGG TGAGGTAATA AGGAAAGAAG AATGCCTTTA ATGGAAACCT GTATCGTTTT TATCGTGTCT   
  
  
+ TACTGCCTTT TTAGATTGTC AATGCTGCAC AGTGGGAATT TTCCGCTCAA CCTATAACCG TATGAGCTTA   
  
  
+ CCTCAAAGTA CAACTGGTGT GTAGTTCTAG CTGCCGACGT CGACCACCAA GCACACACCG TGATTGCACA   
  
  
+ TTAACTGGTC TCTCCTCCGC CTCTCGCACC GCCACGTCCT CTATTCCTCC ACTCCACCGG TCTACTGTCG   
  
  
+ CTCGGCTAAA ACACCCCAGG TGCAAGAAAA CGGTTTTCCC TACGGCAAAC TGTCGTAGAG CACCGAGCAC   
  
  
+ TAGAGTTACG GTTGGTCGAC ACGAGGGAAG AGCGTAGCAT ACTAAGTCCT AGGTTGCCGG CTTTTGCCAA   
  
  
+ GTGGAATAGT AGGTCAGTCG AATTACTAGT TTATAAAAGT TGAGAGGTGC CGACTCTACG CGACTGTCTC   
  
  
+ AGAGACATAT ATATTCGCTC TCATGCTTTT GGTAACTCCT AAATCTTCAT CCTCACCCGT AGTGCTCTGT   
  
  
+ ACCCTAACGT TCGCACACCC CAGTGTGAAA ATAAAATTAA ACAGACAAAT CCTGATTATT CAACCTTCAC   
  
  
+ CGCTCAGAAC ACAACCTGGG TTAAAGTCCG GGGGGTAGTA CCAAAGAGGG CGGTGTATTA ATGTCACAGA   
  
  
+ CTGGCTTAAT GTAATAACAA TAAGAACAA  

- GACCCTTTTT GTATCTCTCG CTCTACGGCC GTTTCAGCGA CAAGGCTCTG TGAATCCGTA ACAGACCTCC   
  
  
- GCAGGGACTG TTAGAGAAGA GACAGAGAGA GAAAGAGAGA AGACAAAAGA ATGCGCTGAC TTTTGAGTAA   
  
  
- AGAGAGAGAA AATGGTAGGG GCTAGTAATG AGTGGGGTTA CCAATATATA TAGAGAGAGA GAGAGACGGC   
  
  
- ACAGTACACA TGCACCTGTA TGTAGAGAGA GAGAGCCGAA GTGGTTATTT AGTATTCACA AGTCTAGATA   
  
  
- GAGAGAGAAA CTAGAGAAGT TGGCATCTCA ATGCCCATTG TTGTTCTTTC TCTCTCTGCT ATCCGACAAT   
  
  
- TGAAAACCAG AAACGGTTTC TGGTTTTGGG GTTGACTTCT CGCACATCTC GTTGGGTTCG CCCTCTCCAA   
  
  
- TTCTTTTTTC ACACAAAAAG TCCACGCGCT CTCTTTTTCT CTCTCTCTCT CGTGGGCGCT CGTGAATCAC   
  
  
- CGACAAAAGA GAGGAATTAA AAGATAGCTT CTGCAAAGAG GATTAAACGA AAGTGGACGC CAGTTAACGA   
  
  
- GGGCGCTCCT ATCAATTTCA ACTGTGTTTT TACGATTTTA CCCATAACAA AAGAAGAAAG GATTTAAATC   
  
  
- AATTTAGTTG TTGGTTGCTT CCGTAGTTAA TTATGTGCCT CTTTTTATGT CGCCGGGTTT GTTCGTCCCG   
  
  
- TGCATGACAC GTGCCGAGTC ACTGGCAGTT CGATTAAACC ACACCGGTTC CTACTTTCAC TCCCTGTGTT   
  
  
- TGCTTCTTCC ACTCCATTAT TCCTTTCTTC TTACGGAAAT TACCTTTGGA CATAGCAAAA ATAGCACAGA   
  
  
- ATGACGGAAA AATCTAACAG TTACGACGTG TCACCCTTAA AAGGCGAGTT GGATATTGGC ATACTCGAAT   
  
  
- GGAGTTTCAT GTTGACCACA CATCAAGATC GACGGCTGCA GCTGGTGGTT CGTGTGTGGC ACTAACGTGT   
  
  
- AATTGACCAG AGAGGAGGCG GAGAGCGTGG CGGTGCAGGA GATAAGGAGG TGAGGTGGCC AGATGACAGC   
  
  
- GAGCCGATTT TGTGGGGTCC ACGTTCTTTT GCCAAAAGGG ATGCCGTTTG ACAGCATCTC GTGGCTCGTG   
  
  
- ATCTCAATGC CAACCAGCTG TGCTCCCTTC TCGCATCGTA TGATTCAGGA TCCAACGGCC GAAAACGGTT   
  
  
- CACCTTATCA TCCAGTCAGC TTAATGATCA AATATTTTCA ACTCTCCACG GCTGAGATGC GCTGACAGAG   
  
  
- TCTCTGTATA TATAAGCGAG AGTACGAAAA CCATTGAGGA TTTAGAAGTA GGAGTGGGCA TCACGAGACA   
  
  
- TGGGATTGCA AGCGTGTGGG GTCACACTTT TATTTTAATT TGTCTGTTTA GGACTAATAA GTTGGAAGTG   
  
  
- GCGAGTCTTG TGTTGGACCC AATTTCAGGC CCCCCATCAT GGTTTCTCCC GCCACATAAT TACAGTGTCT   
  
  
- GACCGAATTA CATTATTGTT ATTCTTGTT

+     GCN4\_motif

| Site Name | Organism | Position | Strand | Matrix score. | sequence | function |
| --- | --- | --- | --- | --- | --- | --- |
| GCN4\_motif | Oryza sativa | 581 | - | 7 | TGTGTCA | cis-regulatory element involved in endosperm expression |

> 2018/04/13 10:10:12  
+ CTGGGAAAAA CATAGAGAGC GAGATGCCGG CAAAGTCGCT GTTCCGAGAC ACTTAGGCAT TGTCTGGAGG   
  
  
+ CGTCCCTGAC AATCTCTTCT CTGTCTCTCT CTTTCTCTCT TCTGTTTTCT TACGCGACTG AAAACTCATT   
  
  
+ TCTCTCTCTT TTACCATCCC CGATCATTAC TCACCCCAAT GGTTATATAT ATCTCTCTCT CTCTCTGCCG   
  
  
+ TGTCATGTGT ACGTGGACAT ACATCTCTCT CTCTCGGCTT CACCAATAAA TCATAAGTGT TCAGATCTAT   
  
  
+ CTCTCTCTTT GATCTCTTCA ACCGTAGAGT TACGGGTAAC AACAAGAAAG AGAGAGACGA TAGGCTGTTA   
  
  
+ ACTTTTGGTC TTTGCCAAAG ACCAAAACCC CAACTGAAGA GCGTGTAGAG CAACCCAAGC GGGAGAGGTT   
  
  
+ AAGAAAAAAG TGTGTTTTTC AGGTGCGCGA GAGAAAAAGA GAGAGAGAGA GCACCCGCGA GCACTTAGTG   
  
  
+ GCTGTTTTCT CTCCTTAATT TTCTATCGAA GACGTTTCTC CTAATTTGCT TTCACCTGCG GTCAATTGCT   
  
  
+ CCCGCGAGGA TAGTTAAAGT TGACACAAAA ATGCTAAAAT GGGTATTGTT TTCTTCTTTC CTAAATTTAG   
  
  
+ TTAAATCAAC AACCAACGAA GGCATCAATT AATACACGGA GAAAAATACA GCGGCCCAAA CAAGCAGGGC   
  
  
+ ACGTACTGTG CACGGCTCAG TGACCGTCAA GCTAATTTGG TGTGGCCAAG GATGAAAGTG AGGGACACAA   
  
  
+ ACGAAGAAGG TGAGGTAATA AGGAAAGAAG AATGCCTTTA ATGGAAACCT GTATCGTTTT TATCGTGTCT   
  
  
+ TACTGCCTTT TTAGATTGTC AATGCTGCAC AGTGGGAATT TTCCGCTCAA CCTATAACCG TATGAGCTTA   
  
  
+ CCTCAAAGTA CAACTGGTGT GTAGTTCTAG CTGCCGACGT CGACCACCAA GCACACACCG TGATTGCACA   
  
  
+ TTAACTGGTC TCTCCTCCGC CTCTCGCACC GCCACGTCCT CTATTCCTCC ACTCCACCGG TCTACTGTCG   
  
  
+ CTCGGCTAAA ACACCCCAGG TGCAAGAAAA CGGTTTTCCC TACGGCAAAC TGTCGTAGAG CACCGAGCAC   
  
  
+ TAGAGTTACG GTTGGTCGAC ACGAGGGAAG AGCGTAGCAT ACTAAGTCCT AGGTTGCCGG CTTTTGCCAA   
  
  
+ GTGGAATAGT AGGTCAGTCG AATTACTAGT TTATAAAAGT TGAGAGGTGC CGACTCTACG CGACTGTCTC   
  
  
+ AGAGACATAT ATATTCGCTC TCATGCTTTT GGTAACTCCT AAATCTTCAT CCTCACCCGT AGTGCTCTGT   
  
  
+ ACCCTAACGT TCGCACACCC CAGTGTGAAA ATAAAATTAA ACAGACAAAT CCTGATTATT CAACCTTCAC   
  
  
+ CGCTCAGAAC ACAACCTGGG TTAAAGTCCG GGGGGTAGTA CCAAAGAGGG CGGTGTATTA ATGTCACAGA   
  
  
+ CTGGCTTAAT GTAATAACAA TAAGAACAA  

- GACCCTTTTT GTATCTCTCG CTCTACGGCC GTTTCAGCGA CAAGGCTCTG TGAATCCGTA ACAGACCTCC   
  
  
- GCAGGGACTG TTAGAGAAGA GACAGAGAGA GAAAGAGAGA AGACAAAAGA ATGCGCTGAC TTTTGAGTAA   
  
  
- AGAGAGAGAA AATGGTAGGG GCTAGTAATG AGTGGGGTTA CCAATATATA TAGAGAGAGA GAGAGACGGC   
  
  
- ACAGTACACA TGCACCTGTA TGTAGAGAGA GAGAGCCGAA GTGGTTATTT AGTATTCACA AGTCTAGATA   
  
  
- GAGAGAGAAA CTAGAGAAGT TGGCATCTCA ATGCCCATTG TTGTTCTTTC TCTCTCTGCT ATCCGACAAT   
  
  
- TGAAAACCAG AAACGGTTTC TGGTTTTGGG GTTGACTTCT CGCACATCTC GTTGGGTTCG CCCTCTCCAA   
  
  
- TTCTTTTTTC ACACAAAAAG TCCACGCGCT CTCTTTTTCT CTCTCTCTCT CGTGGGCGCT CGTGAATCAC   
  
  
- CGACAAAAGA GAGGAATTAA AAGATAGCTT CTGCAAAGAG GATTAAACGA AAGTGGACGC CAGTTAACGA   
  
  
- GGGCGCTCCT ATCAATTTCA ACTGTGTTTT TACGATTTTA CCCATAACAA AAGAAGAAAG GATTTAAATC   
  
  
- AATTTAGTTG TTGGTTGCTT CCGTAGTTAA TTATGTGCCT CTTTTTATGT CGCCGGGTTT GTTCGTCCCG   
  
  
- TGCATGACAC GTGCCGAGTC ACTGGCAGTT CGATTAAACC ACACCGGTTC CTACTTTCAC TCCCTGTGTT   
  
  
- TGCTTCTTCC ACTCCATTAT TCCTTTCTTC TTACGGAAAT TACCTTTGGA CATAGCAAAA ATAGCACAGA   
  
  
- ATGACGGAAA AATCTAACAG TTACGACGTG TCACCCTTAA AAGGCGAGTT GGATATTGGC ATACTCGAAT   
  
  
- GGAGTTTCAT GTTGACCACA CATCAAGATC GACGGCTGCA GCTGGTGGTT CGTGTGTGGC ACTAACGTGT   
  
  
- AATTGACCAG AGAGGAGGCG GAGAGCGTGG CGGTGCAGGA GATAAGGAGG TGAGGTGGCC AGATGACAGC   
  
  
- GAGCCGATTT TGTGGGGTCC ACGTTCTTTT GCCAAAAGGG ATGCCGTTTG ACAGCATCTC GTGGCTCGTG   
  
  
- ATCTCAATGC CAACCAGCTG TGCTCCCTTC TCGCATCGTA TGATTCAGGA TCCAACGGCC GAAAACGGTT   
  
  
- CACCTTATCA TCCAGTCAGC TTAATGATCA AATATTTTCA ACTCTCCACG GCTGAGATGC GCTGACAGAG   
  
  
- TCTCTGTATA TATAAGCGAG AGTACGAAAA CCATTGAGGA TTTAGAAGTA GGAGTGGGCA TCACGAGACA   
  
  
- TGGGATTGCA AGCGTGTGGG GTCACACTTT TATTTTAATT TGTCTGTTTA GGACTAATAA GTTGGAAGTG   
  
  
- GCGAGTCTTG TGTTGGACCC AATTTCAGGC CCCCCATCAT GGTTTCTCCC GCCACATAAT TACAGTGTCT   
  
  
- GACCGAATTA CATTATTGTT ATTCTTGTT

+     GT1-motif

| Site Name | Organism | Position | Strand | Matrix score. | sequence | function |
| --- | --- | --- | --- | --- | --- | --- |
| GT1-motif | Arabidopsis thaliana | 1419 | + | 6 | GGTTAA | light responsive element |
| GT1-motif | Arabidopsis thaliana | 417 | + | 6 | GGTTAA | light responsive element |

> 2018/04/13 10:10:12  
+ CTGGGAAAAA CATAGAGAGC GAGATGCCGG CAAAGTCGCT GTTCCGAGAC ACTTAGGCAT TGTCTGGAGG   
  
  
+ CGTCCCTGAC AATCTCTTCT CTGTCTCTCT CTTTCTCTCT TCTGTTTTCT TACGCGACTG AAAACTCATT   
  
  
+ TCTCTCTCTT TTACCATCCC CGATCATTAC TCACCCCAAT GGTTATATAT ATCTCTCTCT CTCTCTGCCG   
  
  
+ TGTCATGTGT ACGTGGACAT ACATCTCTCT CTCTCGGCTT CACCAATAAA TCATAAGTGT TCAGATCTAT   
  
  
+ CTCTCTCTTT GATCTCTTCA ACCGTAGAGT TACGGGTAAC AACAAGAAAG AGAGAGACGA TAGGCTGTTA   
  
  
+ ACTTTTGGTC TTTGCCAAAG ACCAAAACCC CAACTGAAGA GCGTGTAGAG CAACCCAAGC GGGAGAGGTT   
  
  
+ AAGAAAAAAG TGTGTTTTTC AGGTGCGCGA GAGAAAAAGA GAGAGAGAGA GCACCCGCGA GCACTTAGTG   
  
  
+ GCTGTTTTCT CTCCTTAATT TTCTATCGAA GACGTTTCTC CTAATTTGCT TTCACCTGCG GTCAATTGCT   
  
  
+ CCCGCGAGGA TAGTTAAAGT TGACACAAAA ATGCTAAAAT GGGTATTGTT TTCTTCTTTC CTAAATTTAG   
  
  
+ TTAAATCAAC AACCAACGAA GGCATCAATT AATACACGGA GAAAAATACA GCGGCCCAAA CAAGCAGGGC   
  
  
+ ACGTACTGTG CACGGCTCAG TGACCGTCAA GCTAATTTGG TGTGGCCAAG GATGAAAGTG AGGGACACAA   
  
  
+ ACGAAGAAGG TGAGGTAATA AGGAAAGAAG AATGCCTTTA ATGGAAACCT GTATCGTTTT TATCGTGTCT   
  
  
+ TACTGCCTTT TTAGATTGTC AATGCTGCAC AGTGGGAATT TTCCGCTCAA CCTATAACCG TATGAGCTTA   
  
  
+ CCTCAAAGTA CAACTGGTGT GTAGTTCTAG CTGCCGACGT CGACCACCAA GCACACACCG TGATTGCACA   
  
  
+ TTAACTGGTC TCTCCTCCGC CTCTCGCACC GCCACGTCCT CTATTCCTCC ACTCCACCGG TCTACTGTCG   
  
  
+ CTCGGCTAAA ACACCCCAGG TGCAAGAAAA CGGTTTTCCC TACGGCAAAC TGTCGTAGAG CACCGAGCAC   
  
  
+ TAGAGTTACG GTTGGTCGAC ACGAGGGAAG AGCGTAGCAT ACTAAGTCCT AGGTTGCCGG CTTTTGCCAA   
  
  
+ GTGGAATAGT AGGTCAGTCG AATTACTAGT TTATAAAAGT TGAGAGGTGC CGACTCTACG CGACTGTCTC   
  
  
+ AGAGACATAT ATATTCGCTC TCATGCTTTT GGTAACTCCT AAATCTTCAT CCTCACCCGT AGTGCTCTGT   
  
  
+ ACCCTAACGT TCGCACACCC CAGTGTGAAA ATAAAATTAA ACAGACAAAT CCTGATTATT CAACCTTCAC   
  
  
+ CGCTCAGAAC ACAACCTGGG TTAAAGTCCG GGGGGTAGTA CCAAAGAGGG CGGTGTATTA ATGTCACAGA   
  
  
+ CTGGCTTAAT GTAATAACAA TAAGAACAA  

- GACCCTTTTT GTATCTCTCG CTCTACGGCC GTTTCAGCGA CAAGGCTCTG TGAATCCGTA ACAGACCTCC   
  
  
- GCAGGGACTG TTAGAGAAGA GACAGAGAGA GAAAGAGAGA AGACAAAAGA ATGCGCTGAC TTTTGAGTAA   
  
  
- AGAGAGAGAA AATGGTAGGG GCTAGTAATG AGTGGGGTTA CCAATATATA TAGAGAGAGA GAGAGACGGC   
  
  
- ACAGTACACA TGCACCTGTA TGTAGAGAGA GAGAGCCGAA GTGGTTATTT AGTATTCACA AGTCTAGATA   
  
  
- GAGAGAGAAA CTAGAGAAGT TGGCATCTCA ATGCCCATTG TTGTTCTTTC TCTCTCTGCT ATCCGACAAT   
  
  
- TGAAAACCAG AAACGGTTTC TGGTTTTGGG GTTGACTTCT CGCACATCTC GTTGGGTTCG CCCTCTCCAA   
  
  
- TTCTTTTTTC ACACAAAAAG TCCACGCGCT CTCTTTTTCT CTCTCTCTCT CGTGGGCGCT CGTGAATCAC   
  
  
- CGACAAAAGA GAGGAATTAA AAGATAGCTT CTGCAAAGAG GATTAAACGA AAGTGGACGC CAGTTAACGA   
  
  
- GGGCGCTCCT ATCAATTTCA ACTGTGTTTT TACGATTTTA CCCATAACAA AAGAAGAAAG GATTTAAATC   
  
  
- AATTTAGTTG TTGGTTGCTT CCGTAGTTAA TTATGTGCCT CTTTTTATGT CGCCGGGTTT GTTCGTCCCG   
  
  
- TGCATGACAC GTGCCGAGTC ACTGGCAGTT CGATTAAACC ACACCGGTTC CTACTTTCAC TCCCTGTGTT   
  
  
- TGCTTCTTCC ACTCCATTAT TCCTTTCTTC TTACGGAAAT TACCTTTGGA CATAGCAAAA ATAGCACAGA   
  
  
- ATGACGGAAA AATCTAACAG TTACGACGTG TCACCCTTAA AAGGCGAGTT GGATATTGGC ATACTCGAAT   
  
  
- GGAGTTTCAT GTTGACCACA CATCAAGATC GACGGCTGCA GCTGGTGGTT CGTGTGTGGC ACTAACGTGT   
  
  
- AATTGACCAG AGAGGAGGCG GAGAGCGTGG CGGTGCAGGA GATAAGGAGG TGAGGTGGCC AGATGACAGC   
  
  
- GAGCCGATTT TGTGGGGTCC ACGTTCTTTT GCCAAAAGGG ATGCCGTTTG ACAGCATCTC GTGGCTCGTG   
  
  
- ATCTCAATGC CAACCAGCTG TGCTCCCTTC TCGCATCGTA TGATTCAGGA TCCAACGGCC GAAAACGGTT   
  
  
- CACCTTATCA TCCAGTCAGC TTAATGATCA AATATTTTCA ACTCTCCACG GCTGAGATGC GCTGACAGAG   
  
  
- TCTCTGTATA TATAAGCGAG AGTACGAAAA CCATTGAGGA TTTAGAAGTA GGAGTGGGCA TCACGAGACA   
  
  
- TGGGATTGCA AGCGTGTGGG GTCACACTTT TATTTTAATT TGTCTGTTTA GGACTAATAA GTTGGAAGTG   
  
  
- GCGAGTCTTG TGTTGGACCC AATTTCAGGC CCCCCATCAT GGTTTCTCCC GCCACATAAT TACAGTGTCT   
  
  
- GACCGAATTA CATTATTGTT ATTCTTGTT

+     I-box

| Site Name | Organism | Position | Strand | Matrix score. | sequence | function |
| --- | --- | --- | --- | --- | --- | --- |
| I-box | Nicotiana plumbaginifolia | 1278 | + | 9 | CTCTTATGCT | part of a light responsive element |

> 2018/04/13 10:10:12  
+ CTGGGAAAAA CATAGAGAGC GAGATGCCGG CAAAGTCGCT GTTCCGAGAC ACTTAGGCAT TGTCTGGAGG   
  
  
+ CGTCCCTGAC AATCTCTTCT CTGTCTCTCT CTTTCTCTCT TCTGTTTTCT TACGCGACTG AAAACTCATT   
  
  
+ TCTCTCTCTT TTACCATCCC CGATCATTAC TCACCCCAAT GGTTATATAT ATCTCTCTCT CTCTCTGCCG   
  
  
+ TGTCATGTGT ACGTGGACAT ACATCTCTCT CTCTCGGCTT CACCAATAAA TCATAAGTGT TCAGATCTAT   
  
  
+ CTCTCTCTTT GATCTCTTCA ACCGTAGAGT TACGGGTAAC AACAAGAAAG AGAGAGACGA TAGGCTGTTA   
  
  
+ ACTTTTGGTC TTTGCCAAAG ACCAAAACCC CAACTGAAGA GCGTGTAGAG CAACCCAAGC GGGAGAGGTT   
  
  
+ AAGAAAAAAG TGTGTTTTTC AGGTGCGCGA GAGAAAAAGA GAGAGAGAGA GCACCCGCGA GCACTTAGTG   
  
  
+ GCTGTTTTCT CTCCTTAATT TTCTATCGAA GACGTTTCTC CTAATTTGCT TTCACCTGCG GTCAATTGCT   
  
  
+ CCCGCGAGGA TAGTTAAAGT TGACACAAAA ATGCTAAAAT GGGTATTGTT TTCTTCTTTC CTAAATTTAG   
  
  
+ TTAAATCAAC AACCAACGAA GGCATCAATT AATACACGGA GAAAAATACA GCGGCCCAAA CAAGCAGGGC   
  
  
+ ACGTACTGTG CACGGCTCAG TGACCGTCAA GCTAATTTGG TGTGGCCAAG GATGAAAGTG AGGGACACAA   
  
  
+ ACGAAGAAGG TGAGGTAATA AGGAAAGAAG AATGCCTTTA ATGGAAACCT GTATCGTTTT TATCGTGTCT   
  
  
+ TACTGCCTTT TTAGATTGTC AATGCTGCAC AGTGGGAATT TTCCGCTCAA CCTATAACCG TATGAGCTTA   
  
  
+ CCTCAAAGTA CAACTGGTGT GTAGTTCTAG CTGCCGACGT CGACCACCAA GCACACACCG TGATTGCACA   
  
  
+ TTAACTGGTC TCTCCTCCGC CTCTCGCACC GCCACGTCCT CTATTCCTCC ACTCCACCGG TCTACTGTCG   
  
  
+ CTCGGCTAAA ACACCCCAGG TGCAAGAAAA CGGTTTTCCC TACGGCAAAC TGTCGTAGAG CACCGAGCAC   
  
  
+ TAGAGTTACG GTTGGTCGAC ACGAGGGAAG AGCGTAGCAT ACTAAGTCCT AGGTTGCCGG CTTTTGCCAA   
  
  
+ GTGGAATAGT AGGTCAGTCG AATTACTAGT TTATAAAAGT TGAGAGGTGC CGACTCTACG CGACTGTCTC   
  
  
+ AGAGACATAT ATATTCGCTC TCATGCTTTT GGTAACTCCT AAATCTTCAT CCTCACCCGT AGTGCTCTGT   
  
  
+ ACCCTAACGT TCGCACACCC CAGTGTGAAA ATAAAATTAA ACAGACAAAT CCTGATTATT CAACCTTCAC   
  
  
+ CGCTCAGAAC ACAACCTGGG TTAAAGTCCG GGGGGTAGTA CCAAAGAGGG CGGTGTATTA ATGTCACAGA   
  
  
+ CTGGCTTAAT GTAATAACAA TAAGAACAA  

- GACCCTTTTT GTATCTCTCG CTCTACGGCC GTTTCAGCGA CAAGGCTCTG TGAATCCGTA ACAGACCTCC   
  
  
- GCAGGGACTG TTAGAGAAGA GACAGAGAGA GAAAGAGAGA AGACAAAAGA ATGCGCTGAC TTTTGAGTAA   
  
  
- AGAGAGAGAA AATGGTAGGG GCTAGTAATG AGTGGGGTTA CCAATATATA TAGAGAGAGA GAGAGACGGC   
  
  
- ACAGTACACA TGCACCTGTA TGTAGAGAGA GAGAGCCGAA GTGGTTATTT AGTATTCACA AGTCTAGATA   
  
  
- GAGAGAGAAA CTAGAGAAGT TGGCATCTCA ATGCCCATTG TTGTTCTTTC TCTCTCTGCT ATCCGACAAT   
  
  
- TGAAAACCAG AAACGGTTTC TGGTTTTGGG GTTGACTTCT CGCACATCTC GTTGGGTTCG CCCTCTCCAA   
  
  
- TTCTTTTTTC ACACAAAAAG TCCACGCGCT CTCTTTTTCT CTCTCTCTCT CGTGGGCGCT CGTGAATCAC   
  
  
- CGACAAAAGA GAGGAATTAA AAGATAGCTT CTGCAAAGAG GATTAAACGA AAGTGGACGC CAGTTAACGA   
  
  
- GGGCGCTCCT ATCAATTTCA ACTGTGTTTT TACGATTTTA CCCATAACAA AAGAAGAAAG GATTTAAATC   
  
  
- AATTTAGTTG TTGGTTGCTT CCGTAGTTAA TTATGTGCCT CTTTTTATGT CGCCGGGTTT GTTCGTCCCG   
  
  
- TGCATGACAC GTGCCGAGTC ACTGGCAGTT CGATTAAACC ACACCGGTTC CTACTTTCAC TCCCTGTGTT   
  
  
- TGCTTCTTCC ACTCCATTAT TCCTTTCTTC TTACGGAAAT TACCTTTGGA CATAGCAAAA ATAGCACAGA   
  
  
- ATGACGGAAA AATCTAACAG TTACGACGTG TCACCCTTAA AAGGCGAGTT GGATATTGGC ATACTCGAAT   
  
  
- GGAGTTTCAT GTTGACCACA CATCAAGATC GACGGCTGCA GCTGGTGGTT CGTGTGTGGC ACTAACGTGT   
  
  
- AATTGACCAG AGAGGAGGCG GAGAGCGTGG CGGTGCAGGA GATAAGGAGG TGAGGTGGCC AGATGACAGC   
  
  
- GAGCCGATTT TGTGGGGTCC ACGTTCTTTT GCCAAAAGGG ATGCCGTTTG ACAGCATCTC GTGGCTCGTG   
  
  
- ATCTCAATGC CAACCAGCTG TGCTCCCTTC TCGCATCGTA TGATTCAGGA TCCAACGGCC GAAAACGGTT   
  
  
- CACCTTATCA TCCAGTCAGC TTAATGATCA AATATTTTCA ACTCTCCACG GCTGAGATGC GCTGACAGAG   
  
  
- TCTCTGTATA TATAAGCGAG AGTACGAAAA CCATTGAGGA TTTAGAAGTA GGAGTGGGCA TCACGAGACA   
  
  
- TGGGATTGCA AGCGTGTGGG GTCACACTTT TATTTTAATT TGTCTGTTTA GGACTAATAA GTTGGAAGTG   
  
  
- GCGAGTCTTG TGTTGGACCC AATTTCAGGC CCCCCATCAT GGTTTCTCCC GCCACATAAT TACAGTGTCT   
  
  
- GACCGAATTA CATTATTGTT ATTCTTGTT

+     MBS

| Site Name | Organism | Position | Strand | Matrix score. | sequence | function |
| --- | --- | --- | --- | --- | --- | --- |
| MBS | Zea mays | 549 | + | 6 | CGGTCA | MYB Binding Site |
| MBS | Arabidopsis thaliana | 982 | + | 6 | TAACTG | MYB binding site involved in drought-inducibility |
| MBS | Arabidopsis thaliana | 921 | + | 6 | CAACTG | MYB binding site involved in drought-inducibility |
| MBS | Zea mays | 721 | - | 6 | CGGTCA | MYB Binding Site |
| MBS | Arabidopsis thaliana | 381 | + | 6 | CAACTG | MYB binding site involved in drought-inducibility |

> 2018/04/13 10:10:12  
+ CTGGGAAAAA CATAGAGAGC GAGATGCCGG CAAAGTCGCT GTTCCGAGAC ACTTAGGCAT TGTCTGGAGG   
  
  
+ CGTCCCTGAC AATCTCTTCT CTGTCTCTCT CTTTCTCTCT TCTGTTTTCT TACGCGACTG AAAACTCATT   
  
  
+ TCTCTCTCTT TTACCATCCC CGATCATTAC TCACCCCAAT GGTTATATAT ATCTCTCTCT CTCTCTGCCG   
  
  
+ TGTCATGTGT ACGTGGACAT ACATCTCTCT CTCTCGGCTT CACCAATAAA TCATAAGTGT TCAGATCTAT   
  
  
+ CTCTCTCTTT GATCTCTTCA ACCGTAGAGT TACGGGTAAC AACAAGAAAG AGAGAGACGA TAGGCTGTTA   
  
  
+ ACTTTTGGTC TTTGCCAAAG ACCAAAACCC CAACTGAAGA GCGTGTAGAG CAACCCAAGC GGGAGAGGTT   
  
  
+ AAGAAAAAAG TGTGTTTTTC AGGTGCGCGA GAGAAAAAGA GAGAGAGAGA GCACCCGCGA GCACTTAGTG   
  
  
+ GCTGTTTTCT CTCCTTAATT TTCTATCGAA GACGTTTCTC CTAATTTGCT TTCACCTGCG GTCAATTGCT   
  
  
+ CCCGCGAGGA TAGTTAAAGT TGACACAAAA ATGCTAAAAT GGGTATTGTT TTCTTCTTTC CTAAATTTAG   
  
  
+ TTAAATCAAC AACCAACGAA GGCATCAATT AATACACGGA GAAAAATACA GCGGCCCAAA CAAGCAGGGC   
  
  
+ ACGTACTGTG CACGGCTCAG TGACCGTCAA GCTAATTTGG TGTGGCCAAG GATGAAAGTG AGGGACACAA   
  
  
+ ACGAAGAAGG TGAGGTAATA AGGAAAGAAG AATGCCTTTA ATGGAAACCT GTATCGTTTT TATCGTGTCT   
  
  
+ TACTGCCTTT TTAGATTGTC AATGCTGCAC AGTGGGAATT TTCCGCTCAA CCTATAACCG TATGAGCTTA   
  
  
+ CCTCAAAGTA CAACTGGTGT GTAGTTCTAG CTGCCGACGT CGACCACCAA GCACACACCG TGATTGCACA   
  
  
+ TTAACTGGTC TCTCCTCCGC CTCTCGCACC GCCACGTCCT CTATTCCTCC ACTCCACCGG TCTACTGTCG   
  
  
+ CTCGGCTAAA ACACCCCAGG TGCAAGAAAA CGGTTTTCCC TACGGCAAAC TGTCGTAGAG CACCGAGCAC   
  
  
+ TAGAGTTACG GTTGGTCGAC ACGAGGGAAG AGCGTAGCAT ACTAAGTCCT AGGTTGCCGG CTTTTGCCAA   
  
  
+ GTGGAATAGT AGGTCAGTCG AATTACTAGT TTATAAAAGT TGAGAGGTGC CGACTCTACG CGACTGTCTC   
  
  
+ AGAGACATAT ATATTCGCTC TCATGCTTTT GGTAACTCCT AAATCTTCAT CCTCACCCGT AGTGCTCTGT   
  
  
+ ACCCTAACGT TCGCACACCC CAGTGTGAAA ATAAAATTAA ACAGACAAAT CCTGATTATT CAACCTTCAC   
  
  
+ CGCTCAGAAC ACAACCTGGG TTAAAGTCCG GGGGGTAGTA CCAAAGAGGG CGGTGTATTA ATGTCACAGA   
  
  
+ CTGGCTTAAT GTAATAACAA TAAGAACAA  

- GACCCTTTTT GTATCTCTCG CTCTACGGCC GTTTCAGCGA CAAGGCTCTG TGAATCCGTA ACAGACCTCC   
  
  
- GCAGGGACTG TTAGAGAAGA GACAGAGAGA GAAAGAGAGA AGACAAAAGA ATGCGCTGAC TTTTGAGTAA   
  
  
- AGAGAGAGAA AATGGTAGGG GCTAGTAATG AGTGGGGTTA CCAATATATA TAGAGAGAGA GAGAGACGGC   
  
  
- ACAGTACACA TGCACCTGTA TGTAGAGAGA GAGAGCCGAA GTGGTTATTT AGTATTCACA AGTCTAGATA   
  
  
- GAGAGAGAAA CTAGAGAAGT TGGCATCTCA ATGCCCATTG TTGTTCTTTC TCTCTCTGCT ATCCGACAAT   
  
  
- TGAAAACCAG AAACGGTTTC TGGTTTTGGG GTTGACTTCT CGCACATCTC GTTGGGTTCG CCCTCTCCAA   
  
  
- TTCTTTTTTC ACACAAAAAG TCCACGCGCT CTCTTTTTCT CTCTCTCTCT CGTGGGCGCT CGTGAATCAC   
  
  
- CGACAAAAGA GAGGAATTAA AAGATAGCTT CTGCAAAGAG GATTAAACGA AAGTGGACGC CAGTTAACGA   
  
  
- GGGCGCTCCT ATCAATTTCA ACTGTGTTTT TACGATTTTA CCCATAACAA AAGAAGAAAG GATTTAAATC   
  
  
- AATTTAGTTG TTGGTTGCTT CCGTAGTTAA TTATGTGCCT CTTTTTATGT CGCCGGGTTT GTTCGTCCCG   
  
  
- TGCATGACAC GTGCCGAGTC ACTGGCAGTT CGATTAAACC ACACCGGTTC CTACTTTCAC TCCCTGTGTT   
  
  
- TGCTTCTTCC ACTCCATTAT TCCTTTCTTC TTACGGAAAT TACCTTTGGA CATAGCAAAA ATAGCACAGA   
  
  
- ATGACGGAAA AATCTAACAG TTACGACGTG TCACCCTTAA AAGGCGAGTT GGATATTGGC ATACTCGAAT   
  
  
- GGAGTTTCAT GTTGACCACA CATCAAGATC GACGGCTGCA GCTGGTGGTT CGTGTGTGGC ACTAACGTGT   
  
  
- AATTGACCAG AGAGGAGGCG GAGAGCGTGG CGGTGCAGGA GATAAGGAGG TGAGGTGGCC AGATGACAGC   
  
  
- GAGCCGATTT TGTGGGGTCC ACGTTCTTTT GCCAAAAGGG ATGCCGTTTG ACAGCATCTC GTGGCTCGTG   
  
  
- ATCTCAATGC CAACCAGCTG TGCTCCCTTC TCGCATCGTA TGATTCAGGA TCCAACGGCC GAAAACGGTT   
  
  
- CACCTTATCA TCCAGTCAGC TTAATGATCA AATATTTTCA ACTCTCCACG GCTGAGATGC GCTGACAGAG   
  
  
- TCTCTGTATA TATAAGCGAG AGTACGAAAA CCATTGAGGA TTTAGAAGTA GGAGTGGGCA TCACGAGACA   
  
  
- TGGGATTGCA AGCGTGTGGG GTCACACTTT TATTTTAATT TGTCTGTTTA GGACTAATAA GTTGGAAGTG   
  
  
- GCGAGTCTTG TGTTGGACCC AATTTCAGGC CCCCCATCAT GGTTTCTCCC GCCACATAAT TACAGTGTCT   
  
  
- GACCGAATTA CATTATTGTT ATTCTTGTT

+     MNF1

| Site Name | Organism | Position | Strand | Matrix score. | sequence | function |
| --- | --- | --- | --- | --- | --- | --- |
| MNF1 | Zea mays | 695 | - | 6.5 | GTGCCC(A/T)(A/T) | light responsive element |

> 2018/04/13 10:10:12  
+ CTGGGAAAAA CATAGAGAGC GAGATGCCGG CAAAGTCGCT GTTCCGAGAC ACTTAGGCAT TGTCTGGAGG   
  
  
+ CGTCCCTGAC AATCTCTTCT CTGTCTCTCT CTTTCTCTCT TCTGTTTTCT TACGCGACTG AAAACTCATT   
  
  
+ TCTCTCTCTT TTACCATCCC CGATCATTAC TCACCCCAAT GGTTATATAT ATCTCTCTCT CTCTCTGCCG   
  
  
+ TGTCATGTGT ACGTGGACAT ACATCTCTCT CTCTCGGCTT CACCAATAAA TCATAAGTGT TCAGATCTAT   
  
  
+ CTCTCTCTTT GATCTCTTCA ACCGTAGAGT TACGGGTAAC AACAAGAAAG AGAGAGACGA TAGGCTGTTA   
  
  
+ ACTTTTGGTC TTTGCCAAAG ACCAAAACCC CAACTGAAGA GCGTGTAGAG CAACCCAAGC GGGAGAGGTT   
  
  
+ AAGAAAAAAG TGTGTTTTTC AGGTGCGCGA GAGAAAAAGA GAGAGAGAGA GCACCCGCGA GCACTTAGTG   
  
  
+ GCTGTTTTCT CTCCTTAATT TTCTATCGAA GACGTTTCTC CTAATTTGCT TTCACCTGCG GTCAATTGCT   
  
  
+ CCCGCGAGGA TAGTTAAAGT TGACACAAAA ATGCTAAAAT GGGTATTGTT TTCTTCTTTC CTAAATTTAG   
  
  
+ TTAAATCAAC AACCAACGAA GGCATCAATT AATACACGGA GAAAAATACA GCGGCCCAAA CAAGCAGGGC   
  
  
+ ACGTACTGTG CACGGCTCAG TGACCGTCAA GCTAATTTGG TGTGGCCAAG GATGAAAGTG AGGGACACAA   
  
  
+ ACGAAGAAGG TGAGGTAATA AGGAAAGAAG AATGCCTTTA ATGGAAACCT GTATCGTTTT TATCGTGTCT   
  
  
+ TACTGCCTTT TTAGATTGTC AATGCTGCAC AGTGGGAATT TTCCGCTCAA CCTATAACCG TATGAGCTTA   
  
  
+ CCTCAAAGTA CAACTGGTGT GTAGTTCTAG CTGCCGACGT CGACCACCAA GCACACACCG TGATTGCACA   
  
  
+ TTAACTGGTC TCTCCTCCGC CTCTCGCACC GCCACGTCCT CTATTCCTCC ACTCCACCGG TCTACTGTCG   
  
  
+ CTCGGCTAAA ACACCCCAGG TGCAAGAAAA CGGTTTTCCC TACGGCAAAC TGTCGTAGAG CACCGAGCAC   
  
  
+ TAGAGTTACG GTTGGTCGAC ACGAGGGAAG AGCGTAGCAT ACTAAGTCCT AGGTTGCCGG CTTTTGCCAA   
  
  
+ GTGGAATAGT AGGTCAGTCG AATTACTAGT TTATAAAAGT TGAGAGGTGC CGACTCTACG CGACTGTCTC   
  
  
+ AGAGACATAT ATATTCGCTC TCATGCTTTT GGTAACTCCT AAATCTTCAT CCTCACCCGT AGTGCTCTGT   
  
  
+ ACCCTAACGT TCGCACACCC CAGTGTGAAA ATAAAATTAA ACAGACAAAT CCTGATTATT CAACCTTCAC   
  
  
+ CGCTCAGAAC ACAACCTGGG TTAAAGTCCG GGGGGTAGTA CCAAAGAGGG CGGTGTATTA ATGTCACAGA   
  
  
+ CTGGCTTAAT GTAATAACAA TAAGAACAA  

- GACCCTTTTT GTATCTCTCG CTCTACGGCC GTTTCAGCGA CAAGGCTCTG TGAATCCGTA ACAGACCTCC   
  
  
- GCAGGGACTG TTAGAGAAGA GACAGAGAGA GAAAGAGAGA AGACAAAAGA ATGCGCTGAC TTTTGAGTAA   
  
  
- AGAGAGAGAA AATGGTAGGG GCTAGTAATG AGTGGGGTTA CCAATATATA TAGAGAGAGA GAGAGACGGC   
  
  
- ACAGTACACA TGCACCTGTA TGTAGAGAGA GAGAGCCGAA GTGGTTATTT AGTATTCACA AGTCTAGATA   
  
  
- GAGAGAGAAA CTAGAGAAGT TGGCATCTCA ATGCCCATTG TTGTTCTTTC TCTCTCTGCT ATCCGACAAT   
  
  
- TGAAAACCAG AAACGGTTTC TGGTTTTGGG GTTGACTTCT CGCACATCTC GTTGGGTTCG CCCTCTCCAA   
  
  
- TTCTTTTTTC ACACAAAAAG TCCACGCGCT CTCTTTTTCT CTCTCTCTCT CGTGGGCGCT CGTGAATCAC   
  
  
- CGACAAAAGA GAGGAATTAA AAGATAGCTT CTGCAAAGAG GATTAAACGA AAGTGGACGC CAGTTAACGA   
  
  
- GGGCGCTCCT ATCAATTTCA ACTGTGTTTT TACGATTTTA CCCATAACAA AAGAAGAAAG GATTTAAATC   
  
  
- AATTTAGTTG TTGGTTGCTT CCGTAGTTAA TTATGTGCCT CTTTTTATGT CGCCGGGTTT GTTCGTCCCG   
  
  
- TGCATGACAC GTGCCGAGTC ACTGGCAGTT CGATTAAACC ACACCGGTTC CTACTTTCAC TCCCTGTGTT   
  
  
- TGCTTCTTCC ACTCCATTAT TCCTTTCTTC TTACGGAAAT TACCTTTGGA CATAGCAAAA ATAGCACAGA   
  
  
- ATGACGGAAA AATCTAACAG TTACGACGTG TCACCCTTAA AAGGCGAGTT GGATATTGGC ATACTCGAAT   
  
  
- GGAGTTTCAT GTTGACCACA CATCAAGATC GACGGCTGCA GCTGGTGGTT CGTGTGTGGC ACTAACGTGT   
  
  
- AATTGACCAG AGAGGAGGCG GAGAGCGTGG CGGTGCAGGA GATAAGGAGG TGAGGTGGCC AGATGACAGC   
  
  
- GAGCCGATTT TGTGGGGTCC ACGTTCTTTT GCCAAAAGGG ATGCCGTTTG ACAGCATCTC GTGGCTCGTG   
  
  
- ATCTCAATGC CAACCAGCTG TGCTCCCTTC TCGCATCGTA TGATTCAGGA TCCAACGGCC GAAAACGGTT   
  
  
- CACCTTATCA TCCAGTCAGC TTAATGATCA AATATTTTCA ACTCTCCACG GCTGAGATGC GCTGACAGAG   
  
  
- TCTCTGTATA TATAAGCGAG AGTACGAAAA CCATTGAGGA TTTAGAAGTA GGAGTGGGCA TCACGAGACA   
  
  
- TGGGATTGCA AGCGTGTGGG GTCACACTTT TATTTTAATT TGTCTGTTTA GGACTAATAA GTTGGAAGTG   
  
  
- GCGAGTCTTG TGTTGGACCC AATTTCAGGC CCCCCATCAT GGTTTCTCCC GCCACATAAT TACAGTGTCT   
  
  
- GACCGAATTA CATTATTGTT ATTCTTGTT

+     Skn-1\_motif

| Site Name | Organism | Position | Strand | Matrix score. | sequence | function |
| --- | --- | --- | --- | --- | --- | --- |
| Skn-1\_motif | Oryza sativa | 212 | + | 5 | GTCAT | cis-acting regulatory element required for endosperm expression |

> 2018/04/13 10:10:12  
+ CTGGGAAAAA CATAGAGAGC GAGATGCCGG CAAAGTCGCT GTTCCGAGAC ACTTAGGCAT TGTCTGGAGG   
  
  
+ CGTCCCTGAC AATCTCTTCT CTGTCTCTCT CTTTCTCTCT TCTGTTTTCT TACGCGACTG AAAACTCATT   
  
  
+ TCTCTCTCTT TTACCATCCC CGATCATTAC TCACCCCAAT GGTTATATAT ATCTCTCTCT CTCTCTGCCG   
  
  
+ TGTCATGTGT ACGTGGACAT ACATCTCTCT CTCTCGGCTT CACCAATAAA TCATAAGTGT TCAGATCTAT   
  
  
+ CTCTCTCTTT GATCTCTTCA ACCGTAGAGT TACGGGTAAC AACAAGAAAG AGAGAGACGA TAGGCTGTTA   
  
  
+ ACTTTTGGTC TTTGCCAAAG ACCAAAACCC CAACTGAAGA GCGTGTAGAG CAACCCAAGC GGGAGAGGTT   
  
  
+ AAGAAAAAAG TGTGTTTTTC AGGTGCGCGA GAGAAAAAGA GAGAGAGAGA GCACCCGCGA GCACTTAGTG   
  
  
+ GCTGTTTTCT CTCCTTAATT TTCTATCGAA GACGTTTCTC CTAATTTGCT TTCACCTGCG GTCAATTGCT   
  
  
+ CCCGCGAGGA TAGTTAAAGT TGACACAAAA ATGCTAAAAT GGGTATTGTT TTCTTCTTTC CTAAATTTAG   
  
  
+ TTAAATCAAC AACCAACGAA GGCATCAATT AATACACGGA GAAAAATACA GCGGCCCAAA CAAGCAGGGC   
  
  
+ ACGTACTGTG CACGGCTCAG TGACCGTCAA GCTAATTTGG TGTGGCCAAG GATGAAAGTG AGGGACACAA   
  
  
+ ACGAAGAAGG TGAGGTAATA AGGAAAGAAG AATGCCTTTA ATGGAAACCT GTATCGTTTT TATCGTGTCT   
  
  
+ TACTGCCTTT TTAGATTGTC AATGCTGCAC AGTGGGAATT TTCCGCTCAA CCTATAACCG TATGAGCTTA   
  
  
+ CCTCAAAGTA CAACTGGTGT GTAGTTCTAG CTGCCGACGT CGACCACCAA GCACACACCG TGATTGCACA   
  
  
+ TTAACTGGTC TCTCCTCCGC CTCTCGCACC GCCACGTCCT CTATTCCTCC ACTCCACCGG TCTACTGTCG   
  
  
+ CTCGGCTAAA ACACCCCAGG TGCAAGAAAA CGGTTTTCCC TACGGCAAAC TGTCGTAGAG CACCGAGCAC   
  
  
+ TAGAGTTACG GTTGGTCGAC ACGAGGGAAG AGCGTAGCAT ACTAAGTCCT AGGTTGCCGG CTTTTGCCAA   
  
  
+ GTGGAATAGT AGGTCAGTCG AATTACTAGT TTATAAAAGT TGAGAGGTGC CGACTCTACG CGACTGTCTC   
  
  
+ AGAGACATAT ATATTCGCTC TCATGCTTTT GGTAACTCCT AAATCTTCAT CCTCACCCGT AGTGCTCTGT   
  
  
+ ACCCTAACGT TCGCACACCC CAGTGTGAAA ATAAAATTAA ACAGACAAAT CCTGATTATT CAACCTTCAC   
  
  
+ CGCTCAGAAC ACAACCTGGG TTAAAGTCCG GGGGGTAGTA CCAAAGAGGG CGGTGTATTA ATGTCACAGA   
  
  
+ CTGGCTTAAT GTAATAACAA TAAGAACAA  

- GACCCTTTTT GTATCTCTCG CTCTACGGCC GTTTCAGCGA CAAGGCTCTG TGAATCCGTA ACAGACCTCC   
  
  
- GCAGGGACTG TTAGAGAAGA GACAGAGAGA GAAAGAGAGA AGACAAAAGA ATGCGCTGAC TTTTGAGTAA   
  
  
- AGAGAGAGAA AATGGTAGGG GCTAGTAATG AGTGGGGTTA CCAATATATA TAGAGAGAGA GAGAGACGGC   
  
  
- ACAGTACACA TGCACCTGTA TGTAGAGAGA GAGAGCCGAA GTGGTTATTT AGTATTCACA AGTCTAGATA   
  
  
- GAGAGAGAAA CTAGAGAAGT TGGCATCTCA ATGCCCATTG TTGTTCTTTC TCTCTCTGCT ATCCGACAAT   
  
  
- TGAAAACCAG AAACGGTTTC TGGTTTTGGG GTTGACTTCT CGCACATCTC GTTGGGTTCG CCCTCTCCAA   
  
  
- TTCTTTTTTC ACACAAAAAG TCCACGCGCT CTCTTTTTCT CTCTCTCTCT CGTGGGCGCT CGTGAATCAC   
  
  
- CGACAAAAGA GAGGAATTAA AAGATAGCTT CTGCAAAGAG GATTAAACGA AAGTGGACGC CAGTTAACGA   
  
  
- GGGCGCTCCT ATCAATTTCA ACTGTGTTTT TACGATTTTA CCCATAACAA AAGAAGAAAG GATTTAAATC   
  
  
- AATTTAGTTG TTGGTTGCTT CCGTAGTTAA TTATGTGCCT CTTTTTATGT CGCCGGGTTT GTTCGTCCCG   
  
  
- TGCATGACAC GTGCCGAGTC ACTGGCAGTT CGATTAAACC ACACCGGTTC CTACTTTCAC TCCCTGTGTT   
  
  
- TGCTTCTTCC ACTCCATTAT TCCTTTCTTC TTACGGAAAT TACCTTTGGA CATAGCAAAA ATAGCACAGA   
  
  
- ATGACGGAAA AATCTAACAG TTACGACGTG TCACCCTTAA AAGGCGAGTT GGATATTGGC ATACTCGAAT   
  
  
- GGAGTTTCAT GTTGACCACA CATCAAGATC GACGGCTGCA GCTGGTGGTT CGTGTGTGGC ACTAACGTGT   
  
  
- AATTGACCAG AGAGGAGGCG GAGAGCGTGG CGGTGCAGGA GATAAGGAGG TGAGGTGGCC AGATGACAGC   
  
  
- GAGCCGATTT TGTGGGGTCC ACGTTCTTTT GCCAAAAGGG ATGCCGTTTG ACAGCATCTC GTGGCTCGTG   
  
  
- ATCTCAATGC CAACCAGCTG TGCTCCCTTC TCGCATCGTA TGATTCAGGA TCCAACGGCC GAAAACGGTT   
  
  
- CACCTTATCA TCCAGTCAGC TTAATGATCA AATATTTTCA ACTCTCCACG GCTGAGATGC GCTGACAGAG   
  
  
- TCTCTGTATA TATAAGCGAG AGTACGAAAA CCATTGAGGA TTTAGAAGTA GGAGTGGGCA TCACGAGACA   
  
  
- TGGGATTGCA AGCGTGTGGG GTCACACTTT TATTTTAATT TGTCTGTTTA GGACTAATAA GTTGGAAGTG   
  
  
- GCGAGTCTTG TGTTGGACCC AATTTCAGGC CCCCCATCAT GGTTTCTCCC GCCACATAAT TACAGTGTCT   
  
  
- GACCGAATTA CATTATTGTT ATTCTTGTT

+     Sp1

| Site Name | Organism | Position | Strand | Matrix score. | sequence | function |
| --- | --- | --- | --- | --- | --- | --- |
| Sp1 | Oryza sativa | 1448 | + | 6 | GGGCGG | light responsive element |
| Sp1 | Zea mays | 1430 | - | 5 | CC(G/A)CCC | light responsive element |

> 2018/04/13 10:10:12  
+ CTGGGAAAAA CATAGAGAGC GAGATGCCGG CAAAGTCGCT GTTCCGAGAC ACTTAGGCAT TGTCTGGAGG   
  
  
+ CGTCCCTGAC AATCTCTTCT CTGTCTCTCT CTTTCTCTCT TCTGTTTTCT TACGCGACTG AAAACTCATT   
  
  
+ TCTCTCTCTT TTACCATCCC CGATCATTAC TCACCCCAAT GGTTATATAT ATCTCTCTCT CTCTCTGCCG   
  
  
+ TGTCATGTGT ACGTGGACAT ACATCTCTCT CTCTCGGCTT CACCAATAAA TCATAAGTGT TCAGATCTAT   
  
  
+ CTCTCTCTTT GATCTCTTCA ACCGTAGAGT TACGGGTAAC AACAAGAAAG AGAGAGACGA TAGGCTGTTA   
  
  
+ ACTTTTGGTC TTTGCCAAAG ACCAAAACCC CAACTGAAGA GCGTGTAGAG CAACCCAAGC GGGAGAGGTT   
  
  
+ AAGAAAAAAG TGTGTTTTTC AGGTGCGCGA GAGAAAAAGA GAGAGAGAGA GCACCCGCGA GCACTTAGTG   
  
  
+ GCTGTTTTCT CTCCTTAATT TTCTATCGAA GACGTTTCTC CTAATTTGCT TTCACCTGCG GTCAATTGCT   
  
  
+ CCCGCGAGGA TAGTTAAAGT TGACACAAAA ATGCTAAAAT GGGTATTGTT TTCTTCTTTC CTAAATTTAG   
  
  
+ TTAAATCAAC AACCAACGAA GGCATCAATT AATACACGGA GAAAAATACA GCGGCCCAAA CAAGCAGGGC   
  
  
+ ACGTACTGTG CACGGCTCAG TGACCGTCAA GCTAATTTGG TGTGGCCAAG GATGAAAGTG AGGGACACAA   
  
  
+ ACGAAGAAGG TGAGGTAATA AGGAAAGAAG AATGCCTTTA ATGGAAACCT GTATCGTTTT TATCGTGTCT   
  
  
+ TACTGCCTTT TTAGATTGTC AATGCTGCAC AGTGGGAATT TTCCGCTCAA CCTATAACCG TATGAGCTTA   
  
  
+ CCTCAAAGTA CAACTGGTGT GTAGTTCTAG CTGCCGACGT CGACCACCAA GCACACACCG TGATTGCACA   
  
  
+ TTAACTGGTC TCTCCTCCGC CTCTCGCACC GCCACGTCCT CTATTCCTCC ACTCCACCGG TCTACTGTCG   
  
  
+ CTCGGCTAAA ACACCCCAGG TGCAAGAAAA CGGTTTTCCC TACGGCAAAC TGTCGTAGAG CACCGAGCAC   
  
  
+ TAGAGTTACG GTTGGTCGAC ACGAGGGAAG AGCGTAGCAT ACTAAGTCCT AGGTTGCCGG CTTTTGCCAA   
  
  
+ GTGGAATAGT AGGTCAGTCG AATTACTAGT TTATAAAAGT TGAGAGGTGC CGACTCTACG CGACTGTCTC   
  
  
+ AGAGACATAT ATATTCGCTC TCATGCTTTT GGTAACTCCT AAATCTTCAT CCTCACCCGT AGTGCTCTGT   
  
  
+ ACCCTAACGT TCGCACACCC CAGTGTGAAA ATAAAATTAA ACAGACAAAT CCTGATTATT CAACCTTCAC   
  
  
+ CGCTCAGAAC ACAACCTGGG TTAAAGTCCG GGGGGTAGTA CCAAAGAGGG CGGTGTATTA ATGTCACAGA   
  
  
+ CTGGCTTAAT GTAATAACAA TAAGAACAA  

- GACCCTTTTT GTATCTCTCG CTCTACGGCC GTTTCAGCGA CAAGGCTCTG TGAATCCGTA ACAGACCTCC   
  
  
- GCAGGGACTG TTAGAGAAGA GACAGAGAGA GAAAGAGAGA AGACAAAAGA ATGCGCTGAC TTTTGAGTAA   
  
  
- AGAGAGAGAA AATGGTAGGG GCTAGTAATG AGTGGGGTTA CCAATATATA TAGAGAGAGA GAGAGACGGC   
  
  
- ACAGTACACA TGCACCTGTA TGTAGAGAGA GAGAGCCGAA GTGGTTATTT AGTATTCACA AGTCTAGATA   
  
  
- GAGAGAGAAA CTAGAGAAGT TGGCATCTCA ATGCCCATTG TTGTTCTTTC TCTCTCTGCT ATCCGACAAT   
  
  
- TGAAAACCAG AAACGGTTTC TGGTTTTGGG GTTGACTTCT CGCACATCTC GTTGGGTTCG CCCTCTCCAA   
  
  
- TTCTTTTTTC ACACAAAAAG TCCACGCGCT CTCTTTTTCT CTCTCTCTCT CGTGGGCGCT CGTGAATCAC   
  
  
- CGACAAAAGA GAGGAATTAA AAGATAGCTT CTGCAAAGAG GATTAAACGA AAGTGGACGC CAGTTAACGA   
  
  
- GGGCGCTCCT ATCAATTTCA ACTGTGTTTT TACGATTTTA CCCATAACAA AAGAAGAAAG GATTTAAATC   
  
  
- AATTTAGTTG TTGGTTGCTT CCGTAGTTAA TTATGTGCCT CTTTTTATGT CGCCGGGTTT GTTCGTCCCG   
  
  
- TGCATGACAC GTGCCGAGTC ACTGGCAGTT CGATTAAACC ACACCGGTTC CTACTTTCAC TCCCTGTGTT   
  
  
- TGCTTCTTCC ACTCCATTAT TCCTTTCTTC TTACGGAAAT TACCTTTGGA CATAGCAAAA ATAGCACAGA   
  
  
- ATGACGGAAA AATCTAACAG TTACGACGTG TCACCCTTAA AAGGCGAGTT GGATATTGGC ATACTCGAAT   
  
  
- GGAGTTTCAT GTTGACCACA CATCAAGATC GACGGCTGCA GCTGGTGGTT CGTGTGTGGC ACTAACGTGT   
  
  
- AATTGACCAG AGAGGAGGCG GAGAGCGTGG CGGTGCAGGA GATAAGGAGG TGAGGTGGCC AGATGACAGC   
  
  
- GAGCCGATTT TGTGGGGTCC ACGTTCTTTT GCCAAAAGGG ATGCCGTTTG ACAGCATCTC GTGGCTCGTG   
  
  
- ATCTCAATGC CAACCAGCTG TGCTCCCTTC TCGCATCGTA TGATTCAGGA TCCAACGGCC GAAAACGGTT   
  
  
- CACCTTATCA TCCAGTCAGC TTAATGATCA AATATTTTCA ACTCTCCACG GCTGAGATGC GCTGACAGAG   
  
  
- TCTCTGTATA TATAAGCGAG AGTACGAAAA CCATTGAGGA TTTAGAAGTA GGAGTGGGCA TCACGAGACA   
  
  
- TGGGATTGCA AGCGTGTGGG GTCACACTTT TATTTTAATT TGTCTGTTTA GGACTAATAA GTTGGAAGTG   
  
  
- GCGAGTCTTG TGTTGGACCC AATTTCAGGC CCCCCATCAT GGTTTCTCCC GCCACATAAT TACAGTGTCT   
  
  
- GACCGAATTA CATTATTGTT ATTCTTGTT

+     TATA-box

| Site Name | Organism | Position | Strand | Matrix score. | sequence | function |
| --- | --- | --- | --- | --- | --- | --- |
| TATA-box | Glycine max | 1482 | + | 5 | TAATA | core promoter element around -30 of transcription start |
| TATA-box | Pisum sativum | 1265 | - | 7 | TATATGT | core promoter element around -30 of transcription start |
| TATA-box | Arabidopsis thaliana | 1222 | + | 6 | TATAAA | core promoter element around -30 of transcription start |
| TATA-box | Lycopersicon esculentum | 149 | + | 5 | TTTTA | core promoter element around -30 of transcription start |
| TATA-box | Arabidopsis thaliana | 1270 | - | 4 | TATA | core promoter element around -30 of transcription start |
| TATA-box | Glycine max | 660 | + | 5 | TAATA | core promoter element around -30 of transcription start |
| TATA-box | Arabidopsis thaliana | 186 | + | 4 | TATA | core promoter element around -30 of transcription start |
| TATA-box | Arabidopsis thaliana | 1220 | - | 6 | TATAAA | core promoter element around -30 of transcription start |
| TATA-box | Arabidopsis thaliana | 1221 | - | 5 | TATAA | core promoter element around -30 of transcription start |
| TATA-box | Arabidopsis thaliana | 1268 | - | 4 | TATA | core promoter element around -30 of transcription start |
| TATA-box | Lycopersicon esculentum | 1224 | - | 5 | TTTTA | core promoter element around -30 of transcription start |
| TATA-box | Arabidopsis thaliana | 893 | - | 4 | TATA | core promoter element around -30 of transcription start |
| TATA-box | Arabidopsis thaliana | 183 | - | 7 | TATATAA | core promoter element around -30 of transcription start |
| TATA-box | Glycine max | 786 | + | 5 | TAATA | core promoter element around -30 of transcription start |
| TATA-box | Glycine max | 1456 | - | 5 | TAATA | core promoter element around -30 of transcription start |
| TATA-box | Brassica napus | 1267 | - | 6 | ATATAT | core promoter element around -30 of transcription start |
| TATA-box | Arabidopsis thaliana | 188 | + | 4 | TATA | core promoter element around -30 of transcription start |
| TATA-box | Lycopersicon esculentum | 828 | + | 5 | TTTTA | core promoter element around -30 of transcription start |
| TATA-box | Brassica napus | 1269 | - | 6 | ATATAT | core promoter element around -30 of transcription start |
| TATA-box | Lycopersicon esculentum | 1362 | - | 5 | TTTTA | core promoter element around -30 of transcription start |
| TATA-box | Arabidopsis thaliana | 184 | + | 8 | TATATATA | core promoter element around -30 of transcription start |
| TATA-box | Lycopersicon esculentum | 849 | + | 5 | TTTTA | core promoter element around -30 of transcription start |
| TATA-box | Brassica napus | 187 | + | 6 | ATATAT | core promoter element around -30 of transcription start |
| TATA-box | Lycopersicon esculentum | 1057 | - | 5 | TTTTA | core promoter element around -30 of transcription start |
| TATA-box | Lycopersicon esculentum | 595 | - | 5 | TTTTA | core promoter element around -30 of transcription start |
| TATA-box | Brassica napus | 185 | + | 6 | ATATAT | core promoter element around -30 of transcription start |

> 2018/04/13 10:10:12  
+ CTGGGAAAAA CATAGAGAGC GAGATGCCGG CAAAGTCGCT GTTCCGAGAC ACTTAGGCAT TGTCTGGAGG   
  
  
+ CGTCCCTGAC AATCTCTTCT CTGTCTCTCT CTTTCTCTCT TCTGTTTTCT TACGCGACTG AAAACTCATT   
  
  
+ TCTCTCTCTT TTACCATCCC CGATCATTAC TCACCCCAAT GGTTATATAT ATCTCTCTCT CTCTCTGCCG   
  
  
+ TGTCATGTGT ACGTGGACAT ACATCTCTCT CTCTCGGCTT CACCAATAAA TCATAAGTGT TCAGATCTAT   
  
  
+ CTCTCTCTTT GATCTCTTCA ACCGTAGAGT TACGGGTAAC AACAAGAAAG AGAGAGACGA TAGGCTGTTA   
  
  
+ ACTTTTGGTC TTTGCCAAAG ACCAAAACCC CAACTGAAGA GCGTGTAGAG CAACCCAAGC GGGAGAGGTT   
  
  
+ AAGAAAAAAG TGTGTTTTTC AGGTGCGCGA GAGAAAAAGA GAGAGAGAGA GCACCCGCGA GCACTTAGTG   
  
  
+ GCTGTTTTCT CTCCTTAATT TTCTATCGAA GACGTTTCTC CTAATTTGCT TTCACCTGCG GTCAATTGCT   
  
  
+ CCCGCGAGGA TAGTTAAAGT TGACACAAAA ATGCTAAAAT GGGTATTGTT TTCTTCTTTC CTAAATTTAG   
  
  
+ TTAAATCAAC AACCAACGAA GGCATCAATT AATACACGGA GAAAAATACA GCGGCCCAAA CAAGCAGGGC   
  
  
+ ACGTACTGTG CACGGCTCAG TGACCGTCAA GCTAATTTGG TGTGGCCAAG GATGAAAGTG AGGGACACAA   
  
  
+ ACGAAGAAGG TGAGGTAATA AGGAAAGAAG AATGCCTTTA ATGGAAACCT GTATCGTTTT TATCGTGTCT   
  
  
+ TACTGCCTTT TTAGATTGTC AATGCTGCAC AGTGGGAATT TTCCGCTCAA CCTATAACCG TATGAGCTTA   
  
  
+ CCTCAAAGTA CAACTGGTGT GTAGTTCTAG CTGCCGACGT CGACCACCAA GCACACACCG TGATTGCACA   
  
  
+ TTAACTGGTC TCTCCTCCGC CTCTCGCACC GCCACGTCCT CTATTCCTCC ACTCCACCGG TCTACTGTCG   
  
  
+ CTCGGCTAAA ACACCCCAGG TGCAAGAAAA CGGTTTTCCC TACGGCAAAC TGTCGTAGAG CACCGAGCAC   
  
  
+ TAGAGTTACG GTTGGTCGAC ACGAGGGAAG AGCGTAGCAT ACTAAGTCCT AGGTTGCCGG CTTTTGCCAA   
  
  
+ GTGGAATAGT AGGTCAGTCG AATTACTAGT TTATAAAAGT TGAGAGGTGC CGACTCTACG CGACTGTCTC   
  
  
+ AGAGACATAT ATATTCGCTC TCATGCTTTT GGTAACTCCT AAATCTTCAT CCTCACCCGT AGTGCTCTGT   
  
  
+ ACCCTAACGT TCGCACACCC CAGTGTGAAA ATAAAATTAA ACAGACAAAT CCTGATTATT CAACCTTCAC   
  
  
+ CGCTCAGAAC ACAACCTGGG TTAAAGTCCG GGGGGTAGTA CCAAAGAGGG CGGTGTATTA ATGTCACAGA   
  
  
+ CTGGCTTAAT GTAATAACAA TAAGAACAA  

- GACCCTTTTT GTATCTCTCG CTCTACGGCC GTTTCAGCGA CAAGGCTCTG TGAATCCGTA ACAGACCTCC   
  
  
- GCAGGGACTG TTAGAGAAGA GACAGAGAGA GAAAGAGAGA AGACAAAAGA ATGCGCTGAC TTTTGAGTAA   
  
  
- AGAGAGAGAA AATGGTAGGG GCTAGTAATG AGTGGGGTTA CCAATATATA TAGAGAGAGA GAGAGACGGC   
  
  
- ACAGTACACA TGCACCTGTA TGTAGAGAGA GAGAGCCGAA GTGGTTATTT AGTATTCACA AGTCTAGATA   
  
  
- GAGAGAGAAA CTAGAGAAGT TGGCATCTCA ATGCCCATTG TTGTTCTTTC TCTCTCTGCT ATCCGACAAT   
  
  
- TGAAAACCAG AAACGGTTTC TGGTTTTGGG GTTGACTTCT CGCACATCTC GTTGGGTTCG CCCTCTCCAA   
  
  
- TTCTTTTTTC ACACAAAAAG TCCACGCGCT CTCTTTTTCT CTCTCTCTCT CGTGGGCGCT CGTGAATCAC   
  
  
- CGACAAAAGA GAGGAATTAA AAGATAGCTT CTGCAAAGAG GATTAAACGA AAGTGGACGC CAGTTAACGA   
  
  
- GGGCGCTCCT ATCAATTTCA ACTGTGTTTT TACGATTTTA CCCATAACAA AAGAAGAAAG GATTTAAATC   
  
  
- AATTTAGTTG TTGGTTGCTT CCGTAGTTAA TTATGTGCCT CTTTTTATGT CGCCGGGTTT GTTCGTCCCG   
  
  
- TGCATGACAC GTGCCGAGTC ACTGGCAGTT CGATTAAACC ACACCGGTTC CTACTTTCAC TCCCTGTGTT   
  
  
- TGCTTCTTCC ACTCCATTAT TCCTTTCTTC TTACGGAAAT TACCTTTGGA CATAGCAAAA ATAGCACAGA   
  
  
- ATGACGGAAA AATCTAACAG TTACGACGTG TCACCCTTAA AAGGCGAGTT GGATATTGGC ATACTCGAAT   
  
  
- GGAGTTTCAT GTTGACCACA CATCAAGATC GACGGCTGCA GCTGGTGGTT CGTGTGTGGC ACTAACGTGT   
  
  
- AATTGACCAG AGAGGAGGCG GAGAGCGTGG CGGTGCAGGA GATAAGGAGG TGAGGTGGCC AGATGACAGC   
  
  
- GAGCCGATTT TGTGGGGTCC ACGTTCTTTT GCCAAAAGGG ATGCCGTTTG ACAGCATCTC GTGGCTCGTG   
  
  
- ATCTCAATGC CAACCAGCTG TGCTCCCTTC TCGCATCGTA TGATTCAGGA TCCAACGGCC GAAAACGGTT   
  
  
- CACCTTATCA TCCAGTCAGC TTAATGATCA AATATTTTCA ACTCTCCACG GCTGAGATGC GCTGACAGAG   
  
  
- TCTCTGTATA TATAAGCGAG AGTACGAAAA CCATTGAGGA TTTAGAAGTA GGAGTGGGCA TCACGAGACA   
  
  
- TGGGATTGCA AGCGTGTGGG GTCACACTTT TATTTTAATT TGTCTGTTTA GGACTAATAA GTTGGAAGTG   
  
  
- GCGAGTCTTG TGTTGGACCC AATTTCAGGC CCCCCATCAT GGTTTCTCCC GCCACATAAT TACAGTGTCT   
  
  
- GACCGAATTA CATTATTGTT ATTCTTGTT

+     TC-rich repeats

| Site Name | Organism | Position | Strand | Matrix score. | sequence | function |
| --- | --- | --- | --- | --- | --- | --- |
| TC-rich repeats | Nicotiana tabacum | 1072 | - | 9 | GTTTTCTTAC | cis-acting element involved in defense and stress responsiveness |
| TC-rich repeats | Nicotiana tabacum | 114 | + | 10 | GTTTTCTTAC | cis-acting element involved in defense and stress responsiveness |

> 2018/04/13 10:10:12  
+ CTGGGAAAAA CATAGAGAGC GAGATGCCGG CAAAGTCGCT GTTCCGAGAC ACTTAGGCAT TGTCTGGAGG   
  
  
+ CGTCCCTGAC AATCTCTTCT CTGTCTCTCT CTTTCTCTCT TCTGTTTTCT TACGCGACTG AAAACTCATT   
  
  
+ TCTCTCTCTT TTACCATCCC CGATCATTAC TCACCCCAAT GGTTATATAT ATCTCTCTCT CTCTCTGCCG   
  
  
+ TGTCATGTGT ACGTGGACAT ACATCTCTCT CTCTCGGCTT CACCAATAAA TCATAAGTGT TCAGATCTAT   
  
  
+ CTCTCTCTTT GATCTCTTCA ACCGTAGAGT TACGGGTAAC AACAAGAAAG AGAGAGACGA TAGGCTGTTA   
  
  
+ ACTTTTGGTC TTTGCCAAAG ACCAAAACCC CAACTGAAGA GCGTGTAGAG CAACCCAAGC GGGAGAGGTT   
  
  
+ AAGAAAAAAG TGTGTTTTTC AGGTGCGCGA GAGAAAAAGA GAGAGAGAGA GCACCCGCGA GCACTTAGTG   
  
  
+ GCTGTTTTCT CTCCTTAATT TTCTATCGAA GACGTTTCTC CTAATTTGCT TTCACCTGCG GTCAATTGCT   
  
  
+ CCCGCGAGGA TAGTTAAAGT TGACACAAAA ATGCTAAAAT GGGTATTGTT TTCTTCTTTC CTAAATTTAG   
  
  
+ TTAAATCAAC AACCAACGAA GGCATCAATT AATACACGGA GAAAAATACA GCGGCCCAAA CAAGCAGGGC   
  
  
+ ACGTACTGTG CACGGCTCAG TGACCGTCAA GCTAATTTGG TGTGGCCAAG GATGAAAGTG AGGGACACAA   
  
  
+ ACGAAGAAGG TGAGGTAATA AGGAAAGAAG AATGCCTTTA ATGGAAACCT GTATCGTTTT TATCGTGTCT   
  
  
+ TACTGCCTTT TTAGATTGTC AATGCTGCAC AGTGGGAATT TTCCGCTCAA CCTATAACCG TATGAGCTTA   
  
  
+ CCTCAAAGTA CAACTGGTGT GTAGTTCTAG CTGCCGACGT CGACCACCAA GCACACACCG TGATTGCACA   
  
  
+ TTAACTGGTC TCTCCTCCGC CTCTCGCACC GCCACGTCCT CTATTCCTCC ACTCCACCGG TCTACTGTCG   
  
  
+ CTCGGCTAAA ACACCCCAGG TGCAAGAAAA CGGTTTTCCC TACGGCAAAC TGTCGTAGAG CACCGAGCAC   
  
  
+ TAGAGTTACG GTTGGTCGAC ACGAGGGAAG AGCGTAGCAT ACTAAGTCCT AGGTTGCCGG CTTTTGCCAA   
  
  
+ GTGGAATAGT AGGTCAGTCG AATTACTAGT TTATAAAAGT TGAGAGGTGC CGACTCTACG CGACTGTCTC   
  
  
+ AGAGACATAT ATATTCGCTC TCATGCTTTT GGTAACTCCT AAATCTTCAT CCTCACCCGT AGTGCTCTGT   
  
  
+ ACCCTAACGT TCGCACACCC CAGTGTGAAA ATAAAATTAA ACAGACAAAT CCTGATTATT CAACCTTCAC   
  
  
+ CGCTCAGAAC ACAACCTGGG TTAAAGTCCG GGGGGTAGTA CCAAAGAGGG CGGTGTATTA ATGTCACAGA   
  
  
+ CTGGCTTAAT GTAATAACAA TAAGAACAA  

- GACCCTTTTT GTATCTCTCG CTCTACGGCC GTTTCAGCGA CAAGGCTCTG TGAATCCGTA ACAGACCTCC   
  
  
- GCAGGGACTG TTAGAGAAGA GACAGAGAGA GAAAGAGAGA AGACAAAAGA ATGCGCTGAC TTTTGAGTAA   
  
  
- AGAGAGAGAA AATGGTAGGG GCTAGTAATG AGTGGGGTTA CCAATATATA TAGAGAGAGA GAGAGACGGC   
  
  
- ACAGTACACA TGCACCTGTA TGTAGAGAGA GAGAGCCGAA GTGGTTATTT AGTATTCACA AGTCTAGATA   
  
  
- GAGAGAGAAA CTAGAGAAGT TGGCATCTCA ATGCCCATTG TTGTTCTTTC TCTCTCTGCT ATCCGACAAT   
  
  
- TGAAAACCAG AAACGGTTTC TGGTTTTGGG GTTGACTTCT CGCACATCTC GTTGGGTTCG CCCTCTCCAA   
  
  
- TTCTTTTTTC ACACAAAAAG TCCACGCGCT CTCTTTTTCT CTCTCTCTCT CGTGGGCGCT CGTGAATCAC   
  
  
- CGACAAAAGA GAGGAATTAA AAGATAGCTT CTGCAAAGAG GATTAAACGA AAGTGGACGC CAGTTAACGA   
  
  
- GGGCGCTCCT ATCAATTTCA ACTGTGTTTT TACGATTTTA CCCATAACAA AAGAAGAAAG GATTTAAATC   
  
  
- AATTTAGTTG TTGGTTGCTT CCGTAGTTAA TTATGTGCCT CTTTTTATGT CGCCGGGTTT GTTCGTCCCG   
  
  
- TGCATGACAC GTGCCGAGTC ACTGGCAGTT CGATTAAACC ACACCGGTTC CTACTTTCAC TCCCTGTGTT   
  
  
- TGCTTCTTCC ACTCCATTAT TCCTTTCTTC TTACGGAAAT TACCTTTGGA CATAGCAAAA ATAGCACAGA   
  
  
- ATGACGGAAA AATCTAACAG TTACGACGTG TCACCCTTAA AAGGCGAGTT GGATATTGGC ATACTCGAAT   
  
  
- GGAGTTTCAT GTTGACCACA CATCAAGATC GACGGCTGCA GCTGGTGGTT CGTGTGTGGC ACTAACGTGT   
  
  
- AATTGACCAG AGAGGAGGCG GAGAGCGTGG CGGTGCAGGA GATAAGGAGG TGAGGTGGCC AGATGACAGC   
  
  
- GAGCCGATTT TGTGGGGTCC ACGTTCTTTT GCCAAAAGGG ATGCCGTTTG ACAGCATCTC GTGGCTCGTG   
  
  
- ATCTCAATGC CAACCAGCTG TGCTCCCTTC TCGCATCGTA TGATTCAGGA TCCAACGGCC GAAAACGGTT   
  
  
- CACCTTATCA TCCAGTCAGC TTAATGATCA AATATTTTCA ACTCTCCACG GCTGAGATGC GCTGACAGAG   
  
  
- TCTCTGTATA TATAAGCGAG AGTACGAAAA CCATTGAGGA TTTAGAAGTA GGAGTGGGCA TCACGAGACA   
  
  
- TGGGATTGCA AGCGTGTGGG GTCACACTTT TATTTTAATT TGTCTGTTTA GGACTAATAA GTTGGAAGTG   
  
  
- GCGAGTCTTG TGTTGGACCC AATTTCAGGC CCCCCATCAT GGTTTCTCCC GCCACATAAT TACAGTGTCT   
  
  
- GACCGAATTA CATTATTGTT ATTCTTGTT

+     TCA-element

| Site Name | Organism | Position | Strand | Matrix score. | sequence | function |
| --- | --- | --- | --- | --- | --- | --- |
| TCA-element | Brassica oleracea | 669 | + | 9 | GAGAAGAATA | cis-acting element involved in salicylic acid responsiveness |

> 2018/04/13 10:10:12  
+ CTGGGAAAAA CATAGAGAGC GAGATGCCGG CAAAGTCGCT GTTCCGAGAC ACTTAGGCAT TGTCTGGAGG   
  
  
+ CGTCCCTGAC AATCTCTTCT CTGTCTCTCT CTTTCTCTCT TCTGTTTTCT TACGCGACTG AAAACTCATT   
  
  
+ TCTCTCTCTT TTACCATCCC CGATCATTAC TCACCCCAAT GGTTATATAT ATCTCTCTCT CTCTCTGCCG   
  
  
+ TGTCATGTGT ACGTGGACAT ACATCTCTCT CTCTCGGCTT CACCAATAAA TCATAAGTGT TCAGATCTAT   
  
  
+ CTCTCTCTTT GATCTCTTCA ACCGTAGAGT TACGGGTAAC AACAAGAAAG AGAGAGACGA TAGGCTGTTA   
  
  
+ ACTTTTGGTC TTTGCCAAAG ACCAAAACCC CAACTGAAGA GCGTGTAGAG CAACCCAAGC GGGAGAGGTT   
  
  
+ AAGAAAAAAG TGTGTTTTTC AGGTGCGCGA GAGAAAAAGA GAGAGAGAGA GCACCCGCGA GCACTTAGTG   
  
  
+ GCTGTTTTCT CTCCTTAATT TTCTATCGAA GACGTTTCTC CTAATTTGCT TTCACCTGCG GTCAATTGCT   
  
  
+ CCCGCGAGGA TAGTTAAAGT TGACACAAAA ATGCTAAAAT GGGTATTGTT TTCTTCTTTC CTAAATTTAG   
  
  
+ TTAAATCAAC AACCAACGAA GGCATCAATT AATACACGGA GAAAAATACA GCGGCCCAAA CAAGCAGGGC   
  
  
+ ACGTACTGTG CACGGCTCAG TGACCGTCAA GCTAATTTGG TGTGGCCAAG GATGAAAGTG AGGGACACAA   
  
  
+ ACGAAGAAGG TGAGGTAATA AGGAAAGAAG AATGCCTTTA ATGGAAACCT GTATCGTTTT TATCGTGTCT   
  
  
+ TACTGCCTTT TTAGATTGTC AATGCTGCAC AGTGGGAATT TTCCGCTCAA CCTATAACCG TATGAGCTTA   
  
  
+ CCTCAAAGTA CAACTGGTGT GTAGTTCTAG CTGCCGACGT CGACCACCAA GCACACACCG TGATTGCACA   
  
  
+ TTAACTGGTC TCTCCTCCGC CTCTCGCACC GCCACGTCCT CTATTCCTCC ACTCCACCGG TCTACTGTCG   
  
  
+ CTCGGCTAAA ACACCCCAGG TGCAAGAAAA CGGTTTTCCC TACGGCAAAC TGTCGTAGAG CACCGAGCAC   
  
  
+ TAGAGTTACG GTTGGTCGAC ACGAGGGAAG AGCGTAGCAT ACTAAGTCCT AGGTTGCCGG CTTTTGCCAA   
  
  
+ GTGGAATAGT AGGTCAGTCG AATTACTAGT TTATAAAAGT TGAGAGGTGC CGACTCTACG CGACTGTCTC   
  
  
+ AGAGACATAT ATATTCGCTC TCATGCTTTT GGTAACTCCT AAATCTTCAT CCTCACCCGT AGTGCTCTGT   
  
  
+ ACCCTAACGT TCGCACACCC CAGTGTGAAA ATAAAATTAA ACAGACAAAT CCTGATTATT CAACCTTCAC   
  
  
+ CGCTCAGAAC ACAACCTGGG TTAAAGTCCG GGGGGTAGTA CCAAAGAGGG CGGTGTATTA ATGTCACAGA   
  
  
+ CTGGCTTAAT GTAATAACAA TAAGAACAA  

- GACCCTTTTT GTATCTCTCG CTCTACGGCC GTTTCAGCGA CAAGGCTCTG TGAATCCGTA ACAGACCTCC   
  
  
- GCAGGGACTG TTAGAGAAGA GACAGAGAGA GAAAGAGAGA AGACAAAAGA ATGCGCTGAC TTTTGAGTAA   
  
  
- AGAGAGAGAA AATGGTAGGG GCTAGTAATG AGTGGGGTTA CCAATATATA TAGAGAGAGA GAGAGACGGC   
  
  
- ACAGTACACA TGCACCTGTA TGTAGAGAGA GAGAGCCGAA GTGGTTATTT AGTATTCACA AGTCTAGATA   
  
  
- GAGAGAGAAA CTAGAGAAGT TGGCATCTCA ATGCCCATTG TTGTTCTTTC TCTCTCTGCT ATCCGACAAT   
  
  
- TGAAAACCAG AAACGGTTTC TGGTTTTGGG GTTGACTTCT CGCACATCTC GTTGGGTTCG CCCTCTCCAA   
  
  
- TTCTTTTTTC ACACAAAAAG TCCACGCGCT CTCTTTTTCT CTCTCTCTCT CGTGGGCGCT CGTGAATCAC   
  
  
- CGACAAAAGA GAGGAATTAA AAGATAGCTT CTGCAAAGAG GATTAAACGA AAGTGGACGC CAGTTAACGA   
  
  
- GGGCGCTCCT ATCAATTTCA ACTGTGTTTT TACGATTTTA CCCATAACAA AAGAAGAAAG GATTTAAATC   
  
  
- AATTTAGTTG TTGGTTGCTT CCGTAGTTAA TTATGTGCCT CTTTTTATGT CGCCGGGTTT GTTCGTCCCG   
  
  
- TGCATGACAC GTGCCGAGTC ACTGGCAGTT CGATTAAACC ACACCGGTTC CTACTTTCAC TCCCTGTGTT   
  
  
- TGCTTCTTCC ACTCCATTAT TCCTTTCTTC TTACGGAAAT TACCTTTGGA CATAGCAAAA ATAGCACAGA   
  
  
- ATGACGGAAA AATCTAACAG TTACGACGTG TCACCCTTAA AAGGCGAGTT GGATATTGGC ATACTCGAAT   
  
  
- GGAGTTTCAT GTTGACCACA CATCAAGATC GACGGCTGCA GCTGGTGGTT CGTGTGTGGC ACTAACGTGT   
  
  
- AATTGACCAG AGAGGAGGCG GAGAGCGTGG CGGTGCAGGA GATAAGGAGG TGAGGTGGCC AGATGACAGC   
  
  
- GAGCCGATTT TGTGGGGTCC ACGTTCTTTT GCCAAAAGGG ATGCCGTTTG ACAGCATCTC GTGGCTCGTG   
  
  
- ATCTCAATGC CAACCAGCTG TGCTCCCTTC TCGCATCGTA TGATTCAGGA TCCAACGGCC GAAAACGGTT   
  
  
- CACCTTATCA TCCAGTCAGC TTAATGATCA AATATTTTCA ACTCTCCACG GCTGAGATGC GCTGACAGAG   
  
  
- TCTCTGTATA TATAAGCGAG AGTACGAAAA CCATTGAGGA TTTAGAAGTA GGAGTGGGCA TCACGAGACA   
  
  
- TGGGATTGCA AGCGTGTGGG GTCACACTTT TATTTTAATT TGTCTGTTTA GGACTAATAA GTTGGAAGTG   
  
  
- GCGAGTCTTG TGTTGGACCC AATTTCAGGC CCCCCATCAT GGTTTCTCCC GCCACATAAT TACAGTGTCT   
  
  
- GACCGAATTA CATTATTGTT ATTCTTGTT

+     TCT-motif

| Site Name | Organism | Position | Strand | Matrix score. | sequence | function |
| --- | --- | --- | --- | --- | --- | --- |
| TCT-motif | Arabidopsis thaliana | 118 | + | 6 | TCTTAC | part of a light responsive element |
| TCT-motif | Arabidopsis thaliana | 838 | + | 6 | TCTTAC | part of a light responsive element |

> 2018/04/13 10:10:12  
+ CTGGGAAAAA CATAGAGAGC GAGATGCCGG CAAAGTCGCT GTTCCGAGAC ACTTAGGCAT TGTCTGGAGG   
  
  
+ CGTCCCTGAC AATCTCTTCT CTGTCTCTCT CTTTCTCTCT TCTGTTTTCT TACGCGACTG AAAACTCATT   
  
  
+ TCTCTCTCTT TTACCATCCC CGATCATTAC TCACCCCAAT GGTTATATAT ATCTCTCTCT CTCTCTGCCG   
  
  
+ TGTCATGTGT ACGTGGACAT ACATCTCTCT CTCTCGGCTT CACCAATAAA TCATAAGTGT TCAGATCTAT   
  
  
+ CTCTCTCTTT GATCTCTTCA ACCGTAGAGT TACGGGTAAC AACAAGAAAG AGAGAGACGA TAGGCTGTTA   
  
  
+ ACTTTTGGTC TTTGCCAAAG ACCAAAACCC CAACTGAAGA GCGTGTAGAG CAACCCAAGC GGGAGAGGTT   
  
  
+ AAGAAAAAAG TGTGTTTTTC AGGTGCGCGA GAGAAAAAGA GAGAGAGAGA GCACCCGCGA GCACTTAGTG   
  
  
+ GCTGTTTTCT CTCCTTAATT TTCTATCGAA GACGTTTCTC CTAATTTGCT TTCACCTGCG GTCAATTGCT   
  
  
+ CCCGCGAGGA TAGTTAAAGT TGACACAAAA ATGCTAAAAT GGGTATTGTT TTCTTCTTTC CTAAATTTAG   
  
  
+ TTAAATCAAC AACCAACGAA GGCATCAATT AATACACGGA GAAAAATACA GCGGCCCAAA CAAGCAGGGC   
  
  
+ ACGTACTGTG CACGGCTCAG TGACCGTCAA GCTAATTTGG TGTGGCCAAG GATGAAAGTG AGGGACACAA   
  
  
+ ACGAAGAAGG TGAGGTAATA AGGAAAGAAG AATGCCTTTA ATGGAAACCT GTATCGTTTT TATCGTGTCT   
  
  
+ TACTGCCTTT TTAGATTGTC AATGCTGCAC AGTGGGAATT TTCCGCTCAA CCTATAACCG TATGAGCTTA   
  
  
+ CCTCAAAGTA CAACTGGTGT GTAGTTCTAG CTGCCGACGT CGACCACCAA GCACACACCG TGATTGCACA   
  
  
+ TTAACTGGTC TCTCCTCCGC CTCTCGCACC GCCACGTCCT CTATTCCTCC ACTCCACCGG TCTACTGTCG   
  
  
+ CTCGGCTAAA ACACCCCAGG TGCAAGAAAA CGGTTTTCCC TACGGCAAAC TGTCGTAGAG CACCGAGCAC   
  
  
+ TAGAGTTACG GTTGGTCGAC ACGAGGGAAG AGCGTAGCAT ACTAAGTCCT AGGTTGCCGG CTTTTGCCAA   
  
  
+ GTGGAATAGT AGGTCAGTCG AATTACTAGT TTATAAAAGT TGAGAGGTGC CGACTCTACG CGACTGTCTC   
  
  
+ AGAGACATAT ATATTCGCTC TCATGCTTTT GGTAACTCCT AAATCTTCAT CCTCACCCGT AGTGCTCTGT   
  
  
+ ACCCTAACGT TCGCACACCC CAGTGTGAAA ATAAAATTAA ACAGACAAAT CCTGATTATT CAACCTTCAC   
  
  
+ CGCTCAGAAC ACAACCTGGG TTAAAGTCCG GGGGGTAGTA CCAAAGAGGG CGGTGTATTA ATGTCACAGA   
  
  
+ CTGGCTTAAT GTAATAACAA TAAGAACAA  

- GACCCTTTTT GTATCTCTCG CTCTACGGCC GTTTCAGCGA CAAGGCTCTG TGAATCCGTA ACAGACCTCC   
  
  
- GCAGGGACTG TTAGAGAAGA GACAGAGAGA GAAAGAGAGA AGACAAAAGA ATGCGCTGAC TTTTGAGTAA   
  
  
- AGAGAGAGAA AATGGTAGGG GCTAGTAATG AGTGGGGTTA CCAATATATA TAGAGAGAGA GAGAGACGGC   
  
  
- ACAGTACACA TGCACCTGTA TGTAGAGAGA GAGAGCCGAA GTGGTTATTT AGTATTCACA AGTCTAGATA   
  
  
- GAGAGAGAAA CTAGAGAAGT TGGCATCTCA ATGCCCATTG TTGTTCTTTC TCTCTCTGCT ATCCGACAAT   
  
  
- TGAAAACCAG AAACGGTTTC TGGTTTTGGG GTTGACTTCT CGCACATCTC GTTGGGTTCG CCCTCTCCAA   
  
  
- TTCTTTTTTC ACACAAAAAG TCCACGCGCT CTCTTTTTCT CTCTCTCTCT CGTGGGCGCT CGTGAATCAC   
  
  
- CGACAAAAGA GAGGAATTAA AAGATAGCTT CTGCAAAGAG GATTAAACGA AAGTGGACGC CAGTTAACGA   
  
  
- GGGCGCTCCT ATCAATTTCA ACTGTGTTTT TACGATTTTA CCCATAACAA AAGAAGAAAG GATTTAAATC   
  
  
- AATTTAGTTG TTGGTTGCTT CCGTAGTTAA TTATGTGCCT CTTTTTATGT CGCCGGGTTT GTTCGTCCCG   
  
  
- TGCATGACAC GTGCCGAGTC ACTGGCAGTT CGATTAAACC ACACCGGTTC CTACTTTCAC TCCCTGTGTT   
  
  
- TGCTTCTTCC ACTCCATTAT TCCTTTCTTC TTACGGAAAT TACCTTTGGA CATAGCAAAA ATAGCACAGA   
  
  
- ATGACGGAAA AATCTAACAG TTACGACGTG TCACCCTTAA AAGGCGAGTT GGATATTGGC ATACTCGAAT   
  
  
- GGAGTTTCAT GTTGACCACA CATCAAGATC GACGGCTGCA GCTGGTGGTT CGTGTGTGGC ACTAACGTGT   
  
  
- AATTGACCAG AGAGGAGGCG GAGAGCGTGG CGGTGCAGGA GATAAGGAGG TGAGGTGGCC AGATGACAGC   
  
  
- GAGCCGATTT TGTGGGGTCC ACGTTCTTTT GCCAAAAGGG ATGCCGTTTG ACAGCATCTC GTGGCTCGTG   
  
  
- ATCTCAATGC CAACCAGCTG TGCTCCCTTC TCGCATCGTA TGATTCAGGA TCCAACGGCC GAAAACGGTT   
  
  
- CACCTTATCA TCCAGTCAGC TTAATGATCA AATATTTTCA ACTCTCCACG GCTGAGATGC GCTGACAGAG   
  
  
- TCTCTGTATA TATAAGCGAG AGTACGAAAA CCATTGAGGA TTTAGAAGTA GGAGTGGGCA TCACGAGACA   
  
  
- TGGGATTGCA AGCGTGTGGG GTCACACTTT TATTTTAATT TGTCTGTTTA GGACTAATAA GTTGGAAGTG   
  
  
- GCGAGTCTTG TGTTGGACCC AATTTCAGGC CCCCCATCAT GGTTTCTCCC GCCACATAAT TACAGTGTCT   
  
  
- GACCGAATTA CATTATTGTT ATTCTTGTT

+     TGACG-motif

| Site Name | Organism | Position | Strand | Matrix score. | sequence | function |
| --- | --- | --- | --- | --- | --- | --- |
| TGACG-motif | Hordeum vulgare | 725 | - | 5 | TGACG | cis-acting regulatory element involved in the MeJA-responsiveness |

> 2018/04/13 10:10:12  
+ CTGGGAAAAA CATAGAGAGC GAGATGCCGG CAAAGTCGCT GTTCCGAGAC ACTTAGGCAT TGTCTGGAGG   
  
  
+ CGTCCCTGAC AATCTCTTCT CTGTCTCTCT CTTTCTCTCT TCTGTTTTCT TACGCGACTG AAAACTCATT   
  
  
+ TCTCTCTCTT TTACCATCCC CGATCATTAC TCACCCCAAT GGTTATATAT ATCTCTCTCT CTCTCTGCCG   
  
  
+ TGTCATGTGT ACGTGGACAT ACATCTCTCT CTCTCGGCTT CACCAATAAA TCATAAGTGT TCAGATCTAT   
  
  
+ CTCTCTCTTT GATCTCTTCA ACCGTAGAGT TACGGGTAAC AACAAGAAAG AGAGAGACGA TAGGCTGTTA   
  
  
+ ACTTTTGGTC TTTGCCAAAG ACCAAAACCC CAACTGAAGA GCGTGTAGAG CAACCCAAGC GGGAGAGGTT   
  
  
+ AAGAAAAAAG TGTGTTTTTC AGGTGCGCGA GAGAAAAAGA GAGAGAGAGA GCACCCGCGA GCACTTAGTG   
  
  
+ GCTGTTTTCT CTCCTTAATT TTCTATCGAA GACGTTTCTC CTAATTTGCT TTCACCTGCG GTCAATTGCT   
  
  
+ CCCGCGAGGA TAGTTAAAGT TGACACAAAA ATGCTAAAAT GGGTATTGTT TTCTTCTTTC CTAAATTTAG   
  
  
+ TTAAATCAAC AACCAACGAA GGCATCAATT AATACACGGA GAAAAATACA GCGGCCCAAA CAAGCAGGGC   
  
  
+ ACGTACTGTG CACGGCTCAG TGACCGTCAA GCTAATTTGG TGTGGCCAAG GATGAAAGTG AGGGACACAA   
  
  
+ ACGAAGAAGG TGAGGTAATA AGGAAAGAAG AATGCCTTTA ATGGAAACCT GTATCGTTTT TATCGTGTCT   
  
  
+ TACTGCCTTT TTAGATTGTC AATGCTGCAC AGTGGGAATT TTCCGCTCAA CCTATAACCG TATGAGCTTA   
  
  
+ CCTCAAAGTA CAACTGGTGT GTAGTTCTAG CTGCCGACGT CGACCACCAA GCACACACCG TGATTGCACA   
  
  
+ TTAACTGGTC TCTCCTCCGC CTCTCGCACC GCCACGTCCT CTATTCCTCC ACTCCACCGG TCTACTGTCG   
  
  
+ CTCGGCTAAA ACACCCCAGG TGCAAGAAAA CGGTTTTCCC TACGGCAAAC TGTCGTAGAG CACCGAGCAC   
  
  
+ TAGAGTTACG GTTGGTCGAC ACGAGGGAAG AGCGTAGCAT ACTAAGTCCT AGGTTGCCGG CTTTTGCCAA   
  
  
+ GTGGAATAGT AGGTCAGTCG AATTACTAGT TTATAAAAGT TGAGAGGTGC CGACTCTACG CGACTGTCTC   
  
  
+ AGAGACATAT ATATTCGCTC TCATGCTTTT GGTAACTCCT AAATCTTCAT CCTCACCCGT AGTGCTCTGT   
  
  
+ ACCCTAACGT TCGCACACCC CAGTGTGAAA ATAAAATTAA ACAGACAAAT CCTGATTATT CAACCTTCAC   
  
  
+ CGCTCAGAAC ACAACCTGGG TTAAAGTCCG GGGGGTAGTA CCAAAGAGGG CGGTGTATTA ATGTCACAGA   
  
  
+ CTGGCTTAAT GTAATAACAA TAAGAACAA  

- GACCCTTTTT GTATCTCTCG CTCTACGGCC GTTTCAGCGA CAAGGCTCTG TGAATCCGTA ACAGACCTCC   
  
  
- GCAGGGACTG TTAGAGAAGA GACAGAGAGA GAAAGAGAGA AGACAAAAGA ATGCGCTGAC TTTTGAGTAA   
  
  
- AGAGAGAGAA AATGGTAGGG GCTAGTAATG AGTGGGGTTA CCAATATATA TAGAGAGAGA GAGAGACGGC   
  
  
- ACAGTACACA TGCACCTGTA TGTAGAGAGA GAGAGCCGAA GTGGTTATTT AGTATTCACA AGTCTAGATA   
  
  
- GAGAGAGAAA CTAGAGAAGT TGGCATCTCA ATGCCCATTG TTGTTCTTTC TCTCTCTGCT ATCCGACAAT   
  
  
- TGAAAACCAG AAACGGTTTC TGGTTTTGGG GTTGACTTCT CGCACATCTC GTTGGGTTCG CCCTCTCCAA   
  
  
- TTCTTTTTTC ACACAAAAAG TCCACGCGCT CTCTTTTTCT CTCTCTCTCT CGTGGGCGCT CGTGAATCAC   
  
  
- CGACAAAAGA GAGGAATTAA AAGATAGCTT CTGCAAAGAG GATTAAACGA AAGTGGACGC CAGTTAACGA   
  
  
- GGGCGCTCCT ATCAATTTCA ACTGTGTTTT TACGATTTTA CCCATAACAA AAGAAGAAAG GATTTAAATC   
  
  
- AATTTAGTTG TTGGTTGCTT CCGTAGTTAA TTATGTGCCT CTTTTTATGT CGCCGGGTTT GTTCGTCCCG   
  
  
- TGCATGACAC GTGCCGAGTC ACTGGCAGTT CGATTAAACC ACACCGGTTC CTACTTTCAC TCCCTGTGTT   
  
  
- TGCTTCTTCC ACTCCATTAT TCCTTTCTTC TTACGGAAAT TACCTTTGGA CATAGCAAAA ATAGCACAGA   
  
  
- ATGACGGAAA AATCTAACAG TTACGACGTG TCACCCTTAA AAGGCGAGTT GGATATTGGC ATACTCGAAT   
  
  
- GGAGTTTCAT GTTGACCACA CATCAAGATC GACGGCTGCA GCTGGTGGTT CGTGTGTGGC ACTAACGTGT   
  
  
- AATTGACCAG AGAGGAGGCG GAGAGCGTGG CGGTGCAGGA GATAAGGAGG TGAGGTGGCC AGATGACAGC   
  
  
- GAGCCGATTT TGTGGGGTCC ACGTTCTTTT GCCAAAAGGG ATGCCGTTTG ACAGCATCTC GTGGCTCGTG   
  
  
- ATCTCAATGC CAACCAGCTG TGCTCCCTTC TCGCATCGTA TGATTCAGGA TCCAACGGCC GAAAACGGTT   
  
  
- CACCTTATCA TCCAGTCAGC TTAATGATCA AATATTTTCA ACTCTCCACG GCTGAGATGC GCTGACAGAG   
  
  
- TCTCTGTATA TATAAGCGAG AGTACGAAAA CCATTGAGGA TTTAGAAGTA GGAGTGGGCA TCACGAGACA   
  
  
- TGGGATTGCA AGCGTGTGGG GTCACACTTT TATTTTAATT TGTCTGTTTA GGACTAATAA GTTGGAAGTG   
  
  
- GCGAGTCTTG TGTTGGACCC AATTTCAGGC CCCCCATCAT GGTTTCTCCC GCCACATAAT TACAGTGTCT   
  
  
- GACCGAATTA CATTATTGTT ATTCTTGTT

+     Unnamed\_\_1

| Site Name | Organism | Position | Strand | Matrix score. | sequence | function |
| --- | --- | --- | --- | --- | --- | --- |
| Unnamed\_\_1 | Zea mays | 1012 | - | 5 | CGTGG |  |
| Unnamed\_\_1 | Zea mays | 222 | + | 5 | CGTGG |  |

> 2018/04/13 10:10:12  
+ CTGGGAAAAA CATAGAGAGC GAGATGCCGG CAAAGTCGCT GTTCCGAGAC ACTTAGGCAT TGTCTGGAGG   
  
  
+ CGTCCCTGAC AATCTCTTCT CTGTCTCTCT CTTTCTCTCT TCTGTTTTCT TACGCGACTG AAAACTCATT   
  
  
+ TCTCTCTCTT TTACCATCCC CGATCATTAC TCACCCCAAT GGTTATATAT ATCTCTCTCT CTCTCTGCCG   
  
  
+ TGTCATGTGT ACGTGGACAT ACATCTCTCT CTCTCGGCTT CACCAATAAA TCATAAGTGT TCAGATCTAT   
  
  
+ CTCTCTCTTT GATCTCTTCA ACCGTAGAGT TACGGGTAAC AACAAGAAAG AGAGAGACGA TAGGCTGTTA   
  
  
+ ACTTTTGGTC TTTGCCAAAG ACCAAAACCC CAACTGAAGA GCGTGTAGAG CAACCCAAGC GGGAGAGGTT   
  
  
+ AAGAAAAAAG TGTGTTTTTC AGGTGCGCGA GAGAAAAAGA GAGAGAGAGA GCACCCGCGA GCACTTAGTG   
  
  
+ GCTGTTTTCT CTCCTTAATT TTCTATCGAA GACGTTTCTC CTAATTTGCT TTCACCTGCG GTCAATTGCT   
  
  
+ CCCGCGAGGA TAGTTAAAGT TGACACAAAA ATGCTAAAAT GGGTATTGTT TTCTTCTTTC CTAAATTTAG   
  
  
+ TTAAATCAAC AACCAACGAA GGCATCAATT AATACACGGA GAAAAATACA GCGGCCCAAA CAAGCAGGGC   
  
  
+ ACGTACTGTG CACGGCTCAG TGACCGTCAA GCTAATTTGG TGTGGCCAAG GATGAAAGTG AGGGACACAA   
  
  
+ ACGAAGAAGG TGAGGTAATA AGGAAAGAAG AATGCCTTTA ATGGAAACCT GTATCGTTTT TATCGTGTCT   
  
  
+ TACTGCCTTT TTAGATTGTC AATGCTGCAC AGTGGGAATT TTCCGCTCAA CCTATAACCG TATGAGCTTA   
  
  
+ CCTCAAAGTA CAACTGGTGT GTAGTTCTAG CTGCCGACGT CGACCACCAA GCACACACCG TGATTGCACA   
  
  
+ TTAACTGGTC TCTCCTCCGC CTCTCGCACC GCCACGTCCT CTATTCCTCC ACTCCACCGG TCTACTGTCG   
  
  
+ CTCGGCTAAA ACACCCCAGG TGCAAGAAAA CGGTTTTCCC TACGGCAAAC TGTCGTAGAG CACCGAGCAC   
  
  
+ TAGAGTTACG GTTGGTCGAC ACGAGGGAAG AGCGTAGCAT ACTAAGTCCT AGGTTGCCGG CTTTTGCCAA   
  
  
+ GTGGAATAGT AGGTCAGTCG AATTACTAGT TTATAAAAGT TGAGAGGTGC CGACTCTACG CGACTGTCTC   
  
  
+ AGAGACATAT ATATTCGCTC TCATGCTTTT GGTAACTCCT AAATCTTCAT CCTCACCCGT AGTGCTCTGT   
  
  
+ ACCCTAACGT TCGCACACCC CAGTGTGAAA ATAAAATTAA ACAGACAAAT CCTGATTATT CAACCTTCAC   
  
  
+ CGCTCAGAAC ACAACCTGGG TTAAAGTCCG GGGGGTAGTA CCAAAGAGGG CGGTGTATTA ATGTCACAGA   
  
  
+ CTGGCTTAAT GTAATAACAA TAAGAACAA  

- GACCCTTTTT GTATCTCTCG CTCTACGGCC GTTTCAGCGA CAAGGCTCTG TGAATCCGTA ACAGACCTCC   
  
  
- GCAGGGACTG TTAGAGAAGA GACAGAGAGA GAAAGAGAGA AGACAAAAGA ATGCGCTGAC TTTTGAGTAA   
  
  
- AGAGAGAGAA AATGGTAGGG GCTAGTAATG AGTGGGGTTA CCAATATATA TAGAGAGAGA GAGAGACGGC   
  
  
- ACAGTACACA TGCACCTGTA TGTAGAGAGA GAGAGCCGAA GTGGTTATTT AGTATTCACA AGTCTAGATA   
  
  
- GAGAGAGAAA CTAGAGAAGT TGGCATCTCA ATGCCCATTG TTGTTCTTTC TCTCTCTGCT ATCCGACAAT   
  
  
- TGAAAACCAG AAACGGTTTC TGGTTTTGGG GTTGACTTCT CGCACATCTC GTTGGGTTCG CCCTCTCCAA   
  
  
- TTCTTTTTTC ACACAAAAAG TCCACGCGCT CTCTTTTTCT CTCTCTCTCT CGTGGGCGCT CGTGAATCAC   
  
  
- CGACAAAAGA GAGGAATTAA AAGATAGCTT CTGCAAAGAG GATTAAACGA AAGTGGACGC CAGTTAACGA   
  
  
- GGGCGCTCCT ATCAATTTCA ACTGTGTTTT TACGATTTTA CCCATAACAA AAGAAGAAAG GATTTAAATC   
  
  
- AATTTAGTTG TTGGTTGCTT CCGTAGTTAA TTATGTGCCT CTTTTTATGT CGCCGGGTTT GTTCGTCCCG   
  
  
- TGCATGACAC GTGCCGAGTC ACTGGCAGTT CGATTAAACC ACACCGGTTC CTACTTTCAC TCCCTGTGTT   
  
  
- TGCTTCTTCC ACTCCATTAT TCCTTTCTTC TTACGGAAAT TACCTTTGGA CATAGCAAAA ATAGCACAGA   
  
  
- ATGACGGAAA AATCTAACAG TTACGACGTG TCACCCTTAA AAGGCGAGTT GGATATTGGC ATACTCGAAT   
  
  
- GGAGTTTCAT GTTGACCACA CATCAAGATC GACGGCTGCA GCTGGTGGTT CGTGTGTGGC ACTAACGTGT   
  
  
- AATTGACCAG AGAGGAGGCG GAGAGCGTGG CGGTGCAGGA GATAAGGAGG TGAGGTGGCC AGATGACAGC   
  
  
- GAGCCGATTT TGTGGGGTCC ACGTTCTTTT GCCAAAAGGG ATGCCGTTTG ACAGCATCTC GTGGCTCGTG   
  
  
- ATCTCAATGC CAACCAGCTG TGCTCCCTTC TCGCATCGTA TGATTCAGGA TCCAACGGCC GAAAACGGTT   
  
  
- CACCTTATCA TCCAGTCAGC TTAATGATCA AATATTTTCA ACTCTCCACG GCTGAGATGC GCTGACAGAG   
  
  
- TCTCTGTATA TATAAGCGAG AGTACGAAAA CCATTGAGGA TTTAGAAGTA GGAGTGGGCA TCACGAGACA   
  
  
- TGGGATTGCA AGCGTGTGGG GTCACACTTT TATTTTAATT TGTCTGTTTA GGACTAATAA GTTGGAAGTG   
  
  
- GCGAGTCTTG TGTTGGACCC AATTTCAGGC CCCCCATCAT GGTTTCTCCC GCCACATAAT TACAGTGTCT   
  
  
- GACCGAATTA CATTATTGTT ATTCTTGTT

+     Unnamed\_\_10

| Site Name | Organism | Position | Strand | Matrix score. | sequence | function |
| --- | --- | --- | --- | --- | --- | --- |
| Unnamed\_\_10 | Zea mays | 218 | - | 9 | TCCACGTAGA |  |

> 2018/04/13 10:10:12  
+ CTGGGAAAAA CATAGAGAGC GAGATGCCGG CAAAGTCGCT GTTCCGAGAC ACTTAGGCAT TGTCTGGAGG   
  
  
+ CGTCCCTGAC AATCTCTTCT CTGTCTCTCT CTTTCTCTCT TCTGTTTTCT TACGCGACTG AAAACTCATT   
  
  
+ TCTCTCTCTT TTACCATCCC CGATCATTAC TCACCCCAAT GGTTATATAT ATCTCTCTCT CTCTCTGCCG   
  
  
+ TGTCATGTGT ACGTGGACAT ACATCTCTCT CTCTCGGCTT CACCAATAAA TCATAAGTGT TCAGATCTAT   
  
  
+ CTCTCTCTTT GATCTCTTCA ACCGTAGAGT TACGGGTAAC AACAAGAAAG AGAGAGACGA TAGGCTGTTA   
  
  
+ ACTTTTGGTC TTTGCCAAAG ACCAAAACCC CAACTGAAGA GCGTGTAGAG CAACCCAAGC GGGAGAGGTT   
  
  
+ AAGAAAAAAG TGTGTTTTTC AGGTGCGCGA GAGAAAAAGA GAGAGAGAGA GCACCCGCGA GCACTTAGTG   
  
  
+ GCTGTTTTCT CTCCTTAATT TTCTATCGAA GACGTTTCTC CTAATTTGCT TTCACCTGCG GTCAATTGCT   
  
  
+ CCCGCGAGGA TAGTTAAAGT TGACACAAAA ATGCTAAAAT GGGTATTGTT TTCTTCTTTC CTAAATTTAG   
  
  
+ TTAAATCAAC AACCAACGAA GGCATCAATT AATACACGGA GAAAAATACA GCGGCCCAAA CAAGCAGGGC   
  
  
+ ACGTACTGTG CACGGCTCAG TGACCGTCAA GCTAATTTGG TGTGGCCAAG GATGAAAGTG AGGGACACAA   
  
  
+ ACGAAGAAGG TGAGGTAATA AGGAAAGAAG AATGCCTTTA ATGGAAACCT GTATCGTTTT TATCGTGTCT   
  
  
+ TACTGCCTTT TTAGATTGTC AATGCTGCAC AGTGGGAATT TTCCGCTCAA CCTATAACCG TATGAGCTTA   
  
  
+ CCTCAAAGTA CAACTGGTGT GTAGTTCTAG CTGCCGACGT CGACCACCAA GCACACACCG TGATTGCACA   
  
  
+ TTAACTGGTC TCTCCTCCGC CTCTCGCACC GCCACGTCCT CTATTCCTCC ACTCCACCGG TCTACTGTCG   
  
  
+ CTCGGCTAAA ACACCCCAGG TGCAAGAAAA CGGTTTTCCC TACGGCAAAC TGTCGTAGAG CACCGAGCAC   
  
  
+ TAGAGTTACG GTTGGTCGAC ACGAGGGAAG AGCGTAGCAT ACTAAGTCCT AGGTTGCCGG CTTTTGCCAA   
  
  
+ GTGGAATAGT AGGTCAGTCG AATTACTAGT TTATAAAAGT TGAGAGGTGC CGACTCTACG CGACTGTCTC   
  
  
+ AGAGACATAT ATATTCGCTC TCATGCTTTT GGTAACTCCT AAATCTTCAT CCTCACCCGT AGTGCTCTGT   
  
  
+ ACCCTAACGT TCGCACACCC CAGTGTGAAA ATAAAATTAA ACAGACAAAT CCTGATTATT CAACCTTCAC   
  
  
+ CGCTCAGAAC ACAACCTGGG TTAAAGTCCG GGGGGTAGTA CCAAAGAGGG CGGTGTATTA ATGTCACAGA   
  
  
+ CTGGCTTAAT GTAATAACAA TAAGAACAA  

- GACCCTTTTT GTATCTCTCG CTCTACGGCC GTTTCAGCGA CAAGGCTCTG TGAATCCGTA ACAGACCTCC   
  
  
- GCAGGGACTG TTAGAGAAGA GACAGAGAGA GAAAGAGAGA AGACAAAAGA ATGCGCTGAC TTTTGAGTAA   
  
  
- AGAGAGAGAA AATGGTAGGG GCTAGTAATG AGTGGGGTTA CCAATATATA TAGAGAGAGA GAGAGACGGC   
  
  
- ACAGTACACA TGCACCTGTA TGTAGAGAGA GAGAGCCGAA GTGGTTATTT AGTATTCACA AGTCTAGATA   
  
  
- GAGAGAGAAA CTAGAGAAGT TGGCATCTCA ATGCCCATTG TTGTTCTTTC TCTCTCTGCT ATCCGACAAT   
  
  
- TGAAAACCAG AAACGGTTTC TGGTTTTGGG GTTGACTTCT CGCACATCTC GTTGGGTTCG CCCTCTCCAA   
  
  
- TTCTTTTTTC ACACAAAAAG TCCACGCGCT CTCTTTTTCT CTCTCTCTCT CGTGGGCGCT CGTGAATCAC   
  
  
- CGACAAAAGA GAGGAATTAA AAGATAGCTT CTGCAAAGAG GATTAAACGA AAGTGGACGC CAGTTAACGA   
  
  
- GGGCGCTCCT ATCAATTTCA ACTGTGTTTT TACGATTTTA CCCATAACAA AAGAAGAAAG GATTTAAATC   
  
  
- AATTTAGTTG TTGGTTGCTT CCGTAGTTAA TTATGTGCCT CTTTTTATGT CGCCGGGTTT GTTCGTCCCG   
  
  
- TGCATGACAC GTGCCGAGTC ACTGGCAGTT CGATTAAACC ACACCGGTTC CTACTTTCAC TCCCTGTGTT   
  
  
- TGCTTCTTCC ACTCCATTAT TCCTTTCTTC TTACGGAAAT TACCTTTGGA CATAGCAAAA ATAGCACAGA   
  
  
- ATGACGGAAA AATCTAACAG TTACGACGTG TCACCCTTAA AAGGCGAGTT GGATATTGGC ATACTCGAAT   
  
  
- GGAGTTTCAT GTTGACCACA CATCAAGATC GACGGCTGCA GCTGGTGGTT CGTGTGTGGC ACTAACGTGT   
  
  
- AATTGACCAG AGAGGAGGCG GAGAGCGTGG CGGTGCAGGA GATAAGGAGG TGAGGTGGCC AGATGACAGC   
  
  
- GAGCCGATTT TGTGGGGTCC ACGTTCTTTT GCCAAAAGGG ATGCCGTTTG ACAGCATCTC GTGGCTCGTG   
  
  
- ATCTCAATGC CAACCAGCTG TGCTCCCTTC TCGCATCGTA TGATTCAGGA TCCAACGGCC GAAAACGGTT   
  
  
- CACCTTATCA TCCAGTCAGC TTAATGATCA AATATTTTCA ACTCTCCACG GCTGAGATGC GCTGACAGAG   
  
  
- TCTCTGTATA TATAAGCGAG AGTACGAAAA CCATTGAGGA TTTAGAAGTA GGAGTGGGCA TCACGAGACA   
  
  
- TGGGATTGCA AGCGTGTGGG GTCACACTTT TATTTTAATT TGTCTGTTTA GGACTAATAA GTTGGAAGTG   
  
  
- GCGAGTCTTG TGTTGGACCC AATTTCAGGC CCCCCATCAT GGTTTCTCCC GCCACATAAT TACAGTGTCT   
  
  
- GACCGAATTA CATTATTGTT ATTCTTGTT

+     Unnamed\_\_12

| Site Name | Organism | Position | Strand | Matrix score. | sequence | function |
| --- | --- | --- | --- | --- | --- | --- |
| Unnamed\_\_12 | Zea mays | 218 | - | 9 | TCCACGTAGA |  |

> 2018/04/13 10:10:12  
+ CTGGGAAAAA CATAGAGAGC GAGATGCCGG CAAAGTCGCT GTTCCGAGAC ACTTAGGCAT TGTCTGGAGG   
  
  
+ CGTCCCTGAC AATCTCTTCT CTGTCTCTCT CTTTCTCTCT TCTGTTTTCT TACGCGACTG AAAACTCATT   
  
  
+ TCTCTCTCTT TTACCATCCC CGATCATTAC TCACCCCAAT GGTTATATAT ATCTCTCTCT CTCTCTGCCG   
  
  
+ TGTCATGTGT ACGTGGACAT ACATCTCTCT CTCTCGGCTT CACCAATAAA TCATAAGTGT TCAGATCTAT   
  
  
+ CTCTCTCTTT GATCTCTTCA ACCGTAGAGT TACGGGTAAC AACAAGAAAG AGAGAGACGA TAGGCTGTTA   
  
  
+ ACTTTTGGTC TTTGCCAAAG ACCAAAACCC CAACTGAAGA GCGTGTAGAG CAACCCAAGC GGGAGAGGTT   
  
  
+ AAGAAAAAAG TGTGTTTTTC AGGTGCGCGA GAGAAAAAGA GAGAGAGAGA GCACCCGCGA GCACTTAGTG   
  
  
+ GCTGTTTTCT CTCCTTAATT TTCTATCGAA GACGTTTCTC CTAATTTGCT TTCACCTGCG GTCAATTGCT   
  
  
+ CCCGCGAGGA TAGTTAAAGT TGACACAAAA ATGCTAAAAT GGGTATTGTT TTCTTCTTTC CTAAATTTAG   
  
  
+ TTAAATCAAC AACCAACGAA GGCATCAATT AATACACGGA GAAAAATACA GCGGCCCAAA CAAGCAGGGC   
  
  
+ ACGTACTGTG CACGGCTCAG TGACCGTCAA GCTAATTTGG TGTGGCCAAG GATGAAAGTG AGGGACACAA   
  
  
+ ACGAAGAAGG TGAGGTAATA AGGAAAGAAG AATGCCTTTA ATGGAAACCT GTATCGTTTT TATCGTGTCT   
  
  
+ TACTGCCTTT TTAGATTGTC AATGCTGCAC AGTGGGAATT TTCCGCTCAA CCTATAACCG TATGAGCTTA   
  
  
+ CCTCAAAGTA CAACTGGTGT GTAGTTCTAG CTGCCGACGT CGACCACCAA GCACACACCG TGATTGCACA   
  
  
+ TTAACTGGTC TCTCCTCCGC CTCTCGCACC GCCACGTCCT CTATTCCTCC ACTCCACCGG TCTACTGTCG   
  
  
+ CTCGGCTAAA ACACCCCAGG TGCAAGAAAA CGGTTTTCCC TACGGCAAAC TGTCGTAGAG CACCGAGCAC   
  
  
+ TAGAGTTACG GTTGGTCGAC ACGAGGGAAG AGCGTAGCAT ACTAAGTCCT AGGTTGCCGG CTTTTGCCAA   
  
  
+ GTGGAATAGT AGGTCAGTCG AATTACTAGT TTATAAAAGT TGAGAGGTGC CGACTCTACG CGACTGTCTC   
  
  
+ AGAGACATAT ATATTCGCTC TCATGCTTTT GGTAACTCCT AAATCTTCAT CCTCACCCGT AGTGCTCTGT   
  
  
+ ACCCTAACGT TCGCACACCC CAGTGTGAAA ATAAAATTAA ACAGACAAAT CCTGATTATT CAACCTTCAC   
  
  
+ CGCTCAGAAC ACAACCTGGG TTAAAGTCCG GGGGGTAGTA CCAAAGAGGG CGGTGTATTA ATGTCACAGA   
  
  
+ CTGGCTTAAT GTAATAACAA TAAGAACAA  

- GACCCTTTTT GTATCTCTCG CTCTACGGCC GTTTCAGCGA CAAGGCTCTG TGAATCCGTA ACAGACCTCC   
  
  
- GCAGGGACTG TTAGAGAAGA GACAGAGAGA GAAAGAGAGA AGACAAAAGA ATGCGCTGAC TTTTGAGTAA   
  
  
- AGAGAGAGAA AATGGTAGGG GCTAGTAATG AGTGGGGTTA CCAATATATA TAGAGAGAGA GAGAGACGGC   
  
  
- ACAGTACACA TGCACCTGTA TGTAGAGAGA GAGAGCCGAA GTGGTTATTT AGTATTCACA AGTCTAGATA   
  
  
- GAGAGAGAAA CTAGAGAAGT TGGCATCTCA ATGCCCATTG TTGTTCTTTC TCTCTCTGCT ATCCGACAAT   
  
  
- TGAAAACCAG AAACGGTTTC TGGTTTTGGG GTTGACTTCT CGCACATCTC GTTGGGTTCG CCCTCTCCAA   
  
  
- TTCTTTTTTC ACACAAAAAG TCCACGCGCT CTCTTTTTCT CTCTCTCTCT CGTGGGCGCT CGTGAATCAC   
  
  
- CGACAAAAGA GAGGAATTAA AAGATAGCTT CTGCAAAGAG GATTAAACGA AAGTGGACGC CAGTTAACGA   
  
  
- GGGCGCTCCT ATCAATTTCA ACTGTGTTTT TACGATTTTA CCCATAACAA AAGAAGAAAG GATTTAAATC   
  
  
- AATTTAGTTG TTGGTTGCTT CCGTAGTTAA TTATGTGCCT CTTTTTATGT CGCCGGGTTT GTTCGTCCCG   
  
  
- TGCATGACAC GTGCCGAGTC ACTGGCAGTT CGATTAAACC ACACCGGTTC CTACTTTCAC TCCCTGTGTT   
  
  
- TGCTTCTTCC ACTCCATTAT TCCTTTCTTC TTACGGAAAT TACCTTTGGA CATAGCAAAA ATAGCACAGA   
  
  
- ATGACGGAAA AATCTAACAG TTACGACGTG TCACCCTTAA AAGGCGAGTT GGATATTGGC ATACTCGAAT   
  
  
- GGAGTTTCAT GTTGACCACA CATCAAGATC GACGGCTGCA GCTGGTGGTT CGTGTGTGGC ACTAACGTGT   
  
  
- AATTGACCAG AGAGGAGGCG GAGAGCGTGG CGGTGCAGGA GATAAGGAGG TGAGGTGGCC AGATGACAGC   
  
  
- GAGCCGATTT TGTGGGGTCC ACGTTCTTTT GCCAAAAGGG ATGCCGTTTG ACAGCATCTC GTGGCTCGTG   
  
  
- ATCTCAATGC CAACCAGCTG TGCTCCCTTC TCGCATCGTA TGATTCAGGA TCCAACGGCC GAAAACGGTT   
  
  
- CACCTTATCA TCCAGTCAGC TTAATGATCA AATATTTTCA ACTCTCCACG GCTGAGATGC GCTGACAGAG   
  
  
- TCTCTGTATA TATAAGCGAG AGTACGAAAA CCATTGAGGA TTTAGAAGTA GGAGTGGGCA TCACGAGACA   
  
  
- TGGGATTGCA AGCGTGTGGG GTCACACTTT TATTTTAATT TGTCTGTTTA GGACTAATAA GTTGGAAGTG   
  
  
- GCGAGTCTTG TGTTGGACCC AATTTCAGGC CCCCCATCAT GGTTTCTCCC GCCACATAAT TACAGTGTCT   
  
  
- GACCGAATTA CATTATTGTT ATTCTTGTT

+     Unnamed\_\_14

| Site Name | Organism | Position | Strand | Matrix score. | sequence | function |
| --- | --- | --- | --- | --- | --- | --- |
| Unnamed\_\_14 | Zea mays | 218 | - | 9 | TCCACGTAGA |  |

> 2018/04/13 10:10:12  
+ CTGGGAAAAA CATAGAGAGC GAGATGCCGG CAAAGTCGCT GTTCCGAGAC ACTTAGGCAT TGTCTGGAGG   
  
  
+ CGTCCCTGAC AATCTCTTCT CTGTCTCTCT CTTTCTCTCT TCTGTTTTCT TACGCGACTG AAAACTCATT   
  
  
+ TCTCTCTCTT TTACCATCCC CGATCATTAC TCACCCCAAT GGTTATATAT ATCTCTCTCT CTCTCTGCCG   
  
  
+ TGTCATGTGT ACGTGGACAT ACATCTCTCT CTCTCGGCTT CACCAATAAA TCATAAGTGT TCAGATCTAT   
  
  
+ CTCTCTCTTT GATCTCTTCA ACCGTAGAGT TACGGGTAAC AACAAGAAAG AGAGAGACGA TAGGCTGTTA   
  
  
+ ACTTTTGGTC TTTGCCAAAG ACCAAAACCC CAACTGAAGA GCGTGTAGAG CAACCCAAGC GGGAGAGGTT   
  
  
+ AAGAAAAAAG TGTGTTTTTC AGGTGCGCGA GAGAAAAAGA GAGAGAGAGA GCACCCGCGA GCACTTAGTG   
  
  
+ GCTGTTTTCT CTCCTTAATT TTCTATCGAA GACGTTTCTC CTAATTTGCT TTCACCTGCG GTCAATTGCT   
  
  
+ CCCGCGAGGA TAGTTAAAGT TGACACAAAA ATGCTAAAAT GGGTATTGTT TTCTTCTTTC CTAAATTTAG   
  
  
+ TTAAATCAAC AACCAACGAA GGCATCAATT AATACACGGA GAAAAATACA GCGGCCCAAA CAAGCAGGGC   
  
  
+ ACGTACTGTG CACGGCTCAG TGACCGTCAA GCTAATTTGG TGTGGCCAAG GATGAAAGTG AGGGACACAA   
  
  
+ ACGAAGAAGG TGAGGTAATA AGGAAAGAAG AATGCCTTTA ATGGAAACCT GTATCGTTTT TATCGTGTCT   
  
  
+ TACTGCCTTT TTAGATTGTC AATGCTGCAC AGTGGGAATT TTCCGCTCAA CCTATAACCG TATGAGCTTA   
  
  
+ CCTCAAAGTA CAACTGGTGT GTAGTTCTAG CTGCCGACGT CGACCACCAA GCACACACCG TGATTGCACA   
  
  
+ TTAACTGGTC TCTCCTCCGC CTCTCGCACC GCCACGTCCT CTATTCCTCC ACTCCACCGG TCTACTGTCG   
  
  
+ CTCGGCTAAA ACACCCCAGG TGCAAGAAAA CGGTTTTCCC TACGGCAAAC TGTCGTAGAG CACCGAGCAC   
  
  
+ TAGAGTTACG GTTGGTCGAC ACGAGGGAAG AGCGTAGCAT ACTAAGTCCT AGGTTGCCGG CTTTTGCCAA   
  
  
+ GTGGAATAGT AGGTCAGTCG AATTACTAGT TTATAAAAGT TGAGAGGTGC CGACTCTACG CGACTGTCTC   
  
  
+ AGAGACATAT ATATTCGCTC TCATGCTTTT GGTAACTCCT AAATCTTCAT CCTCACCCGT AGTGCTCTGT   
  
  
+ ACCCTAACGT TCGCACACCC CAGTGTGAAA ATAAAATTAA ACAGACAAAT CCTGATTATT CAACCTTCAC   
  
  
+ CGCTCAGAAC ACAACCTGGG TTAAAGTCCG GGGGGTAGTA CCAAAGAGGG CGGTGTATTA ATGTCACAGA   
  
  
+ CTGGCTTAAT GTAATAACAA TAAGAACAA  

- GACCCTTTTT GTATCTCTCG CTCTACGGCC GTTTCAGCGA CAAGGCTCTG TGAATCCGTA ACAGACCTCC   
  
  
- GCAGGGACTG TTAGAGAAGA GACAGAGAGA GAAAGAGAGA AGACAAAAGA ATGCGCTGAC TTTTGAGTAA   
  
  
- AGAGAGAGAA AATGGTAGGG GCTAGTAATG AGTGGGGTTA CCAATATATA TAGAGAGAGA GAGAGACGGC   
  
  
- ACAGTACACA TGCACCTGTA TGTAGAGAGA GAGAGCCGAA GTGGTTATTT AGTATTCACA AGTCTAGATA   
  
  
- GAGAGAGAAA CTAGAGAAGT TGGCATCTCA ATGCCCATTG TTGTTCTTTC TCTCTCTGCT ATCCGACAAT   
  
  
- TGAAAACCAG AAACGGTTTC TGGTTTTGGG GTTGACTTCT CGCACATCTC GTTGGGTTCG CCCTCTCCAA   
  
  
- TTCTTTTTTC ACACAAAAAG TCCACGCGCT CTCTTTTTCT CTCTCTCTCT CGTGGGCGCT CGTGAATCAC   
  
  
- CGACAAAAGA GAGGAATTAA AAGATAGCTT CTGCAAAGAG GATTAAACGA AAGTGGACGC CAGTTAACGA   
  
  
- GGGCGCTCCT ATCAATTTCA ACTGTGTTTT TACGATTTTA CCCATAACAA AAGAAGAAAG GATTTAAATC   
  
  
- AATTTAGTTG TTGGTTGCTT CCGTAGTTAA TTATGTGCCT CTTTTTATGT CGCCGGGTTT GTTCGTCCCG   
  
  
- TGCATGACAC GTGCCGAGTC ACTGGCAGTT CGATTAAACC ACACCGGTTC CTACTTTCAC TCCCTGTGTT   
  
  
- TGCTTCTTCC ACTCCATTAT TCCTTTCTTC TTACGGAAAT TACCTTTGGA CATAGCAAAA ATAGCACAGA   
  
  
- ATGACGGAAA AATCTAACAG TTACGACGTG TCACCCTTAA AAGGCGAGTT GGATATTGGC ATACTCGAAT   
  
  
- GGAGTTTCAT GTTGACCACA CATCAAGATC GACGGCTGCA GCTGGTGGTT CGTGTGTGGC ACTAACGTGT   
  
  
- AATTGACCAG AGAGGAGGCG GAGAGCGTGG CGGTGCAGGA GATAAGGAGG TGAGGTGGCC AGATGACAGC   
  
  
- GAGCCGATTT TGTGGGGTCC ACGTTCTTTT GCCAAAAGGG ATGCCGTTTG ACAGCATCTC GTGGCTCGTG   
  
  
- ATCTCAATGC CAACCAGCTG TGCTCCCTTC TCGCATCGTA TGATTCAGGA TCCAACGGCC GAAAACGGTT   
  
  
- CACCTTATCA TCCAGTCAGC TTAATGATCA AATATTTTCA ACTCTCCACG GCTGAGATGC GCTGACAGAG   
  
  
- TCTCTGTATA TATAAGCGAG AGTACGAAAA CCATTGAGGA TTTAGAAGTA GGAGTGGGCA TCACGAGACA   
  
  
- TGGGATTGCA AGCGTGTGGG GTCACACTTT TATTTTAATT TGTCTGTTTA GGACTAATAA GTTGGAAGTG   
  
  
- GCGAGTCTTG TGTTGGACCC AATTTCAGGC CCCCCATCAT GGTTTCTCCC GCCACATAAT TACAGTGTCT   
  
  
- GACCGAATTA CATTATTGTT ATTCTTGTT

+     Unnamed\_\_2

| Site Name | Organism | Position | Strand | Matrix score. | sequence | function |
| --- | --- | --- | --- | --- | --- | --- |
| Unnamed\_\_2 | Zea mays | 1428 | - | 6 | CCCCGG |  |

> 2018/04/13 10:10:12  
+ CTGGGAAAAA CATAGAGAGC GAGATGCCGG CAAAGTCGCT GTTCCGAGAC ACTTAGGCAT TGTCTGGAGG   
  
  
+ CGTCCCTGAC AATCTCTTCT CTGTCTCTCT CTTTCTCTCT TCTGTTTTCT TACGCGACTG AAAACTCATT   
  
  
+ TCTCTCTCTT TTACCATCCC CGATCATTAC TCACCCCAAT GGTTATATAT ATCTCTCTCT CTCTCTGCCG   
  
  
+ TGTCATGTGT ACGTGGACAT ACATCTCTCT CTCTCGGCTT CACCAATAAA TCATAAGTGT TCAGATCTAT   
  
  
+ CTCTCTCTTT GATCTCTTCA ACCGTAGAGT TACGGGTAAC AACAAGAAAG AGAGAGACGA TAGGCTGTTA   
  
  
+ ACTTTTGGTC TTTGCCAAAG ACCAAAACCC CAACTGAAGA GCGTGTAGAG CAACCCAAGC GGGAGAGGTT   
  
  
+ AAGAAAAAAG TGTGTTTTTC AGGTGCGCGA GAGAAAAAGA GAGAGAGAGA GCACCCGCGA GCACTTAGTG   
  
  
+ GCTGTTTTCT CTCCTTAATT TTCTATCGAA GACGTTTCTC CTAATTTGCT TTCACCTGCG GTCAATTGCT   
  
  
+ CCCGCGAGGA TAGTTAAAGT TGACACAAAA ATGCTAAAAT GGGTATTGTT TTCTTCTTTC CTAAATTTAG   
  
  
+ TTAAATCAAC AACCAACGAA GGCATCAATT AATACACGGA GAAAAATACA GCGGCCCAAA CAAGCAGGGC   
  
  
+ ACGTACTGTG CACGGCTCAG TGACCGTCAA GCTAATTTGG TGTGGCCAAG GATGAAAGTG AGGGACACAA   
  
  
+ ACGAAGAAGG TGAGGTAATA AGGAAAGAAG AATGCCTTTA ATGGAAACCT GTATCGTTTT TATCGTGTCT   
  
  
+ TACTGCCTTT TTAGATTGTC AATGCTGCAC AGTGGGAATT TTCCGCTCAA CCTATAACCG TATGAGCTTA   
  
  
+ CCTCAAAGTA CAACTGGTGT GTAGTTCTAG CTGCCGACGT CGACCACCAA GCACACACCG TGATTGCACA   
  
  
+ TTAACTGGTC TCTCCTCCGC CTCTCGCACC GCCACGTCCT CTATTCCTCC ACTCCACCGG TCTACTGTCG   
  
  
+ CTCGGCTAAA ACACCCCAGG TGCAAGAAAA CGGTTTTCCC TACGGCAAAC TGTCGTAGAG CACCGAGCAC   
  
  
+ TAGAGTTACG GTTGGTCGAC ACGAGGGAAG AGCGTAGCAT ACTAAGTCCT AGGTTGCCGG CTTTTGCCAA   
  
  
+ GTGGAATAGT AGGTCAGTCG AATTACTAGT TTATAAAAGT TGAGAGGTGC CGACTCTACG CGACTGTCTC   
  
  
+ AGAGACATAT ATATTCGCTC TCATGCTTTT GGTAACTCCT AAATCTTCAT CCTCACCCGT AGTGCTCTGT   
  
  
+ ACCCTAACGT TCGCACACCC CAGTGTGAAA ATAAAATTAA ACAGACAAAT CCTGATTATT CAACCTTCAC   
  
  
+ CGCTCAGAAC ACAACCTGGG TTAAAGTCCG GGGGGTAGTA CCAAAGAGGG CGGTGTATTA ATGTCACAGA   
  
  
+ CTGGCTTAAT GTAATAACAA TAAGAACAA  

- GACCCTTTTT GTATCTCTCG CTCTACGGCC GTTTCAGCGA CAAGGCTCTG TGAATCCGTA ACAGACCTCC   
  
  
- GCAGGGACTG TTAGAGAAGA GACAGAGAGA GAAAGAGAGA AGACAAAAGA ATGCGCTGAC TTTTGAGTAA   
  
  
- AGAGAGAGAA AATGGTAGGG GCTAGTAATG AGTGGGGTTA CCAATATATA TAGAGAGAGA GAGAGACGGC   
  
  
- ACAGTACACA TGCACCTGTA TGTAGAGAGA GAGAGCCGAA GTGGTTATTT AGTATTCACA AGTCTAGATA   
  
  
- GAGAGAGAAA CTAGAGAAGT TGGCATCTCA ATGCCCATTG TTGTTCTTTC TCTCTCTGCT ATCCGACAAT   
  
  
- TGAAAACCAG AAACGGTTTC TGGTTTTGGG GTTGACTTCT CGCACATCTC GTTGGGTTCG CCCTCTCCAA   
  
  
- TTCTTTTTTC ACACAAAAAG TCCACGCGCT CTCTTTTTCT CTCTCTCTCT CGTGGGCGCT CGTGAATCAC   
  
  
- CGACAAAAGA GAGGAATTAA AAGATAGCTT CTGCAAAGAG GATTAAACGA AAGTGGACGC CAGTTAACGA   
  
  
- GGGCGCTCCT ATCAATTTCA ACTGTGTTTT TACGATTTTA CCCATAACAA AAGAAGAAAG GATTTAAATC   
  
  
- AATTTAGTTG TTGGTTGCTT CCGTAGTTAA TTATGTGCCT CTTTTTATGT CGCCGGGTTT GTTCGTCCCG   
  
  
- TGCATGACAC GTGCCGAGTC ACTGGCAGTT CGATTAAACC ACACCGGTTC CTACTTTCAC TCCCTGTGTT   
  
  
- TGCTTCTTCC ACTCCATTAT TCCTTTCTTC TTACGGAAAT TACCTTTGGA CATAGCAAAA ATAGCACAGA   
  
  
- ATGACGGAAA AATCTAACAG TTACGACGTG TCACCCTTAA AAGGCGAGTT GGATATTGGC ATACTCGAAT   
  
  
- GGAGTTTCAT GTTGACCACA CATCAAGATC GACGGCTGCA GCTGGTGGTT CGTGTGTGGC ACTAACGTGT   
  
  
- AATTGACCAG AGAGGAGGCG GAGAGCGTGG CGGTGCAGGA GATAAGGAGG TGAGGTGGCC AGATGACAGC   
  
  
- GAGCCGATTT TGTGGGGTCC ACGTTCTTTT GCCAAAAGGG ATGCCGTTTG ACAGCATCTC GTGGCTCGTG   
  
  
- ATCTCAATGC CAACCAGCTG TGCTCCCTTC TCGCATCGTA TGATTCAGGA TCCAACGGCC GAAAACGGTT   
  
  
- CACCTTATCA TCCAGTCAGC TTAATGATCA AATATTTTCA ACTCTCCACG GCTGAGATGC GCTGACAGAG   
  
  
- TCTCTGTATA TATAAGCGAG AGTACGAAAA CCATTGAGGA TTTAGAAGTA GGAGTGGGCA TCACGAGACA   
  
  
- TGGGATTGCA AGCGTGTGGG GTCACACTTT TATTTTAATT TGTCTGTTTA GGACTAATAA GTTGGAAGTG   
  
  
- GCGAGTCTTG TGTTGGACCC AATTTCAGGC CCCCCATCAT GGTTTCTCCC GCCACATAAT TACAGTGTCT   
  
  
- GACCGAATTA CATTATTGTT ATTCTTGTT

+     Unnamed\_\_3

| Site Name | Organism | Position | Strand | Matrix score. | sequence | function |
| --- | --- | --- | --- | --- | --- | --- |
| Unnamed\_\_3 | Zea mays | 222 | + | 5 | CGTGG |  |
| Unnamed\_\_3 | Zea mays | 1012 | - | 5 | CGTGG |  |

> 2018/04/13 10:10:12  
+ CTGGGAAAAA CATAGAGAGC GAGATGCCGG CAAAGTCGCT GTTCCGAGAC ACTTAGGCAT TGTCTGGAGG   
  
  
+ CGTCCCTGAC AATCTCTTCT CTGTCTCTCT CTTTCTCTCT TCTGTTTTCT TACGCGACTG AAAACTCATT   
  
  
+ TCTCTCTCTT TTACCATCCC CGATCATTAC TCACCCCAAT GGTTATATAT ATCTCTCTCT CTCTCTGCCG   
  
  
+ TGTCATGTGT ACGTGGACAT ACATCTCTCT CTCTCGGCTT CACCAATAAA TCATAAGTGT TCAGATCTAT   
  
  
+ CTCTCTCTTT GATCTCTTCA ACCGTAGAGT TACGGGTAAC AACAAGAAAG AGAGAGACGA TAGGCTGTTA   
  
  
+ ACTTTTGGTC TTTGCCAAAG ACCAAAACCC CAACTGAAGA GCGTGTAGAG CAACCCAAGC GGGAGAGGTT   
  
  
+ AAGAAAAAAG TGTGTTTTTC AGGTGCGCGA GAGAAAAAGA GAGAGAGAGA GCACCCGCGA GCACTTAGTG   
  
  
+ GCTGTTTTCT CTCCTTAATT TTCTATCGAA GACGTTTCTC CTAATTTGCT TTCACCTGCG GTCAATTGCT   
  
  
+ CCCGCGAGGA TAGTTAAAGT TGACACAAAA ATGCTAAAAT GGGTATTGTT TTCTTCTTTC CTAAATTTAG   
  
  
+ TTAAATCAAC AACCAACGAA GGCATCAATT AATACACGGA GAAAAATACA GCGGCCCAAA CAAGCAGGGC   
  
  
+ ACGTACTGTG CACGGCTCAG TGACCGTCAA GCTAATTTGG TGTGGCCAAG GATGAAAGTG AGGGACACAA   
  
  
+ ACGAAGAAGG TGAGGTAATA AGGAAAGAAG AATGCCTTTA ATGGAAACCT GTATCGTTTT TATCGTGTCT   
  
  
+ TACTGCCTTT TTAGATTGTC AATGCTGCAC AGTGGGAATT TTCCGCTCAA CCTATAACCG TATGAGCTTA   
  
  
+ CCTCAAAGTA CAACTGGTGT GTAGTTCTAG CTGCCGACGT CGACCACCAA GCACACACCG TGATTGCACA   
  
  
+ TTAACTGGTC TCTCCTCCGC CTCTCGCACC GCCACGTCCT CTATTCCTCC ACTCCACCGG TCTACTGTCG   
  
  
+ CTCGGCTAAA ACACCCCAGG TGCAAGAAAA CGGTTTTCCC TACGGCAAAC TGTCGTAGAG CACCGAGCAC   
  
  
+ TAGAGTTACG GTTGGTCGAC ACGAGGGAAG AGCGTAGCAT ACTAAGTCCT AGGTTGCCGG CTTTTGCCAA   
  
  
+ GTGGAATAGT AGGTCAGTCG AATTACTAGT TTATAAAAGT TGAGAGGTGC CGACTCTACG CGACTGTCTC   
  
  
+ AGAGACATAT ATATTCGCTC TCATGCTTTT GGTAACTCCT AAATCTTCAT CCTCACCCGT AGTGCTCTGT   
  
  
+ ACCCTAACGT TCGCACACCC CAGTGTGAAA ATAAAATTAA ACAGACAAAT CCTGATTATT CAACCTTCAC   
  
  
+ CGCTCAGAAC ACAACCTGGG TTAAAGTCCG GGGGGTAGTA CCAAAGAGGG CGGTGTATTA ATGTCACAGA   
  
  
+ CTGGCTTAAT GTAATAACAA TAAGAACAA  

- GACCCTTTTT GTATCTCTCG CTCTACGGCC GTTTCAGCGA CAAGGCTCTG TGAATCCGTA ACAGACCTCC   
  
  
- GCAGGGACTG TTAGAGAAGA GACAGAGAGA GAAAGAGAGA AGACAAAAGA ATGCGCTGAC TTTTGAGTAA   
  
  
- AGAGAGAGAA AATGGTAGGG GCTAGTAATG AGTGGGGTTA CCAATATATA TAGAGAGAGA GAGAGACGGC   
  
  
- ACAGTACACA TGCACCTGTA TGTAGAGAGA GAGAGCCGAA GTGGTTATTT AGTATTCACA AGTCTAGATA   
  
  
- GAGAGAGAAA CTAGAGAAGT TGGCATCTCA ATGCCCATTG TTGTTCTTTC TCTCTCTGCT ATCCGACAAT   
  
  
- TGAAAACCAG AAACGGTTTC TGGTTTTGGG GTTGACTTCT CGCACATCTC GTTGGGTTCG CCCTCTCCAA   
  
  
- TTCTTTTTTC ACACAAAAAG TCCACGCGCT CTCTTTTTCT CTCTCTCTCT CGTGGGCGCT CGTGAATCAC   
  
  
- CGACAAAAGA GAGGAATTAA AAGATAGCTT CTGCAAAGAG GATTAAACGA AAGTGGACGC CAGTTAACGA   
  
  
- GGGCGCTCCT ATCAATTTCA ACTGTGTTTT TACGATTTTA CCCATAACAA AAGAAGAAAG GATTTAAATC   
  
  
- AATTTAGTTG TTGGTTGCTT CCGTAGTTAA TTATGTGCCT CTTTTTATGT CGCCGGGTTT GTTCGTCCCG   
  
  
- TGCATGACAC GTGCCGAGTC ACTGGCAGTT CGATTAAACC ACACCGGTTC CTACTTTCAC TCCCTGTGTT   
  
  
- TGCTTCTTCC ACTCCATTAT TCCTTTCTTC TTACGGAAAT TACCTTTGGA CATAGCAAAA ATAGCACAGA   
  
  
- ATGACGGAAA AATCTAACAG TTACGACGTG TCACCCTTAA AAGGCGAGTT GGATATTGGC ATACTCGAAT   
  
  
- GGAGTTTCAT GTTGACCACA CATCAAGATC GACGGCTGCA GCTGGTGGTT CGTGTGTGGC ACTAACGTGT   
  
  
- AATTGACCAG AGAGGAGGCG GAGAGCGTGG CGGTGCAGGA GATAAGGAGG TGAGGTGGCC AGATGACAGC   
  
  
- GAGCCGATTT TGTGGGGTCC ACGTTCTTTT GCCAAAAGGG ATGCCGTTTG ACAGCATCTC GTGGCTCGTG   
  
  
- ATCTCAATGC CAACCAGCTG TGCTCCCTTC TCGCATCGTA TGATTCAGGA TCCAACGGCC GAAAACGGTT   
  
  
- CACCTTATCA TCCAGTCAGC TTAATGATCA AATATTTTCA ACTCTCCACG GCTGAGATGC GCTGACAGAG   
  
  
- TCTCTGTATA TATAAGCGAG AGTACGAAAA CCATTGAGGA TTTAGAAGTA GGAGTGGGCA TCACGAGACA   
  
  
- TGGGATTGCA AGCGTGTGGG GTCACACTTT TATTTTAATT TGTCTGTTTA GGACTAATAA GTTGGAAGTG   
  
  
- GCGAGTCTTG TGTTGGACCC AATTTCAGGC CCCCCATCAT GGTTTCTCCC GCCACATAAT TACAGTGTCT   
  
  
- GACCGAATTA CATTATTGTT ATTCTTGTT

+     Unnamed\_\_4

| Site Name | Organism | Position | Strand | Matrix score. | sequence | function |
| --- | --- | --- | --- | --- | --- | --- |
| Unnamed\_\_4 | Petroselinum hortense | 528 | + | 4 | CTCC |  |
| Unnamed\_\_4 | Petroselinum hortense | 501 | + | 4 | CTCC |  |
| Unnamed\_\_4 | Petroselinum hortense | 1027 | + | 4 | CTCC |  |
| Unnamed\_\_4 | Petroselinum hortense | 559 | + | 4 | CTCC |  |
| Unnamed\_\_4 | Petroselinum hortense | 1296 | + | 4 | CTCC |  |
| Unnamed\_\_4 | Petroselinum hortense | 668 | - | 4 | CTCC |  |
| Unnamed\_\_4 | Petroselinum hortense | 412 | - | 4 | CTCC |  |
| Unnamed\_\_4 | Petroselinum hortense | 66 | - | 4 | CTCC |  |
| Unnamed\_\_4 | Petroselinum hortense | 1032 | + | 4 | CTCC |  |
| Unnamed\_\_4 | Petroselinum hortense | 992 | + | 4 | CTCC |  |
| Unnamed\_\_4 | Petroselinum hortense | 995 | + | 4 | CTCC |  |

> 2018/04/13 10:10:12  
+ CTGGGAAAAA CATAGAGAGC GAGATGCCGG CAAAGTCGCT GTTCCGAGAC ACTTAGGCAT TGTCTGGAGG   
  
  
+ CGTCCCTGAC AATCTCTTCT CTGTCTCTCT CTTTCTCTCT TCTGTTTTCT TACGCGACTG AAAACTCATT   
  
  
+ TCTCTCTCTT TTACCATCCC CGATCATTAC TCACCCCAAT GGTTATATAT ATCTCTCTCT CTCTCTGCCG   
  
  
+ TGTCATGTGT ACGTGGACAT ACATCTCTCT CTCTCGGCTT CACCAATAAA TCATAAGTGT TCAGATCTAT   
  
  
+ CTCTCTCTTT GATCTCTTCA ACCGTAGAGT TACGGGTAAC AACAAGAAAG AGAGAGACGA TAGGCTGTTA   
  
  
+ ACTTTTGGTC TTTGCCAAAG ACCAAAACCC CAACTGAAGA GCGTGTAGAG CAACCCAAGC GGGAGAGGTT   
  
  
+ AAGAAAAAAG TGTGTTTTTC AGGTGCGCGA GAGAAAAAGA GAGAGAGAGA GCACCCGCGA GCACTTAGTG   
  
  
+ GCTGTTTTCT CTCCTTAATT TTCTATCGAA GACGTTTCTC CTAATTTGCT TTCACCTGCG GTCAATTGCT   
  
  
+ CCCGCGAGGA TAGTTAAAGT TGACACAAAA ATGCTAAAAT GGGTATTGTT TTCTTCTTTC CTAAATTTAG   
  
  
+ TTAAATCAAC AACCAACGAA GGCATCAATT AATACACGGA GAAAAATACA GCGGCCCAAA CAAGCAGGGC   
  
  
+ ACGTACTGTG CACGGCTCAG TGACCGTCAA GCTAATTTGG TGTGGCCAAG GATGAAAGTG AGGGACACAA   
  
  
+ ACGAAGAAGG TGAGGTAATA AGGAAAGAAG AATGCCTTTA ATGGAAACCT GTATCGTTTT TATCGTGTCT   
  
  
+ TACTGCCTTT TTAGATTGTC AATGCTGCAC AGTGGGAATT TTCCGCTCAA CCTATAACCG TATGAGCTTA   
  
  
+ CCTCAAAGTA CAACTGGTGT GTAGTTCTAG CTGCCGACGT CGACCACCAA GCACACACCG TGATTGCACA   
  
  
+ TTAACTGGTC TCTCCTCCGC CTCTCGCACC GCCACGTCCT CTATTCCTCC ACTCCACCGG TCTACTGTCG   
  
  
+ CTCGGCTAAA ACACCCCAGG TGCAAGAAAA CGGTTTTCCC TACGGCAAAC TGTCGTAGAG CACCGAGCAC   
  
  
+ TAGAGTTACG GTTGGTCGAC ACGAGGGAAG AGCGTAGCAT ACTAAGTCCT AGGTTGCCGG CTTTTGCCAA   
  
  
+ GTGGAATAGT AGGTCAGTCG AATTACTAGT TTATAAAAGT TGAGAGGTGC CGACTCTACG CGACTGTCTC   
  
  
+ AGAGACATAT ATATTCGCTC TCATGCTTTT GGTAACTCCT AAATCTTCAT CCTCACCCGT AGTGCTCTGT   
  
  
+ ACCCTAACGT TCGCACACCC CAGTGTGAAA ATAAAATTAA ACAGACAAAT CCTGATTATT CAACCTTCAC   
  
  
+ CGCTCAGAAC ACAACCTGGG TTAAAGTCCG GGGGGTAGTA CCAAAGAGGG CGGTGTATTA ATGTCACAGA   
  
  
+ CTGGCTTAAT GTAATAACAA TAAGAACAA  

- GACCCTTTTT GTATCTCTCG CTCTACGGCC GTTTCAGCGA CAAGGCTCTG TGAATCCGTA ACAGACCTCC   
  
  
- GCAGGGACTG TTAGAGAAGA GACAGAGAGA GAAAGAGAGA AGACAAAAGA ATGCGCTGAC TTTTGAGTAA   
  
  
- AGAGAGAGAA AATGGTAGGG GCTAGTAATG AGTGGGGTTA CCAATATATA TAGAGAGAGA GAGAGACGGC   
  
  
- ACAGTACACA TGCACCTGTA TGTAGAGAGA GAGAGCCGAA GTGGTTATTT AGTATTCACA AGTCTAGATA   
  
  
- GAGAGAGAAA CTAGAGAAGT TGGCATCTCA ATGCCCATTG TTGTTCTTTC TCTCTCTGCT ATCCGACAAT   
  
  
- TGAAAACCAG AAACGGTTTC TGGTTTTGGG GTTGACTTCT CGCACATCTC GTTGGGTTCG CCCTCTCCAA   
  
  
- TTCTTTTTTC ACACAAAAAG TCCACGCGCT CTCTTTTTCT CTCTCTCTCT CGTGGGCGCT CGTGAATCAC   
  
  
- CGACAAAAGA GAGGAATTAA AAGATAGCTT CTGCAAAGAG GATTAAACGA AAGTGGACGC CAGTTAACGA   
  
  
- GGGCGCTCCT ATCAATTTCA ACTGTGTTTT TACGATTTTA CCCATAACAA AAGAAGAAAG GATTTAAATC   
  
  
- AATTTAGTTG TTGGTTGCTT CCGTAGTTAA TTATGTGCCT CTTTTTATGT CGCCGGGTTT GTTCGTCCCG   
  
  
- TGCATGACAC GTGCCGAGTC ACTGGCAGTT CGATTAAACC ACACCGGTTC CTACTTTCAC TCCCTGTGTT   
  
  
- TGCTTCTTCC ACTCCATTAT TCCTTTCTTC TTACGGAAAT TACCTTTGGA CATAGCAAAA ATAGCACAGA   
  
  
- ATGACGGAAA AATCTAACAG TTACGACGTG TCACCCTTAA AAGGCGAGTT GGATATTGGC ATACTCGAAT   
  
  
- GGAGTTTCAT GTTGACCACA CATCAAGATC GACGGCTGCA GCTGGTGGTT CGTGTGTGGC ACTAACGTGT   
  
  
- AATTGACCAG AGAGGAGGCG GAGAGCGTGG CGGTGCAGGA GATAAGGAGG TGAGGTGGCC AGATGACAGC   
  
  
- GAGCCGATTT TGTGGGGTCC ACGTTCTTTT GCCAAAAGGG ATGCCGTTTG ACAGCATCTC GTGGCTCGTG   
  
  
- ATCTCAATGC CAACCAGCTG TGCTCCCTTC TCGCATCGTA TGATTCAGGA TCCAACGGCC GAAAACGGTT   
  
  
- CACCTTATCA TCCAGTCAGC TTAATGATCA AATATTTTCA ACTCTCCACG GCTGAGATGC GCTGACAGAG   
  
  
- TCTCTGTATA TATAAGCGAG AGTACGAAAA CCATTGAGGA TTTAGAAGTA GGAGTGGGCA TCACGAGACA   
  
  
- TGGGATTGCA AGCGTGTGGG GTCACACTTT TATTTTAATT TGTCTGTTTA GGACTAATAA GTTGGAAGTG   
  
  
- GCGAGTCTTG TGTTGGACCC AATTTCAGGC CCCCCATCAT GGTTTCTCCC GCCACATAAT TACAGTGTCT   
  
  
- GACCGAATTA CATTATTGTT ATTCTTGTT

+     Unnamed\_\_6

| Site Name | Organism | Position | Strand | Matrix score. | sequence | function |
| --- | --- | --- | --- | --- | --- | --- |
| Unnamed\_\_6 | Zea mays | 184 | + | 10 | taTAAATATct |  |

> 2018/04/13 10:10:12  
+ CTGGGAAAAA CATAGAGAGC GAGATGCCGG CAAAGTCGCT GTTCCGAGAC ACTTAGGCAT TGTCTGGAGG   
  
  
+ CGTCCCTGAC AATCTCTTCT CTGTCTCTCT CTTTCTCTCT TCTGTTTTCT TACGCGACTG AAAACTCATT   
  
  
+ TCTCTCTCTT TTACCATCCC CGATCATTAC TCACCCCAAT GGTTATATAT ATCTCTCTCT CTCTCTGCCG   
  
  
+ TGTCATGTGT ACGTGGACAT ACATCTCTCT CTCTCGGCTT CACCAATAAA TCATAAGTGT TCAGATCTAT   
  
  
+ CTCTCTCTTT GATCTCTTCA ACCGTAGAGT TACGGGTAAC AACAAGAAAG AGAGAGACGA TAGGCTGTTA   
  
  
+ ACTTTTGGTC TTTGCCAAAG ACCAAAACCC CAACTGAAGA GCGTGTAGAG CAACCCAAGC GGGAGAGGTT   
  
  
+ AAGAAAAAAG TGTGTTTTTC AGGTGCGCGA GAGAAAAAGA GAGAGAGAGA GCACCCGCGA GCACTTAGTG   
  
  
+ GCTGTTTTCT CTCCTTAATT TTCTATCGAA GACGTTTCTC CTAATTTGCT TTCACCTGCG GTCAATTGCT   
  
  
+ CCCGCGAGGA TAGTTAAAGT TGACACAAAA ATGCTAAAAT GGGTATTGTT TTCTTCTTTC CTAAATTTAG   
  
  
+ TTAAATCAAC AACCAACGAA GGCATCAATT AATACACGGA GAAAAATACA GCGGCCCAAA CAAGCAGGGC   
  
  
+ ACGTACTGTG CACGGCTCAG TGACCGTCAA GCTAATTTGG TGTGGCCAAG GATGAAAGTG AGGGACACAA   
  
  
+ ACGAAGAAGG TGAGGTAATA AGGAAAGAAG AATGCCTTTA ATGGAAACCT GTATCGTTTT TATCGTGTCT   
  
  
+ TACTGCCTTT TTAGATTGTC AATGCTGCAC AGTGGGAATT TTCCGCTCAA CCTATAACCG TATGAGCTTA   
  
  
+ CCTCAAAGTA CAACTGGTGT GTAGTTCTAG CTGCCGACGT CGACCACCAA GCACACACCG TGATTGCACA   
  
  
+ TTAACTGGTC TCTCCTCCGC CTCTCGCACC GCCACGTCCT CTATTCCTCC ACTCCACCGG TCTACTGTCG   
  
  
+ CTCGGCTAAA ACACCCCAGG TGCAAGAAAA CGGTTTTCCC TACGGCAAAC TGTCGTAGAG CACCGAGCAC   
  
  
+ TAGAGTTACG GTTGGTCGAC ACGAGGGAAG AGCGTAGCAT ACTAAGTCCT AGGTTGCCGG CTTTTGCCAA   
  
  
+ GTGGAATAGT AGGTCAGTCG AATTACTAGT TTATAAAAGT TGAGAGGTGC CGACTCTACG CGACTGTCTC   
  
  
+ AGAGACATAT ATATTCGCTC TCATGCTTTT GGTAACTCCT AAATCTTCAT CCTCACCCGT AGTGCTCTGT   
  
  
+ ACCCTAACGT TCGCACACCC CAGTGTGAAA ATAAAATTAA ACAGACAAAT CCTGATTATT CAACCTTCAC   
  
  
+ CGCTCAGAAC ACAACCTGGG TTAAAGTCCG GGGGGTAGTA CCAAAGAGGG CGGTGTATTA ATGTCACAGA   
  
  
+ CTGGCTTAAT GTAATAACAA TAAGAACAA  

- GACCCTTTTT GTATCTCTCG CTCTACGGCC GTTTCAGCGA CAAGGCTCTG TGAATCCGTA ACAGACCTCC   
  
  
- GCAGGGACTG TTAGAGAAGA GACAGAGAGA GAAAGAGAGA AGACAAAAGA ATGCGCTGAC TTTTGAGTAA   
  
  
- AGAGAGAGAA AATGGTAGGG GCTAGTAATG AGTGGGGTTA CCAATATATA TAGAGAGAGA GAGAGACGGC   
  
  
- ACAGTACACA TGCACCTGTA TGTAGAGAGA GAGAGCCGAA GTGGTTATTT AGTATTCACA AGTCTAGATA   
  
  
- GAGAGAGAAA CTAGAGAAGT TGGCATCTCA ATGCCCATTG TTGTTCTTTC TCTCTCTGCT ATCCGACAAT   
  
  
- TGAAAACCAG AAACGGTTTC TGGTTTTGGG GTTGACTTCT CGCACATCTC GTTGGGTTCG CCCTCTCCAA   
  
  
- TTCTTTTTTC ACACAAAAAG TCCACGCGCT CTCTTTTTCT CTCTCTCTCT CGTGGGCGCT CGTGAATCAC   
  
  
- CGACAAAAGA GAGGAATTAA AAGATAGCTT CTGCAAAGAG GATTAAACGA AAGTGGACGC CAGTTAACGA   
  
  
- GGGCGCTCCT ATCAATTTCA ACTGTGTTTT TACGATTTTA CCCATAACAA AAGAAGAAAG GATTTAAATC   
  
  
- AATTTAGTTG TTGGTTGCTT CCGTAGTTAA TTATGTGCCT CTTTTTATGT CGCCGGGTTT GTTCGTCCCG   
  
  
- TGCATGACAC GTGCCGAGTC ACTGGCAGTT CGATTAAACC ACACCGGTTC CTACTTTCAC TCCCTGTGTT   
  
  
- TGCTTCTTCC ACTCCATTAT TCCTTTCTTC TTACGGAAAT TACCTTTGGA CATAGCAAAA ATAGCACAGA   
  
  
- ATGACGGAAA AATCTAACAG TTACGACGTG TCACCCTTAA AAGGCGAGTT GGATATTGGC ATACTCGAAT   
  
  
- GGAGTTTCAT GTTGACCACA CATCAAGATC GACGGCTGCA GCTGGTGGTT CGTGTGTGGC ACTAACGTGT   
  
  
- AATTGACCAG AGAGGAGGCG GAGAGCGTGG CGGTGCAGGA GATAAGGAGG TGAGGTGGCC AGATGACAGC   
  
  
- GAGCCGATTT TGTGGGGTCC ACGTTCTTTT GCCAAAAGGG ATGCCGTTTG ACAGCATCTC GTGGCTCGTG   
  
  
- ATCTCAATGC CAACCAGCTG TGCTCCCTTC TCGCATCGTA TGATTCAGGA TCCAACGGCC GAAAACGGTT   
  
  
- CACCTTATCA TCCAGTCAGC TTAATGATCA AATATTTTCA ACTCTCCACG GCTGAGATGC GCTGACAGAG   
  
  
- TCTCTGTATA TATAAGCGAG AGTACGAAAA CCATTGAGGA TTTAGAAGTA GGAGTGGGCA TCACGAGACA   
  
  
- TGGGATTGCA AGCGTGTGGG GTCACACTTT TATTTTAATT TGTCTGTTTA GGACTAATAA GTTGGAAGTG   
  
  
- GCGAGTCTTG TGTTGGACCC AATTTCAGGC CCCCCATCAT GGTTTCTCCC GCCACATAAT TACAGTGTCT   
  
  
- GACCGAATTA CATTATTGTT ATTCTTGTT

+     Unnamed\_\_8

| Site Name | Organism | Position | Strand | Matrix score. | sequence | function |
| --- | --- | --- | --- | --- | --- | --- |
| Unnamed\_\_8 | Glycine max cv. Provar | 585 | - | 9 | CATTTTTGT |  |
| Unnamed\_\_8 | Zea mays | 218 | - | 9 | TCCACGTAGA |  |

> 2018/04/13 10:10:12  
+ CTGGGAAAAA CATAGAGAGC GAGATGCCGG CAAAGTCGCT GTTCCGAGAC ACTTAGGCAT TGTCTGGAGG   
  
  
+ CGTCCCTGAC AATCTCTTCT CTGTCTCTCT CTTTCTCTCT TCTGTTTTCT TACGCGACTG AAAACTCATT   
  
  
+ TCTCTCTCTT TTACCATCCC CGATCATTAC TCACCCCAAT GGTTATATAT ATCTCTCTCT CTCTCTGCCG   
  
  
+ TGTCATGTGT ACGTGGACAT ACATCTCTCT CTCTCGGCTT CACCAATAAA TCATAAGTGT TCAGATCTAT   
  
  
+ CTCTCTCTTT GATCTCTTCA ACCGTAGAGT TACGGGTAAC AACAAGAAAG AGAGAGACGA TAGGCTGTTA   
  
  
+ ACTTTTGGTC TTTGCCAAAG ACCAAAACCC CAACTGAAGA GCGTGTAGAG CAACCCAAGC GGGAGAGGTT   
  
  
+ AAGAAAAAAG TGTGTTTTTC AGGTGCGCGA GAGAAAAAGA GAGAGAGAGA GCACCCGCGA GCACTTAGTG   
  
  
+ GCTGTTTTCT CTCCTTAATT TTCTATCGAA GACGTTTCTC CTAATTTGCT TTCACCTGCG GTCAATTGCT   
  
  
+ CCCGCGAGGA TAGTTAAAGT TGACACAAAA ATGCTAAAAT GGGTATTGTT TTCTTCTTTC CTAAATTTAG   
  
  
+ TTAAATCAAC AACCAACGAA GGCATCAATT AATACACGGA GAAAAATACA GCGGCCCAAA CAAGCAGGGC   
  
  
+ ACGTACTGTG CACGGCTCAG TGACCGTCAA GCTAATTTGG TGTGGCCAAG GATGAAAGTG AGGGACACAA   
  
  
+ ACGAAGAAGG TGAGGTAATA AGGAAAGAAG AATGCCTTTA ATGGAAACCT GTATCGTTTT TATCGTGTCT   
  
  
+ TACTGCCTTT TTAGATTGTC AATGCTGCAC AGTGGGAATT TTCCGCTCAA CCTATAACCG TATGAGCTTA   
  
  
+ CCTCAAAGTA CAACTGGTGT GTAGTTCTAG CTGCCGACGT CGACCACCAA GCACACACCG TGATTGCACA   
  
  
+ TTAACTGGTC TCTCCTCCGC CTCTCGCACC GCCACGTCCT CTATTCCTCC ACTCCACCGG TCTACTGTCG   
  
  
+ CTCGGCTAAA ACACCCCAGG TGCAAGAAAA CGGTTTTCCC TACGGCAAAC TGTCGTAGAG CACCGAGCAC   
  
  
+ TAGAGTTACG GTTGGTCGAC ACGAGGGAAG AGCGTAGCAT ACTAAGTCCT AGGTTGCCGG CTTTTGCCAA   
  
  
+ GTGGAATAGT AGGTCAGTCG AATTACTAGT TTATAAAAGT TGAGAGGTGC CGACTCTACG CGACTGTCTC   
  
  
+ AGAGACATAT ATATTCGCTC TCATGCTTTT GGTAACTCCT AAATCTTCAT CCTCACCCGT AGTGCTCTGT   
  
  
+ ACCCTAACGT TCGCACACCC CAGTGTGAAA ATAAAATTAA ACAGACAAAT CCTGATTATT CAACCTTCAC   
  
  
+ CGCTCAGAAC ACAACCTGGG TTAAAGTCCG GGGGGTAGTA CCAAAGAGGG CGGTGTATTA ATGTCACAGA   
  
  
+ CTGGCTTAAT GTAATAACAA TAAGAACAA  

- GACCCTTTTT GTATCTCTCG CTCTACGGCC GTTTCAGCGA CAAGGCTCTG TGAATCCGTA ACAGACCTCC   
  
  
- GCAGGGACTG TTAGAGAAGA GACAGAGAGA GAAAGAGAGA AGACAAAAGA ATGCGCTGAC TTTTGAGTAA   
  
  
- AGAGAGAGAA AATGGTAGGG GCTAGTAATG AGTGGGGTTA CCAATATATA TAGAGAGAGA GAGAGACGGC   
  
  
- ACAGTACACA TGCACCTGTA TGTAGAGAGA GAGAGCCGAA GTGGTTATTT AGTATTCACA AGTCTAGATA   
  
  
- GAGAGAGAAA CTAGAGAAGT TGGCATCTCA ATGCCCATTG TTGTTCTTTC TCTCTCTGCT ATCCGACAAT   
  
  
- TGAAAACCAG AAACGGTTTC TGGTTTTGGG GTTGACTTCT CGCACATCTC GTTGGGTTCG CCCTCTCCAA   
  
  
- TTCTTTTTTC ACACAAAAAG TCCACGCGCT CTCTTTTTCT CTCTCTCTCT CGTGGGCGCT CGTGAATCAC   
  
  
- CGACAAAAGA GAGGAATTAA AAGATAGCTT CTGCAAAGAG GATTAAACGA AAGTGGACGC CAGTTAACGA   
  
  
- GGGCGCTCCT ATCAATTTCA ACTGTGTTTT TACGATTTTA CCCATAACAA AAGAAGAAAG GATTTAAATC   
  
  
- AATTTAGTTG TTGGTTGCTT CCGTAGTTAA TTATGTGCCT CTTTTTATGT CGCCGGGTTT GTTCGTCCCG   
  
  
- TGCATGACAC GTGCCGAGTC ACTGGCAGTT CGATTAAACC ACACCGGTTC CTACTTTCAC TCCCTGTGTT   
  
  
- TGCTTCTTCC ACTCCATTAT TCCTTTCTTC TTACGGAAAT TACCTTTGGA CATAGCAAAA ATAGCACAGA   
  
  
- ATGACGGAAA AATCTAACAG TTACGACGTG TCACCCTTAA AAGGCGAGTT GGATATTGGC ATACTCGAAT   
  
  
- GGAGTTTCAT GTTGACCACA CATCAAGATC GACGGCTGCA GCTGGTGGTT CGTGTGTGGC ACTAACGTGT   
  
  
- AATTGACCAG AGAGGAGGCG GAGAGCGTGG CGGTGCAGGA GATAAGGAGG TGAGGTGGCC AGATGACAGC   
  
  
- GAGCCGATTT TGTGGGGTCC ACGTTCTTTT GCCAAAAGGG ATGCCGTTTG ACAGCATCTC GTGGCTCGTG   
  
  
- ATCTCAATGC CAACCAGCTG TGCTCCCTTC TCGCATCGTA TGATTCAGGA TCCAACGGCC GAAAACGGTT   
  
  
- CACCTTATCA TCCAGTCAGC TTAATGATCA AATATTTTCA ACTCTCCACG GCTGAGATGC GCTGACAGAG   
  
  
- TCTCTGTATA TATAAGCGAG AGTACGAAAA CCATTGAGGA TTTAGAAGTA GGAGTGGGCA TCACGAGACA   
  
  
- TGGGATTGCA AGCGTGTGGG GTCACACTTT TATTTTAATT TGTCTGTTTA GGACTAATAA GTTGGAAGTG   
  
  
- GCGAGTCTTG TGTTGGACCC AATTTCAGGC CCCCCATCAT GGTTTCTCCC GCCACATAAT TACAGTGTCT   
  
  
- GACCGAATTA CATTATTGTT ATTCTTGTT

+     Unnamed\_\_9

| Site Name | Organism | Position | Strand | Matrix score. | sequence | function |
| --- | --- | --- | --- | --- | --- | --- |
| Unnamed\_\_9 | Zea mays | 218 | - | 9 | TCCACGTAGA |  |

> 2018/04/13 10:10:12  
+ CTGGGAAAAA CATAGAGAGC GAGATGCCGG CAAAGTCGCT GTTCCGAGAC ACTTAGGCAT TGTCTGGAGG   
  
  
+ CGTCCCTGAC AATCTCTTCT CTGTCTCTCT CTTTCTCTCT TCTGTTTTCT TACGCGACTG AAAACTCATT   
  
  
+ TCTCTCTCTT TTACCATCCC CGATCATTAC TCACCCCAAT GGTTATATAT ATCTCTCTCT CTCTCTGCCG   
  
  
+ TGTCATGTGT ACGTGGACAT ACATCTCTCT CTCTCGGCTT CACCAATAAA TCATAAGTGT TCAGATCTAT   
  
  
+ CTCTCTCTTT GATCTCTTCA ACCGTAGAGT TACGGGTAAC AACAAGAAAG AGAGAGACGA TAGGCTGTTA   
  
  
+ ACTTTTGGTC TTTGCCAAAG ACCAAAACCC CAACTGAAGA GCGTGTAGAG CAACCCAAGC GGGAGAGGTT   
  
  
+ AAGAAAAAAG TGTGTTTTTC AGGTGCGCGA GAGAAAAAGA GAGAGAGAGA GCACCCGCGA GCACTTAGTG   
  
  
+ GCTGTTTTCT CTCCTTAATT TTCTATCGAA GACGTTTCTC CTAATTTGCT TTCACCTGCG GTCAATTGCT   
  
  
+ CCCGCGAGGA TAGTTAAAGT TGACACAAAA ATGCTAAAAT GGGTATTGTT TTCTTCTTTC CTAAATTTAG   
  
  
+ TTAAATCAAC AACCAACGAA GGCATCAATT AATACACGGA GAAAAATACA GCGGCCCAAA CAAGCAGGGC   
  
  
+ ACGTACTGTG CACGGCTCAG TGACCGTCAA GCTAATTTGG TGTGGCCAAG GATGAAAGTG AGGGACACAA   
  
  
+ ACGAAGAAGG TGAGGTAATA AGGAAAGAAG AATGCCTTTA ATGGAAACCT GTATCGTTTT TATCGTGTCT   
  
  
+ TACTGCCTTT TTAGATTGTC AATGCTGCAC AGTGGGAATT TTCCGCTCAA CCTATAACCG TATGAGCTTA   
  
  
+ CCTCAAAGTA CAACTGGTGT GTAGTTCTAG CTGCCGACGT CGACCACCAA GCACACACCG TGATTGCACA   
  
  
+ TTAACTGGTC TCTCCTCCGC CTCTCGCACC GCCACGTCCT CTATTCCTCC ACTCCACCGG TCTACTGTCG   
  
  
+ CTCGGCTAAA ACACCCCAGG TGCAAGAAAA CGGTTTTCCC TACGGCAAAC TGTCGTAGAG CACCGAGCAC   
  
  
+ TAGAGTTACG GTTGGTCGAC ACGAGGGAAG AGCGTAGCAT ACTAAGTCCT AGGTTGCCGG CTTTTGCCAA   
  
  
+ GTGGAATAGT AGGTCAGTCG AATTACTAGT TTATAAAAGT TGAGAGGTGC CGACTCTACG CGACTGTCTC   
  
  
+ AGAGACATAT ATATTCGCTC TCATGCTTTT GGTAACTCCT AAATCTTCAT CCTCACCCGT AGTGCTCTGT   
  
  
+ ACCCTAACGT TCGCACACCC CAGTGTGAAA ATAAAATTAA ACAGACAAAT CCTGATTATT CAACCTTCAC   
  
  
+ CGCTCAGAAC ACAACCTGGG TTAAAGTCCG GGGGGTAGTA CCAAAGAGGG CGGTGTATTA ATGTCACAGA   
  
  
+ CTGGCTTAAT GTAATAACAA TAAGAACAA  

- GACCCTTTTT GTATCTCTCG CTCTACGGCC GTTTCAGCGA CAAGGCTCTG TGAATCCGTA ACAGACCTCC   
  
  
- GCAGGGACTG TTAGAGAAGA GACAGAGAGA GAAAGAGAGA AGACAAAAGA ATGCGCTGAC TTTTGAGTAA   
  
  
- AGAGAGAGAA AATGGTAGGG GCTAGTAATG AGTGGGGTTA CCAATATATA TAGAGAGAGA GAGAGACGGC   
  
  
- ACAGTACACA TGCACCTGTA TGTAGAGAGA GAGAGCCGAA GTGGTTATTT AGTATTCACA AGTCTAGATA   
  
  
- GAGAGAGAAA CTAGAGAAGT TGGCATCTCA ATGCCCATTG TTGTTCTTTC TCTCTCTGCT ATCCGACAAT   
  
  
- TGAAAACCAG AAACGGTTTC TGGTTTTGGG GTTGACTTCT CGCACATCTC GTTGGGTTCG CCCTCTCCAA   
  
  
- TTCTTTTTTC ACACAAAAAG TCCACGCGCT CTCTTTTTCT CTCTCTCTCT CGTGGGCGCT CGTGAATCAC   
  
  
- CGACAAAAGA GAGGAATTAA AAGATAGCTT CTGCAAAGAG GATTAAACGA AAGTGGACGC CAGTTAACGA   
  
  
- GGGCGCTCCT ATCAATTTCA ACTGTGTTTT TACGATTTTA CCCATAACAA AAGAAGAAAG GATTTAAATC   
  
  
- AATTTAGTTG TTGGTTGCTT CCGTAGTTAA TTATGTGCCT CTTTTTATGT CGCCGGGTTT GTTCGTCCCG   
  
  
- TGCATGACAC GTGCCGAGTC ACTGGCAGTT CGATTAAACC ACACCGGTTC CTACTTTCAC TCCCTGTGTT   
  
  
- TGCTTCTTCC ACTCCATTAT TCCTTTCTTC TTACGGAAAT TACCTTTGGA CATAGCAAAA ATAGCACAGA   
  
  
- ATGACGGAAA AATCTAACAG TTACGACGTG TCACCCTTAA AAGGCGAGTT GGATATTGGC ATACTCGAAT   
  
  
- GGAGTTTCAT GTTGACCACA CATCAAGATC GACGGCTGCA GCTGGTGGTT CGTGTGTGGC ACTAACGTGT   
  
  
- AATTGACCAG AGAGGAGGCG GAGAGCGTGG CGGTGCAGGA GATAAGGAGG TGAGGTGGCC AGATGACAGC   
  
  
- GAGCCGATTT TGTGGGGTCC ACGTTCTTTT GCCAAAAGGG ATGCCGTTTG ACAGCATCTC GTGGCTCGTG   
  
  
- ATCTCAATGC CAACCAGCTG TGCTCCCTTC TCGCATCGTA TGATTCAGGA TCCAACGGCC GAAAACGGTT   
  
  
- CACCTTATCA TCCAGTCAGC TTAATGATCA AATATTTTCA ACTCTCCACG GCTGAGATGC GCTGACAGAG   
  
  
- TCTCTGTATA TATAAGCGAG AGTACGAAAA CCATTGAGGA TTTAGAAGTA GGAGTGGGCA TCACGAGACA   
  
  
- TGGGATTGCA AGCGTGTGGG GTCACACTTT TATTTTAATT TGTCTGTTTA GGACTAATAA GTTGGAAGTG   
  
  
- GCGAGTCTTG TGTTGGACCC AATTTCAGGC CCCCCATCAT GGTTTCTCCC GCCACATAAT TACAGTGTCT   
  
  
- GACCGAATTA CATTATTGTT ATTCTTGTT

+     W box

| Site Name | Organism | Position | Strand | Matrix score. | sequence | function |
| --- | --- | --- | --- | --- | --- | --- |
| W box | Arabidopsis thaliana | 550 | - | 6 | TTGACC |  |

> 2018/04/13 10:10:12  
+ CTGGGAAAAA CATAGAGAGC GAGATGCCGG CAAAGTCGCT GTTCCGAGAC ACTTAGGCAT TGTCTGGAGG   
  
  
+ CGTCCCTGAC AATCTCTTCT CTGTCTCTCT CTTTCTCTCT TCTGTTTTCT TACGCGACTG AAAACTCATT   
  
  
+ TCTCTCTCTT TTACCATCCC CGATCATTAC TCACCCCAAT GGTTATATAT ATCTCTCTCT CTCTCTGCCG   
  
  
+ TGTCATGTGT ACGTGGACAT ACATCTCTCT CTCTCGGCTT CACCAATAAA TCATAAGTGT TCAGATCTAT   
  
  
+ CTCTCTCTTT GATCTCTTCA ACCGTAGAGT TACGGGTAAC AACAAGAAAG AGAGAGACGA TAGGCTGTTA   
  
  
+ ACTTTTGGTC TTTGCCAAAG ACCAAAACCC CAACTGAAGA GCGTGTAGAG CAACCCAAGC GGGAGAGGTT   
  
  
+ AAGAAAAAAG TGTGTTTTTC AGGTGCGCGA GAGAAAAAGA GAGAGAGAGA GCACCCGCGA GCACTTAGTG   
  
  
+ GCTGTTTTCT CTCCTTAATT TTCTATCGAA GACGTTTCTC CTAATTTGCT TTCACCTGCG GTCAATTGCT   
  
  
+ CCCGCGAGGA TAGTTAAAGT TGACACAAAA ATGCTAAAAT GGGTATTGTT TTCTTCTTTC CTAAATTTAG   
  
  
+ TTAAATCAAC AACCAACGAA GGCATCAATT AATACACGGA GAAAAATACA GCGGCCCAAA CAAGCAGGGC   
  
  
+ ACGTACTGTG CACGGCTCAG TGACCGTCAA GCTAATTTGG TGTGGCCAAG GATGAAAGTG AGGGACACAA   
  
  
+ ACGAAGAAGG TGAGGTAATA AGGAAAGAAG AATGCCTTTA ATGGAAACCT GTATCGTTTT TATCGTGTCT   
  
  
+ TACTGCCTTT TTAGATTGTC AATGCTGCAC AGTGGGAATT TTCCGCTCAA CCTATAACCG TATGAGCTTA   
  
  
+ CCTCAAAGTA CAACTGGTGT GTAGTTCTAG CTGCCGACGT CGACCACCAA GCACACACCG TGATTGCACA   
  
  
+ TTAACTGGTC TCTCCTCCGC CTCTCGCACC GCCACGTCCT CTATTCCTCC ACTCCACCGG TCTACTGTCG   
  
  
+ CTCGGCTAAA ACACCCCAGG TGCAAGAAAA CGGTTTTCCC TACGGCAAAC TGTCGTAGAG CACCGAGCAC   
  
  
+ TAGAGTTACG GTTGGTCGAC ACGAGGGAAG AGCGTAGCAT ACTAAGTCCT AGGTTGCCGG CTTTTGCCAA   
  
  
+ GTGGAATAGT AGGTCAGTCG AATTACTAGT TTATAAAAGT TGAGAGGTGC CGACTCTACG CGACTGTCTC   
  
  
+ AGAGACATAT ATATTCGCTC TCATGCTTTT GGTAACTCCT AAATCTTCAT CCTCACCCGT AGTGCTCTGT   
  
  
+ ACCCTAACGT TCGCACACCC CAGTGTGAAA ATAAAATTAA ACAGACAAAT CCTGATTATT CAACCTTCAC   
  
  
+ CGCTCAGAAC ACAACCTGGG TTAAAGTCCG GGGGGTAGTA CCAAAGAGGG CGGTGTATTA ATGTCACAGA   
  
  
+ CTGGCTTAAT GTAATAACAA TAAGAACAA  

- GACCCTTTTT GTATCTCTCG CTCTACGGCC GTTTCAGCGA CAAGGCTCTG TGAATCCGTA ACAGACCTCC   
  
  
- GCAGGGACTG TTAGAGAAGA GACAGAGAGA GAAAGAGAGA AGACAAAAGA ATGCGCTGAC TTTTGAGTAA   
  
  
- AGAGAGAGAA AATGGTAGGG GCTAGTAATG AGTGGGGTTA CCAATATATA TAGAGAGAGA GAGAGACGGC   
  
  
- ACAGTACACA TGCACCTGTA TGTAGAGAGA GAGAGCCGAA GTGGTTATTT AGTATTCACA AGTCTAGATA   
  
  
- GAGAGAGAAA CTAGAGAAGT TGGCATCTCA ATGCCCATTG TTGTTCTTTC TCTCTCTGCT ATCCGACAAT   
  
  
- TGAAAACCAG AAACGGTTTC TGGTTTTGGG GTTGACTTCT CGCACATCTC GTTGGGTTCG CCCTCTCCAA   
  
  
- TTCTTTTTTC ACACAAAAAG TCCACGCGCT CTCTTTTTCT CTCTCTCTCT CGTGGGCGCT CGTGAATCAC   
  
  
- CGACAAAAGA GAGGAATTAA AAGATAGCTT CTGCAAAGAG GATTAAACGA AAGTGGACGC CAGTTAACGA   
  
  
- GGGCGCTCCT ATCAATTTCA ACTGTGTTTT TACGATTTTA CCCATAACAA AAGAAGAAAG GATTTAAATC   
  
  
- AATTTAGTTG TTGGTTGCTT CCGTAGTTAA TTATGTGCCT CTTTTTATGT CGCCGGGTTT GTTCGTCCCG   
  
  
- TGCATGACAC GTGCCGAGTC ACTGGCAGTT CGATTAAACC ACACCGGTTC CTACTTTCAC TCCCTGTGTT   
  
  
- TGCTTCTTCC ACTCCATTAT TCCTTTCTTC TTACGGAAAT TACCTTTGGA CATAGCAAAA ATAGCACAGA   
  
  
- ATGACGGAAA AATCTAACAG TTACGACGTG TCACCCTTAA AAGGCGAGTT GGATATTGGC ATACTCGAAT   
  
  
- GGAGTTTCAT GTTGACCACA CATCAAGATC GACGGCTGCA GCTGGTGGTT CGTGTGTGGC ACTAACGTGT   
  
  
- AATTGACCAG AGAGGAGGCG GAGAGCGTGG CGGTGCAGGA GATAAGGAGG TGAGGTGGCC AGATGACAGC   
  
  
- GAGCCGATTT TGTGGGGTCC ACGTTCTTTT GCCAAAAGGG ATGCCGTTTG ACAGCATCTC GTGGCTCGTG   
  
  
- ATCTCAATGC CAACCAGCTG TGCTCCCTTC TCGCATCGTA TGATTCAGGA TCCAACGGCC GAAAACGGTT   
  
  
- CACCTTATCA TCCAGTCAGC TTAATGATCA AATATTTTCA ACTCTCCACG GCTGAGATGC GCTGACAGAG   
  
  
- TCTCTGTATA TATAAGCGAG AGTACGAAAA CCATTGAGGA TTTAGAAGTA GGAGTGGGCA TCACGAGACA   
  
  
- TGGGATTGCA AGCGTGTGGG GTCACACTTT TATTTTAATT TGTCTGTTTA GGACTAATAA GTTGGAAGTG   
  
  
- GCGAGTCTTG TGTTGGACCC AATTTCAGGC CCCCCATCAT GGTTTCTCCC GCCACATAAT TACAGTGTCT   
  
  
- GACCGAATTA CATTATTGTT ATTCTTGTT

+     box II

| Site Name | Organism | Position | Strand | Matrix score. | sequence | function |
| --- | --- | --- | --- | --- | --- | --- |
| box II | Petroselinum hortense | 1186 | - | 9 | TCCACGTGGC | part of a light responsive element |

> 2018/04/13 10:10:12  
+ CTGGGAAAAA CATAGAGAGC GAGATGCCGG CAAAGTCGCT GTTCCGAGAC ACTTAGGCAT TGTCTGGAGG   
  
  
+ CGTCCCTGAC AATCTCTTCT CTGTCTCTCT CTTTCTCTCT TCTGTTTTCT TACGCGACTG AAAACTCATT   
  
  
+ TCTCTCTCTT TTACCATCCC CGATCATTAC TCACCCCAAT GGTTATATAT ATCTCTCTCT CTCTCTGCCG   
  
  
+ TGTCATGTGT ACGTGGACAT ACATCTCTCT CTCTCGGCTT CACCAATAAA TCATAAGTGT TCAGATCTAT   
  
  
+ CTCTCTCTTT GATCTCTTCA ACCGTAGAGT TACGGGTAAC AACAAGAAAG AGAGAGACGA TAGGCTGTTA   
  
  
+ ACTTTTGGTC TTTGCCAAAG ACCAAAACCC CAACTGAAGA GCGTGTAGAG CAACCCAAGC GGGAGAGGTT   
  
  
+ AAGAAAAAAG TGTGTTTTTC AGGTGCGCGA GAGAAAAAGA GAGAGAGAGA GCACCCGCGA GCACTTAGTG   
  
  
+ GCTGTTTTCT CTCCTTAATT TTCTATCGAA GACGTTTCTC CTAATTTGCT TTCACCTGCG GTCAATTGCT   
  
  
+ CCCGCGAGGA TAGTTAAAGT TGACACAAAA ATGCTAAAAT GGGTATTGTT TTCTTCTTTC CTAAATTTAG   
  
  
+ TTAAATCAAC AACCAACGAA GGCATCAATT AATACACGGA GAAAAATACA GCGGCCCAAA CAAGCAGGGC   
  
  
+ ACGTACTGTG CACGGCTCAG TGACCGTCAA GCTAATTTGG TGTGGCCAAG GATGAAAGTG AGGGACACAA   
  
  
+ ACGAAGAAGG TGAGGTAATA AGGAAAGAAG AATGCCTTTA ATGGAAACCT GTATCGTTTT TATCGTGTCT   
  
  
+ TACTGCCTTT TTAGATTGTC AATGCTGCAC AGTGGGAATT TTCCGCTCAA CCTATAACCG TATGAGCTTA   
  
  
+ CCTCAAAGTA CAACTGGTGT GTAGTTCTAG CTGCCGACGT CGACCACCAA GCACACACCG TGATTGCACA   
  
  
+ TTAACTGGTC TCTCCTCCGC CTCTCGCACC GCCACGTCCT CTATTCCTCC ACTCCACCGG TCTACTGTCG   
  
  
+ CTCGGCTAAA ACACCCCAGG TGCAAGAAAA CGGTTTTCCC TACGGCAAAC TGTCGTAGAG CACCGAGCAC   
  
  
+ TAGAGTTACG GTTGGTCGAC ACGAGGGAAG AGCGTAGCAT ACTAAGTCCT AGGTTGCCGG CTTTTGCCAA   
  
  
+ GTGGAATAGT AGGTCAGTCG AATTACTAGT TTATAAAAGT TGAGAGGTGC CGACTCTACG CGACTGTCTC   
  
  
+ AGAGACATAT ATATTCGCTC TCATGCTTTT GGTAACTCCT AAATCTTCAT CCTCACCCGT AGTGCTCTGT   
  
  
+ ACCCTAACGT TCGCACACCC CAGTGTGAAA ATAAAATTAA ACAGACAAAT CCTGATTATT CAACCTTCAC   
  
  
+ CGCTCAGAAC ACAACCTGGG TTAAAGTCCG GGGGGTAGTA CCAAAGAGGG CGGTGTATTA ATGTCACAGA   
  
  
+ CTGGCTTAAT GTAATAACAA TAAGAACAA  

- GACCCTTTTT GTATCTCTCG CTCTACGGCC GTTTCAGCGA CAAGGCTCTG TGAATCCGTA ACAGACCTCC   
  
  
- GCAGGGACTG TTAGAGAAGA GACAGAGAGA GAAAGAGAGA AGACAAAAGA ATGCGCTGAC TTTTGAGTAA   
  
  
- AGAGAGAGAA AATGGTAGGG GCTAGTAATG AGTGGGGTTA CCAATATATA TAGAGAGAGA GAGAGACGGC   
  
  
- ACAGTACACA TGCACCTGTA TGTAGAGAGA GAGAGCCGAA GTGGTTATTT AGTATTCACA AGTCTAGATA   
  
  
- GAGAGAGAAA CTAGAGAAGT TGGCATCTCA ATGCCCATTG TTGTTCTTTC TCTCTCTGCT ATCCGACAAT   
  
  
- TGAAAACCAG AAACGGTTTC TGGTTTTGGG GTTGACTTCT CGCACATCTC GTTGGGTTCG CCCTCTCCAA   
  
  
- TTCTTTTTTC ACACAAAAAG TCCACGCGCT CTCTTTTTCT CTCTCTCTCT CGTGGGCGCT CGTGAATCAC   
  
  
- CGACAAAAGA GAGGAATTAA AAGATAGCTT CTGCAAAGAG GATTAAACGA AAGTGGACGC CAGTTAACGA   
  
  
- GGGCGCTCCT ATCAATTTCA ACTGTGTTTT TACGATTTTA CCCATAACAA AAGAAGAAAG GATTTAAATC   
  
  
- AATTTAGTTG TTGGTTGCTT CCGTAGTTAA TTATGTGCCT CTTTTTATGT CGCCGGGTTT GTTCGTCCCG   
  
  
- TGCATGACAC GTGCCGAGTC ACTGGCAGTT CGATTAAACC ACACCGGTTC CTACTTTCAC TCCCTGTGTT   
  
  
- TGCTTCTTCC ACTCCATTAT TCCTTTCTTC TTACGGAAAT TACCTTTGGA CATAGCAAAA ATAGCACAGA   
  
  
- ATGACGGAAA AATCTAACAG TTACGACGTG TCACCCTTAA AAGGCGAGTT GGATATTGGC ATACTCGAAT   
  
  
- GGAGTTTCAT GTTGACCACA CATCAAGATC GACGGCTGCA GCTGGTGGTT CGTGTGTGGC ACTAACGTGT   
  
  
- AATTGACCAG AGAGGAGGCG GAGAGCGTGG CGGTGCAGGA GATAAGGAGG TGAGGTGGCC AGATGACAGC   
  
  
- GAGCCGATTT TGTGGGGTCC ACGTTCTTTT GCCAAAAGGG ATGCCGTTTG ACAGCATCTC GTGGCTCGTG   
  
  
- ATCTCAATGC CAACCAGCTG TGCTCCCTTC TCGCATCGTA TGATTCAGGA TCCAACGGCC GAAAACGGTT   
  
  
- CACCTTATCA TCCAGTCAGC TTAATGATCA AATATTTTCA ACTCTCCACG GCTGAGATGC GCTGACAGAG   
  
  
- TCTCTGTATA TATAAGCGAG AGTACGAAAA CCATTGAGGA TTTAGAAGTA GGAGTGGGCA TCACGAGACA   
  
  
- TGGGATTGCA AGCGTGTGGG GTCACACTTT TATTTTAATT TGTCTGTTTA GGACTAATAA GTTGGAAGTG   
  
  
- GCGAGTCTTG TGTTGGACCC AATTTCAGGC CCCCCATCAT GGTTTCTCCC GCCACATAAT TACAGTGTCT   
  
  
- GACCGAATTA CATTATTGTT ATTCTTGTT
