# Supplementary material for: Genome-wide analysis of the WRKY gene family in drumstick (Moringa oleifera Lam.)
Source: PeerJ. 2019 Jun 10;7:e7063. doi: 10.7717/peerj.7063 (PMC6563795; doi:10.7717/peerj.7063)
Supplement: Supplemental Information 1 [file peerj-07-7063-s003.gz › MoWRKY49_plantcare.html]

Content-Type: text/html; charset=ISO-8859-1


CallMat\_Firefox


Webmaster Firefox specific output  
To save the result:
click on the frame with the right mouse button and save the source code as a text file with extension .html  
REFERENCE:PlantCARE: a database of plant cis-acting regulatory elements and a portal to tools for in silico analysis of promoter sequences.  
Lescot, M., Déhais, P., Moreau, Y., De Moor, B., Rouzé ,P.,and Rombauts, S.  
Nucleic Acids Res., Database issue(2002), 30(1):325-327.   


---

> 2018/04/13 10:10:12  
+ ATTTGAATAT ATTAATTATT TATTATAAAA TTTTAAATAT TTAATATTAA AACAATTTAA AAATATTTGT   
  
  
+ AATACTAAAA AAATTTTAAA ATTTTTTGTG ATAATATCTT GAATATTTTT TTACAATTAT AAAAAGTTTT   
  
  
+ AAATAAAAAT TCGTAATATA AAATTTTTAA TTTGAATACT TTTATTTTAA AATTTTTTAA TAAAATATAA   
  
  
+ TTTGATTATA TTTCATCAAT CAGCTATGTT ACTTCAAAAT TTTAAATAAT ATTTGCTTTT AAATTTATTT   
  
  
+ TATTATTTCA TTTAATTATT ATATATTAAC ATTTATTTTA AAATAATATA TCTTTAAAAA ATATTTTTAA   
  
  
+ TTAAGCACTT ACTGTTAACA AGAAGTCTCA GGAAGGCAGT AGTGTCCAGG CCGGTGCGAA GTGCGTTCGC   
  
  
+ GAGTCTACGG CTCATGGCTC ACATTTCGGT CACAGCCTCG TAGGGGACCA CAAAAGTCTG GATCATACAT   
  
  
+ AGCGGCTCCT TGCGTCCCCG TTACGTTTTT CCTCAACGAC CCAGACACGG ATCTGGCATT TGAATCGGTC   
  
  
+ TATTTTTCGG CTTTTAACAT TTATTTATTT ATTATATTTC CCTAAAAATG GACCAAAATT ATCTTGTTAC   
  
  
+ TTTACCAAGT GTCTTAATTT TATATATTCC TCAATAAAAT AATATAAATA GAACGACATC GTTTACACGT   
  
  
+ AAGCAAGGGT CATGGAACTA ACGTCACACT TACTTTAAGA TCGTTTCTTC CTCTTTTTTT CTCACCTTTC   
  
  
+ TGTAGTAGTA ACGTGGAATT TGCCAAGTTT GAAGATTCTT TGGTCTTCTG GTTCCCAACA TCTTCCATTG   
  
  
+ GCTTCCGTTC TTTCCTCTAG AATCCTTTAT TACACTCGAC CAATGAGGAT TCAGGAACCT CAACGAAGAG   
  
  
+ CACTTGACAG AGAGATGGAG TTTCCTGTCT GGACTGAACA TAGAGAAGTG GTCAGCCGTT GGACTTCCCT   
  
  
+ TTTTTTTTTT TTTTATGTTG AACTCGAATT AGTATGACCA ACGACTGGTC TAATGAAGAA AACCCACTTC   
  
  
+ AGCTTTTCTC CAGTCAACCA TCTTCCTTCA ATTTTCTACG TTCAAAAGGA GGCAAATCCT TTAAAATCAC   
  
  
+ ATAACCTTAT TCTCCTTCTT CCACAGGTTA AGTCCCAATA CCTCTAAGTA TTCATCTGCT TAGTTTCTCG   
  
  
+ CCATTACAGA ACAAGGGAAA CTCATTGAAC CCTTTTAGTC ATTCAAACTC TTTTGCATCT TTTGTTTTTT   
  
  
+ CAACCCAATT TCGAAAGTTT TCAAGCGTAG AAGTAACAGA AAGCTGATTC TTTCAAGGGA ATGCTGAATA   
  
  
+ CCCAGTTCAA ATTGAGCATT TCCAGCTTCT CATATCTTTC TCTCTGCCGT TGTCACCTTC TTCGCTTCTC   
  
  
+ CAGTTCCGTC GTAAACCAGT CATTTTCATG GCCTGATACC ATCCGGCCTT GCTTTTGAGA ACCCATTGGC   
  
  
+ CCATTAATTA TTCGTGTTGC TTACAAATC  

- TAAACTTATA TAATTAATAA ATAATATTTT AAAATTTATA AATTATAATT TTGTTAAATT TTTATAAACA   
  
  
- TTATGATTTT TTTAAAATTT TAAAAAACAC TATTATAGAA CTTATAAAAA AATGTTAATA TTTTTCAAAA   
  
  
- TTTATTTTTA AGCATTATAT TTTAAAAATT AAACTTATGA AAATAAAATT TTAAAAAATT ATTTTATATT   
  
  
- AAACTAATAT AAAGTAGTTA GTCGATACAA TGAAGTTTTA AAATTTATTA TAAACGAAAA TTTAAATAAA   
  
  
- ATAATAAAGT AAATTAATAA TATATAATTG TAAATAAAAT TTTATTATAT AGAAATTTTT TATAAAAATT   
  
  
- AATTCGTGAA TGACAATTGT TCTTCAGAGT CCTTCCGTCA TCACAGGTCC GGCCACGCTT CACGCAAGCG   
  
  
- CTCAGATGCC GAGTACCGAG TGTAAAGCCA GTGTCGGAGC ATCCCCTGGT GTTTTCAGAC CTAGTATGTA   
  
  
- TCGCCGAGGA ACGCAGGGGC AATGCAAAAA GGAGTTGCTG GGTCTGTGCC TAGACCGTAA ACTTAGCCAG   
  
  
- ATAAAAAGCC GAAAATTGTA AATAAATAAA TAATATAAAG GGATTTTTAC CTGGTTTTAA TAGAACAATG   
  
  
- AAATGGTTCA CAGAATTAAA ATATATAAGG AGTTATTTTA TTATATTTAT CTTGCTGTAG CAAATGTGCA   
  
  
- TTCGTTCCCA GTACCTTGAT TGCAGTGTGA ATGAAATTCT AGCAAAGAAG GAGAAAAAAA GAGTGGAAAG   
  
  
- ACATCATCAT TGCACCTTAA ACGGTTCAAA CTTCTAAGAA ACCAGAAGAC CAAGGGTTGT AGAAGGTAAC   
  
  
- CGAAGGCAAG AAAGGAGATC TTAGGAAATA ATGTGAGCTG GTTACTCCTA AGTCCTTGGA GTTGCTTCTC   
  
  
- GTGAACTGTC TCTCTACCTC AAAGGACAGA CCTGACTTGT ATCTCTTCAC CAGTCGGCAA CCTGAAGGGA   
  
  
- AAAAAAAAAA AAAATACAAC TTGAGCTTAA TCATACTGGT TGCTGACCAG ATTACTTCTT TTGGGTGAAG   
  
  
- TCGAAAAGAG GTCAGTTGGT AGAAGGAAGT TAAAAGATGC AAGTTTTCCT CCGTTTAGGA AATTTTAGTG   
  
  
- TATTGGAATA AGAGGAAGAA GGTGTCCAAT TCAGGGTTAT GGAGATTCAT AAGTAGACGA ATCAAAGAGC   
  
  
- GGTAATGTCT TGTTCCCTTT GAGTAACTTG GGAAAATCAG TAAGTTTGAG AAAACGTAGA AAACAAAAAA   
  
  
- GTTGGGTTAA AGCTTTCAAA AGTTCGCATC TTCATTGTCT TTCGACTAAG AAAGTTCCCT TACGACTTAT   
  
  
- GGGTCAAGTT TAACTCGTAA AGGTCGAAGA GTATAGAAAG AGAGACGGCA ACAGTGGAAG AAGCGAAGAG   
  
  
- GTCAAGGCAG CATTTGGTCA GTAAAAGTAC CGGACTATGG TAGGCCGGAA CGAAAACTCT TGGGTAACCG   
  
  
- GGTAATTAAT AAGCACAACG AATGTTTAG

  
  
Motifs Found  

+     AAGAA-motif

| Site Name | Organism | Position | Strand | Matrix score. | sequence | function |
| --- | --- | --- | --- | --- | --- | --- |
| AAGAA-motif | Avena sativa | 848 | - | 7 | GAAAGAA |  |
| AAGAA-motif | Avena sativa | 627 | - | 9 | gGTAAAGAAA |  |
| AAGAA-motif | Avena sativa | 1308 | - | 7 | GAAAGAA |  |

> 2018/04/13 10:10:12  
+ ATTTGAATAT ATTAATTATT TATTATAAAA TTTTAAATAT TTAATATTAA AACAATTTAA AAATATTTGT   
  
  
+ AATACTAAAA AAATTTTAAA ATTTTTTGTG ATAATATCTT GAATATTTTT TTACAATTAT AAAAAGTTTT   
  
  
+ AAATAAAAAT TCGTAATATA AAATTTTTAA TTTGAATACT TTTATTTTAA AATTTTTTAA TAAAATATAA   
  
  
+ TTTGATTATA TTTCATCAAT CAGCTATGTT ACTTCAAAAT TTTAAATAAT ATTTGCTTTT AAATTTATTT   
  
  
+ TATTATTTCA TTTAATTATT ATATATTAAC ATTTATTTTA AAATAATATA TCTTTAAAAA ATATTTTTAA   
  
  
+ TTAAGCACTT ACTGTTAACA AGAAGTCTCA GGAAGGCAGT AGTGTCCAGG CCGGTGCGAA GTGCGTTCGC   
  
  
+ GAGTCTACGG CTCATGGCTC ACATTTCGGT CACAGCCTCG TAGGGGACCA CAAAAGTCTG GATCATACAT   
  
  
+ AGCGGCTCCT TGCGTCCCCG TTACGTTTTT CCTCAACGAC CCAGACACGG ATCTGGCATT TGAATCGGTC   
  
  
+ TATTTTTCGG CTTTTAACAT TTATTTATTT ATTATATTTC CCTAAAAATG GACCAAAATT ATCTTGTTAC   
  
  
+ TTTACCAAGT GTCTTAATTT TATATATTCC TCAATAAAAT AATATAAATA GAACGACATC GTTTACACGT   
  
  
+ AAGCAAGGGT CATGGAACTA ACGTCACACT TACTTTAAGA TCGTTTCTTC CTCTTTTTTT CTCACCTTTC   
  
  
+ TGTAGTAGTA ACGTGGAATT TGCCAAGTTT GAAGATTCTT TGGTCTTCTG GTTCCCAACA TCTTCCATTG   
  
  
+ GCTTCCGTTC TTTCCTCTAG AATCCTTTAT TACACTCGAC CAATGAGGAT TCAGGAACCT CAACGAAGAG   
  
  
+ CACTTGACAG AGAGATGGAG TTTCCTGTCT GGACTGAACA TAGAGAAGTG GTCAGCCGTT GGACTTCCCT   
  
  
+ TTTTTTTTTT TTTTATGTTG AACTCGAATT AGTATGACCA ACGACTGGTC TAATGAAGAA AACCCACTTC   
  
  
+ AGCTTTTCTC CAGTCAACCA TCTTCCTTCA ATTTTCTACG TTCAAAAGGA GGCAAATCCT TTAAAATCAC   
  
  
+ ATAACCTTAT TCTCCTTCTT CCACAGGTTA AGTCCCAATA CCTCTAAGTA TTCATCTGCT TAGTTTCTCG   
  
  
+ CCATTACAGA ACAAGGGAAA CTCATTGAAC CCTTTTAGTC ATTCAAACTC TTTTGCATCT TTTGTTTTTT   
  
  
+ CAACCCAATT TCGAAAGTTT TCAAGCGTAG AAGTAACAGA AAGCTGATTC TTTCAAGGGA ATGCTGAATA   
  
  
+ CCCAGTTCAA ATTGAGCATT TCCAGCTTCT CATATCTTTC TCTCTGCCGT TGTCACCTTC TTCGCTTCTC   
  
  
+ CAGTTCCGTC GTAAACCAGT CATTTTCATG GCCTGATACC ATCCGGCCTT GCTTTTGAGA ACCCATTGGC   
  
  
+ CCATTAATTA TTCGTGTTGC TTACAAATC  

- TAAACTTATA TAATTAATAA ATAATATTTT AAAATTTATA AATTATAATT TTGTTAAATT TTTATAAACA   
  
  
- TTATGATTTT TTTAAAATTT TAAAAAACAC TATTATAGAA CTTATAAAAA AATGTTAATA TTTTTCAAAA   
  
  
- TTTATTTTTA AGCATTATAT TTTAAAAATT AAACTTATGA AAATAAAATT TTAAAAAATT ATTTTATATT   
  
  
- AAACTAATAT AAAGTAGTTA GTCGATACAA TGAAGTTTTA AAATTTATTA TAAACGAAAA TTTAAATAAA   
  
  
- ATAATAAAGT AAATTAATAA TATATAATTG TAAATAAAAT TTTATTATAT AGAAATTTTT TATAAAAATT   
  
  
- AATTCGTGAA TGACAATTGT TCTTCAGAGT CCTTCCGTCA TCACAGGTCC GGCCACGCTT CACGCAAGCG   
  
  
- CTCAGATGCC GAGTACCGAG TGTAAAGCCA GTGTCGGAGC ATCCCCTGGT GTTTTCAGAC CTAGTATGTA   
  
  
- TCGCCGAGGA ACGCAGGGGC AATGCAAAAA GGAGTTGCTG GGTCTGTGCC TAGACCGTAA ACTTAGCCAG   
  
  
- ATAAAAAGCC GAAAATTGTA AATAAATAAA TAATATAAAG GGATTTTTAC CTGGTTTTAA TAGAACAATG   
  
  
- AAATGGTTCA CAGAATTAAA ATATATAAGG AGTTATTTTA TTATATTTAT CTTGCTGTAG CAAATGTGCA   
  
  
- TTCGTTCCCA GTACCTTGAT TGCAGTGTGA ATGAAATTCT AGCAAAGAAG GAGAAAAAAA GAGTGGAAAG   
  
  
- ACATCATCAT TGCACCTTAA ACGGTTCAAA CTTCTAAGAA ACCAGAAGAC CAAGGGTTGT AGAAGGTAAC   
  
  
- CGAAGGCAAG AAAGGAGATC TTAGGAAATA ATGTGAGCTG GTTACTCCTA AGTCCTTGGA GTTGCTTCTC   
  
  
- GTGAACTGTC TCTCTACCTC AAAGGACAGA CCTGACTTGT ATCTCTTCAC CAGTCGGCAA CCTGAAGGGA   
  
  
- AAAAAAAAAA AAAATACAAC TTGAGCTTAA TCATACTGGT TGCTGACCAG ATTACTTCTT TTGGGTGAAG   
  
  
- TCGAAAAGAG GTCAGTTGGT AGAAGGAAGT TAAAAGATGC AAGTTTTCCT CCGTTTAGGA AATTTTAGTG   
  
  
- TATTGGAATA AGAGGAAGAA GGTGTCCAAT TCAGGGTTAT GGAGATTCAT AAGTAGACGA ATCAAAGAGC   
  
  
- GGTAATGTCT TGTTCCCTTT GAGTAACTTG GGAAAATCAG TAAGTTTGAG AAAACGTAGA AAACAAAAAA   
  
  
- GTTGGGTTAA AGCTTTCAAA AGTTCGCATC TTCATTGTCT TTCGACTAAG AAAGTTCCCT TACGACTTAT   
  
  
- GGGTCAAGTT TAACTCGTAA AGGTCGAAGA GTATAGAAAG AGAGACGGCA ACAGTGGAAG AAGCGAAGAG   
  
  
- GTCAAGGCAG CATTTGGTCA GTAAAAGTAC CGGACTATGG TAGGCCGGAA CGAAAACTCT TGGGTAACCG   
  
  
- GGTAATTAAT AAGCACAACG AATGTTTAG

+     ABRE

| Site Name | Organism | Position | Strand | Matrix score. | sequence | function |
| --- | --- | --- | --- | --- | --- | --- |
| ABRE | Arabidopsis thaliana | 696 | - | 6 | TACGTG | cis-acting element involved in the abscisic acid responsiveness |
| ABRE | Hordeum vulgare | 455 | - | 9 | CCTACGTGGC | cis-acting element involved in the abscisic acid responsiveness |

> 2018/04/13 10:10:12  
+ ATTTGAATAT ATTAATTATT TATTATAAAA TTTTAAATAT TTAATATTAA AACAATTTAA AAATATTTGT   
  
  
+ AATACTAAAA AAATTTTAAA ATTTTTTGTG ATAATATCTT GAATATTTTT TTACAATTAT AAAAAGTTTT   
  
  
+ AAATAAAAAT TCGTAATATA AAATTTTTAA TTTGAATACT TTTATTTTAA AATTTTTTAA TAAAATATAA   
  
  
+ TTTGATTATA TTTCATCAAT CAGCTATGTT ACTTCAAAAT TTTAAATAAT ATTTGCTTTT AAATTTATTT   
  
  
+ TATTATTTCA TTTAATTATT ATATATTAAC ATTTATTTTA AAATAATATA TCTTTAAAAA ATATTTTTAA   
  
  
+ TTAAGCACTT ACTGTTAACA AGAAGTCTCA GGAAGGCAGT AGTGTCCAGG CCGGTGCGAA GTGCGTTCGC   
  
  
+ GAGTCTACGG CTCATGGCTC ACATTTCGGT CACAGCCTCG TAGGGGACCA CAAAAGTCTG GATCATACAT   
  
  
+ AGCGGCTCCT TGCGTCCCCG TTACGTTTTT CCTCAACGAC CCAGACACGG ATCTGGCATT TGAATCGGTC   
  
  
+ TATTTTTCGG CTTTTAACAT TTATTTATTT ATTATATTTC CCTAAAAATG GACCAAAATT ATCTTGTTAC   
  
  
+ TTTACCAAGT GTCTTAATTT TATATATTCC TCAATAAAAT AATATAAATA GAACGACATC GTTTACACGT   
  
  
+ AAGCAAGGGT CATGGAACTA ACGTCACACT TACTTTAAGA TCGTTTCTTC CTCTTTTTTT CTCACCTTTC   
  
  
+ TGTAGTAGTA ACGTGGAATT TGCCAAGTTT GAAGATTCTT TGGTCTTCTG GTTCCCAACA TCTTCCATTG   
  
  
+ GCTTCCGTTC TTTCCTCTAG AATCCTTTAT TACACTCGAC CAATGAGGAT TCAGGAACCT CAACGAAGAG   
  
  
+ CACTTGACAG AGAGATGGAG TTTCCTGTCT GGACTGAACA TAGAGAAGTG GTCAGCCGTT GGACTTCCCT   
  
  
+ TTTTTTTTTT TTTTATGTTG AACTCGAATT AGTATGACCA ACGACTGGTC TAATGAAGAA AACCCACTTC   
  
  
+ AGCTTTTCTC CAGTCAACCA TCTTCCTTCA ATTTTCTACG TTCAAAAGGA GGCAAATCCT TTAAAATCAC   
  
  
+ ATAACCTTAT TCTCCTTCTT CCACAGGTTA AGTCCCAATA CCTCTAAGTA TTCATCTGCT TAGTTTCTCG   
  
  
+ CCATTACAGA ACAAGGGAAA CTCATTGAAC CCTTTTAGTC ATTCAAACTC TTTTGCATCT TTTGTTTTTT   
  
  
+ CAACCCAATT TCGAAAGTTT TCAAGCGTAG AAGTAACAGA AAGCTGATTC TTTCAAGGGA ATGCTGAATA   
  
  
+ CCCAGTTCAA ATTGAGCATT TCCAGCTTCT CATATCTTTC TCTCTGCCGT TGTCACCTTC TTCGCTTCTC   
  
  
+ CAGTTCCGTC GTAAACCAGT CATTTTCATG GCCTGATACC ATCCGGCCTT GCTTTTGAGA ACCCATTGGC   
  
  
+ CCATTAATTA TTCGTGTTGC TTACAAATC  

- TAAACTTATA TAATTAATAA ATAATATTTT AAAATTTATA AATTATAATT TTGTTAAATT TTTATAAACA   
  
  
- TTATGATTTT TTTAAAATTT TAAAAAACAC TATTATAGAA CTTATAAAAA AATGTTAATA TTTTTCAAAA   
  
  
- TTTATTTTTA AGCATTATAT TTTAAAAATT AAACTTATGA AAATAAAATT TTAAAAAATT ATTTTATATT   
  
  
- AAACTAATAT AAAGTAGTTA GTCGATACAA TGAAGTTTTA AAATTTATTA TAAACGAAAA TTTAAATAAA   
  
  
- ATAATAAAGT AAATTAATAA TATATAATTG TAAATAAAAT TTTATTATAT AGAAATTTTT TATAAAAATT   
  
  
- AATTCGTGAA TGACAATTGT TCTTCAGAGT CCTTCCGTCA TCACAGGTCC GGCCACGCTT CACGCAAGCG   
  
  
- CTCAGATGCC GAGTACCGAG TGTAAAGCCA GTGTCGGAGC ATCCCCTGGT GTTTTCAGAC CTAGTATGTA   
  
  
- TCGCCGAGGA ACGCAGGGGC AATGCAAAAA GGAGTTGCTG GGTCTGTGCC TAGACCGTAA ACTTAGCCAG   
  
  
- ATAAAAAGCC GAAAATTGTA AATAAATAAA TAATATAAAG GGATTTTTAC CTGGTTTTAA TAGAACAATG   
  
  
- AAATGGTTCA CAGAATTAAA ATATATAAGG AGTTATTTTA TTATATTTAT CTTGCTGTAG CAAATGTGCA   
  
  
- TTCGTTCCCA GTACCTTGAT TGCAGTGTGA ATGAAATTCT AGCAAAGAAG GAGAAAAAAA GAGTGGAAAG   
  
  
- ACATCATCAT TGCACCTTAA ACGGTTCAAA CTTCTAAGAA ACCAGAAGAC CAAGGGTTGT AGAAGGTAAC   
  
  
- CGAAGGCAAG AAAGGAGATC TTAGGAAATA ATGTGAGCTG GTTACTCCTA AGTCCTTGGA GTTGCTTCTC   
  
  
- GTGAACTGTC TCTCTACCTC AAAGGACAGA CCTGACTTGT ATCTCTTCAC CAGTCGGCAA CCTGAAGGGA   
  
  
- AAAAAAAAAA AAAATACAAC TTGAGCTTAA TCATACTGGT TGCTGACCAG ATTACTTCTT TTGGGTGAAG   
  
  
- TCGAAAAGAG GTCAGTTGGT AGAAGGAAGT TAAAAGATGC AAGTTTTCCT CCGTTTAGGA AATTTTAGTG   
  
  
- TATTGGAATA AGAGGAAGAA GGTGTCCAAT TCAGGGTTAT GGAGATTCAT AAGTAGACGA ATCAAAGAGC   
  
  
- GGTAATGTCT TGTTCCCTTT GAGTAACTTG GGAAAATCAG TAAGTTTGAG AAAACGTAGA AAACAAAAAA   
  
  
- GTTGGGTTAA AGCTTTCAAA AGTTCGCATC TTCATTGTCT TTCGACTAAG AAAGTTCCCT TACGACTTAT   
  
  
- GGGTCAAGTT TAACTCGTAA AGGTCGAAGA GTATAGAAAG AGAGACGGCA ACAGTGGAAG AAGCGAAGAG   
  
  
- GTCAAGGCAG CATTTGGTCA GTAAAAGTAC CGGACTATGG TAGGCCGGAA CGAAAACTCT TGGGTAACCG   
  
  
- GGTAATTAAT AAGCACAACG AATGTTTAG

+     ACE

| Site Name | Organism | Position | Strand | Matrix score. | sequence | function |
| --- | --- | --- | --- | --- | --- | --- |
| ACE | Petroselinum hortense | 781 | + | 7 | ACGTGGA | cis-acting element involved in light responsiveness |

> 2018/04/13 10:10:12  
+ ATTTGAATAT ATTAATTATT TATTATAAAA TTTTAAATAT TTAATATTAA AACAATTTAA AAATATTTGT   
  
  
+ AATACTAAAA AAATTTTAAA ATTTTTTGTG ATAATATCTT GAATATTTTT TTACAATTAT AAAAAGTTTT   
  
  
+ AAATAAAAAT TCGTAATATA AAATTTTTAA TTTGAATACT TTTATTTTAA AATTTTTTAA TAAAATATAA   
  
  
+ TTTGATTATA TTTCATCAAT CAGCTATGTT ACTTCAAAAT TTTAAATAAT ATTTGCTTTT AAATTTATTT   
  
  
+ TATTATTTCA TTTAATTATT ATATATTAAC ATTTATTTTA AAATAATATA TCTTTAAAAA ATATTTTTAA   
  
  
+ TTAAGCACTT ACTGTTAACA AGAAGTCTCA GGAAGGCAGT AGTGTCCAGG CCGGTGCGAA GTGCGTTCGC   
  
  
+ GAGTCTACGG CTCATGGCTC ACATTTCGGT CACAGCCTCG TAGGGGACCA CAAAAGTCTG GATCATACAT   
  
  
+ AGCGGCTCCT TGCGTCCCCG TTACGTTTTT CCTCAACGAC CCAGACACGG ATCTGGCATT TGAATCGGTC   
  
  
+ TATTTTTCGG CTTTTAACAT TTATTTATTT ATTATATTTC CCTAAAAATG GACCAAAATT ATCTTGTTAC   
  
  
+ TTTACCAAGT GTCTTAATTT TATATATTCC TCAATAAAAT AATATAAATA GAACGACATC GTTTACACGT   
  
  
+ AAGCAAGGGT CATGGAACTA ACGTCACACT TACTTTAAGA TCGTTTCTTC CTCTTTTTTT CTCACCTTTC   
  
  
+ TGTAGTAGTA ACGTGGAATT TGCCAAGTTT GAAGATTCTT TGGTCTTCTG GTTCCCAACA TCTTCCATTG   
  
  
+ GCTTCCGTTC TTTCCTCTAG AATCCTTTAT TACACTCGAC CAATGAGGAT TCAGGAACCT CAACGAAGAG   
  
  
+ CACTTGACAG AGAGATGGAG TTTCCTGTCT GGACTGAACA TAGAGAAGTG GTCAGCCGTT GGACTTCCCT   
  
  
+ TTTTTTTTTT TTTTATGTTG AACTCGAATT AGTATGACCA ACGACTGGTC TAATGAAGAA AACCCACTTC   
  
  
+ AGCTTTTCTC CAGTCAACCA TCTTCCTTCA ATTTTCTACG TTCAAAAGGA GGCAAATCCT TTAAAATCAC   
  
  
+ ATAACCTTAT TCTCCTTCTT CCACAGGTTA AGTCCCAATA CCTCTAAGTA TTCATCTGCT TAGTTTCTCG   
  
  
+ CCATTACAGA ACAAGGGAAA CTCATTGAAC CCTTTTAGTC ATTCAAACTC TTTTGCATCT TTTGTTTTTT   
  
  
+ CAACCCAATT TCGAAAGTTT TCAAGCGTAG AAGTAACAGA AAGCTGATTC TTTCAAGGGA ATGCTGAATA   
  
  
+ CCCAGTTCAA ATTGAGCATT TCCAGCTTCT CATATCTTTC TCTCTGCCGT TGTCACCTTC TTCGCTTCTC   
  
  
+ CAGTTCCGTC GTAAACCAGT CATTTTCATG GCCTGATACC ATCCGGCCTT GCTTTTGAGA ACCCATTGGC   
  
  
+ CCATTAATTA TTCGTGTTGC TTACAAATC  

- TAAACTTATA TAATTAATAA ATAATATTTT AAAATTTATA AATTATAATT TTGTTAAATT TTTATAAACA   
  
  
- TTATGATTTT TTTAAAATTT TAAAAAACAC TATTATAGAA CTTATAAAAA AATGTTAATA TTTTTCAAAA   
  
  
- TTTATTTTTA AGCATTATAT TTTAAAAATT AAACTTATGA AAATAAAATT TTAAAAAATT ATTTTATATT   
  
  
- AAACTAATAT AAAGTAGTTA GTCGATACAA TGAAGTTTTA AAATTTATTA TAAACGAAAA TTTAAATAAA   
  
  
- ATAATAAAGT AAATTAATAA TATATAATTG TAAATAAAAT TTTATTATAT AGAAATTTTT TATAAAAATT   
  
  
- AATTCGTGAA TGACAATTGT TCTTCAGAGT CCTTCCGTCA TCACAGGTCC GGCCACGCTT CACGCAAGCG   
  
  
- CTCAGATGCC GAGTACCGAG TGTAAAGCCA GTGTCGGAGC ATCCCCTGGT GTTTTCAGAC CTAGTATGTA   
  
  
- TCGCCGAGGA ACGCAGGGGC AATGCAAAAA GGAGTTGCTG GGTCTGTGCC TAGACCGTAA ACTTAGCCAG   
  
  
- ATAAAAAGCC GAAAATTGTA AATAAATAAA TAATATAAAG GGATTTTTAC CTGGTTTTAA TAGAACAATG   
  
  
- AAATGGTTCA CAGAATTAAA ATATATAAGG AGTTATTTTA TTATATTTAT CTTGCTGTAG CAAATGTGCA   
  
  
- TTCGTTCCCA GTACCTTGAT TGCAGTGTGA ATGAAATTCT AGCAAAGAAG GAGAAAAAAA GAGTGGAAAG   
  
  
- ACATCATCAT TGCACCTTAA ACGGTTCAAA CTTCTAAGAA ACCAGAAGAC CAAGGGTTGT AGAAGGTAAC   
  
  
- CGAAGGCAAG AAAGGAGATC TTAGGAAATA ATGTGAGCTG GTTACTCCTA AGTCCTTGGA GTTGCTTCTC   
  
  
- GTGAACTGTC TCTCTACCTC AAAGGACAGA CCTGACTTGT ATCTCTTCAC CAGTCGGCAA CCTGAAGGGA   
  
  
- AAAAAAAAAA AAAATACAAC TTGAGCTTAA TCATACTGGT TGCTGACCAG ATTACTTCTT TTGGGTGAAG   
  
  
- TCGAAAAGAG GTCAGTTGGT AGAAGGAAGT TAAAAGATGC AAGTTTTCCT CCGTTTAGGA AATTTTAGTG   
  
  
- TATTGGAATA AGAGGAAGAA GGTGTCCAAT TCAGGGTTAT GGAGATTCAT AAGTAGACGA ATCAAAGAGC   
  
  
- GGTAATGTCT TGTTCCCTTT GAGTAACTTG GGAAAATCAG TAAGTTTGAG AAAACGTAGA AAACAAAAAA   
  
  
- GTTGGGTTAA AGCTTTCAAA AGTTCGCATC TTCATTGTCT TTCGACTAAG AAAGTTCCCT TACGACTTAT   
  
  
- GGGTCAAGTT TAACTCGTAA AGGTCGAAGA GTATAGAAAG AGAGACGGCA ACAGTGGAAG AAGCGAAGAG   
  
  
- GTCAAGGCAG CATTTGGTCA GTAAAAGTAC CGGACTATGG TAGGCCGGAA CGAAAACTCT TGGGTAACCG   
  
  
- GGTAATTAAT AAGCACAACG AATGTTTAG

+     ARE

| Site Name | Organism | Position | Strand | Matrix score. | sequence | function |
| --- | --- | --- | --- | --- | --- | --- |
| ARE | Zea mays | 1413 | - | 6 | TGGTTT | cis-acting regulatory element essential for the anaerobic induction |

> 2018/04/13 10:10:12  
+ ATTTGAATAT ATTAATTATT TATTATAAAA TTTTAAATAT TTAATATTAA AACAATTTAA AAATATTTGT   
  
  
+ AATACTAAAA AAATTTTAAA ATTTTTTGTG ATAATATCTT GAATATTTTT TTACAATTAT AAAAAGTTTT   
  
  
+ AAATAAAAAT TCGTAATATA AAATTTTTAA TTTGAATACT TTTATTTTAA AATTTTTTAA TAAAATATAA   
  
  
+ TTTGATTATA TTTCATCAAT CAGCTATGTT ACTTCAAAAT TTTAAATAAT ATTTGCTTTT AAATTTATTT   
  
  
+ TATTATTTCA TTTAATTATT ATATATTAAC ATTTATTTTA AAATAATATA TCTTTAAAAA ATATTTTTAA   
  
  
+ TTAAGCACTT ACTGTTAACA AGAAGTCTCA GGAAGGCAGT AGTGTCCAGG CCGGTGCGAA GTGCGTTCGC   
  
  
+ GAGTCTACGG CTCATGGCTC ACATTTCGGT CACAGCCTCG TAGGGGACCA CAAAAGTCTG GATCATACAT   
  
  
+ AGCGGCTCCT TGCGTCCCCG TTACGTTTTT CCTCAACGAC CCAGACACGG ATCTGGCATT TGAATCGGTC   
  
  
+ TATTTTTCGG CTTTTAACAT TTATTTATTT ATTATATTTC CCTAAAAATG GACCAAAATT ATCTTGTTAC   
  
  
+ TTTACCAAGT GTCTTAATTT TATATATTCC TCAATAAAAT AATATAAATA GAACGACATC GTTTACACGT   
  
  
+ AAGCAAGGGT CATGGAACTA ACGTCACACT TACTTTAAGA TCGTTTCTTC CTCTTTTTTT CTCACCTTTC   
  
  
+ TGTAGTAGTA ACGTGGAATT TGCCAAGTTT GAAGATTCTT TGGTCTTCTG GTTCCCAACA TCTTCCATTG   
  
  
+ GCTTCCGTTC TTTCCTCTAG AATCCTTTAT TACACTCGAC CAATGAGGAT TCAGGAACCT CAACGAAGAG   
  
  
+ CACTTGACAG AGAGATGGAG TTTCCTGTCT GGACTGAACA TAGAGAAGTG GTCAGCCGTT GGACTTCCCT   
  
  
+ TTTTTTTTTT TTTTATGTTG AACTCGAATT AGTATGACCA ACGACTGGTC TAATGAAGAA AACCCACTTC   
  
  
+ AGCTTTTCTC CAGTCAACCA TCTTCCTTCA ATTTTCTACG TTCAAAAGGA GGCAAATCCT TTAAAATCAC   
  
  
+ ATAACCTTAT TCTCCTTCTT CCACAGGTTA AGTCCCAATA CCTCTAAGTA TTCATCTGCT TAGTTTCTCG   
  
  
+ CCATTACAGA ACAAGGGAAA CTCATTGAAC CCTTTTAGTC ATTCAAACTC TTTTGCATCT TTTGTTTTTT   
  
  
+ CAACCCAATT TCGAAAGTTT TCAAGCGTAG AAGTAACAGA AAGCTGATTC TTTCAAGGGA ATGCTGAATA   
  
  
+ CCCAGTTCAA ATTGAGCATT TCCAGCTTCT CATATCTTTC TCTCTGCCGT TGTCACCTTC TTCGCTTCTC   
  
  
+ CAGTTCCGTC GTAAACCAGT CATTTTCATG GCCTGATACC ATCCGGCCTT GCTTTTGAGA ACCCATTGGC   
  
  
+ CCATTAATTA TTCGTGTTGC TTACAAATC  

- TAAACTTATA TAATTAATAA ATAATATTTT AAAATTTATA AATTATAATT TTGTTAAATT TTTATAAACA   
  
  
- TTATGATTTT TTTAAAATTT TAAAAAACAC TATTATAGAA CTTATAAAAA AATGTTAATA TTTTTCAAAA   
  
  
- TTTATTTTTA AGCATTATAT TTTAAAAATT AAACTTATGA AAATAAAATT TTAAAAAATT ATTTTATATT   
  
  
- AAACTAATAT AAAGTAGTTA GTCGATACAA TGAAGTTTTA AAATTTATTA TAAACGAAAA TTTAAATAAA   
  
  
- ATAATAAAGT AAATTAATAA TATATAATTG TAAATAAAAT TTTATTATAT AGAAATTTTT TATAAAAATT   
  
  
- AATTCGTGAA TGACAATTGT TCTTCAGAGT CCTTCCGTCA TCACAGGTCC GGCCACGCTT CACGCAAGCG   
  
  
- CTCAGATGCC GAGTACCGAG TGTAAAGCCA GTGTCGGAGC ATCCCCTGGT GTTTTCAGAC CTAGTATGTA   
  
  
- TCGCCGAGGA ACGCAGGGGC AATGCAAAAA GGAGTTGCTG GGTCTGTGCC TAGACCGTAA ACTTAGCCAG   
  
  
- ATAAAAAGCC GAAAATTGTA AATAAATAAA TAATATAAAG GGATTTTTAC CTGGTTTTAA TAGAACAATG   
  
  
- AAATGGTTCA CAGAATTAAA ATATATAAGG AGTTATTTTA TTATATTTAT CTTGCTGTAG CAAATGTGCA   
  
  
- TTCGTTCCCA GTACCTTGAT TGCAGTGTGA ATGAAATTCT AGCAAAGAAG GAGAAAAAAA GAGTGGAAAG   
  
  
- ACATCATCAT TGCACCTTAA ACGGTTCAAA CTTCTAAGAA ACCAGAAGAC CAAGGGTTGT AGAAGGTAAC   
  
  
- CGAAGGCAAG AAAGGAGATC TTAGGAAATA ATGTGAGCTG GTTACTCCTA AGTCCTTGGA GTTGCTTCTC   
  
  
- GTGAACTGTC TCTCTACCTC AAAGGACAGA CCTGACTTGT ATCTCTTCAC CAGTCGGCAA CCTGAAGGGA   
  
  
- AAAAAAAAAA AAAATACAAC TTGAGCTTAA TCATACTGGT TGCTGACCAG ATTACTTCTT TTGGGTGAAG   
  
  
- TCGAAAAGAG GTCAGTTGGT AGAAGGAAGT TAAAAGATGC AAGTTTTCCT CCGTTTAGGA AATTTTAGTG   
  
  
- TATTGGAATA AGAGGAAGAA GGTGTCCAAT TCAGGGTTAT GGAGATTCAT AAGTAGACGA ATCAAAGAGC   
  
  
- GGTAATGTCT TGTTCCCTTT GAGTAACTTG GGAAAATCAG TAAGTTTGAG AAAACGTAGA AAACAAAAAA   
  
  
- GTTGGGTTAA AGCTTTCAAA AGTTCGCATC TTCATTGTCT TTCGACTAAG AAAGTTCCCT TACGACTTAT   
  
  
- GGGTCAAGTT TAACTCGTAA AGGTCGAAGA GTATAGAAAG AGAGACGGCA ACAGTGGAAG AAGCGAAGAG   
  
  
- GTCAAGGCAG CATTTGGTCA GTAAAAGTAC CGGACTATGG TAGGCCGGAA CGAAAACTCT TGGGTAACCG   
  
  
- GGTAATTAAT AAGCACAACG AATGTTTAG

+     AT1-motif

| Site Name | Organism | Position | Strand | Matrix score. | sequence | function |
| --- | --- | --- | --- | --- | --- | --- |
| AT1-motif | Solanum tuberosum | 199 | - | 13 | AATTATTTTTTATT | part of a light responsive module |

> 2018/04/13 10:10:12  
+ ATTTGAATAT ATTAATTATT TATTATAAAA TTTTAAATAT TTAATATTAA AACAATTTAA AAATATTTGT   
  
  
+ AATACTAAAA AAATTTTAAA ATTTTTTGTG ATAATATCTT GAATATTTTT TTACAATTAT AAAAAGTTTT   
  
  
+ AAATAAAAAT TCGTAATATA AAATTTTTAA TTTGAATACT TTTATTTTAA AATTTTTTAA TAAAATATAA   
  
  
+ TTTGATTATA TTTCATCAAT CAGCTATGTT ACTTCAAAAT TTTAAATAAT ATTTGCTTTT AAATTTATTT   
  
  
+ TATTATTTCA TTTAATTATT ATATATTAAC ATTTATTTTA AAATAATATA TCTTTAAAAA ATATTTTTAA   
  
  
+ TTAAGCACTT ACTGTTAACA AGAAGTCTCA GGAAGGCAGT AGTGTCCAGG CCGGTGCGAA GTGCGTTCGC   
  
  
+ GAGTCTACGG CTCATGGCTC ACATTTCGGT CACAGCCTCG TAGGGGACCA CAAAAGTCTG GATCATACAT   
  
  
+ AGCGGCTCCT TGCGTCCCCG TTACGTTTTT CCTCAACGAC CCAGACACGG ATCTGGCATT TGAATCGGTC   
  
  
+ TATTTTTCGG CTTTTAACAT TTATTTATTT ATTATATTTC CCTAAAAATG GACCAAAATT ATCTTGTTAC   
  
  
+ TTTACCAAGT GTCTTAATTT TATATATTCC TCAATAAAAT AATATAAATA GAACGACATC GTTTACACGT   
  
  
+ AAGCAAGGGT CATGGAACTA ACGTCACACT TACTTTAAGA TCGTTTCTTC CTCTTTTTTT CTCACCTTTC   
  
  
+ TGTAGTAGTA ACGTGGAATT TGCCAAGTTT GAAGATTCTT TGGTCTTCTG GTTCCCAACA TCTTCCATTG   
  
  
+ GCTTCCGTTC TTTCCTCTAG AATCCTTTAT TACACTCGAC CAATGAGGAT TCAGGAACCT CAACGAAGAG   
  
  
+ CACTTGACAG AGAGATGGAG TTTCCTGTCT GGACTGAACA TAGAGAAGTG GTCAGCCGTT GGACTTCCCT   
  
  
+ TTTTTTTTTT TTTTATGTTG AACTCGAATT AGTATGACCA ACGACTGGTC TAATGAAGAA AACCCACTTC   
  
  
+ AGCTTTTCTC CAGTCAACCA TCTTCCTTCA ATTTTCTACG TTCAAAAGGA GGCAAATCCT TTAAAATCAC   
  
  
+ ATAACCTTAT TCTCCTTCTT CCACAGGTTA AGTCCCAATA CCTCTAAGTA TTCATCTGCT TAGTTTCTCG   
  
  
+ CCATTACAGA ACAAGGGAAA CTCATTGAAC CCTTTTAGTC ATTCAAACTC TTTTGCATCT TTTGTTTTTT   
  
  
+ CAACCCAATT TCGAAAGTTT TCAAGCGTAG AAGTAACAGA AAGCTGATTC TTTCAAGGGA ATGCTGAATA   
  
  
+ CCCAGTTCAA ATTGAGCATT TCCAGCTTCT CATATCTTTC TCTCTGCCGT TGTCACCTTC TTCGCTTCTC   
  
  
+ CAGTTCCGTC GTAAACCAGT CATTTTCATG GCCTGATACC ATCCGGCCTT GCTTTTGAGA ACCCATTGGC   
  
  
+ CCATTAATTA TTCGTGTTGC TTACAAATC  

- TAAACTTATA TAATTAATAA ATAATATTTT AAAATTTATA AATTATAATT TTGTTAAATT TTTATAAACA   
  
  
- TTATGATTTT TTTAAAATTT TAAAAAACAC TATTATAGAA CTTATAAAAA AATGTTAATA TTTTTCAAAA   
  
  
- TTTATTTTTA AGCATTATAT TTTAAAAATT AAACTTATGA AAATAAAATT TTAAAAAATT ATTTTATATT   
  
  
- AAACTAATAT AAAGTAGTTA GTCGATACAA TGAAGTTTTA AAATTTATTA TAAACGAAAA TTTAAATAAA   
  
  
- ATAATAAAGT AAATTAATAA TATATAATTG TAAATAAAAT TTTATTATAT AGAAATTTTT TATAAAAATT   
  
  
- AATTCGTGAA TGACAATTGT TCTTCAGAGT CCTTCCGTCA TCACAGGTCC GGCCACGCTT CACGCAAGCG   
  
  
- CTCAGATGCC GAGTACCGAG TGTAAAGCCA GTGTCGGAGC ATCCCCTGGT GTTTTCAGAC CTAGTATGTA   
  
  
- TCGCCGAGGA ACGCAGGGGC AATGCAAAAA GGAGTTGCTG GGTCTGTGCC TAGACCGTAA ACTTAGCCAG   
  
  
- ATAAAAAGCC GAAAATTGTA AATAAATAAA TAATATAAAG GGATTTTTAC CTGGTTTTAA TAGAACAATG   
  
  
- AAATGGTTCA CAGAATTAAA ATATATAAGG AGTTATTTTA TTATATTTAT CTTGCTGTAG CAAATGTGCA   
  
  
- TTCGTTCCCA GTACCTTGAT TGCAGTGTGA ATGAAATTCT AGCAAAGAAG GAGAAAAAAA GAGTGGAAAG   
  
  
- ACATCATCAT TGCACCTTAA ACGGTTCAAA CTTCTAAGAA ACCAGAAGAC CAAGGGTTGT AGAAGGTAAC   
  
  
- CGAAGGCAAG AAAGGAGATC TTAGGAAATA ATGTGAGCTG GTTACTCCTA AGTCCTTGGA GTTGCTTCTC   
  
  
- GTGAACTGTC TCTCTACCTC AAAGGACAGA CCTGACTTGT ATCTCTTCAC CAGTCGGCAA CCTGAAGGGA   
  
  
- AAAAAAAAAA AAAATACAAC TTGAGCTTAA TCATACTGGT TGCTGACCAG ATTACTTCTT TTGGGTGAAG   
  
  
- TCGAAAAGAG GTCAGTTGGT AGAAGGAAGT TAAAAGATGC AAGTTTTCCT CCGTTTAGGA AATTTTAGTG   
  
  
- TATTGGAATA AGAGGAAGAA GGTGTCCAAT TCAGGGTTAT GGAGATTCAT AAGTAGACGA ATCAAAGAGC   
  
  
- GGTAATGTCT TGTTCCCTTT GAGTAACTTG GGAAAATCAG TAAGTTTGAG AAAACGTAGA AAACAAAAAA   
  
  
- GTTGGGTTAA AGCTTTCAAA AGTTCGCATC TTCATTGTCT TTCGACTAAG AAAGTTCCCT TACGACTTAT   
  
  
- GGGTCAAGTT TAACTCGTAA AGGTCGAAGA GTATAGAAAG AGAGACGGCA ACAGTGGAAG AAGCGAAGAG   
  
  
- GTCAAGGCAG CATTTGGTCA GTAAAAGTAC CGGACTATGG TAGGCCGGAA CGAAAACTCT TGGGTAACCG   
  
  
- GGTAATTAAT AAGCACAACG AATGTTTAG

+     AuxRR-core

| Site Name | Organism | Position | Strand | Matrix score. | sequence | function |
| --- | --- | --- | --- | --- | --- | --- |
| AuxRR-core | Nicotiana tabacum | 608 | - | 7 | GGTCCAT | cis-acting regulatory element involved in auxin responsiveness |

> 2018/04/13 10:10:12  
+ ATTTGAATAT ATTAATTATT TATTATAAAA TTTTAAATAT TTAATATTAA AACAATTTAA AAATATTTGT   
  
  
+ AATACTAAAA AAATTTTAAA ATTTTTTGTG ATAATATCTT GAATATTTTT TTACAATTAT AAAAAGTTTT   
  
  
+ AAATAAAAAT TCGTAATATA AAATTTTTAA TTTGAATACT TTTATTTTAA AATTTTTTAA TAAAATATAA   
  
  
+ TTTGATTATA TTTCATCAAT CAGCTATGTT ACTTCAAAAT TTTAAATAAT ATTTGCTTTT AAATTTATTT   
  
  
+ TATTATTTCA TTTAATTATT ATATATTAAC ATTTATTTTA AAATAATATA TCTTTAAAAA ATATTTTTAA   
  
  
+ TTAAGCACTT ACTGTTAACA AGAAGTCTCA GGAAGGCAGT AGTGTCCAGG CCGGTGCGAA GTGCGTTCGC   
  
  
+ GAGTCTACGG CTCATGGCTC ACATTTCGGT CACAGCCTCG TAGGGGACCA CAAAAGTCTG GATCATACAT   
  
  
+ AGCGGCTCCT TGCGTCCCCG TTACGTTTTT CCTCAACGAC CCAGACACGG ATCTGGCATT TGAATCGGTC   
  
  
+ TATTTTTCGG CTTTTAACAT TTATTTATTT ATTATATTTC CCTAAAAATG GACCAAAATT ATCTTGTTAC   
  
  
+ TTTACCAAGT GTCTTAATTT TATATATTCC TCAATAAAAT AATATAAATA GAACGACATC GTTTACACGT   
  
  
+ AAGCAAGGGT CATGGAACTA ACGTCACACT TACTTTAAGA TCGTTTCTTC CTCTTTTTTT CTCACCTTTC   
  
  
+ TGTAGTAGTA ACGTGGAATT TGCCAAGTTT GAAGATTCTT TGGTCTTCTG GTTCCCAACA TCTTCCATTG   
  
  
+ GCTTCCGTTC TTTCCTCTAG AATCCTTTAT TACACTCGAC CAATGAGGAT TCAGGAACCT CAACGAAGAG   
  
  
+ CACTTGACAG AGAGATGGAG TTTCCTGTCT GGACTGAACA TAGAGAAGTG GTCAGCCGTT GGACTTCCCT   
  
  
+ TTTTTTTTTT TTTTATGTTG AACTCGAATT AGTATGACCA ACGACTGGTC TAATGAAGAA AACCCACTTC   
  
  
+ AGCTTTTCTC CAGTCAACCA TCTTCCTTCA ATTTTCTACG TTCAAAAGGA GGCAAATCCT TTAAAATCAC   
  
  
+ ATAACCTTAT TCTCCTTCTT CCACAGGTTA AGTCCCAATA CCTCTAAGTA TTCATCTGCT TAGTTTCTCG   
  
  
+ CCATTACAGA ACAAGGGAAA CTCATTGAAC CCTTTTAGTC ATTCAAACTC TTTTGCATCT TTTGTTTTTT   
  
  
+ CAACCCAATT TCGAAAGTTT TCAAGCGTAG AAGTAACAGA AAGCTGATTC TTTCAAGGGA ATGCTGAATA   
  
  
+ CCCAGTTCAA ATTGAGCATT TCCAGCTTCT CATATCTTTC TCTCTGCCGT TGTCACCTTC TTCGCTTCTC   
  
  
+ CAGTTCCGTC GTAAACCAGT CATTTTCATG GCCTGATACC ATCCGGCCTT GCTTTTGAGA ACCCATTGGC   
  
  
+ CCATTAATTA TTCGTGTTGC TTACAAATC  

- TAAACTTATA TAATTAATAA ATAATATTTT AAAATTTATA AATTATAATT TTGTTAAATT TTTATAAACA   
  
  
- TTATGATTTT TTTAAAATTT TAAAAAACAC TATTATAGAA CTTATAAAAA AATGTTAATA TTTTTCAAAA   
  
  
- TTTATTTTTA AGCATTATAT TTTAAAAATT AAACTTATGA AAATAAAATT TTAAAAAATT ATTTTATATT   
  
  
- AAACTAATAT AAAGTAGTTA GTCGATACAA TGAAGTTTTA AAATTTATTA TAAACGAAAA TTTAAATAAA   
  
  
- ATAATAAAGT AAATTAATAA TATATAATTG TAAATAAAAT TTTATTATAT AGAAATTTTT TATAAAAATT   
  
  
- AATTCGTGAA TGACAATTGT TCTTCAGAGT CCTTCCGTCA TCACAGGTCC GGCCACGCTT CACGCAAGCG   
  
  
- CTCAGATGCC GAGTACCGAG TGTAAAGCCA GTGTCGGAGC ATCCCCTGGT GTTTTCAGAC CTAGTATGTA   
  
  
- TCGCCGAGGA ACGCAGGGGC AATGCAAAAA GGAGTTGCTG GGTCTGTGCC TAGACCGTAA ACTTAGCCAG   
  
  
- ATAAAAAGCC GAAAATTGTA AATAAATAAA TAATATAAAG GGATTTTTAC CTGGTTTTAA TAGAACAATG   
  
  
- AAATGGTTCA CAGAATTAAA ATATATAAGG AGTTATTTTA TTATATTTAT CTTGCTGTAG CAAATGTGCA   
  
  
- TTCGTTCCCA GTACCTTGAT TGCAGTGTGA ATGAAATTCT AGCAAAGAAG GAGAAAAAAA GAGTGGAAAG   
  
  
- ACATCATCAT TGCACCTTAA ACGGTTCAAA CTTCTAAGAA ACCAGAAGAC CAAGGGTTGT AGAAGGTAAC   
  
  
- CGAAGGCAAG AAAGGAGATC TTAGGAAATA ATGTGAGCTG GTTACTCCTA AGTCCTTGGA GTTGCTTCTC   
  
  
- GTGAACTGTC TCTCTACCTC AAAGGACAGA CCTGACTTGT ATCTCTTCAC CAGTCGGCAA CCTGAAGGGA   
  
  
- AAAAAAAAAA AAAATACAAC TTGAGCTTAA TCATACTGGT TGCTGACCAG ATTACTTCTT TTGGGTGAAG   
  
  
- TCGAAAAGAG GTCAGTTGGT AGAAGGAAGT TAAAAGATGC AAGTTTTCCT CCGTTTAGGA AATTTTAGTG   
  
  
- TATTGGAATA AGAGGAAGAA GGTGTCCAAT TCAGGGTTAT GGAGATTCAT AAGTAGACGA ATCAAAGAGC   
  
  
- GGTAATGTCT TGTTCCCTTT GAGTAACTTG GGAAAATCAG TAAGTTTGAG AAAACGTAGA AAACAAAAAA   
  
  
- GTTGGGTTAA AGCTTTCAAA AGTTCGCATC TTCATTGTCT TTCGACTAAG AAAGTTCCCT TACGACTTAT   
  
  
- GGGTCAAGTT TAACTCGTAA AGGTCGAAGA GTATAGAAAG AGAGACGGCA ACAGTGGAAG AAGCGAAGAG   
  
  
- GTCAAGGCAG CATTTGGTCA GTAAAAGTAC CGGACTATGG TAGGCCGGAA CGAAAACTCT TGGGTAACCG   
  
  
- GGTAATTAAT AAGCACAACG AATGTTTAG

+     Box 4

| Site Name | Organism | Position | Strand | Matrix score. | sequence | function |
| --- | --- | --- | --- | --- | --- | --- |
| Box 4 | Petroselinum crispum | 1473 | - | 6 | ATTAAT | part of a conserved DNA module involved in light responsiveness |
| Box 4 | Petroselinum crispum | 11 | + | 6 | ATTAAT | part of a conserved DNA module involved in light responsiveness |

> 2018/04/13 10:10:12  
+ ATTTGAATAT ATTAATTATT TATTATAAAA TTTTAAATAT TTAATATTAA AACAATTTAA AAATATTTGT   
  
  
+ AATACTAAAA AAATTTTAAA ATTTTTTGTG ATAATATCTT GAATATTTTT TTACAATTAT AAAAAGTTTT   
  
  
+ AAATAAAAAT TCGTAATATA AAATTTTTAA TTTGAATACT TTTATTTTAA AATTTTTTAA TAAAATATAA   
  
  
+ TTTGATTATA TTTCATCAAT CAGCTATGTT ACTTCAAAAT TTTAAATAAT ATTTGCTTTT AAATTTATTT   
  
  
+ TATTATTTCA TTTAATTATT ATATATTAAC ATTTATTTTA AAATAATATA TCTTTAAAAA ATATTTTTAA   
  
  
+ TTAAGCACTT ACTGTTAACA AGAAGTCTCA GGAAGGCAGT AGTGTCCAGG CCGGTGCGAA GTGCGTTCGC   
  
  
+ GAGTCTACGG CTCATGGCTC ACATTTCGGT CACAGCCTCG TAGGGGACCA CAAAAGTCTG GATCATACAT   
  
  
+ AGCGGCTCCT TGCGTCCCCG TTACGTTTTT CCTCAACGAC CCAGACACGG ATCTGGCATT TGAATCGGTC   
  
  
+ TATTTTTCGG CTTTTAACAT TTATTTATTT ATTATATTTC CCTAAAAATG GACCAAAATT ATCTTGTTAC   
  
  
+ TTTACCAAGT GTCTTAATTT TATATATTCC TCAATAAAAT AATATAAATA GAACGACATC GTTTACACGT   
  
  
+ AAGCAAGGGT CATGGAACTA ACGTCACACT TACTTTAAGA TCGTTTCTTC CTCTTTTTTT CTCACCTTTC   
  
  
+ TGTAGTAGTA ACGTGGAATT TGCCAAGTTT GAAGATTCTT TGGTCTTCTG GTTCCCAACA TCTTCCATTG   
  
  
+ GCTTCCGTTC TTTCCTCTAG AATCCTTTAT TACACTCGAC CAATGAGGAT TCAGGAACCT CAACGAAGAG   
  
  
+ CACTTGACAG AGAGATGGAG TTTCCTGTCT GGACTGAACA TAGAGAAGTG GTCAGCCGTT GGACTTCCCT   
  
  
+ TTTTTTTTTT TTTTATGTTG AACTCGAATT AGTATGACCA ACGACTGGTC TAATGAAGAA AACCCACTTC   
  
  
+ AGCTTTTCTC CAGTCAACCA TCTTCCTTCA ATTTTCTACG TTCAAAAGGA GGCAAATCCT TTAAAATCAC   
  
  
+ ATAACCTTAT TCTCCTTCTT CCACAGGTTA AGTCCCAATA CCTCTAAGTA TTCATCTGCT TAGTTTCTCG   
  
  
+ CCATTACAGA ACAAGGGAAA CTCATTGAAC CCTTTTAGTC ATTCAAACTC TTTTGCATCT TTTGTTTTTT   
  
  
+ CAACCCAATT TCGAAAGTTT TCAAGCGTAG AAGTAACAGA AAGCTGATTC TTTCAAGGGA ATGCTGAATA   
  
  
+ CCCAGTTCAA ATTGAGCATT TCCAGCTTCT CATATCTTTC TCTCTGCCGT TGTCACCTTC TTCGCTTCTC   
  
  
+ CAGTTCCGTC GTAAACCAGT CATTTTCATG GCCTGATACC ATCCGGCCTT GCTTTTGAGA ACCCATTGGC   
  
  
+ CCATTAATTA TTCGTGTTGC TTACAAATC  

- TAAACTTATA TAATTAATAA ATAATATTTT AAAATTTATA AATTATAATT TTGTTAAATT TTTATAAACA   
  
  
- TTATGATTTT TTTAAAATTT TAAAAAACAC TATTATAGAA CTTATAAAAA AATGTTAATA TTTTTCAAAA   
  
  
- TTTATTTTTA AGCATTATAT TTTAAAAATT AAACTTATGA AAATAAAATT TTAAAAAATT ATTTTATATT   
  
  
- AAACTAATAT AAAGTAGTTA GTCGATACAA TGAAGTTTTA AAATTTATTA TAAACGAAAA TTTAAATAAA   
  
  
- ATAATAAAGT AAATTAATAA TATATAATTG TAAATAAAAT TTTATTATAT AGAAATTTTT TATAAAAATT   
  
  
- AATTCGTGAA TGACAATTGT TCTTCAGAGT CCTTCCGTCA TCACAGGTCC GGCCACGCTT CACGCAAGCG   
  
  
- CTCAGATGCC GAGTACCGAG TGTAAAGCCA GTGTCGGAGC ATCCCCTGGT GTTTTCAGAC CTAGTATGTA   
  
  
- TCGCCGAGGA ACGCAGGGGC AATGCAAAAA GGAGTTGCTG GGTCTGTGCC TAGACCGTAA ACTTAGCCAG   
  
  
- ATAAAAAGCC GAAAATTGTA AATAAATAAA TAATATAAAG GGATTTTTAC CTGGTTTTAA TAGAACAATG   
  
  
- AAATGGTTCA CAGAATTAAA ATATATAAGG AGTTATTTTA TTATATTTAT CTTGCTGTAG CAAATGTGCA   
  
  
- TTCGTTCCCA GTACCTTGAT TGCAGTGTGA ATGAAATTCT AGCAAAGAAG GAGAAAAAAA GAGTGGAAAG   
  
  
- ACATCATCAT TGCACCTTAA ACGGTTCAAA CTTCTAAGAA ACCAGAAGAC CAAGGGTTGT AGAAGGTAAC   
  
  
- CGAAGGCAAG AAAGGAGATC TTAGGAAATA ATGTGAGCTG GTTACTCCTA AGTCCTTGGA GTTGCTTCTC   
  
  
- GTGAACTGTC TCTCTACCTC AAAGGACAGA CCTGACTTGT ATCTCTTCAC CAGTCGGCAA CCTGAAGGGA   
  
  
- AAAAAAAAAA AAAATACAAC TTGAGCTTAA TCATACTGGT TGCTGACCAG ATTACTTCTT TTGGGTGAAG   
  
  
- TCGAAAAGAG GTCAGTTGGT AGAAGGAAGT TAAAAGATGC AAGTTTTCCT CCGTTTAGGA AATTTTAGTG   
  
  
- TATTGGAATA AGAGGAAGAA GGTGTCCAAT TCAGGGTTAT GGAGATTCAT AAGTAGACGA ATCAAAGAGC   
  
  
- GGTAATGTCT TGTTCCCTTT GAGTAACTTG GGAAAATCAG TAAGTTTGAG AAAACGTAGA AAACAAAAAA   
  
  
- GTTGGGTTAA AGCTTTCAAA AGTTCGCATC TTCATTGTCT TTCGACTAAG AAAGTTCCCT TACGACTTAT   
  
  
- GGGTCAAGTT TAACTCGTAA AGGTCGAAGA GTATAGAAAG AGAGACGGCA ACAGTGGAAG AAGCGAAGAG   
  
  
- GTCAAGGCAG CATTTGGTCA GTAAAAGTAC CGGACTATGG TAGGCCGGAA CGAAAACTCT TGGGTAACCG   
  
  
- GGTAATTAAT AAGCACAACG AATGTTTAG

+     CAAT-box

| Site Name | Organism | Position | Strand | Matrix score. | sequence | function |
| --- | --- | --- | --- | --- | --- | --- |
| CAAT-box | Brassica rapa | 261 | - | 5 | CAAAT | common cis-acting element in promoter and enhancer regions |
| CAAT-box | Brassica rapa | 210 | - | 5 | CAAAT | common cis-acting element in promoter and enhancer regions |
| CAAT-box | Brassica rapa | 1494 | + | 5 | CAAAT | common cis-acting element in promoter and enhancer regions |
| CAAT-box | Arabidopsis thaliana | 1264 | + | 8 | CCCAATTT | common cis-acting element in promoter and enhancer regions |
| CAAT-box | Brassica rapa | 1338 | + | 5 | CAAAT | common cis-acting element in promoter and enhancer regions |
| CAAT-box | Brassica rapa | 1103 | + | 5 | CAAAT | common cis-acting element in promoter and enhancer regions |
| CAAT-box | Brassica rapa | 788 | - | 5 | CAAAT | common cis-acting element in promoter and enhancer regions |
| CAAT-box | Hordeum vulgare | 1214 | - | 4 | CAAT | common cis-acting element in promoter and enhancer regions |
| CAAT-box | Glycine max | 53 | + | 5 | CAATT | common cis-acting element in promoter and enhancer regions |
| CAAT-box | Glycine max | 1266 | + | 5 | CAATT | common cis-acting element in promoter and enhancer regions |
| CAAT-box | Brassica rapa | 65 | - | 5 | CAAAT | common cis-acting element in promoter and enhancer regions |
| CAAT-box | Brassica rapa | 548 | - | 5 | CAAAT | common cis-acting element in promoter and enhancer regions |
| CAAT-box | Glycine max | 124 | + | 5 | CAATT | common cis-acting element in promoter and enhancer regions |
| CAAT-box | Arabidopsis thaliana | 837 | - | 5 | CCAAT | common cis-acting element in promoter and enhancer regions |
| CAAT-box | Hordeum vulgare | 227 | + | 4 | CAAT | common cis-acting element in promoter and enhancer regions |
| CAAT-box | Glycine max | 1340 | - | 5 | CAATT | common cis-acting element in promoter and enhancer regions |
| CAAT-box | Hordeum vulgare | 1156 | + | 4 | CAAT | common cis-acting element in promoter and enhancer regions |
| CAAT-box | Arabidopsis thaliana | 1155 | + | 5 | CCAAT | common cis-acting element in promoter and enhancer regions |
| CAAT-box | Arabidopsis thaliana | 1465 | - | 5 | CCAAT | common cis-acting element in promoter and enhancer regions |
| CAAT-box | Hordeum vulgare | 881 | + | 4 | CAAT | common cis-acting element in promoter and enhancer regions |
| CAAT-box | Arabidopsis thaliana | 1265 | + | 5 | CCAAT | common cis-acting element in promoter and enhancer regions |
| CAAT-box | Brassica rapa | 170 | - | 5 | CAAAT | common cis-acting element in promoter and enhancer regions |
| CAAT-box | Hordeum vulgare | 662 | + | 4 | CAAT | common cis-acting element in promoter and enhancer regions |
| CAAT-box | Hordeum vulgare | 1341 | - | 4 | CAAT | common cis-acting element in promoter and enhancer regions |
| CAAT-box | Brassica rapa | 1 | - | 5 | CAAAT | common cis-acting element in promoter and enhancer regions |
| CAAT-box | Arabidopsis thaliana | 880 | + | 5 | CCAAT | common cis-acting element in promoter and enhancer regions |
| CAAT-box | Glycine max | 1079 | + | 5 | CAATT | common cis-acting element in promoter and enhancer regions |

> 2018/04/13 10:10:12  
+ ATTTGAATAT ATTAATTATT TATTATAAAA TTTTAAATAT TTAATATTAA AACAATTTAA AAATATTTGT   
  
  
+ AATACTAAAA AAATTTTAAA ATTTTTTGTG ATAATATCTT GAATATTTTT TTACAATTAT AAAAAGTTTT   
  
  
+ AAATAAAAAT TCGTAATATA AAATTTTTAA TTTGAATACT TTTATTTTAA AATTTTTTAA TAAAATATAA   
  
  
+ TTTGATTATA TTTCATCAAT CAGCTATGTT ACTTCAAAAT TTTAAATAAT ATTTGCTTTT AAATTTATTT   
  
  
+ TATTATTTCA TTTAATTATT ATATATTAAC ATTTATTTTA AAATAATATA TCTTTAAAAA ATATTTTTAA   
  
  
+ TTAAGCACTT ACTGTTAACA AGAAGTCTCA GGAAGGCAGT AGTGTCCAGG CCGGTGCGAA GTGCGTTCGC   
  
  
+ GAGTCTACGG CTCATGGCTC ACATTTCGGT CACAGCCTCG TAGGGGACCA CAAAAGTCTG GATCATACAT   
  
  
+ AGCGGCTCCT TGCGTCCCCG TTACGTTTTT CCTCAACGAC CCAGACACGG ATCTGGCATT TGAATCGGTC   
  
  
+ TATTTTTCGG CTTTTAACAT TTATTTATTT ATTATATTTC CCTAAAAATG GACCAAAATT ATCTTGTTAC   
  
  
+ TTTACCAAGT GTCTTAATTT TATATATTCC TCAATAAAAT AATATAAATA GAACGACATC GTTTACACGT   
  
  
+ AAGCAAGGGT CATGGAACTA ACGTCACACT TACTTTAAGA TCGTTTCTTC CTCTTTTTTT CTCACCTTTC   
  
  
+ TGTAGTAGTA ACGTGGAATT TGCCAAGTTT GAAGATTCTT TGGTCTTCTG GTTCCCAACA TCTTCCATTG   
  
  
+ GCTTCCGTTC TTTCCTCTAG AATCCTTTAT TACACTCGAC CAATGAGGAT TCAGGAACCT CAACGAAGAG   
  
  
+ CACTTGACAG AGAGATGGAG TTTCCTGTCT GGACTGAACA TAGAGAAGTG GTCAGCCGTT GGACTTCCCT   
  
  
+ TTTTTTTTTT TTTTATGTTG AACTCGAATT AGTATGACCA ACGACTGGTC TAATGAAGAA AACCCACTTC   
  
  
+ AGCTTTTCTC CAGTCAACCA TCTTCCTTCA ATTTTCTACG TTCAAAAGGA GGCAAATCCT TTAAAATCAC   
  
  
+ ATAACCTTAT TCTCCTTCTT CCACAGGTTA AGTCCCAATA CCTCTAAGTA TTCATCTGCT TAGTTTCTCG   
  
  
+ CCATTACAGA ACAAGGGAAA CTCATTGAAC CCTTTTAGTC ATTCAAACTC TTTTGCATCT TTTGTTTTTT   
  
  
+ CAACCCAATT TCGAAAGTTT TCAAGCGTAG AAGTAACAGA AAGCTGATTC TTTCAAGGGA ATGCTGAATA   
  
  
+ CCCAGTTCAA ATTGAGCATT TCCAGCTTCT CATATCTTTC TCTCTGCCGT TGTCACCTTC TTCGCTTCTC   
  
  
+ CAGTTCCGTC GTAAACCAGT CATTTTCATG GCCTGATACC ATCCGGCCTT GCTTTTGAGA ACCCATTGGC   
  
  
+ CCATTAATTA TTCGTGTTGC TTACAAATC  

- TAAACTTATA TAATTAATAA ATAATATTTT AAAATTTATA AATTATAATT TTGTTAAATT TTTATAAACA   
  
  
- TTATGATTTT TTTAAAATTT TAAAAAACAC TATTATAGAA CTTATAAAAA AATGTTAATA TTTTTCAAAA   
  
  
- TTTATTTTTA AGCATTATAT TTTAAAAATT AAACTTATGA AAATAAAATT TTAAAAAATT ATTTTATATT   
  
  
- AAACTAATAT AAAGTAGTTA GTCGATACAA TGAAGTTTTA AAATTTATTA TAAACGAAAA TTTAAATAAA   
  
  
- ATAATAAAGT AAATTAATAA TATATAATTG TAAATAAAAT TTTATTATAT AGAAATTTTT TATAAAAATT   
  
  
- AATTCGTGAA TGACAATTGT TCTTCAGAGT CCTTCCGTCA TCACAGGTCC GGCCACGCTT CACGCAAGCG   
  
  
- CTCAGATGCC GAGTACCGAG TGTAAAGCCA GTGTCGGAGC ATCCCCTGGT GTTTTCAGAC CTAGTATGTA   
  
  
- TCGCCGAGGA ACGCAGGGGC AATGCAAAAA GGAGTTGCTG GGTCTGTGCC TAGACCGTAA ACTTAGCCAG   
  
  
- ATAAAAAGCC GAAAATTGTA AATAAATAAA TAATATAAAG GGATTTTTAC CTGGTTTTAA TAGAACAATG   
  
  
- AAATGGTTCA CAGAATTAAA ATATATAAGG AGTTATTTTA TTATATTTAT CTTGCTGTAG CAAATGTGCA   
  
  
- TTCGTTCCCA GTACCTTGAT TGCAGTGTGA ATGAAATTCT AGCAAAGAAG GAGAAAAAAA GAGTGGAAAG   
  
  
- ACATCATCAT TGCACCTTAA ACGGTTCAAA CTTCTAAGAA ACCAGAAGAC CAAGGGTTGT AGAAGGTAAC   
  
  
- CGAAGGCAAG AAAGGAGATC TTAGGAAATA ATGTGAGCTG GTTACTCCTA AGTCCTTGGA GTTGCTTCTC   
  
  
- GTGAACTGTC TCTCTACCTC AAAGGACAGA CCTGACTTGT ATCTCTTCAC CAGTCGGCAA CCTGAAGGGA   
  
  
- AAAAAAAAAA AAAATACAAC TTGAGCTTAA TCATACTGGT TGCTGACCAG ATTACTTCTT TTGGGTGAAG   
  
  
- TCGAAAAGAG GTCAGTTGGT AGAAGGAAGT TAAAAGATGC AAGTTTTCCT CCGTTTAGGA AATTTTAGTG   
  
  
- TATTGGAATA AGAGGAAGAA GGTGTCCAAT TCAGGGTTAT GGAGATTCAT AAGTAGACGA ATCAAAGAGC   
  
  
- GGTAATGTCT TGTTCCCTTT GAGTAACTTG GGAAAATCAG TAAGTTTGAG AAAACGTAGA AAACAAAAAA   
  
  
- GTTGGGTTAA AGCTTTCAAA AGTTCGCATC TTCATTGTCT TTCGACTAAG AAAGTTCCCT TACGACTTAT   
  
  
- GGGTCAAGTT TAACTCGTAA AGGTCGAAGA GTATAGAAAG AGAGACGGCA ACAGTGGAAG AAGCGAAGAG   
  
  
- GTCAAGGCAG CATTTGGTCA GTAAAAGTAC CGGACTATGG TAGGCCGGAA CGAAAACTCT TGGGTAACCG   
  
  
- GGTAATTAAT AAGCACAACG AATGTTTAG

+     CATT-motif

| Site Name | Organism | Position | Strand | Matrix score. | sequence | function |
| --- | --- | --- | --- | --- | --- | --- |
| CATT-motif | Zea mays | 1319 | - | 6 | GCATTC | part of a light responsive element |

> 2018/04/13 10:10:12  
+ ATTTGAATAT ATTAATTATT TATTATAAAA TTTTAAATAT TTAATATTAA AACAATTTAA AAATATTTGT   
  
  
+ AATACTAAAA AAATTTTAAA ATTTTTTGTG ATAATATCTT GAATATTTTT TTACAATTAT AAAAAGTTTT   
  
  
+ AAATAAAAAT TCGTAATATA AAATTTTTAA TTTGAATACT TTTATTTTAA AATTTTTTAA TAAAATATAA   
  
  
+ TTTGATTATA TTTCATCAAT CAGCTATGTT ACTTCAAAAT TTTAAATAAT ATTTGCTTTT AAATTTATTT   
  
  
+ TATTATTTCA TTTAATTATT ATATATTAAC ATTTATTTTA AAATAATATA TCTTTAAAAA ATATTTTTAA   
  
  
+ TTAAGCACTT ACTGTTAACA AGAAGTCTCA GGAAGGCAGT AGTGTCCAGG CCGGTGCGAA GTGCGTTCGC   
  
  
+ GAGTCTACGG CTCATGGCTC ACATTTCGGT CACAGCCTCG TAGGGGACCA CAAAAGTCTG GATCATACAT   
  
  
+ AGCGGCTCCT TGCGTCCCCG TTACGTTTTT CCTCAACGAC CCAGACACGG ATCTGGCATT TGAATCGGTC   
  
  
+ TATTTTTCGG CTTTTAACAT TTATTTATTT ATTATATTTC CCTAAAAATG GACCAAAATT ATCTTGTTAC   
  
  
+ TTTACCAAGT GTCTTAATTT TATATATTCC TCAATAAAAT AATATAAATA GAACGACATC GTTTACACGT   
  
  
+ AAGCAAGGGT CATGGAACTA ACGTCACACT TACTTTAAGA TCGTTTCTTC CTCTTTTTTT CTCACCTTTC   
  
  
+ TGTAGTAGTA ACGTGGAATT TGCCAAGTTT GAAGATTCTT TGGTCTTCTG GTTCCCAACA TCTTCCATTG   
  
  
+ GCTTCCGTTC TTTCCTCTAG AATCCTTTAT TACACTCGAC CAATGAGGAT TCAGGAACCT CAACGAAGAG   
  
  
+ CACTTGACAG AGAGATGGAG TTTCCTGTCT GGACTGAACA TAGAGAAGTG GTCAGCCGTT GGACTTCCCT   
  
  
+ TTTTTTTTTT TTTTATGTTG AACTCGAATT AGTATGACCA ACGACTGGTC TAATGAAGAA AACCCACTTC   
  
  
+ AGCTTTTCTC CAGTCAACCA TCTTCCTTCA ATTTTCTACG TTCAAAAGGA GGCAAATCCT TTAAAATCAC   
  
  
+ ATAACCTTAT TCTCCTTCTT CCACAGGTTA AGTCCCAATA CCTCTAAGTA TTCATCTGCT TAGTTTCTCG   
  
  
+ CCATTACAGA ACAAGGGAAA CTCATTGAAC CCTTTTAGTC ATTCAAACTC TTTTGCATCT TTTGTTTTTT   
  
  
+ CAACCCAATT TCGAAAGTTT TCAAGCGTAG AAGTAACAGA AAGCTGATTC TTTCAAGGGA ATGCTGAATA   
  
  
+ CCCAGTTCAA ATTGAGCATT TCCAGCTTCT CATATCTTTC TCTCTGCCGT TGTCACCTTC TTCGCTTCTC   
  
  
+ CAGTTCCGTC GTAAACCAGT CATTTTCATG GCCTGATACC ATCCGGCCTT GCTTTTGAGA ACCCATTGGC   
  
  
+ CCATTAATTA TTCGTGTTGC TTACAAATC  

- TAAACTTATA TAATTAATAA ATAATATTTT AAAATTTATA AATTATAATT TTGTTAAATT TTTATAAACA   
  
  
- TTATGATTTT TTTAAAATTT TAAAAAACAC TATTATAGAA CTTATAAAAA AATGTTAATA TTTTTCAAAA   
  
  
- TTTATTTTTA AGCATTATAT TTTAAAAATT AAACTTATGA AAATAAAATT TTAAAAAATT ATTTTATATT   
  
  
- AAACTAATAT AAAGTAGTTA GTCGATACAA TGAAGTTTTA AAATTTATTA TAAACGAAAA TTTAAATAAA   
  
  
- ATAATAAAGT AAATTAATAA TATATAATTG TAAATAAAAT TTTATTATAT AGAAATTTTT TATAAAAATT   
  
  
- AATTCGTGAA TGACAATTGT TCTTCAGAGT CCTTCCGTCA TCACAGGTCC GGCCACGCTT CACGCAAGCG   
  
  
- CTCAGATGCC GAGTACCGAG TGTAAAGCCA GTGTCGGAGC ATCCCCTGGT GTTTTCAGAC CTAGTATGTA   
  
  
- TCGCCGAGGA ACGCAGGGGC AATGCAAAAA GGAGTTGCTG GGTCTGTGCC TAGACCGTAA ACTTAGCCAG   
  
  
- ATAAAAAGCC GAAAATTGTA AATAAATAAA TAATATAAAG GGATTTTTAC CTGGTTTTAA TAGAACAATG   
  
  
- AAATGGTTCA CAGAATTAAA ATATATAAGG AGTTATTTTA TTATATTTAT CTTGCTGTAG CAAATGTGCA   
  
  
- TTCGTTCCCA GTACCTTGAT TGCAGTGTGA ATGAAATTCT AGCAAAGAAG GAGAAAAAAA GAGTGGAAAG   
  
  
- ACATCATCAT TGCACCTTAA ACGGTTCAAA CTTCTAAGAA ACCAGAAGAC CAAGGGTTGT AGAAGGTAAC   
  
  
- CGAAGGCAAG AAAGGAGATC TTAGGAAATA ATGTGAGCTG GTTACTCCTA AGTCCTTGGA GTTGCTTCTC   
  
  
- GTGAACTGTC TCTCTACCTC AAAGGACAGA CCTGACTTGT ATCTCTTCAC CAGTCGGCAA CCTGAAGGGA   
  
  
- AAAAAAAAAA AAAATACAAC TTGAGCTTAA TCATACTGGT TGCTGACCAG ATTACTTCTT TTGGGTGAAG   
  
  
- TCGAAAAGAG GTCAGTTGGT AGAAGGAAGT TAAAAGATGC AAGTTTTCCT CCGTTTAGGA AATTTTAGTG   
  
  
- TATTGGAATA AGAGGAAGAA GGTGTCCAAT TCAGGGTTAT GGAGATTCAT AAGTAGACGA ATCAAAGAGC   
  
  
- GGTAATGTCT TGTTCCCTTT GAGTAACTTG GGAAAATCAG TAAGTTTGAG AAAACGTAGA AAACAAAAAA   
  
  
- GTTGGGTTAA AGCTTTCAAA AGTTCGCATC TTCATTGTCT TTCGACTAAG AAAGTTCCCT TACGACTTAT   
  
  
- GGGTCAAGTT TAACTCGTAA AGGTCGAAGA GTATAGAAAG AGAGACGGCA ACAGTGGAAG AAGCGAAGAG   
  
  
- GTCAAGGCAG CATTTGGTCA GTAAAAGTAC CGGACTATGG TAGGCCGGAA CGAAAACTCT TGGGTAACCG   
  
  
- GGTAATTAAT AAGCACAACG AATGTTTAG

+     CCAAT-box

| Site Name | Organism | Position | Strand | Matrix score. | sequence | function |
| --- | --- | --- | --- | --- | --- | --- |
| CCAAT-box | Hordeum vulgare | 966 | - | 6 | CAACGG | MYBHv1 binding site |
| CCAAT-box | Hordeum vulgare | 1377 | - | 6 | CAACGG | MYBHv1 binding site |

> 2018/04/13 10:10:12  
+ ATTTGAATAT ATTAATTATT TATTATAAAA TTTTAAATAT TTAATATTAA AACAATTTAA AAATATTTGT   
  
  
+ AATACTAAAA AAATTTTAAA ATTTTTTGTG ATAATATCTT GAATATTTTT TTACAATTAT AAAAAGTTTT   
  
  
+ AAATAAAAAT TCGTAATATA AAATTTTTAA TTTGAATACT TTTATTTTAA AATTTTTTAA TAAAATATAA   
  
  
+ TTTGATTATA TTTCATCAAT CAGCTATGTT ACTTCAAAAT TTTAAATAAT ATTTGCTTTT AAATTTATTT   
  
  
+ TATTATTTCA TTTAATTATT ATATATTAAC ATTTATTTTA AAATAATATA TCTTTAAAAA ATATTTTTAA   
  
  
+ TTAAGCACTT ACTGTTAACA AGAAGTCTCA GGAAGGCAGT AGTGTCCAGG CCGGTGCGAA GTGCGTTCGC   
  
  
+ GAGTCTACGG CTCATGGCTC ACATTTCGGT CACAGCCTCG TAGGGGACCA CAAAAGTCTG GATCATACAT   
  
  
+ AGCGGCTCCT TGCGTCCCCG TTACGTTTTT CCTCAACGAC CCAGACACGG ATCTGGCATT TGAATCGGTC   
  
  
+ TATTTTTCGG CTTTTAACAT TTATTTATTT ATTATATTTC CCTAAAAATG GACCAAAATT ATCTTGTTAC   
  
  
+ TTTACCAAGT GTCTTAATTT TATATATTCC TCAATAAAAT AATATAAATA GAACGACATC GTTTACACGT   
  
  
+ AAGCAAGGGT CATGGAACTA ACGTCACACT TACTTTAAGA TCGTTTCTTC CTCTTTTTTT CTCACCTTTC   
  
  
+ TGTAGTAGTA ACGTGGAATT TGCCAAGTTT GAAGATTCTT TGGTCTTCTG GTTCCCAACA TCTTCCATTG   
  
  
+ GCTTCCGTTC TTTCCTCTAG AATCCTTTAT TACACTCGAC CAATGAGGAT TCAGGAACCT CAACGAAGAG   
  
  
+ CACTTGACAG AGAGATGGAG TTTCCTGTCT GGACTGAACA TAGAGAAGTG GTCAGCCGTT GGACTTCCCT   
  
  
+ TTTTTTTTTT TTTTATGTTG AACTCGAATT AGTATGACCA ACGACTGGTC TAATGAAGAA AACCCACTTC   
  
  
+ AGCTTTTCTC CAGTCAACCA TCTTCCTTCA ATTTTCTACG TTCAAAAGGA GGCAAATCCT TTAAAATCAC   
  
  
+ ATAACCTTAT TCTCCTTCTT CCACAGGTTA AGTCCCAATA CCTCTAAGTA TTCATCTGCT TAGTTTCTCG   
  
  
+ CCATTACAGA ACAAGGGAAA CTCATTGAAC CCTTTTAGTC ATTCAAACTC TTTTGCATCT TTTGTTTTTT   
  
  
+ CAACCCAATT TCGAAAGTTT TCAAGCGTAG AAGTAACAGA AAGCTGATTC TTTCAAGGGA ATGCTGAATA   
  
  
+ CCCAGTTCAA ATTGAGCATT TCCAGCTTCT CATATCTTTC TCTCTGCCGT TGTCACCTTC TTCGCTTCTC   
  
  
+ CAGTTCCGTC GTAAACCAGT CATTTTCATG GCCTGATACC ATCCGGCCTT GCTTTTGAGA ACCCATTGGC   
  
  
+ CCATTAATTA TTCGTGTTGC TTACAAATC  

- TAAACTTATA TAATTAATAA ATAATATTTT AAAATTTATA AATTATAATT TTGTTAAATT TTTATAAACA   
  
  
- TTATGATTTT TTTAAAATTT TAAAAAACAC TATTATAGAA CTTATAAAAA AATGTTAATA TTTTTCAAAA   
  
  
- TTTATTTTTA AGCATTATAT TTTAAAAATT AAACTTATGA AAATAAAATT TTAAAAAATT ATTTTATATT   
  
  
- AAACTAATAT AAAGTAGTTA GTCGATACAA TGAAGTTTTA AAATTTATTA TAAACGAAAA TTTAAATAAA   
  
  
- ATAATAAAGT AAATTAATAA TATATAATTG TAAATAAAAT TTTATTATAT AGAAATTTTT TATAAAAATT   
  
  
- AATTCGTGAA TGACAATTGT TCTTCAGAGT CCTTCCGTCA TCACAGGTCC GGCCACGCTT CACGCAAGCG   
  
  
- CTCAGATGCC GAGTACCGAG TGTAAAGCCA GTGTCGGAGC ATCCCCTGGT GTTTTCAGAC CTAGTATGTA   
  
  
- TCGCCGAGGA ACGCAGGGGC AATGCAAAAA GGAGTTGCTG GGTCTGTGCC TAGACCGTAA ACTTAGCCAG   
  
  
- ATAAAAAGCC GAAAATTGTA AATAAATAAA TAATATAAAG GGATTTTTAC CTGGTTTTAA TAGAACAATG   
  
  
- AAATGGTTCA CAGAATTAAA ATATATAAGG AGTTATTTTA TTATATTTAT CTTGCTGTAG CAAATGTGCA   
  
  
- TTCGTTCCCA GTACCTTGAT TGCAGTGTGA ATGAAATTCT AGCAAAGAAG GAGAAAAAAA GAGTGGAAAG   
  
  
- ACATCATCAT TGCACCTTAA ACGGTTCAAA CTTCTAAGAA ACCAGAAGAC CAAGGGTTGT AGAAGGTAAC   
  
  
- CGAAGGCAAG AAAGGAGATC TTAGGAAATA ATGTGAGCTG GTTACTCCTA AGTCCTTGGA GTTGCTTCTC   
  
  
- GTGAACTGTC TCTCTACCTC AAAGGACAGA CCTGACTTGT ATCTCTTCAC CAGTCGGCAA CCTGAAGGGA   
  
  
- AAAAAAAAAA AAAATACAAC TTGAGCTTAA TCATACTGGT TGCTGACCAG ATTACTTCTT TTGGGTGAAG   
  
  
- TCGAAAAGAG GTCAGTTGGT AGAAGGAAGT TAAAAGATGC AAGTTTTCCT CCGTTTAGGA AATTTTAGTG   
  
  
- TATTGGAATA AGAGGAAGAA GGTGTCCAAT TCAGGGTTAT GGAGATTCAT AAGTAGACGA ATCAAAGAGC   
  
  
- GGTAATGTCT TGTTCCCTTT GAGTAACTTG GGAAAATCAG TAAGTTTGAG AAAACGTAGA AAACAAAAAA   
  
  
- GTTGGGTTAA AGCTTTCAAA AGTTCGCATC TTCATTGTCT TTCGACTAAG AAAGTTCCCT TACGACTTAT   
  
  
- GGGTCAAGTT TAACTCGTAA AGGTCGAAGA GTATAGAAAG AGAGACGGCA ACAGTGGAAG AAGCGAAGAG   
  
  
- GTCAAGGCAG CATTTGGTCA GTAAAAGTAC CGGACTATGG TAGGCCGGAA CGAAAACTCT TGGGTAACCG   
  
  
- GGTAATTAAT AAGCACAACG AATGTTTAG

+     CGTCA-motif

| Site Name | Organism | Position | Strand | Matrix score. | sequence | function |
| --- | --- | --- | --- | --- | --- | --- |
| CGTCA-motif | Hordeum vulgare | 722 | + | 5 | CGTCA | cis-acting regulatory element involved in the MeJA-responsiveness |

> 2018/04/13 10:10:12  
+ ATTTGAATAT ATTAATTATT TATTATAAAA TTTTAAATAT TTAATATTAA AACAATTTAA AAATATTTGT   
  
  
+ AATACTAAAA AAATTTTAAA ATTTTTTGTG ATAATATCTT GAATATTTTT TTACAATTAT AAAAAGTTTT   
  
  
+ AAATAAAAAT TCGTAATATA AAATTTTTAA TTTGAATACT TTTATTTTAA AATTTTTTAA TAAAATATAA   
  
  
+ TTTGATTATA TTTCATCAAT CAGCTATGTT ACTTCAAAAT TTTAAATAAT ATTTGCTTTT AAATTTATTT   
  
  
+ TATTATTTCA TTTAATTATT ATATATTAAC ATTTATTTTA AAATAATATA TCTTTAAAAA ATATTTTTAA   
  
  
+ TTAAGCACTT ACTGTTAACA AGAAGTCTCA GGAAGGCAGT AGTGTCCAGG CCGGTGCGAA GTGCGTTCGC   
  
  
+ GAGTCTACGG CTCATGGCTC ACATTTCGGT CACAGCCTCG TAGGGGACCA CAAAAGTCTG GATCATACAT   
  
  
+ AGCGGCTCCT TGCGTCCCCG TTACGTTTTT CCTCAACGAC CCAGACACGG ATCTGGCATT TGAATCGGTC   
  
  
+ TATTTTTCGG CTTTTAACAT TTATTTATTT ATTATATTTC CCTAAAAATG GACCAAAATT ATCTTGTTAC   
  
  
+ TTTACCAAGT GTCTTAATTT TATATATTCC TCAATAAAAT AATATAAATA GAACGACATC GTTTACACGT   
  
  
+ AAGCAAGGGT CATGGAACTA ACGTCACACT TACTTTAAGA TCGTTTCTTC CTCTTTTTTT CTCACCTTTC   
  
  
+ TGTAGTAGTA ACGTGGAATT TGCCAAGTTT GAAGATTCTT TGGTCTTCTG GTTCCCAACA TCTTCCATTG   
  
  
+ GCTTCCGTTC TTTCCTCTAG AATCCTTTAT TACACTCGAC CAATGAGGAT TCAGGAACCT CAACGAAGAG   
  
  
+ CACTTGACAG AGAGATGGAG TTTCCTGTCT GGACTGAACA TAGAGAAGTG GTCAGCCGTT GGACTTCCCT   
  
  
+ TTTTTTTTTT TTTTATGTTG AACTCGAATT AGTATGACCA ACGACTGGTC TAATGAAGAA AACCCACTTC   
  
  
+ AGCTTTTCTC CAGTCAACCA TCTTCCTTCA ATTTTCTACG TTCAAAAGGA GGCAAATCCT TTAAAATCAC   
  
  
+ ATAACCTTAT TCTCCTTCTT CCACAGGTTA AGTCCCAATA CCTCTAAGTA TTCATCTGCT TAGTTTCTCG   
  
  
+ CCATTACAGA ACAAGGGAAA CTCATTGAAC CCTTTTAGTC ATTCAAACTC TTTTGCATCT TTTGTTTTTT   
  
  
+ CAACCCAATT TCGAAAGTTT TCAAGCGTAG AAGTAACAGA AAGCTGATTC TTTCAAGGGA ATGCTGAATA   
  
  
+ CCCAGTTCAA ATTGAGCATT TCCAGCTTCT CATATCTTTC TCTCTGCCGT TGTCACCTTC TTCGCTTCTC   
  
  
+ CAGTTCCGTC GTAAACCAGT CATTTTCATG GCCTGATACC ATCCGGCCTT GCTTTTGAGA ACCCATTGGC   
  
  
+ CCATTAATTA TTCGTGTTGC TTACAAATC  

- TAAACTTATA TAATTAATAA ATAATATTTT AAAATTTATA AATTATAATT TTGTTAAATT TTTATAAACA   
  
  
- TTATGATTTT TTTAAAATTT TAAAAAACAC TATTATAGAA CTTATAAAAA AATGTTAATA TTTTTCAAAA   
  
  
- TTTATTTTTA AGCATTATAT TTTAAAAATT AAACTTATGA AAATAAAATT TTAAAAAATT ATTTTATATT   
  
  
- AAACTAATAT AAAGTAGTTA GTCGATACAA TGAAGTTTTA AAATTTATTA TAAACGAAAA TTTAAATAAA   
  
  
- ATAATAAAGT AAATTAATAA TATATAATTG TAAATAAAAT TTTATTATAT AGAAATTTTT TATAAAAATT   
  
  
- AATTCGTGAA TGACAATTGT TCTTCAGAGT CCTTCCGTCA TCACAGGTCC GGCCACGCTT CACGCAAGCG   
  
  
- CTCAGATGCC GAGTACCGAG TGTAAAGCCA GTGTCGGAGC ATCCCCTGGT GTTTTCAGAC CTAGTATGTA   
  
  
- TCGCCGAGGA ACGCAGGGGC AATGCAAAAA GGAGTTGCTG GGTCTGTGCC TAGACCGTAA ACTTAGCCAG   
  
  
- ATAAAAAGCC GAAAATTGTA AATAAATAAA TAATATAAAG GGATTTTTAC CTGGTTTTAA TAGAACAATG   
  
  
- AAATGGTTCA CAGAATTAAA ATATATAAGG AGTTATTTTA TTATATTTAT CTTGCTGTAG CAAATGTGCA   
  
  
- TTCGTTCCCA GTACCTTGAT TGCAGTGTGA ATGAAATTCT AGCAAAGAAG GAGAAAAAAA GAGTGGAAAG   
  
  
- ACATCATCAT TGCACCTTAA ACGGTTCAAA CTTCTAAGAA ACCAGAAGAC CAAGGGTTGT AGAAGGTAAC   
  
  
- CGAAGGCAAG AAAGGAGATC TTAGGAAATA ATGTGAGCTG GTTACTCCTA AGTCCTTGGA GTTGCTTCTC   
  
  
- GTGAACTGTC TCTCTACCTC AAAGGACAGA CCTGACTTGT ATCTCTTCAC CAGTCGGCAA CCTGAAGGGA   
  
  
- AAAAAAAAAA AAAATACAAC TTGAGCTTAA TCATACTGGT TGCTGACCAG ATTACTTCTT TTGGGTGAAG   
  
  
- TCGAAAAGAG GTCAGTTGGT AGAAGGAAGT TAAAAGATGC AAGTTTTCCT CCGTTTAGGA AATTTTAGTG   
  
  
- TATTGGAATA AGAGGAAGAA GGTGTCCAAT TCAGGGTTAT GGAGATTCAT AAGTAGACGA ATCAAAGAGC   
  
  
- GGTAATGTCT TGTTCCCTTT GAGTAACTTG GGAAAATCAG TAAGTTTGAG AAAACGTAGA AAACAAAAAA   
  
  
- GTTGGGTTAA AGCTTTCAAA AGTTCGCATC TTCATTGTCT TTCGACTAAG AAAGTTCCCT TACGACTTAT   
  
  
- GGGTCAAGTT TAACTCGTAA AGGTCGAAGA GTATAGAAAG AGAGACGGCA ACAGTGGAAG AAGCGAAGAG   
  
  
- GTCAAGGCAG CATTTGGTCA GTAAAAGTAC CGGACTATGG TAGGCCGGAA CGAAAACTCT TGGGTAACCG   
  
  
- GGTAATTAAT AAGCACAACG AATGTTTAG

+     G-Box

| Site Name | Organism | Position | Strand | Matrix score. | sequence | function |
| --- | --- | --- | --- | --- | --- | --- |
| G-Box | Pisum sativum | 780 | - | 6 | CACGTT | cis-acting regulatory element involved in light responsiveness |
| G-Box | Antirrhinum majus | 696 | + | 6 | CACGTA | cis-acting regulatory element involved in light responsiveness |

> 2018/04/13 10:10:12  
+ ATTTGAATAT ATTAATTATT TATTATAAAA TTTTAAATAT TTAATATTAA AACAATTTAA AAATATTTGT   
  
  
+ AATACTAAAA AAATTTTAAA ATTTTTTGTG ATAATATCTT GAATATTTTT TTACAATTAT AAAAAGTTTT   
  
  
+ AAATAAAAAT TCGTAATATA AAATTTTTAA TTTGAATACT TTTATTTTAA AATTTTTTAA TAAAATATAA   
  
  
+ TTTGATTATA TTTCATCAAT CAGCTATGTT ACTTCAAAAT TTTAAATAAT ATTTGCTTTT AAATTTATTT   
  
  
+ TATTATTTCA TTTAATTATT ATATATTAAC ATTTATTTTA AAATAATATA TCTTTAAAAA ATATTTTTAA   
  
  
+ TTAAGCACTT ACTGTTAACA AGAAGTCTCA GGAAGGCAGT AGTGTCCAGG CCGGTGCGAA GTGCGTTCGC   
  
  
+ GAGTCTACGG CTCATGGCTC ACATTTCGGT CACAGCCTCG TAGGGGACCA CAAAAGTCTG GATCATACAT   
  
  
+ AGCGGCTCCT TGCGTCCCCG TTACGTTTTT CCTCAACGAC CCAGACACGG ATCTGGCATT TGAATCGGTC   
  
  
+ TATTTTTCGG CTTTTAACAT TTATTTATTT ATTATATTTC CCTAAAAATG GACCAAAATT ATCTTGTTAC   
  
  
+ TTTACCAAGT GTCTTAATTT TATATATTCC TCAATAAAAT AATATAAATA GAACGACATC GTTTACACGT   
  
  
+ AAGCAAGGGT CATGGAACTA ACGTCACACT TACTTTAAGA TCGTTTCTTC CTCTTTTTTT CTCACCTTTC   
  
  
+ TGTAGTAGTA ACGTGGAATT TGCCAAGTTT GAAGATTCTT TGGTCTTCTG GTTCCCAACA TCTTCCATTG   
  
  
+ GCTTCCGTTC TTTCCTCTAG AATCCTTTAT TACACTCGAC CAATGAGGAT TCAGGAACCT CAACGAAGAG   
  
  
+ CACTTGACAG AGAGATGGAG TTTCCTGTCT GGACTGAACA TAGAGAAGTG GTCAGCCGTT GGACTTCCCT   
  
  
+ TTTTTTTTTT TTTTATGTTG AACTCGAATT AGTATGACCA ACGACTGGTC TAATGAAGAA AACCCACTTC   
  
  
+ AGCTTTTCTC CAGTCAACCA TCTTCCTTCA ATTTTCTACG TTCAAAAGGA GGCAAATCCT TTAAAATCAC   
  
  
+ ATAACCTTAT TCTCCTTCTT CCACAGGTTA AGTCCCAATA CCTCTAAGTA TTCATCTGCT TAGTTTCTCG   
  
  
+ CCATTACAGA ACAAGGGAAA CTCATTGAAC CCTTTTAGTC ATTCAAACTC TTTTGCATCT TTTGTTTTTT   
  
  
+ CAACCCAATT TCGAAAGTTT TCAAGCGTAG AAGTAACAGA AAGCTGATTC TTTCAAGGGA ATGCTGAATA   
  
  
+ CCCAGTTCAA ATTGAGCATT TCCAGCTTCT CATATCTTTC TCTCTGCCGT TGTCACCTTC TTCGCTTCTC   
  
  
+ CAGTTCCGTC GTAAACCAGT CATTTTCATG GCCTGATACC ATCCGGCCTT GCTTTTGAGA ACCCATTGGC   
  
  
+ CCATTAATTA TTCGTGTTGC TTACAAATC  

- TAAACTTATA TAATTAATAA ATAATATTTT AAAATTTATA AATTATAATT TTGTTAAATT TTTATAAACA   
  
  
- TTATGATTTT TTTAAAATTT TAAAAAACAC TATTATAGAA CTTATAAAAA AATGTTAATA TTTTTCAAAA   
  
  
- TTTATTTTTA AGCATTATAT TTTAAAAATT AAACTTATGA AAATAAAATT TTAAAAAATT ATTTTATATT   
  
  
- AAACTAATAT AAAGTAGTTA GTCGATACAA TGAAGTTTTA AAATTTATTA TAAACGAAAA TTTAAATAAA   
  
  
- ATAATAAAGT AAATTAATAA TATATAATTG TAAATAAAAT TTTATTATAT AGAAATTTTT TATAAAAATT   
  
  
- AATTCGTGAA TGACAATTGT TCTTCAGAGT CCTTCCGTCA TCACAGGTCC GGCCACGCTT CACGCAAGCG   
  
  
- CTCAGATGCC GAGTACCGAG TGTAAAGCCA GTGTCGGAGC ATCCCCTGGT GTTTTCAGAC CTAGTATGTA   
  
  
- TCGCCGAGGA ACGCAGGGGC AATGCAAAAA GGAGTTGCTG GGTCTGTGCC TAGACCGTAA ACTTAGCCAG   
  
  
- ATAAAAAGCC GAAAATTGTA AATAAATAAA TAATATAAAG GGATTTTTAC CTGGTTTTAA TAGAACAATG   
  
  
- AAATGGTTCA CAGAATTAAA ATATATAAGG AGTTATTTTA TTATATTTAT CTTGCTGTAG CAAATGTGCA   
  
  
- TTCGTTCCCA GTACCTTGAT TGCAGTGTGA ATGAAATTCT AGCAAAGAAG GAGAAAAAAA GAGTGGAAAG   
  
  
- ACATCATCAT TGCACCTTAA ACGGTTCAAA CTTCTAAGAA ACCAGAAGAC CAAGGGTTGT AGAAGGTAAC   
  
  
- CGAAGGCAAG AAAGGAGATC TTAGGAAATA ATGTGAGCTG GTTACTCCTA AGTCCTTGGA GTTGCTTCTC   
  
  
- GTGAACTGTC TCTCTACCTC AAAGGACAGA CCTGACTTGT ATCTCTTCAC CAGTCGGCAA CCTGAAGGGA   
  
  
- AAAAAAAAAA AAAATACAAC TTGAGCTTAA TCATACTGGT TGCTGACCAG ATTACTTCTT TTGGGTGAAG   
  
  
- TCGAAAAGAG GTCAGTTGGT AGAAGGAAGT TAAAAGATGC AAGTTTTCCT CCGTTTAGGA AATTTTAGTG   
  
  
- TATTGGAATA AGAGGAAGAA GGTGTCCAAT TCAGGGTTAT GGAGATTCAT AAGTAGACGA ATCAAAGAGC   
  
  
- GGTAATGTCT TGTTCCCTTT GAGTAACTTG GGAAAATCAG TAAGTTTGAG AAAACGTAGA AAACAAAAAA   
  
  
- GTTGGGTTAA AGCTTTCAAA AGTTCGCATC TTCATTGTCT TTCGACTAAG AAAGTTCCCT TACGACTTAT   
  
  
- GGGTCAAGTT TAACTCGTAA AGGTCGAAGA GTATAGAAAG AGAGACGGCA ACAGTGGAAG AAGCGAAGAG   
  
  
- GTCAAGGCAG CATTTGGTCA GTAAAAGTAC CGGACTATGG TAGGCCGGAA CGAAAACTCT TGGGTAACCG   
  
  
- GGTAATTAAT AAGCACAACG AATGTTTAG

+     G-box

| Site Name | Organism | Position | Strand | Matrix score. | sequence | function |
| --- | --- | --- | --- | --- | --- | --- |
| G-box | Daucus carota | 696 | - | 6 | TACGTG | cis-acting regulatory element involved in light responsiveness |
| G-box | Zea mays | 780 | - | 6 | CACGTT | cis-acting regulatory element involved in light responsiveness |

> 2018/04/13 10:10:12  
+ ATTTGAATAT ATTAATTATT TATTATAAAA TTTTAAATAT TTAATATTAA AACAATTTAA AAATATTTGT   
  
  
+ AATACTAAAA AAATTTTAAA ATTTTTTGTG ATAATATCTT GAATATTTTT TTACAATTAT AAAAAGTTTT   
  
  
+ AAATAAAAAT TCGTAATATA AAATTTTTAA TTTGAATACT TTTATTTTAA AATTTTTTAA TAAAATATAA   
  
  
+ TTTGATTATA TTTCATCAAT CAGCTATGTT ACTTCAAAAT TTTAAATAAT ATTTGCTTTT AAATTTATTT   
  
  
+ TATTATTTCA TTTAATTATT ATATATTAAC ATTTATTTTA AAATAATATA TCTTTAAAAA ATATTTTTAA   
  
  
+ TTAAGCACTT ACTGTTAACA AGAAGTCTCA GGAAGGCAGT AGTGTCCAGG CCGGTGCGAA GTGCGTTCGC   
  
  
+ GAGTCTACGG CTCATGGCTC ACATTTCGGT CACAGCCTCG TAGGGGACCA CAAAAGTCTG GATCATACAT   
  
  
+ AGCGGCTCCT TGCGTCCCCG TTACGTTTTT CCTCAACGAC CCAGACACGG ATCTGGCATT TGAATCGGTC   
  
  
+ TATTTTTCGG CTTTTAACAT TTATTTATTT ATTATATTTC CCTAAAAATG GACCAAAATT ATCTTGTTAC   
  
  
+ TTTACCAAGT GTCTTAATTT TATATATTCC TCAATAAAAT AATATAAATA GAACGACATC GTTTACACGT   
  
  
+ AAGCAAGGGT CATGGAACTA ACGTCACACT TACTTTAAGA TCGTTTCTTC CTCTTTTTTT CTCACCTTTC   
  
  
+ TGTAGTAGTA ACGTGGAATT TGCCAAGTTT GAAGATTCTT TGGTCTTCTG GTTCCCAACA TCTTCCATTG   
  
  
+ GCTTCCGTTC TTTCCTCTAG AATCCTTTAT TACACTCGAC CAATGAGGAT TCAGGAACCT CAACGAAGAG   
  
  
+ CACTTGACAG AGAGATGGAG TTTCCTGTCT GGACTGAACA TAGAGAAGTG GTCAGCCGTT GGACTTCCCT   
  
  
+ TTTTTTTTTT TTTTATGTTG AACTCGAATT AGTATGACCA ACGACTGGTC TAATGAAGAA AACCCACTTC   
  
  
+ AGCTTTTCTC CAGTCAACCA TCTTCCTTCA ATTTTCTACG TTCAAAAGGA GGCAAATCCT TTAAAATCAC   
  
  
+ ATAACCTTAT TCTCCTTCTT CCACAGGTTA AGTCCCAATA CCTCTAAGTA TTCATCTGCT TAGTTTCTCG   
  
  
+ CCATTACAGA ACAAGGGAAA CTCATTGAAC CCTTTTAGTC ATTCAAACTC TTTTGCATCT TTTGTTTTTT   
  
  
+ CAACCCAATT TCGAAAGTTT TCAAGCGTAG AAGTAACAGA AAGCTGATTC TTTCAAGGGA ATGCTGAATA   
  
  
+ CCCAGTTCAA ATTGAGCATT TCCAGCTTCT CATATCTTTC TCTCTGCCGT TGTCACCTTC TTCGCTTCTC   
  
  
+ CAGTTCCGTC GTAAACCAGT CATTTTCATG GCCTGATACC ATCCGGCCTT GCTTTTGAGA ACCCATTGGC   
  
  
+ CCATTAATTA TTCGTGTTGC TTACAAATC  

- TAAACTTATA TAATTAATAA ATAATATTTT AAAATTTATA AATTATAATT TTGTTAAATT TTTATAAACA   
  
  
- TTATGATTTT TTTAAAATTT TAAAAAACAC TATTATAGAA CTTATAAAAA AATGTTAATA TTTTTCAAAA   
  
  
- TTTATTTTTA AGCATTATAT TTTAAAAATT AAACTTATGA AAATAAAATT TTAAAAAATT ATTTTATATT   
  
  
- AAACTAATAT AAAGTAGTTA GTCGATACAA TGAAGTTTTA AAATTTATTA TAAACGAAAA TTTAAATAAA   
  
  
- ATAATAAAGT AAATTAATAA TATATAATTG TAAATAAAAT TTTATTATAT AGAAATTTTT TATAAAAATT   
  
  
- AATTCGTGAA TGACAATTGT TCTTCAGAGT CCTTCCGTCA TCACAGGTCC GGCCACGCTT CACGCAAGCG   
  
  
- CTCAGATGCC GAGTACCGAG TGTAAAGCCA GTGTCGGAGC ATCCCCTGGT GTTTTCAGAC CTAGTATGTA   
  
  
- TCGCCGAGGA ACGCAGGGGC AATGCAAAAA GGAGTTGCTG GGTCTGTGCC TAGACCGTAA ACTTAGCCAG   
  
  
- ATAAAAAGCC GAAAATTGTA AATAAATAAA TAATATAAAG GGATTTTTAC CTGGTTTTAA TAGAACAATG   
  
  
- AAATGGTTCA CAGAATTAAA ATATATAAGG AGTTATTTTA TTATATTTAT CTTGCTGTAG CAAATGTGCA   
  
  
- TTCGTTCCCA GTACCTTGAT TGCAGTGTGA ATGAAATTCT AGCAAAGAAG GAGAAAAAAA GAGTGGAAAG   
  
  
- ACATCATCAT TGCACCTTAA ACGGTTCAAA CTTCTAAGAA ACCAGAAGAC CAAGGGTTGT AGAAGGTAAC   
  
  
- CGAAGGCAAG AAAGGAGATC TTAGGAAATA ATGTGAGCTG GTTACTCCTA AGTCCTTGGA GTTGCTTCTC   
  
  
- GTGAACTGTC TCTCTACCTC AAAGGACAGA CCTGACTTGT ATCTCTTCAC CAGTCGGCAA CCTGAAGGGA   
  
  
- AAAAAAAAAA AAAATACAAC TTGAGCTTAA TCATACTGGT TGCTGACCAG ATTACTTCTT TTGGGTGAAG   
  
  
- TCGAAAAGAG GTCAGTTGGT AGAAGGAAGT TAAAAGATGC AAGTTTTCCT CCGTTTAGGA AATTTTAGTG   
  
  
- TATTGGAATA AGAGGAAGAA GGTGTCCAAT TCAGGGTTAT GGAGATTCAT AAGTAGACGA ATCAAAGAGC   
  
  
- GGTAATGTCT TGTTCCCTTT GAGTAACTTG GGAAAATCAG TAAGTTTGAG AAAACGTAGA AAACAAAAAA   
  
  
- GTTGGGTTAA AGCTTTCAAA AGTTCGCATC TTCATTGTCT TTCGACTAAG AAAGTTCCCT TACGACTTAT   
  
  
- GGGTCAAGTT TAACTCGTAA AGGTCGAAGA GTATAGAAAG AGAGACGGCA ACAGTGGAAG AAGCGAAGAG   
  
  
- GTCAAGGCAG CATTTGGTCA GTAAAAGTAC CGGACTATGG TAGGCCGGAA CGAAAACTCT TGGGTAACCG   
  
  
- GGTAATTAAT AAGCACAACG AATGTTTAG

+     GA-motif

| Site Name | Organism | Position | Strand | Matrix score. | sequence | function |
| --- | --- | --- | --- | --- | --- | --- |
| GA-motif | Glycine max | 1071 | - | 8 | AAGGAAGA | part of a light responsive element |

> 2018/04/13 10:10:12  
+ ATTTGAATAT ATTAATTATT TATTATAAAA TTTTAAATAT TTAATATTAA AACAATTTAA AAATATTTGT   
  
  
+ AATACTAAAA AAATTTTAAA ATTTTTTGTG ATAATATCTT GAATATTTTT TTACAATTAT AAAAAGTTTT   
  
  
+ AAATAAAAAT TCGTAATATA AAATTTTTAA TTTGAATACT TTTATTTTAA AATTTTTTAA TAAAATATAA   
  
  
+ TTTGATTATA TTTCATCAAT CAGCTATGTT ACTTCAAAAT TTTAAATAAT ATTTGCTTTT AAATTTATTT   
  
  
+ TATTATTTCA TTTAATTATT ATATATTAAC ATTTATTTTA AAATAATATA TCTTTAAAAA ATATTTTTAA   
  
  
+ TTAAGCACTT ACTGTTAACA AGAAGTCTCA GGAAGGCAGT AGTGTCCAGG CCGGTGCGAA GTGCGTTCGC   
  
  
+ GAGTCTACGG CTCATGGCTC ACATTTCGGT CACAGCCTCG TAGGGGACCA CAAAAGTCTG GATCATACAT   
  
  
+ AGCGGCTCCT TGCGTCCCCG TTACGTTTTT CCTCAACGAC CCAGACACGG ATCTGGCATT TGAATCGGTC   
  
  
+ TATTTTTCGG CTTTTAACAT TTATTTATTT ATTATATTTC CCTAAAAATG GACCAAAATT ATCTTGTTAC   
  
  
+ TTTACCAAGT GTCTTAATTT TATATATTCC TCAATAAAAT AATATAAATA GAACGACATC GTTTACACGT   
  
  
+ AAGCAAGGGT CATGGAACTA ACGTCACACT TACTTTAAGA TCGTTTCTTC CTCTTTTTTT CTCACCTTTC   
  
  
+ TGTAGTAGTA ACGTGGAATT TGCCAAGTTT GAAGATTCTT TGGTCTTCTG GTTCCCAACA TCTTCCATTG   
  
  
+ GCTTCCGTTC TTTCCTCTAG AATCCTTTAT TACACTCGAC CAATGAGGAT TCAGGAACCT CAACGAAGAG   
  
  
+ CACTTGACAG AGAGATGGAG TTTCCTGTCT GGACTGAACA TAGAGAAGTG GTCAGCCGTT GGACTTCCCT   
  
  
+ TTTTTTTTTT TTTTATGTTG AACTCGAATT AGTATGACCA ACGACTGGTC TAATGAAGAA AACCCACTTC   
  
  
+ AGCTTTTCTC CAGTCAACCA TCTTCCTTCA ATTTTCTACG TTCAAAAGGA GGCAAATCCT TTAAAATCAC   
  
  
+ ATAACCTTAT TCTCCTTCTT CCACAGGTTA AGTCCCAATA CCTCTAAGTA TTCATCTGCT TAGTTTCTCG   
  
  
+ CCATTACAGA ACAAGGGAAA CTCATTGAAC CCTTTTAGTC ATTCAAACTC TTTTGCATCT TTTGTTTTTT   
  
  
+ CAACCCAATT TCGAAAGTTT TCAAGCGTAG AAGTAACAGA AAGCTGATTC TTTCAAGGGA ATGCTGAATA   
  
  
+ CCCAGTTCAA ATTGAGCATT TCCAGCTTCT CATATCTTTC TCTCTGCCGT TGTCACCTTC TTCGCTTCTC   
  
  
+ CAGTTCCGTC GTAAACCAGT CATTTTCATG GCCTGATACC ATCCGGCCTT GCTTTTGAGA ACCCATTGGC   
  
  
+ CCATTAATTA TTCGTGTTGC TTACAAATC  

- TAAACTTATA TAATTAATAA ATAATATTTT AAAATTTATA AATTATAATT TTGTTAAATT TTTATAAACA   
  
  
- TTATGATTTT TTTAAAATTT TAAAAAACAC TATTATAGAA CTTATAAAAA AATGTTAATA TTTTTCAAAA   
  
  
- TTTATTTTTA AGCATTATAT TTTAAAAATT AAACTTATGA AAATAAAATT TTAAAAAATT ATTTTATATT   
  
  
- AAACTAATAT AAAGTAGTTA GTCGATACAA TGAAGTTTTA AAATTTATTA TAAACGAAAA TTTAAATAAA   
  
  
- ATAATAAAGT AAATTAATAA TATATAATTG TAAATAAAAT TTTATTATAT AGAAATTTTT TATAAAAATT   
  
  
- AATTCGTGAA TGACAATTGT TCTTCAGAGT CCTTCCGTCA TCACAGGTCC GGCCACGCTT CACGCAAGCG   
  
  
- CTCAGATGCC GAGTACCGAG TGTAAAGCCA GTGTCGGAGC ATCCCCTGGT GTTTTCAGAC CTAGTATGTA   
  
  
- TCGCCGAGGA ACGCAGGGGC AATGCAAAAA GGAGTTGCTG GGTCTGTGCC TAGACCGTAA ACTTAGCCAG   
  
  
- ATAAAAAGCC GAAAATTGTA AATAAATAAA TAATATAAAG GGATTTTTAC CTGGTTTTAA TAGAACAATG   
  
  
- AAATGGTTCA CAGAATTAAA ATATATAAGG AGTTATTTTA TTATATTTAT CTTGCTGTAG CAAATGTGCA   
  
  
- TTCGTTCCCA GTACCTTGAT TGCAGTGTGA ATGAAATTCT AGCAAAGAAG GAGAAAAAAA GAGTGGAAAG   
  
  
- ACATCATCAT TGCACCTTAA ACGGTTCAAA CTTCTAAGAA ACCAGAAGAC CAAGGGTTGT AGAAGGTAAC   
  
  
- CGAAGGCAAG AAAGGAGATC TTAGGAAATA ATGTGAGCTG GTTACTCCTA AGTCCTTGGA GTTGCTTCTC   
  
  
- GTGAACTGTC TCTCTACCTC AAAGGACAGA CCTGACTTGT ATCTCTTCAC CAGTCGGCAA CCTGAAGGGA   
  
  
- AAAAAAAAAA AAAATACAAC TTGAGCTTAA TCATACTGGT TGCTGACCAG ATTACTTCTT TTGGGTGAAG   
  
  
- TCGAAAAGAG GTCAGTTGGT AGAAGGAAGT TAAAAGATGC AAGTTTTCCT CCGTTTAGGA AATTTTAGTG   
  
  
- TATTGGAATA AGAGGAAGAA GGTGTCCAAT TCAGGGTTAT GGAGATTCAT AAGTAGACGA ATCAAAGAGC   
  
  
- GGTAATGTCT TGTTCCCTTT GAGTAACTTG GGAAAATCAG TAAGTTTGAG AAAACGTAGA AAACAAAAAA   
  
  
- GTTGGGTTAA AGCTTTCAAA AGTTCGCATC TTCATTGTCT TTCGACTAAG AAAGTTCCCT TACGACTTAT   
  
  
- GGGTCAAGTT TAACTCGTAA AGGTCGAAGA GTATAGAAAG AGAGACGGCA ACAGTGGAAG AAGCGAAGAG   
  
  
- GTCAAGGCAG CATTTGGTCA GTAAAAGTAC CGGACTATGG TAGGCCGGAA CGAAAACTCT TGGGTAACCG   
  
  
- GGTAATTAAT AAGCACAACG AATGTTTAG

+     GAG-motif

| Site Name | Organism | Position | Strand | Matrix score. | sequence | function |
| --- | --- | --- | --- | --- | --- | --- |
| GAG-motif | Spinacia oleracea | 921 | + | 7 | AGAGATG | part of a light responsive element |

> 2018/04/13 10:10:12  
+ ATTTGAATAT ATTAATTATT TATTATAAAA TTTTAAATAT TTAATATTAA AACAATTTAA AAATATTTGT   
  
  
+ AATACTAAAA AAATTTTAAA ATTTTTTGTG ATAATATCTT GAATATTTTT TTACAATTAT AAAAAGTTTT   
  
  
+ AAATAAAAAT TCGTAATATA AAATTTTTAA TTTGAATACT TTTATTTTAA AATTTTTTAA TAAAATATAA   
  
  
+ TTTGATTATA TTTCATCAAT CAGCTATGTT ACTTCAAAAT TTTAAATAAT ATTTGCTTTT AAATTTATTT   
  
  
+ TATTATTTCA TTTAATTATT ATATATTAAC ATTTATTTTA AAATAATATA TCTTTAAAAA ATATTTTTAA   
  
  
+ TTAAGCACTT ACTGTTAACA AGAAGTCTCA GGAAGGCAGT AGTGTCCAGG CCGGTGCGAA GTGCGTTCGC   
  
  
+ GAGTCTACGG CTCATGGCTC ACATTTCGGT CACAGCCTCG TAGGGGACCA CAAAAGTCTG GATCATACAT   
  
  
+ AGCGGCTCCT TGCGTCCCCG TTACGTTTTT CCTCAACGAC CCAGACACGG ATCTGGCATT TGAATCGGTC   
  
  
+ TATTTTTCGG CTTTTAACAT TTATTTATTT ATTATATTTC CCTAAAAATG GACCAAAATT ATCTTGTTAC   
  
  
+ TTTACCAAGT GTCTTAATTT TATATATTCC TCAATAAAAT AATATAAATA GAACGACATC GTTTACACGT   
  
  
+ AAGCAAGGGT CATGGAACTA ACGTCACACT TACTTTAAGA TCGTTTCTTC CTCTTTTTTT CTCACCTTTC   
  
  
+ TGTAGTAGTA ACGTGGAATT TGCCAAGTTT GAAGATTCTT TGGTCTTCTG GTTCCCAACA TCTTCCATTG   
  
  
+ GCTTCCGTTC TTTCCTCTAG AATCCTTTAT TACACTCGAC CAATGAGGAT TCAGGAACCT CAACGAAGAG   
  
  
+ CACTTGACAG AGAGATGGAG TTTCCTGTCT GGACTGAACA TAGAGAAGTG GTCAGCCGTT GGACTTCCCT   
  
  
+ TTTTTTTTTT TTTTATGTTG AACTCGAATT AGTATGACCA ACGACTGGTC TAATGAAGAA AACCCACTTC   
  
  
+ AGCTTTTCTC CAGTCAACCA TCTTCCTTCA ATTTTCTACG TTCAAAAGGA GGCAAATCCT TTAAAATCAC   
  
  
+ ATAACCTTAT TCTCCTTCTT CCACAGGTTA AGTCCCAATA CCTCTAAGTA TTCATCTGCT TAGTTTCTCG   
  
  
+ CCATTACAGA ACAAGGGAAA CTCATTGAAC CCTTTTAGTC ATTCAAACTC TTTTGCATCT TTTGTTTTTT   
  
  
+ CAACCCAATT TCGAAAGTTT TCAAGCGTAG AAGTAACAGA AAGCTGATTC TTTCAAGGGA ATGCTGAATA   
  
  
+ CCCAGTTCAA ATTGAGCATT TCCAGCTTCT CATATCTTTC TCTCTGCCGT TGTCACCTTC TTCGCTTCTC   
  
  
+ CAGTTCCGTC GTAAACCAGT CATTTTCATG GCCTGATACC ATCCGGCCTT GCTTTTGAGA ACCCATTGGC   
  
  
+ CCATTAATTA TTCGTGTTGC TTACAAATC  

- TAAACTTATA TAATTAATAA ATAATATTTT AAAATTTATA AATTATAATT TTGTTAAATT TTTATAAACA   
  
  
- TTATGATTTT TTTAAAATTT TAAAAAACAC TATTATAGAA CTTATAAAAA AATGTTAATA TTTTTCAAAA   
  
  
- TTTATTTTTA AGCATTATAT TTTAAAAATT AAACTTATGA AAATAAAATT TTAAAAAATT ATTTTATATT   
  
  
- AAACTAATAT AAAGTAGTTA GTCGATACAA TGAAGTTTTA AAATTTATTA TAAACGAAAA TTTAAATAAA   
  
  
- ATAATAAAGT AAATTAATAA TATATAATTG TAAATAAAAT TTTATTATAT AGAAATTTTT TATAAAAATT   
  
  
- AATTCGTGAA TGACAATTGT TCTTCAGAGT CCTTCCGTCA TCACAGGTCC GGCCACGCTT CACGCAAGCG   
  
  
- CTCAGATGCC GAGTACCGAG TGTAAAGCCA GTGTCGGAGC ATCCCCTGGT GTTTTCAGAC CTAGTATGTA   
  
  
- TCGCCGAGGA ACGCAGGGGC AATGCAAAAA GGAGTTGCTG GGTCTGTGCC TAGACCGTAA ACTTAGCCAG   
  
  
- ATAAAAAGCC GAAAATTGTA AATAAATAAA TAATATAAAG GGATTTTTAC CTGGTTTTAA TAGAACAATG   
  
  
- AAATGGTTCA CAGAATTAAA ATATATAAGG AGTTATTTTA TTATATTTAT CTTGCTGTAG CAAATGTGCA   
  
  
- TTCGTTCCCA GTACCTTGAT TGCAGTGTGA ATGAAATTCT AGCAAAGAAG GAGAAAAAAA GAGTGGAAAG   
  
  
- ACATCATCAT TGCACCTTAA ACGGTTCAAA CTTCTAAGAA ACCAGAAGAC CAAGGGTTGT AGAAGGTAAC   
  
  
- CGAAGGCAAG AAAGGAGATC TTAGGAAATA ATGTGAGCTG GTTACTCCTA AGTCCTTGGA GTTGCTTCTC   
  
  
- GTGAACTGTC TCTCTACCTC AAAGGACAGA CCTGACTTGT ATCTCTTCAC CAGTCGGCAA CCTGAAGGGA   
  
  
- AAAAAAAAAA AAAATACAAC TTGAGCTTAA TCATACTGGT TGCTGACCAG ATTACTTCTT TTGGGTGAAG   
  
  
- TCGAAAAGAG GTCAGTTGGT AGAAGGAAGT TAAAAGATGC AAGTTTTCCT CCGTTTAGGA AATTTTAGTG   
  
  
- TATTGGAATA AGAGGAAGAA GGTGTCCAAT TCAGGGTTAT GGAGATTCAT AAGTAGACGA ATCAAAGAGC   
  
  
- GGTAATGTCT TGTTCCCTTT GAGTAACTTG GGAAAATCAG TAAGTTTGAG AAAACGTAGA AAACAAAAAA   
  
  
- GTTGGGTTAA AGCTTTCAAA AGTTCGCATC TTCATTGTCT TTCGACTAAG AAAGTTCCCT TACGACTTAT   
  
  
- GGGTCAAGTT TAACTCGTAA AGGTCGAAGA GTATAGAAAG AGAGACGGCA ACAGTGGAAG AAGCGAAGAG   
  
  
- GTCAAGGCAG CATTTGGTCA GTAAAAGTAC CGGACTATGG TAGGCCGGAA CGAAAACTCT TGGGTAACCG   
  
  
- GGTAATTAAT AAGCACAACG AATGTTTAG

+     GT1-motif

| Site Name | Organism | Position | Strand | Matrix score. | sequence | function |
| --- | --- | --- | --- | --- | --- | --- |
| GT1-motif | Arabidopsis thaliana | 1146 | + | 6 | GGTTAA | light responsive element |

> 2018/04/13 10:10:12  
+ ATTTGAATAT ATTAATTATT TATTATAAAA TTTTAAATAT TTAATATTAA AACAATTTAA AAATATTTGT   
  
  
+ AATACTAAAA AAATTTTAAA ATTTTTTGTG ATAATATCTT GAATATTTTT TTACAATTAT AAAAAGTTTT   
  
  
+ AAATAAAAAT TCGTAATATA AAATTTTTAA TTTGAATACT TTTATTTTAA AATTTTTTAA TAAAATATAA   
  
  
+ TTTGATTATA TTTCATCAAT CAGCTATGTT ACTTCAAAAT TTTAAATAAT ATTTGCTTTT AAATTTATTT   
  
  
+ TATTATTTCA TTTAATTATT ATATATTAAC ATTTATTTTA AAATAATATA TCTTTAAAAA ATATTTTTAA   
  
  
+ TTAAGCACTT ACTGTTAACA AGAAGTCTCA GGAAGGCAGT AGTGTCCAGG CCGGTGCGAA GTGCGTTCGC   
  
  
+ GAGTCTACGG CTCATGGCTC ACATTTCGGT CACAGCCTCG TAGGGGACCA CAAAAGTCTG GATCATACAT   
  
  
+ AGCGGCTCCT TGCGTCCCCG TTACGTTTTT CCTCAACGAC CCAGACACGG ATCTGGCATT TGAATCGGTC   
  
  
+ TATTTTTCGG CTTTTAACAT TTATTTATTT ATTATATTTC CCTAAAAATG GACCAAAATT ATCTTGTTAC   
  
  
+ TTTACCAAGT GTCTTAATTT TATATATTCC TCAATAAAAT AATATAAATA GAACGACATC GTTTACACGT   
  
  
+ AAGCAAGGGT CATGGAACTA ACGTCACACT TACTTTAAGA TCGTTTCTTC CTCTTTTTTT CTCACCTTTC   
  
  
+ TGTAGTAGTA ACGTGGAATT TGCCAAGTTT GAAGATTCTT TGGTCTTCTG GTTCCCAACA TCTTCCATTG   
  
  
+ GCTTCCGTTC TTTCCTCTAG AATCCTTTAT TACACTCGAC CAATGAGGAT TCAGGAACCT CAACGAAGAG   
  
  
+ CACTTGACAG AGAGATGGAG TTTCCTGTCT GGACTGAACA TAGAGAAGTG GTCAGCCGTT GGACTTCCCT   
  
  
+ TTTTTTTTTT TTTTATGTTG AACTCGAATT AGTATGACCA ACGACTGGTC TAATGAAGAA AACCCACTTC   
  
  
+ AGCTTTTCTC CAGTCAACCA TCTTCCTTCA ATTTTCTACG TTCAAAAGGA GGCAAATCCT TTAAAATCAC   
  
  
+ ATAACCTTAT TCTCCTTCTT CCACAGGTTA AGTCCCAATA CCTCTAAGTA TTCATCTGCT TAGTTTCTCG   
  
  
+ CCATTACAGA ACAAGGGAAA CTCATTGAAC CCTTTTAGTC ATTCAAACTC TTTTGCATCT TTTGTTTTTT   
  
  
+ CAACCCAATT TCGAAAGTTT TCAAGCGTAG AAGTAACAGA AAGCTGATTC TTTCAAGGGA ATGCTGAATA   
  
  
+ CCCAGTTCAA ATTGAGCATT TCCAGCTTCT CATATCTTTC TCTCTGCCGT TGTCACCTTC TTCGCTTCTC   
  
  
+ CAGTTCCGTC GTAAACCAGT CATTTTCATG GCCTGATACC ATCCGGCCTT GCTTTTGAGA ACCCATTGGC   
  
  
+ CCATTAATTA TTCGTGTTGC TTACAAATC  

- TAAACTTATA TAATTAATAA ATAATATTTT AAAATTTATA AATTATAATT TTGTTAAATT TTTATAAACA   
  
  
- TTATGATTTT TTTAAAATTT TAAAAAACAC TATTATAGAA CTTATAAAAA AATGTTAATA TTTTTCAAAA   
  
  
- TTTATTTTTA AGCATTATAT TTTAAAAATT AAACTTATGA AAATAAAATT TTAAAAAATT ATTTTATATT   
  
  
- AAACTAATAT AAAGTAGTTA GTCGATACAA TGAAGTTTTA AAATTTATTA TAAACGAAAA TTTAAATAAA   
  
  
- ATAATAAAGT AAATTAATAA TATATAATTG TAAATAAAAT TTTATTATAT AGAAATTTTT TATAAAAATT   
  
  
- AATTCGTGAA TGACAATTGT TCTTCAGAGT CCTTCCGTCA TCACAGGTCC GGCCACGCTT CACGCAAGCG   
  
  
- CTCAGATGCC GAGTACCGAG TGTAAAGCCA GTGTCGGAGC ATCCCCTGGT GTTTTCAGAC CTAGTATGTA   
  
  
- TCGCCGAGGA ACGCAGGGGC AATGCAAAAA GGAGTTGCTG GGTCTGTGCC TAGACCGTAA ACTTAGCCAG   
  
  
- ATAAAAAGCC GAAAATTGTA AATAAATAAA TAATATAAAG GGATTTTTAC CTGGTTTTAA TAGAACAATG   
  
  
- AAATGGTTCA CAGAATTAAA ATATATAAGG AGTTATTTTA TTATATTTAT CTTGCTGTAG CAAATGTGCA   
  
  
- TTCGTTCCCA GTACCTTGAT TGCAGTGTGA ATGAAATTCT AGCAAAGAAG GAGAAAAAAA GAGTGGAAAG   
  
  
- ACATCATCAT TGCACCTTAA ACGGTTCAAA CTTCTAAGAA ACCAGAAGAC CAAGGGTTGT AGAAGGTAAC   
  
  
- CGAAGGCAAG AAAGGAGATC TTAGGAAATA ATGTGAGCTG GTTACTCCTA AGTCCTTGGA GTTGCTTCTC   
  
  
- GTGAACTGTC TCTCTACCTC AAAGGACAGA CCTGACTTGT ATCTCTTCAC CAGTCGGCAA CCTGAAGGGA   
  
  
- AAAAAAAAAA AAAATACAAC TTGAGCTTAA TCATACTGGT TGCTGACCAG ATTACTTCTT TTGGGTGAAG   
  
  
- TCGAAAAGAG GTCAGTTGGT AGAAGGAAGT TAAAAGATGC AAGTTTTCCT CCGTTTAGGA AATTTTAGTG   
  
  
- TATTGGAATA AGAGGAAGAA GGTGTCCAAT TCAGGGTTAT GGAGATTCAT AAGTAGACGA ATCAAAGAGC   
  
  
- GGTAATGTCT TGTTCCCTTT GAGTAACTTG GGAAAATCAG TAAGTTTGAG AAAACGTAGA AAACAAAAAA   
  
  
- GTTGGGTTAA AGCTTTCAAA AGTTCGCATC TTCATTGTCT TTCGACTAAG AAAGTTCCCT TACGACTTAT   
  
  
- GGGTCAAGTT TAACTCGTAA AGGTCGAAGA GTATAGAAAG AGAGACGGCA ACAGTGGAAG AAGCGAAGAG   
  
  
- GTCAAGGCAG CATTTGGTCA GTAAAAGTAC CGGACTATGG TAGGCCGGAA CGAAAACTCT TGGGTAACCG   
  
  
- GGTAATTAAT AAGCACAACG AATGTTTAG

+     HSE

| Site Name | Organism | Position | Strand | Matrix score. | sequence | function |
| --- | --- | --- | --- | --- | --- | --- |
| HSE | Brassica oleracea | 189 | - | 9 | AAAAAATTTC | cis-acting element involved in heat stress responsiveness |
| HSE | Brassica oleracea | 88 | - | 9 | AAAAAATTTC | cis-acting element involved in heat stress responsiveness |
| HSE | Brassica oleracea | 78 | + | 9 | AAAAAATTTC | cis-acting element involved in heat stress responsiveness |

> 2018/04/13 10:10:12  
+ ATTTGAATAT ATTAATTATT TATTATAAAA TTTTAAATAT TTAATATTAA AACAATTTAA AAATATTTGT   
  
  
+ AATACTAAAA AAATTTTAAA ATTTTTTGTG ATAATATCTT GAATATTTTT TTACAATTAT AAAAAGTTTT   
  
  
+ AAATAAAAAT TCGTAATATA AAATTTTTAA TTTGAATACT TTTATTTTAA AATTTTTTAA TAAAATATAA   
  
  
+ TTTGATTATA TTTCATCAAT CAGCTATGTT ACTTCAAAAT TTTAAATAAT ATTTGCTTTT AAATTTATTT   
  
  
+ TATTATTTCA TTTAATTATT ATATATTAAC ATTTATTTTA AAATAATATA TCTTTAAAAA ATATTTTTAA   
  
  
+ TTAAGCACTT ACTGTTAACA AGAAGTCTCA GGAAGGCAGT AGTGTCCAGG CCGGTGCGAA GTGCGTTCGC   
  
  
+ GAGTCTACGG CTCATGGCTC ACATTTCGGT CACAGCCTCG TAGGGGACCA CAAAAGTCTG GATCATACAT   
  
  
+ AGCGGCTCCT TGCGTCCCCG TTACGTTTTT CCTCAACGAC CCAGACACGG ATCTGGCATT TGAATCGGTC   
  
  
+ TATTTTTCGG CTTTTAACAT TTATTTATTT ATTATATTTC CCTAAAAATG GACCAAAATT ATCTTGTTAC   
  
  
+ TTTACCAAGT GTCTTAATTT TATATATTCC TCAATAAAAT AATATAAATA GAACGACATC GTTTACACGT   
  
  
+ AAGCAAGGGT CATGGAACTA ACGTCACACT TACTTTAAGA TCGTTTCTTC CTCTTTTTTT CTCACCTTTC   
  
  
+ TGTAGTAGTA ACGTGGAATT TGCCAAGTTT GAAGATTCTT TGGTCTTCTG GTTCCCAACA TCTTCCATTG   
  
  
+ GCTTCCGTTC TTTCCTCTAG AATCCTTTAT TACACTCGAC CAATGAGGAT TCAGGAACCT CAACGAAGAG   
  
  
+ CACTTGACAG AGAGATGGAG TTTCCTGTCT GGACTGAACA TAGAGAAGTG GTCAGCCGTT GGACTTCCCT   
  
  
+ TTTTTTTTTT TTTTATGTTG AACTCGAATT AGTATGACCA ACGACTGGTC TAATGAAGAA AACCCACTTC   
  
  
+ AGCTTTTCTC CAGTCAACCA TCTTCCTTCA ATTTTCTACG TTCAAAAGGA GGCAAATCCT TTAAAATCAC   
  
  
+ ATAACCTTAT TCTCCTTCTT CCACAGGTTA AGTCCCAATA CCTCTAAGTA TTCATCTGCT TAGTTTCTCG   
  
  
+ CCATTACAGA ACAAGGGAAA CTCATTGAAC CCTTTTAGTC ATTCAAACTC TTTTGCATCT TTTGTTTTTT   
  
  
+ CAACCCAATT TCGAAAGTTT TCAAGCGTAG AAGTAACAGA AAGCTGATTC TTTCAAGGGA ATGCTGAATA   
  
  
+ CCCAGTTCAA ATTGAGCATT TCCAGCTTCT CATATCTTTC TCTCTGCCGT TGTCACCTTC TTCGCTTCTC   
  
  
+ CAGTTCCGTC GTAAACCAGT CATTTTCATG GCCTGATACC ATCCGGCCTT GCTTTTGAGA ACCCATTGGC   
  
  
+ CCATTAATTA TTCGTGTTGC TTACAAATC  

- TAAACTTATA TAATTAATAA ATAATATTTT AAAATTTATA AATTATAATT TTGTTAAATT TTTATAAACA   
  
  
- TTATGATTTT TTTAAAATTT TAAAAAACAC TATTATAGAA CTTATAAAAA AATGTTAATA TTTTTCAAAA   
  
  
- TTTATTTTTA AGCATTATAT TTTAAAAATT AAACTTATGA AAATAAAATT TTAAAAAATT ATTTTATATT   
  
  
- AAACTAATAT AAAGTAGTTA GTCGATACAA TGAAGTTTTA AAATTTATTA TAAACGAAAA TTTAAATAAA   
  
  
- ATAATAAAGT AAATTAATAA TATATAATTG TAAATAAAAT TTTATTATAT AGAAATTTTT TATAAAAATT   
  
  
- AATTCGTGAA TGACAATTGT TCTTCAGAGT CCTTCCGTCA TCACAGGTCC GGCCACGCTT CACGCAAGCG   
  
  
- CTCAGATGCC GAGTACCGAG TGTAAAGCCA GTGTCGGAGC ATCCCCTGGT GTTTTCAGAC CTAGTATGTA   
  
  
- TCGCCGAGGA ACGCAGGGGC AATGCAAAAA GGAGTTGCTG GGTCTGTGCC TAGACCGTAA ACTTAGCCAG   
  
  
- ATAAAAAGCC GAAAATTGTA AATAAATAAA TAATATAAAG GGATTTTTAC CTGGTTTTAA TAGAACAATG   
  
  
- AAATGGTTCA CAGAATTAAA ATATATAAGG AGTTATTTTA TTATATTTAT CTTGCTGTAG CAAATGTGCA   
  
  
- TTCGTTCCCA GTACCTTGAT TGCAGTGTGA ATGAAATTCT AGCAAAGAAG GAGAAAAAAA GAGTGGAAAG   
  
  
- ACATCATCAT TGCACCTTAA ACGGTTCAAA CTTCTAAGAA ACCAGAAGAC CAAGGGTTGT AGAAGGTAAC   
  
  
- CGAAGGCAAG AAAGGAGATC TTAGGAAATA ATGTGAGCTG GTTACTCCTA AGTCCTTGGA GTTGCTTCTC   
  
  
- GTGAACTGTC TCTCTACCTC AAAGGACAGA CCTGACTTGT ATCTCTTCAC CAGTCGGCAA CCTGAAGGGA   
  
  
- AAAAAAAAAA AAAATACAAC TTGAGCTTAA TCATACTGGT TGCTGACCAG ATTACTTCTT TTGGGTGAAG   
  
  
- TCGAAAAGAG GTCAGTTGGT AGAAGGAAGT TAAAAGATGC AAGTTTTCCT CCGTTTAGGA AATTTTAGTG   
  
  
- TATTGGAATA AGAGGAAGAA GGTGTCCAAT TCAGGGTTAT GGAGATTCAT AAGTAGACGA ATCAAAGAGC   
  
  
- GGTAATGTCT TGTTCCCTTT GAGTAACTTG GGAAAATCAG TAAGTTTGAG AAAACGTAGA AAACAAAAAA   
  
  
- GTTGGGTTAA AGCTTTCAAA AGTTCGCATC TTCATTGTCT TTCGACTAAG AAAGTTCCCT TACGACTTAT   
  
  
- GGGTCAAGTT TAACTCGTAA AGGTCGAAGA GTATAGAAAG AGAGACGGCA ACAGTGGAAG AAGCGAAGAG   
  
  
- GTCAAGGCAG CATTTGGTCA GTAAAAGTAC CGGACTATGG TAGGCCGGAA CGAAAACTCT TGGGTAACCG   
  
  
- GGTAATTAAT AAGCACAACG AATGTTTAG

+     I-box

| Site Name | Organism | Position | Strand | Matrix score. | sequence | function |
| --- | --- | --- | --- | --- | --- | --- |
| I-box | Larix laricina | 1430 | - | 9 | GTATAAGGCC | part of a light responsive element |

> 2018/04/13 10:10:12  
+ ATTTGAATAT ATTAATTATT TATTATAAAA TTTTAAATAT TTAATATTAA AACAATTTAA AAATATTTGT   
  
  
+ AATACTAAAA AAATTTTAAA ATTTTTTGTG ATAATATCTT GAATATTTTT TTACAATTAT AAAAAGTTTT   
  
  
+ AAATAAAAAT TCGTAATATA AAATTTTTAA TTTGAATACT TTTATTTTAA AATTTTTTAA TAAAATATAA   
  
  
+ TTTGATTATA TTTCATCAAT CAGCTATGTT ACTTCAAAAT TTTAAATAAT ATTTGCTTTT AAATTTATTT   
  
  
+ TATTATTTCA TTTAATTATT ATATATTAAC ATTTATTTTA AAATAATATA TCTTTAAAAA ATATTTTTAA   
  
  
+ TTAAGCACTT ACTGTTAACA AGAAGTCTCA GGAAGGCAGT AGTGTCCAGG CCGGTGCGAA GTGCGTTCGC   
  
  
+ GAGTCTACGG CTCATGGCTC ACATTTCGGT CACAGCCTCG TAGGGGACCA CAAAAGTCTG GATCATACAT   
  
  
+ AGCGGCTCCT TGCGTCCCCG TTACGTTTTT CCTCAACGAC CCAGACACGG ATCTGGCATT TGAATCGGTC   
  
  
+ TATTTTTCGG CTTTTAACAT TTATTTATTT ATTATATTTC CCTAAAAATG GACCAAAATT ATCTTGTTAC   
  
  
+ TTTACCAAGT GTCTTAATTT TATATATTCC TCAATAAAAT AATATAAATA GAACGACATC GTTTACACGT   
  
  
+ AAGCAAGGGT CATGGAACTA ACGTCACACT TACTTTAAGA TCGTTTCTTC CTCTTTTTTT CTCACCTTTC   
  
  
+ TGTAGTAGTA ACGTGGAATT TGCCAAGTTT GAAGATTCTT TGGTCTTCTG GTTCCCAACA TCTTCCATTG   
  
  
+ GCTTCCGTTC TTTCCTCTAG AATCCTTTAT TACACTCGAC CAATGAGGAT TCAGGAACCT CAACGAAGAG   
  
  
+ CACTTGACAG AGAGATGGAG TTTCCTGTCT GGACTGAACA TAGAGAAGTG GTCAGCCGTT GGACTTCCCT   
  
  
+ TTTTTTTTTT TTTTATGTTG AACTCGAATT AGTATGACCA ACGACTGGTC TAATGAAGAA AACCCACTTC   
  
  
+ AGCTTTTCTC CAGTCAACCA TCTTCCTTCA ATTTTCTACG TTCAAAAGGA GGCAAATCCT TTAAAATCAC   
  
  
+ ATAACCTTAT TCTCCTTCTT CCACAGGTTA AGTCCCAATA CCTCTAAGTA TTCATCTGCT TAGTTTCTCG   
  
  
+ CCATTACAGA ACAAGGGAAA CTCATTGAAC CCTTTTAGTC ATTCAAACTC TTTTGCATCT TTTGTTTTTT   
  
  
+ CAACCCAATT TCGAAAGTTT TCAAGCGTAG AAGTAACAGA AAGCTGATTC TTTCAAGGGA ATGCTGAATA   
  
  
+ CCCAGTTCAA ATTGAGCATT TCCAGCTTCT CATATCTTTC TCTCTGCCGT TGTCACCTTC TTCGCTTCTC   
  
  
+ CAGTTCCGTC GTAAACCAGT CATTTTCATG GCCTGATACC ATCCGGCCTT GCTTTTGAGA ACCCATTGGC   
  
  
+ CCATTAATTA TTCGTGTTGC TTACAAATC  

- TAAACTTATA TAATTAATAA ATAATATTTT AAAATTTATA AATTATAATT TTGTTAAATT TTTATAAACA   
  
  
- TTATGATTTT TTTAAAATTT TAAAAAACAC TATTATAGAA CTTATAAAAA AATGTTAATA TTTTTCAAAA   
  
  
- TTTATTTTTA AGCATTATAT TTTAAAAATT AAACTTATGA AAATAAAATT TTAAAAAATT ATTTTATATT   
  
  
- AAACTAATAT AAAGTAGTTA GTCGATACAA TGAAGTTTTA AAATTTATTA TAAACGAAAA TTTAAATAAA   
  
  
- ATAATAAAGT AAATTAATAA TATATAATTG TAAATAAAAT TTTATTATAT AGAAATTTTT TATAAAAATT   
  
  
- AATTCGTGAA TGACAATTGT TCTTCAGAGT CCTTCCGTCA TCACAGGTCC GGCCACGCTT CACGCAAGCG   
  
  
- CTCAGATGCC GAGTACCGAG TGTAAAGCCA GTGTCGGAGC ATCCCCTGGT GTTTTCAGAC CTAGTATGTA   
  
  
- TCGCCGAGGA ACGCAGGGGC AATGCAAAAA GGAGTTGCTG GGTCTGTGCC TAGACCGTAA ACTTAGCCAG   
  
  
- ATAAAAAGCC GAAAATTGTA AATAAATAAA TAATATAAAG GGATTTTTAC CTGGTTTTAA TAGAACAATG   
  
  
- AAATGGTTCA CAGAATTAAA ATATATAAGG AGTTATTTTA TTATATTTAT CTTGCTGTAG CAAATGTGCA   
  
  
- TTCGTTCCCA GTACCTTGAT TGCAGTGTGA ATGAAATTCT AGCAAAGAAG GAGAAAAAAA GAGTGGAAAG   
  
  
- ACATCATCAT TGCACCTTAA ACGGTTCAAA CTTCTAAGAA ACCAGAAGAC CAAGGGTTGT AGAAGGTAAC   
  
  
- CGAAGGCAAG AAAGGAGATC TTAGGAAATA ATGTGAGCTG GTTACTCCTA AGTCCTTGGA GTTGCTTCTC   
  
  
- GTGAACTGTC TCTCTACCTC AAAGGACAGA CCTGACTTGT ATCTCTTCAC CAGTCGGCAA CCTGAAGGGA   
  
  
- AAAAAAAAAA AAAATACAAC TTGAGCTTAA TCATACTGGT TGCTGACCAG ATTACTTCTT TTGGGTGAAG   
  
  
- TCGAAAAGAG GTCAGTTGGT AGAAGGAAGT TAAAAGATGC AAGTTTTCCT CCGTTTAGGA AATTTTAGTG   
  
  
- TATTGGAATA AGAGGAAGAA GGTGTCCAAT TCAGGGTTAT GGAGATTCAT AAGTAGACGA ATCAAAGAGC   
  
  
- GGTAATGTCT TGTTCCCTTT GAGTAACTTG GGAAAATCAG TAAGTTTGAG AAAACGTAGA AAACAAAAAA   
  
  
- GTTGGGTTAA AGCTTTCAAA AGTTCGCATC TTCATTGTCT TTCGACTAAG AAAGTTCCCT TACGACTTAT   
  
  
- GGGTCAAGTT TAACTCGTAA AGGTCGAAGA GTATAGAAAG AGAGACGGCA ACAGTGGAAG AAGCGAAGAG   
  
  
- GTCAAGGCAG CATTTGGTCA GTAAAAGTAC CGGACTATGG TAGGCCGGAA CGAAAACTCT TGGGTAACCG   
  
  
- GGTAATTAAT AAGCACAACG AATGTTTAG

+     LTR

| Site Name | Organism | Position | Strand | Matrix score. | sequence | function |
| --- | --- | --- | --- | --- | --- | --- |
| LTR | Hordeum vulgare | 565 | - | 6 | CCGAAA | cis-acting element involved in low-temperature responsiveness |
| LTR | Hordeum vulgare | 444 | - | 6 | CCGAAA | cis-acting element involved in low-temperature responsiveness |

> 2018/04/13 10:10:12  
+ ATTTGAATAT ATTAATTATT TATTATAAAA TTTTAAATAT TTAATATTAA AACAATTTAA AAATATTTGT   
  
  
+ AATACTAAAA AAATTTTAAA ATTTTTTGTG ATAATATCTT GAATATTTTT TTACAATTAT AAAAAGTTTT   
  
  
+ AAATAAAAAT TCGTAATATA AAATTTTTAA TTTGAATACT TTTATTTTAA AATTTTTTAA TAAAATATAA   
  
  
+ TTTGATTATA TTTCATCAAT CAGCTATGTT ACTTCAAAAT TTTAAATAAT ATTTGCTTTT AAATTTATTT   
  
  
+ TATTATTTCA TTTAATTATT ATATATTAAC ATTTATTTTA AAATAATATA TCTTTAAAAA ATATTTTTAA   
  
  
+ TTAAGCACTT ACTGTTAACA AGAAGTCTCA GGAAGGCAGT AGTGTCCAGG CCGGTGCGAA GTGCGTTCGC   
  
  
+ GAGTCTACGG CTCATGGCTC ACATTTCGGT CACAGCCTCG TAGGGGACCA CAAAAGTCTG GATCATACAT   
  
  
+ AGCGGCTCCT TGCGTCCCCG TTACGTTTTT CCTCAACGAC CCAGACACGG ATCTGGCATT TGAATCGGTC   
  
  
+ TATTTTTCGG CTTTTAACAT TTATTTATTT ATTATATTTC CCTAAAAATG GACCAAAATT ATCTTGTTAC   
  
  
+ TTTACCAAGT GTCTTAATTT TATATATTCC TCAATAAAAT AATATAAATA GAACGACATC GTTTACACGT   
  
  
+ AAGCAAGGGT CATGGAACTA ACGTCACACT TACTTTAAGA TCGTTTCTTC CTCTTTTTTT CTCACCTTTC   
  
  
+ TGTAGTAGTA ACGTGGAATT TGCCAAGTTT GAAGATTCTT TGGTCTTCTG GTTCCCAACA TCTTCCATTG   
  
  
+ GCTTCCGTTC TTTCCTCTAG AATCCTTTAT TACACTCGAC CAATGAGGAT TCAGGAACCT CAACGAAGAG   
  
  
+ CACTTGACAG AGAGATGGAG TTTCCTGTCT GGACTGAACA TAGAGAAGTG GTCAGCCGTT GGACTTCCCT   
  
  
+ TTTTTTTTTT TTTTATGTTG AACTCGAATT AGTATGACCA ACGACTGGTC TAATGAAGAA AACCCACTTC   
  
  
+ AGCTTTTCTC CAGTCAACCA TCTTCCTTCA ATTTTCTACG TTCAAAAGGA GGCAAATCCT TTAAAATCAC   
  
  
+ ATAACCTTAT TCTCCTTCTT CCACAGGTTA AGTCCCAATA CCTCTAAGTA TTCATCTGCT TAGTTTCTCG   
  
  
+ CCATTACAGA ACAAGGGAAA CTCATTGAAC CCTTTTAGTC ATTCAAACTC TTTTGCATCT TTTGTTTTTT   
  
  
+ CAACCCAATT TCGAAAGTTT TCAAGCGTAG AAGTAACAGA AAGCTGATTC TTTCAAGGGA ATGCTGAATA   
  
  
+ CCCAGTTCAA ATTGAGCATT TCCAGCTTCT CATATCTTTC TCTCTGCCGT TGTCACCTTC TTCGCTTCTC   
  
  
+ CAGTTCCGTC GTAAACCAGT CATTTTCATG GCCTGATACC ATCCGGCCTT GCTTTTGAGA ACCCATTGGC   
  
  
+ CCATTAATTA TTCGTGTTGC TTACAAATC  

- TAAACTTATA TAATTAATAA ATAATATTTT AAAATTTATA AATTATAATT TTGTTAAATT TTTATAAACA   
  
  
- TTATGATTTT TTTAAAATTT TAAAAAACAC TATTATAGAA CTTATAAAAA AATGTTAATA TTTTTCAAAA   
  
  
- TTTATTTTTA AGCATTATAT TTTAAAAATT AAACTTATGA AAATAAAATT TTAAAAAATT ATTTTATATT   
  
  
- AAACTAATAT AAAGTAGTTA GTCGATACAA TGAAGTTTTA AAATTTATTA TAAACGAAAA TTTAAATAAA   
  
  
- ATAATAAAGT AAATTAATAA TATATAATTG TAAATAAAAT TTTATTATAT AGAAATTTTT TATAAAAATT   
  
  
- AATTCGTGAA TGACAATTGT TCTTCAGAGT CCTTCCGTCA TCACAGGTCC GGCCACGCTT CACGCAAGCG   
  
  
- CTCAGATGCC GAGTACCGAG TGTAAAGCCA GTGTCGGAGC ATCCCCTGGT GTTTTCAGAC CTAGTATGTA   
  
  
- TCGCCGAGGA ACGCAGGGGC AATGCAAAAA GGAGTTGCTG GGTCTGTGCC TAGACCGTAA ACTTAGCCAG   
  
  
- ATAAAAAGCC GAAAATTGTA AATAAATAAA TAATATAAAG GGATTTTTAC CTGGTTTTAA TAGAACAATG   
  
  
- AAATGGTTCA CAGAATTAAA ATATATAAGG AGTTATTTTA TTATATTTAT CTTGCTGTAG CAAATGTGCA   
  
  
- TTCGTTCCCA GTACCTTGAT TGCAGTGTGA ATGAAATTCT AGCAAAGAAG GAGAAAAAAA GAGTGGAAAG   
  
  
- ACATCATCAT TGCACCTTAA ACGGTTCAAA CTTCTAAGAA ACCAGAAGAC CAAGGGTTGT AGAAGGTAAC   
  
  
- CGAAGGCAAG AAAGGAGATC TTAGGAAATA ATGTGAGCTG GTTACTCCTA AGTCCTTGGA GTTGCTTCTC   
  
  
- GTGAACTGTC TCTCTACCTC AAAGGACAGA CCTGACTTGT ATCTCTTCAC CAGTCGGCAA CCTGAAGGGA   
  
  
- AAAAAAAAAA AAAATACAAC TTGAGCTTAA TCATACTGGT TGCTGACCAG ATTACTTCTT TTGGGTGAAG   
  
  
- TCGAAAAGAG GTCAGTTGGT AGAAGGAAGT TAAAAGATGC AAGTTTTCCT CCGTTTAGGA AATTTTAGTG   
  
  
- TATTGGAATA AGAGGAAGAA GGTGTCCAAT TCAGGGTTAT GGAGATTCAT AAGTAGACGA ATCAAAGAGC   
  
  
- GGTAATGTCT TGTTCCCTTT GAGTAACTTG GGAAAATCAG TAAGTTTGAG AAAACGTAGA AAACAAAAAA   
  
  
- GTTGGGTTAA AGCTTTCAAA AGTTCGCATC TTCATTGTCT TTCGACTAAG AAAGTTCCCT TACGACTTAT   
  
  
- GGGTCAAGTT TAACTCGTAA AGGTCGAAGA GTATAGAAAG AGAGACGGCA ACAGTGGAAG AAGCGAAGAG   
  
  
- GTCAAGGCAG CATTTGGTCA GTAAAAGTAC CGGACTATGG TAGGCCGGAA CGAAAACTCT TGGGTAACCG   
  
  
- GGTAATTAAT AAGCACAACG AATGTTTAG

+     MBS

| Site Name | Organism | Position | Strand | Matrix score. | sequence | function |
| --- | --- | --- | --- | --- | --- | --- |
| MBS | Zea mays | 447 | + | 6 | CGGTCA | MYB Binding Site |

> 2018/04/13 10:10:12  
+ ATTTGAATAT ATTAATTATT TATTATAAAA TTTTAAATAT TTAATATTAA AACAATTTAA AAATATTTGT   
  
  
+ AATACTAAAA AAATTTTAAA ATTTTTTGTG ATAATATCTT GAATATTTTT TTACAATTAT AAAAAGTTTT   
  
  
+ AAATAAAAAT TCGTAATATA AAATTTTTAA TTTGAATACT TTTATTTTAA AATTTTTTAA TAAAATATAA   
  
  
+ TTTGATTATA TTTCATCAAT CAGCTATGTT ACTTCAAAAT TTTAAATAAT ATTTGCTTTT AAATTTATTT   
  
  
+ TATTATTTCA TTTAATTATT ATATATTAAC ATTTATTTTA AAATAATATA TCTTTAAAAA ATATTTTTAA   
  
  
+ TTAAGCACTT ACTGTTAACA AGAAGTCTCA GGAAGGCAGT AGTGTCCAGG CCGGTGCGAA GTGCGTTCGC   
  
  
+ GAGTCTACGG CTCATGGCTC ACATTTCGGT CACAGCCTCG TAGGGGACCA CAAAAGTCTG GATCATACAT   
  
  
+ AGCGGCTCCT TGCGTCCCCG TTACGTTTTT CCTCAACGAC CCAGACACGG ATCTGGCATT TGAATCGGTC   
  
  
+ TATTTTTCGG CTTTTAACAT TTATTTATTT ATTATATTTC CCTAAAAATG GACCAAAATT ATCTTGTTAC   
  
  
+ TTTACCAAGT GTCTTAATTT TATATATTCC TCAATAAAAT AATATAAATA GAACGACATC GTTTACACGT   
  
  
+ AAGCAAGGGT CATGGAACTA ACGTCACACT TACTTTAAGA TCGTTTCTTC CTCTTTTTTT CTCACCTTTC   
  
  
+ TGTAGTAGTA ACGTGGAATT TGCCAAGTTT GAAGATTCTT TGGTCTTCTG GTTCCCAACA TCTTCCATTG   
  
  
+ GCTTCCGTTC TTTCCTCTAG AATCCTTTAT TACACTCGAC CAATGAGGAT TCAGGAACCT CAACGAAGAG   
  
  
+ CACTTGACAG AGAGATGGAG TTTCCTGTCT GGACTGAACA TAGAGAAGTG GTCAGCCGTT GGACTTCCCT   
  
  
+ TTTTTTTTTT TTTTATGTTG AACTCGAATT AGTATGACCA ACGACTGGTC TAATGAAGAA AACCCACTTC   
  
  
+ AGCTTTTCTC CAGTCAACCA TCTTCCTTCA ATTTTCTACG TTCAAAAGGA GGCAAATCCT TTAAAATCAC   
  
  
+ ATAACCTTAT TCTCCTTCTT CCACAGGTTA AGTCCCAATA CCTCTAAGTA TTCATCTGCT TAGTTTCTCG   
  
  
+ CCATTACAGA ACAAGGGAAA CTCATTGAAC CCTTTTAGTC ATTCAAACTC TTTTGCATCT TTTGTTTTTT   
  
  
+ CAACCCAATT TCGAAAGTTT TCAAGCGTAG AAGTAACAGA AAGCTGATTC TTTCAAGGGA ATGCTGAATA   
  
  
+ CCCAGTTCAA ATTGAGCATT TCCAGCTTCT CATATCTTTC TCTCTGCCGT TGTCACCTTC TTCGCTTCTC   
  
  
+ CAGTTCCGTC GTAAACCAGT CATTTTCATG GCCTGATACC ATCCGGCCTT GCTTTTGAGA ACCCATTGGC   
  
  
+ CCATTAATTA TTCGTGTTGC TTACAAATC  

- TAAACTTATA TAATTAATAA ATAATATTTT AAAATTTATA AATTATAATT TTGTTAAATT TTTATAAACA   
  
  
- TTATGATTTT TTTAAAATTT TAAAAAACAC TATTATAGAA CTTATAAAAA AATGTTAATA TTTTTCAAAA   
  
  
- TTTATTTTTA AGCATTATAT TTTAAAAATT AAACTTATGA AAATAAAATT TTAAAAAATT ATTTTATATT   
  
  
- AAACTAATAT AAAGTAGTTA GTCGATACAA TGAAGTTTTA AAATTTATTA TAAACGAAAA TTTAAATAAA   
  
  
- ATAATAAAGT AAATTAATAA TATATAATTG TAAATAAAAT TTTATTATAT AGAAATTTTT TATAAAAATT   
  
  
- AATTCGTGAA TGACAATTGT TCTTCAGAGT CCTTCCGTCA TCACAGGTCC GGCCACGCTT CACGCAAGCG   
  
  
- CTCAGATGCC GAGTACCGAG TGTAAAGCCA GTGTCGGAGC ATCCCCTGGT GTTTTCAGAC CTAGTATGTA   
  
  
- TCGCCGAGGA ACGCAGGGGC AATGCAAAAA GGAGTTGCTG GGTCTGTGCC TAGACCGTAA ACTTAGCCAG   
  
  
- ATAAAAAGCC GAAAATTGTA AATAAATAAA TAATATAAAG GGATTTTTAC CTGGTTTTAA TAGAACAATG   
  
  
- AAATGGTTCA CAGAATTAAA ATATATAAGG AGTTATTTTA TTATATTTAT CTTGCTGTAG CAAATGTGCA   
  
  
- TTCGTTCCCA GTACCTTGAT TGCAGTGTGA ATGAAATTCT AGCAAAGAAG GAGAAAAAAA GAGTGGAAAG   
  
  
- ACATCATCAT TGCACCTTAA ACGGTTCAAA CTTCTAAGAA ACCAGAAGAC CAAGGGTTGT AGAAGGTAAC   
  
  
- CGAAGGCAAG AAAGGAGATC TTAGGAAATA ATGTGAGCTG GTTACTCCTA AGTCCTTGGA GTTGCTTCTC   
  
  
- GTGAACTGTC TCTCTACCTC AAAGGACAGA CCTGACTTGT ATCTCTTCAC CAGTCGGCAA CCTGAAGGGA   
  
  
- AAAAAAAAAA AAAATACAAC TTGAGCTTAA TCATACTGGT TGCTGACCAG ATTACTTCTT TTGGGTGAAG   
  
  
- TCGAAAAGAG GTCAGTTGGT AGAAGGAAGT TAAAAGATGC AAGTTTTCCT CCGTTTAGGA AATTTTAGTG   
  
  
- TATTGGAATA AGAGGAAGAA GGTGTCCAAT TCAGGGTTAT GGAGATTCAT AAGTAGACGA ATCAAAGAGC   
  
  
- GGTAATGTCT TGTTCCCTTT GAGTAACTTG GGAAAATCAG TAAGTTTGAG AAAACGTAGA AAACAAAAAA   
  
  
- GTTGGGTTAA AGCTTTCAAA AGTTCGCATC TTCATTGTCT TTCGACTAAG AAAGTTCCCT TACGACTTAT   
  
  
- GGGTCAAGTT TAACTCGTAA AGGTCGAAGA GTATAGAAAG AGAGACGGCA ACAGTGGAAG AAGCGAAGAG   
  
  
- GTCAAGGCAG CATTTGGTCA GTAAAAGTAC CGGACTATGG TAGGCCGGAA CGAAAACTCT TGGGTAACCG   
  
  
- GGTAATTAAT AAGCACAACG AATGTTTAG

+     P-box

| Site Name | Organism | Position | Strand | Matrix score. | sequence | function |
| --- | --- | --- | --- | --- | --- | --- |
| P-box | Oryza sativa | 1093 | - | 7 | CCTTTTG | gibberellin-responsive element |

> 2018/04/13 10:10:12  
+ ATTTGAATAT ATTAATTATT TATTATAAAA TTTTAAATAT TTAATATTAA AACAATTTAA AAATATTTGT   
  
  
+ AATACTAAAA AAATTTTAAA ATTTTTTGTG ATAATATCTT GAATATTTTT TTACAATTAT AAAAAGTTTT   
  
  
+ AAATAAAAAT TCGTAATATA AAATTTTTAA TTTGAATACT TTTATTTTAA AATTTTTTAA TAAAATATAA   
  
  
+ TTTGATTATA TTTCATCAAT CAGCTATGTT ACTTCAAAAT TTTAAATAAT ATTTGCTTTT AAATTTATTT   
  
  
+ TATTATTTCA TTTAATTATT ATATATTAAC ATTTATTTTA AAATAATATA TCTTTAAAAA ATATTTTTAA   
  
  
+ TTAAGCACTT ACTGTTAACA AGAAGTCTCA GGAAGGCAGT AGTGTCCAGG CCGGTGCGAA GTGCGTTCGC   
  
  
+ GAGTCTACGG CTCATGGCTC ACATTTCGGT CACAGCCTCG TAGGGGACCA CAAAAGTCTG GATCATACAT   
  
  
+ AGCGGCTCCT TGCGTCCCCG TTACGTTTTT CCTCAACGAC CCAGACACGG ATCTGGCATT TGAATCGGTC   
  
  
+ TATTTTTCGG CTTTTAACAT TTATTTATTT ATTATATTTC CCTAAAAATG GACCAAAATT ATCTTGTTAC   
  
  
+ TTTACCAAGT GTCTTAATTT TATATATTCC TCAATAAAAT AATATAAATA GAACGACATC GTTTACACGT   
  
  
+ AAGCAAGGGT CATGGAACTA ACGTCACACT TACTTTAAGA TCGTTTCTTC CTCTTTTTTT CTCACCTTTC   
  
  
+ TGTAGTAGTA ACGTGGAATT TGCCAAGTTT GAAGATTCTT TGGTCTTCTG GTTCCCAACA TCTTCCATTG   
  
  
+ GCTTCCGTTC TTTCCTCTAG AATCCTTTAT TACACTCGAC CAATGAGGAT TCAGGAACCT CAACGAAGAG   
  
  
+ CACTTGACAG AGAGATGGAG TTTCCTGTCT GGACTGAACA TAGAGAAGTG GTCAGCCGTT GGACTTCCCT   
  
  
+ TTTTTTTTTT TTTTATGTTG AACTCGAATT AGTATGACCA ACGACTGGTC TAATGAAGAA AACCCACTTC   
  
  
+ AGCTTTTCTC CAGTCAACCA TCTTCCTTCA ATTTTCTACG TTCAAAAGGA GGCAAATCCT TTAAAATCAC   
  
  
+ ATAACCTTAT TCTCCTTCTT CCACAGGTTA AGTCCCAATA CCTCTAAGTA TTCATCTGCT TAGTTTCTCG   
  
  
+ CCATTACAGA ACAAGGGAAA CTCATTGAAC CCTTTTAGTC ATTCAAACTC TTTTGCATCT TTTGTTTTTT   
  
  
+ CAACCCAATT TCGAAAGTTT TCAAGCGTAG AAGTAACAGA AAGCTGATTC TTTCAAGGGA ATGCTGAATA   
  
  
+ CCCAGTTCAA ATTGAGCATT TCCAGCTTCT CATATCTTTC TCTCTGCCGT TGTCACCTTC TTCGCTTCTC   
  
  
+ CAGTTCCGTC GTAAACCAGT CATTTTCATG GCCTGATACC ATCCGGCCTT GCTTTTGAGA ACCCATTGGC   
  
  
+ CCATTAATTA TTCGTGTTGC TTACAAATC  

- TAAACTTATA TAATTAATAA ATAATATTTT AAAATTTATA AATTATAATT TTGTTAAATT TTTATAAACA   
  
  
- TTATGATTTT TTTAAAATTT TAAAAAACAC TATTATAGAA CTTATAAAAA AATGTTAATA TTTTTCAAAA   
  
  
- TTTATTTTTA AGCATTATAT TTTAAAAATT AAACTTATGA AAATAAAATT TTAAAAAATT ATTTTATATT   
  
  
- AAACTAATAT AAAGTAGTTA GTCGATACAA TGAAGTTTTA AAATTTATTA TAAACGAAAA TTTAAATAAA   
  
  
- ATAATAAAGT AAATTAATAA TATATAATTG TAAATAAAAT TTTATTATAT AGAAATTTTT TATAAAAATT   
  
  
- AATTCGTGAA TGACAATTGT TCTTCAGAGT CCTTCCGTCA TCACAGGTCC GGCCACGCTT CACGCAAGCG   
  
  
- CTCAGATGCC GAGTACCGAG TGTAAAGCCA GTGTCGGAGC ATCCCCTGGT GTTTTCAGAC CTAGTATGTA   
  
  
- TCGCCGAGGA ACGCAGGGGC AATGCAAAAA GGAGTTGCTG GGTCTGTGCC TAGACCGTAA ACTTAGCCAG   
  
  
- ATAAAAAGCC GAAAATTGTA AATAAATAAA TAATATAAAG GGATTTTTAC CTGGTTTTAA TAGAACAATG   
  
  
- AAATGGTTCA CAGAATTAAA ATATATAAGG AGTTATTTTA TTATATTTAT CTTGCTGTAG CAAATGTGCA   
  
  
- TTCGTTCCCA GTACCTTGAT TGCAGTGTGA ATGAAATTCT AGCAAAGAAG GAGAAAAAAA GAGTGGAAAG   
  
  
- ACATCATCAT TGCACCTTAA ACGGTTCAAA CTTCTAAGAA ACCAGAAGAC CAAGGGTTGT AGAAGGTAAC   
  
  
- CGAAGGCAAG AAAGGAGATC TTAGGAAATA ATGTGAGCTG GTTACTCCTA AGTCCTTGGA GTTGCTTCTC   
  
  
- GTGAACTGTC TCTCTACCTC AAAGGACAGA CCTGACTTGT ATCTCTTCAC CAGTCGGCAA CCTGAAGGGA   
  
  
- AAAAAAAAAA AAAATACAAC TTGAGCTTAA TCATACTGGT TGCTGACCAG ATTACTTCTT TTGGGTGAAG   
  
  
- TCGAAAAGAG GTCAGTTGGT AGAAGGAAGT TAAAAGATGC AAGTTTTCCT CCGTTTAGGA AATTTTAGTG   
  
  
- TATTGGAATA AGAGGAAGAA GGTGTCCAAT TCAGGGTTAT GGAGATTCAT AAGTAGACGA ATCAAAGAGC   
  
  
- GGTAATGTCT TGTTCCCTTT GAGTAACTTG GGAAAATCAG TAAGTTTGAG AAAACGTAGA AAACAAAAAA   
  
  
- GTTGGGTTAA AGCTTTCAAA AGTTCGCATC TTCATTGTCT TTCGACTAAG AAAGTTCCCT TACGACTTAT   
  
  
- GGGTCAAGTT TAACTCGTAA AGGTCGAAGA GTATAGAAAG AGAGACGGCA ACAGTGGAAG AAGCGAAGAG   
  
  
- GTCAAGGCAG CATTTGGTCA GTAAAAGTAC CGGACTATGG TAGGCCGGAA CGAAAACTCT TGGGTAACCG   
  
  
- GGTAATTAAT AAGCACAACG AATGTTTAG

+     Skn-1\_motif

| Site Name | Organism | Position | Strand | Matrix score. | sequence | function |
| --- | --- | --- | --- | --- | --- | --- |
| Skn-1\_motif | Oryza sativa | 1419 | + | 5 | GTCAT | cis-acting regulatory element required for endosperm expression |
| Skn-1\_motif | Oryza sativa | 1228 | + | 5 | GTCAT | cis-acting regulatory element required for endosperm expression |
| Skn-1\_motif | Oryza sativa | 1014 | - | 5 | GTCAT | cis-acting regulatory element required for endosperm expression |
| Skn-1\_motif | Oryza sativa | 709 | + | 5 | GTCAT | cis-acting regulatory element required for endosperm expression |

> 2018/04/13 10:10:12  
+ ATTTGAATAT ATTAATTATT TATTATAAAA TTTTAAATAT TTAATATTAA AACAATTTAA AAATATTTGT   
  
  
+ AATACTAAAA AAATTTTAAA ATTTTTTGTG ATAATATCTT GAATATTTTT TTACAATTAT AAAAAGTTTT   
  
  
+ AAATAAAAAT TCGTAATATA AAATTTTTAA TTTGAATACT TTTATTTTAA AATTTTTTAA TAAAATATAA   
  
  
+ TTTGATTATA TTTCATCAAT CAGCTATGTT ACTTCAAAAT TTTAAATAAT ATTTGCTTTT AAATTTATTT   
  
  
+ TATTATTTCA TTTAATTATT ATATATTAAC ATTTATTTTA AAATAATATA TCTTTAAAAA ATATTTTTAA   
  
  
+ TTAAGCACTT ACTGTTAACA AGAAGTCTCA GGAAGGCAGT AGTGTCCAGG CCGGTGCGAA GTGCGTTCGC   
  
  
+ GAGTCTACGG CTCATGGCTC ACATTTCGGT CACAGCCTCG TAGGGGACCA CAAAAGTCTG GATCATACAT   
  
  
+ AGCGGCTCCT TGCGTCCCCG TTACGTTTTT CCTCAACGAC CCAGACACGG ATCTGGCATT TGAATCGGTC   
  
  
+ TATTTTTCGG CTTTTAACAT TTATTTATTT ATTATATTTC CCTAAAAATG GACCAAAATT ATCTTGTTAC   
  
  
+ TTTACCAAGT GTCTTAATTT TATATATTCC TCAATAAAAT AATATAAATA GAACGACATC GTTTACACGT   
  
  
+ AAGCAAGGGT CATGGAACTA ACGTCACACT TACTTTAAGA TCGTTTCTTC CTCTTTTTTT CTCACCTTTC   
  
  
+ TGTAGTAGTA ACGTGGAATT TGCCAAGTTT GAAGATTCTT TGGTCTTCTG GTTCCCAACA TCTTCCATTG   
  
  
+ GCTTCCGTTC TTTCCTCTAG AATCCTTTAT TACACTCGAC CAATGAGGAT TCAGGAACCT CAACGAAGAG   
  
  
+ CACTTGACAG AGAGATGGAG TTTCCTGTCT GGACTGAACA TAGAGAAGTG GTCAGCCGTT GGACTTCCCT   
  
  
+ TTTTTTTTTT TTTTATGTTG AACTCGAATT AGTATGACCA ACGACTGGTC TAATGAAGAA AACCCACTTC   
  
  
+ AGCTTTTCTC CAGTCAACCA TCTTCCTTCA ATTTTCTACG TTCAAAAGGA GGCAAATCCT TTAAAATCAC   
  
  
+ ATAACCTTAT TCTCCTTCTT CCACAGGTTA AGTCCCAATA CCTCTAAGTA TTCATCTGCT TAGTTTCTCG   
  
  
+ CCATTACAGA ACAAGGGAAA CTCATTGAAC CCTTTTAGTC ATTCAAACTC TTTTGCATCT TTTGTTTTTT   
  
  
+ CAACCCAATT TCGAAAGTTT TCAAGCGTAG AAGTAACAGA AAGCTGATTC TTTCAAGGGA ATGCTGAATA   
  
  
+ CCCAGTTCAA ATTGAGCATT TCCAGCTTCT CATATCTTTC TCTCTGCCGT TGTCACCTTC TTCGCTTCTC   
  
  
+ CAGTTCCGTC GTAAACCAGT CATTTTCATG GCCTGATACC ATCCGGCCTT GCTTTTGAGA ACCCATTGGC   
  
  
+ CCATTAATTA TTCGTGTTGC TTACAAATC  

- TAAACTTATA TAATTAATAA ATAATATTTT AAAATTTATA AATTATAATT TTGTTAAATT TTTATAAACA   
  
  
- TTATGATTTT TTTAAAATTT TAAAAAACAC TATTATAGAA CTTATAAAAA AATGTTAATA TTTTTCAAAA   
  
  
- TTTATTTTTA AGCATTATAT TTTAAAAATT AAACTTATGA AAATAAAATT TTAAAAAATT ATTTTATATT   
  
  
- AAACTAATAT AAAGTAGTTA GTCGATACAA TGAAGTTTTA AAATTTATTA TAAACGAAAA TTTAAATAAA   
  
  
- ATAATAAAGT AAATTAATAA TATATAATTG TAAATAAAAT TTTATTATAT AGAAATTTTT TATAAAAATT   
  
  
- AATTCGTGAA TGACAATTGT TCTTCAGAGT CCTTCCGTCA TCACAGGTCC GGCCACGCTT CACGCAAGCG   
  
  
- CTCAGATGCC GAGTACCGAG TGTAAAGCCA GTGTCGGAGC ATCCCCTGGT GTTTTCAGAC CTAGTATGTA   
  
  
- TCGCCGAGGA ACGCAGGGGC AATGCAAAAA GGAGTTGCTG GGTCTGTGCC TAGACCGTAA ACTTAGCCAG   
  
  
- ATAAAAAGCC GAAAATTGTA AATAAATAAA TAATATAAAG GGATTTTTAC CTGGTTTTAA TAGAACAATG   
  
  
- AAATGGTTCA CAGAATTAAA ATATATAAGG AGTTATTTTA TTATATTTAT CTTGCTGTAG CAAATGTGCA   
  
  
- TTCGTTCCCA GTACCTTGAT TGCAGTGTGA ATGAAATTCT AGCAAAGAAG GAGAAAAAAA GAGTGGAAAG   
  
  
- ACATCATCAT TGCACCTTAA ACGGTTCAAA CTTCTAAGAA ACCAGAAGAC CAAGGGTTGT AGAAGGTAAC   
  
  
- CGAAGGCAAG AAAGGAGATC TTAGGAAATA ATGTGAGCTG GTTACTCCTA AGTCCTTGGA GTTGCTTCTC   
  
  
- GTGAACTGTC TCTCTACCTC AAAGGACAGA CCTGACTTGT ATCTCTTCAC CAGTCGGCAA CCTGAAGGGA   
  
  
- AAAAAAAAAA AAAATACAAC TTGAGCTTAA TCATACTGGT TGCTGACCAG ATTACTTCTT TTGGGTGAAG   
  
  
- TCGAAAAGAG GTCAGTTGGT AGAAGGAAGT TAAAAGATGC AAGTTTTCCT CCGTTTAGGA AATTTTAGTG   
  
  
- TATTGGAATA AGAGGAAGAA GGTGTCCAAT TCAGGGTTAT GGAGATTCAT AAGTAGACGA ATCAAAGAGC   
  
  
- GGTAATGTCT TGTTCCCTTT GAGTAACTTG GGAAAATCAG TAAGTTTGAG AAAACGTAGA AAACAAAAAA   
  
  
- GTTGGGTTAA AGCTTTCAAA AGTTCGCATC TTCATTGTCT TTCGACTAAG AAAGTTCCCT TACGACTTAT   
  
  
- GGGTCAAGTT TAACTCGTAA AGGTCGAAGA GTATAGAAAG AGAGACGGCA ACAGTGGAAG AAGCGAAGAG   
  
  
- GTCAAGGCAG CATTTGGTCA GTAAAAGTAC CGGACTATGG TAGGCCGGAA CGAAAACTCT TGGGTAACCG   
  
  
- GGTAATTAAT AAGCACAACG AATGTTTAG

+     TATA-box

| Site Name | Organism | Position | Strand | Matrix score. | sequence | function |
| --- | --- | --- | --- | --- | --- | --- |
| TATA-box | Lycopersicon esculentum | 1223 | + | 5 | TTTTA | core promoter element around -30 of transcription start |
| TATA-box | Brassica oleracea | 672 | + | 6 | ATATAA | core promoter element around -30 of transcription start |
| TATA-box | Brassica napus | 215 | + | 6 | ATTATA | core promoter element around -30 of transcription start |
| TATA-box | Arabidopsis thaliana | 648 | - | 7 | TATAAAA | core promoter element around -30 of transcription start |
| TATA-box | Arabidopsis thaliana | 157 | + | 6 | TATAAA | core promoter element around -30 of transcription start |
| TATA-box | Brassica oleracea | 156 | + | 6 | ATATAA | core promoter element around -30 of transcription start |
| TATA-box | Glycine max | 304 | - | 5 | TAATA | core promoter element around -30 of transcription start |
| TATA-box | Arabidopsis thaliana | 8 | + | 4 | TATA | core promoter element around -30 of transcription start |
| TATA-box | Glycine max | 10 | - | 5 | TAATA | core promoter element around -30 of transcription start |
| TATA-box | Lycopersicon esculentum | 1112 | - | 5 | TTTTA | core promoter element around -30 of transcription start |
| TATA-box | Lycopersicon esculentum | 603 | - | 5 | TTTTA | core promoter element around -30 of transcription start |
| TATA-box | Pisum sativum | 647 | - | 8 | TATAAAAT | core promoter element around -30 of transcription start |
| TATA-box | Lycopersicon esculentum | 991 | + | 5 | TTTTA | core promoter element around -30 of transcription start |
| TATA-box | Arabidopsis thaliana | 650 | - | 7 | TATATAA | core promoter element around -30 of transcription start |
| TATA-box | Arabidopsis thaliana | 651 | + | 4 | TATA | core promoter element around -30 of transcription start |
| TATA-box | Arabidopsis thaliana | 23 | - | 5 | TATAA | core promoter element around -30 of transcription start |
| TATA-box | Lycopersicon esculentum | 26 | - | 5 | TTTTA | core promoter element around -30 of transcription start |
| TATA-box | Lycopersicon esculentum | 76 | - | 5 | TTTTA | core promoter element around -30 of transcription start |
| TATA-box | Glycine max | 70 | + | 5 | TAATA | core promoter element around -30 of transcription start |
| TATA-box | Lycopersicon esculentum | 144 | - | 5 | TTTTA | core promoter element around -30 of transcription start |
| TATA-box | Arabidopsis thaliana | 217 | + | 4 | TATA | core promoter element around -30 of transcription start |
| TATA-box | Arabidopsis thaliana | 206 | + | 4 | TATA | core promoter element around -30 of transcription start |
| TATA-box | Arabidopsis thaliana | 216 | - | 5 | TATAA | core promoter element around -30 of transcription start |
| TATA-box | Arabidopsis thaliana | 138 | - | 8 | TATTTAAA | core promoter element around -30 of transcription start |
| TATA-box | Glycine max | 198 | + | 5 | TAATA | core promoter element around -30 of transcription start |
| TATA-box | Glycine max | 868 | - | 5 | TAATA | core promoter element around -30 of transcription start |
| TATA-box | Lycopersicon esculentum | 195 | + | 5 | TTTTA | core promoter element around -30 of transcription start |
| TATA-box | Brassica oleracea | 205 | + | 7 | ATATAAT | core promoter element around -30 of transcription start |
| TATA-box | Arabidopsis thaliana | 128 | + | 6 | TATAAA | core promoter element around -30 of transcription start |
| TATA-box | Glycine max | 590 | - | 5 | TAATA | core promoter element around -30 of transcription start |
| TATA-box | Arabidopsis thaliana | 593 | + | 4 | TATA | core promoter element around -30 of transcription start |
| TATA-box | Arabidopsis thaliana | 592 | - | 5 | TATAA | core promoter element around -30 of transcription start |
| TATA-box | Brassica napus | 7 | + | 6 | ATATAT | core promoter element around -30 of transcription start |
| TATA-box | Lycopersicon esculentum | 665 | - | 5 | TTTTA | core promoter element around -30 of transcription start |
| TATA-box | Brassica napus | 326 | + | 6 | ATATAT | core promoter element around -30 of transcription start |
| TATA-box | Lycopersicon esculentum | 572 | + | 5 | TTTTA | core promoter element around -30 of transcription start |
| TATA-box | Brassica napus | 591 | + | 6 | ATTATA | core promoter element around -30 of transcription start |
| TATA-box | Arabidopsis thaliana | 302 | + | 4 | TATA | core promoter element around -30 of transcription start |
| TATA-box | Brassica napus | 301 | + | 6 | ATATAT | core promoter element around -30 of transcription start |
| TATA-box | Glycine max | 281 | - | 5 | TAATA | core promoter element around -30 of transcription start |
| TATA-box | Glycine max | 42 | + | 5 | TAATA | core promoter element around -30 of transcription start |
| TATA-box | Zea mays | 333 | + | 8 | TTTAAAAA | core promoter element around -30 of transcription start |
| TATA-box | Glycine max | 45 | - | 5 | TAATA | core promoter element around -30 of transcription start |
| TATA-box | Arabidopsis thaliana | 649 | - | 6 | TATAAA | core promoter element around -30 of transcription start |
| TATA-box | Lycopersicon esculentum | 165 | + | 5 | TTTTA | core promoter element around -30 of transcription start |
| TATA-box | Brassica napus | 22 | + | 6 | ATTATA | core promoter element around -30 of transcription start |
| TATA-box | Lycopersicon esculentum | 278 | + | 5 | TTTTA | core promoter element around -30 of transcription start |
| TATA-box | Arabidopsis thaliana | 251 | - | 8 | TATTTAAA | core promoter element around -30 of transcription start |
| TATA-box | Lycopersicon esculentum | 201 | - | 5 | TTTTA | core promoter element around -30 of transcription start |
| TATA-box | Arabidopsis thaliana | 24 | + | 6 | TATAAA | core promoter element around -30 of transcription start |
| TATA-box | Lycopersicon esculentum | 188 | - | 5 | TTTTA | core promoter element around -30 of transcription start |
| TATA-box | Lycopersicon esculentum | 335 | - | 5 | TTTTA | core promoter element around -30 of transcription start |
| TATA-box | Lycopersicon esculentum | 250 | + | 5 | TTTTA | core promoter element around -30 of transcription start |
| TATA-box | Arabidopsis thaliana | 653 | + | 4 | TATA | core promoter element around -30 of transcription start |
| TATA-box | Lycopersicon esculentum | 87 | - | 5 | TTTTA | core promoter element around -30 of transcription start |
| TATA-box | Lycopersicon esculentum | 84 | + | 5 | TTTTA | core promoter element around -30 of transcription start |
| TATA-box | Lycopersicon esculentum | 31 | + | 5 | TTTTA | core promoter element around -30 of transcription start |
| TATA-box | Lycopersicon esculentum | 319 | - | 5 | TTTTA | core promoter element around -30 of transcription start |
| TATA-box | Ac | 673 | + | 7 | TATAAAT | core promoter element around -30 of transcription start |
| TATA-box | Brassica napus | 652 | + | 6 | ATATAT | core promoter element around -30 of transcription start |
| TATA-box | Lycopersicon esculentum | 119 | + | 5 | TTTTA | core promoter element around -30 of transcription start |
| TATA-box | Lycopersicon esculentum | 58 | - | 5 | TTTTA | core promoter element around -30 of transcription start |
| TATA-box | Lycopersicon esculentum | 185 | + | 5 | TTTTA | core promoter element around -30 of transcription start |
| TATA-box | Zea mays | 56 | + | 8 | TTTAAAAA | core promoter element around -30 of transcription start |
| TATA-box | Glycine max | 257 | + | 5 | TAATA | core promoter element around -30 of transcription start |
| TATA-box | Lycopersicon esculentum | 48 | - | 5 | TTTTA | core promoter element around -30 of transcription start |
| TATA-box | Lycopersicon esculentum | 267 | + | 5 | TTTTA | core promoter element around -30 of transcription start |
| TATA-box | Glycine max | 21 | - | 5 | TAATA | core promoter element around -30 of transcription start |
| TATA-box | Glycine max | 670 | + | 5 | TAATA | core promoter element around -30 of transcription start |
| TATA-box | Lycopersicon esculentum | 130 | - | 5 | TTTTA | core promoter element around -30 of transcription start |
| TATA-box | Arabidopsis thaliana | 32 | - | 8 | TATTTAAA | core promoter element around -30 of transcription start |
| TATA-box | Lycopersicon esculentum | 345 | + | 5 | TTTTA | core promoter element around -30 of transcription start |
| TATA-box | Glycine max | 154 | + | 5 | TAATA | core promoter element around -30 of transcription start |
| TATA-box | Arabidopsis thaliana | 327 | + | 4 | TATA | core promoter element around -30 of transcription start |
| TATA-box | Glycine max | 102 | + | 5 | TAATA | core promoter element around -30 of transcription start |
| TATA-box | Arabidopsis thaliana | 299 | - | 7 | TATATAA | core promoter element around -30 of transcription start |
| TATA-box | Lycopersicon esculentum | 180 | + | 5 | TTTTA | core promoter element around -30 of transcription start |
| TATA-box | Arabidopsis thaliana | 127 | - | 5 | TATAA | core promoter element around -30 of transcription start |
| TATA-box | Glycine max | 297 | - | 5 | TAATA | core promoter element around -30 of transcription start |
| TATA-box | Brassica napus | 126 | + | 6 | ATTATA | core promoter element around -30 of transcription start |
| TATA-box | Lycopersicon esculentum | 137 | + | 5 | TTTTA | core promoter element around -30 of transcription start |
| TATA-box | Lycopersicon esculentum | 316 | + | 5 | TTTTA | core promoter element around -30 of transcription start |
| TATA-box | Brassica napus | 298 | + | 6 | ATTATA | core promoter element around -30 of transcription start |
| TATA-box | Lycopersicon esculentum | 159 | - | 5 | TTTTA | core promoter element around -30 of transcription start |
| TATA-box | Arabidopsis thaliana | 300 | + | 4 | TATA | core promoter element around -30 of transcription start |
| TATA-box | Glycine max | 324 | + | 5 | TAATA | core promoter element around -30 of transcription start |

> 2018/04/13 10:10:12  
+ ATTTGAATAT ATTAATTATT TATTATAAAA TTTTAAATAT TTAATATTAA AACAATTTAA AAATATTTGT   
  
  
+ AATACTAAAA AAATTTTAAA ATTTTTTGTG ATAATATCTT GAATATTTTT TTACAATTAT AAAAAGTTTT   
  
  
+ AAATAAAAAT TCGTAATATA AAATTTTTAA TTTGAATACT TTTATTTTAA AATTTTTTAA TAAAATATAA   
  
  
+ TTTGATTATA TTTCATCAAT CAGCTATGTT ACTTCAAAAT TTTAAATAAT ATTTGCTTTT AAATTTATTT   
  
  
+ TATTATTTCA TTTAATTATT ATATATTAAC ATTTATTTTA AAATAATATA TCTTTAAAAA ATATTTTTAA   
  
  
+ TTAAGCACTT ACTGTTAACA AGAAGTCTCA GGAAGGCAGT AGTGTCCAGG CCGGTGCGAA GTGCGTTCGC   
  
  
+ GAGTCTACGG CTCATGGCTC ACATTTCGGT CACAGCCTCG TAGGGGACCA CAAAAGTCTG GATCATACAT   
  
  
+ AGCGGCTCCT TGCGTCCCCG TTACGTTTTT CCTCAACGAC CCAGACACGG ATCTGGCATT TGAATCGGTC   
  
  
+ TATTTTTCGG CTTTTAACAT TTATTTATTT ATTATATTTC CCTAAAAATG GACCAAAATT ATCTTGTTAC   
  
  
+ TTTACCAAGT GTCTTAATTT TATATATTCC TCAATAAAAT AATATAAATA GAACGACATC GTTTACACGT   
  
  
+ AAGCAAGGGT CATGGAACTA ACGTCACACT TACTTTAAGA TCGTTTCTTC CTCTTTTTTT CTCACCTTTC   
  
  
+ TGTAGTAGTA ACGTGGAATT TGCCAAGTTT GAAGATTCTT TGGTCTTCTG GTTCCCAACA TCTTCCATTG   
  
  
+ GCTTCCGTTC TTTCCTCTAG AATCCTTTAT TACACTCGAC CAATGAGGAT TCAGGAACCT CAACGAAGAG   
  
  
+ CACTTGACAG AGAGATGGAG TTTCCTGTCT GGACTGAACA TAGAGAAGTG GTCAGCCGTT GGACTTCCCT   
  
  
+ TTTTTTTTTT TTTTATGTTG AACTCGAATT AGTATGACCA ACGACTGGTC TAATGAAGAA AACCCACTTC   
  
  
+ AGCTTTTCTC CAGTCAACCA TCTTCCTTCA ATTTTCTACG TTCAAAAGGA GGCAAATCCT TTAAAATCAC   
  
  
+ ATAACCTTAT TCTCCTTCTT CCACAGGTTA AGTCCCAATA CCTCTAAGTA TTCATCTGCT TAGTTTCTCG   
  
  
+ CCATTACAGA ACAAGGGAAA CTCATTGAAC CCTTTTAGTC ATTCAAACTC TTTTGCATCT TTTGTTTTTT   
  
  
+ CAACCCAATT TCGAAAGTTT TCAAGCGTAG AAGTAACAGA AAGCTGATTC TTTCAAGGGA ATGCTGAATA   
  
  
+ CCCAGTTCAA ATTGAGCATT TCCAGCTTCT CATATCTTTC TCTCTGCCGT TGTCACCTTC TTCGCTTCTC   
  
  
+ CAGTTCCGTC GTAAACCAGT CATTTTCATG GCCTGATACC ATCCGGCCTT GCTTTTGAGA ACCCATTGGC   
  
  
+ CCATTAATTA TTCGTGTTGC TTACAAATC  

- TAAACTTATA TAATTAATAA ATAATATTTT AAAATTTATA AATTATAATT TTGTTAAATT TTTATAAACA   
  
  
- TTATGATTTT TTTAAAATTT TAAAAAACAC TATTATAGAA CTTATAAAAA AATGTTAATA TTTTTCAAAA   
  
  
- TTTATTTTTA AGCATTATAT TTTAAAAATT AAACTTATGA AAATAAAATT TTAAAAAATT ATTTTATATT   
  
  
- AAACTAATAT AAAGTAGTTA GTCGATACAA TGAAGTTTTA AAATTTATTA TAAACGAAAA TTTAAATAAA   
  
  
- ATAATAAAGT AAATTAATAA TATATAATTG TAAATAAAAT TTTATTATAT AGAAATTTTT TATAAAAATT   
  
  
- AATTCGTGAA TGACAATTGT TCTTCAGAGT CCTTCCGTCA TCACAGGTCC GGCCACGCTT CACGCAAGCG   
  
  
- CTCAGATGCC GAGTACCGAG TGTAAAGCCA GTGTCGGAGC ATCCCCTGGT GTTTTCAGAC CTAGTATGTA   
  
  
- TCGCCGAGGA ACGCAGGGGC AATGCAAAAA GGAGTTGCTG GGTCTGTGCC TAGACCGTAA ACTTAGCCAG   
  
  
- ATAAAAAGCC GAAAATTGTA AATAAATAAA TAATATAAAG GGATTTTTAC CTGGTTTTAA TAGAACAATG   
  
  
- AAATGGTTCA CAGAATTAAA ATATATAAGG AGTTATTTTA TTATATTTAT CTTGCTGTAG CAAATGTGCA   
  
  
- TTCGTTCCCA GTACCTTGAT TGCAGTGTGA ATGAAATTCT AGCAAAGAAG GAGAAAAAAA GAGTGGAAAG   
  
  
- ACATCATCAT TGCACCTTAA ACGGTTCAAA CTTCTAAGAA ACCAGAAGAC CAAGGGTTGT AGAAGGTAAC   
  
  
- CGAAGGCAAG AAAGGAGATC TTAGGAAATA ATGTGAGCTG GTTACTCCTA AGTCCTTGGA GTTGCTTCTC   
  
  
- GTGAACTGTC TCTCTACCTC AAAGGACAGA CCTGACTTGT ATCTCTTCAC CAGTCGGCAA CCTGAAGGGA   
  
  
- AAAAAAAAAA AAAATACAAC TTGAGCTTAA TCATACTGGT TGCTGACCAG ATTACTTCTT TTGGGTGAAG   
  
  
- TCGAAAAGAG GTCAGTTGGT AGAAGGAAGT TAAAAGATGC AAGTTTTCCT CCGTTTAGGA AATTTTAGTG   
  
  
- TATTGGAATA AGAGGAAGAA GGTGTCCAAT TCAGGGTTAT GGAGATTCAT AAGTAGACGA ATCAAAGAGC   
  
  
- GGTAATGTCT TGTTCCCTTT GAGTAACTTG GGAAAATCAG TAAGTTTGAG AAAACGTAGA AAACAAAAAA   
  
  
- GTTGGGTTAA AGCTTTCAAA AGTTCGCATC TTCATTGTCT TTCGACTAAG AAAGTTCCCT TACGACTTAT   
  
  
- GGGTCAAGTT TAACTCGTAA AGGTCGAAGA GTATAGAAAG AGAGACGGCA ACAGTGGAAG AAGCGAAGAG   
  
  
- GTCAAGGCAG CATTTGGTCA GTAAAAGTAC CGGACTATGG TAGGCCGGAA CGAAAACTCT TGGGTAACCG   
  
  
- GGTAATTAAT AAGCACAACG AATGTTTAG

+     TC-rich repeats

| Site Name | Organism | Position | Strand | Matrix score. | sequence | function |
| --- | --- | --- | --- | --- | --- | --- |
| TC-rich repeats | Nicotiana tabacum | 1034 | - | 9 | ATTTTCTTCA | cis-acting element involved in defense and stress responsiveness |
| TC-rich repeats | Nicotiana tabacum | 1053 | + | 9 | ATTTTCTCCA | cis-acting element involved in defense and stress responsiveness |

> 2018/04/13 10:10:12  
+ ATTTGAATAT ATTAATTATT TATTATAAAA TTTTAAATAT TTAATATTAA AACAATTTAA AAATATTTGT   
  
  
+ AATACTAAAA AAATTTTAAA ATTTTTTGTG ATAATATCTT GAATATTTTT TTACAATTAT AAAAAGTTTT   
  
  
+ AAATAAAAAT TCGTAATATA AAATTTTTAA TTTGAATACT TTTATTTTAA AATTTTTTAA TAAAATATAA   
  
  
+ TTTGATTATA TTTCATCAAT CAGCTATGTT ACTTCAAAAT TTTAAATAAT ATTTGCTTTT AAATTTATTT   
  
  
+ TATTATTTCA TTTAATTATT ATATATTAAC ATTTATTTTA AAATAATATA TCTTTAAAAA ATATTTTTAA   
  
  
+ TTAAGCACTT ACTGTTAACA AGAAGTCTCA GGAAGGCAGT AGTGTCCAGG CCGGTGCGAA GTGCGTTCGC   
  
  
+ GAGTCTACGG CTCATGGCTC ACATTTCGGT CACAGCCTCG TAGGGGACCA CAAAAGTCTG GATCATACAT   
  
  
+ AGCGGCTCCT TGCGTCCCCG TTACGTTTTT CCTCAACGAC CCAGACACGG ATCTGGCATT TGAATCGGTC   
  
  
+ TATTTTTCGG CTTTTAACAT TTATTTATTT ATTATATTTC CCTAAAAATG GACCAAAATT ATCTTGTTAC   
  
  
+ TTTACCAAGT GTCTTAATTT TATATATTCC TCAATAAAAT AATATAAATA GAACGACATC GTTTACACGT   
  
  
+ AAGCAAGGGT CATGGAACTA ACGTCACACT TACTTTAAGA TCGTTTCTTC CTCTTTTTTT CTCACCTTTC   
  
  
+ TGTAGTAGTA ACGTGGAATT TGCCAAGTTT GAAGATTCTT TGGTCTTCTG GTTCCCAACA TCTTCCATTG   
  
  
+ GCTTCCGTTC TTTCCTCTAG AATCCTTTAT TACACTCGAC CAATGAGGAT TCAGGAACCT CAACGAAGAG   
  
  
+ CACTTGACAG AGAGATGGAG TTTCCTGTCT GGACTGAACA TAGAGAAGTG GTCAGCCGTT GGACTTCCCT   
  
  
+ TTTTTTTTTT TTTTATGTTG AACTCGAATT AGTATGACCA ACGACTGGTC TAATGAAGAA AACCCACTTC   
  
  
+ AGCTTTTCTC CAGTCAACCA TCTTCCTTCA ATTTTCTACG TTCAAAAGGA GGCAAATCCT TTAAAATCAC   
  
  
+ ATAACCTTAT TCTCCTTCTT CCACAGGTTA AGTCCCAATA CCTCTAAGTA TTCATCTGCT TAGTTTCTCG   
  
  
+ CCATTACAGA ACAAGGGAAA CTCATTGAAC CCTTTTAGTC ATTCAAACTC TTTTGCATCT TTTGTTTTTT   
  
  
+ CAACCCAATT TCGAAAGTTT TCAAGCGTAG AAGTAACAGA AAGCTGATTC TTTCAAGGGA ATGCTGAATA   
  
  
+ CCCAGTTCAA ATTGAGCATT TCCAGCTTCT CATATCTTTC TCTCTGCCGT TGTCACCTTC TTCGCTTCTC   
  
  
+ CAGTTCCGTC GTAAACCAGT CATTTTCATG GCCTGATACC ATCCGGCCTT GCTTTTGAGA ACCCATTGGC   
  
  
+ CCATTAATTA TTCGTGTTGC TTACAAATC  

- TAAACTTATA TAATTAATAA ATAATATTTT AAAATTTATA AATTATAATT TTGTTAAATT TTTATAAACA   
  
  
- TTATGATTTT TTTAAAATTT TAAAAAACAC TATTATAGAA CTTATAAAAA AATGTTAATA TTTTTCAAAA   
  
  
- TTTATTTTTA AGCATTATAT TTTAAAAATT AAACTTATGA AAATAAAATT TTAAAAAATT ATTTTATATT   
  
  
- AAACTAATAT AAAGTAGTTA GTCGATACAA TGAAGTTTTA AAATTTATTA TAAACGAAAA TTTAAATAAA   
  
  
- ATAATAAAGT AAATTAATAA TATATAATTG TAAATAAAAT TTTATTATAT AGAAATTTTT TATAAAAATT   
  
  
- AATTCGTGAA TGACAATTGT TCTTCAGAGT CCTTCCGTCA TCACAGGTCC GGCCACGCTT CACGCAAGCG   
  
  
- CTCAGATGCC GAGTACCGAG TGTAAAGCCA GTGTCGGAGC ATCCCCTGGT GTTTTCAGAC CTAGTATGTA   
  
  
- TCGCCGAGGA ACGCAGGGGC AATGCAAAAA GGAGTTGCTG GGTCTGTGCC TAGACCGTAA ACTTAGCCAG   
  
  
- ATAAAAAGCC GAAAATTGTA AATAAATAAA TAATATAAAG GGATTTTTAC CTGGTTTTAA TAGAACAATG   
  
  
- AAATGGTTCA CAGAATTAAA ATATATAAGG AGTTATTTTA TTATATTTAT CTTGCTGTAG CAAATGTGCA   
  
  
- TTCGTTCCCA GTACCTTGAT TGCAGTGTGA ATGAAATTCT AGCAAAGAAG GAGAAAAAAA GAGTGGAAAG   
  
  
- ACATCATCAT TGCACCTTAA ACGGTTCAAA CTTCTAAGAA ACCAGAAGAC CAAGGGTTGT AGAAGGTAAC   
  
  
- CGAAGGCAAG AAAGGAGATC TTAGGAAATA ATGTGAGCTG GTTACTCCTA AGTCCTTGGA GTTGCTTCTC   
  
  
- GTGAACTGTC TCTCTACCTC AAAGGACAGA CCTGACTTGT ATCTCTTCAC CAGTCGGCAA CCTGAAGGGA   
  
  
- AAAAAAAAAA AAAATACAAC TTGAGCTTAA TCATACTGGT TGCTGACCAG ATTACTTCTT TTGGGTGAAG   
  
  
- TCGAAAAGAG GTCAGTTGGT AGAAGGAAGT TAAAAGATGC AAGTTTTCCT CCGTTTAGGA AATTTTAGTG   
  
  
- TATTGGAATA AGAGGAAGAA GGTGTCCAAT TCAGGGTTAT GGAGATTCAT AAGTAGACGA ATCAAAGAGC   
  
  
- GGTAATGTCT TGTTCCCTTT GAGTAACTTG GGAAAATCAG TAAGTTTGAG AAAACGTAGA AAACAAAAAA   
  
  
- GTTGGGTTAA AGCTTTCAAA AGTTCGCATC TTCATTGTCT TTCGACTAAG AAAGTTCCCT TACGACTTAT   
  
  
- GGGTCAAGTT TAACTCGTAA AGGTCGAAGA GTATAGAAAG AGAGACGGCA ACAGTGGAAG AAGCGAAGAG   
  
  
- GTCAAGGCAG CATTTGGTCA GTAAAAGTAC CGGACTATGG TAGGCCGGAA CGAAAACTCT TGGGTAACCG   
  
  
- GGTAATTAAT AAGCACAACG AATGTTTAG

+     TGA-element

| Site Name | Organism | Position | Strand | Matrix score. | sequence | function |
| --- | --- | --- | --- | --- | --- | --- |
| TGA-element | Brassica oleracea | 1020 | + | 6 | AACGAC | auxin-responsive element |
| TGA-element | Brassica oleracea | 682 | + | 6 | AACGAC | auxin-responsive element |
| TGA-element | Brassica oleracea | 525 | + | 6 | AACGAC | auxin-responsive element |

> 2018/04/13 10:10:12  
+ ATTTGAATAT ATTAATTATT TATTATAAAA TTTTAAATAT TTAATATTAA AACAATTTAA AAATATTTGT   
  
  
+ AATACTAAAA AAATTTTAAA ATTTTTTGTG ATAATATCTT GAATATTTTT TTACAATTAT AAAAAGTTTT   
  
  
+ AAATAAAAAT TCGTAATATA AAATTTTTAA TTTGAATACT TTTATTTTAA AATTTTTTAA TAAAATATAA   
  
  
+ TTTGATTATA TTTCATCAAT CAGCTATGTT ACTTCAAAAT TTTAAATAAT ATTTGCTTTT AAATTTATTT   
  
  
+ TATTATTTCA TTTAATTATT ATATATTAAC ATTTATTTTA AAATAATATA TCTTTAAAAA ATATTTTTAA   
  
  
+ TTAAGCACTT ACTGTTAACA AGAAGTCTCA GGAAGGCAGT AGTGTCCAGG CCGGTGCGAA GTGCGTTCGC   
  
  
+ GAGTCTACGG CTCATGGCTC ACATTTCGGT CACAGCCTCG TAGGGGACCA CAAAAGTCTG GATCATACAT   
  
  
+ AGCGGCTCCT TGCGTCCCCG TTACGTTTTT CCTCAACGAC CCAGACACGG ATCTGGCATT TGAATCGGTC   
  
  
+ TATTTTTCGG CTTTTAACAT TTATTTATTT ATTATATTTC CCTAAAAATG GACCAAAATT ATCTTGTTAC   
  
  
+ TTTACCAAGT GTCTTAATTT TATATATTCC TCAATAAAAT AATATAAATA GAACGACATC GTTTACACGT   
  
  
+ AAGCAAGGGT CATGGAACTA ACGTCACACT TACTTTAAGA TCGTTTCTTC CTCTTTTTTT CTCACCTTTC   
  
  
+ TGTAGTAGTA ACGTGGAATT TGCCAAGTTT GAAGATTCTT TGGTCTTCTG GTTCCCAACA TCTTCCATTG   
  
  
+ GCTTCCGTTC TTTCCTCTAG AATCCTTTAT TACACTCGAC CAATGAGGAT TCAGGAACCT CAACGAAGAG   
  
  
+ CACTTGACAG AGAGATGGAG TTTCCTGTCT GGACTGAACA TAGAGAAGTG GTCAGCCGTT GGACTTCCCT   
  
  
+ TTTTTTTTTT TTTTATGTTG AACTCGAATT AGTATGACCA ACGACTGGTC TAATGAAGAA AACCCACTTC   
  
  
+ AGCTTTTCTC CAGTCAACCA TCTTCCTTCA ATTTTCTACG TTCAAAAGGA GGCAAATCCT TTAAAATCAC   
  
  
+ ATAACCTTAT TCTCCTTCTT CCACAGGTTA AGTCCCAATA CCTCTAAGTA TTCATCTGCT TAGTTTCTCG   
  
  
+ CCATTACAGA ACAAGGGAAA CTCATTGAAC CCTTTTAGTC ATTCAAACTC TTTTGCATCT TTTGTTTTTT   
  
  
+ CAACCCAATT TCGAAAGTTT TCAAGCGTAG AAGTAACAGA AAGCTGATTC TTTCAAGGGA ATGCTGAATA   
  
  
+ CCCAGTTCAA ATTGAGCATT TCCAGCTTCT CATATCTTTC TCTCTGCCGT TGTCACCTTC TTCGCTTCTC   
  
  
+ CAGTTCCGTC GTAAACCAGT CATTTTCATG GCCTGATACC ATCCGGCCTT GCTTTTGAGA ACCCATTGGC   
  
  
+ CCATTAATTA TTCGTGTTGC TTACAAATC  

- TAAACTTATA TAATTAATAA ATAATATTTT AAAATTTATA AATTATAATT TTGTTAAATT TTTATAAACA   
  
  
- TTATGATTTT TTTAAAATTT TAAAAAACAC TATTATAGAA CTTATAAAAA AATGTTAATA TTTTTCAAAA   
  
  
- TTTATTTTTA AGCATTATAT TTTAAAAATT AAACTTATGA AAATAAAATT TTAAAAAATT ATTTTATATT   
  
  
- AAACTAATAT AAAGTAGTTA GTCGATACAA TGAAGTTTTA AAATTTATTA TAAACGAAAA TTTAAATAAA   
  
  
- ATAATAAAGT AAATTAATAA TATATAATTG TAAATAAAAT TTTATTATAT AGAAATTTTT TATAAAAATT   
  
  
- AATTCGTGAA TGACAATTGT TCTTCAGAGT CCTTCCGTCA TCACAGGTCC GGCCACGCTT CACGCAAGCG   
  
  
- CTCAGATGCC GAGTACCGAG TGTAAAGCCA GTGTCGGAGC ATCCCCTGGT GTTTTCAGAC CTAGTATGTA   
  
  
- TCGCCGAGGA ACGCAGGGGC AATGCAAAAA GGAGTTGCTG GGTCTGTGCC TAGACCGTAA ACTTAGCCAG   
  
  
- ATAAAAAGCC GAAAATTGTA AATAAATAAA TAATATAAAG GGATTTTTAC CTGGTTTTAA TAGAACAATG   
  
  
- AAATGGTTCA CAGAATTAAA ATATATAAGG AGTTATTTTA TTATATTTAT CTTGCTGTAG CAAATGTGCA   
  
  
- TTCGTTCCCA GTACCTTGAT TGCAGTGTGA ATGAAATTCT AGCAAAGAAG GAGAAAAAAA GAGTGGAAAG   
  
  
- ACATCATCAT TGCACCTTAA ACGGTTCAAA CTTCTAAGAA ACCAGAAGAC CAAGGGTTGT AGAAGGTAAC   
  
  
- CGAAGGCAAG AAAGGAGATC TTAGGAAATA ATGTGAGCTG GTTACTCCTA AGTCCTTGGA GTTGCTTCTC   
  
  
- GTGAACTGTC TCTCTACCTC AAAGGACAGA CCTGACTTGT ATCTCTTCAC CAGTCGGCAA CCTGAAGGGA   
  
  
- AAAAAAAAAA AAAATACAAC TTGAGCTTAA TCATACTGGT TGCTGACCAG ATTACTTCTT TTGGGTGAAG   
  
  
- TCGAAAAGAG GTCAGTTGGT AGAAGGAAGT TAAAAGATGC AAGTTTTCCT CCGTTTAGGA AATTTTAGTG   
  
  
- TATTGGAATA AGAGGAAGAA GGTGTCCAAT TCAGGGTTAT GGAGATTCAT AAGTAGACGA ATCAAAGAGC   
  
  
- GGTAATGTCT TGTTCCCTTT GAGTAACTTG GGAAAATCAG TAAGTTTGAG AAAACGTAGA AAACAAAAAA   
  
  
- GTTGGGTTAA AGCTTTCAAA AGTTCGCATC TTCATTGTCT TTCGACTAAG AAAGTTCCCT TACGACTTAT   
  
  
- GGGTCAAGTT TAACTCGTAA AGGTCGAAGA GTATAGAAAG AGAGACGGCA ACAGTGGAAG AAGCGAAGAG   
  
  
- GTCAAGGCAG CATTTGGTCA GTAAAAGTAC CGGACTATGG TAGGCCGGAA CGAAAACTCT TGGGTAACCG   
  
  
- GGTAATTAAT AAGCACAACG AATGTTTAG

+     TGACG-motif

| Site Name | Organism | Position | Strand | Matrix score. | sequence | function |
| --- | --- | --- | --- | --- | --- | --- |
| TGACG-motif | Hordeum vulgare | 722 | - | 5 | TGACG | cis-acting regulatory element involved in the MeJA-responsiveness |

> 2018/04/13 10:10:12  
+ ATTTGAATAT ATTAATTATT TATTATAAAA TTTTAAATAT TTAATATTAA AACAATTTAA AAATATTTGT   
  
  
+ AATACTAAAA AAATTTTAAA ATTTTTTGTG ATAATATCTT GAATATTTTT TTACAATTAT AAAAAGTTTT   
  
  
+ AAATAAAAAT TCGTAATATA AAATTTTTAA TTTGAATACT TTTATTTTAA AATTTTTTAA TAAAATATAA   
  
  
+ TTTGATTATA TTTCATCAAT CAGCTATGTT ACTTCAAAAT TTTAAATAAT ATTTGCTTTT AAATTTATTT   
  
  
+ TATTATTTCA TTTAATTATT ATATATTAAC ATTTATTTTA AAATAATATA TCTTTAAAAA ATATTTTTAA   
  
  
+ TTAAGCACTT ACTGTTAACA AGAAGTCTCA GGAAGGCAGT AGTGTCCAGG CCGGTGCGAA GTGCGTTCGC   
  
  
+ GAGTCTACGG CTCATGGCTC ACATTTCGGT CACAGCCTCG TAGGGGACCA CAAAAGTCTG GATCATACAT   
  
  
+ AGCGGCTCCT TGCGTCCCCG TTACGTTTTT CCTCAACGAC CCAGACACGG ATCTGGCATT TGAATCGGTC   
  
  
+ TATTTTTCGG CTTTTAACAT TTATTTATTT ATTATATTTC CCTAAAAATG GACCAAAATT ATCTTGTTAC   
  
  
+ TTTACCAAGT GTCTTAATTT TATATATTCC TCAATAAAAT AATATAAATA GAACGACATC GTTTACACGT   
  
  
+ AAGCAAGGGT CATGGAACTA ACGTCACACT TACTTTAAGA TCGTTTCTTC CTCTTTTTTT CTCACCTTTC   
  
  
+ TGTAGTAGTA ACGTGGAATT TGCCAAGTTT GAAGATTCTT TGGTCTTCTG GTTCCCAACA TCTTCCATTG   
  
  
+ GCTTCCGTTC TTTCCTCTAG AATCCTTTAT TACACTCGAC CAATGAGGAT TCAGGAACCT CAACGAAGAG   
  
  
+ CACTTGACAG AGAGATGGAG TTTCCTGTCT GGACTGAACA TAGAGAAGTG GTCAGCCGTT GGACTTCCCT   
  
  
+ TTTTTTTTTT TTTTATGTTG AACTCGAATT AGTATGACCA ACGACTGGTC TAATGAAGAA AACCCACTTC   
  
  
+ AGCTTTTCTC CAGTCAACCA TCTTCCTTCA ATTTTCTACG TTCAAAAGGA GGCAAATCCT TTAAAATCAC   
  
  
+ ATAACCTTAT TCTCCTTCTT CCACAGGTTA AGTCCCAATA CCTCTAAGTA TTCATCTGCT TAGTTTCTCG   
  
  
+ CCATTACAGA ACAAGGGAAA CTCATTGAAC CCTTTTAGTC ATTCAAACTC TTTTGCATCT TTTGTTTTTT   
  
  
+ CAACCCAATT TCGAAAGTTT TCAAGCGTAG AAGTAACAGA AAGCTGATTC TTTCAAGGGA ATGCTGAATA   
  
  
+ CCCAGTTCAA ATTGAGCATT TCCAGCTTCT CATATCTTTC TCTCTGCCGT TGTCACCTTC TTCGCTTCTC   
  
  
+ CAGTTCCGTC GTAAACCAGT CATTTTCATG GCCTGATACC ATCCGGCCTT GCTTTTGAGA ACCCATTGGC   
  
  
+ CCATTAATTA TTCGTGTTGC TTACAAATC  

- TAAACTTATA TAATTAATAA ATAATATTTT AAAATTTATA AATTATAATT TTGTTAAATT TTTATAAACA   
  
  
- TTATGATTTT TTTAAAATTT TAAAAAACAC TATTATAGAA CTTATAAAAA AATGTTAATA TTTTTCAAAA   
  
  
- TTTATTTTTA AGCATTATAT TTTAAAAATT AAACTTATGA AAATAAAATT TTAAAAAATT ATTTTATATT   
  
  
- AAACTAATAT AAAGTAGTTA GTCGATACAA TGAAGTTTTA AAATTTATTA TAAACGAAAA TTTAAATAAA   
  
  
- ATAATAAAGT AAATTAATAA TATATAATTG TAAATAAAAT TTTATTATAT AGAAATTTTT TATAAAAATT   
  
  
- AATTCGTGAA TGACAATTGT TCTTCAGAGT CCTTCCGTCA TCACAGGTCC GGCCACGCTT CACGCAAGCG   
  
  
- CTCAGATGCC GAGTACCGAG TGTAAAGCCA GTGTCGGAGC ATCCCCTGGT GTTTTCAGAC CTAGTATGTA   
  
  
- TCGCCGAGGA ACGCAGGGGC AATGCAAAAA GGAGTTGCTG GGTCTGTGCC TAGACCGTAA ACTTAGCCAG   
  
  
- ATAAAAAGCC GAAAATTGTA AATAAATAAA TAATATAAAG GGATTTTTAC CTGGTTTTAA TAGAACAATG   
  
  
- AAATGGTTCA CAGAATTAAA ATATATAAGG AGTTATTTTA TTATATTTAT CTTGCTGTAG CAAATGTGCA   
  
  
- TTCGTTCCCA GTACCTTGAT TGCAGTGTGA ATGAAATTCT AGCAAAGAAG GAGAAAAAAA GAGTGGAAAG   
  
  
- ACATCATCAT TGCACCTTAA ACGGTTCAAA CTTCTAAGAA ACCAGAAGAC CAAGGGTTGT AGAAGGTAAC   
  
  
- CGAAGGCAAG AAAGGAGATC TTAGGAAATA ATGTGAGCTG GTTACTCCTA AGTCCTTGGA GTTGCTTCTC   
  
  
- GTGAACTGTC TCTCTACCTC AAAGGACAGA CCTGACTTGT ATCTCTTCAC CAGTCGGCAA CCTGAAGGGA   
  
  
- AAAAAAAAAA AAAATACAAC TTGAGCTTAA TCATACTGGT TGCTGACCAG ATTACTTCTT TTGGGTGAAG   
  
  
- TCGAAAAGAG GTCAGTTGGT AGAAGGAAGT TAAAAGATGC AAGTTTTCCT CCGTTTAGGA AATTTTAGTG   
  
  
- TATTGGAATA AGAGGAAGAA GGTGTCCAAT TCAGGGTTAT GGAGATTCAT AAGTAGACGA ATCAAAGAGC   
  
  
- GGTAATGTCT TGTTCCCTTT GAGTAACTTG GGAAAATCAG TAAGTTTGAG AAAACGTAGA AAACAAAAAA   
  
  
- GTTGGGTTAA AGCTTTCAAA AGTTCGCATC TTCATTGTCT TTCGACTAAG AAAGTTCCCT TACGACTTAT   
  
  
- GGGTCAAGTT TAACTCGTAA AGGTCGAAGA GTATAGAAAG AGAGACGGCA ACAGTGGAAG AAGCGAAGAG   
  
  
- GTCAAGGCAG CATTTGGTCA GTAAAAGTAC CGGACTATGG TAGGCCGGAA CGAAAACTCT TGGGTAACCG   
  
  
- GGTAATTAAT AAGCACAACG AATGTTTAG

+     Unnamed\_\_1

| Site Name | Organism | Position | Strand | Matrix score. | sequence | function |
| --- | --- | --- | --- | --- | --- | --- |
| Unnamed\_\_1 | Zea mays | 782 | + | 5 | CGTGG |  |

> 2018/04/13 10:10:12  
+ ATTTGAATAT ATTAATTATT TATTATAAAA TTTTAAATAT TTAATATTAA AACAATTTAA AAATATTTGT   
  
  
+ AATACTAAAA AAATTTTAAA ATTTTTTGTG ATAATATCTT GAATATTTTT TTACAATTAT AAAAAGTTTT   
  
  
+ AAATAAAAAT TCGTAATATA AAATTTTTAA TTTGAATACT TTTATTTTAA AATTTTTTAA TAAAATATAA   
  
  
+ TTTGATTATA TTTCATCAAT CAGCTATGTT ACTTCAAAAT TTTAAATAAT ATTTGCTTTT AAATTTATTT   
  
  
+ TATTATTTCA TTTAATTATT ATATATTAAC ATTTATTTTA AAATAATATA TCTTTAAAAA ATATTTTTAA   
  
  
+ TTAAGCACTT ACTGTTAACA AGAAGTCTCA GGAAGGCAGT AGTGTCCAGG CCGGTGCGAA GTGCGTTCGC   
  
  
+ GAGTCTACGG CTCATGGCTC ACATTTCGGT CACAGCCTCG TAGGGGACCA CAAAAGTCTG GATCATACAT   
  
  
+ AGCGGCTCCT TGCGTCCCCG TTACGTTTTT CCTCAACGAC CCAGACACGG ATCTGGCATT TGAATCGGTC   
  
  
+ TATTTTTCGG CTTTTAACAT TTATTTATTT ATTATATTTC CCTAAAAATG GACCAAAATT ATCTTGTTAC   
  
  
+ TTTACCAAGT GTCTTAATTT TATATATTCC TCAATAAAAT AATATAAATA GAACGACATC GTTTACACGT   
  
  
+ AAGCAAGGGT CATGGAACTA ACGTCACACT TACTTTAAGA TCGTTTCTTC CTCTTTTTTT CTCACCTTTC   
  
  
+ TGTAGTAGTA ACGTGGAATT TGCCAAGTTT GAAGATTCTT TGGTCTTCTG GTTCCCAACA TCTTCCATTG   
  
  
+ GCTTCCGTTC TTTCCTCTAG AATCCTTTAT TACACTCGAC CAATGAGGAT TCAGGAACCT CAACGAAGAG   
  
  
+ CACTTGACAG AGAGATGGAG TTTCCTGTCT GGACTGAACA TAGAGAAGTG GTCAGCCGTT GGACTTCCCT   
  
  
+ TTTTTTTTTT TTTTATGTTG AACTCGAATT AGTATGACCA ACGACTGGTC TAATGAAGAA AACCCACTTC   
  
  
+ AGCTTTTCTC CAGTCAACCA TCTTCCTTCA ATTTTCTACG TTCAAAAGGA GGCAAATCCT TTAAAATCAC   
  
  
+ ATAACCTTAT TCTCCTTCTT CCACAGGTTA AGTCCCAATA CCTCTAAGTA TTCATCTGCT TAGTTTCTCG   
  
  
+ CCATTACAGA ACAAGGGAAA CTCATTGAAC CCTTTTAGTC ATTCAAACTC TTTTGCATCT TTTGTTTTTT   
  
  
+ CAACCCAATT TCGAAAGTTT TCAAGCGTAG AAGTAACAGA AAGCTGATTC TTTCAAGGGA ATGCTGAATA   
  
  
+ CCCAGTTCAA ATTGAGCATT TCCAGCTTCT CATATCTTTC TCTCTGCCGT TGTCACCTTC TTCGCTTCTC   
  
  
+ CAGTTCCGTC GTAAACCAGT CATTTTCATG GCCTGATACC ATCCGGCCTT GCTTTTGAGA ACCCATTGGC   
  
  
+ CCATTAATTA TTCGTGTTGC TTACAAATC  

- TAAACTTATA TAATTAATAA ATAATATTTT AAAATTTATA AATTATAATT TTGTTAAATT TTTATAAACA   
  
  
- TTATGATTTT TTTAAAATTT TAAAAAACAC TATTATAGAA CTTATAAAAA AATGTTAATA TTTTTCAAAA   
  
  
- TTTATTTTTA AGCATTATAT TTTAAAAATT AAACTTATGA AAATAAAATT TTAAAAAATT ATTTTATATT   
  
  
- AAACTAATAT AAAGTAGTTA GTCGATACAA TGAAGTTTTA AAATTTATTA TAAACGAAAA TTTAAATAAA   
  
  
- ATAATAAAGT AAATTAATAA TATATAATTG TAAATAAAAT TTTATTATAT AGAAATTTTT TATAAAAATT   
  
  
- AATTCGTGAA TGACAATTGT TCTTCAGAGT CCTTCCGTCA TCACAGGTCC GGCCACGCTT CACGCAAGCG   
  
  
- CTCAGATGCC GAGTACCGAG TGTAAAGCCA GTGTCGGAGC ATCCCCTGGT GTTTTCAGAC CTAGTATGTA   
  
  
- TCGCCGAGGA ACGCAGGGGC AATGCAAAAA GGAGTTGCTG GGTCTGTGCC TAGACCGTAA ACTTAGCCAG   
  
  
- ATAAAAAGCC GAAAATTGTA AATAAATAAA TAATATAAAG GGATTTTTAC CTGGTTTTAA TAGAACAATG   
  
  
- AAATGGTTCA CAGAATTAAA ATATATAAGG AGTTATTTTA TTATATTTAT CTTGCTGTAG CAAATGTGCA   
  
  
- TTCGTTCCCA GTACCTTGAT TGCAGTGTGA ATGAAATTCT AGCAAAGAAG GAGAAAAAAA GAGTGGAAAG   
  
  
- ACATCATCAT TGCACCTTAA ACGGTTCAAA CTTCTAAGAA ACCAGAAGAC CAAGGGTTGT AGAAGGTAAC   
  
  
- CGAAGGCAAG AAAGGAGATC TTAGGAAATA ATGTGAGCTG GTTACTCCTA AGTCCTTGGA GTTGCTTCTC   
  
  
- GTGAACTGTC TCTCTACCTC AAAGGACAGA CCTGACTTGT ATCTCTTCAC CAGTCGGCAA CCTGAAGGGA   
  
  
- AAAAAAAAAA AAAATACAAC TTGAGCTTAA TCATACTGGT TGCTGACCAG ATTACTTCTT TTGGGTGAAG   
  
  
- TCGAAAAGAG GTCAGTTGGT AGAAGGAAGT TAAAAGATGC AAGTTTTCCT CCGTTTAGGA AATTTTAGTG   
  
  
- TATTGGAATA AGAGGAAGAA GGTGTCCAAT TCAGGGTTAT GGAGATTCAT AAGTAGACGA ATCAAAGAGC   
  
  
- GGTAATGTCT TGTTCCCTTT GAGTAACTTG GGAAAATCAG TAAGTTTGAG AAAACGTAGA AAACAAAAAA   
  
  
- GTTGGGTTAA AGCTTTCAAA AGTTCGCATC TTCATTGTCT TTCGACTAAG AAAGTTCCCT TACGACTTAT   
  
  
- GGGTCAAGTT TAACTCGTAA AGGTCGAAGA GTATAGAAAG AGAGACGGCA ACAGTGGAAG AAGCGAAGAG   
  
  
- GTCAAGGCAG CATTTGGTCA GTAAAAGTAC CGGACTATGG TAGGCCGGAA CGAAAACTCT TGGGTAACCG   
  
  
- GGTAATTAAT AAGCACAACG AATGTTTAG

+     Unnamed\_\_3

| Site Name | Organism | Position | Strand | Matrix score. | sequence | function |
| --- | --- | --- | --- | --- | --- | --- |
| Unnamed\_\_3 | Zea mays | 782 | + | 5 | CGTGG |  |

> 2018/04/13 10:10:12  
+ ATTTGAATAT ATTAATTATT TATTATAAAA TTTTAAATAT TTAATATTAA AACAATTTAA AAATATTTGT   
  
  
+ AATACTAAAA AAATTTTAAA ATTTTTTGTG ATAATATCTT GAATATTTTT TTACAATTAT AAAAAGTTTT   
  
  
+ AAATAAAAAT TCGTAATATA AAATTTTTAA TTTGAATACT TTTATTTTAA AATTTTTTAA TAAAATATAA   
  
  
+ TTTGATTATA TTTCATCAAT CAGCTATGTT ACTTCAAAAT TTTAAATAAT ATTTGCTTTT AAATTTATTT   
  
  
+ TATTATTTCA TTTAATTATT ATATATTAAC ATTTATTTTA AAATAATATA TCTTTAAAAA ATATTTTTAA   
  
  
+ TTAAGCACTT ACTGTTAACA AGAAGTCTCA GGAAGGCAGT AGTGTCCAGG CCGGTGCGAA GTGCGTTCGC   
  
  
+ GAGTCTACGG CTCATGGCTC ACATTTCGGT CACAGCCTCG TAGGGGACCA CAAAAGTCTG GATCATACAT   
  
  
+ AGCGGCTCCT TGCGTCCCCG TTACGTTTTT CCTCAACGAC CCAGACACGG ATCTGGCATT TGAATCGGTC   
  
  
+ TATTTTTCGG CTTTTAACAT TTATTTATTT ATTATATTTC CCTAAAAATG GACCAAAATT ATCTTGTTAC   
  
  
+ TTTACCAAGT GTCTTAATTT TATATATTCC TCAATAAAAT AATATAAATA GAACGACATC GTTTACACGT   
  
  
+ AAGCAAGGGT CATGGAACTA ACGTCACACT TACTTTAAGA TCGTTTCTTC CTCTTTTTTT CTCACCTTTC   
  
  
+ TGTAGTAGTA ACGTGGAATT TGCCAAGTTT GAAGATTCTT TGGTCTTCTG GTTCCCAACA TCTTCCATTG   
  
  
+ GCTTCCGTTC TTTCCTCTAG AATCCTTTAT TACACTCGAC CAATGAGGAT TCAGGAACCT CAACGAAGAG   
  
  
+ CACTTGACAG AGAGATGGAG TTTCCTGTCT GGACTGAACA TAGAGAAGTG GTCAGCCGTT GGACTTCCCT   
  
  
+ TTTTTTTTTT TTTTATGTTG AACTCGAATT AGTATGACCA ACGACTGGTC TAATGAAGAA AACCCACTTC   
  
  
+ AGCTTTTCTC CAGTCAACCA TCTTCCTTCA ATTTTCTACG TTCAAAAGGA GGCAAATCCT TTAAAATCAC   
  
  
+ ATAACCTTAT TCTCCTTCTT CCACAGGTTA AGTCCCAATA CCTCTAAGTA TTCATCTGCT TAGTTTCTCG   
  
  
+ CCATTACAGA ACAAGGGAAA CTCATTGAAC CCTTTTAGTC ATTCAAACTC TTTTGCATCT TTTGTTTTTT   
  
  
+ CAACCCAATT TCGAAAGTTT TCAAGCGTAG AAGTAACAGA AAGCTGATTC TTTCAAGGGA ATGCTGAATA   
  
  
+ CCCAGTTCAA ATTGAGCATT TCCAGCTTCT CATATCTTTC TCTCTGCCGT TGTCACCTTC TTCGCTTCTC   
  
  
+ CAGTTCCGTC GTAAACCAGT CATTTTCATG GCCTGATACC ATCCGGCCTT GCTTTTGAGA ACCCATTGGC   
  
  
+ CCATTAATTA TTCGTGTTGC TTACAAATC  

- TAAACTTATA TAATTAATAA ATAATATTTT AAAATTTATA AATTATAATT TTGTTAAATT TTTATAAACA   
  
  
- TTATGATTTT TTTAAAATTT TAAAAAACAC TATTATAGAA CTTATAAAAA AATGTTAATA TTTTTCAAAA   
  
  
- TTTATTTTTA AGCATTATAT TTTAAAAATT AAACTTATGA AAATAAAATT TTAAAAAATT ATTTTATATT   
  
  
- AAACTAATAT AAAGTAGTTA GTCGATACAA TGAAGTTTTA AAATTTATTA TAAACGAAAA TTTAAATAAA   
  
  
- ATAATAAAGT AAATTAATAA TATATAATTG TAAATAAAAT TTTATTATAT AGAAATTTTT TATAAAAATT   
  
  
- AATTCGTGAA TGACAATTGT TCTTCAGAGT CCTTCCGTCA TCACAGGTCC GGCCACGCTT CACGCAAGCG   
  
  
- CTCAGATGCC GAGTACCGAG TGTAAAGCCA GTGTCGGAGC ATCCCCTGGT GTTTTCAGAC CTAGTATGTA   
  
  
- TCGCCGAGGA ACGCAGGGGC AATGCAAAAA GGAGTTGCTG GGTCTGTGCC TAGACCGTAA ACTTAGCCAG   
  
  
- ATAAAAAGCC GAAAATTGTA AATAAATAAA TAATATAAAG GGATTTTTAC CTGGTTTTAA TAGAACAATG   
  
  
- AAATGGTTCA CAGAATTAAA ATATATAAGG AGTTATTTTA TTATATTTAT CTTGCTGTAG CAAATGTGCA   
  
  
- TTCGTTCCCA GTACCTTGAT TGCAGTGTGA ATGAAATTCT AGCAAAGAAG GAGAAAAAAA GAGTGGAAAG   
  
  
- ACATCATCAT TGCACCTTAA ACGGTTCAAA CTTCTAAGAA ACCAGAAGAC CAAGGGTTGT AGAAGGTAAC   
  
  
- CGAAGGCAAG AAAGGAGATC TTAGGAAATA ATGTGAGCTG GTTACTCCTA AGTCCTTGGA GTTGCTTCTC   
  
  
- GTGAACTGTC TCTCTACCTC AAAGGACAGA CCTGACTTGT ATCTCTTCAC CAGTCGGCAA CCTGAAGGGA   
  
  
- AAAAAAAAAA AAAATACAAC TTGAGCTTAA TCATACTGGT TGCTGACCAG ATTACTTCTT TTGGGTGAAG   
  
  
- TCGAAAAGAG GTCAGTTGGT AGAAGGAAGT TAAAAGATGC AAGTTTTCCT CCGTTTAGGA AATTTTAGTG   
  
  
- TATTGGAATA AGAGGAAGAA GGTGTCCAAT TCAGGGTTAT GGAGATTCAT AAGTAGACGA ATCAAAGAGC   
  
  
- GGTAATGTCT TGTTCCCTTT GAGTAACTTG GGAAAATCAG TAAGTTTGAG AAAACGTAGA AAACAAAAAA   
  
  
- GTTGGGTTAA AGCTTTCAAA AGTTCGCATC TTCATTGTCT TTCGACTAAG AAAGTTCCCT TACGACTTAT   
  
  
- GGGTCAAGTT TAACTCGTAA AGGTCGAAGA GTATAGAAAG AGAGACGGCA ACAGTGGAAG AAGCGAAGAG   
  
  
- GTCAAGGCAG CATTTGGTCA GTAAAAGTAC CGGACTATGG TAGGCCGGAA CGAAAACTCT TGGGTAACCG   
  
  
- GGTAATTAAT AAGCACAACG AATGTTTAG

+     Unnamed\_\_4

| Site Name | Organism | Position | Strand | Matrix score. | sequence | function |
| --- | --- | --- | --- | --- | --- | --- |
| Unnamed\_\_4 | Petroselinum hortense | 1398 | + | 4 | CTCC |  |
| Unnamed\_\_4 | Petroselinum hortense | 927 | - | 4 | CTCC |  |
| Unnamed\_\_4 | Petroselinum hortense | 496 | + | 4 | CTCC |  |
| Unnamed\_\_4 | Petroselinum hortense | 1098 | - | 4 | CTCC |  |
| Unnamed\_\_4 | Petroselinum hortense | 1132 | + | 4 | CTCC |  |
| Unnamed\_\_4 | Petroselinum hortense | 1058 | + | 4 | CTCC |  |

> 2018/04/13 10:10:12  
+ ATTTGAATAT ATTAATTATT TATTATAAAA TTTTAAATAT TTAATATTAA AACAATTTAA AAATATTTGT   
  
  
+ AATACTAAAA AAATTTTAAA ATTTTTTGTG ATAATATCTT GAATATTTTT TTACAATTAT AAAAAGTTTT   
  
  
+ AAATAAAAAT TCGTAATATA AAATTTTTAA TTTGAATACT TTTATTTTAA AATTTTTTAA TAAAATATAA   
  
  
+ TTTGATTATA TTTCATCAAT CAGCTATGTT ACTTCAAAAT TTTAAATAAT ATTTGCTTTT AAATTTATTT   
  
  
+ TATTATTTCA TTTAATTATT ATATATTAAC ATTTATTTTA AAATAATATA TCTTTAAAAA ATATTTTTAA   
  
  
+ TTAAGCACTT ACTGTTAACA AGAAGTCTCA GGAAGGCAGT AGTGTCCAGG CCGGTGCGAA GTGCGTTCGC   
  
  
+ GAGTCTACGG CTCATGGCTC ACATTTCGGT CACAGCCTCG TAGGGGACCA CAAAAGTCTG GATCATACAT   
  
  
+ AGCGGCTCCT TGCGTCCCCG TTACGTTTTT CCTCAACGAC CCAGACACGG ATCTGGCATT TGAATCGGTC   
  
  
+ TATTTTTCGG CTTTTAACAT TTATTTATTT ATTATATTTC CCTAAAAATG GACCAAAATT ATCTTGTTAC   
  
  
+ TTTACCAAGT GTCTTAATTT TATATATTCC TCAATAAAAT AATATAAATA GAACGACATC GTTTACACGT   
  
  
+ AAGCAAGGGT CATGGAACTA ACGTCACACT TACTTTAAGA TCGTTTCTTC CTCTTTTTTT CTCACCTTTC   
  
  
+ TGTAGTAGTA ACGTGGAATT TGCCAAGTTT GAAGATTCTT TGGTCTTCTG GTTCCCAACA TCTTCCATTG   
  
  
+ GCTTCCGTTC TTTCCTCTAG AATCCTTTAT TACACTCGAC CAATGAGGAT TCAGGAACCT CAACGAAGAG   
  
  
+ CACTTGACAG AGAGATGGAG TTTCCTGTCT GGACTGAACA TAGAGAAGTG GTCAGCCGTT GGACTTCCCT   
  
  
+ TTTTTTTTTT TTTTATGTTG AACTCGAATT AGTATGACCA ACGACTGGTC TAATGAAGAA AACCCACTTC   
  
  
+ AGCTTTTCTC CAGTCAACCA TCTTCCTTCA ATTTTCTACG TTCAAAAGGA GGCAAATCCT TTAAAATCAC   
  
  
+ ATAACCTTAT TCTCCTTCTT CCACAGGTTA AGTCCCAATA CCTCTAAGTA TTCATCTGCT TAGTTTCTCG   
  
  
+ CCATTACAGA ACAAGGGAAA CTCATTGAAC CCTTTTAGTC ATTCAAACTC TTTTGCATCT TTTGTTTTTT   
  
  
+ CAACCCAATT TCGAAAGTTT TCAAGCGTAG AAGTAACAGA AAGCTGATTC TTTCAAGGGA ATGCTGAATA   
  
  
+ CCCAGTTCAA ATTGAGCATT TCCAGCTTCT CATATCTTTC TCTCTGCCGT TGTCACCTTC TTCGCTTCTC   
  
  
+ CAGTTCCGTC GTAAACCAGT CATTTTCATG GCCTGATACC ATCCGGCCTT GCTTTTGAGA ACCCATTGGC   
  
  
+ CCATTAATTA TTCGTGTTGC TTACAAATC  

- TAAACTTATA TAATTAATAA ATAATATTTT AAAATTTATA AATTATAATT TTGTTAAATT TTTATAAACA   
  
  
- TTATGATTTT TTTAAAATTT TAAAAAACAC TATTATAGAA CTTATAAAAA AATGTTAATA TTTTTCAAAA   
  
  
- TTTATTTTTA AGCATTATAT TTTAAAAATT AAACTTATGA AAATAAAATT TTAAAAAATT ATTTTATATT   
  
  
- AAACTAATAT AAAGTAGTTA GTCGATACAA TGAAGTTTTA AAATTTATTA TAAACGAAAA TTTAAATAAA   
  
  
- ATAATAAAGT AAATTAATAA TATATAATTG TAAATAAAAT TTTATTATAT AGAAATTTTT TATAAAAATT   
  
  
- AATTCGTGAA TGACAATTGT TCTTCAGAGT CCTTCCGTCA TCACAGGTCC GGCCACGCTT CACGCAAGCG   
  
  
- CTCAGATGCC GAGTACCGAG TGTAAAGCCA GTGTCGGAGC ATCCCCTGGT GTTTTCAGAC CTAGTATGTA   
  
  
- TCGCCGAGGA ACGCAGGGGC AATGCAAAAA GGAGTTGCTG GGTCTGTGCC TAGACCGTAA ACTTAGCCAG   
  
  
- ATAAAAAGCC GAAAATTGTA AATAAATAAA TAATATAAAG GGATTTTTAC CTGGTTTTAA TAGAACAATG   
  
  
- AAATGGTTCA CAGAATTAAA ATATATAAGG AGTTATTTTA TTATATTTAT CTTGCTGTAG CAAATGTGCA   
  
  
- TTCGTTCCCA GTACCTTGAT TGCAGTGTGA ATGAAATTCT AGCAAAGAAG GAGAAAAAAA GAGTGGAAAG   
  
  
- ACATCATCAT TGCACCTTAA ACGGTTCAAA CTTCTAAGAA ACCAGAAGAC CAAGGGTTGT AGAAGGTAAC   
  
  
- CGAAGGCAAG AAAGGAGATC TTAGGAAATA ATGTGAGCTG GTTACTCCTA AGTCCTTGGA GTTGCTTCTC   
  
  
- GTGAACTGTC TCTCTACCTC AAAGGACAGA CCTGACTTGT ATCTCTTCAC CAGTCGGCAA CCTGAAGGGA   
  
  
- AAAAAAAAAA AAAATACAAC TTGAGCTTAA TCATACTGGT TGCTGACCAG ATTACTTCTT TTGGGTGAAG   
  
  
- TCGAAAAGAG GTCAGTTGGT AGAAGGAAGT TAAAAGATGC AAGTTTTCCT CCGTTTAGGA AATTTTAGTG   
  
  
- TATTGGAATA AGAGGAAGAA GGTGTCCAAT TCAGGGTTAT GGAGATTCAT AAGTAGACGA ATCAAAGAGC   
  
  
- GGTAATGTCT TGTTCCCTTT GAGTAACTTG GGAAAATCAG TAAGTTTGAG AAAACGTAGA AAACAAAAAA   
  
  
- GTTGGGTTAA AGCTTTCAAA AGTTCGCATC TTCATTGTCT TTCGACTAAG AAAGTTCCCT TACGACTTAT   
  
  
- GGGTCAAGTT TAACTCGTAA AGGTCGAAGA GTATAGAAAG AGAGACGGCA ACAGTGGAAG AAGCGAAGAG   
  
  
- GTCAAGGCAG CATTTGGTCA GTAAAAGTAC CGGACTATGG TAGGCCGGAA CGAAAACTCT TGGGTAACCG   
  
  
- GGTAATTAAT AAGCACAACG AATGTTTAG

+     circadian

| Site Name | Organism | Position | Strand | Matrix score. | sequence | function |
| --- | --- | --- | --- | --- | --- | --- |
| circadian | Lycopersicon esculentum | 614 | + | 6 | CAANNNNATC | cis-acting regulatory element involved in circadian control |

> 2018/04/13 10:10:12  
+ ATTTGAATAT ATTAATTATT TATTATAAAA TTTTAAATAT TTAATATTAA AACAATTTAA AAATATTTGT   
  
  
+ AATACTAAAA AAATTTTAAA ATTTTTTGTG ATAATATCTT GAATATTTTT TTACAATTAT AAAAAGTTTT   
  
  
+ AAATAAAAAT TCGTAATATA AAATTTTTAA TTTGAATACT TTTATTTTAA AATTTTTTAA TAAAATATAA   
  
  
+ TTTGATTATA TTTCATCAAT CAGCTATGTT ACTTCAAAAT TTTAAATAAT ATTTGCTTTT AAATTTATTT   
  
  
+ TATTATTTCA TTTAATTATT ATATATTAAC ATTTATTTTA AAATAATATA TCTTTAAAAA ATATTTTTAA   
  
  
+ TTAAGCACTT ACTGTTAACA AGAAGTCTCA GGAAGGCAGT AGTGTCCAGG CCGGTGCGAA GTGCGTTCGC   
  
  
+ GAGTCTACGG CTCATGGCTC ACATTTCGGT CACAGCCTCG TAGGGGACCA CAAAAGTCTG GATCATACAT   
  
  
+ AGCGGCTCCT TGCGTCCCCG TTACGTTTTT CCTCAACGAC CCAGACACGG ATCTGGCATT TGAATCGGTC   
  
  
+ TATTTTTCGG CTTTTAACAT TTATTTATTT ATTATATTTC CCTAAAAATG GACCAAAATT ATCTTGTTAC   
  
  
+ TTTACCAAGT GTCTTAATTT TATATATTCC TCAATAAAAT AATATAAATA GAACGACATC GTTTACACGT   
  
  
+ AAGCAAGGGT CATGGAACTA ACGTCACACT TACTTTAAGA TCGTTTCTTC CTCTTTTTTT CTCACCTTTC   
  
  
+ TGTAGTAGTA ACGTGGAATT TGCCAAGTTT GAAGATTCTT TGGTCTTCTG GTTCCCAACA TCTTCCATTG   
  
  
+ GCTTCCGTTC TTTCCTCTAG AATCCTTTAT TACACTCGAC CAATGAGGAT TCAGGAACCT CAACGAAGAG   
  
  
+ CACTTGACAG AGAGATGGAG TTTCCTGTCT GGACTGAACA TAGAGAAGTG GTCAGCCGTT GGACTTCCCT   
  
  
+ TTTTTTTTTT TTTTATGTTG AACTCGAATT AGTATGACCA ACGACTGGTC TAATGAAGAA AACCCACTTC   
  
  
+ AGCTTTTCTC CAGTCAACCA TCTTCCTTCA ATTTTCTACG TTCAAAAGGA GGCAAATCCT TTAAAATCAC   
  
  
+ ATAACCTTAT TCTCCTTCTT CCACAGGTTA AGTCCCAATA CCTCTAAGTA TTCATCTGCT TAGTTTCTCG   
  
  
+ CCATTACAGA ACAAGGGAAA CTCATTGAAC CCTTTTAGTC ATTCAAACTC TTTTGCATCT TTTGTTTTTT   
  
  
+ CAACCCAATT TCGAAAGTTT TCAAGCGTAG AAGTAACAGA AAGCTGATTC TTTCAAGGGA ATGCTGAATA   
  
  
+ CCCAGTTCAA ATTGAGCATT TCCAGCTTCT CATATCTTTC TCTCTGCCGT TGTCACCTTC TTCGCTTCTC   
  
  
+ CAGTTCCGTC GTAAACCAGT CATTTTCATG GCCTGATACC ATCCGGCCTT GCTTTTGAGA ACCCATTGGC   
  
  
+ CCATTAATTA TTCGTGTTGC TTACAAATC  

- TAAACTTATA TAATTAATAA ATAATATTTT AAAATTTATA AATTATAATT TTGTTAAATT TTTATAAACA   
  
  
- TTATGATTTT TTTAAAATTT TAAAAAACAC TATTATAGAA CTTATAAAAA AATGTTAATA TTTTTCAAAA   
  
  
- TTTATTTTTA AGCATTATAT TTTAAAAATT AAACTTATGA AAATAAAATT TTAAAAAATT ATTTTATATT   
  
  
- AAACTAATAT AAAGTAGTTA GTCGATACAA TGAAGTTTTA AAATTTATTA TAAACGAAAA TTTAAATAAA   
  
  
- ATAATAAAGT AAATTAATAA TATATAATTG TAAATAAAAT TTTATTATAT AGAAATTTTT TATAAAAATT   
  
  
- AATTCGTGAA TGACAATTGT TCTTCAGAGT CCTTCCGTCA TCACAGGTCC GGCCACGCTT CACGCAAGCG   
  
  
- CTCAGATGCC GAGTACCGAG TGTAAAGCCA GTGTCGGAGC ATCCCCTGGT GTTTTCAGAC CTAGTATGTA   
  
  
- TCGCCGAGGA ACGCAGGGGC AATGCAAAAA GGAGTTGCTG GGTCTGTGCC TAGACCGTAA ACTTAGCCAG   
  
  
- ATAAAAAGCC GAAAATTGTA AATAAATAAA TAATATAAAG GGATTTTTAC CTGGTTTTAA TAGAACAATG   
  
  
- AAATGGTTCA CAGAATTAAA ATATATAAGG AGTTATTTTA TTATATTTAT CTTGCTGTAG CAAATGTGCA   
  
  
- TTCGTTCCCA GTACCTTGAT TGCAGTGTGA ATGAAATTCT AGCAAAGAAG GAGAAAAAAA GAGTGGAAAG   
  
  
- ACATCATCAT TGCACCTTAA ACGGTTCAAA CTTCTAAGAA ACCAGAAGAC CAAGGGTTGT AGAAGGTAAC   
  
  
- CGAAGGCAAG AAAGGAGATC TTAGGAAATA ATGTGAGCTG GTTACTCCTA AGTCCTTGGA GTTGCTTCTC   
  
  
- GTGAACTGTC TCTCTACCTC AAAGGACAGA CCTGACTTGT ATCTCTTCAC CAGTCGGCAA CCTGAAGGGA   
  
  
- AAAAAAAAAA AAAATACAAC TTGAGCTTAA TCATACTGGT TGCTGACCAG ATTACTTCTT TTGGGTGAAG   
  
  
- TCGAAAAGAG GTCAGTTGGT AGAAGGAAGT TAAAAGATGC AAGTTTTCCT CCGTTTAGGA AATTTTAGTG   
  
  
- TATTGGAATA AGAGGAAGAA GGTGTCCAAT TCAGGGTTAT GGAGATTCAT AAGTAGACGA ATCAAAGAGC   
  
  
- GGTAATGTCT TGTTCCCTTT GAGTAACTTG GGAAAATCAG TAAGTTTGAG AAAACGTAGA AAACAAAAAA   
  
  
- GTTGGGTTAA AGCTTTCAAA AGTTCGCATC TTCATTGTCT TTCGACTAAG AAAGTTCCCT TACGACTTAT   
  
  
- GGGTCAAGTT TAACTCGTAA AGGTCGAAGA GTATAGAAAG AGAGACGGCA ACAGTGGAAG AAGCGAAGAG   
  
  
- GTCAAGGCAG CATTTGGTCA GTAAAAGTAC CGGACTATGG TAGGCCGGAA CGAAAACTCT TGGGTAACCG   
  
  
- GGTAATTAAT AAGCACAACG AATGTTTAG

+     dOCT

| Site Name | Organism | Position | Strand | Matrix score. | sequence | function |
| --- | --- | --- | --- | --- | --- | --- |
| dOCT | Arabidopsis thaliana | 536 | + | 8 | CaCGGATC | cis-acting regulatory element related to meristem specific activation |

> 2018/04/13 10:10:12  
+ ATTTGAATAT ATTAATTATT TATTATAAAA TTTTAAATAT TTAATATTAA AACAATTTAA AAATATTTGT   
  
  
+ AATACTAAAA AAATTTTAAA ATTTTTTGTG ATAATATCTT GAATATTTTT TTACAATTAT AAAAAGTTTT   
  
  
+ AAATAAAAAT TCGTAATATA AAATTTTTAA TTTGAATACT TTTATTTTAA AATTTTTTAA TAAAATATAA   
  
  
+ TTTGATTATA TTTCATCAAT CAGCTATGTT ACTTCAAAAT TTTAAATAAT ATTTGCTTTT AAATTTATTT   
  
  
+ TATTATTTCA TTTAATTATT ATATATTAAC ATTTATTTTA AAATAATATA TCTTTAAAAA ATATTTTTAA   
  
  
+ TTAAGCACTT ACTGTTAACA AGAAGTCTCA GGAAGGCAGT AGTGTCCAGG CCGGTGCGAA GTGCGTTCGC   
  
  
+ GAGTCTACGG CTCATGGCTC ACATTTCGGT CACAGCCTCG TAGGGGACCA CAAAAGTCTG GATCATACAT   
  
  
+ AGCGGCTCCT TGCGTCCCCG TTACGTTTTT CCTCAACGAC CCAGACACGG ATCTGGCATT TGAATCGGTC   
  
  
+ TATTTTTCGG CTTTTAACAT TTATTTATTT ATTATATTTC CCTAAAAATG GACCAAAATT ATCTTGTTAC   
  
  
+ TTTACCAAGT GTCTTAATTT TATATATTCC TCAATAAAAT AATATAAATA GAACGACATC GTTTACACGT   
  
  
+ AAGCAAGGGT CATGGAACTA ACGTCACACT TACTTTAAGA TCGTTTCTTC CTCTTTTTTT CTCACCTTTC   
  
  
+ TGTAGTAGTA ACGTGGAATT TGCCAAGTTT GAAGATTCTT TGGTCTTCTG GTTCCCAACA TCTTCCATTG   
  
  
+ GCTTCCGTTC TTTCCTCTAG AATCCTTTAT TACACTCGAC CAATGAGGAT TCAGGAACCT CAACGAAGAG   
  
  
+ CACTTGACAG AGAGATGGAG TTTCCTGTCT GGACTGAACA TAGAGAAGTG GTCAGCCGTT GGACTTCCCT   
  
  
+ TTTTTTTTTT TTTTATGTTG AACTCGAATT AGTATGACCA ACGACTGGTC TAATGAAGAA AACCCACTTC   
  
  
+ AGCTTTTCTC CAGTCAACCA TCTTCCTTCA ATTTTCTACG TTCAAAAGGA GGCAAATCCT TTAAAATCAC   
  
  
+ ATAACCTTAT TCTCCTTCTT CCACAGGTTA AGTCCCAATA CCTCTAAGTA TTCATCTGCT TAGTTTCTCG   
  
  
+ CCATTACAGA ACAAGGGAAA CTCATTGAAC CCTTTTAGTC ATTCAAACTC TTTTGCATCT TTTGTTTTTT   
  
  
+ CAACCCAATT TCGAAAGTTT TCAAGCGTAG AAGTAACAGA AAGCTGATTC TTTCAAGGGA ATGCTGAATA   
  
  
+ CCCAGTTCAA ATTGAGCATT TCCAGCTTCT CATATCTTTC TCTCTGCCGT TGTCACCTTC TTCGCTTCTC   
  
  
+ CAGTTCCGTC GTAAACCAGT CATTTTCATG GCCTGATACC ATCCGGCCTT GCTTTTGAGA ACCCATTGGC   
  
  
+ CCATTAATTA TTCGTGTTGC TTACAAATC  

- TAAACTTATA TAATTAATAA ATAATATTTT AAAATTTATA AATTATAATT TTGTTAAATT TTTATAAACA   
  
  
- TTATGATTTT TTTAAAATTT TAAAAAACAC TATTATAGAA CTTATAAAAA AATGTTAATA TTTTTCAAAA   
  
  
- TTTATTTTTA AGCATTATAT TTTAAAAATT AAACTTATGA AAATAAAATT TTAAAAAATT ATTTTATATT   
  
  
- AAACTAATAT AAAGTAGTTA GTCGATACAA TGAAGTTTTA AAATTTATTA TAAACGAAAA TTTAAATAAA   
  
  
- ATAATAAAGT AAATTAATAA TATATAATTG TAAATAAAAT TTTATTATAT AGAAATTTTT TATAAAAATT   
  
  
- AATTCGTGAA TGACAATTGT TCTTCAGAGT CCTTCCGTCA TCACAGGTCC GGCCACGCTT CACGCAAGCG   
  
  
- CTCAGATGCC GAGTACCGAG TGTAAAGCCA GTGTCGGAGC ATCCCCTGGT GTTTTCAGAC CTAGTATGTA   
  
  
- TCGCCGAGGA ACGCAGGGGC AATGCAAAAA GGAGTTGCTG GGTCTGTGCC TAGACCGTAA ACTTAGCCAG   
  
  
- ATAAAAAGCC GAAAATTGTA AATAAATAAA TAATATAAAG GGATTTTTAC CTGGTTTTAA TAGAACAATG   
  
  
- AAATGGTTCA CAGAATTAAA ATATATAAGG AGTTATTTTA TTATATTTAT CTTGCTGTAG CAAATGTGCA   
  
  
- TTCGTTCCCA GTACCTTGAT TGCAGTGTGA ATGAAATTCT AGCAAAGAAG GAGAAAAAAA GAGTGGAAAG   
  
  
- ACATCATCAT TGCACCTTAA ACGGTTCAAA CTTCTAAGAA ACCAGAAGAC CAAGGGTTGT AGAAGGTAAC   
  
  
- CGAAGGCAAG AAAGGAGATC TTAGGAAATA ATGTGAGCTG GTTACTCCTA AGTCCTTGGA GTTGCTTCTC   
  
  
- GTGAACTGTC TCTCTACCTC AAAGGACAGA CCTGACTTGT ATCTCTTCAC CAGTCGGCAA CCTGAAGGGA   
  
  
- AAAAAAAAAA AAAATACAAC TTGAGCTTAA TCATACTGGT TGCTGACCAG ATTACTTCTT TTGGGTGAAG   
  
  
- TCGAAAAGAG GTCAGTTGGT AGAAGGAAGT TAAAAGATGC AAGTTTTCCT CCGTTTAGGA AATTTTAGTG   
  
  
- TATTGGAATA AGAGGAAGAA GGTGTCCAAT TCAGGGTTAT GGAGATTCAT AAGTAGACGA ATCAAAGAGC   
  
  
- GGTAATGTCT TGTTCCCTTT GAGTAACTTG GGAAAATCAG TAAGTTTGAG AAAACGTAGA AAACAAAAAA   
  
  
- GTTGGGTTAA AGCTTTCAAA AGTTCGCATC TTCATTGTCT TTCGACTAAG AAAGTTCCCT TACGACTTAT   
  
  
- GGGTCAAGTT TAACTCGTAA AGGTCGAAGA GTATAGAAAG AGAGACGGCA ACAGTGGAAG AAGCGAAGAG   
  
  
- GTCAAGGCAG CATTTGGTCA GTAAAAGTAC CGGACTATGG TAGGCCGGAA CGAAAACTCT TGGGTAACCG   
  
  
- GGTAATTAAT AAGCACAACG AATGTTTAG
