# Supplementary material for: Genome-wide analysis of the WRKY gene family in drumstick (Moringa oleifera Lam.)
Source: PeerJ. 2019 Jun 10;7:e7063. doi: 10.7717/peerj.7063 (PMC6563795; doi:10.7717/peerj.7063)
Supplement: Supplemental Information 1 [file peerj-07-7063-s003.gz › MoWRKY4_plantcare.html]

Content-Type: text/html; charset=ISO-8859-1


CallMat\_Firefox


Webmaster Firefox specific output  
To save the result:
click on the frame with the right mouse button and save the source code as a text file with extension .html  
REFERENCE:PlantCARE: a database of plant cis-acting regulatory elements and a portal to tools for in silico analysis of promoter sequences.  
Lescot, M., Déhais, P., Moreau, Y., De Moor, B., Rouzé ,P.,and Rombauts, S.  
Nucleic Acids Res., Database issue(2002), 30(1):325-327.   


---

> 2018/04/13 10:10:12  
+ ATTATCGCAC TTTATCCACT AAGAACCTAA TTACCCAACT AGTGGGTTAA CTTGTAGGCT AAGCTTACAT   
  
  
+ ATCTGCTTAT GTTCTCTCCA TTTTGTGTGA AAACGTGTAG ACTTAATCTA ACCAAATCAT GATTGTGACT   
  
  
+ TTGATTATGA AATAAAAGGT CTATGGTTTA ATTAAATTAA TTACAATATG ACGTAAACTA AACATATACC   
  
  
+ TTTAATGGCT CTGGAGCTTT GTGTCATTTT TTATTTCCAC GATCATTAGG GCAAAAATTG GTTGAGAAAT   
  
  
+ TATAATTTAG AGTATAAGAT ACTCTGGCTA GCTTTTGGAT TTCATTGACT AAAAAGGCAT TACCCCTTTT   
  
  
+ ACACTTTAGG ACTCCTAGAA TCTTCTAAGC TAGACTTAAT TATATCTTTT AATAACTTTT TTAATGCTTG   
  
  
+ TTTGACACTA TTGCATGTTA GCCTCTAAGC TTTATTTTAT CGAGATCCAT ATAAATGTTT TTTTTTGTTT   
  
  
+ TTTCCGGTTT TACATTGAGA TAAAAATCAT TTACTAATTT GAAATCAAAT TGATTTTTTC ATTAAAAAAT   
  
  
+ TTAATGGTTA TTTTTATAAT TTAAAATAAC AATATAAAAT AAAAAAATTA ATTATTGATT TTATTAAATT   
  
  
+ GTGGTGCGTT TATTTGAAAG TGTATATGTC ATATTTCATA ATTTTACATT ATAAATAAAA ATATATAAAA   
  
  
+ TACATATAAT TAGTTGTGTT ATTTCTGGAC ATCACTTACA AAATGTAAAT ATGAAACAAA AATTTTGCGC   
  
  
+ ATTTAAAATA TATAAAATAC ATATAATTAG TTGTGTTATT TCTGGACATC ACTTACAAAA TGTAAATATG   
  
  
+ AAACAAAAAT TTTGCGCATT TAAGTAATTA TTATTAACTA TTTTTAATGA TTATAAGATA AATACATTTA   
  
  
+ TTTAATTAAT AATTTTTTAG AAGAAATATA TTTAATCTAA ACTTTTATGG GATATGCATG GATGGCATGA   
  
  
+ AATTTGCACT TGTCATATGC CTCCCTCCTA CACTAAAGCC AATAGGCATC AGCAGCCATT TACGGCACTG   
  
  
+ AGAAGGATAA ATATTAGATA TGTGATTGGG CCTCAGCCGC CCGCTGGTCA AAGTCACGTG CTTTGACCAA   
  
  
+ ACCACGCGGG CCAAAACACA CACACGCCCA CACACACTCT CTCTCTCTCT CTTTCTTCCC GTATTTTTCA   
  
  
+ CGGTCAAACT TCTCGTTACC TTCGCAGAAA CTGCCTTGCA AAGAAAAAAC ACACTCTCTC TCTCTCTCTT   
  
  
+ TCTTCCCGTA TTTTTCACGG TCAAACTTCT CGTTACCTTC GCAGAAACTG CCTTGCAAAG AAAAAGAAGA   
  
  
+ AAACTAGAAA AAAAAGAGAG AAAAACCAAA GAAGCAGAGA CGCCTTGTAC CTTACGTGAC CGTCTTCTCC   
  
  
+ GTTCTCTCTC TCTCTCTCTC CCCCCCTTGA TCCTTATATT CATCGTATAA GCTGTCTTCT TTCTGTACCT   
  
  
+ GTAACCTTAG TTCTCTCTCT GTCGCTTTT  

- TAATAGCGTG AAATAGGTGA TTCTTGGATT AATGGGTTGA TCACCCAATT GAACATCCGA TTCGAATGTA   
  
  
- TAGACGAATA CAAGAGAGGT AAAACACACT TTTGCACATC TGAATTAGAT TGGTTTAGTA CTAACACTGA   
  
  
- AACTAATACT TTATTTTCCA GATACCAAAT TAATTTAATT AATGTTATAC TGCATTTGAT TTGTATATGG   
  
  
- AAATTACCGA GACCTCGAAA CACAGTAAAA AATAAAGGTG CTAGTAATCC CGTTTTTAAC CAACTCTTTA   
  
  
- ATATTAAATC TCATATTCTA TGAGACCGAT CGAAAACCTA AAGTAACTGA TTTTTCCGTA ATGGGGAAAA   
  
  
- TGTGAAATCC TGAGGATCTT AGAAGATTCG ATCTGAATTA ATATAGAAAA TTATTGAAAA AATTACGAAC   
  
  
- AAACTGTGAT AACGTACAAT CGGAGATTCG AAATAAAATA GCTCTAGGTA TATTTACAAA AAAAAACAAA   
  
  
- AAAGGCCAAA ATGTAACTCT ATTTTTAGTA AATGATTAAA CTTTAGTTTA ACTAAAAAAG TAATTTTTTA   
  
  
- AATTACCAAT AAAAATATTA AATTTTATTG TTATATTTTA TTTTTTTAAT TAATAACTAA AATAATTTAA   
  
  
- CACCACGCAA ATAAACTTTC ACATATACAG TATAAAGTAT TAAAATGTAA TATTTATTTT TATATATTTT   
  
  
- ATGTATATTA ATCAACACAA TAAAGACCTG TAGTGAATGT TTTACATTTA TACTTTGTTT TTAAAACGCG   
  
  
- TAAATTTTAT ATATTTTATG TATATTAATC AACACAATAA AGACCTGTAG TGAATGTTTT ACATTTATAC   
  
  
- TTTGTTTTTA AAACGCGTAA ATTCATTAAT AATAATTGAT AAAAATTACT AATATTCTAT TTATGTAAAT   
  
  
- AAATTAATTA TTAAAAAATC TTCTTTATAT AAATTAGATT TGAAAATACC CTATACGTAC CTACCGTACT   
  
  
- TTAAACGTGA ACAGTATACG GAGGGAGGAT GTGATTTCGG TTATCCGTAG TCGTCGGTAA ATGCCGTGAC   
  
  
- TCTTCCTATT TATAATCTAT ACACTAACCC GGAGTCGGCG GGCGACCAGT TTCAGTGCAC GAAACTGGTT   
  
  
- TGGTGCGCCC GGTTTTGTGT GTGTGCGGGT GTGTGTGAGA GAGAGAGAGA GAAAGAAGGG CATAAAAAGT   
  
  
- GCCAGTTTGA AGAGCAATGG AAGCGTCTTT GACGGAACGT TTCTTTTTTG TGTGAGAGAG AGAGAGAGAA   
  
  
- AGAAGGGCAT AAAAAGTGCC AGTTTGAAGA GCAATGGAAG CGTCTTTGAC GGAACGTTTC TTTTTCTTCT   
  
  
- TTTGATCTTT TTTTTCTCTC TTTTTGGTTT CTTCGTCTCT GCGGAACATG GAATGCACTG GCAGAAGAGG   
  
  
- CAAGAGAGAG AGAGAGAGAG GGGGGGAACT AGGAATATAA GTAGCATATT CGACAGAAGA AAGACATGGA   
  
  
- CATTGGAATC AAGAGAGAGA CAGCGAAAA

  
  
Motifs Found  

+     5UTR Py-rich stretch

| Site Name | Organism | Position | Strand | Matrix score. | sequence | function |
| --- | --- | --- | --- | --- | --- | --- |
| 5UTR Py-rich stretch | Lycopersicon esculentum | 1407 | + | 13 | TTTCTCTCTCTCTC | cis-acting element conferring high transcription levels |
| 5UTR Py-rich stretch | Lycopersicon esculentum | 1158 | + | 13 | TTTCTCTCTCTCTC | cis-acting element conferring high transcription levels |
| 5UTR Py-rich stretch | Lycopersicon esculentum | 1323 | - | 9 | TTTCTTCTCT | cis-acting element conferring high transcription levels |
| 5UTR Py-rich stretch | Lycopersicon esculentum | 1245 | + | 13 | TTTCTCTCTCTCTC | cis-acting element conferring high transcription levels |
| 5UTR Py-rich stretch | Lycopersicon esculentum | 1401 | + | 13 | TTTCTCTCTCTCTC | cis-acting element conferring high transcription levels |
| 5UTR Py-rich stretch | Lycopersicon esculentum | 1403 | + | 13 | TTTCTCTCTCTCTC | cis-acting element conferring high transcription levels |
| 5UTR Py-rich stretch | Lycopersicon esculentum | 1405 | + | 13 | TTTCTCTCTCTCTC | cis-acting element conferring high transcription levels |

> 2018/04/13 10:10:12  
+ ATTATCGCAC TTTATCCACT AAGAACCTAA TTACCCAACT AGTGGGTTAA CTTGTAGGCT AAGCTTACAT   
  
  
+ ATCTGCTTAT GTTCTCTCCA TTTTGTGTGA AAACGTGTAG ACTTAATCTA ACCAAATCAT GATTGTGACT   
  
  
+ TTGATTATGA AATAAAAGGT CTATGGTTTA ATTAAATTAA TTACAATATG ACGTAAACTA AACATATACC   
  
  
+ TTTAATGGCT CTGGAGCTTT GTGTCATTTT TTATTTCCAC GATCATTAGG GCAAAAATTG GTTGAGAAAT   
  
  
+ TATAATTTAG AGTATAAGAT ACTCTGGCTA GCTTTTGGAT TTCATTGACT AAAAAGGCAT TACCCCTTTT   
  
  
+ ACACTTTAGG ACTCCTAGAA TCTTCTAAGC TAGACTTAAT TATATCTTTT AATAACTTTT TTAATGCTTG   
  
  
+ TTTGACACTA TTGCATGTTA GCCTCTAAGC TTTATTTTAT CGAGATCCAT ATAAATGTTT TTTTTTGTTT   
  
  
+ TTTCCGGTTT TACATTGAGA TAAAAATCAT TTACTAATTT GAAATCAAAT TGATTTTTTC ATTAAAAAAT   
  
  
+ TTAATGGTTA TTTTTATAAT TTAAAATAAC AATATAAAAT AAAAAAATTA ATTATTGATT TTATTAAATT   
  
  
+ GTGGTGCGTT TATTTGAAAG TGTATATGTC ATATTTCATA ATTTTACATT ATAAATAAAA ATATATAAAA   
  
  
+ TACATATAAT TAGTTGTGTT ATTTCTGGAC ATCACTTACA AAATGTAAAT ATGAAACAAA AATTTTGCGC   
  
  
+ ATTTAAAATA TATAAAATAC ATATAATTAG TTGTGTTATT TCTGGACATC ACTTACAAAA TGTAAATATG   
  
  
+ AAACAAAAAT TTTGCGCATT TAAGTAATTA TTATTAACTA TTTTTAATGA TTATAAGATA AATACATTTA   
  
  
+ TTTAATTAAT AATTTTTTAG AAGAAATATA TTTAATCTAA ACTTTTATGG GATATGCATG GATGGCATGA   
  
  
+ AATTTGCACT TGTCATATGC CTCCCTCCTA CACTAAAGCC AATAGGCATC AGCAGCCATT TACGGCACTG   
  
  
+ AGAAGGATAA ATATTAGATA TGTGATTGGG CCTCAGCCGC CCGCTGGTCA AAGTCACGTG CTTTGACCAA   
  
  
+ ACCACGCGGG CCAAAACACA CACACGCCCA CACACACTCT CTCTCTCTCT CTTTCTTCCC GTATTTTTCA   
  
  
+ CGGTCAAACT TCTCGTTACC TTCGCAGAAA CTGCCTTGCA AAGAAAAAAC ACACTCTCTC TCTCTCTCTT   
  
  
+ TCTTCCCGTA TTTTTCACGG TCAAACTTCT CGTTACCTTC GCAGAAACTG CCTTGCAAAG AAAAAGAAGA   
  
  
+ AAACTAGAAA AAAAAGAGAG AAAAACCAAA GAAGCAGAGA CGCCTTGTAC CTTACGTGAC CGTCTTCTCC   
  
  
+ GTTCTCTCTC TCTCTCTCTC CCCCCCTTGA TCCTTATATT CATCGTATAA GCTGTCTTCT TTCTGTACCT   
  
  
+ GTAACCTTAG TTCTCTCTCT GTCGCTTTT  

- TAATAGCGTG AAATAGGTGA TTCTTGGATT AATGGGTTGA TCACCCAATT GAACATCCGA TTCGAATGTA   
  
  
- TAGACGAATA CAAGAGAGGT AAAACACACT TTTGCACATC TGAATTAGAT TGGTTTAGTA CTAACACTGA   
  
  
- AACTAATACT TTATTTTCCA GATACCAAAT TAATTTAATT AATGTTATAC TGCATTTGAT TTGTATATGG   
  
  
- AAATTACCGA GACCTCGAAA CACAGTAAAA AATAAAGGTG CTAGTAATCC CGTTTTTAAC CAACTCTTTA   
  
  
- ATATTAAATC TCATATTCTA TGAGACCGAT CGAAAACCTA AAGTAACTGA TTTTTCCGTA ATGGGGAAAA   
  
  
- TGTGAAATCC TGAGGATCTT AGAAGATTCG ATCTGAATTA ATATAGAAAA TTATTGAAAA AATTACGAAC   
  
  
- AAACTGTGAT AACGTACAAT CGGAGATTCG AAATAAAATA GCTCTAGGTA TATTTACAAA AAAAAACAAA   
  
  
- AAAGGCCAAA ATGTAACTCT ATTTTTAGTA AATGATTAAA CTTTAGTTTA ACTAAAAAAG TAATTTTTTA   
  
  
- AATTACCAAT AAAAATATTA AATTTTATTG TTATATTTTA TTTTTTTAAT TAATAACTAA AATAATTTAA   
  
  
- CACCACGCAA ATAAACTTTC ACATATACAG TATAAAGTAT TAAAATGTAA TATTTATTTT TATATATTTT   
  
  
- ATGTATATTA ATCAACACAA TAAAGACCTG TAGTGAATGT TTTACATTTA TACTTTGTTT TTAAAACGCG   
  
  
- TAAATTTTAT ATATTTTATG TATATTAATC AACACAATAA AGACCTGTAG TGAATGTTTT ACATTTATAC   
  
  
- TTTGTTTTTA AAACGCGTAA ATTCATTAAT AATAATTGAT AAAAATTACT AATATTCTAT TTATGTAAAT   
  
  
- AAATTAATTA TTAAAAAATC TTCTTTATAT AAATTAGATT TGAAAATACC CTATACGTAC CTACCGTACT   
  
  
- TTAAACGTGA ACAGTATACG GAGGGAGGAT GTGATTTCGG TTATCCGTAG TCGTCGGTAA ATGCCGTGAC   
  
  
- TCTTCCTATT TATAATCTAT ACACTAACCC GGAGTCGGCG GGCGACCAGT TTCAGTGCAC GAAACTGGTT   
  
  
- TGGTGCGCCC GGTTTTGTGT GTGTGCGGGT GTGTGTGAGA GAGAGAGAGA GAAAGAAGGG CATAAAAAGT   
  
  
- GCCAGTTTGA AGAGCAATGG AAGCGTCTTT GACGGAACGT TTCTTTTTTG TGTGAGAGAG AGAGAGAGAA   
  
  
- AGAAGGGCAT AAAAAGTGCC AGTTTGAAGA GCAATGGAAG CGTCTTTGAC GGAACGTTTC TTTTTCTTCT   
  
  
- TTTGATCTTT TTTTTCTCTC TTTTTGGTTT CTTCGTCTCT GCGGAACATG GAATGCACTG GCAGAAGAGG   
  
  
- CAAGAGAGAG AGAGAGAGAG GGGGGGAACT AGGAATATAA GTAGCATATT CGACAGAAGA AAGACATGGA   
  
  
- CATTGGAATC AAGAGAGAGA CAGCGAAAA

+     AAGAA-motif

| Site Name | Organism | Position | Strand | Matrix score. | sequence | function |
| --- | --- | --- | --- | --- | --- | --- |
| AAGAA-motif | Avena sativa | 1460 | - | 9 | gGTAAAGAAA |  |
| AAGAA-motif | Avena sativa | 1457 | - | 7 | GAAAGAA |  |

> 2018/04/13 10:10:12  
+ ATTATCGCAC TTTATCCACT AAGAACCTAA TTACCCAACT AGTGGGTTAA CTTGTAGGCT AAGCTTACAT   
  
  
+ ATCTGCTTAT GTTCTCTCCA TTTTGTGTGA AAACGTGTAG ACTTAATCTA ACCAAATCAT GATTGTGACT   
  
  
+ TTGATTATGA AATAAAAGGT CTATGGTTTA ATTAAATTAA TTACAATATG ACGTAAACTA AACATATACC   
  
  
+ TTTAATGGCT CTGGAGCTTT GTGTCATTTT TTATTTCCAC GATCATTAGG GCAAAAATTG GTTGAGAAAT   
  
  
+ TATAATTTAG AGTATAAGAT ACTCTGGCTA GCTTTTGGAT TTCATTGACT AAAAAGGCAT TACCCCTTTT   
  
  
+ ACACTTTAGG ACTCCTAGAA TCTTCTAAGC TAGACTTAAT TATATCTTTT AATAACTTTT TTAATGCTTG   
  
  
+ TTTGACACTA TTGCATGTTA GCCTCTAAGC TTTATTTTAT CGAGATCCAT ATAAATGTTT TTTTTTGTTT   
  
  
+ TTTCCGGTTT TACATTGAGA TAAAAATCAT TTACTAATTT GAAATCAAAT TGATTTTTTC ATTAAAAAAT   
  
  
+ TTAATGGTTA TTTTTATAAT TTAAAATAAC AATATAAAAT AAAAAAATTA ATTATTGATT TTATTAAATT   
  
  
+ GTGGTGCGTT TATTTGAAAG TGTATATGTC ATATTTCATA ATTTTACATT ATAAATAAAA ATATATAAAA   
  
  
+ TACATATAAT TAGTTGTGTT ATTTCTGGAC ATCACTTACA AAATGTAAAT ATGAAACAAA AATTTTGCGC   
  
  
+ ATTTAAAATA TATAAAATAC ATATAATTAG TTGTGTTATT TCTGGACATC ACTTACAAAA TGTAAATATG   
  
  
+ AAACAAAAAT TTTGCGCATT TAAGTAATTA TTATTAACTA TTTTTAATGA TTATAAGATA AATACATTTA   
  
  
+ TTTAATTAAT AATTTTTTAG AAGAAATATA TTTAATCTAA ACTTTTATGG GATATGCATG GATGGCATGA   
  
  
+ AATTTGCACT TGTCATATGC CTCCCTCCTA CACTAAAGCC AATAGGCATC AGCAGCCATT TACGGCACTG   
  
  
+ AGAAGGATAA ATATTAGATA TGTGATTGGG CCTCAGCCGC CCGCTGGTCA AAGTCACGTG CTTTGACCAA   
  
  
+ ACCACGCGGG CCAAAACACA CACACGCCCA CACACACTCT CTCTCTCTCT CTTTCTTCCC GTATTTTTCA   
  
  
+ CGGTCAAACT TCTCGTTACC TTCGCAGAAA CTGCCTTGCA AAGAAAAAAC ACACTCTCTC TCTCTCTCTT   
  
  
+ TCTTCCCGTA TTTTTCACGG TCAAACTTCT CGTTACCTTC GCAGAAACTG CCTTGCAAAG AAAAAGAAGA   
  
  
+ AAACTAGAAA AAAAAGAGAG AAAAACCAAA GAAGCAGAGA CGCCTTGTAC CTTACGTGAC CGTCTTCTCC   
  
  
+ GTTCTCTCTC TCTCTCTCTC CCCCCCTTGA TCCTTATATT CATCGTATAA GCTGTCTTCT TTCTGTACCT   
  
  
+ GTAACCTTAG TTCTCTCTCT GTCGCTTTT  

- TAATAGCGTG AAATAGGTGA TTCTTGGATT AATGGGTTGA TCACCCAATT GAACATCCGA TTCGAATGTA   
  
  
- TAGACGAATA CAAGAGAGGT AAAACACACT TTTGCACATC TGAATTAGAT TGGTTTAGTA CTAACACTGA   
  
  
- AACTAATACT TTATTTTCCA GATACCAAAT TAATTTAATT AATGTTATAC TGCATTTGAT TTGTATATGG   
  
  
- AAATTACCGA GACCTCGAAA CACAGTAAAA AATAAAGGTG CTAGTAATCC CGTTTTTAAC CAACTCTTTA   
  
  
- ATATTAAATC TCATATTCTA TGAGACCGAT CGAAAACCTA AAGTAACTGA TTTTTCCGTA ATGGGGAAAA   
  
  
- TGTGAAATCC TGAGGATCTT AGAAGATTCG ATCTGAATTA ATATAGAAAA TTATTGAAAA AATTACGAAC   
  
  
- AAACTGTGAT AACGTACAAT CGGAGATTCG AAATAAAATA GCTCTAGGTA TATTTACAAA AAAAAACAAA   
  
  
- AAAGGCCAAA ATGTAACTCT ATTTTTAGTA AATGATTAAA CTTTAGTTTA ACTAAAAAAG TAATTTTTTA   
  
  
- AATTACCAAT AAAAATATTA AATTTTATTG TTATATTTTA TTTTTTTAAT TAATAACTAA AATAATTTAA   
  
  
- CACCACGCAA ATAAACTTTC ACATATACAG TATAAAGTAT TAAAATGTAA TATTTATTTT TATATATTTT   
  
  
- ATGTATATTA ATCAACACAA TAAAGACCTG TAGTGAATGT TTTACATTTA TACTTTGTTT TTAAAACGCG   
  
  
- TAAATTTTAT ATATTTTATG TATATTAATC AACACAATAA AGACCTGTAG TGAATGTTTT ACATTTATAC   
  
  
- TTTGTTTTTA AAACGCGTAA ATTCATTAAT AATAATTGAT AAAAATTACT AATATTCTAT TTATGTAAAT   
  
  
- AAATTAATTA TTAAAAAATC TTCTTTATAT AAATTAGATT TGAAAATACC CTATACGTAC CTACCGTACT   
  
  
- TTAAACGTGA ACAGTATACG GAGGGAGGAT GTGATTTCGG TTATCCGTAG TCGTCGGTAA ATGCCGTGAC   
  
  
- TCTTCCTATT TATAATCTAT ACACTAACCC GGAGTCGGCG GGCGACCAGT TTCAGTGCAC GAAACTGGTT   
  
  
- TGGTGCGCCC GGTTTTGTGT GTGTGCGGGT GTGTGTGAGA GAGAGAGAGA GAAAGAAGGG CATAAAAAGT   
  
  
- GCCAGTTTGA AGAGCAATGG AAGCGTCTTT GACGGAACGT TTCTTTTTTG TGTGAGAGAG AGAGAGAGAA   
  
  
- AGAAGGGCAT AAAAAGTGCC AGTTTGAAGA GCAATGGAAG CGTCTTTGAC GGAACGTTTC TTTTTCTTCT   
  
  
- TTTGATCTTT TTTTTCTCTC TTTTTGGTTT CTTCGTCTCT GCGGAACATG GAATGCACTG GCAGAAGAGG   
  
  
- CAAGAGAGAG AGAGAGAGAG GGGGGGAACT AGGAATATAA GTAGCATATT CGACAGAAGA AAGACATGGA   
  
  
- CATTGGAATC AAGAGAGAGA CAGCGAAAA

+     ABRE

| Site Name | Organism | Position | Strand | Matrix score. | sequence | function |
| --- | --- | --- | --- | --- | --- | --- |
| ABRE | Arabidopsis thaliana | 1105 | - | 6 | CACGTG | cis-acting element involved in the abscisic acid responsiveness |
| ABRE | Arabidopsis thaliana | 1383 | + | 6 | TACGTG | cis-acting element involved in the abscisic acid responsiveness |

> 2018/04/13 10:10:12  
+ ATTATCGCAC TTTATCCACT AAGAACCTAA TTACCCAACT AGTGGGTTAA CTTGTAGGCT AAGCTTACAT   
  
  
+ ATCTGCTTAT GTTCTCTCCA TTTTGTGTGA AAACGTGTAG ACTTAATCTA ACCAAATCAT GATTGTGACT   
  
  
+ TTGATTATGA AATAAAAGGT CTATGGTTTA ATTAAATTAA TTACAATATG ACGTAAACTA AACATATACC   
  
  
+ TTTAATGGCT CTGGAGCTTT GTGTCATTTT TTATTTCCAC GATCATTAGG GCAAAAATTG GTTGAGAAAT   
  
  
+ TATAATTTAG AGTATAAGAT ACTCTGGCTA GCTTTTGGAT TTCATTGACT AAAAAGGCAT TACCCCTTTT   
  
  
+ ACACTTTAGG ACTCCTAGAA TCTTCTAAGC TAGACTTAAT TATATCTTTT AATAACTTTT TTAATGCTTG   
  
  
+ TTTGACACTA TTGCATGTTA GCCTCTAAGC TTTATTTTAT CGAGATCCAT ATAAATGTTT TTTTTTGTTT   
  
  
+ TTTCCGGTTT TACATTGAGA TAAAAATCAT TTACTAATTT GAAATCAAAT TGATTTTTTC ATTAAAAAAT   
  
  
+ TTAATGGTTA TTTTTATAAT TTAAAATAAC AATATAAAAT AAAAAAATTA ATTATTGATT TTATTAAATT   
  
  
+ GTGGTGCGTT TATTTGAAAG TGTATATGTC ATATTTCATA ATTTTACATT ATAAATAAAA ATATATAAAA   
  
  
+ TACATATAAT TAGTTGTGTT ATTTCTGGAC ATCACTTACA AAATGTAAAT ATGAAACAAA AATTTTGCGC   
  
  
+ ATTTAAAATA TATAAAATAC ATATAATTAG TTGTGTTATT TCTGGACATC ACTTACAAAA TGTAAATATG   
  
  
+ AAACAAAAAT TTTGCGCATT TAAGTAATTA TTATTAACTA TTTTTAATGA TTATAAGATA AATACATTTA   
  
  
+ TTTAATTAAT AATTTTTTAG AAGAAATATA TTTAATCTAA ACTTTTATGG GATATGCATG GATGGCATGA   
  
  
+ AATTTGCACT TGTCATATGC CTCCCTCCTA CACTAAAGCC AATAGGCATC AGCAGCCATT TACGGCACTG   
  
  
+ AGAAGGATAA ATATTAGATA TGTGATTGGG CCTCAGCCGC CCGCTGGTCA AAGTCACGTG CTTTGACCAA   
  
  
+ ACCACGCGGG CCAAAACACA CACACGCCCA CACACACTCT CTCTCTCTCT CTTTCTTCCC GTATTTTTCA   
  
  
+ CGGTCAAACT TCTCGTTACC TTCGCAGAAA CTGCCTTGCA AAGAAAAAAC ACACTCTCTC TCTCTCTCTT   
  
  
+ TCTTCCCGTA TTTTTCACGG TCAAACTTCT CGTTACCTTC GCAGAAACTG CCTTGCAAAG AAAAAGAAGA   
  
  
+ AAACTAGAAA AAAAAGAGAG AAAAACCAAA GAAGCAGAGA CGCCTTGTAC CTTACGTGAC CGTCTTCTCC   
  
  
+ GTTCTCTCTC TCTCTCTCTC CCCCCCTTGA TCCTTATATT CATCGTATAA GCTGTCTTCT TTCTGTACCT   
  
  
+ GTAACCTTAG TTCTCTCTCT GTCGCTTTT  

- TAATAGCGTG AAATAGGTGA TTCTTGGATT AATGGGTTGA TCACCCAATT GAACATCCGA TTCGAATGTA   
  
  
- TAGACGAATA CAAGAGAGGT AAAACACACT TTTGCACATC TGAATTAGAT TGGTTTAGTA CTAACACTGA   
  
  
- AACTAATACT TTATTTTCCA GATACCAAAT TAATTTAATT AATGTTATAC TGCATTTGAT TTGTATATGG   
  
  
- AAATTACCGA GACCTCGAAA CACAGTAAAA AATAAAGGTG CTAGTAATCC CGTTTTTAAC CAACTCTTTA   
  
  
- ATATTAAATC TCATATTCTA TGAGACCGAT CGAAAACCTA AAGTAACTGA TTTTTCCGTA ATGGGGAAAA   
  
  
- TGTGAAATCC TGAGGATCTT AGAAGATTCG ATCTGAATTA ATATAGAAAA TTATTGAAAA AATTACGAAC   
  
  
- AAACTGTGAT AACGTACAAT CGGAGATTCG AAATAAAATA GCTCTAGGTA TATTTACAAA AAAAAACAAA   
  
  
- AAAGGCCAAA ATGTAACTCT ATTTTTAGTA AATGATTAAA CTTTAGTTTA ACTAAAAAAG TAATTTTTTA   
  
  
- AATTACCAAT AAAAATATTA AATTTTATTG TTATATTTTA TTTTTTTAAT TAATAACTAA AATAATTTAA   
  
  
- CACCACGCAA ATAAACTTTC ACATATACAG TATAAAGTAT TAAAATGTAA TATTTATTTT TATATATTTT   
  
  
- ATGTATATTA ATCAACACAA TAAAGACCTG TAGTGAATGT TTTACATTTA TACTTTGTTT TTAAAACGCG   
  
  
- TAAATTTTAT ATATTTTATG TATATTAATC AACACAATAA AGACCTGTAG TGAATGTTTT ACATTTATAC   
  
  
- TTTGTTTTTA AAACGCGTAA ATTCATTAAT AATAATTGAT AAAAATTACT AATATTCTAT TTATGTAAAT   
  
  
- AAATTAATTA TTAAAAAATC TTCTTTATAT AAATTAGATT TGAAAATACC CTATACGTAC CTACCGTACT   
  
  
- TTAAACGTGA ACAGTATACG GAGGGAGGAT GTGATTTCGG TTATCCGTAG TCGTCGGTAA ATGCCGTGAC   
  
  
- TCTTCCTATT TATAATCTAT ACACTAACCC GGAGTCGGCG GGCGACCAGT TTCAGTGCAC GAAACTGGTT   
  
  
- TGGTGCGCCC GGTTTTGTGT GTGTGCGGGT GTGTGTGAGA GAGAGAGAGA GAAAGAAGGG CATAAAAAGT   
  
  
- GCCAGTTTGA AGAGCAATGG AAGCGTCTTT GACGGAACGT TTCTTTTTTG TGTGAGAGAG AGAGAGAGAA   
  
  
- AGAAGGGCAT AAAAAGTGCC AGTTTGAAGA GCAATGGAAG CGTCTTTGAC GGAACGTTTC TTTTTCTTCT   
  
  
- TTTGATCTTT TTTTTCTCTC TTTTTGGTTT CTTCGTCTCT GCGGAACATG GAATGCACTG GCAGAAGAGG   
  
  
- CAAGAGAGAG AGAGAGAGAG GGGGGGAACT AGGAATATAA GTAGCATATT CGACAGAAGA AAGACATGGA   
  
  
- CATTGGAATC AAGAGAGAGA CAGCGAAAA

+     AC-II

| Site Name | Organism | Position | Strand | Matrix score. | sequence | function |
| --- | --- | --- | --- | --- | --- | --- |
| AC-II | Phaseolus vulgaris | 1112 | + | 9 | (C/T)T(T/C)(C/T)(A/C)(A/C)C(A/C)A(A/C)C(C/A)(C/A)C |  |

> 2018/04/13 10:10:12  
+ ATTATCGCAC TTTATCCACT AAGAACCTAA TTACCCAACT AGTGGGTTAA CTTGTAGGCT AAGCTTACAT   
  
  
+ ATCTGCTTAT GTTCTCTCCA TTTTGTGTGA AAACGTGTAG ACTTAATCTA ACCAAATCAT GATTGTGACT   
  
  
+ TTGATTATGA AATAAAAGGT CTATGGTTTA ATTAAATTAA TTACAATATG ACGTAAACTA AACATATACC   
  
  
+ TTTAATGGCT CTGGAGCTTT GTGTCATTTT TTATTTCCAC GATCATTAGG GCAAAAATTG GTTGAGAAAT   
  
  
+ TATAATTTAG AGTATAAGAT ACTCTGGCTA GCTTTTGGAT TTCATTGACT AAAAAGGCAT TACCCCTTTT   
  
  
+ ACACTTTAGG ACTCCTAGAA TCTTCTAAGC TAGACTTAAT TATATCTTTT AATAACTTTT TTAATGCTTG   
  
  
+ TTTGACACTA TTGCATGTTA GCCTCTAAGC TTTATTTTAT CGAGATCCAT ATAAATGTTT TTTTTTGTTT   
  
  
+ TTTCCGGTTT TACATTGAGA TAAAAATCAT TTACTAATTT GAAATCAAAT TGATTTTTTC ATTAAAAAAT   
  
  
+ TTAATGGTTA TTTTTATAAT TTAAAATAAC AATATAAAAT AAAAAAATTA ATTATTGATT TTATTAAATT   
  
  
+ GTGGTGCGTT TATTTGAAAG TGTATATGTC ATATTTCATA ATTTTACATT ATAAATAAAA ATATATAAAA   
  
  
+ TACATATAAT TAGTTGTGTT ATTTCTGGAC ATCACTTACA AAATGTAAAT ATGAAACAAA AATTTTGCGC   
  
  
+ ATTTAAAATA TATAAAATAC ATATAATTAG TTGTGTTATT TCTGGACATC ACTTACAAAA TGTAAATATG   
  
  
+ AAACAAAAAT TTTGCGCATT TAAGTAATTA TTATTAACTA TTTTTAATGA TTATAAGATA AATACATTTA   
  
  
+ TTTAATTAAT AATTTTTTAG AAGAAATATA TTTAATCTAA ACTTTTATGG GATATGCATG GATGGCATGA   
  
  
+ AATTTGCACT TGTCATATGC CTCCCTCCTA CACTAAAGCC AATAGGCATC AGCAGCCATT TACGGCACTG   
  
  
+ AGAAGGATAA ATATTAGATA TGTGATTGGG CCTCAGCCGC CCGCTGGTCA AAGTCACGTG CTTTGACCAA   
  
  
+ ACCACGCGGG CCAAAACACA CACACGCCCA CACACACTCT CTCTCTCTCT CTTTCTTCCC GTATTTTTCA   
  
  
+ CGGTCAAACT TCTCGTTACC TTCGCAGAAA CTGCCTTGCA AAGAAAAAAC ACACTCTCTC TCTCTCTCTT   
  
  
+ TCTTCCCGTA TTTTTCACGG TCAAACTTCT CGTTACCTTC GCAGAAACTG CCTTGCAAAG AAAAAGAAGA   
  
  
+ AAACTAGAAA AAAAAGAGAG AAAAACCAAA GAAGCAGAGA CGCCTTGTAC CTTACGTGAC CGTCTTCTCC   
  
  
+ GTTCTCTCTC TCTCTCTCTC CCCCCCTTGA TCCTTATATT CATCGTATAA GCTGTCTTCT TTCTGTACCT   
  
  
+ GTAACCTTAG TTCTCTCTCT GTCGCTTTT  

- TAATAGCGTG AAATAGGTGA TTCTTGGATT AATGGGTTGA TCACCCAATT GAACATCCGA TTCGAATGTA   
  
  
- TAGACGAATA CAAGAGAGGT AAAACACACT TTTGCACATC TGAATTAGAT TGGTTTAGTA CTAACACTGA   
  
  
- AACTAATACT TTATTTTCCA GATACCAAAT TAATTTAATT AATGTTATAC TGCATTTGAT TTGTATATGG   
  
  
- AAATTACCGA GACCTCGAAA CACAGTAAAA AATAAAGGTG CTAGTAATCC CGTTTTTAAC CAACTCTTTA   
  
  
- ATATTAAATC TCATATTCTA TGAGACCGAT CGAAAACCTA AAGTAACTGA TTTTTCCGTA ATGGGGAAAA   
  
  
- TGTGAAATCC TGAGGATCTT AGAAGATTCG ATCTGAATTA ATATAGAAAA TTATTGAAAA AATTACGAAC   
  
  
- AAACTGTGAT AACGTACAAT CGGAGATTCG AAATAAAATA GCTCTAGGTA TATTTACAAA AAAAAACAAA   
  
  
- AAAGGCCAAA ATGTAACTCT ATTTTTAGTA AATGATTAAA CTTTAGTTTA ACTAAAAAAG TAATTTTTTA   
  
  
- AATTACCAAT AAAAATATTA AATTTTATTG TTATATTTTA TTTTTTTAAT TAATAACTAA AATAATTTAA   
  
  
- CACCACGCAA ATAAACTTTC ACATATACAG TATAAAGTAT TAAAATGTAA TATTTATTTT TATATATTTT   
  
  
- ATGTATATTA ATCAACACAA TAAAGACCTG TAGTGAATGT TTTACATTTA TACTTTGTTT TTAAAACGCG   
  
  
- TAAATTTTAT ATATTTTATG TATATTAATC AACACAATAA AGACCTGTAG TGAATGTTTT ACATTTATAC   
  
  
- TTTGTTTTTA AAACGCGTAA ATTCATTAAT AATAATTGAT AAAAATTACT AATATTCTAT TTATGTAAAT   
  
  
- AAATTAATTA TTAAAAAATC TTCTTTATAT AAATTAGATT TGAAAATACC CTATACGTAC CTACCGTACT   
  
  
- TTAAACGTGA ACAGTATACG GAGGGAGGAT GTGATTTCGG TTATCCGTAG TCGTCGGTAA ATGCCGTGAC   
  
  
- TCTTCCTATT TATAATCTAT ACACTAACCC GGAGTCGGCG GGCGACCAGT TTCAGTGCAC GAAACTGGTT   
  
  
- TGGTGCGCCC GGTTTTGTGT GTGTGCGGGT GTGTGTGAGA GAGAGAGAGA GAAAGAAGGG CATAAAAAGT   
  
  
- GCCAGTTTGA AGAGCAATGG AAGCGTCTTT GACGGAACGT TTCTTTTTTG TGTGAGAGAG AGAGAGAGAA   
  
  
- AGAAGGGCAT AAAAAGTGCC AGTTTGAAGA GCAATGGAAG CGTCTTTGAC GGAACGTTTC TTTTTCTTCT   
  
  
- TTTGATCTTT TTTTTCTCTC TTTTTGGTTT CTTCGTCTCT GCGGAACATG GAATGCACTG GCAGAAGAGG   
  
  
- CAAGAGAGAG AGAGAGAGAG GGGGGGAACT AGGAATATAA GTAGCATATT CGACAGAAGA AAGACATGGA   
  
  
- CATTGGAATC AAGAGAGAGA CAGCGAAAA

+     ACE

| Site Name | Organism | Position | Strand | Matrix score. | sequence | function |
| --- | --- | --- | --- | --- | --- | --- |
| ACE | Petroselinum crispum | 472 | - | 9 | AAAACGTTTA | cis-acting element involved in light responsiveness |
| ACE | Petroselinum crispum | 100 | + | 9 | AAAACGTTTA | cis-acting element involved in light responsiveness |

> 2018/04/13 10:10:12  
+ ATTATCGCAC TTTATCCACT AAGAACCTAA TTACCCAACT AGTGGGTTAA CTTGTAGGCT AAGCTTACAT   
  
  
+ ATCTGCTTAT GTTCTCTCCA TTTTGTGTGA AAACGTGTAG ACTTAATCTA ACCAAATCAT GATTGTGACT   
  
  
+ TTGATTATGA AATAAAAGGT CTATGGTTTA ATTAAATTAA TTACAATATG ACGTAAACTA AACATATACC   
  
  
+ TTTAATGGCT CTGGAGCTTT GTGTCATTTT TTATTTCCAC GATCATTAGG GCAAAAATTG GTTGAGAAAT   
  
  
+ TATAATTTAG AGTATAAGAT ACTCTGGCTA GCTTTTGGAT TTCATTGACT AAAAAGGCAT TACCCCTTTT   
  
  
+ ACACTTTAGG ACTCCTAGAA TCTTCTAAGC TAGACTTAAT TATATCTTTT AATAACTTTT TTAATGCTTG   
  
  
+ TTTGACACTA TTGCATGTTA GCCTCTAAGC TTTATTTTAT CGAGATCCAT ATAAATGTTT TTTTTTGTTT   
  
  
+ TTTCCGGTTT TACATTGAGA TAAAAATCAT TTACTAATTT GAAATCAAAT TGATTTTTTC ATTAAAAAAT   
  
  
+ TTAATGGTTA TTTTTATAAT TTAAAATAAC AATATAAAAT AAAAAAATTA ATTATTGATT TTATTAAATT   
  
  
+ GTGGTGCGTT TATTTGAAAG TGTATATGTC ATATTTCATA ATTTTACATT ATAAATAAAA ATATATAAAA   
  
  
+ TACATATAAT TAGTTGTGTT ATTTCTGGAC ATCACTTACA AAATGTAAAT ATGAAACAAA AATTTTGCGC   
  
  
+ ATTTAAAATA TATAAAATAC ATATAATTAG TTGTGTTATT TCTGGACATC ACTTACAAAA TGTAAATATG   
  
  
+ AAACAAAAAT TTTGCGCATT TAAGTAATTA TTATTAACTA TTTTTAATGA TTATAAGATA AATACATTTA   
  
  
+ TTTAATTAAT AATTTTTTAG AAGAAATATA TTTAATCTAA ACTTTTATGG GATATGCATG GATGGCATGA   
  
  
+ AATTTGCACT TGTCATATGC CTCCCTCCTA CACTAAAGCC AATAGGCATC AGCAGCCATT TACGGCACTG   
  
  
+ AGAAGGATAA ATATTAGATA TGTGATTGGG CCTCAGCCGC CCGCTGGTCA AAGTCACGTG CTTTGACCAA   
  
  
+ ACCACGCGGG CCAAAACACA CACACGCCCA CACACACTCT CTCTCTCTCT CTTTCTTCCC GTATTTTTCA   
  
  
+ CGGTCAAACT TCTCGTTACC TTCGCAGAAA CTGCCTTGCA AAGAAAAAAC ACACTCTCTC TCTCTCTCTT   
  
  
+ TCTTCCCGTA TTTTTCACGG TCAAACTTCT CGTTACCTTC GCAGAAACTG CCTTGCAAAG AAAAAGAAGA   
  
  
+ AAACTAGAAA AAAAAGAGAG AAAAACCAAA GAAGCAGAGA CGCCTTGTAC CTTACGTGAC CGTCTTCTCC   
  
  
+ GTTCTCTCTC TCTCTCTCTC CCCCCCTTGA TCCTTATATT CATCGTATAA GCTGTCTTCT TTCTGTACCT   
  
  
+ GTAACCTTAG TTCTCTCTCT GTCGCTTTT  

- TAATAGCGTG AAATAGGTGA TTCTTGGATT AATGGGTTGA TCACCCAATT GAACATCCGA TTCGAATGTA   
  
  
- TAGACGAATA CAAGAGAGGT AAAACACACT TTTGCACATC TGAATTAGAT TGGTTTAGTA CTAACACTGA   
  
  
- AACTAATACT TTATTTTCCA GATACCAAAT TAATTTAATT AATGTTATAC TGCATTTGAT TTGTATATGG   
  
  
- AAATTACCGA GACCTCGAAA CACAGTAAAA AATAAAGGTG CTAGTAATCC CGTTTTTAAC CAACTCTTTA   
  
  
- ATATTAAATC TCATATTCTA TGAGACCGAT CGAAAACCTA AAGTAACTGA TTTTTCCGTA ATGGGGAAAA   
  
  
- TGTGAAATCC TGAGGATCTT AGAAGATTCG ATCTGAATTA ATATAGAAAA TTATTGAAAA AATTACGAAC   
  
  
- AAACTGTGAT AACGTACAAT CGGAGATTCG AAATAAAATA GCTCTAGGTA TATTTACAAA AAAAAACAAA   
  
  
- AAAGGCCAAA ATGTAACTCT ATTTTTAGTA AATGATTAAA CTTTAGTTTA ACTAAAAAAG TAATTTTTTA   
  
  
- AATTACCAAT AAAAATATTA AATTTTATTG TTATATTTTA TTTTTTTAAT TAATAACTAA AATAATTTAA   
  
  
- CACCACGCAA ATAAACTTTC ACATATACAG TATAAAGTAT TAAAATGTAA TATTTATTTT TATATATTTT   
  
  
- ATGTATATTA ATCAACACAA TAAAGACCTG TAGTGAATGT TTTACATTTA TACTTTGTTT TTAAAACGCG   
  
  
- TAAATTTTAT ATATTTTATG TATATTAATC AACACAATAA AGACCTGTAG TGAATGTTTT ACATTTATAC   
  
  
- TTTGTTTTTA AAACGCGTAA ATTCATTAAT AATAATTGAT AAAAATTACT AATATTCTAT TTATGTAAAT   
  
  
- AAATTAATTA TTAAAAAATC TTCTTTATAT AAATTAGATT TGAAAATACC CTATACGTAC CTACCGTACT   
  
  
- TTAAACGTGA ACAGTATACG GAGGGAGGAT GTGATTTCGG TTATCCGTAG TCGTCGGTAA ATGCCGTGAC   
  
  
- TCTTCCTATT TATAATCTAT ACACTAACCC GGAGTCGGCG GGCGACCAGT TTCAGTGCAC GAAACTGGTT   
  
  
- TGGTGCGCCC GGTTTTGTGT GTGTGCGGGT GTGTGTGAGA GAGAGAGAGA GAAAGAAGGG CATAAAAAGT   
  
  
- GCCAGTTTGA AGAGCAATGG AAGCGTCTTT GACGGAACGT TTCTTTTTTG TGTGAGAGAG AGAGAGAGAA   
  
  
- AGAAGGGCAT AAAAAGTGCC AGTTTGAAGA GCAATGGAAG CGTCTTTGAC GGAACGTTTC TTTTTCTTCT   
  
  
- TTTGATCTTT TTTTTCTCTC TTTTTGGTTT CTTCGTCTCT GCGGAACATG GAATGCACTG GCAGAAGAGG   
  
  
- CAAGAGAGAG AGAGAGAGAG GGGGGGAACT AGGAATATAA GTAGCATATT CGACAGAAGA AAGACATGGA   
  
  
- CATTGGAATC AAGAGAGAGA CAGCGAAAA

+     ARE

| Site Name | Organism | Position | Strand | Matrix score. | sequence | function |
| --- | --- | --- | --- | --- | --- | --- |
| ARE | Zea mays | 1119 | - | 6 | TGGTTT | cis-acting regulatory element essential for the anaerobic induction |
| ARE | Zea mays | 1353 | - | 6 | TGGTTT | cis-acting regulatory element essential for the anaerobic induction |
| ARE | Zea mays | 164 | + | 6 | TGGTTT | cis-acting regulatory element essential for the anaerobic induction |

> 2018/04/13 10:10:12  
+ ATTATCGCAC TTTATCCACT AAGAACCTAA TTACCCAACT AGTGGGTTAA CTTGTAGGCT AAGCTTACAT   
  
  
+ ATCTGCTTAT GTTCTCTCCA TTTTGTGTGA AAACGTGTAG ACTTAATCTA ACCAAATCAT GATTGTGACT   
  
  
+ TTGATTATGA AATAAAAGGT CTATGGTTTA ATTAAATTAA TTACAATATG ACGTAAACTA AACATATACC   
  
  
+ TTTAATGGCT CTGGAGCTTT GTGTCATTTT TTATTTCCAC GATCATTAGG GCAAAAATTG GTTGAGAAAT   
  
  
+ TATAATTTAG AGTATAAGAT ACTCTGGCTA GCTTTTGGAT TTCATTGACT AAAAAGGCAT TACCCCTTTT   
  
  
+ ACACTTTAGG ACTCCTAGAA TCTTCTAAGC TAGACTTAAT TATATCTTTT AATAACTTTT TTAATGCTTG   
  
  
+ TTTGACACTA TTGCATGTTA GCCTCTAAGC TTTATTTTAT CGAGATCCAT ATAAATGTTT TTTTTTGTTT   
  
  
+ TTTCCGGTTT TACATTGAGA TAAAAATCAT TTACTAATTT GAAATCAAAT TGATTTTTTC ATTAAAAAAT   
  
  
+ TTAATGGTTA TTTTTATAAT TTAAAATAAC AATATAAAAT AAAAAAATTA ATTATTGATT TTATTAAATT   
  
  
+ GTGGTGCGTT TATTTGAAAG TGTATATGTC ATATTTCATA ATTTTACATT ATAAATAAAA ATATATAAAA   
  
  
+ TACATATAAT TAGTTGTGTT ATTTCTGGAC ATCACTTACA AAATGTAAAT ATGAAACAAA AATTTTGCGC   
  
  
+ ATTTAAAATA TATAAAATAC ATATAATTAG TTGTGTTATT TCTGGACATC ACTTACAAAA TGTAAATATG   
  
  
+ AAACAAAAAT TTTGCGCATT TAAGTAATTA TTATTAACTA TTTTTAATGA TTATAAGATA AATACATTTA   
  
  
+ TTTAATTAAT AATTTTTTAG AAGAAATATA TTTAATCTAA ACTTTTATGG GATATGCATG GATGGCATGA   
  
  
+ AATTTGCACT TGTCATATGC CTCCCTCCTA CACTAAAGCC AATAGGCATC AGCAGCCATT TACGGCACTG   
  
  
+ AGAAGGATAA ATATTAGATA TGTGATTGGG CCTCAGCCGC CCGCTGGTCA AAGTCACGTG CTTTGACCAA   
  
  
+ ACCACGCGGG CCAAAACACA CACACGCCCA CACACACTCT CTCTCTCTCT CTTTCTTCCC GTATTTTTCA   
  
  
+ CGGTCAAACT TCTCGTTACC TTCGCAGAAA CTGCCTTGCA AAGAAAAAAC ACACTCTCTC TCTCTCTCTT   
  
  
+ TCTTCCCGTA TTTTTCACGG TCAAACTTCT CGTTACCTTC GCAGAAACTG CCTTGCAAAG AAAAAGAAGA   
  
  
+ AAACTAGAAA AAAAAGAGAG AAAAACCAAA GAAGCAGAGA CGCCTTGTAC CTTACGTGAC CGTCTTCTCC   
  
  
+ GTTCTCTCTC TCTCTCTCTC CCCCCCTTGA TCCTTATATT CATCGTATAA GCTGTCTTCT TTCTGTACCT   
  
  
+ GTAACCTTAG TTCTCTCTCT GTCGCTTTT  

- TAATAGCGTG AAATAGGTGA TTCTTGGATT AATGGGTTGA TCACCCAATT GAACATCCGA TTCGAATGTA   
  
  
- TAGACGAATA CAAGAGAGGT AAAACACACT TTTGCACATC TGAATTAGAT TGGTTTAGTA CTAACACTGA   
  
  
- AACTAATACT TTATTTTCCA GATACCAAAT TAATTTAATT AATGTTATAC TGCATTTGAT TTGTATATGG   
  
  
- AAATTACCGA GACCTCGAAA CACAGTAAAA AATAAAGGTG CTAGTAATCC CGTTTTTAAC CAACTCTTTA   
  
  
- ATATTAAATC TCATATTCTA TGAGACCGAT CGAAAACCTA AAGTAACTGA TTTTTCCGTA ATGGGGAAAA   
  
  
- TGTGAAATCC TGAGGATCTT AGAAGATTCG ATCTGAATTA ATATAGAAAA TTATTGAAAA AATTACGAAC   
  
  
- AAACTGTGAT AACGTACAAT CGGAGATTCG AAATAAAATA GCTCTAGGTA TATTTACAAA AAAAAACAAA   
  
  
- AAAGGCCAAA ATGTAACTCT ATTTTTAGTA AATGATTAAA CTTTAGTTTA ACTAAAAAAG TAATTTTTTA   
  
  
- AATTACCAAT AAAAATATTA AATTTTATTG TTATATTTTA TTTTTTTAAT TAATAACTAA AATAATTTAA   
  
  
- CACCACGCAA ATAAACTTTC ACATATACAG TATAAAGTAT TAAAATGTAA TATTTATTTT TATATATTTT   
  
  
- ATGTATATTA ATCAACACAA TAAAGACCTG TAGTGAATGT TTTACATTTA TACTTTGTTT TTAAAACGCG   
  
  
- TAAATTTTAT ATATTTTATG TATATTAATC AACACAATAA AGACCTGTAG TGAATGTTTT ACATTTATAC   
  
  
- TTTGTTTTTA AAACGCGTAA ATTCATTAAT AATAATTGAT AAAAATTACT AATATTCTAT TTATGTAAAT   
  
  
- AAATTAATTA TTAAAAAATC TTCTTTATAT AAATTAGATT TGAAAATACC CTATACGTAC CTACCGTACT   
  
  
- TTAAACGTGA ACAGTATACG GAGGGAGGAT GTGATTTCGG TTATCCGTAG TCGTCGGTAA ATGCCGTGAC   
  
  
- TCTTCCTATT TATAATCTAT ACACTAACCC GGAGTCGGCG GGCGACCAGT TTCAGTGCAC GAAACTGGTT   
  
  
- TGGTGCGCCC GGTTTTGTGT GTGTGCGGGT GTGTGTGAGA GAGAGAGAGA GAAAGAAGGG CATAAAAAGT   
  
  
- GCCAGTTTGA AGAGCAATGG AAGCGTCTTT GACGGAACGT TTCTTTTTTG TGTGAGAGAG AGAGAGAGAA   
  
  
- AGAAGGGCAT AAAAAGTGCC AGTTTGAAGA GCAATGGAAG CGTCTTTGAC GGAACGTTTC TTTTTCTTCT   
  
  
- TTTGATCTTT TTTTTCTCTC TTTTTGGTTT CTTCGTCTCT GCGGAACATG GAATGCACTG GCAGAAGAGG   
  
  
- CAAGAGAGAG AGAGAGAGAG GGGGGGAACT AGGAATATAA GTAGCATATT CGACAGAAGA AAGACATGGA   
  
  
- CATTGGAATC AAGAGAGAGA CAGCGAAAA

+     ATCT-motif

| Site Name | Organism | Position | Strand | Matrix score. | sequence | function |
| --- | --- | --- | --- | --- | --- | --- |
| ATCT-motif | Arabidopsis thaliana | 944 | + | 9 | AATCTAATCT | part of a conserved DNA module involved in light responsiveness |

> 2018/04/13 10:10:12  
+ ATTATCGCAC TTTATCCACT AAGAACCTAA TTACCCAACT AGTGGGTTAA CTTGTAGGCT AAGCTTACAT   
  
  
+ ATCTGCTTAT GTTCTCTCCA TTTTGTGTGA AAACGTGTAG ACTTAATCTA ACCAAATCAT GATTGTGACT   
  
  
+ TTGATTATGA AATAAAAGGT CTATGGTTTA ATTAAATTAA TTACAATATG ACGTAAACTA AACATATACC   
  
  
+ TTTAATGGCT CTGGAGCTTT GTGTCATTTT TTATTTCCAC GATCATTAGG GCAAAAATTG GTTGAGAAAT   
  
  
+ TATAATTTAG AGTATAAGAT ACTCTGGCTA GCTTTTGGAT TTCATTGACT AAAAAGGCAT TACCCCTTTT   
  
  
+ ACACTTTAGG ACTCCTAGAA TCTTCTAAGC TAGACTTAAT TATATCTTTT AATAACTTTT TTAATGCTTG   
  
  
+ TTTGACACTA TTGCATGTTA GCCTCTAAGC TTTATTTTAT CGAGATCCAT ATAAATGTTT TTTTTTGTTT   
  
  
+ TTTCCGGTTT TACATTGAGA TAAAAATCAT TTACTAATTT GAAATCAAAT TGATTTTTTC ATTAAAAAAT   
  
  
+ TTAATGGTTA TTTTTATAAT TTAAAATAAC AATATAAAAT AAAAAAATTA ATTATTGATT TTATTAAATT   
  
  
+ GTGGTGCGTT TATTTGAAAG TGTATATGTC ATATTTCATA ATTTTACATT ATAAATAAAA ATATATAAAA   
  
  
+ TACATATAAT TAGTTGTGTT ATTTCTGGAC ATCACTTACA AAATGTAAAT ATGAAACAAA AATTTTGCGC   
  
  
+ ATTTAAAATA TATAAAATAC ATATAATTAG TTGTGTTATT TCTGGACATC ACTTACAAAA TGTAAATATG   
  
  
+ AAACAAAAAT TTTGCGCATT TAAGTAATTA TTATTAACTA TTTTTAATGA TTATAAGATA AATACATTTA   
  
  
+ TTTAATTAAT AATTTTTTAG AAGAAATATA TTTAATCTAA ACTTTTATGG GATATGCATG GATGGCATGA   
  
  
+ AATTTGCACT TGTCATATGC CTCCCTCCTA CACTAAAGCC AATAGGCATC AGCAGCCATT TACGGCACTG   
  
  
+ AGAAGGATAA ATATTAGATA TGTGATTGGG CCTCAGCCGC CCGCTGGTCA AAGTCACGTG CTTTGACCAA   
  
  
+ ACCACGCGGG CCAAAACACA CACACGCCCA CACACACTCT CTCTCTCTCT CTTTCTTCCC GTATTTTTCA   
  
  
+ CGGTCAAACT TCTCGTTACC TTCGCAGAAA CTGCCTTGCA AAGAAAAAAC ACACTCTCTC TCTCTCTCTT   
  
  
+ TCTTCCCGTA TTTTTCACGG TCAAACTTCT CGTTACCTTC GCAGAAACTG CCTTGCAAAG AAAAAGAAGA   
  
  
+ AAACTAGAAA AAAAAGAGAG AAAAACCAAA GAAGCAGAGA CGCCTTGTAC CTTACGTGAC CGTCTTCTCC   
  
  
+ GTTCTCTCTC TCTCTCTCTC CCCCCCTTGA TCCTTATATT CATCGTATAA GCTGTCTTCT TTCTGTACCT   
  
  
+ GTAACCTTAG TTCTCTCTCT GTCGCTTTT  

- TAATAGCGTG AAATAGGTGA TTCTTGGATT AATGGGTTGA TCACCCAATT GAACATCCGA TTCGAATGTA   
  
  
- TAGACGAATA CAAGAGAGGT AAAACACACT TTTGCACATC TGAATTAGAT TGGTTTAGTA CTAACACTGA   
  
  
- AACTAATACT TTATTTTCCA GATACCAAAT TAATTTAATT AATGTTATAC TGCATTTGAT TTGTATATGG   
  
  
- AAATTACCGA GACCTCGAAA CACAGTAAAA AATAAAGGTG CTAGTAATCC CGTTTTTAAC CAACTCTTTA   
  
  
- ATATTAAATC TCATATTCTA TGAGACCGAT CGAAAACCTA AAGTAACTGA TTTTTCCGTA ATGGGGAAAA   
  
  
- TGTGAAATCC TGAGGATCTT AGAAGATTCG ATCTGAATTA ATATAGAAAA TTATTGAAAA AATTACGAAC   
  
  
- AAACTGTGAT AACGTACAAT CGGAGATTCG AAATAAAATA GCTCTAGGTA TATTTACAAA AAAAAACAAA   
  
  
- AAAGGCCAAA ATGTAACTCT ATTTTTAGTA AATGATTAAA CTTTAGTTTA ACTAAAAAAG TAATTTTTTA   
  
  
- AATTACCAAT AAAAATATTA AATTTTATTG TTATATTTTA TTTTTTTAAT TAATAACTAA AATAATTTAA   
  
  
- CACCACGCAA ATAAACTTTC ACATATACAG TATAAAGTAT TAAAATGTAA TATTTATTTT TATATATTTT   
  
  
- ATGTATATTA ATCAACACAA TAAAGACCTG TAGTGAATGT TTTACATTTA TACTTTGTTT TTAAAACGCG   
  
  
- TAAATTTTAT ATATTTTATG TATATTAATC AACACAATAA AGACCTGTAG TGAATGTTTT ACATTTATAC   
  
  
- TTTGTTTTTA AAACGCGTAA ATTCATTAAT AATAATTGAT AAAAATTACT AATATTCTAT TTATGTAAAT   
  
  
- AAATTAATTA TTAAAAAATC TTCTTTATAT AAATTAGATT TGAAAATACC CTATACGTAC CTACCGTACT   
  
  
- TTAAACGTGA ACAGTATACG GAGGGAGGAT GTGATTTCGG TTATCCGTAG TCGTCGGTAA ATGCCGTGAC   
  
  
- TCTTCCTATT TATAATCTAT ACACTAACCC GGAGTCGGCG GGCGACCAGT TTCAGTGCAC GAAACTGGTT   
  
  
- TGGTGCGCCC GGTTTTGTGT GTGTGCGGGT GTGTGTGAGA GAGAGAGAGA GAAAGAAGGG CATAAAAAGT   
  
  
- GCCAGTTTGA AGAGCAATGG AAGCGTCTTT GACGGAACGT TTCTTTTTTG TGTGAGAGAG AGAGAGAGAA   
  
  
- AGAAGGGCAT AAAAAGTGCC AGTTTGAAGA GCAATGGAAG CGTCTTTGAC GGAACGTTTC TTTTTCTTCT   
  
  
- TTTGATCTTT TTTTTCTCTC TTTTTGGTTT CTTCGTCTCT GCGGAACATG GAATGCACTG GCAGAAGAGG   
  
  
- CAAGAGAGAG AGAGAGAGAG GGGGGGAACT AGGAATATAA GTAGCATATT CGACAGAAGA AAGACATGGA   
  
  
- CATTGGAATC AAGAGAGAGA CAGCGAAAA

+     Box 4

| Site Name | Organism | Position | Strand | Matrix score. | sequence | function |
| --- | --- | --- | --- | --- | --- | --- |
| Box 4 | Petroselinum crispum | 607 | + | 6 | ATTAAT | part of a conserved DNA module involved in light responsiveness |
| Box 4 | Petroselinum crispum | 176 | + | 6 | ATTAAT | part of a conserved DNA module involved in light responsiveness |
| Box 4 | Petroselinum crispum | 915 | - | 6 | ATTAAT | part of a conserved DNA module involved in light responsiveness |

> 2018/04/13 10:10:12  
+ ATTATCGCAC TTTATCCACT AAGAACCTAA TTACCCAACT AGTGGGTTAA CTTGTAGGCT AAGCTTACAT   
  
  
+ ATCTGCTTAT GTTCTCTCCA TTTTGTGTGA AAACGTGTAG ACTTAATCTA ACCAAATCAT GATTGTGACT   
  
  
+ TTGATTATGA AATAAAAGGT CTATGGTTTA ATTAAATTAA TTACAATATG ACGTAAACTA AACATATACC   
  
  
+ TTTAATGGCT CTGGAGCTTT GTGTCATTTT TTATTTCCAC GATCATTAGG GCAAAAATTG GTTGAGAAAT   
  
  
+ TATAATTTAG AGTATAAGAT ACTCTGGCTA GCTTTTGGAT TTCATTGACT AAAAAGGCAT TACCCCTTTT   
  
  
+ ACACTTTAGG ACTCCTAGAA TCTTCTAAGC TAGACTTAAT TATATCTTTT AATAACTTTT TTAATGCTTG   
  
  
+ TTTGACACTA TTGCATGTTA GCCTCTAAGC TTTATTTTAT CGAGATCCAT ATAAATGTTT TTTTTTGTTT   
  
  
+ TTTCCGGTTT TACATTGAGA TAAAAATCAT TTACTAATTT GAAATCAAAT TGATTTTTTC ATTAAAAAAT   
  
  
+ TTAATGGTTA TTTTTATAAT TTAAAATAAC AATATAAAAT AAAAAAATTA ATTATTGATT TTATTAAATT   
  
  
+ GTGGTGCGTT TATTTGAAAG TGTATATGTC ATATTTCATA ATTTTACATT ATAAATAAAA ATATATAAAA   
  
  
+ TACATATAAT TAGTTGTGTT ATTTCTGGAC ATCACTTACA AAATGTAAAT ATGAAACAAA AATTTTGCGC   
  
  
+ ATTTAAAATA TATAAAATAC ATATAATTAG TTGTGTTATT TCTGGACATC ACTTACAAAA TGTAAATATG   
  
  
+ AAACAAAAAT TTTGCGCATT TAAGTAATTA TTATTAACTA TTTTTAATGA TTATAAGATA AATACATTTA   
  
  
+ TTTAATTAAT AATTTTTTAG AAGAAATATA TTTAATCTAA ACTTTTATGG GATATGCATG GATGGCATGA   
  
  
+ AATTTGCACT TGTCATATGC CTCCCTCCTA CACTAAAGCC AATAGGCATC AGCAGCCATT TACGGCACTG   
  
  
+ AGAAGGATAA ATATTAGATA TGTGATTGGG CCTCAGCCGC CCGCTGGTCA AAGTCACGTG CTTTGACCAA   
  
  
+ ACCACGCGGG CCAAAACACA CACACGCCCA CACACACTCT CTCTCTCTCT CTTTCTTCCC GTATTTTTCA   
  
  
+ CGGTCAAACT TCTCGTTACC TTCGCAGAAA CTGCCTTGCA AAGAAAAAAC ACACTCTCTC TCTCTCTCTT   
  
  
+ TCTTCCCGTA TTTTTCACGG TCAAACTTCT CGTTACCTTC GCAGAAACTG CCTTGCAAAG AAAAAGAAGA   
  
  
+ AAACTAGAAA AAAAAGAGAG AAAAACCAAA GAAGCAGAGA CGCCTTGTAC CTTACGTGAC CGTCTTCTCC   
  
  
+ GTTCTCTCTC TCTCTCTCTC CCCCCCTTGA TCCTTATATT CATCGTATAA GCTGTCTTCT TTCTGTACCT   
  
  
+ GTAACCTTAG TTCTCTCTCT GTCGCTTTT  

- TAATAGCGTG AAATAGGTGA TTCTTGGATT AATGGGTTGA TCACCCAATT GAACATCCGA TTCGAATGTA   
  
  
- TAGACGAATA CAAGAGAGGT AAAACACACT TTTGCACATC TGAATTAGAT TGGTTTAGTA CTAACACTGA   
  
  
- AACTAATACT TTATTTTCCA GATACCAAAT TAATTTAATT AATGTTATAC TGCATTTGAT TTGTATATGG   
  
  
- AAATTACCGA GACCTCGAAA CACAGTAAAA AATAAAGGTG CTAGTAATCC CGTTTTTAAC CAACTCTTTA   
  
  
- ATATTAAATC TCATATTCTA TGAGACCGAT CGAAAACCTA AAGTAACTGA TTTTTCCGTA ATGGGGAAAA   
  
  
- TGTGAAATCC TGAGGATCTT AGAAGATTCG ATCTGAATTA ATATAGAAAA TTATTGAAAA AATTACGAAC   
  
  
- AAACTGTGAT AACGTACAAT CGGAGATTCG AAATAAAATA GCTCTAGGTA TATTTACAAA AAAAAACAAA   
  
  
- AAAGGCCAAA ATGTAACTCT ATTTTTAGTA AATGATTAAA CTTTAGTTTA ACTAAAAAAG TAATTTTTTA   
  
  
- AATTACCAAT AAAAATATTA AATTTTATTG TTATATTTTA TTTTTTTAAT TAATAACTAA AATAATTTAA   
  
  
- CACCACGCAA ATAAACTTTC ACATATACAG TATAAAGTAT TAAAATGTAA TATTTATTTT TATATATTTT   
  
  
- ATGTATATTA ATCAACACAA TAAAGACCTG TAGTGAATGT TTTACATTTA TACTTTGTTT TTAAAACGCG   
  
  
- TAAATTTTAT ATATTTTATG TATATTAATC AACACAATAA AGACCTGTAG TGAATGTTTT ACATTTATAC   
  
  
- TTTGTTTTTA AAACGCGTAA ATTCATTAAT AATAATTGAT AAAAATTACT AATATTCTAT TTATGTAAAT   
  
  
- AAATTAATTA TTAAAAAATC TTCTTTATAT AAATTAGATT TGAAAATACC CTATACGTAC CTACCGTACT   
  
  
- TTAAACGTGA ACAGTATACG GAGGGAGGAT GTGATTTCGG TTATCCGTAG TCGTCGGTAA ATGCCGTGAC   
  
  
- TCTTCCTATT TATAATCTAT ACACTAACCC GGAGTCGGCG GGCGACCAGT TTCAGTGCAC GAAACTGGTT   
  
  
- TGGTGCGCCC GGTTTTGTGT GTGTGCGGGT GTGTGTGAGA GAGAGAGAGA GAAAGAAGGG CATAAAAAGT   
  
  
- GCCAGTTTGA AGAGCAATGG AAGCGTCTTT GACGGAACGT TTCTTTTTTG TGTGAGAGAG AGAGAGAGAA   
  
  
- AGAAGGGCAT AAAAAGTGCC AGTTTGAAGA GCAATGGAAG CGTCTTTGAC GGAACGTTTC TTTTTCTTCT   
  
  
- TTTGATCTTT TTTTTCTCTC TTTTTGGTTT CTTCGTCTCT GCGGAACATG GAATGCACTG GCAGAAGAGG   
  
  
- CAAGAGAGAG AGAGAGAGAG GGGGGGAACT AGGAATATAA GTAGCATATT CGACAGAAGA AAGACATGGA   
  
  
- CATTGGAATC AAGAGAGAGA CAGCGAAAA

+     Box I

| Site Name | Organism | Position | Strand | Matrix score. | sequence | function |
| --- | --- | --- | --- | --- | --- | --- |
| Box I | Pisum sativum | 528 | - | 7 | TTTCAAA | light responsive element |
| Box I | Pisum sativum | 643 | - | 7 | TTTCAAA | light responsive element |

> 2018/04/13 10:10:12  
+ ATTATCGCAC TTTATCCACT AAGAACCTAA TTACCCAACT AGTGGGTTAA CTTGTAGGCT AAGCTTACAT   
  
  
+ ATCTGCTTAT GTTCTCTCCA TTTTGTGTGA AAACGTGTAG ACTTAATCTA ACCAAATCAT GATTGTGACT   
  
  
+ TTGATTATGA AATAAAAGGT CTATGGTTTA ATTAAATTAA TTACAATATG ACGTAAACTA AACATATACC   
  
  
+ TTTAATGGCT CTGGAGCTTT GTGTCATTTT TTATTTCCAC GATCATTAGG GCAAAAATTG GTTGAGAAAT   
  
  
+ TATAATTTAG AGTATAAGAT ACTCTGGCTA GCTTTTGGAT TTCATTGACT AAAAAGGCAT TACCCCTTTT   
  
  
+ ACACTTTAGG ACTCCTAGAA TCTTCTAAGC TAGACTTAAT TATATCTTTT AATAACTTTT TTAATGCTTG   
  
  
+ TTTGACACTA TTGCATGTTA GCCTCTAAGC TTTATTTTAT CGAGATCCAT ATAAATGTTT TTTTTTGTTT   
  
  
+ TTTCCGGTTT TACATTGAGA TAAAAATCAT TTACTAATTT GAAATCAAAT TGATTTTTTC ATTAAAAAAT   
  
  
+ TTAATGGTTA TTTTTATAAT TTAAAATAAC AATATAAAAT AAAAAAATTA ATTATTGATT TTATTAAATT   
  
  
+ GTGGTGCGTT TATTTGAAAG TGTATATGTC ATATTTCATA ATTTTACATT ATAAATAAAA ATATATAAAA   
  
  
+ TACATATAAT TAGTTGTGTT ATTTCTGGAC ATCACTTACA AAATGTAAAT ATGAAACAAA AATTTTGCGC   
  
  
+ ATTTAAAATA TATAAAATAC ATATAATTAG TTGTGTTATT TCTGGACATC ACTTACAAAA TGTAAATATG   
  
  
+ AAACAAAAAT TTTGCGCATT TAAGTAATTA TTATTAACTA TTTTTAATGA TTATAAGATA AATACATTTA   
  
  
+ TTTAATTAAT AATTTTTTAG AAGAAATATA TTTAATCTAA ACTTTTATGG GATATGCATG GATGGCATGA   
  
  
+ AATTTGCACT TGTCATATGC CTCCCTCCTA CACTAAAGCC AATAGGCATC AGCAGCCATT TACGGCACTG   
  
  
+ AGAAGGATAA ATATTAGATA TGTGATTGGG CCTCAGCCGC CCGCTGGTCA AAGTCACGTG CTTTGACCAA   
  
  
+ ACCACGCGGG CCAAAACACA CACACGCCCA CACACACTCT CTCTCTCTCT CTTTCTTCCC GTATTTTTCA   
  
  
+ CGGTCAAACT TCTCGTTACC TTCGCAGAAA CTGCCTTGCA AAGAAAAAAC ACACTCTCTC TCTCTCTCTT   
  
  
+ TCTTCCCGTA TTTTTCACGG TCAAACTTCT CGTTACCTTC GCAGAAACTG CCTTGCAAAG AAAAAGAAGA   
  
  
+ AAACTAGAAA AAAAAGAGAG AAAAACCAAA GAAGCAGAGA CGCCTTGTAC CTTACGTGAC CGTCTTCTCC   
  
  
+ GTTCTCTCTC TCTCTCTCTC CCCCCCTTGA TCCTTATATT CATCGTATAA GCTGTCTTCT TTCTGTACCT   
  
  
+ GTAACCTTAG TTCTCTCTCT GTCGCTTTT  

- TAATAGCGTG AAATAGGTGA TTCTTGGATT AATGGGTTGA TCACCCAATT GAACATCCGA TTCGAATGTA   
  
  
- TAGACGAATA CAAGAGAGGT AAAACACACT TTTGCACATC TGAATTAGAT TGGTTTAGTA CTAACACTGA   
  
  
- AACTAATACT TTATTTTCCA GATACCAAAT TAATTTAATT AATGTTATAC TGCATTTGAT TTGTATATGG   
  
  
- AAATTACCGA GACCTCGAAA CACAGTAAAA AATAAAGGTG CTAGTAATCC CGTTTTTAAC CAACTCTTTA   
  
  
- ATATTAAATC TCATATTCTA TGAGACCGAT CGAAAACCTA AAGTAACTGA TTTTTCCGTA ATGGGGAAAA   
  
  
- TGTGAAATCC TGAGGATCTT AGAAGATTCG ATCTGAATTA ATATAGAAAA TTATTGAAAA AATTACGAAC   
  
  
- AAACTGTGAT AACGTACAAT CGGAGATTCG AAATAAAATA GCTCTAGGTA TATTTACAAA AAAAAACAAA   
  
  
- AAAGGCCAAA ATGTAACTCT ATTTTTAGTA AATGATTAAA CTTTAGTTTA ACTAAAAAAG TAATTTTTTA   
  
  
- AATTACCAAT AAAAATATTA AATTTTATTG TTATATTTTA TTTTTTTAAT TAATAACTAA AATAATTTAA   
  
  
- CACCACGCAA ATAAACTTTC ACATATACAG TATAAAGTAT TAAAATGTAA TATTTATTTT TATATATTTT   
  
  
- ATGTATATTA ATCAACACAA TAAAGACCTG TAGTGAATGT TTTACATTTA TACTTTGTTT TTAAAACGCG   
  
  
- TAAATTTTAT ATATTTTATG TATATTAATC AACACAATAA AGACCTGTAG TGAATGTTTT ACATTTATAC   
  
  
- TTTGTTTTTA AAACGCGTAA ATTCATTAAT AATAATTGAT AAAAATTACT AATATTCTAT TTATGTAAAT   
  
  
- AAATTAATTA TTAAAAAATC TTCTTTATAT AAATTAGATT TGAAAATACC CTATACGTAC CTACCGTACT   
  
  
- TTAAACGTGA ACAGTATACG GAGGGAGGAT GTGATTTCGG TTATCCGTAG TCGTCGGTAA ATGCCGTGAC   
  
  
- TCTTCCTATT TATAATCTAT ACACTAACCC GGAGTCGGCG GGCGACCAGT TTCAGTGCAC GAAACTGGTT   
  
  
- TGGTGCGCCC GGTTTTGTGT GTGTGCGGGT GTGTGTGAGA GAGAGAGAGA GAAAGAAGGG CATAAAAAGT   
  
  
- GCCAGTTTGA AGAGCAATGG AAGCGTCTTT GACGGAACGT TTCTTTTTTG TGTGAGAGAG AGAGAGAGAA   
  
  
- AGAAGGGCAT AAAAAGTGCC AGTTTGAAGA GCAATGGAAG CGTCTTTGAC GGAACGTTTC TTTTTCTTCT   
  
  
- TTTGATCTTT TTTTTCTCTC TTTTTGGTTT CTTCGTCTCT GCGGAACATG GAATGCACTG GCAGAAGAGG   
  
  
- CAAGAGAGAG AGAGAGAGAG GGGGGGAACT AGGAATATAA GTAGCATATT CGACAGAAGA AAGACATGGA   
  
  
- CATTGGAATC AAGAGAGAGA CAGCGAAAA

+     Box III

| Site Name | Organism | Position | Strand | Matrix score. | sequence | function |
| --- | --- | --- | --- | --- | --- | --- |
| Box III | Pisum sativum | 346 | + | 9 | CATTTACACT | protein binding site |
| Box III | Pisum sativum | 649 | - | 9 | CATTTACACT | protein binding site |

> 2018/04/13 10:10:12  
+ ATTATCGCAC TTTATCCACT AAGAACCTAA TTACCCAACT AGTGGGTTAA CTTGTAGGCT AAGCTTACAT   
  
  
+ ATCTGCTTAT GTTCTCTCCA TTTTGTGTGA AAACGTGTAG ACTTAATCTA ACCAAATCAT GATTGTGACT   
  
  
+ TTGATTATGA AATAAAAGGT CTATGGTTTA ATTAAATTAA TTACAATATG ACGTAAACTA AACATATACC   
  
  
+ TTTAATGGCT CTGGAGCTTT GTGTCATTTT TTATTTCCAC GATCATTAGG GCAAAAATTG GTTGAGAAAT   
  
  
+ TATAATTTAG AGTATAAGAT ACTCTGGCTA GCTTTTGGAT TTCATTGACT AAAAAGGCAT TACCCCTTTT   
  
  
+ ACACTTTAGG ACTCCTAGAA TCTTCTAAGC TAGACTTAAT TATATCTTTT AATAACTTTT TTAATGCTTG   
  
  
+ TTTGACACTA TTGCATGTTA GCCTCTAAGC TTTATTTTAT CGAGATCCAT ATAAATGTTT TTTTTTGTTT   
  
  
+ TTTCCGGTTT TACATTGAGA TAAAAATCAT TTACTAATTT GAAATCAAAT TGATTTTTTC ATTAAAAAAT   
  
  
+ TTAATGGTTA TTTTTATAAT TTAAAATAAC AATATAAAAT AAAAAAATTA ATTATTGATT TTATTAAATT   
  
  
+ GTGGTGCGTT TATTTGAAAG TGTATATGTC ATATTTCATA ATTTTACATT ATAAATAAAA ATATATAAAA   
  
  
+ TACATATAAT TAGTTGTGTT ATTTCTGGAC ATCACTTACA AAATGTAAAT ATGAAACAAA AATTTTGCGC   
  
  
+ ATTTAAAATA TATAAAATAC ATATAATTAG TTGTGTTATT TCTGGACATC ACTTACAAAA TGTAAATATG   
  
  
+ AAACAAAAAT TTTGCGCATT TAAGTAATTA TTATTAACTA TTTTTAATGA TTATAAGATA AATACATTTA   
  
  
+ TTTAATTAAT AATTTTTTAG AAGAAATATA TTTAATCTAA ACTTTTATGG GATATGCATG GATGGCATGA   
  
  
+ AATTTGCACT TGTCATATGC CTCCCTCCTA CACTAAAGCC AATAGGCATC AGCAGCCATT TACGGCACTG   
  
  
+ AGAAGGATAA ATATTAGATA TGTGATTGGG CCTCAGCCGC CCGCTGGTCA AAGTCACGTG CTTTGACCAA   
  
  
+ ACCACGCGGG CCAAAACACA CACACGCCCA CACACACTCT CTCTCTCTCT CTTTCTTCCC GTATTTTTCA   
  
  
+ CGGTCAAACT TCTCGTTACC TTCGCAGAAA CTGCCTTGCA AAGAAAAAAC ACACTCTCTC TCTCTCTCTT   
  
  
+ TCTTCCCGTA TTTTTCACGG TCAAACTTCT CGTTACCTTC GCAGAAACTG CCTTGCAAAG AAAAAGAAGA   
  
  
+ AAACTAGAAA AAAAAGAGAG AAAAACCAAA GAAGCAGAGA CGCCTTGTAC CTTACGTGAC CGTCTTCTCC   
  
  
+ GTTCTCTCTC TCTCTCTCTC CCCCCCTTGA TCCTTATATT CATCGTATAA GCTGTCTTCT TTCTGTACCT   
  
  
+ GTAACCTTAG TTCTCTCTCT GTCGCTTTT  

- TAATAGCGTG AAATAGGTGA TTCTTGGATT AATGGGTTGA TCACCCAATT GAACATCCGA TTCGAATGTA   
  
  
- TAGACGAATA CAAGAGAGGT AAAACACACT TTTGCACATC TGAATTAGAT TGGTTTAGTA CTAACACTGA   
  
  
- AACTAATACT TTATTTTCCA GATACCAAAT TAATTTAATT AATGTTATAC TGCATTTGAT TTGTATATGG   
  
  
- AAATTACCGA GACCTCGAAA CACAGTAAAA AATAAAGGTG CTAGTAATCC CGTTTTTAAC CAACTCTTTA   
  
  
- ATATTAAATC TCATATTCTA TGAGACCGAT CGAAAACCTA AAGTAACTGA TTTTTCCGTA ATGGGGAAAA   
  
  
- TGTGAAATCC TGAGGATCTT AGAAGATTCG ATCTGAATTA ATATAGAAAA TTATTGAAAA AATTACGAAC   
  
  
- AAACTGTGAT AACGTACAAT CGGAGATTCG AAATAAAATA GCTCTAGGTA TATTTACAAA AAAAAACAAA   
  
  
- AAAGGCCAAA ATGTAACTCT ATTTTTAGTA AATGATTAAA CTTTAGTTTA ACTAAAAAAG TAATTTTTTA   
  
  
- AATTACCAAT AAAAATATTA AATTTTATTG TTATATTTTA TTTTTTTAAT TAATAACTAA AATAATTTAA   
  
  
- CACCACGCAA ATAAACTTTC ACATATACAG TATAAAGTAT TAAAATGTAA TATTTATTTT TATATATTTT   
  
  
- ATGTATATTA ATCAACACAA TAAAGACCTG TAGTGAATGT TTTACATTTA TACTTTGTTT TTAAAACGCG   
  
  
- TAAATTTTAT ATATTTTATG TATATTAATC AACACAATAA AGACCTGTAG TGAATGTTTT ACATTTATAC   
  
  
- TTTGTTTTTA AAACGCGTAA ATTCATTAAT AATAATTGAT AAAAATTACT AATATTCTAT TTATGTAAAT   
  
  
- AAATTAATTA TTAAAAAATC TTCTTTATAT AAATTAGATT TGAAAATACC CTATACGTAC CTACCGTACT   
  
  
- TTAAACGTGA ACAGTATACG GAGGGAGGAT GTGATTTCGG TTATCCGTAG TCGTCGGTAA ATGCCGTGAC   
  
  
- TCTTCCTATT TATAATCTAT ACACTAACCC GGAGTCGGCG GGCGACCAGT TTCAGTGCAC GAAACTGGTT   
  
  
- TGGTGCGCCC GGTTTTGTGT GTGTGCGGGT GTGTGTGAGA GAGAGAGAGA GAAAGAAGGG CATAAAAAGT   
  
  
- GCCAGTTTGA AGAGCAATGG AAGCGTCTTT GACGGAACGT TTCTTTTTTG TGTGAGAGAG AGAGAGAGAA   
  
  
- AGAAGGGCAT AAAAAGTGCC AGTTTGAAGA GCAATGGAAG CGTCTTTGAC GGAACGTTTC TTTTTCTTCT   
  
  
- TTTGATCTTT TTTTTCTCTC TTTTTGGTTT CTTCGTCTCT GCGGAACATG GAATGCACTG GCAGAAGAGG   
  
  
- CAAGAGAGAG AGAGAGAGAG GGGGGGAACT AGGAATATAA GTAGCATATT CGACAGAAGA AAGACATGGA   
  
  
- CATTGGAATC AAGAGAGAGA CAGCGAAAA

+     Box-W1

| Site Name | Organism | Position | Strand | Matrix score. | sequence | function |
| --- | --- | --- | --- | --- | --- | --- |
| Box-W1 | Petroselinum crispum | 1279 | - | 6 | TTGACC | fungal elicitor responsive element |
| Box-W1 | Petroselinum crispum | 1113 | + | 6 | TTGACC | fungal elicitor responsive element |
| Box-W1 | Petroselinum crispum | 1192 | - | 6 | TTGACC | fungal elicitor responsive element |
| Box-W1 | Petroselinum crispum | 1096 | - | 6 | TTGACC | fungal elicitor responsive element |

> 2018/04/13 10:10:12  
+ ATTATCGCAC TTTATCCACT AAGAACCTAA TTACCCAACT AGTGGGTTAA CTTGTAGGCT AAGCTTACAT   
  
  
+ ATCTGCTTAT GTTCTCTCCA TTTTGTGTGA AAACGTGTAG ACTTAATCTA ACCAAATCAT GATTGTGACT   
  
  
+ TTGATTATGA AATAAAAGGT CTATGGTTTA ATTAAATTAA TTACAATATG ACGTAAACTA AACATATACC   
  
  
+ TTTAATGGCT CTGGAGCTTT GTGTCATTTT TTATTTCCAC GATCATTAGG GCAAAAATTG GTTGAGAAAT   
  
  
+ TATAATTTAG AGTATAAGAT ACTCTGGCTA GCTTTTGGAT TTCATTGACT AAAAAGGCAT TACCCCTTTT   
  
  
+ ACACTTTAGG ACTCCTAGAA TCTTCTAAGC TAGACTTAAT TATATCTTTT AATAACTTTT TTAATGCTTG   
  
  
+ TTTGACACTA TTGCATGTTA GCCTCTAAGC TTTATTTTAT CGAGATCCAT ATAAATGTTT TTTTTTGTTT   
  
  
+ TTTCCGGTTT TACATTGAGA TAAAAATCAT TTACTAATTT GAAATCAAAT TGATTTTTTC ATTAAAAAAT   
  
  
+ TTAATGGTTA TTTTTATAAT TTAAAATAAC AATATAAAAT AAAAAAATTA ATTATTGATT TTATTAAATT   
  
  
+ GTGGTGCGTT TATTTGAAAG TGTATATGTC ATATTTCATA ATTTTACATT ATAAATAAAA ATATATAAAA   
  
  
+ TACATATAAT TAGTTGTGTT ATTTCTGGAC ATCACTTACA AAATGTAAAT ATGAAACAAA AATTTTGCGC   
  
  
+ ATTTAAAATA TATAAAATAC ATATAATTAG TTGTGTTATT TCTGGACATC ACTTACAAAA TGTAAATATG   
  
  
+ AAACAAAAAT TTTGCGCATT TAAGTAATTA TTATTAACTA TTTTTAATGA TTATAAGATA AATACATTTA   
  
  
+ TTTAATTAAT AATTTTTTAG AAGAAATATA TTTAATCTAA ACTTTTATGG GATATGCATG GATGGCATGA   
  
  
+ AATTTGCACT TGTCATATGC CTCCCTCCTA CACTAAAGCC AATAGGCATC AGCAGCCATT TACGGCACTG   
  
  
+ AGAAGGATAA ATATTAGATA TGTGATTGGG CCTCAGCCGC CCGCTGGTCA AAGTCACGTG CTTTGACCAA   
  
  
+ ACCACGCGGG CCAAAACACA CACACGCCCA CACACACTCT CTCTCTCTCT CTTTCTTCCC GTATTTTTCA   
  
  
+ CGGTCAAACT TCTCGTTACC TTCGCAGAAA CTGCCTTGCA AAGAAAAAAC ACACTCTCTC TCTCTCTCTT   
  
  
+ TCTTCCCGTA TTTTTCACGG TCAAACTTCT CGTTACCTTC GCAGAAACTG CCTTGCAAAG AAAAAGAAGA   
  
  
+ AAACTAGAAA AAAAAGAGAG AAAAACCAAA GAAGCAGAGA CGCCTTGTAC CTTACGTGAC CGTCTTCTCC   
  
  
+ GTTCTCTCTC TCTCTCTCTC CCCCCCTTGA TCCTTATATT CATCGTATAA GCTGTCTTCT TTCTGTACCT   
  
  
+ GTAACCTTAG TTCTCTCTCT GTCGCTTTT  

- TAATAGCGTG AAATAGGTGA TTCTTGGATT AATGGGTTGA TCACCCAATT GAACATCCGA TTCGAATGTA   
  
  
- TAGACGAATA CAAGAGAGGT AAAACACACT TTTGCACATC TGAATTAGAT TGGTTTAGTA CTAACACTGA   
  
  
- AACTAATACT TTATTTTCCA GATACCAAAT TAATTTAATT AATGTTATAC TGCATTTGAT TTGTATATGG   
  
  
- AAATTACCGA GACCTCGAAA CACAGTAAAA AATAAAGGTG CTAGTAATCC CGTTTTTAAC CAACTCTTTA   
  
  
- ATATTAAATC TCATATTCTA TGAGACCGAT CGAAAACCTA AAGTAACTGA TTTTTCCGTA ATGGGGAAAA   
  
  
- TGTGAAATCC TGAGGATCTT AGAAGATTCG ATCTGAATTA ATATAGAAAA TTATTGAAAA AATTACGAAC   
  
  
- AAACTGTGAT AACGTACAAT CGGAGATTCG AAATAAAATA GCTCTAGGTA TATTTACAAA AAAAAACAAA   
  
  
- AAAGGCCAAA ATGTAACTCT ATTTTTAGTA AATGATTAAA CTTTAGTTTA ACTAAAAAAG TAATTTTTTA   
  
  
- AATTACCAAT AAAAATATTA AATTTTATTG TTATATTTTA TTTTTTTAAT TAATAACTAA AATAATTTAA   
  
  
- CACCACGCAA ATAAACTTTC ACATATACAG TATAAAGTAT TAAAATGTAA TATTTATTTT TATATATTTT   
  
  
- ATGTATATTA ATCAACACAA TAAAGACCTG TAGTGAATGT TTTACATTTA TACTTTGTTT TTAAAACGCG   
  
  
- TAAATTTTAT ATATTTTATG TATATTAATC AACACAATAA AGACCTGTAG TGAATGTTTT ACATTTATAC   
  
  
- TTTGTTTTTA AAACGCGTAA ATTCATTAAT AATAATTGAT AAAAATTACT AATATTCTAT TTATGTAAAT   
  
  
- AAATTAATTA TTAAAAAATC TTCTTTATAT AAATTAGATT TGAAAATACC CTATACGTAC CTACCGTACT   
  
  
- TTAAACGTGA ACAGTATACG GAGGGAGGAT GTGATTTCGG TTATCCGTAG TCGTCGGTAA ATGCCGTGAC   
  
  
- TCTTCCTATT TATAATCTAT ACACTAACCC GGAGTCGGCG GGCGACCAGT TTCAGTGCAC GAAACTGGTT   
  
  
- TGGTGCGCCC GGTTTTGTGT GTGTGCGGGT GTGTGTGAGA GAGAGAGAGA GAAAGAAGGG CATAAAAAGT   
  
  
- GCCAGTTTGA AGAGCAATGG AAGCGTCTTT GACGGAACGT TTCTTTTTTG TGTGAGAGAG AGAGAGAGAA   
  
  
- AGAAGGGCAT AAAAAGTGCC AGTTTGAAGA GCAATGGAAG CGTCTTTGAC GGAACGTTTC TTTTTCTTCT   
  
  
- TTTGATCTTT TTTTTCTCTC TTTTTGGTTT CTTCGTCTCT GCGGAACATG GAATGCACTG GCAGAAGAGG   
  
  
- CAAGAGAGAG AGAGAGAGAG GGGGGGAACT AGGAATATAA GTAGCATATT CGACAGAAGA AAGACATGGA   
  
  
- CATTGGAATC AAGAGAGAGA CAGCGAAAA

+     CAAT-box

| Site Name | Organism | Position | Strand | Matrix score. | sequence | function |
| --- | --- | --- | --- | --- | --- | --- |
| CAAT-box | Brassica rapa | 527 | - | 5 | CAAAT | common cis-acting element in promoter and enhancer regions |
| CAAT-box | Glycine max | 627 | - | 5 | CAATT | common cis-acting element in promoter and enhancer regions |
| CAAT-box | Hordeum vulgare | 324 | - | 4 | CAAT | common cis-acting element in promoter and enhancer regions |
| CAAT-box | Arabidopsis thaliana | 1019 | + | 5 | CCAAT | common cis-acting element in promoter and enhancer regions |
| CAAT-box | Brassica rapa | 123 | + | 5 | CAAAT | common cis-acting element in promoter and enhancer regions |
| CAAT-box | Brassica rapa | 642 | - | 5 | CAAAT | common cis-acting element in promoter and enhancer regions |
| CAAT-box | Glycine max | 266 | - | 5 | CAATT | common cis-acting element in promoter and enhancer regions |
| CAAT-box | Arabidopsis thaliana | 1075 | - | 5 | CCAAT | common cis-acting element in promoter and enhancer regions |
| CAAT-box | Brassica rapa | 536 | + | 5 | CAAAT | common cis-acting element in promoter and enhancer regions |
| CAAT-box | Glycine max | 538 | - | 5 | CAATT | common cis-acting element in promoter and enhancer regions |
| CAAT-box | Hordeum vulgare | 590 | + | 4 | CAAT | common cis-acting element in promoter and enhancer regions |
| CAAT-box | Arabidopsis thaliana | 267 | - | 5 | CCAAT | common cis-acting element in promoter and enhancer regions |
| CAAT-box | Hordeum vulgare | 1020 | + | 4 | CAAT | common cis-acting element in promoter and enhancer regions |
| CAAT-box | Hordeum vulgare | 504 | - | 4 | CAAT | common cis-acting element in promoter and enhancer regions |
| CAAT-box | Brassica rapa | 982 | - | 5 | CAAAT | common cis-acting element in promoter and enhancer regions |
| CAAT-box | Hordeum vulgare | 539 | - | 4 | CAAT | common cis-acting element in promoter and enhancer regions |
| CAAT-box | Hordeum vulgare | 614 | - | 4 | CAAT | common cis-acting element in promoter and enhancer regions |
| CAAT-box | Hordeum vulgare | 132 | - | 4 | CAAT | common cis-acting element in promoter and enhancer regions |
| CAAT-box | Hordeum vulgare | 628 | - | 4 | CAAT | common cis-acting element in promoter and enhancer regions |
| CAAT-box | Hordeum vulgare | 184 | + | 4 | CAAT | common cis-acting element in promoter and enhancer regions |
| CAAT-box | Hordeum vulgare | 430 | - | 4 | CAAT | common cis-acting element in promoter and enhancer regions |

> 2018/04/13 10:10:12  
+ ATTATCGCAC TTTATCCACT AAGAACCTAA TTACCCAACT AGTGGGTTAA CTTGTAGGCT AAGCTTACAT   
  
  
+ ATCTGCTTAT GTTCTCTCCA TTTTGTGTGA AAACGTGTAG ACTTAATCTA ACCAAATCAT GATTGTGACT   
  
  
+ TTGATTATGA AATAAAAGGT CTATGGTTTA ATTAAATTAA TTACAATATG ACGTAAACTA AACATATACC   
  
  
+ TTTAATGGCT CTGGAGCTTT GTGTCATTTT TTATTTCCAC GATCATTAGG GCAAAAATTG GTTGAGAAAT   
  
  
+ TATAATTTAG AGTATAAGAT ACTCTGGCTA GCTTTTGGAT TTCATTGACT AAAAAGGCAT TACCCCTTTT   
  
  
+ ACACTTTAGG ACTCCTAGAA TCTTCTAAGC TAGACTTAAT TATATCTTTT AATAACTTTT TTAATGCTTG   
  
  
+ TTTGACACTA TTGCATGTTA GCCTCTAAGC TTTATTTTAT CGAGATCCAT ATAAATGTTT TTTTTTGTTT   
  
  
+ TTTCCGGTTT TACATTGAGA TAAAAATCAT TTACTAATTT GAAATCAAAT TGATTTTTTC ATTAAAAAAT   
  
  
+ TTAATGGTTA TTTTTATAAT TTAAAATAAC AATATAAAAT AAAAAAATTA ATTATTGATT TTATTAAATT   
  
  
+ GTGGTGCGTT TATTTGAAAG TGTATATGTC ATATTTCATA ATTTTACATT ATAAATAAAA ATATATAAAA   
  
  
+ TACATATAAT TAGTTGTGTT ATTTCTGGAC ATCACTTACA AAATGTAAAT ATGAAACAAA AATTTTGCGC   
  
  
+ ATTTAAAATA TATAAAATAC ATATAATTAG TTGTGTTATT TCTGGACATC ACTTACAAAA TGTAAATATG   
  
  
+ AAACAAAAAT TTTGCGCATT TAAGTAATTA TTATTAACTA TTTTTAATGA TTATAAGATA AATACATTTA   
  
  
+ TTTAATTAAT AATTTTTTAG AAGAAATATA TTTAATCTAA ACTTTTATGG GATATGCATG GATGGCATGA   
  
  
+ AATTTGCACT TGTCATATGC CTCCCTCCTA CACTAAAGCC AATAGGCATC AGCAGCCATT TACGGCACTG   
  
  
+ AGAAGGATAA ATATTAGATA TGTGATTGGG CCTCAGCCGC CCGCTGGTCA AAGTCACGTG CTTTGACCAA   
  
  
+ ACCACGCGGG CCAAAACACA CACACGCCCA CACACACTCT CTCTCTCTCT CTTTCTTCCC GTATTTTTCA   
  
  
+ CGGTCAAACT TCTCGTTACC TTCGCAGAAA CTGCCTTGCA AAGAAAAAAC ACACTCTCTC TCTCTCTCTT   
  
  
+ TCTTCCCGTA TTTTTCACGG TCAAACTTCT CGTTACCTTC GCAGAAACTG CCTTGCAAAG AAAAAGAAGA   
  
  
+ AAACTAGAAA AAAAAGAGAG AAAAACCAAA GAAGCAGAGA CGCCTTGTAC CTTACGTGAC CGTCTTCTCC   
  
  
+ GTTCTCTCTC TCTCTCTCTC CCCCCCTTGA TCCTTATATT CATCGTATAA GCTGTCTTCT TTCTGTACCT   
  
  
+ GTAACCTTAG TTCTCTCTCT GTCGCTTTT  

- TAATAGCGTG AAATAGGTGA TTCTTGGATT AATGGGTTGA TCACCCAATT GAACATCCGA TTCGAATGTA   
  
  
- TAGACGAATA CAAGAGAGGT AAAACACACT TTTGCACATC TGAATTAGAT TGGTTTAGTA CTAACACTGA   
  
  
- AACTAATACT TTATTTTCCA GATACCAAAT TAATTTAATT AATGTTATAC TGCATTTGAT TTGTATATGG   
  
  
- AAATTACCGA GACCTCGAAA CACAGTAAAA AATAAAGGTG CTAGTAATCC CGTTTTTAAC CAACTCTTTA   
  
  
- ATATTAAATC TCATATTCTA TGAGACCGAT CGAAAACCTA AAGTAACTGA TTTTTCCGTA ATGGGGAAAA   
  
  
- TGTGAAATCC TGAGGATCTT AGAAGATTCG ATCTGAATTA ATATAGAAAA TTATTGAAAA AATTACGAAC   
  
  
- AAACTGTGAT AACGTACAAT CGGAGATTCG AAATAAAATA GCTCTAGGTA TATTTACAAA AAAAAACAAA   
  
  
- AAAGGCCAAA ATGTAACTCT ATTTTTAGTA AATGATTAAA CTTTAGTTTA ACTAAAAAAG TAATTTTTTA   
  
  
- AATTACCAAT AAAAATATTA AATTTTATTG TTATATTTTA TTTTTTTAAT TAATAACTAA AATAATTTAA   
  
  
- CACCACGCAA ATAAACTTTC ACATATACAG TATAAAGTAT TAAAATGTAA TATTTATTTT TATATATTTT   
  
  
- ATGTATATTA ATCAACACAA TAAAGACCTG TAGTGAATGT TTTACATTTA TACTTTGTTT TTAAAACGCG   
  
  
- TAAATTTTAT ATATTTTATG TATATTAATC AACACAATAA AGACCTGTAG TGAATGTTTT ACATTTATAC   
  
  
- TTTGTTTTTA AAACGCGTAA ATTCATTAAT AATAATTGAT AAAAATTACT AATATTCTAT TTATGTAAAT   
  
  
- AAATTAATTA TTAAAAAATC TTCTTTATAT AAATTAGATT TGAAAATACC CTATACGTAC CTACCGTACT   
  
  
- TTAAACGTGA ACAGTATACG GAGGGAGGAT GTGATTTCGG TTATCCGTAG TCGTCGGTAA ATGCCGTGAC   
  
  
- TCTTCCTATT TATAATCTAT ACACTAACCC GGAGTCGGCG GGCGACCAGT TTCAGTGCAC GAAACTGGTT   
  
  
- TGGTGCGCCC GGTTTTGTGT GTGTGCGGGT GTGTGTGAGA GAGAGAGAGA GAAAGAAGGG CATAAAAAGT   
  
  
- GCCAGTTTGA AGAGCAATGG AAGCGTCTTT GACGGAACGT TTCTTTTTTG TGTGAGAGAG AGAGAGAGAA   
  
  
- AGAAGGGCAT AAAAAGTGCC AGTTTGAAGA GCAATGGAAG CGTCTTTGAC GGAACGTTTC TTTTTCTTCT   
  
  
- TTTGATCTTT TTTTTCTCTC TTTTTGGTTT CTTCGTCTCT GCGGAACATG GAATGCACTG GCAGAAGAGG   
  
  
- CAAGAGAGAG AGAGAGAGAG GGGGGGAACT AGGAATATAA GTAGCATATT CGACAGAAGA AAGACATGGA   
  
  
- CATTGGAATC AAGAGAGAGA CAGCGAAAA

+     CGTCA-motif

| Site Name | Organism | Position | Strand | Matrix score. | sequence | function |
| --- | --- | --- | --- | --- | --- | --- |
| CGTCA-motif | Hordeum vulgare | 189 | - | 5 | CGTCA | cis-acting regulatory element involved in the MeJA-responsiveness |

> 2018/04/13 10:10:12  
+ ATTATCGCAC TTTATCCACT AAGAACCTAA TTACCCAACT AGTGGGTTAA CTTGTAGGCT AAGCTTACAT   
  
  
+ ATCTGCTTAT GTTCTCTCCA TTTTGTGTGA AAACGTGTAG ACTTAATCTA ACCAAATCAT GATTGTGACT   
  
  
+ TTGATTATGA AATAAAAGGT CTATGGTTTA ATTAAATTAA TTACAATATG ACGTAAACTA AACATATACC   
  
  
+ TTTAATGGCT CTGGAGCTTT GTGTCATTTT TTATTTCCAC GATCATTAGG GCAAAAATTG GTTGAGAAAT   
  
  
+ TATAATTTAG AGTATAAGAT ACTCTGGCTA GCTTTTGGAT TTCATTGACT AAAAAGGCAT TACCCCTTTT   
  
  
+ ACACTTTAGG ACTCCTAGAA TCTTCTAAGC TAGACTTAAT TATATCTTTT AATAACTTTT TTAATGCTTG   
  
  
+ TTTGACACTA TTGCATGTTA GCCTCTAAGC TTTATTTTAT CGAGATCCAT ATAAATGTTT TTTTTTGTTT   
  
  
+ TTTCCGGTTT TACATTGAGA TAAAAATCAT TTACTAATTT GAAATCAAAT TGATTTTTTC ATTAAAAAAT   
  
  
+ TTAATGGTTA TTTTTATAAT TTAAAATAAC AATATAAAAT AAAAAAATTA ATTATTGATT TTATTAAATT   
  
  
+ GTGGTGCGTT TATTTGAAAG TGTATATGTC ATATTTCATA ATTTTACATT ATAAATAAAA ATATATAAAA   
  
  
+ TACATATAAT TAGTTGTGTT ATTTCTGGAC ATCACTTACA AAATGTAAAT ATGAAACAAA AATTTTGCGC   
  
  
+ ATTTAAAATA TATAAAATAC ATATAATTAG TTGTGTTATT TCTGGACATC ACTTACAAAA TGTAAATATG   
  
  
+ AAACAAAAAT TTTGCGCATT TAAGTAATTA TTATTAACTA TTTTTAATGA TTATAAGATA AATACATTTA   
  
  
+ TTTAATTAAT AATTTTTTAG AAGAAATATA TTTAATCTAA ACTTTTATGG GATATGCATG GATGGCATGA   
  
  
+ AATTTGCACT TGTCATATGC CTCCCTCCTA CACTAAAGCC AATAGGCATC AGCAGCCATT TACGGCACTG   
  
  
+ AGAAGGATAA ATATTAGATA TGTGATTGGG CCTCAGCCGC CCGCTGGTCA AAGTCACGTG CTTTGACCAA   
  
  
+ ACCACGCGGG CCAAAACACA CACACGCCCA CACACACTCT CTCTCTCTCT CTTTCTTCCC GTATTTTTCA   
  
  
+ CGGTCAAACT TCTCGTTACC TTCGCAGAAA CTGCCTTGCA AAGAAAAAAC ACACTCTCTC TCTCTCTCTT   
  
  
+ TCTTCCCGTA TTTTTCACGG TCAAACTTCT CGTTACCTTC GCAGAAACTG CCTTGCAAAG AAAAAGAAGA   
  
  
+ AAACTAGAAA AAAAAGAGAG AAAAACCAAA GAAGCAGAGA CGCCTTGTAC CTTACGTGAC CGTCTTCTCC   
  
  
+ GTTCTCTCTC TCTCTCTCTC CCCCCCTTGA TCCTTATATT CATCGTATAA GCTGTCTTCT TTCTGTACCT   
  
  
+ GTAACCTTAG TTCTCTCTCT GTCGCTTTT  

- TAATAGCGTG AAATAGGTGA TTCTTGGATT AATGGGTTGA TCACCCAATT GAACATCCGA TTCGAATGTA   
  
  
- TAGACGAATA CAAGAGAGGT AAAACACACT TTTGCACATC TGAATTAGAT TGGTTTAGTA CTAACACTGA   
  
  
- AACTAATACT TTATTTTCCA GATACCAAAT TAATTTAATT AATGTTATAC TGCATTTGAT TTGTATATGG   
  
  
- AAATTACCGA GACCTCGAAA CACAGTAAAA AATAAAGGTG CTAGTAATCC CGTTTTTAAC CAACTCTTTA   
  
  
- ATATTAAATC TCATATTCTA TGAGACCGAT CGAAAACCTA AAGTAACTGA TTTTTCCGTA ATGGGGAAAA   
  
  
- TGTGAAATCC TGAGGATCTT AGAAGATTCG ATCTGAATTA ATATAGAAAA TTATTGAAAA AATTACGAAC   
  
  
- AAACTGTGAT AACGTACAAT CGGAGATTCG AAATAAAATA GCTCTAGGTA TATTTACAAA AAAAAACAAA   
  
  
- AAAGGCCAAA ATGTAACTCT ATTTTTAGTA AATGATTAAA CTTTAGTTTA ACTAAAAAAG TAATTTTTTA   
  
  
- AATTACCAAT AAAAATATTA AATTTTATTG TTATATTTTA TTTTTTTAAT TAATAACTAA AATAATTTAA   
  
  
- CACCACGCAA ATAAACTTTC ACATATACAG TATAAAGTAT TAAAATGTAA TATTTATTTT TATATATTTT   
  
  
- ATGTATATTA ATCAACACAA TAAAGACCTG TAGTGAATGT TTTACATTTA TACTTTGTTT TTAAAACGCG   
  
  
- TAAATTTTAT ATATTTTATG TATATTAATC AACACAATAA AGACCTGTAG TGAATGTTTT ACATTTATAC   
  
  
- TTTGTTTTTA AAACGCGTAA ATTCATTAAT AATAATTGAT AAAAATTACT AATATTCTAT TTATGTAAAT   
  
  
- AAATTAATTA TTAAAAAATC TTCTTTATAT AAATTAGATT TGAAAATACC CTATACGTAC CTACCGTACT   
  
  
- TTAAACGTGA ACAGTATACG GAGGGAGGAT GTGATTTCGG TTATCCGTAG TCGTCGGTAA ATGCCGTGAC   
  
  
- TCTTCCTATT TATAATCTAT ACACTAACCC GGAGTCGGCG GGCGACCAGT TTCAGTGCAC GAAACTGGTT   
  
  
- TGGTGCGCCC GGTTTTGTGT GTGTGCGGGT GTGTGTGAGA GAGAGAGAGA GAAAGAAGGG CATAAAAAGT   
  
  
- GCCAGTTTGA AGAGCAATGG AAGCGTCTTT GACGGAACGT TTCTTTTTTG TGTGAGAGAG AGAGAGAGAA   
  
  
- AGAAGGGCAT AAAAAGTGCC AGTTTGAAGA GCAATGGAAG CGTCTTTGAC GGAACGTTTC TTTTTCTTCT   
  
  
- TTTGATCTTT TTTTTCTCTC TTTTTGGTTT CTTCGTCTCT GCGGAACATG GAATGCACTG GCAGAAGAGG   
  
  
- CAAGAGAGAG AGAGAGAGAG GGGGGGAACT AGGAATATAA GTAGCATATT CGACAGAAGA AAGACATGGA   
  
  
- CATTGGAATC AAGAGAGAGA CAGCGAAAA

+     ERE

| Site Name | Organism | Position | Strand | Matrix score. | sequence | function |
| --- | --- | --- | --- | --- | --- | --- |
| ERE | Dianthus caryophyllus | 528 | - | 8 | ATTTCAAA | ethylene-responsive element |

> 2018/04/13 10:10:12  
+ ATTATCGCAC TTTATCCACT AAGAACCTAA TTACCCAACT AGTGGGTTAA CTTGTAGGCT AAGCTTACAT   
  
  
+ ATCTGCTTAT GTTCTCTCCA TTTTGTGTGA AAACGTGTAG ACTTAATCTA ACCAAATCAT GATTGTGACT   
  
  
+ TTGATTATGA AATAAAAGGT CTATGGTTTA ATTAAATTAA TTACAATATG ACGTAAACTA AACATATACC   
  
  
+ TTTAATGGCT CTGGAGCTTT GTGTCATTTT TTATTTCCAC GATCATTAGG GCAAAAATTG GTTGAGAAAT   
  
  
+ TATAATTTAG AGTATAAGAT ACTCTGGCTA GCTTTTGGAT TTCATTGACT AAAAAGGCAT TACCCCTTTT   
  
  
+ ACACTTTAGG ACTCCTAGAA TCTTCTAAGC TAGACTTAAT TATATCTTTT AATAACTTTT TTAATGCTTG   
  
  
+ TTTGACACTA TTGCATGTTA GCCTCTAAGC TTTATTTTAT CGAGATCCAT ATAAATGTTT TTTTTTGTTT   
  
  
+ TTTCCGGTTT TACATTGAGA TAAAAATCAT TTACTAATTT GAAATCAAAT TGATTTTTTC ATTAAAAAAT   
  
  
+ TTAATGGTTA TTTTTATAAT TTAAAATAAC AATATAAAAT AAAAAAATTA ATTATTGATT TTATTAAATT   
  
  
+ GTGGTGCGTT TATTTGAAAG TGTATATGTC ATATTTCATA ATTTTACATT ATAAATAAAA ATATATAAAA   
  
  
+ TACATATAAT TAGTTGTGTT ATTTCTGGAC ATCACTTACA AAATGTAAAT ATGAAACAAA AATTTTGCGC   
  
  
+ ATTTAAAATA TATAAAATAC ATATAATTAG TTGTGTTATT TCTGGACATC ACTTACAAAA TGTAAATATG   
  
  
+ AAACAAAAAT TTTGCGCATT TAAGTAATTA TTATTAACTA TTTTTAATGA TTATAAGATA AATACATTTA   
  
  
+ TTTAATTAAT AATTTTTTAG AAGAAATATA TTTAATCTAA ACTTTTATGG GATATGCATG GATGGCATGA   
  
  
+ AATTTGCACT TGTCATATGC CTCCCTCCTA CACTAAAGCC AATAGGCATC AGCAGCCATT TACGGCACTG   
  
  
+ AGAAGGATAA ATATTAGATA TGTGATTGGG CCTCAGCCGC CCGCTGGTCA AAGTCACGTG CTTTGACCAA   
  
  
+ ACCACGCGGG CCAAAACACA CACACGCCCA CACACACTCT CTCTCTCTCT CTTTCTTCCC GTATTTTTCA   
  
  
+ CGGTCAAACT TCTCGTTACC TTCGCAGAAA CTGCCTTGCA AAGAAAAAAC ACACTCTCTC TCTCTCTCTT   
  
  
+ TCTTCCCGTA TTTTTCACGG TCAAACTTCT CGTTACCTTC GCAGAAACTG CCTTGCAAAG AAAAAGAAGA   
  
  
+ AAACTAGAAA AAAAAGAGAG AAAAACCAAA GAAGCAGAGA CGCCTTGTAC CTTACGTGAC CGTCTTCTCC   
  
  
+ GTTCTCTCTC TCTCTCTCTC CCCCCCTTGA TCCTTATATT CATCGTATAA GCTGTCTTCT TTCTGTACCT   
  
  
+ GTAACCTTAG TTCTCTCTCT GTCGCTTTT  

- TAATAGCGTG AAATAGGTGA TTCTTGGATT AATGGGTTGA TCACCCAATT GAACATCCGA TTCGAATGTA   
  
  
- TAGACGAATA CAAGAGAGGT AAAACACACT TTTGCACATC TGAATTAGAT TGGTTTAGTA CTAACACTGA   
  
  
- AACTAATACT TTATTTTCCA GATACCAAAT TAATTTAATT AATGTTATAC TGCATTTGAT TTGTATATGG   
  
  
- AAATTACCGA GACCTCGAAA CACAGTAAAA AATAAAGGTG CTAGTAATCC CGTTTTTAAC CAACTCTTTA   
  
  
- ATATTAAATC TCATATTCTA TGAGACCGAT CGAAAACCTA AAGTAACTGA TTTTTCCGTA ATGGGGAAAA   
  
  
- TGTGAAATCC TGAGGATCTT AGAAGATTCG ATCTGAATTA ATATAGAAAA TTATTGAAAA AATTACGAAC   
  
  
- AAACTGTGAT AACGTACAAT CGGAGATTCG AAATAAAATA GCTCTAGGTA TATTTACAAA AAAAAACAAA   
  
  
- AAAGGCCAAA ATGTAACTCT ATTTTTAGTA AATGATTAAA CTTTAGTTTA ACTAAAAAAG TAATTTTTTA   
  
  
- AATTACCAAT AAAAATATTA AATTTTATTG TTATATTTTA TTTTTTTAAT TAATAACTAA AATAATTTAA   
  
  
- CACCACGCAA ATAAACTTTC ACATATACAG TATAAAGTAT TAAAATGTAA TATTTATTTT TATATATTTT   
  
  
- ATGTATATTA ATCAACACAA TAAAGACCTG TAGTGAATGT TTTACATTTA TACTTTGTTT TTAAAACGCG   
  
  
- TAAATTTTAT ATATTTTATG TATATTAATC AACACAATAA AGACCTGTAG TGAATGTTTT ACATTTATAC   
  
  
- TTTGTTTTTA AAACGCGTAA ATTCATTAAT AATAATTGAT AAAAATTACT AATATTCTAT TTATGTAAAT   
  
  
- AAATTAATTA TTAAAAAATC TTCTTTATAT AAATTAGATT TGAAAATACC CTATACGTAC CTACCGTACT   
  
  
- TTAAACGTGA ACAGTATACG GAGGGAGGAT GTGATTTCGG TTATCCGTAG TCGTCGGTAA ATGCCGTGAC   
  
  
- TCTTCCTATT TATAATCTAT ACACTAACCC GGAGTCGGCG GGCGACCAGT TTCAGTGCAC GAAACTGGTT   
  
  
- TGGTGCGCCC GGTTTTGTGT GTGTGCGGGT GTGTGTGAGA GAGAGAGAGA GAAAGAAGGG CATAAAAAGT   
  
  
- GCCAGTTTGA AGAGCAATGG AAGCGTCTTT GACGGAACGT TTCTTTTTTG TGTGAGAGAG AGAGAGAGAA   
  
  
- AGAAGGGCAT AAAAAGTGCC AGTTTGAAGA GCAATGGAAG CGTCTTTGAC GGAACGTTTC TTTTTCTTCT   
  
  
- TTTGATCTTT TTTTTCTCTC TTTTTGGTTT CTTCGTCTCT GCGGAACATG GAATGCACTG GCAGAAGAGG   
  
  
- CAAGAGAGAG AGAGAGAGAG GGGGGGAACT AGGAATATAA GTAGCATATT CGACAGAAGA AAGACATGGA   
  
  
- CATTGGAATC AAGAGAGAGA CAGCGAAAA

+     G-Box

| Site Name | Organism | Position | Strand | Matrix score. | sequence | function |
| --- | --- | --- | --- | --- | --- | --- |
| G-Box | Antirrhinum majus | 1383 | - | 6 | CACGTA | cis-acting regulatory element involved in light responsiveness |
| G-Box | Pisum sativum | 102 | - | 6 | CACGTT | cis-acting regulatory element involved in light responsiveness |
| G-Box | Pisum sativum | 1105 | - | 6 | CACGTG | cis-acting regulatory element involved in light responsiveness |

> 2018/04/13 10:10:12  
+ ATTATCGCAC TTTATCCACT AAGAACCTAA TTACCCAACT AGTGGGTTAA CTTGTAGGCT AAGCTTACAT   
  
  
+ ATCTGCTTAT GTTCTCTCCA TTTTGTGTGA AAACGTGTAG ACTTAATCTA ACCAAATCAT GATTGTGACT   
  
  
+ TTGATTATGA AATAAAAGGT CTATGGTTTA ATTAAATTAA TTACAATATG ACGTAAACTA AACATATACC   
  
  
+ TTTAATGGCT CTGGAGCTTT GTGTCATTTT TTATTTCCAC GATCATTAGG GCAAAAATTG GTTGAGAAAT   
  
  
+ TATAATTTAG AGTATAAGAT ACTCTGGCTA GCTTTTGGAT TTCATTGACT AAAAAGGCAT TACCCCTTTT   
  
  
+ ACACTTTAGG ACTCCTAGAA TCTTCTAAGC TAGACTTAAT TATATCTTTT AATAACTTTT TTAATGCTTG   
  
  
+ TTTGACACTA TTGCATGTTA GCCTCTAAGC TTTATTTTAT CGAGATCCAT ATAAATGTTT TTTTTTGTTT   
  
  
+ TTTCCGGTTT TACATTGAGA TAAAAATCAT TTACTAATTT GAAATCAAAT TGATTTTTTC ATTAAAAAAT   
  
  
+ TTAATGGTTA TTTTTATAAT TTAAAATAAC AATATAAAAT AAAAAAATTA ATTATTGATT TTATTAAATT   
  
  
+ GTGGTGCGTT TATTTGAAAG TGTATATGTC ATATTTCATA ATTTTACATT ATAAATAAAA ATATATAAAA   
  
  
+ TACATATAAT TAGTTGTGTT ATTTCTGGAC ATCACTTACA AAATGTAAAT ATGAAACAAA AATTTTGCGC   
  
  
+ ATTTAAAATA TATAAAATAC ATATAATTAG TTGTGTTATT TCTGGACATC ACTTACAAAA TGTAAATATG   
  
  
+ AAACAAAAAT TTTGCGCATT TAAGTAATTA TTATTAACTA TTTTTAATGA TTATAAGATA AATACATTTA   
  
  
+ TTTAATTAAT AATTTTTTAG AAGAAATATA TTTAATCTAA ACTTTTATGG GATATGCATG GATGGCATGA   
  
  
+ AATTTGCACT TGTCATATGC CTCCCTCCTA CACTAAAGCC AATAGGCATC AGCAGCCATT TACGGCACTG   
  
  
+ AGAAGGATAA ATATTAGATA TGTGATTGGG CCTCAGCCGC CCGCTGGTCA AAGTCACGTG CTTTGACCAA   
  
  
+ ACCACGCGGG CCAAAACACA CACACGCCCA CACACACTCT CTCTCTCTCT CTTTCTTCCC GTATTTTTCA   
  
  
+ CGGTCAAACT TCTCGTTACC TTCGCAGAAA CTGCCTTGCA AAGAAAAAAC ACACTCTCTC TCTCTCTCTT   
  
  
+ TCTTCCCGTA TTTTTCACGG TCAAACTTCT CGTTACCTTC GCAGAAACTG CCTTGCAAAG AAAAAGAAGA   
  
  
+ AAACTAGAAA AAAAAGAGAG AAAAACCAAA GAAGCAGAGA CGCCTTGTAC CTTACGTGAC CGTCTTCTCC   
  
  
+ GTTCTCTCTC TCTCTCTCTC CCCCCCTTGA TCCTTATATT CATCGTATAA GCTGTCTTCT TTCTGTACCT   
  
  
+ GTAACCTTAG TTCTCTCTCT GTCGCTTTT  

- TAATAGCGTG AAATAGGTGA TTCTTGGATT AATGGGTTGA TCACCCAATT GAACATCCGA TTCGAATGTA   
  
  
- TAGACGAATA CAAGAGAGGT AAAACACACT TTTGCACATC TGAATTAGAT TGGTTTAGTA CTAACACTGA   
  
  
- AACTAATACT TTATTTTCCA GATACCAAAT TAATTTAATT AATGTTATAC TGCATTTGAT TTGTATATGG   
  
  
- AAATTACCGA GACCTCGAAA CACAGTAAAA AATAAAGGTG CTAGTAATCC CGTTTTTAAC CAACTCTTTA   
  
  
- ATATTAAATC TCATATTCTA TGAGACCGAT CGAAAACCTA AAGTAACTGA TTTTTCCGTA ATGGGGAAAA   
  
  
- TGTGAAATCC TGAGGATCTT AGAAGATTCG ATCTGAATTA ATATAGAAAA TTATTGAAAA AATTACGAAC   
  
  
- AAACTGTGAT AACGTACAAT CGGAGATTCG AAATAAAATA GCTCTAGGTA TATTTACAAA AAAAAACAAA   
  
  
- AAAGGCCAAA ATGTAACTCT ATTTTTAGTA AATGATTAAA CTTTAGTTTA ACTAAAAAAG TAATTTTTTA   
  
  
- AATTACCAAT AAAAATATTA AATTTTATTG TTATATTTTA TTTTTTTAAT TAATAACTAA AATAATTTAA   
  
  
- CACCACGCAA ATAAACTTTC ACATATACAG TATAAAGTAT TAAAATGTAA TATTTATTTT TATATATTTT   
  
  
- ATGTATATTA ATCAACACAA TAAAGACCTG TAGTGAATGT TTTACATTTA TACTTTGTTT TTAAAACGCG   
  
  
- TAAATTTTAT ATATTTTATG TATATTAATC AACACAATAA AGACCTGTAG TGAATGTTTT ACATTTATAC   
  
  
- TTTGTTTTTA AAACGCGTAA ATTCATTAAT AATAATTGAT AAAAATTACT AATATTCTAT TTATGTAAAT   
  
  
- AAATTAATTA TTAAAAAATC TTCTTTATAT AAATTAGATT TGAAAATACC CTATACGTAC CTACCGTACT   
  
  
- TTAAACGTGA ACAGTATACG GAGGGAGGAT GTGATTTCGG TTATCCGTAG TCGTCGGTAA ATGCCGTGAC   
  
  
- TCTTCCTATT TATAATCTAT ACACTAACCC GGAGTCGGCG GGCGACCAGT TTCAGTGCAC GAAACTGGTT   
  
  
- TGGTGCGCCC GGTTTTGTGT GTGTGCGGGT GTGTGTGAGA GAGAGAGAGA GAAAGAAGGG CATAAAAAGT   
  
  
- GCCAGTTTGA AGAGCAATGG AAGCGTCTTT GACGGAACGT TTCTTTTTTG TGTGAGAGAG AGAGAGAGAA   
  
  
- AGAAGGGCAT AAAAAGTGCC AGTTTGAAGA GCAATGGAAG CGTCTTTGAC GGAACGTTTC TTTTTCTTCT   
  
  
- TTTGATCTTT TTTTTCTCTC TTTTTGGTTT CTTCGTCTCT GCGGAACATG GAATGCACTG GCAGAAGAGG   
  
  
- CAAGAGAGAG AGAGAGAGAG GGGGGGAACT AGGAATATAA GTAGCATATT CGACAGAAGA AAGACATGGA   
  
  
- CATTGGAATC AAGAGAGAGA CAGCGAAAA

+     G-box

| Site Name | Organism | Position | Strand | Matrix score. | sequence | function |
| --- | --- | --- | --- | --- | --- | --- |
| G-box | Zea mays | 102 | - | 6 | CACGTT | cis-acting regulatory element involved in light responsiveness |
| G-box | Daucus carota | 1383 | + | 6 | TACGTG | cis-acting regulatory element involved in light responsiveness |
| G-box | Arabidopsis thaliana | 1105 | - | 6 | CACGTG | cis-acting regulatory element involved in light responsiveness |

> 2018/04/13 10:10:12  
+ ATTATCGCAC TTTATCCACT AAGAACCTAA TTACCCAACT AGTGGGTTAA CTTGTAGGCT AAGCTTACAT   
  
  
+ ATCTGCTTAT GTTCTCTCCA TTTTGTGTGA AAACGTGTAG ACTTAATCTA ACCAAATCAT GATTGTGACT   
  
  
+ TTGATTATGA AATAAAAGGT CTATGGTTTA ATTAAATTAA TTACAATATG ACGTAAACTA AACATATACC   
  
  
+ TTTAATGGCT CTGGAGCTTT GTGTCATTTT TTATTTCCAC GATCATTAGG GCAAAAATTG GTTGAGAAAT   
  
  
+ TATAATTTAG AGTATAAGAT ACTCTGGCTA GCTTTTGGAT TTCATTGACT AAAAAGGCAT TACCCCTTTT   
  
  
+ ACACTTTAGG ACTCCTAGAA TCTTCTAAGC TAGACTTAAT TATATCTTTT AATAACTTTT TTAATGCTTG   
  
  
+ TTTGACACTA TTGCATGTTA GCCTCTAAGC TTTATTTTAT CGAGATCCAT ATAAATGTTT TTTTTTGTTT   
  
  
+ TTTCCGGTTT TACATTGAGA TAAAAATCAT TTACTAATTT GAAATCAAAT TGATTTTTTC ATTAAAAAAT   
  
  
+ TTAATGGTTA TTTTTATAAT TTAAAATAAC AATATAAAAT AAAAAAATTA ATTATTGATT TTATTAAATT   
  
  
+ GTGGTGCGTT TATTTGAAAG TGTATATGTC ATATTTCATA ATTTTACATT ATAAATAAAA ATATATAAAA   
  
  
+ TACATATAAT TAGTTGTGTT ATTTCTGGAC ATCACTTACA AAATGTAAAT ATGAAACAAA AATTTTGCGC   
  
  
+ ATTTAAAATA TATAAAATAC ATATAATTAG TTGTGTTATT TCTGGACATC ACTTACAAAA TGTAAATATG   
  
  
+ AAACAAAAAT TTTGCGCATT TAAGTAATTA TTATTAACTA TTTTTAATGA TTATAAGATA AATACATTTA   
  
  
+ TTTAATTAAT AATTTTTTAG AAGAAATATA TTTAATCTAA ACTTTTATGG GATATGCATG GATGGCATGA   
  
  
+ AATTTGCACT TGTCATATGC CTCCCTCCTA CACTAAAGCC AATAGGCATC AGCAGCCATT TACGGCACTG   
  
  
+ AGAAGGATAA ATATTAGATA TGTGATTGGG CCTCAGCCGC CCGCTGGTCA AAGTCACGTG CTTTGACCAA   
  
  
+ ACCACGCGGG CCAAAACACA CACACGCCCA CACACACTCT CTCTCTCTCT CTTTCTTCCC GTATTTTTCA   
  
  
+ CGGTCAAACT TCTCGTTACC TTCGCAGAAA CTGCCTTGCA AAGAAAAAAC ACACTCTCTC TCTCTCTCTT   
  
  
+ TCTTCCCGTA TTTTTCACGG TCAAACTTCT CGTTACCTTC GCAGAAACTG CCTTGCAAAG AAAAAGAAGA   
  
  
+ AAACTAGAAA AAAAAGAGAG AAAAACCAAA GAAGCAGAGA CGCCTTGTAC CTTACGTGAC CGTCTTCTCC   
  
  
+ GTTCTCTCTC TCTCTCTCTC CCCCCCTTGA TCCTTATATT CATCGTATAA GCTGTCTTCT TTCTGTACCT   
  
  
+ GTAACCTTAG TTCTCTCTCT GTCGCTTTT  

- TAATAGCGTG AAATAGGTGA TTCTTGGATT AATGGGTTGA TCACCCAATT GAACATCCGA TTCGAATGTA   
  
  
- TAGACGAATA CAAGAGAGGT AAAACACACT TTTGCACATC TGAATTAGAT TGGTTTAGTA CTAACACTGA   
  
  
- AACTAATACT TTATTTTCCA GATACCAAAT TAATTTAATT AATGTTATAC TGCATTTGAT TTGTATATGG   
  
  
- AAATTACCGA GACCTCGAAA CACAGTAAAA AATAAAGGTG CTAGTAATCC CGTTTTTAAC CAACTCTTTA   
  
  
- ATATTAAATC TCATATTCTA TGAGACCGAT CGAAAACCTA AAGTAACTGA TTTTTCCGTA ATGGGGAAAA   
  
  
- TGTGAAATCC TGAGGATCTT AGAAGATTCG ATCTGAATTA ATATAGAAAA TTATTGAAAA AATTACGAAC   
  
  
- AAACTGTGAT AACGTACAAT CGGAGATTCG AAATAAAATA GCTCTAGGTA TATTTACAAA AAAAAACAAA   
  
  
- AAAGGCCAAA ATGTAACTCT ATTTTTAGTA AATGATTAAA CTTTAGTTTA ACTAAAAAAG TAATTTTTTA   
  
  
- AATTACCAAT AAAAATATTA AATTTTATTG TTATATTTTA TTTTTTTAAT TAATAACTAA AATAATTTAA   
  
  
- CACCACGCAA ATAAACTTTC ACATATACAG TATAAAGTAT TAAAATGTAA TATTTATTTT TATATATTTT   
  
  
- ATGTATATTA ATCAACACAA TAAAGACCTG TAGTGAATGT TTTACATTTA TACTTTGTTT TTAAAACGCG   
  
  
- TAAATTTTAT ATATTTTATG TATATTAATC AACACAATAA AGACCTGTAG TGAATGTTTT ACATTTATAC   
  
  
- TTTGTTTTTA AAACGCGTAA ATTCATTAAT AATAATTGAT AAAAATTACT AATATTCTAT TTATGTAAAT   
  
  
- AAATTAATTA TTAAAAAATC TTCTTTATAT AAATTAGATT TGAAAATACC CTATACGTAC CTACCGTACT   
  
  
- TTAAACGTGA ACAGTATACG GAGGGAGGAT GTGATTTCGG TTATCCGTAG TCGTCGGTAA ATGCCGTGAC   
  
  
- TCTTCCTATT TATAATCTAT ACACTAACCC GGAGTCGGCG GGCGACCAGT TTCAGTGCAC GAAACTGGTT   
  
  
- TGGTGCGCCC GGTTTTGTGT GTGTGCGGGT GTGTGTGAGA GAGAGAGAGA GAAAGAAGGG CATAAAAAGT   
  
  
- GCCAGTTTGA AGAGCAATGG AAGCGTCTTT GACGGAACGT TTCTTTTTTG TGTGAGAGAG AGAGAGAGAA   
  
  
- AGAAGGGCAT AAAAAGTGCC AGTTTGAAGA GCAATGGAAG CGTCTTTGAC GGAACGTTTC TTTTTCTTCT   
  
  
- TTTGATCTTT TTTTTCTCTC TTTTTGGTTT CTTCGTCTCT GCGGAACATG GAATGCACTG GCAGAAGAGG   
  
  
- CAAGAGAGAG AGAGAGAGAG GGGGGGAACT AGGAATATAA GTAGCATATT CGACAGAAGA AAGACATGGA   
  
  
- CATTGGAATC AAGAGAGAGA CAGCGAAAA

+     GAG-motif

| Site Name | Organism | Position | Strand | Matrix score. | sequence | function |
| --- | --- | --- | --- | --- | --- | --- |
| GAG-motif | Arabidopsis thaliana | 1243 | - | 7 | AGAGAGT | part of a light responsive element |
| GAG-motif | Arabidopsis thaliana | 1156 | - | 7 | AGAGAGT | part of a light responsive element |

> 2018/04/13 10:10:12  
+ ATTATCGCAC TTTATCCACT AAGAACCTAA TTACCCAACT AGTGGGTTAA CTTGTAGGCT AAGCTTACAT   
  
  
+ ATCTGCTTAT GTTCTCTCCA TTTTGTGTGA AAACGTGTAG ACTTAATCTA ACCAAATCAT GATTGTGACT   
  
  
+ TTGATTATGA AATAAAAGGT CTATGGTTTA ATTAAATTAA TTACAATATG ACGTAAACTA AACATATACC   
  
  
+ TTTAATGGCT CTGGAGCTTT GTGTCATTTT TTATTTCCAC GATCATTAGG GCAAAAATTG GTTGAGAAAT   
  
  
+ TATAATTTAG AGTATAAGAT ACTCTGGCTA GCTTTTGGAT TTCATTGACT AAAAAGGCAT TACCCCTTTT   
  
  
+ ACACTTTAGG ACTCCTAGAA TCTTCTAAGC TAGACTTAAT TATATCTTTT AATAACTTTT TTAATGCTTG   
  
  
+ TTTGACACTA TTGCATGTTA GCCTCTAAGC TTTATTTTAT CGAGATCCAT ATAAATGTTT TTTTTTGTTT   
  
  
+ TTTCCGGTTT TACATTGAGA TAAAAATCAT TTACTAATTT GAAATCAAAT TGATTTTTTC ATTAAAAAAT   
  
  
+ TTAATGGTTA TTTTTATAAT TTAAAATAAC AATATAAAAT AAAAAAATTA ATTATTGATT TTATTAAATT   
  
  
+ GTGGTGCGTT TATTTGAAAG TGTATATGTC ATATTTCATA ATTTTACATT ATAAATAAAA ATATATAAAA   
  
  
+ TACATATAAT TAGTTGTGTT ATTTCTGGAC ATCACTTACA AAATGTAAAT ATGAAACAAA AATTTTGCGC   
  
  
+ ATTTAAAATA TATAAAATAC ATATAATTAG TTGTGTTATT TCTGGACATC ACTTACAAAA TGTAAATATG   
  
  
+ AAACAAAAAT TTTGCGCATT TAAGTAATTA TTATTAACTA TTTTTAATGA TTATAAGATA AATACATTTA   
  
  
+ TTTAATTAAT AATTTTTTAG AAGAAATATA TTTAATCTAA ACTTTTATGG GATATGCATG GATGGCATGA   
  
  
+ AATTTGCACT TGTCATATGC CTCCCTCCTA CACTAAAGCC AATAGGCATC AGCAGCCATT TACGGCACTG   
  
  
+ AGAAGGATAA ATATTAGATA TGTGATTGGG CCTCAGCCGC CCGCTGGTCA AAGTCACGTG CTTTGACCAA   
  
  
+ ACCACGCGGG CCAAAACACA CACACGCCCA CACACACTCT CTCTCTCTCT CTTTCTTCCC GTATTTTTCA   
  
  
+ CGGTCAAACT TCTCGTTACC TTCGCAGAAA CTGCCTTGCA AAGAAAAAAC ACACTCTCTC TCTCTCTCTT   
  
  
+ TCTTCCCGTA TTTTTCACGG TCAAACTTCT CGTTACCTTC GCAGAAACTG CCTTGCAAAG AAAAAGAAGA   
  
  
+ AAACTAGAAA AAAAAGAGAG AAAAACCAAA GAAGCAGAGA CGCCTTGTAC CTTACGTGAC CGTCTTCTCC   
  
  
+ GTTCTCTCTC TCTCTCTCTC CCCCCCTTGA TCCTTATATT CATCGTATAA GCTGTCTTCT TTCTGTACCT   
  
  
+ GTAACCTTAG TTCTCTCTCT GTCGCTTTT  

- TAATAGCGTG AAATAGGTGA TTCTTGGATT AATGGGTTGA TCACCCAATT GAACATCCGA TTCGAATGTA   
  
  
- TAGACGAATA CAAGAGAGGT AAAACACACT TTTGCACATC TGAATTAGAT TGGTTTAGTA CTAACACTGA   
  
  
- AACTAATACT TTATTTTCCA GATACCAAAT TAATTTAATT AATGTTATAC TGCATTTGAT TTGTATATGG   
  
  
- AAATTACCGA GACCTCGAAA CACAGTAAAA AATAAAGGTG CTAGTAATCC CGTTTTTAAC CAACTCTTTA   
  
  
- ATATTAAATC TCATATTCTA TGAGACCGAT CGAAAACCTA AAGTAACTGA TTTTTCCGTA ATGGGGAAAA   
  
  
- TGTGAAATCC TGAGGATCTT AGAAGATTCG ATCTGAATTA ATATAGAAAA TTATTGAAAA AATTACGAAC   
  
  
- AAACTGTGAT AACGTACAAT CGGAGATTCG AAATAAAATA GCTCTAGGTA TATTTACAAA AAAAAACAAA   
  
  
- AAAGGCCAAA ATGTAACTCT ATTTTTAGTA AATGATTAAA CTTTAGTTTA ACTAAAAAAG TAATTTTTTA   
  
  
- AATTACCAAT AAAAATATTA AATTTTATTG TTATATTTTA TTTTTTTAAT TAATAACTAA AATAATTTAA   
  
  
- CACCACGCAA ATAAACTTTC ACATATACAG TATAAAGTAT TAAAATGTAA TATTTATTTT TATATATTTT   
  
  
- ATGTATATTA ATCAACACAA TAAAGACCTG TAGTGAATGT TTTACATTTA TACTTTGTTT TTAAAACGCG   
  
  
- TAAATTTTAT ATATTTTATG TATATTAATC AACACAATAA AGACCTGTAG TGAATGTTTT ACATTTATAC   
  
  
- TTTGTTTTTA AAACGCGTAA ATTCATTAAT AATAATTGAT AAAAATTACT AATATTCTAT TTATGTAAAT   
  
  
- AAATTAATTA TTAAAAAATC TTCTTTATAT AAATTAGATT TGAAAATACC CTATACGTAC CTACCGTACT   
  
  
- TTAAACGTGA ACAGTATACG GAGGGAGGAT GTGATTTCGG TTATCCGTAG TCGTCGGTAA ATGCCGTGAC   
  
  
- TCTTCCTATT TATAATCTAT ACACTAACCC GGAGTCGGCG GGCGACCAGT TTCAGTGCAC GAAACTGGTT   
  
  
- TGGTGCGCCC GGTTTTGTGT GTGTGCGGGT GTGTGTGAGA GAGAGAGAGA GAAAGAAGGG CATAAAAAGT   
  
  
- GCCAGTTTGA AGAGCAATGG AAGCGTCTTT GACGGAACGT TTCTTTTTTG TGTGAGAGAG AGAGAGAGAA   
  
  
- AGAAGGGCAT AAAAAGTGCC AGTTTGAAGA GCAATGGAAG CGTCTTTGAC GGAACGTTTC TTTTTCTTCT   
  
  
- TTTGATCTTT TTTTTCTCTC TTTTTGGTTT CTTCGTCTCT GCGGAACATG GAATGCACTG GCAGAAGAGG   
  
  
- CAAGAGAGAG AGAGAGAGAG GGGGGGAACT AGGAATATAA GTAGCATATT CGACAGAAGA AAGACATGGA   
  
  
- CATTGGAATC AAGAGAGAGA CAGCGAAAA

+     GCC box

| Site Name | Organism | Position | Strand | Matrix score. | sequence | function |
| --- | --- | --- | --- | --- | --- | --- |
| GCC box | Arabidopsis thaliana | 1085 | + | 7 | AGCCGCC |  |

> 2018/04/13 10:10:12  
+ ATTATCGCAC TTTATCCACT AAGAACCTAA TTACCCAACT AGTGGGTTAA CTTGTAGGCT AAGCTTACAT   
  
  
+ ATCTGCTTAT GTTCTCTCCA TTTTGTGTGA AAACGTGTAG ACTTAATCTA ACCAAATCAT GATTGTGACT   
  
  
+ TTGATTATGA AATAAAAGGT CTATGGTTTA ATTAAATTAA TTACAATATG ACGTAAACTA AACATATACC   
  
  
+ TTTAATGGCT CTGGAGCTTT GTGTCATTTT TTATTTCCAC GATCATTAGG GCAAAAATTG GTTGAGAAAT   
  
  
+ TATAATTTAG AGTATAAGAT ACTCTGGCTA GCTTTTGGAT TTCATTGACT AAAAAGGCAT TACCCCTTTT   
  
  
+ ACACTTTAGG ACTCCTAGAA TCTTCTAAGC TAGACTTAAT TATATCTTTT AATAACTTTT TTAATGCTTG   
  
  
+ TTTGACACTA TTGCATGTTA GCCTCTAAGC TTTATTTTAT CGAGATCCAT ATAAATGTTT TTTTTTGTTT   
  
  
+ TTTCCGGTTT TACATTGAGA TAAAAATCAT TTACTAATTT GAAATCAAAT TGATTTTTTC ATTAAAAAAT   
  
  
+ TTAATGGTTA TTTTTATAAT TTAAAATAAC AATATAAAAT AAAAAAATTA ATTATTGATT TTATTAAATT   
  
  
+ GTGGTGCGTT TATTTGAAAG TGTATATGTC ATATTTCATA ATTTTACATT ATAAATAAAA ATATATAAAA   
  
  
+ TACATATAAT TAGTTGTGTT ATTTCTGGAC ATCACTTACA AAATGTAAAT ATGAAACAAA AATTTTGCGC   
  
  
+ ATTTAAAATA TATAAAATAC ATATAATTAG TTGTGTTATT TCTGGACATC ACTTACAAAA TGTAAATATG   
  
  
+ AAACAAAAAT TTTGCGCATT TAAGTAATTA TTATTAACTA TTTTTAATGA TTATAAGATA AATACATTTA   
  
  
+ TTTAATTAAT AATTTTTTAG AAGAAATATA TTTAATCTAA ACTTTTATGG GATATGCATG GATGGCATGA   
  
  
+ AATTTGCACT TGTCATATGC CTCCCTCCTA CACTAAAGCC AATAGGCATC AGCAGCCATT TACGGCACTG   
  
  
+ AGAAGGATAA ATATTAGATA TGTGATTGGG CCTCAGCCGC CCGCTGGTCA AAGTCACGTG CTTTGACCAA   
  
  
+ ACCACGCGGG CCAAAACACA CACACGCCCA CACACACTCT CTCTCTCTCT CTTTCTTCCC GTATTTTTCA   
  
  
+ CGGTCAAACT TCTCGTTACC TTCGCAGAAA CTGCCTTGCA AAGAAAAAAC ACACTCTCTC TCTCTCTCTT   
  
  
+ TCTTCCCGTA TTTTTCACGG TCAAACTTCT CGTTACCTTC GCAGAAACTG CCTTGCAAAG AAAAAGAAGA   
  
  
+ AAACTAGAAA AAAAAGAGAG AAAAACCAAA GAAGCAGAGA CGCCTTGTAC CTTACGTGAC CGTCTTCTCC   
  
  
+ GTTCTCTCTC TCTCTCTCTC CCCCCCTTGA TCCTTATATT CATCGTATAA GCTGTCTTCT TTCTGTACCT   
  
  
+ GTAACCTTAG TTCTCTCTCT GTCGCTTTT  

- TAATAGCGTG AAATAGGTGA TTCTTGGATT AATGGGTTGA TCACCCAATT GAACATCCGA TTCGAATGTA   
  
  
- TAGACGAATA CAAGAGAGGT AAAACACACT TTTGCACATC TGAATTAGAT TGGTTTAGTA CTAACACTGA   
  
  
- AACTAATACT TTATTTTCCA GATACCAAAT TAATTTAATT AATGTTATAC TGCATTTGAT TTGTATATGG   
  
  
- AAATTACCGA GACCTCGAAA CACAGTAAAA AATAAAGGTG CTAGTAATCC CGTTTTTAAC CAACTCTTTA   
  
  
- ATATTAAATC TCATATTCTA TGAGACCGAT CGAAAACCTA AAGTAACTGA TTTTTCCGTA ATGGGGAAAA   
  
  
- TGTGAAATCC TGAGGATCTT AGAAGATTCG ATCTGAATTA ATATAGAAAA TTATTGAAAA AATTACGAAC   
  
  
- AAACTGTGAT AACGTACAAT CGGAGATTCG AAATAAAATA GCTCTAGGTA TATTTACAAA AAAAAACAAA   
  
  
- AAAGGCCAAA ATGTAACTCT ATTTTTAGTA AATGATTAAA CTTTAGTTTA ACTAAAAAAG TAATTTTTTA   
  
  
- AATTACCAAT AAAAATATTA AATTTTATTG TTATATTTTA TTTTTTTAAT TAATAACTAA AATAATTTAA   
  
  
- CACCACGCAA ATAAACTTTC ACATATACAG TATAAAGTAT TAAAATGTAA TATTTATTTT TATATATTTT   
  
  
- ATGTATATTA ATCAACACAA TAAAGACCTG TAGTGAATGT TTTACATTTA TACTTTGTTT TTAAAACGCG   
  
  
- TAAATTTTAT ATATTTTATG TATATTAATC AACACAATAA AGACCTGTAG TGAATGTTTT ACATTTATAC   
  
  
- TTTGTTTTTA AAACGCGTAA ATTCATTAAT AATAATTGAT AAAAATTACT AATATTCTAT TTATGTAAAT   
  
  
- AAATTAATTA TTAAAAAATC TTCTTTATAT AAATTAGATT TGAAAATACC CTATACGTAC CTACCGTACT   
  
  
- TTAAACGTGA ACAGTATACG GAGGGAGGAT GTGATTTCGG TTATCCGTAG TCGTCGGTAA ATGCCGTGAC   
  
  
- TCTTCCTATT TATAATCTAT ACACTAACCC GGAGTCGGCG GGCGACCAGT TTCAGTGCAC GAAACTGGTT   
  
  
- TGGTGCGCCC GGTTTTGTGT GTGTGCGGGT GTGTGTGAGA GAGAGAGAGA GAAAGAAGGG CATAAAAAGT   
  
  
- GCCAGTTTGA AGAGCAATGG AAGCGTCTTT GACGGAACGT TTCTTTTTTG TGTGAGAGAG AGAGAGAGAA   
  
  
- AGAAGGGCAT AAAAAGTGCC AGTTTGAAGA GCAATGGAAG CGTCTTTGAC GGAACGTTTC TTTTTCTTCT   
  
  
- TTTGATCTTT TTTTTCTCTC TTTTTGGTTT CTTCGTCTCT GCGGAACATG GAATGCACTG GCAGAAGAGG   
  
  
- CAAGAGAGAG AGAGAGAGAG GGGGGGAACT AGGAATATAA GTAGCATATT CGACAGAAGA AAGACATGGA   
  
  
- CATTGGAATC AAGAGAGAGA CAGCGAAAA

+     GCN4\_motif

| Site Name | Organism | Position | Strand | Matrix score. | sequence | function |
| --- | --- | --- | --- | --- | --- | --- |
| GCN4\_motif | Oryza sativa | 230 | + | 7 | TGTGTCA | cis-regulatory element involved in endosperm expression |

> 2018/04/13 10:10:12  
+ ATTATCGCAC TTTATCCACT AAGAACCTAA TTACCCAACT AGTGGGTTAA CTTGTAGGCT AAGCTTACAT   
  
  
+ ATCTGCTTAT GTTCTCTCCA TTTTGTGTGA AAACGTGTAG ACTTAATCTA ACCAAATCAT GATTGTGACT   
  
  
+ TTGATTATGA AATAAAAGGT CTATGGTTTA ATTAAATTAA TTACAATATG ACGTAAACTA AACATATACC   
  
  
+ TTTAATGGCT CTGGAGCTTT GTGTCATTTT TTATTTCCAC GATCATTAGG GCAAAAATTG GTTGAGAAAT   
  
  
+ TATAATTTAG AGTATAAGAT ACTCTGGCTA GCTTTTGGAT TTCATTGACT AAAAAGGCAT TACCCCTTTT   
  
  
+ ACACTTTAGG ACTCCTAGAA TCTTCTAAGC TAGACTTAAT TATATCTTTT AATAACTTTT TTAATGCTTG   
  
  
+ TTTGACACTA TTGCATGTTA GCCTCTAAGC TTTATTTTAT CGAGATCCAT ATAAATGTTT TTTTTTGTTT   
  
  
+ TTTCCGGTTT TACATTGAGA TAAAAATCAT TTACTAATTT GAAATCAAAT TGATTTTTTC ATTAAAAAAT   
  
  
+ TTAATGGTTA TTTTTATAAT TTAAAATAAC AATATAAAAT AAAAAAATTA ATTATTGATT TTATTAAATT   
  
  
+ GTGGTGCGTT TATTTGAAAG TGTATATGTC ATATTTCATA ATTTTACATT ATAAATAAAA ATATATAAAA   
  
  
+ TACATATAAT TAGTTGTGTT ATTTCTGGAC ATCACTTACA AAATGTAAAT ATGAAACAAA AATTTTGCGC   
  
  
+ ATTTAAAATA TATAAAATAC ATATAATTAG TTGTGTTATT TCTGGACATC ACTTACAAAA TGTAAATATG   
  
  
+ AAACAAAAAT TTTGCGCATT TAAGTAATTA TTATTAACTA TTTTTAATGA TTATAAGATA AATACATTTA   
  
  
+ TTTAATTAAT AATTTTTTAG AAGAAATATA TTTAATCTAA ACTTTTATGG GATATGCATG GATGGCATGA   
  
  
+ AATTTGCACT TGTCATATGC CTCCCTCCTA CACTAAAGCC AATAGGCATC AGCAGCCATT TACGGCACTG   
  
  
+ AGAAGGATAA ATATTAGATA TGTGATTGGG CCTCAGCCGC CCGCTGGTCA AAGTCACGTG CTTTGACCAA   
  
  
+ ACCACGCGGG CCAAAACACA CACACGCCCA CACACACTCT CTCTCTCTCT CTTTCTTCCC GTATTTTTCA   
  
  
+ CGGTCAAACT TCTCGTTACC TTCGCAGAAA CTGCCTTGCA AAGAAAAAAC ACACTCTCTC TCTCTCTCTT   
  
  
+ TCTTCCCGTA TTTTTCACGG TCAAACTTCT CGTTACCTTC GCAGAAACTG CCTTGCAAAG AAAAAGAAGA   
  
  
+ AAACTAGAAA AAAAAGAGAG AAAAACCAAA GAAGCAGAGA CGCCTTGTAC CTTACGTGAC CGTCTTCTCC   
  
  
+ GTTCTCTCTC TCTCTCTCTC CCCCCCTTGA TCCTTATATT CATCGTATAA GCTGTCTTCT TTCTGTACCT   
  
  
+ GTAACCTTAG TTCTCTCTCT GTCGCTTTT  

- TAATAGCGTG AAATAGGTGA TTCTTGGATT AATGGGTTGA TCACCCAATT GAACATCCGA TTCGAATGTA   
  
  
- TAGACGAATA CAAGAGAGGT AAAACACACT TTTGCACATC TGAATTAGAT TGGTTTAGTA CTAACACTGA   
  
  
- AACTAATACT TTATTTTCCA GATACCAAAT TAATTTAATT AATGTTATAC TGCATTTGAT TTGTATATGG   
  
  
- AAATTACCGA GACCTCGAAA CACAGTAAAA AATAAAGGTG CTAGTAATCC CGTTTTTAAC CAACTCTTTA   
  
  
- ATATTAAATC TCATATTCTA TGAGACCGAT CGAAAACCTA AAGTAACTGA TTTTTCCGTA ATGGGGAAAA   
  
  
- TGTGAAATCC TGAGGATCTT AGAAGATTCG ATCTGAATTA ATATAGAAAA TTATTGAAAA AATTACGAAC   
  
  
- AAACTGTGAT AACGTACAAT CGGAGATTCG AAATAAAATA GCTCTAGGTA TATTTACAAA AAAAAACAAA   
  
  
- AAAGGCCAAA ATGTAACTCT ATTTTTAGTA AATGATTAAA CTTTAGTTTA ACTAAAAAAG TAATTTTTTA   
  
  
- AATTACCAAT AAAAATATTA AATTTTATTG TTATATTTTA TTTTTTTAAT TAATAACTAA AATAATTTAA   
  
  
- CACCACGCAA ATAAACTTTC ACATATACAG TATAAAGTAT TAAAATGTAA TATTTATTTT TATATATTTT   
  
  
- ATGTATATTA ATCAACACAA TAAAGACCTG TAGTGAATGT TTTACATTTA TACTTTGTTT TTAAAACGCG   
  
  
- TAAATTTTAT ATATTTTATG TATATTAATC AACACAATAA AGACCTGTAG TGAATGTTTT ACATTTATAC   
  
  
- TTTGTTTTTA AAACGCGTAA ATTCATTAAT AATAATTGAT AAAAATTACT AATATTCTAT TTATGTAAAT   
  
  
- AAATTAATTA TTAAAAAATC TTCTTTATAT AAATTAGATT TGAAAATACC CTATACGTAC CTACCGTACT   
  
  
- TTAAACGTGA ACAGTATACG GAGGGAGGAT GTGATTTCGG TTATCCGTAG TCGTCGGTAA ATGCCGTGAC   
  
  
- TCTTCCTATT TATAATCTAT ACACTAACCC GGAGTCGGCG GGCGACCAGT TTCAGTGCAC GAAACTGGTT   
  
  
- TGGTGCGCCC GGTTTTGTGT GTGTGCGGGT GTGTGTGAGA GAGAGAGAGA GAAAGAAGGG CATAAAAAGT   
  
  
- GCCAGTTTGA AGAGCAATGG AAGCGTCTTT GACGGAACGT TTCTTTTTTG TGTGAGAGAG AGAGAGAGAA   
  
  
- AGAAGGGCAT AAAAAGTGCC AGTTTGAAGA GCAATGGAAG CGTCTTTGAC GGAACGTTTC TTTTTCTTCT   
  
  
- TTTGATCTTT TTTTTCTCTC TTTTTGGTTT CTTCGTCTCT GCGGAACATG GAATGCACTG GCAGAAGAGG   
  
  
- CAAGAGAGAG AGAGAGAGAG GGGGGGAACT AGGAATATAA GTAGCATATT CGACAGAAGA AAGACATGGA   
  
  
- CATTGGAATC AAGAGAGAGA CAGCGAAAA

+     GT1-motif

| Site Name | Organism | Position | Strand | Matrix score. | sequence | function |
| --- | --- | --- | --- | --- | --- | --- |
| GT1-motif | Arabidopsis thaliana | 45 | + | 6 | GGTTAA | light responsive element |

> 2018/04/13 10:10:12  
+ ATTATCGCAC TTTATCCACT AAGAACCTAA TTACCCAACT AGTGGGTTAA CTTGTAGGCT AAGCTTACAT   
  
  
+ ATCTGCTTAT GTTCTCTCCA TTTTGTGTGA AAACGTGTAG ACTTAATCTA ACCAAATCAT GATTGTGACT   
  
  
+ TTGATTATGA AATAAAAGGT CTATGGTTTA ATTAAATTAA TTACAATATG ACGTAAACTA AACATATACC   
  
  
+ TTTAATGGCT CTGGAGCTTT GTGTCATTTT TTATTTCCAC GATCATTAGG GCAAAAATTG GTTGAGAAAT   
  
  
+ TATAATTTAG AGTATAAGAT ACTCTGGCTA GCTTTTGGAT TTCATTGACT AAAAAGGCAT TACCCCTTTT   
  
  
+ ACACTTTAGG ACTCCTAGAA TCTTCTAAGC TAGACTTAAT TATATCTTTT AATAACTTTT TTAATGCTTG   
  
  
+ TTTGACACTA TTGCATGTTA GCCTCTAAGC TTTATTTTAT CGAGATCCAT ATAAATGTTT TTTTTTGTTT   
  
  
+ TTTCCGGTTT TACATTGAGA TAAAAATCAT TTACTAATTT GAAATCAAAT TGATTTTTTC ATTAAAAAAT   
  
  
+ TTAATGGTTA TTTTTATAAT TTAAAATAAC AATATAAAAT AAAAAAATTA ATTATTGATT TTATTAAATT   
  
  
+ GTGGTGCGTT TATTTGAAAG TGTATATGTC ATATTTCATA ATTTTACATT ATAAATAAAA ATATATAAAA   
  
  
+ TACATATAAT TAGTTGTGTT ATTTCTGGAC ATCACTTACA AAATGTAAAT ATGAAACAAA AATTTTGCGC   
  
  
+ ATTTAAAATA TATAAAATAC ATATAATTAG TTGTGTTATT TCTGGACATC ACTTACAAAA TGTAAATATG   
  
  
+ AAACAAAAAT TTTGCGCATT TAAGTAATTA TTATTAACTA TTTTTAATGA TTATAAGATA AATACATTTA   
  
  
+ TTTAATTAAT AATTTTTTAG AAGAAATATA TTTAATCTAA ACTTTTATGG GATATGCATG GATGGCATGA   
  
  
+ AATTTGCACT TGTCATATGC CTCCCTCCTA CACTAAAGCC AATAGGCATC AGCAGCCATT TACGGCACTG   
  
  
+ AGAAGGATAA ATATTAGATA TGTGATTGGG CCTCAGCCGC CCGCTGGTCA AAGTCACGTG CTTTGACCAA   
  
  
+ ACCACGCGGG CCAAAACACA CACACGCCCA CACACACTCT CTCTCTCTCT CTTTCTTCCC GTATTTTTCA   
  
  
+ CGGTCAAACT TCTCGTTACC TTCGCAGAAA CTGCCTTGCA AAGAAAAAAC ACACTCTCTC TCTCTCTCTT   
  
  
+ TCTTCCCGTA TTTTTCACGG TCAAACTTCT CGTTACCTTC GCAGAAACTG CCTTGCAAAG AAAAAGAAGA   
  
  
+ AAACTAGAAA AAAAAGAGAG AAAAACCAAA GAAGCAGAGA CGCCTTGTAC CTTACGTGAC CGTCTTCTCC   
  
  
+ GTTCTCTCTC TCTCTCTCTC CCCCCCTTGA TCCTTATATT CATCGTATAA GCTGTCTTCT TTCTGTACCT   
  
  
+ GTAACCTTAG TTCTCTCTCT GTCGCTTTT  

- TAATAGCGTG AAATAGGTGA TTCTTGGATT AATGGGTTGA TCACCCAATT GAACATCCGA TTCGAATGTA   
  
  
- TAGACGAATA CAAGAGAGGT AAAACACACT TTTGCACATC TGAATTAGAT TGGTTTAGTA CTAACACTGA   
  
  
- AACTAATACT TTATTTTCCA GATACCAAAT TAATTTAATT AATGTTATAC TGCATTTGAT TTGTATATGG   
  
  
- AAATTACCGA GACCTCGAAA CACAGTAAAA AATAAAGGTG CTAGTAATCC CGTTTTTAAC CAACTCTTTA   
  
  
- ATATTAAATC TCATATTCTA TGAGACCGAT CGAAAACCTA AAGTAACTGA TTTTTCCGTA ATGGGGAAAA   
  
  
- TGTGAAATCC TGAGGATCTT AGAAGATTCG ATCTGAATTA ATATAGAAAA TTATTGAAAA AATTACGAAC   
  
  
- AAACTGTGAT AACGTACAAT CGGAGATTCG AAATAAAATA GCTCTAGGTA TATTTACAAA AAAAAACAAA   
  
  
- AAAGGCCAAA ATGTAACTCT ATTTTTAGTA AATGATTAAA CTTTAGTTTA ACTAAAAAAG TAATTTTTTA   
  
  
- AATTACCAAT AAAAATATTA AATTTTATTG TTATATTTTA TTTTTTTAAT TAATAACTAA AATAATTTAA   
  
  
- CACCACGCAA ATAAACTTTC ACATATACAG TATAAAGTAT TAAAATGTAA TATTTATTTT TATATATTTT   
  
  
- ATGTATATTA ATCAACACAA TAAAGACCTG TAGTGAATGT TTTACATTTA TACTTTGTTT TTAAAACGCG   
  
  
- TAAATTTTAT ATATTTTATG TATATTAATC AACACAATAA AGACCTGTAG TGAATGTTTT ACATTTATAC   
  
  
- TTTGTTTTTA AAACGCGTAA ATTCATTAAT AATAATTGAT AAAAATTACT AATATTCTAT TTATGTAAAT   
  
  
- AAATTAATTA TTAAAAAATC TTCTTTATAT AAATTAGATT TGAAAATACC CTATACGTAC CTACCGTACT   
  
  
- TTAAACGTGA ACAGTATACG GAGGGAGGAT GTGATTTCGG TTATCCGTAG TCGTCGGTAA ATGCCGTGAC   
  
  
- TCTTCCTATT TATAATCTAT ACACTAACCC GGAGTCGGCG GGCGACCAGT TTCAGTGCAC GAAACTGGTT   
  
  
- TGGTGCGCCC GGTTTTGTGT GTGTGCGGGT GTGTGTGAGA GAGAGAGAGA GAAAGAAGGG CATAAAAAGT   
  
  
- GCCAGTTTGA AGAGCAATGG AAGCGTCTTT GACGGAACGT TTCTTTTTTG TGTGAGAGAG AGAGAGAGAA   
  
  
- AGAAGGGCAT AAAAAGTGCC AGTTTGAAGA GCAATGGAAG CGTCTTTGAC GGAACGTTTC TTTTTCTTCT   
  
  
- TTTGATCTTT TTTTTCTCTC TTTTTGGTTT CTTCGTCTCT GCGGAACATG GAATGCACTG GCAGAAGAGG   
  
  
- CAAGAGAGAG AGAGAGAGAG GGGGGGAACT AGGAATATAA GTAGCATATT CGACAGAAGA AAGACATGGA   
  
  
- CATTGGAATC AAGAGAGAGA CAGCGAAAA

+     Gap-box

| Site Name | Organism | Position | Strand | Matrix score. | sequence | function |
| --- | --- | --- | --- | --- | --- | --- |
| Gap-box | Arabidopsis thaliana | 85 | - | 9 | AAATGGAGA | part of a light responsive element |

> 2018/04/13 10:10:12  
+ ATTATCGCAC TTTATCCACT AAGAACCTAA TTACCCAACT AGTGGGTTAA CTTGTAGGCT AAGCTTACAT   
  
  
+ ATCTGCTTAT GTTCTCTCCA TTTTGTGTGA AAACGTGTAG ACTTAATCTA ACCAAATCAT GATTGTGACT   
  
  
+ TTGATTATGA AATAAAAGGT CTATGGTTTA ATTAAATTAA TTACAATATG ACGTAAACTA AACATATACC   
  
  
+ TTTAATGGCT CTGGAGCTTT GTGTCATTTT TTATTTCCAC GATCATTAGG GCAAAAATTG GTTGAGAAAT   
  
  
+ TATAATTTAG AGTATAAGAT ACTCTGGCTA GCTTTTGGAT TTCATTGACT AAAAAGGCAT TACCCCTTTT   
  
  
+ ACACTTTAGG ACTCCTAGAA TCTTCTAAGC TAGACTTAAT TATATCTTTT AATAACTTTT TTAATGCTTG   
  
  
+ TTTGACACTA TTGCATGTTA GCCTCTAAGC TTTATTTTAT CGAGATCCAT ATAAATGTTT TTTTTTGTTT   
  
  
+ TTTCCGGTTT TACATTGAGA TAAAAATCAT TTACTAATTT GAAATCAAAT TGATTTTTTC ATTAAAAAAT   
  
  
+ TTAATGGTTA TTTTTATAAT TTAAAATAAC AATATAAAAT AAAAAAATTA ATTATTGATT TTATTAAATT   
  
  
+ GTGGTGCGTT TATTTGAAAG TGTATATGTC ATATTTCATA ATTTTACATT ATAAATAAAA ATATATAAAA   
  
  
+ TACATATAAT TAGTTGTGTT ATTTCTGGAC ATCACTTACA AAATGTAAAT ATGAAACAAA AATTTTGCGC   
  
  
+ ATTTAAAATA TATAAAATAC ATATAATTAG TTGTGTTATT TCTGGACATC ACTTACAAAA TGTAAATATG   
  
  
+ AAACAAAAAT TTTGCGCATT TAAGTAATTA TTATTAACTA TTTTTAATGA TTATAAGATA AATACATTTA   
  
  
+ TTTAATTAAT AATTTTTTAG AAGAAATATA TTTAATCTAA ACTTTTATGG GATATGCATG GATGGCATGA   
  
  
+ AATTTGCACT TGTCATATGC CTCCCTCCTA CACTAAAGCC AATAGGCATC AGCAGCCATT TACGGCACTG   
  
  
+ AGAAGGATAA ATATTAGATA TGTGATTGGG CCTCAGCCGC CCGCTGGTCA AAGTCACGTG CTTTGACCAA   
  
  
+ ACCACGCGGG CCAAAACACA CACACGCCCA CACACACTCT CTCTCTCTCT CTTTCTTCCC GTATTTTTCA   
  
  
+ CGGTCAAACT TCTCGTTACC TTCGCAGAAA CTGCCTTGCA AAGAAAAAAC ACACTCTCTC TCTCTCTCTT   
  
  
+ TCTTCCCGTA TTTTTCACGG TCAAACTTCT CGTTACCTTC GCAGAAACTG CCTTGCAAAG AAAAAGAAGA   
  
  
+ AAACTAGAAA AAAAAGAGAG AAAAACCAAA GAAGCAGAGA CGCCTTGTAC CTTACGTGAC CGTCTTCTCC   
  
  
+ GTTCTCTCTC TCTCTCTCTC CCCCCCTTGA TCCTTATATT CATCGTATAA GCTGTCTTCT TTCTGTACCT   
  
  
+ GTAACCTTAG TTCTCTCTCT GTCGCTTTT  

- TAATAGCGTG AAATAGGTGA TTCTTGGATT AATGGGTTGA TCACCCAATT GAACATCCGA TTCGAATGTA   
  
  
- TAGACGAATA CAAGAGAGGT AAAACACACT TTTGCACATC TGAATTAGAT TGGTTTAGTA CTAACACTGA   
  
  
- AACTAATACT TTATTTTCCA GATACCAAAT TAATTTAATT AATGTTATAC TGCATTTGAT TTGTATATGG   
  
  
- AAATTACCGA GACCTCGAAA CACAGTAAAA AATAAAGGTG CTAGTAATCC CGTTTTTAAC CAACTCTTTA   
  
  
- ATATTAAATC TCATATTCTA TGAGACCGAT CGAAAACCTA AAGTAACTGA TTTTTCCGTA ATGGGGAAAA   
  
  
- TGTGAAATCC TGAGGATCTT AGAAGATTCG ATCTGAATTA ATATAGAAAA TTATTGAAAA AATTACGAAC   
  
  
- AAACTGTGAT AACGTACAAT CGGAGATTCG AAATAAAATA GCTCTAGGTA TATTTACAAA AAAAAACAAA   
  
  
- AAAGGCCAAA ATGTAACTCT ATTTTTAGTA AATGATTAAA CTTTAGTTTA ACTAAAAAAG TAATTTTTTA   
  
  
- AATTACCAAT AAAAATATTA AATTTTATTG TTATATTTTA TTTTTTTAAT TAATAACTAA AATAATTTAA   
  
  
- CACCACGCAA ATAAACTTTC ACATATACAG TATAAAGTAT TAAAATGTAA TATTTATTTT TATATATTTT   
  
  
- ATGTATATTA ATCAACACAA TAAAGACCTG TAGTGAATGT TTTACATTTA TACTTTGTTT TTAAAACGCG   
  
  
- TAAATTTTAT ATATTTTATG TATATTAATC AACACAATAA AGACCTGTAG TGAATGTTTT ACATTTATAC   
  
  
- TTTGTTTTTA AAACGCGTAA ATTCATTAAT AATAATTGAT AAAAATTACT AATATTCTAT TTATGTAAAT   
  
  
- AAATTAATTA TTAAAAAATC TTCTTTATAT AAATTAGATT TGAAAATACC CTATACGTAC CTACCGTACT   
  
  
- TTAAACGTGA ACAGTATACG GAGGGAGGAT GTGATTTCGG TTATCCGTAG TCGTCGGTAA ATGCCGTGAC   
  
  
- TCTTCCTATT TATAATCTAT ACACTAACCC GGAGTCGGCG GGCGACCAGT TTCAGTGCAC GAAACTGGTT   
  
  
- TGGTGCGCCC GGTTTTGTGT GTGTGCGGGT GTGTGTGAGA GAGAGAGAGA GAAAGAAGGG CATAAAAAGT   
  
  
- GCCAGTTTGA AGAGCAATGG AAGCGTCTTT GACGGAACGT TTCTTTTTTG TGTGAGAGAG AGAGAGAGAA   
  
  
- AGAAGGGCAT AAAAAGTGCC AGTTTGAAGA GCAATGGAAG CGTCTTTGAC GGAACGTTTC TTTTTCTTCT   
  
  
- TTTGATCTTT TTTTTCTCTC TTTTTGGTTT CTTCGTCTCT GCGGAACATG GAATGCACTG GCAGAAGAGG   
  
  
- CAAGAGAGAG AGAGAGAGAG GGGGGGAACT AGGAATATAA GTAGCATATT CGACAGAAGA AAGACATGGA   
  
  
- CATTGGAATC AAGAGAGAGA CAGCGAAAA

+     HSE

| Site Name | Organism | Position | Strand | Matrix score. | sequence | function |
| --- | --- | --- | --- | --- | --- | --- |
| HSE | Brassica oleracea | 554 | + | 9 | AAAAAATTTC | cis-acting element involved in heat stress responsiveness |

> 2018/04/13 10:10:12  
+ ATTATCGCAC TTTATCCACT AAGAACCTAA TTACCCAACT AGTGGGTTAA CTTGTAGGCT AAGCTTACAT   
  
  
+ ATCTGCTTAT GTTCTCTCCA TTTTGTGTGA AAACGTGTAG ACTTAATCTA ACCAAATCAT GATTGTGACT   
  
  
+ TTGATTATGA AATAAAAGGT CTATGGTTTA ATTAAATTAA TTACAATATG ACGTAAACTA AACATATACC   
  
  
+ TTTAATGGCT CTGGAGCTTT GTGTCATTTT TTATTTCCAC GATCATTAGG GCAAAAATTG GTTGAGAAAT   
  
  
+ TATAATTTAG AGTATAAGAT ACTCTGGCTA GCTTTTGGAT TTCATTGACT AAAAAGGCAT TACCCCTTTT   
  
  
+ ACACTTTAGG ACTCCTAGAA TCTTCTAAGC TAGACTTAAT TATATCTTTT AATAACTTTT TTAATGCTTG   
  
  
+ TTTGACACTA TTGCATGTTA GCCTCTAAGC TTTATTTTAT CGAGATCCAT ATAAATGTTT TTTTTTGTTT   
  
  
+ TTTCCGGTTT TACATTGAGA TAAAAATCAT TTACTAATTT GAAATCAAAT TGATTTTTTC ATTAAAAAAT   
  
  
+ TTAATGGTTA TTTTTATAAT TTAAAATAAC AATATAAAAT AAAAAAATTA ATTATTGATT TTATTAAATT   
  
  
+ GTGGTGCGTT TATTTGAAAG TGTATATGTC ATATTTCATA ATTTTACATT ATAAATAAAA ATATATAAAA   
  
  
+ TACATATAAT TAGTTGTGTT ATTTCTGGAC ATCACTTACA AAATGTAAAT ATGAAACAAA AATTTTGCGC   
  
  
+ ATTTAAAATA TATAAAATAC ATATAATTAG TTGTGTTATT TCTGGACATC ACTTACAAAA TGTAAATATG   
  
  
+ AAACAAAAAT TTTGCGCATT TAAGTAATTA TTATTAACTA TTTTTAATGA TTATAAGATA AATACATTTA   
  
  
+ TTTAATTAAT AATTTTTTAG AAGAAATATA TTTAATCTAA ACTTTTATGG GATATGCATG GATGGCATGA   
  
  
+ AATTTGCACT TGTCATATGC CTCCCTCCTA CACTAAAGCC AATAGGCATC AGCAGCCATT TACGGCACTG   
  
  
+ AGAAGGATAA ATATTAGATA TGTGATTGGG CCTCAGCCGC CCGCTGGTCA AAGTCACGTG CTTTGACCAA   
  
  
+ ACCACGCGGG CCAAAACACA CACACGCCCA CACACACTCT CTCTCTCTCT CTTTCTTCCC GTATTTTTCA   
  
  
+ CGGTCAAACT TCTCGTTACC TTCGCAGAAA CTGCCTTGCA AAGAAAAAAC ACACTCTCTC TCTCTCTCTT   
  
  
+ TCTTCCCGTA TTTTTCACGG TCAAACTTCT CGTTACCTTC GCAGAAACTG CCTTGCAAAG AAAAAGAAGA   
  
  
+ AAACTAGAAA AAAAAGAGAG AAAAACCAAA GAAGCAGAGA CGCCTTGTAC CTTACGTGAC CGTCTTCTCC   
  
  
+ GTTCTCTCTC TCTCTCTCTC CCCCCCTTGA TCCTTATATT CATCGTATAA GCTGTCTTCT TTCTGTACCT   
  
  
+ GTAACCTTAG TTCTCTCTCT GTCGCTTTT  

- TAATAGCGTG AAATAGGTGA TTCTTGGATT AATGGGTTGA TCACCCAATT GAACATCCGA TTCGAATGTA   
  
  
- TAGACGAATA CAAGAGAGGT AAAACACACT TTTGCACATC TGAATTAGAT TGGTTTAGTA CTAACACTGA   
  
  
- AACTAATACT TTATTTTCCA GATACCAAAT TAATTTAATT AATGTTATAC TGCATTTGAT TTGTATATGG   
  
  
- AAATTACCGA GACCTCGAAA CACAGTAAAA AATAAAGGTG CTAGTAATCC CGTTTTTAAC CAACTCTTTA   
  
  
- ATATTAAATC TCATATTCTA TGAGACCGAT CGAAAACCTA AAGTAACTGA TTTTTCCGTA ATGGGGAAAA   
  
  
- TGTGAAATCC TGAGGATCTT AGAAGATTCG ATCTGAATTA ATATAGAAAA TTATTGAAAA AATTACGAAC   
  
  
- AAACTGTGAT AACGTACAAT CGGAGATTCG AAATAAAATA GCTCTAGGTA TATTTACAAA AAAAAACAAA   
  
  
- AAAGGCCAAA ATGTAACTCT ATTTTTAGTA AATGATTAAA CTTTAGTTTA ACTAAAAAAG TAATTTTTTA   
  
  
- AATTACCAAT AAAAATATTA AATTTTATTG TTATATTTTA TTTTTTTAAT TAATAACTAA AATAATTTAA   
  
  
- CACCACGCAA ATAAACTTTC ACATATACAG TATAAAGTAT TAAAATGTAA TATTTATTTT TATATATTTT   
  
  
- ATGTATATTA ATCAACACAA TAAAGACCTG TAGTGAATGT TTTACATTTA TACTTTGTTT TTAAAACGCG   
  
  
- TAAATTTTAT ATATTTTATG TATATTAATC AACACAATAA AGACCTGTAG TGAATGTTTT ACATTTATAC   
  
  
- TTTGTTTTTA AAACGCGTAA ATTCATTAAT AATAATTGAT AAAAATTACT AATATTCTAT TTATGTAAAT   
  
  
- AAATTAATTA TTAAAAAATC TTCTTTATAT AAATTAGATT TGAAAATACC CTATACGTAC CTACCGTACT   
  
  
- TTAAACGTGA ACAGTATACG GAGGGAGGAT GTGATTTCGG TTATCCGTAG TCGTCGGTAA ATGCCGTGAC   
  
  
- TCTTCCTATT TATAATCTAT ACACTAACCC GGAGTCGGCG GGCGACCAGT TTCAGTGCAC GAAACTGGTT   
  
  
- TGGTGCGCCC GGTTTTGTGT GTGTGCGGGT GTGTGTGAGA GAGAGAGAGA GAAAGAAGGG CATAAAAAGT   
  
  
- GCCAGTTTGA AGAGCAATGG AAGCGTCTTT GACGGAACGT TTCTTTTTTG TGTGAGAGAG AGAGAGAGAA   
  
  
- AGAAGGGCAT AAAAAGTGCC AGTTTGAAGA GCAATGGAAG CGTCTTTGAC GGAACGTTTC TTTTTCTTCT   
  
  
- TTTGATCTTT TTTTTCTCTC TTTTTGGTTT CTTCGTCTCT GCGGAACATG GAATGCACTG GCAGAAGAGG   
  
  
- CAAGAGAGAG AGAGAGAGAG GGGGGGAACT AGGAATATAA GTAGCATATT CGACAGAAGA AAGACATGGA   
  
  
- CATTGGAATC AAGAGAGAGA CAGCGAAAA

+     I-box

| Site Name | Organism | Position | Strand | Matrix score. | sequence | function |
| --- | --- | --- | --- | --- | --- | --- |
| I-box | Zea mays | 8 | - | 9 | gGATAAGGTG | part of a light responsive element |

> 2018/04/13 10:10:12  
+ ATTATCGCAC TTTATCCACT AAGAACCTAA TTACCCAACT AGTGGGTTAA CTTGTAGGCT AAGCTTACAT   
  
  
+ ATCTGCTTAT GTTCTCTCCA TTTTGTGTGA AAACGTGTAG ACTTAATCTA ACCAAATCAT GATTGTGACT   
  
  
+ TTGATTATGA AATAAAAGGT CTATGGTTTA ATTAAATTAA TTACAATATG ACGTAAACTA AACATATACC   
  
  
+ TTTAATGGCT CTGGAGCTTT GTGTCATTTT TTATTTCCAC GATCATTAGG GCAAAAATTG GTTGAGAAAT   
  
  
+ TATAATTTAG AGTATAAGAT ACTCTGGCTA GCTTTTGGAT TTCATTGACT AAAAAGGCAT TACCCCTTTT   
  
  
+ ACACTTTAGG ACTCCTAGAA TCTTCTAAGC TAGACTTAAT TATATCTTTT AATAACTTTT TTAATGCTTG   
  
  
+ TTTGACACTA TTGCATGTTA GCCTCTAAGC TTTATTTTAT CGAGATCCAT ATAAATGTTT TTTTTTGTTT   
  
  
+ TTTCCGGTTT TACATTGAGA TAAAAATCAT TTACTAATTT GAAATCAAAT TGATTTTTTC ATTAAAAAAT   
  
  
+ TTAATGGTTA TTTTTATAAT TTAAAATAAC AATATAAAAT AAAAAAATTA ATTATTGATT TTATTAAATT   
  
  
+ GTGGTGCGTT TATTTGAAAG TGTATATGTC ATATTTCATA ATTTTACATT ATAAATAAAA ATATATAAAA   
  
  
+ TACATATAAT TAGTTGTGTT ATTTCTGGAC ATCACTTACA AAATGTAAAT ATGAAACAAA AATTTTGCGC   
  
  
+ ATTTAAAATA TATAAAATAC ATATAATTAG TTGTGTTATT TCTGGACATC ACTTACAAAA TGTAAATATG   
  
  
+ AAACAAAAAT TTTGCGCATT TAAGTAATTA TTATTAACTA TTTTTAATGA TTATAAGATA AATACATTTA   
  
  
+ TTTAATTAAT AATTTTTTAG AAGAAATATA TTTAATCTAA ACTTTTATGG GATATGCATG GATGGCATGA   
  
  
+ AATTTGCACT TGTCATATGC CTCCCTCCTA CACTAAAGCC AATAGGCATC AGCAGCCATT TACGGCACTG   
  
  
+ AGAAGGATAA ATATTAGATA TGTGATTGGG CCTCAGCCGC CCGCTGGTCA AAGTCACGTG CTTTGACCAA   
  
  
+ ACCACGCGGG CCAAAACACA CACACGCCCA CACACACTCT CTCTCTCTCT CTTTCTTCCC GTATTTTTCA   
  
  
+ CGGTCAAACT TCTCGTTACC TTCGCAGAAA CTGCCTTGCA AAGAAAAAAC ACACTCTCTC TCTCTCTCTT   
  
  
+ TCTTCCCGTA TTTTTCACGG TCAAACTTCT CGTTACCTTC GCAGAAACTG CCTTGCAAAG AAAAAGAAGA   
  
  
+ AAACTAGAAA AAAAAGAGAG AAAAACCAAA GAAGCAGAGA CGCCTTGTAC CTTACGTGAC CGTCTTCTCC   
  
  
+ GTTCTCTCTC TCTCTCTCTC CCCCCCTTGA TCCTTATATT CATCGTATAA GCTGTCTTCT TTCTGTACCT   
  
  
+ GTAACCTTAG TTCTCTCTCT GTCGCTTTT  

- TAATAGCGTG AAATAGGTGA TTCTTGGATT AATGGGTTGA TCACCCAATT GAACATCCGA TTCGAATGTA   
  
  
- TAGACGAATA CAAGAGAGGT AAAACACACT TTTGCACATC TGAATTAGAT TGGTTTAGTA CTAACACTGA   
  
  
- AACTAATACT TTATTTTCCA GATACCAAAT TAATTTAATT AATGTTATAC TGCATTTGAT TTGTATATGG   
  
  
- AAATTACCGA GACCTCGAAA CACAGTAAAA AATAAAGGTG CTAGTAATCC CGTTTTTAAC CAACTCTTTA   
  
  
- ATATTAAATC TCATATTCTA TGAGACCGAT CGAAAACCTA AAGTAACTGA TTTTTCCGTA ATGGGGAAAA   
  
  
- TGTGAAATCC TGAGGATCTT AGAAGATTCG ATCTGAATTA ATATAGAAAA TTATTGAAAA AATTACGAAC   
  
  
- AAACTGTGAT AACGTACAAT CGGAGATTCG AAATAAAATA GCTCTAGGTA TATTTACAAA AAAAAACAAA   
  
  
- AAAGGCCAAA ATGTAACTCT ATTTTTAGTA AATGATTAAA CTTTAGTTTA ACTAAAAAAG TAATTTTTTA   
  
  
- AATTACCAAT AAAAATATTA AATTTTATTG TTATATTTTA TTTTTTTAAT TAATAACTAA AATAATTTAA   
  
  
- CACCACGCAA ATAAACTTTC ACATATACAG TATAAAGTAT TAAAATGTAA TATTTATTTT TATATATTTT   
  
  
- ATGTATATTA ATCAACACAA TAAAGACCTG TAGTGAATGT TTTACATTTA TACTTTGTTT TTAAAACGCG   
  
  
- TAAATTTTAT ATATTTTATG TATATTAATC AACACAATAA AGACCTGTAG TGAATGTTTT ACATTTATAC   
  
  
- TTTGTTTTTA AAACGCGTAA ATTCATTAAT AATAATTGAT AAAAATTACT AATATTCTAT TTATGTAAAT   
  
  
- AAATTAATTA TTAAAAAATC TTCTTTATAT AAATTAGATT TGAAAATACC CTATACGTAC CTACCGTACT   
  
  
- TTAAACGTGA ACAGTATACG GAGGGAGGAT GTGATTTCGG TTATCCGTAG TCGTCGGTAA ATGCCGTGAC   
  
  
- TCTTCCTATT TATAATCTAT ACACTAACCC GGAGTCGGCG GGCGACCAGT TTCAGTGCAC GAAACTGGTT   
  
  
- TGGTGCGCCC GGTTTTGTGT GTGTGCGGGT GTGTGTGAGA GAGAGAGAGA GAAAGAAGGG CATAAAAAGT   
  
  
- GCCAGTTTGA AGAGCAATGG AAGCGTCTTT GACGGAACGT TTCTTTTTTG TGTGAGAGAG AGAGAGAGAA   
  
  
- AGAAGGGCAT AAAAAGTGCC AGTTTGAAGA GCAATGGAAG CGTCTTTGAC GGAACGTTTC TTTTTCTTCT   
  
  
- TTTGATCTTT TTTTTCTCTC TTTTTGGTTT CTTCGTCTCT GCGGAACATG GAATGCACTG GCAGAAGAGG   
  
  
- CAAGAGAGAG AGAGAGAGAG GGGGGGAACT AGGAATATAA GTAGCATATT CGACAGAAGA AAGACATGGA   
  
  
- CATTGGAATC AAGAGAGAGA CAGCGAAAA

+     MBS

| Site Name | Organism | Position | Strand | Matrix score. | sequence | function |
| --- | --- | --- | --- | --- | --- | --- |
| MBS | Zea mays | 1278 | + | 6 | CGGTCA | MYB Binding Site |
| MBS | Zea mays | 1191 | + | 6 | CGGTCA | MYB Binding Site |
| MBS | Zea mays | 1387 | - | 6 | CGGTCA | MYB Binding Site |

> 2018/04/13 10:10:12  
+ ATTATCGCAC TTTATCCACT AAGAACCTAA TTACCCAACT AGTGGGTTAA CTTGTAGGCT AAGCTTACAT   
  
  
+ ATCTGCTTAT GTTCTCTCCA TTTTGTGTGA AAACGTGTAG ACTTAATCTA ACCAAATCAT GATTGTGACT   
  
  
+ TTGATTATGA AATAAAAGGT CTATGGTTTA ATTAAATTAA TTACAATATG ACGTAAACTA AACATATACC   
  
  
+ TTTAATGGCT CTGGAGCTTT GTGTCATTTT TTATTTCCAC GATCATTAGG GCAAAAATTG GTTGAGAAAT   
  
  
+ TATAATTTAG AGTATAAGAT ACTCTGGCTA GCTTTTGGAT TTCATTGACT AAAAAGGCAT TACCCCTTTT   
  
  
+ ACACTTTAGG ACTCCTAGAA TCTTCTAAGC TAGACTTAAT TATATCTTTT AATAACTTTT TTAATGCTTG   
  
  
+ TTTGACACTA TTGCATGTTA GCCTCTAAGC TTTATTTTAT CGAGATCCAT ATAAATGTTT TTTTTTGTTT   
  
  
+ TTTCCGGTTT TACATTGAGA TAAAAATCAT TTACTAATTT GAAATCAAAT TGATTTTTTC ATTAAAAAAT   
  
  
+ TTAATGGTTA TTTTTATAAT TTAAAATAAC AATATAAAAT AAAAAAATTA ATTATTGATT TTATTAAATT   
  
  
+ GTGGTGCGTT TATTTGAAAG TGTATATGTC ATATTTCATA ATTTTACATT ATAAATAAAA ATATATAAAA   
  
  
+ TACATATAAT TAGTTGTGTT ATTTCTGGAC ATCACTTACA AAATGTAAAT ATGAAACAAA AATTTTGCGC   
  
  
+ ATTTAAAATA TATAAAATAC ATATAATTAG TTGTGTTATT TCTGGACATC ACTTACAAAA TGTAAATATG   
  
  
+ AAACAAAAAT TTTGCGCATT TAAGTAATTA TTATTAACTA TTTTTAATGA TTATAAGATA AATACATTTA   
  
  
+ TTTAATTAAT AATTTTTTAG AAGAAATATA TTTAATCTAA ACTTTTATGG GATATGCATG GATGGCATGA   
  
  
+ AATTTGCACT TGTCATATGC CTCCCTCCTA CACTAAAGCC AATAGGCATC AGCAGCCATT TACGGCACTG   
  
  
+ AGAAGGATAA ATATTAGATA TGTGATTGGG CCTCAGCCGC CCGCTGGTCA AAGTCACGTG CTTTGACCAA   
  
  
+ ACCACGCGGG CCAAAACACA CACACGCCCA CACACACTCT CTCTCTCTCT CTTTCTTCCC GTATTTTTCA   
  
  
+ CGGTCAAACT TCTCGTTACC TTCGCAGAAA CTGCCTTGCA AAGAAAAAAC ACACTCTCTC TCTCTCTCTT   
  
  
+ TCTTCCCGTA TTTTTCACGG TCAAACTTCT CGTTACCTTC GCAGAAACTG CCTTGCAAAG AAAAAGAAGA   
  
  
+ AAACTAGAAA AAAAAGAGAG AAAAACCAAA GAAGCAGAGA CGCCTTGTAC CTTACGTGAC CGTCTTCTCC   
  
  
+ GTTCTCTCTC TCTCTCTCTC CCCCCCTTGA TCCTTATATT CATCGTATAA GCTGTCTTCT TTCTGTACCT   
  
  
+ GTAACCTTAG TTCTCTCTCT GTCGCTTTT  

- TAATAGCGTG AAATAGGTGA TTCTTGGATT AATGGGTTGA TCACCCAATT GAACATCCGA TTCGAATGTA   
  
  
- TAGACGAATA CAAGAGAGGT AAAACACACT TTTGCACATC TGAATTAGAT TGGTTTAGTA CTAACACTGA   
  
  
- AACTAATACT TTATTTTCCA GATACCAAAT TAATTTAATT AATGTTATAC TGCATTTGAT TTGTATATGG   
  
  
- AAATTACCGA GACCTCGAAA CACAGTAAAA AATAAAGGTG CTAGTAATCC CGTTTTTAAC CAACTCTTTA   
  
  
- ATATTAAATC TCATATTCTA TGAGACCGAT CGAAAACCTA AAGTAACTGA TTTTTCCGTA ATGGGGAAAA   
  
  
- TGTGAAATCC TGAGGATCTT AGAAGATTCG ATCTGAATTA ATATAGAAAA TTATTGAAAA AATTACGAAC   
  
  
- AAACTGTGAT AACGTACAAT CGGAGATTCG AAATAAAATA GCTCTAGGTA TATTTACAAA AAAAAACAAA   
  
  
- AAAGGCCAAA ATGTAACTCT ATTTTTAGTA AATGATTAAA CTTTAGTTTA ACTAAAAAAG TAATTTTTTA   
  
  
- AATTACCAAT AAAAATATTA AATTTTATTG TTATATTTTA TTTTTTTAAT TAATAACTAA AATAATTTAA   
  
  
- CACCACGCAA ATAAACTTTC ACATATACAG TATAAAGTAT TAAAATGTAA TATTTATTTT TATATATTTT   
  
  
- ATGTATATTA ATCAACACAA TAAAGACCTG TAGTGAATGT TTTACATTTA TACTTTGTTT TTAAAACGCG   
  
  
- TAAATTTTAT ATATTTTATG TATATTAATC AACACAATAA AGACCTGTAG TGAATGTTTT ACATTTATAC   
  
  
- TTTGTTTTTA AAACGCGTAA ATTCATTAAT AATAATTGAT AAAAATTACT AATATTCTAT TTATGTAAAT   
  
  
- AAATTAATTA TTAAAAAATC TTCTTTATAT AAATTAGATT TGAAAATACC CTATACGTAC CTACCGTACT   
  
  
- TTAAACGTGA ACAGTATACG GAGGGAGGAT GTGATTTCGG TTATCCGTAG TCGTCGGTAA ATGCCGTGAC   
  
  
- TCTTCCTATT TATAATCTAT ACACTAACCC GGAGTCGGCG GGCGACCAGT TTCAGTGCAC GAAACTGGTT   
  
  
- TGGTGCGCCC GGTTTTGTGT GTGTGCGGGT GTGTGTGAGA GAGAGAGAGA GAAAGAAGGG CATAAAAAGT   
  
  
- GCCAGTTTGA AGAGCAATGG AAGCGTCTTT GACGGAACGT TTCTTTTTTG TGTGAGAGAG AGAGAGAGAA   
  
  
- AGAAGGGCAT AAAAAGTGCC AGTTTGAAGA GCAATGGAAG CGTCTTTGAC GGAACGTTTC TTTTTCTTCT   
  
  
- TTTGATCTTT TTTTTCTCTC TTTTTGGTTT CTTCGTCTCT GCGGAACATG GAATGCACTG GCAGAAGAGG   
  
  
- CAAGAGAGAG AGAGAGAGAG GGGGGGAACT AGGAATATAA GTAGCATATT CGACAGAAGA AAGACATGGA   
  
  
- CATTGGAATC AAGAGAGAGA CAGCGAAAA

+     MRE

| Site Name | Organism | Position | Strand | Matrix score. | sequence | function |
| --- | --- | --- | --- | --- | --- | --- |
| MRE | Petroselinum crispum | 24 | + | 7 | AACCTAA | MYB binding site involved in light responsiveness |

> 2018/04/13 10:10:12  
+ ATTATCGCAC TTTATCCACT AAGAACCTAA TTACCCAACT AGTGGGTTAA CTTGTAGGCT AAGCTTACAT   
  
  
+ ATCTGCTTAT GTTCTCTCCA TTTTGTGTGA AAACGTGTAG ACTTAATCTA ACCAAATCAT GATTGTGACT   
  
  
+ TTGATTATGA AATAAAAGGT CTATGGTTTA ATTAAATTAA TTACAATATG ACGTAAACTA AACATATACC   
  
  
+ TTTAATGGCT CTGGAGCTTT GTGTCATTTT TTATTTCCAC GATCATTAGG GCAAAAATTG GTTGAGAAAT   
  
  
+ TATAATTTAG AGTATAAGAT ACTCTGGCTA GCTTTTGGAT TTCATTGACT AAAAAGGCAT TACCCCTTTT   
  
  
+ ACACTTTAGG ACTCCTAGAA TCTTCTAAGC TAGACTTAAT TATATCTTTT AATAACTTTT TTAATGCTTG   
  
  
+ TTTGACACTA TTGCATGTTA GCCTCTAAGC TTTATTTTAT CGAGATCCAT ATAAATGTTT TTTTTTGTTT   
  
  
+ TTTCCGGTTT TACATTGAGA TAAAAATCAT TTACTAATTT GAAATCAAAT TGATTTTTTC ATTAAAAAAT   
  
  
+ TTAATGGTTA TTTTTATAAT TTAAAATAAC AATATAAAAT AAAAAAATTA ATTATTGATT TTATTAAATT   
  
  
+ GTGGTGCGTT TATTTGAAAG TGTATATGTC ATATTTCATA ATTTTACATT ATAAATAAAA ATATATAAAA   
  
  
+ TACATATAAT TAGTTGTGTT ATTTCTGGAC ATCACTTACA AAATGTAAAT ATGAAACAAA AATTTTGCGC   
  
  
+ ATTTAAAATA TATAAAATAC ATATAATTAG TTGTGTTATT TCTGGACATC ACTTACAAAA TGTAAATATG   
  
  
+ AAACAAAAAT TTTGCGCATT TAAGTAATTA TTATTAACTA TTTTTAATGA TTATAAGATA AATACATTTA   
  
  
+ TTTAATTAAT AATTTTTTAG AAGAAATATA TTTAATCTAA ACTTTTATGG GATATGCATG GATGGCATGA   
  
  
+ AATTTGCACT TGTCATATGC CTCCCTCCTA CACTAAAGCC AATAGGCATC AGCAGCCATT TACGGCACTG   
  
  
+ AGAAGGATAA ATATTAGATA TGTGATTGGG CCTCAGCCGC CCGCTGGTCA AAGTCACGTG CTTTGACCAA   
  
  
+ ACCACGCGGG CCAAAACACA CACACGCCCA CACACACTCT CTCTCTCTCT CTTTCTTCCC GTATTTTTCA   
  
  
+ CGGTCAAACT TCTCGTTACC TTCGCAGAAA CTGCCTTGCA AAGAAAAAAC ACACTCTCTC TCTCTCTCTT   
  
  
+ TCTTCCCGTA TTTTTCACGG TCAAACTTCT CGTTACCTTC GCAGAAACTG CCTTGCAAAG AAAAAGAAGA   
  
  
+ AAACTAGAAA AAAAAGAGAG AAAAACCAAA GAAGCAGAGA CGCCTTGTAC CTTACGTGAC CGTCTTCTCC   
  
  
+ GTTCTCTCTC TCTCTCTCTC CCCCCCTTGA TCCTTATATT CATCGTATAA GCTGTCTTCT TTCTGTACCT   
  
  
+ GTAACCTTAG TTCTCTCTCT GTCGCTTTT  

- TAATAGCGTG AAATAGGTGA TTCTTGGATT AATGGGTTGA TCACCCAATT GAACATCCGA TTCGAATGTA   
  
  
- TAGACGAATA CAAGAGAGGT AAAACACACT TTTGCACATC TGAATTAGAT TGGTTTAGTA CTAACACTGA   
  
  
- AACTAATACT TTATTTTCCA GATACCAAAT TAATTTAATT AATGTTATAC TGCATTTGAT TTGTATATGG   
  
  
- AAATTACCGA GACCTCGAAA CACAGTAAAA AATAAAGGTG CTAGTAATCC CGTTTTTAAC CAACTCTTTA   
  
  
- ATATTAAATC TCATATTCTA TGAGACCGAT CGAAAACCTA AAGTAACTGA TTTTTCCGTA ATGGGGAAAA   
  
  
- TGTGAAATCC TGAGGATCTT AGAAGATTCG ATCTGAATTA ATATAGAAAA TTATTGAAAA AATTACGAAC   
  
  
- AAACTGTGAT AACGTACAAT CGGAGATTCG AAATAAAATA GCTCTAGGTA TATTTACAAA AAAAAACAAA   
  
  
- AAAGGCCAAA ATGTAACTCT ATTTTTAGTA AATGATTAAA CTTTAGTTTA ACTAAAAAAG TAATTTTTTA   
  
  
- AATTACCAAT AAAAATATTA AATTTTATTG TTATATTTTA TTTTTTTAAT TAATAACTAA AATAATTTAA   
  
  
- CACCACGCAA ATAAACTTTC ACATATACAG TATAAAGTAT TAAAATGTAA TATTTATTTT TATATATTTT   
  
  
- ATGTATATTA ATCAACACAA TAAAGACCTG TAGTGAATGT TTTACATTTA TACTTTGTTT TTAAAACGCG   
  
  
- TAAATTTTAT ATATTTTATG TATATTAATC AACACAATAA AGACCTGTAG TGAATGTTTT ACATTTATAC   
  
  
- TTTGTTTTTA AAACGCGTAA ATTCATTAAT AATAATTGAT AAAAATTACT AATATTCTAT TTATGTAAAT   
  
  
- AAATTAATTA TTAAAAAATC TTCTTTATAT AAATTAGATT TGAAAATACC CTATACGTAC CTACCGTACT   
  
  
- TTAAACGTGA ACAGTATACG GAGGGAGGAT GTGATTTCGG TTATCCGTAG TCGTCGGTAA ATGCCGTGAC   
  
  
- TCTTCCTATT TATAATCTAT ACACTAACCC GGAGTCGGCG GGCGACCAGT TTCAGTGCAC GAAACTGGTT   
  
  
- TGGTGCGCCC GGTTTTGTGT GTGTGCGGGT GTGTGTGAGA GAGAGAGAGA GAAAGAAGGG CATAAAAAGT   
  
  
- GCCAGTTTGA AGAGCAATGG AAGCGTCTTT GACGGAACGT TTCTTTTTTG TGTGAGAGAG AGAGAGAGAA   
  
  
- AGAAGGGCAT AAAAAGTGCC AGTTTGAAGA GCAATGGAAG CGTCTTTGAC GGAACGTTTC TTTTTCTTCT   
  
  
- TTTGATCTTT TTTTTCTCTC TTTTTGGTTT CTTCGTCTCT GCGGAACATG GAATGCACTG GCAGAAGAGG   
  
  
- CAAGAGAGAG AGAGAGAGAG GGGGGGAACT AGGAATATAA GTAGCATATT CGACAGAAGA AAGACATGGA   
  
  
- CATTGGAATC AAGAGAGAGA CAGCGAAAA

+     O2-site

| Site Name | Organism | Position | Strand | Matrix score. | sequence | function |
| --- | --- | --- | --- | --- | --- | --- |
| O2-site | Zea mays | 971 | + | 9 | GATGACATGA | cis-acting regulatory element involved in zein metabolism regulation |

> 2018/04/13 10:10:12  
+ ATTATCGCAC TTTATCCACT AAGAACCTAA TTACCCAACT AGTGGGTTAA CTTGTAGGCT AAGCTTACAT   
  
  
+ ATCTGCTTAT GTTCTCTCCA TTTTGTGTGA AAACGTGTAG ACTTAATCTA ACCAAATCAT GATTGTGACT   
  
  
+ TTGATTATGA AATAAAAGGT CTATGGTTTA ATTAAATTAA TTACAATATG ACGTAAACTA AACATATACC   
  
  
+ TTTAATGGCT CTGGAGCTTT GTGTCATTTT TTATTTCCAC GATCATTAGG GCAAAAATTG GTTGAGAAAT   
  
  
+ TATAATTTAG AGTATAAGAT ACTCTGGCTA GCTTTTGGAT TTCATTGACT AAAAAGGCAT TACCCCTTTT   
  
  
+ ACACTTTAGG ACTCCTAGAA TCTTCTAAGC TAGACTTAAT TATATCTTTT AATAACTTTT TTAATGCTTG   
  
  
+ TTTGACACTA TTGCATGTTA GCCTCTAAGC TTTATTTTAT CGAGATCCAT ATAAATGTTT TTTTTTGTTT   
  
  
+ TTTCCGGTTT TACATTGAGA TAAAAATCAT TTACTAATTT GAAATCAAAT TGATTTTTTC ATTAAAAAAT   
  
  
+ TTAATGGTTA TTTTTATAAT TTAAAATAAC AATATAAAAT AAAAAAATTA ATTATTGATT TTATTAAATT   
  
  
+ GTGGTGCGTT TATTTGAAAG TGTATATGTC ATATTTCATA ATTTTACATT ATAAATAAAA ATATATAAAA   
  
  
+ TACATATAAT TAGTTGTGTT ATTTCTGGAC ATCACTTACA AAATGTAAAT ATGAAACAAA AATTTTGCGC   
  
  
+ ATTTAAAATA TATAAAATAC ATATAATTAG TTGTGTTATT TCTGGACATC ACTTACAAAA TGTAAATATG   
  
  
+ AAACAAAAAT TTTGCGCATT TAAGTAATTA TTATTAACTA TTTTTAATGA TTATAAGATA AATACATTTA   
  
  
+ TTTAATTAAT AATTTTTTAG AAGAAATATA TTTAATCTAA ACTTTTATGG GATATGCATG GATGGCATGA   
  
  
+ AATTTGCACT TGTCATATGC CTCCCTCCTA CACTAAAGCC AATAGGCATC AGCAGCCATT TACGGCACTG   
  
  
+ AGAAGGATAA ATATTAGATA TGTGATTGGG CCTCAGCCGC CCGCTGGTCA AAGTCACGTG CTTTGACCAA   
  
  
+ ACCACGCGGG CCAAAACACA CACACGCCCA CACACACTCT CTCTCTCTCT CTTTCTTCCC GTATTTTTCA   
  
  
+ CGGTCAAACT TCTCGTTACC TTCGCAGAAA CTGCCTTGCA AAGAAAAAAC ACACTCTCTC TCTCTCTCTT   
  
  
+ TCTTCCCGTA TTTTTCACGG TCAAACTTCT CGTTACCTTC GCAGAAACTG CCTTGCAAAG AAAAAGAAGA   
  
  
+ AAACTAGAAA AAAAAGAGAG AAAAACCAAA GAAGCAGAGA CGCCTTGTAC CTTACGTGAC CGTCTTCTCC   
  
  
+ GTTCTCTCTC TCTCTCTCTC CCCCCCTTGA TCCTTATATT CATCGTATAA GCTGTCTTCT TTCTGTACCT   
  
  
+ GTAACCTTAG TTCTCTCTCT GTCGCTTTT  

- TAATAGCGTG AAATAGGTGA TTCTTGGATT AATGGGTTGA TCACCCAATT GAACATCCGA TTCGAATGTA   
  
  
- TAGACGAATA CAAGAGAGGT AAAACACACT TTTGCACATC TGAATTAGAT TGGTTTAGTA CTAACACTGA   
  
  
- AACTAATACT TTATTTTCCA GATACCAAAT TAATTTAATT AATGTTATAC TGCATTTGAT TTGTATATGG   
  
  
- AAATTACCGA GACCTCGAAA CACAGTAAAA AATAAAGGTG CTAGTAATCC CGTTTTTAAC CAACTCTTTA   
  
  
- ATATTAAATC TCATATTCTA TGAGACCGAT CGAAAACCTA AAGTAACTGA TTTTTCCGTA ATGGGGAAAA   
  
  
- TGTGAAATCC TGAGGATCTT AGAAGATTCG ATCTGAATTA ATATAGAAAA TTATTGAAAA AATTACGAAC   
  
  
- AAACTGTGAT AACGTACAAT CGGAGATTCG AAATAAAATA GCTCTAGGTA TATTTACAAA AAAAAACAAA   
  
  
- AAAGGCCAAA ATGTAACTCT ATTTTTAGTA AATGATTAAA CTTTAGTTTA ACTAAAAAAG TAATTTTTTA   
  
  
- AATTACCAAT AAAAATATTA AATTTTATTG TTATATTTTA TTTTTTTAAT TAATAACTAA AATAATTTAA   
  
  
- CACCACGCAA ATAAACTTTC ACATATACAG TATAAAGTAT TAAAATGTAA TATTTATTTT TATATATTTT   
  
  
- ATGTATATTA ATCAACACAA TAAAGACCTG TAGTGAATGT TTTACATTTA TACTTTGTTT TTAAAACGCG   
  
  
- TAAATTTTAT ATATTTTATG TATATTAATC AACACAATAA AGACCTGTAG TGAATGTTTT ACATTTATAC   
  
  
- TTTGTTTTTA AAACGCGTAA ATTCATTAAT AATAATTGAT AAAAATTACT AATATTCTAT TTATGTAAAT   
  
  
- AAATTAATTA TTAAAAAATC TTCTTTATAT AAATTAGATT TGAAAATACC CTATACGTAC CTACCGTACT   
  
  
- TTAAACGTGA ACAGTATACG GAGGGAGGAT GTGATTTCGG TTATCCGTAG TCGTCGGTAA ATGCCGTGAC   
  
  
- TCTTCCTATT TATAATCTAT ACACTAACCC GGAGTCGGCG GGCGACCAGT TTCAGTGCAC GAAACTGGTT   
  
  
- TGGTGCGCCC GGTTTTGTGT GTGTGCGGGT GTGTGTGAGA GAGAGAGAGA GAAAGAAGGG CATAAAAAGT   
  
  
- GCCAGTTTGA AGAGCAATGG AAGCGTCTTT GACGGAACGT TTCTTTTTTG TGTGAGAGAG AGAGAGAGAA   
  
  
- AGAAGGGCAT AAAAAGTGCC AGTTTGAAGA GCAATGGAAG CGTCTTTGAC GGAACGTTTC TTTTTCTTCT   
  
  
- TTTGATCTTT TTTTTCTCTC TTTTTGGTTT CTTCGTCTCT GCGGAACATG GAATGCACTG GCAGAAGAGG   
  
  
- CAAGAGAGAG AGAGAGAGAG GGGGGGAACT AGGAATATAA GTAGCATATT CGACAGAAGA AAGACATGGA   
  
  
- CATTGGAATC AAGAGAGAGA CAGCGAAAA

+     OBP-1 site

| Site Name | Organism | Position | Strand | Matrix score. | sequence | function |
| --- | --- | --- | --- | --- | --- | --- |
| OBP-1 site | Arabidopsis thaliana | 350 | + | 10 | TACACTTTTGG | cis-acting regulatory element |

> 2018/04/13 10:10:12  
+ ATTATCGCAC TTTATCCACT AAGAACCTAA TTACCCAACT AGTGGGTTAA CTTGTAGGCT AAGCTTACAT   
  
  
+ ATCTGCTTAT GTTCTCTCCA TTTTGTGTGA AAACGTGTAG ACTTAATCTA ACCAAATCAT GATTGTGACT   
  
  
+ TTGATTATGA AATAAAAGGT CTATGGTTTA ATTAAATTAA TTACAATATG ACGTAAACTA AACATATACC   
  
  
+ TTTAATGGCT CTGGAGCTTT GTGTCATTTT TTATTTCCAC GATCATTAGG GCAAAAATTG GTTGAGAAAT   
  
  
+ TATAATTTAG AGTATAAGAT ACTCTGGCTA GCTTTTGGAT TTCATTGACT AAAAAGGCAT TACCCCTTTT   
  
  
+ ACACTTTAGG ACTCCTAGAA TCTTCTAAGC TAGACTTAAT TATATCTTTT AATAACTTTT TTAATGCTTG   
  
  
+ TTTGACACTA TTGCATGTTA GCCTCTAAGC TTTATTTTAT CGAGATCCAT ATAAATGTTT TTTTTTGTTT   
  
  
+ TTTCCGGTTT TACATTGAGA TAAAAATCAT TTACTAATTT GAAATCAAAT TGATTTTTTC ATTAAAAAAT   
  
  
+ TTAATGGTTA TTTTTATAAT TTAAAATAAC AATATAAAAT AAAAAAATTA ATTATTGATT TTATTAAATT   
  
  
+ GTGGTGCGTT TATTTGAAAG TGTATATGTC ATATTTCATA ATTTTACATT ATAAATAAAA ATATATAAAA   
  
  
+ TACATATAAT TAGTTGTGTT ATTTCTGGAC ATCACTTACA AAATGTAAAT ATGAAACAAA AATTTTGCGC   
  
  
+ ATTTAAAATA TATAAAATAC ATATAATTAG TTGTGTTATT TCTGGACATC ACTTACAAAA TGTAAATATG   
  
  
+ AAACAAAAAT TTTGCGCATT TAAGTAATTA TTATTAACTA TTTTTAATGA TTATAAGATA AATACATTTA   
  
  
+ TTTAATTAAT AATTTTTTAG AAGAAATATA TTTAATCTAA ACTTTTATGG GATATGCATG GATGGCATGA   
  
  
+ AATTTGCACT TGTCATATGC CTCCCTCCTA CACTAAAGCC AATAGGCATC AGCAGCCATT TACGGCACTG   
  
  
+ AGAAGGATAA ATATTAGATA TGTGATTGGG CCTCAGCCGC CCGCTGGTCA AAGTCACGTG CTTTGACCAA   
  
  
+ ACCACGCGGG CCAAAACACA CACACGCCCA CACACACTCT CTCTCTCTCT CTTTCTTCCC GTATTTTTCA   
  
  
+ CGGTCAAACT TCTCGTTACC TTCGCAGAAA CTGCCTTGCA AAGAAAAAAC ACACTCTCTC TCTCTCTCTT   
  
  
+ TCTTCCCGTA TTTTTCACGG TCAAACTTCT CGTTACCTTC GCAGAAACTG CCTTGCAAAG AAAAAGAAGA   
  
  
+ AAACTAGAAA AAAAAGAGAG AAAAACCAAA GAAGCAGAGA CGCCTTGTAC CTTACGTGAC CGTCTTCTCC   
  
  
+ GTTCTCTCTC TCTCTCTCTC CCCCCCTTGA TCCTTATATT CATCGTATAA GCTGTCTTCT TTCTGTACCT   
  
  
+ GTAACCTTAG TTCTCTCTCT GTCGCTTTT  

- TAATAGCGTG AAATAGGTGA TTCTTGGATT AATGGGTTGA TCACCCAATT GAACATCCGA TTCGAATGTA   
  
  
- TAGACGAATA CAAGAGAGGT AAAACACACT TTTGCACATC TGAATTAGAT TGGTTTAGTA CTAACACTGA   
  
  
- AACTAATACT TTATTTTCCA GATACCAAAT TAATTTAATT AATGTTATAC TGCATTTGAT TTGTATATGG   
  
  
- AAATTACCGA GACCTCGAAA CACAGTAAAA AATAAAGGTG CTAGTAATCC CGTTTTTAAC CAACTCTTTA   
  
  
- ATATTAAATC TCATATTCTA TGAGACCGAT CGAAAACCTA AAGTAACTGA TTTTTCCGTA ATGGGGAAAA   
  
  
- TGTGAAATCC TGAGGATCTT AGAAGATTCG ATCTGAATTA ATATAGAAAA TTATTGAAAA AATTACGAAC   
  
  
- AAACTGTGAT AACGTACAAT CGGAGATTCG AAATAAAATA GCTCTAGGTA TATTTACAAA AAAAAACAAA   
  
  
- AAAGGCCAAA ATGTAACTCT ATTTTTAGTA AATGATTAAA CTTTAGTTTA ACTAAAAAAG TAATTTTTTA   
  
  
- AATTACCAAT AAAAATATTA AATTTTATTG TTATATTTTA TTTTTTTAAT TAATAACTAA AATAATTTAA   
  
  
- CACCACGCAA ATAAACTTTC ACATATACAG TATAAAGTAT TAAAATGTAA TATTTATTTT TATATATTTT   
  
  
- ATGTATATTA ATCAACACAA TAAAGACCTG TAGTGAATGT TTTACATTTA TACTTTGTTT TTAAAACGCG   
  
  
- TAAATTTTAT ATATTTTATG TATATTAATC AACACAATAA AGACCTGTAG TGAATGTTTT ACATTTATAC   
  
  
- TTTGTTTTTA AAACGCGTAA ATTCATTAAT AATAATTGAT AAAAATTACT AATATTCTAT TTATGTAAAT   
  
  
- AAATTAATTA TTAAAAAATC TTCTTTATAT AAATTAGATT TGAAAATACC CTATACGTAC CTACCGTACT   
  
  
- TTAAACGTGA ACAGTATACG GAGGGAGGAT GTGATTTCGG TTATCCGTAG TCGTCGGTAA ATGCCGTGAC   
  
  
- TCTTCCTATT TATAATCTAT ACACTAACCC GGAGTCGGCG GGCGACCAGT TTCAGTGCAC GAAACTGGTT   
  
  
- TGGTGCGCCC GGTTTTGTGT GTGTGCGGGT GTGTGTGAGA GAGAGAGAGA GAAAGAAGGG CATAAAAAGT   
  
  
- GCCAGTTTGA AGAGCAATGG AAGCGTCTTT GACGGAACGT TTCTTTTTTG TGTGAGAGAG AGAGAGAGAA   
  
  
- AGAAGGGCAT AAAAAGTGCC AGTTTGAAGA GCAATGGAAG CGTCTTTGAC GGAACGTTTC TTTTTCTTCT   
  
  
- TTTGATCTTT TTTTTCTCTC TTTTTGGTTT CTTCGTCTCT GCGGAACATG GAATGCACTG GCAGAAGAGG   
  
  
- CAAGAGAGAG AGAGAGAGAG GGGGGGAACT AGGAATATAA GTAGCATATT CGACAGAAGA AAGACATGGA   
  
  
- CATTGGAATC AAGAGAGAGA CAGCGAAAA

+     P-box

| Site Name | Organism | Position | Strand | Matrix score. | sequence | function |
| --- | --- | --- | --- | --- | --- | --- |
| P-box | Oryza sativa | 328 | - | 10 | GCCTTTTGAGT | gibberellin-responsive element |

> 2018/04/13 10:10:12  
+ ATTATCGCAC TTTATCCACT AAGAACCTAA TTACCCAACT AGTGGGTTAA CTTGTAGGCT AAGCTTACAT   
  
  
+ ATCTGCTTAT GTTCTCTCCA TTTTGTGTGA AAACGTGTAG ACTTAATCTA ACCAAATCAT GATTGTGACT   
  
  
+ TTGATTATGA AATAAAAGGT CTATGGTTTA ATTAAATTAA TTACAATATG ACGTAAACTA AACATATACC   
  
  
+ TTTAATGGCT CTGGAGCTTT GTGTCATTTT TTATTTCCAC GATCATTAGG GCAAAAATTG GTTGAGAAAT   
  
  
+ TATAATTTAG AGTATAAGAT ACTCTGGCTA GCTTTTGGAT TTCATTGACT AAAAAGGCAT TACCCCTTTT   
  
  
+ ACACTTTAGG ACTCCTAGAA TCTTCTAAGC TAGACTTAAT TATATCTTTT AATAACTTTT TTAATGCTTG   
  
  
+ TTTGACACTA TTGCATGTTA GCCTCTAAGC TTTATTTTAT CGAGATCCAT ATAAATGTTT TTTTTTGTTT   
  
  
+ TTTCCGGTTT TACATTGAGA TAAAAATCAT TTACTAATTT GAAATCAAAT TGATTTTTTC ATTAAAAAAT   
  
  
+ TTAATGGTTA TTTTTATAAT TTAAAATAAC AATATAAAAT AAAAAAATTA ATTATTGATT TTATTAAATT   
  
  
+ GTGGTGCGTT TATTTGAAAG TGTATATGTC ATATTTCATA ATTTTACATT ATAAATAAAA ATATATAAAA   
  
  
+ TACATATAAT TAGTTGTGTT ATTTCTGGAC ATCACTTACA AAATGTAAAT ATGAAACAAA AATTTTGCGC   
  
  
+ ATTTAAAATA TATAAAATAC ATATAATTAG TTGTGTTATT TCTGGACATC ACTTACAAAA TGTAAATATG   
  
  
+ AAACAAAAAT TTTGCGCATT TAAGTAATTA TTATTAACTA TTTTTAATGA TTATAAGATA AATACATTTA   
  
  
+ TTTAATTAAT AATTTTTTAG AAGAAATATA TTTAATCTAA ACTTTTATGG GATATGCATG GATGGCATGA   
  
  
+ AATTTGCACT TGTCATATGC CTCCCTCCTA CACTAAAGCC AATAGGCATC AGCAGCCATT TACGGCACTG   
  
  
+ AGAAGGATAA ATATTAGATA TGTGATTGGG CCTCAGCCGC CCGCTGGTCA AAGTCACGTG CTTTGACCAA   
  
  
+ ACCACGCGGG CCAAAACACA CACACGCCCA CACACACTCT CTCTCTCTCT CTTTCTTCCC GTATTTTTCA   
  
  
+ CGGTCAAACT TCTCGTTACC TTCGCAGAAA CTGCCTTGCA AAGAAAAAAC ACACTCTCTC TCTCTCTCTT   
  
  
+ TCTTCCCGTA TTTTTCACGG TCAAACTTCT CGTTACCTTC GCAGAAACTG CCTTGCAAAG AAAAAGAAGA   
  
  
+ AAACTAGAAA AAAAAGAGAG AAAAACCAAA GAAGCAGAGA CGCCTTGTAC CTTACGTGAC CGTCTTCTCC   
  
  
+ GTTCTCTCTC TCTCTCTCTC CCCCCCTTGA TCCTTATATT CATCGTATAA GCTGTCTTCT TTCTGTACCT   
  
  
+ GTAACCTTAG TTCTCTCTCT GTCGCTTTT  

- TAATAGCGTG AAATAGGTGA TTCTTGGATT AATGGGTTGA TCACCCAATT GAACATCCGA TTCGAATGTA   
  
  
- TAGACGAATA CAAGAGAGGT AAAACACACT TTTGCACATC TGAATTAGAT TGGTTTAGTA CTAACACTGA   
  
  
- AACTAATACT TTATTTTCCA GATACCAAAT TAATTTAATT AATGTTATAC TGCATTTGAT TTGTATATGG   
  
  
- AAATTACCGA GACCTCGAAA CACAGTAAAA AATAAAGGTG CTAGTAATCC CGTTTTTAAC CAACTCTTTA   
  
  
- ATATTAAATC TCATATTCTA TGAGACCGAT CGAAAACCTA AAGTAACTGA TTTTTCCGTA ATGGGGAAAA   
  
  
- TGTGAAATCC TGAGGATCTT AGAAGATTCG ATCTGAATTA ATATAGAAAA TTATTGAAAA AATTACGAAC   
  
  
- AAACTGTGAT AACGTACAAT CGGAGATTCG AAATAAAATA GCTCTAGGTA TATTTACAAA AAAAAACAAA   
  
  
- AAAGGCCAAA ATGTAACTCT ATTTTTAGTA AATGATTAAA CTTTAGTTTA ACTAAAAAAG TAATTTTTTA   
  
  
- AATTACCAAT AAAAATATTA AATTTTATTG TTATATTTTA TTTTTTTAAT TAATAACTAA AATAATTTAA   
  
  
- CACCACGCAA ATAAACTTTC ACATATACAG TATAAAGTAT TAAAATGTAA TATTTATTTT TATATATTTT   
  
  
- ATGTATATTA ATCAACACAA TAAAGACCTG TAGTGAATGT TTTACATTTA TACTTTGTTT TTAAAACGCG   
  
  
- TAAATTTTAT ATATTTTATG TATATTAATC AACACAATAA AGACCTGTAG TGAATGTTTT ACATTTATAC   
  
  
- TTTGTTTTTA AAACGCGTAA ATTCATTAAT AATAATTGAT AAAAATTACT AATATTCTAT TTATGTAAAT   
  
  
- AAATTAATTA TTAAAAAATC TTCTTTATAT AAATTAGATT TGAAAATACC CTATACGTAC CTACCGTACT   
  
  
- TTAAACGTGA ACAGTATACG GAGGGAGGAT GTGATTTCGG TTATCCGTAG TCGTCGGTAA ATGCCGTGAC   
  
  
- TCTTCCTATT TATAATCTAT ACACTAACCC GGAGTCGGCG GGCGACCAGT TTCAGTGCAC GAAACTGGTT   
  
  
- TGGTGCGCCC GGTTTTGTGT GTGTGCGGGT GTGTGTGAGA GAGAGAGAGA GAAAGAAGGG CATAAAAAGT   
  
  
- GCCAGTTTGA AGAGCAATGG AAGCGTCTTT GACGGAACGT TTCTTTTTTG TGTGAGAGAG AGAGAGAGAA   
  
  
- AGAAGGGCAT AAAAAGTGCC AGTTTGAAGA GCAATGGAAG CGTCTTTGAC GGAACGTTTC TTTTTCTTCT   
  
  
- TTTGATCTTT TTTTTCTCTC TTTTTGGTTT CTTCGTCTCT GCGGAACATG GAATGCACTG GCAGAAGAGG   
  
  
- CAAGAGAGAG AGAGAGAGAG GGGGGGAACT AGGAATATAA GTAGCATATT CGACAGAAGA AAGACATGGA   
  
  
- CATTGGAATC AAGAGAGAGA CAGCGAAAA

+     Pc-CMA2c

| Site Name | Organism | Position | Strand | Matrix score. | sequence | function |
| --- | --- | --- | --- | --- | --- | --- |
| Pc-CMA2c | Spinacia oleracea | 1146 | + | 9 | GCCCACACA | part of a light responsive element |

> 2018/04/13 10:10:12  
+ ATTATCGCAC TTTATCCACT AAGAACCTAA TTACCCAACT AGTGGGTTAA CTTGTAGGCT AAGCTTACAT   
  
  
+ ATCTGCTTAT GTTCTCTCCA TTTTGTGTGA AAACGTGTAG ACTTAATCTA ACCAAATCAT GATTGTGACT   
  
  
+ TTGATTATGA AATAAAAGGT CTATGGTTTA ATTAAATTAA TTACAATATG ACGTAAACTA AACATATACC   
  
  
+ TTTAATGGCT CTGGAGCTTT GTGTCATTTT TTATTTCCAC GATCATTAGG GCAAAAATTG GTTGAGAAAT   
  
  
+ TATAATTTAG AGTATAAGAT ACTCTGGCTA GCTTTTGGAT TTCATTGACT AAAAAGGCAT TACCCCTTTT   
  
  
+ ACACTTTAGG ACTCCTAGAA TCTTCTAAGC TAGACTTAAT TATATCTTTT AATAACTTTT TTAATGCTTG   
  
  
+ TTTGACACTA TTGCATGTTA GCCTCTAAGC TTTATTTTAT CGAGATCCAT ATAAATGTTT TTTTTTGTTT   
  
  
+ TTTCCGGTTT TACATTGAGA TAAAAATCAT TTACTAATTT GAAATCAAAT TGATTTTTTC ATTAAAAAAT   
  
  
+ TTAATGGTTA TTTTTATAAT TTAAAATAAC AATATAAAAT AAAAAAATTA ATTATTGATT TTATTAAATT   
  
  
+ GTGGTGCGTT TATTTGAAAG TGTATATGTC ATATTTCATA ATTTTACATT ATAAATAAAA ATATATAAAA   
  
  
+ TACATATAAT TAGTTGTGTT ATTTCTGGAC ATCACTTACA AAATGTAAAT ATGAAACAAA AATTTTGCGC   
  
  
+ ATTTAAAATA TATAAAATAC ATATAATTAG TTGTGTTATT TCTGGACATC ACTTACAAAA TGTAAATATG   
  
  
+ AAACAAAAAT TTTGCGCATT TAAGTAATTA TTATTAACTA TTTTTAATGA TTATAAGATA AATACATTTA   
  
  
+ TTTAATTAAT AATTTTTTAG AAGAAATATA TTTAATCTAA ACTTTTATGG GATATGCATG GATGGCATGA   
  
  
+ AATTTGCACT TGTCATATGC CTCCCTCCTA CACTAAAGCC AATAGGCATC AGCAGCCATT TACGGCACTG   
  
  
+ AGAAGGATAA ATATTAGATA TGTGATTGGG CCTCAGCCGC CCGCTGGTCA AAGTCACGTG CTTTGACCAA   
  
  
+ ACCACGCGGG CCAAAACACA CACACGCCCA CACACACTCT CTCTCTCTCT CTTTCTTCCC GTATTTTTCA   
  
  
+ CGGTCAAACT TCTCGTTACC TTCGCAGAAA CTGCCTTGCA AAGAAAAAAC ACACTCTCTC TCTCTCTCTT   
  
  
+ TCTTCCCGTA TTTTTCACGG TCAAACTTCT CGTTACCTTC GCAGAAACTG CCTTGCAAAG AAAAAGAAGA   
  
  
+ AAACTAGAAA AAAAAGAGAG AAAAACCAAA GAAGCAGAGA CGCCTTGTAC CTTACGTGAC CGTCTTCTCC   
  
  
+ GTTCTCTCTC TCTCTCTCTC CCCCCCTTGA TCCTTATATT CATCGTATAA GCTGTCTTCT TTCTGTACCT   
  
  
+ GTAACCTTAG TTCTCTCTCT GTCGCTTTT  

- TAATAGCGTG AAATAGGTGA TTCTTGGATT AATGGGTTGA TCACCCAATT GAACATCCGA TTCGAATGTA   
  
  
- TAGACGAATA CAAGAGAGGT AAAACACACT TTTGCACATC TGAATTAGAT TGGTTTAGTA CTAACACTGA   
  
  
- AACTAATACT TTATTTTCCA GATACCAAAT TAATTTAATT AATGTTATAC TGCATTTGAT TTGTATATGG   
  
  
- AAATTACCGA GACCTCGAAA CACAGTAAAA AATAAAGGTG CTAGTAATCC CGTTTTTAAC CAACTCTTTA   
  
  
- ATATTAAATC TCATATTCTA TGAGACCGAT CGAAAACCTA AAGTAACTGA TTTTTCCGTA ATGGGGAAAA   
  
  
- TGTGAAATCC TGAGGATCTT AGAAGATTCG ATCTGAATTA ATATAGAAAA TTATTGAAAA AATTACGAAC   
  
  
- AAACTGTGAT AACGTACAAT CGGAGATTCG AAATAAAATA GCTCTAGGTA TATTTACAAA AAAAAACAAA   
  
  
- AAAGGCCAAA ATGTAACTCT ATTTTTAGTA AATGATTAAA CTTTAGTTTA ACTAAAAAAG TAATTTTTTA   
  
  
- AATTACCAAT AAAAATATTA AATTTTATTG TTATATTTTA TTTTTTTAAT TAATAACTAA AATAATTTAA   
  
  
- CACCACGCAA ATAAACTTTC ACATATACAG TATAAAGTAT TAAAATGTAA TATTTATTTT TATATATTTT   
  
  
- ATGTATATTA ATCAACACAA TAAAGACCTG TAGTGAATGT TTTACATTTA TACTTTGTTT TTAAAACGCG   
  
  
- TAAATTTTAT ATATTTTATG TATATTAATC AACACAATAA AGACCTGTAG TGAATGTTTT ACATTTATAC   
  
  
- TTTGTTTTTA AAACGCGTAA ATTCATTAAT AATAATTGAT AAAAATTACT AATATTCTAT TTATGTAAAT   
  
  
- AAATTAATTA TTAAAAAATC TTCTTTATAT AAATTAGATT TGAAAATACC CTATACGTAC CTACCGTACT   
  
  
- TTAAACGTGA ACAGTATACG GAGGGAGGAT GTGATTTCGG TTATCCGTAG TCGTCGGTAA ATGCCGTGAC   
  
  
- TCTTCCTATT TATAATCTAT ACACTAACCC GGAGTCGGCG GGCGACCAGT TTCAGTGCAC GAAACTGGTT   
  
  
- TGGTGCGCCC GGTTTTGTGT GTGTGCGGGT GTGTGTGAGA GAGAGAGAGA GAAAGAAGGG CATAAAAAGT   
  
  
- GCCAGTTTGA AGAGCAATGG AAGCGTCTTT GACGGAACGT TTCTTTTTTG TGTGAGAGAG AGAGAGAGAA   
  
  
- AGAAGGGCAT AAAAAGTGCC AGTTTGAAGA GCAATGGAAG CGTCTTTGAC GGAACGTTTC TTTTTCTTCT   
  
  
- TTTGATCTTT TTTTTCTCTC TTTTTGGTTT CTTCGTCTCT GCGGAACATG GAATGCACTG GCAGAAGAGG   
  
  
- CAAGAGAGAG AGAGAGAGAG GGGGGGAACT AGGAATATAA GTAGCATATT CGACAGAAGA AAGACATGGA   
  
  
- CATTGGAATC AAGAGAGAGA CAGCGAAAA

+     Skn-1\_motif

| Site Name | Organism | Position | Strand | Matrix score. | sequence | function |
| --- | --- | --- | --- | --- | --- | --- |
| Skn-1\_motif | Oryza sativa | 992 | + | 5 | GTCAT | cis-acting regulatory element required for endosperm expression |
| Skn-1\_motif | Oryza sativa | 658 | + | 5 | GTCAT | cis-acting regulatory element required for endosperm expression |
| Skn-1\_motif | Oryza sativa | 188 | - | 5 | GTCAT | cis-acting regulatory element required for endosperm expression |
| Skn-1\_motif | Oryza sativa | 233 | + | 5 | GTCAT | cis-acting regulatory element required for endosperm expression |

> 2018/04/13 10:10:12  
+ ATTATCGCAC TTTATCCACT AAGAACCTAA TTACCCAACT AGTGGGTTAA CTTGTAGGCT AAGCTTACAT   
  
  
+ ATCTGCTTAT GTTCTCTCCA TTTTGTGTGA AAACGTGTAG ACTTAATCTA ACCAAATCAT GATTGTGACT   
  
  
+ TTGATTATGA AATAAAAGGT CTATGGTTTA ATTAAATTAA TTACAATATG ACGTAAACTA AACATATACC   
  
  
+ TTTAATGGCT CTGGAGCTTT GTGTCATTTT TTATTTCCAC GATCATTAGG GCAAAAATTG GTTGAGAAAT   
  
  
+ TATAATTTAG AGTATAAGAT ACTCTGGCTA GCTTTTGGAT TTCATTGACT AAAAAGGCAT TACCCCTTTT   
  
  
+ ACACTTTAGG ACTCCTAGAA TCTTCTAAGC TAGACTTAAT TATATCTTTT AATAACTTTT TTAATGCTTG   
  
  
+ TTTGACACTA TTGCATGTTA GCCTCTAAGC TTTATTTTAT CGAGATCCAT ATAAATGTTT TTTTTTGTTT   
  
  
+ TTTCCGGTTT TACATTGAGA TAAAAATCAT TTACTAATTT GAAATCAAAT TGATTTTTTC ATTAAAAAAT   
  
  
+ TTAATGGTTA TTTTTATAAT TTAAAATAAC AATATAAAAT AAAAAAATTA ATTATTGATT TTATTAAATT   
  
  
+ GTGGTGCGTT TATTTGAAAG TGTATATGTC ATATTTCATA ATTTTACATT ATAAATAAAA ATATATAAAA   
  
  
+ TACATATAAT TAGTTGTGTT ATTTCTGGAC ATCACTTACA AAATGTAAAT ATGAAACAAA AATTTTGCGC   
  
  
+ ATTTAAAATA TATAAAATAC ATATAATTAG TTGTGTTATT TCTGGACATC ACTTACAAAA TGTAAATATG   
  
  
+ AAACAAAAAT TTTGCGCATT TAAGTAATTA TTATTAACTA TTTTTAATGA TTATAAGATA AATACATTTA   
  
  
+ TTTAATTAAT AATTTTTTAG AAGAAATATA TTTAATCTAA ACTTTTATGG GATATGCATG GATGGCATGA   
  
  
+ AATTTGCACT TGTCATATGC CTCCCTCCTA CACTAAAGCC AATAGGCATC AGCAGCCATT TACGGCACTG   
  
  
+ AGAAGGATAA ATATTAGATA TGTGATTGGG CCTCAGCCGC CCGCTGGTCA AAGTCACGTG CTTTGACCAA   
  
  
+ ACCACGCGGG CCAAAACACA CACACGCCCA CACACACTCT CTCTCTCTCT CTTTCTTCCC GTATTTTTCA   
  
  
+ CGGTCAAACT TCTCGTTACC TTCGCAGAAA CTGCCTTGCA AAGAAAAAAC ACACTCTCTC TCTCTCTCTT   
  
  
+ TCTTCCCGTA TTTTTCACGG TCAAACTTCT CGTTACCTTC GCAGAAACTG CCTTGCAAAG AAAAAGAAGA   
  
  
+ AAACTAGAAA AAAAAGAGAG AAAAACCAAA GAAGCAGAGA CGCCTTGTAC CTTACGTGAC CGTCTTCTCC   
  
  
+ GTTCTCTCTC TCTCTCTCTC CCCCCCTTGA TCCTTATATT CATCGTATAA GCTGTCTTCT TTCTGTACCT   
  
  
+ GTAACCTTAG TTCTCTCTCT GTCGCTTTT  

- TAATAGCGTG AAATAGGTGA TTCTTGGATT AATGGGTTGA TCACCCAATT GAACATCCGA TTCGAATGTA   
  
  
- TAGACGAATA CAAGAGAGGT AAAACACACT TTTGCACATC TGAATTAGAT TGGTTTAGTA CTAACACTGA   
  
  
- AACTAATACT TTATTTTCCA GATACCAAAT TAATTTAATT AATGTTATAC TGCATTTGAT TTGTATATGG   
  
  
- AAATTACCGA GACCTCGAAA CACAGTAAAA AATAAAGGTG CTAGTAATCC CGTTTTTAAC CAACTCTTTA   
  
  
- ATATTAAATC TCATATTCTA TGAGACCGAT CGAAAACCTA AAGTAACTGA TTTTTCCGTA ATGGGGAAAA   
  
  
- TGTGAAATCC TGAGGATCTT AGAAGATTCG ATCTGAATTA ATATAGAAAA TTATTGAAAA AATTACGAAC   
  
  
- AAACTGTGAT AACGTACAAT CGGAGATTCG AAATAAAATA GCTCTAGGTA TATTTACAAA AAAAAACAAA   
  
  
- AAAGGCCAAA ATGTAACTCT ATTTTTAGTA AATGATTAAA CTTTAGTTTA ACTAAAAAAG TAATTTTTTA   
  
  
- AATTACCAAT AAAAATATTA AATTTTATTG TTATATTTTA TTTTTTTAAT TAATAACTAA AATAATTTAA   
  
  
- CACCACGCAA ATAAACTTTC ACATATACAG TATAAAGTAT TAAAATGTAA TATTTATTTT TATATATTTT   
  
  
- ATGTATATTA ATCAACACAA TAAAGACCTG TAGTGAATGT TTTACATTTA TACTTTGTTT TTAAAACGCG   
  
  
- TAAATTTTAT ATATTTTATG TATATTAATC AACACAATAA AGACCTGTAG TGAATGTTTT ACATTTATAC   
  
  
- TTTGTTTTTA AAACGCGTAA ATTCATTAAT AATAATTGAT AAAAATTACT AATATTCTAT TTATGTAAAT   
  
  
- AAATTAATTA TTAAAAAATC TTCTTTATAT AAATTAGATT TGAAAATACC CTATACGTAC CTACCGTACT   
  
  
- TTAAACGTGA ACAGTATACG GAGGGAGGAT GTGATTTCGG TTATCCGTAG TCGTCGGTAA ATGCCGTGAC   
  
  
- TCTTCCTATT TATAATCTAT ACACTAACCC GGAGTCGGCG GGCGACCAGT TTCAGTGCAC GAAACTGGTT   
  
  
- TGGTGCGCCC GGTTTTGTGT GTGTGCGGGT GTGTGTGAGA GAGAGAGAGA GAAAGAAGGG CATAAAAAGT   
  
  
- GCCAGTTTGA AGAGCAATGG AAGCGTCTTT GACGGAACGT TTCTTTTTTG TGTGAGAGAG AGAGAGAGAA   
  
  
- AGAAGGGCAT AAAAAGTGCC AGTTTGAAGA GCAATGGAAG CGTCTTTGAC GGAACGTTTC TTTTTCTTCT   
  
  
- TTTGATCTTT TTTTTCTCTC TTTTTGGTTT CTTCGTCTCT GCGGAACATG GAATGCACTG GCAGAAGAGG   
  
  
- CAAGAGAGAG AGAGAGAGAG GGGGGGAACT AGGAATATAA GTAGCATATT CGACAGAAGA AAGACATGGA   
  
  
- CATTGGAATC AAGAGAGAGA CAGCGAAAA

+     Sp1

| Site Name | Organism | Position | Strand | Matrix score. | sequence | function |
| --- | --- | --- | --- | --- | --- | --- |
| Sp1 | Zea mays | 1420 | + | 5 | CC(G/A)CCC | light responsive element |
| Sp1 | Zea mays | 1000 | + | 5 | CC(G/A)CCC | light responsive element |
| Sp1 | Oryza sativa | 1087 | - | 6 | GGGCGG | light responsive element |
| Sp1 | Zea mays | 1421 | + | 5 | CC(G/A)CCC | light responsive element |

> 2018/04/13 10:10:12  
+ ATTATCGCAC TTTATCCACT AAGAACCTAA TTACCCAACT AGTGGGTTAA CTTGTAGGCT AAGCTTACAT   
  
  
+ ATCTGCTTAT GTTCTCTCCA TTTTGTGTGA AAACGTGTAG ACTTAATCTA ACCAAATCAT GATTGTGACT   
  
  
+ TTGATTATGA AATAAAAGGT CTATGGTTTA ATTAAATTAA TTACAATATG ACGTAAACTA AACATATACC   
  
  
+ TTTAATGGCT CTGGAGCTTT GTGTCATTTT TTATTTCCAC GATCATTAGG GCAAAAATTG GTTGAGAAAT   
  
  
+ TATAATTTAG AGTATAAGAT ACTCTGGCTA GCTTTTGGAT TTCATTGACT AAAAAGGCAT TACCCCTTTT   
  
  
+ ACACTTTAGG ACTCCTAGAA TCTTCTAAGC TAGACTTAAT TATATCTTTT AATAACTTTT TTAATGCTTG   
  
  
+ TTTGACACTA TTGCATGTTA GCCTCTAAGC TTTATTTTAT CGAGATCCAT ATAAATGTTT TTTTTTGTTT   
  
  
+ TTTCCGGTTT TACATTGAGA TAAAAATCAT TTACTAATTT GAAATCAAAT TGATTTTTTC ATTAAAAAAT   
  
  
+ TTAATGGTTA TTTTTATAAT TTAAAATAAC AATATAAAAT AAAAAAATTA ATTATTGATT TTATTAAATT   
  
  
+ GTGGTGCGTT TATTTGAAAG TGTATATGTC ATATTTCATA ATTTTACATT ATAAATAAAA ATATATAAAA   
  
  
+ TACATATAAT TAGTTGTGTT ATTTCTGGAC ATCACTTACA AAATGTAAAT ATGAAACAAA AATTTTGCGC   
  
  
+ ATTTAAAATA TATAAAATAC ATATAATTAG TTGTGTTATT TCTGGACATC ACTTACAAAA TGTAAATATG   
  
  
+ AAACAAAAAT TTTGCGCATT TAAGTAATTA TTATTAACTA TTTTTAATGA TTATAAGATA AATACATTTA   
  
  
+ TTTAATTAAT AATTTTTTAG AAGAAATATA TTTAATCTAA ACTTTTATGG GATATGCATG GATGGCATGA   
  
  
+ AATTTGCACT TGTCATATGC CTCCCTCCTA CACTAAAGCC AATAGGCATC AGCAGCCATT TACGGCACTG   
  
  
+ AGAAGGATAA ATATTAGATA TGTGATTGGG CCTCAGCCGC CCGCTGGTCA AAGTCACGTG CTTTGACCAA   
  
  
+ ACCACGCGGG CCAAAACACA CACACGCCCA CACACACTCT CTCTCTCTCT CTTTCTTCCC GTATTTTTCA   
  
  
+ CGGTCAAACT TCTCGTTACC TTCGCAGAAA CTGCCTTGCA AAGAAAAAAC ACACTCTCTC TCTCTCTCTT   
  
  
+ TCTTCCCGTA TTTTTCACGG TCAAACTTCT CGTTACCTTC GCAGAAACTG CCTTGCAAAG AAAAAGAAGA   
  
  
+ AAACTAGAAA AAAAAGAGAG AAAAACCAAA GAAGCAGAGA CGCCTTGTAC CTTACGTGAC CGTCTTCTCC   
  
  
+ GTTCTCTCTC TCTCTCTCTC CCCCCCTTGA TCCTTATATT CATCGTATAA GCTGTCTTCT TTCTGTACCT   
  
  
+ GTAACCTTAG TTCTCTCTCT GTCGCTTTT  

- TAATAGCGTG AAATAGGTGA TTCTTGGATT AATGGGTTGA TCACCCAATT GAACATCCGA TTCGAATGTA   
  
  
- TAGACGAATA CAAGAGAGGT AAAACACACT TTTGCACATC TGAATTAGAT TGGTTTAGTA CTAACACTGA   
  
  
- AACTAATACT TTATTTTCCA GATACCAAAT TAATTTAATT AATGTTATAC TGCATTTGAT TTGTATATGG   
  
  
- AAATTACCGA GACCTCGAAA CACAGTAAAA AATAAAGGTG CTAGTAATCC CGTTTTTAAC CAACTCTTTA   
  
  
- ATATTAAATC TCATATTCTA TGAGACCGAT CGAAAACCTA AAGTAACTGA TTTTTCCGTA ATGGGGAAAA   
  
  
- TGTGAAATCC TGAGGATCTT AGAAGATTCG ATCTGAATTA ATATAGAAAA TTATTGAAAA AATTACGAAC   
  
  
- AAACTGTGAT AACGTACAAT CGGAGATTCG AAATAAAATA GCTCTAGGTA TATTTACAAA AAAAAACAAA   
  
  
- AAAGGCCAAA ATGTAACTCT ATTTTTAGTA AATGATTAAA CTTTAGTTTA ACTAAAAAAG TAATTTTTTA   
  
  
- AATTACCAAT AAAAATATTA AATTTTATTG TTATATTTTA TTTTTTTAAT TAATAACTAA AATAATTTAA   
  
  
- CACCACGCAA ATAAACTTTC ACATATACAG TATAAAGTAT TAAAATGTAA TATTTATTTT TATATATTTT   
  
  
- ATGTATATTA ATCAACACAA TAAAGACCTG TAGTGAATGT TTTACATTTA TACTTTGTTT TTAAAACGCG   
  
  
- TAAATTTTAT ATATTTTATG TATATTAATC AACACAATAA AGACCTGTAG TGAATGTTTT ACATTTATAC   
  
  
- TTTGTTTTTA AAACGCGTAA ATTCATTAAT AATAATTGAT AAAAATTACT AATATTCTAT TTATGTAAAT   
  
  
- AAATTAATTA TTAAAAAATC TTCTTTATAT AAATTAGATT TGAAAATACC CTATACGTAC CTACCGTACT   
  
  
- TTAAACGTGA ACAGTATACG GAGGGAGGAT GTGATTTCGG TTATCCGTAG TCGTCGGTAA ATGCCGTGAC   
  
  
- TCTTCCTATT TATAATCTAT ACACTAACCC GGAGTCGGCG GGCGACCAGT TTCAGTGCAC GAAACTGGTT   
  
  
- TGGTGCGCCC GGTTTTGTGT GTGTGCGGGT GTGTGTGAGA GAGAGAGAGA GAAAGAAGGG CATAAAAAGT   
  
  
- GCCAGTTTGA AGAGCAATGG AAGCGTCTTT GACGGAACGT TTCTTTTTTG TGTGAGAGAG AGAGAGAGAA   
  
  
- AGAAGGGCAT AAAAAGTGCC AGTTTGAAGA GCAATGGAAG CGTCTTTGAC GGAACGTTTC TTTTTCTTCT   
  
  
- TTTGATCTTT TTTTTCTCTC TTTTTGGTTT CTTCGTCTCT GCGGAACATG GAATGCACTG GCAGAAGAGG   
  
  
- CAAGAGAGAG AGAGAGAGAG GGGGGGAACT AGGAATATAA GTAGCATATT CGACAGAAGA AAGACATGGA   
  
  
- CATTGGAATC AAGAGAGAGA CAGCGAAAA

+     TATA-box

| Site Name | Organism | Position | Strand | Matrix score. | sequence | function |
| --- | --- | --- | --- | --- | --- | --- |
| TATA-box | Glycine max | 622 | - | 5 | TAATA | core promoter element around -30 of transcription start |
| TATA-box | Lycopersicon esculentum | 498 | + | 5 | TTTTA | core promoter element around -30 of transcription start |
| TATA-box | Glycine max | 400 | + | 5 | TAATA | core promoter element around -30 of transcription start |
| TATA-box | Glycine max | 872 | - | 5 | TAATA | core promoter element around -30 of transcription start |
| TATA-box | Glycine max | 1062 | - | 5 | TAATA | core promoter element around -30 of transcription start |
| TATA-box | Lycopersicon esculentum | 511 | - | 5 | TTTTA | core promoter element around -30 of transcription start |
| TATA-box | Lycopersicon esculentum | 595 | - | 5 | TTTTA | core promoter element around -30 of transcription start |
| TATA-box | Arabidopsis thaliana | 575 | + | 4 | TATA | core promoter element around -30 of transcription start |
| TATA-box | Lycopersicon esculentum | 455 | + | 5 | TTTTA | core promoter element around -30 of transcription start |
| TATA-box | Arabidopsis thaliana | 574 | - | 5 | TATAA | core promoter element around -30 of transcription start |
| TATA-box | Brassica oleracea | 469 | + | 6 | ATATAA | core promoter element around -30 of transcription start |
| TATA-box | Arabidopsis thaliana | 792 | - | 4 | TATA | core promoter element around -30 of transcription start |
| TATA-box | Lycopersicon esculentum | 409 | + | 5 | TTTTA | core promoter element around -30 of transcription start |
| TATA-box | Arabidopsis thaliana | 779 | - | 4 | TATA | core promoter element around -30 of transcription start |
| TATA-box | Lycopersicon esculentum | 672 | + | 5 | TTTTA | core promoter element around -30 of transcription start |
| TATA-box | Arabidopsis thaliana | 281 | + | 4 | TATA | core promoter element around -30 of transcription start |
| TATA-box | Arabidopsis thaliana | 390 | - | 5 | TATAA | core promoter element around -30 of transcription start |
| TATA-box | Lycopersicon esculentum | 239 | + | 5 | TTTTA | core promoter element around -30 of transcription start |
| TATA-box | Arabidopsis thaliana | 692 | + | 4 | TATA | core promoter element around -30 of transcription start |
| TATA-box | Lycopersicon esculentum | 696 | - | 5 | TTTTA | core promoter element around -30 of transcription start |
| TATA-box | Pisum sativum | 202 | - | 7 | TATATGT | core promoter element around -30 of transcription start |
| TATA-box | Pisum sativum | 789 | - | 7 | TATATGT | core promoter element around -30 of transcription start |
| TATA-box | Arabidopsis thaliana | 1435 | - | 4 | TATA | core promoter element around -30 of transcription start |
| TATA-box | Brassica oleracea | 693 | + | 6 | ATATAA | core promoter element around -30 of transcription start |
| TATA-box | Lycopersicon esculentum | 153 | - | 5 | TTTTA | core promoter element around -30 of transcription start |
| TATA-box | Brassica oleracea | 592 | + | 6 | ATATAA | core promoter element around -30 of transcription start |
| TATA-box | Lycopersicon esculentum | 330 | - | 5 | TTTTA | core promoter element around -30 of transcription start |
| TATA-box | Arabidopsis thaliana | 653 | + | 4 | TATA | core promoter element around -30 of transcription start |
| TATA-box | Arabidopsis thaliana | 679 | - | 5 | TATAA | core promoter element around -30 of transcription start |
| TATA-box | Arabidopsis thaliana | 892 | - | 4 | TATA | core promoter element around -30 of transcription start |
| TATA-box | Lycopersicon esculentum | 1496 | + | 5 | TTTTA | core promoter element around -30 of transcription start |
| TATA-box | Glycine max | 917 | + | 5 | TAATA | core promoter element around -30 of transcription start |
| TATA-box | Brassica oleracea | 780 | + | 6 | ATATAA | core promoter element around -30 of transcription start |
| TATA-box | Ac | 470 | + | 7 | TATAAAT | core promoter element around -30 of transcription start |
| TATA-box | Lycopersicon esculentum | 397 | + | 5 | TTTTA | core promoter element around -30 of transcription start |
| TATA-box | Brassica napus | 778 | - | 6 | ATATAT | core promoter element around -30 of transcription start |
| TATA-box | Oryza sativa | 737 | + | 7 | TACAAAA | core promoter element around -30 of transcription start |
| TATA-box | Lycopersicon esculentum | 774 | - | 5 | TTTTA | core promoter element around -30 of transcription start |
| TATA-box | Arabidopsis thaliana | 568 | - | 9 | TAAAAATAA | core promoter element around -30 of transcription start |
| TATA-box | Arabidopsis thaliana | 293 | + | 4 | TATA | core promoter element around -30 of transcription start |
| TATA-box | Lycopersicon esculentum | 582 | - | 5 | TTTTA | core promoter element around -30 of transcription start |
| TATA-box | Lycopersicon esculentum | 619 | + | 5 | TTTTA | core promoter element around -30 of transcription start |
| TATA-box | Brassica napus | 279 | + | 6 | ATTATA | core promoter element around -30 of transcription start |
| TATA-box | Brassica napus | 678 | + | 6 | ATTATA | core promoter element around -30 of transcription start |
| TATA-box | Arabidopsis thaliana | 1446 | - | 4 | TATA | core promoter element around -30 of transcription start |
| TATA-box | Brassica napus | 890 | + | 6 | ATTATA | core promoter element around -30 of transcription start |
| TATA-box | Arabidopsis thaliana | 391 | + | 4 | TATA | core promoter element around -30 of transcription start |
| TATA-box | Arabidopsis thaliana | 573 | - | 6 | TATAAA | core promoter element around -30 of transcription start |
| TATA-box | Arabidopsis thaliana | 205 | + | 4 | TATA | core promoter element around -30 of transcription start |
| TATA-box | Lycopersicon esculentum | 925 | + | 5 | TTTTA | core promoter element around -30 of transcription start |
| TATA-box | Arabidopsis thaliana | 280 | - | 5 | TATAA | core promoter element around -30 of transcription start |
| TATA-box | Lycopersicon esculentum | 882 | + | 5 | TTTTA | core promoter element around -30 of transcription start |
| TATA-box | Arabidopsis thaliana | 593 | + | 6 | TATAAA | core promoter element around -30 of transcription start |
| TATA-box | Lycopersicon esculentum | 686 | - | 5 | TTTTA | core promoter element around -30 of transcription start |
| TATA-box | Brassica napus | 389 | + | 6 | ATTATA | core promoter element around -30 of transcription start |
| TATA-box | Pisum sativum | 702 | - | 7 | TATATGT | core promoter element around -30 of transcription start |
| TATA-box | Helianthus annuus | 651 | - | 6 | TATACA | core promoter element around -30 of transcription start |
| TATA-box | Lycopersicon esculentum | 953 | + | 5 | TTTTA | core promoter element around -30 of transcription start |
| TATA-box | Lycopersicon esculentum | 347 | + | 5 | TTTTA | core promoter element around -30 of transcription start |
| TATA-box | Glycine max | 869 | - | 5 | TAATA | core promoter element around -30 of transcription start |
| TATA-box | Lycopersicon esculentum | 553 | - | 5 | TTTTA | core promoter element around -30 of transcription start |
| TATA-box | Arabidopsis thaliana | 781 | + | 6 | TATAAA | core promoter element around -30 of transcription start |
| TATA-box | Arabidopsis thaliana | 705 | + | 4 | TATA | core promoter element around -30 of transcription start |
| TATA-box | Ac | 680 | + | 7 | TATAAAT | core promoter element around -30 of transcription start |
| TATA-box | Arabidopsis thaliana | 572 | - | 7 | TATAAAA | core promoter element around -30 of transcription start |
| TATA-box | Arabidopsis thaliana | 1434 | - | 5 | TATAA | core promoter element around -30 of transcription start |
| TATA-box | Avena sativa | 683 | - | 12 | TATATTTATATTT | core promoter element around -30 of transcription start |
| TATA-box | Arabidopsis thaliana | 690 | - | 9 | tcTATATAtt | core promoter element around -30 of transcription start |
| TATA-box | Arabidopsis thaliana | 937 | - | 4 | TATA | core promoter element around -30 of transcription start |
| TATA-box | Lycopersicon esculentum | 600 | - | 5 | TTTTA | core promoter element around -30 of transcription start |
| TATA-box | Brassica napus | 691 | + | 6 | ATATAT | core promoter element around -30 of transcription start |
| TATA-box | Brassica oleracea | 791 | + | 7 | ATATAAT | core promoter element around -30 of transcription start |
| TATA-box | Lycopersicon esculentum | 783 | - | 5 | TTTTA | core promoter element around -30 of transcription start |
| TATA-box | Arabidopsis thaliana | 694 | + | 6 | TATAAA | core promoter element around -30 of transcription start |
| TATA-box | Arabidopsis thaliana | 777 | - | 9 | tcTATATAtt | core promoter element around -30 of transcription start |
| TATA-box | Brassica oleracea | 704 | + | 7 | ATATAAT | core promoter element around -30 of transcription start |
| TATA-box | Brassica napus | 936 | - | 6 | ATATAT | core promoter element around -30 of transcription start |
| TATA-box | Arabidopsis thaliana | 891 | - | 5 | TATAA | core promoter element around -30 of transcription start |
| TATA-box | Oryza sativa | 824 | + | 7 | TACAAAA | core promoter element around -30 of transcription start |

> 2018/04/13 10:10:12  
+ ATTATCGCAC TTTATCCACT AAGAACCTAA TTACCCAACT AGTGGGTTAA CTTGTAGGCT AAGCTTACAT   
  
  
+ ATCTGCTTAT GTTCTCTCCA TTTTGTGTGA AAACGTGTAG ACTTAATCTA ACCAAATCAT GATTGTGACT   
  
  
+ TTGATTATGA AATAAAAGGT CTATGGTTTA ATTAAATTAA TTACAATATG ACGTAAACTA AACATATACC   
  
  
+ TTTAATGGCT CTGGAGCTTT GTGTCATTTT TTATTTCCAC GATCATTAGG GCAAAAATTG GTTGAGAAAT   
  
  
+ TATAATTTAG AGTATAAGAT ACTCTGGCTA GCTTTTGGAT TTCATTGACT AAAAAGGCAT TACCCCTTTT   
  
  
+ ACACTTTAGG ACTCCTAGAA TCTTCTAAGC TAGACTTAAT TATATCTTTT AATAACTTTT TTAATGCTTG   
  
  
+ TTTGACACTA TTGCATGTTA GCCTCTAAGC TTTATTTTAT CGAGATCCAT ATAAATGTTT TTTTTTGTTT   
  
  
+ TTTCCGGTTT TACATTGAGA TAAAAATCAT TTACTAATTT GAAATCAAAT TGATTTTTTC ATTAAAAAAT   
  
  
+ TTAATGGTTA TTTTTATAAT TTAAAATAAC AATATAAAAT AAAAAAATTA ATTATTGATT TTATTAAATT   
  
  
+ GTGGTGCGTT TATTTGAAAG TGTATATGTC ATATTTCATA ATTTTACATT ATAAATAAAA ATATATAAAA   
  
  
+ TACATATAAT TAGTTGTGTT ATTTCTGGAC ATCACTTACA AAATGTAAAT ATGAAACAAA AATTTTGCGC   
  
  
+ ATTTAAAATA TATAAAATAC ATATAATTAG TTGTGTTATT TCTGGACATC ACTTACAAAA TGTAAATATG   
  
  
+ AAACAAAAAT TTTGCGCATT TAAGTAATTA TTATTAACTA TTTTTAATGA TTATAAGATA AATACATTTA   
  
  
+ TTTAATTAAT AATTTTTTAG AAGAAATATA TTTAATCTAA ACTTTTATGG GATATGCATG GATGGCATGA   
  
  
+ AATTTGCACT TGTCATATGC CTCCCTCCTA CACTAAAGCC AATAGGCATC AGCAGCCATT TACGGCACTG   
  
  
+ AGAAGGATAA ATATTAGATA TGTGATTGGG CCTCAGCCGC CCGCTGGTCA AAGTCACGTG CTTTGACCAA   
  
  
+ ACCACGCGGG CCAAAACACA CACACGCCCA CACACACTCT CTCTCTCTCT CTTTCTTCCC GTATTTTTCA   
  
  
+ CGGTCAAACT TCTCGTTACC TTCGCAGAAA CTGCCTTGCA AAGAAAAAAC ACACTCTCTC TCTCTCTCTT   
  
  
+ TCTTCCCGTA TTTTTCACGG TCAAACTTCT CGTTACCTTC GCAGAAACTG CCTTGCAAAG AAAAAGAAGA   
  
  
+ AAACTAGAAA AAAAAGAGAG AAAAACCAAA GAAGCAGAGA CGCCTTGTAC CTTACGTGAC CGTCTTCTCC   
  
  
+ GTTCTCTCTC TCTCTCTCTC CCCCCCTTGA TCCTTATATT CATCGTATAA GCTGTCTTCT TTCTGTACCT   
  
  
+ GTAACCTTAG TTCTCTCTCT GTCGCTTTTA   

- TAATAGCGTG AAATAGGTGA TTCTTGGATT AATGGGTTGA TCACCCAATT GAACATCCGA TTCGAATGTA   
  
  
- TAGACGAATA CAAGAGAGGT AAAACACACT TTTGCACATC TGAATTAGAT TGGTTTAGTA CTAACACTGA   
  
  
- AACTAATACT TTATTTTCCA GATACCAAAT TAATTTAATT AATGTTATAC TGCATTTGAT TTGTATATGG   
  
  
- AAATTACCGA GACCTCGAAA CACAGTAAAA AATAAAGGTG CTAGTAATCC CGTTTTTAAC CAACTCTTTA   
  
  
- ATATTAAATC TCATATTCTA TGAGACCGAT CGAAAACCTA AAGTAACTGA TTTTTCCGTA ATGGGGAAAA   
  
  
- TGTGAAATCC TGAGGATCTT AGAAGATTCG ATCTGAATTA ATATAGAAAA TTATTGAAAA AATTACGAAC   
  
  
- AAACTGTGAT AACGTACAAT CGGAGATTCG AAATAAAATA GCTCTAGGTA TATTTACAAA AAAAAACAAA   
  
  
- AAAGGCCAAA ATGTAACTCT ATTTTTAGTA AATGATTAAA CTTTAGTTTA ACTAAAAAAG TAATTTTTTA   
  
  
- AATTACCAAT AAAAATATTA AATTTTATTG TTATATTTTA TTTTTTTAAT TAATAACTAA AATAATTTAA   
  
  
- CACCACGCAA ATAAACTTTC ACATATACAG TATAAAGTAT TAAAATGTAA TATTTATTTT TATATATTTT   
  
  
- ATGTATATTA ATCAACACAA TAAAGACCTG TAGTGAATGT TTTACATTTA TACTTTGTTT TTAAAACGCG   
  
  
- TAAATTTTAT ATATTTTATG TATATTAATC AACACAATAA AGACCTGTAG TGAATGTTTT ACATTTATAC   
  
  
- TTTGTTTTTA AAACGCGTAA ATTCATTAAT AATAATTGAT AAAAATTACT AATATTCTAT TTATGTAAAT   
  
  
- AAATTAATTA TTAAAAAATC TTCTTTATAT AAATTAGATT TGAAAATACC CTATACGTAC CTACCGTACT   
  
  
- TTAAACGTGA ACAGTATACG GAGGGAGGAT GTGATTTCGG TTATCCGTAG TCGTCGGTAA ATGCCGTGAC   
  
  
- TCTTCCTATT TATAATCTAT ACACTAACCC GGAGTCGGCG GGCGACCAGT TTCAGTGCAC GAAACTGGTT   
  
  
- TGGTGCGCCC GGTTTTGTGT GTGTGCGGGT GTGTGTGAGA GAGAGAGAGA GAAAGAAGGG CATAAAAAGT   
  
  
- GCCAGTTTGA AGAGCAATGG AAGCGTCTTT GACGGAACGT TTCTTTTTTG TGTGAGAGAG AGAGAGAGAA   
  
  
- AGAAGGGCAT AAAAAGTGCC AGTTTGAAGA GCAATGGAAG CGTCTTTGAC GGAACGTTTC TTTTTCTTCT   
  
  
- TTTGATCTTT TTTTTCTCTC TTTTTGGTTT CTTCGTCTCT GCGGAACATG GAATGCACTG GCAGAAGAGG   
  
  
- CAAGAGAGAG AGAGAGAGAG GGGGGGAACT AGGAATATAA GTAGCATATT CGACAGAAGA AAGACATGGA   
  
  
- CATTGGAATC AAGAGAGAGA CAGCGAAAA

+     TATC-box

| Site Name | Organism | Position | Strand | Matrix score. | sequence | function |
| --- | --- | --- | --- | --- | --- | --- |
| TATC-box | Oryza sativa | 958 | - | 7 | TATCCCA | cis-acting element involved in gibberellin-responsiveness |

> 2018/04/13 10:10:12  
+ ATTATCGCAC TTTATCCACT AAGAACCTAA TTACCCAACT AGTGGGTTAA CTTGTAGGCT AAGCTTACAT   
  
  
+ ATCTGCTTAT GTTCTCTCCA TTTTGTGTGA AAACGTGTAG ACTTAATCTA ACCAAATCAT GATTGTGACT   
  
  
+ TTGATTATGA AATAAAAGGT CTATGGTTTA ATTAAATTAA TTACAATATG ACGTAAACTA AACATATACC   
  
  
+ TTTAATGGCT CTGGAGCTTT GTGTCATTTT TTATTTCCAC GATCATTAGG GCAAAAATTG GTTGAGAAAT   
  
  
+ TATAATTTAG AGTATAAGAT ACTCTGGCTA GCTTTTGGAT TTCATTGACT AAAAAGGCAT TACCCCTTTT   
  
  
+ ACACTTTAGG ACTCCTAGAA TCTTCTAAGC TAGACTTAAT TATATCTTTT AATAACTTTT TTAATGCTTG   
  
  
+ TTTGACACTA TTGCATGTTA GCCTCTAAGC TTTATTTTAT CGAGATCCAT ATAAATGTTT TTTTTTGTTT   
  
  
+ TTTCCGGTTT TACATTGAGA TAAAAATCAT TTACTAATTT GAAATCAAAT TGATTTTTTC ATTAAAAAAT   
  
  
+ TTAATGGTTA TTTTTATAAT TTAAAATAAC AATATAAAAT AAAAAAATTA ATTATTGATT TTATTAAATT   
  
  
+ GTGGTGCGTT TATTTGAAAG TGTATATGTC ATATTTCATA ATTTTACATT ATAAATAAAA ATATATAAAA   
  
  
+ TACATATAAT TAGTTGTGTT ATTTCTGGAC ATCACTTACA AAATGTAAAT ATGAAACAAA AATTTTGCGC   
  
  
+ ATTTAAAATA TATAAAATAC ATATAATTAG TTGTGTTATT TCTGGACATC ACTTACAAAA TGTAAATATG   
  
  
+ AAACAAAAAT TTTGCGCATT TAAGTAATTA TTATTAACTA TTTTTAATGA TTATAAGATA AATACATTTA   
  
  
+ TTTAATTAAT AATTTTTTAG AAGAAATATA TTTAATCTAA ACTTTTATGG GATATGCATG GATGGCATGA   
  
  
+ AATTTGCACT TGTCATATGC CTCCCTCCTA CACTAAAGCC AATAGGCATC AGCAGCCATT TACGGCACTG   
  
  
+ AGAAGGATAA ATATTAGATA TGTGATTGGG CCTCAGCCGC CCGCTGGTCA AAGTCACGTG CTTTGACCAA   
  
  
+ ACCACGCGGG CCAAAACACA CACACGCCCA CACACACTCT CTCTCTCTCT CTTTCTTCCC GTATTTTTCA   
  
  
+ CGGTCAAACT TCTCGTTACC TTCGCAGAAA CTGCCTTGCA AAGAAAAAAC ACACTCTCTC TCTCTCTCTT   
  
  
+ TCTTCCCGTA TTTTTCACGG TCAAACTTCT CGTTACCTTC GCAGAAACTG CCTTGCAAAG AAAAAGAAGA   
  
  
+ AAACTAGAAA AAAAAGAGAG AAAAACCAAA GAAGCAGAGA CGCCTTGTAC CTTACGTGAC CGTCTTCTCC   
  
  
+ GTTCTCTCTC TCTCTCTCTC CCCCCCTTGA TCCTTATATT CATCGTATAA GCTGTCTTCT TTCTGTACCT   
  
  
+ GTAACCTTAG TTCTCTCTCT GTCGCTTTT  

- TAATAGCGTG AAATAGGTGA TTCTTGGATT AATGGGTTGA TCACCCAATT GAACATCCGA TTCGAATGTA   
  
  
- TAGACGAATA CAAGAGAGGT AAAACACACT TTTGCACATC TGAATTAGAT TGGTTTAGTA CTAACACTGA   
  
  
- AACTAATACT TTATTTTCCA GATACCAAAT TAATTTAATT AATGTTATAC TGCATTTGAT TTGTATATGG   
  
  
- AAATTACCGA GACCTCGAAA CACAGTAAAA AATAAAGGTG CTAGTAATCC CGTTTTTAAC CAACTCTTTA   
  
  
- ATATTAAATC TCATATTCTA TGAGACCGAT CGAAAACCTA AAGTAACTGA TTTTTCCGTA ATGGGGAAAA   
  
  
- TGTGAAATCC TGAGGATCTT AGAAGATTCG ATCTGAATTA ATATAGAAAA TTATTGAAAA AATTACGAAC   
  
  
- AAACTGTGAT AACGTACAAT CGGAGATTCG AAATAAAATA GCTCTAGGTA TATTTACAAA AAAAAACAAA   
  
  
- AAAGGCCAAA ATGTAACTCT ATTTTTAGTA AATGATTAAA CTTTAGTTTA ACTAAAAAAG TAATTTTTTA   
  
  
- AATTACCAAT AAAAATATTA AATTTTATTG TTATATTTTA TTTTTTTAAT TAATAACTAA AATAATTTAA   
  
  
- CACCACGCAA ATAAACTTTC ACATATACAG TATAAAGTAT TAAAATGTAA TATTTATTTT TATATATTTT   
  
  
- ATGTATATTA ATCAACACAA TAAAGACCTG TAGTGAATGT TTTACATTTA TACTTTGTTT TTAAAACGCG   
  
  
- TAAATTTTAT ATATTTTATG TATATTAATC AACACAATAA AGACCTGTAG TGAATGTTTT ACATTTATAC   
  
  
- TTTGTTTTTA AAACGCGTAA ATTCATTAAT AATAATTGAT AAAAATTACT AATATTCTAT TTATGTAAAT   
  
  
- AAATTAATTA TTAAAAAATC TTCTTTATAT AAATTAGATT TGAAAATACC CTATACGTAC CTACCGTACT   
  
  
- TTAAACGTGA ACAGTATACG GAGGGAGGAT GTGATTTCGG TTATCCGTAG TCGTCGGTAA ATGCCGTGAC   
  
  
- TCTTCCTATT TATAATCTAT ACACTAACCC GGAGTCGGCG GGCGACCAGT TTCAGTGCAC GAAACTGGTT   
  
  
- TGGTGCGCCC GGTTTTGTGT GTGTGCGGGT GTGTGTGAGA GAGAGAGAGA GAAAGAAGGG CATAAAAAGT   
  
  
- GCCAGTTTGA AGAGCAATGG AAGCGTCTTT GACGGAACGT TTCTTTTTTG TGTGAGAGAG AGAGAGAGAA   
  
  
- AGAAGGGCAT AAAAAGTGCC AGTTTGAAGA GCAATGGAAG CGTCTTTGAC GGAACGTTTC TTTTTCTTCT   
  
  
- TTTGATCTTT TTTTTCTCTC TTTTTGGTTT CTTCGTCTCT GCGGAACATG GAATGCACTG GCAGAAGAGG   
  
  
- CAAGAGAGAG AGAGAGAGAG GGGGGGAACT AGGAATATAA GTAGCATATT CGACAGAAGA AAGACATGGA   
  
  
- CATTGGAATC AAGAGAGAGA CAGCGAAAA

+     TCA-element

| Site Name | Organism | Position | Strand | Matrix score. | sequence | function |
| --- | --- | --- | --- | --- | --- | --- |
| TCA-element | Brassica oleracea | 1050 | + | 9 | GAGAAGAATA | cis-acting element involved in salicylic acid responsiveness |

> 2018/04/13 10:10:12  
+ ATTATCGCAC TTTATCCACT AAGAACCTAA TTACCCAACT AGTGGGTTAA CTTGTAGGCT AAGCTTACAT   
  
  
+ ATCTGCTTAT GTTCTCTCCA TTTTGTGTGA AAACGTGTAG ACTTAATCTA ACCAAATCAT GATTGTGACT   
  
  
+ TTGATTATGA AATAAAAGGT CTATGGTTTA ATTAAATTAA TTACAATATG ACGTAAACTA AACATATACC   
  
  
+ TTTAATGGCT CTGGAGCTTT GTGTCATTTT TTATTTCCAC GATCATTAGG GCAAAAATTG GTTGAGAAAT   
  
  
+ TATAATTTAG AGTATAAGAT ACTCTGGCTA GCTTTTGGAT TTCATTGACT AAAAAGGCAT TACCCCTTTT   
  
  
+ ACACTTTAGG ACTCCTAGAA TCTTCTAAGC TAGACTTAAT TATATCTTTT AATAACTTTT TTAATGCTTG   
  
  
+ TTTGACACTA TTGCATGTTA GCCTCTAAGC TTTATTTTAT CGAGATCCAT ATAAATGTTT TTTTTTGTTT   
  
  
+ TTTCCGGTTT TACATTGAGA TAAAAATCAT TTACTAATTT GAAATCAAAT TGATTTTTTC ATTAAAAAAT   
  
  
+ TTAATGGTTA TTTTTATAAT TTAAAATAAC AATATAAAAT AAAAAAATTA ATTATTGATT TTATTAAATT   
  
  
+ GTGGTGCGTT TATTTGAAAG TGTATATGTC ATATTTCATA ATTTTACATT ATAAATAAAA ATATATAAAA   
  
  
+ TACATATAAT TAGTTGTGTT ATTTCTGGAC ATCACTTACA AAATGTAAAT ATGAAACAAA AATTTTGCGC   
  
  
+ ATTTAAAATA TATAAAATAC ATATAATTAG TTGTGTTATT TCTGGACATC ACTTACAAAA TGTAAATATG   
  
  
+ AAACAAAAAT TTTGCGCATT TAAGTAATTA TTATTAACTA TTTTTAATGA TTATAAGATA AATACATTTA   
  
  
+ TTTAATTAAT AATTTTTTAG AAGAAATATA TTTAATCTAA ACTTTTATGG GATATGCATG GATGGCATGA   
  
  
+ AATTTGCACT TGTCATATGC CTCCCTCCTA CACTAAAGCC AATAGGCATC AGCAGCCATT TACGGCACTG   
  
  
+ AGAAGGATAA ATATTAGATA TGTGATTGGG CCTCAGCCGC CCGCTGGTCA AAGTCACGTG CTTTGACCAA   
  
  
+ ACCACGCGGG CCAAAACACA CACACGCCCA CACACACTCT CTCTCTCTCT CTTTCTTCCC GTATTTTTCA   
  
  
+ CGGTCAAACT TCTCGTTACC TTCGCAGAAA CTGCCTTGCA AAGAAAAAAC ACACTCTCTC TCTCTCTCTT   
  
  
+ TCTTCCCGTA TTTTTCACGG TCAAACTTCT CGTTACCTTC GCAGAAACTG CCTTGCAAAG AAAAAGAAGA   
  
  
+ AAACTAGAAA AAAAAGAGAG AAAAACCAAA GAAGCAGAGA CGCCTTGTAC CTTACGTGAC CGTCTTCTCC   
  
  
+ GTTCTCTCTC TCTCTCTCTC CCCCCCTTGA TCCTTATATT CATCGTATAA GCTGTCTTCT TTCTGTACCT   
  
  
+ GTAACCTTAG TTCTCTCTCT GTCGCTTTT  

- TAATAGCGTG AAATAGGTGA TTCTTGGATT AATGGGTTGA TCACCCAATT GAACATCCGA TTCGAATGTA   
  
  
- TAGACGAATA CAAGAGAGGT AAAACACACT TTTGCACATC TGAATTAGAT TGGTTTAGTA CTAACACTGA   
  
  
- AACTAATACT TTATTTTCCA GATACCAAAT TAATTTAATT AATGTTATAC TGCATTTGAT TTGTATATGG   
  
  
- AAATTACCGA GACCTCGAAA CACAGTAAAA AATAAAGGTG CTAGTAATCC CGTTTTTAAC CAACTCTTTA   
  
  
- ATATTAAATC TCATATTCTA TGAGACCGAT CGAAAACCTA AAGTAACTGA TTTTTCCGTA ATGGGGAAAA   
  
  
- TGTGAAATCC TGAGGATCTT AGAAGATTCG ATCTGAATTA ATATAGAAAA TTATTGAAAA AATTACGAAC   
  
  
- AAACTGTGAT AACGTACAAT CGGAGATTCG AAATAAAATA GCTCTAGGTA TATTTACAAA AAAAAACAAA   
  
  
- AAAGGCCAAA ATGTAACTCT ATTTTTAGTA AATGATTAAA CTTTAGTTTA ACTAAAAAAG TAATTTTTTA   
  
  
- AATTACCAAT AAAAATATTA AATTTTATTG TTATATTTTA TTTTTTTAAT TAATAACTAA AATAATTTAA   
  
  
- CACCACGCAA ATAAACTTTC ACATATACAG TATAAAGTAT TAAAATGTAA TATTTATTTT TATATATTTT   
  
  
- ATGTATATTA ATCAACACAA TAAAGACCTG TAGTGAATGT TTTACATTTA TACTTTGTTT TTAAAACGCG   
  
  
- TAAATTTTAT ATATTTTATG TATATTAATC AACACAATAA AGACCTGTAG TGAATGTTTT ACATTTATAC   
  
  
- TTTGTTTTTA AAACGCGTAA ATTCATTAAT AATAATTGAT AAAAATTACT AATATTCTAT TTATGTAAAT   
  
  
- AAATTAATTA TTAAAAAATC TTCTTTATAT AAATTAGATT TGAAAATACC CTATACGTAC CTACCGTACT   
  
  
- TTAAACGTGA ACAGTATACG GAGGGAGGAT GTGATTTCGG TTATCCGTAG TCGTCGGTAA ATGCCGTGAC   
  
  
- TCTTCCTATT TATAATCTAT ACACTAACCC GGAGTCGGCG GGCGACCAGT TTCAGTGCAC GAAACTGGTT   
  
  
- TGGTGCGCCC GGTTTTGTGT GTGTGCGGGT GTGTGTGAGA GAGAGAGAGA GAAAGAAGGG CATAAAAAGT   
  
  
- GCCAGTTTGA AGAGCAATGG AAGCGTCTTT GACGGAACGT TTCTTTTTTG TGTGAGAGAG AGAGAGAGAA   
  
  
- AGAAGGGCAT AAAAAGTGCC AGTTTGAAGA GCAATGGAAG CGTCTTTGAC GGAACGTTTC TTTTTCTTCT   
  
  
- TTTGATCTTT TTTTTCTCTC TTTTTGGTTT CTTCGTCTCT GCGGAACATG GAATGCACTG GCAGAAGAGG   
  
  
- CAAGAGAGAG AGAGAGAGAG GGGGGGAACT AGGAATATAA GTAGCATATT CGACAGAAGA AAGACATGGA   
  
  
- CATTGGAATC AAGAGAGAGA CAGCGAAAA

+     TGA-box

| Site Name | Organism | Position | Strand | Matrix score. | sequence | function |
| --- | --- | --- | --- | --- | --- | --- |
| TGA-box | Glycine max | 189 | + | 8 | TGACGTAA | part of an auxin-responsive element |

> 2018/04/13 10:10:12  
+ ATTATCGCAC TTTATCCACT AAGAACCTAA TTACCCAACT AGTGGGTTAA CTTGTAGGCT AAGCTTACAT   
  
  
+ ATCTGCTTAT GTTCTCTCCA TTTTGTGTGA AAACGTGTAG ACTTAATCTA ACCAAATCAT GATTGTGACT   
  
  
+ TTGATTATGA AATAAAAGGT CTATGGTTTA ATTAAATTAA TTACAATATG ACGTAAACTA AACATATACC   
  
  
+ TTTAATGGCT CTGGAGCTTT GTGTCATTTT TTATTTCCAC GATCATTAGG GCAAAAATTG GTTGAGAAAT   
  
  
+ TATAATTTAG AGTATAAGAT ACTCTGGCTA GCTTTTGGAT TTCATTGACT AAAAAGGCAT TACCCCTTTT   
  
  
+ ACACTTTAGG ACTCCTAGAA TCTTCTAAGC TAGACTTAAT TATATCTTTT AATAACTTTT TTAATGCTTG   
  
  
+ TTTGACACTA TTGCATGTTA GCCTCTAAGC TTTATTTTAT CGAGATCCAT ATAAATGTTT TTTTTTGTTT   
  
  
+ TTTCCGGTTT TACATTGAGA TAAAAATCAT TTACTAATTT GAAATCAAAT TGATTTTTTC ATTAAAAAAT   
  
  
+ TTAATGGTTA TTTTTATAAT TTAAAATAAC AATATAAAAT AAAAAAATTA ATTATTGATT TTATTAAATT   
  
  
+ GTGGTGCGTT TATTTGAAAG TGTATATGTC ATATTTCATA ATTTTACATT ATAAATAAAA ATATATAAAA   
  
  
+ TACATATAAT TAGTTGTGTT ATTTCTGGAC ATCACTTACA AAATGTAAAT ATGAAACAAA AATTTTGCGC   
  
  
+ ATTTAAAATA TATAAAATAC ATATAATTAG TTGTGTTATT TCTGGACATC ACTTACAAAA TGTAAATATG   
  
  
+ AAACAAAAAT TTTGCGCATT TAAGTAATTA TTATTAACTA TTTTTAATGA TTATAAGATA AATACATTTA   
  
  
+ TTTAATTAAT AATTTTTTAG AAGAAATATA TTTAATCTAA ACTTTTATGG GATATGCATG GATGGCATGA   
  
  
+ AATTTGCACT TGTCATATGC CTCCCTCCTA CACTAAAGCC AATAGGCATC AGCAGCCATT TACGGCACTG   
  
  
+ AGAAGGATAA ATATTAGATA TGTGATTGGG CCTCAGCCGC CCGCTGGTCA AAGTCACGTG CTTTGACCAA   
  
  
+ ACCACGCGGG CCAAAACACA CACACGCCCA CACACACTCT CTCTCTCTCT CTTTCTTCCC GTATTTTTCA   
  
  
+ CGGTCAAACT TCTCGTTACC TTCGCAGAAA CTGCCTTGCA AAGAAAAAAC ACACTCTCTC TCTCTCTCTT   
  
  
+ TCTTCCCGTA TTTTTCACGG TCAAACTTCT CGTTACCTTC GCAGAAACTG CCTTGCAAAG AAAAAGAAGA   
  
  
+ AAACTAGAAA AAAAAGAGAG AAAAACCAAA GAAGCAGAGA CGCCTTGTAC CTTACGTGAC CGTCTTCTCC   
  
  
+ GTTCTCTCTC TCTCTCTCTC CCCCCCTTGA TCCTTATATT CATCGTATAA GCTGTCTTCT TTCTGTACCT   
  
  
+ GTAACCTTAG TTCTCTCTCT GTCGCTTTT  

- TAATAGCGTG AAATAGGTGA TTCTTGGATT AATGGGTTGA TCACCCAATT GAACATCCGA TTCGAATGTA   
  
  
- TAGACGAATA CAAGAGAGGT AAAACACACT TTTGCACATC TGAATTAGAT TGGTTTAGTA CTAACACTGA   
  
  
- AACTAATACT TTATTTTCCA GATACCAAAT TAATTTAATT AATGTTATAC TGCATTTGAT TTGTATATGG   
  
  
- AAATTACCGA GACCTCGAAA CACAGTAAAA AATAAAGGTG CTAGTAATCC CGTTTTTAAC CAACTCTTTA   
  
  
- ATATTAAATC TCATATTCTA TGAGACCGAT CGAAAACCTA AAGTAACTGA TTTTTCCGTA ATGGGGAAAA   
  
  
- TGTGAAATCC TGAGGATCTT AGAAGATTCG ATCTGAATTA ATATAGAAAA TTATTGAAAA AATTACGAAC   
  
  
- AAACTGTGAT AACGTACAAT CGGAGATTCG AAATAAAATA GCTCTAGGTA TATTTACAAA AAAAAACAAA   
  
  
- AAAGGCCAAA ATGTAACTCT ATTTTTAGTA AATGATTAAA CTTTAGTTTA ACTAAAAAAG TAATTTTTTA   
  
  
- AATTACCAAT AAAAATATTA AATTTTATTG TTATATTTTA TTTTTTTAAT TAATAACTAA AATAATTTAA   
  
  
- CACCACGCAA ATAAACTTTC ACATATACAG TATAAAGTAT TAAAATGTAA TATTTATTTT TATATATTTT   
  
  
- ATGTATATTA ATCAACACAA TAAAGACCTG TAGTGAATGT TTTACATTTA TACTTTGTTT TTAAAACGCG   
  
  
- TAAATTTTAT ATATTTTATG TATATTAATC AACACAATAA AGACCTGTAG TGAATGTTTT ACATTTATAC   
  
  
- TTTGTTTTTA AAACGCGTAA ATTCATTAAT AATAATTGAT AAAAATTACT AATATTCTAT TTATGTAAAT   
  
  
- AAATTAATTA TTAAAAAATC TTCTTTATAT AAATTAGATT TGAAAATACC CTATACGTAC CTACCGTACT   
  
  
- TTAAACGTGA ACAGTATACG GAGGGAGGAT GTGATTTCGG TTATCCGTAG TCGTCGGTAA ATGCCGTGAC   
  
  
- TCTTCCTATT TATAATCTAT ACACTAACCC GGAGTCGGCG GGCGACCAGT TTCAGTGCAC GAAACTGGTT   
  
  
- TGGTGCGCCC GGTTTTGTGT GTGTGCGGGT GTGTGTGAGA GAGAGAGAGA GAAAGAAGGG CATAAAAAGT   
  
  
- GCCAGTTTGA AGAGCAATGG AAGCGTCTTT GACGGAACGT TTCTTTTTTG TGTGAGAGAG AGAGAGAGAA   
  
  
- AGAAGGGCAT AAAAAGTGCC AGTTTGAAGA GCAATGGAAG CGTCTTTGAC GGAACGTTTC TTTTTCTTCT   
  
  
- TTTGATCTTT TTTTTCTCTC TTTTTGGTTT CTTCGTCTCT GCGGAACATG GAATGCACTG GCAGAAGAGG   
  
  
- CAAGAGAGAG AGAGAGAGAG GGGGGGAACT AGGAATATAA GTAGCATATT CGACAGAAGA AAGACATGGA   
  
  
- CATTGGAATC AAGAGAGAGA CAGCGAAAA

+     TGACG-motif

| Site Name | Organism | Position | Strand | Matrix score. | sequence | function |
| --- | --- | --- | --- | --- | --- | --- |
| TGACG-motif | Hordeum vulgare | 189 | + | 5 | TGACG | cis-acting regulatory element involved in the MeJA-responsiveness |

> 2018/04/13 10:10:12  
+ ATTATCGCAC TTTATCCACT AAGAACCTAA TTACCCAACT AGTGGGTTAA CTTGTAGGCT AAGCTTACAT   
  
  
+ ATCTGCTTAT GTTCTCTCCA TTTTGTGTGA AAACGTGTAG ACTTAATCTA ACCAAATCAT GATTGTGACT   
  
  
+ TTGATTATGA AATAAAAGGT CTATGGTTTA ATTAAATTAA TTACAATATG ACGTAAACTA AACATATACC   
  
  
+ TTTAATGGCT CTGGAGCTTT GTGTCATTTT TTATTTCCAC GATCATTAGG GCAAAAATTG GTTGAGAAAT   
  
  
+ TATAATTTAG AGTATAAGAT ACTCTGGCTA GCTTTTGGAT TTCATTGACT AAAAAGGCAT TACCCCTTTT   
  
  
+ ACACTTTAGG ACTCCTAGAA TCTTCTAAGC TAGACTTAAT TATATCTTTT AATAACTTTT TTAATGCTTG   
  
  
+ TTTGACACTA TTGCATGTTA GCCTCTAAGC TTTATTTTAT CGAGATCCAT ATAAATGTTT TTTTTTGTTT   
  
  
+ TTTCCGGTTT TACATTGAGA TAAAAATCAT TTACTAATTT GAAATCAAAT TGATTTTTTC ATTAAAAAAT   
  
  
+ TTAATGGTTA TTTTTATAAT TTAAAATAAC AATATAAAAT AAAAAAATTA ATTATTGATT TTATTAAATT   
  
  
+ GTGGTGCGTT TATTTGAAAG TGTATATGTC ATATTTCATA ATTTTACATT ATAAATAAAA ATATATAAAA   
  
  
+ TACATATAAT TAGTTGTGTT ATTTCTGGAC ATCACTTACA AAATGTAAAT ATGAAACAAA AATTTTGCGC   
  
  
+ ATTTAAAATA TATAAAATAC ATATAATTAG TTGTGTTATT TCTGGACATC ACTTACAAAA TGTAAATATG   
  
  
+ AAACAAAAAT TTTGCGCATT TAAGTAATTA TTATTAACTA TTTTTAATGA TTATAAGATA AATACATTTA   
  
  
+ TTTAATTAAT AATTTTTTAG AAGAAATATA TTTAATCTAA ACTTTTATGG GATATGCATG GATGGCATGA   
  
  
+ AATTTGCACT TGTCATATGC CTCCCTCCTA CACTAAAGCC AATAGGCATC AGCAGCCATT TACGGCACTG   
  
  
+ AGAAGGATAA ATATTAGATA TGTGATTGGG CCTCAGCCGC CCGCTGGTCA AAGTCACGTG CTTTGACCAA   
  
  
+ ACCACGCGGG CCAAAACACA CACACGCCCA CACACACTCT CTCTCTCTCT CTTTCTTCCC GTATTTTTCA   
  
  
+ CGGTCAAACT TCTCGTTACC TTCGCAGAAA CTGCCTTGCA AAGAAAAAAC ACACTCTCTC TCTCTCTCTT   
  
  
+ TCTTCCCGTA TTTTTCACGG TCAAACTTCT CGTTACCTTC GCAGAAACTG CCTTGCAAAG AAAAAGAAGA   
  
  
+ AAACTAGAAA AAAAAGAGAG AAAAACCAAA GAAGCAGAGA CGCCTTGTAC CTTACGTGAC CGTCTTCTCC   
  
  
+ GTTCTCTCTC TCTCTCTCTC CCCCCCTTGA TCCTTATATT CATCGTATAA GCTGTCTTCT TTCTGTACCT   
  
  
+ GTAACCTTAG TTCTCTCTCT GTCGCTTTT  

- TAATAGCGTG AAATAGGTGA TTCTTGGATT AATGGGTTGA TCACCCAATT GAACATCCGA TTCGAATGTA   
  
  
- TAGACGAATA CAAGAGAGGT AAAACACACT TTTGCACATC TGAATTAGAT TGGTTTAGTA CTAACACTGA   
  
  
- AACTAATACT TTATTTTCCA GATACCAAAT TAATTTAATT AATGTTATAC TGCATTTGAT TTGTATATGG   
  
  
- AAATTACCGA GACCTCGAAA CACAGTAAAA AATAAAGGTG CTAGTAATCC CGTTTTTAAC CAACTCTTTA   
  
  
- ATATTAAATC TCATATTCTA TGAGACCGAT CGAAAACCTA AAGTAACTGA TTTTTCCGTA ATGGGGAAAA   
  
  
- TGTGAAATCC TGAGGATCTT AGAAGATTCG ATCTGAATTA ATATAGAAAA TTATTGAAAA AATTACGAAC   
  
  
- AAACTGTGAT AACGTACAAT CGGAGATTCG AAATAAAATA GCTCTAGGTA TATTTACAAA AAAAAACAAA   
  
  
- AAAGGCCAAA ATGTAACTCT ATTTTTAGTA AATGATTAAA CTTTAGTTTA ACTAAAAAAG TAATTTTTTA   
  
  
- AATTACCAAT AAAAATATTA AATTTTATTG TTATATTTTA TTTTTTTAAT TAATAACTAA AATAATTTAA   
  
  
- CACCACGCAA ATAAACTTTC ACATATACAG TATAAAGTAT TAAAATGTAA TATTTATTTT TATATATTTT   
  
  
- ATGTATATTA ATCAACACAA TAAAGACCTG TAGTGAATGT TTTACATTTA TACTTTGTTT TTAAAACGCG   
  
  
- TAAATTTTAT ATATTTTATG TATATTAATC AACACAATAA AGACCTGTAG TGAATGTTTT ACATTTATAC   
  
  
- TTTGTTTTTA AAACGCGTAA ATTCATTAAT AATAATTGAT AAAAATTACT AATATTCTAT TTATGTAAAT   
  
  
- AAATTAATTA TTAAAAAATC TTCTTTATAT AAATTAGATT TGAAAATACC CTATACGTAC CTACCGTACT   
  
  
- TTAAACGTGA ACAGTATACG GAGGGAGGAT GTGATTTCGG TTATCCGTAG TCGTCGGTAA ATGCCGTGAC   
  
  
- TCTTCCTATT TATAATCTAT ACACTAACCC GGAGTCGGCG GGCGACCAGT TTCAGTGCAC GAAACTGGTT   
  
  
- TGGTGCGCCC GGTTTTGTGT GTGTGCGGGT GTGTGTGAGA GAGAGAGAGA GAAAGAAGGG CATAAAAAGT   
  
  
- GCCAGTTTGA AGAGCAATGG AAGCGTCTTT GACGGAACGT TTCTTTTTTG TGTGAGAGAG AGAGAGAGAA   
  
  
- AGAAGGGCAT AAAAAGTGCC AGTTTGAAGA GCAATGGAAG CGTCTTTGAC GGAACGTTTC TTTTTCTTCT   
  
  
- TTTGATCTTT TTTTTCTCTC TTTTTGGTTT CTTCGTCTCT GCGGAACATG GAATGCACTG GCAGAAGAGG   
  
  
- CAAGAGAGAG AGAGAGAGAG GGGGGGAACT AGGAATATAA GTAGCATATT CGACAGAAGA AAGACATGGA   
  
  
- CATTGGAATC AAGAGAGAGA CAGCGAAAA

+     Unnamed\_\_1

| Site Name | Organism | Position | Strand | Matrix score. | sequence | function |
| --- | --- | --- | --- | --- | --- | --- |
| Unnamed\_\_1 | Zea mays | 1122 | - | 5 | CGTGG |  |
| Unnamed\_\_1 | Glycine max | 168 | - | 11 | GAATTTAATTAA | 60K protein binding site |
| Unnamed\_\_1 | Zea mays | 247 | - | 5 | CGTGG |  |

> 2018/04/13 10:10:12  
+ ATTATCGCAC TTTATCCACT AAGAACCTAA TTACCCAACT AGTGGGTTAA CTTGTAGGCT AAGCTTACAT   
  
  
+ ATCTGCTTAT GTTCTCTCCA TTTTGTGTGA AAACGTGTAG ACTTAATCTA ACCAAATCAT GATTGTGACT   
  
  
+ TTGATTATGA AATAAAAGGT CTATGGTTTA ATTAAATTAA TTACAATATG ACGTAAACTA AACATATACC   
  
  
+ TTTAATGGCT CTGGAGCTTT GTGTCATTTT TTATTTCCAC GATCATTAGG GCAAAAATTG GTTGAGAAAT   
  
  
+ TATAATTTAG AGTATAAGAT ACTCTGGCTA GCTTTTGGAT TTCATTGACT AAAAAGGCAT TACCCCTTTT   
  
  
+ ACACTTTAGG ACTCCTAGAA TCTTCTAAGC TAGACTTAAT TATATCTTTT AATAACTTTT TTAATGCTTG   
  
  
+ TTTGACACTA TTGCATGTTA GCCTCTAAGC TTTATTTTAT CGAGATCCAT ATAAATGTTT TTTTTTGTTT   
  
  
+ TTTCCGGTTT TACATTGAGA TAAAAATCAT TTACTAATTT GAAATCAAAT TGATTTTTTC ATTAAAAAAT   
  
  
+ TTAATGGTTA TTTTTATAAT TTAAAATAAC AATATAAAAT AAAAAAATTA ATTATTGATT TTATTAAATT   
  
  
+ GTGGTGCGTT TATTTGAAAG TGTATATGTC ATATTTCATA ATTTTACATT ATAAATAAAA ATATATAAAA   
  
  
+ TACATATAAT TAGTTGTGTT ATTTCTGGAC ATCACTTACA AAATGTAAAT ATGAAACAAA AATTTTGCGC   
  
  
+ ATTTAAAATA TATAAAATAC ATATAATTAG TTGTGTTATT TCTGGACATC ACTTACAAAA TGTAAATATG   
  
  
+ AAACAAAAAT TTTGCGCATT TAAGTAATTA TTATTAACTA TTTTTAATGA TTATAAGATA AATACATTTA   
  
  
+ TTTAATTAAT AATTTTTTAG AAGAAATATA TTTAATCTAA ACTTTTATGG GATATGCATG GATGGCATGA   
  
  
+ AATTTGCACT TGTCATATGC CTCCCTCCTA CACTAAAGCC AATAGGCATC AGCAGCCATT TACGGCACTG   
  
  
+ AGAAGGATAA ATATTAGATA TGTGATTGGG CCTCAGCCGC CCGCTGGTCA AAGTCACGTG CTTTGACCAA   
  
  
+ ACCACGCGGG CCAAAACACA CACACGCCCA CACACACTCT CTCTCTCTCT CTTTCTTCCC GTATTTTTCA   
  
  
+ CGGTCAAACT TCTCGTTACC TTCGCAGAAA CTGCCTTGCA AAGAAAAAAC ACACTCTCTC TCTCTCTCTT   
  
  
+ TCTTCCCGTA TTTTTCACGG TCAAACTTCT CGTTACCTTC GCAGAAACTG CCTTGCAAAG AAAAAGAAGA   
  
  
+ AAACTAGAAA AAAAAGAGAG AAAAACCAAA GAAGCAGAGA CGCCTTGTAC CTTACGTGAC CGTCTTCTCC   
  
  
+ GTTCTCTCTC TCTCTCTCTC CCCCCCTTGA TCCTTATATT CATCGTATAA GCTGTCTTCT TTCTGTACCT   
  
  
+ GTAACCTTAG TTCTCTCTCT GTCGCTTTT  

- TAATAGCGTG AAATAGGTGA TTCTTGGATT AATGGGTTGA TCACCCAATT GAACATCCGA TTCGAATGTA   
  
  
- TAGACGAATA CAAGAGAGGT AAAACACACT TTTGCACATC TGAATTAGAT TGGTTTAGTA CTAACACTGA   
  
  
- AACTAATACT TTATTTTCCA GATACCAAAT TAATTTAATT AATGTTATAC TGCATTTGAT TTGTATATGG   
  
  
- AAATTACCGA GACCTCGAAA CACAGTAAAA AATAAAGGTG CTAGTAATCC CGTTTTTAAC CAACTCTTTA   
  
  
- ATATTAAATC TCATATTCTA TGAGACCGAT CGAAAACCTA AAGTAACTGA TTTTTCCGTA ATGGGGAAAA   
  
  
- TGTGAAATCC TGAGGATCTT AGAAGATTCG ATCTGAATTA ATATAGAAAA TTATTGAAAA AATTACGAAC   
  
  
- AAACTGTGAT AACGTACAAT CGGAGATTCG AAATAAAATA GCTCTAGGTA TATTTACAAA AAAAAACAAA   
  
  
- AAAGGCCAAA ATGTAACTCT ATTTTTAGTA AATGATTAAA CTTTAGTTTA ACTAAAAAAG TAATTTTTTA   
  
  
- AATTACCAAT AAAAATATTA AATTTTATTG TTATATTTTA TTTTTTTAAT TAATAACTAA AATAATTTAA   
  
  
- CACCACGCAA ATAAACTTTC ACATATACAG TATAAAGTAT TAAAATGTAA TATTTATTTT TATATATTTT   
  
  
- ATGTATATTA ATCAACACAA TAAAGACCTG TAGTGAATGT TTTACATTTA TACTTTGTTT TTAAAACGCG   
  
  
- TAAATTTTAT ATATTTTATG TATATTAATC AACACAATAA AGACCTGTAG TGAATGTTTT ACATTTATAC   
  
  
- TTTGTTTTTA AAACGCGTAA ATTCATTAAT AATAATTGAT AAAAATTACT AATATTCTAT TTATGTAAAT   
  
  
- AAATTAATTA TTAAAAAATC TTCTTTATAT AAATTAGATT TGAAAATACC CTATACGTAC CTACCGTACT   
  
  
- TTAAACGTGA ACAGTATACG GAGGGAGGAT GTGATTTCGG TTATCCGTAG TCGTCGGTAA ATGCCGTGAC   
  
  
- TCTTCCTATT TATAATCTAT ACACTAACCC GGAGTCGGCG GGCGACCAGT TTCAGTGCAC GAAACTGGTT   
  
  
- TGGTGCGCCC GGTTTTGTGT GTGTGCGGGT GTGTGTGAGA GAGAGAGAGA GAAAGAAGGG CATAAAAAGT   
  
  
- GCCAGTTTGA AGAGCAATGG AAGCGTCTTT GACGGAACGT TTCTTTTTTG TGTGAGAGAG AGAGAGAGAA   
  
  
- AGAAGGGCAT AAAAAGTGCC AGTTTGAAGA GCAATGGAAG CGTCTTTGAC GGAACGTTTC TTTTTCTTCT   
  
  
- TTTGATCTTT TTTTTCTCTC TTTTTGGTTT CTTCGTCTCT GCGGAACATG GAATGCACTG GCAGAAGAGG   
  
  
- CAAGAGAGAG AGAGAGAGAG GGGGGGAACT AGGAATATAA GTAGCATATT CGACAGAAGA AAGACATGGA   
  
  
- CATTGGAATC AAGAGAGAGA CAGCGAAAA

+     Unnamed\_\_3

| Site Name | Organism | Position | Strand | Matrix score. | sequence | function |
| --- | --- | --- | --- | --- | --- | --- |
| Unnamed\_\_3 | Zea mays | 247 | - | 5 | CGTGG |  |
| Unnamed\_\_3 | Zea mays | 1122 | - | 5 | CGTGG |  |

> 2018/04/13 10:10:12  
+ ATTATCGCAC TTTATCCACT AAGAACCTAA TTACCCAACT AGTGGGTTAA CTTGTAGGCT AAGCTTACAT   
  
  
+ ATCTGCTTAT GTTCTCTCCA TTTTGTGTGA AAACGTGTAG ACTTAATCTA ACCAAATCAT GATTGTGACT   
  
  
+ TTGATTATGA AATAAAAGGT CTATGGTTTA ATTAAATTAA TTACAATATG ACGTAAACTA AACATATACC   
  
  
+ TTTAATGGCT CTGGAGCTTT GTGTCATTTT TTATTTCCAC GATCATTAGG GCAAAAATTG GTTGAGAAAT   
  
  
+ TATAATTTAG AGTATAAGAT ACTCTGGCTA GCTTTTGGAT TTCATTGACT AAAAAGGCAT TACCCCTTTT   
  
  
+ ACACTTTAGG ACTCCTAGAA TCTTCTAAGC TAGACTTAAT TATATCTTTT AATAACTTTT TTAATGCTTG   
  
  
+ TTTGACACTA TTGCATGTTA GCCTCTAAGC TTTATTTTAT CGAGATCCAT ATAAATGTTT TTTTTTGTTT   
  
  
+ TTTCCGGTTT TACATTGAGA TAAAAATCAT TTACTAATTT GAAATCAAAT TGATTTTTTC ATTAAAAAAT   
  
  
+ TTAATGGTTA TTTTTATAAT TTAAAATAAC AATATAAAAT AAAAAAATTA ATTATTGATT TTATTAAATT   
  
  
+ GTGGTGCGTT TATTTGAAAG TGTATATGTC ATATTTCATA ATTTTACATT ATAAATAAAA ATATATAAAA   
  
  
+ TACATATAAT TAGTTGTGTT ATTTCTGGAC ATCACTTACA AAATGTAAAT ATGAAACAAA AATTTTGCGC   
  
  
+ ATTTAAAATA TATAAAATAC ATATAATTAG TTGTGTTATT TCTGGACATC ACTTACAAAA TGTAAATATG   
  
  
+ AAACAAAAAT TTTGCGCATT TAAGTAATTA TTATTAACTA TTTTTAATGA TTATAAGATA AATACATTTA   
  
  
+ TTTAATTAAT AATTTTTTAG AAGAAATATA TTTAATCTAA ACTTTTATGG GATATGCATG GATGGCATGA   
  
  
+ AATTTGCACT TGTCATATGC CTCCCTCCTA CACTAAAGCC AATAGGCATC AGCAGCCATT TACGGCACTG   
  
  
+ AGAAGGATAA ATATTAGATA TGTGATTGGG CCTCAGCCGC CCGCTGGTCA AAGTCACGTG CTTTGACCAA   
  
  
+ ACCACGCGGG CCAAAACACA CACACGCCCA CACACACTCT CTCTCTCTCT CTTTCTTCCC GTATTTTTCA   
  
  
+ CGGTCAAACT TCTCGTTACC TTCGCAGAAA CTGCCTTGCA AAGAAAAAAC ACACTCTCTC TCTCTCTCTT   
  
  
+ TCTTCCCGTA TTTTTCACGG TCAAACTTCT CGTTACCTTC GCAGAAACTG CCTTGCAAAG AAAAAGAAGA   
  
  
+ AAACTAGAAA AAAAAGAGAG AAAAACCAAA GAAGCAGAGA CGCCTTGTAC CTTACGTGAC CGTCTTCTCC   
  
  
+ GTTCTCTCTC TCTCTCTCTC CCCCCCTTGA TCCTTATATT CATCGTATAA GCTGTCTTCT TTCTGTACCT   
  
  
+ GTAACCTTAG TTCTCTCTCT GTCGCTTTT  

- TAATAGCGTG AAATAGGTGA TTCTTGGATT AATGGGTTGA TCACCCAATT GAACATCCGA TTCGAATGTA   
  
  
- TAGACGAATA CAAGAGAGGT AAAACACACT TTTGCACATC TGAATTAGAT TGGTTTAGTA CTAACACTGA   
  
  
- AACTAATACT TTATTTTCCA GATACCAAAT TAATTTAATT AATGTTATAC TGCATTTGAT TTGTATATGG   
  
  
- AAATTACCGA GACCTCGAAA CACAGTAAAA AATAAAGGTG CTAGTAATCC CGTTTTTAAC CAACTCTTTA   
  
  
- ATATTAAATC TCATATTCTA TGAGACCGAT CGAAAACCTA AAGTAACTGA TTTTTCCGTA ATGGGGAAAA   
  
  
- TGTGAAATCC TGAGGATCTT AGAAGATTCG ATCTGAATTA ATATAGAAAA TTATTGAAAA AATTACGAAC   
  
  
- AAACTGTGAT AACGTACAAT CGGAGATTCG AAATAAAATA GCTCTAGGTA TATTTACAAA AAAAAACAAA   
  
  
- AAAGGCCAAA ATGTAACTCT ATTTTTAGTA AATGATTAAA CTTTAGTTTA ACTAAAAAAG TAATTTTTTA   
  
  
- AATTACCAAT AAAAATATTA AATTTTATTG TTATATTTTA TTTTTTTAAT TAATAACTAA AATAATTTAA   
  
  
- CACCACGCAA ATAAACTTTC ACATATACAG TATAAAGTAT TAAAATGTAA TATTTATTTT TATATATTTT   
  
  
- ATGTATATTA ATCAACACAA TAAAGACCTG TAGTGAATGT TTTACATTTA TACTTTGTTT TTAAAACGCG   
  
  
- TAAATTTTAT ATATTTTATG TATATTAATC AACACAATAA AGACCTGTAG TGAATGTTTT ACATTTATAC   
  
  
- TTTGTTTTTA AAACGCGTAA ATTCATTAAT AATAATTGAT AAAAATTACT AATATTCTAT TTATGTAAAT   
  
  
- AAATTAATTA TTAAAAAATC TTCTTTATAT AAATTAGATT TGAAAATACC CTATACGTAC CTACCGTACT   
  
  
- TTAAACGTGA ACAGTATACG GAGGGAGGAT GTGATTTCGG TTATCCGTAG TCGTCGGTAA ATGCCGTGAC   
  
  
- TCTTCCTATT TATAATCTAT ACACTAACCC GGAGTCGGCG GGCGACCAGT TTCAGTGCAC GAAACTGGTT   
  
  
- TGGTGCGCCC GGTTTTGTGT GTGTGCGGGT GTGTGTGAGA GAGAGAGAGA GAAAGAAGGG CATAAAAAGT   
  
  
- GCCAGTTTGA AGAGCAATGG AAGCGTCTTT GACGGAACGT TTCTTTTTTG TGTGAGAGAG AGAGAGAGAA   
  
  
- AGAAGGGCAT AAAAAGTGCC AGTTTGAAGA GCAATGGAAG CGTCTTTGAC GGAACGTTTC TTTTTCTTCT   
  
  
- TTTGATCTTT TTTTTCTCTC TTTTTGGTTT CTTCGTCTCT GCGGAACATG GAATGCACTG GCAGAAGAGG   
  
  
- CAAGAGAGAG AGAGAGAGAG GGGGGGAACT AGGAATATAA GTAGCATATT CGACAGAAGA AAGACATGGA   
  
  
- CATTGGAATC AAGAGAGAGA CAGCGAAAA

+     Unnamed\_\_4

| Site Name | Organism | Position | Strand | Matrix score. | sequence | function |
| --- | --- | --- | --- | --- | --- | --- |
| Unnamed\_\_4 | Petroselinum hortense | 362 | + | 4 | CTCC |  |
| Unnamed\_\_4 | Petroselinum hortense | 86 | + | 4 | CTCC |  |
| Unnamed\_\_4 | Petroselinum hortense | 1397 | + | 4 | CTCC |  |
| Unnamed\_\_4 | Petroselinum hortense | 223 | - | 4 | CTCC |  |
| Unnamed\_\_4 | Petroselinum hortense | 1005 | + | 4 | CTCC |  |
| Unnamed\_\_4 | Petroselinum hortense | 1418 | + | 4 | CTCC |  |
| Unnamed\_\_4 | Petroselinum hortense | 1001 | + | 4 | CTCC |  |

> 2018/04/13 10:10:12  
+ ATTATCGCAC TTTATCCACT AAGAACCTAA TTACCCAACT AGTGGGTTAA CTTGTAGGCT AAGCTTACAT   
  
  
+ ATCTGCTTAT GTTCTCTCCA TTTTGTGTGA AAACGTGTAG ACTTAATCTA ACCAAATCAT GATTGTGACT   
  
  
+ TTGATTATGA AATAAAAGGT CTATGGTTTA ATTAAATTAA TTACAATATG ACGTAAACTA AACATATACC   
  
  
+ TTTAATGGCT CTGGAGCTTT GTGTCATTTT TTATTTCCAC GATCATTAGG GCAAAAATTG GTTGAGAAAT   
  
  
+ TATAATTTAG AGTATAAGAT ACTCTGGCTA GCTTTTGGAT TTCATTGACT AAAAAGGCAT TACCCCTTTT   
  
  
+ ACACTTTAGG ACTCCTAGAA TCTTCTAAGC TAGACTTAAT TATATCTTTT AATAACTTTT TTAATGCTTG   
  
  
+ TTTGACACTA TTGCATGTTA GCCTCTAAGC TTTATTTTAT CGAGATCCAT ATAAATGTTT TTTTTTGTTT   
  
  
+ TTTCCGGTTT TACATTGAGA TAAAAATCAT TTACTAATTT GAAATCAAAT TGATTTTTTC ATTAAAAAAT   
  
  
+ TTAATGGTTA TTTTTATAAT TTAAAATAAC AATATAAAAT AAAAAAATTA ATTATTGATT TTATTAAATT   
  
  
+ GTGGTGCGTT TATTTGAAAG TGTATATGTC ATATTTCATA ATTTTACATT ATAAATAAAA ATATATAAAA   
  
  
+ TACATATAAT TAGTTGTGTT ATTTCTGGAC ATCACTTACA AAATGTAAAT ATGAAACAAA AATTTTGCGC   
  
  
+ ATTTAAAATA TATAAAATAC ATATAATTAG TTGTGTTATT TCTGGACATC ACTTACAAAA TGTAAATATG   
  
  
+ AAACAAAAAT TTTGCGCATT TAAGTAATTA TTATTAACTA TTTTTAATGA TTATAAGATA AATACATTTA   
  
  
+ TTTAATTAAT AATTTTTTAG AAGAAATATA TTTAATCTAA ACTTTTATGG GATATGCATG GATGGCATGA   
  
  
+ AATTTGCACT TGTCATATGC CTCCCTCCTA CACTAAAGCC AATAGGCATC AGCAGCCATT TACGGCACTG   
  
  
+ AGAAGGATAA ATATTAGATA TGTGATTGGG CCTCAGCCGC CCGCTGGTCA AAGTCACGTG CTTTGACCAA   
  
  
+ ACCACGCGGG CCAAAACACA CACACGCCCA CACACACTCT CTCTCTCTCT CTTTCTTCCC GTATTTTTCA   
  
  
+ CGGTCAAACT TCTCGTTACC TTCGCAGAAA CTGCCTTGCA AAGAAAAAAC ACACTCTCTC TCTCTCTCTT   
  
  
+ TCTTCCCGTA TTTTTCACGG TCAAACTTCT CGTTACCTTC GCAGAAACTG CCTTGCAAAG AAAAAGAAGA   
  
  
+ AAACTAGAAA AAAAAGAGAG AAAAACCAAA GAAGCAGAGA CGCCTTGTAC CTTACGTGAC CGTCTTCTCC   
  
  
+ GTTCTCTCTC TCTCTCTCTC CCCCCCTTGA TCCTTATATT CATCGTATAA GCTGTCTTCT TTCTGTACCT   
  
  
+ GTAACCTTAG TTCTCTCTCT GTCGCTTTT  

- TAATAGCGTG AAATAGGTGA TTCTTGGATT AATGGGTTGA TCACCCAATT GAACATCCGA TTCGAATGTA   
  
  
- TAGACGAATA CAAGAGAGGT AAAACACACT TTTGCACATC TGAATTAGAT TGGTTTAGTA CTAACACTGA   
  
  
- AACTAATACT TTATTTTCCA GATACCAAAT TAATTTAATT AATGTTATAC TGCATTTGAT TTGTATATGG   
  
  
- AAATTACCGA GACCTCGAAA CACAGTAAAA AATAAAGGTG CTAGTAATCC CGTTTTTAAC CAACTCTTTA   
  
  
- ATATTAAATC TCATATTCTA TGAGACCGAT CGAAAACCTA AAGTAACTGA TTTTTCCGTA ATGGGGAAAA   
  
  
- TGTGAAATCC TGAGGATCTT AGAAGATTCG ATCTGAATTA ATATAGAAAA TTATTGAAAA AATTACGAAC   
  
  
- AAACTGTGAT AACGTACAAT CGGAGATTCG AAATAAAATA GCTCTAGGTA TATTTACAAA AAAAAACAAA   
  
  
- AAAGGCCAAA ATGTAACTCT ATTTTTAGTA AATGATTAAA CTTTAGTTTA ACTAAAAAAG TAATTTTTTA   
  
  
- AATTACCAAT AAAAATATTA AATTTTATTG TTATATTTTA TTTTTTTAAT TAATAACTAA AATAATTTAA   
  
  
- CACCACGCAA ATAAACTTTC ACATATACAG TATAAAGTAT TAAAATGTAA TATTTATTTT TATATATTTT   
  
  
- ATGTATATTA ATCAACACAA TAAAGACCTG TAGTGAATGT TTTACATTTA TACTTTGTTT TTAAAACGCG   
  
  
- TAAATTTTAT ATATTTTATG TATATTAATC AACACAATAA AGACCTGTAG TGAATGTTTT ACATTTATAC   
  
  
- TTTGTTTTTA AAACGCGTAA ATTCATTAAT AATAATTGAT AAAAATTACT AATATTCTAT TTATGTAAAT   
  
  
- AAATTAATTA TTAAAAAATC TTCTTTATAT AAATTAGATT TGAAAATACC CTATACGTAC CTACCGTACT   
  
  
- TTAAACGTGA ACAGTATACG GAGGGAGGAT GTGATTTCGG TTATCCGTAG TCGTCGGTAA ATGCCGTGAC   
  
  
- TCTTCCTATT TATAATCTAT ACACTAACCC GGAGTCGGCG GGCGACCAGT TTCAGTGCAC GAAACTGGTT   
  
  
- TGGTGCGCCC GGTTTTGTGT GTGTGCGGGT GTGTGTGAGA GAGAGAGAGA GAAAGAAGGG CATAAAAAGT   
  
  
- GCCAGTTTGA AGAGCAATGG AAGCGTCTTT GACGGAACGT TTCTTTTTTG TGTGAGAGAG AGAGAGAGAA   
  
  
- AGAAGGGCAT AAAAAGTGCC AGTTTGAAGA GCAATGGAAG CGTCTTTGAC GGAACGTTTC TTTTTCTTCT   
  
  
- TTTGATCTTT TTTTTCTCTC TTTTTGGTTT CTTCGTCTCT GCGGAACATG GAATGCACTG GCAGAAGAGG   
  
  
- CAAGAGAGAG AGAGAGAGAG GGGGGGAACT AGGAATATAA GTAGCATATT CGACAGAAGA AAGACATGGA   
  
  
- CATTGGAATC AAGAGAGAGA CAGCGAAAA

+     W box

| Site Name | Organism | Position | Strand | Matrix score. | sequence | function |
| --- | --- | --- | --- | --- | --- | --- |
| W box | Arabidopsis thaliana | 1096 | - | 6 | TTGACC |  |
| W box | Arabidopsis thaliana | 1113 | + | 6 | TTGACC |  |
| W box | Arabidopsis thaliana | 1279 | - | 6 | TTGACC |  |
| W box | Arabidopsis thaliana | 1192 | - | 6 | TTGACC |  |

> 2018/04/13 10:10:12  
+ ATTATCGCAC TTTATCCACT AAGAACCTAA TTACCCAACT AGTGGGTTAA CTTGTAGGCT AAGCTTACAT   
  
  
+ ATCTGCTTAT GTTCTCTCCA TTTTGTGTGA AAACGTGTAG ACTTAATCTA ACCAAATCAT GATTGTGACT   
  
  
+ TTGATTATGA AATAAAAGGT CTATGGTTTA ATTAAATTAA TTACAATATG ACGTAAACTA AACATATACC   
  
  
+ TTTAATGGCT CTGGAGCTTT GTGTCATTTT TTATTTCCAC GATCATTAGG GCAAAAATTG GTTGAGAAAT   
  
  
+ TATAATTTAG AGTATAAGAT ACTCTGGCTA GCTTTTGGAT TTCATTGACT AAAAAGGCAT TACCCCTTTT   
  
  
+ ACACTTTAGG ACTCCTAGAA TCTTCTAAGC TAGACTTAAT TATATCTTTT AATAACTTTT TTAATGCTTG   
  
  
+ TTTGACACTA TTGCATGTTA GCCTCTAAGC TTTATTTTAT CGAGATCCAT ATAAATGTTT TTTTTTGTTT   
  
  
+ TTTCCGGTTT TACATTGAGA TAAAAATCAT TTACTAATTT GAAATCAAAT TGATTTTTTC ATTAAAAAAT   
  
  
+ TTAATGGTTA TTTTTATAAT TTAAAATAAC AATATAAAAT AAAAAAATTA ATTATTGATT TTATTAAATT   
  
  
+ GTGGTGCGTT TATTTGAAAG TGTATATGTC ATATTTCATA ATTTTACATT ATAAATAAAA ATATATAAAA   
  
  
+ TACATATAAT TAGTTGTGTT ATTTCTGGAC ATCACTTACA AAATGTAAAT ATGAAACAAA AATTTTGCGC   
  
  
+ ATTTAAAATA TATAAAATAC ATATAATTAG TTGTGTTATT TCTGGACATC ACTTACAAAA TGTAAATATG   
  
  
+ AAACAAAAAT TTTGCGCATT TAAGTAATTA TTATTAACTA TTTTTAATGA TTATAAGATA AATACATTTA   
  
  
+ TTTAATTAAT AATTTTTTAG AAGAAATATA TTTAATCTAA ACTTTTATGG GATATGCATG GATGGCATGA   
  
  
+ AATTTGCACT TGTCATATGC CTCCCTCCTA CACTAAAGCC AATAGGCATC AGCAGCCATT TACGGCACTG   
  
  
+ AGAAGGATAA ATATTAGATA TGTGATTGGG CCTCAGCCGC CCGCTGGTCA AAGTCACGTG CTTTGACCAA   
  
  
+ ACCACGCGGG CCAAAACACA CACACGCCCA CACACACTCT CTCTCTCTCT CTTTCTTCCC GTATTTTTCA   
  
  
+ CGGTCAAACT TCTCGTTACC TTCGCAGAAA CTGCCTTGCA AAGAAAAAAC ACACTCTCTC TCTCTCTCTT   
  
  
+ TCTTCCCGTA TTTTTCACGG TCAAACTTCT CGTTACCTTC GCAGAAACTG CCTTGCAAAG AAAAAGAAGA   
  
  
+ AAACTAGAAA AAAAAGAGAG AAAAACCAAA GAAGCAGAGA CGCCTTGTAC CTTACGTGAC CGTCTTCTCC   
  
  
+ GTTCTCTCTC TCTCTCTCTC CCCCCCTTGA TCCTTATATT CATCGTATAA GCTGTCTTCT TTCTGTACCT   
  
  
+ GTAACCTTAG TTCTCTCTCT GTCGCTTTT  

- TAATAGCGTG AAATAGGTGA TTCTTGGATT AATGGGTTGA TCACCCAATT GAACATCCGA TTCGAATGTA   
  
  
- TAGACGAATA CAAGAGAGGT AAAACACACT TTTGCACATC TGAATTAGAT TGGTTTAGTA CTAACACTGA   
  
  
- AACTAATACT TTATTTTCCA GATACCAAAT TAATTTAATT AATGTTATAC TGCATTTGAT TTGTATATGG   
  
  
- AAATTACCGA GACCTCGAAA CACAGTAAAA AATAAAGGTG CTAGTAATCC CGTTTTTAAC CAACTCTTTA   
  
  
- ATATTAAATC TCATATTCTA TGAGACCGAT CGAAAACCTA AAGTAACTGA TTTTTCCGTA ATGGGGAAAA   
  
  
- TGTGAAATCC TGAGGATCTT AGAAGATTCG ATCTGAATTA ATATAGAAAA TTATTGAAAA AATTACGAAC   
  
  
- AAACTGTGAT AACGTACAAT CGGAGATTCG AAATAAAATA GCTCTAGGTA TATTTACAAA AAAAAACAAA   
  
  
- AAAGGCCAAA ATGTAACTCT ATTTTTAGTA AATGATTAAA CTTTAGTTTA ACTAAAAAAG TAATTTTTTA   
  
  
- AATTACCAAT AAAAATATTA AATTTTATTG TTATATTTTA TTTTTTTAAT TAATAACTAA AATAATTTAA   
  
  
- CACCACGCAA ATAAACTTTC ACATATACAG TATAAAGTAT TAAAATGTAA TATTTATTTT TATATATTTT   
  
  
- ATGTATATTA ATCAACACAA TAAAGACCTG TAGTGAATGT TTTACATTTA TACTTTGTTT TTAAAACGCG   
  
  
- TAAATTTTAT ATATTTTATG TATATTAATC AACACAATAA AGACCTGTAG TGAATGTTTT ACATTTATAC   
  
  
- TTTGTTTTTA AAACGCGTAA ATTCATTAAT AATAATTGAT AAAAATTACT AATATTCTAT TTATGTAAAT   
  
  
- AAATTAATTA TTAAAAAATC TTCTTTATAT AAATTAGATT TGAAAATACC CTATACGTAC CTACCGTACT   
  
  
- TTAAACGTGA ACAGTATACG GAGGGAGGAT GTGATTTCGG TTATCCGTAG TCGTCGGTAA ATGCCGTGAC   
  
  
- TCTTCCTATT TATAATCTAT ACACTAACCC GGAGTCGGCG GGCGACCAGT TTCAGTGCAC GAAACTGGTT   
  
  
- TGGTGCGCCC GGTTTTGTGT GTGTGCGGGT GTGTGTGAGA GAGAGAGAGA GAAAGAAGGG CATAAAAAGT   
  
  
- GCCAGTTTGA AGAGCAATGG AAGCGTCTTT GACGGAACGT TTCTTTTTTG TGTGAGAGAG AGAGAGAGAA   
  
  
- AGAAGGGCAT AAAAAGTGCC AGTTTGAAGA GCAATGGAAG CGTCTTTGAC GGAACGTTTC TTTTTCTTCT   
  
  
- TTTGATCTTT TTTTTCTCTC TTTTTGGTTT CTTCGTCTCT GCGGAACATG GAATGCACTG GCAGAAGAGG   
  
  
- CAAGAGAGAG AGAGAGAGAG GGGGGGAACT AGGAATATAA GTAGCATATT CGACAGAAGA AAGACATGGA   
  
  
- CATTGGAATC AAGAGAGAGA CAGCGAAAA

+     chs-CMA1a

| Site Name | Organism | Position | Strand | Matrix score. | sequence | function |
| --- | --- | --- | --- | --- | --- | --- |
| chs-CMA1a | Daucus carota | 860 | - | 8 | TTACTTAA | part of a light responsive element |

> 2018/04/13 10:10:12  
+ ATTATCGCAC TTTATCCACT AAGAACCTAA TTACCCAACT AGTGGGTTAA CTTGTAGGCT AAGCTTACAT   
  
  
+ ATCTGCTTAT GTTCTCTCCA TTTTGTGTGA AAACGTGTAG ACTTAATCTA ACCAAATCAT GATTGTGACT   
  
  
+ TTGATTATGA AATAAAAGGT CTATGGTTTA ATTAAATTAA TTACAATATG ACGTAAACTA AACATATACC   
  
  
+ TTTAATGGCT CTGGAGCTTT GTGTCATTTT TTATTTCCAC GATCATTAGG GCAAAAATTG GTTGAGAAAT   
  
  
+ TATAATTTAG AGTATAAGAT ACTCTGGCTA GCTTTTGGAT TTCATTGACT AAAAAGGCAT TACCCCTTTT   
  
  
+ ACACTTTAGG ACTCCTAGAA TCTTCTAAGC TAGACTTAAT TATATCTTTT AATAACTTTT TTAATGCTTG   
  
  
+ TTTGACACTA TTGCATGTTA GCCTCTAAGC TTTATTTTAT CGAGATCCAT ATAAATGTTT TTTTTTGTTT   
  
  
+ TTTCCGGTTT TACATTGAGA TAAAAATCAT TTACTAATTT GAAATCAAAT TGATTTTTTC ATTAAAAAAT   
  
  
+ TTAATGGTTA TTTTTATAAT TTAAAATAAC AATATAAAAT AAAAAAATTA ATTATTGATT TTATTAAATT   
  
  
+ GTGGTGCGTT TATTTGAAAG TGTATATGTC ATATTTCATA ATTTTACATT ATAAATAAAA ATATATAAAA   
  
  
+ TACATATAAT TAGTTGTGTT ATTTCTGGAC ATCACTTACA AAATGTAAAT ATGAAACAAA AATTTTGCGC   
  
  
+ ATTTAAAATA TATAAAATAC ATATAATTAG TTGTGTTATT TCTGGACATC ACTTACAAAA TGTAAATATG   
  
  
+ AAACAAAAAT TTTGCGCATT TAAGTAATTA TTATTAACTA TTTTTAATGA TTATAAGATA AATACATTTA   
  
  
+ TTTAATTAAT AATTTTTTAG AAGAAATATA TTTAATCTAA ACTTTTATGG GATATGCATG GATGGCATGA   
  
  
+ AATTTGCACT TGTCATATGC CTCCCTCCTA CACTAAAGCC AATAGGCATC AGCAGCCATT TACGGCACTG   
  
  
+ AGAAGGATAA ATATTAGATA TGTGATTGGG CCTCAGCCGC CCGCTGGTCA AAGTCACGTG CTTTGACCAA   
  
  
+ ACCACGCGGG CCAAAACACA CACACGCCCA CACACACTCT CTCTCTCTCT CTTTCTTCCC GTATTTTTCA   
  
  
+ CGGTCAAACT TCTCGTTACC TTCGCAGAAA CTGCCTTGCA AAGAAAAAAC ACACTCTCTC TCTCTCTCTT   
  
  
+ TCTTCCCGTA TTTTTCACGG TCAAACTTCT CGTTACCTTC GCAGAAACTG CCTTGCAAAG AAAAAGAAGA   
  
  
+ AAACTAGAAA AAAAAGAGAG AAAAACCAAA GAAGCAGAGA CGCCTTGTAC CTTACGTGAC CGTCTTCTCC   
  
  
+ GTTCTCTCTC TCTCTCTCTC CCCCCCTTGA TCCTTATATT CATCGTATAA GCTGTCTTCT TTCTGTACCT   
  
  
+ GTAACCTTAG TTCTCTCTCT GTCGCTTTT  

- TAATAGCGTG AAATAGGTGA TTCTTGGATT AATGGGTTGA TCACCCAATT GAACATCCGA TTCGAATGTA   
  
  
- TAGACGAATA CAAGAGAGGT AAAACACACT TTTGCACATC TGAATTAGAT TGGTTTAGTA CTAACACTGA   
  
  
- AACTAATACT TTATTTTCCA GATACCAAAT TAATTTAATT AATGTTATAC TGCATTTGAT TTGTATATGG   
  
  
- AAATTACCGA GACCTCGAAA CACAGTAAAA AATAAAGGTG CTAGTAATCC CGTTTTTAAC CAACTCTTTA   
  
  
- ATATTAAATC TCATATTCTA TGAGACCGAT CGAAAACCTA AAGTAACTGA TTTTTCCGTA ATGGGGAAAA   
  
  
- TGTGAAATCC TGAGGATCTT AGAAGATTCG ATCTGAATTA ATATAGAAAA TTATTGAAAA AATTACGAAC   
  
  
- AAACTGTGAT AACGTACAAT CGGAGATTCG AAATAAAATA GCTCTAGGTA TATTTACAAA AAAAAACAAA   
  
  
- AAAGGCCAAA ATGTAACTCT ATTTTTAGTA AATGATTAAA CTTTAGTTTA ACTAAAAAAG TAATTTTTTA   
  
  
- AATTACCAAT AAAAATATTA AATTTTATTG TTATATTTTA TTTTTTTAAT TAATAACTAA AATAATTTAA   
  
  
- CACCACGCAA ATAAACTTTC ACATATACAG TATAAAGTAT TAAAATGTAA TATTTATTTT TATATATTTT   
  
  
- ATGTATATTA ATCAACACAA TAAAGACCTG TAGTGAATGT TTTACATTTA TACTTTGTTT TTAAAACGCG   
  
  
- TAAATTTTAT ATATTTTATG TATATTAATC AACACAATAA AGACCTGTAG TGAATGTTTT ACATTTATAC   
  
  
- TTTGTTTTTA AAACGCGTAA ATTCATTAAT AATAATTGAT AAAAATTACT AATATTCTAT TTATGTAAAT   
  
  
- AAATTAATTA TTAAAAAATC TTCTTTATAT AAATTAGATT TGAAAATACC CTATACGTAC CTACCGTACT   
  
  
- TTAAACGTGA ACAGTATACG GAGGGAGGAT GTGATTTCGG TTATCCGTAG TCGTCGGTAA ATGCCGTGAC   
  
  
- TCTTCCTATT TATAATCTAT ACACTAACCC GGAGTCGGCG GGCGACCAGT TTCAGTGCAC GAAACTGGTT   
  
  
- TGGTGCGCCC GGTTTTGTGT GTGTGCGGGT GTGTGTGAGA GAGAGAGAGA GAAAGAAGGG CATAAAAAGT   
  
  
- GCCAGTTTGA AGAGCAATGG AAGCGTCTTT GACGGAACGT TTCTTTTTTG TGTGAGAGAG AGAGAGAGAA   
  
  
- AGAAGGGCAT AAAAAGTGCC AGTTTGAAGA GCAATGGAAG CGTCTTTGAC GGAACGTTTC TTTTTCTTCT   
  
  
- TTTGATCTTT TTTTTCTCTC TTTTTGGTTT CTTCGTCTCT GCGGAACATG GAATGCACTG GCAGAAGAGG   
  
  
- CAAGAGAGAG AGAGAGAGAG GGGGGGAACT AGGAATATAA GTAGCATATT CGACAGAAGA AAGACATGGA   
  
  
- CATTGGAATC AAGAGAGAGA CAGCGAAAA

+     circadian

| Site Name | Organism | Position | Strand | Matrix score. | sequence | function |
| --- | --- | --- | --- | --- | --- | --- |
| circadian | Lycopersicon esculentum | 318 | - | 6 | CAANNNNATC | cis-acting regulatory element involved in circadian control |

> 2018/04/13 10:10:12  
+ ATTATCGCAC TTTATCCACT AAGAACCTAA TTACCCAACT AGTGGGTTAA CTTGTAGGCT AAGCTTACAT   
  
  
+ ATCTGCTTAT GTTCTCTCCA TTTTGTGTGA AAACGTGTAG ACTTAATCTA ACCAAATCAT GATTGTGACT   
  
  
+ TTGATTATGA AATAAAAGGT CTATGGTTTA ATTAAATTAA TTACAATATG ACGTAAACTA AACATATACC   
  
  
+ TTTAATGGCT CTGGAGCTTT GTGTCATTTT TTATTTCCAC GATCATTAGG GCAAAAATTG GTTGAGAAAT   
  
  
+ TATAATTTAG AGTATAAGAT ACTCTGGCTA GCTTTTGGAT TTCATTGACT AAAAAGGCAT TACCCCTTTT   
  
  
+ ACACTTTAGG ACTCCTAGAA TCTTCTAAGC TAGACTTAAT TATATCTTTT AATAACTTTT TTAATGCTTG   
  
  
+ TTTGACACTA TTGCATGTTA GCCTCTAAGC TTTATTTTAT CGAGATCCAT ATAAATGTTT TTTTTTGTTT   
  
  
+ TTTCCGGTTT TACATTGAGA TAAAAATCAT TTACTAATTT GAAATCAAAT TGATTTTTTC ATTAAAAAAT   
  
  
+ TTAATGGTTA TTTTTATAAT TTAAAATAAC AATATAAAAT AAAAAAATTA ATTATTGATT TTATTAAATT   
  
  
+ GTGGTGCGTT TATTTGAAAG TGTATATGTC ATATTTCATA ATTTTACATT ATAAATAAAA ATATATAAAA   
  
  
+ TACATATAAT TAGTTGTGTT ATTTCTGGAC ATCACTTACA AAATGTAAAT ATGAAACAAA AATTTTGCGC   
  
  
+ ATTTAAAATA TATAAAATAC ATATAATTAG TTGTGTTATT TCTGGACATC ACTTACAAAA TGTAAATATG   
  
  
+ AAACAAAAAT TTTGCGCATT TAAGTAATTA TTATTAACTA TTTTTAATGA TTATAAGATA AATACATTTA   
  
  
+ TTTAATTAAT AATTTTTTAG AAGAAATATA TTTAATCTAA ACTTTTATGG GATATGCATG GATGGCATGA   
  
  
+ AATTTGCACT TGTCATATGC CTCCCTCCTA CACTAAAGCC AATAGGCATC AGCAGCCATT TACGGCACTG   
  
  
+ AGAAGGATAA ATATTAGATA TGTGATTGGG CCTCAGCCGC CCGCTGGTCA AAGTCACGTG CTTTGACCAA   
  
  
+ ACCACGCGGG CCAAAACACA CACACGCCCA CACACACTCT CTCTCTCTCT CTTTCTTCCC GTATTTTTCA   
  
  
+ CGGTCAAACT TCTCGTTACC TTCGCAGAAA CTGCCTTGCA AAGAAAAAAC ACACTCTCTC TCTCTCTCTT   
  
  
+ TCTTCCCGTA TTTTTCACGG TCAAACTTCT CGTTACCTTC GCAGAAACTG CCTTGCAAAG AAAAAGAAGA   
  
  
+ AAACTAGAAA AAAAAGAGAG AAAAACCAAA GAAGCAGAGA CGCCTTGTAC CTTACGTGAC CGTCTTCTCC   
  
  
+ GTTCTCTCTC TCTCTCTCTC CCCCCCTTGA TCCTTATATT CATCGTATAA GCTGTCTTCT TTCTGTACCT   
  
  
+ GTAACCTTAG TTCTCTCTCT GTCGCTTTT  

- TAATAGCGTG AAATAGGTGA TTCTTGGATT AATGGGTTGA TCACCCAATT GAACATCCGA TTCGAATGTA   
  
  
- TAGACGAATA CAAGAGAGGT AAAACACACT TTTGCACATC TGAATTAGAT TGGTTTAGTA CTAACACTGA   
  
  
- AACTAATACT TTATTTTCCA GATACCAAAT TAATTTAATT AATGTTATAC TGCATTTGAT TTGTATATGG   
  
  
- AAATTACCGA GACCTCGAAA CACAGTAAAA AATAAAGGTG CTAGTAATCC CGTTTTTAAC CAACTCTTTA   
  
  
- ATATTAAATC TCATATTCTA TGAGACCGAT CGAAAACCTA AAGTAACTGA TTTTTCCGTA ATGGGGAAAA   
  
  
- TGTGAAATCC TGAGGATCTT AGAAGATTCG ATCTGAATTA ATATAGAAAA TTATTGAAAA AATTACGAAC   
  
  
- AAACTGTGAT AACGTACAAT CGGAGATTCG AAATAAAATA GCTCTAGGTA TATTTACAAA AAAAAACAAA   
  
  
- AAAGGCCAAA ATGTAACTCT ATTTTTAGTA AATGATTAAA CTTTAGTTTA ACTAAAAAAG TAATTTTTTA   
  
  
- AATTACCAAT AAAAATATTA AATTTTATTG TTATATTTTA TTTTTTTAAT TAATAACTAA AATAATTTAA   
  
  
- CACCACGCAA ATAAACTTTC ACATATACAG TATAAAGTAT TAAAATGTAA TATTTATTTT TATATATTTT   
  
  
- ATGTATATTA ATCAACACAA TAAAGACCTG TAGTGAATGT TTTACATTTA TACTTTGTTT TTAAAACGCG   
  
  
- TAAATTTTAT ATATTTTATG TATATTAATC AACACAATAA AGACCTGTAG TGAATGTTTT ACATTTATAC   
  
  
- TTTGTTTTTA AAACGCGTAA ATTCATTAAT AATAATTGAT AAAAATTACT AATATTCTAT TTATGTAAAT   
  
  
- AAATTAATTA TTAAAAAATC TTCTTTATAT AAATTAGATT TGAAAATACC CTATACGTAC CTACCGTACT   
  
  
- TTAAACGTGA ACAGTATACG GAGGGAGGAT GTGATTTCGG TTATCCGTAG TCGTCGGTAA ATGCCGTGAC   
  
  
- TCTTCCTATT TATAATCTAT ACACTAACCC GGAGTCGGCG GGCGACCAGT TTCAGTGCAC GAAACTGGTT   
  
  
- TGGTGCGCCC GGTTTTGTGT GTGTGCGGGT GTGTGTGAGA GAGAGAGAGA GAAAGAAGGG CATAAAAAGT   
  
  
- GCCAGTTTGA AGAGCAATGG AAGCGTCTTT GACGGAACGT TTCTTTTTTG TGTGAGAGAG AGAGAGAGAA   
  
  
- AGAAGGGCAT AAAAAGTGCC AGTTTGAAGA GCAATGGAAG CGTCTTTGAC GGAACGTTTC TTTTTCTTCT   
  
  
- TTTGATCTTT TTTTTCTCTC TTTTTGGTTT CTTCGTCTCT GCGGAACATG GAATGCACTG GCAGAAGAGG   
  
  
- CAAGAGAGAG AGAGAGAGAG GGGGGGAACT AGGAATATAA GTAGCATATT CGACAGAAGA AAGACATGGA   
  
  
- CATTGGAATC AAGAGAGAGA CAGCGAAAA
