# Supplementary material for: Genome-wide analysis of the WRKY gene family in drumstick (Moringa oleifera Lam.)
Source: PeerJ. 2019 Jun 10;7:e7063. doi: 10.7717/peerj.7063 (PMC6563795; doi:10.7717/peerj.7063)
Supplement: Supplemental Information 1 [file peerj-07-7063-s003.gz › MoWRKY50_plantcare.html]

Content-Type: text/html; charset=ISO-8859-1


CallMat\_Firefox


Webmaster Firefox specific output  
To save the result:
click on the frame with the right mouse button and save the source code as a text file with extension .html  
REFERENCE:PlantCARE: a database of plant cis-acting regulatory elements and a portal to tools for in silico analysis of promoter sequences.  
Lescot, M., Déhais, P., Moreau, Y., De Moor, B., Rouzé ,P.,and Rombauts, S.  
Nucleic Acids Res., Database issue(2002), 30(1):325-327.   


---

> 2018/04/13 10:10:12  
+ CCAAAGCCAT TCCATCGACT TTAATCCCAA TCCCAAACGT TCCATTAGGT AACGGTCTTT CGTTCTCCTC   
  
  
+ AACCTGTACC ATCGCACTCT CTACCCATGA AAACATACAA TCCGAACGAG GGTGATATAT ATGTTTCTTA   
  
  
+ ATTTTTATGA ATTATTTTCT TATTTCTGTA ATATTTTAAT TTTTATAATA GAAGTAAAAC TTGACGACGA   
  
  
+ AGTACCTGTG GAAGGGCCTC CCCATGATGC TTCGATGGAA CACCACGGTG CACACACCGG GTGTACGATA   
  
  
+ CTGCAGTAGC ATTACTGCAT CCGGACCGAG TGAACTTTCG GTATGACCGG GTGAACTCAT TATTAACGGG   
  
  
+ CTACAGAACA AAAACTATCC GCCTCCATGA AAACACCCTG GTGTTGCGGA ACCTAAATGA GGATTATTCA   
  
  
+ GGACAGACAC TAGTGTTAAG GACTATGGCA GTCTACTAGA GCCACCAGAG TAAGTTCGTA TTTTAATAAA   
  
  
+ AATAATATTT TAATTTTAGA AGGTGGTCTT ATTTTTACAA GTCACTCATA CCCTTATAAA CATCTAAATA   
  
  
+ TAATAAAAAA ATTAATGAAG ATCAAATTTT TATAAACTTA AAATGTTATT ATACTACCAA ATTTATTTCT   
  
  
+ AAAATAGTAT TTCATTGCAT ACAATTAAAC AAATTAATAC TAGCATATTA AAATAAATTA ATTTTTTGTA   
  
  
+ AAGTTTTTGG TAGGGTAGTT CTATATATTA CCGTGCTATT TTTAATCAAC TTCACCTTTG TAAAATTCAT   
  
  
+ GTGAATAGTT AACCTGTGTC GATATTTTTA CTTAAATATT CTAGGGTTGG TTATTGAAAT TTTTAATTAT   
  
  
+ TAAAAAATTA TTAAATATTA TTTAATACAT ATAGAAAATT AATTTAATAA AATGTCAATA AAAAAACCTT   
  
  
+ TACTTATTTT TTTCCTCTTT AATTTCAACA ACTTAACGTT TAAATTACAA CACACCTAAC TTATCCGTCG   
  
  
+ ACGGTTCTCT CAGTTACTCA GTTGTTTATA CTAACTTAGT TTATTTAATC ATCAACCCCG CTCCACATTA   
  
  
+ ATTCGGTTAA TTTTGGAAGC AGTTTGTCAC AGTAGGAATC ATTTATACTG AGTTAACTAT TACCAGAACT   
  
  
+ AGTACTTAAC ACATAAATCT ATTACGCGTT ATATACTAAC GTAAACTGTT TAATTAATTC TATCTACAAA   
  
  
+ GTTCAATTCG TATCACAAAC TGACTGAATC AATTAAGTTA ATTCAAGGCA CTGAATCTCT AGTTTAATAT   
  
  
+ TAGTAGTGAG TGTCATGCGC TTTACTTTAT GTATTATAAT GTAATGAGAT TTGGGTCTTA ATGGGGGGGT   
  
  
+ TTTTTTTTTG TGTGTTTTTT TTTTCCACCG AATAGGAATT TTTTTTTTAG AAGTTCATTT TACAGAATAA   
  
  
+ ATTTTTATTC TTTTTCTGTT TTTTTTTCTG TTTCCCGCCC TTTCCCCAGT TTTAACATTC TAATGAAATT   
  
  
+ AGTGTAGTAC AGTATCGGTT TAACACAAA  

- GGTTTCGGTA AGGTAGCTGA AATTAGGGTT AGGGTTTGCA AGGTAATCCA TTGCCAGAAA GCAAGAGGAG   
  
  
- TTGGACATGG TAGCGTGAGA GATGGGTACT TTTGTATGTT AGGCTTGCTC CCACTATATA TACAAAGAAT   
  
  
- TAAAAATACT TAATAAAAGA ATAAAGACAT TATAAAATTA AAAATATTAT CTTCATTTTG AACTGCTGCT   
  
  
- TCATGGACAC CTTCCCGGAG GGGTACTACG AAGCTACCTT GTGGTGCCAC GTGTGTGGCC CACATGCTAT   
  
  
- GACGTCATCG TAATGACGTA GGCCTGGCTC ACTTGAAAGC CATACTGGCC CACTTGAGTA ATAATTGCCC   
  
  
- GATGTCTTGT TTTTGATAGG CGGAGGTACT TTTGTGGGAC CACAACGCCT TGGATTTACT CCTAATAAGT   
  
  
- CCTGTCTGTG ATCACAATTC CTGATACCGT CAGATGATCT CGGTGGTCTC ATTCAAGCAT AAAATTATTT   
  
  
- TTATTATAAA ATTAAAATCT TCCACCAGAA TAAAAATGTT CAGTGAGTAT GGGAATATTT GTAGATTTAT   
  
  
- ATTATTTTTT TAATTACTTC TAGTTTAAAA ATATTTGAAT TTTACAATAA TATGATGGTT TAAATAAAGA   
  
  
- TTTTATCATA AAGTAACGTA TGTTAATTTG TTTAATTATG ATCGTATAAT TTTATTTAAT TAAAAAACAT   
  
  
- TTCAAAAACC ATCCCATCAA GATATATAAT GGCACGATAA AAATTAGTTG AAGTGGAAAC ATTTTAAGTA   
  
  
- CACTTATCAA TTGGACACAG CTATAAAAAT GAATTTATAA GATCCCAACC AATAACTTTA AAAATTAATA   
  
  
- ATTTTTTAAT AATTTATAAT AAATTATGTA TATCTTTTAA TTAAATTATT TTACAGTTAT TTTTTTGGAA   
  
  
- ATGAATAAAA AAAGGAGAAA TTAAAGTTGT TGAATTGCAA ATTTAATGTT GTGTGGATTG AATAGGCAGC   
  
  
- TGCCAAGAGA GTCAATGAGT CAACAAATAT GATTGAATCA AATAAATTAG TAGTTGGGGC GAGGTGTAAT   
  
  
- TAAGCCAATT AAAACCTTCG TCAAACAGTG TCATCCTTAG TAAATATGAC TCAATTGATA ATGGTCTTGA   
  
  
- TCATGAATTG TGTATTTAGA TAATGCGCAA TATATGATTG CATTTGACAA ATTAATTAAG ATAGATGTTT   
  
  
- CAAGTTAAGC ATAGTGTTTG ACTGACTTAG TTAATTCAAT TAAGTTCCGT GACTTAGAGA TCAAATTATA   
  
  
- ATCATCACTC ACAGTACGCG AAATGAAATA CATAATATTA CATTACTCTA AACCCAGAAT TACCCCCCCA   
  
  
- AAAAAAAAAC ACACAAAAAA AAAAGGTGGC TTATCCTTAA AAAAAAAATC TTCAAGTAAA ATGTCTTATT   
  
  
- TAAAAATAAG AAAAAGACAA AAAAAAAGAC AAAGGGCGGG AAAGGGGTCA AAATTGTAAG ATTACTTTAA   
  
  
- TCACATCATG TCATAGCCAA ATTGTGTTT

  
  
Motifs Found  

+     ACE

| Site Name | Organism | Position | Strand | Matrix score. | sequence | function |
| --- | --- | --- | --- | --- | --- | --- |
| ACE | Petroselinum crispum | 903 | + | 9 | AAAACGTTTA | cis-acting element involved in light responsiveness |

> 2018/04/13 10:10:12  
+ CCAAAGCCAT TCCATCGACT TTAATCCCAA TCCCAAACGT TCCATTAGGT AACGGTCTTT CGTTCTCCTC   
  
  
+ AACCTGTACC ATCGCACTCT CTACCCATGA AAACATACAA TCCGAACGAG GGTGATATAT ATGTTTCTTA   
  
  
+ ATTTTTATGA ATTATTTTCT TATTTCTGTA ATATTTTAAT TTTTATAATA GAAGTAAAAC TTGACGACGA   
  
  
+ AGTACCTGTG GAAGGGCCTC CCCATGATGC TTCGATGGAA CACCACGGTG CACACACCGG GTGTACGATA   
  
  
+ CTGCAGTAGC ATTACTGCAT CCGGACCGAG TGAACTTTCG GTATGACCGG GTGAACTCAT TATTAACGGG   
  
  
+ CTACAGAACA AAAACTATCC GCCTCCATGA AAACACCCTG GTGTTGCGGA ACCTAAATGA GGATTATTCA   
  
  
+ GGACAGACAC TAGTGTTAAG GACTATGGCA GTCTACTAGA GCCACCAGAG TAAGTTCGTA TTTTAATAAA   
  
  
+ AATAATATTT TAATTTTAGA AGGTGGTCTT ATTTTTACAA GTCACTCATA CCCTTATAAA CATCTAAATA   
  
  
+ TAATAAAAAA ATTAATGAAG ATCAAATTTT TATAAACTTA AAATGTTATT ATACTACCAA ATTTATTTCT   
  
  
+ AAAATAGTAT TTCATTGCAT ACAATTAAAC AAATTAATAC TAGCATATTA AAATAAATTA ATTTTTTGTA   
  
  
+ AAGTTTTTGG TAGGGTAGTT CTATATATTA CCGTGCTATT TTTAATCAAC TTCACCTTTG TAAAATTCAT   
  
  
+ GTGAATAGTT AACCTGTGTC GATATTTTTA CTTAAATATT CTAGGGTTGG TTATTGAAAT TTTTAATTAT   
  
  
+ TAAAAAATTA TTAAATATTA TTTAATACAT ATAGAAAATT AATTTAATAA AATGTCAATA AAAAAACCTT   
  
  
+ TACTTATTTT TTTCCTCTTT AATTTCAACA ACTTAACGTT TAAATTACAA CACACCTAAC TTATCCGTCG   
  
  
+ ACGGTTCTCT CAGTTACTCA GTTGTTTATA CTAACTTAGT TTATTTAATC ATCAACCCCG CTCCACATTA   
  
  
+ ATTCGGTTAA TTTTGGAAGC AGTTTGTCAC AGTAGGAATC ATTTATACTG AGTTAACTAT TACCAGAACT   
  
  
+ AGTACTTAAC ACATAAATCT ATTACGCGTT ATATACTAAC GTAAACTGTT TAATTAATTC TATCTACAAA   
  
  
+ GTTCAATTCG TATCACAAAC TGACTGAATC AATTAAGTTA ATTCAAGGCA CTGAATCTCT AGTTTAATAT   
  
  
+ TAGTAGTGAG TGTCATGCGC TTTACTTTAT GTATTATAAT GTAATGAGAT TTGGGTCTTA ATGGGGGGGT   
  
  
+ TTTTTTTTTG TGTGTTTTTT TTTTCCACCG AATAGGAATT TTTTTTTTAG AAGTTCATTT TACAGAATAA   
  
  
+ ATTTTTATTC TTTTTCTGTT TTTTTTTCTG TTTCCCGCCC TTTCCCCAGT TTTAACATTC TAATGAAATT   
  
  
+ AGTGTAGTAC AGTATCGGTT TAACACAAA  

- GGTTTCGGTA AGGTAGCTGA AATTAGGGTT AGGGTTTGCA AGGTAATCCA TTGCCAGAAA GCAAGAGGAG   
  
  
- TTGGACATGG TAGCGTGAGA GATGGGTACT TTTGTATGTT AGGCTTGCTC CCACTATATA TACAAAGAAT   
  
  
- TAAAAATACT TAATAAAAGA ATAAAGACAT TATAAAATTA AAAATATTAT CTTCATTTTG AACTGCTGCT   
  
  
- TCATGGACAC CTTCCCGGAG GGGTACTACG AAGCTACCTT GTGGTGCCAC GTGTGTGGCC CACATGCTAT   
  
  
- GACGTCATCG TAATGACGTA GGCCTGGCTC ACTTGAAAGC CATACTGGCC CACTTGAGTA ATAATTGCCC   
  
  
- GATGTCTTGT TTTTGATAGG CGGAGGTACT TTTGTGGGAC CACAACGCCT TGGATTTACT CCTAATAAGT   
  
  
- CCTGTCTGTG ATCACAATTC CTGATACCGT CAGATGATCT CGGTGGTCTC ATTCAAGCAT AAAATTATTT   
  
  
- TTATTATAAA ATTAAAATCT TCCACCAGAA TAAAAATGTT CAGTGAGTAT GGGAATATTT GTAGATTTAT   
  
  
- ATTATTTTTT TAATTACTTC TAGTTTAAAA ATATTTGAAT TTTACAATAA TATGATGGTT TAAATAAAGA   
  
  
- TTTTATCATA AAGTAACGTA TGTTAATTTG TTTAATTATG ATCGTATAAT TTTATTTAAT TAAAAAACAT   
  
  
- TTCAAAAACC ATCCCATCAA GATATATAAT GGCACGATAA AAATTAGTTG AAGTGGAAAC ATTTTAAGTA   
  
  
- CACTTATCAA TTGGACACAG CTATAAAAAT GAATTTATAA GATCCCAACC AATAACTTTA AAAATTAATA   
  
  
- ATTTTTTAAT AATTTATAAT AAATTATGTA TATCTTTTAA TTAAATTATT TTACAGTTAT TTTTTTGGAA   
  
  
- ATGAATAAAA AAAGGAGAAA TTAAAGTTGT TGAATTGCAA ATTTAATGTT GTGTGGATTG AATAGGCAGC   
  
  
- TGCCAAGAGA GTCAATGAGT CAACAAATAT GATTGAATCA AATAAATTAG TAGTTGGGGC GAGGTGTAAT   
  
  
- TAAGCCAATT AAAACCTTCG TCAAACAGTG TCATCCTTAG TAAATATGAC TCAATTGATA ATGGTCTTGA   
  
  
- TCATGAATTG TGTATTTAGA TAATGCGCAA TATATGATTG CATTTGACAA ATTAATTAAG ATAGATGTTT   
  
  
- CAAGTTAAGC ATAGTGTTTG ACTGACTTAG TTAATTCAAT TAAGTTCCGT GACTTAGAGA TCAAATTATA   
  
  
- ATCATCACTC ACAGTACGCG AAATGAAATA CATAATATTA CATTACTCTA AACCCAGAAT TACCCCCCCA   
  
  
- AAAAAAAAAC ACACAAAAAA AAAAGGTGGC TTATCCTTAA AAAAAAAATC TTCAAGTAAA ATGTCTTATT   
  
  
- TAAAAATAAG AAAAAGACAA AAAAAAAGAC AAAGGGCGGG AAAGGGGTCA AAATTGTAAG ATTACTTTAA   
  
  
- TCACATCATG TCATAGCCAA ATTGTGTTT

+     AE-box

| Site Name | Organism | Position | Strand | Matrix score. | sequence | function |
| --- | --- | --- | --- | --- | --- | --- |
| AE-box | Arabidopsis thaliana | 131 | - | 8 | AGAAACAT | part of a module for light response |

> 2018/04/13 10:10:12  
+ CCAAAGCCAT TCCATCGACT TTAATCCCAA TCCCAAACGT TCCATTAGGT AACGGTCTTT CGTTCTCCTC   
  
  
+ AACCTGTACC ATCGCACTCT CTACCCATGA AAACATACAA TCCGAACGAG GGTGATATAT ATGTTTCTTA   
  
  
+ ATTTTTATGA ATTATTTTCT TATTTCTGTA ATATTTTAAT TTTTATAATA GAAGTAAAAC TTGACGACGA   
  
  
+ AGTACCTGTG GAAGGGCCTC CCCATGATGC TTCGATGGAA CACCACGGTG CACACACCGG GTGTACGATA   
  
  
+ CTGCAGTAGC ATTACTGCAT CCGGACCGAG TGAACTTTCG GTATGACCGG GTGAACTCAT TATTAACGGG   
  
  
+ CTACAGAACA AAAACTATCC GCCTCCATGA AAACACCCTG GTGTTGCGGA ACCTAAATGA GGATTATTCA   
  
  
+ GGACAGACAC TAGTGTTAAG GACTATGGCA GTCTACTAGA GCCACCAGAG TAAGTTCGTA TTTTAATAAA   
  
  
+ AATAATATTT TAATTTTAGA AGGTGGTCTT ATTTTTACAA GTCACTCATA CCCTTATAAA CATCTAAATA   
  
  
+ TAATAAAAAA ATTAATGAAG ATCAAATTTT TATAAACTTA AAATGTTATT ATACTACCAA ATTTATTTCT   
  
  
+ AAAATAGTAT TTCATTGCAT ACAATTAAAC AAATTAATAC TAGCATATTA AAATAAATTA ATTTTTTGTA   
  
  
+ AAGTTTTTGG TAGGGTAGTT CTATATATTA CCGTGCTATT TTTAATCAAC TTCACCTTTG TAAAATTCAT   
  
  
+ GTGAATAGTT AACCTGTGTC GATATTTTTA CTTAAATATT CTAGGGTTGG TTATTGAAAT TTTTAATTAT   
  
  
+ TAAAAAATTA TTAAATATTA TTTAATACAT ATAGAAAATT AATTTAATAA AATGTCAATA AAAAAACCTT   
  
  
+ TACTTATTTT TTTCCTCTTT AATTTCAACA ACTTAACGTT TAAATTACAA CACACCTAAC TTATCCGTCG   
  
  
+ ACGGTTCTCT CAGTTACTCA GTTGTTTATA CTAACTTAGT TTATTTAATC ATCAACCCCG CTCCACATTA   
  
  
+ ATTCGGTTAA TTTTGGAAGC AGTTTGTCAC AGTAGGAATC ATTTATACTG AGTTAACTAT TACCAGAACT   
  
  
+ AGTACTTAAC ACATAAATCT ATTACGCGTT ATATACTAAC GTAAACTGTT TAATTAATTC TATCTACAAA   
  
  
+ GTTCAATTCG TATCACAAAC TGACTGAATC AATTAAGTTA ATTCAAGGCA CTGAATCTCT AGTTTAATAT   
  
  
+ TAGTAGTGAG TGTCATGCGC TTTACTTTAT GTATTATAAT GTAATGAGAT TTGGGTCTTA ATGGGGGGGT   
  
  
+ TTTTTTTTTG TGTGTTTTTT TTTTCCACCG AATAGGAATT TTTTTTTTAG AAGTTCATTT TACAGAATAA   
  
  
+ ATTTTTATTC TTTTTCTGTT TTTTTTTCTG TTTCCCGCCC TTTCCCCAGT TTTAACATTC TAATGAAATT   
  
  
+ AGTGTAGTAC AGTATCGGTT TAACACAAA  

- GGTTTCGGTA AGGTAGCTGA AATTAGGGTT AGGGTTTGCA AGGTAATCCA TTGCCAGAAA GCAAGAGGAG   
  
  
- TTGGACATGG TAGCGTGAGA GATGGGTACT TTTGTATGTT AGGCTTGCTC CCACTATATA TACAAAGAAT   
  
  
- TAAAAATACT TAATAAAAGA ATAAAGACAT TATAAAATTA AAAATATTAT CTTCATTTTG AACTGCTGCT   
  
  
- TCATGGACAC CTTCCCGGAG GGGTACTACG AAGCTACCTT GTGGTGCCAC GTGTGTGGCC CACATGCTAT   
  
  
- GACGTCATCG TAATGACGTA GGCCTGGCTC ACTTGAAAGC CATACTGGCC CACTTGAGTA ATAATTGCCC   
  
  
- GATGTCTTGT TTTTGATAGG CGGAGGTACT TTTGTGGGAC CACAACGCCT TGGATTTACT CCTAATAAGT   
  
  
- CCTGTCTGTG ATCACAATTC CTGATACCGT CAGATGATCT CGGTGGTCTC ATTCAAGCAT AAAATTATTT   
  
  
- TTATTATAAA ATTAAAATCT TCCACCAGAA TAAAAATGTT CAGTGAGTAT GGGAATATTT GTAGATTTAT   
  
  
- ATTATTTTTT TAATTACTTC TAGTTTAAAA ATATTTGAAT TTTACAATAA TATGATGGTT TAAATAAAGA   
  
  
- TTTTATCATA AAGTAACGTA TGTTAATTTG TTTAATTATG ATCGTATAAT TTTATTTAAT TAAAAAACAT   
  
  
- TTCAAAAACC ATCCCATCAA GATATATAAT GGCACGATAA AAATTAGTTG AAGTGGAAAC ATTTTAAGTA   
  
  
- CACTTATCAA TTGGACACAG CTATAAAAAT GAATTTATAA GATCCCAACC AATAACTTTA AAAATTAATA   
  
  
- ATTTTTTAAT AATTTATAAT AAATTATGTA TATCTTTTAA TTAAATTATT TTACAGTTAT TTTTTTGGAA   
  
  
- ATGAATAAAA AAAGGAGAAA TTAAAGTTGT TGAATTGCAA ATTTAATGTT GTGTGGATTG AATAGGCAGC   
  
  
- TGCCAAGAGA GTCAATGAGT CAACAAATAT GATTGAATCA AATAAATTAG TAGTTGGGGC GAGGTGTAAT   
  
  
- TAAGCCAATT AAAACCTTCG TCAAACAGTG TCATCCTTAG TAAATATGAC TCAATTGATA ATGGTCTTGA   
  
  
- TCATGAATTG TGTATTTAGA TAATGCGCAA TATATGATTG CATTTGACAA ATTAATTAAG ATAGATGTTT   
  
  
- CAAGTTAAGC ATAGTGTTTG ACTGACTTAG TTAATTCAAT TAAGTTCCGT GACTTAGAGA TCAAATTATA   
  
  
- ATCATCACTC ACAGTACGCG AAATGAAATA CATAATATTA CATTACTCTA AACCCAGAAT TACCCCCCCA   
  
  
- AAAAAAAAAC ACACAAAAAA AAAAGGTGGC TTATCCTTAA AAAAAAAATC TTCAAGTAAA ATGTCTTATT   
  
  
- TAAAAATAAG AAAAAGACAA AAAAAAAGAC AAAGGGCGGG AAAGGGGTCA AAATTGTAAG ATTACTTTAA   
  
  
- TCACATCATG TCATAGCCAA ATTGTGTTT

+     AT1-motif

| Site Name | Organism | Position | Strand | Matrix score. | sequence | function |
| --- | --- | --- | --- | --- | --- | --- |
| AT1-motif | Solanum tuberosum | 759 | - | 11 | ATTAATTTTACA | part of a light responsive module |

> 2018/04/13 10:10:12  
+ CCAAAGCCAT TCCATCGACT TTAATCCCAA TCCCAAACGT TCCATTAGGT AACGGTCTTT CGTTCTCCTC   
  
  
+ AACCTGTACC ATCGCACTCT CTACCCATGA AAACATACAA TCCGAACGAG GGTGATATAT ATGTTTCTTA   
  
  
+ ATTTTTATGA ATTATTTTCT TATTTCTGTA ATATTTTAAT TTTTATAATA GAAGTAAAAC TTGACGACGA   
  
  
+ AGTACCTGTG GAAGGGCCTC CCCATGATGC TTCGATGGAA CACCACGGTG CACACACCGG GTGTACGATA   
  
  
+ CTGCAGTAGC ATTACTGCAT CCGGACCGAG TGAACTTTCG GTATGACCGG GTGAACTCAT TATTAACGGG   
  
  
+ CTACAGAACA AAAACTATCC GCCTCCATGA AAACACCCTG GTGTTGCGGA ACCTAAATGA GGATTATTCA   
  
  
+ GGACAGACAC TAGTGTTAAG GACTATGGCA GTCTACTAGA GCCACCAGAG TAAGTTCGTA TTTTAATAAA   
  
  
+ AATAATATTT TAATTTTAGA AGGTGGTCTT ATTTTTACAA GTCACTCATA CCCTTATAAA CATCTAAATA   
  
  
+ TAATAAAAAA ATTAATGAAG ATCAAATTTT TATAAACTTA AAATGTTATT ATACTACCAA ATTTATTTCT   
  
  
+ AAAATAGTAT TTCATTGCAT ACAATTAAAC AAATTAATAC TAGCATATTA AAATAAATTA ATTTTTTGTA   
  
  
+ AAGTTTTTGG TAGGGTAGTT CTATATATTA CCGTGCTATT TTTAATCAAC TTCACCTTTG TAAAATTCAT   
  
  
+ GTGAATAGTT AACCTGTGTC GATATTTTTA CTTAAATATT CTAGGGTTGG TTATTGAAAT TTTTAATTAT   
  
  
+ TAAAAAATTA TTAAATATTA TTTAATACAT ATAGAAAATT AATTTAATAA AATGTCAATA AAAAAACCTT   
  
  
+ TACTTATTTT TTTCCTCTTT AATTTCAACA ACTTAACGTT TAAATTACAA CACACCTAAC TTATCCGTCG   
  
  
+ ACGGTTCTCT CAGTTACTCA GTTGTTTATA CTAACTTAGT TTATTTAATC ATCAACCCCG CTCCACATTA   
  
  
+ ATTCGGTTAA TTTTGGAAGC AGTTTGTCAC AGTAGGAATC ATTTATACTG AGTTAACTAT TACCAGAACT   
  
  
+ AGTACTTAAC ACATAAATCT ATTACGCGTT ATATACTAAC GTAAACTGTT TAATTAATTC TATCTACAAA   
  
  
+ GTTCAATTCG TATCACAAAC TGACTGAATC AATTAAGTTA ATTCAAGGCA CTGAATCTCT AGTTTAATAT   
  
  
+ TAGTAGTGAG TGTCATGCGC TTTACTTTAT GTATTATAAT GTAATGAGAT TTGGGTCTTA ATGGGGGGGT   
  
  
+ TTTTTTTTTG TGTGTTTTTT TTTTCCACCG AATAGGAATT TTTTTTTTAG AAGTTCATTT TACAGAATAA   
  
  
+ ATTTTTATTC TTTTTCTGTT TTTTTTTCTG TTTCCCGCCC TTTCCCCAGT TTTAACATTC TAATGAAATT   
  
  
+ AGTGTAGTAC AGTATCGGTT TAACACAAA  

- GGTTTCGGTA AGGTAGCTGA AATTAGGGTT AGGGTTTGCA AGGTAATCCA TTGCCAGAAA GCAAGAGGAG   
  
  
- TTGGACATGG TAGCGTGAGA GATGGGTACT TTTGTATGTT AGGCTTGCTC CCACTATATA TACAAAGAAT   
  
  
- TAAAAATACT TAATAAAAGA ATAAAGACAT TATAAAATTA AAAATATTAT CTTCATTTTG AACTGCTGCT   
  
  
- TCATGGACAC CTTCCCGGAG GGGTACTACG AAGCTACCTT GTGGTGCCAC GTGTGTGGCC CACATGCTAT   
  
  
- GACGTCATCG TAATGACGTA GGCCTGGCTC ACTTGAAAGC CATACTGGCC CACTTGAGTA ATAATTGCCC   
  
  
- GATGTCTTGT TTTTGATAGG CGGAGGTACT TTTGTGGGAC CACAACGCCT TGGATTTACT CCTAATAAGT   
  
  
- CCTGTCTGTG ATCACAATTC CTGATACCGT CAGATGATCT CGGTGGTCTC ATTCAAGCAT AAAATTATTT   
  
  
- TTATTATAAA ATTAAAATCT TCCACCAGAA TAAAAATGTT CAGTGAGTAT GGGAATATTT GTAGATTTAT   
  
  
- ATTATTTTTT TAATTACTTC TAGTTTAAAA ATATTTGAAT TTTACAATAA TATGATGGTT TAAATAAAGA   
  
  
- TTTTATCATA AAGTAACGTA TGTTAATTTG TTTAATTATG ATCGTATAAT TTTATTTAAT TAAAAAACAT   
  
  
- TTCAAAAACC ATCCCATCAA GATATATAAT GGCACGATAA AAATTAGTTG AAGTGGAAAC ATTTTAAGTA   
  
  
- CACTTATCAA TTGGACACAG CTATAAAAAT GAATTTATAA GATCCCAACC AATAACTTTA AAAATTAATA   
  
  
- ATTTTTTAAT AATTTATAAT AAATTATGTA TATCTTTTAA TTAAATTATT TTACAGTTAT TTTTTTGGAA   
  
  
- ATGAATAAAA AAAGGAGAAA TTAAAGTTGT TGAATTGCAA ATTTAATGTT GTGTGGATTG AATAGGCAGC   
  
  
- TGCCAAGAGA GTCAATGAGT CAACAAATAT GATTGAATCA AATAAATTAG TAGTTGGGGC GAGGTGTAAT   
  
  
- TAAGCCAATT AAAACCTTCG TCAAACAGTG TCATCCTTAG TAAATATGAC TCAATTGATA ATGGTCTTGA   
  
  
- TCATGAATTG TGTATTTAGA TAATGCGCAA TATATGATTG CATTTGACAA ATTAATTAAG ATAGATGTTT   
  
  
- CAAGTTAAGC ATAGTGTTTG ACTGACTTAG TTAATTCAAT TAAGTTCCGT GACTTAGAGA TCAAATTATA   
  
  
- ATCATCACTC ACAGTACGCG AAATGAAATA CATAATATTA CATTACTCTA AACCCAGAAT TACCCCCCCA   
  
  
- AAAAAAAAAC ACACAAAAAA AAAAGGTGGC TTATCCTTAA AAAAAAAATC TTCAAGTAAA ATGTCTTATT   
  
  
- TAAAAATAAG AAAAAGACAA AAAAAAAGAC AAAGGGCGGG AAAGGGGTCA AAATTGTAAG ATTACTTTAA   
  
  
- TCACATCATG TCATAGCCAA ATTGTGTTT

+     Box 4

| Site Name | Organism | Position | Strand | Matrix score. | sequence | function |
| --- | --- | --- | --- | --- | --- | --- |
| Box 4 | Petroselinum crispum | 1173 | - | 6 | ATTAAT | part of a conserved DNA module involved in light responsiveness |
| Box 4 | Petroselinum crispum | 878 | - | 6 | ATTAAT | part of a conserved DNA module involved in light responsiveness |
| Box 4 | Petroselinum crispum | 1047 | - | 6 | ATTAAT | part of a conserved DNA module involved in light responsiveness |
| Box 4 | Petroselinum crispum | 663 | + | 6 | ATTAAT | part of a conserved DNA module involved in light responsiveness |
| Box 4 | Petroselinum crispum | 571 | + | 6 | ATTAAT | part of a conserved DNA module involved in light responsiveness |
| Box 4 | Petroselinum crispum | 687 | + | 6 | ATTAAT | part of a conserved DNA module involved in light responsiveness |

> 2018/04/13 10:10:12  
+ CCAAAGCCAT TCCATCGACT TTAATCCCAA TCCCAAACGT TCCATTAGGT AACGGTCTTT CGTTCTCCTC   
  
  
+ AACCTGTACC ATCGCACTCT CTACCCATGA AAACATACAA TCCGAACGAG GGTGATATAT ATGTTTCTTA   
  
  
+ ATTTTTATGA ATTATTTTCT TATTTCTGTA ATATTTTAAT TTTTATAATA GAAGTAAAAC TTGACGACGA   
  
  
+ AGTACCTGTG GAAGGGCCTC CCCATGATGC TTCGATGGAA CACCACGGTG CACACACCGG GTGTACGATA   
  
  
+ CTGCAGTAGC ATTACTGCAT CCGGACCGAG TGAACTTTCG GTATGACCGG GTGAACTCAT TATTAACGGG   
  
  
+ CTACAGAACA AAAACTATCC GCCTCCATGA AAACACCCTG GTGTTGCGGA ACCTAAATGA GGATTATTCA   
  
  
+ GGACAGACAC TAGTGTTAAG GACTATGGCA GTCTACTAGA GCCACCAGAG TAAGTTCGTA TTTTAATAAA   
  
  
+ AATAATATTT TAATTTTAGA AGGTGGTCTT ATTTTTACAA GTCACTCATA CCCTTATAAA CATCTAAATA   
  
  
+ TAATAAAAAA ATTAATGAAG ATCAAATTTT TATAAACTTA AAATGTTATT ATACTACCAA ATTTATTTCT   
  
  
+ AAAATAGTAT TTCATTGCAT ACAATTAAAC AAATTAATAC TAGCATATTA AAATAAATTA ATTTTTTGTA   
  
  
+ AAGTTTTTGG TAGGGTAGTT CTATATATTA CCGTGCTATT TTTAATCAAC TTCACCTTTG TAAAATTCAT   
  
  
+ GTGAATAGTT AACCTGTGTC GATATTTTTA CTTAAATATT CTAGGGTTGG TTATTGAAAT TTTTAATTAT   
  
  
+ TAAAAAATTA TTAAATATTA TTTAATACAT ATAGAAAATT AATTTAATAA AATGTCAATA AAAAAACCTT   
  
  
+ TACTTATTTT TTTCCTCTTT AATTTCAACA ACTTAACGTT TAAATTACAA CACACCTAAC TTATCCGTCG   
  
  
+ ACGGTTCTCT CAGTTACTCA GTTGTTTATA CTAACTTAGT TTATTTAATC ATCAACCCCG CTCCACATTA   
  
  
+ ATTCGGTTAA TTTTGGAAGC AGTTTGTCAC AGTAGGAATC ATTTATACTG AGTTAACTAT TACCAGAACT   
  
  
+ AGTACTTAAC ACATAAATCT ATTACGCGTT ATATACTAAC GTAAACTGTT TAATTAATTC TATCTACAAA   
  
  
+ GTTCAATTCG TATCACAAAC TGACTGAATC AATTAAGTTA ATTCAAGGCA CTGAATCTCT AGTTTAATAT   
  
  
+ TAGTAGTGAG TGTCATGCGC TTTACTTTAT GTATTATAAT GTAATGAGAT TTGGGTCTTA ATGGGGGGGT   
  
  
+ TTTTTTTTTG TGTGTTTTTT TTTTCCACCG AATAGGAATT TTTTTTTTAG AAGTTCATTT TACAGAATAA   
  
  
+ ATTTTTATTC TTTTTCTGTT TTTTTTTCTG TTTCCCGCCC TTTCCCCAGT TTTAACATTC TAATGAAATT   
  
  
+ AGTGTAGTAC AGTATCGGTT TAACACAAA  

- GGTTTCGGTA AGGTAGCTGA AATTAGGGTT AGGGTTTGCA AGGTAATCCA TTGCCAGAAA GCAAGAGGAG   
  
  
- TTGGACATGG TAGCGTGAGA GATGGGTACT TTTGTATGTT AGGCTTGCTC CCACTATATA TACAAAGAAT   
  
  
- TAAAAATACT TAATAAAAGA ATAAAGACAT TATAAAATTA AAAATATTAT CTTCATTTTG AACTGCTGCT   
  
  
- TCATGGACAC CTTCCCGGAG GGGTACTACG AAGCTACCTT GTGGTGCCAC GTGTGTGGCC CACATGCTAT   
  
  
- GACGTCATCG TAATGACGTA GGCCTGGCTC ACTTGAAAGC CATACTGGCC CACTTGAGTA ATAATTGCCC   
  
  
- GATGTCTTGT TTTTGATAGG CGGAGGTACT TTTGTGGGAC CACAACGCCT TGGATTTACT CCTAATAAGT   
  
  
- CCTGTCTGTG ATCACAATTC CTGATACCGT CAGATGATCT CGGTGGTCTC ATTCAAGCAT AAAATTATTT   
  
  
- TTATTATAAA ATTAAAATCT TCCACCAGAA TAAAAATGTT CAGTGAGTAT GGGAATATTT GTAGATTTAT   
  
  
- ATTATTTTTT TAATTACTTC TAGTTTAAAA ATATTTGAAT TTTACAATAA TATGATGGTT TAAATAAAGA   
  
  
- TTTTATCATA AAGTAACGTA TGTTAATTTG TTTAATTATG ATCGTATAAT TTTATTTAAT TAAAAAACAT   
  
  
- TTCAAAAACC ATCCCATCAA GATATATAAT GGCACGATAA AAATTAGTTG AAGTGGAAAC ATTTTAAGTA   
  
  
- CACTTATCAA TTGGACACAG CTATAAAAAT GAATTTATAA GATCCCAACC AATAACTTTA AAAATTAATA   
  
  
- ATTTTTTAAT AATTTATAAT AAATTATGTA TATCTTTTAA TTAAATTATT TTACAGTTAT TTTTTTGGAA   
  
  
- ATGAATAAAA AAAGGAGAAA TTAAAGTTGT TGAATTGCAA ATTTAATGTT GTGTGGATTG AATAGGCAGC   
  
  
- TGCCAAGAGA GTCAATGAGT CAACAAATAT GATTGAATCA AATAAATTAG TAGTTGGGGC GAGGTGTAAT   
  
  
- TAAGCCAATT AAAACCTTCG TCAAACAGTG TCATCCTTAG TAAATATGAC TCAATTGATA ATGGTCTTGA   
  
  
- TCATGAATTG TGTATTTAGA TAATGCGCAA TATATGATTG CATTTGACAA ATTAATTAAG ATAGATGTTT   
  
  
- CAAGTTAAGC ATAGTGTTTG ACTGACTTAG TTAATTCAAT TAAGTTCCGT GACTTAGAGA TCAAATTATA   
  
  
- ATCATCACTC ACAGTACGCG AAATGAAATA CATAATATTA CATTACTCTA AACCCAGAAT TACCCCCCCA   
  
  
- AAAAAAAAAC ACACAAAAAA AAAAGGTGGC TTATCCTTAA AAAAAAAATC TTCAAGTAAA ATGTCTTATT   
  
  
- TAAAAATAAG AAAAAGACAA AAAAAAAGAC AAAGGGCGGG AAAGGGGTCA AAATTGTAAG ATTACTTTAA   
  
  
- TCACATCATG TCATAGCCAA ATTGTGTTT

+     Box III

| Site Name | Organism | Position | Strand | Matrix score. | sequence | function |
| --- | --- | --- | --- | --- | --- | --- |
| Box III | Pisum sativum | 1090 | + | 9 | CATTTACACT | protein binding site |

> 2018/04/13 10:10:12  
+ CCAAAGCCAT TCCATCGACT TTAATCCCAA TCCCAAACGT TCCATTAGGT AACGGTCTTT CGTTCTCCTC   
  
  
+ AACCTGTACC ATCGCACTCT CTACCCATGA AAACATACAA TCCGAACGAG GGTGATATAT ATGTTTCTTA   
  
  
+ ATTTTTATGA ATTATTTTCT TATTTCTGTA ATATTTTAAT TTTTATAATA GAAGTAAAAC TTGACGACGA   
  
  
+ AGTACCTGTG GAAGGGCCTC CCCATGATGC TTCGATGGAA CACCACGGTG CACACACCGG GTGTACGATA   
  
  
+ CTGCAGTAGC ATTACTGCAT CCGGACCGAG TGAACTTTCG GTATGACCGG GTGAACTCAT TATTAACGGG   
  
  
+ CTACAGAACA AAAACTATCC GCCTCCATGA AAACACCCTG GTGTTGCGGA ACCTAAATGA GGATTATTCA   
  
  
+ GGACAGACAC TAGTGTTAAG GACTATGGCA GTCTACTAGA GCCACCAGAG TAAGTTCGTA TTTTAATAAA   
  
  
+ AATAATATTT TAATTTTAGA AGGTGGTCTT ATTTTTACAA GTCACTCATA CCCTTATAAA CATCTAAATA   
  
  
+ TAATAAAAAA ATTAATGAAG ATCAAATTTT TATAAACTTA AAATGTTATT ATACTACCAA ATTTATTTCT   
  
  
+ AAAATAGTAT TTCATTGCAT ACAATTAAAC AAATTAATAC TAGCATATTA AAATAAATTA ATTTTTTGTA   
  
  
+ AAGTTTTTGG TAGGGTAGTT CTATATATTA CCGTGCTATT TTTAATCAAC TTCACCTTTG TAAAATTCAT   
  
  
+ GTGAATAGTT AACCTGTGTC GATATTTTTA CTTAAATATT CTAGGGTTGG TTATTGAAAT TTTTAATTAT   
  
  
+ TAAAAAATTA TTAAATATTA TTTAATACAT ATAGAAAATT AATTTAATAA AATGTCAATA AAAAAACCTT   
  
  
+ TACTTATTTT TTTCCTCTTT AATTTCAACA ACTTAACGTT TAAATTACAA CACACCTAAC TTATCCGTCG   
  
  
+ ACGGTTCTCT CAGTTACTCA GTTGTTTATA CTAACTTAGT TTATTTAATC ATCAACCCCG CTCCACATTA   
  
  
+ ATTCGGTTAA TTTTGGAAGC AGTTTGTCAC AGTAGGAATC ATTTATACTG AGTTAACTAT TACCAGAACT   
  
  
+ AGTACTTAAC ACATAAATCT ATTACGCGTT ATATACTAAC GTAAACTGTT TAATTAATTC TATCTACAAA   
  
  
+ GTTCAATTCG TATCACAAAC TGACTGAATC AATTAAGTTA ATTCAAGGCA CTGAATCTCT AGTTTAATAT   
  
  
+ TAGTAGTGAG TGTCATGCGC TTTACTTTAT GTATTATAAT GTAATGAGAT TTGGGTCTTA ATGGGGGGGT   
  
  
+ TTTTTTTTTG TGTGTTTTTT TTTTCCACCG AATAGGAATT TTTTTTTTAG AAGTTCATTT TACAGAATAA   
  
  
+ ATTTTTATTC TTTTTCTGTT TTTTTTTCTG TTTCCCGCCC TTTCCCCAGT TTTAACATTC TAATGAAATT   
  
  
+ AGTGTAGTAC AGTATCGGTT TAACACAAA  

- GGTTTCGGTA AGGTAGCTGA AATTAGGGTT AGGGTTTGCA AGGTAATCCA TTGCCAGAAA GCAAGAGGAG   
  
  
- TTGGACATGG TAGCGTGAGA GATGGGTACT TTTGTATGTT AGGCTTGCTC CCACTATATA TACAAAGAAT   
  
  
- TAAAAATACT TAATAAAAGA ATAAAGACAT TATAAAATTA AAAATATTAT CTTCATTTTG AACTGCTGCT   
  
  
- TCATGGACAC CTTCCCGGAG GGGTACTACG AAGCTACCTT GTGGTGCCAC GTGTGTGGCC CACATGCTAT   
  
  
- GACGTCATCG TAATGACGTA GGCCTGGCTC ACTTGAAAGC CATACTGGCC CACTTGAGTA ATAATTGCCC   
  
  
- GATGTCTTGT TTTTGATAGG CGGAGGTACT TTTGTGGGAC CACAACGCCT TGGATTTACT CCTAATAAGT   
  
  
- CCTGTCTGTG ATCACAATTC CTGATACCGT CAGATGATCT CGGTGGTCTC ATTCAAGCAT AAAATTATTT   
  
  
- TTATTATAAA ATTAAAATCT TCCACCAGAA TAAAAATGTT CAGTGAGTAT GGGAATATTT GTAGATTTAT   
  
  
- ATTATTTTTT TAATTACTTC TAGTTTAAAA ATATTTGAAT TTTACAATAA TATGATGGTT TAAATAAAGA   
  
  
- TTTTATCATA AAGTAACGTA TGTTAATTTG TTTAATTATG ATCGTATAAT TTTATTTAAT TAAAAAACAT   
  
  
- TTCAAAAACC ATCCCATCAA GATATATAAT GGCACGATAA AAATTAGTTG AAGTGGAAAC ATTTTAAGTA   
  
  
- CACTTATCAA TTGGACACAG CTATAAAAAT GAATTTATAA GATCCCAACC AATAACTTTA AAAATTAATA   
  
  
- ATTTTTTAAT AATTTATAAT AAATTATGTA TATCTTTTAA TTAAATTATT TTACAGTTAT TTTTTTGGAA   
  
  
- ATGAATAAAA AAAGGAGAAA TTAAAGTTGT TGAATTGCAA ATTTAATGTT GTGTGGATTG AATAGGCAGC   
  
  
- TGCCAAGAGA GTCAATGAGT CAACAAATAT GATTGAATCA AATAAATTAG TAGTTGGGGC GAGGTGTAAT   
  
  
- TAAGCCAATT AAAACCTTCG TCAAACAGTG TCATCCTTAG TAAATATGAC TCAATTGATA ATGGTCTTGA   
  
  
- TCATGAATTG TGTATTTAGA TAATGCGCAA TATATGATTG CATTTGACAA ATTAATTAAG ATAGATGTTT   
  
  
- CAAGTTAAGC ATAGTGTTTG ACTGACTTAG TTAATTCAAT TAAGTTCCGT GACTTAGAGA TCAAATTATA   
  
  
- ATCATCACTC ACAGTACGCG AAATGAAATA CATAATATTA CATTACTCTA AACCCAGAAT TACCCCCCCA   
  
  
- AAAAAAAAAC ACACAAAAAA AAAAGGTGGC TTATCCTTAA AAAAAAAATC TTCAAGTAAA ATGTCTTATT   
  
  
- TAAAAATAAG AAAAAGACAA AAAAAAAGAC AAAGGGCGGG AAAGGGGTCA AAATTGTAAG ATTACTTTAA   
  
  
- TCACATCATG TCATAGCCAA ATTGTGTTT

+     CAAT-box

| Site Name | Organism | Position | Strand | Matrix score. | sequence | function |
| --- | --- | --- | --- | --- | --- | --- |
| CAAT-box | Brassica rapa | 660 | + | 5 | CAAAT | common cis-acting element in promoter and enhancer regions |
| CAAT-box | Brassica rapa | 618 | + | 5 | CAAAT | common cis-acting element in promoter and enhancer regions |
| CAAT-box | Arabidopsis thaliana | 27 | + | 5 | CCAAT | common cis-acting element in promoter and enhancer regions |
| CAAT-box | Brassica rapa | 1309 | - | 5 | CAAAT | common cis-acting element in promoter and enhancer regions |
| CAAT-box | Glycine max | 1220 | + | 5 | CAATT | common cis-acting element in promoter and enhancer regions |
| CAAT-box | Hordeum vulgare | 644 | - | 4 | CAAT | common cis-acting element in promoter and enhancer regions |
| CAAT-box | Glycine max | 1194 | + | 5 | CAATT | common cis-acting element in promoter and enhancer regions |
| CAAT-box | Hordeum vulgare | 108 | + | 4 | CAAT | common cis-acting element in promoter and enhancer regions |
| CAAT-box | Glycine max | 652 | + | 5 | CAATT | common cis-acting element in promoter and enhancer regions |
| CAAT-box | Hordeum vulgare | 823 | - | 4 | CAAT | common cis-acting element in promoter and enhancer regions |
| CAAT-box | Hordeum vulgare | 896 | + | 4 | CAAT | common cis-acting element in promoter and enhancer regions |
| CAAT-box | Hordeum vulgare | 28 | + | 4 | CAAT | common cis-acting element in promoter and enhancer regions |
| CAAT-box | Brassica rapa | 583 | + | 5 | CAAAT | common cis-acting element in promoter and enhancer regions |

> 2018/04/13 10:10:12  
+ CCAAAGCCAT TCCATCGACT TTAATCCCAA TCCCAAACGT TCCATTAGGT AACGGTCTTT CGTTCTCCTC   
  
  
+ AACCTGTACC ATCGCACTCT CTACCCATGA AAACATACAA TCCGAACGAG GGTGATATAT ATGTTTCTTA   
  
  
+ ATTTTTATGA ATTATTTTCT TATTTCTGTA ATATTTTAAT TTTTATAATA GAAGTAAAAC TTGACGACGA   
  
  
+ AGTACCTGTG GAAGGGCCTC CCCATGATGC TTCGATGGAA CACCACGGTG CACACACCGG GTGTACGATA   
  
  
+ CTGCAGTAGC ATTACTGCAT CCGGACCGAG TGAACTTTCG GTATGACCGG GTGAACTCAT TATTAACGGG   
  
  
+ CTACAGAACA AAAACTATCC GCCTCCATGA AAACACCCTG GTGTTGCGGA ACCTAAATGA GGATTATTCA   
  
  
+ GGACAGACAC TAGTGTTAAG GACTATGGCA GTCTACTAGA GCCACCAGAG TAAGTTCGTA TTTTAATAAA   
  
  
+ AATAATATTT TAATTTTAGA AGGTGGTCTT ATTTTTACAA GTCACTCATA CCCTTATAAA CATCTAAATA   
  
  
+ TAATAAAAAA ATTAATGAAG ATCAAATTTT TATAAACTTA AAATGTTATT ATACTACCAA ATTTATTTCT   
  
  
+ AAAATAGTAT TTCATTGCAT ACAATTAAAC AAATTAATAC TAGCATATTA AAATAAATTA ATTTTTTGTA   
  
  
+ AAGTTTTTGG TAGGGTAGTT CTATATATTA CCGTGCTATT TTTAATCAAC TTCACCTTTG TAAAATTCAT   
  
  
+ GTGAATAGTT AACCTGTGTC GATATTTTTA CTTAAATATT CTAGGGTTGG TTATTGAAAT TTTTAATTAT   
  
  
+ TAAAAAATTA TTAAATATTA TTTAATACAT ATAGAAAATT AATTTAATAA AATGTCAATA AAAAAACCTT   
  
  
+ TACTTATTTT TTTCCTCTTT AATTTCAACA ACTTAACGTT TAAATTACAA CACACCTAAC TTATCCGTCG   
  
  
+ ACGGTTCTCT CAGTTACTCA GTTGTTTATA CTAACTTAGT TTATTTAATC ATCAACCCCG CTCCACATTA   
  
  
+ ATTCGGTTAA TTTTGGAAGC AGTTTGTCAC AGTAGGAATC ATTTATACTG AGTTAACTAT TACCAGAACT   
  
  
+ AGTACTTAAC ACATAAATCT ATTACGCGTT ATATACTAAC GTAAACTGTT TAATTAATTC TATCTACAAA   
  
  
+ GTTCAATTCG TATCACAAAC TGACTGAATC AATTAAGTTA ATTCAAGGCA CTGAATCTCT AGTTTAATAT   
  
  
+ TAGTAGTGAG TGTCATGCGC TTTACTTTAT GTATTATAAT GTAATGAGAT TTGGGTCTTA ATGGGGGGGT   
  
  
+ TTTTTTTTTG TGTGTTTTTT TTTTCCACCG AATAGGAATT TTTTTTTTAG AAGTTCATTT TACAGAATAA   
  
  
+ ATTTTTATTC TTTTTCTGTT TTTTTTTCTG TTTCCCGCCC TTTCCCCAGT TTTAACATTC TAATGAAATT   
  
  
+ AGTGTAGTAC AGTATCGGTT TAACACAAA  

- GGTTTCGGTA AGGTAGCTGA AATTAGGGTT AGGGTTTGCA AGGTAATCCA TTGCCAGAAA GCAAGAGGAG   
  
  
- TTGGACATGG TAGCGTGAGA GATGGGTACT TTTGTATGTT AGGCTTGCTC CCACTATATA TACAAAGAAT   
  
  
- TAAAAATACT TAATAAAAGA ATAAAGACAT TATAAAATTA AAAATATTAT CTTCATTTTG AACTGCTGCT   
  
  
- TCATGGACAC CTTCCCGGAG GGGTACTACG AAGCTACCTT GTGGTGCCAC GTGTGTGGCC CACATGCTAT   
  
  
- GACGTCATCG TAATGACGTA GGCCTGGCTC ACTTGAAAGC CATACTGGCC CACTTGAGTA ATAATTGCCC   
  
  
- GATGTCTTGT TTTTGATAGG CGGAGGTACT TTTGTGGGAC CACAACGCCT TGGATTTACT CCTAATAAGT   
  
  
- CCTGTCTGTG ATCACAATTC CTGATACCGT CAGATGATCT CGGTGGTCTC ATTCAAGCAT AAAATTATTT   
  
  
- TTATTATAAA ATTAAAATCT TCCACCAGAA TAAAAATGTT CAGTGAGTAT GGGAATATTT GTAGATTTAT   
  
  
- ATTATTTTTT TAATTACTTC TAGTTTAAAA ATATTTGAAT TTTACAATAA TATGATGGTT TAAATAAAGA   
  
  
- TTTTATCATA AAGTAACGTA TGTTAATTTG TTTAATTATG ATCGTATAAT TTTATTTAAT TAAAAAACAT   
  
  
- TTCAAAAACC ATCCCATCAA GATATATAAT GGCACGATAA AAATTAGTTG AAGTGGAAAC ATTTTAAGTA   
  
  
- CACTTATCAA TTGGACACAG CTATAAAAAT GAATTTATAA GATCCCAACC AATAACTTTA AAAATTAATA   
  
  
- ATTTTTTAAT AATTTATAAT AAATTATGTA TATCTTTTAA TTAAATTATT TTACAGTTAT TTTTTTGGAA   
  
  
- ATGAATAAAA AAAGGAGAAA TTAAAGTTGT TGAATTGCAA ATTTAATGTT GTGTGGATTG AATAGGCAGC   
  
  
- TGCCAAGAGA GTCAATGAGT CAACAAATAT GATTGAATCA AATAAATTAG TAGTTGGGGC GAGGTGTAAT   
  
  
- TAAGCCAATT AAAACCTTCG TCAAACAGTG TCATCCTTAG TAAATATGAC TCAATTGATA ATGGTCTTGA   
  
  
- TCATGAATTG TGTATTTAGA TAATGCGCAA TATATGATTG CATTTGACAA ATTAATTAAG ATAGATGTTT   
  
  
- CAAGTTAAGC ATAGTGTTTG ACTGACTTAG TTAATTCAAT TAAGTTCCGT GACTTAGAGA TCAAATTATA   
  
  
- ATCATCACTC ACAGTACGCG AAATGAAATA CATAATATTA CATTACTCTA AACCCAGAAT TACCCCCCCA   
  
  
- AAAAAAAAAC ACACAAAAAA AAAAGGTGGC TTATCCTTAA AAAAAAAATC TTCAAGTAAA ATGTCTTATT   
  
  
- TAAAAATAAG AAAAAGACAA AAAAAAAGAC AAAGGGCGGG AAAGGGGTCA AAATTGTAAG ATTACTTTAA   
  
  
- TCACATCATG TCATAGCCAA ATTGTGTTT

+     CGTCA-motif

| Site Name | Organism | Position | Strand | Matrix score. | sequence | function |
| --- | --- | --- | --- | --- | --- | --- |
| CGTCA-motif | Hordeum vulgare | 202 | - | 5 | CGTCA | cis-acting regulatory element involved in the MeJA-responsiveness |

> 2018/04/13 10:10:12  
+ CCAAAGCCAT TCCATCGACT TTAATCCCAA TCCCAAACGT TCCATTAGGT AACGGTCTTT CGTTCTCCTC   
  
  
+ AACCTGTACC ATCGCACTCT CTACCCATGA AAACATACAA TCCGAACGAG GGTGATATAT ATGTTTCTTA   
  
  
+ ATTTTTATGA ATTATTTTCT TATTTCTGTA ATATTTTAAT TTTTATAATA GAAGTAAAAC TTGACGACGA   
  
  
+ AGTACCTGTG GAAGGGCCTC CCCATGATGC TTCGATGGAA CACCACGGTG CACACACCGG GTGTACGATA   
  
  
+ CTGCAGTAGC ATTACTGCAT CCGGACCGAG TGAACTTTCG GTATGACCGG GTGAACTCAT TATTAACGGG   
  
  
+ CTACAGAACA AAAACTATCC GCCTCCATGA AAACACCCTG GTGTTGCGGA ACCTAAATGA GGATTATTCA   
  
  
+ GGACAGACAC TAGTGTTAAG GACTATGGCA GTCTACTAGA GCCACCAGAG TAAGTTCGTA TTTTAATAAA   
  
  
+ AATAATATTT TAATTTTAGA AGGTGGTCTT ATTTTTACAA GTCACTCATA CCCTTATAAA CATCTAAATA   
  
  
+ TAATAAAAAA ATTAATGAAG ATCAAATTTT TATAAACTTA AAATGTTATT ATACTACCAA ATTTATTTCT   
  
  
+ AAAATAGTAT TTCATTGCAT ACAATTAAAC AAATTAATAC TAGCATATTA AAATAAATTA ATTTTTTGTA   
  
  
+ AAGTTTTTGG TAGGGTAGTT CTATATATTA CCGTGCTATT TTTAATCAAC TTCACCTTTG TAAAATTCAT   
  
  
+ GTGAATAGTT AACCTGTGTC GATATTTTTA CTTAAATATT CTAGGGTTGG TTATTGAAAT TTTTAATTAT   
  
  
+ TAAAAAATTA TTAAATATTA TTTAATACAT ATAGAAAATT AATTTAATAA AATGTCAATA AAAAAACCTT   
  
  
+ TACTTATTTT TTTCCTCTTT AATTTCAACA ACTTAACGTT TAAATTACAA CACACCTAAC TTATCCGTCG   
  
  
+ ACGGTTCTCT CAGTTACTCA GTTGTTTATA CTAACTTAGT TTATTTAATC ATCAACCCCG CTCCACATTA   
  
  
+ ATTCGGTTAA TTTTGGAAGC AGTTTGTCAC AGTAGGAATC ATTTATACTG AGTTAACTAT TACCAGAACT   
  
  
+ AGTACTTAAC ACATAAATCT ATTACGCGTT ATATACTAAC GTAAACTGTT TAATTAATTC TATCTACAAA   
  
  
+ GTTCAATTCG TATCACAAAC TGACTGAATC AATTAAGTTA ATTCAAGGCA CTGAATCTCT AGTTTAATAT   
  
  
+ TAGTAGTGAG TGTCATGCGC TTTACTTTAT GTATTATAAT GTAATGAGAT TTGGGTCTTA ATGGGGGGGT   
  
  
+ TTTTTTTTTG TGTGTTTTTT TTTTCCACCG AATAGGAATT TTTTTTTTAG AAGTTCATTT TACAGAATAA   
  
  
+ ATTTTTATTC TTTTTCTGTT TTTTTTTCTG TTTCCCGCCC TTTCCCCAGT TTTAACATTC TAATGAAATT   
  
  
+ AGTGTAGTAC AGTATCGGTT TAACACAAA  

- GGTTTCGGTA AGGTAGCTGA AATTAGGGTT AGGGTTTGCA AGGTAATCCA TTGCCAGAAA GCAAGAGGAG   
  
  
- TTGGACATGG TAGCGTGAGA GATGGGTACT TTTGTATGTT AGGCTTGCTC CCACTATATA TACAAAGAAT   
  
  
- TAAAAATACT TAATAAAAGA ATAAAGACAT TATAAAATTA AAAATATTAT CTTCATTTTG AACTGCTGCT   
  
  
- TCATGGACAC CTTCCCGGAG GGGTACTACG AAGCTACCTT GTGGTGCCAC GTGTGTGGCC CACATGCTAT   
  
  
- GACGTCATCG TAATGACGTA GGCCTGGCTC ACTTGAAAGC CATACTGGCC CACTTGAGTA ATAATTGCCC   
  
  
- GATGTCTTGT TTTTGATAGG CGGAGGTACT TTTGTGGGAC CACAACGCCT TGGATTTACT CCTAATAAGT   
  
  
- CCTGTCTGTG ATCACAATTC CTGATACCGT CAGATGATCT CGGTGGTCTC ATTCAAGCAT AAAATTATTT   
  
  
- TTATTATAAA ATTAAAATCT TCCACCAGAA TAAAAATGTT CAGTGAGTAT GGGAATATTT GTAGATTTAT   
  
  
- ATTATTTTTT TAATTACTTC TAGTTTAAAA ATATTTGAAT TTTACAATAA TATGATGGTT TAAATAAAGA   
  
  
- TTTTATCATA AAGTAACGTA TGTTAATTTG TTTAATTATG ATCGTATAAT TTTATTTAAT TAAAAAACAT   
  
  
- TTCAAAAACC ATCCCATCAA GATATATAAT GGCACGATAA AAATTAGTTG AAGTGGAAAC ATTTTAAGTA   
  
  
- CACTTATCAA TTGGACACAG CTATAAAAAT GAATTTATAA GATCCCAACC AATAACTTTA AAAATTAATA   
  
  
- ATTTTTTAAT AATTTATAAT AAATTATGTA TATCTTTTAA TTAAATTATT TTACAGTTAT TTTTTTGGAA   
  
  
- ATGAATAAAA AAAGGAGAAA TTAAAGTTGT TGAATTGCAA ATTTAATGTT GTGTGGATTG AATAGGCAGC   
  
  
- TGCCAAGAGA GTCAATGAGT CAACAAATAT GATTGAATCA AATAAATTAG TAGTTGGGGC GAGGTGTAAT   
  
  
- TAAGCCAATT AAAACCTTCG TCAAACAGTG TCATCCTTAG TAAATATGAC TCAATTGATA ATGGTCTTGA   
  
  
- TCATGAATTG TGTATTTAGA TAATGCGCAA TATATGATTG CATTTGACAA ATTAATTAAG ATAGATGTTT   
  
  
- CAAGTTAAGC ATAGTGTTTG ACTGACTTAG TTAATTCAAT TAAGTTCCGT GACTTAGAGA TCAAATTATA   
  
  
- ATCATCACTC ACAGTACGCG AAATGAAATA CATAATATTA CATTACTCTA AACCCAGAAT TACCCCCCCA   
  
  
- AAAAAAAAAC ACACAAAAAA AAAAGGTGGC TTATCCTTAA AAAAAAAATC TTCAAGTAAA ATGTCTTATT   
  
  
- TAAAAATAAG AAAAAGACAA AAAAAAAGAC AAAGGGCGGG AAAGGGGTCA AAATTGTAAG ATTACTTTAA   
  
  
- TCACATCATG TCATAGCCAA ATTGTGTTT

+     E2Fa

| Site Name | Organism | Position | Strand | Matrix score. | sequence | function |
| --- | --- | --- | --- | --- | --- | --- |
| E2Fa | Nicotiana tabacum | 1431 | + | 8 | TTTCCCGC |  |

> 2018/04/13 10:10:12  
+ CCAAAGCCAT TCCATCGACT TTAATCCCAA TCCCAAACGT TCCATTAGGT AACGGTCTTT CGTTCTCCTC   
  
  
+ AACCTGTACC ATCGCACTCT CTACCCATGA AAACATACAA TCCGAACGAG GGTGATATAT ATGTTTCTTA   
  
  
+ ATTTTTATGA ATTATTTTCT TATTTCTGTA ATATTTTAAT TTTTATAATA GAAGTAAAAC TTGACGACGA   
  
  
+ AGTACCTGTG GAAGGGCCTC CCCATGATGC TTCGATGGAA CACCACGGTG CACACACCGG GTGTACGATA   
  
  
+ CTGCAGTAGC ATTACTGCAT CCGGACCGAG TGAACTTTCG GTATGACCGG GTGAACTCAT TATTAACGGG   
  
  
+ CTACAGAACA AAAACTATCC GCCTCCATGA AAACACCCTG GTGTTGCGGA ACCTAAATGA GGATTATTCA   
  
  
+ GGACAGACAC TAGTGTTAAG GACTATGGCA GTCTACTAGA GCCACCAGAG TAAGTTCGTA TTTTAATAAA   
  
  
+ AATAATATTT TAATTTTAGA AGGTGGTCTT ATTTTTACAA GTCACTCATA CCCTTATAAA CATCTAAATA   
  
  
+ TAATAAAAAA ATTAATGAAG ATCAAATTTT TATAAACTTA AAATGTTATT ATACTACCAA ATTTATTTCT   
  
  
+ AAAATAGTAT TTCATTGCAT ACAATTAAAC AAATTAATAC TAGCATATTA AAATAAATTA ATTTTTTGTA   
  
  
+ AAGTTTTTGG TAGGGTAGTT CTATATATTA CCGTGCTATT TTTAATCAAC TTCACCTTTG TAAAATTCAT   
  
  
+ GTGAATAGTT AACCTGTGTC GATATTTTTA CTTAAATATT CTAGGGTTGG TTATTGAAAT TTTTAATTAT   
  
  
+ TAAAAAATTA TTAAATATTA TTTAATACAT ATAGAAAATT AATTTAATAA AATGTCAATA AAAAAACCTT   
  
  
+ TACTTATTTT TTTCCTCTTT AATTTCAACA ACTTAACGTT TAAATTACAA CACACCTAAC TTATCCGTCG   
  
  
+ ACGGTTCTCT CAGTTACTCA GTTGTTTATA CTAACTTAGT TTATTTAATC ATCAACCCCG CTCCACATTA   
  
  
+ ATTCGGTTAA TTTTGGAAGC AGTTTGTCAC AGTAGGAATC ATTTATACTG AGTTAACTAT TACCAGAACT   
  
  
+ AGTACTTAAC ACATAAATCT ATTACGCGTT ATATACTAAC GTAAACTGTT TAATTAATTC TATCTACAAA   
  
  
+ GTTCAATTCG TATCACAAAC TGACTGAATC AATTAAGTTA ATTCAAGGCA CTGAATCTCT AGTTTAATAT   
  
  
+ TAGTAGTGAG TGTCATGCGC TTTACTTTAT GTATTATAAT GTAATGAGAT TTGGGTCTTA ATGGGGGGGT   
  
  
+ TTTTTTTTTG TGTGTTTTTT TTTTCCACCG AATAGGAATT TTTTTTTTAG AAGTTCATTT TACAGAATAA   
  
  
+ ATTTTTATTC TTTTTCTGTT TTTTTTTCTG TTTCCCGCCC TTTCCCCAGT TTTAACATTC TAATGAAATT   
  
  
+ AGTGTAGTAC AGTATCGGTT TAACACAAA  

- GGTTTCGGTA AGGTAGCTGA AATTAGGGTT AGGGTTTGCA AGGTAATCCA TTGCCAGAAA GCAAGAGGAG   
  
  
- TTGGACATGG TAGCGTGAGA GATGGGTACT TTTGTATGTT AGGCTTGCTC CCACTATATA TACAAAGAAT   
  
  
- TAAAAATACT TAATAAAAGA ATAAAGACAT TATAAAATTA AAAATATTAT CTTCATTTTG AACTGCTGCT   
  
  
- TCATGGACAC CTTCCCGGAG GGGTACTACG AAGCTACCTT GTGGTGCCAC GTGTGTGGCC CACATGCTAT   
  
  
- GACGTCATCG TAATGACGTA GGCCTGGCTC ACTTGAAAGC CATACTGGCC CACTTGAGTA ATAATTGCCC   
  
  
- GATGTCTTGT TTTTGATAGG CGGAGGTACT TTTGTGGGAC CACAACGCCT TGGATTTACT CCTAATAAGT   
  
  
- CCTGTCTGTG ATCACAATTC CTGATACCGT CAGATGATCT CGGTGGTCTC ATTCAAGCAT AAAATTATTT   
  
  
- TTATTATAAA ATTAAAATCT TCCACCAGAA TAAAAATGTT CAGTGAGTAT GGGAATATTT GTAGATTTAT   
  
  
- ATTATTTTTT TAATTACTTC TAGTTTAAAA ATATTTGAAT TTTACAATAA TATGATGGTT TAAATAAAGA   
  
  
- TTTTATCATA AAGTAACGTA TGTTAATTTG TTTAATTATG ATCGTATAAT TTTATTTAAT TAAAAAACAT   
  
  
- TTCAAAAACC ATCCCATCAA GATATATAAT GGCACGATAA AAATTAGTTG AAGTGGAAAC ATTTTAAGTA   
  
  
- CACTTATCAA TTGGACACAG CTATAAAAAT GAATTTATAA GATCCCAACC AATAACTTTA AAAATTAATA   
  
  
- ATTTTTTAAT AATTTATAAT AAATTATGTA TATCTTTTAA TTAAATTATT TTACAGTTAT TTTTTTGGAA   
  
  
- ATGAATAAAA AAAGGAGAAA TTAAAGTTGT TGAATTGCAA ATTTAATGTT GTGTGGATTG AATAGGCAGC   
  
  
- TGCCAAGAGA GTCAATGAGT CAACAAATAT GATTGAATCA AATAAATTAG TAGTTGGGGC GAGGTGTAAT   
  
  
- TAAGCCAATT AAAACCTTCG TCAAACAGTG TCATCCTTAG TAAATATGAC TCAATTGATA ATGGTCTTGA   
  
  
- TCATGAATTG TGTATTTAGA TAATGCGCAA TATATGATTG CATTTGACAA ATTAATTAAG ATAGATGTTT   
  
  
- CAAGTTAAGC ATAGTGTTTG ACTGACTTAG TTAATTCAAT TAAGTTCCGT GACTTAGAGA TCAAATTATA   
  
  
- ATCATCACTC ACAGTACGCG AAATGAAATA CATAATATTA CATTACTCTA AACCCAGAAT TACCCCCCCA   
  
  
- AAAAAAAAAC ACACAAAAAA AAAAGGTGGC TTATCCTTAA AAAAAAAATC TTCAAGTAAA ATGTCTTATT   
  
  
- TAAAAATAAG AAAAAGACAA AAAAAAAGAC AAAGGGCGGG AAAGGGGTCA AAATTGTAAG ATTACTTTAA   
  
  
- TCACATCATG TCATAGCCAA ATTGTGTTT

+     GAG-motif

| Site Name | Organism | Position | Strand | Matrix score. | sequence | function |
| --- | --- | --- | --- | --- | --- | --- |
| GAG-motif | Arabidopsis thaliana | 86 | - | 7 | AGAGAGT | part of a light responsive element |

> 2018/04/13 10:10:12  
+ CCAAAGCCAT TCCATCGACT TTAATCCCAA TCCCAAACGT TCCATTAGGT AACGGTCTTT CGTTCTCCTC   
  
  
+ AACCTGTACC ATCGCACTCT CTACCCATGA AAACATACAA TCCGAACGAG GGTGATATAT ATGTTTCTTA   
  
  
+ ATTTTTATGA ATTATTTTCT TATTTCTGTA ATATTTTAAT TTTTATAATA GAAGTAAAAC TTGACGACGA   
  
  
+ AGTACCTGTG GAAGGGCCTC CCCATGATGC TTCGATGGAA CACCACGGTG CACACACCGG GTGTACGATA   
  
  
+ CTGCAGTAGC ATTACTGCAT CCGGACCGAG TGAACTTTCG GTATGACCGG GTGAACTCAT TATTAACGGG   
  
  
+ CTACAGAACA AAAACTATCC GCCTCCATGA AAACACCCTG GTGTTGCGGA ACCTAAATGA GGATTATTCA   
  
  
+ GGACAGACAC TAGTGTTAAG GACTATGGCA GTCTACTAGA GCCACCAGAG TAAGTTCGTA TTTTAATAAA   
  
  
+ AATAATATTT TAATTTTAGA AGGTGGTCTT ATTTTTACAA GTCACTCATA CCCTTATAAA CATCTAAATA   
  
  
+ TAATAAAAAA ATTAATGAAG ATCAAATTTT TATAAACTTA AAATGTTATT ATACTACCAA ATTTATTTCT   
  
  
+ AAAATAGTAT TTCATTGCAT ACAATTAAAC AAATTAATAC TAGCATATTA AAATAAATTA ATTTTTTGTA   
  
  
+ AAGTTTTTGG TAGGGTAGTT CTATATATTA CCGTGCTATT TTTAATCAAC TTCACCTTTG TAAAATTCAT   
  
  
+ GTGAATAGTT AACCTGTGTC GATATTTTTA CTTAAATATT CTAGGGTTGG TTATTGAAAT TTTTAATTAT   
  
  
+ TAAAAAATTA TTAAATATTA TTTAATACAT ATAGAAAATT AATTTAATAA AATGTCAATA AAAAAACCTT   
  
  
+ TACTTATTTT TTTCCTCTTT AATTTCAACA ACTTAACGTT TAAATTACAA CACACCTAAC TTATCCGTCG   
  
  
+ ACGGTTCTCT CAGTTACTCA GTTGTTTATA CTAACTTAGT TTATTTAATC ATCAACCCCG CTCCACATTA   
  
  
+ ATTCGGTTAA TTTTGGAAGC AGTTTGTCAC AGTAGGAATC ATTTATACTG AGTTAACTAT TACCAGAACT   
  
  
+ AGTACTTAAC ACATAAATCT ATTACGCGTT ATATACTAAC GTAAACTGTT TAATTAATTC TATCTACAAA   
  
  
+ GTTCAATTCG TATCACAAAC TGACTGAATC AATTAAGTTA ATTCAAGGCA CTGAATCTCT AGTTTAATAT   
  
  
+ TAGTAGTGAG TGTCATGCGC TTTACTTTAT GTATTATAAT GTAATGAGAT TTGGGTCTTA ATGGGGGGGT   
  
  
+ TTTTTTTTTG TGTGTTTTTT TTTTCCACCG AATAGGAATT TTTTTTTTAG AAGTTCATTT TACAGAATAA   
  
  
+ ATTTTTATTC TTTTTCTGTT TTTTTTTCTG TTTCCCGCCC TTTCCCCAGT TTTAACATTC TAATGAAATT   
  
  
+ AGTGTAGTAC AGTATCGGTT TAACACAAA  

- GGTTTCGGTA AGGTAGCTGA AATTAGGGTT AGGGTTTGCA AGGTAATCCA TTGCCAGAAA GCAAGAGGAG   
  
  
- TTGGACATGG TAGCGTGAGA GATGGGTACT TTTGTATGTT AGGCTTGCTC CCACTATATA TACAAAGAAT   
  
  
- TAAAAATACT TAATAAAAGA ATAAAGACAT TATAAAATTA AAAATATTAT CTTCATTTTG AACTGCTGCT   
  
  
- TCATGGACAC CTTCCCGGAG GGGTACTACG AAGCTACCTT GTGGTGCCAC GTGTGTGGCC CACATGCTAT   
  
  
- GACGTCATCG TAATGACGTA GGCCTGGCTC ACTTGAAAGC CATACTGGCC CACTTGAGTA ATAATTGCCC   
  
  
- GATGTCTTGT TTTTGATAGG CGGAGGTACT TTTGTGGGAC CACAACGCCT TGGATTTACT CCTAATAAGT   
  
  
- CCTGTCTGTG ATCACAATTC CTGATACCGT CAGATGATCT CGGTGGTCTC ATTCAAGCAT AAAATTATTT   
  
  
- TTATTATAAA ATTAAAATCT TCCACCAGAA TAAAAATGTT CAGTGAGTAT GGGAATATTT GTAGATTTAT   
  
  
- ATTATTTTTT TAATTACTTC TAGTTTAAAA ATATTTGAAT TTTACAATAA TATGATGGTT TAAATAAAGA   
  
  
- TTTTATCATA AAGTAACGTA TGTTAATTTG TTTAATTATG ATCGTATAAT TTTATTTAAT TAAAAAACAT   
  
  
- TTCAAAAACC ATCCCATCAA GATATATAAT GGCACGATAA AAATTAGTTG AAGTGGAAAC ATTTTAAGTA   
  
  
- CACTTATCAA TTGGACACAG CTATAAAAAT GAATTTATAA GATCCCAACC AATAACTTTA AAAATTAATA   
  
  
- ATTTTTTAAT AATTTATAAT AAATTATGTA TATCTTTTAA TTAAATTATT TTACAGTTAT TTTTTTGGAA   
  
  
- ATGAATAAAA AAAGGAGAAA TTAAAGTTGT TGAATTGCAA ATTTAATGTT GTGTGGATTG AATAGGCAGC   
  
  
- TGCCAAGAGA GTCAATGAGT CAACAAATAT GATTGAATCA AATAAATTAG TAGTTGGGGC GAGGTGTAAT   
  
  
- TAAGCCAATT AAAACCTTCG TCAAACAGTG TCATCCTTAG TAAATATGAC TCAATTGATA ATGGTCTTGA   
  
  
- TCATGAATTG TGTATTTAGA TAATGCGCAA TATATGATTG CATTTGACAA ATTAATTAAG ATAGATGTTT   
  
  
- CAAGTTAAGC ATAGTGTTTG ACTGACTTAG TTAATTCAAT TAAGTTCCGT GACTTAGAGA TCAAATTATA   
  
  
- ATCATCACTC ACAGTACGCG AAATGAAATA CATAATATTA CATTACTCTA AACCCAGAAT TACCCCCCCA   
  
  
- AAAAAAAAAC ACACAAAAAA AAAAGGTGGC TTATCCTTAA AAAAAAAATC TTCAAGTAAA ATGTCTTATT   
  
  
- TAAAAATAAG AAAAAGACAA AAAAAAAGAC AAAGGGCGGG AAAGGGGTCA AAATTGTAAG ATTACTTTAA   
  
  
- TCACATCATG TCATAGCCAA ATTGTGTTT

+     GARE-motif

| Site Name | Organism | Position | Strand | Matrix score. | sequence | function |
| --- | --- | --- | --- | --- | --- | --- |
| GARE-motif | Brassica oleracea | 1427 | - | 7 | AAACAGA | gibberellin-responsive element |
| GARE-motif | Brassica oleracea | 1415 | - | 7 | AAACAGA | gibberellin-responsive element |

> 2018/04/13 10:10:12  
+ CCAAAGCCAT TCCATCGACT TTAATCCCAA TCCCAAACGT TCCATTAGGT AACGGTCTTT CGTTCTCCTC   
  
  
+ AACCTGTACC ATCGCACTCT CTACCCATGA AAACATACAA TCCGAACGAG GGTGATATAT ATGTTTCTTA   
  
  
+ ATTTTTATGA ATTATTTTCT TATTTCTGTA ATATTTTAAT TTTTATAATA GAAGTAAAAC TTGACGACGA   
  
  
+ AGTACCTGTG GAAGGGCCTC CCCATGATGC TTCGATGGAA CACCACGGTG CACACACCGG GTGTACGATA   
  
  
+ CTGCAGTAGC ATTACTGCAT CCGGACCGAG TGAACTTTCG GTATGACCGG GTGAACTCAT TATTAACGGG   
  
  
+ CTACAGAACA AAAACTATCC GCCTCCATGA AAACACCCTG GTGTTGCGGA ACCTAAATGA GGATTATTCA   
  
  
+ GGACAGACAC TAGTGTTAAG GACTATGGCA GTCTACTAGA GCCACCAGAG TAAGTTCGTA TTTTAATAAA   
  
  
+ AATAATATTT TAATTTTAGA AGGTGGTCTT ATTTTTACAA GTCACTCATA CCCTTATAAA CATCTAAATA   
  
  
+ TAATAAAAAA ATTAATGAAG ATCAAATTTT TATAAACTTA AAATGTTATT ATACTACCAA ATTTATTTCT   
  
  
+ AAAATAGTAT TTCATTGCAT ACAATTAAAC AAATTAATAC TAGCATATTA AAATAAATTA ATTTTTTGTA   
  
  
+ AAGTTTTTGG TAGGGTAGTT CTATATATTA CCGTGCTATT TTTAATCAAC TTCACCTTTG TAAAATTCAT   
  
  
+ GTGAATAGTT AACCTGTGTC GATATTTTTA CTTAAATATT CTAGGGTTGG TTATTGAAAT TTTTAATTAT   
  
  
+ TAAAAAATTA TTAAATATTA TTTAATACAT ATAGAAAATT AATTTAATAA AATGTCAATA AAAAAACCTT   
  
  
+ TACTTATTTT TTTCCTCTTT AATTTCAACA ACTTAACGTT TAAATTACAA CACACCTAAC TTATCCGTCG   
  
  
+ ACGGTTCTCT CAGTTACTCA GTTGTTTATA CTAACTTAGT TTATTTAATC ATCAACCCCG CTCCACATTA   
  
  
+ ATTCGGTTAA TTTTGGAAGC AGTTTGTCAC AGTAGGAATC ATTTATACTG AGTTAACTAT TACCAGAACT   
  
  
+ AGTACTTAAC ACATAAATCT ATTACGCGTT ATATACTAAC GTAAACTGTT TAATTAATTC TATCTACAAA   
  
  
+ GTTCAATTCG TATCACAAAC TGACTGAATC AATTAAGTTA ATTCAAGGCA CTGAATCTCT AGTTTAATAT   
  
  
+ TAGTAGTGAG TGTCATGCGC TTTACTTTAT GTATTATAAT GTAATGAGAT TTGGGTCTTA ATGGGGGGGT   
  
  
+ TTTTTTTTTG TGTGTTTTTT TTTTCCACCG AATAGGAATT TTTTTTTTAG AAGTTCATTT TACAGAATAA   
  
  
+ ATTTTTATTC TTTTTCTGTT TTTTTTTCTG TTTCCCGCCC TTTCCCCAGT TTTAACATTC TAATGAAATT   
  
  
+ AGTGTAGTAC AGTATCGGTT TAACACAAA  

- GGTTTCGGTA AGGTAGCTGA AATTAGGGTT AGGGTTTGCA AGGTAATCCA TTGCCAGAAA GCAAGAGGAG   
  
  
- TTGGACATGG TAGCGTGAGA GATGGGTACT TTTGTATGTT AGGCTTGCTC CCACTATATA TACAAAGAAT   
  
  
- TAAAAATACT TAATAAAAGA ATAAAGACAT TATAAAATTA AAAATATTAT CTTCATTTTG AACTGCTGCT   
  
  
- TCATGGACAC CTTCCCGGAG GGGTACTACG AAGCTACCTT GTGGTGCCAC GTGTGTGGCC CACATGCTAT   
  
  
- GACGTCATCG TAATGACGTA GGCCTGGCTC ACTTGAAAGC CATACTGGCC CACTTGAGTA ATAATTGCCC   
  
  
- GATGTCTTGT TTTTGATAGG CGGAGGTACT TTTGTGGGAC CACAACGCCT TGGATTTACT CCTAATAAGT   
  
  
- CCTGTCTGTG ATCACAATTC CTGATACCGT CAGATGATCT CGGTGGTCTC ATTCAAGCAT AAAATTATTT   
  
  
- TTATTATAAA ATTAAAATCT TCCACCAGAA TAAAAATGTT CAGTGAGTAT GGGAATATTT GTAGATTTAT   
  
  
- ATTATTTTTT TAATTACTTC TAGTTTAAAA ATATTTGAAT TTTACAATAA TATGATGGTT TAAATAAAGA   
  
  
- TTTTATCATA AAGTAACGTA TGTTAATTTG TTTAATTATG ATCGTATAAT TTTATTTAAT TAAAAAACAT   
  
  
- TTCAAAAACC ATCCCATCAA GATATATAAT GGCACGATAA AAATTAGTTG AAGTGGAAAC ATTTTAAGTA   
  
  
- CACTTATCAA TTGGACACAG CTATAAAAAT GAATTTATAA GATCCCAACC AATAACTTTA AAAATTAATA   
  
  
- ATTTTTTAAT AATTTATAAT AAATTATGTA TATCTTTTAA TTAAATTATT TTACAGTTAT TTTTTTGGAA   
  
  
- ATGAATAAAA AAAGGAGAAA TTAAAGTTGT TGAATTGCAA ATTTAATGTT GTGTGGATTG AATAGGCAGC   
  
  
- TGCCAAGAGA GTCAATGAGT CAACAAATAT GATTGAATCA AATAAATTAG TAGTTGGGGC GAGGTGTAAT   
  
  
- TAAGCCAATT AAAACCTTCG TCAAACAGTG TCATCCTTAG TAAATATGAC TCAATTGATA ATGGTCTTGA   
  
  
- TCATGAATTG TGTATTTAGA TAATGCGCAA TATATGATTG CATTTGACAA ATTAATTAAG ATAGATGTTT   
  
  
- CAAGTTAAGC ATAGTGTTTG ACTGACTTAG TTAATTCAAT TAAGTTCCGT GACTTAGAGA TCAAATTATA   
  
  
- ATCATCACTC ACAGTACGCG AAATGAAATA CATAATATTA CATTACTCTA AACCCAGAAT TACCCCCCCA   
  
  
- AAAAAAAAAC ACACAAAAAA AAAAGGTGGC TTATCCTTAA AAAAAAAATC TTCAAGTAAA ATGTCTTATT   
  
  
- TAAAAATAAG AAAAAGACAA AAAAAAAGAC AAAGGGCGGG AAAGGGGTCA AAATTGTAAG ATTACTTTAA   
  
  
- TCACATCATG TCATAGCCAA ATTGTGTTT

+     GT1-motif

| Site Name | Organism | Position | Strand | Matrix score. | sequence | function |
| --- | --- | --- | --- | --- | --- | --- |
| GT1-motif | Arabidopsis thaliana | 779 | - | 6 | GGTTAA | light responsive element |
| GT1-motif | Avena sativa | 1055 | + | 7 | GGTTAAT | light responsive element |

> 2018/04/13 10:10:12  
+ CCAAAGCCAT TCCATCGACT TTAATCCCAA TCCCAAACGT TCCATTAGGT AACGGTCTTT CGTTCTCCTC   
  
  
+ AACCTGTACC ATCGCACTCT CTACCCATGA AAACATACAA TCCGAACGAG GGTGATATAT ATGTTTCTTA   
  
  
+ ATTTTTATGA ATTATTTTCT TATTTCTGTA ATATTTTAAT TTTTATAATA GAAGTAAAAC TTGACGACGA   
  
  
+ AGTACCTGTG GAAGGGCCTC CCCATGATGC TTCGATGGAA CACCACGGTG CACACACCGG GTGTACGATA   
  
  
+ CTGCAGTAGC ATTACTGCAT CCGGACCGAG TGAACTTTCG GTATGACCGG GTGAACTCAT TATTAACGGG   
  
  
+ CTACAGAACA AAAACTATCC GCCTCCATGA AAACACCCTG GTGTTGCGGA ACCTAAATGA GGATTATTCA   
  
  
+ GGACAGACAC TAGTGTTAAG GACTATGGCA GTCTACTAGA GCCACCAGAG TAAGTTCGTA TTTTAATAAA   
  
  
+ AATAATATTT TAATTTTAGA AGGTGGTCTT ATTTTTACAA GTCACTCATA CCCTTATAAA CATCTAAATA   
  
  
+ TAATAAAAAA ATTAATGAAG ATCAAATTTT TATAAACTTA AAATGTTATT ATACTACCAA ATTTATTTCT   
  
  
+ AAAATAGTAT TTCATTGCAT ACAATTAAAC AAATTAATAC TAGCATATTA AAATAAATTA ATTTTTTGTA   
  
  
+ AAGTTTTTGG TAGGGTAGTT CTATATATTA CCGTGCTATT TTTAATCAAC TTCACCTTTG TAAAATTCAT   
  
  
+ GTGAATAGTT AACCTGTGTC GATATTTTTA CTTAAATATT CTAGGGTTGG TTATTGAAAT TTTTAATTAT   
  
  
+ TAAAAAATTA TTAAATATTA TTTAATACAT ATAGAAAATT AATTTAATAA AATGTCAATA AAAAAACCTT   
  
  
+ TACTTATTTT TTTCCTCTTT AATTTCAACA ACTTAACGTT TAAATTACAA CACACCTAAC TTATCCGTCG   
  
  
+ ACGGTTCTCT CAGTTACTCA GTTGTTTATA CTAACTTAGT TTATTTAATC ATCAACCCCG CTCCACATTA   
  
  
+ ATTCGGTTAA TTTTGGAAGC AGTTTGTCAC AGTAGGAATC ATTTATACTG AGTTAACTAT TACCAGAACT   
  
  
+ AGTACTTAAC ACATAAATCT ATTACGCGTT ATATACTAAC GTAAACTGTT TAATTAATTC TATCTACAAA   
  
  
+ GTTCAATTCG TATCACAAAC TGACTGAATC AATTAAGTTA ATTCAAGGCA CTGAATCTCT AGTTTAATAT   
  
  
+ TAGTAGTGAG TGTCATGCGC TTTACTTTAT GTATTATAAT GTAATGAGAT TTGGGTCTTA ATGGGGGGGT   
  
  
+ TTTTTTTTTG TGTGTTTTTT TTTTCCACCG AATAGGAATT TTTTTTTTAG AAGTTCATTT TACAGAATAA   
  
  
+ ATTTTTATTC TTTTTCTGTT TTTTTTTCTG TTTCCCGCCC TTTCCCCAGT TTTAACATTC TAATGAAATT   
  
  
+ AGTGTAGTAC AGTATCGGTT TAACACAAA  

- GGTTTCGGTA AGGTAGCTGA AATTAGGGTT AGGGTTTGCA AGGTAATCCA TTGCCAGAAA GCAAGAGGAG   
  
  
- TTGGACATGG TAGCGTGAGA GATGGGTACT TTTGTATGTT AGGCTTGCTC CCACTATATA TACAAAGAAT   
  
  
- TAAAAATACT TAATAAAAGA ATAAAGACAT TATAAAATTA AAAATATTAT CTTCATTTTG AACTGCTGCT   
  
  
- TCATGGACAC CTTCCCGGAG GGGTACTACG AAGCTACCTT GTGGTGCCAC GTGTGTGGCC CACATGCTAT   
  
  
- GACGTCATCG TAATGACGTA GGCCTGGCTC ACTTGAAAGC CATACTGGCC CACTTGAGTA ATAATTGCCC   
  
  
- GATGTCTTGT TTTTGATAGG CGGAGGTACT TTTGTGGGAC CACAACGCCT TGGATTTACT CCTAATAAGT   
  
  
- CCTGTCTGTG ATCACAATTC CTGATACCGT CAGATGATCT CGGTGGTCTC ATTCAAGCAT AAAATTATTT   
  
  
- TTATTATAAA ATTAAAATCT TCCACCAGAA TAAAAATGTT CAGTGAGTAT GGGAATATTT GTAGATTTAT   
  
  
- ATTATTTTTT TAATTACTTC TAGTTTAAAA ATATTTGAAT TTTACAATAA TATGATGGTT TAAATAAAGA   
  
  
- TTTTATCATA AAGTAACGTA TGTTAATTTG TTTAATTATG ATCGTATAAT TTTATTTAAT TAAAAAACAT   
  
  
- TTCAAAAACC ATCCCATCAA GATATATAAT GGCACGATAA AAATTAGTTG AAGTGGAAAC ATTTTAAGTA   
  
  
- CACTTATCAA TTGGACACAG CTATAAAAAT GAATTTATAA GATCCCAACC AATAACTTTA AAAATTAATA   
  
  
- ATTTTTTAAT AATTTATAAT AAATTATGTA TATCTTTTAA TTAAATTATT TTACAGTTAT TTTTTTGGAA   
  
  
- ATGAATAAAA AAAGGAGAAA TTAAAGTTGT TGAATTGCAA ATTTAATGTT GTGTGGATTG AATAGGCAGC   
  
  
- TGCCAAGAGA GTCAATGAGT CAACAAATAT GATTGAATCA AATAAATTAG TAGTTGGGGC GAGGTGTAAT   
  
  
- TAAGCCAATT AAAACCTTCG TCAAACAGTG TCATCCTTAG TAAATATGAC TCAATTGATA ATGGTCTTGA   
  
  
- TCATGAATTG TGTATTTAGA TAATGCGCAA TATATGATTG CATTTGACAA ATTAATTAAG ATAGATGTTT   
  
  
- CAAGTTAAGC ATAGTGTTTG ACTGACTTAG TTAATTCAAT TAAGTTCCGT GACTTAGAGA TCAAATTATA   
  
  
- ATCATCACTC ACAGTACGCG AAATGAAATA CATAATATTA CATTACTCTA AACCCAGAAT TACCCCCCCA   
  
  
- AAAAAAAAAC ACACAAAAAA AAAAGGTGGC TTATCCTTAA AAAAAAAATC TTCAAGTAAA ATGTCTTATT   
  
  
- TAAAAATAAG AAAAAGACAA AAAAAAAGAC AAAGGGCGGG AAAGGGGTCA AAATTGTAAG ATTACTTTAA   
  
  
- TCACATCATG TCATAGCCAA ATTGTGTTT

+     HSE

| Site Name | Organism | Position | Strand | Matrix score. | sequence | function |
| --- | --- | --- | --- | --- | --- | --- |
| HSE | Brassica oleracea | 1366 | - | 9 | AAAAAATTTC | cis-acting element involved in heat stress responsiveness |
| HSE | Brassica oleracea | 1365 | - | 9 | AAAAAATTTC | cis-acting element involved in heat stress responsiveness |
| HSE | Brassica oleracea | 826 | - | 9 | AAAAAATTTC | cis-acting element involved in heat stress responsiveness |

> 2018/04/13 10:10:12  
+ CCAAAGCCAT TCCATCGACT TTAATCCCAA TCCCAAACGT TCCATTAGGT AACGGTCTTT CGTTCTCCTC   
  
  
+ AACCTGTACC ATCGCACTCT CTACCCATGA AAACATACAA TCCGAACGAG GGTGATATAT ATGTTTCTTA   
  
  
+ ATTTTTATGA ATTATTTTCT TATTTCTGTA ATATTTTAAT TTTTATAATA GAAGTAAAAC TTGACGACGA   
  
  
+ AGTACCTGTG GAAGGGCCTC CCCATGATGC TTCGATGGAA CACCACGGTG CACACACCGG GTGTACGATA   
  
  
+ CTGCAGTAGC ATTACTGCAT CCGGACCGAG TGAACTTTCG GTATGACCGG GTGAACTCAT TATTAACGGG   
  
  
+ CTACAGAACA AAAACTATCC GCCTCCATGA AAACACCCTG GTGTTGCGGA ACCTAAATGA GGATTATTCA   
  
  
+ GGACAGACAC TAGTGTTAAG GACTATGGCA GTCTACTAGA GCCACCAGAG TAAGTTCGTA TTTTAATAAA   
  
  
+ AATAATATTT TAATTTTAGA AGGTGGTCTT ATTTTTACAA GTCACTCATA CCCTTATAAA CATCTAAATA   
  
  
+ TAATAAAAAA ATTAATGAAG ATCAAATTTT TATAAACTTA AAATGTTATT ATACTACCAA ATTTATTTCT   
  
  
+ AAAATAGTAT TTCATTGCAT ACAATTAAAC AAATTAATAC TAGCATATTA AAATAAATTA ATTTTTTGTA   
  
  
+ AAGTTTTTGG TAGGGTAGTT CTATATATTA CCGTGCTATT TTTAATCAAC TTCACCTTTG TAAAATTCAT   
  
  
+ GTGAATAGTT AACCTGTGTC GATATTTTTA CTTAAATATT CTAGGGTTGG TTATTGAAAT TTTTAATTAT   
  
  
+ TAAAAAATTA TTAAATATTA TTTAATACAT ATAGAAAATT AATTTAATAA AATGTCAATA AAAAAACCTT   
  
  
+ TACTTATTTT TTTCCTCTTT AATTTCAACA ACTTAACGTT TAAATTACAA CACACCTAAC TTATCCGTCG   
  
  
+ ACGGTTCTCT CAGTTACTCA GTTGTTTATA CTAACTTAGT TTATTTAATC ATCAACCCCG CTCCACATTA   
  
  
+ ATTCGGTTAA TTTTGGAAGC AGTTTGTCAC AGTAGGAATC ATTTATACTG AGTTAACTAT TACCAGAACT   
  
  
+ AGTACTTAAC ACATAAATCT ATTACGCGTT ATATACTAAC GTAAACTGTT TAATTAATTC TATCTACAAA   
  
  
+ GTTCAATTCG TATCACAAAC TGACTGAATC AATTAAGTTA ATTCAAGGCA CTGAATCTCT AGTTTAATAT   
  
  
+ TAGTAGTGAG TGTCATGCGC TTTACTTTAT GTATTATAAT GTAATGAGAT TTGGGTCTTA ATGGGGGGGT   
  
  
+ TTTTTTTTTG TGTGTTTTTT TTTTCCACCG AATAGGAATT TTTTTTTTAG AAGTTCATTT TACAGAATAA   
  
  
+ ATTTTTATTC TTTTTCTGTT TTTTTTTCTG TTTCCCGCCC TTTCCCCAGT TTTAACATTC TAATGAAATT   
  
  
+ AGTGTAGTAC AGTATCGGTT TAACACAAA  

- GGTTTCGGTA AGGTAGCTGA AATTAGGGTT AGGGTTTGCA AGGTAATCCA TTGCCAGAAA GCAAGAGGAG   
  
  
- TTGGACATGG TAGCGTGAGA GATGGGTACT TTTGTATGTT AGGCTTGCTC CCACTATATA TACAAAGAAT   
  
  
- TAAAAATACT TAATAAAAGA ATAAAGACAT TATAAAATTA AAAATATTAT CTTCATTTTG AACTGCTGCT   
  
  
- TCATGGACAC CTTCCCGGAG GGGTACTACG AAGCTACCTT GTGGTGCCAC GTGTGTGGCC CACATGCTAT   
  
  
- GACGTCATCG TAATGACGTA GGCCTGGCTC ACTTGAAAGC CATACTGGCC CACTTGAGTA ATAATTGCCC   
  
  
- GATGTCTTGT TTTTGATAGG CGGAGGTACT TTTGTGGGAC CACAACGCCT TGGATTTACT CCTAATAAGT   
  
  
- CCTGTCTGTG ATCACAATTC CTGATACCGT CAGATGATCT CGGTGGTCTC ATTCAAGCAT AAAATTATTT   
  
  
- TTATTATAAA ATTAAAATCT TCCACCAGAA TAAAAATGTT CAGTGAGTAT GGGAATATTT GTAGATTTAT   
  
  
- ATTATTTTTT TAATTACTTC TAGTTTAAAA ATATTTGAAT TTTACAATAA TATGATGGTT TAAATAAAGA   
  
  
- TTTTATCATA AAGTAACGTA TGTTAATTTG TTTAATTATG ATCGTATAAT TTTATTTAAT TAAAAAACAT   
  
  
- TTCAAAAACC ATCCCATCAA GATATATAAT GGCACGATAA AAATTAGTTG AAGTGGAAAC ATTTTAAGTA   
  
  
- CACTTATCAA TTGGACACAG CTATAAAAAT GAATTTATAA GATCCCAACC AATAACTTTA AAAATTAATA   
  
  
- ATTTTTTAAT AATTTATAAT AAATTATGTA TATCTTTTAA TTAAATTATT TTACAGTTAT TTTTTTGGAA   
  
  
- ATGAATAAAA AAAGGAGAAA TTAAAGTTGT TGAATTGCAA ATTTAATGTT GTGTGGATTG AATAGGCAGC   
  
  
- TGCCAAGAGA GTCAATGAGT CAACAAATAT GATTGAATCA AATAAATTAG TAGTTGGGGC GAGGTGTAAT   
  
  
- TAAGCCAATT AAAACCTTCG TCAAACAGTG TCATCCTTAG TAAATATGAC TCAATTGATA ATGGTCTTGA   
  
  
- TCATGAATTG TGTATTTAGA TAATGCGCAA TATATGATTG CATTTGACAA ATTAATTAAG ATAGATGTTT   
  
  
- CAAGTTAAGC ATAGTGTTTG ACTGACTTAG TTAATTCAAT TAAGTTCCGT GACTTAGAGA TCAAATTATA   
  
  
- ATCATCACTC ACAGTACGCG AAATGAAATA CATAATATTA CATTACTCTA AACCCAGAAT TACCCCCCCA   
  
  
- AAAAAAAAAC ACACAAAAAA AAAAGGTGGC TTATCCTTAA AAAAAAAATC TTCAAGTAAA ATGTCTTATT   
  
  
- TAAAAATAAG AAAAAGACAA AAAAAAAGAC AAAGGGCGGG AAAGGGGTCA AAATTGTAAG ATTACTTTAA   
  
  
- TCACATCATG TCATAGCCAA ATTGTGTTT

+     LTR

| Site Name | Organism | Position | Strand | Matrix score. | sequence | function |
| --- | --- | --- | --- | --- | --- | --- |
| LTR | Hordeum vulgare | 316 | - | 6 | CCGAAA | cis-acting element involved in low-temperature responsiveness |

> 2018/04/13 10:10:12  
+ CCAAAGCCAT TCCATCGACT TTAATCCCAA TCCCAAACGT TCCATTAGGT AACGGTCTTT CGTTCTCCTC   
  
  
+ AACCTGTACC ATCGCACTCT CTACCCATGA AAACATACAA TCCGAACGAG GGTGATATAT ATGTTTCTTA   
  
  
+ ATTTTTATGA ATTATTTTCT TATTTCTGTA ATATTTTAAT TTTTATAATA GAAGTAAAAC TTGACGACGA   
  
  
+ AGTACCTGTG GAAGGGCCTC CCCATGATGC TTCGATGGAA CACCACGGTG CACACACCGG GTGTACGATA   
  
  
+ CTGCAGTAGC ATTACTGCAT CCGGACCGAG TGAACTTTCG GTATGACCGG GTGAACTCAT TATTAACGGG   
  
  
+ CTACAGAACA AAAACTATCC GCCTCCATGA AAACACCCTG GTGTTGCGGA ACCTAAATGA GGATTATTCA   
  
  
+ GGACAGACAC TAGTGTTAAG GACTATGGCA GTCTACTAGA GCCACCAGAG TAAGTTCGTA TTTTAATAAA   
  
  
+ AATAATATTT TAATTTTAGA AGGTGGTCTT ATTTTTACAA GTCACTCATA CCCTTATAAA CATCTAAATA   
  
  
+ TAATAAAAAA ATTAATGAAG ATCAAATTTT TATAAACTTA AAATGTTATT ATACTACCAA ATTTATTTCT   
  
  
+ AAAATAGTAT TTCATTGCAT ACAATTAAAC AAATTAATAC TAGCATATTA AAATAAATTA ATTTTTTGTA   
  
  
+ AAGTTTTTGG TAGGGTAGTT CTATATATTA CCGTGCTATT TTTAATCAAC TTCACCTTTG TAAAATTCAT   
  
  
+ GTGAATAGTT AACCTGTGTC GATATTTTTA CTTAAATATT CTAGGGTTGG TTATTGAAAT TTTTAATTAT   
  
  
+ TAAAAAATTA TTAAATATTA TTTAATACAT ATAGAAAATT AATTTAATAA AATGTCAATA AAAAAACCTT   
  
  
+ TACTTATTTT TTTCCTCTTT AATTTCAACA ACTTAACGTT TAAATTACAA CACACCTAAC TTATCCGTCG   
  
  
+ ACGGTTCTCT CAGTTACTCA GTTGTTTATA CTAACTTAGT TTATTTAATC ATCAACCCCG CTCCACATTA   
  
  
+ ATTCGGTTAA TTTTGGAAGC AGTTTGTCAC AGTAGGAATC ATTTATACTG AGTTAACTAT TACCAGAACT   
  
  
+ AGTACTTAAC ACATAAATCT ATTACGCGTT ATATACTAAC GTAAACTGTT TAATTAATTC TATCTACAAA   
  
  
+ GTTCAATTCG TATCACAAAC TGACTGAATC AATTAAGTTA ATTCAAGGCA CTGAATCTCT AGTTTAATAT   
  
  
+ TAGTAGTGAG TGTCATGCGC TTTACTTTAT GTATTATAAT GTAATGAGAT TTGGGTCTTA ATGGGGGGGT   
  
  
+ TTTTTTTTTG TGTGTTTTTT TTTTCCACCG AATAGGAATT TTTTTTTTAG AAGTTCATTT TACAGAATAA   
  
  
+ ATTTTTATTC TTTTTCTGTT TTTTTTTCTG TTTCCCGCCC TTTCCCCAGT TTTAACATTC TAATGAAATT   
  
  
+ AGTGTAGTAC AGTATCGGTT TAACACAAA  

- GGTTTCGGTA AGGTAGCTGA AATTAGGGTT AGGGTTTGCA AGGTAATCCA TTGCCAGAAA GCAAGAGGAG   
  
  
- TTGGACATGG TAGCGTGAGA GATGGGTACT TTTGTATGTT AGGCTTGCTC CCACTATATA TACAAAGAAT   
  
  
- TAAAAATACT TAATAAAAGA ATAAAGACAT TATAAAATTA AAAATATTAT CTTCATTTTG AACTGCTGCT   
  
  
- TCATGGACAC CTTCCCGGAG GGGTACTACG AAGCTACCTT GTGGTGCCAC GTGTGTGGCC CACATGCTAT   
  
  
- GACGTCATCG TAATGACGTA GGCCTGGCTC ACTTGAAAGC CATACTGGCC CACTTGAGTA ATAATTGCCC   
  
  
- GATGTCTTGT TTTTGATAGG CGGAGGTACT TTTGTGGGAC CACAACGCCT TGGATTTACT CCTAATAAGT   
  
  
- CCTGTCTGTG ATCACAATTC CTGATACCGT CAGATGATCT CGGTGGTCTC ATTCAAGCAT AAAATTATTT   
  
  
- TTATTATAAA ATTAAAATCT TCCACCAGAA TAAAAATGTT CAGTGAGTAT GGGAATATTT GTAGATTTAT   
  
  
- ATTATTTTTT TAATTACTTC TAGTTTAAAA ATATTTGAAT TTTACAATAA TATGATGGTT TAAATAAAGA   
  
  
- TTTTATCATA AAGTAACGTA TGTTAATTTG TTTAATTATG ATCGTATAAT TTTATTTAAT TAAAAAACAT   
  
  
- TTCAAAAACC ATCCCATCAA GATATATAAT GGCACGATAA AAATTAGTTG AAGTGGAAAC ATTTTAAGTA   
  
  
- CACTTATCAA TTGGACACAG CTATAAAAAT GAATTTATAA GATCCCAACC AATAACTTTA AAAATTAATA   
  
  
- ATTTTTTAAT AATTTATAAT AAATTATGTA TATCTTTTAA TTAAATTATT TTACAGTTAT TTTTTTGGAA   
  
  
- ATGAATAAAA AAAGGAGAAA TTAAAGTTGT TGAATTGCAA ATTTAATGTT GTGTGGATTG AATAGGCAGC   
  
  
- TGCCAAGAGA GTCAATGAGT CAACAAATAT GATTGAATCA AATAAATTAG TAGTTGGGGC GAGGTGTAAT   
  
  
- TAAGCCAATT AAAACCTTCG TCAAACAGTG TCATCCTTAG TAAATATGAC TCAATTGATA ATGGTCTTGA   
  
  
- TCATGAATTG TGTATTTAGA TAATGCGCAA TATATGATTG CATTTGACAA ATTAATTAAG ATAGATGTTT   
  
  
- CAAGTTAAGC ATAGTGTTTG ACTGACTTAG TTAATTCAAT TAAGTTCCGT GACTTAGAGA TCAAATTATA   
  
  
- ATCATCACTC ACAGTACGCG AAATGAAATA CATAATATTA CATTACTCTA AACCCAGAAT TACCCCCCCA   
  
  
- AAAAAAAAAC ACACAAAAAA AAAAGGTGGC TTATCCTTAA AAAAAAAATC TTCAAGTAAA ATGTCTTATT   
  
  
- TAAAAATAAG AAAAAGACAA AAAAAAAGAC AAAGGGCGGG AAAGGGGTCA AAATTGTAAG ATTACTTTAA   
  
  
- TCACATCATG TCATAGCCAA ATTGTGTTT

+     MBS

| Site Name | Organism | Position | Strand | Matrix score. | sequence | function |
| --- | --- | --- | --- | --- | --- | --- |
| MBS | Arabidopsis thaliana | 999 | - | 6 | CAACTG | MYB binding site involved in drought-inducibility |
| MBS | Zea mays | 324 | - | 6 | CGGTCA | MYB Binding Site |
| MBS | Arabidopsis thaliana | 991 | - | 6 | TAACTG | MYB binding site involved in drought-inducibility |

> 2018/04/13 10:10:12  
+ CCAAAGCCAT TCCATCGACT TTAATCCCAA TCCCAAACGT TCCATTAGGT AACGGTCTTT CGTTCTCCTC   
  
  
+ AACCTGTACC ATCGCACTCT CTACCCATGA AAACATACAA TCCGAACGAG GGTGATATAT ATGTTTCTTA   
  
  
+ ATTTTTATGA ATTATTTTCT TATTTCTGTA ATATTTTAAT TTTTATAATA GAAGTAAAAC TTGACGACGA   
  
  
+ AGTACCTGTG GAAGGGCCTC CCCATGATGC TTCGATGGAA CACCACGGTG CACACACCGG GTGTACGATA   
  
  
+ CTGCAGTAGC ATTACTGCAT CCGGACCGAG TGAACTTTCG GTATGACCGG GTGAACTCAT TATTAACGGG   
  
  
+ CTACAGAACA AAAACTATCC GCCTCCATGA AAACACCCTG GTGTTGCGGA ACCTAAATGA GGATTATTCA   
  
  
+ GGACAGACAC TAGTGTTAAG GACTATGGCA GTCTACTAGA GCCACCAGAG TAAGTTCGTA TTTTAATAAA   
  
  
+ AATAATATTT TAATTTTAGA AGGTGGTCTT ATTTTTACAA GTCACTCATA CCCTTATAAA CATCTAAATA   
  
  
+ TAATAAAAAA ATTAATGAAG ATCAAATTTT TATAAACTTA AAATGTTATT ATACTACCAA ATTTATTTCT   
  
  
+ AAAATAGTAT TTCATTGCAT ACAATTAAAC AAATTAATAC TAGCATATTA AAATAAATTA ATTTTTTGTA   
  
  
+ AAGTTTTTGG TAGGGTAGTT CTATATATTA CCGTGCTATT TTTAATCAAC TTCACCTTTG TAAAATTCAT   
  
  
+ GTGAATAGTT AACCTGTGTC GATATTTTTA CTTAAATATT CTAGGGTTGG TTATTGAAAT TTTTAATTAT   
  
  
+ TAAAAAATTA TTAAATATTA TTTAATACAT ATAGAAAATT AATTTAATAA AATGTCAATA AAAAAACCTT   
  
  
+ TACTTATTTT TTTCCTCTTT AATTTCAACA ACTTAACGTT TAAATTACAA CACACCTAAC TTATCCGTCG   
  
  
+ ACGGTTCTCT CAGTTACTCA GTTGTTTATA CTAACTTAGT TTATTTAATC ATCAACCCCG CTCCACATTA   
  
  
+ ATTCGGTTAA TTTTGGAAGC AGTTTGTCAC AGTAGGAATC ATTTATACTG AGTTAACTAT TACCAGAACT   
  
  
+ AGTACTTAAC ACATAAATCT ATTACGCGTT ATATACTAAC GTAAACTGTT TAATTAATTC TATCTACAAA   
  
  
+ GTTCAATTCG TATCACAAAC TGACTGAATC AATTAAGTTA ATTCAAGGCA CTGAATCTCT AGTTTAATAT   
  
  
+ TAGTAGTGAG TGTCATGCGC TTTACTTTAT GTATTATAAT GTAATGAGAT TTGGGTCTTA ATGGGGGGGT   
  
  
+ TTTTTTTTTG TGTGTTTTTT TTTTCCACCG AATAGGAATT TTTTTTTTAG AAGTTCATTT TACAGAATAA   
  
  
+ ATTTTTATTC TTTTTCTGTT TTTTTTTCTG TTTCCCGCCC TTTCCCCAGT TTTAACATTC TAATGAAATT   
  
  
+ AGTGTAGTAC AGTATCGGTT TAACACAAA  

- GGTTTCGGTA AGGTAGCTGA AATTAGGGTT AGGGTTTGCA AGGTAATCCA TTGCCAGAAA GCAAGAGGAG   
  
  
- TTGGACATGG TAGCGTGAGA GATGGGTACT TTTGTATGTT AGGCTTGCTC CCACTATATA TACAAAGAAT   
  
  
- TAAAAATACT TAATAAAAGA ATAAAGACAT TATAAAATTA AAAATATTAT CTTCATTTTG AACTGCTGCT   
  
  
- TCATGGACAC CTTCCCGGAG GGGTACTACG AAGCTACCTT GTGGTGCCAC GTGTGTGGCC CACATGCTAT   
  
  
- GACGTCATCG TAATGACGTA GGCCTGGCTC ACTTGAAAGC CATACTGGCC CACTTGAGTA ATAATTGCCC   
  
  
- GATGTCTTGT TTTTGATAGG CGGAGGTACT TTTGTGGGAC CACAACGCCT TGGATTTACT CCTAATAAGT   
  
  
- CCTGTCTGTG ATCACAATTC CTGATACCGT CAGATGATCT CGGTGGTCTC ATTCAAGCAT AAAATTATTT   
  
  
- TTATTATAAA ATTAAAATCT TCCACCAGAA TAAAAATGTT CAGTGAGTAT GGGAATATTT GTAGATTTAT   
  
  
- ATTATTTTTT TAATTACTTC TAGTTTAAAA ATATTTGAAT TTTACAATAA TATGATGGTT TAAATAAAGA   
  
  
- TTTTATCATA AAGTAACGTA TGTTAATTTG TTTAATTATG ATCGTATAAT TTTATTTAAT TAAAAAACAT   
  
  
- TTCAAAAACC ATCCCATCAA GATATATAAT GGCACGATAA AAATTAGTTG AAGTGGAAAC ATTTTAAGTA   
  
  
- CACTTATCAA TTGGACACAG CTATAAAAAT GAATTTATAA GATCCCAACC AATAACTTTA AAAATTAATA   
  
  
- ATTTTTTAAT AATTTATAAT AAATTATGTA TATCTTTTAA TTAAATTATT TTACAGTTAT TTTTTTGGAA   
  
  
- ATGAATAAAA AAAGGAGAAA TTAAAGTTGT TGAATTGCAA ATTTAATGTT GTGTGGATTG AATAGGCAGC   
  
  
- TGCCAAGAGA GTCAATGAGT CAACAAATAT GATTGAATCA AATAAATTAG TAGTTGGGGC GAGGTGTAAT   
  
  
- TAAGCCAATT AAAACCTTCG TCAAACAGTG TCATCCTTAG TAAATATGAC TCAATTGATA ATGGTCTTGA   
  
  
- TCATGAATTG TGTATTTAGA TAATGCGCAA TATATGATTG CATTTGACAA ATTAATTAAG ATAGATGTTT   
  
  
- CAAGTTAAGC ATAGTGTTTG ACTGACTTAG TTAATTCAAT TAAGTTCCGT GACTTAGAGA TCAAATTATA   
  
  
- ATCATCACTC ACAGTACGCG AAATGAAATA CATAATATTA CATTACTCTA AACCCAGAAT TACCCCCCCA   
  
  
- AAAAAAAAAC ACACAAAAAA AAAAGGTGGC TTATCCTTAA AAAAAAAATC TTCAAGTAAA ATGTCTTATT   
  
  
- TAAAAATAAG AAAAAGACAA AAAAAAAGAC AAAGGGCGGG AAAGGGGTCA AAATTGTAAG ATTACTTTAA   
  
  
- TCACATCATG TCATAGCCAA ATTGTGTTT

+     MBSI

| Site Name | Organism | Position | Strand | Matrix score. | sequence | function |
| --- | --- | --- | --- | --- | --- | --- |
| MBSI | Petunia hybrida | 901 | + | 10.5 | aaaAaaC(G/C)GTTA | MYB binding site involved in flavonoid biosynthetic genes regulation |

> 2018/04/13 10:10:12  
+ CCAAAGCCAT TCCATCGACT TTAATCCCAA TCCCAAACGT TCCATTAGGT AACGGTCTTT CGTTCTCCTC   
  
  
+ AACCTGTACC ATCGCACTCT CTACCCATGA AAACATACAA TCCGAACGAG GGTGATATAT ATGTTTCTTA   
  
  
+ ATTTTTATGA ATTATTTTCT TATTTCTGTA ATATTTTAAT TTTTATAATA GAAGTAAAAC TTGACGACGA   
  
  
+ AGTACCTGTG GAAGGGCCTC CCCATGATGC TTCGATGGAA CACCACGGTG CACACACCGG GTGTACGATA   
  
  
+ CTGCAGTAGC ATTACTGCAT CCGGACCGAG TGAACTTTCG GTATGACCGG GTGAACTCAT TATTAACGGG   
  
  
+ CTACAGAACA AAAACTATCC GCCTCCATGA AAACACCCTG GTGTTGCGGA ACCTAAATGA GGATTATTCA   
  
  
+ GGACAGACAC TAGTGTTAAG GACTATGGCA GTCTACTAGA GCCACCAGAG TAAGTTCGTA TTTTAATAAA   
  
  
+ AATAATATTT TAATTTTAGA AGGTGGTCTT ATTTTTACAA GTCACTCATA CCCTTATAAA CATCTAAATA   
  
  
+ TAATAAAAAA ATTAATGAAG ATCAAATTTT TATAAACTTA AAATGTTATT ATACTACCAA ATTTATTTCT   
  
  
+ AAAATAGTAT TTCATTGCAT ACAATTAAAC AAATTAATAC TAGCATATTA AAATAAATTA ATTTTTTGTA   
  
  
+ AAGTTTTTGG TAGGGTAGTT CTATATATTA CCGTGCTATT TTTAATCAAC TTCACCTTTG TAAAATTCAT   
  
  
+ GTGAATAGTT AACCTGTGTC GATATTTTTA CTTAAATATT CTAGGGTTGG TTATTGAAAT TTTTAATTAT   
  
  
+ TAAAAAATTA TTAAATATTA TTTAATACAT ATAGAAAATT AATTTAATAA AATGTCAATA AAAAAACCTT   
  
  
+ TACTTATTTT TTTCCTCTTT AATTTCAACA ACTTAACGTT TAAATTACAA CACACCTAAC TTATCCGTCG   
  
  
+ ACGGTTCTCT CAGTTACTCA GTTGTTTATA CTAACTTAGT TTATTTAATC ATCAACCCCG CTCCACATTA   
  
  
+ ATTCGGTTAA TTTTGGAAGC AGTTTGTCAC AGTAGGAATC ATTTATACTG AGTTAACTAT TACCAGAACT   
  
  
+ AGTACTTAAC ACATAAATCT ATTACGCGTT ATATACTAAC GTAAACTGTT TAATTAATTC TATCTACAAA   
  
  
+ GTTCAATTCG TATCACAAAC TGACTGAATC AATTAAGTTA ATTCAAGGCA CTGAATCTCT AGTTTAATAT   
  
  
+ TAGTAGTGAG TGTCATGCGC TTTACTTTAT GTATTATAAT GTAATGAGAT TTGGGTCTTA ATGGGGGGGT   
  
  
+ TTTTTTTTTG TGTGTTTTTT TTTTCCACCG AATAGGAATT TTTTTTTTAG AAGTTCATTT TACAGAATAA   
  
  
+ ATTTTTATTC TTTTTCTGTT TTTTTTTCTG TTTCCCGCCC TTTCCCCAGT TTTAACATTC TAATGAAATT   
  
  
+ AGTGTAGTAC AGTATCGGTT TAACACAAA  

- GGTTTCGGTA AGGTAGCTGA AATTAGGGTT AGGGTTTGCA AGGTAATCCA TTGCCAGAAA GCAAGAGGAG   
  
  
- TTGGACATGG TAGCGTGAGA GATGGGTACT TTTGTATGTT AGGCTTGCTC CCACTATATA TACAAAGAAT   
  
  
- TAAAAATACT TAATAAAAGA ATAAAGACAT TATAAAATTA AAAATATTAT CTTCATTTTG AACTGCTGCT   
  
  
- TCATGGACAC CTTCCCGGAG GGGTACTACG AAGCTACCTT GTGGTGCCAC GTGTGTGGCC CACATGCTAT   
  
  
- GACGTCATCG TAATGACGTA GGCCTGGCTC ACTTGAAAGC CATACTGGCC CACTTGAGTA ATAATTGCCC   
  
  
- GATGTCTTGT TTTTGATAGG CGGAGGTACT TTTGTGGGAC CACAACGCCT TGGATTTACT CCTAATAAGT   
  
  
- CCTGTCTGTG ATCACAATTC CTGATACCGT CAGATGATCT CGGTGGTCTC ATTCAAGCAT AAAATTATTT   
  
  
- TTATTATAAA ATTAAAATCT TCCACCAGAA TAAAAATGTT CAGTGAGTAT GGGAATATTT GTAGATTTAT   
  
  
- ATTATTTTTT TAATTACTTC TAGTTTAAAA ATATTTGAAT TTTACAATAA TATGATGGTT TAAATAAAGA   
  
  
- TTTTATCATA AAGTAACGTA TGTTAATTTG TTTAATTATG ATCGTATAAT TTTATTTAAT TAAAAAACAT   
  
  
- TTCAAAAACC ATCCCATCAA GATATATAAT GGCACGATAA AAATTAGTTG AAGTGGAAAC ATTTTAAGTA   
  
  
- CACTTATCAA TTGGACACAG CTATAAAAAT GAATTTATAA GATCCCAACC AATAACTTTA AAAATTAATA   
  
  
- ATTTTTTAAT AATTTATAAT AAATTATGTA TATCTTTTAA TTAAATTATT TTACAGTTAT TTTTTTGGAA   
  
  
- ATGAATAAAA AAAGGAGAAA TTAAAGTTGT TGAATTGCAA ATTTAATGTT GTGTGGATTG AATAGGCAGC   
  
  
- TGCCAAGAGA GTCAATGAGT CAACAAATAT GATTGAATCA AATAAATTAG TAGTTGGGGC GAGGTGTAAT   
  
  
- TAAGCCAATT AAAACCTTCG TCAAACAGTG TCATCCTTAG TAAATATGAC TCAATTGATA ATGGTCTTGA   
  
  
- TCATGAATTG TGTATTTAGA TAATGCGCAA TATATGATTG CATTTGACAA ATTAATTAAG ATAGATGTTT   
  
  
- CAAGTTAAGC ATAGTGTTTG ACTGACTTAG TTAATTCAAT TAAGTTCCGT GACTTAGAGA TCAAATTATA   
  
  
- ATCATCACTC ACAGTACGCG AAATGAAATA CATAATATTA CATTACTCTA AACCCAGAAT TACCCCCCCA   
  
  
- AAAAAAAAAC ACACAAAAAA AAAAGGTGGC TTATCCTTAA AAAAAAAATC TTCAAGTAAA ATGTCTTATT   
  
  
- TAAAAATAAG AAAAAGACAA AAAAAAAGAC AAAGGGCGGG AAAGGGGTCA AAATTGTAAG ATTACTTTAA   
  
  
- TCACATCATG TCATAGCCAA ATTGTGTTT

+     MRE

| Site Name | Organism | Position | Strand | Matrix score. | sequence | function |
| --- | --- | --- | --- | --- | --- | --- |
| MRE | Petroselinum crispum | 400 | + | 7 | AACCTAA | MYB binding site involved in light responsiveness |

> 2018/04/13 10:10:12  
+ CCAAAGCCAT TCCATCGACT TTAATCCCAA TCCCAAACGT TCCATTAGGT AACGGTCTTT CGTTCTCCTC   
  
  
+ AACCTGTACC ATCGCACTCT CTACCCATGA AAACATACAA TCCGAACGAG GGTGATATAT ATGTTTCTTA   
  
  
+ ATTTTTATGA ATTATTTTCT TATTTCTGTA ATATTTTAAT TTTTATAATA GAAGTAAAAC TTGACGACGA   
  
  
+ AGTACCTGTG GAAGGGCCTC CCCATGATGC TTCGATGGAA CACCACGGTG CACACACCGG GTGTACGATA   
  
  
+ CTGCAGTAGC ATTACTGCAT CCGGACCGAG TGAACTTTCG GTATGACCGG GTGAACTCAT TATTAACGGG   
  
  
+ CTACAGAACA AAAACTATCC GCCTCCATGA AAACACCCTG GTGTTGCGGA ACCTAAATGA GGATTATTCA   
  
  
+ GGACAGACAC TAGTGTTAAG GACTATGGCA GTCTACTAGA GCCACCAGAG TAAGTTCGTA TTTTAATAAA   
  
  
+ AATAATATTT TAATTTTAGA AGGTGGTCTT ATTTTTACAA GTCACTCATA CCCTTATAAA CATCTAAATA   
  
  
+ TAATAAAAAA ATTAATGAAG ATCAAATTTT TATAAACTTA AAATGTTATT ATACTACCAA ATTTATTTCT   
  
  
+ AAAATAGTAT TTCATTGCAT ACAATTAAAC AAATTAATAC TAGCATATTA AAATAAATTA ATTTTTTGTA   
  
  
+ AAGTTTTTGG TAGGGTAGTT CTATATATTA CCGTGCTATT TTTAATCAAC TTCACCTTTG TAAAATTCAT   
  
  
+ GTGAATAGTT AACCTGTGTC GATATTTTTA CTTAAATATT CTAGGGTTGG TTATTGAAAT TTTTAATTAT   
  
  
+ TAAAAAATTA TTAAATATTA TTTAATACAT ATAGAAAATT AATTTAATAA AATGTCAATA AAAAAACCTT   
  
  
+ TACTTATTTT TTTCCTCTTT AATTTCAACA ACTTAACGTT TAAATTACAA CACACCTAAC TTATCCGTCG   
  
  
+ ACGGTTCTCT CAGTTACTCA GTTGTTTATA CTAACTTAGT TTATTTAATC ATCAACCCCG CTCCACATTA   
  
  
+ ATTCGGTTAA TTTTGGAAGC AGTTTGTCAC AGTAGGAATC ATTTATACTG AGTTAACTAT TACCAGAACT   
  
  
+ AGTACTTAAC ACATAAATCT ATTACGCGTT ATATACTAAC GTAAACTGTT TAATTAATTC TATCTACAAA   
  
  
+ GTTCAATTCG TATCACAAAC TGACTGAATC AATTAAGTTA ATTCAAGGCA CTGAATCTCT AGTTTAATAT   
  
  
+ TAGTAGTGAG TGTCATGCGC TTTACTTTAT GTATTATAAT GTAATGAGAT TTGGGTCTTA ATGGGGGGGT   
  
  
+ TTTTTTTTTG TGTGTTTTTT TTTTCCACCG AATAGGAATT TTTTTTTTAG AAGTTCATTT TACAGAATAA   
  
  
+ ATTTTTATTC TTTTTCTGTT TTTTTTTCTG TTTCCCGCCC TTTCCCCAGT TTTAACATTC TAATGAAATT   
  
  
+ AGTGTAGTAC AGTATCGGTT TAACACAAA  

- GGTTTCGGTA AGGTAGCTGA AATTAGGGTT AGGGTTTGCA AGGTAATCCA TTGCCAGAAA GCAAGAGGAG   
  
  
- TTGGACATGG TAGCGTGAGA GATGGGTACT TTTGTATGTT AGGCTTGCTC CCACTATATA TACAAAGAAT   
  
  
- TAAAAATACT TAATAAAAGA ATAAAGACAT TATAAAATTA AAAATATTAT CTTCATTTTG AACTGCTGCT   
  
  
- TCATGGACAC CTTCCCGGAG GGGTACTACG AAGCTACCTT GTGGTGCCAC GTGTGTGGCC CACATGCTAT   
  
  
- GACGTCATCG TAATGACGTA GGCCTGGCTC ACTTGAAAGC CATACTGGCC CACTTGAGTA ATAATTGCCC   
  
  
- GATGTCTTGT TTTTGATAGG CGGAGGTACT TTTGTGGGAC CACAACGCCT TGGATTTACT CCTAATAAGT   
  
  
- CCTGTCTGTG ATCACAATTC CTGATACCGT CAGATGATCT CGGTGGTCTC ATTCAAGCAT AAAATTATTT   
  
  
- TTATTATAAA ATTAAAATCT TCCACCAGAA TAAAAATGTT CAGTGAGTAT GGGAATATTT GTAGATTTAT   
  
  
- ATTATTTTTT TAATTACTTC TAGTTTAAAA ATATTTGAAT TTTACAATAA TATGATGGTT TAAATAAAGA   
  
  
- TTTTATCATA AAGTAACGTA TGTTAATTTG TTTAATTATG ATCGTATAAT TTTATTTAAT TAAAAAACAT   
  
  
- TTCAAAAACC ATCCCATCAA GATATATAAT GGCACGATAA AAATTAGTTG AAGTGGAAAC ATTTTAAGTA   
  
  
- CACTTATCAA TTGGACACAG CTATAAAAAT GAATTTATAA GATCCCAACC AATAACTTTA AAAATTAATA   
  
  
- ATTTTTTAAT AATTTATAAT AAATTATGTA TATCTTTTAA TTAAATTATT TTACAGTTAT TTTTTTGGAA   
  
  
- ATGAATAAAA AAAGGAGAAA TTAAAGTTGT TGAATTGCAA ATTTAATGTT GTGTGGATTG AATAGGCAGC   
  
  
- TGCCAAGAGA GTCAATGAGT CAACAAATAT GATTGAATCA AATAAATTAG TAGTTGGGGC GAGGTGTAAT   
  
  
- TAAGCCAATT AAAACCTTCG TCAAACAGTG TCATCCTTAG TAAATATGAC TCAATTGATA ATGGTCTTGA   
  
  
- TCATGAATTG TGTATTTAGA TAATGCGCAA TATATGATTG CATTTGACAA ATTAATTAAG ATAGATGTTT   
  
  
- CAAGTTAAGC ATAGTGTTTG ACTGACTTAG TTAATTCAAT TAAGTTCCGT GACTTAGAGA TCAAATTATA   
  
  
- ATCATCACTC ACAGTACGCG AAATGAAATA CATAATATTA CATTACTCTA AACCCAGAAT TACCCCCCCA   
  
  
- AAAAAAAAAC ACACAAAAAA AAAAGGTGGC TTATCCTTAA AAAAAAAATC TTCAAGTAAA ATGTCTTATT   
  
  
- TAAAAATAAG AAAAAGACAA AAAAAAAGAC AAAGGGCGGG AAAGGGGTCA AAATTGTAAG ATTACTTTAA   
  
  
- TCACATCATG TCATAGCCAA ATTGTGTTT

+     Skn-1\_motif

| Site Name | Organism | Position | Strand | Matrix score. | sequence | function |
| --- | --- | --- | --- | --- | --- | --- |
| Skn-1\_motif | Oryza sativa | 323 | - | 5 | GTCAT | cis-acting regulatory element required for endosperm expression |
| Skn-1\_motif | Oryza sativa | 1272 | + | 5 | GTCAT | cis-acting regulatory element required for endosperm expression |

> 2018/04/13 10:10:12  
+ CCAAAGCCAT TCCATCGACT TTAATCCCAA TCCCAAACGT TCCATTAGGT AACGGTCTTT CGTTCTCCTC   
  
  
+ AACCTGTACC ATCGCACTCT CTACCCATGA AAACATACAA TCCGAACGAG GGTGATATAT ATGTTTCTTA   
  
  
+ ATTTTTATGA ATTATTTTCT TATTTCTGTA ATATTTTAAT TTTTATAATA GAAGTAAAAC TTGACGACGA   
  
  
+ AGTACCTGTG GAAGGGCCTC CCCATGATGC TTCGATGGAA CACCACGGTG CACACACCGG GTGTACGATA   
  
  
+ CTGCAGTAGC ATTACTGCAT CCGGACCGAG TGAACTTTCG GTATGACCGG GTGAACTCAT TATTAACGGG   
  
  
+ CTACAGAACA AAAACTATCC GCCTCCATGA AAACACCCTG GTGTTGCGGA ACCTAAATGA GGATTATTCA   
  
  
+ GGACAGACAC TAGTGTTAAG GACTATGGCA GTCTACTAGA GCCACCAGAG TAAGTTCGTA TTTTAATAAA   
  
  
+ AATAATATTT TAATTTTAGA AGGTGGTCTT ATTTTTACAA GTCACTCATA CCCTTATAAA CATCTAAATA   
  
  
+ TAATAAAAAA ATTAATGAAG ATCAAATTTT TATAAACTTA AAATGTTATT ATACTACCAA ATTTATTTCT   
  
  
+ AAAATAGTAT TTCATTGCAT ACAATTAAAC AAATTAATAC TAGCATATTA AAATAAATTA ATTTTTTGTA   
  
  
+ AAGTTTTTGG TAGGGTAGTT CTATATATTA CCGTGCTATT TTTAATCAAC TTCACCTTTG TAAAATTCAT   
  
  
+ GTGAATAGTT AACCTGTGTC GATATTTTTA CTTAAATATT CTAGGGTTGG TTATTGAAAT TTTTAATTAT   
  
  
+ TAAAAAATTA TTAAATATTA TTTAATACAT ATAGAAAATT AATTTAATAA AATGTCAATA AAAAAACCTT   
  
  
+ TACTTATTTT TTTCCTCTTT AATTTCAACA ACTTAACGTT TAAATTACAA CACACCTAAC TTATCCGTCG   
  
  
+ ACGGTTCTCT CAGTTACTCA GTTGTTTATA CTAACTTAGT TTATTTAATC ATCAACCCCG CTCCACATTA   
  
  
+ ATTCGGTTAA TTTTGGAAGC AGTTTGTCAC AGTAGGAATC ATTTATACTG AGTTAACTAT TACCAGAACT   
  
  
+ AGTACTTAAC ACATAAATCT ATTACGCGTT ATATACTAAC GTAAACTGTT TAATTAATTC TATCTACAAA   
  
  
+ GTTCAATTCG TATCACAAAC TGACTGAATC AATTAAGTTA ATTCAAGGCA CTGAATCTCT AGTTTAATAT   
  
  
+ TAGTAGTGAG TGTCATGCGC TTTACTTTAT GTATTATAAT GTAATGAGAT TTGGGTCTTA ATGGGGGGGT   
  
  
+ TTTTTTTTTG TGTGTTTTTT TTTTCCACCG AATAGGAATT TTTTTTTTAG AAGTTCATTT TACAGAATAA   
  
  
+ ATTTTTATTC TTTTTCTGTT TTTTTTTCTG TTTCCCGCCC TTTCCCCAGT TTTAACATTC TAATGAAATT   
  
  
+ AGTGTAGTAC AGTATCGGTT TAACACAAA  

- GGTTTCGGTA AGGTAGCTGA AATTAGGGTT AGGGTTTGCA AGGTAATCCA TTGCCAGAAA GCAAGAGGAG   
  
  
- TTGGACATGG TAGCGTGAGA GATGGGTACT TTTGTATGTT AGGCTTGCTC CCACTATATA TACAAAGAAT   
  
  
- TAAAAATACT TAATAAAAGA ATAAAGACAT TATAAAATTA AAAATATTAT CTTCATTTTG AACTGCTGCT   
  
  
- TCATGGACAC CTTCCCGGAG GGGTACTACG AAGCTACCTT GTGGTGCCAC GTGTGTGGCC CACATGCTAT   
  
  
- GACGTCATCG TAATGACGTA GGCCTGGCTC ACTTGAAAGC CATACTGGCC CACTTGAGTA ATAATTGCCC   
  
  
- GATGTCTTGT TTTTGATAGG CGGAGGTACT TTTGTGGGAC CACAACGCCT TGGATTTACT CCTAATAAGT   
  
  
- CCTGTCTGTG ATCACAATTC CTGATACCGT CAGATGATCT CGGTGGTCTC ATTCAAGCAT AAAATTATTT   
  
  
- TTATTATAAA ATTAAAATCT TCCACCAGAA TAAAAATGTT CAGTGAGTAT GGGAATATTT GTAGATTTAT   
  
  
- ATTATTTTTT TAATTACTTC TAGTTTAAAA ATATTTGAAT TTTACAATAA TATGATGGTT TAAATAAAGA   
  
  
- TTTTATCATA AAGTAACGTA TGTTAATTTG TTTAATTATG ATCGTATAAT TTTATTTAAT TAAAAAACAT   
  
  
- TTCAAAAACC ATCCCATCAA GATATATAAT GGCACGATAA AAATTAGTTG AAGTGGAAAC ATTTTAAGTA   
  
  
- CACTTATCAA TTGGACACAG CTATAAAAAT GAATTTATAA GATCCCAACC AATAACTTTA AAAATTAATA   
  
  
- ATTTTTTAAT AATTTATAAT AAATTATGTA TATCTTTTAA TTAAATTATT TTACAGTTAT TTTTTTGGAA   
  
  
- ATGAATAAAA AAAGGAGAAA TTAAAGTTGT TGAATTGCAA ATTTAATGTT GTGTGGATTG AATAGGCAGC   
  
  
- TGCCAAGAGA GTCAATGAGT CAACAAATAT GATTGAATCA AATAAATTAG TAGTTGGGGC GAGGTGTAAT   
  
  
- TAAGCCAATT AAAACCTTCG TCAAACAGTG TCATCCTTAG TAAATATGAC TCAATTGATA ATGGTCTTGA   
  
  
- TCATGAATTG TGTATTTAGA TAATGCGCAA TATATGATTG CATTTGACAA ATTAATTAAG ATAGATGTTT   
  
  
- CAAGTTAAGC ATAGTGTTTG ACTGACTTAG TTAATTCAAT TAAGTTCCGT GACTTAGAGA TCAAATTATA   
  
  
- ATCATCACTC ACAGTACGCG AAATGAAATA CATAATATTA CATTACTCTA AACCCAGAAT TACCCCCCCA   
  
  
- AAAAAAAAAC ACACAAAAAA AAAAGGTGGC TTATCCTTAA AAAAAAAATC TTCAAGTAAA ATGTCTTATT   
  
  
- TAAAAATAAG AAAAAGACAA AAAAAAAGAC AAAGGGCGGG AAAGGGGTCA AAATTGTAAG ATTACTTTAA   
  
  
- TCACATCATG TCATAGCCAA ATTGTGTTT

+     Sp1

| Site Name | Organism | Position | Strand | Matrix score. | sequence | function |
| --- | --- | --- | --- | --- | --- | --- |
| Sp1 | Oryza sativa | 1435 | - | 6 | GGGCGG | light responsive element |
| Sp1 | Zea mays | 227 | + | 5 | CC(G/A)CCC | light responsive element |
| Sp1 | Zea mays | 1323 | - | 5 | CC(G/A)CCC | light responsive element |
| Sp1 | Zea mays | 1324 | - | 5 | CC(G/A)CCC | light responsive element |

> 2018/04/13 10:10:12  
+ CCAAAGCCAT TCCATCGACT TTAATCCCAA TCCCAAACGT TCCATTAGGT AACGGTCTTT CGTTCTCCTC   
  
  
+ AACCTGTACC ATCGCACTCT CTACCCATGA AAACATACAA TCCGAACGAG GGTGATATAT ATGTTTCTTA   
  
  
+ ATTTTTATGA ATTATTTTCT TATTTCTGTA ATATTTTAAT TTTTATAATA GAAGTAAAAC TTGACGACGA   
  
  
+ AGTACCTGTG GAAGGGCCTC CCCATGATGC TTCGATGGAA CACCACGGTG CACACACCGG GTGTACGATA   
  
  
+ CTGCAGTAGC ATTACTGCAT CCGGACCGAG TGAACTTTCG GTATGACCGG GTGAACTCAT TATTAACGGG   
  
  
+ CTACAGAACA AAAACTATCC GCCTCCATGA AAACACCCTG GTGTTGCGGA ACCTAAATGA GGATTATTCA   
  
  
+ GGACAGACAC TAGTGTTAAG GACTATGGCA GTCTACTAGA GCCACCAGAG TAAGTTCGTA TTTTAATAAA   
  
  
+ AATAATATTT TAATTTTAGA AGGTGGTCTT ATTTTTACAA GTCACTCATA CCCTTATAAA CATCTAAATA   
  
  
+ TAATAAAAAA ATTAATGAAG ATCAAATTTT TATAAACTTA AAATGTTATT ATACTACCAA ATTTATTTCT   
  
  
+ AAAATAGTAT TTCATTGCAT ACAATTAAAC AAATTAATAC TAGCATATTA AAATAAATTA ATTTTTTGTA   
  
  
+ AAGTTTTTGG TAGGGTAGTT CTATATATTA CCGTGCTATT TTTAATCAAC TTCACCTTTG TAAAATTCAT   
  
  
+ GTGAATAGTT AACCTGTGTC GATATTTTTA CTTAAATATT CTAGGGTTGG TTATTGAAAT TTTTAATTAT   
  
  
+ TAAAAAATTA TTAAATATTA TTTAATACAT ATAGAAAATT AATTTAATAA AATGTCAATA AAAAAACCTT   
  
  
+ TACTTATTTT TTTCCTCTTT AATTTCAACA ACTTAACGTT TAAATTACAA CACACCTAAC TTATCCGTCG   
  
  
+ ACGGTTCTCT CAGTTACTCA GTTGTTTATA CTAACTTAGT TTATTTAATC ATCAACCCCG CTCCACATTA   
  
  
+ ATTCGGTTAA TTTTGGAAGC AGTTTGTCAC AGTAGGAATC ATTTATACTG AGTTAACTAT TACCAGAACT   
  
  
+ AGTACTTAAC ACATAAATCT ATTACGCGTT ATATACTAAC GTAAACTGTT TAATTAATTC TATCTACAAA   
  
  
+ GTTCAATTCG TATCACAAAC TGACTGAATC AATTAAGTTA ATTCAAGGCA CTGAATCTCT AGTTTAATAT   
  
  
+ TAGTAGTGAG TGTCATGCGC TTTACTTTAT GTATTATAAT GTAATGAGAT TTGGGTCTTA ATGGGGGGGT   
  
  
+ TTTTTTTTTG TGTGTTTTTT TTTTCCACCG AATAGGAATT TTTTTTTTAG AAGTTCATTT TACAGAATAA   
  
  
+ ATTTTTATTC TTTTTCTGTT TTTTTTTCTG TTTCCCGCCC TTTCCCCAGT TTTAACATTC TAATGAAATT   
  
  
+ AGTGTAGTAC AGTATCGGTT TAACACAAA  

- GGTTTCGGTA AGGTAGCTGA AATTAGGGTT AGGGTTTGCA AGGTAATCCA TTGCCAGAAA GCAAGAGGAG   
  
  
- TTGGACATGG TAGCGTGAGA GATGGGTACT TTTGTATGTT AGGCTTGCTC CCACTATATA TACAAAGAAT   
  
  
- TAAAAATACT TAATAAAAGA ATAAAGACAT TATAAAATTA AAAATATTAT CTTCATTTTG AACTGCTGCT   
  
  
- TCATGGACAC CTTCCCGGAG GGGTACTACG AAGCTACCTT GTGGTGCCAC GTGTGTGGCC CACATGCTAT   
  
  
- GACGTCATCG TAATGACGTA GGCCTGGCTC ACTTGAAAGC CATACTGGCC CACTTGAGTA ATAATTGCCC   
  
  
- GATGTCTTGT TTTTGATAGG CGGAGGTACT TTTGTGGGAC CACAACGCCT TGGATTTACT CCTAATAAGT   
  
  
- CCTGTCTGTG ATCACAATTC CTGATACCGT CAGATGATCT CGGTGGTCTC ATTCAAGCAT AAAATTATTT   
  
  
- TTATTATAAA ATTAAAATCT TCCACCAGAA TAAAAATGTT CAGTGAGTAT GGGAATATTT GTAGATTTAT   
  
  
- ATTATTTTTT TAATTACTTC TAGTTTAAAA ATATTTGAAT TTTACAATAA TATGATGGTT TAAATAAAGA   
  
  
- TTTTATCATA AAGTAACGTA TGTTAATTTG TTTAATTATG ATCGTATAAT TTTATTTAAT TAAAAAACAT   
  
  
- TTCAAAAACC ATCCCATCAA GATATATAAT GGCACGATAA AAATTAGTTG AAGTGGAAAC ATTTTAAGTA   
  
  
- CACTTATCAA TTGGACACAG CTATAAAAAT GAATTTATAA GATCCCAACC AATAACTTTA AAAATTAATA   
  
  
- ATTTTTTAAT AATTTATAAT AAATTATGTA TATCTTTTAA TTAAATTATT TTACAGTTAT TTTTTTGGAA   
  
  
- ATGAATAAAA AAAGGAGAAA TTAAAGTTGT TGAATTGCAA ATTTAATGTT GTGTGGATTG AATAGGCAGC   
  
  
- TGCCAAGAGA GTCAATGAGT CAACAAATAT GATTGAATCA AATAAATTAG TAGTTGGGGC GAGGTGTAAT   
  
  
- TAAGCCAATT AAAACCTTCG TCAAACAGTG TCATCCTTAG TAAATATGAC TCAATTGATA ATGGTCTTGA   
  
  
- TCATGAATTG TGTATTTAGA TAATGCGCAA TATATGATTG CATTTGACAA ATTAATTAAG ATAGATGTTT   
  
  
- CAAGTTAAGC ATAGTGTTTG ACTGACTTAG TTAATTCAAT TAAGTTCCGT GACTTAGAGA TCAAATTATA   
  
  
- ATCATCACTC ACAGTACGCG AAATGAAATA CATAATATTA CATTACTCTA AACCCAGAAT TACCCCCCCA   
  
  
- AAAAAAAAAC ACACAAAAAA AAAAGGTGGC TTATCCTTAA AAAAAAAATC TTCAAGTAAA ATGTCTTATT   
  
  
- TAAAAATAAG AAAAAGACAA AAAAAAAGAC AAAGGGCGGG AAAGGGGTCA AAATTGTAAG ATTACTTTAA   
  
  
- TCACATCATG TCATAGCCAA ATTGTGTTT

+     TATA-box

| Site Name | Organism | Position | Strand | Matrix score. | sequence | function |
| --- | --- | --- | --- | --- | --- | --- |
| TATA-box | Arabidopsis thaliana | 1294 | - | 5 | TATAA | core promoter element around -30 of transcription start |
| TATA-box | Glycine max | 1292 | - | 5 | TAATA | core promoter element around -30 of transcription start |
| TATA-box | Arabidopsis thaliana | 1150 | - | 4 | TATA | core promoter element around -30 of transcription start |
| TATA-box | Arabidopsis thaliana | 590 | - | 5 | TATAA | core promoter element around -30 of transcription start |
| TATA-box | Oryza sativa | 694 | - | 7 | TACAAAA | core promoter element around -30 of transcription start |
| TATA-box | Lycopersicon esculentum | 679 | - | 5 | TTTTA | core promoter element around -30 of transcription start |
| TATA-box | Lycopersicon esculentum | 1375 | + | 5 | TTTTA | core promoter element around -30 of transcription start |
| TATA-box | Lycopersicon esculentum | 888 | - | 5 | TTTTA | core promoter element around -30 of transcription start |
| TATA-box | Glycine max | 856 | - | 5 | TAATA | core promoter element around -30 of transcription start |
| TATA-box | Glycine max | 561 | + | 5 | TAATA | core promoter element around -30 of transcription start |
| TATA-box | Lycopersicon esculentum | 599 | - | 5 | TTTTA | core promoter element around -30 of transcription start |
| TATA-box | Glycine max | 863 | + | 5 | TAATA | core promoter element around -30 of transcription start |
| TATA-box | Lycopersicon esculentum | 564 | - | 5 | TTTTA | core promoter element around -30 of transcription start |
| TATA-box | Arabidopsis thaliana | 559 | + | 4 | TATA | core promoter element around -30 of transcription start |
| TATA-box | Glycine max | 1140 | - | 5 | TAATA | core promoter element around -30 of transcription start |
| TATA-box | Arabidopsis thaliana | 1006 | - | 5 | TATAA | core promoter element around -30 of transcription start |
| TATA-box | Glycine max | 665 | + | 5 | TAATA | core promoter element around -30 of transcription start |
| TATA-box | Arabidopsis thaliana | 184 | + | 4 | TATA | core promoter element around -30 of transcription start |
| TATA-box | Lycopersicon esculentum | 195 | - | 5 | TTTTA | core promoter element around -30 of transcription start |
| TATA-box | Arabidopsis thaliana | 487 | + | 9 | TAAAAATAA | core promoter element around -30 of transcription start |
| TATA-box | Lycopersicon esculentum | 761 | - | 5 | TTTTA | core promoter element around -30 of transcription start |
| TATA-box | Arabidopsis thaliana | 519 | - | 9 | TAAAAATAA | core promoter element around -30 of transcription start |
| TATA-box | Glycine max | 676 | - | 5 | TAATA | core promoter element around -30 of transcription start |
| TATA-box | Brassica oleracea | 558 | + | 7 | ATATAAT | core promoter element around -30 of transcription start |
| TATA-box | Glycine max | 186 | + | 5 | TAATA | core promoter element around -30 of transcription start |
| TATA-box | Lycopersicon esculentum | 630 | - | 5 | TTTTA | core promoter element around -30 of transcription start |
| TATA-box | Arabidopsis thaliana | 544 | - | 5 | TATAA | core promoter element around -30 of transcription start |
| TATA-box | Glycine max | 341 | - | 5 | TAATA | core promoter element around -30 of transcription start |
| TATA-box | Glycine max | 726 | - | 5 | TAATA | core promoter element around -30 of transcription start |
| TATA-box | Glycine max | 493 | + | 5 | TAATA | core promoter element around -30 of transcription start |
| TATA-box | Lycopersicon esculentum | 481 | + | 5 | TTTTA | core promoter element around -30 of transcription start |
| TATA-box | Glycine max | 1255 | + | 5 | TAATA | core promoter element around -30 of transcription start |
| TATA-box | Arabidopsis thaliana | 545 | + | 6 | TATAAA | core promoter element around -30 of transcription start |
| TATA-box | Pisum sativum | 867 | - | 7 | TATATGT | core promoter element around -30 of transcription start |
| TATA-box | Glycine max | 849 | - | 5 | TAATA | core promoter element around -30 of transcription start |
| TATA-box | Glycine max | 607 | - | 5 | TAATA | core promoter element around -30 of transcription start |
| TATA-box | Arabidopsis thaliana | 1007 | - | 4 | TATA | core promoter element around -30 of transcription start |
| TATA-box | Glycine max | 484 | + | 5 | TAATA | core promoter element around -30 of transcription start |
| TATA-box | Lycopersicon esculentum | 740 | + | 5 | TTTTA | core promoter element around -30 of transcription start |
| TATA-box | Brassica napus | 1293 | + | 6 | ATTATA | core promoter element around -30 of transcription start |
| TATA-box | Glycine max | 1258 | - | 5 | TAATA | core promoter element around -30 of transcription start |
| TATA-box | Arabidopsis thaliana | 609 | - | 5 | TATAA | core promoter element around -30 of transcription start |
| TATA-box | Lycopersicon esculentum | 1403 | + | 5 | TTTTA | core promoter element around -30 of transcription start |
| TATA-box | Lycopersicon esculentum | 1388 | + | 5 | TTTTA | core promoter element around -30 of transcription start |
| TATA-box | Arabidopsis thaliana | 591 | + | 6 | TATAAA | core promoter element around -30 of transcription start |
| TATA-box | Arabidopsis thaliana | 589 | - | 6 | TATAAA | core promoter element around -30 of transcription start |
| TATA-box | Arabidopsis thaliana | 183 | - | 5 | TATAA | core promoter element around -30 of transcription start |
| TATA-box | Arabidopsis thaliana | 610 | + | 4 | TATA | core promoter element around -30 of transcription start |
| TATA-box | Lycopersicon esculentum | 796 | + | 5 | TTTTA | core promoter element around -30 of transcription start |
| TATA-box | Arabidopsis thaliana | 1295 | - | 4 | TATA | core promoter element around -30 of transcription start |
| TATA-box | Arabidopsis thaliana | 1152 | - | 4 | TATA | core promoter element around -30 of transcription start |
| TATA-box | Arabidopsis thaliana | 1149 | - | 7 | TATATAA | core promoter element around -30 of transcription start |
| TATA-box | Brassica napus | 608 | + | 6 | ATTATA | core promoter element around -30 of transcription start |
| TATA-box | Lycopersicon esculentum | 1450 | + | 5 | TTTTA | core promoter element around -30 of transcription start |
| TATA-box | Arabidopsis thaliana | 588 | - | 7 | TATAAAA | core promoter element around -30 of transcription start |
| TATA-box | Lycopersicon esculentum | 841 | - | 5 | TTTTA | core promoter element around -30 of transcription start |
| TATA-box | Lycopersicon esculentum | 498 | + | 5 | TTTTA | core promoter element around -30 of transcription start |
| TATA-box | Lycopersicon esculentum | 831 | + | 5 | TTTTA | core promoter element around -30 of transcription start |
| TATA-box | Arabidopsis thaliana | 182 | - | 6 | TATAAA | core promoter element around -30 of transcription start |
| TATA-box | Lycopersicon esculentum | 504 | + | 5 | TTTTA | core promoter element around -30 of transcription start |
| TATA-box | Lycopersicon esculentum | 523 | + | 5 | TTTTA | core promoter element around -30 of transcription start |
| TATA-box | Arabidopsis thaliana | 181 | - | 7 | TATAAAA | core promoter element around -30 of transcription start |
| TATA-box | Arabidopsis thaliana | 1092 | - | 6 | TATAAA | core promoter element around -30 of transcription start |
| TATA-box | Lycopersicon esculentum | 174 | + | 5 | TTTTA | core promoter element around -30 of transcription start |
| TATA-box | Glycine max | 169 | + | 5 | TAATA | core promoter element around -30 of transcription start |
| TATA-box | Arabidopsis thaliana | 128 | + | 4 | TATA | core promoter element around -30 of transcription start |
| TATA-box | Lycopersicon esculentum | 143 | + | 5 | TTTTA | core promoter element around -30 of transcription start |
| TATA-box | Arabidopsis thaliana | 126 | + | 4 | TATA | core promoter element around -30 of transcription start |
| TATA-box | Brassica napus | 127 | + | 6 | ATATAT | core promoter element around -30 of transcription start |
| TATA-box | Brassica napus | 125 | + | 6 | ATATAT | core promoter element around -30 of transcription start |
| TATA-box | Glycine max | 838 | - | 5 | TAATA | core promoter element around -30 of transcription start |
| TATA-box | Arabidopsis thaliana | 720 | + | 10 | tcTATATAtt | core promoter element around -30 of transcription start |
| TATA-box | Glycine max | 1108 | - | 5 | TAATA | core promoter element around -30 of transcription start |
| TATA-box | Ac | 1091 | - | 7 | TATAAAT | core promoter element around -30 of transcription start |
| TATA-box | Arabidopsis thaliana | 722 | + | 4 | TATA | core promoter element around -30 of transcription start |
| TATA-box | Oryza sativa | 1286 | - | 8 | TACATAAA | core promoter element around -30 of transcription start |
| TATA-box | Brassica napus | 723 | + | 6 | ATATAT | core promoter element around -30 of transcription start |
| TATA-box | Arabidopsis thaliana | 724 | + | 4 | TATA | core promoter element around -30 of transcription start |
| TATA-box | Arabidopsis thaliana | 1005 | - | 6 | TATAAA | core promoter element around -30 of transcription start |
| TATA-box | Lycopersicon esculentum | 899 | - | 5 | TTTTA | core promoter element around -30 of transcription start |
| TATA-box | Arabidopsis thaliana | 1094 | - | 4 | TATA | core promoter element around -30 of transcription start |
| TATA-box | Arabidopsis thaliana | 1093 | - | 5 | TATAA | core promoter element around -30 of transcription start |
| TATA-box | Arabidopsis thaliana | 870 | - | 4 | TATA | core promoter element around -30 of transcription start |
| TATA-box | Glycine max | 885 | + | 5 | TAATA | core promoter element around -30 of transcription start |

> 2018/04/13 10:10:12  
+ CCAAAGCCAT TCCATCGACT TTAATCCCAA TCCCAAACGT TCCATTAGGT AACGGTCTTT CGTTCTCCTC   
  
  
+ AACCTGTACC ATCGCACTCT CTACCCATGA AAACATACAA TCCGAACGAG GGTGATATAT ATGTTTCTTA   
  
  
+ ATTTTTATGA ATTATTTTCT TATTTCTGTA ATATTTTAAT TTTTATAATA GAAGTAAAAC TTGACGACGA   
  
  
+ AGTACCTGTG GAAGGGCCTC CCCATGATGC TTCGATGGAA CACCACGGTG CACACACCGG GTGTACGATA   
  
  
+ CTGCAGTAGC ATTACTGCAT CCGGACCGAG TGAACTTTCG GTATGACCGG GTGAACTCAT TATTAACGGG   
  
  
+ CTACAGAACA AAAACTATCC GCCTCCATGA AAACACCCTG GTGTTGCGGA ACCTAAATGA GGATTATTCA   
  
  
+ GGACAGACAC TAGTGTTAAG GACTATGGCA GTCTACTAGA GCCACCAGAG TAAGTTCGTA TTTTAATAAA   
  
  
+ AATAATATTT TAATTTTAGA AGGTGGTCTT ATTTTTACAA GTCACTCATA CCCTTATAAA CATCTAAATA   
  
  
+ TAATAAAAAA ATTAATGAAG ATCAAATTTT TATAAACTTA AAATGTTATT ATACTACCAA ATTTATTTCT   
  
  
+ AAAATAGTAT TTCATTGCAT ACAATTAAAC AAATTAATAC TAGCATATTA AAATAAATTA ATTTTTTGTA   
  
  
+ AAGTTTTTGG TAGGGTAGTT CTATATATTA CCGTGCTATT TTTAATCAAC TTCACCTTTG TAAAATTCAT   
  
  
+ GTGAATAGTT AACCTGTGTC GATATTTTTA CTTAAATATT CTAGGGTTGG TTATTGAAAT TTTTAATTAT   
  
  
+ TAAAAAATTA TTAAATATTA TTTAATACAT ATAGAAAATT AATTTAATAA AATGTCAATA AAAAAACCTT   
  
  
+ TACTTATTTT TTTCCTCTTT AATTTCAACA ACTTAACGTT TAAATTACAA CACACCTAAC TTATCCGTCG   
  
  
+ ACGGTTCTCT CAGTTACTCA GTTGTTTATA CTAACTTAGT TTATTTAATC ATCAACCCCG CTCCACATTA   
  
  
+ ATTCGGTTAA TTTTGGAAGC AGTTTGTCAC AGTAGGAATC ATTTATACTG AGTTAACTAT TACCAGAACT   
  
  
+ AGTACTTAAC ACATAAATCT ATTACGCGTT ATATACTAAC GTAAACTGTT TAATTAATTC TATCTACAAA   
  
  
+ GTTCAATTCG TATCACAAAC TGACTGAATC AATTAAGTTA ATTCAAGGCA CTGAATCTCT AGTTTAATAT   
  
  
+ TAGTAGTGAG TGTCATGCGC TTTACTTTAT GTATTATAAT GTAATGAGAT TTGGGTCTTA ATGGGGGGGT   
  
  
+ TTTTTTTTTG TGTGTTTTTT TTTTCCACCG AATAGGAATT TTTTTTTTAG AAGTTCATTT TACAGAATAA   
  
  
+ ATTTTTATTC TTTTTCTGTT TTTTTTTCTG TTTCCCGCCC TTTCCCCAGT TTTAACATTC TAATGAAATT   
  
  
+ AGTGTAGTAC AGTATCGGTT TAACACAAA  

- GGTTTCGGTA AGGTAGCTGA AATTAGGGTT AGGGTTTGCA AGGTAATCCA TTGCCAGAAA GCAAGAGGAG   
  
  
- TTGGACATGG TAGCGTGAGA GATGGGTACT TTTGTATGTT AGGCTTGCTC CCACTATATA TACAAAGAAT   
  
  
- TAAAAATACT TAATAAAAGA ATAAAGACAT TATAAAATTA AAAATATTAT CTTCATTTTG AACTGCTGCT   
  
  
- TCATGGACAC CTTCCCGGAG GGGTACTACG AAGCTACCTT GTGGTGCCAC GTGTGTGGCC CACATGCTAT   
  
  
- GACGTCATCG TAATGACGTA GGCCTGGCTC ACTTGAAAGC CATACTGGCC CACTTGAGTA ATAATTGCCC   
  
  
- GATGTCTTGT TTTTGATAGG CGGAGGTACT TTTGTGGGAC CACAACGCCT TGGATTTACT CCTAATAAGT   
  
  
- CCTGTCTGTG ATCACAATTC CTGATACCGT CAGATGATCT CGGTGGTCTC ATTCAAGCAT AAAATTATTT   
  
  
- TTATTATAAA ATTAAAATCT TCCACCAGAA TAAAAATGTT CAGTGAGTAT GGGAATATTT GTAGATTTAT   
  
  
- ATTATTTTTT TAATTACTTC TAGTTTAAAA ATATTTGAAT TTTACAATAA TATGATGGTT TAAATAAAGA   
  
  
- TTTTATCATA AAGTAACGTA TGTTAATTTG TTTAATTATG ATCGTATAAT TTTATTTAAT TAAAAAACAT   
  
  
- TTCAAAAACC ATCCCATCAA GATATATAAT GGCACGATAA AAATTAGTTG AAGTGGAAAC ATTTTAAGTA   
  
  
- CACTTATCAA TTGGACACAG CTATAAAAAT GAATTTATAA GATCCCAACC AATAACTTTA AAAATTAATA   
  
  
- ATTTTTTAAT AATTTATAAT AAATTATGTA TATCTTTTAA TTAAATTATT TTACAGTTAT TTTTTTGGAA   
  
  
- ATGAATAAAA AAAGGAGAAA TTAAAGTTGT TGAATTGCAA ATTTAATGTT GTGTGGATTG AATAGGCAGC   
  
  
- TGCCAAGAGA GTCAATGAGT CAACAAATAT GATTGAATCA AATAAATTAG TAGTTGGGGC GAGGTGTAAT   
  
  
- TAAGCCAATT AAAACCTTCG TCAAACAGTG TCATCCTTAG TAAATATGAC TCAATTGATA ATGGTCTTGA   
  
  
- TCATGAATTG TGTATTTAGA TAATGCGCAA TATATGATTG CATTTGACAA ATTAATTAAG ATAGATGTTT   
  
  
- CAAGTTAAGC ATAGTGTTTG ACTGACTTAG TTAATTCAAT TAAGTTCCGT GACTTAGAGA TCAAATTATA   
  
  
- ATCATCACTC ACAGTACGCG AAATGAAATA CATAATATTA CATTACTCTA AACCCAGAAT TACCCCCCCA   
  
  
- AAAAAAAAAC ACACAAAAAA AAAAGGTGGC TTATCCTTAA AAAAAAAATC TTCAAGTAAA ATGTCTTATT   
  
  
- TAAAAATAAG AAAAAGACAA AAAAAAAGAC AAAGGGCGGG AAAGGGGTCA AAATTGTAAG ATTACTTTAA   
  
  
- TCACATCATG TCATAGCCAA ATTGTGTTT

+     TCA-element

| Site Name | Organism | Position | Strand | Matrix score. | sequence | function |
| --- | --- | --- | --- | --- | --- | --- |
| TCA-element | Brassica oleracea | 1409 | - | 9 | CAGAAAAGGA | cis-acting element involved in salicylic acid responsiveness |

> 2018/04/13 10:10:12  
+ CCAAAGCCAT TCCATCGACT TTAATCCCAA TCCCAAACGT TCCATTAGGT AACGGTCTTT CGTTCTCCTC   
  
  
+ AACCTGTACC ATCGCACTCT CTACCCATGA AAACATACAA TCCGAACGAG GGTGATATAT ATGTTTCTTA   
  
  
+ ATTTTTATGA ATTATTTTCT TATTTCTGTA ATATTTTAAT TTTTATAATA GAAGTAAAAC TTGACGACGA   
  
  
+ AGTACCTGTG GAAGGGCCTC CCCATGATGC TTCGATGGAA CACCACGGTG CACACACCGG GTGTACGATA   
  
  
+ CTGCAGTAGC ATTACTGCAT CCGGACCGAG TGAACTTTCG GTATGACCGG GTGAACTCAT TATTAACGGG   
  
  
+ CTACAGAACA AAAACTATCC GCCTCCATGA AAACACCCTG GTGTTGCGGA ACCTAAATGA GGATTATTCA   
  
  
+ GGACAGACAC TAGTGTTAAG GACTATGGCA GTCTACTAGA GCCACCAGAG TAAGTTCGTA TTTTAATAAA   
  
  
+ AATAATATTT TAATTTTAGA AGGTGGTCTT ATTTTTACAA GTCACTCATA CCCTTATAAA CATCTAAATA   
  
  
+ TAATAAAAAA ATTAATGAAG ATCAAATTTT TATAAACTTA AAATGTTATT ATACTACCAA ATTTATTTCT   
  
  
+ AAAATAGTAT TTCATTGCAT ACAATTAAAC AAATTAATAC TAGCATATTA AAATAAATTA ATTTTTTGTA   
  
  
+ AAGTTTTTGG TAGGGTAGTT CTATATATTA CCGTGCTATT TTTAATCAAC TTCACCTTTG TAAAATTCAT   
  
  
+ GTGAATAGTT AACCTGTGTC GATATTTTTA CTTAAATATT CTAGGGTTGG TTATTGAAAT TTTTAATTAT   
  
  
+ TAAAAAATTA TTAAATATTA TTTAATACAT ATAGAAAATT AATTTAATAA AATGTCAATA AAAAAACCTT   
  
  
+ TACTTATTTT TTTCCTCTTT AATTTCAACA ACTTAACGTT TAAATTACAA CACACCTAAC TTATCCGTCG   
  
  
+ ACGGTTCTCT CAGTTACTCA GTTGTTTATA CTAACTTAGT TTATTTAATC ATCAACCCCG CTCCACATTA   
  
  
+ ATTCGGTTAA TTTTGGAAGC AGTTTGTCAC AGTAGGAATC ATTTATACTG AGTTAACTAT TACCAGAACT   
  
  
+ AGTACTTAAC ACATAAATCT ATTACGCGTT ATATACTAAC GTAAACTGTT TAATTAATTC TATCTACAAA   
  
  
+ GTTCAATTCG TATCACAAAC TGACTGAATC AATTAAGTTA ATTCAAGGCA CTGAATCTCT AGTTTAATAT   
  
  
+ TAGTAGTGAG TGTCATGCGC TTTACTTTAT GTATTATAAT GTAATGAGAT TTGGGTCTTA ATGGGGGGGT   
  
  
+ TTTTTTTTTG TGTGTTTTTT TTTTCCACCG AATAGGAATT TTTTTTTTAG AAGTTCATTT TACAGAATAA   
  
  
+ ATTTTTATTC TTTTTCTGTT TTTTTTTCTG TTTCCCGCCC TTTCCCCAGT TTTAACATTC TAATGAAATT   
  
  
+ AGTGTAGTAC AGTATCGGTT TAACACAAA  

- GGTTTCGGTA AGGTAGCTGA AATTAGGGTT AGGGTTTGCA AGGTAATCCA TTGCCAGAAA GCAAGAGGAG   
  
  
- TTGGACATGG TAGCGTGAGA GATGGGTACT TTTGTATGTT AGGCTTGCTC CCACTATATA TACAAAGAAT   
  
  
- TAAAAATACT TAATAAAAGA ATAAAGACAT TATAAAATTA AAAATATTAT CTTCATTTTG AACTGCTGCT   
  
  
- TCATGGACAC CTTCCCGGAG GGGTACTACG AAGCTACCTT GTGGTGCCAC GTGTGTGGCC CACATGCTAT   
  
  
- GACGTCATCG TAATGACGTA GGCCTGGCTC ACTTGAAAGC CATACTGGCC CACTTGAGTA ATAATTGCCC   
  
  
- GATGTCTTGT TTTTGATAGG CGGAGGTACT TTTGTGGGAC CACAACGCCT TGGATTTACT CCTAATAAGT   
  
  
- CCTGTCTGTG ATCACAATTC CTGATACCGT CAGATGATCT CGGTGGTCTC ATTCAAGCAT AAAATTATTT   
  
  
- TTATTATAAA ATTAAAATCT TCCACCAGAA TAAAAATGTT CAGTGAGTAT GGGAATATTT GTAGATTTAT   
  
  
- ATTATTTTTT TAATTACTTC TAGTTTAAAA ATATTTGAAT TTTACAATAA TATGATGGTT TAAATAAAGA   
  
  
- TTTTATCATA AAGTAACGTA TGTTAATTTG TTTAATTATG ATCGTATAAT TTTATTTAAT TAAAAAACAT   
  
  
- TTCAAAAACC ATCCCATCAA GATATATAAT GGCACGATAA AAATTAGTTG AAGTGGAAAC ATTTTAAGTA   
  
  
- CACTTATCAA TTGGACACAG CTATAAAAAT GAATTTATAA GATCCCAACC AATAACTTTA AAAATTAATA   
  
  
- ATTTTTTAAT AATTTATAAT AAATTATGTA TATCTTTTAA TTAAATTATT TTACAGTTAT TTTTTTGGAA   
  
  
- ATGAATAAAA AAAGGAGAAA TTAAAGTTGT TGAATTGCAA ATTTAATGTT GTGTGGATTG AATAGGCAGC   
  
  
- TGCCAAGAGA GTCAATGAGT CAACAAATAT GATTGAATCA AATAAATTAG TAGTTGGGGC GAGGTGTAAT   
  
  
- TAAGCCAATT AAAACCTTCG TCAAACAGTG TCATCCTTAG TAAATATGAC TCAATTGATA ATGGTCTTGA   
  
  
- TCATGAATTG TGTATTTAGA TAATGCGCAA TATATGATTG CATTTGACAA ATTAATTAAG ATAGATGTTT   
  
  
- CAAGTTAAGC ATAGTGTTTG ACTGACTTAG TTAATTCAAT TAAGTTCCGT GACTTAGAGA TCAAATTATA   
  
  
- ATCATCACTC ACAGTACGCG AAATGAAATA CATAATATTA CATTACTCTA AACCCAGAAT TACCCCCCCA   
  
  
- AAAAAAAAAC ACACAAAAAA AAAAGGTGGC TTATCCTTAA AAAAAAAATC TTCAAGTAAA ATGTCTTATT   
  
  
- TAAAAATAAG AAAAAGACAA AAAAAAAGAC AAAGGGCGGG AAAGGGGTCA AAATTGTAAG ATTACTTTAA   
  
  
- TCACATCATG TCATAGCCAA ATTGTGTTT

+     TGACG-motif

| Site Name | Organism | Position | Strand | Matrix score. | sequence | function |
| --- | --- | --- | --- | --- | --- | --- |
| TGACG-motif | Hordeum vulgare | 202 | + | 5 | TGACG | cis-acting regulatory element involved in the MeJA-responsiveness |

> 2018/04/13 10:10:12  
+ CCAAAGCCAT TCCATCGACT TTAATCCCAA TCCCAAACGT TCCATTAGGT AACGGTCTTT CGTTCTCCTC   
  
  
+ AACCTGTACC ATCGCACTCT CTACCCATGA AAACATACAA TCCGAACGAG GGTGATATAT ATGTTTCTTA   
  
  
+ ATTTTTATGA ATTATTTTCT TATTTCTGTA ATATTTTAAT TTTTATAATA GAAGTAAAAC TTGACGACGA   
  
  
+ AGTACCTGTG GAAGGGCCTC CCCATGATGC TTCGATGGAA CACCACGGTG CACACACCGG GTGTACGATA   
  
  
+ CTGCAGTAGC ATTACTGCAT CCGGACCGAG TGAACTTTCG GTATGACCGG GTGAACTCAT TATTAACGGG   
  
  
+ CTACAGAACA AAAACTATCC GCCTCCATGA AAACACCCTG GTGTTGCGGA ACCTAAATGA GGATTATTCA   
  
  
+ GGACAGACAC TAGTGTTAAG GACTATGGCA GTCTACTAGA GCCACCAGAG TAAGTTCGTA TTTTAATAAA   
  
  
+ AATAATATTT TAATTTTAGA AGGTGGTCTT ATTTTTACAA GTCACTCATA CCCTTATAAA CATCTAAATA   
  
  
+ TAATAAAAAA ATTAATGAAG ATCAAATTTT TATAAACTTA AAATGTTATT ATACTACCAA ATTTATTTCT   
  
  
+ AAAATAGTAT TTCATTGCAT ACAATTAAAC AAATTAATAC TAGCATATTA AAATAAATTA ATTTTTTGTA   
  
  
+ AAGTTTTTGG TAGGGTAGTT CTATATATTA CCGTGCTATT TTTAATCAAC TTCACCTTTG TAAAATTCAT   
  
  
+ GTGAATAGTT AACCTGTGTC GATATTTTTA CTTAAATATT CTAGGGTTGG TTATTGAAAT TTTTAATTAT   
  
  
+ TAAAAAATTA TTAAATATTA TTTAATACAT ATAGAAAATT AATTTAATAA AATGTCAATA AAAAAACCTT   
  
  
+ TACTTATTTT TTTCCTCTTT AATTTCAACA ACTTAACGTT TAAATTACAA CACACCTAAC TTATCCGTCG   
  
  
+ ACGGTTCTCT CAGTTACTCA GTTGTTTATA CTAACTTAGT TTATTTAATC ATCAACCCCG CTCCACATTA   
  
  
+ ATTCGGTTAA TTTTGGAAGC AGTTTGTCAC AGTAGGAATC ATTTATACTG AGTTAACTAT TACCAGAACT   
  
  
+ AGTACTTAAC ACATAAATCT ATTACGCGTT ATATACTAAC GTAAACTGTT TAATTAATTC TATCTACAAA   
  
  
+ GTTCAATTCG TATCACAAAC TGACTGAATC AATTAAGTTA ATTCAAGGCA CTGAATCTCT AGTTTAATAT   
  
  
+ TAGTAGTGAG TGTCATGCGC TTTACTTTAT GTATTATAAT GTAATGAGAT TTGGGTCTTA ATGGGGGGGT   
  
  
+ TTTTTTTTTG TGTGTTTTTT TTTTCCACCG AATAGGAATT TTTTTTTTAG AAGTTCATTT TACAGAATAA   
  
  
+ ATTTTTATTC TTTTTCTGTT TTTTTTTCTG TTTCCCGCCC TTTCCCCAGT TTTAACATTC TAATGAAATT   
  
  
+ AGTGTAGTAC AGTATCGGTT TAACACAAA  

- GGTTTCGGTA AGGTAGCTGA AATTAGGGTT AGGGTTTGCA AGGTAATCCA TTGCCAGAAA GCAAGAGGAG   
  
  
- TTGGACATGG TAGCGTGAGA GATGGGTACT TTTGTATGTT AGGCTTGCTC CCACTATATA TACAAAGAAT   
  
  
- TAAAAATACT TAATAAAAGA ATAAAGACAT TATAAAATTA AAAATATTAT CTTCATTTTG AACTGCTGCT   
  
  
- TCATGGACAC CTTCCCGGAG GGGTACTACG AAGCTACCTT GTGGTGCCAC GTGTGTGGCC CACATGCTAT   
  
  
- GACGTCATCG TAATGACGTA GGCCTGGCTC ACTTGAAAGC CATACTGGCC CACTTGAGTA ATAATTGCCC   
  
  
- GATGTCTTGT TTTTGATAGG CGGAGGTACT TTTGTGGGAC CACAACGCCT TGGATTTACT CCTAATAAGT   
  
  
- CCTGTCTGTG ATCACAATTC CTGATACCGT CAGATGATCT CGGTGGTCTC ATTCAAGCAT AAAATTATTT   
  
  
- TTATTATAAA ATTAAAATCT TCCACCAGAA TAAAAATGTT CAGTGAGTAT GGGAATATTT GTAGATTTAT   
  
  
- ATTATTTTTT TAATTACTTC TAGTTTAAAA ATATTTGAAT TTTACAATAA TATGATGGTT TAAATAAAGA   
  
  
- TTTTATCATA AAGTAACGTA TGTTAATTTG TTTAATTATG ATCGTATAAT TTTATTTAAT TAAAAAACAT   
  
  
- TTCAAAAACC ATCCCATCAA GATATATAAT GGCACGATAA AAATTAGTTG AAGTGGAAAC ATTTTAAGTA   
  
  
- CACTTATCAA TTGGACACAG CTATAAAAAT GAATTTATAA GATCCCAACC AATAACTTTA AAAATTAATA   
  
  
- ATTTTTTAAT AATTTATAAT AAATTATGTA TATCTTTTAA TTAAATTATT TTACAGTTAT TTTTTTGGAA   
  
  
- ATGAATAAAA AAAGGAGAAA TTAAAGTTGT TGAATTGCAA ATTTAATGTT GTGTGGATTG AATAGGCAGC   
  
  
- TGCCAAGAGA GTCAATGAGT CAACAAATAT GATTGAATCA AATAAATTAG TAGTTGGGGC GAGGTGTAAT   
  
  
- TAAGCCAATT AAAACCTTCG TCAAACAGTG TCATCCTTAG TAAATATGAC TCAATTGATA ATGGTCTTGA   
  
  
- TCATGAATTG TGTATTTAGA TAATGCGCAA TATATGATTG CATTTGACAA ATTAATTAAG ATAGATGTTT   
  
  
- CAAGTTAAGC ATAGTGTTTG ACTGACTTAG TTAATTCAAT TAAGTTCCGT GACTTAGAGA TCAAATTATA   
  
  
- ATCATCACTC ACAGTACGCG AAATGAAATA CATAATATTA CATTACTCTA AACCCAGAAT TACCCCCCCA   
  
  
- AAAAAAAAAC ACACAAAAAA AAAAGGTGGC TTATCCTTAA AAAAAAAATC TTCAAGTAAA ATGTCTTATT   
  
  
- TAAAAATAAG AAAAAGACAA AAAAAAAGAC AAAGGGCGGG AAAGGGGTCA AAATTGTAAG ATTACTTTAA   
  
  
- TCACATCATG TCATAGCCAA ATTGTGTTT

+     Unnamed\_\_1

| Site Name | Organism | Position | Strand | Matrix score. | sequence | function |
| --- | --- | --- | --- | --- | --- | --- |
| Unnamed\_\_1 | Zea mays | 253 | - | 5 | CGTGG |  |

> 2018/04/13 10:10:12  
+ CCAAAGCCAT TCCATCGACT TTAATCCCAA TCCCAAACGT TCCATTAGGT AACGGTCTTT CGTTCTCCTC   
  
  
+ AACCTGTACC ATCGCACTCT CTACCCATGA AAACATACAA TCCGAACGAG GGTGATATAT ATGTTTCTTA   
  
  
+ ATTTTTATGA ATTATTTTCT TATTTCTGTA ATATTTTAAT TTTTATAATA GAAGTAAAAC TTGACGACGA   
  
  
+ AGTACCTGTG GAAGGGCCTC CCCATGATGC TTCGATGGAA CACCACGGTG CACACACCGG GTGTACGATA   
  
  
+ CTGCAGTAGC ATTACTGCAT CCGGACCGAG TGAACTTTCG GTATGACCGG GTGAACTCAT TATTAACGGG   
  
  
+ CTACAGAACA AAAACTATCC GCCTCCATGA AAACACCCTG GTGTTGCGGA ACCTAAATGA GGATTATTCA   
  
  
+ GGACAGACAC TAGTGTTAAG GACTATGGCA GTCTACTAGA GCCACCAGAG TAAGTTCGTA TTTTAATAAA   
  
  
+ AATAATATTT TAATTTTAGA AGGTGGTCTT ATTTTTACAA GTCACTCATA CCCTTATAAA CATCTAAATA   
  
  
+ TAATAAAAAA ATTAATGAAG ATCAAATTTT TATAAACTTA AAATGTTATT ATACTACCAA ATTTATTTCT   
  
  
+ AAAATAGTAT TTCATTGCAT ACAATTAAAC AAATTAATAC TAGCATATTA AAATAAATTA ATTTTTTGTA   
  
  
+ AAGTTTTTGG TAGGGTAGTT CTATATATTA CCGTGCTATT TTTAATCAAC TTCACCTTTG TAAAATTCAT   
  
  
+ GTGAATAGTT AACCTGTGTC GATATTTTTA CTTAAATATT CTAGGGTTGG TTATTGAAAT TTTTAATTAT   
  
  
+ TAAAAAATTA TTAAATATTA TTTAATACAT ATAGAAAATT AATTTAATAA AATGTCAATA AAAAAACCTT   
  
  
+ TACTTATTTT TTTCCTCTTT AATTTCAACA ACTTAACGTT TAAATTACAA CACACCTAAC TTATCCGTCG   
  
  
+ ACGGTTCTCT CAGTTACTCA GTTGTTTATA CTAACTTAGT TTATTTAATC ATCAACCCCG CTCCACATTA   
  
  
+ ATTCGGTTAA TTTTGGAAGC AGTTTGTCAC AGTAGGAATC ATTTATACTG AGTTAACTAT TACCAGAACT   
  
  
+ AGTACTTAAC ACATAAATCT ATTACGCGTT ATATACTAAC GTAAACTGTT TAATTAATTC TATCTACAAA   
  
  
+ GTTCAATTCG TATCACAAAC TGACTGAATC AATTAAGTTA ATTCAAGGCA CTGAATCTCT AGTTTAATAT   
  
  
+ TAGTAGTGAG TGTCATGCGC TTTACTTTAT GTATTATAAT GTAATGAGAT TTGGGTCTTA ATGGGGGGGT   
  
  
+ TTTTTTTTTG TGTGTTTTTT TTTTCCACCG AATAGGAATT TTTTTTTTAG AAGTTCATTT TACAGAATAA   
  
  
+ ATTTTTATTC TTTTTCTGTT TTTTTTTCTG TTTCCCGCCC TTTCCCCAGT TTTAACATTC TAATGAAATT   
  
  
+ AGTGTAGTAC AGTATCGGTT TAACACAAA  

- GGTTTCGGTA AGGTAGCTGA AATTAGGGTT AGGGTTTGCA AGGTAATCCA TTGCCAGAAA GCAAGAGGAG   
  
  
- TTGGACATGG TAGCGTGAGA GATGGGTACT TTTGTATGTT AGGCTTGCTC CCACTATATA TACAAAGAAT   
  
  
- TAAAAATACT TAATAAAAGA ATAAAGACAT TATAAAATTA AAAATATTAT CTTCATTTTG AACTGCTGCT   
  
  
- TCATGGACAC CTTCCCGGAG GGGTACTACG AAGCTACCTT GTGGTGCCAC GTGTGTGGCC CACATGCTAT   
  
  
- GACGTCATCG TAATGACGTA GGCCTGGCTC ACTTGAAAGC CATACTGGCC CACTTGAGTA ATAATTGCCC   
  
  
- GATGTCTTGT TTTTGATAGG CGGAGGTACT TTTGTGGGAC CACAACGCCT TGGATTTACT CCTAATAAGT   
  
  
- CCTGTCTGTG ATCACAATTC CTGATACCGT CAGATGATCT CGGTGGTCTC ATTCAAGCAT AAAATTATTT   
  
  
- TTATTATAAA ATTAAAATCT TCCACCAGAA TAAAAATGTT CAGTGAGTAT GGGAATATTT GTAGATTTAT   
  
  
- ATTATTTTTT TAATTACTTC TAGTTTAAAA ATATTTGAAT TTTACAATAA TATGATGGTT TAAATAAAGA   
  
  
- TTTTATCATA AAGTAACGTA TGTTAATTTG TTTAATTATG ATCGTATAAT TTTATTTAAT TAAAAAACAT   
  
  
- TTCAAAAACC ATCCCATCAA GATATATAAT GGCACGATAA AAATTAGTTG AAGTGGAAAC ATTTTAAGTA   
  
  
- CACTTATCAA TTGGACACAG CTATAAAAAT GAATTTATAA GATCCCAACC AATAACTTTA AAAATTAATA   
  
  
- ATTTTTTAAT AATTTATAAT AAATTATGTA TATCTTTTAA TTAAATTATT TTACAGTTAT TTTTTTGGAA   
  
  
- ATGAATAAAA AAAGGAGAAA TTAAAGTTGT TGAATTGCAA ATTTAATGTT GTGTGGATTG AATAGGCAGC   
  
  
- TGCCAAGAGA GTCAATGAGT CAACAAATAT GATTGAATCA AATAAATTAG TAGTTGGGGC GAGGTGTAAT   
  
  
- TAAGCCAATT AAAACCTTCG TCAAACAGTG TCATCCTTAG TAAATATGAC TCAATTGATA ATGGTCTTGA   
  
  
- TCATGAATTG TGTATTTAGA TAATGCGCAA TATATGATTG CATTTGACAA ATTAATTAAG ATAGATGTTT   
  
  
- CAAGTTAAGC ATAGTGTTTG ACTGACTTAG TTAATTCAAT TAAGTTCCGT GACTTAGAGA TCAAATTATA   
  
  
- ATCATCACTC ACAGTACGCG AAATGAAATA CATAATATTA CATTACTCTA AACCCAGAAT TACCCCCCCA   
  
  
- AAAAAAAAAC ACACAAAAAA AAAAGGTGGC TTATCCTTAA AAAAAAAATC TTCAAGTAAA ATGTCTTATT   
  
  
- TAAAAATAAG AAAAAGACAA AAAAAAAGAC AAAGGGCGGG AAAGGGGTCA AAATTGTAAG ATTACTTTAA   
  
  
- TCACATCATG TCATAGCCAA ATTGTGTTT

+     Unnamed\_\_3

| Site Name | Organism | Position | Strand | Matrix score. | sequence | function |
| --- | --- | --- | --- | --- | --- | --- |
| Unnamed\_\_3 | Zea mays | 253 | - | 5 | CGTGG |  |

> 2018/04/13 10:10:12  
+ CCAAAGCCAT TCCATCGACT TTAATCCCAA TCCCAAACGT TCCATTAGGT AACGGTCTTT CGTTCTCCTC   
  
  
+ AACCTGTACC ATCGCACTCT CTACCCATGA AAACATACAA TCCGAACGAG GGTGATATAT ATGTTTCTTA   
  
  
+ ATTTTTATGA ATTATTTTCT TATTTCTGTA ATATTTTAAT TTTTATAATA GAAGTAAAAC TTGACGACGA   
  
  
+ AGTACCTGTG GAAGGGCCTC CCCATGATGC TTCGATGGAA CACCACGGTG CACACACCGG GTGTACGATA   
  
  
+ CTGCAGTAGC ATTACTGCAT CCGGACCGAG TGAACTTTCG GTATGACCGG GTGAACTCAT TATTAACGGG   
  
  
+ CTACAGAACA AAAACTATCC GCCTCCATGA AAACACCCTG GTGTTGCGGA ACCTAAATGA GGATTATTCA   
  
  
+ GGACAGACAC TAGTGTTAAG GACTATGGCA GTCTACTAGA GCCACCAGAG TAAGTTCGTA TTTTAATAAA   
  
  
+ AATAATATTT TAATTTTAGA AGGTGGTCTT ATTTTTACAA GTCACTCATA CCCTTATAAA CATCTAAATA   
  
  
+ TAATAAAAAA ATTAATGAAG ATCAAATTTT TATAAACTTA AAATGTTATT ATACTACCAA ATTTATTTCT   
  
  
+ AAAATAGTAT TTCATTGCAT ACAATTAAAC AAATTAATAC TAGCATATTA AAATAAATTA ATTTTTTGTA   
  
  
+ AAGTTTTTGG TAGGGTAGTT CTATATATTA CCGTGCTATT TTTAATCAAC TTCACCTTTG TAAAATTCAT   
  
  
+ GTGAATAGTT AACCTGTGTC GATATTTTTA CTTAAATATT CTAGGGTTGG TTATTGAAAT TTTTAATTAT   
  
  
+ TAAAAAATTA TTAAATATTA TTTAATACAT ATAGAAAATT AATTTAATAA AATGTCAATA AAAAAACCTT   
  
  
+ TACTTATTTT TTTCCTCTTT AATTTCAACA ACTTAACGTT TAAATTACAA CACACCTAAC TTATCCGTCG   
  
  
+ ACGGTTCTCT CAGTTACTCA GTTGTTTATA CTAACTTAGT TTATTTAATC ATCAACCCCG CTCCACATTA   
  
  
+ ATTCGGTTAA TTTTGGAAGC AGTTTGTCAC AGTAGGAATC ATTTATACTG AGTTAACTAT TACCAGAACT   
  
  
+ AGTACTTAAC ACATAAATCT ATTACGCGTT ATATACTAAC GTAAACTGTT TAATTAATTC TATCTACAAA   
  
  
+ GTTCAATTCG TATCACAAAC TGACTGAATC AATTAAGTTA ATTCAAGGCA CTGAATCTCT AGTTTAATAT   
  
  
+ TAGTAGTGAG TGTCATGCGC TTTACTTTAT GTATTATAAT GTAATGAGAT TTGGGTCTTA ATGGGGGGGT   
  
  
+ TTTTTTTTTG TGTGTTTTTT TTTTCCACCG AATAGGAATT TTTTTTTTAG AAGTTCATTT TACAGAATAA   
  
  
+ ATTTTTATTC TTTTTCTGTT TTTTTTTCTG TTTCCCGCCC TTTCCCCAGT TTTAACATTC TAATGAAATT   
  
  
+ AGTGTAGTAC AGTATCGGTT TAACACAAA  

- GGTTTCGGTA AGGTAGCTGA AATTAGGGTT AGGGTTTGCA AGGTAATCCA TTGCCAGAAA GCAAGAGGAG   
  
  
- TTGGACATGG TAGCGTGAGA GATGGGTACT TTTGTATGTT AGGCTTGCTC CCACTATATA TACAAAGAAT   
  
  
- TAAAAATACT TAATAAAAGA ATAAAGACAT TATAAAATTA AAAATATTAT CTTCATTTTG AACTGCTGCT   
  
  
- TCATGGACAC CTTCCCGGAG GGGTACTACG AAGCTACCTT GTGGTGCCAC GTGTGTGGCC CACATGCTAT   
  
  
- GACGTCATCG TAATGACGTA GGCCTGGCTC ACTTGAAAGC CATACTGGCC CACTTGAGTA ATAATTGCCC   
  
  
- GATGTCTTGT TTTTGATAGG CGGAGGTACT TTTGTGGGAC CACAACGCCT TGGATTTACT CCTAATAAGT   
  
  
- CCTGTCTGTG ATCACAATTC CTGATACCGT CAGATGATCT CGGTGGTCTC ATTCAAGCAT AAAATTATTT   
  
  
- TTATTATAAA ATTAAAATCT TCCACCAGAA TAAAAATGTT CAGTGAGTAT GGGAATATTT GTAGATTTAT   
  
  
- ATTATTTTTT TAATTACTTC TAGTTTAAAA ATATTTGAAT TTTACAATAA TATGATGGTT TAAATAAAGA   
  
  
- TTTTATCATA AAGTAACGTA TGTTAATTTG TTTAATTATG ATCGTATAAT TTTATTTAAT TAAAAAACAT   
  
  
- TTCAAAAACC ATCCCATCAA GATATATAAT GGCACGATAA AAATTAGTTG AAGTGGAAAC ATTTTAAGTA   
  
  
- CACTTATCAA TTGGACACAG CTATAAAAAT GAATTTATAA GATCCCAACC AATAACTTTA AAAATTAATA   
  
  
- ATTTTTTAAT AATTTATAAT AAATTATGTA TATCTTTTAA TTAAATTATT TTACAGTTAT TTTTTTGGAA   
  
  
- ATGAATAAAA AAAGGAGAAA TTAAAGTTGT TGAATTGCAA ATTTAATGTT GTGTGGATTG AATAGGCAGC   
  
  
- TGCCAAGAGA GTCAATGAGT CAACAAATAT GATTGAATCA AATAAATTAG TAGTTGGGGC GAGGTGTAAT   
  
  
- TAAGCCAATT AAAACCTTCG TCAAACAGTG TCATCCTTAG TAAATATGAC TCAATTGATA ATGGTCTTGA   
  
  
- TCATGAATTG TGTATTTAGA TAATGCGCAA TATATGATTG CATTTGACAA ATTAATTAAG ATAGATGTTT   
  
  
- CAAGTTAAGC ATAGTGTTTG ACTGACTTAG TTAATTCAAT TAAGTTCCGT GACTTAGAGA TCAAATTATA   
  
  
- ATCATCACTC ACAGTACGCG AAATGAAATA CATAATATTA CATTACTCTA AACCCAGAAT TACCCCCCCA   
  
  
- AAAAAAAAAC ACACAAAAAA AAAAGGTGGC TTATCCTTAA AAAAAAAATC TTCAAGTAAA ATGTCTTATT   
  
  
- TAAAAATAAG AAAAAGACAA AAAAAAAGAC AAAGGGCGGG AAAGGGGTCA AAATTGTAAG ATTACTTTAA   
  
  
- TCACATCATG TCATAGCCAA ATTGTGTTT

+     Unnamed\_\_4

| Site Name | Organism | Position | Strand | Matrix score. | sequence | function |
| --- | --- | --- | --- | --- | --- | --- |
| Unnamed\_\_4 | Petroselinum hortense | 228 | + | 4 | CTCC |  |
| Unnamed\_\_4 | Petroselinum hortense | 373 | + | 4 | CTCC |  |
| Unnamed\_\_4 | Petroselinum hortense | 65 | + | 4 | CTCC |  |
| Unnamed\_\_4 | Petroselinum hortense | 1041 | + | 4 | CTCC |  |

> 2018/04/13 10:10:12  
+ CCAAAGCCAT TCCATCGACT TTAATCCCAA TCCCAAACGT TCCATTAGGT AACGGTCTTT CGTTCTCCTC   
  
  
+ AACCTGTACC ATCGCACTCT CTACCCATGA AAACATACAA TCCGAACGAG GGTGATATAT ATGTTTCTTA   
  
  
+ ATTTTTATGA ATTATTTTCT TATTTCTGTA ATATTTTAAT TTTTATAATA GAAGTAAAAC TTGACGACGA   
  
  
+ AGTACCTGTG GAAGGGCCTC CCCATGATGC TTCGATGGAA CACCACGGTG CACACACCGG GTGTACGATA   
  
  
+ CTGCAGTAGC ATTACTGCAT CCGGACCGAG TGAACTTTCG GTATGACCGG GTGAACTCAT TATTAACGGG   
  
  
+ CTACAGAACA AAAACTATCC GCCTCCATGA AAACACCCTG GTGTTGCGGA ACCTAAATGA GGATTATTCA   
  
  
+ GGACAGACAC TAGTGTTAAG GACTATGGCA GTCTACTAGA GCCACCAGAG TAAGTTCGTA TTTTAATAAA   
  
  
+ AATAATATTT TAATTTTAGA AGGTGGTCTT ATTTTTACAA GTCACTCATA CCCTTATAAA CATCTAAATA   
  
  
+ TAATAAAAAA ATTAATGAAG ATCAAATTTT TATAAACTTA AAATGTTATT ATACTACCAA ATTTATTTCT   
  
  
+ AAAATAGTAT TTCATTGCAT ACAATTAAAC AAATTAATAC TAGCATATTA AAATAAATTA ATTTTTTGTA   
  
  
+ AAGTTTTTGG TAGGGTAGTT CTATATATTA CCGTGCTATT TTTAATCAAC TTCACCTTTG TAAAATTCAT   
  
  
+ GTGAATAGTT AACCTGTGTC GATATTTTTA CTTAAATATT CTAGGGTTGG TTATTGAAAT TTTTAATTAT   
  
  
+ TAAAAAATTA TTAAATATTA TTTAATACAT ATAGAAAATT AATTTAATAA AATGTCAATA AAAAAACCTT   
  
  
+ TACTTATTTT TTTCCTCTTT AATTTCAACA ACTTAACGTT TAAATTACAA CACACCTAAC TTATCCGTCG   
  
  
+ ACGGTTCTCT CAGTTACTCA GTTGTTTATA CTAACTTAGT TTATTTAATC ATCAACCCCG CTCCACATTA   
  
  
+ ATTCGGTTAA TTTTGGAAGC AGTTTGTCAC AGTAGGAATC ATTTATACTG AGTTAACTAT TACCAGAACT   
  
  
+ AGTACTTAAC ACATAAATCT ATTACGCGTT ATATACTAAC GTAAACTGTT TAATTAATTC TATCTACAAA   
  
  
+ GTTCAATTCG TATCACAAAC TGACTGAATC AATTAAGTTA ATTCAAGGCA CTGAATCTCT AGTTTAATAT   
  
  
+ TAGTAGTGAG TGTCATGCGC TTTACTTTAT GTATTATAAT GTAATGAGAT TTGGGTCTTA ATGGGGGGGT   
  
  
+ TTTTTTTTTG TGTGTTTTTT TTTTCCACCG AATAGGAATT TTTTTTTTAG AAGTTCATTT TACAGAATAA   
  
  
+ ATTTTTATTC TTTTTCTGTT TTTTTTTCTG TTTCCCGCCC TTTCCCCAGT TTTAACATTC TAATGAAATT   
  
  
+ AGTGTAGTAC AGTATCGGTT TAACACAAA  

- GGTTTCGGTA AGGTAGCTGA AATTAGGGTT AGGGTTTGCA AGGTAATCCA TTGCCAGAAA GCAAGAGGAG   
  
  
- TTGGACATGG TAGCGTGAGA GATGGGTACT TTTGTATGTT AGGCTTGCTC CCACTATATA TACAAAGAAT   
  
  
- TAAAAATACT TAATAAAAGA ATAAAGACAT TATAAAATTA AAAATATTAT CTTCATTTTG AACTGCTGCT   
  
  
- TCATGGACAC CTTCCCGGAG GGGTACTACG AAGCTACCTT GTGGTGCCAC GTGTGTGGCC CACATGCTAT   
  
  
- GACGTCATCG TAATGACGTA GGCCTGGCTC ACTTGAAAGC CATACTGGCC CACTTGAGTA ATAATTGCCC   
  
  
- GATGTCTTGT TTTTGATAGG CGGAGGTACT TTTGTGGGAC CACAACGCCT TGGATTTACT CCTAATAAGT   
  
  
- CCTGTCTGTG ATCACAATTC CTGATACCGT CAGATGATCT CGGTGGTCTC ATTCAAGCAT AAAATTATTT   
  
  
- TTATTATAAA ATTAAAATCT TCCACCAGAA TAAAAATGTT CAGTGAGTAT GGGAATATTT GTAGATTTAT   
  
  
- ATTATTTTTT TAATTACTTC TAGTTTAAAA ATATTTGAAT TTTACAATAA TATGATGGTT TAAATAAAGA   
  
  
- TTTTATCATA AAGTAACGTA TGTTAATTTG TTTAATTATG ATCGTATAAT TTTATTTAAT TAAAAAACAT   
  
  
- TTCAAAAACC ATCCCATCAA GATATATAAT GGCACGATAA AAATTAGTTG AAGTGGAAAC ATTTTAAGTA   
  
  
- CACTTATCAA TTGGACACAG CTATAAAAAT GAATTTATAA GATCCCAACC AATAACTTTA AAAATTAATA   
  
  
- ATTTTTTAAT AATTTATAAT AAATTATGTA TATCTTTTAA TTAAATTATT TTACAGTTAT TTTTTTGGAA   
  
  
- ATGAATAAAA AAAGGAGAAA TTAAAGTTGT TGAATTGCAA ATTTAATGTT GTGTGGATTG AATAGGCAGC   
  
  
- TGCCAAGAGA GTCAATGAGT CAACAAATAT GATTGAATCA AATAAATTAG TAGTTGGGGC GAGGTGTAAT   
  
  
- TAAGCCAATT AAAACCTTCG TCAAACAGTG TCATCCTTAG TAAATATGAC TCAATTGATA ATGGTCTTGA   
  
  
- TCATGAATTG TGTATTTAGA TAATGCGCAA TATATGATTG CATTTGACAA ATTAATTAAG ATAGATGTTT   
  
  
- CAAGTTAAGC ATAGTGTTTG ACTGACTTAG TTAATTCAAT TAAGTTCCGT GACTTAGAGA TCAAATTATA   
  
  
- ATCATCACTC ACAGTACGCG AAATGAAATA CATAATATTA CATTACTCTA AACCCAGAAT TACCCCCCCA   
  
  
- AAAAAAAAAC ACACAAAAAA AAAAGGTGGC TTATCCTTAA AAAAAAAATC TTCAAGTAAA ATGTCTTATT   
  
  
- TAAAAATAAG AAAAAGACAA AAAAAAAGAC AAAGGGCGGG AAAGGGGTCA AAATTGTAAG ATTACTTTAA   
  
  
- TCACATCATG TCATAGCCAA ATTGTGTTT

+     Unnamed\_\_6

| Site Name | Organism | Position | Strand | Matrix score. | sequence | function |
| --- | --- | --- | --- | --- | --- | --- |
| Unnamed\_\_6 | Zea mays | 545 | + | 10 | taTAAATATct |  |

> 2018/04/13 10:10:12  
+ CCAAAGCCAT TCCATCGACT TTAATCCCAA TCCCAAACGT TCCATTAGGT AACGGTCTTT CGTTCTCCTC   
  
  
+ AACCTGTACC ATCGCACTCT CTACCCATGA AAACATACAA TCCGAACGAG GGTGATATAT ATGTTTCTTA   
  
  
+ ATTTTTATGA ATTATTTTCT TATTTCTGTA ATATTTTAAT TTTTATAATA GAAGTAAAAC TTGACGACGA   
  
  
+ AGTACCTGTG GAAGGGCCTC CCCATGATGC TTCGATGGAA CACCACGGTG CACACACCGG GTGTACGATA   
  
  
+ CTGCAGTAGC ATTACTGCAT CCGGACCGAG TGAACTTTCG GTATGACCGG GTGAACTCAT TATTAACGGG   
  
  
+ CTACAGAACA AAAACTATCC GCCTCCATGA AAACACCCTG GTGTTGCGGA ACCTAAATGA GGATTATTCA   
  
  
+ GGACAGACAC TAGTGTTAAG GACTATGGCA GTCTACTAGA GCCACCAGAG TAAGTTCGTA TTTTAATAAA   
  
  
+ AATAATATTT TAATTTTAGA AGGTGGTCTT ATTTTTACAA GTCACTCATA CCCTTATAAA CATCTAAATA   
  
  
+ TAATAAAAAA ATTAATGAAG ATCAAATTTT TATAAACTTA AAATGTTATT ATACTACCAA ATTTATTTCT   
  
  
+ AAAATAGTAT TTCATTGCAT ACAATTAAAC AAATTAATAC TAGCATATTA AAATAAATTA ATTTTTTGTA   
  
  
+ AAGTTTTTGG TAGGGTAGTT CTATATATTA CCGTGCTATT TTTAATCAAC TTCACCTTTG TAAAATTCAT   
  
  
+ GTGAATAGTT AACCTGTGTC GATATTTTTA CTTAAATATT CTAGGGTTGG TTATTGAAAT TTTTAATTAT   
  
  
+ TAAAAAATTA TTAAATATTA TTTAATACAT ATAGAAAATT AATTTAATAA AATGTCAATA AAAAAACCTT   
  
  
+ TACTTATTTT TTTCCTCTTT AATTTCAACA ACTTAACGTT TAAATTACAA CACACCTAAC TTATCCGTCG   
  
  
+ ACGGTTCTCT CAGTTACTCA GTTGTTTATA CTAACTTAGT TTATTTAATC ATCAACCCCG CTCCACATTA   
  
  
+ ATTCGGTTAA TTTTGGAAGC AGTTTGTCAC AGTAGGAATC ATTTATACTG AGTTAACTAT TACCAGAACT   
  
  
+ AGTACTTAAC ACATAAATCT ATTACGCGTT ATATACTAAC GTAAACTGTT TAATTAATTC TATCTACAAA   
  
  
+ GTTCAATTCG TATCACAAAC TGACTGAATC AATTAAGTTA ATTCAAGGCA CTGAATCTCT AGTTTAATAT   
  
  
+ TAGTAGTGAG TGTCATGCGC TTTACTTTAT GTATTATAAT GTAATGAGAT TTGGGTCTTA ATGGGGGGGT   
  
  
+ TTTTTTTTTG TGTGTTTTTT TTTTCCACCG AATAGGAATT TTTTTTTTAG AAGTTCATTT TACAGAATAA   
  
  
+ ATTTTTATTC TTTTTCTGTT TTTTTTTCTG TTTCCCGCCC TTTCCCCAGT TTTAACATTC TAATGAAATT   
  
  
+ AGTGTAGTAC AGTATCGGTT TAACACAAA  

- GGTTTCGGTA AGGTAGCTGA AATTAGGGTT AGGGTTTGCA AGGTAATCCA TTGCCAGAAA GCAAGAGGAG   
  
  
- TTGGACATGG TAGCGTGAGA GATGGGTACT TTTGTATGTT AGGCTTGCTC CCACTATATA TACAAAGAAT   
  
  
- TAAAAATACT TAATAAAAGA ATAAAGACAT TATAAAATTA AAAATATTAT CTTCATTTTG AACTGCTGCT   
  
  
- TCATGGACAC CTTCCCGGAG GGGTACTACG AAGCTACCTT GTGGTGCCAC GTGTGTGGCC CACATGCTAT   
  
  
- GACGTCATCG TAATGACGTA GGCCTGGCTC ACTTGAAAGC CATACTGGCC CACTTGAGTA ATAATTGCCC   
  
  
- GATGTCTTGT TTTTGATAGG CGGAGGTACT TTTGTGGGAC CACAACGCCT TGGATTTACT CCTAATAAGT   
  
  
- CCTGTCTGTG ATCACAATTC CTGATACCGT CAGATGATCT CGGTGGTCTC ATTCAAGCAT AAAATTATTT   
  
  
- TTATTATAAA ATTAAAATCT TCCACCAGAA TAAAAATGTT CAGTGAGTAT GGGAATATTT GTAGATTTAT   
  
  
- ATTATTTTTT TAATTACTTC TAGTTTAAAA ATATTTGAAT TTTACAATAA TATGATGGTT TAAATAAAGA   
  
  
- TTTTATCATA AAGTAACGTA TGTTAATTTG TTTAATTATG ATCGTATAAT TTTATTTAAT TAAAAAACAT   
  
  
- TTCAAAAACC ATCCCATCAA GATATATAAT GGCACGATAA AAATTAGTTG AAGTGGAAAC ATTTTAAGTA   
  
  
- CACTTATCAA TTGGACACAG CTATAAAAAT GAATTTATAA GATCCCAACC AATAACTTTA AAAATTAATA   
  
  
- ATTTTTTAAT AATTTATAAT AAATTATGTA TATCTTTTAA TTAAATTATT TTACAGTTAT TTTTTTGGAA   
  
  
- ATGAATAAAA AAAGGAGAAA TTAAAGTTGT TGAATTGCAA ATTTAATGTT GTGTGGATTG AATAGGCAGC   
  
  
- TGCCAAGAGA GTCAATGAGT CAACAAATAT GATTGAATCA AATAAATTAG TAGTTGGGGC GAGGTGTAAT   
  
  
- TAAGCCAATT AAAACCTTCG TCAAACAGTG TCATCCTTAG TAAATATGAC TCAATTGATA ATGGTCTTGA   
  
  
- TCATGAATTG TGTATTTAGA TAATGCGCAA TATATGATTG CATTTGACAA ATTAATTAAG ATAGATGTTT   
  
  
- CAAGTTAAGC ATAGTGTTTG ACTGACTTAG TTAATTCAAT TAAGTTCCGT GACTTAGAGA TCAAATTATA   
  
  
- ATCATCACTC ACAGTACGCG AAATGAAATA CATAATATTA CATTACTCTA AACCCAGAAT TACCCCCCCA   
  
  
- AAAAAAAAAC ACACAAAAAA AAAAGGTGGC TTATCCTTAA AAAAAAAATC TTCAAGTAAA ATGTCTTATT   
  
  
- TAAAAATAAG AAAAAGACAA AAAAAAAGAC AAAGGGCGGG AAAGGGGTCA AAATTGTAAG ATTACTTTAA   
  
  
- TCACATCATG TCATAGCCAA ATTGTGTTT

+     box S

| Site Name | Organism | Position | Strand | Matrix score. | sequence | function |
| --- | --- | --- | --- | --- | --- | --- |
| box S | Arabidopsis thaliana | 460 | + | 7 | AGCCACC |  |

> 2018/04/13 10:10:12  
+ CCAAAGCCAT TCCATCGACT TTAATCCCAA TCCCAAACGT TCCATTAGGT AACGGTCTTT CGTTCTCCTC   
  
  
+ AACCTGTACC ATCGCACTCT CTACCCATGA AAACATACAA TCCGAACGAG GGTGATATAT ATGTTTCTTA   
  
  
+ ATTTTTATGA ATTATTTTCT TATTTCTGTA ATATTTTAAT TTTTATAATA GAAGTAAAAC TTGACGACGA   
  
  
+ AGTACCTGTG GAAGGGCCTC CCCATGATGC TTCGATGGAA CACCACGGTG CACACACCGG GTGTACGATA   
  
  
+ CTGCAGTAGC ATTACTGCAT CCGGACCGAG TGAACTTTCG GTATGACCGG GTGAACTCAT TATTAACGGG   
  
  
+ CTACAGAACA AAAACTATCC GCCTCCATGA AAACACCCTG GTGTTGCGGA ACCTAAATGA GGATTATTCA   
  
  
+ GGACAGACAC TAGTGTTAAG GACTATGGCA GTCTACTAGA GCCACCAGAG TAAGTTCGTA TTTTAATAAA   
  
  
+ AATAATATTT TAATTTTAGA AGGTGGTCTT ATTTTTACAA GTCACTCATA CCCTTATAAA CATCTAAATA   
  
  
+ TAATAAAAAA ATTAATGAAG ATCAAATTTT TATAAACTTA AAATGTTATT ATACTACCAA ATTTATTTCT   
  
  
+ AAAATAGTAT TTCATTGCAT ACAATTAAAC AAATTAATAC TAGCATATTA AAATAAATTA ATTTTTTGTA   
  
  
+ AAGTTTTTGG TAGGGTAGTT CTATATATTA CCGTGCTATT TTTAATCAAC TTCACCTTTG TAAAATTCAT   
  
  
+ GTGAATAGTT AACCTGTGTC GATATTTTTA CTTAAATATT CTAGGGTTGG TTATTGAAAT TTTTAATTAT   
  
  
+ TAAAAAATTA TTAAATATTA TTTAATACAT ATAGAAAATT AATTTAATAA AATGTCAATA AAAAAACCTT   
  
  
+ TACTTATTTT TTTCCTCTTT AATTTCAACA ACTTAACGTT TAAATTACAA CACACCTAAC TTATCCGTCG   
  
  
+ ACGGTTCTCT CAGTTACTCA GTTGTTTATA CTAACTTAGT TTATTTAATC ATCAACCCCG CTCCACATTA   
  
  
+ ATTCGGTTAA TTTTGGAAGC AGTTTGTCAC AGTAGGAATC ATTTATACTG AGTTAACTAT TACCAGAACT   
  
  
+ AGTACTTAAC ACATAAATCT ATTACGCGTT ATATACTAAC GTAAACTGTT TAATTAATTC TATCTACAAA   
  
  
+ GTTCAATTCG TATCACAAAC TGACTGAATC AATTAAGTTA ATTCAAGGCA CTGAATCTCT AGTTTAATAT   
  
  
+ TAGTAGTGAG TGTCATGCGC TTTACTTTAT GTATTATAAT GTAATGAGAT TTGGGTCTTA ATGGGGGGGT   
  
  
+ TTTTTTTTTG TGTGTTTTTT TTTTCCACCG AATAGGAATT TTTTTTTTAG AAGTTCATTT TACAGAATAA   
  
  
+ ATTTTTATTC TTTTTCTGTT TTTTTTTCTG TTTCCCGCCC TTTCCCCAGT TTTAACATTC TAATGAAATT   
  
  
+ AGTGTAGTAC AGTATCGGTT TAACACAAA  

- GGTTTCGGTA AGGTAGCTGA AATTAGGGTT AGGGTTTGCA AGGTAATCCA TTGCCAGAAA GCAAGAGGAG   
  
  
- TTGGACATGG TAGCGTGAGA GATGGGTACT TTTGTATGTT AGGCTTGCTC CCACTATATA TACAAAGAAT   
  
  
- TAAAAATACT TAATAAAAGA ATAAAGACAT TATAAAATTA AAAATATTAT CTTCATTTTG AACTGCTGCT   
  
  
- TCATGGACAC CTTCCCGGAG GGGTACTACG AAGCTACCTT GTGGTGCCAC GTGTGTGGCC CACATGCTAT   
  
  
- GACGTCATCG TAATGACGTA GGCCTGGCTC ACTTGAAAGC CATACTGGCC CACTTGAGTA ATAATTGCCC   
  
  
- GATGTCTTGT TTTTGATAGG CGGAGGTACT TTTGTGGGAC CACAACGCCT TGGATTTACT CCTAATAAGT   
  
  
- CCTGTCTGTG ATCACAATTC CTGATACCGT CAGATGATCT CGGTGGTCTC ATTCAAGCAT AAAATTATTT   
  
  
- TTATTATAAA ATTAAAATCT TCCACCAGAA TAAAAATGTT CAGTGAGTAT GGGAATATTT GTAGATTTAT   
  
  
- ATTATTTTTT TAATTACTTC TAGTTTAAAA ATATTTGAAT TTTACAATAA TATGATGGTT TAAATAAAGA   
  
  
- TTTTATCATA AAGTAACGTA TGTTAATTTG TTTAATTATG ATCGTATAAT TTTATTTAAT TAAAAAACAT   
  
  
- TTCAAAAACC ATCCCATCAA GATATATAAT GGCACGATAA AAATTAGTTG AAGTGGAAAC ATTTTAAGTA   
  
  
- CACTTATCAA TTGGACACAG CTATAAAAAT GAATTTATAA GATCCCAACC AATAACTTTA AAAATTAATA   
  
  
- ATTTTTTAAT AATTTATAAT AAATTATGTA TATCTTTTAA TTAAATTATT TTACAGTTAT TTTTTTGGAA   
  
  
- ATGAATAAAA AAAGGAGAAA TTAAAGTTGT TGAATTGCAA ATTTAATGTT GTGTGGATTG AATAGGCAGC   
  
  
- TGCCAAGAGA GTCAATGAGT CAACAAATAT GATTGAATCA AATAAATTAG TAGTTGGGGC GAGGTGTAAT   
  
  
- TAAGCCAATT AAAACCTTCG TCAAACAGTG TCATCCTTAG TAAATATGAC TCAATTGATA ATGGTCTTGA   
  
  
- TCATGAATTG TGTATTTAGA TAATGCGCAA TATATGATTG CATTTGACAA ATTAATTAAG ATAGATGTTT   
  
  
- CAAGTTAAGC ATAGTGTTTG ACTGACTTAG TTAATTCAAT TAAGTTCCGT GACTTAGAGA TCAAATTATA   
  
  
- ATCATCACTC ACAGTACGCG AAATGAAATA CATAATATTA CATTACTCTA AACCCAGAAT TACCCCCCCA   
  
  
- AAAAAAAAAC ACACAAAAAA AAAAGGTGGC TTATCCTTAA AAAAAAAATC TTCAAGTAAA ATGTCTTATT   
  
  
- TAAAAATAAG AAAAAGACAA AAAAAAAGAC AAAGGGCGGG AAAGGGGTCA AAATTGTAAG ATTACTTTAA   
  
  
- TCACATCATG TCATAGCCAA ATTGTGTTT

+     chs-CMA1a

| Site Name | Organism | Position | Strand | Matrix score. | sequence | function |
| --- | --- | --- | --- | --- | --- | --- |
| chs-CMA1a | Daucus carota | 798 | + | 8 | TTACTTAA | part of a light responsive element |

> 2018/04/13 10:10:12  
+ CCAAAGCCAT TCCATCGACT TTAATCCCAA TCCCAAACGT TCCATTAGGT AACGGTCTTT CGTTCTCCTC   
  
  
+ AACCTGTACC ATCGCACTCT CTACCCATGA AAACATACAA TCCGAACGAG GGTGATATAT ATGTTTCTTA   
  
  
+ ATTTTTATGA ATTATTTTCT TATTTCTGTA ATATTTTAAT TTTTATAATA GAAGTAAAAC TTGACGACGA   
  
  
+ AGTACCTGTG GAAGGGCCTC CCCATGATGC TTCGATGGAA CACCACGGTG CACACACCGG GTGTACGATA   
  
  
+ CTGCAGTAGC ATTACTGCAT CCGGACCGAG TGAACTTTCG GTATGACCGG GTGAACTCAT TATTAACGGG   
  
  
+ CTACAGAACA AAAACTATCC GCCTCCATGA AAACACCCTG GTGTTGCGGA ACCTAAATGA GGATTATTCA   
  
  
+ GGACAGACAC TAGTGTTAAG GACTATGGCA GTCTACTAGA GCCACCAGAG TAAGTTCGTA TTTTAATAAA   
  
  
+ AATAATATTT TAATTTTAGA AGGTGGTCTT ATTTTTACAA GTCACTCATA CCCTTATAAA CATCTAAATA   
  
  
+ TAATAAAAAA ATTAATGAAG ATCAAATTTT TATAAACTTA AAATGTTATT ATACTACCAA ATTTATTTCT   
  
  
+ AAAATAGTAT TTCATTGCAT ACAATTAAAC AAATTAATAC TAGCATATTA AAATAAATTA ATTTTTTGTA   
  
  
+ AAGTTTTTGG TAGGGTAGTT CTATATATTA CCGTGCTATT TTTAATCAAC TTCACCTTTG TAAAATTCAT   
  
  
+ GTGAATAGTT AACCTGTGTC GATATTTTTA CTTAAATATT CTAGGGTTGG TTATTGAAAT TTTTAATTAT   
  
  
+ TAAAAAATTA TTAAATATTA TTTAATACAT ATAGAAAATT AATTTAATAA AATGTCAATA AAAAAACCTT   
  
  
+ TACTTATTTT TTTCCTCTTT AATTTCAACA ACTTAACGTT TAAATTACAA CACACCTAAC TTATCCGTCG   
  
  
+ ACGGTTCTCT CAGTTACTCA GTTGTTTATA CTAACTTAGT TTATTTAATC ATCAACCCCG CTCCACATTA   
  
  
+ ATTCGGTTAA TTTTGGAAGC AGTTTGTCAC AGTAGGAATC ATTTATACTG AGTTAACTAT TACCAGAACT   
  
  
+ AGTACTTAAC ACATAAATCT ATTACGCGTT ATATACTAAC GTAAACTGTT TAATTAATTC TATCTACAAA   
  
  
+ GTTCAATTCG TATCACAAAC TGACTGAATC AATTAAGTTA ATTCAAGGCA CTGAATCTCT AGTTTAATAT   
  
  
+ TAGTAGTGAG TGTCATGCGC TTTACTTTAT GTATTATAAT GTAATGAGAT TTGGGTCTTA ATGGGGGGGT   
  
  
+ TTTTTTTTTG TGTGTTTTTT TTTTCCACCG AATAGGAATT TTTTTTTTAG AAGTTCATTT TACAGAATAA   
  
  
+ ATTTTTATTC TTTTTCTGTT TTTTTTTCTG TTTCCCGCCC TTTCCCCAGT TTTAACATTC TAATGAAATT   
  
  
+ AGTGTAGTAC AGTATCGGTT TAACACAAA  

- GGTTTCGGTA AGGTAGCTGA AATTAGGGTT AGGGTTTGCA AGGTAATCCA TTGCCAGAAA GCAAGAGGAG   
  
  
- TTGGACATGG TAGCGTGAGA GATGGGTACT TTTGTATGTT AGGCTTGCTC CCACTATATA TACAAAGAAT   
  
  
- TAAAAATACT TAATAAAAGA ATAAAGACAT TATAAAATTA AAAATATTAT CTTCATTTTG AACTGCTGCT   
  
  
- TCATGGACAC CTTCCCGGAG GGGTACTACG AAGCTACCTT GTGGTGCCAC GTGTGTGGCC CACATGCTAT   
  
  
- GACGTCATCG TAATGACGTA GGCCTGGCTC ACTTGAAAGC CATACTGGCC CACTTGAGTA ATAATTGCCC   
  
  
- GATGTCTTGT TTTTGATAGG CGGAGGTACT TTTGTGGGAC CACAACGCCT TGGATTTACT CCTAATAAGT   
  
  
- CCTGTCTGTG ATCACAATTC CTGATACCGT CAGATGATCT CGGTGGTCTC ATTCAAGCAT AAAATTATTT   
  
  
- TTATTATAAA ATTAAAATCT TCCACCAGAA TAAAAATGTT CAGTGAGTAT GGGAATATTT GTAGATTTAT   
  
  
- ATTATTTTTT TAATTACTTC TAGTTTAAAA ATATTTGAAT TTTACAATAA TATGATGGTT TAAATAAAGA   
  
  
- TTTTATCATA AAGTAACGTA TGTTAATTTG TTTAATTATG ATCGTATAAT TTTATTTAAT TAAAAAACAT   
  
  
- TTCAAAAACC ATCCCATCAA GATATATAAT GGCACGATAA AAATTAGTTG AAGTGGAAAC ATTTTAAGTA   
  
  
- CACTTATCAA TTGGACACAG CTATAAAAAT GAATTTATAA GATCCCAACC AATAACTTTA AAAATTAATA   
  
  
- ATTTTTTAAT AATTTATAAT AAATTATGTA TATCTTTTAA TTAAATTATT TTACAGTTAT TTTTTTGGAA   
  
  
- ATGAATAAAA AAAGGAGAAA TTAAAGTTGT TGAATTGCAA ATTTAATGTT GTGTGGATTG AATAGGCAGC   
  
  
- TGCCAAGAGA GTCAATGAGT CAACAAATAT GATTGAATCA AATAAATTAG TAGTTGGGGC GAGGTGTAAT   
  
  
- TAAGCCAATT AAAACCTTCG TCAAACAGTG TCATCCTTAG TAAATATGAC TCAATTGATA ATGGTCTTGA   
  
  
- TCATGAATTG TGTATTTAGA TAATGCGCAA TATATGATTG CATTTGACAA ATTAATTAAG ATAGATGTTT   
  
  
- CAAGTTAAGC ATAGTGTTTG ACTGACTTAG TTAATTCAAT TAAGTTCCGT GACTTAGAGA TCAAATTATA   
  
  
- ATCATCACTC ACAGTACGCG AAATGAAATA CATAATATTA CATTACTCTA AACCCAGAAT TACCCCCCCA   
  
  
- AAAAAAAAAC ACACAAAAAA AAAAGGTGGC TTATCCTTAA AAAAAAAATC TTCAAGTAAA ATGTCTTATT   
  
  
- TAAAAATAAG AAAAAGACAA AAAAAAAGAC AAAGGGCGGG AAAGGGGTCA AAATTGTAAG ATTACTTTAA   
  
  
- TCACATCATG TCATAGCCAA ATTGTGTTT
