# Supplementary material for: Genome-wide analysis of the WRKY gene family in drumstick (Moringa oleifera Lam.)
Source: PeerJ. 2019 Jun 10;7:e7063. doi: 10.7717/peerj.7063 (PMC6563795; doi:10.7717/peerj.7063)
Supplement: Supplemental Information 1 [file peerj-07-7063-s003.gz › MoWRKY51_plantcare.html]

Content-Type: text/html; charset=ISO-8859-1


CallMat\_Firefox


Webmaster Firefox specific output  
To save the result:
click on the frame with the right mouse button and save the source code as a text file with extension .html  
REFERENCE:PlantCARE: a database of plant cis-acting regulatory elements and a portal to tools for in silico analysis of promoter sequences.  
Lescot, M., Déhais, P., Moreau, Y., De Moor, B., Rouzé ,P.,and Rombauts, S.  
Nucleic Acids Res., Database issue(2002), 30(1):325-327.   


---

> 2018/04/13 10:10:12  
+ CAGAAATTGT AAGTTTTTAT TGGTGTCACT ATAATTTAAT TTGTTTCCTT GACGATAGTA CTATTTGTAC   
  
  
+ GACATGAAAG TTTGGTCCGA TTCAACTAAC TAATTAATTG ATTAAGATAA ATTTAATTTG TTTCCTTGAC   
  
  
+ GATAGTACTA TTTGTACGAC ATGAAAGTTT GGTCCGATTC AACTAACTAA TTAATTGATT AAGATATCAA   
  
  
+ TGCGATCTTA ATCAAAGCAA GCTAGCCTAG CCTTAATCAT GAAGCCTCTA ATATTTTCTC ATTGCTTTGA   
  
  
+ TACTTTTTTT TTAAAATTTT ATTTAAAATT TAAAATTATA TTTTTACCTT GAAGTGGAGT CCAATGTTTT   
  
  
+ AAAGATGACA ATTCATATCA TAATTTATTA AATTAAATTT AATTATTTAA AAAATTAAAA ATTAAATTTT   
  
  
+ ATTGTATAAA AAAATATTTA AACCATTATT ATAAATAAAA TATATATTTT TAGATATTAT TTATTTTAAA   
  
  
+ ATTGTACTGT CAAAATTTTA AAATATTTAT AAAAAGTAAT TTACAAGTCA TATGAATGAA TTGAAAATAA   
  
  
+ AATGTAAAAT TTATTTACTT TATTTTATAA CAAAAAGTTT TTAAATTTTT ATAATCATTT GAACTAAATT   
  
  
+ TAATTAATAA ATACAAATTT GATTTCTTTT TCTAAAAATT AAATCTAATT CACTATTTTC AAATATTAAA   
  
  
+ ATTTAATTTA ATGCATGAAA CACTTTAAGA TCAAAATTAT ATAAAATTAT CATATTTACT TTATATTATC   
  
  
+ AATCAATTTT TTTGTTAAAA AGGTGACAAA TTGAATTTTG ATCTACTGGT TTAATATCAA ATTAAATCTT   
  
  
+ AATTAAAATA ATCATAACAA TTAAAAATAT TTTTGTTATT AAAATATTTA AACTATTTGA ATTATTTTTT   
  
  
+ ATTTTATTTC AAGTTCATTC TTTAAATGTA AATAATTTTT GATGCAAGAT CTGGTTTTTC TAAATTTTTA   
  
  
+ AAATTTTGAT TATATTTAAC AATACATTTT TAATACTCAA AAAATTTTAA AATTTTTATG ATAATATTTT   
  
  
+ AAATATTAAA TTTGAAATTA AAATTTATTA TATAAAATTT TTAATTTAAT TATTTTAAAA TTTAAATTTT   
  
  
+ TTAATAACAA TTAATTTAAT TTAATTTAAT AATATAAAAT AAATATTAAA ATTTTATATA ATTTTTATTC   
  
  
+ AAAATTTTAA ACACATTTTT TATTTTTTCT TGTGGCGGGC TTCCGAATCA GCTACCTTTT TGTAGAGGTA   
  
  
+ TCACTATATA AAATTTACGT GCCGTTTACG ACACCAAAGA TACCATTAGG ATAAGAAGCT GCCTCACAGA   
  
  
+ CAGGCCGCCT CCGCCACTGT TCTGAATTTC AACCACACTC AGTACTCCTC CTATATATTA CCACACAAAC   
  
  
+ CATATTCATT GATTCTTATT TATTCTTCTT GTCCTTCTTC GTCGTATGAT CCGACGCTCA CTTTTCTCTC   
  
  
+ AGACTTTCTC TCGTTTTCTC GGGAAAATA  

- GTCTTTAACA TTCAAAAATA ACCACAGTGA TATTAAATTA AACAAAGGAA CTGCTATCAT GATAAACATG   
  
  
- CTGTACTTTC AAACCAGGCT AAGTTGATTG ATTAATTAAC TAATTCTATT TAAATTAAAC AAAGGAACTG   
  
  
- CTATCATGAT AAACATGCTG TACTTTCAAA CCAGGCTAAG TTGATTGATT AATTAACTAA TTCTATAGTT   
  
  
- ACGCTAGAAT TAGTTTCGTT CGATCGGATC GGAATTAGTA CTTCGGAGAT TATAAAAGAG TAACGAAACT   
  
  
- ATGAAAAAAA AATTTTAAAA TAAATTTTAA ATTTTAATAT AAAAATGGAA CTTCACCTCA GGTTACAAAA   
  
  
- TTTCTACTGT TAAGTATAGT ATTAAATAAT TTAATTTAAA TTAATAAATT TTTTAATTTT TAATTTAAAA   
  
  
- TAACATATTT TTTTATAAAT TTGGTAATAA TATTTATTTT ATATATAAAA ATCTATAATA AATAAAATTT   
  
  
- TAACATGACA GTTTTAAAAT TTTATAAATA TTTTTCATTA AATGTTCAGT ATACTTACTT AACTTTTATT   
  
  
- TTACATTTTA AATAAATGAA ATAAAATATT GTTTTTCAAA AATTTAAAAA TATTAGTAAA CTTGATTTAA   
  
  
- ATTAATTATT TATGTTTAAA CTAAAGAAAA AGATTTTTAA TTTAGATTAA GTGATAAAAG TTTATAATTT   
  
  
- TAAATTAAAT TACGTACTTT GTGAAATTCT AGTTTTAATA TATTTTAATA GTATAAATGA AATATAATAG   
  
  
- TTAGTTAAAA AAACAATTTT TCCACTGTTT AACTTAAAAC TAGATGACCA AATTATAGTT TAATTTAGAA   
  
  
- TTAATTTTAT TAGTATTGTT AATTTTTATA AAAACAATAA TTTTATAAAT TTGATAAACT TAATAAAAAA   
  
  
- TAAAATAAAG TTCAAGTAAG AAATTTACAT TTATTAAAAA CTACGTTCTA GACCAAAAAG ATTTAAAAAT   
  
  
- TTTAAAACTA ATATAAATTG TTATGTAAAA ATTATGAGTT TTTTAAAATT TTAAAAATAC TATTATAAAA   
  
  
- TTTATAATTT AAACTTTAAT TTTAAATAAT ATATTTTAAA AATTAAATTA ATAAAATTTT AAATTTAAAA   
  
  
- AATTATTGTT AATTAAATTA AATTAAATTA TTATATTTTA TTTATAATTT TAAAATATAT TAAAAATAAG   
  
  
- TTTTAAAATT TGTGTAAAAA ATAAAAAAGA ACACCGCCCG AAGGCTTAGT CGATGGAAAA ACATCTCCAT   
  
  
- AGTGATATAT TTTAAATGCA CGGCAAATGC TGTGGTTTCT ATGGTAATCC TATTCTTCGA CGGAGTGTCT   
  
  
- GTCCGGCGGA GGCGGTGACA AGACTTAAAG TTGGTGTGAG TCATGAGGAG GATATATAAT GGTGTGTTTG   
  
  
- GTATAAGTAA CTAAGAATAA ATAAGAAGAA CAGGAAGAAG CAGCATACTA GGCTGCGAGT GAAAAGAGAG   
  
  
- TCTGAAAGAG AGCAAAAGAG CCCTTTTAT

  
  
Motifs Found  

+     ABRE

| Site Name | Organism | Position | Strand | Matrix score. | sequence | function |
| --- | --- | --- | --- | --- | --- | --- |
| ABRE | Arabidopsis thaliana | 1276 | + | 6 | TACGTG | cis-acting element involved in the abscisic acid responsiveness |

> 2018/04/13 10:10:12  
+ CAGAAATTGT AAGTTTTTAT TGGTGTCACT ATAATTTAAT TTGTTTCCTT GACGATAGTA CTATTTGTAC   
  
  
+ GACATGAAAG TTTGGTCCGA TTCAACTAAC TAATTAATTG ATTAAGATAA ATTTAATTTG TTTCCTTGAC   
  
  
+ GATAGTACTA TTTGTACGAC ATGAAAGTTT GGTCCGATTC AACTAACTAA TTAATTGATT AAGATATCAA   
  
  
+ TGCGATCTTA ATCAAAGCAA GCTAGCCTAG CCTTAATCAT GAAGCCTCTA ATATTTTCTC ATTGCTTTGA   
  
  
+ TACTTTTTTT TTAAAATTTT ATTTAAAATT TAAAATTATA TTTTTACCTT GAAGTGGAGT CCAATGTTTT   
  
  
+ AAAGATGACA ATTCATATCA TAATTTATTA AATTAAATTT AATTATTTAA AAAATTAAAA ATTAAATTTT   
  
  
+ ATTGTATAAA AAAATATTTA AACCATTATT ATAAATAAAA TATATATTTT TAGATATTAT TTATTTTAAA   
  
  
+ ATTGTACTGT CAAAATTTTA AAATATTTAT AAAAAGTAAT TTACAAGTCA TATGAATGAA TTGAAAATAA   
  
  
+ AATGTAAAAT TTATTTACTT TATTTTATAA CAAAAAGTTT TTAAATTTTT ATAATCATTT GAACTAAATT   
  
  
+ TAATTAATAA ATACAAATTT GATTTCTTTT TCTAAAAATT AAATCTAATT CACTATTTTC AAATATTAAA   
  
  
+ ATTTAATTTA ATGCATGAAA CACTTTAAGA TCAAAATTAT ATAAAATTAT CATATTTACT TTATATTATC   
  
  
+ AATCAATTTT TTTGTTAAAA AGGTGACAAA TTGAATTTTG ATCTACTGGT TTAATATCAA ATTAAATCTT   
  
  
+ AATTAAAATA ATCATAACAA TTAAAAATAT TTTTGTTATT AAAATATTTA AACTATTTGA ATTATTTTTT   
  
  
+ ATTTTATTTC AAGTTCATTC TTTAAATGTA AATAATTTTT GATGCAAGAT CTGGTTTTTC TAAATTTTTA   
  
  
+ AAATTTTGAT TATATTTAAC AATACATTTT TAATACTCAA AAAATTTTAA AATTTTTATG ATAATATTTT   
  
  
+ AAATATTAAA TTTGAAATTA AAATTTATTA TATAAAATTT TTAATTTAAT TATTTTAAAA TTTAAATTTT   
  
  
+ TTAATAACAA TTAATTTAAT TTAATTTAAT AATATAAAAT AAATATTAAA ATTTTATATA ATTTTTATTC   
  
  
+ AAAATTTTAA ACACATTTTT TATTTTTTCT TGTGGCGGGC TTCCGAATCA GCTACCTTTT TGTAGAGGTA   
  
  
+ TCACTATATA AAATTTACGT GCCGTTTACG ACACCAAAGA TACCATTAGG ATAAGAAGCT GCCTCACAGA   
  
  
+ CAGGCCGCCT CCGCCACTGT TCTGAATTTC AACCACACTC AGTACTCCTC CTATATATTA CCACACAAAC   
  
  
+ CATATTCATT GATTCTTATT TATTCTTCTT GTCCTTCTTC GTCGTATGAT CCGACGCTCA CTTTTCTCTC   
  
  
+ AGACTTTCTC TCGTTTTCTC GGGAAAATA  

- GTCTTTAACA TTCAAAAATA ACCACAGTGA TATTAAATTA AACAAAGGAA CTGCTATCAT GATAAACATG   
  
  
- CTGTACTTTC AAACCAGGCT AAGTTGATTG ATTAATTAAC TAATTCTATT TAAATTAAAC AAAGGAACTG   
  
  
- CTATCATGAT AAACATGCTG TACTTTCAAA CCAGGCTAAG TTGATTGATT AATTAACTAA TTCTATAGTT   
  
  
- ACGCTAGAAT TAGTTTCGTT CGATCGGATC GGAATTAGTA CTTCGGAGAT TATAAAAGAG TAACGAAACT   
  
  
- ATGAAAAAAA AATTTTAAAA TAAATTTTAA ATTTTAATAT AAAAATGGAA CTTCACCTCA GGTTACAAAA   
  
  
- TTTCTACTGT TAAGTATAGT ATTAAATAAT TTAATTTAAA TTAATAAATT TTTTAATTTT TAATTTAAAA   
  
  
- TAACATATTT TTTTATAAAT TTGGTAATAA TATTTATTTT ATATATAAAA ATCTATAATA AATAAAATTT   
  
  
- TAACATGACA GTTTTAAAAT TTTATAAATA TTTTTCATTA AATGTTCAGT ATACTTACTT AACTTTTATT   
  
  
- TTACATTTTA AATAAATGAA ATAAAATATT GTTTTTCAAA AATTTAAAAA TATTAGTAAA CTTGATTTAA   
  
  
- ATTAATTATT TATGTTTAAA CTAAAGAAAA AGATTTTTAA TTTAGATTAA GTGATAAAAG TTTATAATTT   
  
  
- TAAATTAAAT TACGTACTTT GTGAAATTCT AGTTTTAATA TATTTTAATA GTATAAATGA AATATAATAG   
  
  
- TTAGTTAAAA AAACAATTTT TCCACTGTTT AACTTAAAAC TAGATGACCA AATTATAGTT TAATTTAGAA   
  
  
- TTAATTTTAT TAGTATTGTT AATTTTTATA AAAACAATAA TTTTATAAAT TTGATAAACT TAATAAAAAA   
  
  
- TAAAATAAAG TTCAAGTAAG AAATTTACAT TTATTAAAAA CTACGTTCTA GACCAAAAAG ATTTAAAAAT   
  
  
- TTTAAAACTA ATATAAATTG TTATGTAAAA ATTATGAGTT TTTTAAAATT TTAAAAATAC TATTATAAAA   
  
  
- TTTATAATTT AAACTTTAAT TTTAAATAAT ATATTTTAAA AATTAAATTA ATAAAATTTT AAATTTAAAA   
  
  
- AATTATTGTT AATTAAATTA AATTAAATTA TTATATTTTA TTTATAATTT TAAAATATAT TAAAAATAAG   
  
  
- TTTTAAAATT TGTGTAAAAA ATAAAAAAGA ACACCGCCCG AAGGCTTAGT CGATGGAAAA ACATCTCCAT   
  
  
- AGTGATATAT TTTAAATGCA CGGCAAATGC TGTGGTTTCT ATGGTAATCC TATTCTTCGA CGGAGTGTCT   
  
  
- GTCCGGCGGA GGCGGTGACA AGACTTAAAG TTGGTGTGAG TCATGAGGAG GATATATAAT GGTGTGTTTG   
  
  
- GTATAAGTAA CTAAGAATAA ATAAGAAGAA CAGGAAGAAG CAGCATACTA GGCTGCGAGT GAAAAGAGAG   
  
  
- TCTGAAAGAG AGCAAAAGAG CCCTTTTAT

+     ARE

| Site Name | Organism | Position | Strand | Matrix score. | sequence | function |
| --- | --- | --- | --- | --- | --- | --- |
| ARE | Zea mays | 440 | - | 6 | TGGTTT | cis-acting regulatory element essential for the anaerobic induction |
| ARE | Zea mays | 962 | + | 6 | TGGTTT | cis-acting regulatory element essential for the anaerobic induction |
| ARE | Zea mays | 817 | + | 6 | TGGTTT | cis-acting regulatory element essential for the anaerobic induction |
| ARE | Zea mays | 1397 | - | 6 | TGGTTT | cis-acting regulatory element essential for the anaerobic induction |

> 2018/04/13 10:10:12  
+ CAGAAATTGT AAGTTTTTAT TGGTGTCACT ATAATTTAAT TTGTTTCCTT GACGATAGTA CTATTTGTAC   
  
  
+ GACATGAAAG TTTGGTCCGA TTCAACTAAC TAATTAATTG ATTAAGATAA ATTTAATTTG TTTCCTTGAC   
  
  
+ GATAGTACTA TTTGTACGAC ATGAAAGTTT GGTCCGATTC AACTAACTAA TTAATTGATT AAGATATCAA   
  
  
+ TGCGATCTTA ATCAAAGCAA GCTAGCCTAG CCTTAATCAT GAAGCCTCTA ATATTTTCTC ATTGCTTTGA   
  
  
+ TACTTTTTTT TTAAAATTTT ATTTAAAATT TAAAATTATA TTTTTACCTT GAAGTGGAGT CCAATGTTTT   
  
  
+ AAAGATGACA ATTCATATCA TAATTTATTA AATTAAATTT AATTATTTAA AAAATTAAAA ATTAAATTTT   
  
  
+ ATTGTATAAA AAAATATTTA AACCATTATT ATAAATAAAA TATATATTTT TAGATATTAT TTATTTTAAA   
  
  
+ ATTGTACTGT CAAAATTTTA AAATATTTAT AAAAAGTAAT TTACAAGTCA TATGAATGAA TTGAAAATAA   
  
  
+ AATGTAAAAT TTATTTACTT TATTTTATAA CAAAAAGTTT TTAAATTTTT ATAATCATTT GAACTAAATT   
  
  
+ TAATTAATAA ATACAAATTT GATTTCTTTT TCTAAAAATT AAATCTAATT CACTATTTTC AAATATTAAA   
  
  
+ ATTTAATTTA ATGCATGAAA CACTTTAAGA TCAAAATTAT ATAAAATTAT CATATTTACT TTATATTATC   
  
  
+ AATCAATTTT TTTGTTAAAA AGGTGACAAA TTGAATTTTG ATCTACTGGT TTAATATCAA ATTAAATCTT   
  
  
+ AATTAAAATA ATCATAACAA TTAAAAATAT TTTTGTTATT AAAATATTTA AACTATTTGA ATTATTTTTT   
  
  
+ ATTTTATTTC AAGTTCATTC TTTAAATGTA AATAATTTTT GATGCAAGAT CTGGTTTTTC TAAATTTTTA   
  
  
+ AAATTTTGAT TATATTTAAC AATACATTTT TAATACTCAA AAAATTTTAA AATTTTTATG ATAATATTTT   
  
  
+ AAATATTAAA TTTGAAATTA AAATTTATTA TATAAAATTT TTAATTTAAT TATTTTAAAA TTTAAATTTT   
  
  
+ TTAATAACAA TTAATTTAAT TTAATTTAAT AATATAAAAT AAATATTAAA ATTTTATATA ATTTTTATTC   
  
  
+ AAAATTTTAA ACACATTTTT TATTTTTTCT TGTGGCGGGC TTCCGAATCA GCTACCTTTT TGTAGAGGTA   
  
  
+ TCACTATATA AAATTTACGT GCCGTTTACG ACACCAAAGA TACCATTAGG ATAAGAAGCT GCCTCACAGA   
  
  
+ CAGGCCGCCT CCGCCACTGT TCTGAATTTC AACCACACTC AGTACTCCTC CTATATATTA CCACACAAAC   
  
  
+ CATATTCATT GATTCTTATT TATTCTTCTT GTCCTTCTTC GTCGTATGAT CCGACGCTCA CTTTTCTCTC   
  
  
+ AGACTTTCTC TCGTTTTCTC GGGAAAATA  

- GTCTTTAACA TTCAAAAATA ACCACAGTGA TATTAAATTA AACAAAGGAA CTGCTATCAT GATAAACATG   
  
  
- CTGTACTTTC AAACCAGGCT AAGTTGATTG ATTAATTAAC TAATTCTATT TAAATTAAAC AAAGGAACTG   
  
  
- CTATCATGAT AAACATGCTG TACTTTCAAA CCAGGCTAAG TTGATTGATT AATTAACTAA TTCTATAGTT   
  
  
- ACGCTAGAAT TAGTTTCGTT CGATCGGATC GGAATTAGTA CTTCGGAGAT TATAAAAGAG TAACGAAACT   
  
  
- ATGAAAAAAA AATTTTAAAA TAAATTTTAA ATTTTAATAT AAAAATGGAA CTTCACCTCA GGTTACAAAA   
  
  
- TTTCTACTGT TAAGTATAGT ATTAAATAAT TTAATTTAAA TTAATAAATT TTTTAATTTT TAATTTAAAA   
  
  
- TAACATATTT TTTTATAAAT TTGGTAATAA TATTTATTTT ATATATAAAA ATCTATAATA AATAAAATTT   
  
  
- TAACATGACA GTTTTAAAAT TTTATAAATA TTTTTCATTA AATGTTCAGT ATACTTACTT AACTTTTATT   
  
  
- TTACATTTTA AATAAATGAA ATAAAATATT GTTTTTCAAA AATTTAAAAA TATTAGTAAA CTTGATTTAA   
  
  
- ATTAATTATT TATGTTTAAA CTAAAGAAAA AGATTTTTAA TTTAGATTAA GTGATAAAAG TTTATAATTT   
  
  
- TAAATTAAAT TACGTACTTT GTGAAATTCT AGTTTTAATA TATTTTAATA GTATAAATGA AATATAATAG   
  
  
- TTAGTTAAAA AAACAATTTT TCCACTGTTT AACTTAAAAC TAGATGACCA AATTATAGTT TAATTTAGAA   
  
  
- TTAATTTTAT TAGTATTGTT AATTTTTATA AAAACAATAA TTTTATAAAT TTGATAAACT TAATAAAAAA   
  
  
- TAAAATAAAG TTCAAGTAAG AAATTTACAT TTATTAAAAA CTACGTTCTA GACCAAAAAG ATTTAAAAAT   
  
  
- TTTAAAACTA ATATAAATTG TTATGTAAAA ATTATGAGTT TTTTAAAATT TTAAAAATAC TATTATAAAA   
  
  
- TTTATAATTT AAACTTTAAT TTTAAATAAT ATATTTTAAA AATTAAATTA ATAAAATTTT AAATTTAAAA   
  
  
- AATTATTGTT AATTAAATTA AATTAAATTA TTATATTTTA TTTATAATTT TAAAATATAT TAAAAATAAG   
  
  
- TTTTAAAATT TGTGTAAAAA ATAAAAAAGA ACACCGCCCG AAGGCTTAGT CGATGGAAAA ACATCTCCAT   
  
  
- AGTGATATAT TTTAAATGCA CGGCAAATGC TGTGGTTTCT ATGGTAATCC TATTCTTCGA CGGAGTGTCT   
  
  
- GTCCGGCGGA GGCGGTGACA AGACTTAAAG TTGGTGTGAG TCATGAGGAG GATATATAAT GGTGTGTTTG   
  
  
- GTATAAGTAA CTAAGAATAA ATAAGAAGAA CAGGAAGAAG CAGCATACTA GGCTGCGAGT GAAAAGAGAG   
  
  
- TCTGAAAGAG AGCAAAAGAG CCCTTTTAT

+     AT-rich element

| Site Name | Organism | Position | Strand | Matrix score. | sequence | function |
| --- | --- | --- | --- | --- | --- | --- |
| AT-rich element | Glycine max | 649 | - | 10 | ATAGAAATCAA | binding site of AT-rich DNA binding protein (ATBP-1) |

> 2018/04/13 10:10:12  
+ CAGAAATTGT AAGTTTTTAT TGGTGTCACT ATAATTTAAT TTGTTTCCTT GACGATAGTA CTATTTGTAC   
  
  
+ GACATGAAAG TTTGGTCCGA TTCAACTAAC TAATTAATTG ATTAAGATAA ATTTAATTTG TTTCCTTGAC   
  
  
+ GATAGTACTA TTTGTACGAC ATGAAAGTTT GGTCCGATTC AACTAACTAA TTAATTGATT AAGATATCAA   
  
  
+ TGCGATCTTA ATCAAAGCAA GCTAGCCTAG CCTTAATCAT GAAGCCTCTA ATATTTTCTC ATTGCTTTGA   
  
  
+ TACTTTTTTT TTAAAATTTT ATTTAAAATT TAAAATTATA TTTTTACCTT GAAGTGGAGT CCAATGTTTT   
  
  
+ AAAGATGACA ATTCATATCA TAATTTATTA AATTAAATTT AATTATTTAA AAAATTAAAA ATTAAATTTT   
  
  
+ ATTGTATAAA AAAATATTTA AACCATTATT ATAAATAAAA TATATATTTT TAGATATTAT TTATTTTAAA   
  
  
+ ATTGTACTGT CAAAATTTTA AAATATTTAT AAAAAGTAAT TTACAAGTCA TATGAATGAA TTGAAAATAA   
  
  
+ AATGTAAAAT TTATTTACTT TATTTTATAA CAAAAAGTTT TTAAATTTTT ATAATCATTT GAACTAAATT   
  
  
+ TAATTAATAA ATACAAATTT GATTTCTTTT TCTAAAAATT AAATCTAATT CACTATTTTC AAATATTAAA   
  
  
+ ATTTAATTTA ATGCATGAAA CACTTTAAGA TCAAAATTAT ATAAAATTAT CATATTTACT TTATATTATC   
  
  
+ AATCAATTTT TTTGTTAAAA AGGTGACAAA TTGAATTTTG ATCTACTGGT TTAATATCAA ATTAAATCTT   
  
  
+ AATTAAAATA ATCATAACAA TTAAAAATAT TTTTGTTATT AAAATATTTA AACTATTTGA ATTATTTTTT   
  
  
+ ATTTTATTTC AAGTTCATTC TTTAAATGTA AATAATTTTT GATGCAAGAT CTGGTTTTTC TAAATTTTTA   
  
  
+ AAATTTTGAT TATATTTAAC AATACATTTT TAATACTCAA AAAATTTTAA AATTTTTATG ATAATATTTT   
  
  
+ AAATATTAAA TTTGAAATTA AAATTTATTA TATAAAATTT TTAATTTAAT TATTTTAAAA TTTAAATTTT   
  
  
+ TTAATAACAA TTAATTTAAT TTAATTTAAT AATATAAAAT AAATATTAAA ATTTTATATA ATTTTTATTC   
  
  
+ AAAATTTTAA ACACATTTTT TATTTTTTCT TGTGGCGGGC TTCCGAATCA GCTACCTTTT TGTAGAGGTA   
  
  
+ TCACTATATA AAATTTACGT GCCGTTTACG ACACCAAAGA TACCATTAGG ATAAGAAGCT GCCTCACAGA   
  
  
+ CAGGCCGCCT CCGCCACTGT TCTGAATTTC AACCACACTC AGTACTCCTC CTATATATTA CCACACAAAC   
  
  
+ CATATTCATT GATTCTTATT TATTCTTCTT GTCCTTCTTC GTCGTATGAT CCGACGCTCA CTTTTCTCTC   
  
  
+ AGACTTTCTC TCGTTTTCTC GGGAAAATA  

- GTCTTTAACA TTCAAAAATA ACCACAGTGA TATTAAATTA AACAAAGGAA CTGCTATCAT GATAAACATG   
  
  
- CTGTACTTTC AAACCAGGCT AAGTTGATTG ATTAATTAAC TAATTCTATT TAAATTAAAC AAAGGAACTG   
  
  
- CTATCATGAT AAACATGCTG TACTTTCAAA CCAGGCTAAG TTGATTGATT AATTAACTAA TTCTATAGTT   
  
  
- ACGCTAGAAT TAGTTTCGTT CGATCGGATC GGAATTAGTA CTTCGGAGAT TATAAAAGAG TAACGAAACT   
  
  
- ATGAAAAAAA AATTTTAAAA TAAATTTTAA ATTTTAATAT AAAAATGGAA CTTCACCTCA GGTTACAAAA   
  
  
- TTTCTACTGT TAAGTATAGT ATTAAATAAT TTAATTTAAA TTAATAAATT TTTTAATTTT TAATTTAAAA   
  
  
- TAACATATTT TTTTATAAAT TTGGTAATAA TATTTATTTT ATATATAAAA ATCTATAATA AATAAAATTT   
  
  
- TAACATGACA GTTTTAAAAT TTTATAAATA TTTTTCATTA AATGTTCAGT ATACTTACTT AACTTTTATT   
  
  
- TTACATTTTA AATAAATGAA ATAAAATATT GTTTTTCAAA AATTTAAAAA TATTAGTAAA CTTGATTTAA   
  
  
- ATTAATTATT TATGTTTAAA CTAAAGAAAA AGATTTTTAA TTTAGATTAA GTGATAAAAG TTTATAATTT   
  
  
- TAAATTAAAT TACGTACTTT GTGAAATTCT AGTTTTAATA TATTTTAATA GTATAAATGA AATATAATAG   
  
  
- TTAGTTAAAA AAACAATTTT TCCACTGTTT AACTTAAAAC TAGATGACCA AATTATAGTT TAATTTAGAA   
  
  
- TTAATTTTAT TAGTATTGTT AATTTTTATA AAAACAATAA TTTTATAAAT TTGATAAACT TAATAAAAAA   
  
  
- TAAAATAAAG TTCAAGTAAG AAATTTACAT TTATTAAAAA CTACGTTCTA GACCAAAAAG ATTTAAAAAT   
  
  
- TTTAAAACTA ATATAAATTG TTATGTAAAA ATTATGAGTT TTTTAAAATT TTAAAAATAC TATTATAAAA   
  
  
- TTTATAATTT AAACTTTAAT TTTAAATAAT ATATTTTAAA AATTAAATTA ATAAAATTTT AAATTTAAAA   
  
  
- AATTATTGTT AATTAAATTA AATTAAATTA TTATATTTTA TTTATAATTT TAAAATATAT TAAAAATAAG   
  
  
- TTTTAAAATT TGTGTAAAAA ATAAAAAAGA ACACCGCCCG AAGGCTTAGT CGATGGAAAA ACATCTCCAT   
  
  
- AGTGATATAT TTTAAATGCA CGGCAAATGC TGTGGTTTCT ATGGTAATCC TATTCTTCGA CGGAGTGTCT   
  
  
- GTCCGGCGGA GGCGGTGACA AGACTTAAAG TTGGTGTGAG TCATGAGGAG GATATATAAT GGTGTGTTTG   
  
  
- GTATAAGTAA CTAAGAATAA ATAAGAAGAA CAGGAAGAAG CAGCATACTA GGCTGCGAGT GAAAAGAGAG   
  
  
- TCTGAAAGAG AGCAAAAGAG CCCTTTTAT

+     AT1-motif

| Site Name | Organism | Position | Strand | Matrix score. | sequence | function |
| --- | --- | --- | --- | --- | --- | --- |
| AT1-motif | Solanum tuberosum | 563 | - | 11 | ATTAATTTTACA | part of a light responsive module |
| AT1-motif | Solanum tuberosum | 900 | + | 14 | AATTATTTTTTATT | part of a light responsive module |

> 2018/04/13 10:10:12  
+ CAGAAATTGT AAGTTTTTAT TGGTGTCACT ATAATTTAAT TTGTTTCCTT GACGATAGTA CTATTTGTAC   
  
  
+ GACATGAAAG TTTGGTCCGA TTCAACTAAC TAATTAATTG ATTAAGATAA ATTTAATTTG TTTCCTTGAC   
  
  
+ GATAGTACTA TTTGTACGAC ATGAAAGTTT GGTCCGATTC AACTAACTAA TTAATTGATT AAGATATCAA   
  
  
+ TGCGATCTTA ATCAAAGCAA GCTAGCCTAG CCTTAATCAT GAAGCCTCTA ATATTTTCTC ATTGCTTTGA   
  
  
+ TACTTTTTTT TTAAAATTTT ATTTAAAATT TAAAATTATA TTTTTACCTT GAAGTGGAGT CCAATGTTTT   
  
  
+ AAAGATGACA ATTCATATCA TAATTTATTA AATTAAATTT AATTATTTAA AAAATTAAAA ATTAAATTTT   
  
  
+ ATTGTATAAA AAAATATTTA AACCATTATT ATAAATAAAA TATATATTTT TAGATATTAT TTATTTTAAA   
  
  
+ ATTGTACTGT CAAAATTTTA AAATATTTAT AAAAAGTAAT TTACAAGTCA TATGAATGAA TTGAAAATAA   
  
  
+ AATGTAAAAT TTATTTACTT TATTTTATAA CAAAAAGTTT TTAAATTTTT ATAATCATTT GAACTAAATT   
  
  
+ TAATTAATAA ATACAAATTT GATTTCTTTT TCTAAAAATT AAATCTAATT CACTATTTTC AAATATTAAA   
  
  
+ ATTTAATTTA ATGCATGAAA CACTTTAAGA TCAAAATTAT ATAAAATTAT CATATTTACT TTATATTATC   
  
  
+ AATCAATTTT TTTGTTAAAA AGGTGACAAA TTGAATTTTG ATCTACTGGT TTAATATCAA ATTAAATCTT   
  
  
+ AATTAAAATA ATCATAACAA TTAAAAATAT TTTTGTTATT AAAATATTTA AACTATTTGA ATTATTTTTT   
  
  
+ ATTTTATTTC AAGTTCATTC TTTAAATGTA AATAATTTTT GATGCAAGAT CTGGTTTTTC TAAATTTTTA   
  
  
+ AAATTTTGAT TATATTTAAC AATACATTTT TAATACTCAA AAAATTTTAA AATTTTTATG ATAATATTTT   
  
  
+ AAATATTAAA TTTGAAATTA AAATTTATTA TATAAAATTT TTAATTTAAT TATTTTAAAA TTTAAATTTT   
  
  
+ TTAATAACAA TTAATTTAAT TTAATTTAAT AATATAAAAT AAATATTAAA ATTTTATATA ATTTTTATTC   
  
  
+ AAAATTTTAA ACACATTTTT TATTTTTTCT TGTGGCGGGC TTCCGAATCA GCTACCTTTT TGTAGAGGTA   
  
  
+ TCACTATATA AAATTTACGT GCCGTTTACG ACACCAAAGA TACCATTAGG ATAAGAAGCT GCCTCACAGA   
  
  
+ CAGGCCGCCT CCGCCACTGT TCTGAATTTC AACCACACTC AGTACTCCTC CTATATATTA CCACACAAAC   
  
  
+ CATATTCATT GATTCTTATT TATTCTTCTT GTCCTTCTTC GTCGTATGAT CCGACGCTCA CTTTTCTCTC   
  
  
+ AGACTTTCTC TCGTTTTCTC GGGAAAATA  

- GTCTTTAACA TTCAAAAATA ACCACAGTGA TATTAAATTA AACAAAGGAA CTGCTATCAT GATAAACATG   
  
  
- CTGTACTTTC AAACCAGGCT AAGTTGATTG ATTAATTAAC TAATTCTATT TAAATTAAAC AAAGGAACTG   
  
  
- CTATCATGAT AAACATGCTG TACTTTCAAA CCAGGCTAAG TTGATTGATT AATTAACTAA TTCTATAGTT   
  
  
- ACGCTAGAAT TAGTTTCGTT CGATCGGATC GGAATTAGTA CTTCGGAGAT TATAAAAGAG TAACGAAACT   
  
  
- ATGAAAAAAA AATTTTAAAA TAAATTTTAA ATTTTAATAT AAAAATGGAA CTTCACCTCA GGTTACAAAA   
  
  
- TTTCTACTGT TAAGTATAGT ATTAAATAAT TTAATTTAAA TTAATAAATT TTTTAATTTT TAATTTAAAA   
  
  
- TAACATATTT TTTTATAAAT TTGGTAATAA TATTTATTTT ATATATAAAA ATCTATAATA AATAAAATTT   
  
  
- TAACATGACA GTTTTAAAAT TTTATAAATA TTTTTCATTA AATGTTCAGT ATACTTACTT AACTTTTATT   
  
  
- TTACATTTTA AATAAATGAA ATAAAATATT GTTTTTCAAA AATTTAAAAA TATTAGTAAA CTTGATTTAA   
  
  
- ATTAATTATT TATGTTTAAA CTAAAGAAAA AGATTTTTAA TTTAGATTAA GTGATAAAAG TTTATAATTT   
  
  
- TAAATTAAAT TACGTACTTT GTGAAATTCT AGTTTTAATA TATTTTAATA GTATAAATGA AATATAATAG   
  
  
- TTAGTTAAAA AAACAATTTT TCCACTGTTT AACTTAAAAC TAGATGACCA AATTATAGTT TAATTTAGAA   
  
  
- TTAATTTTAT TAGTATTGTT AATTTTTATA AAAACAATAA TTTTATAAAT TTGATAAACT TAATAAAAAA   
  
  
- TAAAATAAAG TTCAAGTAAG AAATTTACAT TTATTAAAAA CTACGTTCTA GACCAAAAAG ATTTAAAAAT   
  
  
- TTTAAAACTA ATATAAATTG TTATGTAAAA ATTATGAGTT TTTTAAAATT TTAAAAATAC TATTATAAAA   
  
  
- TTTATAATTT AAACTTTAAT TTTAAATAAT ATATTTTAAA AATTAAATTA ATAAAATTTT AAATTTAAAA   
  
  
- AATTATTGTT AATTAAATTA AATTAAATTA TTATATTTTA TTTATAATTT TAAAATATAT TAAAAATAAG   
  
  
- TTTTAAAATT TGTGTAAAAA ATAAAAAAGA ACACCGCCCG AAGGCTTAGT CGATGGAAAA ACATCTCCAT   
  
  
- AGTGATATAT TTTAAATGCA CGGCAAATGC TGTGGTTTCT ATGGTAATCC TATTCTTCGA CGGAGTGTCT   
  
  
- GTCCGGCGGA GGCGGTGACA AGACTTAAAG TTGGTGTGAG TCATGAGGAG GATATATAAT GGTGTGTTTG   
  
  
- GTATAAGTAA CTAAGAATAA ATAAGAAGAA CAGGAAGAAG CAGCATACTA GGCTGCGAGT GAAAAGAGAG   
  
  
- TCTGAAAGAG AGCAAAAGAG CCCTTTTAT

+     ATCT-motif

| Site Name | Organism | Position | Strand | Matrix score. | sequence | function |
| --- | --- | --- | --- | --- | --- | --- |
| ATCT-motif | Pisum sativum | 672 | + | 9 | AATCTAATCC | part of a conserved DNA module involved in light responsiveness |

> 2018/04/13 10:10:12  
+ CAGAAATTGT AAGTTTTTAT TGGTGTCACT ATAATTTAAT TTGTTTCCTT GACGATAGTA CTATTTGTAC   
  
  
+ GACATGAAAG TTTGGTCCGA TTCAACTAAC TAATTAATTG ATTAAGATAA ATTTAATTTG TTTCCTTGAC   
  
  
+ GATAGTACTA TTTGTACGAC ATGAAAGTTT GGTCCGATTC AACTAACTAA TTAATTGATT AAGATATCAA   
  
  
+ TGCGATCTTA ATCAAAGCAA GCTAGCCTAG CCTTAATCAT GAAGCCTCTA ATATTTTCTC ATTGCTTTGA   
  
  
+ TACTTTTTTT TTAAAATTTT ATTTAAAATT TAAAATTATA TTTTTACCTT GAAGTGGAGT CCAATGTTTT   
  
  
+ AAAGATGACA ATTCATATCA TAATTTATTA AATTAAATTT AATTATTTAA AAAATTAAAA ATTAAATTTT   
  
  
+ ATTGTATAAA AAAATATTTA AACCATTATT ATAAATAAAA TATATATTTT TAGATATTAT TTATTTTAAA   
  
  
+ ATTGTACTGT CAAAATTTTA AAATATTTAT AAAAAGTAAT TTACAAGTCA TATGAATGAA TTGAAAATAA   
  
  
+ AATGTAAAAT TTATTTACTT TATTTTATAA CAAAAAGTTT TTAAATTTTT ATAATCATTT GAACTAAATT   
  
  
+ TAATTAATAA ATACAAATTT GATTTCTTTT TCTAAAAATT AAATCTAATT CACTATTTTC AAATATTAAA   
  
  
+ ATTTAATTTA ATGCATGAAA CACTTTAAGA TCAAAATTAT ATAAAATTAT CATATTTACT TTATATTATC   
  
  
+ AATCAATTTT TTTGTTAAAA AGGTGACAAA TTGAATTTTG ATCTACTGGT TTAATATCAA ATTAAATCTT   
  
  
+ AATTAAAATA ATCATAACAA TTAAAAATAT TTTTGTTATT AAAATATTTA AACTATTTGA ATTATTTTTT   
  
  
+ ATTTTATTTC AAGTTCATTC TTTAAATGTA AATAATTTTT GATGCAAGAT CTGGTTTTTC TAAATTTTTA   
  
  
+ AAATTTTGAT TATATTTAAC AATACATTTT TAATACTCAA AAAATTTTAA AATTTTTATG ATAATATTTT   
  
  
+ AAATATTAAA TTTGAAATTA AAATTTATTA TATAAAATTT TTAATTTAAT TATTTTAAAA TTTAAATTTT   
  
  
+ TTAATAACAA TTAATTTAAT TTAATTTAAT AATATAAAAT AAATATTAAA ATTTTATATA ATTTTTATTC   
  
  
+ AAAATTTTAA ACACATTTTT TATTTTTTCT TGTGGCGGGC TTCCGAATCA GCTACCTTTT TGTAGAGGTA   
  
  
+ TCACTATATA AAATTTACGT GCCGTTTACG ACACCAAAGA TACCATTAGG ATAAGAAGCT GCCTCACAGA   
  
  
+ CAGGCCGCCT CCGCCACTGT TCTGAATTTC AACCACACTC AGTACTCCTC CTATATATTA CCACACAAAC   
  
  
+ CATATTCATT GATTCTTATT TATTCTTCTT GTCCTTCTTC GTCGTATGAT CCGACGCTCA CTTTTCTCTC   
  
  
+ AGACTTTCTC TCGTTTTCTC GGGAAAATA  

- GTCTTTAACA TTCAAAAATA ACCACAGTGA TATTAAATTA AACAAAGGAA CTGCTATCAT GATAAACATG   
  
  
- CTGTACTTTC AAACCAGGCT AAGTTGATTG ATTAATTAAC TAATTCTATT TAAATTAAAC AAAGGAACTG   
  
  
- CTATCATGAT AAACATGCTG TACTTTCAAA CCAGGCTAAG TTGATTGATT AATTAACTAA TTCTATAGTT   
  
  
- ACGCTAGAAT TAGTTTCGTT CGATCGGATC GGAATTAGTA CTTCGGAGAT TATAAAAGAG TAACGAAACT   
  
  
- ATGAAAAAAA AATTTTAAAA TAAATTTTAA ATTTTAATAT AAAAATGGAA CTTCACCTCA GGTTACAAAA   
  
  
- TTTCTACTGT TAAGTATAGT ATTAAATAAT TTAATTTAAA TTAATAAATT TTTTAATTTT TAATTTAAAA   
  
  
- TAACATATTT TTTTATAAAT TTGGTAATAA TATTTATTTT ATATATAAAA ATCTATAATA AATAAAATTT   
  
  
- TAACATGACA GTTTTAAAAT TTTATAAATA TTTTTCATTA AATGTTCAGT ATACTTACTT AACTTTTATT   
  
  
- TTACATTTTA AATAAATGAA ATAAAATATT GTTTTTCAAA AATTTAAAAA TATTAGTAAA CTTGATTTAA   
  
  
- ATTAATTATT TATGTTTAAA CTAAAGAAAA AGATTTTTAA TTTAGATTAA GTGATAAAAG TTTATAATTT   
  
  
- TAAATTAAAT TACGTACTTT GTGAAATTCT AGTTTTAATA TATTTTAATA GTATAAATGA AATATAATAG   
  
  
- TTAGTTAAAA AAACAATTTT TCCACTGTTT AACTTAAAAC TAGATGACCA AATTATAGTT TAATTTAGAA   
  
  
- TTAATTTTAT TAGTATTGTT AATTTTTATA AAAACAATAA TTTTATAAAT TTGATAAACT TAATAAAAAA   
  
  
- TAAAATAAAG TTCAAGTAAG AAATTTACAT TTATTAAAAA CTACGTTCTA GACCAAAAAG ATTTAAAAAT   
  
  
- TTTAAAACTA ATATAAATTG TTATGTAAAA ATTATGAGTT TTTTAAAATT TTAAAAATAC TATTATAAAA   
  
  
- TTTATAATTT AAACTTTAAT TTTAAATAAT ATATTTTAAA AATTAAATTA ATAAAATTTT AAATTTAAAA   
  
  
- AATTATTGTT AATTAAATTA AATTAAATTA TTATATTTTA TTTATAATTT TAAAATATAT TAAAAATAAG   
  
  
- TTTTAAAATT TGTGTAAAAA ATAAAAAAGA ACACCGCCCG AAGGCTTAGT CGATGGAAAA ACATCTCCAT   
  
  
- AGTGATATAT TTTAAATGCA CGGCAAATGC TGTGGTTTCT ATGGTAATCC TATTCTTCGA CGGAGTGTCT   
  
  
- GTCCGGCGGA GGCGGTGACA AGACTTAAAG TTGGTGTGAG TCATGAGGAG GATATATAAT GGTGTGTTTG   
  
  
- GTATAAGTAA CTAAGAATAA ATAAGAAGAA CAGGAAGAAG CAGCATACTA GGCTGCGAGT GAAAAGAGAG   
  
  
- TCTGAAAGAG AGCAAAAGAG CCCTTTTAT

+     ATGCAAAT motif

| Site Name | Organism | Position | Strand | Matrix score. | sequence | function |
| --- | --- | --- | --- | --- | --- | --- |
| ATGCAAAT motif | Oryza sativa | 641 | + | 8 | ATACAAAT | cis-acting regulatory element associated to the TGAGTCA motif |

> 2018/04/13 10:10:12  
+ CAGAAATTGT AAGTTTTTAT TGGTGTCACT ATAATTTAAT TTGTTTCCTT GACGATAGTA CTATTTGTAC   
  
  
+ GACATGAAAG TTTGGTCCGA TTCAACTAAC TAATTAATTG ATTAAGATAA ATTTAATTTG TTTCCTTGAC   
  
  
+ GATAGTACTA TTTGTACGAC ATGAAAGTTT GGTCCGATTC AACTAACTAA TTAATTGATT AAGATATCAA   
  
  
+ TGCGATCTTA ATCAAAGCAA GCTAGCCTAG CCTTAATCAT GAAGCCTCTA ATATTTTCTC ATTGCTTTGA   
  
  
+ TACTTTTTTT TTAAAATTTT ATTTAAAATT TAAAATTATA TTTTTACCTT GAAGTGGAGT CCAATGTTTT   
  
  
+ AAAGATGACA ATTCATATCA TAATTTATTA AATTAAATTT AATTATTTAA AAAATTAAAA ATTAAATTTT   
  
  
+ ATTGTATAAA AAAATATTTA AACCATTATT ATAAATAAAA TATATATTTT TAGATATTAT TTATTTTAAA   
  
  
+ ATTGTACTGT CAAAATTTTA AAATATTTAT AAAAAGTAAT TTACAAGTCA TATGAATGAA TTGAAAATAA   
  
  
+ AATGTAAAAT TTATTTACTT TATTTTATAA CAAAAAGTTT TTAAATTTTT ATAATCATTT GAACTAAATT   
  
  
+ TAATTAATAA ATACAAATTT GATTTCTTTT TCTAAAAATT AAATCTAATT CACTATTTTC AAATATTAAA   
  
  
+ ATTTAATTTA ATGCATGAAA CACTTTAAGA TCAAAATTAT ATAAAATTAT CATATTTACT TTATATTATC   
  
  
+ AATCAATTTT TTTGTTAAAA AGGTGACAAA TTGAATTTTG ATCTACTGGT TTAATATCAA ATTAAATCTT   
  
  
+ AATTAAAATA ATCATAACAA TTAAAAATAT TTTTGTTATT AAAATATTTA AACTATTTGA ATTATTTTTT   
  
  
+ ATTTTATTTC AAGTTCATTC TTTAAATGTA AATAATTTTT GATGCAAGAT CTGGTTTTTC TAAATTTTTA   
  
  
+ AAATTTTGAT TATATTTAAC AATACATTTT TAATACTCAA AAAATTTTAA AATTTTTATG ATAATATTTT   
  
  
+ AAATATTAAA TTTGAAATTA AAATTTATTA TATAAAATTT TTAATTTAAT TATTTTAAAA TTTAAATTTT   
  
  
+ TTAATAACAA TTAATTTAAT TTAATTTAAT AATATAAAAT AAATATTAAA ATTTTATATA ATTTTTATTC   
  
  
+ AAAATTTTAA ACACATTTTT TATTTTTTCT TGTGGCGGGC TTCCGAATCA GCTACCTTTT TGTAGAGGTA   
  
  
+ TCACTATATA AAATTTACGT GCCGTTTACG ACACCAAAGA TACCATTAGG ATAAGAAGCT GCCTCACAGA   
  
  
+ CAGGCCGCCT CCGCCACTGT TCTGAATTTC AACCACACTC AGTACTCCTC CTATATATTA CCACACAAAC   
  
  
+ CATATTCATT GATTCTTATT TATTCTTCTT GTCCTTCTTC GTCGTATGAT CCGACGCTCA CTTTTCTCTC   
  
  
+ AGACTTTCTC TCGTTTTCTC GGGAAAATA  

- GTCTTTAACA TTCAAAAATA ACCACAGTGA TATTAAATTA AACAAAGGAA CTGCTATCAT GATAAACATG   
  
  
- CTGTACTTTC AAACCAGGCT AAGTTGATTG ATTAATTAAC TAATTCTATT TAAATTAAAC AAAGGAACTG   
  
  
- CTATCATGAT AAACATGCTG TACTTTCAAA CCAGGCTAAG TTGATTGATT AATTAACTAA TTCTATAGTT   
  
  
- ACGCTAGAAT TAGTTTCGTT CGATCGGATC GGAATTAGTA CTTCGGAGAT TATAAAAGAG TAACGAAACT   
  
  
- ATGAAAAAAA AATTTTAAAA TAAATTTTAA ATTTTAATAT AAAAATGGAA CTTCACCTCA GGTTACAAAA   
  
  
- TTTCTACTGT TAAGTATAGT ATTAAATAAT TTAATTTAAA TTAATAAATT TTTTAATTTT TAATTTAAAA   
  
  
- TAACATATTT TTTTATAAAT TTGGTAATAA TATTTATTTT ATATATAAAA ATCTATAATA AATAAAATTT   
  
  
- TAACATGACA GTTTTAAAAT TTTATAAATA TTTTTCATTA AATGTTCAGT ATACTTACTT AACTTTTATT   
  
  
- TTACATTTTA AATAAATGAA ATAAAATATT GTTTTTCAAA AATTTAAAAA TATTAGTAAA CTTGATTTAA   
  
  
- ATTAATTATT TATGTTTAAA CTAAAGAAAA AGATTTTTAA TTTAGATTAA GTGATAAAAG TTTATAATTT   
  
  
- TAAATTAAAT TACGTACTTT GTGAAATTCT AGTTTTAATA TATTTTAATA GTATAAATGA AATATAATAG   
  
  
- TTAGTTAAAA AAACAATTTT TCCACTGTTT AACTTAAAAC TAGATGACCA AATTATAGTT TAATTTAGAA   
  
  
- TTAATTTTAT TAGTATTGTT AATTTTTATA AAAACAATAA TTTTATAAAT TTGATAAACT TAATAAAAAA   
  
  
- TAAAATAAAG TTCAAGTAAG AAATTTACAT TTATTAAAAA CTACGTTCTA GACCAAAAAG ATTTAAAAAT   
  
  
- TTTAAAACTA ATATAAATTG TTATGTAAAA ATTATGAGTT TTTTAAAATT TTAAAAATAC TATTATAAAA   
  
  
- TTTATAATTT AAACTTTAAT TTTAAATAAT ATATTTTAAA AATTAAATTA ATAAAATTTT AAATTTAAAA   
  
  
- AATTATTGTT AATTAAATTA AATTAAATTA TTATATTTTA TTTATAATTT TAAAATATAT TAAAAATAAG   
  
  
- TTTTAAAATT TGTGTAAAAA ATAAAAAAGA ACACCGCCCG AAGGCTTAGT CGATGGAAAA ACATCTCCAT   
  
  
- AGTGATATAT TTTAAATGCA CGGCAAATGC TGTGGTTTCT ATGGTAATCC TATTCTTCGA CGGAGTGTCT   
  
  
- GTCCGGCGGA GGCGGTGACA AGACTTAAAG TTGGTGTGAG TCATGAGGAG GATATATAAT GGTGTGTTTG   
  
  
- GTATAAGTAA CTAAGAATAA ATAAGAAGAA CAGGAAGAAG CAGCATACTA GGCTGCGAGT GAAAAGAGAG   
  
  
- TCTGAAAGAG AGCAAAAGAG CCCTTTTAT

+     Box 4

| Site Name | Organism | Position | Strand | Matrix score. | sequence | function |
| --- | --- | --- | --- | --- | --- | --- |
| Box 4 | Petroselinum crispum | 103 | + | 6 | ATTAAT | part of a conserved DNA module involved in light responsiveness |
| Box 4 | Petroselinum crispum | 633 | + | 6 | ATTAAT | part of a conserved DNA module involved in light responsiveness |
| Box 4 | Petroselinum crispum | 190 | + | 6 | ATTAAT | part of a conserved DNA module involved in light responsiveness |
| Box 4 | Petroselinum crispum | 1130 | - | 6 | ATTAAT | part of a conserved DNA module involved in light responsiveness |

> 2018/04/13 10:10:12  
+ CAGAAATTGT AAGTTTTTAT TGGTGTCACT ATAATTTAAT TTGTTTCCTT GACGATAGTA CTATTTGTAC   
  
  
+ GACATGAAAG TTTGGTCCGA TTCAACTAAC TAATTAATTG ATTAAGATAA ATTTAATTTG TTTCCTTGAC   
  
  
+ GATAGTACTA TTTGTACGAC ATGAAAGTTT GGTCCGATTC AACTAACTAA TTAATTGATT AAGATATCAA   
  
  
+ TGCGATCTTA ATCAAAGCAA GCTAGCCTAG CCTTAATCAT GAAGCCTCTA ATATTTTCTC ATTGCTTTGA   
  
  
+ TACTTTTTTT TTAAAATTTT ATTTAAAATT TAAAATTATA TTTTTACCTT GAAGTGGAGT CCAATGTTTT   
  
  
+ AAAGATGACA ATTCATATCA TAATTTATTA AATTAAATTT AATTATTTAA AAAATTAAAA ATTAAATTTT   
  
  
+ ATTGTATAAA AAAATATTTA AACCATTATT ATAAATAAAA TATATATTTT TAGATATTAT TTATTTTAAA   
  
  
+ ATTGTACTGT CAAAATTTTA AAATATTTAT AAAAAGTAAT TTACAAGTCA TATGAATGAA TTGAAAATAA   
  
  
+ AATGTAAAAT TTATTTACTT TATTTTATAA CAAAAAGTTT TTAAATTTTT ATAATCATTT GAACTAAATT   
  
  
+ TAATTAATAA ATACAAATTT GATTTCTTTT TCTAAAAATT AAATCTAATT CACTATTTTC AAATATTAAA   
  
  
+ ATTTAATTTA ATGCATGAAA CACTTTAAGA TCAAAATTAT ATAAAATTAT CATATTTACT TTATATTATC   
  
  
+ AATCAATTTT TTTGTTAAAA AGGTGACAAA TTGAATTTTG ATCTACTGGT TTAATATCAA ATTAAATCTT   
  
  
+ AATTAAAATA ATCATAACAA TTAAAAATAT TTTTGTTATT AAAATATTTA AACTATTTGA ATTATTTTTT   
  
  
+ ATTTTATTTC AAGTTCATTC TTTAAATGTA AATAATTTTT GATGCAAGAT CTGGTTTTTC TAAATTTTTA   
  
  
+ AAATTTTGAT TATATTTAAC AATACATTTT TAATACTCAA AAAATTTTAA AATTTTTATG ATAATATTTT   
  
  
+ AAATATTAAA TTTGAAATTA AAATTTATTA TATAAAATTT TTAATTTAAT TATTTTAAAA TTTAAATTTT   
  
  
+ TTAATAACAA TTAATTTAAT TTAATTTAAT AATATAAAAT AAATATTAAA ATTTTATATA ATTTTTATTC   
  
  
+ AAAATTTTAA ACACATTTTT TATTTTTTCT TGTGGCGGGC TTCCGAATCA GCTACCTTTT TGTAGAGGTA   
  
  
+ TCACTATATA AAATTTACGT GCCGTTTACG ACACCAAAGA TACCATTAGG ATAAGAAGCT GCCTCACAGA   
  
  
+ CAGGCCGCCT CCGCCACTGT TCTGAATTTC AACCACACTC AGTACTCCTC CTATATATTA CCACACAAAC   
  
  
+ CATATTCATT GATTCTTATT TATTCTTCTT GTCCTTCTTC GTCGTATGAT CCGACGCTCA CTTTTCTCTC   
  
  
+ AGACTTTCTC TCGTTTTCTC GGGAAAATA  

- GTCTTTAACA TTCAAAAATA ACCACAGTGA TATTAAATTA AACAAAGGAA CTGCTATCAT GATAAACATG   
  
  
- CTGTACTTTC AAACCAGGCT AAGTTGATTG ATTAATTAAC TAATTCTATT TAAATTAAAC AAAGGAACTG   
  
  
- CTATCATGAT AAACATGCTG TACTTTCAAA CCAGGCTAAG TTGATTGATT AATTAACTAA TTCTATAGTT   
  
  
- ACGCTAGAAT TAGTTTCGTT CGATCGGATC GGAATTAGTA CTTCGGAGAT TATAAAAGAG TAACGAAACT   
  
  
- ATGAAAAAAA AATTTTAAAA TAAATTTTAA ATTTTAATAT AAAAATGGAA CTTCACCTCA GGTTACAAAA   
  
  
- TTTCTACTGT TAAGTATAGT ATTAAATAAT TTAATTTAAA TTAATAAATT TTTTAATTTT TAATTTAAAA   
  
  
- TAACATATTT TTTTATAAAT TTGGTAATAA TATTTATTTT ATATATAAAA ATCTATAATA AATAAAATTT   
  
  
- TAACATGACA GTTTTAAAAT TTTATAAATA TTTTTCATTA AATGTTCAGT ATACTTACTT AACTTTTATT   
  
  
- TTACATTTTA AATAAATGAA ATAAAATATT GTTTTTCAAA AATTTAAAAA TATTAGTAAA CTTGATTTAA   
  
  
- ATTAATTATT TATGTTTAAA CTAAAGAAAA AGATTTTTAA TTTAGATTAA GTGATAAAAG TTTATAATTT   
  
  
- TAAATTAAAT TACGTACTTT GTGAAATTCT AGTTTTAATA TATTTTAATA GTATAAATGA AATATAATAG   
  
  
- TTAGTTAAAA AAACAATTTT TCCACTGTTT AACTTAAAAC TAGATGACCA AATTATAGTT TAATTTAGAA   
  
  
- TTAATTTTAT TAGTATTGTT AATTTTTATA AAAACAATAA TTTTATAAAT TTGATAAACT TAATAAAAAA   
  
  
- TAAAATAAAG TTCAAGTAAG AAATTTACAT TTATTAAAAA CTACGTTCTA GACCAAAAAG ATTTAAAAAT   
  
  
- TTTAAAACTA ATATAAATTG TTATGTAAAA ATTATGAGTT TTTTAAAATT TTAAAAATAC TATTATAAAA   
  
  
- TTTATAATTT AAACTTTAAT TTTAAATAAT ATATTTTAAA AATTAAATTA ATAAAATTTT AAATTTAAAA   
  
  
- AATTATTGTT AATTAAATTA AATTAAATTA TTATATTTTA TTTATAATTT TAAAATATAT TAAAAATAAG   
  
  
- TTTTAAAATT TGTGTAAAAA ATAAAAAAGA ACACCGCCCG AAGGCTTAGT CGATGGAAAA ACATCTCCAT   
  
  
- AGTGATATAT TTTAAATGCA CGGCAAATGC TGTGGTTTCT ATGGTAATCC TATTCTTCGA CGGAGTGTCT   
  
  
- GTCCGGCGGA GGCGGTGACA AGACTTAAAG TTGGTGTGAG TCATGAGGAG GATATATAAT GGTGTGTTTG   
  
  
- GTATAAGTAA CTAAGAATAA ATAAGAAGAA CAGGAAGAAG CAGCATACTA GGCTGCGAGT GAAAAGAGAG   
  
  
- TCTGAAAGAG AGCAAAAGAG CCCTTTTAT

+     Box I

| Site Name | Organism | Position | Strand | Matrix score. | sequence | function |
| --- | --- | --- | --- | --- | --- | --- |
| Box I | Pisum sativum | 687 | + | 7 | TTTCAAA | light responsive element |
| Box I | Pisum sativum | 1061 | - | 7 | TTTCAAA | light responsive element |

> 2018/04/13 10:10:12  
+ CAGAAATTGT AAGTTTTTAT TGGTGTCACT ATAATTTAAT TTGTTTCCTT GACGATAGTA CTATTTGTAC   
  
  
+ GACATGAAAG TTTGGTCCGA TTCAACTAAC TAATTAATTG ATTAAGATAA ATTTAATTTG TTTCCTTGAC   
  
  
+ GATAGTACTA TTTGTACGAC ATGAAAGTTT GGTCCGATTC AACTAACTAA TTAATTGATT AAGATATCAA   
  
  
+ TGCGATCTTA ATCAAAGCAA GCTAGCCTAG CCTTAATCAT GAAGCCTCTA ATATTTTCTC ATTGCTTTGA   
  
  
+ TACTTTTTTT TTAAAATTTT ATTTAAAATT TAAAATTATA TTTTTACCTT GAAGTGGAGT CCAATGTTTT   
  
  
+ AAAGATGACA ATTCATATCA TAATTTATTA AATTAAATTT AATTATTTAA AAAATTAAAA ATTAAATTTT   
  
  
+ ATTGTATAAA AAAATATTTA AACCATTATT ATAAATAAAA TATATATTTT TAGATATTAT TTATTTTAAA   
  
  
+ ATTGTACTGT CAAAATTTTA AAATATTTAT AAAAAGTAAT TTACAAGTCA TATGAATGAA TTGAAAATAA   
  
  
+ AATGTAAAAT TTATTTACTT TATTTTATAA CAAAAAGTTT TTAAATTTTT ATAATCATTT GAACTAAATT   
  
  
+ TAATTAATAA ATACAAATTT GATTTCTTTT TCTAAAAATT AAATCTAATT CACTATTTTC AAATATTAAA   
  
  
+ ATTTAATTTA ATGCATGAAA CACTTTAAGA TCAAAATTAT ATAAAATTAT CATATTTACT TTATATTATC   
  
  
+ AATCAATTTT TTTGTTAAAA AGGTGACAAA TTGAATTTTG ATCTACTGGT TTAATATCAA ATTAAATCTT   
  
  
+ AATTAAAATA ATCATAACAA TTAAAAATAT TTTTGTTATT AAAATATTTA AACTATTTGA ATTATTTTTT   
  
  
+ ATTTTATTTC AAGTTCATTC TTTAAATGTA AATAATTTTT GATGCAAGAT CTGGTTTTTC TAAATTTTTA   
  
  
+ AAATTTTGAT TATATTTAAC AATACATTTT TAATACTCAA AAAATTTTAA AATTTTTATG ATAATATTTT   
  
  
+ AAATATTAAA TTTGAAATTA AAATTTATTA TATAAAATTT TTAATTTAAT TATTTTAAAA TTTAAATTTT   
  
  
+ TTAATAACAA TTAATTTAAT TTAATTTAAT AATATAAAAT AAATATTAAA ATTTTATATA ATTTTTATTC   
  
  
+ AAAATTTTAA ACACATTTTT TATTTTTTCT TGTGGCGGGC TTCCGAATCA GCTACCTTTT TGTAGAGGTA   
  
  
+ TCACTATATA AAATTTACGT GCCGTTTACG ACACCAAAGA TACCATTAGG ATAAGAAGCT GCCTCACAGA   
  
  
+ CAGGCCGCCT CCGCCACTGT TCTGAATTTC AACCACACTC AGTACTCCTC CTATATATTA CCACACAAAC   
  
  
+ CATATTCATT GATTCTTATT TATTCTTCTT GTCCTTCTTC GTCGTATGAT CCGACGCTCA CTTTTCTCTC   
  
  
+ AGACTTTCTC TCGTTTTCTC GGGAAAATA  

- GTCTTTAACA TTCAAAAATA ACCACAGTGA TATTAAATTA AACAAAGGAA CTGCTATCAT GATAAACATG   
  
  
- CTGTACTTTC AAACCAGGCT AAGTTGATTG ATTAATTAAC TAATTCTATT TAAATTAAAC AAAGGAACTG   
  
  
- CTATCATGAT AAACATGCTG TACTTTCAAA CCAGGCTAAG TTGATTGATT AATTAACTAA TTCTATAGTT   
  
  
- ACGCTAGAAT TAGTTTCGTT CGATCGGATC GGAATTAGTA CTTCGGAGAT TATAAAAGAG TAACGAAACT   
  
  
- ATGAAAAAAA AATTTTAAAA TAAATTTTAA ATTTTAATAT AAAAATGGAA CTTCACCTCA GGTTACAAAA   
  
  
- TTTCTACTGT TAAGTATAGT ATTAAATAAT TTAATTTAAA TTAATAAATT TTTTAATTTT TAATTTAAAA   
  
  
- TAACATATTT TTTTATAAAT TTGGTAATAA TATTTATTTT ATATATAAAA ATCTATAATA AATAAAATTT   
  
  
- TAACATGACA GTTTTAAAAT TTTATAAATA TTTTTCATTA AATGTTCAGT ATACTTACTT AACTTTTATT   
  
  
- TTACATTTTA AATAAATGAA ATAAAATATT GTTTTTCAAA AATTTAAAAA TATTAGTAAA CTTGATTTAA   
  
  
- ATTAATTATT TATGTTTAAA CTAAAGAAAA AGATTTTTAA TTTAGATTAA GTGATAAAAG TTTATAATTT   
  
  
- TAAATTAAAT TACGTACTTT GTGAAATTCT AGTTTTAATA TATTTTAATA GTATAAATGA AATATAATAG   
  
  
- TTAGTTAAAA AAACAATTTT TCCACTGTTT AACTTAAAAC TAGATGACCA AATTATAGTT TAATTTAGAA   
  
  
- TTAATTTTAT TAGTATTGTT AATTTTTATA AAAACAATAA TTTTATAAAT TTGATAAACT TAATAAAAAA   
  
  
- TAAAATAAAG TTCAAGTAAG AAATTTACAT TTATTAAAAA CTACGTTCTA GACCAAAAAG ATTTAAAAAT   
  
  
- TTTAAAACTA ATATAAATTG TTATGTAAAA ATTATGAGTT TTTTAAAATT TTAAAAATAC TATTATAAAA   
  
  
- TTTATAATTT AAACTTTAAT TTTAAATAAT ATATTTTAAA AATTAAATTA ATAAAATTTT AAATTTAAAA   
  
  
- AATTATTGTT AATTAAATTA AATTAAATTA TTATATTTTA TTTATAATTT TAAAATATAT TAAAAATAAG   
  
  
- TTTTAAAATT TGTGTAAAAA ATAAAAAAGA ACACCGCCCG AAGGCTTAGT CGATGGAAAA ACATCTCCAT   
  
  
- AGTGATATAT TTTAAATGCA CGGCAAATGC TGTGGTTTCT ATGGTAATCC TATTCTTCGA CGGAGTGTCT   
  
  
- GTCCGGCGGA GGCGGTGACA AGACTTAAAG TTGGTGTGAG TCATGAGGAG GATATATAAT GGTGTGTTTG   
  
  
- GTATAAGTAA CTAAGAATAA ATAAGAAGAA CAGGAAGAAG CAGCATACTA GGCTGCGAGT GAAAAGAGAG   
  
  
- TCTGAAAGAG AGCAAAAGAG CCCTTTTAT

+     Box II

| Site Name | Organism | Position | Strand | Matrix score. | sequence | function |
| --- | --- | --- | --- | --- | --- | --- |
| Box II | Pisum sativum | 1385 | - | 11 | GTGAGGTAATAT | part of a light responsive element |

> 2018/04/13 10:10:12  
+ CAGAAATTGT AAGTTTTTAT TGGTGTCACT ATAATTTAAT TTGTTTCCTT GACGATAGTA CTATTTGTAC   
  
  
+ GACATGAAAG TTTGGTCCGA TTCAACTAAC TAATTAATTG ATTAAGATAA ATTTAATTTG TTTCCTTGAC   
  
  
+ GATAGTACTA TTTGTACGAC ATGAAAGTTT GGTCCGATTC AACTAACTAA TTAATTGATT AAGATATCAA   
  
  
+ TGCGATCTTA ATCAAAGCAA GCTAGCCTAG CCTTAATCAT GAAGCCTCTA ATATTTTCTC ATTGCTTTGA   
  
  
+ TACTTTTTTT TTAAAATTTT ATTTAAAATT TAAAATTATA TTTTTACCTT GAAGTGGAGT CCAATGTTTT   
  
  
+ AAAGATGACA ATTCATATCA TAATTTATTA AATTAAATTT AATTATTTAA AAAATTAAAA ATTAAATTTT   
  
  
+ ATTGTATAAA AAAATATTTA AACCATTATT ATAAATAAAA TATATATTTT TAGATATTAT TTATTTTAAA   
  
  
+ ATTGTACTGT CAAAATTTTA AAATATTTAT AAAAAGTAAT TTACAAGTCA TATGAATGAA TTGAAAATAA   
  
  
+ AATGTAAAAT TTATTTACTT TATTTTATAA CAAAAAGTTT TTAAATTTTT ATAATCATTT GAACTAAATT   
  
  
+ TAATTAATAA ATACAAATTT GATTTCTTTT TCTAAAAATT AAATCTAATT CACTATTTTC AAATATTAAA   
  
  
+ ATTTAATTTA ATGCATGAAA CACTTTAAGA TCAAAATTAT ATAAAATTAT CATATTTACT TTATATTATC   
  
  
+ AATCAATTTT TTTGTTAAAA AGGTGACAAA TTGAATTTTG ATCTACTGGT TTAATATCAA ATTAAATCTT   
  
  
+ AATTAAAATA ATCATAACAA TTAAAAATAT TTTTGTTATT AAAATATTTA AACTATTTGA ATTATTTTTT   
  
  
+ ATTTTATTTC AAGTTCATTC TTTAAATGTA AATAATTTTT GATGCAAGAT CTGGTTTTTC TAAATTTTTA   
  
  
+ AAATTTTGAT TATATTTAAC AATACATTTT TAATACTCAA AAAATTTTAA AATTTTTATG ATAATATTTT   
  
  
+ AAATATTAAA TTTGAAATTA AAATTTATTA TATAAAATTT TTAATTTAAT TATTTTAAAA TTTAAATTTT   
  
  
+ TTAATAACAA TTAATTTAAT TTAATTTAAT AATATAAAAT AAATATTAAA ATTTTATATA ATTTTTATTC   
  
  
+ AAAATTTTAA ACACATTTTT TATTTTTTCT TGTGGCGGGC TTCCGAATCA GCTACCTTTT TGTAGAGGTA   
  
  
+ TCACTATATA AAATTTACGT GCCGTTTACG ACACCAAAGA TACCATTAGG ATAAGAAGCT GCCTCACAGA   
  
  
+ CAGGCCGCCT CCGCCACTGT TCTGAATTTC AACCACACTC AGTACTCCTC CTATATATTA CCACACAAAC   
  
  
+ CATATTCATT GATTCTTATT TATTCTTCTT GTCCTTCTTC GTCGTATGAT CCGACGCTCA CTTTTCTCTC   
  
  
+ AGACTTTCTC TCGTTTTCTC GGGAAAATA  

- GTCTTTAACA TTCAAAAATA ACCACAGTGA TATTAAATTA AACAAAGGAA CTGCTATCAT GATAAACATG   
  
  
- CTGTACTTTC AAACCAGGCT AAGTTGATTG ATTAATTAAC TAATTCTATT TAAATTAAAC AAAGGAACTG   
  
  
- CTATCATGAT AAACATGCTG TACTTTCAAA CCAGGCTAAG TTGATTGATT AATTAACTAA TTCTATAGTT   
  
  
- ACGCTAGAAT TAGTTTCGTT CGATCGGATC GGAATTAGTA CTTCGGAGAT TATAAAAGAG TAACGAAACT   
  
  
- ATGAAAAAAA AATTTTAAAA TAAATTTTAA ATTTTAATAT AAAAATGGAA CTTCACCTCA GGTTACAAAA   
  
  
- TTTCTACTGT TAAGTATAGT ATTAAATAAT TTAATTTAAA TTAATAAATT TTTTAATTTT TAATTTAAAA   
  
  
- TAACATATTT TTTTATAAAT TTGGTAATAA TATTTATTTT ATATATAAAA ATCTATAATA AATAAAATTT   
  
  
- TAACATGACA GTTTTAAAAT TTTATAAATA TTTTTCATTA AATGTTCAGT ATACTTACTT AACTTTTATT   
  
  
- TTACATTTTA AATAAATGAA ATAAAATATT GTTTTTCAAA AATTTAAAAA TATTAGTAAA CTTGATTTAA   
  
  
- ATTAATTATT TATGTTTAAA CTAAAGAAAA AGATTTTTAA TTTAGATTAA GTGATAAAAG TTTATAATTT   
  
  
- TAAATTAAAT TACGTACTTT GTGAAATTCT AGTTTTAATA TATTTTAATA GTATAAATGA AATATAATAG   
  
  
- TTAGTTAAAA AAACAATTTT TCCACTGTTT AACTTAAAAC TAGATGACCA AATTATAGTT TAATTTAGAA   
  
  
- TTAATTTTAT TAGTATTGTT AATTTTTATA AAAACAATAA TTTTATAAAT TTGATAAACT TAATAAAAAA   
  
  
- TAAAATAAAG TTCAAGTAAG AAATTTACAT TTATTAAAAA CTACGTTCTA GACCAAAAAG ATTTAAAAAT   
  
  
- TTTAAAACTA ATATAAATTG TTATGTAAAA ATTATGAGTT TTTTAAAATT TTAAAAATAC TATTATAAAA   
  
  
- TTTATAATTT AAACTTTAAT TTTAAATAAT ATATTTTAAA AATTAAATTA ATAAAATTTT AAATTTAAAA   
  
  
- AATTATTGTT AATTAAATTA AATTAAATTA TTATATTTTA TTTATAATTT TAAAATATAT TAAAAATAAG   
  
  
- TTTTAAAATT TGTGTAAAAA ATAAAAAAGA ACACCGCCCG AAGGCTTAGT CGATGGAAAA ACATCTCCAT   
  
  
- AGTGATATAT TTTAAATGCA CGGCAAATGC TGTGGTTTCT ATGGTAATCC TATTCTTCGA CGGAGTGTCT   
  
  
- GTCCGGCGGA GGCGGTGACA AGACTTAAAG TTGGTGTGAG TCATGAGGAG GATATATAAT GGTGTGTTTG   
  
  
- GTATAAGTAA CTAAGAATAA ATAAGAAGAA CAGGAAGAAG CAGCATACTA GGCTGCGAGT GAAAAGAGAG   
  
  
- TCTGAAAGAG AGCAAAAGAG CCCTTTTAT

+     CAAT-box

| Site Name | Organism | Position | Strand | Matrix score. | sequence | function |
| --- | --- | --- | --- | --- | --- | --- |
| CAAT-box | Arabidopsis thaliana | 341 | + | 5 | CCAAT | common cis-acting element in promoter and enhancer regions |
| CAAT-box | Arabidopsis thaliana | 19 | - | 5 | CCAAT | common cis-acting element in promoter and enhancer regions |
| CAAT-box | Brassica rapa | 63 | - | 5 | CAAAT | common cis-acting element in promoter and enhancer regions |
| CAAT-box | Brassica rapa | 150 | - | 5 | CAAAT | common cis-acting element in promoter and enhancer regions |
| CAAT-box | Glycine max | 5 | - | 5 | CAATT | common cis-acting element in promoter and enhancer regions |
| CAAT-box | Hordeum vulgare | 194 | - | 4 | CAAT | common cis-acting element in promoter and enhancer regions |
| CAAT-box | Hordeum vulgare | 107 | - | 4 | CAAT | common cis-acting element in promoter and enhancer regions |
| CAAT-box | Hordeum vulgare | 342 | + | 4 | CAAT | common cis-acting element in promoter and enhancer regions |
| CAAT-box | Hordeum vulgare | 6 | - | 4 | CAAT | common cis-acting element in promoter and enhancer regions |
| CAAT-box | Glycine max | 193 | - | 5 | CAATT | common cis-acting element in promoter and enhancer regions |
| CAAT-box | Glycine max | 106 | - | 5 | CAATT | common cis-acting element in promoter and enhancer regions |
| CAAT-box | Hordeum vulgare | 271 | - | 4 | CAAT | common cis-acting element in promoter and enhancer regions |
| CAAT-box | Brassica rapa | 39 | - | 5 | CAAAT | common cis-acting element in promoter and enhancer regions |
| CAAT-box | Hordeum vulgare | 208 | + | 4 | CAAT | common cis-acting element in promoter and enhancer regions |
| CAAT-box | Brassica rapa | 126 | - | 5 | CAAAT | common cis-acting element in promoter and enhancer regions |
| CAAT-box | Glycine max | 359 | + | 5 | CAATT | common cis-acting element in promoter and enhancer regions |
| CAAT-box | Hordeum vulgare | 421 | - | 4 | CAAT | common cis-acting element in promoter and enhancer regions |
| CAAT-box | Glycine max | 490 | - | 5 | CAATT | common cis-acting element in promoter and enhancer regions |
| CAAT-box | Hordeum vulgare | 491 | - | 4 | CAAT | common cis-acting element in promoter and enhancer regions |
| CAAT-box | Glycine max | 549 | - | 5 | CAATT | common cis-acting element in promoter and enhancer regions |
| CAAT-box | Hordeum vulgare | 550 | - | 4 | CAAT | common cis-acting element in promoter and enhancer regions |
| CAAT-box | Brassica rapa | 617 | - | 5 | CAAAT | common cis-acting element in promoter and enhancer regions |
| CAAT-box | Brassica rapa | 644 | + | 5 | CAAAT | common cis-acting element in promoter and enhancer regions |
| CAAT-box | Brassica rapa | 647 | - | 5 | CAAAT | common cis-acting element in promoter and enhancer regions |
| CAAT-box | Brassica rapa | 690 | + | 5 | CAAAT | common cis-acting element in promoter and enhancer regions |
| CAAT-box | Hordeum vulgare | 770 | + | 4 | CAAT | common cis-acting element in promoter and enhancer regions |
| CAAT-box | Glycine max | 774 | + | 5 | CAATT | common cis-acting element in promoter and enhancer regions |
| CAAT-box | Brassica rapa | 797 | + | 5 | CAAAT | common cis-acting element in promoter and enhancer regions |
| CAAT-box | Glycine max | 799 | - | 5 | CAATT | common cis-acting element in promoter and enhancer regions |
| CAAT-box | Hordeum vulgare | 800 | - | 4 | CAAT | common cis-acting element in promoter and enhancer regions |
| CAAT-box | Brassica rapa | 828 | + | 5 | CAAAT | common cis-acting element in promoter and enhancer regions |
| CAAT-box | Glycine max | 858 | + | 5 | CAATT | common cis-acting element in promoter and enhancer regions |
| CAAT-box | Brassica rapa | 895 | - | 5 | CAAAT | common cis-acting element in promoter and enhancer regions |
| CAAT-box | Hordeum vulgare | 1000 | + | 4 | CAAT | common cis-acting element in promoter and enhancer regions |
| CAAT-box | Brassica rapa | 1060 | - | 5 | CAAAT | common cis-acting element in promoter and enhancer regions |
| CAAT-box | Glycine max | 1128 | + | 5 | CAATT | common cis-acting element in promoter and enhancer regions |
| CAAT-box | Hordeum vulgare | 1408 | - | 4 | CAAT | common cis-acting element in promoter and enhancer regions |

> 2018/04/13 10:10:12  
+ CAGAAATTGT AAGTTTTTAT TGGTGTCACT ATAATTTAAT TTGTTTCCTT GACGATAGTA CTATTTGTAC   
  
  
+ GACATGAAAG TTTGGTCCGA TTCAACTAAC TAATTAATTG ATTAAGATAA ATTTAATTTG TTTCCTTGAC   
  
  
+ GATAGTACTA TTTGTACGAC ATGAAAGTTT GGTCCGATTC AACTAACTAA TTAATTGATT AAGATATCAA   
  
  
+ TGCGATCTTA ATCAAAGCAA GCTAGCCTAG CCTTAATCAT GAAGCCTCTA ATATTTTCTC ATTGCTTTGA   
  
  
+ TACTTTTTTT TTAAAATTTT ATTTAAAATT TAAAATTATA TTTTTACCTT GAAGTGGAGT CCAATGTTTT   
  
  
+ AAAGATGACA ATTCATATCA TAATTTATTA AATTAAATTT AATTATTTAA AAAATTAAAA ATTAAATTTT   
  
  
+ ATTGTATAAA AAAATATTTA AACCATTATT ATAAATAAAA TATATATTTT TAGATATTAT TTATTTTAAA   
  
  
+ ATTGTACTGT CAAAATTTTA AAATATTTAT AAAAAGTAAT TTACAAGTCA TATGAATGAA TTGAAAATAA   
  
  
+ AATGTAAAAT TTATTTACTT TATTTTATAA CAAAAAGTTT TTAAATTTTT ATAATCATTT GAACTAAATT   
  
  
+ TAATTAATAA ATACAAATTT GATTTCTTTT TCTAAAAATT AAATCTAATT CACTATTTTC AAATATTAAA   
  
  
+ ATTTAATTTA ATGCATGAAA CACTTTAAGA TCAAAATTAT ATAAAATTAT CATATTTACT TTATATTATC   
  
  
+ AATCAATTTT TTTGTTAAAA AGGTGACAAA TTGAATTTTG ATCTACTGGT TTAATATCAA ATTAAATCTT   
  
  
+ AATTAAAATA ATCATAACAA TTAAAAATAT TTTTGTTATT AAAATATTTA AACTATTTGA ATTATTTTTT   
  
  
+ ATTTTATTTC AAGTTCATTC TTTAAATGTA AATAATTTTT GATGCAAGAT CTGGTTTTTC TAAATTTTTA   
  
  
+ AAATTTTGAT TATATTTAAC AATACATTTT TAATACTCAA AAAATTTTAA AATTTTTATG ATAATATTTT   
  
  
+ AAATATTAAA TTTGAAATTA AAATTTATTA TATAAAATTT TTAATTTAAT TATTTTAAAA TTTAAATTTT   
  
  
+ TTAATAACAA TTAATTTAAT TTAATTTAAT AATATAAAAT AAATATTAAA ATTTTATATA ATTTTTATTC   
  
  
+ AAAATTTTAA ACACATTTTT TATTTTTTCT TGTGGCGGGC TTCCGAATCA GCTACCTTTT TGTAGAGGTA   
  
  
+ TCACTATATA AAATTTACGT GCCGTTTACG ACACCAAAGA TACCATTAGG ATAAGAAGCT GCCTCACAGA   
  
  
+ CAGGCCGCCT CCGCCACTGT TCTGAATTTC AACCACACTC AGTACTCCTC CTATATATTA CCACACAAAC   
  
  
+ CATATTCATT GATTCTTATT TATTCTTCTT GTCCTTCTTC GTCGTATGAT CCGACGCTCA CTTTTCTCTC   
  
  
+ AGACTTTCTC TCGTTTTCTC GGGAAAATA  

- GTCTTTAACA TTCAAAAATA ACCACAGTGA TATTAAATTA AACAAAGGAA CTGCTATCAT GATAAACATG   
  
  
- CTGTACTTTC AAACCAGGCT AAGTTGATTG ATTAATTAAC TAATTCTATT TAAATTAAAC AAAGGAACTG   
  
  
- CTATCATGAT AAACATGCTG TACTTTCAAA CCAGGCTAAG TTGATTGATT AATTAACTAA TTCTATAGTT   
  
  
- ACGCTAGAAT TAGTTTCGTT CGATCGGATC GGAATTAGTA CTTCGGAGAT TATAAAAGAG TAACGAAACT   
  
  
- ATGAAAAAAA AATTTTAAAA TAAATTTTAA ATTTTAATAT AAAAATGGAA CTTCACCTCA GGTTACAAAA   
  
  
- TTTCTACTGT TAAGTATAGT ATTAAATAAT TTAATTTAAA TTAATAAATT TTTTAATTTT TAATTTAAAA   
  
  
- TAACATATTT TTTTATAAAT TTGGTAATAA TATTTATTTT ATATATAAAA ATCTATAATA AATAAAATTT   
  
  
- TAACATGACA GTTTTAAAAT TTTATAAATA TTTTTCATTA AATGTTCAGT ATACTTACTT AACTTTTATT   
  
  
- TTACATTTTA AATAAATGAA ATAAAATATT GTTTTTCAAA AATTTAAAAA TATTAGTAAA CTTGATTTAA   
  
  
- ATTAATTATT TATGTTTAAA CTAAAGAAAA AGATTTTTAA TTTAGATTAA GTGATAAAAG TTTATAATTT   
  
  
- TAAATTAAAT TACGTACTTT GTGAAATTCT AGTTTTAATA TATTTTAATA GTATAAATGA AATATAATAG   
  
  
- TTAGTTAAAA AAACAATTTT TCCACTGTTT AACTTAAAAC TAGATGACCA AATTATAGTT TAATTTAGAA   
  
  
- TTAATTTTAT TAGTATTGTT AATTTTTATA AAAACAATAA TTTTATAAAT TTGATAAACT TAATAAAAAA   
  
  
- TAAAATAAAG TTCAAGTAAG AAATTTACAT TTATTAAAAA CTACGTTCTA GACCAAAAAG ATTTAAAAAT   
  
  
- TTTAAAACTA ATATAAATTG TTATGTAAAA ATTATGAGTT TTTTAAAATT TTAAAAATAC TATTATAAAA   
  
  
- TTTATAATTT AAACTTTAAT TTTAAATAAT ATATTTTAAA AATTAAATTA ATAAAATTTT AAATTTAAAA   
  
  
- AATTATTGTT AATTAAATTA AATTAAATTA TTATATTTTA TTTATAATTT TAAAATATAT TAAAAATAAG   
  
  
- TTTTAAAATT TGTGTAAAAA ATAAAAAAGA ACACCGCCCG AAGGCTTAGT CGATGGAAAA ACATCTCCAT   
  
  
- AGTGATATAT TTTAAATGCA CGGCAAATGC TGTGGTTTCT ATGGTAATCC TATTCTTCGA CGGAGTGTCT   
  
  
- GTCCGGCGGA GGCGGTGACA AGACTTAAAG TTGGTGTGAG TCATGAGGAG GATATATAAT GGTGTGTTTG   
  
  
- GTATAAGTAA CTAAGAATAA ATAAGAAGAA CAGGAAGAAG CAGCATACTA GGCTGCGAGT GAAAAGAGAG   
  
  
- TCTGAAAGAG AGCAAAAGAG CCCTTTTAT

+     CAT-box

| Site Name | Organism | Position | Strand | Matrix score. | sequence | function |
| --- | --- | --- | --- | --- | --- | --- |
| CAT-box | Arabidopsis thaliana | 1343 | + | 6 | GCCACT | cis-acting regulatory element related to meristem expression |

> 2018/04/13 10:10:12  
+ CAGAAATTGT AAGTTTTTAT TGGTGTCACT ATAATTTAAT TTGTTTCCTT GACGATAGTA CTATTTGTAC   
  
  
+ GACATGAAAG TTTGGTCCGA TTCAACTAAC TAATTAATTG ATTAAGATAA ATTTAATTTG TTTCCTTGAC   
  
  
+ GATAGTACTA TTTGTACGAC ATGAAAGTTT GGTCCGATTC AACTAACTAA TTAATTGATT AAGATATCAA   
  
  
+ TGCGATCTTA ATCAAAGCAA GCTAGCCTAG CCTTAATCAT GAAGCCTCTA ATATTTTCTC ATTGCTTTGA   
  
  
+ TACTTTTTTT TTAAAATTTT ATTTAAAATT TAAAATTATA TTTTTACCTT GAAGTGGAGT CCAATGTTTT   
  
  
+ AAAGATGACA ATTCATATCA TAATTTATTA AATTAAATTT AATTATTTAA AAAATTAAAA ATTAAATTTT   
  
  
+ ATTGTATAAA AAAATATTTA AACCATTATT ATAAATAAAA TATATATTTT TAGATATTAT TTATTTTAAA   
  
  
+ ATTGTACTGT CAAAATTTTA AAATATTTAT AAAAAGTAAT TTACAAGTCA TATGAATGAA TTGAAAATAA   
  
  
+ AATGTAAAAT TTATTTACTT TATTTTATAA CAAAAAGTTT TTAAATTTTT ATAATCATTT GAACTAAATT   
  
  
+ TAATTAATAA ATACAAATTT GATTTCTTTT TCTAAAAATT AAATCTAATT CACTATTTTC AAATATTAAA   
  
  
+ ATTTAATTTA ATGCATGAAA CACTTTAAGA TCAAAATTAT ATAAAATTAT CATATTTACT TTATATTATC   
  
  
+ AATCAATTTT TTTGTTAAAA AGGTGACAAA TTGAATTTTG ATCTACTGGT TTAATATCAA ATTAAATCTT   
  
  
+ AATTAAAATA ATCATAACAA TTAAAAATAT TTTTGTTATT AAAATATTTA AACTATTTGA ATTATTTTTT   
  
  
+ ATTTTATTTC AAGTTCATTC TTTAAATGTA AATAATTTTT GATGCAAGAT CTGGTTTTTC TAAATTTTTA   
  
  
+ AAATTTTGAT TATATTTAAC AATACATTTT TAATACTCAA AAAATTTTAA AATTTTTATG ATAATATTTT   
  
  
+ AAATATTAAA TTTGAAATTA AAATTTATTA TATAAAATTT TTAATTTAAT TATTTTAAAA TTTAAATTTT   
  
  
+ TTAATAACAA TTAATTTAAT TTAATTTAAT AATATAAAAT AAATATTAAA ATTTTATATA ATTTTTATTC   
  
  
+ AAAATTTTAA ACACATTTTT TATTTTTTCT TGTGGCGGGC TTCCGAATCA GCTACCTTTT TGTAGAGGTA   
  
  
+ TCACTATATA AAATTTACGT GCCGTTTACG ACACCAAAGA TACCATTAGG ATAAGAAGCT GCCTCACAGA   
  
  
+ CAGGCCGCCT CCGCCACTGT TCTGAATTTC AACCACACTC AGTACTCCTC CTATATATTA CCACACAAAC   
  
  
+ CATATTCATT GATTCTTATT TATTCTTCTT GTCCTTCTTC GTCGTATGAT CCGACGCTCA CTTTTCTCTC   
  
  
+ AGACTTTCTC TCGTTTTCTC GGGAAAATA  

- GTCTTTAACA TTCAAAAATA ACCACAGTGA TATTAAATTA AACAAAGGAA CTGCTATCAT GATAAACATG   
  
  
- CTGTACTTTC AAACCAGGCT AAGTTGATTG ATTAATTAAC TAATTCTATT TAAATTAAAC AAAGGAACTG   
  
  
- CTATCATGAT AAACATGCTG TACTTTCAAA CCAGGCTAAG TTGATTGATT AATTAACTAA TTCTATAGTT   
  
  
- ACGCTAGAAT TAGTTTCGTT CGATCGGATC GGAATTAGTA CTTCGGAGAT TATAAAAGAG TAACGAAACT   
  
  
- ATGAAAAAAA AATTTTAAAA TAAATTTTAA ATTTTAATAT AAAAATGGAA CTTCACCTCA GGTTACAAAA   
  
  
- TTTCTACTGT TAAGTATAGT ATTAAATAAT TTAATTTAAA TTAATAAATT TTTTAATTTT TAATTTAAAA   
  
  
- TAACATATTT TTTTATAAAT TTGGTAATAA TATTTATTTT ATATATAAAA ATCTATAATA AATAAAATTT   
  
  
- TAACATGACA GTTTTAAAAT TTTATAAATA TTTTTCATTA AATGTTCAGT ATACTTACTT AACTTTTATT   
  
  
- TTACATTTTA AATAAATGAA ATAAAATATT GTTTTTCAAA AATTTAAAAA TATTAGTAAA CTTGATTTAA   
  
  
- ATTAATTATT TATGTTTAAA CTAAAGAAAA AGATTTTTAA TTTAGATTAA GTGATAAAAG TTTATAATTT   
  
  
- TAAATTAAAT TACGTACTTT GTGAAATTCT AGTTTTAATA TATTTTAATA GTATAAATGA AATATAATAG   
  
  
- TTAGTTAAAA AAACAATTTT TCCACTGTTT AACTTAAAAC TAGATGACCA AATTATAGTT TAATTTAGAA   
  
  
- TTAATTTTAT TAGTATTGTT AATTTTTATA AAAACAATAA TTTTATAAAT TTGATAAACT TAATAAAAAA   
  
  
- TAAAATAAAG TTCAAGTAAG AAATTTACAT TTATTAAAAA CTACGTTCTA GACCAAAAAG ATTTAAAAAT   
  
  
- TTTAAAACTA ATATAAATTG TTATGTAAAA ATTATGAGTT TTTTAAAATT TTAAAAATAC TATTATAAAA   
  
  
- TTTATAATTT AAACTTTAAT TTTAAATAAT ATATTTTAAA AATTAAATTA ATAAAATTTT AAATTTAAAA   
  
  
- AATTATTGTT AATTAAATTA AATTAAATTA TTATATTTTA TTTATAATTT TAAAATATAT TAAAAATAAG   
  
  
- TTTTAAAATT TGTGTAAAAA ATAAAAAAGA ACACCGCCCG AAGGCTTAGT CGATGGAAAA ACATCTCCAT   
  
  
- AGTGATATAT TTTAAATGCA CGGCAAATGC TGTGGTTTCT ATGGTAATCC TATTCTTCGA CGGAGTGTCT   
  
  
- GTCCGGCGGA GGCGGTGACA AGACTTAAAG TTGGTGTGAG TCATGAGGAG GATATATAAT GGTGTGTTTG   
  
  
- GTATAAGTAA CTAAGAATAA ATAAGAAGAA CAGGAAGAAG CAGCATACTA GGCTGCGAGT GAAAAGAGAG   
  
  
- TCTGAAAGAG AGCAAAAGAG CCCTTTTAT

+     CGTCA-motif

| Site Name | Organism | Position | Strand | Matrix score. | sequence | function |
| --- | --- | --- | --- | --- | --- | --- |
| CGTCA-motif | Hordeum vulgare | 50 | - | 5 | CGTCA | cis-acting regulatory element involved in the MeJA-responsiveness |
| CGTCA-motif | Hordeum vulgare | 137 | - | 5 | CGTCA | cis-acting regulatory element involved in the MeJA-responsiveness |

> 2018/04/13 10:10:12  
+ CAGAAATTGT AAGTTTTTAT TGGTGTCACT ATAATTTAAT TTGTTTCCTT GACGATAGTA CTATTTGTAC   
  
  
+ GACATGAAAG TTTGGTCCGA TTCAACTAAC TAATTAATTG ATTAAGATAA ATTTAATTTG TTTCCTTGAC   
  
  
+ GATAGTACTA TTTGTACGAC ATGAAAGTTT GGTCCGATTC AACTAACTAA TTAATTGATT AAGATATCAA   
  
  
+ TGCGATCTTA ATCAAAGCAA GCTAGCCTAG CCTTAATCAT GAAGCCTCTA ATATTTTCTC ATTGCTTTGA   
  
  
+ TACTTTTTTT TTAAAATTTT ATTTAAAATT TAAAATTATA TTTTTACCTT GAAGTGGAGT CCAATGTTTT   
  
  
+ AAAGATGACA ATTCATATCA TAATTTATTA AATTAAATTT AATTATTTAA AAAATTAAAA ATTAAATTTT   
  
  
+ ATTGTATAAA AAAATATTTA AACCATTATT ATAAATAAAA TATATATTTT TAGATATTAT TTATTTTAAA   
  
  
+ ATTGTACTGT CAAAATTTTA AAATATTTAT AAAAAGTAAT TTACAAGTCA TATGAATGAA TTGAAAATAA   
  
  
+ AATGTAAAAT TTATTTACTT TATTTTATAA CAAAAAGTTT TTAAATTTTT ATAATCATTT GAACTAAATT   
  
  
+ TAATTAATAA ATACAAATTT GATTTCTTTT TCTAAAAATT AAATCTAATT CACTATTTTC AAATATTAAA   
  
  
+ ATTTAATTTA ATGCATGAAA CACTTTAAGA TCAAAATTAT ATAAAATTAT CATATTTACT TTATATTATC   
  
  
+ AATCAATTTT TTTGTTAAAA AGGTGACAAA TTGAATTTTG ATCTACTGGT TTAATATCAA ATTAAATCTT   
  
  
+ AATTAAAATA ATCATAACAA TTAAAAATAT TTTTGTTATT AAAATATTTA AACTATTTGA ATTATTTTTT   
  
  
+ ATTTTATTTC AAGTTCATTC TTTAAATGTA AATAATTTTT GATGCAAGAT CTGGTTTTTC TAAATTTTTA   
  
  
+ AAATTTTGAT TATATTTAAC AATACATTTT TAATACTCAA AAAATTTTAA AATTTTTATG ATAATATTTT   
  
  
+ AAATATTAAA TTTGAAATTA AAATTTATTA TATAAAATTT TTAATTTAAT TATTTTAAAA TTTAAATTTT   
  
  
+ TTAATAACAA TTAATTTAAT TTAATTTAAT AATATAAAAT AAATATTAAA ATTTTATATA ATTTTTATTC   
  
  
+ AAAATTTTAA ACACATTTTT TATTTTTTCT TGTGGCGGGC TTCCGAATCA GCTACCTTTT TGTAGAGGTA   
  
  
+ TCACTATATA AAATTTACGT GCCGTTTACG ACACCAAAGA TACCATTAGG ATAAGAAGCT GCCTCACAGA   
  
  
+ CAGGCCGCCT CCGCCACTGT TCTGAATTTC AACCACACTC AGTACTCCTC CTATATATTA CCACACAAAC   
  
  
+ CATATTCATT GATTCTTATT TATTCTTCTT GTCCTTCTTC GTCGTATGAT CCGACGCTCA CTTTTCTCTC   
  
  
+ AGACTTTCTC TCGTTTTCTC GGGAAAATA  

- GTCTTTAACA TTCAAAAATA ACCACAGTGA TATTAAATTA AACAAAGGAA CTGCTATCAT GATAAACATG   
  
  
- CTGTACTTTC AAACCAGGCT AAGTTGATTG ATTAATTAAC TAATTCTATT TAAATTAAAC AAAGGAACTG   
  
  
- CTATCATGAT AAACATGCTG TACTTTCAAA CCAGGCTAAG TTGATTGATT AATTAACTAA TTCTATAGTT   
  
  
- ACGCTAGAAT TAGTTTCGTT CGATCGGATC GGAATTAGTA CTTCGGAGAT TATAAAAGAG TAACGAAACT   
  
  
- ATGAAAAAAA AATTTTAAAA TAAATTTTAA ATTTTAATAT AAAAATGGAA CTTCACCTCA GGTTACAAAA   
  
  
- TTTCTACTGT TAAGTATAGT ATTAAATAAT TTAATTTAAA TTAATAAATT TTTTAATTTT TAATTTAAAA   
  
  
- TAACATATTT TTTTATAAAT TTGGTAATAA TATTTATTTT ATATATAAAA ATCTATAATA AATAAAATTT   
  
  
- TAACATGACA GTTTTAAAAT TTTATAAATA TTTTTCATTA AATGTTCAGT ATACTTACTT AACTTTTATT   
  
  
- TTACATTTTA AATAAATGAA ATAAAATATT GTTTTTCAAA AATTTAAAAA TATTAGTAAA CTTGATTTAA   
  
  
- ATTAATTATT TATGTTTAAA CTAAAGAAAA AGATTTTTAA TTTAGATTAA GTGATAAAAG TTTATAATTT   
  
  
- TAAATTAAAT TACGTACTTT GTGAAATTCT AGTTTTAATA TATTTTAATA GTATAAATGA AATATAATAG   
  
  
- TTAGTTAAAA AAACAATTTT TCCACTGTTT AACTTAAAAC TAGATGACCA AATTATAGTT TAATTTAGAA   
  
  
- TTAATTTTAT TAGTATTGTT AATTTTTATA AAAACAATAA TTTTATAAAT TTGATAAACT TAATAAAAAA   
  
  
- TAAAATAAAG TTCAAGTAAG AAATTTACAT TTATTAAAAA CTACGTTCTA GACCAAAAAG ATTTAAAAAT   
  
  
- TTTAAAACTA ATATAAATTG TTATGTAAAA ATTATGAGTT TTTTAAAATT TTAAAAATAC TATTATAAAA   
  
  
- TTTATAATTT AAACTTTAAT TTTAAATAAT ATATTTTAAA AATTAAATTA ATAAAATTTT AAATTTAAAA   
  
  
- AATTATTGTT AATTAAATTA AATTAAATTA TTATATTTTA TTTATAATTT TAAAATATAT TAAAAATAAG   
  
  
- TTTTAAAATT TGTGTAAAAA ATAAAAAAGA ACACCGCCCG AAGGCTTAGT CGATGGAAAA ACATCTCCAT   
  
  
- AGTGATATAT TTTAAATGCA CGGCAAATGC TGTGGTTTCT ATGGTAATCC TATTCTTCGA CGGAGTGTCT   
  
  
- GTCCGGCGGA GGCGGTGACA AGACTTAAAG TTGGTGTGAG TCATGAGGAG GATATATAAT GGTGTGTTTG   
  
  
- GTATAAGTAA CTAAGAATAA ATAAGAAGAA CAGGAAGAAG CAGCATACTA GGCTGCGAGT GAAAAGAGAG   
  
  
- TCTGAAAGAG AGCAAAAGAG CCCTTTTAT

+     ERE

| Site Name | Organism | Position | Strand | Matrix score. | sequence | function |
| --- | --- | --- | --- | --- | --- | --- |
| ERE | Dianthus caryophyllus | 1061 | - | 8 | ATTTCAAA | ethylene-responsive element |

> 2018/04/13 10:10:12  
+ CAGAAATTGT AAGTTTTTAT TGGTGTCACT ATAATTTAAT TTGTTTCCTT GACGATAGTA CTATTTGTAC   
  
  
+ GACATGAAAG TTTGGTCCGA TTCAACTAAC TAATTAATTG ATTAAGATAA ATTTAATTTG TTTCCTTGAC   
  
  
+ GATAGTACTA TTTGTACGAC ATGAAAGTTT GGTCCGATTC AACTAACTAA TTAATTGATT AAGATATCAA   
  
  
+ TGCGATCTTA ATCAAAGCAA GCTAGCCTAG CCTTAATCAT GAAGCCTCTA ATATTTTCTC ATTGCTTTGA   
  
  
+ TACTTTTTTT TTAAAATTTT ATTTAAAATT TAAAATTATA TTTTTACCTT GAAGTGGAGT CCAATGTTTT   
  
  
+ AAAGATGACA ATTCATATCA TAATTTATTA AATTAAATTT AATTATTTAA AAAATTAAAA ATTAAATTTT   
  
  
+ ATTGTATAAA AAAATATTTA AACCATTATT ATAAATAAAA TATATATTTT TAGATATTAT TTATTTTAAA   
  
  
+ ATTGTACTGT CAAAATTTTA AAATATTTAT AAAAAGTAAT TTACAAGTCA TATGAATGAA TTGAAAATAA   
  
  
+ AATGTAAAAT TTATTTACTT TATTTTATAA CAAAAAGTTT TTAAATTTTT ATAATCATTT GAACTAAATT   
  
  
+ TAATTAATAA ATACAAATTT GATTTCTTTT TCTAAAAATT AAATCTAATT CACTATTTTC AAATATTAAA   
  
  
+ ATTTAATTTA ATGCATGAAA CACTTTAAGA TCAAAATTAT ATAAAATTAT CATATTTACT TTATATTATC   
  
  
+ AATCAATTTT TTTGTTAAAA AGGTGACAAA TTGAATTTTG ATCTACTGGT TTAATATCAA ATTAAATCTT   
  
  
+ AATTAAAATA ATCATAACAA TTAAAAATAT TTTTGTTATT AAAATATTTA AACTATTTGA ATTATTTTTT   
  
  
+ ATTTTATTTC AAGTTCATTC TTTAAATGTA AATAATTTTT GATGCAAGAT CTGGTTTTTC TAAATTTTTA   
  
  
+ AAATTTTGAT TATATTTAAC AATACATTTT TAATACTCAA AAAATTTTAA AATTTTTATG ATAATATTTT   
  
  
+ AAATATTAAA TTTGAAATTA AAATTTATTA TATAAAATTT TTAATTTAAT TATTTTAAAA TTTAAATTTT   
  
  
+ TTAATAACAA TTAATTTAAT TTAATTTAAT AATATAAAAT AAATATTAAA ATTTTATATA ATTTTTATTC   
  
  
+ AAAATTTTAA ACACATTTTT TATTTTTTCT TGTGGCGGGC TTCCGAATCA GCTACCTTTT TGTAGAGGTA   
  
  
+ TCACTATATA AAATTTACGT GCCGTTTACG ACACCAAAGA TACCATTAGG ATAAGAAGCT GCCTCACAGA   
  
  
+ CAGGCCGCCT CCGCCACTGT TCTGAATTTC AACCACACTC AGTACTCCTC CTATATATTA CCACACAAAC   
  
  
+ CATATTCATT GATTCTTATT TATTCTTCTT GTCCTTCTTC GTCGTATGAT CCGACGCTCA CTTTTCTCTC   
  
  
+ AGACTTTCTC TCGTTTTCTC GGGAAAATA  

- GTCTTTAACA TTCAAAAATA ACCACAGTGA TATTAAATTA AACAAAGGAA CTGCTATCAT GATAAACATG   
  
  
- CTGTACTTTC AAACCAGGCT AAGTTGATTG ATTAATTAAC TAATTCTATT TAAATTAAAC AAAGGAACTG   
  
  
- CTATCATGAT AAACATGCTG TACTTTCAAA CCAGGCTAAG TTGATTGATT AATTAACTAA TTCTATAGTT   
  
  
- ACGCTAGAAT TAGTTTCGTT CGATCGGATC GGAATTAGTA CTTCGGAGAT TATAAAAGAG TAACGAAACT   
  
  
- ATGAAAAAAA AATTTTAAAA TAAATTTTAA ATTTTAATAT AAAAATGGAA CTTCACCTCA GGTTACAAAA   
  
  
- TTTCTACTGT TAAGTATAGT ATTAAATAAT TTAATTTAAA TTAATAAATT TTTTAATTTT TAATTTAAAA   
  
  
- TAACATATTT TTTTATAAAT TTGGTAATAA TATTTATTTT ATATATAAAA ATCTATAATA AATAAAATTT   
  
  
- TAACATGACA GTTTTAAAAT TTTATAAATA TTTTTCATTA AATGTTCAGT ATACTTACTT AACTTTTATT   
  
  
- TTACATTTTA AATAAATGAA ATAAAATATT GTTTTTCAAA AATTTAAAAA TATTAGTAAA CTTGATTTAA   
  
  
- ATTAATTATT TATGTTTAAA CTAAAGAAAA AGATTTTTAA TTTAGATTAA GTGATAAAAG TTTATAATTT   
  
  
- TAAATTAAAT TACGTACTTT GTGAAATTCT AGTTTTAATA TATTTTAATA GTATAAATGA AATATAATAG   
  
  
- TTAGTTAAAA AAACAATTTT TCCACTGTTT AACTTAAAAC TAGATGACCA AATTATAGTT TAATTTAGAA   
  
  
- TTAATTTTAT TAGTATTGTT AATTTTTATA AAAACAATAA TTTTATAAAT TTGATAAACT TAATAAAAAA   
  
  
- TAAAATAAAG TTCAAGTAAG AAATTTACAT TTATTAAAAA CTACGTTCTA GACCAAAAAG ATTTAAAAAT   
  
  
- TTTAAAACTA ATATAAATTG TTATGTAAAA ATTATGAGTT TTTTAAAATT TTAAAAATAC TATTATAAAA   
  
  
- TTTATAATTT AAACTTTAAT TTTAAATAAT ATATTTTAAA AATTAAATTA ATAAAATTTT AAATTTAAAA   
  
  
- AATTATTGTT AATTAAATTA AATTAAATTA TTATATTTTA TTTATAATTT TAAAATATAT TAAAAATAAG   
  
  
- TTTTAAAATT TGTGTAAAAA ATAAAAAAGA ACACCGCCCG AAGGCTTAGT CGATGGAAAA ACATCTCCAT   
  
  
- AGTGATATAT TTTAAATGCA CGGCAAATGC TGTGGTTTCT ATGGTAATCC TATTCTTCGA CGGAGTGTCT   
  
  
- GTCCGGCGGA GGCGGTGACA AGACTTAAAG TTGGTGTGAG TCATGAGGAG GATATATAAT GGTGTGTTTG   
  
  
- GTATAAGTAA CTAAGAATAA ATAAGAAGAA CAGGAAGAAG CAGCATACTA GGCTGCGAGT GAAAAGAGAG   
  
  
- TCTGAAAGAG AGCAAAAGAG CCCTTTTAT

+     G-Box

| Site Name | Organism | Position | Strand | Matrix score. | sequence | function |
| --- | --- | --- | --- | --- | --- | --- |
| G-Box | Antirrhinum majus | 1276 | - | 6 | CACGTA | cis-acting regulatory element involved in light responsiveness |

> 2018/04/13 10:10:12  
+ CAGAAATTGT AAGTTTTTAT TGGTGTCACT ATAATTTAAT TTGTTTCCTT GACGATAGTA CTATTTGTAC   
  
  
+ GACATGAAAG TTTGGTCCGA TTCAACTAAC TAATTAATTG ATTAAGATAA ATTTAATTTG TTTCCTTGAC   
  
  
+ GATAGTACTA TTTGTACGAC ATGAAAGTTT GGTCCGATTC AACTAACTAA TTAATTGATT AAGATATCAA   
  
  
+ TGCGATCTTA ATCAAAGCAA GCTAGCCTAG CCTTAATCAT GAAGCCTCTA ATATTTTCTC ATTGCTTTGA   
  
  
+ TACTTTTTTT TTAAAATTTT ATTTAAAATT TAAAATTATA TTTTTACCTT GAAGTGGAGT CCAATGTTTT   
  
  
+ AAAGATGACA ATTCATATCA TAATTTATTA AATTAAATTT AATTATTTAA AAAATTAAAA ATTAAATTTT   
  
  
+ ATTGTATAAA AAAATATTTA AACCATTATT ATAAATAAAA TATATATTTT TAGATATTAT TTATTTTAAA   
  
  
+ ATTGTACTGT CAAAATTTTA AAATATTTAT AAAAAGTAAT TTACAAGTCA TATGAATGAA TTGAAAATAA   
  
  
+ AATGTAAAAT TTATTTACTT TATTTTATAA CAAAAAGTTT TTAAATTTTT ATAATCATTT GAACTAAATT   
  
  
+ TAATTAATAA ATACAAATTT GATTTCTTTT TCTAAAAATT AAATCTAATT CACTATTTTC AAATATTAAA   
  
  
+ ATTTAATTTA ATGCATGAAA CACTTTAAGA TCAAAATTAT ATAAAATTAT CATATTTACT TTATATTATC   
  
  
+ AATCAATTTT TTTGTTAAAA AGGTGACAAA TTGAATTTTG ATCTACTGGT TTAATATCAA ATTAAATCTT   
  
  
+ AATTAAAATA ATCATAACAA TTAAAAATAT TTTTGTTATT AAAATATTTA AACTATTTGA ATTATTTTTT   
  
  
+ ATTTTATTTC AAGTTCATTC TTTAAATGTA AATAATTTTT GATGCAAGAT CTGGTTTTTC TAAATTTTTA   
  
  
+ AAATTTTGAT TATATTTAAC AATACATTTT TAATACTCAA AAAATTTTAA AATTTTTATG ATAATATTTT   
  
  
+ AAATATTAAA TTTGAAATTA AAATTTATTA TATAAAATTT TTAATTTAAT TATTTTAAAA TTTAAATTTT   
  
  
+ TTAATAACAA TTAATTTAAT TTAATTTAAT AATATAAAAT AAATATTAAA ATTTTATATA ATTTTTATTC   
  
  
+ AAAATTTTAA ACACATTTTT TATTTTTTCT TGTGGCGGGC TTCCGAATCA GCTACCTTTT TGTAGAGGTA   
  
  
+ TCACTATATA AAATTTACGT GCCGTTTACG ACACCAAAGA TACCATTAGG ATAAGAAGCT GCCTCACAGA   
  
  
+ CAGGCCGCCT CCGCCACTGT TCTGAATTTC AACCACACTC AGTACTCCTC CTATATATTA CCACACAAAC   
  
  
+ CATATTCATT GATTCTTATT TATTCTTCTT GTCCTTCTTC GTCGTATGAT CCGACGCTCA CTTTTCTCTC   
  
  
+ AGACTTTCTC TCGTTTTCTC GGGAAAATA  

- GTCTTTAACA TTCAAAAATA ACCACAGTGA TATTAAATTA AACAAAGGAA CTGCTATCAT GATAAACATG   
  
  
- CTGTACTTTC AAACCAGGCT AAGTTGATTG ATTAATTAAC TAATTCTATT TAAATTAAAC AAAGGAACTG   
  
  
- CTATCATGAT AAACATGCTG TACTTTCAAA CCAGGCTAAG TTGATTGATT AATTAACTAA TTCTATAGTT   
  
  
- ACGCTAGAAT TAGTTTCGTT CGATCGGATC GGAATTAGTA CTTCGGAGAT TATAAAAGAG TAACGAAACT   
  
  
- ATGAAAAAAA AATTTTAAAA TAAATTTTAA ATTTTAATAT AAAAATGGAA CTTCACCTCA GGTTACAAAA   
  
  
- TTTCTACTGT TAAGTATAGT ATTAAATAAT TTAATTTAAA TTAATAAATT TTTTAATTTT TAATTTAAAA   
  
  
- TAACATATTT TTTTATAAAT TTGGTAATAA TATTTATTTT ATATATAAAA ATCTATAATA AATAAAATTT   
  
  
- TAACATGACA GTTTTAAAAT TTTATAAATA TTTTTCATTA AATGTTCAGT ATACTTACTT AACTTTTATT   
  
  
- TTACATTTTA AATAAATGAA ATAAAATATT GTTTTTCAAA AATTTAAAAA TATTAGTAAA CTTGATTTAA   
  
  
- ATTAATTATT TATGTTTAAA CTAAAGAAAA AGATTTTTAA TTTAGATTAA GTGATAAAAG TTTATAATTT   
  
  
- TAAATTAAAT TACGTACTTT GTGAAATTCT AGTTTTAATA TATTTTAATA GTATAAATGA AATATAATAG   
  
  
- TTAGTTAAAA AAACAATTTT TCCACTGTTT AACTTAAAAC TAGATGACCA AATTATAGTT TAATTTAGAA   
  
  
- TTAATTTTAT TAGTATTGTT AATTTTTATA AAAACAATAA TTTTATAAAT TTGATAAACT TAATAAAAAA   
  
  
- TAAAATAAAG TTCAAGTAAG AAATTTACAT TTATTAAAAA CTACGTTCTA GACCAAAAAG ATTTAAAAAT   
  
  
- TTTAAAACTA ATATAAATTG TTATGTAAAA ATTATGAGTT TTTTAAAATT TTAAAAATAC TATTATAAAA   
  
  
- TTTATAATTT AAACTTTAAT TTTAAATAAT ATATTTTAAA AATTAAATTA ATAAAATTTT AAATTTAAAA   
  
  
- AATTATTGTT AATTAAATTA AATTAAATTA TTATATTTTA TTTATAATTT TAAAATATAT TAAAAATAAG   
  
  
- TTTTAAAATT TGTGTAAAAA ATAAAAAAGA ACACCGCCCG AAGGCTTAGT CGATGGAAAA ACATCTCCAT   
  
  
- AGTGATATAT TTTAAATGCA CGGCAAATGC TGTGGTTTCT ATGGTAATCC TATTCTTCGA CGGAGTGTCT   
  
  
- GTCCGGCGGA GGCGGTGACA AGACTTAAAG TTGGTGTGAG TCATGAGGAG GATATATAAT GGTGTGTTTG   
  
  
- GTATAAGTAA CTAAGAATAA ATAAGAAGAA CAGGAAGAAG CAGCATACTA GGCTGCGAGT GAAAAGAGAG   
  
  
- TCTGAAAGAG AGCAAAAGAG CCCTTTTAT

+     G-box

| Site Name | Organism | Position | Strand | Matrix score. | sequence | function |
| --- | --- | --- | --- | --- | --- | --- |
| G-box | Daucus carota | 1276 | + | 6 | TACGTG | cis-acting regulatory element involved in light responsiveness |

> 2018/04/13 10:10:12  
+ CAGAAATTGT AAGTTTTTAT TGGTGTCACT ATAATTTAAT TTGTTTCCTT GACGATAGTA CTATTTGTAC   
  
  
+ GACATGAAAG TTTGGTCCGA TTCAACTAAC TAATTAATTG ATTAAGATAA ATTTAATTTG TTTCCTTGAC   
  
  
+ GATAGTACTA TTTGTACGAC ATGAAAGTTT GGTCCGATTC AACTAACTAA TTAATTGATT AAGATATCAA   
  
  
+ TGCGATCTTA ATCAAAGCAA GCTAGCCTAG CCTTAATCAT GAAGCCTCTA ATATTTTCTC ATTGCTTTGA   
  
  
+ TACTTTTTTT TTAAAATTTT ATTTAAAATT TAAAATTATA TTTTTACCTT GAAGTGGAGT CCAATGTTTT   
  
  
+ AAAGATGACA ATTCATATCA TAATTTATTA AATTAAATTT AATTATTTAA AAAATTAAAA ATTAAATTTT   
  
  
+ ATTGTATAAA AAAATATTTA AACCATTATT ATAAATAAAA TATATATTTT TAGATATTAT TTATTTTAAA   
  
  
+ ATTGTACTGT CAAAATTTTA AAATATTTAT AAAAAGTAAT TTACAAGTCA TATGAATGAA TTGAAAATAA   
  
  
+ AATGTAAAAT TTATTTACTT TATTTTATAA CAAAAAGTTT TTAAATTTTT ATAATCATTT GAACTAAATT   
  
  
+ TAATTAATAA ATACAAATTT GATTTCTTTT TCTAAAAATT AAATCTAATT CACTATTTTC AAATATTAAA   
  
  
+ ATTTAATTTA ATGCATGAAA CACTTTAAGA TCAAAATTAT ATAAAATTAT CATATTTACT TTATATTATC   
  
  
+ AATCAATTTT TTTGTTAAAA AGGTGACAAA TTGAATTTTG ATCTACTGGT TTAATATCAA ATTAAATCTT   
  
  
+ AATTAAAATA ATCATAACAA TTAAAAATAT TTTTGTTATT AAAATATTTA AACTATTTGA ATTATTTTTT   
  
  
+ ATTTTATTTC AAGTTCATTC TTTAAATGTA AATAATTTTT GATGCAAGAT CTGGTTTTTC TAAATTTTTA   
  
  
+ AAATTTTGAT TATATTTAAC AATACATTTT TAATACTCAA AAAATTTTAA AATTTTTATG ATAATATTTT   
  
  
+ AAATATTAAA TTTGAAATTA AAATTTATTA TATAAAATTT TTAATTTAAT TATTTTAAAA TTTAAATTTT   
  
  
+ TTAATAACAA TTAATTTAAT TTAATTTAAT AATATAAAAT AAATATTAAA ATTTTATATA ATTTTTATTC   
  
  
+ AAAATTTTAA ACACATTTTT TATTTTTTCT TGTGGCGGGC TTCCGAATCA GCTACCTTTT TGTAGAGGTA   
  
  
+ TCACTATATA AAATTTACGT GCCGTTTACG ACACCAAAGA TACCATTAGG ATAAGAAGCT GCCTCACAGA   
  
  
+ CAGGCCGCCT CCGCCACTGT TCTGAATTTC AACCACACTC AGTACTCCTC CTATATATTA CCACACAAAC   
  
  
+ CATATTCATT GATTCTTATT TATTCTTCTT GTCCTTCTTC GTCGTATGAT CCGACGCTCA CTTTTCTCTC   
  
  
+ AGACTTTCTC TCGTTTTCTC GGGAAAATA  

- GTCTTTAACA TTCAAAAATA ACCACAGTGA TATTAAATTA AACAAAGGAA CTGCTATCAT GATAAACATG   
  
  
- CTGTACTTTC AAACCAGGCT AAGTTGATTG ATTAATTAAC TAATTCTATT TAAATTAAAC AAAGGAACTG   
  
  
- CTATCATGAT AAACATGCTG TACTTTCAAA CCAGGCTAAG TTGATTGATT AATTAACTAA TTCTATAGTT   
  
  
- ACGCTAGAAT TAGTTTCGTT CGATCGGATC GGAATTAGTA CTTCGGAGAT TATAAAAGAG TAACGAAACT   
  
  
- ATGAAAAAAA AATTTTAAAA TAAATTTTAA ATTTTAATAT AAAAATGGAA CTTCACCTCA GGTTACAAAA   
  
  
- TTTCTACTGT TAAGTATAGT ATTAAATAAT TTAATTTAAA TTAATAAATT TTTTAATTTT TAATTTAAAA   
  
  
- TAACATATTT TTTTATAAAT TTGGTAATAA TATTTATTTT ATATATAAAA ATCTATAATA AATAAAATTT   
  
  
- TAACATGACA GTTTTAAAAT TTTATAAATA TTTTTCATTA AATGTTCAGT ATACTTACTT AACTTTTATT   
  
  
- TTACATTTTA AATAAATGAA ATAAAATATT GTTTTTCAAA AATTTAAAAA TATTAGTAAA CTTGATTTAA   
  
  
- ATTAATTATT TATGTTTAAA CTAAAGAAAA AGATTTTTAA TTTAGATTAA GTGATAAAAG TTTATAATTT   
  
  
- TAAATTAAAT TACGTACTTT GTGAAATTCT AGTTTTAATA TATTTTAATA GTATAAATGA AATATAATAG   
  
  
- TTAGTTAAAA AAACAATTTT TCCACTGTTT AACTTAAAAC TAGATGACCA AATTATAGTT TAATTTAGAA   
  
  
- TTAATTTTAT TAGTATTGTT AATTTTTATA AAAACAATAA TTTTATAAAT TTGATAAACT TAATAAAAAA   
  
  
- TAAAATAAAG TTCAAGTAAG AAATTTACAT TTATTAAAAA CTACGTTCTA GACCAAAAAG ATTTAAAAAT   
  
  
- TTTAAAACTA ATATAAATTG TTATGTAAAA ATTATGAGTT TTTTAAAATT TTAAAAATAC TATTATAAAA   
  
  
- TTTATAATTT AAACTTTAAT TTTAAATAAT ATATTTTAAA AATTAAATTA ATAAAATTTT AAATTTAAAA   
  
  
- AATTATTGTT AATTAAATTA AATTAAATTA TTATATTTTA TTTATAATTT TAAAATATAT TAAAAATAAG   
  
  
- TTTTAAAATT TGTGTAAAAA ATAAAAAAGA ACACCGCCCG AAGGCTTAGT CGATGGAAAA ACATCTCCAT   
  
  
- AGTGATATAT TTTAAATGCA CGGCAAATGC TGTGGTTTCT ATGGTAATCC TATTCTTCGA CGGAGTGTCT   
  
  
- GTCCGGCGGA GGCGGTGACA AGACTTAAAG TTGGTGTGAG TCATGAGGAG GATATATAAT GGTGTGTTTG   
  
  
- GTATAAGTAA CTAAGAATAA ATAAGAAGAA CAGGAAGAAG CAGCATACTA GGCTGCGAGT GAAAAGAGAG   
  
  
- TCTGAAAGAG AGCAAAAGAG CCCTTTTAT

+     GA-motif

| Site Name | Organism | Position | Strand | Matrix score. | sequence | function |
| --- | --- | --- | --- | --- | --- | --- |
| GA-motif | Helianthus annuus | 351 | + | 8 | AAAGATGA | part of a light responsive element |

> 2018/04/13 10:10:12  
+ CAGAAATTGT AAGTTTTTAT TGGTGTCACT ATAATTTAAT TTGTTTCCTT GACGATAGTA CTATTTGTAC   
  
  
+ GACATGAAAG TTTGGTCCGA TTCAACTAAC TAATTAATTG ATTAAGATAA ATTTAATTTG TTTCCTTGAC   
  
  
+ GATAGTACTA TTTGTACGAC ATGAAAGTTT GGTCCGATTC AACTAACTAA TTAATTGATT AAGATATCAA   
  
  
+ TGCGATCTTA ATCAAAGCAA GCTAGCCTAG CCTTAATCAT GAAGCCTCTA ATATTTTCTC ATTGCTTTGA   
  
  
+ TACTTTTTTT TTAAAATTTT ATTTAAAATT TAAAATTATA TTTTTACCTT GAAGTGGAGT CCAATGTTTT   
  
  
+ AAAGATGACA ATTCATATCA TAATTTATTA AATTAAATTT AATTATTTAA AAAATTAAAA ATTAAATTTT   
  
  
+ ATTGTATAAA AAAATATTTA AACCATTATT ATAAATAAAA TATATATTTT TAGATATTAT TTATTTTAAA   
  
  
+ ATTGTACTGT CAAAATTTTA AAATATTTAT AAAAAGTAAT TTACAAGTCA TATGAATGAA TTGAAAATAA   
  
  
+ AATGTAAAAT TTATTTACTT TATTTTATAA CAAAAAGTTT TTAAATTTTT ATAATCATTT GAACTAAATT   
  
  
+ TAATTAATAA ATACAAATTT GATTTCTTTT TCTAAAAATT AAATCTAATT CACTATTTTC AAATATTAAA   
  
  
+ ATTTAATTTA ATGCATGAAA CACTTTAAGA TCAAAATTAT ATAAAATTAT CATATTTACT TTATATTATC   
  
  
+ AATCAATTTT TTTGTTAAAA AGGTGACAAA TTGAATTTTG ATCTACTGGT TTAATATCAA ATTAAATCTT   
  
  
+ AATTAAAATA ATCATAACAA TTAAAAATAT TTTTGTTATT AAAATATTTA AACTATTTGA ATTATTTTTT   
  
  
+ ATTTTATTTC AAGTTCATTC TTTAAATGTA AATAATTTTT GATGCAAGAT CTGGTTTTTC TAAATTTTTA   
  
  
+ AAATTTTGAT TATATTTAAC AATACATTTT TAATACTCAA AAAATTTTAA AATTTTTATG ATAATATTTT   
  
  
+ AAATATTAAA TTTGAAATTA AAATTTATTA TATAAAATTT TTAATTTAAT TATTTTAAAA TTTAAATTTT   
  
  
+ TTAATAACAA TTAATTTAAT TTAATTTAAT AATATAAAAT AAATATTAAA ATTTTATATA ATTTTTATTC   
  
  
+ AAAATTTTAA ACACATTTTT TATTTTTTCT TGTGGCGGGC TTCCGAATCA GCTACCTTTT TGTAGAGGTA   
  
  
+ TCACTATATA AAATTTACGT GCCGTTTACG ACACCAAAGA TACCATTAGG ATAAGAAGCT GCCTCACAGA   
  
  
+ CAGGCCGCCT CCGCCACTGT TCTGAATTTC AACCACACTC AGTACTCCTC CTATATATTA CCACACAAAC   
  
  
+ CATATTCATT GATTCTTATT TATTCTTCTT GTCCTTCTTC GTCGTATGAT CCGACGCTCA CTTTTCTCTC   
  
  
+ AGACTTTCTC TCGTTTTCTC GGGAAAATA  

- GTCTTTAACA TTCAAAAATA ACCACAGTGA TATTAAATTA AACAAAGGAA CTGCTATCAT GATAAACATG   
  
  
- CTGTACTTTC AAACCAGGCT AAGTTGATTG ATTAATTAAC TAATTCTATT TAAATTAAAC AAAGGAACTG   
  
  
- CTATCATGAT AAACATGCTG TACTTTCAAA CCAGGCTAAG TTGATTGATT AATTAACTAA TTCTATAGTT   
  
  
- ACGCTAGAAT TAGTTTCGTT CGATCGGATC GGAATTAGTA CTTCGGAGAT TATAAAAGAG TAACGAAACT   
  
  
- ATGAAAAAAA AATTTTAAAA TAAATTTTAA ATTTTAATAT AAAAATGGAA CTTCACCTCA GGTTACAAAA   
  
  
- TTTCTACTGT TAAGTATAGT ATTAAATAAT TTAATTTAAA TTAATAAATT TTTTAATTTT TAATTTAAAA   
  
  
- TAACATATTT TTTTATAAAT TTGGTAATAA TATTTATTTT ATATATAAAA ATCTATAATA AATAAAATTT   
  
  
- TAACATGACA GTTTTAAAAT TTTATAAATA TTTTTCATTA AATGTTCAGT ATACTTACTT AACTTTTATT   
  
  
- TTACATTTTA AATAAATGAA ATAAAATATT GTTTTTCAAA AATTTAAAAA TATTAGTAAA CTTGATTTAA   
  
  
- ATTAATTATT TATGTTTAAA CTAAAGAAAA AGATTTTTAA TTTAGATTAA GTGATAAAAG TTTATAATTT   
  
  
- TAAATTAAAT TACGTACTTT GTGAAATTCT AGTTTTAATA TATTTTAATA GTATAAATGA AATATAATAG   
  
  
- TTAGTTAAAA AAACAATTTT TCCACTGTTT AACTTAAAAC TAGATGACCA AATTATAGTT TAATTTAGAA   
  
  
- TTAATTTTAT TAGTATTGTT AATTTTTATA AAAACAATAA TTTTATAAAT TTGATAAACT TAATAAAAAA   
  
  
- TAAAATAAAG TTCAAGTAAG AAATTTACAT TTATTAAAAA CTACGTTCTA GACCAAAAAG ATTTAAAAAT   
  
  
- TTTAAAACTA ATATAAATTG TTATGTAAAA ATTATGAGTT TTTTAAAATT TTAAAAATAC TATTATAAAA   
  
  
- TTTATAATTT AAACTTTAAT TTTAAATAAT ATATTTTAAA AATTAAATTA ATAAAATTTT AAATTTAAAA   
  
  
- AATTATTGTT AATTAAATTA AATTAAATTA TTATATTTTA TTTATAATTT TAAAATATAT TAAAAATAAG   
  
  
- TTTTAAAATT TGTGTAAAAA ATAAAAAAGA ACACCGCCCG AAGGCTTAGT CGATGGAAAA ACATCTCCAT   
  
  
- AGTGATATAT TTTAAATGCA CGGCAAATGC TGTGGTTTCT ATGGTAATCC TATTCTTCGA CGGAGTGTCT   
  
  
- GTCCGGCGGA GGCGGTGACA AGACTTAAAG TTGGTGTGAG TCATGAGGAG GATATATAAT GGTGTGTTTG   
  
  
- GTATAAGTAA CTAAGAATAA ATAAGAAGAA CAGGAAGAAG CAGCATACTA GGCTGCGAGT GAAAAGAGAG   
  
  
- TCTGAAAGAG AGCAAAAGAG CCCTTTTAT

+     HSE

| Site Name | Organism | Position | Strand | Matrix score. | sequence | function |
| --- | --- | --- | --- | --- | --- | --- |
| HSE | Brassica oleracea | 1019 | + | 9 | AAAAAATTTC | cis-acting element involved in heat stress responsiveness |
| HSE | Brassica oleracea | 1113 | - | 9 | AAAAAATTTC | cis-acting element involved in heat stress responsiveness |

> 2018/04/13 10:10:12  
+ CAGAAATTGT AAGTTTTTAT TGGTGTCACT ATAATTTAAT TTGTTTCCTT GACGATAGTA CTATTTGTAC   
  
  
+ GACATGAAAG TTTGGTCCGA TTCAACTAAC TAATTAATTG ATTAAGATAA ATTTAATTTG TTTCCTTGAC   
  
  
+ GATAGTACTA TTTGTACGAC ATGAAAGTTT GGTCCGATTC AACTAACTAA TTAATTGATT AAGATATCAA   
  
  
+ TGCGATCTTA ATCAAAGCAA GCTAGCCTAG CCTTAATCAT GAAGCCTCTA ATATTTTCTC ATTGCTTTGA   
  
  
+ TACTTTTTTT TTAAAATTTT ATTTAAAATT TAAAATTATA TTTTTACCTT GAAGTGGAGT CCAATGTTTT   
  
  
+ AAAGATGACA ATTCATATCA TAATTTATTA AATTAAATTT AATTATTTAA AAAATTAAAA ATTAAATTTT   
  
  
+ ATTGTATAAA AAAATATTTA AACCATTATT ATAAATAAAA TATATATTTT TAGATATTAT TTATTTTAAA   
  
  
+ ATTGTACTGT CAAAATTTTA AAATATTTAT AAAAAGTAAT TTACAAGTCA TATGAATGAA TTGAAAATAA   
  
  
+ AATGTAAAAT TTATTTACTT TATTTTATAA CAAAAAGTTT TTAAATTTTT ATAATCATTT GAACTAAATT   
  
  
+ TAATTAATAA ATACAAATTT GATTTCTTTT TCTAAAAATT AAATCTAATT CACTATTTTC AAATATTAAA   
  
  
+ ATTTAATTTA ATGCATGAAA CACTTTAAGA TCAAAATTAT ATAAAATTAT CATATTTACT TTATATTATC   
  
  
+ AATCAATTTT TTTGTTAAAA AGGTGACAAA TTGAATTTTG ATCTACTGGT TTAATATCAA ATTAAATCTT   
  
  
+ AATTAAAATA ATCATAACAA TTAAAAATAT TTTTGTTATT AAAATATTTA AACTATTTGA ATTATTTTTT   
  
  
+ ATTTTATTTC AAGTTCATTC TTTAAATGTA AATAATTTTT GATGCAAGAT CTGGTTTTTC TAAATTTTTA   
  
  
+ AAATTTTGAT TATATTTAAC AATACATTTT TAATACTCAA AAAATTTTAA AATTTTTATG ATAATATTTT   
  
  
+ AAATATTAAA TTTGAAATTA AAATTTATTA TATAAAATTT TTAATTTAAT TATTTTAAAA TTTAAATTTT   
  
  
+ TTAATAACAA TTAATTTAAT TTAATTTAAT AATATAAAAT AAATATTAAA ATTTTATATA ATTTTTATTC   
  
  
+ AAAATTTTAA ACACATTTTT TATTTTTTCT TGTGGCGGGC TTCCGAATCA GCTACCTTTT TGTAGAGGTA   
  
  
+ TCACTATATA AAATTTACGT GCCGTTTACG ACACCAAAGA TACCATTAGG ATAAGAAGCT GCCTCACAGA   
  
  
+ CAGGCCGCCT CCGCCACTGT TCTGAATTTC AACCACACTC AGTACTCCTC CTATATATTA CCACACAAAC   
  
  
+ CATATTCATT GATTCTTATT TATTCTTCTT GTCCTTCTTC GTCGTATGAT CCGACGCTCA CTTTTCTCTC   
  
  
+ AGACTTTCTC TCGTTTTCTC GGGAAAATA  

- GTCTTTAACA TTCAAAAATA ACCACAGTGA TATTAAATTA AACAAAGGAA CTGCTATCAT GATAAACATG   
  
  
- CTGTACTTTC AAACCAGGCT AAGTTGATTG ATTAATTAAC TAATTCTATT TAAATTAAAC AAAGGAACTG   
  
  
- CTATCATGAT AAACATGCTG TACTTTCAAA CCAGGCTAAG TTGATTGATT AATTAACTAA TTCTATAGTT   
  
  
- ACGCTAGAAT TAGTTTCGTT CGATCGGATC GGAATTAGTA CTTCGGAGAT TATAAAAGAG TAACGAAACT   
  
  
- ATGAAAAAAA AATTTTAAAA TAAATTTTAA ATTTTAATAT AAAAATGGAA CTTCACCTCA GGTTACAAAA   
  
  
- TTTCTACTGT TAAGTATAGT ATTAAATAAT TTAATTTAAA TTAATAAATT TTTTAATTTT TAATTTAAAA   
  
  
- TAACATATTT TTTTATAAAT TTGGTAATAA TATTTATTTT ATATATAAAA ATCTATAATA AATAAAATTT   
  
  
- TAACATGACA GTTTTAAAAT TTTATAAATA TTTTTCATTA AATGTTCAGT ATACTTACTT AACTTTTATT   
  
  
- TTACATTTTA AATAAATGAA ATAAAATATT GTTTTTCAAA AATTTAAAAA TATTAGTAAA CTTGATTTAA   
  
  
- ATTAATTATT TATGTTTAAA CTAAAGAAAA AGATTTTTAA TTTAGATTAA GTGATAAAAG TTTATAATTT   
  
  
- TAAATTAAAT TACGTACTTT GTGAAATTCT AGTTTTAATA TATTTTAATA GTATAAATGA AATATAATAG   
  
  
- TTAGTTAAAA AAACAATTTT TCCACTGTTT AACTTAAAAC TAGATGACCA AATTATAGTT TAATTTAGAA   
  
  
- TTAATTTTAT TAGTATTGTT AATTTTTATA AAAACAATAA TTTTATAAAT TTGATAAACT TAATAAAAAA   
  
  
- TAAAATAAAG TTCAAGTAAG AAATTTACAT TTATTAAAAA CTACGTTCTA GACCAAAAAG ATTTAAAAAT   
  
  
- TTTAAAACTA ATATAAATTG TTATGTAAAA ATTATGAGTT TTTTAAAATT TTAAAAATAC TATTATAAAA   
  
  
- TTTATAATTT AAACTTTAAT TTTAAATAAT ATATTTTAAA AATTAAATTA ATAAAATTTT AAATTTAAAA   
  
  
- AATTATTGTT AATTAAATTA AATTAAATTA TTATATTTTA TTTATAATTT TAAAATATAT TAAAAATAAG   
  
  
- TTTTAAAATT TGTGTAAAAA ATAAAAAAGA ACACCGCCCG AAGGCTTAGT CGATGGAAAA ACATCTCCAT   
  
  
- AGTGATATAT TTTAAATGCA CGGCAAATGC TGTGGTTTCT ATGGTAATCC TATTCTTCGA CGGAGTGTCT   
  
  
- GTCCGGCGGA GGCGGTGACA AGACTTAAAG TTGGTGTGAG TCATGAGGAG GATATATAAT GGTGTGTTTG   
  
  
- GTATAAGTAA CTAAGAATAA ATAAGAAGAA CAGGAAGAAG CAGCATACTA GGCTGCGAGT GAAAAGAGAG   
  
  
- TCTGAAAGAG AGCAAAAGAG CCCTTTTAT

+     I-box

| Site Name | Organism | Position | Strand | Matrix score. | sequence | function |
| --- | --- | --- | --- | --- | --- | --- |
| I-box | Pisum sativum | 363 | - | 9 | ATGATATGA | part of a light responsive element |

> 2018/04/13 10:10:12  
+ CAGAAATTGT AAGTTTTTAT TGGTGTCACT ATAATTTAAT TTGTTTCCTT GACGATAGTA CTATTTGTAC   
  
  
+ GACATGAAAG TTTGGTCCGA TTCAACTAAC TAATTAATTG ATTAAGATAA ATTTAATTTG TTTCCTTGAC   
  
  
+ GATAGTACTA TTTGTACGAC ATGAAAGTTT GGTCCGATTC AACTAACTAA TTAATTGATT AAGATATCAA   
  
  
+ TGCGATCTTA ATCAAAGCAA GCTAGCCTAG CCTTAATCAT GAAGCCTCTA ATATTTTCTC ATTGCTTTGA   
  
  
+ TACTTTTTTT TTAAAATTTT ATTTAAAATT TAAAATTATA TTTTTACCTT GAAGTGGAGT CCAATGTTTT   
  
  
+ AAAGATGACA ATTCATATCA TAATTTATTA AATTAAATTT AATTATTTAA AAAATTAAAA ATTAAATTTT   
  
  
+ ATTGTATAAA AAAATATTTA AACCATTATT ATAAATAAAA TATATATTTT TAGATATTAT TTATTTTAAA   
  
  
+ ATTGTACTGT CAAAATTTTA AAATATTTAT AAAAAGTAAT TTACAAGTCA TATGAATGAA TTGAAAATAA   
  
  
+ AATGTAAAAT TTATTTACTT TATTTTATAA CAAAAAGTTT TTAAATTTTT ATAATCATTT GAACTAAATT   
  
  
+ TAATTAATAA ATACAAATTT GATTTCTTTT TCTAAAAATT AAATCTAATT CACTATTTTC AAATATTAAA   
  
  
+ ATTTAATTTA ATGCATGAAA CACTTTAAGA TCAAAATTAT ATAAAATTAT CATATTTACT TTATATTATC   
  
  
+ AATCAATTTT TTTGTTAAAA AGGTGACAAA TTGAATTTTG ATCTACTGGT TTAATATCAA ATTAAATCTT   
  
  
+ AATTAAAATA ATCATAACAA TTAAAAATAT TTTTGTTATT AAAATATTTA AACTATTTGA ATTATTTTTT   
  
  
+ ATTTTATTTC AAGTTCATTC TTTAAATGTA AATAATTTTT GATGCAAGAT CTGGTTTTTC TAAATTTTTA   
  
  
+ AAATTTTGAT TATATTTAAC AATACATTTT TAATACTCAA AAAATTTTAA AATTTTTATG ATAATATTTT   
  
  
+ AAATATTAAA TTTGAAATTA AAATTTATTA TATAAAATTT TTAATTTAAT TATTTTAAAA TTTAAATTTT   
  
  
+ TTAATAACAA TTAATTTAAT TTAATTTAAT AATATAAAAT AAATATTAAA ATTTTATATA ATTTTTATTC   
  
  
+ AAAATTTTAA ACACATTTTT TATTTTTTCT TGTGGCGGGC TTCCGAATCA GCTACCTTTT TGTAGAGGTA   
  
  
+ TCACTATATA AAATTTACGT GCCGTTTACG ACACCAAAGA TACCATTAGG ATAAGAAGCT GCCTCACAGA   
  
  
+ CAGGCCGCCT CCGCCACTGT TCTGAATTTC AACCACACTC AGTACTCCTC CTATATATTA CCACACAAAC   
  
  
+ CATATTCATT GATTCTTATT TATTCTTCTT GTCCTTCTTC GTCGTATGAT CCGACGCTCA CTTTTCTCTC   
  
  
+ AGACTTTCTC TCGTTTTCTC GGGAAAATA  

- GTCTTTAACA TTCAAAAATA ACCACAGTGA TATTAAATTA AACAAAGGAA CTGCTATCAT GATAAACATG   
  
  
- CTGTACTTTC AAACCAGGCT AAGTTGATTG ATTAATTAAC TAATTCTATT TAAATTAAAC AAAGGAACTG   
  
  
- CTATCATGAT AAACATGCTG TACTTTCAAA CCAGGCTAAG TTGATTGATT AATTAACTAA TTCTATAGTT   
  
  
- ACGCTAGAAT TAGTTTCGTT CGATCGGATC GGAATTAGTA CTTCGGAGAT TATAAAAGAG TAACGAAACT   
  
  
- ATGAAAAAAA AATTTTAAAA TAAATTTTAA ATTTTAATAT AAAAATGGAA CTTCACCTCA GGTTACAAAA   
  
  
- TTTCTACTGT TAAGTATAGT ATTAAATAAT TTAATTTAAA TTAATAAATT TTTTAATTTT TAATTTAAAA   
  
  
- TAACATATTT TTTTATAAAT TTGGTAATAA TATTTATTTT ATATATAAAA ATCTATAATA AATAAAATTT   
  
  
- TAACATGACA GTTTTAAAAT TTTATAAATA TTTTTCATTA AATGTTCAGT ATACTTACTT AACTTTTATT   
  
  
- TTACATTTTA AATAAATGAA ATAAAATATT GTTTTTCAAA AATTTAAAAA TATTAGTAAA CTTGATTTAA   
  
  
- ATTAATTATT TATGTTTAAA CTAAAGAAAA AGATTTTTAA TTTAGATTAA GTGATAAAAG TTTATAATTT   
  
  
- TAAATTAAAT TACGTACTTT GTGAAATTCT AGTTTTAATA TATTTTAATA GTATAAATGA AATATAATAG   
  
  
- TTAGTTAAAA AAACAATTTT TCCACTGTTT AACTTAAAAC TAGATGACCA AATTATAGTT TAATTTAGAA   
  
  
- TTAATTTTAT TAGTATTGTT AATTTTTATA AAAACAATAA TTTTATAAAT TTGATAAACT TAATAAAAAA   
  
  
- TAAAATAAAG TTCAAGTAAG AAATTTACAT TTATTAAAAA CTACGTTCTA GACCAAAAAG ATTTAAAAAT   
  
  
- TTTAAAACTA ATATAAATTG TTATGTAAAA ATTATGAGTT TTTTAAAATT TTAAAAATAC TATTATAAAA   
  
  
- TTTATAATTT AAACTTTAAT TTTAAATAAT ATATTTTAAA AATTAAATTA ATAAAATTTT AAATTTAAAA   
  
  
- AATTATTGTT AATTAAATTA AATTAAATTA TTATATTTTA TTTATAATTT TAAAATATAT TAAAAATAAG   
  
  
- TTTTAAAATT TGTGTAAAAA ATAAAAAAGA ACACCGCCCG AAGGCTTAGT CGATGGAAAA ACATCTCCAT   
  
  
- AGTGATATAT TTTAAATGCA CGGCAAATGC TGTGGTTTCT ATGGTAATCC TATTCTTCGA CGGAGTGTCT   
  
  
- GTCCGGCGGA GGCGGTGACA AGACTTAAAG TTGGTGTGAG TCATGAGGAG GATATATAAT GGTGTGTTTG   
  
  
- GTATAAGTAA CTAAGAATAA ATAAGAAGAA CAGGAAGAAG CAGCATACTA GGCTGCGAGT GAAAAGAGAG   
  
  
- TCTGAAAGAG AGCAAAAGAG CCCTTTTAT

+     Skn-1\_motif

| Site Name | Organism | Position | Strand | Matrix score. | sequence | function |
| --- | --- | --- | --- | --- | --- | --- |
| Skn-1\_motif | Oryza sativa | 355 | - | 5 | GTCAT | cis-acting regulatory element required for endosperm expression |
| Skn-1\_motif | Oryza sativa | 537 | + | 5 | GTCAT | cis-acting regulatory element required for endosperm expression |

> 2018/04/13 10:10:12  
+ CAGAAATTGT AAGTTTTTAT TGGTGTCACT ATAATTTAAT TTGTTTCCTT GACGATAGTA CTATTTGTAC   
  
  
+ GACATGAAAG TTTGGTCCGA TTCAACTAAC TAATTAATTG ATTAAGATAA ATTTAATTTG TTTCCTTGAC   
  
  
+ GATAGTACTA TTTGTACGAC ATGAAAGTTT GGTCCGATTC AACTAACTAA TTAATTGATT AAGATATCAA   
  
  
+ TGCGATCTTA ATCAAAGCAA GCTAGCCTAG CCTTAATCAT GAAGCCTCTA ATATTTTCTC ATTGCTTTGA   
  
  
+ TACTTTTTTT TTAAAATTTT ATTTAAAATT TAAAATTATA TTTTTACCTT GAAGTGGAGT CCAATGTTTT   
  
  
+ AAAGATGACA ATTCATATCA TAATTTATTA AATTAAATTT AATTATTTAA AAAATTAAAA ATTAAATTTT   
  
  
+ ATTGTATAAA AAAATATTTA AACCATTATT ATAAATAAAA TATATATTTT TAGATATTAT TTATTTTAAA   
  
  
+ ATTGTACTGT CAAAATTTTA AAATATTTAT AAAAAGTAAT TTACAAGTCA TATGAATGAA TTGAAAATAA   
  
  
+ AATGTAAAAT TTATTTACTT TATTTTATAA CAAAAAGTTT TTAAATTTTT ATAATCATTT GAACTAAATT   
  
  
+ TAATTAATAA ATACAAATTT GATTTCTTTT TCTAAAAATT AAATCTAATT CACTATTTTC AAATATTAAA   
  
  
+ ATTTAATTTA ATGCATGAAA CACTTTAAGA TCAAAATTAT ATAAAATTAT CATATTTACT TTATATTATC   
  
  
+ AATCAATTTT TTTGTTAAAA AGGTGACAAA TTGAATTTTG ATCTACTGGT TTAATATCAA ATTAAATCTT   
  
  
+ AATTAAAATA ATCATAACAA TTAAAAATAT TTTTGTTATT AAAATATTTA AACTATTTGA ATTATTTTTT   
  
  
+ ATTTTATTTC AAGTTCATTC TTTAAATGTA AATAATTTTT GATGCAAGAT CTGGTTTTTC TAAATTTTTA   
  
  
+ AAATTTTGAT TATATTTAAC AATACATTTT TAATACTCAA AAAATTTTAA AATTTTTATG ATAATATTTT   
  
  
+ AAATATTAAA TTTGAAATTA AAATTTATTA TATAAAATTT TTAATTTAAT TATTTTAAAA TTTAAATTTT   
  
  
+ TTAATAACAA TTAATTTAAT TTAATTTAAT AATATAAAAT AAATATTAAA ATTTTATATA ATTTTTATTC   
  
  
+ AAAATTTTAA ACACATTTTT TATTTTTTCT TGTGGCGGGC TTCCGAATCA GCTACCTTTT TGTAGAGGTA   
  
  
+ TCACTATATA AAATTTACGT GCCGTTTACG ACACCAAAGA TACCATTAGG ATAAGAAGCT GCCTCACAGA   
  
  
+ CAGGCCGCCT CCGCCACTGT TCTGAATTTC AACCACACTC AGTACTCCTC CTATATATTA CCACACAAAC   
  
  
+ CATATTCATT GATTCTTATT TATTCTTCTT GTCCTTCTTC GTCGTATGAT CCGACGCTCA CTTTTCTCTC   
  
  
+ AGACTTTCTC TCGTTTTCTC GGGAAAATA  

- GTCTTTAACA TTCAAAAATA ACCACAGTGA TATTAAATTA AACAAAGGAA CTGCTATCAT GATAAACATG   
  
  
- CTGTACTTTC AAACCAGGCT AAGTTGATTG ATTAATTAAC TAATTCTATT TAAATTAAAC AAAGGAACTG   
  
  
- CTATCATGAT AAACATGCTG TACTTTCAAA CCAGGCTAAG TTGATTGATT AATTAACTAA TTCTATAGTT   
  
  
- ACGCTAGAAT TAGTTTCGTT CGATCGGATC GGAATTAGTA CTTCGGAGAT TATAAAAGAG TAACGAAACT   
  
  
- ATGAAAAAAA AATTTTAAAA TAAATTTTAA ATTTTAATAT AAAAATGGAA CTTCACCTCA GGTTACAAAA   
  
  
- TTTCTACTGT TAAGTATAGT ATTAAATAAT TTAATTTAAA TTAATAAATT TTTTAATTTT TAATTTAAAA   
  
  
- TAACATATTT TTTTATAAAT TTGGTAATAA TATTTATTTT ATATATAAAA ATCTATAATA AATAAAATTT   
  
  
- TAACATGACA GTTTTAAAAT TTTATAAATA TTTTTCATTA AATGTTCAGT ATACTTACTT AACTTTTATT   
  
  
- TTACATTTTA AATAAATGAA ATAAAATATT GTTTTTCAAA AATTTAAAAA TATTAGTAAA CTTGATTTAA   
  
  
- ATTAATTATT TATGTTTAAA CTAAAGAAAA AGATTTTTAA TTTAGATTAA GTGATAAAAG TTTATAATTT   
  
  
- TAAATTAAAT TACGTACTTT GTGAAATTCT AGTTTTAATA TATTTTAATA GTATAAATGA AATATAATAG   
  
  
- TTAGTTAAAA AAACAATTTT TCCACTGTTT AACTTAAAAC TAGATGACCA AATTATAGTT TAATTTAGAA   
  
  
- TTAATTTTAT TAGTATTGTT AATTTTTATA AAAACAATAA TTTTATAAAT TTGATAAACT TAATAAAAAA   
  
  
- TAAAATAAAG TTCAAGTAAG AAATTTACAT TTATTAAAAA CTACGTTCTA GACCAAAAAG ATTTAAAAAT   
  
  
- TTTAAAACTA ATATAAATTG TTATGTAAAA ATTATGAGTT TTTTAAAATT TTAAAAATAC TATTATAAAA   
  
  
- TTTATAATTT AAACTTTAAT TTTAAATAAT ATATTTTAAA AATTAAATTA ATAAAATTTT AAATTTAAAA   
  
  
- AATTATTGTT AATTAAATTA AATTAAATTA TTATATTTTA TTTATAATTT TAAAATATAT TAAAAATAAG   
  
  
- TTTTAAAATT TGTGTAAAAA ATAAAAAAGA ACACCGCCCG AAGGCTTAGT CGATGGAAAA ACATCTCCAT   
  
  
- AGTGATATAT TTTAAATGCA CGGCAAATGC TGTGGTTTCT ATGGTAATCC TATTCTTCGA CGGAGTGTCT   
  
  
- GTCCGGCGGA GGCGGTGACA AGACTTAAAG TTGGTGTGAG TCATGAGGAG GATATATAAT GGTGTGTTTG   
  
  
- GTATAAGTAA CTAAGAATAA ATAAGAAGAA CAGGAAGAAG CAGCATACTA GGCTGCGAGT GAAAAGAGAG   
  
  
- TCTGAAAGAG AGCAAAAGAG CCCTTTTAT

+     TATA-box

| Site Name | Organism | Position | Strand | Matrix score. | sequence | function |
| --- | --- | --- | --- | --- | --- | --- |
| TATA-box | Arabidopsis thaliana | 300 | + | 8 | TATTTAAA | core promoter element around -30 of transcription start |
| TATA-box | Lycopersicon esculentum | 742 | - | 5 | TTTTA | core promoter element around -30 of transcription start |
| TATA-box | Lycopersicon esculentum | 468 | + | 5 | TTTTA | core promoter element around -30 of transcription start |
| TATA-box | Arabidopsis thaliana | 435 | + | 8 | TATTTAAA | core promoter element around -30 of transcription start |
| TATA-box | Arabidopsis thaliana | 586 | + | 4 | TATA | core promoter element around -30 of transcription start |
| TATA-box | Arabidopsis thaliana | 30 | + | 4 | TATA | core promoter element around -30 of transcription start |
| TATA-box | Arabidopsis thaliana | 518 | + | 6 | TATAAA | core promoter element around -30 of transcription start |
| TATA-box | Arabidopsis thaliana | 463 | + | 4 | TATA | core promoter element around -30 of transcription start |
| TATA-box | Lycopersicon esculentum | 1083 | - | 5 | TTTTA | core promoter element around -30 of transcription start |
| TATA-box | Lycopersicon esculentum | 663 | - | 5 | TTTTA | core promoter element around -30 of transcription start |
| TATA-box | Brassica napus | 315 | + | 6 | ATTATA | core promoter element around -30 of transcription start |
| TATA-box | Ac | 515 | - | 7 | TATAAAT | core promoter element around -30 of transcription start |
| TATA-box | Arabidopsis thaliana | 425 | + | 6 | TATAAA | core promoter element around -30 of transcription start |
| TATA-box | Arabidopsis thaliana | 608 | - | 6 | TATAAA | core promoter element around -30 of transcription start |
| TATA-box | Arabidopsis thaliana | 317 | + | 4 | TATA | core promoter element around -30 of transcription start |
| TATA-box | Arabidopsis thaliana | 990 | - | 5 | TATAA | core promoter element around -30 of transcription start |
| TATA-box | Arabidopsis thaliana | 1177 | - | 4 | TATA | core promoter element around -30 of transcription start |
| TATA-box | Arabidopsis thaliana | 885 | + | 8 | TATTTAAA | core promoter element around -30 of transcription start |
| TATA-box | Pisum sativum | 582 | - | 8 | TATAAAAT | core promoter element around -30 of transcription start |
| TATA-box | Arabidopsis thaliana | 461 | + | 4 | TATA | core promoter element around -30 of transcription start |
| TATA-box | Arabidopsis thaliana | 738 | + | 4 | TATA | core promoter element around -30 of transcription start |
| TATA-box | Arabidopsis thaliana | 394 | + | 8 | TATTTAAA | core promoter element around -30 of transcription start |
| TATA-box | Lycopersicon esculentum | 1155 | - | 5 | TTTTA | core promoter element around -30 of transcription start |
| TATA-box | Lycopersicon esculentum | 506 | + | 5 | TTTTA | core promoter element around -30 of transcription start |
| TATA-box | Glycine max | 1054 | - | 5 | TAATA | core promoter element around -30 of transcription start |
| TATA-box | Arabidopsis thaliana | 1267 | + | 6 | TATAAA | core promoter element around -30 of transcription start |
| TATA-box | Zea mays | 396 | + | 8 | TTTAAAAA | core promoter element around -30 of transcription start |
| TATA-box | Lycopersicon esculentum | 599 | + | 5 | TTTTA | core promoter element around -30 of transcription start |
| TATA-box | Glycine max | 822 | + | 5 | TAATA | core promoter element around -30 of transcription start |
| TATA-box | Zea mays | 288 | - | 8 | TTTAAAAA | core promoter element around -30 of transcription start |
| TATA-box | Glycine max | 1147 | + | 5 | TAATA | core promoter element around -30 of transcription start |
| TATA-box | Lycopersicon esculentum | 558 | - | 5 | TTTTA | core promoter element around -30 of transcription start |
| TATA-box | Ac | 450 | + | 7 | TATAAAT | core promoter element around -30 of transcription start |
| TATA-box | Lycopersicon esculentum | 1034 | + | 5 | TTTTA | core promoter element around -30 of transcription start |
| TATA-box | Lycopersicon esculentum | 697 | - | 5 | TTTTA | core promoter element around -30 of transcription start |
| TATA-box | Lycopersicon esculentum | 1208 | + | 5 | TTTTA | core promoter element around -30 of transcription start |
| TATA-box | Lycopersicon esculentum | 292 | - | 5 | TTTTA | core promoter element around -30 of transcription start |
| TATA-box | Arabidopsis thaliana | 516 | - | 6 | TATAAA | core promoter element around -30 of transcription start |
| TATA-box | Zea mays | 975 | - | 8 | TTTAAAAA | core promoter element around -30 of transcription start |
| TATA-box | Arabidopsis thaliana | 1173 | - | 6 | TATAAA | core promoter element around -30 of transcription start |
| TATA-box | Arabidopsis thaliana | 1079 | - | 4 | TATA | core promoter element around -30 of transcription start |
| TATA-box | Arabidopsis thaliana | 1382 | - | 4 | TATA | core promoter element around -30 of transcription start |
| TATA-box | Glycine max | 447 | - | 5 | TAATA | core promoter element around -30 of transcription start |
| TATA-box | Arabidopsis thaliana | 610 | + | 4 | TATA | core promoter element around -30 of transcription start |
| TATA-box | Glycine max | 764 | - | 5 | TAATA | core promoter element around -30 of transcription start |
| TATA-box | Lycopersicon esculentum | 311 | - | 5 | TTTTA | core promoter element around -30 of transcription start |
| TATA-box | Arabidopsis thaliana | 584 | - | 6 | TATAAA | core promoter element around -30 of transcription start |
| TATA-box | Lycopersicon esculentum | 1119 | + | 5 | TTTTA | core promoter element around -30 of transcription start |
| TATA-box | Lycopersicon esculentum | 456 | - | 5 | TTTTA | core promoter element around -30 of transcription start |
| TATA-box | Arabidopsis thaliana | 740 | + | 6 | TATAAA | core promoter element around -30 of transcription start |
| TATA-box | Lycopersicon esculentum | 1025 | + | 5 | TTTTA | core promoter element around -30 of transcription start |
| TATA-box | Arabidopsis thaliana | 14 | - | 9 | ccTATAAAaa | core promoter element around -30 of transcription start |
| TATA-box | Lycopersicon esculentum | 1183 | + | 5 | TTTTA | core promoter element around -30 of transcription start |
| TATA-box | Lycopersicon esculentum | 484 | + | 5 | TTTTA | core promoter element around -30 of transcription start |
| TATA-box | Lycopersicon esculentum | 907 | + | 5 | TTTTA | core promoter element around -30 of transcription start |
| TATA-box | Lycopersicon esculentum | 406 | - | 5 | TTTTA | core promoter element around -30 of transcription start |
| TATA-box | Lycopersicon esculentum | 1167 | - | 5 | TTTTA | core promoter element around -30 of transcription start |
| TATA-box | Zea mays | 598 | - | 8 | TTTAAAAA | core promoter element around -30 of transcription start |
| TATA-box | Glycine max | 1076 | - | 5 | TAATA | core promoter element around -30 of transcription start |
| TATA-box | Arabidopsis thaliana | 1380 | + | 9 | tcTATATAtt | core promoter element around -30 of transcription start |
| TATA-box | Arabidopsis thaliana | 1153 | + | 6 | TATAAA | core promoter element around -30 of transcription start |
| TATA-box | Lycopersicon esculentum | 322 | + | 5 | TTTTA | core promoter element around -30 of transcription start |
| TATA-box | Lycopersicon esculentum | 520 | - | 5 | TTTTA | core promoter element around -30 of transcription start |
| TATA-box | Lycopersicon esculentum | 862 | - | 5 | TTTTA | core promoter element around -30 of transcription start |
| TATA-box | Arabidopsis thaliana | 1048 | - | 8 | TATTTAAA | core promoter element around -30 of transcription start |
| TATA-box | Brassica napus | 462 | + | 6 | ATATAT | core promoter element around -30 of transcription start |
| TATA-box | Glycine max | 694 | - | 5 | TAATA | core promoter element around -30 of transcription start |
| TATA-box | Brassica napus | 1262 | + | 11 | CACTATATAAAG | core promoter element around -30 of transcription start |
| TATA-box | Arabidopsis thaliana | 1174 | - | 7 | TATATAA | core promoter element around -30 of transcription start |
| TATA-box | Arabidopsis thaliana | 1384 | - | 4 | TATA | core promoter element around -30 of transcription start |
| TATA-box | Lycopersicon esculentum | 297 | + | 5 | TTTTA | core promoter element around -30 of transcription start |
| TATA-box | Daucus carota | 514 | - | 8 | TATAAATA | core promoter element around -30 of transcription start |
| TATA-box | Lycopersicon esculentum | 979 | - | 5 | TTTTA | core promoter element around -30 of transcription start |
| TATA-box | Arabidopsis thaliana | 1081 | + | 6 | TATAAA | core promoter element around -30 of transcription start |
| TATA-box | Arabidopsis thaliana | 762 | - | 4 | TATA | core promoter element around -30 of transcription start |
| TATA-box | Lycopersicon esculentum | 427 | - | 5 | TTTTA | core promoter element around -30 of transcription start |
| TATA-box | Arabidopsis thaliana | 609 | - | 5 | TATAA | core promoter element around -30 of transcription start |
| TATA-box | Lycopersicon esculentum | 1103 | + | 5 | TTTTA | core promoter element around -30 of transcription start |
| TATA-box | Arabidopsis thaliana | 316 | - | 5 | TATAA | core promoter element around -30 of transcription start |
| TATA-box | Lycopersicon esculentum | 1008 | + | 5 | TTTTA | core promoter element around -30 of transcription start |
| TATA-box | Arabidopsis thaliana | 583 | - | 7 | TATAAAA | core promoter element around -30 of transcription start |
| TATA-box | Brassica oleracea | 1176 | + | 7 | ATATAAT | core promoter element around -30 of transcription start |
| TATA-box | Arabidopsis thaliana | 449 | - | 5 | TATAA | core promoter element around -30 of transcription start |
| TATA-box | Arabidopsis thaliana | 737 | - | 7 | TATATAA | core promoter element around -30 of transcription start |
| TATA-box | Lycopersicon esculentum | 880 | - | 5 | TTTTA | core promoter element around -30 of transcription start |
| TATA-box | Lycopersicon esculentum | 289 | + | 5 | TTTTA | core promoter element around -30 of transcription start |
| TATA-box | Glycine max | 1164 | - | 5 | TAATA | core promoter element around -30 of transcription start |
| TATA-box | Lycopersicon esculentum | 509 | - | 5 | TTTTA | core promoter element around -30 of transcription start |
| TATA-box | Lycopersicon esculentum | 1069 | - | 5 | TTTTA | core promoter element around -30 of transcription start |
| TATA-box | Helianthus annuus | 423 | - | 6 | TATACA | core promoter element around -30 of transcription start |
| TATA-box | Brassica oleracea | 1266 | + | 6 | ATATAA | core promoter element around -30 of transcription start |
| TATA-box | Arabidopsis thaliana | 607 | - | 7 | TATAAAA | core promoter element around -30 of transcription start |
| TATA-box | Lycopersicon esculentum | 844 | - | 5 | TTTTA | core promoter element around -30 of transcription start |
| TATA-box | Lycopersicon esculentum | 347 | + | 5 | TTTTA | core promoter element around -30 of transcription start |
| TATA-box | Glycine max | 1150 | + | 5 | TAATA | core promoter element around -30 of transcription start |
| TATA-box | Lycopersicon esculentum | 565 | - | 5 | TTTTA | core promoter element around -30 of transcription start |
| TATA-box | Glycine max | 1042 | + | 5 | TAATA | core promoter element around -30 of transcription start |
| TATA-box | Nicotiana tabacum | 465 | - | 9 | tcTATAAAta | core promoter element around -30 of transcription start |
| TATA-box | Oryza sativa | 1248 | - | 7 | TACAAAA | core promoter element around -30 of transcription start |
| TATA-box | Brassica napus | 736 | + | 6 | ATTATA | core promoter element around -30 of transcription start |
| TATA-box | Lycopersicon esculentum | 976 | + | 5 | TTTTA | core promoter element around -30 of transcription start |
| TATA-box | Lycopersicon esculentum | 304 | - | 5 | TTTTA | core promoter element around -30 of transcription start |
| TATA-box | Arabidopsis thaliana | 1078 | - | 7 | TATATAA | core promoter element around -30 of transcription start |
| TATA-box | Arabidopsis thaliana | 517 | - | 5 | TATAA | core promoter element around -30 of transcription start |
| TATA-box | Arabidopsis thaliana | 1172 | - | 7 | TATAAAA | core promoter element around -30 of transcription start |
| TATA-box | Brassica napus | 448 | + | 6 | ATTATA | core promoter element around -30 of transcription start |
| TATA-box | Lycopersicon esculentum | 786 | - | 5 | TTTTA | core promoter element around -30 of transcription start |
| TATA-box | Glycine max | 635 | + | 5 | TAATA | core promoter element around -30 of transcription start |
| TATA-box | Brassica napus | 1383 | - | 6 | ATATAT | core promoter element around -30 of transcription start |
| TATA-box | Glycine max | 376 | - | 5 | TAATA | core promoter element around -30 of transcription start |
| TATA-box | Lycopersicon esculentum | 1028 | - | 5 | TTTTA | core promoter element around -30 of transcription start |
| TATA-box | Arabidopsis thaliana | 585 | - | 5 | TATAA | core promoter element around -30 of transcription start |
| TATA-box | Glycine max | 1122 | + | 5 | TAATA | core promoter element around -30 of transcription start |
| TATA-box | Brassica napus | 460 | + | 6 | ATATAT | core promoter element around -30 of transcription start |
| TATA-box | Lycopersicon esculentum | 912 | + | 5 | TTTTA | core promoter element around -30 of transcription start |
| TATA-box | Brassica oleracea | 739 | + | 6 | ATATAA | core promoter element around -30 of transcription start |
| TATA-box | Lycopersicon esculentum | 1195 | + | 5 | TTTTA | core promoter element around -30 of transcription start |
| TATA-box | Lycopersicon esculentum | 15 | + | 5 | TTTTA | core promoter element around -30 of transcription start |
| TATA-box | Brassica napus | 1077 | + | 6 | ATTATA | core promoter element around -30 of transcription start |
| TATA-box | Glycine max | 475 | - | 5 | TAATA | core promoter element around -30 of transcription start |
| TATA-box | Pisum sativum | 1171 | - | 8 | TATAAAAT | core promoter element around -30 of transcription start |
| TATA-box | Lycopersicon esculentum | 398 | - | 5 | TTTTA | core promoter element around -30 of transcription start |
| TATA-box | Glycine max | 877 | - | 5 | TAATA | core promoter element around -30 of transcription start |
| TATA-box | Lycopersicon esculentum | 487 | - | 5 | TTTTA | core promoter element around -30 of transcription start |
| TATA-box | Lycopersicon esculentum | 1269 | - | 5 | TTTTA | core promoter element around -30 of transcription start |
| TATA-box | Glycine max | 259 | + | 5 | TAATA | core promoter element around -30 of transcription start |
| TATA-box | Lycopersicon esculentum | 1047 | + | 5 | TTTTA | core promoter element around -30 of transcription start |
| TATA-box | Lycopersicon esculentum | 417 | + | 5 | TTTTA | core promoter element around -30 of transcription start |
| TATA-box | Brassica oleracea | 1152 | + | 6 | ATATAA | core promoter element around -30 of transcription start |
| TATA-box | Arabidopsis thaliana | 1265 | - | 4 | TATA | core promoter element around -30 of transcription start |
| TATA-box | Arabidopsis thaliana | 1175 | - | 4 | TATA | core promoter element around -30 of transcription start |
| TATA-box | Brassica napus | 989 | + | 6 | ATTATA | core promoter element around -30 of transcription start |
| TATA-box | Brassica oleracea | 1080 | + | 6 | ATATAA | core promoter element around -30 of transcription start |
| TATA-box | Arabidopsis thaliana | 760 | - | 6 | TATAAA | core promoter element around -30 of transcription start |
| TATA-box | Arabidopsis thaliana | 991 | - | 4 | TATA | core promoter element around -30 of transcription start |
| TATA-box | Glycine max | 1386 | - | 5 | TAATA | core promoter element around -30 of transcription start |
| TATA-box | Arabidopsis thaliana | 761 | - | 5 | TATAA | core promoter element around -30 of transcription start |
| TATA-box | Lycopersicon esculentum | 1089 | + | 5 | TTTTA | core promoter element around -30 of transcription start |
| TATA-box | Glycine max | 1011 | + | 5 | TAATA | core promoter element around -30 of transcription start |
| TATA-box | Lycopersicon esculentum | 1106 | - | 5 | TTTTA | core promoter element around -30 of transcription start |

> 2018/04/13 10:10:12  
+ CAGAAATTGT AAGTTTTTAT TGGTGTCACT ATAATTTAAT TTGTTTCCTT GACGATAGTA CTATTTGTAC   
  
  
+ GACATGAAAG TTTGGTCCGA TTCAACTAAC TAATTAATTG ATTAAGATAA ATTTAATTTG TTTCCTTGAC   
  
  
+ GATAGTACTA TTTGTACGAC ATGAAAGTTT GGTCCGATTC AACTAACTAA TTAATTGATT AAGATATCAA   
  
  
+ TGCGATCTTA ATCAAAGCAA GCTAGCCTAG CCTTAATCAT GAAGCCTCTA ATATTTTCTC ATTGCTTTGA   
  
  
+ TACTTTTTTT TTAAAATTTT ATTTAAAATT TAAAATTATA TTTTTACCTT GAAGTGGAGT CCAATGTTTT   
  
  
+ AAAGATGACA ATTCATATCA TAATTTATTA AATTAAATTT AATTATTTAA AAAATTAAAA ATTAAATTTT   
  
  
+ ATTGTATAAA AAAATATTTA AACCATTATT ATAAATAAAA TATATATTTT TAGATATTAT TTATTTTAAA   
  
  
+ ATTGTACTGT CAAAATTTTA AAATATTTAT AAAAAGTAAT TTACAAGTCA TATGAATGAA TTGAAAATAA   
  
  
+ AATGTAAAAT TTATTTACTT TATTTTATAA CAAAAAGTTT TTAAATTTTT ATAATCATTT GAACTAAATT   
  
  
+ TAATTAATAA ATACAAATTT GATTTCTTTT TCTAAAAATT AAATCTAATT CACTATTTTC AAATATTAAA   
  
  
+ ATTTAATTTA ATGCATGAAA CACTTTAAGA TCAAAATTAT ATAAAATTAT CATATTTACT TTATATTATC   
  
  
+ AATCAATTTT TTTGTTAAAA AGGTGACAAA TTGAATTTTG ATCTACTGGT TTAATATCAA ATTAAATCTT   
  
  
+ AATTAAAATA ATCATAACAA TTAAAAATAT TTTTGTTATT AAAATATTTA AACTATTTGA ATTATTTTTT   
  
  
+ ATTTTATTTC AAGTTCATTC TTTAAATGTA AATAATTTTT GATGCAAGAT CTGGTTTTTC TAAATTTTTA   
  
  
+ AAATTTTGAT TATATTTAAC AATACATTTT TAATACTCAA AAAATTTTAA AATTTTTATG ATAATATTTT   
  
  
+ AAATATTAAA TTTGAAATTA AAATTTATTA TATAAAATTT TTAATTTAAT TATTTTAAAA TTTAAATTTT   
  
  
+ TTAATAACAA TTAATTTAAT TTAATTTAAT AATATAAAAT AAATATTAAA ATTTTATATA ATTTTTATTC   
  
  
+ AAAATTTTAA ACACATTTTT TATTTTTTCT TGTGGCGGGC TTCCGAATCA GCTACCTTTT TGTAGAGGTA   
  
  
+ TCACTATATA AAATTTACGT GCCGTTTACG ACACCAAAGA TACCATTAGG ATAAGAAGCT GCCTCACAGA   
  
  
+ CAGGCCGCCT CCGCCACTGT TCTGAATTTC AACCACACTC AGTACTCCTC CTATATATTA CCACACAAAC   
  
  
+ CATATTCATT GATTCTTATT TATTCTTCTT GTCCTTCTTC GTCGTATGAT CCGACGCTCA CTTTTCTCTC   
  
  
+ AGACTTTCTC TCGTTTTCTC GGGAAAATA  

- GTCTTTAACA TTCAAAAATA ACCACAGTGA TATTAAATTA AACAAAGGAA CTGCTATCAT GATAAACATG   
  
  
- CTGTACTTTC AAACCAGGCT AAGTTGATTG ATTAATTAAC TAATTCTATT TAAATTAAAC AAAGGAACTG   
  
  
- CTATCATGAT AAACATGCTG TACTTTCAAA CCAGGCTAAG TTGATTGATT AATTAACTAA TTCTATAGTT   
  
  
- ACGCTAGAAT TAGTTTCGTT CGATCGGATC GGAATTAGTA CTTCGGAGAT TATAAAAGAG TAACGAAACT   
  
  
- ATGAAAAAAA AATTTTAAAA TAAATTTTAA ATTTTAATAT AAAAATGGAA CTTCACCTCA GGTTACAAAA   
  
  
- TTTCTACTGT TAAGTATAGT ATTAAATAAT TTAATTTAAA TTAATAAATT TTTTAATTTT TAATTTAAAA   
  
  
- TAACATATTT TTTTATAAAT TTGGTAATAA TATTTATTTT ATATATAAAA ATCTATAATA AATAAAATTT   
  
  
- TAACATGACA GTTTTAAAAT TTTATAAATA TTTTTCATTA AATGTTCAGT ATACTTACTT AACTTTTATT   
  
  
- TTACATTTTA AATAAATGAA ATAAAATATT GTTTTTCAAA AATTTAAAAA TATTAGTAAA CTTGATTTAA   
  
  
- ATTAATTATT TATGTTTAAA CTAAAGAAAA AGATTTTTAA TTTAGATTAA GTGATAAAAG TTTATAATTT   
  
  
- TAAATTAAAT TACGTACTTT GTGAAATTCT AGTTTTAATA TATTTTAATA GTATAAATGA AATATAATAG   
  
  
- TTAGTTAAAA AAACAATTTT TCCACTGTTT AACTTAAAAC TAGATGACCA AATTATAGTT TAATTTAGAA   
  
  
- TTAATTTTAT TAGTATTGTT AATTTTTATA AAAACAATAA TTTTATAAAT TTGATAAACT TAATAAAAAA   
  
  
- TAAAATAAAG TTCAAGTAAG AAATTTACAT TTATTAAAAA CTACGTTCTA GACCAAAAAG ATTTAAAAAT   
  
  
- TTTAAAACTA ATATAAATTG TTATGTAAAA ATTATGAGTT TTTTAAAATT TTAAAAATAC TATTATAAAA   
  
  
- TTTATAATTT AAACTTTAAT TTTAAATAAT ATATTTTAAA AATTAAATTA ATAAAATTTT AAATTTAAAA   
  
  
- AATTATTGTT AATTAAATTA AATTAAATTA TTATATTTTA TTTATAATTT TAAAATATAT TAAAAATAAG   
  
  
- TTTTAAAATT TGTGTAAAAA ATAAAAAAGA ACACCGCCCG AAGGCTTAGT CGATGGAAAA ACATCTCCAT   
  
  
- AGTGATATAT TTTAAATGCA CGGCAAATGC TGTGGTTTCT ATGGTAATCC TATTCTTCGA CGGAGTGTCT   
  
  
- GTCCGGCGGA GGCGGTGACA AGACTTAAAG TTGGTGTGAG TCATGAGGAG GATATATAAT GGTGTGTTTG   
  
  
- GTATAAGTAA CTAAGAATAA ATAAGAAGAA CAGGAAGAAG CAGCATACTA GGCTGCGAGT GAAAAGAGAG   
  
  
- TCTGAAAGAG AGCAAAAGAG CCCTTTTAT

+     TCA-element

| Site Name | Organism | Position | Strand | Matrix score. | sequence | function |
| --- | --- | --- | --- | --- | --- | --- |
| TCA-element | Brassica oleracea | 1421 | - | 9 | GAGAAGAATA | cis-acting element involved in salicylic acid responsiveness |

> 2018/04/13 10:10:12  
+ CAGAAATTGT AAGTTTTTAT TGGTGTCACT ATAATTTAAT TTGTTTCCTT GACGATAGTA CTATTTGTAC   
  
  
+ GACATGAAAG TTTGGTCCGA TTCAACTAAC TAATTAATTG ATTAAGATAA ATTTAATTTG TTTCCTTGAC   
  
  
+ GATAGTACTA TTTGTACGAC ATGAAAGTTT GGTCCGATTC AACTAACTAA TTAATTGATT AAGATATCAA   
  
  
+ TGCGATCTTA ATCAAAGCAA GCTAGCCTAG CCTTAATCAT GAAGCCTCTA ATATTTTCTC ATTGCTTTGA   
  
  
+ TACTTTTTTT TTAAAATTTT ATTTAAAATT TAAAATTATA TTTTTACCTT GAAGTGGAGT CCAATGTTTT   
  
  
+ AAAGATGACA ATTCATATCA TAATTTATTA AATTAAATTT AATTATTTAA AAAATTAAAA ATTAAATTTT   
  
  
+ ATTGTATAAA AAAATATTTA AACCATTATT ATAAATAAAA TATATATTTT TAGATATTAT TTATTTTAAA   
  
  
+ ATTGTACTGT CAAAATTTTA AAATATTTAT AAAAAGTAAT TTACAAGTCA TATGAATGAA TTGAAAATAA   
  
  
+ AATGTAAAAT TTATTTACTT TATTTTATAA CAAAAAGTTT TTAAATTTTT ATAATCATTT GAACTAAATT   
  
  
+ TAATTAATAA ATACAAATTT GATTTCTTTT TCTAAAAATT AAATCTAATT CACTATTTTC AAATATTAAA   
  
  
+ ATTTAATTTA ATGCATGAAA CACTTTAAGA TCAAAATTAT ATAAAATTAT CATATTTACT TTATATTATC   
  
  
+ AATCAATTTT TTTGTTAAAA AGGTGACAAA TTGAATTTTG ATCTACTGGT TTAATATCAA ATTAAATCTT   
  
  
+ AATTAAAATA ATCATAACAA TTAAAAATAT TTTTGTTATT AAAATATTTA AACTATTTGA ATTATTTTTT   
  
  
+ ATTTTATTTC AAGTTCATTC TTTAAATGTA AATAATTTTT GATGCAAGAT CTGGTTTTTC TAAATTTTTA   
  
  
+ AAATTTTGAT TATATTTAAC AATACATTTT TAATACTCAA AAAATTTTAA AATTTTTATG ATAATATTTT   
  
  
+ AAATATTAAA TTTGAAATTA AAATTTATTA TATAAAATTT TTAATTTAAT TATTTTAAAA TTTAAATTTT   
  
  
+ TTAATAACAA TTAATTTAAT TTAATTTAAT AATATAAAAT AAATATTAAA ATTTTATATA ATTTTTATTC   
  
  
+ AAAATTTTAA ACACATTTTT TATTTTTTCT TGTGGCGGGC TTCCGAATCA GCTACCTTTT TGTAGAGGTA   
  
  
+ TCACTATATA AAATTTACGT GCCGTTTACG ACACCAAAGA TACCATTAGG ATAAGAAGCT GCCTCACAGA   
  
  
+ CAGGCCGCCT CCGCCACTGT TCTGAATTTC AACCACACTC AGTACTCCTC CTATATATTA CCACACAAAC   
  
  
+ CATATTCATT GATTCTTATT TATTCTTCTT GTCCTTCTTC GTCGTATGAT CCGACGCTCA CTTTTCTCTC   
  
  
+ AGACTTTCTC TCGTTTTCTC GGGAAAATA  

- GTCTTTAACA TTCAAAAATA ACCACAGTGA TATTAAATTA AACAAAGGAA CTGCTATCAT GATAAACATG   
  
  
- CTGTACTTTC AAACCAGGCT AAGTTGATTG ATTAATTAAC TAATTCTATT TAAATTAAAC AAAGGAACTG   
  
  
- CTATCATGAT AAACATGCTG TACTTTCAAA CCAGGCTAAG TTGATTGATT AATTAACTAA TTCTATAGTT   
  
  
- ACGCTAGAAT TAGTTTCGTT CGATCGGATC GGAATTAGTA CTTCGGAGAT TATAAAAGAG TAACGAAACT   
  
  
- ATGAAAAAAA AATTTTAAAA TAAATTTTAA ATTTTAATAT AAAAATGGAA CTTCACCTCA GGTTACAAAA   
  
  
- TTTCTACTGT TAAGTATAGT ATTAAATAAT TTAATTTAAA TTAATAAATT TTTTAATTTT TAATTTAAAA   
  
  
- TAACATATTT TTTTATAAAT TTGGTAATAA TATTTATTTT ATATATAAAA ATCTATAATA AATAAAATTT   
  
  
- TAACATGACA GTTTTAAAAT TTTATAAATA TTTTTCATTA AATGTTCAGT ATACTTACTT AACTTTTATT   
  
  
- TTACATTTTA AATAAATGAA ATAAAATATT GTTTTTCAAA AATTTAAAAA TATTAGTAAA CTTGATTTAA   
  
  
- ATTAATTATT TATGTTTAAA CTAAAGAAAA AGATTTTTAA TTTAGATTAA GTGATAAAAG TTTATAATTT   
  
  
- TAAATTAAAT TACGTACTTT GTGAAATTCT AGTTTTAATA TATTTTAATA GTATAAATGA AATATAATAG   
  
  
- TTAGTTAAAA AAACAATTTT TCCACTGTTT AACTTAAAAC TAGATGACCA AATTATAGTT TAATTTAGAA   
  
  
- TTAATTTTAT TAGTATTGTT AATTTTTATA AAAACAATAA TTTTATAAAT TTGATAAACT TAATAAAAAA   
  
  
- TAAAATAAAG TTCAAGTAAG AAATTTACAT TTATTAAAAA CTACGTTCTA GACCAAAAAG ATTTAAAAAT   
  
  
- TTTAAAACTA ATATAAATTG TTATGTAAAA ATTATGAGTT TTTTAAAATT TTAAAAATAC TATTATAAAA   
  
  
- TTTATAATTT AAACTTTAAT TTTAAATAAT ATATTTTAAA AATTAAATTA ATAAAATTTT AAATTTAAAA   
  
  
- AATTATTGTT AATTAAATTA AATTAAATTA TTATATTTTA TTTATAATTT TAAAATATAT TAAAAATAAG   
  
  
- TTTTAAAATT TGTGTAAAAA ATAAAAAAGA ACACCGCCCG AAGGCTTAGT CGATGGAAAA ACATCTCCAT   
  
  
- AGTGATATAT TTTAAATGCA CGGCAAATGC TGTGGTTTCT ATGGTAATCC TATTCTTCGA CGGAGTGTCT   
  
  
- GTCCGGCGGA GGCGGTGACA AGACTTAAAG TTGGTGTGAG TCATGAGGAG GATATATAAT GGTGTGTTTG   
  
  
- GTATAAGTAA CTAAGAATAA ATAAGAAGAA CAGGAAGAAG CAGCATACTA GGCTGCGAGT GAAAAGAGAG   
  
  
- TCTGAAAGAG AGCAAAAGAG CCCTTTTAT

+     TGACG-motif

| Site Name | Organism | Position | Strand | Matrix score. | sequence | function |
| --- | --- | --- | --- | --- | --- | --- |
| TGACG-motif | Hordeum vulgare | 50 | + | 5 | TGACG | cis-acting regulatory element involved in the MeJA-responsiveness |
| TGACG-motif | Hordeum vulgare | 137 | + | 5 | TGACG | cis-acting regulatory element involved in the MeJA-responsiveness |

> 2018/04/13 10:10:12  
+ CAGAAATTGT AAGTTTTTAT TGGTGTCACT ATAATTTAAT TTGTTTCCTT GACGATAGTA CTATTTGTAC   
  
  
+ GACATGAAAG TTTGGTCCGA TTCAACTAAC TAATTAATTG ATTAAGATAA ATTTAATTTG TTTCCTTGAC   
  
  
+ GATAGTACTA TTTGTACGAC ATGAAAGTTT GGTCCGATTC AACTAACTAA TTAATTGATT AAGATATCAA   
  
  
+ TGCGATCTTA ATCAAAGCAA GCTAGCCTAG CCTTAATCAT GAAGCCTCTA ATATTTTCTC ATTGCTTTGA   
  
  
+ TACTTTTTTT TTAAAATTTT ATTTAAAATT TAAAATTATA TTTTTACCTT GAAGTGGAGT CCAATGTTTT   
  
  
+ AAAGATGACA ATTCATATCA TAATTTATTA AATTAAATTT AATTATTTAA AAAATTAAAA ATTAAATTTT   
  
  
+ ATTGTATAAA AAAATATTTA AACCATTATT ATAAATAAAA TATATATTTT TAGATATTAT TTATTTTAAA   
  
  
+ ATTGTACTGT CAAAATTTTA AAATATTTAT AAAAAGTAAT TTACAAGTCA TATGAATGAA TTGAAAATAA   
  
  
+ AATGTAAAAT TTATTTACTT TATTTTATAA CAAAAAGTTT TTAAATTTTT ATAATCATTT GAACTAAATT   
  
  
+ TAATTAATAA ATACAAATTT GATTTCTTTT TCTAAAAATT AAATCTAATT CACTATTTTC AAATATTAAA   
  
  
+ ATTTAATTTA ATGCATGAAA CACTTTAAGA TCAAAATTAT ATAAAATTAT CATATTTACT TTATATTATC   
  
  
+ AATCAATTTT TTTGTTAAAA AGGTGACAAA TTGAATTTTG ATCTACTGGT TTAATATCAA ATTAAATCTT   
  
  
+ AATTAAAATA ATCATAACAA TTAAAAATAT TTTTGTTATT AAAATATTTA AACTATTTGA ATTATTTTTT   
  
  
+ ATTTTATTTC AAGTTCATTC TTTAAATGTA AATAATTTTT GATGCAAGAT CTGGTTTTTC TAAATTTTTA   
  
  
+ AAATTTTGAT TATATTTAAC AATACATTTT TAATACTCAA AAAATTTTAA AATTTTTATG ATAATATTTT   
  
  
+ AAATATTAAA TTTGAAATTA AAATTTATTA TATAAAATTT TTAATTTAAT TATTTTAAAA TTTAAATTTT   
  
  
+ TTAATAACAA TTAATTTAAT TTAATTTAAT AATATAAAAT AAATATTAAA ATTTTATATA ATTTTTATTC   
  
  
+ AAAATTTTAA ACACATTTTT TATTTTTTCT TGTGGCGGGC TTCCGAATCA GCTACCTTTT TGTAGAGGTA   
  
  
+ TCACTATATA AAATTTACGT GCCGTTTACG ACACCAAAGA TACCATTAGG ATAAGAAGCT GCCTCACAGA   
  
  
+ CAGGCCGCCT CCGCCACTGT TCTGAATTTC AACCACACTC AGTACTCCTC CTATATATTA CCACACAAAC   
  
  
+ CATATTCATT GATTCTTATT TATTCTTCTT GTCCTTCTTC GTCGTATGAT CCGACGCTCA CTTTTCTCTC   
  
  
+ AGACTTTCTC TCGTTTTCTC GGGAAAATA  

- GTCTTTAACA TTCAAAAATA ACCACAGTGA TATTAAATTA AACAAAGGAA CTGCTATCAT GATAAACATG   
  
  
- CTGTACTTTC AAACCAGGCT AAGTTGATTG ATTAATTAAC TAATTCTATT TAAATTAAAC AAAGGAACTG   
  
  
- CTATCATGAT AAACATGCTG TACTTTCAAA CCAGGCTAAG TTGATTGATT AATTAACTAA TTCTATAGTT   
  
  
- ACGCTAGAAT TAGTTTCGTT CGATCGGATC GGAATTAGTA CTTCGGAGAT TATAAAAGAG TAACGAAACT   
  
  
- ATGAAAAAAA AATTTTAAAA TAAATTTTAA ATTTTAATAT AAAAATGGAA CTTCACCTCA GGTTACAAAA   
  
  
- TTTCTACTGT TAAGTATAGT ATTAAATAAT TTAATTTAAA TTAATAAATT TTTTAATTTT TAATTTAAAA   
  
  
- TAACATATTT TTTTATAAAT TTGGTAATAA TATTTATTTT ATATATAAAA ATCTATAATA AATAAAATTT   
  
  
- TAACATGACA GTTTTAAAAT TTTATAAATA TTTTTCATTA AATGTTCAGT ATACTTACTT AACTTTTATT   
  
  
- TTACATTTTA AATAAATGAA ATAAAATATT GTTTTTCAAA AATTTAAAAA TATTAGTAAA CTTGATTTAA   
  
  
- ATTAATTATT TATGTTTAAA CTAAAGAAAA AGATTTTTAA TTTAGATTAA GTGATAAAAG TTTATAATTT   
  
  
- TAAATTAAAT TACGTACTTT GTGAAATTCT AGTTTTAATA TATTTTAATA GTATAAATGA AATATAATAG   
  
  
- TTAGTTAAAA AAACAATTTT TCCACTGTTT AACTTAAAAC TAGATGACCA AATTATAGTT TAATTTAGAA   
  
  
- TTAATTTTAT TAGTATTGTT AATTTTTATA AAAACAATAA TTTTATAAAT TTGATAAACT TAATAAAAAA   
  
  
- TAAAATAAAG TTCAAGTAAG AAATTTACAT TTATTAAAAA CTACGTTCTA GACCAAAAAG ATTTAAAAAT   
  
  
- TTTAAAACTA ATATAAATTG TTATGTAAAA ATTATGAGTT TTTTAAAATT TTAAAAATAC TATTATAAAA   
  
  
- TTTATAATTT AAACTTTAAT TTTAAATAAT ATATTTTAAA AATTAAATTA ATAAAATTTT AAATTTAAAA   
  
  
- AATTATTGTT AATTAAATTA AATTAAATTA TTATATTTTA TTTATAATTT TAAAATATAT TAAAAATAAG   
  
  
- TTTTAAAATT TGTGTAAAAA ATAAAAAAGA ACACCGCCCG AAGGCTTAGT CGATGGAAAA ACATCTCCAT   
  
  
- AGTGATATAT TTTAAATGCA CGGCAAATGC TGTGGTTTCT ATGGTAATCC TATTCTTCGA CGGAGTGTCT   
  
  
- GTCCGGCGGA GGCGGTGACA AGACTTAAAG TTGGTGTGAG TCATGAGGAG GATATATAAT GGTGTGTTTG   
  
  
- GTATAAGTAA CTAAGAATAA ATAAGAAGAA CAGGAAGAAG CAGCATACTA GGCTGCGAGT GAAAAGAGAG   
  
  
- TCTGAAAGAG AGCAAAAGAG CCCTTTTAT

+     Unnamed\_\_1

| Site Name | Organism | Position | Strand | Matrix score. | sequence | function |
| --- | --- | --- | --- | --- | --- | --- |
| Unnamed\_\_1 | Glycine max | 626 | + | 11 | GAATTTAATTAA | 60K protein binding site |

> 2018/04/13 10:10:12  
+ CAGAAATTGT AAGTTTTTAT TGGTGTCACT ATAATTTAAT TTGTTTCCTT GACGATAGTA CTATTTGTAC   
  
  
+ GACATGAAAG TTTGGTCCGA TTCAACTAAC TAATTAATTG ATTAAGATAA ATTTAATTTG TTTCCTTGAC   
  
  
+ GATAGTACTA TTTGTACGAC ATGAAAGTTT GGTCCGATTC AACTAACTAA TTAATTGATT AAGATATCAA   
  
  
+ TGCGATCTTA ATCAAAGCAA GCTAGCCTAG CCTTAATCAT GAAGCCTCTA ATATTTTCTC ATTGCTTTGA   
  
  
+ TACTTTTTTT TTAAAATTTT ATTTAAAATT TAAAATTATA TTTTTACCTT GAAGTGGAGT CCAATGTTTT   
  
  
+ AAAGATGACA ATTCATATCA TAATTTATTA AATTAAATTT AATTATTTAA AAAATTAAAA ATTAAATTTT   
  
  
+ ATTGTATAAA AAAATATTTA AACCATTATT ATAAATAAAA TATATATTTT TAGATATTAT TTATTTTAAA   
  
  
+ ATTGTACTGT CAAAATTTTA AAATATTTAT AAAAAGTAAT TTACAAGTCA TATGAATGAA TTGAAAATAA   
  
  
+ AATGTAAAAT TTATTTACTT TATTTTATAA CAAAAAGTTT TTAAATTTTT ATAATCATTT GAACTAAATT   
  
  
+ TAATTAATAA ATACAAATTT GATTTCTTTT TCTAAAAATT AAATCTAATT CACTATTTTC AAATATTAAA   
  
  
+ ATTTAATTTA ATGCATGAAA CACTTTAAGA TCAAAATTAT ATAAAATTAT CATATTTACT TTATATTATC   
  
  
+ AATCAATTTT TTTGTTAAAA AGGTGACAAA TTGAATTTTG ATCTACTGGT TTAATATCAA ATTAAATCTT   
  
  
+ AATTAAAATA ATCATAACAA TTAAAAATAT TTTTGTTATT AAAATATTTA AACTATTTGA ATTATTTTTT   
  
  
+ ATTTTATTTC AAGTTCATTC TTTAAATGTA AATAATTTTT GATGCAAGAT CTGGTTTTTC TAAATTTTTA   
  
  
+ AAATTTTGAT TATATTTAAC AATACATTTT TAATACTCAA AAAATTTTAA AATTTTTATG ATAATATTTT   
  
  
+ AAATATTAAA TTTGAAATTA AAATTTATTA TATAAAATTT TTAATTTAAT TATTTTAAAA TTTAAATTTT   
  
  
+ TTAATAACAA TTAATTTAAT TTAATTTAAT AATATAAAAT AAATATTAAA ATTTTATATA ATTTTTATTC   
  
  
+ AAAATTTTAA ACACATTTTT TATTTTTTCT TGTGGCGGGC TTCCGAATCA GCTACCTTTT TGTAGAGGTA   
  
  
+ TCACTATATA AAATTTACGT GCCGTTTACG ACACCAAAGA TACCATTAGG ATAAGAAGCT GCCTCACAGA   
  
  
+ CAGGCCGCCT CCGCCACTGT TCTGAATTTC AACCACACTC AGTACTCCTC CTATATATTA CCACACAAAC   
  
  
+ CATATTCATT GATTCTTATT TATTCTTCTT GTCCTTCTTC GTCGTATGAT CCGACGCTCA CTTTTCTCTC   
  
  
+ AGACTTTCTC TCGTTTTCTC GGGAAAATA  

- GTCTTTAACA TTCAAAAATA ACCACAGTGA TATTAAATTA AACAAAGGAA CTGCTATCAT GATAAACATG   
  
  
- CTGTACTTTC AAACCAGGCT AAGTTGATTG ATTAATTAAC TAATTCTATT TAAATTAAAC AAAGGAACTG   
  
  
- CTATCATGAT AAACATGCTG TACTTTCAAA CCAGGCTAAG TTGATTGATT AATTAACTAA TTCTATAGTT   
  
  
- ACGCTAGAAT TAGTTTCGTT CGATCGGATC GGAATTAGTA CTTCGGAGAT TATAAAAGAG TAACGAAACT   
  
  
- ATGAAAAAAA AATTTTAAAA TAAATTTTAA ATTTTAATAT AAAAATGGAA CTTCACCTCA GGTTACAAAA   
  
  
- TTTCTACTGT TAAGTATAGT ATTAAATAAT TTAATTTAAA TTAATAAATT TTTTAATTTT TAATTTAAAA   
  
  
- TAACATATTT TTTTATAAAT TTGGTAATAA TATTTATTTT ATATATAAAA ATCTATAATA AATAAAATTT   
  
  
- TAACATGACA GTTTTAAAAT TTTATAAATA TTTTTCATTA AATGTTCAGT ATACTTACTT AACTTTTATT   
  
  
- TTACATTTTA AATAAATGAA ATAAAATATT GTTTTTCAAA AATTTAAAAA TATTAGTAAA CTTGATTTAA   
  
  
- ATTAATTATT TATGTTTAAA CTAAAGAAAA AGATTTTTAA TTTAGATTAA GTGATAAAAG TTTATAATTT   
  
  
- TAAATTAAAT TACGTACTTT GTGAAATTCT AGTTTTAATA TATTTTAATA GTATAAATGA AATATAATAG   
  
  
- TTAGTTAAAA AAACAATTTT TCCACTGTTT AACTTAAAAC TAGATGACCA AATTATAGTT TAATTTAGAA   
  
  
- TTAATTTTAT TAGTATTGTT AATTTTTATA AAAACAATAA TTTTATAAAT TTGATAAACT TAATAAAAAA   
  
  
- TAAAATAAAG TTCAAGTAAG AAATTTACAT TTATTAAAAA CTACGTTCTA GACCAAAAAG ATTTAAAAAT   
  
  
- TTTAAAACTA ATATAAATTG TTATGTAAAA ATTATGAGTT TTTTAAAATT TTAAAAATAC TATTATAAAA   
  
  
- TTTATAATTT AAACTTTAAT TTTAAATAAT ATATTTTAAA AATTAAATTA ATAAAATTTT AAATTTAAAA   
  
  
- AATTATTGTT AATTAAATTA AATTAAATTA TTATATTTTA TTTATAATTT TAAAATATAT TAAAAATAAG   
  
  
- TTTTAAAATT TGTGTAAAAA ATAAAAAAGA ACACCGCCCG AAGGCTTAGT CGATGGAAAA ACATCTCCAT   
  
  
- AGTGATATAT TTTAAATGCA CGGCAAATGC TGTGGTTTCT ATGGTAATCC TATTCTTCGA CGGAGTGTCT   
  
  
- GTCCGGCGGA GGCGGTGACA AGACTTAAAG TTGGTGTGAG TCATGAGGAG GATATATAAT GGTGTGTTTG   
  
  
- GTATAAGTAA CTAAGAATAA ATAAGAAGAA CAGGAAGAAG CAGCATACTA GGCTGCGAGT GAAAAGAGAG   
  
  
- TCTGAAAGAG AGCAAAAGAG CCCTTTTAT

+     Unnamed\_\_2

| Site Name | Organism | Position | Strand | Matrix score. | sequence | function |
| --- | --- | --- | --- | --- | --- | --- |
| Unnamed\_\_2 | Glycine max | 1055 | + | 14 | ATTAAATTTTAAATT |  |

> 2018/04/13 10:10:12  
+ CAGAAATTGT AAGTTTTTAT TGGTGTCACT ATAATTTAAT TTGTTTCCTT GACGATAGTA CTATTTGTAC   
  
  
+ GACATGAAAG TTTGGTCCGA TTCAACTAAC TAATTAATTG ATTAAGATAA ATTTAATTTG TTTCCTTGAC   
  
  
+ GATAGTACTA TTTGTACGAC ATGAAAGTTT GGTCCGATTC AACTAACTAA TTAATTGATT AAGATATCAA   
  
  
+ TGCGATCTTA ATCAAAGCAA GCTAGCCTAG CCTTAATCAT GAAGCCTCTA ATATTTTCTC ATTGCTTTGA   
  
  
+ TACTTTTTTT TTAAAATTTT ATTTAAAATT TAAAATTATA TTTTTACCTT GAAGTGGAGT CCAATGTTTT   
  
  
+ AAAGATGACA ATTCATATCA TAATTTATTA AATTAAATTT AATTATTTAA AAAATTAAAA ATTAAATTTT   
  
  
+ ATTGTATAAA AAAATATTTA AACCATTATT ATAAATAAAA TATATATTTT TAGATATTAT TTATTTTAAA   
  
  
+ ATTGTACTGT CAAAATTTTA AAATATTTAT AAAAAGTAAT TTACAAGTCA TATGAATGAA TTGAAAATAA   
  
  
+ AATGTAAAAT TTATTTACTT TATTTTATAA CAAAAAGTTT TTAAATTTTT ATAATCATTT GAACTAAATT   
  
  
+ TAATTAATAA ATACAAATTT GATTTCTTTT TCTAAAAATT AAATCTAATT CACTATTTTC AAATATTAAA   
  
  
+ ATTTAATTTA ATGCATGAAA CACTTTAAGA TCAAAATTAT ATAAAATTAT CATATTTACT TTATATTATC   
  
  
+ AATCAATTTT TTTGTTAAAA AGGTGACAAA TTGAATTTTG ATCTACTGGT TTAATATCAA ATTAAATCTT   
  
  
+ AATTAAAATA ATCATAACAA TTAAAAATAT TTTTGTTATT AAAATATTTA AACTATTTGA ATTATTTTTT   
  
  
+ ATTTTATTTC AAGTTCATTC TTTAAATGTA AATAATTTTT GATGCAAGAT CTGGTTTTTC TAAATTTTTA   
  
  
+ AAATTTTGAT TATATTTAAC AATACATTTT TAATACTCAA AAAATTTTAA AATTTTTATG ATAATATTTT   
  
  
+ AAATATTAAA TTTGAAATTA AAATTTATTA TATAAAATTT TTAATTTAAT TATTTTAAAA TTTAAATTTT   
  
  
+ TTAATAACAA TTAATTTAAT TTAATTTAAT AATATAAAAT AAATATTAAA ATTTTATATA ATTTTTATTC   
  
  
+ AAAATTTTAA ACACATTTTT TATTTTTTCT TGTGGCGGGC TTCCGAATCA GCTACCTTTT TGTAGAGGTA   
  
  
+ TCACTATATA AAATTTACGT GCCGTTTACG ACACCAAAGA TACCATTAGG ATAAGAAGCT GCCTCACAGA   
  
  
+ CAGGCCGCCT CCGCCACTGT TCTGAATTTC AACCACACTC AGTACTCCTC CTATATATTA CCACACAAAC   
  
  
+ CATATTCATT GATTCTTATT TATTCTTCTT GTCCTTCTTC GTCGTATGAT CCGACGCTCA CTTTTCTCTC   
  
  
+ AGACTTTCTC TCGTTTTCTC GGGAAAATA  

- GTCTTTAACA TTCAAAAATA ACCACAGTGA TATTAAATTA AACAAAGGAA CTGCTATCAT GATAAACATG   
  
  
- CTGTACTTTC AAACCAGGCT AAGTTGATTG ATTAATTAAC TAATTCTATT TAAATTAAAC AAAGGAACTG   
  
  
- CTATCATGAT AAACATGCTG TACTTTCAAA CCAGGCTAAG TTGATTGATT AATTAACTAA TTCTATAGTT   
  
  
- ACGCTAGAAT TAGTTTCGTT CGATCGGATC GGAATTAGTA CTTCGGAGAT TATAAAAGAG TAACGAAACT   
  
  
- ATGAAAAAAA AATTTTAAAA TAAATTTTAA ATTTTAATAT AAAAATGGAA CTTCACCTCA GGTTACAAAA   
  
  
- TTTCTACTGT TAAGTATAGT ATTAAATAAT TTAATTTAAA TTAATAAATT TTTTAATTTT TAATTTAAAA   
  
  
- TAACATATTT TTTTATAAAT TTGGTAATAA TATTTATTTT ATATATAAAA ATCTATAATA AATAAAATTT   
  
  
- TAACATGACA GTTTTAAAAT TTTATAAATA TTTTTCATTA AATGTTCAGT ATACTTACTT AACTTTTATT   
  
  
- TTACATTTTA AATAAATGAA ATAAAATATT GTTTTTCAAA AATTTAAAAA TATTAGTAAA CTTGATTTAA   
  
  
- ATTAATTATT TATGTTTAAA CTAAAGAAAA AGATTTTTAA TTTAGATTAA GTGATAAAAG TTTATAATTT   
  
  
- TAAATTAAAT TACGTACTTT GTGAAATTCT AGTTTTAATA TATTTTAATA GTATAAATGA AATATAATAG   
  
  
- TTAGTTAAAA AAACAATTTT TCCACTGTTT AACTTAAAAC TAGATGACCA AATTATAGTT TAATTTAGAA   
  
  
- TTAATTTTAT TAGTATTGTT AATTTTTATA AAAACAATAA TTTTATAAAT TTGATAAACT TAATAAAAAA   
  
  
- TAAAATAAAG TTCAAGTAAG AAATTTACAT TTATTAAAAA CTACGTTCTA GACCAAAAAG ATTTAAAAAT   
  
  
- TTTAAAACTA ATATAAATTG TTATGTAAAA ATTATGAGTT TTTTAAAATT TTAAAAATAC TATTATAAAA   
  
  
- TTTATAATTT AAACTTTAAT TTTAAATAAT ATATTTTAAA AATTAAATTA ATAAAATTTT AAATTTAAAA   
  
  
- AATTATTGTT AATTAAATTA AATTAAATTA TTATATTTTA TTTATAATTT TAAAATATAT TAAAAATAAG   
  
  
- TTTTAAAATT TGTGTAAAAA ATAAAAAAGA ACACCGCCCG AAGGCTTAGT CGATGGAAAA ACATCTCCAT   
  
  
- AGTGATATAT TTTAAATGCA CGGCAAATGC TGTGGTTTCT ATGGTAATCC TATTCTTCGA CGGAGTGTCT   
  
  
- GTCCGGCGGA GGCGGTGACA AGACTTAAAG TTGGTGTGAG TCATGAGGAG GATATATAAT GGTGTGTTTG   
  
  
- GTATAAGTAA CTAAGAATAA ATAAGAAGAA CAGGAAGAAG CAGCATACTA GGCTGCGAGT GAAAAGAGAG   
  
  
- TCTGAAAGAG AGCAAAAGAG CCCTTTTAT

+     Unnamed\_\_4

| Site Name | Organism | Position | Strand | Matrix score. | sequence | function |
| --- | --- | --- | --- | --- | --- | --- |
| Unnamed\_\_4 | Petroselinum hortense | 336 | - | 4 | CTCC |  |
| Unnamed\_\_4 | Petroselinum hortense | 1375 | + | 4 | CTCC |  |
| Unnamed\_\_4 | Petroselinum hortense | 1339 | + | 4 | CTCC |  |
| Unnamed\_\_4 | Petroselinum hortense | 1378 | + | 4 | CTCC |  |

> 2018/04/13 10:10:12  
+ CAGAAATTGT AAGTTTTTAT TGGTGTCACT ATAATTTAAT TTGTTTCCTT GACGATAGTA CTATTTGTAC   
  
  
+ GACATGAAAG TTTGGTCCGA TTCAACTAAC TAATTAATTG ATTAAGATAA ATTTAATTTG TTTCCTTGAC   
  
  
+ GATAGTACTA TTTGTACGAC ATGAAAGTTT GGTCCGATTC AACTAACTAA TTAATTGATT AAGATATCAA   
  
  
+ TGCGATCTTA ATCAAAGCAA GCTAGCCTAG CCTTAATCAT GAAGCCTCTA ATATTTTCTC ATTGCTTTGA   
  
  
+ TACTTTTTTT TTAAAATTTT ATTTAAAATT TAAAATTATA TTTTTACCTT GAAGTGGAGT CCAATGTTTT   
  
  
+ AAAGATGACA ATTCATATCA TAATTTATTA AATTAAATTT AATTATTTAA AAAATTAAAA ATTAAATTTT   
  
  
+ ATTGTATAAA AAAATATTTA AACCATTATT ATAAATAAAA TATATATTTT TAGATATTAT TTATTTTAAA   
  
  
+ ATTGTACTGT CAAAATTTTA AAATATTTAT AAAAAGTAAT TTACAAGTCA TATGAATGAA TTGAAAATAA   
  
  
+ AATGTAAAAT TTATTTACTT TATTTTATAA CAAAAAGTTT TTAAATTTTT ATAATCATTT GAACTAAATT   
  
  
+ TAATTAATAA ATACAAATTT GATTTCTTTT TCTAAAAATT AAATCTAATT CACTATTTTC AAATATTAAA   
  
  
+ ATTTAATTTA ATGCATGAAA CACTTTAAGA TCAAAATTAT ATAAAATTAT CATATTTACT TTATATTATC   
  
  
+ AATCAATTTT TTTGTTAAAA AGGTGACAAA TTGAATTTTG ATCTACTGGT TTAATATCAA ATTAAATCTT   
  
  
+ AATTAAAATA ATCATAACAA TTAAAAATAT TTTTGTTATT AAAATATTTA AACTATTTGA ATTATTTTTT   
  
  
+ ATTTTATTTC AAGTTCATTC TTTAAATGTA AATAATTTTT GATGCAAGAT CTGGTTTTTC TAAATTTTTA   
  
  
+ AAATTTTGAT TATATTTAAC AATACATTTT TAATACTCAA AAAATTTTAA AATTTTTATG ATAATATTTT   
  
  
+ AAATATTAAA TTTGAAATTA AAATTTATTA TATAAAATTT TTAATTTAAT TATTTTAAAA TTTAAATTTT   
  
  
+ TTAATAACAA TTAATTTAAT TTAATTTAAT AATATAAAAT AAATATTAAA ATTTTATATA ATTTTTATTC   
  
  
+ AAAATTTTAA ACACATTTTT TATTTTTTCT TGTGGCGGGC TTCCGAATCA GCTACCTTTT TGTAGAGGTA   
  
  
+ TCACTATATA AAATTTACGT GCCGTTTACG ACACCAAAGA TACCATTAGG ATAAGAAGCT GCCTCACAGA   
  
  
+ CAGGCCGCCT CCGCCACTGT TCTGAATTTC AACCACACTC AGTACTCCTC CTATATATTA CCACACAAAC   
  
  
+ CATATTCATT GATTCTTATT TATTCTTCTT GTCCTTCTTC GTCGTATGAT CCGACGCTCA CTTTTCTCTC   
  
  
+ AGACTTTCTC TCGTTTTCTC GGGAAAATA  

- GTCTTTAACA TTCAAAAATA ACCACAGTGA TATTAAATTA AACAAAGGAA CTGCTATCAT GATAAACATG   
  
  
- CTGTACTTTC AAACCAGGCT AAGTTGATTG ATTAATTAAC TAATTCTATT TAAATTAAAC AAAGGAACTG   
  
  
- CTATCATGAT AAACATGCTG TACTTTCAAA CCAGGCTAAG TTGATTGATT AATTAACTAA TTCTATAGTT   
  
  
- ACGCTAGAAT TAGTTTCGTT CGATCGGATC GGAATTAGTA CTTCGGAGAT TATAAAAGAG TAACGAAACT   
  
  
- ATGAAAAAAA AATTTTAAAA TAAATTTTAA ATTTTAATAT AAAAATGGAA CTTCACCTCA GGTTACAAAA   
  
  
- TTTCTACTGT TAAGTATAGT ATTAAATAAT TTAATTTAAA TTAATAAATT TTTTAATTTT TAATTTAAAA   
  
  
- TAACATATTT TTTTATAAAT TTGGTAATAA TATTTATTTT ATATATAAAA ATCTATAATA AATAAAATTT   
  
  
- TAACATGACA GTTTTAAAAT TTTATAAATA TTTTTCATTA AATGTTCAGT ATACTTACTT AACTTTTATT   
  
  
- TTACATTTTA AATAAATGAA ATAAAATATT GTTTTTCAAA AATTTAAAAA TATTAGTAAA CTTGATTTAA   
  
  
- ATTAATTATT TATGTTTAAA CTAAAGAAAA AGATTTTTAA TTTAGATTAA GTGATAAAAG TTTATAATTT   
  
  
- TAAATTAAAT TACGTACTTT GTGAAATTCT AGTTTTAATA TATTTTAATA GTATAAATGA AATATAATAG   
  
  
- TTAGTTAAAA AAACAATTTT TCCACTGTTT AACTTAAAAC TAGATGACCA AATTATAGTT TAATTTAGAA   
  
  
- TTAATTTTAT TAGTATTGTT AATTTTTATA AAAACAATAA TTTTATAAAT TTGATAAACT TAATAAAAAA   
  
  
- TAAAATAAAG TTCAAGTAAG AAATTTACAT TTATTAAAAA CTACGTTCTA GACCAAAAAG ATTTAAAAAT   
  
  
- TTTAAAACTA ATATAAATTG TTATGTAAAA ATTATGAGTT TTTTAAAATT TTAAAAATAC TATTATAAAA   
  
  
- TTTATAATTT AAACTTTAAT TTTAAATAAT ATATTTTAAA AATTAAATTA ATAAAATTTT AAATTTAAAA   
  
  
- AATTATTGTT AATTAAATTA AATTAAATTA TTATATTTTA TTTATAATTT TAAAATATAT TAAAAATAAG   
  
  
- TTTTAAAATT TGTGTAAAAA ATAAAAAAGA ACACCGCCCG AAGGCTTAGT CGATGGAAAA ACATCTCCAT   
  
  
- AGTGATATAT TTTAAATGCA CGGCAAATGC TGTGGTTTCT ATGGTAATCC TATTCTTCGA CGGAGTGTCT   
  
  
- GTCCGGCGGA GGCGGTGACA AGACTTAAAG TTGGTGTGAG TCATGAGGAG GATATATAAT GGTGTGTTTG   
  
  
- GTATAAGTAA CTAAGAATAA ATAAGAAGAA CAGGAAGAAG CAGCATACTA GGCTGCGAGT GAAAAGAGAG   
  
  
- TCTGAAAGAG AGCAAAAGAG CCCTTTTAT

+     Unnamed\_\_6

| Site Name | Organism | Position | Strand | Matrix score. | sequence | function |
| --- | --- | --- | --- | --- | --- | --- |
| Unnamed\_\_6 | Zea mays | 511 | - | 10 | taTAAATATct |  |

> 2018/04/13 10:10:12  
+ CAGAAATTGT AAGTTTTTAT TGGTGTCACT ATAATTTAAT TTGTTTCCTT GACGATAGTA CTATTTGTAC   
  
  
+ GACATGAAAG TTTGGTCCGA TTCAACTAAC TAATTAATTG ATTAAGATAA ATTTAATTTG TTTCCTTGAC   
  
  
+ GATAGTACTA TTTGTACGAC ATGAAAGTTT GGTCCGATTC AACTAACTAA TTAATTGATT AAGATATCAA   
  
  
+ TGCGATCTTA ATCAAAGCAA GCTAGCCTAG CCTTAATCAT GAAGCCTCTA ATATTTTCTC ATTGCTTTGA   
  
  
+ TACTTTTTTT TTAAAATTTT ATTTAAAATT TAAAATTATA TTTTTACCTT GAAGTGGAGT CCAATGTTTT   
  
  
+ AAAGATGACA ATTCATATCA TAATTTATTA AATTAAATTT AATTATTTAA AAAATTAAAA ATTAAATTTT   
  
  
+ ATTGTATAAA AAAATATTTA AACCATTATT ATAAATAAAA TATATATTTT TAGATATTAT TTATTTTAAA   
  
  
+ ATTGTACTGT CAAAATTTTA AAATATTTAT AAAAAGTAAT TTACAAGTCA TATGAATGAA TTGAAAATAA   
  
  
+ AATGTAAAAT TTATTTACTT TATTTTATAA CAAAAAGTTT TTAAATTTTT ATAATCATTT GAACTAAATT   
  
  
+ TAATTAATAA ATACAAATTT GATTTCTTTT TCTAAAAATT AAATCTAATT CACTATTTTC AAATATTAAA   
  
  
+ ATTTAATTTA ATGCATGAAA CACTTTAAGA TCAAAATTAT ATAAAATTAT CATATTTACT TTATATTATC   
  
  
+ AATCAATTTT TTTGTTAAAA AGGTGACAAA TTGAATTTTG ATCTACTGGT TTAATATCAA ATTAAATCTT   
  
  
+ AATTAAAATA ATCATAACAA TTAAAAATAT TTTTGTTATT AAAATATTTA AACTATTTGA ATTATTTTTT   
  
  
+ ATTTTATTTC AAGTTCATTC TTTAAATGTA AATAATTTTT GATGCAAGAT CTGGTTTTTC TAAATTTTTA   
  
  
+ AAATTTTGAT TATATTTAAC AATACATTTT TAATACTCAA AAAATTTTAA AATTTTTATG ATAATATTTT   
  
  
+ AAATATTAAA TTTGAAATTA AAATTTATTA TATAAAATTT TTAATTTAAT TATTTTAAAA TTTAAATTTT   
  
  
+ TTAATAACAA TTAATTTAAT TTAATTTAAT AATATAAAAT AAATATTAAA ATTTTATATA ATTTTTATTC   
  
  
+ AAAATTTTAA ACACATTTTT TATTTTTTCT TGTGGCGGGC TTCCGAATCA GCTACCTTTT TGTAGAGGTA   
  
  
+ TCACTATATA AAATTTACGT GCCGTTTACG ACACCAAAGA TACCATTAGG ATAAGAAGCT GCCTCACAGA   
  
  
+ CAGGCCGCCT CCGCCACTGT TCTGAATTTC AACCACACTC AGTACTCCTC CTATATATTA CCACACAAAC   
  
  
+ CATATTCATT GATTCTTATT TATTCTTCTT GTCCTTCTTC GTCGTATGAT CCGACGCTCA CTTTTCTCTC   
  
  
+ AGACTTTCTC TCGTTTTCTC GGGAAAATA  

- GTCTTTAACA TTCAAAAATA ACCACAGTGA TATTAAATTA AACAAAGGAA CTGCTATCAT GATAAACATG   
  
  
- CTGTACTTTC AAACCAGGCT AAGTTGATTG ATTAATTAAC TAATTCTATT TAAATTAAAC AAAGGAACTG   
  
  
- CTATCATGAT AAACATGCTG TACTTTCAAA CCAGGCTAAG TTGATTGATT AATTAACTAA TTCTATAGTT   
  
  
- ACGCTAGAAT TAGTTTCGTT CGATCGGATC GGAATTAGTA CTTCGGAGAT TATAAAAGAG TAACGAAACT   
  
  
- ATGAAAAAAA AATTTTAAAA TAAATTTTAA ATTTTAATAT AAAAATGGAA CTTCACCTCA GGTTACAAAA   
  
  
- TTTCTACTGT TAAGTATAGT ATTAAATAAT TTAATTTAAA TTAATAAATT TTTTAATTTT TAATTTAAAA   
  
  
- TAACATATTT TTTTATAAAT TTGGTAATAA TATTTATTTT ATATATAAAA ATCTATAATA AATAAAATTT   
  
  
- TAACATGACA GTTTTAAAAT TTTATAAATA TTTTTCATTA AATGTTCAGT ATACTTACTT AACTTTTATT   
  
  
- TTACATTTTA AATAAATGAA ATAAAATATT GTTTTTCAAA AATTTAAAAA TATTAGTAAA CTTGATTTAA   
  
  
- ATTAATTATT TATGTTTAAA CTAAAGAAAA AGATTTTTAA TTTAGATTAA GTGATAAAAG TTTATAATTT   
  
  
- TAAATTAAAT TACGTACTTT GTGAAATTCT AGTTTTAATA TATTTTAATA GTATAAATGA AATATAATAG   
  
  
- TTAGTTAAAA AAACAATTTT TCCACTGTTT AACTTAAAAC TAGATGACCA AATTATAGTT TAATTTAGAA   
  
  
- TTAATTTTAT TAGTATTGTT AATTTTTATA AAAACAATAA TTTTATAAAT TTGATAAACT TAATAAAAAA   
  
  
- TAAAATAAAG TTCAAGTAAG AAATTTACAT TTATTAAAAA CTACGTTCTA GACCAAAAAG ATTTAAAAAT   
  
  
- TTTAAAACTA ATATAAATTG TTATGTAAAA ATTATGAGTT TTTTAAAATT TTAAAAATAC TATTATAAAA   
  
  
- TTTATAATTT AAACTTTAAT TTTAAATAAT ATATTTTAAA AATTAAATTA ATAAAATTTT AAATTTAAAA   
  
  
- AATTATTGTT AATTAAATTA AATTAAATTA TTATATTTTA TTTATAATTT TAAAATATAT TAAAAATAAG   
  
  
- TTTTAAAATT TGTGTAAAAA ATAAAAAAGA ACACCGCCCG AAGGCTTAGT CGATGGAAAA ACATCTCCAT   
  
  
- AGTGATATAT TTTAAATGCA CGGCAAATGC TGTGGTTTCT ATGGTAATCC TATTCTTCGA CGGAGTGTCT   
  
  
- GTCCGGCGGA GGCGGTGACA AGACTTAAAG TTGGTGTGAG TCATGAGGAG GATATATAAT GGTGTGTTTG   
  
  
- GTATAAGTAA CTAAGAATAA ATAAGAAGAA CAGGAAGAAG CAGCATACTA GGCTGCGAGT GAAAAGAGAG   
  
  
- TCTGAAAGAG AGCAAAAGAG CCCTTTTAT

+     circadian

| Site Name | Organism | Position | Strand | Matrix score. | sequence | function |
| --- | --- | --- | --- | --- | --- | --- |
| circadian | Lycopersicon esculentum | 208 | + | 6 | CAANNNNATC | cis-acting regulatory element involved in circadian control |
| circadian | Lycopersicon esculentum | 1295 | + | 9 | CAAAGATATC | cis-acting regulatory element involved in circadian control |

> 2018/04/13 10:10:12  
+ CAGAAATTGT AAGTTTTTAT TGGTGTCACT ATAATTTAAT TTGTTTCCTT GACGATAGTA CTATTTGTAC   
  
  
+ GACATGAAAG TTTGGTCCGA TTCAACTAAC TAATTAATTG ATTAAGATAA ATTTAATTTG TTTCCTTGAC   
  
  
+ GATAGTACTA TTTGTACGAC ATGAAAGTTT GGTCCGATTC AACTAACTAA TTAATTGATT AAGATATCAA   
  
  
+ TGCGATCTTA ATCAAAGCAA GCTAGCCTAG CCTTAATCAT GAAGCCTCTA ATATTTTCTC ATTGCTTTGA   
  
  
+ TACTTTTTTT TTAAAATTTT ATTTAAAATT TAAAATTATA TTTTTACCTT GAAGTGGAGT CCAATGTTTT   
  
  
+ AAAGATGACA ATTCATATCA TAATTTATTA AATTAAATTT AATTATTTAA AAAATTAAAA ATTAAATTTT   
  
  
+ ATTGTATAAA AAAATATTTA AACCATTATT ATAAATAAAA TATATATTTT TAGATATTAT TTATTTTAAA   
  
  
+ ATTGTACTGT CAAAATTTTA AAATATTTAT AAAAAGTAAT TTACAAGTCA TATGAATGAA TTGAAAATAA   
  
  
+ AATGTAAAAT TTATTTACTT TATTTTATAA CAAAAAGTTT TTAAATTTTT ATAATCATTT GAACTAAATT   
  
  
+ TAATTAATAA ATACAAATTT GATTTCTTTT TCTAAAAATT AAATCTAATT CACTATTTTC AAATATTAAA   
  
  
+ ATTTAATTTA ATGCATGAAA CACTTTAAGA TCAAAATTAT ATAAAATTAT CATATTTACT TTATATTATC   
  
  
+ AATCAATTTT TTTGTTAAAA AGGTGACAAA TTGAATTTTG ATCTACTGGT TTAATATCAA ATTAAATCTT   
  
  
+ AATTAAAATA ATCATAACAA TTAAAAATAT TTTTGTTATT AAAATATTTA AACTATTTGA ATTATTTTTT   
  
  
+ ATTTTATTTC AAGTTCATTC TTTAAATGTA AATAATTTTT GATGCAAGAT CTGGTTTTTC TAAATTTTTA   
  
  
+ AAATTTTGAT TATATTTAAC AATACATTTT TAATACTCAA AAAATTTTAA AATTTTTATG ATAATATTTT   
  
  
+ AAATATTAAA TTTGAAATTA AAATTTATTA TATAAAATTT TTAATTTAAT TATTTTAAAA TTTAAATTTT   
  
  
+ TTAATAACAA TTAATTTAAT TTAATTTAAT AATATAAAAT AAATATTAAA ATTTTATATA ATTTTTATTC   
  
  
+ AAAATTTTAA ACACATTTTT TATTTTTTCT TGTGGCGGGC TTCCGAATCA GCTACCTTTT TGTAGAGGTA   
  
  
+ TCACTATATA AAATTTACGT GCCGTTTACG ACACCAAAGA TACCATTAGG ATAAGAAGCT GCCTCACAGA   
  
  
+ CAGGCCGCCT CCGCCACTGT TCTGAATTTC AACCACACTC AGTACTCCTC CTATATATTA CCACACAAAC   
  
  
+ CATATTCATT GATTCTTATT TATTCTTCTT GTCCTTCTTC GTCGTATGAT CCGACGCTCA CTTTTCTCTC   
  
  
+ AGACTTTCTC TCGTTTTCTC GGGAAAATA  

- GTCTTTAACA TTCAAAAATA ACCACAGTGA TATTAAATTA AACAAAGGAA CTGCTATCAT GATAAACATG   
  
  
- CTGTACTTTC AAACCAGGCT AAGTTGATTG ATTAATTAAC TAATTCTATT TAAATTAAAC AAAGGAACTG   
  
  
- CTATCATGAT AAACATGCTG TACTTTCAAA CCAGGCTAAG TTGATTGATT AATTAACTAA TTCTATAGTT   
  
  
- ACGCTAGAAT TAGTTTCGTT CGATCGGATC GGAATTAGTA CTTCGGAGAT TATAAAAGAG TAACGAAACT   
  
  
- ATGAAAAAAA AATTTTAAAA TAAATTTTAA ATTTTAATAT AAAAATGGAA CTTCACCTCA GGTTACAAAA   
  
  
- TTTCTACTGT TAAGTATAGT ATTAAATAAT TTAATTTAAA TTAATAAATT TTTTAATTTT TAATTTAAAA   
  
  
- TAACATATTT TTTTATAAAT TTGGTAATAA TATTTATTTT ATATATAAAA ATCTATAATA AATAAAATTT   
  
  
- TAACATGACA GTTTTAAAAT TTTATAAATA TTTTTCATTA AATGTTCAGT ATACTTACTT AACTTTTATT   
  
  
- TTACATTTTA AATAAATGAA ATAAAATATT GTTTTTCAAA AATTTAAAAA TATTAGTAAA CTTGATTTAA   
  
  
- ATTAATTATT TATGTTTAAA CTAAAGAAAA AGATTTTTAA TTTAGATTAA GTGATAAAAG TTTATAATTT   
  
  
- TAAATTAAAT TACGTACTTT GTGAAATTCT AGTTTTAATA TATTTTAATA GTATAAATGA AATATAATAG   
  
  
- TTAGTTAAAA AAACAATTTT TCCACTGTTT AACTTAAAAC TAGATGACCA AATTATAGTT TAATTTAGAA   
  
  
- TTAATTTTAT TAGTATTGTT AATTTTTATA AAAACAATAA TTTTATAAAT TTGATAAACT TAATAAAAAA   
  
  
- TAAAATAAAG TTCAAGTAAG AAATTTACAT TTATTAAAAA CTACGTTCTA GACCAAAAAG ATTTAAAAAT   
  
  
- TTTAAAACTA ATATAAATTG TTATGTAAAA ATTATGAGTT TTTTAAAATT TTAAAAATAC TATTATAAAA   
  
  
- TTTATAATTT AAACTTTAAT TTTAAATAAT ATATTTTAAA AATTAAATTA ATAAAATTTT AAATTTAAAA   
  
  
- AATTATTGTT AATTAAATTA AATTAAATTA TTATATTTTA TTTATAATTT TAAAATATAT TAAAAATAAG   
  
  
- TTTTAAAATT TGTGTAAAAA ATAAAAAAGA ACACCGCCCG AAGGCTTAGT CGATGGAAAA ACATCTCCAT   
  
  
- AGTGATATAT TTTAAATGCA CGGCAAATGC TGTGGTTTCT ATGGTAATCC TATTCTTCGA CGGAGTGTCT   
  
  
- GTCCGGCGGA GGCGGTGACA AGACTTAAAG TTGGTGTGAG TCATGAGGAG GATATATAAT GGTGTGTTTG   
  
  
- GTATAAGTAA CTAAGAATAA ATAAGAAGAA CAGGAAGAAG CAGCATACTA GGCTGCGAGT GAAAAGAGAG   
  
  
- TCTGAAAGAG AGCAAAAGAG CCCTTTTAT
