# Supplementary material for: Genome-wide analysis of the WRKY gene family in drumstick (Moringa oleifera Lam.)
Source: PeerJ. 2019 Jun 10;7:e7063. doi: 10.7717/peerj.7063 (PMC6563795; doi:10.7717/peerj.7063)
Supplement: Supplemental Information 1 [file peerj-07-7063-s003.gz › MoWRKY52_plantcare.html]

Content-Type: text/html; charset=ISO-8859-1


CallMat\_Firefox


Webmaster Firefox specific output  
To save the result:
click on the frame with the right mouse button and save the source code as a text file with extension .html  
REFERENCE:PlantCARE: a database of plant cis-acting regulatory elements and a portal to tools for in silico analysis of promoter sequences.  
Lescot, M., Déhais, P., Moreau, Y., De Moor, B., Rouzé ,P.,and Rombauts, S.  
Nucleic Acids Res., Database issue(2002), 30(1):325-327.   


---

> 2018/04/13 10:10:12  
+ AAGCTATTAT TGCCAAGCCA GAGACTCAAT GTATTTTAGT CAGAACAAGT TGCTAAGCTC TTGAATTCTG   
  
  
+ AATTATCAGT CACTAAACCA CTTATTGTGA TGTAAATATG TTATCATGTG TAAGCAATGA AGCAGTAAGC   
  
  
+ CAAGATTAAG GAGAACCACA GTAATTGGCC TCAAAGAAAG AAAGAAAGAA AAAACAAAAA TAGTAGAGAT   
  
  
+ CAAGCCTAAA CACAGAAATT TCTAGAGTGA AGTGAAAACT GAACAAAACG ACAGAGAGAA AGAGAGGGAG   
  
  
+ GATGGGGGAG ATGAGAAAAA GAGGGGATTG CAAGTTACCT TGGAGGTGCC CCGAAAGTGA GACCAGGAGC   
  
  
+ CCCAAGCGGA GGGATAAGGA TCGCCTATGT TGTAAGGGAG ATCCTTGAGT CCCGTACCAG ATCCACCCAC   
  
  
+ CACTCCCTTC AAGAATTTGA ACATGTTGTT TTTGTCCTCT CGTCTCCTTT TTTTCGGGTG TTTATGGGTT   
  
  
+ TATGTATGCA TATAAGGTTG TATCCTCTGA TGCGTATAGG CGAATGACCC CAAACAGTCT CTCTCTCTCT   
  
  
+ CTCTCTCTCT CTCTCTCTCT CTCCCTCTGC ATGGATGATG GAGAGGGCGG GGAACAGGTT CGATCTGCGA   
  
  
+ GAGAGTGTAA GAGAGAGAGA GATGCAGATG CAAGGTGTCA AAAACGGAAA AGAAAATTAA ATTGATGTTT   
  
  
+ TCGGGTGCAA CCCGAAGTAT CGAGCAGCCT GGTTCTTCAA ATAAAGACCG TTAAGATTGC CTCCTTATTC   
  
  
+ AACTTCCTTA AATGGGCCAG TGGGGTGTCA AGCCCTTCTC TGTTCCAGCC CATAAGTACG CCTCTTTCTG   
  
  
+ TCTGTTTTAT TTGGGCCCTC CGATTGCCCG ACTTTTCATT TGTAATTACT TGCTTAATTA ATGGTTATAT   
  
  
+ GCTGTGCTTT ATTATTAAAA TTCCACTTAC AGAGAGTTAC ATCAATATCG CGAAACAGAA AGAAATTCAG   
  
  
+ TGTTGTATAC ATTTCATTTG GTAATAATAC CCATGGGCCA CGGAAACTTG ACTCCCTGTG TCATGACTCA   
  
  
+ AGAACCATGA CGTACGGGTA AAGGGTTGGT TCAAACCGCG GTGACCATGA ACGAGTAAAA CAGAGTGCTC   
  
  
+ CCATGCAAGT TCAGAACAAG ACGCTTTCTC CTCCACAGTC AATAACATCA AAGCAAGAGA AACTTCTTCA   
  
  
+ AGGTCTAAGC AATTGGTTTC AATGGGACAA GGTCTTCTTT TAGACTCAGA CCGGAAAAAG TTGAAAACCC   
  
  
+ CGCACTCATT AACAAGACCG TGACTTGGTC ATATTTCCAA GTTGCTTCTT CTTTGTCTTT TCGTTTTAAT   
  
  
+ TAATTAACTT CTTGCACCGT GTAAAAGGAA CACGAATACT TTGAATGACA TATGGAAAGC GTTATGATTA   
  
  
+ TATATACCAA CGATCAAAGA ACCAACGAAC CCCCATTTCA GATTTTGTCC TTCCAACCCA ATAAGAGAAG   
  
  
+ CCAAACAGAC CAAGAACGCC CACACACAC  

- TTCGATAATA ACGGTTCGGT CTCTGAGTTA CATAAAATCA GTCTTGTTCA ACGATTCGAG AACTTAAGAC   
  
  
- TTAATAGTCA GTGATTTGGT GAATAACACT ACATTTATAC AATAGTACAC ATTCGTTACT TCGTCATTCG   
  
  
- GTTCTAATTC CTCTTGGTGT CATTAACCGG AGTTTCTTTC TTTCTTTCTT TTTTGTTTTT ATCATCTCTA   
  
  
- GTTCGGATTT GTGTCTTTAA AGATCTCACT TCACTTTTGA CTTGTTTTGC TGTCTCTCTT TCTCTCCCTC   
  
  
- CTACCCCCTC TACTCTTTTT CTCCCCTAAC GTTCAATGGA ACCTCCACGG GGCTTTCACT CTGGTCCTCG   
  
  
- GGGTTCGCCT CCCTATTCCT AGCGGATACA ACATTCCCTC TAGGAACTCA GGGCATGGTC TAGGTGGGTG   
  
  
- GTGAGGGAAG TTCTTAAACT TGTACAACAA AAACAGGAGA GCAGAGGAAA AAAAGCCCAC AAATACCCAA   
  
  
- ATACATACGT ATATTCCAAC ATAGGAGACT ACGCATATCC GCTTACTGGG GTTTGTCAGA GAGAGAGAGA   
  
  
- GAGAGAGAGA GAGAGAGAGA GAGGGAGACG TACCTACTAC CTCTCCCGCC CCTTGTCCAA GCTAGACGCT   
  
  
- CTCTCACATT CTCTCTCTCT CTACGTCTAC GTTCCACAGT TTTTGCCTTT TCTTTTAATT TAACTACAAA   
  
  
- AGCCCACGTT GGGCTTCATA GCTCGTCGGA CCAAGAAGTT TATTTCTGGC AATTCTAACG GAGGAATAAG   
  
  
- TTGAAGGAAT TTACCCGGTC ACCCCACAGT TCGGGAAGAG ACAAGGTCGG GTATTCATGC GGAGAAAGAC   
  
  
- AGACAAAATA AACCCGGGAG GCTAACGGGC TGAAAAGTAA ACATTAATGA ACGAATTAAT TACCAATATA   
  
  
- CGACACGAAA TAATAATTTT AAGGTGAATG TCTCTCAATG TAGTTATAGC GCTTTGTCTT TCTTTAAGTC   
  
  
- ACAACATATG TAAAGTAAAC CATTATTATG GGTACCCGGT GCCTTTGAAC TGAGGGACAC AGTACTGAGT   
  
  
- TCTTGGTACT GCATGCCCAT TTCCCAACCA AGTTTGGCGC CACTGGTACT TGCTCATTTT GTCTCACGAG   
  
  
- GGTACGTTCA AGTCTTGTTC TGCGAAAGAG GAGGTGTCAG TTATTGTAGT TTCGTTCTCT TTGAAGAAGT   
  
  
- TCCAGATTCG TTAACCAAAG TTACCCTGTT CCAGAAGAAA ATCTGAGTCT GGCCTTTTTC AACTTTTGGG   
  
  
- GCGTGAGTAA TTGTTCTGGC ACTGAACCAG TATAAAGGTT CAACGAAGAA GAAACAGAAA AGCAAAATTA   
  
  
- ATTAATTGAA GAACGTGGCA CATTTTCCTT GTGCTTATGA AACTTACTGT ATACCTTTCG CAATACTAAT   
  
  
- ATATATGGTT GCTAGTTTCT TGGTTGCTTG GGGGTAAAGT CTAAAACAGG AAGGTTGGGT TATTCTCTTC   
  
  
- GGTTTGTCTG GTTCTTGCGG GTGTGTGTG

  
  
Motifs Found  

+     5UTR Py-rich stretch

| Site Name | Organism | Position | Strand | Matrix score. | sequence | function |
| --- | --- | --- | --- | --- | --- | --- |
| 5UTR Py-rich stretch | Lycopersicon esculentum | 566 | + | 13 | TTTCTCTCTCTCTC | cis-acting element conferring high transcription levels |
| 5UTR Py-rich stretch | Lycopersicon esculentum | 564 | + | 13 | TTTCTCTCTCTCTC | cis-acting element conferring high transcription levels |
| 5UTR Py-rich stretch | Lycopersicon esculentum | 554 | + | 13 | TTTCTCTCTCTCTC | cis-acting element conferring high transcription levels |
| 5UTR Py-rich stretch | Lycopersicon esculentum | 568 | + | 13 | TTTCTCTCTCTCTC | cis-acting element conferring high transcription levels |
| 5UTR Py-rich stretch | Lycopersicon esculentum | 552 | + | 13 | TTTCTCTCTCTCTC | cis-acting element conferring high transcription levels |
| 5UTR Py-rich stretch | Lycopersicon esculentum | 556 | + | 13 | TTTCTCTCTCTCTC | cis-acting element conferring high transcription levels |
| 5UTR Py-rich stretch | Lycopersicon esculentum | 558 | + | 13 | TTTCTCTCTCTCTC | cis-acting element conferring high transcription levels |
| 5UTR Py-rich stretch | Lycopersicon esculentum | 548 | + | 13 | TTTCTCTCTCTCTC | cis-acting element conferring high transcription levels |
| 5UTR Py-rich stretch | Lycopersicon esculentum | 560 | + | 13 | TTTCTCTCTCTCTC | cis-acting element conferring high transcription levels |
| 5UTR Py-rich stretch | Lycopersicon esculentum | 550 | + | 13 | TTTCTCTCTCTCTC | cis-acting element conferring high transcription levels |
| 5UTR Py-rich stretch | Lycopersicon esculentum | 562 | + | 13 | TTTCTCTCTCTCTC | cis-acting element conferring high transcription levels |
| 5UTR Py-rich stretch | Lycopersicon esculentum | 570 | + | 13 | TTTCTCTCTCTCTC | cis-acting element conferring high transcription levels |

> 2018/04/13 10:10:12  
+ AAGCTATTAT TGCCAAGCCA GAGACTCAAT GTATTTTAGT CAGAACAAGT TGCTAAGCTC TTGAATTCTG   
  
  
+ AATTATCAGT CACTAAACCA CTTATTGTGA TGTAAATATG TTATCATGTG TAAGCAATGA AGCAGTAAGC   
  
  
+ CAAGATTAAG GAGAACCACA GTAATTGGCC TCAAAGAAAG AAAGAAAGAA AAAACAAAAA TAGTAGAGAT   
  
  
+ CAAGCCTAAA CACAGAAATT TCTAGAGTGA AGTGAAAACT GAACAAAACG ACAGAGAGAA AGAGAGGGAG   
  
  
+ GATGGGGGAG ATGAGAAAAA GAGGGGATTG CAAGTTACCT TGGAGGTGCC CCGAAAGTGA GACCAGGAGC   
  
  
+ CCCAAGCGGA GGGATAAGGA TCGCCTATGT TGTAAGGGAG ATCCTTGAGT CCCGTACCAG ATCCACCCAC   
  
  
+ CACTCCCTTC AAGAATTTGA ACATGTTGTT TTTGTCCTCT CGTCTCCTTT TTTTCGGGTG TTTATGGGTT   
  
  
+ TATGTATGCA TATAAGGTTG TATCCTCTGA TGCGTATAGG CGAATGACCC CAAACAGTCT CTCTCTCTCT   
  
  
+ CTCTCTCTCT CTCTCTCTCT CTCCCTCTGC ATGGATGATG GAGAGGGCGG GGAACAGGTT CGATCTGCGA   
  
  
+ GAGAGTGTAA GAGAGAGAGA GATGCAGATG CAAGGTGTCA AAAACGGAAA AGAAAATTAA ATTGATGTTT   
  
  
+ TCGGGTGCAA CCCGAAGTAT CGAGCAGCCT GGTTCTTCAA ATAAAGACCG TTAAGATTGC CTCCTTATTC   
  
  
+ AACTTCCTTA AATGGGCCAG TGGGGTGTCA AGCCCTTCTC TGTTCCAGCC CATAAGTACG CCTCTTTCTG   
  
  
+ TCTGTTTTAT TTGGGCCCTC CGATTGCCCG ACTTTTCATT TGTAATTACT TGCTTAATTA ATGGTTATAT   
  
  
+ GCTGTGCTTT ATTATTAAAA TTCCACTTAC AGAGAGTTAC ATCAATATCG CGAAACAGAA AGAAATTCAG   
  
  
+ TGTTGTATAC ATTTCATTTG GTAATAATAC CCATGGGCCA CGGAAACTTG ACTCCCTGTG TCATGACTCA   
  
  
+ AGAACCATGA CGTACGGGTA AAGGGTTGGT TCAAACCGCG GTGACCATGA ACGAGTAAAA CAGAGTGCTC   
  
  
+ CCATGCAAGT TCAGAACAAG ACGCTTTCTC CTCCACAGTC AATAACATCA AAGCAAGAGA AACTTCTTCA   
  
  
+ AGGTCTAAGC AATTGGTTTC AATGGGACAA GGTCTTCTTT TAGACTCAGA CCGGAAAAAG TTGAAAACCC   
  
  
+ CGCACTCATT AACAAGACCG TGACTTGGTC ATATTTCCAA GTTGCTTCTT CTTTGTCTTT TCGTTTTAAT   
  
  
+ TAATTAACTT CTTGCACCGT GTAAAAGGAA CACGAATACT TTGAATGACA TATGGAAAGC GTTATGATTA   
  
  
+ TATATACCAA CGATCAAAGA ACCAACGAAC CCCCATTTCA GATTTTGTCC TTCCAACCCA ATAAGAGAAG   
  
  
+ CCAAACAGAC CAAGAACGCC CACACACAC  

- TTCGATAATA ACGGTTCGGT CTCTGAGTTA CATAAAATCA GTCTTGTTCA ACGATTCGAG AACTTAAGAC   
  
  
- TTAATAGTCA GTGATTTGGT GAATAACACT ACATTTATAC AATAGTACAC ATTCGTTACT TCGTCATTCG   
  
  
- GTTCTAATTC CTCTTGGTGT CATTAACCGG AGTTTCTTTC TTTCTTTCTT TTTTGTTTTT ATCATCTCTA   
  
  
- GTTCGGATTT GTGTCTTTAA AGATCTCACT TCACTTTTGA CTTGTTTTGC TGTCTCTCTT TCTCTCCCTC   
  
  
- CTACCCCCTC TACTCTTTTT CTCCCCTAAC GTTCAATGGA ACCTCCACGG GGCTTTCACT CTGGTCCTCG   
  
  
- GGGTTCGCCT CCCTATTCCT AGCGGATACA ACATTCCCTC TAGGAACTCA GGGCATGGTC TAGGTGGGTG   
  
  
- GTGAGGGAAG TTCTTAAACT TGTACAACAA AAACAGGAGA GCAGAGGAAA AAAAGCCCAC AAATACCCAA   
  
  
- ATACATACGT ATATTCCAAC ATAGGAGACT ACGCATATCC GCTTACTGGG GTTTGTCAGA GAGAGAGAGA   
  
  
- GAGAGAGAGA GAGAGAGAGA GAGGGAGACG TACCTACTAC CTCTCCCGCC CCTTGTCCAA GCTAGACGCT   
  
  
- CTCTCACATT CTCTCTCTCT CTACGTCTAC GTTCCACAGT TTTTGCCTTT TCTTTTAATT TAACTACAAA   
  
  
- AGCCCACGTT GGGCTTCATA GCTCGTCGGA CCAAGAAGTT TATTTCTGGC AATTCTAACG GAGGAATAAG   
  
  
- TTGAAGGAAT TTACCCGGTC ACCCCACAGT TCGGGAAGAG ACAAGGTCGG GTATTCATGC GGAGAAAGAC   
  
  
- AGACAAAATA AACCCGGGAG GCTAACGGGC TGAAAAGTAA ACATTAATGA ACGAATTAAT TACCAATATA   
  
  
- CGACACGAAA TAATAATTTT AAGGTGAATG TCTCTCAATG TAGTTATAGC GCTTTGTCTT TCTTTAAGTC   
  
  
- ACAACATATG TAAAGTAAAC CATTATTATG GGTACCCGGT GCCTTTGAAC TGAGGGACAC AGTACTGAGT   
  
  
- TCTTGGTACT GCATGCCCAT TTCCCAACCA AGTTTGGCGC CACTGGTACT TGCTCATTTT GTCTCACGAG   
  
  
- GGTACGTTCA AGTCTTGTTC TGCGAAAGAG GAGGTGTCAG TTATTGTAGT TTCGTTCTCT TTGAAGAAGT   
  
  
- TCCAGATTCG TTAACCAAAG TTACCCTGTT CCAGAAGAAA ATCTGAGTCT GGCCTTTTTC AACTTTTGGG   
  
  
- GCGTGAGTAA TTGTTCTGGC ACTGAACCAG TATAAAGGTT CAACGAAGAA GAAACAGAAA AGCAAAATTA   
  
  
- ATTAATTGAA GAACGTGGCA CATTTTCCTT GTGCTTATGA AACTTACTGT ATACCTTTCG CAATACTAAT   
  
  
- ATATATGGTT GCTAGTTTCT TGGTTGCTTG GGGGTAAAGT CTAAAACAGG AAGGTTGGGT TATTCTCTTC   
  
  
- GGTTTGTCTG GTTCTTGCGG GTGTGTGTG

+     AAGAA-motif

| Site Name | Organism | Position | Strand | Matrix score. | sequence | function |
| --- | --- | --- | --- | --- | --- | --- |
| AAGAA-motif | Avena sativa | 176 | + | 7 | GAAAGAA |  |
| AAGAA-motif | Avena sativa | 968 | + | 7 | GAAAGAA |  |
| AAGAA-motif | Avena sativa | 184 | + | 7 | GAAAGAA |  |
| AAGAA-motif | Avena sativa | 676 | + | 9 | gGTAAAGAAA |  |
| AAGAA-motif | Avena sativa | 180 | + | 7 | GAAAGAA |  |

> 2018/04/13 10:10:12  
+ AAGCTATTAT TGCCAAGCCA GAGACTCAAT GTATTTTAGT CAGAACAAGT TGCTAAGCTC TTGAATTCTG   
  
  
+ AATTATCAGT CACTAAACCA CTTATTGTGA TGTAAATATG TTATCATGTG TAAGCAATGA AGCAGTAAGC   
  
  
+ CAAGATTAAG GAGAACCACA GTAATTGGCC TCAAAGAAAG AAAGAAAGAA AAAACAAAAA TAGTAGAGAT   
  
  
+ CAAGCCTAAA CACAGAAATT TCTAGAGTGA AGTGAAAACT GAACAAAACG ACAGAGAGAA AGAGAGGGAG   
  
  
+ GATGGGGGAG ATGAGAAAAA GAGGGGATTG CAAGTTACCT TGGAGGTGCC CCGAAAGTGA GACCAGGAGC   
  
  
+ CCCAAGCGGA GGGATAAGGA TCGCCTATGT TGTAAGGGAG ATCCTTGAGT CCCGTACCAG ATCCACCCAC   
  
  
+ CACTCCCTTC AAGAATTTGA ACATGTTGTT TTTGTCCTCT CGTCTCCTTT TTTTCGGGTG TTTATGGGTT   
  
  
+ TATGTATGCA TATAAGGTTG TATCCTCTGA TGCGTATAGG CGAATGACCC CAAACAGTCT CTCTCTCTCT   
  
  
+ CTCTCTCTCT CTCTCTCTCT CTCCCTCTGC ATGGATGATG GAGAGGGCGG GGAACAGGTT CGATCTGCGA   
  
  
+ GAGAGTGTAA GAGAGAGAGA GATGCAGATG CAAGGTGTCA AAAACGGAAA AGAAAATTAA ATTGATGTTT   
  
  
+ TCGGGTGCAA CCCGAAGTAT CGAGCAGCCT GGTTCTTCAA ATAAAGACCG TTAAGATTGC CTCCTTATTC   
  
  
+ AACTTCCTTA AATGGGCCAG TGGGGTGTCA AGCCCTTCTC TGTTCCAGCC CATAAGTACG CCTCTTTCTG   
  
  
+ TCTGTTTTAT TTGGGCCCTC CGATTGCCCG ACTTTTCATT TGTAATTACT TGCTTAATTA ATGGTTATAT   
  
  
+ GCTGTGCTTT ATTATTAAAA TTCCACTTAC AGAGAGTTAC ATCAATATCG CGAAACAGAA AGAAATTCAG   
  
  
+ TGTTGTATAC ATTTCATTTG GTAATAATAC CCATGGGCCA CGGAAACTTG ACTCCCTGTG TCATGACTCA   
  
  
+ AGAACCATGA CGTACGGGTA AAGGGTTGGT TCAAACCGCG GTGACCATGA ACGAGTAAAA CAGAGTGCTC   
  
  
+ CCATGCAAGT TCAGAACAAG ACGCTTTCTC CTCCACAGTC AATAACATCA AAGCAAGAGA AACTTCTTCA   
  
  
+ AGGTCTAAGC AATTGGTTTC AATGGGACAA GGTCTTCTTT TAGACTCAGA CCGGAAAAAG TTGAAAACCC   
  
  
+ CGCACTCATT AACAAGACCG TGACTTGGTC ATATTTCCAA GTTGCTTCTT CTTTGTCTTT TCGTTTTAAT   
  
  
+ TAATTAACTT CTTGCACCGT GTAAAAGGAA CACGAATACT TTGAATGACA TATGGAAAGC GTTATGATTA   
  
  
+ TATATACCAA CGATCAAAGA ACCAACGAAC CCCCATTTCA GATTTTGTCC TTCCAACCCA ATAAGAGAAG   
  
  
+ CCAAACAGAC CAAGAACGCC CACACACAC  

- TTCGATAATA ACGGTTCGGT CTCTGAGTTA CATAAAATCA GTCTTGTTCA ACGATTCGAG AACTTAAGAC   
  
  
- TTAATAGTCA GTGATTTGGT GAATAACACT ACATTTATAC AATAGTACAC ATTCGTTACT TCGTCATTCG   
  
  
- GTTCTAATTC CTCTTGGTGT CATTAACCGG AGTTTCTTTC TTTCTTTCTT TTTTGTTTTT ATCATCTCTA   
  
  
- GTTCGGATTT GTGTCTTTAA AGATCTCACT TCACTTTTGA CTTGTTTTGC TGTCTCTCTT TCTCTCCCTC   
  
  
- CTACCCCCTC TACTCTTTTT CTCCCCTAAC GTTCAATGGA ACCTCCACGG GGCTTTCACT CTGGTCCTCG   
  
  
- GGGTTCGCCT CCCTATTCCT AGCGGATACA ACATTCCCTC TAGGAACTCA GGGCATGGTC TAGGTGGGTG   
  
  
- GTGAGGGAAG TTCTTAAACT TGTACAACAA AAACAGGAGA GCAGAGGAAA AAAAGCCCAC AAATACCCAA   
  
  
- ATACATACGT ATATTCCAAC ATAGGAGACT ACGCATATCC GCTTACTGGG GTTTGTCAGA GAGAGAGAGA   
  
  
- GAGAGAGAGA GAGAGAGAGA GAGGGAGACG TACCTACTAC CTCTCCCGCC CCTTGTCCAA GCTAGACGCT   
  
  
- CTCTCACATT CTCTCTCTCT CTACGTCTAC GTTCCACAGT TTTTGCCTTT TCTTTTAATT TAACTACAAA   
  
  
- AGCCCACGTT GGGCTTCATA GCTCGTCGGA CCAAGAAGTT TATTTCTGGC AATTCTAACG GAGGAATAAG   
  
  
- TTGAAGGAAT TTACCCGGTC ACCCCACAGT TCGGGAAGAG ACAAGGTCGG GTATTCATGC GGAGAAAGAC   
  
  
- AGACAAAATA AACCCGGGAG GCTAACGGGC TGAAAAGTAA ACATTAATGA ACGAATTAAT TACCAATATA   
  
  
- CGACACGAAA TAATAATTTT AAGGTGAATG TCTCTCAATG TAGTTATAGC GCTTTGTCTT TCTTTAAGTC   
  
  
- ACAACATATG TAAAGTAAAC CATTATTATG GGTACCCGGT GCCTTTGAAC TGAGGGACAC AGTACTGAGT   
  
  
- TCTTGGTACT GCATGCCCAT TTCCCAACCA AGTTTGGCGC CACTGGTACT TGCTCATTTT GTCTCACGAG   
  
  
- GGTACGTTCA AGTCTTGTTC TGCGAAAGAG GAGGTGTCAG TTATTGTAGT TTCGTTCTCT TTGAAGAAGT   
  
  
- TCCAGATTCG TTAACCAAAG TTACCCTGTT CCAGAAGAAA ATCTGAGTCT GGCCTTTTTC AACTTTTGGG   
  
  
- GCGTGAGTAA TTGTTCTGGC ACTGAACCAG TATAAAGGTT CAACGAAGAA GAAACAGAAA AGCAAAATTA   
  
  
- ATTAATTGAA GAACGTGGCA CATTTTCCTT GTGCTTATGA AACTTACTGT ATACCTTTCG CAATACTAAT   
  
  
- ATATATGGTT GCTAGTTTCT TGGTTGCTTG GGGGTAAAGT CTAAAACAGG AAGGTTGGGT TATTCTCTTC   
  
  
- GGTTTGTCTG GTTCTTGCGG GTGTGTGTG

+     ABRE

| Site Name | Organism | Position | Strand | Matrix score. | sequence | function |
| --- | --- | --- | --- | --- | --- | --- |
| ABRE | Hordeum vulgare | 660 | + | 9 | GCAACGTGTC | cis-acting element involved in the abscisic acid responsiveness |

> 2018/04/13 10:10:12  
+ AAGCTATTAT TGCCAAGCCA GAGACTCAAT GTATTTTAGT CAGAACAAGT TGCTAAGCTC TTGAATTCTG   
  
  
+ AATTATCAGT CACTAAACCA CTTATTGTGA TGTAAATATG TTATCATGTG TAAGCAATGA AGCAGTAAGC   
  
  
+ CAAGATTAAG GAGAACCACA GTAATTGGCC TCAAAGAAAG AAAGAAAGAA AAAACAAAAA TAGTAGAGAT   
  
  
+ CAAGCCTAAA CACAGAAATT TCTAGAGTGA AGTGAAAACT GAACAAAACG ACAGAGAGAA AGAGAGGGAG   
  
  
+ GATGGGGGAG ATGAGAAAAA GAGGGGATTG CAAGTTACCT TGGAGGTGCC CCGAAAGTGA GACCAGGAGC   
  
  
+ CCCAAGCGGA GGGATAAGGA TCGCCTATGT TGTAAGGGAG ATCCTTGAGT CCCGTACCAG ATCCACCCAC   
  
  
+ CACTCCCTTC AAGAATTTGA ACATGTTGTT TTTGTCCTCT CGTCTCCTTT TTTTCGGGTG TTTATGGGTT   
  
  
+ TATGTATGCA TATAAGGTTG TATCCTCTGA TGCGTATAGG CGAATGACCC CAAACAGTCT CTCTCTCTCT   
  
  
+ CTCTCTCTCT CTCTCTCTCT CTCCCTCTGC ATGGATGATG GAGAGGGCGG GGAACAGGTT CGATCTGCGA   
  
  
+ GAGAGTGTAA GAGAGAGAGA GATGCAGATG CAAGGTGTCA AAAACGGAAA AGAAAATTAA ATTGATGTTT   
  
  
+ TCGGGTGCAA CCCGAAGTAT CGAGCAGCCT GGTTCTTCAA ATAAAGACCG TTAAGATTGC CTCCTTATTC   
  
  
+ AACTTCCTTA AATGGGCCAG TGGGGTGTCA AGCCCTTCTC TGTTCCAGCC CATAAGTACG CCTCTTTCTG   
  
  
+ TCTGTTTTAT TTGGGCCCTC CGATTGCCCG ACTTTTCATT TGTAATTACT TGCTTAATTA ATGGTTATAT   
  
  
+ GCTGTGCTTT ATTATTAAAA TTCCACTTAC AGAGAGTTAC ATCAATATCG CGAAACAGAA AGAAATTCAG   
  
  
+ TGTTGTATAC ATTTCATTTG GTAATAATAC CCATGGGCCA CGGAAACTTG ACTCCCTGTG TCATGACTCA   
  
  
+ AGAACCATGA CGTACGGGTA AAGGGTTGGT TCAAACCGCG GTGACCATGA ACGAGTAAAA CAGAGTGCTC   
  
  
+ CCATGCAAGT TCAGAACAAG ACGCTTTCTC CTCCACAGTC AATAACATCA AAGCAAGAGA AACTTCTTCA   
  
  
+ AGGTCTAAGC AATTGGTTTC AATGGGACAA GGTCTTCTTT TAGACTCAGA CCGGAAAAAG TTGAAAACCC   
  
  
+ CGCACTCATT AACAAGACCG TGACTTGGTC ATATTTCCAA GTTGCTTCTT CTTTGTCTTT TCGTTTTAAT   
  
  
+ TAATTAACTT CTTGCACCGT GTAAAAGGAA CACGAATACT TTGAATGACA TATGGAAAGC GTTATGATTA   
  
  
+ TATATACCAA CGATCAAAGA ACCAACGAAC CCCCATTTCA GATTTTGTCC TTCCAACCCA ATAAGAGAAG   
  
  
+ CCAAACAGAC CAAGAACGCC CACACACAC  

- TTCGATAATA ACGGTTCGGT CTCTGAGTTA CATAAAATCA GTCTTGTTCA ACGATTCGAG AACTTAAGAC   
  
  
- TTAATAGTCA GTGATTTGGT GAATAACACT ACATTTATAC AATAGTACAC ATTCGTTACT TCGTCATTCG   
  
  
- GTTCTAATTC CTCTTGGTGT CATTAACCGG AGTTTCTTTC TTTCTTTCTT TTTTGTTTTT ATCATCTCTA   
  
  
- GTTCGGATTT GTGTCTTTAA AGATCTCACT TCACTTTTGA CTTGTTTTGC TGTCTCTCTT TCTCTCCCTC   
  
  
- CTACCCCCTC TACTCTTTTT CTCCCCTAAC GTTCAATGGA ACCTCCACGG GGCTTTCACT CTGGTCCTCG   
  
  
- GGGTTCGCCT CCCTATTCCT AGCGGATACA ACATTCCCTC TAGGAACTCA GGGCATGGTC TAGGTGGGTG   
  
  
- GTGAGGGAAG TTCTTAAACT TGTACAACAA AAACAGGAGA GCAGAGGAAA AAAAGCCCAC AAATACCCAA   
  
  
- ATACATACGT ATATTCCAAC ATAGGAGACT ACGCATATCC GCTTACTGGG GTTTGTCAGA GAGAGAGAGA   
  
  
- GAGAGAGAGA GAGAGAGAGA GAGGGAGACG TACCTACTAC CTCTCCCGCC CCTTGTCCAA GCTAGACGCT   
  
  
- CTCTCACATT CTCTCTCTCT CTACGTCTAC GTTCCACAGT TTTTGCCTTT TCTTTTAATT TAACTACAAA   
  
  
- AGCCCACGTT GGGCTTCATA GCTCGTCGGA CCAAGAAGTT TATTTCTGGC AATTCTAACG GAGGAATAAG   
  
  
- TTGAAGGAAT TTACCCGGTC ACCCCACAGT TCGGGAAGAG ACAAGGTCGG GTATTCATGC GGAGAAAGAC   
  
  
- AGACAAAATA AACCCGGGAG GCTAACGGGC TGAAAAGTAA ACATTAATGA ACGAATTAAT TACCAATATA   
  
  
- CGACACGAAA TAATAATTTT AAGGTGAATG TCTCTCAATG TAGTTATAGC GCTTTGTCTT TCTTTAAGTC   
  
  
- ACAACATATG TAAAGTAAAC CATTATTATG GGTACCCGGT GCCTTTGAAC TGAGGGACAC AGTACTGAGT   
  
  
- TCTTGGTACT GCATGCCCAT TTCCCAACCA AGTTTGGCGC CACTGGTACT TGCTCATTTT GTCTCACGAG   
  
  
- GGTACGTTCA AGTCTTGTTC TGCGAAAGAG GAGGTGTCAG TTATTGTAGT TTCGTTCTCT TTGAAGAAGT   
  
  
- TCCAGATTCG TTAACCAAAG TTACCCTGTT CCAGAAGAAA ATCTGAGTCT GGCCTTTTTC AACTTTTGGG   
  
  
- GCGTGAGTAA TTGTTCTGGC ACTGAACCAG TATAAAGGTT CAACGAAGAA GAAACAGAAA AGCAAAATTA   
  
  
- ATTAATTGAA GAACGTGGCA CATTTTCCTT GTGCTTATGA AACTTACTGT ATACCTTTCG CAATACTAAT   
  
  
- ATATATGGTT GCTAGTTTCT TGGTTGCTTG GGGGTAAAGT CTAAAACAGG AAGGTTGGGT TATTCTCTTC   
  
  
- GGTTTGTCTG GTTCTTGCGG GTGTGTGTG

+     AE-box

| Site Name | Organism | Position | Strand | Matrix score. | sequence | function |
| --- | --- | --- | --- | --- | --- | --- |
| AE-box | Arabidopsis thaliana | 1178 | + | 8 | AGAAACTT | part of a module for light response |

> 2018/04/13 10:10:12  
+ AAGCTATTAT TGCCAAGCCA GAGACTCAAT GTATTTTAGT CAGAACAAGT TGCTAAGCTC TTGAATTCTG   
  
  
+ AATTATCAGT CACTAAACCA CTTATTGTGA TGTAAATATG TTATCATGTG TAAGCAATGA AGCAGTAAGC   
  
  
+ CAAGATTAAG GAGAACCACA GTAATTGGCC TCAAAGAAAG AAAGAAAGAA AAAACAAAAA TAGTAGAGAT   
  
  
+ CAAGCCTAAA CACAGAAATT TCTAGAGTGA AGTGAAAACT GAACAAAACG ACAGAGAGAA AGAGAGGGAG   
  
  
+ GATGGGGGAG ATGAGAAAAA GAGGGGATTG CAAGTTACCT TGGAGGTGCC CCGAAAGTGA GACCAGGAGC   
  
  
+ CCCAAGCGGA GGGATAAGGA TCGCCTATGT TGTAAGGGAG ATCCTTGAGT CCCGTACCAG ATCCACCCAC   
  
  
+ CACTCCCTTC AAGAATTTGA ACATGTTGTT TTTGTCCTCT CGTCTCCTTT TTTTCGGGTG TTTATGGGTT   
  
  
+ TATGTATGCA TATAAGGTTG TATCCTCTGA TGCGTATAGG CGAATGACCC CAAACAGTCT CTCTCTCTCT   
  
  
+ CTCTCTCTCT CTCTCTCTCT CTCCCTCTGC ATGGATGATG GAGAGGGCGG GGAACAGGTT CGATCTGCGA   
  
  
+ GAGAGTGTAA GAGAGAGAGA GATGCAGATG CAAGGTGTCA AAAACGGAAA AGAAAATTAA ATTGATGTTT   
  
  
+ TCGGGTGCAA CCCGAAGTAT CGAGCAGCCT GGTTCTTCAA ATAAAGACCG TTAAGATTGC CTCCTTATTC   
  
  
+ AACTTCCTTA AATGGGCCAG TGGGGTGTCA AGCCCTTCTC TGTTCCAGCC CATAAGTACG CCTCTTTCTG   
  
  
+ TCTGTTTTAT TTGGGCCCTC CGATTGCCCG ACTTTTCATT TGTAATTACT TGCTTAATTA ATGGTTATAT   
  
  
+ GCTGTGCTTT ATTATTAAAA TTCCACTTAC AGAGAGTTAC ATCAATATCG CGAAACAGAA AGAAATTCAG   
  
  
+ TGTTGTATAC ATTTCATTTG GTAATAATAC CCATGGGCCA CGGAAACTTG ACTCCCTGTG TCATGACTCA   
  
  
+ AGAACCATGA CGTACGGGTA AAGGGTTGGT TCAAACCGCG GTGACCATGA ACGAGTAAAA CAGAGTGCTC   
  
  
+ CCATGCAAGT TCAGAACAAG ACGCTTTCTC CTCCACAGTC AATAACATCA AAGCAAGAGA AACTTCTTCA   
  
  
+ AGGTCTAAGC AATTGGTTTC AATGGGACAA GGTCTTCTTT TAGACTCAGA CCGGAAAAAG TTGAAAACCC   
  
  
+ CGCACTCATT AACAAGACCG TGACTTGGTC ATATTTCCAA GTTGCTTCTT CTTTGTCTTT TCGTTTTAAT   
  
  
+ TAATTAACTT CTTGCACCGT GTAAAAGGAA CACGAATACT TTGAATGACA TATGGAAAGC GTTATGATTA   
  
  
+ TATATACCAA CGATCAAAGA ACCAACGAAC CCCCATTTCA GATTTTGTCC TTCCAACCCA ATAAGAGAAG   
  
  
+ CCAAACAGAC CAAGAACGCC CACACACAC  

- TTCGATAATA ACGGTTCGGT CTCTGAGTTA CATAAAATCA GTCTTGTTCA ACGATTCGAG AACTTAAGAC   
  
  
- TTAATAGTCA GTGATTTGGT GAATAACACT ACATTTATAC AATAGTACAC ATTCGTTACT TCGTCATTCG   
  
  
- GTTCTAATTC CTCTTGGTGT CATTAACCGG AGTTTCTTTC TTTCTTTCTT TTTTGTTTTT ATCATCTCTA   
  
  
- GTTCGGATTT GTGTCTTTAA AGATCTCACT TCACTTTTGA CTTGTTTTGC TGTCTCTCTT TCTCTCCCTC   
  
  
- CTACCCCCTC TACTCTTTTT CTCCCCTAAC GTTCAATGGA ACCTCCACGG GGCTTTCACT CTGGTCCTCG   
  
  
- GGGTTCGCCT CCCTATTCCT AGCGGATACA ACATTCCCTC TAGGAACTCA GGGCATGGTC TAGGTGGGTG   
  
  
- GTGAGGGAAG TTCTTAAACT TGTACAACAA AAACAGGAGA GCAGAGGAAA AAAAGCCCAC AAATACCCAA   
  
  
- ATACATACGT ATATTCCAAC ATAGGAGACT ACGCATATCC GCTTACTGGG GTTTGTCAGA GAGAGAGAGA   
  
  
- GAGAGAGAGA GAGAGAGAGA GAGGGAGACG TACCTACTAC CTCTCCCGCC CCTTGTCCAA GCTAGACGCT   
  
  
- CTCTCACATT CTCTCTCTCT CTACGTCTAC GTTCCACAGT TTTTGCCTTT TCTTTTAATT TAACTACAAA   
  
  
- AGCCCACGTT GGGCTTCATA GCTCGTCGGA CCAAGAAGTT TATTTCTGGC AATTCTAACG GAGGAATAAG   
  
  
- TTGAAGGAAT TTACCCGGTC ACCCCACAGT TCGGGAAGAG ACAAGGTCGG GTATTCATGC GGAGAAAGAC   
  
  
- AGACAAAATA AACCCGGGAG GCTAACGGGC TGAAAAGTAA ACATTAATGA ACGAATTAAT TACCAATATA   
  
  
- CGACACGAAA TAATAATTTT AAGGTGAATG TCTCTCAATG TAGTTATAGC GCTTTGTCTT TCTTTAAGTC   
  
  
- ACAACATATG TAAAGTAAAC CATTATTATG GGTACCCGGT GCCTTTGAAC TGAGGGACAC AGTACTGAGT   
  
  
- TCTTGGTACT GCATGCCCAT TTCCCAACCA AGTTTGGCGC CACTGGTACT TGCTCATTTT GTCTCACGAG   
  
  
- GGTACGTTCA AGTCTTGTTC TGCGAAAGAG GAGGTGTCAG TTATTGTAGT TTCGTTCTCT TTGAAGAAGT   
  
  
- TCCAGATTCG TTAACCAAAG TTACCCTGTT CCAGAAGAAA ATCTGAGTCT GGCCTTTTTC AACTTTTGGG   
  
  
- GCGTGAGTAA TTGTTCTGGC ACTGAACCAG TATAAAGGTT CAACGAAGAA GAAACAGAAA AGCAAAATTA   
  
  
- ATTAATTGAA GAACGTGGCA CATTTTCCTT GTGCTTATGA AACTTACTGT ATACCTTTCG CAATACTAAT   
  
  
- ATATATGGTT GCTAGTTTCT TGGTTGCTTG GGGGTAAAGT CTAAAACAGG AAGGTTGGGT TATTCTCTTC   
  
  
- GGTTTGTCTG GTTCTTGCGG GTGTGTGTG

+     ARE

| Site Name | Organism | Position | Strand | Matrix score. | sequence | function |
| --- | --- | --- | --- | --- | --- | --- |
| ARE | Zea mays | 1204 | + | 6 | TGGTTT | cis-acting regulatory element essential for the anaerobic induction |
| ARE | Zea mays | 85 | - | 6 | TGGTTT | cis-acting regulatory element essential for the anaerobic induction |

> 2018/04/13 10:10:12  
+ AAGCTATTAT TGCCAAGCCA GAGACTCAAT GTATTTTAGT CAGAACAAGT TGCTAAGCTC TTGAATTCTG   
  
  
+ AATTATCAGT CACTAAACCA CTTATTGTGA TGTAAATATG TTATCATGTG TAAGCAATGA AGCAGTAAGC   
  
  
+ CAAGATTAAG GAGAACCACA GTAATTGGCC TCAAAGAAAG AAAGAAAGAA AAAACAAAAA TAGTAGAGAT   
  
  
+ CAAGCCTAAA CACAGAAATT TCTAGAGTGA AGTGAAAACT GAACAAAACG ACAGAGAGAA AGAGAGGGAG   
  
  
+ GATGGGGGAG ATGAGAAAAA GAGGGGATTG CAAGTTACCT TGGAGGTGCC CCGAAAGTGA GACCAGGAGC   
  
  
+ CCCAAGCGGA GGGATAAGGA TCGCCTATGT TGTAAGGGAG ATCCTTGAGT CCCGTACCAG ATCCACCCAC   
  
  
+ CACTCCCTTC AAGAATTTGA ACATGTTGTT TTTGTCCTCT CGTCTCCTTT TTTTCGGGTG TTTATGGGTT   
  
  
+ TATGTATGCA TATAAGGTTG TATCCTCTGA TGCGTATAGG CGAATGACCC CAAACAGTCT CTCTCTCTCT   
  
  
+ CTCTCTCTCT CTCTCTCTCT CTCCCTCTGC ATGGATGATG GAGAGGGCGG GGAACAGGTT CGATCTGCGA   
  
  
+ GAGAGTGTAA GAGAGAGAGA GATGCAGATG CAAGGTGTCA AAAACGGAAA AGAAAATTAA ATTGATGTTT   
  
  
+ TCGGGTGCAA CCCGAAGTAT CGAGCAGCCT GGTTCTTCAA ATAAAGACCG TTAAGATTGC CTCCTTATTC   
  
  
+ AACTTCCTTA AATGGGCCAG TGGGGTGTCA AGCCCTTCTC TGTTCCAGCC CATAAGTACG CCTCTTTCTG   
  
  
+ TCTGTTTTAT TTGGGCCCTC CGATTGCCCG ACTTTTCATT TGTAATTACT TGCTTAATTA ATGGTTATAT   
  
  
+ GCTGTGCTTT ATTATTAAAA TTCCACTTAC AGAGAGTTAC ATCAATATCG CGAAACAGAA AGAAATTCAG   
  
  
+ TGTTGTATAC ATTTCATTTG GTAATAATAC CCATGGGCCA CGGAAACTTG ACTCCCTGTG TCATGACTCA   
  
  
+ AGAACCATGA CGTACGGGTA AAGGGTTGGT TCAAACCGCG GTGACCATGA ACGAGTAAAA CAGAGTGCTC   
  
  
+ CCATGCAAGT TCAGAACAAG ACGCTTTCTC CTCCACAGTC AATAACATCA AAGCAAGAGA AACTTCTTCA   
  
  
+ AGGTCTAAGC AATTGGTTTC AATGGGACAA GGTCTTCTTT TAGACTCAGA CCGGAAAAAG TTGAAAACCC   
  
  
+ CGCACTCATT AACAAGACCG TGACTTGGTC ATATTTCCAA GTTGCTTCTT CTTTGTCTTT TCGTTTTAAT   
  
  
+ TAATTAACTT CTTGCACCGT GTAAAAGGAA CACGAATACT TTGAATGACA TATGGAAAGC GTTATGATTA   
  
  
+ TATATACCAA CGATCAAAGA ACCAACGAAC CCCCATTTCA GATTTTGTCC TTCCAACCCA ATAAGAGAAG   
  
  
+ CCAAACAGAC CAAGAACGCC CACACACAC  

- TTCGATAATA ACGGTTCGGT CTCTGAGTTA CATAAAATCA GTCTTGTTCA ACGATTCGAG AACTTAAGAC   
  
  
- TTAATAGTCA GTGATTTGGT GAATAACACT ACATTTATAC AATAGTACAC ATTCGTTACT TCGTCATTCG   
  
  
- GTTCTAATTC CTCTTGGTGT CATTAACCGG AGTTTCTTTC TTTCTTTCTT TTTTGTTTTT ATCATCTCTA   
  
  
- GTTCGGATTT GTGTCTTTAA AGATCTCACT TCACTTTTGA CTTGTTTTGC TGTCTCTCTT TCTCTCCCTC   
  
  
- CTACCCCCTC TACTCTTTTT CTCCCCTAAC GTTCAATGGA ACCTCCACGG GGCTTTCACT CTGGTCCTCG   
  
  
- GGGTTCGCCT CCCTATTCCT AGCGGATACA ACATTCCCTC TAGGAACTCA GGGCATGGTC TAGGTGGGTG   
  
  
- GTGAGGGAAG TTCTTAAACT TGTACAACAA AAACAGGAGA GCAGAGGAAA AAAAGCCCAC AAATACCCAA   
  
  
- ATACATACGT ATATTCCAAC ATAGGAGACT ACGCATATCC GCTTACTGGG GTTTGTCAGA GAGAGAGAGA   
  
  
- GAGAGAGAGA GAGAGAGAGA GAGGGAGACG TACCTACTAC CTCTCCCGCC CCTTGTCCAA GCTAGACGCT   
  
  
- CTCTCACATT CTCTCTCTCT CTACGTCTAC GTTCCACAGT TTTTGCCTTT TCTTTTAATT TAACTACAAA   
  
  
- AGCCCACGTT GGGCTTCATA GCTCGTCGGA CCAAGAAGTT TATTTCTGGC AATTCTAACG GAGGAATAAG   
  
  
- TTGAAGGAAT TTACCCGGTC ACCCCACAGT TCGGGAAGAG ACAAGGTCGG GTATTCATGC GGAGAAAGAC   
  
  
- AGACAAAATA AACCCGGGAG GCTAACGGGC TGAAAAGTAA ACATTAATGA ACGAATTAAT TACCAATATA   
  
  
- CGACACGAAA TAATAATTTT AAGGTGAATG TCTCTCAATG TAGTTATAGC GCTTTGTCTT TCTTTAAGTC   
  
  
- ACAACATATG TAAAGTAAAC CATTATTATG GGTACCCGGT GCCTTTGAAC TGAGGGACAC AGTACTGAGT   
  
  
- TCTTGGTACT GCATGCCCAT TTCCCAACCA AGTTTGGCGC CACTGGTACT TGCTCATTTT GTCTCACGAG   
  
  
- GGTACGTTCA AGTCTTGTTC TGCGAAAGAG GAGGTGTCAG TTATTGTAGT TTCGTTCTCT TTGAAGAAGT   
  
  
- TCCAGATTCG TTAACCAAAG TTACCCTGTT CCAGAAGAAA ATCTGAGTCT GGCCTTTTTC AACTTTTGGG   
  
  
- GCGTGAGTAA TTGTTCTGGC ACTGAACCAG TATAAAGGTT CAACGAAGAA GAAACAGAAA AGCAAAATTA   
  
  
- ATTAATTGAA GAACGTGGCA CATTTTCCTT GTGCTTATGA AACTTACTGT ATACCTTTCG CAATACTAAT   
  
  
- ATATATGGTT GCTAGTTTCT TGGTTGCTTG GGGGTAAAGT CTAAAACAGG AAGGTTGGGT TATTCTCTTC   
  
  
- GGTTTGTCTG GTTCTTGCGG GTGTGTGTG

+     Box 4

| Site Name | Organism | Position | Strand | Matrix score. | sequence | function |
| --- | --- | --- | --- | --- | --- | --- |
| Box 4 | Petroselinum crispum | 897 | - | 6 | ATTAAT | part of a conserved DNA module involved in light responsiveness |
| Box 4 | Petroselinum crispum | 1329 | - | 6 | ATTAAT | part of a conserved DNA module involved in light responsiveness |

> 2018/04/13 10:10:12  
+ AAGCTATTAT TGCCAAGCCA GAGACTCAAT GTATTTTAGT CAGAACAAGT TGCTAAGCTC TTGAATTCTG   
  
  
+ AATTATCAGT CACTAAACCA CTTATTGTGA TGTAAATATG TTATCATGTG TAAGCAATGA AGCAGTAAGC   
  
  
+ CAAGATTAAG GAGAACCACA GTAATTGGCC TCAAAGAAAG AAAGAAAGAA AAAACAAAAA TAGTAGAGAT   
  
  
+ CAAGCCTAAA CACAGAAATT TCTAGAGTGA AGTGAAAACT GAACAAAACG ACAGAGAGAA AGAGAGGGAG   
  
  
+ GATGGGGGAG ATGAGAAAAA GAGGGGATTG CAAGTTACCT TGGAGGTGCC CCGAAAGTGA GACCAGGAGC   
  
  
+ CCCAAGCGGA GGGATAAGGA TCGCCTATGT TGTAAGGGAG ATCCTTGAGT CCCGTACCAG ATCCACCCAC   
  
  
+ CACTCCCTTC AAGAATTTGA ACATGTTGTT TTTGTCCTCT CGTCTCCTTT TTTTCGGGTG TTTATGGGTT   
  
  
+ TATGTATGCA TATAAGGTTG TATCCTCTGA TGCGTATAGG CGAATGACCC CAAACAGTCT CTCTCTCTCT   
  
  
+ CTCTCTCTCT CTCTCTCTCT CTCCCTCTGC ATGGATGATG GAGAGGGCGG GGAACAGGTT CGATCTGCGA   
  
  
+ GAGAGTGTAA GAGAGAGAGA GATGCAGATG CAAGGTGTCA AAAACGGAAA AGAAAATTAA ATTGATGTTT   
  
  
+ TCGGGTGCAA CCCGAAGTAT CGAGCAGCCT GGTTCTTCAA ATAAAGACCG TTAAGATTGC CTCCTTATTC   
  
  
+ AACTTCCTTA AATGGGCCAG TGGGGTGTCA AGCCCTTCTC TGTTCCAGCC CATAAGTACG CCTCTTTCTG   
  
  
+ TCTGTTTTAT TTGGGCCCTC CGATTGCCCG ACTTTTCATT TGTAATTACT TGCTTAATTA ATGGTTATAT   
  
  
+ GCTGTGCTTT ATTATTAAAA TTCCACTTAC AGAGAGTTAC ATCAATATCG CGAAACAGAA AGAAATTCAG   
  
  
+ TGTTGTATAC ATTTCATTTG GTAATAATAC CCATGGGCCA CGGAAACTTG ACTCCCTGTG TCATGACTCA   
  
  
+ AGAACCATGA CGTACGGGTA AAGGGTTGGT TCAAACCGCG GTGACCATGA ACGAGTAAAA CAGAGTGCTC   
  
  
+ CCATGCAAGT TCAGAACAAG ACGCTTTCTC CTCCACAGTC AATAACATCA AAGCAAGAGA AACTTCTTCA   
  
  
+ AGGTCTAAGC AATTGGTTTC AATGGGACAA GGTCTTCTTT TAGACTCAGA CCGGAAAAAG TTGAAAACCC   
  
  
+ CGCACTCATT AACAAGACCG TGACTTGGTC ATATTTCCAA GTTGCTTCTT CTTTGTCTTT TCGTTTTAAT   
  
  
+ TAATTAACTT CTTGCACCGT GTAAAAGGAA CACGAATACT TTGAATGACA TATGGAAAGC GTTATGATTA   
  
  
+ TATATACCAA CGATCAAAGA ACCAACGAAC CCCCATTTCA GATTTTGTCC TTCCAACCCA ATAAGAGAAG   
  
  
+ CCAAACAGAC CAAGAACGCC CACACACAC  

- TTCGATAATA ACGGTTCGGT CTCTGAGTTA CATAAAATCA GTCTTGTTCA ACGATTCGAG AACTTAAGAC   
  
  
- TTAATAGTCA GTGATTTGGT GAATAACACT ACATTTATAC AATAGTACAC ATTCGTTACT TCGTCATTCG   
  
  
- GTTCTAATTC CTCTTGGTGT CATTAACCGG AGTTTCTTTC TTTCTTTCTT TTTTGTTTTT ATCATCTCTA   
  
  
- GTTCGGATTT GTGTCTTTAA AGATCTCACT TCACTTTTGA CTTGTTTTGC TGTCTCTCTT TCTCTCCCTC   
  
  
- CTACCCCCTC TACTCTTTTT CTCCCCTAAC GTTCAATGGA ACCTCCACGG GGCTTTCACT CTGGTCCTCG   
  
  
- GGGTTCGCCT CCCTATTCCT AGCGGATACA ACATTCCCTC TAGGAACTCA GGGCATGGTC TAGGTGGGTG   
  
  
- GTGAGGGAAG TTCTTAAACT TGTACAACAA AAACAGGAGA GCAGAGGAAA AAAAGCCCAC AAATACCCAA   
  
  
- ATACATACGT ATATTCCAAC ATAGGAGACT ACGCATATCC GCTTACTGGG GTTTGTCAGA GAGAGAGAGA   
  
  
- GAGAGAGAGA GAGAGAGAGA GAGGGAGACG TACCTACTAC CTCTCCCGCC CCTTGTCCAA GCTAGACGCT   
  
  
- CTCTCACATT CTCTCTCTCT CTACGTCTAC GTTCCACAGT TTTTGCCTTT TCTTTTAATT TAACTACAAA   
  
  
- AGCCCACGTT GGGCTTCATA GCTCGTCGGA CCAAGAAGTT TATTTCTGGC AATTCTAACG GAGGAATAAG   
  
  
- TTGAAGGAAT TTACCCGGTC ACCCCACAGT TCGGGAAGAG ACAAGGTCGG GTATTCATGC GGAGAAAGAC   
  
  
- AGACAAAATA AACCCGGGAG GCTAACGGGC TGAAAAGTAA ACATTAATGA ACGAATTAAT TACCAATATA   
  
  
- CGACACGAAA TAATAATTTT AAGGTGAATG TCTCTCAATG TAGTTATAGC GCTTTGTCTT TCTTTAAGTC   
  
  
- ACAACATATG TAAAGTAAAC CATTATTATG GGTACCCGGT GCCTTTGAAC TGAGGGACAC AGTACTGAGT   
  
  
- TCTTGGTACT GCATGCCCAT TTCCCAACCA AGTTTGGCGC CACTGGTACT TGCTCATTTT GTCTCACGAG   
  
  
- GGTACGTTCA AGTCTTGTTC TGCGAAAGAG GAGGTGTCAG TTATTGTAGT TTCGTTCTCT TTGAAGAAGT   
  
  
- TCCAGATTCG TTAACCAAAG TTACCCTGTT CCAGAAGAAA ATCTGAGTCT GGCCTTTTTC AACTTTTGGG   
  
  
- GCGTGAGTAA TTGTTCTGGC ACTGAACCAG TATAAAGGTT CAACGAAGAA GAAACAGAAA AGCAAAATTA   
  
  
- ATTAATTGAA GAACGTGGCA CATTTTCCTT GTGCTTATGA AACTTACTGT ATACCTTTCG CAATACTAAT   
  
  
- ATATATGGTT GCTAGTTTCT TGGTTGCTTG GGGGTAAAGT CTAAAACAGG AAGGTTGGGT TATTCTCTTC   
  
  
- GGTTTGTCTG GTTCTTGCGG GTGTGTGTG

+     Box II

| Site Name | Organism | Position | Strand | Matrix score. | sequence | function |
| --- | --- | --- | --- | --- | --- | --- |
| Box II | Solanum tuberosum | 999 | + | 9 | TGGTAATAA | part of a light responsive element |

> 2018/04/13 10:10:12  
+ AAGCTATTAT TGCCAAGCCA GAGACTCAAT GTATTTTAGT CAGAACAAGT TGCTAAGCTC TTGAATTCTG   
  
  
+ AATTATCAGT CACTAAACCA CTTATTGTGA TGTAAATATG TTATCATGTG TAAGCAATGA AGCAGTAAGC   
  
  
+ CAAGATTAAG GAGAACCACA GTAATTGGCC TCAAAGAAAG AAAGAAAGAA AAAACAAAAA TAGTAGAGAT   
  
  
+ CAAGCCTAAA CACAGAAATT TCTAGAGTGA AGTGAAAACT GAACAAAACG ACAGAGAGAA AGAGAGGGAG   
  
  
+ GATGGGGGAG ATGAGAAAAA GAGGGGATTG CAAGTTACCT TGGAGGTGCC CCGAAAGTGA GACCAGGAGC   
  
  
+ CCCAAGCGGA GGGATAAGGA TCGCCTATGT TGTAAGGGAG ATCCTTGAGT CCCGTACCAG ATCCACCCAC   
  
  
+ CACTCCCTTC AAGAATTTGA ACATGTTGTT TTTGTCCTCT CGTCTCCTTT TTTTCGGGTG TTTATGGGTT   
  
  
+ TATGTATGCA TATAAGGTTG TATCCTCTGA TGCGTATAGG CGAATGACCC CAAACAGTCT CTCTCTCTCT   
  
  
+ CTCTCTCTCT CTCTCTCTCT CTCCCTCTGC ATGGATGATG GAGAGGGCGG GGAACAGGTT CGATCTGCGA   
  
  
+ GAGAGTGTAA GAGAGAGAGA GATGCAGATG CAAGGTGTCA AAAACGGAAA AGAAAATTAA ATTGATGTTT   
  
  
+ TCGGGTGCAA CCCGAAGTAT CGAGCAGCCT GGTTCTTCAA ATAAAGACCG TTAAGATTGC CTCCTTATTC   
  
  
+ AACTTCCTTA AATGGGCCAG TGGGGTGTCA AGCCCTTCTC TGTTCCAGCC CATAAGTACG CCTCTTTCTG   
  
  
+ TCTGTTTTAT TTGGGCCCTC CGATTGCCCG ACTTTTCATT TGTAATTACT TGCTTAATTA ATGGTTATAT   
  
  
+ GCTGTGCTTT ATTATTAAAA TTCCACTTAC AGAGAGTTAC ATCAATATCG CGAAACAGAA AGAAATTCAG   
  
  
+ TGTTGTATAC ATTTCATTTG GTAATAATAC CCATGGGCCA CGGAAACTTG ACTCCCTGTG TCATGACTCA   
  
  
+ AGAACCATGA CGTACGGGTA AAGGGTTGGT TCAAACCGCG GTGACCATGA ACGAGTAAAA CAGAGTGCTC   
  
  
+ CCATGCAAGT TCAGAACAAG ACGCTTTCTC CTCCACAGTC AATAACATCA AAGCAAGAGA AACTTCTTCA   
  
  
+ AGGTCTAAGC AATTGGTTTC AATGGGACAA GGTCTTCTTT TAGACTCAGA CCGGAAAAAG TTGAAAACCC   
  
  
+ CGCACTCATT AACAAGACCG TGACTTGGTC ATATTTCCAA GTTGCTTCTT CTTTGTCTTT TCGTTTTAAT   
  
  
+ TAATTAACTT CTTGCACCGT GTAAAAGGAA CACGAATACT TTGAATGACA TATGGAAAGC GTTATGATTA   
  
  
+ TATATACCAA CGATCAAAGA ACCAACGAAC CCCCATTTCA GATTTTGTCC TTCCAACCCA ATAAGAGAAG   
  
  
+ CCAAACAGAC CAAGAACGCC CACACACAC  

- TTCGATAATA ACGGTTCGGT CTCTGAGTTA CATAAAATCA GTCTTGTTCA ACGATTCGAG AACTTAAGAC   
  
  
- TTAATAGTCA GTGATTTGGT GAATAACACT ACATTTATAC AATAGTACAC ATTCGTTACT TCGTCATTCG   
  
  
- GTTCTAATTC CTCTTGGTGT CATTAACCGG AGTTTCTTTC TTTCTTTCTT TTTTGTTTTT ATCATCTCTA   
  
  
- GTTCGGATTT GTGTCTTTAA AGATCTCACT TCACTTTTGA CTTGTTTTGC TGTCTCTCTT TCTCTCCCTC   
  
  
- CTACCCCCTC TACTCTTTTT CTCCCCTAAC GTTCAATGGA ACCTCCACGG GGCTTTCACT CTGGTCCTCG   
  
  
- GGGTTCGCCT CCCTATTCCT AGCGGATACA ACATTCCCTC TAGGAACTCA GGGCATGGTC TAGGTGGGTG   
  
  
- GTGAGGGAAG TTCTTAAACT TGTACAACAA AAACAGGAGA GCAGAGGAAA AAAAGCCCAC AAATACCCAA   
  
  
- ATACATACGT ATATTCCAAC ATAGGAGACT ACGCATATCC GCTTACTGGG GTTTGTCAGA GAGAGAGAGA   
  
  
- GAGAGAGAGA GAGAGAGAGA GAGGGAGACG TACCTACTAC CTCTCCCGCC CCTTGTCCAA GCTAGACGCT   
  
  
- CTCTCACATT CTCTCTCTCT CTACGTCTAC GTTCCACAGT TTTTGCCTTT TCTTTTAATT TAACTACAAA   
  
  
- AGCCCACGTT GGGCTTCATA GCTCGTCGGA CCAAGAAGTT TATTTCTGGC AATTCTAACG GAGGAATAAG   
  
  
- TTGAAGGAAT TTACCCGGTC ACCCCACAGT TCGGGAAGAG ACAAGGTCGG GTATTCATGC GGAGAAAGAC   
  
  
- AGACAAAATA AACCCGGGAG GCTAACGGGC TGAAAAGTAA ACATTAATGA ACGAATTAAT TACCAATATA   
  
  
- CGACACGAAA TAATAATTTT AAGGTGAATG TCTCTCAATG TAGTTATAGC GCTTTGTCTT TCTTTAAGTC   
  
  
- ACAACATATG TAAAGTAAAC CATTATTATG GGTACCCGGT GCCTTTGAAC TGAGGGACAC AGTACTGAGT   
  
  
- TCTTGGTACT GCATGCCCAT TTCCCAACCA AGTTTGGCGC CACTGGTACT TGCTCATTTT GTCTCACGAG   
  
  
- GGTACGTTCA AGTCTTGTTC TGCGAAAGAG GAGGTGTCAG TTATTGTAGT TTCGTTCTCT TTGAAGAAGT   
  
  
- TCCAGATTCG TTAACCAAAG TTACCCTGTT CCAGAAGAAA ATCTGAGTCT GGCCTTTTTC AACTTTTGGG   
  
  
- GCGTGAGTAA TTGTTCTGGC ACTGAACCAG TATAAAGGTT CAACGAAGAA GAAACAGAAA AGCAAAATTA   
  
  
- ATTAATTGAA GAACGTGGCA CATTTTCCTT GTGCTTATGA AACTTACTGT ATACCTTTCG CAATACTAAT   
  
  
- ATATATGGTT GCTAGTTTCT TGGTTGCTTG GGGGTAAAGT CTAAAACAGG AAGGTTGGGT TATTCTCTTC   
  
  
- GGTTTGTCTG GTTCTTGCGG GTGTGTGTG

+     CAAT-box

| Site Name | Organism | Position | Strand | Matrix score. | sequence | function |
| --- | --- | --- | --- | --- | --- | --- |
| CAAT-box | Arabidopsis thaliana | 1458 | + | 5 | CCAAT | common cis-acting element in promoter and enhancer regions |
| CAAT-box | Hordeum vulgare | 1160 | + | 4 | CAAT | common cis-acting element in promoter and enhancer regions |
| CAAT-box | Brassica rapa | 996 | - | 5 | CAAAT | common cis-acting element in promoter and enhancer regions |
| CAAT-box | Hordeum vulgare | 953 | + | 4 | CAAT | common cis-acting element in promoter and enhancer regions |
| CAAT-box | Brassica rapa | 849 | - | 5 | CAAAT | common cis-acting element in promoter and enhancer regions |
| CAAT-box | Brassica rapa | 738 | + | 5 | CAAAT | common cis-acting element in promoter and enhancer regions |
| CAAT-box | Hordeum vulgare | 1459 | + | 4 | CAAT | common cis-acting element in promoter and enhancer regions |
| CAAT-box | Brassica rapa | 878 | - | 5 | CAAAT | common cis-acting element in promoter and enhancer regions |
| CAAT-box | Arabidopsis thaliana | 863 | - | 6 | gGCAAT | common cis-acting element in promoter and enhancer regions |
| CAAT-box | Hordeum vulgare | 94 | - | 4 | CAAT | common cis-acting element in promoter and enhancer regions |
| CAAT-box | Glycine max | 690 | - | 5 | CAATT | common cis-acting element in promoter and enhancer regions |
| CAAT-box | Arabidopsis thaliana | 9 | - | 6 | gGCAAT | common cis-acting element in promoter and enhancer regions |
| CAAT-box | Arabidopsis thaliana | 1202 | - | 5 | CCAAT | common cis-acting element in promoter and enhancer regions |
| CAAT-box | Glycine max | 1200 | + | 5 | CAATT | common cis-acting element in promoter and enhancer regions |
| CAAT-box | Brassica rapa | 435 | - | 5 | CAAAT | common cis-acting element in promoter and enhancer regions |
| CAAT-box | Arabidopsis thaliana | 756 | - | 6 | gGCAAT | common cis-acting element in promoter and enhancer regions |
| CAAT-box | Glycine max | 1201 | - | 5 | CAATT | common cis-acting element in promoter and enhancer regions |
| CAAT-box | Hordeum vulgare | 307 | - | 4 | CAAT | common cis-acting element in promoter and enhancer regions |
| CAAT-box | Glycine max | 163 | - | 5 | CAATT | common cis-acting element in promoter and enhancer regions |
| CAAT-box | Hordeum vulgare | 1210 | + | 4 | CAAT | common cis-acting element in promoter and enhancer regions |
| CAAT-box | Hordeum vulgare | 125 | + | 4 | CAAT | common cis-acting element in promoter and enhancer regions |
| CAAT-box | Hordeum vulgare | 691 | - | 4 | CAAT | common cis-acting element in promoter and enhancer regions |
| CAAT-box | Arabidopsis thaliana | 164 | - | 5 | CCAAT | common cis-acting element in promoter and enhancer regions |
| CAAT-box | Hordeum vulgare | 27 | + | 4 | CAAT | common cis-acting element in promoter and enhancer regions |

> 2018/04/13 10:10:12  
+ AAGCTATTAT TGCCAAGCCA GAGACTCAAT GTATTTTAGT CAGAACAAGT TGCTAAGCTC TTGAATTCTG   
  
  
+ AATTATCAGT CACTAAACCA CTTATTGTGA TGTAAATATG TTATCATGTG TAAGCAATGA AGCAGTAAGC   
  
  
+ CAAGATTAAG GAGAACCACA GTAATTGGCC TCAAAGAAAG AAAGAAAGAA AAAACAAAAA TAGTAGAGAT   
  
  
+ CAAGCCTAAA CACAGAAATT TCTAGAGTGA AGTGAAAACT GAACAAAACG ACAGAGAGAA AGAGAGGGAG   
  
  
+ GATGGGGGAG ATGAGAAAAA GAGGGGATTG CAAGTTACCT TGGAGGTGCC CCGAAAGTGA GACCAGGAGC   
  
  
+ CCCAAGCGGA GGGATAAGGA TCGCCTATGT TGTAAGGGAG ATCCTTGAGT CCCGTACCAG ATCCACCCAC   
  
  
+ CACTCCCTTC AAGAATTTGA ACATGTTGTT TTTGTCCTCT CGTCTCCTTT TTTTCGGGTG TTTATGGGTT   
  
  
+ TATGTATGCA TATAAGGTTG TATCCTCTGA TGCGTATAGG CGAATGACCC CAAACAGTCT CTCTCTCTCT   
  
  
+ CTCTCTCTCT CTCTCTCTCT CTCCCTCTGC ATGGATGATG GAGAGGGCGG GGAACAGGTT CGATCTGCGA   
  
  
+ GAGAGTGTAA GAGAGAGAGA GATGCAGATG CAAGGTGTCA AAAACGGAAA AGAAAATTAA ATTGATGTTT   
  
  
+ TCGGGTGCAA CCCGAAGTAT CGAGCAGCCT GGTTCTTCAA ATAAAGACCG TTAAGATTGC CTCCTTATTC   
  
  
+ AACTTCCTTA AATGGGCCAG TGGGGTGTCA AGCCCTTCTC TGTTCCAGCC CATAAGTACG CCTCTTTCTG   
  
  
+ TCTGTTTTAT TTGGGCCCTC CGATTGCCCG ACTTTTCATT TGTAATTACT TGCTTAATTA ATGGTTATAT   
  
  
+ GCTGTGCTTT ATTATTAAAA TTCCACTTAC AGAGAGTTAC ATCAATATCG CGAAACAGAA AGAAATTCAG   
  
  
+ TGTTGTATAC ATTTCATTTG GTAATAATAC CCATGGGCCA CGGAAACTTG ACTCCCTGTG TCATGACTCA   
  
  
+ AGAACCATGA CGTACGGGTA AAGGGTTGGT TCAAACCGCG GTGACCATGA ACGAGTAAAA CAGAGTGCTC   
  
  
+ CCATGCAAGT TCAGAACAAG ACGCTTTCTC CTCCACAGTC AATAACATCA AAGCAAGAGA AACTTCTTCA   
  
  
+ AGGTCTAAGC AATTGGTTTC AATGGGACAA GGTCTTCTTT TAGACTCAGA CCGGAAAAAG TTGAAAACCC   
  
  
+ CGCACTCATT AACAAGACCG TGACTTGGTC ATATTTCCAA GTTGCTTCTT CTTTGTCTTT TCGTTTTAAT   
  
  
+ TAATTAACTT CTTGCACCGT GTAAAAGGAA CACGAATACT TTGAATGACA TATGGAAAGC GTTATGATTA   
  
  
+ TATATACCAA CGATCAAAGA ACCAACGAAC CCCCATTTCA GATTTTGTCC TTCCAACCCA ATAAGAGAAG   
  
  
+ CCAAACAGAC CAAGAACGCC CACACACAC  

- TTCGATAATA ACGGTTCGGT CTCTGAGTTA CATAAAATCA GTCTTGTTCA ACGATTCGAG AACTTAAGAC   
  
  
- TTAATAGTCA GTGATTTGGT GAATAACACT ACATTTATAC AATAGTACAC ATTCGTTACT TCGTCATTCG   
  
  
- GTTCTAATTC CTCTTGGTGT CATTAACCGG AGTTTCTTTC TTTCTTTCTT TTTTGTTTTT ATCATCTCTA   
  
  
- GTTCGGATTT GTGTCTTTAA AGATCTCACT TCACTTTTGA CTTGTTTTGC TGTCTCTCTT TCTCTCCCTC   
  
  
- CTACCCCCTC TACTCTTTTT CTCCCCTAAC GTTCAATGGA ACCTCCACGG GGCTTTCACT CTGGTCCTCG   
  
  
- GGGTTCGCCT CCCTATTCCT AGCGGATACA ACATTCCCTC TAGGAACTCA GGGCATGGTC TAGGTGGGTG   
  
  
- GTGAGGGAAG TTCTTAAACT TGTACAACAA AAACAGGAGA GCAGAGGAAA AAAAGCCCAC AAATACCCAA   
  
  
- ATACATACGT ATATTCCAAC ATAGGAGACT ACGCATATCC GCTTACTGGG GTTTGTCAGA GAGAGAGAGA   
  
  
- GAGAGAGAGA GAGAGAGAGA GAGGGAGACG TACCTACTAC CTCTCCCGCC CCTTGTCCAA GCTAGACGCT   
  
  
- CTCTCACATT CTCTCTCTCT CTACGTCTAC GTTCCACAGT TTTTGCCTTT TCTTTTAATT TAACTACAAA   
  
  
- AGCCCACGTT GGGCTTCATA GCTCGTCGGA CCAAGAAGTT TATTTCTGGC AATTCTAACG GAGGAATAAG   
  
  
- TTGAAGGAAT TTACCCGGTC ACCCCACAGT TCGGGAAGAG ACAAGGTCGG GTATTCATGC GGAGAAAGAC   
  
  
- AGACAAAATA AACCCGGGAG GCTAACGGGC TGAAAAGTAA ACATTAATGA ACGAATTAAT TACCAATATA   
  
  
- CGACACGAAA TAATAATTTT AAGGTGAATG TCTCTCAATG TAGTTATAGC GCTTTGTCTT TCTTTAAGTC   
  
  
- ACAACATATG TAAAGTAAAC CATTATTATG GGTACCCGGT GCCTTTGAAC TGAGGGACAC AGTACTGAGT   
  
  
- TCTTGGTACT GCATGCCCAT TTCCCAACCA AGTTTGGCGC CACTGGTACT TGCTCATTTT GTCTCACGAG   
  
  
- GGTACGTTCA AGTCTTGTTC TGCGAAAGAG GAGGTGTCAG TTATTGTAGT TTCGTTCTCT TTGAAGAAGT   
  
  
- TCCAGATTCG TTAACCAAAG TTACCCTGTT CCAGAAGAAA ATCTGAGTCT GGCCTTTTTC AACTTTTGGG   
  
  
- GCGTGAGTAA TTGTTCTGGC ACTGAACCAG TATAAAGGTT CAACGAAGAA GAAACAGAAA AGCAAAATTA   
  
  
- ATTAATTGAA GAACGTGGCA CATTTTCCTT GTGCTTATGA AACTTACTGT ATACCTTTCG CAATACTAAT   
  
  
- ATATATGGTT GCTAGTTTCT TGGTTGCTTG GGGGTAAAGT CTAAAACAGG AAGGTTGGGT TATTCTCTTC   
  
  
- GGTTTGTCTG GTTCTTGCGG GTGTGTGTG

+     CGTCA-motif

| Site Name | Organism | Position | Strand | Matrix score. | sequence | function |
| --- | --- | --- | --- | --- | --- | --- |
| CGTCA-motif | Hordeum vulgare | 1058 | - | 5 | CGTCA | cis-acting regulatory element involved in the MeJA-responsiveness |

> 2018/04/13 10:10:12  
+ AAGCTATTAT TGCCAAGCCA GAGACTCAAT GTATTTTAGT CAGAACAAGT TGCTAAGCTC TTGAATTCTG   
  
  
+ AATTATCAGT CACTAAACCA CTTATTGTGA TGTAAATATG TTATCATGTG TAAGCAATGA AGCAGTAAGC   
  
  
+ CAAGATTAAG GAGAACCACA GTAATTGGCC TCAAAGAAAG AAAGAAAGAA AAAACAAAAA TAGTAGAGAT   
  
  
+ CAAGCCTAAA CACAGAAATT TCTAGAGTGA AGTGAAAACT GAACAAAACG ACAGAGAGAA AGAGAGGGAG   
  
  
+ GATGGGGGAG ATGAGAAAAA GAGGGGATTG CAAGTTACCT TGGAGGTGCC CCGAAAGTGA GACCAGGAGC   
  
  
+ CCCAAGCGGA GGGATAAGGA TCGCCTATGT TGTAAGGGAG ATCCTTGAGT CCCGTACCAG ATCCACCCAC   
  
  
+ CACTCCCTTC AAGAATTTGA ACATGTTGTT TTTGTCCTCT CGTCTCCTTT TTTTCGGGTG TTTATGGGTT   
  
  
+ TATGTATGCA TATAAGGTTG TATCCTCTGA TGCGTATAGG CGAATGACCC CAAACAGTCT CTCTCTCTCT   
  
  
+ CTCTCTCTCT CTCTCTCTCT CTCCCTCTGC ATGGATGATG GAGAGGGCGG GGAACAGGTT CGATCTGCGA   
  
  
+ GAGAGTGTAA GAGAGAGAGA GATGCAGATG CAAGGTGTCA AAAACGGAAA AGAAAATTAA ATTGATGTTT   
  
  
+ TCGGGTGCAA CCCGAAGTAT CGAGCAGCCT GGTTCTTCAA ATAAAGACCG TTAAGATTGC CTCCTTATTC   
  
  
+ AACTTCCTTA AATGGGCCAG TGGGGTGTCA AGCCCTTCTC TGTTCCAGCC CATAAGTACG CCTCTTTCTG   
  
  
+ TCTGTTTTAT TTGGGCCCTC CGATTGCCCG ACTTTTCATT TGTAATTACT TGCTTAATTA ATGGTTATAT   
  
  
+ GCTGTGCTTT ATTATTAAAA TTCCACTTAC AGAGAGTTAC ATCAATATCG CGAAACAGAA AGAAATTCAG   
  
  
+ TGTTGTATAC ATTTCATTTG GTAATAATAC CCATGGGCCA CGGAAACTTG ACTCCCTGTG TCATGACTCA   
  
  
+ AGAACCATGA CGTACGGGTA AAGGGTTGGT TCAAACCGCG GTGACCATGA ACGAGTAAAA CAGAGTGCTC   
  
  
+ CCATGCAAGT TCAGAACAAG ACGCTTTCTC CTCCACAGTC AATAACATCA AAGCAAGAGA AACTTCTTCA   
  
  
+ AGGTCTAAGC AATTGGTTTC AATGGGACAA GGTCTTCTTT TAGACTCAGA CCGGAAAAAG TTGAAAACCC   
  
  
+ CGCACTCATT AACAAGACCG TGACTTGGTC ATATTTCCAA GTTGCTTCTT CTTTGTCTTT TCGTTTTAAT   
  
  
+ TAATTAACTT CTTGCACCGT GTAAAAGGAA CACGAATACT TTGAATGACA TATGGAAAGC GTTATGATTA   
  
  
+ TATATACCAA CGATCAAAGA ACCAACGAAC CCCCATTTCA GATTTTGTCC TTCCAACCCA ATAAGAGAAG   
  
  
+ CCAAACAGAC CAAGAACGCC CACACACAC  

- TTCGATAATA ACGGTTCGGT CTCTGAGTTA CATAAAATCA GTCTTGTTCA ACGATTCGAG AACTTAAGAC   
  
  
- TTAATAGTCA GTGATTTGGT GAATAACACT ACATTTATAC AATAGTACAC ATTCGTTACT TCGTCATTCG   
  
  
- GTTCTAATTC CTCTTGGTGT CATTAACCGG AGTTTCTTTC TTTCTTTCTT TTTTGTTTTT ATCATCTCTA   
  
  
- GTTCGGATTT GTGTCTTTAA AGATCTCACT TCACTTTTGA CTTGTTTTGC TGTCTCTCTT TCTCTCCCTC   
  
  
- CTACCCCCTC TACTCTTTTT CTCCCCTAAC GTTCAATGGA ACCTCCACGG GGCTTTCACT CTGGTCCTCG   
  
  
- GGGTTCGCCT CCCTATTCCT AGCGGATACA ACATTCCCTC TAGGAACTCA GGGCATGGTC TAGGTGGGTG   
  
  
- GTGAGGGAAG TTCTTAAACT TGTACAACAA AAACAGGAGA GCAGAGGAAA AAAAGCCCAC AAATACCCAA   
  
  
- ATACATACGT ATATTCCAAC ATAGGAGACT ACGCATATCC GCTTACTGGG GTTTGTCAGA GAGAGAGAGA   
  
  
- GAGAGAGAGA GAGAGAGAGA GAGGGAGACG TACCTACTAC CTCTCCCGCC CCTTGTCCAA GCTAGACGCT   
  
  
- CTCTCACATT CTCTCTCTCT CTACGTCTAC GTTCCACAGT TTTTGCCTTT TCTTTTAATT TAACTACAAA   
  
  
- AGCCCACGTT GGGCTTCATA GCTCGTCGGA CCAAGAAGTT TATTTCTGGC AATTCTAACG GAGGAATAAG   
  
  
- TTGAAGGAAT TTACCCGGTC ACCCCACAGT TCGGGAAGAG ACAAGGTCGG GTATTCATGC GGAGAAAGAC   
  
  
- AGACAAAATA AACCCGGGAG GCTAACGGGC TGAAAAGTAA ACATTAATGA ACGAATTAAT TACCAATATA   
  
  
- CGACACGAAA TAATAATTTT AAGGTGAATG TCTCTCAATG TAGTTATAGC GCTTTGTCTT TCTTTAAGTC   
  
  
- ACAACATATG TAAAGTAAAC CATTATTATG GGTACCCGGT GCCTTTGAAC TGAGGGACAC AGTACTGAGT   
  
  
- TCTTGGTACT GCATGCCCAT TTCCCAACCA AGTTTGGCGC CACTGGTACT TGCTCATTTT GTCTCACGAG   
  
  
- GGTACGTTCA AGTCTTGTTC TGCGAAAGAG GAGGTGTCAG TTATTGTAGT TTCGTTCTCT TTGAAGAAGT   
  
  
- TCCAGATTCG TTAACCAAAG TTACCCTGTT CCAGAAGAAA ATCTGAGTCT GGCCTTTTTC AACTTTTGGG   
  
  
- GCGTGAGTAA TTGTTCTGGC ACTGAACCAG TATAAAGGTT CAACGAAGAA GAAACAGAAA AGCAAAATTA   
  
  
- ATTAATTGAA GAACGTGGCA CATTTTCCTT GTGCTTATGA AACTTACTGT ATACCTTTCG CAATACTAAT   
  
  
- ATATATGGTT GCTAGTTTCT TGGTTGCTTG GGGGTAAAGT CTAAAACAGG AAGGTTGGGT TATTCTCTTC   
  
  
- GGTTTGTCTG GTTCTTGCGG GTGTGTGTG

+     ELI-box3

| Site Name | Organism | Position | Strand | Matrix score. | sequence | function |
| --- | --- | --- | --- | --- | --- | --- |
| ELI-box3 | Brassica oleracea | 1201 | - | 9 | AAACCAATT | elicitor-responsive element |

> 2018/04/13 10:10:12  
+ AAGCTATTAT TGCCAAGCCA GAGACTCAAT GTATTTTAGT CAGAACAAGT TGCTAAGCTC TTGAATTCTG   
  
  
+ AATTATCAGT CACTAAACCA CTTATTGTGA TGTAAATATG TTATCATGTG TAAGCAATGA AGCAGTAAGC   
  
  
+ CAAGATTAAG GAGAACCACA GTAATTGGCC TCAAAGAAAG AAAGAAAGAA AAAACAAAAA TAGTAGAGAT   
  
  
+ CAAGCCTAAA CACAGAAATT TCTAGAGTGA AGTGAAAACT GAACAAAACG ACAGAGAGAA AGAGAGGGAG   
  
  
+ GATGGGGGAG ATGAGAAAAA GAGGGGATTG CAAGTTACCT TGGAGGTGCC CCGAAAGTGA GACCAGGAGC   
  
  
+ CCCAAGCGGA GGGATAAGGA TCGCCTATGT TGTAAGGGAG ATCCTTGAGT CCCGTACCAG ATCCACCCAC   
  
  
+ CACTCCCTTC AAGAATTTGA ACATGTTGTT TTTGTCCTCT CGTCTCCTTT TTTTCGGGTG TTTATGGGTT   
  
  
+ TATGTATGCA TATAAGGTTG TATCCTCTGA TGCGTATAGG CGAATGACCC CAAACAGTCT CTCTCTCTCT   
  
  
+ CTCTCTCTCT CTCTCTCTCT CTCCCTCTGC ATGGATGATG GAGAGGGCGG GGAACAGGTT CGATCTGCGA   
  
  
+ GAGAGTGTAA GAGAGAGAGA GATGCAGATG CAAGGTGTCA AAAACGGAAA AGAAAATTAA ATTGATGTTT   
  
  
+ TCGGGTGCAA CCCGAAGTAT CGAGCAGCCT GGTTCTTCAA ATAAAGACCG TTAAGATTGC CTCCTTATTC   
  
  
+ AACTTCCTTA AATGGGCCAG TGGGGTGTCA AGCCCTTCTC TGTTCCAGCC CATAAGTACG CCTCTTTCTG   
  
  
+ TCTGTTTTAT TTGGGCCCTC CGATTGCCCG ACTTTTCATT TGTAATTACT TGCTTAATTA ATGGTTATAT   
  
  
+ GCTGTGCTTT ATTATTAAAA TTCCACTTAC AGAGAGTTAC ATCAATATCG CGAAACAGAA AGAAATTCAG   
  
  
+ TGTTGTATAC ATTTCATTTG GTAATAATAC CCATGGGCCA CGGAAACTTG ACTCCCTGTG TCATGACTCA   
  
  
+ AGAACCATGA CGTACGGGTA AAGGGTTGGT TCAAACCGCG GTGACCATGA ACGAGTAAAA CAGAGTGCTC   
  
  
+ CCATGCAAGT TCAGAACAAG ACGCTTTCTC CTCCACAGTC AATAACATCA AAGCAAGAGA AACTTCTTCA   
  
  
+ AGGTCTAAGC AATTGGTTTC AATGGGACAA GGTCTTCTTT TAGACTCAGA CCGGAAAAAG TTGAAAACCC   
  
  
+ CGCACTCATT AACAAGACCG TGACTTGGTC ATATTTCCAA GTTGCTTCTT CTTTGTCTTT TCGTTTTAAT   
  
  
+ TAATTAACTT CTTGCACCGT GTAAAAGGAA CACGAATACT TTGAATGACA TATGGAAAGC GTTATGATTA   
  
  
+ TATATACCAA CGATCAAAGA ACCAACGAAC CCCCATTTCA GATTTTGTCC TTCCAACCCA ATAAGAGAAG   
  
  
+ CCAAACAGAC CAAGAACGCC CACACACAC  

- TTCGATAATA ACGGTTCGGT CTCTGAGTTA CATAAAATCA GTCTTGTTCA ACGATTCGAG AACTTAAGAC   
  
  
- TTAATAGTCA GTGATTTGGT GAATAACACT ACATTTATAC AATAGTACAC ATTCGTTACT TCGTCATTCG   
  
  
- GTTCTAATTC CTCTTGGTGT CATTAACCGG AGTTTCTTTC TTTCTTTCTT TTTTGTTTTT ATCATCTCTA   
  
  
- GTTCGGATTT GTGTCTTTAA AGATCTCACT TCACTTTTGA CTTGTTTTGC TGTCTCTCTT TCTCTCCCTC   
  
  
- CTACCCCCTC TACTCTTTTT CTCCCCTAAC GTTCAATGGA ACCTCCACGG GGCTTTCACT CTGGTCCTCG   
  
  
- GGGTTCGCCT CCCTATTCCT AGCGGATACA ACATTCCCTC TAGGAACTCA GGGCATGGTC TAGGTGGGTG   
  
  
- GTGAGGGAAG TTCTTAAACT TGTACAACAA AAACAGGAGA GCAGAGGAAA AAAAGCCCAC AAATACCCAA   
  
  
- ATACATACGT ATATTCCAAC ATAGGAGACT ACGCATATCC GCTTACTGGG GTTTGTCAGA GAGAGAGAGA   
  
  
- GAGAGAGAGA GAGAGAGAGA GAGGGAGACG TACCTACTAC CTCTCCCGCC CCTTGTCCAA GCTAGACGCT   
  
  
- CTCTCACATT CTCTCTCTCT CTACGTCTAC GTTCCACAGT TTTTGCCTTT TCTTTTAATT TAACTACAAA   
  
  
- AGCCCACGTT GGGCTTCATA GCTCGTCGGA CCAAGAAGTT TATTTCTGGC AATTCTAACG GAGGAATAAG   
  
  
- TTGAAGGAAT TTACCCGGTC ACCCCACAGT TCGGGAAGAG ACAAGGTCGG GTATTCATGC GGAGAAAGAC   
  
  
- AGACAAAATA AACCCGGGAG GCTAACGGGC TGAAAAGTAA ACATTAATGA ACGAATTAAT TACCAATATA   
  
  
- CGACACGAAA TAATAATTTT AAGGTGAATG TCTCTCAATG TAGTTATAGC GCTTTGTCTT TCTTTAAGTC   
  
  
- ACAACATATG TAAAGTAAAC CATTATTATG GGTACCCGGT GCCTTTGAAC TGAGGGACAC AGTACTGAGT   
  
  
- TCTTGGTACT GCATGCCCAT TTCCCAACCA AGTTTGGCGC CACTGGTACT TGCTCATTTT GTCTCACGAG   
  
  
- GGTACGTTCA AGTCTTGTTC TGCGAAAGAG GAGGTGTCAG TTATTGTAGT TTCGTTCTCT TTGAAGAAGT   
  
  
- TCCAGATTCG TTAACCAAAG TTACCCTGTT CCAGAAGAAA ATCTGAGTCT GGCCTTTTTC AACTTTTGGG   
  
  
- GCGTGAGTAA TTGTTCTGGC ACTGAACCAG TATAAAGGTT CAACGAAGAA GAAACAGAAA AGCAAAATTA   
  
  
- ATTAATTGAA GAACGTGGCA CATTTTCCTT GTGCTTATGA AACTTACTGT ATACCTTTCG CAATACTAAT   
  
  
- ATATATGGTT GCTAGTTTCT TGGTTGCTTG GGGGTAAAGT CTAAAACAGG AAGGTTGGGT TATTCTCTTC   
  
  
- GGTTTGTCTG GTTCTTGCGG GTGTGTGTG

+     GAG-motif

| Site Name | Organism | Position | Strand | Matrix score. | sequence | function |
| --- | --- | --- | --- | --- | --- | --- |
| GAG-motif | Spinacia oleracea | 648 | + | 7 | AGAGATG | part of a light responsive element |
| GAG-motif | Hordeum vulgare | 287 | + | 7 | GGAGATG | part of a light responsive element |
| GAG-motif | Arabidopsis thaliana | 630 | + | 7 | AGAGAGT | part of a light responsive element |
| GAG-motif | Arabidopsis thaliana | 941 | + | 7 | AGAGAGT | part of a light responsive element |

> 2018/04/13 10:10:12  
+ AAGCTATTAT TGCCAAGCCA GAGACTCAAT GTATTTTAGT CAGAACAAGT TGCTAAGCTC TTGAATTCTG   
  
  
+ AATTATCAGT CACTAAACCA CTTATTGTGA TGTAAATATG TTATCATGTG TAAGCAATGA AGCAGTAAGC   
  
  
+ CAAGATTAAG GAGAACCACA GTAATTGGCC TCAAAGAAAG AAAGAAAGAA AAAACAAAAA TAGTAGAGAT   
  
  
+ CAAGCCTAAA CACAGAAATT TCTAGAGTGA AGTGAAAACT GAACAAAACG ACAGAGAGAA AGAGAGGGAG   
  
  
+ GATGGGGGAG ATGAGAAAAA GAGGGGATTG CAAGTTACCT TGGAGGTGCC CCGAAAGTGA GACCAGGAGC   
  
  
+ CCCAAGCGGA GGGATAAGGA TCGCCTATGT TGTAAGGGAG ATCCTTGAGT CCCGTACCAG ATCCACCCAC   
  
  
+ CACTCCCTTC AAGAATTTGA ACATGTTGTT TTTGTCCTCT CGTCTCCTTT TTTTCGGGTG TTTATGGGTT   
  
  
+ TATGTATGCA TATAAGGTTG TATCCTCTGA TGCGTATAGG CGAATGACCC CAAACAGTCT CTCTCTCTCT   
  
  
+ CTCTCTCTCT CTCTCTCTCT CTCCCTCTGC ATGGATGATG GAGAGGGCGG GGAACAGGTT CGATCTGCGA   
  
  
+ GAGAGTGTAA GAGAGAGAGA GATGCAGATG CAAGGTGTCA AAAACGGAAA AGAAAATTAA ATTGATGTTT   
  
  
+ TCGGGTGCAA CCCGAAGTAT CGAGCAGCCT GGTTCTTCAA ATAAAGACCG TTAAGATTGC CTCCTTATTC   
  
  
+ AACTTCCTTA AATGGGCCAG TGGGGTGTCA AGCCCTTCTC TGTTCCAGCC CATAAGTACG CCTCTTTCTG   
  
  
+ TCTGTTTTAT TTGGGCCCTC CGATTGCCCG ACTTTTCATT TGTAATTACT TGCTTAATTA ATGGTTATAT   
  
  
+ GCTGTGCTTT ATTATTAAAA TTCCACTTAC AGAGAGTTAC ATCAATATCG CGAAACAGAA AGAAATTCAG   
  
  
+ TGTTGTATAC ATTTCATTTG GTAATAATAC CCATGGGCCA CGGAAACTTG ACTCCCTGTG TCATGACTCA   
  
  
+ AGAACCATGA CGTACGGGTA AAGGGTTGGT TCAAACCGCG GTGACCATGA ACGAGTAAAA CAGAGTGCTC   
  
  
+ CCATGCAAGT TCAGAACAAG ACGCTTTCTC CTCCACAGTC AATAACATCA AAGCAAGAGA AACTTCTTCA   
  
  
+ AGGTCTAAGC AATTGGTTTC AATGGGACAA GGTCTTCTTT TAGACTCAGA CCGGAAAAAG TTGAAAACCC   
  
  
+ CGCACTCATT AACAAGACCG TGACTTGGTC ATATTTCCAA GTTGCTTCTT CTTTGTCTTT TCGTTTTAAT   
  
  
+ TAATTAACTT CTTGCACCGT GTAAAAGGAA CACGAATACT TTGAATGACA TATGGAAAGC GTTATGATTA   
  
  
+ TATATACCAA CGATCAAAGA ACCAACGAAC CCCCATTTCA GATTTTGTCC TTCCAACCCA ATAAGAGAAG   
  
  
+ CCAAACAGAC CAAGAACGCC CACACACAC  

- TTCGATAATA ACGGTTCGGT CTCTGAGTTA CATAAAATCA GTCTTGTTCA ACGATTCGAG AACTTAAGAC   
  
  
- TTAATAGTCA GTGATTTGGT GAATAACACT ACATTTATAC AATAGTACAC ATTCGTTACT TCGTCATTCG   
  
  
- GTTCTAATTC CTCTTGGTGT CATTAACCGG AGTTTCTTTC TTTCTTTCTT TTTTGTTTTT ATCATCTCTA   
  
  
- GTTCGGATTT GTGTCTTTAA AGATCTCACT TCACTTTTGA CTTGTTTTGC TGTCTCTCTT TCTCTCCCTC   
  
  
- CTACCCCCTC TACTCTTTTT CTCCCCTAAC GTTCAATGGA ACCTCCACGG GGCTTTCACT CTGGTCCTCG   
  
  
- GGGTTCGCCT CCCTATTCCT AGCGGATACA ACATTCCCTC TAGGAACTCA GGGCATGGTC TAGGTGGGTG   
  
  
- GTGAGGGAAG TTCTTAAACT TGTACAACAA AAACAGGAGA GCAGAGGAAA AAAAGCCCAC AAATACCCAA   
  
  
- ATACATACGT ATATTCCAAC ATAGGAGACT ACGCATATCC GCTTACTGGG GTTTGTCAGA GAGAGAGAGA   
  
  
- GAGAGAGAGA GAGAGAGAGA GAGGGAGACG TACCTACTAC CTCTCCCGCC CCTTGTCCAA GCTAGACGCT   
  
  
- CTCTCACATT CTCTCTCTCT CTACGTCTAC GTTCCACAGT TTTTGCCTTT TCTTTTAATT TAACTACAAA   
  
  
- AGCCCACGTT GGGCTTCATA GCTCGTCGGA CCAAGAAGTT TATTTCTGGC AATTCTAACG GAGGAATAAG   
  
  
- TTGAAGGAAT TTACCCGGTC ACCCCACAGT TCGGGAAGAG ACAAGGTCGG GTATTCATGC GGAGAAAGAC   
  
  
- AGACAAAATA AACCCGGGAG GCTAACGGGC TGAAAAGTAA ACATTAATGA ACGAATTAAT TACCAATATA   
  
  
- CGACACGAAA TAATAATTTT AAGGTGAATG TCTCTCAATG TAGTTATAGC GCTTTGTCTT TCTTTAAGTC   
  
  
- ACAACATATG TAAAGTAAAC CATTATTATG GGTACCCGGT GCCTTTGAAC TGAGGGACAC AGTACTGAGT   
  
  
- TCTTGGTACT GCATGCCCAT TTCCCAACCA AGTTTGGCGC CACTGGTACT TGCTCATTTT GTCTCACGAG   
  
  
- GGTACGTTCA AGTCTTGTTC TGCGAAAGAG GAGGTGTCAG TTATTGTAGT TTCGTTCTCT TTGAAGAAGT   
  
  
- TCCAGATTCG TTAACCAAAG TTACCCTGTT CCAGAAGAAA ATCTGAGTCT GGCCTTTTTC AACTTTTGGG   
  
  
- GCGTGAGTAA TTGTTCTGGC ACTGAACCAG TATAAAGGTT CAACGAAGAA GAAACAGAAA AGCAAAATTA   
  
  
- ATTAATTGAA GAACGTGGCA CATTTTCCTT GTGCTTATGA AACTTACTGT ATACCTTTCG CAATACTAAT   
  
  
- ATATATGGTT GCTAGTTTCT TGGTTGCTTG GGGGTAAAGT CTAAAACAGG AAGGTTGGGT TATTCTCTTC   
  
  
- GGTTTGTCTG GTTCTTGCGG GTGTGTGTG

+     GARE-motif

| Site Name | Organism | Position | Strand | Matrix score. | sequence | function |
| --- | --- | --- | --- | --- | --- | --- |
| GARE-motif | Brassica oleracea | 963 | + | 7 | AAACAGA | gibberellin-responsive element |
| GARE-motif | Brassica oleracea | 1473 | + | 7 | AAACAGA | gibberellin-responsive element |
| GARE-motif | Brassica oleracea | 841 | - | 7 | AAACAGA | gibberellin-responsive element |
| GARE-motif | Brassica oleracea | 1108 | + | 7 | AAACAGA | gibberellin-responsive element |

> 2018/04/13 10:10:12  
+ AAGCTATTAT TGCCAAGCCA GAGACTCAAT GTATTTTAGT CAGAACAAGT TGCTAAGCTC TTGAATTCTG   
  
  
+ AATTATCAGT CACTAAACCA CTTATTGTGA TGTAAATATG TTATCATGTG TAAGCAATGA AGCAGTAAGC   
  
  
+ CAAGATTAAG GAGAACCACA GTAATTGGCC TCAAAGAAAG AAAGAAAGAA AAAACAAAAA TAGTAGAGAT   
  
  
+ CAAGCCTAAA CACAGAAATT TCTAGAGTGA AGTGAAAACT GAACAAAACG ACAGAGAGAA AGAGAGGGAG   
  
  
+ GATGGGGGAG ATGAGAAAAA GAGGGGATTG CAAGTTACCT TGGAGGTGCC CCGAAAGTGA GACCAGGAGC   
  
  
+ CCCAAGCGGA GGGATAAGGA TCGCCTATGT TGTAAGGGAG ATCCTTGAGT CCCGTACCAG ATCCACCCAC   
  
  
+ CACTCCCTTC AAGAATTTGA ACATGTTGTT TTTGTCCTCT CGTCTCCTTT TTTTCGGGTG TTTATGGGTT   
  
  
+ TATGTATGCA TATAAGGTTG TATCCTCTGA TGCGTATAGG CGAATGACCC CAAACAGTCT CTCTCTCTCT   
  
  
+ CTCTCTCTCT CTCTCTCTCT CTCCCTCTGC ATGGATGATG GAGAGGGCGG GGAACAGGTT CGATCTGCGA   
  
  
+ GAGAGTGTAA GAGAGAGAGA GATGCAGATG CAAGGTGTCA AAAACGGAAA AGAAAATTAA ATTGATGTTT   
  
  
+ TCGGGTGCAA CCCGAAGTAT CGAGCAGCCT GGTTCTTCAA ATAAAGACCG TTAAGATTGC CTCCTTATTC   
  
  
+ AACTTCCTTA AATGGGCCAG TGGGGTGTCA AGCCCTTCTC TGTTCCAGCC CATAAGTACG CCTCTTTCTG   
  
  
+ TCTGTTTTAT TTGGGCCCTC CGATTGCCCG ACTTTTCATT TGTAATTACT TGCTTAATTA ATGGTTATAT   
  
  
+ GCTGTGCTTT ATTATTAAAA TTCCACTTAC AGAGAGTTAC ATCAATATCG CGAAACAGAA AGAAATTCAG   
  
  
+ TGTTGTATAC ATTTCATTTG GTAATAATAC CCATGGGCCA CGGAAACTTG ACTCCCTGTG TCATGACTCA   
  
  
+ AGAACCATGA CGTACGGGTA AAGGGTTGGT TCAAACCGCG GTGACCATGA ACGAGTAAAA CAGAGTGCTC   
  
  
+ CCATGCAAGT TCAGAACAAG ACGCTTTCTC CTCCACAGTC AATAACATCA AAGCAAGAGA AACTTCTTCA   
  
  
+ AGGTCTAAGC AATTGGTTTC AATGGGACAA GGTCTTCTTT TAGACTCAGA CCGGAAAAAG TTGAAAACCC   
  
  
+ CGCACTCATT AACAAGACCG TGACTTGGTC ATATTTCCAA GTTGCTTCTT CTTTGTCTTT TCGTTTTAAT   
  
  
+ TAATTAACTT CTTGCACCGT GTAAAAGGAA CACGAATACT TTGAATGACA TATGGAAAGC GTTATGATTA   
  
  
+ TATATACCAA CGATCAAAGA ACCAACGAAC CCCCATTTCA GATTTTGTCC TTCCAACCCA ATAAGAGAAG   
  
  
+ CCAAACAGAC CAAGAACGCC CACACACAC  

- TTCGATAATA ACGGTTCGGT CTCTGAGTTA CATAAAATCA GTCTTGTTCA ACGATTCGAG AACTTAAGAC   
  
  
- TTAATAGTCA GTGATTTGGT GAATAACACT ACATTTATAC AATAGTACAC ATTCGTTACT TCGTCATTCG   
  
  
- GTTCTAATTC CTCTTGGTGT CATTAACCGG AGTTTCTTTC TTTCTTTCTT TTTTGTTTTT ATCATCTCTA   
  
  
- GTTCGGATTT GTGTCTTTAA AGATCTCACT TCACTTTTGA CTTGTTTTGC TGTCTCTCTT TCTCTCCCTC   
  
  
- CTACCCCCTC TACTCTTTTT CTCCCCTAAC GTTCAATGGA ACCTCCACGG GGCTTTCACT CTGGTCCTCG   
  
  
- GGGTTCGCCT CCCTATTCCT AGCGGATACA ACATTCCCTC TAGGAACTCA GGGCATGGTC TAGGTGGGTG   
  
  
- GTGAGGGAAG TTCTTAAACT TGTACAACAA AAACAGGAGA GCAGAGGAAA AAAAGCCCAC AAATACCCAA   
  
  
- ATACATACGT ATATTCCAAC ATAGGAGACT ACGCATATCC GCTTACTGGG GTTTGTCAGA GAGAGAGAGA   
  
  
- GAGAGAGAGA GAGAGAGAGA GAGGGAGACG TACCTACTAC CTCTCCCGCC CCTTGTCCAA GCTAGACGCT   
  
  
- CTCTCACATT CTCTCTCTCT CTACGTCTAC GTTCCACAGT TTTTGCCTTT TCTTTTAATT TAACTACAAA   
  
  
- AGCCCACGTT GGGCTTCATA GCTCGTCGGA CCAAGAAGTT TATTTCTGGC AATTCTAACG GAGGAATAAG   
  
  
- TTGAAGGAAT TTACCCGGTC ACCCCACAGT TCGGGAAGAG ACAAGGTCGG GTATTCATGC GGAGAAAGAC   
  
  
- AGACAAAATA AACCCGGGAG GCTAACGGGC TGAAAAGTAA ACATTAATGA ACGAATTAAT TACCAATATA   
  
  
- CGACACGAAA TAATAATTTT AAGGTGAATG TCTCTCAATG TAGTTATAGC GCTTTGTCTT TCTTTAAGTC   
  
  
- ACAACATATG TAAAGTAAAC CATTATTATG GGTACCCGGT GCCTTTGAAC TGAGGGACAC AGTACTGAGT   
  
  
- TCTTGGTACT GCATGCCCAT TTCCCAACCA AGTTTGGCGC CACTGGTACT TGCTCATTTT GTCTCACGAG   
  
  
- GGTACGTTCA AGTCTTGTTC TGCGAAAGAG GAGGTGTCAG TTATTGTAGT TTCGTTCTCT TTGAAGAAGT   
  
  
- TCCAGATTCG TTAACCAAAG TTACCCTGTT CCAGAAGAAA ATCTGAGTCT GGCCTTTTTC AACTTTTGGG   
  
  
- GCGTGAGTAA TTGTTCTGGC ACTGAACCAG TATAAAGGTT CAACGAAGAA GAAACAGAAA AGCAAAATTA   
  
  
- ATTAATTGAA GAACGTGGCA CATTTTCCTT GTGCTTATGA AACTTACTGT ATACCTTTCG CAATACTAAT   
  
  
- ATATATGGTT GCTAGTTTCT TGGTTGCTTG GGGGTAAAGT CTAAAACAGG AAGGTTGGGT TATTCTCTTC   
  
  
- GGTTTGTCTG GTTCTTGCGG GTGTGTGTG

+     GATA-motif

| Site Name | Organism | Position | Strand | Matrix score. | sequence | function |
| --- | --- | --- | --- | --- | --- | --- |
| GATA-motif | Solanum tuberosum | 360 | + | 9 | AAGGATAAGG | part of a light responsive element |

> 2018/04/13 10:10:12  
+ AAGCTATTAT TGCCAAGCCA GAGACTCAAT GTATTTTAGT CAGAACAAGT TGCTAAGCTC TTGAATTCTG   
  
  
+ AATTATCAGT CACTAAACCA CTTATTGTGA TGTAAATATG TTATCATGTG TAAGCAATGA AGCAGTAAGC   
  
  
+ CAAGATTAAG GAGAACCACA GTAATTGGCC TCAAAGAAAG AAAGAAAGAA AAAACAAAAA TAGTAGAGAT   
  
  
+ CAAGCCTAAA CACAGAAATT TCTAGAGTGA AGTGAAAACT GAACAAAACG ACAGAGAGAA AGAGAGGGAG   
  
  
+ GATGGGGGAG ATGAGAAAAA GAGGGGATTG CAAGTTACCT TGGAGGTGCC CCGAAAGTGA GACCAGGAGC   
  
  
+ CCCAAGCGGA GGGATAAGGA TCGCCTATGT TGTAAGGGAG ATCCTTGAGT CCCGTACCAG ATCCACCCAC   
  
  
+ CACTCCCTTC AAGAATTTGA ACATGTTGTT TTTGTCCTCT CGTCTCCTTT TTTTCGGGTG TTTATGGGTT   
  
  
+ TATGTATGCA TATAAGGTTG TATCCTCTGA TGCGTATAGG CGAATGACCC CAAACAGTCT CTCTCTCTCT   
  
  
+ CTCTCTCTCT CTCTCTCTCT CTCCCTCTGC ATGGATGATG GAGAGGGCGG GGAACAGGTT CGATCTGCGA   
  
  
+ GAGAGTGTAA GAGAGAGAGA GATGCAGATG CAAGGTGTCA AAAACGGAAA AGAAAATTAA ATTGATGTTT   
  
  
+ TCGGGTGCAA CCCGAAGTAT CGAGCAGCCT GGTTCTTCAA ATAAAGACCG TTAAGATTGC CTCCTTATTC   
  
  
+ AACTTCCTTA AATGGGCCAG TGGGGTGTCA AGCCCTTCTC TGTTCCAGCC CATAAGTACG CCTCTTTCTG   
  
  
+ TCTGTTTTAT TTGGGCCCTC CGATTGCCCG ACTTTTCATT TGTAATTACT TGCTTAATTA ATGGTTATAT   
  
  
+ GCTGTGCTTT ATTATTAAAA TTCCACTTAC AGAGAGTTAC ATCAATATCG CGAAACAGAA AGAAATTCAG   
  
  
+ TGTTGTATAC ATTTCATTTG GTAATAATAC CCATGGGCCA CGGAAACTTG ACTCCCTGTG TCATGACTCA   
  
  
+ AGAACCATGA CGTACGGGTA AAGGGTTGGT TCAAACCGCG GTGACCATGA ACGAGTAAAA CAGAGTGCTC   
  
  
+ CCATGCAAGT TCAGAACAAG ACGCTTTCTC CTCCACAGTC AATAACATCA AAGCAAGAGA AACTTCTTCA   
  
  
+ AGGTCTAAGC AATTGGTTTC AATGGGACAA GGTCTTCTTT TAGACTCAGA CCGGAAAAAG TTGAAAACCC   
  
  
+ CGCACTCATT AACAAGACCG TGACTTGGTC ATATTTCCAA GTTGCTTCTT CTTTGTCTTT TCGTTTTAAT   
  
  
+ TAATTAACTT CTTGCACCGT GTAAAAGGAA CACGAATACT TTGAATGACA TATGGAAAGC GTTATGATTA   
  
  
+ TATATACCAA CGATCAAAGA ACCAACGAAC CCCCATTTCA GATTTTGTCC TTCCAACCCA ATAAGAGAAG   
  
  
+ CCAAACAGAC CAAGAACGCC CACACACAC  

- TTCGATAATA ACGGTTCGGT CTCTGAGTTA CATAAAATCA GTCTTGTTCA ACGATTCGAG AACTTAAGAC   
  
  
- TTAATAGTCA GTGATTTGGT GAATAACACT ACATTTATAC AATAGTACAC ATTCGTTACT TCGTCATTCG   
  
  
- GTTCTAATTC CTCTTGGTGT CATTAACCGG AGTTTCTTTC TTTCTTTCTT TTTTGTTTTT ATCATCTCTA   
  
  
- GTTCGGATTT GTGTCTTTAA AGATCTCACT TCACTTTTGA CTTGTTTTGC TGTCTCTCTT TCTCTCCCTC   
  
  
- CTACCCCCTC TACTCTTTTT CTCCCCTAAC GTTCAATGGA ACCTCCACGG GGCTTTCACT CTGGTCCTCG   
  
  
- GGGTTCGCCT CCCTATTCCT AGCGGATACA ACATTCCCTC TAGGAACTCA GGGCATGGTC TAGGTGGGTG   
  
  
- GTGAGGGAAG TTCTTAAACT TGTACAACAA AAACAGGAGA GCAGAGGAAA AAAAGCCCAC AAATACCCAA   
  
  
- ATACATACGT ATATTCCAAC ATAGGAGACT ACGCATATCC GCTTACTGGG GTTTGTCAGA GAGAGAGAGA   
  
  
- GAGAGAGAGA GAGAGAGAGA GAGGGAGACG TACCTACTAC CTCTCCCGCC CCTTGTCCAA GCTAGACGCT   
  
  
- CTCTCACATT CTCTCTCTCT CTACGTCTAC GTTCCACAGT TTTTGCCTTT TCTTTTAATT TAACTACAAA   
  
  
- AGCCCACGTT GGGCTTCATA GCTCGTCGGA CCAAGAAGTT TATTTCTGGC AATTCTAACG GAGGAATAAG   
  
  
- TTGAAGGAAT TTACCCGGTC ACCCCACAGT TCGGGAAGAG ACAAGGTCGG GTATTCATGC GGAGAAAGAC   
  
  
- AGACAAAATA AACCCGGGAG GCTAACGGGC TGAAAAGTAA ACATTAATGA ACGAATTAAT TACCAATATA   
  
  
- CGACACGAAA TAATAATTTT AAGGTGAATG TCTCTCAATG TAGTTATAGC GCTTTGTCTT TCTTTAAGTC   
  
  
- ACAACATATG TAAAGTAAAC CATTATTATG GGTACCCGGT GCCTTTGAAC TGAGGGACAC AGTACTGAGT   
  
  
- TCTTGGTACT GCATGCCCAT TTCCCAACCA AGTTTGGCGC CACTGGTACT TGCTCATTTT GTCTCACGAG   
  
  
- GGTACGTTCA AGTCTTGTTC TGCGAAAGAG GAGGTGTCAG TTATTGTAGT TTCGTTCTCT TTGAAGAAGT   
  
  
- TCCAGATTCG TTAACCAAAG TTACCCTGTT CCAGAAGAAA ATCTGAGTCT GGCCTTTTTC AACTTTTGGG   
  
  
- GCGTGAGTAA TTGTTCTGGC ACTGAACCAG TATAAAGGTT CAACGAAGAA GAAACAGAAA AGCAAAATTA   
  
  
- ATTAATTGAA GAACGTGGCA CATTTTCCTT GTGCTTATGA AACTTACTGT ATACCTTTCG CAATACTAAT   
  
  
- ATATATGGTT GCTAGTTTCT TGGTTGCTTG GGGGTAAAGT CTAAAACAGG AAGGTTGGGT TATTCTCTTC   
  
  
- GGTTTGTCTG GTTCTTGCGG GTGTGTGTG

+     GCN4\_motif

| Site Name | Organism | Position | Strand | Matrix score. | sequence | function |
| --- | --- | --- | --- | --- | --- | --- |
| GCN4\_motif | Oryza sativa | 1037 | + | 7 | TGTGTCA | cis-regulatory element involved in endosperm expression |
| GCN4\_motif | Oryza sativa | 14 | + | 7 | CAAGCCA | cis-regulatory element involved in endosperm expression |
| GCN4\_motif | Oryza sativa | 1044 | - | 7 | TGAGTCA | cis-regulatory element involved in endosperm expression |

> 2018/04/13 10:10:12  
+ AAGCTATTAT TGCCAAGCCA GAGACTCAAT GTATTTTAGT CAGAACAAGT TGCTAAGCTC TTGAATTCTG   
  
  
+ AATTATCAGT CACTAAACCA CTTATTGTGA TGTAAATATG TTATCATGTG TAAGCAATGA AGCAGTAAGC   
  
  
+ CAAGATTAAG GAGAACCACA GTAATTGGCC TCAAAGAAAG AAAGAAAGAA AAAACAAAAA TAGTAGAGAT   
  
  
+ CAAGCCTAAA CACAGAAATT TCTAGAGTGA AGTGAAAACT GAACAAAACG ACAGAGAGAA AGAGAGGGAG   
  
  
+ GATGGGGGAG ATGAGAAAAA GAGGGGATTG CAAGTTACCT TGGAGGTGCC CCGAAAGTGA GACCAGGAGC   
  
  
+ CCCAAGCGGA GGGATAAGGA TCGCCTATGT TGTAAGGGAG ATCCTTGAGT CCCGTACCAG ATCCACCCAC   
  
  
+ CACTCCCTTC AAGAATTTGA ACATGTTGTT TTTGTCCTCT CGTCTCCTTT TTTTCGGGTG TTTATGGGTT   
  
  
+ TATGTATGCA TATAAGGTTG TATCCTCTGA TGCGTATAGG CGAATGACCC CAAACAGTCT CTCTCTCTCT   
  
  
+ CTCTCTCTCT CTCTCTCTCT CTCCCTCTGC ATGGATGATG GAGAGGGCGG GGAACAGGTT CGATCTGCGA   
  
  
+ GAGAGTGTAA GAGAGAGAGA GATGCAGATG CAAGGTGTCA AAAACGGAAA AGAAAATTAA ATTGATGTTT   
  
  
+ TCGGGTGCAA CCCGAAGTAT CGAGCAGCCT GGTTCTTCAA ATAAAGACCG TTAAGATTGC CTCCTTATTC   
  
  
+ AACTTCCTTA AATGGGCCAG TGGGGTGTCA AGCCCTTCTC TGTTCCAGCC CATAAGTACG CCTCTTTCTG   
  
  
+ TCTGTTTTAT TTGGGCCCTC CGATTGCCCG ACTTTTCATT TGTAATTACT TGCTTAATTA ATGGTTATAT   
  
  
+ GCTGTGCTTT ATTATTAAAA TTCCACTTAC AGAGAGTTAC ATCAATATCG CGAAACAGAA AGAAATTCAG   
  
  
+ TGTTGTATAC ATTTCATTTG GTAATAATAC CCATGGGCCA CGGAAACTTG ACTCCCTGTG TCATGACTCA   
  
  
+ AGAACCATGA CGTACGGGTA AAGGGTTGGT TCAAACCGCG GTGACCATGA ACGAGTAAAA CAGAGTGCTC   
  
  
+ CCATGCAAGT TCAGAACAAG ACGCTTTCTC CTCCACAGTC AATAACATCA AAGCAAGAGA AACTTCTTCA   
  
  
+ AGGTCTAAGC AATTGGTTTC AATGGGACAA GGTCTTCTTT TAGACTCAGA CCGGAAAAAG TTGAAAACCC   
  
  
+ CGCACTCATT AACAAGACCG TGACTTGGTC ATATTTCCAA GTTGCTTCTT CTTTGTCTTT TCGTTTTAAT   
  
  
+ TAATTAACTT CTTGCACCGT GTAAAAGGAA CACGAATACT TTGAATGACA TATGGAAAGC GTTATGATTA   
  
  
+ TATATACCAA CGATCAAAGA ACCAACGAAC CCCCATTTCA GATTTTGTCC TTCCAACCCA ATAAGAGAAG   
  
  
+ CCAAACAGAC CAAGAACGCC CACACACAC  

- TTCGATAATA ACGGTTCGGT CTCTGAGTTA CATAAAATCA GTCTTGTTCA ACGATTCGAG AACTTAAGAC   
  
  
- TTAATAGTCA GTGATTTGGT GAATAACACT ACATTTATAC AATAGTACAC ATTCGTTACT TCGTCATTCG   
  
  
- GTTCTAATTC CTCTTGGTGT CATTAACCGG AGTTTCTTTC TTTCTTTCTT TTTTGTTTTT ATCATCTCTA   
  
  
- GTTCGGATTT GTGTCTTTAA AGATCTCACT TCACTTTTGA CTTGTTTTGC TGTCTCTCTT TCTCTCCCTC   
  
  
- CTACCCCCTC TACTCTTTTT CTCCCCTAAC GTTCAATGGA ACCTCCACGG GGCTTTCACT CTGGTCCTCG   
  
  
- GGGTTCGCCT CCCTATTCCT AGCGGATACA ACATTCCCTC TAGGAACTCA GGGCATGGTC TAGGTGGGTG   
  
  
- GTGAGGGAAG TTCTTAAACT TGTACAACAA AAACAGGAGA GCAGAGGAAA AAAAGCCCAC AAATACCCAA   
  
  
- ATACATACGT ATATTCCAAC ATAGGAGACT ACGCATATCC GCTTACTGGG GTTTGTCAGA GAGAGAGAGA   
  
  
- GAGAGAGAGA GAGAGAGAGA GAGGGAGACG TACCTACTAC CTCTCCCGCC CCTTGTCCAA GCTAGACGCT   
  
  
- CTCTCACATT CTCTCTCTCT CTACGTCTAC GTTCCACAGT TTTTGCCTTT TCTTTTAATT TAACTACAAA   
  
  
- AGCCCACGTT GGGCTTCATA GCTCGTCGGA CCAAGAAGTT TATTTCTGGC AATTCTAACG GAGGAATAAG   
  
  
- TTGAAGGAAT TTACCCGGTC ACCCCACAGT TCGGGAAGAG ACAAGGTCGG GTATTCATGC GGAGAAAGAC   
  
  
- AGACAAAATA AACCCGGGAG GCTAACGGGC TGAAAAGTAA ACATTAATGA ACGAATTAAT TACCAATATA   
  
  
- CGACACGAAA TAATAATTTT AAGGTGAATG TCTCTCAATG TAGTTATAGC GCTTTGTCTT TCTTTAAGTC   
  
  
- ACAACATATG TAAAGTAAAC CATTATTATG GGTACCCGGT GCCTTTGAAC TGAGGGACAC AGTACTGAGT   
  
  
- TCTTGGTACT GCATGCCCAT TTCCCAACCA AGTTTGGCGC CACTGGTACT TGCTCATTTT GTCTCACGAG   
  
  
- GGTACGTTCA AGTCTTGTTC TGCGAAAGAG GAGGTGTCAG TTATTGTAGT TTCGTTCTCT TTGAAGAAGT   
  
  
- TCCAGATTCG TTAACCAAAG TTACCCTGTT CCAGAAGAAA ATCTGAGTCT GGCCTTTTTC AACTTTTGGG   
  
  
- GCGTGAGTAA TTGTTCTGGC ACTGAACCAG TATAAAGGTT CAACGAAGAA GAAACAGAAA AGCAAAATTA   
  
  
- ATTAATTGAA GAACGTGGCA CATTTTCCTT GTGCTTATGA AACTTACTGT ATACCTTTCG CAATACTAAT   
  
  
- ATATATGGTT GCTAGTTTCT TGGTTGCTTG GGGGTAAAGT CTAAAACAGG AAGGTTGGGT TATTCTCTTC   
  
  
- GGTTTGTCTG GTTCTTGCGG GTGTGTGTG

+     Gap-box

| Site Name | Organism | Position | Strand | Matrix score. | sequence | function |
| --- | --- | --- | --- | --- | --- | --- |
| Gap-box | Arabidopsis thaliana | 873 | - | 9.5 | CAAATGAA(A/G)A | part of a light responsive element |

> 2018/04/13 10:10:12  
+ AAGCTATTAT TGCCAAGCCA GAGACTCAAT GTATTTTAGT CAGAACAAGT TGCTAAGCTC TTGAATTCTG   
  
  
+ AATTATCAGT CACTAAACCA CTTATTGTGA TGTAAATATG TTATCATGTG TAAGCAATGA AGCAGTAAGC   
  
  
+ CAAGATTAAG GAGAACCACA GTAATTGGCC TCAAAGAAAG AAAGAAAGAA AAAACAAAAA TAGTAGAGAT   
  
  
+ CAAGCCTAAA CACAGAAATT TCTAGAGTGA AGTGAAAACT GAACAAAACG ACAGAGAGAA AGAGAGGGAG   
  
  
+ GATGGGGGAG ATGAGAAAAA GAGGGGATTG CAAGTTACCT TGGAGGTGCC CCGAAAGTGA GACCAGGAGC   
  
  
+ CCCAAGCGGA GGGATAAGGA TCGCCTATGT TGTAAGGGAG ATCCTTGAGT CCCGTACCAG ATCCACCCAC   
  
  
+ CACTCCCTTC AAGAATTTGA ACATGTTGTT TTTGTCCTCT CGTCTCCTTT TTTTCGGGTG TTTATGGGTT   
  
  
+ TATGTATGCA TATAAGGTTG TATCCTCTGA TGCGTATAGG CGAATGACCC CAAACAGTCT CTCTCTCTCT   
  
  
+ CTCTCTCTCT CTCTCTCTCT CTCCCTCTGC ATGGATGATG GAGAGGGCGG GGAACAGGTT CGATCTGCGA   
  
  
+ GAGAGTGTAA GAGAGAGAGA GATGCAGATG CAAGGTGTCA AAAACGGAAA AGAAAATTAA ATTGATGTTT   
  
  
+ TCGGGTGCAA CCCGAAGTAT CGAGCAGCCT GGTTCTTCAA ATAAAGACCG TTAAGATTGC CTCCTTATTC   
  
  
+ AACTTCCTTA AATGGGCCAG TGGGGTGTCA AGCCCTTCTC TGTTCCAGCC CATAAGTACG CCTCTTTCTG   
  
  
+ TCTGTTTTAT TTGGGCCCTC CGATTGCCCG ACTTTTCATT TGTAATTACT TGCTTAATTA ATGGTTATAT   
  
  
+ GCTGTGCTTT ATTATTAAAA TTCCACTTAC AGAGAGTTAC ATCAATATCG CGAAACAGAA AGAAATTCAG   
  
  
+ TGTTGTATAC ATTTCATTTG GTAATAATAC CCATGGGCCA CGGAAACTTG ACTCCCTGTG TCATGACTCA   
  
  
+ AGAACCATGA CGTACGGGTA AAGGGTTGGT TCAAACCGCG GTGACCATGA ACGAGTAAAA CAGAGTGCTC   
  
  
+ CCATGCAAGT TCAGAACAAG ACGCTTTCTC CTCCACAGTC AATAACATCA AAGCAAGAGA AACTTCTTCA   
  
  
+ AGGTCTAAGC AATTGGTTTC AATGGGACAA GGTCTTCTTT TAGACTCAGA CCGGAAAAAG TTGAAAACCC   
  
  
+ CGCACTCATT AACAAGACCG TGACTTGGTC ATATTTCCAA GTTGCTTCTT CTTTGTCTTT TCGTTTTAAT   
  
  
+ TAATTAACTT CTTGCACCGT GTAAAAGGAA CACGAATACT TTGAATGACA TATGGAAAGC GTTATGATTA   
  
  
+ TATATACCAA CGATCAAAGA ACCAACGAAC CCCCATTTCA GATTTTGTCC TTCCAACCCA ATAAGAGAAG   
  
  
+ CCAAACAGAC CAAGAACGCC CACACACAC  

- TTCGATAATA ACGGTTCGGT CTCTGAGTTA CATAAAATCA GTCTTGTTCA ACGATTCGAG AACTTAAGAC   
  
  
- TTAATAGTCA GTGATTTGGT GAATAACACT ACATTTATAC AATAGTACAC ATTCGTTACT TCGTCATTCG   
  
  
- GTTCTAATTC CTCTTGGTGT CATTAACCGG AGTTTCTTTC TTTCTTTCTT TTTTGTTTTT ATCATCTCTA   
  
  
- GTTCGGATTT GTGTCTTTAA AGATCTCACT TCACTTTTGA CTTGTTTTGC TGTCTCTCTT TCTCTCCCTC   
  
  
- CTACCCCCTC TACTCTTTTT CTCCCCTAAC GTTCAATGGA ACCTCCACGG GGCTTTCACT CTGGTCCTCG   
  
  
- GGGTTCGCCT CCCTATTCCT AGCGGATACA ACATTCCCTC TAGGAACTCA GGGCATGGTC TAGGTGGGTG   
  
  
- GTGAGGGAAG TTCTTAAACT TGTACAACAA AAACAGGAGA GCAGAGGAAA AAAAGCCCAC AAATACCCAA   
  
  
- ATACATACGT ATATTCCAAC ATAGGAGACT ACGCATATCC GCTTACTGGG GTTTGTCAGA GAGAGAGAGA   
  
  
- GAGAGAGAGA GAGAGAGAGA GAGGGAGACG TACCTACTAC CTCTCCCGCC CCTTGTCCAA GCTAGACGCT   
  
  
- CTCTCACATT CTCTCTCTCT CTACGTCTAC GTTCCACAGT TTTTGCCTTT TCTTTTAATT TAACTACAAA   
  
  
- AGCCCACGTT GGGCTTCATA GCTCGTCGGA CCAAGAAGTT TATTTCTGGC AATTCTAACG GAGGAATAAG   
  
  
- TTGAAGGAAT TTACCCGGTC ACCCCACAGT TCGGGAAGAG ACAAGGTCGG GTATTCATGC GGAGAAAGAC   
  
  
- AGACAAAATA AACCCGGGAG GCTAACGGGC TGAAAAGTAA ACATTAATGA ACGAATTAAT TACCAATATA   
  
  
- CGACACGAAA TAATAATTTT AAGGTGAATG TCTCTCAATG TAGTTATAGC GCTTTGTCTT TCTTTAAGTC   
  
  
- ACAACATATG TAAAGTAAAC CATTATTATG GGTACCCGGT GCCTTTGAAC TGAGGGACAC AGTACTGAGT   
  
  
- TCTTGGTACT GCATGCCCAT TTCCCAACCA AGTTTGGCGC CACTGGTACT TGCTCATTTT GTCTCACGAG   
  
  
- GGTACGTTCA AGTCTTGTTC TGCGAAAGAG GAGGTGTCAG TTATTGTAGT TTCGTTCTCT TTGAAGAAGT   
  
  
- TCCAGATTCG TTAACCAAAG TTACCCTGTT CCAGAAGAAA ATCTGAGTCT GGCCTTTTTC AACTTTTGGG   
  
  
- GCGTGAGTAA TTGTTCTGGC ACTGAACCAG TATAAAGGTT CAACGAAGAA GAAACAGAAA AGCAAAATTA   
  
  
- ATTAATTGAA GAACGTGGCA CATTTTCCTT GTGCTTATGA AACTTACTGT ATACCTTTCG CAATACTAAT   
  
  
- ATATATGGTT GCTAGTTTCT TGGTTGCTTG GGGGTAAAGT CTAAAACAGG AAGGTTGGGT TATTCTCTTC   
  
  
- GGTTTGTCTG GTTCTTGCGG GTGTGTGTG

+     LTR

| Site Name | Organism | Position | Strand | Matrix score. | sequence | function |
| --- | --- | --- | --- | --- | --- | --- |
| LTR | Hordeum vulgare | 331 | + | 6 | CCGAAA | cis-acting element involved in low-temperature responsiveness |
| LTR | Hordeum vulgare | 699 | - | 6 | CCGAAA | cis-acting element involved in low-temperature responsiveness |
| LTR | Hordeum vulgare | 472 | - | 6 | CCGAAA | cis-acting element involved in low-temperature responsiveness |

> 2018/04/13 10:10:12  
+ AAGCTATTAT TGCCAAGCCA GAGACTCAAT GTATTTTAGT CAGAACAAGT TGCTAAGCTC TTGAATTCTG   
  
  
+ AATTATCAGT CACTAAACCA CTTATTGTGA TGTAAATATG TTATCATGTG TAAGCAATGA AGCAGTAAGC   
  
  
+ CAAGATTAAG GAGAACCACA GTAATTGGCC TCAAAGAAAG AAAGAAAGAA AAAACAAAAA TAGTAGAGAT   
  
  
+ CAAGCCTAAA CACAGAAATT TCTAGAGTGA AGTGAAAACT GAACAAAACG ACAGAGAGAA AGAGAGGGAG   
  
  
+ GATGGGGGAG ATGAGAAAAA GAGGGGATTG CAAGTTACCT TGGAGGTGCC CCGAAAGTGA GACCAGGAGC   
  
  
+ CCCAAGCGGA GGGATAAGGA TCGCCTATGT TGTAAGGGAG ATCCTTGAGT CCCGTACCAG ATCCACCCAC   
  
  
+ CACTCCCTTC AAGAATTTGA ACATGTTGTT TTTGTCCTCT CGTCTCCTTT TTTTCGGGTG TTTATGGGTT   
  
  
+ TATGTATGCA TATAAGGTTG TATCCTCTGA TGCGTATAGG CGAATGACCC CAAACAGTCT CTCTCTCTCT   
  
  
+ CTCTCTCTCT CTCTCTCTCT CTCCCTCTGC ATGGATGATG GAGAGGGCGG GGAACAGGTT CGATCTGCGA   
  
  
+ GAGAGTGTAA GAGAGAGAGA GATGCAGATG CAAGGTGTCA AAAACGGAAA AGAAAATTAA ATTGATGTTT   
  
  
+ TCGGGTGCAA CCCGAAGTAT CGAGCAGCCT GGTTCTTCAA ATAAAGACCG TTAAGATTGC CTCCTTATTC   
  
  
+ AACTTCCTTA AATGGGCCAG TGGGGTGTCA AGCCCTTCTC TGTTCCAGCC CATAAGTACG CCTCTTTCTG   
  
  
+ TCTGTTTTAT TTGGGCCCTC CGATTGCCCG ACTTTTCATT TGTAATTACT TGCTTAATTA ATGGTTATAT   
  
  
+ GCTGTGCTTT ATTATTAAAA TTCCACTTAC AGAGAGTTAC ATCAATATCG CGAAACAGAA AGAAATTCAG   
  
  
+ TGTTGTATAC ATTTCATTTG GTAATAATAC CCATGGGCCA CGGAAACTTG ACTCCCTGTG TCATGACTCA   
  
  
+ AGAACCATGA CGTACGGGTA AAGGGTTGGT TCAAACCGCG GTGACCATGA ACGAGTAAAA CAGAGTGCTC   
  
  
+ CCATGCAAGT TCAGAACAAG ACGCTTTCTC CTCCACAGTC AATAACATCA AAGCAAGAGA AACTTCTTCA   
  
  
+ AGGTCTAAGC AATTGGTTTC AATGGGACAA GGTCTTCTTT TAGACTCAGA CCGGAAAAAG TTGAAAACCC   
  
  
+ CGCACTCATT AACAAGACCG TGACTTGGTC ATATTTCCAA GTTGCTTCTT CTTTGTCTTT TCGTTTTAAT   
  
  
+ TAATTAACTT CTTGCACCGT GTAAAAGGAA CACGAATACT TTGAATGACA TATGGAAAGC GTTATGATTA   
  
  
+ TATATACCAA CGATCAAAGA ACCAACGAAC CCCCATTTCA GATTTTGTCC TTCCAACCCA ATAAGAGAAG   
  
  
+ CCAAACAGAC CAAGAACGCC CACACACAC  

- TTCGATAATA ACGGTTCGGT CTCTGAGTTA CATAAAATCA GTCTTGTTCA ACGATTCGAG AACTTAAGAC   
  
  
- TTAATAGTCA GTGATTTGGT GAATAACACT ACATTTATAC AATAGTACAC ATTCGTTACT TCGTCATTCG   
  
  
- GTTCTAATTC CTCTTGGTGT CATTAACCGG AGTTTCTTTC TTTCTTTCTT TTTTGTTTTT ATCATCTCTA   
  
  
- GTTCGGATTT GTGTCTTTAA AGATCTCACT TCACTTTTGA CTTGTTTTGC TGTCTCTCTT TCTCTCCCTC   
  
  
- CTACCCCCTC TACTCTTTTT CTCCCCTAAC GTTCAATGGA ACCTCCACGG GGCTTTCACT CTGGTCCTCG   
  
  
- GGGTTCGCCT CCCTATTCCT AGCGGATACA ACATTCCCTC TAGGAACTCA GGGCATGGTC TAGGTGGGTG   
  
  
- GTGAGGGAAG TTCTTAAACT TGTACAACAA AAACAGGAGA GCAGAGGAAA AAAAGCCCAC AAATACCCAA   
  
  
- ATACATACGT ATATTCCAAC ATAGGAGACT ACGCATATCC GCTTACTGGG GTTTGTCAGA GAGAGAGAGA   
  
  
- GAGAGAGAGA GAGAGAGAGA GAGGGAGACG TACCTACTAC CTCTCCCGCC CCTTGTCCAA GCTAGACGCT   
  
  
- CTCTCACATT CTCTCTCTCT CTACGTCTAC GTTCCACAGT TTTTGCCTTT TCTTTTAATT TAACTACAAA   
  
  
- AGCCCACGTT GGGCTTCATA GCTCGTCGGA CCAAGAAGTT TATTTCTGGC AATTCTAACG GAGGAATAAG   
  
  
- TTGAAGGAAT TTACCCGGTC ACCCCACAGT TCGGGAAGAG ACAAGGTCGG GTATTCATGC GGAGAAAGAC   
  
  
- AGACAAAATA AACCCGGGAG GCTAACGGGC TGAAAAGTAA ACATTAATGA ACGAATTAAT TACCAATATA   
  
  
- CGACACGAAA TAATAATTTT AAGGTGAATG TCTCTCAATG TAGTTATAGC GCTTTGTCTT TCTTTAAGTC   
  
  
- ACAACATATG TAAAGTAAAC CATTATTATG GGTACCCGGT GCCTTTGAAC TGAGGGACAC AGTACTGAGT   
  
  
- TCTTGGTACT GCATGCCCAT TTCCCAACCA AGTTTGGCGC CACTGGTACT TGCTCATTTT GTCTCACGAG   
  
  
- GGTACGTTCA AGTCTTGTTC TGCGAAAGAG GAGGTGTCAG TTATTGTAGT TTCGTTCTCT TTGAAGAAGT   
  
  
- TCCAGATTCG TTAACCAAAG TTACCCTGTT CCAGAAGAAA ATCTGAGTCT GGCCTTTTTC AACTTTTGGG   
  
  
- GCGTGAGTAA TTGTTCTGGC ACTGAACCAG TATAAAGGTT CAACGAAGAA GAAACAGAAA AGCAAAATTA   
  
  
- ATTAATTGAA GAACGTGGCA CATTTTCCTT GTGCTTATGA AACTTACTGT ATACCTTTCG CAATACTAAT   
  
  
- ATATATGGTT GCTAGTTTCT TGGTTGCTTG GGGGTAAAGT CTAAAACAGG AAGGTTGGGT TATTCTCTTC   
  
  
- GGTTTGTCTG GTTCTTGCGG GTGTGTGTG

+     Pc-CMA2c

| Site Name | Organism | Position | Strand | Matrix score. | sequence | function |
| --- | --- | --- | --- | --- | --- | --- |
| Pc-CMA2c | Spinacia oleracea | 1488 | + | 9 | GCCCACACA | part of a light responsive element |

> 2018/04/13 10:10:12  
+ AAGCTATTAT TGCCAAGCCA GAGACTCAAT GTATTTTAGT CAGAACAAGT TGCTAAGCTC TTGAATTCTG   
  
  
+ AATTATCAGT CACTAAACCA CTTATTGTGA TGTAAATATG TTATCATGTG TAAGCAATGA AGCAGTAAGC   
  
  
+ CAAGATTAAG GAGAACCACA GTAATTGGCC TCAAAGAAAG AAAGAAAGAA AAAACAAAAA TAGTAGAGAT   
  
  
+ CAAGCCTAAA CACAGAAATT TCTAGAGTGA AGTGAAAACT GAACAAAACG ACAGAGAGAA AGAGAGGGAG   
  
  
+ GATGGGGGAG ATGAGAAAAA GAGGGGATTG CAAGTTACCT TGGAGGTGCC CCGAAAGTGA GACCAGGAGC   
  
  
+ CCCAAGCGGA GGGATAAGGA TCGCCTATGT TGTAAGGGAG ATCCTTGAGT CCCGTACCAG ATCCACCCAC   
  
  
+ CACTCCCTTC AAGAATTTGA ACATGTTGTT TTTGTCCTCT CGTCTCCTTT TTTTCGGGTG TTTATGGGTT   
  
  
+ TATGTATGCA TATAAGGTTG TATCCTCTGA TGCGTATAGG CGAATGACCC CAAACAGTCT CTCTCTCTCT   
  
  
+ CTCTCTCTCT CTCTCTCTCT CTCCCTCTGC ATGGATGATG GAGAGGGCGG GGAACAGGTT CGATCTGCGA   
  
  
+ GAGAGTGTAA GAGAGAGAGA GATGCAGATG CAAGGTGTCA AAAACGGAAA AGAAAATTAA ATTGATGTTT   
  
  
+ TCGGGTGCAA CCCGAAGTAT CGAGCAGCCT GGTTCTTCAA ATAAAGACCG TTAAGATTGC CTCCTTATTC   
  
  
+ AACTTCCTTA AATGGGCCAG TGGGGTGTCA AGCCCTTCTC TGTTCCAGCC CATAAGTACG CCTCTTTCTG   
  
  
+ TCTGTTTTAT TTGGGCCCTC CGATTGCCCG ACTTTTCATT TGTAATTACT TGCTTAATTA ATGGTTATAT   
  
  
+ GCTGTGCTTT ATTATTAAAA TTCCACTTAC AGAGAGTTAC ATCAATATCG CGAAACAGAA AGAAATTCAG   
  
  
+ TGTTGTATAC ATTTCATTTG GTAATAATAC CCATGGGCCA CGGAAACTTG ACTCCCTGTG TCATGACTCA   
  
  
+ AGAACCATGA CGTACGGGTA AAGGGTTGGT TCAAACCGCG GTGACCATGA ACGAGTAAAA CAGAGTGCTC   
  
  
+ CCATGCAAGT TCAGAACAAG ACGCTTTCTC CTCCACAGTC AATAACATCA AAGCAAGAGA AACTTCTTCA   
  
  
+ AGGTCTAAGC AATTGGTTTC AATGGGACAA GGTCTTCTTT TAGACTCAGA CCGGAAAAAG TTGAAAACCC   
  
  
+ CGCACTCATT AACAAGACCG TGACTTGGTC ATATTTCCAA GTTGCTTCTT CTTTGTCTTT TCGTTTTAAT   
  
  
+ TAATTAACTT CTTGCACCGT GTAAAAGGAA CACGAATACT TTGAATGACA TATGGAAAGC GTTATGATTA   
  
  
+ TATATACCAA CGATCAAAGA ACCAACGAAC CCCCATTTCA GATTTTGTCC TTCCAACCCA ATAAGAGAAG   
  
  
+ CCAAACAGAC CAAGAACGCC CACACACAC  

- TTCGATAATA ACGGTTCGGT CTCTGAGTTA CATAAAATCA GTCTTGTTCA ACGATTCGAG AACTTAAGAC   
  
  
- TTAATAGTCA GTGATTTGGT GAATAACACT ACATTTATAC AATAGTACAC ATTCGTTACT TCGTCATTCG   
  
  
- GTTCTAATTC CTCTTGGTGT CATTAACCGG AGTTTCTTTC TTTCTTTCTT TTTTGTTTTT ATCATCTCTA   
  
  
- GTTCGGATTT GTGTCTTTAA AGATCTCACT TCACTTTTGA CTTGTTTTGC TGTCTCTCTT TCTCTCCCTC   
  
  
- CTACCCCCTC TACTCTTTTT CTCCCCTAAC GTTCAATGGA ACCTCCACGG GGCTTTCACT CTGGTCCTCG   
  
  
- GGGTTCGCCT CCCTATTCCT AGCGGATACA ACATTCCCTC TAGGAACTCA GGGCATGGTC TAGGTGGGTG   
  
  
- GTGAGGGAAG TTCTTAAACT TGTACAACAA AAACAGGAGA GCAGAGGAAA AAAAGCCCAC AAATACCCAA   
  
  
- ATACATACGT ATATTCCAAC ATAGGAGACT ACGCATATCC GCTTACTGGG GTTTGTCAGA GAGAGAGAGA   
  
  
- GAGAGAGAGA GAGAGAGAGA GAGGGAGACG TACCTACTAC CTCTCCCGCC CCTTGTCCAA GCTAGACGCT   
  
  
- CTCTCACATT CTCTCTCTCT CTACGTCTAC GTTCCACAGT TTTTGCCTTT TCTTTTAATT TAACTACAAA   
  
  
- AGCCCACGTT GGGCTTCATA GCTCGTCGGA CCAAGAAGTT TATTTCTGGC AATTCTAACG GAGGAATAAG   
  
  
- TTGAAGGAAT TTACCCGGTC ACCCCACAGT TCGGGAAGAG ACAAGGTCGG GTATTCATGC GGAGAAAGAC   
  
  
- AGACAAAATA AACCCGGGAG GCTAACGGGC TGAAAAGTAA ACATTAATGA ACGAATTAAT TACCAATATA   
  
  
- CGACACGAAA TAATAATTTT AAGGTGAATG TCTCTCAATG TAGTTATAGC GCTTTGTCTT TCTTTAAGTC   
  
  
- ACAACATATG TAAAGTAAAC CATTATTATG GGTACCCGGT GCCTTTGAAC TGAGGGACAC AGTACTGAGT   
  
  
- TCTTGGTACT GCATGCCCAT TTCCCAACCA AGTTTGGCGC CACTGGTACT TGCTCATTTT GTCTCACGAG   
  
  
- GGTACGTTCA AGTCTTGTTC TGCGAAAGAG GAGGTGTCAG TTATTGTAGT TTCGTTCTCT TTGAAGAAGT   
  
  
- TCCAGATTCG TTAACCAAAG TTACCCTGTT CCAGAAGAAA ATCTGAGTCT GGCCTTTTTC AACTTTTGGG   
  
  
- GCGTGAGTAA TTGTTCTGGC ACTGAACCAG TATAAAGGTT CAACGAAGAA GAAACAGAAA AGCAAAATTA   
  
  
- ATTAATTGAA GAACGTGGCA CATTTTCCTT GTGCTTATGA AACTTACTGT ATACCTTTCG CAATACTAAT   
  
  
- ATATATGGTT GCTAGTTTCT TGGTTGCTTG GGGGTAAAGT CTAAAACAGG AAGGTTGGGT TATTCTCTTC   
  
  
- GGTTTGTCTG GTTCTTGCGG GTGTGTGTG

+     Skn-1\_motif

| Site Name | Organism | Position | Strand | Matrix score. | sequence | function |
| --- | --- | --- | --- | --- | --- | --- |
| Skn-1\_motif | Oryza sativa | 1288 | + | 5 | GTCAT | cis-acting regulatory element required for endosperm expression |
| Skn-1\_motif | Oryza sativa | 1057 | - | 5 | GTCAT | cis-acting regulatory element required for endosperm expression |
| Skn-1\_motif | Oryza sativa | 1043 | - | 5 | GTCAT | cis-acting regulatory element required for endosperm expression |
| Skn-1\_motif | Oryza sativa | 534 | - | 5 | GTCAT | cis-acting regulatory element required for endosperm expression |
| Skn-1\_motif | Oryza sativa | 1375 | - | 5 | GTCAT | cis-acting regulatory element required for endosperm expression |
| Skn-1\_motif | Oryza sativa | 1040 | + | 5 | GTCAT | cis-acting regulatory element required for endosperm expression |

> 2018/04/13 10:10:12  
+ AAGCTATTAT TGCCAAGCCA GAGACTCAAT GTATTTTAGT CAGAACAAGT TGCTAAGCTC TTGAATTCTG   
  
  
+ AATTATCAGT CACTAAACCA CTTATTGTGA TGTAAATATG TTATCATGTG TAAGCAATGA AGCAGTAAGC   
  
  
+ CAAGATTAAG GAGAACCACA GTAATTGGCC TCAAAGAAAG AAAGAAAGAA AAAACAAAAA TAGTAGAGAT   
  
  
+ CAAGCCTAAA CACAGAAATT TCTAGAGTGA AGTGAAAACT GAACAAAACG ACAGAGAGAA AGAGAGGGAG   
  
  
+ GATGGGGGAG ATGAGAAAAA GAGGGGATTG CAAGTTACCT TGGAGGTGCC CCGAAAGTGA GACCAGGAGC   
  
  
+ CCCAAGCGGA GGGATAAGGA TCGCCTATGT TGTAAGGGAG ATCCTTGAGT CCCGTACCAG ATCCACCCAC   
  
  
+ CACTCCCTTC AAGAATTTGA ACATGTTGTT TTTGTCCTCT CGTCTCCTTT TTTTCGGGTG TTTATGGGTT   
  
  
+ TATGTATGCA TATAAGGTTG TATCCTCTGA TGCGTATAGG CGAATGACCC CAAACAGTCT CTCTCTCTCT   
  
  
+ CTCTCTCTCT CTCTCTCTCT CTCCCTCTGC ATGGATGATG GAGAGGGCGG GGAACAGGTT CGATCTGCGA   
  
  
+ GAGAGTGTAA GAGAGAGAGA GATGCAGATG CAAGGTGTCA AAAACGGAAA AGAAAATTAA ATTGATGTTT   
  
  
+ TCGGGTGCAA CCCGAAGTAT CGAGCAGCCT GGTTCTTCAA ATAAAGACCG TTAAGATTGC CTCCTTATTC   
  
  
+ AACTTCCTTA AATGGGCCAG TGGGGTGTCA AGCCCTTCTC TGTTCCAGCC CATAAGTACG CCTCTTTCTG   
  
  
+ TCTGTTTTAT TTGGGCCCTC CGATTGCCCG ACTTTTCATT TGTAATTACT TGCTTAATTA ATGGTTATAT   
  
  
+ GCTGTGCTTT ATTATTAAAA TTCCACTTAC AGAGAGTTAC ATCAATATCG CGAAACAGAA AGAAATTCAG   
  
  
+ TGTTGTATAC ATTTCATTTG GTAATAATAC CCATGGGCCA CGGAAACTTG ACTCCCTGTG TCATGACTCA   
  
  
+ AGAACCATGA CGTACGGGTA AAGGGTTGGT TCAAACCGCG GTGACCATGA ACGAGTAAAA CAGAGTGCTC   
  
  
+ CCATGCAAGT TCAGAACAAG ACGCTTTCTC CTCCACAGTC AATAACATCA AAGCAAGAGA AACTTCTTCA   
  
  
+ AGGTCTAAGC AATTGGTTTC AATGGGACAA GGTCTTCTTT TAGACTCAGA CCGGAAAAAG TTGAAAACCC   
  
  
+ CGCACTCATT AACAAGACCG TGACTTGGTC ATATTTCCAA GTTGCTTCTT CTTTGTCTTT TCGTTTTAAT   
  
  
+ TAATTAACTT CTTGCACCGT GTAAAAGGAA CACGAATACT TTGAATGACA TATGGAAAGC GTTATGATTA   
  
  
+ TATATACCAA CGATCAAAGA ACCAACGAAC CCCCATTTCA GATTTTGTCC TTCCAACCCA ATAAGAGAAG   
  
  
+ CCAAACAGAC CAAGAACGCC CACACACAC  

- TTCGATAATA ACGGTTCGGT CTCTGAGTTA CATAAAATCA GTCTTGTTCA ACGATTCGAG AACTTAAGAC   
  
  
- TTAATAGTCA GTGATTTGGT GAATAACACT ACATTTATAC AATAGTACAC ATTCGTTACT TCGTCATTCG   
  
  
- GTTCTAATTC CTCTTGGTGT CATTAACCGG AGTTTCTTTC TTTCTTTCTT TTTTGTTTTT ATCATCTCTA   
  
  
- GTTCGGATTT GTGTCTTTAA AGATCTCACT TCACTTTTGA CTTGTTTTGC TGTCTCTCTT TCTCTCCCTC   
  
  
- CTACCCCCTC TACTCTTTTT CTCCCCTAAC GTTCAATGGA ACCTCCACGG GGCTTTCACT CTGGTCCTCG   
  
  
- GGGTTCGCCT CCCTATTCCT AGCGGATACA ACATTCCCTC TAGGAACTCA GGGCATGGTC TAGGTGGGTG   
  
  
- GTGAGGGAAG TTCTTAAACT TGTACAACAA AAACAGGAGA GCAGAGGAAA AAAAGCCCAC AAATACCCAA   
  
  
- ATACATACGT ATATTCCAAC ATAGGAGACT ACGCATATCC GCTTACTGGG GTTTGTCAGA GAGAGAGAGA   
  
  
- GAGAGAGAGA GAGAGAGAGA GAGGGAGACG TACCTACTAC CTCTCCCGCC CCTTGTCCAA GCTAGACGCT   
  
  
- CTCTCACATT CTCTCTCTCT CTACGTCTAC GTTCCACAGT TTTTGCCTTT TCTTTTAATT TAACTACAAA   
  
  
- AGCCCACGTT GGGCTTCATA GCTCGTCGGA CCAAGAAGTT TATTTCTGGC AATTCTAACG GAGGAATAAG   
  
  
- TTGAAGGAAT TTACCCGGTC ACCCCACAGT TCGGGAAGAG ACAAGGTCGG GTATTCATGC GGAGAAAGAC   
  
  
- AGACAAAATA AACCCGGGAG GCTAACGGGC TGAAAAGTAA ACATTAATGA ACGAATTAAT TACCAATATA   
  
  
- CGACACGAAA TAATAATTTT AAGGTGAATG TCTCTCAATG TAGTTATAGC GCTTTGTCTT TCTTTAAGTC   
  
  
- ACAACATATG TAAAGTAAAC CATTATTATG GGTACCCGGT GCCTTTGAAC TGAGGGACAC AGTACTGAGT   
  
  
- TCTTGGTACT GCATGCCCAT TTCCCAACCA AGTTTGGCGC CACTGGTACT TGCTCATTTT GTCTCACGAG   
  
  
- GGTACGTTCA AGTCTTGTTC TGCGAAAGAG GAGGTGTCAG TTATTGTAGT TTCGTTCTCT TTGAAGAAGT   
  
  
- TCCAGATTCG TTAACCAAAG TTACCCTGTT CCAGAAGAAA ATCTGAGTCT GGCCTTTTTC AACTTTTGGG   
  
  
- GCGTGAGTAA TTGTTCTGGC ACTGAACCAG TATAAAGGTT CAACGAAGAA GAAACAGAAA AGCAAAATTA   
  
  
- ATTAATTGAA GAACGTGGCA CATTTTCCTT GTGCTTATGA AACTTACTGT ATACCTTTCG CAATACTAAT   
  
  
- ATATATGGTT GCTAGTTTCT TGGTTGCTTG GGGGTAAAGT CTAAAACAGG AAGGTTGGGT TATTCTCTTC   
  
  
- GGTTTGTCTG GTTCTTGCGG GTGTGTGTG

+     Sp1

| Site Name | Organism | Position | Strand | Matrix score. | sequence | function |
| --- | --- | --- | --- | --- | --- | --- |
| Sp1 | Zea mays | 276 | - | 5 | CC(G/A)CCC | light responsive element |
| Sp1 | Zea mays | 413 | + | 5.5 | CC(G/A)CCC | light responsive element |
| Sp1 | Oryza sativa | 605 | + | 6 | GGGCGG | light responsive element |

> 2018/04/13 10:10:12  
+ AAGCTATTAT TGCCAAGCCA GAGACTCAAT GTATTTTAGT CAGAACAAGT TGCTAAGCTC TTGAATTCTG   
  
  
+ AATTATCAGT CACTAAACCA CTTATTGTGA TGTAAATATG TTATCATGTG TAAGCAATGA AGCAGTAAGC   
  
  
+ CAAGATTAAG GAGAACCACA GTAATTGGCC TCAAAGAAAG AAAGAAAGAA AAAACAAAAA TAGTAGAGAT   
  
  
+ CAAGCCTAAA CACAGAAATT TCTAGAGTGA AGTGAAAACT GAACAAAACG ACAGAGAGAA AGAGAGGGAG   
  
  
+ GATGGGGGAG ATGAGAAAAA GAGGGGATTG CAAGTTACCT TGGAGGTGCC CCGAAAGTGA GACCAGGAGC   
  
  
+ CCCAAGCGGA GGGATAAGGA TCGCCTATGT TGTAAGGGAG ATCCTTGAGT CCCGTACCAG ATCCACCCAC   
  
  
+ CACTCCCTTC AAGAATTTGA ACATGTTGTT TTTGTCCTCT CGTCTCCTTT TTTTCGGGTG TTTATGGGTT   
  
  
+ TATGTATGCA TATAAGGTTG TATCCTCTGA TGCGTATAGG CGAATGACCC CAAACAGTCT CTCTCTCTCT   
  
  
+ CTCTCTCTCT CTCTCTCTCT CTCCCTCTGC ATGGATGATG GAGAGGGCGG GGAACAGGTT CGATCTGCGA   
  
  
+ GAGAGTGTAA GAGAGAGAGA GATGCAGATG CAAGGTGTCA AAAACGGAAA AGAAAATTAA ATTGATGTTT   
  
  
+ TCGGGTGCAA CCCGAAGTAT CGAGCAGCCT GGTTCTTCAA ATAAAGACCG TTAAGATTGC CTCCTTATTC   
  
  
+ AACTTCCTTA AATGGGCCAG TGGGGTGTCA AGCCCTTCTC TGTTCCAGCC CATAAGTACG CCTCTTTCTG   
  
  
+ TCTGTTTTAT TTGGGCCCTC CGATTGCCCG ACTTTTCATT TGTAATTACT TGCTTAATTA ATGGTTATAT   
  
  
+ GCTGTGCTTT ATTATTAAAA TTCCACTTAC AGAGAGTTAC ATCAATATCG CGAAACAGAA AGAAATTCAG   
  
  
+ TGTTGTATAC ATTTCATTTG GTAATAATAC CCATGGGCCA CGGAAACTTG ACTCCCTGTG TCATGACTCA   
  
  
+ AGAACCATGA CGTACGGGTA AAGGGTTGGT TCAAACCGCG GTGACCATGA ACGAGTAAAA CAGAGTGCTC   
  
  
+ CCATGCAAGT TCAGAACAAG ACGCTTTCTC CTCCACAGTC AATAACATCA AAGCAAGAGA AACTTCTTCA   
  
  
+ AGGTCTAAGC AATTGGTTTC AATGGGACAA GGTCTTCTTT TAGACTCAGA CCGGAAAAAG TTGAAAACCC   
  
  
+ CGCACTCATT AACAAGACCG TGACTTGGTC ATATTTCCAA GTTGCTTCTT CTTTGTCTTT TCGTTTTAAT   
  
  
+ TAATTAACTT CTTGCACCGT GTAAAAGGAA CACGAATACT TTGAATGACA TATGGAAAGC GTTATGATTA   
  
  
+ TATATACCAA CGATCAAAGA ACCAACGAAC CCCCATTTCA GATTTTGTCC TTCCAACCCA ATAAGAGAAG   
  
  
+ CCAAACAGAC CAAGAACGCC CACACACAC  

- TTCGATAATA ACGGTTCGGT CTCTGAGTTA CATAAAATCA GTCTTGTTCA ACGATTCGAG AACTTAAGAC   
  
  
- TTAATAGTCA GTGATTTGGT GAATAACACT ACATTTATAC AATAGTACAC ATTCGTTACT TCGTCATTCG   
  
  
- GTTCTAATTC CTCTTGGTGT CATTAACCGG AGTTTCTTTC TTTCTTTCTT TTTTGTTTTT ATCATCTCTA   
  
  
- GTTCGGATTT GTGTCTTTAA AGATCTCACT TCACTTTTGA CTTGTTTTGC TGTCTCTCTT TCTCTCCCTC   
  
  
- CTACCCCCTC TACTCTTTTT CTCCCCTAAC GTTCAATGGA ACCTCCACGG GGCTTTCACT CTGGTCCTCG   
  
  
- GGGTTCGCCT CCCTATTCCT AGCGGATACA ACATTCCCTC TAGGAACTCA GGGCATGGTC TAGGTGGGTG   
  
  
- GTGAGGGAAG TTCTTAAACT TGTACAACAA AAACAGGAGA GCAGAGGAAA AAAAGCCCAC AAATACCCAA   
  
  
- ATACATACGT ATATTCCAAC ATAGGAGACT ACGCATATCC GCTTACTGGG GTTTGTCAGA GAGAGAGAGA   
  
  
- GAGAGAGAGA GAGAGAGAGA GAGGGAGACG TACCTACTAC CTCTCCCGCC CCTTGTCCAA GCTAGACGCT   
  
  
- CTCTCACATT CTCTCTCTCT CTACGTCTAC GTTCCACAGT TTTTGCCTTT TCTTTTAATT TAACTACAAA   
  
  
- AGCCCACGTT GGGCTTCATA GCTCGTCGGA CCAAGAAGTT TATTTCTGGC AATTCTAACG GAGGAATAAG   
  
  
- TTGAAGGAAT TTACCCGGTC ACCCCACAGT TCGGGAAGAG ACAAGGTCGG GTATTCATGC GGAGAAAGAC   
  
  
- AGACAAAATA AACCCGGGAG GCTAACGGGC TGAAAAGTAA ACATTAATGA ACGAATTAAT TACCAATATA   
  
  
- CGACACGAAA TAATAATTTT AAGGTGAATG TCTCTCAATG TAGTTATAGC GCTTTGTCTT TCTTTAAGTC   
  
  
- ACAACATATG TAAAGTAAAC CATTATTATG GGTACCCGGT GCCTTTGAAC TGAGGGACAC AGTACTGAGT   
  
  
- TCTTGGTACT GCATGCCCAT TTCCCAACCA AGTTTGGCGC CACTGGTACT TGCTCATTTT GTCTCACGAG   
  
  
- GGTACGTTCA AGTCTTGTTC TGCGAAAGAG GAGGTGTCAG TTATTGTAGT TTCGTTCTCT TTGAAGAAGT   
  
  
- TCCAGATTCG TTAACCAAAG TTACCCTGTT CCAGAAGAAA ATCTGAGTCT GGCCTTTTTC AACTTTTGGG   
  
  
- GCGTGAGTAA TTGTTCTGGC ACTGAACCAG TATAAAGGTT CAACGAAGAA GAAACAGAAA AGCAAAATTA   
  
  
- ATTAATTGAA GAACGTGGCA CATTTTCCTT GTGCTTATGA AACTTACTGT ATACCTTTCG CAATACTAAT   
  
  
- ATATATGGTT GCTAGTTTCT TGGTTGCTTG GGGGTAAAGT CTAAAACAGG AAGGTTGGGT TATTCTCTTC   
  
  
- GGTTTGTCTG GTTCTTGCGG GTGTGTGTG

+     TATA-box

| Site Name | Organism | Position | Strand | Matrix score. | sequence | function |
| --- | --- | --- | --- | --- | --- | --- |
| TATA-box | Arabidopsis thaliana | 1403 | - | 4 | TATA | core promoter element around -30 of transcription start |
| TATA-box | Lycopersicon esculentum | 1324 | + | 5 | TTTTA | core promoter element around -30 of transcription start |
| TATA-box | Lycopersicon esculentum | 34 | + | 5 | TTTTA | core promoter element around -30 of transcription start |
| TATA-box | Arabidopsis thaliana | 1401 | - | 4 | TATA | core promoter element around -30 of transcription start |
| TATA-box | Arabidopsis thaliana | 1399 | - | 8 | TATATATA | core promoter element around -30 of transcription start |
| TATA-box | Lycopersicon esculentum | 1106 | - | 5 | TTTTA | core promoter element around -30 of transcription start |
| TATA-box | Glycine max | 5 | - | 5 | TAATA | core promoter element around -30 of transcription start |
| TATA-box | Arabidopsis thaliana | 986 | - | 4 | TATA | core promoter element around -30 of transcription start |
| TATA-box | Glycine max | 1005 | + | 5 | TAATA | core promoter element around -30 of transcription start |
| TATA-box | Arabidopsis thaliana | 501 | + | 4 | TATA | core promoter element around -30 of transcription start |
| TATA-box | Lycopersicon esculentum | 926 | - | 5 | TTTTA | core promoter element around -30 of transcription start |
| TATA-box | Brassica napus | 1397 | + | 6 | ATTATA | core promoter element around -30 of transcription start |
| TATA-box | Oryza sativa | 489 | - | 8 | TACATAAA | core promoter element around -30 of transcription start |
| TATA-box | Lycopersicon esculentum | 1352 | - | 5 | TTTTA | core promoter element around -30 of transcription start |
| TATA-box | Brassica oleracea | 500 | + | 6 | ATATAA | core promoter element around -30 of transcription start |
| TATA-box | Arabidopsis thaliana | 905 | - | 5 | TATAA | core promoter element around -30 of transcription start |
| TATA-box | Glycine max | 1002 | + | 5 | TAATA | core promoter element around -30 of transcription start |
| TATA-box | Lycopersicon esculentum | 845 | + | 5 | TTTTA | core promoter element around -30 of transcription start |
| TATA-box | Lycopersicon esculentum | 1228 | + | 5 | TTTTA | core promoter element around -30 of transcription start |
| TATA-box | Glycine max | 923 | - | 5 | TAATA | core promoter element around -30 of transcription start |
| TATA-box | Brassica napus | 1400 | - | 6 | ATATAT | core promoter element around -30 of transcription start |
| TATA-box | Arabidopsis thaliana | 525 | + | 4 | TATA | core promoter element around -30 of transcription start |
| TATA-box | Arabidopsis thaliana | 1398 | - | 7 | TATATAA | core promoter element around -30 of transcription start |
| TATA-box | Glycine max | 920 | - | 5 | TAATA | core promoter element around -30 of transcription start |
| TATA-box | Helianthus annuus | 984 | - | 6 | TATACA | core promoter element around -30 of transcription start |
| TATA-box | Arabidopsis thaliana | 906 | - | 4 | TATA | core promoter element around -30 of transcription start |

> 2018/04/13 10:10:12  
+ AAGCTATTAT TGCCAAGCCA GAGACTCAAT GTATTTTAGT CAGAACAAGT TGCTAAGCTC TTGAATTCTG   
  
  
+ AATTATCAGT CACTAAACCA CTTATTGTGA TGTAAATATG TTATCATGTG TAAGCAATGA AGCAGTAAGC   
  
  
+ CAAGATTAAG GAGAACCACA GTAATTGGCC TCAAAGAAAG AAAGAAAGAA AAAACAAAAA TAGTAGAGAT   
  
  
+ CAAGCCTAAA CACAGAAATT TCTAGAGTGA AGTGAAAACT GAACAAAACG ACAGAGAGAA AGAGAGGGAG   
  
  
+ GATGGGGGAG ATGAGAAAAA GAGGGGATTG CAAGTTACCT TGGAGGTGCC CCGAAAGTGA GACCAGGAGC   
  
  
+ CCCAAGCGGA GGGATAAGGA TCGCCTATGT TGTAAGGGAG ATCCTTGAGT CCCGTACCAG ATCCACCCAC   
  
  
+ CACTCCCTTC AAGAATTTGA ACATGTTGTT TTTGTCCTCT CGTCTCCTTT TTTTCGGGTG TTTATGGGTT   
  
  
+ TATGTATGCA TATAAGGTTG TATCCTCTGA TGCGTATAGG CGAATGACCC CAAACAGTCT CTCTCTCTCT   
  
  
+ CTCTCTCTCT CTCTCTCTCT CTCCCTCTGC ATGGATGATG GAGAGGGCGG GGAACAGGTT CGATCTGCGA   
  
  
+ GAGAGTGTAA GAGAGAGAGA GATGCAGATG CAAGGTGTCA AAAACGGAAA AGAAAATTAA ATTGATGTTT   
  
  
+ TCGGGTGCAA CCCGAAGTAT CGAGCAGCCT GGTTCTTCAA ATAAAGACCG TTAAGATTGC CTCCTTATTC   
  
  
+ AACTTCCTTA AATGGGCCAG TGGGGTGTCA AGCCCTTCTC TGTTCCAGCC CATAAGTACG CCTCTTTCTG   
  
  
+ TCTGTTTTAT TTGGGCCCTC CGATTGCCCG ACTTTTCATT TGTAATTACT TGCTTAATTA ATGGTTATAT   
  
  
+ GCTGTGCTTT ATTATTAAAA TTCCACTTAC AGAGAGTTAC ATCAATATCG CGAAACAGAA AGAAATTCAG   
  
  
+ TGTTGTATAC ATTTCATTTG GTAATAATAC CCATGGGCCA CGGAAACTTG ACTCCCTGTG TCATGACTCA   
  
  
+ AGAACCATGA CGTACGGGTA AAGGGTTGGT TCAAACCGCG GTGACCATGA ACGAGTAAAA CAGAGTGCTC   
  
  
+ CCATGCAAGT TCAGAACAAG ACGCTTTCTC CTCCACAGTC AATAACATCA AAGCAAGAGA AACTTCTTCA   
  
  
+ AGGTCTAAGC AATTGGTTTC AATGGGACAA GGTCTTCTTT TAGACTCAGA CCGGAAAAAG TTGAAAACCC   
  
  
+ CGCACTCATT AACAAGACCG TGACTTGGTC ATATTTCCAA GTTGCTTCTT CTTTGTCTTT TCGTTTTAAT   
  
  
+ TAATTAACTT CTTGCACCGT GTAAAAGGAA CACGAATACT TTGAATGACA TATGGAAAGC GTTATGATTA   
  
  
+ TATATACCAA CGATCAAAGA ACCAACGAAC CCCCATTTCA GATTTTGTCC TTCCAACCCA ATAAGAGAAG   
  
  
+ CCAAACAGAC CAAGAACGCC CACACACAC  

- TTCGATAATA ACGGTTCGGT CTCTGAGTTA CATAAAATCA GTCTTGTTCA ACGATTCGAG AACTTAAGAC   
  
  
- TTAATAGTCA GTGATTTGGT GAATAACACT ACATTTATAC AATAGTACAC ATTCGTTACT TCGTCATTCG   
  
  
- GTTCTAATTC CTCTTGGTGT CATTAACCGG AGTTTCTTTC TTTCTTTCTT TTTTGTTTTT ATCATCTCTA   
  
  
- GTTCGGATTT GTGTCTTTAA AGATCTCACT TCACTTTTGA CTTGTTTTGC TGTCTCTCTT TCTCTCCCTC   
  
  
- CTACCCCCTC TACTCTTTTT CTCCCCTAAC GTTCAATGGA ACCTCCACGG GGCTTTCACT CTGGTCCTCG   
  
  
- GGGTTCGCCT CCCTATTCCT AGCGGATACA ACATTCCCTC TAGGAACTCA GGGCATGGTC TAGGTGGGTG   
  
  
- GTGAGGGAAG TTCTTAAACT TGTACAACAA AAACAGGAGA GCAGAGGAAA AAAAGCCCAC AAATACCCAA   
  
  
- ATACATACGT ATATTCCAAC ATAGGAGACT ACGCATATCC GCTTACTGGG GTTTGTCAGA GAGAGAGAGA   
  
  
- GAGAGAGAGA GAGAGAGAGA GAGGGAGACG TACCTACTAC CTCTCCCGCC CCTTGTCCAA GCTAGACGCT   
  
  
- CTCTCACATT CTCTCTCTCT CTACGTCTAC GTTCCACAGT TTTTGCCTTT TCTTTTAATT TAACTACAAA   
  
  
- AGCCCACGTT GGGCTTCATA GCTCGTCGGA CCAAGAAGTT TATTTCTGGC AATTCTAACG GAGGAATAAG   
  
  
- TTGAAGGAAT TTACCCGGTC ACCCCACAGT TCGGGAAGAG ACAAGGTCGG GTATTCATGC GGAGAAAGAC   
  
  
- AGACAAAATA AACCCGGGAG GCTAACGGGC TGAAAAGTAA ACATTAATGA ACGAATTAAT TACCAATATA   
  
  
- CGACACGAAA TAATAATTTT AAGGTGAATG TCTCTCAATG TAGTTATAGC GCTTTGTCTT TCTTTAAGTC   
  
  
- ACAACATATG TAAAGTAAAC CATTATTATG GGTACCCGGT GCCTTTGAAC TGAGGGACAC AGTACTGAGT   
  
  
- TCTTGGTACT GCATGCCCAT TTCCCAACCA AGTTTGGCGC CACTGGTACT TGCTCATTTT GTCTCACGAG   
  
  
- GGTACGTTCA AGTCTTGTTC TGCGAAAGAG GAGGTGTCAG TTATTGTAGT TTCGTTCTCT TTGAAGAAGT   
  
  
- TCCAGATTCG TTAACCAAAG TTACCCTGTT CCAGAAGAAA ATCTGAGTCT GGCCTTTTTC AACTTTTGGG   
  
  
- GCGTGAGTAA TTGTTCTGGC ACTGAACCAG TATAAAGGTT CAACGAAGAA GAAACAGAAA AGCAAAATTA   
  
  
- ATTAATTGAA GAACGTGGCA CATTTTCCTT GTGCTTATGA AACTTACTGT ATACCTTTCG CAATACTAAT   
  
  
- ATATATGGTT GCTAGTTTCT TGGTTGCTTG GGGGTAAAGT CTAAAACAGG AAGGTTGGGT TATTCTCTTC   
  
  
- GGTTTGTCTG GTTCTTGCGG GTGTGTGTG

+     TCA-element

| Site Name | Organism | Position | Strand | Matrix score. | sequence | function |
| --- | --- | --- | --- | --- | --- | --- |
| TCA-element | Nicotiana tabacum | 296 | - | 9 | CCATCTTTTT | cis-acting element involved in salicylic acid responsiveness |

> 2018/04/13 10:10:12  
+ AAGCTATTAT TGCCAAGCCA GAGACTCAAT GTATTTTAGT CAGAACAAGT TGCTAAGCTC TTGAATTCTG   
  
  
+ AATTATCAGT CACTAAACCA CTTATTGTGA TGTAAATATG TTATCATGTG TAAGCAATGA AGCAGTAAGC   
  
  
+ CAAGATTAAG GAGAACCACA GTAATTGGCC TCAAAGAAAG AAAGAAAGAA AAAACAAAAA TAGTAGAGAT   
  
  
+ CAAGCCTAAA CACAGAAATT TCTAGAGTGA AGTGAAAACT GAACAAAACG ACAGAGAGAA AGAGAGGGAG   
  
  
+ GATGGGGGAG ATGAGAAAAA GAGGGGATTG CAAGTTACCT TGGAGGTGCC CCGAAAGTGA GACCAGGAGC   
  
  
+ CCCAAGCGGA GGGATAAGGA TCGCCTATGT TGTAAGGGAG ATCCTTGAGT CCCGTACCAG ATCCACCCAC   
  
  
+ CACTCCCTTC AAGAATTTGA ACATGTTGTT TTTGTCCTCT CGTCTCCTTT TTTTCGGGTG TTTATGGGTT   
  
  
+ TATGTATGCA TATAAGGTTG TATCCTCTGA TGCGTATAGG CGAATGACCC CAAACAGTCT CTCTCTCTCT   
  
  
+ CTCTCTCTCT CTCTCTCTCT CTCCCTCTGC ATGGATGATG GAGAGGGCGG GGAACAGGTT CGATCTGCGA   
  
  
+ GAGAGTGTAA GAGAGAGAGA GATGCAGATG CAAGGTGTCA AAAACGGAAA AGAAAATTAA ATTGATGTTT   
  
  
+ TCGGGTGCAA CCCGAAGTAT CGAGCAGCCT GGTTCTTCAA ATAAAGACCG TTAAGATTGC CTCCTTATTC   
  
  
+ AACTTCCTTA AATGGGCCAG TGGGGTGTCA AGCCCTTCTC TGTTCCAGCC CATAAGTACG CCTCTTTCTG   
  
  
+ TCTGTTTTAT TTGGGCCCTC CGATTGCCCG ACTTTTCATT TGTAATTACT TGCTTAATTA ATGGTTATAT   
  
  
+ GCTGTGCTTT ATTATTAAAA TTCCACTTAC AGAGAGTTAC ATCAATATCG CGAAACAGAA AGAAATTCAG   
  
  
+ TGTTGTATAC ATTTCATTTG GTAATAATAC CCATGGGCCA CGGAAACTTG ACTCCCTGTG TCATGACTCA   
  
  
+ AGAACCATGA CGTACGGGTA AAGGGTTGGT TCAAACCGCG GTGACCATGA ACGAGTAAAA CAGAGTGCTC   
  
  
+ CCATGCAAGT TCAGAACAAG ACGCTTTCTC CTCCACAGTC AATAACATCA AAGCAAGAGA AACTTCTTCA   
  
  
+ AGGTCTAAGC AATTGGTTTC AATGGGACAA GGTCTTCTTT TAGACTCAGA CCGGAAAAAG TTGAAAACCC   
  
  
+ CGCACTCATT AACAAGACCG TGACTTGGTC ATATTTCCAA GTTGCTTCTT CTTTGTCTTT TCGTTTTAAT   
  
  
+ TAATTAACTT CTTGCACCGT GTAAAAGGAA CACGAATACT TTGAATGACA TATGGAAAGC GTTATGATTA   
  
  
+ TATATACCAA CGATCAAAGA ACCAACGAAC CCCCATTTCA GATTTTGTCC TTCCAACCCA ATAAGAGAAG   
  
  
+ CCAAACAGAC CAAGAACGCC CACACACAC  

- TTCGATAATA ACGGTTCGGT CTCTGAGTTA CATAAAATCA GTCTTGTTCA ACGATTCGAG AACTTAAGAC   
  
  
- TTAATAGTCA GTGATTTGGT GAATAACACT ACATTTATAC AATAGTACAC ATTCGTTACT TCGTCATTCG   
  
  
- GTTCTAATTC CTCTTGGTGT CATTAACCGG AGTTTCTTTC TTTCTTTCTT TTTTGTTTTT ATCATCTCTA   
  
  
- GTTCGGATTT GTGTCTTTAA AGATCTCACT TCACTTTTGA CTTGTTTTGC TGTCTCTCTT TCTCTCCCTC   
  
  
- CTACCCCCTC TACTCTTTTT CTCCCCTAAC GTTCAATGGA ACCTCCACGG GGCTTTCACT CTGGTCCTCG   
  
  
- GGGTTCGCCT CCCTATTCCT AGCGGATACA ACATTCCCTC TAGGAACTCA GGGCATGGTC TAGGTGGGTG   
  
  
- GTGAGGGAAG TTCTTAAACT TGTACAACAA AAACAGGAGA GCAGAGGAAA AAAAGCCCAC AAATACCCAA   
  
  
- ATACATACGT ATATTCCAAC ATAGGAGACT ACGCATATCC GCTTACTGGG GTTTGTCAGA GAGAGAGAGA   
  
  
- GAGAGAGAGA GAGAGAGAGA GAGGGAGACG TACCTACTAC CTCTCCCGCC CCTTGTCCAA GCTAGACGCT   
  
  
- CTCTCACATT CTCTCTCTCT CTACGTCTAC GTTCCACAGT TTTTGCCTTT TCTTTTAATT TAACTACAAA   
  
  
- AGCCCACGTT GGGCTTCATA GCTCGTCGGA CCAAGAAGTT TATTTCTGGC AATTCTAACG GAGGAATAAG   
  
  
- TTGAAGGAAT TTACCCGGTC ACCCCACAGT TCGGGAAGAG ACAAGGTCGG GTATTCATGC GGAGAAAGAC   
  
  
- AGACAAAATA AACCCGGGAG GCTAACGGGC TGAAAAGTAA ACATTAATGA ACGAATTAAT TACCAATATA   
  
  
- CGACACGAAA TAATAATTTT AAGGTGAATG TCTCTCAATG TAGTTATAGC GCTTTGTCTT TCTTTAAGTC   
  
  
- ACAACATATG TAAAGTAAAC CATTATTATG GGTACCCGGT GCCTTTGAAC TGAGGGACAC AGTACTGAGT   
  
  
- TCTTGGTACT GCATGCCCAT TTCCCAACCA AGTTTGGCGC CACTGGTACT TGCTCATTTT GTCTCACGAG   
  
  
- GGTACGTTCA AGTCTTGTTC TGCGAAAGAG GAGGTGTCAG TTATTGTAGT TTCGTTCTCT TTGAAGAAGT   
  
  
- TCCAGATTCG TTAACCAAAG TTACCCTGTT CCAGAAGAAA ATCTGAGTCT GGCCTTTTTC AACTTTTGGG   
  
  
- GCGTGAGTAA TTGTTCTGGC ACTGAACCAG TATAAAGGTT CAACGAAGAA GAAACAGAAA AGCAAAATTA   
  
  
- ATTAATTGAA GAACGTGGCA CATTTTCCTT GTGCTTATGA AACTTACTGT ATACCTTTCG CAATACTAAT   
  
  
- ATATATGGTT GCTAGTTTCT TGGTTGCTTG GGGGTAAAGT CTAAAACAGG AAGGTTGGGT TATTCTCTTC   
  
  
- GGTTTGTCTG GTTCTTGCGG GTGTGTGTG

+     TCCC-motif

| Site Name | Organism | Position | Strand | Matrix score. | sequence | function |
| --- | --- | --- | --- | --- | --- | --- |
| TCCC-motif | Spinacia oleracea | 580 | + | 7 | TCTCCCT | part of a light responsive element |
| TCCC-motif | Spinacia oleracea | 385 | - | 7 | TCTCCCT | part of a light responsive element |

> 2018/04/13 10:10:12  
+ AAGCTATTAT TGCCAAGCCA GAGACTCAAT GTATTTTAGT CAGAACAAGT TGCTAAGCTC TTGAATTCTG   
  
  
+ AATTATCAGT CACTAAACCA CTTATTGTGA TGTAAATATG TTATCATGTG TAAGCAATGA AGCAGTAAGC   
  
  
+ CAAGATTAAG GAGAACCACA GTAATTGGCC TCAAAGAAAG AAAGAAAGAA AAAACAAAAA TAGTAGAGAT   
  
  
+ CAAGCCTAAA CACAGAAATT TCTAGAGTGA AGTGAAAACT GAACAAAACG ACAGAGAGAA AGAGAGGGAG   
  
  
+ GATGGGGGAG ATGAGAAAAA GAGGGGATTG CAAGTTACCT TGGAGGTGCC CCGAAAGTGA GACCAGGAGC   
  
  
+ CCCAAGCGGA GGGATAAGGA TCGCCTATGT TGTAAGGGAG ATCCTTGAGT CCCGTACCAG ATCCACCCAC   
  
  
+ CACTCCCTTC AAGAATTTGA ACATGTTGTT TTTGTCCTCT CGTCTCCTTT TTTTCGGGTG TTTATGGGTT   
  
  
+ TATGTATGCA TATAAGGTTG TATCCTCTGA TGCGTATAGG CGAATGACCC CAAACAGTCT CTCTCTCTCT   
  
  
+ CTCTCTCTCT CTCTCTCTCT CTCCCTCTGC ATGGATGATG GAGAGGGCGG GGAACAGGTT CGATCTGCGA   
  
  
+ GAGAGTGTAA GAGAGAGAGA GATGCAGATG CAAGGTGTCA AAAACGGAAA AGAAAATTAA ATTGATGTTT   
  
  
+ TCGGGTGCAA CCCGAAGTAT CGAGCAGCCT GGTTCTTCAA ATAAAGACCG TTAAGATTGC CTCCTTATTC   
  
  
+ AACTTCCTTA AATGGGCCAG TGGGGTGTCA AGCCCTTCTC TGTTCCAGCC CATAAGTACG CCTCTTTCTG   
  
  
+ TCTGTTTTAT TTGGGCCCTC CGATTGCCCG ACTTTTCATT TGTAATTACT TGCTTAATTA ATGGTTATAT   
  
  
+ GCTGTGCTTT ATTATTAAAA TTCCACTTAC AGAGAGTTAC ATCAATATCG CGAAACAGAA AGAAATTCAG   
  
  
+ TGTTGTATAC ATTTCATTTG GTAATAATAC CCATGGGCCA CGGAAACTTG ACTCCCTGTG TCATGACTCA   
  
  
+ AGAACCATGA CGTACGGGTA AAGGGTTGGT TCAAACCGCG GTGACCATGA ACGAGTAAAA CAGAGTGCTC   
  
  
+ CCATGCAAGT TCAGAACAAG ACGCTTTCTC CTCCACAGTC AATAACATCA AAGCAAGAGA AACTTCTTCA   
  
  
+ AGGTCTAAGC AATTGGTTTC AATGGGACAA GGTCTTCTTT TAGACTCAGA CCGGAAAAAG TTGAAAACCC   
  
  
+ CGCACTCATT AACAAGACCG TGACTTGGTC ATATTTCCAA GTTGCTTCTT CTTTGTCTTT TCGTTTTAAT   
  
  
+ TAATTAACTT CTTGCACCGT GTAAAAGGAA CACGAATACT TTGAATGACA TATGGAAAGC GTTATGATTA   
  
  
+ TATATACCAA CGATCAAAGA ACCAACGAAC CCCCATTTCA GATTTTGTCC TTCCAACCCA ATAAGAGAAG   
  
  
+ CCAAACAGAC CAAGAACGCC CACACACAC  

- TTCGATAATA ACGGTTCGGT CTCTGAGTTA CATAAAATCA GTCTTGTTCA ACGATTCGAG AACTTAAGAC   
  
  
- TTAATAGTCA GTGATTTGGT GAATAACACT ACATTTATAC AATAGTACAC ATTCGTTACT TCGTCATTCG   
  
  
- GTTCTAATTC CTCTTGGTGT CATTAACCGG AGTTTCTTTC TTTCTTTCTT TTTTGTTTTT ATCATCTCTA   
  
  
- GTTCGGATTT GTGTCTTTAA AGATCTCACT TCACTTTTGA CTTGTTTTGC TGTCTCTCTT TCTCTCCCTC   
  
  
- CTACCCCCTC TACTCTTTTT CTCCCCTAAC GTTCAATGGA ACCTCCACGG GGCTTTCACT CTGGTCCTCG   
  
  
- GGGTTCGCCT CCCTATTCCT AGCGGATACA ACATTCCCTC TAGGAACTCA GGGCATGGTC TAGGTGGGTG   
  
  
- GTGAGGGAAG TTCTTAAACT TGTACAACAA AAACAGGAGA GCAGAGGAAA AAAAGCCCAC AAATACCCAA   
  
  
- ATACATACGT ATATTCCAAC ATAGGAGACT ACGCATATCC GCTTACTGGG GTTTGTCAGA GAGAGAGAGA   
  
  
- GAGAGAGAGA GAGAGAGAGA GAGGGAGACG TACCTACTAC CTCTCCCGCC CCTTGTCCAA GCTAGACGCT   
  
  
- CTCTCACATT CTCTCTCTCT CTACGTCTAC GTTCCACAGT TTTTGCCTTT TCTTTTAATT TAACTACAAA   
  
  
- AGCCCACGTT GGGCTTCATA GCTCGTCGGA CCAAGAAGTT TATTTCTGGC AATTCTAACG GAGGAATAAG   
  
  
- TTGAAGGAAT TTACCCGGTC ACCCCACAGT TCGGGAAGAG ACAAGGTCGG GTATTCATGC GGAGAAAGAC   
  
  
- AGACAAAATA AACCCGGGAG GCTAACGGGC TGAAAAGTAA ACATTAATGA ACGAATTAAT TACCAATATA   
  
  
- CGACACGAAA TAATAATTTT AAGGTGAATG TCTCTCAATG TAGTTATAGC GCTTTGTCTT TCTTTAAGTC   
  
  
- ACAACATATG TAAAGTAAAC CATTATTATG GGTACCCGGT GCCTTTGAAC TGAGGGACAC AGTACTGAGT   
  
  
- TCTTGGTACT GCATGCCCAT TTCCCAACCA AGTTTGGCGC CACTGGTACT TGCTCATTTT GTCTCACGAG   
  
  
- GGTACGTTCA AGTCTTGTTC TGCGAAAGAG GAGGTGTCAG TTATTGTAGT TTCGTTCTCT TTGAAGAAGT   
  
  
- TCCAGATTCG TTAACCAAAG TTACCCTGTT CCAGAAGAAA ATCTGAGTCT GGCCTTTTTC AACTTTTGGG   
  
  
- GCGTGAGTAA TTGTTCTGGC ACTGAACCAG TATAAAGGTT CAACGAAGAA GAAACAGAAA AGCAAAATTA   
  
  
- ATTAATTGAA GAACGTGGCA CATTTTCCTT GTGCTTATGA AACTTACTGT ATACCTTTCG CAATACTAAT   
  
  
- ATATATGGTT GCTAGTTTCT TGGTTGCTTG GGGGTAAAGT CTAAAACAGG AAGGTTGGGT TATTCTCTTC   
  
  
- GGTTTGTCTG GTTCTTGCGG GTGTGTGTG

+     TCT-motif

| Site Name | Organism | Position | Strand | Matrix score. | sequence | function |
| --- | --- | --- | --- | --- | --- | --- |
| TCT-motif | Arabidopsis thaliana | 637 | - | 6 | TCTTAC | part of a light responsive element |

> 2018/04/13 10:10:12  
+ AAGCTATTAT TGCCAAGCCA GAGACTCAAT GTATTTTAGT CAGAACAAGT TGCTAAGCTC TTGAATTCTG   
  
  
+ AATTATCAGT CACTAAACCA CTTATTGTGA TGTAAATATG TTATCATGTG TAAGCAATGA AGCAGTAAGC   
  
  
+ CAAGATTAAG GAGAACCACA GTAATTGGCC TCAAAGAAAG AAAGAAAGAA AAAACAAAAA TAGTAGAGAT   
  
  
+ CAAGCCTAAA CACAGAAATT TCTAGAGTGA AGTGAAAACT GAACAAAACG ACAGAGAGAA AGAGAGGGAG   
  
  
+ GATGGGGGAG ATGAGAAAAA GAGGGGATTG CAAGTTACCT TGGAGGTGCC CCGAAAGTGA GACCAGGAGC   
  
  
+ CCCAAGCGGA GGGATAAGGA TCGCCTATGT TGTAAGGGAG ATCCTTGAGT CCCGTACCAG ATCCACCCAC   
  
  
+ CACTCCCTTC AAGAATTTGA ACATGTTGTT TTTGTCCTCT CGTCTCCTTT TTTTCGGGTG TTTATGGGTT   
  
  
+ TATGTATGCA TATAAGGTTG TATCCTCTGA TGCGTATAGG CGAATGACCC CAAACAGTCT CTCTCTCTCT   
  
  
+ CTCTCTCTCT CTCTCTCTCT CTCCCTCTGC ATGGATGATG GAGAGGGCGG GGAACAGGTT CGATCTGCGA   
  
  
+ GAGAGTGTAA GAGAGAGAGA GATGCAGATG CAAGGTGTCA AAAACGGAAA AGAAAATTAA ATTGATGTTT   
  
  
+ TCGGGTGCAA CCCGAAGTAT CGAGCAGCCT GGTTCTTCAA ATAAAGACCG TTAAGATTGC CTCCTTATTC   
  
  
+ AACTTCCTTA AATGGGCCAG TGGGGTGTCA AGCCCTTCTC TGTTCCAGCC CATAAGTACG CCTCTTTCTG   
  
  
+ TCTGTTTTAT TTGGGCCCTC CGATTGCCCG ACTTTTCATT TGTAATTACT TGCTTAATTA ATGGTTATAT   
  
  
+ GCTGTGCTTT ATTATTAAAA TTCCACTTAC AGAGAGTTAC ATCAATATCG CGAAACAGAA AGAAATTCAG   
  
  
+ TGTTGTATAC ATTTCATTTG GTAATAATAC CCATGGGCCA CGGAAACTTG ACTCCCTGTG TCATGACTCA   
  
  
+ AGAACCATGA CGTACGGGTA AAGGGTTGGT TCAAACCGCG GTGACCATGA ACGAGTAAAA CAGAGTGCTC   
  
  
+ CCATGCAAGT TCAGAACAAG ACGCTTTCTC CTCCACAGTC AATAACATCA AAGCAAGAGA AACTTCTTCA   
  
  
+ AGGTCTAAGC AATTGGTTTC AATGGGACAA GGTCTTCTTT TAGACTCAGA CCGGAAAAAG TTGAAAACCC   
  
  
+ CGCACTCATT AACAAGACCG TGACTTGGTC ATATTTCCAA GTTGCTTCTT CTTTGTCTTT TCGTTTTAAT   
  
  
+ TAATTAACTT CTTGCACCGT GTAAAAGGAA CACGAATACT TTGAATGACA TATGGAAAGC GTTATGATTA   
  
  
+ TATATACCAA CGATCAAAGA ACCAACGAAC CCCCATTTCA GATTTTGTCC TTCCAACCCA ATAAGAGAAG   
  
  
+ CCAAACAGAC CAAGAACGCC CACACACAC  

- TTCGATAATA ACGGTTCGGT CTCTGAGTTA CATAAAATCA GTCTTGTTCA ACGATTCGAG AACTTAAGAC   
  
  
- TTAATAGTCA GTGATTTGGT GAATAACACT ACATTTATAC AATAGTACAC ATTCGTTACT TCGTCATTCG   
  
  
- GTTCTAATTC CTCTTGGTGT CATTAACCGG AGTTTCTTTC TTTCTTTCTT TTTTGTTTTT ATCATCTCTA   
  
  
- GTTCGGATTT GTGTCTTTAA AGATCTCACT TCACTTTTGA CTTGTTTTGC TGTCTCTCTT TCTCTCCCTC   
  
  
- CTACCCCCTC TACTCTTTTT CTCCCCTAAC GTTCAATGGA ACCTCCACGG GGCTTTCACT CTGGTCCTCG   
  
  
- GGGTTCGCCT CCCTATTCCT AGCGGATACA ACATTCCCTC TAGGAACTCA GGGCATGGTC TAGGTGGGTG   
  
  
- GTGAGGGAAG TTCTTAAACT TGTACAACAA AAACAGGAGA GCAGAGGAAA AAAAGCCCAC AAATACCCAA   
  
  
- ATACATACGT ATATTCCAAC ATAGGAGACT ACGCATATCC GCTTACTGGG GTTTGTCAGA GAGAGAGAGA   
  
  
- GAGAGAGAGA GAGAGAGAGA GAGGGAGACG TACCTACTAC CTCTCCCGCC CCTTGTCCAA GCTAGACGCT   
  
  
- CTCTCACATT CTCTCTCTCT CTACGTCTAC GTTCCACAGT TTTTGCCTTT TCTTTTAATT TAACTACAAA   
  
  
- AGCCCACGTT GGGCTTCATA GCTCGTCGGA CCAAGAAGTT TATTTCTGGC AATTCTAACG GAGGAATAAG   
  
  
- TTGAAGGAAT TTACCCGGTC ACCCCACAGT TCGGGAAGAG ACAAGGTCGG GTATTCATGC GGAGAAAGAC   
  
  
- AGACAAAATA AACCCGGGAG GCTAACGGGC TGAAAAGTAA ACATTAATGA ACGAATTAAT TACCAATATA   
  
  
- CGACACGAAA TAATAATTTT AAGGTGAATG TCTCTCAATG TAGTTATAGC GCTTTGTCTT TCTTTAAGTC   
  
  
- ACAACATATG TAAAGTAAAC CATTATTATG GGTACCCGGT GCCTTTGAAC TGAGGGACAC AGTACTGAGT   
  
  
- TCTTGGTACT GCATGCCCAT TTCCCAACCA AGTTTGGCGC CACTGGTACT TGCTCATTTT GTCTCACGAG   
  
  
- GGTACGTTCA AGTCTTGTTC TGCGAAAGAG GAGGTGTCAG TTATTGTAGT TTCGTTCTCT TTGAAGAAGT   
  
  
- TCCAGATTCG TTAACCAAAG TTACCCTGTT CCAGAAGAAA ATCTGAGTCT GGCCTTTTTC AACTTTTGGG   
  
  
- GCGTGAGTAA TTGTTCTGGC ACTGAACCAG TATAAAGGTT CAACGAAGAA GAAACAGAAA AGCAAAATTA   
  
  
- ATTAATTGAA GAACGTGGCA CATTTTCCTT GTGCTTATGA AACTTACTGT ATACCTTTCG CAATACTAAT   
  
  
- ATATATGGTT GCTAGTTTCT TGGTTGCTTG GGGGTAAAGT CTAAAACAGG AAGGTTGGGT TATTCTCTTC   
  
  
- GGTTTGTCTG GTTCTTGCGG GTGTGTGTG

+     TGA-element

| Site Name | Organism | Position | Strand | Matrix score. | sequence | function |
| --- | --- | --- | --- | --- | --- | --- |
| TGA-element | Brassica oleracea | 257 | + | 6 | AACGAC | auxin-responsive element |

> 2018/04/13 10:10:12  
+ AAGCTATTAT TGCCAAGCCA GAGACTCAAT GTATTTTAGT CAGAACAAGT TGCTAAGCTC TTGAATTCTG   
  
  
+ AATTATCAGT CACTAAACCA CTTATTGTGA TGTAAATATG TTATCATGTG TAAGCAATGA AGCAGTAAGC   
  
  
+ CAAGATTAAG GAGAACCACA GTAATTGGCC TCAAAGAAAG AAAGAAAGAA AAAACAAAAA TAGTAGAGAT   
  
  
+ CAAGCCTAAA CACAGAAATT TCTAGAGTGA AGTGAAAACT GAACAAAACG ACAGAGAGAA AGAGAGGGAG   
  
  
+ GATGGGGGAG ATGAGAAAAA GAGGGGATTG CAAGTTACCT TGGAGGTGCC CCGAAAGTGA GACCAGGAGC   
  
  
+ CCCAAGCGGA GGGATAAGGA TCGCCTATGT TGTAAGGGAG ATCCTTGAGT CCCGTACCAG ATCCACCCAC   
  
  
+ CACTCCCTTC AAGAATTTGA ACATGTTGTT TTTGTCCTCT CGTCTCCTTT TTTTCGGGTG TTTATGGGTT   
  
  
+ TATGTATGCA TATAAGGTTG TATCCTCTGA TGCGTATAGG CGAATGACCC CAAACAGTCT CTCTCTCTCT   
  
  
+ CTCTCTCTCT CTCTCTCTCT CTCCCTCTGC ATGGATGATG GAGAGGGCGG GGAACAGGTT CGATCTGCGA   
  
  
+ GAGAGTGTAA GAGAGAGAGA GATGCAGATG CAAGGTGTCA AAAACGGAAA AGAAAATTAA ATTGATGTTT   
  
  
+ TCGGGTGCAA CCCGAAGTAT CGAGCAGCCT GGTTCTTCAA ATAAAGACCG TTAAGATTGC CTCCTTATTC   
  
  
+ AACTTCCTTA AATGGGCCAG TGGGGTGTCA AGCCCTTCTC TGTTCCAGCC CATAAGTACG CCTCTTTCTG   
  
  
+ TCTGTTTTAT TTGGGCCCTC CGATTGCCCG ACTTTTCATT TGTAATTACT TGCTTAATTA ATGGTTATAT   
  
  
+ GCTGTGCTTT ATTATTAAAA TTCCACTTAC AGAGAGTTAC ATCAATATCG CGAAACAGAA AGAAATTCAG   
  
  
+ TGTTGTATAC ATTTCATTTG GTAATAATAC CCATGGGCCA CGGAAACTTG ACTCCCTGTG TCATGACTCA   
  
  
+ AGAACCATGA CGTACGGGTA AAGGGTTGGT TCAAACCGCG GTGACCATGA ACGAGTAAAA CAGAGTGCTC   
  
  
+ CCATGCAAGT TCAGAACAAG ACGCTTTCTC CTCCACAGTC AATAACATCA AAGCAAGAGA AACTTCTTCA   
  
  
+ AGGTCTAAGC AATTGGTTTC AATGGGACAA GGTCTTCTTT TAGACTCAGA CCGGAAAAAG TTGAAAACCC   
  
  
+ CGCACTCATT AACAAGACCG TGACTTGGTC ATATTTCCAA GTTGCTTCTT CTTTGTCTTT TCGTTTTAAT   
  
  
+ TAATTAACTT CTTGCACCGT GTAAAAGGAA CACGAATACT TTGAATGACA TATGGAAAGC GTTATGATTA   
  
  
+ TATATACCAA CGATCAAAGA ACCAACGAAC CCCCATTTCA GATTTTGTCC TTCCAACCCA ATAAGAGAAG   
  
  
+ CCAAACAGAC CAAGAACGCC CACACACAC  

- TTCGATAATA ACGGTTCGGT CTCTGAGTTA CATAAAATCA GTCTTGTTCA ACGATTCGAG AACTTAAGAC   
  
  
- TTAATAGTCA GTGATTTGGT GAATAACACT ACATTTATAC AATAGTACAC ATTCGTTACT TCGTCATTCG   
  
  
- GTTCTAATTC CTCTTGGTGT CATTAACCGG AGTTTCTTTC TTTCTTTCTT TTTTGTTTTT ATCATCTCTA   
  
  
- GTTCGGATTT GTGTCTTTAA AGATCTCACT TCACTTTTGA CTTGTTTTGC TGTCTCTCTT TCTCTCCCTC   
  
  
- CTACCCCCTC TACTCTTTTT CTCCCCTAAC GTTCAATGGA ACCTCCACGG GGCTTTCACT CTGGTCCTCG   
  
  
- GGGTTCGCCT CCCTATTCCT AGCGGATACA ACATTCCCTC TAGGAACTCA GGGCATGGTC TAGGTGGGTG   
  
  
- GTGAGGGAAG TTCTTAAACT TGTACAACAA AAACAGGAGA GCAGAGGAAA AAAAGCCCAC AAATACCCAA   
  
  
- ATACATACGT ATATTCCAAC ATAGGAGACT ACGCATATCC GCTTACTGGG GTTTGTCAGA GAGAGAGAGA   
  
  
- GAGAGAGAGA GAGAGAGAGA GAGGGAGACG TACCTACTAC CTCTCCCGCC CCTTGTCCAA GCTAGACGCT   
  
  
- CTCTCACATT CTCTCTCTCT CTACGTCTAC GTTCCACAGT TTTTGCCTTT TCTTTTAATT TAACTACAAA   
  
  
- AGCCCACGTT GGGCTTCATA GCTCGTCGGA CCAAGAAGTT TATTTCTGGC AATTCTAACG GAGGAATAAG   
  
  
- TTGAAGGAAT TTACCCGGTC ACCCCACAGT TCGGGAAGAG ACAAGGTCGG GTATTCATGC GGAGAAAGAC   
  
  
- AGACAAAATA AACCCGGGAG GCTAACGGGC TGAAAAGTAA ACATTAATGA ACGAATTAAT TACCAATATA   
  
  
- CGACACGAAA TAATAATTTT AAGGTGAATG TCTCTCAATG TAGTTATAGC GCTTTGTCTT TCTTTAAGTC   
  
  
- ACAACATATG TAAAGTAAAC CATTATTATG GGTACCCGGT GCCTTTGAAC TGAGGGACAC AGTACTGAGT   
  
  
- TCTTGGTACT GCATGCCCAT TTCCCAACCA AGTTTGGCGC CACTGGTACT TGCTCATTTT GTCTCACGAG   
  
  
- GGTACGTTCA AGTCTTGTTC TGCGAAAGAG GAGGTGTCAG TTATTGTAGT TTCGTTCTCT TTGAAGAAGT   
  
  
- TCCAGATTCG TTAACCAAAG TTACCCTGTT CCAGAAGAAA ATCTGAGTCT GGCCTTTTTC AACTTTTGGG   
  
  
- GCGTGAGTAA TTGTTCTGGC ACTGAACCAG TATAAAGGTT CAACGAAGAA GAAACAGAAA AGCAAAATTA   
  
  
- ATTAATTGAA GAACGTGGCA CATTTTCCTT GTGCTTATGA AACTTACTGT ATACCTTTCG CAATACTAAT   
  
  
- ATATATGGTT GCTAGTTTCT TGGTTGCTTG GGGGTAAAGT CTAAAACAGG AAGGTTGGGT TATTCTCTTC   
  
  
- GGTTTGTCTG GTTCTTGCGG GTGTGTGTG

+     TGACG-motif

| Site Name | Organism | Position | Strand | Matrix score. | sequence | function |
| --- | --- | --- | --- | --- | --- | --- |
| TGACG-motif | Hordeum vulgare | 1058 | + | 5 | TGACG | cis-acting regulatory element involved in the MeJA-responsiveness |

> 2018/04/13 10:10:12  
+ AAGCTATTAT TGCCAAGCCA GAGACTCAAT GTATTTTAGT CAGAACAAGT TGCTAAGCTC TTGAATTCTG   
  
  
+ AATTATCAGT CACTAAACCA CTTATTGTGA TGTAAATATG TTATCATGTG TAAGCAATGA AGCAGTAAGC   
  
  
+ CAAGATTAAG GAGAACCACA GTAATTGGCC TCAAAGAAAG AAAGAAAGAA AAAACAAAAA TAGTAGAGAT   
  
  
+ CAAGCCTAAA CACAGAAATT TCTAGAGTGA AGTGAAAACT GAACAAAACG ACAGAGAGAA AGAGAGGGAG   
  
  
+ GATGGGGGAG ATGAGAAAAA GAGGGGATTG CAAGTTACCT TGGAGGTGCC CCGAAAGTGA GACCAGGAGC   
  
  
+ CCCAAGCGGA GGGATAAGGA TCGCCTATGT TGTAAGGGAG ATCCTTGAGT CCCGTACCAG ATCCACCCAC   
  
  
+ CACTCCCTTC AAGAATTTGA ACATGTTGTT TTTGTCCTCT CGTCTCCTTT TTTTCGGGTG TTTATGGGTT   
  
  
+ TATGTATGCA TATAAGGTTG TATCCTCTGA TGCGTATAGG CGAATGACCC CAAACAGTCT CTCTCTCTCT   
  
  
+ CTCTCTCTCT CTCTCTCTCT CTCCCTCTGC ATGGATGATG GAGAGGGCGG GGAACAGGTT CGATCTGCGA   
  
  
+ GAGAGTGTAA GAGAGAGAGA GATGCAGATG CAAGGTGTCA AAAACGGAAA AGAAAATTAA ATTGATGTTT   
  
  
+ TCGGGTGCAA CCCGAAGTAT CGAGCAGCCT GGTTCTTCAA ATAAAGACCG TTAAGATTGC CTCCTTATTC   
  
  
+ AACTTCCTTA AATGGGCCAG TGGGGTGTCA AGCCCTTCTC TGTTCCAGCC CATAAGTACG CCTCTTTCTG   
  
  
+ TCTGTTTTAT TTGGGCCCTC CGATTGCCCG ACTTTTCATT TGTAATTACT TGCTTAATTA ATGGTTATAT   
  
  
+ GCTGTGCTTT ATTATTAAAA TTCCACTTAC AGAGAGTTAC ATCAATATCG CGAAACAGAA AGAAATTCAG   
  
  
+ TGTTGTATAC ATTTCATTTG GTAATAATAC CCATGGGCCA CGGAAACTTG ACTCCCTGTG TCATGACTCA   
  
  
+ AGAACCATGA CGTACGGGTA AAGGGTTGGT TCAAACCGCG GTGACCATGA ACGAGTAAAA CAGAGTGCTC   
  
  
+ CCATGCAAGT TCAGAACAAG ACGCTTTCTC CTCCACAGTC AATAACATCA AAGCAAGAGA AACTTCTTCA   
  
  
+ AGGTCTAAGC AATTGGTTTC AATGGGACAA GGTCTTCTTT TAGACTCAGA CCGGAAAAAG TTGAAAACCC   
  
  
+ CGCACTCATT AACAAGACCG TGACTTGGTC ATATTTCCAA GTTGCTTCTT CTTTGTCTTT TCGTTTTAAT   
  
  
+ TAATTAACTT CTTGCACCGT GTAAAAGGAA CACGAATACT TTGAATGACA TATGGAAAGC GTTATGATTA   
  
  
+ TATATACCAA CGATCAAAGA ACCAACGAAC CCCCATTTCA GATTTTGTCC TTCCAACCCA ATAAGAGAAG   
  
  
+ CCAAACAGAC CAAGAACGCC CACACACAC  

- TTCGATAATA ACGGTTCGGT CTCTGAGTTA CATAAAATCA GTCTTGTTCA ACGATTCGAG AACTTAAGAC   
  
  
- TTAATAGTCA GTGATTTGGT GAATAACACT ACATTTATAC AATAGTACAC ATTCGTTACT TCGTCATTCG   
  
  
- GTTCTAATTC CTCTTGGTGT CATTAACCGG AGTTTCTTTC TTTCTTTCTT TTTTGTTTTT ATCATCTCTA   
  
  
- GTTCGGATTT GTGTCTTTAA AGATCTCACT TCACTTTTGA CTTGTTTTGC TGTCTCTCTT TCTCTCCCTC   
  
  
- CTACCCCCTC TACTCTTTTT CTCCCCTAAC GTTCAATGGA ACCTCCACGG GGCTTTCACT CTGGTCCTCG   
  
  
- GGGTTCGCCT CCCTATTCCT AGCGGATACA ACATTCCCTC TAGGAACTCA GGGCATGGTC TAGGTGGGTG   
  
  
- GTGAGGGAAG TTCTTAAACT TGTACAACAA AAACAGGAGA GCAGAGGAAA AAAAGCCCAC AAATACCCAA   
  
  
- ATACATACGT ATATTCCAAC ATAGGAGACT ACGCATATCC GCTTACTGGG GTTTGTCAGA GAGAGAGAGA   
  
  
- GAGAGAGAGA GAGAGAGAGA GAGGGAGACG TACCTACTAC CTCTCCCGCC CCTTGTCCAA GCTAGACGCT   
  
  
- CTCTCACATT CTCTCTCTCT CTACGTCTAC GTTCCACAGT TTTTGCCTTT TCTTTTAATT TAACTACAAA   
  
  
- AGCCCACGTT GGGCTTCATA GCTCGTCGGA CCAAGAAGTT TATTTCTGGC AATTCTAACG GAGGAATAAG   
  
  
- TTGAAGGAAT TTACCCGGTC ACCCCACAGT TCGGGAAGAG ACAAGGTCGG GTATTCATGC GGAGAAAGAC   
  
  
- AGACAAAATA AACCCGGGAG GCTAACGGGC TGAAAAGTAA ACATTAATGA ACGAATTAAT TACCAATATA   
  
  
- CGACACGAAA TAATAATTTT AAGGTGAATG TCTCTCAATG TAGTTATAGC GCTTTGTCTT TCTTTAAGTC   
  
  
- ACAACATATG TAAAGTAAAC CATTATTATG GGTACCCGGT GCCTTTGAAC TGAGGGACAC AGTACTGAGT   
  
  
- TCTTGGTACT GCATGCCCAT TTCCCAACCA AGTTTGGCGC CACTGGTACT TGCTCATTTT GTCTCACGAG   
  
  
- GGTACGTTCA AGTCTTGTTC TGCGAAAGAG GAGGTGTCAG TTATTGTAGT TTCGTTCTCT TTGAAGAAGT   
  
  
- TCCAGATTCG TTAACCAAAG TTACCCTGTT CCAGAAGAAA ATCTGAGTCT GGCCTTTTTC AACTTTTGGG   
  
  
- GCGTGAGTAA TTGTTCTGGC ACTGAACCAG TATAAAGGTT CAACGAAGAA GAAACAGAAA AGCAAAATTA   
  
  
- ATTAATTGAA GAACGTGGCA CATTTTCCTT GTGCTTATGA AACTTACTGT ATACCTTTCG CAATACTAAT   
  
  
- ATATATGGTT GCTAGTTTCT TGGTTGCTTG GGGGTAAAGT CTAAAACAGG AAGGTTGGGT TATTCTCTTC   
  
  
- GGTTTGTCTG GTTCTTGCGG GTGTGTGTG

+     Unnamed\_\_1

| Site Name | Organism | Position | Strand | Matrix score. | sequence | function |
| --- | --- | --- | --- | --- | --- | --- |
| Unnamed\_\_1 | Zea mays | 1018 | - | 5 | CGTGG |  |
| Unnamed\_\_1 | Glycine max | 1330 | - | 11 | GAATTTAATTAA | 60K protein binding site |

> 2018/04/13 10:10:12  
+ AAGCTATTAT TGCCAAGCCA GAGACTCAAT GTATTTTAGT CAGAACAAGT TGCTAAGCTC TTGAATTCTG   
  
  
+ AATTATCAGT CACTAAACCA CTTATTGTGA TGTAAATATG TTATCATGTG TAAGCAATGA AGCAGTAAGC   
  
  
+ CAAGATTAAG GAGAACCACA GTAATTGGCC TCAAAGAAAG AAAGAAAGAA AAAACAAAAA TAGTAGAGAT   
  
  
+ CAAGCCTAAA CACAGAAATT TCTAGAGTGA AGTGAAAACT GAACAAAACG ACAGAGAGAA AGAGAGGGAG   
  
  
+ GATGGGGGAG ATGAGAAAAA GAGGGGATTG CAAGTTACCT TGGAGGTGCC CCGAAAGTGA GACCAGGAGC   
  
  
+ CCCAAGCGGA GGGATAAGGA TCGCCTATGT TGTAAGGGAG ATCCTTGAGT CCCGTACCAG ATCCACCCAC   
  
  
+ CACTCCCTTC AAGAATTTGA ACATGTTGTT TTTGTCCTCT CGTCTCCTTT TTTTCGGGTG TTTATGGGTT   
  
  
+ TATGTATGCA TATAAGGTTG TATCCTCTGA TGCGTATAGG CGAATGACCC CAAACAGTCT CTCTCTCTCT   
  
  
+ CTCTCTCTCT CTCTCTCTCT CTCCCTCTGC ATGGATGATG GAGAGGGCGG GGAACAGGTT CGATCTGCGA   
  
  
+ GAGAGTGTAA GAGAGAGAGA GATGCAGATG CAAGGTGTCA AAAACGGAAA AGAAAATTAA ATTGATGTTT   
  
  
+ TCGGGTGCAA CCCGAAGTAT CGAGCAGCCT GGTTCTTCAA ATAAAGACCG TTAAGATTGC CTCCTTATTC   
  
  
+ AACTTCCTTA AATGGGCCAG TGGGGTGTCA AGCCCTTCTC TGTTCCAGCC CATAAGTACG CCTCTTTCTG   
  
  
+ TCTGTTTTAT TTGGGCCCTC CGATTGCCCG ACTTTTCATT TGTAATTACT TGCTTAATTA ATGGTTATAT   
  
  
+ GCTGTGCTTT ATTATTAAAA TTCCACTTAC AGAGAGTTAC ATCAATATCG CGAAACAGAA AGAAATTCAG   
  
  
+ TGTTGTATAC ATTTCATTTG GTAATAATAC CCATGGGCCA CGGAAACTTG ACTCCCTGTG TCATGACTCA   
  
  
+ AGAACCATGA CGTACGGGTA AAGGGTTGGT TCAAACCGCG GTGACCATGA ACGAGTAAAA CAGAGTGCTC   
  
  
+ CCATGCAAGT TCAGAACAAG ACGCTTTCTC CTCCACAGTC AATAACATCA AAGCAAGAGA AACTTCTTCA   
  
  
+ AGGTCTAAGC AATTGGTTTC AATGGGACAA GGTCTTCTTT TAGACTCAGA CCGGAAAAAG TTGAAAACCC   
  
  
+ CGCACTCATT AACAAGACCG TGACTTGGTC ATATTTCCAA GTTGCTTCTT CTTTGTCTTT TCGTTTTAAT   
  
  
+ TAATTAACTT CTTGCACCGT GTAAAAGGAA CACGAATACT TTGAATGACA TATGGAAAGC GTTATGATTA   
  
  
+ TATATACCAA CGATCAAAGA ACCAACGAAC CCCCATTTCA GATTTTGTCC TTCCAACCCA ATAAGAGAAG   
  
  
+ CCAAACAGAC CAAGAACGCC CACACACAC  

- TTCGATAATA ACGGTTCGGT CTCTGAGTTA CATAAAATCA GTCTTGTTCA ACGATTCGAG AACTTAAGAC   
  
  
- TTAATAGTCA GTGATTTGGT GAATAACACT ACATTTATAC AATAGTACAC ATTCGTTACT TCGTCATTCG   
  
  
- GTTCTAATTC CTCTTGGTGT CATTAACCGG AGTTTCTTTC TTTCTTTCTT TTTTGTTTTT ATCATCTCTA   
  
  
- GTTCGGATTT GTGTCTTTAA AGATCTCACT TCACTTTTGA CTTGTTTTGC TGTCTCTCTT TCTCTCCCTC   
  
  
- CTACCCCCTC TACTCTTTTT CTCCCCTAAC GTTCAATGGA ACCTCCACGG GGCTTTCACT CTGGTCCTCG   
  
  
- GGGTTCGCCT CCCTATTCCT AGCGGATACA ACATTCCCTC TAGGAACTCA GGGCATGGTC TAGGTGGGTG   
  
  
- GTGAGGGAAG TTCTTAAACT TGTACAACAA AAACAGGAGA GCAGAGGAAA AAAAGCCCAC AAATACCCAA   
  
  
- ATACATACGT ATATTCCAAC ATAGGAGACT ACGCATATCC GCTTACTGGG GTTTGTCAGA GAGAGAGAGA   
  
  
- GAGAGAGAGA GAGAGAGAGA GAGGGAGACG TACCTACTAC CTCTCCCGCC CCTTGTCCAA GCTAGACGCT   
  
  
- CTCTCACATT CTCTCTCTCT CTACGTCTAC GTTCCACAGT TTTTGCCTTT TCTTTTAATT TAACTACAAA   
  
  
- AGCCCACGTT GGGCTTCATA GCTCGTCGGA CCAAGAAGTT TATTTCTGGC AATTCTAACG GAGGAATAAG   
  
  
- TTGAAGGAAT TTACCCGGTC ACCCCACAGT TCGGGAAGAG ACAAGGTCGG GTATTCATGC GGAGAAAGAC   
  
  
- AGACAAAATA AACCCGGGAG GCTAACGGGC TGAAAAGTAA ACATTAATGA ACGAATTAAT TACCAATATA   
  
  
- CGACACGAAA TAATAATTTT AAGGTGAATG TCTCTCAATG TAGTTATAGC GCTTTGTCTT TCTTTAAGTC   
  
  
- ACAACATATG TAAAGTAAAC CATTATTATG GGTACCCGGT GCCTTTGAAC TGAGGGACAC AGTACTGAGT   
  
  
- TCTTGGTACT GCATGCCCAT TTCCCAACCA AGTTTGGCGC CACTGGTACT TGCTCATTTT GTCTCACGAG   
  
  
- GGTACGTTCA AGTCTTGTTC TGCGAAAGAG GAGGTGTCAG TTATTGTAGT TTCGTTCTCT TTGAAGAAGT   
  
  
- TCCAGATTCG TTAACCAAAG TTACCCTGTT CCAGAAGAAA ATCTGAGTCT GGCCTTTTTC AACTTTTGGG   
  
  
- GCGTGAGTAA TTGTTCTGGC ACTGAACCAG TATAAAGGTT CAACGAAGAA GAAACAGAAA AGCAAAATTA   
  
  
- ATTAATTGAA GAACGTGGCA CATTTTCCTT GTGCTTATGA AACTTACTGT ATACCTTTCG CAATACTAAT   
  
  
- ATATATGGTT GCTAGTTTCT TGGTTGCTTG GGGGTAAAGT CTAAAACAGG AAGGTTGGGT TATTCTCTTC   
  
  
- GGTTTGTCTG GTTCTTGCGG GTGTGTGTG

+     Unnamed\_\_3

| Site Name | Organism | Position | Strand | Matrix score. | sequence | function |
| --- | --- | --- | --- | --- | --- | --- |
| Unnamed\_\_3 | Zea mays | 1018 | - | 5 | CGTGG |  |

> 2018/04/13 10:10:12  
+ AAGCTATTAT TGCCAAGCCA GAGACTCAAT GTATTTTAGT CAGAACAAGT TGCTAAGCTC TTGAATTCTG   
  
  
+ AATTATCAGT CACTAAACCA CTTATTGTGA TGTAAATATG TTATCATGTG TAAGCAATGA AGCAGTAAGC   
  
  
+ CAAGATTAAG GAGAACCACA GTAATTGGCC TCAAAGAAAG AAAGAAAGAA AAAACAAAAA TAGTAGAGAT   
  
  
+ CAAGCCTAAA CACAGAAATT TCTAGAGTGA AGTGAAAACT GAACAAAACG ACAGAGAGAA AGAGAGGGAG   
  
  
+ GATGGGGGAG ATGAGAAAAA GAGGGGATTG CAAGTTACCT TGGAGGTGCC CCGAAAGTGA GACCAGGAGC   
  
  
+ CCCAAGCGGA GGGATAAGGA TCGCCTATGT TGTAAGGGAG ATCCTTGAGT CCCGTACCAG ATCCACCCAC   
  
  
+ CACTCCCTTC AAGAATTTGA ACATGTTGTT TTTGTCCTCT CGTCTCCTTT TTTTCGGGTG TTTATGGGTT   
  
  
+ TATGTATGCA TATAAGGTTG TATCCTCTGA TGCGTATAGG CGAATGACCC CAAACAGTCT CTCTCTCTCT   
  
  
+ CTCTCTCTCT CTCTCTCTCT CTCCCTCTGC ATGGATGATG GAGAGGGCGG GGAACAGGTT CGATCTGCGA   
  
  
+ GAGAGTGTAA GAGAGAGAGA GATGCAGATG CAAGGTGTCA AAAACGGAAA AGAAAATTAA ATTGATGTTT   
  
  
+ TCGGGTGCAA CCCGAAGTAT CGAGCAGCCT GGTTCTTCAA ATAAAGACCG TTAAGATTGC CTCCTTATTC   
  
  
+ AACTTCCTTA AATGGGCCAG TGGGGTGTCA AGCCCTTCTC TGTTCCAGCC CATAAGTACG CCTCTTTCTG   
  
  
+ TCTGTTTTAT TTGGGCCCTC CGATTGCCCG ACTTTTCATT TGTAATTACT TGCTTAATTA ATGGTTATAT   
  
  
+ GCTGTGCTTT ATTATTAAAA TTCCACTTAC AGAGAGTTAC ATCAATATCG CGAAACAGAA AGAAATTCAG   
  
  
+ TGTTGTATAC ATTTCATTTG GTAATAATAC CCATGGGCCA CGGAAACTTG ACTCCCTGTG TCATGACTCA   
  
  
+ AGAACCATGA CGTACGGGTA AAGGGTTGGT TCAAACCGCG GTGACCATGA ACGAGTAAAA CAGAGTGCTC   
  
  
+ CCATGCAAGT TCAGAACAAG ACGCTTTCTC CTCCACAGTC AATAACATCA AAGCAAGAGA AACTTCTTCA   
  
  
+ AGGTCTAAGC AATTGGTTTC AATGGGACAA GGTCTTCTTT TAGACTCAGA CCGGAAAAAG TTGAAAACCC   
  
  
+ CGCACTCATT AACAAGACCG TGACTTGGTC ATATTTCCAA GTTGCTTCTT CTTTGTCTTT TCGTTTTAAT   
  
  
+ TAATTAACTT CTTGCACCGT GTAAAAGGAA CACGAATACT TTGAATGACA TATGGAAAGC GTTATGATTA   
  
  
+ TATATACCAA CGATCAAAGA ACCAACGAAC CCCCATTTCA GATTTTGTCC TTCCAACCCA ATAAGAGAAG   
  
  
+ CCAAACAGAC CAAGAACGCC CACACACAC  

- TTCGATAATA ACGGTTCGGT CTCTGAGTTA CATAAAATCA GTCTTGTTCA ACGATTCGAG AACTTAAGAC   
  
  
- TTAATAGTCA GTGATTTGGT GAATAACACT ACATTTATAC AATAGTACAC ATTCGTTACT TCGTCATTCG   
  
  
- GTTCTAATTC CTCTTGGTGT CATTAACCGG AGTTTCTTTC TTTCTTTCTT TTTTGTTTTT ATCATCTCTA   
  
  
- GTTCGGATTT GTGTCTTTAA AGATCTCACT TCACTTTTGA CTTGTTTTGC TGTCTCTCTT TCTCTCCCTC   
  
  
- CTACCCCCTC TACTCTTTTT CTCCCCTAAC GTTCAATGGA ACCTCCACGG GGCTTTCACT CTGGTCCTCG   
  
  
- GGGTTCGCCT CCCTATTCCT AGCGGATACA ACATTCCCTC TAGGAACTCA GGGCATGGTC TAGGTGGGTG   
  
  
- GTGAGGGAAG TTCTTAAACT TGTACAACAA AAACAGGAGA GCAGAGGAAA AAAAGCCCAC AAATACCCAA   
  
  
- ATACATACGT ATATTCCAAC ATAGGAGACT ACGCATATCC GCTTACTGGG GTTTGTCAGA GAGAGAGAGA   
  
  
- GAGAGAGAGA GAGAGAGAGA GAGGGAGACG TACCTACTAC CTCTCCCGCC CCTTGTCCAA GCTAGACGCT   
  
  
- CTCTCACATT CTCTCTCTCT CTACGTCTAC GTTCCACAGT TTTTGCCTTT TCTTTTAATT TAACTACAAA   
  
  
- AGCCCACGTT GGGCTTCATA GCTCGTCGGA CCAAGAAGTT TATTTCTGGC AATTCTAACG GAGGAATAAG   
  
  
- TTGAAGGAAT TTACCCGGTC ACCCCACAGT TCGGGAAGAG ACAAGGTCGG GTATTCATGC GGAGAAAGAC   
  
  
- AGACAAAATA AACCCGGGAG GCTAACGGGC TGAAAAGTAA ACATTAATGA ACGAATTAAT TACCAATATA   
  
  
- CGACACGAAA TAATAATTTT AAGGTGAATG TCTCTCAATG TAGTTATAGC GCTTTGTCTT TCTTTAAGTC   
  
  
- ACAACATATG TAAAGTAAAC CATTATTATG GGTACCCGGT GCCTTTGAAC TGAGGGACAC AGTACTGAGT   
  
  
- TCTTGGTACT GCATGCCCAT TTCCCAACCA AGTTTGGCGC CACTGGTACT TGCTCATTTT GTCTCACGAG   
  
  
- GGTACGTTCA AGTCTTGTTC TGCGAAAGAG GAGGTGTCAG TTATTGTAGT TTCGTTCTCT TTGAAGAAGT   
  
  
- TCCAGATTCG TTAACCAAAG TTACCCTGTT CCAGAAGAAA ATCTGAGTCT GGCCTTTTTC AACTTTTGGG   
  
  
- GCGTGAGTAA TTGTTCTGGC ACTGAACCAG TATAAAGGTT CAACGAAGAA GAAACAGAAA AGCAAAATTA   
  
  
- ATTAATTGAA GAACGTGGCA CATTTTCCTT GTGCTTATGA AACTTACTGT ATACCTTTCG CAATACTAAT   
  
  
- ATATATGGTT GCTAGTTTCT TGGTTGCTTG GGGGTAAAGT CTAAAACAGG AAGGTTGGGT TATTCTCTTC   
  
  
- GGTTTGTCTG GTTCTTGCGG GTGTGTGTG

+     Unnamed\_\_4

| Site Name | Organism | Position | Strand | Matrix score. | sequence | function |
| --- | --- | --- | --- | --- | --- | --- |
| Unnamed\_\_4 | Petroselinum hortense | 387 | - | 4 | CTCC |  |
| Unnamed\_\_4 | Petroselinum hortense | 600 | - | 4 | CTCC |  |
| Unnamed\_\_4 | Petroselinum hortense | 322 | - | 4 | CTCC |  |
| Unnamed\_\_4 | Petroselinum hortense | 150 | - | 4 | CTCC |  |
| Unnamed\_\_4 | Petroselinum hortense | 464 | + | 4 | CTCC |  |
| Unnamed\_\_4 | Petroselinum hortense | 1118 | + | 4 | CTCC |  |
| Unnamed\_\_4 | Petroselinum hortense | 1148 | + | 4 | CTCC |  |
| Unnamed\_\_4 | Petroselinum hortense | 346 | - | 4 | CTCC |  |
| Unnamed\_\_4 | Petroselinum hortense | 277 | - | 4 | CTCC |  |
| Unnamed\_\_4 | Petroselinum hortense | 358 | - | 4 | CTCC |  |
| Unnamed\_\_4 | Petroselinum hortense | 761 | + | 4 | CTCC |  |
| Unnamed\_\_4 | Petroselinum hortense | 858 | + | 4 | CTCC |  |
| Unnamed\_\_4 | Petroselinum hortense | 1151 | + | 4 | CTCC |  |
| Unnamed\_\_4 | Petroselinum hortense | 1032 | + | 4 | CTCC |  |
| Unnamed\_\_4 | Petroselinum hortense | 423 | + | 4 | CTCC |  |
| Unnamed\_\_4 | Petroselinum hortense | 581 | + | 4 | CTCC |  |
| Unnamed\_\_4 | Petroselinum hortense | 287 | - | 4 | CTCC |  |

> 2018/04/13 10:10:12  
+ AAGCTATTAT TGCCAAGCCA GAGACTCAAT GTATTTTAGT CAGAACAAGT TGCTAAGCTC TTGAATTCTG   
  
  
+ AATTATCAGT CACTAAACCA CTTATTGTGA TGTAAATATG TTATCATGTG TAAGCAATGA AGCAGTAAGC   
  
  
+ CAAGATTAAG GAGAACCACA GTAATTGGCC TCAAAGAAAG AAAGAAAGAA AAAACAAAAA TAGTAGAGAT   
  
  
+ CAAGCCTAAA CACAGAAATT TCTAGAGTGA AGTGAAAACT GAACAAAACG ACAGAGAGAA AGAGAGGGAG   
  
  
+ GATGGGGGAG ATGAGAAAAA GAGGGGATTG CAAGTTACCT TGGAGGTGCC CCGAAAGTGA GACCAGGAGC   
  
  
+ CCCAAGCGGA GGGATAAGGA TCGCCTATGT TGTAAGGGAG ATCCTTGAGT CCCGTACCAG ATCCACCCAC   
  
  
+ CACTCCCTTC AAGAATTTGA ACATGTTGTT TTTGTCCTCT CGTCTCCTTT TTTTCGGGTG TTTATGGGTT   
  
  
+ TATGTATGCA TATAAGGTTG TATCCTCTGA TGCGTATAGG CGAATGACCC CAAACAGTCT CTCTCTCTCT   
  
  
+ CTCTCTCTCT CTCTCTCTCT CTCCCTCTGC ATGGATGATG GAGAGGGCGG GGAACAGGTT CGATCTGCGA   
  
  
+ GAGAGTGTAA GAGAGAGAGA GATGCAGATG CAAGGTGTCA AAAACGGAAA AGAAAATTAA ATTGATGTTT   
  
  
+ TCGGGTGCAA CCCGAAGTAT CGAGCAGCCT GGTTCTTCAA ATAAAGACCG TTAAGATTGC CTCCTTATTC   
  
  
+ AACTTCCTTA AATGGGCCAG TGGGGTGTCA AGCCCTTCTC TGTTCCAGCC CATAAGTACG CCTCTTTCTG   
  
  
+ TCTGTTTTAT TTGGGCCCTC CGATTGCCCG ACTTTTCATT TGTAATTACT TGCTTAATTA ATGGTTATAT   
  
  
+ GCTGTGCTTT ATTATTAAAA TTCCACTTAC AGAGAGTTAC ATCAATATCG CGAAACAGAA AGAAATTCAG   
  
  
+ TGTTGTATAC ATTTCATTTG GTAATAATAC CCATGGGCCA CGGAAACTTG ACTCCCTGTG TCATGACTCA   
  
  
+ AGAACCATGA CGTACGGGTA AAGGGTTGGT TCAAACCGCG GTGACCATGA ACGAGTAAAA CAGAGTGCTC   
  
  
+ CCATGCAAGT TCAGAACAAG ACGCTTTCTC CTCCACAGTC AATAACATCA AAGCAAGAGA AACTTCTTCA   
  
  
+ AGGTCTAAGC AATTGGTTTC AATGGGACAA GGTCTTCTTT TAGACTCAGA CCGGAAAAAG TTGAAAACCC   
  
  
+ CGCACTCATT AACAAGACCG TGACTTGGTC ATATTTCCAA GTTGCTTCTT CTTTGTCTTT TCGTTTTAAT   
  
  
+ TAATTAACTT CTTGCACCGT GTAAAAGGAA CACGAATACT TTGAATGACA TATGGAAAGC GTTATGATTA   
  
  
+ TATATACCAA CGATCAAAGA ACCAACGAAC CCCCATTTCA GATTTTGTCC TTCCAACCCA ATAAGAGAAG   
  
  
+ CCAAACAGAC CAAGAACGCC CACACACAC  

- TTCGATAATA ACGGTTCGGT CTCTGAGTTA CATAAAATCA GTCTTGTTCA ACGATTCGAG AACTTAAGAC   
  
  
- TTAATAGTCA GTGATTTGGT GAATAACACT ACATTTATAC AATAGTACAC ATTCGTTACT TCGTCATTCG   
  
  
- GTTCTAATTC CTCTTGGTGT CATTAACCGG AGTTTCTTTC TTTCTTTCTT TTTTGTTTTT ATCATCTCTA   
  
  
- GTTCGGATTT GTGTCTTTAA AGATCTCACT TCACTTTTGA CTTGTTTTGC TGTCTCTCTT TCTCTCCCTC   
  
  
- CTACCCCCTC TACTCTTTTT CTCCCCTAAC GTTCAATGGA ACCTCCACGG GGCTTTCACT CTGGTCCTCG   
  
  
- GGGTTCGCCT CCCTATTCCT AGCGGATACA ACATTCCCTC TAGGAACTCA GGGCATGGTC TAGGTGGGTG   
  
  
- GTGAGGGAAG TTCTTAAACT TGTACAACAA AAACAGGAGA GCAGAGGAAA AAAAGCCCAC AAATACCCAA   
  
  
- ATACATACGT ATATTCCAAC ATAGGAGACT ACGCATATCC GCTTACTGGG GTTTGTCAGA GAGAGAGAGA   
  
  
- GAGAGAGAGA GAGAGAGAGA GAGGGAGACG TACCTACTAC CTCTCCCGCC CCTTGTCCAA GCTAGACGCT   
  
  
- CTCTCACATT CTCTCTCTCT CTACGTCTAC GTTCCACAGT TTTTGCCTTT TCTTTTAATT TAACTACAAA   
  
  
- AGCCCACGTT GGGCTTCATA GCTCGTCGGA CCAAGAAGTT TATTTCTGGC AATTCTAACG GAGGAATAAG   
  
  
- TTGAAGGAAT TTACCCGGTC ACCCCACAGT TCGGGAAGAG ACAAGGTCGG GTATTCATGC GGAGAAAGAC   
  
  
- AGACAAAATA AACCCGGGAG GCTAACGGGC TGAAAAGTAA ACATTAATGA ACGAATTAAT TACCAATATA   
  
  
- CGACACGAAA TAATAATTTT AAGGTGAATG TCTCTCAATG TAGTTATAGC GCTTTGTCTT TCTTTAAGTC   
  
  
- ACAACATATG TAAAGTAAAC CATTATTATG GGTACCCGGT GCCTTTGAAC TGAGGGACAC AGTACTGAGT   
  
  
- TCTTGGTACT GCATGCCCAT TTCCCAACCA AGTTTGGCGC CACTGGTACT TGCTCATTTT GTCTCACGAG   
  
  
- GGTACGTTCA AGTCTTGTTC TGCGAAAGAG GAGGTGTCAG TTATTGTAGT TTCGTTCTCT TTGAAGAAGT   
  
  
- TCCAGATTCG TTAACCAAAG TTACCCTGTT CCAGAAGAAA ATCTGAGTCT GGCCTTTTTC AACTTTTGGG   
  
  
- GCGTGAGTAA TTGTTCTGGC ACTGAACCAG TATAAAGGTT CAACGAAGAA GAAACAGAAA AGCAAAATTA   
  
  
- ATTAATTGAA GAACGTGGCA CATTTTCCTT GTGCTTATGA AACTTACTGT ATACCTTTCG CAATACTAAT   
  
  
- ATATATGGTT GCTAGTTTCT TGGTTGCTTG GGGGTAAAGT CTAAAACAGG AAGGTTGGGT TATTCTCTTC   
  
  
- GGTTTGTCTG GTTCTTGCGG GTGTGTGTG

+     Y-box

| Site Name | Organism | Position | Strand | Matrix score. | sequence | function |
| --- | --- | --- | --- | --- | --- | --- |
| Y-box | Lemna gibba | 1146 | - | 11 | TGTGGAGGAGCA | ? |

> 2018/04/13 10:10:12  
+ AAGCTATTAT TGCCAAGCCA GAGACTCAAT GTATTTTAGT CAGAACAAGT TGCTAAGCTC TTGAATTCTG   
  
  
+ AATTATCAGT CACTAAACCA CTTATTGTGA TGTAAATATG TTATCATGTG TAAGCAATGA AGCAGTAAGC   
  
  
+ CAAGATTAAG GAGAACCACA GTAATTGGCC TCAAAGAAAG AAAGAAAGAA AAAACAAAAA TAGTAGAGAT   
  
  
+ CAAGCCTAAA CACAGAAATT TCTAGAGTGA AGTGAAAACT GAACAAAACG ACAGAGAGAA AGAGAGGGAG   
  
  
+ GATGGGGGAG ATGAGAAAAA GAGGGGATTG CAAGTTACCT TGGAGGTGCC CCGAAAGTGA GACCAGGAGC   
  
  
+ CCCAAGCGGA GGGATAAGGA TCGCCTATGT TGTAAGGGAG ATCCTTGAGT CCCGTACCAG ATCCACCCAC   
  
  
+ CACTCCCTTC AAGAATTTGA ACATGTTGTT TTTGTCCTCT CGTCTCCTTT TTTTCGGGTG TTTATGGGTT   
  
  
+ TATGTATGCA TATAAGGTTG TATCCTCTGA TGCGTATAGG CGAATGACCC CAAACAGTCT CTCTCTCTCT   
  
  
+ CTCTCTCTCT CTCTCTCTCT CTCCCTCTGC ATGGATGATG GAGAGGGCGG GGAACAGGTT CGATCTGCGA   
  
  
+ GAGAGTGTAA GAGAGAGAGA GATGCAGATG CAAGGTGTCA AAAACGGAAA AGAAAATTAA ATTGATGTTT   
  
  
+ TCGGGTGCAA CCCGAAGTAT CGAGCAGCCT GGTTCTTCAA ATAAAGACCG TTAAGATTGC CTCCTTATTC   
  
  
+ AACTTCCTTA AATGGGCCAG TGGGGTGTCA AGCCCTTCTC TGTTCCAGCC CATAAGTACG CCTCTTTCTG   
  
  
+ TCTGTTTTAT TTGGGCCCTC CGATTGCCCG ACTTTTCATT TGTAATTACT TGCTTAATTA ATGGTTATAT   
  
  
+ GCTGTGCTTT ATTATTAAAA TTCCACTTAC AGAGAGTTAC ATCAATATCG CGAAACAGAA AGAAATTCAG   
  
  
+ TGTTGTATAC ATTTCATTTG GTAATAATAC CCATGGGCCA CGGAAACTTG ACTCCCTGTG TCATGACTCA   
  
  
+ AGAACCATGA CGTACGGGTA AAGGGTTGGT TCAAACCGCG GTGACCATGA ACGAGTAAAA CAGAGTGCTC   
  
  
+ CCATGCAAGT TCAGAACAAG ACGCTTTCTC CTCCACAGTC AATAACATCA AAGCAAGAGA AACTTCTTCA   
  
  
+ AGGTCTAAGC AATTGGTTTC AATGGGACAA GGTCTTCTTT TAGACTCAGA CCGGAAAAAG TTGAAAACCC   
  
  
+ CGCACTCATT AACAAGACCG TGACTTGGTC ATATTTCCAA GTTGCTTCTT CTTTGTCTTT TCGTTTTAAT   
  
  
+ TAATTAACTT CTTGCACCGT GTAAAAGGAA CACGAATACT TTGAATGACA TATGGAAAGC GTTATGATTA   
  
  
+ TATATACCAA CGATCAAAGA ACCAACGAAC CCCCATTTCA GATTTTGTCC TTCCAACCCA ATAAGAGAAG   
  
  
+ CCAAACAGAC CAAGAACGCC CACACACAC  

- TTCGATAATA ACGGTTCGGT CTCTGAGTTA CATAAAATCA GTCTTGTTCA ACGATTCGAG AACTTAAGAC   
  
  
- TTAATAGTCA GTGATTTGGT GAATAACACT ACATTTATAC AATAGTACAC ATTCGTTACT TCGTCATTCG   
  
  
- GTTCTAATTC CTCTTGGTGT CATTAACCGG AGTTTCTTTC TTTCTTTCTT TTTTGTTTTT ATCATCTCTA   
  
  
- GTTCGGATTT GTGTCTTTAA AGATCTCACT TCACTTTTGA CTTGTTTTGC TGTCTCTCTT TCTCTCCCTC   
  
  
- CTACCCCCTC TACTCTTTTT CTCCCCTAAC GTTCAATGGA ACCTCCACGG GGCTTTCACT CTGGTCCTCG   
  
  
- GGGTTCGCCT CCCTATTCCT AGCGGATACA ACATTCCCTC TAGGAACTCA GGGCATGGTC TAGGTGGGTG   
  
  
- GTGAGGGAAG TTCTTAAACT TGTACAACAA AAACAGGAGA GCAGAGGAAA AAAAGCCCAC AAATACCCAA   
  
  
- ATACATACGT ATATTCCAAC ATAGGAGACT ACGCATATCC GCTTACTGGG GTTTGTCAGA GAGAGAGAGA   
  
  
- GAGAGAGAGA GAGAGAGAGA GAGGGAGACG TACCTACTAC CTCTCCCGCC CCTTGTCCAA GCTAGACGCT   
  
  
- CTCTCACATT CTCTCTCTCT CTACGTCTAC GTTCCACAGT TTTTGCCTTT TCTTTTAATT TAACTACAAA   
  
  
- AGCCCACGTT GGGCTTCATA GCTCGTCGGA CCAAGAAGTT TATTTCTGGC AATTCTAACG GAGGAATAAG   
  
  
- TTGAAGGAAT TTACCCGGTC ACCCCACAGT TCGGGAAGAG ACAAGGTCGG GTATTCATGC GGAGAAAGAC   
  
  
- AGACAAAATA AACCCGGGAG GCTAACGGGC TGAAAAGTAA ACATTAATGA ACGAATTAAT TACCAATATA   
  
  
- CGACACGAAA TAATAATTTT AAGGTGAATG TCTCTCAATG TAGTTATAGC GCTTTGTCTT TCTTTAAGTC   
  
  
- ACAACATATG TAAAGTAAAC CATTATTATG GGTACCCGGT GCCTTTGAAC TGAGGGACAC AGTACTGAGT   
  
  
- TCTTGGTACT GCATGCCCAT TTCCCAACCA AGTTTGGCGC CACTGGTACT TGCTCATTTT GTCTCACGAG   
  
  
- GGTACGTTCA AGTCTTGTTC TGCGAAAGAG GAGGTGTCAG TTATTGTAGT TTCGTTCTCT TTGAAGAAGT   
  
  
- TCCAGATTCG TTAACCAAAG TTACCCTGTT CCAGAAGAAA ATCTGAGTCT GGCCTTTTTC AACTTTTGGG   
  
  
- GCGTGAGTAA TTGTTCTGGC ACTGAACCAG TATAAAGGTT CAACGAAGAA GAAACAGAAA AGCAAAATTA   
  
  
- ATTAATTGAA GAACGTGGCA CATTTTCCTT GTGCTTATGA AACTTACTGT ATACCTTTCG CAATACTAAT   
  
  
- ATATATGGTT GCTAGTTTCT TGGTTGCTTG GGGGTAAAGT CTAAAACAGG AAGGTTGGGT TATTCTCTTC   
  
  
- GGTTTGTCTG GTTCTTGCGG GTGTGTGTG

+     as-2-box

| Site Name | Organism | Position | Strand | Matrix score. | sequence | function |
| --- | --- | --- | --- | --- | --- | --- |
| as-2-box | Nicotiana tabacum | 950 | - | 9 | GATAatGATG | involved in shoot-specific expression and light responsiveness |

> 2018/04/13 10:10:12  
+ AAGCTATTAT TGCCAAGCCA GAGACTCAAT GTATTTTAGT CAGAACAAGT TGCTAAGCTC TTGAATTCTG   
  
  
+ AATTATCAGT CACTAAACCA CTTATTGTGA TGTAAATATG TTATCATGTG TAAGCAATGA AGCAGTAAGC   
  
  
+ CAAGATTAAG GAGAACCACA GTAATTGGCC TCAAAGAAAG AAAGAAAGAA AAAACAAAAA TAGTAGAGAT   
  
  
+ CAAGCCTAAA CACAGAAATT TCTAGAGTGA AGTGAAAACT GAACAAAACG ACAGAGAGAA AGAGAGGGAG   
  
  
+ GATGGGGGAG ATGAGAAAAA GAGGGGATTG CAAGTTACCT TGGAGGTGCC CCGAAAGTGA GACCAGGAGC   
  
  
+ CCCAAGCGGA GGGATAAGGA TCGCCTATGT TGTAAGGGAG ATCCTTGAGT CCCGTACCAG ATCCACCCAC   
  
  
+ CACTCCCTTC AAGAATTTGA ACATGTTGTT TTTGTCCTCT CGTCTCCTTT TTTTCGGGTG TTTATGGGTT   
  
  
+ TATGTATGCA TATAAGGTTG TATCCTCTGA TGCGTATAGG CGAATGACCC CAAACAGTCT CTCTCTCTCT   
  
  
+ CTCTCTCTCT CTCTCTCTCT CTCCCTCTGC ATGGATGATG GAGAGGGCGG GGAACAGGTT CGATCTGCGA   
  
  
+ GAGAGTGTAA GAGAGAGAGA GATGCAGATG CAAGGTGTCA AAAACGGAAA AGAAAATTAA ATTGATGTTT   
  
  
+ TCGGGTGCAA CCCGAAGTAT CGAGCAGCCT GGTTCTTCAA ATAAAGACCG TTAAGATTGC CTCCTTATTC   
  
  
+ AACTTCCTTA AATGGGCCAG TGGGGTGTCA AGCCCTTCTC TGTTCCAGCC CATAAGTACG CCTCTTTCTG   
  
  
+ TCTGTTTTAT TTGGGCCCTC CGATTGCCCG ACTTTTCATT TGTAATTACT TGCTTAATTA ATGGTTATAT   
  
  
+ GCTGTGCTTT ATTATTAAAA TTCCACTTAC AGAGAGTTAC ATCAATATCG CGAAACAGAA AGAAATTCAG   
  
  
+ TGTTGTATAC ATTTCATTTG GTAATAATAC CCATGGGCCA CGGAAACTTG ACTCCCTGTG TCATGACTCA   
  
  
+ AGAACCATGA CGTACGGGTA AAGGGTTGGT TCAAACCGCG GTGACCATGA ACGAGTAAAA CAGAGTGCTC   
  
  
+ CCATGCAAGT TCAGAACAAG ACGCTTTCTC CTCCACAGTC AATAACATCA AAGCAAGAGA AACTTCTTCA   
  
  
+ AGGTCTAAGC AATTGGTTTC AATGGGACAA GGTCTTCTTT TAGACTCAGA CCGGAAAAAG TTGAAAACCC   
  
  
+ CGCACTCATT AACAAGACCG TGACTTGGTC ATATTTCCAA GTTGCTTCTT CTTTGTCTTT TCGTTTTAAT   
  
  
+ TAATTAACTT CTTGCACCGT GTAAAAGGAA CACGAATACT TTGAATGACA TATGGAAAGC GTTATGATTA   
  
  
+ TATATACCAA CGATCAAAGA ACCAACGAAC CCCCATTTCA GATTTTGTCC TTCCAACCCA ATAAGAGAAG   
  
  
+ CCAAACAGAC CAAGAACGCC CACACACAC  

- TTCGATAATA ACGGTTCGGT CTCTGAGTTA CATAAAATCA GTCTTGTTCA ACGATTCGAG AACTTAAGAC   
  
  
- TTAATAGTCA GTGATTTGGT GAATAACACT ACATTTATAC AATAGTACAC ATTCGTTACT TCGTCATTCG   
  
  
- GTTCTAATTC CTCTTGGTGT CATTAACCGG AGTTTCTTTC TTTCTTTCTT TTTTGTTTTT ATCATCTCTA   
  
  
- GTTCGGATTT GTGTCTTTAA AGATCTCACT TCACTTTTGA CTTGTTTTGC TGTCTCTCTT TCTCTCCCTC   
  
  
- CTACCCCCTC TACTCTTTTT CTCCCCTAAC GTTCAATGGA ACCTCCACGG GGCTTTCACT CTGGTCCTCG   
  
  
- GGGTTCGCCT CCCTATTCCT AGCGGATACA ACATTCCCTC TAGGAACTCA GGGCATGGTC TAGGTGGGTG   
  
  
- GTGAGGGAAG TTCTTAAACT TGTACAACAA AAACAGGAGA GCAGAGGAAA AAAAGCCCAC AAATACCCAA   
  
  
- ATACATACGT ATATTCCAAC ATAGGAGACT ACGCATATCC GCTTACTGGG GTTTGTCAGA GAGAGAGAGA   
  
  
- GAGAGAGAGA GAGAGAGAGA GAGGGAGACG TACCTACTAC CTCTCCCGCC CCTTGTCCAA GCTAGACGCT   
  
  
- CTCTCACATT CTCTCTCTCT CTACGTCTAC GTTCCACAGT TTTTGCCTTT TCTTTTAATT TAACTACAAA   
  
  
- AGCCCACGTT GGGCTTCATA GCTCGTCGGA CCAAGAAGTT TATTTCTGGC AATTCTAACG GAGGAATAAG   
  
  
- TTGAAGGAAT TTACCCGGTC ACCCCACAGT TCGGGAAGAG ACAAGGTCGG GTATTCATGC GGAGAAAGAC   
  
  
- AGACAAAATA AACCCGGGAG GCTAACGGGC TGAAAAGTAA ACATTAATGA ACGAATTAAT TACCAATATA   
  
  
- CGACACGAAA TAATAATTTT AAGGTGAATG TCTCTCAATG TAGTTATAGC GCTTTGTCTT TCTTTAAGTC   
  
  
- ACAACATATG TAAAGTAAAC CATTATTATG GGTACCCGGT GCCTTTGAAC TGAGGGACAC AGTACTGAGT   
  
  
- TCTTGGTACT GCATGCCCAT TTCCCAACCA AGTTTGGCGC CACTGGTACT TGCTCATTTT GTCTCACGAG   
  
  
- GGTACGTTCA AGTCTTGTTC TGCGAAAGAG GAGGTGTCAG TTATTGTAGT TTCGTTCTCT TTGAAGAAGT   
  
  
- TCCAGATTCG TTAACCAAAG TTACCCTGTT CCAGAAGAAA ATCTGAGTCT GGCCTTTTTC AACTTTTGGG   
  
  
- GCGTGAGTAA TTGTTCTGGC ACTGAACCAG TATAAAGGTT CAACGAAGAA GAAACAGAAA AGCAAAATTA   
  
  
- ATTAATTGAA GAACGTGGCA CATTTTCCTT GTGCTTATGA AACTTACTGT ATACCTTTCG CAATACTAAT   
  
  
- ATATATGGTT GCTAGTTTCT TGGTTGCTTG GGGGTAAAGT CTAAAACAGG AAGGTTGGGT TATTCTCTTC   
  
  
- GGTTTGTCTG GTTCTTGCGG GTGTGTGTG

+     circadian

| Site Name | Organism | Position | Strand | Matrix score. | sequence | function |
| --- | --- | --- | --- | --- | --- | --- |
| circadian | Lycopersicon esculentum | 1160 | + | 6 | CAANNNNATC | cis-acting regulatory element involved in circadian control |

> 2018/04/13 10:10:12  
+ AAGCTATTAT TGCCAAGCCA GAGACTCAAT GTATTTTAGT CAGAACAAGT TGCTAAGCTC TTGAATTCTG   
  
  
+ AATTATCAGT CACTAAACCA CTTATTGTGA TGTAAATATG TTATCATGTG TAAGCAATGA AGCAGTAAGC   
  
  
+ CAAGATTAAG GAGAACCACA GTAATTGGCC TCAAAGAAAG AAAGAAAGAA AAAACAAAAA TAGTAGAGAT   
  
  
+ CAAGCCTAAA CACAGAAATT TCTAGAGTGA AGTGAAAACT GAACAAAACG ACAGAGAGAA AGAGAGGGAG   
  
  
+ GATGGGGGAG ATGAGAAAAA GAGGGGATTG CAAGTTACCT TGGAGGTGCC CCGAAAGTGA GACCAGGAGC   
  
  
+ CCCAAGCGGA GGGATAAGGA TCGCCTATGT TGTAAGGGAG ATCCTTGAGT CCCGTACCAG ATCCACCCAC   
  
  
+ CACTCCCTTC AAGAATTTGA ACATGTTGTT TTTGTCCTCT CGTCTCCTTT TTTTCGGGTG TTTATGGGTT   
  
  
+ TATGTATGCA TATAAGGTTG TATCCTCTGA TGCGTATAGG CGAATGACCC CAAACAGTCT CTCTCTCTCT   
  
  
+ CTCTCTCTCT CTCTCTCTCT CTCCCTCTGC ATGGATGATG GAGAGGGCGG GGAACAGGTT CGATCTGCGA   
  
  
+ GAGAGTGTAA GAGAGAGAGA GATGCAGATG CAAGGTGTCA AAAACGGAAA AGAAAATTAA ATTGATGTTT   
  
  
+ TCGGGTGCAA CCCGAAGTAT CGAGCAGCCT GGTTCTTCAA ATAAAGACCG TTAAGATTGC CTCCTTATTC   
  
  
+ AACTTCCTTA AATGGGCCAG TGGGGTGTCA AGCCCTTCTC TGTTCCAGCC CATAAGTACG CCTCTTTCTG   
  
  
+ TCTGTTTTAT TTGGGCCCTC CGATTGCCCG ACTTTTCATT TGTAATTACT TGCTTAATTA ATGGTTATAT   
  
  
+ GCTGTGCTTT ATTATTAAAA TTCCACTTAC AGAGAGTTAC ATCAATATCG CGAAACAGAA AGAAATTCAG   
  
  
+ TGTTGTATAC ATTTCATTTG GTAATAATAC CCATGGGCCA CGGAAACTTG ACTCCCTGTG TCATGACTCA   
  
  
+ AGAACCATGA CGTACGGGTA AAGGGTTGGT TCAAACCGCG GTGACCATGA ACGAGTAAAA CAGAGTGCTC   
  
  
+ CCATGCAAGT TCAGAACAAG ACGCTTTCTC CTCCACAGTC AATAACATCA AAGCAAGAGA AACTTCTTCA   
  
  
+ AGGTCTAAGC AATTGGTTTC AATGGGACAA GGTCTTCTTT TAGACTCAGA CCGGAAAAAG TTGAAAACCC   
  
  
+ CGCACTCATT AACAAGACCG TGACTTGGTC ATATTTCCAA GTTGCTTCTT CTTTGTCTTT TCGTTTTAAT   
  
  
+ TAATTAACTT CTTGCACCGT GTAAAAGGAA CACGAATACT TTGAATGACA TATGGAAAGC GTTATGATTA   
  
  
+ TATATACCAA CGATCAAAGA ACCAACGAAC CCCCATTTCA GATTTTGTCC TTCCAACCCA ATAAGAGAAG   
  
  
+ CCAAACAGAC CAAGAACGCC CACACACAC  

- TTCGATAATA ACGGTTCGGT CTCTGAGTTA CATAAAATCA GTCTTGTTCA ACGATTCGAG AACTTAAGAC   
  
  
- TTAATAGTCA GTGATTTGGT GAATAACACT ACATTTATAC AATAGTACAC ATTCGTTACT TCGTCATTCG   
  
  
- GTTCTAATTC CTCTTGGTGT CATTAACCGG AGTTTCTTTC TTTCTTTCTT TTTTGTTTTT ATCATCTCTA   
  
  
- GTTCGGATTT GTGTCTTTAA AGATCTCACT TCACTTTTGA CTTGTTTTGC TGTCTCTCTT TCTCTCCCTC   
  
  
- CTACCCCCTC TACTCTTTTT CTCCCCTAAC GTTCAATGGA ACCTCCACGG GGCTTTCACT CTGGTCCTCG   
  
  
- GGGTTCGCCT CCCTATTCCT AGCGGATACA ACATTCCCTC TAGGAACTCA GGGCATGGTC TAGGTGGGTG   
  
  
- GTGAGGGAAG TTCTTAAACT TGTACAACAA AAACAGGAGA GCAGAGGAAA AAAAGCCCAC AAATACCCAA   
  
  
- ATACATACGT ATATTCCAAC ATAGGAGACT ACGCATATCC GCTTACTGGG GTTTGTCAGA GAGAGAGAGA   
  
  
- GAGAGAGAGA GAGAGAGAGA GAGGGAGACG TACCTACTAC CTCTCCCGCC CCTTGTCCAA GCTAGACGCT   
  
  
- CTCTCACATT CTCTCTCTCT CTACGTCTAC GTTCCACAGT TTTTGCCTTT TCTTTTAATT TAACTACAAA   
  
  
- AGCCCACGTT GGGCTTCATA GCTCGTCGGA CCAAGAAGTT TATTTCTGGC AATTCTAACG GAGGAATAAG   
  
  
- TTGAAGGAAT TTACCCGGTC ACCCCACAGT TCGGGAAGAG ACAAGGTCGG GTATTCATGC GGAGAAAGAC   
  
  
- AGACAAAATA AACCCGGGAG GCTAACGGGC TGAAAAGTAA ACATTAATGA ACGAATTAAT TACCAATATA   
  
  
- CGACACGAAA TAATAATTTT AAGGTGAATG TCTCTCAATG TAGTTATAGC GCTTTGTCTT TCTTTAAGTC   
  
  
- ACAACATATG TAAAGTAAAC CATTATTATG GGTACCCGGT GCCTTTGAAC TGAGGGACAC AGTACTGAGT   
  
  
- TCTTGGTACT GCATGCCCAT TTCCCAACCA AGTTTGGCGC CACTGGTACT TGCTCATTTT GTCTCACGAG   
  
  
- GGTACGTTCA AGTCTTGTTC TGCGAAAGAG GAGGTGTCAG TTATTGTAGT TTCGTTCTCT TTGAAGAAGT   
  
  
- TCCAGATTCG TTAACCAAAG TTACCCTGTT CCAGAAGAAA ATCTGAGTCT GGCCTTTTTC AACTTTTGGG   
  
  
- GCGTGAGTAA TTGTTCTGGC ACTGAACCAG TATAAAGGTT CAACGAAGAA GAAACAGAAA AGCAAAATTA   
  
  
- ATTAATTGAA GAACGTGGCA CATTTTCCTT GTGCTTATGA AACTTACTGT ATACCTTTCG CAATACTAAT   
  
  
- ATATATGGTT GCTAGTTTCT TGGTTGCTTG GGGGTAAAGT CTAAAACAGG AAGGTTGGGT TATTCTCTTC   
  
  
- GGTTTGTCTG GTTCTTGCGG GTGTGTGTG
