# Supplementary material for: Genome-wide analysis of the WRKY gene family in drumstick (Moringa oleifera Lam.)
Source: PeerJ. 2019 Jun 10;7:e7063. doi: 10.7717/peerj.7063 (PMC6563795; doi:10.7717/peerj.7063)
Supplement: Supplemental Information 1 [file peerj-07-7063-s003.gz › MoWRKY53_plantcare.html]

Content-Type: text/html; charset=ISO-8859-1


CallMat\_Firefox


Webmaster Firefox specific output  
To save the result:
click on the frame with the right mouse button and save the source code as a text file with extension .html  
REFERENCE:PlantCARE: a database of plant cis-acting regulatory elements and a portal to tools for in silico analysis of promoter sequences.  
Lescot, M., Déhais, P., Moreau, Y., De Moor, B., Rouzé ,P.,and Rombauts, S.  
Nucleic Acids Res., Database issue(2002), 30(1):325-327.   


---

> 2018/04/13 10:10:12  
+ CTAGCTAGCT ACTATCCCAG TGACGGGTGT CTTGTGTCAC CACGACAAAT TAAAAAACCG GAACTAAAGC   
  
  
+ AACCCATCTT CTCGATCCCC TATAATATTC ACCCCACCCC CCTCTCCTTC GCTTCGTCTA TCTCTATTCG   
  
  
+ AACATTGATT ACTACGCAAA CCTGATTTTC TTTAATTTCC TCTCTACTCC TTTCCCTCTT CGTTTACTTT   
  
  
+ CCTTCTCCAC TCCTTTCTCC CTCATTCAAC TTCCCTTTTT CCCTTCTCTC TTTTTGGCGT CCTTTTCAAT   
  
  
+ CACTGCTTCG TTGGTCACTG CAGAGAGTAT ACTACGTTAC CACTACGGTT CGGTTCGAAA GAGAAAGAAA   
  
  
+ GAAAGGAAAG AAAGAAAGAA AGAAGAAAAT AGAGCCGTAC GAATTAAAAG GAGTGGTGTT TATTTGTCTT   
  
  
+ ACTTTAGTTC ACCCGAACTA TGACTATCAC ATGACACAAA CCCCCACACG TGTTGTTAAG TTTCAGATTC   
  
  
+ TTAGTTAGAG CATCGATCTC CCGACGTTAC GGCTCTACTA CCTAAAATTT TTTCTAGTTT AACGTTTTTT   
  
  
+ GTTTTTTGTT TTTTGGTTTT TACCAACCGT AATGACAATC ATGCTACGTT CACCGATGAA AATAAGATAA   
  
  
+ GATTACGTTG ACCTAGTAAT GCACCGTGTC CGGAAAGGTG AAAAGAAAGA TTAGGCTTAT TACATTAGTC   
  
  
+ ATTAGTGTTA TGTTCTCACC GTACTAACTA CTACTTAAGG TCTTTAGCAT AACCAACGCA GGCAGGGTAC   
  
  
+ TACGAGAGAA AACTTTTCAG TTACGAAGTT TGGATTGGAA GGTAGTAAAA GGTACAAACG TGAAGGGTAG   
  
  
+ ATTTGTTGAC CGAGTTTTAG TTTAGAAAAA AAAAAAAAAA AATTTTTTTT TTTTCCCTTC GCGAGTAAAC   
  
  
+ TTTGAAATAT TTCACATACA TATATCTTTT ATTTAAAACT ATTGTTCTCT CTCATTTTAT TGTTCGAAAA   
  
  
+ TAGTACTACT TGTTGTATGG AGTTGTTTTA ATTAATTTTT TTGTTGTTCG TAAGTAAAAC TAAAAATGAA   
  
  
+ GATTTAGATT TTCCTAATGT TAAATTATTT TATTTTGTTT ATATAAAAAT TTTATAAAAA GCAAAATGAA   
  
  
+ AGTTATTTTT GTCATTTTAA AATTAAATTC CTAATGTTAA ATTATTTTAT TTTGTTTATA TAAAAATTTT   
  
  
+ ATAAAAAGCA AAATGAAAGT TATTTTTGTC ATTTTAAAAT TAAAACTTCG TTTAACTAGT CTACTGCAAT   
  
  
+ TTTCTAGATC AATGTAGGTG ATCTGAATTA GGTGTATTTC GTTAGAGGTA GACGGTGCAG AATTGTGGTC   
  
  
+ CTGTATTGAC ATTATTGGTG CGGAGACCTA TTTAGGAAGG TGTCTCTGGT CTTCCTTCGA TGACTCATAC   
  
  
+ TAATGACATG GTGTGACATG AGATGACATT ATTGGTGCGG AGACCTATTT AGGAAGGTGT CTCTGGTCTT   
  
  
+ CCTTCGATGA CTCATACTAA TGACATGGT  

- GATCGATCGA TGATAGGGTC ACTGCCCACA GAACACAGTG GTGCTGTTTA ATTTTTTGGC CTTGATTTCG   
  
  
- TTGGGTAGAA GAGCTAGGGG ATATTATAAG TGGGGTGGGG GGAGAGGAAG CGAAGCAGAT AGAGATAAGC   
  
  
- TTGTAACTAA TGATGCGTTT GGACTAAAAG AAATTAAAGG AGAGATGAGG AAAGGGAGAA GCAAATGAAA   
  
  
- GGAAGAGGTG AGGAAAGAGG GAGTAAGTTG AAGGGAAAAA GGGAAGAGAG AAAAACCGCA GGAAAAGTTA   
  
  
- GTGACGAAGC AACCAGTGAC GTCTCTCATA TGATGCAATG GTGATGCCAA GCCAAGCTTT CTCTTTCTTT   
  
  
- CTTTCCTTTC TTTCTTTCTT TCTTCTTTTA TCTCGGCATG CTTAATTTTC CTCACCACAA ATAAACAGAA   
  
  
- TGAAATCAAG TGGGCTTGAT ACTGATAGTG TACTGTGTTT GGGGGTGTGC ACAACAATTC AAAGTCTAAG   
  
  
- AATCAATCTC GTAGCTAGAG GGCTGCAATG CCGAGATGAT GGATTTTAAA AAAGATCAAA TTGCAAAAAA   
  
  
- CAAAAAACAA AAAACCAAAA ATGGTTGGCA TTACTGTTAG TACGATGCAA GTGGCTACTT TTATTCTATT   
  
  
- CTAATGCAAC TGGATCATTA CGTGGCACAG GCCTTTCCAC TTTTCTTTCT AATCCGAATA ATGTAATCAG   
  
  
- TAATCACAAT ACAAGAGTGG CATGATTGAT GATGAATTCC AGAAATCGTA TTGGTTGCGT CCGTCCCATG   
  
  
- ATGCTCTCTT TTGAAAAGTC AATGCTTCAA ACCTAACCTT CCATCATTTT CCATGTTTGC ACTTCCCATC   
  
  
- TAAACAACTG GCTCAAAATC AAATCTTTTT TTTTTTTTTT TTAAAAAAAA AAAAGGGAAG CGCTCATTTG   
  
  
- AAACTTTATA AAGTGTATGT ATATAGAAAA TAAATTTTGA TAACAAGAGA GAGTAAAATA ACAAGCTTTT   
  
  
- ATCATGATGA ACAACATACC TCAACAAAAT TAATTAAAAA AACAACAAGC ATTCATTTTG ATTTTTACTT   
  
  
- CTAAATCTAA AAGGATTACA ATTTAATAAA ATAAAACAAA TATATTTTTA AAATATTTTT CGTTTTACTT   
  
  
- TCAATAAAAA CAGTAAAATT TTAATTTAAG GATTACAATT TAATAAAATA AAACAAATAT ATTTTTAAAA   
  
  
- TATTTTTCGT TTTACTTTCA ATAAAAACAG TAAAATTTTA ATTTTGAAGC AAATTGATCA GATGACGTTA   
  
  
- AAAGATCTAG TTACATCCAC TAGACTTAAT CCACATAAAG CAATCTCCAT CTGCCACGTC TTAACACCAG   
  
  
- GACATAACTG TAATAACCAC GCCTCTGGAT AAATCCTTCC ACAGAGACCA GAAGGAAGCT ACTGAGTATG   
  
  
- ATTACTGTAC CACACTGTAC TCTACTGTAA TAACCACGCC TCTGGATAAA TCCTTCCACA GAGACCAGAA   
  
  
- GGAAGCTACT GAGTATGATT ACTGTACCA

  
  
Motifs Found  

+     5UTR Py-rich stretch

| Site Name | Organism | Position | Strand | Matrix score. | sequence | function |
| --- | --- | --- | --- | --- | --- | --- |
| 5UTR Py-rich stretch | Lycopersicon esculentum | 369 | - | 9 | TTTCTTCTCT | cis-acting element conferring high transcription levels |
| 5UTR Py-rich stretch | Lycopersicon esculentum | 176 | + | 9 | TTTCTTCTCT | cis-acting element conferring high transcription levels |

> 2018/04/13 10:10:12  
+ CTAGCTAGCT ACTATCCCAG TGACGGGTGT CTTGTGTCAC CACGACAAAT TAAAAAACCG GAACTAAAGC   
  
  
+ AACCCATCTT CTCGATCCCC TATAATATTC ACCCCACCCC CCTCTCCTTC GCTTCGTCTA TCTCTATTCG   
  
  
+ AACATTGATT ACTACGCAAA CCTGATTTTC TTTAATTTCC TCTCTACTCC TTTCCCTCTT CGTTTACTTT   
  
  
+ CCTTCTCCAC TCCTTTCTCC CTCATTCAAC TTCCCTTTTT CCCTTCTCTC TTTTTGGCGT CCTTTTCAAT   
  
  
+ CACTGCTTCG TTGGTCACTG CAGAGAGTAT ACTACGTTAC CACTACGGTT CGGTTCGAAA GAGAAAGAAA   
  
  
+ GAAAGGAAAG AAAGAAAGAA AGAAGAAAAT AGAGCCGTAC GAATTAAAAG GAGTGGTGTT TATTTGTCTT   
  
  
+ ACTTTAGTTC ACCCGAACTA TGACTATCAC ATGACACAAA CCCCCACACG TGTTGTTAAG TTTCAGATTC   
  
  
+ TTAGTTAGAG CATCGATCTC CCGACGTTAC GGCTCTACTA CCTAAAATTT TTTCTAGTTT AACGTTTTTT   
  
  
+ GTTTTTTGTT TTTTGGTTTT TACCAACCGT AATGACAATC ATGCTACGTT CACCGATGAA AATAAGATAA   
  
  
+ GATTACGTTG ACCTAGTAAT GCACCGTGTC CGGAAAGGTG AAAAGAAAGA TTAGGCTTAT TACATTAGTC   
  
  
+ ATTAGTGTTA TGTTCTCACC GTACTAACTA CTACTTAAGG TCTTTAGCAT AACCAACGCA GGCAGGGTAC   
  
  
+ TACGAGAGAA AACTTTTCAG TTACGAAGTT TGGATTGGAA GGTAGTAAAA GGTACAAACG TGAAGGGTAG   
  
  
+ ATTTGTTGAC CGAGTTTTAG TTTAGAAAAA AAAAAAAAAA AATTTTTTTT TTTTCCCTTC GCGAGTAAAC   
  
  
+ TTTGAAATAT TTCACATACA TATATCTTTT ATTTAAAACT ATTGTTCTCT CTCATTTTAT TGTTCGAAAA   
  
  
+ TAGTACTACT TGTTGTATGG AGTTGTTTTA ATTAATTTTT TTGTTGTTCG TAAGTAAAAC TAAAAATGAA   
  
  
+ GATTTAGATT TTCCTAATGT TAAATTATTT TATTTTGTTT ATATAAAAAT TTTATAAAAA GCAAAATGAA   
  
  
+ AGTTATTTTT GTCATTTTAA AATTAAATTC CTAATGTTAA ATTATTTTAT TTTGTTTATA TAAAAATTTT   
  
  
+ ATAAAAAGCA AAATGAAAGT TATTTTTGTC ATTTTAAAAT TAAAACTTCG TTTAACTAGT CTACTGCAAT   
  
  
+ TTTCTAGATC AATGTAGGTG ATCTGAATTA GGTGTATTTC GTTAGAGGTA GACGGTGCAG AATTGTGGTC   
  
  
+ CTGTATTGAC ATTATTGGTG CGGAGACCTA TTTAGGAAGG TGTCTCTGGT CTTCCTTCGA TGACTCATAC   
  
  
+ TAATGACATG GTGTGACATG AGATGACATT ATTGGTGCGG AGACCTATTT AGGAAGGTGT CTCTGGTCTT   
  
  
+ CCTTCGATGA CTCATACTAA TGACATGGT  

- GATCGATCGA TGATAGGGTC ACTGCCCACA GAACACAGTG GTGCTGTTTA ATTTTTTGGC CTTGATTTCG   
  
  
- TTGGGTAGAA GAGCTAGGGG ATATTATAAG TGGGGTGGGG GGAGAGGAAG CGAAGCAGAT AGAGATAAGC   
  
  
- TTGTAACTAA TGATGCGTTT GGACTAAAAG AAATTAAAGG AGAGATGAGG AAAGGGAGAA GCAAATGAAA   
  
  
- GGAAGAGGTG AGGAAAGAGG GAGTAAGTTG AAGGGAAAAA GGGAAGAGAG AAAAACCGCA GGAAAAGTTA   
  
  
- GTGACGAAGC AACCAGTGAC GTCTCTCATA TGATGCAATG GTGATGCCAA GCCAAGCTTT CTCTTTCTTT   
  
  
- CTTTCCTTTC TTTCTTTCTT TCTTCTTTTA TCTCGGCATG CTTAATTTTC CTCACCACAA ATAAACAGAA   
  
  
- TGAAATCAAG TGGGCTTGAT ACTGATAGTG TACTGTGTTT GGGGGTGTGC ACAACAATTC AAAGTCTAAG   
  
  
- AATCAATCTC GTAGCTAGAG GGCTGCAATG CCGAGATGAT GGATTTTAAA AAAGATCAAA TTGCAAAAAA   
  
  
- CAAAAAACAA AAAACCAAAA ATGGTTGGCA TTACTGTTAG TACGATGCAA GTGGCTACTT TTATTCTATT   
  
  
- CTAATGCAAC TGGATCATTA CGTGGCACAG GCCTTTCCAC TTTTCTTTCT AATCCGAATA ATGTAATCAG   
  
  
- TAATCACAAT ACAAGAGTGG CATGATTGAT GATGAATTCC AGAAATCGTA TTGGTTGCGT CCGTCCCATG   
  
  
- ATGCTCTCTT TTGAAAAGTC AATGCTTCAA ACCTAACCTT CCATCATTTT CCATGTTTGC ACTTCCCATC   
  
  
- TAAACAACTG GCTCAAAATC AAATCTTTTT TTTTTTTTTT TTAAAAAAAA AAAAGGGAAG CGCTCATTTG   
  
  
- AAACTTTATA AAGTGTATGT ATATAGAAAA TAAATTTTGA TAACAAGAGA GAGTAAAATA ACAAGCTTTT   
  
  
- ATCATGATGA ACAACATACC TCAACAAAAT TAATTAAAAA AACAACAAGC ATTCATTTTG ATTTTTACTT   
  
  
- CTAAATCTAA AAGGATTACA ATTTAATAAA ATAAAACAAA TATATTTTTA AAATATTTTT CGTTTTACTT   
  
  
- TCAATAAAAA CAGTAAAATT TTAATTTAAG GATTACAATT TAATAAAATA AAACAAATAT ATTTTTAAAA   
  
  
- TATTTTTCGT TTTACTTTCA ATAAAAACAG TAAAATTTTA ATTTTGAAGC AAATTGATCA GATGACGTTA   
  
  
- AAAGATCTAG TTACATCCAC TAGACTTAAT CCACATAAAG CAATCTCCAT CTGCCACGTC TTAACACCAG   
  
  
- GACATAACTG TAATAACCAC GCCTCTGGAT AAATCCTTCC ACAGAGACCA GAAGGAAGCT ACTGAGTATG   
  
  
- ATTACTGTAC CACACTGTAC TCTACTGTAA TAACCACGCC TCTGGATAAA TCCTTCCACA GAGACCAGAA   
  
  
- GGAAGCTACT GAGTATGATT ACTGTACCA

+     AAGAA-motif

| Site Name | Organism | Position | Strand | Matrix score. | sequence | function |
| --- | --- | --- | --- | --- | --- | --- |
| AAGAA-motif | Avena sativa | 368 | + | 7 | GAAAGAA |  |
| AAGAA-motif | Avena sativa | 360 | + | 7 | GAAAGAA |  |
| AAGAA-motif | Avena sativa | 364 | + | 7 | GAAAGAA |  |
| AAGAA-motif | Avena sativa | 356 | + | 7 | GAAAGAA |  |
| AAGAA-motif | Avena sativa | 347 | + | 7 | GAAAGAA |  |
| AAGAA-motif | Avena sativa | 343 | + | 7 | GAAAGAA |  |

> 2018/04/13 10:10:12  
+ CTAGCTAGCT ACTATCCCAG TGACGGGTGT CTTGTGTCAC CACGACAAAT TAAAAAACCG GAACTAAAGC   
  
  
+ AACCCATCTT CTCGATCCCC TATAATATTC ACCCCACCCC CCTCTCCTTC GCTTCGTCTA TCTCTATTCG   
  
  
+ AACATTGATT ACTACGCAAA CCTGATTTTC TTTAATTTCC TCTCTACTCC TTTCCCTCTT CGTTTACTTT   
  
  
+ CCTTCTCCAC TCCTTTCTCC CTCATTCAAC TTCCCTTTTT CCCTTCTCTC TTTTTGGCGT CCTTTTCAAT   
  
  
+ CACTGCTTCG TTGGTCACTG CAGAGAGTAT ACTACGTTAC CACTACGGTT CGGTTCGAAA GAGAAAGAAA   
  
  
+ GAAAGGAAAG AAAGAAAGAA AGAAGAAAAT AGAGCCGTAC GAATTAAAAG GAGTGGTGTT TATTTGTCTT   
  
  
+ ACTTTAGTTC ACCCGAACTA TGACTATCAC ATGACACAAA CCCCCACACG TGTTGTTAAG TTTCAGATTC   
  
  
+ TTAGTTAGAG CATCGATCTC CCGACGTTAC GGCTCTACTA CCTAAAATTT TTTCTAGTTT AACGTTTTTT   
  
  
+ GTTTTTTGTT TTTTGGTTTT TACCAACCGT AATGACAATC ATGCTACGTT CACCGATGAA AATAAGATAA   
  
  
+ GATTACGTTG ACCTAGTAAT GCACCGTGTC CGGAAAGGTG AAAAGAAAGA TTAGGCTTAT TACATTAGTC   
  
  
+ ATTAGTGTTA TGTTCTCACC GTACTAACTA CTACTTAAGG TCTTTAGCAT AACCAACGCA GGCAGGGTAC   
  
  
+ TACGAGAGAA AACTTTTCAG TTACGAAGTT TGGATTGGAA GGTAGTAAAA GGTACAAACG TGAAGGGTAG   
  
  
+ ATTTGTTGAC CGAGTTTTAG TTTAGAAAAA AAAAAAAAAA AATTTTTTTT TTTTCCCTTC GCGAGTAAAC   
  
  
+ TTTGAAATAT TTCACATACA TATATCTTTT ATTTAAAACT ATTGTTCTCT CTCATTTTAT TGTTCGAAAA   
  
  
+ TAGTACTACT TGTTGTATGG AGTTGTTTTA ATTAATTTTT TTGTTGTTCG TAAGTAAAAC TAAAAATGAA   
  
  
+ GATTTAGATT TTCCTAATGT TAAATTATTT TATTTTGTTT ATATAAAAAT TTTATAAAAA GCAAAATGAA   
  
  
+ AGTTATTTTT GTCATTTTAA AATTAAATTC CTAATGTTAA ATTATTTTAT TTTGTTTATA TAAAAATTTT   
  
  
+ ATAAAAAGCA AAATGAAAGT TATTTTTGTC ATTTTAAAAT TAAAACTTCG TTTAACTAGT CTACTGCAAT   
  
  
+ TTTCTAGATC AATGTAGGTG ATCTGAATTA GGTGTATTTC GTTAGAGGTA GACGGTGCAG AATTGTGGTC   
  
  
+ CTGTATTGAC ATTATTGGTG CGGAGACCTA TTTAGGAAGG TGTCTCTGGT CTTCCTTCGA TGACTCATAC   
  
  
+ TAATGACATG GTGTGACATG AGATGACATT ATTGGTGCGG AGACCTATTT AGGAAGGTGT CTCTGGTCTT   
  
  
+ CCTTCGATGA CTCATACTAA TGACATGGT  

- GATCGATCGA TGATAGGGTC ACTGCCCACA GAACACAGTG GTGCTGTTTA ATTTTTTGGC CTTGATTTCG   
  
  
- TTGGGTAGAA GAGCTAGGGG ATATTATAAG TGGGGTGGGG GGAGAGGAAG CGAAGCAGAT AGAGATAAGC   
  
  
- TTGTAACTAA TGATGCGTTT GGACTAAAAG AAATTAAAGG AGAGATGAGG AAAGGGAGAA GCAAATGAAA   
  
  
- GGAAGAGGTG AGGAAAGAGG GAGTAAGTTG AAGGGAAAAA GGGAAGAGAG AAAAACCGCA GGAAAAGTTA   
  
  
- GTGACGAAGC AACCAGTGAC GTCTCTCATA TGATGCAATG GTGATGCCAA GCCAAGCTTT CTCTTTCTTT   
  
  
- CTTTCCTTTC TTTCTTTCTT TCTTCTTTTA TCTCGGCATG CTTAATTTTC CTCACCACAA ATAAACAGAA   
  
  
- TGAAATCAAG TGGGCTTGAT ACTGATAGTG TACTGTGTTT GGGGGTGTGC ACAACAATTC AAAGTCTAAG   
  
  
- AATCAATCTC GTAGCTAGAG GGCTGCAATG CCGAGATGAT GGATTTTAAA AAAGATCAAA TTGCAAAAAA   
  
  
- CAAAAAACAA AAAACCAAAA ATGGTTGGCA TTACTGTTAG TACGATGCAA GTGGCTACTT TTATTCTATT   
  
  
- CTAATGCAAC TGGATCATTA CGTGGCACAG GCCTTTCCAC TTTTCTTTCT AATCCGAATA ATGTAATCAG   
  
  
- TAATCACAAT ACAAGAGTGG CATGATTGAT GATGAATTCC AGAAATCGTA TTGGTTGCGT CCGTCCCATG   
  
  
- ATGCTCTCTT TTGAAAAGTC AATGCTTCAA ACCTAACCTT CCATCATTTT CCATGTTTGC ACTTCCCATC   
  
  
- TAAACAACTG GCTCAAAATC AAATCTTTTT TTTTTTTTTT TTAAAAAAAA AAAAGGGAAG CGCTCATTTG   
  
  
- AAACTTTATA AAGTGTATGT ATATAGAAAA TAAATTTTGA TAACAAGAGA GAGTAAAATA ACAAGCTTTT   
  
  
- ATCATGATGA ACAACATACC TCAACAAAAT TAATTAAAAA AACAACAAGC ATTCATTTTG ATTTTTACTT   
  
  
- CTAAATCTAA AAGGATTACA ATTTAATAAA ATAAAACAAA TATATTTTTA AAATATTTTT CGTTTTACTT   
  
  
- TCAATAAAAA CAGTAAAATT TTAATTTAAG GATTACAATT TAATAAAATA AAACAAATAT ATTTTTAAAA   
  
  
- TATTTTTCGT TTTACTTTCA ATAAAAACAG TAAAATTTTA ATTTTGAAGC AAATTGATCA GATGACGTTA   
  
  
- AAAGATCTAG TTACATCCAC TAGACTTAAT CCACATAAAG CAATCTCCAT CTGCCACGTC TTAACACCAG   
  
  
- GACATAACTG TAATAACCAC GCCTCTGGAT AAATCCTTCC ACAGAGACCA GAAGGAAGCT ACTGAGTATG   
  
  
- ATTACTGTAC CACACTGTAC TCTACTGTAA TAACCACGCC TCTGGATAAA TCCTTCCACA GAGACCAGAA   
  
  
- GGAAGCTACT GAGTATGATT ACTGTACCA

+     ABRE

| Site Name | Organism | Position | Strand | Matrix score. | sequence | function |
| --- | --- | --- | --- | --- | --- | --- |
| ABRE | Hordeum vulgare | 651 | + | 9 | GCAACGTGTC | cis-acting element involved in the abscisic acid responsiveness |
| ABRE | Arabidopsis thaliana | 467 | + | 6 | CACGTG | cis-acting element involved in the abscisic acid responsiveness |

> 2018/04/13 10:10:12  
+ CTAGCTAGCT ACTATCCCAG TGACGGGTGT CTTGTGTCAC CACGACAAAT TAAAAAACCG GAACTAAAGC   
  
  
+ AACCCATCTT CTCGATCCCC TATAATATTC ACCCCACCCC CCTCTCCTTC GCTTCGTCTA TCTCTATTCG   
  
  
+ AACATTGATT ACTACGCAAA CCTGATTTTC TTTAATTTCC TCTCTACTCC TTTCCCTCTT CGTTTACTTT   
  
  
+ CCTTCTCCAC TCCTTTCTCC CTCATTCAAC TTCCCTTTTT CCCTTCTCTC TTTTTGGCGT CCTTTTCAAT   
  
  
+ CACTGCTTCG TTGGTCACTG CAGAGAGTAT ACTACGTTAC CACTACGGTT CGGTTCGAAA GAGAAAGAAA   
  
  
+ GAAAGGAAAG AAAGAAAGAA AGAAGAAAAT AGAGCCGTAC GAATTAAAAG GAGTGGTGTT TATTTGTCTT   
  
  
+ ACTTTAGTTC ACCCGAACTA TGACTATCAC ATGACACAAA CCCCCACACG TGTTGTTAAG TTTCAGATTC   
  
  
+ TTAGTTAGAG CATCGATCTC CCGACGTTAC GGCTCTACTA CCTAAAATTT TTTCTAGTTT AACGTTTTTT   
  
  
+ GTTTTTTGTT TTTTGGTTTT TACCAACCGT AATGACAATC ATGCTACGTT CACCGATGAA AATAAGATAA   
  
  
+ GATTACGTTG ACCTAGTAAT GCACCGTGTC CGGAAAGGTG AAAAGAAAGA TTAGGCTTAT TACATTAGTC   
  
  
+ ATTAGTGTTA TGTTCTCACC GTACTAACTA CTACTTAAGG TCTTTAGCAT AACCAACGCA GGCAGGGTAC   
  
  
+ TACGAGAGAA AACTTTTCAG TTACGAAGTT TGGATTGGAA GGTAGTAAAA GGTACAAACG TGAAGGGTAG   
  
  
+ ATTTGTTGAC CGAGTTTTAG TTTAGAAAAA AAAAAAAAAA AATTTTTTTT TTTTCCCTTC GCGAGTAAAC   
  
  
+ TTTGAAATAT TTCACATACA TATATCTTTT ATTTAAAACT ATTGTTCTCT CTCATTTTAT TGTTCGAAAA   
  
  
+ TAGTACTACT TGTTGTATGG AGTTGTTTTA ATTAATTTTT TTGTTGTTCG TAAGTAAAAC TAAAAATGAA   
  
  
+ GATTTAGATT TTCCTAATGT TAAATTATTT TATTTTGTTT ATATAAAAAT TTTATAAAAA GCAAAATGAA   
  
  
+ AGTTATTTTT GTCATTTTAA AATTAAATTC CTAATGTTAA ATTATTTTAT TTTGTTTATA TAAAAATTTT   
  
  
+ ATAAAAAGCA AAATGAAAGT TATTTTTGTC ATTTTAAAAT TAAAACTTCG TTTAACTAGT CTACTGCAAT   
  
  
+ TTTCTAGATC AATGTAGGTG ATCTGAATTA GGTGTATTTC GTTAGAGGTA GACGGTGCAG AATTGTGGTC   
  
  
+ CTGTATTGAC ATTATTGGTG CGGAGACCTA TTTAGGAAGG TGTCTCTGGT CTTCCTTCGA TGACTCATAC   
  
  
+ TAATGACATG GTGTGACATG AGATGACATT ATTGGTGCGG AGACCTATTT AGGAAGGTGT CTCTGGTCTT   
  
  
+ CCTTCGATGA CTCATACTAA TGACATGGT  

- GATCGATCGA TGATAGGGTC ACTGCCCACA GAACACAGTG GTGCTGTTTA ATTTTTTGGC CTTGATTTCG   
  
  
- TTGGGTAGAA GAGCTAGGGG ATATTATAAG TGGGGTGGGG GGAGAGGAAG CGAAGCAGAT AGAGATAAGC   
  
  
- TTGTAACTAA TGATGCGTTT GGACTAAAAG AAATTAAAGG AGAGATGAGG AAAGGGAGAA GCAAATGAAA   
  
  
- GGAAGAGGTG AGGAAAGAGG GAGTAAGTTG AAGGGAAAAA GGGAAGAGAG AAAAACCGCA GGAAAAGTTA   
  
  
- GTGACGAAGC AACCAGTGAC GTCTCTCATA TGATGCAATG GTGATGCCAA GCCAAGCTTT CTCTTTCTTT   
  
  
- CTTTCCTTTC TTTCTTTCTT TCTTCTTTTA TCTCGGCATG CTTAATTTTC CTCACCACAA ATAAACAGAA   
  
  
- TGAAATCAAG TGGGCTTGAT ACTGATAGTG TACTGTGTTT GGGGGTGTGC ACAACAATTC AAAGTCTAAG   
  
  
- AATCAATCTC GTAGCTAGAG GGCTGCAATG CCGAGATGAT GGATTTTAAA AAAGATCAAA TTGCAAAAAA   
  
  
- CAAAAAACAA AAAACCAAAA ATGGTTGGCA TTACTGTTAG TACGATGCAA GTGGCTACTT TTATTCTATT   
  
  
- CTAATGCAAC TGGATCATTA CGTGGCACAG GCCTTTCCAC TTTTCTTTCT AATCCGAATA ATGTAATCAG   
  
  
- TAATCACAAT ACAAGAGTGG CATGATTGAT GATGAATTCC AGAAATCGTA TTGGTTGCGT CCGTCCCATG   
  
  
- ATGCTCTCTT TTGAAAAGTC AATGCTTCAA ACCTAACCTT CCATCATTTT CCATGTTTGC ACTTCCCATC   
  
  
- TAAACAACTG GCTCAAAATC AAATCTTTTT TTTTTTTTTT TTAAAAAAAA AAAAGGGAAG CGCTCATTTG   
  
  
- AAACTTTATA AAGTGTATGT ATATAGAAAA TAAATTTTGA TAACAAGAGA GAGTAAAATA ACAAGCTTTT   
  
  
- ATCATGATGA ACAACATACC TCAACAAAAT TAATTAAAAA AACAACAAGC ATTCATTTTG ATTTTTACTT   
  
  
- CTAAATCTAA AAGGATTACA ATTTAATAAA ATAAAACAAA TATATTTTTA AAATATTTTT CGTTTTACTT   
  
  
- TCAATAAAAA CAGTAAAATT TTAATTTAAG GATTACAATT TAATAAAATA AAACAAATAT ATTTTTAAAA   
  
  
- TATTTTTCGT TTTACTTTCA ATAAAAACAG TAAAATTTTA ATTTTGAAGC AAATTGATCA GATGACGTTA   
  
  
- AAAGATCTAG TTACATCCAC TAGACTTAAT CCACATAAAG CAATCTCCAT CTGCCACGTC TTAACACCAG   
  
  
- GACATAACTG TAATAACCAC GCCTCTGGAT AAATCCTTCC ACAGAGACCA GAAGGAAGCT ACTGAGTATG   
  
  
- ATTACTGTAC CACACTGTAC TCTACTGTAA TAACCACGCC TCTGGATAAA TCCTTCCACA GAGACCAGAA   
  
  
- GGAAGCTACT GAGTATGATT ACTGTACCA

+     AC-II

| Site Name | Organism | Position | Strand | Matrix score. | sequence | function |
| --- | --- | --- | --- | --- | --- | --- |
| AC-II | Phaseolus vulgaris | 98 | + | 9 | (C/T)T(T/C)(C/T)(A/C)(A/C)C(A/C)A(A/C)C(C/A)(C/A)C |  |

> 2018/04/13 10:10:12  
+ CTAGCTAGCT ACTATCCCAG TGACGGGTGT CTTGTGTCAC CACGACAAAT TAAAAAACCG GAACTAAAGC   
  
  
+ AACCCATCTT CTCGATCCCC TATAATATTC ACCCCACCCC CCTCTCCTTC GCTTCGTCTA TCTCTATTCG   
  
  
+ AACATTGATT ACTACGCAAA CCTGATTTTC TTTAATTTCC TCTCTACTCC TTTCCCTCTT CGTTTACTTT   
  
  
+ CCTTCTCCAC TCCTTTCTCC CTCATTCAAC TTCCCTTTTT CCCTTCTCTC TTTTTGGCGT CCTTTTCAAT   
  
  
+ CACTGCTTCG TTGGTCACTG CAGAGAGTAT ACTACGTTAC CACTACGGTT CGGTTCGAAA GAGAAAGAAA   
  
  
+ GAAAGGAAAG AAAGAAAGAA AGAAGAAAAT AGAGCCGTAC GAATTAAAAG GAGTGGTGTT TATTTGTCTT   
  
  
+ ACTTTAGTTC ACCCGAACTA TGACTATCAC ATGACACAAA CCCCCACACG TGTTGTTAAG TTTCAGATTC   
  
  
+ TTAGTTAGAG CATCGATCTC CCGACGTTAC GGCTCTACTA CCTAAAATTT TTTCTAGTTT AACGTTTTTT   
  
  
+ GTTTTTTGTT TTTTGGTTTT TACCAACCGT AATGACAATC ATGCTACGTT CACCGATGAA AATAAGATAA   
  
  
+ GATTACGTTG ACCTAGTAAT GCACCGTGTC CGGAAAGGTG AAAAGAAAGA TTAGGCTTAT TACATTAGTC   
  
  
+ ATTAGTGTTA TGTTCTCACC GTACTAACTA CTACTTAAGG TCTTTAGCAT AACCAACGCA GGCAGGGTAC   
  
  
+ TACGAGAGAA AACTTTTCAG TTACGAAGTT TGGATTGGAA GGTAGTAAAA GGTACAAACG TGAAGGGTAG   
  
  
+ ATTTGTTGAC CGAGTTTTAG TTTAGAAAAA AAAAAAAAAA AATTTTTTTT TTTTCCCTTC GCGAGTAAAC   
  
  
+ TTTGAAATAT TTCACATACA TATATCTTTT ATTTAAAACT ATTGTTCTCT CTCATTTTAT TGTTCGAAAA   
  
  
+ TAGTACTACT TGTTGTATGG AGTTGTTTTA ATTAATTTTT TTGTTGTTCG TAAGTAAAAC TAAAAATGAA   
  
  
+ GATTTAGATT TTCCTAATGT TAAATTATTT TATTTTGTTT ATATAAAAAT TTTATAAAAA GCAAAATGAA   
  
  
+ AGTTATTTTT GTCATTTTAA AATTAAATTC CTAATGTTAA ATTATTTTAT TTTGTTTATA TAAAAATTTT   
  
  
+ ATAAAAAGCA AAATGAAAGT TATTTTTGTC ATTTTAAAAT TAAAACTTCG TTTAACTAGT CTACTGCAAT   
  
  
+ TTTCTAGATC AATGTAGGTG ATCTGAATTA GGTGTATTTC GTTAGAGGTA GACGGTGCAG AATTGTGGTC   
  
  
+ CTGTATTGAC ATTATTGGTG CGGAGACCTA TTTAGGAAGG TGTCTCTGGT CTTCCTTCGA TGACTCATAC   
  
  
+ TAATGACATG GTGTGACATG AGATGACATT ATTGGTGCGG AGACCTATTT AGGAAGGTGT CTCTGGTCTT   
  
  
+ CCTTCGATGA CTCATACTAA TGACATGGT  

- GATCGATCGA TGATAGGGTC ACTGCCCACA GAACACAGTG GTGCTGTTTA ATTTTTTGGC CTTGATTTCG   
  
  
- TTGGGTAGAA GAGCTAGGGG ATATTATAAG TGGGGTGGGG GGAGAGGAAG CGAAGCAGAT AGAGATAAGC   
  
  
- TTGTAACTAA TGATGCGTTT GGACTAAAAG AAATTAAAGG AGAGATGAGG AAAGGGAGAA GCAAATGAAA   
  
  
- GGAAGAGGTG AGGAAAGAGG GAGTAAGTTG AAGGGAAAAA GGGAAGAGAG AAAAACCGCA GGAAAAGTTA   
  
  
- GTGACGAAGC AACCAGTGAC GTCTCTCATA TGATGCAATG GTGATGCCAA GCCAAGCTTT CTCTTTCTTT   
  
  
- CTTTCCTTTC TTTCTTTCTT TCTTCTTTTA TCTCGGCATG CTTAATTTTC CTCACCACAA ATAAACAGAA   
  
  
- TGAAATCAAG TGGGCTTGAT ACTGATAGTG TACTGTGTTT GGGGGTGTGC ACAACAATTC AAAGTCTAAG   
  
  
- AATCAATCTC GTAGCTAGAG GGCTGCAATG CCGAGATGAT GGATTTTAAA AAAGATCAAA TTGCAAAAAA   
  
  
- CAAAAAACAA AAAACCAAAA ATGGTTGGCA TTACTGTTAG TACGATGCAA GTGGCTACTT TTATTCTATT   
  
  
- CTAATGCAAC TGGATCATTA CGTGGCACAG GCCTTTCCAC TTTTCTTTCT AATCCGAATA ATGTAATCAG   
  
  
- TAATCACAAT ACAAGAGTGG CATGATTGAT GATGAATTCC AGAAATCGTA TTGGTTGCGT CCGTCCCATG   
  
  
- ATGCTCTCTT TTGAAAAGTC AATGCTTCAA ACCTAACCTT CCATCATTTT CCATGTTTGC ACTTCCCATC   
  
  
- TAAACAACTG GCTCAAAATC AAATCTTTTT TTTTTTTTTT TTAAAAAAAA AAAAGGGAAG CGCTCATTTG   
  
  
- AAACTTTATA AAGTGTATGT ATATAGAAAA TAAATTTTGA TAACAAGAGA GAGTAAAATA ACAAGCTTTT   
  
  
- ATCATGATGA ACAACATACC TCAACAAAAT TAATTAAAAA AACAACAAGC ATTCATTTTG ATTTTTACTT   
  
  
- CTAAATCTAA AAGGATTACA ATTTAATAAA ATAAAACAAA TATATTTTTA AAATATTTTT CGTTTTACTT   
  
  
- TCAATAAAAA CAGTAAAATT TTAATTTAAG GATTACAATT TAATAAAATA AAACAAATAT ATTTTTAAAA   
  
  
- TATTTTTCGT TTTACTTTCA ATAAAAACAG TAAAATTTTA ATTTTGAAGC AAATTGATCA GATGACGTTA   
  
  
- AAAGATCTAG TTACATCCAC TAGACTTAAT CCACATAAAG CAATCTCCAT CTGCCACGTC TTAACACCAG   
  
  
- GACATAACTG TAATAACCAC GCCTCTGGAT AAATCCTTCC ACAGAGACCA GAAGGAAGCT ACTGAGTATG   
  
  
- ATTACTGTAC CACACTGTAC TCTACTGTAA TAACCACGCC TCTGGATAAA TCCTTCCACA GAGACCAGAA   
  
  
- GGAAGCTACT GAGTATGATT ACTGTACCA

+     ACE

| Site Name | Organism | Position | Strand | Matrix score. | sequence | function |
| --- | --- | --- | --- | --- | --- | --- |
| ACE | Petroselinum crispum | 549 | - | 9 | AAAACGTTTA | cis-acting element involved in light responsiveness |

> 2018/04/13 10:10:12  
+ CTAGCTAGCT ACTATCCCAG TGACGGGTGT CTTGTGTCAC CACGACAAAT TAAAAAACCG GAACTAAAGC   
  
  
+ AACCCATCTT CTCGATCCCC TATAATATTC ACCCCACCCC CCTCTCCTTC GCTTCGTCTA TCTCTATTCG   
  
  
+ AACATTGATT ACTACGCAAA CCTGATTTTC TTTAATTTCC TCTCTACTCC TTTCCCTCTT CGTTTACTTT   
  
  
+ CCTTCTCCAC TCCTTTCTCC CTCATTCAAC TTCCCTTTTT CCCTTCTCTC TTTTTGGCGT CCTTTTCAAT   
  
  
+ CACTGCTTCG TTGGTCACTG CAGAGAGTAT ACTACGTTAC CACTACGGTT CGGTTCGAAA GAGAAAGAAA   
  
  
+ GAAAGGAAAG AAAGAAAGAA AGAAGAAAAT AGAGCCGTAC GAATTAAAAG GAGTGGTGTT TATTTGTCTT   
  
  
+ ACTTTAGTTC ACCCGAACTA TGACTATCAC ATGACACAAA CCCCCACACG TGTTGTTAAG TTTCAGATTC   
  
  
+ TTAGTTAGAG CATCGATCTC CCGACGTTAC GGCTCTACTA CCTAAAATTT TTTCTAGTTT AACGTTTTTT   
  
  
+ GTTTTTTGTT TTTTGGTTTT TACCAACCGT AATGACAATC ATGCTACGTT CACCGATGAA AATAAGATAA   
  
  
+ GATTACGTTG ACCTAGTAAT GCACCGTGTC CGGAAAGGTG AAAAGAAAGA TTAGGCTTAT TACATTAGTC   
  
  
+ ATTAGTGTTA TGTTCTCACC GTACTAACTA CTACTTAAGG TCTTTAGCAT AACCAACGCA GGCAGGGTAC   
  
  
+ TACGAGAGAA AACTTTTCAG TTACGAAGTT TGGATTGGAA GGTAGTAAAA GGTACAAACG TGAAGGGTAG   
  
  
+ ATTTGTTGAC CGAGTTTTAG TTTAGAAAAA AAAAAAAAAA AATTTTTTTT TTTTCCCTTC GCGAGTAAAC   
  
  
+ TTTGAAATAT TTCACATACA TATATCTTTT ATTTAAAACT ATTGTTCTCT CTCATTTTAT TGTTCGAAAA   
  
  
+ TAGTACTACT TGTTGTATGG AGTTGTTTTA ATTAATTTTT TTGTTGTTCG TAAGTAAAAC TAAAAATGAA   
  
  
+ GATTTAGATT TTCCTAATGT TAAATTATTT TATTTTGTTT ATATAAAAAT TTTATAAAAA GCAAAATGAA   
  
  
+ AGTTATTTTT GTCATTTTAA AATTAAATTC CTAATGTTAA ATTATTTTAT TTTGTTTATA TAAAAATTTT   
  
  
+ ATAAAAAGCA AAATGAAAGT TATTTTTGTC ATTTTAAAAT TAAAACTTCG TTTAACTAGT CTACTGCAAT   
  
  
+ TTTCTAGATC AATGTAGGTG ATCTGAATTA GGTGTATTTC GTTAGAGGTA GACGGTGCAG AATTGTGGTC   
  
  
+ CTGTATTGAC ATTATTGGTG CGGAGACCTA TTTAGGAAGG TGTCTCTGGT CTTCCTTCGA TGACTCATAC   
  
  
+ TAATGACATG GTGTGACATG AGATGACATT ATTGGTGCGG AGACCTATTT AGGAAGGTGT CTCTGGTCTT   
  
  
+ CCTTCGATGA CTCATACTAA TGACATGGT  

- GATCGATCGA TGATAGGGTC ACTGCCCACA GAACACAGTG GTGCTGTTTA ATTTTTTGGC CTTGATTTCG   
  
  
- TTGGGTAGAA GAGCTAGGGG ATATTATAAG TGGGGTGGGG GGAGAGGAAG CGAAGCAGAT AGAGATAAGC   
  
  
- TTGTAACTAA TGATGCGTTT GGACTAAAAG AAATTAAAGG AGAGATGAGG AAAGGGAGAA GCAAATGAAA   
  
  
- GGAAGAGGTG AGGAAAGAGG GAGTAAGTTG AAGGGAAAAA GGGAAGAGAG AAAAACCGCA GGAAAAGTTA   
  
  
- GTGACGAAGC AACCAGTGAC GTCTCTCATA TGATGCAATG GTGATGCCAA GCCAAGCTTT CTCTTTCTTT   
  
  
- CTTTCCTTTC TTTCTTTCTT TCTTCTTTTA TCTCGGCATG CTTAATTTTC CTCACCACAA ATAAACAGAA   
  
  
- TGAAATCAAG TGGGCTTGAT ACTGATAGTG TACTGTGTTT GGGGGTGTGC ACAACAATTC AAAGTCTAAG   
  
  
- AATCAATCTC GTAGCTAGAG GGCTGCAATG CCGAGATGAT GGATTTTAAA AAAGATCAAA TTGCAAAAAA   
  
  
- CAAAAAACAA AAAACCAAAA ATGGTTGGCA TTACTGTTAG TACGATGCAA GTGGCTACTT TTATTCTATT   
  
  
- CTAATGCAAC TGGATCATTA CGTGGCACAG GCCTTTCCAC TTTTCTTTCT AATCCGAATA ATGTAATCAG   
  
  
- TAATCACAAT ACAAGAGTGG CATGATTGAT GATGAATTCC AGAAATCGTA TTGGTTGCGT CCGTCCCATG   
  
  
- ATGCTCTCTT TTGAAAAGTC AATGCTTCAA ACCTAACCTT CCATCATTTT CCATGTTTGC ACTTCCCATC   
  
  
- TAAACAACTG GCTCAAAATC AAATCTTTTT TTTTTTTTTT TTAAAAAAAA AAAAGGGAAG CGCTCATTTG   
  
  
- AAACTTTATA AAGTGTATGT ATATAGAAAA TAAATTTTGA TAACAAGAGA GAGTAAAATA ACAAGCTTTT   
  
  
- ATCATGATGA ACAACATACC TCAACAAAAT TAATTAAAAA AACAACAAGC ATTCATTTTG ATTTTTACTT   
  
  
- CTAAATCTAA AAGGATTACA ATTTAATAAA ATAAAACAAA TATATTTTTA AAATATTTTT CGTTTTACTT   
  
  
- TCAATAAAAA CAGTAAAATT TTAATTTAAG GATTACAATT TAATAAAATA AAACAAATAT ATTTTTAAAA   
  
  
- TATTTTTCGT TTTACTTTCA ATAAAAACAG TAAAATTTTA ATTTTGAAGC AAATTGATCA GATGACGTTA   
  
  
- AAAGATCTAG TTACATCCAC TAGACTTAAT CCACATAAAG CAATCTCCAT CTGCCACGTC TTAACACCAG   
  
  
- GACATAACTG TAATAACCAC GCCTCTGGAT AAATCCTTCC ACAGAGACCA GAAGGAAGCT ACTGAGTATG   
  
  
- ATTACTGTAC CACACTGTAC TCTACTGTAA TAACCACGCC TCTGGATAAA TCCTTCCACA GAGACCAGAA   
  
  
- GGAAGCTACT GAGTATGATT ACTGTACCA

+     ARE

| Site Name | Organism | Position | Strand | Matrix score. | sequence | function |
| --- | --- | --- | --- | --- | --- | --- |
| ARE | Zea mays | 574 | + | 6 | TGGTTT | cis-acting regulatory element essential for the anaerobic induction |

> 2018/04/13 10:10:12  
+ CTAGCTAGCT ACTATCCCAG TGACGGGTGT CTTGTGTCAC CACGACAAAT TAAAAAACCG GAACTAAAGC   
  
  
+ AACCCATCTT CTCGATCCCC TATAATATTC ACCCCACCCC CCTCTCCTTC GCTTCGTCTA TCTCTATTCG   
  
  
+ AACATTGATT ACTACGCAAA CCTGATTTTC TTTAATTTCC TCTCTACTCC TTTCCCTCTT CGTTTACTTT   
  
  
+ CCTTCTCCAC TCCTTTCTCC CTCATTCAAC TTCCCTTTTT CCCTTCTCTC TTTTTGGCGT CCTTTTCAAT   
  
  
+ CACTGCTTCG TTGGTCACTG CAGAGAGTAT ACTACGTTAC CACTACGGTT CGGTTCGAAA GAGAAAGAAA   
  
  
+ GAAAGGAAAG AAAGAAAGAA AGAAGAAAAT AGAGCCGTAC GAATTAAAAG GAGTGGTGTT TATTTGTCTT   
  
  
+ ACTTTAGTTC ACCCGAACTA TGACTATCAC ATGACACAAA CCCCCACACG TGTTGTTAAG TTTCAGATTC   
  
  
+ TTAGTTAGAG CATCGATCTC CCGACGTTAC GGCTCTACTA CCTAAAATTT TTTCTAGTTT AACGTTTTTT   
  
  
+ GTTTTTTGTT TTTTGGTTTT TACCAACCGT AATGACAATC ATGCTACGTT CACCGATGAA AATAAGATAA   
  
  
+ GATTACGTTG ACCTAGTAAT GCACCGTGTC CGGAAAGGTG AAAAGAAAGA TTAGGCTTAT TACATTAGTC   
  
  
+ ATTAGTGTTA TGTTCTCACC GTACTAACTA CTACTTAAGG TCTTTAGCAT AACCAACGCA GGCAGGGTAC   
  
  
+ TACGAGAGAA AACTTTTCAG TTACGAAGTT TGGATTGGAA GGTAGTAAAA GGTACAAACG TGAAGGGTAG   
  
  
+ ATTTGTTGAC CGAGTTTTAG TTTAGAAAAA AAAAAAAAAA AATTTTTTTT TTTTCCCTTC GCGAGTAAAC   
  
  
+ TTTGAAATAT TTCACATACA TATATCTTTT ATTTAAAACT ATTGTTCTCT CTCATTTTAT TGTTCGAAAA   
  
  
+ TAGTACTACT TGTTGTATGG AGTTGTTTTA ATTAATTTTT TTGTTGTTCG TAAGTAAAAC TAAAAATGAA   
  
  
+ GATTTAGATT TTCCTAATGT TAAATTATTT TATTTTGTTT ATATAAAAAT TTTATAAAAA GCAAAATGAA   
  
  
+ AGTTATTTTT GTCATTTTAA AATTAAATTC CTAATGTTAA ATTATTTTAT TTTGTTTATA TAAAAATTTT   
  
  
+ ATAAAAAGCA AAATGAAAGT TATTTTTGTC ATTTTAAAAT TAAAACTTCG TTTAACTAGT CTACTGCAAT   
  
  
+ TTTCTAGATC AATGTAGGTG ATCTGAATTA GGTGTATTTC GTTAGAGGTA GACGGTGCAG AATTGTGGTC   
  
  
+ CTGTATTGAC ATTATTGGTG CGGAGACCTA TTTAGGAAGG TGTCTCTGGT CTTCCTTCGA TGACTCATAC   
  
  
+ TAATGACATG GTGTGACATG AGATGACATT ATTGGTGCGG AGACCTATTT AGGAAGGTGT CTCTGGTCTT   
  
  
+ CCTTCGATGA CTCATACTAA TGACATGGT  

- GATCGATCGA TGATAGGGTC ACTGCCCACA GAACACAGTG GTGCTGTTTA ATTTTTTGGC CTTGATTTCG   
  
  
- TTGGGTAGAA GAGCTAGGGG ATATTATAAG TGGGGTGGGG GGAGAGGAAG CGAAGCAGAT AGAGATAAGC   
  
  
- TTGTAACTAA TGATGCGTTT GGACTAAAAG AAATTAAAGG AGAGATGAGG AAAGGGAGAA GCAAATGAAA   
  
  
- GGAAGAGGTG AGGAAAGAGG GAGTAAGTTG AAGGGAAAAA GGGAAGAGAG AAAAACCGCA GGAAAAGTTA   
  
  
- GTGACGAAGC AACCAGTGAC GTCTCTCATA TGATGCAATG GTGATGCCAA GCCAAGCTTT CTCTTTCTTT   
  
  
- CTTTCCTTTC TTTCTTTCTT TCTTCTTTTA TCTCGGCATG CTTAATTTTC CTCACCACAA ATAAACAGAA   
  
  
- TGAAATCAAG TGGGCTTGAT ACTGATAGTG TACTGTGTTT GGGGGTGTGC ACAACAATTC AAAGTCTAAG   
  
  
- AATCAATCTC GTAGCTAGAG GGCTGCAATG CCGAGATGAT GGATTTTAAA AAAGATCAAA TTGCAAAAAA   
  
  
- CAAAAAACAA AAAACCAAAA ATGGTTGGCA TTACTGTTAG TACGATGCAA GTGGCTACTT TTATTCTATT   
  
  
- CTAATGCAAC TGGATCATTA CGTGGCACAG GCCTTTCCAC TTTTCTTTCT AATCCGAATA ATGTAATCAG   
  
  
- TAATCACAAT ACAAGAGTGG CATGATTGAT GATGAATTCC AGAAATCGTA TTGGTTGCGT CCGTCCCATG   
  
  
- ATGCTCTCTT TTGAAAAGTC AATGCTTCAA ACCTAACCTT CCATCATTTT CCATGTTTGC ACTTCCCATC   
  
  
- TAAACAACTG GCTCAAAATC AAATCTTTTT TTTTTTTTTT TTAAAAAAAA AAAAGGGAAG CGCTCATTTG   
  
  
- AAACTTTATA AAGTGTATGT ATATAGAAAA TAAATTTTGA TAACAAGAGA GAGTAAAATA ACAAGCTTTT   
  
  
- ATCATGATGA ACAACATACC TCAACAAAAT TAATTAAAAA AACAACAAGC ATTCATTTTG ATTTTTACTT   
  
  
- CTAAATCTAA AAGGATTACA ATTTAATAAA ATAAAACAAA TATATTTTTA AAATATTTTT CGTTTTACTT   
  
  
- TCAATAAAAA CAGTAAAATT TTAATTTAAG GATTACAATT TAATAAAATA AAACAAATAT ATTTTTAAAA   
  
  
- TATTTTTCGT TTTACTTTCA ATAAAAACAG TAAAATTTTA ATTTTGAAGC AAATTGATCA GATGACGTTA   
  
  
- AAAGATCTAG TTACATCCAC TAGACTTAAT CCACATAAAG CAATCTCCAT CTGCCACGTC TTAACACCAG   
  
  
- GACATAACTG TAATAACCAC GCCTCTGGAT AAATCCTTCC ACAGAGACCA GAAGGAAGCT ACTGAGTATG   
  
  
- ATTACTGTAC CACACTGTAC TCTACTGTAA TAACCACGCC TCTGGATAAA TCCTTCCACA GAGACCAGAA   
  
  
- GGAAGCTACT GAGTATGATT ACTGTACCA

+     ATCT-motif

| Site Name | Organism | Position | Strand | Matrix score. | sequence | function |
| --- | --- | --- | --- | --- | --- | --- |
| ATCT-motif | Arabidopsis thaliana | 625 | - | 9 | AATCTAATCT | part of a conserved DNA module involved in light responsiveness |

> 2018/04/13 10:10:12  
+ CTAGCTAGCT ACTATCCCAG TGACGGGTGT CTTGTGTCAC CACGACAAAT TAAAAAACCG GAACTAAAGC   
  
  
+ AACCCATCTT CTCGATCCCC TATAATATTC ACCCCACCCC CCTCTCCTTC GCTTCGTCTA TCTCTATTCG   
  
  
+ AACATTGATT ACTACGCAAA CCTGATTTTC TTTAATTTCC TCTCTACTCC TTTCCCTCTT CGTTTACTTT   
  
  
+ CCTTCTCCAC TCCTTTCTCC CTCATTCAAC TTCCCTTTTT CCCTTCTCTC TTTTTGGCGT CCTTTTCAAT   
  
  
+ CACTGCTTCG TTGGTCACTG CAGAGAGTAT ACTACGTTAC CACTACGGTT CGGTTCGAAA GAGAAAGAAA   
  
  
+ GAAAGGAAAG AAAGAAAGAA AGAAGAAAAT AGAGCCGTAC GAATTAAAAG GAGTGGTGTT TATTTGTCTT   
  
  
+ ACTTTAGTTC ACCCGAACTA TGACTATCAC ATGACACAAA CCCCCACACG TGTTGTTAAG TTTCAGATTC   
  
  
+ TTAGTTAGAG CATCGATCTC CCGACGTTAC GGCTCTACTA CCTAAAATTT TTTCTAGTTT AACGTTTTTT   
  
  
+ GTTTTTTGTT TTTTGGTTTT TACCAACCGT AATGACAATC ATGCTACGTT CACCGATGAA AATAAGATAA   
  
  
+ GATTACGTTG ACCTAGTAAT GCACCGTGTC CGGAAAGGTG AAAAGAAAGA TTAGGCTTAT TACATTAGTC   
  
  
+ ATTAGTGTTA TGTTCTCACC GTACTAACTA CTACTTAAGG TCTTTAGCAT AACCAACGCA GGCAGGGTAC   
  
  
+ TACGAGAGAA AACTTTTCAG TTACGAAGTT TGGATTGGAA GGTAGTAAAA GGTACAAACG TGAAGGGTAG   
  
  
+ ATTTGTTGAC CGAGTTTTAG TTTAGAAAAA AAAAAAAAAA AATTTTTTTT TTTTCCCTTC GCGAGTAAAC   
  
  
+ TTTGAAATAT TTCACATACA TATATCTTTT ATTTAAAACT ATTGTTCTCT CTCATTTTAT TGTTCGAAAA   
  
  
+ TAGTACTACT TGTTGTATGG AGTTGTTTTA ATTAATTTTT TTGTTGTTCG TAAGTAAAAC TAAAAATGAA   
  
  
+ GATTTAGATT TTCCTAATGT TAAATTATTT TATTTTGTTT ATATAAAAAT TTTATAAAAA GCAAAATGAA   
  
  
+ AGTTATTTTT GTCATTTTAA AATTAAATTC CTAATGTTAA ATTATTTTAT TTTGTTTATA TAAAAATTTT   
  
  
+ ATAAAAAGCA AAATGAAAGT TATTTTTGTC ATTTTAAAAT TAAAACTTCG TTTAACTAGT CTACTGCAAT   
  
  
+ TTTCTAGATC AATGTAGGTG ATCTGAATTA GGTGTATTTC GTTAGAGGTA GACGGTGCAG AATTGTGGTC   
  
  
+ CTGTATTGAC ATTATTGGTG CGGAGACCTA TTTAGGAAGG TGTCTCTGGT CTTCCTTCGA TGACTCATAC   
  
  
+ TAATGACATG GTGTGACATG AGATGACATT ATTGGTGCGG AGACCTATTT AGGAAGGTGT CTCTGGTCTT   
  
  
+ CCTTCGATGA CTCATACTAA TGACATGGT  

- GATCGATCGA TGATAGGGTC ACTGCCCACA GAACACAGTG GTGCTGTTTA ATTTTTTGGC CTTGATTTCG   
  
  
- TTGGGTAGAA GAGCTAGGGG ATATTATAAG TGGGGTGGGG GGAGAGGAAG CGAAGCAGAT AGAGATAAGC   
  
  
- TTGTAACTAA TGATGCGTTT GGACTAAAAG AAATTAAAGG AGAGATGAGG AAAGGGAGAA GCAAATGAAA   
  
  
- GGAAGAGGTG AGGAAAGAGG GAGTAAGTTG AAGGGAAAAA GGGAAGAGAG AAAAACCGCA GGAAAAGTTA   
  
  
- GTGACGAAGC AACCAGTGAC GTCTCTCATA TGATGCAATG GTGATGCCAA GCCAAGCTTT CTCTTTCTTT   
  
  
- CTTTCCTTTC TTTCTTTCTT TCTTCTTTTA TCTCGGCATG CTTAATTTTC CTCACCACAA ATAAACAGAA   
  
  
- TGAAATCAAG TGGGCTTGAT ACTGATAGTG TACTGTGTTT GGGGGTGTGC ACAACAATTC AAAGTCTAAG   
  
  
- AATCAATCTC GTAGCTAGAG GGCTGCAATG CCGAGATGAT GGATTTTAAA AAAGATCAAA TTGCAAAAAA   
  
  
- CAAAAAACAA AAAACCAAAA ATGGTTGGCA TTACTGTTAG TACGATGCAA GTGGCTACTT TTATTCTATT   
  
  
- CTAATGCAAC TGGATCATTA CGTGGCACAG GCCTTTCCAC TTTTCTTTCT AATCCGAATA ATGTAATCAG   
  
  
- TAATCACAAT ACAAGAGTGG CATGATTGAT GATGAATTCC AGAAATCGTA TTGGTTGCGT CCGTCCCATG   
  
  
- ATGCTCTCTT TTGAAAAGTC AATGCTTCAA ACCTAACCTT CCATCATTTT CCATGTTTGC ACTTCCCATC   
  
  
- TAAACAACTG GCTCAAAATC AAATCTTTTT TTTTTTTTTT TTAAAAAAAA AAAAGGGAAG CGCTCATTTG   
  
  
- AAACTTTATA AAGTGTATGT ATATAGAAAA TAAATTTTGA TAACAAGAGA GAGTAAAATA ACAAGCTTTT   
  
  
- ATCATGATGA ACAACATACC TCAACAAAAT TAATTAAAAA AACAACAAGC ATTCATTTTG ATTTTTACTT   
  
  
- CTAAATCTAA AAGGATTACA ATTTAATAAA ATAAAACAAA TATATTTTTA AAATATTTTT CGTTTTACTT   
  
  
- TCAATAAAAA CAGTAAAATT TTAATTTAAG GATTACAATT TAATAAAATA AAACAAATAT ATTTTTAAAA   
  
  
- TATTTTTCGT TTTACTTTCA ATAAAAACAG TAAAATTTTA ATTTTGAAGC AAATTGATCA GATGACGTTA   
  
  
- AAAGATCTAG TTACATCCAC TAGACTTAAT CCACATAAAG CAATCTCCAT CTGCCACGTC TTAACACCAG   
  
  
- GACATAACTG TAATAACCAC GCCTCTGGAT AAATCCTTCC ACAGAGACCA GAAGGAAGCT ACTGAGTATG   
  
  
- ATTACTGTAC CACACTGTAC TCTACTGTAA TAACCACGCC TCTGGATAAA TCCTTCCACA GAGACCAGAA   
  
  
- GGAAGCTACT GAGTATGATT ACTGTACCA

+     Box 4

| Site Name | Organism | Position | Strand | Matrix score. | sequence | function |
| --- | --- | --- | --- | --- | --- | --- |
| Box 4 | Petroselinum crispum | 1011 | - | 6 | ATTAAT | part of a conserved DNA module involved in light responsiveness |

> 2018/04/13 10:10:12  
+ CTAGCTAGCT ACTATCCCAG TGACGGGTGT CTTGTGTCAC CACGACAAAT TAAAAAACCG GAACTAAAGC   
  
  
+ AACCCATCTT CTCGATCCCC TATAATATTC ACCCCACCCC CCTCTCCTTC GCTTCGTCTA TCTCTATTCG   
  
  
+ AACATTGATT ACTACGCAAA CCTGATTTTC TTTAATTTCC TCTCTACTCC TTTCCCTCTT CGTTTACTTT   
  
  
+ CCTTCTCCAC TCCTTTCTCC CTCATTCAAC TTCCCTTTTT CCCTTCTCTC TTTTTGGCGT CCTTTTCAAT   
  
  
+ CACTGCTTCG TTGGTCACTG CAGAGAGTAT ACTACGTTAC CACTACGGTT CGGTTCGAAA GAGAAAGAAA   
  
  
+ GAAAGGAAAG AAAGAAAGAA AGAAGAAAAT AGAGCCGTAC GAATTAAAAG GAGTGGTGTT TATTTGTCTT   
  
  
+ ACTTTAGTTC ACCCGAACTA TGACTATCAC ATGACACAAA CCCCCACACG TGTTGTTAAG TTTCAGATTC   
  
  
+ TTAGTTAGAG CATCGATCTC CCGACGTTAC GGCTCTACTA CCTAAAATTT TTTCTAGTTT AACGTTTTTT   
  
  
+ GTTTTTTGTT TTTTGGTTTT TACCAACCGT AATGACAATC ATGCTACGTT CACCGATGAA AATAAGATAA   
  
  
+ GATTACGTTG ACCTAGTAAT GCACCGTGTC CGGAAAGGTG AAAAGAAAGA TTAGGCTTAT TACATTAGTC   
  
  
+ ATTAGTGTTA TGTTCTCACC GTACTAACTA CTACTTAAGG TCTTTAGCAT AACCAACGCA GGCAGGGTAC   
  
  
+ TACGAGAGAA AACTTTTCAG TTACGAAGTT TGGATTGGAA GGTAGTAAAA GGTACAAACG TGAAGGGTAG   
  
  
+ ATTTGTTGAC CGAGTTTTAG TTTAGAAAAA AAAAAAAAAA AATTTTTTTT TTTTCCCTTC GCGAGTAAAC   
  
  
+ TTTGAAATAT TTCACATACA TATATCTTTT ATTTAAAACT ATTGTTCTCT CTCATTTTAT TGTTCGAAAA   
  
  
+ TAGTACTACT TGTTGTATGG AGTTGTTTTA ATTAATTTTT TTGTTGTTCG TAAGTAAAAC TAAAAATGAA   
  
  
+ GATTTAGATT TTCCTAATGT TAAATTATTT TATTTTGTTT ATATAAAAAT TTTATAAAAA GCAAAATGAA   
  
  
+ AGTTATTTTT GTCATTTTAA AATTAAATTC CTAATGTTAA ATTATTTTAT TTTGTTTATA TAAAAATTTT   
  
  
+ ATAAAAAGCA AAATGAAAGT TATTTTTGTC ATTTTAAAAT TAAAACTTCG TTTAACTAGT CTACTGCAAT   
  
  
+ TTTCTAGATC AATGTAGGTG ATCTGAATTA GGTGTATTTC GTTAGAGGTA GACGGTGCAG AATTGTGGTC   
  
  
+ CTGTATTGAC ATTATTGGTG CGGAGACCTA TTTAGGAAGG TGTCTCTGGT CTTCCTTCGA TGACTCATAC   
  
  
+ TAATGACATG GTGTGACATG AGATGACATT ATTGGTGCGG AGACCTATTT AGGAAGGTGT CTCTGGTCTT   
  
  
+ CCTTCGATGA CTCATACTAA TGACATGGT  

- GATCGATCGA TGATAGGGTC ACTGCCCACA GAACACAGTG GTGCTGTTTA ATTTTTTGGC CTTGATTTCG   
  
  
- TTGGGTAGAA GAGCTAGGGG ATATTATAAG TGGGGTGGGG GGAGAGGAAG CGAAGCAGAT AGAGATAAGC   
  
  
- TTGTAACTAA TGATGCGTTT GGACTAAAAG AAATTAAAGG AGAGATGAGG AAAGGGAGAA GCAAATGAAA   
  
  
- GGAAGAGGTG AGGAAAGAGG GAGTAAGTTG AAGGGAAAAA GGGAAGAGAG AAAAACCGCA GGAAAAGTTA   
  
  
- GTGACGAAGC AACCAGTGAC GTCTCTCATA TGATGCAATG GTGATGCCAA GCCAAGCTTT CTCTTTCTTT   
  
  
- CTTTCCTTTC TTTCTTTCTT TCTTCTTTTA TCTCGGCATG CTTAATTTTC CTCACCACAA ATAAACAGAA   
  
  
- TGAAATCAAG TGGGCTTGAT ACTGATAGTG TACTGTGTTT GGGGGTGTGC ACAACAATTC AAAGTCTAAG   
  
  
- AATCAATCTC GTAGCTAGAG GGCTGCAATG CCGAGATGAT GGATTTTAAA AAAGATCAAA TTGCAAAAAA   
  
  
- CAAAAAACAA AAAACCAAAA ATGGTTGGCA TTACTGTTAG TACGATGCAA GTGGCTACTT TTATTCTATT   
  
  
- CTAATGCAAC TGGATCATTA CGTGGCACAG GCCTTTCCAC TTTTCTTTCT AATCCGAATA ATGTAATCAG   
  
  
- TAATCACAAT ACAAGAGTGG CATGATTGAT GATGAATTCC AGAAATCGTA TTGGTTGCGT CCGTCCCATG   
  
  
- ATGCTCTCTT TTGAAAAGTC AATGCTTCAA ACCTAACCTT CCATCATTTT CCATGTTTGC ACTTCCCATC   
  
  
- TAAACAACTG GCTCAAAATC AAATCTTTTT TTTTTTTTTT TTAAAAAAAA AAAAGGGAAG CGCTCATTTG   
  
  
- AAACTTTATA AAGTGTATGT ATATAGAAAA TAAATTTTGA TAACAAGAGA GAGTAAAATA ACAAGCTTTT   
  
  
- ATCATGATGA ACAACATACC TCAACAAAAT TAATTAAAAA AACAACAAGC ATTCATTTTG ATTTTTACTT   
  
  
- CTAAATCTAA AAGGATTACA ATTTAATAAA ATAAAACAAA TATATTTTTA AAATATTTTT CGTTTTACTT   
  
  
- TCAATAAAAA CAGTAAAATT TTAATTTAAG GATTACAATT TAATAAAATA AAACAAATAT ATTTTTAAAA   
  
  
- TATTTTTCGT TTTACTTTCA ATAAAAACAG TAAAATTTTA ATTTTGAAGC AAATTGATCA GATGACGTTA   
  
  
- AAAGATCTAG TTACATCCAC TAGACTTAAT CCACATAAAG CAATCTCCAT CTGCCACGTC TTAACACCAG   
  
  
- GACATAACTG TAATAACCAC GCCTCTGGAT AAATCCTTCC ACAGAGACCA GAAGGAAGCT ACTGAGTATG   
  
  
- ATTACTGTAC CACACTGTAC TCTACTGTAA TAACCACGCC TCTGGATAAA TCCTTCCACA GAGACCAGAA   
  
  
- GGAAGCTACT GAGTATGATT ACTGTACCA

+     Box I

| Site Name | Organism | Position | Strand | Matrix score. | sequence | function |
| --- | --- | --- | --- | --- | --- | --- |
| Box I | Pisum sativum | 911 | - | 7 | TTTCAAA | light responsive element |

> 2018/04/13 10:10:12  
+ CTAGCTAGCT ACTATCCCAG TGACGGGTGT CTTGTGTCAC CACGACAAAT TAAAAAACCG GAACTAAAGC   
  
  
+ AACCCATCTT CTCGATCCCC TATAATATTC ACCCCACCCC CCTCTCCTTC GCTTCGTCTA TCTCTATTCG   
  
  
+ AACATTGATT ACTACGCAAA CCTGATTTTC TTTAATTTCC TCTCTACTCC TTTCCCTCTT CGTTTACTTT   
  
  
+ CCTTCTCCAC TCCTTTCTCC CTCATTCAAC TTCCCTTTTT CCCTTCTCTC TTTTTGGCGT CCTTTTCAAT   
  
  
+ CACTGCTTCG TTGGTCACTG CAGAGAGTAT ACTACGTTAC CACTACGGTT CGGTTCGAAA GAGAAAGAAA   
  
  
+ GAAAGGAAAG AAAGAAAGAA AGAAGAAAAT AGAGCCGTAC GAATTAAAAG GAGTGGTGTT TATTTGTCTT   
  
  
+ ACTTTAGTTC ACCCGAACTA TGACTATCAC ATGACACAAA CCCCCACACG TGTTGTTAAG TTTCAGATTC   
  
  
+ TTAGTTAGAG CATCGATCTC CCGACGTTAC GGCTCTACTA CCTAAAATTT TTTCTAGTTT AACGTTTTTT   
  
  
+ GTTTTTTGTT TTTTGGTTTT TACCAACCGT AATGACAATC ATGCTACGTT CACCGATGAA AATAAGATAA   
  
  
+ GATTACGTTG ACCTAGTAAT GCACCGTGTC CGGAAAGGTG AAAAGAAAGA TTAGGCTTAT TACATTAGTC   
  
  
+ ATTAGTGTTA TGTTCTCACC GTACTAACTA CTACTTAAGG TCTTTAGCAT AACCAACGCA GGCAGGGTAC   
  
  
+ TACGAGAGAA AACTTTTCAG TTACGAAGTT TGGATTGGAA GGTAGTAAAA GGTACAAACG TGAAGGGTAG   
  
  
+ ATTTGTTGAC CGAGTTTTAG TTTAGAAAAA AAAAAAAAAA AATTTTTTTT TTTTCCCTTC GCGAGTAAAC   
  
  
+ TTTGAAATAT TTCACATACA TATATCTTTT ATTTAAAACT ATTGTTCTCT CTCATTTTAT TGTTCGAAAA   
  
  
+ TAGTACTACT TGTTGTATGG AGTTGTTTTA ATTAATTTTT TTGTTGTTCG TAAGTAAAAC TAAAAATGAA   
  
  
+ GATTTAGATT TTCCTAATGT TAAATTATTT TATTTTGTTT ATATAAAAAT TTTATAAAAA GCAAAATGAA   
  
  
+ AGTTATTTTT GTCATTTTAA AATTAAATTC CTAATGTTAA ATTATTTTAT TTTGTTTATA TAAAAATTTT   
  
  
+ ATAAAAAGCA AAATGAAAGT TATTTTTGTC ATTTTAAAAT TAAAACTTCG TTTAACTAGT CTACTGCAAT   
  
  
+ TTTCTAGATC AATGTAGGTG ATCTGAATTA GGTGTATTTC GTTAGAGGTA GACGGTGCAG AATTGTGGTC   
  
  
+ CTGTATTGAC ATTATTGGTG CGGAGACCTA TTTAGGAAGG TGTCTCTGGT CTTCCTTCGA TGACTCATAC   
  
  
+ TAATGACATG GTGTGACATG AGATGACATT ATTGGTGCGG AGACCTATTT AGGAAGGTGT CTCTGGTCTT   
  
  
+ CCTTCGATGA CTCATACTAA TGACATGGT  

- GATCGATCGA TGATAGGGTC ACTGCCCACA GAACACAGTG GTGCTGTTTA ATTTTTTGGC CTTGATTTCG   
  
  
- TTGGGTAGAA GAGCTAGGGG ATATTATAAG TGGGGTGGGG GGAGAGGAAG CGAAGCAGAT AGAGATAAGC   
  
  
- TTGTAACTAA TGATGCGTTT GGACTAAAAG AAATTAAAGG AGAGATGAGG AAAGGGAGAA GCAAATGAAA   
  
  
- GGAAGAGGTG AGGAAAGAGG GAGTAAGTTG AAGGGAAAAA GGGAAGAGAG AAAAACCGCA GGAAAAGTTA   
  
  
- GTGACGAAGC AACCAGTGAC GTCTCTCATA TGATGCAATG GTGATGCCAA GCCAAGCTTT CTCTTTCTTT   
  
  
- CTTTCCTTTC TTTCTTTCTT TCTTCTTTTA TCTCGGCATG CTTAATTTTC CTCACCACAA ATAAACAGAA   
  
  
- TGAAATCAAG TGGGCTTGAT ACTGATAGTG TACTGTGTTT GGGGGTGTGC ACAACAATTC AAAGTCTAAG   
  
  
- AATCAATCTC GTAGCTAGAG GGCTGCAATG CCGAGATGAT GGATTTTAAA AAAGATCAAA TTGCAAAAAA   
  
  
- CAAAAAACAA AAAACCAAAA ATGGTTGGCA TTACTGTTAG TACGATGCAA GTGGCTACTT TTATTCTATT   
  
  
- CTAATGCAAC TGGATCATTA CGTGGCACAG GCCTTTCCAC TTTTCTTTCT AATCCGAATA ATGTAATCAG   
  
  
- TAATCACAAT ACAAGAGTGG CATGATTGAT GATGAATTCC AGAAATCGTA TTGGTTGCGT CCGTCCCATG   
  
  
- ATGCTCTCTT TTGAAAAGTC AATGCTTCAA ACCTAACCTT CCATCATTTT CCATGTTTGC ACTTCCCATC   
  
  
- TAAACAACTG GCTCAAAATC AAATCTTTTT TTTTTTTTTT TTAAAAAAAA AAAAGGGAAG CGCTCATTTG   
  
  
- AAACTTTATA AAGTGTATGT ATATAGAAAA TAAATTTTGA TAACAAGAGA GAGTAAAATA ACAAGCTTTT   
  
  
- ATCATGATGA ACAACATACC TCAACAAAAT TAATTAAAAA AACAACAAGC ATTCATTTTG ATTTTTACTT   
  
  
- CTAAATCTAA AAGGATTACA ATTTAATAAA ATAAAACAAA TATATTTTTA AAATATTTTT CGTTTTACTT   
  
  
- TCAATAAAAA CAGTAAAATT TTAATTTAAG GATTACAATT TAATAAAATA AAACAAATAT ATTTTTAAAA   
  
  
- TATTTTTCGT TTTACTTTCA ATAAAAACAG TAAAATTTTA ATTTTGAAGC AAATTGATCA GATGACGTTA   
  
  
- AAAGATCTAG TTACATCCAC TAGACTTAAT CCACATAAAG CAATCTCCAT CTGCCACGTC TTAACACCAG   
  
  
- GACATAACTG TAATAACCAC GCCTCTGGAT AAATCCTTCC ACAGAGACCA GAAGGAAGCT ACTGAGTATG   
  
  
- ATTACTGTAC CACACTGTAC TCTACTGTAA TAACCACGCC TCTGGATAAA TCCTTCCACA GAGACCAGAA   
  
  
- GGAAGCTACT GAGTATGATT ACTGTACCA

+     Box-W1

| Site Name | Organism | Position | Strand | Matrix score. | sequence | function |
| --- | --- | --- | --- | --- | --- | --- |
| Box-W1 | Petroselinum crispum | 846 | + | 6 | TTGACC | fungal elicitor responsive element |
| Box-W1 | Petroselinum crispum | 638 | + | 6 | TTGACC | fungal elicitor responsive element |

> 2018/04/13 10:10:12  
+ CTAGCTAGCT ACTATCCCAG TGACGGGTGT CTTGTGTCAC CACGACAAAT TAAAAAACCG GAACTAAAGC   
  
  
+ AACCCATCTT CTCGATCCCC TATAATATTC ACCCCACCCC CCTCTCCTTC GCTTCGTCTA TCTCTATTCG   
  
  
+ AACATTGATT ACTACGCAAA CCTGATTTTC TTTAATTTCC TCTCTACTCC TTTCCCTCTT CGTTTACTTT   
  
  
+ CCTTCTCCAC TCCTTTCTCC CTCATTCAAC TTCCCTTTTT CCCTTCTCTC TTTTTGGCGT CCTTTTCAAT   
  
  
+ CACTGCTTCG TTGGTCACTG CAGAGAGTAT ACTACGTTAC CACTACGGTT CGGTTCGAAA GAGAAAGAAA   
  
  
+ GAAAGGAAAG AAAGAAAGAA AGAAGAAAAT AGAGCCGTAC GAATTAAAAG GAGTGGTGTT TATTTGTCTT   
  
  
+ ACTTTAGTTC ACCCGAACTA TGACTATCAC ATGACACAAA CCCCCACACG TGTTGTTAAG TTTCAGATTC   
  
  
+ TTAGTTAGAG CATCGATCTC CCGACGTTAC GGCTCTACTA CCTAAAATTT TTTCTAGTTT AACGTTTTTT   
  
  
+ GTTTTTTGTT TTTTGGTTTT TACCAACCGT AATGACAATC ATGCTACGTT CACCGATGAA AATAAGATAA   
  
  
+ GATTACGTTG ACCTAGTAAT GCACCGTGTC CGGAAAGGTG AAAAGAAAGA TTAGGCTTAT TACATTAGTC   
  
  
+ ATTAGTGTTA TGTTCTCACC GTACTAACTA CTACTTAAGG TCTTTAGCAT AACCAACGCA GGCAGGGTAC   
  
  
+ TACGAGAGAA AACTTTTCAG TTACGAAGTT TGGATTGGAA GGTAGTAAAA GGTACAAACG TGAAGGGTAG   
  
  
+ ATTTGTTGAC CGAGTTTTAG TTTAGAAAAA AAAAAAAAAA AATTTTTTTT TTTTCCCTTC GCGAGTAAAC   
  
  
+ TTTGAAATAT TTCACATACA TATATCTTTT ATTTAAAACT ATTGTTCTCT CTCATTTTAT TGTTCGAAAA   
  
  
+ TAGTACTACT TGTTGTATGG AGTTGTTTTA ATTAATTTTT TTGTTGTTCG TAAGTAAAAC TAAAAATGAA   
  
  
+ GATTTAGATT TTCCTAATGT TAAATTATTT TATTTTGTTT ATATAAAAAT TTTATAAAAA GCAAAATGAA   
  
  
+ AGTTATTTTT GTCATTTTAA AATTAAATTC CTAATGTTAA ATTATTTTAT TTTGTTTATA TAAAAATTTT   
  
  
+ ATAAAAAGCA AAATGAAAGT TATTTTTGTC ATTTTAAAAT TAAAACTTCG TTTAACTAGT CTACTGCAAT   
  
  
+ TTTCTAGATC AATGTAGGTG ATCTGAATTA GGTGTATTTC GTTAGAGGTA GACGGTGCAG AATTGTGGTC   
  
  
+ CTGTATTGAC ATTATTGGTG CGGAGACCTA TTTAGGAAGG TGTCTCTGGT CTTCCTTCGA TGACTCATAC   
  
  
+ TAATGACATG GTGTGACATG AGATGACATT ATTGGTGCGG AGACCTATTT AGGAAGGTGT CTCTGGTCTT   
  
  
+ CCTTCGATGA CTCATACTAA TGACATGGT  

- GATCGATCGA TGATAGGGTC ACTGCCCACA GAACACAGTG GTGCTGTTTA ATTTTTTGGC CTTGATTTCG   
  
  
- TTGGGTAGAA GAGCTAGGGG ATATTATAAG TGGGGTGGGG GGAGAGGAAG CGAAGCAGAT AGAGATAAGC   
  
  
- TTGTAACTAA TGATGCGTTT GGACTAAAAG AAATTAAAGG AGAGATGAGG AAAGGGAGAA GCAAATGAAA   
  
  
- GGAAGAGGTG AGGAAAGAGG GAGTAAGTTG AAGGGAAAAA GGGAAGAGAG AAAAACCGCA GGAAAAGTTA   
  
  
- GTGACGAAGC AACCAGTGAC GTCTCTCATA TGATGCAATG GTGATGCCAA GCCAAGCTTT CTCTTTCTTT   
  
  
- CTTTCCTTTC TTTCTTTCTT TCTTCTTTTA TCTCGGCATG CTTAATTTTC CTCACCACAA ATAAACAGAA   
  
  
- TGAAATCAAG TGGGCTTGAT ACTGATAGTG TACTGTGTTT GGGGGTGTGC ACAACAATTC AAAGTCTAAG   
  
  
- AATCAATCTC GTAGCTAGAG GGCTGCAATG CCGAGATGAT GGATTTTAAA AAAGATCAAA TTGCAAAAAA   
  
  
- CAAAAAACAA AAAACCAAAA ATGGTTGGCA TTACTGTTAG TACGATGCAA GTGGCTACTT TTATTCTATT   
  
  
- CTAATGCAAC TGGATCATTA CGTGGCACAG GCCTTTCCAC TTTTCTTTCT AATCCGAATA ATGTAATCAG   
  
  
- TAATCACAAT ACAAGAGTGG CATGATTGAT GATGAATTCC AGAAATCGTA TTGGTTGCGT CCGTCCCATG   
  
  
- ATGCTCTCTT TTGAAAAGTC AATGCTTCAA ACCTAACCTT CCATCATTTT CCATGTTTGC ACTTCCCATC   
  
  
- TAAACAACTG GCTCAAAATC AAATCTTTTT TTTTTTTTTT TTAAAAAAAA AAAAGGGAAG CGCTCATTTG   
  
  
- AAACTTTATA AAGTGTATGT ATATAGAAAA TAAATTTTGA TAACAAGAGA GAGTAAAATA ACAAGCTTTT   
  
  
- ATCATGATGA ACAACATACC TCAACAAAAT TAATTAAAAA AACAACAAGC ATTCATTTTG ATTTTTACTT   
  
  
- CTAAATCTAA AAGGATTACA ATTTAATAAA ATAAAACAAA TATATTTTTA AAATATTTTT CGTTTTACTT   
  
  
- TCAATAAAAA CAGTAAAATT TTAATTTAAG GATTACAATT TAATAAAATA AAACAAATAT ATTTTTAAAA   
  
  
- TATTTTTCGT TTTACTTTCA ATAAAAACAG TAAAATTTTA ATTTTGAAGC AAATTGATCA GATGACGTTA   
  
  
- AAAGATCTAG TTACATCCAC TAGACTTAAT CCACATAAAG CAATCTCCAT CTGCCACGTC TTAACACCAG   
  
  
- GACATAACTG TAATAACCAC GCCTCTGGAT AAATCCTTCC ACAGAGACCA GAAGGAAGCT ACTGAGTATG   
  
  
- ATTACTGTAC CACACTGTAC TCTACTGTAA TAACCACGCC TCTGGATAAA TCCTTCCACA GAGACCAGAA   
  
  
- GGAAGCTACT GAGTATGATT ACTGTACCA

+     CAAT-box

| Site Name | Organism | Position | Strand | Matrix score. | sequence | function |
| --- | --- | --- | --- | --- | --- | --- |
| CAAT-box | Arabidopsis thaliana | 1431 | - | 5 | CCAAT | common cis-acting element in promoter and enhancer regions |
| CAAT-box | Hordeum vulgare | 1270 | + | 4 | CAAT | common cis-acting element in promoter and enhancer regions |
| CAAT-box | Glycine max | 1321 | - | 5 | CAATT | common cis-acting element in promoter and enhancer regions |
| CAAT-box | Glycine max | 1257 | + | 5 | CAATT | common cis-acting element in promoter and enhancer regions |
| CAAT-box | Hordeum vulgare | 969 | - | 4 | CAAT | common cis-acting element in promoter and enhancer regions |
| CAAT-box | Hordeum vulgare | 951 | - | 4 | CAAT | common cis-acting element in promoter and enhancer regions |
| CAAT-box | Arabidopsis thaliana | 1344 | - | 5 | CCAAT | common cis-acting element in promoter and enhancer regions |
| CAAT-box | Hordeum vulgare | 1322 | - | 4 | CAAT | common cis-acting element in promoter and enhancer regions |
| CAAT-box | Hordeum vulgare | 1335 | - | 4 | CAAT | common cis-acting element in promoter and enhancer regions |
| CAAT-box | Brassica rapa | 412 | - | 5 | CAAAT | common cis-acting element in promoter and enhancer regions |
| CAAT-box | Brassica rapa | 841 | - | 5 | CAAAT | common cis-acting element in promoter and enhancer regions |
| CAAT-box | Hordeum vulgare | 596 | + | 4 | CAAT | common cis-acting element in promoter and enhancer regions |
| CAAT-box | Arabidopsis thaliana | 804 | - | 5 | CCAAT | common cis-acting element in promoter and enhancer regions |
| CAAT-box | Hordeum vulgare | 277 | + | 4 | CAAT | common cis-acting element in promoter and enhancer regions |
| CAAT-box | Hordeum vulgare | 144 | - | 4 | CAAT | common cis-acting element in promoter and enhancer regions |
| CAAT-box | Brassica rapa | 46 | + | 5 | CAAAT | common cis-acting element in promoter and enhancer regions |

> 2018/04/13 10:10:12  
+ CTAGCTAGCT ACTATCCCAG TGACGGGTGT CTTGTGTCAC CACGACAAAT TAAAAAACCG GAACTAAAGC   
  
  
+ AACCCATCTT CTCGATCCCC TATAATATTC ACCCCACCCC CCTCTCCTTC GCTTCGTCTA TCTCTATTCG   
  
  
+ AACATTGATT ACTACGCAAA CCTGATTTTC TTTAATTTCC TCTCTACTCC TTTCCCTCTT CGTTTACTTT   
  
  
+ CCTTCTCCAC TCCTTTCTCC CTCATTCAAC TTCCCTTTTT CCCTTCTCTC TTTTTGGCGT CCTTTTCAAT   
  
  
+ CACTGCTTCG TTGGTCACTG CAGAGAGTAT ACTACGTTAC CACTACGGTT CGGTTCGAAA GAGAAAGAAA   
  
  
+ GAAAGGAAAG AAAGAAAGAA AGAAGAAAAT AGAGCCGTAC GAATTAAAAG GAGTGGTGTT TATTTGTCTT   
  
  
+ ACTTTAGTTC ACCCGAACTA TGACTATCAC ATGACACAAA CCCCCACACG TGTTGTTAAG TTTCAGATTC   
  
  
+ TTAGTTAGAG CATCGATCTC CCGACGTTAC GGCTCTACTA CCTAAAATTT TTTCTAGTTT AACGTTTTTT   
  
  
+ GTTTTTTGTT TTTTGGTTTT TACCAACCGT AATGACAATC ATGCTACGTT CACCGATGAA AATAAGATAA   
  
  
+ GATTACGTTG ACCTAGTAAT GCACCGTGTC CGGAAAGGTG AAAAGAAAGA TTAGGCTTAT TACATTAGTC   
  
  
+ ATTAGTGTTA TGTTCTCACC GTACTAACTA CTACTTAAGG TCTTTAGCAT AACCAACGCA GGCAGGGTAC   
  
  
+ TACGAGAGAA AACTTTTCAG TTACGAAGTT TGGATTGGAA GGTAGTAAAA GGTACAAACG TGAAGGGTAG   
  
  
+ ATTTGTTGAC CGAGTTTTAG TTTAGAAAAA AAAAAAAAAA AATTTTTTTT TTTTCCCTTC GCGAGTAAAC   
  
  
+ TTTGAAATAT TTCACATACA TATATCTTTT ATTTAAAACT ATTGTTCTCT CTCATTTTAT TGTTCGAAAA   
  
  
+ TAGTACTACT TGTTGTATGG AGTTGTTTTA ATTAATTTTT TTGTTGTTCG TAAGTAAAAC TAAAAATGAA   
  
  
+ GATTTAGATT TTCCTAATGT TAAATTATTT TATTTTGTTT ATATAAAAAT TTTATAAAAA GCAAAATGAA   
  
  
+ AGTTATTTTT GTCATTTTAA AATTAAATTC CTAATGTTAA ATTATTTTAT TTTGTTTATA TAAAAATTTT   
  
  
+ ATAAAAAGCA AAATGAAAGT TATTTTTGTC ATTTTAAAAT TAAAACTTCG TTTAACTAGT CTACTGCAAT   
  
  
+ TTTCTAGATC AATGTAGGTG ATCTGAATTA GGTGTATTTC GTTAGAGGTA GACGGTGCAG AATTGTGGTC   
  
  
+ CTGTATTGAC ATTATTGGTG CGGAGACCTA TTTAGGAAGG TGTCTCTGGT CTTCCTTCGA TGACTCATAC   
  
  
+ TAATGACATG GTGTGACATG AGATGACATT ATTGGTGCGG AGACCTATTT AGGAAGGTGT CTCTGGTCTT   
  
  
+ CCTTCGATGA CTCATACTAA TGACATGGT  

- GATCGATCGA TGATAGGGTC ACTGCCCACA GAACACAGTG GTGCTGTTTA ATTTTTTGGC CTTGATTTCG   
  
  
- TTGGGTAGAA GAGCTAGGGG ATATTATAAG TGGGGTGGGG GGAGAGGAAG CGAAGCAGAT AGAGATAAGC   
  
  
- TTGTAACTAA TGATGCGTTT GGACTAAAAG AAATTAAAGG AGAGATGAGG AAAGGGAGAA GCAAATGAAA   
  
  
- GGAAGAGGTG AGGAAAGAGG GAGTAAGTTG AAGGGAAAAA GGGAAGAGAG AAAAACCGCA GGAAAAGTTA   
  
  
- GTGACGAAGC AACCAGTGAC GTCTCTCATA TGATGCAATG GTGATGCCAA GCCAAGCTTT CTCTTTCTTT   
  
  
- CTTTCCTTTC TTTCTTTCTT TCTTCTTTTA TCTCGGCATG CTTAATTTTC CTCACCACAA ATAAACAGAA   
  
  
- TGAAATCAAG TGGGCTTGAT ACTGATAGTG TACTGTGTTT GGGGGTGTGC ACAACAATTC AAAGTCTAAG   
  
  
- AATCAATCTC GTAGCTAGAG GGCTGCAATG CCGAGATGAT GGATTTTAAA AAAGATCAAA TTGCAAAAAA   
  
  
- CAAAAAACAA AAAACCAAAA ATGGTTGGCA TTACTGTTAG TACGATGCAA GTGGCTACTT TTATTCTATT   
  
  
- CTAATGCAAC TGGATCATTA CGTGGCACAG GCCTTTCCAC TTTTCTTTCT AATCCGAATA ATGTAATCAG   
  
  
- TAATCACAAT ACAAGAGTGG CATGATTGAT GATGAATTCC AGAAATCGTA TTGGTTGCGT CCGTCCCATG   
  
  
- ATGCTCTCTT TTGAAAAGTC AATGCTTCAA ACCTAACCTT CCATCATTTT CCATGTTTGC ACTTCCCATC   
  
  
- TAAACAACTG GCTCAAAATC AAATCTTTTT TTTTTTTTTT TTAAAAAAAA AAAAGGGAAG CGCTCATTTG   
  
  
- AAACTTTATA AAGTGTATGT ATATAGAAAA TAAATTTTGA TAACAAGAGA GAGTAAAATA ACAAGCTTTT   
  
  
- ATCATGATGA ACAACATACC TCAACAAAAT TAATTAAAAA AACAACAAGC ATTCATTTTG ATTTTTACTT   
  
  
- CTAAATCTAA AAGGATTACA ATTTAATAAA ATAAAACAAA TATATTTTTA AAATATTTTT CGTTTTACTT   
  
  
- TCAATAAAAA CAGTAAAATT TTAATTTAAG GATTACAATT TAATAAAATA AAACAAATAT ATTTTTAAAA   
  
  
- TATTTTTCGT TTTACTTTCA ATAAAAACAG TAAAATTTTA ATTTTGAAGC AAATTGATCA GATGACGTTA   
  
  
- AAAGATCTAG TTACATCCAC TAGACTTAAT CCACATAAAG CAATCTCCAT CTGCCACGTC TTAACACCAG   
  
  
- GACATAACTG TAATAACCAC GCCTCTGGAT AAATCCTTCC ACAGAGACCA GAAGGAAGCT ACTGAGTATG   
  
  
- ATTACTGTAC CACACTGTAC TCTACTGTAA TAACCACGCC TCTGGATAAA TCCTTCCACA GAGACCAGAA   
  
  
- GGAAGCTACT GAGTATGATT ACTGTACCA

+     CE3

| Site Name | Organism | Position | Strand | Matrix score. | sequence | function |
| --- | --- | --- | --- | --- | --- | --- |
| CE3 | Oryza sativa | 22 | + | 9 | GACGCGTGTC | cis-acting element involved in ABA and VP1 responsiveness |

> 2018/04/13 10:10:12  
+ CTAGCTAGCT ACTATCCCAG TGACGGGTGT CTTGTGTCAC CACGACAAAT TAAAAAACCG GAACTAAAGC   
  
  
+ AACCCATCTT CTCGATCCCC TATAATATTC ACCCCACCCC CCTCTCCTTC GCTTCGTCTA TCTCTATTCG   
  
  
+ AACATTGATT ACTACGCAAA CCTGATTTTC TTTAATTTCC TCTCTACTCC TTTCCCTCTT CGTTTACTTT   
  
  
+ CCTTCTCCAC TCCTTTCTCC CTCATTCAAC TTCCCTTTTT CCCTTCTCTC TTTTTGGCGT CCTTTTCAAT   
  
  
+ CACTGCTTCG TTGGTCACTG CAGAGAGTAT ACTACGTTAC CACTACGGTT CGGTTCGAAA GAGAAAGAAA   
  
  
+ GAAAGGAAAG AAAGAAAGAA AGAAGAAAAT AGAGCCGTAC GAATTAAAAG GAGTGGTGTT TATTTGTCTT   
  
  
+ ACTTTAGTTC ACCCGAACTA TGACTATCAC ATGACACAAA CCCCCACACG TGTTGTTAAG TTTCAGATTC   
  
  
+ TTAGTTAGAG CATCGATCTC CCGACGTTAC GGCTCTACTA CCTAAAATTT TTTCTAGTTT AACGTTTTTT   
  
  
+ GTTTTTTGTT TTTTGGTTTT TACCAACCGT AATGACAATC ATGCTACGTT CACCGATGAA AATAAGATAA   
  
  
+ GATTACGTTG ACCTAGTAAT GCACCGTGTC CGGAAAGGTG AAAAGAAAGA TTAGGCTTAT TACATTAGTC   
  
  
+ ATTAGTGTTA TGTTCTCACC GTACTAACTA CTACTTAAGG TCTTTAGCAT AACCAACGCA GGCAGGGTAC   
  
  
+ TACGAGAGAA AACTTTTCAG TTACGAAGTT TGGATTGGAA GGTAGTAAAA GGTACAAACG TGAAGGGTAG   
  
  
+ ATTTGTTGAC CGAGTTTTAG TTTAGAAAAA AAAAAAAAAA AATTTTTTTT TTTTCCCTTC GCGAGTAAAC   
  
  
+ TTTGAAATAT TTCACATACA TATATCTTTT ATTTAAAACT ATTGTTCTCT CTCATTTTAT TGTTCGAAAA   
  
  
+ TAGTACTACT TGTTGTATGG AGTTGTTTTA ATTAATTTTT TTGTTGTTCG TAAGTAAAAC TAAAAATGAA   
  
  
+ GATTTAGATT TTCCTAATGT TAAATTATTT TATTTTGTTT ATATAAAAAT TTTATAAAAA GCAAAATGAA   
  
  
+ AGTTATTTTT GTCATTTTAA AATTAAATTC CTAATGTTAA ATTATTTTAT TTTGTTTATA TAAAAATTTT   
  
  
+ ATAAAAAGCA AAATGAAAGT TATTTTTGTC ATTTTAAAAT TAAAACTTCG TTTAACTAGT CTACTGCAAT   
  
  
+ TTTCTAGATC AATGTAGGTG ATCTGAATTA GGTGTATTTC GTTAGAGGTA GACGGTGCAG AATTGTGGTC   
  
  
+ CTGTATTGAC ATTATTGGTG CGGAGACCTA TTTAGGAAGG TGTCTCTGGT CTTCCTTCGA TGACTCATAC   
  
  
+ TAATGACATG GTGTGACATG AGATGACATT ATTGGTGCGG AGACCTATTT AGGAAGGTGT CTCTGGTCTT   
  
  
+ CCTTCGATGA CTCATACTAA TGACATGGT  

- GATCGATCGA TGATAGGGTC ACTGCCCACA GAACACAGTG GTGCTGTTTA ATTTTTTGGC CTTGATTTCG   
  
  
- TTGGGTAGAA GAGCTAGGGG ATATTATAAG TGGGGTGGGG GGAGAGGAAG CGAAGCAGAT AGAGATAAGC   
  
  
- TTGTAACTAA TGATGCGTTT GGACTAAAAG AAATTAAAGG AGAGATGAGG AAAGGGAGAA GCAAATGAAA   
  
  
- GGAAGAGGTG AGGAAAGAGG GAGTAAGTTG AAGGGAAAAA GGGAAGAGAG AAAAACCGCA GGAAAAGTTA   
  
  
- GTGACGAAGC AACCAGTGAC GTCTCTCATA TGATGCAATG GTGATGCCAA GCCAAGCTTT CTCTTTCTTT   
  
  
- CTTTCCTTTC TTTCTTTCTT TCTTCTTTTA TCTCGGCATG CTTAATTTTC CTCACCACAA ATAAACAGAA   
  
  
- TGAAATCAAG TGGGCTTGAT ACTGATAGTG TACTGTGTTT GGGGGTGTGC ACAACAATTC AAAGTCTAAG   
  
  
- AATCAATCTC GTAGCTAGAG GGCTGCAATG CCGAGATGAT GGATTTTAAA AAAGATCAAA TTGCAAAAAA   
  
  
- CAAAAAACAA AAAACCAAAA ATGGTTGGCA TTACTGTTAG TACGATGCAA GTGGCTACTT TTATTCTATT   
  
  
- CTAATGCAAC TGGATCATTA CGTGGCACAG GCCTTTCCAC TTTTCTTTCT AATCCGAATA ATGTAATCAG   
  
  
- TAATCACAAT ACAAGAGTGG CATGATTGAT GATGAATTCC AGAAATCGTA TTGGTTGCGT CCGTCCCATG   
  
  
- ATGCTCTCTT TTGAAAAGTC AATGCTTCAA ACCTAACCTT CCATCATTTT CCATGTTTGC ACTTCCCATC   
  
  
- TAAACAACTG GCTCAAAATC AAATCTTTTT TTTTTTTTTT TTAAAAAAAA AAAAGGGAAG CGCTCATTTG   
  
  
- AAACTTTATA AAGTGTATGT ATATAGAAAA TAAATTTTGA TAACAAGAGA GAGTAAAATA ACAAGCTTTT   
  
  
- ATCATGATGA ACAACATACC TCAACAAAAT TAATTAAAAA AACAACAAGC ATTCATTTTG ATTTTTACTT   
  
  
- CTAAATCTAA AAGGATTACA ATTTAATAAA ATAAAACAAA TATATTTTTA AAATATTTTT CGTTTTACTT   
  
  
- TCAATAAAAA CAGTAAAATT TTAATTTAAG GATTACAATT TAATAAAATA AAACAAATAT ATTTTTAAAA   
  
  
- TATTTTTCGT TTTACTTTCA ATAAAAACAG TAAAATTTTA ATTTTGAAGC AAATTGATCA GATGACGTTA   
  
  
- AAAGATCTAG TTACATCCAC TAGACTTAAT CCACATAAAG CAATCTCCAT CTGCCACGTC TTAACACCAG   
  
  
- GACATAACTG TAATAACCAC GCCTCTGGAT AAATCCTTCC ACAGAGACCA GAAGGAAGCT ACTGAGTATG   
  
  
- ATTACTGTAC CACACTGTAC TCTACTGTAA TAACCACGCC TCTGGATAAA TCCTTCCACA GAGACCAGAA   
  
  
- GGAAGCTACT GAGTATGATT ACTGTACCA

+     CGTCA-motif

| Site Name | Organism | Position | Strand | Matrix score. | sequence | function |
| --- | --- | --- | --- | --- | --- | --- |
| CGTCA-motif | Hordeum vulgare | 21 | - | 5 | CGTCA | cis-acting regulatory element involved in the MeJA-responsiveness |

> 2018/04/13 10:10:12  
+ CTAGCTAGCT ACTATCCCAG TGACGGGTGT CTTGTGTCAC CACGACAAAT TAAAAAACCG GAACTAAAGC   
  
  
+ AACCCATCTT CTCGATCCCC TATAATATTC ACCCCACCCC CCTCTCCTTC GCTTCGTCTA TCTCTATTCG   
  
  
+ AACATTGATT ACTACGCAAA CCTGATTTTC TTTAATTTCC TCTCTACTCC TTTCCCTCTT CGTTTACTTT   
  
  
+ CCTTCTCCAC TCCTTTCTCC CTCATTCAAC TTCCCTTTTT CCCTTCTCTC TTTTTGGCGT CCTTTTCAAT   
  
  
+ CACTGCTTCG TTGGTCACTG CAGAGAGTAT ACTACGTTAC CACTACGGTT CGGTTCGAAA GAGAAAGAAA   
  
  
+ GAAAGGAAAG AAAGAAAGAA AGAAGAAAAT AGAGCCGTAC GAATTAAAAG GAGTGGTGTT TATTTGTCTT   
  
  
+ ACTTTAGTTC ACCCGAACTA TGACTATCAC ATGACACAAA CCCCCACACG TGTTGTTAAG TTTCAGATTC   
  
  
+ TTAGTTAGAG CATCGATCTC CCGACGTTAC GGCTCTACTA CCTAAAATTT TTTCTAGTTT AACGTTTTTT   
  
  
+ GTTTTTTGTT TTTTGGTTTT TACCAACCGT AATGACAATC ATGCTACGTT CACCGATGAA AATAAGATAA   
  
  
+ GATTACGTTG ACCTAGTAAT GCACCGTGTC CGGAAAGGTG AAAAGAAAGA TTAGGCTTAT TACATTAGTC   
  
  
+ ATTAGTGTTA TGTTCTCACC GTACTAACTA CTACTTAAGG TCTTTAGCAT AACCAACGCA GGCAGGGTAC   
  
  
+ TACGAGAGAA AACTTTTCAG TTACGAAGTT TGGATTGGAA GGTAGTAAAA GGTACAAACG TGAAGGGTAG   
  
  
+ ATTTGTTGAC CGAGTTTTAG TTTAGAAAAA AAAAAAAAAA AATTTTTTTT TTTTCCCTTC GCGAGTAAAC   
  
  
+ TTTGAAATAT TTCACATACA TATATCTTTT ATTTAAAACT ATTGTTCTCT CTCATTTTAT TGTTCGAAAA   
  
  
+ TAGTACTACT TGTTGTATGG AGTTGTTTTA ATTAATTTTT TTGTTGTTCG TAAGTAAAAC TAAAAATGAA   
  
  
+ GATTTAGATT TTCCTAATGT TAAATTATTT TATTTTGTTT ATATAAAAAT TTTATAAAAA GCAAAATGAA   
  
  
+ AGTTATTTTT GTCATTTTAA AATTAAATTC CTAATGTTAA ATTATTTTAT TTTGTTTATA TAAAAATTTT   
  
  
+ ATAAAAAGCA AAATGAAAGT TATTTTTGTC ATTTTAAAAT TAAAACTTCG TTTAACTAGT CTACTGCAAT   
  
  
+ TTTCTAGATC AATGTAGGTG ATCTGAATTA GGTGTATTTC GTTAGAGGTA GACGGTGCAG AATTGTGGTC   
  
  
+ CTGTATTGAC ATTATTGGTG CGGAGACCTA TTTAGGAAGG TGTCTCTGGT CTTCCTTCGA TGACTCATAC   
  
  
+ TAATGACATG GTGTGACATG AGATGACATT ATTGGTGCGG AGACCTATTT AGGAAGGTGT CTCTGGTCTT   
  
  
+ CCTTCGATGA CTCATACTAA TGACATGGT  

- GATCGATCGA TGATAGGGTC ACTGCCCACA GAACACAGTG GTGCTGTTTA ATTTTTTGGC CTTGATTTCG   
  
  
- TTGGGTAGAA GAGCTAGGGG ATATTATAAG TGGGGTGGGG GGAGAGGAAG CGAAGCAGAT AGAGATAAGC   
  
  
- TTGTAACTAA TGATGCGTTT GGACTAAAAG AAATTAAAGG AGAGATGAGG AAAGGGAGAA GCAAATGAAA   
  
  
- GGAAGAGGTG AGGAAAGAGG GAGTAAGTTG AAGGGAAAAA GGGAAGAGAG AAAAACCGCA GGAAAAGTTA   
  
  
- GTGACGAAGC AACCAGTGAC GTCTCTCATA TGATGCAATG GTGATGCCAA GCCAAGCTTT CTCTTTCTTT   
  
  
- CTTTCCTTTC TTTCTTTCTT TCTTCTTTTA TCTCGGCATG CTTAATTTTC CTCACCACAA ATAAACAGAA   
  
  
- TGAAATCAAG TGGGCTTGAT ACTGATAGTG TACTGTGTTT GGGGGTGTGC ACAACAATTC AAAGTCTAAG   
  
  
- AATCAATCTC GTAGCTAGAG GGCTGCAATG CCGAGATGAT GGATTTTAAA AAAGATCAAA TTGCAAAAAA   
  
  
- CAAAAAACAA AAAACCAAAA ATGGTTGGCA TTACTGTTAG TACGATGCAA GTGGCTACTT TTATTCTATT   
  
  
- CTAATGCAAC TGGATCATTA CGTGGCACAG GCCTTTCCAC TTTTCTTTCT AATCCGAATA ATGTAATCAG   
  
  
- TAATCACAAT ACAAGAGTGG CATGATTGAT GATGAATTCC AGAAATCGTA TTGGTTGCGT CCGTCCCATG   
  
  
- ATGCTCTCTT TTGAAAAGTC AATGCTTCAA ACCTAACCTT CCATCATTTT CCATGTTTGC ACTTCCCATC   
  
  
- TAAACAACTG GCTCAAAATC AAATCTTTTT TTTTTTTTTT TTAAAAAAAA AAAAGGGAAG CGCTCATTTG   
  
  
- AAACTTTATA AAGTGTATGT ATATAGAAAA TAAATTTTGA TAACAAGAGA GAGTAAAATA ACAAGCTTTT   
  
  
- ATCATGATGA ACAACATACC TCAACAAAAT TAATTAAAAA AACAACAAGC ATTCATTTTG ATTTTTACTT   
  
  
- CTAAATCTAA AAGGATTACA ATTTAATAAA ATAAAACAAA TATATTTTTA AAATATTTTT CGTTTTACTT   
  
  
- TCAATAAAAA CAGTAAAATT TTAATTTAAG GATTACAATT TAATAAAATA AAACAAATAT ATTTTTAAAA   
  
  
- TATTTTTCGT TTTACTTTCA ATAAAAACAG TAAAATTTTA ATTTTGAAGC AAATTGATCA GATGACGTTA   
  
  
- AAAGATCTAG TTACATCCAC TAGACTTAAT CCACATAAAG CAATCTCCAT CTGCCACGTC TTAACACCAG   
  
  
- GACATAACTG TAATAACCAC GCCTCTGGAT AAATCCTTCC ACAGAGACCA GAAGGAAGCT ACTGAGTATG   
  
  
- ATTACTGTAC CACACTGTAC TCTACTGTAA TAACCACGCC TCTGGATAAA TCCTTCCACA GAGACCAGAA   
  
  
- GGAAGCTACT GAGTATGATT ACTGTACCA

+     ERE

| Site Name | Organism | Position | Strand | Matrix score. | sequence | function |
| --- | --- | --- | --- | --- | --- | --- |
| ERE | Dianthus caryophyllus | 911 | - | 8 | ATTTCAAA | ethylene-responsive element |

> 2018/04/13 10:10:12  
+ CTAGCTAGCT ACTATCCCAG TGACGGGTGT CTTGTGTCAC CACGACAAAT TAAAAAACCG GAACTAAAGC   
  
  
+ AACCCATCTT CTCGATCCCC TATAATATTC ACCCCACCCC CCTCTCCTTC GCTTCGTCTA TCTCTATTCG   
  
  
+ AACATTGATT ACTACGCAAA CCTGATTTTC TTTAATTTCC TCTCTACTCC TTTCCCTCTT CGTTTACTTT   
  
  
+ CCTTCTCCAC TCCTTTCTCC CTCATTCAAC TTCCCTTTTT CCCTTCTCTC TTTTTGGCGT CCTTTTCAAT   
  
  
+ CACTGCTTCG TTGGTCACTG CAGAGAGTAT ACTACGTTAC CACTACGGTT CGGTTCGAAA GAGAAAGAAA   
  
  
+ GAAAGGAAAG AAAGAAAGAA AGAAGAAAAT AGAGCCGTAC GAATTAAAAG GAGTGGTGTT TATTTGTCTT   
  
  
+ ACTTTAGTTC ACCCGAACTA TGACTATCAC ATGACACAAA CCCCCACACG TGTTGTTAAG TTTCAGATTC   
  
  
+ TTAGTTAGAG CATCGATCTC CCGACGTTAC GGCTCTACTA CCTAAAATTT TTTCTAGTTT AACGTTTTTT   
  
  
+ GTTTTTTGTT TTTTGGTTTT TACCAACCGT AATGACAATC ATGCTACGTT CACCGATGAA AATAAGATAA   
  
  
+ GATTACGTTG ACCTAGTAAT GCACCGTGTC CGGAAAGGTG AAAAGAAAGA TTAGGCTTAT TACATTAGTC   
  
  
+ ATTAGTGTTA TGTTCTCACC GTACTAACTA CTACTTAAGG TCTTTAGCAT AACCAACGCA GGCAGGGTAC   
  
  
+ TACGAGAGAA AACTTTTCAG TTACGAAGTT TGGATTGGAA GGTAGTAAAA GGTACAAACG TGAAGGGTAG   
  
  
+ ATTTGTTGAC CGAGTTTTAG TTTAGAAAAA AAAAAAAAAA AATTTTTTTT TTTTCCCTTC GCGAGTAAAC   
  
  
+ TTTGAAATAT TTCACATACA TATATCTTTT ATTTAAAACT ATTGTTCTCT CTCATTTTAT TGTTCGAAAA   
  
  
+ TAGTACTACT TGTTGTATGG AGTTGTTTTA ATTAATTTTT TTGTTGTTCG TAAGTAAAAC TAAAAATGAA   
  
  
+ GATTTAGATT TTCCTAATGT TAAATTATTT TATTTTGTTT ATATAAAAAT TTTATAAAAA GCAAAATGAA   
  
  
+ AGTTATTTTT GTCATTTTAA AATTAAATTC CTAATGTTAA ATTATTTTAT TTTGTTTATA TAAAAATTTT   
  
  
+ ATAAAAAGCA AAATGAAAGT TATTTTTGTC ATTTTAAAAT TAAAACTTCG TTTAACTAGT CTACTGCAAT   
  
  
+ TTTCTAGATC AATGTAGGTG ATCTGAATTA GGTGTATTTC GTTAGAGGTA GACGGTGCAG AATTGTGGTC   
  
  
+ CTGTATTGAC ATTATTGGTG CGGAGACCTA TTTAGGAAGG TGTCTCTGGT CTTCCTTCGA TGACTCATAC   
  
  
+ TAATGACATG GTGTGACATG AGATGACATT ATTGGTGCGG AGACCTATTT AGGAAGGTGT CTCTGGTCTT   
  
  
+ CCTTCGATGA CTCATACTAA TGACATGGT  

- GATCGATCGA TGATAGGGTC ACTGCCCACA GAACACAGTG GTGCTGTTTA ATTTTTTGGC CTTGATTTCG   
  
  
- TTGGGTAGAA GAGCTAGGGG ATATTATAAG TGGGGTGGGG GGAGAGGAAG CGAAGCAGAT AGAGATAAGC   
  
  
- TTGTAACTAA TGATGCGTTT GGACTAAAAG AAATTAAAGG AGAGATGAGG AAAGGGAGAA GCAAATGAAA   
  
  
- GGAAGAGGTG AGGAAAGAGG GAGTAAGTTG AAGGGAAAAA GGGAAGAGAG AAAAACCGCA GGAAAAGTTA   
  
  
- GTGACGAAGC AACCAGTGAC GTCTCTCATA TGATGCAATG GTGATGCCAA GCCAAGCTTT CTCTTTCTTT   
  
  
- CTTTCCTTTC TTTCTTTCTT TCTTCTTTTA TCTCGGCATG CTTAATTTTC CTCACCACAA ATAAACAGAA   
  
  
- TGAAATCAAG TGGGCTTGAT ACTGATAGTG TACTGTGTTT GGGGGTGTGC ACAACAATTC AAAGTCTAAG   
  
  
- AATCAATCTC GTAGCTAGAG GGCTGCAATG CCGAGATGAT GGATTTTAAA AAAGATCAAA TTGCAAAAAA   
  
  
- CAAAAAACAA AAAACCAAAA ATGGTTGGCA TTACTGTTAG TACGATGCAA GTGGCTACTT TTATTCTATT   
  
  
- CTAATGCAAC TGGATCATTA CGTGGCACAG GCCTTTCCAC TTTTCTTTCT AATCCGAATA ATGTAATCAG   
  
  
- TAATCACAAT ACAAGAGTGG CATGATTGAT GATGAATTCC AGAAATCGTA TTGGTTGCGT CCGTCCCATG   
  
  
- ATGCTCTCTT TTGAAAAGTC AATGCTTCAA ACCTAACCTT CCATCATTTT CCATGTTTGC ACTTCCCATC   
  
  
- TAAACAACTG GCTCAAAATC AAATCTTTTT TTTTTTTTTT TTAAAAAAAA AAAAGGGAAG CGCTCATTTG   
  
  
- AAACTTTATA AAGTGTATGT ATATAGAAAA TAAATTTTGA TAACAAGAGA GAGTAAAATA ACAAGCTTTT   
  
  
- ATCATGATGA ACAACATACC TCAACAAAAT TAATTAAAAA AACAACAAGC ATTCATTTTG ATTTTTACTT   
  
  
- CTAAATCTAA AAGGATTACA ATTTAATAAA ATAAAACAAA TATATTTTTA AAATATTTTT CGTTTTACTT   
  
  
- TCAATAAAAA CAGTAAAATT TTAATTTAAG GATTACAATT TAATAAAATA AAACAAATAT ATTTTTAAAA   
  
  
- TATTTTTCGT TTTACTTTCA ATAAAAACAG TAAAATTTTA ATTTTGAAGC AAATTGATCA GATGACGTTA   
  
  
- AAAGATCTAG TTACATCCAC TAGACTTAAT CCACATAAAG CAATCTCCAT CTGCCACGTC TTAACACCAG   
  
  
- GACATAACTG TAATAACCAC GCCTCTGGAT AAATCCTTCC ACAGAGACCA GAAGGAAGCT ACTGAGTATG   
  
  
- ATTACTGTAC CACACTGTAC TCTACTGTAA TAACCACGCC TCTGGATAAA TCCTTCCACA GAGACCAGAA   
  
  
- GGAAGCTACT GAGTATGATT ACTGTACCA

+     G-Box

| Site Name | Organism | Position | Strand | Matrix score. | sequence | function |
| --- | --- | --- | --- | --- | --- | --- |
| G-Box | Pisum sativum | 827 | - | 6 | CACGTT | cis-acting regulatory element involved in light responsiveness |
| G-Box | Pisum sativum | 467 | + | 6 | CACGTG | cis-acting regulatory element involved in light responsiveness |

> 2018/04/13 10:10:12  
+ CTAGCTAGCT ACTATCCCAG TGACGGGTGT CTTGTGTCAC CACGACAAAT TAAAAAACCG GAACTAAAGC   
  
  
+ AACCCATCTT CTCGATCCCC TATAATATTC ACCCCACCCC CCTCTCCTTC GCTTCGTCTA TCTCTATTCG   
  
  
+ AACATTGATT ACTACGCAAA CCTGATTTTC TTTAATTTCC TCTCTACTCC TTTCCCTCTT CGTTTACTTT   
  
  
+ CCTTCTCCAC TCCTTTCTCC CTCATTCAAC TTCCCTTTTT CCCTTCTCTC TTTTTGGCGT CCTTTTCAAT   
  
  
+ CACTGCTTCG TTGGTCACTG CAGAGAGTAT ACTACGTTAC CACTACGGTT CGGTTCGAAA GAGAAAGAAA   
  
  
+ GAAAGGAAAG AAAGAAAGAA AGAAGAAAAT AGAGCCGTAC GAATTAAAAG GAGTGGTGTT TATTTGTCTT   
  
  
+ ACTTTAGTTC ACCCGAACTA TGACTATCAC ATGACACAAA CCCCCACACG TGTTGTTAAG TTTCAGATTC   
  
  
+ TTAGTTAGAG CATCGATCTC CCGACGTTAC GGCTCTACTA CCTAAAATTT TTTCTAGTTT AACGTTTTTT   
  
  
+ GTTTTTTGTT TTTTGGTTTT TACCAACCGT AATGACAATC ATGCTACGTT CACCGATGAA AATAAGATAA   
  
  
+ GATTACGTTG ACCTAGTAAT GCACCGTGTC CGGAAAGGTG AAAAGAAAGA TTAGGCTTAT TACATTAGTC   
  
  
+ ATTAGTGTTA TGTTCTCACC GTACTAACTA CTACTTAAGG TCTTTAGCAT AACCAACGCA GGCAGGGTAC   
  
  
+ TACGAGAGAA AACTTTTCAG TTACGAAGTT TGGATTGGAA GGTAGTAAAA GGTACAAACG TGAAGGGTAG   
  
  
+ ATTTGTTGAC CGAGTTTTAG TTTAGAAAAA AAAAAAAAAA AATTTTTTTT TTTTCCCTTC GCGAGTAAAC   
  
  
+ TTTGAAATAT TTCACATACA TATATCTTTT ATTTAAAACT ATTGTTCTCT CTCATTTTAT TGTTCGAAAA   
  
  
+ TAGTACTACT TGTTGTATGG AGTTGTTTTA ATTAATTTTT TTGTTGTTCG TAAGTAAAAC TAAAAATGAA   
  
  
+ GATTTAGATT TTCCTAATGT TAAATTATTT TATTTTGTTT ATATAAAAAT TTTATAAAAA GCAAAATGAA   
  
  
+ AGTTATTTTT GTCATTTTAA AATTAAATTC CTAATGTTAA ATTATTTTAT TTTGTTTATA TAAAAATTTT   
  
  
+ ATAAAAAGCA AAATGAAAGT TATTTTTGTC ATTTTAAAAT TAAAACTTCG TTTAACTAGT CTACTGCAAT   
  
  
+ TTTCTAGATC AATGTAGGTG ATCTGAATTA GGTGTATTTC GTTAGAGGTA GACGGTGCAG AATTGTGGTC   
  
  
+ CTGTATTGAC ATTATTGGTG CGGAGACCTA TTTAGGAAGG TGTCTCTGGT CTTCCTTCGA TGACTCATAC   
  
  
+ TAATGACATG GTGTGACATG AGATGACATT ATTGGTGCGG AGACCTATTT AGGAAGGTGT CTCTGGTCTT   
  
  
+ CCTTCGATGA CTCATACTAA TGACATGGT  

- GATCGATCGA TGATAGGGTC ACTGCCCACA GAACACAGTG GTGCTGTTTA ATTTTTTGGC CTTGATTTCG   
  
  
- TTGGGTAGAA GAGCTAGGGG ATATTATAAG TGGGGTGGGG GGAGAGGAAG CGAAGCAGAT AGAGATAAGC   
  
  
- TTGTAACTAA TGATGCGTTT GGACTAAAAG AAATTAAAGG AGAGATGAGG AAAGGGAGAA GCAAATGAAA   
  
  
- GGAAGAGGTG AGGAAAGAGG GAGTAAGTTG AAGGGAAAAA GGGAAGAGAG AAAAACCGCA GGAAAAGTTA   
  
  
- GTGACGAAGC AACCAGTGAC GTCTCTCATA TGATGCAATG GTGATGCCAA GCCAAGCTTT CTCTTTCTTT   
  
  
- CTTTCCTTTC TTTCTTTCTT TCTTCTTTTA TCTCGGCATG CTTAATTTTC CTCACCACAA ATAAACAGAA   
  
  
- TGAAATCAAG TGGGCTTGAT ACTGATAGTG TACTGTGTTT GGGGGTGTGC ACAACAATTC AAAGTCTAAG   
  
  
- AATCAATCTC GTAGCTAGAG GGCTGCAATG CCGAGATGAT GGATTTTAAA AAAGATCAAA TTGCAAAAAA   
  
  
- CAAAAAACAA AAAACCAAAA ATGGTTGGCA TTACTGTTAG TACGATGCAA GTGGCTACTT TTATTCTATT   
  
  
- CTAATGCAAC TGGATCATTA CGTGGCACAG GCCTTTCCAC TTTTCTTTCT AATCCGAATA ATGTAATCAG   
  
  
- TAATCACAAT ACAAGAGTGG CATGATTGAT GATGAATTCC AGAAATCGTA TTGGTTGCGT CCGTCCCATG   
  
  
- ATGCTCTCTT TTGAAAAGTC AATGCTTCAA ACCTAACCTT CCATCATTTT CCATGTTTGC ACTTCCCATC   
  
  
- TAAACAACTG GCTCAAAATC AAATCTTTTT TTTTTTTTTT TTAAAAAAAA AAAAGGGAAG CGCTCATTTG   
  
  
- AAACTTTATA AAGTGTATGT ATATAGAAAA TAAATTTTGA TAACAAGAGA GAGTAAAATA ACAAGCTTTT   
  
  
- ATCATGATGA ACAACATACC TCAACAAAAT TAATTAAAAA AACAACAAGC ATTCATTTTG ATTTTTACTT   
  
  
- CTAAATCTAA AAGGATTACA ATTTAATAAA ATAAAACAAA TATATTTTTA AAATATTTTT CGTTTTACTT   
  
  
- TCAATAAAAA CAGTAAAATT TTAATTTAAG GATTACAATT TAATAAAATA AAACAAATAT ATTTTTAAAA   
  
  
- TATTTTTCGT TTTACTTTCA ATAAAAACAG TAAAATTTTA ATTTTGAAGC AAATTGATCA GATGACGTTA   
  
  
- AAAGATCTAG TTACATCCAC TAGACTTAAT CCACATAAAG CAATCTCCAT CTGCCACGTC TTAACACCAG   
  
  
- GACATAACTG TAATAACCAC GCCTCTGGAT AAATCCTTCC ACAGAGACCA GAAGGAAGCT ACTGAGTATG   
  
  
- ATTACTGTAC CACACTGTAC TCTACTGTAA TAACCACGCC TCTGGATAAA TCCTTCCACA GAGACCAGAA   
  
  
- GGAAGCTACT GAGTATGATT ACTGTACCA

+     G-box

| Site Name | Organism | Position | Strand | Matrix score. | sequence | function |
| --- | --- | --- | --- | --- | --- | --- |
| G-box | Brassica napus | 466 | + | 8 | ACACGTGT | cis-acting regulatory element involved in light responsiveness |
| G-box | Zea mays | 41 | + | 6 | CACGAC | cis-acting regulatory element involved in light responsiveness |
| G-box | Zea mays | 827 | - | 6 | CACGTT | cis-acting regulatory element involved in light responsiveness |
| G-box | Arabidopsis thaliana | 467 | + | 6 | CACGTG | cis-acting regulatory element involved in light responsiveness |

> 2018/04/13 10:10:12  
+ CTAGCTAGCT ACTATCCCAG TGACGGGTGT CTTGTGTCAC CACGACAAAT TAAAAAACCG GAACTAAAGC   
  
  
+ AACCCATCTT CTCGATCCCC TATAATATTC ACCCCACCCC CCTCTCCTTC GCTTCGTCTA TCTCTATTCG   
  
  
+ AACATTGATT ACTACGCAAA CCTGATTTTC TTTAATTTCC TCTCTACTCC TTTCCCTCTT CGTTTACTTT   
  
  
+ CCTTCTCCAC TCCTTTCTCC CTCATTCAAC TTCCCTTTTT CCCTTCTCTC TTTTTGGCGT CCTTTTCAAT   
  
  
+ CACTGCTTCG TTGGTCACTG CAGAGAGTAT ACTACGTTAC CACTACGGTT CGGTTCGAAA GAGAAAGAAA   
  
  
+ GAAAGGAAAG AAAGAAAGAA AGAAGAAAAT AGAGCCGTAC GAATTAAAAG GAGTGGTGTT TATTTGTCTT   
  
  
+ ACTTTAGTTC ACCCGAACTA TGACTATCAC ATGACACAAA CCCCCACACG TGTTGTTAAG TTTCAGATTC   
  
  
+ TTAGTTAGAG CATCGATCTC CCGACGTTAC GGCTCTACTA CCTAAAATTT TTTCTAGTTT AACGTTTTTT   
  
  
+ GTTTTTTGTT TTTTGGTTTT TACCAACCGT AATGACAATC ATGCTACGTT CACCGATGAA AATAAGATAA   
  
  
+ GATTACGTTG ACCTAGTAAT GCACCGTGTC CGGAAAGGTG AAAAGAAAGA TTAGGCTTAT TACATTAGTC   
  
  
+ ATTAGTGTTA TGTTCTCACC GTACTAACTA CTACTTAAGG TCTTTAGCAT AACCAACGCA GGCAGGGTAC   
  
  
+ TACGAGAGAA AACTTTTCAG TTACGAAGTT TGGATTGGAA GGTAGTAAAA GGTACAAACG TGAAGGGTAG   
  
  
+ ATTTGTTGAC CGAGTTTTAG TTTAGAAAAA AAAAAAAAAA AATTTTTTTT TTTTCCCTTC GCGAGTAAAC   
  
  
+ TTTGAAATAT TTCACATACA TATATCTTTT ATTTAAAACT ATTGTTCTCT CTCATTTTAT TGTTCGAAAA   
  
  
+ TAGTACTACT TGTTGTATGG AGTTGTTTTA ATTAATTTTT TTGTTGTTCG TAAGTAAAAC TAAAAATGAA   
  
  
+ GATTTAGATT TTCCTAATGT TAAATTATTT TATTTTGTTT ATATAAAAAT TTTATAAAAA GCAAAATGAA   
  
  
+ AGTTATTTTT GTCATTTTAA AATTAAATTC CTAATGTTAA ATTATTTTAT TTTGTTTATA TAAAAATTTT   
  
  
+ ATAAAAAGCA AAATGAAAGT TATTTTTGTC ATTTTAAAAT TAAAACTTCG TTTAACTAGT CTACTGCAAT   
  
  
+ TTTCTAGATC AATGTAGGTG ATCTGAATTA GGTGTATTTC GTTAGAGGTA GACGGTGCAG AATTGTGGTC   
  
  
+ CTGTATTGAC ATTATTGGTG CGGAGACCTA TTTAGGAAGG TGTCTCTGGT CTTCCTTCGA TGACTCATAC   
  
  
+ TAATGACATG GTGTGACATG AGATGACATT ATTGGTGCGG AGACCTATTT AGGAAGGTGT CTCTGGTCTT   
  
  
+ CCTTCGATGA CTCATACTAA TGACATGGT  

- GATCGATCGA TGATAGGGTC ACTGCCCACA GAACACAGTG GTGCTGTTTA ATTTTTTGGC CTTGATTTCG   
  
  
- TTGGGTAGAA GAGCTAGGGG ATATTATAAG TGGGGTGGGG GGAGAGGAAG CGAAGCAGAT AGAGATAAGC   
  
  
- TTGTAACTAA TGATGCGTTT GGACTAAAAG AAATTAAAGG AGAGATGAGG AAAGGGAGAA GCAAATGAAA   
  
  
- GGAAGAGGTG AGGAAAGAGG GAGTAAGTTG AAGGGAAAAA GGGAAGAGAG AAAAACCGCA GGAAAAGTTA   
  
  
- GTGACGAAGC AACCAGTGAC GTCTCTCATA TGATGCAATG GTGATGCCAA GCCAAGCTTT CTCTTTCTTT   
  
  
- CTTTCCTTTC TTTCTTTCTT TCTTCTTTTA TCTCGGCATG CTTAATTTTC CTCACCACAA ATAAACAGAA   
  
  
- TGAAATCAAG TGGGCTTGAT ACTGATAGTG TACTGTGTTT GGGGGTGTGC ACAACAATTC AAAGTCTAAG   
  
  
- AATCAATCTC GTAGCTAGAG GGCTGCAATG CCGAGATGAT GGATTTTAAA AAAGATCAAA TTGCAAAAAA   
  
  
- CAAAAAACAA AAAACCAAAA ATGGTTGGCA TTACTGTTAG TACGATGCAA GTGGCTACTT TTATTCTATT   
  
  
- CTAATGCAAC TGGATCATTA CGTGGCACAG GCCTTTCCAC TTTTCTTTCT AATCCGAATA ATGTAATCAG   
  
  
- TAATCACAAT ACAAGAGTGG CATGATTGAT GATGAATTCC AGAAATCGTA TTGGTTGCGT CCGTCCCATG   
  
  
- ATGCTCTCTT TTGAAAAGTC AATGCTTCAA ACCTAACCTT CCATCATTTT CCATGTTTGC ACTTCCCATC   
  
  
- TAAACAACTG GCTCAAAATC AAATCTTTTT TTTTTTTTTT TTAAAAAAAA AAAAGGGAAG CGCTCATTTG   
  
  
- AAACTTTATA AAGTGTATGT ATATAGAAAA TAAATTTTGA TAACAAGAGA GAGTAAAATA ACAAGCTTTT   
  
  
- ATCATGATGA ACAACATACC TCAACAAAAT TAATTAAAAA AACAACAAGC ATTCATTTTG ATTTTTACTT   
  
  
- CTAAATCTAA AAGGATTACA ATTTAATAAA ATAAAACAAA TATATTTTTA AAATATTTTT CGTTTTACTT   
  
  
- TCAATAAAAA CAGTAAAATT TTAATTTAAG GATTACAATT TAATAAAATA AAACAAATAT ATTTTTAAAA   
  
  
- TATTTTTCGT TTTACTTTCA ATAAAAACAG TAAAATTTTA ATTTTGAAGC AAATTGATCA GATGACGTTA   
  
  
- AAAGATCTAG TTACATCCAC TAGACTTAAT CCACATAAAG CAATCTCCAT CTGCCACGTC TTAACACCAG   
  
  
- GACATAACTG TAATAACCAC GCCTCTGGAT AAATCCTTCC ACAGAGACCA GAAGGAAGCT ACTGAGTATG   
  
  
- ATTACTGTAC CACACTGTAC TCTACTGTAA TAACCACGCC TCTGGATAAA TCCTTCCACA GAGACCAGAA   
  
  
- GGAAGCTACT GAGTATGATT ACTGTACCA

+     GA-motif

| Site Name | Organism | Position | Strand | Matrix score. | sequence | function |
| --- | --- | --- | --- | --- | --- | --- |
| GA-motif | Glycine max | 1467 | - | 8 | AAGGAAGA | part of a light responsive element |
| GA-motif | Glycine max | 1380 | - | 8 | AAGGAAGA | part of a light responsive element |

> 2018/04/13 10:10:12  
+ CTAGCTAGCT ACTATCCCAG TGACGGGTGT CTTGTGTCAC CACGACAAAT TAAAAAACCG GAACTAAAGC   
  
  
+ AACCCATCTT CTCGATCCCC TATAATATTC ACCCCACCCC CCTCTCCTTC GCTTCGTCTA TCTCTATTCG   
  
  
+ AACATTGATT ACTACGCAAA CCTGATTTTC TTTAATTTCC TCTCTACTCC TTTCCCTCTT CGTTTACTTT   
  
  
+ CCTTCTCCAC TCCTTTCTCC CTCATTCAAC TTCCCTTTTT CCCTTCTCTC TTTTTGGCGT CCTTTTCAAT   
  
  
+ CACTGCTTCG TTGGTCACTG CAGAGAGTAT ACTACGTTAC CACTACGGTT CGGTTCGAAA GAGAAAGAAA   
  
  
+ GAAAGGAAAG AAAGAAAGAA AGAAGAAAAT AGAGCCGTAC GAATTAAAAG GAGTGGTGTT TATTTGTCTT   
  
  
+ ACTTTAGTTC ACCCGAACTA TGACTATCAC ATGACACAAA CCCCCACACG TGTTGTTAAG TTTCAGATTC   
  
  
+ TTAGTTAGAG CATCGATCTC CCGACGTTAC GGCTCTACTA CCTAAAATTT TTTCTAGTTT AACGTTTTTT   
  
  
+ GTTTTTTGTT TTTTGGTTTT TACCAACCGT AATGACAATC ATGCTACGTT CACCGATGAA AATAAGATAA   
  
  
+ GATTACGTTG ACCTAGTAAT GCACCGTGTC CGGAAAGGTG AAAAGAAAGA TTAGGCTTAT TACATTAGTC   
  
  
+ ATTAGTGTTA TGTTCTCACC GTACTAACTA CTACTTAAGG TCTTTAGCAT AACCAACGCA GGCAGGGTAC   
  
  
+ TACGAGAGAA AACTTTTCAG TTACGAAGTT TGGATTGGAA GGTAGTAAAA GGTACAAACG TGAAGGGTAG   
  
  
+ ATTTGTTGAC CGAGTTTTAG TTTAGAAAAA AAAAAAAAAA AATTTTTTTT TTTTCCCTTC GCGAGTAAAC   
  
  
+ TTTGAAATAT TTCACATACA TATATCTTTT ATTTAAAACT ATTGTTCTCT CTCATTTTAT TGTTCGAAAA   
  
  
+ TAGTACTACT TGTTGTATGG AGTTGTTTTA ATTAATTTTT TTGTTGTTCG TAAGTAAAAC TAAAAATGAA   
  
  
+ GATTTAGATT TTCCTAATGT TAAATTATTT TATTTTGTTT ATATAAAAAT TTTATAAAAA GCAAAATGAA   
  
  
+ AGTTATTTTT GTCATTTTAA AATTAAATTC CTAATGTTAA ATTATTTTAT TTTGTTTATA TAAAAATTTT   
  
  
+ ATAAAAAGCA AAATGAAAGT TATTTTTGTC ATTTTAAAAT TAAAACTTCG TTTAACTAGT CTACTGCAAT   
  
  
+ TTTCTAGATC AATGTAGGTG ATCTGAATTA GGTGTATTTC GTTAGAGGTA GACGGTGCAG AATTGTGGTC   
  
  
+ CTGTATTGAC ATTATTGGTG CGGAGACCTA TTTAGGAAGG TGTCTCTGGT CTTCCTTCGA TGACTCATAC   
  
  
+ TAATGACATG GTGTGACATG AGATGACATT ATTGGTGCGG AGACCTATTT AGGAAGGTGT CTCTGGTCTT   
  
  
+ CCTTCGATGA CTCATACTAA TGACATGGT  

- GATCGATCGA TGATAGGGTC ACTGCCCACA GAACACAGTG GTGCTGTTTA ATTTTTTGGC CTTGATTTCG   
  
  
- TTGGGTAGAA GAGCTAGGGG ATATTATAAG TGGGGTGGGG GGAGAGGAAG CGAAGCAGAT AGAGATAAGC   
  
  
- TTGTAACTAA TGATGCGTTT GGACTAAAAG AAATTAAAGG AGAGATGAGG AAAGGGAGAA GCAAATGAAA   
  
  
- GGAAGAGGTG AGGAAAGAGG GAGTAAGTTG AAGGGAAAAA GGGAAGAGAG AAAAACCGCA GGAAAAGTTA   
  
  
- GTGACGAAGC AACCAGTGAC GTCTCTCATA TGATGCAATG GTGATGCCAA GCCAAGCTTT CTCTTTCTTT   
  
  
- CTTTCCTTTC TTTCTTTCTT TCTTCTTTTA TCTCGGCATG CTTAATTTTC CTCACCACAA ATAAACAGAA   
  
  
- TGAAATCAAG TGGGCTTGAT ACTGATAGTG TACTGTGTTT GGGGGTGTGC ACAACAATTC AAAGTCTAAG   
  
  
- AATCAATCTC GTAGCTAGAG GGCTGCAATG CCGAGATGAT GGATTTTAAA AAAGATCAAA TTGCAAAAAA   
  
  
- CAAAAAACAA AAAACCAAAA ATGGTTGGCA TTACTGTTAG TACGATGCAA GTGGCTACTT TTATTCTATT   
  
  
- CTAATGCAAC TGGATCATTA CGTGGCACAG GCCTTTCCAC TTTTCTTTCT AATCCGAATA ATGTAATCAG   
  
  
- TAATCACAAT ACAAGAGTGG CATGATTGAT GATGAATTCC AGAAATCGTA TTGGTTGCGT CCGTCCCATG   
  
  
- ATGCTCTCTT TTGAAAAGTC AATGCTTCAA ACCTAACCTT CCATCATTTT CCATGTTTGC ACTTCCCATC   
  
  
- TAAACAACTG GCTCAAAATC AAATCTTTTT TTTTTTTTTT TTAAAAAAAA AAAAGGGAAG CGCTCATTTG   
  
  
- AAACTTTATA AAGTGTATGT ATATAGAAAA TAAATTTTGA TAACAAGAGA GAGTAAAATA ACAAGCTTTT   
  
  
- ATCATGATGA ACAACATACC TCAACAAAAT TAATTAAAAA AACAACAAGC ATTCATTTTG ATTTTTACTT   
  
  
- CTAAATCTAA AAGGATTACA ATTTAATAAA ATAAAACAAA TATATTTTTA AAATATTTTT CGTTTTACTT   
  
  
- TCAATAAAAA CAGTAAAATT TTAATTTAAG GATTACAATT TAATAAAATA AAACAAATAT ATTTTTAAAA   
  
  
- TATTTTTCGT TTTACTTTCA ATAAAAACAG TAAAATTTTA ATTTTGAAGC AAATTGATCA GATGACGTTA   
  
  
- AAAGATCTAG TTACATCCAC TAGACTTAAT CCACATAAAG CAATCTCCAT CTGCCACGTC TTAACACCAG   
  
  
- GACATAACTG TAATAACCAC GCCTCTGGAT AAATCCTTCC ACAGAGACCA GAAGGAAGCT ACTGAGTATG   
  
  
- ATTACTGTAC CACACTGTAC TCTACTGTAA TAACCACGCC TCTGGATAAA TCCTTCCACA GAGACCAGAA   
  
  
- GGAAGCTACT GAGTATGATT ACTGTACCA

+     GAG-motif

| Site Name | Organism | Position | Strand | Matrix score. | sequence | function |
| --- | --- | --- | --- | --- | --- | --- |
| GAG-motif | Arabidopsis thaliana | 302 | + | 7 | AGAGAGT | part of a light responsive element |

> 2018/04/13 10:10:12  
+ CTAGCTAGCT ACTATCCCAG TGACGGGTGT CTTGTGTCAC CACGACAAAT TAAAAAACCG GAACTAAAGC   
  
  
+ AACCCATCTT CTCGATCCCC TATAATATTC ACCCCACCCC CCTCTCCTTC GCTTCGTCTA TCTCTATTCG   
  
  
+ AACATTGATT ACTACGCAAA CCTGATTTTC TTTAATTTCC TCTCTACTCC TTTCCCTCTT CGTTTACTTT   
  
  
+ CCTTCTCCAC TCCTTTCTCC CTCATTCAAC TTCCCTTTTT CCCTTCTCTC TTTTTGGCGT CCTTTTCAAT   
  
  
+ CACTGCTTCG TTGGTCACTG CAGAGAGTAT ACTACGTTAC CACTACGGTT CGGTTCGAAA GAGAAAGAAA   
  
  
+ GAAAGGAAAG AAAGAAAGAA AGAAGAAAAT AGAGCCGTAC GAATTAAAAG GAGTGGTGTT TATTTGTCTT   
  
  
+ ACTTTAGTTC ACCCGAACTA TGACTATCAC ATGACACAAA CCCCCACACG TGTTGTTAAG TTTCAGATTC   
  
  
+ TTAGTTAGAG CATCGATCTC CCGACGTTAC GGCTCTACTA CCTAAAATTT TTTCTAGTTT AACGTTTTTT   
  
  
+ GTTTTTTGTT TTTTGGTTTT TACCAACCGT AATGACAATC ATGCTACGTT CACCGATGAA AATAAGATAA   
  
  
+ GATTACGTTG ACCTAGTAAT GCACCGTGTC CGGAAAGGTG AAAAGAAAGA TTAGGCTTAT TACATTAGTC   
  
  
+ ATTAGTGTTA TGTTCTCACC GTACTAACTA CTACTTAAGG TCTTTAGCAT AACCAACGCA GGCAGGGTAC   
  
  
+ TACGAGAGAA AACTTTTCAG TTACGAAGTT TGGATTGGAA GGTAGTAAAA GGTACAAACG TGAAGGGTAG   
  
  
+ ATTTGTTGAC CGAGTTTTAG TTTAGAAAAA AAAAAAAAAA AATTTTTTTT TTTTCCCTTC GCGAGTAAAC   
  
  
+ TTTGAAATAT TTCACATACA TATATCTTTT ATTTAAAACT ATTGTTCTCT CTCATTTTAT TGTTCGAAAA   
  
  
+ TAGTACTACT TGTTGTATGG AGTTGTTTTA ATTAATTTTT TTGTTGTTCG TAAGTAAAAC TAAAAATGAA   
  
  
+ GATTTAGATT TTCCTAATGT TAAATTATTT TATTTTGTTT ATATAAAAAT TTTATAAAAA GCAAAATGAA   
  
  
+ AGTTATTTTT GTCATTTTAA AATTAAATTC CTAATGTTAA ATTATTTTAT TTTGTTTATA TAAAAATTTT   
  
  
+ ATAAAAAGCA AAATGAAAGT TATTTTTGTC ATTTTAAAAT TAAAACTTCG TTTAACTAGT CTACTGCAAT   
  
  
+ TTTCTAGATC AATGTAGGTG ATCTGAATTA GGTGTATTTC GTTAGAGGTA GACGGTGCAG AATTGTGGTC   
  
  
+ CTGTATTGAC ATTATTGGTG CGGAGACCTA TTTAGGAAGG TGTCTCTGGT CTTCCTTCGA TGACTCATAC   
  
  
+ TAATGACATG GTGTGACATG AGATGACATT ATTGGTGCGG AGACCTATTT AGGAAGGTGT CTCTGGTCTT   
  
  
+ CCTTCGATGA CTCATACTAA TGACATGGT  

- GATCGATCGA TGATAGGGTC ACTGCCCACA GAACACAGTG GTGCTGTTTA ATTTTTTGGC CTTGATTTCG   
  
  
- TTGGGTAGAA GAGCTAGGGG ATATTATAAG TGGGGTGGGG GGAGAGGAAG CGAAGCAGAT AGAGATAAGC   
  
  
- TTGTAACTAA TGATGCGTTT GGACTAAAAG AAATTAAAGG AGAGATGAGG AAAGGGAGAA GCAAATGAAA   
  
  
- GGAAGAGGTG AGGAAAGAGG GAGTAAGTTG AAGGGAAAAA GGGAAGAGAG AAAAACCGCA GGAAAAGTTA   
  
  
- GTGACGAAGC AACCAGTGAC GTCTCTCATA TGATGCAATG GTGATGCCAA GCCAAGCTTT CTCTTTCTTT   
  
  
- CTTTCCTTTC TTTCTTTCTT TCTTCTTTTA TCTCGGCATG CTTAATTTTC CTCACCACAA ATAAACAGAA   
  
  
- TGAAATCAAG TGGGCTTGAT ACTGATAGTG TACTGTGTTT GGGGGTGTGC ACAACAATTC AAAGTCTAAG   
  
  
- AATCAATCTC GTAGCTAGAG GGCTGCAATG CCGAGATGAT GGATTTTAAA AAAGATCAAA TTGCAAAAAA   
  
  
- CAAAAAACAA AAAACCAAAA ATGGTTGGCA TTACTGTTAG TACGATGCAA GTGGCTACTT TTATTCTATT   
  
  
- CTAATGCAAC TGGATCATTA CGTGGCACAG GCCTTTCCAC TTTTCTTTCT AATCCGAATA ATGTAATCAG   
  
  
- TAATCACAAT ACAAGAGTGG CATGATTGAT GATGAATTCC AGAAATCGTA TTGGTTGCGT CCGTCCCATG   
  
  
- ATGCTCTCTT TTGAAAAGTC AATGCTTCAA ACCTAACCTT CCATCATTTT CCATGTTTGC ACTTCCCATC   
  
  
- TAAACAACTG GCTCAAAATC AAATCTTTTT TTTTTTTTTT TTAAAAAAAA AAAAGGGAAG CGCTCATTTG   
  
  
- AAACTTTATA AAGTGTATGT ATATAGAAAA TAAATTTTGA TAACAAGAGA GAGTAAAATA ACAAGCTTTT   
  
  
- ATCATGATGA ACAACATACC TCAACAAAAT TAATTAAAAA AACAACAAGC ATTCATTTTG ATTTTTACTT   
  
  
- CTAAATCTAA AAGGATTACA ATTTAATAAA ATAAAACAAA TATATTTTTA AAATATTTTT CGTTTTACTT   
  
  
- TCAATAAAAA CAGTAAAATT TTAATTTAAG GATTACAATT TAATAAAATA AAACAAATAT ATTTTTAAAA   
  
  
- TATTTTTCGT TTTACTTTCA ATAAAAACAG TAAAATTTTA ATTTTGAAGC AAATTGATCA GATGACGTTA   
  
  
- AAAGATCTAG TTACATCCAC TAGACTTAAT CCACATAAAG CAATCTCCAT CTGCCACGTC TTAACACCAG   
  
  
- GACATAACTG TAATAACCAC GCCTCTGGAT AAATCCTTCC ACAGAGACCA GAAGGAAGCT ACTGAGTATG   
  
  
- ATTACTGTAC CACACTGTAC TCTACTGTAA TAACCACGCC TCTGGATAAA TCCTTCCACA GAGACCAGAA   
  
  
- GGAAGCTACT GAGTATGATT ACTGTACCA

+     GATA-motif

| Site Name | Organism | Position | Strand | Matrix score. | sequence | function |
| --- | --- | --- | --- | --- | --- | --- |
| GATA-motif | Arabidopsis thaliana | 624 | + | 11 | AAGATAAGATT | part of a light responsive element |

> 2018/04/13 10:10:12  
+ CTAGCTAGCT ACTATCCCAG TGACGGGTGT CTTGTGTCAC CACGACAAAT TAAAAAACCG GAACTAAAGC   
  
  
+ AACCCATCTT CTCGATCCCC TATAATATTC ACCCCACCCC CCTCTCCTTC GCTTCGTCTA TCTCTATTCG   
  
  
+ AACATTGATT ACTACGCAAA CCTGATTTTC TTTAATTTCC TCTCTACTCC TTTCCCTCTT CGTTTACTTT   
  
  
+ CCTTCTCCAC TCCTTTCTCC CTCATTCAAC TTCCCTTTTT CCCTTCTCTC TTTTTGGCGT CCTTTTCAAT   
  
  
+ CACTGCTTCG TTGGTCACTG CAGAGAGTAT ACTACGTTAC CACTACGGTT CGGTTCGAAA GAGAAAGAAA   
  
  
+ GAAAGGAAAG AAAGAAAGAA AGAAGAAAAT AGAGCCGTAC GAATTAAAAG GAGTGGTGTT TATTTGTCTT   
  
  
+ ACTTTAGTTC ACCCGAACTA TGACTATCAC ATGACACAAA CCCCCACACG TGTTGTTAAG TTTCAGATTC   
  
  
+ TTAGTTAGAG CATCGATCTC CCGACGTTAC GGCTCTACTA CCTAAAATTT TTTCTAGTTT AACGTTTTTT   
  
  
+ GTTTTTTGTT TTTTGGTTTT TACCAACCGT AATGACAATC ATGCTACGTT CACCGATGAA AATAAGATAA   
  
  
+ GATTACGTTG ACCTAGTAAT GCACCGTGTC CGGAAAGGTG AAAAGAAAGA TTAGGCTTAT TACATTAGTC   
  
  
+ ATTAGTGTTA TGTTCTCACC GTACTAACTA CTACTTAAGG TCTTTAGCAT AACCAACGCA GGCAGGGTAC   
  
  
+ TACGAGAGAA AACTTTTCAG TTACGAAGTT TGGATTGGAA GGTAGTAAAA GGTACAAACG TGAAGGGTAG   
  
  
+ ATTTGTTGAC CGAGTTTTAG TTTAGAAAAA AAAAAAAAAA AATTTTTTTT TTTTCCCTTC GCGAGTAAAC   
  
  
+ TTTGAAATAT TTCACATACA TATATCTTTT ATTTAAAACT ATTGTTCTCT CTCATTTTAT TGTTCGAAAA   
  
  
+ TAGTACTACT TGTTGTATGG AGTTGTTTTA ATTAATTTTT TTGTTGTTCG TAAGTAAAAC TAAAAATGAA   
  
  
+ GATTTAGATT TTCCTAATGT TAAATTATTT TATTTTGTTT ATATAAAAAT TTTATAAAAA GCAAAATGAA   
  
  
+ AGTTATTTTT GTCATTTTAA AATTAAATTC CTAATGTTAA ATTATTTTAT TTTGTTTATA TAAAAATTTT   
  
  
+ ATAAAAAGCA AAATGAAAGT TATTTTTGTC ATTTTAAAAT TAAAACTTCG TTTAACTAGT CTACTGCAAT   
  
  
+ TTTCTAGATC AATGTAGGTG ATCTGAATTA GGTGTATTTC GTTAGAGGTA GACGGTGCAG AATTGTGGTC   
  
  
+ CTGTATTGAC ATTATTGGTG CGGAGACCTA TTTAGGAAGG TGTCTCTGGT CTTCCTTCGA TGACTCATAC   
  
  
+ TAATGACATG GTGTGACATG AGATGACATT ATTGGTGCGG AGACCTATTT AGGAAGGTGT CTCTGGTCTT   
  
  
+ CCTTCGATGA CTCATACTAA TGACATGGT  

- GATCGATCGA TGATAGGGTC ACTGCCCACA GAACACAGTG GTGCTGTTTA ATTTTTTGGC CTTGATTTCG   
  
  
- TTGGGTAGAA GAGCTAGGGG ATATTATAAG TGGGGTGGGG GGAGAGGAAG CGAAGCAGAT AGAGATAAGC   
  
  
- TTGTAACTAA TGATGCGTTT GGACTAAAAG AAATTAAAGG AGAGATGAGG AAAGGGAGAA GCAAATGAAA   
  
  
- GGAAGAGGTG AGGAAAGAGG GAGTAAGTTG AAGGGAAAAA GGGAAGAGAG AAAAACCGCA GGAAAAGTTA   
  
  
- GTGACGAAGC AACCAGTGAC GTCTCTCATA TGATGCAATG GTGATGCCAA GCCAAGCTTT CTCTTTCTTT   
  
  
- CTTTCCTTTC TTTCTTTCTT TCTTCTTTTA TCTCGGCATG CTTAATTTTC CTCACCACAA ATAAACAGAA   
  
  
- TGAAATCAAG TGGGCTTGAT ACTGATAGTG TACTGTGTTT GGGGGTGTGC ACAACAATTC AAAGTCTAAG   
  
  
- AATCAATCTC GTAGCTAGAG GGCTGCAATG CCGAGATGAT GGATTTTAAA AAAGATCAAA TTGCAAAAAA   
  
  
- CAAAAAACAA AAAACCAAAA ATGGTTGGCA TTACTGTTAG TACGATGCAA GTGGCTACTT TTATTCTATT   
  
  
- CTAATGCAAC TGGATCATTA CGTGGCACAG GCCTTTCCAC TTTTCTTTCT AATCCGAATA ATGTAATCAG   
  
  
- TAATCACAAT ACAAGAGTGG CATGATTGAT GATGAATTCC AGAAATCGTA TTGGTTGCGT CCGTCCCATG   
  
  
- ATGCTCTCTT TTGAAAAGTC AATGCTTCAA ACCTAACCTT CCATCATTTT CCATGTTTGC ACTTCCCATC   
  
  
- TAAACAACTG GCTCAAAATC AAATCTTTTT TTTTTTTTTT TTAAAAAAAA AAAAGGGAAG CGCTCATTTG   
  
  
- AAACTTTATA AAGTGTATGT ATATAGAAAA TAAATTTTGA TAACAAGAGA GAGTAAAATA ACAAGCTTTT   
  
  
- ATCATGATGA ACAACATACC TCAACAAAAT TAATTAAAAA AACAACAAGC ATTCATTTTG ATTTTTACTT   
  
  
- CTAAATCTAA AAGGATTACA ATTTAATAAA ATAAAACAAA TATATTTTTA AAATATTTTT CGTTTTACTT   
  
  
- TCAATAAAAA CAGTAAAATT TTAATTTAAG GATTACAATT TAATAAAATA AAACAAATAT ATTTTTAAAA   
  
  
- TATTTTTCGT TTTACTTTCA ATAAAAACAG TAAAATTTTA ATTTTGAAGC AAATTGATCA GATGACGTTA   
  
  
- AAAGATCTAG TTACATCCAC TAGACTTAAT CCACATAAAG CAATCTCCAT CTGCCACGTC TTAACACCAG   
  
  
- GACATAACTG TAATAACCAC GCCTCTGGAT AAATCCTTCC ACAGAGACCA GAAGGAAGCT ACTGAGTATG   
  
  
- ATTACTGTAC CACACTGTAC TCTACTGTAA TAACCACGCC TCTGGATAAA TCCTTCCACA GAGACCAGAA   
  
  
- GGAAGCTACT GAGTATGATT ACTGTACCA

+     GCN4\_motif

| Site Name | Organism | Position | Strand | Matrix score. | sequence | function |
| --- | --- | --- | --- | --- | --- | --- |
| GCN4\_motif | Oryza sativa | 1478 | - | 7 | TGAGTCA | cis-regulatory element involved in endosperm expression |
| GCN4\_motif | Oryza sativa | 452 | - | 7 | TGTGTCA | cis-regulatory element involved in endosperm expression |
| GCN4\_motif | Oryza sativa | 1391 | - | 7 | TGAGTCA | cis-regulatory element involved in endosperm expression |
| GCN4\_motif | Oryza sativa | 33 | + | 7 | TGTGTCA | cis-regulatory element involved in endosperm expression |

> 2018/04/13 10:10:12  
+ CTAGCTAGCT ACTATCCCAG TGACGGGTGT CTTGTGTCAC CACGACAAAT TAAAAAACCG GAACTAAAGC   
  
  
+ AACCCATCTT CTCGATCCCC TATAATATTC ACCCCACCCC CCTCTCCTTC GCTTCGTCTA TCTCTATTCG   
  
  
+ AACATTGATT ACTACGCAAA CCTGATTTTC TTTAATTTCC TCTCTACTCC TTTCCCTCTT CGTTTACTTT   
  
  
+ CCTTCTCCAC TCCTTTCTCC CTCATTCAAC TTCCCTTTTT CCCTTCTCTC TTTTTGGCGT CCTTTTCAAT   
  
  
+ CACTGCTTCG TTGGTCACTG CAGAGAGTAT ACTACGTTAC CACTACGGTT CGGTTCGAAA GAGAAAGAAA   
  
  
+ GAAAGGAAAG AAAGAAAGAA AGAAGAAAAT AGAGCCGTAC GAATTAAAAG GAGTGGTGTT TATTTGTCTT   
  
  
+ ACTTTAGTTC ACCCGAACTA TGACTATCAC ATGACACAAA CCCCCACACG TGTTGTTAAG TTTCAGATTC   
  
  
+ TTAGTTAGAG CATCGATCTC CCGACGTTAC GGCTCTACTA CCTAAAATTT TTTCTAGTTT AACGTTTTTT   
  
  
+ GTTTTTTGTT TTTTGGTTTT TACCAACCGT AATGACAATC ATGCTACGTT CACCGATGAA AATAAGATAA   
  
  
+ GATTACGTTG ACCTAGTAAT GCACCGTGTC CGGAAAGGTG AAAAGAAAGA TTAGGCTTAT TACATTAGTC   
  
  
+ ATTAGTGTTA TGTTCTCACC GTACTAACTA CTACTTAAGG TCTTTAGCAT AACCAACGCA GGCAGGGTAC   
  
  
+ TACGAGAGAA AACTTTTCAG TTACGAAGTT TGGATTGGAA GGTAGTAAAA GGTACAAACG TGAAGGGTAG   
  
  
+ ATTTGTTGAC CGAGTTTTAG TTTAGAAAAA AAAAAAAAAA AATTTTTTTT TTTTCCCTTC GCGAGTAAAC   
  
  
+ TTTGAAATAT TTCACATACA TATATCTTTT ATTTAAAACT ATTGTTCTCT CTCATTTTAT TGTTCGAAAA   
  
  
+ TAGTACTACT TGTTGTATGG AGTTGTTTTA ATTAATTTTT TTGTTGTTCG TAAGTAAAAC TAAAAATGAA   
  
  
+ GATTTAGATT TTCCTAATGT TAAATTATTT TATTTTGTTT ATATAAAAAT TTTATAAAAA GCAAAATGAA   
  
  
+ AGTTATTTTT GTCATTTTAA AATTAAATTC CTAATGTTAA ATTATTTTAT TTTGTTTATA TAAAAATTTT   
  
  
+ ATAAAAAGCA AAATGAAAGT TATTTTTGTC ATTTTAAAAT TAAAACTTCG TTTAACTAGT CTACTGCAAT   
  
  
+ TTTCTAGATC AATGTAGGTG ATCTGAATTA GGTGTATTTC GTTAGAGGTA GACGGTGCAG AATTGTGGTC   
  
  
+ CTGTATTGAC ATTATTGGTG CGGAGACCTA TTTAGGAAGG TGTCTCTGGT CTTCCTTCGA TGACTCATAC   
  
  
+ TAATGACATG GTGTGACATG AGATGACATT ATTGGTGCGG AGACCTATTT AGGAAGGTGT CTCTGGTCTT   
  
  
+ CCTTCGATGA CTCATACTAA TGACATGGT  

- GATCGATCGA TGATAGGGTC ACTGCCCACA GAACACAGTG GTGCTGTTTA ATTTTTTGGC CTTGATTTCG   
  
  
- TTGGGTAGAA GAGCTAGGGG ATATTATAAG TGGGGTGGGG GGAGAGGAAG CGAAGCAGAT AGAGATAAGC   
  
  
- TTGTAACTAA TGATGCGTTT GGACTAAAAG AAATTAAAGG AGAGATGAGG AAAGGGAGAA GCAAATGAAA   
  
  
- GGAAGAGGTG AGGAAAGAGG GAGTAAGTTG AAGGGAAAAA GGGAAGAGAG AAAAACCGCA GGAAAAGTTA   
  
  
- GTGACGAAGC AACCAGTGAC GTCTCTCATA TGATGCAATG GTGATGCCAA GCCAAGCTTT CTCTTTCTTT   
  
  
- CTTTCCTTTC TTTCTTTCTT TCTTCTTTTA TCTCGGCATG CTTAATTTTC CTCACCACAA ATAAACAGAA   
  
  
- TGAAATCAAG TGGGCTTGAT ACTGATAGTG TACTGTGTTT GGGGGTGTGC ACAACAATTC AAAGTCTAAG   
  
  
- AATCAATCTC GTAGCTAGAG GGCTGCAATG CCGAGATGAT GGATTTTAAA AAAGATCAAA TTGCAAAAAA   
  
  
- CAAAAAACAA AAAACCAAAA ATGGTTGGCA TTACTGTTAG TACGATGCAA GTGGCTACTT TTATTCTATT   
  
  
- CTAATGCAAC TGGATCATTA CGTGGCACAG GCCTTTCCAC TTTTCTTTCT AATCCGAATA ATGTAATCAG   
  
  
- TAATCACAAT ACAAGAGTGG CATGATTGAT GATGAATTCC AGAAATCGTA TTGGTTGCGT CCGTCCCATG   
  
  
- ATGCTCTCTT TTGAAAAGTC AATGCTTCAA ACCTAACCTT CCATCATTTT CCATGTTTGC ACTTCCCATC   
  
  
- TAAACAACTG GCTCAAAATC AAATCTTTTT TTTTTTTTTT TTAAAAAAAA AAAAGGGAAG CGCTCATTTG   
  
  
- AAACTTTATA AAGTGTATGT ATATAGAAAA TAAATTTTGA TAACAAGAGA GAGTAAAATA ACAAGCTTTT   
  
  
- ATCATGATGA ACAACATACC TCAACAAAAT TAATTAAAAA AACAACAAGC ATTCATTTTG ATTTTTACTT   
  
  
- CTAAATCTAA AAGGATTACA ATTTAATAAA ATAAAACAAA TATATTTTTA AAATATTTTT CGTTTTACTT   
  
  
- TCAATAAAAA CAGTAAAATT TTAATTTAAG GATTACAATT TAATAAAATA AAACAAATAT ATTTTTAAAA   
  
  
- TATTTTTCGT TTTACTTTCA ATAAAAACAG TAAAATTTTA ATTTTGAAGC AAATTGATCA GATGACGTTA   
  
  
- AAAGATCTAG TTACATCCAC TAGACTTAAT CCACATAAAG CAATCTCCAT CTGCCACGTC TTAACACCAG   
  
  
- GACATAACTG TAATAACCAC GCCTCTGGAT AAATCCTTCC ACAGAGACCA GAAGGAAGCT ACTGAGTATG   
  
  
- ATTACTGTAC CACACTGTAC TCTACTGTAA TAACCACGCC TCTGGATAAA TCCTTCCACA GAGACCAGAA   
  
  
- GGAAGCTACT GAGTATGATT ACTGTACCA

+     HSE

| Site Name | Organism | Position | Strand | Matrix score. | sequence | function |
| --- | --- | --- | --- | --- | --- | --- |
| HSE | Brassica oleracea | 877 | + | 9 | AAAAAATTTC | cis-acting element involved in heat stress responsiveness |
| HSE | Brassica oleracea | 534 | - | 9 | AAAAAATTTC | cis-acting element involved in heat stress responsiveness |
| HSE | Brassica oleracea | 879 | - | 9 | AAAAAATTTC | cis-acting element involved in heat stress responsiveness |

> 2018/04/13 10:10:12  
+ CTAGCTAGCT ACTATCCCAG TGACGGGTGT CTTGTGTCAC CACGACAAAT TAAAAAACCG GAACTAAAGC   
  
  
+ AACCCATCTT CTCGATCCCC TATAATATTC ACCCCACCCC CCTCTCCTTC GCTTCGTCTA TCTCTATTCG   
  
  
+ AACATTGATT ACTACGCAAA CCTGATTTTC TTTAATTTCC TCTCTACTCC TTTCCCTCTT CGTTTACTTT   
  
  
+ CCTTCTCCAC TCCTTTCTCC CTCATTCAAC TTCCCTTTTT CCCTTCTCTC TTTTTGGCGT CCTTTTCAAT   
  
  
+ CACTGCTTCG TTGGTCACTG CAGAGAGTAT ACTACGTTAC CACTACGGTT CGGTTCGAAA GAGAAAGAAA   
  
  
+ GAAAGGAAAG AAAGAAAGAA AGAAGAAAAT AGAGCCGTAC GAATTAAAAG GAGTGGTGTT TATTTGTCTT   
  
  
+ ACTTTAGTTC ACCCGAACTA TGACTATCAC ATGACACAAA CCCCCACACG TGTTGTTAAG TTTCAGATTC   
  
  
+ TTAGTTAGAG CATCGATCTC CCGACGTTAC GGCTCTACTA CCTAAAATTT TTTCTAGTTT AACGTTTTTT   
  
  
+ GTTTTTTGTT TTTTGGTTTT TACCAACCGT AATGACAATC ATGCTACGTT CACCGATGAA AATAAGATAA   
  
  
+ GATTACGTTG ACCTAGTAAT GCACCGTGTC CGGAAAGGTG AAAAGAAAGA TTAGGCTTAT TACATTAGTC   
  
  
+ ATTAGTGTTA TGTTCTCACC GTACTAACTA CTACTTAAGG TCTTTAGCAT AACCAACGCA GGCAGGGTAC   
  
  
+ TACGAGAGAA AACTTTTCAG TTACGAAGTT TGGATTGGAA GGTAGTAAAA GGTACAAACG TGAAGGGTAG   
  
  
+ ATTTGTTGAC CGAGTTTTAG TTTAGAAAAA AAAAAAAAAA AATTTTTTTT TTTTCCCTTC GCGAGTAAAC   
  
  
+ TTTGAAATAT TTCACATACA TATATCTTTT ATTTAAAACT ATTGTTCTCT CTCATTTTAT TGTTCGAAAA   
  
  
+ TAGTACTACT TGTTGTATGG AGTTGTTTTA ATTAATTTTT TTGTTGTTCG TAAGTAAAAC TAAAAATGAA   
  
  
+ GATTTAGATT TTCCTAATGT TAAATTATTT TATTTTGTTT ATATAAAAAT TTTATAAAAA GCAAAATGAA   
  
  
+ AGTTATTTTT GTCATTTTAA AATTAAATTC CTAATGTTAA ATTATTTTAT TTTGTTTATA TAAAAATTTT   
  
  
+ ATAAAAAGCA AAATGAAAGT TATTTTTGTC ATTTTAAAAT TAAAACTTCG TTTAACTAGT CTACTGCAAT   
  
  
+ TTTCTAGATC AATGTAGGTG ATCTGAATTA GGTGTATTTC GTTAGAGGTA GACGGTGCAG AATTGTGGTC   
  
  
+ CTGTATTGAC ATTATTGGTG CGGAGACCTA TTTAGGAAGG TGTCTCTGGT CTTCCTTCGA TGACTCATAC   
  
  
+ TAATGACATG GTGTGACATG AGATGACATT ATTGGTGCGG AGACCTATTT AGGAAGGTGT CTCTGGTCTT   
  
  
+ CCTTCGATGA CTCATACTAA TGACATGGT  

- GATCGATCGA TGATAGGGTC ACTGCCCACA GAACACAGTG GTGCTGTTTA ATTTTTTGGC CTTGATTTCG   
  
  
- TTGGGTAGAA GAGCTAGGGG ATATTATAAG TGGGGTGGGG GGAGAGGAAG CGAAGCAGAT AGAGATAAGC   
  
  
- TTGTAACTAA TGATGCGTTT GGACTAAAAG AAATTAAAGG AGAGATGAGG AAAGGGAGAA GCAAATGAAA   
  
  
- GGAAGAGGTG AGGAAAGAGG GAGTAAGTTG AAGGGAAAAA GGGAAGAGAG AAAAACCGCA GGAAAAGTTA   
  
  
- GTGACGAAGC AACCAGTGAC GTCTCTCATA TGATGCAATG GTGATGCCAA GCCAAGCTTT CTCTTTCTTT   
  
  
- CTTTCCTTTC TTTCTTTCTT TCTTCTTTTA TCTCGGCATG CTTAATTTTC CTCACCACAA ATAAACAGAA   
  
  
- TGAAATCAAG TGGGCTTGAT ACTGATAGTG TACTGTGTTT GGGGGTGTGC ACAACAATTC AAAGTCTAAG   
  
  
- AATCAATCTC GTAGCTAGAG GGCTGCAATG CCGAGATGAT GGATTTTAAA AAAGATCAAA TTGCAAAAAA   
  
  
- CAAAAAACAA AAAACCAAAA ATGGTTGGCA TTACTGTTAG TACGATGCAA GTGGCTACTT TTATTCTATT   
  
  
- CTAATGCAAC TGGATCATTA CGTGGCACAG GCCTTTCCAC TTTTCTTTCT AATCCGAATA ATGTAATCAG   
  
  
- TAATCACAAT ACAAGAGTGG CATGATTGAT GATGAATTCC AGAAATCGTA TTGGTTGCGT CCGTCCCATG   
  
  
- ATGCTCTCTT TTGAAAAGTC AATGCTTCAA ACCTAACCTT CCATCATTTT CCATGTTTGC ACTTCCCATC   
  
  
- TAAACAACTG GCTCAAAATC AAATCTTTTT TTTTTTTTTT TTAAAAAAAA AAAAGGGAAG CGCTCATTTG   
  
  
- AAACTTTATA AAGTGTATGT ATATAGAAAA TAAATTTTGA TAACAAGAGA GAGTAAAATA ACAAGCTTTT   
  
  
- ATCATGATGA ACAACATACC TCAACAAAAT TAATTAAAAA AACAACAAGC ATTCATTTTG ATTTTTACTT   
  
  
- CTAAATCTAA AAGGATTACA ATTTAATAAA ATAAAACAAA TATATTTTTA AAATATTTTT CGTTTTACTT   
  
  
- TCAATAAAAA CAGTAAAATT TTAATTTAAG GATTACAATT TAATAAAATA AAACAAATAT ATTTTTAAAA   
  
  
- TATTTTTCGT TTTACTTTCA ATAAAAACAG TAAAATTTTA ATTTTGAAGC AAATTGATCA GATGACGTTA   
  
  
- AAAGATCTAG TTACATCCAC TAGACTTAAT CCACATAAAG CAATCTCCAT CTGCCACGTC TTAACACCAG   
  
  
- GACATAACTG TAATAACCAC GCCTCTGGAT AAATCCTTCC ACAGAGACCA GAAGGAAGCT ACTGAGTATG   
  
  
- ATTACTGTAC CACACTGTAC TCTACTGTAA TAACCACGCC TCTGGATAAA TCCTTCCACA GAGACCAGAA   
  
  
- GGAAGCTACT GAGTATGATT ACTGTACCA

+     I-box

| Site Name | Organism | Position | Strand | Matrix score. | sequence | function |
| --- | --- | --- | --- | --- | --- | --- |
| I-box | Gossypium hirsutum | 677 | + | 10 | AAGATAAGGCT | part of a light responsive element |
| I-box | Arabidopsis thaliana | 626 | + | 9 | GATAAGATT | part of a light responsive element |
| I-box | Triticum aestivum | 624 | + | 9 | aAGATAAGA | part of a light responsive element |

> 2018/04/13 10:10:12  
+ CTAGCTAGCT ACTATCCCAG TGACGGGTGT CTTGTGTCAC CACGACAAAT TAAAAAACCG GAACTAAAGC   
  
  
+ AACCCATCTT CTCGATCCCC TATAATATTC ACCCCACCCC CCTCTCCTTC GCTTCGTCTA TCTCTATTCG   
  
  
+ AACATTGATT ACTACGCAAA CCTGATTTTC TTTAATTTCC TCTCTACTCC TTTCCCTCTT CGTTTACTTT   
  
  
+ CCTTCTCCAC TCCTTTCTCC CTCATTCAAC TTCCCTTTTT CCCTTCTCTC TTTTTGGCGT CCTTTTCAAT   
  
  
+ CACTGCTTCG TTGGTCACTG CAGAGAGTAT ACTACGTTAC CACTACGGTT CGGTTCGAAA GAGAAAGAAA   
  
  
+ GAAAGGAAAG AAAGAAAGAA AGAAGAAAAT AGAGCCGTAC GAATTAAAAG GAGTGGTGTT TATTTGTCTT   
  
  
+ ACTTTAGTTC ACCCGAACTA TGACTATCAC ATGACACAAA CCCCCACACG TGTTGTTAAG TTTCAGATTC   
  
  
+ TTAGTTAGAG CATCGATCTC CCGACGTTAC GGCTCTACTA CCTAAAATTT TTTCTAGTTT AACGTTTTTT   
  
  
+ GTTTTTTGTT TTTTGGTTTT TACCAACCGT AATGACAATC ATGCTACGTT CACCGATGAA AATAAGATAA   
  
  
+ GATTACGTTG ACCTAGTAAT GCACCGTGTC CGGAAAGGTG AAAAGAAAGA TTAGGCTTAT TACATTAGTC   
  
  
+ ATTAGTGTTA TGTTCTCACC GTACTAACTA CTACTTAAGG TCTTTAGCAT AACCAACGCA GGCAGGGTAC   
  
  
+ TACGAGAGAA AACTTTTCAG TTACGAAGTT TGGATTGGAA GGTAGTAAAA GGTACAAACG TGAAGGGTAG   
  
  
+ ATTTGTTGAC CGAGTTTTAG TTTAGAAAAA AAAAAAAAAA AATTTTTTTT TTTTCCCTTC GCGAGTAAAC   
  
  
+ TTTGAAATAT TTCACATACA TATATCTTTT ATTTAAAACT ATTGTTCTCT CTCATTTTAT TGTTCGAAAA   
  
  
+ TAGTACTACT TGTTGTATGG AGTTGTTTTA ATTAATTTTT TTGTTGTTCG TAAGTAAAAC TAAAAATGAA   
  
  
+ GATTTAGATT TTCCTAATGT TAAATTATTT TATTTTGTTT ATATAAAAAT TTTATAAAAA GCAAAATGAA   
  
  
+ AGTTATTTTT GTCATTTTAA AATTAAATTC CTAATGTTAA ATTATTTTAT TTTGTTTATA TAAAAATTTT   
  
  
+ ATAAAAAGCA AAATGAAAGT TATTTTTGTC ATTTTAAAAT TAAAACTTCG TTTAACTAGT CTACTGCAAT   
  
  
+ TTTCTAGATC AATGTAGGTG ATCTGAATTA GGTGTATTTC GTTAGAGGTA GACGGTGCAG AATTGTGGTC   
  
  
+ CTGTATTGAC ATTATTGGTG CGGAGACCTA TTTAGGAAGG TGTCTCTGGT CTTCCTTCGA TGACTCATAC   
  
  
+ TAATGACATG GTGTGACATG AGATGACATT ATTGGTGCGG AGACCTATTT AGGAAGGTGT CTCTGGTCTT   
  
  
+ CCTTCGATGA CTCATACTAA TGACATGGT  

- GATCGATCGA TGATAGGGTC ACTGCCCACA GAACACAGTG GTGCTGTTTA ATTTTTTGGC CTTGATTTCG   
  
  
- TTGGGTAGAA GAGCTAGGGG ATATTATAAG TGGGGTGGGG GGAGAGGAAG CGAAGCAGAT AGAGATAAGC   
  
  
- TTGTAACTAA TGATGCGTTT GGACTAAAAG AAATTAAAGG AGAGATGAGG AAAGGGAGAA GCAAATGAAA   
  
  
- GGAAGAGGTG AGGAAAGAGG GAGTAAGTTG AAGGGAAAAA GGGAAGAGAG AAAAACCGCA GGAAAAGTTA   
  
  
- GTGACGAAGC AACCAGTGAC GTCTCTCATA TGATGCAATG GTGATGCCAA GCCAAGCTTT CTCTTTCTTT   
  
  
- CTTTCCTTTC TTTCTTTCTT TCTTCTTTTA TCTCGGCATG CTTAATTTTC CTCACCACAA ATAAACAGAA   
  
  
- TGAAATCAAG TGGGCTTGAT ACTGATAGTG TACTGTGTTT GGGGGTGTGC ACAACAATTC AAAGTCTAAG   
  
  
- AATCAATCTC GTAGCTAGAG GGCTGCAATG CCGAGATGAT GGATTTTAAA AAAGATCAAA TTGCAAAAAA   
  
  
- CAAAAAACAA AAAACCAAAA ATGGTTGGCA TTACTGTTAG TACGATGCAA GTGGCTACTT TTATTCTATT   
  
  
- CTAATGCAAC TGGATCATTA CGTGGCACAG GCCTTTCCAC TTTTCTTTCT AATCCGAATA ATGTAATCAG   
  
  
- TAATCACAAT ACAAGAGTGG CATGATTGAT GATGAATTCC AGAAATCGTA TTGGTTGCGT CCGTCCCATG   
  
  
- ATGCTCTCTT TTGAAAAGTC AATGCTTCAA ACCTAACCTT CCATCATTTT CCATGTTTGC ACTTCCCATC   
  
  
- TAAACAACTG GCTCAAAATC AAATCTTTTT TTTTTTTTTT TTAAAAAAAA AAAAGGGAAG CGCTCATTTG   
  
  
- AAACTTTATA AAGTGTATGT ATATAGAAAA TAAATTTTGA TAACAAGAGA GAGTAAAATA ACAAGCTTTT   
  
  
- ATCATGATGA ACAACATACC TCAACAAAAT TAATTAAAAA AACAACAAGC ATTCATTTTG ATTTTTACTT   
  
  
- CTAAATCTAA AAGGATTACA ATTTAATAAA ATAAAACAAA TATATTTTTA AAATATTTTT CGTTTTACTT   
  
  
- TCAATAAAAA CAGTAAAATT TTAATTTAAG GATTACAATT TAATAAAATA AAACAAATAT ATTTTTAAAA   
  
  
- TATTTTTCGT TTTACTTTCA ATAAAAACAG TAAAATTTTA ATTTTGAAGC AAATTGATCA GATGACGTTA   
  
  
- AAAGATCTAG TTACATCCAC TAGACTTAAT CCACATAAAG CAATCTCCAT CTGCCACGTC TTAACACCAG   
  
  
- GACATAACTG TAATAACCAC GCCTCTGGAT AAATCCTTCC ACAGAGACCA GAAGGAAGCT ACTGAGTATG   
  
  
- ATTACTGTAC CACACTGTAC TCTACTGTAA TAACCACGCC TCTGGATAAA TCCTTCCACA GAGACCAGAA   
  
  
- GGAAGCTACT GAGTATGATT ACTGTACCA

+     MBS

| Site Name | Organism | Position | Strand | Matrix score. | sequence | function |
| --- | --- | --- | --- | --- | --- | --- |
| MBS | Zea mays | 847 | - | 6 | CGGTCA | MYB Binding Site |
| MBS | Arabidopsis thaliana | 788 | - | 6 | TAACTG | MYB binding site involved in drought-inducibility |

> 2018/04/13 10:10:12  
+ CTAGCTAGCT ACTATCCCAG TGACGGGTGT CTTGTGTCAC CACGACAAAT TAAAAAACCG GAACTAAAGC   
  
  
+ AACCCATCTT CTCGATCCCC TATAATATTC ACCCCACCCC CCTCTCCTTC GCTTCGTCTA TCTCTATTCG   
  
  
+ AACATTGATT ACTACGCAAA CCTGATTTTC TTTAATTTCC TCTCTACTCC TTTCCCTCTT CGTTTACTTT   
  
  
+ CCTTCTCCAC TCCTTTCTCC CTCATTCAAC TTCCCTTTTT CCCTTCTCTC TTTTTGGCGT CCTTTTCAAT   
  
  
+ CACTGCTTCG TTGGTCACTG CAGAGAGTAT ACTACGTTAC CACTACGGTT CGGTTCGAAA GAGAAAGAAA   
  
  
+ GAAAGGAAAG AAAGAAAGAA AGAAGAAAAT AGAGCCGTAC GAATTAAAAG GAGTGGTGTT TATTTGTCTT   
  
  
+ ACTTTAGTTC ACCCGAACTA TGACTATCAC ATGACACAAA CCCCCACACG TGTTGTTAAG TTTCAGATTC   
  
  
+ TTAGTTAGAG CATCGATCTC CCGACGTTAC GGCTCTACTA CCTAAAATTT TTTCTAGTTT AACGTTTTTT   
  
  
+ GTTTTTTGTT TTTTGGTTTT TACCAACCGT AATGACAATC ATGCTACGTT CACCGATGAA AATAAGATAA   
  
  
+ GATTACGTTG ACCTAGTAAT GCACCGTGTC CGGAAAGGTG AAAAGAAAGA TTAGGCTTAT TACATTAGTC   
  
  
+ ATTAGTGTTA TGTTCTCACC GTACTAACTA CTACTTAAGG TCTTTAGCAT AACCAACGCA GGCAGGGTAC   
  
  
+ TACGAGAGAA AACTTTTCAG TTACGAAGTT TGGATTGGAA GGTAGTAAAA GGTACAAACG TGAAGGGTAG   
  
  
+ ATTTGTTGAC CGAGTTTTAG TTTAGAAAAA AAAAAAAAAA AATTTTTTTT TTTTCCCTTC GCGAGTAAAC   
  
  
+ TTTGAAATAT TTCACATACA TATATCTTTT ATTTAAAACT ATTGTTCTCT CTCATTTTAT TGTTCGAAAA   
  
  
+ TAGTACTACT TGTTGTATGG AGTTGTTTTA ATTAATTTTT TTGTTGTTCG TAAGTAAAAC TAAAAATGAA   
  
  
+ GATTTAGATT TTCCTAATGT TAAATTATTT TATTTTGTTT ATATAAAAAT TTTATAAAAA GCAAAATGAA   
  
  
+ AGTTATTTTT GTCATTTTAA AATTAAATTC CTAATGTTAA ATTATTTTAT TTTGTTTATA TAAAAATTTT   
  
  
+ ATAAAAAGCA AAATGAAAGT TATTTTTGTC ATTTTAAAAT TAAAACTTCG TTTAACTAGT CTACTGCAAT   
  
  
+ TTTCTAGATC AATGTAGGTG ATCTGAATTA GGTGTATTTC GTTAGAGGTA GACGGTGCAG AATTGTGGTC   
  
  
+ CTGTATTGAC ATTATTGGTG CGGAGACCTA TTTAGGAAGG TGTCTCTGGT CTTCCTTCGA TGACTCATAC   
  
  
+ TAATGACATG GTGTGACATG AGATGACATT ATTGGTGCGG AGACCTATTT AGGAAGGTGT CTCTGGTCTT   
  
  
+ CCTTCGATGA CTCATACTAA TGACATGGT  

- GATCGATCGA TGATAGGGTC ACTGCCCACA GAACACAGTG GTGCTGTTTA ATTTTTTGGC CTTGATTTCG   
  
  
- TTGGGTAGAA GAGCTAGGGG ATATTATAAG TGGGGTGGGG GGAGAGGAAG CGAAGCAGAT AGAGATAAGC   
  
  
- TTGTAACTAA TGATGCGTTT GGACTAAAAG AAATTAAAGG AGAGATGAGG AAAGGGAGAA GCAAATGAAA   
  
  
- GGAAGAGGTG AGGAAAGAGG GAGTAAGTTG AAGGGAAAAA GGGAAGAGAG AAAAACCGCA GGAAAAGTTA   
  
  
- GTGACGAAGC AACCAGTGAC GTCTCTCATA TGATGCAATG GTGATGCCAA GCCAAGCTTT CTCTTTCTTT   
  
  
- CTTTCCTTTC TTTCTTTCTT TCTTCTTTTA TCTCGGCATG CTTAATTTTC CTCACCACAA ATAAACAGAA   
  
  
- TGAAATCAAG TGGGCTTGAT ACTGATAGTG TACTGTGTTT GGGGGTGTGC ACAACAATTC AAAGTCTAAG   
  
  
- AATCAATCTC GTAGCTAGAG GGCTGCAATG CCGAGATGAT GGATTTTAAA AAAGATCAAA TTGCAAAAAA   
  
  
- CAAAAAACAA AAAACCAAAA ATGGTTGGCA TTACTGTTAG TACGATGCAA GTGGCTACTT TTATTCTATT   
  
  
- CTAATGCAAC TGGATCATTA CGTGGCACAG GCCTTTCCAC TTTTCTTTCT AATCCGAATA ATGTAATCAG   
  
  
- TAATCACAAT ACAAGAGTGG CATGATTGAT GATGAATTCC AGAAATCGTA TTGGTTGCGT CCGTCCCATG   
  
  
- ATGCTCTCTT TTGAAAAGTC AATGCTTCAA ACCTAACCTT CCATCATTTT CCATGTTTGC ACTTCCCATC   
  
  
- TAAACAACTG GCTCAAAATC AAATCTTTTT TTTTTTTTTT TTAAAAAAAA AAAAGGGAAG CGCTCATTTG   
  
  
- AAACTTTATA AAGTGTATGT ATATAGAAAA TAAATTTTGA TAACAAGAGA GAGTAAAATA ACAAGCTTTT   
  
  
- ATCATGATGA ACAACATACC TCAACAAAAT TAATTAAAAA AACAACAAGC ATTCATTTTG ATTTTTACTT   
  
  
- CTAAATCTAA AAGGATTACA ATTTAATAAA ATAAAACAAA TATATTTTTA AAATATTTTT CGTTTTACTT   
  
  
- TCAATAAAAA CAGTAAAATT TTAATTTAAG GATTACAATT TAATAAAATA AAACAAATAT ATTTTTAAAA   
  
  
- TATTTTTCGT TTTACTTTCA ATAAAAACAG TAAAATTTTA ATTTTGAAGC AAATTGATCA GATGACGTTA   
  
  
- AAAGATCTAG TTACATCCAC TAGACTTAAT CCACATAAAG CAATCTCCAT CTGCCACGTC TTAACACCAG   
  
  
- GACATAACTG TAATAACCAC GCCTCTGGAT AAATCCTTCC ACAGAGACCA GAAGGAAGCT ACTGAGTATG   
  
  
- ATTACTGTAC CACACTGTAC TCTACTGTAA TAACCACGCC TCTGGATAAA TCCTTCCACA GAGACCAGAA   
  
  
- GGAAGCTACT GAGTATGATT ACTGTACCA

+     O2-site

| Site Name | Organism | Position | Strand | Matrix score. | sequence | function |
| --- | --- | --- | --- | --- | --- | --- |
| O2-site | Zea mays | 1489 | + | 9 | GATGACATGG | cis-acting regulatory element involved in zein metabolism regulation |
| O2-site | Zea mays | 1422 | + | 9 | GATGACATGA | cis-acting regulatory element involved in zein metabolism regulation |
| O2-site | Zea mays | 1402 | + | 9 | GATGACATGG | cis-acting regulatory element involved in zein metabolism regulation |

> 2018/04/13 10:10:12  
+ CTAGCTAGCT ACTATCCCAG TGACGGGTGT CTTGTGTCAC CACGACAAAT TAAAAAACCG GAACTAAAGC   
  
  
+ AACCCATCTT CTCGATCCCC TATAATATTC ACCCCACCCC CCTCTCCTTC GCTTCGTCTA TCTCTATTCG   
  
  
+ AACATTGATT ACTACGCAAA CCTGATTTTC TTTAATTTCC TCTCTACTCC TTTCCCTCTT CGTTTACTTT   
  
  
+ CCTTCTCCAC TCCTTTCTCC CTCATTCAAC TTCCCTTTTT CCCTTCTCTC TTTTTGGCGT CCTTTTCAAT   
  
  
+ CACTGCTTCG TTGGTCACTG CAGAGAGTAT ACTACGTTAC CACTACGGTT CGGTTCGAAA GAGAAAGAAA   
  
  
+ GAAAGGAAAG AAAGAAAGAA AGAAGAAAAT AGAGCCGTAC GAATTAAAAG GAGTGGTGTT TATTTGTCTT   
  
  
+ ACTTTAGTTC ACCCGAACTA TGACTATCAC ATGACACAAA CCCCCACACG TGTTGTTAAG TTTCAGATTC   
  
  
+ TTAGTTAGAG CATCGATCTC CCGACGTTAC GGCTCTACTA CCTAAAATTT TTTCTAGTTT AACGTTTTTT   
  
  
+ GTTTTTTGTT TTTTGGTTTT TACCAACCGT AATGACAATC ATGCTACGTT CACCGATGAA AATAAGATAA   
  
  
+ GATTACGTTG ACCTAGTAAT GCACCGTGTC CGGAAAGGTG AAAAGAAAGA TTAGGCTTAT TACATTAGTC   
  
  
+ ATTAGTGTTA TGTTCTCACC GTACTAACTA CTACTTAAGG TCTTTAGCAT AACCAACGCA GGCAGGGTAC   
  
  
+ TACGAGAGAA AACTTTTCAG TTACGAAGTT TGGATTGGAA GGTAGTAAAA GGTACAAACG TGAAGGGTAG   
  
  
+ ATTTGTTGAC CGAGTTTTAG TTTAGAAAAA AAAAAAAAAA AATTTTTTTT TTTTCCCTTC GCGAGTAAAC   
  
  
+ TTTGAAATAT TTCACATACA TATATCTTTT ATTTAAAACT ATTGTTCTCT CTCATTTTAT TGTTCGAAAA   
  
  
+ TAGTACTACT TGTTGTATGG AGTTGTTTTA ATTAATTTTT TTGTTGTTCG TAAGTAAAAC TAAAAATGAA   
  
  
+ GATTTAGATT TTCCTAATGT TAAATTATTT TATTTTGTTT ATATAAAAAT TTTATAAAAA GCAAAATGAA   
  
  
+ AGTTATTTTT GTCATTTTAA AATTAAATTC CTAATGTTAA ATTATTTTAT TTTGTTTATA TAAAAATTTT   
  
  
+ ATAAAAAGCA AAATGAAAGT TATTTTTGTC ATTTTAAAAT TAAAACTTCG TTTAACTAGT CTACTGCAAT   
  
  
+ TTTCTAGATC AATGTAGGTG ATCTGAATTA GGTGTATTTC GTTAGAGGTA GACGGTGCAG AATTGTGGTC   
  
  
+ CTGTATTGAC ATTATTGGTG CGGAGACCTA TTTAGGAAGG TGTCTCTGGT CTTCCTTCGA TGACTCATAC   
  
  
+ TAATGACATG GTGTGACATG AGATGACATT ATTGGTGCGG AGACCTATTT AGGAAGGTGT CTCTGGTCTT   
  
  
+ CCTTCGATGA CTCATACTAA TGACATGGT  

- GATCGATCGA TGATAGGGTC ACTGCCCACA GAACACAGTG GTGCTGTTTA ATTTTTTGGC CTTGATTTCG   
  
  
- TTGGGTAGAA GAGCTAGGGG ATATTATAAG TGGGGTGGGG GGAGAGGAAG CGAAGCAGAT AGAGATAAGC   
  
  
- TTGTAACTAA TGATGCGTTT GGACTAAAAG AAATTAAAGG AGAGATGAGG AAAGGGAGAA GCAAATGAAA   
  
  
- GGAAGAGGTG AGGAAAGAGG GAGTAAGTTG AAGGGAAAAA GGGAAGAGAG AAAAACCGCA GGAAAAGTTA   
  
  
- GTGACGAAGC AACCAGTGAC GTCTCTCATA TGATGCAATG GTGATGCCAA GCCAAGCTTT CTCTTTCTTT   
  
  
- CTTTCCTTTC TTTCTTTCTT TCTTCTTTTA TCTCGGCATG CTTAATTTTC CTCACCACAA ATAAACAGAA   
  
  
- TGAAATCAAG TGGGCTTGAT ACTGATAGTG TACTGTGTTT GGGGGTGTGC ACAACAATTC AAAGTCTAAG   
  
  
- AATCAATCTC GTAGCTAGAG GGCTGCAATG CCGAGATGAT GGATTTTAAA AAAGATCAAA TTGCAAAAAA   
  
  
- CAAAAAACAA AAAACCAAAA ATGGTTGGCA TTACTGTTAG TACGATGCAA GTGGCTACTT TTATTCTATT   
  
  
- CTAATGCAAC TGGATCATTA CGTGGCACAG GCCTTTCCAC TTTTCTTTCT AATCCGAATA ATGTAATCAG   
  
  
- TAATCACAAT ACAAGAGTGG CATGATTGAT GATGAATTCC AGAAATCGTA TTGGTTGCGT CCGTCCCATG   
  
  
- ATGCTCTCTT TTGAAAAGTC AATGCTTCAA ACCTAACCTT CCATCATTTT CCATGTTTGC ACTTCCCATC   
  
  
- TAAACAACTG GCTCAAAATC AAATCTTTTT TTTTTTTTTT TTAAAAAAAA AAAAGGGAAG CGCTCATTTG   
  
  
- AAACTTTATA AAGTGTATGT ATATAGAAAA TAAATTTTGA TAACAAGAGA GAGTAAAATA ACAAGCTTTT   
  
  
- ATCATGATGA ACAACATACC TCAACAAAAT TAATTAAAAA AACAACAAGC ATTCATTTTG ATTTTTACTT   
  
  
- CTAAATCTAA AAGGATTACA ATTTAATAAA ATAAAACAAA TATATTTTTA AAATATTTTT CGTTTTACTT   
  
  
- TCAATAAAAA CAGTAAAATT TTAATTTAAG GATTACAATT TAATAAAATA AAACAAATAT ATTTTTAAAA   
  
  
- TATTTTTCGT TTTACTTTCA ATAAAAACAG TAAAATTTTA ATTTTGAAGC AAATTGATCA GATGACGTTA   
  
  
- AAAGATCTAG TTACATCCAC TAGACTTAAT CCACATAAAG CAATCTCCAT CTGCCACGTC TTAACACCAG   
  
  
- GACATAACTG TAATAACCAC GCCTCTGGAT AAATCCTTCC ACAGAGACCA GAAGGAAGCT ACTGAGTATG   
  
  
- ATTACTGTAC CACACTGTAC TCTACTGTAA TAACCACGCC TCTGGATAAA TCCTTCCACA GAGACCAGAA   
  
  
- GGAAGCTACT GAGTATGATT ACTGTACCA

+     Skn-1\_motif

| Site Name | Organism | Position | Strand | Matrix score. | sequence | function |
| --- | --- | --- | --- | --- | --- | --- |
| Skn-1\_motif | Oryza sativa | 1490 | - | 5 | GTCAT | cis-acting regulatory element required for endosperm expression |
| Skn-1\_motif | Oryza sativa | 1423 | - | 5 | GTCAT | cis-acting regulatory element required for endosperm expression |
| Skn-1\_motif | Oryza sativa | 1403 | - | 5 | GTCAT | cis-acting regulatory element required for endosperm expression |
| Skn-1\_motif | Oryza sativa | 1477 | - | 5 | GTCAT | cis-acting regulatory element required for endosperm expression |
| Skn-1\_motif | Oryza sativa | 1390 | - | 5 | GTCAT | cis-acting regulatory element required for endosperm expression |
| Skn-1\_motif | Oryza sativa | 1218 | + | 5 | GTCAT | cis-acting regulatory element required for endosperm expression |
| Skn-1\_motif | Oryza sativa | 1131 | + | 5 | GTCAT | cis-acting regulatory element required for endosperm expression |
| Skn-1\_motif | Oryza sativa | 698 | + | 5 | GTCAT | cis-acting regulatory element required for endosperm expression |
| Skn-1\_motif | Oryza sativa | 592 | - | 5 | GTCAT | cis-acting regulatory element required for endosperm expression |
| Skn-1\_motif | Oryza sativa | 451 | - | 5 | GTCAT | cis-acting regulatory element required for endosperm expression |
| Skn-1\_motif | Oryza sativa | 440 | - | 5 | GTCAT | cis-acting regulatory element required for endosperm expression |

> 2018/04/13 10:10:12  
+ CTAGCTAGCT ACTATCCCAG TGACGGGTGT CTTGTGTCAC CACGACAAAT TAAAAAACCG GAACTAAAGC   
  
  
+ AACCCATCTT CTCGATCCCC TATAATATTC ACCCCACCCC CCTCTCCTTC GCTTCGTCTA TCTCTATTCG   
  
  
+ AACATTGATT ACTACGCAAA CCTGATTTTC TTTAATTTCC TCTCTACTCC TTTCCCTCTT CGTTTACTTT   
  
  
+ CCTTCTCCAC TCCTTTCTCC CTCATTCAAC TTCCCTTTTT CCCTTCTCTC TTTTTGGCGT CCTTTTCAAT   
  
  
+ CACTGCTTCG TTGGTCACTG CAGAGAGTAT ACTACGTTAC CACTACGGTT CGGTTCGAAA GAGAAAGAAA   
  
  
+ GAAAGGAAAG AAAGAAAGAA AGAAGAAAAT AGAGCCGTAC GAATTAAAAG GAGTGGTGTT TATTTGTCTT   
  
  
+ ACTTTAGTTC ACCCGAACTA TGACTATCAC ATGACACAAA CCCCCACACG TGTTGTTAAG TTTCAGATTC   
  
  
+ TTAGTTAGAG CATCGATCTC CCGACGTTAC GGCTCTACTA CCTAAAATTT TTTCTAGTTT AACGTTTTTT   
  
  
+ GTTTTTTGTT TTTTGGTTTT TACCAACCGT AATGACAATC ATGCTACGTT CACCGATGAA AATAAGATAA   
  
  
+ GATTACGTTG ACCTAGTAAT GCACCGTGTC CGGAAAGGTG AAAAGAAAGA TTAGGCTTAT TACATTAGTC   
  
  
+ ATTAGTGTTA TGTTCTCACC GTACTAACTA CTACTTAAGG TCTTTAGCAT AACCAACGCA GGCAGGGTAC   
  
  
+ TACGAGAGAA AACTTTTCAG TTACGAAGTT TGGATTGGAA GGTAGTAAAA GGTACAAACG TGAAGGGTAG   
  
  
+ ATTTGTTGAC CGAGTTTTAG TTTAGAAAAA AAAAAAAAAA AATTTTTTTT TTTTCCCTTC GCGAGTAAAC   
  
  
+ TTTGAAATAT TTCACATACA TATATCTTTT ATTTAAAACT ATTGTTCTCT CTCATTTTAT TGTTCGAAAA   
  
  
+ TAGTACTACT TGTTGTATGG AGTTGTTTTA ATTAATTTTT TTGTTGTTCG TAAGTAAAAC TAAAAATGAA   
  
  
+ GATTTAGATT TTCCTAATGT TAAATTATTT TATTTTGTTT ATATAAAAAT TTTATAAAAA GCAAAATGAA   
  
  
+ AGTTATTTTT GTCATTTTAA AATTAAATTC CTAATGTTAA ATTATTTTAT TTTGTTTATA TAAAAATTTT   
  
  
+ ATAAAAAGCA AAATGAAAGT TATTTTTGTC ATTTTAAAAT TAAAACTTCG TTTAACTAGT CTACTGCAAT   
  
  
+ TTTCTAGATC AATGTAGGTG ATCTGAATTA GGTGTATTTC GTTAGAGGTA GACGGTGCAG AATTGTGGTC   
  
  
+ CTGTATTGAC ATTATTGGTG CGGAGACCTA TTTAGGAAGG TGTCTCTGGT CTTCCTTCGA TGACTCATAC   
  
  
+ TAATGACATG GTGTGACATG AGATGACATT ATTGGTGCGG AGACCTATTT AGGAAGGTGT CTCTGGTCTT   
  
  
+ CCTTCGATGA CTCATACTAA TGACATGGT  

- GATCGATCGA TGATAGGGTC ACTGCCCACA GAACACAGTG GTGCTGTTTA ATTTTTTGGC CTTGATTTCG   
  
  
- TTGGGTAGAA GAGCTAGGGG ATATTATAAG TGGGGTGGGG GGAGAGGAAG CGAAGCAGAT AGAGATAAGC   
  
  
- TTGTAACTAA TGATGCGTTT GGACTAAAAG AAATTAAAGG AGAGATGAGG AAAGGGAGAA GCAAATGAAA   
  
  
- GGAAGAGGTG AGGAAAGAGG GAGTAAGTTG AAGGGAAAAA GGGAAGAGAG AAAAACCGCA GGAAAAGTTA   
  
  
- GTGACGAAGC AACCAGTGAC GTCTCTCATA TGATGCAATG GTGATGCCAA GCCAAGCTTT CTCTTTCTTT   
  
  
- CTTTCCTTTC TTTCTTTCTT TCTTCTTTTA TCTCGGCATG CTTAATTTTC CTCACCACAA ATAAACAGAA   
  
  
- TGAAATCAAG TGGGCTTGAT ACTGATAGTG TACTGTGTTT GGGGGTGTGC ACAACAATTC AAAGTCTAAG   
  
  
- AATCAATCTC GTAGCTAGAG GGCTGCAATG CCGAGATGAT GGATTTTAAA AAAGATCAAA TTGCAAAAAA   
  
  
- CAAAAAACAA AAAACCAAAA ATGGTTGGCA TTACTGTTAG TACGATGCAA GTGGCTACTT TTATTCTATT   
  
  
- CTAATGCAAC TGGATCATTA CGTGGCACAG GCCTTTCCAC TTTTCTTTCT AATCCGAATA ATGTAATCAG   
  
  
- TAATCACAAT ACAAGAGTGG CATGATTGAT GATGAATTCC AGAAATCGTA TTGGTTGCGT CCGTCCCATG   
  
  
- ATGCTCTCTT TTGAAAAGTC AATGCTTCAA ACCTAACCTT CCATCATTTT CCATGTTTGC ACTTCCCATC   
  
  
- TAAACAACTG GCTCAAAATC AAATCTTTTT TTTTTTTTTT TTAAAAAAAA AAAAGGGAAG CGCTCATTTG   
  
  
- AAACTTTATA AAGTGTATGT ATATAGAAAA TAAATTTTGA TAACAAGAGA GAGTAAAATA ACAAGCTTTT   
  
  
- ATCATGATGA ACAACATACC TCAACAAAAT TAATTAAAAA AACAACAAGC ATTCATTTTG ATTTTTACTT   
  
  
- CTAAATCTAA AAGGATTACA ATTTAATAAA ATAAAACAAA TATATTTTTA AAATATTTTT CGTTTTACTT   
  
  
- TCAATAAAAA CAGTAAAATT TTAATTTAAG GATTACAATT TAATAAAATA AAACAAATAT ATTTTTAAAA   
  
  
- TATTTTTCGT TTTACTTTCA ATAAAAACAG TAAAATTTTA ATTTTGAAGC AAATTGATCA GATGACGTTA   
  
  
- AAAGATCTAG TTACATCCAC TAGACTTAAT CCACATAAAG CAATCTCCAT CTGCCACGTC TTAACACCAG   
  
  
- GACATAACTG TAATAACCAC GCCTCTGGAT AAATCCTTCC ACAGAGACCA GAAGGAAGCT ACTGAGTATG   
  
  
- ATTACTGTAC CACACTGTAC TCTACTGTAA TAACCACGCC TCTGGATAAA TCCTTCCACA GAGACCAGAA   
  
  
- GGAAGCTACT GAGTATGATT ACTGTACCA

+     Sp1

| Site Name | Organism | Position | Strand | Matrix score. | sequence | function |
| --- | --- | --- | --- | --- | --- | --- |
| Sp1 | Zea mays | 107 | + | 5 | CC(G/A)CCC | light responsive element |
| Sp1 | Zea mays | 104 | + | 5.5 | CC(G/A)CCC | light responsive element |

> 2018/04/13 10:10:12  
+ CTAGCTAGCT ACTATCCCAG TGACGGGTGT CTTGTGTCAC CACGACAAAT TAAAAAACCG GAACTAAAGC   
  
  
+ AACCCATCTT CTCGATCCCC TATAATATTC ACCCCACCCC CCTCTCCTTC GCTTCGTCTA TCTCTATTCG   
  
  
+ AACATTGATT ACTACGCAAA CCTGATTTTC TTTAATTTCC TCTCTACTCC TTTCCCTCTT CGTTTACTTT   
  
  
+ CCTTCTCCAC TCCTTTCTCC CTCATTCAAC TTCCCTTTTT CCCTTCTCTC TTTTTGGCGT CCTTTTCAAT   
  
  
+ CACTGCTTCG TTGGTCACTG CAGAGAGTAT ACTACGTTAC CACTACGGTT CGGTTCGAAA GAGAAAGAAA   
  
  
+ GAAAGGAAAG AAAGAAAGAA AGAAGAAAAT AGAGCCGTAC GAATTAAAAG GAGTGGTGTT TATTTGTCTT   
  
  
+ ACTTTAGTTC ACCCGAACTA TGACTATCAC ATGACACAAA CCCCCACACG TGTTGTTAAG TTTCAGATTC   
  
  
+ TTAGTTAGAG CATCGATCTC CCGACGTTAC GGCTCTACTA CCTAAAATTT TTTCTAGTTT AACGTTTTTT   
  
  
+ GTTTTTTGTT TTTTGGTTTT TACCAACCGT AATGACAATC ATGCTACGTT CACCGATGAA AATAAGATAA   
  
  
+ GATTACGTTG ACCTAGTAAT GCACCGTGTC CGGAAAGGTG AAAAGAAAGA TTAGGCTTAT TACATTAGTC   
  
  
+ ATTAGTGTTA TGTTCTCACC GTACTAACTA CTACTTAAGG TCTTTAGCAT AACCAACGCA GGCAGGGTAC   
  
  
+ TACGAGAGAA AACTTTTCAG TTACGAAGTT TGGATTGGAA GGTAGTAAAA GGTACAAACG TGAAGGGTAG   
  
  
+ ATTTGTTGAC CGAGTTTTAG TTTAGAAAAA AAAAAAAAAA AATTTTTTTT TTTTCCCTTC GCGAGTAAAC   
  
  
+ TTTGAAATAT TTCACATACA TATATCTTTT ATTTAAAACT ATTGTTCTCT CTCATTTTAT TGTTCGAAAA   
  
  
+ TAGTACTACT TGTTGTATGG AGTTGTTTTA ATTAATTTTT TTGTTGTTCG TAAGTAAAAC TAAAAATGAA   
  
  
+ GATTTAGATT TTCCTAATGT TAAATTATTT TATTTTGTTT ATATAAAAAT TTTATAAAAA GCAAAATGAA   
  
  
+ AGTTATTTTT GTCATTTTAA AATTAAATTC CTAATGTTAA ATTATTTTAT TTTGTTTATA TAAAAATTTT   
  
  
+ ATAAAAAGCA AAATGAAAGT TATTTTTGTC ATTTTAAAAT TAAAACTTCG TTTAACTAGT CTACTGCAAT   
  
  
+ TTTCTAGATC AATGTAGGTG ATCTGAATTA GGTGTATTTC GTTAGAGGTA GACGGTGCAG AATTGTGGTC   
  
  
+ CTGTATTGAC ATTATTGGTG CGGAGACCTA TTTAGGAAGG TGTCTCTGGT CTTCCTTCGA TGACTCATAC   
  
  
+ TAATGACATG GTGTGACATG AGATGACATT ATTGGTGCGG AGACCTATTT AGGAAGGTGT CTCTGGTCTT   
  
  
+ CCTTCGATGA CTCATACTAA TGACATGGT  

- GATCGATCGA TGATAGGGTC ACTGCCCACA GAACACAGTG GTGCTGTTTA ATTTTTTGGC CTTGATTTCG   
  
  
- TTGGGTAGAA GAGCTAGGGG ATATTATAAG TGGGGTGGGG GGAGAGGAAG CGAAGCAGAT AGAGATAAGC   
  
  
- TTGTAACTAA TGATGCGTTT GGACTAAAAG AAATTAAAGG AGAGATGAGG AAAGGGAGAA GCAAATGAAA   
  
  
- GGAAGAGGTG AGGAAAGAGG GAGTAAGTTG AAGGGAAAAA GGGAAGAGAG AAAAACCGCA GGAAAAGTTA   
  
  
- GTGACGAAGC AACCAGTGAC GTCTCTCATA TGATGCAATG GTGATGCCAA GCCAAGCTTT CTCTTTCTTT   
  
  
- CTTTCCTTTC TTTCTTTCTT TCTTCTTTTA TCTCGGCATG CTTAATTTTC CTCACCACAA ATAAACAGAA   
  
  
- TGAAATCAAG TGGGCTTGAT ACTGATAGTG TACTGTGTTT GGGGGTGTGC ACAACAATTC AAAGTCTAAG   
  
  
- AATCAATCTC GTAGCTAGAG GGCTGCAATG CCGAGATGAT GGATTTTAAA AAAGATCAAA TTGCAAAAAA   
  
  
- CAAAAAACAA AAAACCAAAA ATGGTTGGCA TTACTGTTAG TACGATGCAA GTGGCTACTT TTATTCTATT   
  
  
- CTAATGCAAC TGGATCATTA CGTGGCACAG GCCTTTCCAC TTTTCTTTCT AATCCGAATA ATGTAATCAG   
  
  
- TAATCACAAT ACAAGAGTGG CATGATTGAT GATGAATTCC AGAAATCGTA TTGGTTGCGT CCGTCCCATG   
  
  
- ATGCTCTCTT TTGAAAAGTC AATGCTTCAA ACCTAACCTT CCATCATTTT CCATGTTTGC ACTTCCCATC   
  
  
- TAAACAACTG GCTCAAAATC AAATCTTTTT TTTTTTTTTT TTAAAAAAAA AAAAGGGAAG CGCTCATTTG   
  
  
- AAACTTTATA AAGTGTATGT ATATAGAAAA TAAATTTTGA TAACAAGAGA GAGTAAAATA ACAAGCTTTT   
  
  
- ATCATGATGA ACAACATACC TCAACAAAAT TAATTAAAAA AACAACAAGC ATTCATTTTG ATTTTTACTT   
  
  
- CTAAATCTAA AAGGATTACA ATTTAATAAA ATAAAACAAA TATATTTTTA AAATATTTTT CGTTTTACTT   
  
  
- TCAATAAAAA CAGTAAAATT TTAATTTAAG GATTACAATT TAATAAAATA AAACAAATAT ATTTTTAAAA   
  
  
- TATTTTTCGT TTTACTTTCA ATAAAAACAG TAAAATTTTA ATTTTGAAGC AAATTGATCA GATGACGTTA   
  
  
- AAAGATCTAG TTACATCCAC TAGACTTAAT CCACATAAAG CAATCTCCAT CTGCCACGTC TTAACACCAG   
  
  
- GACATAACTG TAATAACCAC GCCTCTGGAT AAATCCTTCC ACAGAGACCA GAAGGAAGCT ACTGAGTATG   
  
  
- ATTACTGTAC CACACTGTAC TCTACTGTAA TAACCACGCC TCTGGATAAA TCCTTCCACA GAGACCAGAA   
  
  
- GGAAGCTACT GAGTATGATT ACTGTACCA

+     TATA-box

| Site Name | Organism | Position | Strand | Matrix score. | sequence | function |
| --- | --- | --- | --- | --- | --- | --- |
| TATA-box | Lycopersicon esculentum | 1225 | - | 5 | TTTTA | core promoter element around -30 of transcription start |
| TATA-box | Lycopersicon esculentum | 1222 | + | 5 | TTTTA | core promoter element around -30 of transcription start |
| TATA-box | Lycopersicon esculentum | 1192 | - | 5 | TTTTA | core promoter element around -30 of transcription start |
| TATA-box | Lycopersicon esculentum | 944 | - | 5 | TTTTA | core promoter element around -30 of transcription start |
| TATA-box | Lycopersicon esculentum | 1231 | - | 5 | TTTTA | core promoter element around -30 of transcription start |
| TATA-box | Arabidopsis thaliana | 1190 | + | 6 | TATAAA | core promoter element around -30 of transcription start |
| TATA-box | Arabidopsis thaliana | 1189 | - | 5 | TATAA | core promoter element around -30 of transcription start |
| TATA-box | Arabidopsis thaliana | 940 | + | 8 | TATTTAAA | core promoter element around -30 of transcription start |
| TATA-box | Arabidopsis thaliana | 1188 | - | 6 | TATAAA | core promoter element around -30 of transcription start |
| TATA-box | Arabidopsis thaliana | 1187 | - | 7 | TATAAAA | core promoter element around -30 of transcription start |
| TATA-box | Pisum sativum | 1186 | - | 8 | TATAAAAT | core promoter element around -30 of transcription start |
| TATA-box | Lycopersicon esculentum | 1181 | - | 5 | TTTTA | core promoter element around -30 of transcription start |
| TATA-box | Brassica oleracea | 1178 | + | 6 | ATATAA | core promoter element around -30 of transcription start |
| TATA-box | Arabidopsis thaliana | 1179 | + | 6 | TATAAA | core promoter element around -30 of transcription start |
| TATA-box | Arabidopsis thaliana | 1177 | - | 4 | TATA | core promoter element around -30 of transcription start |
| TATA-box | Arabidopsis thaliana | 1176 | - | 7 | TATATAA | core promoter element around -30 of transcription start |
| TATA-box | Arabidopsis thaliana | 1175 | - | 6 | TATAAA | core promoter element around -30 of transcription start |
| TATA-box | Lycopersicon esculentum | 1165 | + | 5 | TTTTA | core promoter element around -30 of transcription start |
| TATA-box | Lycopersicon esculentum | 1138 | - | 5 | TTTTA | core promoter element around -30 of transcription start |
| TATA-box | Lycopersicon esculentum | 1135 | + | 5 | TTTTA | core promoter element around -30 of transcription start |
| TATA-box | Lycopersicon esculentum | 1105 | - | 5 | TTTTA | core promoter element around -30 of transcription start |
| TATA-box | Arabidopsis thaliana | 1103 | + | 6 | TATAAA | core promoter element around -30 of transcription start |
| TATA-box | Arabidopsis thaliana | 1102 | - | 5 | TATAA | core promoter element around -30 of transcription start |
| TATA-box | Arabidopsis thaliana | 1101 | - | 6 | TATAAA | core promoter element around -30 of transcription start |
| TATA-box | Arabidopsis thaliana | 1100 | - | 7 | TATAAAA | core promoter element around -30 of transcription start |
| TATA-box | Pisum sativum | 1099 | - | 8 | TATAAAAT | core promoter element around -30 of transcription start |
| TATA-box | Lycopersicon esculentum | 1094 | - | 5 | TTTTA | core promoter element around -30 of transcription start |
| TATA-box | Arabidopsis thaliana | 1092 | + | 6 | TATAAA | core promoter element around -30 of transcription start |
| TATA-box | Brassica oleracea | 1091 | + | 6 | ATATAA | core promoter element around -30 of transcription start |
| TATA-box | Arabidopsis thaliana | 1090 | - | 4 | TATA | core promoter element around -30 of transcription start |
| TATA-box | Arabidopsis thaliana | 1089 | - | 7 | TATATAA | core promoter element around -30 of transcription start |
| TATA-box | Arabidopsis thaliana | 1088 | - | 6 | TATAAA | core promoter element around -30 of transcription start |
| TATA-box | Lycopersicon esculentum | 1078 | + | 5 | TTTTA | core promoter element around -30 of transcription start |
| TATA-box | Lycopersicon esculentum | 1041 | - | 5 | TTTTA | core promoter element around -30 of transcription start |
| TATA-box | Lycopersicon esculentum | 1035 | - | 5 | TTTTA | core promoter element around -30 of transcription start |
| TATA-box | Lycopersicon esculentum | 1006 | + | 5 | TTTTA | core promoter element around -30 of transcription start |
| TATA-box | Lycopersicon esculentum | 965 | + | 5 | TTTTA | core promoter element around -30 of transcription start |
| TATA-box | Lycopersicon esculentum | 937 | + | 5 | TTTTA | core promoter element around -30 of transcription start |
| TATA-box | Arabidopsis thaliana | 931 | - | 4 | TATA | core promoter element around -30 of transcription start |
| TATA-box | Lycopersicon esculentum | 533 | - | 5 | TTTTA | core promoter element around -30 of transcription start |
| TATA-box | Brassica napus | 930 | - | 6 | ATATAT | core promoter element around -30 of transcription start |
| TATA-box | Pisum sativum | 928 | - | 7 | TATATGT | core promoter element around -30 of transcription start |
| TATA-box | Lycopersicon esculentum | 855 | + | 5 | TTTTA | core promoter element around -30 of transcription start |
| TATA-box | Lycopersicon esculentum | 816 | - | 5 | TTTTA | core promoter element around -30 of transcription start |
| TATA-box | Glycine max | 688 | - | 5 | TAATA | core promoter element around -30 of transcription start |
| TATA-box | Lycopersicon esculentum | 578 | + | 5 | TTTTA | core promoter element around -30 of transcription start |
| TATA-box | Lycopersicon esculentum | 395 | - | 5 | TTTTA | core promoter element around -30 of transcription start |
| TATA-box | Glycine max | 93 | + | 5 | TAATA | core promoter element around -30 of transcription start |
| TATA-box | Arabidopsis thaliana | 308 | + | 4 | TATA | core promoter element around -30 of transcription start |
| TATA-box | Arabidopsis thaliana | 91 | + | 4 | TATA | core promoter element around -30 of transcription start |
| TATA-box | Lycopersicon esculentum | 51 | - | 5 | TTTTA | core promoter element around -30 of transcription start |

> 2018/04/13 10:10:12  
+ CTAGCTAGCT ACTATCCCAG TGACGGGTGT CTTGTGTCAC CACGACAAAT TAAAAAACCG GAACTAAAGC   
  
  
+ AACCCATCTT CTCGATCCCC TATAATATTC ACCCCACCCC CCTCTCCTTC GCTTCGTCTA TCTCTATTCG   
  
  
+ AACATTGATT ACTACGCAAA CCTGATTTTC TTTAATTTCC TCTCTACTCC TTTCCCTCTT CGTTTACTTT   
  
  
+ CCTTCTCCAC TCCTTTCTCC CTCATTCAAC TTCCCTTTTT CCCTTCTCTC TTTTTGGCGT CCTTTTCAAT   
  
  
+ CACTGCTTCG TTGGTCACTG CAGAGAGTAT ACTACGTTAC CACTACGGTT CGGTTCGAAA GAGAAAGAAA   
  
  
+ GAAAGGAAAG AAAGAAAGAA AGAAGAAAAT AGAGCCGTAC GAATTAAAAG GAGTGGTGTT TATTTGTCTT   
  
  
+ ACTTTAGTTC ACCCGAACTA TGACTATCAC ATGACACAAA CCCCCACACG TGTTGTTAAG TTTCAGATTC   
  
  
+ TTAGTTAGAG CATCGATCTC CCGACGTTAC GGCTCTACTA CCTAAAATTT TTTCTAGTTT AACGTTTTTT   
  
  
+ GTTTTTTGTT TTTTGGTTTT TACCAACCGT AATGACAATC ATGCTACGTT CACCGATGAA AATAAGATAA   
  
  
+ GATTACGTTG ACCTAGTAAT GCACCGTGTC CGGAAAGGTG AAAAGAAAGA TTAGGCTTAT TACATTAGTC   
  
  
+ ATTAGTGTTA TGTTCTCACC GTACTAACTA CTACTTAAGG TCTTTAGCAT AACCAACGCA GGCAGGGTAC   
  
  
+ TACGAGAGAA AACTTTTCAG TTACGAAGTT TGGATTGGAA GGTAGTAAAA GGTACAAACG TGAAGGGTAG   
  
  
+ ATTTGTTGAC CGAGTTTTAG TTTAGAAAAA AAAAAAAAAA AATTTTTTTT TTTTCCCTTC GCGAGTAAAC   
  
  
+ TTTGAAATAT TTCACATACA TATATCTTTT ATTTAAAACT ATTGTTCTCT CTCATTTTAT TGTTCGAAAA   
  
  
+ TAGTACTACT TGTTGTATGG AGTTGTTTTA ATTAATTTTT TTGTTGTTCG TAAGTAAAAC TAAAAATGAA   
  
  
+ GATTTAGATT TTCCTAATGT TAAATTATTT TATTTTGTTT ATATAAAAAT TTTATAAAAA GCAAAATGAA   
  
  
+ AGTTATTTTT GTCATTTTAA AATTAAATTC CTAATGTTAA ATTATTTTAT TTTGTTTATA TAAAAATTTT   
  
  
+ ATAAAAAGCA AAATGAAAGT TATTTTTGTC ATTTTAAAAT TAAAACTTCG TTTAACTAGT CTACTGCAAT   
  
  
+ TTTCTAGATC AATGTAGGTG ATCTGAATTA GGTGTATTTC GTTAGAGGTA GACGGTGCAG AATTGTGGTC   
  
  
+ CTGTATTGAC ATTATTGGTG CGGAGACCTA TTTAGGAAGG TGTCTCTGGT CTTCCTTCGA TGACTCATAC   
  
  
+ TAATGACATG GTGTGACATG AGATGACATT ATTGGTGCGG AGACCTATTT AGGAAGGTGT CTCTGGTCTT   
  
  
+ CCTTCGATGA CTCATACTAA TGACATGGT  

- GATCGATCGA TGATAGGGTC ACTGCCCACA GAACACAGTG GTGCTGTTTA ATTTTTTGGC CTTGATTTCG   
  
  
- TTGGGTAGAA GAGCTAGGGG ATATTATAAG TGGGGTGGGG GGAGAGGAAG CGAAGCAGAT AGAGATAAGC   
  
  
- TTGTAACTAA TGATGCGTTT GGACTAAAAG AAATTAAAGG AGAGATGAGG AAAGGGAGAA GCAAATGAAA   
  
  
- GGAAGAGGTG AGGAAAGAGG GAGTAAGTTG AAGGGAAAAA GGGAAGAGAG AAAAACCGCA GGAAAAGTTA   
  
  
- GTGACGAAGC AACCAGTGAC GTCTCTCATA TGATGCAATG GTGATGCCAA GCCAAGCTTT CTCTTTCTTT   
  
  
- CTTTCCTTTC TTTCTTTCTT TCTTCTTTTA TCTCGGCATG CTTAATTTTC CTCACCACAA ATAAACAGAA   
  
  
- TGAAATCAAG TGGGCTTGAT ACTGATAGTG TACTGTGTTT GGGGGTGTGC ACAACAATTC AAAGTCTAAG   
  
  
- AATCAATCTC GTAGCTAGAG GGCTGCAATG CCGAGATGAT GGATTTTAAA AAAGATCAAA TTGCAAAAAA   
  
  
- CAAAAAACAA AAAACCAAAA ATGGTTGGCA TTACTGTTAG TACGATGCAA GTGGCTACTT TTATTCTATT   
  
  
- CTAATGCAAC TGGATCATTA CGTGGCACAG GCCTTTCCAC TTTTCTTTCT AATCCGAATA ATGTAATCAG   
  
  
- TAATCACAAT ACAAGAGTGG CATGATTGAT GATGAATTCC AGAAATCGTA TTGGTTGCGT CCGTCCCATG   
  
  
- ATGCTCTCTT TTGAAAAGTC AATGCTTCAA ACCTAACCTT CCATCATTTT CCATGTTTGC ACTTCCCATC   
  
  
- TAAACAACTG GCTCAAAATC AAATCTTTTT TTTTTTTTTT TTAAAAAAAA AAAAGGGAAG CGCTCATTTG   
  
  
- AAACTTTATA AAGTGTATGT ATATAGAAAA TAAATTTTGA TAACAAGAGA GAGTAAAATA ACAAGCTTTT   
  
  
- ATCATGATGA ACAACATACC TCAACAAAAT TAATTAAAAA AACAACAAGC ATTCATTTTG ATTTTTACTT   
  
  
- CTAAATCTAA AAGGATTACA ATTTAATAAA ATAAAACAAA TATATTTTTA AAATATTTTT CGTTTTACTT   
  
  
- TCAATAAAAA CAGTAAAATT TTAATTTAAG GATTACAATT TAATAAAATA AAACAAATAT ATTTTTAAAA   
  
  
- TATTTTTCGT TTTACTTTCA ATAAAAACAG TAAAATTTTA ATTTTGAAGC AAATTGATCA GATGACGTTA   
  
  
- AAAGATCTAG TTACATCCAC TAGACTTAAT CCACATAAAG CAATCTCCAT CTGCCACGTC TTAACACCAG   
  
  
- GACATAACTG TAATAACCAC GCCTCTGGAT AAATCCTTCC ACAGAGACCA GAAGGAAGCT ACTGAGTATG   
  
  
- ATTACTGTAC CACACTGTAC TCTACTGTAA TAACCACGCC TCTGGATAAA TCCTTCCACA GAGACCAGAA   
  
  
- GGAAGCTACT GAGTATGATT ACTGTACCA

+     TATC-box

| Site Name | Organism | Position | Strand | Matrix score. | sequence | function |
| --- | --- | --- | --- | --- | --- | --- |
| TATC-box | Oryza sativa | 13 | + | 7 | TATCCCA | cis-acting element involved in gibberellin-responsiveness |

> 2018/04/13 10:10:12  
+ CTAGCTAGCT ACTATCCCAG TGACGGGTGT CTTGTGTCAC CACGACAAAT TAAAAAACCG GAACTAAAGC   
  
  
+ AACCCATCTT CTCGATCCCC TATAATATTC ACCCCACCCC CCTCTCCTTC GCTTCGTCTA TCTCTATTCG   
  
  
+ AACATTGATT ACTACGCAAA CCTGATTTTC TTTAATTTCC TCTCTACTCC TTTCCCTCTT CGTTTACTTT   
  
  
+ CCTTCTCCAC TCCTTTCTCC CTCATTCAAC TTCCCTTTTT CCCTTCTCTC TTTTTGGCGT CCTTTTCAAT   
  
  
+ CACTGCTTCG TTGGTCACTG CAGAGAGTAT ACTACGTTAC CACTACGGTT CGGTTCGAAA GAGAAAGAAA   
  
  
+ GAAAGGAAAG AAAGAAAGAA AGAAGAAAAT AGAGCCGTAC GAATTAAAAG GAGTGGTGTT TATTTGTCTT   
  
  
+ ACTTTAGTTC ACCCGAACTA TGACTATCAC ATGACACAAA CCCCCACACG TGTTGTTAAG TTTCAGATTC   
  
  
+ TTAGTTAGAG CATCGATCTC CCGACGTTAC GGCTCTACTA CCTAAAATTT TTTCTAGTTT AACGTTTTTT   
  
  
+ GTTTTTTGTT TTTTGGTTTT TACCAACCGT AATGACAATC ATGCTACGTT CACCGATGAA AATAAGATAA   
  
  
+ GATTACGTTG ACCTAGTAAT GCACCGTGTC CGGAAAGGTG AAAAGAAAGA TTAGGCTTAT TACATTAGTC   
  
  
+ ATTAGTGTTA TGTTCTCACC GTACTAACTA CTACTTAAGG TCTTTAGCAT AACCAACGCA GGCAGGGTAC   
  
  
+ TACGAGAGAA AACTTTTCAG TTACGAAGTT TGGATTGGAA GGTAGTAAAA GGTACAAACG TGAAGGGTAG   
  
  
+ ATTTGTTGAC CGAGTTTTAG TTTAGAAAAA AAAAAAAAAA AATTTTTTTT TTTTCCCTTC GCGAGTAAAC   
  
  
+ TTTGAAATAT TTCACATACA TATATCTTTT ATTTAAAACT ATTGTTCTCT CTCATTTTAT TGTTCGAAAA   
  
  
+ TAGTACTACT TGTTGTATGG AGTTGTTTTA ATTAATTTTT TTGTTGTTCG TAAGTAAAAC TAAAAATGAA   
  
  
+ GATTTAGATT TTCCTAATGT TAAATTATTT TATTTTGTTT ATATAAAAAT TTTATAAAAA GCAAAATGAA   
  
  
+ AGTTATTTTT GTCATTTTAA AATTAAATTC CTAATGTTAA ATTATTTTAT TTTGTTTATA TAAAAATTTT   
  
  
+ ATAAAAAGCA AAATGAAAGT TATTTTTGTC ATTTTAAAAT TAAAACTTCG TTTAACTAGT CTACTGCAAT   
  
  
+ TTTCTAGATC AATGTAGGTG ATCTGAATTA GGTGTATTTC GTTAGAGGTA GACGGTGCAG AATTGTGGTC   
  
  
+ CTGTATTGAC ATTATTGGTG CGGAGACCTA TTTAGGAAGG TGTCTCTGGT CTTCCTTCGA TGACTCATAC   
  
  
+ TAATGACATG GTGTGACATG AGATGACATT ATTGGTGCGG AGACCTATTT AGGAAGGTGT CTCTGGTCTT   
  
  
+ CCTTCGATGA CTCATACTAA TGACATGGT  

- GATCGATCGA TGATAGGGTC ACTGCCCACA GAACACAGTG GTGCTGTTTA ATTTTTTGGC CTTGATTTCG   
  
  
- TTGGGTAGAA GAGCTAGGGG ATATTATAAG TGGGGTGGGG GGAGAGGAAG CGAAGCAGAT AGAGATAAGC   
  
  
- TTGTAACTAA TGATGCGTTT GGACTAAAAG AAATTAAAGG AGAGATGAGG AAAGGGAGAA GCAAATGAAA   
  
  
- GGAAGAGGTG AGGAAAGAGG GAGTAAGTTG AAGGGAAAAA GGGAAGAGAG AAAAACCGCA GGAAAAGTTA   
  
  
- GTGACGAAGC AACCAGTGAC GTCTCTCATA TGATGCAATG GTGATGCCAA GCCAAGCTTT CTCTTTCTTT   
  
  
- CTTTCCTTTC TTTCTTTCTT TCTTCTTTTA TCTCGGCATG CTTAATTTTC CTCACCACAA ATAAACAGAA   
  
  
- TGAAATCAAG TGGGCTTGAT ACTGATAGTG TACTGTGTTT GGGGGTGTGC ACAACAATTC AAAGTCTAAG   
  
  
- AATCAATCTC GTAGCTAGAG GGCTGCAATG CCGAGATGAT GGATTTTAAA AAAGATCAAA TTGCAAAAAA   
  
  
- CAAAAAACAA AAAACCAAAA ATGGTTGGCA TTACTGTTAG TACGATGCAA GTGGCTACTT TTATTCTATT   
  
  
- CTAATGCAAC TGGATCATTA CGTGGCACAG GCCTTTCCAC TTTTCTTTCT AATCCGAATA ATGTAATCAG   
  
  
- TAATCACAAT ACAAGAGTGG CATGATTGAT GATGAATTCC AGAAATCGTA TTGGTTGCGT CCGTCCCATG   
  
  
- ATGCTCTCTT TTGAAAAGTC AATGCTTCAA ACCTAACCTT CCATCATTTT CCATGTTTGC ACTTCCCATC   
  
  
- TAAACAACTG GCTCAAAATC AAATCTTTTT TTTTTTTTTT TTAAAAAAAA AAAAGGGAAG CGCTCATTTG   
  
  
- AAACTTTATA AAGTGTATGT ATATAGAAAA TAAATTTTGA TAACAAGAGA GAGTAAAATA ACAAGCTTTT   
  
  
- ATCATGATGA ACAACATACC TCAACAAAAT TAATTAAAAA AACAACAAGC ATTCATTTTG ATTTTTACTT   
  
  
- CTAAATCTAA AAGGATTACA ATTTAATAAA ATAAAACAAA TATATTTTTA AAATATTTTT CGTTTTACTT   
  
  
- TCAATAAAAA CAGTAAAATT TTAATTTAAG GATTACAATT TAATAAAATA AAACAAATAT ATTTTTAAAA   
  
  
- TATTTTTCGT TTTACTTTCA ATAAAAACAG TAAAATTTTA ATTTTGAAGC AAATTGATCA GATGACGTTA   
  
  
- AAAGATCTAG TTACATCCAC TAGACTTAAT CCACATAAAG CAATCTCCAT CTGCCACGTC TTAACACCAG   
  
  
- GACATAACTG TAATAACCAC GCCTCTGGAT AAATCCTTCC ACAGAGACCA GAAGGAAGCT ACTGAGTATG   
  
  
- ATTACTGTAC CACACTGTAC TCTACTGTAA TAACCACGCC TCTGGATAAA TCCTTCCACA GAGACCAGAA   
  
  
- GGAAGCTACT GAGTATGATT ACTGTACCA

+     TC-rich repeats

| Site Name | Organism | Position | Strand | Matrix score. | sequence | function |
| --- | --- | --- | --- | --- | --- | --- |
| TC-rich repeats | Nicotiana tabacum | 494 | - | 9 | ATTCTCTAAC | cis-acting element involved in defense and stress responsiveness |
| TC-rich repeats | Nicotiana tabacum | 371 | - | 9 | ATTTTCTTCA | cis-acting element involved in defense and stress responsiveness |
| TC-rich repeats | Nicotiana tabacum | 165 | + | 9 | ATTTTCTTCA | cis-acting element involved in defense and stress responsiveness |

> 2018/04/13 10:10:12  
+ CTAGCTAGCT ACTATCCCAG TGACGGGTGT CTTGTGTCAC CACGACAAAT TAAAAAACCG GAACTAAAGC   
  
  
+ AACCCATCTT CTCGATCCCC TATAATATTC ACCCCACCCC CCTCTCCTTC GCTTCGTCTA TCTCTATTCG   
  
  
+ AACATTGATT ACTACGCAAA CCTGATTTTC TTTAATTTCC TCTCTACTCC TTTCCCTCTT CGTTTACTTT   
  
  
+ CCTTCTCCAC TCCTTTCTCC CTCATTCAAC TTCCCTTTTT CCCTTCTCTC TTTTTGGCGT CCTTTTCAAT   
  
  
+ CACTGCTTCG TTGGTCACTG CAGAGAGTAT ACTACGTTAC CACTACGGTT CGGTTCGAAA GAGAAAGAAA   
  
  
+ GAAAGGAAAG AAAGAAAGAA AGAAGAAAAT AGAGCCGTAC GAATTAAAAG GAGTGGTGTT TATTTGTCTT   
  
  
+ ACTTTAGTTC ACCCGAACTA TGACTATCAC ATGACACAAA CCCCCACACG TGTTGTTAAG TTTCAGATTC   
  
  
+ TTAGTTAGAG CATCGATCTC CCGACGTTAC GGCTCTACTA CCTAAAATTT TTTCTAGTTT AACGTTTTTT   
  
  
+ GTTTTTTGTT TTTTGGTTTT TACCAACCGT AATGACAATC ATGCTACGTT CACCGATGAA AATAAGATAA   
  
  
+ GATTACGTTG ACCTAGTAAT GCACCGTGTC CGGAAAGGTG AAAAGAAAGA TTAGGCTTAT TACATTAGTC   
  
  
+ ATTAGTGTTA TGTTCTCACC GTACTAACTA CTACTTAAGG TCTTTAGCAT AACCAACGCA GGCAGGGTAC   
  
  
+ TACGAGAGAA AACTTTTCAG TTACGAAGTT TGGATTGGAA GGTAGTAAAA GGTACAAACG TGAAGGGTAG   
  
  
+ ATTTGTTGAC CGAGTTTTAG TTTAGAAAAA AAAAAAAAAA AATTTTTTTT TTTTCCCTTC GCGAGTAAAC   
  
  
+ TTTGAAATAT TTCACATACA TATATCTTTT ATTTAAAACT ATTGTTCTCT CTCATTTTAT TGTTCGAAAA   
  
  
+ TAGTACTACT TGTTGTATGG AGTTGTTTTA ATTAATTTTT TTGTTGTTCG TAAGTAAAAC TAAAAATGAA   
  
  
+ GATTTAGATT TTCCTAATGT TAAATTATTT TATTTTGTTT ATATAAAAAT TTTATAAAAA GCAAAATGAA   
  
  
+ AGTTATTTTT GTCATTTTAA AATTAAATTC CTAATGTTAA ATTATTTTAT TTTGTTTATA TAAAAATTTT   
  
  
+ ATAAAAAGCA AAATGAAAGT TATTTTTGTC ATTTTAAAAT TAAAACTTCG TTTAACTAGT CTACTGCAAT   
  
  
+ TTTCTAGATC AATGTAGGTG ATCTGAATTA GGTGTATTTC GTTAGAGGTA GACGGTGCAG AATTGTGGTC   
  
  
+ CTGTATTGAC ATTATTGGTG CGGAGACCTA TTTAGGAAGG TGTCTCTGGT CTTCCTTCGA TGACTCATAC   
  
  
+ TAATGACATG GTGTGACATG AGATGACATT ATTGGTGCGG AGACCTATTT AGGAAGGTGT CTCTGGTCTT   
  
  
+ CCTTCGATGA CTCATACTAA TGACATGGT  

- GATCGATCGA TGATAGGGTC ACTGCCCACA GAACACAGTG GTGCTGTTTA ATTTTTTGGC CTTGATTTCG   
  
  
- TTGGGTAGAA GAGCTAGGGG ATATTATAAG TGGGGTGGGG GGAGAGGAAG CGAAGCAGAT AGAGATAAGC   
  
  
- TTGTAACTAA TGATGCGTTT GGACTAAAAG AAATTAAAGG AGAGATGAGG AAAGGGAGAA GCAAATGAAA   
  
  
- GGAAGAGGTG AGGAAAGAGG GAGTAAGTTG AAGGGAAAAA GGGAAGAGAG AAAAACCGCA GGAAAAGTTA   
  
  
- GTGACGAAGC AACCAGTGAC GTCTCTCATA TGATGCAATG GTGATGCCAA GCCAAGCTTT CTCTTTCTTT   
  
  
- CTTTCCTTTC TTTCTTTCTT TCTTCTTTTA TCTCGGCATG CTTAATTTTC CTCACCACAA ATAAACAGAA   
  
  
- TGAAATCAAG TGGGCTTGAT ACTGATAGTG TACTGTGTTT GGGGGTGTGC ACAACAATTC AAAGTCTAAG   
  
  
- AATCAATCTC GTAGCTAGAG GGCTGCAATG CCGAGATGAT GGATTTTAAA AAAGATCAAA TTGCAAAAAA   
  
  
- CAAAAAACAA AAAACCAAAA ATGGTTGGCA TTACTGTTAG TACGATGCAA GTGGCTACTT TTATTCTATT   
  
  
- CTAATGCAAC TGGATCATTA CGTGGCACAG GCCTTTCCAC TTTTCTTTCT AATCCGAATA ATGTAATCAG   
  
  
- TAATCACAAT ACAAGAGTGG CATGATTGAT GATGAATTCC AGAAATCGTA TTGGTTGCGT CCGTCCCATG   
  
  
- ATGCTCTCTT TTGAAAAGTC AATGCTTCAA ACCTAACCTT CCATCATTTT CCATGTTTGC ACTTCCCATC   
  
  
- TAAACAACTG GCTCAAAATC AAATCTTTTT TTTTTTTTTT TTAAAAAAAA AAAAGGGAAG CGCTCATTTG   
  
  
- AAACTTTATA AAGTGTATGT ATATAGAAAA TAAATTTTGA TAACAAGAGA GAGTAAAATA ACAAGCTTTT   
  
  
- ATCATGATGA ACAACATACC TCAACAAAAT TAATTAAAAA AACAACAAGC ATTCATTTTG ATTTTTACTT   
  
  
- CTAAATCTAA AAGGATTACA ATTTAATAAA ATAAAACAAA TATATTTTTA AAATATTTTT CGTTTTACTT   
  
  
- TCAATAAAAA CAGTAAAATT TTAATTTAAG GATTACAATT TAATAAAATA AAACAAATAT ATTTTTAAAA   
  
  
- TATTTTTCGT TTTACTTTCA ATAAAAACAG TAAAATTTTA ATTTTGAAGC AAATTGATCA GATGACGTTA   
  
  
- AAAGATCTAG TTACATCCAC TAGACTTAAT CCACATAAAG CAATCTCCAT CTGCCACGTC TTAACACCAG   
  
  
- GACATAACTG TAATAACCAC GCCTCTGGAT AAATCCTTCC ACAGAGACCA GAAGGAAGCT ACTGAGTATG   
  
  
- ATTACTGTAC CACACTGTAC TCTACTGTAA TAACCACGCC TCTGGATAAA TCCTTCCACA GAGACCAGAA   
  
  
- GGAAGCTACT GAGTATGATT ACTGTACCA

+     TCA-element

| Site Name | Organism | Position | Strand | Matrix score. | sequence | function |
| --- | --- | --- | --- | --- | --- | --- |
| TCA-element | Brassica oleracea | 950 | - | 9 | GAGAAGAATA | cis-acting element involved in salicylic acid responsiveness |

> 2018/04/13 10:10:12  
+ CTAGCTAGCT ACTATCCCAG TGACGGGTGT CTTGTGTCAC CACGACAAAT TAAAAAACCG GAACTAAAGC   
  
  
+ AACCCATCTT CTCGATCCCC TATAATATTC ACCCCACCCC CCTCTCCTTC GCTTCGTCTA TCTCTATTCG   
  
  
+ AACATTGATT ACTACGCAAA CCTGATTTTC TTTAATTTCC TCTCTACTCC TTTCCCTCTT CGTTTACTTT   
  
  
+ CCTTCTCCAC TCCTTTCTCC CTCATTCAAC TTCCCTTTTT CCCTTCTCTC TTTTTGGCGT CCTTTTCAAT   
  
  
+ CACTGCTTCG TTGGTCACTG CAGAGAGTAT ACTACGTTAC CACTACGGTT CGGTTCGAAA GAGAAAGAAA   
  
  
+ GAAAGGAAAG AAAGAAAGAA AGAAGAAAAT AGAGCCGTAC GAATTAAAAG GAGTGGTGTT TATTTGTCTT   
  
  
+ ACTTTAGTTC ACCCGAACTA TGACTATCAC ATGACACAAA CCCCCACACG TGTTGTTAAG TTTCAGATTC   
  
  
+ TTAGTTAGAG CATCGATCTC CCGACGTTAC GGCTCTACTA CCTAAAATTT TTTCTAGTTT AACGTTTTTT   
  
  
+ GTTTTTTGTT TTTTGGTTTT TACCAACCGT AATGACAATC ATGCTACGTT CACCGATGAA AATAAGATAA   
  
  
+ GATTACGTTG ACCTAGTAAT GCACCGTGTC CGGAAAGGTG AAAAGAAAGA TTAGGCTTAT TACATTAGTC   
  
  
+ ATTAGTGTTA TGTTCTCACC GTACTAACTA CTACTTAAGG TCTTTAGCAT AACCAACGCA GGCAGGGTAC   
  
  
+ TACGAGAGAA AACTTTTCAG TTACGAAGTT TGGATTGGAA GGTAGTAAAA GGTACAAACG TGAAGGGTAG   
  
  
+ ATTTGTTGAC CGAGTTTTAG TTTAGAAAAA AAAAAAAAAA AATTTTTTTT TTTTCCCTTC GCGAGTAAAC   
  
  
+ TTTGAAATAT TTCACATACA TATATCTTTT ATTTAAAACT ATTGTTCTCT CTCATTTTAT TGTTCGAAAA   
  
  
+ TAGTACTACT TGTTGTATGG AGTTGTTTTA ATTAATTTTT TTGTTGTTCG TAAGTAAAAC TAAAAATGAA   
  
  
+ GATTTAGATT TTCCTAATGT TAAATTATTT TATTTTGTTT ATATAAAAAT TTTATAAAAA GCAAAATGAA   
  
  
+ AGTTATTTTT GTCATTTTAA AATTAAATTC CTAATGTTAA ATTATTTTAT TTTGTTTATA TAAAAATTTT   
  
  
+ ATAAAAAGCA AAATGAAAGT TATTTTTGTC ATTTTAAAAT TAAAACTTCG TTTAACTAGT CTACTGCAAT   
  
  
+ TTTCTAGATC AATGTAGGTG ATCTGAATTA GGTGTATTTC GTTAGAGGTA GACGGTGCAG AATTGTGGTC   
  
  
+ CTGTATTGAC ATTATTGGTG CGGAGACCTA TTTAGGAAGG TGTCTCTGGT CTTCCTTCGA TGACTCATAC   
  
  
+ TAATGACATG GTGTGACATG AGATGACATT ATTGGTGCGG AGACCTATTT AGGAAGGTGT CTCTGGTCTT   
  
  
+ CCTTCGATGA CTCATACTAA TGACATGGT  

- GATCGATCGA TGATAGGGTC ACTGCCCACA GAACACAGTG GTGCTGTTTA ATTTTTTGGC CTTGATTTCG   
  
  
- TTGGGTAGAA GAGCTAGGGG ATATTATAAG TGGGGTGGGG GGAGAGGAAG CGAAGCAGAT AGAGATAAGC   
  
  
- TTGTAACTAA TGATGCGTTT GGACTAAAAG AAATTAAAGG AGAGATGAGG AAAGGGAGAA GCAAATGAAA   
  
  
- GGAAGAGGTG AGGAAAGAGG GAGTAAGTTG AAGGGAAAAA GGGAAGAGAG AAAAACCGCA GGAAAAGTTA   
  
  
- GTGACGAAGC AACCAGTGAC GTCTCTCATA TGATGCAATG GTGATGCCAA GCCAAGCTTT CTCTTTCTTT   
  
  
- CTTTCCTTTC TTTCTTTCTT TCTTCTTTTA TCTCGGCATG CTTAATTTTC CTCACCACAA ATAAACAGAA   
  
  
- TGAAATCAAG TGGGCTTGAT ACTGATAGTG TACTGTGTTT GGGGGTGTGC ACAACAATTC AAAGTCTAAG   
  
  
- AATCAATCTC GTAGCTAGAG GGCTGCAATG CCGAGATGAT GGATTTTAAA AAAGATCAAA TTGCAAAAAA   
  
  
- CAAAAAACAA AAAACCAAAA ATGGTTGGCA TTACTGTTAG TACGATGCAA GTGGCTACTT TTATTCTATT   
  
  
- CTAATGCAAC TGGATCATTA CGTGGCACAG GCCTTTCCAC TTTTCTTTCT AATCCGAATA ATGTAATCAG   
  
  
- TAATCACAAT ACAAGAGTGG CATGATTGAT GATGAATTCC AGAAATCGTA TTGGTTGCGT CCGTCCCATG   
  
  
- ATGCTCTCTT TTGAAAAGTC AATGCTTCAA ACCTAACCTT CCATCATTTT CCATGTTTGC ACTTCCCATC   
  
  
- TAAACAACTG GCTCAAAATC AAATCTTTTT TTTTTTTTTT TTAAAAAAAA AAAAGGGAAG CGCTCATTTG   
  
  
- AAACTTTATA AAGTGTATGT ATATAGAAAA TAAATTTTGA TAACAAGAGA GAGTAAAATA ACAAGCTTTT   
  
  
- ATCATGATGA ACAACATACC TCAACAAAAT TAATTAAAAA AACAACAAGC ATTCATTTTG ATTTTTACTT   
  
  
- CTAAATCTAA AAGGATTACA ATTTAATAAA ATAAAACAAA TATATTTTTA AAATATTTTT CGTTTTACTT   
  
  
- TCAATAAAAA CAGTAAAATT TTAATTTAAG GATTACAATT TAATAAAATA AAACAAATAT ATTTTTAAAA   
  
  
- TATTTTTCGT TTTACTTTCA ATAAAAACAG TAAAATTTTA ATTTTGAAGC AAATTGATCA GATGACGTTA   
  
  
- AAAGATCTAG TTACATCCAC TAGACTTAAT CCACATAAAG CAATCTCCAT CTGCCACGTC TTAACACCAG   
  
  
- GACATAACTG TAATAACCAC GCCTCTGGAT AAATCCTTCC ACAGAGACCA GAAGGAAGCT ACTGAGTATG   
  
  
- ATTACTGTAC CACACTGTAC TCTACTGTAA TAACCACGCC TCTGGATAAA TCCTTCCACA GAGACCAGAA   
  
  
- GGAAGCTACT GAGTATGATT ACTGTACCA

+     TCCC-motif

| Site Name | Organism | Position | Strand | Matrix score. | sequence | function |
| --- | --- | --- | --- | --- | --- | --- |
| TCCC-motif | Spinacia oleracea | 226 | + | 7 | TCTCCCT | part of a light responsive element |

> 2018/04/13 10:10:12  
+ CTAGCTAGCT ACTATCCCAG TGACGGGTGT CTTGTGTCAC CACGACAAAT TAAAAAACCG GAACTAAAGC   
  
  
+ AACCCATCTT CTCGATCCCC TATAATATTC ACCCCACCCC CCTCTCCTTC GCTTCGTCTA TCTCTATTCG   
  
  
+ AACATTGATT ACTACGCAAA CCTGATTTTC TTTAATTTCC TCTCTACTCC TTTCCCTCTT CGTTTACTTT   
  
  
+ CCTTCTCCAC TCCTTTCTCC CTCATTCAAC TTCCCTTTTT CCCTTCTCTC TTTTTGGCGT CCTTTTCAAT   
  
  
+ CACTGCTTCG TTGGTCACTG CAGAGAGTAT ACTACGTTAC CACTACGGTT CGGTTCGAAA GAGAAAGAAA   
  
  
+ GAAAGGAAAG AAAGAAAGAA AGAAGAAAAT AGAGCCGTAC GAATTAAAAG GAGTGGTGTT TATTTGTCTT   
  
  
+ ACTTTAGTTC ACCCGAACTA TGACTATCAC ATGACACAAA CCCCCACACG TGTTGTTAAG TTTCAGATTC   
  
  
+ TTAGTTAGAG CATCGATCTC CCGACGTTAC GGCTCTACTA CCTAAAATTT TTTCTAGTTT AACGTTTTTT   
  
  
+ GTTTTTTGTT TTTTGGTTTT TACCAACCGT AATGACAATC ATGCTACGTT CACCGATGAA AATAAGATAA   
  
  
+ GATTACGTTG ACCTAGTAAT GCACCGTGTC CGGAAAGGTG AAAAGAAAGA TTAGGCTTAT TACATTAGTC   
  
  
+ ATTAGTGTTA TGTTCTCACC GTACTAACTA CTACTTAAGG TCTTTAGCAT AACCAACGCA GGCAGGGTAC   
  
  
+ TACGAGAGAA AACTTTTCAG TTACGAAGTT TGGATTGGAA GGTAGTAAAA GGTACAAACG TGAAGGGTAG   
  
  
+ ATTTGTTGAC CGAGTTTTAG TTTAGAAAAA AAAAAAAAAA AATTTTTTTT TTTTCCCTTC GCGAGTAAAC   
  
  
+ TTTGAAATAT TTCACATACA TATATCTTTT ATTTAAAACT ATTGTTCTCT CTCATTTTAT TGTTCGAAAA   
  
  
+ TAGTACTACT TGTTGTATGG AGTTGTTTTA ATTAATTTTT TTGTTGTTCG TAAGTAAAAC TAAAAATGAA   
  
  
+ GATTTAGATT TTCCTAATGT TAAATTATTT TATTTTGTTT ATATAAAAAT TTTATAAAAA GCAAAATGAA   
  
  
+ AGTTATTTTT GTCATTTTAA AATTAAATTC CTAATGTTAA ATTATTTTAT TTTGTTTATA TAAAAATTTT   
  
  
+ ATAAAAAGCA AAATGAAAGT TATTTTTGTC ATTTTAAAAT TAAAACTTCG TTTAACTAGT CTACTGCAAT   
  
  
+ TTTCTAGATC AATGTAGGTG ATCTGAATTA GGTGTATTTC GTTAGAGGTA GACGGTGCAG AATTGTGGTC   
  
  
+ CTGTATTGAC ATTATTGGTG CGGAGACCTA TTTAGGAAGG TGTCTCTGGT CTTCCTTCGA TGACTCATAC   
  
  
+ TAATGACATG GTGTGACATG AGATGACATT ATTGGTGCGG AGACCTATTT AGGAAGGTGT CTCTGGTCTT   
  
  
+ CCTTCGATGA CTCATACTAA TGACATGGT  

- GATCGATCGA TGATAGGGTC ACTGCCCACA GAACACAGTG GTGCTGTTTA ATTTTTTGGC CTTGATTTCG   
  
  
- TTGGGTAGAA GAGCTAGGGG ATATTATAAG TGGGGTGGGG GGAGAGGAAG CGAAGCAGAT AGAGATAAGC   
  
  
- TTGTAACTAA TGATGCGTTT GGACTAAAAG AAATTAAAGG AGAGATGAGG AAAGGGAGAA GCAAATGAAA   
  
  
- GGAAGAGGTG AGGAAAGAGG GAGTAAGTTG AAGGGAAAAA GGGAAGAGAG AAAAACCGCA GGAAAAGTTA   
  
  
- GTGACGAAGC AACCAGTGAC GTCTCTCATA TGATGCAATG GTGATGCCAA GCCAAGCTTT CTCTTTCTTT   
  
  
- CTTTCCTTTC TTTCTTTCTT TCTTCTTTTA TCTCGGCATG CTTAATTTTC CTCACCACAA ATAAACAGAA   
  
  
- TGAAATCAAG TGGGCTTGAT ACTGATAGTG TACTGTGTTT GGGGGTGTGC ACAACAATTC AAAGTCTAAG   
  
  
- AATCAATCTC GTAGCTAGAG GGCTGCAATG CCGAGATGAT GGATTTTAAA AAAGATCAAA TTGCAAAAAA   
  
  
- CAAAAAACAA AAAACCAAAA ATGGTTGGCA TTACTGTTAG TACGATGCAA GTGGCTACTT TTATTCTATT   
  
  
- CTAATGCAAC TGGATCATTA CGTGGCACAG GCCTTTCCAC TTTTCTTTCT AATCCGAATA ATGTAATCAG   
  
  
- TAATCACAAT ACAAGAGTGG CATGATTGAT GATGAATTCC AGAAATCGTA TTGGTTGCGT CCGTCCCATG   
  
  
- ATGCTCTCTT TTGAAAAGTC AATGCTTCAA ACCTAACCTT CCATCATTTT CCATGTTTGC ACTTCCCATC   
  
  
- TAAACAACTG GCTCAAAATC AAATCTTTTT TTTTTTTTTT TTAAAAAAAA AAAAGGGAAG CGCTCATTTG   
  
  
- AAACTTTATA AAGTGTATGT ATATAGAAAA TAAATTTTGA TAACAAGAGA GAGTAAAATA ACAAGCTTTT   
  
  
- ATCATGATGA ACAACATACC TCAACAAAAT TAATTAAAAA AACAACAAGC ATTCATTTTG ATTTTTACTT   
  
  
- CTAAATCTAA AAGGATTACA ATTTAATAAA ATAAAACAAA TATATTTTTA AAATATTTTT CGTTTTACTT   
  
  
- TCAATAAAAA CAGTAAAATT TTAATTTAAG GATTACAATT TAATAAAATA AAACAAATAT ATTTTTAAAA   
  
  
- TATTTTTCGT TTTACTTTCA ATAAAAACAG TAAAATTTTA ATTTTGAAGC AAATTGATCA GATGACGTTA   
  
  
- AAAGATCTAG TTACATCCAC TAGACTTAAT CCACATAAAG CAATCTCCAT CTGCCACGTC TTAACACCAG   
  
  
- GACATAACTG TAATAACCAC GCCTCTGGAT AAATCCTTCC ACAGAGACCA GAAGGAAGCT ACTGAGTATG   
  
  
- ATTACTGTAC CACACTGTAC TCTACTGTAA TAACCACGCC TCTGGATAAA TCCTTCCACA GAGACCAGAA   
  
  
- GGAAGCTACT GAGTATGATT ACTGTACCA

+     TCT-motif

| Site Name | Organism | Position | Strand | Matrix score. | sequence | function |
| --- | --- | --- | --- | --- | --- | --- |
| TCT-motif | Arabidopsis thaliana | 417 | + | 6 | TCTTAC | part of a light responsive element |

> 2018/04/13 10:10:12  
+ CTAGCTAGCT ACTATCCCAG TGACGGGTGT CTTGTGTCAC CACGACAAAT TAAAAAACCG GAACTAAAGC   
  
  
+ AACCCATCTT CTCGATCCCC TATAATATTC ACCCCACCCC CCTCTCCTTC GCTTCGTCTA TCTCTATTCG   
  
  
+ AACATTGATT ACTACGCAAA CCTGATTTTC TTTAATTTCC TCTCTACTCC TTTCCCTCTT CGTTTACTTT   
  
  
+ CCTTCTCCAC TCCTTTCTCC CTCATTCAAC TTCCCTTTTT CCCTTCTCTC TTTTTGGCGT CCTTTTCAAT   
  
  
+ CACTGCTTCG TTGGTCACTG CAGAGAGTAT ACTACGTTAC CACTACGGTT CGGTTCGAAA GAGAAAGAAA   
  
  
+ GAAAGGAAAG AAAGAAAGAA AGAAGAAAAT AGAGCCGTAC GAATTAAAAG GAGTGGTGTT TATTTGTCTT   
  
  
+ ACTTTAGTTC ACCCGAACTA TGACTATCAC ATGACACAAA CCCCCACACG TGTTGTTAAG TTTCAGATTC   
  
  
+ TTAGTTAGAG CATCGATCTC CCGACGTTAC GGCTCTACTA CCTAAAATTT TTTCTAGTTT AACGTTTTTT   
  
  
+ GTTTTTTGTT TTTTGGTTTT TACCAACCGT AATGACAATC ATGCTACGTT CACCGATGAA AATAAGATAA   
  
  
+ GATTACGTTG ACCTAGTAAT GCACCGTGTC CGGAAAGGTG AAAAGAAAGA TTAGGCTTAT TACATTAGTC   
  
  
+ ATTAGTGTTA TGTTCTCACC GTACTAACTA CTACTTAAGG TCTTTAGCAT AACCAACGCA GGCAGGGTAC   
  
  
+ TACGAGAGAA AACTTTTCAG TTACGAAGTT TGGATTGGAA GGTAGTAAAA GGTACAAACG TGAAGGGTAG   
  
  
+ ATTTGTTGAC CGAGTTTTAG TTTAGAAAAA AAAAAAAAAA AATTTTTTTT TTTTCCCTTC GCGAGTAAAC   
  
  
+ TTTGAAATAT TTCACATACA TATATCTTTT ATTTAAAACT ATTGTTCTCT CTCATTTTAT TGTTCGAAAA   
  
  
+ TAGTACTACT TGTTGTATGG AGTTGTTTTA ATTAATTTTT TTGTTGTTCG TAAGTAAAAC TAAAAATGAA   
  
  
+ GATTTAGATT TTCCTAATGT TAAATTATTT TATTTTGTTT ATATAAAAAT TTTATAAAAA GCAAAATGAA   
  
  
+ AGTTATTTTT GTCATTTTAA AATTAAATTC CTAATGTTAA ATTATTTTAT TTTGTTTATA TAAAAATTTT   
  
  
+ ATAAAAAGCA AAATGAAAGT TATTTTTGTC ATTTTAAAAT TAAAACTTCG TTTAACTAGT CTACTGCAAT   
  
  
+ TTTCTAGATC AATGTAGGTG ATCTGAATTA GGTGTATTTC GTTAGAGGTA GACGGTGCAG AATTGTGGTC   
  
  
+ CTGTATTGAC ATTATTGGTG CGGAGACCTA TTTAGGAAGG TGTCTCTGGT CTTCCTTCGA TGACTCATAC   
  
  
+ TAATGACATG GTGTGACATG AGATGACATT ATTGGTGCGG AGACCTATTT AGGAAGGTGT CTCTGGTCTT   
  
  
+ CCTTCGATGA CTCATACTAA TGACATGGT  

- GATCGATCGA TGATAGGGTC ACTGCCCACA GAACACAGTG GTGCTGTTTA ATTTTTTGGC CTTGATTTCG   
  
  
- TTGGGTAGAA GAGCTAGGGG ATATTATAAG TGGGGTGGGG GGAGAGGAAG CGAAGCAGAT AGAGATAAGC   
  
  
- TTGTAACTAA TGATGCGTTT GGACTAAAAG AAATTAAAGG AGAGATGAGG AAAGGGAGAA GCAAATGAAA   
  
  
- GGAAGAGGTG AGGAAAGAGG GAGTAAGTTG AAGGGAAAAA GGGAAGAGAG AAAAACCGCA GGAAAAGTTA   
  
  
- GTGACGAAGC AACCAGTGAC GTCTCTCATA TGATGCAATG GTGATGCCAA GCCAAGCTTT CTCTTTCTTT   
  
  
- CTTTCCTTTC TTTCTTTCTT TCTTCTTTTA TCTCGGCATG CTTAATTTTC CTCACCACAA ATAAACAGAA   
  
  
- TGAAATCAAG TGGGCTTGAT ACTGATAGTG TACTGTGTTT GGGGGTGTGC ACAACAATTC AAAGTCTAAG   
  
  
- AATCAATCTC GTAGCTAGAG GGCTGCAATG CCGAGATGAT GGATTTTAAA AAAGATCAAA TTGCAAAAAA   
  
  
- CAAAAAACAA AAAACCAAAA ATGGTTGGCA TTACTGTTAG TACGATGCAA GTGGCTACTT TTATTCTATT   
  
  
- CTAATGCAAC TGGATCATTA CGTGGCACAG GCCTTTCCAC TTTTCTTTCT AATCCGAATA ATGTAATCAG   
  
  
- TAATCACAAT ACAAGAGTGG CATGATTGAT GATGAATTCC AGAAATCGTA TTGGTTGCGT CCGTCCCATG   
  
  
- ATGCTCTCTT TTGAAAAGTC AATGCTTCAA ACCTAACCTT CCATCATTTT CCATGTTTGC ACTTCCCATC   
  
  
- TAAACAACTG GCTCAAAATC AAATCTTTTT TTTTTTTTTT TTAAAAAAAA AAAAGGGAAG CGCTCATTTG   
  
  
- AAACTTTATA AAGTGTATGT ATATAGAAAA TAAATTTTGA TAACAAGAGA GAGTAAAATA ACAAGCTTTT   
  
  
- ATCATGATGA ACAACATACC TCAACAAAAT TAATTAAAAA AACAACAAGC ATTCATTTTG ATTTTTACTT   
  
  
- CTAAATCTAA AAGGATTACA ATTTAATAAA ATAAAACAAA TATATTTTTA AAATATTTTT CGTTTTACTT   
  
  
- TCAATAAAAA CAGTAAAATT TTAATTTAAG GATTACAATT TAATAAAATA AAACAAATAT ATTTTTAAAA   
  
  
- TATTTTTCGT TTTACTTTCA ATAAAAACAG TAAAATTTTA ATTTTGAAGC AAATTGATCA GATGACGTTA   
  
  
- AAAGATCTAG TTACATCCAC TAGACTTAAT CCACATAAAG CAATCTCCAT CTGCCACGTC TTAACACCAG   
  
  
- GACATAACTG TAATAACCAC GCCTCTGGAT AAATCCTTCC ACAGAGACCA GAAGGAAGCT ACTGAGTATG   
  
  
- ATTACTGTAC CACACTGTAC TCTACTGTAA TAACCACGCC TCTGGATAAA TCCTTCCACA GAGACCAGAA   
  
  
- GGAAGCTACT GAGTATGATT ACTGTACCA

+     TGACG-motif

| Site Name | Organism | Position | Strand | Matrix score. | sequence | function |
| --- | --- | --- | --- | --- | --- | --- |
| TGACG-motif | Hordeum vulgare | 21 | + | 5 | TGACG | cis-acting regulatory element involved in the MeJA-responsiveness |

> 2018/04/13 10:10:12  
+ CTAGCTAGCT ACTATCCCAG TGACGGGTGT CTTGTGTCAC CACGACAAAT TAAAAAACCG GAACTAAAGC   
  
  
+ AACCCATCTT CTCGATCCCC TATAATATTC ACCCCACCCC CCTCTCCTTC GCTTCGTCTA TCTCTATTCG   
  
  
+ AACATTGATT ACTACGCAAA CCTGATTTTC TTTAATTTCC TCTCTACTCC TTTCCCTCTT CGTTTACTTT   
  
  
+ CCTTCTCCAC TCCTTTCTCC CTCATTCAAC TTCCCTTTTT CCCTTCTCTC TTTTTGGCGT CCTTTTCAAT   
  
  
+ CACTGCTTCG TTGGTCACTG CAGAGAGTAT ACTACGTTAC CACTACGGTT CGGTTCGAAA GAGAAAGAAA   
  
  
+ GAAAGGAAAG AAAGAAAGAA AGAAGAAAAT AGAGCCGTAC GAATTAAAAG GAGTGGTGTT TATTTGTCTT   
  
  
+ ACTTTAGTTC ACCCGAACTA TGACTATCAC ATGACACAAA CCCCCACACG TGTTGTTAAG TTTCAGATTC   
  
  
+ TTAGTTAGAG CATCGATCTC CCGACGTTAC GGCTCTACTA CCTAAAATTT TTTCTAGTTT AACGTTTTTT   
  
  
+ GTTTTTTGTT TTTTGGTTTT TACCAACCGT AATGACAATC ATGCTACGTT CACCGATGAA AATAAGATAA   
  
  
+ GATTACGTTG ACCTAGTAAT GCACCGTGTC CGGAAAGGTG AAAAGAAAGA TTAGGCTTAT TACATTAGTC   
  
  
+ ATTAGTGTTA TGTTCTCACC GTACTAACTA CTACTTAAGG TCTTTAGCAT AACCAACGCA GGCAGGGTAC   
  
  
+ TACGAGAGAA AACTTTTCAG TTACGAAGTT TGGATTGGAA GGTAGTAAAA GGTACAAACG TGAAGGGTAG   
  
  
+ ATTTGTTGAC CGAGTTTTAG TTTAGAAAAA AAAAAAAAAA AATTTTTTTT TTTTCCCTTC GCGAGTAAAC   
  
  
+ TTTGAAATAT TTCACATACA TATATCTTTT ATTTAAAACT ATTGTTCTCT CTCATTTTAT TGTTCGAAAA   
  
  
+ TAGTACTACT TGTTGTATGG AGTTGTTTTA ATTAATTTTT TTGTTGTTCG TAAGTAAAAC TAAAAATGAA   
  
  
+ GATTTAGATT TTCCTAATGT TAAATTATTT TATTTTGTTT ATATAAAAAT TTTATAAAAA GCAAAATGAA   
  
  
+ AGTTATTTTT GTCATTTTAA AATTAAATTC CTAATGTTAA ATTATTTTAT TTTGTTTATA TAAAAATTTT   
  
  
+ ATAAAAAGCA AAATGAAAGT TATTTTTGTC ATTTTAAAAT TAAAACTTCG TTTAACTAGT CTACTGCAAT   
  
  
+ TTTCTAGATC AATGTAGGTG ATCTGAATTA GGTGTATTTC GTTAGAGGTA GACGGTGCAG AATTGTGGTC   
  
  
+ CTGTATTGAC ATTATTGGTG CGGAGACCTA TTTAGGAAGG TGTCTCTGGT CTTCCTTCGA TGACTCATAC   
  
  
+ TAATGACATG GTGTGACATG AGATGACATT ATTGGTGCGG AGACCTATTT AGGAAGGTGT CTCTGGTCTT   
  
  
+ CCTTCGATGA CTCATACTAA TGACATGGT  

- GATCGATCGA TGATAGGGTC ACTGCCCACA GAACACAGTG GTGCTGTTTA ATTTTTTGGC CTTGATTTCG   
  
  
- TTGGGTAGAA GAGCTAGGGG ATATTATAAG TGGGGTGGGG GGAGAGGAAG CGAAGCAGAT AGAGATAAGC   
  
  
- TTGTAACTAA TGATGCGTTT GGACTAAAAG AAATTAAAGG AGAGATGAGG AAAGGGAGAA GCAAATGAAA   
  
  
- GGAAGAGGTG AGGAAAGAGG GAGTAAGTTG AAGGGAAAAA GGGAAGAGAG AAAAACCGCA GGAAAAGTTA   
  
  
- GTGACGAAGC AACCAGTGAC GTCTCTCATA TGATGCAATG GTGATGCCAA GCCAAGCTTT CTCTTTCTTT   
  
  
- CTTTCCTTTC TTTCTTTCTT TCTTCTTTTA TCTCGGCATG CTTAATTTTC CTCACCACAA ATAAACAGAA   
  
  
- TGAAATCAAG TGGGCTTGAT ACTGATAGTG TACTGTGTTT GGGGGTGTGC ACAACAATTC AAAGTCTAAG   
  
  
- AATCAATCTC GTAGCTAGAG GGCTGCAATG CCGAGATGAT GGATTTTAAA AAAGATCAAA TTGCAAAAAA   
  
  
- CAAAAAACAA AAAACCAAAA ATGGTTGGCA TTACTGTTAG TACGATGCAA GTGGCTACTT TTATTCTATT   
  
  
- CTAATGCAAC TGGATCATTA CGTGGCACAG GCCTTTCCAC TTTTCTTTCT AATCCGAATA ATGTAATCAG   
  
  
- TAATCACAAT ACAAGAGTGG CATGATTGAT GATGAATTCC AGAAATCGTA TTGGTTGCGT CCGTCCCATG   
  
  
- ATGCTCTCTT TTGAAAAGTC AATGCTTCAA ACCTAACCTT CCATCATTTT CCATGTTTGC ACTTCCCATC   
  
  
- TAAACAACTG GCTCAAAATC AAATCTTTTT TTTTTTTTTT TTAAAAAAAA AAAAGGGAAG CGCTCATTTG   
  
  
- AAACTTTATA AAGTGTATGT ATATAGAAAA TAAATTTTGA TAACAAGAGA GAGTAAAATA ACAAGCTTTT   
  
  
- ATCATGATGA ACAACATACC TCAACAAAAT TAATTAAAAA AACAACAAGC ATTCATTTTG ATTTTTACTT   
  
  
- CTAAATCTAA AAGGATTACA ATTTAATAAA ATAAAACAAA TATATTTTTA AAATATTTTT CGTTTTACTT   
  
  
- TCAATAAAAA CAGTAAAATT TTAATTTAAG GATTACAATT TAATAAAATA AAACAAATAT ATTTTTAAAA   
  
  
- TATTTTTCGT TTTACTTTCA ATAAAAACAG TAAAATTTTA ATTTTGAAGC AAATTGATCA GATGACGTTA   
  
  
- AAAGATCTAG TTACATCCAC TAGACTTAAT CCACATAAAG CAATCTCCAT CTGCCACGTC TTAACACCAG   
  
  
- GACATAACTG TAATAACCAC GCCTCTGGAT AAATCCTTCC ACAGAGACCA GAAGGAAGCT ACTGAGTATG   
  
  
- ATTACTGTAC CACACTGTAC TCTACTGTAA TAACCACGCC TCTGGATAAA TCCTTCCACA GAGACCAGAA   
  
  
- GGAAGCTACT GAGTATGATT ACTGTACCA

+     Unnamed\_\_1

| Site Name | Organism | Position | Strand | Matrix score. | sequence | function |
| --- | --- | --- | --- | --- | --- | --- |
| Unnamed\_\_1 | Zea mays | 40 | - | 5 | CGTGG |  |

> 2018/04/13 10:10:12  
+ CTAGCTAGCT ACTATCCCAG TGACGGGTGT CTTGTGTCAC CACGACAAAT TAAAAAACCG GAACTAAAGC   
  
  
+ AACCCATCTT CTCGATCCCC TATAATATTC ACCCCACCCC CCTCTCCTTC GCTTCGTCTA TCTCTATTCG   
  
  
+ AACATTGATT ACTACGCAAA CCTGATTTTC TTTAATTTCC TCTCTACTCC TTTCCCTCTT CGTTTACTTT   
  
  
+ CCTTCTCCAC TCCTTTCTCC CTCATTCAAC TTCCCTTTTT CCCTTCTCTC TTTTTGGCGT CCTTTTCAAT   
  
  
+ CACTGCTTCG TTGGTCACTG CAGAGAGTAT ACTACGTTAC CACTACGGTT CGGTTCGAAA GAGAAAGAAA   
  
  
+ GAAAGGAAAG AAAGAAAGAA AGAAGAAAAT AGAGCCGTAC GAATTAAAAG GAGTGGTGTT TATTTGTCTT   
  
  
+ ACTTTAGTTC ACCCGAACTA TGACTATCAC ATGACACAAA CCCCCACACG TGTTGTTAAG TTTCAGATTC   
  
  
+ TTAGTTAGAG CATCGATCTC CCGACGTTAC GGCTCTACTA CCTAAAATTT TTTCTAGTTT AACGTTTTTT   
  
  
+ GTTTTTTGTT TTTTGGTTTT TACCAACCGT AATGACAATC ATGCTACGTT CACCGATGAA AATAAGATAA   
  
  
+ GATTACGTTG ACCTAGTAAT GCACCGTGTC CGGAAAGGTG AAAAGAAAGA TTAGGCTTAT TACATTAGTC   
  
  
+ ATTAGTGTTA TGTTCTCACC GTACTAACTA CTACTTAAGG TCTTTAGCAT AACCAACGCA GGCAGGGTAC   
  
  
+ TACGAGAGAA AACTTTTCAG TTACGAAGTT TGGATTGGAA GGTAGTAAAA GGTACAAACG TGAAGGGTAG   
  
  
+ ATTTGTTGAC CGAGTTTTAG TTTAGAAAAA AAAAAAAAAA AATTTTTTTT TTTTCCCTTC GCGAGTAAAC   
  
  
+ TTTGAAATAT TTCACATACA TATATCTTTT ATTTAAAACT ATTGTTCTCT CTCATTTTAT TGTTCGAAAA   
  
  
+ TAGTACTACT TGTTGTATGG AGTTGTTTTA ATTAATTTTT TTGTTGTTCG TAAGTAAAAC TAAAAATGAA   
  
  
+ GATTTAGATT TTCCTAATGT TAAATTATTT TATTTTGTTT ATATAAAAAT TTTATAAAAA GCAAAATGAA   
  
  
+ AGTTATTTTT GTCATTTTAA AATTAAATTC CTAATGTTAA ATTATTTTAT TTTGTTTATA TAAAAATTTT   
  
  
+ ATAAAAAGCA AAATGAAAGT TATTTTTGTC ATTTTAAAAT TAAAACTTCG TTTAACTAGT CTACTGCAAT   
  
  
+ TTTCTAGATC AATGTAGGTG ATCTGAATTA GGTGTATTTC GTTAGAGGTA GACGGTGCAG AATTGTGGTC   
  
  
+ CTGTATTGAC ATTATTGGTG CGGAGACCTA TTTAGGAAGG TGTCTCTGGT CTTCCTTCGA TGACTCATAC   
  
  
+ TAATGACATG GTGTGACATG AGATGACATT ATTGGTGCGG AGACCTATTT AGGAAGGTGT CTCTGGTCTT   
  
  
+ CCTTCGATGA CTCATACTAA TGACATGGT  

- GATCGATCGA TGATAGGGTC ACTGCCCACA GAACACAGTG GTGCTGTTTA ATTTTTTGGC CTTGATTTCG   
  
  
- TTGGGTAGAA GAGCTAGGGG ATATTATAAG TGGGGTGGGG GGAGAGGAAG CGAAGCAGAT AGAGATAAGC   
  
  
- TTGTAACTAA TGATGCGTTT GGACTAAAAG AAATTAAAGG AGAGATGAGG AAAGGGAGAA GCAAATGAAA   
  
  
- GGAAGAGGTG AGGAAAGAGG GAGTAAGTTG AAGGGAAAAA GGGAAGAGAG AAAAACCGCA GGAAAAGTTA   
  
  
- GTGACGAAGC AACCAGTGAC GTCTCTCATA TGATGCAATG GTGATGCCAA GCCAAGCTTT CTCTTTCTTT   
  
  
- CTTTCCTTTC TTTCTTTCTT TCTTCTTTTA TCTCGGCATG CTTAATTTTC CTCACCACAA ATAAACAGAA   
  
  
- TGAAATCAAG TGGGCTTGAT ACTGATAGTG TACTGTGTTT GGGGGTGTGC ACAACAATTC AAAGTCTAAG   
  
  
- AATCAATCTC GTAGCTAGAG GGCTGCAATG CCGAGATGAT GGATTTTAAA AAAGATCAAA TTGCAAAAAA   
  
  
- CAAAAAACAA AAAACCAAAA ATGGTTGGCA TTACTGTTAG TACGATGCAA GTGGCTACTT TTATTCTATT   
  
  
- CTAATGCAAC TGGATCATTA CGTGGCACAG GCCTTTCCAC TTTTCTTTCT AATCCGAATA ATGTAATCAG   
  
  
- TAATCACAAT ACAAGAGTGG CATGATTGAT GATGAATTCC AGAAATCGTA TTGGTTGCGT CCGTCCCATG   
  
  
- ATGCTCTCTT TTGAAAAGTC AATGCTTCAA ACCTAACCTT CCATCATTTT CCATGTTTGC ACTTCCCATC   
  
  
- TAAACAACTG GCTCAAAATC AAATCTTTTT TTTTTTTTTT TTAAAAAAAA AAAAGGGAAG CGCTCATTTG   
  
  
- AAACTTTATA AAGTGTATGT ATATAGAAAA TAAATTTTGA TAACAAGAGA GAGTAAAATA ACAAGCTTTT   
  
  
- ATCATGATGA ACAACATACC TCAACAAAAT TAATTAAAAA AACAACAAGC ATTCATTTTG ATTTTTACTT   
  
  
- CTAAATCTAA AAGGATTACA ATTTAATAAA ATAAAACAAA TATATTTTTA AAATATTTTT CGTTTTACTT   
  
  
- TCAATAAAAA CAGTAAAATT TTAATTTAAG GATTACAATT TAATAAAATA AAACAAATAT ATTTTTAAAA   
  
  
- TATTTTTCGT TTTACTTTCA ATAAAAACAG TAAAATTTTA ATTTTGAAGC AAATTGATCA GATGACGTTA   
  
  
- AAAGATCTAG TTACATCCAC TAGACTTAAT CCACATAAAG CAATCTCCAT CTGCCACGTC TTAACACCAG   
  
  
- GACATAACTG TAATAACCAC GCCTCTGGAT AAATCCTTCC ACAGAGACCA GAAGGAAGCT ACTGAGTATG   
  
  
- ATTACTGTAC CACACTGTAC TCTACTGTAA TAACCACGCC TCTGGATAAA TCCTTCCACA GAGACCAGAA   
  
  
- GGAAGCTACT GAGTATGATT ACTGTACCA

+     Unnamed\_\_3

| Site Name | Organism | Position | Strand | Matrix score. | sequence | function |
| --- | --- | --- | --- | --- | --- | --- |
| Unnamed\_\_3 | Zea mays | 40 | - | 5 | CGTGG |  |

> 2018/04/13 10:10:12  
+ CTAGCTAGCT ACTATCCCAG TGACGGGTGT CTTGTGTCAC CACGACAAAT TAAAAAACCG GAACTAAAGC   
  
  
+ AACCCATCTT CTCGATCCCC TATAATATTC ACCCCACCCC CCTCTCCTTC GCTTCGTCTA TCTCTATTCG   
  
  
+ AACATTGATT ACTACGCAAA CCTGATTTTC TTTAATTTCC TCTCTACTCC TTTCCCTCTT CGTTTACTTT   
  
  
+ CCTTCTCCAC TCCTTTCTCC CTCATTCAAC TTCCCTTTTT CCCTTCTCTC TTTTTGGCGT CCTTTTCAAT   
  
  
+ CACTGCTTCG TTGGTCACTG CAGAGAGTAT ACTACGTTAC CACTACGGTT CGGTTCGAAA GAGAAAGAAA   
  
  
+ GAAAGGAAAG AAAGAAAGAA AGAAGAAAAT AGAGCCGTAC GAATTAAAAG GAGTGGTGTT TATTTGTCTT   
  
  
+ ACTTTAGTTC ACCCGAACTA TGACTATCAC ATGACACAAA CCCCCACACG TGTTGTTAAG TTTCAGATTC   
  
  
+ TTAGTTAGAG CATCGATCTC CCGACGTTAC GGCTCTACTA CCTAAAATTT TTTCTAGTTT AACGTTTTTT   
  
  
+ GTTTTTTGTT TTTTGGTTTT TACCAACCGT AATGACAATC ATGCTACGTT CACCGATGAA AATAAGATAA   
  
  
+ GATTACGTTG ACCTAGTAAT GCACCGTGTC CGGAAAGGTG AAAAGAAAGA TTAGGCTTAT TACATTAGTC   
  
  
+ ATTAGTGTTA TGTTCTCACC GTACTAACTA CTACTTAAGG TCTTTAGCAT AACCAACGCA GGCAGGGTAC   
  
  
+ TACGAGAGAA AACTTTTCAG TTACGAAGTT TGGATTGGAA GGTAGTAAAA GGTACAAACG TGAAGGGTAG   
  
  
+ ATTTGTTGAC CGAGTTTTAG TTTAGAAAAA AAAAAAAAAA AATTTTTTTT TTTTCCCTTC GCGAGTAAAC   
  
  
+ TTTGAAATAT TTCACATACA TATATCTTTT ATTTAAAACT ATTGTTCTCT CTCATTTTAT TGTTCGAAAA   
  
  
+ TAGTACTACT TGTTGTATGG AGTTGTTTTA ATTAATTTTT TTGTTGTTCG TAAGTAAAAC TAAAAATGAA   
  
  
+ GATTTAGATT TTCCTAATGT TAAATTATTT TATTTTGTTT ATATAAAAAT TTTATAAAAA GCAAAATGAA   
  
  
+ AGTTATTTTT GTCATTTTAA AATTAAATTC CTAATGTTAA ATTATTTTAT TTTGTTTATA TAAAAATTTT   
  
  
+ ATAAAAAGCA AAATGAAAGT TATTTTTGTC ATTTTAAAAT TAAAACTTCG TTTAACTAGT CTACTGCAAT   
  
  
+ TTTCTAGATC AATGTAGGTG ATCTGAATTA GGTGTATTTC GTTAGAGGTA GACGGTGCAG AATTGTGGTC   
  
  
+ CTGTATTGAC ATTATTGGTG CGGAGACCTA TTTAGGAAGG TGTCTCTGGT CTTCCTTCGA TGACTCATAC   
  
  
+ TAATGACATG GTGTGACATG AGATGACATT ATTGGTGCGG AGACCTATTT AGGAAGGTGT CTCTGGTCTT   
  
  
+ CCTTCGATGA CTCATACTAA TGACATGGT  

- GATCGATCGA TGATAGGGTC ACTGCCCACA GAACACAGTG GTGCTGTTTA ATTTTTTGGC CTTGATTTCG   
  
  
- TTGGGTAGAA GAGCTAGGGG ATATTATAAG TGGGGTGGGG GGAGAGGAAG CGAAGCAGAT AGAGATAAGC   
  
  
- TTGTAACTAA TGATGCGTTT GGACTAAAAG AAATTAAAGG AGAGATGAGG AAAGGGAGAA GCAAATGAAA   
  
  
- GGAAGAGGTG AGGAAAGAGG GAGTAAGTTG AAGGGAAAAA GGGAAGAGAG AAAAACCGCA GGAAAAGTTA   
  
  
- GTGACGAAGC AACCAGTGAC GTCTCTCATA TGATGCAATG GTGATGCCAA GCCAAGCTTT CTCTTTCTTT   
  
  
- CTTTCCTTTC TTTCTTTCTT TCTTCTTTTA TCTCGGCATG CTTAATTTTC CTCACCACAA ATAAACAGAA   
  
  
- TGAAATCAAG TGGGCTTGAT ACTGATAGTG TACTGTGTTT GGGGGTGTGC ACAACAATTC AAAGTCTAAG   
  
  
- AATCAATCTC GTAGCTAGAG GGCTGCAATG CCGAGATGAT GGATTTTAAA AAAGATCAAA TTGCAAAAAA   
  
  
- CAAAAAACAA AAAACCAAAA ATGGTTGGCA TTACTGTTAG TACGATGCAA GTGGCTACTT TTATTCTATT   
  
  
- CTAATGCAAC TGGATCATTA CGTGGCACAG GCCTTTCCAC TTTTCTTTCT AATCCGAATA ATGTAATCAG   
  
  
- TAATCACAAT ACAAGAGTGG CATGATTGAT GATGAATTCC AGAAATCGTA TTGGTTGCGT CCGTCCCATG   
  
  
- ATGCTCTCTT TTGAAAAGTC AATGCTTCAA ACCTAACCTT CCATCATTTT CCATGTTTGC ACTTCCCATC   
  
  
- TAAACAACTG GCTCAAAATC AAATCTTTTT TTTTTTTTTT TTAAAAAAAA AAAAGGGAAG CGCTCATTTG   
  
  
- AAACTTTATA AAGTGTATGT ATATAGAAAA TAAATTTTGA TAACAAGAGA GAGTAAAATA ACAAGCTTTT   
  
  
- ATCATGATGA ACAACATACC TCAACAAAAT TAATTAAAAA AACAACAAGC ATTCATTTTG ATTTTTACTT   
  
  
- CTAAATCTAA AAGGATTACA ATTTAATAAA ATAAAACAAA TATATTTTTA AAATATTTTT CGTTTTACTT   
  
  
- TCAATAAAAA CAGTAAAATT TTAATTTAAG GATTACAATT TAATAAAATA AAACAAATAT ATTTTTAAAA   
  
  
- TATTTTTCGT TTTACTTTCA ATAAAAACAG TAAAATTTTA ATTTTGAAGC AAATTGATCA GATGACGTTA   
  
  
- AAAGATCTAG TTACATCCAC TAGACTTAAT CCACATAAAG CAATCTCCAT CTGCCACGTC TTAACACCAG   
  
  
- GACATAACTG TAATAACCAC GCCTCTGGAT AAATCCTTCC ACAGAGACCA GAAGGAAGCT ACTGAGTATG   
  
  
- ATTACTGTAC CACACTGTAC TCTACTGTAA TAACCACGCC TCTGGATAAA TCCTTCCACA GAGACCAGAA   
  
  
- GGAAGCTACT GAGTATGATT ACTGTACCA

+     Unnamed\_\_4

| Site Name | Organism | Position | Strand | Matrix score. | sequence | function |
| --- | --- | --- | --- | --- | --- | --- |
| Unnamed\_\_4 | Petroselinum hortense | 1439 | - | 4 | CTCC |  |
| Unnamed\_\_4 | Petroselinum hortense | 1352 | - | 4 | CTCC |  |
| Unnamed\_\_4 | Petroselinum hortense | 508 | + | 4 | CTCC |  |
| Unnamed\_\_4 | Petroselinum hortense | 999 | - | 4 | CTCC |  |
| Unnamed\_\_4 | Petroselinum hortense | 227 | + | 4 | CTCC |  |
| Unnamed\_\_4 | Petroselinum hortense | 400 | - | 4 | CTCC |  |
| Unnamed\_\_4 | Petroselinum hortense | 220 | + | 4 | CTCC |  |
| Unnamed\_\_4 | Petroselinum hortense | 215 | + | 4 | CTCC |  |
| Unnamed\_\_4 | Petroselinum hortense | 187 | + | 4 | CTCC |  |
| Unnamed\_\_4 | Petroselinum hortense | 114 | + | 4 | CTCC |  |

> 2018/04/13 10:10:12  
+ CTAGCTAGCT ACTATCCCAG TGACGGGTGT CTTGTGTCAC CACGACAAAT TAAAAAACCG GAACTAAAGC   
  
  
+ AACCCATCTT CTCGATCCCC TATAATATTC ACCCCACCCC CCTCTCCTTC GCTTCGTCTA TCTCTATTCG   
  
  
+ AACATTGATT ACTACGCAAA CCTGATTTTC TTTAATTTCC TCTCTACTCC TTTCCCTCTT CGTTTACTTT   
  
  
+ CCTTCTCCAC TCCTTTCTCC CTCATTCAAC TTCCCTTTTT CCCTTCTCTC TTTTTGGCGT CCTTTTCAAT   
  
  
+ CACTGCTTCG TTGGTCACTG CAGAGAGTAT ACTACGTTAC CACTACGGTT CGGTTCGAAA GAGAAAGAAA   
  
  
+ GAAAGGAAAG AAAGAAAGAA AGAAGAAAAT AGAGCCGTAC GAATTAAAAG GAGTGGTGTT TATTTGTCTT   
  
  
+ ACTTTAGTTC ACCCGAACTA TGACTATCAC ATGACACAAA CCCCCACACG TGTTGTTAAG TTTCAGATTC   
  
  
+ TTAGTTAGAG CATCGATCTC CCGACGTTAC GGCTCTACTA CCTAAAATTT TTTCTAGTTT AACGTTTTTT   
  
  
+ GTTTTTTGTT TTTTGGTTTT TACCAACCGT AATGACAATC ATGCTACGTT CACCGATGAA AATAAGATAA   
  
  
+ GATTACGTTG ACCTAGTAAT GCACCGTGTC CGGAAAGGTG AAAAGAAAGA TTAGGCTTAT TACATTAGTC   
  
  
+ ATTAGTGTTA TGTTCTCACC GTACTAACTA CTACTTAAGG TCTTTAGCAT AACCAACGCA GGCAGGGTAC   
  
  
+ TACGAGAGAA AACTTTTCAG TTACGAAGTT TGGATTGGAA GGTAGTAAAA GGTACAAACG TGAAGGGTAG   
  
  
+ ATTTGTTGAC CGAGTTTTAG TTTAGAAAAA AAAAAAAAAA AATTTTTTTT TTTTCCCTTC GCGAGTAAAC   
  
  
+ TTTGAAATAT TTCACATACA TATATCTTTT ATTTAAAACT ATTGTTCTCT CTCATTTTAT TGTTCGAAAA   
  
  
+ TAGTACTACT TGTTGTATGG AGTTGTTTTA ATTAATTTTT TTGTTGTTCG TAAGTAAAAC TAAAAATGAA   
  
  
+ GATTTAGATT TTCCTAATGT TAAATTATTT TATTTTGTTT ATATAAAAAT TTTATAAAAA GCAAAATGAA   
  
  
+ AGTTATTTTT GTCATTTTAA AATTAAATTC CTAATGTTAA ATTATTTTAT TTTGTTTATA TAAAAATTTT   
  
  
+ ATAAAAAGCA AAATGAAAGT TATTTTTGTC ATTTTAAAAT TAAAACTTCG TTTAACTAGT CTACTGCAAT   
  
  
+ TTTCTAGATC AATGTAGGTG ATCTGAATTA GGTGTATTTC GTTAGAGGTA GACGGTGCAG AATTGTGGTC   
  
  
+ CTGTATTGAC ATTATTGGTG CGGAGACCTA TTTAGGAAGG TGTCTCTGGT CTTCCTTCGA TGACTCATAC   
  
  
+ TAATGACATG GTGTGACATG AGATGACATT ATTGGTGCGG AGACCTATTT AGGAAGGTGT CTCTGGTCTT   
  
  
+ CCTTCGATGA CTCATACTAA TGACATGGT  

- GATCGATCGA TGATAGGGTC ACTGCCCACA GAACACAGTG GTGCTGTTTA ATTTTTTGGC CTTGATTTCG   
  
  
- TTGGGTAGAA GAGCTAGGGG ATATTATAAG TGGGGTGGGG GGAGAGGAAG CGAAGCAGAT AGAGATAAGC   
  
  
- TTGTAACTAA TGATGCGTTT GGACTAAAAG AAATTAAAGG AGAGATGAGG AAAGGGAGAA GCAAATGAAA   
  
  
- GGAAGAGGTG AGGAAAGAGG GAGTAAGTTG AAGGGAAAAA GGGAAGAGAG AAAAACCGCA GGAAAAGTTA   
  
  
- GTGACGAAGC AACCAGTGAC GTCTCTCATA TGATGCAATG GTGATGCCAA GCCAAGCTTT CTCTTTCTTT   
  
  
- CTTTCCTTTC TTTCTTTCTT TCTTCTTTTA TCTCGGCATG CTTAATTTTC CTCACCACAA ATAAACAGAA   
  
  
- TGAAATCAAG TGGGCTTGAT ACTGATAGTG TACTGTGTTT GGGGGTGTGC ACAACAATTC AAAGTCTAAG   
  
  
- AATCAATCTC GTAGCTAGAG GGCTGCAATG CCGAGATGAT GGATTTTAAA AAAGATCAAA TTGCAAAAAA   
  
  
- CAAAAAACAA AAAACCAAAA ATGGTTGGCA TTACTGTTAG TACGATGCAA GTGGCTACTT TTATTCTATT   
  
  
- CTAATGCAAC TGGATCATTA CGTGGCACAG GCCTTTCCAC TTTTCTTTCT AATCCGAATA ATGTAATCAG   
  
  
- TAATCACAAT ACAAGAGTGG CATGATTGAT GATGAATTCC AGAAATCGTA TTGGTTGCGT CCGTCCCATG   
  
  
- ATGCTCTCTT TTGAAAAGTC AATGCTTCAA ACCTAACCTT CCATCATTTT CCATGTTTGC ACTTCCCATC   
  
  
- TAAACAACTG GCTCAAAATC AAATCTTTTT TTTTTTTTTT TTAAAAAAAA AAAAGGGAAG CGCTCATTTG   
  
  
- AAACTTTATA AAGTGTATGT ATATAGAAAA TAAATTTTGA TAACAAGAGA GAGTAAAATA ACAAGCTTTT   
  
  
- ATCATGATGA ACAACATACC TCAACAAAAT TAATTAAAAA AACAACAAGC ATTCATTTTG ATTTTTACTT   
  
  
- CTAAATCTAA AAGGATTACA ATTTAATAAA ATAAAACAAA TATATTTTTA AAATATTTTT CGTTTTACTT   
  
  
- TCAATAAAAA CAGTAAAATT TTAATTTAAG GATTACAATT TAATAAAATA AAACAAATAT ATTTTTAAAA   
  
  
- TATTTTTCGT TTTACTTTCA ATAAAAACAG TAAAATTTTA ATTTTGAAGC AAATTGATCA GATGACGTTA   
  
  
- AAAGATCTAG TTACATCCAC TAGACTTAAT CCACATAAAG CAATCTCCAT CTGCCACGTC TTAACACCAG   
  
  
- GACATAACTG TAATAACCAC GCCTCTGGAT AAATCCTTCC ACAGAGACCA GAAGGAAGCT ACTGAGTATG   
  
  
- ATTACTGTAC CACACTGTAC TCTACTGTAA TAACCACGCC TCTGGATAAA TCCTTCCACA GAGACCAGAA   
  
  
- GGAAGCTACT GAGTATGATT ACTGTACCA

+     W box

| Site Name | Organism | Position | Strand | Matrix score. | sequence | function |
| --- | --- | --- | --- | --- | --- | --- |
| W box | Arabidopsis thaliana | 846 | + | 6 | TTGACC |  |
| W box | Arabidopsis thaliana | 638 | + | 6 | TTGACC |  |

> 2018/04/13 10:10:12  
+ CTAGCTAGCT ACTATCCCAG TGACGGGTGT CTTGTGTCAC CACGACAAAT TAAAAAACCG GAACTAAAGC   
  
  
+ AACCCATCTT CTCGATCCCC TATAATATTC ACCCCACCCC CCTCTCCTTC GCTTCGTCTA TCTCTATTCG   
  
  
+ AACATTGATT ACTACGCAAA CCTGATTTTC TTTAATTTCC TCTCTACTCC TTTCCCTCTT CGTTTACTTT   
  
  
+ CCTTCTCCAC TCCTTTCTCC CTCATTCAAC TTCCCTTTTT CCCTTCTCTC TTTTTGGCGT CCTTTTCAAT   
  
  
+ CACTGCTTCG TTGGTCACTG CAGAGAGTAT ACTACGTTAC CACTACGGTT CGGTTCGAAA GAGAAAGAAA   
  
  
+ GAAAGGAAAG AAAGAAAGAA AGAAGAAAAT AGAGCCGTAC GAATTAAAAG GAGTGGTGTT TATTTGTCTT   
  
  
+ ACTTTAGTTC ACCCGAACTA TGACTATCAC ATGACACAAA CCCCCACACG TGTTGTTAAG TTTCAGATTC   
  
  
+ TTAGTTAGAG CATCGATCTC CCGACGTTAC GGCTCTACTA CCTAAAATTT TTTCTAGTTT AACGTTTTTT   
  
  
+ GTTTTTTGTT TTTTGGTTTT TACCAACCGT AATGACAATC ATGCTACGTT CACCGATGAA AATAAGATAA   
  
  
+ GATTACGTTG ACCTAGTAAT GCACCGTGTC CGGAAAGGTG AAAAGAAAGA TTAGGCTTAT TACATTAGTC   
  
  
+ ATTAGTGTTA TGTTCTCACC GTACTAACTA CTACTTAAGG TCTTTAGCAT AACCAACGCA GGCAGGGTAC   
  
  
+ TACGAGAGAA AACTTTTCAG TTACGAAGTT TGGATTGGAA GGTAGTAAAA GGTACAAACG TGAAGGGTAG   
  
  
+ ATTTGTTGAC CGAGTTTTAG TTTAGAAAAA AAAAAAAAAA AATTTTTTTT TTTTCCCTTC GCGAGTAAAC   
  
  
+ TTTGAAATAT TTCACATACA TATATCTTTT ATTTAAAACT ATTGTTCTCT CTCATTTTAT TGTTCGAAAA   
  
  
+ TAGTACTACT TGTTGTATGG AGTTGTTTTA ATTAATTTTT TTGTTGTTCG TAAGTAAAAC TAAAAATGAA   
  
  
+ GATTTAGATT TTCCTAATGT TAAATTATTT TATTTTGTTT ATATAAAAAT TTTATAAAAA GCAAAATGAA   
  
  
+ AGTTATTTTT GTCATTTTAA AATTAAATTC CTAATGTTAA ATTATTTTAT TTTGTTTATA TAAAAATTTT   
  
  
+ ATAAAAAGCA AAATGAAAGT TATTTTTGTC ATTTTAAAAT TAAAACTTCG TTTAACTAGT CTACTGCAAT   
  
  
+ TTTCTAGATC AATGTAGGTG ATCTGAATTA GGTGTATTTC GTTAGAGGTA GACGGTGCAG AATTGTGGTC   
  
  
+ CTGTATTGAC ATTATTGGTG CGGAGACCTA TTTAGGAAGG TGTCTCTGGT CTTCCTTCGA TGACTCATAC   
  
  
+ TAATGACATG GTGTGACATG AGATGACATT ATTGGTGCGG AGACCTATTT AGGAAGGTGT CTCTGGTCTT   
  
  
+ CCTTCGATGA CTCATACTAA TGACATGGT  

- GATCGATCGA TGATAGGGTC ACTGCCCACA GAACACAGTG GTGCTGTTTA ATTTTTTGGC CTTGATTTCG   
  
  
- TTGGGTAGAA GAGCTAGGGG ATATTATAAG TGGGGTGGGG GGAGAGGAAG CGAAGCAGAT AGAGATAAGC   
  
  
- TTGTAACTAA TGATGCGTTT GGACTAAAAG AAATTAAAGG AGAGATGAGG AAAGGGAGAA GCAAATGAAA   
  
  
- GGAAGAGGTG AGGAAAGAGG GAGTAAGTTG AAGGGAAAAA GGGAAGAGAG AAAAACCGCA GGAAAAGTTA   
  
  
- GTGACGAAGC AACCAGTGAC GTCTCTCATA TGATGCAATG GTGATGCCAA GCCAAGCTTT CTCTTTCTTT   
  
  
- CTTTCCTTTC TTTCTTTCTT TCTTCTTTTA TCTCGGCATG CTTAATTTTC CTCACCACAA ATAAACAGAA   
  
  
- TGAAATCAAG TGGGCTTGAT ACTGATAGTG TACTGTGTTT GGGGGTGTGC ACAACAATTC AAAGTCTAAG   
  
  
- AATCAATCTC GTAGCTAGAG GGCTGCAATG CCGAGATGAT GGATTTTAAA AAAGATCAAA TTGCAAAAAA   
  
  
- CAAAAAACAA AAAACCAAAA ATGGTTGGCA TTACTGTTAG TACGATGCAA GTGGCTACTT TTATTCTATT   
  
  
- CTAATGCAAC TGGATCATTA CGTGGCACAG GCCTTTCCAC TTTTCTTTCT AATCCGAATA ATGTAATCAG   
  
  
- TAATCACAAT ACAAGAGTGG CATGATTGAT GATGAATTCC AGAAATCGTA TTGGTTGCGT CCGTCCCATG   
  
  
- ATGCTCTCTT TTGAAAAGTC AATGCTTCAA ACCTAACCTT CCATCATTTT CCATGTTTGC ACTTCCCATC   
  
  
- TAAACAACTG GCTCAAAATC AAATCTTTTT TTTTTTTTTT TTAAAAAAAA AAAAGGGAAG CGCTCATTTG   
  
  
- AAACTTTATA AAGTGTATGT ATATAGAAAA TAAATTTTGA TAACAAGAGA GAGTAAAATA ACAAGCTTTT   
  
  
- ATCATGATGA ACAACATACC TCAACAAAAT TAATTAAAAA AACAACAAGC ATTCATTTTG ATTTTTACTT   
  
  
- CTAAATCTAA AAGGATTACA ATTTAATAAA ATAAAACAAA TATATTTTTA AAATATTTTT CGTTTTACTT   
  
  
- TCAATAAAAA CAGTAAAATT TTAATTTAAG GATTACAATT TAATAAAATA AAACAAATAT ATTTTTAAAA   
  
  
- TATTTTTCGT TTTACTTTCA ATAAAAACAG TAAAATTTTA ATTTTGAAGC AAATTGATCA GATGACGTTA   
  
  
- AAAGATCTAG TTACATCCAC TAGACTTAAT CCACATAAAG CAATCTCCAT CTGCCACGTC TTAACACCAG   
  
  
- GACATAACTG TAATAACCAC GCCTCTGGAT AAATCCTTCC ACAGAGACCA GAAGGAAGCT ACTGAGTATG   
  
  
- ATTACTGTAC CACACTGTAC TCTACTGTAA TAACCACGCC TCTGGATAAA TCCTTCCACA GAGACCAGAA   
  
  
- GGAAGCTACT GAGTATGATT ACTGTACCA

+     circadian

| Site Name | Organism | Position | Strand | Matrix score. | sequence | function |
| --- | --- | --- | --- | --- | --- | --- |
| circadian | Lycopersicon esculentum | 631 | - | 6 | CAANNNNATC | cis-acting regulatory element involved in circadian control |

> 2018/04/13 10:10:12  
+ CTAGCTAGCT ACTATCCCAG TGACGGGTGT CTTGTGTCAC CACGACAAAT TAAAAAACCG GAACTAAAGC   
  
  
+ AACCCATCTT CTCGATCCCC TATAATATTC ACCCCACCCC CCTCTCCTTC GCTTCGTCTA TCTCTATTCG   
  
  
+ AACATTGATT ACTACGCAAA CCTGATTTTC TTTAATTTCC TCTCTACTCC TTTCCCTCTT CGTTTACTTT   
  
  
+ CCTTCTCCAC TCCTTTCTCC CTCATTCAAC TTCCCTTTTT CCCTTCTCTC TTTTTGGCGT CCTTTTCAAT   
  
  
+ CACTGCTTCG TTGGTCACTG CAGAGAGTAT ACTACGTTAC CACTACGGTT CGGTTCGAAA GAGAAAGAAA   
  
  
+ GAAAGGAAAG AAAGAAAGAA AGAAGAAAAT AGAGCCGTAC GAATTAAAAG GAGTGGTGTT TATTTGTCTT   
  
  
+ ACTTTAGTTC ACCCGAACTA TGACTATCAC ATGACACAAA CCCCCACACG TGTTGTTAAG TTTCAGATTC   
  
  
+ TTAGTTAGAG CATCGATCTC CCGACGTTAC GGCTCTACTA CCTAAAATTT TTTCTAGTTT AACGTTTTTT   
  
  
+ GTTTTTTGTT TTTTGGTTTT TACCAACCGT AATGACAATC ATGCTACGTT CACCGATGAA AATAAGATAA   
  
  
+ GATTACGTTG ACCTAGTAAT GCACCGTGTC CGGAAAGGTG AAAAGAAAGA TTAGGCTTAT TACATTAGTC   
  
  
+ ATTAGTGTTA TGTTCTCACC GTACTAACTA CTACTTAAGG TCTTTAGCAT AACCAACGCA GGCAGGGTAC   
  
  
+ TACGAGAGAA AACTTTTCAG TTACGAAGTT TGGATTGGAA GGTAGTAAAA GGTACAAACG TGAAGGGTAG   
  
  
+ ATTTGTTGAC CGAGTTTTAG TTTAGAAAAA AAAAAAAAAA AATTTTTTTT TTTTCCCTTC GCGAGTAAAC   
  
  
+ TTTGAAATAT TTCACATACA TATATCTTTT ATTTAAAACT ATTGTTCTCT CTCATTTTAT TGTTCGAAAA   
  
  
+ TAGTACTACT TGTTGTATGG AGTTGTTTTA ATTAATTTTT TTGTTGTTCG TAAGTAAAAC TAAAAATGAA   
  
  
+ GATTTAGATT TTCCTAATGT TAAATTATTT TATTTTGTTT ATATAAAAAT TTTATAAAAA GCAAAATGAA   
  
  
+ AGTTATTTTT GTCATTTTAA AATTAAATTC CTAATGTTAA ATTATTTTAT TTTGTTTATA TAAAAATTTT   
  
  
+ ATAAAAAGCA AAATGAAAGT TATTTTTGTC ATTTTAAAAT TAAAACTTCG TTTAACTAGT CTACTGCAAT   
  
  
+ TTTCTAGATC AATGTAGGTG ATCTGAATTA GGTGTATTTC GTTAGAGGTA GACGGTGCAG AATTGTGGTC   
  
  
+ CTGTATTGAC ATTATTGGTG CGGAGACCTA TTTAGGAAGG TGTCTCTGGT CTTCCTTCGA TGACTCATAC   
  
  
+ TAATGACATG GTGTGACATG AGATGACATT ATTGGTGCGG AGACCTATTT AGGAAGGTGT CTCTGGTCTT   
  
  
+ CCTTCGATGA CTCATACTAA TGACATGGT  

- GATCGATCGA TGATAGGGTC ACTGCCCACA GAACACAGTG GTGCTGTTTA ATTTTTTGGC CTTGATTTCG   
  
  
- TTGGGTAGAA GAGCTAGGGG ATATTATAAG TGGGGTGGGG GGAGAGGAAG CGAAGCAGAT AGAGATAAGC   
  
  
- TTGTAACTAA TGATGCGTTT GGACTAAAAG AAATTAAAGG AGAGATGAGG AAAGGGAGAA GCAAATGAAA   
  
  
- GGAAGAGGTG AGGAAAGAGG GAGTAAGTTG AAGGGAAAAA GGGAAGAGAG AAAAACCGCA GGAAAAGTTA   
  
  
- GTGACGAAGC AACCAGTGAC GTCTCTCATA TGATGCAATG GTGATGCCAA GCCAAGCTTT CTCTTTCTTT   
  
  
- CTTTCCTTTC TTTCTTTCTT TCTTCTTTTA TCTCGGCATG CTTAATTTTC CTCACCACAA ATAAACAGAA   
  
  
- TGAAATCAAG TGGGCTTGAT ACTGATAGTG TACTGTGTTT GGGGGTGTGC ACAACAATTC AAAGTCTAAG   
  
  
- AATCAATCTC GTAGCTAGAG GGCTGCAATG CCGAGATGAT GGATTTTAAA AAAGATCAAA TTGCAAAAAA   
  
  
- CAAAAAACAA AAAACCAAAA ATGGTTGGCA TTACTGTTAG TACGATGCAA GTGGCTACTT TTATTCTATT   
  
  
- CTAATGCAAC TGGATCATTA CGTGGCACAG GCCTTTCCAC TTTTCTTTCT AATCCGAATA ATGTAATCAG   
  
  
- TAATCACAAT ACAAGAGTGG CATGATTGAT GATGAATTCC AGAAATCGTA TTGGTTGCGT CCGTCCCATG   
  
  
- ATGCTCTCTT TTGAAAAGTC AATGCTTCAA ACCTAACCTT CCATCATTTT CCATGTTTGC ACTTCCCATC   
  
  
- TAAACAACTG GCTCAAAATC AAATCTTTTT TTTTTTTTTT TTAAAAAAAA AAAAGGGAAG CGCTCATTTG   
  
  
- AAACTTTATA AAGTGTATGT ATATAGAAAA TAAATTTTGA TAACAAGAGA GAGTAAAATA ACAAGCTTTT   
  
  
- ATCATGATGA ACAACATACC TCAACAAAAT TAATTAAAAA AACAACAAGC ATTCATTTTG ATTTTTACTT   
  
  
- CTAAATCTAA AAGGATTACA ATTTAATAAA ATAAAACAAA TATATTTTTA AAATATTTTT CGTTTTACTT   
  
  
- TCAATAAAAA CAGTAAAATT TTAATTTAAG GATTACAATT TAATAAAATA AAACAAATAT ATTTTTAAAA   
  
  
- TATTTTTCGT TTTACTTTCA ATAAAAACAG TAAAATTTTA ATTTTGAAGC AAATTGATCA GATGACGTTA   
  
  
- AAAGATCTAG TTACATCCAC TAGACTTAAT CCACATAAAG CAATCTCCAT CTGCCACGTC TTAACACCAG   
  
  
- GACATAACTG TAATAACCAC GCCTCTGGAT AAATCCTTCC ACAGAGACCA GAAGGAAGCT ACTGAGTATG   
  
  
- ATTACTGTAC CACACTGTAC TCTACTGTAA TAACCACGCC TCTGGATAAA TCCTTCCACA GAGACCAGAA   
  
  
- GGAAGCTACT GAGTATGATT ACTGTACCA
