# Supplementary material for: Genome-wide analysis of the WRKY gene family in drumstick (Moringa oleifera Lam.)
Source: PeerJ. 2019 Jun 10;7:e7063. doi: 10.7717/peerj.7063 (PMC6563795; doi:10.7717/peerj.7063)
Supplement: Supplemental Information 1 [file peerj-07-7063-s003.gz › MoWRKY54_plantcare.html]

Content-Type: text/html; charset=ISO-8859-1


CallMat\_Firefox


Webmaster Firefox specific output  
To save the result:
click on the frame with the right mouse button and save the source code as a text file with extension .html  
REFERENCE:PlantCARE: a database of plant cis-acting regulatory elements and a portal to tools for in silico analysis of promoter sequences.  
Lescot, M., Déhais, P., Moreau, Y., De Moor, B., Rouzé ,P.,and Rombauts, S.  
Nucleic Acids Res., Database issue(2002), 30(1):325-327.   


---

> 2018/04/13 10:10:12  
+ AAAGACAAAA GCAAACACTT TGACACGCAA ACAACATATG TGTTCACGCC ACTTTCCTCT CAAGTCCGTT   
  
  
+ TCGTTTCGGT TCTCCCCCGA AAAATACAAA TCTTCTTTCT CACGAGACTT TAGTGTAACG TAAACGGACC   
  
  
+ TTCTAAATAA CTTGGATCGA CCCTTTTAAG GAGTAGAAAA CCTGAATTGT GGTGGGGGTG CGCGTCGATT   
  
  
+ CCGTCGCAGG TGCGACGATT GCCTTGTGTA CAGGGGACGA CGACCACCCT TCTTTGCCTT AACGTGGAGT   
  
  
+ TTTCAACTTT AAAATTGCCT CCCGAAACCG CGCAGCTGCT ACTTCTCGCA TTCCCCCGGG TTATACCGAA   
  
  
+ GAATGCCTCA ATTTACTCCA GTGAGACGGT TACAGTACCA CCACTGTAGT TAAAGGACTA CGGTGTACAG   
  
  
+ TGGGAATCAA AATTTATAGG GTACAACTGT CGTTACCATA AAAAGAATAG GACTGTTTTG ACCTACTACT   
  
  
+ TTATTGCTTA TATCGGAGCT CTGAGATCCC GAAACTTCAC TTCATTTCCT CCGTTGCCGT CCCCGTAAAT   
  
  
+ CCTGTTGTTC TCGACTATCG TCGGCCCATC AATATAATAG CTTCAGGAGA GGAGGAGAAT AAAATAAGAT   
  
  
+ GACTGTACCT ATTTGTGCGT CAACCTCTAC TACAGTACAC GGGTCACTGG TCCCAAACGG TTCATTTCGT   
  
  
+ CCGTATTCCT GGCAACTTAA TACTAACTCA ATCGCACTAC CCTCTTGATT GCCCAGACTA AATCCTAGAT   
  
  
+ CGATCACGAT CACCTCTACT TAGAATGACA TTTTCGTCAC TGTTCATCCT CACCAAACAG AAAATGTTCA   
  
  
+ CCGCCTAGAG CATTAACCTC ATTTCGTCAC CCTTTGCATC TAAATCTAGA CACTGGTCAC TGATGATCGG   
  
  
+ GCTTGTTCTT AACTTCTTTT ATTTTTTAAT TTTTTTCTTT TCCCTACGAG TCTAGAGACT ACGTATTAAC   
  
  
+ CGAGATTCTT GTTGCCTATC CCGTCACTTG AACCAGTCCA CGAAACGCGT ACTCACTACT ACGAGAGTAG   
  
  
+ ACTAACCGAG GCGTCGGAGG AAGAACAGGC CTCGCTACTG AACAAGAAGT TTGATCTATT TGTGTAACAA   
  
  
+ CGTAGAGTAG TTGACAATAA TAAACCAAGG AAGACAAAAC ACAAAAAAAA AAAGGGACAC TTTGCGTTAA   
  
  
+ ACTACCTAAG AAACTTTACT CGTAATAACC TACGCCCTCG AAGTTTCATA CAGAATGTCC ATCTATAAAC   
  
  
+ GTACCGCTCC GACGTAGAGA TGAATGGTAA AACCTTATAC ATGTTTTCAA AACTTTGAAA AAGTTCAGTA   
  
  
+ ATTACCCGTA TTGCTATATC GGTTGATTAT ATGTATACAA GAAACGGCAG AAGAAGGCTC TTACAGTAGA   
  
  
+ TCGAAGAACC TTACCGTTAA CCCGTTGACG ATATATTATA AAAAACTACT ATCAAATTTG CATTACTCTA   
  
  
+ ATCCACTTAA ATGAATAACT ATAACACCT  

- TTTCTGTTTT CGTTTGTGAA ACTGTGCGTT TGTTGTATAC ACAAGTGCGG TGAAAGGAGA GTTCAGGCAA   
  
  
- AGCAAAGCCA AGAGGGGGCT TTTTATGTTT AGAAGAAAGA GTGCTCTGAA ATCACATTGC ATTTGCCTGG   
  
  
- AAGATTTATT GAACCTAGCT GGGAAAATTC CTCATCTTTT GGACTTAACA CCACCCCCAC GCGCAGCTAA   
  
  
- GGCAGCGTCC ACGCTGCTAA CGGAACACAT GTCCCCTGCT GCTGGTGGGA AGAAACGGAA TTGCACCTCA   
  
  
- AAAGTTGAAA TTTTAACGGA GGGCTTTGGC GCGTCGACGA TGAAGAGCGT AAGGGGGCCC AATATGGCTT   
  
  
- CTTACGGAGT TAAATGAGGT CACTCTGCCA ATGTCATGGT GGTGACATCA ATTTCCTGAT GCCACATGTC   
  
  
- ACCCTTAGTT TTAAATATCC CATGTTGACA GCAATGGTAT TTTTCTTATC CTGACAAAAC TGGATGATGA   
  
  
- AATAACGAAT ATAGCCTCGA GACTCTAGGG CTTTGAAGTG AAGTAAAGGA GGCAACGGCA GGGGCATTTA   
  
  
- GGACAACAAG AGCTGATAGC AGCCGGGTAG TTATATTATC GAAGTCCTCT CCTCCTCTTA TTTTATTCTA   
  
  
- CTGACATGGA TAAACACGCA GTTGGAGATG ATGTCATGTG CCCAGTGACC AGGGTTTGCC AAGTAAAGCA   
  
  
- GGCATAAGGA CCGTTGAATT ATGATTGAGT TAGCGTGATG GGAGAACTAA CGGGTCTGAT TTAGGATCTA   
  
  
- GCTAGTGCTA GTGGAGATGA ATCTTACTGT AAAAGCAGTG ACAAGTAGGA GTGGTTTGTC TTTTACAAGT   
  
  
- GGCGGATCTC GTAATTGGAG TAAAGCAGTG GGAAACGTAG ATTTAGATCT GTGACCAGTG ACTACTAGCC   
  
  
- CGAACAAGAA TTGAAGAAAA TAAAAAATTA AAAAAAGAAA AGGGATGCTC AGATCTCTGA TGCATAATTG   
  
  
- GCTCTAAGAA CAACGGATAG GGCAGTGAAC TTGGTCAGGT GCTTTGCGCA TGAGTGATGA TGCTCTCATC   
  
  
- TGATTGGCTC CGCAGCCTCC TTCTTGTCCG GAGCGATGAC TTGTTCTTCA AACTAGATAA ACACATTGTT   
  
  
- GCATCTCATC AACTGTTATT ATTTGGTTCC TTCTGTTTTG TGTTTTTTTT TTTCCCTGTG AAACGCAATT   
  
  
- TGATGGATTC TTTGAAATGA GCATTATTGG ATGCGGGAGC TTCAAAGTAT GTCTTACAGG TAGATATTTG   
  
  
- CATGGCGAGG CTGCATCTCT ACTTACCATT TTGGAATATG TACAAAAGTT TTGAAACTTT TTCAAGTCAT   
  
  
- TAATGGGCAT AACGATATAG CCAACTAATA TACATATGTT CTTTGCCGTC TTCTTCCGAG AATGTCATCT   
  
  
- AGCTTCTTGG AATGGCAATT GGGCAACTGC TATATAATAT TTTTTGATGA TAGTTTAAAC GTAATGAGAT   
  
  
- TAGGTGAATT TACTTATTGA TATTGTGGA

  
  
Motifs Found  

+     A-box

| Site Name | Organism | Position | Strand | Matrix score. | sequence | function |
| --- | --- | --- | --- | --- | --- | --- |
| A-box | Petroselinum crispum | 547 | + | 6 | CCGTCC | cis-acting regulatory element |

> 2018/04/13 10:10:12  
+ AAAGACAAAA GCAAACACTT TGACACGCAA ACAACATATG TGTTCACGCC ACTTTCCTCT CAAGTCCGTT   
  
  
+ TCGTTTCGGT TCTCCCCCGA AAAATACAAA TCTTCTTTCT CACGAGACTT TAGTGTAACG TAAACGGACC   
  
  
+ TTCTAAATAA CTTGGATCGA CCCTTTTAAG GAGTAGAAAA CCTGAATTGT GGTGGGGGTG CGCGTCGATT   
  
  
+ CCGTCGCAGG TGCGACGATT GCCTTGTGTA CAGGGGACGA CGACCACCCT TCTTTGCCTT AACGTGGAGT   
  
  
+ TTTCAACTTT AAAATTGCCT CCCGAAACCG CGCAGCTGCT ACTTCTCGCA TTCCCCCGGG TTATACCGAA   
  
  
+ GAATGCCTCA ATTTACTCCA GTGAGACGGT TACAGTACCA CCACTGTAGT TAAAGGACTA CGGTGTACAG   
  
  
+ TGGGAATCAA AATTTATAGG GTACAACTGT CGTTACCATA AAAAGAATAG GACTGTTTTG ACCTACTACT   
  
  
+ TTATTGCTTA TATCGGAGCT CTGAGATCCC GAAACTTCAC TTCATTTCCT CCGTTGCCGT CCCCGTAAAT   
  
  
+ CCTGTTGTTC TCGACTATCG TCGGCCCATC AATATAATAG CTTCAGGAGA GGAGGAGAAT AAAATAAGAT   
  
  
+ GACTGTACCT ATTTGTGCGT CAACCTCTAC TACAGTACAC GGGTCACTGG TCCCAAACGG TTCATTTCGT   
  
  
+ CCGTATTCCT GGCAACTTAA TACTAACTCA ATCGCACTAC CCTCTTGATT GCCCAGACTA AATCCTAGAT   
  
  
+ CGATCACGAT CACCTCTACT TAGAATGACA TTTTCGTCAC TGTTCATCCT CACCAAACAG AAAATGTTCA   
  
  
+ CCGCCTAGAG CATTAACCTC ATTTCGTCAC CCTTTGCATC TAAATCTAGA CACTGGTCAC TGATGATCGG   
  
  
+ GCTTGTTCTT AACTTCTTTT ATTTTTTAAT TTTTTTCTTT TCCCTACGAG TCTAGAGACT ACGTATTAAC   
  
  
+ CGAGATTCTT GTTGCCTATC CCGTCACTTG AACCAGTCCA CGAAACGCGT ACTCACTACT ACGAGAGTAG   
  
  
+ ACTAACCGAG GCGTCGGAGG AAGAACAGGC CTCGCTACTG AACAAGAAGT TTGATCTATT TGTGTAACAA   
  
  
+ CGTAGAGTAG TTGACAATAA TAAACCAAGG AAGACAAAAC ACAAAAAAAA AAAGGGACAC TTTGCGTTAA   
  
  
+ ACTACCTAAG AAACTTTACT CGTAATAACC TACGCCCTCG AAGTTTCATA CAGAATGTCC ATCTATAAAC   
  
  
+ GTACCGCTCC GACGTAGAGA TGAATGGTAA AACCTTATAC ATGTTTTCAA AACTTTGAAA AAGTTCAGTA   
  
  
+ ATTACCCGTA TTGCTATATC GGTTGATTAT ATGTATACAA GAAACGGCAG AAGAAGGCTC TTACAGTAGA   
  
  
+ TCGAAGAACC TTACCGTTAA CCCGTTGACG ATATATTATA AAAAACTACT ATCAAATTTG CATTACTCTA   
  
  
+ ATCCACTTAA ATGAATAACT ATAACACCT  

- TTTCTGTTTT CGTTTGTGAA ACTGTGCGTT TGTTGTATAC ACAAGTGCGG TGAAAGGAGA GTTCAGGCAA   
  
  
- AGCAAAGCCA AGAGGGGGCT TTTTATGTTT AGAAGAAAGA GTGCTCTGAA ATCACATTGC ATTTGCCTGG   
  
  
- AAGATTTATT GAACCTAGCT GGGAAAATTC CTCATCTTTT GGACTTAACA CCACCCCCAC GCGCAGCTAA   
  
  
- GGCAGCGTCC ACGCTGCTAA CGGAACACAT GTCCCCTGCT GCTGGTGGGA AGAAACGGAA TTGCACCTCA   
  
  
- AAAGTTGAAA TTTTAACGGA GGGCTTTGGC GCGTCGACGA TGAAGAGCGT AAGGGGGCCC AATATGGCTT   
  
  
- CTTACGGAGT TAAATGAGGT CACTCTGCCA ATGTCATGGT GGTGACATCA ATTTCCTGAT GCCACATGTC   
  
  
- ACCCTTAGTT TTAAATATCC CATGTTGACA GCAATGGTAT TTTTCTTATC CTGACAAAAC TGGATGATGA   
  
  
- AATAACGAAT ATAGCCTCGA GACTCTAGGG CTTTGAAGTG AAGTAAAGGA GGCAACGGCA GGGGCATTTA   
  
  
- GGACAACAAG AGCTGATAGC AGCCGGGTAG TTATATTATC GAAGTCCTCT CCTCCTCTTA TTTTATTCTA   
  
  
- CTGACATGGA TAAACACGCA GTTGGAGATG ATGTCATGTG CCCAGTGACC AGGGTTTGCC AAGTAAAGCA   
  
  
- GGCATAAGGA CCGTTGAATT ATGATTGAGT TAGCGTGATG GGAGAACTAA CGGGTCTGAT TTAGGATCTA   
  
  
- GCTAGTGCTA GTGGAGATGA ATCTTACTGT AAAAGCAGTG ACAAGTAGGA GTGGTTTGTC TTTTACAAGT   
  
  
- GGCGGATCTC GTAATTGGAG TAAAGCAGTG GGAAACGTAG ATTTAGATCT GTGACCAGTG ACTACTAGCC   
  
  
- CGAACAAGAA TTGAAGAAAA TAAAAAATTA AAAAAAGAAA AGGGATGCTC AGATCTCTGA TGCATAATTG   
  
  
- GCTCTAAGAA CAACGGATAG GGCAGTGAAC TTGGTCAGGT GCTTTGCGCA TGAGTGATGA TGCTCTCATC   
  
  
- TGATTGGCTC CGCAGCCTCC TTCTTGTCCG GAGCGATGAC TTGTTCTTCA AACTAGATAA ACACATTGTT   
  
  
- GCATCTCATC AACTGTTATT ATTTGGTTCC TTCTGTTTTG TGTTTTTTTT TTTCCCTGTG AAACGCAATT   
  
  
- TGATGGATTC TTTGAAATGA GCATTATTGG ATGCGGGAGC TTCAAAGTAT GTCTTACAGG TAGATATTTG   
  
  
- CATGGCGAGG CTGCATCTCT ACTTACCATT TTGGAATATG TACAAAAGTT TTGAAACTTT TTCAAGTCAT   
  
  
- TAATGGGCAT AACGATATAG CCAACTAATA TACATATGTT CTTTGCCGTC TTCTTCCGAG AATGTCATCT   
  
  
- AGCTTCTTGG AATGGCAATT GGGCAACTGC TATATAATAT TTTTTGATGA TAGTTTAAAC GTAATGAGAT   
  
  
- TAGGTGAATT TACTTATTGA TATTGTGGA

+     AAGAA-motif

| Site Name | Organism | Position | Strand | Matrix score. | sequence | function |
| --- | --- | --- | --- | --- | --- | --- |
| AAGAA-motif | Avena sativa | 103 | - | 7 | GAAAGAA |  |
| AAGAA-motif | Avena sativa | 944 | - | 9 | gGTAAAGAAA |  |

> 2018/04/13 10:10:12  
+ AAAGACAAAA GCAAACACTT TGACACGCAA ACAACATATG TGTTCACGCC ACTTTCCTCT CAAGTCCGTT   
  
  
+ TCGTTTCGGT TCTCCCCCGA AAAATACAAA TCTTCTTTCT CACGAGACTT TAGTGTAACG TAAACGGACC   
  
  
+ TTCTAAATAA CTTGGATCGA CCCTTTTAAG GAGTAGAAAA CCTGAATTGT GGTGGGGGTG CGCGTCGATT   
  
  
+ CCGTCGCAGG TGCGACGATT GCCTTGTGTA CAGGGGACGA CGACCACCCT TCTTTGCCTT AACGTGGAGT   
  
  
+ TTTCAACTTT AAAATTGCCT CCCGAAACCG CGCAGCTGCT ACTTCTCGCA TTCCCCCGGG TTATACCGAA   
  
  
+ GAATGCCTCA ATTTACTCCA GTGAGACGGT TACAGTACCA CCACTGTAGT TAAAGGACTA CGGTGTACAG   
  
  
+ TGGGAATCAA AATTTATAGG GTACAACTGT CGTTACCATA AAAAGAATAG GACTGTTTTG ACCTACTACT   
  
  
+ TTATTGCTTA TATCGGAGCT CTGAGATCCC GAAACTTCAC TTCATTTCCT CCGTTGCCGT CCCCGTAAAT   
  
  
+ CCTGTTGTTC TCGACTATCG TCGGCCCATC AATATAATAG CTTCAGGAGA GGAGGAGAAT AAAATAAGAT   
  
  
+ GACTGTACCT ATTTGTGCGT CAACCTCTAC TACAGTACAC GGGTCACTGG TCCCAAACGG TTCATTTCGT   
  
  
+ CCGTATTCCT GGCAACTTAA TACTAACTCA ATCGCACTAC CCTCTTGATT GCCCAGACTA AATCCTAGAT   
  
  
+ CGATCACGAT CACCTCTACT TAGAATGACA TTTTCGTCAC TGTTCATCCT CACCAAACAG AAAATGTTCA   
  
  
+ CCGCCTAGAG CATTAACCTC ATTTCGTCAC CCTTTGCATC TAAATCTAGA CACTGGTCAC TGATGATCGG   
  
  
+ GCTTGTTCTT AACTTCTTTT ATTTTTTAAT TTTTTTCTTT TCCCTACGAG TCTAGAGACT ACGTATTAAC   
  
  
+ CGAGATTCTT GTTGCCTATC CCGTCACTTG AACCAGTCCA CGAAACGCGT ACTCACTACT ACGAGAGTAG   
  
  
+ ACTAACCGAG GCGTCGGAGG AAGAACAGGC CTCGCTACTG AACAAGAAGT TTGATCTATT TGTGTAACAA   
  
  
+ CGTAGAGTAG TTGACAATAA TAAACCAAGG AAGACAAAAC ACAAAAAAAA AAAGGGACAC TTTGCGTTAA   
  
  
+ ACTACCTAAG AAACTTTACT CGTAATAACC TACGCCCTCG AAGTTTCATA CAGAATGTCC ATCTATAAAC   
  
  
+ GTACCGCTCC GACGTAGAGA TGAATGGTAA AACCTTATAC ATGTTTTCAA AACTTTGAAA AAGTTCAGTA   
  
  
+ ATTACCCGTA TTGCTATATC GGTTGATTAT ATGTATACAA GAAACGGCAG AAGAAGGCTC TTACAGTAGA   
  
  
+ TCGAAGAACC TTACCGTTAA CCCGTTGACG ATATATTATA AAAAACTACT ATCAAATTTG CATTACTCTA   
  
  
+ ATCCACTTAA ATGAATAACT ATAACACCT  

- TTTCTGTTTT CGTTTGTGAA ACTGTGCGTT TGTTGTATAC ACAAGTGCGG TGAAAGGAGA GTTCAGGCAA   
  
  
- AGCAAAGCCA AGAGGGGGCT TTTTATGTTT AGAAGAAAGA GTGCTCTGAA ATCACATTGC ATTTGCCTGG   
  
  
- AAGATTTATT GAACCTAGCT GGGAAAATTC CTCATCTTTT GGACTTAACA CCACCCCCAC GCGCAGCTAA   
  
  
- GGCAGCGTCC ACGCTGCTAA CGGAACACAT GTCCCCTGCT GCTGGTGGGA AGAAACGGAA TTGCACCTCA   
  
  
- AAAGTTGAAA TTTTAACGGA GGGCTTTGGC GCGTCGACGA TGAAGAGCGT AAGGGGGCCC AATATGGCTT   
  
  
- CTTACGGAGT TAAATGAGGT CACTCTGCCA ATGTCATGGT GGTGACATCA ATTTCCTGAT GCCACATGTC   
  
  
- ACCCTTAGTT TTAAATATCC CATGTTGACA GCAATGGTAT TTTTCTTATC CTGACAAAAC TGGATGATGA   
  
  
- AATAACGAAT ATAGCCTCGA GACTCTAGGG CTTTGAAGTG AAGTAAAGGA GGCAACGGCA GGGGCATTTA   
  
  
- GGACAACAAG AGCTGATAGC AGCCGGGTAG TTATATTATC GAAGTCCTCT CCTCCTCTTA TTTTATTCTA   
  
  
- CTGACATGGA TAAACACGCA GTTGGAGATG ATGTCATGTG CCCAGTGACC AGGGTTTGCC AAGTAAAGCA   
  
  
- GGCATAAGGA CCGTTGAATT ATGATTGAGT TAGCGTGATG GGAGAACTAA CGGGTCTGAT TTAGGATCTA   
  
  
- GCTAGTGCTA GTGGAGATGA ATCTTACTGT AAAAGCAGTG ACAAGTAGGA GTGGTTTGTC TTTTACAAGT   
  
  
- GGCGGATCTC GTAATTGGAG TAAAGCAGTG GGAAACGTAG ATTTAGATCT GTGACCAGTG ACTACTAGCC   
  
  
- CGAACAAGAA TTGAAGAAAA TAAAAAATTA AAAAAAGAAA AGGGATGCTC AGATCTCTGA TGCATAATTG   
  
  
- GCTCTAAGAA CAACGGATAG GGCAGTGAAC TTGGTCAGGT GCTTTGCGCA TGAGTGATGA TGCTCTCATC   
  
  
- TGATTGGCTC CGCAGCCTCC TTCTTGTCCG GAGCGATGAC TTGTTCTTCA AACTAGATAA ACACATTGTT   
  
  
- GCATCTCATC AACTGTTATT ATTTGGTTCC TTCTGTTTTG TGTTTTTTTT TTTCCCTGTG AAACGCAATT   
  
  
- TGATGGATTC TTTGAAATGA GCATTATTGG ATGCGGGAGC TTCAAAGTAT GTCTTACAGG TAGATATTTG   
  
  
- CATGGCGAGG CTGCATCTCT ACTTACCATT TTGGAATATG TACAAAAGTT TTGAAACTTT TTCAAGTCAT   
  
  
- TAATGGGCAT AACGATATAG CCAACTAATA TACATATGTT CTTTGCCGTC TTCTTCCGAG AATGTCATCT   
  
  
- AGCTTCTTGG AATGGCAATT GGGCAACTGC TATATAATAT TTTTTGATGA TAGTTTAAAC GTAATGAGAT   
  
  
- TAGGTGAATT TACTTATTGA TATTGTGGA

+     ABRE

| Site Name | Organism | Position | Strand | Matrix score. | sequence | function |
| --- | --- | --- | --- | --- | --- | --- |
| ABRE | Hordeum vulgare | 1176 | - | 9 | GCAACGTGTC | cis-acting element involved in the abscisic acid responsiveness |

> 2018/04/13 10:10:12  
+ AAAGACAAAA GCAAACACTT TGACACGCAA ACAACATATG TGTTCACGCC ACTTTCCTCT CAAGTCCGTT   
  
  
+ TCGTTTCGGT TCTCCCCCGA AAAATACAAA TCTTCTTTCT CACGAGACTT TAGTGTAACG TAAACGGACC   
  
  
+ TTCTAAATAA CTTGGATCGA CCCTTTTAAG GAGTAGAAAA CCTGAATTGT GGTGGGGGTG CGCGTCGATT   
  
  
+ CCGTCGCAGG TGCGACGATT GCCTTGTGTA CAGGGGACGA CGACCACCCT TCTTTGCCTT AACGTGGAGT   
  
  
+ TTTCAACTTT AAAATTGCCT CCCGAAACCG CGCAGCTGCT ACTTCTCGCA TTCCCCCGGG TTATACCGAA   
  
  
+ GAATGCCTCA ATTTACTCCA GTGAGACGGT TACAGTACCA CCACTGTAGT TAAAGGACTA CGGTGTACAG   
  
  
+ TGGGAATCAA AATTTATAGG GTACAACTGT CGTTACCATA AAAAGAATAG GACTGTTTTG ACCTACTACT   
  
  
+ TTATTGCTTA TATCGGAGCT CTGAGATCCC GAAACTTCAC TTCATTTCCT CCGTTGCCGT CCCCGTAAAT   
  
  
+ CCTGTTGTTC TCGACTATCG TCGGCCCATC AATATAATAG CTTCAGGAGA GGAGGAGAAT AAAATAAGAT   
  
  
+ GACTGTACCT ATTTGTGCGT CAACCTCTAC TACAGTACAC GGGTCACTGG TCCCAAACGG TTCATTTCGT   
  
  
+ CCGTATTCCT GGCAACTTAA TACTAACTCA ATCGCACTAC CCTCTTGATT GCCCAGACTA AATCCTAGAT   
  
  
+ CGATCACGAT CACCTCTACT TAGAATGACA TTTTCGTCAC TGTTCATCCT CACCAAACAG AAAATGTTCA   
  
  
+ CCGCCTAGAG CATTAACCTC ATTTCGTCAC CCTTTGCATC TAAATCTAGA CACTGGTCAC TGATGATCGG   
  
  
+ GCTTGTTCTT AACTTCTTTT ATTTTTTAAT TTTTTTCTTT TCCCTACGAG TCTAGAGACT ACGTATTAAC   
  
  
+ CGAGATTCTT GTTGCCTATC CCGTCACTTG AACCAGTCCA CGAAACGCGT ACTCACTACT ACGAGAGTAG   
  
  
+ ACTAACCGAG GCGTCGGAGG AAGAACAGGC CTCGCTACTG AACAAGAAGT TTGATCTATT TGTGTAACAA   
  
  
+ CGTAGAGTAG TTGACAATAA TAAACCAAGG AAGACAAAAC ACAAAAAAAA AAAGGGACAC TTTGCGTTAA   
  
  
+ ACTACCTAAG AAACTTTACT CGTAATAACC TACGCCCTCG AAGTTTCATA CAGAATGTCC ATCTATAAAC   
  
  
+ GTACCGCTCC GACGTAGAGA TGAATGGTAA AACCTTATAC ATGTTTTCAA AACTTTGAAA AAGTTCAGTA   
  
  
+ ATTACCCGTA TTGCTATATC GGTTGATTAT ATGTATACAA GAAACGGCAG AAGAAGGCTC TTACAGTAGA   
  
  
+ TCGAAGAACC TTACCGTTAA CCCGTTGACG ATATATTATA AAAAACTACT ATCAAATTTG CATTACTCTA   
  
  
+ ATCCACTTAA ATGAATAACT ATAACACCT  

- TTTCTGTTTT CGTTTGTGAA ACTGTGCGTT TGTTGTATAC ACAAGTGCGG TGAAAGGAGA GTTCAGGCAA   
  
  
- AGCAAAGCCA AGAGGGGGCT TTTTATGTTT AGAAGAAAGA GTGCTCTGAA ATCACATTGC ATTTGCCTGG   
  
  
- AAGATTTATT GAACCTAGCT GGGAAAATTC CTCATCTTTT GGACTTAACA CCACCCCCAC GCGCAGCTAA   
  
  
- GGCAGCGTCC ACGCTGCTAA CGGAACACAT GTCCCCTGCT GCTGGTGGGA AGAAACGGAA TTGCACCTCA   
  
  
- AAAGTTGAAA TTTTAACGGA GGGCTTTGGC GCGTCGACGA TGAAGAGCGT AAGGGGGCCC AATATGGCTT   
  
  
- CTTACGGAGT TAAATGAGGT CACTCTGCCA ATGTCATGGT GGTGACATCA ATTTCCTGAT GCCACATGTC   
  
  
- ACCCTTAGTT TTAAATATCC CATGTTGACA GCAATGGTAT TTTTCTTATC CTGACAAAAC TGGATGATGA   
  
  
- AATAACGAAT ATAGCCTCGA GACTCTAGGG CTTTGAAGTG AAGTAAAGGA GGCAACGGCA GGGGCATTTA   
  
  
- GGACAACAAG AGCTGATAGC AGCCGGGTAG TTATATTATC GAAGTCCTCT CCTCCTCTTA TTTTATTCTA   
  
  
- CTGACATGGA TAAACACGCA GTTGGAGATG ATGTCATGTG CCCAGTGACC AGGGTTTGCC AAGTAAAGCA   
  
  
- GGCATAAGGA CCGTTGAATT ATGATTGAGT TAGCGTGATG GGAGAACTAA CGGGTCTGAT TTAGGATCTA   
  
  
- GCTAGTGCTA GTGGAGATGA ATCTTACTGT AAAAGCAGTG ACAAGTAGGA GTGGTTTGTC TTTTACAAGT   
  
  
- GGCGGATCTC GTAATTGGAG TAAAGCAGTG GGAAACGTAG ATTTAGATCT GTGACCAGTG ACTACTAGCC   
  
  
- CGAACAAGAA TTGAAGAAAA TAAAAAATTA AAAAAAGAAA AGGGATGCTC AGATCTCTGA TGCATAATTG   
  
  
- GCTCTAAGAA CAACGGATAG GGCAGTGAAC TTGGTCAGGT GCTTTGCGCA TGAGTGATGA TGCTCTCATC   
  
  
- TGATTGGCTC CGCAGCCTCC TTCTTGTCCG GAGCGATGAC TTGTTCTTCA AACTAGATAA ACACATTGTT   
  
  
- GCATCTCATC AACTGTTATT ATTTGGTTCC TTCTGTTTTG TGTTTTTTTT TTTCCCTGTG AAACGCAATT   
  
  
- TGATGGATTC TTTGAAATGA GCATTATTGG ATGCGGGAGC TTCAAAGTAT GTCTTACAGG TAGATATTTG   
  
  
- CATGGCGAGG CTGCATCTCT ACTTACCATT TTGGAATATG TACAAAAGTT TTGAAACTTT TTCAAGTCAT   
  
  
- TAATGGGCAT AACGATATAG CCAACTAATA TACATATGTT CTTTGCCGTC TTCTTCCGAG AATGTCATCT   
  
  
- AGCTTCTTGG AATGGCAATT GGGCAACTGC TATATAATAT TTTTTGATGA TAGTTTAAAC GTAATGAGAT   
  
  
- TAGGTGAATT TACTTATTGA TATTGTGGA

+     ACE

| Site Name | Organism | Position | Strand | Matrix score. | sequence | function |
| --- | --- | --- | --- | --- | --- | --- |
| ACE | Petroselinum hortense | 272 | + | 7 | ACGTGGA | cis-acting element involved in light responsiveness |

> 2018/04/13 10:10:12  
+ AAAGACAAAA GCAAACACTT TGACACGCAA ACAACATATG TGTTCACGCC ACTTTCCTCT CAAGTCCGTT   
  
  
+ TCGTTTCGGT TCTCCCCCGA AAAATACAAA TCTTCTTTCT CACGAGACTT TAGTGTAACG TAAACGGACC   
  
  
+ TTCTAAATAA CTTGGATCGA CCCTTTTAAG GAGTAGAAAA CCTGAATTGT GGTGGGGGTG CGCGTCGATT   
  
  
+ CCGTCGCAGG TGCGACGATT GCCTTGTGTA CAGGGGACGA CGACCACCCT TCTTTGCCTT AACGTGGAGT   
  
  
+ TTTCAACTTT AAAATTGCCT CCCGAAACCG CGCAGCTGCT ACTTCTCGCA TTCCCCCGGG TTATACCGAA   
  
  
+ GAATGCCTCA ATTTACTCCA GTGAGACGGT TACAGTACCA CCACTGTAGT TAAAGGACTA CGGTGTACAG   
  
  
+ TGGGAATCAA AATTTATAGG GTACAACTGT CGTTACCATA AAAAGAATAG GACTGTTTTG ACCTACTACT   
  
  
+ TTATTGCTTA TATCGGAGCT CTGAGATCCC GAAACTTCAC TTCATTTCCT CCGTTGCCGT CCCCGTAAAT   
  
  
+ CCTGTTGTTC TCGACTATCG TCGGCCCATC AATATAATAG CTTCAGGAGA GGAGGAGAAT AAAATAAGAT   
  
  
+ GACTGTACCT ATTTGTGCGT CAACCTCTAC TACAGTACAC GGGTCACTGG TCCCAAACGG TTCATTTCGT   
  
  
+ CCGTATTCCT GGCAACTTAA TACTAACTCA ATCGCACTAC CCTCTTGATT GCCCAGACTA AATCCTAGAT   
  
  
+ CGATCACGAT CACCTCTACT TAGAATGACA TTTTCGTCAC TGTTCATCCT CACCAAACAG AAAATGTTCA   
  
  
+ CCGCCTAGAG CATTAACCTC ATTTCGTCAC CCTTTGCATC TAAATCTAGA CACTGGTCAC TGATGATCGG   
  
  
+ GCTTGTTCTT AACTTCTTTT ATTTTTTAAT TTTTTTCTTT TCCCTACGAG TCTAGAGACT ACGTATTAAC   
  
  
+ CGAGATTCTT GTTGCCTATC CCGTCACTTG AACCAGTCCA CGAAACGCGT ACTCACTACT ACGAGAGTAG   
  
  
+ ACTAACCGAG GCGTCGGAGG AAGAACAGGC CTCGCTACTG AACAAGAAGT TTGATCTATT TGTGTAACAA   
  
  
+ CGTAGAGTAG TTGACAATAA TAAACCAAGG AAGACAAAAC ACAAAAAAAA AAAGGGACAC TTTGCGTTAA   
  
  
+ ACTACCTAAG AAACTTTACT CGTAATAACC TACGCCCTCG AAGTTTCATA CAGAATGTCC ATCTATAAAC   
  
  
+ GTACCGCTCC GACGTAGAGA TGAATGGTAA AACCTTATAC ATGTTTTCAA AACTTTGAAA AAGTTCAGTA   
  
  
+ ATTACCCGTA TTGCTATATC GGTTGATTAT ATGTATACAA GAAACGGCAG AAGAAGGCTC TTACAGTAGA   
  
  
+ TCGAAGAACC TTACCGTTAA CCCGTTGACG ATATATTATA AAAAACTACT ATCAAATTTG CATTACTCTA   
  
  
+ ATCCACTTAA ATGAATAACT ATAACACCT  

- TTTCTGTTTT CGTTTGTGAA ACTGTGCGTT TGTTGTATAC ACAAGTGCGG TGAAAGGAGA GTTCAGGCAA   
  
  
- AGCAAAGCCA AGAGGGGGCT TTTTATGTTT AGAAGAAAGA GTGCTCTGAA ATCACATTGC ATTTGCCTGG   
  
  
- AAGATTTATT GAACCTAGCT GGGAAAATTC CTCATCTTTT GGACTTAACA CCACCCCCAC GCGCAGCTAA   
  
  
- GGCAGCGTCC ACGCTGCTAA CGGAACACAT GTCCCCTGCT GCTGGTGGGA AGAAACGGAA TTGCACCTCA   
  
  
- AAAGTTGAAA TTTTAACGGA GGGCTTTGGC GCGTCGACGA TGAAGAGCGT AAGGGGGCCC AATATGGCTT   
  
  
- CTTACGGAGT TAAATGAGGT CACTCTGCCA ATGTCATGGT GGTGACATCA ATTTCCTGAT GCCACATGTC   
  
  
- ACCCTTAGTT TTAAATATCC CATGTTGACA GCAATGGTAT TTTTCTTATC CTGACAAAAC TGGATGATGA   
  
  
- AATAACGAAT ATAGCCTCGA GACTCTAGGG CTTTGAAGTG AAGTAAAGGA GGCAACGGCA GGGGCATTTA   
  
  
- GGACAACAAG AGCTGATAGC AGCCGGGTAG TTATATTATC GAAGTCCTCT CCTCCTCTTA TTTTATTCTA   
  
  
- CTGACATGGA TAAACACGCA GTTGGAGATG ATGTCATGTG CCCAGTGACC AGGGTTTGCC AAGTAAAGCA   
  
  
- GGCATAAGGA CCGTTGAATT ATGATTGAGT TAGCGTGATG GGAGAACTAA CGGGTCTGAT TTAGGATCTA   
  
  
- GCTAGTGCTA GTGGAGATGA ATCTTACTGT AAAAGCAGTG ACAAGTAGGA GTGGTTTGTC TTTTACAAGT   
  
  
- GGCGGATCTC GTAATTGGAG TAAAGCAGTG GGAAACGTAG ATTTAGATCT GTGACCAGTG ACTACTAGCC   
  
  
- CGAACAAGAA TTGAAGAAAA TAAAAAATTA AAAAAAGAAA AGGGATGCTC AGATCTCTGA TGCATAATTG   
  
  
- GCTCTAAGAA CAACGGATAG GGCAGTGAAC TTGGTCAGGT GCTTTGCGCA TGAGTGATGA TGCTCTCATC   
  
  
- TGATTGGCTC CGCAGCCTCC TTCTTGTCCG GAGCGATGAC TTGTTCTTCA AACTAGATAA ACACATTGTT   
  
  
- GCATCTCATC AACTGTTATT ATTTGGTTCC TTCTGTTTTG TGTTTTTTTT TTTCCCTGTG AAACGCAATT   
  
  
- TGATGGATTC TTTGAAATGA GCATTATTGG ATGCGGGAGC TTCAAAGTAT GTCTTACAGG TAGATATTTG   
  
  
- CATGGCGAGG CTGCATCTCT ACTTACCATT TTGGAATATG TACAAAAGTT TTGAAACTTT TTCAAGTCAT   
  
  
- TAATGGGCAT AACGATATAG CCAACTAATA TACATATGTT CTTTGCCGTC TTCTTCCGAG AATGTCATCT   
  
  
- AGCTTCTTGG AATGGCAATT GGGCAACTGC TATATAATAT TTTTTGATGA TAGTTTAAAC GTAATGAGAT   
  
  
- TAGGTGAATT TACTTATTGA TATTGTGGA

+     AE-box

| Site Name | Organism | Position | Strand | Matrix score. | sequence | function |
| --- | --- | --- | --- | --- | --- | --- |
| AE-box | Arabidopsis thaliana | 1199 | + | 8 | AGAAACTT | part of a module for light response |

> 2018/04/13 10:10:12  
+ AAAGACAAAA GCAAACACTT TGACACGCAA ACAACATATG TGTTCACGCC ACTTTCCTCT CAAGTCCGTT   
  
  
+ TCGTTTCGGT TCTCCCCCGA AAAATACAAA TCTTCTTTCT CACGAGACTT TAGTGTAACG TAAACGGACC   
  
  
+ TTCTAAATAA CTTGGATCGA CCCTTTTAAG GAGTAGAAAA CCTGAATTGT GGTGGGGGTG CGCGTCGATT   
  
  
+ CCGTCGCAGG TGCGACGATT GCCTTGTGTA CAGGGGACGA CGACCACCCT TCTTTGCCTT AACGTGGAGT   
  
  
+ TTTCAACTTT AAAATTGCCT CCCGAAACCG CGCAGCTGCT ACTTCTCGCA TTCCCCCGGG TTATACCGAA   
  
  
+ GAATGCCTCA ATTTACTCCA GTGAGACGGT TACAGTACCA CCACTGTAGT TAAAGGACTA CGGTGTACAG   
  
  
+ TGGGAATCAA AATTTATAGG GTACAACTGT CGTTACCATA AAAAGAATAG GACTGTTTTG ACCTACTACT   
  
  
+ TTATTGCTTA TATCGGAGCT CTGAGATCCC GAAACTTCAC TTCATTTCCT CCGTTGCCGT CCCCGTAAAT   
  
  
+ CCTGTTGTTC TCGACTATCG TCGGCCCATC AATATAATAG CTTCAGGAGA GGAGGAGAAT AAAATAAGAT   
  
  
+ GACTGTACCT ATTTGTGCGT CAACCTCTAC TACAGTACAC GGGTCACTGG TCCCAAACGG TTCATTTCGT   
  
  
+ CCGTATTCCT GGCAACTTAA TACTAACTCA ATCGCACTAC CCTCTTGATT GCCCAGACTA AATCCTAGAT   
  
  
+ CGATCACGAT CACCTCTACT TAGAATGACA TTTTCGTCAC TGTTCATCCT CACCAAACAG AAAATGTTCA   
  
  
+ CCGCCTAGAG CATTAACCTC ATTTCGTCAC CCTTTGCATC TAAATCTAGA CACTGGTCAC TGATGATCGG   
  
  
+ GCTTGTTCTT AACTTCTTTT ATTTTTTAAT TTTTTTCTTT TCCCTACGAG TCTAGAGACT ACGTATTAAC   
  
  
+ CGAGATTCTT GTTGCCTATC CCGTCACTTG AACCAGTCCA CGAAACGCGT ACTCACTACT ACGAGAGTAG   
  
  
+ ACTAACCGAG GCGTCGGAGG AAGAACAGGC CTCGCTACTG AACAAGAAGT TTGATCTATT TGTGTAACAA   
  
  
+ CGTAGAGTAG TTGACAATAA TAAACCAAGG AAGACAAAAC ACAAAAAAAA AAAGGGACAC TTTGCGTTAA   
  
  
+ ACTACCTAAG AAACTTTACT CGTAATAACC TACGCCCTCG AAGTTTCATA CAGAATGTCC ATCTATAAAC   
  
  
+ GTACCGCTCC GACGTAGAGA TGAATGGTAA AACCTTATAC ATGTTTTCAA AACTTTGAAA AAGTTCAGTA   
  
  
+ ATTACCCGTA TTGCTATATC GGTTGATTAT ATGTATACAA GAAACGGCAG AAGAAGGCTC TTACAGTAGA   
  
  
+ TCGAAGAACC TTACCGTTAA CCCGTTGACG ATATATTATA AAAAACTACT ATCAAATTTG CATTACTCTA   
  
  
+ ATCCACTTAA ATGAATAACT ATAACACCT  

- TTTCTGTTTT CGTTTGTGAA ACTGTGCGTT TGTTGTATAC ACAAGTGCGG TGAAAGGAGA GTTCAGGCAA   
  
  
- AGCAAAGCCA AGAGGGGGCT TTTTATGTTT AGAAGAAAGA GTGCTCTGAA ATCACATTGC ATTTGCCTGG   
  
  
- AAGATTTATT GAACCTAGCT GGGAAAATTC CTCATCTTTT GGACTTAACA CCACCCCCAC GCGCAGCTAA   
  
  
- GGCAGCGTCC ACGCTGCTAA CGGAACACAT GTCCCCTGCT GCTGGTGGGA AGAAACGGAA TTGCACCTCA   
  
  
- AAAGTTGAAA TTTTAACGGA GGGCTTTGGC GCGTCGACGA TGAAGAGCGT AAGGGGGCCC AATATGGCTT   
  
  
- CTTACGGAGT TAAATGAGGT CACTCTGCCA ATGTCATGGT GGTGACATCA ATTTCCTGAT GCCACATGTC   
  
  
- ACCCTTAGTT TTAAATATCC CATGTTGACA GCAATGGTAT TTTTCTTATC CTGACAAAAC TGGATGATGA   
  
  
- AATAACGAAT ATAGCCTCGA GACTCTAGGG CTTTGAAGTG AAGTAAAGGA GGCAACGGCA GGGGCATTTA   
  
  
- GGACAACAAG AGCTGATAGC AGCCGGGTAG TTATATTATC GAAGTCCTCT CCTCCTCTTA TTTTATTCTA   
  
  
- CTGACATGGA TAAACACGCA GTTGGAGATG ATGTCATGTG CCCAGTGACC AGGGTTTGCC AAGTAAAGCA   
  
  
- GGCATAAGGA CCGTTGAATT ATGATTGAGT TAGCGTGATG GGAGAACTAA CGGGTCTGAT TTAGGATCTA   
  
  
- GCTAGTGCTA GTGGAGATGA ATCTTACTGT AAAAGCAGTG ACAAGTAGGA GTGGTTTGTC TTTTACAAGT   
  
  
- GGCGGATCTC GTAATTGGAG TAAAGCAGTG GGAAACGTAG ATTTAGATCT GTGACCAGTG ACTACTAGCC   
  
  
- CGAACAAGAA TTGAAGAAAA TAAAAAATTA AAAAAAGAAA AGGGATGCTC AGATCTCTGA TGCATAATTG   
  
  
- GCTCTAAGAA CAACGGATAG GGCAGTGAAC TTGGTCAGGT GCTTTGCGCA TGAGTGATGA TGCTCTCATC   
  
  
- TGATTGGCTC CGCAGCCTCC TTCTTGTCCG GAGCGATGAC TTGTTCTTCA AACTAGATAA ACACATTGTT   
  
  
- GCATCTCATC AACTGTTATT ATTTGGTTCC TTCTGTTTTG TGTTTTTTTT TTTCCCTGTG AAACGCAATT   
  
  
- TGATGGATTC TTTGAAATGA GCATTATTGG ATGCGGGAGC TTCAAAGTAT GTCTTACAGG TAGATATTTG   
  
  
- CATGGCGAGG CTGCATCTCT ACTTACCATT TTGGAATATG TACAAAAGTT TTGAAACTTT TTCAAGTCAT   
  
  
- TAATGGGCAT AACGATATAG CCAACTAATA TACATATGTT CTTTGCCGTC TTCTTCCGAG AATGTCATCT   
  
  
- AGCTTCTTGG AATGGCAATT GGGCAACTGC TATATAATAT TTTTTGATGA TAGTTTAAAC GTAATGAGAT   
  
  
- TAGGTGAATT TACTTATTGA TATTGTGGA

+     ARE

| Site Name | Organism | Position | Strand | Matrix score. | sequence | function |
| --- | --- | --- | --- | --- | --- | --- |
| ARE | Zea mays | 1142 | - | 6 | TGGTTT | cis-acting regulatory element essential for the anaerobic induction |

> 2018/04/13 10:10:12  
+ AAAGACAAAA GCAAACACTT TGACACGCAA ACAACATATG TGTTCACGCC ACTTTCCTCT CAAGTCCGTT   
  
  
+ TCGTTTCGGT TCTCCCCCGA AAAATACAAA TCTTCTTTCT CACGAGACTT TAGTGTAACG TAAACGGACC   
  
  
+ TTCTAAATAA CTTGGATCGA CCCTTTTAAG GAGTAGAAAA CCTGAATTGT GGTGGGGGTG CGCGTCGATT   
  
  
+ CCGTCGCAGG TGCGACGATT GCCTTGTGTA CAGGGGACGA CGACCACCCT TCTTTGCCTT AACGTGGAGT   
  
  
+ TTTCAACTTT AAAATTGCCT CCCGAAACCG CGCAGCTGCT ACTTCTCGCA TTCCCCCGGG TTATACCGAA   
  
  
+ GAATGCCTCA ATTTACTCCA GTGAGACGGT TACAGTACCA CCACTGTAGT TAAAGGACTA CGGTGTACAG   
  
  
+ TGGGAATCAA AATTTATAGG GTACAACTGT CGTTACCATA AAAAGAATAG GACTGTTTTG ACCTACTACT   
  
  
+ TTATTGCTTA TATCGGAGCT CTGAGATCCC GAAACTTCAC TTCATTTCCT CCGTTGCCGT CCCCGTAAAT   
  
  
+ CCTGTTGTTC TCGACTATCG TCGGCCCATC AATATAATAG CTTCAGGAGA GGAGGAGAAT AAAATAAGAT   
  
  
+ GACTGTACCT ATTTGTGCGT CAACCTCTAC TACAGTACAC GGGTCACTGG TCCCAAACGG TTCATTTCGT   
  
  
+ CCGTATTCCT GGCAACTTAA TACTAACTCA ATCGCACTAC CCTCTTGATT GCCCAGACTA AATCCTAGAT   
  
  
+ CGATCACGAT CACCTCTACT TAGAATGACA TTTTCGTCAC TGTTCATCCT CACCAAACAG AAAATGTTCA   
  
  
+ CCGCCTAGAG CATTAACCTC ATTTCGTCAC CCTTTGCATC TAAATCTAGA CACTGGTCAC TGATGATCGG   
  
  
+ GCTTGTTCTT AACTTCTTTT ATTTTTTAAT TTTTTTCTTT TCCCTACGAG TCTAGAGACT ACGTATTAAC   
  
  
+ CGAGATTCTT GTTGCCTATC CCGTCACTTG AACCAGTCCA CGAAACGCGT ACTCACTACT ACGAGAGTAG   
  
  
+ ACTAACCGAG GCGTCGGAGG AAGAACAGGC CTCGCTACTG AACAAGAAGT TTGATCTATT TGTGTAACAA   
  
  
+ CGTAGAGTAG TTGACAATAA TAAACCAAGG AAGACAAAAC ACAAAAAAAA AAAGGGACAC TTTGCGTTAA   
  
  
+ ACTACCTAAG AAACTTTACT CGTAATAACC TACGCCCTCG AAGTTTCATA CAGAATGTCC ATCTATAAAC   
  
  
+ GTACCGCTCC GACGTAGAGA TGAATGGTAA AACCTTATAC ATGTTTTCAA AACTTTGAAA AAGTTCAGTA   
  
  
+ ATTACCCGTA TTGCTATATC GGTTGATTAT ATGTATACAA GAAACGGCAG AAGAAGGCTC TTACAGTAGA   
  
  
+ TCGAAGAACC TTACCGTTAA CCCGTTGACG ATATATTATA AAAAACTACT ATCAAATTTG CATTACTCTA   
  
  
+ ATCCACTTAA ATGAATAACT ATAACACCT  

- TTTCTGTTTT CGTTTGTGAA ACTGTGCGTT TGTTGTATAC ACAAGTGCGG TGAAAGGAGA GTTCAGGCAA   
  
  
- AGCAAAGCCA AGAGGGGGCT TTTTATGTTT AGAAGAAAGA GTGCTCTGAA ATCACATTGC ATTTGCCTGG   
  
  
- AAGATTTATT GAACCTAGCT GGGAAAATTC CTCATCTTTT GGACTTAACA CCACCCCCAC GCGCAGCTAA   
  
  
- GGCAGCGTCC ACGCTGCTAA CGGAACACAT GTCCCCTGCT GCTGGTGGGA AGAAACGGAA TTGCACCTCA   
  
  
- AAAGTTGAAA TTTTAACGGA GGGCTTTGGC GCGTCGACGA TGAAGAGCGT AAGGGGGCCC AATATGGCTT   
  
  
- CTTACGGAGT TAAATGAGGT CACTCTGCCA ATGTCATGGT GGTGACATCA ATTTCCTGAT GCCACATGTC   
  
  
- ACCCTTAGTT TTAAATATCC CATGTTGACA GCAATGGTAT TTTTCTTATC CTGACAAAAC TGGATGATGA   
  
  
- AATAACGAAT ATAGCCTCGA GACTCTAGGG CTTTGAAGTG AAGTAAAGGA GGCAACGGCA GGGGCATTTA   
  
  
- GGACAACAAG AGCTGATAGC AGCCGGGTAG TTATATTATC GAAGTCCTCT CCTCCTCTTA TTTTATTCTA   
  
  
- CTGACATGGA TAAACACGCA GTTGGAGATG ATGTCATGTG CCCAGTGACC AGGGTTTGCC AAGTAAAGCA   
  
  
- GGCATAAGGA CCGTTGAATT ATGATTGAGT TAGCGTGATG GGAGAACTAA CGGGTCTGAT TTAGGATCTA   
  
  
- GCTAGTGCTA GTGGAGATGA ATCTTACTGT AAAAGCAGTG ACAAGTAGGA GTGGTTTGTC TTTTACAAGT   
  
  
- GGCGGATCTC GTAATTGGAG TAAAGCAGTG GGAAACGTAG ATTTAGATCT GTGACCAGTG ACTACTAGCC   
  
  
- CGAACAAGAA TTGAAGAAAA TAAAAAATTA AAAAAAGAAA AGGGATGCTC AGATCTCTGA TGCATAATTG   
  
  
- GCTCTAAGAA CAACGGATAG GGCAGTGAAC TTGGTCAGGT GCTTTGCGCA TGAGTGATGA TGCTCTCATC   
  
  
- TGATTGGCTC CGCAGCCTCC TTCTTGTCCG GAGCGATGAC TTGTTCTTCA AACTAGATAA ACACATTGTT   
  
  
- GCATCTCATC AACTGTTATT ATTTGGTTCC TTCTGTTTTG TGTTTTTTTT TTTCCCTGTG AAACGCAATT   
  
  
- TGATGGATTC TTTGAAATGA GCATTATTGG ATGCGGGAGC TTCAAAGTAT GTCTTACAGG TAGATATTTG   
  
  
- CATGGCGAGG CTGCATCTCT ACTTACCATT TTGGAATATG TACAAAAGTT TTGAAACTTT TTCAAGTCAT   
  
  
- TAATGGGCAT AACGATATAG CCAACTAATA TACATATGTT CTTTGCCGTC TTCTTCCGAG AATGTCATCT   
  
  
- AGCTTCTTGG AATGGCAATT GGGCAACTGC TATATAATAT TTTTTGATGA TAGTTTAAAC GTAATGAGAT   
  
  
- TAGGTGAATT TACTTATTGA TATTGTGGA

+     ATCT-motif

| Site Name | Organism | Position | Strand | Matrix score. | sequence | function |
| --- | --- | --- | --- | --- | --- | --- |
| ATCT-motif | Pisum sativum | 1465 | + | 9 | AATCTAATCC | part of a conserved DNA module involved in light responsiveness |

> 2018/04/13 10:10:12  
+ AAAGACAAAA GCAAACACTT TGACACGCAA ACAACATATG TGTTCACGCC ACTTTCCTCT CAAGTCCGTT   
  
  
+ TCGTTTCGGT TCTCCCCCGA AAAATACAAA TCTTCTTTCT CACGAGACTT TAGTGTAACG TAAACGGACC   
  
  
+ TTCTAAATAA CTTGGATCGA CCCTTTTAAG GAGTAGAAAA CCTGAATTGT GGTGGGGGTG CGCGTCGATT   
  
  
+ CCGTCGCAGG TGCGACGATT GCCTTGTGTA CAGGGGACGA CGACCACCCT TCTTTGCCTT AACGTGGAGT   
  
  
+ TTTCAACTTT AAAATTGCCT CCCGAAACCG CGCAGCTGCT ACTTCTCGCA TTCCCCCGGG TTATACCGAA   
  
  
+ GAATGCCTCA ATTTACTCCA GTGAGACGGT TACAGTACCA CCACTGTAGT TAAAGGACTA CGGTGTACAG   
  
  
+ TGGGAATCAA AATTTATAGG GTACAACTGT CGTTACCATA AAAAGAATAG GACTGTTTTG ACCTACTACT   
  
  
+ TTATTGCTTA TATCGGAGCT CTGAGATCCC GAAACTTCAC TTCATTTCCT CCGTTGCCGT CCCCGTAAAT   
  
  
+ CCTGTTGTTC TCGACTATCG TCGGCCCATC AATATAATAG CTTCAGGAGA GGAGGAGAAT AAAATAAGAT   
  
  
+ GACTGTACCT ATTTGTGCGT CAACCTCTAC TACAGTACAC GGGTCACTGG TCCCAAACGG TTCATTTCGT   
  
  
+ CCGTATTCCT GGCAACTTAA TACTAACTCA ATCGCACTAC CCTCTTGATT GCCCAGACTA AATCCTAGAT   
  
  
+ CGATCACGAT CACCTCTACT TAGAATGACA TTTTCGTCAC TGTTCATCCT CACCAAACAG AAAATGTTCA   
  
  
+ CCGCCTAGAG CATTAACCTC ATTTCGTCAC CCTTTGCATC TAAATCTAGA CACTGGTCAC TGATGATCGG   
  
  
+ GCTTGTTCTT AACTTCTTTT ATTTTTTAAT TTTTTTCTTT TCCCTACGAG TCTAGAGACT ACGTATTAAC   
  
  
+ CGAGATTCTT GTTGCCTATC CCGTCACTTG AACCAGTCCA CGAAACGCGT ACTCACTACT ACGAGAGTAG   
  
  
+ ACTAACCGAG GCGTCGGAGG AAGAACAGGC CTCGCTACTG AACAAGAAGT TTGATCTATT TGTGTAACAA   
  
  
+ CGTAGAGTAG TTGACAATAA TAAACCAAGG AAGACAAAAC ACAAAAAAAA AAAGGGACAC TTTGCGTTAA   
  
  
+ ACTACCTAAG AAACTTTACT CGTAATAACC TACGCCCTCG AAGTTTCATA CAGAATGTCC ATCTATAAAC   
  
  
+ GTACCGCTCC GACGTAGAGA TGAATGGTAA AACCTTATAC ATGTTTTCAA AACTTTGAAA AAGTTCAGTA   
  
  
+ ATTACCCGTA TTGCTATATC GGTTGATTAT ATGTATACAA GAAACGGCAG AAGAAGGCTC TTACAGTAGA   
  
  
+ TCGAAGAACC TTACCGTTAA CCCGTTGACG ATATATTATA AAAAACTACT ATCAAATTTG CATTACTCTA   
  
  
+ ATCCACTTAA ATGAATAACT ATAACACCT  

- TTTCTGTTTT CGTTTGTGAA ACTGTGCGTT TGTTGTATAC ACAAGTGCGG TGAAAGGAGA GTTCAGGCAA   
  
  
- AGCAAAGCCA AGAGGGGGCT TTTTATGTTT AGAAGAAAGA GTGCTCTGAA ATCACATTGC ATTTGCCTGG   
  
  
- AAGATTTATT GAACCTAGCT GGGAAAATTC CTCATCTTTT GGACTTAACA CCACCCCCAC GCGCAGCTAA   
  
  
- GGCAGCGTCC ACGCTGCTAA CGGAACACAT GTCCCCTGCT GCTGGTGGGA AGAAACGGAA TTGCACCTCA   
  
  
- AAAGTTGAAA TTTTAACGGA GGGCTTTGGC GCGTCGACGA TGAAGAGCGT AAGGGGGCCC AATATGGCTT   
  
  
- CTTACGGAGT TAAATGAGGT CACTCTGCCA ATGTCATGGT GGTGACATCA ATTTCCTGAT GCCACATGTC   
  
  
- ACCCTTAGTT TTAAATATCC CATGTTGACA GCAATGGTAT TTTTCTTATC CTGACAAAAC TGGATGATGA   
  
  
- AATAACGAAT ATAGCCTCGA GACTCTAGGG CTTTGAAGTG AAGTAAAGGA GGCAACGGCA GGGGCATTTA   
  
  
- GGACAACAAG AGCTGATAGC AGCCGGGTAG TTATATTATC GAAGTCCTCT CCTCCTCTTA TTTTATTCTA   
  
  
- CTGACATGGA TAAACACGCA GTTGGAGATG ATGTCATGTG CCCAGTGACC AGGGTTTGCC AAGTAAAGCA   
  
  
- GGCATAAGGA CCGTTGAATT ATGATTGAGT TAGCGTGATG GGAGAACTAA CGGGTCTGAT TTAGGATCTA   
  
  
- GCTAGTGCTA GTGGAGATGA ATCTTACTGT AAAAGCAGTG ACAAGTAGGA GTGGTTTGTC TTTTACAAGT   
  
  
- GGCGGATCTC GTAATTGGAG TAAAGCAGTG GGAAACGTAG ATTTAGATCT GTGACCAGTG ACTACTAGCC   
  
  
- CGAACAAGAA TTGAAGAAAA TAAAAAATTA AAAAAAGAAA AGGGATGCTC AGATCTCTGA TGCATAATTG   
  
  
- GCTCTAAGAA CAACGGATAG GGCAGTGAAC TTGGTCAGGT GCTTTGCGCA TGAGTGATGA TGCTCTCATC   
  
  
- TGATTGGCTC CGCAGCCTCC TTCTTGTCCG GAGCGATGAC TTGTTCTTCA AACTAGATAA ACACATTGTT   
  
  
- GCATCTCATC AACTGTTATT ATTTGGTTCC TTCTGTTTTG TGTTTTTTTT TTTCCCTGTG AAACGCAATT   
  
  
- TGATGGATTC TTTGAAATGA GCATTATTGG ATGCGGGAGC TTCAAAGTAT GTCTTACAGG TAGATATTTG   
  
  
- CATGGCGAGG CTGCATCTCT ACTTACCATT TTGGAATATG TACAAAAGTT TTGAAACTTT TTCAAGTCAT   
  
  
- TAATGGGCAT AACGATATAG CCAACTAATA TACATATGTT CTTTGCCGTC TTCTTCCGAG AATGTCATCT   
  
  
- AGCTTCTTGG AATGGCAATT GGGCAACTGC TATATAATAT TTTTTGATGA TAGTTTAAAC GTAATGAGAT   
  
  
- TAGGTGAATT TACTTATTGA TATTGTGGA

+     ATGCAAAT motif

| Site Name | Organism | Position | Strand | Matrix score. | sequence | function |
| --- | --- | --- | --- | --- | --- | --- |
| ATGCAAAT motif | Oryza sativa | 94 | + | 8 | ATACAAAT | cis-acting regulatory element associated to the TGAGTCA motif |

> 2018/04/13 10:10:12  
+ AAAGACAAAA GCAAACACTT TGACACGCAA ACAACATATG TGTTCACGCC ACTTTCCTCT CAAGTCCGTT   
  
  
+ TCGTTTCGGT TCTCCCCCGA AAAATACAAA TCTTCTTTCT CACGAGACTT TAGTGTAACG TAAACGGACC   
  
  
+ TTCTAAATAA CTTGGATCGA CCCTTTTAAG GAGTAGAAAA CCTGAATTGT GGTGGGGGTG CGCGTCGATT   
  
  
+ CCGTCGCAGG TGCGACGATT GCCTTGTGTA CAGGGGACGA CGACCACCCT TCTTTGCCTT AACGTGGAGT   
  
  
+ TTTCAACTTT AAAATTGCCT CCCGAAACCG CGCAGCTGCT ACTTCTCGCA TTCCCCCGGG TTATACCGAA   
  
  
+ GAATGCCTCA ATTTACTCCA GTGAGACGGT TACAGTACCA CCACTGTAGT TAAAGGACTA CGGTGTACAG   
  
  
+ TGGGAATCAA AATTTATAGG GTACAACTGT CGTTACCATA AAAAGAATAG GACTGTTTTG ACCTACTACT   
  
  
+ TTATTGCTTA TATCGGAGCT CTGAGATCCC GAAACTTCAC TTCATTTCCT CCGTTGCCGT CCCCGTAAAT   
  
  
+ CCTGTTGTTC TCGACTATCG TCGGCCCATC AATATAATAG CTTCAGGAGA GGAGGAGAAT AAAATAAGAT   
  
  
+ GACTGTACCT ATTTGTGCGT CAACCTCTAC TACAGTACAC GGGTCACTGG TCCCAAACGG TTCATTTCGT   
  
  
+ CCGTATTCCT GGCAACTTAA TACTAACTCA ATCGCACTAC CCTCTTGATT GCCCAGACTA AATCCTAGAT   
  
  
+ CGATCACGAT CACCTCTACT TAGAATGACA TTTTCGTCAC TGTTCATCCT CACCAAACAG AAAATGTTCA   
  
  
+ CCGCCTAGAG CATTAACCTC ATTTCGTCAC CCTTTGCATC TAAATCTAGA CACTGGTCAC TGATGATCGG   
  
  
+ GCTTGTTCTT AACTTCTTTT ATTTTTTAAT TTTTTTCTTT TCCCTACGAG TCTAGAGACT ACGTATTAAC   
  
  
+ CGAGATTCTT GTTGCCTATC CCGTCACTTG AACCAGTCCA CGAAACGCGT ACTCACTACT ACGAGAGTAG   
  
  
+ ACTAACCGAG GCGTCGGAGG AAGAACAGGC CTCGCTACTG AACAAGAAGT TTGATCTATT TGTGTAACAA   
  
  
+ CGTAGAGTAG TTGACAATAA TAAACCAAGG AAGACAAAAC ACAAAAAAAA AAAGGGACAC TTTGCGTTAA   
  
  
+ ACTACCTAAG AAACTTTACT CGTAATAACC TACGCCCTCG AAGTTTCATA CAGAATGTCC ATCTATAAAC   
  
  
+ GTACCGCTCC GACGTAGAGA TGAATGGTAA AACCTTATAC ATGTTTTCAA AACTTTGAAA AAGTTCAGTA   
  
  
+ ATTACCCGTA TTGCTATATC GGTTGATTAT ATGTATACAA GAAACGGCAG AAGAAGGCTC TTACAGTAGA   
  
  
+ TCGAAGAACC TTACCGTTAA CCCGTTGACG ATATATTATA AAAAACTACT ATCAAATTTG CATTACTCTA   
  
  
+ ATCCACTTAA ATGAATAACT ATAACACCT  

- TTTCTGTTTT CGTTTGTGAA ACTGTGCGTT TGTTGTATAC ACAAGTGCGG TGAAAGGAGA GTTCAGGCAA   
  
  
- AGCAAAGCCA AGAGGGGGCT TTTTATGTTT AGAAGAAAGA GTGCTCTGAA ATCACATTGC ATTTGCCTGG   
  
  
- AAGATTTATT GAACCTAGCT GGGAAAATTC CTCATCTTTT GGACTTAACA CCACCCCCAC GCGCAGCTAA   
  
  
- GGCAGCGTCC ACGCTGCTAA CGGAACACAT GTCCCCTGCT GCTGGTGGGA AGAAACGGAA TTGCACCTCA   
  
  
- AAAGTTGAAA TTTTAACGGA GGGCTTTGGC GCGTCGACGA TGAAGAGCGT AAGGGGGCCC AATATGGCTT   
  
  
- CTTACGGAGT TAAATGAGGT CACTCTGCCA ATGTCATGGT GGTGACATCA ATTTCCTGAT GCCACATGTC   
  
  
- ACCCTTAGTT TTAAATATCC CATGTTGACA GCAATGGTAT TTTTCTTATC CTGACAAAAC TGGATGATGA   
  
  
- AATAACGAAT ATAGCCTCGA GACTCTAGGG CTTTGAAGTG AAGTAAAGGA GGCAACGGCA GGGGCATTTA   
  
  
- GGACAACAAG AGCTGATAGC AGCCGGGTAG TTATATTATC GAAGTCCTCT CCTCCTCTTA TTTTATTCTA   
  
  
- CTGACATGGA TAAACACGCA GTTGGAGATG ATGTCATGTG CCCAGTGACC AGGGTTTGCC AAGTAAAGCA   
  
  
- GGCATAAGGA CCGTTGAATT ATGATTGAGT TAGCGTGATG GGAGAACTAA CGGGTCTGAT TTAGGATCTA   
  
  
- GCTAGTGCTA GTGGAGATGA ATCTTACTGT AAAAGCAGTG ACAAGTAGGA GTGGTTTGTC TTTTACAAGT   
  
  
- GGCGGATCTC GTAATTGGAG TAAAGCAGTG GGAAACGTAG ATTTAGATCT GTGACCAGTG ACTACTAGCC   
  
  
- CGAACAAGAA TTGAAGAAAA TAAAAAATTA AAAAAAGAAA AGGGATGCTC AGATCTCTGA TGCATAATTG   
  
  
- GCTCTAAGAA CAACGGATAG GGCAGTGAAC TTGGTCAGGT GCTTTGCGCA TGAGTGATGA TGCTCTCATC   
  
  
- TGATTGGCTC CGCAGCCTCC TTCTTGTCCG GAGCGATGAC TTGTTCTTCA AACTAGATAA ACACATTGTT   
  
  
- GCATCTCATC AACTGTTATT ATTTGGTTCC TTCTGTTTTG TGTTTTTTTT TTTCCCTGTG AAACGCAATT   
  
  
- TGATGGATTC TTTGAAATGA GCATTATTGG ATGCGGGAGC TTCAAAGTAT GTCTTACAGG TAGATATTTG   
  
  
- CATGGCGAGG CTGCATCTCT ACTTACCATT TTGGAATATG TACAAAAGTT TTGAAACTTT TTCAAGTCAT   
  
  
- TAATGGGCAT AACGATATAG CCAACTAATA TACATATGTT CTTTGCCGTC TTCTTCCGAG AATGTCATCT   
  
  
- AGCTTCTTGG AATGGCAATT GGGCAACTGC TATATAATAT TTTTTGATGA TAGTTTAAAC GTAATGAGAT   
  
  
- TAGGTGAATT TACTTATTGA TATTGTGGA

+     Box I

| Site Name | Organism | Position | Strand | Matrix score. | sequence | function |
| --- | --- | --- | --- | --- | --- | --- |
| Box I | Pisum sativum | 1314 | - | 7 | TTTCAAA | light responsive element |
| Box I | Pisum sativum | 1305 | + | 7 | TTTCAAA | light responsive element |

> 2018/04/13 10:10:12  
+ AAAGACAAAA GCAAACACTT TGACACGCAA ACAACATATG TGTTCACGCC ACTTTCCTCT CAAGTCCGTT   
  
  
+ TCGTTTCGGT TCTCCCCCGA AAAATACAAA TCTTCTTTCT CACGAGACTT TAGTGTAACG TAAACGGACC   
  
  
+ TTCTAAATAA CTTGGATCGA CCCTTTTAAG GAGTAGAAAA CCTGAATTGT GGTGGGGGTG CGCGTCGATT   
  
  
+ CCGTCGCAGG TGCGACGATT GCCTTGTGTA CAGGGGACGA CGACCACCCT TCTTTGCCTT AACGTGGAGT   
  
  
+ TTTCAACTTT AAAATTGCCT CCCGAAACCG CGCAGCTGCT ACTTCTCGCA TTCCCCCGGG TTATACCGAA   
  
  
+ GAATGCCTCA ATTTACTCCA GTGAGACGGT TACAGTACCA CCACTGTAGT TAAAGGACTA CGGTGTACAG   
  
  
+ TGGGAATCAA AATTTATAGG GTACAACTGT CGTTACCATA AAAAGAATAG GACTGTTTTG ACCTACTACT   
  
  
+ TTATTGCTTA TATCGGAGCT CTGAGATCCC GAAACTTCAC TTCATTTCCT CCGTTGCCGT CCCCGTAAAT   
  
  
+ CCTGTTGTTC TCGACTATCG TCGGCCCATC AATATAATAG CTTCAGGAGA GGAGGAGAAT AAAATAAGAT   
  
  
+ GACTGTACCT ATTTGTGCGT CAACCTCTAC TACAGTACAC GGGTCACTGG TCCCAAACGG TTCATTTCGT   
  
  
+ CCGTATTCCT GGCAACTTAA TACTAACTCA ATCGCACTAC CCTCTTGATT GCCCAGACTA AATCCTAGAT   
  
  
+ CGATCACGAT CACCTCTACT TAGAATGACA TTTTCGTCAC TGTTCATCCT CACCAAACAG AAAATGTTCA   
  
  
+ CCGCCTAGAG CATTAACCTC ATTTCGTCAC CCTTTGCATC TAAATCTAGA CACTGGTCAC TGATGATCGG   
  
  
+ GCTTGTTCTT AACTTCTTTT ATTTTTTAAT TTTTTTCTTT TCCCTACGAG TCTAGAGACT ACGTATTAAC   
  
  
+ CGAGATTCTT GTTGCCTATC CCGTCACTTG AACCAGTCCA CGAAACGCGT ACTCACTACT ACGAGAGTAG   
  
  
+ ACTAACCGAG GCGTCGGAGG AAGAACAGGC CTCGCTACTG AACAAGAAGT TTGATCTATT TGTGTAACAA   
  
  
+ CGTAGAGTAG TTGACAATAA TAAACCAAGG AAGACAAAAC ACAAAAAAAA AAAGGGACAC TTTGCGTTAA   
  
  
+ ACTACCTAAG AAACTTTACT CGTAATAACC TACGCCCTCG AAGTTTCATA CAGAATGTCC ATCTATAAAC   
  
  
+ GTACCGCTCC GACGTAGAGA TGAATGGTAA AACCTTATAC ATGTTTTCAA AACTTTGAAA AAGTTCAGTA   
  
  
+ ATTACCCGTA TTGCTATATC GGTTGATTAT ATGTATACAA GAAACGGCAG AAGAAGGCTC TTACAGTAGA   
  
  
+ TCGAAGAACC TTACCGTTAA CCCGTTGACG ATATATTATA AAAAACTACT ATCAAATTTG CATTACTCTA   
  
  
+ ATCCACTTAA ATGAATAACT ATAACACCT  

- TTTCTGTTTT CGTTTGTGAA ACTGTGCGTT TGTTGTATAC ACAAGTGCGG TGAAAGGAGA GTTCAGGCAA   
  
  
- AGCAAAGCCA AGAGGGGGCT TTTTATGTTT AGAAGAAAGA GTGCTCTGAA ATCACATTGC ATTTGCCTGG   
  
  
- AAGATTTATT GAACCTAGCT GGGAAAATTC CTCATCTTTT GGACTTAACA CCACCCCCAC GCGCAGCTAA   
  
  
- GGCAGCGTCC ACGCTGCTAA CGGAACACAT GTCCCCTGCT GCTGGTGGGA AGAAACGGAA TTGCACCTCA   
  
  
- AAAGTTGAAA TTTTAACGGA GGGCTTTGGC GCGTCGACGA TGAAGAGCGT AAGGGGGCCC AATATGGCTT   
  
  
- CTTACGGAGT TAAATGAGGT CACTCTGCCA ATGTCATGGT GGTGACATCA ATTTCCTGAT GCCACATGTC   
  
  
- ACCCTTAGTT TTAAATATCC CATGTTGACA GCAATGGTAT TTTTCTTATC CTGACAAAAC TGGATGATGA   
  
  
- AATAACGAAT ATAGCCTCGA GACTCTAGGG CTTTGAAGTG AAGTAAAGGA GGCAACGGCA GGGGCATTTA   
  
  
- GGACAACAAG AGCTGATAGC AGCCGGGTAG TTATATTATC GAAGTCCTCT CCTCCTCTTA TTTTATTCTA   
  
  
- CTGACATGGA TAAACACGCA GTTGGAGATG ATGTCATGTG CCCAGTGACC AGGGTTTGCC AAGTAAAGCA   
  
  
- GGCATAAGGA CCGTTGAATT ATGATTGAGT TAGCGTGATG GGAGAACTAA CGGGTCTGAT TTAGGATCTA   
  
  
- GCTAGTGCTA GTGGAGATGA ATCTTACTGT AAAAGCAGTG ACAAGTAGGA GTGGTTTGTC TTTTACAAGT   
  
  
- GGCGGATCTC GTAATTGGAG TAAAGCAGTG GGAAACGTAG ATTTAGATCT GTGACCAGTG ACTACTAGCC   
  
  
- CGAACAAGAA TTGAAGAAAA TAAAAAATTA AAAAAAGAAA AGGGATGCTC AGATCTCTGA TGCATAATTG   
  
  
- GCTCTAAGAA CAACGGATAG GGCAGTGAAC TTGGTCAGGT GCTTTGCGCA TGAGTGATGA TGCTCTCATC   
  
  
- TGATTGGCTC CGCAGCCTCC TTCTTGTCCG GAGCGATGAC TTGTTCTTCA AACTAGATAA ACACATTGTT   
  
  
- GCATCTCATC AACTGTTATT ATTTGGTTCC TTCTGTTTTG TGTTTTTTTT TTTCCCTGTG AAACGCAATT   
  
  
- TGATGGATTC TTTGAAATGA GCATTATTGG ATGCGGGAGC TTCAAAGTAT GTCTTACAGG TAGATATTTG   
  
  
- CATGGCGAGG CTGCATCTCT ACTTACCATT TTGGAATATG TACAAAAGTT TTGAAACTTT TTCAAGTCAT   
  
  
- TAATGGGCAT AACGATATAG CCAACTAATA TACATATGTT CTTTGCCGTC TTCTTCCGAG AATGTCATCT   
  
  
- AGCTTCTTGG AATGGCAATT GGGCAACTGC TATATAATAT TTTTTGATGA TAGTTTAAAC GTAATGAGAT   
  
  
- TAGGTGAATT TACTTATTGA TATTGTGGA

+     Box-W1

| Site Name | Organism | Position | Strand | Matrix score. | sequence | function |
| --- | --- | --- | --- | --- | --- | --- |
| Box-W1 | Petroselinum crispum | 478 | + | 6 | TTGACC | fungal elicitor responsive element |

> 2018/04/13 10:10:12  
+ AAAGACAAAA GCAAACACTT TGACACGCAA ACAACATATG TGTTCACGCC ACTTTCCTCT CAAGTCCGTT   
  
  
+ TCGTTTCGGT TCTCCCCCGA AAAATACAAA TCTTCTTTCT CACGAGACTT TAGTGTAACG TAAACGGACC   
  
  
+ TTCTAAATAA CTTGGATCGA CCCTTTTAAG GAGTAGAAAA CCTGAATTGT GGTGGGGGTG CGCGTCGATT   
  
  
+ CCGTCGCAGG TGCGACGATT GCCTTGTGTA CAGGGGACGA CGACCACCCT TCTTTGCCTT AACGTGGAGT   
  
  
+ TTTCAACTTT AAAATTGCCT CCCGAAACCG CGCAGCTGCT ACTTCTCGCA TTCCCCCGGG TTATACCGAA   
  
  
+ GAATGCCTCA ATTTACTCCA GTGAGACGGT TACAGTACCA CCACTGTAGT TAAAGGACTA CGGTGTACAG   
  
  
+ TGGGAATCAA AATTTATAGG GTACAACTGT CGTTACCATA AAAAGAATAG GACTGTTTTG ACCTACTACT   
  
  
+ TTATTGCTTA TATCGGAGCT CTGAGATCCC GAAACTTCAC TTCATTTCCT CCGTTGCCGT CCCCGTAAAT   
  
  
+ CCTGTTGTTC TCGACTATCG TCGGCCCATC AATATAATAG CTTCAGGAGA GGAGGAGAAT AAAATAAGAT   
  
  
+ GACTGTACCT ATTTGTGCGT CAACCTCTAC TACAGTACAC GGGTCACTGG TCCCAAACGG TTCATTTCGT   
  
  
+ CCGTATTCCT GGCAACTTAA TACTAACTCA ATCGCACTAC CCTCTTGATT GCCCAGACTA AATCCTAGAT   
  
  
+ CGATCACGAT CACCTCTACT TAGAATGACA TTTTCGTCAC TGTTCATCCT CACCAAACAG AAAATGTTCA   
  
  
+ CCGCCTAGAG CATTAACCTC ATTTCGTCAC CCTTTGCATC TAAATCTAGA CACTGGTCAC TGATGATCGG   
  
  
+ GCTTGTTCTT AACTTCTTTT ATTTTTTAAT TTTTTTCTTT TCCCTACGAG TCTAGAGACT ACGTATTAAC   
  
  
+ CGAGATTCTT GTTGCCTATC CCGTCACTTG AACCAGTCCA CGAAACGCGT ACTCACTACT ACGAGAGTAG   
  
  
+ ACTAACCGAG GCGTCGGAGG AAGAACAGGC CTCGCTACTG AACAAGAAGT TTGATCTATT TGTGTAACAA   
  
  
+ CGTAGAGTAG TTGACAATAA TAAACCAAGG AAGACAAAAC ACAAAAAAAA AAAGGGACAC TTTGCGTTAA   
  
  
+ ACTACCTAAG AAACTTTACT CGTAATAACC TACGCCCTCG AAGTTTCATA CAGAATGTCC ATCTATAAAC   
  
  
+ GTACCGCTCC GACGTAGAGA TGAATGGTAA AACCTTATAC ATGTTTTCAA AACTTTGAAA AAGTTCAGTA   
  
  
+ ATTACCCGTA TTGCTATATC GGTTGATTAT ATGTATACAA GAAACGGCAG AAGAAGGCTC TTACAGTAGA   
  
  
+ TCGAAGAACC TTACCGTTAA CCCGTTGACG ATATATTATA AAAAACTACT ATCAAATTTG CATTACTCTA   
  
  
+ ATCCACTTAA ATGAATAACT ATAACACCT  

- TTTCTGTTTT CGTTTGTGAA ACTGTGCGTT TGTTGTATAC ACAAGTGCGG TGAAAGGAGA GTTCAGGCAA   
  
  
- AGCAAAGCCA AGAGGGGGCT TTTTATGTTT AGAAGAAAGA GTGCTCTGAA ATCACATTGC ATTTGCCTGG   
  
  
- AAGATTTATT GAACCTAGCT GGGAAAATTC CTCATCTTTT GGACTTAACA CCACCCCCAC GCGCAGCTAA   
  
  
- GGCAGCGTCC ACGCTGCTAA CGGAACACAT GTCCCCTGCT GCTGGTGGGA AGAAACGGAA TTGCACCTCA   
  
  
- AAAGTTGAAA TTTTAACGGA GGGCTTTGGC GCGTCGACGA TGAAGAGCGT AAGGGGGCCC AATATGGCTT   
  
  
- CTTACGGAGT TAAATGAGGT CACTCTGCCA ATGTCATGGT GGTGACATCA ATTTCCTGAT GCCACATGTC   
  
  
- ACCCTTAGTT TTAAATATCC CATGTTGACA GCAATGGTAT TTTTCTTATC CTGACAAAAC TGGATGATGA   
  
  
- AATAACGAAT ATAGCCTCGA GACTCTAGGG CTTTGAAGTG AAGTAAAGGA GGCAACGGCA GGGGCATTTA   
  
  
- GGACAACAAG AGCTGATAGC AGCCGGGTAG TTATATTATC GAAGTCCTCT CCTCCTCTTA TTTTATTCTA   
  
  
- CTGACATGGA TAAACACGCA GTTGGAGATG ATGTCATGTG CCCAGTGACC AGGGTTTGCC AAGTAAAGCA   
  
  
- GGCATAAGGA CCGTTGAATT ATGATTGAGT TAGCGTGATG GGAGAACTAA CGGGTCTGAT TTAGGATCTA   
  
  
- GCTAGTGCTA GTGGAGATGA ATCTTACTGT AAAAGCAGTG ACAAGTAGGA GTGGTTTGTC TTTTACAAGT   
  
  
- GGCGGATCTC GTAATTGGAG TAAAGCAGTG GGAAACGTAG ATTTAGATCT GTGACCAGTG ACTACTAGCC   
  
  
- CGAACAAGAA TTGAAGAAAA TAAAAAATTA AAAAAAGAAA AGGGATGCTC AGATCTCTGA TGCATAATTG   
  
  
- GCTCTAAGAA CAACGGATAG GGCAGTGAAC TTGGTCAGGT GCTTTGCGCA TGAGTGATGA TGCTCTCATC   
  
  
- TGATTGGCTC CGCAGCCTCC TTCTTGTCCG GAGCGATGAC TTGTTCTTCA AACTAGATAA ACACATTGTT   
  
  
- GCATCTCATC AACTGTTATT ATTTGGTTCC TTCTGTTTTG TGTTTTTTTT TTTCCCTGTG AAACGCAATT   
  
  
- TGATGGATTC TTTGAAATGA GCATTATTGG ATGCGGGAGC TTCAAAGTAT GTCTTACAGG TAGATATTTG   
  
  
- CATGGCGAGG CTGCATCTCT ACTTACCATT TTGGAATATG TACAAAAGTT TTGAAACTTT TTCAAGTCAT   
  
  
- TAATGGGCAT AACGATATAG CCAACTAATA TACATATGTT CTTTGCCGTC TTCTTCCGAG AATGTCATCT   
  
  
- AGCTTCTTGG AATGGCAATT GGGCAACTGC TATATAATAT TTTTTGATGA TAGTTTAAAC GTAATGAGAT   
  
  
- TAGGTGAATT TACTTATTGA TATTGTGGA

+     CAAT-box

| Site Name | Organism | Position | Strand | Matrix score. | sequence | function |
| --- | --- | --- | --- | --- | --- | --- |
| CAAT-box | Hordeum vulgare | 1340 | - | 4 | CAAT | common cis-acting element in promoter and enhancer regions |
| CAAT-box | Hordeum vulgare | 590 | + | 4 | CAAT | common cis-acting element in promoter and enhancer regions |
| CAAT-box | Brassica rapa | 1456 | - | 5 | CAAAT | common cis-acting element in promoter and enhancer regions |
| CAAT-box | Brassica rapa | 1453 | + | 5 | CAAAT | common cis-acting element in promoter and enhancer regions |
| CAAT-box | Brassica rapa | 1108 | - | 5 | CAAAT | common cis-acting element in promoter and enhancer regions |
| CAAT-box | Glycine max | 359 | + | 5 | CAATT | common cis-acting element in promoter and enhancer regions |
| CAAT-box | Hordeum vulgare | 493 | - | 4 | CAAT | common cis-acting element in promoter and enhancer regions |
| CAAT-box | Arabidopsis thaliana | 748 | - | 6 | gGCAAT | common cis-acting element in promoter and enhancer regions |
| CAAT-box | Brassica rapa | 97 | + | 5 | CAAAT | common cis-acting element in promoter and enhancer regions |
| CAAT-box | Arabidopsis thaliana | 294 | - | 6 | gGCAAT | common cis-acting element in promoter and enhancer regions |
| CAAT-box | Hordeum vulgare | 729 | + | 4 | CAAT | common cis-acting element in promoter and enhancer regions |
| CAAT-box | Brassica rapa | 641 | - | 5 | CAAAT | common cis-acting element in promoter and enhancer regions |
| CAAT-box | Glycine max | 293 | - | 5 | CAATT | common cis-acting element in promoter and enhancer regions |
| CAAT-box | Hordeum vulgare | 1135 | + | 4 | CAAT | common cis-acting element in promoter and enhancer regions |
| CAAT-box | Glycine max | 185 | - | 5 | CAATT | common cis-acting element in promoter and enhancer regions |
| CAAT-box | Hordeum vulgare | 186 | - | 4 | CAAT | common cis-acting element in promoter and enhancer regions |
| CAAT-box | Arabidopsis thaliana | 228 | - | 6 | gGCAAT | common cis-acting element in promoter and enhancer regions |

> 2018/04/13 10:10:12  
+ AAAGACAAAA GCAAACACTT TGACACGCAA ACAACATATG TGTTCACGCC ACTTTCCTCT CAAGTCCGTT   
  
  
+ TCGTTTCGGT TCTCCCCCGA AAAATACAAA TCTTCTTTCT CACGAGACTT TAGTGTAACG TAAACGGACC   
  
  
+ TTCTAAATAA CTTGGATCGA CCCTTTTAAG GAGTAGAAAA CCTGAATTGT GGTGGGGGTG CGCGTCGATT   
  
  
+ CCGTCGCAGG TGCGACGATT GCCTTGTGTA CAGGGGACGA CGACCACCCT TCTTTGCCTT AACGTGGAGT   
  
  
+ TTTCAACTTT AAAATTGCCT CCCGAAACCG CGCAGCTGCT ACTTCTCGCA TTCCCCCGGG TTATACCGAA   
  
  
+ GAATGCCTCA ATTTACTCCA GTGAGACGGT TACAGTACCA CCACTGTAGT TAAAGGACTA CGGTGTACAG   
  
  
+ TGGGAATCAA AATTTATAGG GTACAACTGT CGTTACCATA AAAAGAATAG GACTGTTTTG ACCTACTACT   
  
  
+ TTATTGCTTA TATCGGAGCT CTGAGATCCC GAAACTTCAC TTCATTTCCT CCGTTGCCGT CCCCGTAAAT   
  
  
+ CCTGTTGTTC TCGACTATCG TCGGCCCATC AATATAATAG CTTCAGGAGA GGAGGAGAAT AAAATAAGAT   
  
  
+ GACTGTACCT ATTTGTGCGT CAACCTCTAC TACAGTACAC GGGTCACTGG TCCCAAACGG TTCATTTCGT   
  
  
+ CCGTATTCCT GGCAACTTAA TACTAACTCA ATCGCACTAC CCTCTTGATT GCCCAGACTA AATCCTAGAT   
  
  
+ CGATCACGAT CACCTCTACT TAGAATGACA TTTTCGTCAC TGTTCATCCT CACCAAACAG AAAATGTTCA   
  
  
+ CCGCCTAGAG CATTAACCTC ATTTCGTCAC CCTTTGCATC TAAATCTAGA CACTGGTCAC TGATGATCGG   
  
  
+ GCTTGTTCTT AACTTCTTTT ATTTTTTAAT TTTTTTCTTT TCCCTACGAG TCTAGAGACT ACGTATTAAC   
  
  
+ CGAGATTCTT GTTGCCTATC CCGTCACTTG AACCAGTCCA CGAAACGCGT ACTCACTACT ACGAGAGTAG   
  
  
+ ACTAACCGAG GCGTCGGAGG AAGAACAGGC CTCGCTACTG AACAAGAAGT TTGATCTATT TGTGTAACAA   
  
  
+ CGTAGAGTAG TTGACAATAA TAAACCAAGG AAGACAAAAC ACAAAAAAAA AAAGGGACAC TTTGCGTTAA   
  
  
+ ACTACCTAAG AAACTTTACT CGTAATAACC TACGCCCTCG AAGTTTCATA CAGAATGTCC ATCTATAAAC   
  
  
+ GTACCGCTCC GACGTAGAGA TGAATGGTAA AACCTTATAC ATGTTTTCAA AACTTTGAAA AAGTTCAGTA   
  
  
+ ATTACCCGTA TTGCTATATC GGTTGATTAT ATGTATACAA GAAACGGCAG AAGAAGGCTC TTACAGTAGA   
  
  
+ TCGAAGAACC TTACCGTTAA CCCGTTGACG ATATATTATA AAAAACTACT ATCAAATTTG CATTACTCTA   
  
  
+ ATCCACTTAA ATGAATAACT ATAACACCT  

- TTTCTGTTTT CGTTTGTGAA ACTGTGCGTT TGTTGTATAC ACAAGTGCGG TGAAAGGAGA GTTCAGGCAA   
  
  
- AGCAAAGCCA AGAGGGGGCT TTTTATGTTT AGAAGAAAGA GTGCTCTGAA ATCACATTGC ATTTGCCTGG   
  
  
- AAGATTTATT GAACCTAGCT GGGAAAATTC CTCATCTTTT GGACTTAACA CCACCCCCAC GCGCAGCTAA   
  
  
- GGCAGCGTCC ACGCTGCTAA CGGAACACAT GTCCCCTGCT GCTGGTGGGA AGAAACGGAA TTGCACCTCA   
  
  
- AAAGTTGAAA TTTTAACGGA GGGCTTTGGC GCGTCGACGA TGAAGAGCGT AAGGGGGCCC AATATGGCTT   
  
  
- CTTACGGAGT TAAATGAGGT CACTCTGCCA ATGTCATGGT GGTGACATCA ATTTCCTGAT GCCACATGTC   
  
  
- ACCCTTAGTT TTAAATATCC CATGTTGACA GCAATGGTAT TTTTCTTATC CTGACAAAAC TGGATGATGA   
  
  
- AATAACGAAT ATAGCCTCGA GACTCTAGGG CTTTGAAGTG AAGTAAAGGA GGCAACGGCA GGGGCATTTA   
  
  
- GGACAACAAG AGCTGATAGC AGCCGGGTAG TTATATTATC GAAGTCCTCT CCTCCTCTTA TTTTATTCTA   
  
  
- CTGACATGGA TAAACACGCA GTTGGAGATG ATGTCATGTG CCCAGTGACC AGGGTTTGCC AAGTAAAGCA   
  
  
- GGCATAAGGA CCGTTGAATT ATGATTGAGT TAGCGTGATG GGAGAACTAA CGGGTCTGAT TTAGGATCTA   
  
  
- GCTAGTGCTA GTGGAGATGA ATCTTACTGT AAAAGCAGTG ACAAGTAGGA GTGGTTTGTC TTTTACAAGT   
  
  
- GGCGGATCTC GTAATTGGAG TAAAGCAGTG GGAAACGTAG ATTTAGATCT GTGACCAGTG ACTACTAGCC   
  
  
- CGAACAAGAA TTGAAGAAAA TAAAAAATTA AAAAAAGAAA AGGGATGCTC AGATCTCTGA TGCATAATTG   
  
  
- GCTCTAAGAA CAACGGATAG GGCAGTGAAC TTGGTCAGGT GCTTTGCGCA TGAGTGATGA TGCTCTCATC   
  
  
- TGATTGGCTC CGCAGCCTCC TTCTTGTCCG GAGCGATGAC TTGTTCTTCA AACTAGATAA ACACATTGTT   
  
  
- GCATCTCATC AACTGTTATT ATTTGGTTCC TTCTGTTTTG TGTTTTTTTT TTTCCCTGTG AAACGCAATT   
  
  
- TGATGGATTC TTTGAAATGA GCATTATTGG ATGCGGGAGC TTCAAAGTAT GTCTTACAGG TAGATATTTG   
  
  
- CATGGCGAGG CTGCATCTCT ACTTACCATT TTGGAATATG TACAAAAGTT TTGAAACTTT TTCAAGTCAT   
  
  
- TAATGGGCAT AACGATATAG CCAACTAATA TACATATGTT CTTTGCCGTC TTCTTCCGAG AATGTCATCT   
  
  
- AGCTTCTTGG AATGGCAATT GGGCAACTGC TATATAATAT TTTTTGATGA TAGTTTAAAC GTAATGAGAT   
  
  
- TAGGTGAATT TACTTATTGA TATTGTGGA

+     CAT-box

| Site Name | Organism | Position | Strand | Matrix score. | sequence | function |
| --- | --- | --- | --- | --- | --- | --- |
| CAT-box | Arabidopsis thaliana | 48 | + | 6 | GCCACT | cis-acting regulatory element related to meristem expression |

> 2018/04/13 10:10:12  
+ AAAGACAAAA GCAAACACTT TGACACGCAA ACAACATATG TGTTCACGCC ACTTTCCTCT CAAGTCCGTT   
  
  
+ TCGTTTCGGT TCTCCCCCGA AAAATACAAA TCTTCTTTCT CACGAGACTT TAGTGTAACG TAAACGGACC   
  
  
+ TTCTAAATAA CTTGGATCGA CCCTTTTAAG GAGTAGAAAA CCTGAATTGT GGTGGGGGTG CGCGTCGATT   
  
  
+ CCGTCGCAGG TGCGACGATT GCCTTGTGTA CAGGGGACGA CGACCACCCT TCTTTGCCTT AACGTGGAGT   
  
  
+ TTTCAACTTT AAAATTGCCT CCCGAAACCG CGCAGCTGCT ACTTCTCGCA TTCCCCCGGG TTATACCGAA   
  
  
+ GAATGCCTCA ATTTACTCCA GTGAGACGGT TACAGTACCA CCACTGTAGT TAAAGGACTA CGGTGTACAG   
  
  
+ TGGGAATCAA AATTTATAGG GTACAACTGT CGTTACCATA AAAAGAATAG GACTGTTTTG ACCTACTACT   
  
  
+ TTATTGCTTA TATCGGAGCT CTGAGATCCC GAAACTTCAC TTCATTTCCT CCGTTGCCGT CCCCGTAAAT   
  
  
+ CCTGTTGTTC TCGACTATCG TCGGCCCATC AATATAATAG CTTCAGGAGA GGAGGAGAAT AAAATAAGAT   
  
  
+ GACTGTACCT ATTTGTGCGT CAACCTCTAC TACAGTACAC GGGTCACTGG TCCCAAACGG TTCATTTCGT   
  
  
+ CCGTATTCCT GGCAACTTAA TACTAACTCA ATCGCACTAC CCTCTTGATT GCCCAGACTA AATCCTAGAT   
  
  
+ CGATCACGAT CACCTCTACT TAGAATGACA TTTTCGTCAC TGTTCATCCT CACCAAACAG AAAATGTTCA   
  
  
+ CCGCCTAGAG CATTAACCTC ATTTCGTCAC CCTTTGCATC TAAATCTAGA CACTGGTCAC TGATGATCGG   
  
  
+ GCTTGTTCTT AACTTCTTTT ATTTTTTAAT TTTTTTCTTT TCCCTACGAG TCTAGAGACT ACGTATTAAC   
  
  
+ CGAGATTCTT GTTGCCTATC CCGTCACTTG AACCAGTCCA CGAAACGCGT ACTCACTACT ACGAGAGTAG   
  
  
+ ACTAACCGAG GCGTCGGAGG AAGAACAGGC CTCGCTACTG AACAAGAAGT TTGATCTATT TGTGTAACAA   
  
  
+ CGTAGAGTAG TTGACAATAA TAAACCAAGG AAGACAAAAC ACAAAAAAAA AAAGGGACAC TTTGCGTTAA   
  
  
+ ACTACCTAAG AAACTTTACT CGTAATAACC TACGCCCTCG AAGTTTCATA CAGAATGTCC ATCTATAAAC   
  
  
+ GTACCGCTCC GACGTAGAGA TGAATGGTAA AACCTTATAC ATGTTTTCAA AACTTTGAAA AAGTTCAGTA   
  
  
+ ATTACCCGTA TTGCTATATC GGTTGATTAT ATGTATACAA GAAACGGCAG AAGAAGGCTC TTACAGTAGA   
  
  
+ TCGAAGAACC TTACCGTTAA CCCGTTGACG ATATATTATA AAAAACTACT ATCAAATTTG CATTACTCTA   
  
  
+ ATCCACTTAA ATGAATAACT ATAACACCT  

- TTTCTGTTTT CGTTTGTGAA ACTGTGCGTT TGTTGTATAC ACAAGTGCGG TGAAAGGAGA GTTCAGGCAA   
  
  
- AGCAAAGCCA AGAGGGGGCT TTTTATGTTT AGAAGAAAGA GTGCTCTGAA ATCACATTGC ATTTGCCTGG   
  
  
- AAGATTTATT GAACCTAGCT GGGAAAATTC CTCATCTTTT GGACTTAACA CCACCCCCAC GCGCAGCTAA   
  
  
- GGCAGCGTCC ACGCTGCTAA CGGAACACAT GTCCCCTGCT GCTGGTGGGA AGAAACGGAA TTGCACCTCA   
  
  
- AAAGTTGAAA TTTTAACGGA GGGCTTTGGC GCGTCGACGA TGAAGAGCGT AAGGGGGCCC AATATGGCTT   
  
  
- CTTACGGAGT TAAATGAGGT CACTCTGCCA ATGTCATGGT GGTGACATCA ATTTCCTGAT GCCACATGTC   
  
  
- ACCCTTAGTT TTAAATATCC CATGTTGACA GCAATGGTAT TTTTCTTATC CTGACAAAAC TGGATGATGA   
  
  
- AATAACGAAT ATAGCCTCGA GACTCTAGGG CTTTGAAGTG AAGTAAAGGA GGCAACGGCA GGGGCATTTA   
  
  
- GGACAACAAG AGCTGATAGC AGCCGGGTAG TTATATTATC GAAGTCCTCT CCTCCTCTTA TTTTATTCTA   
  
  
- CTGACATGGA TAAACACGCA GTTGGAGATG ATGTCATGTG CCCAGTGACC AGGGTTTGCC AAGTAAAGCA   
  
  
- GGCATAAGGA CCGTTGAATT ATGATTGAGT TAGCGTGATG GGAGAACTAA CGGGTCTGAT TTAGGATCTA   
  
  
- GCTAGTGCTA GTGGAGATGA ATCTTACTGT AAAAGCAGTG ACAAGTAGGA GTGGTTTGTC TTTTACAAGT   
  
  
- GGCGGATCTC GTAATTGGAG TAAAGCAGTG GGAAACGTAG ATTTAGATCT GTGACCAGTG ACTACTAGCC   
  
  
- CGAACAAGAA TTGAAGAAAA TAAAAAATTA AAAAAAGAAA AGGGATGCTC AGATCTCTGA TGCATAATTG   
  
  
- GCTCTAAGAA CAACGGATAG GGCAGTGAAC TTGGTCAGGT GCTTTGCGCA TGAGTGATGA TGCTCTCATC   
  
  
- TGATTGGCTC CGCAGCCTCC TTCTTGTCCG GAGCGATGAC TTGTTCTTCA AACTAGATAA ACACATTGTT   
  
  
- GCATCTCATC AACTGTTATT ATTTGGTTCC TTCTGTTTTG TGTTTTTTTT TTTCCCTGTG AAACGCAATT   
  
  
- TGATGGATTC TTTGAAATGA GCATTATTGG ATGCGGGAGC TTCAAAGTAT GTCTTACAGG TAGATATTTG   
  
  
- CATGGCGAGG CTGCATCTCT ACTTACCATT TTGGAATATG TACAAAAGTT TTGAAACTTT TTCAAGTCAT   
  
  
- TAATGGGCAT AACGATATAG CCAACTAATA TACATATGTT CTTTGCCGTC TTCTTCCGAG AATGTCATCT   
  
  
- AGCTTCTTGG AATGGCAATT GGGCAACTGC TATATAATAT TTTTTGATGA TAGTTTAAAC GTAATGAGAT   
  
  
- TAGGTGAATT TACTTATTGA TATTGTGGA

+     CATT-motif

| Site Name | Organism | Position | Strand | Matrix score. | sequence | function |
| --- | --- | --- | --- | --- | --- | --- |
| CATT-motif | Zea mays | 351 | - | 6 | GCATTC | part of a light responsive element |
| CATT-motif | Zea mays | 328 | + | 6 | GCATTC | part of a light responsive element |

> 2018/04/13 10:10:12  
+ AAAGACAAAA GCAAACACTT TGACACGCAA ACAACATATG TGTTCACGCC ACTTTCCTCT CAAGTCCGTT   
  
  
+ TCGTTTCGGT TCTCCCCCGA AAAATACAAA TCTTCTTTCT CACGAGACTT TAGTGTAACG TAAACGGACC   
  
  
+ TTCTAAATAA CTTGGATCGA CCCTTTTAAG GAGTAGAAAA CCTGAATTGT GGTGGGGGTG CGCGTCGATT   
  
  
+ CCGTCGCAGG TGCGACGATT GCCTTGTGTA CAGGGGACGA CGACCACCCT TCTTTGCCTT AACGTGGAGT   
  
  
+ TTTCAACTTT AAAATTGCCT CCCGAAACCG CGCAGCTGCT ACTTCTCGCA TTCCCCCGGG TTATACCGAA   
  
  
+ GAATGCCTCA ATTTACTCCA GTGAGACGGT TACAGTACCA CCACTGTAGT TAAAGGACTA CGGTGTACAG   
  
  
+ TGGGAATCAA AATTTATAGG GTACAACTGT CGTTACCATA AAAAGAATAG GACTGTTTTG ACCTACTACT   
  
  
+ TTATTGCTTA TATCGGAGCT CTGAGATCCC GAAACTTCAC TTCATTTCCT CCGTTGCCGT CCCCGTAAAT   
  
  
+ CCTGTTGTTC TCGACTATCG TCGGCCCATC AATATAATAG CTTCAGGAGA GGAGGAGAAT AAAATAAGAT   
  
  
+ GACTGTACCT ATTTGTGCGT CAACCTCTAC TACAGTACAC GGGTCACTGG TCCCAAACGG TTCATTTCGT   
  
  
+ CCGTATTCCT GGCAACTTAA TACTAACTCA ATCGCACTAC CCTCTTGATT GCCCAGACTA AATCCTAGAT   
  
  
+ CGATCACGAT CACCTCTACT TAGAATGACA TTTTCGTCAC TGTTCATCCT CACCAAACAG AAAATGTTCA   
  
  
+ CCGCCTAGAG CATTAACCTC ATTTCGTCAC CCTTTGCATC TAAATCTAGA CACTGGTCAC TGATGATCGG   
  
  
+ GCTTGTTCTT AACTTCTTTT ATTTTTTAAT TTTTTTCTTT TCCCTACGAG TCTAGAGACT ACGTATTAAC   
  
  
+ CGAGATTCTT GTTGCCTATC CCGTCACTTG AACCAGTCCA CGAAACGCGT ACTCACTACT ACGAGAGTAG   
  
  
+ ACTAACCGAG GCGTCGGAGG AAGAACAGGC CTCGCTACTG AACAAGAAGT TTGATCTATT TGTGTAACAA   
  
  
+ CGTAGAGTAG TTGACAATAA TAAACCAAGG AAGACAAAAC ACAAAAAAAA AAAGGGACAC TTTGCGTTAA   
  
  
+ ACTACCTAAG AAACTTTACT CGTAATAACC TACGCCCTCG AAGTTTCATA CAGAATGTCC ATCTATAAAC   
  
  
+ GTACCGCTCC GACGTAGAGA TGAATGGTAA AACCTTATAC ATGTTTTCAA AACTTTGAAA AAGTTCAGTA   
  
  
+ ATTACCCGTA TTGCTATATC GGTTGATTAT ATGTATACAA GAAACGGCAG AAGAAGGCTC TTACAGTAGA   
  
  
+ TCGAAGAACC TTACCGTTAA CCCGTTGACG ATATATTATA AAAAACTACT ATCAAATTTG CATTACTCTA   
  
  
+ ATCCACTTAA ATGAATAACT ATAACACCT  

- TTTCTGTTTT CGTTTGTGAA ACTGTGCGTT TGTTGTATAC ACAAGTGCGG TGAAAGGAGA GTTCAGGCAA   
  
  
- AGCAAAGCCA AGAGGGGGCT TTTTATGTTT AGAAGAAAGA GTGCTCTGAA ATCACATTGC ATTTGCCTGG   
  
  
- AAGATTTATT GAACCTAGCT GGGAAAATTC CTCATCTTTT GGACTTAACA CCACCCCCAC GCGCAGCTAA   
  
  
- GGCAGCGTCC ACGCTGCTAA CGGAACACAT GTCCCCTGCT GCTGGTGGGA AGAAACGGAA TTGCACCTCA   
  
  
- AAAGTTGAAA TTTTAACGGA GGGCTTTGGC GCGTCGACGA TGAAGAGCGT AAGGGGGCCC AATATGGCTT   
  
  
- CTTACGGAGT TAAATGAGGT CACTCTGCCA ATGTCATGGT GGTGACATCA ATTTCCTGAT GCCACATGTC   
  
  
- ACCCTTAGTT TTAAATATCC CATGTTGACA GCAATGGTAT TTTTCTTATC CTGACAAAAC TGGATGATGA   
  
  
- AATAACGAAT ATAGCCTCGA GACTCTAGGG CTTTGAAGTG AAGTAAAGGA GGCAACGGCA GGGGCATTTA   
  
  
- GGACAACAAG AGCTGATAGC AGCCGGGTAG TTATATTATC GAAGTCCTCT CCTCCTCTTA TTTTATTCTA   
  
  
- CTGACATGGA TAAACACGCA GTTGGAGATG ATGTCATGTG CCCAGTGACC AGGGTTTGCC AAGTAAAGCA   
  
  
- GGCATAAGGA CCGTTGAATT ATGATTGAGT TAGCGTGATG GGAGAACTAA CGGGTCTGAT TTAGGATCTA   
  
  
- GCTAGTGCTA GTGGAGATGA ATCTTACTGT AAAAGCAGTG ACAAGTAGGA GTGGTTTGTC TTTTACAAGT   
  
  
- GGCGGATCTC GTAATTGGAG TAAAGCAGTG GGAAACGTAG ATTTAGATCT GTGACCAGTG ACTACTAGCC   
  
  
- CGAACAAGAA TTGAAGAAAA TAAAAAATTA AAAAAAGAAA AGGGATGCTC AGATCTCTGA TGCATAATTG   
  
  
- GCTCTAAGAA CAACGGATAG GGCAGTGAAC TTGGTCAGGT GCTTTGCGCA TGAGTGATGA TGCTCTCATC   
  
  
- TGATTGGCTC CGCAGCCTCC TTCTTGTCCG GAGCGATGAC TTGTTCTTCA AACTAGATAA ACACATTGTT   
  
  
- GCATCTCATC AACTGTTATT ATTTGGTTCC TTCTGTTTTG TGTTTTTTTT TTTCCCTGTG AAACGCAATT   
  
  
- TGATGGATTC TTTGAAATGA GCATTATTGG ATGCGGGAGC TTCAAAGTAT GTCTTACAGG TAGATATTTG   
  
  
- CATGGCGAGG CTGCATCTCT ACTTACCATT TTGGAATATG TACAAAAGTT TTGAAACTTT TTCAAGTCAT   
  
  
- TAATGGGCAT AACGATATAG CCAACTAATA TACATATGTT CTTTGCCGTC TTCTTCCGAG AATGTCATCT   
  
  
- AGCTTCTTGG AATGGCAATT GGGCAACTGC TATATAATAT TTTTTGATGA TAGTTTAAAC GTAATGAGAT   
  
  
- TAGGTGAATT TACTTATTGA TATTGTGGA

+     CCAAT-box

| Site Name | Organism | Position | Strand | Matrix score. | sequence | function |
| --- | --- | --- | --- | --- | --- | --- |
| CCAAT-box | Hordeum vulgare | 541 | - | 6 | CAACGG | MYBHv1 binding site |
| CCAAT-box | Hordeum vulgare | 1422 | - | 6 | CAACGG | MYBHv1 binding site |

> 2018/04/13 10:10:12  
+ AAAGACAAAA GCAAACACTT TGACACGCAA ACAACATATG TGTTCACGCC ACTTTCCTCT CAAGTCCGTT   
  
  
+ TCGTTTCGGT TCTCCCCCGA AAAATACAAA TCTTCTTTCT CACGAGACTT TAGTGTAACG TAAACGGACC   
  
  
+ TTCTAAATAA CTTGGATCGA CCCTTTTAAG GAGTAGAAAA CCTGAATTGT GGTGGGGGTG CGCGTCGATT   
  
  
+ CCGTCGCAGG TGCGACGATT GCCTTGTGTA CAGGGGACGA CGACCACCCT TCTTTGCCTT AACGTGGAGT   
  
  
+ TTTCAACTTT AAAATTGCCT CCCGAAACCG CGCAGCTGCT ACTTCTCGCA TTCCCCCGGG TTATACCGAA   
  
  
+ GAATGCCTCA ATTTACTCCA GTGAGACGGT TACAGTACCA CCACTGTAGT TAAAGGACTA CGGTGTACAG   
  
  
+ TGGGAATCAA AATTTATAGG GTACAACTGT CGTTACCATA AAAAGAATAG GACTGTTTTG ACCTACTACT   
  
  
+ TTATTGCTTA TATCGGAGCT CTGAGATCCC GAAACTTCAC TTCATTTCCT CCGTTGCCGT CCCCGTAAAT   
  
  
+ CCTGTTGTTC TCGACTATCG TCGGCCCATC AATATAATAG CTTCAGGAGA GGAGGAGAAT AAAATAAGAT   
  
  
+ GACTGTACCT ATTTGTGCGT CAACCTCTAC TACAGTACAC GGGTCACTGG TCCCAAACGG TTCATTTCGT   
  
  
+ CCGTATTCCT GGCAACTTAA TACTAACTCA ATCGCACTAC CCTCTTGATT GCCCAGACTA AATCCTAGAT   
  
  
+ CGATCACGAT CACCTCTACT TAGAATGACA TTTTCGTCAC TGTTCATCCT CACCAAACAG AAAATGTTCA   
  
  
+ CCGCCTAGAG CATTAACCTC ATTTCGTCAC CCTTTGCATC TAAATCTAGA CACTGGTCAC TGATGATCGG   
  
  
+ GCTTGTTCTT AACTTCTTTT ATTTTTTAAT TTTTTTCTTT TCCCTACGAG TCTAGAGACT ACGTATTAAC   
  
  
+ CGAGATTCTT GTTGCCTATC CCGTCACTTG AACCAGTCCA CGAAACGCGT ACTCACTACT ACGAGAGTAG   
  
  
+ ACTAACCGAG GCGTCGGAGG AAGAACAGGC CTCGCTACTG AACAAGAAGT TTGATCTATT TGTGTAACAA   
  
  
+ CGTAGAGTAG TTGACAATAA TAAACCAAGG AAGACAAAAC ACAAAAAAAA AAAGGGACAC TTTGCGTTAA   
  
  
+ ACTACCTAAG AAACTTTACT CGTAATAACC TACGCCCTCG AAGTTTCATA CAGAATGTCC ATCTATAAAC   
  
  
+ GTACCGCTCC GACGTAGAGA TGAATGGTAA AACCTTATAC ATGTTTTCAA AACTTTGAAA AAGTTCAGTA   
  
  
+ ATTACCCGTA TTGCTATATC GGTTGATTAT ATGTATACAA GAAACGGCAG AAGAAGGCTC TTACAGTAGA   
  
  
+ TCGAAGAACC TTACCGTTAA CCCGTTGACG ATATATTATA AAAAACTACT ATCAAATTTG CATTACTCTA   
  
  
+ ATCCACTTAA ATGAATAACT ATAACACCT  

- TTTCTGTTTT CGTTTGTGAA ACTGTGCGTT TGTTGTATAC ACAAGTGCGG TGAAAGGAGA GTTCAGGCAA   
  
  
- AGCAAAGCCA AGAGGGGGCT TTTTATGTTT AGAAGAAAGA GTGCTCTGAA ATCACATTGC ATTTGCCTGG   
  
  
- AAGATTTATT GAACCTAGCT GGGAAAATTC CTCATCTTTT GGACTTAACA CCACCCCCAC GCGCAGCTAA   
  
  
- GGCAGCGTCC ACGCTGCTAA CGGAACACAT GTCCCCTGCT GCTGGTGGGA AGAAACGGAA TTGCACCTCA   
  
  
- AAAGTTGAAA TTTTAACGGA GGGCTTTGGC GCGTCGACGA TGAAGAGCGT AAGGGGGCCC AATATGGCTT   
  
  
- CTTACGGAGT TAAATGAGGT CACTCTGCCA ATGTCATGGT GGTGACATCA ATTTCCTGAT GCCACATGTC   
  
  
- ACCCTTAGTT TTAAATATCC CATGTTGACA GCAATGGTAT TTTTCTTATC CTGACAAAAC TGGATGATGA   
  
  
- AATAACGAAT ATAGCCTCGA GACTCTAGGG CTTTGAAGTG AAGTAAAGGA GGCAACGGCA GGGGCATTTA   
  
  
- GGACAACAAG AGCTGATAGC AGCCGGGTAG TTATATTATC GAAGTCCTCT CCTCCTCTTA TTTTATTCTA   
  
  
- CTGACATGGA TAAACACGCA GTTGGAGATG ATGTCATGTG CCCAGTGACC AGGGTTTGCC AAGTAAAGCA   
  
  
- GGCATAAGGA CCGTTGAATT ATGATTGAGT TAGCGTGATG GGAGAACTAA CGGGTCTGAT TTAGGATCTA   
  
  
- GCTAGTGCTA GTGGAGATGA ATCTTACTGT AAAAGCAGTG ACAAGTAGGA GTGGTTTGTC TTTTACAAGT   
  
  
- GGCGGATCTC GTAATTGGAG TAAAGCAGTG GGAAACGTAG ATTTAGATCT GTGACCAGTG ACTACTAGCC   
  
  
- CGAACAAGAA TTGAAGAAAA TAAAAAATTA AAAAAAGAAA AGGGATGCTC AGATCTCTGA TGCATAATTG   
  
  
- GCTCTAAGAA CAACGGATAG GGCAGTGAAC TTGGTCAGGT GCTTTGCGCA TGAGTGATGA TGCTCTCATC   
  
  
- TGATTGGCTC CGCAGCCTCC TTCTTGTCCG GAGCGATGAC TTGTTCTTCA AACTAGATAA ACACATTGTT   
  
  
- GCATCTCATC AACTGTTATT ATTTGGTTCC TTCTGTTTTG TGTTTTTTTT TTTCCCTGTG AAACGCAATT   
  
  
- TGATGGATTC TTTGAAATGA GCATTATTGG ATGCGGGAGC TTCAAAGTAT GTCTTACAGG TAGATATTTG   
  
  
- CATGGCGAGG CTGCATCTCT ACTTACCATT TTGGAATATG TACAAAAGTT TTGAAACTTT TTCAAGTCAT   
  
  
- TAATGGGCAT AACGATATAG CCAACTAATA TACATATGTT CTTTGCCGTC TTCTTCCGAG AATGTCATCT   
  
  
- AGCTTCTTGG AATGGCAATT GGGCAACTGC TATATAATAT TTTTTGATGA TAGTTTAAAC GTAATGAGAT   
  
  
- TAGGTGAATT TACTTATTGA TATTGTGGA

+     CCGTCC-box

| Site Name | Organism | Position | Strand | Matrix score. | sequence | function |
| --- | --- | --- | --- | --- | --- | --- |
| CCGTCC-box | Arabidopsis thaliana | 547 | + | 6 | CCGTCC | cis-acting regulatory element related to meristem specific activation |

> 2018/04/13 10:10:12  
+ AAAGACAAAA GCAAACACTT TGACACGCAA ACAACATATG TGTTCACGCC ACTTTCCTCT CAAGTCCGTT   
  
  
+ TCGTTTCGGT TCTCCCCCGA AAAATACAAA TCTTCTTTCT CACGAGACTT TAGTGTAACG TAAACGGACC   
  
  
+ TTCTAAATAA CTTGGATCGA CCCTTTTAAG GAGTAGAAAA CCTGAATTGT GGTGGGGGTG CGCGTCGATT   
  
  
+ CCGTCGCAGG TGCGACGATT GCCTTGTGTA CAGGGGACGA CGACCACCCT TCTTTGCCTT AACGTGGAGT   
  
  
+ TTTCAACTTT AAAATTGCCT CCCGAAACCG CGCAGCTGCT ACTTCTCGCA TTCCCCCGGG TTATACCGAA   
  
  
+ GAATGCCTCA ATTTACTCCA GTGAGACGGT TACAGTACCA CCACTGTAGT TAAAGGACTA CGGTGTACAG   
  
  
+ TGGGAATCAA AATTTATAGG GTACAACTGT CGTTACCATA AAAAGAATAG GACTGTTTTG ACCTACTACT   
  
  
+ TTATTGCTTA TATCGGAGCT CTGAGATCCC GAAACTTCAC TTCATTTCCT CCGTTGCCGT CCCCGTAAAT   
  
  
+ CCTGTTGTTC TCGACTATCG TCGGCCCATC AATATAATAG CTTCAGGAGA GGAGGAGAAT AAAATAAGAT   
  
  
+ GACTGTACCT ATTTGTGCGT CAACCTCTAC TACAGTACAC GGGTCACTGG TCCCAAACGG TTCATTTCGT   
  
  
+ CCGTATTCCT GGCAACTTAA TACTAACTCA ATCGCACTAC CCTCTTGATT GCCCAGACTA AATCCTAGAT   
  
  
+ CGATCACGAT CACCTCTACT TAGAATGACA TTTTCGTCAC TGTTCATCCT CACCAAACAG AAAATGTTCA   
  
  
+ CCGCCTAGAG CATTAACCTC ATTTCGTCAC CCTTTGCATC TAAATCTAGA CACTGGTCAC TGATGATCGG   
  
  
+ GCTTGTTCTT AACTTCTTTT ATTTTTTAAT TTTTTTCTTT TCCCTACGAG TCTAGAGACT ACGTATTAAC   
  
  
+ CGAGATTCTT GTTGCCTATC CCGTCACTTG AACCAGTCCA CGAAACGCGT ACTCACTACT ACGAGAGTAG   
  
  
+ ACTAACCGAG GCGTCGGAGG AAGAACAGGC CTCGCTACTG AACAAGAAGT TTGATCTATT TGTGTAACAA   
  
  
+ CGTAGAGTAG TTGACAATAA TAAACCAAGG AAGACAAAAC ACAAAAAAAA AAAGGGACAC TTTGCGTTAA   
  
  
+ ACTACCTAAG AAACTTTACT CGTAATAACC TACGCCCTCG AAGTTTCATA CAGAATGTCC ATCTATAAAC   
  
  
+ GTACCGCTCC GACGTAGAGA TGAATGGTAA AACCTTATAC ATGTTTTCAA AACTTTGAAA AAGTTCAGTA   
  
  
+ ATTACCCGTA TTGCTATATC GGTTGATTAT ATGTATACAA GAAACGGCAG AAGAAGGCTC TTACAGTAGA   
  
  
+ TCGAAGAACC TTACCGTTAA CCCGTTGACG ATATATTATA AAAAACTACT ATCAAATTTG CATTACTCTA   
  
  
+ ATCCACTTAA ATGAATAACT ATAACACCT  

- TTTCTGTTTT CGTTTGTGAA ACTGTGCGTT TGTTGTATAC ACAAGTGCGG TGAAAGGAGA GTTCAGGCAA   
  
  
- AGCAAAGCCA AGAGGGGGCT TTTTATGTTT AGAAGAAAGA GTGCTCTGAA ATCACATTGC ATTTGCCTGG   
  
  
- AAGATTTATT GAACCTAGCT GGGAAAATTC CTCATCTTTT GGACTTAACA CCACCCCCAC GCGCAGCTAA   
  
  
- GGCAGCGTCC ACGCTGCTAA CGGAACACAT GTCCCCTGCT GCTGGTGGGA AGAAACGGAA TTGCACCTCA   
  
  
- AAAGTTGAAA TTTTAACGGA GGGCTTTGGC GCGTCGACGA TGAAGAGCGT AAGGGGGCCC AATATGGCTT   
  
  
- CTTACGGAGT TAAATGAGGT CACTCTGCCA ATGTCATGGT GGTGACATCA ATTTCCTGAT GCCACATGTC   
  
  
- ACCCTTAGTT TTAAATATCC CATGTTGACA GCAATGGTAT TTTTCTTATC CTGACAAAAC TGGATGATGA   
  
  
- AATAACGAAT ATAGCCTCGA GACTCTAGGG CTTTGAAGTG AAGTAAAGGA GGCAACGGCA GGGGCATTTA   
  
  
- GGACAACAAG AGCTGATAGC AGCCGGGTAG TTATATTATC GAAGTCCTCT CCTCCTCTTA TTTTATTCTA   
  
  
- CTGACATGGA TAAACACGCA GTTGGAGATG ATGTCATGTG CCCAGTGACC AGGGTTTGCC AAGTAAAGCA   
  
  
- GGCATAAGGA CCGTTGAATT ATGATTGAGT TAGCGTGATG GGAGAACTAA CGGGTCTGAT TTAGGATCTA   
  
  
- GCTAGTGCTA GTGGAGATGA ATCTTACTGT AAAAGCAGTG ACAAGTAGGA GTGGTTTGTC TTTTACAAGT   
  
  
- GGCGGATCTC GTAATTGGAG TAAAGCAGTG GGAAACGTAG ATTTAGATCT GTGACCAGTG ACTACTAGCC   
  
  
- CGAACAAGAA TTGAAGAAAA TAAAAAATTA AAAAAAGAAA AGGGATGCTC AGATCTCTGA TGCATAATTG   
  
  
- GCTCTAAGAA CAACGGATAG GGCAGTGAAC TTGGTCAGGT GCTTTGCGCA TGAGTGATGA TGCTCTCATC   
  
  
- TGATTGGCTC CGCAGCCTCC TTCTTGTCCG GAGCGATGAC TTGTTCTTCA AACTAGATAA ACACATTGTT   
  
  
- GCATCTCATC AACTGTTATT ATTTGGTTCC TTCTGTTTTG TGTTTTTTTT TTTCCCTGTG AAACGCAATT   
  
  
- TGATGGATTC TTTGAAATGA GCATTATTGG ATGCGGGAGC TTCAAAGTAT GTCTTACAGG TAGATATTTG   
  
  
- CATGGCGAGG CTGCATCTCT ACTTACCATT TTGGAATATG TACAAAAGTT TTGAAACTTT TTCAAGTCAT   
  
  
- TAATGGGCAT AACGATATAG CCAACTAATA TACATATGTT CTTTGCCGTC TTCTTCCGAG AATGTCATCT   
  
  
- AGCTTCTTGG AATGGCAATT GGGCAACTGC TATATAATAT TTTTTGATGA TAGTTTAAAC GTAATGAGAT   
  
  
- TAGGTGAATT TACTTATTGA TATTGTGGA

+     CGTCA-motif

| Site Name | Organism | Position | Strand | Matrix score. | sequence | function |
| --- | --- | --- | --- | --- | --- | --- |
| CGTCA-motif | Hordeum vulgare | 1426 | - | 5 | CGTCA | cis-acting regulatory element involved in the MeJA-responsiveness |
| CGTCA-motif | Hordeum vulgare | 805 | + | 5 | CGTCA | cis-acting regulatory element involved in the MeJA-responsiveness |
| CGTCA-motif | Hordeum vulgare | 865 | + | 5 | CGTCA | cis-acting regulatory element involved in the MeJA-responsiveness |
| CGTCA-motif | Hordeum vulgare | 648 | + | 5 | CGTCA | cis-acting regulatory element involved in the MeJA-responsiveness |
| CGTCA-motif | Hordeum vulgare | 1002 | + | 5 | CGTCA | cis-acting regulatory element involved in the MeJA-responsiveness |

> 2018/04/13 10:10:12  
+ AAAGACAAAA GCAAACACTT TGACACGCAA ACAACATATG TGTTCACGCC ACTTTCCTCT CAAGTCCGTT   
  
  
+ TCGTTTCGGT TCTCCCCCGA AAAATACAAA TCTTCTTTCT CACGAGACTT TAGTGTAACG TAAACGGACC   
  
  
+ TTCTAAATAA CTTGGATCGA CCCTTTTAAG GAGTAGAAAA CCTGAATTGT GGTGGGGGTG CGCGTCGATT   
  
  
+ CCGTCGCAGG TGCGACGATT GCCTTGTGTA CAGGGGACGA CGACCACCCT TCTTTGCCTT AACGTGGAGT   
  
  
+ TTTCAACTTT AAAATTGCCT CCCGAAACCG CGCAGCTGCT ACTTCTCGCA TTCCCCCGGG TTATACCGAA   
  
  
+ GAATGCCTCA ATTTACTCCA GTGAGACGGT TACAGTACCA CCACTGTAGT TAAAGGACTA CGGTGTACAG   
  
  
+ TGGGAATCAA AATTTATAGG GTACAACTGT CGTTACCATA AAAAGAATAG GACTGTTTTG ACCTACTACT   
  
  
+ TTATTGCTTA TATCGGAGCT CTGAGATCCC GAAACTTCAC TTCATTTCCT CCGTTGCCGT CCCCGTAAAT   
  
  
+ CCTGTTGTTC TCGACTATCG TCGGCCCATC AATATAATAG CTTCAGGAGA GGAGGAGAAT AAAATAAGAT   
  
  
+ GACTGTACCT ATTTGTGCGT CAACCTCTAC TACAGTACAC GGGTCACTGG TCCCAAACGG TTCATTTCGT   
  
  
+ CCGTATTCCT GGCAACTTAA TACTAACTCA ATCGCACTAC CCTCTTGATT GCCCAGACTA AATCCTAGAT   
  
  
+ CGATCACGAT CACCTCTACT TAGAATGACA TTTTCGTCAC TGTTCATCCT CACCAAACAG AAAATGTTCA   
  
  
+ CCGCCTAGAG CATTAACCTC ATTTCGTCAC CCTTTGCATC TAAATCTAGA CACTGGTCAC TGATGATCGG   
  
  
+ GCTTGTTCTT AACTTCTTTT ATTTTTTAAT TTTTTTCTTT TCCCTACGAG TCTAGAGACT ACGTATTAAC   
  
  
+ CGAGATTCTT GTTGCCTATC CCGTCACTTG AACCAGTCCA CGAAACGCGT ACTCACTACT ACGAGAGTAG   
  
  
+ ACTAACCGAG GCGTCGGAGG AAGAACAGGC CTCGCTACTG AACAAGAAGT TTGATCTATT TGTGTAACAA   
  
  
+ CGTAGAGTAG TTGACAATAA TAAACCAAGG AAGACAAAAC ACAAAAAAAA AAAGGGACAC TTTGCGTTAA   
  
  
+ ACTACCTAAG AAACTTTACT CGTAATAACC TACGCCCTCG AAGTTTCATA CAGAATGTCC ATCTATAAAC   
  
  
+ GTACCGCTCC GACGTAGAGA TGAATGGTAA AACCTTATAC ATGTTTTCAA AACTTTGAAA AAGTTCAGTA   
  
  
+ ATTACCCGTA TTGCTATATC GGTTGATTAT ATGTATACAA GAAACGGCAG AAGAAGGCTC TTACAGTAGA   
  
  
+ TCGAAGAACC TTACCGTTAA CCCGTTGACG ATATATTATA AAAAACTACT ATCAAATTTG CATTACTCTA   
  
  
+ ATCCACTTAA ATGAATAACT ATAACACCT  

- TTTCTGTTTT CGTTTGTGAA ACTGTGCGTT TGTTGTATAC ACAAGTGCGG TGAAAGGAGA GTTCAGGCAA   
  
  
- AGCAAAGCCA AGAGGGGGCT TTTTATGTTT AGAAGAAAGA GTGCTCTGAA ATCACATTGC ATTTGCCTGG   
  
  
- AAGATTTATT GAACCTAGCT GGGAAAATTC CTCATCTTTT GGACTTAACA CCACCCCCAC GCGCAGCTAA   
  
  
- GGCAGCGTCC ACGCTGCTAA CGGAACACAT GTCCCCTGCT GCTGGTGGGA AGAAACGGAA TTGCACCTCA   
  
  
- AAAGTTGAAA TTTTAACGGA GGGCTTTGGC GCGTCGACGA TGAAGAGCGT AAGGGGGCCC AATATGGCTT   
  
  
- CTTACGGAGT TAAATGAGGT CACTCTGCCA ATGTCATGGT GGTGACATCA ATTTCCTGAT GCCACATGTC   
  
  
- ACCCTTAGTT TTAAATATCC CATGTTGACA GCAATGGTAT TTTTCTTATC CTGACAAAAC TGGATGATGA   
  
  
- AATAACGAAT ATAGCCTCGA GACTCTAGGG CTTTGAAGTG AAGTAAAGGA GGCAACGGCA GGGGCATTTA   
  
  
- GGACAACAAG AGCTGATAGC AGCCGGGTAG TTATATTATC GAAGTCCTCT CCTCCTCTTA TTTTATTCTA   
  
  
- CTGACATGGA TAAACACGCA GTTGGAGATG ATGTCATGTG CCCAGTGACC AGGGTTTGCC AAGTAAAGCA   
  
  
- GGCATAAGGA CCGTTGAATT ATGATTGAGT TAGCGTGATG GGAGAACTAA CGGGTCTGAT TTAGGATCTA   
  
  
- GCTAGTGCTA GTGGAGATGA ATCTTACTGT AAAAGCAGTG ACAAGTAGGA GTGGTTTGTC TTTTACAAGT   
  
  
- GGCGGATCTC GTAATTGGAG TAAAGCAGTG GGAAACGTAG ATTTAGATCT GTGACCAGTG ACTACTAGCC   
  
  
- CGAACAAGAA TTGAAGAAAA TAAAAAATTA AAAAAAGAAA AGGGATGCTC AGATCTCTGA TGCATAATTG   
  
  
- GCTCTAAGAA CAACGGATAG GGCAGTGAAC TTGGTCAGGT GCTTTGCGCA TGAGTGATGA TGCTCTCATC   
  
  
- TGATTGGCTC CGCAGCCTCC TTCTTGTCCG GAGCGATGAC TTGTTCTTCA AACTAGATAA ACACATTGTT   
  
  
- GCATCTCATC AACTGTTATT ATTTGGTTCC TTCTGTTTTG TGTTTTTTTT TTTCCCTGTG AAACGCAATT   
  
  
- TGATGGATTC TTTGAAATGA GCATTATTGG ATGCGGGAGC TTCAAAGTAT GTCTTACAGG TAGATATTTG   
  
  
- CATGGCGAGG CTGCATCTCT ACTTACCATT TTGGAATATG TACAAAAGTT TTGAAACTTT TTCAAGTCAT   
  
  
- TAATGGGCAT AACGATATAG CCAACTAATA TACATATGTT CTTTGCCGTC TTCTTCCGAG AATGTCATCT   
  
  
- AGCTTCTTGG AATGGCAATT GGGCAACTGC TATATAATAT TTTTTGATGA TAGTTTAAAC GTAATGAGAT   
  
  
- TAGGTGAATT TACTTATTGA TATTGTGGA

+     G-Box

| Site Name | Organism | Position | Strand | Matrix score. | sequence | function |
| --- | --- | --- | --- | --- | --- | --- |
| G-Box | Pisum sativum | 271 | - | 6 | CACGTT | cis-acting regulatory element involved in light responsiveness |

> 2018/04/13 10:10:12  
+ AAAGACAAAA GCAAACACTT TGACACGCAA ACAACATATG TGTTCACGCC ACTTTCCTCT CAAGTCCGTT   
  
  
+ TCGTTTCGGT TCTCCCCCGA AAAATACAAA TCTTCTTTCT CACGAGACTT TAGTGTAACG TAAACGGACC   
  
  
+ TTCTAAATAA CTTGGATCGA CCCTTTTAAG GAGTAGAAAA CCTGAATTGT GGTGGGGGTG CGCGTCGATT   
  
  
+ CCGTCGCAGG TGCGACGATT GCCTTGTGTA CAGGGGACGA CGACCACCCT TCTTTGCCTT AACGTGGAGT   
  
  
+ TTTCAACTTT AAAATTGCCT CCCGAAACCG CGCAGCTGCT ACTTCTCGCA TTCCCCCGGG TTATACCGAA   
  
  
+ GAATGCCTCA ATTTACTCCA GTGAGACGGT TACAGTACCA CCACTGTAGT TAAAGGACTA CGGTGTACAG   
  
  
+ TGGGAATCAA AATTTATAGG GTACAACTGT CGTTACCATA AAAAGAATAG GACTGTTTTG ACCTACTACT   
  
  
+ TTATTGCTTA TATCGGAGCT CTGAGATCCC GAAACTTCAC TTCATTTCCT CCGTTGCCGT CCCCGTAAAT   
  
  
+ CCTGTTGTTC TCGACTATCG TCGGCCCATC AATATAATAG CTTCAGGAGA GGAGGAGAAT AAAATAAGAT   
  
  
+ GACTGTACCT ATTTGTGCGT CAACCTCTAC TACAGTACAC GGGTCACTGG TCCCAAACGG TTCATTTCGT   
  
  
+ CCGTATTCCT GGCAACTTAA TACTAACTCA ATCGCACTAC CCTCTTGATT GCCCAGACTA AATCCTAGAT   
  
  
+ CGATCACGAT CACCTCTACT TAGAATGACA TTTTCGTCAC TGTTCATCCT CACCAAACAG AAAATGTTCA   
  
  
+ CCGCCTAGAG CATTAACCTC ATTTCGTCAC CCTTTGCATC TAAATCTAGA CACTGGTCAC TGATGATCGG   
  
  
+ GCTTGTTCTT AACTTCTTTT ATTTTTTAAT TTTTTTCTTT TCCCTACGAG TCTAGAGACT ACGTATTAAC   
  
  
+ CGAGATTCTT GTTGCCTATC CCGTCACTTG AACCAGTCCA CGAAACGCGT ACTCACTACT ACGAGAGTAG   
  
  
+ ACTAACCGAG GCGTCGGAGG AAGAACAGGC CTCGCTACTG AACAAGAAGT TTGATCTATT TGTGTAACAA   
  
  
+ CGTAGAGTAG TTGACAATAA TAAACCAAGG AAGACAAAAC ACAAAAAAAA AAAGGGACAC TTTGCGTTAA   
  
  
+ ACTACCTAAG AAACTTTACT CGTAATAACC TACGCCCTCG AAGTTTCATA CAGAATGTCC ATCTATAAAC   
  
  
+ GTACCGCTCC GACGTAGAGA TGAATGGTAA AACCTTATAC ATGTTTTCAA AACTTTGAAA AAGTTCAGTA   
  
  
+ ATTACCCGTA TTGCTATATC GGTTGATTAT ATGTATACAA GAAACGGCAG AAGAAGGCTC TTACAGTAGA   
  
  
+ TCGAAGAACC TTACCGTTAA CCCGTTGACG ATATATTATA AAAAACTACT ATCAAATTTG CATTACTCTA   
  
  
+ ATCCACTTAA ATGAATAACT ATAACACCT  

- TTTCTGTTTT CGTTTGTGAA ACTGTGCGTT TGTTGTATAC ACAAGTGCGG TGAAAGGAGA GTTCAGGCAA   
  
  
- AGCAAAGCCA AGAGGGGGCT TTTTATGTTT AGAAGAAAGA GTGCTCTGAA ATCACATTGC ATTTGCCTGG   
  
  
- AAGATTTATT GAACCTAGCT GGGAAAATTC CTCATCTTTT GGACTTAACA CCACCCCCAC GCGCAGCTAA   
  
  
- GGCAGCGTCC ACGCTGCTAA CGGAACACAT GTCCCCTGCT GCTGGTGGGA AGAAACGGAA TTGCACCTCA   
  
  
- AAAGTTGAAA TTTTAACGGA GGGCTTTGGC GCGTCGACGA TGAAGAGCGT AAGGGGGCCC AATATGGCTT   
  
  
- CTTACGGAGT TAAATGAGGT CACTCTGCCA ATGTCATGGT GGTGACATCA ATTTCCTGAT GCCACATGTC   
  
  
- ACCCTTAGTT TTAAATATCC CATGTTGACA GCAATGGTAT TTTTCTTATC CTGACAAAAC TGGATGATGA   
  
  
- AATAACGAAT ATAGCCTCGA GACTCTAGGG CTTTGAAGTG AAGTAAAGGA GGCAACGGCA GGGGCATTTA   
  
  
- GGACAACAAG AGCTGATAGC AGCCGGGTAG TTATATTATC GAAGTCCTCT CCTCCTCTTA TTTTATTCTA   
  
  
- CTGACATGGA TAAACACGCA GTTGGAGATG ATGTCATGTG CCCAGTGACC AGGGTTTGCC AAGTAAAGCA   
  
  
- GGCATAAGGA CCGTTGAATT ATGATTGAGT TAGCGTGATG GGAGAACTAA CGGGTCTGAT TTAGGATCTA   
  
  
- GCTAGTGCTA GTGGAGATGA ATCTTACTGT AAAAGCAGTG ACAAGTAGGA GTGGTTTGTC TTTTACAAGT   
  
  
- GGCGGATCTC GTAATTGGAG TAAAGCAGTG GGAAACGTAG ATTTAGATCT GTGACCAGTG ACTACTAGCC   
  
  
- CGAACAAGAA TTGAAGAAAA TAAAAAATTA AAAAAAGAAA AGGGATGCTC AGATCTCTGA TGCATAATTG   
  
  
- GCTCTAAGAA CAACGGATAG GGCAGTGAAC TTGGTCAGGT GCTTTGCGCA TGAGTGATGA TGCTCTCATC   
  
  
- TGATTGGCTC CGCAGCCTCC TTCTTGTCCG GAGCGATGAC TTGTTCTTCA AACTAGATAA ACACATTGTT   
  
  
- GCATCTCATC AACTGTTATT ATTTGGTTCC TTCTGTTTTG TGTTTTTTTT TTTCCCTGTG AAACGCAATT   
  
  
- TGATGGATTC TTTGAAATGA GCATTATTGG ATGCGGGAGC TTCAAAGTAT GTCTTACAGG TAGATATTTG   
  
  
- CATGGCGAGG CTGCATCTCT ACTTACCATT TTGGAATATG TACAAAAGTT TTGAAACTTT TTCAAGTCAT   
  
  
- TAATGGGCAT AACGATATAG CCAACTAATA TACATATGTT CTTTGCCGTC TTCTTCCGAG AATGTCATCT   
  
  
- AGCTTCTTGG AATGGCAATT GGGCAACTGC TATATAATAT TTTTTGATGA TAGTTTAAAC GTAATGAGAT   
  
  
- TAGGTGAATT TACTTATTGA TATTGTGGA

+     G-box

| Site Name | Organism | Position | Strand | Matrix score. | sequence | function |
| --- | --- | --- | --- | --- | --- | --- |
| G-box | Brassica oleracea | 969 | - | 9 | TAACACGTAG | cis-acting regulatory element involved in light responsiveness |
| G-box | Hordeum vulgare | 231 | + | 10 | GCCTTGTGTAG | cis-acting regulatory element involved in light responsiveness |
| G-box | Zea mays | 271 | - | 6 | CACGTT | cis-acting regulatory element involved in light responsiveness |

> 2018/04/13 10:10:12  
+ AAAGACAAAA GCAAACACTT TGACACGCAA ACAACATATG TGTTCACGCC ACTTTCCTCT CAAGTCCGTT   
  
  
+ TCGTTTCGGT TCTCCCCCGA AAAATACAAA TCTTCTTTCT CACGAGACTT TAGTGTAACG TAAACGGACC   
  
  
+ TTCTAAATAA CTTGGATCGA CCCTTTTAAG GAGTAGAAAA CCTGAATTGT GGTGGGGGTG CGCGTCGATT   
  
  
+ CCGTCGCAGG TGCGACGATT GCCTTGTGTA CAGGGGACGA CGACCACCCT TCTTTGCCTT AACGTGGAGT   
  
  
+ TTTCAACTTT AAAATTGCCT CCCGAAACCG CGCAGCTGCT ACTTCTCGCA TTCCCCCGGG TTATACCGAA   
  
  
+ GAATGCCTCA ATTTACTCCA GTGAGACGGT TACAGTACCA CCACTGTAGT TAAAGGACTA CGGTGTACAG   
  
  
+ TGGGAATCAA AATTTATAGG GTACAACTGT CGTTACCATA AAAAGAATAG GACTGTTTTG ACCTACTACT   
  
  
+ TTATTGCTTA TATCGGAGCT CTGAGATCCC GAAACTTCAC TTCATTTCCT CCGTTGCCGT CCCCGTAAAT   
  
  
+ CCTGTTGTTC TCGACTATCG TCGGCCCATC AATATAATAG CTTCAGGAGA GGAGGAGAAT AAAATAAGAT   
  
  
+ GACTGTACCT ATTTGTGCGT CAACCTCTAC TACAGTACAC GGGTCACTGG TCCCAAACGG TTCATTTCGT   
  
  
+ CCGTATTCCT GGCAACTTAA TACTAACTCA ATCGCACTAC CCTCTTGATT GCCCAGACTA AATCCTAGAT   
  
  
+ CGATCACGAT CACCTCTACT TAGAATGACA TTTTCGTCAC TGTTCATCCT CACCAAACAG AAAATGTTCA   
  
  
+ CCGCCTAGAG CATTAACCTC ATTTCGTCAC CCTTTGCATC TAAATCTAGA CACTGGTCAC TGATGATCGG   
  
  
+ GCTTGTTCTT AACTTCTTTT ATTTTTTAAT TTTTTTCTTT TCCCTACGAG TCTAGAGACT ACGTATTAAC   
  
  
+ CGAGATTCTT GTTGCCTATC CCGTCACTTG AACCAGTCCA CGAAACGCGT ACTCACTACT ACGAGAGTAG   
  
  
+ ACTAACCGAG GCGTCGGAGG AAGAACAGGC CTCGCTACTG AACAAGAAGT TTGATCTATT TGTGTAACAA   
  
  
+ CGTAGAGTAG TTGACAATAA TAAACCAAGG AAGACAAAAC ACAAAAAAAA AAAGGGACAC TTTGCGTTAA   
  
  
+ ACTACCTAAG AAACTTTACT CGTAATAACC TACGCCCTCG AAGTTTCATA CAGAATGTCC ATCTATAAAC   
  
  
+ GTACCGCTCC GACGTAGAGA TGAATGGTAA AACCTTATAC ATGTTTTCAA AACTTTGAAA AAGTTCAGTA   
  
  
+ ATTACCCGTA TTGCTATATC GGTTGATTAT ATGTATACAA GAAACGGCAG AAGAAGGCTC TTACAGTAGA   
  
  
+ TCGAAGAACC TTACCGTTAA CCCGTTGACG ATATATTATA AAAAACTACT ATCAAATTTG CATTACTCTA   
  
  
+ ATCCACTTAA ATGAATAACT ATAACACCT  

- TTTCTGTTTT CGTTTGTGAA ACTGTGCGTT TGTTGTATAC ACAAGTGCGG TGAAAGGAGA GTTCAGGCAA   
  
  
- AGCAAAGCCA AGAGGGGGCT TTTTATGTTT AGAAGAAAGA GTGCTCTGAA ATCACATTGC ATTTGCCTGG   
  
  
- AAGATTTATT GAACCTAGCT GGGAAAATTC CTCATCTTTT GGACTTAACA CCACCCCCAC GCGCAGCTAA   
  
  
- GGCAGCGTCC ACGCTGCTAA CGGAACACAT GTCCCCTGCT GCTGGTGGGA AGAAACGGAA TTGCACCTCA   
  
  
- AAAGTTGAAA TTTTAACGGA GGGCTTTGGC GCGTCGACGA TGAAGAGCGT AAGGGGGCCC AATATGGCTT   
  
  
- CTTACGGAGT TAAATGAGGT CACTCTGCCA ATGTCATGGT GGTGACATCA ATTTCCTGAT GCCACATGTC   
  
  
- ACCCTTAGTT TTAAATATCC CATGTTGACA GCAATGGTAT TTTTCTTATC CTGACAAAAC TGGATGATGA   
  
  
- AATAACGAAT ATAGCCTCGA GACTCTAGGG CTTTGAAGTG AAGTAAAGGA GGCAACGGCA GGGGCATTTA   
  
  
- GGACAACAAG AGCTGATAGC AGCCGGGTAG TTATATTATC GAAGTCCTCT CCTCCTCTTA TTTTATTCTA   
  
  
- CTGACATGGA TAAACACGCA GTTGGAGATG ATGTCATGTG CCCAGTGACC AGGGTTTGCC AAGTAAAGCA   
  
  
- GGCATAAGGA CCGTTGAATT ATGATTGAGT TAGCGTGATG GGAGAACTAA CGGGTCTGAT TTAGGATCTA   
  
  
- GCTAGTGCTA GTGGAGATGA ATCTTACTGT AAAAGCAGTG ACAAGTAGGA GTGGTTTGTC TTTTACAAGT   
  
  
- GGCGGATCTC GTAATTGGAG TAAAGCAGTG GGAAACGTAG ATTTAGATCT GTGACCAGTG ACTACTAGCC   
  
  
- CGAACAAGAA TTGAAGAAAA TAAAAAATTA AAAAAAGAAA AGGGATGCTC AGATCTCTGA TGCATAATTG   
  
  
- GCTCTAAGAA CAACGGATAG GGCAGTGAAC TTGGTCAGGT GCTTTGCGCA TGAGTGATGA TGCTCTCATC   
  
  
- TGATTGGCTC CGCAGCCTCC TTCTTGTCCG GAGCGATGAC TTGTTCTTCA AACTAGATAA ACACATTGTT   
  
  
- GCATCTCATC AACTGTTATT ATTTGGTTCC TTCTGTTTTG TGTTTTTTTT TTTCCCTGTG AAACGCAATT   
  
  
- TGATGGATTC TTTGAAATGA GCATTATTGG ATGCGGGAGC TTCAAAGTAT GTCTTACAGG TAGATATTTG   
  
  
- CATGGCGAGG CTGCATCTCT ACTTACCATT TTGGAATATG TACAAAAGTT TTGAAACTTT TTCAAGTCAT   
  
  
- TAATGGGCAT AACGATATAG CCAACTAATA TACATATGTT CTTTGCCGTC TTCTTCCGAG AATGTCATCT   
  
  
- AGCTTCTTGG AATGGCAATT GGGCAACTGC TATATAATAT TTTTTGATGA TAGTTTAAAC GTAATGAGAT   
  
  
- TAGGTGAATT TACTTATTGA TATTGTGGA

+     GA-motif

| Site Name | Organism | Position | Strand | Matrix score. | sequence | function |
| --- | --- | --- | --- | --- | --- | --- |
| GA-motif | Glycine max | 1147 | + | 8 | AAGGAAGA | part of a light responsive element |

> 2018/04/13 10:10:12  
+ AAAGACAAAA GCAAACACTT TGACACGCAA ACAACATATG TGTTCACGCC ACTTTCCTCT CAAGTCCGTT   
  
  
+ TCGTTTCGGT TCTCCCCCGA AAAATACAAA TCTTCTTTCT CACGAGACTT TAGTGTAACG TAAACGGACC   
  
  
+ TTCTAAATAA CTTGGATCGA CCCTTTTAAG GAGTAGAAAA CCTGAATTGT GGTGGGGGTG CGCGTCGATT   
  
  
+ CCGTCGCAGG TGCGACGATT GCCTTGTGTA CAGGGGACGA CGACCACCCT TCTTTGCCTT AACGTGGAGT   
  
  
+ TTTCAACTTT AAAATTGCCT CCCGAAACCG CGCAGCTGCT ACTTCTCGCA TTCCCCCGGG TTATACCGAA   
  
  
+ GAATGCCTCA ATTTACTCCA GTGAGACGGT TACAGTACCA CCACTGTAGT TAAAGGACTA CGGTGTACAG   
  
  
+ TGGGAATCAA AATTTATAGG GTACAACTGT CGTTACCATA AAAAGAATAG GACTGTTTTG ACCTACTACT   
  
  
+ TTATTGCTTA TATCGGAGCT CTGAGATCCC GAAACTTCAC TTCATTTCCT CCGTTGCCGT CCCCGTAAAT   
  
  
+ CCTGTTGTTC TCGACTATCG TCGGCCCATC AATATAATAG CTTCAGGAGA GGAGGAGAAT AAAATAAGAT   
  
  
+ GACTGTACCT ATTTGTGCGT CAACCTCTAC TACAGTACAC GGGTCACTGG TCCCAAACGG TTCATTTCGT   
  
  
+ CCGTATTCCT GGCAACTTAA TACTAACTCA ATCGCACTAC CCTCTTGATT GCCCAGACTA AATCCTAGAT   
  
  
+ CGATCACGAT CACCTCTACT TAGAATGACA TTTTCGTCAC TGTTCATCCT CACCAAACAG AAAATGTTCA   
  
  
+ CCGCCTAGAG CATTAACCTC ATTTCGTCAC CCTTTGCATC TAAATCTAGA CACTGGTCAC TGATGATCGG   
  
  
+ GCTTGTTCTT AACTTCTTTT ATTTTTTAAT TTTTTTCTTT TCCCTACGAG TCTAGAGACT ACGTATTAAC   
  
  
+ CGAGATTCTT GTTGCCTATC CCGTCACTTG AACCAGTCCA CGAAACGCGT ACTCACTACT ACGAGAGTAG   
  
  
+ ACTAACCGAG GCGTCGGAGG AAGAACAGGC CTCGCTACTG AACAAGAAGT TTGATCTATT TGTGTAACAA   
  
  
+ CGTAGAGTAG TTGACAATAA TAAACCAAGG AAGACAAAAC ACAAAAAAAA AAAGGGACAC TTTGCGTTAA   
  
  
+ ACTACCTAAG AAACTTTACT CGTAATAACC TACGCCCTCG AAGTTTCATA CAGAATGTCC ATCTATAAAC   
  
  
+ GTACCGCTCC GACGTAGAGA TGAATGGTAA AACCTTATAC ATGTTTTCAA AACTTTGAAA AAGTTCAGTA   
  
  
+ ATTACCCGTA TTGCTATATC GGTTGATTAT ATGTATACAA GAAACGGCAG AAGAAGGCTC TTACAGTAGA   
  
  
+ TCGAAGAACC TTACCGTTAA CCCGTTGACG ATATATTATA AAAAACTACT ATCAAATTTG CATTACTCTA   
  
  
+ ATCCACTTAA ATGAATAACT ATAACACCT  

- TTTCTGTTTT CGTTTGTGAA ACTGTGCGTT TGTTGTATAC ACAAGTGCGG TGAAAGGAGA GTTCAGGCAA   
  
  
- AGCAAAGCCA AGAGGGGGCT TTTTATGTTT AGAAGAAAGA GTGCTCTGAA ATCACATTGC ATTTGCCTGG   
  
  
- AAGATTTATT GAACCTAGCT GGGAAAATTC CTCATCTTTT GGACTTAACA CCACCCCCAC GCGCAGCTAA   
  
  
- GGCAGCGTCC ACGCTGCTAA CGGAACACAT GTCCCCTGCT GCTGGTGGGA AGAAACGGAA TTGCACCTCA   
  
  
- AAAGTTGAAA TTTTAACGGA GGGCTTTGGC GCGTCGACGA TGAAGAGCGT AAGGGGGCCC AATATGGCTT   
  
  
- CTTACGGAGT TAAATGAGGT CACTCTGCCA ATGTCATGGT GGTGACATCA ATTTCCTGAT GCCACATGTC   
  
  
- ACCCTTAGTT TTAAATATCC CATGTTGACA GCAATGGTAT TTTTCTTATC CTGACAAAAC TGGATGATGA   
  
  
- AATAACGAAT ATAGCCTCGA GACTCTAGGG CTTTGAAGTG AAGTAAAGGA GGCAACGGCA GGGGCATTTA   
  
  
- GGACAACAAG AGCTGATAGC AGCCGGGTAG TTATATTATC GAAGTCCTCT CCTCCTCTTA TTTTATTCTA   
  
  
- CTGACATGGA TAAACACGCA GTTGGAGATG ATGTCATGTG CCCAGTGACC AGGGTTTGCC AAGTAAAGCA   
  
  
- GGCATAAGGA CCGTTGAATT ATGATTGAGT TAGCGTGATG GGAGAACTAA CGGGTCTGAT TTAGGATCTA   
  
  
- GCTAGTGCTA GTGGAGATGA ATCTTACTGT AAAAGCAGTG ACAAGTAGGA GTGGTTTGTC TTTTACAAGT   
  
  
- GGCGGATCTC GTAATTGGAG TAAAGCAGTG GGAAACGTAG ATTTAGATCT GTGACCAGTG ACTACTAGCC   
  
  
- CGAACAAGAA TTGAAGAAAA TAAAAAATTA AAAAAAGAAA AGGGATGCTC AGATCTCTGA TGCATAATTG   
  
  
- GCTCTAAGAA CAACGGATAG GGCAGTGAAC TTGGTCAGGT GCTTTGCGCA TGAGTGATGA TGCTCTCATC   
  
  
- TGATTGGCTC CGCAGCCTCC TTCTTGTCCG GAGCGATGAC TTGTTCTTCA AACTAGATAA ACACATTGTT   
  
  
- GCATCTCATC AACTGTTATT ATTTGGTTCC TTCTGTTTTG TGTTTTTTTT TTTCCCTGTG AAACGCAATT   
  
  
- TGATGGATTC TTTGAAATGA GCATTATTGG ATGCGGGAGC TTCAAAGTAT GTCTTACAGG TAGATATTTG   
  
  
- CATGGCGAGG CTGCATCTCT ACTTACCATT TTGGAATATG TACAAAAGTT TTGAAACTTT TTCAAGTCAT   
  
  
- TAATGGGCAT AACGATATAG CCAACTAATA TACATATGTT CTTTGCCGTC TTCTTCCGAG AATGTCATCT   
  
  
- AGCTTCTTGG AATGGCAATT GGGCAACTGC TATATAATAT TTTTTGATGA TAGTTTAAAC GTAATGAGAT   
  
  
- TAGGTGAATT TACTTATTGA TATTGTGGA

+     GAG-motif

| Site Name | Organism | Position | Strand | Matrix score. | sequence | function |
| --- | --- | --- | --- | --- | --- | --- |
| GAG-motif | Spinacia oleracea | 1276 | + | 7 | AGAGATG | part of a light responsive element |

> 2018/04/13 10:10:12  
+ AAAGACAAAA GCAAACACTT TGACACGCAA ACAACATATG TGTTCACGCC ACTTTCCTCT CAAGTCCGTT   
  
  
+ TCGTTTCGGT TCTCCCCCGA AAAATACAAA TCTTCTTTCT CACGAGACTT TAGTGTAACG TAAACGGACC   
  
  
+ TTCTAAATAA CTTGGATCGA CCCTTTTAAG GAGTAGAAAA CCTGAATTGT GGTGGGGGTG CGCGTCGATT   
  
  
+ CCGTCGCAGG TGCGACGATT GCCTTGTGTA CAGGGGACGA CGACCACCCT TCTTTGCCTT AACGTGGAGT   
  
  
+ TTTCAACTTT AAAATTGCCT CCCGAAACCG CGCAGCTGCT ACTTCTCGCA TTCCCCCGGG TTATACCGAA   
  
  
+ GAATGCCTCA ATTTACTCCA GTGAGACGGT TACAGTACCA CCACTGTAGT TAAAGGACTA CGGTGTACAG   
  
  
+ TGGGAATCAA AATTTATAGG GTACAACTGT CGTTACCATA AAAAGAATAG GACTGTTTTG ACCTACTACT   
  
  
+ TTATTGCTTA TATCGGAGCT CTGAGATCCC GAAACTTCAC TTCATTTCCT CCGTTGCCGT CCCCGTAAAT   
  
  
+ CCTGTTGTTC TCGACTATCG TCGGCCCATC AATATAATAG CTTCAGGAGA GGAGGAGAAT AAAATAAGAT   
  
  
+ GACTGTACCT ATTTGTGCGT CAACCTCTAC TACAGTACAC GGGTCACTGG TCCCAAACGG TTCATTTCGT   
  
  
+ CCGTATTCCT GGCAACTTAA TACTAACTCA ATCGCACTAC CCTCTTGATT GCCCAGACTA AATCCTAGAT   
  
  
+ CGATCACGAT CACCTCTACT TAGAATGACA TTTTCGTCAC TGTTCATCCT CACCAAACAG AAAATGTTCA   
  
  
+ CCGCCTAGAG CATTAACCTC ATTTCGTCAC CCTTTGCATC TAAATCTAGA CACTGGTCAC TGATGATCGG   
  
  
+ GCTTGTTCTT AACTTCTTTT ATTTTTTAAT TTTTTTCTTT TCCCTACGAG TCTAGAGACT ACGTATTAAC   
  
  
+ CGAGATTCTT GTTGCCTATC CCGTCACTTG AACCAGTCCA CGAAACGCGT ACTCACTACT ACGAGAGTAG   
  
  
+ ACTAACCGAG GCGTCGGAGG AAGAACAGGC CTCGCTACTG AACAAGAAGT TTGATCTATT TGTGTAACAA   
  
  
+ CGTAGAGTAG TTGACAATAA TAAACCAAGG AAGACAAAAC ACAAAAAAAA AAAGGGACAC TTTGCGTTAA   
  
  
+ ACTACCTAAG AAACTTTACT CGTAATAACC TACGCCCTCG AAGTTTCATA CAGAATGTCC ATCTATAAAC   
  
  
+ GTACCGCTCC GACGTAGAGA TGAATGGTAA AACCTTATAC ATGTTTTCAA AACTTTGAAA AAGTTCAGTA   
  
  
+ ATTACCCGTA TTGCTATATC GGTTGATTAT ATGTATACAA GAAACGGCAG AAGAAGGCTC TTACAGTAGA   
  
  
+ TCGAAGAACC TTACCGTTAA CCCGTTGACG ATATATTATA AAAAACTACT ATCAAATTTG CATTACTCTA   
  
  
+ ATCCACTTAA ATGAATAACT ATAACACCT  

- TTTCTGTTTT CGTTTGTGAA ACTGTGCGTT TGTTGTATAC ACAAGTGCGG TGAAAGGAGA GTTCAGGCAA   
  
  
- AGCAAAGCCA AGAGGGGGCT TTTTATGTTT AGAAGAAAGA GTGCTCTGAA ATCACATTGC ATTTGCCTGG   
  
  
- AAGATTTATT GAACCTAGCT GGGAAAATTC CTCATCTTTT GGACTTAACA CCACCCCCAC GCGCAGCTAA   
  
  
- GGCAGCGTCC ACGCTGCTAA CGGAACACAT GTCCCCTGCT GCTGGTGGGA AGAAACGGAA TTGCACCTCA   
  
  
- AAAGTTGAAA TTTTAACGGA GGGCTTTGGC GCGTCGACGA TGAAGAGCGT AAGGGGGCCC AATATGGCTT   
  
  
- CTTACGGAGT TAAATGAGGT CACTCTGCCA ATGTCATGGT GGTGACATCA ATTTCCTGAT GCCACATGTC   
  
  
- ACCCTTAGTT TTAAATATCC CATGTTGACA GCAATGGTAT TTTTCTTATC CTGACAAAAC TGGATGATGA   
  
  
- AATAACGAAT ATAGCCTCGA GACTCTAGGG CTTTGAAGTG AAGTAAAGGA GGCAACGGCA GGGGCATTTA   
  
  
- GGACAACAAG AGCTGATAGC AGCCGGGTAG TTATATTATC GAAGTCCTCT CCTCCTCTTA TTTTATTCTA   
  
  
- CTGACATGGA TAAACACGCA GTTGGAGATG ATGTCATGTG CCCAGTGACC AGGGTTTGCC AAGTAAAGCA   
  
  
- GGCATAAGGA CCGTTGAATT ATGATTGAGT TAGCGTGATG GGAGAACTAA CGGGTCTGAT TTAGGATCTA   
  
  
- GCTAGTGCTA GTGGAGATGA ATCTTACTGT AAAAGCAGTG ACAAGTAGGA GTGGTTTGTC TTTTACAAGT   
  
  
- GGCGGATCTC GTAATTGGAG TAAAGCAGTG GGAAACGTAG ATTTAGATCT GTGACCAGTG ACTACTAGCC   
  
  
- CGAACAAGAA TTGAAGAAAA TAAAAAATTA AAAAAAGAAA AGGGATGCTC AGATCTCTGA TGCATAATTG   
  
  
- GCTCTAAGAA CAACGGATAG GGCAGTGAAC TTGGTCAGGT GCTTTGCGCA TGAGTGATGA TGCTCTCATC   
  
  
- TGATTGGCTC CGCAGCCTCC TTCTTGTCCG GAGCGATGAC TTGTTCTTCA AACTAGATAA ACACATTGTT   
  
  
- GCATCTCATC AACTGTTATT ATTTGGTTCC TTCTGTTTTG TGTTTTTTTT TTTCCCTGTG AAACGCAATT   
  
  
- TGATGGATTC TTTGAAATGA GCATTATTGG ATGCGGGAGC TTCAAAGTAT GTCTTACAGG TAGATATTTG   
  
  
- CATGGCGAGG CTGCATCTCT ACTTACCATT TTGGAATATG TACAAAAGTT TTGAAACTTT TTCAAGTCAT   
  
  
- TAATGGGCAT AACGATATAG CCAACTAATA TACATATGTT CTTTGCCGTC TTCTTCCGAG AATGTCATCT   
  
  
- AGCTTCTTGG AATGGCAATT GGGCAACTGC TATATAATAT TTTTTGATGA TAGTTTAAAC GTAATGAGAT   
  
  
- TAGGTGAATT TACTTATTGA TATTGTGGA

+     GARE-motif

| Site Name | Organism | Position | Strand | Matrix score. | sequence | function |
| --- | --- | --- | --- | --- | --- | --- |
| GARE-motif | Brassica oleracea | 825 | + | 7 | AAACAGA | gibberellin-responsive element |

> 2018/04/13 10:10:12  
+ AAAGACAAAA GCAAACACTT TGACACGCAA ACAACATATG TGTTCACGCC ACTTTCCTCT CAAGTCCGTT   
  
  
+ TCGTTTCGGT TCTCCCCCGA AAAATACAAA TCTTCTTTCT CACGAGACTT TAGTGTAACG TAAACGGACC   
  
  
+ TTCTAAATAA CTTGGATCGA CCCTTTTAAG GAGTAGAAAA CCTGAATTGT GGTGGGGGTG CGCGTCGATT   
  
  
+ CCGTCGCAGG TGCGACGATT GCCTTGTGTA CAGGGGACGA CGACCACCCT TCTTTGCCTT AACGTGGAGT   
  
  
+ TTTCAACTTT AAAATTGCCT CCCGAAACCG CGCAGCTGCT ACTTCTCGCA TTCCCCCGGG TTATACCGAA   
  
  
+ GAATGCCTCA ATTTACTCCA GTGAGACGGT TACAGTACCA CCACTGTAGT TAAAGGACTA CGGTGTACAG   
  
  
+ TGGGAATCAA AATTTATAGG GTACAACTGT CGTTACCATA AAAAGAATAG GACTGTTTTG ACCTACTACT   
  
  
+ TTATTGCTTA TATCGGAGCT CTGAGATCCC GAAACTTCAC TTCATTTCCT CCGTTGCCGT CCCCGTAAAT   
  
  
+ CCTGTTGTTC TCGACTATCG TCGGCCCATC AATATAATAG CTTCAGGAGA GGAGGAGAAT AAAATAAGAT   
  
  
+ GACTGTACCT ATTTGTGCGT CAACCTCTAC TACAGTACAC GGGTCACTGG TCCCAAACGG TTCATTTCGT   
  
  
+ CCGTATTCCT GGCAACTTAA TACTAACTCA ATCGCACTAC CCTCTTGATT GCCCAGACTA AATCCTAGAT   
  
  
+ CGATCACGAT CACCTCTACT TAGAATGACA TTTTCGTCAC TGTTCATCCT CACCAAACAG AAAATGTTCA   
  
  
+ CCGCCTAGAG CATTAACCTC ATTTCGTCAC CCTTTGCATC TAAATCTAGA CACTGGTCAC TGATGATCGG   
  
  
+ GCTTGTTCTT AACTTCTTTT ATTTTTTAAT TTTTTTCTTT TCCCTACGAG TCTAGAGACT ACGTATTAAC   
  
  
+ CGAGATTCTT GTTGCCTATC CCGTCACTTG AACCAGTCCA CGAAACGCGT ACTCACTACT ACGAGAGTAG   
  
  
+ ACTAACCGAG GCGTCGGAGG AAGAACAGGC CTCGCTACTG AACAAGAAGT TTGATCTATT TGTGTAACAA   
  
  
+ CGTAGAGTAG TTGACAATAA TAAACCAAGG AAGACAAAAC ACAAAAAAAA AAAGGGACAC TTTGCGTTAA   
  
  
+ ACTACCTAAG AAACTTTACT CGTAATAACC TACGCCCTCG AAGTTTCATA CAGAATGTCC ATCTATAAAC   
  
  
+ GTACCGCTCC GACGTAGAGA TGAATGGTAA AACCTTATAC ATGTTTTCAA AACTTTGAAA AAGTTCAGTA   
  
  
+ ATTACCCGTA TTGCTATATC GGTTGATTAT ATGTATACAA GAAACGGCAG AAGAAGGCTC TTACAGTAGA   
  
  
+ TCGAAGAACC TTACCGTTAA CCCGTTGACG ATATATTATA AAAAACTACT ATCAAATTTG CATTACTCTA   
  
  
+ ATCCACTTAA ATGAATAACT ATAACACCT  

- TTTCTGTTTT CGTTTGTGAA ACTGTGCGTT TGTTGTATAC ACAAGTGCGG TGAAAGGAGA GTTCAGGCAA   
  
  
- AGCAAAGCCA AGAGGGGGCT TTTTATGTTT AGAAGAAAGA GTGCTCTGAA ATCACATTGC ATTTGCCTGG   
  
  
- AAGATTTATT GAACCTAGCT GGGAAAATTC CTCATCTTTT GGACTTAACA CCACCCCCAC GCGCAGCTAA   
  
  
- GGCAGCGTCC ACGCTGCTAA CGGAACACAT GTCCCCTGCT GCTGGTGGGA AGAAACGGAA TTGCACCTCA   
  
  
- AAAGTTGAAA TTTTAACGGA GGGCTTTGGC GCGTCGACGA TGAAGAGCGT AAGGGGGCCC AATATGGCTT   
  
  
- CTTACGGAGT TAAATGAGGT CACTCTGCCA ATGTCATGGT GGTGACATCA ATTTCCTGAT GCCACATGTC   
  
  
- ACCCTTAGTT TTAAATATCC CATGTTGACA GCAATGGTAT TTTTCTTATC CTGACAAAAC TGGATGATGA   
  
  
- AATAACGAAT ATAGCCTCGA GACTCTAGGG CTTTGAAGTG AAGTAAAGGA GGCAACGGCA GGGGCATTTA   
  
  
- GGACAACAAG AGCTGATAGC AGCCGGGTAG TTATATTATC GAAGTCCTCT CCTCCTCTTA TTTTATTCTA   
  
  
- CTGACATGGA TAAACACGCA GTTGGAGATG ATGTCATGTG CCCAGTGACC AGGGTTTGCC AAGTAAAGCA   
  
  
- GGCATAAGGA CCGTTGAATT ATGATTGAGT TAGCGTGATG GGAGAACTAA CGGGTCTGAT TTAGGATCTA   
  
  
- GCTAGTGCTA GTGGAGATGA ATCTTACTGT AAAAGCAGTG ACAAGTAGGA GTGGTTTGTC TTTTACAAGT   
  
  
- GGCGGATCTC GTAATTGGAG TAAAGCAGTG GGAAACGTAG ATTTAGATCT GTGACCAGTG ACTACTAGCC   
  
  
- CGAACAAGAA TTGAAGAAAA TAAAAAATTA AAAAAAGAAA AGGGATGCTC AGATCTCTGA TGCATAATTG   
  
  
- GCTCTAAGAA CAACGGATAG GGCAGTGAAC TTGGTCAGGT GCTTTGCGCA TGAGTGATGA TGCTCTCATC   
  
  
- TGATTGGCTC CGCAGCCTCC TTCTTGTCCG GAGCGATGAC TTGTTCTTCA AACTAGATAA ACACATTGTT   
  
  
- GCATCTCATC AACTGTTATT ATTTGGTTCC TTCTGTTTTG TGTTTTTTTT TTTCCCTGTG AAACGCAATT   
  
  
- TGATGGATTC TTTGAAATGA GCATTATTGG ATGCGGGAGC TTCAAAGTAT GTCTTACAGG TAGATATTTG   
  
  
- CATGGCGAGG CTGCATCTCT ACTTACCATT TTGGAATATG TACAAAAGTT TTGAAACTTT TTCAAGTCAT   
  
  
- TAATGGGCAT AACGATATAG CCAACTAATA TACATATGTT CTTTGCCGTC TTCTTCCGAG AATGTCATCT   
  
  
- AGCTTCTTGG AATGGCAATT GGGCAACTGC TATATAATAT TTTTTGATGA TAGTTTAAAC GTAATGAGAT   
  
  
- TAGGTGAATT TACTTATTGA TATTGTGGA

+     GC-motif

| Site Name | Organism | Position | Strand | Matrix score. | sequence | function |
| --- | --- | --- | --- | --- | --- | --- |
| GC-motif | Zea mays | 84 | + | 6 | CCCCCG | enhancer-like element involved in anoxic specific inducibility |
| GC-motif | Zea mays | 333 | + | 6 | CCCCCG | enhancer-like element involved in anoxic specific inducibility |

> 2018/04/13 10:10:12  
+ AAAGACAAAA GCAAACACTT TGACACGCAA ACAACATATG TGTTCACGCC ACTTTCCTCT CAAGTCCGTT   
  
  
+ TCGTTTCGGT TCTCCCCCGA AAAATACAAA TCTTCTTTCT CACGAGACTT TAGTGTAACG TAAACGGACC   
  
  
+ TTCTAAATAA CTTGGATCGA CCCTTTTAAG GAGTAGAAAA CCTGAATTGT GGTGGGGGTG CGCGTCGATT   
  
  
+ CCGTCGCAGG TGCGACGATT GCCTTGTGTA CAGGGGACGA CGACCACCCT TCTTTGCCTT AACGTGGAGT   
  
  
+ TTTCAACTTT AAAATTGCCT CCCGAAACCG CGCAGCTGCT ACTTCTCGCA TTCCCCCGGG TTATACCGAA   
  
  
+ GAATGCCTCA ATTTACTCCA GTGAGACGGT TACAGTACCA CCACTGTAGT TAAAGGACTA CGGTGTACAG   
  
  
+ TGGGAATCAA AATTTATAGG GTACAACTGT CGTTACCATA AAAAGAATAG GACTGTTTTG ACCTACTACT   
  
  
+ TTATTGCTTA TATCGGAGCT CTGAGATCCC GAAACTTCAC TTCATTTCCT CCGTTGCCGT CCCCGTAAAT   
  
  
+ CCTGTTGTTC TCGACTATCG TCGGCCCATC AATATAATAG CTTCAGGAGA GGAGGAGAAT AAAATAAGAT   
  
  
+ GACTGTACCT ATTTGTGCGT CAACCTCTAC TACAGTACAC GGGTCACTGG TCCCAAACGG TTCATTTCGT   
  
  
+ CCGTATTCCT GGCAACTTAA TACTAACTCA ATCGCACTAC CCTCTTGATT GCCCAGACTA AATCCTAGAT   
  
  
+ CGATCACGAT CACCTCTACT TAGAATGACA TTTTCGTCAC TGTTCATCCT CACCAAACAG AAAATGTTCA   
  
  
+ CCGCCTAGAG CATTAACCTC ATTTCGTCAC CCTTTGCATC TAAATCTAGA CACTGGTCAC TGATGATCGG   
  
  
+ GCTTGTTCTT AACTTCTTTT ATTTTTTAAT TTTTTTCTTT TCCCTACGAG TCTAGAGACT ACGTATTAAC   
  
  
+ CGAGATTCTT GTTGCCTATC CCGTCACTTG AACCAGTCCA CGAAACGCGT ACTCACTACT ACGAGAGTAG   
  
  
+ ACTAACCGAG GCGTCGGAGG AAGAACAGGC CTCGCTACTG AACAAGAAGT TTGATCTATT TGTGTAACAA   
  
  
+ CGTAGAGTAG TTGACAATAA TAAACCAAGG AAGACAAAAC ACAAAAAAAA AAAGGGACAC TTTGCGTTAA   
  
  
+ ACTACCTAAG AAACTTTACT CGTAATAACC TACGCCCTCG AAGTTTCATA CAGAATGTCC ATCTATAAAC   
  
  
+ GTACCGCTCC GACGTAGAGA TGAATGGTAA AACCTTATAC ATGTTTTCAA AACTTTGAAA AAGTTCAGTA   
  
  
+ ATTACCCGTA TTGCTATATC GGTTGATTAT ATGTATACAA GAAACGGCAG AAGAAGGCTC TTACAGTAGA   
  
  
+ TCGAAGAACC TTACCGTTAA CCCGTTGACG ATATATTATA AAAAACTACT ATCAAATTTG CATTACTCTA   
  
  
+ ATCCACTTAA ATGAATAACT ATAACACCT  

- TTTCTGTTTT CGTTTGTGAA ACTGTGCGTT TGTTGTATAC ACAAGTGCGG TGAAAGGAGA GTTCAGGCAA   
  
  
- AGCAAAGCCA AGAGGGGGCT TTTTATGTTT AGAAGAAAGA GTGCTCTGAA ATCACATTGC ATTTGCCTGG   
  
  
- AAGATTTATT GAACCTAGCT GGGAAAATTC CTCATCTTTT GGACTTAACA CCACCCCCAC GCGCAGCTAA   
  
  
- GGCAGCGTCC ACGCTGCTAA CGGAACACAT GTCCCCTGCT GCTGGTGGGA AGAAACGGAA TTGCACCTCA   
  
  
- AAAGTTGAAA TTTTAACGGA GGGCTTTGGC GCGTCGACGA TGAAGAGCGT AAGGGGGCCC AATATGGCTT   
  
  
- CTTACGGAGT TAAATGAGGT CACTCTGCCA ATGTCATGGT GGTGACATCA ATTTCCTGAT GCCACATGTC   
  
  
- ACCCTTAGTT TTAAATATCC CATGTTGACA GCAATGGTAT TTTTCTTATC CTGACAAAAC TGGATGATGA   
  
  
- AATAACGAAT ATAGCCTCGA GACTCTAGGG CTTTGAAGTG AAGTAAAGGA GGCAACGGCA GGGGCATTTA   
  
  
- GGACAACAAG AGCTGATAGC AGCCGGGTAG TTATATTATC GAAGTCCTCT CCTCCTCTTA TTTTATTCTA   
  
  
- CTGACATGGA TAAACACGCA GTTGGAGATG ATGTCATGTG CCCAGTGACC AGGGTTTGCC AAGTAAAGCA   
  
  
- GGCATAAGGA CCGTTGAATT ATGATTGAGT TAGCGTGATG GGAGAACTAA CGGGTCTGAT TTAGGATCTA   
  
  
- GCTAGTGCTA GTGGAGATGA ATCTTACTGT AAAAGCAGTG ACAAGTAGGA GTGGTTTGTC TTTTACAAGT   
  
  
- GGCGGATCTC GTAATTGGAG TAAAGCAGTG GGAAACGTAG ATTTAGATCT GTGACCAGTG ACTACTAGCC   
  
  
- CGAACAAGAA TTGAAGAAAA TAAAAAATTA AAAAAAGAAA AGGGATGCTC AGATCTCTGA TGCATAATTG   
  
  
- GCTCTAAGAA CAACGGATAG GGCAGTGAAC TTGGTCAGGT GCTTTGCGCA TGAGTGATGA TGCTCTCATC   
  
  
- TGATTGGCTC CGCAGCCTCC TTCTTGTCCG GAGCGATGAC TTGTTCTTCA AACTAGATAA ACACATTGTT   
  
  
- GCATCTCATC AACTGTTATT ATTTGGTTCC TTCTGTTTTG TGTTTTTTTT TTTCCCTGTG AAACGCAATT   
  
  
- TGATGGATTC TTTGAAATGA GCATTATTGG ATGCGGGAGC TTCAAAGTAT GTCTTACAGG TAGATATTTG   
  
  
- CATGGCGAGG CTGCATCTCT ACTTACCATT TTGGAATATG TACAAAAGTT TTGAAACTTT TTCAAGTCAT   
  
  
- TAATGGGCAT AACGATATAG CCAACTAATA TACATATGTT CTTTGCCGTC TTCTTCCGAG AATGTCATCT   
  
  
- AGCTTCTTGG AATGGCAATT GGGCAACTGC TATATAATAT TTTTTGATGA TAGTTTAAAC GTAATGAGAT   
  
  
- TAGGTGAATT TACTTATTGA TATTGTGGA

+     GT1-motif

| Site Name | Organism | Position | Strand | Matrix score. | sequence | function |
| --- | --- | --- | --- | --- | --- | --- |
| GT1-motif | Avena sativa | 975 | - | 7 | GGTTAAT | light responsive element |
| GT1-motif | Arabidopsis thaliana | 1417 | - | 6 | GGTTAA | light responsive element |
| GT1-motif | Avena sativa | 852 | - | 7 | GGTTAAT | light responsive element |
| GT1-motif | Arabidopsis thaliana | 853 | - | 6 | GGTTAA | light responsive element |
| GT1-motif | Arabidopsis thaliana | 976 | - | 6 | GGTTAA | light responsive element |

> 2018/04/13 10:10:12  
+ AAAGACAAAA GCAAACACTT TGACACGCAA ACAACATATG TGTTCACGCC ACTTTCCTCT CAAGTCCGTT   
  
  
+ TCGTTTCGGT TCTCCCCCGA AAAATACAAA TCTTCTTTCT CACGAGACTT TAGTGTAACG TAAACGGACC   
  
  
+ TTCTAAATAA CTTGGATCGA CCCTTTTAAG GAGTAGAAAA CCTGAATTGT GGTGGGGGTG CGCGTCGATT   
  
  
+ CCGTCGCAGG TGCGACGATT GCCTTGTGTA CAGGGGACGA CGACCACCCT TCTTTGCCTT AACGTGGAGT   
  
  
+ TTTCAACTTT AAAATTGCCT CCCGAAACCG CGCAGCTGCT ACTTCTCGCA TTCCCCCGGG TTATACCGAA   
  
  
+ GAATGCCTCA ATTTACTCCA GTGAGACGGT TACAGTACCA CCACTGTAGT TAAAGGACTA CGGTGTACAG   
  
  
+ TGGGAATCAA AATTTATAGG GTACAACTGT CGTTACCATA AAAAGAATAG GACTGTTTTG ACCTACTACT   
  
  
+ TTATTGCTTA TATCGGAGCT CTGAGATCCC GAAACTTCAC TTCATTTCCT CCGTTGCCGT CCCCGTAAAT   
  
  
+ CCTGTTGTTC TCGACTATCG TCGGCCCATC AATATAATAG CTTCAGGAGA GGAGGAGAAT AAAATAAGAT   
  
  
+ GACTGTACCT ATTTGTGCGT CAACCTCTAC TACAGTACAC GGGTCACTGG TCCCAAACGG TTCATTTCGT   
  
  
+ CCGTATTCCT GGCAACTTAA TACTAACTCA ATCGCACTAC CCTCTTGATT GCCCAGACTA AATCCTAGAT   
  
  
+ CGATCACGAT CACCTCTACT TAGAATGACA TTTTCGTCAC TGTTCATCCT CACCAAACAG AAAATGTTCA   
  
  
+ CCGCCTAGAG CATTAACCTC ATTTCGTCAC CCTTTGCATC TAAATCTAGA CACTGGTCAC TGATGATCGG   
  
  
+ GCTTGTTCTT AACTTCTTTT ATTTTTTAAT TTTTTTCTTT TCCCTACGAG TCTAGAGACT ACGTATTAAC   
  
  
+ CGAGATTCTT GTTGCCTATC CCGTCACTTG AACCAGTCCA CGAAACGCGT ACTCACTACT ACGAGAGTAG   
  
  
+ ACTAACCGAG GCGTCGGAGG AAGAACAGGC CTCGCTACTG AACAAGAAGT TTGATCTATT TGTGTAACAA   
  
  
+ CGTAGAGTAG TTGACAATAA TAAACCAAGG AAGACAAAAC ACAAAAAAAA AAAGGGACAC TTTGCGTTAA   
  
  
+ ACTACCTAAG AAACTTTACT CGTAATAACC TACGCCCTCG AAGTTTCATA CAGAATGTCC ATCTATAAAC   
  
  
+ GTACCGCTCC GACGTAGAGA TGAATGGTAA AACCTTATAC ATGTTTTCAA AACTTTGAAA AAGTTCAGTA   
  
  
+ ATTACCCGTA TTGCTATATC GGTTGATTAT ATGTATACAA GAAACGGCAG AAGAAGGCTC TTACAGTAGA   
  
  
+ TCGAAGAACC TTACCGTTAA CCCGTTGACG ATATATTATA AAAAACTACT ATCAAATTTG CATTACTCTA   
  
  
+ ATCCACTTAA ATGAATAACT ATAACACCT  

- TTTCTGTTTT CGTTTGTGAA ACTGTGCGTT TGTTGTATAC ACAAGTGCGG TGAAAGGAGA GTTCAGGCAA   
  
  
- AGCAAAGCCA AGAGGGGGCT TTTTATGTTT AGAAGAAAGA GTGCTCTGAA ATCACATTGC ATTTGCCTGG   
  
  
- AAGATTTATT GAACCTAGCT GGGAAAATTC CTCATCTTTT GGACTTAACA CCACCCCCAC GCGCAGCTAA   
  
  
- GGCAGCGTCC ACGCTGCTAA CGGAACACAT GTCCCCTGCT GCTGGTGGGA AGAAACGGAA TTGCACCTCA   
  
  
- AAAGTTGAAA TTTTAACGGA GGGCTTTGGC GCGTCGACGA TGAAGAGCGT AAGGGGGCCC AATATGGCTT   
  
  
- CTTACGGAGT TAAATGAGGT CACTCTGCCA ATGTCATGGT GGTGACATCA ATTTCCTGAT GCCACATGTC   
  
  
- ACCCTTAGTT TTAAATATCC CATGTTGACA GCAATGGTAT TTTTCTTATC CTGACAAAAC TGGATGATGA   
  
  
- AATAACGAAT ATAGCCTCGA GACTCTAGGG CTTTGAAGTG AAGTAAAGGA GGCAACGGCA GGGGCATTTA   
  
  
- GGACAACAAG AGCTGATAGC AGCCGGGTAG TTATATTATC GAAGTCCTCT CCTCCTCTTA TTTTATTCTA   
  
  
- CTGACATGGA TAAACACGCA GTTGGAGATG ATGTCATGTG CCCAGTGACC AGGGTTTGCC AAGTAAAGCA   
  
  
- GGCATAAGGA CCGTTGAATT ATGATTGAGT TAGCGTGATG GGAGAACTAA CGGGTCTGAT TTAGGATCTA   
  
  
- GCTAGTGCTA GTGGAGATGA ATCTTACTGT AAAAGCAGTG ACAAGTAGGA GTGGTTTGTC TTTTACAAGT   
  
  
- GGCGGATCTC GTAATTGGAG TAAAGCAGTG GGAAACGTAG ATTTAGATCT GTGACCAGTG ACTACTAGCC   
  
  
- CGAACAAGAA TTGAAGAAAA TAAAAAATTA AAAAAAGAAA AGGGATGCTC AGATCTCTGA TGCATAATTG   
  
  
- GCTCTAAGAA CAACGGATAG GGCAGTGAAC TTGGTCAGGT GCTTTGCGCA TGAGTGATGA TGCTCTCATC   
  
  
- TGATTGGCTC CGCAGCCTCC TTCTTGTCCG GAGCGATGAC TTGTTCTTCA AACTAGATAA ACACATTGTT   
  
  
- GCATCTCATC AACTGTTATT ATTTGGTTCC TTCTGTTTTG TGTTTTTTTT TTTCCCTGTG AAACGCAATT   
  
  
- TGATGGATTC TTTGAAATGA GCATTATTGG ATGCGGGAGC TTCAAAGTAT GTCTTACAGG TAGATATTTG   
  
  
- CATGGCGAGG CTGCATCTCT ACTTACCATT TTGGAATATG TACAAAAGTT TTGAAACTTT TTCAAGTCAT   
  
  
- TAATGGGCAT AACGATATAG CCAACTAATA TACATATGTT CTTTGCCGTC TTCTTCCGAG AATGTCATCT   
  
  
- AGCTTCTTGG AATGGCAATT GGGCAACTGC TATATAATAT TTTTTGATGA TAGTTTAAAC GTAATGAGAT   
  
  
- TAGGTGAATT TACTTATTGA TATTGTGGA

+     LTR

| Site Name | Organism | Position | Strand | Matrix score. | sequence | function |
| --- | --- | --- | --- | --- | --- | --- |
| LTR | Hordeum vulgare | 519 | + | 6 | CCGAAA | cis-acting element involved in low-temperature responsiveness |
| LTR | Hordeum vulgare | 74 | - | 6 | CCGAAA | cis-acting element involved in low-temperature responsiveness |
| LTR | Hordeum vulgare | 87 | + | 6 | CCGAAA | cis-acting element involved in low-temperature responsiveness |
| LTR | Hordeum vulgare | 302 | + | 6 | CCGAAA | cis-acting element involved in low-temperature responsiveness |

> 2018/04/13 10:10:12  
+ AAAGACAAAA GCAAACACTT TGACACGCAA ACAACATATG TGTTCACGCC ACTTTCCTCT CAAGTCCGTT   
  
  
+ TCGTTTCGGT TCTCCCCCGA AAAATACAAA TCTTCTTTCT CACGAGACTT TAGTGTAACG TAAACGGACC   
  
  
+ TTCTAAATAA CTTGGATCGA CCCTTTTAAG GAGTAGAAAA CCTGAATTGT GGTGGGGGTG CGCGTCGATT   
  
  
+ CCGTCGCAGG TGCGACGATT GCCTTGTGTA CAGGGGACGA CGACCACCCT TCTTTGCCTT AACGTGGAGT   
  
  
+ TTTCAACTTT AAAATTGCCT CCCGAAACCG CGCAGCTGCT ACTTCTCGCA TTCCCCCGGG TTATACCGAA   
  
  
+ GAATGCCTCA ATTTACTCCA GTGAGACGGT TACAGTACCA CCACTGTAGT TAAAGGACTA CGGTGTACAG   
  
  
+ TGGGAATCAA AATTTATAGG GTACAACTGT CGTTACCATA AAAAGAATAG GACTGTTTTG ACCTACTACT   
  
  
+ TTATTGCTTA TATCGGAGCT CTGAGATCCC GAAACTTCAC TTCATTTCCT CCGTTGCCGT CCCCGTAAAT   
  
  
+ CCTGTTGTTC TCGACTATCG TCGGCCCATC AATATAATAG CTTCAGGAGA GGAGGAGAAT AAAATAAGAT   
  
  
+ GACTGTACCT ATTTGTGCGT CAACCTCTAC TACAGTACAC GGGTCACTGG TCCCAAACGG TTCATTTCGT   
  
  
+ CCGTATTCCT GGCAACTTAA TACTAACTCA ATCGCACTAC CCTCTTGATT GCCCAGACTA AATCCTAGAT   
  
  
+ CGATCACGAT CACCTCTACT TAGAATGACA TTTTCGTCAC TGTTCATCCT CACCAAACAG AAAATGTTCA   
  
  
+ CCGCCTAGAG CATTAACCTC ATTTCGTCAC CCTTTGCATC TAAATCTAGA CACTGGTCAC TGATGATCGG   
  
  
+ GCTTGTTCTT AACTTCTTTT ATTTTTTAAT TTTTTTCTTT TCCCTACGAG TCTAGAGACT ACGTATTAAC   
  
  
+ CGAGATTCTT GTTGCCTATC CCGTCACTTG AACCAGTCCA CGAAACGCGT ACTCACTACT ACGAGAGTAG   
  
  
+ ACTAACCGAG GCGTCGGAGG AAGAACAGGC CTCGCTACTG AACAAGAAGT TTGATCTATT TGTGTAACAA   
  
  
+ CGTAGAGTAG TTGACAATAA TAAACCAAGG AAGACAAAAC ACAAAAAAAA AAAGGGACAC TTTGCGTTAA   
  
  
+ ACTACCTAAG AAACTTTACT CGTAATAACC TACGCCCTCG AAGTTTCATA CAGAATGTCC ATCTATAAAC   
  
  
+ GTACCGCTCC GACGTAGAGA TGAATGGTAA AACCTTATAC ATGTTTTCAA AACTTTGAAA AAGTTCAGTA   
  
  
+ ATTACCCGTA TTGCTATATC GGTTGATTAT ATGTATACAA GAAACGGCAG AAGAAGGCTC TTACAGTAGA   
  
  
+ TCGAAGAACC TTACCGTTAA CCCGTTGACG ATATATTATA AAAAACTACT ATCAAATTTG CATTACTCTA   
  
  
+ ATCCACTTAA ATGAATAACT ATAACACCT  

- TTTCTGTTTT CGTTTGTGAA ACTGTGCGTT TGTTGTATAC ACAAGTGCGG TGAAAGGAGA GTTCAGGCAA   
  
  
- AGCAAAGCCA AGAGGGGGCT TTTTATGTTT AGAAGAAAGA GTGCTCTGAA ATCACATTGC ATTTGCCTGG   
  
  
- AAGATTTATT GAACCTAGCT GGGAAAATTC CTCATCTTTT GGACTTAACA CCACCCCCAC GCGCAGCTAA   
  
  
- GGCAGCGTCC ACGCTGCTAA CGGAACACAT GTCCCCTGCT GCTGGTGGGA AGAAACGGAA TTGCACCTCA   
  
  
- AAAGTTGAAA TTTTAACGGA GGGCTTTGGC GCGTCGACGA TGAAGAGCGT AAGGGGGCCC AATATGGCTT   
  
  
- CTTACGGAGT TAAATGAGGT CACTCTGCCA ATGTCATGGT GGTGACATCA ATTTCCTGAT GCCACATGTC   
  
  
- ACCCTTAGTT TTAAATATCC CATGTTGACA GCAATGGTAT TTTTCTTATC CTGACAAAAC TGGATGATGA   
  
  
- AATAACGAAT ATAGCCTCGA GACTCTAGGG CTTTGAAGTG AAGTAAAGGA GGCAACGGCA GGGGCATTTA   
  
  
- GGACAACAAG AGCTGATAGC AGCCGGGTAG TTATATTATC GAAGTCCTCT CCTCCTCTTA TTTTATTCTA   
  
  
- CTGACATGGA TAAACACGCA GTTGGAGATG ATGTCATGTG CCCAGTGACC AGGGTTTGCC AAGTAAAGCA   
  
  
- GGCATAAGGA CCGTTGAATT ATGATTGAGT TAGCGTGATG GGAGAACTAA CGGGTCTGAT TTAGGATCTA   
  
  
- GCTAGTGCTA GTGGAGATGA ATCTTACTGT AAAAGCAGTG ACAAGTAGGA GTGGTTTGTC TTTTACAAGT   
  
  
- GGCGGATCTC GTAATTGGAG TAAAGCAGTG GGAAACGTAG ATTTAGATCT GTGACCAGTG ACTACTAGCC   
  
  
- CGAACAAGAA TTGAAGAAAA TAAAAAATTA AAAAAAGAAA AGGGATGCTC AGATCTCTGA TGCATAATTG   
  
  
- GCTCTAAGAA CAACGGATAG GGCAGTGAAC TTGGTCAGGT GCTTTGCGCA TGAGTGATGA TGCTCTCATC   
  
  
- TGATTGGCTC CGCAGCCTCC TTCTTGTCCG GAGCGATGAC TTGTTCTTCA AACTAGATAA ACACATTGTT   
  
  
- GCATCTCATC AACTGTTATT ATTTGGTTCC TTCTGTTTTG TGTTTTTTTT TTTCCCTGTG AAACGCAATT   
  
  
- TGATGGATTC TTTGAAATGA GCATTATTGG ATGCGGGAGC TTCAAAGTAT GTCTTACAGG TAGATATTTG   
  
  
- CATGGCGAGG CTGCATCTCT ACTTACCATT TTGGAATATG TACAAAAGTT TTGAAACTTT TTCAAGTCAT   
  
  
- TAATGGGCAT AACGATATAG CCAACTAATA TACATATGTT CTTTGCCGTC TTCTTCCGAG AATGTCATCT   
  
  
- AGCTTCTTGG AATGGCAATT GGGCAACTGC TATATAATAT TTTTTGATGA TAGTTTAAAC GTAATGAGAT   
  
  
- TAGGTGAATT TACTTATTGA TATTGTGGA

+     MBS

| Site Name | Organism | Position | Strand | Matrix score. | sequence | function |
| --- | --- | --- | --- | --- | --- | --- |
| MBS | Arabidopsis thaliana | 444 | + | 6 | CAACTG | MYB binding site involved in drought-inducibility |

> 2018/04/13 10:10:12  
+ AAAGACAAAA GCAAACACTT TGACACGCAA ACAACATATG TGTTCACGCC ACTTTCCTCT CAAGTCCGTT   
  
  
+ TCGTTTCGGT TCTCCCCCGA AAAATACAAA TCTTCTTTCT CACGAGACTT TAGTGTAACG TAAACGGACC   
  
  
+ TTCTAAATAA CTTGGATCGA CCCTTTTAAG GAGTAGAAAA CCTGAATTGT GGTGGGGGTG CGCGTCGATT   
  
  
+ CCGTCGCAGG TGCGACGATT GCCTTGTGTA CAGGGGACGA CGACCACCCT TCTTTGCCTT AACGTGGAGT   
  
  
+ TTTCAACTTT AAAATTGCCT CCCGAAACCG CGCAGCTGCT ACTTCTCGCA TTCCCCCGGG TTATACCGAA   
  
  
+ GAATGCCTCA ATTTACTCCA GTGAGACGGT TACAGTACCA CCACTGTAGT TAAAGGACTA CGGTGTACAG   
  
  
+ TGGGAATCAA AATTTATAGG GTACAACTGT CGTTACCATA AAAAGAATAG GACTGTTTTG ACCTACTACT   
  
  
+ TTATTGCTTA TATCGGAGCT CTGAGATCCC GAAACTTCAC TTCATTTCCT CCGTTGCCGT CCCCGTAAAT   
  
  
+ CCTGTTGTTC TCGACTATCG TCGGCCCATC AATATAATAG CTTCAGGAGA GGAGGAGAAT AAAATAAGAT   
  
  
+ GACTGTACCT ATTTGTGCGT CAACCTCTAC TACAGTACAC GGGTCACTGG TCCCAAACGG TTCATTTCGT   
  
  
+ CCGTATTCCT GGCAACTTAA TACTAACTCA ATCGCACTAC CCTCTTGATT GCCCAGACTA AATCCTAGAT   
  
  
+ CGATCACGAT CACCTCTACT TAGAATGACA TTTTCGTCAC TGTTCATCCT CACCAAACAG AAAATGTTCA   
  
  
+ CCGCCTAGAG CATTAACCTC ATTTCGTCAC CCTTTGCATC TAAATCTAGA CACTGGTCAC TGATGATCGG   
  
  
+ GCTTGTTCTT AACTTCTTTT ATTTTTTAAT TTTTTTCTTT TCCCTACGAG TCTAGAGACT ACGTATTAAC   
  
  
+ CGAGATTCTT GTTGCCTATC CCGTCACTTG AACCAGTCCA CGAAACGCGT ACTCACTACT ACGAGAGTAG   
  
  
+ ACTAACCGAG GCGTCGGAGG AAGAACAGGC CTCGCTACTG AACAAGAAGT TTGATCTATT TGTGTAACAA   
  
  
+ CGTAGAGTAG TTGACAATAA TAAACCAAGG AAGACAAAAC ACAAAAAAAA AAAGGGACAC TTTGCGTTAA   
  
  
+ ACTACCTAAG AAACTTTACT CGTAATAACC TACGCCCTCG AAGTTTCATA CAGAATGTCC ATCTATAAAC   
  
  
+ GTACCGCTCC GACGTAGAGA TGAATGGTAA AACCTTATAC ATGTTTTCAA AACTTTGAAA AAGTTCAGTA   
  
  
+ ATTACCCGTA TTGCTATATC GGTTGATTAT ATGTATACAA GAAACGGCAG AAGAAGGCTC TTACAGTAGA   
  
  
+ TCGAAGAACC TTACCGTTAA CCCGTTGACG ATATATTATA AAAAACTACT ATCAAATTTG CATTACTCTA   
  
  
+ ATCCACTTAA ATGAATAACT ATAACACCT  

- TTTCTGTTTT CGTTTGTGAA ACTGTGCGTT TGTTGTATAC ACAAGTGCGG TGAAAGGAGA GTTCAGGCAA   
  
  
- AGCAAAGCCA AGAGGGGGCT TTTTATGTTT AGAAGAAAGA GTGCTCTGAA ATCACATTGC ATTTGCCTGG   
  
  
- AAGATTTATT GAACCTAGCT GGGAAAATTC CTCATCTTTT GGACTTAACA CCACCCCCAC GCGCAGCTAA   
  
  
- GGCAGCGTCC ACGCTGCTAA CGGAACACAT GTCCCCTGCT GCTGGTGGGA AGAAACGGAA TTGCACCTCA   
  
  
- AAAGTTGAAA TTTTAACGGA GGGCTTTGGC GCGTCGACGA TGAAGAGCGT AAGGGGGCCC AATATGGCTT   
  
  
- CTTACGGAGT TAAATGAGGT CACTCTGCCA ATGTCATGGT GGTGACATCA ATTTCCTGAT GCCACATGTC   
  
  
- ACCCTTAGTT TTAAATATCC CATGTTGACA GCAATGGTAT TTTTCTTATC CTGACAAAAC TGGATGATGA   
  
  
- AATAACGAAT ATAGCCTCGA GACTCTAGGG CTTTGAAGTG AAGTAAAGGA GGCAACGGCA GGGGCATTTA   
  
  
- GGACAACAAG AGCTGATAGC AGCCGGGTAG TTATATTATC GAAGTCCTCT CCTCCTCTTA TTTTATTCTA   
  
  
- CTGACATGGA TAAACACGCA GTTGGAGATG ATGTCATGTG CCCAGTGACC AGGGTTTGCC AAGTAAAGCA   
  
  
- GGCATAAGGA CCGTTGAATT ATGATTGAGT TAGCGTGATG GGAGAACTAA CGGGTCTGAT TTAGGATCTA   
  
  
- GCTAGTGCTA GTGGAGATGA ATCTTACTGT AAAAGCAGTG ACAAGTAGGA GTGGTTTGTC TTTTACAAGT   
  
  
- GGCGGATCTC GTAATTGGAG TAAAGCAGTG GGAAACGTAG ATTTAGATCT GTGACCAGTG ACTACTAGCC   
  
  
- CGAACAAGAA TTGAAGAAAA TAAAAAATTA AAAAAAGAAA AGGGATGCTC AGATCTCTGA TGCATAATTG   
  
  
- GCTCTAAGAA CAACGGATAG GGCAGTGAAC TTGGTCAGGT GCTTTGCGCA TGAGTGATGA TGCTCTCATC   
  
  
- TGATTGGCTC CGCAGCCTCC TTCTTGTCCG GAGCGATGAC TTGTTCTTCA AACTAGATAA ACACATTGTT   
  
  
- GCATCTCATC AACTGTTATT ATTTGGTTCC TTCTGTTTTG TGTTTTTTTT TTTCCCTGTG AAACGCAATT   
  
  
- TGATGGATTC TTTGAAATGA GCATTATTGG ATGCGGGAGC TTCAAAGTAT GTCTTACAGG TAGATATTTG   
  
  
- CATGGCGAGG CTGCATCTCT ACTTACCATT TTGGAATATG TACAAAAGTT TTGAAACTTT TTCAAGTCAT   
  
  
- TAATGGGCAT AACGATATAG CCAACTAATA TACATATGTT CTTTGCCGTC TTCTTCCGAG AATGTCATCT   
  
  
- AGCTTCTTGG AATGGCAATT GGGCAACTGC TATATAATAT TTTTTGATGA TAGTTTAAAC GTAATGAGAT   
  
  
- TAGGTGAATT TACTTATTGA TATTGTGGA

+     Skn-1\_motif

| Site Name | Organism | Position | Strand | Matrix score. | sequence | function |
| --- | --- | --- | --- | --- | --- | --- |
| Skn-1\_motif | Oryza sativa | 795 | - | 5 | GTCAT | cis-acting regulatory element required for endosperm expression |
| Skn-1\_motif | Oryza sativa | 629 | - | 5 | GTCAT | cis-acting regulatory element required for endosperm expression |

> 2018/04/13 10:10:12  
+ AAAGACAAAA GCAAACACTT TGACACGCAA ACAACATATG TGTTCACGCC ACTTTCCTCT CAAGTCCGTT   
  
  
+ TCGTTTCGGT TCTCCCCCGA AAAATACAAA TCTTCTTTCT CACGAGACTT TAGTGTAACG TAAACGGACC   
  
  
+ TTCTAAATAA CTTGGATCGA CCCTTTTAAG GAGTAGAAAA CCTGAATTGT GGTGGGGGTG CGCGTCGATT   
  
  
+ CCGTCGCAGG TGCGACGATT GCCTTGTGTA CAGGGGACGA CGACCACCCT TCTTTGCCTT AACGTGGAGT   
  
  
+ TTTCAACTTT AAAATTGCCT CCCGAAACCG CGCAGCTGCT ACTTCTCGCA TTCCCCCGGG TTATACCGAA   
  
  
+ GAATGCCTCA ATTTACTCCA GTGAGACGGT TACAGTACCA CCACTGTAGT TAAAGGACTA CGGTGTACAG   
  
  
+ TGGGAATCAA AATTTATAGG GTACAACTGT CGTTACCATA AAAAGAATAG GACTGTTTTG ACCTACTACT   
  
  
+ TTATTGCTTA TATCGGAGCT CTGAGATCCC GAAACTTCAC TTCATTTCCT CCGTTGCCGT CCCCGTAAAT   
  
  
+ CCTGTTGTTC TCGACTATCG TCGGCCCATC AATATAATAG CTTCAGGAGA GGAGGAGAAT AAAATAAGAT   
  
  
+ GACTGTACCT ATTTGTGCGT CAACCTCTAC TACAGTACAC GGGTCACTGG TCCCAAACGG TTCATTTCGT   
  
  
+ CCGTATTCCT GGCAACTTAA TACTAACTCA ATCGCACTAC CCTCTTGATT GCCCAGACTA AATCCTAGAT   
  
  
+ CGATCACGAT CACCTCTACT TAGAATGACA TTTTCGTCAC TGTTCATCCT CACCAAACAG AAAATGTTCA   
  
  
+ CCGCCTAGAG CATTAACCTC ATTTCGTCAC CCTTTGCATC TAAATCTAGA CACTGGTCAC TGATGATCGG   
  
  
+ GCTTGTTCTT AACTTCTTTT ATTTTTTAAT TTTTTTCTTT TCCCTACGAG TCTAGAGACT ACGTATTAAC   
  
  
+ CGAGATTCTT GTTGCCTATC CCGTCACTTG AACCAGTCCA CGAAACGCGT ACTCACTACT ACGAGAGTAG   
  
  
+ ACTAACCGAG GCGTCGGAGG AAGAACAGGC CTCGCTACTG AACAAGAAGT TTGATCTATT TGTGTAACAA   
  
  
+ CGTAGAGTAG TTGACAATAA TAAACCAAGG AAGACAAAAC ACAAAAAAAA AAAGGGACAC TTTGCGTTAA   
  
  
+ ACTACCTAAG AAACTTTACT CGTAATAACC TACGCCCTCG AAGTTTCATA CAGAATGTCC ATCTATAAAC   
  
  
+ GTACCGCTCC GACGTAGAGA TGAATGGTAA AACCTTATAC ATGTTTTCAA AACTTTGAAA AAGTTCAGTA   
  
  
+ ATTACCCGTA TTGCTATATC GGTTGATTAT ATGTATACAA GAAACGGCAG AAGAAGGCTC TTACAGTAGA   
  
  
+ TCGAAGAACC TTACCGTTAA CCCGTTGACG ATATATTATA AAAAACTACT ATCAAATTTG CATTACTCTA   
  
  
+ ATCCACTTAA ATGAATAACT ATAACACCT  

- TTTCTGTTTT CGTTTGTGAA ACTGTGCGTT TGTTGTATAC ACAAGTGCGG TGAAAGGAGA GTTCAGGCAA   
  
  
- AGCAAAGCCA AGAGGGGGCT TTTTATGTTT AGAAGAAAGA GTGCTCTGAA ATCACATTGC ATTTGCCTGG   
  
  
- AAGATTTATT GAACCTAGCT GGGAAAATTC CTCATCTTTT GGACTTAACA CCACCCCCAC GCGCAGCTAA   
  
  
- GGCAGCGTCC ACGCTGCTAA CGGAACACAT GTCCCCTGCT GCTGGTGGGA AGAAACGGAA TTGCACCTCA   
  
  
- AAAGTTGAAA TTTTAACGGA GGGCTTTGGC GCGTCGACGA TGAAGAGCGT AAGGGGGCCC AATATGGCTT   
  
  
- CTTACGGAGT TAAATGAGGT CACTCTGCCA ATGTCATGGT GGTGACATCA ATTTCCTGAT GCCACATGTC   
  
  
- ACCCTTAGTT TTAAATATCC CATGTTGACA GCAATGGTAT TTTTCTTATC CTGACAAAAC TGGATGATGA   
  
  
- AATAACGAAT ATAGCCTCGA GACTCTAGGG CTTTGAAGTG AAGTAAAGGA GGCAACGGCA GGGGCATTTA   
  
  
- GGACAACAAG AGCTGATAGC AGCCGGGTAG TTATATTATC GAAGTCCTCT CCTCCTCTTA TTTTATTCTA   
  
  
- CTGACATGGA TAAACACGCA GTTGGAGATG ATGTCATGTG CCCAGTGACC AGGGTTTGCC AAGTAAAGCA   
  
  
- GGCATAAGGA CCGTTGAATT ATGATTGAGT TAGCGTGATG GGAGAACTAA CGGGTCTGAT TTAGGATCTA   
  
  
- GCTAGTGCTA GTGGAGATGA ATCTTACTGT AAAAGCAGTG ACAAGTAGGA GTGGTTTGTC TTTTACAAGT   
  
  
- GGCGGATCTC GTAATTGGAG TAAAGCAGTG GGAAACGTAG ATTTAGATCT GTGACCAGTG ACTACTAGCC   
  
  
- CGAACAAGAA TTGAAGAAAA TAAAAAATTA AAAAAAGAAA AGGGATGCTC AGATCTCTGA TGCATAATTG   
  
  
- GCTCTAAGAA CAACGGATAG GGCAGTGAAC TTGGTCAGGT GCTTTGCGCA TGAGTGATGA TGCTCTCATC   
  
  
- TGATTGGCTC CGCAGCCTCC TTCTTGTCCG GAGCGATGAC TTGTTCTTCA AACTAGATAA ACACATTGTT   
  
  
- GCATCTCATC AACTGTTATT ATTTGGTTCC TTCTGTTTTG TGTTTTTTTT TTTCCCTGTG AAACGCAATT   
  
  
- TGATGGATTC TTTGAAATGA GCATTATTGG ATGCGGGAGC TTCAAAGTAT GTCTTACAGG TAGATATTTG   
  
  
- CATGGCGAGG CTGCATCTCT ACTTACCATT TTGGAATATG TACAAAAGTT TTGAAACTTT TTCAAGTCAT   
  
  
- TAATGGGCAT AACGATATAG CCAACTAATA TACATATGTT CTTTGCCGTC TTCTTCCGAG AATGTCATCT   
  
  
- AGCTTCTTGG AATGGCAATT GGGCAACTGC TATATAATAT TTTTTGATGA TAGTTTAAAC GTAATGAGAT   
  
  
- TAGGTGAATT TACTTATTGA TATTGTGGA

+     Sp1

| Site Name | Organism | Position | Strand | Matrix score. | sequence | function |
| --- | --- | --- | --- | --- | --- | --- |
| Sp1 | Zea mays | 254 | + | 5.5 | CC(G/A)CCC | light responsive element |
| Sp1 | Zea mays | 298 | + | 5 | CC(G/A)CCC | light responsive element |

> 2018/04/13 10:10:12  
+ AAAGACAAAA GCAAACACTT TGACACGCAA ACAACATATG TGTTCACGCC ACTTTCCTCT CAAGTCCGTT   
  
  
+ TCGTTTCGGT TCTCCCCCGA AAAATACAAA TCTTCTTTCT CACGAGACTT TAGTGTAACG TAAACGGACC   
  
  
+ TTCTAAATAA CTTGGATCGA CCCTTTTAAG GAGTAGAAAA CCTGAATTGT GGTGGGGGTG CGCGTCGATT   
  
  
+ CCGTCGCAGG TGCGACGATT GCCTTGTGTA CAGGGGACGA CGACCACCCT TCTTTGCCTT AACGTGGAGT   
  
  
+ TTTCAACTTT AAAATTGCCT CCCGAAACCG CGCAGCTGCT ACTTCTCGCA TTCCCCCGGG TTATACCGAA   
  
  
+ GAATGCCTCA ATTTACTCCA GTGAGACGGT TACAGTACCA CCACTGTAGT TAAAGGACTA CGGTGTACAG   
  
  
+ TGGGAATCAA AATTTATAGG GTACAACTGT CGTTACCATA AAAAGAATAG GACTGTTTTG ACCTACTACT   
  
  
+ TTATTGCTTA TATCGGAGCT CTGAGATCCC GAAACTTCAC TTCATTTCCT CCGTTGCCGT CCCCGTAAAT   
  
  
+ CCTGTTGTTC TCGACTATCG TCGGCCCATC AATATAATAG CTTCAGGAGA GGAGGAGAAT AAAATAAGAT   
  
  
+ GACTGTACCT ATTTGTGCGT CAACCTCTAC TACAGTACAC GGGTCACTGG TCCCAAACGG TTCATTTCGT   
  
  
+ CCGTATTCCT GGCAACTTAA TACTAACTCA ATCGCACTAC CCTCTTGATT GCCCAGACTA AATCCTAGAT   
  
  
+ CGATCACGAT CACCTCTACT TAGAATGACA TTTTCGTCAC TGTTCATCCT CACCAAACAG AAAATGTTCA   
  
  
+ CCGCCTAGAG CATTAACCTC ATTTCGTCAC CCTTTGCATC TAAATCTAGA CACTGGTCAC TGATGATCGG   
  
  
+ GCTTGTTCTT AACTTCTTTT ATTTTTTAAT TTTTTTCTTT TCCCTACGAG TCTAGAGACT ACGTATTAAC   
  
  
+ CGAGATTCTT GTTGCCTATC CCGTCACTTG AACCAGTCCA CGAAACGCGT ACTCACTACT ACGAGAGTAG   
  
  
+ ACTAACCGAG GCGTCGGAGG AAGAACAGGC CTCGCTACTG AACAAGAAGT TTGATCTATT TGTGTAACAA   
  
  
+ CGTAGAGTAG TTGACAATAA TAAACCAAGG AAGACAAAAC ACAAAAAAAA AAAGGGACAC TTTGCGTTAA   
  
  
+ ACTACCTAAG AAACTTTACT CGTAATAACC TACGCCCTCG AAGTTTCATA CAGAATGTCC ATCTATAAAC   
  
  
+ GTACCGCTCC GACGTAGAGA TGAATGGTAA AACCTTATAC ATGTTTTCAA AACTTTGAAA AAGTTCAGTA   
  
  
+ ATTACCCGTA TTGCTATATC GGTTGATTAT ATGTATACAA GAAACGGCAG AAGAAGGCTC TTACAGTAGA   
  
  
+ TCGAAGAACC TTACCGTTAA CCCGTTGACG ATATATTATA AAAAACTACT ATCAAATTTG CATTACTCTA   
  
  
+ ATCCACTTAA ATGAATAACT ATAACACCT  

- TTTCTGTTTT CGTTTGTGAA ACTGTGCGTT TGTTGTATAC ACAAGTGCGG TGAAAGGAGA GTTCAGGCAA   
  
  
- AGCAAAGCCA AGAGGGGGCT TTTTATGTTT AGAAGAAAGA GTGCTCTGAA ATCACATTGC ATTTGCCTGG   
  
  
- AAGATTTATT GAACCTAGCT GGGAAAATTC CTCATCTTTT GGACTTAACA CCACCCCCAC GCGCAGCTAA   
  
  
- GGCAGCGTCC ACGCTGCTAA CGGAACACAT GTCCCCTGCT GCTGGTGGGA AGAAACGGAA TTGCACCTCA   
  
  
- AAAGTTGAAA TTTTAACGGA GGGCTTTGGC GCGTCGACGA TGAAGAGCGT AAGGGGGCCC AATATGGCTT   
  
  
- CTTACGGAGT TAAATGAGGT CACTCTGCCA ATGTCATGGT GGTGACATCA ATTTCCTGAT GCCACATGTC   
  
  
- ACCCTTAGTT TTAAATATCC CATGTTGACA GCAATGGTAT TTTTCTTATC CTGACAAAAC TGGATGATGA   
  
  
- AATAACGAAT ATAGCCTCGA GACTCTAGGG CTTTGAAGTG AAGTAAAGGA GGCAACGGCA GGGGCATTTA   
  
  
- GGACAACAAG AGCTGATAGC AGCCGGGTAG TTATATTATC GAAGTCCTCT CCTCCTCTTA TTTTATTCTA   
  
  
- CTGACATGGA TAAACACGCA GTTGGAGATG ATGTCATGTG CCCAGTGACC AGGGTTTGCC AAGTAAAGCA   
  
  
- GGCATAAGGA CCGTTGAATT ATGATTGAGT TAGCGTGATG GGAGAACTAA CGGGTCTGAT TTAGGATCTA   
  
  
- GCTAGTGCTA GTGGAGATGA ATCTTACTGT AAAAGCAGTG ACAAGTAGGA GTGGTTTGTC TTTTACAAGT   
  
  
- GGCGGATCTC GTAATTGGAG TAAAGCAGTG GGAAACGTAG ATTTAGATCT GTGACCAGTG ACTACTAGCC   
  
  
- CGAACAAGAA TTGAAGAAAA TAAAAAATTA AAAAAAGAAA AGGGATGCTC AGATCTCTGA TGCATAATTG   
  
  
- GCTCTAAGAA CAACGGATAG GGCAGTGAAC TTGGTCAGGT GCTTTGCGCA TGAGTGATGA TGCTCTCATC   
  
  
- TGATTGGCTC CGCAGCCTCC TTCTTGTCCG GAGCGATGAC TTGTTCTTCA AACTAGATAA ACACATTGTT   
  
  
- GCATCTCATC AACTGTTATT ATTTGGTTCC TTCTGTTTTG TGTTTTTTTT TTTCCCTGTG AAACGCAATT   
  
  
- TGATGGATTC TTTGAAATGA GCATTATTGG ATGCGGGAGC TTCAAAGTAT GTCTTACAGG TAGATATTTG   
  
  
- CATGGCGAGG CTGCATCTCT ACTTACCATT TTGGAATATG TACAAAAGTT TTGAAACTTT TTCAAGTCAT   
  
  
- TAATGGGCAT AACGATATAG CCAACTAATA TACATATGTT CTTTGCCGTC TTCTTCCGAG AATGTCATCT   
  
  
- AGCTTCTTGG AATGGCAATT GGGCAACTGC TATATAATAT TTTTTGATGA TAGTTTAAAC GTAATGAGAT   
  
  
- TAGGTGAATT TACTTATTGA TATTGTGGA

+     TATA-box

| Site Name | Organism | Position | Strand | Matrix score. | sequence | function |
| --- | --- | --- | --- | --- | --- | --- |
| TATA-box | Arabidopsis thaliana | 1490 | - | 4 | TATA | core promoter element around -30 of transcription start |
| TATA-box | Glycine max | 1434 | - | 5 | TAATA | core promoter element around -30 of transcription start |
| TATA-box | Brassica napus | 1435 | + | 6 | ATTATA | core promoter element around -30 of transcription start |
| TATA-box | Arabidopsis thaliana | 593 | + | 4 | TATA | core promoter element around -30 of transcription start |
| TATA-box | Lycopersicon esculentum | 459 | - | 5 | TTTTA | core promoter element around -30 of transcription start |
| TATA-box | Ac | 432 | - | 7 | TATAAAT | core promoter element around -30 of transcription start |
| TATA-box | Brassica napus | 1431 | - | 6 | ATATAT | core promoter element around -30 of transcription start |
| TATA-box | Arabidopsis thaliana | 1364 | - | 4 | TATA | core promoter element around -30 of transcription start |
| TATA-box | Arabidopsis thaliana | 1296 | - | 4 | TATA | core promoter element around -30 of transcription start |
| TATA-box | Antirrhinum majus | 431 | - | 8 | TATAAATT | core promoter element around -30 of transcription start |
| TATA-box | Arabidopsis thaliana | 341 | - | 5 | TATAA | core promoter element around -30 of transcription start |
| TATA-box | Arabidopsis thaliana | 1432 | - | 4 | TATA | core promoter element around -30 of transcription start |
| TATA-box | Glycine max | 718 | + | 5 | TAATA | core promoter element around -30 of transcription start |
| TATA-box | Lycopersicon esculentum | 164 | + | 5 | TTTTA | core promoter element around -30 of transcription start |
| TATA-box | Brassica oleracea | 592 | + | 7 | ATATAAT | core promoter element around -30 of transcription start |
| TATA-box | Arabidopsis thaliana | 434 | - | 5 | TATAA | core promoter element around -30 of transcription start |
| TATA-box | Arabidopsis thaliana | 435 | + | 4 | TATA | core promoter element around -30 of transcription start |
| TATA-box | Arabidopsis thaliana | 1357 | - | 5 | TATAA | core promoter element around -30 of transcription start |
| TATA-box | Arabidopsis thaliana | 1295 | - | 5 | TATAA | core promoter element around -30 of transcription start |
| TATA-box | Glycine max | 595 | + | 5 | TAATA | core promoter element around -30 of transcription start |
| TATA-box | Lycopersicon esculentum | 290 | - | 5 | TTTTA | core promoter element around -30 of transcription start |
| TATA-box | Arabidopsis thaliana | 1436 | - | 5 | TATAA | core promoter element around -30 of transcription start |
| TATA-box | Arabidopsis thaliana | 342 | + | 4 | TATA | core promoter element around -30 of transcription start |
| TATA-box | Lycopersicon esculentum | 934 | + | 5 | TTTTA | core promoter element around -30 of transcription start |
| TATA-box | Lycopersicon esculentum | 927 | + | 5 | TTTTA | core promoter element around -30 of transcription start |
| TATA-box | Glycine max | 1213 | + | 5 | TAATA | core promoter element around -30 of transcription start |
| TATA-box | Arabidopsis thaliana | 433 | - | 6 | TATAAA | core promoter element around -30 of transcription start |
| TATA-box | Brassica napus | 1356 | + | 6 | ATTATA | core promoter element around -30 of transcription start |
| TATA-box | Glycine max | 974 | - | 5 | TAATA | core promoter element around -30 of transcription start |
| TATA-box | Arabidopsis thaliana | 498 | - | 5 | TATAA | core promoter element around -30 of transcription start |
| TATA-box | Lycopersicon esculentum | 620 | - | 5 | TTTTA | core promoter element around -30 of transcription start |
| TATA-box | Helianthus annuus | 1362 | - | 6 | TATACA | core promoter element around -30 of transcription start |
| TATA-box | Arabidopsis thaliana | 499 | + | 4 | TATA | core promoter element around -30 of transcription start |
| TATA-box | Arabidopsis thaliana | 1345 | - | 4 | TATA | core promoter element around -30 of transcription start |
| TATA-box | Arabidopsis thaliana | 1254 | + | 6 | TATAAA | core promoter element around -30 of transcription start |
| TATA-box | Arabidopsis thaliana | 1437 | + | 6 | TATAAA | core promoter element around -30 of transcription start |
| TATA-box | Glycine max | 1138 | + | 5 | TAATA | core promoter element around -30 of transcription start |
| TATA-box | Lycopersicon esculentum | 1439 | - | 5 | TTTTA | core promoter element around -30 of transcription start |
| TATA-box | Lycopersicon esculentum | 1288 | - | 5 | TTTTA | core promoter element around -30 of transcription start |
| TATA-box | Arabidopsis thaliana | 1358 | - | 4 | TATA | core promoter element around -30 of transcription start |

> 2018/04/13 10:10:12  
+ AAAGACAAAA GCAAACACTT TGACACGCAA ACAACATATG TGTTCACGCC ACTTTCCTCT CAAGTCCGTT   
  
  
+ TCGTTTCGGT TCTCCCCCGA AAAATACAAA TCTTCTTTCT CACGAGACTT TAGTGTAACG TAAACGGACC   
  
  
+ TTCTAAATAA CTTGGATCGA CCCTTTTAAG GAGTAGAAAA CCTGAATTGT GGTGGGGGTG CGCGTCGATT   
  
  
+ CCGTCGCAGG TGCGACGATT GCCTTGTGTA CAGGGGACGA CGACCACCCT TCTTTGCCTT AACGTGGAGT   
  
  
+ TTTCAACTTT AAAATTGCCT CCCGAAACCG CGCAGCTGCT ACTTCTCGCA TTCCCCCGGG TTATACCGAA   
  
  
+ GAATGCCTCA ATTTACTCCA GTGAGACGGT TACAGTACCA CCACTGTAGT TAAAGGACTA CGGTGTACAG   
  
  
+ TGGGAATCAA AATTTATAGG GTACAACTGT CGTTACCATA AAAAGAATAG GACTGTTTTG ACCTACTACT   
  
  
+ TTATTGCTTA TATCGGAGCT CTGAGATCCC GAAACTTCAC TTCATTTCCT CCGTTGCCGT CCCCGTAAAT   
  
  
+ CCTGTTGTTC TCGACTATCG TCGGCCCATC AATATAATAG CTTCAGGAGA GGAGGAGAAT AAAATAAGAT   
  
  
+ GACTGTACCT ATTTGTGCGT CAACCTCTAC TACAGTACAC GGGTCACTGG TCCCAAACGG TTCATTTCGT   
  
  
+ CCGTATTCCT GGCAACTTAA TACTAACTCA ATCGCACTAC CCTCTTGATT GCCCAGACTA AATCCTAGAT   
  
  
+ CGATCACGAT CACCTCTACT TAGAATGACA TTTTCGTCAC TGTTCATCCT CACCAAACAG AAAATGTTCA   
  
  
+ CCGCCTAGAG CATTAACCTC ATTTCGTCAC CCTTTGCATC TAAATCTAGA CACTGGTCAC TGATGATCGG   
  
  
+ GCTTGTTCTT AACTTCTTTT ATTTTTTAAT TTTTTTCTTT TCCCTACGAG TCTAGAGACT ACGTATTAAC   
  
  
+ CGAGATTCTT GTTGCCTATC CCGTCACTTG AACCAGTCCA CGAAACGCGT ACTCACTACT ACGAGAGTAG   
  
  
+ ACTAACCGAG GCGTCGGAGG AAGAACAGGC CTCGCTACTG AACAAGAAGT TTGATCTATT TGTGTAACAA   
  
  
+ CGTAGAGTAG TTGACAATAA TAAACCAAGG AAGACAAAAC ACAAAAAAAA AAAGGGACAC TTTGCGTTAA   
  
  
+ ACTACCTAAG AAACTTTACT CGTAATAACC TACGCCCTCG AAGTTTCATA CAGAATGTCC ATCTATAAAC   
  
  
+ GTACCGCTCC GACGTAGAGA TGAATGGTAA AACCTTATAC ATGTTTTCAA AACTTTGAAA AAGTTCAGTA   
  
  
+ ATTACCCGTA TTGCTATATC GGTTGATTAT ATGTATACAA GAAACGGCAG AAGAAGGCTC TTACAGTAGA   
  
  
+ TCGAAGAACC TTACCGTTAA CCCGTTGACG ATATATTATA AAAAACTACT ATCAAATTTG CATTACTCTA   
  
  
+ ATCCACTTAA ATGAATAACT ATAACACCT  

- TTTCTGTTTT CGTTTGTGAA ACTGTGCGTT TGTTGTATAC ACAAGTGCGG TGAAAGGAGA GTTCAGGCAA   
  
  
- AGCAAAGCCA AGAGGGGGCT TTTTATGTTT AGAAGAAAGA GTGCTCTGAA ATCACATTGC ATTTGCCTGG   
  
  
- AAGATTTATT GAACCTAGCT GGGAAAATTC CTCATCTTTT GGACTTAACA CCACCCCCAC GCGCAGCTAA   
  
  
- GGCAGCGTCC ACGCTGCTAA CGGAACACAT GTCCCCTGCT GCTGGTGGGA AGAAACGGAA TTGCACCTCA   
  
  
- AAAGTTGAAA TTTTAACGGA GGGCTTTGGC GCGTCGACGA TGAAGAGCGT AAGGGGGCCC AATATGGCTT   
  
  
- CTTACGGAGT TAAATGAGGT CACTCTGCCA ATGTCATGGT GGTGACATCA ATTTCCTGAT GCCACATGTC   
  
  
- ACCCTTAGTT TTAAATATCC CATGTTGACA GCAATGGTAT TTTTCTTATC CTGACAAAAC TGGATGATGA   
  
  
- AATAACGAAT ATAGCCTCGA GACTCTAGGG CTTTGAAGTG AAGTAAAGGA GGCAACGGCA GGGGCATTTA   
  
  
- GGACAACAAG AGCTGATAGC AGCCGGGTAG TTATATTATC GAAGTCCTCT CCTCCTCTTA TTTTATTCTA   
  
  
- CTGACATGGA TAAACACGCA GTTGGAGATG ATGTCATGTG CCCAGTGACC AGGGTTTGCC AAGTAAAGCA   
  
  
- GGCATAAGGA CCGTTGAATT ATGATTGAGT TAGCGTGATG GGAGAACTAA CGGGTCTGAT TTAGGATCTA   
  
  
- GCTAGTGCTA GTGGAGATGA ATCTTACTGT AAAAGCAGTG ACAAGTAGGA GTGGTTTGTC TTTTACAAGT   
  
  
- GGCGGATCTC GTAATTGGAG TAAAGCAGTG GGAAACGTAG ATTTAGATCT GTGACCAGTG ACTACTAGCC   
  
  
- CGAACAAGAA TTGAAGAAAA TAAAAAATTA AAAAAAGAAA AGGGATGCTC AGATCTCTGA TGCATAATTG   
  
  
- GCTCTAAGAA CAACGGATAG GGCAGTGAAC TTGGTCAGGT GCTTTGCGCA TGAGTGATGA TGCTCTCATC   
  
  
- TGATTGGCTC CGCAGCCTCC TTCTTGTCCG GAGCGATGAC TTGTTCTTCA AACTAGATAA ACACATTGTT   
  
  
- GCATCTCATC AACTGTTATT ATTTGGTTCC TTCTGTTTTG TGTTTTTTTT TTTCCCTGTG AAACGCAATT   
  
  
- TGATGGATTC TTTGAAATGA GCATTATTGG ATGCGGGAGC TTCAAAGTAT GTCTTACAGG TAGATATTTG   
  
  
- CATGGCGAGG CTGCATCTCT ACTTACCATT TTGGAATATG TACAAAAGTT TTGAAACTTT TTCAAGTCAT   
  
  
- TAATGGGCAT AACGATATAG CCAACTAATA TACATATGTT CTTTGCCGTC TTCTTCCGAG AATGTCATCT   
  
  
- AGCTTCTTGG AATGGCAATT GGGCAACTGC TATATAATAT TTTTTGATGA TAGTTTAAAC GTAATGAGAT   
  
  
- TAGGTGAATT TACTTATTGA TATTGTGGA

+     TC-rich repeats

| Site Name | Organism | Position | Strand | Matrix score. | sequence | function |
| --- | --- | --- | --- | --- | --- | --- |
| TC-rich repeats | Nicotiana tabacum | 361 | + | 9 | ATTTTCTCCA | cis-acting element involved in defense and stress responsiveness |
| TC-rich repeats | Nicotiana tabacum | 800 | + | 9 | ATTTTCTTCA | cis-acting element involved in defense and stress responsiveness |

> 2018/04/13 10:10:12  
+ AAAGACAAAA GCAAACACTT TGACACGCAA ACAACATATG TGTTCACGCC ACTTTCCTCT CAAGTCCGTT   
  
  
+ TCGTTTCGGT TCTCCCCCGA AAAATACAAA TCTTCTTTCT CACGAGACTT TAGTGTAACG TAAACGGACC   
  
  
+ TTCTAAATAA CTTGGATCGA CCCTTTTAAG GAGTAGAAAA CCTGAATTGT GGTGGGGGTG CGCGTCGATT   
  
  
+ CCGTCGCAGG TGCGACGATT GCCTTGTGTA CAGGGGACGA CGACCACCCT TCTTTGCCTT AACGTGGAGT   
  
  
+ TTTCAACTTT AAAATTGCCT CCCGAAACCG CGCAGCTGCT ACTTCTCGCA TTCCCCCGGG TTATACCGAA   
  
  
+ GAATGCCTCA ATTTACTCCA GTGAGACGGT TACAGTACCA CCACTGTAGT TAAAGGACTA CGGTGTACAG   
  
  
+ TGGGAATCAA AATTTATAGG GTACAACTGT CGTTACCATA AAAAGAATAG GACTGTTTTG ACCTACTACT   
  
  
+ TTATTGCTTA TATCGGAGCT CTGAGATCCC GAAACTTCAC TTCATTTCCT CCGTTGCCGT CCCCGTAAAT   
  
  
+ CCTGTTGTTC TCGACTATCG TCGGCCCATC AATATAATAG CTTCAGGAGA GGAGGAGAAT AAAATAAGAT   
  
  
+ GACTGTACCT ATTTGTGCGT CAACCTCTAC TACAGTACAC GGGTCACTGG TCCCAAACGG TTCATTTCGT   
  
  
+ CCGTATTCCT GGCAACTTAA TACTAACTCA ATCGCACTAC CCTCTTGATT GCCCAGACTA AATCCTAGAT   
  
  
+ CGATCACGAT CACCTCTACT TAGAATGACA TTTTCGTCAC TGTTCATCCT CACCAAACAG AAAATGTTCA   
  
  
+ CCGCCTAGAG CATTAACCTC ATTTCGTCAC CCTTTGCATC TAAATCTAGA CACTGGTCAC TGATGATCGG   
  
  
+ GCTTGTTCTT AACTTCTTTT ATTTTTTAAT TTTTTTCTTT TCCCTACGAG TCTAGAGACT ACGTATTAAC   
  
  
+ CGAGATTCTT GTTGCCTATC CCGTCACTTG AACCAGTCCA CGAAACGCGT ACTCACTACT ACGAGAGTAG   
  
  
+ ACTAACCGAG GCGTCGGAGG AAGAACAGGC CTCGCTACTG AACAAGAAGT TTGATCTATT TGTGTAACAA   
  
  
+ CGTAGAGTAG TTGACAATAA TAAACCAAGG AAGACAAAAC ACAAAAAAAA AAAGGGACAC TTTGCGTTAA   
  
  
+ ACTACCTAAG AAACTTTACT CGTAATAACC TACGCCCTCG AAGTTTCATA CAGAATGTCC ATCTATAAAC   
  
  
+ GTACCGCTCC GACGTAGAGA TGAATGGTAA AACCTTATAC ATGTTTTCAA AACTTTGAAA AAGTTCAGTA   
  
  
+ ATTACCCGTA TTGCTATATC GGTTGATTAT ATGTATACAA GAAACGGCAG AAGAAGGCTC TTACAGTAGA   
  
  
+ TCGAAGAACC TTACCGTTAA CCCGTTGACG ATATATTATA AAAAACTACT ATCAAATTTG CATTACTCTA   
  
  
+ ATCCACTTAA ATGAATAACT ATAACACCT  

- TTTCTGTTTT CGTTTGTGAA ACTGTGCGTT TGTTGTATAC ACAAGTGCGG TGAAAGGAGA GTTCAGGCAA   
  
  
- AGCAAAGCCA AGAGGGGGCT TTTTATGTTT AGAAGAAAGA GTGCTCTGAA ATCACATTGC ATTTGCCTGG   
  
  
- AAGATTTATT GAACCTAGCT GGGAAAATTC CTCATCTTTT GGACTTAACA CCACCCCCAC GCGCAGCTAA   
  
  
- GGCAGCGTCC ACGCTGCTAA CGGAACACAT GTCCCCTGCT GCTGGTGGGA AGAAACGGAA TTGCACCTCA   
  
  
- AAAGTTGAAA TTTTAACGGA GGGCTTTGGC GCGTCGACGA TGAAGAGCGT AAGGGGGCCC AATATGGCTT   
  
  
- CTTACGGAGT TAAATGAGGT CACTCTGCCA ATGTCATGGT GGTGACATCA ATTTCCTGAT GCCACATGTC   
  
  
- ACCCTTAGTT TTAAATATCC CATGTTGACA GCAATGGTAT TTTTCTTATC CTGACAAAAC TGGATGATGA   
  
  
- AATAACGAAT ATAGCCTCGA GACTCTAGGG CTTTGAAGTG AAGTAAAGGA GGCAACGGCA GGGGCATTTA   
  
  
- GGACAACAAG AGCTGATAGC AGCCGGGTAG TTATATTATC GAAGTCCTCT CCTCCTCTTA TTTTATTCTA   
  
  
- CTGACATGGA TAAACACGCA GTTGGAGATG ATGTCATGTG CCCAGTGACC AGGGTTTGCC AAGTAAAGCA   
  
  
- GGCATAAGGA CCGTTGAATT ATGATTGAGT TAGCGTGATG GGAGAACTAA CGGGTCTGAT TTAGGATCTA   
  
  
- GCTAGTGCTA GTGGAGATGA ATCTTACTGT AAAAGCAGTG ACAAGTAGGA GTGGTTTGTC TTTTACAAGT   
  
  
- GGCGGATCTC GTAATTGGAG TAAAGCAGTG GGAAACGTAG ATTTAGATCT GTGACCAGTG ACTACTAGCC   
  
  
- CGAACAAGAA TTGAAGAAAA TAAAAAATTA AAAAAAGAAA AGGGATGCTC AGATCTCTGA TGCATAATTG   
  
  
- GCTCTAAGAA CAACGGATAG GGCAGTGAAC TTGGTCAGGT GCTTTGCGCA TGAGTGATGA TGCTCTCATC   
  
  
- TGATTGGCTC CGCAGCCTCC TTCTTGTCCG GAGCGATGAC TTGTTCTTCA AACTAGATAA ACACATTGTT   
  
  
- GCATCTCATC AACTGTTATT ATTTGGTTCC TTCTGTTTTG TGTTTTTTTT TTTCCCTGTG AAACGCAATT   
  
  
- TGATGGATTC TTTGAAATGA GCATTATTGG ATGCGGGAGC TTCAAAGTAT GTCTTACAGG TAGATATTTG   
  
  
- CATGGCGAGG CTGCATCTCT ACTTACCATT TTGGAATATG TACAAAAGTT TTGAAACTTT TTCAAGTCAT   
  
  
- TAATGGGCAT AACGATATAG CCAACTAATA TACATATGTT CTTTGCCGTC TTCTTCCGAG AATGTCATCT   
  
  
- AGCTTCTTGG AATGGCAATT GGGCAACTGC TATATAATAT TTTTTGATGA TAGTTTAAAC GTAATGAGAT   
  
  
- TAGGTGAATT TACTTATTGA TATTGTGGA

+     TCA-element

| Site Name | Organism | Position | Strand | Matrix score. | sequence | function |
| --- | --- | --- | --- | --- | --- | --- |
| TCA-element | Brassica oleracea | 603 | + | 9 | TCAGAAGAGG | cis-acting element involved in salicylic acid responsiveness |
| TCA-element | Brassica oleracea | 612 | + | 9 | GAGAAGAATA | cis-acting element involved in salicylic acid responsiveness |

> 2018/04/13 10:10:12  
+ AAAGACAAAA GCAAACACTT TGACACGCAA ACAACATATG TGTTCACGCC ACTTTCCTCT CAAGTCCGTT   
  
  
+ TCGTTTCGGT TCTCCCCCGA AAAATACAAA TCTTCTTTCT CACGAGACTT TAGTGTAACG TAAACGGACC   
  
  
+ TTCTAAATAA CTTGGATCGA CCCTTTTAAG GAGTAGAAAA CCTGAATTGT GGTGGGGGTG CGCGTCGATT   
  
  
+ CCGTCGCAGG TGCGACGATT GCCTTGTGTA CAGGGGACGA CGACCACCCT TCTTTGCCTT AACGTGGAGT   
  
  
+ TTTCAACTTT AAAATTGCCT CCCGAAACCG CGCAGCTGCT ACTTCTCGCA TTCCCCCGGG TTATACCGAA   
  
  
+ GAATGCCTCA ATTTACTCCA GTGAGACGGT TACAGTACCA CCACTGTAGT TAAAGGACTA CGGTGTACAG   
  
  
+ TGGGAATCAA AATTTATAGG GTACAACTGT CGTTACCATA AAAAGAATAG GACTGTTTTG ACCTACTACT   
  
  
+ TTATTGCTTA TATCGGAGCT CTGAGATCCC GAAACTTCAC TTCATTTCCT CCGTTGCCGT CCCCGTAAAT   
  
  
+ CCTGTTGTTC TCGACTATCG TCGGCCCATC AATATAATAG CTTCAGGAGA GGAGGAGAAT AAAATAAGAT   
  
  
+ GACTGTACCT ATTTGTGCGT CAACCTCTAC TACAGTACAC GGGTCACTGG TCCCAAACGG TTCATTTCGT   
  
  
+ CCGTATTCCT GGCAACTTAA TACTAACTCA ATCGCACTAC CCTCTTGATT GCCCAGACTA AATCCTAGAT   
  
  
+ CGATCACGAT CACCTCTACT TAGAATGACA TTTTCGTCAC TGTTCATCCT CACCAAACAG AAAATGTTCA   
  
  
+ CCGCCTAGAG CATTAACCTC ATTTCGTCAC CCTTTGCATC TAAATCTAGA CACTGGTCAC TGATGATCGG   
  
  
+ GCTTGTTCTT AACTTCTTTT ATTTTTTAAT TTTTTTCTTT TCCCTACGAG TCTAGAGACT ACGTATTAAC   
  
  
+ CGAGATTCTT GTTGCCTATC CCGTCACTTG AACCAGTCCA CGAAACGCGT ACTCACTACT ACGAGAGTAG   
  
  
+ ACTAACCGAG GCGTCGGAGG AAGAACAGGC CTCGCTACTG AACAAGAAGT TTGATCTATT TGTGTAACAA   
  
  
+ CGTAGAGTAG TTGACAATAA TAAACCAAGG AAGACAAAAC ACAAAAAAAA AAAGGGACAC TTTGCGTTAA   
  
  
+ ACTACCTAAG AAACTTTACT CGTAATAACC TACGCCCTCG AAGTTTCATA CAGAATGTCC ATCTATAAAC   
  
  
+ GTACCGCTCC GACGTAGAGA TGAATGGTAA AACCTTATAC ATGTTTTCAA AACTTTGAAA AAGTTCAGTA   
  
  
+ ATTACCCGTA TTGCTATATC GGTTGATTAT ATGTATACAA GAAACGGCAG AAGAAGGCTC TTACAGTAGA   
  
  
+ TCGAAGAACC TTACCGTTAA CCCGTTGACG ATATATTATA AAAAACTACT ATCAAATTTG CATTACTCTA   
  
  
+ ATCCACTTAA ATGAATAACT ATAACACCT  

- TTTCTGTTTT CGTTTGTGAA ACTGTGCGTT TGTTGTATAC ACAAGTGCGG TGAAAGGAGA GTTCAGGCAA   
  
  
- AGCAAAGCCA AGAGGGGGCT TTTTATGTTT AGAAGAAAGA GTGCTCTGAA ATCACATTGC ATTTGCCTGG   
  
  
- AAGATTTATT GAACCTAGCT GGGAAAATTC CTCATCTTTT GGACTTAACA CCACCCCCAC GCGCAGCTAA   
  
  
- GGCAGCGTCC ACGCTGCTAA CGGAACACAT GTCCCCTGCT GCTGGTGGGA AGAAACGGAA TTGCACCTCA   
  
  
- AAAGTTGAAA TTTTAACGGA GGGCTTTGGC GCGTCGACGA TGAAGAGCGT AAGGGGGCCC AATATGGCTT   
  
  
- CTTACGGAGT TAAATGAGGT CACTCTGCCA ATGTCATGGT GGTGACATCA ATTTCCTGAT GCCACATGTC   
  
  
- ACCCTTAGTT TTAAATATCC CATGTTGACA GCAATGGTAT TTTTCTTATC CTGACAAAAC TGGATGATGA   
  
  
- AATAACGAAT ATAGCCTCGA GACTCTAGGG CTTTGAAGTG AAGTAAAGGA GGCAACGGCA GGGGCATTTA   
  
  
- GGACAACAAG AGCTGATAGC AGCCGGGTAG TTATATTATC GAAGTCCTCT CCTCCTCTTA TTTTATTCTA   
  
  
- CTGACATGGA TAAACACGCA GTTGGAGATG ATGTCATGTG CCCAGTGACC AGGGTTTGCC AAGTAAAGCA   
  
  
- GGCATAAGGA CCGTTGAATT ATGATTGAGT TAGCGTGATG GGAGAACTAA CGGGTCTGAT TTAGGATCTA   
  
  
- GCTAGTGCTA GTGGAGATGA ATCTTACTGT AAAAGCAGTG ACAAGTAGGA GTGGTTTGTC TTTTACAAGT   
  
  
- GGCGGATCTC GTAATTGGAG TAAAGCAGTG GGAAACGTAG ATTTAGATCT GTGACCAGTG ACTACTAGCC   
  
  
- CGAACAAGAA TTGAAGAAAA TAAAAAATTA AAAAAAGAAA AGGGATGCTC AGATCTCTGA TGCATAATTG   
  
  
- GCTCTAAGAA CAACGGATAG GGCAGTGAAC TTGGTCAGGT GCTTTGCGCA TGAGTGATGA TGCTCTCATC   
  
  
- TGATTGGCTC CGCAGCCTCC TTCTTGTCCG GAGCGATGAC TTGTTCTTCA AACTAGATAA ACACATTGTT   
  
  
- GCATCTCATC AACTGTTATT ATTTGGTTCC TTCTGTTTTG TGTTTTTTTT TTTCCCTGTG AAACGCAATT   
  
  
- TGATGGATTC TTTGAAATGA GCATTATTGG ATGCGGGAGC TTCAAAGTAT GTCTTACAGG TAGATATTTG   
  
  
- CATGGCGAGG CTGCATCTCT ACTTACCATT TTGGAATATG TACAAAAGTT TTGAAACTTT TTCAAGTCAT   
  
  
- TAATGGGCAT AACGATATAG CCAACTAATA TACATATGTT CTTTGCCGTC TTCTTCCGAG AATGTCATCT   
  
  
- AGCTTCTTGG AATGGCAATT GGGCAACTGC TATATAATAT TTTTTGATGA TAGTTTAAAC GTAATGAGAT   
  
  
- TAGGTGAATT TACTTATTGA TATTGTGGA

+     TCT-motif

| Site Name | Organism | Position | Strand | Matrix score. | sequence | function |
| --- | --- | --- | --- | --- | --- | --- |
| TCT-motif | Arabidopsis thaliana | 1389 | + | 6 | TCTTAC | part of a light responsive element |

> 2018/04/13 10:10:12  
+ AAAGACAAAA GCAAACACTT TGACACGCAA ACAACATATG TGTTCACGCC ACTTTCCTCT CAAGTCCGTT   
  
  
+ TCGTTTCGGT TCTCCCCCGA AAAATACAAA TCTTCTTTCT CACGAGACTT TAGTGTAACG TAAACGGACC   
  
  
+ TTCTAAATAA CTTGGATCGA CCCTTTTAAG GAGTAGAAAA CCTGAATTGT GGTGGGGGTG CGCGTCGATT   
  
  
+ CCGTCGCAGG TGCGACGATT GCCTTGTGTA CAGGGGACGA CGACCACCCT TCTTTGCCTT AACGTGGAGT   
  
  
+ TTTCAACTTT AAAATTGCCT CCCGAAACCG CGCAGCTGCT ACTTCTCGCA TTCCCCCGGG TTATACCGAA   
  
  
+ GAATGCCTCA ATTTACTCCA GTGAGACGGT TACAGTACCA CCACTGTAGT TAAAGGACTA CGGTGTACAG   
  
  
+ TGGGAATCAA AATTTATAGG GTACAACTGT CGTTACCATA AAAAGAATAG GACTGTTTTG ACCTACTACT   
  
  
+ TTATTGCTTA TATCGGAGCT CTGAGATCCC GAAACTTCAC TTCATTTCCT CCGTTGCCGT CCCCGTAAAT   
  
  
+ CCTGTTGTTC TCGACTATCG TCGGCCCATC AATATAATAG CTTCAGGAGA GGAGGAGAAT AAAATAAGAT   
  
  
+ GACTGTACCT ATTTGTGCGT CAACCTCTAC TACAGTACAC GGGTCACTGG TCCCAAACGG TTCATTTCGT   
  
  
+ CCGTATTCCT GGCAACTTAA TACTAACTCA ATCGCACTAC CCTCTTGATT GCCCAGACTA AATCCTAGAT   
  
  
+ CGATCACGAT CACCTCTACT TAGAATGACA TTTTCGTCAC TGTTCATCCT CACCAAACAG AAAATGTTCA   
  
  
+ CCGCCTAGAG CATTAACCTC ATTTCGTCAC CCTTTGCATC TAAATCTAGA CACTGGTCAC TGATGATCGG   
  
  
+ GCTTGTTCTT AACTTCTTTT ATTTTTTAAT TTTTTTCTTT TCCCTACGAG TCTAGAGACT ACGTATTAAC   
  
  
+ CGAGATTCTT GTTGCCTATC CCGTCACTTG AACCAGTCCA CGAAACGCGT ACTCACTACT ACGAGAGTAG   
  
  
+ ACTAACCGAG GCGTCGGAGG AAGAACAGGC CTCGCTACTG AACAAGAAGT TTGATCTATT TGTGTAACAA   
  
  
+ CGTAGAGTAG TTGACAATAA TAAACCAAGG AAGACAAAAC ACAAAAAAAA AAAGGGACAC TTTGCGTTAA   
  
  
+ ACTACCTAAG AAACTTTACT CGTAATAACC TACGCCCTCG AAGTTTCATA CAGAATGTCC ATCTATAAAC   
  
  
+ GTACCGCTCC GACGTAGAGA TGAATGGTAA AACCTTATAC ATGTTTTCAA AACTTTGAAA AAGTTCAGTA   
  
  
+ ATTACCCGTA TTGCTATATC GGTTGATTAT ATGTATACAA GAAACGGCAG AAGAAGGCTC TTACAGTAGA   
  
  
+ TCGAAGAACC TTACCGTTAA CCCGTTGACG ATATATTATA AAAAACTACT ATCAAATTTG CATTACTCTA   
  
  
+ ATCCACTTAA ATGAATAACT ATAACACCT  

- TTTCTGTTTT CGTTTGTGAA ACTGTGCGTT TGTTGTATAC ACAAGTGCGG TGAAAGGAGA GTTCAGGCAA   
  
  
- AGCAAAGCCA AGAGGGGGCT TTTTATGTTT AGAAGAAAGA GTGCTCTGAA ATCACATTGC ATTTGCCTGG   
  
  
- AAGATTTATT GAACCTAGCT GGGAAAATTC CTCATCTTTT GGACTTAACA CCACCCCCAC GCGCAGCTAA   
  
  
- GGCAGCGTCC ACGCTGCTAA CGGAACACAT GTCCCCTGCT GCTGGTGGGA AGAAACGGAA TTGCACCTCA   
  
  
- AAAGTTGAAA TTTTAACGGA GGGCTTTGGC GCGTCGACGA TGAAGAGCGT AAGGGGGCCC AATATGGCTT   
  
  
- CTTACGGAGT TAAATGAGGT CACTCTGCCA ATGTCATGGT GGTGACATCA ATTTCCTGAT GCCACATGTC   
  
  
- ACCCTTAGTT TTAAATATCC CATGTTGACA GCAATGGTAT TTTTCTTATC CTGACAAAAC TGGATGATGA   
  
  
- AATAACGAAT ATAGCCTCGA GACTCTAGGG CTTTGAAGTG AAGTAAAGGA GGCAACGGCA GGGGCATTTA   
  
  
- GGACAACAAG AGCTGATAGC AGCCGGGTAG TTATATTATC GAAGTCCTCT CCTCCTCTTA TTTTATTCTA   
  
  
- CTGACATGGA TAAACACGCA GTTGGAGATG ATGTCATGTG CCCAGTGACC AGGGTTTGCC AAGTAAAGCA   
  
  
- GGCATAAGGA CCGTTGAATT ATGATTGAGT TAGCGTGATG GGAGAACTAA CGGGTCTGAT TTAGGATCTA   
  
  
- GCTAGTGCTA GTGGAGATGA ATCTTACTGT AAAAGCAGTG ACAAGTAGGA GTGGTTTGTC TTTTACAAGT   
  
  
- GGCGGATCTC GTAATTGGAG TAAAGCAGTG GGAAACGTAG ATTTAGATCT GTGACCAGTG ACTACTAGCC   
  
  
- CGAACAAGAA TTGAAGAAAA TAAAAAATTA AAAAAAGAAA AGGGATGCTC AGATCTCTGA TGCATAATTG   
  
  
- GCTCTAAGAA CAACGGATAG GGCAGTGAAC TTGGTCAGGT GCTTTGCGCA TGAGTGATGA TGCTCTCATC   
  
  
- TGATTGGCTC CGCAGCCTCC TTCTTGTCCG GAGCGATGAC TTGTTCTTCA AACTAGATAA ACACATTGTT   
  
  
- GCATCTCATC AACTGTTATT ATTTGGTTCC TTCTGTTTTG TGTTTTTTTT TTTCCCTGTG AAACGCAATT   
  
  
- TGATGGATTC TTTGAAATGA GCATTATTGG ATGCGGGAGC TTCAAAGTAT GTCTTACAGG TAGATATTTG   
  
  
- CATGGCGAGG CTGCATCTCT ACTTACCATT TTGGAATATG TACAAAAGTT TTGAAACTTT TTCAAGTCAT   
  
  
- TAATGGGCAT AACGATATAG CCAACTAATA TACATATGTT CTTTGCCGTC TTCTTCCGAG AATGTCATCT   
  
  
- AGCTTCTTGG AATGGCAATT GGGCAACTGC TATATAATAT TTTTTGATGA TAGTTTAAAC GTAATGAGAT   
  
  
- TAGGTGAATT TACTTATTGA TATTGTGGA

+     TGA-element

| Site Name | Organism | Position | Strand | Matrix score. | sequence | function |
| --- | --- | --- | --- | --- | --- | --- |
| TGA-element | Brassica oleracea | 449 | - | 6 | AACGAC | auxin-responsive element |

> 2018/04/13 10:10:12  
+ AAAGACAAAA GCAAACACTT TGACACGCAA ACAACATATG TGTTCACGCC ACTTTCCTCT CAAGTCCGTT   
  
  
+ TCGTTTCGGT TCTCCCCCGA AAAATACAAA TCTTCTTTCT CACGAGACTT TAGTGTAACG TAAACGGACC   
  
  
+ TTCTAAATAA CTTGGATCGA CCCTTTTAAG GAGTAGAAAA CCTGAATTGT GGTGGGGGTG CGCGTCGATT   
  
  
+ CCGTCGCAGG TGCGACGATT GCCTTGTGTA CAGGGGACGA CGACCACCCT TCTTTGCCTT AACGTGGAGT   
  
  
+ TTTCAACTTT AAAATTGCCT CCCGAAACCG CGCAGCTGCT ACTTCTCGCA TTCCCCCGGG TTATACCGAA   
  
  
+ GAATGCCTCA ATTTACTCCA GTGAGACGGT TACAGTACCA CCACTGTAGT TAAAGGACTA CGGTGTACAG   
  
  
+ TGGGAATCAA AATTTATAGG GTACAACTGT CGTTACCATA AAAAGAATAG GACTGTTTTG ACCTACTACT   
  
  
+ TTATTGCTTA TATCGGAGCT CTGAGATCCC GAAACTTCAC TTCATTTCCT CCGTTGCCGT CCCCGTAAAT   
  
  
+ CCTGTTGTTC TCGACTATCG TCGGCCCATC AATATAATAG CTTCAGGAGA GGAGGAGAAT AAAATAAGAT   
  
  
+ GACTGTACCT ATTTGTGCGT CAACCTCTAC TACAGTACAC GGGTCACTGG TCCCAAACGG TTCATTTCGT   
  
  
+ CCGTATTCCT GGCAACTTAA TACTAACTCA ATCGCACTAC CCTCTTGATT GCCCAGACTA AATCCTAGAT   
  
  
+ CGATCACGAT CACCTCTACT TAGAATGACA TTTTCGTCAC TGTTCATCCT CACCAAACAG AAAATGTTCA   
  
  
+ CCGCCTAGAG CATTAACCTC ATTTCGTCAC CCTTTGCATC TAAATCTAGA CACTGGTCAC TGATGATCGG   
  
  
+ GCTTGTTCTT AACTTCTTTT ATTTTTTAAT TTTTTTCTTT TCCCTACGAG TCTAGAGACT ACGTATTAAC   
  
  
+ CGAGATTCTT GTTGCCTATC CCGTCACTTG AACCAGTCCA CGAAACGCGT ACTCACTACT ACGAGAGTAG   
  
  
+ ACTAACCGAG GCGTCGGAGG AAGAACAGGC CTCGCTACTG AACAAGAAGT TTGATCTATT TGTGTAACAA   
  
  
+ CGTAGAGTAG TTGACAATAA TAAACCAAGG AAGACAAAAC ACAAAAAAAA AAAGGGACAC TTTGCGTTAA   
  
  
+ ACTACCTAAG AAACTTTACT CGTAATAACC TACGCCCTCG AAGTTTCATA CAGAATGTCC ATCTATAAAC   
  
  
+ GTACCGCTCC GACGTAGAGA TGAATGGTAA AACCTTATAC ATGTTTTCAA AACTTTGAAA AAGTTCAGTA   
  
  
+ ATTACCCGTA TTGCTATATC GGTTGATTAT ATGTATACAA GAAACGGCAG AAGAAGGCTC TTACAGTAGA   
  
  
+ TCGAAGAACC TTACCGTTAA CCCGTTGACG ATATATTATA AAAAACTACT ATCAAATTTG CATTACTCTA   
  
  
+ ATCCACTTAA ATGAATAACT ATAACACCT  

- TTTCTGTTTT CGTTTGTGAA ACTGTGCGTT TGTTGTATAC ACAAGTGCGG TGAAAGGAGA GTTCAGGCAA   
  
  
- AGCAAAGCCA AGAGGGGGCT TTTTATGTTT AGAAGAAAGA GTGCTCTGAA ATCACATTGC ATTTGCCTGG   
  
  
- AAGATTTATT GAACCTAGCT GGGAAAATTC CTCATCTTTT GGACTTAACA CCACCCCCAC GCGCAGCTAA   
  
  
- GGCAGCGTCC ACGCTGCTAA CGGAACACAT GTCCCCTGCT GCTGGTGGGA AGAAACGGAA TTGCACCTCA   
  
  
- AAAGTTGAAA TTTTAACGGA GGGCTTTGGC GCGTCGACGA TGAAGAGCGT AAGGGGGCCC AATATGGCTT   
  
  
- CTTACGGAGT TAAATGAGGT CACTCTGCCA ATGTCATGGT GGTGACATCA ATTTCCTGAT GCCACATGTC   
  
  
- ACCCTTAGTT TTAAATATCC CATGTTGACA GCAATGGTAT TTTTCTTATC CTGACAAAAC TGGATGATGA   
  
  
- AATAACGAAT ATAGCCTCGA GACTCTAGGG CTTTGAAGTG AAGTAAAGGA GGCAACGGCA GGGGCATTTA   
  
  
- GGACAACAAG AGCTGATAGC AGCCGGGTAG TTATATTATC GAAGTCCTCT CCTCCTCTTA TTTTATTCTA   
  
  
- CTGACATGGA TAAACACGCA GTTGGAGATG ATGTCATGTG CCCAGTGACC AGGGTTTGCC AAGTAAAGCA   
  
  
- GGCATAAGGA CCGTTGAATT ATGATTGAGT TAGCGTGATG GGAGAACTAA CGGGTCTGAT TTAGGATCTA   
  
  
- GCTAGTGCTA GTGGAGATGA ATCTTACTGT AAAAGCAGTG ACAAGTAGGA GTGGTTTGTC TTTTACAAGT   
  
  
- GGCGGATCTC GTAATTGGAG TAAAGCAGTG GGAAACGTAG ATTTAGATCT GTGACCAGTG ACTACTAGCC   
  
  
- CGAACAAGAA TTGAAGAAAA TAAAAAATTA AAAAAAGAAA AGGGATGCTC AGATCTCTGA TGCATAATTG   
  
  
- GCTCTAAGAA CAACGGATAG GGCAGTGAAC TTGGTCAGGT GCTTTGCGCA TGAGTGATGA TGCTCTCATC   
  
  
- TGATTGGCTC CGCAGCCTCC TTCTTGTCCG GAGCGATGAC TTGTTCTTCA AACTAGATAA ACACATTGTT   
  
  
- GCATCTCATC AACTGTTATT ATTTGGTTCC TTCTGTTTTG TGTTTTTTTT TTTCCCTGTG AAACGCAATT   
  
  
- TGATGGATTC TTTGAAATGA GCATTATTGG ATGCGGGAGC TTCAAAGTAT GTCTTACAGG TAGATATTTG   
  
  
- CATGGCGAGG CTGCATCTCT ACTTACCATT TTGGAATATG TACAAAAGTT TTGAAACTTT TTCAAGTCAT   
  
  
- TAATGGGCAT AACGATATAG CCAACTAATA TACATATGTT CTTTGCCGTC TTCTTCCGAG AATGTCATCT   
  
  
- AGCTTCTTGG AATGGCAATT GGGCAACTGC TATATAATAT TTTTTGATGA TAGTTTAAAC GTAATGAGAT   
  
  
- TAGGTGAATT TACTTATTGA TATTGTGGA

+     TGACG-motif

| Site Name | Organism | Position | Strand | Matrix score. | sequence | function |
| --- | --- | --- | --- | --- | --- | --- |
| TGACG-motif | Hordeum vulgare | 1426 | + | 5 | TGACG | cis-acting regulatory element involved in the MeJA-responsiveness |
| TGACG-motif | Hordeum vulgare | 1002 | - | 5 | TGACG | cis-acting regulatory element involved in the MeJA-responsiveness |
| TGACG-motif | Hordeum vulgare | 865 | - | 5 | TGACG | cis-acting regulatory element involved in the MeJA-responsiveness |
| TGACG-motif | Hordeum vulgare | 805 | - | 5 | TGACG | cis-acting regulatory element involved in the MeJA-responsiveness |
| TGACG-motif | Hordeum vulgare | 648 | - | 5 | TGACG | cis-acting regulatory element involved in the MeJA-responsiveness |

> 2018/04/13 10:10:12  
+ AAAGACAAAA GCAAACACTT TGACACGCAA ACAACATATG TGTTCACGCC ACTTTCCTCT CAAGTCCGTT   
  
  
+ TCGTTTCGGT TCTCCCCCGA AAAATACAAA TCTTCTTTCT CACGAGACTT TAGTGTAACG TAAACGGACC   
  
  
+ TTCTAAATAA CTTGGATCGA CCCTTTTAAG GAGTAGAAAA CCTGAATTGT GGTGGGGGTG CGCGTCGATT   
  
  
+ CCGTCGCAGG TGCGACGATT GCCTTGTGTA CAGGGGACGA CGACCACCCT TCTTTGCCTT AACGTGGAGT   
  
  
+ TTTCAACTTT AAAATTGCCT CCCGAAACCG CGCAGCTGCT ACTTCTCGCA TTCCCCCGGG TTATACCGAA   
  
  
+ GAATGCCTCA ATTTACTCCA GTGAGACGGT TACAGTACCA CCACTGTAGT TAAAGGACTA CGGTGTACAG   
  
  
+ TGGGAATCAA AATTTATAGG GTACAACTGT CGTTACCATA AAAAGAATAG GACTGTTTTG ACCTACTACT   
  
  
+ TTATTGCTTA TATCGGAGCT CTGAGATCCC GAAACTTCAC TTCATTTCCT CCGTTGCCGT CCCCGTAAAT   
  
  
+ CCTGTTGTTC TCGACTATCG TCGGCCCATC AATATAATAG CTTCAGGAGA GGAGGAGAAT AAAATAAGAT   
  
  
+ GACTGTACCT ATTTGTGCGT CAACCTCTAC TACAGTACAC GGGTCACTGG TCCCAAACGG TTCATTTCGT   
  
  
+ CCGTATTCCT GGCAACTTAA TACTAACTCA ATCGCACTAC CCTCTTGATT GCCCAGACTA AATCCTAGAT   
  
  
+ CGATCACGAT CACCTCTACT TAGAATGACA TTTTCGTCAC TGTTCATCCT CACCAAACAG AAAATGTTCA   
  
  
+ CCGCCTAGAG CATTAACCTC ATTTCGTCAC CCTTTGCATC TAAATCTAGA CACTGGTCAC TGATGATCGG   
  
  
+ GCTTGTTCTT AACTTCTTTT ATTTTTTAAT TTTTTTCTTT TCCCTACGAG TCTAGAGACT ACGTATTAAC   
  
  
+ CGAGATTCTT GTTGCCTATC CCGTCACTTG AACCAGTCCA CGAAACGCGT ACTCACTACT ACGAGAGTAG   
  
  
+ ACTAACCGAG GCGTCGGAGG AAGAACAGGC CTCGCTACTG AACAAGAAGT TTGATCTATT TGTGTAACAA   
  
  
+ CGTAGAGTAG TTGACAATAA TAAACCAAGG AAGACAAAAC ACAAAAAAAA AAAGGGACAC TTTGCGTTAA   
  
  
+ ACTACCTAAG AAACTTTACT CGTAATAACC TACGCCCTCG AAGTTTCATA CAGAATGTCC ATCTATAAAC   
  
  
+ GTACCGCTCC GACGTAGAGA TGAATGGTAA AACCTTATAC ATGTTTTCAA AACTTTGAAA AAGTTCAGTA   
  
  
+ ATTACCCGTA TTGCTATATC GGTTGATTAT ATGTATACAA GAAACGGCAG AAGAAGGCTC TTACAGTAGA   
  
  
+ TCGAAGAACC TTACCGTTAA CCCGTTGACG ATATATTATA AAAAACTACT ATCAAATTTG CATTACTCTA   
  
  
+ ATCCACTTAA ATGAATAACT ATAACACCT  

- TTTCTGTTTT CGTTTGTGAA ACTGTGCGTT TGTTGTATAC ACAAGTGCGG TGAAAGGAGA GTTCAGGCAA   
  
  
- AGCAAAGCCA AGAGGGGGCT TTTTATGTTT AGAAGAAAGA GTGCTCTGAA ATCACATTGC ATTTGCCTGG   
  
  
- AAGATTTATT GAACCTAGCT GGGAAAATTC CTCATCTTTT GGACTTAACA CCACCCCCAC GCGCAGCTAA   
  
  
- GGCAGCGTCC ACGCTGCTAA CGGAACACAT GTCCCCTGCT GCTGGTGGGA AGAAACGGAA TTGCACCTCA   
  
  
- AAAGTTGAAA TTTTAACGGA GGGCTTTGGC GCGTCGACGA TGAAGAGCGT AAGGGGGCCC AATATGGCTT   
  
  
- CTTACGGAGT TAAATGAGGT CACTCTGCCA ATGTCATGGT GGTGACATCA ATTTCCTGAT GCCACATGTC   
  
  
- ACCCTTAGTT TTAAATATCC CATGTTGACA GCAATGGTAT TTTTCTTATC CTGACAAAAC TGGATGATGA   
  
  
- AATAACGAAT ATAGCCTCGA GACTCTAGGG CTTTGAAGTG AAGTAAAGGA GGCAACGGCA GGGGCATTTA   
  
  
- GGACAACAAG AGCTGATAGC AGCCGGGTAG TTATATTATC GAAGTCCTCT CCTCCTCTTA TTTTATTCTA   
  
  
- CTGACATGGA TAAACACGCA GTTGGAGATG ATGTCATGTG CCCAGTGACC AGGGTTTGCC AAGTAAAGCA   
  
  
- GGCATAAGGA CCGTTGAATT ATGATTGAGT TAGCGTGATG GGAGAACTAA CGGGTCTGAT TTAGGATCTA   
  
  
- GCTAGTGCTA GTGGAGATGA ATCTTACTGT AAAAGCAGTG ACAAGTAGGA GTGGTTTGTC TTTTACAAGT   
  
  
- GGCGGATCTC GTAATTGGAG TAAAGCAGTG GGAAACGTAG ATTTAGATCT GTGACCAGTG ACTACTAGCC   
  
  
- CGAACAAGAA TTGAAGAAAA TAAAAAATTA AAAAAAGAAA AGGGATGCTC AGATCTCTGA TGCATAATTG   
  
  
- GCTCTAAGAA CAACGGATAG GGCAGTGAAC TTGGTCAGGT GCTTTGCGCA TGAGTGATGA TGCTCTCATC   
  
  
- TGATTGGCTC CGCAGCCTCC TTCTTGTCCG GAGCGATGAC TTGTTCTTCA AACTAGATAA ACACATTGTT   
  
  
- GCATCTCATC AACTGTTATT ATTTGGTTCC TTCTGTTTTG TGTTTTTTTT TTTCCCTGTG AAACGCAATT   
  
  
- TGATGGATTC TTTGAAATGA GCATTATTGG ATGCGGGAGC TTCAAAGTAT GTCTTACAGG TAGATATTTG   
  
  
- CATGGCGAGG CTGCATCTCT ACTTACCATT TTGGAATATG TACAAAAGTT TTGAAACTTT TTCAAGTCAT   
  
  
- TAATGGGCAT AACGATATAG CCAACTAATA TACATATGTT CTTTGCCGTC TTCTTCCGAG AATGTCATCT   
  
  
- AGCTTCTTGG AATGGCAATT GGGCAACTGC TATATAATAT TTTTTGATGA TAGTTTAAAC GTAATGAGAT   
  
  
- TAGGTGAATT TACTTATTGA TATTGTGGA

+     Unnamed\_\_1

| Site Name | Organism | Position | Strand | Matrix score. | sequence | function |
| --- | --- | --- | --- | --- | --- | --- |
| Unnamed\_\_1 | Zea mays | 1018 | - | 5 | CGTGG |  |
| Unnamed\_\_1 | Zea mays | 273 | + | 5 | CGTGG |  |

> 2018/04/13 10:10:12  
+ AAAGACAAAA GCAAACACTT TGACACGCAA ACAACATATG TGTTCACGCC ACTTTCCTCT CAAGTCCGTT   
  
  
+ TCGTTTCGGT TCTCCCCCGA AAAATACAAA TCTTCTTTCT CACGAGACTT TAGTGTAACG TAAACGGACC   
  
  
+ TTCTAAATAA CTTGGATCGA CCCTTTTAAG GAGTAGAAAA CCTGAATTGT GGTGGGGGTG CGCGTCGATT   
  
  
+ CCGTCGCAGG TGCGACGATT GCCTTGTGTA CAGGGGACGA CGACCACCCT TCTTTGCCTT AACGTGGAGT   
  
  
+ TTTCAACTTT AAAATTGCCT CCCGAAACCG CGCAGCTGCT ACTTCTCGCA TTCCCCCGGG TTATACCGAA   
  
  
+ GAATGCCTCA ATTTACTCCA GTGAGACGGT TACAGTACCA CCACTGTAGT TAAAGGACTA CGGTGTACAG   
  
  
+ TGGGAATCAA AATTTATAGG GTACAACTGT CGTTACCATA AAAAGAATAG GACTGTTTTG ACCTACTACT   
  
  
+ TTATTGCTTA TATCGGAGCT CTGAGATCCC GAAACTTCAC TTCATTTCCT CCGTTGCCGT CCCCGTAAAT   
  
  
+ CCTGTTGTTC TCGACTATCG TCGGCCCATC AATATAATAG CTTCAGGAGA GGAGGAGAAT AAAATAAGAT   
  
  
+ GACTGTACCT ATTTGTGCGT CAACCTCTAC TACAGTACAC GGGTCACTGG TCCCAAACGG TTCATTTCGT   
  
  
+ CCGTATTCCT GGCAACTTAA TACTAACTCA ATCGCACTAC CCTCTTGATT GCCCAGACTA AATCCTAGAT   
  
  
+ CGATCACGAT CACCTCTACT TAGAATGACA TTTTCGTCAC TGTTCATCCT CACCAAACAG AAAATGTTCA   
  
  
+ CCGCCTAGAG CATTAACCTC ATTTCGTCAC CCTTTGCATC TAAATCTAGA CACTGGTCAC TGATGATCGG   
  
  
+ GCTTGTTCTT AACTTCTTTT ATTTTTTAAT TTTTTTCTTT TCCCTACGAG TCTAGAGACT ACGTATTAAC   
  
  
+ CGAGATTCTT GTTGCCTATC CCGTCACTTG AACCAGTCCA CGAAACGCGT ACTCACTACT ACGAGAGTAG   
  
  
+ ACTAACCGAG GCGTCGGAGG AAGAACAGGC CTCGCTACTG AACAAGAAGT TTGATCTATT TGTGTAACAA   
  
  
+ CGTAGAGTAG TTGACAATAA TAAACCAAGG AAGACAAAAC ACAAAAAAAA AAAGGGACAC TTTGCGTTAA   
  
  
+ ACTACCTAAG AAACTTTACT CGTAATAACC TACGCCCTCG AAGTTTCATA CAGAATGTCC ATCTATAAAC   
  
  
+ GTACCGCTCC GACGTAGAGA TGAATGGTAA AACCTTATAC ATGTTTTCAA AACTTTGAAA AAGTTCAGTA   
  
  
+ ATTACCCGTA TTGCTATATC GGTTGATTAT ATGTATACAA GAAACGGCAG AAGAAGGCTC TTACAGTAGA   
  
  
+ TCGAAGAACC TTACCGTTAA CCCGTTGACG ATATATTATA AAAAACTACT ATCAAATTTG CATTACTCTA   
  
  
+ ATCCACTTAA ATGAATAACT ATAACACCT  

- TTTCTGTTTT CGTTTGTGAA ACTGTGCGTT TGTTGTATAC ACAAGTGCGG TGAAAGGAGA GTTCAGGCAA   
  
  
- AGCAAAGCCA AGAGGGGGCT TTTTATGTTT AGAAGAAAGA GTGCTCTGAA ATCACATTGC ATTTGCCTGG   
  
  
- AAGATTTATT GAACCTAGCT GGGAAAATTC CTCATCTTTT GGACTTAACA CCACCCCCAC GCGCAGCTAA   
  
  
- GGCAGCGTCC ACGCTGCTAA CGGAACACAT GTCCCCTGCT GCTGGTGGGA AGAAACGGAA TTGCACCTCA   
  
  
- AAAGTTGAAA TTTTAACGGA GGGCTTTGGC GCGTCGACGA TGAAGAGCGT AAGGGGGCCC AATATGGCTT   
  
  
- CTTACGGAGT TAAATGAGGT CACTCTGCCA ATGTCATGGT GGTGACATCA ATTTCCTGAT GCCACATGTC   
  
  
- ACCCTTAGTT TTAAATATCC CATGTTGACA GCAATGGTAT TTTTCTTATC CTGACAAAAC TGGATGATGA   
  
  
- AATAACGAAT ATAGCCTCGA GACTCTAGGG CTTTGAAGTG AAGTAAAGGA GGCAACGGCA GGGGCATTTA   
  
  
- GGACAACAAG AGCTGATAGC AGCCGGGTAG TTATATTATC GAAGTCCTCT CCTCCTCTTA TTTTATTCTA   
  
  
- CTGACATGGA TAAACACGCA GTTGGAGATG ATGTCATGTG CCCAGTGACC AGGGTTTGCC AAGTAAAGCA   
  
  
- GGCATAAGGA CCGTTGAATT ATGATTGAGT TAGCGTGATG GGAGAACTAA CGGGTCTGAT TTAGGATCTA   
  
  
- GCTAGTGCTA GTGGAGATGA ATCTTACTGT AAAAGCAGTG ACAAGTAGGA GTGGTTTGTC TTTTACAAGT   
  
  
- GGCGGATCTC GTAATTGGAG TAAAGCAGTG GGAAACGTAG ATTTAGATCT GTGACCAGTG ACTACTAGCC   
  
  
- CGAACAAGAA TTGAAGAAAA TAAAAAATTA AAAAAAGAAA AGGGATGCTC AGATCTCTGA TGCATAATTG   
  
  
- GCTCTAAGAA CAACGGATAG GGCAGTGAAC TTGGTCAGGT GCTTTGCGCA TGAGTGATGA TGCTCTCATC   
  
  
- TGATTGGCTC CGCAGCCTCC TTCTTGTCCG GAGCGATGAC TTGTTCTTCA AACTAGATAA ACACATTGTT   
  
  
- GCATCTCATC AACTGTTATT ATTTGGTTCC TTCTGTTTTG TGTTTTTTTT TTTCCCTGTG AAACGCAATT   
  
  
- TGATGGATTC TTTGAAATGA GCATTATTGG ATGCGGGAGC TTCAAAGTAT GTCTTACAGG TAGATATTTG   
  
  
- CATGGCGAGG CTGCATCTCT ACTTACCATT TTGGAATATG TACAAAAGTT TTGAAACTTT TTCAAGTCAT   
  
  
- TAATGGGCAT AACGATATAG CCAACTAATA TACATATGTT CTTTGCCGTC TTCTTCCGAG AATGTCATCT   
  
  
- AGCTTCTTGG AATGGCAATT GGGCAACTGC TATATAATAT TTTTTGATGA TAGTTTAAAC GTAATGAGAT   
  
  
- TAGGTGAATT TACTTATTGA TATTGTGGA

+     Unnamed\_\_15

| Site Name | Organism | Position | Strand | Matrix score. | sequence | function |
| --- | --- | --- | --- | --- | --- | --- |
| Unnamed\_\_15 | Zea mays | 995 | + | 10 | CCTCTCCCGTC |  |

> 2018/04/13 10:10:12  
+ AAAGACAAAA GCAAACACTT TGACACGCAA ACAACATATG TGTTCACGCC ACTTTCCTCT CAAGTCCGTT   
  
  
+ TCGTTTCGGT TCTCCCCCGA AAAATACAAA TCTTCTTTCT CACGAGACTT TAGTGTAACG TAAACGGACC   
  
  
+ TTCTAAATAA CTTGGATCGA CCCTTTTAAG GAGTAGAAAA CCTGAATTGT GGTGGGGGTG CGCGTCGATT   
  
  
+ CCGTCGCAGG TGCGACGATT GCCTTGTGTA CAGGGGACGA CGACCACCCT TCTTTGCCTT AACGTGGAGT   
  
  
+ TTTCAACTTT AAAATTGCCT CCCGAAACCG CGCAGCTGCT ACTTCTCGCA TTCCCCCGGG TTATACCGAA   
  
  
+ GAATGCCTCA ATTTACTCCA GTGAGACGGT TACAGTACCA CCACTGTAGT TAAAGGACTA CGGTGTACAG   
  
  
+ TGGGAATCAA AATTTATAGG GTACAACTGT CGTTACCATA AAAAGAATAG GACTGTTTTG ACCTACTACT   
  
  
+ TTATTGCTTA TATCGGAGCT CTGAGATCCC GAAACTTCAC TTCATTTCCT CCGTTGCCGT CCCCGTAAAT   
  
  
+ CCTGTTGTTC TCGACTATCG TCGGCCCATC AATATAATAG CTTCAGGAGA GGAGGAGAAT AAAATAAGAT   
  
  
+ GACTGTACCT ATTTGTGCGT CAACCTCTAC TACAGTACAC GGGTCACTGG TCCCAAACGG TTCATTTCGT   
  
  
+ CCGTATTCCT GGCAACTTAA TACTAACTCA ATCGCACTAC CCTCTTGATT GCCCAGACTA AATCCTAGAT   
  
  
+ CGATCACGAT CACCTCTACT TAGAATGACA TTTTCGTCAC TGTTCATCCT CACCAAACAG AAAATGTTCA   
  
  
+ CCGCCTAGAG CATTAACCTC ATTTCGTCAC CCTTTGCATC TAAATCTAGA CACTGGTCAC TGATGATCGG   
  
  
+ GCTTGTTCTT AACTTCTTTT ATTTTTTAAT TTTTTTCTTT TCCCTACGAG TCTAGAGACT ACGTATTAAC   
  
  
+ CGAGATTCTT GTTGCCTATC CCGTCACTTG AACCAGTCCA CGAAACGCGT ACTCACTACT ACGAGAGTAG   
  
  
+ ACTAACCGAG GCGTCGGAGG AAGAACAGGC CTCGCTACTG AACAAGAAGT TTGATCTATT TGTGTAACAA   
  
  
+ CGTAGAGTAG TTGACAATAA TAAACCAAGG AAGACAAAAC ACAAAAAAAA AAAGGGACAC TTTGCGTTAA   
  
  
+ ACTACCTAAG AAACTTTACT CGTAATAACC TACGCCCTCG AAGTTTCATA CAGAATGTCC ATCTATAAAC   
  
  
+ GTACCGCTCC GACGTAGAGA TGAATGGTAA AACCTTATAC ATGTTTTCAA AACTTTGAAA AAGTTCAGTA   
  
  
+ ATTACCCGTA TTGCTATATC GGTTGATTAT ATGTATACAA GAAACGGCAG AAGAAGGCTC TTACAGTAGA   
  
  
+ TCGAAGAACC TTACCGTTAA CCCGTTGACG ATATATTATA AAAAACTACT ATCAAATTTG CATTACTCTA   
  
  
+ ATCCACTTAA ATGAATAACT ATAACACCT  

- TTTCTGTTTT CGTTTGTGAA ACTGTGCGTT TGTTGTATAC ACAAGTGCGG TGAAAGGAGA GTTCAGGCAA   
  
  
- AGCAAAGCCA AGAGGGGGCT TTTTATGTTT AGAAGAAAGA GTGCTCTGAA ATCACATTGC ATTTGCCTGG   
  
  
- AAGATTTATT GAACCTAGCT GGGAAAATTC CTCATCTTTT GGACTTAACA CCACCCCCAC GCGCAGCTAA   
  
  
- GGCAGCGTCC ACGCTGCTAA CGGAACACAT GTCCCCTGCT GCTGGTGGGA AGAAACGGAA TTGCACCTCA   
  
  
- AAAGTTGAAA TTTTAACGGA GGGCTTTGGC GCGTCGACGA TGAAGAGCGT AAGGGGGCCC AATATGGCTT   
  
  
- CTTACGGAGT TAAATGAGGT CACTCTGCCA ATGTCATGGT GGTGACATCA ATTTCCTGAT GCCACATGTC   
  
  
- ACCCTTAGTT TTAAATATCC CATGTTGACA GCAATGGTAT TTTTCTTATC CTGACAAAAC TGGATGATGA   
  
  
- AATAACGAAT ATAGCCTCGA GACTCTAGGG CTTTGAAGTG AAGTAAAGGA GGCAACGGCA GGGGCATTTA   
  
  
- GGACAACAAG AGCTGATAGC AGCCGGGTAG TTATATTATC GAAGTCCTCT CCTCCTCTTA TTTTATTCTA   
  
  
- CTGACATGGA TAAACACGCA GTTGGAGATG ATGTCATGTG CCCAGTGACC AGGGTTTGCC AAGTAAAGCA   
  
  
- GGCATAAGGA CCGTTGAATT ATGATTGAGT TAGCGTGATG GGAGAACTAA CGGGTCTGAT TTAGGATCTA   
  
  
- GCTAGTGCTA GTGGAGATGA ATCTTACTGT AAAAGCAGTG ACAAGTAGGA GTGGTTTGTC TTTTACAAGT   
  
  
- GGCGGATCTC GTAATTGGAG TAAAGCAGTG GGAAACGTAG ATTTAGATCT GTGACCAGTG ACTACTAGCC   
  
  
- CGAACAAGAA TTGAAGAAAA TAAAAAATTA AAAAAAGAAA AGGGATGCTC AGATCTCTGA TGCATAATTG   
  
  
- GCTCTAAGAA CAACGGATAG GGCAGTGAAC TTGGTCAGGT GCTTTGCGCA TGAGTGATGA TGCTCTCATC   
  
  
- TGATTGGCTC CGCAGCCTCC TTCTTGTCCG GAGCGATGAC TTGTTCTTCA AACTAGATAA ACACATTGTT   
  
  
- GCATCTCATC AACTGTTATT ATTTGGTTCC TTCTGTTTTG TGTTTTTTTT TTTCCCTGTG AAACGCAATT   
  
  
- TGATGGATTC TTTGAAATGA GCATTATTGG ATGCGGGAGC TTCAAAGTAT GTCTTACAGG TAGATATTTG   
  
  
- CATGGCGAGG CTGCATCTCT ACTTACCATT TTGGAATATG TACAAAAGTT TTGAAACTTT TTCAAGTCAT   
  
  
- TAATGGGCAT AACGATATAG CCAACTAATA TACATATGTT CTTTGCCGTC TTCTTCCGAG AATGTCATCT   
  
  
- AGCTTCTTGG AATGGCAATT GGGCAACTGC TATATAATAT TTTTTGATGA TAGTTTAAAC GTAATGAGAT   
  
  
- TAGGTGAATT TACTTATTGA TATTGTGGA

+     Unnamed\_\_2

| Site Name | Organism | Position | Strand | Matrix score. | sequence | function |
| --- | --- | --- | --- | --- | --- | --- |
| Unnamed\_\_2 | Zea mays | 334 | + | 6 | CCCCGG |  |

> 2018/04/13 10:10:12  
+ AAAGACAAAA GCAAACACTT TGACACGCAA ACAACATATG TGTTCACGCC ACTTTCCTCT CAAGTCCGTT   
  
  
+ TCGTTTCGGT TCTCCCCCGA AAAATACAAA TCTTCTTTCT CACGAGACTT TAGTGTAACG TAAACGGACC   
  
  
+ TTCTAAATAA CTTGGATCGA CCCTTTTAAG GAGTAGAAAA CCTGAATTGT GGTGGGGGTG CGCGTCGATT   
  
  
+ CCGTCGCAGG TGCGACGATT GCCTTGTGTA CAGGGGACGA CGACCACCCT TCTTTGCCTT AACGTGGAGT   
  
  
+ TTTCAACTTT AAAATTGCCT CCCGAAACCG CGCAGCTGCT ACTTCTCGCA TTCCCCCGGG TTATACCGAA   
  
  
+ GAATGCCTCA ATTTACTCCA GTGAGACGGT TACAGTACCA CCACTGTAGT TAAAGGACTA CGGTGTACAG   
  
  
+ TGGGAATCAA AATTTATAGG GTACAACTGT CGTTACCATA AAAAGAATAG GACTGTTTTG ACCTACTACT   
  
  
+ TTATTGCTTA TATCGGAGCT CTGAGATCCC GAAACTTCAC TTCATTTCCT CCGTTGCCGT CCCCGTAAAT   
  
  
+ CCTGTTGTTC TCGACTATCG TCGGCCCATC AATATAATAG CTTCAGGAGA GGAGGAGAAT AAAATAAGAT   
  
  
+ GACTGTACCT ATTTGTGCGT CAACCTCTAC TACAGTACAC GGGTCACTGG TCCCAAACGG TTCATTTCGT   
  
  
+ CCGTATTCCT GGCAACTTAA TACTAACTCA ATCGCACTAC CCTCTTGATT GCCCAGACTA AATCCTAGAT   
  
  
+ CGATCACGAT CACCTCTACT TAGAATGACA TTTTCGTCAC TGTTCATCCT CACCAAACAG AAAATGTTCA   
  
  
+ CCGCCTAGAG CATTAACCTC ATTTCGTCAC CCTTTGCATC TAAATCTAGA CACTGGTCAC TGATGATCGG   
  
  
+ GCTTGTTCTT AACTTCTTTT ATTTTTTAAT TTTTTTCTTT TCCCTACGAG TCTAGAGACT ACGTATTAAC   
  
  
+ CGAGATTCTT GTTGCCTATC CCGTCACTTG AACCAGTCCA CGAAACGCGT ACTCACTACT ACGAGAGTAG   
  
  
+ ACTAACCGAG GCGTCGGAGG AAGAACAGGC CTCGCTACTG AACAAGAAGT TTGATCTATT TGTGTAACAA   
  
  
+ CGTAGAGTAG TTGACAATAA TAAACCAAGG AAGACAAAAC ACAAAAAAAA AAAGGGACAC TTTGCGTTAA   
  
  
+ ACTACCTAAG AAACTTTACT CGTAATAACC TACGCCCTCG AAGTTTCATA CAGAATGTCC ATCTATAAAC   
  
  
+ GTACCGCTCC GACGTAGAGA TGAATGGTAA AACCTTATAC ATGTTTTCAA AACTTTGAAA AAGTTCAGTA   
  
  
+ ATTACCCGTA TTGCTATATC GGTTGATTAT ATGTATACAA GAAACGGCAG AAGAAGGCTC TTACAGTAGA   
  
  
+ TCGAAGAACC TTACCGTTAA CCCGTTGACG ATATATTATA AAAAACTACT ATCAAATTTG CATTACTCTA   
  
  
+ ATCCACTTAA ATGAATAACT ATAACACCT  

- TTTCTGTTTT CGTTTGTGAA ACTGTGCGTT TGTTGTATAC ACAAGTGCGG TGAAAGGAGA GTTCAGGCAA   
  
  
- AGCAAAGCCA AGAGGGGGCT TTTTATGTTT AGAAGAAAGA GTGCTCTGAA ATCACATTGC ATTTGCCTGG   
  
  
- AAGATTTATT GAACCTAGCT GGGAAAATTC CTCATCTTTT GGACTTAACA CCACCCCCAC GCGCAGCTAA   
  
  
- GGCAGCGTCC ACGCTGCTAA CGGAACACAT GTCCCCTGCT GCTGGTGGGA AGAAACGGAA TTGCACCTCA   
  
  
- AAAGTTGAAA TTTTAACGGA GGGCTTTGGC GCGTCGACGA TGAAGAGCGT AAGGGGGCCC AATATGGCTT   
  
  
- CTTACGGAGT TAAATGAGGT CACTCTGCCA ATGTCATGGT GGTGACATCA ATTTCCTGAT GCCACATGTC   
  
  
- ACCCTTAGTT TTAAATATCC CATGTTGACA GCAATGGTAT TTTTCTTATC CTGACAAAAC TGGATGATGA   
  
  
- AATAACGAAT ATAGCCTCGA GACTCTAGGG CTTTGAAGTG AAGTAAAGGA GGCAACGGCA GGGGCATTTA   
  
  
- GGACAACAAG AGCTGATAGC AGCCGGGTAG TTATATTATC GAAGTCCTCT CCTCCTCTTA TTTTATTCTA   
  
  
- CTGACATGGA TAAACACGCA GTTGGAGATG ATGTCATGTG CCCAGTGACC AGGGTTTGCC AAGTAAAGCA   
  
  
- GGCATAAGGA CCGTTGAATT ATGATTGAGT TAGCGTGATG GGAGAACTAA CGGGTCTGAT TTAGGATCTA   
  
  
- GCTAGTGCTA GTGGAGATGA ATCTTACTGT AAAAGCAGTG ACAAGTAGGA GTGGTTTGTC TTTTACAAGT   
  
  
- GGCGGATCTC GTAATTGGAG TAAAGCAGTG GGAAACGTAG ATTTAGATCT GTGACCAGTG ACTACTAGCC   
  
  
- CGAACAAGAA TTGAAGAAAA TAAAAAATTA AAAAAAGAAA AGGGATGCTC AGATCTCTGA TGCATAATTG   
  
  
- GCTCTAAGAA CAACGGATAG GGCAGTGAAC TTGGTCAGGT GCTTTGCGCA TGAGTGATGA TGCTCTCATC   
  
  
- TGATTGGCTC CGCAGCCTCC TTCTTGTCCG GAGCGATGAC TTGTTCTTCA AACTAGATAA ACACATTGTT   
  
  
- GCATCTCATC AACTGTTATT ATTTGGTTCC TTCTGTTTTG TGTTTTTTTT TTTCCCTGTG AAACGCAATT   
  
  
- TGATGGATTC TTTGAAATGA GCATTATTGG ATGCGGGAGC TTCAAAGTAT GTCTTACAGG TAGATATTTG   
  
  
- CATGGCGAGG CTGCATCTCT ACTTACCATT TTGGAATATG TACAAAAGTT TTGAAACTTT TTCAAGTCAT   
  
  
- TAATGGGCAT AACGATATAG CCAACTAATA TACATATGTT CTTTGCCGTC TTCTTCCGAG AATGTCATCT   
  
  
- AGCTTCTTGG AATGGCAATT GGGCAACTGC TATATAATAT TTTTTGATGA TAGTTTAAAC GTAATGAGAT   
  
  
- TAGGTGAATT TACTTATTGA TATTGTGGA

+     Unnamed\_\_3

| Site Name | Organism | Position | Strand | Matrix score. | sequence | function |
| --- | --- | --- | --- | --- | --- | --- |
| Unnamed\_\_3 | Zea mays | 1018 | - | 5 | CGTGG |  |
| Unnamed\_\_3 | Zea mays | 273 | + | 5 | CGTGG |  |

> 2018/04/13 10:10:12  
+ AAAGACAAAA GCAAACACTT TGACACGCAA ACAACATATG TGTTCACGCC ACTTTCCTCT CAAGTCCGTT   
  
  
+ TCGTTTCGGT TCTCCCCCGA AAAATACAAA TCTTCTTTCT CACGAGACTT TAGTGTAACG TAAACGGACC   
  
  
+ TTCTAAATAA CTTGGATCGA CCCTTTTAAG GAGTAGAAAA CCTGAATTGT GGTGGGGGTG CGCGTCGATT   
  
  
+ CCGTCGCAGG TGCGACGATT GCCTTGTGTA CAGGGGACGA CGACCACCCT TCTTTGCCTT AACGTGGAGT   
  
  
+ TTTCAACTTT AAAATTGCCT CCCGAAACCG CGCAGCTGCT ACTTCTCGCA TTCCCCCGGG TTATACCGAA   
  
  
+ GAATGCCTCA ATTTACTCCA GTGAGACGGT TACAGTACCA CCACTGTAGT TAAAGGACTA CGGTGTACAG   
  
  
+ TGGGAATCAA AATTTATAGG GTACAACTGT CGTTACCATA AAAAGAATAG GACTGTTTTG ACCTACTACT   
  
  
+ TTATTGCTTA TATCGGAGCT CTGAGATCCC GAAACTTCAC TTCATTTCCT CCGTTGCCGT CCCCGTAAAT   
  
  
+ CCTGTTGTTC TCGACTATCG TCGGCCCATC AATATAATAG CTTCAGGAGA GGAGGAGAAT AAAATAAGAT   
  
  
+ GACTGTACCT ATTTGTGCGT CAACCTCTAC TACAGTACAC GGGTCACTGG TCCCAAACGG TTCATTTCGT   
  
  
+ CCGTATTCCT GGCAACTTAA TACTAACTCA ATCGCACTAC CCTCTTGATT GCCCAGACTA AATCCTAGAT   
  
  
+ CGATCACGAT CACCTCTACT TAGAATGACA TTTTCGTCAC TGTTCATCCT CACCAAACAG AAAATGTTCA   
  
  
+ CCGCCTAGAG CATTAACCTC ATTTCGTCAC CCTTTGCATC TAAATCTAGA CACTGGTCAC TGATGATCGG   
  
  
+ GCTTGTTCTT AACTTCTTTT ATTTTTTAAT TTTTTTCTTT TCCCTACGAG TCTAGAGACT ACGTATTAAC   
  
  
+ CGAGATTCTT GTTGCCTATC CCGTCACTTG AACCAGTCCA CGAAACGCGT ACTCACTACT ACGAGAGTAG   
  
  
+ ACTAACCGAG GCGTCGGAGG AAGAACAGGC CTCGCTACTG AACAAGAAGT TTGATCTATT TGTGTAACAA   
  
  
+ CGTAGAGTAG TTGACAATAA TAAACCAAGG AAGACAAAAC ACAAAAAAAA AAAGGGACAC TTTGCGTTAA   
  
  
+ ACTACCTAAG AAACTTTACT CGTAATAACC TACGCCCTCG AAGTTTCATA CAGAATGTCC ATCTATAAAC   
  
  
+ GTACCGCTCC GACGTAGAGA TGAATGGTAA AACCTTATAC ATGTTTTCAA AACTTTGAAA AAGTTCAGTA   
  
  
+ ATTACCCGTA TTGCTATATC GGTTGATTAT ATGTATACAA GAAACGGCAG AAGAAGGCTC TTACAGTAGA   
  
  
+ TCGAAGAACC TTACCGTTAA CCCGTTGACG ATATATTATA AAAAACTACT ATCAAATTTG CATTACTCTA   
  
  
+ ATCCACTTAA ATGAATAACT ATAACACCT  

- TTTCTGTTTT CGTTTGTGAA ACTGTGCGTT TGTTGTATAC ACAAGTGCGG TGAAAGGAGA GTTCAGGCAA   
  
  
- AGCAAAGCCA AGAGGGGGCT TTTTATGTTT AGAAGAAAGA GTGCTCTGAA ATCACATTGC ATTTGCCTGG   
  
  
- AAGATTTATT GAACCTAGCT GGGAAAATTC CTCATCTTTT GGACTTAACA CCACCCCCAC GCGCAGCTAA   
  
  
- GGCAGCGTCC ACGCTGCTAA CGGAACACAT GTCCCCTGCT GCTGGTGGGA AGAAACGGAA TTGCACCTCA   
  
  
- AAAGTTGAAA TTTTAACGGA GGGCTTTGGC GCGTCGACGA TGAAGAGCGT AAGGGGGCCC AATATGGCTT   
  
  
- CTTACGGAGT TAAATGAGGT CACTCTGCCA ATGTCATGGT GGTGACATCA ATTTCCTGAT GCCACATGTC   
  
  
- ACCCTTAGTT TTAAATATCC CATGTTGACA GCAATGGTAT TTTTCTTATC CTGACAAAAC TGGATGATGA   
  
  
- AATAACGAAT ATAGCCTCGA GACTCTAGGG CTTTGAAGTG AAGTAAAGGA GGCAACGGCA GGGGCATTTA   
  
  
- GGACAACAAG AGCTGATAGC AGCCGGGTAG TTATATTATC GAAGTCCTCT CCTCCTCTTA TTTTATTCTA   
  
  
- CTGACATGGA TAAACACGCA GTTGGAGATG ATGTCATGTG CCCAGTGACC AGGGTTTGCC AAGTAAAGCA   
  
  
- GGCATAAGGA CCGTTGAATT ATGATTGAGT TAGCGTGATG GGAGAACTAA CGGGTCTGAT TTAGGATCTA   
  
  
- GCTAGTGCTA GTGGAGATGA ATCTTACTGT AAAAGCAGTG ACAAGTAGGA GTGGTTTGTC TTTTACAAGT   
  
  
- GGCGGATCTC GTAATTGGAG TAAAGCAGTG GGAAACGTAG ATTTAGATCT GTGACCAGTG ACTACTAGCC   
  
  
- CGAACAAGAA TTGAAGAAAA TAAAAAATTA AAAAAAGAAA AGGGATGCTC AGATCTCTGA TGCATAATTG   
  
  
- GCTCTAAGAA CAACGGATAG GGCAGTGAAC TTGGTCAGGT GCTTTGCGCA TGAGTGATGA TGCTCTCATC   
  
  
- TGATTGGCTC CGCAGCCTCC TTCTTGTCCG GAGCGATGAC TTGTTCTTCA AACTAGATAA ACACATTGTT   
  
  
- GCATCTCATC AACTGTTATT ATTTGGTTCC TTCTGTTTTG TGTTTTTTTT TTTCCCTGTG AAACGCAATT   
  
  
- TGATGGATTC TTTGAAATGA GCATTATTGG ATGCGGGAGC TTCAAAGTAT GTCTTACAGG TAGATATTTG   
  
  
- CATGGCGAGG CTGCATCTCT ACTTACCATT TTGGAATATG TACAAAAGTT TTGAAACTTT TTCAAGTCAT   
  
  
- TAATGGGCAT AACGATATAG CCAACTAATA TACATATGTT CTTTGCCGTC TTCTTCCGAG AATGTCATCT   
  
  
- AGCTTCTTGG AATGGCAATT GGGCAACTGC TATATAATAT TTTTTGATGA TAGTTTAAAC GTAATGAGAT   
  
  
- TAGGTGAATT TACTTATTGA TATTGTGGA

+     Unnamed\_\_4

| Site Name | Organism | Position | Strand | Matrix score. | sequence | function |
| --- | --- | --- | --- | --- | --- | --- |
| Unnamed\_\_4 | Petroselinum hortense | 614 | - | 4 | CTCC |  |
| Unnamed\_\_4 | Petroselinum hortense | 611 | - | 4 | CTCC |  |
| Unnamed\_\_4 | Petroselinum hortense | 606 | - | 4 | CTCC |  |
| Unnamed\_\_4 | Petroselinum hortense | 539 | + | 4 | CTCC |  |
| Unnamed\_\_4 | Petroselinum hortense | 1267 | + | 4 | CTCC |  |
| Unnamed\_\_4 | Petroselinum hortense | 276 | - | 4 | CTCC |  |
| Unnamed\_\_4 | Petroselinum hortense | 1066 | - | 4 | CTCC |  |
| Unnamed\_\_4 | Petroselinum hortense | 170 | - | 4 | CTCC |  |
| Unnamed\_\_4 | Petroselinum hortense | 299 | + | 4 | CTCC |  |
| Unnamed\_\_4 | Petroselinum hortense | 366 | + | 4 | CTCC |  |
| Unnamed\_\_4 | Petroselinum hortense | 82 | + | 4 | CTCC |  |
| Unnamed\_\_4 | Petroselinum hortense | 505 | - | 4 | CTCC |  |

> 2018/04/13 10:10:12  
+ AAAGACAAAA GCAAACACTT TGACACGCAA ACAACATATG TGTTCACGCC ACTTTCCTCT CAAGTCCGTT   
  
  
+ TCGTTTCGGT TCTCCCCCGA AAAATACAAA TCTTCTTTCT CACGAGACTT TAGTGTAACG TAAACGGACC   
  
  
+ TTCTAAATAA CTTGGATCGA CCCTTTTAAG GAGTAGAAAA CCTGAATTGT GGTGGGGGTG CGCGTCGATT   
  
  
+ CCGTCGCAGG TGCGACGATT GCCTTGTGTA CAGGGGACGA CGACCACCCT TCTTTGCCTT AACGTGGAGT   
  
  
+ TTTCAACTTT AAAATTGCCT CCCGAAACCG CGCAGCTGCT ACTTCTCGCA TTCCCCCGGG TTATACCGAA   
  
  
+ GAATGCCTCA ATTTACTCCA GTGAGACGGT TACAGTACCA CCACTGTAGT TAAAGGACTA CGGTGTACAG   
  
  
+ TGGGAATCAA AATTTATAGG GTACAACTGT CGTTACCATA AAAAGAATAG GACTGTTTTG ACCTACTACT   
  
  
+ TTATTGCTTA TATCGGAGCT CTGAGATCCC GAAACTTCAC TTCATTTCCT CCGTTGCCGT CCCCGTAAAT   
  
  
+ CCTGTTGTTC TCGACTATCG TCGGCCCATC AATATAATAG CTTCAGGAGA GGAGGAGAAT AAAATAAGAT   
  
  
+ GACTGTACCT ATTTGTGCGT CAACCTCTAC TACAGTACAC GGGTCACTGG TCCCAAACGG TTCATTTCGT   
  
  
+ CCGTATTCCT GGCAACTTAA TACTAACTCA ATCGCACTAC CCTCTTGATT GCCCAGACTA AATCCTAGAT   
  
  
+ CGATCACGAT CACCTCTACT TAGAATGACA TTTTCGTCAC TGTTCATCCT CACCAAACAG AAAATGTTCA   
  
  
+ CCGCCTAGAG CATTAACCTC ATTTCGTCAC CCTTTGCATC TAAATCTAGA CACTGGTCAC TGATGATCGG   
  
  
+ GCTTGTTCTT AACTTCTTTT ATTTTTTAAT TTTTTTCTTT TCCCTACGAG TCTAGAGACT ACGTATTAAC   
  
  
+ CGAGATTCTT GTTGCCTATC CCGTCACTTG AACCAGTCCA CGAAACGCGT ACTCACTACT ACGAGAGTAG   
  
  
+ ACTAACCGAG GCGTCGGAGG AAGAACAGGC CTCGCTACTG AACAAGAAGT TTGATCTATT TGTGTAACAA   
  
  
+ CGTAGAGTAG TTGACAATAA TAAACCAAGG AAGACAAAAC ACAAAAAAAA AAAGGGACAC TTTGCGTTAA   
  
  
+ ACTACCTAAG AAACTTTACT CGTAATAACC TACGCCCTCG AAGTTTCATA CAGAATGTCC ATCTATAAAC   
  
  
+ GTACCGCTCC GACGTAGAGA TGAATGGTAA AACCTTATAC ATGTTTTCAA AACTTTGAAA AAGTTCAGTA   
  
  
+ ATTACCCGTA TTGCTATATC GGTTGATTAT ATGTATACAA GAAACGGCAG AAGAAGGCTC TTACAGTAGA   
  
  
+ TCGAAGAACC TTACCGTTAA CCCGTTGACG ATATATTATA AAAAACTACT ATCAAATTTG CATTACTCTA   
  
  
+ ATCCACTTAA ATGAATAACT ATAACACCT  

- TTTCTGTTTT CGTTTGTGAA ACTGTGCGTT TGTTGTATAC ACAAGTGCGG TGAAAGGAGA GTTCAGGCAA   
  
  
- AGCAAAGCCA AGAGGGGGCT TTTTATGTTT AGAAGAAAGA GTGCTCTGAA ATCACATTGC ATTTGCCTGG   
  
  
- AAGATTTATT GAACCTAGCT GGGAAAATTC CTCATCTTTT GGACTTAACA CCACCCCCAC GCGCAGCTAA   
  
  
- GGCAGCGTCC ACGCTGCTAA CGGAACACAT GTCCCCTGCT GCTGGTGGGA AGAAACGGAA TTGCACCTCA   
  
  
- AAAGTTGAAA TTTTAACGGA GGGCTTTGGC GCGTCGACGA TGAAGAGCGT AAGGGGGCCC AATATGGCTT   
  
  
- CTTACGGAGT TAAATGAGGT CACTCTGCCA ATGTCATGGT GGTGACATCA ATTTCCTGAT GCCACATGTC   
  
  
- ACCCTTAGTT TTAAATATCC CATGTTGACA GCAATGGTAT TTTTCTTATC CTGACAAAAC TGGATGATGA   
  
  
- AATAACGAAT ATAGCCTCGA GACTCTAGGG CTTTGAAGTG AAGTAAAGGA GGCAACGGCA GGGGCATTTA   
  
  
- GGACAACAAG AGCTGATAGC AGCCGGGTAG TTATATTATC GAAGTCCTCT CCTCCTCTTA TTTTATTCTA   
  
  
- CTGACATGGA TAAACACGCA GTTGGAGATG ATGTCATGTG CCCAGTGACC AGGGTTTGCC AAGTAAAGCA   
  
  
- GGCATAAGGA CCGTTGAATT ATGATTGAGT TAGCGTGATG GGAGAACTAA CGGGTCTGAT TTAGGATCTA   
  
  
- GCTAGTGCTA GTGGAGATGA ATCTTACTGT AAAAGCAGTG ACAAGTAGGA GTGGTTTGTC TTTTACAAGT   
  
  
- GGCGGATCTC GTAATTGGAG TAAAGCAGTG GGAAACGTAG ATTTAGATCT GTGACCAGTG ACTACTAGCC   
  
  
- CGAACAAGAA TTGAAGAAAA TAAAAAATTA AAAAAAGAAA AGGGATGCTC AGATCTCTGA TGCATAATTG   
  
  
- GCTCTAAGAA CAACGGATAG GGCAGTGAAC TTGGTCAGGT GCTTTGCGCA TGAGTGATGA TGCTCTCATC   
  
  
- TGATTGGCTC CGCAGCCTCC TTCTTGTCCG GAGCGATGAC TTGTTCTTCA AACTAGATAA ACACATTGTT   
  
  
- GCATCTCATC AACTGTTATT ATTTGGTTCC TTCTGTTTTG TGTTTTTTTT TTTCCCTGTG AAACGCAATT   
  
  
- TGATGGATTC TTTGAAATGA GCATTATTGG ATGCGGGAGC TTCAAAGTAT GTCTTACAGG TAGATATTTG   
  
  
- CATGGCGAGG CTGCATCTCT ACTTACCATT TTGGAATATG TACAAAAGTT TTGAAACTTT TTCAAGTCAT   
  
  
- TAATGGGCAT AACGATATAG CCAACTAATA TACATATGTT CTTTGCCGTC TTCTTCCGAG AATGTCATCT   
  
  
- AGCTTCTTGG AATGGCAATT GGGCAACTGC TATATAATAT TTTTTGATGA TAGTTTAAAC GTAATGAGAT   
  
  
- TAGGTGAATT TACTTATTGA TATTGTGGA

+     W box

| Site Name | Organism | Position | Strand | Matrix score. | sequence | function |
| --- | --- | --- | --- | --- | --- | --- |
| W box | Arabidopsis thaliana | 478 | + | 6 | TTGACC |  |

> 2018/04/13 10:10:12  
+ AAAGACAAAA GCAAACACTT TGACACGCAA ACAACATATG TGTTCACGCC ACTTTCCTCT CAAGTCCGTT   
  
  
+ TCGTTTCGGT TCTCCCCCGA AAAATACAAA TCTTCTTTCT CACGAGACTT TAGTGTAACG TAAACGGACC   
  
  
+ TTCTAAATAA CTTGGATCGA CCCTTTTAAG GAGTAGAAAA CCTGAATTGT GGTGGGGGTG CGCGTCGATT   
  
  
+ CCGTCGCAGG TGCGACGATT GCCTTGTGTA CAGGGGACGA CGACCACCCT TCTTTGCCTT AACGTGGAGT   
  
  
+ TTTCAACTTT AAAATTGCCT CCCGAAACCG CGCAGCTGCT ACTTCTCGCA TTCCCCCGGG TTATACCGAA   
  
  
+ GAATGCCTCA ATTTACTCCA GTGAGACGGT TACAGTACCA CCACTGTAGT TAAAGGACTA CGGTGTACAG   
  
  
+ TGGGAATCAA AATTTATAGG GTACAACTGT CGTTACCATA AAAAGAATAG GACTGTTTTG ACCTACTACT   
  
  
+ TTATTGCTTA TATCGGAGCT CTGAGATCCC GAAACTTCAC TTCATTTCCT CCGTTGCCGT CCCCGTAAAT   
  
  
+ CCTGTTGTTC TCGACTATCG TCGGCCCATC AATATAATAG CTTCAGGAGA GGAGGAGAAT AAAATAAGAT   
  
  
+ GACTGTACCT ATTTGTGCGT CAACCTCTAC TACAGTACAC GGGTCACTGG TCCCAAACGG TTCATTTCGT   
  
  
+ CCGTATTCCT GGCAACTTAA TACTAACTCA ATCGCACTAC CCTCTTGATT GCCCAGACTA AATCCTAGAT   
  
  
+ CGATCACGAT CACCTCTACT TAGAATGACA TTTTCGTCAC TGTTCATCCT CACCAAACAG AAAATGTTCA   
  
  
+ CCGCCTAGAG CATTAACCTC ATTTCGTCAC CCTTTGCATC TAAATCTAGA CACTGGTCAC TGATGATCGG   
  
  
+ GCTTGTTCTT AACTTCTTTT ATTTTTTAAT TTTTTTCTTT TCCCTACGAG TCTAGAGACT ACGTATTAAC   
  
  
+ CGAGATTCTT GTTGCCTATC CCGTCACTTG AACCAGTCCA CGAAACGCGT ACTCACTACT ACGAGAGTAG   
  
  
+ ACTAACCGAG GCGTCGGAGG AAGAACAGGC CTCGCTACTG AACAAGAAGT TTGATCTATT TGTGTAACAA   
  
  
+ CGTAGAGTAG TTGACAATAA TAAACCAAGG AAGACAAAAC ACAAAAAAAA AAAGGGACAC TTTGCGTTAA   
  
  
+ ACTACCTAAG AAACTTTACT CGTAATAACC TACGCCCTCG AAGTTTCATA CAGAATGTCC ATCTATAAAC   
  
  
+ GTACCGCTCC GACGTAGAGA TGAATGGTAA AACCTTATAC ATGTTTTCAA AACTTTGAAA AAGTTCAGTA   
  
  
+ ATTACCCGTA TTGCTATATC GGTTGATTAT ATGTATACAA GAAACGGCAG AAGAAGGCTC TTACAGTAGA   
  
  
+ TCGAAGAACC TTACCGTTAA CCCGTTGACG ATATATTATA AAAAACTACT ATCAAATTTG CATTACTCTA   
  
  
+ ATCCACTTAA ATGAATAACT ATAACACCT  

- TTTCTGTTTT CGTTTGTGAA ACTGTGCGTT TGTTGTATAC ACAAGTGCGG TGAAAGGAGA GTTCAGGCAA   
  
  
- AGCAAAGCCA AGAGGGGGCT TTTTATGTTT AGAAGAAAGA GTGCTCTGAA ATCACATTGC ATTTGCCTGG   
  
  
- AAGATTTATT GAACCTAGCT GGGAAAATTC CTCATCTTTT GGACTTAACA CCACCCCCAC GCGCAGCTAA   
  
  
- GGCAGCGTCC ACGCTGCTAA CGGAACACAT GTCCCCTGCT GCTGGTGGGA AGAAACGGAA TTGCACCTCA   
  
  
- AAAGTTGAAA TTTTAACGGA GGGCTTTGGC GCGTCGACGA TGAAGAGCGT AAGGGGGCCC AATATGGCTT   
  
  
- CTTACGGAGT TAAATGAGGT CACTCTGCCA ATGTCATGGT GGTGACATCA ATTTCCTGAT GCCACATGTC   
  
  
- ACCCTTAGTT TTAAATATCC CATGTTGACA GCAATGGTAT TTTTCTTATC CTGACAAAAC TGGATGATGA   
  
  
- AATAACGAAT ATAGCCTCGA GACTCTAGGG CTTTGAAGTG AAGTAAAGGA GGCAACGGCA GGGGCATTTA   
  
  
- GGACAACAAG AGCTGATAGC AGCCGGGTAG TTATATTATC GAAGTCCTCT CCTCCTCTTA TTTTATTCTA   
  
  
- CTGACATGGA TAAACACGCA GTTGGAGATG ATGTCATGTG CCCAGTGACC AGGGTTTGCC AAGTAAAGCA   
  
  
- GGCATAAGGA CCGTTGAATT ATGATTGAGT TAGCGTGATG GGAGAACTAA CGGGTCTGAT TTAGGATCTA   
  
  
- GCTAGTGCTA GTGGAGATGA ATCTTACTGT AAAAGCAGTG ACAAGTAGGA GTGGTTTGTC TTTTACAAGT   
  
  
- GGCGGATCTC GTAATTGGAG TAAAGCAGTG GGAAACGTAG ATTTAGATCT GTGACCAGTG ACTACTAGCC   
  
  
- CGAACAAGAA TTGAAGAAAA TAAAAAATTA AAAAAAGAAA AGGGATGCTC AGATCTCTGA TGCATAATTG   
  
  
- GCTCTAAGAA CAACGGATAG GGCAGTGAAC TTGGTCAGGT GCTTTGCGCA TGAGTGATGA TGCTCTCATC   
  
  
- TGATTGGCTC CGCAGCCTCC TTCTTGTCCG GAGCGATGAC TTGTTCTTCA AACTAGATAA ACACATTGTT   
  
  
- GCATCTCATC AACTGTTATT ATTTGGTTCC TTCTGTTTTG TGTTTTTTTT TTTCCCTGTG AAACGCAATT   
  
  
- TGATGGATTC TTTGAAATGA GCATTATTGG ATGCGGGAGC TTCAAAGTAT GTCTTACAGG TAGATATTTG   
  
  
- CATGGCGAGG CTGCATCTCT ACTTACCATT TTGGAATATG TACAAAAGTT TTGAAACTTT TTCAAGTCAT   
  
  
- TAATGGGCAT AACGATATAG CCAACTAATA TACATATGTT CTTTGCCGTC TTCTTCCGAG AATGTCATCT   
  
  
- AGCTTCTTGG AATGGCAATT GGGCAACTGC TATATAATAT TTTTTGATGA TAGTTTAAAC GTAATGAGAT   
  
  
- TAGGTGAATT TACTTATTGA TATTGTGGA

+     chs-CMA2a

| Site Name | Organism | Position | Strand | Matrix score. | sequence | function |
| --- | --- | --- | --- | --- | --- | --- |
| chs-CMA2a | Petroselinum crispum | 1004 | + | 8 | TCACTTGA | part of a light responsive element |

> 2018/04/13 10:10:12  
+ AAAGACAAAA GCAAACACTT TGACACGCAA ACAACATATG TGTTCACGCC ACTTTCCTCT CAAGTCCGTT   
  
  
+ TCGTTTCGGT TCTCCCCCGA AAAATACAAA TCTTCTTTCT CACGAGACTT TAGTGTAACG TAAACGGACC   
  
  
+ TTCTAAATAA CTTGGATCGA CCCTTTTAAG GAGTAGAAAA CCTGAATTGT GGTGGGGGTG CGCGTCGATT   
  
  
+ CCGTCGCAGG TGCGACGATT GCCTTGTGTA CAGGGGACGA CGACCACCCT TCTTTGCCTT AACGTGGAGT   
  
  
+ TTTCAACTTT AAAATTGCCT CCCGAAACCG CGCAGCTGCT ACTTCTCGCA TTCCCCCGGG TTATACCGAA   
  
  
+ GAATGCCTCA ATTTACTCCA GTGAGACGGT TACAGTACCA CCACTGTAGT TAAAGGACTA CGGTGTACAG   
  
  
+ TGGGAATCAA AATTTATAGG GTACAACTGT CGTTACCATA AAAAGAATAG GACTGTTTTG ACCTACTACT   
  
  
+ TTATTGCTTA TATCGGAGCT CTGAGATCCC GAAACTTCAC TTCATTTCCT CCGTTGCCGT CCCCGTAAAT   
  
  
+ CCTGTTGTTC TCGACTATCG TCGGCCCATC AATATAATAG CTTCAGGAGA GGAGGAGAAT AAAATAAGAT   
  
  
+ GACTGTACCT ATTTGTGCGT CAACCTCTAC TACAGTACAC GGGTCACTGG TCCCAAACGG TTCATTTCGT   
  
  
+ CCGTATTCCT GGCAACTTAA TACTAACTCA ATCGCACTAC CCTCTTGATT GCCCAGACTA AATCCTAGAT   
  
  
+ CGATCACGAT CACCTCTACT TAGAATGACA TTTTCGTCAC TGTTCATCCT CACCAAACAG AAAATGTTCA   
  
  
+ CCGCCTAGAG CATTAACCTC ATTTCGTCAC CCTTTGCATC TAAATCTAGA CACTGGTCAC TGATGATCGG   
  
  
+ GCTTGTTCTT AACTTCTTTT ATTTTTTAAT TTTTTTCTTT TCCCTACGAG TCTAGAGACT ACGTATTAAC   
  
  
+ CGAGATTCTT GTTGCCTATC CCGTCACTTG AACCAGTCCA CGAAACGCGT ACTCACTACT ACGAGAGTAG   
  
  
+ ACTAACCGAG GCGTCGGAGG AAGAACAGGC CTCGCTACTG AACAAGAAGT TTGATCTATT TGTGTAACAA   
  
  
+ CGTAGAGTAG TTGACAATAA TAAACCAAGG AAGACAAAAC ACAAAAAAAA AAAGGGACAC TTTGCGTTAA   
  
  
+ ACTACCTAAG AAACTTTACT CGTAATAACC TACGCCCTCG AAGTTTCATA CAGAATGTCC ATCTATAAAC   
  
  
+ GTACCGCTCC GACGTAGAGA TGAATGGTAA AACCTTATAC ATGTTTTCAA AACTTTGAAA AAGTTCAGTA   
  
  
+ ATTACCCGTA TTGCTATATC GGTTGATTAT ATGTATACAA GAAACGGCAG AAGAAGGCTC TTACAGTAGA   
  
  
+ TCGAAGAACC TTACCGTTAA CCCGTTGACG ATATATTATA AAAAACTACT ATCAAATTTG CATTACTCTA   
  
  
+ ATCCACTTAA ATGAATAACT ATAACACCT  

- TTTCTGTTTT CGTTTGTGAA ACTGTGCGTT TGTTGTATAC ACAAGTGCGG TGAAAGGAGA GTTCAGGCAA   
  
  
- AGCAAAGCCA AGAGGGGGCT TTTTATGTTT AGAAGAAAGA GTGCTCTGAA ATCACATTGC ATTTGCCTGG   
  
  
- AAGATTTATT GAACCTAGCT GGGAAAATTC CTCATCTTTT GGACTTAACA CCACCCCCAC GCGCAGCTAA   
  
  
- GGCAGCGTCC ACGCTGCTAA CGGAACACAT GTCCCCTGCT GCTGGTGGGA AGAAACGGAA TTGCACCTCA   
  
  
- AAAGTTGAAA TTTTAACGGA GGGCTTTGGC GCGTCGACGA TGAAGAGCGT AAGGGGGCCC AATATGGCTT   
  
  
- CTTACGGAGT TAAATGAGGT CACTCTGCCA ATGTCATGGT GGTGACATCA ATTTCCTGAT GCCACATGTC   
  
  
- ACCCTTAGTT TTAAATATCC CATGTTGACA GCAATGGTAT TTTTCTTATC CTGACAAAAC TGGATGATGA   
  
  
- AATAACGAAT ATAGCCTCGA GACTCTAGGG CTTTGAAGTG AAGTAAAGGA GGCAACGGCA GGGGCATTTA   
  
  
- GGACAACAAG AGCTGATAGC AGCCGGGTAG TTATATTATC GAAGTCCTCT CCTCCTCTTA TTTTATTCTA   
  
  
- CTGACATGGA TAAACACGCA GTTGGAGATG ATGTCATGTG CCCAGTGACC AGGGTTTGCC AAGTAAAGCA   
  
  
- GGCATAAGGA CCGTTGAATT ATGATTGAGT TAGCGTGATG GGAGAACTAA CGGGTCTGAT TTAGGATCTA   
  
  
- GCTAGTGCTA GTGGAGATGA ATCTTACTGT AAAAGCAGTG ACAAGTAGGA GTGGTTTGTC TTTTACAAGT   
  
  
- GGCGGATCTC GTAATTGGAG TAAAGCAGTG GGAAACGTAG ATTTAGATCT GTGACCAGTG ACTACTAGCC   
  
  
- CGAACAAGAA TTGAAGAAAA TAAAAAATTA AAAAAAGAAA AGGGATGCTC AGATCTCTGA TGCATAATTG   
  
  
- GCTCTAAGAA CAACGGATAG GGCAGTGAAC TTGGTCAGGT GCTTTGCGCA TGAGTGATGA TGCTCTCATC   
  
  
- TGATTGGCTC CGCAGCCTCC TTCTTGTCCG GAGCGATGAC TTGTTCTTCA AACTAGATAA ACACATTGTT   
  
  
- GCATCTCATC AACTGTTATT ATTTGGTTCC TTCTGTTTTG TGTTTTTTTT TTTCCCTGTG AAACGCAATT   
  
  
- TGATGGATTC TTTGAAATGA GCATTATTGG ATGCGGGAGC TTCAAAGTAT GTCTTACAGG TAGATATTTG   
  
  
- CATGGCGAGG CTGCATCTCT ACTTACCATT TTGGAATATG TACAAAAGTT TTGAAACTTT TTCAAGTCAT   
  
  
- TAATGGGCAT AACGATATAG CCAACTAATA TACATATGTT CTTTGCCGTC TTCTTCCGAG AATGTCATCT   
  
  
- AGCTTCTTGG AATGGCAATT GGGCAACTGC TATATAATAT TTTTTGATGA TAGTTTAAAC GTAATGAGAT   
  
  
- TAGGTGAATT TACTTATTGA TATTGTGGA

+     circadian

| Site Name | Organism | Position | Strand | Matrix score. | sequence | function |
| --- | --- | --- | --- | --- | --- | --- |
| circadian | Lycopersicon esculentum | 1103 | - | 6 | CAANNNNATC | cis-acting regulatory element involved in circadian control |
| circadian | Lycopersicon esculentum | 227 | - | 6 | CAANNNNATC | cis-acting regulatory element involved in circadian control |

> 2018/04/13 10:10:12  
+ AAAGACAAAA GCAAACACTT TGACACGCAA ACAACATATG TGTTCACGCC ACTTTCCTCT CAAGTCCGTT   
  
  
+ TCGTTTCGGT TCTCCCCCGA AAAATACAAA TCTTCTTTCT CACGAGACTT TAGTGTAACG TAAACGGACC   
  
  
+ TTCTAAATAA CTTGGATCGA CCCTTTTAAG GAGTAGAAAA CCTGAATTGT GGTGGGGGTG CGCGTCGATT   
  
  
+ CCGTCGCAGG TGCGACGATT GCCTTGTGTA CAGGGGACGA CGACCACCCT TCTTTGCCTT AACGTGGAGT   
  
  
+ TTTCAACTTT AAAATTGCCT CCCGAAACCG CGCAGCTGCT ACTTCTCGCA TTCCCCCGGG TTATACCGAA   
  
  
+ GAATGCCTCA ATTTACTCCA GTGAGACGGT TACAGTACCA CCACTGTAGT TAAAGGACTA CGGTGTACAG   
  
  
+ TGGGAATCAA AATTTATAGG GTACAACTGT CGTTACCATA AAAAGAATAG GACTGTTTTG ACCTACTACT   
  
  
+ TTATTGCTTA TATCGGAGCT CTGAGATCCC GAAACTTCAC TTCATTTCCT CCGTTGCCGT CCCCGTAAAT   
  
  
+ CCTGTTGTTC TCGACTATCG TCGGCCCATC AATATAATAG CTTCAGGAGA GGAGGAGAAT AAAATAAGAT   
  
  
+ GACTGTACCT ATTTGTGCGT CAACCTCTAC TACAGTACAC GGGTCACTGG TCCCAAACGG TTCATTTCGT   
  
  
+ CCGTATTCCT GGCAACTTAA TACTAACTCA ATCGCACTAC CCTCTTGATT GCCCAGACTA AATCCTAGAT   
  
  
+ CGATCACGAT CACCTCTACT TAGAATGACA TTTTCGTCAC TGTTCATCCT CACCAAACAG AAAATGTTCA   
  
  
+ CCGCCTAGAG CATTAACCTC ATTTCGTCAC CCTTTGCATC TAAATCTAGA CACTGGTCAC TGATGATCGG   
  
  
+ GCTTGTTCTT AACTTCTTTT ATTTTTTAAT TTTTTTCTTT TCCCTACGAG TCTAGAGACT ACGTATTAAC   
  
  
+ CGAGATTCTT GTTGCCTATC CCGTCACTTG AACCAGTCCA CGAAACGCGT ACTCACTACT ACGAGAGTAG   
  
  
+ ACTAACCGAG GCGTCGGAGG AAGAACAGGC CTCGCTACTG AACAAGAAGT TTGATCTATT TGTGTAACAA   
  
  
+ CGTAGAGTAG TTGACAATAA TAAACCAAGG AAGACAAAAC ACAAAAAAAA AAAGGGACAC TTTGCGTTAA   
  
  
+ ACTACCTAAG AAACTTTACT CGTAATAACC TACGCCCTCG AAGTTTCATA CAGAATGTCC ATCTATAAAC   
  
  
+ GTACCGCTCC GACGTAGAGA TGAATGGTAA AACCTTATAC ATGTTTTCAA AACTTTGAAA AAGTTCAGTA   
  
  
+ ATTACCCGTA TTGCTATATC GGTTGATTAT ATGTATACAA GAAACGGCAG AAGAAGGCTC TTACAGTAGA   
  
  
+ TCGAAGAACC TTACCGTTAA CCCGTTGACG ATATATTATA AAAAACTACT ATCAAATTTG CATTACTCTA   
  
  
+ ATCCACTTAA ATGAATAACT ATAACACCT  

- TTTCTGTTTT CGTTTGTGAA ACTGTGCGTT TGTTGTATAC ACAAGTGCGG TGAAAGGAGA GTTCAGGCAA   
  
  
- AGCAAAGCCA AGAGGGGGCT TTTTATGTTT AGAAGAAAGA GTGCTCTGAA ATCACATTGC ATTTGCCTGG   
  
  
- AAGATTTATT GAACCTAGCT GGGAAAATTC CTCATCTTTT GGACTTAACA CCACCCCCAC GCGCAGCTAA   
  
  
- GGCAGCGTCC ACGCTGCTAA CGGAACACAT GTCCCCTGCT GCTGGTGGGA AGAAACGGAA TTGCACCTCA   
  
  
- AAAGTTGAAA TTTTAACGGA GGGCTTTGGC GCGTCGACGA TGAAGAGCGT AAGGGGGCCC AATATGGCTT   
  
  
- CTTACGGAGT TAAATGAGGT CACTCTGCCA ATGTCATGGT GGTGACATCA ATTTCCTGAT GCCACATGTC   
  
  
- ACCCTTAGTT TTAAATATCC CATGTTGACA GCAATGGTAT TTTTCTTATC CTGACAAAAC TGGATGATGA   
  
  
- AATAACGAAT ATAGCCTCGA GACTCTAGGG CTTTGAAGTG AAGTAAAGGA GGCAACGGCA GGGGCATTTA   
  
  
- GGACAACAAG AGCTGATAGC AGCCGGGTAG TTATATTATC GAAGTCCTCT CCTCCTCTTA TTTTATTCTA   
  
  
- CTGACATGGA TAAACACGCA GTTGGAGATG ATGTCATGTG CCCAGTGACC AGGGTTTGCC AAGTAAAGCA   
  
  
- GGCATAAGGA CCGTTGAATT ATGATTGAGT TAGCGTGATG GGAGAACTAA CGGGTCTGAT TTAGGATCTA   
  
  
- GCTAGTGCTA GTGGAGATGA ATCTTACTGT AAAAGCAGTG ACAAGTAGGA GTGGTTTGTC TTTTACAAGT   
  
  
- GGCGGATCTC GTAATTGGAG TAAAGCAGTG GGAAACGTAG ATTTAGATCT GTGACCAGTG ACTACTAGCC   
  
  
- CGAACAAGAA TTGAAGAAAA TAAAAAATTA AAAAAAGAAA AGGGATGCTC AGATCTCTGA TGCATAATTG   
  
  
- GCTCTAAGAA CAACGGATAG GGCAGTGAAC TTGGTCAGGT GCTTTGCGCA TGAGTGATGA TGCTCTCATC   
  
  
- TGATTGGCTC CGCAGCCTCC TTCTTGTCCG GAGCGATGAC TTGTTCTTCA AACTAGATAA ACACATTGTT   
  
  
- GCATCTCATC AACTGTTATT ATTTGGTTCC TTCTGTTTTG TGTTTTTTTT TTTCCCTGTG AAACGCAATT   
  
  
- TGATGGATTC TTTGAAATGA GCATTATTGG ATGCGGGAGC TTCAAAGTAT GTCTTACAGG TAGATATTTG   
  
  
- CATGGCGAGG CTGCATCTCT ACTTACCATT TTGGAATATG TACAAAAGTT TTGAAACTTT TTCAAGTCAT   
  
  
- TAATGGGCAT AACGATATAG CCAACTAATA TACATATGTT CTTTGCCGTC TTCTTCCGAG AATGTCATCT   
  
  
- AGCTTCTTGG AATGGCAATT GGGCAACTGC TATATAATAT TTTTTGATGA TAGTTTAAAC GTAATGAGAT   
  
  
- TAGGTGAATT TACTTATTGA TATTGTGGA
