# Supplementary material for: Genome-wide analysis of the WRKY gene family in drumstick (Moringa oleifera Lam.)
Source: PeerJ. 2019 Jun 10;7:e7063. doi: 10.7717/peerj.7063 (PMC6563795; doi:10.7717/peerj.7063)
Supplement: Supplemental Information 1 [file peerj-07-7063-s003.gz › MoWRKY5_plantcare.html]

Content-Type: text/html; charset=ISO-8859-1


CallMat\_Firefox


Webmaster Firefox specific output  
To save the result:
click on the frame with the right mouse button and save the source code as a text file with extension .html  
REFERENCE:PlantCARE: a database of plant cis-acting regulatory elements and a portal to tools for in silico analysis of promoter sequences.  
Lescot, M., Déhais, P., Moreau, Y., De Moor, B., Rouzé ,P.,and Rombauts, S.  
Nucleic Acids Res., Database issue(2002), 30(1):325-327.   


---

> 2018/04/13 10:10:12  
+ CTATAAAGTT TCTGAACCGA GTCTCTGTCC AGCGATTATT ACTTCCACCG ACTTAAGGAC TTTCCAAGTT   
  
  
+ TTGTTTGTAC CATATGTTAT TTTACTTACC GGGTGGATTT AGTTCCGATC GTGTCTGTCT CTTCGTTGTG   
  
  
+ ACTTAGATCG TGGAAAGTTA ACGTCTTCTA TGCGTTTGAG ACCTGTCTAA TTTAATATAC TTTTACTTGG   
  
  
+ AAGGTAAGTT CAGTCTCATT GAGATCAGGA GAGTGGTCCC GTGAGAAAGT ATTTTCCTTA GGATTTGATC   
  
  
+ TTTTGTTTTA GTATTGTAGG TTTGTTTAAG TTAAAAAAAA AAGAGGGTCA ACTCGTAGAC TTTCGTCCAA   
  
  
+ ATCTTCATTT TCCATTTCCG AAAACCGGGG ACCAACTCTT TCTTAAAAGC TTTATTACTA TTTCTCTAGT   
  
  
+ TACTAAAGTA ATGTAGTCGA GAAATGGAAG GGTTAGTCGA GAATGTTGCC ATCGGTTGTT AGTTGGTTTC   
  
  
+ CTTTAACTAA ACCTTGTCTA AGTCGTGTCT AAGCGCCTTT GTTCTTTGGA AAAGGAACAA ACAAAAGTAA   
  
  
+ TAGTTTAACA GAGCCAAGAA GAGTTTTGTT TCGTTCGGTA TTAGTGCATT CATCTCTAAT GGCTATAGGG   
  
  
+ TTTAAACGGC TTCGATACAT TATTCCTTCG TTAGCCTTAC TTCGTTACTG GAGATATTTA AGTCTTTGAC   
  
  
+ TTCTTCTAGA TTCCTTTTTT TATAAACTTC AAGTTCTCTA ATAGCTACTT TTTCCTTTGC TTTGAAGGTC   
  
  
+ CTAGTTCCCT TTTACTTATT TTCGTGAAAG TTCATTTGTT ATTATACTTG CTCTCGTATC TAGTTCAACT   
  
  
+ TAATTAGTGT TGGAGAGTTT TTTTTTTAGT GTCTTTGTTT CACGTCGCGG ATTTAAAATC GACCCTCCGG   
  
  
+ TCTTCAATTC GAACAATAAT AATTTTTTTC TTTTTTCTAA TTCTCTATTT ATTGCAGAAC TCATTTTCGT   
  
  
+ TCTGTTTCTT ATATCTGTGT CTCTCTCTCT CTCTCTCTCT CTTTTTCTTT TTTCTAATTC TCTATTTATT   
  
  
+ GCAGAACTCA TTTTCGTTCT GTTTCTTATA TCTGTGTCTC TCTCTCTCTC TCTCTCTCTT TCTTTCTTTC   
  
  
+ TCTCTTACTA ATATTTACTA ATGGACTAAC TAATTTCTCG ATTTCCACGT ACTGCTTCTG GGTGGTGGTG   
  
  
+ CCGGTGGTAG TGGTGGTTAG ACTGGACTGG GCTCAAGATT TGATTGGCGT TTGCCTGGGC TAGTCTAACA   
  
  
+ AGGCAGCTCA TATGCTACTT AAGTTACCTC TAGCTTTGTT CGTGGGCAGG CAACACCCGT AGTCTCTCGT   
  
  
+ TTTTGTTTTC CCGTTTTCCG TTGGAGAGAT AGTCAGAAGA GAGAGAAGGG CGAACAGAAA AGAATATGGG   
  
  
+ TTTTACAAGG TCAACTAATA AAAAGTTTTC TTTGATGATT TTGCTTGTTT ACGTAGAATA AGGTATTCTA   
  
  
+ CTTAAGGTTA CTTGCAGCCT GTAATTTTT  

- GATATTTCAA AGACTTGGCT CAGAGACAGG TCGCTAATAA TGAAGGTGGC TGAATTCCTG AAAGGTTCAA   
  
  
- AACAAACATG GTATACAATA AAATGAATGG CCCACCTAAA TCAAGGCTAG CACAGACAGA GAAGCAACAC   
  
  
- TGAATCTAGC ACCTTTCAAT TGCAGAAGAT ACGCAAACTC TGGACAGATT AAATTATATG AAAATGAACC   
  
  
- TTCCATTCAA GTCAGAGTAA CTCTAGTCCT CTCACCAGGG CACTCTTTCA TAAAAGGAAT CCTAAACTAG   
  
  
- AAAACAAAAT CATAACATCC AAACAAATTC AATTTTTTTT TTCTCCCAGT TGAGCATCTG AAAGCAGGTT   
  
  
- TAGAAGTAAA AGGTAAAGGC TTTTGGCCCC TGGTTGAGAA AGAATTTTCG AAATAATGAT AAAGAGATCA   
  
  
- ATGATTTCAT TACATCAGCT CTTTACCTTC CCAATCAGCT CTTACAACGG TAGCCAACAA TCAACCAAAG   
  
  
- GAAATTGATT TGGAACAGAT TCAGCACAGA TTCGCGGAAA CAAGAAACCT TTTCCTTGTT TGTTTTCATT   
  
  
- ATCAAATTGT CTCGGTTCTT CTCAAAACAA AGCAAGCCAT AATCACGTAA GTAGAGATTA CCGATATCCC   
  
  
- AAATTTGCCG AAGCTATGTA ATAAGGAAGC AATCGGAATG AAGCAATGAC CTCTATAAAT TCAGAAACTG   
  
  
- AAGAAGATCT AAGGAAAAAA ATATTTGAAG TTCAAGAGAT TATCGATGAA AAAGGAAACG AAACTTCCAG   
  
  
- GATCAAGGGA AAATGAATAA AAGCACTTTC AAGTAAACAA TAATATGAAC GAGAGCATAG ATCAAGTTGA   
  
  
- ATTAATCACA ACCTCTCAAA AAAAAAATCA CAGAAACAAA GTGCAGCGCC TAAATTTTAG CTGGGAGGCC   
  
  
- AGAAGTTAAG CTTGTTATTA TTAAAAAAAG AAAAAAGATT AAGAGATAAA TAACGTCTTG AGTAAAAGCA   
  
  
- AGACAAAGAA TATAGACACA GAGAGAGAGA GAGAGAGAGA GAAAAAGAAA AAAGATTAAG AGATAAATAA   
  
  
- CGTCTTGAGT AAAAGCAAGA CAAAGAATAT AGACACAGAG AGAGAGAGAG AGAGAGAGAA AGAAAGAAAG   
  
  
- AGAGAATGAT TATAAATGAT TACCTGATTG ATTAAAGAGC TAAAGGTGCA TGACGAAGAC CCACCACCAC   
  
  
- GGCCACCATC ACCACCAATC TGACCTGACC CGAGTTCTAA ACTAACCGCA AACGGACCCG ATCAGATTGT   
  
  
- TCCGTCGAGT ATACGATGAA TTCAATGGAG ATCGAAACAA GCACCCGTCC GTTGTGGGCA TCAGAGAGCA   
  
  
- AAAACAAAAG GGCAAAAGGC AACCTCTCTA TCAGTCTTCT CTCTCTTCCC GCTTGTCTTT TCTTATACCC   
  
  
- AAAATGTTCC AGTTGATTAT TTTTCAAAAG AAACTACTAA AACGAACAAA TGCATCTTAT TCCATAAGAT   
  
  
- GAATTCCAAT GAACGTCGGA CATTAAAAA

  
  
Motifs Found  

+     3-AF1 binding site

| Site Name | Organism | Position | Strand | Matrix score. | sequence | function |
| --- | --- | --- | --- | --- | --- | --- |
| 3-AF1 binding site | Solanum tuberosum | 1117 | - | 10 | TAAGAGAGGAA | light responsive element |

> 2018/04/13 10:10:12  
+ CTATAAAGTT TCTGAACCGA GTCTCTGTCC AGCGATTATT ACTTCCACCG ACTTAAGGAC TTTCCAAGTT   
  
  
+ TTGTTTGTAC CATATGTTAT TTTACTTACC GGGTGGATTT AGTTCCGATC GTGTCTGTCT CTTCGTTGTG   
  
  
+ ACTTAGATCG TGGAAAGTTA ACGTCTTCTA TGCGTTTGAG ACCTGTCTAA TTTAATATAC TTTTACTTGG   
  
  
+ AAGGTAAGTT CAGTCTCATT GAGATCAGGA GAGTGGTCCC GTGAGAAAGT ATTTTCCTTA GGATTTGATC   
  
  
+ TTTTGTTTTA GTATTGTAGG TTTGTTTAAG TTAAAAAAAA AAGAGGGTCA ACTCGTAGAC TTTCGTCCAA   
  
  
+ ATCTTCATTT TCCATTTCCG AAAACCGGGG ACCAACTCTT TCTTAAAAGC TTTATTACTA TTTCTCTAGT   
  
  
+ TACTAAAGTA ATGTAGTCGA GAAATGGAAG GGTTAGTCGA GAATGTTGCC ATCGGTTGTT AGTTGGTTTC   
  
  
+ CTTTAACTAA ACCTTGTCTA AGTCGTGTCT AAGCGCCTTT GTTCTTTGGA AAAGGAACAA ACAAAAGTAA   
  
  
+ TAGTTTAACA GAGCCAAGAA GAGTTTTGTT TCGTTCGGTA TTAGTGCATT CATCTCTAAT GGCTATAGGG   
  
  
+ TTTAAACGGC TTCGATACAT TATTCCTTCG TTAGCCTTAC TTCGTTACTG GAGATATTTA AGTCTTTGAC   
  
  
+ TTCTTCTAGA TTCCTTTTTT TATAAACTTC AAGTTCTCTA ATAGCTACTT TTTCCTTTGC TTTGAAGGTC   
  
  
+ CTAGTTCCCT TTTACTTATT TTCGTGAAAG TTCATTTGTT ATTATACTTG CTCTCGTATC TAGTTCAACT   
  
  
+ TAATTAGTGT TGGAGAGTTT TTTTTTTAGT GTCTTTGTTT CACGTCGCGG ATTTAAAATC GACCCTCCGG   
  
  
+ TCTTCAATTC GAACAATAAT AATTTTTTTC TTTTTTCTAA TTCTCTATTT ATTGCAGAAC TCATTTTCGT   
  
  
+ TCTGTTTCTT ATATCTGTGT CTCTCTCTCT CTCTCTCTCT CTTTTTCTTT TTTCTAATTC TCTATTTATT   
  
  
+ GCAGAACTCA TTTTCGTTCT GTTTCTTATA TCTGTGTCTC TCTCTCTCTC TCTCTCTCTT TCTTTCTTTC   
  
  
+ TCTCTTACTA ATATTTACTA ATGGACTAAC TAATTTCTCG ATTTCCACGT ACTGCTTCTG GGTGGTGGTG   
  
  
+ CCGGTGGTAG TGGTGGTTAG ACTGGACTGG GCTCAAGATT TGATTGGCGT TTGCCTGGGC TAGTCTAACA   
  
  
+ AGGCAGCTCA TATGCTACTT AAGTTACCTC TAGCTTTGTT CGTGGGCAGG CAACACCCGT AGTCTCTCGT   
  
  
+ TTTTGTTTTC CCGTTTTCCG TTGGAGAGAT AGTCAGAAGA GAGAGAAGGG CGAACAGAAA AGAATATGGG   
  
  
+ TTTTACAAGG TCAACTAATA AAAAGTTTTC TTTGATGATT TTGCTTGTTT ACGTAGAATA AGGTATTCTA   
  
  
+ CTTAAGGTTA CTTGCAGCCT GTAATTTTT  

- GATATTTCAA AGACTTGGCT CAGAGACAGG TCGCTAATAA TGAAGGTGGC TGAATTCCTG AAAGGTTCAA   
  
  
- AACAAACATG GTATACAATA AAATGAATGG CCCACCTAAA TCAAGGCTAG CACAGACAGA GAAGCAACAC   
  
  
- TGAATCTAGC ACCTTTCAAT TGCAGAAGAT ACGCAAACTC TGGACAGATT AAATTATATG AAAATGAACC   
  
  
- TTCCATTCAA GTCAGAGTAA CTCTAGTCCT CTCACCAGGG CACTCTTTCA TAAAAGGAAT CCTAAACTAG   
  
  
- AAAACAAAAT CATAACATCC AAACAAATTC AATTTTTTTT TTCTCCCAGT TGAGCATCTG AAAGCAGGTT   
  
  
- TAGAAGTAAA AGGTAAAGGC TTTTGGCCCC TGGTTGAGAA AGAATTTTCG AAATAATGAT AAAGAGATCA   
  
  
- ATGATTTCAT TACATCAGCT CTTTACCTTC CCAATCAGCT CTTACAACGG TAGCCAACAA TCAACCAAAG   
  
  
- GAAATTGATT TGGAACAGAT TCAGCACAGA TTCGCGGAAA CAAGAAACCT TTTCCTTGTT TGTTTTCATT   
  
  
- ATCAAATTGT CTCGGTTCTT CTCAAAACAA AGCAAGCCAT AATCACGTAA GTAGAGATTA CCGATATCCC   
  
  
- AAATTTGCCG AAGCTATGTA ATAAGGAAGC AATCGGAATG AAGCAATGAC CTCTATAAAT TCAGAAACTG   
  
  
- AAGAAGATCT AAGGAAAAAA ATATTTGAAG TTCAAGAGAT TATCGATGAA AAAGGAAACG AAACTTCCAG   
  
  
- GATCAAGGGA AAATGAATAA AAGCACTTTC AAGTAAACAA TAATATGAAC GAGAGCATAG ATCAAGTTGA   
  
  
- ATTAATCACA ACCTCTCAAA AAAAAAATCA CAGAAACAAA GTGCAGCGCC TAAATTTTAG CTGGGAGGCC   
  
  
- AGAAGTTAAG CTTGTTATTA TTAAAAAAAG AAAAAAGATT AAGAGATAAA TAACGTCTTG AGTAAAAGCA   
  
  
- AGACAAAGAA TATAGACACA GAGAGAGAGA GAGAGAGAGA GAAAAAGAAA AAAGATTAAG AGATAAATAA   
  
  
- CGTCTTGAGT AAAAGCAAGA CAAAGAATAT AGACACAGAG AGAGAGAGAG AGAGAGAGAA AGAAAGAAAG   
  
  
- AGAGAATGAT TATAAATGAT TACCTGATTG ATTAAAGAGC TAAAGGTGCA TGACGAAGAC CCACCACCAC   
  
  
- GGCCACCATC ACCACCAATC TGACCTGACC CGAGTTCTAA ACTAACCGCA AACGGACCCG ATCAGATTGT   
  
  
- TCCGTCGAGT ATACGATGAA TTCAATGGAG ATCGAAACAA GCACCCGTCC GTTGTGGGCA TCAGAGAGCA   
  
  
- AAAACAAAAG GGCAAAAGGC AACCTCTCTA TCAGTCTTCT CTCTCTTCCC GCTTGTCTTT TCTTATACCC   
  
  
- AAAATGTTCC AGTTGATTAT TTTTCAAAAG AAACTACTAA AACGAACAAA TGCATCTTAT TCCATAAGAT   
  
  
- GAATTCCAAT GAACGTCGGA CATTAAAAA

+     5UTR Py-rich stretch

| Site Name | Organism | Position | Strand | Matrix score. | sequence | function |
| --- | --- | --- | --- | --- | --- | --- |
| 5UTR Py-rich stretch | Lycopersicon esculentum | 1091 | + | 13 | TTTCTCTCTCTCTC | cis-acting element conferring high transcription levels |
| 5UTR Py-rich stretch | Lycopersicon esculentum | 1089 | + | 13 | TTTCTCTCTCTCTC | cis-acting element conferring high transcription levels |
| 5UTR Py-rich stretch | Lycopersicon esculentum | 1008 | + | 13 | TTTCTCTCTCTCTC | cis-acting element conferring high transcription levels |
| 5UTR Py-rich stretch | Lycopersicon esculentum | 1093 | + | 13 | TTTCTCTCTCTCTC | cis-acting element conferring high transcription levels |
| 5UTR Py-rich stretch | Lycopersicon esculentum | 1000 | + | 13 | TTTCTCTCTCTCTC | cis-acting element conferring high transcription levels |
| 5UTR Py-rich stretch | Lycopersicon esculentum | 998 | + | 13 | TTTCTCTCTCTCTC | cis-acting element conferring high transcription levels |
| 5UTR Py-rich stretch | Lycopersicon esculentum | 1087 | + | 13 | TTTCTCTCTCTCTC | cis-acting element conferring high transcription levels |
| 5UTR Py-rich stretch | Lycopersicon esculentum | 1095 | + | 13 | TTTCTCTCTCTCTC | cis-acting element conferring high transcription levels |
| 5UTR Py-rich stretch | Lycopersicon esculentum | 1085 | + | 13 | TTTCTCTCTCTCTC | cis-acting element conferring high transcription levels |
| 5UTR Py-rich stretch | Lycopersicon esculentum | 1006 | + | 13 | TTTCTCTCTCTCTC | cis-acting element conferring high transcription levels |
| 5UTR Py-rich stretch | Lycopersicon esculentum | 1004 | + | 13 | TTTCTCTCTCTCTC | cis-acting element conferring high transcription levels |
| 5UTR Py-rich stretch | Lycopersicon esculentum | 1002 | + | 13 | TTTCTCTCTCTCTC | cis-acting element conferring high transcription levels |

> 2018/04/13 10:10:12  
+ CTATAAAGTT TCTGAACCGA GTCTCTGTCC AGCGATTATT ACTTCCACCG ACTTAAGGAC TTTCCAAGTT   
  
  
+ TTGTTTGTAC CATATGTTAT TTTACTTACC GGGTGGATTT AGTTCCGATC GTGTCTGTCT CTTCGTTGTG   
  
  
+ ACTTAGATCG TGGAAAGTTA ACGTCTTCTA TGCGTTTGAG ACCTGTCTAA TTTAATATAC TTTTACTTGG   
  
  
+ AAGGTAAGTT CAGTCTCATT GAGATCAGGA GAGTGGTCCC GTGAGAAAGT ATTTTCCTTA GGATTTGATC   
  
  
+ TTTTGTTTTA GTATTGTAGG TTTGTTTAAG TTAAAAAAAA AAGAGGGTCA ACTCGTAGAC TTTCGTCCAA   
  
  
+ ATCTTCATTT TCCATTTCCG AAAACCGGGG ACCAACTCTT TCTTAAAAGC TTTATTACTA TTTCTCTAGT   
  
  
+ TACTAAAGTA ATGTAGTCGA GAAATGGAAG GGTTAGTCGA GAATGTTGCC ATCGGTTGTT AGTTGGTTTC   
  
  
+ CTTTAACTAA ACCTTGTCTA AGTCGTGTCT AAGCGCCTTT GTTCTTTGGA AAAGGAACAA ACAAAAGTAA   
  
  
+ TAGTTTAACA GAGCCAAGAA GAGTTTTGTT TCGTTCGGTA TTAGTGCATT CATCTCTAAT GGCTATAGGG   
  
  
+ TTTAAACGGC TTCGATACAT TATTCCTTCG TTAGCCTTAC TTCGTTACTG GAGATATTTA AGTCTTTGAC   
  
  
+ TTCTTCTAGA TTCCTTTTTT TATAAACTTC AAGTTCTCTA ATAGCTACTT TTTCCTTTGC TTTGAAGGTC   
  
  
+ CTAGTTCCCT TTTACTTATT TTCGTGAAAG TTCATTTGTT ATTATACTTG CTCTCGTATC TAGTTCAACT   
  
  
+ TAATTAGTGT TGGAGAGTTT TTTTTTTAGT GTCTTTGTTT CACGTCGCGG ATTTAAAATC GACCCTCCGG   
  
  
+ TCTTCAATTC GAACAATAAT AATTTTTTTC TTTTTTCTAA TTCTCTATTT ATTGCAGAAC TCATTTTCGT   
  
  
+ TCTGTTTCTT ATATCTGTGT CTCTCTCTCT CTCTCTCTCT CTTTTTCTTT TTTCTAATTC TCTATTTATT   
  
  
+ GCAGAACTCA TTTTCGTTCT GTTTCTTATA TCTGTGTCTC TCTCTCTCTC TCTCTCTCTT TCTTTCTTTC   
  
  
+ TCTCTTACTA ATATTTACTA ATGGACTAAC TAATTTCTCG ATTTCCACGT ACTGCTTCTG GGTGGTGGTG   
  
  
+ CCGGTGGTAG TGGTGGTTAG ACTGGACTGG GCTCAAGATT TGATTGGCGT TTGCCTGGGC TAGTCTAACA   
  
  
+ AGGCAGCTCA TATGCTACTT AAGTTACCTC TAGCTTTGTT CGTGGGCAGG CAACACCCGT AGTCTCTCGT   
  
  
+ TTTTGTTTTC CCGTTTTCCG TTGGAGAGAT AGTCAGAAGA GAGAGAAGGG CGAACAGAAA AGAATATGGG   
  
  
+ TTTTACAAGG TCAACTAATA AAAAGTTTTC TTTGATGATT TTGCTTGTTT ACGTAGAATA AGGTATTCTA   
  
  
+ CTTAAGGTTA CTTGCAGCCT GTAATTTTT  

- GATATTTCAA AGACTTGGCT CAGAGACAGG TCGCTAATAA TGAAGGTGGC TGAATTCCTG AAAGGTTCAA   
  
  
- AACAAACATG GTATACAATA AAATGAATGG CCCACCTAAA TCAAGGCTAG CACAGACAGA GAAGCAACAC   
  
  
- TGAATCTAGC ACCTTTCAAT TGCAGAAGAT ACGCAAACTC TGGACAGATT AAATTATATG AAAATGAACC   
  
  
- TTCCATTCAA GTCAGAGTAA CTCTAGTCCT CTCACCAGGG CACTCTTTCA TAAAAGGAAT CCTAAACTAG   
  
  
- AAAACAAAAT CATAACATCC AAACAAATTC AATTTTTTTT TTCTCCCAGT TGAGCATCTG AAAGCAGGTT   
  
  
- TAGAAGTAAA AGGTAAAGGC TTTTGGCCCC TGGTTGAGAA AGAATTTTCG AAATAATGAT AAAGAGATCA   
  
  
- ATGATTTCAT TACATCAGCT CTTTACCTTC CCAATCAGCT CTTACAACGG TAGCCAACAA TCAACCAAAG   
  
  
- GAAATTGATT TGGAACAGAT TCAGCACAGA TTCGCGGAAA CAAGAAACCT TTTCCTTGTT TGTTTTCATT   
  
  
- ATCAAATTGT CTCGGTTCTT CTCAAAACAA AGCAAGCCAT AATCACGTAA GTAGAGATTA CCGATATCCC   
  
  
- AAATTTGCCG AAGCTATGTA ATAAGGAAGC AATCGGAATG AAGCAATGAC CTCTATAAAT TCAGAAACTG   
  
  
- AAGAAGATCT AAGGAAAAAA ATATTTGAAG TTCAAGAGAT TATCGATGAA AAAGGAAACG AAACTTCCAG   
  
  
- GATCAAGGGA AAATGAATAA AAGCACTTTC AAGTAAACAA TAATATGAAC GAGAGCATAG ATCAAGTTGA   
  
  
- ATTAATCACA ACCTCTCAAA AAAAAAATCA CAGAAACAAA GTGCAGCGCC TAAATTTTAG CTGGGAGGCC   
  
  
- AGAAGTTAAG CTTGTTATTA TTAAAAAAAG AAAAAAGATT AAGAGATAAA TAACGTCTTG AGTAAAAGCA   
  
  
- AGACAAAGAA TATAGACACA GAGAGAGAGA GAGAGAGAGA GAAAAAGAAA AAAGATTAAG AGATAAATAA   
  
  
- CGTCTTGAGT AAAAGCAAGA CAAAGAATAT AGACACAGAG AGAGAGAGAG AGAGAGAGAA AGAAAGAAAG   
  
  
- AGAGAATGAT TATAAATGAT TACCTGATTG ATTAAAGAGC TAAAGGTGCA TGACGAAGAC CCACCACCAC   
  
  
- GGCCACCATC ACCACCAATC TGACCTGACC CGAGTTCTAA ACTAACCGCA AACGGACCCG ATCAGATTGT   
  
  
- TCCGTCGAGT ATACGATGAA TTCAATGGAG ATCGAAACAA GCACCCGTCC GTTGTGGGCA TCAGAGAGCA   
  
  
- AAAACAAAAG GGCAAAAGGC AACCTCTCTA TCAGTCTTCT CTCTCTTCCC GCTTGTCTTT TCTTATACCC   
  
  
- AAAATGTTCC AGTTGATTAT TTTTCAAAAG AAACTACTAA AACGAACAAA TGCATCTTAT TCCATAAGAT   
  
  
- GAATTCCAAT GAACGTCGGA CATTAAAAA

+     AAGAA-motif

| Site Name | Organism | Position | Strand | Matrix score. | sequence | function |
| --- | --- | --- | --- | --- | --- | --- |
| AAGAA-motif | Avena sativa | 1114 | - | 7 | GAAAGAA |  |
| AAGAA-motif | Avena sativa | 1110 | - | 7 | GAAAGAA |  |

> 2018/04/13 10:10:12  
+ CTATAAAGTT TCTGAACCGA GTCTCTGTCC AGCGATTATT ACTTCCACCG ACTTAAGGAC TTTCCAAGTT   
  
  
+ TTGTTTGTAC CATATGTTAT TTTACTTACC GGGTGGATTT AGTTCCGATC GTGTCTGTCT CTTCGTTGTG   
  
  
+ ACTTAGATCG TGGAAAGTTA ACGTCTTCTA TGCGTTTGAG ACCTGTCTAA TTTAATATAC TTTTACTTGG   
  
  
+ AAGGTAAGTT CAGTCTCATT GAGATCAGGA GAGTGGTCCC GTGAGAAAGT ATTTTCCTTA GGATTTGATC   
  
  
+ TTTTGTTTTA GTATTGTAGG TTTGTTTAAG TTAAAAAAAA AAGAGGGTCA ACTCGTAGAC TTTCGTCCAA   
  
  
+ ATCTTCATTT TCCATTTCCG AAAACCGGGG ACCAACTCTT TCTTAAAAGC TTTATTACTA TTTCTCTAGT   
  
  
+ TACTAAAGTA ATGTAGTCGA GAAATGGAAG GGTTAGTCGA GAATGTTGCC ATCGGTTGTT AGTTGGTTTC   
  
  
+ CTTTAACTAA ACCTTGTCTA AGTCGTGTCT AAGCGCCTTT GTTCTTTGGA AAAGGAACAA ACAAAAGTAA   
  
  
+ TAGTTTAACA GAGCCAAGAA GAGTTTTGTT TCGTTCGGTA TTAGTGCATT CATCTCTAAT GGCTATAGGG   
  
  
+ TTTAAACGGC TTCGATACAT TATTCCTTCG TTAGCCTTAC TTCGTTACTG GAGATATTTA AGTCTTTGAC   
  
  
+ TTCTTCTAGA TTCCTTTTTT TATAAACTTC AAGTTCTCTA ATAGCTACTT TTTCCTTTGC TTTGAAGGTC   
  
  
+ CTAGTTCCCT TTTACTTATT TTCGTGAAAG TTCATTTGTT ATTATACTTG CTCTCGTATC TAGTTCAACT   
  
  
+ TAATTAGTGT TGGAGAGTTT TTTTTTTAGT GTCTTTGTTT CACGTCGCGG ATTTAAAATC GACCCTCCGG   
  
  
+ TCTTCAATTC GAACAATAAT AATTTTTTTC TTTTTTCTAA TTCTCTATTT ATTGCAGAAC TCATTTTCGT   
  
  
+ TCTGTTTCTT ATATCTGTGT CTCTCTCTCT CTCTCTCTCT CTTTTTCTTT TTTCTAATTC TCTATTTATT   
  
  
+ GCAGAACTCA TTTTCGTTCT GTTTCTTATA TCTGTGTCTC TCTCTCTCTC TCTCTCTCTT TCTTTCTTTC   
  
  
+ TCTCTTACTA ATATTTACTA ATGGACTAAC TAATTTCTCG ATTTCCACGT ACTGCTTCTG GGTGGTGGTG   
  
  
+ CCGGTGGTAG TGGTGGTTAG ACTGGACTGG GCTCAAGATT TGATTGGCGT TTGCCTGGGC TAGTCTAACA   
  
  
+ AGGCAGCTCA TATGCTACTT AAGTTACCTC TAGCTTTGTT CGTGGGCAGG CAACACCCGT AGTCTCTCGT   
  
  
+ TTTTGTTTTC CCGTTTTCCG TTGGAGAGAT AGTCAGAAGA GAGAGAAGGG CGAACAGAAA AGAATATGGG   
  
  
+ TTTTACAAGG TCAACTAATA AAAAGTTTTC TTTGATGATT TTGCTTGTTT ACGTAGAATA AGGTATTCTA   
  
  
+ CTTAAGGTTA CTTGCAGCCT GTAATTTTT  

- GATATTTCAA AGACTTGGCT CAGAGACAGG TCGCTAATAA TGAAGGTGGC TGAATTCCTG AAAGGTTCAA   
  
  
- AACAAACATG GTATACAATA AAATGAATGG CCCACCTAAA TCAAGGCTAG CACAGACAGA GAAGCAACAC   
  
  
- TGAATCTAGC ACCTTTCAAT TGCAGAAGAT ACGCAAACTC TGGACAGATT AAATTATATG AAAATGAACC   
  
  
- TTCCATTCAA GTCAGAGTAA CTCTAGTCCT CTCACCAGGG CACTCTTTCA TAAAAGGAAT CCTAAACTAG   
  
  
- AAAACAAAAT CATAACATCC AAACAAATTC AATTTTTTTT TTCTCCCAGT TGAGCATCTG AAAGCAGGTT   
  
  
- TAGAAGTAAA AGGTAAAGGC TTTTGGCCCC TGGTTGAGAA AGAATTTTCG AAATAATGAT AAAGAGATCA   
  
  
- ATGATTTCAT TACATCAGCT CTTTACCTTC CCAATCAGCT CTTACAACGG TAGCCAACAA TCAACCAAAG   
  
  
- GAAATTGATT TGGAACAGAT TCAGCACAGA TTCGCGGAAA CAAGAAACCT TTTCCTTGTT TGTTTTCATT   
  
  
- ATCAAATTGT CTCGGTTCTT CTCAAAACAA AGCAAGCCAT AATCACGTAA GTAGAGATTA CCGATATCCC   
  
  
- AAATTTGCCG AAGCTATGTA ATAAGGAAGC AATCGGAATG AAGCAATGAC CTCTATAAAT TCAGAAACTG   
  
  
- AAGAAGATCT AAGGAAAAAA ATATTTGAAG TTCAAGAGAT TATCGATGAA AAAGGAAACG AAACTTCCAG   
  
  
- GATCAAGGGA AAATGAATAA AAGCACTTTC AAGTAAACAA TAATATGAAC GAGAGCATAG ATCAAGTTGA   
  
  
- ATTAATCACA ACCTCTCAAA AAAAAAATCA CAGAAACAAA GTGCAGCGCC TAAATTTTAG CTGGGAGGCC   
  
  
- AGAAGTTAAG CTTGTTATTA TTAAAAAAAG AAAAAAGATT AAGAGATAAA TAACGTCTTG AGTAAAAGCA   
  
  
- AGACAAAGAA TATAGACACA GAGAGAGAGA GAGAGAGAGA GAAAAAGAAA AAAGATTAAG AGATAAATAA   
  
  
- CGTCTTGAGT AAAAGCAAGA CAAAGAATAT AGACACAGAG AGAGAGAGAG AGAGAGAGAA AGAAAGAAAG   
  
  
- AGAGAATGAT TATAAATGAT TACCTGATTG ATTAAAGAGC TAAAGGTGCA TGACGAAGAC CCACCACCAC   
  
  
- GGCCACCATC ACCACCAATC TGACCTGACC CGAGTTCTAA ACTAACCGCA AACGGACCCG ATCAGATTGT   
  
  
- TCCGTCGAGT ATACGATGAA TTCAATGGAG ATCGAAACAA GCACCCGTCC GTTGTGGGCA TCAGAGAGCA   
  
  
- AAAACAAAAG GGCAAAAGGC AACCTCTCTA TCAGTCTTCT CTCTCTTCCC GCTTGTCTTT TCTTATACCC   
  
  
- AAAATGTTCC AGTTGATTAT TTTTCAAAAG AAACTACTAA AACGAACAAA TGCATCTTAT TCCATAAGAT   
  
  
- GAATTCCAAT GAACGTCGGA CATTAAAAA

+     ABRE

| Site Name | Organism | Position | Strand | Matrix score. | sequence | function |
| --- | --- | --- | --- | --- | --- | --- |
| ABRE | Arabidopsis thaliana | 1166 | - | 6 | TACGTG | cis-acting element involved in the abscisic acid responsiveness |
| ABRE | Oryza sativa | 1164 | - | 9 | AGTACGTGGC | cis-acting element involved in the abscisic acid responsiveness |

> 2018/04/13 10:10:12  
+ CTATAAAGTT TCTGAACCGA GTCTCTGTCC AGCGATTATT ACTTCCACCG ACTTAAGGAC TTTCCAAGTT   
  
  
+ TTGTTTGTAC CATATGTTAT TTTACTTACC GGGTGGATTT AGTTCCGATC GTGTCTGTCT CTTCGTTGTG   
  
  
+ ACTTAGATCG TGGAAAGTTA ACGTCTTCTA TGCGTTTGAG ACCTGTCTAA TTTAATATAC TTTTACTTGG   
  
  
+ AAGGTAAGTT CAGTCTCATT GAGATCAGGA GAGTGGTCCC GTGAGAAAGT ATTTTCCTTA GGATTTGATC   
  
  
+ TTTTGTTTTA GTATTGTAGG TTTGTTTAAG TTAAAAAAAA AAGAGGGTCA ACTCGTAGAC TTTCGTCCAA   
  
  
+ ATCTTCATTT TCCATTTCCG AAAACCGGGG ACCAACTCTT TCTTAAAAGC TTTATTACTA TTTCTCTAGT   
  
  
+ TACTAAAGTA ATGTAGTCGA GAAATGGAAG GGTTAGTCGA GAATGTTGCC ATCGGTTGTT AGTTGGTTTC   
  
  
+ CTTTAACTAA ACCTTGTCTA AGTCGTGTCT AAGCGCCTTT GTTCTTTGGA AAAGGAACAA ACAAAAGTAA   
  
  
+ TAGTTTAACA GAGCCAAGAA GAGTTTTGTT TCGTTCGGTA TTAGTGCATT CATCTCTAAT GGCTATAGGG   
  
  
+ TTTAAACGGC TTCGATACAT TATTCCTTCG TTAGCCTTAC TTCGTTACTG GAGATATTTA AGTCTTTGAC   
  
  
+ TTCTTCTAGA TTCCTTTTTT TATAAACTTC AAGTTCTCTA ATAGCTACTT TTTCCTTTGC TTTGAAGGTC   
  
  
+ CTAGTTCCCT TTTACTTATT TTCGTGAAAG TTCATTTGTT ATTATACTTG CTCTCGTATC TAGTTCAACT   
  
  
+ TAATTAGTGT TGGAGAGTTT TTTTTTTAGT GTCTTTGTTT CACGTCGCGG ATTTAAAATC GACCCTCCGG   
  
  
+ TCTTCAATTC GAACAATAAT AATTTTTTTC TTTTTTCTAA TTCTCTATTT ATTGCAGAAC TCATTTTCGT   
  
  
+ TCTGTTTCTT ATATCTGTGT CTCTCTCTCT CTCTCTCTCT CTTTTTCTTT TTTCTAATTC TCTATTTATT   
  
  
+ GCAGAACTCA TTTTCGTTCT GTTTCTTATA TCTGTGTCTC TCTCTCTCTC TCTCTCTCTT TCTTTCTTTC   
  
  
+ TCTCTTACTA ATATTTACTA ATGGACTAAC TAATTTCTCG ATTTCCACGT ACTGCTTCTG GGTGGTGGTG   
  
  
+ CCGGTGGTAG TGGTGGTTAG ACTGGACTGG GCTCAAGATT TGATTGGCGT TTGCCTGGGC TAGTCTAACA   
  
  
+ AGGCAGCTCA TATGCTACTT AAGTTACCTC TAGCTTTGTT CGTGGGCAGG CAACACCCGT AGTCTCTCGT   
  
  
+ TTTTGTTTTC CCGTTTTCCG TTGGAGAGAT AGTCAGAAGA GAGAGAAGGG CGAACAGAAA AGAATATGGG   
  
  
+ TTTTACAAGG TCAACTAATA AAAAGTTTTC TTTGATGATT TTGCTTGTTT ACGTAGAATA AGGTATTCTA   
  
  
+ CTTAAGGTTA CTTGCAGCCT GTAATTTTT  

- GATATTTCAA AGACTTGGCT CAGAGACAGG TCGCTAATAA TGAAGGTGGC TGAATTCCTG AAAGGTTCAA   
  
  
- AACAAACATG GTATACAATA AAATGAATGG CCCACCTAAA TCAAGGCTAG CACAGACAGA GAAGCAACAC   
  
  
- TGAATCTAGC ACCTTTCAAT TGCAGAAGAT ACGCAAACTC TGGACAGATT AAATTATATG AAAATGAACC   
  
  
- TTCCATTCAA GTCAGAGTAA CTCTAGTCCT CTCACCAGGG CACTCTTTCA TAAAAGGAAT CCTAAACTAG   
  
  
- AAAACAAAAT CATAACATCC AAACAAATTC AATTTTTTTT TTCTCCCAGT TGAGCATCTG AAAGCAGGTT   
  
  
- TAGAAGTAAA AGGTAAAGGC TTTTGGCCCC TGGTTGAGAA AGAATTTTCG AAATAATGAT AAAGAGATCA   
  
  
- ATGATTTCAT TACATCAGCT CTTTACCTTC CCAATCAGCT CTTACAACGG TAGCCAACAA TCAACCAAAG   
  
  
- GAAATTGATT TGGAACAGAT TCAGCACAGA TTCGCGGAAA CAAGAAACCT TTTCCTTGTT TGTTTTCATT   
  
  
- ATCAAATTGT CTCGGTTCTT CTCAAAACAA AGCAAGCCAT AATCACGTAA GTAGAGATTA CCGATATCCC   
  
  
- AAATTTGCCG AAGCTATGTA ATAAGGAAGC AATCGGAATG AAGCAATGAC CTCTATAAAT TCAGAAACTG   
  
  
- AAGAAGATCT AAGGAAAAAA ATATTTGAAG TTCAAGAGAT TATCGATGAA AAAGGAAACG AAACTTCCAG   
  
  
- GATCAAGGGA AAATGAATAA AAGCACTTTC AAGTAAACAA TAATATGAAC GAGAGCATAG ATCAAGTTGA   
  
  
- ATTAATCACA ACCTCTCAAA AAAAAAATCA CAGAAACAAA GTGCAGCGCC TAAATTTTAG CTGGGAGGCC   
  
  
- AGAAGTTAAG CTTGTTATTA TTAAAAAAAG AAAAAAGATT AAGAGATAAA TAACGTCTTG AGTAAAAGCA   
  
  
- AGACAAAGAA TATAGACACA GAGAGAGAGA GAGAGAGAGA GAAAAAGAAA AAAGATTAAG AGATAAATAA   
  
  
- CGTCTTGAGT AAAAGCAAGA CAAAGAATAT AGACACAGAG AGAGAGAGAG AGAGAGAGAA AGAAAGAAAG   
  
  
- AGAGAATGAT TATAAATGAT TACCTGATTG ATTAAAGAGC TAAAGGTGCA TGACGAAGAC CCACCACCAC   
  
  
- GGCCACCATC ACCACCAATC TGACCTGACC CGAGTTCTAA ACTAACCGCA AACGGACCCG ATCAGATTGT   
  
  
- TCCGTCGAGT ATACGATGAA TTCAATGGAG ATCGAAACAA GCACCCGTCC GTTGTGGGCA TCAGAGAGCA   
  
  
- AAAACAAAAG GGCAAAAGGC AACCTCTCTA TCAGTCTTCT CTCTCTTCCC GCTTGTCTTT TCTTATACCC   
  
  
- AAAATGTTCC AGTTGATTAT TTTTCAAAAG AAACTACTAA AACGAACAAA TGCATCTTAT TCCATAAGAT   
  
  
- GAATTCCAAT GAACGTCGGA CATTAAAAA

+     ACE

| Site Name | Organism | Position | Strand | Matrix score. | sequence | function |
| --- | --- | --- | --- | --- | --- | --- |
| ACE | Petroselinum hortense | 1164 | - | 7 | ACGTGGA | cis-acting element involved in light responsiveness |

> 2018/04/13 10:10:12  
+ CTATAAAGTT TCTGAACCGA GTCTCTGTCC AGCGATTATT ACTTCCACCG ACTTAAGGAC TTTCCAAGTT   
  
  
+ TTGTTTGTAC CATATGTTAT TTTACTTACC GGGTGGATTT AGTTCCGATC GTGTCTGTCT CTTCGTTGTG   
  
  
+ ACTTAGATCG TGGAAAGTTA ACGTCTTCTA TGCGTTTGAG ACCTGTCTAA TTTAATATAC TTTTACTTGG   
  
  
+ AAGGTAAGTT CAGTCTCATT GAGATCAGGA GAGTGGTCCC GTGAGAAAGT ATTTTCCTTA GGATTTGATC   
  
  
+ TTTTGTTTTA GTATTGTAGG TTTGTTTAAG TTAAAAAAAA AAGAGGGTCA ACTCGTAGAC TTTCGTCCAA   
  
  
+ ATCTTCATTT TCCATTTCCG AAAACCGGGG ACCAACTCTT TCTTAAAAGC TTTATTACTA TTTCTCTAGT   
  
  
+ TACTAAAGTA ATGTAGTCGA GAAATGGAAG GGTTAGTCGA GAATGTTGCC ATCGGTTGTT AGTTGGTTTC   
  
  
+ CTTTAACTAA ACCTTGTCTA AGTCGTGTCT AAGCGCCTTT GTTCTTTGGA AAAGGAACAA ACAAAAGTAA   
  
  
+ TAGTTTAACA GAGCCAAGAA GAGTTTTGTT TCGTTCGGTA TTAGTGCATT CATCTCTAAT GGCTATAGGG   
  
  
+ TTTAAACGGC TTCGATACAT TATTCCTTCG TTAGCCTTAC TTCGTTACTG GAGATATTTA AGTCTTTGAC   
  
  
+ TTCTTCTAGA TTCCTTTTTT TATAAACTTC AAGTTCTCTA ATAGCTACTT TTTCCTTTGC TTTGAAGGTC   
  
  
+ CTAGTTCCCT TTTACTTATT TTCGTGAAAG TTCATTTGTT ATTATACTTG CTCTCGTATC TAGTTCAACT   
  
  
+ TAATTAGTGT TGGAGAGTTT TTTTTTTAGT GTCTTTGTTT CACGTCGCGG ATTTAAAATC GACCCTCCGG   
  
  
+ TCTTCAATTC GAACAATAAT AATTTTTTTC TTTTTTCTAA TTCTCTATTT ATTGCAGAAC TCATTTTCGT   
  
  
+ TCTGTTTCTT ATATCTGTGT CTCTCTCTCT CTCTCTCTCT CTTTTTCTTT TTTCTAATTC TCTATTTATT   
  
  
+ GCAGAACTCA TTTTCGTTCT GTTTCTTATA TCTGTGTCTC TCTCTCTCTC TCTCTCTCTT TCTTTCTTTC   
  
  
+ TCTCTTACTA ATATTTACTA ATGGACTAAC TAATTTCTCG ATTTCCACGT ACTGCTTCTG GGTGGTGGTG   
  
  
+ CCGGTGGTAG TGGTGGTTAG ACTGGACTGG GCTCAAGATT TGATTGGCGT TTGCCTGGGC TAGTCTAACA   
  
  
+ AGGCAGCTCA TATGCTACTT AAGTTACCTC TAGCTTTGTT CGTGGGCAGG CAACACCCGT AGTCTCTCGT   
  
  
+ TTTTGTTTTC CCGTTTTCCG TTGGAGAGAT AGTCAGAAGA GAGAGAAGGG CGAACAGAAA AGAATATGGG   
  
  
+ TTTTACAAGG TCAACTAATA AAAAGTTTTC TTTGATGATT TTGCTTGTTT ACGTAGAATA AGGTATTCTA   
  
  
+ CTTAAGGTTA CTTGCAGCCT GTAATTTTT  

- GATATTTCAA AGACTTGGCT CAGAGACAGG TCGCTAATAA TGAAGGTGGC TGAATTCCTG AAAGGTTCAA   
  
  
- AACAAACATG GTATACAATA AAATGAATGG CCCACCTAAA TCAAGGCTAG CACAGACAGA GAAGCAACAC   
  
  
- TGAATCTAGC ACCTTTCAAT TGCAGAAGAT ACGCAAACTC TGGACAGATT AAATTATATG AAAATGAACC   
  
  
- TTCCATTCAA GTCAGAGTAA CTCTAGTCCT CTCACCAGGG CACTCTTTCA TAAAAGGAAT CCTAAACTAG   
  
  
- AAAACAAAAT CATAACATCC AAACAAATTC AATTTTTTTT TTCTCCCAGT TGAGCATCTG AAAGCAGGTT   
  
  
- TAGAAGTAAA AGGTAAAGGC TTTTGGCCCC TGGTTGAGAA AGAATTTTCG AAATAATGAT AAAGAGATCA   
  
  
- ATGATTTCAT TACATCAGCT CTTTACCTTC CCAATCAGCT CTTACAACGG TAGCCAACAA TCAACCAAAG   
  
  
- GAAATTGATT TGGAACAGAT TCAGCACAGA TTCGCGGAAA CAAGAAACCT TTTCCTTGTT TGTTTTCATT   
  
  
- ATCAAATTGT CTCGGTTCTT CTCAAAACAA AGCAAGCCAT AATCACGTAA GTAGAGATTA CCGATATCCC   
  
  
- AAATTTGCCG AAGCTATGTA ATAAGGAAGC AATCGGAATG AAGCAATGAC CTCTATAAAT TCAGAAACTG   
  
  
- AAGAAGATCT AAGGAAAAAA ATATTTGAAG TTCAAGAGAT TATCGATGAA AAAGGAAACG AAACTTCCAG   
  
  
- GATCAAGGGA AAATGAATAA AAGCACTTTC AAGTAAACAA TAATATGAAC GAGAGCATAG ATCAAGTTGA   
  
  
- ATTAATCACA ACCTCTCAAA AAAAAAATCA CAGAAACAAA GTGCAGCGCC TAAATTTTAG CTGGGAGGCC   
  
  
- AGAAGTTAAG CTTGTTATTA TTAAAAAAAG AAAAAAGATT AAGAGATAAA TAACGTCTTG AGTAAAAGCA   
  
  
- AGACAAAGAA TATAGACACA GAGAGAGAGA GAGAGAGAGA GAAAAAGAAA AAAGATTAAG AGATAAATAA   
  
  
- CGTCTTGAGT AAAAGCAAGA CAAAGAATAT AGACACAGAG AGAGAGAGAG AGAGAGAGAA AGAAAGAAAG   
  
  
- AGAGAATGAT TATAAATGAT TACCTGATTG ATTAAAGAGC TAAAGGTGCA TGACGAAGAC CCACCACCAC   
  
  
- GGCCACCATC ACCACCAATC TGACCTGACC CGAGTTCTAA ACTAACCGCA AACGGACCCG ATCAGATTGT   
  
  
- TCCGTCGAGT ATACGATGAA TTCAATGGAG ATCGAAACAA GCACCCGTCC GTTGTGGGCA TCAGAGAGCA   
  
  
- AAAACAAAAG GGCAAAAGGC AACCTCTCTA TCAGTCTTCT CTCTCTTCCC GCTTGTCTTT TCTTATACCC   
  
  
- AAAATGTTCC AGTTGATTAT TTTTCAAAAG AAACTACTAA AACGAACAAA TGCATCTTAT TCCATAAGAT   
  
  
- GAATTCCAAT GAACGTCGGA CATTAAAAA

+     AE-box

| Site Name | Organism | Position | Strand | Matrix score. | sequence | function |
| --- | --- | --- | --- | --- | --- | --- |
| AE-box | Arabidopsis thaliana | 6 | - | 8 | AGAAACTT | part of a module for light response |

> 2018/04/13 10:10:12  
+ CTATAAAGTT TCTGAACCGA GTCTCTGTCC AGCGATTATT ACTTCCACCG ACTTAAGGAC TTTCCAAGTT   
  
  
+ TTGTTTGTAC CATATGTTAT TTTACTTACC GGGTGGATTT AGTTCCGATC GTGTCTGTCT CTTCGTTGTG   
  
  
+ ACTTAGATCG TGGAAAGTTA ACGTCTTCTA TGCGTTTGAG ACCTGTCTAA TTTAATATAC TTTTACTTGG   
  
  
+ AAGGTAAGTT CAGTCTCATT GAGATCAGGA GAGTGGTCCC GTGAGAAAGT ATTTTCCTTA GGATTTGATC   
  
  
+ TTTTGTTTTA GTATTGTAGG TTTGTTTAAG TTAAAAAAAA AAGAGGGTCA ACTCGTAGAC TTTCGTCCAA   
  
  
+ ATCTTCATTT TCCATTTCCG AAAACCGGGG ACCAACTCTT TCTTAAAAGC TTTATTACTA TTTCTCTAGT   
  
  
+ TACTAAAGTA ATGTAGTCGA GAAATGGAAG GGTTAGTCGA GAATGTTGCC ATCGGTTGTT AGTTGGTTTC   
  
  
+ CTTTAACTAA ACCTTGTCTA AGTCGTGTCT AAGCGCCTTT GTTCTTTGGA AAAGGAACAA ACAAAAGTAA   
  
  
+ TAGTTTAACA GAGCCAAGAA GAGTTTTGTT TCGTTCGGTA TTAGTGCATT CATCTCTAAT GGCTATAGGG   
  
  
+ TTTAAACGGC TTCGATACAT TATTCCTTCG TTAGCCTTAC TTCGTTACTG GAGATATTTA AGTCTTTGAC   
  
  
+ TTCTTCTAGA TTCCTTTTTT TATAAACTTC AAGTTCTCTA ATAGCTACTT TTTCCTTTGC TTTGAAGGTC   
  
  
+ CTAGTTCCCT TTTACTTATT TTCGTGAAAG TTCATTTGTT ATTATACTTG CTCTCGTATC TAGTTCAACT   
  
  
+ TAATTAGTGT TGGAGAGTTT TTTTTTTAGT GTCTTTGTTT CACGTCGCGG ATTTAAAATC GACCCTCCGG   
  
  
+ TCTTCAATTC GAACAATAAT AATTTTTTTC TTTTTTCTAA TTCTCTATTT ATTGCAGAAC TCATTTTCGT   
  
  
+ TCTGTTTCTT ATATCTGTGT CTCTCTCTCT CTCTCTCTCT CTTTTTCTTT TTTCTAATTC TCTATTTATT   
  
  
+ GCAGAACTCA TTTTCGTTCT GTTTCTTATA TCTGTGTCTC TCTCTCTCTC TCTCTCTCTT TCTTTCTTTC   
  
  
+ TCTCTTACTA ATATTTACTA ATGGACTAAC TAATTTCTCG ATTTCCACGT ACTGCTTCTG GGTGGTGGTG   
  
  
+ CCGGTGGTAG TGGTGGTTAG ACTGGACTGG GCTCAAGATT TGATTGGCGT TTGCCTGGGC TAGTCTAACA   
  
  
+ AGGCAGCTCA TATGCTACTT AAGTTACCTC TAGCTTTGTT CGTGGGCAGG CAACACCCGT AGTCTCTCGT   
  
  
+ TTTTGTTTTC CCGTTTTCCG TTGGAGAGAT AGTCAGAAGA GAGAGAAGGG CGAACAGAAA AGAATATGGG   
  
  
+ TTTTACAAGG TCAACTAATA AAAAGTTTTC TTTGATGATT TTGCTTGTTT ACGTAGAATA AGGTATTCTA   
  
  
+ CTTAAGGTTA CTTGCAGCCT GTAATTTTT  

- GATATTTCAA AGACTTGGCT CAGAGACAGG TCGCTAATAA TGAAGGTGGC TGAATTCCTG AAAGGTTCAA   
  
  
- AACAAACATG GTATACAATA AAATGAATGG CCCACCTAAA TCAAGGCTAG CACAGACAGA GAAGCAACAC   
  
  
- TGAATCTAGC ACCTTTCAAT TGCAGAAGAT ACGCAAACTC TGGACAGATT AAATTATATG AAAATGAACC   
  
  
- TTCCATTCAA GTCAGAGTAA CTCTAGTCCT CTCACCAGGG CACTCTTTCA TAAAAGGAAT CCTAAACTAG   
  
  
- AAAACAAAAT CATAACATCC AAACAAATTC AATTTTTTTT TTCTCCCAGT TGAGCATCTG AAAGCAGGTT   
  
  
- TAGAAGTAAA AGGTAAAGGC TTTTGGCCCC TGGTTGAGAA AGAATTTTCG AAATAATGAT AAAGAGATCA   
  
  
- ATGATTTCAT TACATCAGCT CTTTACCTTC CCAATCAGCT CTTACAACGG TAGCCAACAA TCAACCAAAG   
  
  
- GAAATTGATT TGGAACAGAT TCAGCACAGA TTCGCGGAAA CAAGAAACCT TTTCCTTGTT TGTTTTCATT   
  
  
- ATCAAATTGT CTCGGTTCTT CTCAAAACAA AGCAAGCCAT AATCACGTAA GTAGAGATTA CCGATATCCC   
  
  
- AAATTTGCCG AAGCTATGTA ATAAGGAAGC AATCGGAATG AAGCAATGAC CTCTATAAAT TCAGAAACTG   
  
  
- AAGAAGATCT AAGGAAAAAA ATATTTGAAG TTCAAGAGAT TATCGATGAA AAAGGAAACG AAACTTCCAG   
  
  
- GATCAAGGGA AAATGAATAA AAGCACTTTC AAGTAAACAA TAATATGAAC GAGAGCATAG ATCAAGTTGA   
  
  
- ATTAATCACA ACCTCTCAAA AAAAAAATCA CAGAAACAAA GTGCAGCGCC TAAATTTTAG CTGGGAGGCC   
  
  
- AGAAGTTAAG CTTGTTATTA TTAAAAAAAG AAAAAAGATT AAGAGATAAA TAACGTCTTG AGTAAAAGCA   
  
  
- AGACAAAGAA TATAGACACA GAGAGAGAGA GAGAGAGAGA GAAAAAGAAA AAAGATTAAG AGATAAATAA   
  
  
- CGTCTTGAGT AAAAGCAAGA CAAAGAATAT AGACACAGAG AGAGAGAGAG AGAGAGAGAA AGAAAGAAAG   
  
  
- AGAGAATGAT TATAAATGAT TACCTGATTG ATTAAAGAGC TAAAGGTGCA TGACGAAGAC CCACCACCAC   
  
  
- GGCCACCATC ACCACCAATC TGACCTGACC CGAGTTCTAA ACTAACCGCA AACGGACCCG ATCAGATTGT   
  
  
- TCCGTCGAGT ATACGATGAA TTCAATGGAG ATCGAAACAA GCACCCGTCC GTTGTGGGCA TCAGAGAGCA   
  
  
- AAAACAAAAG GGCAAAAGGC AACCTCTCTA TCAGTCTTCT CTCTCTTCCC GCTTGTCTTT TCTTATACCC   
  
  
- AAAATGTTCC AGTTGATTAT TTTTCAAAAG AAACTACTAA AACGAACAAA TGCATCTTAT TCCATAAGAT   
  
  
- GAATTCCAAT GAACGTCGGA CATTAAAAA

+     ARE

| Site Name | Organism | Position | Strand | Matrix score. | sequence | function |
| --- | --- | --- | --- | --- | --- | --- |
| ARE | Zea mays | 484 | + | 6 | TGGTTT | cis-acting regulatory element essential for the anaerobic induction |

> 2018/04/13 10:10:12  
+ CTATAAAGTT TCTGAACCGA GTCTCTGTCC AGCGATTATT ACTTCCACCG ACTTAAGGAC TTTCCAAGTT   
  
  
+ TTGTTTGTAC CATATGTTAT TTTACTTACC GGGTGGATTT AGTTCCGATC GTGTCTGTCT CTTCGTTGTG   
  
  
+ ACTTAGATCG TGGAAAGTTA ACGTCTTCTA TGCGTTTGAG ACCTGTCTAA TTTAATATAC TTTTACTTGG   
  
  
+ AAGGTAAGTT CAGTCTCATT GAGATCAGGA GAGTGGTCCC GTGAGAAAGT ATTTTCCTTA GGATTTGATC   
  
  
+ TTTTGTTTTA GTATTGTAGG TTTGTTTAAG TTAAAAAAAA AAGAGGGTCA ACTCGTAGAC TTTCGTCCAA   
  
  
+ ATCTTCATTT TCCATTTCCG AAAACCGGGG ACCAACTCTT TCTTAAAAGC TTTATTACTA TTTCTCTAGT   
  
  
+ TACTAAAGTA ATGTAGTCGA GAAATGGAAG GGTTAGTCGA GAATGTTGCC ATCGGTTGTT AGTTGGTTTC   
  
  
+ CTTTAACTAA ACCTTGTCTA AGTCGTGTCT AAGCGCCTTT GTTCTTTGGA AAAGGAACAA ACAAAAGTAA   
  
  
+ TAGTTTAACA GAGCCAAGAA GAGTTTTGTT TCGTTCGGTA TTAGTGCATT CATCTCTAAT GGCTATAGGG   
  
  
+ TTTAAACGGC TTCGATACAT TATTCCTTCG TTAGCCTTAC TTCGTTACTG GAGATATTTA AGTCTTTGAC   
  
  
+ TTCTTCTAGA TTCCTTTTTT TATAAACTTC AAGTTCTCTA ATAGCTACTT TTTCCTTTGC TTTGAAGGTC   
  
  
+ CTAGTTCCCT TTTACTTATT TTCGTGAAAG TTCATTTGTT ATTATACTTG CTCTCGTATC TAGTTCAACT   
  
  
+ TAATTAGTGT TGGAGAGTTT TTTTTTTAGT GTCTTTGTTT CACGTCGCGG ATTTAAAATC GACCCTCCGG   
  
  
+ TCTTCAATTC GAACAATAAT AATTTTTTTC TTTTTTCTAA TTCTCTATTT ATTGCAGAAC TCATTTTCGT   
  
  
+ TCTGTTTCTT ATATCTGTGT CTCTCTCTCT CTCTCTCTCT CTTTTTCTTT TTTCTAATTC TCTATTTATT   
  
  
+ GCAGAACTCA TTTTCGTTCT GTTTCTTATA TCTGTGTCTC TCTCTCTCTC TCTCTCTCTT TCTTTCTTTC   
  
  
+ TCTCTTACTA ATATTTACTA ATGGACTAAC TAATTTCTCG ATTTCCACGT ACTGCTTCTG GGTGGTGGTG   
  
  
+ CCGGTGGTAG TGGTGGTTAG ACTGGACTGG GCTCAAGATT TGATTGGCGT TTGCCTGGGC TAGTCTAACA   
  
  
+ AGGCAGCTCA TATGCTACTT AAGTTACCTC TAGCTTTGTT CGTGGGCAGG CAACACCCGT AGTCTCTCGT   
  
  
+ TTTTGTTTTC CCGTTTTCCG TTGGAGAGAT AGTCAGAAGA GAGAGAAGGG CGAACAGAAA AGAATATGGG   
  
  
+ TTTTACAAGG TCAACTAATA AAAAGTTTTC TTTGATGATT TTGCTTGTTT ACGTAGAATA AGGTATTCTA   
  
  
+ CTTAAGGTTA CTTGCAGCCT GTAATTTTT  

- GATATTTCAA AGACTTGGCT CAGAGACAGG TCGCTAATAA TGAAGGTGGC TGAATTCCTG AAAGGTTCAA   
  
  
- AACAAACATG GTATACAATA AAATGAATGG CCCACCTAAA TCAAGGCTAG CACAGACAGA GAAGCAACAC   
  
  
- TGAATCTAGC ACCTTTCAAT TGCAGAAGAT ACGCAAACTC TGGACAGATT AAATTATATG AAAATGAACC   
  
  
- TTCCATTCAA GTCAGAGTAA CTCTAGTCCT CTCACCAGGG CACTCTTTCA TAAAAGGAAT CCTAAACTAG   
  
  
- AAAACAAAAT CATAACATCC AAACAAATTC AATTTTTTTT TTCTCCCAGT TGAGCATCTG AAAGCAGGTT   
  
  
- TAGAAGTAAA AGGTAAAGGC TTTTGGCCCC TGGTTGAGAA AGAATTTTCG AAATAATGAT AAAGAGATCA   
  
  
- ATGATTTCAT TACATCAGCT CTTTACCTTC CCAATCAGCT CTTACAACGG TAGCCAACAA TCAACCAAAG   
  
  
- GAAATTGATT TGGAACAGAT TCAGCACAGA TTCGCGGAAA CAAGAAACCT TTTCCTTGTT TGTTTTCATT   
  
  
- ATCAAATTGT CTCGGTTCTT CTCAAAACAA AGCAAGCCAT AATCACGTAA GTAGAGATTA CCGATATCCC   
  
  
- AAATTTGCCG AAGCTATGTA ATAAGGAAGC AATCGGAATG AAGCAATGAC CTCTATAAAT TCAGAAACTG   
  
  
- AAGAAGATCT AAGGAAAAAA ATATTTGAAG TTCAAGAGAT TATCGATGAA AAAGGAAACG AAACTTCCAG   
  
  
- GATCAAGGGA AAATGAATAA AAGCACTTTC AAGTAAACAA TAATATGAAC GAGAGCATAG ATCAAGTTGA   
  
  
- ATTAATCACA ACCTCTCAAA AAAAAAATCA CAGAAACAAA GTGCAGCGCC TAAATTTTAG CTGGGAGGCC   
  
  
- AGAAGTTAAG CTTGTTATTA TTAAAAAAAG AAAAAAGATT AAGAGATAAA TAACGTCTTG AGTAAAAGCA   
  
  
- AGACAAAGAA TATAGACACA GAGAGAGAGA GAGAGAGAGA GAAAAAGAAA AAAGATTAAG AGATAAATAA   
  
  
- CGTCTTGAGT AAAAGCAAGA CAAAGAATAT AGACACAGAG AGAGAGAGAG AGAGAGAGAA AGAAAGAAAG   
  
  
- AGAGAATGAT TATAAATGAT TACCTGATTG ATTAAAGAGC TAAAGGTGCA TGACGAAGAC CCACCACCAC   
  
  
- GGCCACCATC ACCACCAATC TGACCTGACC CGAGTTCTAA ACTAACCGCA AACGGACCCG ATCAGATTGT   
  
  
- TCCGTCGAGT ATACGATGAA TTCAATGGAG ATCGAAACAA GCACCCGTCC GTTGTGGGCA TCAGAGAGCA   
  
  
- AAAACAAAAG GGCAAAAGGC AACCTCTCTA TCAGTCTTCT CTCTCTTCCC GCTTGTCTTT TCTTATACCC   
  
  
- AAAATGTTCC AGTTGATTAT TTTTCAAAAG AAACTACTAA AACGAACAAA TGCATCTTAT TCCATAAGAT   
  
  
- GAATTCCAAT GAACGTCGGA CATTAAAAA

+     ATCT-motif

| Site Name | Organism | Position | Strand | Matrix score. | sequence | function |
| --- | --- | --- | --- | --- | --- | --- |
| ATCT-motif | Arabidopsis thaliana | 1226 | - | 9 | AATCTAATCT | part of a conserved DNA module involved in light responsiveness |

> 2018/04/13 10:10:12  
+ CTATAAAGTT TCTGAACCGA GTCTCTGTCC AGCGATTATT ACTTCCACCG ACTTAAGGAC TTTCCAAGTT   
  
  
+ TTGTTTGTAC CATATGTTAT TTTACTTACC GGGTGGATTT AGTTCCGATC GTGTCTGTCT CTTCGTTGTG   
  
  
+ ACTTAGATCG TGGAAAGTTA ACGTCTTCTA TGCGTTTGAG ACCTGTCTAA TTTAATATAC TTTTACTTGG   
  
  
+ AAGGTAAGTT CAGTCTCATT GAGATCAGGA GAGTGGTCCC GTGAGAAAGT ATTTTCCTTA GGATTTGATC   
  
  
+ TTTTGTTTTA GTATTGTAGG TTTGTTTAAG TTAAAAAAAA AAGAGGGTCA ACTCGTAGAC TTTCGTCCAA   
  
  
+ ATCTTCATTT TCCATTTCCG AAAACCGGGG ACCAACTCTT TCTTAAAAGC TTTATTACTA TTTCTCTAGT   
  
  
+ TACTAAAGTA ATGTAGTCGA GAAATGGAAG GGTTAGTCGA GAATGTTGCC ATCGGTTGTT AGTTGGTTTC   
  
  
+ CTTTAACTAA ACCTTGTCTA AGTCGTGTCT AAGCGCCTTT GTTCTTTGGA AAAGGAACAA ACAAAAGTAA   
  
  
+ TAGTTTAACA GAGCCAAGAA GAGTTTTGTT TCGTTCGGTA TTAGTGCATT CATCTCTAAT GGCTATAGGG   
  
  
+ TTTAAACGGC TTCGATACAT TATTCCTTCG TTAGCCTTAC TTCGTTACTG GAGATATTTA AGTCTTTGAC   
  
  
+ TTCTTCTAGA TTCCTTTTTT TATAAACTTC AAGTTCTCTA ATAGCTACTT TTTCCTTTGC TTTGAAGGTC   
  
  
+ CTAGTTCCCT TTTACTTATT TTCGTGAAAG TTCATTTGTT ATTATACTTG CTCTCGTATC TAGTTCAACT   
  
  
+ TAATTAGTGT TGGAGAGTTT TTTTTTTAGT GTCTTTGTTT CACGTCGCGG ATTTAAAATC GACCCTCCGG   
  
  
+ TCTTCAATTC GAACAATAAT AATTTTTTTC TTTTTTCTAA TTCTCTATTT ATTGCAGAAC TCATTTTCGT   
  
  
+ TCTGTTTCTT ATATCTGTGT CTCTCTCTCT CTCTCTCTCT CTTTTTCTTT TTTCTAATTC TCTATTTATT   
  
  
+ GCAGAACTCA TTTTCGTTCT GTTTCTTATA TCTGTGTCTC TCTCTCTCTC TCTCTCTCTT TCTTTCTTTC   
  
  
+ TCTCTTACTA ATATTTACTA ATGGACTAAC TAATTTCTCG ATTTCCACGT ACTGCTTCTG GGTGGTGGTG   
  
  
+ CCGGTGGTAG TGGTGGTTAG ACTGGACTGG GCTCAAGATT TGATTGGCGT TTGCCTGGGC TAGTCTAACA   
  
  
+ AGGCAGCTCA TATGCTACTT AAGTTACCTC TAGCTTTGTT CGTGGGCAGG CAACACCCGT AGTCTCTCGT   
  
  
+ TTTTGTTTTC CCGTTTTCCG TTGGAGAGAT AGTCAGAAGA GAGAGAAGGG CGAACAGAAA AGAATATGGG   
  
  
+ TTTTACAAGG TCAACTAATA AAAAGTTTTC TTTGATGATT TTGCTTGTTT ACGTAGAATA AGGTATTCTA   
  
  
+ CTTAAGGTTA CTTGCAGCCT GTAATTTTT  

- GATATTTCAA AGACTTGGCT CAGAGACAGG TCGCTAATAA TGAAGGTGGC TGAATTCCTG AAAGGTTCAA   
  
  
- AACAAACATG GTATACAATA AAATGAATGG CCCACCTAAA TCAAGGCTAG CACAGACAGA GAAGCAACAC   
  
  
- TGAATCTAGC ACCTTTCAAT TGCAGAAGAT ACGCAAACTC TGGACAGATT AAATTATATG AAAATGAACC   
  
  
- TTCCATTCAA GTCAGAGTAA CTCTAGTCCT CTCACCAGGG CACTCTTTCA TAAAAGGAAT CCTAAACTAG   
  
  
- AAAACAAAAT CATAACATCC AAACAAATTC AATTTTTTTT TTCTCCCAGT TGAGCATCTG AAAGCAGGTT   
  
  
- TAGAAGTAAA AGGTAAAGGC TTTTGGCCCC TGGTTGAGAA AGAATTTTCG AAATAATGAT AAAGAGATCA   
  
  
- ATGATTTCAT TACATCAGCT CTTTACCTTC CCAATCAGCT CTTACAACGG TAGCCAACAA TCAACCAAAG   
  
  
- GAAATTGATT TGGAACAGAT TCAGCACAGA TTCGCGGAAA CAAGAAACCT TTTCCTTGTT TGTTTTCATT   
  
  
- ATCAAATTGT CTCGGTTCTT CTCAAAACAA AGCAAGCCAT AATCACGTAA GTAGAGATTA CCGATATCCC   
  
  
- AAATTTGCCG AAGCTATGTA ATAAGGAAGC AATCGGAATG AAGCAATGAC CTCTATAAAT TCAGAAACTG   
  
  
- AAGAAGATCT AAGGAAAAAA ATATTTGAAG TTCAAGAGAT TATCGATGAA AAAGGAAACG AAACTTCCAG   
  
  
- GATCAAGGGA AAATGAATAA AAGCACTTTC AAGTAAACAA TAATATGAAC GAGAGCATAG ATCAAGTTGA   
  
  
- ATTAATCACA ACCTCTCAAA AAAAAAATCA CAGAAACAAA GTGCAGCGCC TAAATTTTAG CTGGGAGGCC   
  
  
- AGAAGTTAAG CTTGTTATTA TTAAAAAAAG AAAAAAGATT AAGAGATAAA TAACGTCTTG AGTAAAAGCA   
  
  
- AGACAAAGAA TATAGACACA GAGAGAGAGA GAGAGAGAGA GAAAAAGAAA AAAGATTAAG AGATAAATAA   
  
  
- CGTCTTGAGT AAAAGCAAGA CAAAGAATAT AGACACAGAG AGAGAGAGAG AGAGAGAGAA AGAAAGAAAG   
  
  
- AGAGAATGAT TATAAATGAT TACCTGATTG ATTAAAGAGC TAAAGGTGCA TGACGAAGAC CCACCACCAC   
  
  
- GGCCACCATC ACCACCAATC TGACCTGACC CGAGTTCTAA ACTAACCGCA AACGGACCCG ATCAGATTGT   
  
  
- TCCGTCGAGT ATACGATGAA TTCAATGGAG ATCGAAACAA GCACCCGTCC GTTGTGGGCA TCAGAGAGCA   
  
  
- AAAACAAAAG GGCAAAAGGC AACCTCTCTA TCAGTCTTCT CTCTCTTCCC GCTTGTCTTT TCTTATACCC   
  
  
- AAAATGTTCC AGTTGATTAT TTTTCAAAAG AAACTACTAA AACGAACAAA TGCATCTTAT TCCATAAGAT   
  
  
- GAATTCCAAT GAACGTCGGA CATTAAAAA

+     Box-W1

| Site Name | Organism | Position | Strand | Matrix score. | sequence | function |
| --- | --- | --- | --- | --- | --- | --- |
| Box-W1 | Petroselinum crispum | 1409 | - | 6 | TTGACC | fungal elicitor responsive element |
| Box-W1 | Petroselinum crispum | 326 | - | 6 | TTGACC | fungal elicitor responsive element |

> 2018/04/13 10:10:12  
+ CTATAAAGTT TCTGAACCGA GTCTCTGTCC AGCGATTATT ACTTCCACCG ACTTAAGGAC TTTCCAAGTT   
  
  
+ TTGTTTGTAC CATATGTTAT TTTACTTACC GGGTGGATTT AGTTCCGATC GTGTCTGTCT CTTCGTTGTG   
  
  
+ ACTTAGATCG TGGAAAGTTA ACGTCTTCTA TGCGTTTGAG ACCTGTCTAA TTTAATATAC TTTTACTTGG   
  
  
+ AAGGTAAGTT CAGTCTCATT GAGATCAGGA GAGTGGTCCC GTGAGAAAGT ATTTTCCTTA GGATTTGATC   
  
  
+ TTTTGTTTTA GTATTGTAGG TTTGTTTAAG TTAAAAAAAA AAGAGGGTCA ACTCGTAGAC TTTCGTCCAA   
  
  
+ ATCTTCATTT TCCATTTCCG AAAACCGGGG ACCAACTCTT TCTTAAAAGC TTTATTACTA TTTCTCTAGT   
  
  
+ TACTAAAGTA ATGTAGTCGA GAAATGGAAG GGTTAGTCGA GAATGTTGCC ATCGGTTGTT AGTTGGTTTC   
  
  
+ CTTTAACTAA ACCTTGTCTA AGTCGTGTCT AAGCGCCTTT GTTCTTTGGA AAAGGAACAA ACAAAAGTAA   
  
  
+ TAGTTTAACA GAGCCAAGAA GAGTTTTGTT TCGTTCGGTA TTAGTGCATT CATCTCTAAT GGCTATAGGG   
  
  
+ TTTAAACGGC TTCGATACAT TATTCCTTCG TTAGCCTTAC TTCGTTACTG GAGATATTTA AGTCTTTGAC   
  
  
+ TTCTTCTAGA TTCCTTTTTT TATAAACTTC AAGTTCTCTA ATAGCTACTT TTTCCTTTGC TTTGAAGGTC   
  
  
+ CTAGTTCCCT TTTACTTATT TTCGTGAAAG TTCATTTGTT ATTATACTTG CTCTCGTATC TAGTTCAACT   
  
  
+ TAATTAGTGT TGGAGAGTTT TTTTTTTAGT GTCTTTGTTT CACGTCGCGG ATTTAAAATC GACCCTCCGG   
  
  
+ TCTTCAATTC GAACAATAAT AATTTTTTTC TTTTTTCTAA TTCTCTATTT ATTGCAGAAC TCATTTTCGT   
  
  
+ TCTGTTTCTT ATATCTGTGT CTCTCTCTCT CTCTCTCTCT CTTTTTCTTT TTTCTAATTC TCTATTTATT   
  
  
+ GCAGAACTCA TTTTCGTTCT GTTTCTTATA TCTGTGTCTC TCTCTCTCTC TCTCTCTCTT TCTTTCTTTC   
  
  
+ TCTCTTACTA ATATTTACTA ATGGACTAAC TAATTTCTCG ATTTCCACGT ACTGCTTCTG GGTGGTGGTG   
  
  
+ CCGGTGGTAG TGGTGGTTAG ACTGGACTGG GCTCAAGATT TGATTGGCGT TTGCCTGGGC TAGTCTAACA   
  
  
+ AGGCAGCTCA TATGCTACTT AAGTTACCTC TAGCTTTGTT CGTGGGCAGG CAACACCCGT AGTCTCTCGT   
  
  
+ TTTTGTTTTC CCGTTTTCCG TTGGAGAGAT AGTCAGAAGA GAGAGAAGGG CGAACAGAAA AGAATATGGG   
  
  
+ TTTTACAAGG TCAACTAATA AAAAGTTTTC TTTGATGATT TTGCTTGTTT ACGTAGAATA AGGTATTCTA   
  
  
+ CTTAAGGTTA CTTGCAGCCT GTAATTTTT  

- GATATTTCAA AGACTTGGCT CAGAGACAGG TCGCTAATAA TGAAGGTGGC TGAATTCCTG AAAGGTTCAA   
  
  
- AACAAACATG GTATACAATA AAATGAATGG CCCACCTAAA TCAAGGCTAG CACAGACAGA GAAGCAACAC   
  
  
- TGAATCTAGC ACCTTTCAAT TGCAGAAGAT ACGCAAACTC TGGACAGATT AAATTATATG AAAATGAACC   
  
  
- TTCCATTCAA GTCAGAGTAA CTCTAGTCCT CTCACCAGGG CACTCTTTCA TAAAAGGAAT CCTAAACTAG   
  
  
- AAAACAAAAT CATAACATCC AAACAAATTC AATTTTTTTT TTCTCCCAGT TGAGCATCTG AAAGCAGGTT   
  
  
- TAGAAGTAAA AGGTAAAGGC TTTTGGCCCC TGGTTGAGAA AGAATTTTCG AAATAATGAT AAAGAGATCA   
  
  
- ATGATTTCAT TACATCAGCT CTTTACCTTC CCAATCAGCT CTTACAACGG TAGCCAACAA TCAACCAAAG   
  
  
- GAAATTGATT TGGAACAGAT TCAGCACAGA TTCGCGGAAA CAAGAAACCT TTTCCTTGTT TGTTTTCATT   
  
  
- ATCAAATTGT CTCGGTTCTT CTCAAAACAA AGCAAGCCAT AATCACGTAA GTAGAGATTA CCGATATCCC   
  
  
- AAATTTGCCG AAGCTATGTA ATAAGGAAGC AATCGGAATG AAGCAATGAC CTCTATAAAT TCAGAAACTG   
  
  
- AAGAAGATCT AAGGAAAAAA ATATTTGAAG TTCAAGAGAT TATCGATGAA AAAGGAAACG AAACTTCCAG   
  
  
- GATCAAGGGA AAATGAATAA AAGCACTTTC AAGTAAACAA TAATATGAAC GAGAGCATAG ATCAAGTTGA   
  
  
- ATTAATCACA ACCTCTCAAA AAAAAAATCA CAGAAACAAA GTGCAGCGCC TAAATTTTAG CTGGGAGGCC   
  
  
- AGAAGTTAAG CTTGTTATTA TTAAAAAAAG AAAAAAGATT AAGAGATAAA TAACGTCTTG AGTAAAAGCA   
  
  
- AGACAAAGAA TATAGACACA GAGAGAGAGA GAGAGAGAGA GAAAAAGAAA AAAGATTAAG AGATAAATAA   
  
  
- CGTCTTGAGT AAAAGCAAGA CAAAGAATAT AGACACAGAG AGAGAGAGAG AGAGAGAGAA AGAAAGAAAG   
  
  
- AGAGAATGAT TATAAATGAT TACCTGATTG ATTAAAGAGC TAAAGGTGCA TGACGAAGAC CCACCACCAC   
  
  
- GGCCACCATC ACCACCAATC TGACCTGACC CGAGTTCTAA ACTAACCGCA AACGGACCCG ATCAGATTGT   
  
  
- TCCGTCGAGT ATACGATGAA TTCAATGGAG ATCGAAACAA GCACCCGTCC GTTGTGGGCA TCAGAGAGCA   
  
  
- AAAACAAAAG GGCAAAAGGC AACCTCTCTA TCAGTCTTCT CTCTCTTCCC GCTTGTCTTT TCTTATACCC   
  
  
- AAAATGTTCC AGTTGATTAT TTTTCAAAAG AAACTACTAA AACGAACAAA TGCATCTTAT TCCATAAGAT   
  
  
- GAATTCCAAT GAACGTCGGA CATTAAAAA

+     CAAT-box

| Site Name | Organism | Position | Strand | Matrix score. | sequence | function |
| --- | --- | --- | --- | --- | --- | --- |
| CAAT-box | Glycine max | 915 | + | 5 | CAATT | common cis-acting element in promoter and enhancer regions |
| CAAT-box | Brassica rapa | 804 | - | 5 | CAAAT | common cis-acting element in promoter and enhancer regions |
| CAAT-box | Brassica rapa | 273 | - | 5 | CAAAT | common cis-acting element in promoter and enhancer regions |
| CAAT-box | Hordeum vulgare | 228 | - | 4 | CAAT | common cis-acting element in promoter and enhancer regions |
| CAAT-box | Brassica rapa | 348 | + | 5 | CAAAT | common cis-acting element in promoter and enhancer regions |
| CAAT-box | Hordeum vulgare | 293 | - | 4 | CAAT | common cis-acting element in promoter and enhancer regions |
| CAAT-box | Arabidopsis thaliana | 1233 | - | 5 | CCAAT | common cis-acting element in promoter and enhancer regions |
| CAAT-box | Hordeum vulgare | 961 | - | 4 | CAAT | common cis-acting element in promoter and enhancer regions |
| CAAT-box | Hordeum vulgare | 924 | + | 4 | CAAT | common cis-acting element in promoter and enhancer regions |
| CAAT-box | Brassica rapa | 1228 | - | 5 | CAAAT | common cis-acting element in promoter and enhancer regions |
| CAAT-box | Hordeum vulgare | 1048 | - | 4 | CAAT | common cis-acting element in promoter and enhancer regions |

> 2018/04/13 10:10:12  
+ CTATAAAGTT TCTGAACCGA GTCTCTGTCC AGCGATTATT ACTTCCACCG ACTTAAGGAC TTTCCAAGTT   
  
  
+ TTGTTTGTAC CATATGTTAT TTTACTTACC GGGTGGATTT AGTTCCGATC GTGTCTGTCT CTTCGTTGTG   
  
  
+ ACTTAGATCG TGGAAAGTTA ACGTCTTCTA TGCGTTTGAG ACCTGTCTAA TTTAATATAC TTTTACTTGG   
  
  
+ AAGGTAAGTT CAGTCTCATT GAGATCAGGA GAGTGGTCCC GTGAGAAAGT ATTTTCCTTA GGATTTGATC   
  
  
+ TTTTGTTTTA GTATTGTAGG TTTGTTTAAG TTAAAAAAAA AAGAGGGTCA ACTCGTAGAC TTTCGTCCAA   
  
  
+ ATCTTCATTT TCCATTTCCG AAAACCGGGG ACCAACTCTT TCTTAAAAGC TTTATTACTA TTTCTCTAGT   
  
  
+ TACTAAAGTA ATGTAGTCGA GAAATGGAAG GGTTAGTCGA GAATGTTGCC ATCGGTTGTT AGTTGGTTTC   
  
  
+ CTTTAACTAA ACCTTGTCTA AGTCGTGTCT AAGCGCCTTT GTTCTTTGGA AAAGGAACAA ACAAAAGTAA   
  
  
+ TAGTTTAACA GAGCCAAGAA GAGTTTTGTT TCGTTCGGTA TTAGTGCATT CATCTCTAAT GGCTATAGGG   
  
  
+ TTTAAACGGC TTCGATACAT TATTCCTTCG TTAGCCTTAC TTCGTTACTG GAGATATTTA AGTCTTTGAC   
  
  
+ TTCTTCTAGA TTCCTTTTTT TATAAACTTC AAGTTCTCTA ATAGCTACTT TTTCCTTTGC TTTGAAGGTC   
  
  
+ CTAGTTCCCT TTTACTTATT TTCGTGAAAG TTCATTTGTT ATTATACTTG CTCTCGTATC TAGTTCAACT   
  
  
+ TAATTAGTGT TGGAGAGTTT TTTTTTTAGT GTCTTTGTTT CACGTCGCGG ATTTAAAATC GACCCTCCGG   
  
  
+ TCTTCAATTC GAACAATAAT AATTTTTTTC TTTTTTCTAA TTCTCTATTT ATTGCAGAAC TCATTTTCGT   
  
  
+ TCTGTTTCTT ATATCTGTGT CTCTCTCTCT CTCTCTCTCT CTTTTTCTTT TTTCTAATTC TCTATTTATT   
  
  
+ GCAGAACTCA TTTTCGTTCT GTTTCTTATA TCTGTGTCTC TCTCTCTCTC TCTCTCTCTT TCTTTCTTTC   
  
  
+ TCTCTTACTA ATATTTACTA ATGGACTAAC TAATTTCTCG ATTTCCACGT ACTGCTTCTG GGTGGTGGTG   
  
  
+ CCGGTGGTAG TGGTGGTTAG ACTGGACTGG GCTCAAGATT TGATTGGCGT TTGCCTGGGC TAGTCTAACA   
  
  
+ AGGCAGCTCA TATGCTACTT AAGTTACCTC TAGCTTTGTT CGTGGGCAGG CAACACCCGT AGTCTCTCGT   
  
  
+ TTTTGTTTTC CCGTTTTCCG TTGGAGAGAT AGTCAGAAGA GAGAGAAGGG CGAACAGAAA AGAATATGGG   
  
  
+ TTTTACAAGG TCAACTAATA AAAAGTTTTC TTTGATGATT TTGCTTGTTT ACGTAGAATA AGGTATTCTA   
  
  
+ CTTAAGGTTA CTTGCAGCCT GTAATTTTT  

- GATATTTCAA AGACTTGGCT CAGAGACAGG TCGCTAATAA TGAAGGTGGC TGAATTCCTG AAAGGTTCAA   
  
  
- AACAAACATG GTATACAATA AAATGAATGG CCCACCTAAA TCAAGGCTAG CACAGACAGA GAAGCAACAC   
  
  
- TGAATCTAGC ACCTTTCAAT TGCAGAAGAT ACGCAAACTC TGGACAGATT AAATTATATG AAAATGAACC   
  
  
- TTCCATTCAA GTCAGAGTAA CTCTAGTCCT CTCACCAGGG CACTCTTTCA TAAAAGGAAT CCTAAACTAG   
  
  
- AAAACAAAAT CATAACATCC AAACAAATTC AATTTTTTTT TTCTCCCAGT TGAGCATCTG AAAGCAGGTT   
  
  
- TAGAAGTAAA AGGTAAAGGC TTTTGGCCCC TGGTTGAGAA AGAATTTTCG AAATAATGAT AAAGAGATCA   
  
  
- ATGATTTCAT TACATCAGCT CTTTACCTTC CCAATCAGCT CTTACAACGG TAGCCAACAA TCAACCAAAG   
  
  
- GAAATTGATT TGGAACAGAT TCAGCACAGA TTCGCGGAAA CAAGAAACCT TTTCCTTGTT TGTTTTCATT   
  
  
- ATCAAATTGT CTCGGTTCTT CTCAAAACAA AGCAAGCCAT AATCACGTAA GTAGAGATTA CCGATATCCC   
  
  
- AAATTTGCCG AAGCTATGTA ATAAGGAAGC AATCGGAATG AAGCAATGAC CTCTATAAAT TCAGAAACTG   
  
  
- AAGAAGATCT AAGGAAAAAA ATATTTGAAG TTCAAGAGAT TATCGATGAA AAAGGAAACG AAACTTCCAG   
  
  
- GATCAAGGGA AAATGAATAA AAGCACTTTC AAGTAAACAA TAATATGAAC GAGAGCATAG ATCAAGTTGA   
  
  
- ATTAATCACA ACCTCTCAAA AAAAAAATCA CAGAAACAAA GTGCAGCGCC TAAATTTTAG CTGGGAGGCC   
  
  
- AGAAGTTAAG CTTGTTATTA TTAAAAAAAG AAAAAAGATT AAGAGATAAA TAACGTCTTG AGTAAAAGCA   
  
  
- AGACAAAGAA TATAGACACA GAGAGAGAGA GAGAGAGAGA GAAAAAGAAA AAAGATTAAG AGATAAATAA   
  
  
- CGTCTTGAGT AAAAGCAAGA CAAAGAATAT AGACACAGAG AGAGAGAGAG AGAGAGAGAA AGAAAGAAAG   
  
  
- AGAGAATGAT TATAAATGAT TACCTGATTG ATTAAAGAGC TAAAGGTGCA TGACGAAGAC CCACCACCAC   
  
  
- GGCCACCATC ACCACCAATC TGACCTGACC CGAGTTCTAA ACTAACCGCA AACGGACCCG ATCAGATTGT   
  
  
- TCCGTCGAGT ATACGATGAA TTCAATGGAG ATCGAAACAA GCACCCGTCC GTTGTGGGCA TCAGAGAGCA   
  
  
- AAAACAAAAG GGCAAAAGGC AACCTCTCTA TCAGTCTTCT CTCTCTTCCC GCTTGTCTTT TCTTATACCC   
  
  
- AAAATGTTCC AGTTGATTAT TTTTCAAAAG AAACTACTAA AACGAACAAA TGCATCTTAT TCCATAAGAT   
  
  
- GAATTCCAAT GAACGTCGGA CATTAAAAA

+     CATT-motif

| Site Name | Organism | Position | Strand | Matrix score. | sequence | function |
| --- | --- | --- | --- | --- | --- | --- |
| CATT-motif | Zea mays | 606 | + | 6 | GCATTC | part of a light responsive element |

> 2018/04/13 10:10:12  
+ CTATAAAGTT TCTGAACCGA GTCTCTGTCC AGCGATTATT ACTTCCACCG ACTTAAGGAC TTTCCAAGTT   
  
  
+ TTGTTTGTAC CATATGTTAT TTTACTTACC GGGTGGATTT AGTTCCGATC GTGTCTGTCT CTTCGTTGTG   
  
  
+ ACTTAGATCG TGGAAAGTTA ACGTCTTCTA TGCGTTTGAG ACCTGTCTAA TTTAATATAC TTTTACTTGG   
  
  
+ AAGGTAAGTT CAGTCTCATT GAGATCAGGA GAGTGGTCCC GTGAGAAAGT ATTTTCCTTA GGATTTGATC   
  
  
+ TTTTGTTTTA GTATTGTAGG TTTGTTTAAG TTAAAAAAAA AAGAGGGTCA ACTCGTAGAC TTTCGTCCAA   
  
  
+ ATCTTCATTT TCCATTTCCG AAAACCGGGG ACCAACTCTT TCTTAAAAGC TTTATTACTA TTTCTCTAGT   
  
  
+ TACTAAAGTA ATGTAGTCGA GAAATGGAAG GGTTAGTCGA GAATGTTGCC ATCGGTTGTT AGTTGGTTTC   
  
  
+ CTTTAACTAA ACCTTGTCTA AGTCGTGTCT AAGCGCCTTT GTTCTTTGGA AAAGGAACAA ACAAAAGTAA   
  
  
+ TAGTTTAACA GAGCCAAGAA GAGTTTTGTT TCGTTCGGTA TTAGTGCATT CATCTCTAAT GGCTATAGGG   
  
  
+ TTTAAACGGC TTCGATACAT TATTCCTTCG TTAGCCTTAC TTCGTTACTG GAGATATTTA AGTCTTTGAC   
  
  
+ TTCTTCTAGA TTCCTTTTTT TATAAACTTC AAGTTCTCTA ATAGCTACTT TTTCCTTTGC TTTGAAGGTC   
  
  
+ CTAGTTCCCT TTTACTTATT TTCGTGAAAG TTCATTTGTT ATTATACTTG CTCTCGTATC TAGTTCAACT   
  
  
+ TAATTAGTGT TGGAGAGTTT TTTTTTTAGT GTCTTTGTTT CACGTCGCGG ATTTAAAATC GACCCTCCGG   
  
  
+ TCTTCAATTC GAACAATAAT AATTTTTTTC TTTTTTCTAA TTCTCTATTT ATTGCAGAAC TCATTTTCGT   
  
  
+ TCTGTTTCTT ATATCTGTGT CTCTCTCTCT CTCTCTCTCT CTTTTTCTTT TTTCTAATTC TCTATTTATT   
  
  
+ GCAGAACTCA TTTTCGTTCT GTTTCTTATA TCTGTGTCTC TCTCTCTCTC TCTCTCTCTT TCTTTCTTTC   
  
  
+ TCTCTTACTA ATATTTACTA ATGGACTAAC TAATTTCTCG ATTTCCACGT ACTGCTTCTG GGTGGTGGTG   
  
  
+ CCGGTGGTAG TGGTGGTTAG ACTGGACTGG GCTCAAGATT TGATTGGCGT TTGCCTGGGC TAGTCTAACA   
  
  
+ AGGCAGCTCA TATGCTACTT AAGTTACCTC TAGCTTTGTT CGTGGGCAGG CAACACCCGT AGTCTCTCGT   
  
  
+ TTTTGTTTTC CCGTTTTCCG TTGGAGAGAT AGTCAGAAGA GAGAGAAGGG CGAACAGAAA AGAATATGGG   
  
  
+ TTTTACAAGG TCAACTAATA AAAAGTTTTC TTTGATGATT TTGCTTGTTT ACGTAGAATA AGGTATTCTA   
  
  
+ CTTAAGGTTA CTTGCAGCCT GTAATTTTT  

- GATATTTCAA AGACTTGGCT CAGAGACAGG TCGCTAATAA TGAAGGTGGC TGAATTCCTG AAAGGTTCAA   
  
  
- AACAAACATG GTATACAATA AAATGAATGG CCCACCTAAA TCAAGGCTAG CACAGACAGA GAAGCAACAC   
  
  
- TGAATCTAGC ACCTTTCAAT TGCAGAAGAT ACGCAAACTC TGGACAGATT AAATTATATG AAAATGAACC   
  
  
- TTCCATTCAA GTCAGAGTAA CTCTAGTCCT CTCACCAGGG CACTCTTTCA TAAAAGGAAT CCTAAACTAG   
  
  
- AAAACAAAAT CATAACATCC AAACAAATTC AATTTTTTTT TTCTCCCAGT TGAGCATCTG AAAGCAGGTT   
  
  
- TAGAAGTAAA AGGTAAAGGC TTTTGGCCCC TGGTTGAGAA AGAATTTTCG AAATAATGAT AAAGAGATCA   
  
  
- ATGATTTCAT TACATCAGCT CTTTACCTTC CCAATCAGCT CTTACAACGG TAGCCAACAA TCAACCAAAG   
  
  
- GAAATTGATT TGGAACAGAT TCAGCACAGA TTCGCGGAAA CAAGAAACCT TTTCCTTGTT TGTTTTCATT   
  
  
- ATCAAATTGT CTCGGTTCTT CTCAAAACAA AGCAAGCCAT AATCACGTAA GTAGAGATTA CCGATATCCC   
  
  
- AAATTTGCCG AAGCTATGTA ATAAGGAAGC AATCGGAATG AAGCAATGAC CTCTATAAAT TCAGAAACTG   
  
  
- AAGAAGATCT AAGGAAAAAA ATATTTGAAG TTCAAGAGAT TATCGATGAA AAAGGAAACG AAACTTCCAG   
  
  
- GATCAAGGGA AAATGAATAA AAGCACTTTC AAGTAAACAA TAATATGAAC GAGAGCATAG ATCAAGTTGA   
  
  
- ATTAATCACA ACCTCTCAAA AAAAAAATCA CAGAAACAAA GTGCAGCGCC TAAATTTTAG CTGGGAGGCC   
  
  
- AGAAGTTAAG CTTGTTATTA TTAAAAAAAG AAAAAAGATT AAGAGATAAA TAACGTCTTG AGTAAAAGCA   
  
  
- AGACAAAGAA TATAGACACA GAGAGAGAGA GAGAGAGAGA GAAAAAGAAA AAAGATTAAG AGATAAATAA   
  
  
- CGTCTTGAGT AAAAGCAAGA CAAAGAATAT AGACACAGAG AGAGAGAGAG AGAGAGAGAA AGAAAGAAAG   
  
  
- AGAGAATGAT TATAAATGAT TACCTGATTG ATTAAAGAGC TAAAGGTGCA TGACGAAGAC CCACCACCAC   
  
  
- GGCCACCATC ACCACCAATC TGACCTGACC CGAGTTCTAA ACTAACCGCA AACGGACCCG ATCAGATTGT   
  
  
- TCCGTCGAGT ATACGATGAA TTCAATGGAG ATCGAAACAA GCACCCGTCC GTTGTGGGCA TCAGAGAGCA   
  
  
- AAAACAAAAG GGCAAAAGGC AACCTCTCTA TCAGTCTTCT CTCTCTTCCC GCTTGTCTTT TCTTATACCC   
  
  
- AAAATGTTCC AGTTGATTAT TTTTCAAAAG AAACTACTAA AACGAACAAA TGCATCTTAT TCCATAAGAT   
  
  
- GAATTCCAAT GAACGTCGGA CATTAAAAA

+     CCAAT-box

| Site Name | Organism | Position | Strand | Matrix score. | sequence | function |
| --- | --- | --- | --- | --- | --- | --- |
| CCAAT-box | Hordeum vulgare | 1348 | - | 6 | CAACGG | MYBHv1 binding site |

> 2018/04/13 10:10:12  
+ CTATAAAGTT TCTGAACCGA GTCTCTGTCC AGCGATTATT ACTTCCACCG ACTTAAGGAC TTTCCAAGTT   
  
  
+ TTGTTTGTAC CATATGTTAT TTTACTTACC GGGTGGATTT AGTTCCGATC GTGTCTGTCT CTTCGTTGTG   
  
  
+ ACTTAGATCG TGGAAAGTTA ACGTCTTCTA TGCGTTTGAG ACCTGTCTAA TTTAATATAC TTTTACTTGG   
  
  
+ AAGGTAAGTT CAGTCTCATT GAGATCAGGA GAGTGGTCCC GTGAGAAAGT ATTTTCCTTA GGATTTGATC   
  
  
+ TTTTGTTTTA GTATTGTAGG TTTGTTTAAG TTAAAAAAAA AAGAGGGTCA ACTCGTAGAC TTTCGTCCAA   
  
  
+ ATCTTCATTT TCCATTTCCG AAAACCGGGG ACCAACTCTT TCTTAAAAGC TTTATTACTA TTTCTCTAGT   
  
  
+ TACTAAAGTA ATGTAGTCGA GAAATGGAAG GGTTAGTCGA GAATGTTGCC ATCGGTTGTT AGTTGGTTTC   
  
  
+ CTTTAACTAA ACCTTGTCTA AGTCGTGTCT AAGCGCCTTT GTTCTTTGGA AAAGGAACAA ACAAAAGTAA   
  
  
+ TAGTTTAACA GAGCCAAGAA GAGTTTTGTT TCGTTCGGTA TTAGTGCATT CATCTCTAAT GGCTATAGGG   
  
  
+ TTTAAACGGC TTCGATACAT TATTCCTTCG TTAGCCTTAC TTCGTTACTG GAGATATTTA AGTCTTTGAC   
  
  
+ TTCTTCTAGA TTCCTTTTTT TATAAACTTC AAGTTCTCTA ATAGCTACTT TTTCCTTTGC TTTGAAGGTC   
  
  
+ CTAGTTCCCT TTTACTTATT TTCGTGAAAG TTCATTTGTT ATTATACTTG CTCTCGTATC TAGTTCAACT   
  
  
+ TAATTAGTGT TGGAGAGTTT TTTTTTTAGT GTCTTTGTTT CACGTCGCGG ATTTAAAATC GACCCTCCGG   
  
  
+ TCTTCAATTC GAACAATAAT AATTTTTTTC TTTTTTCTAA TTCTCTATTT ATTGCAGAAC TCATTTTCGT   
  
  
+ TCTGTTTCTT ATATCTGTGT CTCTCTCTCT CTCTCTCTCT CTTTTTCTTT TTTCTAATTC TCTATTTATT   
  
  
+ GCAGAACTCA TTTTCGTTCT GTTTCTTATA TCTGTGTCTC TCTCTCTCTC TCTCTCTCTT TCTTTCTTTC   
  
  
+ TCTCTTACTA ATATTTACTA ATGGACTAAC TAATTTCTCG ATTTCCACGT ACTGCTTCTG GGTGGTGGTG   
  
  
+ CCGGTGGTAG TGGTGGTTAG ACTGGACTGG GCTCAAGATT TGATTGGCGT TTGCCTGGGC TAGTCTAACA   
  
  
+ AGGCAGCTCA TATGCTACTT AAGTTACCTC TAGCTTTGTT CGTGGGCAGG CAACACCCGT AGTCTCTCGT   
  
  
+ TTTTGTTTTC CCGTTTTCCG TTGGAGAGAT AGTCAGAAGA GAGAGAAGGG CGAACAGAAA AGAATATGGG   
  
  
+ TTTTACAAGG TCAACTAATA AAAAGTTTTC TTTGATGATT TTGCTTGTTT ACGTAGAATA AGGTATTCTA   
  
  
+ CTTAAGGTTA CTTGCAGCCT GTAATTTTT  

- GATATTTCAA AGACTTGGCT CAGAGACAGG TCGCTAATAA TGAAGGTGGC TGAATTCCTG AAAGGTTCAA   
  
  
- AACAAACATG GTATACAATA AAATGAATGG CCCACCTAAA TCAAGGCTAG CACAGACAGA GAAGCAACAC   
  
  
- TGAATCTAGC ACCTTTCAAT TGCAGAAGAT ACGCAAACTC TGGACAGATT AAATTATATG AAAATGAACC   
  
  
- TTCCATTCAA GTCAGAGTAA CTCTAGTCCT CTCACCAGGG CACTCTTTCA TAAAAGGAAT CCTAAACTAG   
  
  
- AAAACAAAAT CATAACATCC AAACAAATTC AATTTTTTTT TTCTCCCAGT TGAGCATCTG AAAGCAGGTT   
  
  
- TAGAAGTAAA AGGTAAAGGC TTTTGGCCCC TGGTTGAGAA AGAATTTTCG AAATAATGAT AAAGAGATCA   
  
  
- ATGATTTCAT TACATCAGCT CTTTACCTTC CCAATCAGCT CTTACAACGG TAGCCAACAA TCAACCAAAG   
  
  
- GAAATTGATT TGGAACAGAT TCAGCACAGA TTCGCGGAAA CAAGAAACCT TTTCCTTGTT TGTTTTCATT   
  
  
- ATCAAATTGT CTCGGTTCTT CTCAAAACAA AGCAAGCCAT AATCACGTAA GTAGAGATTA CCGATATCCC   
  
  
- AAATTTGCCG AAGCTATGTA ATAAGGAAGC AATCGGAATG AAGCAATGAC CTCTATAAAT TCAGAAACTG   
  
  
- AAGAAGATCT AAGGAAAAAA ATATTTGAAG TTCAAGAGAT TATCGATGAA AAAGGAAACG AAACTTCCAG   
  
  
- GATCAAGGGA AAATGAATAA AAGCACTTTC AAGTAAACAA TAATATGAAC GAGAGCATAG ATCAAGTTGA   
  
  
- ATTAATCACA ACCTCTCAAA AAAAAAATCA CAGAAACAAA GTGCAGCGCC TAAATTTTAG CTGGGAGGCC   
  
  
- AGAAGTTAAG CTTGTTATTA TTAAAAAAAG AAAAAAGATT AAGAGATAAA TAACGTCTTG AGTAAAAGCA   
  
  
- AGACAAAGAA TATAGACACA GAGAGAGAGA GAGAGAGAGA GAAAAAGAAA AAAGATTAAG AGATAAATAA   
  
  
- CGTCTTGAGT AAAAGCAAGA CAAAGAATAT AGACACAGAG AGAGAGAGAG AGAGAGAGAA AGAAAGAAAG   
  
  
- AGAGAATGAT TATAAATGAT TACCTGATTG ATTAAAGAGC TAAAGGTGCA TGACGAAGAC CCACCACCAC   
  
  
- GGCCACCATC ACCACCAATC TGACCTGACC CGAGTTCTAA ACTAACCGCA AACGGACCCG ATCAGATTGT   
  
  
- TCCGTCGAGT ATACGATGAA TTCAATGGAG ATCGAAACAA GCACCCGTCC GTTGTGGGCA TCAGAGAGCA   
  
  
- AAAACAAAAG GGCAAAAGGC AACCTCTCTA TCAGTCTTCT CTCTCTTCCC GCTTGTCTTT TCTTATACCC   
  
  
- AAAATGTTCC AGTTGATTAT TTTTCAAAAG AAACTACTAA AACGAACAAA TGCATCTTAT TCCATAAGAT   
  
  
- GAATTCCAAT GAACGTCGGA CATTAAAAA

+     G-Box

| Site Name | Organism | Position | Strand | Matrix score. | sequence | function |
| --- | --- | --- | --- | --- | --- | --- |
| G-Box | Antirrhinum majus | 1166 | + | 6 | CACGTA | cis-acting regulatory element involved in light responsiveness |

> 2018/04/13 10:10:12  
+ CTATAAAGTT TCTGAACCGA GTCTCTGTCC AGCGATTATT ACTTCCACCG ACTTAAGGAC TTTCCAAGTT   
  
  
+ TTGTTTGTAC CATATGTTAT TTTACTTACC GGGTGGATTT AGTTCCGATC GTGTCTGTCT CTTCGTTGTG   
  
  
+ ACTTAGATCG TGGAAAGTTA ACGTCTTCTA TGCGTTTGAG ACCTGTCTAA TTTAATATAC TTTTACTTGG   
  
  
+ AAGGTAAGTT CAGTCTCATT GAGATCAGGA GAGTGGTCCC GTGAGAAAGT ATTTTCCTTA GGATTTGATC   
  
  
+ TTTTGTTTTA GTATTGTAGG TTTGTTTAAG TTAAAAAAAA AAGAGGGTCA ACTCGTAGAC TTTCGTCCAA   
  
  
+ ATCTTCATTT TCCATTTCCG AAAACCGGGG ACCAACTCTT TCTTAAAAGC TTTATTACTA TTTCTCTAGT   
  
  
+ TACTAAAGTA ATGTAGTCGA GAAATGGAAG GGTTAGTCGA GAATGTTGCC ATCGGTTGTT AGTTGGTTTC   
  
  
+ CTTTAACTAA ACCTTGTCTA AGTCGTGTCT AAGCGCCTTT GTTCTTTGGA AAAGGAACAA ACAAAAGTAA   
  
  
+ TAGTTTAACA GAGCCAAGAA GAGTTTTGTT TCGTTCGGTA TTAGTGCATT CATCTCTAAT GGCTATAGGG   
  
  
+ TTTAAACGGC TTCGATACAT TATTCCTTCG TTAGCCTTAC TTCGTTACTG GAGATATTTA AGTCTTTGAC   
  
  
+ TTCTTCTAGA TTCCTTTTTT TATAAACTTC AAGTTCTCTA ATAGCTACTT TTTCCTTTGC TTTGAAGGTC   
  
  
+ CTAGTTCCCT TTTACTTATT TTCGTGAAAG TTCATTTGTT ATTATACTTG CTCTCGTATC TAGTTCAACT   
  
  
+ TAATTAGTGT TGGAGAGTTT TTTTTTTAGT GTCTTTGTTT CACGTCGCGG ATTTAAAATC GACCCTCCGG   
  
  
+ TCTTCAATTC GAACAATAAT AATTTTTTTC TTTTTTCTAA TTCTCTATTT ATTGCAGAAC TCATTTTCGT   
  
  
+ TCTGTTTCTT ATATCTGTGT CTCTCTCTCT CTCTCTCTCT CTTTTTCTTT TTTCTAATTC TCTATTTATT   
  
  
+ GCAGAACTCA TTTTCGTTCT GTTTCTTATA TCTGTGTCTC TCTCTCTCTC TCTCTCTCTT TCTTTCTTTC   
  
  
+ TCTCTTACTA ATATTTACTA ATGGACTAAC TAATTTCTCG ATTTCCACGT ACTGCTTCTG GGTGGTGGTG   
  
  
+ CCGGTGGTAG TGGTGGTTAG ACTGGACTGG GCTCAAGATT TGATTGGCGT TTGCCTGGGC TAGTCTAACA   
  
  
+ AGGCAGCTCA TATGCTACTT AAGTTACCTC TAGCTTTGTT CGTGGGCAGG CAACACCCGT AGTCTCTCGT   
  
  
+ TTTTGTTTTC CCGTTTTCCG TTGGAGAGAT AGTCAGAAGA GAGAGAAGGG CGAACAGAAA AGAATATGGG   
  
  
+ TTTTACAAGG TCAACTAATA AAAAGTTTTC TTTGATGATT TTGCTTGTTT ACGTAGAATA AGGTATTCTA   
  
  
+ CTTAAGGTTA CTTGCAGCCT GTAATTTTT  

- GATATTTCAA AGACTTGGCT CAGAGACAGG TCGCTAATAA TGAAGGTGGC TGAATTCCTG AAAGGTTCAA   
  
  
- AACAAACATG GTATACAATA AAATGAATGG CCCACCTAAA TCAAGGCTAG CACAGACAGA GAAGCAACAC   
  
  
- TGAATCTAGC ACCTTTCAAT TGCAGAAGAT ACGCAAACTC TGGACAGATT AAATTATATG AAAATGAACC   
  
  
- TTCCATTCAA GTCAGAGTAA CTCTAGTCCT CTCACCAGGG CACTCTTTCA TAAAAGGAAT CCTAAACTAG   
  
  
- AAAACAAAAT CATAACATCC AAACAAATTC AATTTTTTTT TTCTCCCAGT TGAGCATCTG AAAGCAGGTT   
  
  
- TAGAAGTAAA AGGTAAAGGC TTTTGGCCCC TGGTTGAGAA AGAATTTTCG AAATAATGAT AAAGAGATCA   
  
  
- ATGATTTCAT TACATCAGCT CTTTACCTTC CCAATCAGCT CTTACAACGG TAGCCAACAA TCAACCAAAG   
  
  
- GAAATTGATT TGGAACAGAT TCAGCACAGA TTCGCGGAAA CAAGAAACCT TTTCCTTGTT TGTTTTCATT   
  
  
- ATCAAATTGT CTCGGTTCTT CTCAAAACAA AGCAAGCCAT AATCACGTAA GTAGAGATTA CCGATATCCC   
  
  
- AAATTTGCCG AAGCTATGTA ATAAGGAAGC AATCGGAATG AAGCAATGAC CTCTATAAAT TCAGAAACTG   
  
  
- AAGAAGATCT AAGGAAAAAA ATATTTGAAG TTCAAGAGAT TATCGATGAA AAAGGAAACG AAACTTCCAG   
  
  
- GATCAAGGGA AAATGAATAA AAGCACTTTC AAGTAAACAA TAATATGAAC GAGAGCATAG ATCAAGTTGA   
  
  
- ATTAATCACA ACCTCTCAAA AAAAAAATCA CAGAAACAAA GTGCAGCGCC TAAATTTTAG CTGGGAGGCC   
  
  
- AGAAGTTAAG CTTGTTATTA TTAAAAAAAG AAAAAAGATT AAGAGATAAA TAACGTCTTG AGTAAAAGCA   
  
  
- AGACAAAGAA TATAGACACA GAGAGAGAGA GAGAGAGAGA GAAAAAGAAA AAAGATTAAG AGATAAATAA   
  
  
- CGTCTTGAGT AAAAGCAAGA CAAAGAATAT AGACACAGAG AGAGAGAGAG AGAGAGAGAA AGAAAGAAAG   
  
  
- AGAGAATGAT TATAAATGAT TACCTGATTG ATTAAAGAGC TAAAGGTGCA TGACGAAGAC CCACCACCAC   
  
  
- GGCCACCATC ACCACCAATC TGACCTGACC CGAGTTCTAA ACTAACCGCA AACGGACCCG ATCAGATTGT   
  
  
- TCCGTCGAGT ATACGATGAA TTCAATGGAG ATCGAAACAA GCACCCGTCC GTTGTGGGCA TCAGAGAGCA   
  
  
- AAAACAAAAG GGCAAAAGGC AACCTCTCTA TCAGTCTTCT CTCTCTTCCC GCTTGTCTTT TCTTATACCC   
  
  
- AAAATGTTCC AGTTGATTAT TTTTCAAAAG AAACTACTAA AACGAACAAA TGCATCTTAT TCCATAAGAT   
  
  
- GAATTCCAAT GAACGTCGGA CATTAAAAA

+     G-box

| Site Name | Organism | Position | Strand | Matrix score. | sequence | function |
| --- | --- | --- | --- | --- | --- | --- |
| G-box | Zea mays | 881 | + | 6 | CACGTC | cis-acting regulatory element involved in light responsiveness |
| G-box | Daucus carota | 1166 | - | 6 | TACGTG | cis-acting regulatory element involved in light responsiveness |
| G-box | Zea mays | 512 | - | 6 | CACGAC | cis-acting regulatory element involved in light responsiveness |

> 2018/04/13 10:10:12  
+ CTATAAAGTT TCTGAACCGA GTCTCTGTCC AGCGATTATT ACTTCCACCG ACTTAAGGAC TTTCCAAGTT   
  
  
+ TTGTTTGTAC CATATGTTAT TTTACTTACC GGGTGGATTT AGTTCCGATC GTGTCTGTCT CTTCGTTGTG   
  
  
+ ACTTAGATCG TGGAAAGTTA ACGTCTTCTA TGCGTTTGAG ACCTGTCTAA TTTAATATAC TTTTACTTGG   
  
  
+ AAGGTAAGTT CAGTCTCATT GAGATCAGGA GAGTGGTCCC GTGAGAAAGT ATTTTCCTTA GGATTTGATC   
  
  
+ TTTTGTTTTA GTATTGTAGG TTTGTTTAAG TTAAAAAAAA AAGAGGGTCA ACTCGTAGAC TTTCGTCCAA   
  
  
+ ATCTTCATTT TCCATTTCCG AAAACCGGGG ACCAACTCTT TCTTAAAAGC TTTATTACTA TTTCTCTAGT   
  
  
+ TACTAAAGTA ATGTAGTCGA GAAATGGAAG GGTTAGTCGA GAATGTTGCC ATCGGTTGTT AGTTGGTTTC   
  
  
+ CTTTAACTAA ACCTTGTCTA AGTCGTGTCT AAGCGCCTTT GTTCTTTGGA AAAGGAACAA ACAAAAGTAA   
  
  
+ TAGTTTAACA GAGCCAAGAA GAGTTTTGTT TCGTTCGGTA TTAGTGCATT CATCTCTAAT GGCTATAGGG   
  
  
+ TTTAAACGGC TTCGATACAT TATTCCTTCG TTAGCCTTAC TTCGTTACTG GAGATATTTA AGTCTTTGAC   
  
  
+ TTCTTCTAGA TTCCTTTTTT TATAAACTTC AAGTTCTCTA ATAGCTACTT TTTCCTTTGC TTTGAAGGTC   
  
  
+ CTAGTTCCCT TTTACTTATT TTCGTGAAAG TTCATTTGTT ATTATACTTG CTCTCGTATC TAGTTCAACT   
  
  
+ TAATTAGTGT TGGAGAGTTT TTTTTTTAGT GTCTTTGTTT CACGTCGCGG ATTTAAAATC GACCCTCCGG   
  
  
+ TCTTCAATTC GAACAATAAT AATTTTTTTC TTTTTTCTAA TTCTCTATTT ATTGCAGAAC TCATTTTCGT   
  
  
+ TCTGTTTCTT ATATCTGTGT CTCTCTCTCT CTCTCTCTCT CTTTTTCTTT TTTCTAATTC TCTATTTATT   
  
  
+ GCAGAACTCA TTTTCGTTCT GTTTCTTATA TCTGTGTCTC TCTCTCTCTC TCTCTCTCTT TCTTTCTTTC   
  
  
+ TCTCTTACTA ATATTTACTA ATGGACTAAC TAATTTCTCG ATTTCCACGT ACTGCTTCTG GGTGGTGGTG   
  
  
+ CCGGTGGTAG TGGTGGTTAG ACTGGACTGG GCTCAAGATT TGATTGGCGT TTGCCTGGGC TAGTCTAACA   
  
  
+ AGGCAGCTCA TATGCTACTT AAGTTACCTC TAGCTTTGTT CGTGGGCAGG CAACACCCGT AGTCTCTCGT   
  
  
+ TTTTGTTTTC CCGTTTTCCG TTGGAGAGAT AGTCAGAAGA GAGAGAAGGG CGAACAGAAA AGAATATGGG   
  
  
+ TTTTACAAGG TCAACTAATA AAAAGTTTTC TTTGATGATT TTGCTTGTTT ACGTAGAATA AGGTATTCTA   
  
  
+ CTTAAGGTTA CTTGCAGCCT GTAATTTTT  

- GATATTTCAA AGACTTGGCT CAGAGACAGG TCGCTAATAA TGAAGGTGGC TGAATTCCTG AAAGGTTCAA   
  
  
- AACAAACATG GTATACAATA AAATGAATGG CCCACCTAAA TCAAGGCTAG CACAGACAGA GAAGCAACAC   
  
  
- TGAATCTAGC ACCTTTCAAT TGCAGAAGAT ACGCAAACTC TGGACAGATT AAATTATATG AAAATGAACC   
  
  
- TTCCATTCAA GTCAGAGTAA CTCTAGTCCT CTCACCAGGG CACTCTTTCA TAAAAGGAAT CCTAAACTAG   
  
  
- AAAACAAAAT CATAACATCC AAACAAATTC AATTTTTTTT TTCTCCCAGT TGAGCATCTG AAAGCAGGTT   
  
  
- TAGAAGTAAA AGGTAAAGGC TTTTGGCCCC TGGTTGAGAA AGAATTTTCG AAATAATGAT AAAGAGATCA   
  
  
- ATGATTTCAT TACATCAGCT CTTTACCTTC CCAATCAGCT CTTACAACGG TAGCCAACAA TCAACCAAAG   
  
  
- GAAATTGATT TGGAACAGAT TCAGCACAGA TTCGCGGAAA CAAGAAACCT TTTCCTTGTT TGTTTTCATT   
  
  
- ATCAAATTGT CTCGGTTCTT CTCAAAACAA AGCAAGCCAT AATCACGTAA GTAGAGATTA CCGATATCCC   
  
  
- AAATTTGCCG AAGCTATGTA ATAAGGAAGC AATCGGAATG AAGCAATGAC CTCTATAAAT TCAGAAACTG   
  
  
- AAGAAGATCT AAGGAAAAAA ATATTTGAAG TTCAAGAGAT TATCGATGAA AAAGGAAACG AAACTTCCAG   
  
  
- GATCAAGGGA AAATGAATAA AAGCACTTTC AAGTAAACAA TAATATGAAC GAGAGCATAG ATCAAGTTGA   
  
  
- ATTAATCACA ACCTCTCAAA AAAAAAATCA CAGAAACAAA GTGCAGCGCC TAAATTTTAG CTGGGAGGCC   
  
  
- AGAAGTTAAG CTTGTTATTA TTAAAAAAAG AAAAAAGATT AAGAGATAAA TAACGTCTTG AGTAAAAGCA   
  
  
- AGACAAAGAA TATAGACACA GAGAGAGAGA GAGAGAGAGA GAAAAAGAAA AAAGATTAAG AGATAAATAA   
  
  
- CGTCTTGAGT AAAAGCAAGA CAAAGAATAT AGACACAGAG AGAGAGAGAG AGAGAGAGAA AGAAAGAAAG   
  
  
- AGAGAATGAT TATAAATGAT TACCTGATTG ATTAAAGAGC TAAAGGTGCA TGACGAAGAC CCACCACCAC   
  
  
- GGCCACCATC ACCACCAATC TGACCTGACC CGAGTTCTAA ACTAACCGCA AACGGACCCG ATCAGATTGT   
  
  
- TCCGTCGAGT ATACGATGAA TTCAATGGAG ATCGAAACAA GCACCCGTCC GTTGTGGGCA TCAGAGAGCA   
  
  
- AAAACAAAAG GGCAAAAGGC AACCTCTCTA TCAGTCTTCT CTCTCTTCCC GCTTGTCTTT TCTTATACCC   
  
  
- AAAATGTTCC AGTTGATTAT TTTTCAAAAG AAACTACTAA AACGAACAAA TGCATCTTAT TCCATAAGAT   
  
  
- GAATTCCAAT GAACGTCGGA CATTAAAAA

+     GAG-motif

| Site Name | Organism | Position | Strand | Matrix score. | sequence | function |
| --- | --- | --- | --- | --- | --- | --- |
| GAG-motif | Spinacia oleracea | 611 | - | 7 | AGAGATG | part of a light responsive element |

> 2018/04/13 10:10:12  
+ CTATAAAGTT TCTGAACCGA GTCTCTGTCC AGCGATTATT ACTTCCACCG ACTTAAGGAC TTTCCAAGTT   
  
  
+ TTGTTTGTAC CATATGTTAT TTTACTTACC GGGTGGATTT AGTTCCGATC GTGTCTGTCT CTTCGTTGTG   
  
  
+ ACTTAGATCG TGGAAAGTTA ACGTCTTCTA TGCGTTTGAG ACCTGTCTAA TTTAATATAC TTTTACTTGG   
  
  
+ AAGGTAAGTT CAGTCTCATT GAGATCAGGA GAGTGGTCCC GTGAGAAAGT ATTTTCCTTA GGATTTGATC   
  
  
+ TTTTGTTTTA GTATTGTAGG TTTGTTTAAG TTAAAAAAAA AAGAGGGTCA ACTCGTAGAC TTTCGTCCAA   
  
  
+ ATCTTCATTT TCCATTTCCG AAAACCGGGG ACCAACTCTT TCTTAAAAGC TTTATTACTA TTTCTCTAGT   
  
  
+ TACTAAAGTA ATGTAGTCGA GAAATGGAAG GGTTAGTCGA GAATGTTGCC ATCGGTTGTT AGTTGGTTTC   
  
  
+ CTTTAACTAA ACCTTGTCTA AGTCGTGTCT AAGCGCCTTT GTTCTTTGGA AAAGGAACAA ACAAAAGTAA   
  
  
+ TAGTTTAACA GAGCCAAGAA GAGTTTTGTT TCGTTCGGTA TTAGTGCATT CATCTCTAAT GGCTATAGGG   
  
  
+ TTTAAACGGC TTCGATACAT TATTCCTTCG TTAGCCTTAC TTCGTTACTG GAGATATTTA AGTCTTTGAC   
  
  
+ TTCTTCTAGA TTCCTTTTTT TATAAACTTC AAGTTCTCTA ATAGCTACTT TTTCCTTTGC TTTGAAGGTC   
  
  
+ CTAGTTCCCT TTTACTTATT TTCGTGAAAG TTCATTTGTT ATTATACTTG CTCTCGTATC TAGTTCAACT   
  
  
+ TAATTAGTGT TGGAGAGTTT TTTTTTTAGT GTCTTTGTTT CACGTCGCGG ATTTAAAATC GACCCTCCGG   
  
  
+ TCTTCAATTC GAACAATAAT AATTTTTTTC TTTTTTCTAA TTCTCTATTT ATTGCAGAAC TCATTTTCGT   
  
  
+ TCTGTTTCTT ATATCTGTGT CTCTCTCTCT CTCTCTCTCT CTTTTTCTTT TTTCTAATTC TCTATTTATT   
  
  
+ GCAGAACTCA TTTTCGTTCT GTTTCTTATA TCTGTGTCTC TCTCTCTCTC TCTCTCTCTT TCTTTCTTTC   
  
  
+ TCTCTTACTA ATATTTACTA ATGGACTAAC TAATTTCTCG ATTTCCACGT ACTGCTTCTG GGTGGTGGTG   
  
  
+ CCGGTGGTAG TGGTGGTTAG ACTGGACTGG GCTCAAGATT TGATTGGCGT TTGCCTGGGC TAGTCTAACA   
  
  
+ AGGCAGCTCA TATGCTACTT AAGTTACCTC TAGCTTTGTT CGTGGGCAGG CAACACCCGT AGTCTCTCGT   
  
  
+ TTTTGTTTTC CCGTTTTCCG TTGGAGAGAT AGTCAGAAGA GAGAGAAGGG CGAACAGAAA AGAATATGGG   
  
  
+ TTTTACAAGG TCAACTAATA AAAAGTTTTC TTTGATGATT TTGCTTGTTT ACGTAGAATA AGGTATTCTA   
  
  
+ CTTAAGGTTA CTTGCAGCCT GTAATTTTT  

- GATATTTCAA AGACTTGGCT CAGAGACAGG TCGCTAATAA TGAAGGTGGC TGAATTCCTG AAAGGTTCAA   
  
  
- AACAAACATG GTATACAATA AAATGAATGG CCCACCTAAA TCAAGGCTAG CACAGACAGA GAAGCAACAC   
  
  
- TGAATCTAGC ACCTTTCAAT TGCAGAAGAT ACGCAAACTC TGGACAGATT AAATTATATG AAAATGAACC   
  
  
- TTCCATTCAA GTCAGAGTAA CTCTAGTCCT CTCACCAGGG CACTCTTTCA TAAAAGGAAT CCTAAACTAG   
  
  
- AAAACAAAAT CATAACATCC AAACAAATTC AATTTTTTTT TTCTCCCAGT TGAGCATCTG AAAGCAGGTT   
  
  
- TAGAAGTAAA AGGTAAAGGC TTTTGGCCCC TGGTTGAGAA AGAATTTTCG AAATAATGAT AAAGAGATCA   
  
  
- ATGATTTCAT TACATCAGCT CTTTACCTTC CCAATCAGCT CTTACAACGG TAGCCAACAA TCAACCAAAG   
  
  
- GAAATTGATT TGGAACAGAT TCAGCACAGA TTCGCGGAAA CAAGAAACCT TTTCCTTGTT TGTTTTCATT   
  
  
- ATCAAATTGT CTCGGTTCTT CTCAAAACAA AGCAAGCCAT AATCACGTAA GTAGAGATTA CCGATATCCC   
  
  
- AAATTTGCCG AAGCTATGTA ATAAGGAAGC AATCGGAATG AAGCAATGAC CTCTATAAAT TCAGAAACTG   
  
  
- AAGAAGATCT AAGGAAAAAA ATATTTGAAG TTCAAGAGAT TATCGATGAA AAAGGAAACG AAACTTCCAG   
  
  
- GATCAAGGGA AAATGAATAA AAGCACTTTC AAGTAAACAA TAATATGAAC GAGAGCATAG ATCAAGTTGA   
  
  
- ATTAATCACA ACCTCTCAAA AAAAAAATCA CAGAAACAAA GTGCAGCGCC TAAATTTTAG CTGGGAGGCC   
  
  
- AGAAGTTAAG CTTGTTATTA TTAAAAAAAG AAAAAAGATT AAGAGATAAA TAACGTCTTG AGTAAAAGCA   
  
  
- AGACAAAGAA TATAGACACA GAGAGAGAGA GAGAGAGAGA GAAAAAGAAA AAAGATTAAG AGATAAATAA   
  
  
- CGTCTTGAGT AAAAGCAAGA CAAAGAATAT AGACACAGAG AGAGAGAGAG AGAGAGAGAA AGAAAGAAAG   
  
  
- AGAGAATGAT TATAAATGAT TACCTGATTG ATTAAAGAGC TAAAGGTGCA TGACGAAGAC CCACCACCAC   
  
  
- GGCCACCATC ACCACCAATC TGACCTGACC CGAGTTCTAA ACTAACCGCA AACGGACCCG ATCAGATTGT   
  
  
- TCCGTCGAGT ATACGATGAA TTCAATGGAG ATCGAAACAA GCACCCGTCC GTTGTGGGCA TCAGAGAGCA   
  
  
- AAAACAAAAG GGCAAAAGGC AACCTCTCTA TCAGTCTTCT CTCTCTTCCC GCTTGTCTTT TCTTATACCC   
  
  
- AAAATGTTCC AGTTGATTAT TTTTCAAAAG AAACTACTAA AACGAACAAA TGCATCTTAT TCCATAAGAT   
  
  
- GAATTCCAAT GAACGTCGGA CATTAAAAA

+     GARE-motif

| Site Name | Organism | Position | Strand | Matrix score. | sequence | function |
| --- | --- | --- | --- | --- | --- | --- |
| GARE-motif | Brassica oleracea | 1068 | - | 7 | AAACAGA | gibberellin-responsive element |
| GARE-motif | Brassica oleracea | 981 | - | 7 | AAACAGA | gibberellin-responsive element |

> 2018/04/13 10:10:12  
+ CTATAAAGTT TCTGAACCGA GTCTCTGTCC AGCGATTATT ACTTCCACCG ACTTAAGGAC TTTCCAAGTT   
  
  
+ TTGTTTGTAC CATATGTTAT TTTACTTACC GGGTGGATTT AGTTCCGATC GTGTCTGTCT CTTCGTTGTG   
  
  
+ ACTTAGATCG TGGAAAGTTA ACGTCTTCTA TGCGTTTGAG ACCTGTCTAA TTTAATATAC TTTTACTTGG   
  
  
+ AAGGTAAGTT CAGTCTCATT GAGATCAGGA GAGTGGTCCC GTGAGAAAGT ATTTTCCTTA GGATTTGATC   
  
  
+ TTTTGTTTTA GTATTGTAGG TTTGTTTAAG TTAAAAAAAA AAGAGGGTCA ACTCGTAGAC TTTCGTCCAA   
  
  
+ ATCTTCATTT TCCATTTCCG AAAACCGGGG ACCAACTCTT TCTTAAAAGC TTTATTACTA TTTCTCTAGT   
  
  
+ TACTAAAGTA ATGTAGTCGA GAAATGGAAG GGTTAGTCGA GAATGTTGCC ATCGGTTGTT AGTTGGTTTC   
  
  
+ CTTTAACTAA ACCTTGTCTA AGTCGTGTCT AAGCGCCTTT GTTCTTTGGA AAAGGAACAA ACAAAAGTAA   
  
  
+ TAGTTTAACA GAGCCAAGAA GAGTTTTGTT TCGTTCGGTA TTAGTGCATT CATCTCTAAT GGCTATAGGG   
  
  
+ TTTAAACGGC TTCGATACAT TATTCCTTCG TTAGCCTTAC TTCGTTACTG GAGATATTTA AGTCTTTGAC   
  
  
+ TTCTTCTAGA TTCCTTTTTT TATAAACTTC AAGTTCTCTA ATAGCTACTT TTTCCTTTGC TTTGAAGGTC   
  
  
+ CTAGTTCCCT TTTACTTATT TTCGTGAAAG TTCATTTGTT ATTATACTTG CTCTCGTATC TAGTTCAACT   
  
  
+ TAATTAGTGT TGGAGAGTTT TTTTTTTAGT GTCTTTGTTT CACGTCGCGG ATTTAAAATC GACCCTCCGG   
  
  
+ TCTTCAATTC GAACAATAAT AATTTTTTTC TTTTTTCTAA TTCTCTATTT ATTGCAGAAC TCATTTTCGT   
  
  
+ TCTGTTTCTT ATATCTGTGT CTCTCTCTCT CTCTCTCTCT CTTTTTCTTT TTTCTAATTC TCTATTTATT   
  
  
+ GCAGAACTCA TTTTCGTTCT GTTTCTTATA TCTGTGTCTC TCTCTCTCTC TCTCTCTCTT TCTTTCTTTC   
  
  
+ TCTCTTACTA ATATTTACTA ATGGACTAAC TAATTTCTCG ATTTCCACGT ACTGCTTCTG GGTGGTGGTG   
  
  
+ CCGGTGGTAG TGGTGGTTAG ACTGGACTGG GCTCAAGATT TGATTGGCGT TTGCCTGGGC TAGTCTAACA   
  
  
+ AGGCAGCTCA TATGCTACTT AAGTTACCTC TAGCTTTGTT CGTGGGCAGG CAACACCCGT AGTCTCTCGT   
  
  
+ TTTTGTTTTC CCGTTTTCCG TTGGAGAGAT AGTCAGAAGA GAGAGAAGGG CGAACAGAAA AGAATATGGG   
  
  
+ TTTTACAAGG TCAACTAATA AAAAGTTTTC TTTGATGATT TTGCTTGTTT ACGTAGAATA AGGTATTCTA   
  
  
+ CTTAAGGTTA CTTGCAGCCT GTAATTTTT  

- GATATTTCAA AGACTTGGCT CAGAGACAGG TCGCTAATAA TGAAGGTGGC TGAATTCCTG AAAGGTTCAA   
  
  
- AACAAACATG GTATACAATA AAATGAATGG CCCACCTAAA TCAAGGCTAG CACAGACAGA GAAGCAACAC   
  
  
- TGAATCTAGC ACCTTTCAAT TGCAGAAGAT ACGCAAACTC TGGACAGATT AAATTATATG AAAATGAACC   
  
  
- TTCCATTCAA GTCAGAGTAA CTCTAGTCCT CTCACCAGGG CACTCTTTCA TAAAAGGAAT CCTAAACTAG   
  
  
- AAAACAAAAT CATAACATCC AAACAAATTC AATTTTTTTT TTCTCCCAGT TGAGCATCTG AAAGCAGGTT   
  
  
- TAGAAGTAAA AGGTAAAGGC TTTTGGCCCC TGGTTGAGAA AGAATTTTCG AAATAATGAT AAAGAGATCA   
  
  
- ATGATTTCAT TACATCAGCT CTTTACCTTC CCAATCAGCT CTTACAACGG TAGCCAACAA TCAACCAAAG   
  
  
- GAAATTGATT TGGAACAGAT TCAGCACAGA TTCGCGGAAA CAAGAAACCT TTTCCTTGTT TGTTTTCATT   
  
  
- ATCAAATTGT CTCGGTTCTT CTCAAAACAA AGCAAGCCAT AATCACGTAA GTAGAGATTA CCGATATCCC   
  
  
- AAATTTGCCG AAGCTATGTA ATAAGGAAGC AATCGGAATG AAGCAATGAC CTCTATAAAT TCAGAAACTG   
  
  
- AAGAAGATCT AAGGAAAAAA ATATTTGAAG TTCAAGAGAT TATCGATGAA AAAGGAAACG AAACTTCCAG   
  
  
- GATCAAGGGA AAATGAATAA AAGCACTTTC AAGTAAACAA TAATATGAAC GAGAGCATAG ATCAAGTTGA   
  
  
- ATTAATCACA ACCTCTCAAA AAAAAAATCA CAGAAACAAA GTGCAGCGCC TAAATTTTAG CTGGGAGGCC   
  
  
- AGAAGTTAAG CTTGTTATTA TTAAAAAAAG AAAAAAGATT AAGAGATAAA TAACGTCTTG AGTAAAAGCA   
  
  
- AGACAAAGAA TATAGACACA GAGAGAGAGA GAGAGAGAGA GAAAAAGAAA AAAGATTAAG AGATAAATAA   
  
  
- CGTCTTGAGT AAAAGCAAGA CAAAGAATAT AGACACAGAG AGAGAGAGAG AGAGAGAGAA AGAAAGAAAG   
  
  
- AGAGAATGAT TATAAATGAT TACCTGATTG ATTAAAGAGC TAAAGGTGCA TGACGAAGAC CCACCACCAC   
  
  
- GGCCACCATC ACCACCAATC TGACCTGACC CGAGTTCTAA ACTAACCGCA AACGGACCCG ATCAGATTGT   
  
  
- TCCGTCGAGT ATACGATGAA TTCAATGGAG ATCGAAACAA GCACCCGTCC GTTGTGGGCA TCAGAGAGCA   
  
  
- AAAACAAAAG GGCAAAAGGC AACCTCTCTA TCAGTCTTCT CTCTCTTCCC GCTTGTCTTT TCTTATACCC   
  
  
- AAAATGTTCC AGTTGATTAT TTTTCAAAAG AAACTACTAA AACGAACAAA TGCATCTTAT TCCATAAGAT   
  
  
- GAATTCCAAT GAACGTCGGA CATTAAAAA

+     HSE

| Site Name | Organism | Position | Strand | Matrix score. | sequence | function |
| --- | --- | --- | --- | --- | --- | --- |
| HSE | Brassica oleracea | 1491 | - | 9 | AAAAAATTTC | cis-acting element involved in heat stress responsiveness |

> 2018/04/13 10:10:12  
+ CTATAAAGTT TCTGAACCGA GTCTCTGTCC AGCGATTATT ACTTCCACCG ACTTAAGGAC TTTCCAAGTT   
  
  
+ TTGTTTGTAC CATATGTTAT TTTACTTACC GGGTGGATTT AGTTCCGATC GTGTCTGTCT CTTCGTTGTG   
  
  
+ ACTTAGATCG TGGAAAGTTA ACGTCTTCTA TGCGTTTGAG ACCTGTCTAA TTTAATATAC TTTTACTTGG   
  
  
+ AAGGTAAGTT CAGTCTCATT GAGATCAGGA GAGTGGTCCC GTGAGAAAGT ATTTTCCTTA GGATTTGATC   
  
  
+ TTTTGTTTTA GTATTGTAGG TTTGTTTAAG TTAAAAAAAA AAGAGGGTCA ACTCGTAGAC TTTCGTCCAA   
  
  
+ ATCTTCATTT TCCATTTCCG AAAACCGGGG ACCAACTCTT TCTTAAAAGC TTTATTACTA TTTCTCTAGT   
  
  
+ TACTAAAGTA ATGTAGTCGA GAAATGGAAG GGTTAGTCGA GAATGTTGCC ATCGGTTGTT AGTTGGTTTC   
  
  
+ CTTTAACTAA ACCTTGTCTA AGTCGTGTCT AAGCGCCTTT GTTCTTTGGA AAAGGAACAA ACAAAAGTAA   
  
  
+ TAGTTTAACA GAGCCAAGAA GAGTTTTGTT TCGTTCGGTA TTAGTGCATT CATCTCTAAT GGCTATAGGG   
  
  
+ TTTAAACGGC TTCGATACAT TATTCCTTCG TTAGCCTTAC TTCGTTACTG GAGATATTTA AGTCTTTGAC   
  
  
+ TTCTTCTAGA TTCCTTTTTT TATAAACTTC AAGTTCTCTA ATAGCTACTT TTTCCTTTGC TTTGAAGGTC   
  
  
+ CTAGTTCCCT TTTACTTATT TTCGTGAAAG TTCATTTGTT ATTATACTTG CTCTCGTATC TAGTTCAACT   
  
  
+ TAATTAGTGT TGGAGAGTTT TTTTTTTAGT GTCTTTGTTT CACGTCGCGG ATTTAAAATC GACCCTCCGG   
  
  
+ TCTTCAATTC GAACAATAAT AATTTTTTTC TTTTTTCTAA TTCTCTATTT ATTGCAGAAC TCATTTTCGT   
  
  
+ TCTGTTTCTT ATATCTGTGT CTCTCTCTCT CTCTCTCTCT CTTTTTCTTT TTTCTAATTC TCTATTTATT   
  
  
+ GCAGAACTCA TTTTCGTTCT GTTTCTTATA TCTGTGTCTC TCTCTCTCTC TCTCTCTCTT TCTTTCTTTC   
  
  
+ TCTCTTACTA ATATTTACTA ATGGACTAAC TAATTTCTCG ATTTCCACGT ACTGCTTCTG GGTGGTGGTG   
  
  
+ CCGGTGGTAG TGGTGGTTAG ACTGGACTGG GCTCAAGATT TGATTGGCGT TTGCCTGGGC TAGTCTAACA   
  
  
+ AGGCAGCTCA TATGCTACTT AAGTTACCTC TAGCTTTGTT CGTGGGCAGG CAACACCCGT AGTCTCTCGT   
  
  
+ TTTTGTTTTC CCGTTTTCCG TTGGAGAGAT AGTCAGAAGA GAGAGAAGGG CGAACAGAAA AGAATATGGG   
  
  
+ TTTTACAAGG TCAACTAATA AAAAGTTTTC TTTGATGATT TTGCTTGTTT ACGTAGAATA AGGTATTCTA   
  
  
+ CTTAAGGTTA CTTGCAGCCT GTAATTTTT  

- GATATTTCAA AGACTTGGCT CAGAGACAGG TCGCTAATAA TGAAGGTGGC TGAATTCCTG AAAGGTTCAA   
  
  
- AACAAACATG GTATACAATA AAATGAATGG CCCACCTAAA TCAAGGCTAG CACAGACAGA GAAGCAACAC   
  
  
- TGAATCTAGC ACCTTTCAAT TGCAGAAGAT ACGCAAACTC TGGACAGATT AAATTATATG AAAATGAACC   
  
  
- TTCCATTCAA GTCAGAGTAA CTCTAGTCCT CTCACCAGGG CACTCTTTCA TAAAAGGAAT CCTAAACTAG   
  
  
- AAAACAAAAT CATAACATCC AAACAAATTC AATTTTTTTT TTCTCCCAGT TGAGCATCTG AAAGCAGGTT   
  
  
- TAGAAGTAAA AGGTAAAGGC TTTTGGCCCC TGGTTGAGAA AGAATTTTCG AAATAATGAT AAAGAGATCA   
  
  
- ATGATTTCAT TACATCAGCT CTTTACCTTC CCAATCAGCT CTTACAACGG TAGCCAACAA TCAACCAAAG   
  
  
- GAAATTGATT TGGAACAGAT TCAGCACAGA TTCGCGGAAA CAAGAAACCT TTTCCTTGTT TGTTTTCATT   
  
  
- ATCAAATTGT CTCGGTTCTT CTCAAAACAA AGCAAGCCAT AATCACGTAA GTAGAGATTA CCGATATCCC   
  
  
- AAATTTGCCG AAGCTATGTA ATAAGGAAGC AATCGGAATG AAGCAATGAC CTCTATAAAT TCAGAAACTG   
  
  
- AAGAAGATCT AAGGAAAAAA ATATTTGAAG TTCAAGAGAT TATCGATGAA AAAGGAAACG AAACTTCCAG   
  
  
- GATCAAGGGA AAATGAATAA AAGCACTTTC AAGTAAACAA TAATATGAAC GAGAGCATAG ATCAAGTTGA   
  
  
- ATTAATCACA ACCTCTCAAA AAAAAAATCA CAGAAACAAA GTGCAGCGCC TAAATTTTAG CTGGGAGGCC   
  
  
- AGAAGTTAAG CTTGTTATTA TTAAAAAAAG AAAAAAGATT AAGAGATAAA TAACGTCTTG AGTAAAAGCA   
  
  
- AGACAAAGAA TATAGACACA GAGAGAGAGA GAGAGAGAGA GAAAAAGAAA AAAGATTAAG AGATAAATAA   
  
  
- CGTCTTGAGT AAAAGCAAGA CAAAGAATAT AGACACAGAG AGAGAGAGAG AGAGAGAGAA AGAAAGAAAG   
  
  
- AGAGAATGAT TATAAATGAT TACCTGATTG ATTAAAGAGC TAAAGGTGCA TGACGAAGAC CCACCACCAC   
  
  
- GGCCACCATC ACCACCAATC TGACCTGACC CGAGTTCTAA ACTAACCGCA AACGGACCCG ATCAGATTGT   
  
  
- TCCGTCGAGT ATACGATGAA TTCAATGGAG ATCGAAACAA GCACCCGTCC GTTGTGGGCA TCAGAGAGCA   
  
  
- AAAACAAAAG GGCAAAAGGC AACCTCTCTA TCAGTCTTCT CTCTCTTCCC GCTTGTCTTT TCTTATACCC   
  
  
- AAAATGTTCC AGTTGATTAT TTTTCAAAAG AAACTACTAA AACGAACAAA TGCATCTTAT TCCATAAGAT   
  
  
- GAATTCCAAT GAACGTCGGA CATTAAAAAA

+     I-box

| Site Name | Organism | Position | Strand | Matrix score. | sequence | function |
| --- | --- | --- | --- | --- | --- | --- |
| I-box | Nicotiana plumbaginifolia | 1267 | + | 9 | CTCTTATGCT | part of a light responsive element |

> 2018/04/13 10:10:12  
+ CTATAAAGTT TCTGAACCGA GTCTCTGTCC AGCGATTATT ACTTCCACCG ACTTAAGGAC TTTCCAAGTT   
  
  
+ TTGTTTGTAC CATATGTTAT TTTACTTACC GGGTGGATTT AGTTCCGATC GTGTCTGTCT CTTCGTTGTG   
  
  
+ ACTTAGATCG TGGAAAGTTA ACGTCTTCTA TGCGTTTGAG ACCTGTCTAA TTTAATATAC TTTTACTTGG   
  
  
+ AAGGTAAGTT CAGTCTCATT GAGATCAGGA GAGTGGTCCC GTGAGAAAGT ATTTTCCTTA GGATTTGATC   
  
  
+ TTTTGTTTTA GTATTGTAGG TTTGTTTAAG TTAAAAAAAA AAGAGGGTCA ACTCGTAGAC TTTCGTCCAA   
  
  
+ ATCTTCATTT TCCATTTCCG AAAACCGGGG ACCAACTCTT TCTTAAAAGC TTTATTACTA TTTCTCTAGT   
  
  
+ TACTAAAGTA ATGTAGTCGA GAAATGGAAG GGTTAGTCGA GAATGTTGCC ATCGGTTGTT AGTTGGTTTC   
  
  
+ CTTTAACTAA ACCTTGTCTA AGTCGTGTCT AAGCGCCTTT GTTCTTTGGA AAAGGAACAA ACAAAAGTAA   
  
  
+ TAGTTTAACA GAGCCAAGAA GAGTTTTGTT TCGTTCGGTA TTAGTGCATT CATCTCTAAT GGCTATAGGG   
  
  
+ TTTAAACGGC TTCGATACAT TATTCCTTCG TTAGCCTTAC TTCGTTACTG GAGATATTTA AGTCTTTGAC   
  
  
+ TTCTTCTAGA TTCCTTTTTT TATAAACTTC AAGTTCTCTA ATAGCTACTT TTTCCTTTGC TTTGAAGGTC   
  
  
+ CTAGTTCCCT TTTACTTATT TTCGTGAAAG TTCATTTGTT ATTATACTTG CTCTCGTATC TAGTTCAACT   
  
  
+ TAATTAGTGT TGGAGAGTTT TTTTTTTAGT GTCTTTGTTT CACGTCGCGG ATTTAAAATC GACCCTCCGG   
  
  
+ TCTTCAATTC GAACAATAAT AATTTTTTTC TTTTTTCTAA TTCTCTATTT ATTGCAGAAC TCATTTTCGT   
  
  
+ TCTGTTTCTT ATATCTGTGT CTCTCTCTCT CTCTCTCTCT CTTTTTCTTT TTTCTAATTC TCTATTTATT   
  
  
+ GCAGAACTCA TTTTCGTTCT GTTTCTTATA TCTGTGTCTC TCTCTCTCTC TCTCTCTCTT TCTTTCTTTC   
  
  
+ TCTCTTACTA ATATTTACTA ATGGACTAAC TAATTTCTCG ATTTCCACGT ACTGCTTCTG GGTGGTGGTG   
  
  
+ CCGGTGGTAG TGGTGGTTAG ACTGGACTGG GCTCAAGATT TGATTGGCGT TTGCCTGGGC TAGTCTAACA   
  
  
+ AGGCAGCTCA TATGCTACTT AAGTTACCTC TAGCTTTGTT CGTGGGCAGG CAACACCCGT AGTCTCTCGT   
  
  
+ TTTTGTTTTC CCGTTTTCCG TTGGAGAGAT AGTCAGAAGA GAGAGAAGGG CGAACAGAAA AGAATATGGG   
  
  
+ TTTTACAAGG TCAACTAATA AAAAGTTTTC TTTGATGATT TTGCTTGTTT ACGTAGAATA AGGTATTCTA   
  
  
+ CTTAAGGTTA CTTGCAGCCT GTAATTTTT  

- GATATTTCAA AGACTTGGCT CAGAGACAGG TCGCTAATAA TGAAGGTGGC TGAATTCCTG AAAGGTTCAA   
  
  
- AACAAACATG GTATACAATA AAATGAATGG CCCACCTAAA TCAAGGCTAG CACAGACAGA GAAGCAACAC   
  
  
- TGAATCTAGC ACCTTTCAAT TGCAGAAGAT ACGCAAACTC TGGACAGATT AAATTATATG AAAATGAACC   
  
  
- TTCCATTCAA GTCAGAGTAA CTCTAGTCCT CTCACCAGGG CACTCTTTCA TAAAAGGAAT CCTAAACTAG   
  
  
- AAAACAAAAT CATAACATCC AAACAAATTC AATTTTTTTT TTCTCCCAGT TGAGCATCTG AAAGCAGGTT   
  
  
- TAGAAGTAAA AGGTAAAGGC TTTTGGCCCC TGGTTGAGAA AGAATTTTCG AAATAATGAT AAAGAGATCA   
  
  
- ATGATTTCAT TACATCAGCT CTTTACCTTC CCAATCAGCT CTTACAACGG TAGCCAACAA TCAACCAAAG   
  
  
- GAAATTGATT TGGAACAGAT TCAGCACAGA TTCGCGGAAA CAAGAAACCT TTTCCTTGTT TGTTTTCATT   
  
  
- ATCAAATTGT CTCGGTTCTT CTCAAAACAA AGCAAGCCAT AATCACGTAA GTAGAGATTA CCGATATCCC   
  
  
- AAATTTGCCG AAGCTATGTA ATAAGGAAGC AATCGGAATG AAGCAATGAC CTCTATAAAT TCAGAAACTG   
  
  
- AAGAAGATCT AAGGAAAAAA ATATTTGAAG TTCAAGAGAT TATCGATGAA AAAGGAAACG AAACTTCCAG   
  
  
- GATCAAGGGA AAATGAATAA AAGCACTTTC AAGTAAACAA TAATATGAAC GAGAGCATAG ATCAAGTTGA   
  
  
- ATTAATCACA ACCTCTCAAA AAAAAAATCA CAGAAACAAA GTGCAGCGCC TAAATTTTAG CTGGGAGGCC   
  
  
- AGAAGTTAAG CTTGTTATTA TTAAAAAAAG AAAAAAGATT AAGAGATAAA TAACGTCTTG AGTAAAAGCA   
  
  
- AGACAAAGAA TATAGACACA GAGAGAGAGA GAGAGAGAGA GAAAAAGAAA AAAGATTAAG AGATAAATAA   
  
  
- CGTCTTGAGT AAAAGCAAGA CAAAGAATAT AGACACAGAG AGAGAGAGAG AGAGAGAGAA AGAAAGAAAG   
  
  
- AGAGAATGAT TATAAATGAT TACCTGATTG ATTAAAGAGC TAAAGGTGCA TGACGAAGAC CCACCACCAC   
  
  
- GGCCACCATC ACCACCAATC TGACCTGACC CGAGTTCTAA ACTAACCGCA AACGGACCCG ATCAGATTGT   
  
  
- TCCGTCGAGT ATACGATGAA TTCAATGGAG ATCGAAACAA GCACCCGTCC GTTGTGGGCA TCAGAGAGCA   
  
  
- AAAACAAAAG GGCAAAAGGC AACCTCTCTA TCAGTCTTCT CTCTCTTCCC GCTTGTCTTT TCTTATACCC   
  
  
- AAAATGTTCC AGTTGATTAT TTTTCAAAAG AAACTACTAA AACGAACAAA TGCATCTTAT TCCATAAGAT   
  
  
- GAATTCCAAT GAACGTCGGA CATTAAAAA

+     LTR

| Site Name | Organism | Position | Strand | Matrix score. | sequence | function |
| --- | --- | --- | --- | --- | --- | --- |
| LTR | Hordeum vulgare | 368 | + | 6 | CCGAAA | cis-acting element involved in low-temperature responsiveness |

> 2018/04/13 10:10:12  
+ CTATAAAGTT TCTGAACCGA GTCTCTGTCC AGCGATTATT ACTTCCACCG ACTTAAGGAC TTTCCAAGTT   
  
  
+ TTGTTTGTAC CATATGTTAT TTTACTTACC GGGTGGATTT AGTTCCGATC GTGTCTGTCT CTTCGTTGTG   
  
  
+ ACTTAGATCG TGGAAAGTTA ACGTCTTCTA TGCGTTTGAG ACCTGTCTAA TTTAATATAC TTTTACTTGG   
  
  
+ AAGGTAAGTT CAGTCTCATT GAGATCAGGA GAGTGGTCCC GTGAGAAAGT ATTTTCCTTA GGATTTGATC   
  
  
+ TTTTGTTTTA GTATTGTAGG TTTGTTTAAG TTAAAAAAAA AAGAGGGTCA ACTCGTAGAC TTTCGTCCAA   
  
  
+ ATCTTCATTT TCCATTTCCG AAAACCGGGG ACCAACTCTT TCTTAAAAGC TTTATTACTA TTTCTCTAGT   
  
  
+ TACTAAAGTA ATGTAGTCGA GAAATGGAAG GGTTAGTCGA GAATGTTGCC ATCGGTTGTT AGTTGGTTTC   
  
  
+ CTTTAACTAA ACCTTGTCTA AGTCGTGTCT AAGCGCCTTT GTTCTTTGGA AAAGGAACAA ACAAAAGTAA   
  
  
+ TAGTTTAACA GAGCCAAGAA GAGTTTTGTT TCGTTCGGTA TTAGTGCATT CATCTCTAAT GGCTATAGGG   
  
  
+ TTTAAACGGC TTCGATACAT TATTCCTTCG TTAGCCTTAC TTCGTTACTG GAGATATTTA AGTCTTTGAC   
  
  
+ TTCTTCTAGA TTCCTTTTTT TATAAACTTC AAGTTCTCTA ATAGCTACTT TTTCCTTTGC TTTGAAGGTC   
  
  
+ CTAGTTCCCT TTTACTTATT TTCGTGAAAG TTCATTTGTT ATTATACTTG CTCTCGTATC TAGTTCAACT   
  
  
+ TAATTAGTGT TGGAGAGTTT TTTTTTTAGT GTCTTTGTTT CACGTCGCGG ATTTAAAATC GACCCTCCGG   
  
  
+ TCTTCAATTC GAACAATAAT AATTTTTTTC TTTTTTCTAA TTCTCTATTT ATTGCAGAAC TCATTTTCGT   
  
  
+ TCTGTTTCTT ATATCTGTGT CTCTCTCTCT CTCTCTCTCT CTTTTTCTTT TTTCTAATTC TCTATTTATT   
  
  
+ GCAGAACTCA TTTTCGTTCT GTTTCTTATA TCTGTGTCTC TCTCTCTCTC TCTCTCTCTT TCTTTCTTTC   
  
  
+ TCTCTTACTA ATATTTACTA ATGGACTAAC TAATTTCTCG ATTTCCACGT ACTGCTTCTG GGTGGTGGTG   
  
  
+ CCGGTGGTAG TGGTGGTTAG ACTGGACTGG GCTCAAGATT TGATTGGCGT TTGCCTGGGC TAGTCTAACA   
  
  
+ AGGCAGCTCA TATGCTACTT AAGTTACCTC TAGCTTTGTT CGTGGGCAGG CAACACCCGT AGTCTCTCGT   
  
  
+ TTTTGTTTTC CCGTTTTCCG TTGGAGAGAT AGTCAGAAGA GAGAGAAGGG CGAACAGAAA AGAATATGGG   
  
  
+ TTTTACAAGG TCAACTAATA AAAAGTTTTC TTTGATGATT TTGCTTGTTT ACGTAGAATA AGGTATTCTA   
  
  
+ CTTAAGGTTA CTTGCAGCCT GTAATTTTT  

- GATATTTCAA AGACTTGGCT CAGAGACAGG TCGCTAATAA TGAAGGTGGC TGAATTCCTG AAAGGTTCAA   
  
  
- AACAAACATG GTATACAATA AAATGAATGG CCCACCTAAA TCAAGGCTAG CACAGACAGA GAAGCAACAC   
  
  
- TGAATCTAGC ACCTTTCAAT TGCAGAAGAT ACGCAAACTC TGGACAGATT AAATTATATG AAAATGAACC   
  
  
- TTCCATTCAA GTCAGAGTAA CTCTAGTCCT CTCACCAGGG CACTCTTTCA TAAAAGGAAT CCTAAACTAG   
  
  
- AAAACAAAAT CATAACATCC AAACAAATTC AATTTTTTTT TTCTCCCAGT TGAGCATCTG AAAGCAGGTT   
  
  
- TAGAAGTAAA AGGTAAAGGC TTTTGGCCCC TGGTTGAGAA AGAATTTTCG AAATAATGAT AAAGAGATCA   
  
  
- ATGATTTCAT TACATCAGCT CTTTACCTTC CCAATCAGCT CTTACAACGG TAGCCAACAA TCAACCAAAG   
  
  
- GAAATTGATT TGGAACAGAT TCAGCACAGA TTCGCGGAAA CAAGAAACCT TTTCCTTGTT TGTTTTCATT   
  
  
- ATCAAATTGT CTCGGTTCTT CTCAAAACAA AGCAAGCCAT AATCACGTAA GTAGAGATTA CCGATATCCC   
  
  
- AAATTTGCCG AAGCTATGTA ATAAGGAAGC AATCGGAATG AAGCAATGAC CTCTATAAAT TCAGAAACTG   
  
  
- AAGAAGATCT AAGGAAAAAA ATATTTGAAG TTCAAGAGAT TATCGATGAA AAAGGAAACG AAACTTCCAG   
  
  
- GATCAAGGGA AAATGAATAA AAGCACTTTC AAGTAAACAA TAATATGAAC GAGAGCATAG ATCAAGTTGA   
  
  
- ATTAATCACA ACCTCTCAAA AAAAAAATCA CAGAAACAAA GTGCAGCGCC TAAATTTTAG CTGGGAGGCC   
  
  
- AGAAGTTAAG CTTGTTATTA TTAAAAAAAG AAAAAAGATT AAGAGATAAA TAACGTCTTG AGTAAAAGCA   
  
  
- AGACAAAGAA TATAGACACA GAGAGAGAGA GAGAGAGAGA GAAAAAGAAA AAAGATTAAG AGATAAATAA   
  
  
- CGTCTTGAGT AAAAGCAAGA CAAAGAATAT AGACACAGAG AGAGAGAGAG AGAGAGAGAA AGAAAGAAAG   
  
  
- AGAGAATGAT TATAAATGAT TACCTGATTG ATTAAAGAGC TAAAGGTGCA TGACGAAGAC CCACCACCAC   
  
  
- GGCCACCATC ACCACCAATC TGACCTGACC CGAGTTCTAA ACTAACCGCA AACGGACCCG ATCAGATTGT   
  
  
- TCCGTCGAGT ATACGATGAA TTCAATGGAG ATCGAAACAA GCACCCGTCC GTTGTGGGCA TCAGAGAGCA   
  
  
- AAAACAAAAG GGCAAAAGGC AACCTCTCTA TCAGTCTTCT CTCTCTTCCC GCTTGTCTTT TCTTATACCC   
  
  
- AAAATGTTCC AGTTGATTAT TTTTCAAAAG AAACTACTAA AACGAACAAA TGCATCTTAT TCCATAAGAT   
  
  
- GAATTCCAAT GAACGTCGGA CATTAAAAA

+     Sp1

| Site Name | Organism | Position | Strand | Matrix score. | sequence | function |
| --- | --- | --- | --- | --- | --- | --- |
| Sp1 | Zea mays | 101 | - | 5.5 | CC(G/A)CCC | light responsive element |
| Sp1 | Zea mays | 1180 | - | 5.5 | CC(G/A)CCC | light responsive element |

> 2018/04/13 10:10:12  
+ CTATAAAGTT TCTGAACCGA GTCTCTGTCC AGCGATTATT ACTTCCACCG ACTTAAGGAC TTTCCAAGTT   
  
  
+ TTGTTTGTAC CATATGTTAT TTTACTTACC GGGTGGATTT AGTTCCGATC GTGTCTGTCT CTTCGTTGTG   
  
  
+ ACTTAGATCG TGGAAAGTTA ACGTCTTCTA TGCGTTTGAG ACCTGTCTAA TTTAATATAC TTTTACTTGG   
  
  
+ AAGGTAAGTT CAGTCTCATT GAGATCAGGA GAGTGGTCCC GTGAGAAAGT ATTTTCCTTA GGATTTGATC   
  
  
+ TTTTGTTTTA GTATTGTAGG TTTGTTTAAG TTAAAAAAAA AAGAGGGTCA ACTCGTAGAC TTTCGTCCAA   
  
  
+ ATCTTCATTT TCCATTTCCG AAAACCGGGG ACCAACTCTT TCTTAAAAGC TTTATTACTA TTTCTCTAGT   
  
  
+ TACTAAAGTA ATGTAGTCGA GAAATGGAAG GGTTAGTCGA GAATGTTGCC ATCGGTTGTT AGTTGGTTTC   
  
  
+ CTTTAACTAA ACCTTGTCTA AGTCGTGTCT AAGCGCCTTT GTTCTTTGGA AAAGGAACAA ACAAAAGTAA   
  
  
+ TAGTTTAACA GAGCCAAGAA GAGTTTTGTT TCGTTCGGTA TTAGTGCATT CATCTCTAAT GGCTATAGGG   
  
  
+ TTTAAACGGC TTCGATACAT TATTCCTTCG TTAGCCTTAC TTCGTTACTG GAGATATTTA AGTCTTTGAC   
  
  
+ TTCTTCTAGA TTCCTTTTTT TATAAACTTC AAGTTCTCTA ATAGCTACTT TTTCCTTTGC TTTGAAGGTC   
  
  
+ CTAGTTCCCT TTTACTTATT TTCGTGAAAG TTCATTTGTT ATTATACTTG CTCTCGTATC TAGTTCAACT   
  
  
+ TAATTAGTGT TGGAGAGTTT TTTTTTTAGT GTCTTTGTTT CACGTCGCGG ATTTAAAATC GACCCTCCGG   
  
  
+ TCTTCAATTC GAACAATAAT AATTTTTTTC TTTTTTCTAA TTCTCTATTT ATTGCAGAAC TCATTTTCGT   
  
  
+ TCTGTTTCTT ATATCTGTGT CTCTCTCTCT CTCTCTCTCT CTTTTTCTTT TTTCTAATTC TCTATTTATT   
  
  
+ GCAGAACTCA TTTTCGTTCT GTTTCTTATA TCTGTGTCTC TCTCTCTCTC TCTCTCTCTT TCTTTCTTTC   
  
  
+ TCTCTTACTA ATATTTACTA ATGGACTAAC TAATTTCTCG ATTTCCACGT ACTGCTTCTG GGTGGTGGTG   
  
  
+ CCGGTGGTAG TGGTGGTTAG ACTGGACTGG GCTCAAGATT TGATTGGCGT TTGCCTGGGC TAGTCTAACA   
  
  
+ AGGCAGCTCA TATGCTACTT AAGTTACCTC TAGCTTTGTT CGTGGGCAGG CAACACCCGT AGTCTCTCGT   
  
  
+ TTTTGTTTTC CCGTTTTCCG TTGGAGAGAT AGTCAGAAGA GAGAGAAGGG CGAACAGAAA AGAATATGGG   
  
  
+ TTTTACAAGG TCAACTAATA AAAAGTTTTC TTTGATGATT TTGCTTGTTT ACGTAGAATA AGGTATTCTA   
  
  
+ CTTAAGGTTA CTTGCAGCCT GTAATTTTT  

- GATATTTCAA AGACTTGGCT CAGAGACAGG TCGCTAATAA TGAAGGTGGC TGAATTCCTG AAAGGTTCAA   
  
  
- AACAAACATG GTATACAATA AAATGAATGG CCCACCTAAA TCAAGGCTAG CACAGACAGA GAAGCAACAC   
  
  
- TGAATCTAGC ACCTTTCAAT TGCAGAAGAT ACGCAAACTC TGGACAGATT AAATTATATG AAAATGAACC   
  
  
- TTCCATTCAA GTCAGAGTAA CTCTAGTCCT CTCACCAGGG CACTCTTTCA TAAAAGGAAT CCTAAACTAG   
  
  
- AAAACAAAAT CATAACATCC AAACAAATTC AATTTTTTTT TTCTCCCAGT TGAGCATCTG AAAGCAGGTT   
  
  
- TAGAAGTAAA AGGTAAAGGC TTTTGGCCCC TGGTTGAGAA AGAATTTTCG AAATAATGAT AAAGAGATCA   
  
  
- ATGATTTCAT TACATCAGCT CTTTACCTTC CCAATCAGCT CTTACAACGG TAGCCAACAA TCAACCAAAG   
  
  
- GAAATTGATT TGGAACAGAT TCAGCACAGA TTCGCGGAAA CAAGAAACCT TTTCCTTGTT TGTTTTCATT   
  
  
- ATCAAATTGT CTCGGTTCTT CTCAAAACAA AGCAAGCCAT AATCACGTAA GTAGAGATTA CCGATATCCC   
  
  
- AAATTTGCCG AAGCTATGTA ATAAGGAAGC AATCGGAATG AAGCAATGAC CTCTATAAAT TCAGAAACTG   
  
  
- AAGAAGATCT AAGGAAAAAA ATATTTGAAG TTCAAGAGAT TATCGATGAA AAAGGAAACG AAACTTCCAG   
  
  
- GATCAAGGGA AAATGAATAA AAGCACTTTC AAGTAAACAA TAATATGAAC GAGAGCATAG ATCAAGTTGA   
  
  
- ATTAATCACA ACCTCTCAAA AAAAAAATCA CAGAAACAAA GTGCAGCGCC TAAATTTTAG CTGGGAGGCC   
  
  
- AGAAGTTAAG CTTGTTATTA TTAAAAAAAG AAAAAAGATT AAGAGATAAA TAACGTCTTG AGTAAAAGCA   
  
  
- AGACAAAGAA TATAGACACA GAGAGAGAGA GAGAGAGAGA GAAAAAGAAA AAAGATTAAG AGATAAATAA   
  
  
- CGTCTTGAGT AAAAGCAAGA CAAAGAATAT AGACACAGAG AGAGAGAGAG AGAGAGAGAA AGAAAGAAAG   
  
  
- AGAGAATGAT TATAAATGAT TACCTGATTG ATTAAAGAGC TAAAGGTGCA TGACGAAGAC CCACCACCAC   
  
  
- GGCCACCATC ACCACCAATC TGACCTGACC CGAGTTCTAA ACTAACCGCA AACGGACCCG ATCAGATTGT   
  
  
- TCCGTCGAGT ATACGATGAA TTCAATGGAG ATCGAAACAA GCACCCGTCC GTTGTGGGCA TCAGAGAGCA   
  
  
- AAAACAAAAG GGCAAAAGGC AACCTCTCTA TCAGTCTTCT CTCTCTTCCC GCTTGTCTTT TCTTATACCC   
  
  
- AAAATGTTCC AGTTGATTAT TTTTCAAAAG AAACTACTAA AACGAACAAA TGCATCTTAT TCCATAAGAT   
  
  
- GAATTCCAAT GAACGTCGGA CATTAAAAA

+     TATA-box

| Site Name | Organism | Position | Strand | Matrix score. | sequence | function |
| --- | --- | --- | --- | --- | --- | --- |
| TATA-box | Lycopersicon esculentum | 1419 | - | 5 | TTTTA | core promoter element around -30 of transcription start |
| TATA-box | Glycine max | 1129 | + | 5 | TAATA | core promoter element around -30 of transcription start |
| TATA-box | Arabidopsis thaliana | 1077 | - | 4 | TATA | core promoter element around -30 of transcription start |
| TATA-box | Arabidopsis thaliana | 720 | - | 5 | TATAA | core promoter element around -30 of transcription start |
| TATA-box | Arabidopsis thaliana | 812 | - | 5 | TATAA | core promoter element around -30 of transcription start |
| TATA-box | Glycine max | 403 | - | 5 | TAATA | core promoter element around -30 of transcription start |
| TATA-box | Glycine max | 1416 | + | 5 | TAATA | core promoter element around -30 of transcription start |
| TATA-box | Arabidopsis thaliana | 1076 | - | 5 | TATAA | core promoter element around -30 of transcription start |
| TATA-box | Glycine max | 37 | - | 5 | TAATA | core promoter element around -30 of transcription start |
| TATA-box | Arabidopsis thaliana | 2 | + | 6 | TATAAA | core promoter element around -30 of transcription start |
| TATA-box | Lycopersicon esculentum | 394 | - | 5 | TTTTA | core promoter element around -30 of transcription start |
| TATA-box | Oryza sativa | 1073 | - | 8 | TATAAGAA | core promoter element around -30 of transcription start |
| TATA-box | Glycine max | 810 | - | 5 | TAATA | core promoter element around -30 of transcription start |
| TATA-box | Lycopersicon esculentum | 90 | + | 5 | TTTTA | core promoter element around -30 of transcription start |
| TATA-box | Arabidopsis thaliana | 1041 | + | 9 | tcTATATAtt | core promoter element around -30 of transcription start |
| TATA-box | Brassica napus | 811 | + | 6 | ATTATA | core promoter element around -30 of transcription start |
| TATA-box | Lycopersicon esculentum | 286 | + | 5 | TTTTA | core promoter element around -30 of transcription start |
| TATA-box | Glycine max | 193 | + | 5 | TAATA | core promoter element around -30 of transcription start |
| TATA-box | Arabidopsis thaliana | 719 | - | 6 | TATAAA | core promoter element around -30 of transcription start |
| TATA-box | Lycopersicon esculentum | 1401 | + | 5 | TTTTA | core promoter element around -30 of transcription start |
| TATA-box | Arabidopsis thaliana | 624 | + | 4 | TATA | core promoter element around -30 of transcription start |
| TATA-box | Arabidopsis thaliana | 196 | + | 4 | TATA | core promoter element around -30 of transcription start |
| TATA-box | Oryza sativa | 986 | - | 8 | TATAAGAA | core promoter element around -30 of transcription start |
| TATA-box | Arabidopsis thaliana | 954 | + | 9 | tcTATATAtt | core promoter element around -30 of transcription start |
| TATA-box | Lycopersicon esculentum | 201 | + | 5 | TTTTA | core promoter element around -30 of transcription start |
| TATA-box | Lycopersicon esculentum | 312 | - | 5 | TTTTA | core promoter element around -30 of transcription start |
| TATA-box | Lycopersicon esculentum | 780 | + | 5 | TTTTA | core promoter element around -30 of transcription start |
| TATA-box | Glycine max | 558 | + | 5 | TAATA | core promoter element around -30 of transcription start |
| TATA-box | Lycopersicon esculentum | 894 | - | 5 | TTTTA | core promoter element around -30 of transcription start |
| TATA-box | Lycopersicon esculentum | 864 | + | 5 | TTTTA | core promoter element around -30 of transcription start |
| TATA-box | Arabidopsis thaliana | 813 | - | 4 | TATA | core promoter element around -30 of transcription start |
| TATA-box | Arabidopsis thaliana | 718 | - | 7 | TATAAAA | core promoter element around -30 of transcription start |
| TATA-box | Glycine max | 599 | - | 5 | TAATA | core promoter element around -30 of transcription start |
| TATA-box | Glycine max | 739 | + | 5 | TAATA | core promoter element around -30 of transcription start |
| TATA-box | Arabidopsis thaliana | 989 | - | 5 | TATAA | core promoter element around -30 of transcription start |
| TATA-box | Arabidopsis thaliana | 721 | + | 6 | TATAAA | core promoter element around -30 of transcription start |
| TATA-box | Glycine max | 927 | + | 5 | TAATA | core promoter element around -30 of transcription start |
| TATA-box | Arabidopsis thaliana | 990 | - | 4 | TATA | core promoter element around -30 of transcription start |

> 2018/04/13 10:10:12  
+ CTATAAAGTT TCTGAACCGA GTCTCTGTCC AGCGATTATT ACTTCCACCG ACTTAAGGAC TTTCCAAGTT   
  
  
+ TTGTTTGTAC CATATGTTAT TTTACTTACC GGGTGGATTT AGTTCCGATC GTGTCTGTCT CTTCGTTGTG   
  
  
+ ACTTAGATCG TGGAAAGTTA ACGTCTTCTA TGCGTTTGAG ACCTGTCTAA TTTAATATAC TTTTACTTGG   
  
  
+ AAGGTAAGTT CAGTCTCATT GAGATCAGGA GAGTGGTCCC GTGAGAAAGT ATTTTCCTTA GGATTTGATC   
  
  
+ TTTTGTTTTA GTATTGTAGG TTTGTTTAAG TTAAAAAAAA AAGAGGGTCA ACTCGTAGAC TTTCGTCCAA   
  
  
+ ATCTTCATTT TCCATTTCCG AAAACCGGGG ACCAACTCTT TCTTAAAAGC TTTATTACTA TTTCTCTAGT   
  
  
+ TACTAAAGTA ATGTAGTCGA GAAATGGAAG GGTTAGTCGA GAATGTTGCC ATCGGTTGTT AGTTGGTTTC   
  
  
+ CTTTAACTAA ACCTTGTCTA AGTCGTGTCT AAGCGCCTTT GTTCTTTGGA AAAGGAACAA ACAAAAGTAA   
  
  
+ TAGTTTAACA GAGCCAAGAA GAGTTTTGTT TCGTTCGGTA TTAGTGCATT CATCTCTAAT GGCTATAGGG   
  
  
+ TTTAAACGGC TTCGATACAT TATTCCTTCG TTAGCCTTAC TTCGTTACTG GAGATATTTA AGTCTTTGAC   
  
  
+ TTCTTCTAGA TTCCTTTTTT TATAAACTTC AAGTTCTCTA ATAGCTACTT TTTCCTTTGC TTTGAAGGTC   
  
  
+ CTAGTTCCCT TTTACTTATT TTCGTGAAAG TTCATTTGTT ATTATACTTG CTCTCGTATC TAGTTCAACT   
  
  
+ TAATTAGTGT TGGAGAGTTT TTTTTTTAGT GTCTTTGTTT CACGTCGCGG ATTTAAAATC GACCCTCCGG   
  
  
+ TCTTCAATTC GAACAATAAT AATTTTTTTC TTTTTTCTAA TTCTCTATTT ATTGCAGAAC TCATTTTCGT   
  
  
+ TCTGTTTCTT ATATCTGTGT CTCTCTCTCT CTCTCTCTCT CTTTTTCTTT TTTCTAATTC TCTATTTATT   
  
  
+ GCAGAACTCA TTTTCGTTCT GTTTCTTATA TCTGTGTCTC TCTCTCTCTC TCTCTCTCTT TCTTTCTTTC   
  
  
+ TCTCTTACTA ATATTTACTA ATGGACTAAC TAATTTCTCG ATTTCCACGT ACTGCTTCTG GGTGGTGGTG   
  
  
+ CCGGTGGTAG TGGTGGTTAG ACTGGACTGG GCTCAAGATT TGATTGGCGT TTGCCTGGGC TAGTCTAACA   
  
  
+ AGGCAGCTCA TATGCTACTT AAGTTACCTC TAGCTTTGTT CGTGGGCAGG CAACACCCGT AGTCTCTCGT   
  
  
+ TTTTGTTTTC CCGTTTTCCG TTGGAGAGAT AGTCAGAAGA GAGAGAAGGG CGAACAGAAA AGAATATGGG   
  
  
+ TTTTACAAGG TCAACTAATA AAAAGTTTTC TTTGATGATT TTGCTTGTTT ACGTAGAATA AGGTATTCTA   
  
  
+ CTTAAGGTTA CTTGCAGCCT GTAATTTTT  

- GATATTTCAA AGACTTGGCT CAGAGACAGG TCGCTAATAA TGAAGGTGGC TGAATTCCTG AAAGGTTCAA   
  
  
- AACAAACATG GTATACAATA AAATGAATGG CCCACCTAAA TCAAGGCTAG CACAGACAGA GAAGCAACAC   
  
  
- TGAATCTAGC ACCTTTCAAT TGCAGAAGAT ACGCAAACTC TGGACAGATT AAATTATATG AAAATGAACC   
  
  
- TTCCATTCAA GTCAGAGTAA CTCTAGTCCT CTCACCAGGG CACTCTTTCA TAAAAGGAAT CCTAAACTAG   
  
  
- AAAACAAAAT CATAACATCC AAACAAATTC AATTTTTTTT TTCTCCCAGT TGAGCATCTG AAAGCAGGTT   
  
  
- TAGAAGTAAA AGGTAAAGGC TTTTGGCCCC TGGTTGAGAA AGAATTTTCG AAATAATGAT AAAGAGATCA   
  
  
- ATGATTTCAT TACATCAGCT CTTTACCTTC CCAATCAGCT CTTACAACGG TAGCCAACAA TCAACCAAAG   
  
  
- GAAATTGATT TGGAACAGAT TCAGCACAGA TTCGCGGAAA CAAGAAACCT TTTCCTTGTT TGTTTTCATT   
  
  
- ATCAAATTGT CTCGGTTCTT CTCAAAACAA AGCAAGCCAT AATCACGTAA GTAGAGATTA CCGATATCCC   
  
  
- AAATTTGCCG AAGCTATGTA ATAAGGAAGC AATCGGAATG AAGCAATGAC CTCTATAAAT TCAGAAACTG   
  
  
- AAGAAGATCT AAGGAAAAAA ATATTTGAAG TTCAAGAGAT TATCGATGAA AAAGGAAACG AAACTTCCAG   
  
  
- GATCAAGGGA AAATGAATAA AAGCACTTTC AAGTAAACAA TAATATGAAC GAGAGCATAG ATCAAGTTGA   
  
  
- ATTAATCACA ACCTCTCAAA AAAAAAATCA CAGAAACAAA GTGCAGCGCC TAAATTTTAG CTGGGAGGCC   
  
  
- AGAAGTTAAG CTTGTTATTA TTAAAAAAAG AAAAAAGATT AAGAGATAAA TAACGTCTTG AGTAAAAGCA   
  
  
- AGACAAAGAA TATAGACACA GAGAGAGAGA GAGAGAGAGA GAAAAAGAAA AAAGATTAAG AGATAAATAA   
  
  
- CGTCTTGAGT AAAAGCAAGA CAAAGAATAT AGACACAGAG AGAGAGAGAG AGAGAGAGAA AGAAAGAAAG   
  
  
- AGAGAATGAT TATAAATGAT TACCTGATTG ATTAAAGAGC TAAAGGTGCA TGACGAAGAC CCACCACCAC   
  
  
- GGCCACCATC ACCACCAATC TGACCTGACC CGAGTTCTAA ACTAACCGCA AACGGACCCG ATCAGATTGT   
  
  
- TCCGTCGAGT ATACGATGAA TTCAATGGAG ATCGAAACAA GCACCCGTCC GTTGTGGGCA TCAGAGAGCA   
  
  
- AAAACAAAAG GGCAAAAGGC AACCTCTCTA TCAGTCTTCT CTCTCTTCCC GCTTGTCTTT TCTTATACCC   
  
  
- AAAATGTTCC AGTTGATTAT TTTTCAAAAG AAACTACTAA AACGAACAAA TGCATCTTAT TCCATAAGAT   
  
  
- GAATTCCAAT GAACGTCGGA CATTAAAAA

+     TCA-element

| Site Name | Organism | Position | Strand | Matrix score. | sequence | function |
| --- | --- | --- | --- | --- | --- | --- |
| TCA-element | Brassica oleracea | 1363 | + | 9 | TCAGAAGAGG | cis-acting element involved in salicylic acid responsiveness |
| TCA-element | Brassica oleracea | 1387 | + | 9 | GAGAAGAATA | cis-acting element involved in salicylic acid responsiveness |
| TCA-element | Nicotiana tabacum | 318 | - | 9 | CCATCTTTTT | cis-acting element involved in salicylic acid responsiveness |
| TCA-element | Brassica oleracea | 1385 | + | 9 | CAGAAAAGGA | cis-acting element involved in salicylic acid responsiveness |

> 2018/04/13 10:10:12  
+ CTATAAAGTT TCTGAACCGA GTCTCTGTCC AGCGATTATT ACTTCCACCG ACTTAAGGAC TTTCCAAGTT   
  
  
+ TTGTTTGTAC CATATGTTAT TTTACTTACC GGGTGGATTT AGTTCCGATC GTGTCTGTCT CTTCGTTGTG   
  
  
+ ACTTAGATCG TGGAAAGTTA ACGTCTTCTA TGCGTTTGAG ACCTGTCTAA TTTAATATAC TTTTACTTGG   
  
  
+ AAGGTAAGTT CAGTCTCATT GAGATCAGGA GAGTGGTCCC GTGAGAAAGT ATTTTCCTTA GGATTTGATC   
  
  
+ TTTTGTTTTA GTATTGTAGG TTTGTTTAAG TTAAAAAAAA AAGAGGGTCA ACTCGTAGAC TTTCGTCCAA   
  
  
+ ATCTTCATTT TCCATTTCCG AAAACCGGGG ACCAACTCTT TCTTAAAAGC TTTATTACTA TTTCTCTAGT   
  
  
+ TACTAAAGTA ATGTAGTCGA GAAATGGAAG GGTTAGTCGA GAATGTTGCC ATCGGTTGTT AGTTGGTTTC   
  
  
+ CTTTAACTAA ACCTTGTCTA AGTCGTGTCT AAGCGCCTTT GTTCTTTGGA AAAGGAACAA ACAAAAGTAA   
  
  
+ TAGTTTAACA GAGCCAAGAA GAGTTTTGTT TCGTTCGGTA TTAGTGCATT CATCTCTAAT GGCTATAGGG   
  
  
+ TTTAAACGGC TTCGATACAT TATTCCTTCG TTAGCCTTAC TTCGTTACTG GAGATATTTA AGTCTTTGAC   
  
  
+ TTCTTCTAGA TTCCTTTTTT TATAAACTTC AAGTTCTCTA ATAGCTACTT TTTCCTTTGC TTTGAAGGTC   
  
  
+ CTAGTTCCCT TTTACTTATT TTCGTGAAAG TTCATTTGTT ATTATACTTG CTCTCGTATC TAGTTCAACT   
  
  
+ TAATTAGTGT TGGAGAGTTT TTTTTTTAGT GTCTTTGTTT CACGTCGCGG ATTTAAAATC GACCCTCCGG   
  
  
+ TCTTCAATTC GAACAATAAT AATTTTTTTC TTTTTTCTAA TTCTCTATTT ATTGCAGAAC TCATTTTCGT   
  
  
+ TCTGTTTCTT ATATCTGTGT CTCTCTCTCT CTCTCTCTCT CTTTTTCTTT TTTCTAATTC TCTATTTATT   
  
  
+ GCAGAACTCA TTTTCGTTCT GTTTCTTATA TCTGTGTCTC TCTCTCTCTC TCTCTCTCTT TCTTTCTTTC   
  
  
+ TCTCTTACTA ATATTTACTA ATGGACTAAC TAATTTCTCG ATTTCCACGT ACTGCTTCTG GGTGGTGGTG   
  
  
+ CCGGTGGTAG TGGTGGTTAG ACTGGACTGG GCTCAAGATT TGATTGGCGT TTGCCTGGGC TAGTCTAACA   
  
  
+ AGGCAGCTCA TATGCTACTT AAGTTACCTC TAGCTTTGTT CGTGGGCAGG CAACACCCGT AGTCTCTCGT   
  
  
+ TTTTGTTTTC CCGTTTTCCG TTGGAGAGAT AGTCAGAAGA GAGAGAAGGG CGAACAGAAA AGAATATGGG   
  
  
+ TTTTACAAGG TCAACTAATA AAAAGTTTTC TTTGATGATT TTGCTTGTTT ACGTAGAATA AGGTATTCTA   
  
  
+ CTTAAGGTTA CTTGCAGCCT GTAATTTTT  

- GATATTTCAA AGACTTGGCT CAGAGACAGG TCGCTAATAA TGAAGGTGGC TGAATTCCTG AAAGGTTCAA   
  
  
- AACAAACATG GTATACAATA AAATGAATGG CCCACCTAAA TCAAGGCTAG CACAGACAGA GAAGCAACAC   
  
  
- TGAATCTAGC ACCTTTCAAT TGCAGAAGAT ACGCAAACTC TGGACAGATT AAATTATATG AAAATGAACC   
  
  
- TTCCATTCAA GTCAGAGTAA CTCTAGTCCT CTCACCAGGG CACTCTTTCA TAAAAGGAAT CCTAAACTAG   
  
  
- AAAACAAAAT CATAACATCC AAACAAATTC AATTTTTTTT TTCTCCCAGT TGAGCATCTG AAAGCAGGTT   
  
  
- TAGAAGTAAA AGGTAAAGGC TTTTGGCCCC TGGTTGAGAA AGAATTTTCG AAATAATGAT AAAGAGATCA   
  
  
- ATGATTTCAT TACATCAGCT CTTTACCTTC CCAATCAGCT CTTACAACGG TAGCCAACAA TCAACCAAAG   
  
  
- GAAATTGATT TGGAACAGAT TCAGCACAGA TTCGCGGAAA CAAGAAACCT TTTCCTTGTT TGTTTTCATT   
  
  
- ATCAAATTGT CTCGGTTCTT CTCAAAACAA AGCAAGCCAT AATCACGTAA GTAGAGATTA CCGATATCCC   
  
  
- AAATTTGCCG AAGCTATGTA ATAAGGAAGC AATCGGAATG AAGCAATGAC CTCTATAAAT TCAGAAACTG   
  
  
- AAGAAGATCT AAGGAAAAAA ATATTTGAAG TTCAAGAGAT TATCGATGAA AAAGGAAACG AAACTTCCAG   
  
  
- GATCAAGGGA AAATGAATAA AAGCACTTTC AAGTAAACAA TAATATGAAC GAGAGCATAG ATCAAGTTGA   
  
  
- ATTAATCACA ACCTCTCAAA AAAAAAATCA CAGAAACAAA GTGCAGCGCC TAAATTTTAG CTGGGAGGCC   
  
  
- AGAAGTTAAG CTTGTTATTA TTAAAAAAAG AAAAAAGATT AAGAGATAAA TAACGTCTTG AGTAAAAGCA   
  
  
- AGACAAAGAA TATAGACACA GAGAGAGAGA GAGAGAGAGA GAAAAAGAAA AAAGATTAAG AGATAAATAA   
  
  
- CGTCTTGAGT AAAAGCAAGA CAAAGAATAT AGACACAGAG AGAGAGAGAG AGAGAGAGAA AGAAAGAAAG   
  
  
- AGAGAATGAT TATAAATGAT TACCTGATTG ATTAAAGAGC TAAAGGTGCA TGACGAAGAC CCACCACCAC   
  
  
- GGCCACCATC ACCACCAATC TGACCTGACC CGAGTTCTAA ACTAACCGCA AACGGACCCG ATCAGATTGT   
  
  
- TCCGTCGAGT ATACGATGAA TTCAATGGAG ATCGAAACAA GCACCCGTCC GTTGTGGGCA TCAGAGAGCA   
  
  
- AAAACAAAAG GGCAAAAGGC AACCTCTCTA TCAGTCTTCT CTCTCTTCCC GCTTGTCTTT TCTTATACCC   
  
  
- AAAATGTTCC AGTTGATTAT TTTTCAAAAG AAACTACTAA AACGAACAAA TGCATCTTAT TCCATAAGAT   
  
  
- GAATTCCAAT GAACGTCGGA CATTAAAAA

+     TCT-motif

| Site Name | Organism | Position | Strand | Matrix score. | sequence | function |
| --- | --- | --- | --- | --- | --- | --- |
| TCT-motif | Arabidopsis thaliana | 1123 | + | 6 | TCTTAC | part of a light responsive element |

> 2018/04/13 10:10:12  
+ CTATAAAGTT TCTGAACCGA GTCTCTGTCC AGCGATTATT ACTTCCACCG ACTTAAGGAC TTTCCAAGTT   
  
  
+ TTGTTTGTAC CATATGTTAT TTTACTTACC GGGTGGATTT AGTTCCGATC GTGTCTGTCT CTTCGTTGTG   
  
  
+ ACTTAGATCG TGGAAAGTTA ACGTCTTCTA TGCGTTTGAG ACCTGTCTAA TTTAATATAC TTTTACTTGG   
  
  
+ AAGGTAAGTT CAGTCTCATT GAGATCAGGA GAGTGGTCCC GTGAGAAAGT ATTTTCCTTA GGATTTGATC   
  
  
+ TTTTGTTTTA GTATTGTAGG TTTGTTTAAG TTAAAAAAAA AAGAGGGTCA ACTCGTAGAC TTTCGTCCAA   
  
  
+ ATCTTCATTT TCCATTTCCG AAAACCGGGG ACCAACTCTT TCTTAAAAGC TTTATTACTA TTTCTCTAGT   
  
  
+ TACTAAAGTA ATGTAGTCGA GAAATGGAAG GGTTAGTCGA GAATGTTGCC ATCGGTTGTT AGTTGGTTTC   
  
  
+ CTTTAACTAA ACCTTGTCTA AGTCGTGTCT AAGCGCCTTT GTTCTTTGGA AAAGGAACAA ACAAAAGTAA   
  
  
+ TAGTTTAACA GAGCCAAGAA GAGTTTTGTT TCGTTCGGTA TTAGTGCATT CATCTCTAAT GGCTATAGGG   
  
  
+ TTTAAACGGC TTCGATACAT TATTCCTTCG TTAGCCTTAC TTCGTTACTG GAGATATTTA AGTCTTTGAC   
  
  
+ TTCTTCTAGA TTCCTTTTTT TATAAACTTC AAGTTCTCTA ATAGCTACTT TTTCCTTTGC TTTGAAGGTC   
  
  
+ CTAGTTCCCT TTTACTTATT TTCGTGAAAG TTCATTTGTT ATTATACTTG CTCTCGTATC TAGTTCAACT   
  
  
+ TAATTAGTGT TGGAGAGTTT TTTTTTTAGT GTCTTTGTTT CACGTCGCGG ATTTAAAATC GACCCTCCGG   
  
  
+ TCTTCAATTC GAACAATAAT AATTTTTTTC TTTTTTCTAA TTCTCTATTT ATTGCAGAAC TCATTTTCGT   
  
  
+ TCTGTTTCTT ATATCTGTGT CTCTCTCTCT CTCTCTCTCT CTTTTTCTTT TTTCTAATTC TCTATTTATT   
  
  
+ GCAGAACTCA TTTTCGTTCT GTTTCTTATA TCTGTGTCTC TCTCTCTCTC TCTCTCTCTT TCTTTCTTTC   
  
  
+ TCTCTTACTA ATATTTACTA ATGGACTAAC TAATTTCTCG ATTTCCACGT ACTGCTTCTG GGTGGTGGTG   
  
  
+ CCGGTGGTAG TGGTGGTTAG ACTGGACTGG GCTCAAGATT TGATTGGCGT TTGCCTGGGC TAGTCTAACA   
  
  
+ AGGCAGCTCA TATGCTACTT AAGTTACCTC TAGCTTTGTT CGTGGGCAGG CAACACCCGT AGTCTCTCGT   
  
  
+ TTTTGTTTTC CCGTTTTCCG TTGGAGAGAT AGTCAGAAGA GAGAGAAGGG CGAACAGAAA AGAATATGGG   
  
  
+ TTTTACAAGG TCAACTAATA AAAAGTTTTC TTTGATGATT TTGCTTGTTT ACGTAGAATA AGGTATTCTA   
  
  
+ CTTAAGGTTA CTTGCAGCCT GTAATTTTT  

- GATATTTCAA AGACTTGGCT CAGAGACAGG TCGCTAATAA TGAAGGTGGC TGAATTCCTG AAAGGTTCAA   
  
  
- AACAAACATG GTATACAATA AAATGAATGG CCCACCTAAA TCAAGGCTAG CACAGACAGA GAAGCAACAC   
  
  
- TGAATCTAGC ACCTTTCAAT TGCAGAAGAT ACGCAAACTC TGGACAGATT AAATTATATG AAAATGAACC   
  
  
- TTCCATTCAA GTCAGAGTAA CTCTAGTCCT CTCACCAGGG CACTCTTTCA TAAAAGGAAT CCTAAACTAG   
  
  
- AAAACAAAAT CATAACATCC AAACAAATTC AATTTTTTTT TTCTCCCAGT TGAGCATCTG AAAGCAGGTT   
  
  
- TAGAAGTAAA AGGTAAAGGC TTTTGGCCCC TGGTTGAGAA AGAATTTTCG AAATAATGAT AAAGAGATCA   
  
  
- ATGATTTCAT TACATCAGCT CTTTACCTTC CCAATCAGCT CTTACAACGG TAGCCAACAA TCAACCAAAG   
  
  
- GAAATTGATT TGGAACAGAT TCAGCACAGA TTCGCGGAAA CAAGAAACCT TTTCCTTGTT TGTTTTCATT   
  
  
- ATCAAATTGT CTCGGTTCTT CTCAAAACAA AGCAAGCCAT AATCACGTAA GTAGAGATTA CCGATATCCC   
  
  
- AAATTTGCCG AAGCTATGTA ATAAGGAAGC AATCGGAATG AAGCAATGAC CTCTATAAAT TCAGAAACTG   
  
  
- AAGAAGATCT AAGGAAAAAA ATATTTGAAG TTCAAGAGAT TATCGATGAA AAAGGAAACG AAACTTCCAG   
  
  
- GATCAAGGGA AAATGAATAA AAGCACTTTC AAGTAAACAA TAATATGAAC GAGAGCATAG ATCAAGTTGA   
  
  
- ATTAATCACA ACCTCTCAAA AAAAAAATCA CAGAAACAAA GTGCAGCGCC TAAATTTTAG CTGGGAGGCC   
  
  
- AGAAGTTAAG CTTGTTATTA TTAAAAAAAG AAAAAAGATT AAGAGATAAA TAACGTCTTG AGTAAAAGCA   
  
  
- AGACAAAGAA TATAGACACA GAGAGAGAGA GAGAGAGAGA GAAAAAGAAA AAAGATTAAG AGATAAATAA   
  
  
- CGTCTTGAGT AAAAGCAAGA CAAAGAATAT AGACACAGAG AGAGAGAGAG AGAGAGAGAA AGAAAGAAAG   
  
  
- AGAGAATGAT TATAAATGAT TACCTGATTG ATTAAAGAGC TAAAGGTGCA TGACGAAGAC CCACCACCAC   
  
  
- GGCCACCATC ACCACCAATC TGACCTGACC CGAGTTCTAA ACTAACCGCA AACGGACCCG ATCAGATTGT   
  
  
- TCCGTCGAGT ATACGATGAA TTCAATGGAG ATCGAAACAA GCACCCGTCC GTTGTGGGCA TCAGAGAGCA   
  
  
- AAAACAAAAG GGCAAAAGGC AACCTCTCTA TCAGTCTTCT CTCTCTTCCC GCTTGTCTTT TCTTATACCC   
  
  
- AAAATGTTCC AGTTGATTAT TTTTCAAAAG AAACTACTAA AACGAACAAA TGCATCTTAT TCCATAAGAT   
  
  
- GAATTCCAAT GAACGTCGGA CATTAAAAA

+     Unnamed\_\_1

| Site Name | Organism | Position | Strand | Matrix score. | sequence | function |
| --- | --- | --- | --- | --- | --- | --- |
| Unnamed\_\_1 | Zea mays | 1301 | + | 5 | CGTGG |  |
| Unnamed\_\_1 | Zea mays | 1165 | - | 5 | CGTGG |  |
| Unnamed\_\_1 | Zea mays | 149 | + | 5 | CGTGG |  |

> 2018/04/13 10:10:12  
+ CTATAAAGTT TCTGAACCGA GTCTCTGTCC AGCGATTATT ACTTCCACCG ACTTAAGGAC TTTCCAAGTT   
  
  
+ TTGTTTGTAC CATATGTTAT TTTACTTACC GGGTGGATTT AGTTCCGATC GTGTCTGTCT CTTCGTTGTG   
  
  
+ ACTTAGATCG TGGAAAGTTA ACGTCTTCTA TGCGTTTGAG ACCTGTCTAA TTTAATATAC TTTTACTTGG   
  
  
+ AAGGTAAGTT CAGTCTCATT GAGATCAGGA GAGTGGTCCC GTGAGAAAGT ATTTTCCTTA GGATTTGATC   
  
  
+ TTTTGTTTTA GTATTGTAGG TTTGTTTAAG TTAAAAAAAA AAGAGGGTCA ACTCGTAGAC TTTCGTCCAA   
  
  
+ ATCTTCATTT TCCATTTCCG AAAACCGGGG ACCAACTCTT TCTTAAAAGC TTTATTACTA TTTCTCTAGT   
  
  
+ TACTAAAGTA ATGTAGTCGA GAAATGGAAG GGTTAGTCGA GAATGTTGCC ATCGGTTGTT AGTTGGTTTC   
  
  
+ CTTTAACTAA ACCTTGTCTA AGTCGTGTCT AAGCGCCTTT GTTCTTTGGA AAAGGAACAA ACAAAAGTAA   
  
  
+ TAGTTTAACA GAGCCAAGAA GAGTTTTGTT TCGTTCGGTA TTAGTGCATT CATCTCTAAT GGCTATAGGG   
  
  
+ TTTAAACGGC TTCGATACAT TATTCCTTCG TTAGCCTTAC TTCGTTACTG GAGATATTTA AGTCTTTGAC   
  
  
+ TTCTTCTAGA TTCCTTTTTT TATAAACTTC AAGTTCTCTA ATAGCTACTT TTTCCTTTGC TTTGAAGGTC   
  
  
+ CTAGTTCCCT TTTACTTATT TTCGTGAAAG TTCATTTGTT ATTATACTTG CTCTCGTATC TAGTTCAACT   
  
  
+ TAATTAGTGT TGGAGAGTTT TTTTTTTAGT GTCTTTGTTT CACGTCGCGG ATTTAAAATC GACCCTCCGG   
  
  
+ TCTTCAATTC GAACAATAAT AATTTTTTTC TTTTTTCTAA TTCTCTATTT ATTGCAGAAC TCATTTTCGT   
  
  
+ TCTGTTTCTT ATATCTGTGT CTCTCTCTCT CTCTCTCTCT CTTTTTCTTT TTTCTAATTC TCTATTTATT   
  
  
+ GCAGAACTCA TTTTCGTTCT GTTTCTTATA TCTGTGTCTC TCTCTCTCTC TCTCTCTCTT TCTTTCTTTC   
  
  
+ TCTCTTACTA ATATTTACTA ATGGACTAAC TAATTTCTCG ATTTCCACGT ACTGCTTCTG GGTGGTGGTG   
  
  
+ CCGGTGGTAG TGGTGGTTAG ACTGGACTGG GCTCAAGATT TGATTGGCGT TTGCCTGGGC TAGTCTAACA   
  
  
+ AGGCAGCTCA TATGCTACTT AAGTTACCTC TAGCTTTGTT CGTGGGCAGG CAACACCCGT AGTCTCTCGT   
  
  
+ TTTTGTTTTC CCGTTTTCCG TTGGAGAGAT AGTCAGAAGA GAGAGAAGGG CGAACAGAAA AGAATATGGG   
  
  
+ TTTTACAAGG TCAACTAATA AAAAGTTTTC TTTGATGATT TTGCTTGTTT ACGTAGAATA AGGTATTCTA   
  
  
+ CTTAAGGTTA CTTGCAGCCT GTAATTTTT  

- GATATTTCAA AGACTTGGCT CAGAGACAGG TCGCTAATAA TGAAGGTGGC TGAATTCCTG AAAGGTTCAA   
  
  
- AACAAACATG GTATACAATA AAATGAATGG CCCACCTAAA TCAAGGCTAG CACAGACAGA GAAGCAACAC   
  
  
- TGAATCTAGC ACCTTTCAAT TGCAGAAGAT ACGCAAACTC TGGACAGATT AAATTATATG AAAATGAACC   
  
  
- TTCCATTCAA GTCAGAGTAA CTCTAGTCCT CTCACCAGGG CACTCTTTCA TAAAAGGAAT CCTAAACTAG   
  
  
- AAAACAAAAT CATAACATCC AAACAAATTC AATTTTTTTT TTCTCCCAGT TGAGCATCTG AAAGCAGGTT   
  
  
- TAGAAGTAAA AGGTAAAGGC TTTTGGCCCC TGGTTGAGAA AGAATTTTCG AAATAATGAT AAAGAGATCA   
  
  
- ATGATTTCAT TACATCAGCT CTTTACCTTC CCAATCAGCT CTTACAACGG TAGCCAACAA TCAACCAAAG   
  
  
- GAAATTGATT TGGAACAGAT TCAGCACAGA TTCGCGGAAA CAAGAAACCT TTTCCTTGTT TGTTTTCATT   
  
  
- ATCAAATTGT CTCGGTTCTT CTCAAAACAA AGCAAGCCAT AATCACGTAA GTAGAGATTA CCGATATCCC   
  
  
- AAATTTGCCG AAGCTATGTA ATAAGGAAGC AATCGGAATG AAGCAATGAC CTCTATAAAT TCAGAAACTG   
  
  
- AAGAAGATCT AAGGAAAAAA ATATTTGAAG TTCAAGAGAT TATCGATGAA AAAGGAAACG AAACTTCCAG   
  
  
- GATCAAGGGA AAATGAATAA AAGCACTTTC AAGTAAACAA TAATATGAAC GAGAGCATAG ATCAAGTTGA   
  
  
- ATTAATCACA ACCTCTCAAA AAAAAAATCA CAGAAACAAA GTGCAGCGCC TAAATTTTAG CTGGGAGGCC   
  
  
- AGAAGTTAAG CTTGTTATTA TTAAAAAAAG AAAAAAGATT AAGAGATAAA TAACGTCTTG AGTAAAAGCA   
  
  
- AGACAAAGAA TATAGACACA GAGAGAGAGA GAGAGAGAGA GAAAAAGAAA AAAGATTAAG AGATAAATAA   
  
  
- CGTCTTGAGT AAAAGCAAGA CAAAGAATAT AGACACAGAG AGAGAGAGAG AGAGAGAGAA AGAAAGAAAG   
  
  
- AGAGAATGAT TATAAATGAT TACCTGATTG ATTAAAGAGC TAAAGGTGCA TGACGAAGAC CCACCACCAC   
  
  
- GGCCACCATC ACCACCAATC TGACCTGACC CGAGTTCTAA ACTAACCGCA AACGGACCCG ATCAGATTGT   
  
  
- TCCGTCGAGT ATACGATGAA TTCAATGGAG ATCGAAACAA GCACCCGTCC GTTGTGGGCA TCAGAGAGCA   
  
  
- AAAACAAAAG GGCAAAAGGC AACCTCTCTA TCAGTCTTCT CTCTCTTCCC GCTTGTCTTT TCTTATACCC   
  
  
- AAAATGTTCC AGTTGATTAT TTTTCAAAAG AAACTACTAA AACGAACAAA TGCATCTTAT TCCATAAGAT   
  
  
- GAATTCCAAT GAACGTCGGA CATTAAAAA

+     Unnamed\_\_13

| Site Name | Organism | Position | Strand | Matrix score. | sequence | function |
| --- | --- | --- | --- | --- | --- | --- |
| Unnamed\_\_13 | Zea mays | 202 | - | 9 | TCCAAGTATA |  |

> 2018/04/13 10:10:12  
+ CTATAAAGTT TCTGAACCGA GTCTCTGTCC AGCGATTATT ACTTCCACCG ACTTAAGGAC TTTCCAAGTT   
  
  
+ TTGTTTGTAC CATATGTTAT TTTACTTACC GGGTGGATTT AGTTCCGATC GTGTCTGTCT CTTCGTTGTG   
  
  
+ ACTTAGATCG TGGAAAGTTA ACGTCTTCTA TGCGTTTGAG ACCTGTCTAA TTTAATATAC TTTTACTTGG   
  
  
+ AAGGTAAGTT CAGTCTCATT GAGATCAGGA GAGTGGTCCC GTGAGAAAGT ATTTTCCTTA GGATTTGATC   
  
  
+ TTTTGTTTTA GTATTGTAGG TTTGTTTAAG TTAAAAAAAA AAGAGGGTCA ACTCGTAGAC TTTCGTCCAA   
  
  
+ ATCTTCATTT TCCATTTCCG AAAACCGGGG ACCAACTCTT TCTTAAAAGC TTTATTACTA TTTCTCTAGT   
  
  
+ TACTAAAGTA ATGTAGTCGA GAAATGGAAG GGTTAGTCGA GAATGTTGCC ATCGGTTGTT AGTTGGTTTC   
  
  
+ CTTTAACTAA ACCTTGTCTA AGTCGTGTCT AAGCGCCTTT GTTCTTTGGA AAAGGAACAA ACAAAAGTAA   
  
  
+ TAGTTTAACA GAGCCAAGAA GAGTTTTGTT TCGTTCGGTA TTAGTGCATT CATCTCTAAT GGCTATAGGG   
  
  
+ TTTAAACGGC TTCGATACAT TATTCCTTCG TTAGCCTTAC TTCGTTACTG GAGATATTTA AGTCTTTGAC   
  
  
+ TTCTTCTAGA TTCCTTTTTT TATAAACTTC AAGTTCTCTA ATAGCTACTT TTTCCTTTGC TTTGAAGGTC   
  
  
+ CTAGTTCCCT TTTACTTATT TTCGTGAAAG TTCATTTGTT ATTATACTTG CTCTCGTATC TAGTTCAACT   
  
  
+ TAATTAGTGT TGGAGAGTTT TTTTTTTAGT GTCTTTGTTT CACGTCGCGG ATTTAAAATC GACCCTCCGG   
  
  
+ TCTTCAATTC GAACAATAAT AATTTTTTTC TTTTTTCTAA TTCTCTATTT ATTGCAGAAC TCATTTTCGT   
  
  
+ TCTGTTTCTT ATATCTGTGT CTCTCTCTCT CTCTCTCTCT CTTTTTCTTT TTTCTAATTC TCTATTTATT   
  
  
+ GCAGAACTCA TTTTCGTTCT GTTTCTTATA TCTGTGTCTC TCTCTCTCTC TCTCTCTCTT TCTTTCTTTC   
  
  
+ TCTCTTACTA ATATTTACTA ATGGACTAAC TAATTTCTCG ATTTCCACGT ACTGCTTCTG GGTGGTGGTG   
  
  
+ CCGGTGGTAG TGGTGGTTAG ACTGGACTGG GCTCAAGATT TGATTGGCGT TTGCCTGGGC TAGTCTAACA   
  
  
+ AGGCAGCTCA TATGCTACTT AAGTTACCTC TAGCTTTGTT CGTGGGCAGG CAACACCCGT AGTCTCTCGT   
  
  
+ TTTTGTTTTC CCGTTTTCCG TTGGAGAGAT AGTCAGAAGA GAGAGAAGGG CGAACAGAAA AGAATATGGG   
  
  
+ TTTTACAAGG TCAACTAATA AAAAGTTTTC TTTGATGATT TTGCTTGTTT ACGTAGAATA AGGTATTCTA   
  
  
+ CTTAAGGTTA CTTGCAGCCT GTAATTTTT  

- GATATTTCAA AGACTTGGCT CAGAGACAGG TCGCTAATAA TGAAGGTGGC TGAATTCCTG AAAGGTTCAA   
  
  
- AACAAACATG GTATACAATA AAATGAATGG CCCACCTAAA TCAAGGCTAG CACAGACAGA GAAGCAACAC   
  
  
- TGAATCTAGC ACCTTTCAAT TGCAGAAGAT ACGCAAACTC TGGACAGATT AAATTATATG AAAATGAACC   
  
  
- TTCCATTCAA GTCAGAGTAA CTCTAGTCCT CTCACCAGGG CACTCTTTCA TAAAAGGAAT CCTAAACTAG   
  
  
- AAAACAAAAT CATAACATCC AAACAAATTC AATTTTTTTT TTCTCCCAGT TGAGCATCTG AAAGCAGGTT   
  
  
- TAGAAGTAAA AGGTAAAGGC TTTTGGCCCC TGGTTGAGAA AGAATTTTCG AAATAATGAT AAAGAGATCA   
  
  
- ATGATTTCAT TACATCAGCT CTTTACCTTC CCAATCAGCT CTTACAACGG TAGCCAACAA TCAACCAAAG   
  
  
- GAAATTGATT TGGAACAGAT TCAGCACAGA TTCGCGGAAA CAAGAAACCT TTTCCTTGTT TGTTTTCATT   
  
  
- ATCAAATTGT CTCGGTTCTT CTCAAAACAA AGCAAGCCAT AATCACGTAA GTAGAGATTA CCGATATCCC   
  
  
- AAATTTGCCG AAGCTATGTA ATAAGGAAGC AATCGGAATG AAGCAATGAC CTCTATAAAT TCAGAAACTG   
  
  
- AAGAAGATCT AAGGAAAAAA ATATTTGAAG TTCAAGAGAT TATCGATGAA AAAGGAAACG AAACTTCCAG   
  
  
- GATCAAGGGA AAATGAATAA AAGCACTTTC AAGTAAACAA TAATATGAAC GAGAGCATAG ATCAAGTTGA   
  
  
- ATTAATCACA ACCTCTCAAA AAAAAAATCA CAGAAACAAA GTGCAGCGCC TAAATTTTAG CTGGGAGGCC   
  
  
- AGAAGTTAAG CTTGTTATTA TTAAAAAAAG AAAAAAGATT AAGAGATAAA TAACGTCTTG AGTAAAAGCA   
  
  
- AGACAAAGAA TATAGACACA GAGAGAGAGA GAGAGAGAGA GAAAAAGAAA AAAGATTAAG AGATAAATAA   
  
  
- CGTCTTGAGT AAAAGCAAGA CAAAGAATAT AGACACAGAG AGAGAGAGAG AGAGAGAGAA AGAAAGAAAG   
  
  
- AGAGAATGAT TATAAATGAT TACCTGATTG ATTAAAGAGC TAAAGGTGCA TGACGAAGAC CCACCACCAC   
  
  
- GGCCACCATC ACCACCAATC TGACCTGACC CGAGTTCTAA ACTAACCGCA AACGGACCCG ATCAGATTGT   
  
  
- TCCGTCGAGT ATACGATGAA TTCAATGGAG ATCGAAACAA GCACCCGTCC GTTGTGGGCA TCAGAGAGCA   
  
  
- AAAACAAAAG GGCAAAAGGC AACCTCTCTA TCAGTCTTCT CTCTCTTCCC GCTTGTCTTT TCTTATACCC   
  
  
- AAAATGTTCC AGTTGATTAT TTTTCAAAAG AAACTACTAA AACGAACAAA TGCATCTTAT TCCATAAGAT   
  
  
- GAATTCCAAT GAACGTCGGA CATTAAAAA

+     Unnamed\_\_2

| Site Name | Organism | Position | Strand | Matrix score. | sequence | function |
| --- | --- | --- | --- | --- | --- | --- |
| Unnamed\_\_2 | Zea mays | 375 | - | 6 | CCCCGG |  |

> 2018/04/13 10:10:12  
+ CTATAAAGTT TCTGAACCGA GTCTCTGTCC AGCGATTATT ACTTCCACCG ACTTAAGGAC TTTCCAAGTT   
  
  
+ TTGTTTGTAC CATATGTTAT TTTACTTACC GGGTGGATTT AGTTCCGATC GTGTCTGTCT CTTCGTTGTG   
  
  
+ ACTTAGATCG TGGAAAGTTA ACGTCTTCTA TGCGTTTGAG ACCTGTCTAA TTTAATATAC TTTTACTTGG   
  
  
+ AAGGTAAGTT CAGTCTCATT GAGATCAGGA GAGTGGTCCC GTGAGAAAGT ATTTTCCTTA GGATTTGATC   
  
  
+ TTTTGTTTTA GTATTGTAGG TTTGTTTAAG TTAAAAAAAA AAGAGGGTCA ACTCGTAGAC TTTCGTCCAA   
  
  
+ ATCTTCATTT TCCATTTCCG AAAACCGGGG ACCAACTCTT TCTTAAAAGC TTTATTACTA TTTCTCTAGT   
  
  
+ TACTAAAGTA ATGTAGTCGA GAAATGGAAG GGTTAGTCGA GAATGTTGCC ATCGGTTGTT AGTTGGTTTC   
  
  
+ CTTTAACTAA ACCTTGTCTA AGTCGTGTCT AAGCGCCTTT GTTCTTTGGA AAAGGAACAA ACAAAAGTAA   
  
  
+ TAGTTTAACA GAGCCAAGAA GAGTTTTGTT TCGTTCGGTA TTAGTGCATT CATCTCTAAT GGCTATAGGG   
  
  
+ TTTAAACGGC TTCGATACAT TATTCCTTCG TTAGCCTTAC TTCGTTACTG GAGATATTTA AGTCTTTGAC   
  
  
+ TTCTTCTAGA TTCCTTTTTT TATAAACTTC AAGTTCTCTA ATAGCTACTT TTTCCTTTGC TTTGAAGGTC   
  
  
+ CTAGTTCCCT TTTACTTATT TTCGTGAAAG TTCATTTGTT ATTATACTTG CTCTCGTATC TAGTTCAACT   
  
  
+ TAATTAGTGT TGGAGAGTTT TTTTTTTAGT GTCTTTGTTT CACGTCGCGG ATTTAAAATC GACCCTCCGG   
  
  
+ TCTTCAATTC GAACAATAAT AATTTTTTTC TTTTTTCTAA TTCTCTATTT ATTGCAGAAC TCATTTTCGT   
  
  
+ TCTGTTTCTT ATATCTGTGT CTCTCTCTCT CTCTCTCTCT CTTTTTCTTT TTTCTAATTC TCTATTTATT   
  
  
+ GCAGAACTCA TTTTCGTTCT GTTTCTTATA TCTGTGTCTC TCTCTCTCTC TCTCTCTCTT TCTTTCTTTC   
  
  
+ TCTCTTACTA ATATTTACTA ATGGACTAAC TAATTTCTCG ATTTCCACGT ACTGCTTCTG GGTGGTGGTG   
  
  
+ CCGGTGGTAG TGGTGGTTAG ACTGGACTGG GCTCAAGATT TGATTGGCGT TTGCCTGGGC TAGTCTAACA   
  
  
+ AGGCAGCTCA TATGCTACTT AAGTTACCTC TAGCTTTGTT CGTGGGCAGG CAACACCCGT AGTCTCTCGT   
  
  
+ TTTTGTTTTC CCGTTTTCCG TTGGAGAGAT AGTCAGAAGA GAGAGAAGGG CGAACAGAAA AGAATATGGG   
  
  
+ TTTTACAAGG TCAACTAATA AAAAGTTTTC TTTGATGATT TTGCTTGTTT ACGTAGAATA AGGTATTCTA   
  
  
+ CTTAAGGTTA CTTGCAGCCT GTAATTTTT  

- GATATTTCAA AGACTTGGCT CAGAGACAGG TCGCTAATAA TGAAGGTGGC TGAATTCCTG AAAGGTTCAA   
  
  
- AACAAACATG GTATACAATA AAATGAATGG CCCACCTAAA TCAAGGCTAG CACAGACAGA GAAGCAACAC   
  
  
- TGAATCTAGC ACCTTTCAAT TGCAGAAGAT ACGCAAACTC TGGACAGATT AAATTATATG AAAATGAACC   
  
  
- TTCCATTCAA GTCAGAGTAA CTCTAGTCCT CTCACCAGGG CACTCTTTCA TAAAAGGAAT CCTAAACTAG   
  
  
- AAAACAAAAT CATAACATCC AAACAAATTC AATTTTTTTT TTCTCCCAGT TGAGCATCTG AAAGCAGGTT   
  
  
- TAGAAGTAAA AGGTAAAGGC TTTTGGCCCC TGGTTGAGAA AGAATTTTCG AAATAATGAT AAAGAGATCA   
  
  
- ATGATTTCAT TACATCAGCT CTTTACCTTC CCAATCAGCT CTTACAACGG TAGCCAACAA TCAACCAAAG   
  
  
- GAAATTGATT TGGAACAGAT TCAGCACAGA TTCGCGGAAA CAAGAAACCT TTTCCTTGTT TGTTTTCATT   
  
  
- ATCAAATTGT CTCGGTTCTT CTCAAAACAA AGCAAGCCAT AATCACGTAA GTAGAGATTA CCGATATCCC   
  
  
- AAATTTGCCG AAGCTATGTA ATAAGGAAGC AATCGGAATG AAGCAATGAC CTCTATAAAT TCAGAAACTG   
  
  
- AAGAAGATCT AAGGAAAAAA ATATTTGAAG TTCAAGAGAT TATCGATGAA AAAGGAAACG AAACTTCCAG   
  
  
- GATCAAGGGA AAATGAATAA AAGCACTTTC AAGTAAACAA TAATATGAAC GAGAGCATAG ATCAAGTTGA   
  
  
- ATTAATCACA ACCTCTCAAA AAAAAAATCA CAGAAACAAA GTGCAGCGCC TAAATTTTAG CTGGGAGGCC   
  
  
- AGAAGTTAAG CTTGTTATTA TTAAAAAAAG AAAAAAGATT AAGAGATAAA TAACGTCTTG AGTAAAAGCA   
  
  
- AGACAAAGAA TATAGACACA GAGAGAGAGA GAGAGAGAGA GAAAAAGAAA AAAGATTAAG AGATAAATAA   
  
  
- CGTCTTGAGT AAAAGCAAGA CAAAGAATAT AGACACAGAG AGAGAGAGAG AGAGAGAGAA AGAAAGAAAG   
  
  
- AGAGAATGAT TATAAATGAT TACCTGATTG ATTAAAGAGC TAAAGGTGCA TGACGAAGAC CCACCACCAC   
  
  
- GGCCACCATC ACCACCAATC TGACCTGACC CGAGTTCTAA ACTAACCGCA AACGGACCCG ATCAGATTGT   
  
  
- TCCGTCGAGT ATACGATGAA TTCAATGGAG ATCGAAACAA GCACCCGTCC GTTGTGGGCA TCAGAGAGCA   
  
  
- AAAACAAAAG GGCAAAAGGC AACCTCTCTA TCAGTCTTCT CTCTCTTCCC GCTTGTCTTT TCTTATACCC   
  
  
- AAAATGTTCC AGTTGATTAT TTTTCAAAAG AAACTACTAA AACGAACAAA TGCATCTTAT TCCATAAGAT   
  
  
- GAATTCCAAT GAACGTCGGA CATTAAAAA

+     Unnamed\_\_3

| Site Name | Organism | Position | Strand | Matrix score. | sequence | function |
| --- | --- | --- | --- | --- | --- | --- |
| Unnamed\_\_3 | Zea mays | 1165 | - | 5 | CGTGG |  |
| Unnamed\_\_3 | Zea mays | 1301 | + | 5 | CGTGG |  |
| Unnamed\_\_3 | Zea mays | 149 | + | 5 | CGTGG |  |

> 2018/04/13 10:10:12  
+ CTATAAAGTT TCTGAACCGA GTCTCTGTCC AGCGATTATT ACTTCCACCG ACTTAAGGAC TTTCCAAGTT   
  
  
+ TTGTTTGTAC CATATGTTAT TTTACTTACC GGGTGGATTT AGTTCCGATC GTGTCTGTCT CTTCGTTGTG   
  
  
+ ACTTAGATCG TGGAAAGTTA ACGTCTTCTA TGCGTTTGAG ACCTGTCTAA TTTAATATAC TTTTACTTGG   
  
  
+ AAGGTAAGTT CAGTCTCATT GAGATCAGGA GAGTGGTCCC GTGAGAAAGT ATTTTCCTTA GGATTTGATC   
  
  
+ TTTTGTTTTA GTATTGTAGG TTTGTTTAAG TTAAAAAAAA AAGAGGGTCA ACTCGTAGAC TTTCGTCCAA   
  
  
+ ATCTTCATTT TCCATTTCCG AAAACCGGGG ACCAACTCTT TCTTAAAAGC TTTATTACTA TTTCTCTAGT   
  
  
+ TACTAAAGTA ATGTAGTCGA GAAATGGAAG GGTTAGTCGA GAATGTTGCC ATCGGTTGTT AGTTGGTTTC   
  
  
+ CTTTAACTAA ACCTTGTCTA AGTCGTGTCT AAGCGCCTTT GTTCTTTGGA AAAGGAACAA ACAAAAGTAA   
  
  
+ TAGTTTAACA GAGCCAAGAA GAGTTTTGTT TCGTTCGGTA TTAGTGCATT CATCTCTAAT GGCTATAGGG   
  
  
+ TTTAAACGGC TTCGATACAT TATTCCTTCG TTAGCCTTAC TTCGTTACTG GAGATATTTA AGTCTTTGAC   
  
  
+ TTCTTCTAGA TTCCTTTTTT TATAAACTTC AAGTTCTCTA ATAGCTACTT TTTCCTTTGC TTTGAAGGTC   
  
  
+ CTAGTTCCCT TTTACTTATT TTCGTGAAAG TTCATTTGTT ATTATACTTG CTCTCGTATC TAGTTCAACT   
  
  
+ TAATTAGTGT TGGAGAGTTT TTTTTTTAGT GTCTTTGTTT CACGTCGCGG ATTTAAAATC GACCCTCCGG   
  
  
+ TCTTCAATTC GAACAATAAT AATTTTTTTC TTTTTTCTAA TTCTCTATTT ATTGCAGAAC TCATTTTCGT   
  
  
+ TCTGTTTCTT ATATCTGTGT CTCTCTCTCT CTCTCTCTCT CTTTTTCTTT TTTCTAATTC TCTATTTATT   
  
  
+ GCAGAACTCA TTTTCGTTCT GTTTCTTATA TCTGTGTCTC TCTCTCTCTC TCTCTCTCTT TCTTTCTTTC   
  
  
+ TCTCTTACTA ATATTTACTA ATGGACTAAC TAATTTCTCG ATTTCCACGT ACTGCTTCTG GGTGGTGGTG   
  
  
+ CCGGTGGTAG TGGTGGTTAG ACTGGACTGG GCTCAAGATT TGATTGGCGT TTGCCTGGGC TAGTCTAACA   
  
  
+ AGGCAGCTCA TATGCTACTT AAGTTACCTC TAGCTTTGTT CGTGGGCAGG CAACACCCGT AGTCTCTCGT   
  
  
+ TTTTGTTTTC CCGTTTTCCG TTGGAGAGAT AGTCAGAAGA GAGAGAAGGG CGAACAGAAA AGAATATGGG   
  
  
+ TTTTACAAGG TCAACTAATA AAAAGTTTTC TTTGATGATT TTGCTTGTTT ACGTAGAATA AGGTATTCTA   
  
  
+ CTTAAGGTTA CTTGCAGCCT GTAATTTTT  

- GATATTTCAA AGACTTGGCT CAGAGACAGG TCGCTAATAA TGAAGGTGGC TGAATTCCTG AAAGGTTCAA   
  
  
- AACAAACATG GTATACAATA AAATGAATGG CCCACCTAAA TCAAGGCTAG CACAGACAGA GAAGCAACAC   
  
  
- TGAATCTAGC ACCTTTCAAT TGCAGAAGAT ACGCAAACTC TGGACAGATT AAATTATATG AAAATGAACC   
  
  
- TTCCATTCAA GTCAGAGTAA CTCTAGTCCT CTCACCAGGG CACTCTTTCA TAAAAGGAAT CCTAAACTAG   
  
  
- AAAACAAAAT CATAACATCC AAACAAATTC AATTTTTTTT TTCTCCCAGT TGAGCATCTG AAAGCAGGTT   
  
  
- TAGAAGTAAA AGGTAAAGGC TTTTGGCCCC TGGTTGAGAA AGAATTTTCG AAATAATGAT AAAGAGATCA   
  
  
- ATGATTTCAT TACATCAGCT CTTTACCTTC CCAATCAGCT CTTACAACGG TAGCCAACAA TCAACCAAAG   
  
  
- GAAATTGATT TGGAACAGAT TCAGCACAGA TTCGCGGAAA CAAGAAACCT TTTCCTTGTT TGTTTTCATT   
  
  
- ATCAAATTGT CTCGGTTCTT CTCAAAACAA AGCAAGCCAT AATCACGTAA GTAGAGATTA CCGATATCCC   
  
  
- AAATTTGCCG AAGCTATGTA ATAAGGAAGC AATCGGAATG AAGCAATGAC CTCTATAAAT TCAGAAACTG   
  
  
- AAGAAGATCT AAGGAAAAAA ATATTTGAAG TTCAAGAGAT TATCGATGAA AAAGGAAACG AAACTTCCAG   
  
  
- GATCAAGGGA AAATGAATAA AAGCACTTTC AAGTAAACAA TAATATGAAC GAGAGCATAG ATCAAGTTGA   
  
  
- ATTAATCACA ACCTCTCAAA AAAAAAATCA CAGAAACAAA GTGCAGCGCC TAAATTTTAG CTGGGAGGCC   
  
  
- AGAAGTTAAG CTTGTTATTA TTAAAAAAAG AAAAAAGATT AAGAGATAAA TAACGTCTTG AGTAAAAGCA   
  
  
- AGACAAAGAA TATAGACACA GAGAGAGAGA GAGAGAGAGA GAAAAAGAAA AAAGATTAAG AGATAAATAA   
  
  
- CGTCTTGAGT AAAAGCAAGA CAAAGAATAT AGACACAGAG AGAGAGAGAG AGAGAGAGAA AGAAAGAAAG   
  
  
- AGAGAATGAT TATAAATGAT TACCTGATTG ATTAAAGAGC TAAAGGTGCA TGACGAAGAC CCACCACCAC   
  
  
- GGCCACCATC ACCACCAATC TGACCTGACC CGAGTTCTAA ACTAACCGCA AACGGACCCG ATCAGATTGT   
  
  
- TCCGTCGAGT ATACGATGAA TTCAATGGAG ATCGAAACAA GCACCCGTCC GTTGTGGGCA TCAGAGAGCA   
  
  
- AAAACAAAAG GGCAAAAGGC AACCTCTCTA TCAGTCTTCT CTCTCTTCCC GCTTGTCTTT TCTTATACCC   
  
  
- AAAATGTTCC AGTTGATTAT TTTTCAAAAG AAACTACTAA AACGAACAAA TGCATCTTAT TCCATAAGAT   
  
  
- GAATTCCAAT GAACGTCGGA CATTAAAAA

+     Unnamed\_\_4

| Site Name | Organism | Position | Strand | Matrix score. | sequence | function |
| --- | --- | --- | --- | --- | --- | --- |
| Unnamed\_\_4 | Petroselinum hortense | 1353 | - | 4 | CTCC |  |
| Unnamed\_\_4 | Petroselinum hortense | 852 | - | 4 | CTCC |  |
| Unnamed\_\_4 | Petroselinum hortense | 905 | + | 4 | CTCC |  |
| Unnamed\_\_4 | Petroselinum hortense | 680 | - | 4 | CTCC |  |
| Unnamed\_\_4 | Petroselinum hortense | 238 | - | 4 | CTCC |  |

> 2018/04/13 10:10:12  
+ CTATAAAGTT TCTGAACCGA GTCTCTGTCC AGCGATTATT ACTTCCACCG ACTTAAGGAC TTTCCAAGTT   
  
  
+ TTGTTTGTAC CATATGTTAT TTTACTTACC GGGTGGATTT AGTTCCGATC GTGTCTGTCT CTTCGTTGTG   
  
  
+ ACTTAGATCG TGGAAAGTTA ACGTCTTCTA TGCGTTTGAG ACCTGTCTAA TTTAATATAC TTTTACTTGG   
  
  
+ AAGGTAAGTT CAGTCTCATT GAGATCAGGA GAGTGGTCCC GTGAGAAAGT ATTTTCCTTA GGATTTGATC   
  
  
+ TTTTGTTTTA GTATTGTAGG TTTGTTTAAG TTAAAAAAAA AAGAGGGTCA ACTCGTAGAC TTTCGTCCAA   
  
  
+ ATCTTCATTT TCCATTTCCG AAAACCGGGG ACCAACTCTT TCTTAAAAGC TTTATTACTA TTTCTCTAGT   
  
  
+ TACTAAAGTA ATGTAGTCGA GAAATGGAAG GGTTAGTCGA GAATGTTGCC ATCGGTTGTT AGTTGGTTTC   
  
  
+ CTTTAACTAA ACCTTGTCTA AGTCGTGTCT AAGCGCCTTT GTTCTTTGGA AAAGGAACAA ACAAAAGTAA   
  
  
+ TAGTTTAACA GAGCCAAGAA GAGTTTTGTT TCGTTCGGTA TTAGTGCATT CATCTCTAAT GGCTATAGGG   
  
  
+ TTTAAACGGC TTCGATACAT TATTCCTTCG TTAGCCTTAC TTCGTTACTG GAGATATTTA AGTCTTTGAC   
  
  
+ TTCTTCTAGA TTCCTTTTTT TATAAACTTC AAGTTCTCTA ATAGCTACTT TTTCCTTTGC TTTGAAGGTC   
  
  
+ CTAGTTCCCT TTTACTTATT TTCGTGAAAG TTCATTTGTT ATTATACTTG CTCTCGTATC TAGTTCAACT   
  
  
+ TAATTAGTGT TGGAGAGTTT TTTTTTTAGT GTCTTTGTTT CACGTCGCGG ATTTAAAATC GACCCTCCGG   
  
  
+ TCTTCAATTC GAACAATAAT AATTTTTTTC TTTTTTCTAA TTCTCTATTT ATTGCAGAAC TCATTTTCGT   
  
  
+ TCTGTTTCTT ATATCTGTGT CTCTCTCTCT CTCTCTCTCT CTTTTTCTTT TTTCTAATTC TCTATTTATT   
  
  
+ GCAGAACTCA TTTTCGTTCT GTTTCTTATA TCTGTGTCTC TCTCTCTCTC TCTCTCTCTT TCTTTCTTTC   
  
  
+ TCTCTTACTA ATATTTACTA ATGGACTAAC TAATTTCTCG ATTTCCACGT ACTGCTTCTG GGTGGTGGTG   
  
  
+ CCGGTGGTAG TGGTGGTTAG ACTGGACTGG GCTCAAGATT TGATTGGCGT TTGCCTGGGC TAGTCTAACA   
  
  
+ AGGCAGCTCA TATGCTACTT AAGTTACCTC TAGCTTTGTT CGTGGGCAGG CAACACCCGT AGTCTCTCGT   
  
  
+ TTTTGTTTTC CCGTTTTCCG TTGGAGAGAT AGTCAGAAGA GAGAGAAGGG CGAACAGAAA AGAATATGGG   
  
  
+ TTTTACAAGG TCAACTAATA AAAAGTTTTC TTTGATGATT TTGCTTGTTT ACGTAGAATA AGGTATTCTA   
  
  
+ CTTAAGGTTA CTTGCAGCCT GTAATTTTT  

- GATATTTCAA AGACTTGGCT CAGAGACAGG TCGCTAATAA TGAAGGTGGC TGAATTCCTG AAAGGTTCAA   
  
  
- AACAAACATG GTATACAATA AAATGAATGG CCCACCTAAA TCAAGGCTAG CACAGACAGA GAAGCAACAC   
  
  
- TGAATCTAGC ACCTTTCAAT TGCAGAAGAT ACGCAAACTC TGGACAGATT AAATTATATG AAAATGAACC   
  
  
- TTCCATTCAA GTCAGAGTAA CTCTAGTCCT CTCACCAGGG CACTCTTTCA TAAAAGGAAT CCTAAACTAG   
  
  
- AAAACAAAAT CATAACATCC AAACAAATTC AATTTTTTTT TTCTCCCAGT TGAGCATCTG AAAGCAGGTT   
  
  
- TAGAAGTAAA AGGTAAAGGC TTTTGGCCCC TGGTTGAGAA AGAATTTTCG AAATAATGAT AAAGAGATCA   
  
  
- ATGATTTCAT TACATCAGCT CTTTACCTTC CCAATCAGCT CTTACAACGG TAGCCAACAA TCAACCAAAG   
  
  
- GAAATTGATT TGGAACAGAT TCAGCACAGA TTCGCGGAAA CAAGAAACCT TTTCCTTGTT TGTTTTCATT   
  
  
- ATCAAATTGT CTCGGTTCTT CTCAAAACAA AGCAAGCCAT AATCACGTAA GTAGAGATTA CCGATATCCC   
  
  
- AAATTTGCCG AAGCTATGTA ATAAGGAAGC AATCGGAATG AAGCAATGAC CTCTATAAAT TCAGAAACTG   
  
  
- AAGAAGATCT AAGGAAAAAA ATATTTGAAG TTCAAGAGAT TATCGATGAA AAAGGAAACG AAACTTCCAG   
  
  
- GATCAAGGGA AAATGAATAA AAGCACTTTC AAGTAAACAA TAATATGAAC GAGAGCATAG ATCAAGTTGA   
  
  
- ATTAATCACA ACCTCTCAAA AAAAAAATCA CAGAAACAAA GTGCAGCGCC TAAATTTTAG CTGGGAGGCC   
  
  
- AGAAGTTAAG CTTGTTATTA TTAAAAAAAG AAAAAAGATT AAGAGATAAA TAACGTCTTG AGTAAAAGCA   
  
  
- AGACAAAGAA TATAGACACA GAGAGAGAGA GAGAGAGAGA GAAAAAGAAA AAAGATTAAG AGATAAATAA   
  
  
- CGTCTTGAGT AAAAGCAAGA CAAAGAATAT AGACACAGAG AGAGAGAGAG AGAGAGAGAA AGAAAGAAAG   
  
  
- AGAGAATGAT TATAAATGAT TACCTGATTG ATTAAAGAGC TAAAGGTGCA TGACGAAGAC CCACCACCAC   
  
  
- GGCCACCATC ACCACCAATC TGACCTGACC CGAGTTCTAA ACTAACCGCA AACGGACCCG ATCAGATTGT   
  
  
- TCCGTCGAGT ATACGATGAA TTCAATGGAG ATCGAAACAA GCACCCGTCC GTTGTGGGCA TCAGAGAGCA   
  
  
- AAAACAAAAG GGCAAAAGGC AACCTCTCTA TCAGTCTTCT CTCTCTTCCC GCTTGTCTTT TCTTATACCC   
  
  
- AAAATGTTCC AGTTGATTAT TTTTCAAAAG AAACTACTAA AACGAACAAA TGCATCTTAT TCCATAAGAT   
  
  
- GAATTCCAAT GAACGTCGGA CATTAAAAA

+     W box

| Site Name | Organism | Position | Strand | Matrix score. | sequence | function |
| --- | --- | --- | --- | --- | --- | --- |
| W box | Arabidopsis thaliana | 1409 | - | 6 | TTGACC |  |
| W box | Arabidopsis thaliana | 326 | - | 6 | TTGACC |  |

> 2018/04/13 10:10:12  
+ CTATAAAGTT TCTGAACCGA GTCTCTGTCC AGCGATTATT ACTTCCACCG ACTTAAGGAC TTTCCAAGTT   
  
  
+ TTGTTTGTAC CATATGTTAT TTTACTTACC GGGTGGATTT AGTTCCGATC GTGTCTGTCT CTTCGTTGTG   
  
  
+ ACTTAGATCG TGGAAAGTTA ACGTCTTCTA TGCGTTTGAG ACCTGTCTAA TTTAATATAC TTTTACTTGG   
  
  
+ AAGGTAAGTT CAGTCTCATT GAGATCAGGA GAGTGGTCCC GTGAGAAAGT ATTTTCCTTA GGATTTGATC   
  
  
+ TTTTGTTTTA GTATTGTAGG TTTGTTTAAG TTAAAAAAAA AAGAGGGTCA ACTCGTAGAC TTTCGTCCAA   
  
  
+ ATCTTCATTT TCCATTTCCG AAAACCGGGG ACCAACTCTT TCTTAAAAGC TTTATTACTA TTTCTCTAGT   
  
  
+ TACTAAAGTA ATGTAGTCGA GAAATGGAAG GGTTAGTCGA GAATGTTGCC ATCGGTTGTT AGTTGGTTTC   
  
  
+ CTTTAACTAA ACCTTGTCTA AGTCGTGTCT AAGCGCCTTT GTTCTTTGGA AAAGGAACAA ACAAAAGTAA   
  
  
+ TAGTTTAACA GAGCCAAGAA GAGTTTTGTT TCGTTCGGTA TTAGTGCATT CATCTCTAAT GGCTATAGGG   
  
  
+ TTTAAACGGC TTCGATACAT TATTCCTTCG TTAGCCTTAC TTCGTTACTG GAGATATTTA AGTCTTTGAC   
  
  
+ TTCTTCTAGA TTCCTTTTTT TATAAACTTC AAGTTCTCTA ATAGCTACTT TTTCCTTTGC TTTGAAGGTC   
  
  
+ CTAGTTCCCT TTTACTTATT TTCGTGAAAG TTCATTTGTT ATTATACTTG CTCTCGTATC TAGTTCAACT   
  
  
+ TAATTAGTGT TGGAGAGTTT TTTTTTTAGT GTCTTTGTTT CACGTCGCGG ATTTAAAATC GACCCTCCGG   
  
  
+ TCTTCAATTC GAACAATAAT AATTTTTTTC TTTTTTCTAA TTCTCTATTT ATTGCAGAAC TCATTTTCGT   
  
  
+ TCTGTTTCTT ATATCTGTGT CTCTCTCTCT CTCTCTCTCT CTTTTTCTTT TTTCTAATTC TCTATTTATT   
  
  
+ GCAGAACTCA TTTTCGTTCT GTTTCTTATA TCTGTGTCTC TCTCTCTCTC TCTCTCTCTT TCTTTCTTTC   
  
  
+ TCTCTTACTA ATATTTACTA ATGGACTAAC TAATTTCTCG ATTTCCACGT ACTGCTTCTG GGTGGTGGTG   
  
  
+ CCGGTGGTAG TGGTGGTTAG ACTGGACTGG GCTCAAGATT TGATTGGCGT TTGCCTGGGC TAGTCTAACA   
  
  
+ AGGCAGCTCA TATGCTACTT AAGTTACCTC TAGCTTTGTT CGTGGGCAGG CAACACCCGT AGTCTCTCGT   
  
  
+ TTTTGTTTTC CCGTTTTCCG TTGGAGAGAT AGTCAGAAGA GAGAGAAGGG CGAACAGAAA AGAATATGGG   
  
  
+ TTTTACAAGG TCAACTAATA AAAAGTTTTC TTTGATGATT TTGCTTGTTT ACGTAGAATA AGGTATTCTA   
  
  
+ CTTAAGGTTA CTTGCAGCCT GTAATTTTT  

- GATATTTCAA AGACTTGGCT CAGAGACAGG TCGCTAATAA TGAAGGTGGC TGAATTCCTG AAAGGTTCAA   
  
  
- AACAAACATG GTATACAATA AAATGAATGG CCCACCTAAA TCAAGGCTAG CACAGACAGA GAAGCAACAC   
  
  
- TGAATCTAGC ACCTTTCAAT TGCAGAAGAT ACGCAAACTC TGGACAGATT AAATTATATG AAAATGAACC   
  
  
- TTCCATTCAA GTCAGAGTAA CTCTAGTCCT CTCACCAGGG CACTCTTTCA TAAAAGGAAT CCTAAACTAG   
  
  
- AAAACAAAAT CATAACATCC AAACAAATTC AATTTTTTTT TTCTCCCAGT TGAGCATCTG AAAGCAGGTT   
  
  
- TAGAAGTAAA AGGTAAAGGC TTTTGGCCCC TGGTTGAGAA AGAATTTTCG AAATAATGAT AAAGAGATCA   
  
  
- ATGATTTCAT TACATCAGCT CTTTACCTTC CCAATCAGCT CTTACAACGG TAGCCAACAA TCAACCAAAG   
  
  
- GAAATTGATT TGGAACAGAT TCAGCACAGA TTCGCGGAAA CAAGAAACCT TTTCCTTGTT TGTTTTCATT   
  
  
- ATCAAATTGT CTCGGTTCTT CTCAAAACAA AGCAAGCCAT AATCACGTAA GTAGAGATTA CCGATATCCC   
  
  
- AAATTTGCCG AAGCTATGTA ATAAGGAAGC AATCGGAATG AAGCAATGAC CTCTATAAAT TCAGAAACTG   
  
  
- AAGAAGATCT AAGGAAAAAA ATATTTGAAG TTCAAGAGAT TATCGATGAA AAAGGAAACG AAACTTCCAG   
  
  
- GATCAAGGGA AAATGAATAA AAGCACTTTC AAGTAAACAA TAATATGAAC GAGAGCATAG ATCAAGTTGA   
  
  
- ATTAATCACA ACCTCTCAAA AAAAAAATCA CAGAAACAAA GTGCAGCGCC TAAATTTTAG CTGGGAGGCC   
  
  
- AGAAGTTAAG CTTGTTATTA TTAAAAAAAG AAAAAAGATT AAGAGATAAA TAACGTCTTG AGTAAAAGCA   
  
  
- AGACAAAGAA TATAGACACA GAGAGAGAGA GAGAGAGAGA GAAAAAGAAA AAAGATTAAG AGATAAATAA   
  
  
- CGTCTTGAGT AAAAGCAAGA CAAAGAATAT AGACACAGAG AGAGAGAGAG AGAGAGAGAA AGAAAGAAAG   
  
  
- AGAGAATGAT TATAAATGAT TACCTGATTG ATTAAAGAGC TAAAGGTGCA TGACGAAGAC CCACCACCAC   
  
  
- GGCCACCATC ACCACCAATC TGACCTGACC CGAGTTCTAA ACTAACCGCA AACGGACCCG ATCAGATTGT   
  
  
- TCCGTCGAGT ATACGATGAA TTCAATGGAG ATCGAAACAA GCACCCGTCC GTTGTGGGCA TCAGAGAGCA   
  
  
- AAAACAAAAG GGCAAAAGGC AACCTCTCTA TCAGTCTTCT CTCTCTTCCC GCTTGTCTTT TCTTATACCC   
  
  
- AAAATGTTCC AGTTGATTAT TTTTCAAAAG AAACTACTAA AACGAACAAA TGCATCTTAT TCCATAAGAT   
  
  
- GAATTCCAAT GAACGTCGGA CATTAAAAA

+     circadian

| Site Name | Organism | Position | Strand | Matrix score. | sequence | function |
| --- | --- | --- | --- | --- | --- | --- |
| circadian | Lycopersicon esculentum | 1434 | - | 6 | CAANNNNATC | cis-acting regulatory element involved in circadian control |
| circadian | Lycopersicon esculentum | 1227 | - | 6 | CAANNNNATC | cis-acting regulatory element involved in circadian control |

> 2018/04/13 10:10:12  
+ CTATAAAGTT TCTGAACCGA GTCTCTGTCC AGCGATTATT ACTTCCACCG ACTTAAGGAC TTTCCAAGTT   
  
  
+ TTGTTTGTAC CATATGTTAT TTTACTTACC GGGTGGATTT AGTTCCGATC GTGTCTGTCT CTTCGTTGTG   
  
  
+ ACTTAGATCG TGGAAAGTTA ACGTCTTCTA TGCGTTTGAG ACCTGTCTAA TTTAATATAC TTTTACTTGG   
  
  
+ AAGGTAAGTT CAGTCTCATT GAGATCAGGA GAGTGGTCCC GTGAGAAAGT ATTTTCCTTA GGATTTGATC   
  
  
+ TTTTGTTTTA GTATTGTAGG TTTGTTTAAG TTAAAAAAAA AAGAGGGTCA ACTCGTAGAC TTTCGTCCAA   
  
  
+ ATCTTCATTT TCCATTTCCG AAAACCGGGG ACCAACTCTT TCTTAAAAGC TTTATTACTA TTTCTCTAGT   
  
  
+ TACTAAAGTA ATGTAGTCGA GAAATGGAAG GGTTAGTCGA GAATGTTGCC ATCGGTTGTT AGTTGGTTTC   
  
  
+ CTTTAACTAA ACCTTGTCTA AGTCGTGTCT AAGCGCCTTT GTTCTTTGGA AAAGGAACAA ACAAAAGTAA   
  
  
+ TAGTTTAACA GAGCCAAGAA GAGTTTTGTT TCGTTCGGTA TTAGTGCATT CATCTCTAAT GGCTATAGGG   
  
  
+ TTTAAACGGC TTCGATACAT TATTCCTTCG TTAGCCTTAC TTCGTTACTG GAGATATTTA AGTCTTTGAC   
  
  
+ TTCTTCTAGA TTCCTTTTTT TATAAACTTC AAGTTCTCTA ATAGCTACTT TTTCCTTTGC TTTGAAGGTC   
  
  
+ CTAGTTCCCT TTTACTTATT TTCGTGAAAG TTCATTTGTT ATTATACTTG CTCTCGTATC TAGTTCAACT   
  
  
+ TAATTAGTGT TGGAGAGTTT TTTTTTTAGT GTCTTTGTTT CACGTCGCGG ATTTAAAATC GACCCTCCGG   
  
  
+ TCTTCAATTC GAACAATAAT AATTTTTTTC TTTTTTCTAA TTCTCTATTT ATTGCAGAAC TCATTTTCGT   
  
  
+ TCTGTTTCTT ATATCTGTGT CTCTCTCTCT CTCTCTCTCT CTTTTTCTTT TTTCTAATTC TCTATTTATT   
  
  
+ GCAGAACTCA TTTTCGTTCT GTTTCTTATA TCTGTGTCTC TCTCTCTCTC TCTCTCTCTT TCTTTCTTTC   
  
  
+ TCTCTTACTA ATATTTACTA ATGGACTAAC TAATTTCTCG ATTTCCACGT ACTGCTTCTG GGTGGTGGTG   
  
  
+ CCGGTGGTAG TGGTGGTTAG ACTGGACTGG GCTCAAGATT TGATTGGCGT TTGCCTGGGC TAGTCTAACA   
  
  
+ AGGCAGCTCA TATGCTACTT AAGTTACCTC TAGCTTTGTT CGTGGGCAGG CAACACCCGT AGTCTCTCGT   
  
  
+ TTTTGTTTTC CCGTTTTCCG TTGGAGAGAT AGTCAGAAGA GAGAGAAGGG CGAACAGAAA AGAATATGGG   
  
  
+ TTTTACAAGG TCAACTAATA AAAAGTTTTC TTTGATGATT TTGCTTGTTT ACGTAGAATA AGGTATTCTA   
  
  
+ CTTAAGGTTA CTTGCAGCCT GTAATTTTT  

- GATATTTCAA AGACTTGGCT CAGAGACAGG TCGCTAATAA TGAAGGTGGC TGAATTCCTG AAAGGTTCAA   
  
  
- AACAAACATG GTATACAATA AAATGAATGG CCCACCTAAA TCAAGGCTAG CACAGACAGA GAAGCAACAC   
  
  
- TGAATCTAGC ACCTTTCAAT TGCAGAAGAT ACGCAAACTC TGGACAGATT AAATTATATG AAAATGAACC   
  
  
- TTCCATTCAA GTCAGAGTAA CTCTAGTCCT CTCACCAGGG CACTCTTTCA TAAAAGGAAT CCTAAACTAG   
  
  
- AAAACAAAAT CATAACATCC AAACAAATTC AATTTTTTTT TTCTCCCAGT TGAGCATCTG AAAGCAGGTT   
  
  
- TAGAAGTAAA AGGTAAAGGC TTTTGGCCCC TGGTTGAGAA AGAATTTTCG AAATAATGAT AAAGAGATCA   
  
  
- ATGATTTCAT TACATCAGCT CTTTACCTTC CCAATCAGCT CTTACAACGG TAGCCAACAA TCAACCAAAG   
  
  
- GAAATTGATT TGGAACAGAT TCAGCACAGA TTCGCGGAAA CAAGAAACCT TTTCCTTGTT TGTTTTCATT   
  
  
- ATCAAATTGT CTCGGTTCTT CTCAAAACAA AGCAAGCCAT AATCACGTAA GTAGAGATTA CCGATATCCC   
  
  
- AAATTTGCCG AAGCTATGTA ATAAGGAAGC AATCGGAATG AAGCAATGAC CTCTATAAAT TCAGAAACTG   
  
  
- AAGAAGATCT AAGGAAAAAA ATATTTGAAG TTCAAGAGAT TATCGATGAA AAAGGAAACG AAACTTCCAG   
  
  
- GATCAAGGGA AAATGAATAA AAGCACTTTC AAGTAAACAA TAATATGAAC GAGAGCATAG ATCAAGTTGA   
  
  
- ATTAATCACA ACCTCTCAAA AAAAAAATCA CAGAAACAAA GTGCAGCGCC TAAATTTTAG CTGGGAGGCC   
  
  
- AGAAGTTAAG CTTGTTATTA TTAAAAAAAG AAAAAAGATT AAGAGATAAA TAACGTCTTG AGTAAAAGCA   
  
  
- AGACAAAGAA TATAGACACA GAGAGAGAGA GAGAGAGAGA GAAAAAGAAA AAAGATTAAG AGATAAATAA   
  
  
- CGTCTTGAGT AAAAGCAAGA CAAAGAATAT AGACACAGAG AGAGAGAGAG AGAGAGAGAA AGAAAGAAAG   
  
  
- AGAGAATGAT TATAAATGAT TACCTGATTG ATTAAAGAGC TAAAGGTGCA TGACGAAGAC CCACCACCAC   
  
  
- GGCCACCATC ACCACCAATC TGACCTGACC CGAGTTCTAA ACTAACCGCA AACGGACCCG ATCAGATTGT   
  
  
- TCCGTCGAGT ATACGATGAA TTCAATGGAG ATCGAAACAA GCACCCGTCC GTTGTGGGCA TCAGAGAGCA   
  
  
- AAAACAAAAG GGCAAAAGGC AACCTCTCTA TCAGTCTTCT CTCTCTTCCC GCTTGTCTTT TCTTATACCC   
  
  
- AAAATGTTCC AGTTGATTAT TTTTCAAAAG AAACTACTAA AACGAACAAA TGCATCTTAT TCCATAAGAT   
  
  
- GAATTCCAAT GAACGTCGGA CATTAAAAA
