# Supplementary material for: Genome-wide analysis of the WRKY gene family in drumstick (Moringa oleifera Lam.)
Source: PeerJ. 2019 Jun 10;7:e7063. doi: 10.7717/peerj.7063 (PMC6563795; doi:10.7717/peerj.7063)
Supplement: Supplemental Information 1 [file peerj-07-7063-s003.gz › MoWRKY6_plantcare.html]

Content-Type: text/html; charset=ISO-8859-1


CallMat\_Firefox


Webmaster Firefox specific output  
To save the result:
click on the frame with the right mouse button and save the source code as a text file with extension .html  
REFERENCE:PlantCARE: a database of plant cis-acting regulatory elements and a portal to tools for in silico analysis of promoter sequences.  
Lescot, M., Déhais, P., Moreau, Y., De Moor, B., Rouzé ,P.,and Rombauts, S.  
Nucleic Acids Res., Database issue(2002), 30(1):325-327.   


---

> 2018/04/13 10:10:12  
+ CTTAACCCAC CAGCTACTCT TTCTCTTCAA CCCATTGCTT AAATATCTTC TTCATAGACT TACCAACCTA   
  
  
+ CTTCGGTACG TGTACTTCTC TCTCTCTCTC TAATCTCTTC CGTTTCCTCT TGTATATTCC TCTCTACAAC   
  
  
+ AGTATCACGC CTACCACTTC CAGGGAGTCG CGAAGAAGAG TCTTTGATTT TCGCTTCAAA CTGGCACCTT   
  
  
+ TTATCAAGAA CCAGTTTGGA ACACCCAAAC TGACCTGACC GGTATGCCCT CCCCCGCTCC GCCGAGAAAT   
  
  
+ CCGTCAAGAC ACTAAAACCA TTATTTTAGG ATGGTAGTCA AAATCGAACG GTGCACATAA CGGTAAAAAG   
  
  
+ GGAGTCAAAA GGAATACAAT TAGTCTATTA TACATATAAA ATTCTACGTT ACACACTAAA ATTCAAAAAT   
  
  
+ GGGTTATTTA TCTATAAAAT ACCGAATTTT TAATATTAAT AAATTTTAAA ATAATTAGTC AATTGTATTA   
  
  
+ ATAATGTACT ACATTTTAAT TTTTGAATTA ATTATACAAT AATATACTAA AATTTTAAAA TTAAATAATA   
  
  
+ TTAAATAATA ATTTTATATT AAAATAAAGT TTAATATACT TATAGCATAG CAAACCTACG GGTACTACAC   
  
  
+ TATTTATAAT AAATAAAATT TTAAAATTAA TAATATTTGT AATACAAACT ATACAAAAAT AAAATATAGT   
  
  
+ ATATTAAAAT TATATATAAA TCTATTTTAT TATTTACCTT TACTTAATAA ATCATAACAT TTAAAATTCA   
  
  
+ CTACTATTTA GATTTTTTTT AAATTAATTA CATATCTATT CAAAACAAAC ACTACACCTT AGGCCACAGG   
  
  
+ AACTTTATAA ACTGGTAGGT TCTTCACTAT TTACGTTTTA TAATTAACAC CCCTTCTTTA CTTACTTCAC   
  
  
+ CCCATTCCCA GTGCACCTAG TTACTCTGAG ACACAAAGAT TCAGTTTCGT TTTTCTGTTT TTCGAGGTTT   
  
  
+ TGGCAAGTAA GGTTTCGAAA ATTTGCGGCT TTGTGAGGAA ACTACAACAG TTTCGGGGGA AGGTGAGGCT   
  
  
+ GTGAGACTAA GAAGGGTTTG TGGGCTGGGC AACTAAAGCC TTAACTGATT TGCAAGGTTA TTTTTTATTA   
  
  
+ TCTTTTTACA TTATAACACG AGAACGTTTT GTTACTCCAT GGAACCCCAT CACCAATCGT GAGACGTGAA   
  
  
+ ACTTAGGTCG CTGGACCCAA GCTGAGGGCC ACCCTGGAAG CAAAGGAACT TACAATAAAT GCAAAAAAGA   
  
  
+ AAACATTGAG TCACTTCGAA CAACACCAAT CCAGTAACTT TCTCTACAAA TTGTGACATT TGTGAAGTGT   
  
  
+ GGCTAAACTA ACACAGTTGG TGTTGAGTCA CTTCGAACAA CACCAATCCA GTAACTTTCT CTACAAATTG   
  
  
+ TGACATTTGT GAAGTGTGGC TAAACTAACA CAGTTGGTGA GCATTAGACA TAATTTCAAT GTTTGGGAAA   
  
  
+ GAAAAAGTTC CTCTTTTTTT TCTTTTCTT  

- GAATTGGGTG GTCGATGAGA AAGAGAAGTT GGGTAACGAA TTTATAGAAG AAGTATCTGA ATGGTTGGAT   
  
  
- GAAGCCATGC ACATGAAGAG AGAGAGAGAG ATTAGAGAAG GCAAAGGAGA ACATATAAGG AGAGATGTTG   
  
  
- TCATAGTGCG GATGGTGAAG GTCCCTCAGC GCTTCTTCTC AGAAACTAAA AGCGAAGTTT GACCGTGGAA   
  
  
- AATAGTTCTT GGTCAAACCT TGTGGGTTTG ACTGGACTGG CCATACGGGA GGGGGCGAGG CGGCTCTTTA   
  
  
- GGCAGTTCTG TGATTTTGGT AATAAAATCC TACCATCAGT TTTAGCTTGC CACGTGTATT GCCATTTTTC   
  
  
- CCTCAGTTTT CCTTATGTTA ATCAGATAAT ATGTATATTT TAAGATGCAA TGTGTGATTT TAAGTTTTTA   
  
  
- CCCAATAAAT AGATATTTTA TGGCTTAAAA ATTATAATTA TTTAAAATTT TATTAATCAG TTAACATAAT   
  
  
- TATTACATGA TGTAAAATTA AAAACTTAAT TAATATGTTA TTATATGATT TTAAAATTTT AATTTATTAT   
  
  
- AATTTATTAT TAAAATATAA TTTTATTTCA AATTATATGA ATATCGTATC GTTTGGATGC CCATGATGTG   
  
  
- ATAAATATTA TTTATTTTAA AATTTTAATT ATTATAAACA TTATGTTTGA TATGTTTTTA TTTTATATCA   
  
  
- TATAATTTTA ATATATATTT AGATAAAATA ATAAATGGAA ATGAATTATT TAGTATTGTA AATTTTAAGT   
  
  
- GATGATAAAT CTAAAAAAAA TTTAATTAAT GTATAGATAA GTTTTGTTTG TGATGTGGAA TCCGGTGTCC   
  
  
- TTGAAATATT TGACCATCCA AGAAGTGATA AATGCAAAAT ATTAATTGTG GGGAAGAAAT GAATGAAGTG   
  
  
- GGGTAAGGGT CACGTGGATC AATGAGACTC TGTGTTTCTA AGTCAAAGCA AAAAGACAAA AAGCTCCAAA   
  
  
- ACCGTTCATT CCAAAGCTTT TAAACGCCGA AACACTCCTT TGATGTTGTC AAAGCCCCCT TCCACTCCGA   
  
  
- CACTCTGATT CTTCCCAAAC ACCCGACCCG TTGATTTCGG AATTGACTAA ACGTTCCAAT AAAAAATAAT   
  
  
- AGAAAAATGT AATATTGTGC TCTTGCAAAA CAATGAGGTA CCTTGGGGTA GTGGTTAGCA CTCTGCACTT   
  
  
- TGAATCCAGC GACCTGGGTT CGACTCCCGG TGGGACCTTC GTTTCCTTGA ATGTTATTTA CGTTTTTTCT   
  
  
- TTTGTAACTC AGTGAAGCTT GTTGTGGTTA GGTCATTGAA AGAGATGTTT AACACTGTAA ACACTTCACA   
  
  
- CCGATTTGAT TGTGTCAACC ACAACTCAGT GAAGCTTGTT GTGGTTAGGT CATTGAAAGA GATGTTTAAC   
  
  
- ACTGTAAACA CTTCACACCG ATTTGATTGT GTCAACCACT CGTAATCTGT ATTAAAGTTA CAAACCCTTT   
  
  
- CTTTTTCAAG GAGAAAAAAA AGAAAAGAA

  
  
Motifs Found  

+     5UTR Py-rich stretch

| Site Name | Organism | Position | Strand | Matrix score. | sequence | function |
| --- | --- | --- | --- | --- | --- | --- |
| 5UTR Py-rich stretch | Lycopersicon esculentum | 85 | + | 13 | TTTCTCTCTCTCTC | cis-acting element conferring high transcription levels |
| 5UTR Py-rich stretch | Lycopersicon esculentum | 1489 | + | 9 | TTTCTTCTCT | cis-acting element conferring high transcription levels |
| 5UTR Py-rich stretch | Lycopersicon esculentum | 87 | + | 13 | TTTCTCTCTCTCTC | cis-acting element conferring high transcription levels |

> 2018/04/13 10:10:12  
+ CTTAACCCAC CAGCTACTCT TTCTCTTCAA CCCATTGCTT AAATATCTTC TTCATAGACT TACCAACCTA   
  
  
+ CTTCGGTACG TGTACTTCTC TCTCTCTCTC TAATCTCTTC CGTTTCCTCT TGTATATTCC TCTCTACAAC   
  
  
+ AGTATCACGC CTACCACTTC CAGGGAGTCG CGAAGAAGAG TCTTTGATTT TCGCTTCAAA CTGGCACCTT   
  
  
+ TTATCAAGAA CCAGTTTGGA ACACCCAAAC TGACCTGACC GGTATGCCCT CCCCCGCTCC GCCGAGAAAT   
  
  
+ CCGTCAAGAC ACTAAAACCA TTATTTTAGG ATGGTAGTCA AAATCGAACG GTGCACATAA CGGTAAAAAG   
  
  
+ GGAGTCAAAA GGAATACAAT TAGTCTATTA TACATATAAA ATTCTACGTT ACACACTAAA ATTCAAAAAT   
  
  
+ GGGTTATTTA TCTATAAAAT ACCGAATTTT TAATATTAAT AAATTTTAAA ATAATTAGTC AATTGTATTA   
  
  
+ ATAATGTACT ACATTTTAAT TTTTGAATTA ATTATACAAT AATATACTAA AATTTTAAAA TTAAATAATA   
  
  
+ TTAAATAATA ATTTTATATT AAAATAAAGT TTAATATACT TATAGCATAG CAAACCTACG GGTACTACAC   
  
  
+ TATTTATAAT AAATAAAATT TTAAAATTAA TAATATTTGT AATACAAACT ATACAAAAAT AAAATATAGT   
  
  
+ ATATTAAAAT TATATATAAA TCTATTTTAT TATTTACCTT TACTTAATAA ATCATAACAT TTAAAATTCA   
  
  
+ CTACTATTTA GATTTTTTTT AAATTAATTA CATATCTATT CAAAACAAAC ACTACACCTT AGGCCACAGG   
  
  
+ AACTTTATAA ACTGGTAGGT TCTTCACTAT TTACGTTTTA TAATTAACAC CCCTTCTTTA CTTACTTCAC   
  
  
+ CCCATTCCCA GTGCACCTAG TTACTCTGAG ACACAAAGAT TCAGTTTCGT TTTTCTGTTT TTCGAGGTTT   
  
  
+ TGGCAAGTAA GGTTTCGAAA ATTTGCGGCT TTGTGAGGAA ACTACAACAG TTTCGGGGGA AGGTGAGGCT   
  
  
+ GTGAGACTAA GAAGGGTTTG TGGGCTGGGC AACTAAAGCC TTAACTGATT TGCAAGGTTA TTTTTTATTA   
  
  
+ TCTTTTTACA TTATAACACG AGAACGTTTT GTTACTCCAT GGAACCCCAT CACCAATCGT GAGACGTGAA   
  
  
+ ACTTAGGTCG CTGGACCCAA GCTGAGGGCC ACCCTGGAAG CAAAGGAACT TACAATAAAT GCAAAAAAGA   
  
  
+ AAACATTGAG TCACTTCGAA CAACACCAAT CCAGTAACTT TCTCTACAAA TTGTGACATT TGTGAAGTGT   
  
  
+ GGCTAAACTA ACACAGTTGG TGTTGAGTCA CTTCGAACAA CACCAATCCA GTAACTTTCT CTACAAATTG   
  
  
+ TGACATTTGT GAAGTGTGGC TAAACTAACA CAGTTGGTGA GCATTAGACA TAATTTCAAT GTTTGGGAAA   
  
  
+ GAAAAAGTTC CTCTTTTTTT TCTTTTCTT  

- GAATTGGGTG GTCGATGAGA AAGAGAAGTT GGGTAACGAA TTTATAGAAG AAGTATCTGA ATGGTTGGAT   
  
  
- GAAGCCATGC ACATGAAGAG AGAGAGAGAG ATTAGAGAAG GCAAAGGAGA ACATATAAGG AGAGATGTTG   
  
  
- TCATAGTGCG GATGGTGAAG GTCCCTCAGC GCTTCTTCTC AGAAACTAAA AGCGAAGTTT GACCGTGGAA   
  
  
- AATAGTTCTT GGTCAAACCT TGTGGGTTTG ACTGGACTGG CCATACGGGA GGGGGCGAGG CGGCTCTTTA   
  
  
- GGCAGTTCTG TGATTTTGGT AATAAAATCC TACCATCAGT TTTAGCTTGC CACGTGTATT GCCATTTTTC   
  
  
- CCTCAGTTTT CCTTATGTTA ATCAGATAAT ATGTATATTT TAAGATGCAA TGTGTGATTT TAAGTTTTTA   
  
  
- CCCAATAAAT AGATATTTTA TGGCTTAAAA ATTATAATTA TTTAAAATTT TATTAATCAG TTAACATAAT   
  
  
- TATTACATGA TGTAAAATTA AAAACTTAAT TAATATGTTA TTATATGATT TTAAAATTTT AATTTATTAT   
  
  
- AATTTATTAT TAAAATATAA TTTTATTTCA AATTATATGA ATATCGTATC GTTTGGATGC CCATGATGTG   
  
  
- ATAAATATTA TTTATTTTAA AATTTTAATT ATTATAAACA TTATGTTTGA TATGTTTTTA TTTTATATCA   
  
  
- TATAATTTTA ATATATATTT AGATAAAATA ATAAATGGAA ATGAATTATT TAGTATTGTA AATTTTAAGT   
  
  
- GATGATAAAT CTAAAAAAAA TTTAATTAAT GTATAGATAA GTTTTGTTTG TGATGTGGAA TCCGGTGTCC   
  
  
- TTGAAATATT TGACCATCCA AGAAGTGATA AATGCAAAAT ATTAATTGTG GGGAAGAAAT GAATGAAGTG   
  
  
- GGGTAAGGGT CACGTGGATC AATGAGACTC TGTGTTTCTA AGTCAAAGCA AAAAGACAAA AAGCTCCAAA   
  
  
- ACCGTTCATT CCAAAGCTTT TAAACGCCGA AACACTCCTT TGATGTTGTC AAAGCCCCCT TCCACTCCGA   
  
  
- CACTCTGATT CTTCCCAAAC ACCCGACCCG TTGATTTCGG AATTGACTAA ACGTTCCAAT AAAAAATAAT   
  
  
- AGAAAAATGT AATATTGTGC TCTTGCAAAA CAATGAGGTA CCTTGGGGTA GTGGTTAGCA CTCTGCACTT   
  
  
- TGAATCCAGC GACCTGGGTT CGACTCCCGG TGGGACCTTC GTTTCCTTGA ATGTTATTTA CGTTTTTTCT   
  
  
- TTTGTAACTC AGTGAAGCTT GTTGTGGTTA GGTCATTGAA AGAGATGTTT AACACTGTAA ACACTTCACA   
  
  
- CCGATTTGAT TGTGTCAACC ACAACTCAGT GAAGCTTGTT GTGGTTAGGT CATTGAAAGA GATGTTTAAC   
  
  
- ACTGTAAACA CTTCACACCG ATTTGATTGT GTCAACCACT CGTAATCTGT ATTAAAGTTA CAAACCCTTT   
  
  
- CTTTTTCAAG GAGAAAAAAA AGAAAAGAA

+     AAGAA-motif

| Site Name | Organism | Position | Strand | Matrix score. | sequence | function |
| --- | --- | --- | --- | --- | --- | --- |
| AAGAA-motif | Avena sativa | 1465 | + | 9 | gGTAAAGAAA |  |
| AAGAA-motif | Avena sativa | 1467 | + | 7 | GAAAGAA |  |

> 2018/04/13 10:10:12  
+ CTTAACCCAC CAGCTACTCT TTCTCTTCAA CCCATTGCTT AAATATCTTC TTCATAGACT TACCAACCTA   
  
  
+ CTTCGGTACG TGTACTTCTC TCTCTCTCTC TAATCTCTTC CGTTTCCTCT TGTATATTCC TCTCTACAAC   
  
  
+ AGTATCACGC CTACCACTTC CAGGGAGTCG CGAAGAAGAG TCTTTGATTT TCGCTTCAAA CTGGCACCTT   
  
  
+ TTATCAAGAA CCAGTTTGGA ACACCCAAAC TGACCTGACC GGTATGCCCT CCCCCGCTCC GCCGAGAAAT   
  
  
+ CCGTCAAGAC ACTAAAACCA TTATTTTAGG ATGGTAGTCA AAATCGAACG GTGCACATAA CGGTAAAAAG   
  
  
+ GGAGTCAAAA GGAATACAAT TAGTCTATTA TACATATAAA ATTCTACGTT ACACACTAAA ATTCAAAAAT   
  
  
+ GGGTTATTTA TCTATAAAAT ACCGAATTTT TAATATTAAT AAATTTTAAA ATAATTAGTC AATTGTATTA   
  
  
+ ATAATGTACT ACATTTTAAT TTTTGAATTA ATTATACAAT AATATACTAA AATTTTAAAA TTAAATAATA   
  
  
+ TTAAATAATA ATTTTATATT AAAATAAAGT TTAATATACT TATAGCATAG CAAACCTACG GGTACTACAC   
  
  
+ TATTTATAAT AAATAAAATT TTAAAATTAA TAATATTTGT AATACAAACT ATACAAAAAT AAAATATAGT   
  
  
+ ATATTAAAAT TATATATAAA TCTATTTTAT TATTTACCTT TACTTAATAA ATCATAACAT TTAAAATTCA   
  
  
+ CTACTATTTA GATTTTTTTT AAATTAATTA CATATCTATT CAAAACAAAC ACTACACCTT AGGCCACAGG   
  
  
+ AACTTTATAA ACTGGTAGGT TCTTCACTAT TTACGTTTTA TAATTAACAC CCCTTCTTTA CTTACTTCAC   
  
  
+ CCCATTCCCA GTGCACCTAG TTACTCTGAG ACACAAAGAT TCAGTTTCGT TTTTCTGTTT TTCGAGGTTT   
  
  
+ TGGCAAGTAA GGTTTCGAAA ATTTGCGGCT TTGTGAGGAA ACTACAACAG TTTCGGGGGA AGGTGAGGCT   
  
  
+ GTGAGACTAA GAAGGGTTTG TGGGCTGGGC AACTAAAGCC TTAACTGATT TGCAAGGTTA TTTTTTATTA   
  
  
+ TCTTTTTACA TTATAACACG AGAACGTTTT GTTACTCCAT GGAACCCCAT CACCAATCGT GAGACGTGAA   
  
  
+ ACTTAGGTCG CTGGACCCAA GCTGAGGGCC ACCCTGGAAG CAAAGGAACT TACAATAAAT GCAAAAAAGA   
  
  
+ AAACATTGAG TCACTTCGAA CAACACCAAT CCAGTAACTT TCTCTACAAA TTGTGACATT TGTGAAGTGT   
  
  
+ GGCTAAACTA ACACAGTTGG TGTTGAGTCA CTTCGAACAA CACCAATCCA GTAACTTTCT CTACAAATTG   
  
  
+ TGACATTTGT GAAGTGTGGC TAAACTAACA CAGTTGGTGA GCATTAGACA TAATTTCAAT GTTTGGGAAA   
  
  
+ GAAAAAGTTC CTCTTTTTTT TCTTTTCTT  

- GAATTGGGTG GTCGATGAGA AAGAGAAGTT GGGTAACGAA TTTATAGAAG AAGTATCTGA ATGGTTGGAT   
  
  
- GAAGCCATGC ACATGAAGAG AGAGAGAGAG ATTAGAGAAG GCAAAGGAGA ACATATAAGG AGAGATGTTG   
  
  
- TCATAGTGCG GATGGTGAAG GTCCCTCAGC GCTTCTTCTC AGAAACTAAA AGCGAAGTTT GACCGTGGAA   
  
  
- AATAGTTCTT GGTCAAACCT TGTGGGTTTG ACTGGACTGG CCATACGGGA GGGGGCGAGG CGGCTCTTTA   
  
  
- GGCAGTTCTG TGATTTTGGT AATAAAATCC TACCATCAGT TTTAGCTTGC CACGTGTATT GCCATTTTTC   
  
  
- CCTCAGTTTT CCTTATGTTA ATCAGATAAT ATGTATATTT TAAGATGCAA TGTGTGATTT TAAGTTTTTA   
  
  
- CCCAATAAAT AGATATTTTA TGGCTTAAAA ATTATAATTA TTTAAAATTT TATTAATCAG TTAACATAAT   
  
  
- TATTACATGA TGTAAAATTA AAAACTTAAT TAATATGTTA TTATATGATT TTAAAATTTT AATTTATTAT   
  
  
- AATTTATTAT TAAAATATAA TTTTATTTCA AATTATATGA ATATCGTATC GTTTGGATGC CCATGATGTG   
  
  
- ATAAATATTA TTTATTTTAA AATTTTAATT ATTATAAACA TTATGTTTGA TATGTTTTTA TTTTATATCA   
  
  
- TATAATTTTA ATATATATTT AGATAAAATA ATAAATGGAA ATGAATTATT TAGTATTGTA AATTTTAAGT   
  
  
- GATGATAAAT CTAAAAAAAA TTTAATTAAT GTATAGATAA GTTTTGTTTG TGATGTGGAA TCCGGTGTCC   
  
  
- TTGAAATATT TGACCATCCA AGAAGTGATA AATGCAAAAT ATTAATTGTG GGGAAGAAAT GAATGAAGTG   
  
  
- GGGTAAGGGT CACGTGGATC AATGAGACTC TGTGTTTCTA AGTCAAAGCA AAAAGACAAA AAGCTCCAAA   
  
  
- ACCGTTCATT CCAAAGCTTT TAAACGCCGA AACACTCCTT TGATGTTGTC AAAGCCCCCT TCCACTCCGA   
  
  
- CACTCTGATT CTTCCCAAAC ACCCGACCCG TTGATTTCGG AATTGACTAA ACGTTCCAAT AAAAAATAAT   
  
  
- AGAAAAATGT AATATTGTGC TCTTGCAAAA CAATGAGGTA CCTTGGGGTA GTGGTTAGCA CTCTGCACTT   
  
  
- TGAATCCAGC GACCTGGGTT CGACTCCCGG TGGGACCTTC GTTTCCTTGA ATGTTATTTA CGTTTTTTCT   
  
  
- TTTGTAACTC AGTGAAGCTT GTTGTGGTTA GGTCATTGAA AGAGATGTTT AACACTGTAA ACACTTCACA   
  
  
- CCGATTTGAT TGTGTCAACC ACAACTCAGT GAAGCTTGTT GTGGTTAGGT CATTGAAAGA GATGTTTAAC   
  
  
- ACTGTAAACA CTTCACACCG ATTTGATTGT GTCAACCACT CGTAATCTGT ATTAAAGTTA CAAACCCTTT   
  
  
- CTTTTTCAAG GAGAAAAAAA AGAAAAGAA

+     ABRE

| Site Name | Organism | Position | Strand | Matrix score. | sequence | function |
| --- | --- | --- | --- | --- | --- | --- |
| ABRE | Arabidopsis thaliana | 77 | + | 6 | TACGTG | cis-acting element involved in the abscisic acid responsiveness |

> 2018/04/13 10:10:12  
+ CTTAACCCAC CAGCTACTCT TTCTCTTCAA CCCATTGCTT AAATATCTTC TTCATAGACT TACCAACCTA   
  
  
+ CTTCGGTACG TGTACTTCTC TCTCTCTCTC TAATCTCTTC CGTTTCCTCT TGTATATTCC TCTCTACAAC   
  
  
+ AGTATCACGC CTACCACTTC CAGGGAGTCG CGAAGAAGAG TCTTTGATTT TCGCTTCAAA CTGGCACCTT   
  
  
+ TTATCAAGAA CCAGTTTGGA ACACCCAAAC TGACCTGACC GGTATGCCCT CCCCCGCTCC GCCGAGAAAT   
  
  
+ CCGTCAAGAC ACTAAAACCA TTATTTTAGG ATGGTAGTCA AAATCGAACG GTGCACATAA CGGTAAAAAG   
  
  
+ GGAGTCAAAA GGAATACAAT TAGTCTATTA TACATATAAA ATTCTACGTT ACACACTAAA ATTCAAAAAT   
  
  
+ GGGTTATTTA TCTATAAAAT ACCGAATTTT TAATATTAAT AAATTTTAAA ATAATTAGTC AATTGTATTA   
  
  
+ ATAATGTACT ACATTTTAAT TTTTGAATTA ATTATACAAT AATATACTAA AATTTTAAAA TTAAATAATA   
  
  
+ TTAAATAATA ATTTTATATT AAAATAAAGT TTAATATACT TATAGCATAG CAAACCTACG GGTACTACAC   
  
  
+ TATTTATAAT AAATAAAATT TTAAAATTAA TAATATTTGT AATACAAACT ATACAAAAAT AAAATATAGT   
  
  
+ ATATTAAAAT TATATATAAA TCTATTTTAT TATTTACCTT TACTTAATAA ATCATAACAT TTAAAATTCA   
  
  
+ CTACTATTTA GATTTTTTTT AAATTAATTA CATATCTATT CAAAACAAAC ACTACACCTT AGGCCACAGG   
  
  
+ AACTTTATAA ACTGGTAGGT TCTTCACTAT TTACGTTTTA TAATTAACAC CCCTTCTTTA CTTACTTCAC   
  
  
+ CCCATTCCCA GTGCACCTAG TTACTCTGAG ACACAAAGAT TCAGTTTCGT TTTTCTGTTT TTCGAGGTTT   
  
  
+ TGGCAAGTAA GGTTTCGAAA ATTTGCGGCT TTGTGAGGAA ACTACAACAG TTTCGGGGGA AGGTGAGGCT   
  
  
+ GTGAGACTAA GAAGGGTTTG TGGGCTGGGC AACTAAAGCC TTAACTGATT TGCAAGGTTA TTTTTTATTA   
  
  
+ TCTTTTTACA TTATAACACG AGAACGTTTT GTTACTCCAT GGAACCCCAT CACCAATCGT GAGACGTGAA   
  
  
+ ACTTAGGTCG CTGGACCCAA GCTGAGGGCC ACCCTGGAAG CAAAGGAACT TACAATAAAT GCAAAAAAGA   
  
  
+ AAACATTGAG TCACTTCGAA CAACACCAAT CCAGTAACTT TCTCTACAAA TTGTGACATT TGTGAAGTGT   
  
  
+ GGCTAAACTA ACACAGTTGG TGTTGAGTCA CTTCGAACAA CACCAATCCA GTAACTTTCT CTACAAATTG   
  
  
+ TGACATTTGT GAAGTGTGGC TAAACTAACA CAGTTGGTGA GCATTAGACA TAATTTCAAT GTTTGGGAAA   
  
  
+ GAAAAAGTTC CTCTTTTTTT TCTTTTCTT  

- GAATTGGGTG GTCGATGAGA AAGAGAAGTT GGGTAACGAA TTTATAGAAG AAGTATCTGA ATGGTTGGAT   
  
  
- GAAGCCATGC ACATGAAGAG AGAGAGAGAG ATTAGAGAAG GCAAAGGAGA ACATATAAGG AGAGATGTTG   
  
  
- TCATAGTGCG GATGGTGAAG GTCCCTCAGC GCTTCTTCTC AGAAACTAAA AGCGAAGTTT GACCGTGGAA   
  
  
- AATAGTTCTT GGTCAAACCT TGTGGGTTTG ACTGGACTGG CCATACGGGA GGGGGCGAGG CGGCTCTTTA   
  
  
- GGCAGTTCTG TGATTTTGGT AATAAAATCC TACCATCAGT TTTAGCTTGC CACGTGTATT GCCATTTTTC   
  
  
- CCTCAGTTTT CCTTATGTTA ATCAGATAAT ATGTATATTT TAAGATGCAA TGTGTGATTT TAAGTTTTTA   
  
  
- CCCAATAAAT AGATATTTTA TGGCTTAAAA ATTATAATTA TTTAAAATTT TATTAATCAG TTAACATAAT   
  
  
- TATTACATGA TGTAAAATTA AAAACTTAAT TAATATGTTA TTATATGATT TTAAAATTTT AATTTATTAT   
  
  
- AATTTATTAT TAAAATATAA TTTTATTTCA AATTATATGA ATATCGTATC GTTTGGATGC CCATGATGTG   
  
  
- ATAAATATTA TTTATTTTAA AATTTTAATT ATTATAAACA TTATGTTTGA TATGTTTTTA TTTTATATCA   
  
  
- TATAATTTTA ATATATATTT AGATAAAATA ATAAATGGAA ATGAATTATT TAGTATTGTA AATTTTAAGT   
  
  
- GATGATAAAT CTAAAAAAAA TTTAATTAAT GTATAGATAA GTTTTGTTTG TGATGTGGAA TCCGGTGTCC   
  
  
- TTGAAATATT TGACCATCCA AGAAGTGATA AATGCAAAAT ATTAATTGTG GGGAAGAAAT GAATGAAGTG   
  
  
- GGGTAAGGGT CACGTGGATC AATGAGACTC TGTGTTTCTA AGTCAAAGCA AAAAGACAAA AAGCTCCAAA   
  
  
- ACCGTTCATT CCAAAGCTTT TAAACGCCGA AACACTCCTT TGATGTTGTC AAAGCCCCCT TCCACTCCGA   
  
  
- CACTCTGATT CTTCCCAAAC ACCCGACCCG TTGATTTCGG AATTGACTAA ACGTTCCAAT AAAAAATAAT   
  
  
- AGAAAAATGT AATATTGTGC TCTTGCAAAA CAATGAGGTA CCTTGGGGTA GTGGTTAGCA CTCTGCACTT   
  
  
- TGAATCCAGC GACCTGGGTT CGACTCCCGG TGGGACCTTC GTTTCCTTGA ATGTTATTTA CGTTTTTTCT   
  
  
- TTTGTAACTC AGTGAAGCTT GTTGTGGTTA GGTCATTGAA AGAGATGTTT AACACTGTAA ACACTTCACA   
  
  
- CCGATTTGAT TGTGTCAACC ACAACTCAGT GAAGCTTGTT GTGGTTAGGT CATTGAAAGA GATGTTTAAC   
  
  
- ACTGTAAACA CTTCACACCG ATTTGATTGT GTCAACCACT CGTAATCTGT ATTAAAGTTA CAAACCCTTT   
  
  
- CTTTTTCAAG GAGAAAAAAA AGAAAAGAA

+     ARE

| Site Name | Organism | Position | Strand | Matrix score. | sequence | function |
| --- | --- | --- | --- | --- | --- | --- |
| ARE | Zea mays | 295 | - | 6 | TGGTTT | cis-acting regulatory element essential for the anaerobic induction |

> 2018/04/13 10:10:12  
+ CTTAACCCAC CAGCTACTCT TTCTCTTCAA CCCATTGCTT AAATATCTTC TTCATAGACT TACCAACCTA   
  
  
+ CTTCGGTACG TGTACTTCTC TCTCTCTCTC TAATCTCTTC CGTTTCCTCT TGTATATTCC TCTCTACAAC   
  
  
+ AGTATCACGC CTACCACTTC CAGGGAGTCG CGAAGAAGAG TCTTTGATTT TCGCTTCAAA CTGGCACCTT   
  
  
+ TTATCAAGAA CCAGTTTGGA ACACCCAAAC TGACCTGACC GGTATGCCCT CCCCCGCTCC GCCGAGAAAT   
  
  
+ CCGTCAAGAC ACTAAAACCA TTATTTTAGG ATGGTAGTCA AAATCGAACG GTGCACATAA CGGTAAAAAG   
  
  
+ GGAGTCAAAA GGAATACAAT TAGTCTATTA TACATATAAA ATTCTACGTT ACACACTAAA ATTCAAAAAT   
  
  
+ GGGTTATTTA TCTATAAAAT ACCGAATTTT TAATATTAAT AAATTTTAAA ATAATTAGTC AATTGTATTA   
  
  
+ ATAATGTACT ACATTTTAAT TTTTGAATTA ATTATACAAT AATATACTAA AATTTTAAAA TTAAATAATA   
  
  
+ TTAAATAATA ATTTTATATT AAAATAAAGT TTAATATACT TATAGCATAG CAAACCTACG GGTACTACAC   
  
  
+ TATTTATAAT AAATAAAATT TTAAAATTAA TAATATTTGT AATACAAACT ATACAAAAAT AAAATATAGT   
  
  
+ ATATTAAAAT TATATATAAA TCTATTTTAT TATTTACCTT TACTTAATAA ATCATAACAT TTAAAATTCA   
  
  
+ CTACTATTTA GATTTTTTTT AAATTAATTA CATATCTATT CAAAACAAAC ACTACACCTT AGGCCACAGG   
  
  
+ AACTTTATAA ACTGGTAGGT TCTTCACTAT TTACGTTTTA TAATTAACAC CCCTTCTTTA CTTACTTCAC   
  
  
+ CCCATTCCCA GTGCACCTAG TTACTCTGAG ACACAAAGAT TCAGTTTCGT TTTTCTGTTT TTCGAGGTTT   
  
  
+ TGGCAAGTAA GGTTTCGAAA ATTTGCGGCT TTGTGAGGAA ACTACAACAG TTTCGGGGGA AGGTGAGGCT   
  
  
+ GTGAGACTAA GAAGGGTTTG TGGGCTGGGC AACTAAAGCC TTAACTGATT TGCAAGGTTA TTTTTTATTA   
  
  
+ TCTTTTTACA TTATAACACG AGAACGTTTT GTTACTCCAT GGAACCCCAT CACCAATCGT GAGACGTGAA   
  
  
+ ACTTAGGTCG CTGGACCCAA GCTGAGGGCC ACCCTGGAAG CAAAGGAACT TACAATAAAT GCAAAAAAGA   
  
  
+ AAACATTGAG TCACTTCGAA CAACACCAAT CCAGTAACTT TCTCTACAAA TTGTGACATT TGTGAAGTGT   
  
  
+ GGCTAAACTA ACACAGTTGG TGTTGAGTCA CTTCGAACAA CACCAATCCA GTAACTTTCT CTACAAATTG   
  
  
+ TGACATTTGT GAAGTGTGGC TAAACTAACA CAGTTGGTGA GCATTAGACA TAATTTCAAT GTTTGGGAAA   
  
  
+ GAAAAAGTTC CTCTTTTTTT TCTTTTCTT  

- GAATTGGGTG GTCGATGAGA AAGAGAAGTT GGGTAACGAA TTTATAGAAG AAGTATCTGA ATGGTTGGAT   
  
  
- GAAGCCATGC ACATGAAGAG AGAGAGAGAG ATTAGAGAAG GCAAAGGAGA ACATATAAGG AGAGATGTTG   
  
  
- TCATAGTGCG GATGGTGAAG GTCCCTCAGC GCTTCTTCTC AGAAACTAAA AGCGAAGTTT GACCGTGGAA   
  
  
- AATAGTTCTT GGTCAAACCT TGTGGGTTTG ACTGGACTGG CCATACGGGA GGGGGCGAGG CGGCTCTTTA   
  
  
- GGCAGTTCTG TGATTTTGGT AATAAAATCC TACCATCAGT TTTAGCTTGC CACGTGTATT GCCATTTTTC   
  
  
- CCTCAGTTTT CCTTATGTTA ATCAGATAAT ATGTATATTT TAAGATGCAA TGTGTGATTT TAAGTTTTTA   
  
  
- CCCAATAAAT AGATATTTTA TGGCTTAAAA ATTATAATTA TTTAAAATTT TATTAATCAG TTAACATAAT   
  
  
- TATTACATGA TGTAAAATTA AAAACTTAAT TAATATGTTA TTATATGATT TTAAAATTTT AATTTATTAT   
  
  
- AATTTATTAT TAAAATATAA TTTTATTTCA AATTATATGA ATATCGTATC GTTTGGATGC CCATGATGTG   
  
  
- ATAAATATTA TTTATTTTAA AATTTTAATT ATTATAAACA TTATGTTTGA TATGTTTTTA TTTTATATCA   
  
  
- TATAATTTTA ATATATATTT AGATAAAATA ATAAATGGAA ATGAATTATT TAGTATTGTA AATTTTAAGT   
  
  
- GATGATAAAT CTAAAAAAAA TTTAATTAAT GTATAGATAA GTTTTGTTTG TGATGTGGAA TCCGGTGTCC   
  
  
- TTGAAATATT TGACCATCCA AGAAGTGATA AATGCAAAAT ATTAATTGTG GGGAAGAAAT GAATGAAGTG   
  
  
- GGGTAAGGGT CACGTGGATC AATGAGACTC TGTGTTTCTA AGTCAAAGCA AAAAGACAAA AAGCTCCAAA   
  
  
- ACCGTTCATT CCAAAGCTTT TAAACGCCGA AACACTCCTT TGATGTTGTC AAAGCCCCCT TCCACTCCGA   
  
  
- CACTCTGATT CTTCCCAAAC ACCCGACCCG TTGATTTCGG AATTGACTAA ACGTTCCAAT AAAAAATAAT   
  
  
- AGAAAAATGT AATATTGTGC TCTTGCAAAA CAATGAGGTA CCTTGGGGTA GTGGTTAGCA CTCTGCACTT   
  
  
- TGAATCCAGC GACCTGGGTT CGACTCCCGG TGGGACCTTC GTTTCCTTGA ATGTTATTTA CGTTTTTTCT   
  
  
- TTTGTAACTC AGTGAAGCTT GTTGTGGTTA GGTCATTGAA AGAGATGTTT AACACTGTAA ACACTTCACA   
  
  
- CCGATTTGAT TGTGTCAACC ACAACTCAGT GAAGCTTGTT GTGGTTAGGT CATTGAAAGA GATGTTTAAC   
  
  
- ACTGTAAACA CTTCACACCG ATTTGATTGT GTCAACCACT CGTAATCTGT ATTAAAGTTA CAAACCCTTT   
  
  
- CTTTTTCAAG GAGAAAAAAA AGAAAAGAA

+     AT1-motif

| Site Name | Organism | Position | Strand | Matrix score. | sequence | function |
| --- | --- | --- | --- | --- | --- | --- |
| AT1-motif | Solanum tuberosum | 650 | - | 11 | ATTAATTTTACA | part of a light responsive module |
| AT1-motif | Solanum tuberosum | 517 | + | 11 | ATTAATTTTACA | part of a light responsive module |

> 2018/04/13 10:10:12  
+ CTTAACCCAC CAGCTACTCT TTCTCTTCAA CCCATTGCTT AAATATCTTC TTCATAGACT TACCAACCTA   
  
  
+ CTTCGGTACG TGTACTTCTC TCTCTCTCTC TAATCTCTTC CGTTTCCTCT TGTATATTCC TCTCTACAAC   
  
  
+ AGTATCACGC CTACCACTTC CAGGGAGTCG CGAAGAAGAG TCTTTGATTT TCGCTTCAAA CTGGCACCTT   
  
  
+ TTATCAAGAA CCAGTTTGGA ACACCCAAAC TGACCTGACC GGTATGCCCT CCCCCGCTCC GCCGAGAAAT   
  
  
+ CCGTCAAGAC ACTAAAACCA TTATTTTAGG ATGGTAGTCA AAATCGAACG GTGCACATAA CGGTAAAAAG   
  
  
+ GGAGTCAAAA GGAATACAAT TAGTCTATTA TACATATAAA ATTCTACGTT ACACACTAAA ATTCAAAAAT   
  
  
+ GGGTTATTTA TCTATAAAAT ACCGAATTTT TAATATTAAT AAATTTTAAA ATAATTAGTC AATTGTATTA   
  
  
+ ATAATGTACT ACATTTTAAT TTTTGAATTA ATTATACAAT AATATACTAA AATTTTAAAA TTAAATAATA   
  
  
+ TTAAATAATA ATTTTATATT AAAATAAAGT TTAATATACT TATAGCATAG CAAACCTACG GGTACTACAC   
  
  
+ TATTTATAAT AAATAAAATT TTAAAATTAA TAATATTTGT AATACAAACT ATACAAAAAT AAAATATAGT   
  
  
+ ATATTAAAAT TATATATAAA TCTATTTTAT TATTTACCTT TACTTAATAA ATCATAACAT TTAAAATTCA   
  
  
+ CTACTATTTA GATTTTTTTT AAATTAATTA CATATCTATT CAAAACAAAC ACTACACCTT AGGCCACAGG   
  
  
+ AACTTTATAA ACTGGTAGGT TCTTCACTAT TTACGTTTTA TAATTAACAC CCCTTCTTTA CTTACTTCAC   
  
  
+ CCCATTCCCA GTGCACCTAG TTACTCTGAG ACACAAAGAT TCAGTTTCGT TTTTCTGTTT TTCGAGGTTT   
  
  
+ TGGCAAGTAA GGTTTCGAAA ATTTGCGGCT TTGTGAGGAA ACTACAACAG TTTCGGGGGA AGGTGAGGCT   
  
  
+ GTGAGACTAA GAAGGGTTTG TGGGCTGGGC AACTAAAGCC TTAACTGATT TGCAAGGTTA TTTTTTATTA   
  
  
+ TCTTTTTACA TTATAACACG AGAACGTTTT GTTACTCCAT GGAACCCCAT CACCAATCGT GAGACGTGAA   
  
  
+ ACTTAGGTCG CTGGACCCAA GCTGAGGGCC ACCCTGGAAG CAAAGGAACT TACAATAAAT GCAAAAAAGA   
  
  
+ AAACATTGAG TCACTTCGAA CAACACCAAT CCAGTAACTT TCTCTACAAA TTGTGACATT TGTGAAGTGT   
  
  
+ GGCTAAACTA ACACAGTTGG TGTTGAGTCA CTTCGAACAA CACCAATCCA GTAACTTTCT CTACAAATTG   
  
  
+ TGACATTTGT GAAGTGTGGC TAAACTAACA CAGTTGGTGA GCATTAGACA TAATTTCAAT GTTTGGGAAA   
  
  
+ GAAAAAGTTC CTCTTTTTTT TCTTTTCTT  

- GAATTGGGTG GTCGATGAGA AAGAGAAGTT GGGTAACGAA TTTATAGAAG AAGTATCTGA ATGGTTGGAT   
  
  
- GAAGCCATGC ACATGAAGAG AGAGAGAGAG ATTAGAGAAG GCAAAGGAGA ACATATAAGG AGAGATGTTG   
  
  
- TCATAGTGCG GATGGTGAAG GTCCCTCAGC GCTTCTTCTC AGAAACTAAA AGCGAAGTTT GACCGTGGAA   
  
  
- AATAGTTCTT GGTCAAACCT TGTGGGTTTG ACTGGACTGG CCATACGGGA GGGGGCGAGG CGGCTCTTTA   
  
  
- GGCAGTTCTG TGATTTTGGT AATAAAATCC TACCATCAGT TTTAGCTTGC CACGTGTATT GCCATTTTTC   
  
  
- CCTCAGTTTT CCTTATGTTA ATCAGATAAT ATGTATATTT TAAGATGCAA TGTGTGATTT TAAGTTTTTA   
  
  
- CCCAATAAAT AGATATTTTA TGGCTTAAAA ATTATAATTA TTTAAAATTT TATTAATCAG TTAACATAAT   
  
  
- TATTACATGA TGTAAAATTA AAAACTTAAT TAATATGTTA TTATATGATT TTAAAATTTT AATTTATTAT   
  
  
- AATTTATTAT TAAAATATAA TTTTATTTCA AATTATATGA ATATCGTATC GTTTGGATGC CCATGATGTG   
  
  
- ATAAATATTA TTTATTTTAA AATTTTAATT ATTATAAACA TTATGTTTGA TATGTTTTTA TTTTATATCA   
  
  
- TATAATTTTA ATATATATTT AGATAAAATA ATAAATGGAA ATGAATTATT TAGTATTGTA AATTTTAAGT   
  
  
- GATGATAAAT CTAAAAAAAA TTTAATTAAT GTATAGATAA GTTTTGTTTG TGATGTGGAA TCCGGTGTCC   
  
  
- TTGAAATATT TGACCATCCA AGAAGTGATA AATGCAAAAT ATTAATTGTG GGGAAGAAAT GAATGAAGTG   
  
  
- GGGTAAGGGT CACGTGGATC AATGAGACTC TGTGTTTCTA AGTCAAAGCA AAAAGACAAA AAGCTCCAAA   
  
  
- ACCGTTCATT CCAAAGCTTT TAAACGCCGA AACACTCCTT TGATGTTGTC AAAGCCCCCT TCCACTCCGA   
  
  
- CACTCTGATT CTTCCCAAAC ACCCGACCCG TTGATTTCGG AATTGACTAA ACGTTCCAAT AAAAAATAAT   
  
  
- AGAAAAATGT AATATTGTGC TCTTGCAAAA CAATGAGGTA CCTTGGGGTA GTGGTTAGCA CTCTGCACTT   
  
  
- TGAATCCAGC GACCTGGGTT CGACTCCCGG TGGGACCTTC GTTTCCTTGA ATGTTATTTA CGTTTTTTCT   
  
  
- TTTGTAACTC AGTGAAGCTT GTTGTGGTTA GGTCATTGAA AGAGATGTTT AACACTGTAA ACACTTCACA   
  
  
- CCGATTTGAT TGTGTCAACC ACAACTCAGT GAAGCTTGTT GTGGTTAGGT CATTGAAAGA GATGTTTAAC   
  
  
- ACTGTAAACA CTTCACACCG ATTTGATTGT GTCAACCACT CGTAATCTGT ATTAAAGTTA CAAACCCTTT   
  
  
- CTTTTTCAAG GAGAAAAAAA AGAAAAGAA

+     Box 4

| Site Name | Organism | Position | Strand | Matrix score. | sequence | function |
| --- | --- | --- | --- | --- | --- | --- |
| Box 4 | Petroselinum crispum | 793 | - | 6 | ATTAAT | part of a conserved DNA module involved in light responsiveness |
| Box 4 | Petroselinum crispum | 487 | + | 6 | ATTAAT | part of a conserved DNA module involved in light responsiveness |
| Box 4 | Petroselinum crispum | 517 | + | 6 | ATTAAT | part of a conserved DNA module involved in light responsiveness |
| Box 4 | Petroselinum crispum | 455 | + | 6 | ATTAAT | part of a conserved DNA module involved in light responsiveness |
| Box 4 | Petroselinum crispum | 656 | + | 6 | ATTAAT | part of a conserved DNA module involved in light responsiveness |

> 2018/04/13 10:10:12  
+ CTTAACCCAC CAGCTACTCT TTCTCTTCAA CCCATTGCTT AAATATCTTC TTCATAGACT TACCAACCTA   
  
  
+ CTTCGGTACG TGTACTTCTC TCTCTCTCTC TAATCTCTTC CGTTTCCTCT TGTATATTCC TCTCTACAAC   
  
  
+ AGTATCACGC CTACCACTTC CAGGGAGTCG CGAAGAAGAG TCTTTGATTT TCGCTTCAAA CTGGCACCTT   
  
  
+ TTATCAAGAA CCAGTTTGGA ACACCCAAAC TGACCTGACC GGTATGCCCT CCCCCGCTCC GCCGAGAAAT   
  
  
+ CCGTCAAGAC ACTAAAACCA TTATTTTAGG ATGGTAGTCA AAATCGAACG GTGCACATAA CGGTAAAAAG   
  
  
+ GGAGTCAAAA GGAATACAAT TAGTCTATTA TACATATAAA ATTCTACGTT ACACACTAAA ATTCAAAAAT   
  
  
+ GGGTTATTTA TCTATAAAAT ACCGAATTTT TAATATTAAT AAATTTTAAA ATAATTAGTC AATTGTATTA   
  
  
+ ATAATGTACT ACATTTTAAT TTTTGAATTA ATTATACAAT AATATACTAA AATTTTAAAA TTAAATAATA   
  
  
+ TTAAATAATA ATTTTATATT AAAATAAAGT TTAATATACT TATAGCATAG CAAACCTACG GGTACTACAC   
  
  
+ TATTTATAAT AAATAAAATT TTAAAATTAA TAATATTTGT AATACAAACT ATACAAAAAT AAAATATAGT   
  
  
+ ATATTAAAAT TATATATAAA TCTATTTTAT TATTTACCTT TACTTAATAA ATCATAACAT TTAAAATTCA   
  
  
+ CTACTATTTA GATTTTTTTT AAATTAATTA CATATCTATT CAAAACAAAC ACTACACCTT AGGCCACAGG   
  
  
+ AACTTTATAA ACTGGTAGGT TCTTCACTAT TTACGTTTTA TAATTAACAC CCCTTCTTTA CTTACTTCAC   
  
  
+ CCCATTCCCA GTGCACCTAG TTACTCTGAG ACACAAAGAT TCAGTTTCGT TTTTCTGTTT TTCGAGGTTT   
  
  
+ TGGCAAGTAA GGTTTCGAAA ATTTGCGGCT TTGTGAGGAA ACTACAACAG TTTCGGGGGA AGGTGAGGCT   
  
  
+ GTGAGACTAA GAAGGGTTTG TGGGCTGGGC AACTAAAGCC TTAACTGATT TGCAAGGTTA TTTTTTATTA   
  
  
+ TCTTTTTACA TTATAACACG AGAACGTTTT GTTACTCCAT GGAACCCCAT CACCAATCGT GAGACGTGAA   
  
  
+ ACTTAGGTCG CTGGACCCAA GCTGAGGGCC ACCCTGGAAG CAAAGGAACT TACAATAAAT GCAAAAAAGA   
  
  
+ AAACATTGAG TCACTTCGAA CAACACCAAT CCAGTAACTT TCTCTACAAA TTGTGACATT TGTGAAGTGT   
  
  
+ GGCTAAACTA ACACAGTTGG TGTTGAGTCA CTTCGAACAA CACCAATCCA GTAACTTTCT CTACAAATTG   
  
  
+ TGACATTTGT GAAGTGTGGC TAAACTAACA CAGTTGGTGA GCATTAGACA TAATTTCAAT GTTTGGGAAA   
  
  
+ GAAAAAGTTC CTCTTTTTTT TCTTTTCTT  

- GAATTGGGTG GTCGATGAGA AAGAGAAGTT GGGTAACGAA TTTATAGAAG AAGTATCTGA ATGGTTGGAT   
  
  
- GAAGCCATGC ACATGAAGAG AGAGAGAGAG ATTAGAGAAG GCAAAGGAGA ACATATAAGG AGAGATGTTG   
  
  
- TCATAGTGCG GATGGTGAAG GTCCCTCAGC GCTTCTTCTC AGAAACTAAA AGCGAAGTTT GACCGTGGAA   
  
  
- AATAGTTCTT GGTCAAACCT TGTGGGTTTG ACTGGACTGG CCATACGGGA GGGGGCGAGG CGGCTCTTTA   
  
  
- GGCAGTTCTG TGATTTTGGT AATAAAATCC TACCATCAGT TTTAGCTTGC CACGTGTATT GCCATTTTTC   
  
  
- CCTCAGTTTT CCTTATGTTA ATCAGATAAT ATGTATATTT TAAGATGCAA TGTGTGATTT TAAGTTTTTA   
  
  
- CCCAATAAAT AGATATTTTA TGGCTTAAAA ATTATAATTA TTTAAAATTT TATTAATCAG TTAACATAAT   
  
  
- TATTACATGA TGTAAAATTA AAAACTTAAT TAATATGTTA TTATATGATT TTAAAATTTT AATTTATTAT   
  
  
- AATTTATTAT TAAAATATAA TTTTATTTCA AATTATATGA ATATCGTATC GTTTGGATGC CCATGATGTG   
  
  
- ATAAATATTA TTTATTTTAA AATTTTAATT ATTATAAACA TTATGTTTGA TATGTTTTTA TTTTATATCA   
  
  
- TATAATTTTA ATATATATTT AGATAAAATA ATAAATGGAA ATGAATTATT TAGTATTGTA AATTTTAAGT   
  
  
- GATGATAAAT CTAAAAAAAA TTTAATTAAT GTATAGATAA GTTTTGTTTG TGATGTGGAA TCCGGTGTCC   
  
  
- TTGAAATATT TGACCATCCA AGAAGTGATA AATGCAAAAT ATTAATTGTG GGGAAGAAAT GAATGAAGTG   
  
  
- GGGTAAGGGT CACGTGGATC AATGAGACTC TGTGTTTCTA AGTCAAAGCA AAAAGACAAA AAGCTCCAAA   
  
  
- ACCGTTCATT CCAAAGCTTT TAAACGCCGA AACACTCCTT TGATGTTGTC AAAGCCCCCT TCCACTCCGA   
  
  
- CACTCTGATT CTTCCCAAAC ACCCGACCCG TTGATTTCGG AATTGACTAA ACGTTCCAAT AAAAAATAAT   
  
  
- AGAAAAATGT AATATTGTGC TCTTGCAAAA CAATGAGGTA CCTTGGGGTA GTGGTTAGCA CTCTGCACTT   
  
  
- TGAATCCAGC GACCTGGGTT CGACTCCCGG TGGGACCTTC GTTTCCTTGA ATGTTATTTA CGTTTTTTCT   
  
  
- TTTGTAACTC AGTGAAGCTT GTTGTGGTTA GGTCATTGAA AGAGATGTTT AACACTGTAA ACACTTCACA   
  
  
- CCGATTTGAT TGTGTCAACC ACAACTCAGT GAAGCTTGTT GTGGTTAGGT CATTGAAAGA GATGTTTAAC   
  
  
- ACTGTAAACA CTTCACACCG ATTTGATTGT GTCAACCACT CGTAATCTGT ATTAAAGTTA CAAACCCTTT   
  
  
- CTTTTTCAAG GAGAAAAAAA AGAAAAGAA

+     CAAT-box

| Site Name | Organism | Position | Strand | Matrix score. | sequence | function |
| --- | --- | --- | --- | --- | --- | --- |
| CAAT-box | Hordeum vulgare | 482 | - | 4 | CAAT | common cis-acting element in promoter and enhancer regions |
| CAAT-box | Glycine max | 481 | - | 5 | CAATT | common cis-acting element in promoter and enhancer regions |
| CAAT-box | Brassica rapa | 1405 | - | 5 | CAAAT | common cis-acting element in promoter and enhancer regions |
| CAAT-box | Glycine max | 1396 | - | 5 | CAATT | common cis-acting element in promoter and enhancer regions |
| CAAT-box | Glycine max | 480 | + | 5 | CAATT | common cis-acting element in promoter and enhancer regions |
| CAAT-box | Hordeum vulgare | 1310 | - | 4 | CAAT | common cis-acting element in promoter and enhancer regions |
| CAAT-box | Glycine max | 1309 | - | 5 | CAATT | common cis-acting element in promoter and enhancer regions |
| CAAT-box | Brassica rapa | 1307 | + | 5 | CAAAT | common cis-acting element in promoter and enhancer regions |
| CAAT-box | Hordeum vulgare | 1287 | + | 4 | CAAT | common cis-acting element in promoter and enhancer regions |
| CAAT-box | Brassica rapa | 665 | - | 5 | CAAAT | common cis-acting element in promoter and enhancer regions |
| CAAT-box | Brassica rapa | 1098 | - | 5 | CAAAT | common cis-acting element in promoter and enhancer regions |
| CAAT-box | Hordeum vulgare | 1265 | - | 4 | CAAT | common cis-acting element in promoter and enhancer regions |
| CAAT-box | Hordeum vulgare | 34 | - | 4 | CAAT | common cis-acting element in promoter and enhancer regions |
| CAAT-box | Brassica rapa | 1318 | - | 5 | CAAAT | common cis-acting element in promoter and enhancer regions |
| CAAT-box | Arabidopsis thaliana | 1173 | + | 5 | CCAAT | common cis-acting element in promoter and enhancer regions |
| CAAT-box | Glycine max | 367 | + | 5 | CAATT | common cis-acting element in promoter and enhancer regions |
| CAAT-box | Hordeum vulgare | 1374 | + | 4 | CAAT | common cis-acting element in promoter and enhancer regions |
| CAAT-box | Hordeum vulgare | 527 | + | 4 | CAAT | common cis-acting element in promoter and enhancer regions |
| CAAT-box | Brassica rapa | 1394 | + | 5 | CAAAT | common cis-acting element in promoter and enhancer regions |
| CAAT-box | Arabidopsis thaliana | 1286 | + | 5 | CCAAT | common cis-acting element in promoter and enhancer regions |
| CAAT-box | Arabidopsis thaliana | 1373 | + | 5 | CCAAT | common cis-acting element in promoter and enhancer regions |
| CAAT-box | Brassica rapa | 1001 | - | 5 | CAAAT | common cis-acting element in promoter and enhancer regions |
| CAAT-box | Hordeum vulgare | 1397 | - | 4 | CAAT | common cis-acting element in promoter and enhancer regions |
| CAAT-box | Hordeum vulgare | 1457 | + | 4 | CAAT | common cis-acting element in promoter and enhancer regions |
| CAAT-box | Hordeum vulgare | 1174 | + | 4 | CAAT | common cis-acting element in promoter and enhancer regions |
| CAAT-box | Hordeum vulgare | 1243 | + | 4 | CAAT | common cis-acting element in promoter and enhancer regions |

> 2018/04/13 10:10:12  
+ CTTAACCCAC CAGCTACTCT TTCTCTTCAA CCCATTGCTT AAATATCTTC TTCATAGACT TACCAACCTA   
  
  
+ CTTCGGTACG TGTACTTCTC TCTCTCTCTC TAATCTCTTC CGTTTCCTCT TGTATATTCC TCTCTACAAC   
  
  
+ AGTATCACGC CTACCACTTC CAGGGAGTCG CGAAGAAGAG TCTTTGATTT TCGCTTCAAA CTGGCACCTT   
  
  
+ TTATCAAGAA CCAGTTTGGA ACACCCAAAC TGACCTGACC GGTATGCCCT CCCCCGCTCC GCCGAGAAAT   
  
  
+ CCGTCAAGAC ACTAAAACCA TTATTTTAGG ATGGTAGTCA AAATCGAACG GTGCACATAA CGGTAAAAAG   
  
  
+ GGAGTCAAAA GGAATACAAT TAGTCTATTA TACATATAAA ATTCTACGTT ACACACTAAA ATTCAAAAAT   
  
  
+ GGGTTATTTA TCTATAAAAT ACCGAATTTT TAATATTAAT AAATTTTAAA ATAATTAGTC AATTGTATTA   
  
  
+ ATAATGTACT ACATTTTAAT TTTTGAATTA ATTATACAAT AATATACTAA AATTTTAAAA TTAAATAATA   
  
  
+ TTAAATAATA ATTTTATATT AAAATAAAGT TTAATATACT TATAGCATAG CAAACCTACG GGTACTACAC   
  
  
+ TATTTATAAT AAATAAAATT TTAAAATTAA TAATATTTGT AATACAAACT ATACAAAAAT AAAATATAGT   
  
  
+ ATATTAAAAT TATATATAAA TCTATTTTAT TATTTACCTT TACTTAATAA ATCATAACAT TTAAAATTCA   
  
  
+ CTACTATTTA GATTTTTTTT AAATTAATTA CATATCTATT CAAAACAAAC ACTACACCTT AGGCCACAGG   
  
  
+ AACTTTATAA ACTGGTAGGT TCTTCACTAT TTACGTTTTA TAATTAACAC CCCTTCTTTA CTTACTTCAC   
  
  
+ CCCATTCCCA GTGCACCTAG TTACTCTGAG ACACAAAGAT TCAGTTTCGT TTTTCTGTTT TTCGAGGTTT   
  
  
+ TGGCAAGTAA GGTTTCGAAA ATTTGCGGCT TTGTGAGGAA ACTACAACAG TTTCGGGGGA AGGTGAGGCT   
  
  
+ GTGAGACTAA GAAGGGTTTG TGGGCTGGGC AACTAAAGCC TTAACTGATT TGCAAGGTTA TTTTTTATTA   
  
  
+ TCTTTTTACA TTATAACACG AGAACGTTTT GTTACTCCAT GGAACCCCAT CACCAATCGT GAGACGTGAA   
  
  
+ ACTTAGGTCG CTGGACCCAA GCTGAGGGCC ACCCTGGAAG CAAAGGAACT TACAATAAAT GCAAAAAAGA   
  
  
+ AAACATTGAG TCACTTCGAA CAACACCAAT CCAGTAACTT TCTCTACAAA TTGTGACATT TGTGAAGTGT   
  
  
+ GGCTAAACTA ACACAGTTGG TGTTGAGTCA CTTCGAACAA CACCAATCCA GTAACTTTCT CTACAAATTG   
  
  
+ TGACATTTGT GAAGTGTGGC TAAACTAACA CAGTTGGTGA GCATTAGACA TAATTTCAAT GTTTGGGAAA   
  
  
+ GAAAAAGTTC CTCTTTTTTT TCTTTTCTT  

- GAATTGGGTG GTCGATGAGA AAGAGAAGTT GGGTAACGAA TTTATAGAAG AAGTATCTGA ATGGTTGGAT   
  
  
- GAAGCCATGC ACATGAAGAG AGAGAGAGAG ATTAGAGAAG GCAAAGGAGA ACATATAAGG AGAGATGTTG   
  
  
- TCATAGTGCG GATGGTGAAG GTCCCTCAGC GCTTCTTCTC AGAAACTAAA AGCGAAGTTT GACCGTGGAA   
  
  
- AATAGTTCTT GGTCAAACCT TGTGGGTTTG ACTGGACTGG CCATACGGGA GGGGGCGAGG CGGCTCTTTA   
  
  
- GGCAGTTCTG TGATTTTGGT AATAAAATCC TACCATCAGT TTTAGCTTGC CACGTGTATT GCCATTTTTC   
  
  
- CCTCAGTTTT CCTTATGTTA ATCAGATAAT ATGTATATTT TAAGATGCAA TGTGTGATTT TAAGTTTTTA   
  
  
- CCCAATAAAT AGATATTTTA TGGCTTAAAA ATTATAATTA TTTAAAATTT TATTAATCAG TTAACATAAT   
  
  
- TATTACATGA TGTAAAATTA AAAACTTAAT TAATATGTTA TTATATGATT TTAAAATTTT AATTTATTAT   
  
  
- AATTTATTAT TAAAATATAA TTTTATTTCA AATTATATGA ATATCGTATC GTTTGGATGC CCATGATGTG   
  
  
- ATAAATATTA TTTATTTTAA AATTTTAATT ATTATAAACA TTATGTTTGA TATGTTTTTA TTTTATATCA   
  
  
- TATAATTTTA ATATATATTT AGATAAAATA ATAAATGGAA ATGAATTATT TAGTATTGTA AATTTTAAGT   
  
  
- GATGATAAAT CTAAAAAAAA TTTAATTAAT GTATAGATAA GTTTTGTTTG TGATGTGGAA TCCGGTGTCC   
  
  
- TTGAAATATT TGACCATCCA AGAAGTGATA AATGCAAAAT ATTAATTGTG GGGAAGAAAT GAATGAAGTG   
  
  
- GGGTAAGGGT CACGTGGATC AATGAGACTC TGTGTTTCTA AGTCAAAGCA AAAAGACAAA AAGCTCCAAA   
  
  
- ACCGTTCATT CCAAAGCTTT TAAACGCCGA AACACTCCTT TGATGTTGTC AAAGCCCCCT TCCACTCCGA   
  
  
- CACTCTGATT CTTCCCAAAC ACCCGACCCG TTGATTTCGG AATTGACTAA ACGTTCCAAT AAAAAATAAT   
  
  
- AGAAAAATGT AATATTGTGC TCTTGCAAAA CAATGAGGTA CCTTGGGGTA GTGGTTAGCA CTCTGCACTT   
  
  
- TGAATCCAGC GACCTGGGTT CGACTCCCGG TGGGACCTTC GTTTCCTTGA ATGTTATTTA CGTTTTTTCT   
  
  
- TTTGTAACTC AGTGAAGCTT GTTGTGGTTA GGTCATTGAA AGAGATGTTT AACACTGTAA ACACTTCACA   
  
  
- CCGATTTGAT TGTGTCAACC ACAACTCAGT GAAGCTTGTT GTGGTTAGGT CATTGAAAGA GATGTTTAAC   
  
  
- ACTGTAAACA CTTCACACCG ATTTGATTGT GTCAACCACT CGTAATCTGT ATTAAAGTTA CAAACCCTTT   
  
  
- CTTTTTCAAG GAGAAAAAAA AGAAAAGAA

+     CGTCA-motif

| Site Name | Organism | Position | Strand | Matrix score. | sequence | function |
| --- | --- | --- | --- | --- | --- | --- |
| CGTCA-motif | Hordeum vulgare | 282 | + | 5 | CGTCA | cis-acting regulatory element involved in the MeJA-responsiveness |

> 2018/04/13 10:10:12  
+ CTTAACCCAC CAGCTACTCT TTCTCTTCAA CCCATTGCTT AAATATCTTC TTCATAGACT TACCAACCTA   
  
  
+ CTTCGGTACG TGTACTTCTC TCTCTCTCTC TAATCTCTTC CGTTTCCTCT TGTATATTCC TCTCTACAAC   
  
  
+ AGTATCACGC CTACCACTTC CAGGGAGTCG CGAAGAAGAG TCTTTGATTT TCGCTTCAAA CTGGCACCTT   
  
  
+ TTATCAAGAA CCAGTTTGGA ACACCCAAAC TGACCTGACC GGTATGCCCT CCCCCGCTCC GCCGAGAAAT   
  
  
+ CCGTCAAGAC ACTAAAACCA TTATTTTAGG ATGGTAGTCA AAATCGAACG GTGCACATAA CGGTAAAAAG   
  
  
+ GGAGTCAAAA GGAATACAAT TAGTCTATTA TACATATAAA ATTCTACGTT ACACACTAAA ATTCAAAAAT   
  
  
+ GGGTTATTTA TCTATAAAAT ACCGAATTTT TAATATTAAT AAATTTTAAA ATAATTAGTC AATTGTATTA   
  
  
+ ATAATGTACT ACATTTTAAT TTTTGAATTA ATTATACAAT AATATACTAA AATTTTAAAA TTAAATAATA   
  
  
+ TTAAATAATA ATTTTATATT AAAATAAAGT TTAATATACT TATAGCATAG CAAACCTACG GGTACTACAC   
  
  
+ TATTTATAAT AAATAAAATT TTAAAATTAA TAATATTTGT AATACAAACT ATACAAAAAT AAAATATAGT   
  
  
+ ATATTAAAAT TATATATAAA TCTATTTTAT TATTTACCTT TACTTAATAA ATCATAACAT TTAAAATTCA   
  
  
+ CTACTATTTA GATTTTTTTT AAATTAATTA CATATCTATT CAAAACAAAC ACTACACCTT AGGCCACAGG   
  
  
+ AACTTTATAA ACTGGTAGGT TCTTCACTAT TTACGTTTTA TAATTAACAC CCCTTCTTTA CTTACTTCAC   
  
  
+ CCCATTCCCA GTGCACCTAG TTACTCTGAG ACACAAAGAT TCAGTTTCGT TTTTCTGTTT TTCGAGGTTT   
  
  
+ TGGCAAGTAA GGTTTCGAAA ATTTGCGGCT TTGTGAGGAA ACTACAACAG TTTCGGGGGA AGGTGAGGCT   
  
  
+ GTGAGACTAA GAAGGGTTTG TGGGCTGGGC AACTAAAGCC TTAACTGATT TGCAAGGTTA TTTTTTATTA   
  
  
+ TCTTTTTACA TTATAACACG AGAACGTTTT GTTACTCCAT GGAACCCCAT CACCAATCGT GAGACGTGAA   
  
  
+ ACTTAGGTCG CTGGACCCAA GCTGAGGGCC ACCCTGGAAG CAAAGGAACT TACAATAAAT GCAAAAAAGA   
  
  
+ AAACATTGAG TCACTTCGAA CAACACCAAT CCAGTAACTT TCTCTACAAA TTGTGACATT TGTGAAGTGT   
  
  
+ GGCTAAACTA ACACAGTTGG TGTTGAGTCA CTTCGAACAA CACCAATCCA GTAACTTTCT CTACAAATTG   
  
  
+ TGACATTTGT GAAGTGTGGC TAAACTAACA CAGTTGGTGA GCATTAGACA TAATTTCAAT GTTTGGGAAA   
  
  
+ GAAAAAGTTC CTCTTTTTTT TCTTTTCTT  

- GAATTGGGTG GTCGATGAGA AAGAGAAGTT GGGTAACGAA TTTATAGAAG AAGTATCTGA ATGGTTGGAT   
  
  
- GAAGCCATGC ACATGAAGAG AGAGAGAGAG ATTAGAGAAG GCAAAGGAGA ACATATAAGG AGAGATGTTG   
  
  
- TCATAGTGCG GATGGTGAAG GTCCCTCAGC GCTTCTTCTC AGAAACTAAA AGCGAAGTTT GACCGTGGAA   
  
  
- AATAGTTCTT GGTCAAACCT TGTGGGTTTG ACTGGACTGG CCATACGGGA GGGGGCGAGG CGGCTCTTTA   
  
  
- GGCAGTTCTG TGATTTTGGT AATAAAATCC TACCATCAGT TTTAGCTTGC CACGTGTATT GCCATTTTTC   
  
  
- CCTCAGTTTT CCTTATGTTA ATCAGATAAT ATGTATATTT TAAGATGCAA TGTGTGATTT TAAGTTTTTA   
  
  
- CCCAATAAAT AGATATTTTA TGGCTTAAAA ATTATAATTA TTTAAAATTT TATTAATCAG TTAACATAAT   
  
  
- TATTACATGA TGTAAAATTA AAAACTTAAT TAATATGTTA TTATATGATT TTAAAATTTT AATTTATTAT   
  
  
- AATTTATTAT TAAAATATAA TTTTATTTCA AATTATATGA ATATCGTATC GTTTGGATGC CCATGATGTG   
  
  
- ATAAATATTA TTTATTTTAA AATTTTAATT ATTATAAACA TTATGTTTGA TATGTTTTTA TTTTATATCA   
  
  
- TATAATTTTA ATATATATTT AGATAAAATA ATAAATGGAA ATGAATTATT TAGTATTGTA AATTTTAAGT   
  
  
- GATGATAAAT CTAAAAAAAA TTTAATTAAT GTATAGATAA GTTTTGTTTG TGATGTGGAA TCCGGTGTCC   
  
  
- TTGAAATATT TGACCATCCA AGAAGTGATA AATGCAAAAT ATTAATTGTG GGGAAGAAAT GAATGAAGTG   
  
  
- GGGTAAGGGT CACGTGGATC AATGAGACTC TGTGTTTCTA AGTCAAAGCA AAAAGACAAA AAGCTCCAAA   
  
  
- ACCGTTCATT CCAAAGCTTT TAAACGCCGA AACACTCCTT TGATGTTGTC AAAGCCCCCT TCCACTCCGA   
  
  
- CACTCTGATT CTTCCCAAAC ACCCGACCCG TTGATTTCGG AATTGACTAA ACGTTCCAAT AAAAAATAAT   
  
  
- AGAAAAATGT AATATTGTGC TCTTGCAAAA CAATGAGGTA CCTTGGGGTA GTGGTTAGCA CTCTGCACTT   
  
  
- TGAATCCAGC GACCTGGGTT CGACTCCCGG TGGGACCTTC GTTTCCTTGA ATGTTATTTA CGTTTTTTCT   
  
  
- TTTGTAACTC AGTGAAGCTT GTTGTGGTTA GGTCATTGAA AGAGATGTTT AACACTGTAA ACACTTCACA   
  
  
- CCGATTTGAT TGTGTCAACC ACAACTCAGT GAAGCTTGTT GTGGTTAGGT CATTGAAAGA GATGTTTAAC   
  
  
- ACTGTAAACA CTTCACACCG ATTTGATTGT GTCAACCACT CGTAATCTGT ATTAAAGTTA CAAACCCTTT   
  
  
- CTTTTTCAAG GAGAAAAAAA AGAAAAGAA

+     G-Box

| Site Name | Organism | Position | Strand | Matrix score. | sequence | function |
| --- | --- | --- | --- | --- | --- | --- |
| G-Box | Antirrhinum majus | 77 | - | 6 | CACGTA | cis-acting regulatory element involved in light responsiveness |

> 2018/04/13 10:10:12  
+ CTTAACCCAC CAGCTACTCT TTCTCTTCAA CCCATTGCTT AAATATCTTC TTCATAGACT TACCAACCTA   
  
  
+ CTTCGGTACG TGTACTTCTC TCTCTCTCTC TAATCTCTTC CGTTTCCTCT TGTATATTCC TCTCTACAAC   
  
  
+ AGTATCACGC CTACCACTTC CAGGGAGTCG CGAAGAAGAG TCTTTGATTT TCGCTTCAAA CTGGCACCTT   
  
  
+ TTATCAAGAA CCAGTTTGGA ACACCCAAAC TGACCTGACC GGTATGCCCT CCCCCGCTCC GCCGAGAAAT   
  
  
+ CCGTCAAGAC ACTAAAACCA TTATTTTAGG ATGGTAGTCA AAATCGAACG GTGCACATAA CGGTAAAAAG   
  
  
+ GGAGTCAAAA GGAATACAAT TAGTCTATTA TACATATAAA ATTCTACGTT ACACACTAAA ATTCAAAAAT   
  
  
+ GGGTTATTTA TCTATAAAAT ACCGAATTTT TAATATTAAT AAATTTTAAA ATAATTAGTC AATTGTATTA   
  
  
+ ATAATGTACT ACATTTTAAT TTTTGAATTA ATTATACAAT AATATACTAA AATTTTAAAA TTAAATAATA   
  
  
+ TTAAATAATA ATTTTATATT AAAATAAAGT TTAATATACT TATAGCATAG CAAACCTACG GGTACTACAC   
  
  
+ TATTTATAAT AAATAAAATT TTAAAATTAA TAATATTTGT AATACAAACT ATACAAAAAT AAAATATAGT   
  
  
+ ATATTAAAAT TATATATAAA TCTATTTTAT TATTTACCTT TACTTAATAA ATCATAACAT TTAAAATTCA   
  
  
+ CTACTATTTA GATTTTTTTT AAATTAATTA CATATCTATT CAAAACAAAC ACTACACCTT AGGCCACAGG   
  
  
+ AACTTTATAA ACTGGTAGGT TCTTCACTAT TTACGTTTTA TAATTAACAC CCCTTCTTTA CTTACTTCAC   
  
  
+ CCCATTCCCA GTGCACCTAG TTACTCTGAG ACACAAAGAT TCAGTTTCGT TTTTCTGTTT TTCGAGGTTT   
  
  
+ TGGCAAGTAA GGTTTCGAAA ATTTGCGGCT TTGTGAGGAA ACTACAACAG TTTCGGGGGA AGGTGAGGCT   
  
  
+ GTGAGACTAA GAAGGGTTTG TGGGCTGGGC AACTAAAGCC TTAACTGATT TGCAAGGTTA TTTTTTATTA   
  
  
+ TCTTTTTACA TTATAACACG AGAACGTTTT GTTACTCCAT GGAACCCCAT CACCAATCGT GAGACGTGAA   
  
  
+ ACTTAGGTCG CTGGACCCAA GCTGAGGGCC ACCCTGGAAG CAAAGGAACT TACAATAAAT GCAAAAAAGA   
  
  
+ AAACATTGAG TCACTTCGAA CAACACCAAT CCAGTAACTT TCTCTACAAA TTGTGACATT TGTGAAGTGT   
  
  
+ GGCTAAACTA ACACAGTTGG TGTTGAGTCA CTTCGAACAA CACCAATCCA GTAACTTTCT CTACAAATTG   
  
  
+ TGACATTTGT GAAGTGTGGC TAAACTAACA CAGTTGGTGA GCATTAGACA TAATTTCAAT GTTTGGGAAA   
  
  
+ GAAAAAGTTC CTCTTTTTTT TCTTTTCTT  

- GAATTGGGTG GTCGATGAGA AAGAGAAGTT GGGTAACGAA TTTATAGAAG AAGTATCTGA ATGGTTGGAT   
  
  
- GAAGCCATGC ACATGAAGAG AGAGAGAGAG ATTAGAGAAG GCAAAGGAGA ACATATAAGG AGAGATGTTG   
  
  
- TCATAGTGCG GATGGTGAAG GTCCCTCAGC GCTTCTTCTC AGAAACTAAA AGCGAAGTTT GACCGTGGAA   
  
  
- AATAGTTCTT GGTCAAACCT TGTGGGTTTG ACTGGACTGG CCATACGGGA GGGGGCGAGG CGGCTCTTTA   
  
  
- GGCAGTTCTG TGATTTTGGT AATAAAATCC TACCATCAGT TTTAGCTTGC CACGTGTATT GCCATTTTTC   
  
  
- CCTCAGTTTT CCTTATGTTA ATCAGATAAT ATGTATATTT TAAGATGCAA TGTGTGATTT TAAGTTTTTA   
  
  
- CCCAATAAAT AGATATTTTA TGGCTTAAAA ATTATAATTA TTTAAAATTT TATTAATCAG TTAACATAAT   
  
  
- TATTACATGA TGTAAAATTA AAAACTTAAT TAATATGTTA TTATATGATT TTAAAATTTT AATTTATTAT   
  
  
- AATTTATTAT TAAAATATAA TTTTATTTCA AATTATATGA ATATCGTATC GTTTGGATGC CCATGATGTG   
  
  
- ATAAATATTA TTTATTTTAA AATTTTAATT ATTATAAACA TTATGTTTGA TATGTTTTTA TTTTATATCA   
  
  
- TATAATTTTA ATATATATTT AGATAAAATA ATAAATGGAA ATGAATTATT TAGTATTGTA AATTTTAAGT   
  
  
- GATGATAAAT CTAAAAAAAA TTTAATTAAT GTATAGATAA GTTTTGTTTG TGATGTGGAA TCCGGTGTCC   
  
  
- TTGAAATATT TGACCATCCA AGAAGTGATA AATGCAAAAT ATTAATTGTG GGGAAGAAAT GAATGAAGTG   
  
  
- GGGTAAGGGT CACGTGGATC AATGAGACTC TGTGTTTCTA AGTCAAAGCA AAAAGACAAA AAGCTCCAAA   
  
  
- ACCGTTCATT CCAAAGCTTT TAAACGCCGA AACACTCCTT TGATGTTGTC AAAGCCCCCT TCCACTCCGA   
  
  
- CACTCTGATT CTTCCCAAAC ACCCGACCCG TTGATTTCGG AATTGACTAA ACGTTCCAAT AAAAAATAAT   
  
  
- AGAAAAATGT AATATTGTGC TCTTGCAAAA CAATGAGGTA CCTTGGGGTA GTGGTTAGCA CTCTGCACTT   
  
  
- TGAATCCAGC GACCTGGGTT CGACTCCCGG TGGGACCTTC GTTTCCTTGA ATGTTATTTA CGTTTTTTCT   
  
  
- TTTGTAACTC AGTGAAGCTT GTTGTGGTTA GGTCATTGAA AGAGATGTTT AACACTGTAA ACACTTCACA   
  
  
- CCGATTTGAT TGTGTCAACC ACAACTCAGT GAAGCTTGTT GTGGTTAGGT CATTGAAAGA GATGTTTAAC   
  
  
- ACTGTAAACA CTTCACACCG ATTTGATTGT GTCAACCACT CGTAATCTGT ATTAAAGTTA CAAACCCTTT   
  
  
- CTTTTTCAAG GAGAAAAAAA AGAAAAGAA

+     G-box

| Site Name | Organism | Position | Strand | Matrix score. | sequence | function |
| --- | --- | --- | --- | --- | --- | --- |
| G-box | Zea mays | 1183 | - | 6 | CACGTC | cis-acting regulatory element involved in light responsiveness |
| G-box | Oryza sativa | 76 | + | 7 | GTACGTG | cis-acting regulatory element involved in light responsiveness |
| G-box | Daucus carota | 77 | + | 6 | TACGTG | cis-acting regulatory element involved in light responsiveness |

> 2018/04/13 10:10:12  
+ CTTAACCCAC CAGCTACTCT TTCTCTTCAA CCCATTGCTT AAATATCTTC TTCATAGACT TACCAACCTA   
  
  
+ CTTCGGTACG TGTACTTCTC TCTCTCTCTC TAATCTCTTC CGTTTCCTCT TGTATATTCC TCTCTACAAC   
  
  
+ AGTATCACGC CTACCACTTC CAGGGAGTCG CGAAGAAGAG TCTTTGATTT TCGCTTCAAA CTGGCACCTT   
  
  
+ TTATCAAGAA CCAGTTTGGA ACACCCAAAC TGACCTGACC GGTATGCCCT CCCCCGCTCC GCCGAGAAAT   
  
  
+ CCGTCAAGAC ACTAAAACCA TTATTTTAGG ATGGTAGTCA AAATCGAACG GTGCACATAA CGGTAAAAAG   
  
  
+ GGAGTCAAAA GGAATACAAT TAGTCTATTA TACATATAAA ATTCTACGTT ACACACTAAA ATTCAAAAAT   
  
  
+ GGGTTATTTA TCTATAAAAT ACCGAATTTT TAATATTAAT AAATTTTAAA ATAATTAGTC AATTGTATTA   
  
  
+ ATAATGTACT ACATTTTAAT TTTTGAATTA ATTATACAAT AATATACTAA AATTTTAAAA TTAAATAATA   
  
  
+ TTAAATAATA ATTTTATATT AAAATAAAGT TTAATATACT TATAGCATAG CAAACCTACG GGTACTACAC   
  
  
+ TATTTATAAT AAATAAAATT TTAAAATTAA TAATATTTGT AATACAAACT ATACAAAAAT AAAATATAGT   
  
  
+ ATATTAAAAT TATATATAAA TCTATTTTAT TATTTACCTT TACTTAATAA ATCATAACAT TTAAAATTCA   
  
  
+ CTACTATTTA GATTTTTTTT AAATTAATTA CATATCTATT CAAAACAAAC ACTACACCTT AGGCCACAGG   
  
  
+ AACTTTATAA ACTGGTAGGT TCTTCACTAT TTACGTTTTA TAATTAACAC CCCTTCTTTA CTTACTTCAC   
  
  
+ CCCATTCCCA GTGCACCTAG TTACTCTGAG ACACAAAGAT TCAGTTTCGT TTTTCTGTTT TTCGAGGTTT   
  
  
+ TGGCAAGTAA GGTTTCGAAA ATTTGCGGCT TTGTGAGGAA ACTACAACAG TTTCGGGGGA AGGTGAGGCT   
  
  
+ GTGAGACTAA GAAGGGTTTG TGGGCTGGGC AACTAAAGCC TTAACTGATT TGCAAGGTTA TTTTTTATTA   
  
  
+ TCTTTTTACA TTATAACACG AGAACGTTTT GTTACTCCAT GGAACCCCAT CACCAATCGT GAGACGTGAA   
  
  
+ ACTTAGGTCG CTGGACCCAA GCTGAGGGCC ACCCTGGAAG CAAAGGAACT TACAATAAAT GCAAAAAAGA   
  
  
+ AAACATTGAG TCACTTCGAA CAACACCAAT CCAGTAACTT TCTCTACAAA TTGTGACATT TGTGAAGTGT   
  
  
+ GGCTAAACTA ACACAGTTGG TGTTGAGTCA CTTCGAACAA CACCAATCCA GTAACTTTCT CTACAAATTG   
  
  
+ TGACATTTGT GAAGTGTGGC TAAACTAACA CAGTTGGTGA GCATTAGACA TAATTTCAAT GTTTGGGAAA   
  
  
+ GAAAAAGTTC CTCTTTTTTT TCTTTTCTT  

- GAATTGGGTG GTCGATGAGA AAGAGAAGTT GGGTAACGAA TTTATAGAAG AAGTATCTGA ATGGTTGGAT   
  
  
- GAAGCCATGC ACATGAAGAG AGAGAGAGAG ATTAGAGAAG GCAAAGGAGA ACATATAAGG AGAGATGTTG   
  
  
- TCATAGTGCG GATGGTGAAG GTCCCTCAGC GCTTCTTCTC AGAAACTAAA AGCGAAGTTT GACCGTGGAA   
  
  
- AATAGTTCTT GGTCAAACCT TGTGGGTTTG ACTGGACTGG CCATACGGGA GGGGGCGAGG CGGCTCTTTA   
  
  
- GGCAGTTCTG TGATTTTGGT AATAAAATCC TACCATCAGT TTTAGCTTGC CACGTGTATT GCCATTTTTC   
  
  
- CCTCAGTTTT CCTTATGTTA ATCAGATAAT ATGTATATTT TAAGATGCAA TGTGTGATTT TAAGTTTTTA   
  
  
- CCCAATAAAT AGATATTTTA TGGCTTAAAA ATTATAATTA TTTAAAATTT TATTAATCAG TTAACATAAT   
  
  
- TATTACATGA TGTAAAATTA AAAACTTAAT TAATATGTTA TTATATGATT TTAAAATTTT AATTTATTAT   
  
  
- AATTTATTAT TAAAATATAA TTTTATTTCA AATTATATGA ATATCGTATC GTTTGGATGC CCATGATGTG   
  
  
- ATAAATATTA TTTATTTTAA AATTTTAATT ATTATAAACA TTATGTTTGA TATGTTTTTA TTTTATATCA   
  
  
- TATAATTTTA ATATATATTT AGATAAAATA ATAAATGGAA ATGAATTATT TAGTATTGTA AATTTTAAGT   
  
  
- GATGATAAAT CTAAAAAAAA TTTAATTAAT GTATAGATAA GTTTTGTTTG TGATGTGGAA TCCGGTGTCC   
  
  
- TTGAAATATT TGACCATCCA AGAAGTGATA AATGCAAAAT ATTAATTGTG GGGAAGAAAT GAATGAAGTG   
  
  
- GGGTAAGGGT CACGTGGATC AATGAGACTC TGTGTTTCTA AGTCAAAGCA AAAAGACAAA AAGCTCCAAA   
  
  
- ACCGTTCATT CCAAAGCTTT TAAACGCCGA AACACTCCTT TGATGTTGTC AAAGCCCCCT TCCACTCCGA   
  
  
- CACTCTGATT CTTCCCAAAC ACCCGACCCG TTGATTTCGG AATTGACTAA ACGTTCCAAT AAAAAATAAT   
  
  
- AGAAAAATGT AATATTGTGC TCTTGCAAAA CAATGAGGTA CCTTGGGGTA GTGGTTAGCA CTCTGCACTT   
  
  
- TGAATCCAGC GACCTGGGTT CGACTCCCGG TGGGACCTTC GTTTCCTTGA ATGTTATTTA CGTTTTTTCT   
  
  
- TTTGTAACTC AGTGAAGCTT GTTGTGGTTA GGTCATTGAA AGAGATGTTT AACACTGTAA ACACTTCACA   
  
  
- CCGATTTGAT TGTGTCAACC ACAACTCAGT GAAGCTTGTT GTGGTTAGGT CATTGAAAGA GATGTTTAAC   
  
  
- ACTGTAAACA CTTCACACCG ATTTGATTGT GTCAACCACT CGTAATCTGT ATTAAAGTTA CAAACCCTTT   
  
  
- CTTTTTCAAG GAGAAAAAAA AGAAAAGAA

+     GA-motif

| Site Name | Organism | Position | Strand | Matrix score. | sequence | function |
| --- | --- | --- | --- | --- | --- | --- |
| GA-motif | Arabidopsis thaliana | 428 | - | 8 | ATAGATAA | part of a light responsive element |

> 2018/04/13 10:10:12  
+ CTTAACCCAC CAGCTACTCT TTCTCTTCAA CCCATTGCTT AAATATCTTC TTCATAGACT TACCAACCTA   
  
  
+ CTTCGGTACG TGTACTTCTC TCTCTCTCTC TAATCTCTTC CGTTTCCTCT TGTATATTCC TCTCTACAAC   
  
  
+ AGTATCACGC CTACCACTTC CAGGGAGTCG CGAAGAAGAG TCTTTGATTT TCGCTTCAAA CTGGCACCTT   
  
  
+ TTATCAAGAA CCAGTTTGGA ACACCCAAAC TGACCTGACC GGTATGCCCT CCCCCGCTCC GCCGAGAAAT   
  
  
+ CCGTCAAGAC ACTAAAACCA TTATTTTAGG ATGGTAGTCA AAATCGAACG GTGCACATAA CGGTAAAAAG   
  
  
+ GGAGTCAAAA GGAATACAAT TAGTCTATTA TACATATAAA ATTCTACGTT ACACACTAAA ATTCAAAAAT   
  
  
+ GGGTTATTTA TCTATAAAAT ACCGAATTTT TAATATTAAT AAATTTTAAA ATAATTAGTC AATTGTATTA   
  
  
+ ATAATGTACT ACATTTTAAT TTTTGAATTA ATTATACAAT AATATACTAA AATTTTAAAA TTAAATAATA   
  
  
+ TTAAATAATA ATTTTATATT AAAATAAAGT TTAATATACT TATAGCATAG CAAACCTACG GGTACTACAC   
  
  
+ TATTTATAAT AAATAAAATT TTAAAATTAA TAATATTTGT AATACAAACT ATACAAAAAT AAAATATAGT   
  
  
+ ATATTAAAAT TATATATAAA TCTATTTTAT TATTTACCTT TACTTAATAA ATCATAACAT TTAAAATTCA   
  
  
+ CTACTATTTA GATTTTTTTT AAATTAATTA CATATCTATT CAAAACAAAC ACTACACCTT AGGCCACAGG   
  
  
+ AACTTTATAA ACTGGTAGGT TCTTCACTAT TTACGTTTTA TAATTAACAC CCCTTCTTTA CTTACTTCAC   
  
  
+ CCCATTCCCA GTGCACCTAG TTACTCTGAG ACACAAAGAT TCAGTTTCGT TTTTCTGTTT TTCGAGGTTT   
  
  
+ TGGCAAGTAA GGTTTCGAAA ATTTGCGGCT TTGTGAGGAA ACTACAACAG TTTCGGGGGA AGGTGAGGCT   
  
  
+ GTGAGACTAA GAAGGGTTTG TGGGCTGGGC AACTAAAGCC TTAACTGATT TGCAAGGTTA TTTTTTATTA   
  
  
+ TCTTTTTACA TTATAACACG AGAACGTTTT GTTACTCCAT GGAACCCCAT CACCAATCGT GAGACGTGAA   
  
  
+ ACTTAGGTCG CTGGACCCAA GCTGAGGGCC ACCCTGGAAG CAAAGGAACT TACAATAAAT GCAAAAAAGA   
  
  
+ AAACATTGAG TCACTTCGAA CAACACCAAT CCAGTAACTT TCTCTACAAA TTGTGACATT TGTGAAGTGT   
  
  
+ GGCTAAACTA ACACAGTTGG TGTTGAGTCA CTTCGAACAA CACCAATCCA GTAACTTTCT CTACAAATTG   
  
  
+ TGACATTTGT GAAGTGTGGC TAAACTAACA CAGTTGGTGA GCATTAGACA TAATTTCAAT GTTTGGGAAA   
  
  
+ GAAAAAGTTC CTCTTTTTTT TCTTTTCTT  

- GAATTGGGTG GTCGATGAGA AAGAGAAGTT GGGTAACGAA TTTATAGAAG AAGTATCTGA ATGGTTGGAT   
  
  
- GAAGCCATGC ACATGAAGAG AGAGAGAGAG ATTAGAGAAG GCAAAGGAGA ACATATAAGG AGAGATGTTG   
  
  
- TCATAGTGCG GATGGTGAAG GTCCCTCAGC GCTTCTTCTC AGAAACTAAA AGCGAAGTTT GACCGTGGAA   
  
  
- AATAGTTCTT GGTCAAACCT TGTGGGTTTG ACTGGACTGG CCATACGGGA GGGGGCGAGG CGGCTCTTTA   
  
  
- GGCAGTTCTG TGATTTTGGT AATAAAATCC TACCATCAGT TTTAGCTTGC CACGTGTATT GCCATTTTTC   
  
  
- CCTCAGTTTT CCTTATGTTA ATCAGATAAT ATGTATATTT TAAGATGCAA TGTGTGATTT TAAGTTTTTA   
  
  
- CCCAATAAAT AGATATTTTA TGGCTTAAAA ATTATAATTA TTTAAAATTT TATTAATCAG TTAACATAAT   
  
  
- TATTACATGA TGTAAAATTA AAAACTTAAT TAATATGTTA TTATATGATT TTAAAATTTT AATTTATTAT   
  
  
- AATTTATTAT TAAAATATAA TTTTATTTCA AATTATATGA ATATCGTATC GTTTGGATGC CCATGATGTG   
  
  
- ATAAATATTA TTTATTTTAA AATTTTAATT ATTATAAACA TTATGTTTGA TATGTTTTTA TTTTATATCA   
  
  
- TATAATTTTA ATATATATTT AGATAAAATA ATAAATGGAA ATGAATTATT TAGTATTGTA AATTTTAAGT   
  
  
- GATGATAAAT CTAAAAAAAA TTTAATTAAT GTATAGATAA GTTTTGTTTG TGATGTGGAA TCCGGTGTCC   
  
  
- TTGAAATATT TGACCATCCA AGAAGTGATA AATGCAAAAT ATTAATTGTG GGGAAGAAAT GAATGAAGTG   
  
  
- GGGTAAGGGT CACGTGGATC AATGAGACTC TGTGTTTCTA AGTCAAAGCA AAAAGACAAA AAGCTCCAAA   
  
  
- ACCGTTCATT CCAAAGCTTT TAAACGCCGA AACACTCCTT TGATGTTGTC AAAGCCCCCT TCCACTCCGA   
  
  
- CACTCTGATT CTTCCCAAAC ACCCGACCCG TTGATTTCGG AATTGACTAA ACGTTCCAAT AAAAAATAAT   
  
  
- AGAAAAATGT AATATTGTGC TCTTGCAAAA CAATGAGGTA CCTTGGGGTA GTGGTTAGCA CTCTGCACTT   
  
  
- TGAATCCAGC GACCTGGGTT CGACTCCCGG TGGGACCTTC GTTTCCTTGA ATGTTATTTA CGTTTTTTCT   
  
  
- TTTGTAACTC AGTGAAGCTT GTTGTGGTTA GGTCATTGAA AGAGATGTTT AACACTGTAA ACACTTCACA   
  
  
- CCGATTTGAT TGTGTCAACC ACAACTCAGT GAAGCTTGTT GTGGTTAGGT CATTGAAAGA GATGTTTAAC   
  
  
- ACTGTAAACA CTTCACACCG ATTTGATTGT GTCAACCACT CGTAATCTGT ATTAAAGTTA CAAACCCTTT   
  
  
- CTTTTTCAAG GAGAAAAAAA AGAAAAGAA

+     GARE-motif

| Site Name | Organism | Position | Strand | Matrix score. | sequence | function |
| --- | --- | --- | --- | --- | --- | --- |
| GARE-motif | Brassica oleracea | 964 | - | 7 | AAACAGA | gibberellin-responsive element |

> 2018/04/13 10:10:12  
+ CTTAACCCAC CAGCTACTCT TTCTCTTCAA CCCATTGCTT AAATATCTTC TTCATAGACT TACCAACCTA   
  
  
+ CTTCGGTACG TGTACTTCTC TCTCTCTCTC TAATCTCTTC CGTTTCCTCT TGTATATTCC TCTCTACAAC   
  
  
+ AGTATCACGC CTACCACTTC CAGGGAGTCG CGAAGAAGAG TCTTTGATTT TCGCTTCAAA CTGGCACCTT   
  
  
+ TTATCAAGAA CCAGTTTGGA ACACCCAAAC TGACCTGACC GGTATGCCCT CCCCCGCTCC GCCGAGAAAT   
  
  
+ CCGTCAAGAC ACTAAAACCA TTATTTTAGG ATGGTAGTCA AAATCGAACG GTGCACATAA CGGTAAAAAG   
  
  
+ GGAGTCAAAA GGAATACAAT TAGTCTATTA TACATATAAA ATTCTACGTT ACACACTAAA ATTCAAAAAT   
  
  
+ GGGTTATTTA TCTATAAAAT ACCGAATTTT TAATATTAAT AAATTTTAAA ATAATTAGTC AATTGTATTA   
  
  
+ ATAATGTACT ACATTTTAAT TTTTGAATTA ATTATACAAT AATATACTAA AATTTTAAAA TTAAATAATA   
  
  
+ TTAAATAATA ATTTTATATT AAAATAAAGT TTAATATACT TATAGCATAG CAAACCTACG GGTACTACAC   
  
  
+ TATTTATAAT AAATAAAATT TTAAAATTAA TAATATTTGT AATACAAACT ATACAAAAAT AAAATATAGT   
  
  
+ ATATTAAAAT TATATATAAA TCTATTTTAT TATTTACCTT TACTTAATAA ATCATAACAT TTAAAATTCA   
  
  
+ CTACTATTTA GATTTTTTTT AAATTAATTA CATATCTATT CAAAACAAAC ACTACACCTT AGGCCACAGG   
  
  
+ AACTTTATAA ACTGGTAGGT TCTTCACTAT TTACGTTTTA TAATTAACAC CCCTTCTTTA CTTACTTCAC   
  
  
+ CCCATTCCCA GTGCACCTAG TTACTCTGAG ACACAAAGAT TCAGTTTCGT TTTTCTGTTT TTCGAGGTTT   
  
  
+ TGGCAAGTAA GGTTTCGAAA ATTTGCGGCT TTGTGAGGAA ACTACAACAG TTTCGGGGGA AGGTGAGGCT   
  
  
+ GTGAGACTAA GAAGGGTTTG TGGGCTGGGC AACTAAAGCC TTAACTGATT TGCAAGGTTA TTTTTTATTA   
  
  
+ TCTTTTTACA TTATAACACG AGAACGTTTT GTTACTCCAT GGAACCCCAT CACCAATCGT GAGACGTGAA   
  
  
+ ACTTAGGTCG CTGGACCCAA GCTGAGGGCC ACCCTGGAAG CAAAGGAACT TACAATAAAT GCAAAAAAGA   
  
  
+ AAACATTGAG TCACTTCGAA CAACACCAAT CCAGTAACTT TCTCTACAAA TTGTGACATT TGTGAAGTGT   
  
  
+ GGCTAAACTA ACACAGTTGG TGTTGAGTCA CTTCGAACAA CACCAATCCA GTAACTTTCT CTACAAATTG   
  
  
+ TGACATTTGT GAAGTGTGGC TAAACTAACA CAGTTGGTGA GCATTAGACA TAATTTCAAT GTTTGGGAAA   
  
  
+ GAAAAAGTTC CTCTTTTTTT TCTTTTCTT  

- GAATTGGGTG GTCGATGAGA AAGAGAAGTT GGGTAACGAA TTTATAGAAG AAGTATCTGA ATGGTTGGAT   
  
  
- GAAGCCATGC ACATGAAGAG AGAGAGAGAG ATTAGAGAAG GCAAAGGAGA ACATATAAGG AGAGATGTTG   
  
  
- TCATAGTGCG GATGGTGAAG GTCCCTCAGC GCTTCTTCTC AGAAACTAAA AGCGAAGTTT GACCGTGGAA   
  
  
- AATAGTTCTT GGTCAAACCT TGTGGGTTTG ACTGGACTGG CCATACGGGA GGGGGCGAGG CGGCTCTTTA   
  
  
- GGCAGTTCTG TGATTTTGGT AATAAAATCC TACCATCAGT TTTAGCTTGC CACGTGTATT GCCATTTTTC   
  
  
- CCTCAGTTTT CCTTATGTTA ATCAGATAAT ATGTATATTT TAAGATGCAA TGTGTGATTT TAAGTTTTTA   
  
  
- CCCAATAAAT AGATATTTTA TGGCTTAAAA ATTATAATTA TTTAAAATTT TATTAATCAG TTAACATAAT   
  
  
- TATTACATGA TGTAAAATTA AAAACTTAAT TAATATGTTA TTATATGATT TTAAAATTTT AATTTATTAT   
  
  
- AATTTATTAT TAAAATATAA TTTTATTTCA AATTATATGA ATATCGTATC GTTTGGATGC CCATGATGTG   
  
  
- ATAAATATTA TTTATTTTAA AATTTTAATT ATTATAAACA TTATGTTTGA TATGTTTTTA TTTTATATCA   
  
  
- TATAATTTTA ATATATATTT AGATAAAATA ATAAATGGAA ATGAATTATT TAGTATTGTA AATTTTAAGT   
  
  
- GATGATAAAT CTAAAAAAAA TTTAATTAAT GTATAGATAA GTTTTGTTTG TGATGTGGAA TCCGGTGTCC   
  
  
- TTGAAATATT TGACCATCCA AGAAGTGATA AATGCAAAAT ATTAATTGTG GGGAAGAAAT GAATGAAGTG   
  
  
- GGGTAAGGGT CACGTGGATC AATGAGACTC TGTGTTTCTA AGTCAAAGCA AAAAGACAAA AAGCTCCAAA   
  
  
- ACCGTTCATT CCAAAGCTTT TAAACGCCGA AACACTCCTT TGATGTTGTC AAAGCCCCCT TCCACTCCGA   
  
  
- CACTCTGATT CTTCCCAAAC ACCCGACCCG TTGATTTCGG AATTGACTAA ACGTTCCAAT AAAAAATAAT   
  
  
- AGAAAAATGT AATATTGTGC TCTTGCAAAA CAATGAGGTA CCTTGGGGTA GTGGTTAGCA CTCTGCACTT   
  
  
- TGAATCCAGC GACCTGGGTT CGACTCCCGG TGGGACCTTC GTTTCCTTGA ATGTTATTTA CGTTTTTTCT   
  
  
- TTTGTAACTC AGTGAAGCTT GTTGTGGTTA GGTCATTGAA AGAGATGTTT AACACTGTAA ACACTTCACA   
  
  
- CCGATTTGAT TGTGTCAACC ACAACTCAGT GAAGCTTGTT GTGGTTAGGT CATTGAAAGA GATGTTTAAC   
  
  
- ACTGTAAACA CTTCACACCG ATTTGATTGT GTCAACCACT CGTAATCTGT ATTAAAGTTA CAAACCCTTT   
  
  
- CTTTTTCAAG GAGAAAAAAA AGAAAAGAA

+     GC-motif

| Site Name | Organism | Position | Strand | Matrix score. | sequence | function |
| --- | --- | --- | --- | --- | --- | --- |
| GC-motif | Zea mays | 1034 | - | 6 | CCCCCG | enhancer-like element involved in anoxic specific inducibility |
| GC-motif | Zea mays | 261 | + | 6 | CCCCCG | enhancer-like element involved in anoxic specific inducibility |

> 2018/04/13 10:10:12  
+ CTTAACCCAC CAGCTACTCT TTCTCTTCAA CCCATTGCTT AAATATCTTC TTCATAGACT TACCAACCTA   
  
  
+ CTTCGGTACG TGTACTTCTC TCTCTCTCTC TAATCTCTTC CGTTTCCTCT TGTATATTCC TCTCTACAAC   
  
  
+ AGTATCACGC CTACCACTTC CAGGGAGTCG CGAAGAAGAG TCTTTGATTT TCGCTTCAAA CTGGCACCTT   
  
  
+ TTATCAAGAA CCAGTTTGGA ACACCCAAAC TGACCTGACC GGTATGCCCT CCCCCGCTCC GCCGAGAAAT   
  
  
+ CCGTCAAGAC ACTAAAACCA TTATTTTAGG ATGGTAGTCA AAATCGAACG GTGCACATAA CGGTAAAAAG   
  
  
+ GGAGTCAAAA GGAATACAAT TAGTCTATTA TACATATAAA ATTCTACGTT ACACACTAAA ATTCAAAAAT   
  
  
+ GGGTTATTTA TCTATAAAAT ACCGAATTTT TAATATTAAT AAATTTTAAA ATAATTAGTC AATTGTATTA   
  
  
+ ATAATGTACT ACATTTTAAT TTTTGAATTA ATTATACAAT AATATACTAA AATTTTAAAA TTAAATAATA   
  
  
+ TTAAATAATA ATTTTATATT AAAATAAAGT TTAATATACT TATAGCATAG CAAACCTACG GGTACTACAC   
  
  
+ TATTTATAAT AAATAAAATT TTAAAATTAA TAATATTTGT AATACAAACT ATACAAAAAT AAAATATAGT   
  
  
+ ATATTAAAAT TATATATAAA TCTATTTTAT TATTTACCTT TACTTAATAA ATCATAACAT TTAAAATTCA   
  
  
+ CTACTATTTA GATTTTTTTT AAATTAATTA CATATCTATT CAAAACAAAC ACTACACCTT AGGCCACAGG   
  
  
+ AACTTTATAA ACTGGTAGGT TCTTCACTAT TTACGTTTTA TAATTAACAC CCCTTCTTTA CTTACTTCAC   
  
  
+ CCCATTCCCA GTGCACCTAG TTACTCTGAG ACACAAAGAT TCAGTTTCGT TTTTCTGTTT TTCGAGGTTT   
  
  
+ TGGCAAGTAA GGTTTCGAAA ATTTGCGGCT TTGTGAGGAA ACTACAACAG TTTCGGGGGA AGGTGAGGCT   
  
  
+ GTGAGACTAA GAAGGGTTTG TGGGCTGGGC AACTAAAGCC TTAACTGATT TGCAAGGTTA TTTTTTATTA   
  
  
+ TCTTTTTACA TTATAACACG AGAACGTTTT GTTACTCCAT GGAACCCCAT CACCAATCGT GAGACGTGAA   
  
  
+ ACTTAGGTCG CTGGACCCAA GCTGAGGGCC ACCCTGGAAG CAAAGGAACT TACAATAAAT GCAAAAAAGA   
  
  
+ AAACATTGAG TCACTTCGAA CAACACCAAT CCAGTAACTT TCTCTACAAA TTGTGACATT TGTGAAGTGT   
  
  
+ GGCTAAACTA ACACAGTTGG TGTTGAGTCA CTTCGAACAA CACCAATCCA GTAACTTTCT CTACAAATTG   
  
  
+ TGACATTTGT GAAGTGTGGC TAAACTAACA CAGTTGGTGA GCATTAGACA TAATTTCAAT GTTTGGGAAA   
  
  
+ GAAAAAGTTC CTCTTTTTTT TCTTTTCTT  

- GAATTGGGTG GTCGATGAGA AAGAGAAGTT GGGTAACGAA TTTATAGAAG AAGTATCTGA ATGGTTGGAT   
  
  
- GAAGCCATGC ACATGAAGAG AGAGAGAGAG ATTAGAGAAG GCAAAGGAGA ACATATAAGG AGAGATGTTG   
  
  
- TCATAGTGCG GATGGTGAAG GTCCCTCAGC GCTTCTTCTC AGAAACTAAA AGCGAAGTTT GACCGTGGAA   
  
  
- AATAGTTCTT GGTCAAACCT TGTGGGTTTG ACTGGACTGG CCATACGGGA GGGGGCGAGG CGGCTCTTTA   
  
  
- GGCAGTTCTG TGATTTTGGT AATAAAATCC TACCATCAGT TTTAGCTTGC CACGTGTATT GCCATTTTTC   
  
  
- CCTCAGTTTT CCTTATGTTA ATCAGATAAT ATGTATATTT TAAGATGCAA TGTGTGATTT TAAGTTTTTA   
  
  
- CCCAATAAAT AGATATTTTA TGGCTTAAAA ATTATAATTA TTTAAAATTT TATTAATCAG TTAACATAAT   
  
  
- TATTACATGA TGTAAAATTA AAAACTTAAT TAATATGTTA TTATATGATT TTAAAATTTT AATTTATTAT   
  
  
- AATTTATTAT TAAAATATAA TTTTATTTCA AATTATATGA ATATCGTATC GTTTGGATGC CCATGATGTG   
  
  
- ATAAATATTA TTTATTTTAA AATTTTAATT ATTATAAACA TTATGTTTGA TATGTTTTTA TTTTATATCA   
  
  
- TATAATTTTA ATATATATTT AGATAAAATA ATAAATGGAA ATGAATTATT TAGTATTGTA AATTTTAAGT   
  
  
- GATGATAAAT CTAAAAAAAA TTTAATTAAT GTATAGATAA GTTTTGTTTG TGATGTGGAA TCCGGTGTCC   
  
  
- TTGAAATATT TGACCATCCA AGAAGTGATA AATGCAAAAT ATTAATTGTG GGGAAGAAAT GAATGAAGTG   
  
  
- GGGTAAGGGT CACGTGGATC AATGAGACTC TGTGTTTCTA AGTCAAAGCA AAAAGACAAA AAGCTCCAAA   
  
  
- ACCGTTCATT CCAAAGCTTT TAAACGCCGA AACACTCCTT TGATGTTGTC AAAGCCCCCT TCCACTCCGA   
  
  
- CACTCTGATT CTTCCCAAAC ACCCGACCCG TTGATTTCGG AATTGACTAA ACGTTCCAAT AAAAAATAAT   
  
  
- AGAAAAATGT AATATTGTGC TCTTGCAAAA CAATGAGGTA CCTTGGGGTA GTGGTTAGCA CTCTGCACTT   
  
  
- TGAATCCAGC GACCTGGGTT CGACTCCCGG TGGGACCTTC GTTTCCTTGA ATGTTATTTA CGTTTTTTCT   
  
  
- TTTGTAACTC AGTGAAGCTT GTTGTGGTTA GGTCATTGAA AGAGATGTTT AACACTGTAA ACACTTCACA   
  
  
- CCGATTTGAT TGTGTCAACC ACAACTCAGT GAAGCTTGTT GTGGTTAGGT CATTGAAAGA GATGTTTAAC   
  
  
- ACTGTAAACA CTTCACACCG ATTTGATTGT GTCAACCACT CGTAATCTGT ATTAAAGTTA CAAACCCTTT   
  
  
- CTTTTTCAAG GAGAAAAAAA AGAAAAGAA

+     GCN4\_motif

| Site Name | Organism | Position | Strand | Matrix score. | sequence | function |
| --- | --- | --- | --- | --- | --- | --- |
| GCN4\_motif | Oryza sativa | 1354 | + | 7 | TGAGTCA | cis-regulatory element involved in endosperm expression |
| GCN4\_motif | Oryza sativa | 1267 | + | 7 | TGAGTCA | cis-regulatory element involved in endosperm expression |

> 2018/04/13 10:10:12  
+ CTTAACCCAC CAGCTACTCT TTCTCTTCAA CCCATTGCTT AAATATCTTC TTCATAGACT TACCAACCTA   
  
  
+ CTTCGGTACG TGTACTTCTC TCTCTCTCTC TAATCTCTTC CGTTTCCTCT TGTATATTCC TCTCTACAAC   
  
  
+ AGTATCACGC CTACCACTTC CAGGGAGTCG CGAAGAAGAG TCTTTGATTT TCGCTTCAAA CTGGCACCTT   
  
  
+ TTATCAAGAA CCAGTTTGGA ACACCCAAAC TGACCTGACC GGTATGCCCT CCCCCGCTCC GCCGAGAAAT   
  
  
+ CCGTCAAGAC ACTAAAACCA TTATTTTAGG ATGGTAGTCA AAATCGAACG GTGCACATAA CGGTAAAAAG   
  
  
+ GGAGTCAAAA GGAATACAAT TAGTCTATTA TACATATAAA ATTCTACGTT ACACACTAAA ATTCAAAAAT   
  
  
+ GGGTTATTTA TCTATAAAAT ACCGAATTTT TAATATTAAT AAATTTTAAA ATAATTAGTC AATTGTATTA   
  
  
+ ATAATGTACT ACATTTTAAT TTTTGAATTA ATTATACAAT AATATACTAA AATTTTAAAA TTAAATAATA   
  
  
+ TTAAATAATA ATTTTATATT AAAATAAAGT TTAATATACT TATAGCATAG CAAACCTACG GGTACTACAC   
  
  
+ TATTTATAAT AAATAAAATT TTAAAATTAA TAATATTTGT AATACAAACT ATACAAAAAT AAAATATAGT   
  
  
+ ATATTAAAAT TATATATAAA TCTATTTTAT TATTTACCTT TACTTAATAA ATCATAACAT TTAAAATTCA   
  
  
+ CTACTATTTA GATTTTTTTT AAATTAATTA CATATCTATT CAAAACAAAC ACTACACCTT AGGCCACAGG   
  
  
+ AACTTTATAA ACTGGTAGGT TCTTCACTAT TTACGTTTTA TAATTAACAC CCCTTCTTTA CTTACTTCAC   
  
  
+ CCCATTCCCA GTGCACCTAG TTACTCTGAG ACACAAAGAT TCAGTTTCGT TTTTCTGTTT TTCGAGGTTT   
  
  
+ TGGCAAGTAA GGTTTCGAAA ATTTGCGGCT TTGTGAGGAA ACTACAACAG TTTCGGGGGA AGGTGAGGCT   
  
  
+ GTGAGACTAA GAAGGGTTTG TGGGCTGGGC AACTAAAGCC TTAACTGATT TGCAAGGTTA TTTTTTATTA   
  
  
+ TCTTTTTACA TTATAACACG AGAACGTTTT GTTACTCCAT GGAACCCCAT CACCAATCGT GAGACGTGAA   
  
  
+ ACTTAGGTCG CTGGACCCAA GCTGAGGGCC ACCCTGGAAG CAAAGGAACT TACAATAAAT GCAAAAAAGA   
  
  
+ AAACATTGAG TCACTTCGAA CAACACCAAT CCAGTAACTT TCTCTACAAA TTGTGACATT TGTGAAGTGT   
  
  
+ GGCTAAACTA ACACAGTTGG TGTTGAGTCA CTTCGAACAA CACCAATCCA GTAACTTTCT CTACAAATTG   
  
  
+ TGACATTTGT GAAGTGTGGC TAAACTAACA CAGTTGGTGA GCATTAGACA TAATTTCAAT GTTTGGGAAA   
  
  
+ GAAAAAGTTC CTCTTTTTTT TCTTTTCTT  

- GAATTGGGTG GTCGATGAGA AAGAGAAGTT GGGTAACGAA TTTATAGAAG AAGTATCTGA ATGGTTGGAT   
  
  
- GAAGCCATGC ACATGAAGAG AGAGAGAGAG ATTAGAGAAG GCAAAGGAGA ACATATAAGG AGAGATGTTG   
  
  
- TCATAGTGCG GATGGTGAAG GTCCCTCAGC GCTTCTTCTC AGAAACTAAA AGCGAAGTTT GACCGTGGAA   
  
  
- AATAGTTCTT GGTCAAACCT TGTGGGTTTG ACTGGACTGG CCATACGGGA GGGGGCGAGG CGGCTCTTTA   
  
  
- GGCAGTTCTG TGATTTTGGT AATAAAATCC TACCATCAGT TTTAGCTTGC CACGTGTATT GCCATTTTTC   
  
  
- CCTCAGTTTT CCTTATGTTA ATCAGATAAT ATGTATATTT TAAGATGCAA TGTGTGATTT TAAGTTTTTA   
  
  
- CCCAATAAAT AGATATTTTA TGGCTTAAAA ATTATAATTA TTTAAAATTT TATTAATCAG TTAACATAAT   
  
  
- TATTACATGA TGTAAAATTA AAAACTTAAT TAATATGTTA TTATATGATT TTAAAATTTT AATTTATTAT   
  
  
- AATTTATTAT TAAAATATAA TTTTATTTCA AATTATATGA ATATCGTATC GTTTGGATGC CCATGATGTG   
  
  
- ATAAATATTA TTTATTTTAA AATTTTAATT ATTATAAACA TTATGTTTGA TATGTTTTTA TTTTATATCA   
  
  
- TATAATTTTA ATATATATTT AGATAAAATA ATAAATGGAA ATGAATTATT TAGTATTGTA AATTTTAAGT   
  
  
- GATGATAAAT CTAAAAAAAA TTTAATTAAT GTATAGATAA GTTTTGTTTG TGATGTGGAA TCCGGTGTCC   
  
  
- TTGAAATATT TGACCATCCA AGAAGTGATA AATGCAAAAT ATTAATTGTG GGGAAGAAAT GAATGAAGTG   
  
  
- GGGTAAGGGT CACGTGGATC AATGAGACTC TGTGTTTCTA AGTCAAAGCA AAAAGACAAA AAGCTCCAAA   
  
  
- ACCGTTCATT CCAAAGCTTT TAAACGCCGA AACACTCCTT TGATGTTGTC AAAGCCCCCT TCCACTCCGA   
  
  
- CACTCTGATT CTTCCCAAAC ACCCGACCCG TTGATTTCGG AATTGACTAA ACGTTCCAAT AAAAAATAAT   
  
  
- AGAAAAATGT AATATTGTGC TCTTGCAAAA CAATGAGGTA CCTTGGGGTA GTGGTTAGCA CTCTGCACTT   
  
  
- TGAATCCAGC GACCTGGGTT CGACTCCCGG TGGGACCTTC GTTTCCTTGA ATGTTATTTA CGTTTTTTCT   
  
  
- TTTGTAACTC AGTGAAGCTT GTTGTGGTTA GGTCATTGAA AGAGATGTTT AACACTGTAA ACACTTCACA   
  
  
- CCGATTTGAT TGTGTCAACC ACAACTCAGT GAAGCTTGTT GTGGTTAGGT CATTGAAAGA GATGTTTAAC   
  
  
- ACTGTAAACA CTTCACACCG ATTTGATTGT GTCAACCACT CGTAATCTGT ATTAAAGTTA CAAACCCTTT   
  
  
- CTTTTTCAAG GAGAAAAAAA AGAAAAGAA

+     GT1-motif

| Site Name | Organism | Position | Strand | Matrix score. | sequence | function |
| --- | --- | --- | --- | --- | --- | --- |
| GT1-motif | Arabidopsis thaliana | 2 | - | 6 | GGTTAA | light responsive element |

> 2018/04/13 10:10:12  
+ CTTAACCCAC CAGCTACTCT TTCTCTTCAA CCCATTGCTT AAATATCTTC TTCATAGACT TACCAACCTA   
  
  
+ CTTCGGTACG TGTACTTCTC TCTCTCTCTC TAATCTCTTC CGTTTCCTCT TGTATATTCC TCTCTACAAC   
  
  
+ AGTATCACGC CTACCACTTC CAGGGAGTCG CGAAGAAGAG TCTTTGATTT TCGCTTCAAA CTGGCACCTT   
  
  
+ TTATCAAGAA CCAGTTTGGA ACACCCAAAC TGACCTGACC GGTATGCCCT CCCCCGCTCC GCCGAGAAAT   
  
  
+ CCGTCAAGAC ACTAAAACCA TTATTTTAGG ATGGTAGTCA AAATCGAACG GTGCACATAA CGGTAAAAAG   
  
  
+ GGAGTCAAAA GGAATACAAT TAGTCTATTA TACATATAAA ATTCTACGTT ACACACTAAA ATTCAAAAAT   
  
  
+ GGGTTATTTA TCTATAAAAT ACCGAATTTT TAATATTAAT AAATTTTAAA ATAATTAGTC AATTGTATTA   
  
  
+ ATAATGTACT ACATTTTAAT TTTTGAATTA ATTATACAAT AATATACTAA AATTTTAAAA TTAAATAATA   
  
  
+ TTAAATAATA ATTTTATATT AAAATAAAGT TTAATATACT TATAGCATAG CAAACCTACG GGTACTACAC   
  
  
+ TATTTATAAT AAATAAAATT TTAAAATTAA TAATATTTGT AATACAAACT ATACAAAAAT AAAATATAGT   
  
  
+ ATATTAAAAT TATATATAAA TCTATTTTAT TATTTACCTT TACTTAATAA ATCATAACAT TTAAAATTCA   
  
  
+ CTACTATTTA GATTTTTTTT AAATTAATTA CATATCTATT CAAAACAAAC ACTACACCTT AGGCCACAGG   
  
  
+ AACTTTATAA ACTGGTAGGT TCTTCACTAT TTACGTTTTA TAATTAACAC CCCTTCTTTA CTTACTTCAC   
  
  
+ CCCATTCCCA GTGCACCTAG TTACTCTGAG ACACAAAGAT TCAGTTTCGT TTTTCTGTTT TTCGAGGTTT   
  
  
+ TGGCAAGTAA GGTTTCGAAA ATTTGCGGCT TTGTGAGGAA ACTACAACAG TTTCGGGGGA AGGTGAGGCT   
  
  
+ GTGAGACTAA GAAGGGTTTG TGGGCTGGGC AACTAAAGCC TTAACTGATT TGCAAGGTTA TTTTTTATTA   
  
  
+ TCTTTTTACA TTATAACACG AGAACGTTTT GTTACTCCAT GGAACCCCAT CACCAATCGT GAGACGTGAA   
  
  
+ ACTTAGGTCG CTGGACCCAA GCTGAGGGCC ACCCTGGAAG CAAAGGAACT TACAATAAAT GCAAAAAAGA   
  
  
+ AAACATTGAG TCACTTCGAA CAACACCAAT CCAGTAACTT TCTCTACAAA TTGTGACATT TGTGAAGTGT   
  
  
+ GGCTAAACTA ACACAGTTGG TGTTGAGTCA CTTCGAACAA CACCAATCCA GTAACTTTCT CTACAAATTG   
  
  
+ TGACATTTGT GAAGTGTGGC TAAACTAACA CAGTTGGTGA GCATTAGACA TAATTTCAAT GTTTGGGAAA   
  
  
+ GAAAAAGTTC CTCTTTTTTT TCTTTTCTT  

- GAATTGGGTG GTCGATGAGA AAGAGAAGTT GGGTAACGAA TTTATAGAAG AAGTATCTGA ATGGTTGGAT   
  
  
- GAAGCCATGC ACATGAAGAG AGAGAGAGAG ATTAGAGAAG GCAAAGGAGA ACATATAAGG AGAGATGTTG   
  
  
- TCATAGTGCG GATGGTGAAG GTCCCTCAGC GCTTCTTCTC AGAAACTAAA AGCGAAGTTT GACCGTGGAA   
  
  
- AATAGTTCTT GGTCAAACCT TGTGGGTTTG ACTGGACTGG CCATACGGGA GGGGGCGAGG CGGCTCTTTA   
  
  
- GGCAGTTCTG TGATTTTGGT AATAAAATCC TACCATCAGT TTTAGCTTGC CACGTGTATT GCCATTTTTC   
  
  
- CCTCAGTTTT CCTTATGTTA ATCAGATAAT ATGTATATTT TAAGATGCAA TGTGTGATTT TAAGTTTTTA   
  
  
- CCCAATAAAT AGATATTTTA TGGCTTAAAA ATTATAATTA TTTAAAATTT TATTAATCAG TTAACATAAT   
  
  
- TATTACATGA TGTAAAATTA AAAACTTAAT TAATATGTTA TTATATGATT TTAAAATTTT AATTTATTAT   
  
  
- AATTTATTAT TAAAATATAA TTTTATTTCA AATTATATGA ATATCGTATC GTTTGGATGC CCATGATGTG   
  
  
- ATAAATATTA TTTATTTTAA AATTTTAATT ATTATAAACA TTATGTTTGA TATGTTTTTA TTTTATATCA   
  
  
- TATAATTTTA ATATATATTT AGATAAAATA ATAAATGGAA ATGAATTATT TAGTATTGTA AATTTTAAGT   
  
  
- GATGATAAAT CTAAAAAAAA TTTAATTAAT GTATAGATAA GTTTTGTTTG TGATGTGGAA TCCGGTGTCC   
  
  
- TTGAAATATT TGACCATCCA AGAAGTGATA AATGCAAAAT ATTAATTGTG GGGAAGAAAT GAATGAAGTG   
  
  
- GGGTAAGGGT CACGTGGATC AATGAGACTC TGTGTTTCTA AGTCAAAGCA AAAAGACAAA AAGCTCCAAA   
  
  
- ACCGTTCATT CCAAAGCTTT TAAACGCCGA AACACTCCTT TGATGTTGTC AAAGCCCCCT TCCACTCCGA   
  
  
- CACTCTGATT CTTCCCAAAC ACCCGACCCG TTGATTTCGG AATTGACTAA ACGTTCCAAT AAAAAATAAT   
  
  
- AGAAAAATGT AATATTGTGC TCTTGCAAAA CAATGAGGTA CCTTGGGGTA GTGGTTAGCA CTCTGCACTT   
  
  
- TGAATCCAGC GACCTGGGTT CGACTCCCGG TGGGACCTTC GTTTCCTTGA ATGTTATTTA CGTTTTTTCT   
  
  
- TTTGTAACTC AGTGAAGCTT GTTGTGGTTA GGTCATTGAA AGAGATGTTT AACACTGTAA ACACTTCACA   
  
  
- CCGATTTGAT TGTGTCAACC ACAACTCAGT GAAGCTTGTT GTGGTTAGGT CATTGAAAGA GATGTTTAAC   
  
  
- ACTGTAAACA CTTCACACCG ATTTGATTGT GTCAACCACT CGTAATCTGT ATTAAAGTTA CAAACCCTTT   
  
  
- CTTTTTCAAG GAGAAAAAAA AGAAAAGAA

+     LTR

| Site Name | Organism | Position | Strand | Matrix score. | sequence | function |
| --- | --- | --- | --- | --- | --- | --- |
| LTR | Hordeum vulgare | 1031 | - | 6 | CCGAAA | cis-acting element involved in low-temperature responsiveness |

> 2018/04/13 10:10:12  
+ CTTAACCCAC CAGCTACTCT TTCTCTTCAA CCCATTGCTT AAATATCTTC TTCATAGACT TACCAACCTA   
  
  
+ CTTCGGTACG TGTACTTCTC TCTCTCTCTC TAATCTCTTC CGTTTCCTCT TGTATATTCC TCTCTACAAC   
  
  
+ AGTATCACGC CTACCACTTC CAGGGAGTCG CGAAGAAGAG TCTTTGATTT TCGCTTCAAA CTGGCACCTT   
  
  
+ TTATCAAGAA CCAGTTTGGA ACACCCAAAC TGACCTGACC GGTATGCCCT CCCCCGCTCC GCCGAGAAAT   
  
  
+ CCGTCAAGAC ACTAAAACCA TTATTTTAGG ATGGTAGTCA AAATCGAACG GTGCACATAA CGGTAAAAAG   
  
  
+ GGAGTCAAAA GGAATACAAT TAGTCTATTA TACATATAAA ATTCTACGTT ACACACTAAA ATTCAAAAAT   
  
  
+ GGGTTATTTA TCTATAAAAT ACCGAATTTT TAATATTAAT AAATTTTAAA ATAATTAGTC AATTGTATTA   
  
  
+ ATAATGTACT ACATTTTAAT TTTTGAATTA ATTATACAAT AATATACTAA AATTTTAAAA TTAAATAATA   
  
  
+ TTAAATAATA ATTTTATATT AAAATAAAGT TTAATATACT TATAGCATAG CAAACCTACG GGTACTACAC   
  
  
+ TATTTATAAT AAATAAAATT TTAAAATTAA TAATATTTGT AATACAAACT ATACAAAAAT AAAATATAGT   
  
  
+ ATATTAAAAT TATATATAAA TCTATTTTAT TATTTACCTT TACTTAATAA ATCATAACAT TTAAAATTCA   
  
  
+ CTACTATTTA GATTTTTTTT AAATTAATTA CATATCTATT CAAAACAAAC ACTACACCTT AGGCCACAGG   
  
  
+ AACTTTATAA ACTGGTAGGT TCTTCACTAT TTACGTTTTA TAATTAACAC CCCTTCTTTA CTTACTTCAC   
  
  
+ CCCATTCCCA GTGCACCTAG TTACTCTGAG ACACAAAGAT TCAGTTTCGT TTTTCTGTTT TTCGAGGTTT   
  
  
+ TGGCAAGTAA GGTTTCGAAA ATTTGCGGCT TTGTGAGGAA ACTACAACAG TTTCGGGGGA AGGTGAGGCT   
  
  
+ GTGAGACTAA GAAGGGTTTG TGGGCTGGGC AACTAAAGCC TTAACTGATT TGCAAGGTTA TTTTTTATTA   
  
  
+ TCTTTTTACA TTATAACACG AGAACGTTTT GTTACTCCAT GGAACCCCAT CACCAATCGT GAGACGTGAA   
  
  
+ ACTTAGGTCG CTGGACCCAA GCTGAGGGCC ACCCTGGAAG CAAAGGAACT TACAATAAAT GCAAAAAAGA   
  
  
+ AAACATTGAG TCACTTCGAA CAACACCAAT CCAGTAACTT TCTCTACAAA TTGTGACATT TGTGAAGTGT   
  
  
+ GGCTAAACTA ACACAGTTGG TGTTGAGTCA CTTCGAACAA CACCAATCCA GTAACTTTCT CTACAAATTG   
  
  
+ TGACATTTGT GAAGTGTGGC TAAACTAACA CAGTTGGTGA GCATTAGACA TAATTTCAAT GTTTGGGAAA   
  
  
+ GAAAAAGTTC CTCTTTTTTT TCTTTTCTT  

- GAATTGGGTG GTCGATGAGA AAGAGAAGTT GGGTAACGAA TTTATAGAAG AAGTATCTGA ATGGTTGGAT   
  
  
- GAAGCCATGC ACATGAAGAG AGAGAGAGAG ATTAGAGAAG GCAAAGGAGA ACATATAAGG AGAGATGTTG   
  
  
- TCATAGTGCG GATGGTGAAG GTCCCTCAGC GCTTCTTCTC AGAAACTAAA AGCGAAGTTT GACCGTGGAA   
  
  
- AATAGTTCTT GGTCAAACCT TGTGGGTTTG ACTGGACTGG CCATACGGGA GGGGGCGAGG CGGCTCTTTA   
  
  
- GGCAGTTCTG TGATTTTGGT AATAAAATCC TACCATCAGT TTTAGCTTGC CACGTGTATT GCCATTTTTC   
  
  
- CCTCAGTTTT CCTTATGTTA ATCAGATAAT ATGTATATTT TAAGATGCAA TGTGTGATTT TAAGTTTTTA   
  
  
- CCCAATAAAT AGATATTTTA TGGCTTAAAA ATTATAATTA TTTAAAATTT TATTAATCAG TTAACATAAT   
  
  
- TATTACATGA TGTAAAATTA AAAACTTAAT TAATATGTTA TTATATGATT TTAAAATTTT AATTTATTAT   
  
  
- AATTTATTAT TAAAATATAA TTTTATTTCA AATTATATGA ATATCGTATC GTTTGGATGC CCATGATGTG   
  
  
- ATAAATATTA TTTATTTTAA AATTTTAATT ATTATAAACA TTATGTTTGA TATGTTTTTA TTTTATATCA   
  
  
- TATAATTTTA ATATATATTT AGATAAAATA ATAAATGGAA ATGAATTATT TAGTATTGTA AATTTTAAGT   
  
  
- GATGATAAAT CTAAAAAAAA TTTAATTAAT GTATAGATAA GTTTTGTTTG TGATGTGGAA TCCGGTGTCC   
  
  
- TTGAAATATT TGACCATCCA AGAAGTGATA AATGCAAAAT ATTAATTGTG GGGAAGAAAT GAATGAAGTG   
  
  
- GGGTAAGGGT CACGTGGATC AATGAGACTC TGTGTTTCTA AGTCAAAGCA AAAAGACAAA AAGCTCCAAA   
  
  
- ACCGTTCATT CCAAAGCTTT TAAACGCCGA AACACTCCTT TGATGTTGTC AAAGCCCCCT TCCACTCCGA   
  
  
- CACTCTGATT CTTCCCAAAC ACCCGACCCG TTGATTTCGG AATTGACTAA ACGTTCCAAT AAAAAATAAT   
  
  
- AGAAAAATGT AATATTGTGC TCTTGCAAAA CAATGAGGTA CCTTGGGGTA GTGGTTAGCA CTCTGCACTT   
  
  
- TGAATCCAGC GACCTGGGTT CGACTCCCGG TGGGACCTTC GTTTCCTTGA ATGTTATTTA CGTTTTTTCT   
  
  
- TTTGTAACTC AGTGAAGCTT GTTGTGGTTA GGTCATTGAA AGAGATGTTT AACACTGTAA ACACTTCACA   
  
  
- CCGATTTGAT TGTGTCAACC ACAACTCAGT GAAGCTTGTT GTGGTTAGGT CATTGAAAGA GATGTTTAAC   
  
  
- ACTGTAAACA CTTCACACCG ATTTGATTGT GTCAACCACT CGTAATCTGT ATTAAAGTTA CAAACCCTTT   
  
  
- CTTTTTCAAG GAGAAAAAAA AGAAAAGAA

+     MBS

| Site Name | Organism | Position | Strand | Matrix score. | sequence | function |
| --- | --- | --- | --- | --- | --- | --- |
| MBS | Arabidopsis thaliana | 1344 | - | 6 | CAACTG | MYB binding site involved in drought-inducibility |
| MBS | Arabidopsis thaliana | 1431 | - | 6 | CAACTG | MYB binding site involved in drought-inducibility |
| MBS | Zea mays | 246 | - | 6 | CGGTCA | MYB Binding Site |
| MBS | Arabidopsis thaliana | 1092 | + | 6 | TAACTG | MYB binding site involved in drought-inducibility |

> 2018/04/13 10:10:12  
+ CTTAACCCAC CAGCTACTCT TTCTCTTCAA CCCATTGCTT AAATATCTTC TTCATAGACT TACCAACCTA   
  
  
+ CTTCGGTACG TGTACTTCTC TCTCTCTCTC TAATCTCTTC CGTTTCCTCT TGTATATTCC TCTCTACAAC   
  
  
+ AGTATCACGC CTACCACTTC CAGGGAGTCG CGAAGAAGAG TCTTTGATTT TCGCTTCAAA CTGGCACCTT   
  
  
+ TTATCAAGAA CCAGTTTGGA ACACCCAAAC TGACCTGACC GGTATGCCCT CCCCCGCTCC GCCGAGAAAT   
  
  
+ CCGTCAAGAC ACTAAAACCA TTATTTTAGG ATGGTAGTCA AAATCGAACG GTGCACATAA CGGTAAAAAG   
  
  
+ GGAGTCAAAA GGAATACAAT TAGTCTATTA TACATATAAA ATTCTACGTT ACACACTAAA ATTCAAAAAT   
  
  
+ GGGTTATTTA TCTATAAAAT ACCGAATTTT TAATATTAAT AAATTTTAAA ATAATTAGTC AATTGTATTA   
  
  
+ ATAATGTACT ACATTTTAAT TTTTGAATTA ATTATACAAT AATATACTAA AATTTTAAAA TTAAATAATA   
  
  
+ TTAAATAATA ATTTTATATT AAAATAAAGT TTAATATACT TATAGCATAG CAAACCTACG GGTACTACAC   
  
  
+ TATTTATAAT AAATAAAATT TTAAAATTAA TAATATTTGT AATACAAACT ATACAAAAAT AAAATATAGT   
  
  
+ ATATTAAAAT TATATATAAA TCTATTTTAT TATTTACCTT TACTTAATAA ATCATAACAT TTAAAATTCA   
  
  
+ CTACTATTTA GATTTTTTTT AAATTAATTA CATATCTATT CAAAACAAAC ACTACACCTT AGGCCACAGG   
  
  
+ AACTTTATAA ACTGGTAGGT TCTTCACTAT TTACGTTTTA TAATTAACAC CCCTTCTTTA CTTACTTCAC   
  
  
+ CCCATTCCCA GTGCACCTAG TTACTCTGAG ACACAAAGAT TCAGTTTCGT TTTTCTGTTT TTCGAGGTTT   
  
  
+ TGGCAAGTAA GGTTTCGAAA ATTTGCGGCT TTGTGAGGAA ACTACAACAG TTTCGGGGGA AGGTGAGGCT   
  
  
+ GTGAGACTAA GAAGGGTTTG TGGGCTGGGC AACTAAAGCC TTAACTGATT TGCAAGGTTA TTTTTTATTA   
  
  
+ TCTTTTTACA TTATAACACG AGAACGTTTT GTTACTCCAT GGAACCCCAT CACCAATCGT GAGACGTGAA   
  
  
+ ACTTAGGTCG CTGGACCCAA GCTGAGGGCC ACCCTGGAAG CAAAGGAACT TACAATAAAT GCAAAAAAGA   
  
  
+ AAACATTGAG TCACTTCGAA CAACACCAAT CCAGTAACTT TCTCTACAAA TTGTGACATT TGTGAAGTGT   
  
  
+ GGCTAAACTA ACACAGTTGG TGTTGAGTCA CTTCGAACAA CACCAATCCA GTAACTTTCT CTACAAATTG   
  
  
+ TGACATTTGT GAAGTGTGGC TAAACTAACA CAGTTGGTGA GCATTAGACA TAATTTCAAT GTTTGGGAAA   
  
  
+ GAAAAAGTTC CTCTTTTTTT TCTTTTCTT  

- GAATTGGGTG GTCGATGAGA AAGAGAAGTT GGGTAACGAA TTTATAGAAG AAGTATCTGA ATGGTTGGAT   
  
  
- GAAGCCATGC ACATGAAGAG AGAGAGAGAG ATTAGAGAAG GCAAAGGAGA ACATATAAGG AGAGATGTTG   
  
  
- TCATAGTGCG GATGGTGAAG GTCCCTCAGC GCTTCTTCTC AGAAACTAAA AGCGAAGTTT GACCGTGGAA   
  
  
- AATAGTTCTT GGTCAAACCT TGTGGGTTTG ACTGGACTGG CCATACGGGA GGGGGCGAGG CGGCTCTTTA   
  
  
- GGCAGTTCTG TGATTTTGGT AATAAAATCC TACCATCAGT TTTAGCTTGC CACGTGTATT GCCATTTTTC   
  
  
- CCTCAGTTTT CCTTATGTTA ATCAGATAAT ATGTATATTT TAAGATGCAA TGTGTGATTT TAAGTTTTTA   
  
  
- CCCAATAAAT AGATATTTTA TGGCTTAAAA ATTATAATTA TTTAAAATTT TATTAATCAG TTAACATAAT   
  
  
- TATTACATGA TGTAAAATTA AAAACTTAAT TAATATGTTA TTATATGATT TTAAAATTTT AATTTATTAT   
  
  
- AATTTATTAT TAAAATATAA TTTTATTTCA AATTATATGA ATATCGTATC GTTTGGATGC CCATGATGTG   
  
  
- ATAAATATTA TTTATTTTAA AATTTTAATT ATTATAAACA TTATGTTTGA TATGTTTTTA TTTTATATCA   
  
  
- TATAATTTTA ATATATATTT AGATAAAATA ATAAATGGAA ATGAATTATT TAGTATTGTA AATTTTAAGT   
  
  
- GATGATAAAT CTAAAAAAAA TTTAATTAAT GTATAGATAA GTTTTGTTTG TGATGTGGAA TCCGGTGTCC   
  
  
- TTGAAATATT TGACCATCCA AGAAGTGATA AATGCAAAAT ATTAATTGTG GGGAAGAAAT GAATGAAGTG   
  
  
- GGGTAAGGGT CACGTGGATC AATGAGACTC TGTGTTTCTA AGTCAAAGCA AAAAGACAAA AAGCTCCAAA   
  
  
- ACCGTTCATT CCAAAGCTTT TAAACGCCGA AACACTCCTT TGATGTTGTC AAAGCCCCCT TCCACTCCGA   
  
  
- CACTCTGATT CTTCCCAAAC ACCCGACCCG TTGATTTCGG AATTGACTAA ACGTTCCAAT AAAAAATAAT   
  
  
- AGAAAAATGT AATATTGTGC TCTTGCAAAA CAATGAGGTA CCTTGGGGTA GTGGTTAGCA CTCTGCACTT   
  
  
- TGAATCCAGC GACCTGGGTT CGACTCCCGG TGGGACCTTC GTTTCCTTGA ATGTTATTTA CGTTTTTTCT   
  
  
- TTTGTAACTC AGTGAAGCTT GTTGTGGTTA GGTCATTGAA AGAGATGTTT AACACTGTAA ACACTTCACA   
  
  
- CCGATTTGAT TGTGTCAACC ACAACTCAGT GAAGCTTGTT GTGGTTAGGT CATTGAAAGA GATGTTTAAC   
  
  
- ACTGTAAACA CTTCACACCG ATTTGATTGT GTCAACCACT CGTAATCTGT ATTAAAGTTA CAAACCCTTT   
  
  
- CTTTTTCAAG GAGAAAAAAA AGAAAAGAA

+     MBSI

| Site Name | Organism | Position | Strand | Matrix score. | sequence | function |
| --- | --- | --- | --- | --- | --- | --- |
| MBSI | Petunia hybrida | 338 | - | 11 | TTTTTACGGTTA | MYB binding site involved in flavonoid biosynthetic genes regulation |

> 2018/04/13 10:10:12  
+ CTTAACCCAC CAGCTACTCT TTCTCTTCAA CCCATTGCTT AAATATCTTC TTCATAGACT TACCAACCTA   
  
  
+ CTTCGGTACG TGTACTTCTC TCTCTCTCTC TAATCTCTTC CGTTTCCTCT TGTATATTCC TCTCTACAAC   
  
  
+ AGTATCACGC CTACCACTTC CAGGGAGTCG CGAAGAAGAG TCTTTGATTT TCGCTTCAAA CTGGCACCTT   
  
  
+ TTATCAAGAA CCAGTTTGGA ACACCCAAAC TGACCTGACC GGTATGCCCT CCCCCGCTCC GCCGAGAAAT   
  
  
+ CCGTCAAGAC ACTAAAACCA TTATTTTAGG ATGGTAGTCA AAATCGAACG GTGCACATAA CGGTAAAAAG   
  
  
+ GGAGTCAAAA GGAATACAAT TAGTCTATTA TACATATAAA ATTCTACGTT ACACACTAAA ATTCAAAAAT   
  
  
+ GGGTTATTTA TCTATAAAAT ACCGAATTTT TAATATTAAT AAATTTTAAA ATAATTAGTC AATTGTATTA   
  
  
+ ATAATGTACT ACATTTTAAT TTTTGAATTA ATTATACAAT AATATACTAA AATTTTAAAA TTAAATAATA   
  
  
+ TTAAATAATA ATTTTATATT AAAATAAAGT TTAATATACT TATAGCATAG CAAACCTACG GGTACTACAC   
  
  
+ TATTTATAAT AAATAAAATT TTAAAATTAA TAATATTTGT AATACAAACT ATACAAAAAT AAAATATAGT   
  
  
+ ATATTAAAAT TATATATAAA TCTATTTTAT TATTTACCTT TACTTAATAA ATCATAACAT TTAAAATTCA   
  
  
+ CTACTATTTA GATTTTTTTT AAATTAATTA CATATCTATT CAAAACAAAC ACTACACCTT AGGCCACAGG   
  
  
+ AACTTTATAA ACTGGTAGGT TCTTCACTAT TTACGTTTTA TAATTAACAC CCCTTCTTTA CTTACTTCAC   
  
  
+ CCCATTCCCA GTGCACCTAG TTACTCTGAG ACACAAAGAT TCAGTTTCGT TTTTCTGTTT TTCGAGGTTT   
  
  
+ TGGCAAGTAA GGTTTCGAAA ATTTGCGGCT TTGTGAGGAA ACTACAACAG TTTCGGGGGA AGGTGAGGCT   
  
  
+ GTGAGACTAA GAAGGGTTTG TGGGCTGGGC AACTAAAGCC TTAACTGATT TGCAAGGTTA TTTTTTATTA   
  
  
+ TCTTTTTACA TTATAACACG AGAACGTTTT GTTACTCCAT GGAACCCCAT CACCAATCGT GAGACGTGAA   
  
  
+ ACTTAGGTCG CTGGACCCAA GCTGAGGGCC ACCCTGGAAG CAAAGGAACT TACAATAAAT GCAAAAAAGA   
  
  
+ AAACATTGAG TCACTTCGAA CAACACCAAT CCAGTAACTT TCTCTACAAA TTGTGACATT TGTGAAGTGT   
  
  
+ GGCTAAACTA ACACAGTTGG TGTTGAGTCA CTTCGAACAA CACCAATCCA GTAACTTTCT CTACAAATTG   
  
  
+ TGACATTTGT GAAGTGTGGC TAAACTAACA CAGTTGGTGA GCATTAGACA TAATTTCAAT GTTTGGGAAA   
  
  
+ GAAAAAGTTC CTCTTTTTTT TCTTTTCTT  

- GAATTGGGTG GTCGATGAGA AAGAGAAGTT GGGTAACGAA TTTATAGAAG AAGTATCTGA ATGGTTGGAT   
  
  
- GAAGCCATGC ACATGAAGAG AGAGAGAGAG ATTAGAGAAG GCAAAGGAGA ACATATAAGG AGAGATGTTG   
  
  
- TCATAGTGCG GATGGTGAAG GTCCCTCAGC GCTTCTTCTC AGAAACTAAA AGCGAAGTTT GACCGTGGAA   
  
  
- AATAGTTCTT GGTCAAACCT TGTGGGTTTG ACTGGACTGG CCATACGGGA GGGGGCGAGG CGGCTCTTTA   
  
  
- GGCAGTTCTG TGATTTTGGT AATAAAATCC TACCATCAGT TTTAGCTTGC CACGTGTATT GCCATTTTTC   
  
  
- CCTCAGTTTT CCTTATGTTA ATCAGATAAT ATGTATATTT TAAGATGCAA TGTGTGATTT TAAGTTTTTA   
  
  
- CCCAATAAAT AGATATTTTA TGGCTTAAAA ATTATAATTA TTTAAAATTT TATTAATCAG TTAACATAAT   
  
  
- TATTACATGA TGTAAAATTA AAAACTTAAT TAATATGTTA TTATATGATT TTAAAATTTT AATTTATTAT   
  
  
- AATTTATTAT TAAAATATAA TTTTATTTCA AATTATATGA ATATCGTATC GTTTGGATGC CCATGATGTG   
  
  
- ATAAATATTA TTTATTTTAA AATTTTAATT ATTATAAACA TTATGTTTGA TATGTTTTTA TTTTATATCA   
  
  
- TATAATTTTA ATATATATTT AGATAAAATA ATAAATGGAA ATGAATTATT TAGTATTGTA AATTTTAAGT   
  
  
- GATGATAAAT CTAAAAAAAA TTTAATTAAT GTATAGATAA GTTTTGTTTG TGATGTGGAA TCCGGTGTCC   
  
  
- TTGAAATATT TGACCATCCA AGAAGTGATA AATGCAAAAT ATTAATTGTG GGGAAGAAAT GAATGAAGTG   
  
  
- GGGTAAGGGT CACGTGGATC AATGAGACTC TGTGTTTCTA AGTCAAAGCA AAAAGACAAA AAGCTCCAAA   
  
  
- ACCGTTCATT CCAAAGCTTT TAAACGCCGA AACACTCCTT TGATGTTGTC AAAGCCCCCT TCCACTCCGA   
  
  
- CACTCTGATT CTTCCCAAAC ACCCGACCCG TTGATTTCGG AATTGACTAA ACGTTCCAAT AAAAAATAAT   
  
  
- AGAAAAATGT AATATTGTGC TCTTGCAAAA CAATGAGGTA CCTTGGGGTA GTGGTTAGCA CTCTGCACTT   
  
  
- TGAATCCAGC GACCTGGGTT CGACTCCCGG TGGGACCTTC GTTTCCTTGA ATGTTATTTA CGTTTTTTCT   
  
  
- TTTGTAACTC AGTGAAGCTT GTTGTGGTTA GGTCATTGAA AGAGATGTTT AACACTGTAA ACACTTCACA   
  
  
- CCGATTTGAT TGTGTCAACC ACAACTCAGT GAAGCTTGTT GTGGTTAGGT CATTGAAAGA GATGTTTAAC   
  
  
- ACTGTAAACA CTTCACACCG ATTTGATTGT GTCAACCACT CGTAATCTGT ATTAAAGTTA CAAACCCTTT   
  
  
- CTTTTTCAAG GAGAAAAAAA AGAAAAGAA

+     P-box

| Site Name | Organism | Position | Strand | Matrix score. | sequence | function |
| --- | --- | --- | --- | --- | --- | --- |
| P-box | Oryza sativa | 356 | - | 7 | CCTTTTG | gibberellin-responsive element |

> 2018/04/13 10:10:12  
+ CTTAACCCAC CAGCTACTCT TTCTCTTCAA CCCATTGCTT AAATATCTTC TTCATAGACT TACCAACCTA   
  
  
+ CTTCGGTACG TGTACTTCTC TCTCTCTCTC TAATCTCTTC CGTTTCCTCT TGTATATTCC TCTCTACAAC   
  
  
+ AGTATCACGC CTACCACTTC CAGGGAGTCG CGAAGAAGAG TCTTTGATTT TCGCTTCAAA CTGGCACCTT   
  
  
+ TTATCAAGAA CCAGTTTGGA ACACCCAAAC TGACCTGACC GGTATGCCCT CCCCCGCTCC GCCGAGAAAT   
  
  
+ CCGTCAAGAC ACTAAAACCA TTATTTTAGG ATGGTAGTCA AAATCGAACG GTGCACATAA CGGTAAAAAG   
  
  
+ GGAGTCAAAA GGAATACAAT TAGTCTATTA TACATATAAA ATTCTACGTT ACACACTAAA ATTCAAAAAT   
  
  
+ GGGTTATTTA TCTATAAAAT ACCGAATTTT TAATATTAAT AAATTTTAAA ATAATTAGTC AATTGTATTA   
  
  
+ ATAATGTACT ACATTTTAAT TTTTGAATTA ATTATACAAT AATATACTAA AATTTTAAAA TTAAATAATA   
  
  
+ TTAAATAATA ATTTTATATT AAAATAAAGT TTAATATACT TATAGCATAG CAAACCTACG GGTACTACAC   
  
  
+ TATTTATAAT AAATAAAATT TTAAAATTAA TAATATTTGT AATACAAACT ATACAAAAAT AAAATATAGT   
  
  
+ ATATTAAAAT TATATATAAA TCTATTTTAT TATTTACCTT TACTTAATAA ATCATAACAT TTAAAATTCA   
  
  
+ CTACTATTTA GATTTTTTTT AAATTAATTA CATATCTATT CAAAACAAAC ACTACACCTT AGGCCACAGG   
  
  
+ AACTTTATAA ACTGGTAGGT TCTTCACTAT TTACGTTTTA TAATTAACAC CCCTTCTTTA CTTACTTCAC   
  
  
+ CCCATTCCCA GTGCACCTAG TTACTCTGAG ACACAAAGAT TCAGTTTCGT TTTTCTGTTT TTCGAGGTTT   
  
  
+ TGGCAAGTAA GGTTTCGAAA ATTTGCGGCT TTGTGAGGAA ACTACAACAG TTTCGGGGGA AGGTGAGGCT   
  
  
+ GTGAGACTAA GAAGGGTTTG TGGGCTGGGC AACTAAAGCC TTAACTGATT TGCAAGGTTA TTTTTTATTA   
  
  
+ TCTTTTTACA TTATAACACG AGAACGTTTT GTTACTCCAT GGAACCCCAT CACCAATCGT GAGACGTGAA   
  
  
+ ACTTAGGTCG CTGGACCCAA GCTGAGGGCC ACCCTGGAAG CAAAGGAACT TACAATAAAT GCAAAAAAGA   
  
  
+ AAACATTGAG TCACTTCGAA CAACACCAAT CCAGTAACTT TCTCTACAAA TTGTGACATT TGTGAAGTGT   
  
  
+ GGCTAAACTA ACACAGTTGG TGTTGAGTCA CTTCGAACAA CACCAATCCA GTAACTTTCT CTACAAATTG   
  
  
+ TGACATTTGT GAAGTGTGGC TAAACTAACA CAGTTGGTGA GCATTAGACA TAATTTCAAT GTTTGGGAAA   
  
  
+ GAAAAAGTTC CTCTTTTTTT TCTTTTCTT  

- GAATTGGGTG GTCGATGAGA AAGAGAAGTT GGGTAACGAA TTTATAGAAG AAGTATCTGA ATGGTTGGAT   
  
  
- GAAGCCATGC ACATGAAGAG AGAGAGAGAG ATTAGAGAAG GCAAAGGAGA ACATATAAGG AGAGATGTTG   
  
  
- TCATAGTGCG GATGGTGAAG GTCCCTCAGC GCTTCTTCTC AGAAACTAAA AGCGAAGTTT GACCGTGGAA   
  
  
- AATAGTTCTT GGTCAAACCT TGTGGGTTTG ACTGGACTGG CCATACGGGA GGGGGCGAGG CGGCTCTTTA   
  
  
- GGCAGTTCTG TGATTTTGGT AATAAAATCC TACCATCAGT TTTAGCTTGC CACGTGTATT GCCATTTTTC   
  
  
- CCTCAGTTTT CCTTATGTTA ATCAGATAAT ATGTATATTT TAAGATGCAA TGTGTGATTT TAAGTTTTTA   
  
  
- CCCAATAAAT AGATATTTTA TGGCTTAAAA ATTATAATTA TTTAAAATTT TATTAATCAG TTAACATAAT   
  
  
- TATTACATGA TGTAAAATTA AAAACTTAAT TAATATGTTA TTATATGATT TTAAAATTTT AATTTATTAT   
  
  
- AATTTATTAT TAAAATATAA TTTTATTTCA AATTATATGA ATATCGTATC GTTTGGATGC CCATGATGTG   
  
  
- ATAAATATTA TTTATTTTAA AATTTTAATT ATTATAAACA TTATGTTTGA TATGTTTTTA TTTTATATCA   
  
  
- TATAATTTTA ATATATATTT AGATAAAATA ATAAATGGAA ATGAATTATT TAGTATTGTA AATTTTAAGT   
  
  
- GATGATAAAT CTAAAAAAAA TTTAATTAAT GTATAGATAA GTTTTGTTTG TGATGTGGAA TCCGGTGTCC   
  
  
- TTGAAATATT TGACCATCCA AGAAGTGATA AATGCAAAAT ATTAATTGTG GGGAAGAAAT GAATGAAGTG   
  
  
- GGGTAAGGGT CACGTGGATC AATGAGACTC TGTGTTTCTA AGTCAAAGCA AAAAGACAAA AAGCTCCAAA   
  
  
- ACCGTTCATT CCAAAGCTTT TAAACGCCGA AACACTCCTT TGATGTTGTC AAAGCCCCCT TCCACTCCGA   
  
  
- CACTCTGATT CTTCCCAAAC ACCCGACCCG TTGATTTCGG AATTGACTAA ACGTTCCAAT AAAAAATAAT   
  
  
- AGAAAAATGT AATATTGTGC TCTTGCAAAA CAATGAGGTA CCTTGGGGTA GTGGTTAGCA CTCTGCACTT   
  
  
- TGAATCCAGC GACCTGGGTT CGACTCCCGG TGGGACCTTC GTTTCCTTGA ATGTTATTTA CGTTTTTTCT   
  
  
- TTTGTAACTC AGTGAAGCTT GTTGTGGTTA GGTCATTGAA AGAGATGTTT AACACTGTAA ACACTTCACA   
  
  
- CCGATTTGAT TGTGTCAACC ACAACTCAGT GAAGCTTGTT GTGGTTAGGT CATTGAAAGA GATGTTTAAC   
  
  
- ACTGTAAACA CTTCACACCG ATTTGATTGT GTCAACCACT CGTAATCTGT ATTAAAGTTA CAAACCCTTT   
  
  
- CTTTTTCAAG GAGAAAAAAA AGAAAAGAA

+     Sp1

| Site Name | Organism | Position | Strand | Matrix score. | sequence | function |
| --- | --- | --- | --- | --- | --- | --- |
| Sp1 | Zea mays | 258 | + | 5 | CC(G/A)CCC | light responsive element |
| Sp1 | Zea mays | 1219 | + | 5.5 | CC(G/A)CCC | light responsive element |

> 2018/04/13 10:10:12  
+ CTTAACCCAC CAGCTACTCT TTCTCTTCAA CCCATTGCTT AAATATCTTC TTCATAGACT TACCAACCTA   
  
  
+ CTTCGGTACG TGTACTTCTC TCTCTCTCTC TAATCTCTTC CGTTTCCTCT TGTATATTCC TCTCTACAAC   
  
  
+ AGTATCACGC CTACCACTTC CAGGGAGTCG CGAAGAAGAG TCTTTGATTT TCGCTTCAAA CTGGCACCTT   
  
  
+ TTATCAAGAA CCAGTTTGGA ACACCCAAAC TGACCTGACC GGTATGCCCT CCCCCGCTCC GCCGAGAAAT   
  
  
+ CCGTCAAGAC ACTAAAACCA TTATTTTAGG ATGGTAGTCA AAATCGAACG GTGCACATAA CGGTAAAAAG   
  
  
+ GGAGTCAAAA GGAATACAAT TAGTCTATTA TACATATAAA ATTCTACGTT ACACACTAAA ATTCAAAAAT   
  
  
+ GGGTTATTTA TCTATAAAAT ACCGAATTTT TAATATTAAT AAATTTTAAA ATAATTAGTC AATTGTATTA   
  
  
+ ATAATGTACT ACATTTTAAT TTTTGAATTA ATTATACAAT AATATACTAA AATTTTAAAA TTAAATAATA   
  
  
+ TTAAATAATA ATTTTATATT AAAATAAAGT TTAATATACT TATAGCATAG CAAACCTACG GGTACTACAC   
  
  
+ TATTTATAAT AAATAAAATT TTAAAATTAA TAATATTTGT AATACAAACT ATACAAAAAT AAAATATAGT   
  
  
+ ATATTAAAAT TATATATAAA TCTATTTTAT TATTTACCTT TACTTAATAA ATCATAACAT TTAAAATTCA   
  
  
+ CTACTATTTA GATTTTTTTT AAATTAATTA CATATCTATT CAAAACAAAC ACTACACCTT AGGCCACAGG   
  
  
+ AACTTTATAA ACTGGTAGGT TCTTCACTAT TTACGTTTTA TAATTAACAC CCCTTCTTTA CTTACTTCAC   
  
  
+ CCCATTCCCA GTGCACCTAG TTACTCTGAG ACACAAAGAT TCAGTTTCGT TTTTCTGTTT TTCGAGGTTT   
  
  
+ TGGCAAGTAA GGTTTCGAAA ATTTGCGGCT TTGTGAGGAA ACTACAACAG TTTCGGGGGA AGGTGAGGCT   
  
  
+ GTGAGACTAA GAAGGGTTTG TGGGCTGGGC AACTAAAGCC TTAACTGATT TGCAAGGTTA TTTTTTATTA   
  
  
+ TCTTTTTACA TTATAACACG AGAACGTTTT GTTACTCCAT GGAACCCCAT CACCAATCGT GAGACGTGAA   
  
  
+ ACTTAGGTCG CTGGACCCAA GCTGAGGGCC ACCCTGGAAG CAAAGGAACT TACAATAAAT GCAAAAAAGA   
  
  
+ AAACATTGAG TCACTTCGAA CAACACCAAT CCAGTAACTT TCTCTACAAA TTGTGACATT TGTGAAGTGT   
  
  
+ GGCTAAACTA ACACAGTTGG TGTTGAGTCA CTTCGAACAA CACCAATCCA GTAACTTTCT CTACAAATTG   
  
  
+ TGACATTTGT GAAGTGTGGC TAAACTAACA CAGTTGGTGA GCATTAGACA TAATTTCAAT GTTTGGGAAA   
  
  
+ GAAAAAGTTC CTCTTTTTTT TCTTTTCTT  

- GAATTGGGTG GTCGATGAGA AAGAGAAGTT GGGTAACGAA TTTATAGAAG AAGTATCTGA ATGGTTGGAT   
  
  
- GAAGCCATGC ACATGAAGAG AGAGAGAGAG ATTAGAGAAG GCAAAGGAGA ACATATAAGG AGAGATGTTG   
  
  
- TCATAGTGCG GATGGTGAAG GTCCCTCAGC GCTTCTTCTC AGAAACTAAA AGCGAAGTTT GACCGTGGAA   
  
  
- AATAGTTCTT GGTCAAACCT TGTGGGTTTG ACTGGACTGG CCATACGGGA GGGGGCGAGG CGGCTCTTTA   
  
  
- GGCAGTTCTG TGATTTTGGT AATAAAATCC TACCATCAGT TTTAGCTTGC CACGTGTATT GCCATTTTTC   
  
  
- CCTCAGTTTT CCTTATGTTA ATCAGATAAT ATGTATATTT TAAGATGCAA TGTGTGATTT TAAGTTTTTA   
  
  
- CCCAATAAAT AGATATTTTA TGGCTTAAAA ATTATAATTA TTTAAAATTT TATTAATCAG TTAACATAAT   
  
  
- TATTACATGA TGTAAAATTA AAAACTTAAT TAATATGTTA TTATATGATT TTAAAATTTT AATTTATTAT   
  
  
- AATTTATTAT TAAAATATAA TTTTATTTCA AATTATATGA ATATCGTATC GTTTGGATGC CCATGATGTG   
  
  
- ATAAATATTA TTTATTTTAA AATTTTAATT ATTATAAACA TTATGTTTGA TATGTTTTTA TTTTATATCA   
  
  
- TATAATTTTA ATATATATTT AGATAAAATA ATAAATGGAA ATGAATTATT TAGTATTGTA AATTTTAAGT   
  
  
- GATGATAAAT CTAAAAAAAA TTTAATTAAT GTATAGATAA GTTTTGTTTG TGATGTGGAA TCCGGTGTCC   
  
  
- TTGAAATATT TGACCATCCA AGAAGTGATA AATGCAAAAT ATTAATTGTG GGGAAGAAAT GAATGAAGTG   
  
  
- GGGTAAGGGT CACGTGGATC AATGAGACTC TGTGTTTCTA AGTCAAAGCA AAAAGACAAA AAGCTCCAAA   
  
  
- ACCGTTCATT CCAAAGCTTT TAAACGCCGA AACACTCCTT TGATGTTGTC AAAGCCCCCT TCCACTCCGA   
  
  
- CACTCTGATT CTTCCCAAAC ACCCGACCCG TTGATTTCGG AATTGACTAA ACGTTCCAAT AAAAAATAAT   
  
  
- AGAAAAATGT AATATTGTGC TCTTGCAAAA CAATGAGGTA CCTTGGGGTA GTGGTTAGCA CTCTGCACTT   
  
  
- TGAATCCAGC GACCTGGGTT CGACTCCCGG TGGGACCTTC GTTTCCTTGA ATGTTATTTA CGTTTTTTCT   
  
  
- TTTGTAACTC AGTGAAGCTT GTTGTGGTTA GGTCATTGAA AGAGATGTTT AACACTGTAA ACACTTCACA   
  
  
- CCGATTTGAT TGTGTCAACC ACAACTCAGT GAAGCTTGTT GTGGTTAGGT CATTGAAAGA GATGTTTAAC   
  
  
- ACTGTAAACA CTTCACACCG ATTTGATTGT GTCAACCACT CGTAATCTGT ATTAAAGTTA CAAACCCTTT   
  
  
- CTTTTTCAAG GAGAAAAAAA AGAAAAGAA

+     TATA-box

| Site Name | Organism | Position | Strand | Matrix score. | sequence | function |
| --- | --- | --- | --- | --- | --- | --- |
| TATA-box | Arabidopsis thaliana | 876 | - | 7 | TATAAAA | core promoter element around -30 of transcription start |
| TATA-box | Glycine max | 745 | + | 5 | TAATA | core promoter element around -30 of transcription start |
| TATA-box | Arabidopsis thaliana | 633 | - | 6 | TATAAA | core promoter element around -30 of transcription start |
| TATA-box | Arabidopsis thaliana | 601 | + | 4 | TATA | core promoter element around -30 of transcription start |
| TATA-box | Zea mays | 786 | - | 8 | TTTAAAAA | core promoter element around -30 of transcription start |
| TATA-box | Lycopersicon esculentum | 787 | + | 5 | TTTTA | core promoter element around -30 of transcription start |
| TATA-box | Ac | 632 | - | 7 | TATAAAT | core promoter element around -30 of transcription start |
| TATA-box | Arabidopsis thaliana | 595 | + | 4 | TATA | core promoter element around -30 of transcription start |
| TATA-box | Glycine max | 556 | + | 5 | TAATA | core promoter element around -30 of transcription start |
| TATA-box | Glycine max | 454 | - | 5 | TAATA | core promoter element around -30 of transcription start |
| TATA-box | Glycine max | 702 | - | 5 | TAATA | core promoter element around -30 of transcription start |
| TATA-box | Arabidopsis thaliana | 878 | - | 5 | TATAA | core promoter element around -30 of transcription start |
| TATA-box | Arabidopsis thaliana | 1131 | - | 5 | TATAA | core promoter element around -30 of transcription start |
| TATA-box | Glycine max | 728 | - | 5 | TAATA | core promoter element around -30 of transcription start |
| TATA-box | Glycine max | 577 | - | 5 | TAATA | core promoter element around -30 of transcription start |
| TATA-box | Glycine max | 451 | + | 5 | TAATA | core promoter element around -30 of transcription start |
| TATA-box | Arabidopsis thaliana | 635 | + | 4 | TATA | core promoter element around -30 of transcription start |
| TATA-box | Brassica napus | 712 | + | 6 | ATATAT | core promoter element around -30 of transcription start |
| TATA-box | Glycine max | 566 | + | 5 | TAATA | core promoter element around -30 of transcription start |
| TATA-box | Arabidopsis thaliana | 574 | - | 5 | TATAA | core promoter element around -30 of transcription start |
| TATA-box | Brassica oleracea | 714 | + | 6 | ATATAA | core promoter element around -30 of transcription start |
| TATA-box | Brassica napus | 521 | + | 6 | ATTATA | core promoter element around -30 of transcription start |
| TATA-box | Arabidopsis thaliana | 379 | + | 11 | TATAAATATAAA | core promoter element around -30 of transcription start |
| TATA-box | Glycine max | 489 | + | 5 | TAATA | core promoter element around -30 of transcription start |
| TATA-box | Lycopersicon esculentum | 304 | + | 5 | TTTTA | core promoter element around -30 of transcription start |
| TATA-box | Brassica napus | 1130 | + | 6 | ATTATA | core promoter element around -30 of transcription start |
| TATA-box | Arabidopsis thaliana | 378 | - | 5 | TATAA | core promoter element around -30 of transcription start |
| TATA-box | Lycopersicon esculentum | 209 | + | 5 | TTTTA | core promoter element around -30 of transcription start |
| TATA-box | Lycopersicon esculentum | 652 | - | 5 | TTTTA | core promoter element around -30 of transcription start |
| TATA-box | Glycine max | 457 | + | 5 | TAATA | core promoter element around -30 of transcription start |
| TATA-box | Arabidopsis thaliana | 710 | - | 7 | TATATAA | core promoter element around -30 of transcription start |
| TATA-box | Brassica napus | 709 | + | 6 | ATTATA | core promoter element around -30 of transcription start |
| TATA-box | Brassica napus | 377 | + | 6 | ATTATA | core promoter element around -30 of transcription start |
| TATA-box | Glycine max | 376 | - | 5 | TAATA | core promoter element around -30 of transcription start |
| TATA-box | Helianthus annuus | 121 | - | 6 | TATACA | core promoter element around -30 of transcription start |
| TATA-box | Arabidopsis thaliana | 846 | + | 6 | TATAAA | core promoter element around -30 of transcription start |
| TATA-box | Brassica oleracea | 384 | + | 6 | ATATAA | core promoter element around -30 of transcription start |
| TATA-box | Pisum sativum | 382 | - | 7 | TATATGT | core promoter element around -30 of transcription start |
| TATA-box | Daucus carota | 631 | - | 8 | TATAAATA | core promoter element around -30 of transcription start |
| TATA-box | Arabidopsis thaliana | 711 | + | 8 | TATATATA | core promoter element around -30 of transcription start |
| TATA-box | Lycopersicon esculentum | 705 | - | 5 | TTTTA | core promoter element around -30 of transcription start |
| TATA-box | Arabidopsis thaliana | 1132 | - | 4 | TATA | core promoter element around -30 of transcription start |
| TATA-box | Glycine max | 486 | - | 5 | TAATA | core promoter element around -30 of transcription start |
| TATA-box | Arabidopsis thaliana | 385 | + | 6 | TATAAA | core promoter element around -30 of transcription start |
| TATA-box | Arabidopsis thaliana | 433 | + | 6 | TATAAA | core promoter element around -30 of transcription start |
| TATA-box | Glycine max | 658 | + | 5 | TAATA | core promoter element around -30 of transcription start |
| TATA-box | Lycopersicon esculentum | 762 | - | 5 | TTTTA | core promoter element around -30 of transcription start |
| TATA-box | Lycopersicon esculentum | 644 | - | 5 | TTTTA | core promoter element around -30 of transcription start |
| TATA-box | Arabidopsis thaliana | 600 | - | 5 | TATAA | core promoter element around -30 of transcription start |
| TATA-box | Glycine max | 559 | - | 5 | TAATA | core promoter element around -30 of transcription start |
| TATA-box | Arabidopsis thaliana | 713 | + | 10 | taTATAAAtc | core promoter element around -30 of transcription start |
| TATA-box | Lycopersicon esculentum | 344 | - | 5 | TTTTA | core promoter element around -30 of transcription start |
| TATA-box | Arabidopsis thaliana | 123 | + | 4 | TATA | core promoter element around -30 of transcription start |
| TATA-box | Arabidopsis thaliana | 700 | + | 4 | TATA | core promoter element around -30 of transcription start |
| TATA-box | Arabidopsis thaliana | 572 | - | 7 | TATAAAA | core promoter element around -30 of transcription start |
| TATA-box | Glycine max | 637 | + | 5 | TAATA | core promoter element around -30 of transcription start |
| TATA-box | Lycopersicon esculentum | 464 | + | 5 | TTTTA | core promoter element around -30 of transcription start |
| TATA-box | Lycopersicon esculentum | 407 | - | 5 | TTTTA | core promoter element around -30 of transcription start |
| TATA-box | Lycopersicon esculentum | 467 | - | 5 | TTTTA | core promoter element around -30 of transcription start |
| TATA-box | Lycopersicon esculentum | 293 | - | 5 | TTTTA | core promoter element around -30 of transcription start |
| TATA-box | Glycine max | 1116 | - | 5 | TAATA | core promoter element around -30 of transcription start |
| TATA-box | Lycopersicon esculentum | 546 | - | 5 | TTTTA | core promoter element around -30 of transcription start |
| TATA-box | Lycopersicon esculentum | 538 | - | 5 | TTTTA | core promoter element around -30 of transcription start |
| TATA-box | Arabidopsis thaliana | 634 | - | 5 | TATAA | core promoter element around -30 of transcription start |
| TATA-box | Arabidopsis thaliana | 879 | - | 4 | TATA | core promoter element around -30 of transcription start |
| TATA-box | Lycopersicon esculentum | 435 | - | 5 | TTTTA | core promoter element around -30 of transcription start |
| TATA-box | Lycopersicon esculentum | 387 | - | 5 | TTTTA | core promoter element around -30 of transcription start |
| TATA-box | Arabidopsis thaliana | 845 | - | 5 | TATAA | core promoter element around -30 of transcription start |
| TATA-box | Lycopersicon esculentum | 1113 | + | 5 | TTTTA | core promoter element around -30 of transcription start |
| TATA-box | Lycopersicon esculentum | 580 | - | 5 | TTTTA | core promoter element around -30 of transcription start |
| TATA-box | Arabidopsis thaliana | 523 | + | 4 | TATA | core promoter element around -30 of transcription start |
| TATA-box | Oryza sativa | 682 | + | 7 | TACAAAA | core promoter element around -30 of transcription start |
| TATA-box | Lycopersicon esculentum | 448 | + | 5 | TTTTA | core promoter element around -30 of transcription start |
| TATA-box | Arabidopsis thaliana | 573 | - | 6 | TATAAA | core promoter element around -30 of transcription start |
| TATA-box | Arabidopsis thaliana | 680 | + | 4 | TATA | core promoter element around -30 of transcription start |
| TATA-box | Glycine max | 592 | + | 5 | TAATA | core promoter element around -30 of transcription start |
| TATA-box | Arabidopsis thaliana | 533 | + | 4 | TATA | core promoter element around -30 of transcription start |
| TATA-box | Glycine max | 661 | + | 5 | TAATA | core promoter element around -30 of transcription start |
| TATA-box | Ac | 715 | + | 7 | TATAAAT | core promoter element around -30 of transcription start |
| TATA-box | Glycine max | 670 | + | 5 | TAATA | core promoter element around -30 of transcription start |
| TATA-box | Glycine max | 530 | + | 5 | TAATA | core promoter element around -30 of transcription start |
| TATA-box | Pisum sativum | 571 | - | 8 | TATAAAAT | core promoter element around -30 of transcription start |
| TATA-box | Arabidopsis thaliana | 844 | - | 6 | TATAAA | core promoter element around -30 of transcription start |
| TATA-box | Arabidopsis thaliana | 522 | - | 5 | TATAA | core promoter element around -30 of transcription start |
| TATA-box | Lycopersicon esculentum | 690 | - | 5 | TTTTA | core promoter element around -30 of transcription start |
| TATA-box | Arabidopsis thaliana | 575 | + | 4 | TATA | core promoter element around -30 of transcription start |
| TATA-box | Lycopersicon esculentum | 1124 | + | 5 | TTTTA | core promoter element around -30 of transcription start |
| TATA-box | Lycopersicon esculentum | 543 | + | 5 | TTTTA | core promoter element around -30 of transcription start |
| TATA-box | Lycopersicon esculentum | 504 | + | 5 | TTTTA | core promoter element around -30 of transcription start |
| TATA-box | Lycopersicon esculentum | 649 | + | 5 | TTTTA | core promoter element around -30 of transcription start |
| TATA-box | Lycopersicon esculentum | 725 | + | 5 | TTTTA | core promoter element around -30 of transcription start |
| TATA-box | Arabidopsis thaliana | 877 | - | 6 | TATAAA | core promoter element around -30 of transcription start |
| TATA-box | Arabidopsis thaliana | 695 | + | 4 | TATA | core promoter element around -30 of transcription start |

> 2018/04/13 10:10:12  
+ CTTAACCCAC CAGCTACTCT TTCTCTTCAA CCCATTGCTT AAATATCTTC TTCATAGACT TACCAACCTA   
  
  
+ CTTCGGTACG TGTACTTCTC TCTCTCTCTC TAATCTCTTC CGTTTCCTCT TGTATATTCC TCTCTACAAC   
  
  
+ AGTATCACGC CTACCACTTC CAGGGAGTCG CGAAGAAGAG TCTTTGATTT TCGCTTCAAA CTGGCACCTT   
  
  
+ TTATCAAGAA CCAGTTTGGA ACACCCAAAC TGACCTGACC GGTATGCCCT CCCCCGCTCC GCCGAGAAAT   
  
  
+ CCGTCAAGAC ACTAAAACCA TTATTTTAGG ATGGTAGTCA AAATCGAACG GTGCACATAA CGGTAAAAAG   
  
  
+ GGAGTCAAAA GGAATACAAT TAGTCTATTA TACATATAAA ATTCTACGTT ACACACTAAA ATTCAAAAAT   
  
  
+ GGGTTATTTA TCTATAAAAT ACCGAATTTT TAATATTAAT AAATTTTAAA ATAATTAGTC AATTGTATTA   
  
  
+ ATAATGTACT ACATTTTAAT TTTTGAATTA ATTATACAAT AATATACTAA AATTTTAAAA TTAAATAATA   
  
  
+ TTAAATAATA ATTTTATATT AAAATAAAGT TTAATATACT TATAGCATAG CAAACCTACG GGTACTACAC   
  
  
+ TATTTATAAT AAATAAAATT TTAAAATTAA TAATATTTGT AATACAAACT ATACAAAAAT AAAATATAGT   
  
  
+ ATATTAAAAT TATATATAAA TCTATTTTAT TATTTACCTT TACTTAATAA ATCATAACAT TTAAAATTCA   
  
  
+ CTACTATTTA GATTTTTTTT AAATTAATTA CATATCTATT CAAAACAAAC ACTACACCTT AGGCCACAGG   
  
  
+ AACTTTATAA ACTGGTAGGT TCTTCACTAT TTACGTTTTA TAATTAACAC CCCTTCTTTA CTTACTTCAC   
  
  
+ CCCATTCCCA GTGCACCTAG TTACTCTGAG ACACAAAGAT TCAGTTTCGT TTTTCTGTTT TTCGAGGTTT   
  
  
+ TGGCAAGTAA GGTTTCGAAA ATTTGCGGCT TTGTGAGGAA ACTACAACAG TTTCGGGGGA AGGTGAGGCT   
  
  
+ GTGAGACTAA GAAGGGTTTG TGGGCTGGGC AACTAAAGCC TTAACTGATT TGCAAGGTTA TTTTTTATTA   
  
  
+ TCTTTTTACA TTATAACACG AGAACGTTTT GTTACTCCAT GGAACCCCAT CACCAATCGT GAGACGTGAA   
  
  
+ ACTTAGGTCG CTGGACCCAA GCTGAGGGCC ACCCTGGAAG CAAAGGAACT TACAATAAAT GCAAAAAAGA   
  
  
+ AAACATTGAG TCACTTCGAA CAACACCAAT CCAGTAACTT TCTCTACAAA TTGTGACATT TGTGAAGTGT   
  
  
+ GGCTAAACTA ACACAGTTGG TGTTGAGTCA CTTCGAACAA CACCAATCCA GTAACTTTCT CTACAAATTG   
  
  
+ TGACATTTGT GAAGTGTGGC TAAACTAACA CAGTTGGTGA GCATTAGACA TAATTTCAAT GTTTGGGAAA   
  
  
+ GAAAAAGTTC CTCTTTTTTT TCTTTTCTT  

- GAATTGGGTG GTCGATGAGA AAGAGAAGTT GGGTAACGAA TTTATAGAAG AAGTATCTGA ATGGTTGGAT   
  
  
- GAAGCCATGC ACATGAAGAG AGAGAGAGAG ATTAGAGAAG GCAAAGGAGA ACATATAAGG AGAGATGTTG   
  
  
- TCATAGTGCG GATGGTGAAG GTCCCTCAGC GCTTCTTCTC AGAAACTAAA AGCGAAGTTT GACCGTGGAA   
  
  
- AATAGTTCTT GGTCAAACCT TGTGGGTTTG ACTGGACTGG CCATACGGGA GGGGGCGAGG CGGCTCTTTA   
  
  
- GGCAGTTCTG TGATTTTGGT AATAAAATCC TACCATCAGT TTTAGCTTGC CACGTGTATT GCCATTTTTC   
  
  
- CCTCAGTTTT CCTTATGTTA ATCAGATAAT ATGTATATTT TAAGATGCAA TGTGTGATTT TAAGTTTTTA   
  
  
- CCCAATAAAT AGATATTTTA TGGCTTAAAA ATTATAATTA TTTAAAATTT TATTAATCAG TTAACATAAT   
  
  
- TATTACATGA TGTAAAATTA AAAACTTAAT TAATATGTTA TTATATGATT TTAAAATTTT AATTTATTAT   
  
  
- AATTTATTAT TAAAATATAA TTTTATTTCA AATTATATGA ATATCGTATC GTTTGGATGC CCATGATGTG   
  
  
- ATAAATATTA TTTATTTTAA AATTTTAATT ATTATAAACA TTATGTTTGA TATGTTTTTA TTTTATATCA   
  
  
- TATAATTTTA ATATATATTT AGATAAAATA ATAAATGGAA ATGAATTATT TAGTATTGTA AATTTTAAGT   
  
  
- GATGATAAAT CTAAAAAAAA TTTAATTAAT GTATAGATAA GTTTTGTTTG TGATGTGGAA TCCGGTGTCC   
  
  
- TTGAAATATT TGACCATCCA AGAAGTGATA AATGCAAAAT ATTAATTGTG GGGAAGAAAT GAATGAAGTG   
  
  
- GGGTAAGGGT CACGTGGATC AATGAGACTC TGTGTTTCTA AGTCAAAGCA AAAAGACAAA AAGCTCCAAA   
  
  
- ACCGTTCATT CCAAAGCTTT TAAACGCCGA AACACTCCTT TGATGTTGTC AAAGCCCCCT TCCACTCCGA   
  
  
- CACTCTGATT CTTCCCAAAC ACCCGACCCG TTGATTTCGG AATTGACTAA ACGTTCCAAT AAAAAATAAT   
  
  
- AGAAAAATGT AATATTGTGC TCTTGCAAAA CAATGAGGTA CCTTGGGGTA GTGGTTAGCA CTCTGCACTT   
  
  
- TGAATCCAGC GACCTGGGTT CGACTCCCGG TGGGACCTTC GTTTCCTTGA ATGTTATTTA CGTTTTTTCT   
  
  
- TTTGTAACTC AGTGAAGCTT GTTGTGGTTA GGTCATTGAA AGAGATGTTT AACACTGTAA ACACTTCACA   
  
  
- CCGATTTGAT TGTGTCAACC ACAACTCAGT GAAGCTTGTT GTGGTTAGGT CATTGAAAGA GATGTTTAAC   
  
  
- ACTGTAAACA CTTCACACCG ATTTGATTGT GTCAACCACT CGTAATCTGT ATTAAAGTTA CAAACCCTTT   
  
  
- CTTTTTCAAG GAGAAAAAAA AGAAAAGAA

+     TC-rich repeats

| Site Name | Organism | Position | Strand | Matrix score. | sequence | function |
| --- | --- | --- | --- | --- | --- | --- |
| TC-rich repeats | Nicotiana tabacum | 45 | + | 9 | ATTTTCTTCA | cis-acting element involved in defense and stress responsiveness |
| TC-rich repeats | Nicotiana tabacum | 1146 | + | 9 | GTTTTCTTAC | cis-acting element involved in defense and stress responsiveness |

> 2018/04/13 10:10:12  
+ CTTAACCCAC CAGCTACTCT TTCTCTTCAA CCCATTGCTT AAATATCTTC TTCATAGACT TACCAACCTA   
  
  
+ CTTCGGTACG TGTACTTCTC TCTCTCTCTC TAATCTCTTC CGTTTCCTCT TGTATATTCC TCTCTACAAC   
  
  
+ AGTATCACGC CTACCACTTC CAGGGAGTCG CGAAGAAGAG TCTTTGATTT TCGCTTCAAA CTGGCACCTT   
  
  
+ TTATCAAGAA CCAGTTTGGA ACACCCAAAC TGACCTGACC GGTATGCCCT CCCCCGCTCC GCCGAGAAAT   
  
  
+ CCGTCAAGAC ACTAAAACCA TTATTTTAGG ATGGTAGTCA AAATCGAACG GTGCACATAA CGGTAAAAAG   
  
  
+ GGAGTCAAAA GGAATACAAT TAGTCTATTA TACATATAAA ATTCTACGTT ACACACTAAA ATTCAAAAAT   
  
  
+ GGGTTATTTA TCTATAAAAT ACCGAATTTT TAATATTAAT AAATTTTAAA ATAATTAGTC AATTGTATTA   
  
  
+ ATAATGTACT ACATTTTAAT TTTTGAATTA ATTATACAAT AATATACTAA AATTTTAAAA TTAAATAATA   
  
  
+ TTAAATAATA ATTTTATATT AAAATAAAGT TTAATATACT TATAGCATAG CAAACCTACG GGTACTACAC   
  
  
+ TATTTATAAT AAATAAAATT TTAAAATTAA TAATATTTGT AATACAAACT ATACAAAAAT AAAATATAGT   
  
  
+ ATATTAAAAT TATATATAAA TCTATTTTAT TATTTACCTT TACTTAATAA ATCATAACAT TTAAAATTCA   
  
  
+ CTACTATTTA GATTTTTTTT AAATTAATTA CATATCTATT CAAAACAAAC ACTACACCTT AGGCCACAGG   
  
  
+ AACTTTATAA ACTGGTAGGT TCTTCACTAT TTACGTTTTA TAATTAACAC CCCTTCTTTA CTTACTTCAC   
  
  
+ CCCATTCCCA GTGCACCTAG TTACTCTGAG ACACAAAGAT TCAGTTTCGT TTTTCTGTTT TTCGAGGTTT   
  
  
+ TGGCAAGTAA GGTTTCGAAA ATTTGCGGCT TTGTGAGGAA ACTACAACAG TTTCGGGGGA AGGTGAGGCT   
  
  
+ GTGAGACTAA GAAGGGTTTG TGGGCTGGGC AACTAAAGCC TTAACTGATT TGCAAGGTTA TTTTTTATTA   
  
  
+ TCTTTTTACA TTATAACACG AGAACGTTTT GTTACTCCAT GGAACCCCAT CACCAATCGT GAGACGTGAA   
  
  
+ ACTTAGGTCG CTGGACCCAA GCTGAGGGCC ACCCTGGAAG CAAAGGAACT TACAATAAAT GCAAAAAAGA   
  
  
+ AAACATTGAG TCACTTCGAA CAACACCAAT CCAGTAACTT TCTCTACAAA TTGTGACATT TGTGAAGTGT   
  
  
+ GGCTAAACTA ACACAGTTGG TGTTGAGTCA CTTCGAACAA CACCAATCCA GTAACTTTCT CTACAAATTG   
  
  
+ TGACATTTGT GAAGTGTGGC TAAACTAACA CAGTTGGTGA GCATTAGACA TAATTTCAAT GTTTGGGAAA   
  
  
+ GAAAAAGTTC CTCTTTTTTT TCTTTTCTT  

- GAATTGGGTG GTCGATGAGA AAGAGAAGTT GGGTAACGAA TTTATAGAAG AAGTATCTGA ATGGTTGGAT   
  
  
- GAAGCCATGC ACATGAAGAG AGAGAGAGAG ATTAGAGAAG GCAAAGGAGA ACATATAAGG AGAGATGTTG   
  
  
- TCATAGTGCG GATGGTGAAG GTCCCTCAGC GCTTCTTCTC AGAAACTAAA AGCGAAGTTT GACCGTGGAA   
  
  
- AATAGTTCTT GGTCAAACCT TGTGGGTTTG ACTGGACTGG CCATACGGGA GGGGGCGAGG CGGCTCTTTA   
  
  
- GGCAGTTCTG TGATTTTGGT AATAAAATCC TACCATCAGT TTTAGCTTGC CACGTGTATT GCCATTTTTC   
  
  
- CCTCAGTTTT CCTTATGTTA ATCAGATAAT ATGTATATTT TAAGATGCAA TGTGTGATTT TAAGTTTTTA   
  
  
- CCCAATAAAT AGATATTTTA TGGCTTAAAA ATTATAATTA TTTAAAATTT TATTAATCAG TTAACATAAT   
  
  
- TATTACATGA TGTAAAATTA AAAACTTAAT TAATATGTTA TTATATGATT TTAAAATTTT AATTTATTAT   
  
  
- AATTTATTAT TAAAATATAA TTTTATTTCA AATTATATGA ATATCGTATC GTTTGGATGC CCATGATGTG   
  
  
- ATAAATATTA TTTATTTTAA AATTTTAATT ATTATAAACA TTATGTTTGA TATGTTTTTA TTTTATATCA   
  
  
- TATAATTTTA ATATATATTT AGATAAAATA ATAAATGGAA ATGAATTATT TAGTATTGTA AATTTTAAGT   
  
  
- GATGATAAAT CTAAAAAAAA TTTAATTAAT GTATAGATAA GTTTTGTTTG TGATGTGGAA TCCGGTGTCC   
  
  
- TTGAAATATT TGACCATCCA AGAAGTGATA AATGCAAAAT ATTAATTGTG GGGAAGAAAT GAATGAAGTG   
  
  
- GGGTAAGGGT CACGTGGATC AATGAGACTC TGTGTTTCTA AGTCAAAGCA AAAAGACAAA AAGCTCCAAA   
  
  
- ACCGTTCATT CCAAAGCTTT TAAACGCCGA AACACTCCTT TGATGTTGTC AAAGCCCCCT TCCACTCCGA   
  
  
- CACTCTGATT CTTCCCAAAC ACCCGACCCG TTGATTTCGG AATTGACTAA ACGTTCCAAT AAAAAATAAT   
  
  
- AGAAAAATGT AATATTGTGC TCTTGCAAAA CAATGAGGTA CCTTGGGGTA GTGGTTAGCA CTCTGCACTT   
  
  
- TGAATCCAGC GACCTGGGTT CGACTCCCGG TGGGACCTTC GTTTCCTTGA ATGTTATTTA CGTTTTTTCT   
  
  
- TTTGTAACTC AGTGAAGCTT GTTGTGGTTA GGTCATTGAA AGAGATGTTT AACACTGTAA ACACTTCACA   
  
  
- CCGATTTGAT TGTGTCAACC ACAACTCAGT GAAGCTTGTT GTGGTTAGGT CATTGAAAGA GATGTTTAAC   
  
  
- ACTGTAAACA CTTCACACCG ATTTGATTGT GTCAACCACT CGTAATCTGT ATTAAAGTTA CAAACCCTTT   
  
  
- CTTTTTCAAG GAGAAAAAAA AGAAAAGAA

+     TCA-element

| Site Name | Organism | Position | Strand | Matrix score. | sequence | function |
| --- | --- | --- | --- | --- | --- | --- |
| TCA-element | Brassica oleracea | 125 | - | 9 | GAGAAGAATA | cis-acting element involved in salicylic acid responsiveness |

> 2018/04/13 10:10:12  
+ CTTAACCCAC CAGCTACTCT TTCTCTTCAA CCCATTGCTT AAATATCTTC TTCATAGACT TACCAACCTA   
  
  
+ CTTCGGTACG TGTACTTCTC TCTCTCTCTC TAATCTCTTC CGTTTCCTCT TGTATATTCC TCTCTACAAC   
  
  
+ AGTATCACGC CTACCACTTC CAGGGAGTCG CGAAGAAGAG TCTTTGATTT TCGCTTCAAA CTGGCACCTT   
  
  
+ TTATCAAGAA CCAGTTTGGA ACACCCAAAC TGACCTGACC GGTATGCCCT CCCCCGCTCC GCCGAGAAAT   
  
  
+ CCGTCAAGAC ACTAAAACCA TTATTTTAGG ATGGTAGTCA AAATCGAACG GTGCACATAA CGGTAAAAAG   
  
  
+ GGAGTCAAAA GGAATACAAT TAGTCTATTA TACATATAAA ATTCTACGTT ACACACTAAA ATTCAAAAAT   
  
  
+ GGGTTATTTA TCTATAAAAT ACCGAATTTT TAATATTAAT AAATTTTAAA ATAATTAGTC AATTGTATTA   
  
  
+ ATAATGTACT ACATTTTAAT TTTTGAATTA ATTATACAAT AATATACTAA AATTTTAAAA TTAAATAATA   
  
  
+ TTAAATAATA ATTTTATATT AAAATAAAGT TTAATATACT TATAGCATAG CAAACCTACG GGTACTACAC   
  
  
+ TATTTATAAT AAATAAAATT TTAAAATTAA TAATATTTGT AATACAAACT ATACAAAAAT AAAATATAGT   
  
  
+ ATATTAAAAT TATATATAAA TCTATTTTAT TATTTACCTT TACTTAATAA ATCATAACAT TTAAAATTCA   
  
  
+ CTACTATTTA GATTTTTTTT AAATTAATTA CATATCTATT CAAAACAAAC ACTACACCTT AGGCCACAGG   
  
  
+ AACTTTATAA ACTGGTAGGT TCTTCACTAT TTACGTTTTA TAATTAACAC CCCTTCTTTA CTTACTTCAC   
  
  
+ CCCATTCCCA GTGCACCTAG TTACTCTGAG ACACAAAGAT TCAGTTTCGT TTTTCTGTTT TTCGAGGTTT   
  
  
+ TGGCAAGTAA GGTTTCGAAA ATTTGCGGCT TTGTGAGGAA ACTACAACAG TTTCGGGGGA AGGTGAGGCT   
  
  
+ GTGAGACTAA GAAGGGTTTG TGGGCTGGGC AACTAAAGCC TTAACTGATT TGCAAGGTTA TTTTTTATTA   
  
  
+ TCTTTTTACA TTATAACACG AGAACGTTTT GTTACTCCAT GGAACCCCAT CACCAATCGT GAGACGTGAA   
  
  
+ ACTTAGGTCG CTGGACCCAA GCTGAGGGCC ACCCTGGAAG CAAAGGAACT TACAATAAAT GCAAAAAAGA   
  
  
+ AAACATTGAG TCACTTCGAA CAACACCAAT CCAGTAACTT TCTCTACAAA TTGTGACATT TGTGAAGTGT   
  
  
+ GGCTAAACTA ACACAGTTGG TGTTGAGTCA CTTCGAACAA CACCAATCCA GTAACTTTCT CTACAAATTG   
  
  
+ TGACATTTGT GAAGTGTGGC TAAACTAACA CAGTTGGTGA GCATTAGACA TAATTTCAAT GTTTGGGAAA   
  
  
+ GAAAAAGTTC CTCTTTTTTT TCTTTTCTT  

- GAATTGGGTG GTCGATGAGA AAGAGAAGTT GGGTAACGAA TTTATAGAAG AAGTATCTGA ATGGTTGGAT   
  
  
- GAAGCCATGC ACATGAAGAG AGAGAGAGAG ATTAGAGAAG GCAAAGGAGA ACATATAAGG AGAGATGTTG   
  
  
- TCATAGTGCG GATGGTGAAG GTCCCTCAGC GCTTCTTCTC AGAAACTAAA AGCGAAGTTT GACCGTGGAA   
  
  
- AATAGTTCTT GGTCAAACCT TGTGGGTTTG ACTGGACTGG CCATACGGGA GGGGGCGAGG CGGCTCTTTA   
  
  
- GGCAGTTCTG TGATTTTGGT AATAAAATCC TACCATCAGT TTTAGCTTGC CACGTGTATT GCCATTTTTC   
  
  
- CCTCAGTTTT CCTTATGTTA ATCAGATAAT ATGTATATTT TAAGATGCAA TGTGTGATTT TAAGTTTTTA   
  
  
- CCCAATAAAT AGATATTTTA TGGCTTAAAA ATTATAATTA TTTAAAATTT TATTAATCAG TTAACATAAT   
  
  
- TATTACATGA TGTAAAATTA AAAACTTAAT TAATATGTTA TTATATGATT TTAAAATTTT AATTTATTAT   
  
  
- AATTTATTAT TAAAATATAA TTTTATTTCA AATTATATGA ATATCGTATC GTTTGGATGC CCATGATGTG   
  
  
- ATAAATATTA TTTATTTTAA AATTTTAATT ATTATAAACA TTATGTTTGA TATGTTTTTA TTTTATATCA   
  
  
- TATAATTTTA ATATATATTT AGATAAAATA ATAAATGGAA ATGAATTATT TAGTATTGTA AATTTTAAGT   
  
  
- GATGATAAAT CTAAAAAAAA TTTAATTAAT GTATAGATAA GTTTTGTTTG TGATGTGGAA TCCGGTGTCC   
  
  
- TTGAAATATT TGACCATCCA AGAAGTGATA AATGCAAAAT ATTAATTGTG GGGAAGAAAT GAATGAAGTG   
  
  
- GGGTAAGGGT CACGTGGATC AATGAGACTC TGTGTTTCTA AGTCAAAGCA AAAAGACAAA AAGCTCCAAA   
  
  
- ACCGTTCATT CCAAAGCTTT TAAACGCCGA AACACTCCTT TGATGTTGTC AAAGCCCCCT TCCACTCCGA   
  
  
- CACTCTGATT CTTCCCAAAC ACCCGACCCG TTGATTTCGG AATTGACTAA ACGTTCCAAT AAAAAATAAT   
  
  
- AGAAAAATGT AATATTGTGC TCTTGCAAAA CAATGAGGTA CCTTGGGGTA GTGGTTAGCA CTCTGCACTT   
  
  
- TGAATCCAGC GACCTGGGTT CGACTCCCGG TGGGACCTTC GTTTCCTTGA ATGTTATTTA CGTTTTTTCT   
  
  
- TTTGTAACTC AGTGAAGCTT GTTGTGGTTA GGTCATTGAA AGAGATGTTT AACACTGTAA ACACTTCACA   
  
  
- CCGATTTGAT TGTGTCAACC ACAACTCAGT GAAGCTTGTT GTGGTTAGGT CATTGAAAGA GATGTTTAAC   
  
  
- ACTGTAAACA CTTCACACCG ATTTGATTGT GTCAACCACT CGTAATCTGT ATTAAAGTTA CAAACCCTTT   
  
  
- CTTTTTCAAG GAGAAAAAAA AGAAAAGAA

+     TGACG-motif

| Site Name | Organism | Position | Strand | Matrix score. | sequence | function |
| --- | --- | --- | --- | --- | --- | --- |
| TGACG-motif | Hordeum vulgare | 282 | - | 5 | TGACG | cis-acting regulatory element involved in the MeJA-responsiveness |

> 2018/04/13 10:10:12  
+ CTTAACCCAC CAGCTACTCT TTCTCTTCAA CCCATTGCTT AAATATCTTC TTCATAGACT TACCAACCTA   
  
  
+ CTTCGGTACG TGTACTTCTC TCTCTCTCTC TAATCTCTTC CGTTTCCTCT TGTATATTCC TCTCTACAAC   
  
  
+ AGTATCACGC CTACCACTTC CAGGGAGTCG CGAAGAAGAG TCTTTGATTT TCGCTTCAAA CTGGCACCTT   
  
  
+ TTATCAAGAA CCAGTTTGGA ACACCCAAAC TGACCTGACC GGTATGCCCT CCCCCGCTCC GCCGAGAAAT   
  
  
+ CCGTCAAGAC ACTAAAACCA TTATTTTAGG ATGGTAGTCA AAATCGAACG GTGCACATAA CGGTAAAAAG   
  
  
+ GGAGTCAAAA GGAATACAAT TAGTCTATTA TACATATAAA ATTCTACGTT ACACACTAAA ATTCAAAAAT   
  
  
+ GGGTTATTTA TCTATAAAAT ACCGAATTTT TAATATTAAT AAATTTTAAA ATAATTAGTC AATTGTATTA   
  
  
+ ATAATGTACT ACATTTTAAT TTTTGAATTA ATTATACAAT AATATACTAA AATTTTAAAA TTAAATAATA   
  
  
+ TTAAATAATA ATTTTATATT AAAATAAAGT TTAATATACT TATAGCATAG CAAACCTACG GGTACTACAC   
  
  
+ TATTTATAAT AAATAAAATT TTAAAATTAA TAATATTTGT AATACAAACT ATACAAAAAT AAAATATAGT   
  
  
+ ATATTAAAAT TATATATAAA TCTATTTTAT TATTTACCTT TACTTAATAA ATCATAACAT TTAAAATTCA   
  
  
+ CTACTATTTA GATTTTTTTT AAATTAATTA CATATCTATT CAAAACAAAC ACTACACCTT AGGCCACAGG   
  
  
+ AACTTTATAA ACTGGTAGGT TCTTCACTAT TTACGTTTTA TAATTAACAC CCCTTCTTTA CTTACTTCAC   
  
  
+ CCCATTCCCA GTGCACCTAG TTACTCTGAG ACACAAAGAT TCAGTTTCGT TTTTCTGTTT TTCGAGGTTT   
  
  
+ TGGCAAGTAA GGTTTCGAAA ATTTGCGGCT TTGTGAGGAA ACTACAACAG TTTCGGGGGA AGGTGAGGCT   
  
  
+ GTGAGACTAA GAAGGGTTTG TGGGCTGGGC AACTAAAGCC TTAACTGATT TGCAAGGTTA TTTTTTATTA   
  
  
+ TCTTTTTACA TTATAACACG AGAACGTTTT GTTACTCCAT GGAACCCCAT CACCAATCGT GAGACGTGAA   
  
  
+ ACTTAGGTCG CTGGACCCAA GCTGAGGGCC ACCCTGGAAG CAAAGGAACT TACAATAAAT GCAAAAAAGA   
  
  
+ AAACATTGAG TCACTTCGAA CAACACCAAT CCAGTAACTT TCTCTACAAA TTGTGACATT TGTGAAGTGT   
  
  
+ GGCTAAACTA ACACAGTTGG TGTTGAGTCA CTTCGAACAA CACCAATCCA GTAACTTTCT CTACAAATTG   
  
  
+ TGACATTTGT GAAGTGTGGC TAAACTAACA CAGTTGGTGA GCATTAGACA TAATTTCAAT GTTTGGGAAA   
  
  
+ GAAAAAGTTC CTCTTTTTTT TCTTTTCTT  

- GAATTGGGTG GTCGATGAGA AAGAGAAGTT GGGTAACGAA TTTATAGAAG AAGTATCTGA ATGGTTGGAT   
  
  
- GAAGCCATGC ACATGAAGAG AGAGAGAGAG ATTAGAGAAG GCAAAGGAGA ACATATAAGG AGAGATGTTG   
  
  
- TCATAGTGCG GATGGTGAAG GTCCCTCAGC GCTTCTTCTC AGAAACTAAA AGCGAAGTTT GACCGTGGAA   
  
  
- AATAGTTCTT GGTCAAACCT TGTGGGTTTG ACTGGACTGG CCATACGGGA GGGGGCGAGG CGGCTCTTTA   
  
  
- GGCAGTTCTG TGATTTTGGT AATAAAATCC TACCATCAGT TTTAGCTTGC CACGTGTATT GCCATTTTTC   
  
  
- CCTCAGTTTT CCTTATGTTA ATCAGATAAT ATGTATATTT TAAGATGCAA TGTGTGATTT TAAGTTTTTA   
  
  
- CCCAATAAAT AGATATTTTA TGGCTTAAAA ATTATAATTA TTTAAAATTT TATTAATCAG TTAACATAAT   
  
  
- TATTACATGA TGTAAAATTA AAAACTTAAT TAATATGTTA TTATATGATT TTAAAATTTT AATTTATTAT   
  
  
- AATTTATTAT TAAAATATAA TTTTATTTCA AATTATATGA ATATCGTATC GTTTGGATGC CCATGATGTG   
  
  
- ATAAATATTA TTTATTTTAA AATTTTAATT ATTATAAACA TTATGTTTGA TATGTTTTTA TTTTATATCA   
  
  
- TATAATTTTA ATATATATTT AGATAAAATA ATAAATGGAA ATGAATTATT TAGTATTGTA AATTTTAAGT   
  
  
- GATGATAAAT CTAAAAAAAA TTTAATTAAT GTATAGATAA GTTTTGTTTG TGATGTGGAA TCCGGTGTCC   
  
  
- TTGAAATATT TGACCATCCA AGAAGTGATA AATGCAAAAT ATTAATTGTG GGGAAGAAAT GAATGAAGTG   
  
  
- GGGTAAGGGT CACGTGGATC AATGAGACTC TGTGTTTCTA AGTCAAAGCA AAAAGACAAA AAGCTCCAAA   
  
  
- ACCGTTCATT CCAAAGCTTT TAAACGCCGA AACACTCCTT TGATGTTGTC AAAGCCCCCT TCCACTCCGA   
  
  
- CACTCTGATT CTTCCCAAAC ACCCGACCCG TTGATTTCGG AATTGACTAA ACGTTCCAAT AAAAAATAAT   
  
  
- AGAAAAATGT AATATTGTGC TCTTGCAAAA CAATGAGGTA CCTTGGGGTA GTGGTTAGCA CTCTGCACTT   
  
  
- TGAATCCAGC GACCTGGGTT CGACTCCCGG TGGGACCTTC GTTTCCTTGA ATGTTATTTA CGTTTTTTCT   
  
  
- TTTGTAACTC AGTGAAGCTT GTTGTGGTTA GGTCATTGAA AGAGATGTTT AACACTGTAA ACACTTCACA   
  
  
- CCGATTTGAT TGTGTCAACC ACAACTCAGT GAAGCTTGTT GTGGTTAGGT CATTGAAAGA GATGTTTAAC   
  
  
- ACTGTAAACA CTTCACACCG ATTTGATTGT GTCAACCACT CGTAATCTGT ATTAAAGTTA CAAACCCTTT   
  
  
- CTTTTTCAAG GAGAAAAAAA AGAAAAGAA

+     Unnamed\_\_4

| Site Name | Organism | Position | Strand | Matrix score. | sequence | function |
| --- | --- | --- | --- | --- | --- | --- |
| Unnamed\_\_4 | Petroselinum hortense | 351 | - | 4 | CTCC |  |
| Unnamed\_\_4 | Petroselinum hortense | 267 | + | 4 | CTCC |  |
| Unnamed\_\_4 | Petroselinum hortense | 259 | + | 4 | CTCC |  |
| Unnamed\_\_4 | Petroselinum hortense | 1155 | + | 4 | CTCC |  |
| Unnamed\_\_4 | Petroselinum hortense | 164 | - | 4 | CTCC |  |

> 2018/04/13 10:10:12  
+ CTTAACCCAC CAGCTACTCT TTCTCTTCAA CCCATTGCTT AAATATCTTC TTCATAGACT TACCAACCTA   
  
  
+ CTTCGGTACG TGTACTTCTC TCTCTCTCTC TAATCTCTTC CGTTTCCTCT TGTATATTCC TCTCTACAAC   
  
  
+ AGTATCACGC CTACCACTTC CAGGGAGTCG CGAAGAAGAG TCTTTGATTT TCGCTTCAAA CTGGCACCTT   
  
  
+ TTATCAAGAA CCAGTTTGGA ACACCCAAAC TGACCTGACC GGTATGCCCT CCCCCGCTCC GCCGAGAAAT   
  
  
+ CCGTCAAGAC ACTAAAACCA TTATTTTAGG ATGGTAGTCA AAATCGAACG GTGCACATAA CGGTAAAAAG   
  
  
+ GGAGTCAAAA GGAATACAAT TAGTCTATTA TACATATAAA ATTCTACGTT ACACACTAAA ATTCAAAAAT   
  
  
+ GGGTTATTTA TCTATAAAAT ACCGAATTTT TAATATTAAT AAATTTTAAA ATAATTAGTC AATTGTATTA   
  
  
+ ATAATGTACT ACATTTTAAT TTTTGAATTA ATTATACAAT AATATACTAA AATTTTAAAA TTAAATAATA   
  
  
+ TTAAATAATA ATTTTATATT AAAATAAAGT TTAATATACT TATAGCATAG CAAACCTACG GGTACTACAC   
  
  
+ TATTTATAAT AAATAAAATT TTAAAATTAA TAATATTTGT AATACAAACT ATACAAAAAT AAAATATAGT   
  
  
+ ATATTAAAAT TATATATAAA TCTATTTTAT TATTTACCTT TACTTAATAA ATCATAACAT TTAAAATTCA   
  
  
+ CTACTATTTA GATTTTTTTT AAATTAATTA CATATCTATT CAAAACAAAC ACTACACCTT AGGCCACAGG   
  
  
+ AACTTTATAA ACTGGTAGGT TCTTCACTAT TTACGTTTTA TAATTAACAC CCCTTCTTTA CTTACTTCAC   
  
  
+ CCCATTCCCA GTGCACCTAG TTACTCTGAG ACACAAAGAT TCAGTTTCGT TTTTCTGTTT TTCGAGGTTT   
  
  
+ TGGCAAGTAA GGTTTCGAAA ATTTGCGGCT TTGTGAGGAA ACTACAACAG TTTCGGGGGA AGGTGAGGCT   
  
  
+ GTGAGACTAA GAAGGGTTTG TGGGCTGGGC AACTAAAGCC TTAACTGATT TGCAAGGTTA TTTTTTATTA   
  
  
+ TCTTTTTACA TTATAACACG AGAACGTTTT GTTACTCCAT GGAACCCCAT CACCAATCGT GAGACGTGAA   
  
  
+ ACTTAGGTCG CTGGACCCAA GCTGAGGGCC ACCCTGGAAG CAAAGGAACT TACAATAAAT GCAAAAAAGA   
  
  
+ AAACATTGAG TCACTTCGAA CAACACCAAT CCAGTAACTT TCTCTACAAA TTGTGACATT TGTGAAGTGT   
  
  
+ GGCTAAACTA ACACAGTTGG TGTTGAGTCA CTTCGAACAA CACCAATCCA GTAACTTTCT CTACAAATTG   
  
  
+ TGACATTTGT GAAGTGTGGC TAAACTAACA CAGTTGGTGA GCATTAGACA TAATTTCAAT GTTTGGGAAA   
  
  
+ GAAAAAGTTC CTCTTTTTTT TCTTTTCTT  

- GAATTGGGTG GTCGATGAGA AAGAGAAGTT GGGTAACGAA TTTATAGAAG AAGTATCTGA ATGGTTGGAT   
  
  
- GAAGCCATGC ACATGAAGAG AGAGAGAGAG ATTAGAGAAG GCAAAGGAGA ACATATAAGG AGAGATGTTG   
  
  
- TCATAGTGCG GATGGTGAAG GTCCCTCAGC GCTTCTTCTC AGAAACTAAA AGCGAAGTTT GACCGTGGAA   
  
  
- AATAGTTCTT GGTCAAACCT TGTGGGTTTG ACTGGACTGG CCATACGGGA GGGGGCGAGG CGGCTCTTTA   
  
  
- GGCAGTTCTG TGATTTTGGT AATAAAATCC TACCATCAGT TTTAGCTTGC CACGTGTATT GCCATTTTTC   
  
  
- CCTCAGTTTT CCTTATGTTA ATCAGATAAT ATGTATATTT TAAGATGCAA TGTGTGATTT TAAGTTTTTA   
  
  
- CCCAATAAAT AGATATTTTA TGGCTTAAAA ATTATAATTA TTTAAAATTT TATTAATCAG TTAACATAAT   
  
  
- TATTACATGA TGTAAAATTA AAAACTTAAT TAATATGTTA TTATATGATT TTAAAATTTT AATTTATTAT   
  
  
- AATTTATTAT TAAAATATAA TTTTATTTCA AATTATATGA ATATCGTATC GTTTGGATGC CCATGATGTG   
  
  
- ATAAATATTA TTTATTTTAA AATTTTAATT ATTATAAACA TTATGTTTGA TATGTTTTTA TTTTATATCA   
  
  
- TATAATTTTA ATATATATTT AGATAAAATA ATAAATGGAA ATGAATTATT TAGTATTGTA AATTTTAAGT   
  
  
- GATGATAAAT CTAAAAAAAA TTTAATTAAT GTATAGATAA GTTTTGTTTG TGATGTGGAA TCCGGTGTCC   
  
  
- TTGAAATATT TGACCATCCA AGAAGTGATA AATGCAAAAT ATTAATTGTG GGGAAGAAAT GAATGAAGTG   
  
  
- GGGTAAGGGT CACGTGGATC AATGAGACTC TGTGTTTCTA AGTCAAAGCA AAAAGACAAA AAGCTCCAAA   
  
  
- ACCGTTCATT CCAAAGCTTT TAAACGCCGA AACACTCCTT TGATGTTGTC AAAGCCCCCT TCCACTCCGA   
  
  
- CACTCTGATT CTTCCCAAAC ACCCGACCCG TTGATTTCGG AATTGACTAA ACGTTCCAAT AAAAAATAAT   
  
  
- AGAAAAATGT AATATTGTGC TCTTGCAAAA CAATGAGGTA CCTTGGGGTA GTGGTTAGCA CTCTGCACTT   
  
  
- TGAATCCAGC GACCTGGGTT CGACTCCCGG TGGGACCTTC GTTTCCTTGA ATGTTATTTA CGTTTTTTCT   
  
  
- TTTGTAACTC AGTGAAGCTT GTTGTGGTTA GGTCATTGAA AGAGATGTTT AACACTGTAA ACACTTCACA   
  
  
- CCGATTTGAT TGTGTCAACC ACAACTCAGT GAAGCTTGTT GTGGTTAGGT CATTGAAAGA GATGTTTAAC   
  
  
- ACTGTAAACA CTTCACACCG ATTTGATTGT GTCAACCACT CGTAATCTGT ATTAAAGTTA CAAACCCTTT   
  
  
- CTTTTTCAAG GAGAAAAAAA AGAAAAGAA

+     chs-CMA1a

| Site Name | Organism | Position | Strand | Matrix score. | sequence | function |
| --- | --- | --- | --- | --- | --- | --- |
| chs-CMA1a | Daucus carota | 740 | + | 8 | TTACTTAA | part of a light responsive element |

> 2018/04/13 10:10:12  
+ CTTAACCCAC CAGCTACTCT TTCTCTTCAA CCCATTGCTT AAATATCTTC TTCATAGACT TACCAACCTA   
  
  
+ CTTCGGTACG TGTACTTCTC TCTCTCTCTC TAATCTCTTC CGTTTCCTCT TGTATATTCC TCTCTACAAC   
  
  
+ AGTATCACGC CTACCACTTC CAGGGAGTCG CGAAGAAGAG TCTTTGATTT TCGCTTCAAA CTGGCACCTT   
  
  
+ TTATCAAGAA CCAGTTTGGA ACACCCAAAC TGACCTGACC GGTATGCCCT CCCCCGCTCC GCCGAGAAAT   
  
  
+ CCGTCAAGAC ACTAAAACCA TTATTTTAGG ATGGTAGTCA AAATCGAACG GTGCACATAA CGGTAAAAAG   
  
  
+ GGAGTCAAAA GGAATACAAT TAGTCTATTA TACATATAAA ATTCTACGTT ACACACTAAA ATTCAAAAAT   
  
  
+ GGGTTATTTA TCTATAAAAT ACCGAATTTT TAATATTAAT AAATTTTAAA ATAATTAGTC AATTGTATTA   
  
  
+ ATAATGTACT ACATTTTAAT TTTTGAATTA ATTATACAAT AATATACTAA AATTTTAAAA TTAAATAATA   
  
  
+ TTAAATAATA ATTTTATATT AAAATAAAGT TTAATATACT TATAGCATAG CAAACCTACG GGTACTACAC   
  
  
+ TATTTATAAT AAATAAAATT TTAAAATTAA TAATATTTGT AATACAAACT ATACAAAAAT AAAATATAGT   
  
  
+ ATATTAAAAT TATATATAAA TCTATTTTAT TATTTACCTT TACTTAATAA ATCATAACAT TTAAAATTCA   
  
  
+ CTACTATTTA GATTTTTTTT AAATTAATTA CATATCTATT CAAAACAAAC ACTACACCTT AGGCCACAGG   
  
  
+ AACTTTATAA ACTGGTAGGT TCTTCACTAT TTACGTTTTA TAATTAACAC CCCTTCTTTA CTTACTTCAC   
  
  
+ CCCATTCCCA GTGCACCTAG TTACTCTGAG ACACAAAGAT TCAGTTTCGT TTTTCTGTTT TTCGAGGTTT   
  
  
+ TGGCAAGTAA GGTTTCGAAA ATTTGCGGCT TTGTGAGGAA ACTACAACAG TTTCGGGGGA AGGTGAGGCT   
  
  
+ GTGAGACTAA GAAGGGTTTG TGGGCTGGGC AACTAAAGCC TTAACTGATT TGCAAGGTTA TTTTTTATTA   
  
  
+ TCTTTTTACA TTATAACACG AGAACGTTTT GTTACTCCAT GGAACCCCAT CACCAATCGT GAGACGTGAA   
  
  
+ ACTTAGGTCG CTGGACCCAA GCTGAGGGCC ACCCTGGAAG CAAAGGAACT TACAATAAAT GCAAAAAAGA   
  
  
+ AAACATTGAG TCACTTCGAA CAACACCAAT CCAGTAACTT TCTCTACAAA TTGTGACATT TGTGAAGTGT   
  
  
+ GGCTAAACTA ACACAGTTGG TGTTGAGTCA CTTCGAACAA CACCAATCCA GTAACTTTCT CTACAAATTG   
  
  
+ TGACATTTGT GAAGTGTGGC TAAACTAACA CAGTTGGTGA GCATTAGACA TAATTTCAAT GTTTGGGAAA   
  
  
+ GAAAAAGTTC CTCTTTTTTT TCTTTTCTT  

- GAATTGGGTG GTCGATGAGA AAGAGAAGTT GGGTAACGAA TTTATAGAAG AAGTATCTGA ATGGTTGGAT   
  
  
- GAAGCCATGC ACATGAAGAG AGAGAGAGAG ATTAGAGAAG GCAAAGGAGA ACATATAAGG AGAGATGTTG   
  
  
- TCATAGTGCG GATGGTGAAG GTCCCTCAGC GCTTCTTCTC AGAAACTAAA AGCGAAGTTT GACCGTGGAA   
  
  
- AATAGTTCTT GGTCAAACCT TGTGGGTTTG ACTGGACTGG CCATACGGGA GGGGGCGAGG CGGCTCTTTA   
  
  
- GGCAGTTCTG TGATTTTGGT AATAAAATCC TACCATCAGT TTTAGCTTGC CACGTGTATT GCCATTTTTC   
  
  
- CCTCAGTTTT CCTTATGTTA ATCAGATAAT ATGTATATTT TAAGATGCAA TGTGTGATTT TAAGTTTTTA   
  
  
- CCCAATAAAT AGATATTTTA TGGCTTAAAA ATTATAATTA TTTAAAATTT TATTAATCAG TTAACATAAT   
  
  
- TATTACATGA TGTAAAATTA AAAACTTAAT TAATATGTTA TTATATGATT TTAAAATTTT AATTTATTAT   
  
  
- AATTTATTAT TAAAATATAA TTTTATTTCA AATTATATGA ATATCGTATC GTTTGGATGC CCATGATGTG   
  
  
- ATAAATATTA TTTATTTTAA AATTTTAATT ATTATAAACA TTATGTTTGA TATGTTTTTA TTTTATATCA   
  
  
- TATAATTTTA ATATATATTT AGATAAAATA ATAAATGGAA ATGAATTATT TAGTATTGTA AATTTTAAGT   
  
  
- GATGATAAAT CTAAAAAAAA TTTAATTAAT GTATAGATAA GTTTTGTTTG TGATGTGGAA TCCGGTGTCC   
  
  
- TTGAAATATT TGACCATCCA AGAAGTGATA AATGCAAAAT ATTAATTGTG GGGAAGAAAT GAATGAAGTG   
  
  
- GGGTAAGGGT CACGTGGATC AATGAGACTC TGTGTTTCTA AGTCAAAGCA AAAAGACAAA AAGCTCCAAA   
  
  
- ACCGTTCATT CCAAAGCTTT TAAACGCCGA AACACTCCTT TGATGTTGTC AAAGCCCCCT TCCACTCCGA   
  
  
- CACTCTGATT CTTCCCAAAC ACCCGACCCG TTGATTTCGG AATTGACTAA ACGTTCCAAT AAAAAATAAT   
  
  
- AGAAAAATGT AATATTGTGC TCTTGCAAAA CAATGAGGTA CCTTGGGGTA GTGGTTAGCA CTCTGCACTT   
  
  
- TGAATCCAGC GACCTGGGTT CGACTCCCGG TGGGACCTTC GTTTCCTTGA ATGTTATTTA CGTTTTTTCT   
  
  
- TTTGTAACTC AGTGAAGCTT GTTGTGGTTA GGTCATTGAA AGAGATGTTT AACACTGTAA ACACTTCACA   
  
  
- CCGATTTGAT TGTGTCAACC ACAACTCAGT GAAGCTTGTT GTGGTTAGGT CATTGAAAGA GATGTTTAAC   
  
  
- ACTGTAAACA CTTCACACCG ATTTGATTGT GTCAACCACT CGTAATCTGT ATTAAAGTTA CAAACCCTTT   
  
  
- CTTTTTCAAG GAGAAAAAAA AGAAAAGAA

+     circadian

| Site Name | Organism | Position | Strand | Matrix score. | sequence | function |
| --- | --- | --- | --- | --- | --- | --- |
| circadian | Lycopersicon esculentum | 137 | + | 6 | CAANNNNATC | cis-acting regulatory element involved in circadian control |

> 2018/04/13 10:10:12  
+ CTTAACCCAC CAGCTACTCT TTCTCTTCAA CCCATTGCTT AAATATCTTC TTCATAGACT TACCAACCTA   
  
  
+ CTTCGGTACG TGTACTTCTC TCTCTCTCTC TAATCTCTTC CGTTTCCTCT TGTATATTCC TCTCTACAAC   
  
  
+ AGTATCACGC CTACCACTTC CAGGGAGTCG CGAAGAAGAG TCTTTGATTT TCGCTTCAAA CTGGCACCTT   
  
  
+ TTATCAAGAA CCAGTTTGGA ACACCCAAAC TGACCTGACC GGTATGCCCT CCCCCGCTCC GCCGAGAAAT   
  
  
+ CCGTCAAGAC ACTAAAACCA TTATTTTAGG ATGGTAGTCA AAATCGAACG GTGCACATAA CGGTAAAAAG   
  
  
+ GGAGTCAAAA GGAATACAAT TAGTCTATTA TACATATAAA ATTCTACGTT ACACACTAAA ATTCAAAAAT   
  
  
+ GGGTTATTTA TCTATAAAAT ACCGAATTTT TAATATTAAT AAATTTTAAA ATAATTAGTC AATTGTATTA   
  
  
+ ATAATGTACT ACATTTTAAT TTTTGAATTA ATTATACAAT AATATACTAA AATTTTAAAA TTAAATAATA   
  
  
+ TTAAATAATA ATTTTATATT AAAATAAAGT TTAATATACT TATAGCATAG CAAACCTACG GGTACTACAC   
  
  
+ TATTTATAAT AAATAAAATT TTAAAATTAA TAATATTTGT AATACAAACT ATACAAAAAT AAAATATAGT   
  
  
+ ATATTAAAAT TATATATAAA TCTATTTTAT TATTTACCTT TACTTAATAA ATCATAACAT TTAAAATTCA   
  
  
+ CTACTATTTA GATTTTTTTT AAATTAATTA CATATCTATT CAAAACAAAC ACTACACCTT AGGCCACAGG   
  
  
+ AACTTTATAA ACTGGTAGGT TCTTCACTAT TTACGTTTTA TAATTAACAC CCCTTCTTTA CTTACTTCAC   
  
  
+ CCCATTCCCA GTGCACCTAG TTACTCTGAG ACACAAAGAT TCAGTTTCGT TTTTCTGTTT TTCGAGGTTT   
  
  
+ TGGCAAGTAA GGTTTCGAAA ATTTGCGGCT TTGTGAGGAA ACTACAACAG TTTCGGGGGA AGGTGAGGCT   
  
  
+ GTGAGACTAA GAAGGGTTTG TGGGCTGGGC AACTAAAGCC TTAACTGATT TGCAAGGTTA TTTTTTATTA   
  
  
+ TCTTTTTACA TTATAACACG AGAACGTTTT GTTACTCCAT GGAACCCCAT CACCAATCGT GAGACGTGAA   
  
  
+ ACTTAGGTCG CTGGACCCAA GCTGAGGGCC ACCCTGGAAG CAAAGGAACT TACAATAAAT GCAAAAAAGA   
  
  
+ AAACATTGAG TCACTTCGAA CAACACCAAT CCAGTAACTT TCTCTACAAA TTGTGACATT TGTGAAGTGT   
  
  
+ GGCTAAACTA ACACAGTTGG TGTTGAGTCA CTTCGAACAA CACCAATCCA GTAACTTTCT CTACAAATTG   
  
  
+ TGACATTTGT GAAGTGTGGC TAAACTAACA CAGTTGGTGA GCATTAGACA TAATTTCAAT GTTTGGGAAA   
  
  
+ GAAAAAGTTC CTCTTTTTTT TCTTTTCTT  

- GAATTGGGTG GTCGATGAGA AAGAGAAGTT GGGTAACGAA TTTATAGAAG AAGTATCTGA ATGGTTGGAT   
  
  
- GAAGCCATGC ACATGAAGAG AGAGAGAGAG ATTAGAGAAG GCAAAGGAGA ACATATAAGG AGAGATGTTG   
  
  
- TCATAGTGCG GATGGTGAAG GTCCCTCAGC GCTTCTTCTC AGAAACTAAA AGCGAAGTTT GACCGTGGAA   
  
  
- AATAGTTCTT GGTCAAACCT TGTGGGTTTG ACTGGACTGG CCATACGGGA GGGGGCGAGG CGGCTCTTTA   
  
  
- GGCAGTTCTG TGATTTTGGT AATAAAATCC TACCATCAGT TTTAGCTTGC CACGTGTATT GCCATTTTTC   
  
  
- CCTCAGTTTT CCTTATGTTA ATCAGATAAT ATGTATATTT TAAGATGCAA TGTGTGATTT TAAGTTTTTA   
  
  
- CCCAATAAAT AGATATTTTA TGGCTTAAAA ATTATAATTA TTTAAAATTT TATTAATCAG TTAACATAAT   
  
  
- TATTACATGA TGTAAAATTA AAAACTTAAT TAATATGTTA TTATATGATT TTAAAATTTT AATTTATTAT   
  
  
- AATTTATTAT TAAAATATAA TTTTATTTCA AATTATATGA ATATCGTATC GTTTGGATGC CCATGATGTG   
  
  
- ATAAATATTA TTTATTTTAA AATTTTAATT ATTATAAACA TTATGTTTGA TATGTTTTTA TTTTATATCA   
  
  
- TATAATTTTA ATATATATTT AGATAAAATA ATAAATGGAA ATGAATTATT TAGTATTGTA AATTTTAAGT   
  
  
- GATGATAAAT CTAAAAAAAA TTTAATTAAT GTATAGATAA GTTTTGTTTG TGATGTGGAA TCCGGTGTCC   
  
  
- TTGAAATATT TGACCATCCA AGAAGTGATA AATGCAAAAT ATTAATTGTG GGGAAGAAAT GAATGAAGTG   
  
  
- GGGTAAGGGT CACGTGGATC AATGAGACTC TGTGTTTCTA AGTCAAAGCA AAAAGACAAA AAGCTCCAAA   
  
  
- ACCGTTCATT CCAAAGCTTT TAAACGCCGA AACACTCCTT TGATGTTGTC AAAGCCCCCT TCCACTCCGA   
  
  
- CACTCTGATT CTTCCCAAAC ACCCGACCCG TTGATTTCGG AATTGACTAA ACGTTCCAAT AAAAAATAAT   
  
  
- AGAAAAATGT AATATTGTGC TCTTGCAAAA CAATGAGGTA CCTTGGGGTA GTGGTTAGCA CTCTGCACTT   
  
  
- TGAATCCAGC GACCTGGGTT CGACTCCCGG TGGGACCTTC GTTTCCTTGA ATGTTATTTA CGTTTTTTCT   
  
  
- TTTGTAACTC AGTGAAGCTT GTTGTGGTTA GGTCATTGAA AGAGATGTTT AACACTGTAA ACACTTCACA   
  
  
- CCGATTTGAT TGTGTCAACC ACAACTCAGT GAAGCTTGTT GTGGTTAGGT CATTGAAAGA GATGTTTAAC   
  
  
- ACTGTAAACA CTTCACACCG ATTTGATTGT GTCAACCACT CGTAATCTGT ATTAAAGTTA CAAACCCTTT   
  
  
- CTTTTTCAAG GAGAAAAAAA AGAAAAGAA
