# Supplementary material for: Genome-wide analysis of the WRKY gene family in drumstick (Moringa oleifera Lam.)
Source: PeerJ. 2019 Jun 10;7:e7063. doi: 10.7717/peerj.7063 (PMC6563795; doi:10.7717/peerj.7063)
Supplement: Supplemental Information 1 [file peerj-07-7063-s003.gz › MoWRKY7_plantcare.html]

Content-Type: text/html; charset=ISO-8859-1


CallMat\_Firefox


Webmaster Firefox specific output  
To save the result:
click on the frame with the right mouse button and save the source code as a text file with extension .html  
REFERENCE:PlantCARE: a database of plant cis-acting regulatory elements and a portal to tools for in silico analysis of promoter sequences.  
Lescot, M., Déhais, P., Moreau, Y., De Moor, B., Rouzé ,P.,and Rombauts, S.  
Nucleic Acids Res., Database issue(2002), 30(1):325-327.   


---

> 2018/04/13 10:10:12  
+ CACACACAGT AAACACTGGT GCATTTATGC ACCTCAAGCA CTGAAAGTTT GCTGGAGAAT GCTAGCCTTA   
  
  
+ TTCAGTGAGA TGCCTTGTTT ATTAAAGAGA AAGAAACCAA GATATAGATG GCTTCTAACC TCTTTTTGTA   
  
  
+ TGAAATGGAT GAAACAAATA GAAATATCGT GCCAAAACAT CCAGGCAGTA TATTTAGATC AGGCAGCATA   
  
  
+ AGAGGAGACA GTACATTTTG CCCGCTTTGG TAGAAAGAAC TAGGATCCTG ACTGTTTTAC CGTCAATTTT   
  
  
+ AGTTGACTGC AAGTTTGTCT TCCCAACCCA TTTAGATTTT CATTTTCTAT TGGACATTCC ATTTCTTCCA   
  
  
+ GAGGTCTAGG AGGTCAAGTT TTTGGAAGAA ATTTATAAGA TTATTGTTGT TTTCTTCCCT GGCTGCAGTT   
  
  
+ ACTTGACTTG TTAGGTGGCA GACAGTTGCT TCGTTCTGTT AGTCCACATC CACATGACAT CAAAACTTAG   
  
  
+ GATTGATTTT AGTTCTTGTT TATGCTCTGA ATTCTCAAAC TGTCTTTAAA AAATTCATTA GTCAGCATTT   
  
  
+ GCTTTTGACT ATTGTTTGCA CTGCTGGCCG CAAGAGGGTT TGTATCTTGA TTATTCGTGT AAAAAGAAAT   
  
  
+ TACAGATGCT CAATTCATAG ATTTGGCCAC ACTGTGGGTT CAAACTAAGA TTGACTGAAG GAAGCCAATA   
  
  
+ AAGATCATCT GTTTTTCCTG CTTAATGGCA CCCATATGAG CAGACAGCCA CTTTTTCTGG ATTGCCCAAT   
  
  
+ TCAATGGAGG CACATTAGTA GTCACAGAGA AGGCAGCCTG TTAAGAAAAT GGTATTTCTA GAAGCCAATT   
  
  
+ AGTACCGTTT CCAGATGATA TGGTTAACAG GTCAATAAAA AAAATTATTT AGGACTCTTT TTGTAGTGTC   
  
  
+ TGCCAACTTA TGGAATGCAG CCCTAATTGA AATATGAGCC CATGTGAGCA TCATGTTATA TCAAAGCACT   
  
  
+ GGCAGGGGCC ACAAAGGACG GTGATTCAAG CTCTGTATAT TAAACATGAA AAAGAGATGA AATGGGTAAG   
  
  
+ AAGTCAGCCT AGCCTTGTGG ATAACAGAGG TAGGCTGGCA GGCATGTCTA AGTTCTGATG TAACAACCAA   
  
  
+ AGAGGCAAAG GAGAAGAAAG CCAAAAAGAA AAAAAAGAAA AAAAGAAAGG TCGAGAAGGC AAGTGACACA   
  
  
+ AACCCAAGAC CCAGATCCAA TGTAACAAGT CAACTTCCTT CTCTTTCTCT CTCTGTCACT GACAATCTTC   
  
  
+ ACTAACACAC ACAAACACGC CCCGCACCGC ATTGGACAAA CACCGAAACC AGAAACACGC TCCCCATCTG   
  
  
+ CGATTCAGTC CCTTCTCTCC TTTAAATTTA CCGTGTTACC CTTCCCTCTC CCCTCTTCAC TGCCCTAATT   
  
  
+ ACAAATCTAC TTCTCTCCTC TTTTGACTCT GTTCTCTCTT CTCTCTCATA CTCGCGGTAA GCACGCCAGA   
  
  
+ CAACGCGCAC GCACACTTTC TCTTGTCTG  

- GTGTGTGTCA TTTGTGACCA CGTAAATACG TGGAGTTCGT GACTTTCAAA CGACCTCTTA CGATCGGAAT   
  
  
- AAGTCACTCT ACGGAACAAA TAATTTCTCT TTCTTTGGTT CTATATCTAC CGAAGATTGG AGAAAAACAT   
  
  
- ACTTTACCTA CTTTGTTTAT CTTTATAGCA CGGTTTTGTA GGTCCGTCAT ATAAATCTAG TCCGTCGTAT   
  
  
- TCTCCTCTGT CATGTAAAAC GGGCGAAACC ATCTTTCTTG ATCCTAGGAC TGACAAAATG GCAGTTAAAA   
  
  
- TCAACTGACG TTCAAACAGA AGGGTTGGGT AAATCTAAAA GTAAAAGATA ACCTGTAAGG TAAAGAAGGT   
  
  
- CTCCAGATCC TCCAGTTCAA AAACCTTCTT TAAATATTCT AATAACAACA AAAGAAGGGA CCGACGTCAA   
  
  
- TGAACTGAAC AATCCACCGT CTGTCAACGA AGCAAGACAA TCAGGTGTAG GTGTACTGTA GTTTTGAATC   
  
  
- CTAACTAAAA TCAAGAACAA ATACGAGACT TAAGAGTTTG ACAGAAATTT TTTAAGTAAT CAGTCGTAAA   
  
  
- CGAAAACTGA TAACAAACGT GACGACCGGC GTTCTCCCAA ACATAGAACT AATAAGCACA TTTTTCTTTA   
  
  
- ATGTCTACGA GTTAAGTATC TAAACCGGTG TGACACCCAA GTTTGATTCT AACTGACTTC CTTCGGTTAT   
  
  
- TTCTAGTAGA CAAAAAGGAC GAATTACCGT GGGTATACTC GTCTGTCGGT GAAAAAGACC TAACGGGTTA   
  
  
- AGTTACCTCC GTGTAATCAT CAGTGTCTCT TCCGTCGGAC AATTCTTTTA CCATAAAGAT CTTCGGTTAA   
  
  
- TCATGGCAAA GGTCTACTAT ACCAATTGTC CAGTTATTTT TTTTAATAAA TCCTGAGAAA AACATCACAG   
  
  
- ACGGTTGAAT ACCTTACGTC GGGATTAACT TTATACTCGG GTACACTCGT AGTACAATAT AGTTTCGTGA   
  
  
- CCGTCCCCGG TGTTTCCTGC CACTAAGTTC GAGACATATA ATTTGTACTT TTTCTCTACT TTACCCATTC   
  
  
- TTCAGTCGGA TCGGAACACC TATTGTCTCC ATCCGACCGT CCGTACAGAT TCAAGACTAC ATTGTTGGTT   
  
  
- TCTCCGTTTC CTCTTCTTTC GGTTTTTCTT TTTTTTCTTT TTTTCTTTCC AGCTCTTCCG TTCACTGTGT   
  
  
- TTGGGTTCTG GGTCTAGGTT ACATTGTTCA GTTGAAGGAA GAGAAAGAGA GAGACAGTGA CTGTTAGAAG   
  
  
- TGATTGTGTG TGTTTGTGCG GGGCGTGGCG TAACCTGTTT GTGGCTTTGG TCTTTGTGCG AGGGGTAGAC   
  
  
- GCTAAGTCAG GGAAGAGAGG AAATTTAAAT GGCACAATGG GAAGGGAGAG GGGAGAAGTG ACGGGATTAA   
  
  
- TGTTTAGATG AAGAGAGGAG AAAACTGAGA CAAGAGAGAA GAGAGAGTAT GAGCGCCATT CGTGCGGTCT   
  
  
- GTTGCGCGTG CGTGTGAAAG AGAACAGAC

  
  
Motifs Found  

+     5UTR Py-rich stretch

| Site Name | Organism | Position | Strand | Matrix score. | sequence | function |
| --- | --- | --- | --- | --- | --- | --- |
| 5UTR Py-rich stretch | Lycopersicon esculentum | 1435 | + | 9 | TTTCTTCTCT | cis-acting element conferring high transcription levels |
| 5UTR Py-rich stretch | Lycopersicon esculentum | 1234 | + | 13 | TTTCTCTCTCTCTC | cis-acting element conferring high transcription levels |
| 5UTR Py-rich stretch | Lycopersicon esculentum | 1225 | + | 9 | TTTCTTCTCT | cis-acting element conferring high transcription levels |
| 5UTR Py-rich stretch | Lycopersicon esculentum | 401 | + | 9 | TTTCTTCTCT | cis-acting element conferring high transcription levels |
| 5UTR Py-rich stretch | Lycopersicon esculentum | 1130 | - | 9 | TTTCTTCTCT | cis-acting element conferring high transcription levels |
| 5UTR Py-rich stretch | Lycopersicon esculentum | 1033 | - | 9 | TTTCTTCTCT | cis-acting element conferring high transcription levels |

> 2018/04/13 10:10:12  
+ CACACACAGT AAACACTGGT GCATTTATGC ACCTCAAGCA CTGAAAGTTT GCTGGAGAAT GCTAGCCTTA   
  
  
+ TTCAGTGAGA TGCCTTGTTT ATTAAAGAGA AAGAAACCAA GATATAGATG GCTTCTAACC TCTTTTTGTA   
  
  
+ TGAAATGGAT GAAACAAATA GAAATATCGT GCCAAAACAT CCAGGCAGTA TATTTAGATC AGGCAGCATA   
  
  
+ AGAGGAGACA GTACATTTTG CCCGCTTTGG TAGAAAGAAC TAGGATCCTG ACTGTTTTAC CGTCAATTTT   
  
  
+ AGTTGACTGC AAGTTTGTCT TCCCAACCCA TTTAGATTTT CATTTTCTAT TGGACATTCC ATTTCTTCCA   
  
  
+ GAGGTCTAGG AGGTCAAGTT TTTGGAAGAA ATTTATAAGA TTATTGTTGT TTTCTTCCCT GGCTGCAGTT   
  
  
+ ACTTGACTTG TTAGGTGGCA GACAGTTGCT TCGTTCTGTT AGTCCACATC CACATGACAT CAAAACTTAG   
  
  
+ GATTGATTTT AGTTCTTGTT TATGCTCTGA ATTCTCAAAC TGTCTTTAAA AAATTCATTA GTCAGCATTT   
  
  
+ GCTTTTGACT ATTGTTTGCA CTGCTGGCCG CAAGAGGGTT TGTATCTTGA TTATTCGTGT AAAAAGAAAT   
  
  
+ TACAGATGCT CAATTCATAG ATTTGGCCAC ACTGTGGGTT CAAACTAAGA TTGACTGAAG GAAGCCAATA   
  
  
+ AAGATCATCT GTTTTTCCTG CTTAATGGCA CCCATATGAG CAGACAGCCA CTTTTTCTGG ATTGCCCAAT   
  
  
+ TCAATGGAGG CACATTAGTA GTCACAGAGA AGGCAGCCTG TTAAGAAAAT GGTATTTCTA GAAGCCAATT   
  
  
+ AGTACCGTTT CCAGATGATA TGGTTAACAG GTCAATAAAA AAAATTATTT AGGACTCTTT TTGTAGTGTC   
  
  
+ TGCCAACTTA TGGAATGCAG CCCTAATTGA AATATGAGCC CATGTGAGCA TCATGTTATA TCAAAGCACT   
  
  
+ GGCAGGGGCC ACAAAGGACG GTGATTCAAG CTCTGTATAT TAAACATGAA AAAGAGATGA AATGGGTAAG   
  
  
+ AAGTCAGCCT AGCCTTGTGG ATAACAGAGG TAGGCTGGCA GGCATGTCTA AGTTCTGATG TAACAACCAA   
  
  
+ AGAGGCAAAG GAGAAGAAAG CCAAAAAGAA AAAAAAGAAA AAAAGAAAGG TCGAGAAGGC AAGTGACACA   
  
  
+ AACCCAAGAC CCAGATCCAA TGTAACAAGT CAACTTCCTT CTCTTTCTCT CTCTGTCACT GACAATCTTC   
  
  
+ ACTAACACAC ACAAACACGC CCCGCACCGC ATTGGACAAA CACCGAAACC AGAAACACGC TCCCCATCTG   
  
  
+ CGATTCAGTC CCTTCTCTCC TTTAAATTTA CCGTGTTACC CTTCCCTCTC CCCTCTTCAC TGCCCTAATT   
  
  
+ ACAAATCTAC TTCTCTCCTC TTTTGACTCT GTTCTCTCTT CTCTCTCATA CTCGCGGTAA GCACGCCAGA   
  
  
+ CAACGCGCAC GCACACTTTC TCTTGTCTG  

- GTGTGTGTCA TTTGTGACCA CGTAAATACG TGGAGTTCGT GACTTTCAAA CGACCTCTTA CGATCGGAAT   
  
  
- AAGTCACTCT ACGGAACAAA TAATTTCTCT TTCTTTGGTT CTATATCTAC CGAAGATTGG AGAAAAACAT   
  
  
- ACTTTACCTA CTTTGTTTAT CTTTATAGCA CGGTTTTGTA GGTCCGTCAT ATAAATCTAG TCCGTCGTAT   
  
  
- TCTCCTCTGT CATGTAAAAC GGGCGAAACC ATCTTTCTTG ATCCTAGGAC TGACAAAATG GCAGTTAAAA   
  
  
- TCAACTGACG TTCAAACAGA AGGGTTGGGT AAATCTAAAA GTAAAAGATA ACCTGTAAGG TAAAGAAGGT   
  
  
- CTCCAGATCC TCCAGTTCAA AAACCTTCTT TAAATATTCT AATAACAACA AAAGAAGGGA CCGACGTCAA   
  
  
- TGAACTGAAC AATCCACCGT CTGTCAACGA AGCAAGACAA TCAGGTGTAG GTGTACTGTA GTTTTGAATC   
  
  
- CTAACTAAAA TCAAGAACAA ATACGAGACT TAAGAGTTTG ACAGAAATTT TTTAAGTAAT CAGTCGTAAA   
  
  
- CGAAAACTGA TAACAAACGT GACGACCGGC GTTCTCCCAA ACATAGAACT AATAAGCACA TTTTTCTTTA   
  
  
- ATGTCTACGA GTTAAGTATC TAAACCGGTG TGACACCCAA GTTTGATTCT AACTGACTTC CTTCGGTTAT   
  
  
- TTCTAGTAGA CAAAAAGGAC GAATTACCGT GGGTATACTC GTCTGTCGGT GAAAAAGACC TAACGGGTTA   
  
  
- AGTTACCTCC GTGTAATCAT CAGTGTCTCT TCCGTCGGAC AATTCTTTTA CCATAAAGAT CTTCGGTTAA   
  
  
- TCATGGCAAA GGTCTACTAT ACCAATTGTC CAGTTATTTT TTTTAATAAA TCCTGAGAAA AACATCACAG   
  
  
- ACGGTTGAAT ACCTTACGTC GGGATTAACT TTATACTCGG GTACACTCGT AGTACAATAT AGTTTCGTGA   
  
  
- CCGTCCCCGG TGTTTCCTGC CACTAAGTTC GAGACATATA ATTTGTACTT TTTCTCTACT TTACCCATTC   
  
  
- TTCAGTCGGA TCGGAACACC TATTGTCTCC ATCCGACCGT CCGTACAGAT TCAAGACTAC ATTGTTGGTT   
  
  
- TCTCCGTTTC CTCTTCTTTC GGTTTTTCTT TTTTTTCTTT TTTTCTTTCC AGCTCTTCCG TTCACTGTGT   
  
  
- TTGGGTTCTG GGTCTAGGTT ACATTGTTCA GTTGAAGGAA GAGAAAGAGA GAGACAGTGA CTGTTAGAAG   
  
  
- TGATTGTGTG TGTTTGTGCG GGGCGTGGCG TAACCTGTTT GTGGCTTTGG TCTTTGTGCG AGGGGTAGAC   
  
  
- GCTAAGTCAG GGAAGAGAGG AAATTTAAAT GGCACAATGG GAAGGGAGAG GGGAGAAGTG ACGGGATTAA   
  
  
- TGTTTAGATG AAGAGAGGAG AAAACTGAGA CAAGAGAGAA GAGAGAGTAT GAGCGCCATT CGTGCGGTCT   
  
  
- GTTGCGCGTG CGTGTGAAAG AGAACAGAC

+     A-box

| Site Name | Organism | Position | Strand | Matrix score. | sequence | function |
| --- | --- | --- | --- | --- | --- | --- |
| A-box | Petroselinum crispum | 996 | - | 6 | CCGTCC | cis-acting regulatory element |

> 2018/04/13 10:10:12  
+ CACACACAGT AAACACTGGT GCATTTATGC ACCTCAAGCA CTGAAAGTTT GCTGGAGAAT GCTAGCCTTA   
  
  
+ TTCAGTGAGA TGCCTTGTTT ATTAAAGAGA AAGAAACCAA GATATAGATG GCTTCTAACC TCTTTTTGTA   
  
  
+ TGAAATGGAT GAAACAAATA GAAATATCGT GCCAAAACAT CCAGGCAGTA TATTTAGATC AGGCAGCATA   
  
  
+ AGAGGAGACA GTACATTTTG CCCGCTTTGG TAGAAAGAAC TAGGATCCTG ACTGTTTTAC CGTCAATTTT   
  
  
+ AGTTGACTGC AAGTTTGTCT TCCCAACCCA TTTAGATTTT CATTTTCTAT TGGACATTCC ATTTCTTCCA   
  
  
+ GAGGTCTAGG AGGTCAAGTT TTTGGAAGAA ATTTATAAGA TTATTGTTGT TTTCTTCCCT GGCTGCAGTT   
  
  
+ ACTTGACTTG TTAGGTGGCA GACAGTTGCT TCGTTCTGTT AGTCCACATC CACATGACAT CAAAACTTAG   
  
  
+ GATTGATTTT AGTTCTTGTT TATGCTCTGA ATTCTCAAAC TGTCTTTAAA AAATTCATTA GTCAGCATTT   
  
  
+ GCTTTTGACT ATTGTTTGCA CTGCTGGCCG CAAGAGGGTT TGTATCTTGA TTATTCGTGT AAAAAGAAAT   
  
  
+ TACAGATGCT CAATTCATAG ATTTGGCCAC ACTGTGGGTT CAAACTAAGA TTGACTGAAG GAAGCCAATA   
  
  
+ AAGATCATCT GTTTTTCCTG CTTAATGGCA CCCATATGAG CAGACAGCCA CTTTTTCTGG ATTGCCCAAT   
  
  
+ TCAATGGAGG CACATTAGTA GTCACAGAGA AGGCAGCCTG TTAAGAAAAT GGTATTTCTA GAAGCCAATT   
  
  
+ AGTACCGTTT CCAGATGATA TGGTTAACAG GTCAATAAAA AAAATTATTT AGGACTCTTT TTGTAGTGTC   
  
  
+ TGCCAACTTA TGGAATGCAG CCCTAATTGA AATATGAGCC CATGTGAGCA TCATGTTATA TCAAAGCACT   
  
  
+ GGCAGGGGCC ACAAAGGACG GTGATTCAAG CTCTGTATAT TAAACATGAA AAAGAGATGA AATGGGTAAG   
  
  
+ AAGTCAGCCT AGCCTTGTGG ATAACAGAGG TAGGCTGGCA GGCATGTCTA AGTTCTGATG TAACAACCAA   
  
  
+ AGAGGCAAAG GAGAAGAAAG CCAAAAAGAA AAAAAAGAAA AAAAGAAAGG TCGAGAAGGC AAGTGACACA   
  
  
+ AACCCAAGAC CCAGATCCAA TGTAACAAGT CAACTTCCTT CTCTTTCTCT CTCTGTCACT GACAATCTTC   
  
  
+ ACTAACACAC ACAAACACGC CCCGCACCGC ATTGGACAAA CACCGAAACC AGAAACACGC TCCCCATCTG   
  
  
+ CGATTCAGTC CCTTCTCTCC TTTAAATTTA CCGTGTTACC CTTCCCTCTC CCCTCTTCAC TGCCCTAATT   
  
  
+ ACAAATCTAC TTCTCTCCTC TTTTGACTCT GTTCTCTCTT CTCTCTCATA CTCGCGGTAA GCACGCCAGA   
  
  
+ CAACGCGCAC GCACACTTTC TCTTGTCTG  

- GTGTGTGTCA TTTGTGACCA CGTAAATACG TGGAGTTCGT GACTTTCAAA CGACCTCTTA CGATCGGAAT   
  
  
- AAGTCACTCT ACGGAACAAA TAATTTCTCT TTCTTTGGTT CTATATCTAC CGAAGATTGG AGAAAAACAT   
  
  
- ACTTTACCTA CTTTGTTTAT CTTTATAGCA CGGTTTTGTA GGTCCGTCAT ATAAATCTAG TCCGTCGTAT   
  
  
- TCTCCTCTGT CATGTAAAAC GGGCGAAACC ATCTTTCTTG ATCCTAGGAC TGACAAAATG GCAGTTAAAA   
  
  
- TCAACTGACG TTCAAACAGA AGGGTTGGGT AAATCTAAAA GTAAAAGATA ACCTGTAAGG TAAAGAAGGT   
  
  
- CTCCAGATCC TCCAGTTCAA AAACCTTCTT TAAATATTCT AATAACAACA AAAGAAGGGA CCGACGTCAA   
  
  
- TGAACTGAAC AATCCACCGT CTGTCAACGA AGCAAGACAA TCAGGTGTAG GTGTACTGTA GTTTTGAATC   
  
  
- CTAACTAAAA TCAAGAACAA ATACGAGACT TAAGAGTTTG ACAGAAATTT TTTAAGTAAT CAGTCGTAAA   
  
  
- CGAAAACTGA TAACAAACGT GACGACCGGC GTTCTCCCAA ACATAGAACT AATAAGCACA TTTTTCTTTA   
  
  
- ATGTCTACGA GTTAAGTATC TAAACCGGTG TGACACCCAA GTTTGATTCT AACTGACTTC CTTCGGTTAT   
  
  
- TTCTAGTAGA CAAAAAGGAC GAATTACCGT GGGTATACTC GTCTGTCGGT GAAAAAGACC TAACGGGTTA   
  
  
- AGTTACCTCC GTGTAATCAT CAGTGTCTCT TCCGTCGGAC AATTCTTTTA CCATAAAGAT CTTCGGTTAA   
  
  
- TCATGGCAAA GGTCTACTAT ACCAATTGTC CAGTTATTTT TTTTAATAAA TCCTGAGAAA AACATCACAG   
  
  
- ACGGTTGAAT ACCTTACGTC GGGATTAACT TTATACTCGG GTACACTCGT AGTACAATAT AGTTTCGTGA   
  
  
- CCGTCCCCGG TGTTTCCTGC CACTAAGTTC GAGACATATA ATTTGTACTT TTTCTCTACT TTACCCATTC   
  
  
- TTCAGTCGGA TCGGAACACC TATTGTCTCC ATCCGACCGT CCGTACAGAT TCAAGACTAC ATTGTTGGTT   
  
  
- TCTCCGTTTC CTCTTCTTTC GGTTTTTCTT TTTTTTCTTT TTTTCTTTCC AGCTCTTCCG TTCACTGTGT   
  
  
- TTGGGTTCTG GGTCTAGGTT ACATTGTTCA GTTGAAGGAA GAGAAAGAGA GAGACAGTGA CTGTTAGAAG   
  
  
- TGATTGTGTG TGTTTGTGCG GGGCGTGGCG TAACCTGTTT GTGGCTTTGG TCTTTGTGCG AGGGGTAGAC   
  
  
- GCTAAGTCAG GGAAGAGAGG AAATTTAAAT GGCACAATGG GAAGGGAGAG GGGAGAAGTG ACGGGATTAA   
  
  
- TGTTTAGATG AAGAGAGGAG AAAACTGAGA CAAGAGAGAA GAGAGAGTAT GAGCGCCATT CGTGCGGTCT   
  
  
- GTTGCGCGTG CGTGTGAAAG AGAACAGAC

+     AAGAA-motif

| Site Name | Organism | Position | Strand | Matrix score. | sequence | function |
| --- | --- | --- | --- | --- | --- | --- |
| AAGAA-motif | Avena sativa | 1045 | + | 8 | gGTAAGAA |  |
| AAGAA-motif | Avena sativa | 243 | + | 7 | GAAAGAA |  |
| AAGAA-motif | Avena sativa | 99 | + | 7 | GAAAGAA |  |

> 2018/04/13 10:10:12  
+ CACACACAGT AAACACTGGT GCATTTATGC ACCTCAAGCA CTGAAAGTTT GCTGGAGAAT GCTAGCCTTA   
  
  
+ TTCAGTGAGA TGCCTTGTTT ATTAAAGAGA AAGAAACCAA GATATAGATG GCTTCTAACC TCTTTTTGTA   
  
  
+ TGAAATGGAT GAAACAAATA GAAATATCGT GCCAAAACAT CCAGGCAGTA TATTTAGATC AGGCAGCATA   
  
  
+ AGAGGAGACA GTACATTTTG CCCGCTTTGG TAGAAAGAAC TAGGATCCTG ACTGTTTTAC CGTCAATTTT   
  
  
+ AGTTGACTGC AAGTTTGTCT TCCCAACCCA TTTAGATTTT CATTTTCTAT TGGACATTCC ATTTCTTCCA   
  
  
+ GAGGTCTAGG AGGTCAAGTT TTTGGAAGAA ATTTATAAGA TTATTGTTGT TTTCTTCCCT GGCTGCAGTT   
  
  
+ ACTTGACTTG TTAGGTGGCA GACAGTTGCT TCGTTCTGTT AGTCCACATC CACATGACAT CAAAACTTAG   
  
  
+ GATTGATTTT AGTTCTTGTT TATGCTCTGA ATTCTCAAAC TGTCTTTAAA AAATTCATTA GTCAGCATTT   
  
  
+ GCTTTTGACT ATTGTTTGCA CTGCTGGCCG CAAGAGGGTT TGTATCTTGA TTATTCGTGT AAAAAGAAAT   
  
  
+ TACAGATGCT CAATTCATAG ATTTGGCCAC ACTGTGGGTT CAAACTAAGA TTGACTGAAG GAAGCCAATA   
  
  
+ AAGATCATCT GTTTTTCCTG CTTAATGGCA CCCATATGAG CAGACAGCCA CTTTTTCTGG ATTGCCCAAT   
  
  
+ TCAATGGAGG CACATTAGTA GTCACAGAGA AGGCAGCCTG TTAAGAAAAT GGTATTTCTA GAAGCCAATT   
  
  
+ AGTACCGTTT CCAGATGATA TGGTTAACAG GTCAATAAAA AAAATTATTT AGGACTCTTT TTGTAGTGTC   
  
  
+ TGCCAACTTA TGGAATGCAG CCCTAATTGA AATATGAGCC CATGTGAGCA TCATGTTATA TCAAAGCACT   
  
  
+ GGCAGGGGCC ACAAAGGACG GTGATTCAAG CTCTGTATAT TAAACATGAA AAAGAGATGA AATGGGTAAG   
  
  
+ AAGTCAGCCT AGCCTTGTGG ATAACAGAGG TAGGCTGGCA GGCATGTCTA AGTTCTGATG TAACAACCAA   
  
  
+ AGAGGCAAAG GAGAAGAAAG CCAAAAAGAA AAAAAAGAAA AAAAGAAAGG TCGAGAAGGC AAGTGACACA   
  
  
+ AACCCAAGAC CCAGATCCAA TGTAACAAGT CAACTTCCTT CTCTTTCTCT CTCTGTCACT GACAATCTTC   
  
  
+ ACTAACACAC ACAAACACGC CCCGCACCGC ATTGGACAAA CACCGAAACC AGAAACACGC TCCCCATCTG   
  
  
+ CGATTCAGTC CCTTCTCTCC TTTAAATTTA CCGTGTTACC CTTCCCTCTC CCCTCTTCAC TGCCCTAATT   
  
  
+ ACAAATCTAC TTCTCTCCTC TTTTGACTCT GTTCTCTCTT CTCTCTCATA CTCGCGGTAA GCACGCCAGA   
  
  
+ CAACGCGCAC GCACACTTTC TCTTGTCTG  

- GTGTGTGTCA TTTGTGACCA CGTAAATACG TGGAGTTCGT GACTTTCAAA CGACCTCTTA CGATCGGAAT   
  
  
- AAGTCACTCT ACGGAACAAA TAATTTCTCT TTCTTTGGTT CTATATCTAC CGAAGATTGG AGAAAAACAT   
  
  
- ACTTTACCTA CTTTGTTTAT CTTTATAGCA CGGTTTTGTA GGTCCGTCAT ATAAATCTAG TCCGTCGTAT   
  
  
- TCTCCTCTGT CATGTAAAAC GGGCGAAACC ATCTTTCTTG ATCCTAGGAC TGACAAAATG GCAGTTAAAA   
  
  
- TCAACTGACG TTCAAACAGA AGGGTTGGGT AAATCTAAAA GTAAAAGATA ACCTGTAAGG TAAAGAAGGT   
  
  
- CTCCAGATCC TCCAGTTCAA AAACCTTCTT TAAATATTCT AATAACAACA AAAGAAGGGA CCGACGTCAA   
  
  
- TGAACTGAAC AATCCACCGT CTGTCAACGA AGCAAGACAA TCAGGTGTAG GTGTACTGTA GTTTTGAATC   
  
  
- CTAACTAAAA TCAAGAACAA ATACGAGACT TAAGAGTTTG ACAGAAATTT TTTAAGTAAT CAGTCGTAAA   
  
  
- CGAAAACTGA TAACAAACGT GACGACCGGC GTTCTCCCAA ACATAGAACT AATAAGCACA TTTTTCTTTA   
  
  
- ATGTCTACGA GTTAAGTATC TAAACCGGTG TGACACCCAA GTTTGATTCT AACTGACTTC CTTCGGTTAT   
  
  
- TTCTAGTAGA CAAAAAGGAC GAATTACCGT GGGTATACTC GTCTGTCGGT GAAAAAGACC TAACGGGTTA   
  
  
- AGTTACCTCC GTGTAATCAT CAGTGTCTCT TCCGTCGGAC AATTCTTTTA CCATAAAGAT CTTCGGTTAA   
  
  
- TCATGGCAAA GGTCTACTAT ACCAATTGTC CAGTTATTTT TTTTAATAAA TCCTGAGAAA AACATCACAG   
  
  
- ACGGTTGAAT ACCTTACGTC GGGATTAACT TTATACTCGG GTACACTCGT AGTACAATAT AGTTTCGTGA   
  
  
- CCGTCCCCGG TGTTTCCTGC CACTAAGTTC GAGACATATA ATTTGTACTT TTTCTCTACT TTACCCATTC   
  
  
- TTCAGTCGGA TCGGAACACC TATTGTCTCC ATCCGACCGT CCGTACAGAT TCAAGACTAC ATTGTTGGTT   
  
  
- TCTCCGTTTC CTCTTCTTTC GGTTTTTCTT TTTTTTCTTT TTTTCTTTCC AGCTCTTCCG TTCACTGTGT   
  
  
- TTGGGTTCTG GGTCTAGGTT ACATTGTTCA GTTGAAGGAA GAGAAAGAGA GAGACAGTGA CTGTTAGAAG   
  
  
- TGATTGTGTG TGTTTGTGCG GGGCGTGGCG TAACCTGTTT GTGGCTTTGG TCTTTGTGCG AGGGGTAGAC   
  
  
- GCTAAGTCAG GGAAGAGAGG AAATTTAAAT GGCACAATGG GAAGGGAGAG GGGAGAAGTG ACGGGATTAA   
  
  
- TGTTTAGATG AAGAGAGGAG AAAACTGAGA CAAGAGAGAA GAGAGAGTAT GAGCGCCATT CGTGCGGTCT   
  
  
- GTTGCGCGTG CGTGTGAAAG AGAACAGAC

+     ARE

| Site Name | Organism | Position | Strand | Matrix score. | sequence | function |
| --- | --- | --- | --- | --- | --- | --- |
| ARE | Zea mays | 1306 | - | 6 | TGGTTT | cis-acting regulatory element essential for the anaerobic induction |
| ARE | Zea mays | 104 | - | 6 | TGGTTT | cis-acting regulatory element essential for the anaerobic induction |

> 2018/04/13 10:10:12  
+ CACACACAGT AAACACTGGT GCATTTATGC ACCTCAAGCA CTGAAAGTTT GCTGGAGAAT GCTAGCCTTA   
  
  
+ TTCAGTGAGA TGCCTTGTTT ATTAAAGAGA AAGAAACCAA GATATAGATG GCTTCTAACC TCTTTTTGTA   
  
  
+ TGAAATGGAT GAAACAAATA GAAATATCGT GCCAAAACAT CCAGGCAGTA TATTTAGATC AGGCAGCATA   
  
  
+ AGAGGAGACA GTACATTTTG CCCGCTTTGG TAGAAAGAAC TAGGATCCTG ACTGTTTTAC CGTCAATTTT   
  
  
+ AGTTGACTGC AAGTTTGTCT TCCCAACCCA TTTAGATTTT CATTTTCTAT TGGACATTCC ATTTCTTCCA   
  
  
+ GAGGTCTAGG AGGTCAAGTT TTTGGAAGAA ATTTATAAGA TTATTGTTGT TTTCTTCCCT GGCTGCAGTT   
  
  
+ ACTTGACTTG TTAGGTGGCA GACAGTTGCT TCGTTCTGTT AGTCCACATC CACATGACAT CAAAACTTAG   
  
  
+ GATTGATTTT AGTTCTTGTT TATGCTCTGA ATTCTCAAAC TGTCTTTAAA AAATTCATTA GTCAGCATTT   
  
  
+ GCTTTTGACT ATTGTTTGCA CTGCTGGCCG CAAGAGGGTT TGTATCTTGA TTATTCGTGT AAAAAGAAAT   
  
  
+ TACAGATGCT CAATTCATAG ATTTGGCCAC ACTGTGGGTT CAAACTAAGA TTGACTGAAG GAAGCCAATA   
  
  
+ AAGATCATCT GTTTTTCCTG CTTAATGGCA CCCATATGAG CAGACAGCCA CTTTTTCTGG ATTGCCCAAT   
  
  
+ TCAATGGAGG CACATTAGTA GTCACAGAGA AGGCAGCCTG TTAAGAAAAT GGTATTTCTA GAAGCCAATT   
  
  
+ AGTACCGTTT CCAGATGATA TGGTTAACAG GTCAATAAAA AAAATTATTT AGGACTCTTT TTGTAGTGTC   
  
  
+ TGCCAACTTA TGGAATGCAG CCCTAATTGA AATATGAGCC CATGTGAGCA TCATGTTATA TCAAAGCACT   
  
  
+ GGCAGGGGCC ACAAAGGACG GTGATTCAAG CTCTGTATAT TAAACATGAA AAAGAGATGA AATGGGTAAG   
  
  
+ AAGTCAGCCT AGCCTTGTGG ATAACAGAGG TAGGCTGGCA GGCATGTCTA AGTTCTGATG TAACAACCAA   
  
  
+ AGAGGCAAAG GAGAAGAAAG CCAAAAAGAA AAAAAAGAAA AAAAGAAAGG TCGAGAAGGC AAGTGACACA   
  
  
+ AACCCAAGAC CCAGATCCAA TGTAACAAGT CAACTTCCTT CTCTTTCTCT CTCTGTCACT GACAATCTTC   
  
  
+ ACTAACACAC ACAAACACGC CCCGCACCGC ATTGGACAAA CACCGAAACC AGAAACACGC TCCCCATCTG   
  
  
+ CGATTCAGTC CCTTCTCTCC TTTAAATTTA CCGTGTTACC CTTCCCTCTC CCCTCTTCAC TGCCCTAATT   
  
  
+ ACAAATCTAC TTCTCTCCTC TTTTGACTCT GTTCTCTCTT CTCTCTCATA CTCGCGGTAA GCACGCCAGA   
  
  
+ CAACGCGCAC GCACACTTTC TCTTGTCTG  

- GTGTGTGTCA TTTGTGACCA CGTAAATACG TGGAGTTCGT GACTTTCAAA CGACCTCTTA CGATCGGAAT   
  
  
- AAGTCACTCT ACGGAACAAA TAATTTCTCT TTCTTTGGTT CTATATCTAC CGAAGATTGG AGAAAAACAT   
  
  
- ACTTTACCTA CTTTGTTTAT CTTTATAGCA CGGTTTTGTA GGTCCGTCAT ATAAATCTAG TCCGTCGTAT   
  
  
- TCTCCTCTGT CATGTAAAAC GGGCGAAACC ATCTTTCTTG ATCCTAGGAC TGACAAAATG GCAGTTAAAA   
  
  
- TCAACTGACG TTCAAACAGA AGGGTTGGGT AAATCTAAAA GTAAAAGATA ACCTGTAAGG TAAAGAAGGT   
  
  
- CTCCAGATCC TCCAGTTCAA AAACCTTCTT TAAATATTCT AATAACAACA AAAGAAGGGA CCGACGTCAA   
  
  
- TGAACTGAAC AATCCACCGT CTGTCAACGA AGCAAGACAA TCAGGTGTAG GTGTACTGTA GTTTTGAATC   
  
  
- CTAACTAAAA TCAAGAACAA ATACGAGACT TAAGAGTTTG ACAGAAATTT TTTAAGTAAT CAGTCGTAAA   
  
  
- CGAAAACTGA TAACAAACGT GACGACCGGC GTTCTCCCAA ACATAGAACT AATAAGCACA TTTTTCTTTA   
  
  
- ATGTCTACGA GTTAAGTATC TAAACCGGTG TGACACCCAA GTTTGATTCT AACTGACTTC CTTCGGTTAT   
  
  
- TTCTAGTAGA CAAAAAGGAC GAATTACCGT GGGTATACTC GTCTGTCGGT GAAAAAGACC TAACGGGTTA   
  
  
- AGTTACCTCC GTGTAATCAT CAGTGTCTCT TCCGTCGGAC AATTCTTTTA CCATAAAGAT CTTCGGTTAA   
  
  
- TCATGGCAAA GGTCTACTAT ACCAATTGTC CAGTTATTTT TTTTAATAAA TCCTGAGAAA AACATCACAG   
  
  
- ACGGTTGAAT ACCTTACGTC GGGATTAACT TTATACTCGG GTACACTCGT AGTACAATAT AGTTTCGTGA   
  
  
- CCGTCCCCGG TGTTTCCTGC CACTAAGTTC GAGACATATA ATTTGTACTT TTTCTCTACT TTACCCATTC   
  
  
- TTCAGTCGGA TCGGAACACC TATTGTCTCC ATCCGACCGT CCGTACAGAT TCAAGACTAC ATTGTTGGTT   
  
  
- TCTCCGTTTC CTCTTCTTTC GGTTTTTCTT TTTTTTCTTT TTTTCTTTCC AGCTCTTCCG TTCACTGTGT   
  
  
- TTGGGTTCTG GGTCTAGGTT ACATTGTTCA GTTGAAGGAA GAGAAAGAGA GAGACAGTGA CTGTTAGAAG   
  
  
- TGATTGTGTG TGTTTGTGCG GGGCGTGGCG TAACCTGTTT GTGGCTTTGG TCTTTGTGCG AGGGGTAGAC   
  
  
- GCTAAGTCAG GGAAGAGAGG AAATTTAAAT GGCACAATGG GAAGGGAGAG GGGAGAAGTG ACGGGATTAA   
  
  
- TGTTTAGATG AAGAGAGGAG AAAACTGAGA CAAGAGAGAA GAGAGAGTAT GAGCGCCATT CGTGCGGTCT   
  
  
- GTTGCGCGTG CGTGTGAAAG AGAACAGAC

+     Box-W1

| Site Name | Organism | Position | Strand | Matrix score. | sequence | function |
| --- | --- | --- | --- | --- | --- | --- |
| Box-W1 | Petroselinum crispum | 362 | - | 6 | TTGACC | fungal elicitor responsive element |
| Box-W1 | Petroselinum crispum | 870 | - | 6 | TTGACC | fungal elicitor responsive element |

> 2018/04/13 10:10:12  
+ CACACACAGT AAACACTGGT GCATTTATGC ACCTCAAGCA CTGAAAGTTT GCTGGAGAAT GCTAGCCTTA   
  
  
+ TTCAGTGAGA TGCCTTGTTT ATTAAAGAGA AAGAAACCAA GATATAGATG GCTTCTAACC TCTTTTTGTA   
  
  
+ TGAAATGGAT GAAACAAATA GAAATATCGT GCCAAAACAT CCAGGCAGTA TATTTAGATC AGGCAGCATA   
  
  
+ AGAGGAGACA GTACATTTTG CCCGCTTTGG TAGAAAGAAC TAGGATCCTG ACTGTTTTAC CGTCAATTTT   
  
  
+ AGTTGACTGC AAGTTTGTCT TCCCAACCCA TTTAGATTTT CATTTTCTAT TGGACATTCC ATTTCTTCCA   
  
  
+ GAGGTCTAGG AGGTCAAGTT TTTGGAAGAA ATTTATAAGA TTATTGTTGT TTTCTTCCCT GGCTGCAGTT   
  
  
+ ACTTGACTTG TTAGGTGGCA GACAGTTGCT TCGTTCTGTT AGTCCACATC CACATGACAT CAAAACTTAG   
  
  
+ GATTGATTTT AGTTCTTGTT TATGCTCTGA ATTCTCAAAC TGTCTTTAAA AAATTCATTA GTCAGCATTT   
  
  
+ GCTTTTGACT ATTGTTTGCA CTGCTGGCCG CAAGAGGGTT TGTATCTTGA TTATTCGTGT AAAAAGAAAT   
  
  
+ TACAGATGCT CAATTCATAG ATTTGGCCAC ACTGTGGGTT CAAACTAAGA TTGACTGAAG GAAGCCAATA   
  
  
+ AAGATCATCT GTTTTTCCTG CTTAATGGCA CCCATATGAG CAGACAGCCA CTTTTTCTGG ATTGCCCAAT   
  
  
+ TCAATGGAGG CACATTAGTA GTCACAGAGA AGGCAGCCTG TTAAGAAAAT GGTATTTCTA GAAGCCAATT   
  
  
+ AGTACCGTTT CCAGATGATA TGGTTAACAG GTCAATAAAA AAAATTATTT AGGACTCTTT TTGTAGTGTC   
  
  
+ TGCCAACTTA TGGAATGCAG CCCTAATTGA AATATGAGCC CATGTGAGCA TCATGTTATA TCAAAGCACT   
  
  
+ GGCAGGGGCC ACAAAGGACG GTGATTCAAG CTCTGTATAT TAAACATGAA AAAGAGATGA AATGGGTAAG   
  
  
+ AAGTCAGCCT AGCCTTGTGG ATAACAGAGG TAGGCTGGCA GGCATGTCTA AGTTCTGATG TAACAACCAA   
  
  
+ AGAGGCAAAG GAGAAGAAAG CCAAAAAGAA AAAAAAGAAA AAAAGAAAGG TCGAGAAGGC AAGTGACACA   
  
  
+ AACCCAAGAC CCAGATCCAA TGTAACAAGT CAACTTCCTT CTCTTTCTCT CTCTGTCACT GACAATCTTC   
  
  
+ ACTAACACAC ACAAACACGC CCCGCACCGC ATTGGACAAA CACCGAAACC AGAAACACGC TCCCCATCTG   
  
  
+ CGATTCAGTC CCTTCTCTCC TTTAAATTTA CCGTGTTACC CTTCCCTCTC CCCTCTTCAC TGCCCTAATT   
  
  
+ ACAAATCTAC TTCTCTCCTC TTTTGACTCT GTTCTCTCTT CTCTCTCATA CTCGCGGTAA GCACGCCAGA   
  
  
+ CAACGCGCAC GCACACTTTC TCTTGTCTG  

- GTGTGTGTCA TTTGTGACCA CGTAAATACG TGGAGTTCGT GACTTTCAAA CGACCTCTTA CGATCGGAAT   
  
  
- AAGTCACTCT ACGGAACAAA TAATTTCTCT TTCTTTGGTT CTATATCTAC CGAAGATTGG AGAAAAACAT   
  
  
- ACTTTACCTA CTTTGTTTAT CTTTATAGCA CGGTTTTGTA GGTCCGTCAT ATAAATCTAG TCCGTCGTAT   
  
  
- TCTCCTCTGT CATGTAAAAC GGGCGAAACC ATCTTTCTTG ATCCTAGGAC TGACAAAATG GCAGTTAAAA   
  
  
- TCAACTGACG TTCAAACAGA AGGGTTGGGT AAATCTAAAA GTAAAAGATA ACCTGTAAGG TAAAGAAGGT   
  
  
- CTCCAGATCC TCCAGTTCAA AAACCTTCTT TAAATATTCT AATAACAACA AAAGAAGGGA CCGACGTCAA   
  
  
- TGAACTGAAC AATCCACCGT CTGTCAACGA AGCAAGACAA TCAGGTGTAG GTGTACTGTA GTTTTGAATC   
  
  
- CTAACTAAAA TCAAGAACAA ATACGAGACT TAAGAGTTTG ACAGAAATTT TTTAAGTAAT CAGTCGTAAA   
  
  
- CGAAAACTGA TAACAAACGT GACGACCGGC GTTCTCCCAA ACATAGAACT AATAAGCACA TTTTTCTTTA   
  
  
- ATGTCTACGA GTTAAGTATC TAAACCGGTG TGACACCCAA GTTTGATTCT AACTGACTTC CTTCGGTTAT   
  
  
- TTCTAGTAGA CAAAAAGGAC GAATTACCGT GGGTATACTC GTCTGTCGGT GAAAAAGACC TAACGGGTTA   
  
  
- AGTTACCTCC GTGTAATCAT CAGTGTCTCT TCCGTCGGAC AATTCTTTTA CCATAAAGAT CTTCGGTTAA   
  
  
- TCATGGCAAA GGTCTACTAT ACCAATTGTC CAGTTATTTT TTTTAATAAA TCCTGAGAAA AACATCACAG   
  
  
- ACGGTTGAAT ACCTTACGTC GGGATTAACT TTATACTCGG GTACACTCGT AGTACAATAT AGTTTCGTGA   
  
  
- CCGTCCCCGG TGTTTCCTGC CACTAAGTTC GAGACATATA ATTTGTACTT TTTCTCTACT TTACCCATTC   
  
  
- TTCAGTCGGA TCGGAACACC TATTGTCTCC ATCCGACCGT CCGTACAGAT TCAAGACTAC ATTGTTGGTT   
  
  
- TCTCCGTTTC CTCTTCTTTC GGTTTTTCTT TTTTTTCTTT TTTTCTTTCC AGCTCTTCCG TTCACTGTGT   
  
  
- TTGGGTTCTG GGTCTAGGTT ACATTGTTCA GTTGAAGGAA GAGAAAGAGA GAGACAGTGA CTGTTAGAAG   
  
  
- TGATTGTGTG TGTTTGTGCG GGGCGTGGCG TAACCTGTTT GTGGCTTTGG TCTTTGTGCG AGGGGTAGAC   
  
  
- GCTAAGTCAG GGAAGAGAGG AAATTTAAAT GGCACAATGG GAAGGGAGAG GGGAGAAGTG ACGGGATTAA   
  
  
- TGTTTAGATG AAGAGAGGAG AAAACTGAGA CAAGAGAGAA GAGAGAGTAT GAGCGCCATT CGTGCGGTCT   
  
  
- GTTGCGCGTG CGTGTGAAAG AGAACAGAC

+     CAAT-box

| Site Name | Organism | Position | Strand | Matrix score. | sequence | function |
| --- | --- | --- | --- | --- | --- | --- |
| CAAT-box | Hordeum vulgare | 936 | - | 4 | CAAT | common cis-acting element in promoter and enhancer regions |
| CAAT-box | Glycine max | 935 | - | 5 | CAATT | common cis-acting element in promoter and enhancer regions |
| CAAT-box | Petunia hybrida | 911 | + | 7 | TGCCAAC | common cis-acting element in promoter and enhancer regions |
| CAAT-box | Hordeum vulgare | 873 | + | 4 | CAAT | common cis-acting element in promoter and enhancer regions |
| CAAT-box | Glycine max | 836 | + | 5 | CAATT | common cis-acting element in promoter and enhancer regions |
| CAAT-box | Arabidopsis thaliana | 835 | + | 5 | CCAAT | common cis-acting element in promoter and enhancer regions |
| CAAT-box | Hordeum vulgare | 772 | + | 4 | CAAT | common cis-acting element in promoter and enhancer regions |
| CAAT-box | Glycine max | 767 | + | 5 | CAATT | common cis-acting element in promoter and enhancer regions |
| CAAT-box | Arabidopsis thaliana | 766 | + | 5 | CCAAT | common cis-acting element in promoter and enhancer regions |
| CAAT-box | Arabidopsis thaliana | 761 | - | 6 | gGCAAT | common cis-acting element in promoter and enhancer regions |
| CAAT-box | Hordeum vulgare | 696 | + | 4 | CAAT | common cis-acting element in promoter and enhancer regions |
| CAAT-box | Arabidopsis thaliana | 695 | + | 5 | CCAAT | common cis-acting element in promoter and enhancer regions |
| CAAT-box | Brassica rapa | 1402 | + | 5 | CAAAT | common cis-acting element in promoter and enhancer regions |
| CAAT-box | Arabidopsis thaliana | 1291 | - | 5 | CCAAT | common cis-acting element in promoter and enhancer regions |
| CAAT-box | Hordeum vulgare | 680 | - | 4 | CAAT | common cis-acting element in promoter and enhancer regions |
| CAAT-box | Hordeum vulgare | 1253 | + | 4 | CAAT | common cis-acting element in promoter and enhancer regions |
| CAAT-box | Arabidopsis thaliana | 1207 | + | 5 | CCAAT | common cis-acting element in promoter and enhancer regions |
| CAAT-box | Brassica rapa | 651 | - | 5 | CAAAT | common cis-acting element in promoter and enhancer regions |
| CAAT-box | Hordeum vulgare | 1208 | + | 4 | CAAT | common cis-acting element in promoter and enhancer regions |
| CAAT-box | Glycine max | 641 | + | 5 | CAATT | common cis-acting element in promoter and enhancer regions |
| CAAT-box | Hordeum vulgare | 571 | - | 4 | CAAT | common cis-acting element in promoter and enhancer regions |
| CAAT-box | Brassica rapa | 557 | - | 5 | CAAAT | common cis-acting element in promoter and enhancer regions |
| CAAT-box | Hordeum vulgare | 492 | - | 4 | CAAT | common cis-acting element in promoter and enhancer regions |
| CAAT-box | Hordeum vulgare | 393 | - | 4 | CAAT | common cis-acting element in promoter and enhancer regions |
| CAAT-box | Arabidopsis thaliana | 329 | - | 5 | CCAAT | common cis-acting element in promoter and enhancer regions |
| CAAT-box | Glycine max | 274 | + | 5 | CAATT | common cis-acting element in promoter and enhancer regions |
| CAAT-box | Brassica rapa | 155 | + | 5 | CAAAT | common cis-acting element in promoter and enhancer regions |

> 2018/04/13 10:10:12  
+ CACACACAGT AAACACTGGT GCATTTATGC ACCTCAAGCA CTGAAAGTTT GCTGGAGAAT GCTAGCCTTA   
  
  
+ TTCAGTGAGA TGCCTTGTTT ATTAAAGAGA AAGAAACCAA GATATAGATG GCTTCTAACC TCTTTTTGTA   
  
  
+ TGAAATGGAT GAAACAAATA GAAATATCGT GCCAAAACAT CCAGGCAGTA TATTTAGATC AGGCAGCATA   
  
  
+ AGAGGAGACA GTACATTTTG CCCGCTTTGG TAGAAAGAAC TAGGATCCTG ACTGTTTTAC CGTCAATTTT   
  
  
+ AGTTGACTGC AAGTTTGTCT TCCCAACCCA TTTAGATTTT CATTTTCTAT TGGACATTCC ATTTCTTCCA   
  
  
+ GAGGTCTAGG AGGTCAAGTT TTTGGAAGAA ATTTATAAGA TTATTGTTGT TTTCTTCCCT GGCTGCAGTT   
  
  
+ ACTTGACTTG TTAGGTGGCA GACAGTTGCT TCGTTCTGTT AGTCCACATC CACATGACAT CAAAACTTAG   
  
  
+ GATTGATTTT AGTTCTTGTT TATGCTCTGA ATTCTCAAAC TGTCTTTAAA AAATTCATTA GTCAGCATTT   
  
  
+ GCTTTTGACT ATTGTTTGCA CTGCTGGCCG CAAGAGGGTT TGTATCTTGA TTATTCGTGT AAAAAGAAAT   
  
  
+ TACAGATGCT CAATTCATAG ATTTGGCCAC ACTGTGGGTT CAAACTAAGA TTGACTGAAG GAAGCCAATA   
  
  
+ AAGATCATCT GTTTTTCCTG CTTAATGGCA CCCATATGAG CAGACAGCCA CTTTTTCTGG ATTGCCCAAT   
  
  
+ TCAATGGAGG CACATTAGTA GTCACAGAGA AGGCAGCCTG TTAAGAAAAT GGTATTTCTA GAAGCCAATT   
  
  
+ AGTACCGTTT CCAGATGATA TGGTTAACAG GTCAATAAAA AAAATTATTT AGGACTCTTT TTGTAGTGTC   
  
  
+ TGCCAACTTA TGGAATGCAG CCCTAATTGA AATATGAGCC CATGTGAGCA TCATGTTATA TCAAAGCACT   
  
  
+ GGCAGGGGCC ACAAAGGACG GTGATTCAAG CTCTGTATAT TAAACATGAA AAAGAGATGA AATGGGTAAG   
  
  
+ AAGTCAGCCT AGCCTTGTGG ATAACAGAGG TAGGCTGGCA GGCATGTCTA AGTTCTGATG TAACAACCAA   
  
  
+ AGAGGCAAAG GAGAAGAAAG CCAAAAAGAA AAAAAAGAAA AAAAGAAAGG TCGAGAAGGC AAGTGACACA   
  
  
+ AACCCAAGAC CCAGATCCAA TGTAACAAGT CAACTTCCTT CTCTTTCTCT CTCTGTCACT GACAATCTTC   
  
  
+ ACTAACACAC ACAAACACGC CCCGCACCGC ATTGGACAAA CACCGAAACC AGAAACACGC TCCCCATCTG   
  
  
+ CGATTCAGTC CCTTCTCTCC TTTAAATTTA CCGTGTTACC CTTCCCTCTC CCCTCTTCAC TGCCCTAATT   
  
  
+ ACAAATCTAC TTCTCTCCTC TTTTGACTCT GTTCTCTCTT CTCTCTCATA CTCGCGGTAA GCACGCCAGA   
  
  
+ CAACGCGCAC GCACACTTTC TCTTGTCTG  

- GTGTGTGTCA TTTGTGACCA CGTAAATACG TGGAGTTCGT GACTTTCAAA CGACCTCTTA CGATCGGAAT   
  
  
- AAGTCACTCT ACGGAACAAA TAATTTCTCT TTCTTTGGTT CTATATCTAC CGAAGATTGG AGAAAAACAT   
  
  
- ACTTTACCTA CTTTGTTTAT CTTTATAGCA CGGTTTTGTA GGTCCGTCAT ATAAATCTAG TCCGTCGTAT   
  
  
- TCTCCTCTGT CATGTAAAAC GGGCGAAACC ATCTTTCTTG ATCCTAGGAC TGACAAAATG GCAGTTAAAA   
  
  
- TCAACTGACG TTCAAACAGA AGGGTTGGGT AAATCTAAAA GTAAAAGATA ACCTGTAAGG TAAAGAAGGT   
  
  
- CTCCAGATCC TCCAGTTCAA AAACCTTCTT TAAATATTCT AATAACAACA AAAGAAGGGA CCGACGTCAA   
  
  
- TGAACTGAAC AATCCACCGT CTGTCAACGA AGCAAGACAA TCAGGTGTAG GTGTACTGTA GTTTTGAATC   
  
  
- CTAACTAAAA TCAAGAACAA ATACGAGACT TAAGAGTTTG ACAGAAATTT TTTAAGTAAT CAGTCGTAAA   
  
  
- CGAAAACTGA TAACAAACGT GACGACCGGC GTTCTCCCAA ACATAGAACT AATAAGCACA TTTTTCTTTA   
  
  
- ATGTCTACGA GTTAAGTATC TAAACCGGTG TGACACCCAA GTTTGATTCT AACTGACTTC CTTCGGTTAT   
  
  
- TTCTAGTAGA CAAAAAGGAC GAATTACCGT GGGTATACTC GTCTGTCGGT GAAAAAGACC TAACGGGTTA   
  
  
- AGTTACCTCC GTGTAATCAT CAGTGTCTCT TCCGTCGGAC AATTCTTTTA CCATAAAGAT CTTCGGTTAA   
  
  
- TCATGGCAAA GGTCTACTAT ACCAATTGTC CAGTTATTTT TTTTAATAAA TCCTGAGAAA AACATCACAG   
  
  
- ACGGTTGAAT ACCTTACGTC GGGATTAACT TTATACTCGG GTACACTCGT AGTACAATAT AGTTTCGTGA   
  
  
- CCGTCCCCGG TGTTTCCTGC CACTAAGTTC GAGACATATA ATTTGTACTT TTTCTCTACT TTACCCATTC   
  
  
- TTCAGTCGGA TCGGAACACC TATTGTCTCC ATCCGACCGT CCGTACAGAT TCAAGACTAC ATTGTTGGTT   
  
  
- TCTCCGTTTC CTCTTCTTTC GGTTTTTCTT TTTTTTCTTT TTTTCTTTCC AGCTCTTCCG TTCACTGTGT   
  
  
- TTGGGTTCTG GGTCTAGGTT ACATTGTTCA GTTGAAGGAA GAGAAAGAGA GAGACAGTGA CTGTTAGAAG   
  
  
- TGATTGTGTG TGTTTGTGCG GGGCGTGGCG TAACCTGTTT GTGGCTTTGG TCTTTGTGCG AGGGGTAGAC   
  
  
- GCTAAGTCAG GGAAGAGAGG AAATTTAAAT GGCACAATGG GAAGGGAGAG GGGAGAAGTG ACGGGATTAA   
  
  
- TGTTTAGATG AAGAGAGGAG AAAACTGAGA CAAGAGAGAA GAGAGAGTAT GAGCGCCATT CGTGCGGTCT   
  
  
- GTTGCGCGTG CGTGTGAAAG AGAACAGAC

+     CAT-box

| Site Name | Organism | Position | Strand | Matrix score. | sequence | function |
| --- | --- | --- | --- | --- | --- | --- |
| CAT-box | Arabidopsis thaliana | 747 | + | 6 | GCCACT | cis-acting regulatory element related to meristem expression |

> 2018/04/13 10:10:12  
+ CACACACAGT AAACACTGGT GCATTTATGC ACCTCAAGCA CTGAAAGTTT GCTGGAGAAT GCTAGCCTTA   
  
  
+ TTCAGTGAGA TGCCTTGTTT ATTAAAGAGA AAGAAACCAA GATATAGATG GCTTCTAACC TCTTTTTGTA   
  
  
+ TGAAATGGAT GAAACAAATA GAAATATCGT GCCAAAACAT CCAGGCAGTA TATTTAGATC AGGCAGCATA   
  
  
+ AGAGGAGACA GTACATTTTG CCCGCTTTGG TAGAAAGAAC TAGGATCCTG ACTGTTTTAC CGTCAATTTT   
  
  
+ AGTTGACTGC AAGTTTGTCT TCCCAACCCA TTTAGATTTT CATTTTCTAT TGGACATTCC ATTTCTTCCA   
  
  
+ GAGGTCTAGG AGGTCAAGTT TTTGGAAGAA ATTTATAAGA TTATTGTTGT TTTCTTCCCT GGCTGCAGTT   
  
  
+ ACTTGACTTG TTAGGTGGCA GACAGTTGCT TCGTTCTGTT AGTCCACATC CACATGACAT CAAAACTTAG   
  
  
+ GATTGATTTT AGTTCTTGTT TATGCTCTGA ATTCTCAAAC TGTCTTTAAA AAATTCATTA GTCAGCATTT   
  
  
+ GCTTTTGACT ATTGTTTGCA CTGCTGGCCG CAAGAGGGTT TGTATCTTGA TTATTCGTGT AAAAAGAAAT   
  
  
+ TACAGATGCT CAATTCATAG ATTTGGCCAC ACTGTGGGTT CAAACTAAGA TTGACTGAAG GAAGCCAATA   
  
  
+ AAGATCATCT GTTTTTCCTG CTTAATGGCA CCCATATGAG CAGACAGCCA CTTTTTCTGG ATTGCCCAAT   
  
  
+ TCAATGGAGG CACATTAGTA GTCACAGAGA AGGCAGCCTG TTAAGAAAAT GGTATTTCTA GAAGCCAATT   
  
  
+ AGTACCGTTT CCAGATGATA TGGTTAACAG GTCAATAAAA AAAATTATTT AGGACTCTTT TTGTAGTGTC   
  
  
+ TGCCAACTTA TGGAATGCAG CCCTAATTGA AATATGAGCC CATGTGAGCA TCATGTTATA TCAAAGCACT   
  
  
+ GGCAGGGGCC ACAAAGGACG GTGATTCAAG CTCTGTATAT TAAACATGAA AAAGAGATGA AATGGGTAAG   
  
  
+ AAGTCAGCCT AGCCTTGTGG ATAACAGAGG TAGGCTGGCA GGCATGTCTA AGTTCTGATG TAACAACCAA   
  
  
+ AGAGGCAAAG GAGAAGAAAG CCAAAAAGAA AAAAAAGAAA AAAAGAAAGG TCGAGAAGGC AAGTGACACA   
  
  
+ AACCCAAGAC CCAGATCCAA TGTAACAAGT CAACTTCCTT CTCTTTCTCT CTCTGTCACT GACAATCTTC   
  
  
+ ACTAACACAC ACAAACACGC CCCGCACCGC ATTGGACAAA CACCGAAACC AGAAACACGC TCCCCATCTG   
  
  
+ CGATTCAGTC CCTTCTCTCC TTTAAATTTA CCGTGTTACC CTTCCCTCTC CCCTCTTCAC TGCCCTAATT   
  
  
+ ACAAATCTAC TTCTCTCCTC TTTTGACTCT GTTCTCTCTT CTCTCTCATA CTCGCGGTAA GCACGCCAGA   
  
  
+ CAACGCGCAC GCACACTTTC TCTTGTCTG  

- GTGTGTGTCA TTTGTGACCA CGTAAATACG TGGAGTTCGT GACTTTCAAA CGACCTCTTA CGATCGGAAT   
  
  
- AAGTCACTCT ACGGAACAAA TAATTTCTCT TTCTTTGGTT CTATATCTAC CGAAGATTGG AGAAAAACAT   
  
  
- ACTTTACCTA CTTTGTTTAT CTTTATAGCA CGGTTTTGTA GGTCCGTCAT ATAAATCTAG TCCGTCGTAT   
  
  
- TCTCCTCTGT CATGTAAAAC GGGCGAAACC ATCTTTCTTG ATCCTAGGAC TGACAAAATG GCAGTTAAAA   
  
  
- TCAACTGACG TTCAAACAGA AGGGTTGGGT AAATCTAAAA GTAAAAGATA ACCTGTAAGG TAAAGAAGGT   
  
  
- CTCCAGATCC TCCAGTTCAA AAACCTTCTT TAAATATTCT AATAACAACA AAAGAAGGGA CCGACGTCAA   
  
  
- TGAACTGAAC AATCCACCGT CTGTCAACGA AGCAAGACAA TCAGGTGTAG GTGTACTGTA GTTTTGAATC   
  
  
- CTAACTAAAA TCAAGAACAA ATACGAGACT TAAGAGTTTG ACAGAAATTT TTTAAGTAAT CAGTCGTAAA   
  
  
- CGAAAACTGA TAACAAACGT GACGACCGGC GTTCTCCCAA ACATAGAACT AATAAGCACA TTTTTCTTTA   
  
  
- ATGTCTACGA GTTAAGTATC TAAACCGGTG TGACACCCAA GTTTGATTCT AACTGACTTC CTTCGGTTAT   
  
  
- TTCTAGTAGA CAAAAAGGAC GAATTACCGT GGGTATACTC GTCTGTCGGT GAAAAAGACC TAACGGGTTA   
  
  
- AGTTACCTCC GTGTAATCAT CAGTGTCTCT TCCGTCGGAC AATTCTTTTA CCATAAAGAT CTTCGGTTAA   
  
  
- TCATGGCAAA GGTCTACTAT ACCAATTGTC CAGTTATTTT TTTTAATAAA TCCTGAGAAA AACATCACAG   
  
  
- ACGGTTGAAT ACCTTACGTC GGGATTAACT TTATACTCGG GTACACTCGT AGTACAATAT AGTTTCGTGA   
  
  
- CCGTCCCCGG TGTTTCCTGC CACTAAGTTC GAGACATATA ATTTGTACTT TTTCTCTACT TTACCCATTC   
  
  
- TTCAGTCGGA TCGGAACACC TATTGTCTCC ATCCGACCGT CCGTACAGAT TCAAGACTAC ATTGTTGGTT   
  
  
- TCTCCGTTTC CTCTTCTTTC GGTTTTTCTT TTTTTTCTTT TTTTCTTTCC AGCTCTTCCG TTCACTGTGT   
  
  
- TTGGGTTCTG GGTCTAGGTT ACATTGTTCA GTTGAAGGAA GAGAAAGAGA GAGACAGTGA CTGTTAGAAG   
  
  
- TGATTGTGTG TGTTTGTGCG GGGCGTGGCG TAACCTGTTT GTGGCTTTGG TCTTTGTGCG AGGGGTAGAC   
  
  
- GCTAAGTCAG GGAAGAGAGG AAATTTAAAT GGCACAATGG GAAGGGAGAG GGGAGAAGTG ACGGGATTAA   
  
  
- TGTTTAGATG AAGAGAGGAG AAAACTGAGA CAAGAGAGAA GAGAGAGTAT GAGCGCCATT CGTGCGGTCT   
  
  
- GTTGCGCGTG CGTGTGAAAG AGAACAGAC

+     CATT-motif

| Site Name | Organism | Position | Strand | Matrix score. | sequence | function |
| --- | --- | --- | --- | --- | --- | --- |
| CATT-motif | Zea mays | 923 | - | 6 | GCATTC | part of a light responsive element |
| CATT-motif | Zea mays | 57 | - | 6 | GCATTC | part of a light responsive element |

> 2018/04/13 10:10:12  
+ CACACACAGT AAACACTGGT GCATTTATGC ACCTCAAGCA CTGAAAGTTT GCTGGAGAAT GCTAGCCTTA   
  
  
+ TTCAGTGAGA TGCCTTGTTT ATTAAAGAGA AAGAAACCAA GATATAGATG GCTTCTAACC TCTTTTTGTA   
  
  
+ TGAAATGGAT GAAACAAATA GAAATATCGT GCCAAAACAT CCAGGCAGTA TATTTAGATC AGGCAGCATA   
  
  
+ AGAGGAGACA GTACATTTTG CCCGCTTTGG TAGAAAGAAC TAGGATCCTG ACTGTTTTAC CGTCAATTTT   
  
  
+ AGTTGACTGC AAGTTTGTCT TCCCAACCCA TTTAGATTTT CATTTTCTAT TGGACATTCC ATTTCTTCCA   
  
  
+ GAGGTCTAGG AGGTCAAGTT TTTGGAAGAA ATTTATAAGA TTATTGTTGT TTTCTTCCCT GGCTGCAGTT   
  
  
+ ACTTGACTTG TTAGGTGGCA GACAGTTGCT TCGTTCTGTT AGTCCACATC CACATGACAT CAAAACTTAG   
  
  
+ GATTGATTTT AGTTCTTGTT TATGCTCTGA ATTCTCAAAC TGTCTTTAAA AAATTCATTA GTCAGCATTT   
  
  
+ GCTTTTGACT ATTGTTTGCA CTGCTGGCCG CAAGAGGGTT TGTATCTTGA TTATTCGTGT AAAAAGAAAT   
  
  
+ TACAGATGCT CAATTCATAG ATTTGGCCAC ACTGTGGGTT CAAACTAAGA TTGACTGAAG GAAGCCAATA   
  
  
+ AAGATCATCT GTTTTTCCTG CTTAATGGCA CCCATATGAG CAGACAGCCA CTTTTTCTGG ATTGCCCAAT   
  
  
+ TCAATGGAGG CACATTAGTA GTCACAGAGA AGGCAGCCTG TTAAGAAAAT GGTATTTCTA GAAGCCAATT   
  
  
+ AGTACCGTTT CCAGATGATA TGGTTAACAG GTCAATAAAA AAAATTATTT AGGACTCTTT TTGTAGTGTC   
  
  
+ TGCCAACTTA TGGAATGCAG CCCTAATTGA AATATGAGCC CATGTGAGCA TCATGTTATA TCAAAGCACT   
  
  
+ GGCAGGGGCC ACAAAGGACG GTGATTCAAG CTCTGTATAT TAAACATGAA AAAGAGATGA AATGGGTAAG   
  
  
+ AAGTCAGCCT AGCCTTGTGG ATAACAGAGG TAGGCTGGCA GGCATGTCTA AGTTCTGATG TAACAACCAA   
  
  
+ AGAGGCAAAG GAGAAGAAAG CCAAAAAGAA AAAAAAGAAA AAAAGAAAGG TCGAGAAGGC AAGTGACACA   
  
  
+ AACCCAAGAC CCAGATCCAA TGTAACAAGT CAACTTCCTT CTCTTTCTCT CTCTGTCACT GACAATCTTC   
  
  
+ ACTAACACAC ACAAACACGC CCCGCACCGC ATTGGACAAA CACCGAAACC AGAAACACGC TCCCCATCTG   
  
  
+ CGATTCAGTC CCTTCTCTCC TTTAAATTTA CCGTGTTACC CTTCCCTCTC CCCTCTTCAC TGCCCTAATT   
  
  
+ ACAAATCTAC TTCTCTCCTC TTTTGACTCT GTTCTCTCTT CTCTCTCATA CTCGCGGTAA GCACGCCAGA   
  
  
+ CAACGCGCAC GCACACTTTC TCTTGTCTG  

- GTGTGTGTCA TTTGTGACCA CGTAAATACG TGGAGTTCGT GACTTTCAAA CGACCTCTTA CGATCGGAAT   
  
  
- AAGTCACTCT ACGGAACAAA TAATTTCTCT TTCTTTGGTT CTATATCTAC CGAAGATTGG AGAAAAACAT   
  
  
- ACTTTACCTA CTTTGTTTAT CTTTATAGCA CGGTTTTGTA GGTCCGTCAT ATAAATCTAG TCCGTCGTAT   
  
  
- TCTCCTCTGT CATGTAAAAC GGGCGAAACC ATCTTTCTTG ATCCTAGGAC TGACAAAATG GCAGTTAAAA   
  
  
- TCAACTGACG TTCAAACAGA AGGGTTGGGT AAATCTAAAA GTAAAAGATA ACCTGTAAGG TAAAGAAGGT   
  
  
- CTCCAGATCC TCCAGTTCAA AAACCTTCTT TAAATATTCT AATAACAACA AAAGAAGGGA CCGACGTCAA   
  
  
- TGAACTGAAC AATCCACCGT CTGTCAACGA AGCAAGACAA TCAGGTGTAG GTGTACTGTA GTTTTGAATC   
  
  
- CTAACTAAAA TCAAGAACAA ATACGAGACT TAAGAGTTTG ACAGAAATTT TTTAAGTAAT CAGTCGTAAA   
  
  
- CGAAAACTGA TAACAAACGT GACGACCGGC GTTCTCCCAA ACATAGAACT AATAAGCACA TTTTTCTTTA   
  
  
- ATGTCTACGA GTTAAGTATC TAAACCGGTG TGACACCCAA GTTTGATTCT AACTGACTTC CTTCGGTTAT   
  
  
- TTCTAGTAGA CAAAAAGGAC GAATTACCGT GGGTATACTC GTCTGTCGGT GAAAAAGACC TAACGGGTTA   
  
  
- AGTTACCTCC GTGTAATCAT CAGTGTCTCT TCCGTCGGAC AATTCTTTTA CCATAAAGAT CTTCGGTTAA   
  
  
- TCATGGCAAA GGTCTACTAT ACCAATTGTC CAGTTATTTT TTTTAATAAA TCCTGAGAAA AACATCACAG   
  
  
- ACGGTTGAAT ACCTTACGTC GGGATTAACT TTATACTCGG GTACACTCGT AGTACAATAT AGTTTCGTGA   
  
  
- CCGTCCCCGG TGTTTCCTGC CACTAAGTTC GAGACATATA ATTTGTACTT TTTCTCTACT TTACCCATTC   
  
  
- TTCAGTCGGA TCGGAACACC TATTGTCTCC ATCCGACCGT CCGTACAGAT TCAAGACTAC ATTGTTGGTT   
  
  
- TCTCCGTTTC CTCTTCTTTC GGTTTTTCTT TTTTTTCTTT TTTTCTTTCC AGCTCTTCCG TTCACTGTGT   
  
  
- TTGGGTTCTG GGTCTAGGTT ACATTGTTCA GTTGAAGGAA GAGAAAGAGA GAGACAGTGA CTGTTAGAAG   
  
  
- TGATTGTGTG TGTTTGTGCG GGGCGTGGCG TAACCTGTTT GTGGCTTTGG TCTTTGTGCG AGGGGTAGAC   
  
  
- GCTAAGTCAG GGAAGAGAGG AAATTTAAAT GGCACAATGG GAAGGGAGAG GGGAGAAGTG ACGGGATTAA   
  
  
- TGTTTAGATG AAGAGAGGAG AAAACTGAGA CAAGAGAGAA GAGAGAGTAT GAGCGCCATT CGTGCGGTCT   
  
  
- GTTGCGCGTG CGTGTGAAAG AGAACAGAC

+     CCGTCC-box

| Site Name | Organism | Position | Strand | Matrix score. | sequence | function |
| --- | --- | --- | --- | --- | --- | --- |
| CCGTCC-box | Arabidopsis thaliana | 996 | - | 6 | CCGTCC | cis-acting regulatory element related to meristem specific activation |

> 2018/04/13 10:10:12  
+ CACACACAGT AAACACTGGT GCATTTATGC ACCTCAAGCA CTGAAAGTTT GCTGGAGAAT GCTAGCCTTA   
  
  
+ TTCAGTGAGA TGCCTTGTTT ATTAAAGAGA AAGAAACCAA GATATAGATG GCTTCTAACC TCTTTTTGTA   
  
  
+ TGAAATGGAT GAAACAAATA GAAATATCGT GCCAAAACAT CCAGGCAGTA TATTTAGATC AGGCAGCATA   
  
  
+ AGAGGAGACA GTACATTTTG CCCGCTTTGG TAGAAAGAAC TAGGATCCTG ACTGTTTTAC CGTCAATTTT   
  
  
+ AGTTGACTGC AAGTTTGTCT TCCCAACCCA TTTAGATTTT CATTTTCTAT TGGACATTCC ATTTCTTCCA   
  
  
+ GAGGTCTAGG AGGTCAAGTT TTTGGAAGAA ATTTATAAGA TTATTGTTGT TTTCTTCCCT GGCTGCAGTT   
  
  
+ ACTTGACTTG TTAGGTGGCA GACAGTTGCT TCGTTCTGTT AGTCCACATC CACATGACAT CAAAACTTAG   
  
  
+ GATTGATTTT AGTTCTTGTT TATGCTCTGA ATTCTCAAAC TGTCTTTAAA AAATTCATTA GTCAGCATTT   
  
  
+ GCTTTTGACT ATTGTTTGCA CTGCTGGCCG CAAGAGGGTT TGTATCTTGA TTATTCGTGT AAAAAGAAAT   
  
  
+ TACAGATGCT CAATTCATAG ATTTGGCCAC ACTGTGGGTT CAAACTAAGA TTGACTGAAG GAAGCCAATA   
  
  
+ AAGATCATCT GTTTTTCCTG CTTAATGGCA CCCATATGAG CAGACAGCCA CTTTTTCTGG ATTGCCCAAT   
  
  
+ TCAATGGAGG CACATTAGTA GTCACAGAGA AGGCAGCCTG TTAAGAAAAT GGTATTTCTA GAAGCCAATT   
  
  
+ AGTACCGTTT CCAGATGATA TGGTTAACAG GTCAATAAAA AAAATTATTT AGGACTCTTT TTGTAGTGTC   
  
  
+ TGCCAACTTA TGGAATGCAG CCCTAATTGA AATATGAGCC CATGTGAGCA TCATGTTATA TCAAAGCACT   
  
  
+ GGCAGGGGCC ACAAAGGACG GTGATTCAAG CTCTGTATAT TAAACATGAA AAAGAGATGA AATGGGTAAG   
  
  
+ AAGTCAGCCT AGCCTTGTGG ATAACAGAGG TAGGCTGGCA GGCATGTCTA AGTTCTGATG TAACAACCAA   
  
  
+ AGAGGCAAAG GAGAAGAAAG CCAAAAAGAA AAAAAAGAAA AAAAGAAAGG TCGAGAAGGC AAGTGACACA   
  
  
+ AACCCAAGAC CCAGATCCAA TGTAACAAGT CAACTTCCTT CTCTTTCTCT CTCTGTCACT GACAATCTTC   
  
  
+ ACTAACACAC ACAAACACGC CCCGCACCGC ATTGGACAAA CACCGAAACC AGAAACACGC TCCCCATCTG   
  
  
+ CGATTCAGTC CCTTCTCTCC TTTAAATTTA CCGTGTTACC CTTCCCTCTC CCCTCTTCAC TGCCCTAATT   
  
  
+ ACAAATCTAC TTCTCTCCTC TTTTGACTCT GTTCTCTCTT CTCTCTCATA CTCGCGGTAA GCACGCCAGA   
  
  
+ CAACGCGCAC GCACACTTTC TCTTGTCTG  

- GTGTGTGTCA TTTGTGACCA CGTAAATACG TGGAGTTCGT GACTTTCAAA CGACCTCTTA CGATCGGAAT   
  
  
- AAGTCACTCT ACGGAACAAA TAATTTCTCT TTCTTTGGTT CTATATCTAC CGAAGATTGG AGAAAAACAT   
  
  
- ACTTTACCTA CTTTGTTTAT CTTTATAGCA CGGTTTTGTA GGTCCGTCAT ATAAATCTAG TCCGTCGTAT   
  
  
- TCTCCTCTGT CATGTAAAAC GGGCGAAACC ATCTTTCTTG ATCCTAGGAC TGACAAAATG GCAGTTAAAA   
  
  
- TCAACTGACG TTCAAACAGA AGGGTTGGGT AAATCTAAAA GTAAAAGATA ACCTGTAAGG TAAAGAAGGT   
  
  
- CTCCAGATCC TCCAGTTCAA AAACCTTCTT TAAATATTCT AATAACAACA AAAGAAGGGA CCGACGTCAA   
  
  
- TGAACTGAAC AATCCACCGT CTGTCAACGA AGCAAGACAA TCAGGTGTAG GTGTACTGTA GTTTTGAATC   
  
  
- CTAACTAAAA TCAAGAACAA ATACGAGACT TAAGAGTTTG ACAGAAATTT TTTAAGTAAT CAGTCGTAAA   
  
  
- CGAAAACTGA TAACAAACGT GACGACCGGC GTTCTCCCAA ACATAGAACT AATAAGCACA TTTTTCTTTA   
  
  
- ATGTCTACGA GTTAAGTATC TAAACCGGTG TGACACCCAA GTTTGATTCT AACTGACTTC CTTCGGTTAT   
  
  
- TTCTAGTAGA CAAAAAGGAC GAATTACCGT GGGTATACTC GTCTGTCGGT GAAAAAGACC TAACGGGTTA   
  
  
- AGTTACCTCC GTGTAATCAT CAGTGTCTCT TCCGTCGGAC AATTCTTTTA CCATAAAGAT CTTCGGTTAA   
  
  
- TCATGGCAAA GGTCTACTAT ACCAATTGTC CAGTTATTTT TTTTAATAAA TCCTGAGAAA AACATCACAG   
  
  
- ACGGTTGAAT ACCTTACGTC GGGATTAACT TTATACTCGG GTACACTCGT AGTACAATAT AGTTTCGTGA   
  
  
- CCGTCCCCGG TGTTTCCTGC CACTAAGTTC GAGACATATA ATTTGTACTT TTTCTCTACT TTACCCATTC   
  
  
- TTCAGTCGGA TCGGAACACC TATTGTCTCC ATCCGACCGT CCGTACAGAT TCAAGACTAC ATTGTTGGTT   
  
  
- TCTCCGTTTC CTCTTCTTTC GGTTTTTCTT TTTTTTCTTT TTTTCTTTCC AGCTCTTCCG TTCACTGTGT   
  
  
- TTGGGTTCTG GGTCTAGGTT ACATTGTTCA GTTGAAGGAA GAGAAAGAGA GAGACAGTGA CTGTTAGAAG   
  
  
- TGATTGTGTG TGTTTGTGCG GGGCGTGGCG TAACCTGTTT GTGGCTTTGG TCTTTGTGCG AGGGGTAGAC   
  
  
- GCTAAGTCAG GGAAGAGAGG AAATTTAAAT GGCACAATGG GAAGGGAGAG GGGAGAAGTG ACGGGATTAA   
  
  
- TGTTTAGATG AAGAGAGGAG AAAACTGAGA CAAGAGAGAA GAGAGAGTAT GAGCGCCATT CGTGCGGTCT   
  
  
- GTTGCGCGTG CGTGTGAAAG AGAACAGAC

+     CGTCA-motif

| Site Name | Organism | Position | Strand | Matrix score. | sequence | function |
| --- | --- | --- | --- | --- | --- | --- |
| CGTCA-motif | Hordeum vulgare | 271 | + | 5 | CGTCA | cis-acting regulatory element involved in the MeJA-responsiveness |

> 2018/04/13 10:10:12  
+ CACACACAGT AAACACTGGT GCATTTATGC ACCTCAAGCA CTGAAAGTTT GCTGGAGAAT GCTAGCCTTA   
  
  
+ TTCAGTGAGA TGCCTTGTTT ATTAAAGAGA AAGAAACCAA GATATAGATG GCTTCTAACC TCTTTTTGTA   
  
  
+ TGAAATGGAT GAAACAAATA GAAATATCGT GCCAAAACAT CCAGGCAGTA TATTTAGATC AGGCAGCATA   
  
  
+ AGAGGAGACA GTACATTTTG CCCGCTTTGG TAGAAAGAAC TAGGATCCTG ACTGTTTTAC CGTCAATTTT   
  
  
+ AGTTGACTGC AAGTTTGTCT TCCCAACCCA TTTAGATTTT CATTTTCTAT TGGACATTCC ATTTCTTCCA   
  
  
+ GAGGTCTAGG AGGTCAAGTT TTTGGAAGAA ATTTATAAGA TTATTGTTGT TTTCTTCCCT GGCTGCAGTT   
  
  
+ ACTTGACTTG TTAGGTGGCA GACAGTTGCT TCGTTCTGTT AGTCCACATC CACATGACAT CAAAACTTAG   
  
  
+ GATTGATTTT AGTTCTTGTT TATGCTCTGA ATTCTCAAAC TGTCTTTAAA AAATTCATTA GTCAGCATTT   
  
  
+ GCTTTTGACT ATTGTTTGCA CTGCTGGCCG CAAGAGGGTT TGTATCTTGA TTATTCGTGT AAAAAGAAAT   
  
  
+ TACAGATGCT CAATTCATAG ATTTGGCCAC ACTGTGGGTT CAAACTAAGA TTGACTGAAG GAAGCCAATA   
  
  
+ AAGATCATCT GTTTTTCCTG CTTAATGGCA CCCATATGAG CAGACAGCCA CTTTTTCTGG ATTGCCCAAT   
  
  
+ TCAATGGAGG CACATTAGTA GTCACAGAGA AGGCAGCCTG TTAAGAAAAT GGTATTTCTA GAAGCCAATT   
  
  
+ AGTACCGTTT CCAGATGATA TGGTTAACAG GTCAATAAAA AAAATTATTT AGGACTCTTT TTGTAGTGTC   
  
  
+ TGCCAACTTA TGGAATGCAG CCCTAATTGA AATATGAGCC CATGTGAGCA TCATGTTATA TCAAAGCACT   
  
  
+ GGCAGGGGCC ACAAAGGACG GTGATTCAAG CTCTGTATAT TAAACATGAA AAAGAGATGA AATGGGTAAG   
  
  
+ AAGTCAGCCT AGCCTTGTGG ATAACAGAGG TAGGCTGGCA GGCATGTCTA AGTTCTGATG TAACAACCAA   
  
  
+ AGAGGCAAAG GAGAAGAAAG CCAAAAAGAA AAAAAAGAAA AAAAGAAAGG TCGAGAAGGC AAGTGACACA   
  
  
+ AACCCAAGAC CCAGATCCAA TGTAACAAGT CAACTTCCTT CTCTTTCTCT CTCTGTCACT GACAATCTTC   
  
  
+ ACTAACACAC ACAAACACGC CCCGCACCGC ATTGGACAAA CACCGAAACC AGAAACACGC TCCCCATCTG   
  
  
+ CGATTCAGTC CCTTCTCTCC TTTAAATTTA CCGTGTTACC CTTCCCTCTC CCCTCTTCAC TGCCCTAATT   
  
  
+ ACAAATCTAC TTCTCTCCTC TTTTGACTCT GTTCTCTCTT CTCTCTCATA CTCGCGGTAA GCACGCCAGA   
  
  
+ CAACGCGCAC GCACACTTTC TCTTGTCTG  

- GTGTGTGTCA TTTGTGACCA CGTAAATACG TGGAGTTCGT GACTTTCAAA CGACCTCTTA CGATCGGAAT   
  
  
- AAGTCACTCT ACGGAACAAA TAATTTCTCT TTCTTTGGTT CTATATCTAC CGAAGATTGG AGAAAAACAT   
  
  
- ACTTTACCTA CTTTGTTTAT CTTTATAGCA CGGTTTTGTA GGTCCGTCAT ATAAATCTAG TCCGTCGTAT   
  
  
- TCTCCTCTGT CATGTAAAAC GGGCGAAACC ATCTTTCTTG ATCCTAGGAC TGACAAAATG GCAGTTAAAA   
  
  
- TCAACTGACG TTCAAACAGA AGGGTTGGGT AAATCTAAAA GTAAAAGATA ACCTGTAAGG TAAAGAAGGT   
  
  
- CTCCAGATCC TCCAGTTCAA AAACCTTCTT TAAATATTCT AATAACAACA AAAGAAGGGA CCGACGTCAA   
  
  
- TGAACTGAAC AATCCACCGT CTGTCAACGA AGCAAGACAA TCAGGTGTAG GTGTACTGTA GTTTTGAATC   
  
  
- CTAACTAAAA TCAAGAACAA ATACGAGACT TAAGAGTTTG ACAGAAATTT TTTAAGTAAT CAGTCGTAAA   
  
  
- CGAAAACTGA TAACAAACGT GACGACCGGC GTTCTCCCAA ACATAGAACT AATAAGCACA TTTTTCTTTA   
  
  
- ATGTCTACGA GTTAAGTATC TAAACCGGTG TGACACCCAA GTTTGATTCT AACTGACTTC CTTCGGTTAT   
  
  
- TTCTAGTAGA CAAAAAGGAC GAATTACCGT GGGTATACTC GTCTGTCGGT GAAAAAGACC TAACGGGTTA   
  
  
- AGTTACCTCC GTGTAATCAT CAGTGTCTCT TCCGTCGGAC AATTCTTTTA CCATAAAGAT CTTCGGTTAA   
  
  
- TCATGGCAAA GGTCTACTAT ACCAATTGTC CAGTTATTTT TTTTAATAAA TCCTGAGAAA AACATCACAG   
  
  
- ACGGTTGAAT ACCTTACGTC GGGATTAACT TTATACTCGG GTACACTCGT AGTACAATAT AGTTTCGTGA   
  
  
- CCGTCCCCGG TGTTTCCTGC CACTAAGTTC GAGACATATA ATTTGTACTT TTTCTCTACT TTACCCATTC   
  
  
- TTCAGTCGGA TCGGAACACC TATTGTCTCC ATCCGACCGT CCGTACAGAT TCAAGACTAC ATTGTTGGTT   
  
  
- TCTCCGTTTC CTCTTCTTTC GGTTTTTCTT TTTTTTCTTT TTTTCTTTCC AGCTCTTCCG TTCACTGTGT   
  
  
- TTGGGTTCTG GGTCTAGGTT ACATTGTTCA GTTGAAGGAA GAGAAAGAGA GAGACAGTGA CTGTTAGAAG   
  
  
- TGATTGTGTG TGTTTGTGCG GGGCGTGGCG TAACCTGTTT GTGGCTTTGG TCTTTGTGCG AGGGGTAGAC   
  
  
- GCTAAGTCAG GGAAGAGAGG AAATTTAAAT GGCACAATGG GAAGGGAGAG GGGAGAAGTG ACGGGATTAA   
  
  
- TGTTTAGATG AAGAGAGGAG AAAACTGAGA CAAGAGAGAA GAGAGAGTAT GAGCGCCATT CGTGCGGTCT   
  
  
- GTTGCGCGTG CGTGTGAAAG AGAACAGAC

+     CTAG-motif

| Site Name | Organism | Position | Strand | Matrix score. | sequence | function |
| --- | --- | --- | --- | --- | --- | --- |
| CTAG-motif | Avena sativa | 454 | - | 9 | ACTAGCAGAA |  |

> 2018/04/13 10:10:12  
+ CACACACAGT AAACACTGGT GCATTTATGC ACCTCAAGCA CTGAAAGTTT GCTGGAGAAT GCTAGCCTTA   
  
  
+ TTCAGTGAGA TGCCTTGTTT ATTAAAGAGA AAGAAACCAA GATATAGATG GCTTCTAACC TCTTTTTGTA   
  
  
+ TGAAATGGAT GAAACAAATA GAAATATCGT GCCAAAACAT CCAGGCAGTA TATTTAGATC AGGCAGCATA   
  
  
+ AGAGGAGACA GTACATTTTG CCCGCTTTGG TAGAAAGAAC TAGGATCCTG ACTGTTTTAC CGTCAATTTT   
  
  
+ AGTTGACTGC AAGTTTGTCT TCCCAACCCA TTTAGATTTT CATTTTCTAT TGGACATTCC ATTTCTTCCA   
  
  
+ GAGGTCTAGG AGGTCAAGTT TTTGGAAGAA ATTTATAAGA TTATTGTTGT TTTCTTCCCT GGCTGCAGTT   
  
  
+ ACTTGACTTG TTAGGTGGCA GACAGTTGCT TCGTTCTGTT AGTCCACATC CACATGACAT CAAAACTTAG   
  
  
+ GATTGATTTT AGTTCTTGTT TATGCTCTGA ATTCTCAAAC TGTCTTTAAA AAATTCATTA GTCAGCATTT   
  
  
+ GCTTTTGACT ATTGTTTGCA CTGCTGGCCG CAAGAGGGTT TGTATCTTGA TTATTCGTGT AAAAAGAAAT   
  
  
+ TACAGATGCT CAATTCATAG ATTTGGCCAC ACTGTGGGTT CAAACTAAGA TTGACTGAAG GAAGCCAATA   
  
  
+ AAGATCATCT GTTTTTCCTG CTTAATGGCA CCCATATGAG CAGACAGCCA CTTTTTCTGG ATTGCCCAAT   
  
  
+ TCAATGGAGG CACATTAGTA GTCACAGAGA AGGCAGCCTG TTAAGAAAAT GGTATTTCTA GAAGCCAATT   
  
  
+ AGTACCGTTT CCAGATGATA TGGTTAACAG GTCAATAAAA AAAATTATTT AGGACTCTTT TTGTAGTGTC   
  
  
+ TGCCAACTTA TGGAATGCAG CCCTAATTGA AATATGAGCC CATGTGAGCA TCATGTTATA TCAAAGCACT   
  
  
+ GGCAGGGGCC ACAAAGGACG GTGATTCAAG CTCTGTATAT TAAACATGAA AAAGAGATGA AATGGGTAAG   
  
  
+ AAGTCAGCCT AGCCTTGTGG ATAACAGAGG TAGGCTGGCA GGCATGTCTA AGTTCTGATG TAACAACCAA   
  
  
+ AGAGGCAAAG GAGAAGAAAG CCAAAAAGAA AAAAAAGAAA AAAAGAAAGG TCGAGAAGGC AAGTGACACA   
  
  
+ AACCCAAGAC CCAGATCCAA TGTAACAAGT CAACTTCCTT CTCTTTCTCT CTCTGTCACT GACAATCTTC   
  
  
+ ACTAACACAC ACAAACACGC CCCGCACCGC ATTGGACAAA CACCGAAACC AGAAACACGC TCCCCATCTG   
  
  
+ CGATTCAGTC CCTTCTCTCC TTTAAATTTA CCGTGTTACC CTTCCCTCTC CCCTCTTCAC TGCCCTAATT   
  
  
+ ACAAATCTAC TTCTCTCCTC TTTTGACTCT GTTCTCTCTT CTCTCTCATA CTCGCGGTAA GCACGCCAGA   
  
  
+ CAACGCGCAC GCACACTTTC TCTTGTCTG  

- GTGTGTGTCA TTTGTGACCA CGTAAATACG TGGAGTTCGT GACTTTCAAA CGACCTCTTA CGATCGGAAT   
  
  
- AAGTCACTCT ACGGAACAAA TAATTTCTCT TTCTTTGGTT CTATATCTAC CGAAGATTGG AGAAAAACAT   
  
  
- ACTTTACCTA CTTTGTTTAT CTTTATAGCA CGGTTTTGTA GGTCCGTCAT ATAAATCTAG TCCGTCGTAT   
  
  
- TCTCCTCTGT CATGTAAAAC GGGCGAAACC ATCTTTCTTG ATCCTAGGAC TGACAAAATG GCAGTTAAAA   
  
  
- TCAACTGACG TTCAAACAGA AGGGTTGGGT AAATCTAAAA GTAAAAGATA ACCTGTAAGG TAAAGAAGGT   
  
  
- CTCCAGATCC TCCAGTTCAA AAACCTTCTT TAAATATTCT AATAACAACA AAAGAAGGGA CCGACGTCAA   
  
  
- TGAACTGAAC AATCCACCGT CTGTCAACGA AGCAAGACAA TCAGGTGTAG GTGTACTGTA GTTTTGAATC   
  
  
- CTAACTAAAA TCAAGAACAA ATACGAGACT TAAGAGTTTG ACAGAAATTT TTTAAGTAAT CAGTCGTAAA   
  
  
- CGAAAACTGA TAACAAACGT GACGACCGGC GTTCTCCCAA ACATAGAACT AATAAGCACA TTTTTCTTTA   
  
  
- ATGTCTACGA GTTAAGTATC TAAACCGGTG TGACACCCAA GTTTGATTCT AACTGACTTC CTTCGGTTAT   
  
  
- TTCTAGTAGA CAAAAAGGAC GAATTACCGT GGGTATACTC GTCTGTCGGT GAAAAAGACC TAACGGGTTA   
  
  
- AGTTACCTCC GTGTAATCAT CAGTGTCTCT TCCGTCGGAC AATTCTTTTA CCATAAAGAT CTTCGGTTAA   
  
  
- TCATGGCAAA GGTCTACTAT ACCAATTGTC CAGTTATTTT TTTTAATAAA TCCTGAGAAA AACATCACAG   
  
  
- ACGGTTGAAT ACCTTACGTC GGGATTAACT TTATACTCGG GTACACTCGT AGTACAATAT AGTTTCGTGA   
  
  
- CCGTCCCCGG TGTTTCCTGC CACTAAGTTC GAGACATATA ATTTGTACTT TTTCTCTACT TTACCCATTC   
  
  
- TTCAGTCGGA TCGGAACACC TATTGTCTCC ATCCGACCGT CCGTACAGAT TCAAGACTAC ATTGTTGGTT   
  
  
- TCTCCGTTTC CTCTTCTTTC GGTTTTTCTT TTTTTTCTTT TTTTCTTTCC AGCTCTTCCG TTCACTGTGT   
  
  
- TTGGGTTCTG GGTCTAGGTT ACATTGTTCA GTTGAAGGAA GAGAAAGAGA GAGACAGTGA CTGTTAGAAG   
  
  
- TGATTGTGTG TGTTTGTGCG GGGCGTGGCG TAACCTGTTT GTGGCTTTGG TCTTTGTGCG AGGGGTAGAC   
  
  
- GCTAAGTCAG GGAAGAGAGG AAATTTAAAT GGCACAATGG GAAGGGAGAG GGGAGAAGTG ACGGGATTAA   
  
  
- TGTTTAGATG AAGAGAGGAG AAAACTGAGA CAAGAGAGAA GAGAGAGTAT GAGCGCCATT CGTGCGGTCT   
  
  
- GTTGCGCGTG CGTGTGAAAG AGAACAGAC

+     G-Box

| Site Name | Organism | Position | Strand | Matrix score. | sequence | function |
| --- | --- | --- | --- | --- | --- | --- |
| G-Box | Triticum aestivum | 469 | + | 10 | TCCACATGGCA | cis-acting regulatory element involved in light responsiveness |

> 2018/04/13 10:10:12  
+ CACACACAGT AAACACTGGT GCATTTATGC ACCTCAAGCA CTGAAAGTTT GCTGGAGAAT GCTAGCCTTA   
  
  
+ TTCAGTGAGA TGCCTTGTTT ATTAAAGAGA AAGAAACCAA GATATAGATG GCTTCTAACC TCTTTTTGTA   
  
  
+ TGAAATGGAT GAAACAAATA GAAATATCGT GCCAAAACAT CCAGGCAGTA TATTTAGATC AGGCAGCATA   
  
  
+ AGAGGAGACA GTACATTTTG CCCGCTTTGG TAGAAAGAAC TAGGATCCTG ACTGTTTTAC CGTCAATTTT   
  
  
+ AGTTGACTGC AAGTTTGTCT TCCCAACCCA TTTAGATTTT CATTTTCTAT TGGACATTCC ATTTCTTCCA   
  
  
+ GAGGTCTAGG AGGTCAAGTT TTTGGAAGAA ATTTATAAGA TTATTGTTGT TTTCTTCCCT GGCTGCAGTT   
  
  
+ ACTTGACTTG TTAGGTGGCA GACAGTTGCT TCGTTCTGTT AGTCCACATC CACATGACAT CAAAACTTAG   
  
  
+ GATTGATTTT AGTTCTTGTT TATGCTCTGA ATTCTCAAAC TGTCTTTAAA AAATTCATTA GTCAGCATTT   
  
  
+ GCTTTTGACT ATTGTTTGCA CTGCTGGCCG CAAGAGGGTT TGTATCTTGA TTATTCGTGT AAAAAGAAAT   
  
  
+ TACAGATGCT CAATTCATAG ATTTGGCCAC ACTGTGGGTT CAAACTAAGA TTGACTGAAG GAAGCCAATA   
  
  
+ AAGATCATCT GTTTTTCCTG CTTAATGGCA CCCATATGAG CAGACAGCCA CTTTTTCTGG ATTGCCCAAT   
  
  
+ TCAATGGAGG CACATTAGTA GTCACAGAGA AGGCAGCCTG TTAAGAAAAT GGTATTTCTA GAAGCCAATT   
  
  
+ AGTACCGTTT CCAGATGATA TGGTTAACAG GTCAATAAAA AAAATTATTT AGGACTCTTT TTGTAGTGTC   
  
  
+ TGCCAACTTA TGGAATGCAG CCCTAATTGA AATATGAGCC CATGTGAGCA TCATGTTATA TCAAAGCACT   
  
  
+ GGCAGGGGCC ACAAAGGACG GTGATTCAAG CTCTGTATAT TAAACATGAA AAAGAGATGA AATGGGTAAG   
  
  
+ AAGTCAGCCT AGCCTTGTGG ATAACAGAGG TAGGCTGGCA GGCATGTCTA AGTTCTGATG TAACAACCAA   
  
  
+ AGAGGCAAAG GAGAAGAAAG CCAAAAAGAA AAAAAAGAAA AAAAGAAAGG TCGAGAAGGC AAGTGACACA   
  
  
+ AACCCAAGAC CCAGATCCAA TGTAACAAGT CAACTTCCTT CTCTTTCTCT CTCTGTCACT GACAATCTTC   
  
  
+ ACTAACACAC ACAAACACGC CCCGCACCGC ATTGGACAAA CACCGAAACC AGAAACACGC TCCCCATCTG   
  
  
+ CGATTCAGTC CCTTCTCTCC TTTAAATTTA CCGTGTTACC CTTCCCTCTC CCCTCTTCAC TGCCCTAATT   
  
  
+ ACAAATCTAC TTCTCTCCTC TTTTGACTCT GTTCTCTCTT CTCTCTCATA CTCGCGGTAA GCACGCCAGA   
  
  
+ CAACGCGCAC GCACACTTTC TCTTGTCTG  

- GTGTGTGTCA TTTGTGACCA CGTAAATACG TGGAGTTCGT GACTTTCAAA CGACCTCTTA CGATCGGAAT   
  
  
- AAGTCACTCT ACGGAACAAA TAATTTCTCT TTCTTTGGTT CTATATCTAC CGAAGATTGG AGAAAAACAT   
  
  
- ACTTTACCTA CTTTGTTTAT CTTTATAGCA CGGTTTTGTA GGTCCGTCAT ATAAATCTAG TCCGTCGTAT   
  
  
- TCTCCTCTGT CATGTAAAAC GGGCGAAACC ATCTTTCTTG ATCCTAGGAC TGACAAAATG GCAGTTAAAA   
  
  
- TCAACTGACG TTCAAACAGA AGGGTTGGGT AAATCTAAAA GTAAAAGATA ACCTGTAAGG TAAAGAAGGT   
  
  
- CTCCAGATCC TCCAGTTCAA AAACCTTCTT TAAATATTCT AATAACAACA AAAGAAGGGA CCGACGTCAA   
  
  
- TGAACTGAAC AATCCACCGT CTGTCAACGA AGCAAGACAA TCAGGTGTAG GTGTACTGTA GTTTTGAATC   
  
  
- CTAACTAAAA TCAAGAACAA ATACGAGACT TAAGAGTTTG ACAGAAATTT TTTAAGTAAT CAGTCGTAAA   
  
  
- CGAAAACTGA TAACAAACGT GACGACCGGC GTTCTCCCAA ACATAGAACT AATAAGCACA TTTTTCTTTA   
  
  
- ATGTCTACGA GTTAAGTATC TAAACCGGTG TGACACCCAA GTTTGATTCT AACTGACTTC CTTCGGTTAT   
  
  
- TTCTAGTAGA CAAAAAGGAC GAATTACCGT GGGTATACTC GTCTGTCGGT GAAAAAGACC TAACGGGTTA   
  
  
- AGTTACCTCC GTGTAATCAT CAGTGTCTCT TCCGTCGGAC AATTCTTTTA CCATAAAGAT CTTCGGTTAA   
  
  
- TCATGGCAAA GGTCTACTAT ACCAATTGTC CAGTTATTTT TTTTAATAAA TCCTGAGAAA AACATCACAG   
  
  
- ACGGTTGAAT ACCTTACGTC GGGATTAACT TTATACTCGG GTACACTCGT AGTACAATAT AGTTTCGTGA   
  
  
- CCGTCCCCGG TGTTTCCTGC CACTAAGTTC GAGACATATA ATTTGTACTT TTTCTCTACT TTACCCATTC   
  
  
- TTCAGTCGGA TCGGAACACC TATTGTCTCC ATCCGACCGT CCGTACAGAT TCAAGACTAC ATTGTTGGTT   
  
  
- TCTCCGTTTC CTCTTCTTTC GGTTTTTCTT TTTTTTCTTT TTTTCTTTCC AGCTCTTCCG TTCACTGTGT   
  
  
- TTGGGTTCTG GGTCTAGGTT ACATTGTTCA GTTGAAGGAA GAGAAAGAGA GAGACAGTGA CTGTTAGAAG   
  
  
- TGATTGTGTG TGTTTGTGCG GGGCGTGGCG TAACCTGTTT GTGGCTTTGG TCTTTGTGCG AGGGGTAGAC   
  
  
- GCTAAGTCAG GGAAGAGAGG AAATTTAAAT GGCACAATGG GAAGGGAGAG GGGAGAAGTG ACGGGATTAA   
  
  
- TGTTTAGATG AAGAGAGGAG AAAACTGAGA CAAGAGAGAA GAGAGAGTAT GAGCGCCATT CGTGCGGTCT   
  
  
- GTTGCGCGTG CGTGTGAAAG AGAACAGAC

+     G-box

| Site Name | Organism | Position | Strand | Matrix score. | sequence | function |
| --- | --- | --- | --- | --- | --- | --- |
| G-box | Solanum tuberosum | 950 | - | 7 | CACATGG | cis-acting regulatory element involved in light responsiveness |

> 2018/04/13 10:10:12  
+ CACACACAGT AAACACTGGT GCATTTATGC ACCTCAAGCA CTGAAAGTTT GCTGGAGAAT GCTAGCCTTA   
  
  
+ TTCAGTGAGA TGCCTTGTTT ATTAAAGAGA AAGAAACCAA GATATAGATG GCTTCTAACC TCTTTTTGTA   
  
  
+ TGAAATGGAT GAAACAAATA GAAATATCGT GCCAAAACAT CCAGGCAGTA TATTTAGATC AGGCAGCATA   
  
  
+ AGAGGAGACA GTACATTTTG CCCGCTTTGG TAGAAAGAAC TAGGATCCTG ACTGTTTTAC CGTCAATTTT   
  
  
+ AGTTGACTGC AAGTTTGTCT TCCCAACCCA TTTAGATTTT CATTTTCTAT TGGACATTCC ATTTCTTCCA   
  
  
+ GAGGTCTAGG AGGTCAAGTT TTTGGAAGAA ATTTATAAGA TTATTGTTGT TTTCTTCCCT GGCTGCAGTT   
  
  
+ ACTTGACTTG TTAGGTGGCA GACAGTTGCT TCGTTCTGTT AGTCCACATC CACATGACAT CAAAACTTAG   
  
  
+ GATTGATTTT AGTTCTTGTT TATGCTCTGA ATTCTCAAAC TGTCTTTAAA AAATTCATTA GTCAGCATTT   
  
  
+ GCTTTTGACT ATTGTTTGCA CTGCTGGCCG CAAGAGGGTT TGTATCTTGA TTATTCGTGT AAAAAGAAAT   
  
  
+ TACAGATGCT CAATTCATAG ATTTGGCCAC ACTGTGGGTT CAAACTAAGA TTGACTGAAG GAAGCCAATA   
  
  
+ AAGATCATCT GTTTTTCCTG CTTAATGGCA CCCATATGAG CAGACAGCCA CTTTTTCTGG ATTGCCCAAT   
  
  
+ TCAATGGAGG CACATTAGTA GTCACAGAGA AGGCAGCCTG TTAAGAAAAT GGTATTTCTA GAAGCCAATT   
  
  
+ AGTACCGTTT CCAGATGATA TGGTTAACAG GTCAATAAAA AAAATTATTT AGGACTCTTT TTGTAGTGTC   
  
  
+ TGCCAACTTA TGGAATGCAG CCCTAATTGA AATATGAGCC CATGTGAGCA TCATGTTATA TCAAAGCACT   
  
  
+ GGCAGGGGCC ACAAAGGACG GTGATTCAAG CTCTGTATAT TAAACATGAA AAAGAGATGA AATGGGTAAG   
  
  
+ AAGTCAGCCT AGCCTTGTGG ATAACAGAGG TAGGCTGGCA GGCATGTCTA AGTTCTGATG TAACAACCAA   
  
  
+ AGAGGCAAAG GAGAAGAAAG CCAAAAAGAA AAAAAAGAAA AAAAGAAAGG TCGAGAAGGC AAGTGACACA   
  
  
+ AACCCAAGAC CCAGATCCAA TGTAACAAGT CAACTTCCTT CTCTTTCTCT CTCTGTCACT GACAATCTTC   
  
  
+ ACTAACACAC ACAAACACGC CCCGCACCGC ATTGGACAAA CACCGAAACC AGAAACACGC TCCCCATCTG   
  
  
+ CGATTCAGTC CCTTCTCTCC TTTAAATTTA CCGTGTTACC CTTCCCTCTC CCCTCTTCAC TGCCCTAATT   
  
  
+ ACAAATCTAC TTCTCTCCTC TTTTGACTCT GTTCTCTCTT CTCTCTCATA CTCGCGGTAA GCACGCCAGA   
  
  
+ CAACGCGCAC GCACACTTTC TCTTGTCTG  

- GTGTGTGTCA TTTGTGACCA CGTAAATACG TGGAGTTCGT GACTTTCAAA CGACCTCTTA CGATCGGAAT   
  
  
- AAGTCACTCT ACGGAACAAA TAATTTCTCT TTCTTTGGTT CTATATCTAC CGAAGATTGG AGAAAAACAT   
  
  
- ACTTTACCTA CTTTGTTTAT CTTTATAGCA CGGTTTTGTA GGTCCGTCAT ATAAATCTAG TCCGTCGTAT   
  
  
- TCTCCTCTGT CATGTAAAAC GGGCGAAACC ATCTTTCTTG ATCCTAGGAC TGACAAAATG GCAGTTAAAA   
  
  
- TCAACTGACG TTCAAACAGA AGGGTTGGGT AAATCTAAAA GTAAAAGATA ACCTGTAAGG TAAAGAAGGT   
  
  
- CTCCAGATCC TCCAGTTCAA AAACCTTCTT TAAATATTCT AATAACAACA AAAGAAGGGA CCGACGTCAA   
  
  
- TGAACTGAAC AATCCACCGT CTGTCAACGA AGCAAGACAA TCAGGTGTAG GTGTACTGTA GTTTTGAATC   
  
  
- CTAACTAAAA TCAAGAACAA ATACGAGACT TAAGAGTTTG ACAGAAATTT TTTAAGTAAT CAGTCGTAAA   
  
  
- CGAAAACTGA TAACAAACGT GACGACCGGC GTTCTCCCAA ACATAGAACT AATAAGCACA TTTTTCTTTA   
  
  
- ATGTCTACGA GTTAAGTATC TAAACCGGTG TGACACCCAA GTTTGATTCT AACTGACTTC CTTCGGTTAT   
  
  
- TTCTAGTAGA CAAAAAGGAC GAATTACCGT GGGTATACTC GTCTGTCGGT GAAAAAGACC TAACGGGTTA   
  
  
- AGTTACCTCC GTGTAATCAT CAGTGTCTCT TCCGTCGGAC AATTCTTTTA CCATAAAGAT CTTCGGTTAA   
  
  
- TCATGGCAAA GGTCTACTAT ACCAATTGTC CAGTTATTTT TTTTAATAAA TCCTGAGAAA AACATCACAG   
  
  
- ACGGTTGAAT ACCTTACGTC GGGATTAACT TTATACTCGG GTACACTCGT AGTACAATAT AGTTTCGTGA   
  
  
- CCGTCCCCGG TGTTTCCTGC CACTAAGTTC GAGACATATA ATTTGTACTT TTTCTCTACT TTACCCATTC   
  
  
- TTCAGTCGGA TCGGAACACC TATTGTCTCC ATCCGACCGT CCGTACAGAT TCAAGACTAC ATTGTTGGTT   
  
  
- TCTCCGTTTC CTCTTCTTTC GGTTTTTCTT TTTTTTCTTT TTTTCTTTCC AGCTCTTCCG TTCACTGTGT   
  
  
- TTGGGTTCTG GGTCTAGGTT ACATTGTTCA GTTGAAGGAA GAGAAAGAGA GAGACAGTGA CTGTTAGAAG   
  
  
- TGATTGTGTG TGTTTGTGCG GGGCGTGGCG TAACCTGTTT GTGGCTTTGG TCTTTGTGCG AGGGGTAGAC   
  
  
- GCTAAGTCAG GGAAGAGAGG AAATTTAAAT GGCACAATGG GAAGGGAGAG GGGAGAAGTG ACGGGATTAA   
  
  
- TGTTTAGATG AAGAGAGGAG AAAACTGAGA CAAGAGAGAA GAGAGAGTAT GAGCGCCATT CGTGCGGTCT   
  
  
- GTTGCGCGTG CGTGTGAAAG AGAACAGAC

+     GAG-motif

| Site Name | Organism | Position | Strand | Matrix score. | sequence | function |
| --- | --- | --- | --- | --- | --- | --- |
| GAG-motif | Spinacia oleracea | 1033 | + | 7 | AGAGATG | part of a light responsive element |

> 2018/04/13 10:10:12  
+ CACACACAGT AAACACTGGT GCATTTATGC ACCTCAAGCA CTGAAAGTTT GCTGGAGAAT GCTAGCCTTA   
  
  
+ TTCAGTGAGA TGCCTTGTTT ATTAAAGAGA AAGAAACCAA GATATAGATG GCTTCTAACC TCTTTTTGTA   
  
  
+ TGAAATGGAT GAAACAAATA GAAATATCGT GCCAAAACAT CCAGGCAGTA TATTTAGATC AGGCAGCATA   
  
  
+ AGAGGAGACA GTACATTTTG CCCGCTTTGG TAGAAAGAAC TAGGATCCTG ACTGTTTTAC CGTCAATTTT   
  
  
+ AGTTGACTGC AAGTTTGTCT TCCCAACCCA TTTAGATTTT CATTTTCTAT TGGACATTCC ATTTCTTCCA   
  
  
+ GAGGTCTAGG AGGTCAAGTT TTTGGAAGAA ATTTATAAGA TTATTGTTGT TTTCTTCCCT GGCTGCAGTT   
  
  
+ ACTTGACTTG TTAGGTGGCA GACAGTTGCT TCGTTCTGTT AGTCCACATC CACATGACAT CAAAACTTAG   
  
  
+ GATTGATTTT AGTTCTTGTT TATGCTCTGA ATTCTCAAAC TGTCTTTAAA AAATTCATTA GTCAGCATTT   
  
  
+ GCTTTTGACT ATTGTTTGCA CTGCTGGCCG CAAGAGGGTT TGTATCTTGA TTATTCGTGT AAAAAGAAAT   
  
  
+ TACAGATGCT CAATTCATAG ATTTGGCCAC ACTGTGGGTT CAAACTAAGA TTGACTGAAG GAAGCCAATA   
  
  
+ AAGATCATCT GTTTTTCCTG CTTAATGGCA CCCATATGAG CAGACAGCCA CTTTTTCTGG ATTGCCCAAT   
  
  
+ TCAATGGAGG CACATTAGTA GTCACAGAGA AGGCAGCCTG TTAAGAAAAT GGTATTTCTA GAAGCCAATT   
  
  
+ AGTACCGTTT CCAGATGATA TGGTTAACAG GTCAATAAAA AAAATTATTT AGGACTCTTT TTGTAGTGTC   
  
  
+ TGCCAACTTA TGGAATGCAG CCCTAATTGA AATATGAGCC CATGTGAGCA TCATGTTATA TCAAAGCACT   
  
  
+ GGCAGGGGCC ACAAAGGACG GTGATTCAAG CTCTGTATAT TAAACATGAA AAAGAGATGA AATGGGTAAG   
  
  
+ AAGTCAGCCT AGCCTTGTGG ATAACAGAGG TAGGCTGGCA GGCATGTCTA AGTTCTGATG TAACAACCAA   
  
  
+ AGAGGCAAAG GAGAAGAAAG CCAAAAAGAA AAAAAAGAAA AAAAGAAAGG TCGAGAAGGC AAGTGACACA   
  
  
+ AACCCAAGAC CCAGATCCAA TGTAACAAGT CAACTTCCTT CTCTTTCTCT CTCTGTCACT GACAATCTTC   
  
  
+ ACTAACACAC ACAAACACGC CCCGCACCGC ATTGGACAAA CACCGAAACC AGAAACACGC TCCCCATCTG   
  
  
+ CGATTCAGTC CCTTCTCTCC TTTAAATTTA CCGTGTTACC CTTCCCTCTC CCCTCTTCAC TGCCCTAATT   
  
  
+ ACAAATCTAC TTCTCTCCTC TTTTGACTCT GTTCTCTCTT CTCTCTCATA CTCGCGGTAA GCACGCCAGA   
  
  
+ CAACGCGCAC GCACACTTTC TCTTGTCTG  

- GTGTGTGTCA TTTGTGACCA CGTAAATACG TGGAGTTCGT GACTTTCAAA CGACCTCTTA CGATCGGAAT   
  
  
- AAGTCACTCT ACGGAACAAA TAATTTCTCT TTCTTTGGTT CTATATCTAC CGAAGATTGG AGAAAAACAT   
  
  
- ACTTTACCTA CTTTGTTTAT CTTTATAGCA CGGTTTTGTA GGTCCGTCAT ATAAATCTAG TCCGTCGTAT   
  
  
- TCTCCTCTGT CATGTAAAAC GGGCGAAACC ATCTTTCTTG ATCCTAGGAC TGACAAAATG GCAGTTAAAA   
  
  
- TCAACTGACG TTCAAACAGA AGGGTTGGGT AAATCTAAAA GTAAAAGATA ACCTGTAAGG TAAAGAAGGT   
  
  
- CTCCAGATCC TCCAGTTCAA AAACCTTCTT TAAATATTCT AATAACAACA AAAGAAGGGA CCGACGTCAA   
  
  
- TGAACTGAAC AATCCACCGT CTGTCAACGA AGCAAGACAA TCAGGTGTAG GTGTACTGTA GTTTTGAATC   
  
  
- CTAACTAAAA TCAAGAACAA ATACGAGACT TAAGAGTTTG ACAGAAATTT TTTAAGTAAT CAGTCGTAAA   
  
  
- CGAAAACTGA TAACAAACGT GACGACCGGC GTTCTCCCAA ACATAGAACT AATAAGCACA TTTTTCTTTA   
  
  
- ATGTCTACGA GTTAAGTATC TAAACCGGTG TGACACCCAA GTTTGATTCT AACTGACTTC CTTCGGTTAT   
  
  
- TTCTAGTAGA CAAAAAGGAC GAATTACCGT GGGTATACTC GTCTGTCGGT GAAAAAGACC TAACGGGTTA   
  
  
- AGTTACCTCC GTGTAATCAT CAGTGTCTCT TCCGTCGGAC AATTCTTTTA CCATAAAGAT CTTCGGTTAA   
  
  
- TCATGGCAAA GGTCTACTAT ACCAATTGTC CAGTTATTTT TTTTAATAAA TCCTGAGAAA AACATCACAG   
  
  
- ACGGTTGAAT ACCTTACGTC GGGATTAACT TTATACTCGG GTACACTCGT AGTACAATAT AGTTTCGTGA   
  
  
- CCGTCCCCGG TGTTTCCTGC CACTAAGTTC GAGACATATA ATTTGTACTT TTTCTCTACT TTACCCATTC   
  
  
- TTCAGTCGGA TCGGAACACC TATTGTCTCC ATCCGACCGT CCGTACAGAT TCAAGACTAC ATTGTTGGTT   
  
  
- TCTCCGTTTC CTCTTCTTTC GGTTTTTCTT TTTTTTCTTT TTTTCTTTCC AGCTCTTCCG TTCACTGTGT   
  
  
- TTGGGTTCTG GGTCTAGGTT ACATTGTTCA GTTGAAGGAA GAGAAAGAGA GAGACAGTGA CTGTTAGAAG   
  
  
- TGATTGTGTG TGTTTGTGCG GGGCGTGGCG TAACCTGTTT GTGGCTTTGG TCTTTGTGCG AGGGGTAGAC   
  
  
- GCTAAGTCAG GGAAGAGAGG AAATTTAAAT GGCACAATGG GAAGGGAGAG GGGAGAAGTG ACGGGATTAA   
  
  
- TGTTTAGATG AAGAGAGGAG AAAACTGAGA CAAGAGAGAA GAGAGAGTAT GAGCGCCATT CGTGCGGTCT   
  
  
- GTTGCGCGTG CGTGTGAAAG AGAACAGAC

+     GARE-motif

| Site Name | Organism | Position | Strand | Matrix score. | sequence | function |
| --- | --- | --- | --- | --- | --- | --- |
| GARE-motif | Brassica oleracea | 708 | - | 7 | AAACAGA | gibberellin-responsive element |

> 2018/04/13 10:10:12  
+ CACACACAGT AAACACTGGT GCATTTATGC ACCTCAAGCA CTGAAAGTTT GCTGGAGAAT GCTAGCCTTA   
  
  
+ TTCAGTGAGA TGCCTTGTTT ATTAAAGAGA AAGAAACCAA GATATAGATG GCTTCTAACC TCTTTTTGTA   
  
  
+ TGAAATGGAT GAAACAAATA GAAATATCGT GCCAAAACAT CCAGGCAGTA TATTTAGATC AGGCAGCATA   
  
  
+ AGAGGAGACA GTACATTTTG CCCGCTTTGG TAGAAAGAAC TAGGATCCTG ACTGTTTTAC CGTCAATTTT   
  
  
+ AGTTGACTGC AAGTTTGTCT TCCCAACCCA TTTAGATTTT CATTTTCTAT TGGACATTCC ATTTCTTCCA   
  
  
+ GAGGTCTAGG AGGTCAAGTT TTTGGAAGAA ATTTATAAGA TTATTGTTGT TTTCTTCCCT GGCTGCAGTT   
  
  
+ ACTTGACTTG TTAGGTGGCA GACAGTTGCT TCGTTCTGTT AGTCCACATC CACATGACAT CAAAACTTAG   
  
  
+ GATTGATTTT AGTTCTTGTT TATGCTCTGA ATTCTCAAAC TGTCTTTAAA AAATTCATTA GTCAGCATTT   
  
  
+ GCTTTTGACT ATTGTTTGCA CTGCTGGCCG CAAGAGGGTT TGTATCTTGA TTATTCGTGT AAAAAGAAAT   
  
  
+ TACAGATGCT CAATTCATAG ATTTGGCCAC ACTGTGGGTT CAAACTAAGA TTGACTGAAG GAAGCCAATA   
  
  
+ AAGATCATCT GTTTTTCCTG CTTAATGGCA CCCATATGAG CAGACAGCCA CTTTTTCTGG ATTGCCCAAT   
  
  
+ TCAATGGAGG CACATTAGTA GTCACAGAGA AGGCAGCCTG TTAAGAAAAT GGTATTTCTA GAAGCCAATT   
  
  
+ AGTACCGTTT CCAGATGATA TGGTTAACAG GTCAATAAAA AAAATTATTT AGGACTCTTT TTGTAGTGTC   
  
  
+ TGCCAACTTA TGGAATGCAG CCCTAATTGA AATATGAGCC CATGTGAGCA TCATGTTATA TCAAAGCACT   
  
  
+ GGCAGGGGCC ACAAAGGACG GTGATTCAAG CTCTGTATAT TAAACATGAA AAAGAGATGA AATGGGTAAG   
  
  
+ AAGTCAGCCT AGCCTTGTGG ATAACAGAGG TAGGCTGGCA GGCATGTCTA AGTTCTGATG TAACAACCAA   
  
  
+ AGAGGCAAAG GAGAAGAAAG CCAAAAAGAA AAAAAAGAAA AAAAGAAAGG TCGAGAAGGC AAGTGACACA   
  
  
+ AACCCAAGAC CCAGATCCAA TGTAACAAGT CAACTTCCTT CTCTTTCTCT CTCTGTCACT GACAATCTTC   
  
  
+ ACTAACACAC ACAAACACGC CCCGCACCGC ATTGGACAAA CACCGAAACC AGAAACACGC TCCCCATCTG   
  
  
+ CGATTCAGTC CCTTCTCTCC TTTAAATTTA CCGTGTTACC CTTCCCTCTC CCCTCTTCAC TGCCCTAATT   
  
  
+ ACAAATCTAC TTCTCTCCTC TTTTGACTCT GTTCTCTCTT CTCTCTCATA CTCGCGGTAA GCACGCCAGA   
  
  
+ CAACGCGCAC GCACACTTTC TCTTGTCTG  

- GTGTGTGTCA TTTGTGACCA CGTAAATACG TGGAGTTCGT GACTTTCAAA CGACCTCTTA CGATCGGAAT   
  
  
- AAGTCACTCT ACGGAACAAA TAATTTCTCT TTCTTTGGTT CTATATCTAC CGAAGATTGG AGAAAAACAT   
  
  
- ACTTTACCTA CTTTGTTTAT CTTTATAGCA CGGTTTTGTA GGTCCGTCAT ATAAATCTAG TCCGTCGTAT   
  
  
- TCTCCTCTGT CATGTAAAAC GGGCGAAACC ATCTTTCTTG ATCCTAGGAC TGACAAAATG GCAGTTAAAA   
  
  
- TCAACTGACG TTCAAACAGA AGGGTTGGGT AAATCTAAAA GTAAAAGATA ACCTGTAAGG TAAAGAAGGT   
  
  
- CTCCAGATCC TCCAGTTCAA AAACCTTCTT TAAATATTCT AATAACAACA AAAGAAGGGA CCGACGTCAA   
  
  
- TGAACTGAAC AATCCACCGT CTGTCAACGA AGCAAGACAA TCAGGTGTAG GTGTACTGTA GTTTTGAATC   
  
  
- CTAACTAAAA TCAAGAACAA ATACGAGACT TAAGAGTTTG ACAGAAATTT TTTAAGTAAT CAGTCGTAAA   
  
  
- CGAAAACTGA TAACAAACGT GACGACCGGC GTTCTCCCAA ACATAGAACT AATAAGCACA TTTTTCTTTA   
  
  
- ATGTCTACGA GTTAAGTATC TAAACCGGTG TGACACCCAA GTTTGATTCT AACTGACTTC CTTCGGTTAT   
  
  
- TTCTAGTAGA CAAAAAGGAC GAATTACCGT GGGTATACTC GTCTGTCGGT GAAAAAGACC TAACGGGTTA   
  
  
- AGTTACCTCC GTGTAATCAT CAGTGTCTCT TCCGTCGGAC AATTCTTTTA CCATAAAGAT CTTCGGTTAA   
  
  
- TCATGGCAAA GGTCTACTAT ACCAATTGTC CAGTTATTTT TTTTAATAAA TCCTGAGAAA AACATCACAG   
  
  
- ACGGTTGAAT ACCTTACGTC GGGATTAACT TTATACTCGG GTACACTCGT AGTACAATAT AGTTTCGTGA   
  
  
- CCGTCCCCGG TGTTTCCTGC CACTAAGTTC GAGACATATA ATTTGTACTT TTTCTCTACT TTACCCATTC   
  
  
- TTCAGTCGGA TCGGAACACC TATTGTCTCC ATCCGACCGT CCGTACAGAT TCAAGACTAC ATTGTTGGTT   
  
  
- TCTCCGTTTC CTCTTCTTTC GGTTTTTCTT TTTTTTCTTT TTTTCTTTCC AGCTCTTCCG TTCACTGTGT   
  
  
- TTGGGTTCTG GGTCTAGGTT ACATTGTTCA GTTGAAGGAA GAGAAAGAGA GAGACAGTGA CTGTTAGAAG   
  
  
- TGATTGTGTG TGTTTGTGCG GGGCGTGGCG TAACCTGTTT GTGGCTTTGG TCTTTGTGCG AGGGGTAGAC   
  
  
- GCTAAGTCAG GGAAGAGAGG AAATTTAAAT GGCACAATGG GAAGGGAGAG GGGAGAAGTG ACGGGATTAA   
  
  
- TGTTTAGATG AAGAGAGGAG AAAACTGAGA CAAGAGAGAA GAGAGAGTAT GAGCGCCATT CGTGCGGTCT   
  
  
- GTTGCGCGTG CGTGTGAAAG AGAACAGAC

+     GCN4\_motif

| Site Name | Organism | Position | Strand | Matrix score. | sequence | function |
| --- | --- | --- | --- | --- | --- | --- |
| GCN4\_motif | Oryza sativa | 1184 | - | 7 | TGTGTCA | cis-regulatory element involved in endosperm expression |

> 2018/04/13 10:10:12  
+ CACACACAGT AAACACTGGT GCATTTATGC ACCTCAAGCA CTGAAAGTTT GCTGGAGAAT GCTAGCCTTA   
  
  
+ TTCAGTGAGA TGCCTTGTTT ATTAAAGAGA AAGAAACCAA GATATAGATG GCTTCTAACC TCTTTTTGTA   
  
  
+ TGAAATGGAT GAAACAAATA GAAATATCGT GCCAAAACAT CCAGGCAGTA TATTTAGATC AGGCAGCATA   
  
  
+ AGAGGAGACA GTACATTTTG CCCGCTTTGG TAGAAAGAAC TAGGATCCTG ACTGTTTTAC CGTCAATTTT   
  
  
+ AGTTGACTGC AAGTTTGTCT TCCCAACCCA TTTAGATTTT CATTTTCTAT TGGACATTCC ATTTCTTCCA   
  
  
+ GAGGTCTAGG AGGTCAAGTT TTTGGAAGAA ATTTATAAGA TTATTGTTGT TTTCTTCCCT GGCTGCAGTT   
  
  
+ ACTTGACTTG TTAGGTGGCA GACAGTTGCT TCGTTCTGTT AGTCCACATC CACATGACAT CAAAACTTAG   
  
  
+ GATTGATTTT AGTTCTTGTT TATGCTCTGA ATTCTCAAAC TGTCTTTAAA AAATTCATTA GTCAGCATTT   
  
  
+ GCTTTTGACT ATTGTTTGCA CTGCTGGCCG CAAGAGGGTT TGTATCTTGA TTATTCGTGT AAAAAGAAAT   
  
  
+ TACAGATGCT CAATTCATAG ATTTGGCCAC ACTGTGGGTT CAAACTAAGA TTGACTGAAG GAAGCCAATA   
  
  
+ AAGATCATCT GTTTTTCCTG CTTAATGGCA CCCATATGAG CAGACAGCCA CTTTTTCTGG ATTGCCCAAT   
  
  
+ TCAATGGAGG CACATTAGTA GTCACAGAGA AGGCAGCCTG TTAAGAAAAT GGTATTTCTA GAAGCCAATT   
  
  
+ AGTACCGTTT CCAGATGATA TGGTTAACAG GTCAATAAAA AAAATTATTT AGGACTCTTT TTGTAGTGTC   
  
  
+ TGCCAACTTA TGGAATGCAG CCCTAATTGA AATATGAGCC CATGTGAGCA TCATGTTATA TCAAAGCACT   
  
  
+ GGCAGGGGCC ACAAAGGACG GTGATTCAAG CTCTGTATAT TAAACATGAA AAAGAGATGA AATGGGTAAG   
  
  
+ AAGTCAGCCT AGCCTTGTGG ATAACAGAGG TAGGCTGGCA GGCATGTCTA AGTTCTGATG TAACAACCAA   
  
  
+ AGAGGCAAAG GAGAAGAAAG CCAAAAAGAA AAAAAAGAAA AAAAGAAAGG TCGAGAAGGC AAGTGACACA   
  
  
+ AACCCAAGAC CCAGATCCAA TGTAACAAGT CAACTTCCTT CTCTTTCTCT CTCTGTCACT GACAATCTTC   
  
  
+ ACTAACACAC ACAAACACGC CCCGCACCGC ATTGGACAAA CACCGAAACC AGAAACACGC TCCCCATCTG   
  
  
+ CGATTCAGTC CCTTCTCTCC TTTAAATTTA CCGTGTTACC CTTCCCTCTC CCCTCTTCAC TGCCCTAATT   
  
  
+ ACAAATCTAC TTCTCTCCTC TTTTGACTCT GTTCTCTCTT CTCTCTCATA CTCGCGGTAA GCACGCCAGA   
  
  
+ CAACGCGCAC GCACACTTTC TCTTGTCTG  

- GTGTGTGTCA TTTGTGACCA CGTAAATACG TGGAGTTCGT GACTTTCAAA CGACCTCTTA CGATCGGAAT   
  
  
- AAGTCACTCT ACGGAACAAA TAATTTCTCT TTCTTTGGTT CTATATCTAC CGAAGATTGG AGAAAAACAT   
  
  
- ACTTTACCTA CTTTGTTTAT CTTTATAGCA CGGTTTTGTA GGTCCGTCAT ATAAATCTAG TCCGTCGTAT   
  
  
- TCTCCTCTGT CATGTAAAAC GGGCGAAACC ATCTTTCTTG ATCCTAGGAC TGACAAAATG GCAGTTAAAA   
  
  
- TCAACTGACG TTCAAACAGA AGGGTTGGGT AAATCTAAAA GTAAAAGATA ACCTGTAAGG TAAAGAAGGT   
  
  
- CTCCAGATCC TCCAGTTCAA AAACCTTCTT TAAATATTCT AATAACAACA AAAGAAGGGA CCGACGTCAA   
  
  
- TGAACTGAAC AATCCACCGT CTGTCAACGA AGCAAGACAA TCAGGTGTAG GTGTACTGTA GTTTTGAATC   
  
  
- CTAACTAAAA TCAAGAACAA ATACGAGACT TAAGAGTTTG ACAGAAATTT TTTAAGTAAT CAGTCGTAAA   
  
  
- CGAAAACTGA TAACAAACGT GACGACCGGC GTTCTCCCAA ACATAGAACT AATAAGCACA TTTTTCTTTA   
  
  
- ATGTCTACGA GTTAAGTATC TAAACCGGTG TGACACCCAA GTTTGATTCT AACTGACTTC CTTCGGTTAT   
  
  
- TTCTAGTAGA CAAAAAGGAC GAATTACCGT GGGTATACTC GTCTGTCGGT GAAAAAGACC TAACGGGTTA   
  
  
- AGTTACCTCC GTGTAATCAT CAGTGTCTCT TCCGTCGGAC AATTCTTTTA CCATAAAGAT CTTCGGTTAA   
  
  
- TCATGGCAAA GGTCTACTAT ACCAATTGTC CAGTTATTTT TTTTAATAAA TCCTGAGAAA AACATCACAG   
  
  
- ACGGTTGAAT ACCTTACGTC GGGATTAACT TTATACTCGG GTACACTCGT AGTACAATAT AGTTTCGTGA   
  
  
- CCGTCCCCGG TGTTTCCTGC CACTAAGTTC GAGACATATA ATTTGTACTT TTTCTCTACT TTACCCATTC   
  
  
- TTCAGTCGGA TCGGAACACC TATTGTCTCC ATCCGACCGT CCGTACAGAT TCAAGACTAC ATTGTTGGTT   
  
  
- TCTCCGTTTC CTCTTCTTTC GGTTTTTCTT TTTTTTCTTT TTTTCTTTCC AGCTCTTCCG TTCACTGTGT   
  
  
- TTGGGTTCTG GGTCTAGGTT ACATTGTTCA GTTGAAGGAA GAGAAAGAGA GAGACAGTGA CTGTTAGAAG   
  
  
- TGATTGTGTG TGTTTGTGCG GGGCGTGGCG TAACCTGTTT GTGGCTTTGG TCTTTGTGCG AGGGGTAGAC   
  
  
- GCTAAGTCAG GGAAGAGAGG AAATTTAAAT GGCACAATGG GAAGGGAGAG GGGAGAAGTG ACGGGATTAA   
  
  
- TGTTTAGATG AAGAGAGGAG AAAACTGAGA CAAGAGAGAA GAGAGAGTAT GAGCGCCATT CGTGCGGTCT   
  
  
- GTTGCGCGTG CGTGTGAAAG AGAACAGAC

+     GT1-motif

| Site Name | Organism | Position | Strand | Matrix score. | sequence | function |
| --- | --- | --- | --- | --- | --- | --- |
| GT1-motif | Arabidopsis thaliana | 862 | + | 6 | GGTTAA | light responsive element |

> 2018/04/13 10:10:12  
+ CACACACAGT AAACACTGGT GCATTTATGC ACCTCAAGCA CTGAAAGTTT GCTGGAGAAT GCTAGCCTTA   
  
  
+ TTCAGTGAGA TGCCTTGTTT ATTAAAGAGA AAGAAACCAA GATATAGATG GCTTCTAACC TCTTTTTGTA   
  
  
+ TGAAATGGAT GAAACAAATA GAAATATCGT GCCAAAACAT CCAGGCAGTA TATTTAGATC AGGCAGCATA   
  
  
+ AGAGGAGACA GTACATTTTG CCCGCTTTGG TAGAAAGAAC TAGGATCCTG ACTGTTTTAC CGTCAATTTT   
  
  
+ AGTTGACTGC AAGTTTGTCT TCCCAACCCA TTTAGATTTT CATTTTCTAT TGGACATTCC ATTTCTTCCA   
  
  
+ GAGGTCTAGG AGGTCAAGTT TTTGGAAGAA ATTTATAAGA TTATTGTTGT TTTCTTCCCT GGCTGCAGTT   
  
  
+ ACTTGACTTG TTAGGTGGCA GACAGTTGCT TCGTTCTGTT AGTCCACATC CACATGACAT CAAAACTTAG   
  
  
+ GATTGATTTT AGTTCTTGTT TATGCTCTGA ATTCTCAAAC TGTCTTTAAA AAATTCATTA GTCAGCATTT   
  
  
+ GCTTTTGACT ATTGTTTGCA CTGCTGGCCG CAAGAGGGTT TGTATCTTGA TTATTCGTGT AAAAAGAAAT   
  
  
+ TACAGATGCT CAATTCATAG ATTTGGCCAC ACTGTGGGTT CAAACTAAGA TTGACTGAAG GAAGCCAATA   
  
  
+ AAGATCATCT GTTTTTCCTG CTTAATGGCA CCCATATGAG CAGACAGCCA CTTTTTCTGG ATTGCCCAAT   
  
  
+ TCAATGGAGG CACATTAGTA GTCACAGAGA AGGCAGCCTG TTAAGAAAAT GGTATTTCTA GAAGCCAATT   
  
  
+ AGTACCGTTT CCAGATGATA TGGTTAACAG GTCAATAAAA AAAATTATTT AGGACTCTTT TTGTAGTGTC   
  
  
+ TGCCAACTTA TGGAATGCAG CCCTAATTGA AATATGAGCC CATGTGAGCA TCATGTTATA TCAAAGCACT   
  
  
+ GGCAGGGGCC ACAAAGGACG GTGATTCAAG CTCTGTATAT TAAACATGAA AAAGAGATGA AATGGGTAAG   
  
  
+ AAGTCAGCCT AGCCTTGTGG ATAACAGAGG TAGGCTGGCA GGCATGTCTA AGTTCTGATG TAACAACCAA   
  
  
+ AGAGGCAAAG GAGAAGAAAG CCAAAAAGAA AAAAAAGAAA AAAAGAAAGG TCGAGAAGGC AAGTGACACA   
  
  
+ AACCCAAGAC CCAGATCCAA TGTAACAAGT CAACTTCCTT CTCTTTCTCT CTCTGTCACT GACAATCTTC   
  
  
+ ACTAACACAC ACAAACACGC CCCGCACCGC ATTGGACAAA CACCGAAACC AGAAACACGC TCCCCATCTG   
  
  
+ CGATTCAGTC CCTTCTCTCC TTTAAATTTA CCGTGTTACC CTTCCCTCTC CCCTCTTCAC TGCCCTAATT   
  
  
+ ACAAATCTAC TTCTCTCCTC TTTTGACTCT GTTCTCTCTT CTCTCTCATA CTCGCGGTAA GCACGCCAGA   
  
  
+ CAACGCGCAC GCACACTTTC TCTTGTCTG  

- GTGTGTGTCA TTTGTGACCA CGTAAATACG TGGAGTTCGT GACTTTCAAA CGACCTCTTA CGATCGGAAT   
  
  
- AAGTCACTCT ACGGAACAAA TAATTTCTCT TTCTTTGGTT CTATATCTAC CGAAGATTGG AGAAAAACAT   
  
  
- ACTTTACCTA CTTTGTTTAT CTTTATAGCA CGGTTTTGTA GGTCCGTCAT ATAAATCTAG TCCGTCGTAT   
  
  
- TCTCCTCTGT CATGTAAAAC GGGCGAAACC ATCTTTCTTG ATCCTAGGAC TGACAAAATG GCAGTTAAAA   
  
  
- TCAACTGACG TTCAAACAGA AGGGTTGGGT AAATCTAAAA GTAAAAGATA ACCTGTAAGG TAAAGAAGGT   
  
  
- CTCCAGATCC TCCAGTTCAA AAACCTTCTT TAAATATTCT AATAACAACA AAAGAAGGGA CCGACGTCAA   
  
  
- TGAACTGAAC AATCCACCGT CTGTCAACGA AGCAAGACAA TCAGGTGTAG GTGTACTGTA GTTTTGAATC   
  
  
- CTAACTAAAA TCAAGAACAA ATACGAGACT TAAGAGTTTG ACAGAAATTT TTTAAGTAAT CAGTCGTAAA   
  
  
- CGAAAACTGA TAACAAACGT GACGACCGGC GTTCTCCCAA ACATAGAACT AATAAGCACA TTTTTCTTTA   
  
  
- ATGTCTACGA GTTAAGTATC TAAACCGGTG TGACACCCAA GTTTGATTCT AACTGACTTC CTTCGGTTAT   
  
  
- TTCTAGTAGA CAAAAAGGAC GAATTACCGT GGGTATACTC GTCTGTCGGT GAAAAAGACC TAACGGGTTA   
  
  
- AGTTACCTCC GTGTAATCAT CAGTGTCTCT TCCGTCGGAC AATTCTTTTA CCATAAAGAT CTTCGGTTAA   
  
  
- TCATGGCAAA GGTCTACTAT ACCAATTGTC CAGTTATTTT TTTTAATAAA TCCTGAGAAA AACATCACAG   
  
  
- ACGGTTGAAT ACCTTACGTC GGGATTAACT TTATACTCGG GTACACTCGT AGTACAATAT AGTTTCGTGA   
  
  
- CCGTCCCCGG TGTTTCCTGC CACTAAGTTC GAGACATATA ATTTGTACTT TTTCTCTACT TTACCCATTC   
  
  
- TTCAGTCGGA TCGGAACACC TATTGTCTCC ATCCGACCGT CCGTACAGAT TCAAGACTAC ATTGTTGGTT   
  
  
- TCTCCGTTTC CTCTTCTTTC GGTTTTTCTT TTTTTTCTTT TTTTCTTTCC AGCTCTTCCG TTCACTGTGT   
  
  
- TTGGGTTCTG GGTCTAGGTT ACATTGTTCA GTTGAAGGAA GAGAAAGAGA GAGACAGTGA CTGTTAGAAG   
  
  
- TGATTGTGTG TGTTTGTGCG GGGCGTGGCG TAACCTGTTT GTGGCTTTGG TCTTTGTGCG AGGGGTAGAC   
  
  
- GCTAAGTCAG GGAAGAGAGG AAATTTAAAT GGCACAATGG GAAGGGAGAG GGGAGAAGTG ACGGGATTAA   
  
  
- TGTTTAGATG AAGAGAGGAG AAAACTGAGA CAAGAGAGAA GAGAGAGTAT GAGCGCCATT CGTGCGGTCT   
  
  
- GTTGCGCGTG CGTGTGAAAG AGAACAGAC

+     I-box

| Site Name | Organism | Position | Strand | Matrix score. | sequence | function |
| --- | --- | --- | --- | --- | --- | --- |
| I-box | Flaveria trinervia | 1201 | + | 10 | cCATATCCAAT | part of a light responsive element |
| I-box | Nicotiana plumbaginifolia | 205 | - | 10 | CTCTTATGCT | part of a light responsive element |
| I-box | Flaveria trinervia | 857 | + | 7 | GATATGG | part of a light responsive element |

> 2018/04/13 10:10:12  
+ CACACACAGT AAACACTGGT GCATTTATGC ACCTCAAGCA CTGAAAGTTT GCTGGAGAAT GCTAGCCTTA   
  
  
+ TTCAGTGAGA TGCCTTGTTT ATTAAAGAGA AAGAAACCAA GATATAGATG GCTTCTAACC TCTTTTTGTA   
  
  
+ TGAAATGGAT GAAACAAATA GAAATATCGT GCCAAAACAT CCAGGCAGTA TATTTAGATC AGGCAGCATA   
  
  
+ AGAGGAGACA GTACATTTTG CCCGCTTTGG TAGAAAGAAC TAGGATCCTG ACTGTTTTAC CGTCAATTTT   
  
  
+ AGTTGACTGC AAGTTTGTCT TCCCAACCCA TTTAGATTTT CATTTTCTAT TGGACATTCC ATTTCTTCCA   
  
  
+ GAGGTCTAGG AGGTCAAGTT TTTGGAAGAA ATTTATAAGA TTATTGTTGT TTTCTTCCCT GGCTGCAGTT   
  
  
+ ACTTGACTTG TTAGGTGGCA GACAGTTGCT TCGTTCTGTT AGTCCACATC CACATGACAT CAAAACTTAG   
  
  
+ GATTGATTTT AGTTCTTGTT TATGCTCTGA ATTCTCAAAC TGTCTTTAAA AAATTCATTA GTCAGCATTT   
  
  
+ GCTTTTGACT ATTGTTTGCA CTGCTGGCCG CAAGAGGGTT TGTATCTTGA TTATTCGTGT AAAAAGAAAT   
  
  
+ TACAGATGCT CAATTCATAG ATTTGGCCAC ACTGTGGGTT CAAACTAAGA TTGACTGAAG GAAGCCAATA   
  
  
+ AAGATCATCT GTTTTTCCTG CTTAATGGCA CCCATATGAG CAGACAGCCA CTTTTTCTGG ATTGCCCAAT   
  
  
+ TCAATGGAGG CACATTAGTA GTCACAGAGA AGGCAGCCTG TTAAGAAAAT GGTATTTCTA GAAGCCAATT   
  
  
+ AGTACCGTTT CCAGATGATA TGGTTAACAG GTCAATAAAA AAAATTATTT AGGACTCTTT TTGTAGTGTC   
  
  
+ TGCCAACTTA TGGAATGCAG CCCTAATTGA AATATGAGCC CATGTGAGCA TCATGTTATA TCAAAGCACT   
  
  
+ GGCAGGGGCC ACAAAGGACG GTGATTCAAG CTCTGTATAT TAAACATGAA AAAGAGATGA AATGGGTAAG   
  
  
+ AAGTCAGCCT AGCCTTGTGG ATAACAGAGG TAGGCTGGCA GGCATGTCTA AGTTCTGATG TAACAACCAA   
  
  
+ AGAGGCAAAG GAGAAGAAAG CCAAAAAGAA AAAAAAGAAA AAAAGAAAGG TCGAGAAGGC AAGTGACACA   
  
  
+ AACCCAAGAC CCAGATCCAA TGTAACAAGT CAACTTCCTT CTCTTTCTCT CTCTGTCACT GACAATCTTC   
  
  
+ ACTAACACAC ACAAACACGC CCCGCACCGC ATTGGACAAA CACCGAAACC AGAAACACGC TCCCCATCTG   
  
  
+ CGATTCAGTC CCTTCTCTCC TTTAAATTTA CCGTGTTACC CTTCCCTCTC CCCTCTTCAC TGCCCTAATT   
  
  
+ ACAAATCTAC TTCTCTCCTC TTTTGACTCT GTTCTCTCTT CTCTCTCATA CTCGCGGTAA GCACGCCAGA   
  
  
+ CAACGCGCAC GCACACTTTC TCTTGTCTG  

- GTGTGTGTCA TTTGTGACCA CGTAAATACG TGGAGTTCGT GACTTTCAAA CGACCTCTTA CGATCGGAAT   
  
  
- AAGTCACTCT ACGGAACAAA TAATTTCTCT TTCTTTGGTT CTATATCTAC CGAAGATTGG AGAAAAACAT   
  
  
- ACTTTACCTA CTTTGTTTAT CTTTATAGCA CGGTTTTGTA GGTCCGTCAT ATAAATCTAG TCCGTCGTAT   
  
  
- TCTCCTCTGT CATGTAAAAC GGGCGAAACC ATCTTTCTTG ATCCTAGGAC TGACAAAATG GCAGTTAAAA   
  
  
- TCAACTGACG TTCAAACAGA AGGGTTGGGT AAATCTAAAA GTAAAAGATA ACCTGTAAGG TAAAGAAGGT   
  
  
- CTCCAGATCC TCCAGTTCAA AAACCTTCTT TAAATATTCT AATAACAACA AAAGAAGGGA CCGACGTCAA   
  
  
- TGAACTGAAC AATCCACCGT CTGTCAACGA AGCAAGACAA TCAGGTGTAG GTGTACTGTA GTTTTGAATC   
  
  
- CTAACTAAAA TCAAGAACAA ATACGAGACT TAAGAGTTTG ACAGAAATTT TTTAAGTAAT CAGTCGTAAA   
  
  
- CGAAAACTGA TAACAAACGT GACGACCGGC GTTCTCCCAA ACATAGAACT AATAAGCACA TTTTTCTTTA   
  
  
- ATGTCTACGA GTTAAGTATC TAAACCGGTG TGACACCCAA GTTTGATTCT AACTGACTTC CTTCGGTTAT   
  
  
- TTCTAGTAGA CAAAAAGGAC GAATTACCGT GGGTATACTC GTCTGTCGGT GAAAAAGACC TAACGGGTTA   
  
  
- AGTTACCTCC GTGTAATCAT CAGTGTCTCT TCCGTCGGAC AATTCTTTTA CCATAAAGAT CTTCGGTTAA   
  
  
- TCATGGCAAA GGTCTACTAT ACCAATTGTC CAGTTATTTT TTTTAATAAA TCCTGAGAAA AACATCACAG   
  
  
- ACGGTTGAAT ACCTTACGTC GGGATTAACT TTATACTCGG GTACACTCGT AGTACAATAT AGTTTCGTGA   
  
  
- CCGTCCCCGG TGTTTCCTGC CACTAAGTTC GAGACATATA ATTTGTACTT TTTCTCTACT TTACCCATTC   
  
  
- TTCAGTCGGA TCGGAACACC TATTGTCTCC ATCCGACCGT CCGTACAGAT TCAAGACTAC ATTGTTGGTT   
  
  
- TCTCCGTTTC CTCTTCTTTC GGTTTTTCTT TTTTTTCTTT TTTTCTTTCC AGCTCTTCCG TTCACTGTGT   
  
  
- TTGGGTTCTG GGTCTAGGTT ACATTGTTCA GTTGAAGGAA GAGAAAGAGA GAGACAGTGA CTGTTAGAAG   
  
  
- TGATTGTGTG TGTTTGTGCG GGGCGTGGCG TAACCTGTTT GTGGCTTTGG TCTTTGTGCG AGGGGTAGAC   
  
  
- GCTAAGTCAG GGAAGAGAGG AAATTTAAAT GGCACAATGG GAAGGGAGAG GGGAGAAGTG ACGGGATTAA   
  
  
- TGTTTAGATG AAGAGAGGAG AAAACTGAGA CAAGAGAGAA GAGAGAGTAT GAGCGCCATT CGTGCGGTCT   
  
  
- GTTGCGCGTG CGTGTGAAAG AGAACAGAC

+     LTR

| Site Name | Organism | Position | Strand | Matrix score. | sequence | function |
| --- | --- | --- | --- | --- | --- | --- |
| LTR | Hordeum vulgare | 1303 | + | 6 | CCGAAA | cis-acting element involved in low-temperature responsiveness |

> 2018/04/13 10:10:12  
+ CACACACAGT AAACACTGGT GCATTTATGC ACCTCAAGCA CTGAAAGTTT GCTGGAGAAT GCTAGCCTTA   
  
  
+ TTCAGTGAGA TGCCTTGTTT ATTAAAGAGA AAGAAACCAA GATATAGATG GCTTCTAACC TCTTTTTGTA   
  
  
+ TGAAATGGAT GAAACAAATA GAAATATCGT GCCAAAACAT CCAGGCAGTA TATTTAGATC AGGCAGCATA   
  
  
+ AGAGGAGACA GTACATTTTG CCCGCTTTGG TAGAAAGAAC TAGGATCCTG ACTGTTTTAC CGTCAATTTT   
  
  
+ AGTTGACTGC AAGTTTGTCT TCCCAACCCA TTTAGATTTT CATTTTCTAT TGGACATTCC ATTTCTTCCA   
  
  
+ GAGGTCTAGG AGGTCAAGTT TTTGGAAGAA ATTTATAAGA TTATTGTTGT TTTCTTCCCT GGCTGCAGTT   
  
  
+ ACTTGACTTG TTAGGTGGCA GACAGTTGCT TCGTTCTGTT AGTCCACATC CACATGACAT CAAAACTTAG   
  
  
+ GATTGATTTT AGTTCTTGTT TATGCTCTGA ATTCTCAAAC TGTCTTTAAA AAATTCATTA GTCAGCATTT   
  
  
+ GCTTTTGACT ATTGTTTGCA CTGCTGGCCG CAAGAGGGTT TGTATCTTGA TTATTCGTGT AAAAAGAAAT   
  
  
+ TACAGATGCT CAATTCATAG ATTTGGCCAC ACTGTGGGTT CAAACTAAGA TTGACTGAAG GAAGCCAATA   
  
  
+ AAGATCATCT GTTTTTCCTG CTTAATGGCA CCCATATGAG CAGACAGCCA CTTTTTCTGG ATTGCCCAAT   
  
  
+ TCAATGGAGG CACATTAGTA GTCACAGAGA AGGCAGCCTG TTAAGAAAAT GGTATTTCTA GAAGCCAATT   
  
  
+ AGTACCGTTT CCAGATGATA TGGTTAACAG GTCAATAAAA AAAATTATTT AGGACTCTTT TTGTAGTGTC   
  
  
+ TGCCAACTTA TGGAATGCAG CCCTAATTGA AATATGAGCC CATGTGAGCA TCATGTTATA TCAAAGCACT   
  
  
+ GGCAGGGGCC ACAAAGGACG GTGATTCAAG CTCTGTATAT TAAACATGAA AAAGAGATGA AATGGGTAAG   
  
  
+ AAGTCAGCCT AGCCTTGTGG ATAACAGAGG TAGGCTGGCA GGCATGTCTA AGTTCTGATG TAACAACCAA   
  
  
+ AGAGGCAAAG GAGAAGAAAG CCAAAAAGAA AAAAAAGAAA AAAAGAAAGG TCGAGAAGGC AAGTGACACA   
  
  
+ AACCCAAGAC CCAGATCCAA TGTAACAAGT CAACTTCCTT CTCTTTCTCT CTCTGTCACT GACAATCTTC   
  
  
+ ACTAACACAC ACAAACACGC CCCGCACCGC ATTGGACAAA CACCGAAACC AGAAACACGC TCCCCATCTG   
  
  
+ CGATTCAGTC CCTTCTCTCC TTTAAATTTA CCGTGTTACC CTTCCCTCTC CCCTCTTCAC TGCCCTAATT   
  
  
+ ACAAATCTAC TTCTCTCCTC TTTTGACTCT GTTCTCTCTT CTCTCTCATA CTCGCGGTAA GCACGCCAGA   
  
  
+ CAACGCGCAC GCACACTTTC TCTTGTCTG  

- GTGTGTGTCA TTTGTGACCA CGTAAATACG TGGAGTTCGT GACTTTCAAA CGACCTCTTA CGATCGGAAT   
  
  
- AAGTCACTCT ACGGAACAAA TAATTTCTCT TTCTTTGGTT CTATATCTAC CGAAGATTGG AGAAAAACAT   
  
  
- ACTTTACCTA CTTTGTTTAT CTTTATAGCA CGGTTTTGTA GGTCCGTCAT ATAAATCTAG TCCGTCGTAT   
  
  
- TCTCCTCTGT CATGTAAAAC GGGCGAAACC ATCTTTCTTG ATCCTAGGAC TGACAAAATG GCAGTTAAAA   
  
  
- TCAACTGACG TTCAAACAGA AGGGTTGGGT AAATCTAAAA GTAAAAGATA ACCTGTAAGG TAAAGAAGGT   
  
  
- CTCCAGATCC TCCAGTTCAA AAACCTTCTT TAAATATTCT AATAACAACA AAAGAAGGGA CCGACGTCAA   
  
  
- TGAACTGAAC AATCCACCGT CTGTCAACGA AGCAAGACAA TCAGGTGTAG GTGTACTGTA GTTTTGAATC   
  
  
- CTAACTAAAA TCAAGAACAA ATACGAGACT TAAGAGTTTG ACAGAAATTT TTTAAGTAAT CAGTCGTAAA   
  
  
- CGAAAACTGA TAACAAACGT GACGACCGGC GTTCTCCCAA ACATAGAACT AATAAGCACA TTTTTCTTTA   
  
  
- ATGTCTACGA GTTAAGTATC TAAACCGGTG TGACACCCAA GTTTGATTCT AACTGACTTC CTTCGGTTAT   
  
  
- TTCTAGTAGA CAAAAAGGAC GAATTACCGT GGGTATACTC GTCTGTCGGT GAAAAAGACC TAACGGGTTA   
  
  
- AGTTACCTCC GTGTAATCAT CAGTGTCTCT TCCGTCGGAC AATTCTTTTA CCATAAAGAT CTTCGGTTAA   
  
  
- TCATGGCAAA GGTCTACTAT ACCAATTGTC CAGTTATTTT TTTTAATAAA TCCTGAGAAA AACATCACAG   
  
  
- ACGGTTGAAT ACCTTACGTC GGGATTAACT TTATACTCGG GTACACTCGT AGTACAATAT AGTTTCGTGA   
  
  
- CCGTCCCCGG TGTTTCCTGC CACTAAGTTC GAGACATATA ATTTGTACTT TTTCTCTACT TTACCCATTC   
  
  
- TTCAGTCGGA TCGGAACACC TATTGTCTCC ATCCGACCGT CCGTACAGAT TCAAGACTAC ATTGTTGGTT   
  
  
- TCTCCGTTTC CTCTTCTTTC GGTTTTTCTT TTTTTTCTTT TTTTCTTTCC AGCTCTTCCG TTCACTGTGT   
  
  
- TTGGGTTCTG GGTCTAGGTT ACATTGTTCA GTTGAAGGAA GAGAAAGAGA GAGACAGTGA CTGTTAGAAG   
  
  
- TGATTGTGTG TGTTTGTGCG GGGCGTGGCG TAACCTGTTT GTGGCTTTGG TCTTTGTGCG AGGGGTAGAC   
  
  
- GCTAAGTCAG GGAAGAGAGG AAATTTAAAT GGCACAATGG GAAGGGAGAG GGGAGAAGTG ACGGGATTAA   
  
  
- TGTTTAGATG AAGAGAGGAG AAAACTGAGA CAAGAGAGAA GAGAGAGTAT GAGCGCCATT CGTGCGGTCT   
  
  
- GTTGCGCGTG CGTGTGAAAG AGAACAGAC

+     MBS

| Site Name | Organism | Position | Strand | Matrix score. | sequence | function |
| --- | --- | --- | --- | --- | --- | --- |
| MBS | Arabidopsis thaliana | 416 | - | 6 | TAACTG | MYB binding site involved in drought-inducibility |
| MBS | Arabidopsis thaliana | 443 | - | 6 | CAACTG | MYB binding site involved in drought-inducibility |

> 2018/04/13 10:10:12  
+ CACACACAGT AAACACTGGT GCATTTATGC ACCTCAAGCA CTGAAAGTTT GCTGGAGAAT GCTAGCCTTA   
  
  
+ TTCAGTGAGA TGCCTTGTTT ATTAAAGAGA AAGAAACCAA GATATAGATG GCTTCTAACC TCTTTTTGTA   
  
  
+ TGAAATGGAT GAAACAAATA GAAATATCGT GCCAAAACAT CCAGGCAGTA TATTTAGATC AGGCAGCATA   
  
  
+ AGAGGAGACA GTACATTTTG CCCGCTTTGG TAGAAAGAAC TAGGATCCTG ACTGTTTTAC CGTCAATTTT   
  
  
+ AGTTGACTGC AAGTTTGTCT TCCCAACCCA TTTAGATTTT CATTTTCTAT TGGACATTCC ATTTCTTCCA   
  
  
+ GAGGTCTAGG AGGTCAAGTT TTTGGAAGAA ATTTATAAGA TTATTGTTGT TTTCTTCCCT GGCTGCAGTT   
  
  
+ ACTTGACTTG TTAGGTGGCA GACAGTTGCT TCGTTCTGTT AGTCCACATC CACATGACAT CAAAACTTAG   
  
  
+ GATTGATTTT AGTTCTTGTT TATGCTCTGA ATTCTCAAAC TGTCTTTAAA AAATTCATTA GTCAGCATTT   
  
  
+ GCTTTTGACT ATTGTTTGCA CTGCTGGCCG CAAGAGGGTT TGTATCTTGA TTATTCGTGT AAAAAGAAAT   
  
  
+ TACAGATGCT CAATTCATAG ATTTGGCCAC ACTGTGGGTT CAAACTAAGA TTGACTGAAG GAAGCCAATA   
  
  
+ AAGATCATCT GTTTTTCCTG CTTAATGGCA CCCATATGAG CAGACAGCCA CTTTTTCTGG ATTGCCCAAT   
  
  
+ TCAATGGAGG CACATTAGTA GTCACAGAGA AGGCAGCCTG TTAAGAAAAT GGTATTTCTA GAAGCCAATT   
  
  
+ AGTACCGTTT CCAGATGATA TGGTTAACAG GTCAATAAAA AAAATTATTT AGGACTCTTT TTGTAGTGTC   
  
  
+ TGCCAACTTA TGGAATGCAG CCCTAATTGA AATATGAGCC CATGTGAGCA TCATGTTATA TCAAAGCACT   
  
  
+ GGCAGGGGCC ACAAAGGACG GTGATTCAAG CTCTGTATAT TAAACATGAA AAAGAGATGA AATGGGTAAG   
  
  
+ AAGTCAGCCT AGCCTTGTGG ATAACAGAGG TAGGCTGGCA GGCATGTCTA AGTTCTGATG TAACAACCAA   
  
  
+ AGAGGCAAAG GAGAAGAAAG CCAAAAAGAA AAAAAAGAAA AAAAGAAAGG TCGAGAAGGC AAGTGACACA   
  
  
+ AACCCAAGAC CCAGATCCAA TGTAACAAGT CAACTTCCTT CTCTTTCTCT CTCTGTCACT GACAATCTTC   
  
  
+ ACTAACACAC ACAAACACGC CCCGCACCGC ATTGGACAAA CACCGAAACC AGAAACACGC TCCCCATCTG   
  
  
+ CGATTCAGTC CCTTCTCTCC TTTAAATTTA CCGTGTTACC CTTCCCTCTC CCCTCTTCAC TGCCCTAATT   
  
  
+ ACAAATCTAC TTCTCTCCTC TTTTGACTCT GTTCTCTCTT CTCTCTCATA CTCGCGGTAA GCACGCCAGA   
  
  
+ CAACGCGCAC GCACACTTTC TCTTGTCTG  

- GTGTGTGTCA TTTGTGACCA CGTAAATACG TGGAGTTCGT GACTTTCAAA CGACCTCTTA CGATCGGAAT   
  
  
- AAGTCACTCT ACGGAACAAA TAATTTCTCT TTCTTTGGTT CTATATCTAC CGAAGATTGG AGAAAAACAT   
  
  
- ACTTTACCTA CTTTGTTTAT CTTTATAGCA CGGTTTTGTA GGTCCGTCAT ATAAATCTAG TCCGTCGTAT   
  
  
- TCTCCTCTGT CATGTAAAAC GGGCGAAACC ATCTTTCTTG ATCCTAGGAC TGACAAAATG GCAGTTAAAA   
  
  
- TCAACTGACG TTCAAACAGA AGGGTTGGGT AAATCTAAAA GTAAAAGATA ACCTGTAAGG TAAAGAAGGT   
  
  
- CTCCAGATCC TCCAGTTCAA AAACCTTCTT TAAATATTCT AATAACAACA AAAGAAGGGA CCGACGTCAA   
  
  
- TGAACTGAAC AATCCACCGT CTGTCAACGA AGCAAGACAA TCAGGTGTAG GTGTACTGTA GTTTTGAATC   
  
  
- CTAACTAAAA TCAAGAACAA ATACGAGACT TAAGAGTTTG ACAGAAATTT TTTAAGTAAT CAGTCGTAAA   
  
  
- CGAAAACTGA TAACAAACGT GACGACCGGC GTTCTCCCAA ACATAGAACT AATAAGCACA TTTTTCTTTA   
  
  
- ATGTCTACGA GTTAAGTATC TAAACCGGTG TGACACCCAA GTTTGATTCT AACTGACTTC CTTCGGTTAT   
  
  
- TTCTAGTAGA CAAAAAGGAC GAATTACCGT GGGTATACTC GTCTGTCGGT GAAAAAGACC TAACGGGTTA   
  
  
- AGTTACCTCC GTGTAATCAT CAGTGTCTCT TCCGTCGGAC AATTCTTTTA CCATAAAGAT CTTCGGTTAA   
  
  
- TCATGGCAAA GGTCTACTAT ACCAATTGTC CAGTTATTTT TTTTAATAAA TCCTGAGAAA AACATCACAG   
  
  
- ACGGTTGAAT ACCTTACGTC GGGATTAACT TTATACTCGG GTACACTCGT AGTACAATAT AGTTTCGTGA   
  
  
- CCGTCCCCGG TGTTTCCTGC CACTAAGTTC GAGACATATA ATTTGTACTT TTTCTCTACT TTACCCATTC   
  
  
- TTCAGTCGGA TCGGAACACC TATTGTCTCC ATCCGACCGT CCGTACAGAT TCAAGACTAC ATTGTTGGTT   
  
  
- TCTCCGTTTC CTCTTCTTTC GGTTTTTCTT TTTTTTCTTT TTTTCTTTCC AGCTCTTCCG TTCACTGTGT   
  
  
- TTGGGTTCTG GGTCTAGGTT ACATTGTTCA GTTGAAGGAA GAGAAAGAGA GAGACAGTGA CTGTTAGAAG   
  
  
- TGATTGTGTG TGTTTGTGCG GGGCGTGGCG TAACCTGTTT GTGGCTTTGG TCTTTGTGCG AGGGGTAGAC   
  
  
- GCTAAGTCAG GGAAGAGAGG AAATTTAAAT GGCACAATGG GAAGGGAGAG GGGAGAAGTG ACGGGATTAA   
  
  
- TGTTTAGATG AAGAGAGGAG AAAACTGAGA CAAGAGAGAA GAGAGAGTAT GAGCGCCATT CGTGCGGTCT   
  
  
- GTTGCGCGTG CGTGTGAAAG AGAACAGAC

+     O2-site

| Site Name | Organism | Position | Strand | Matrix score. | sequence | function |
| --- | --- | --- | --- | --- | --- | --- |
| O2-site | Zea mays | 1036 | + | 9 | GATGACATGG | cis-acting regulatory element involved in zein metabolism regulation |
| O2-site | Zea mays | 854 | + | 9 | GATGACATGG | cis-acting regulatory element involved in zein metabolism regulation |

> 2018/04/13 10:10:12  
+ CACACACAGT AAACACTGGT GCATTTATGC ACCTCAAGCA CTGAAAGTTT GCTGGAGAAT GCTAGCCTTA   
  
  
+ TTCAGTGAGA TGCCTTGTTT ATTAAAGAGA AAGAAACCAA GATATAGATG GCTTCTAACC TCTTTTTGTA   
  
  
+ TGAAATGGAT GAAACAAATA GAAATATCGT GCCAAAACAT CCAGGCAGTA TATTTAGATC AGGCAGCATA   
  
  
+ AGAGGAGACA GTACATTTTG CCCGCTTTGG TAGAAAGAAC TAGGATCCTG ACTGTTTTAC CGTCAATTTT   
  
  
+ AGTTGACTGC AAGTTTGTCT TCCCAACCCA TTTAGATTTT CATTTTCTAT TGGACATTCC ATTTCTTCCA   
  
  
+ GAGGTCTAGG AGGTCAAGTT TTTGGAAGAA ATTTATAAGA TTATTGTTGT TTTCTTCCCT GGCTGCAGTT   
  
  
+ ACTTGACTTG TTAGGTGGCA GACAGTTGCT TCGTTCTGTT AGTCCACATC CACATGACAT CAAAACTTAG   
  
  
+ GATTGATTTT AGTTCTTGTT TATGCTCTGA ATTCTCAAAC TGTCTTTAAA AAATTCATTA GTCAGCATTT   
  
  
+ GCTTTTGACT ATTGTTTGCA CTGCTGGCCG CAAGAGGGTT TGTATCTTGA TTATTCGTGT AAAAAGAAAT   
  
  
+ TACAGATGCT CAATTCATAG ATTTGGCCAC ACTGTGGGTT CAAACTAAGA TTGACTGAAG GAAGCCAATA   
  
  
+ AAGATCATCT GTTTTTCCTG CTTAATGGCA CCCATATGAG CAGACAGCCA CTTTTTCTGG ATTGCCCAAT   
  
  
+ TCAATGGAGG CACATTAGTA GTCACAGAGA AGGCAGCCTG TTAAGAAAAT GGTATTTCTA GAAGCCAATT   
  
  
+ AGTACCGTTT CCAGATGATA TGGTTAACAG GTCAATAAAA AAAATTATTT AGGACTCTTT TTGTAGTGTC   
  
  
+ TGCCAACTTA TGGAATGCAG CCCTAATTGA AATATGAGCC CATGTGAGCA TCATGTTATA TCAAAGCACT   
  
  
+ GGCAGGGGCC ACAAAGGACG GTGATTCAAG CTCTGTATAT TAAACATGAA AAAGAGATGA AATGGGTAAG   
  
  
+ AAGTCAGCCT AGCCTTGTGG ATAACAGAGG TAGGCTGGCA GGCATGTCTA AGTTCTGATG TAACAACCAA   
  
  
+ AGAGGCAAAG GAGAAGAAAG CCAAAAAGAA AAAAAAGAAA AAAAGAAAGG TCGAGAAGGC AAGTGACACA   
  
  
+ AACCCAAGAC CCAGATCCAA TGTAACAAGT CAACTTCCTT CTCTTTCTCT CTCTGTCACT GACAATCTTC   
  
  
+ ACTAACACAC ACAAACACGC CCCGCACCGC ATTGGACAAA CACCGAAACC AGAAACACGC TCCCCATCTG   
  
  
+ CGATTCAGTC CCTTCTCTCC TTTAAATTTA CCGTGTTACC CTTCCCTCTC CCCTCTTCAC TGCCCTAATT   
  
  
+ ACAAATCTAC TTCTCTCCTC TTTTGACTCT GTTCTCTCTT CTCTCTCATA CTCGCGGTAA GCACGCCAGA   
  
  
+ CAACGCGCAC GCACACTTTC TCTTGTCTG  

- GTGTGTGTCA TTTGTGACCA CGTAAATACG TGGAGTTCGT GACTTTCAAA CGACCTCTTA CGATCGGAAT   
  
  
- AAGTCACTCT ACGGAACAAA TAATTTCTCT TTCTTTGGTT CTATATCTAC CGAAGATTGG AGAAAAACAT   
  
  
- ACTTTACCTA CTTTGTTTAT CTTTATAGCA CGGTTTTGTA GGTCCGTCAT ATAAATCTAG TCCGTCGTAT   
  
  
- TCTCCTCTGT CATGTAAAAC GGGCGAAACC ATCTTTCTTG ATCCTAGGAC TGACAAAATG GCAGTTAAAA   
  
  
- TCAACTGACG TTCAAACAGA AGGGTTGGGT AAATCTAAAA GTAAAAGATA ACCTGTAAGG TAAAGAAGGT   
  
  
- CTCCAGATCC TCCAGTTCAA AAACCTTCTT TAAATATTCT AATAACAACA AAAGAAGGGA CCGACGTCAA   
  
  
- TGAACTGAAC AATCCACCGT CTGTCAACGA AGCAAGACAA TCAGGTGTAG GTGTACTGTA GTTTTGAATC   
  
  
- CTAACTAAAA TCAAGAACAA ATACGAGACT TAAGAGTTTG ACAGAAATTT TTTAAGTAAT CAGTCGTAAA   
  
  
- CGAAAACTGA TAACAAACGT GACGACCGGC GTTCTCCCAA ACATAGAACT AATAAGCACA TTTTTCTTTA   
  
  
- ATGTCTACGA GTTAAGTATC TAAACCGGTG TGACACCCAA GTTTGATTCT AACTGACTTC CTTCGGTTAT   
  
  
- TTCTAGTAGA CAAAAAGGAC GAATTACCGT GGGTATACTC GTCTGTCGGT GAAAAAGACC TAACGGGTTA   
  
  
- AGTTACCTCC GTGTAATCAT CAGTGTCTCT TCCGTCGGAC AATTCTTTTA CCATAAAGAT CTTCGGTTAA   
  
  
- TCATGGCAAA GGTCTACTAT ACCAATTGTC CAGTTATTTT TTTTAATAAA TCCTGAGAAA AACATCACAG   
  
  
- ACGGTTGAAT ACCTTACGTC GGGATTAACT TTATACTCGG GTACACTCGT AGTACAATAT AGTTTCGTGA   
  
  
- CCGTCCCCGG TGTTTCCTGC CACTAAGTTC GAGACATATA ATTTGTACTT TTTCTCTACT TTACCCATTC   
  
  
- TTCAGTCGGA TCGGAACACC TATTGTCTCC ATCCGACCGT CCGTACAGAT TCAAGACTAC ATTGTTGGTT   
  
  
- TCTCCGTTTC CTCTTCTTTC GGTTTTTCTT TTTTTTCTTT TTTTCTTTCC AGCTCTTCCG TTCACTGTGT   
  
  
- TTGGGTTCTG GGTCTAGGTT ACATTGTTCA GTTGAAGGAA GAGAAAGAGA GAGACAGTGA CTGTTAGAAG   
  
  
- TGATTGTGTG TGTTTGTGCG GGGCGTGGCG TAACCTGTTT GTGGCTTTGG TCTTTGTGCG AGGGGTAGAC   
  
  
- GCTAAGTCAG GGAAGAGAGG AAATTTAAAT GGCACAATGG GAAGGGAGAG GGGAGAAGTG ACGGGATTAA   
  
  
- TGTTTAGATG AAGAGAGGAG AAAACTGAGA CAAGAGAGAA GAGAGAGTAT GAGCGCCATT CGTGCGGTCT   
  
  
- GTTGCGCGTG CGTGTGAAAG AGAACAGAC

+     Skn-1\_motif

| Site Name | Organism | Position | Strand | Matrix score. | sequence | function |
| --- | --- | --- | --- | --- | --- | --- |
| Skn-1\_motif | Oryza sativa | 474 | - | 5 | GTCAT | cis-acting regulatory element required for endosperm expression |

> 2018/04/13 10:10:12  
+ CACACACAGT AAACACTGGT GCATTTATGC ACCTCAAGCA CTGAAAGTTT GCTGGAGAAT GCTAGCCTTA   
  
  
+ TTCAGTGAGA TGCCTTGTTT ATTAAAGAGA AAGAAACCAA GATATAGATG GCTTCTAACC TCTTTTTGTA   
  
  
+ TGAAATGGAT GAAACAAATA GAAATATCGT GCCAAAACAT CCAGGCAGTA TATTTAGATC AGGCAGCATA   
  
  
+ AGAGGAGACA GTACATTTTG CCCGCTTTGG TAGAAAGAAC TAGGATCCTG ACTGTTTTAC CGTCAATTTT   
  
  
+ AGTTGACTGC AAGTTTGTCT TCCCAACCCA TTTAGATTTT CATTTTCTAT TGGACATTCC ATTTCTTCCA   
  
  
+ GAGGTCTAGG AGGTCAAGTT TTTGGAAGAA ATTTATAAGA TTATTGTTGT TTTCTTCCCT GGCTGCAGTT   
  
  
+ ACTTGACTTG TTAGGTGGCA GACAGTTGCT TCGTTCTGTT AGTCCACATC CACATGACAT CAAAACTTAG   
  
  
+ GATTGATTTT AGTTCTTGTT TATGCTCTGA ATTCTCAAAC TGTCTTTAAA AAATTCATTA GTCAGCATTT   
  
  
+ GCTTTTGACT ATTGTTTGCA CTGCTGGCCG CAAGAGGGTT TGTATCTTGA TTATTCGTGT AAAAAGAAAT   
  
  
+ TACAGATGCT CAATTCATAG ATTTGGCCAC ACTGTGGGTT CAAACTAAGA TTGACTGAAG GAAGCCAATA   
  
  
+ AAGATCATCT GTTTTTCCTG CTTAATGGCA CCCATATGAG CAGACAGCCA CTTTTTCTGG ATTGCCCAAT   
  
  
+ TCAATGGAGG CACATTAGTA GTCACAGAGA AGGCAGCCTG TTAAGAAAAT GGTATTTCTA GAAGCCAATT   
  
  
+ AGTACCGTTT CCAGATGATA TGGTTAACAG GTCAATAAAA AAAATTATTT AGGACTCTTT TTGTAGTGTC   
  
  
+ TGCCAACTTA TGGAATGCAG CCCTAATTGA AATATGAGCC CATGTGAGCA TCATGTTATA TCAAAGCACT   
  
  
+ GGCAGGGGCC ACAAAGGACG GTGATTCAAG CTCTGTATAT TAAACATGAA AAAGAGATGA AATGGGTAAG   
  
  
+ AAGTCAGCCT AGCCTTGTGG ATAACAGAGG TAGGCTGGCA GGCATGTCTA AGTTCTGATG TAACAACCAA   
  
  
+ AGAGGCAAAG GAGAAGAAAG CCAAAAAGAA AAAAAAGAAA AAAAGAAAGG TCGAGAAGGC AAGTGACACA   
  
  
+ AACCCAAGAC CCAGATCCAA TGTAACAAGT CAACTTCCTT CTCTTTCTCT CTCTGTCACT GACAATCTTC   
  
  
+ ACTAACACAC ACAAACACGC CCCGCACCGC ATTGGACAAA CACCGAAACC AGAAACACGC TCCCCATCTG   
  
  
+ CGATTCAGTC CCTTCTCTCC TTTAAATTTA CCGTGTTACC CTTCCCTCTC CCCTCTTCAC TGCCCTAATT   
  
  
+ ACAAATCTAC TTCTCTCCTC TTTTGACTCT GTTCTCTCTT CTCTCTCATA CTCGCGGTAA GCACGCCAGA   
  
  
+ CAACGCGCAC GCACACTTTC TCTTGTCTG  

- GTGTGTGTCA TTTGTGACCA CGTAAATACG TGGAGTTCGT GACTTTCAAA CGACCTCTTA CGATCGGAAT   
  
  
- AAGTCACTCT ACGGAACAAA TAATTTCTCT TTCTTTGGTT CTATATCTAC CGAAGATTGG AGAAAAACAT   
  
  
- ACTTTACCTA CTTTGTTTAT CTTTATAGCA CGGTTTTGTA GGTCCGTCAT ATAAATCTAG TCCGTCGTAT   
  
  
- TCTCCTCTGT CATGTAAAAC GGGCGAAACC ATCTTTCTTG ATCCTAGGAC TGACAAAATG GCAGTTAAAA   
  
  
- TCAACTGACG TTCAAACAGA AGGGTTGGGT AAATCTAAAA GTAAAAGATA ACCTGTAAGG TAAAGAAGGT   
  
  
- CTCCAGATCC TCCAGTTCAA AAACCTTCTT TAAATATTCT AATAACAACA AAAGAAGGGA CCGACGTCAA   
  
  
- TGAACTGAAC AATCCACCGT CTGTCAACGA AGCAAGACAA TCAGGTGTAG GTGTACTGTA GTTTTGAATC   
  
  
- CTAACTAAAA TCAAGAACAA ATACGAGACT TAAGAGTTTG ACAGAAATTT TTTAAGTAAT CAGTCGTAAA   
  
  
- CGAAAACTGA TAACAAACGT GACGACCGGC GTTCTCCCAA ACATAGAACT AATAAGCACA TTTTTCTTTA   
  
  
- ATGTCTACGA GTTAAGTATC TAAACCGGTG TGACACCCAA GTTTGATTCT AACTGACTTC CTTCGGTTAT   
  
  
- TTCTAGTAGA CAAAAAGGAC GAATTACCGT GGGTATACTC GTCTGTCGGT GAAAAAGACC TAACGGGTTA   
  
  
- AGTTACCTCC GTGTAATCAT CAGTGTCTCT TCCGTCGGAC AATTCTTTTA CCATAAAGAT CTTCGGTTAA   
  
  
- TCATGGCAAA GGTCTACTAT ACCAATTGTC CAGTTATTTT TTTTAATAAA TCCTGAGAAA AACATCACAG   
  
  
- ACGGTTGAAT ACCTTACGTC GGGATTAACT TTATACTCGG GTACACTCGT AGTACAATAT AGTTTCGTGA   
  
  
- CCGTCCCCGG TGTTTCCTGC CACTAAGTTC GAGACATATA ATTTGTACTT TTTCTCTACT TTACCCATTC   
  
  
- TTCAGTCGGA TCGGAACACC TATTGTCTCC ATCCGACCGT CCGTACAGAT TCAAGACTAC ATTGTTGGTT   
  
  
- TCTCCGTTTC CTCTTCTTTC GGTTTTTCTT TTTTTTCTTT TTTTCTTTCC AGCTCTTCCG TTCACTGTGT   
  
  
- TTGGGTTCTG GGTCTAGGTT ACATTGTTCA GTTGAAGGAA GAGAAAGAGA GAGACAGTGA CTGTTAGAAG   
  
  
- TGATTGTGTG TGTTTGTGCG GGGCGTGGCG TAACCTGTTT GTGGCTTTGG TCTTTGTGCG AGGGGTAGAC   
  
  
- GCTAAGTCAG GGAAGAGAGG AAATTTAAAT GGCACAATGG GAAGGGAGAG GGGAGAAGTG ACGGGATTAA   
  
  
- TGTTTAGATG AAGAGAGGAG AAAACTGAGA CAAGAGAGAA GAGAGAGTAT GAGCGCCATT CGTGCGGTCT   
  
  
- GTTGCGCGTG CGTGTGAAAG AGAACAGAC

+     TATA-box

| Site Name | Organism | Position | Strand | Matrix score. | sequence | function |
| --- | --- | --- | --- | --- | --- | --- |
| TATA-box | Arabidopsis thaliana | 966 | - | 5 | TATAA | core promoter element around -30 of transcription start |
| TATA-box | Zea mays | 535 | + | 8 | TTTAAAAA | core promoter element around -30 of transcription start |
| TATA-box | Oryza sativa | 899 | - | 7 | TACAAAA | core promoter element around -30 of transcription start |
| TATA-box | Lycopersicon esculentum | 876 | - | 5 | TTTTA | core promoter element around -30 of transcription start |
| TATA-box | Nicotiana tabacum | 823 | - | 9 | tcTATAAAta | core promoter element around -30 of transcription start |
| TATA-box | Lycopersicon esculentum | 620 | - | 5 | TTTTA | core promoter element around -30 of transcription start |
| TATA-box | Lycopersicon esculentum | 537 | - | 5 | TTTTA | core promoter element around -30 of transcription start |
| TATA-box | Daucus carota | 1349 | + | 9 | ccTATAAATT | core promoter element around -30 of transcription start |
| TATA-box | Glycine max | 1018 | - | 5 | TAATA | core promoter element around -30 of transcription start |
| TATA-box | Arabidopsis thaliana | 1016 | - | 4 | TATA | core promoter element around -30 of transcription start |
| TATA-box | Lycopersicon esculentum | 497 | + | 5 | TTTTA | core promoter element around -30 of transcription start |
| TATA-box | Helianthus annuus | 1014 | - | 6 | TATACA | core promoter element around -30 of transcription start |
| TATA-box | Arabidopsis thaliana | 383 | - | 5 | TATAA | core promoter element around -30 of transcription start |
| TATA-box | Arabidopsis thaliana | 384 | + | 4 | TATA | core promoter element around -30 of transcription start |
| TATA-box | Arabidopsis thaliana | 382 | - | 6 | TATAAA | core promoter element around -30 of transcription start |
| TATA-box | Ac | 381 | - | 7 | TATAAAT | core promoter element around -30 of transcription start |
| TATA-box | Lycopersicon esculentum | 277 | + | 5 | TTTTA | core promoter element around -30 of transcription start |
| TATA-box | Arabidopsis thaliana | 1012 | + | 9 | tcTATATAtt | core promoter element around -30 of transcription start |
| TATA-box | Arabidopsis thaliana | 967 | - | 4 | TATA | core promoter element around -30 of transcription start |
| TATA-box | Antirrhinum majus | 380 | - | 8 | TATAAATT | core promoter element around -30 of transcription start |
| TATA-box | Lycopersicon esculentum | 265 | + | 5 | TTTTA | core promoter element around -30 of transcription start |
| TATA-box | Arabidopsis thaliana | 189 | + | 4 | TATA | core promoter element around -30 of transcription start |
| TATA-box | Oryza sativa | 134 | - | 7 | TACAAAA | core promoter element around -30 of transcription start |
| TATA-box | Arabidopsis thaliana | 113 | + | 4 | TATA | core promoter element around -30 of transcription start |
| TATA-box | Glycine max | 90 | - | 5 | TAATA | core promoter element around -30 of transcription start |
| TATA-box | Arabidopsis thaliana | 109 | - | 9 | tcTATATAtt | core promoter element around -30 of transcription start |

> 2018/04/13 10:10:12  
+ CACACACAGT AAACACTGGT GCATTTATGC ACCTCAAGCA CTGAAAGTTT GCTGGAGAAT GCTAGCCTTA   
  
  
+ TTCAGTGAGA TGCCTTGTTT ATTAAAGAGA AAGAAACCAA GATATAGATG GCTTCTAACC TCTTTTTGTA   
  
  
+ TGAAATGGAT GAAACAAATA GAAATATCGT GCCAAAACAT CCAGGCAGTA TATTTAGATC AGGCAGCATA   
  
  
+ AGAGGAGACA GTACATTTTG CCCGCTTTGG TAGAAAGAAC TAGGATCCTG ACTGTTTTAC CGTCAATTTT   
  
  
+ AGTTGACTGC AAGTTTGTCT TCCCAACCCA TTTAGATTTT CATTTTCTAT TGGACATTCC ATTTCTTCCA   
  
  
+ GAGGTCTAGG AGGTCAAGTT TTTGGAAGAA ATTTATAAGA TTATTGTTGT TTTCTTCCCT GGCTGCAGTT   
  
  
+ ACTTGACTTG TTAGGTGGCA GACAGTTGCT TCGTTCTGTT AGTCCACATC CACATGACAT CAAAACTTAG   
  
  
+ GATTGATTTT AGTTCTTGTT TATGCTCTGA ATTCTCAAAC TGTCTTTAAA AAATTCATTA GTCAGCATTT   
  
  
+ GCTTTTGACT ATTGTTTGCA CTGCTGGCCG CAAGAGGGTT TGTATCTTGA TTATTCGTGT AAAAAGAAAT   
  
  
+ TACAGATGCT CAATTCATAG ATTTGGCCAC ACTGTGGGTT CAAACTAAGA TTGACTGAAG GAAGCCAATA   
  
  
+ AAGATCATCT GTTTTTCCTG CTTAATGGCA CCCATATGAG CAGACAGCCA CTTTTTCTGG ATTGCCCAAT   
  
  
+ TCAATGGAGG CACATTAGTA GTCACAGAGA AGGCAGCCTG TTAAGAAAAT GGTATTTCTA GAAGCCAATT   
  
  
+ AGTACCGTTT CCAGATGATA TGGTTAACAG GTCAATAAAA AAAATTATTT AGGACTCTTT TTGTAGTGTC   
  
  
+ TGCCAACTTA TGGAATGCAG CCCTAATTGA AATATGAGCC CATGTGAGCA TCATGTTATA TCAAAGCACT   
  
  
+ GGCAGGGGCC ACAAAGGACG GTGATTCAAG CTCTGTATAT TAAACATGAA AAAGAGATGA AATGGGTAAG   
  
  
+ AAGTCAGCCT AGCCTTGTGG ATAACAGAGG TAGGCTGGCA GGCATGTCTA AGTTCTGATG TAACAACCAA   
  
  
+ AGAGGCAAAG GAGAAGAAAG CCAAAAAGAA AAAAAAGAAA AAAAGAAAGG TCGAGAAGGC AAGTGACACA   
  
  
+ AACCCAAGAC CCAGATCCAA TGTAACAAGT CAACTTCCTT CTCTTTCTCT CTCTGTCACT GACAATCTTC   
  
  
+ ACTAACACAC ACAAACACGC CCCGCACCGC ATTGGACAAA CACCGAAACC AGAAACACGC TCCCCATCTG   
  
  
+ CGATTCAGTC CCTTCTCTCC TTTAAATTTA CCGTGTTACC CTTCCCTCTC CCCTCTTCAC TGCCCTAATT   
  
  
+ ACAAATCTAC TTCTCTCCTC TTTTGACTCT GTTCTCTCTT CTCTCTCATA CTCGCGGTAA GCACGCCAGA   
  
  
+ CAACGCGCAC GCACACTTTC TCTTGTCTG  

- GTGTGTGTCA TTTGTGACCA CGTAAATACG TGGAGTTCGT GACTTTCAAA CGACCTCTTA CGATCGGAAT   
  
  
- AAGTCACTCT ACGGAACAAA TAATTTCTCT TTCTTTGGTT CTATATCTAC CGAAGATTGG AGAAAAACAT   
  
  
- ACTTTACCTA CTTTGTTTAT CTTTATAGCA CGGTTTTGTA GGTCCGTCAT ATAAATCTAG TCCGTCGTAT   
  
  
- TCTCCTCTGT CATGTAAAAC GGGCGAAACC ATCTTTCTTG ATCCTAGGAC TGACAAAATG GCAGTTAAAA   
  
  
- TCAACTGACG TTCAAACAGA AGGGTTGGGT AAATCTAAAA GTAAAAGATA ACCTGTAAGG TAAAGAAGGT   
  
  
- CTCCAGATCC TCCAGTTCAA AAACCTTCTT TAAATATTCT AATAACAACA AAAGAAGGGA CCGACGTCAA   
  
  
- TGAACTGAAC AATCCACCGT CTGTCAACGA AGCAAGACAA TCAGGTGTAG GTGTACTGTA GTTTTGAATC   
  
  
- CTAACTAAAA TCAAGAACAA ATACGAGACT TAAGAGTTTG ACAGAAATTT TTTAAGTAAT CAGTCGTAAA   
  
  
- CGAAAACTGA TAACAAACGT GACGACCGGC GTTCTCCCAA ACATAGAACT AATAAGCACA TTTTTCTTTA   
  
  
- ATGTCTACGA GTTAAGTATC TAAACCGGTG TGACACCCAA GTTTGATTCT AACTGACTTC CTTCGGTTAT   
  
  
- TTCTAGTAGA CAAAAAGGAC GAATTACCGT GGGTATACTC GTCTGTCGGT GAAAAAGACC TAACGGGTTA   
  
  
- AGTTACCTCC GTGTAATCAT CAGTGTCTCT TCCGTCGGAC AATTCTTTTA CCATAAAGAT CTTCGGTTAA   
  
  
- TCATGGCAAA GGTCTACTAT ACCAATTGTC CAGTTATTTT TTTTAATAAA TCCTGAGAAA AACATCACAG   
  
  
- ACGGTTGAAT ACCTTACGTC GGGATTAACT TTATACTCGG GTACACTCGT AGTACAATAT AGTTTCGTGA   
  
  
- CCGTCCCCGG TGTTTCCTGC CACTAAGTTC GAGACATATA ATTTGTACTT TTTCTCTACT TTACCCATTC   
  
  
- TTCAGTCGGA TCGGAACACC TATTGTCTCC ATCCGACCGT CCGTACAGAT TCAAGACTAC ATTGTTGGTT   
  
  
- TCTCCGTTTC CTCTTCTTTC GGTTTTTCTT TTTTTTCTTT TTTTCTTTCC AGCTCTTCCG TTCACTGTGT   
  
  
- TTGGGTTCTG GGTCTAGGTT ACATTGTTCA GTTGAAGGAA GAGAAAGAGA GAGACAGTGA CTGTTAGAAG   
  
  
- TGATTGTGTG TGTTTGTGCG GGGCGTGGCG TAACCTGTTT GTGGCTTTGG TCTTTGTGCG AGGGGTAGAC   
  
  
- GCTAAGTCAG GGAAGAGAGG AAATTTAAAT GGCACAATGG GAAGGGAGAG GGGAGAAGTG ACGGGATTAA   
  
  
- TGTTTAGATG AAGAGAGGAG AAAACTGAGA CAAGAGAGAA GAGAGAGTAT GAGCGCCATT CGTGCGGTCT   
  
  
- GTTGCGCGTG CGTGTGAAAG AGAACAGAC

+     TC-rich repeats

| Site Name | Organism | Position | Strand | Matrix score. | sequence | function |
| --- | --- | --- | --- | --- | --- | --- |
| TC-rich repeats | Nicotiana tabacum | 811 | - | 9 | ATTTTCTTCA | cis-acting element involved in defense and stress responsiveness |
| TC-rich repeats | Nicotiana tabacum | 399 | + | 9 | GTTTTCTTAC | cis-acting element involved in defense and stress responsiveness |
| TC-rich repeats | Nicotiana tabacum | 521 | + | 9 | ATTCTCTAAC | cis-acting element involved in defense and stress responsiveness |

> 2018/04/13 10:10:12  
+ CACACACAGT AAACACTGGT GCATTTATGC ACCTCAAGCA CTGAAAGTTT GCTGGAGAAT GCTAGCCTTA   
  
  
+ TTCAGTGAGA TGCCTTGTTT ATTAAAGAGA AAGAAACCAA GATATAGATG GCTTCTAACC TCTTTTTGTA   
  
  
+ TGAAATGGAT GAAACAAATA GAAATATCGT GCCAAAACAT CCAGGCAGTA TATTTAGATC AGGCAGCATA   
  
  
+ AGAGGAGACA GTACATTTTG CCCGCTTTGG TAGAAAGAAC TAGGATCCTG ACTGTTTTAC CGTCAATTTT   
  
  
+ AGTTGACTGC AAGTTTGTCT TCCCAACCCA TTTAGATTTT CATTTTCTAT TGGACATTCC ATTTCTTCCA   
  
  
+ GAGGTCTAGG AGGTCAAGTT TTTGGAAGAA ATTTATAAGA TTATTGTTGT TTTCTTCCCT GGCTGCAGTT   
  
  
+ ACTTGACTTG TTAGGTGGCA GACAGTTGCT TCGTTCTGTT AGTCCACATC CACATGACAT CAAAACTTAG   
  
  
+ GATTGATTTT AGTTCTTGTT TATGCTCTGA ATTCTCAAAC TGTCTTTAAA AAATTCATTA GTCAGCATTT   
  
  
+ GCTTTTGACT ATTGTTTGCA CTGCTGGCCG CAAGAGGGTT TGTATCTTGA TTATTCGTGT AAAAAGAAAT   
  
  
+ TACAGATGCT CAATTCATAG ATTTGGCCAC ACTGTGGGTT CAAACTAAGA TTGACTGAAG GAAGCCAATA   
  
  
+ AAGATCATCT GTTTTTCCTG CTTAATGGCA CCCATATGAG CAGACAGCCA CTTTTTCTGG ATTGCCCAAT   
  
  
+ TCAATGGAGG CACATTAGTA GTCACAGAGA AGGCAGCCTG TTAAGAAAAT GGTATTTCTA GAAGCCAATT   
  
  
+ AGTACCGTTT CCAGATGATA TGGTTAACAG GTCAATAAAA AAAATTATTT AGGACTCTTT TTGTAGTGTC   
  
  
+ TGCCAACTTA TGGAATGCAG CCCTAATTGA AATATGAGCC CATGTGAGCA TCATGTTATA TCAAAGCACT   
  
  
+ GGCAGGGGCC ACAAAGGACG GTGATTCAAG CTCTGTATAT TAAACATGAA AAAGAGATGA AATGGGTAAG   
  
  
+ AAGTCAGCCT AGCCTTGTGG ATAACAGAGG TAGGCTGGCA GGCATGTCTA AGTTCTGATG TAACAACCAA   
  
  
+ AGAGGCAAAG GAGAAGAAAG CCAAAAAGAA AAAAAAGAAA AAAAGAAAGG TCGAGAAGGC AAGTGACACA   
  
  
+ AACCCAAGAC CCAGATCCAA TGTAACAAGT CAACTTCCTT CTCTTTCTCT CTCTGTCACT GACAATCTTC   
  
  
+ ACTAACACAC ACAAACACGC CCCGCACCGC ATTGGACAAA CACCGAAACC AGAAACACGC TCCCCATCTG   
  
  
+ CGATTCAGTC CCTTCTCTCC TTTAAATTTA CCGTGTTACC CTTCCCTCTC CCCTCTTCAC TGCCCTAATT   
  
  
+ ACAAATCTAC TTCTCTCCTC TTTTGACTCT GTTCTCTCTT CTCTCTCATA CTCGCGGTAA GCACGCCAGA   
  
  
+ CAACGCGCAC GCACACTTTC TCTTGTCTG  

- GTGTGTGTCA TTTGTGACCA CGTAAATACG TGGAGTTCGT GACTTTCAAA CGACCTCTTA CGATCGGAAT   
  
  
- AAGTCACTCT ACGGAACAAA TAATTTCTCT TTCTTTGGTT CTATATCTAC CGAAGATTGG AGAAAAACAT   
  
  
- ACTTTACCTA CTTTGTTTAT CTTTATAGCA CGGTTTTGTA GGTCCGTCAT ATAAATCTAG TCCGTCGTAT   
  
  
- TCTCCTCTGT CATGTAAAAC GGGCGAAACC ATCTTTCTTG ATCCTAGGAC TGACAAAATG GCAGTTAAAA   
  
  
- TCAACTGACG TTCAAACAGA AGGGTTGGGT AAATCTAAAA GTAAAAGATA ACCTGTAAGG TAAAGAAGGT   
  
  
- CTCCAGATCC TCCAGTTCAA AAACCTTCTT TAAATATTCT AATAACAACA AAAGAAGGGA CCGACGTCAA   
  
  
- TGAACTGAAC AATCCACCGT CTGTCAACGA AGCAAGACAA TCAGGTGTAG GTGTACTGTA GTTTTGAATC   
  
  
- CTAACTAAAA TCAAGAACAA ATACGAGACT TAAGAGTTTG ACAGAAATTT TTTAAGTAAT CAGTCGTAAA   
  
  
- CGAAAACTGA TAACAAACGT GACGACCGGC GTTCTCCCAA ACATAGAACT AATAAGCACA TTTTTCTTTA   
  
  
- ATGTCTACGA GTTAAGTATC TAAACCGGTG TGACACCCAA GTTTGATTCT AACTGACTTC CTTCGGTTAT   
  
  
- TTCTAGTAGA CAAAAAGGAC GAATTACCGT GGGTATACTC GTCTGTCGGT GAAAAAGACC TAACGGGTTA   
  
  
- AGTTACCTCC GTGTAATCAT CAGTGTCTCT TCCGTCGGAC AATTCTTTTA CCATAAAGAT CTTCGGTTAA   
  
  
- TCATGGCAAA GGTCTACTAT ACCAATTGTC CAGTTATTTT TTTTAATAAA TCCTGAGAAA AACATCACAG   
  
  
- ACGGTTGAAT ACCTTACGTC GGGATTAACT TTATACTCGG GTACACTCGT AGTACAATAT AGTTTCGTGA   
  
  
- CCGTCCCCGG TGTTTCCTGC CACTAAGTTC GAGACATATA ATTTGTACTT TTTCTCTACT TTACCCATTC   
  
  
- TTCAGTCGGA TCGGAACACC TATTGTCTCC ATCCGACCGT CCGTACAGAT TCAAGACTAC ATTGTTGGTT   
  
  
- TCTCCGTTTC CTCTTCTTTC GGTTTTTCTT TTTTTTCTTT TTTTCTTTCC AGCTCTTCCG TTCACTGTGT   
  
  
- TTGGGTTCTG GGTCTAGGTT ACATTGTTCA GTTGAAGGAA GAGAAAGAGA GAGACAGTGA CTGTTAGAAG   
  
  
- TGATTGTGTG TGTTTGTGCG GGGCGTGGCG TAACCTGTTT GTGGCTTTGG TCTTTGTGCG AGGGGTAGAC   
  
  
- GCTAAGTCAG GGAAGAGAGG AAATTTAAAT GGCACAATGG GAAGGGAGAG GGGAGAAGTG ACGGGATTAA   
  
  
- TGTTTAGATG AAGAGAGGAG AAAACTGAGA CAAGAGAGAA GAGAGAGTAT GAGCGCCATT CGTGCGGTCT   
  
  
- GTTGCGCGTG CGTGTGAAAG AGAACAGAC

+     TCA-element

| Site Name | Organism | Position | Strand | Matrix score. | sequence | function |
| --- | --- | --- | --- | --- | --- | --- |
| TCA-element | Brassica oleracea | 1417 | - | 9 | TCAGAAGAGG | cis-acting element involved in salicylic acid responsiveness |

> 2018/04/13 10:10:12  
+ CACACACAGT AAACACTGGT GCATTTATGC ACCTCAAGCA CTGAAAGTTT GCTGGAGAAT GCTAGCCTTA   
  
  
+ TTCAGTGAGA TGCCTTGTTT ATTAAAGAGA AAGAAACCAA GATATAGATG GCTTCTAACC TCTTTTTGTA   
  
  
+ TGAAATGGAT GAAACAAATA GAAATATCGT GCCAAAACAT CCAGGCAGTA TATTTAGATC AGGCAGCATA   
  
  
+ AGAGGAGACA GTACATTTTG CCCGCTTTGG TAGAAAGAAC TAGGATCCTG ACTGTTTTAC CGTCAATTTT   
  
  
+ AGTTGACTGC AAGTTTGTCT TCCCAACCCA TTTAGATTTT CATTTTCTAT TGGACATTCC ATTTCTTCCA   
  
  
+ GAGGTCTAGG AGGTCAAGTT TTTGGAAGAA ATTTATAAGA TTATTGTTGT TTTCTTCCCT GGCTGCAGTT   
  
  
+ ACTTGACTTG TTAGGTGGCA GACAGTTGCT TCGTTCTGTT AGTCCACATC CACATGACAT CAAAACTTAG   
  
  
+ GATTGATTTT AGTTCTTGTT TATGCTCTGA ATTCTCAAAC TGTCTTTAAA AAATTCATTA GTCAGCATTT   
  
  
+ GCTTTTGACT ATTGTTTGCA CTGCTGGCCG CAAGAGGGTT TGTATCTTGA TTATTCGTGT AAAAAGAAAT   
  
  
+ TACAGATGCT CAATTCATAG ATTTGGCCAC ACTGTGGGTT CAAACTAAGA TTGACTGAAG GAAGCCAATA   
  
  
+ AAGATCATCT GTTTTTCCTG CTTAATGGCA CCCATATGAG CAGACAGCCA CTTTTTCTGG ATTGCCCAAT   
  
  
+ TCAATGGAGG CACATTAGTA GTCACAGAGA AGGCAGCCTG TTAAGAAAAT GGTATTTCTA GAAGCCAATT   
  
  
+ AGTACCGTTT CCAGATGATA TGGTTAACAG GTCAATAAAA AAAATTATTT AGGACTCTTT TTGTAGTGTC   
  
  
+ TGCCAACTTA TGGAATGCAG CCCTAATTGA AATATGAGCC CATGTGAGCA TCATGTTATA TCAAAGCACT   
  
  
+ GGCAGGGGCC ACAAAGGACG GTGATTCAAG CTCTGTATAT TAAACATGAA AAAGAGATGA AATGGGTAAG   
  
  
+ AAGTCAGCCT AGCCTTGTGG ATAACAGAGG TAGGCTGGCA GGCATGTCTA AGTTCTGATG TAACAACCAA   
  
  
+ AGAGGCAAAG GAGAAGAAAG CCAAAAAGAA AAAAAAGAAA AAAAGAAAGG TCGAGAAGGC AAGTGACACA   
  
  
+ AACCCAAGAC CCAGATCCAA TGTAACAAGT CAACTTCCTT CTCTTTCTCT CTCTGTCACT GACAATCTTC   
  
  
+ ACTAACACAC ACAAACACGC CCCGCACCGC ATTGGACAAA CACCGAAACC AGAAACACGC TCCCCATCTG   
  
  
+ CGATTCAGTC CCTTCTCTCC TTTAAATTTA CCGTGTTACC CTTCCCTCTC CCCTCTTCAC TGCCCTAATT   
  
  
+ ACAAATCTAC TTCTCTCCTC TTTTGACTCT GTTCTCTCTT CTCTCTCATA CTCGCGGTAA GCACGCCAGA   
  
  
+ CAACGCGCAC GCACACTTTC TCTTGTCTG  

- GTGTGTGTCA TTTGTGACCA CGTAAATACG TGGAGTTCGT GACTTTCAAA CGACCTCTTA CGATCGGAAT   
  
  
- AAGTCACTCT ACGGAACAAA TAATTTCTCT TTCTTTGGTT CTATATCTAC CGAAGATTGG AGAAAAACAT   
  
  
- ACTTTACCTA CTTTGTTTAT CTTTATAGCA CGGTTTTGTA GGTCCGTCAT ATAAATCTAG TCCGTCGTAT   
  
  
- TCTCCTCTGT CATGTAAAAC GGGCGAAACC ATCTTTCTTG ATCCTAGGAC TGACAAAATG GCAGTTAAAA   
  
  
- TCAACTGACG TTCAAACAGA AGGGTTGGGT AAATCTAAAA GTAAAAGATA ACCTGTAAGG TAAAGAAGGT   
  
  
- CTCCAGATCC TCCAGTTCAA AAACCTTCTT TAAATATTCT AATAACAACA AAAGAAGGGA CCGACGTCAA   
  
  
- TGAACTGAAC AATCCACCGT CTGTCAACGA AGCAAGACAA TCAGGTGTAG GTGTACTGTA GTTTTGAATC   
  
  
- CTAACTAAAA TCAAGAACAA ATACGAGACT TAAGAGTTTG ACAGAAATTT TTTAAGTAAT CAGTCGTAAA   
  
  
- CGAAAACTGA TAACAAACGT GACGACCGGC GTTCTCCCAA ACATAGAACT AATAAGCACA TTTTTCTTTA   
  
  
- ATGTCTACGA GTTAAGTATC TAAACCGGTG TGACACCCAA GTTTGATTCT AACTGACTTC CTTCGGTTAT   
  
  
- TTCTAGTAGA CAAAAAGGAC GAATTACCGT GGGTATACTC GTCTGTCGGT GAAAAAGACC TAACGGGTTA   
  
  
- AGTTACCTCC GTGTAATCAT CAGTGTCTCT TCCGTCGGAC AATTCTTTTA CCATAAAGAT CTTCGGTTAA   
  
  
- TCATGGCAAA GGTCTACTAT ACCAATTGTC CAGTTATTTT TTTTAATAAA TCCTGAGAAA AACATCACAG   
  
  
- ACGGTTGAAT ACCTTACGTC GGGATTAACT TTATACTCGG GTACACTCGT AGTACAATAT AGTTTCGTGA   
  
  
- CCGTCCCCGG TGTTTCCTGC CACTAAGTTC GAGACATATA ATTTGTACTT TTTCTCTACT TTACCCATTC   
  
  
- TTCAGTCGGA TCGGAACACC TATTGTCTCC ATCCGACCGT CCGTACAGAT TCAAGACTAC ATTGTTGGTT   
  
  
- TCTCCGTTTC CTCTTCTTTC GGTTTTTCTT TTTTTTCTTT TTTTCTTTCC AGCTCTTCCG TTCACTGTGT   
  
  
- TTGGGTTCTG GGTCTAGGTT ACATTGTTCA GTTGAAGGAA GAGAAAGAGA GAGACAGTGA CTGTTAGAAG   
  
  
- TGATTGTGTG TGTTTGTGCG GGGCGTGGCG TAACCTGTTT GTGGCTTTGG TCTTTGTGCG AGGGGTAGAC   
  
  
- GCTAAGTCAG GGAAGAGAGG AAATTTAAAT GGCACAATGG GAAGGGAGAG GGGAGAAGTG ACGGGATTAA   
  
  
- TGTTTAGATG AAGAGAGGAG AAAACTGAGA CAAGAGAGAA GAGAGAGTAT GAGCGCCATT CGTGCGGTCT   
  
  
- GTTGCGCGTG CGTGTGAAAG AGAACAGAC

+     TCT-motif

| Site Name | Organism | Position | Strand | Matrix score. | sequence | function |
| --- | --- | --- | --- | --- | --- | --- |
| TCT-motif | Arabidopsis thaliana | 1046 | - | 6 | TCTTAC | part of a light responsive element |

> 2018/04/13 10:10:12  
+ CACACACAGT AAACACTGGT GCATTTATGC ACCTCAAGCA CTGAAAGTTT GCTGGAGAAT GCTAGCCTTA   
  
  
+ TTCAGTGAGA TGCCTTGTTT ATTAAAGAGA AAGAAACCAA GATATAGATG GCTTCTAACC TCTTTTTGTA   
  
  
+ TGAAATGGAT GAAACAAATA GAAATATCGT GCCAAAACAT CCAGGCAGTA TATTTAGATC AGGCAGCATA   
  
  
+ AGAGGAGACA GTACATTTTG CCCGCTTTGG TAGAAAGAAC TAGGATCCTG ACTGTTTTAC CGTCAATTTT   
  
  
+ AGTTGACTGC AAGTTTGTCT TCCCAACCCA TTTAGATTTT CATTTTCTAT TGGACATTCC ATTTCTTCCA   
  
  
+ GAGGTCTAGG AGGTCAAGTT TTTGGAAGAA ATTTATAAGA TTATTGTTGT TTTCTTCCCT GGCTGCAGTT   
  
  
+ ACTTGACTTG TTAGGTGGCA GACAGTTGCT TCGTTCTGTT AGTCCACATC CACATGACAT CAAAACTTAG   
  
  
+ GATTGATTTT AGTTCTTGTT TATGCTCTGA ATTCTCAAAC TGTCTTTAAA AAATTCATTA GTCAGCATTT   
  
  
+ GCTTTTGACT ATTGTTTGCA CTGCTGGCCG CAAGAGGGTT TGTATCTTGA TTATTCGTGT AAAAAGAAAT   
  
  
+ TACAGATGCT CAATTCATAG ATTTGGCCAC ACTGTGGGTT CAAACTAAGA TTGACTGAAG GAAGCCAATA   
  
  
+ AAGATCATCT GTTTTTCCTG CTTAATGGCA CCCATATGAG CAGACAGCCA CTTTTTCTGG ATTGCCCAAT   
  
  
+ TCAATGGAGG CACATTAGTA GTCACAGAGA AGGCAGCCTG TTAAGAAAAT GGTATTTCTA GAAGCCAATT   
  
  
+ AGTACCGTTT CCAGATGATA TGGTTAACAG GTCAATAAAA AAAATTATTT AGGACTCTTT TTGTAGTGTC   
  
  
+ TGCCAACTTA TGGAATGCAG CCCTAATTGA AATATGAGCC CATGTGAGCA TCATGTTATA TCAAAGCACT   
  
  
+ GGCAGGGGCC ACAAAGGACG GTGATTCAAG CTCTGTATAT TAAACATGAA AAAGAGATGA AATGGGTAAG   
  
  
+ AAGTCAGCCT AGCCTTGTGG ATAACAGAGG TAGGCTGGCA GGCATGTCTA AGTTCTGATG TAACAACCAA   
  
  
+ AGAGGCAAAG GAGAAGAAAG CCAAAAAGAA AAAAAAGAAA AAAAGAAAGG TCGAGAAGGC AAGTGACACA   
  
  
+ AACCCAAGAC CCAGATCCAA TGTAACAAGT CAACTTCCTT CTCTTTCTCT CTCTGTCACT GACAATCTTC   
  
  
+ ACTAACACAC ACAAACACGC CCCGCACCGC ATTGGACAAA CACCGAAACC AGAAACACGC TCCCCATCTG   
  
  
+ CGATTCAGTC CCTTCTCTCC TTTAAATTTA CCGTGTTACC CTTCCCTCTC CCCTCTTCAC TGCCCTAATT   
  
  
+ ACAAATCTAC TTCTCTCCTC TTTTGACTCT GTTCTCTCTT CTCTCTCATA CTCGCGGTAA GCACGCCAGA   
  
  
+ CAACGCGCAC GCACACTTTC TCTTGTCTG  

- GTGTGTGTCA TTTGTGACCA CGTAAATACG TGGAGTTCGT GACTTTCAAA CGACCTCTTA CGATCGGAAT   
  
  
- AAGTCACTCT ACGGAACAAA TAATTTCTCT TTCTTTGGTT CTATATCTAC CGAAGATTGG AGAAAAACAT   
  
  
- ACTTTACCTA CTTTGTTTAT CTTTATAGCA CGGTTTTGTA GGTCCGTCAT ATAAATCTAG TCCGTCGTAT   
  
  
- TCTCCTCTGT CATGTAAAAC GGGCGAAACC ATCTTTCTTG ATCCTAGGAC TGACAAAATG GCAGTTAAAA   
  
  
- TCAACTGACG TTCAAACAGA AGGGTTGGGT AAATCTAAAA GTAAAAGATA ACCTGTAAGG TAAAGAAGGT   
  
  
- CTCCAGATCC TCCAGTTCAA AAACCTTCTT TAAATATTCT AATAACAACA AAAGAAGGGA CCGACGTCAA   
  
  
- TGAACTGAAC AATCCACCGT CTGTCAACGA AGCAAGACAA TCAGGTGTAG GTGTACTGTA GTTTTGAATC   
  
  
- CTAACTAAAA TCAAGAACAA ATACGAGACT TAAGAGTTTG ACAGAAATTT TTTAAGTAAT CAGTCGTAAA   
  
  
- CGAAAACTGA TAACAAACGT GACGACCGGC GTTCTCCCAA ACATAGAACT AATAAGCACA TTTTTCTTTA   
  
  
- ATGTCTACGA GTTAAGTATC TAAACCGGTG TGACACCCAA GTTTGATTCT AACTGACTTC CTTCGGTTAT   
  
  
- TTCTAGTAGA CAAAAAGGAC GAATTACCGT GGGTATACTC GTCTGTCGGT GAAAAAGACC TAACGGGTTA   
  
  
- AGTTACCTCC GTGTAATCAT CAGTGTCTCT TCCGTCGGAC AATTCTTTTA CCATAAAGAT CTTCGGTTAA   
  
  
- TCATGGCAAA GGTCTACTAT ACCAATTGTC CAGTTATTTT TTTTAATAAA TCCTGAGAAA AACATCACAG   
  
  
- ACGGTTGAAT ACCTTACGTC GGGATTAACT TTATACTCGG GTACACTCGT AGTACAATAT AGTTTCGTGA   
  
  
- CCGTCCCCGG TGTTTCCTGC CACTAAGTTC GAGACATATA ATTTGTACTT TTTCTCTACT TTACCCATTC   
  
  
- TTCAGTCGGA TCGGAACACC TATTGTCTCC ATCCGACCGT CCGTACAGAT TCAAGACTAC ATTGTTGGTT   
  
  
- TCTCCGTTTC CTCTTCTTTC GGTTTTTCTT TTTTTTCTTT TTTTCTTTCC AGCTCTTCCG TTCACTGTGT   
  
  
- TTGGGTTCTG GGTCTAGGTT ACATTGTTCA GTTGAAGGAA GAGAAAGAGA GAGACAGTGA CTGTTAGAAG   
  
  
- TGATTGTGTG TGTTTGTGCG GGGCGTGGCG TAACCTGTTT GTGGCTTTGG TCTTTGTGCG AGGGGTAGAC   
  
  
- GCTAAGTCAG GGAAGAGAGG AAATTTAAAT GGCACAATGG GAAGGGAGAG GGGAGAAGTG ACGGGATTAA   
  
  
- TGTTTAGATG AAGAGAGGAG AAAACTGAGA CAAGAGAGAA GAGAGAGTAT GAGCGCCATT CGTGCGGTCT   
  
  
- GTTGCGCGTG CGTGTGAAAG AGAACAGAC

+     TGACG-motif

| Site Name | Organism | Position | Strand | Matrix score. | sequence | function |
| --- | --- | --- | --- | --- | --- | --- |
| TGACG-motif | Hordeum vulgare | 271 | - | 5 | TGACG | cis-acting regulatory element involved in the MeJA-responsiveness |

> 2018/04/13 10:10:12  
+ CACACACAGT AAACACTGGT GCATTTATGC ACCTCAAGCA CTGAAAGTTT GCTGGAGAAT GCTAGCCTTA   
  
  
+ TTCAGTGAGA TGCCTTGTTT ATTAAAGAGA AAGAAACCAA GATATAGATG GCTTCTAACC TCTTTTTGTA   
  
  
+ TGAAATGGAT GAAACAAATA GAAATATCGT GCCAAAACAT CCAGGCAGTA TATTTAGATC AGGCAGCATA   
  
  
+ AGAGGAGACA GTACATTTTG CCCGCTTTGG TAGAAAGAAC TAGGATCCTG ACTGTTTTAC CGTCAATTTT   
  
  
+ AGTTGACTGC AAGTTTGTCT TCCCAACCCA TTTAGATTTT CATTTTCTAT TGGACATTCC ATTTCTTCCA   
  
  
+ GAGGTCTAGG AGGTCAAGTT TTTGGAAGAA ATTTATAAGA TTATTGTTGT TTTCTTCCCT GGCTGCAGTT   
  
  
+ ACTTGACTTG TTAGGTGGCA GACAGTTGCT TCGTTCTGTT AGTCCACATC CACATGACAT CAAAACTTAG   
  
  
+ GATTGATTTT AGTTCTTGTT TATGCTCTGA ATTCTCAAAC TGTCTTTAAA AAATTCATTA GTCAGCATTT   
  
  
+ GCTTTTGACT ATTGTTTGCA CTGCTGGCCG CAAGAGGGTT TGTATCTTGA TTATTCGTGT AAAAAGAAAT   
  
  
+ TACAGATGCT CAATTCATAG ATTTGGCCAC ACTGTGGGTT CAAACTAAGA TTGACTGAAG GAAGCCAATA   
  
  
+ AAGATCATCT GTTTTTCCTG CTTAATGGCA CCCATATGAG CAGACAGCCA CTTTTTCTGG ATTGCCCAAT   
  
  
+ TCAATGGAGG CACATTAGTA GTCACAGAGA AGGCAGCCTG TTAAGAAAAT GGTATTTCTA GAAGCCAATT   
  
  
+ AGTACCGTTT CCAGATGATA TGGTTAACAG GTCAATAAAA AAAATTATTT AGGACTCTTT TTGTAGTGTC   
  
  
+ TGCCAACTTA TGGAATGCAG CCCTAATTGA AATATGAGCC CATGTGAGCA TCATGTTATA TCAAAGCACT   
  
  
+ GGCAGGGGCC ACAAAGGACG GTGATTCAAG CTCTGTATAT TAAACATGAA AAAGAGATGA AATGGGTAAG   
  
  
+ AAGTCAGCCT AGCCTTGTGG ATAACAGAGG TAGGCTGGCA GGCATGTCTA AGTTCTGATG TAACAACCAA   
  
  
+ AGAGGCAAAG GAGAAGAAAG CCAAAAAGAA AAAAAAGAAA AAAAGAAAGG TCGAGAAGGC AAGTGACACA   
  
  
+ AACCCAAGAC CCAGATCCAA TGTAACAAGT CAACTTCCTT CTCTTTCTCT CTCTGTCACT GACAATCTTC   
  
  
+ ACTAACACAC ACAAACACGC CCCGCACCGC ATTGGACAAA CACCGAAACC AGAAACACGC TCCCCATCTG   
  
  
+ CGATTCAGTC CCTTCTCTCC TTTAAATTTA CCGTGTTACC CTTCCCTCTC CCCTCTTCAC TGCCCTAATT   
  
  
+ ACAAATCTAC TTCTCTCCTC TTTTGACTCT GTTCTCTCTT CTCTCTCATA CTCGCGGTAA GCACGCCAGA   
  
  
+ CAACGCGCAC GCACACTTTC TCTTGTCTG  

- GTGTGTGTCA TTTGTGACCA CGTAAATACG TGGAGTTCGT GACTTTCAAA CGACCTCTTA CGATCGGAAT   
  
  
- AAGTCACTCT ACGGAACAAA TAATTTCTCT TTCTTTGGTT CTATATCTAC CGAAGATTGG AGAAAAACAT   
  
  
- ACTTTACCTA CTTTGTTTAT CTTTATAGCA CGGTTTTGTA GGTCCGTCAT ATAAATCTAG TCCGTCGTAT   
  
  
- TCTCCTCTGT CATGTAAAAC GGGCGAAACC ATCTTTCTTG ATCCTAGGAC TGACAAAATG GCAGTTAAAA   
  
  
- TCAACTGACG TTCAAACAGA AGGGTTGGGT AAATCTAAAA GTAAAAGATA ACCTGTAAGG TAAAGAAGGT   
  
  
- CTCCAGATCC TCCAGTTCAA AAACCTTCTT TAAATATTCT AATAACAACA AAAGAAGGGA CCGACGTCAA   
  
  
- TGAACTGAAC AATCCACCGT CTGTCAACGA AGCAAGACAA TCAGGTGTAG GTGTACTGTA GTTTTGAATC   
  
  
- CTAACTAAAA TCAAGAACAA ATACGAGACT TAAGAGTTTG ACAGAAATTT TTTAAGTAAT CAGTCGTAAA   
  
  
- CGAAAACTGA TAACAAACGT GACGACCGGC GTTCTCCCAA ACATAGAACT AATAAGCACA TTTTTCTTTA   
  
  
- ATGTCTACGA GTTAAGTATC TAAACCGGTG TGACACCCAA GTTTGATTCT AACTGACTTC CTTCGGTTAT   
  
  
- TTCTAGTAGA CAAAAAGGAC GAATTACCGT GGGTATACTC GTCTGTCGGT GAAAAAGACC TAACGGGTTA   
  
  
- AGTTACCTCC GTGTAATCAT CAGTGTCTCT TCCGTCGGAC AATTCTTTTA CCATAAAGAT CTTCGGTTAA   
  
  
- TCATGGCAAA GGTCTACTAT ACCAATTGTC CAGTTATTTT TTTTAATAAA TCCTGAGAAA AACATCACAG   
  
  
- ACGGTTGAAT ACCTTACGTC GGGATTAACT TTATACTCGG GTACACTCGT AGTACAATAT AGTTTCGTGA   
  
  
- CCGTCCCCGG TGTTTCCTGC CACTAAGTTC GAGACATATA ATTTGTACTT TTTCTCTACT TTACCCATTC   
  
  
- TTCAGTCGGA TCGGAACACC TATTGTCTCC ATCCGACCGT CCGTACAGAT TCAAGACTAC ATTGTTGGTT   
  
  
- TCTCCGTTTC CTCTTCTTTC GGTTTTTCTT TTTTTTCTTT TTTTCTTTCC AGCTCTTCCG TTCACTGTGT   
  
  
- TTGGGTTCTG GGTCTAGGTT ACATTGTTCA GTTGAAGGAA GAGAAAGAGA GAGACAGTGA CTGTTAGAAG   
  
  
- TGATTGTGTG TGTTTGTGCG GGGCGTGGCG TAACCTGTTT GTGGCTTTGG TCTTTGTGCG AGGGGTAGAC   
  
  
- GCTAAGTCAG GGAAGAGAGG AAATTTAAAT GGCACAATGG GAAGGGAGAG GGGAGAAGTG ACGGGATTAA   
  
  
- TGTTTAGATG AAGAGAGGAG AAAACTGAGA CAAGAGAGAA GAGAGAGTAT GAGCGCCATT CGTGCGGTCT   
  
  
- GTTGCGCGTG CGTGTGAAAG AGAACAGAC

+     Unnamed\_\_15

| Site Name | Organism | Position | Strand | Matrix score. | sequence | function |
| --- | --- | --- | --- | --- | --- | --- |
| Unnamed\_\_15 | Zea mays | 1375 | + | 10 | CCTCTCCCGTC |  |

> 2018/04/13 10:10:12  
+ CACACACAGT AAACACTGGT GCATTTATGC ACCTCAAGCA CTGAAAGTTT GCTGGAGAAT GCTAGCCTTA   
  
  
+ TTCAGTGAGA TGCCTTGTTT ATTAAAGAGA AAGAAACCAA GATATAGATG GCTTCTAACC TCTTTTTGTA   
  
  
+ TGAAATGGAT GAAACAAATA GAAATATCGT GCCAAAACAT CCAGGCAGTA TATTTAGATC AGGCAGCATA   
  
  
+ AGAGGAGACA GTACATTTTG CCCGCTTTGG TAGAAAGAAC TAGGATCCTG ACTGTTTTAC CGTCAATTTT   
  
  
+ AGTTGACTGC AAGTTTGTCT TCCCAACCCA TTTAGATTTT CATTTTCTAT TGGACATTCC ATTTCTTCCA   
  
  
+ GAGGTCTAGG AGGTCAAGTT TTTGGAAGAA ATTTATAAGA TTATTGTTGT TTTCTTCCCT GGCTGCAGTT   
  
  
+ ACTTGACTTG TTAGGTGGCA GACAGTTGCT TCGTTCTGTT AGTCCACATC CACATGACAT CAAAACTTAG   
  
  
+ GATTGATTTT AGTTCTTGTT TATGCTCTGA ATTCTCAAAC TGTCTTTAAA AAATTCATTA GTCAGCATTT   
  
  
+ GCTTTTGACT ATTGTTTGCA CTGCTGGCCG CAAGAGGGTT TGTATCTTGA TTATTCGTGT AAAAAGAAAT   
  
  
+ TACAGATGCT CAATTCATAG ATTTGGCCAC ACTGTGGGTT CAAACTAAGA TTGACTGAAG GAAGCCAATA   
  
  
+ AAGATCATCT GTTTTTCCTG CTTAATGGCA CCCATATGAG CAGACAGCCA CTTTTTCTGG ATTGCCCAAT   
  
  
+ TCAATGGAGG CACATTAGTA GTCACAGAGA AGGCAGCCTG TTAAGAAAAT GGTATTTCTA GAAGCCAATT   
  
  
+ AGTACCGTTT CCAGATGATA TGGTTAACAG GTCAATAAAA AAAATTATTT AGGACTCTTT TTGTAGTGTC   
  
  
+ TGCCAACTTA TGGAATGCAG CCCTAATTGA AATATGAGCC CATGTGAGCA TCATGTTATA TCAAAGCACT   
  
  
+ GGCAGGGGCC ACAAAGGACG GTGATTCAAG CTCTGTATAT TAAACATGAA AAAGAGATGA AATGGGTAAG   
  
  
+ AAGTCAGCCT AGCCTTGTGG ATAACAGAGG TAGGCTGGCA GGCATGTCTA AGTTCTGATG TAACAACCAA   
  
  
+ AGAGGCAAAG GAGAAGAAAG CCAAAAAGAA AAAAAAGAAA AAAAGAAAGG TCGAGAAGGC AAGTGACACA   
  
  
+ AACCCAAGAC CCAGATCCAA TGTAACAAGT CAACTTCCTT CTCTTTCTCT CTCTGTCACT GACAATCTTC   
  
  
+ ACTAACACAC ACAAACACGC CCCGCACCGC ATTGGACAAA CACCGAAACC AGAAACACGC TCCCCATCTG   
  
  
+ CGATTCAGTC CCTTCTCTCC TTTAAATTTA CCGTGTTACC CTTCCCTCTC CCCTCTTCAC TGCCCTAATT   
  
  
+ ACAAATCTAC TTCTCTCCTC TTTTGACTCT GTTCTCTCTT CTCTCTCATA CTCGCGGTAA GCACGCCAGA   
  
  
+ CAACGCGCAC GCACACTTTC TCTTGTCTG  

- GTGTGTGTCA TTTGTGACCA CGTAAATACG TGGAGTTCGT GACTTTCAAA CGACCTCTTA CGATCGGAAT   
  
  
- AAGTCACTCT ACGGAACAAA TAATTTCTCT TTCTTTGGTT CTATATCTAC CGAAGATTGG AGAAAAACAT   
  
  
- ACTTTACCTA CTTTGTTTAT CTTTATAGCA CGGTTTTGTA GGTCCGTCAT ATAAATCTAG TCCGTCGTAT   
  
  
- TCTCCTCTGT CATGTAAAAC GGGCGAAACC ATCTTTCTTG ATCCTAGGAC TGACAAAATG GCAGTTAAAA   
  
  
- TCAACTGACG TTCAAACAGA AGGGTTGGGT AAATCTAAAA GTAAAAGATA ACCTGTAAGG TAAAGAAGGT   
  
  
- CTCCAGATCC TCCAGTTCAA AAACCTTCTT TAAATATTCT AATAACAACA AAAGAAGGGA CCGACGTCAA   
  
  
- TGAACTGAAC AATCCACCGT CTGTCAACGA AGCAAGACAA TCAGGTGTAG GTGTACTGTA GTTTTGAATC   
  
  
- CTAACTAAAA TCAAGAACAA ATACGAGACT TAAGAGTTTG ACAGAAATTT TTTAAGTAAT CAGTCGTAAA   
  
  
- CGAAAACTGA TAACAAACGT GACGACCGGC GTTCTCCCAA ACATAGAACT AATAAGCACA TTTTTCTTTA   
  
  
- ATGTCTACGA GTTAAGTATC TAAACCGGTG TGACACCCAA GTTTGATTCT AACTGACTTC CTTCGGTTAT   
  
  
- TTCTAGTAGA CAAAAAGGAC GAATTACCGT GGGTATACTC GTCTGTCGGT GAAAAAGACC TAACGGGTTA   
  
  
- AGTTACCTCC GTGTAATCAT CAGTGTCTCT TCCGTCGGAC AATTCTTTTA CCATAAAGAT CTTCGGTTAA   
  
  
- TCATGGCAAA GGTCTACTAT ACCAATTGTC CAGTTATTTT TTTTAATAAA TCCTGAGAAA AACATCACAG   
  
  
- ACGGTTGAAT ACCTTACGTC GGGATTAACT TTATACTCGG GTACACTCGT AGTACAATAT AGTTTCGTGA   
  
  
- CCGTCCCCGG TGTTTCCTGC CACTAAGTTC GAGACATATA ATTTGTACTT TTTCTCTACT TTACCCATTC   
  
  
- TTCAGTCGGA TCGGAACACC TATTGTCTCC ATCCGACCGT CCGTACAGAT TCAAGACTAC ATTGTTGGTT   
  
  
- TCTCCGTTTC CTCTTCTTTC GGTTTTTCTT TTTTTTCTTT TTTTCTTTCC AGCTCTTCCG TTCACTGTGT   
  
  
- TTGGGTTCTG GGTCTAGGTT ACATTGTTCA GTTGAAGGAA GAGAAAGAGA GAGACAGTGA CTGTTAGAAG   
  
  
- TGATTGTGTG TGTTTGTGCG GGGCGTGGCG TAACCTGTTT GTGGCTTTGG TCTTTGTGCG AGGGGTAGAC   
  
  
- GCTAAGTCAG GGAAGAGAGG AAATTTAAAT GGCACAATGG GAAGGGAGAG GGGAGAAGTG ACGGGATTAA   
  
  
- TGTTTAGATG AAGAGAGGAG AAAACTGAGA CAAGAGAGAA GAGAGAGTAT GAGCGCCATT CGTGCGGTCT   
  
  
- GTTGCGCGTG CGTGTGAAAG AGAACAGAC

+     Unnamed\_\_4

| Site Name | Organism | Position | Strand | Matrix score. | sequence | function |
| --- | --- | --- | --- | --- | --- | --- |
| Unnamed\_\_4 | Petroselinum hortense | 1415 | + | 4 | CTCC |  |
| Unnamed\_\_4 | Petroselinum hortense | 1378 | + | 4 | CTCC |  |
| Unnamed\_\_4 | Petroselinum hortense | 1347 | + | 4 | CTCC |  |
| Unnamed\_\_4 | Petroselinum hortense | 1320 | + | 4 | CTCC |  |
| Unnamed\_\_4 | Petroselinum hortense | 1130 | - | 4 | CTCC |  |
| Unnamed\_\_4 | Petroselinum hortense | 776 | - | 4 | CTCC |  |
| Unnamed\_\_4 | Petroselinum hortense | 359 | - | 4 | CTCC |  |
| Unnamed\_\_4 | Petroselinum hortense | 54 | - | 4 | CTCC |  |
| Unnamed\_\_4 | Petroselinum hortense | 214 | - | 4 | CTCC |  |

> 2018/04/13 10:10:12  
+ CACACACAGT AAACACTGGT GCATTTATGC ACCTCAAGCA CTGAAAGTTT GCTGGAGAAT GCTAGCCTTA   
  
  
+ TTCAGTGAGA TGCCTTGTTT ATTAAAGAGA AAGAAACCAA GATATAGATG GCTTCTAACC TCTTTTTGTA   
  
  
+ TGAAATGGAT GAAACAAATA GAAATATCGT GCCAAAACAT CCAGGCAGTA TATTTAGATC AGGCAGCATA   
  
  
+ AGAGGAGACA GTACATTTTG CCCGCTTTGG TAGAAAGAAC TAGGATCCTG ACTGTTTTAC CGTCAATTTT   
  
  
+ AGTTGACTGC AAGTTTGTCT TCCCAACCCA TTTAGATTTT CATTTTCTAT TGGACATTCC ATTTCTTCCA   
  
  
+ GAGGTCTAGG AGGTCAAGTT TTTGGAAGAA ATTTATAAGA TTATTGTTGT TTTCTTCCCT GGCTGCAGTT   
  
  
+ ACTTGACTTG TTAGGTGGCA GACAGTTGCT TCGTTCTGTT AGTCCACATC CACATGACAT CAAAACTTAG   
  
  
+ GATTGATTTT AGTTCTTGTT TATGCTCTGA ATTCTCAAAC TGTCTTTAAA AAATTCATTA GTCAGCATTT   
  
  
+ GCTTTTGACT ATTGTTTGCA CTGCTGGCCG CAAGAGGGTT TGTATCTTGA TTATTCGTGT AAAAAGAAAT   
  
  
+ TACAGATGCT CAATTCATAG ATTTGGCCAC ACTGTGGGTT CAAACTAAGA TTGACTGAAG GAAGCCAATA   
  
  
+ AAGATCATCT GTTTTTCCTG CTTAATGGCA CCCATATGAG CAGACAGCCA CTTTTTCTGG ATTGCCCAAT   
  
  
+ TCAATGGAGG CACATTAGTA GTCACAGAGA AGGCAGCCTG TTAAGAAAAT GGTATTTCTA GAAGCCAATT   
  
  
+ AGTACCGTTT CCAGATGATA TGGTTAACAG GTCAATAAAA AAAATTATTT AGGACTCTTT TTGTAGTGTC   
  
  
+ TGCCAACTTA TGGAATGCAG CCCTAATTGA AATATGAGCC CATGTGAGCA TCATGTTATA TCAAAGCACT   
  
  
+ GGCAGGGGCC ACAAAGGACG GTGATTCAAG CTCTGTATAT TAAACATGAA AAAGAGATGA AATGGGTAAG   
  
  
+ AAGTCAGCCT AGCCTTGTGG ATAACAGAGG TAGGCTGGCA GGCATGTCTA AGTTCTGATG TAACAACCAA   
  
  
+ AGAGGCAAAG GAGAAGAAAG CCAAAAAGAA AAAAAAGAAA AAAAGAAAGG TCGAGAAGGC AAGTGACACA   
  
  
+ AACCCAAGAC CCAGATCCAA TGTAACAAGT CAACTTCCTT CTCTTTCTCT CTCTGTCACT GACAATCTTC   
  
  
+ ACTAACACAC ACAAACACGC CCCGCACCGC ATTGGACAAA CACCGAAACC AGAAACACGC TCCCCATCTG   
  
  
+ CGATTCAGTC CCTTCTCTCC TTTAAATTTA CCGTGTTACC CTTCCCTCTC CCCTCTTCAC TGCCCTAATT   
  
  
+ ACAAATCTAC TTCTCTCCTC TTTTGACTCT GTTCTCTCTT CTCTCTCATA CTCGCGGTAA GCACGCCAGA   
  
  
+ CAACGCGCAC GCACACTTTC TCTTGTCTG  

- GTGTGTGTCA TTTGTGACCA CGTAAATACG TGGAGTTCGT GACTTTCAAA CGACCTCTTA CGATCGGAAT   
  
  
- AAGTCACTCT ACGGAACAAA TAATTTCTCT TTCTTTGGTT CTATATCTAC CGAAGATTGG AGAAAAACAT   
  
  
- ACTTTACCTA CTTTGTTTAT CTTTATAGCA CGGTTTTGTA GGTCCGTCAT ATAAATCTAG TCCGTCGTAT   
  
  
- TCTCCTCTGT CATGTAAAAC GGGCGAAACC ATCTTTCTTG ATCCTAGGAC TGACAAAATG GCAGTTAAAA   
  
  
- TCAACTGACG TTCAAACAGA AGGGTTGGGT AAATCTAAAA GTAAAAGATA ACCTGTAAGG TAAAGAAGGT   
  
  
- CTCCAGATCC TCCAGTTCAA AAACCTTCTT TAAATATTCT AATAACAACA AAAGAAGGGA CCGACGTCAA   
  
  
- TGAACTGAAC AATCCACCGT CTGTCAACGA AGCAAGACAA TCAGGTGTAG GTGTACTGTA GTTTTGAATC   
  
  
- CTAACTAAAA TCAAGAACAA ATACGAGACT TAAGAGTTTG ACAGAAATTT TTTAAGTAAT CAGTCGTAAA   
  
  
- CGAAAACTGA TAACAAACGT GACGACCGGC GTTCTCCCAA ACATAGAACT AATAAGCACA TTTTTCTTTA   
  
  
- ATGTCTACGA GTTAAGTATC TAAACCGGTG TGACACCCAA GTTTGATTCT AACTGACTTC CTTCGGTTAT   
  
  
- TTCTAGTAGA CAAAAAGGAC GAATTACCGT GGGTATACTC GTCTGTCGGT GAAAAAGACC TAACGGGTTA   
  
  
- AGTTACCTCC GTGTAATCAT CAGTGTCTCT TCCGTCGGAC AATTCTTTTA CCATAAAGAT CTTCGGTTAA   
  
  
- TCATGGCAAA GGTCTACTAT ACCAATTGTC CAGTTATTTT TTTTAATAAA TCCTGAGAAA AACATCACAG   
  
  
- ACGGTTGAAT ACCTTACGTC GGGATTAACT TTATACTCGG GTACACTCGT AGTACAATAT AGTTTCGTGA   
  
  
- CCGTCCCCGG TGTTTCCTGC CACTAAGTTC GAGACATATA ATTTGTACTT TTTCTCTACT TTACCCATTC   
  
  
- TTCAGTCGGA TCGGAACACC TATTGTCTCC ATCCGACCGT CCGTACAGAT TCAAGACTAC ATTGTTGGTT   
  
  
- TCTCCGTTTC CTCTTCTTTC GGTTTTTCTT TTTTTTCTTT TTTTCTTTCC AGCTCTTCCG TTCACTGTGT   
  
  
- TTGGGTTCTG GGTCTAGGTT ACATTGTTCA GTTGAAGGAA GAGAAAGAGA GAGACAGTGA CTGTTAGAAG   
  
  
- TGATTGTGTG TGTTTGTGCG GGGCGTGGCG TAACCTGTTT GTGGCTTTGG TCTTTGTGCG AGGGGTAGAC   
  
  
- GCTAAGTCAG GGAAGAGAGG AAATTTAAAT GGCACAATGG GAAGGGAGAG GGGAGAAGTG ACGGGATTAA   
  
  
- TGTTTAGATG AAGAGAGGAG AAAACTGAGA CAAGAGAGAA GAGAGAGTAT GAGCGCCATT CGTGCGGTCT   
  
  
- GTTGCGCGTG CGTGTGAAAG AGAACAGAC

+     Unnamed\_\_6

| Site Name | Organism | Position | Strand | Matrix score. | sequence | function |
| --- | --- | --- | --- | --- | --- | --- |
| Unnamed\_\_6 | Zea mays | 377 | - | 10 | taTAAATATct |  |

> 2018/04/13 10:10:12  
+ CACACACAGT AAACACTGGT GCATTTATGC ACCTCAAGCA CTGAAAGTTT GCTGGAGAAT GCTAGCCTTA   
  
  
+ TTCAGTGAGA TGCCTTGTTT ATTAAAGAGA AAGAAACCAA GATATAGATG GCTTCTAACC TCTTTTTGTA   
  
  
+ TGAAATGGAT GAAACAAATA GAAATATCGT GCCAAAACAT CCAGGCAGTA TATTTAGATC AGGCAGCATA   
  
  
+ AGAGGAGACA GTACATTTTG CCCGCTTTGG TAGAAAGAAC TAGGATCCTG ACTGTTTTAC CGTCAATTTT   
  
  
+ AGTTGACTGC AAGTTTGTCT TCCCAACCCA TTTAGATTTT CATTTTCTAT TGGACATTCC ATTTCTTCCA   
  
  
+ GAGGTCTAGG AGGTCAAGTT TTTGGAAGAA ATTTATAAGA TTATTGTTGT TTTCTTCCCT GGCTGCAGTT   
  
  
+ ACTTGACTTG TTAGGTGGCA GACAGTTGCT TCGTTCTGTT AGTCCACATC CACATGACAT CAAAACTTAG   
  
  
+ GATTGATTTT AGTTCTTGTT TATGCTCTGA ATTCTCAAAC TGTCTTTAAA AAATTCATTA GTCAGCATTT   
  
  
+ GCTTTTGACT ATTGTTTGCA CTGCTGGCCG CAAGAGGGTT TGTATCTTGA TTATTCGTGT AAAAAGAAAT   
  
  
+ TACAGATGCT CAATTCATAG ATTTGGCCAC ACTGTGGGTT CAAACTAAGA TTGACTGAAG GAAGCCAATA   
  
  
+ AAGATCATCT GTTTTTCCTG CTTAATGGCA CCCATATGAG CAGACAGCCA CTTTTTCTGG ATTGCCCAAT   
  
  
+ TCAATGGAGG CACATTAGTA GTCACAGAGA AGGCAGCCTG TTAAGAAAAT GGTATTTCTA GAAGCCAATT   
  
  
+ AGTACCGTTT CCAGATGATA TGGTTAACAG GTCAATAAAA AAAATTATTT AGGACTCTTT TTGTAGTGTC   
  
  
+ TGCCAACTTA TGGAATGCAG CCCTAATTGA AATATGAGCC CATGTGAGCA TCATGTTATA TCAAAGCACT   
  
  
+ GGCAGGGGCC ACAAAGGACG GTGATTCAAG CTCTGTATAT TAAACATGAA AAAGAGATGA AATGGGTAAG   
  
  
+ AAGTCAGCCT AGCCTTGTGG ATAACAGAGG TAGGCTGGCA GGCATGTCTA AGTTCTGATG TAACAACCAA   
  
  
+ AGAGGCAAAG GAGAAGAAAG CCAAAAAGAA AAAAAAGAAA AAAAGAAAGG TCGAGAAGGC AAGTGACACA   
  
  
+ AACCCAAGAC CCAGATCCAA TGTAACAAGT CAACTTCCTT CTCTTTCTCT CTCTGTCACT GACAATCTTC   
  
  
+ ACTAACACAC ACAAACACGC CCCGCACCGC ATTGGACAAA CACCGAAACC AGAAACACGC TCCCCATCTG   
  
  
+ CGATTCAGTC CCTTCTCTCC TTTAAATTTA CCGTGTTACC CTTCCCTCTC CCCTCTTCAC TGCCCTAATT   
  
  
+ ACAAATCTAC TTCTCTCCTC TTTTGACTCT GTTCTCTCTT CTCTCTCATA CTCGCGGTAA GCACGCCAGA   
  
  
+ CAACGCGCAC GCACACTTTC TCTTGTCTG  

- GTGTGTGTCA TTTGTGACCA CGTAAATACG TGGAGTTCGT GACTTTCAAA CGACCTCTTA CGATCGGAAT   
  
  
- AAGTCACTCT ACGGAACAAA TAATTTCTCT TTCTTTGGTT CTATATCTAC CGAAGATTGG AGAAAAACAT   
  
  
- ACTTTACCTA CTTTGTTTAT CTTTATAGCA CGGTTTTGTA GGTCCGTCAT ATAAATCTAG TCCGTCGTAT   
  
  
- TCTCCTCTGT CATGTAAAAC GGGCGAAACC ATCTTTCTTG ATCCTAGGAC TGACAAAATG GCAGTTAAAA   
  
  
- TCAACTGACG TTCAAACAGA AGGGTTGGGT AAATCTAAAA GTAAAAGATA ACCTGTAAGG TAAAGAAGGT   
  
  
- CTCCAGATCC TCCAGTTCAA AAACCTTCTT TAAATATTCT AATAACAACA AAAGAAGGGA CCGACGTCAA   
  
  
- TGAACTGAAC AATCCACCGT CTGTCAACGA AGCAAGACAA TCAGGTGTAG GTGTACTGTA GTTTTGAATC   
  
  
- CTAACTAAAA TCAAGAACAA ATACGAGACT TAAGAGTTTG ACAGAAATTT TTTAAGTAAT CAGTCGTAAA   
  
  
- CGAAAACTGA TAACAAACGT GACGACCGGC GTTCTCCCAA ACATAGAACT AATAAGCACA TTTTTCTTTA   
  
  
- ATGTCTACGA GTTAAGTATC TAAACCGGTG TGACACCCAA GTTTGATTCT AACTGACTTC CTTCGGTTAT   
  
  
- TTCTAGTAGA CAAAAAGGAC GAATTACCGT GGGTATACTC GTCTGTCGGT GAAAAAGACC TAACGGGTTA   
  
  
- AGTTACCTCC GTGTAATCAT CAGTGTCTCT TCCGTCGGAC AATTCTTTTA CCATAAAGAT CTTCGGTTAA   
  
  
- TCATGGCAAA GGTCTACTAT ACCAATTGTC CAGTTATTTT TTTTAATAAA TCCTGAGAAA AACATCACAG   
  
  
- ACGGTTGAAT ACCTTACGTC GGGATTAACT TTATACTCGG GTACACTCGT AGTACAATAT AGTTTCGTGA   
  
  
- CCGTCCCCGG TGTTTCCTGC CACTAAGTTC GAGACATATA ATTTGTACTT TTTCTCTACT TTACCCATTC   
  
  
- TTCAGTCGGA TCGGAACACC TATTGTCTCC ATCCGACCGT CCGTACAGAT TCAAGACTAC ATTGTTGGTT   
  
  
- TCTCCGTTTC CTCTTCTTTC GGTTTTTCTT TTTTTTCTTT TTTTCTTTCC AGCTCTTCCG TTCACTGTGT   
  
  
- TTGGGTTCTG GGTCTAGGTT ACATTGTTCA GTTGAAGGAA GAGAAAGAGA GAGACAGTGA CTGTTAGAAG   
  
  
- TGATTGTGTG TGTTTGTGCG GGGCGTGGCG TAACCTGTTT GTGGCTTTGG TCTTTGTGCG AGGGGTAGAC   
  
  
- GCTAAGTCAG GGAAGAGAGG AAATTTAAAT GGCACAATGG GAAGGGAGAG GGGAGAAGTG ACGGGATTAA   
  
  
- TGTTTAGATG AAGAGAGGAG AAAACTGAGA CAAGAGAGAA GAGAGAGTAT GAGCGCCATT CGTGCGGTCT   
  
  
- GTTGCGCGTG CGTGTGAAAG AGAACAGAC

+     W box

| Site Name | Organism | Position | Strand | Matrix score. | sequence | function |
| --- | --- | --- | --- | --- | --- | --- |
| W box | Arabidopsis thaliana | 870 | - | 6 | TTGACC |  |
| W box | Arabidopsis thaliana | 362 | - | 6 | TTGACC |  |

> 2018/04/13 10:10:12  
+ CACACACAGT AAACACTGGT GCATTTATGC ACCTCAAGCA CTGAAAGTTT GCTGGAGAAT GCTAGCCTTA   
  
  
+ TTCAGTGAGA TGCCTTGTTT ATTAAAGAGA AAGAAACCAA GATATAGATG GCTTCTAACC TCTTTTTGTA   
  
  
+ TGAAATGGAT GAAACAAATA GAAATATCGT GCCAAAACAT CCAGGCAGTA TATTTAGATC AGGCAGCATA   
  
  
+ AGAGGAGACA GTACATTTTG CCCGCTTTGG TAGAAAGAAC TAGGATCCTG ACTGTTTTAC CGTCAATTTT   
  
  
+ AGTTGACTGC AAGTTTGTCT TCCCAACCCA TTTAGATTTT CATTTTCTAT TGGACATTCC ATTTCTTCCA   
  
  
+ GAGGTCTAGG AGGTCAAGTT TTTGGAAGAA ATTTATAAGA TTATTGTTGT TTTCTTCCCT GGCTGCAGTT   
  
  
+ ACTTGACTTG TTAGGTGGCA GACAGTTGCT TCGTTCTGTT AGTCCACATC CACATGACAT CAAAACTTAG   
  
  
+ GATTGATTTT AGTTCTTGTT TATGCTCTGA ATTCTCAAAC TGTCTTTAAA AAATTCATTA GTCAGCATTT   
  
  
+ GCTTTTGACT ATTGTTTGCA CTGCTGGCCG CAAGAGGGTT TGTATCTTGA TTATTCGTGT AAAAAGAAAT   
  
  
+ TACAGATGCT CAATTCATAG ATTTGGCCAC ACTGTGGGTT CAAACTAAGA TTGACTGAAG GAAGCCAATA   
  
  
+ AAGATCATCT GTTTTTCCTG CTTAATGGCA CCCATATGAG CAGACAGCCA CTTTTTCTGG ATTGCCCAAT   
  
  
+ TCAATGGAGG CACATTAGTA GTCACAGAGA AGGCAGCCTG TTAAGAAAAT GGTATTTCTA GAAGCCAATT   
  
  
+ AGTACCGTTT CCAGATGATA TGGTTAACAG GTCAATAAAA AAAATTATTT AGGACTCTTT TTGTAGTGTC   
  
  
+ TGCCAACTTA TGGAATGCAG CCCTAATTGA AATATGAGCC CATGTGAGCA TCATGTTATA TCAAAGCACT   
  
  
+ GGCAGGGGCC ACAAAGGACG GTGATTCAAG CTCTGTATAT TAAACATGAA AAAGAGATGA AATGGGTAAG   
  
  
+ AAGTCAGCCT AGCCTTGTGG ATAACAGAGG TAGGCTGGCA GGCATGTCTA AGTTCTGATG TAACAACCAA   
  
  
+ AGAGGCAAAG GAGAAGAAAG CCAAAAAGAA AAAAAAGAAA AAAAGAAAGG TCGAGAAGGC AAGTGACACA   
  
  
+ AACCCAAGAC CCAGATCCAA TGTAACAAGT CAACTTCCTT CTCTTTCTCT CTCTGTCACT GACAATCTTC   
  
  
+ ACTAACACAC ACAAACACGC CCCGCACCGC ATTGGACAAA CACCGAAACC AGAAACACGC TCCCCATCTG   
  
  
+ CGATTCAGTC CCTTCTCTCC TTTAAATTTA CCGTGTTACC CTTCCCTCTC CCCTCTTCAC TGCCCTAATT   
  
  
+ ACAAATCTAC TTCTCTCCTC TTTTGACTCT GTTCTCTCTT CTCTCTCATA CTCGCGGTAA GCACGCCAGA   
  
  
+ CAACGCGCAC GCACACTTTC TCTTGTCTG  

- GTGTGTGTCA TTTGTGACCA CGTAAATACG TGGAGTTCGT GACTTTCAAA CGACCTCTTA CGATCGGAAT   
  
  
- AAGTCACTCT ACGGAACAAA TAATTTCTCT TTCTTTGGTT CTATATCTAC CGAAGATTGG AGAAAAACAT   
  
  
- ACTTTACCTA CTTTGTTTAT CTTTATAGCA CGGTTTTGTA GGTCCGTCAT ATAAATCTAG TCCGTCGTAT   
  
  
- TCTCCTCTGT CATGTAAAAC GGGCGAAACC ATCTTTCTTG ATCCTAGGAC TGACAAAATG GCAGTTAAAA   
  
  
- TCAACTGACG TTCAAACAGA AGGGTTGGGT AAATCTAAAA GTAAAAGATA ACCTGTAAGG TAAAGAAGGT   
  
  
- CTCCAGATCC TCCAGTTCAA AAACCTTCTT TAAATATTCT AATAACAACA AAAGAAGGGA CCGACGTCAA   
  
  
- TGAACTGAAC AATCCACCGT CTGTCAACGA AGCAAGACAA TCAGGTGTAG GTGTACTGTA GTTTTGAATC   
  
  
- CTAACTAAAA TCAAGAACAA ATACGAGACT TAAGAGTTTG ACAGAAATTT TTTAAGTAAT CAGTCGTAAA   
  
  
- CGAAAACTGA TAACAAACGT GACGACCGGC GTTCTCCCAA ACATAGAACT AATAAGCACA TTTTTCTTTA   
  
  
- ATGTCTACGA GTTAAGTATC TAAACCGGTG TGACACCCAA GTTTGATTCT AACTGACTTC CTTCGGTTAT   
  
  
- TTCTAGTAGA CAAAAAGGAC GAATTACCGT GGGTATACTC GTCTGTCGGT GAAAAAGACC TAACGGGTTA   
  
  
- AGTTACCTCC GTGTAATCAT CAGTGTCTCT TCCGTCGGAC AATTCTTTTA CCATAAAGAT CTTCGGTTAA   
  
  
- TCATGGCAAA GGTCTACTAT ACCAATTGTC CAGTTATTTT TTTTAATAAA TCCTGAGAAA AACATCACAG   
  
  
- ACGGTTGAAT ACCTTACGTC GGGATTAACT TTATACTCGG GTACACTCGT AGTACAATAT AGTTTCGTGA   
  
  
- CCGTCCCCGG TGTTTCCTGC CACTAAGTTC GAGACATATA ATTTGTACTT TTTCTCTACT TTACCCATTC   
  
  
- TTCAGTCGGA TCGGAACACC TATTGTCTCC ATCCGACCGT CCGTACAGAT TCAAGACTAC ATTGTTGGTT   
  
  
- TCTCCGTTTC CTCTTCTTTC GGTTTTTCTT TTTTTTCTTT TTTTCTTTCC AGCTCTTCCG TTCACTGTGT   
  
  
- TTGGGTTCTG GGTCTAGGTT ACATTGTTCA GTTGAAGGAA GAGAAAGAGA GAGACAGTGA CTGTTAGAAG   
  
  
- TGATTGTGTG TGTTTGTGCG GGGCGTGGCG TAACCTGTTT GTGGCTTTGG TCTTTGTGCG AGGGGTAGAC   
  
  
- GCTAAGTCAG GGAAGAGAGG AAATTTAAAT GGCACAATGG GAAGGGAGAG GGGAGAAGTG ACGGGATTAA   
  
  
- TGTTTAGATG AAGAGAGGAG AAAACTGAGA CAAGAGAGAA GAGAGAGTAT GAGCGCCATT CGTGCGGTCT   
  
  
- GTTGCGCGTG CGTGTGAAAG AGAACAGAC

+     rbcS-CMA7a

| Site Name | Organism | Position | Strand | Matrix score. | sequence | function |
| --- | --- | --- | --- | --- | --- | --- |
| rbcS-CMA7a | Lemna gibba | 1170 | + | 9 | GTCGATAAGG | part of a light responsive element |

> 2018/04/13 10:10:12  
+ CACACACAGT AAACACTGGT GCATTTATGC ACCTCAAGCA CTGAAAGTTT GCTGGAGAAT GCTAGCCTTA   
  
  
+ TTCAGTGAGA TGCCTTGTTT ATTAAAGAGA AAGAAACCAA GATATAGATG GCTTCTAACC TCTTTTTGTA   
  
  
+ TGAAATGGAT GAAACAAATA GAAATATCGT GCCAAAACAT CCAGGCAGTA TATTTAGATC AGGCAGCATA   
  
  
+ AGAGGAGACA GTACATTTTG CCCGCTTTGG TAGAAAGAAC TAGGATCCTG ACTGTTTTAC CGTCAATTTT   
  
  
+ AGTTGACTGC AAGTTTGTCT TCCCAACCCA TTTAGATTTT CATTTTCTAT TGGACATTCC ATTTCTTCCA   
  
  
+ GAGGTCTAGG AGGTCAAGTT TTTGGAAGAA ATTTATAAGA TTATTGTTGT TTTCTTCCCT GGCTGCAGTT   
  
  
+ ACTTGACTTG TTAGGTGGCA GACAGTTGCT TCGTTCTGTT AGTCCACATC CACATGACAT CAAAACTTAG   
  
  
+ GATTGATTTT AGTTCTTGTT TATGCTCTGA ATTCTCAAAC TGTCTTTAAA AAATTCATTA GTCAGCATTT   
  
  
+ GCTTTTGACT ATTGTTTGCA CTGCTGGCCG CAAGAGGGTT TGTATCTTGA TTATTCGTGT AAAAAGAAAT   
  
  
+ TACAGATGCT CAATTCATAG ATTTGGCCAC ACTGTGGGTT CAAACTAAGA TTGACTGAAG GAAGCCAATA   
  
  
+ AAGATCATCT GTTTTTCCTG CTTAATGGCA CCCATATGAG CAGACAGCCA CTTTTTCTGG ATTGCCCAAT   
  
  
+ TCAATGGAGG CACATTAGTA GTCACAGAGA AGGCAGCCTG TTAAGAAAAT GGTATTTCTA GAAGCCAATT   
  
  
+ AGTACCGTTT CCAGATGATA TGGTTAACAG GTCAATAAAA AAAATTATTT AGGACTCTTT TTGTAGTGTC   
  
  
+ TGCCAACTTA TGGAATGCAG CCCTAATTGA AATATGAGCC CATGTGAGCA TCATGTTATA TCAAAGCACT   
  
  
+ GGCAGGGGCC ACAAAGGACG GTGATTCAAG CTCTGTATAT TAAACATGAA AAAGAGATGA AATGGGTAAG   
  
  
+ AAGTCAGCCT AGCCTTGTGG ATAACAGAGG TAGGCTGGCA GGCATGTCTA AGTTCTGATG TAACAACCAA   
  
  
+ AGAGGCAAAG GAGAAGAAAG CCAAAAAGAA AAAAAAGAAA AAAAGAAAGG TCGAGAAGGC AAGTGACACA   
  
  
+ AACCCAAGAC CCAGATCCAA TGTAACAAGT CAACTTCCTT CTCTTTCTCT CTCTGTCACT GACAATCTTC   
  
  
+ ACTAACACAC ACAAACACGC CCCGCACCGC ATTGGACAAA CACCGAAACC AGAAACACGC TCCCCATCTG   
  
  
+ CGATTCAGTC CCTTCTCTCC TTTAAATTTA CCGTGTTACC CTTCCCTCTC CCCTCTTCAC TGCCCTAATT   
  
  
+ ACAAATCTAC TTCTCTCCTC TTTTGACTCT GTTCTCTCTT CTCTCTCATA CTCGCGGTAA GCACGCCAGA   
  
  
+ CAACGCGCAC GCACACTTTC TCTTGTCTG  

- GTGTGTGTCA TTTGTGACCA CGTAAATACG TGGAGTTCGT GACTTTCAAA CGACCTCTTA CGATCGGAAT   
  
  
- AAGTCACTCT ACGGAACAAA TAATTTCTCT TTCTTTGGTT CTATATCTAC CGAAGATTGG AGAAAAACAT   
  
  
- ACTTTACCTA CTTTGTTTAT CTTTATAGCA CGGTTTTGTA GGTCCGTCAT ATAAATCTAG TCCGTCGTAT   
  
  
- TCTCCTCTGT CATGTAAAAC GGGCGAAACC ATCTTTCTTG ATCCTAGGAC TGACAAAATG GCAGTTAAAA   
  
  
- TCAACTGACG TTCAAACAGA AGGGTTGGGT AAATCTAAAA GTAAAAGATA ACCTGTAAGG TAAAGAAGGT   
  
  
- CTCCAGATCC TCCAGTTCAA AAACCTTCTT TAAATATTCT AATAACAACA AAAGAAGGGA CCGACGTCAA   
  
  
- TGAACTGAAC AATCCACCGT CTGTCAACGA AGCAAGACAA TCAGGTGTAG GTGTACTGTA GTTTTGAATC   
  
  
- CTAACTAAAA TCAAGAACAA ATACGAGACT TAAGAGTTTG ACAGAAATTT TTTAAGTAAT CAGTCGTAAA   
  
  
- CGAAAACTGA TAACAAACGT GACGACCGGC GTTCTCCCAA ACATAGAACT AATAAGCACA TTTTTCTTTA   
  
  
- ATGTCTACGA GTTAAGTATC TAAACCGGTG TGACACCCAA GTTTGATTCT AACTGACTTC CTTCGGTTAT   
  
  
- TTCTAGTAGA CAAAAAGGAC GAATTACCGT GGGTATACTC GTCTGTCGGT GAAAAAGACC TAACGGGTTA   
  
  
- AGTTACCTCC GTGTAATCAT CAGTGTCTCT TCCGTCGGAC AATTCTTTTA CCATAAAGAT CTTCGGTTAA   
  
  
- TCATGGCAAA GGTCTACTAT ACCAATTGTC CAGTTATTTT TTTTAATAAA TCCTGAGAAA AACATCACAG   
  
  
- ACGGTTGAAT ACCTTACGTC GGGATTAACT TTATACTCGG GTACACTCGT AGTACAATAT AGTTTCGTGA   
  
  
- CCGTCCCCGG TGTTTCCTGC CACTAAGTTC GAGACATATA ATTTGTACTT TTTCTCTACT TTACCCATTC   
  
  
- TTCAGTCGGA TCGGAACACC TATTGTCTCC ATCCGACCGT CCGTACAGAT TCAAGACTAC ATTGTTGGTT   
  
  
- TCTCCGTTTC CTCTTCTTTC GGTTTTTCTT TTTTTTCTTT TTTTCTTTCC AGCTCTTCCG TTCACTGTGT   
  
  
- TTGGGTTCTG GGTCTAGGTT ACATTGTTCA GTTGAAGGAA GAGAAAGAGA GAGACAGTGA CTGTTAGAAG   
  
  
- TGATTGTGTG TGTTTGTGCG GGGCGTGGCG TAACCTGTTT GTGGCTTTGG TCTTTGTGCG AGGGGTAGAC   
  
  
- GCTAAGTCAG GGAAGAGAGG AAATTTAAAT GGCACAATGG GAAGGGAGAG GGGAGAAGTG ACGGGATTAA   
  
  
- TGTTTAGATG AAGAGAGGAG AAAACTGAGA CAAGAGAGAA GAGAGAGTAT GAGCGCCATT CGTGCGGTCT   
  
  
- GTTGCGCGTG CGTGTGAAAG AGAACAGAC
