# Supplementary material for: Genome-wide analysis of the WRKY gene family in drumstick (Moringa oleifera Lam.)
Source: PeerJ. 2019 Jun 10;7:e7063. doi: 10.7717/peerj.7063 (PMC6563795; doi:10.7717/peerj.7063)
Supplement: Supplemental Information 1 [file peerj-07-7063-s003.gz › MoWRKY8_plantcare.html]

Content-Type: text/html; charset=ISO-8859-1


CallMat\_Firefox


Webmaster Firefox specific output  
To save the result:
click on the frame with the right mouse button and save the source code as a text file with extension .html  
REFERENCE:PlantCARE: a database of plant cis-acting regulatory elements and a portal to tools for in silico analysis of promoter sequences.  
Lescot, M., Déhais, P., Moreau, Y., De Moor, B., Rouzé ,P.,and Rombauts, S.  
Nucleic Acids Res., Database issue(2002), 30(1):325-327.   


---

> 2018/04/13 10:10:12  
+ TTATAATATC GTTTTCTTAT TTTGCACACG AAATTTAAAA TAAAAAGTAA AATGGAAAAA TGAAAAAACA   
  
  
+ TCTGCGGCGG GACCCACTGT CCGGCTTGTA CTCGCCACAG GGATGCCATC ACAGAGGGAG ATCAGAGATT   
  
  
+ CTTCACTGCT TCTCACCGCA CAGAAATCAT TTCTCCTACT CCTCTCTGCT CTTCCCCCAC TCTCTCGACT   
  
  
+ CTGATTATGG TTTAGACTTT TGGACCTCAA CCTTTTCTCT CCCTCTCTTG CTCGGCTTCG CGGTAATTTT   
  
  
+ CTGAAAACCG GACTTCGGGG GTTTATCTTG CTGGTTCGAA TTTCGAGGAC GACGGGATAC GGTCCAGATC   
  
  
+ GAGTCAGAAG TCAACGGCTC CACGTGACGG CGGAGCACTT GATTGGACTT GCTAGGGTTT TTGTGGCCCG   
  
  
+ AGGTCGAAAT TAGAGGCTTG CTGCTCGTGC AGGTAATTCT TTAATAGTTT TGGAGATATT TCTAGCTTAC   
  
  
+ GTGTCAGTTG GACGGAGTTT ATACTTTCTA TATCAAATTT TTGTGGAAAT CGGAGCTCCT AATTGGCTTT   
  
  
+ GGCCTGTTTG TTTCCCGAGA AAATGTAGGA AAAATAATGA AATTTGGAAT TTAATAGCTG TCTAGTGTAA   
  
  
+ ACCCTCCACC ATAATGATCC TAGTACTCCT TTGTCTGTGC AAATTGGATT TTTATTTTTA ATTGATTGGA   
  
  
+ ATTAAAAAAA TAAAGAATCT CAGTGCGAAT GATAGTTAGC TTATGTTTTG ATTTGTCATC GAATCATAGA   
  
  
+ ACTTCATTCA CTTCCTTTTT GTATGGTAAT TAATTTTGGG GCGAAATATC AAGGGAAAAA GAGGACCTAA   
  
  
+ AGGTTTGAAT AGGATAATTT GGAGTTCTGG AACAGAATTA GGCATGTTTG AGAAGCATTC TACAAGAGTT   
  
  
+ GGCATAGTTT TTGCTCTAAA AACAAGAAAG TTTACTTTCT AAAAGGCTTA ATCTTCAATG CAGTGTTCTT   
  
  
+ TTCCATTTTT CTGGGATTAA TTGACGAGTC CGCGTACCCT CTTTTCCAGT TACTTCTTCG AGAAGCCCAT   
  
  
+ TTGCTAAAGT AGAATGGCTT TTTTCCATGA AGATATATTT TTCTTGCTAA GGTAGTCCCT GAAGCTAGTA   
  
  
+ GATTTACTGC TTAAGCTTGC CTGTTGAACG GCTTTTTGCC AAACCATGTT GACCGAAACG GGGAGCCTGA   
  
  
+ CTTGTTGAGG ATTGACATCA AATCGAAGCT GTGTAGCCCT AAGTACGTGG TTGTGTTGGT CTTAGAGACC   
  
  
+ CAGGGATACA CTCTTTGTTT AATTTTCCCA AATTATTTTT TTTCTTGTTT ACTTTCTTAA TTACGTGCTA   
  
  
+ AATTATGGAT TATACAATGG GTAGCAATAT ACATTATGTC ATTGCCTATT TTTTTAATTT TGTGGTGGAT   
  
  
+ CGTTTTTTTC AGGTTTGAAG TAGCTGCATG ATATTGTATC AGTACAGCCG ATATACTCTG ACATGTGAAT   
  
  
+ TCTTCAGTTT CTGATAGAAA ATAAGTTCA  

- AATATTATAG CAAAAGAATA AAACGTGTGC TTTAAATTTT ATTTTTCATT TTACCTTTTT ACTTTTTTGT   
  
  
- AGACGCCGCC CTGGGTGACA GGCCGAACAT GAGCGGTGTC CCTACGGTAG TGTCTCCCTC TAGTCTCTAA   
  
  
- GAAGTGACGA AGAGTGGCGT GTCTTTAGTA AAGAGGATGA GGAGAGACGA GAAGGGGGTG AGAGAGCTGA   
  
  
- GACTAATACC AAATCTGAAA ACCTGGAGTT GGAAAAGAGA GGGAGAGAAC GAGCCGAAGC GCCATTAAAA   
  
  
- GACTTTTGGC CTGAAGCCCC CAAATAGAAC GACCAAGCTT AAAGCTCCTG CTGCCCTATG CCAGGTCTAG   
  
  
- CTCAGTCTTC AGTTGCCGAG GTGCACTGCC GCCTCGTGAA CTAACCTGAA CGATCCCAAA AACACCGGGC   
  
  
- TCCAGCTTTA ATCTCCGAAC GACGAGCACG TCCATTAAGA AATTATCAAA ACCTCTATAA AGATCGAATG   
  
  
- CACAGTCAAC CTGCCTCAAA TATGAAAGAT ATAGTTTAAA AACACCTTTA GCCTCGAGGA TTAACCGAAA   
  
  
- CCGGACAAAC AAAGGGCTCT TTTACATCCT TTTTATTACT TTAAACCTTA AATTATCGAC AGATCACATT   
  
  
- TGGGAGGTGG TATTACTAGG ATCATGAGGA AACAGACACG TTTAACCTAA AAATAAAAAT TAACTAACCT   
  
  
- TAATTTTTTT ATTTCTTAGA GTCACGCTTA CTATCAATCG AATACAAAAC TAAACAGTAG CTTAGTATCT   
  
  
- TGAAGTAAGT GAAGGAAAAA CATACCATTA ATTAAAACCC CGCTTTATAG TTCCCTTTTT CTCCTGGATT   
  
  
- TCCAAACTTA TCCTATTAAA CCTCAAGACC TTGTCTTAAT CCGTACAAAC TCTTCGTAAG ATGTTCTCAA   
  
  
- CCGTATCAAA AACGAGATTT TTGTTCTTTC AAATGAAAGA TTTTCCGAAT TAGAAGTTAC GTCACAAGAA   
  
  
- AAGGTAAAAA GACCCTAATT AACTGCTCAG GCGCATGGGA GAAAAGGTCA ATGAAGAAGC TCTTCGGGTA   
  
  
- AACGATTTCA TCTTACCGAA AAAAGGTACT TCTATATAAA AAGAACGATT CCATCAGGGA CTTCGATCAT   
  
  
- CTAAATGACG AATTCGAACG GACAACTTGC CGAAAAACGG TTTGGTACAA CTGGCTTTGC CCCTCGGACT   
  
  
- GAACAACTCC TAACTGTAGT TTAGCTTCGA CACATCGGGA TTCATGCACC AACACAACCA GAATCTCTGG   
  
  
- GTCCCTATGT GAGAAACAAA TTAAAAGGGT TTAATAAAAA AAAGAACAAA TGAAAGAATT AATGCACGAT   
  
  
- TTAATACCTA ATATGTTACC CATCGTTATA TGTAATACAG TAACGGATAA AAAAATTAAA ACACCACCTA   
  
  
- GCAAAAAAAG TCCAAACTTC ATCGACGTAC TATAACATAG TCATGTCGGC TATATGAGAC TGTACACTTA   
  
  
- AGAAGTCAAA GACTATCTTT TATTCAAGT

  
  
Motifs Found  

+     3-AF1 binding site

| Site Name | Organism | Position | Strand | Matrix score. | sequence | function |
| --- | --- | --- | --- | --- | --- | --- |
| 3-AF1 binding site | Solanum tuberosum | 1080 | + | 10 | AAGAGATATTT | light responsive element |

> 2018/04/13 10:10:12  
+ TTATAATATC GTTTTCTTAT TTTGCACACG AAATTTAAAA TAAAAAGTAA AATGGAAAAA TGAAAAAACA   
  
  
+ TCTGCGGCGG GACCCACTGT CCGGCTTGTA CTCGCCACAG GGATGCCATC ACAGAGGGAG ATCAGAGATT   
  
  
+ CTTCACTGCT TCTCACCGCA CAGAAATCAT TTCTCCTACT CCTCTCTGCT CTTCCCCCAC TCTCTCGACT   
  
  
+ CTGATTATGG TTTAGACTTT TGGACCTCAA CCTTTTCTCT CCCTCTCTTG CTCGGCTTCG CGGTAATTTT   
  
  
+ CTGAAAACCG GACTTCGGGG GTTTATCTTG CTGGTTCGAA TTTCGAGGAC GACGGGATAC GGTCCAGATC   
  
  
+ GAGTCAGAAG TCAACGGCTC CACGTGACGG CGGAGCACTT GATTGGACTT GCTAGGGTTT TTGTGGCCCG   
  
  
+ AGGTCGAAAT TAGAGGCTTG CTGCTCGTGC AGGTAATTCT TTAATAGTTT TGGAGATATT TCTAGCTTAC   
  
  
+ GTGTCAGTTG GACGGAGTTT ATACTTTCTA TATCAAATTT TTGTGGAAAT CGGAGCTCCT AATTGGCTTT   
  
  
+ GGCCTGTTTG TTTCCCGAGA AAATGTAGGA AAAATAATGA AATTTGGAAT TTAATAGCTG TCTAGTGTAA   
  
  
+ ACCCTCCACC ATAATGATCC TAGTACTCCT TTGTCTGTGC AAATTGGATT TTTATTTTTA ATTGATTGGA   
  
  
+ ATTAAAAAAA TAAAGAATCT CAGTGCGAAT GATAGTTAGC TTATGTTTTG ATTTGTCATC GAATCATAGA   
  
  
+ ACTTCATTCA CTTCCTTTTT GTATGGTAAT TAATTTTGGG GCGAAATATC AAGGGAAAAA GAGGACCTAA   
  
  
+ AGGTTTGAAT AGGATAATTT GGAGTTCTGG AACAGAATTA GGCATGTTTG AGAAGCATTC TACAAGAGTT   
  
  
+ GGCATAGTTT TTGCTCTAAA AACAAGAAAG TTTACTTTCT AAAAGGCTTA ATCTTCAATG CAGTGTTCTT   
  
  
+ TTCCATTTTT CTGGGATTAA TTGACGAGTC CGCGTACCCT CTTTTCCAGT TACTTCTTCG AGAAGCCCAT   
  
  
+ TTGCTAAAGT AGAATGGCTT TTTTCCATGA AGATATATTT TTCTTGCTAA GGTAGTCCCT GAAGCTAGTA   
  
  
+ GATTTACTGC TTAAGCTTGC CTGTTGAACG GCTTTTTGCC AAACCATGTT GACCGAAACG GGGAGCCTGA   
  
  
+ CTTGTTGAGG ATTGACATCA AATCGAAGCT GTGTAGCCCT AAGTACGTGG TTGTGTTGGT CTTAGAGACC   
  
  
+ CAGGGATACA CTCTTTGTTT AATTTTCCCA AATTATTTTT TTTCTTGTTT ACTTTCTTAA TTACGTGCTA   
  
  
+ AATTATGGAT TATACAATGG GTAGCAATAT ACATTATGTC ATTGCCTATT TTTTTAATTT TGTGGTGGAT   
  
  
+ CGTTTTTTTC AGGTTTGAAG TAGCTGCATG ATATTGTATC AGTACAGCCG ATATACTCTG ACATGTGAAT   
  
  
+ TCTTCAGTTT CTGATAGAAA ATAAGTTCA  

- AATATTATAG CAAAAGAATA AAACGTGTGC TTTAAATTTT ATTTTTCATT TTACCTTTTT ACTTTTTTGT   
  
  
- AGACGCCGCC CTGGGTGACA GGCCGAACAT GAGCGGTGTC CCTACGGTAG TGTCTCCCTC TAGTCTCTAA   
  
  
- GAAGTGACGA AGAGTGGCGT GTCTTTAGTA AAGAGGATGA GGAGAGACGA GAAGGGGGTG AGAGAGCTGA   
  
  
- GACTAATACC AAATCTGAAA ACCTGGAGTT GGAAAAGAGA GGGAGAGAAC GAGCCGAAGC GCCATTAAAA   
  
  
- GACTTTTGGC CTGAAGCCCC CAAATAGAAC GACCAAGCTT AAAGCTCCTG CTGCCCTATG CCAGGTCTAG   
  
  
- CTCAGTCTTC AGTTGCCGAG GTGCACTGCC GCCTCGTGAA CTAACCTGAA CGATCCCAAA AACACCGGGC   
  
  
- TCCAGCTTTA ATCTCCGAAC GACGAGCACG TCCATTAAGA AATTATCAAA ACCTCTATAA AGATCGAATG   
  
  
- CACAGTCAAC CTGCCTCAAA TATGAAAGAT ATAGTTTAAA AACACCTTTA GCCTCGAGGA TTAACCGAAA   
  
  
- CCGGACAAAC AAAGGGCTCT TTTACATCCT TTTTATTACT TTAAACCTTA AATTATCGAC AGATCACATT   
  
  
- TGGGAGGTGG TATTACTAGG ATCATGAGGA AACAGACACG TTTAACCTAA AAATAAAAAT TAACTAACCT   
  
  
- TAATTTTTTT ATTTCTTAGA GTCACGCTTA CTATCAATCG AATACAAAAC TAAACAGTAG CTTAGTATCT   
  
  
- TGAAGTAAGT GAAGGAAAAA CATACCATTA ATTAAAACCC CGCTTTATAG TTCCCTTTTT CTCCTGGATT   
  
  
- TCCAAACTTA TCCTATTAAA CCTCAAGACC TTGTCTTAAT CCGTACAAAC TCTTCGTAAG ATGTTCTCAA   
  
  
- CCGTATCAAA AACGAGATTT TTGTTCTTTC AAATGAAAGA TTTTCCGAAT TAGAAGTTAC GTCACAAGAA   
  
  
- AAGGTAAAAA GACCCTAATT AACTGCTCAG GCGCATGGGA GAAAAGGTCA ATGAAGAAGC TCTTCGGGTA   
  
  
- AACGATTTCA TCTTACCGAA AAAAGGTACT TCTATATAAA AAGAACGATT CCATCAGGGA CTTCGATCAT   
  
  
- CTAAATGACG AATTCGAACG GACAACTTGC CGAAAAACGG TTTGGTACAA CTGGCTTTGC CCCTCGGACT   
  
  
- GAACAACTCC TAACTGTAGT TTAGCTTCGA CACATCGGGA TTCATGCACC AACACAACCA GAATCTCTGG   
  
  
- GTCCCTATGT GAGAAACAAA TTAAAAGGGT TTAATAAAAA AAAGAACAAA TGAAAGAATT AATGCACGAT   
  
  
- TTAATACCTA ATATGTTACC CATCGTTATA TGTAATACAG TAACGGATAA AAAAATTAAA ACACCACCTA   
  
  
- GCAAAAAAAG TCCAAACTTC ATCGACGTAC TATAACATAG TCATGTCGGC TATATGAGAC TGTACACTTA   
  
  
- AGAAGTCAAA GACTATCTTT TATTCAAGT

+     5UTR Py-rich stretch

| Site Name | Organism | Position | Strand | Matrix score. | sequence | function |
| --- | --- | --- | --- | --- | --- | --- |
| 5UTR Py-rich stretch | Lycopersicon esculentum | 244 | + | 13 | TTTCTCTCTCTCTC | cis-acting element conferring high transcription levels |

> 2018/04/13 10:10:12  
+ TTATAATATC GTTTTCTTAT TTTGCACACG AAATTTAAAA TAAAAAGTAA AATGGAAAAA TGAAAAAACA   
  
  
+ TCTGCGGCGG GACCCACTGT CCGGCTTGTA CTCGCCACAG GGATGCCATC ACAGAGGGAG ATCAGAGATT   
  
  
+ CTTCACTGCT TCTCACCGCA CAGAAATCAT TTCTCCTACT CCTCTCTGCT CTTCCCCCAC TCTCTCGACT   
  
  
+ CTGATTATGG TTTAGACTTT TGGACCTCAA CCTTTTCTCT CCCTCTCTTG CTCGGCTTCG CGGTAATTTT   
  
  
+ CTGAAAACCG GACTTCGGGG GTTTATCTTG CTGGTTCGAA TTTCGAGGAC GACGGGATAC GGTCCAGATC   
  
  
+ GAGTCAGAAG TCAACGGCTC CACGTGACGG CGGAGCACTT GATTGGACTT GCTAGGGTTT TTGTGGCCCG   
  
  
+ AGGTCGAAAT TAGAGGCTTG CTGCTCGTGC AGGTAATTCT TTAATAGTTT TGGAGATATT TCTAGCTTAC   
  
  
+ GTGTCAGTTG GACGGAGTTT ATACTTTCTA TATCAAATTT TTGTGGAAAT CGGAGCTCCT AATTGGCTTT   
  
  
+ GGCCTGTTTG TTTCCCGAGA AAATGTAGGA AAAATAATGA AATTTGGAAT TTAATAGCTG TCTAGTGTAA   
  
  
+ ACCCTCCACC ATAATGATCC TAGTACTCCT TTGTCTGTGC AAATTGGATT TTTATTTTTA ATTGATTGGA   
  
  
+ ATTAAAAAAA TAAAGAATCT CAGTGCGAAT GATAGTTAGC TTATGTTTTG ATTTGTCATC GAATCATAGA   
  
  
+ ACTTCATTCA CTTCCTTTTT GTATGGTAAT TAATTTTGGG GCGAAATATC AAGGGAAAAA GAGGACCTAA   
  
  
+ AGGTTTGAAT AGGATAATTT GGAGTTCTGG AACAGAATTA GGCATGTTTG AGAAGCATTC TACAAGAGTT   
  
  
+ GGCATAGTTT TTGCTCTAAA AACAAGAAAG TTTACTTTCT AAAAGGCTTA ATCTTCAATG CAGTGTTCTT   
  
  
+ TTCCATTTTT CTGGGATTAA TTGACGAGTC CGCGTACCCT CTTTTCCAGT TACTTCTTCG AGAAGCCCAT   
  
  
+ TTGCTAAAGT AGAATGGCTT TTTTCCATGA AGATATATTT TTCTTGCTAA GGTAGTCCCT GAAGCTAGTA   
  
  
+ GATTTACTGC TTAAGCTTGC CTGTTGAACG GCTTTTTGCC AAACCATGTT GACCGAAACG GGGAGCCTGA   
  
  
+ CTTGTTGAGG ATTGACATCA AATCGAAGCT GTGTAGCCCT AAGTACGTGG TTGTGTTGGT CTTAGAGACC   
  
  
+ CAGGGATACA CTCTTTGTTT AATTTTCCCA AATTATTTTT TTTCTTGTTT ACTTTCTTAA TTACGTGCTA   
  
  
+ AATTATGGAT TATACAATGG GTAGCAATAT ACATTATGTC ATTGCCTATT TTTTTAATTT TGTGGTGGAT   
  
  
+ CGTTTTTTTC AGGTTTGAAG TAGCTGCATG ATATTGTATC AGTACAGCCG ATATACTCTG ACATGTGAAT   
  
  
+ TCTTCAGTTT CTGATAGAAA ATAAGTTCA  

- AATATTATAG CAAAAGAATA AAACGTGTGC TTTAAATTTT ATTTTTCATT TTACCTTTTT ACTTTTTTGT   
  
  
- AGACGCCGCC CTGGGTGACA GGCCGAACAT GAGCGGTGTC CCTACGGTAG TGTCTCCCTC TAGTCTCTAA   
  
  
- GAAGTGACGA AGAGTGGCGT GTCTTTAGTA AAGAGGATGA GGAGAGACGA GAAGGGGGTG AGAGAGCTGA   
  
  
- GACTAATACC AAATCTGAAA ACCTGGAGTT GGAAAAGAGA GGGAGAGAAC GAGCCGAAGC GCCATTAAAA   
  
  
- GACTTTTGGC CTGAAGCCCC CAAATAGAAC GACCAAGCTT AAAGCTCCTG CTGCCCTATG CCAGGTCTAG   
  
  
- CTCAGTCTTC AGTTGCCGAG GTGCACTGCC GCCTCGTGAA CTAACCTGAA CGATCCCAAA AACACCGGGC   
  
  
- TCCAGCTTTA ATCTCCGAAC GACGAGCACG TCCATTAAGA AATTATCAAA ACCTCTATAA AGATCGAATG   
  
  
- CACAGTCAAC CTGCCTCAAA TATGAAAGAT ATAGTTTAAA AACACCTTTA GCCTCGAGGA TTAACCGAAA   
  
  
- CCGGACAAAC AAAGGGCTCT TTTACATCCT TTTTATTACT TTAAACCTTA AATTATCGAC AGATCACATT   
  
  
- TGGGAGGTGG TATTACTAGG ATCATGAGGA AACAGACACG TTTAACCTAA AAATAAAAAT TAACTAACCT   
  
  
- TAATTTTTTT ATTTCTTAGA GTCACGCTTA CTATCAATCG AATACAAAAC TAAACAGTAG CTTAGTATCT   
  
  
- TGAAGTAAGT GAAGGAAAAA CATACCATTA ATTAAAACCC CGCTTTATAG TTCCCTTTTT CTCCTGGATT   
  
  
- TCCAAACTTA TCCTATTAAA CCTCAAGACC TTGTCTTAAT CCGTACAAAC TCTTCGTAAG ATGTTCTCAA   
  
  
- CCGTATCAAA AACGAGATTT TTGTTCTTTC AAATGAAAGA TTTTCCGAAT TAGAAGTTAC GTCACAAGAA   
  
  
- AAGGTAAAAA GACCCTAATT AACTGCTCAG GCGCATGGGA GAAAAGGTCA ATGAAGAAGC TCTTCGGGTA   
  
  
- AACGATTTCA TCTTACCGAA AAAAGGTACT TCTATATAAA AAGAACGATT CCATCAGGGA CTTCGATCAT   
  
  
- CTAAATGACG AATTCGAACG GACAACTTGC CGAAAAACGG TTTGGTACAA CTGGCTTTGC CCCTCGGACT   
  
  
- GAACAACTCC TAACTGTAGT TTAGCTTCGA CACATCGGGA TTCATGCACC AACACAACCA GAATCTCTGG   
  
  
- GTCCCTATGT GAGAAACAAA TTAAAAGGGT TTAATAAAAA AAAGAACAAA TGAAAGAATT AATGCACGAT   
  
  
- TTAATACCTA ATATGTTACC CATCGTTATA TGTAATACAG TAACGGATAA AAAAATTAAA ACACCACCTA   
  
  
- GCAAAAAAAG TCCAAACTTC ATCGACGTAC TATAACATAG TCATGTCGGC TATATGAGAC TGTACACTTA   
  
  
- AGAAGTCAAA GACTATCTTT TATTCAAGT

+     A-box

| Site Name | Organism | Position | Strand | Matrix score. | sequence | function |
| --- | --- | --- | --- | --- | --- | --- |
| A-box | Petroselinum crispum | 500 | - | 6 | CCGTCC | cis-acting regulatory element |

> 2018/04/13 10:10:12  
+ TTATAATATC GTTTTCTTAT TTTGCACACG AAATTTAAAA TAAAAAGTAA AATGGAAAAA TGAAAAAACA   
  
  
+ TCTGCGGCGG GACCCACTGT CCGGCTTGTA CTCGCCACAG GGATGCCATC ACAGAGGGAG ATCAGAGATT   
  
  
+ CTTCACTGCT TCTCACCGCA CAGAAATCAT TTCTCCTACT CCTCTCTGCT CTTCCCCCAC TCTCTCGACT   
  
  
+ CTGATTATGG TTTAGACTTT TGGACCTCAA CCTTTTCTCT CCCTCTCTTG CTCGGCTTCG CGGTAATTTT   
  
  
+ CTGAAAACCG GACTTCGGGG GTTTATCTTG CTGGTTCGAA TTTCGAGGAC GACGGGATAC GGTCCAGATC   
  
  
+ GAGTCAGAAG TCAACGGCTC CACGTGACGG CGGAGCACTT GATTGGACTT GCTAGGGTTT TTGTGGCCCG   
  
  
+ AGGTCGAAAT TAGAGGCTTG CTGCTCGTGC AGGTAATTCT TTAATAGTTT TGGAGATATT TCTAGCTTAC   
  
  
+ GTGTCAGTTG GACGGAGTTT ATACTTTCTA TATCAAATTT TTGTGGAAAT CGGAGCTCCT AATTGGCTTT   
  
  
+ GGCCTGTTTG TTTCCCGAGA AAATGTAGGA AAAATAATGA AATTTGGAAT TTAATAGCTG TCTAGTGTAA   
  
  
+ ACCCTCCACC ATAATGATCC TAGTACTCCT TTGTCTGTGC AAATTGGATT TTTATTTTTA ATTGATTGGA   
  
  
+ ATTAAAAAAA TAAAGAATCT CAGTGCGAAT GATAGTTAGC TTATGTTTTG ATTTGTCATC GAATCATAGA   
  
  
+ ACTTCATTCA CTTCCTTTTT GTATGGTAAT TAATTTTGGG GCGAAATATC AAGGGAAAAA GAGGACCTAA   
  
  
+ AGGTTTGAAT AGGATAATTT GGAGTTCTGG AACAGAATTA GGCATGTTTG AGAAGCATTC TACAAGAGTT   
  
  
+ GGCATAGTTT TTGCTCTAAA AACAAGAAAG TTTACTTTCT AAAAGGCTTA ATCTTCAATG CAGTGTTCTT   
  
  
+ TTCCATTTTT CTGGGATTAA TTGACGAGTC CGCGTACCCT CTTTTCCAGT TACTTCTTCG AGAAGCCCAT   
  
  
+ TTGCTAAAGT AGAATGGCTT TTTTCCATGA AGATATATTT TTCTTGCTAA GGTAGTCCCT GAAGCTAGTA   
  
  
+ GATTTACTGC TTAAGCTTGC CTGTTGAACG GCTTTTTGCC AAACCATGTT GACCGAAACG GGGAGCCTGA   
  
  
+ CTTGTTGAGG ATTGACATCA AATCGAAGCT GTGTAGCCCT AAGTACGTGG TTGTGTTGGT CTTAGAGACC   
  
  
+ CAGGGATACA CTCTTTGTTT AATTTTCCCA AATTATTTTT TTTCTTGTTT ACTTTCTTAA TTACGTGCTA   
  
  
+ AATTATGGAT TATACAATGG GTAGCAATAT ACATTATGTC ATTGCCTATT TTTTTAATTT TGTGGTGGAT   
  
  
+ CGTTTTTTTC AGGTTTGAAG TAGCTGCATG ATATTGTATC AGTACAGCCG ATATACTCTG ACATGTGAAT   
  
  
+ TCTTCAGTTT CTGATAGAAA ATAAGTTCA  

- AATATTATAG CAAAAGAATA AAACGTGTGC TTTAAATTTT ATTTTTCATT TTACCTTTTT ACTTTTTTGT   
  
  
- AGACGCCGCC CTGGGTGACA GGCCGAACAT GAGCGGTGTC CCTACGGTAG TGTCTCCCTC TAGTCTCTAA   
  
  
- GAAGTGACGA AGAGTGGCGT GTCTTTAGTA AAGAGGATGA GGAGAGACGA GAAGGGGGTG AGAGAGCTGA   
  
  
- GACTAATACC AAATCTGAAA ACCTGGAGTT GGAAAAGAGA GGGAGAGAAC GAGCCGAAGC GCCATTAAAA   
  
  
- GACTTTTGGC CTGAAGCCCC CAAATAGAAC GACCAAGCTT AAAGCTCCTG CTGCCCTATG CCAGGTCTAG   
  
  
- CTCAGTCTTC AGTTGCCGAG GTGCACTGCC GCCTCGTGAA CTAACCTGAA CGATCCCAAA AACACCGGGC   
  
  
- TCCAGCTTTA ATCTCCGAAC GACGAGCACG TCCATTAAGA AATTATCAAA ACCTCTATAA AGATCGAATG   
  
  
- CACAGTCAAC CTGCCTCAAA TATGAAAGAT ATAGTTTAAA AACACCTTTA GCCTCGAGGA TTAACCGAAA   
  
  
- CCGGACAAAC AAAGGGCTCT TTTACATCCT TTTTATTACT TTAAACCTTA AATTATCGAC AGATCACATT   
  
  
- TGGGAGGTGG TATTACTAGG ATCATGAGGA AACAGACACG TTTAACCTAA AAATAAAAAT TAACTAACCT   
  
  
- TAATTTTTTT ATTTCTTAGA GTCACGCTTA CTATCAATCG AATACAAAAC TAAACAGTAG CTTAGTATCT   
  
  
- TGAAGTAAGT GAAGGAAAAA CATACCATTA ATTAAAACCC CGCTTTATAG TTCCCTTTTT CTCCTGGATT   
  
  
- TCCAAACTTA TCCTATTAAA CCTCAAGACC TTGTCTTAAT CCGTACAAAC TCTTCGTAAG ATGTTCTCAA   
  
  
- CCGTATCAAA AACGAGATTT TTGTTCTTTC AAATGAAAGA TTTTCCGAAT TAGAAGTTAC GTCACAAGAA   
  
  
- AAGGTAAAAA GACCCTAATT AACTGCTCAG GCGCATGGGA GAAAAGGTCA ATGAAGAAGC TCTTCGGGTA   
  
  
- AACGATTTCA TCTTACCGAA AAAAGGTACT TCTATATAAA AAGAACGATT CCATCAGGGA CTTCGATCAT   
  
  
- CTAAATGACG AATTCGAACG GACAACTTGC CGAAAAACGG TTTGGTACAA CTGGCTTTGC CCCTCGGACT   
  
  
- GAACAACTCC TAACTGTAGT TTAGCTTCGA CACATCGGGA TTCATGCACC AACACAACCA GAATCTCTGG   
  
  
- GTCCCTATGT GAGAAACAAA TTAAAAGGGT TTAATAAAAA AAAGAACAAA TGAAAGAATT AATGCACGAT   
  
  
- TTAATACCTA ATATGTTACC CATCGTTATA TGTAATACAG TAACGGATAA AAAAATTAAA ACACCACCTA   
  
  
- GCAAAAAAAG TCCAAACTTC ATCGACGTAC TATAACATAG TCATGTCGGC TATATGAGAC TGTACACTTA   
  
  
- AGAAGTCAAA GACTATCTTT TATTCAAGT

+     ABRE

| Site Name | Organism | Position | Strand | Matrix score. | sequence | function |
| --- | --- | --- | --- | --- | --- | --- |
| ABRE | Arabidopsis thaliana | 1322 | + | 6 | TACGTG | cis-acting element involved in the abscisic acid responsiveness |
| ABRE | Oryza sativa | 1232 | + | 9 | AGTACGTGGC | cis-acting element involved in the abscisic acid responsiveness |
| ABRE | Arabidopsis thaliana | 488 | + | 6 | TACGTG | cis-acting element involved in the abscisic acid responsiveness |
| ABRE | Arabidopsis thaliana | 371 | + | 6 | CACGTG | cis-acting element involved in the abscisic acid responsiveness |
| ABRE | Arabidopsis thaliana | 1234 | + | 6 | TACGTG | cis-acting element involved in the abscisic acid responsiveness |
| ABRE | Arabidopsis thaliana | 338 | + | 7 | TACGGTC | cis-acting element involved in the abscisic acid responsiveness |

> 2018/04/13 10:10:12  
+ TTATAATATC GTTTTCTTAT TTTGCACACG AAATTTAAAA TAAAAAGTAA AATGGAAAAA TGAAAAAACA   
  
  
+ TCTGCGGCGG GACCCACTGT CCGGCTTGTA CTCGCCACAG GGATGCCATC ACAGAGGGAG ATCAGAGATT   
  
  
+ CTTCACTGCT TCTCACCGCA CAGAAATCAT TTCTCCTACT CCTCTCTGCT CTTCCCCCAC TCTCTCGACT   
  
  
+ CTGATTATGG TTTAGACTTT TGGACCTCAA CCTTTTCTCT CCCTCTCTTG CTCGGCTTCG CGGTAATTTT   
  
  
+ CTGAAAACCG GACTTCGGGG GTTTATCTTG CTGGTTCGAA TTTCGAGGAC GACGGGATAC GGTCCAGATC   
  
  
+ GAGTCAGAAG TCAACGGCTC CACGTGACGG CGGAGCACTT GATTGGACTT GCTAGGGTTT TTGTGGCCCG   
  
  
+ AGGTCGAAAT TAGAGGCTTG CTGCTCGTGC AGGTAATTCT TTAATAGTTT TGGAGATATT TCTAGCTTAC   
  
  
+ GTGTCAGTTG GACGGAGTTT ATACTTTCTA TATCAAATTT TTGTGGAAAT CGGAGCTCCT AATTGGCTTT   
  
  
+ GGCCTGTTTG TTTCCCGAGA AAATGTAGGA AAAATAATGA AATTTGGAAT TTAATAGCTG TCTAGTGTAA   
  
  
+ ACCCTCCACC ATAATGATCC TAGTACTCCT TTGTCTGTGC AAATTGGATT TTTATTTTTA ATTGATTGGA   
  
  
+ ATTAAAAAAA TAAAGAATCT CAGTGCGAAT GATAGTTAGC TTATGTTTTG ATTTGTCATC GAATCATAGA   
  
  
+ ACTTCATTCA CTTCCTTTTT GTATGGTAAT TAATTTTGGG GCGAAATATC AAGGGAAAAA GAGGACCTAA   
  
  
+ AGGTTTGAAT AGGATAATTT GGAGTTCTGG AACAGAATTA GGCATGTTTG AGAAGCATTC TACAAGAGTT   
  
  
+ GGCATAGTTT TTGCTCTAAA AACAAGAAAG TTTACTTTCT AAAAGGCTTA ATCTTCAATG CAGTGTTCTT   
  
  
+ TTCCATTTTT CTGGGATTAA TTGACGAGTC CGCGTACCCT CTTTTCCAGT TACTTCTTCG AGAAGCCCAT   
  
  
+ TTGCTAAAGT AGAATGGCTT TTTTCCATGA AGATATATTT TTCTTGCTAA GGTAGTCCCT GAAGCTAGTA   
  
  
+ GATTTACTGC TTAAGCTTGC CTGTTGAACG GCTTTTTGCC AAACCATGTT GACCGAAACG GGGAGCCTGA   
  
  
+ CTTGTTGAGG ATTGACATCA AATCGAAGCT GTGTAGCCCT AAGTACGTGG TTGTGTTGGT CTTAGAGACC   
  
  
+ CAGGGATACA CTCTTTGTTT AATTTTCCCA AATTATTTTT TTTCTTGTTT ACTTTCTTAA TTACGTGCTA   
  
  
+ AATTATGGAT TATACAATGG GTAGCAATAT ACATTATGTC ATTGCCTATT TTTTTAATTT TGTGGTGGAT   
  
  
+ CGTTTTTTTC AGGTTTGAAG TAGCTGCATG ATATTGTATC AGTACAGCCG ATATACTCTG ACATGTGAAT   
  
  
+ TCTTCAGTTT CTGATAGAAA ATAAGTTCA  

- AATATTATAG CAAAAGAATA AAACGTGTGC TTTAAATTTT ATTTTTCATT TTACCTTTTT ACTTTTTTGT   
  
  
- AGACGCCGCC CTGGGTGACA GGCCGAACAT GAGCGGTGTC CCTACGGTAG TGTCTCCCTC TAGTCTCTAA   
  
  
- GAAGTGACGA AGAGTGGCGT GTCTTTAGTA AAGAGGATGA GGAGAGACGA GAAGGGGGTG AGAGAGCTGA   
  
  
- GACTAATACC AAATCTGAAA ACCTGGAGTT GGAAAAGAGA GGGAGAGAAC GAGCCGAAGC GCCATTAAAA   
  
  
- GACTTTTGGC CTGAAGCCCC CAAATAGAAC GACCAAGCTT AAAGCTCCTG CTGCCCTATG CCAGGTCTAG   
  
  
- CTCAGTCTTC AGTTGCCGAG GTGCACTGCC GCCTCGTGAA CTAACCTGAA CGATCCCAAA AACACCGGGC   
  
  
- TCCAGCTTTA ATCTCCGAAC GACGAGCACG TCCATTAAGA AATTATCAAA ACCTCTATAA AGATCGAATG   
  
  
- CACAGTCAAC CTGCCTCAAA TATGAAAGAT ATAGTTTAAA AACACCTTTA GCCTCGAGGA TTAACCGAAA   
  
  
- CCGGACAAAC AAAGGGCTCT TTTACATCCT TTTTATTACT TTAAACCTTA AATTATCGAC AGATCACATT   
  
  
- TGGGAGGTGG TATTACTAGG ATCATGAGGA AACAGACACG TTTAACCTAA AAATAAAAAT TAACTAACCT   
  
  
- TAATTTTTTT ATTTCTTAGA GTCACGCTTA CTATCAATCG AATACAAAAC TAAACAGTAG CTTAGTATCT   
  
  
- TGAAGTAAGT GAAGGAAAAA CATACCATTA ATTAAAACCC CGCTTTATAG TTCCCTTTTT CTCCTGGATT   
  
  
- TCCAAACTTA TCCTATTAAA CCTCAAGACC TTGTCTTAAT CCGTACAAAC TCTTCGTAAG ATGTTCTCAA   
  
  
- CCGTATCAAA AACGAGATTT TTGTTCTTTC AAATGAAAGA TTTTCCGAAT TAGAAGTTAC GTCACAAGAA   
  
  
- AAGGTAAAAA GACCCTAATT AACTGCTCAG GCGCATGGGA GAAAAGGTCA ATGAAGAAGC TCTTCGGGTA   
  
  
- AACGATTTCA TCTTACCGAA AAAAGGTACT TCTATATAAA AAGAACGATT CCATCAGGGA CTTCGATCAT   
  
  
- CTAAATGACG AATTCGAACG GACAACTTGC CGAAAAACGG TTTGGTACAA CTGGCTTTGC CCCTCGGACT   
  
  
- GAACAACTCC TAACTGTAGT TTAGCTTCGA CACATCGGGA TTCATGCACC AACACAACCA GAATCTCTGG   
  
  
- GTCCCTATGT GAGAAACAAA TTAAAAGGGT TTAATAAAAA AAAGAACAAA TGAAAGAATT AATGCACGAT   
  
  
- TTAATACCTA ATATGTTACC CATCGTTATA TGTAATACAG TAACGGATAA AAAAATTAAA ACACCACCTA   
  
  
- GCAAAAAAAG TCCAAACTTC ATCGACGTAC TATAACATAG TCATGTCGGC TATATGAGAC TGTACACTTA   
  
  
- AGAAGTCAAA GACTATCTTT TATTCAAGT

+     ACE

| Site Name | Organism | Position | Strand | Matrix score. | sequence | function |
| --- | --- | --- | --- | --- | --- | --- |
| ACE | Petroselinum crispum | 486 | - | 9 | GACACGTATG | cis-acting element involved in light responsiveness |
| ACE | Petroselinum hortense | 369 | - | 7 | ACGTGGA | cis-acting element involved in light responsiveness |

> 2018/04/13 10:10:12  
+ TTATAATATC GTTTTCTTAT TTTGCACACG AAATTTAAAA TAAAAAGTAA AATGGAAAAA TGAAAAAACA   
  
  
+ TCTGCGGCGG GACCCACTGT CCGGCTTGTA CTCGCCACAG GGATGCCATC ACAGAGGGAG ATCAGAGATT   
  
  
+ CTTCACTGCT TCTCACCGCA CAGAAATCAT TTCTCCTACT CCTCTCTGCT CTTCCCCCAC TCTCTCGACT   
  
  
+ CTGATTATGG TTTAGACTTT TGGACCTCAA CCTTTTCTCT CCCTCTCTTG CTCGGCTTCG CGGTAATTTT   
  
  
+ CTGAAAACCG GACTTCGGGG GTTTATCTTG CTGGTTCGAA TTTCGAGGAC GACGGGATAC GGTCCAGATC   
  
  
+ GAGTCAGAAG TCAACGGCTC CACGTGACGG CGGAGCACTT GATTGGACTT GCTAGGGTTT TTGTGGCCCG   
  
  
+ AGGTCGAAAT TAGAGGCTTG CTGCTCGTGC AGGTAATTCT TTAATAGTTT TGGAGATATT TCTAGCTTAC   
  
  
+ GTGTCAGTTG GACGGAGTTT ATACTTTCTA TATCAAATTT TTGTGGAAAT CGGAGCTCCT AATTGGCTTT   
  
  
+ GGCCTGTTTG TTTCCCGAGA AAATGTAGGA AAAATAATGA AATTTGGAAT TTAATAGCTG TCTAGTGTAA   
  
  
+ ACCCTCCACC ATAATGATCC TAGTACTCCT TTGTCTGTGC AAATTGGATT TTTATTTTTA ATTGATTGGA   
  
  
+ ATTAAAAAAA TAAAGAATCT CAGTGCGAAT GATAGTTAGC TTATGTTTTG ATTTGTCATC GAATCATAGA   
  
  
+ ACTTCATTCA CTTCCTTTTT GTATGGTAAT TAATTTTGGG GCGAAATATC AAGGGAAAAA GAGGACCTAA   
  
  
+ AGGTTTGAAT AGGATAATTT GGAGTTCTGG AACAGAATTA GGCATGTTTG AGAAGCATTC TACAAGAGTT   
  
  
+ GGCATAGTTT TTGCTCTAAA AACAAGAAAG TTTACTTTCT AAAAGGCTTA ATCTTCAATG CAGTGTTCTT   
  
  
+ TTCCATTTTT CTGGGATTAA TTGACGAGTC CGCGTACCCT CTTTTCCAGT TACTTCTTCG AGAAGCCCAT   
  
  
+ TTGCTAAAGT AGAATGGCTT TTTTCCATGA AGATATATTT TTCTTGCTAA GGTAGTCCCT GAAGCTAGTA   
  
  
+ GATTTACTGC TTAAGCTTGC CTGTTGAACG GCTTTTTGCC AAACCATGTT GACCGAAACG GGGAGCCTGA   
  
  
+ CTTGTTGAGG ATTGACATCA AATCGAAGCT GTGTAGCCCT AAGTACGTGG TTGTGTTGGT CTTAGAGACC   
  
  
+ CAGGGATACA CTCTTTGTTT AATTTTCCCA AATTATTTTT TTTCTTGTTT ACTTTCTTAA TTACGTGCTA   
  
  
+ AATTATGGAT TATACAATGG GTAGCAATAT ACATTATGTC ATTGCCTATT TTTTTAATTT TGTGGTGGAT   
  
  
+ CGTTTTTTTC AGGTTTGAAG TAGCTGCATG ATATTGTATC AGTACAGCCG ATATACTCTG ACATGTGAAT   
  
  
+ TCTTCAGTTT CTGATAGAAA ATAAGTTCA  

- AATATTATAG CAAAAGAATA AAACGTGTGC TTTAAATTTT ATTTTTCATT TTACCTTTTT ACTTTTTTGT   
  
  
- AGACGCCGCC CTGGGTGACA GGCCGAACAT GAGCGGTGTC CCTACGGTAG TGTCTCCCTC TAGTCTCTAA   
  
  
- GAAGTGACGA AGAGTGGCGT GTCTTTAGTA AAGAGGATGA GGAGAGACGA GAAGGGGGTG AGAGAGCTGA   
  
  
- GACTAATACC AAATCTGAAA ACCTGGAGTT GGAAAAGAGA GGGAGAGAAC GAGCCGAAGC GCCATTAAAA   
  
  
- GACTTTTGGC CTGAAGCCCC CAAATAGAAC GACCAAGCTT AAAGCTCCTG CTGCCCTATG CCAGGTCTAG   
  
  
- CTCAGTCTTC AGTTGCCGAG GTGCACTGCC GCCTCGTGAA CTAACCTGAA CGATCCCAAA AACACCGGGC   
  
  
- TCCAGCTTTA ATCTCCGAAC GACGAGCACG TCCATTAAGA AATTATCAAA ACCTCTATAA AGATCGAATG   
  
  
- CACAGTCAAC CTGCCTCAAA TATGAAAGAT ATAGTTTAAA AACACCTTTA GCCTCGAGGA TTAACCGAAA   
  
  
- CCGGACAAAC AAAGGGCTCT TTTACATCCT TTTTATTACT TTAAACCTTA AATTATCGAC AGATCACATT   
  
  
- TGGGAGGTGG TATTACTAGG ATCATGAGGA AACAGACACG TTTAACCTAA AAATAAAAAT TAACTAACCT   
  
  
- TAATTTTTTT ATTTCTTAGA GTCACGCTTA CTATCAATCG AATACAAAAC TAAACAGTAG CTTAGTATCT   
  
  
- TGAAGTAAGT GAAGGAAAAA CATACCATTA ATTAAAACCC CGCTTTATAG TTCCCTTTTT CTCCTGGATT   
  
  
- TCCAAACTTA TCCTATTAAA CCTCAAGACC TTGTCTTAAT CCGTACAAAC TCTTCGTAAG ATGTTCTCAA   
  
  
- CCGTATCAAA AACGAGATTT TTGTTCTTTC AAATGAAAGA TTTTCCGAAT TAGAAGTTAC GTCACAAGAA   
  
  
- AAGGTAAAAA GACCCTAATT AACTGCTCAG GCGCATGGGA GAAAAGGTCA ATGAAGAAGC TCTTCGGGTA   
  
  
- AACGATTTCA TCTTACCGAA AAAAGGTACT TCTATATAAA AAGAACGATT CCATCAGGGA CTTCGATCAT   
  
  
- CTAAATGACG AATTCGAACG GACAACTTGC CGAAAAACGG TTTGGTACAA CTGGCTTTGC CCCTCGGACT   
  
  
- GAACAACTCC TAACTGTAGT TTAGCTTCGA CACATCGGGA TTCATGCACC AACACAACCA GAATCTCTGG   
  
  
- GTCCCTATGT GAGAAACAAA TTAAAAGGGT TTAATAAAAA AAAGAACAAA TGAAAGAATT AATGCACGAT   
  
  
- TTAATACCTA ATATGTTACC CATCGTTATA TGTAATACAG TAACGGATAA AAAAATTAAA ACACCACCTA   
  
  
- GCAAAAAAAG TCCAAACTTC ATCGACGTAC TATAACATAG TCATGTCGGC TATATGAGAC TGTACACTTA   
  
  
- AGAAGTCAAA GACTATCTTT TATTCAAGT

+     ARE

| Site Name | Organism | Position | Strand | Matrix score. | sequence | function |
| --- | --- | --- | --- | --- | --- | --- |
| ARE | Zea mays | 218 | + | 6 | TGGTTT | cis-acting regulatory element essential for the anaerobic induction |
| ARE | Zea mays | 1161 | - | 6 | TGGTTT | cis-acting regulatory element essential for the anaerobic induction |

> 2018/04/13 10:10:12  
+ TTATAATATC GTTTTCTTAT TTTGCACACG AAATTTAAAA TAAAAAGTAA AATGGAAAAA TGAAAAAACA   
  
  
+ TCTGCGGCGG GACCCACTGT CCGGCTTGTA CTCGCCACAG GGATGCCATC ACAGAGGGAG ATCAGAGATT   
  
  
+ CTTCACTGCT TCTCACCGCA CAGAAATCAT TTCTCCTACT CCTCTCTGCT CTTCCCCCAC TCTCTCGACT   
  
  
+ CTGATTATGG TTTAGACTTT TGGACCTCAA CCTTTTCTCT CCCTCTCTTG CTCGGCTTCG CGGTAATTTT   
  
  
+ CTGAAAACCG GACTTCGGGG GTTTATCTTG CTGGTTCGAA TTTCGAGGAC GACGGGATAC GGTCCAGATC   
  
  
+ GAGTCAGAAG TCAACGGCTC CACGTGACGG CGGAGCACTT GATTGGACTT GCTAGGGTTT TTGTGGCCCG   
  
  
+ AGGTCGAAAT TAGAGGCTTG CTGCTCGTGC AGGTAATTCT TTAATAGTTT TGGAGATATT TCTAGCTTAC   
  
  
+ GTGTCAGTTG GACGGAGTTT ATACTTTCTA TATCAAATTT TTGTGGAAAT CGGAGCTCCT AATTGGCTTT   
  
  
+ GGCCTGTTTG TTTCCCGAGA AAATGTAGGA AAAATAATGA AATTTGGAAT TTAATAGCTG TCTAGTGTAA   
  
  
+ ACCCTCCACC ATAATGATCC TAGTACTCCT TTGTCTGTGC AAATTGGATT TTTATTTTTA ATTGATTGGA   
  
  
+ ATTAAAAAAA TAAAGAATCT CAGTGCGAAT GATAGTTAGC TTATGTTTTG ATTTGTCATC GAATCATAGA   
  
  
+ ACTTCATTCA CTTCCTTTTT GTATGGTAAT TAATTTTGGG GCGAAATATC AAGGGAAAAA GAGGACCTAA   
  
  
+ AGGTTTGAAT AGGATAATTT GGAGTTCTGG AACAGAATTA GGCATGTTTG AGAAGCATTC TACAAGAGTT   
  
  
+ GGCATAGTTT TTGCTCTAAA AACAAGAAAG TTTACTTTCT AAAAGGCTTA ATCTTCAATG CAGTGTTCTT   
  
  
+ TTCCATTTTT CTGGGATTAA TTGACGAGTC CGCGTACCCT CTTTTCCAGT TACTTCTTCG AGAAGCCCAT   
  
  
+ TTGCTAAAGT AGAATGGCTT TTTTCCATGA AGATATATTT TTCTTGCTAA GGTAGTCCCT GAAGCTAGTA   
  
  
+ GATTTACTGC TTAAGCTTGC CTGTTGAACG GCTTTTTGCC AAACCATGTT GACCGAAACG GGGAGCCTGA   
  
  
+ CTTGTTGAGG ATTGACATCA AATCGAAGCT GTGTAGCCCT AAGTACGTGG TTGTGTTGGT CTTAGAGACC   
  
  
+ CAGGGATACA CTCTTTGTTT AATTTTCCCA AATTATTTTT TTTCTTGTTT ACTTTCTTAA TTACGTGCTA   
  
  
+ AATTATGGAT TATACAATGG GTAGCAATAT ACATTATGTC ATTGCCTATT TTTTTAATTT TGTGGTGGAT   
  
  
+ CGTTTTTTTC AGGTTTGAAG TAGCTGCATG ATATTGTATC AGTACAGCCG ATATACTCTG ACATGTGAAT   
  
  
+ TCTTCAGTTT CTGATAGAAA ATAAGTTCA  

- AATATTATAG CAAAAGAATA AAACGTGTGC TTTAAATTTT ATTTTTCATT TTACCTTTTT ACTTTTTTGT   
  
  
- AGACGCCGCC CTGGGTGACA GGCCGAACAT GAGCGGTGTC CCTACGGTAG TGTCTCCCTC TAGTCTCTAA   
  
  
- GAAGTGACGA AGAGTGGCGT GTCTTTAGTA AAGAGGATGA GGAGAGACGA GAAGGGGGTG AGAGAGCTGA   
  
  
- GACTAATACC AAATCTGAAA ACCTGGAGTT GGAAAAGAGA GGGAGAGAAC GAGCCGAAGC GCCATTAAAA   
  
  
- GACTTTTGGC CTGAAGCCCC CAAATAGAAC GACCAAGCTT AAAGCTCCTG CTGCCCTATG CCAGGTCTAG   
  
  
- CTCAGTCTTC AGTTGCCGAG GTGCACTGCC GCCTCGTGAA CTAACCTGAA CGATCCCAAA AACACCGGGC   
  
  
- TCCAGCTTTA ATCTCCGAAC GACGAGCACG TCCATTAAGA AATTATCAAA ACCTCTATAA AGATCGAATG   
  
  
- CACAGTCAAC CTGCCTCAAA TATGAAAGAT ATAGTTTAAA AACACCTTTA GCCTCGAGGA TTAACCGAAA   
  
  
- CCGGACAAAC AAAGGGCTCT TTTACATCCT TTTTATTACT TTAAACCTTA AATTATCGAC AGATCACATT   
  
  
- TGGGAGGTGG TATTACTAGG ATCATGAGGA AACAGACACG TTTAACCTAA AAATAAAAAT TAACTAACCT   
  
  
- TAATTTTTTT ATTTCTTAGA GTCACGCTTA CTATCAATCG AATACAAAAC TAAACAGTAG CTTAGTATCT   
  
  
- TGAAGTAAGT GAAGGAAAAA CATACCATTA ATTAAAACCC CGCTTTATAG TTCCCTTTTT CTCCTGGATT   
  
  
- TCCAAACTTA TCCTATTAAA CCTCAAGACC TTGTCTTAAT CCGTACAAAC TCTTCGTAAG ATGTTCTCAA   
  
  
- CCGTATCAAA AACGAGATTT TTGTTCTTTC AAATGAAAGA TTTTCCGAAT TAGAAGTTAC GTCACAAGAA   
  
  
- AAGGTAAAAA GACCCTAATT AACTGCTCAG GCGCATGGGA GAAAAGGTCA ATGAAGAAGC TCTTCGGGTA   
  
  
- AACGATTTCA TCTTACCGAA AAAAGGTACT TCTATATAAA AAGAACGATT CCATCAGGGA CTTCGATCAT   
  
  
- CTAAATGACG AATTCGAACG GACAACTTGC CGAAAAACGG TTTGGTACAA CTGGCTTTGC CCCTCGGACT   
  
  
- GAACAACTCC TAACTGTAGT TTAGCTTCGA CACATCGGGA TTCATGCACC AACACAACCA GAATCTCTGG   
  
  
- GTCCCTATGT GAGAAACAAA TTAAAAGGGT TTAATAAAAA AAAGAACAAA TGAAAGAATT AATGCACGAT   
  
  
- TTAATACCTA ATATGTTACC CATCGTTATA TGTAATACAG TAACGGATAA AAAAATTAAA ACACCACCTA   
  
  
- GCAAAAAAAG TCCAAACTTC ATCGACGTAC TATAACATAG TCATGTCGGC TATATGAGAC TGTACACTTA   
  
  
- AGAAGTCAAA GACTATCTTT TATTCAAGT

+     ATCC-motif

| Site Name | Organism | Position | Strand | Matrix score. | sequence | function |
| --- | --- | --- | --- | --- | --- | --- |
| ATCC-motif | Pisum sativum | 1197 | - | 8 | CAATCCTC | part of a conserved DNA module involved in light responsiveness |

> 2018/04/13 10:10:12  
+ TTATAATATC GTTTTCTTAT TTTGCACACG AAATTTAAAA TAAAAAGTAA AATGGAAAAA TGAAAAAACA   
  
  
+ TCTGCGGCGG GACCCACTGT CCGGCTTGTA CTCGCCACAG GGATGCCATC ACAGAGGGAG ATCAGAGATT   
  
  
+ CTTCACTGCT TCTCACCGCA CAGAAATCAT TTCTCCTACT CCTCTCTGCT CTTCCCCCAC TCTCTCGACT   
  
  
+ CTGATTATGG TTTAGACTTT TGGACCTCAA CCTTTTCTCT CCCTCTCTTG CTCGGCTTCG CGGTAATTTT   
  
  
+ CTGAAAACCG GACTTCGGGG GTTTATCTTG CTGGTTCGAA TTTCGAGGAC GACGGGATAC GGTCCAGATC   
  
  
+ GAGTCAGAAG TCAACGGCTC CACGTGACGG CGGAGCACTT GATTGGACTT GCTAGGGTTT TTGTGGCCCG   
  
  
+ AGGTCGAAAT TAGAGGCTTG CTGCTCGTGC AGGTAATTCT TTAATAGTTT TGGAGATATT TCTAGCTTAC   
  
  
+ GTGTCAGTTG GACGGAGTTT ATACTTTCTA TATCAAATTT TTGTGGAAAT CGGAGCTCCT AATTGGCTTT   
  
  
+ GGCCTGTTTG TTTCCCGAGA AAATGTAGGA AAAATAATGA AATTTGGAAT TTAATAGCTG TCTAGTGTAA   
  
  
+ ACCCTCCACC ATAATGATCC TAGTACTCCT TTGTCTGTGC AAATTGGATT TTTATTTTTA ATTGATTGGA   
  
  
+ ATTAAAAAAA TAAAGAATCT CAGTGCGAAT GATAGTTAGC TTATGTTTTG ATTTGTCATC GAATCATAGA   
  
  
+ ACTTCATTCA CTTCCTTTTT GTATGGTAAT TAATTTTGGG GCGAAATATC AAGGGAAAAA GAGGACCTAA   
  
  
+ AGGTTTGAAT AGGATAATTT GGAGTTCTGG AACAGAATTA GGCATGTTTG AGAAGCATTC TACAAGAGTT   
  
  
+ GGCATAGTTT TTGCTCTAAA AACAAGAAAG TTTACTTTCT AAAAGGCTTA ATCTTCAATG CAGTGTTCTT   
  
  
+ TTCCATTTTT CTGGGATTAA TTGACGAGTC CGCGTACCCT CTTTTCCAGT TACTTCTTCG AGAAGCCCAT   
  
  
+ TTGCTAAAGT AGAATGGCTT TTTTCCATGA AGATATATTT TTCTTGCTAA GGTAGTCCCT GAAGCTAGTA   
  
  
+ GATTTACTGC TTAAGCTTGC CTGTTGAACG GCTTTTTGCC AAACCATGTT GACCGAAACG GGGAGCCTGA   
  
  
+ CTTGTTGAGG ATTGACATCA AATCGAAGCT GTGTAGCCCT AAGTACGTGG TTGTGTTGGT CTTAGAGACC   
  
  
+ CAGGGATACA CTCTTTGTTT AATTTTCCCA AATTATTTTT TTTCTTGTTT ACTTTCTTAA TTACGTGCTA   
  
  
+ AATTATGGAT TATACAATGG GTAGCAATAT ACATTATGTC ATTGCCTATT TTTTTAATTT TGTGGTGGAT   
  
  
+ CGTTTTTTTC AGGTTTGAAG TAGCTGCATG ATATTGTATC AGTACAGCCG ATATACTCTG ACATGTGAAT   
  
  
+ TCTTCAGTTT CTGATAGAAA ATAAGTTCA  

- AATATTATAG CAAAAGAATA AAACGTGTGC TTTAAATTTT ATTTTTCATT TTACCTTTTT ACTTTTTTGT   
  
  
- AGACGCCGCC CTGGGTGACA GGCCGAACAT GAGCGGTGTC CCTACGGTAG TGTCTCCCTC TAGTCTCTAA   
  
  
- GAAGTGACGA AGAGTGGCGT GTCTTTAGTA AAGAGGATGA GGAGAGACGA GAAGGGGGTG AGAGAGCTGA   
  
  
- GACTAATACC AAATCTGAAA ACCTGGAGTT GGAAAAGAGA GGGAGAGAAC GAGCCGAAGC GCCATTAAAA   
  
  
- GACTTTTGGC CTGAAGCCCC CAAATAGAAC GACCAAGCTT AAAGCTCCTG CTGCCCTATG CCAGGTCTAG   
  
  
- CTCAGTCTTC AGTTGCCGAG GTGCACTGCC GCCTCGTGAA CTAACCTGAA CGATCCCAAA AACACCGGGC   
  
  
- TCCAGCTTTA ATCTCCGAAC GACGAGCACG TCCATTAAGA AATTATCAAA ACCTCTATAA AGATCGAATG   
  
  
- CACAGTCAAC CTGCCTCAAA TATGAAAGAT ATAGTTTAAA AACACCTTTA GCCTCGAGGA TTAACCGAAA   
  
  
- CCGGACAAAC AAAGGGCTCT TTTACATCCT TTTTATTACT TTAAACCTTA AATTATCGAC AGATCACATT   
  
  
- TGGGAGGTGG TATTACTAGG ATCATGAGGA AACAGACACG TTTAACCTAA AAATAAAAAT TAACTAACCT   
  
  
- TAATTTTTTT ATTTCTTAGA GTCACGCTTA CTATCAATCG AATACAAAAC TAAACAGTAG CTTAGTATCT   
  
  
- TGAAGTAAGT GAAGGAAAAA CATACCATTA ATTAAAACCC CGCTTTATAG TTCCCTTTTT CTCCTGGATT   
  
  
- TCCAAACTTA TCCTATTAAA CCTCAAGACC TTGTCTTAAT CCGTACAAAC TCTTCGTAAG ATGTTCTCAA   
  
  
- CCGTATCAAA AACGAGATTT TTGTTCTTTC AAATGAAAGA TTTTCCGAAT TAGAAGTTAC GTCACAAGAA   
  
  
- AAGGTAAAAA GACCCTAATT AACTGCTCAG GCGCATGGGA GAAAAGGTCA ATGAAGAAGC TCTTCGGGTA   
  
  
- AACGATTTCA TCTTACCGAA AAAAGGTACT TCTATATAAA AAGAACGATT CCATCAGGGA CTTCGATCAT   
  
  
- CTAAATGACG AATTCGAACG GACAACTTGC CGAAAAACGG TTTGGTACAA CTGGCTTTGC CCCTCGGACT   
  
  
- GAACAACTCC TAACTGTAGT TTAGCTTCGA CACATCGGGA TTCATGCACC AACACAACCA GAATCTCTGG   
  
  
- GTCCCTATGT GAGAAACAAA TTAAAAGGGT TTAATAAAAA AAAGAACAAA TGAAAGAATT AATGCACGAT   
  
  
- TTAATACCTA ATATGTTACC CATCGTTATA TGTAATACAG TAACGGATAA AAAAATTAAA ACACCACCTA   
  
  
- GCAAAAAAAG TCCAAACTTC ATCGACGTAC TATAACATAG TCATGTCGGC TATATGAGAC TGTACACTTA   
  
  
- AGAAGTCAAA GACTATCTTT TATTCAAGT

+     Box 4

| Site Name | Organism | Position | Strand | Matrix score. | sequence | function |
| --- | --- | --- | --- | --- | --- | --- |
| Box 4 | Petroselinum crispum | 996 | - | 6 | ATTAAT | part of a conserved DNA module involved in light responsiveness |
| Box 4 | Petroselinum crispum | 799 | - | 6 | ATTAAT | part of a conserved DNA module involved in light responsiveness |

> 2018/04/13 10:10:12  
+ TTATAATATC GTTTTCTTAT TTTGCACACG AAATTTAAAA TAAAAAGTAA AATGGAAAAA TGAAAAAACA   
  
  
+ TCTGCGGCGG GACCCACTGT CCGGCTTGTA CTCGCCACAG GGATGCCATC ACAGAGGGAG ATCAGAGATT   
  
  
+ CTTCACTGCT TCTCACCGCA CAGAAATCAT TTCTCCTACT CCTCTCTGCT CTTCCCCCAC TCTCTCGACT   
  
  
+ CTGATTATGG TTTAGACTTT TGGACCTCAA CCTTTTCTCT CCCTCTCTTG CTCGGCTTCG CGGTAATTTT   
  
  
+ CTGAAAACCG GACTTCGGGG GTTTATCTTG CTGGTTCGAA TTTCGAGGAC GACGGGATAC GGTCCAGATC   
  
  
+ GAGTCAGAAG TCAACGGCTC CACGTGACGG CGGAGCACTT GATTGGACTT GCTAGGGTTT TTGTGGCCCG   
  
  
+ AGGTCGAAAT TAGAGGCTTG CTGCTCGTGC AGGTAATTCT TTAATAGTTT TGGAGATATT TCTAGCTTAC   
  
  
+ GTGTCAGTTG GACGGAGTTT ATACTTTCTA TATCAAATTT TTGTGGAAAT CGGAGCTCCT AATTGGCTTT   
  
  
+ GGCCTGTTTG TTTCCCGAGA AAATGTAGGA AAAATAATGA AATTTGGAAT TTAATAGCTG TCTAGTGTAA   
  
  
+ ACCCTCCACC ATAATGATCC TAGTACTCCT TTGTCTGTGC AAATTGGATT TTTATTTTTA ATTGATTGGA   
  
  
+ ATTAAAAAAA TAAAGAATCT CAGTGCGAAT GATAGTTAGC TTATGTTTTG ATTTGTCATC GAATCATAGA   
  
  
+ ACTTCATTCA CTTCCTTTTT GTATGGTAAT TAATTTTGGG GCGAAATATC AAGGGAAAAA GAGGACCTAA   
  
  
+ AGGTTTGAAT AGGATAATTT GGAGTTCTGG AACAGAATTA GGCATGTTTG AGAAGCATTC TACAAGAGTT   
  
  
+ GGCATAGTTT TTGCTCTAAA AACAAGAAAG TTTACTTTCT AAAAGGCTTA ATCTTCAATG CAGTGTTCTT   
  
  
+ TTCCATTTTT CTGGGATTAA TTGACGAGTC CGCGTACCCT CTTTTCCAGT TACTTCTTCG AGAAGCCCAT   
  
  
+ TTGCTAAAGT AGAATGGCTT TTTTCCATGA AGATATATTT TTCTTGCTAA GGTAGTCCCT GAAGCTAGTA   
  
  
+ GATTTACTGC TTAAGCTTGC CTGTTGAACG GCTTTTTGCC AAACCATGTT GACCGAAACG GGGAGCCTGA   
  
  
+ CTTGTTGAGG ATTGACATCA AATCGAAGCT GTGTAGCCCT AAGTACGTGG TTGTGTTGGT CTTAGAGACC   
  
  
+ CAGGGATACA CTCTTTGTTT AATTTTCCCA AATTATTTTT TTTCTTGTTT ACTTTCTTAA TTACGTGCTA   
  
  
+ AATTATGGAT TATACAATGG GTAGCAATAT ACATTATGTC ATTGCCTATT TTTTTAATTT TGTGGTGGAT   
  
  
+ CGTTTTTTTC AGGTTTGAAG TAGCTGCATG ATATTGTATC AGTACAGCCG ATATACTCTG ACATGTGAAT   
  
  
+ TCTTCAGTTT CTGATAGAAA ATAAGTTCA  

- AATATTATAG CAAAAGAATA AAACGTGTGC TTTAAATTTT ATTTTTCATT TTACCTTTTT ACTTTTTTGT   
  
  
- AGACGCCGCC CTGGGTGACA GGCCGAACAT GAGCGGTGTC CCTACGGTAG TGTCTCCCTC TAGTCTCTAA   
  
  
- GAAGTGACGA AGAGTGGCGT GTCTTTAGTA AAGAGGATGA GGAGAGACGA GAAGGGGGTG AGAGAGCTGA   
  
  
- GACTAATACC AAATCTGAAA ACCTGGAGTT GGAAAAGAGA GGGAGAGAAC GAGCCGAAGC GCCATTAAAA   
  
  
- GACTTTTGGC CTGAAGCCCC CAAATAGAAC GACCAAGCTT AAAGCTCCTG CTGCCCTATG CCAGGTCTAG   
  
  
- CTCAGTCTTC AGTTGCCGAG GTGCACTGCC GCCTCGTGAA CTAACCTGAA CGATCCCAAA AACACCGGGC   
  
  
- TCCAGCTTTA ATCTCCGAAC GACGAGCACG TCCATTAAGA AATTATCAAA ACCTCTATAA AGATCGAATG   
  
  
- CACAGTCAAC CTGCCTCAAA TATGAAAGAT ATAGTTTAAA AACACCTTTA GCCTCGAGGA TTAACCGAAA   
  
  
- CCGGACAAAC AAAGGGCTCT TTTACATCCT TTTTATTACT TTAAACCTTA AATTATCGAC AGATCACATT   
  
  
- TGGGAGGTGG TATTACTAGG ATCATGAGGA AACAGACACG TTTAACCTAA AAATAAAAAT TAACTAACCT   
  
  
- TAATTTTTTT ATTTCTTAGA GTCACGCTTA CTATCAATCG AATACAAAAC TAAACAGTAG CTTAGTATCT   
  
  
- TGAAGTAAGT GAAGGAAAAA CATACCATTA ATTAAAACCC CGCTTTATAG TTCCCTTTTT CTCCTGGATT   
  
  
- TCCAAACTTA TCCTATTAAA CCTCAAGACC TTGTCTTAAT CCGTACAAAC TCTTCGTAAG ATGTTCTCAA   
  
  
- CCGTATCAAA AACGAGATTT TTGTTCTTTC AAATGAAAGA TTTTCCGAAT TAGAAGTTAC GTCACAAGAA   
  
  
- AAGGTAAAAA GACCCTAATT AACTGCTCAG GCGCATGGGA GAAAAGGTCA ATGAAGAAGC TCTTCGGGTA   
  
  
- AACGATTTCA TCTTACCGAA AAAAGGTACT TCTATATAAA AAGAACGATT CCATCAGGGA CTTCGATCAT   
  
  
- CTAAATGACG AATTCGAACG GACAACTTGC CGAAAAACGG TTTGGTACAA CTGGCTTTGC CCCTCGGACT   
  
  
- GAACAACTCC TAACTGTAGT TTAGCTTCGA CACATCGGGA TTCATGCACC AACACAACCA GAATCTCTGG   
  
  
- GTCCCTATGT GAGAAACAAA TTAAAAGGGT TTAATAAAAA AAAGAACAAA TGAAAGAATT AATGCACGAT   
  
  
- TTAATACCTA ATATGTTACC CATCGTTATA TGTAATACAG TAACGGATAA AAAAATTAAA ACACCACCTA   
  
  
- GCAAAAAAAG TCCAAACTTC ATCGACGTAC TATAACATAG TCATGTCGGC TATATGAGAC TGTACACTTA   
  
  
- AGAAGTCAAA GACTATCTTT TATTCAAGT

+     Box-W1

| Site Name | Organism | Position | Strand | Matrix score. | sequence | function |
| --- | --- | --- | --- | --- | --- | --- |
| Box-W1 | Petroselinum crispum | 1169 | + | 6 | TTGACC | fungal elicitor responsive element |

> 2018/04/13 10:10:12  
+ TTATAATATC GTTTTCTTAT TTTGCACACG AAATTTAAAA TAAAAAGTAA AATGGAAAAA TGAAAAAACA   
  
  
+ TCTGCGGCGG GACCCACTGT CCGGCTTGTA CTCGCCACAG GGATGCCATC ACAGAGGGAG ATCAGAGATT   
  
  
+ CTTCACTGCT TCTCACCGCA CAGAAATCAT TTCTCCTACT CCTCTCTGCT CTTCCCCCAC TCTCTCGACT   
  
  
+ CTGATTATGG TTTAGACTTT TGGACCTCAA CCTTTTCTCT CCCTCTCTTG CTCGGCTTCG CGGTAATTTT   
  
  
+ CTGAAAACCG GACTTCGGGG GTTTATCTTG CTGGTTCGAA TTTCGAGGAC GACGGGATAC GGTCCAGATC   
  
  
+ GAGTCAGAAG TCAACGGCTC CACGTGACGG CGGAGCACTT GATTGGACTT GCTAGGGTTT TTGTGGCCCG   
  
  
+ AGGTCGAAAT TAGAGGCTTG CTGCTCGTGC AGGTAATTCT TTAATAGTTT TGGAGATATT TCTAGCTTAC   
  
  
+ GTGTCAGTTG GACGGAGTTT ATACTTTCTA TATCAAATTT TTGTGGAAAT CGGAGCTCCT AATTGGCTTT   
  
  
+ GGCCTGTTTG TTTCCCGAGA AAATGTAGGA AAAATAATGA AATTTGGAAT TTAATAGCTG TCTAGTGTAA   
  
  
+ ACCCTCCACC ATAATGATCC TAGTACTCCT TTGTCTGTGC AAATTGGATT TTTATTTTTA ATTGATTGGA   
  
  
+ ATTAAAAAAA TAAAGAATCT CAGTGCGAAT GATAGTTAGC TTATGTTTTG ATTTGTCATC GAATCATAGA   
  
  
+ ACTTCATTCA CTTCCTTTTT GTATGGTAAT TAATTTTGGG GCGAAATATC AAGGGAAAAA GAGGACCTAA   
  
  
+ AGGTTTGAAT AGGATAATTT GGAGTTCTGG AACAGAATTA GGCATGTTTG AGAAGCATTC TACAAGAGTT   
  
  
+ GGCATAGTTT TTGCTCTAAA AACAAGAAAG TTTACTTTCT AAAAGGCTTA ATCTTCAATG CAGTGTTCTT   
  
  
+ TTCCATTTTT CTGGGATTAA TTGACGAGTC CGCGTACCCT CTTTTCCAGT TACTTCTTCG AGAAGCCCAT   
  
  
+ TTGCTAAAGT AGAATGGCTT TTTTCCATGA AGATATATTT TTCTTGCTAA GGTAGTCCCT GAAGCTAGTA   
  
  
+ GATTTACTGC TTAAGCTTGC CTGTTGAACG GCTTTTTGCC AAACCATGTT GACCGAAACG GGGAGCCTGA   
  
  
+ CTTGTTGAGG ATTGACATCA AATCGAAGCT GTGTAGCCCT AAGTACGTGG TTGTGTTGGT CTTAGAGACC   
  
  
+ CAGGGATACA CTCTTTGTTT AATTTTCCCA AATTATTTTT TTTCTTGTTT ACTTTCTTAA TTACGTGCTA   
  
  
+ AATTATGGAT TATACAATGG GTAGCAATAT ACATTATGTC ATTGCCTATT TTTTTAATTT TGTGGTGGAT   
  
  
+ CGTTTTTTTC AGGTTTGAAG TAGCTGCATG ATATTGTATC AGTACAGCCG ATATACTCTG ACATGTGAAT   
  
  
+ TCTTCAGTTT CTGATAGAAA ATAAGTTCA  

- AATATTATAG CAAAAGAATA AAACGTGTGC TTTAAATTTT ATTTTTCATT TTACCTTTTT ACTTTTTTGT   
  
  
- AGACGCCGCC CTGGGTGACA GGCCGAACAT GAGCGGTGTC CCTACGGTAG TGTCTCCCTC TAGTCTCTAA   
  
  
- GAAGTGACGA AGAGTGGCGT GTCTTTAGTA AAGAGGATGA GGAGAGACGA GAAGGGGGTG AGAGAGCTGA   
  
  
- GACTAATACC AAATCTGAAA ACCTGGAGTT GGAAAAGAGA GGGAGAGAAC GAGCCGAAGC GCCATTAAAA   
  
  
- GACTTTTGGC CTGAAGCCCC CAAATAGAAC GACCAAGCTT AAAGCTCCTG CTGCCCTATG CCAGGTCTAG   
  
  
- CTCAGTCTTC AGTTGCCGAG GTGCACTGCC GCCTCGTGAA CTAACCTGAA CGATCCCAAA AACACCGGGC   
  
  
- TCCAGCTTTA ATCTCCGAAC GACGAGCACG TCCATTAAGA AATTATCAAA ACCTCTATAA AGATCGAATG   
  
  
- CACAGTCAAC CTGCCTCAAA TATGAAAGAT ATAGTTTAAA AACACCTTTA GCCTCGAGGA TTAACCGAAA   
  
  
- CCGGACAAAC AAAGGGCTCT TTTACATCCT TTTTATTACT TTAAACCTTA AATTATCGAC AGATCACATT   
  
  
- TGGGAGGTGG TATTACTAGG ATCATGAGGA AACAGACACG TTTAACCTAA AAATAAAAAT TAACTAACCT   
  
  
- TAATTTTTTT ATTTCTTAGA GTCACGCTTA CTATCAATCG AATACAAAAC TAAACAGTAG CTTAGTATCT   
  
  
- TGAAGTAAGT GAAGGAAAAA CATACCATTA ATTAAAACCC CGCTTTATAG TTCCCTTTTT CTCCTGGATT   
  
  
- TCCAAACTTA TCCTATTAAA CCTCAAGACC TTGTCTTAAT CCGTACAAAC TCTTCGTAAG ATGTTCTCAA   
  
  
- CCGTATCAAA AACGAGATTT TTGTTCTTTC AAATGAAAGA TTTTCCGAAT TAGAAGTTAC GTCACAAGAA   
  
  
- AAGGTAAAAA GACCCTAATT AACTGCTCAG GCGCATGGGA GAAAAGGTCA ATGAAGAAGC TCTTCGGGTA   
  
  
- AACGATTTCA TCTTACCGAA AAAAGGTACT TCTATATAAA AAGAACGATT CCATCAGGGA CTTCGATCAT   
  
  
- CTAAATGACG AATTCGAACG GACAACTTGC CGAAAAACGG TTTGGTACAA CTGGCTTTGC CCCTCGGACT   
  
  
- GAACAACTCC TAACTGTAGT TTAGCTTCGA CACATCGGGA TTCATGCACC AACACAACCA GAATCTCTGG   
  
  
- GTCCCTATGT GAGAAACAAA TTAAAAGGGT TTAATAAAAA AAAGAACAAA TGAAAGAATT AATGCACGAT   
  
  
- TTAATACCTA ATATGTTACC CATCGTTATA TGTAATACAG TAACGGATAA AAAAATTAAA ACACCACCTA   
  
  
- GCAAAAAAAG TCCAAACTTC ATCGACGTAC TATAACATAG TCATGTCGGC TATATGAGAC TGTACACTTA   
  
  
- AGAAGTCAAA GACTATCTTT TATTCAAGT

+     CAAT-box

| Site Name | Organism | Position | Strand | Matrix score. | sequence | function |
| --- | --- | --- | --- | --- | --- | --- |
| CAAT-box | Hordeum vulgare | 1433 | - | 4 | CAAT | common cis-acting element in promoter and enhancer regions |
| CAAT-box | Hordeum vulgare | 1355 | + | 4 | CAAT | common cis-acting element in promoter and enhancer regions |
| CAAT-box | Brassica rapa | 1289 | + | 5 | CAAAT | common cis-acting element in promoter and enhancer regions |
| CAAT-box | Petunia hybrida | 908 | - | 7 | TGCCAAC | common cis-acting element in promoter and enhancer regions |
| CAAT-box | Brassica rapa | 857 | - | 5 | CAAAT | common cis-acting element in promoter and enhancer regions |
| CAAT-box | Glycine max | 690 | - | 5 | CAATT | common cis-acting element in promoter and enhancer regions |
| CAAT-box | Hordeum vulgare | 1201 | - | 4 | CAAT | common cis-acting element in promoter and enhancer regions |
| CAAT-box | Brassica rapa | 1209 | + | 5 | CAAAT | common cis-acting element in promoter and enhancer regions |
| CAAT-box | Hordeum vulgare | 691 | - | 4 | CAAT | common cis-acting element in promoter and enhancer regions |
| CAAT-box | Glycine max | 999 | - | 5 | CAATT | common cis-acting element in promoter and enhancer regions |
| CAAT-box | Arabidopsis thaliana | 673 | - | 5 | CCAAT | common cis-acting element in promoter and enhancer regions |
| CAAT-box | Glycine max | 672 | - | 5 | CAATT | common cis-acting element in promoter and enhancer regions |
| CAAT-box | Hordeum vulgare | 1345 | + | 4 | CAAT | common cis-acting element in promoter and enhancer regions |
| CAAT-box | Arabidopsis thaliana | 695 | - | 5 | CCAAT | common cis-acting element in promoter and enhancer regions |
| CAAT-box | Brassica rapa | 751 | - | 5 | CAAAT | common cis-acting element in promoter and enhancer regions |
| CAAT-box | Hordeum vulgare | 966 | + | 4 | CAAT | common cis-acting element in promoter and enhancer regions |
| CAAT-box | Arabidopsis thaliana | 552 | - | 5 | CCAAT | common cis-acting element in promoter and enhancer regions |
| CAAT-box | Brassica rapa | 1049 | - | 5 | CAAAT | common cis-acting element in promoter and enhancer regions |
| CAAT-box | Brassica rapa | 602 | - | 5 | CAAAT | common cis-acting element in promoter and enhancer regions |
| CAAT-box | Brassica rapa | 670 | + | 5 | CAAAT | common cis-acting element in promoter and enhancer regions |
| CAAT-box | Hordeum vulgare | 1000 | - | 4 | CAAT | common cis-acting element in promoter and enhancer regions |
| CAAT-box | Brassica rapa | 524 | + | 5 | CAAAT | common cis-acting element in promoter and enhancer regions |
| CAAT-box | Arabidopsis thaliana | 1371 | - | 6 | gGCAAT | common cis-acting element in promoter and enhancer regions |
| CAAT-box | Arabidopsis thaliana | 392 | - | 5 | CCAAT | common cis-acting element in promoter and enhancer regions |
| CAAT-box | Glycine max | 551 | - | 5 | CAATT | common cis-acting element in promoter and enhancer regions |

> 2018/04/13 10:10:12  
+ TTATAATATC GTTTTCTTAT TTTGCACACG AAATTTAAAA TAAAAAGTAA AATGGAAAAA TGAAAAAACA   
  
  
+ TCTGCGGCGG GACCCACTGT CCGGCTTGTA CTCGCCACAG GGATGCCATC ACAGAGGGAG ATCAGAGATT   
  
  
+ CTTCACTGCT TCTCACCGCA CAGAAATCAT TTCTCCTACT CCTCTCTGCT CTTCCCCCAC TCTCTCGACT   
  
  
+ CTGATTATGG TTTAGACTTT TGGACCTCAA CCTTTTCTCT CCCTCTCTTG CTCGGCTTCG CGGTAATTTT   
  
  
+ CTGAAAACCG GACTTCGGGG GTTTATCTTG CTGGTTCGAA TTTCGAGGAC GACGGGATAC GGTCCAGATC   
  
  
+ GAGTCAGAAG TCAACGGCTC CACGTGACGG CGGAGCACTT GATTGGACTT GCTAGGGTTT TTGTGGCCCG   
  
  
+ AGGTCGAAAT TAGAGGCTTG CTGCTCGTGC AGGTAATTCT TTAATAGTTT TGGAGATATT TCTAGCTTAC   
  
  
+ GTGTCAGTTG GACGGAGTTT ATACTTTCTA TATCAAATTT TTGTGGAAAT CGGAGCTCCT AATTGGCTTT   
  
  
+ GGCCTGTTTG TTTCCCGAGA AAATGTAGGA AAAATAATGA AATTTGGAAT TTAATAGCTG TCTAGTGTAA   
  
  
+ ACCCTCCACC ATAATGATCC TAGTACTCCT TTGTCTGTGC AAATTGGATT TTTATTTTTA ATTGATTGGA   
  
  
+ ATTAAAAAAA TAAAGAATCT CAGTGCGAAT GATAGTTAGC TTATGTTTTG ATTTGTCATC GAATCATAGA   
  
  
+ ACTTCATTCA CTTCCTTTTT GTATGGTAAT TAATTTTGGG GCGAAATATC AAGGGAAAAA GAGGACCTAA   
  
  
+ AGGTTTGAAT AGGATAATTT GGAGTTCTGG AACAGAATTA GGCATGTTTG AGAAGCATTC TACAAGAGTT   
  
  
+ GGCATAGTTT TTGCTCTAAA AACAAGAAAG TTTACTTTCT AAAAGGCTTA ATCTTCAATG CAGTGTTCTT   
  
  
+ TTCCATTTTT CTGGGATTAA TTGACGAGTC CGCGTACCCT CTTTTCCAGT TACTTCTTCG AGAAGCCCAT   
  
  
+ TTGCTAAAGT AGAATGGCTT TTTTCCATGA AGATATATTT TTCTTGCTAA GGTAGTCCCT GAAGCTAGTA   
  
  
+ GATTTACTGC TTAAGCTTGC CTGTTGAACG GCTTTTTGCC AAACCATGTT GACCGAAACG GGGAGCCTGA   
  
  
+ CTTGTTGAGG ATTGACATCA AATCGAAGCT GTGTAGCCCT AAGTACGTGG TTGTGTTGGT CTTAGAGACC   
  
  
+ CAGGGATACA CTCTTTGTTT AATTTTCCCA AATTATTTTT TTTCTTGTTT ACTTTCTTAA TTACGTGCTA   
  
  
+ AATTATGGAT TATACAATGG GTAGCAATAT ACATTATGTC ATTGCCTATT TTTTTAATTT TGTGGTGGAT   
  
  
+ CGTTTTTTTC AGGTTTGAAG TAGCTGCATG ATATTGTATC AGTACAGCCG ATATACTCTG ACATGTGAAT   
  
  
+ TCTTCAGTTT CTGATAGAAA ATAAGTTCA  

- AATATTATAG CAAAAGAATA AAACGTGTGC TTTAAATTTT ATTTTTCATT TTACCTTTTT ACTTTTTTGT   
  
  
- AGACGCCGCC CTGGGTGACA GGCCGAACAT GAGCGGTGTC CCTACGGTAG TGTCTCCCTC TAGTCTCTAA   
  
  
- GAAGTGACGA AGAGTGGCGT GTCTTTAGTA AAGAGGATGA GGAGAGACGA GAAGGGGGTG AGAGAGCTGA   
  
  
- GACTAATACC AAATCTGAAA ACCTGGAGTT GGAAAAGAGA GGGAGAGAAC GAGCCGAAGC GCCATTAAAA   
  
  
- GACTTTTGGC CTGAAGCCCC CAAATAGAAC GACCAAGCTT AAAGCTCCTG CTGCCCTATG CCAGGTCTAG   
  
  
- CTCAGTCTTC AGTTGCCGAG GTGCACTGCC GCCTCGTGAA CTAACCTGAA CGATCCCAAA AACACCGGGC   
  
  
- TCCAGCTTTA ATCTCCGAAC GACGAGCACG TCCATTAAGA AATTATCAAA ACCTCTATAA AGATCGAATG   
  
  
- CACAGTCAAC CTGCCTCAAA TATGAAAGAT ATAGTTTAAA AACACCTTTA GCCTCGAGGA TTAACCGAAA   
  
  
- CCGGACAAAC AAAGGGCTCT TTTACATCCT TTTTATTACT TTAAACCTTA AATTATCGAC AGATCACATT   
  
  
- TGGGAGGTGG TATTACTAGG ATCATGAGGA AACAGACACG TTTAACCTAA AAATAAAAAT TAACTAACCT   
  
  
- TAATTTTTTT ATTTCTTAGA GTCACGCTTA CTATCAATCG AATACAAAAC TAAACAGTAG CTTAGTATCT   
  
  
- TGAAGTAAGT GAAGGAAAAA CATACCATTA ATTAAAACCC CGCTTTATAG TTCCCTTTTT CTCCTGGATT   
  
  
- TCCAAACTTA TCCTATTAAA CCTCAAGACC TTGTCTTAAT CCGTACAAAC TCTTCGTAAG ATGTTCTCAA   
  
  
- CCGTATCAAA AACGAGATTT TTGTTCTTTC AAATGAAAGA TTTTCCGAAT TAGAAGTTAC GTCACAAGAA   
  
  
- AAGGTAAAAA GACCCTAATT AACTGCTCAG GCGCATGGGA GAAAAGGTCA ATGAAGAAGC TCTTCGGGTA   
  
  
- AACGATTTCA TCTTACCGAA AAAAGGTACT TCTATATAAA AAGAACGATT CCATCAGGGA CTTCGATCAT   
  
  
- CTAAATGACG AATTCGAACG GACAACTTGC CGAAAAACGG TTTGGTACAA CTGGCTTTGC CCCTCGGACT   
  
  
- GAACAACTCC TAACTGTAGT TTAGCTTCGA CACATCGGGA TTCATGCACC AACACAACCA GAATCTCTGG   
  
  
- GTCCCTATGT GAGAAACAAA TTAAAAGGGT TTAATAAAAA AAAGAACAAA TGAAAGAATT AATGCACGAT   
  
  
- TTAATACCTA ATATGTTACC CATCGTTATA TGTAATACAG TAACGGATAA AAAAATTAAA ACACCACCTA   
  
  
- GCAAAAAAAG TCCAAACTTC ATCGACGTAC TATAACATAG TCATGTCGGC TATATGAGAC TGTACACTTA   
  
  
- AGAAGTCAAA GACTATCTTT TATTCAAGT

+     CATT-motif

| Site Name | Organism | Position | Strand | Matrix score. | sequence | function |
| --- | --- | --- | --- | --- | --- | --- |
| CATT-motif | Zea mays | 895 | + | 6 | GCATTC | part of a light responsive element |

> 2018/04/13 10:10:12  
+ TTATAATATC GTTTTCTTAT TTTGCACACG AAATTTAAAA TAAAAAGTAA AATGGAAAAA TGAAAAAACA   
  
  
+ TCTGCGGCGG GACCCACTGT CCGGCTTGTA CTCGCCACAG GGATGCCATC ACAGAGGGAG ATCAGAGATT   
  
  
+ CTTCACTGCT TCTCACCGCA CAGAAATCAT TTCTCCTACT CCTCTCTGCT CTTCCCCCAC TCTCTCGACT   
  
  
+ CTGATTATGG TTTAGACTTT TGGACCTCAA CCTTTTCTCT CCCTCTCTTG CTCGGCTTCG CGGTAATTTT   
  
  
+ CTGAAAACCG GACTTCGGGG GTTTATCTTG CTGGTTCGAA TTTCGAGGAC GACGGGATAC GGTCCAGATC   
  
  
+ GAGTCAGAAG TCAACGGCTC CACGTGACGG CGGAGCACTT GATTGGACTT GCTAGGGTTT TTGTGGCCCG   
  
  
+ AGGTCGAAAT TAGAGGCTTG CTGCTCGTGC AGGTAATTCT TTAATAGTTT TGGAGATATT TCTAGCTTAC   
  
  
+ GTGTCAGTTG GACGGAGTTT ATACTTTCTA TATCAAATTT TTGTGGAAAT CGGAGCTCCT AATTGGCTTT   
  
  
+ GGCCTGTTTG TTTCCCGAGA AAATGTAGGA AAAATAATGA AATTTGGAAT TTAATAGCTG TCTAGTGTAA   
  
  
+ ACCCTCCACC ATAATGATCC TAGTACTCCT TTGTCTGTGC AAATTGGATT TTTATTTTTA ATTGATTGGA   
  
  
+ ATTAAAAAAA TAAAGAATCT CAGTGCGAAT GATAGTTAGC TTATGTTTTG ATTTGTCATC GAATCATAGA   
  
  
+ ACTTCATTCA CTTCCTTTTT GTATGGTAAT TAATTTTGGG GCGAAATATC AAGGGAAAAA GAGGACCTAA   
  
  
+ AGGTTTGAAT AGGATAATTT GGAGTTCTGG AACAGAATTA GGCATGTTTG AGAAGCATTC TACAAGAGTT   
  
  
+ GGCATAGTTT TTGCTCTAAA AACAAGAAAG TTTACTTTCT AAAAGGCTTA ATCTTCAATG CAGTGTTCTT   
  
  
+ TTCCATTTTT CTGGGATTAA TTGACGAGTC CGCGTACCCT CTTTTCCAGT TACTTCTTCG AGAAGCCCAT   
  
  
+ TTGCTAAAGT AGAATGGCTT TTTTCCATGA AGATATATTT TTCTTGCTAA GGTAGTCCCT GAAGCTAGTA   
  
  
+ GATTTACTGC TTAAGCTTGC CTGTTGAACG GCTTTTTGCC AAACCATGTT GACCGAAACG GGGAGCCTGA   
  
  
+ CTTGTTGAGG ATTGACATCA AATCGAAGCT GTGTAGCCCT AAGTACGTGG TTGTGTTGGT CTTAGAGACC   
  
  
+ CAGGGATACA CTCTTTGTTT AATTTTCCCA AATTATTTTT TTTCTTGTTT ACTTTCTTAA TTACGTGCTA   
  
  
+ AATTATGGAT TATACAATGG GTAGCAATAT ACATTATGTC ATTGCCTATT TTTTTAATTT TGTGGTGGAT   
  
  
+ CGTTTTTTTC AGGTTTGAAG TAGCTGCATG ATATTGTATC AGTACAGCCG ATATACTCTG ACATGTGAAT   
  
  
+ TCTTCAGTTT CTGATAGAAA ATAAGTTCA  

- AATATTATAG CAAAAGAATA AAACGTGTGC TTTAAATTTT ATTTTTCATT TTACCTTTTT ACTTTTTTGT   
  
  
- AGACGCCGCC CTGGGTGACA GGCCGAACAT GAGCGGTGTC CCTACGGTAG TGTCTCCCTC TAGTCTCTAA   
  
  
- GAAGTGACGA AGAGTGGCGT GTCTTTAGTA AAGAGGATGA GGAGAGACGA GAAGGGGGTG AGAGAGCTGA   
  
  
- GACTAATACC AAATCTGAAA ACCTGGAGTT GGAAAAGAGA GGGAGAGAAC GAGCCGAAGC GCCATTAAAA   
  
  
- GACTTTTGGC CTGAAGCCCC CAAATAGAAC GACCAAGCTT AAAGCTCCTG CTGCCCTATG CCAGGTCTAG   
  
  
- CTCAGTCTTC AGTTGCCGAG GTGCACTGCC GCCTCGTGAA CTAACCTGAA CGATCCCAAA AACACCGGGC   
  
  
- TCCAGCTTTA ATCTCCGAAC GACGAGCACG TCCATTAAGA AATTATCAAA ACCTCTATAA AGATCGAATG   
  
  
- CACAGTCAAC CTGCCTCAAA TATGAAAGAT ATAGTTTAAA AACACCTTTA GCCTCGAGGA TTAACCGAAA   
  
  
- CCGGACAAAC AAAGGGCTCT TTTACATCCT TTTTATTACT TTAAACCTTA AATTATCGAC AGATCACATT   
  
  
- TGGGAGGTGG TATTACTAGG ATCATGAGGA AACAGACACG TTTAACCTAA AAATAAAAAT TAACTAACCT   
  
  
- TAATTTTTTT ATTTCTTAGA GTCACGCTTA CTATCAATCG AATACAAAAC TAAACAGTAG CTTAGTATCT   
  
  
- TGAAGTAAGT GAAGGAAAAA CATACCATTA ATTAAAACCC CGCTTTATAG TTCCCTTTTT CTCCTGGATT   
  
  
- TCCAAACTTA TCCTATTAAA CCTCAAGACC TTGTCTTAAT CCGTACAAAC TCTTCGTAAG ATGTTCTCAA   
  
  
- CCGTATCAAA AACGAGATTT TTGTTCTTTC AAATGAAAGA TTTTCCGAAT TAGAAGTTAC GTCACAAGAA   
  
  
- AAGGTAAAAA GACCCTAATT AACTGCTCAG GCGCATGGGA GAAAAGGTCA ATGAAGAAGC TCTTCGGGTA   
  
  
- AACGATTTCA TCTTACCGAA AAAAGGTACT TCTATATAAA AAGAACGATT CCATCAGGGA CTTCGATCAT   
  
  
- CTAAATGACG AATTCGAACG GACAACTTGC CGAAAAACGG TTTGGTACAA CTGGCTTTGC CCCTCGGACT   
  
  
- GAACAACTCC TAACTGTAGT TTAGCTTCGA CACATCGGGA TTCATGCACC AACACAACCA GAATCTCTGG   
  
  
- GTCCCTATGT GAGAAACAAA TTAAAAGGGT TTAATAAAAA AAAGAACAAA TGAAAGAATT AATGCACGAT   
  
  
- TTAATACCTA ATATGTTACC CATCGTTATA TGTAATACAG TAACGGATAA AAAAATTAAA ACACCACCTA   
  
  
- GCAAAAAAAG TCCAAACTTC ATCGACGTAC TATAACATAG TCATGTCGGC TATATGAGAC TGTACACTTA   
  
  
- AGAAGTCAAA GACTATCTTT TATTCAAGT

+     CCAAT-box

| Site Name | Organism | Position | Strand | Matrix score. | sequence | function |
| --- | --- | --- | --- | --- | --- | --- |
| CCAAT-box | Hordeum vulgare | 362 | + | 6 | CAACGG | MYBHv1 binding site |

> 2018/04/13 10:10:12  
+ TTATAATATC GTTTTCTTAT TTTGCACACG AAATTTAAAA TAAAAAGTAA AATGGAAAAA TGAAAAAACA   
  
  
+ TCTGCGGCGG GACCCACTGT CCGGCTTGTA CTCGCCACAG GGATGCCATC ACAGAGGGAG ATCAGAGATT   
  
  
+ CTTCACTGCT TCTCACCGCA CAGAAATCAT TTCTCCTACT CCTCTCTGCT CTTCCCCCAC TCTCTCGACT   
  
  
+ CTGATTATGG TTTAGACTTT TGGACCTCAA CCTTTTCTCT CCCTCTCTTG CTCGGCTTCG CGGTAATTTT   
  
  
+ CTGAAAACCG GACTTCGGGG GTTTATCTTG CTGGTTCGAA TTTCGAGGAC GACGGGATAC GGTCCAGATC   
  
  
+ GAGTCAGAAG TCAACGGCTC CACGTGACGG CGGAGCACTT GATTGGACTT GCTAGGGTTT TTGTGGCCCG   
  
  
+ AGGTCGAAAT TAGAGGCTTG CTGCTCGTGC AGGTAATTCT TTAATAGTTT TGGAGATATT TCTAGCTTAC   
  
  
+ GTGTCAGTTG GACGGAGTTT ATACTTTCTA TATCAAATTT TTGTGGAAAT CGGAGCTCCT AATTGGCTTT   
  
  
+ GGCCTGTTTG TTTCCCGAGA AAATGTAGGA AAAATAATGA AATTTGGAAT TTAATAGCTG TCTAGTGTAA   
  
  
+ ACCCTCCACC ATAATGATCC TAGTACTCCT TTGTCTGTGC AAATTGGATT TTTATTTTTA ATTGATTGGA   
  
  
+ ATTAAAAAAA TAAAGAATCT CAGTGCGAAT GATAGTTAGC TTATGTTTTG ATTTGTCATC GAATCATAGA   
  
  
+ ACTTCATTCA CTTCCTTTTT GTATGGTAAT TAATTTTGGG GCGAAATATC AAGGGAAAAA GAGGACCTAA   
  
  
+ AGGTTTGAAT AGGATAATTT GGAGTTCTGG AACAGAATTA GGCATGTTTG AGAAGCATTC TACAAGAGTT   
  
  
+ GGCATAGTTT TTGCTCTAAA AACAAGAAAG TTTACTTTCT AAAAGGCTTA ATCTTCAATG CAGTGTTCTT   
  
  
+ TTCCATTTTT CTGGGATTAA TTGACGAGTC CGCGTACCCT CTTTTCCAGT TACTTCTTCG AGAAGCCCAT   
  
  
+ TTGCTAAAGT AGAATGGCTT TTTTCCATGA AGATATATTT TTCTTGCTAA GGTAGTCCCT GAAGCTAGTA   
  
  
+ GATTTACTGC TTAAGCTTGC CTGTTGAACG GCTTTTTGCC AAACCATGTT GACCGAAACG GGGAGCCTGA   
  
  
+ CTTGTTGAGG ATTGACATCA AATCGAAGCT GTGTAGCCCT AAGTACGTGG TTGTGTTGGT CTTAGAGACC   
  
  
+ CAGGGATACA CTCTTTGTTT AATTTTCCCA AATTATTTTT TTTCTTGTTT ACTTTCTTAA TTACGTGCTA   
  
  
+ AATTATGGAT TATACAATGG GTAGCAATAT ACATTATGTC ATTGCCTATT TTTTTAATTT TGTGGTGGAT   
  
  
+ CGTTTTTTTC AGGTTTGAAG TAGCTGCATG ATATTGTATC AGTACAGCCG ATATACTCTG ACATGTGAAT   
  
  
+ TCTTCAGTTT CTGATAGAAA ATAAGTTCA  

- AATATTATAG CAAAAGAATA AAACGTGTGC TTTAAATTTT ATTTTTCATT TTACCTTTTT ACTTTTTTGT   
  
  
- AGACGCCGCC CTGGGTGACA GGCCGAACAT GAGCGGTGTC CCTACGGTAG TGTCTCCCTC TAGTCTCTAA   
  
  
- GAAGTGACGA AGAGTGGCGT GTCTTTAGTA AAGAGGATGA GGAGAGACGA GAAGGGGGTG AGAGAGCTGA   
  
  
- GACTAATACC AAATCTGAAA ACCTGGAGTT GGAAAAGAGA GGGAGAGAAC GAGCCGAAGC GCCATTAAAA   
  
  
- GACTTTTGGC CTGAAGCCCC CAAATAGAAC GACCAAGCTT AAAGCTCCTG CTGCCCTATG CCAGGTCTAG   
  
  
- CTCAGTCTTC AGTTGCCGAG GTGCACTGCC GCCTCGTGAA CTAACCTGAA CGATCCCAAA AACACCGGGC   
  
  
- TCCAGCTTTA ATCTCCGAAC GACGAGCACG TCCATTAAGA AATTATCAAA ACCTCTATAA AGATCGAATG   
  
  
- CACAGTCAAC CTGCCTCAAA TATGAAAGAT ATAGTTTAAA AACACCTTTA GCCTCGAGGA TTAACCGAAA   
  
  
- CCGGACAAAC AAAGGGCTCT TTTACATCCT TTTTATTACT TTAAACCTTA AATTATCGAC AGATCACATT   
  
  
- TGGGAGGTGG TATTACTAGG ATCATGAGGA AACAGACACG TTTAACCTAA AAATAAAAAT TAACTAACCT   
  
  
- TAATTTTTTT ATTTCTTAGA GTCACGCTTA CTATCAATCG AATACAAAAC TAAACAGTAG CTTAGTATCT   
  
  
- TGAAGTAAGT GAAGGAAAAA CATACCATTA ATTAAAACCC CGCTTTATAG TTCCCTTTTT CTCCTGGATT   
  
  
- TCCAAACTTA TCCTATTAAA CCTCAAGACC TTGTCTTAAT CCGTACAAAC TCTTCGTAAG ATGTTCTCAA   
  
  
- CCGTATCAAA AACGAGATTT TTGTTCTTTC AAATGAAAGA TTTTCCGAAT TAGAAGTTAC GTCACAAGAA   
  
  
- AAGGTAAAAA GACCCTAATT AACTGCTCAG GCGCATGGGA GAAAAGGTCA ATGAAGAAGC TCTTCGGGTA   
  
  
- AACGATTTCA TCTTACCGAA AAAAGGTACT TCTATATAAA AAGAACGATT CCATCAGGGA CTTCGATCAT   
  
  
- CTAAATGACG AATTCGAACG GACAACTTGC CGAAAAACGG TTTGGTACAA CTGGCTTTGC CCCTCGGACT   
  
  
- GAACAACTCC TAACTGTAGT TTAGCTTCGA CACATCGGGA TTCATGCACC AACACAACCA GAATCTCTGG   
  
  
- GTCCCTATGT GAGAAACAAA TTAAAAGGGT TTAATAAAAA AAAGAACAAA TGAAAGAATT AATGCACGAT   
  
  
- TTAATACCTA ATATGTTACC CATCGTTATA TGTAATACAG TAACGGATAA AAAAATTAAA ACACCACCTA   
  
  
- GCAAAAAAAG TCCAAACTTC ATCGACGTAC TATAACATAG TCATGTCGGC TATATGAGAC TGTACACTTA   
  
  
- AGAAGTCAAA GACTATCTTT TATTCAAGT

+     CCGTCC-box

| Site Name | Organism | Position | Strand | Matrix score. | sequence | function |
| --- | --- | --- | --- | --- | --- | --- |
| CCGTCC-box | Arabidopsis thaliana | 500 | - | 6 | CCGTCC | cis-acting regulatory element related to meristem specific activation |

> 2018/04/13 10:10:12  
+ TTATAATATC GTTTTCTTAT TTTGCACACG AAATTTAAAA TAAAAAGTAA AATGGAAAAA TGAAAAAACA   
  
  
+ TCTGCGGCGG GACCCACTGT CCGGCTTGTA CTCGCCACAG GGATGCCATC ACAGAGGGAG ATCAGAGATT   
  
  
+ CTTCACTGCT TCTCACCGCA CAGAAATCAT TTCTCCTACT CCTCTCTGCT CTTCCCCCAC TCTCTCGACT   
  
  
+ CTGATTATGG TTTAGACTTT TGGACCTCAA CCTTTTCTCT CCCTCTCTTG CTCGGCTTCG CGGTAATTTT   
  
  
+ CTGAAAACCG GACTTCGGGG GTTTATCTTG CTGGTTCGAA TTTCGAGGAC GACGGGATAC GGTCCAGATC   
  
  
+ GAGTCAGAAG TCAACGGCTC CACGTGACGG CGGAGCACTT GATTGGACTT GCTAGGGTTT TTGTGGCCCG   
  
  
+ AGGTCGAAAT TAGAGGCTTG CTGCTCGTGC AGGTAATTCT TTAATAGTTT TGGAGATATT TCTAGCTTAC   
  
  
+ GTGTCAGTTG GACGGAGTTT ATACTTTCTA TATCAAATTT TTGTGGAAAT CGGAGCTCCT AATTGGCTTT   
  
  
+ GGCCTGTTTG TTTCCCGAGA AAATGTAGGA AAAATAATGA AATTTGGAAT TTAATAGCTG TCTAGTGTAA   
  
  
+ ACCCTCCACC ATAATGATCC TAGTACTCCT TTGTCTGTGC AAATTGGATT TTTATTTTTA ATTGATTGGA   
  
  
+ ATTAAAAAAA TAAAGAATCT CAGTGCGAAT GATAGTTAGC TTATGTTTTG ATTTGTCATC GAATCATAGA   
  
  
+ ACTTCATTCA CTTCCTTTTT GTATGGTAAT TAATTTTGGG GCGAAATATC AAGGGAAAAA GAGGACCTAA   
  
  
+ AGGTTTGAAT AGGATAATTT GGAGTTCTGG AACAGAATTA GGCATGTTTG AGAAGCATTC TACAAGAGTT   
  
  
+ GGCATAGTTT TTGCTCTAAA AACAAGAAAG TTTACTTTCT AAAAGGCTTA ATCTTCAATG CAGTGTTCTT   
  
  
+ TTCCATTTTT CTGGGATTAA TTGACGAGTC CGCGTACCCT CTTTTCCAGT TACTTCTTCG AGAAGCCCAT   
  
  
+ TTGCTAAAGT AGAATGGCTT TTTTCCATGA AGATATATTT TTCTTGCTAA GGTAGTCCCT GAAGCTAGTA   
  
  
+ GATTTACTGC TTAAGCTTGC CTGTTGAACG GCTTTTTGCC AAACCATGTT GACCGAAACG GGGAGCCTGA   
  
  
+ CTTGTTGAGG ATTGACATCA AATCGAAGCT GTGTAGCCCT AAGTACGTGG TTGTGTTGGT CTTAGAGACC   
  
  
+ CAGGGATACA CTCTTTGTTT AATTTTCCCA AATTATTTTT TTTCTTGTTT ACTTTCTTAA TTACGTGCTA   
  
  
+ AATTATGGAT TATACAATGG GTAGCAATAT ACATTATGTC ATTGCCTATT TTTTTAATTT TGTGGTGGAT   
  
  
+ CGTTTTTTTC AGGTTTGAAG TAGCTGCATG ATATTGTATC AGTACAGCCG ATATACTCTG ACATGTGAAT   
  
  
+ TCTTCAGTTT CTGATAGAAA ATAAGTTCA  

- AATATTATAG CAAAAGAATA AAACGTGTGC TTTAAATTTT ATTTTTCATT TTACCTTTTT ACTTTTTTGT   
  
  
- AGACGCCGCC CTGGGTGACA GGCCGAACAT GAGCGGTGTC CCTACGGTAG TGTCTCCCTC TAGTCTCTAA   
  
  
- GAAGTGACGA AGAGTGGCGT GTCTTTAGTA AAGAGGATGA GGAGAGACGA GAAGGGGGTG AGAGAGCTGA   
  
  
- GACTAATACC AAATCTGAAA ACCTGGAGTT GGAAAAGAGA GGGAGAGAAC GAGCCGAAGC GCCATTAAAA   
  
  
- GACTTTTGGC CTGAAGCCCC CAAATAGAAC GACCAAGCTT AAAGCTCCTG CTGCCCTATG CCAGGTCTAG   
  
  
- CTCAGTCTTC AGTTGCCGAG GTGCACTGCC GCCTCGTGAA CTAACCTGAA CGATCCCAAA AACACCGGGC   
  
  
- TCCAGCTTTA ATCTCCGAAC GACGAGCACG TCCATTAAGA AATTATCAAA ACCTCTATAA AGATCGAATG   
  
  
- CACAGTCAAC CTGCCTCAAA TATGAAAGAT ATAGTTTAAA AACACCTTTA GCCTCGAGGA TTAACCGAAA   
  
  
- CCGGACAAAC AAAGGGCTCT TTTACATCCT TTTTATTACT TTAAACCTTA AATTATCGAC AGATCACATT   
  
  
- TGGGAGGTGG TATTACTAGG ATCATGAGGA AACAGACACG TTTAACCTAA AAATAAAAAT TAACTAACCT   
  
  
- TAATTTTTTT ATTTCTTAGA GTCACGCTTA CTATCAATCG AATACAAAAC TAAACAGTAG CTTAGTATCT   
  
  
- TGAAGTAAGT GAAGGAAAAA CATACCATTA ATTAAAACCC CGCTTTATAG TTCCCTTTTT CTCCTGGATT   
  
  
- TCCAAACTTA TCCTATTAAA CCTCAAGACC TTGTCTTAAT CCGTACAAAC TCTTCGTAAG ATGTTCTCAA   
  
  
- CCGTATCAAA AACGAGATTT TTGTTCTTTC AAATGAAAGA TTTTCCGAAT TAGAAGTTAC GTCACAAGAA   
  
  
- AAGGTAAAAA GACCCTAATT AACTGCTCAG GCGCATGGGA GAAAAGGTCA ATGAAGAAGC TCTTCGGGTA   
  
  
- AACGATTTCA TCTTACCGAA AAAAGGTACT TCTATATAAA AAGAACGATT CCATCAGGGA CTTCGATCAT   
  
  
- CTAAATGACG AATTCGAACG GACAACTTGC CGAAAAACGG TTTGGTACAA CTGGCTTTGC CCCTCGGACT   
  
  
- GAACAACTCC TAACTGTAGT TTAGCTTCGA CACATCGGGA TTCATGCACC AACACAACCA GAATCTCTGG   
  
  
- GTCCCTATGT GAGAAACAAA TTAAAAGGGT TTAATAAAAA AAAGAACAAA TGAAAGAATT AATGCACGAT   
  
  
- TTAATACCTA ATATGTTACC CATCGTTATA TGTAATACAG TAACGGATAA AAAAATTAAA ACACCACCTA   
  
  
- GCAAAAAAAG TCCAAACTTC ATCGACGTAC TATAACATAG TCATGTCGGC TATATGAGAC TGTACACTTA   
  
  
- AGAAGTCAAA GACTATCTTT TATTCAAGT

+     CGTCA-motif

| Site Name | Organism | Position | Strand | Matrix score. | sequence | function |
| --- | --- | --- | --- | --- | --- | --- |
| CGTCA-motif | Hordeum vulgare | 375 | - | 5 | CGTCA | cis-acting regulatory element involved in the MeJA-responsiveness |
| CGTCA-motif | Hordeum vulgare | 1002 | - | 5 | CGTCA | cis-acting regulatory element involved in the MeJA-responsiveness |

> 2018/04/13 10:10:12  
+ TTATAATATC GTTTTCTTAT TTTGCACACG AAATTTAAAA TAAAAAGTAA AATGGAAAAA TGAAAAAACA   
  
  
+ TCTGCGGCGG GACCCACTGT CCGGCTTGTA CTCGCCACAG GGATGCCATC ACAGAGGGAG ATCAGAGATT   
  
  
+ CTTCACTGCT TCTCACCGCA CAGAAATCAT TTCTCCTACT CCTCTCTGCT CTTCCCCCAC TCTCTCGACT   
  
  
+ CTGATTATGG TTTAGACTTT TGGACCTCAA CCTTTTCTCT CCCTCTCTTG CTCGGCTTCG CGGTAATTTT   
  
  
+ CTGAAAACCG GACTTCGGGG GTTTATCTTG CTGGTTCGAA TTTCGAGGAC GACGGGATAC GGTCCAGATC   
  
  
+ GAGTCAGAAG TCAACGGCTC CACGTGACGG CGGAGCACTT GATTGGACTT GCTAGGGTTT TTGTGGCCCG   
  
  
+ AGGTCGAAAT TAGAGGCTTG CTGCTCGTGC AGGTAATTCT TTAATAGTTT TGGAGATATT TCTAGCTTAC   
  
  
+ GTGTCAGTTG GACGGAGTTT ATACTTTCTA TATCAAATTT TTGTGGAAAT CGGAGCTCCT AATTGGCTTT   
  
  
+ GGCCTGTTTG TTTCCCGAGA AAATGTAGGA AAAATAATGA AATTTGGAAT TTAATAGCTG TCTAGTGTAA   
  
  
+ ACCCTCCACC ATAATGATCC TAGTACTCCT TTGTCTGTGC AAATTGGATT TTTATTTTTA ATTGATTGGA   
  
  
+ ATTAAAAAAA TAAAGAATCT CAGTGCGAAT GATAGTTAGC TTATGTTTTG ATTTGTCATC GAATCATAGA   
  
  
+ ACTTCATTCA CTTCCTTTTT GTATGGTAAT TAATTTTGGG GCGAAATATC AAGGGAAAAA GAGGACCTAA   
  
  
+ AGGTTTGAAT AGGATAATTT GGAGTTCTGG AACAGAATTA GGCATGTTTG AGAAGCATTC TACAAGAGTT   
  
  
+ GGCATAGTTT TTGCTCTAAA AACAAGAAAG TTTACTTTCT AAAAGGCTTA ATCTTCAATG CAGTGTTCTT   
  
  
+ TTCCATTTTT CTGGGATTAA TTGACGAGTC CGCGTACCCT CTTTTCCAGT TACTTCTTCG AGAAGCCCAT   
  
  
+ TTGCTAAAGT AGAATGGCTT TTTTCCATGA AGATATATTT TTCTTGCTAA GGTAGTCCCT GAAGCTAGTA   
  
  
+ GATTTACTGC TTAAGCTTGC CTGTTGAACG GCTTTTTGCC AAACCATGTT GACCGAAACG GGGAGCCTGA   
  
  
+ CTTGTTGAGG ATTGACATCA AATCGAAGCT GTGTAGCCCT AAGTACGTGG TTGTGTTGGT CTTAGAGACC   
  
  
+ CAGGGATACA CTCTTTGTTT AATTTTCCCA AATTATTTTT TTTCTTGTTT ACTTTCTTAA TTACGTGCTA   
  
  
+ AATTATGGAT TATACAATGG GTAGCAATAT ACATTATGTC ATTGCCTATT TTTTTAATTT TGTGGTGGAT   
  
  
+ CGTTTTTTTC AGGTTTGAAG TAGCTGCATG ATATTGTATC AGTACAGCCG ATATACTCTG ACATGTGAAT   
  
  
+ TCTTCAGTTT CTGATAGAAA ATAAGTTCA  

- AATATTATAG CAAAAGAATA AAACGTGTGC TTTAAATTTT ATTTTTCATT TTACCTTTTT ACTTTTTTGT   
  
  
- AGACGCCGCC CTGGGTGACA GGCCGAACAT GAGCGGTGTC CCTACGGTAG TGTCTCCCTC TAGTCTCTAA   
  
  
- GAAGTGACGA AGAGTGGCGT GTCTTTAGTA AAGAGGATGA GGAGAGACGA GAAGGGGGTG AGAGAGCTGA   
  
  
- GACTAATACC AAATCTGAAA ACCTGGAGTT GGAAAAGAGA GGGAGAGAAC GAGCCGAAGC GCCATTAAAA   
  
  
- GACTTTTGGC CTGAAGCCCC CAAATAGAAC GACCAAGCTT AAAGCTCCTG CTGCCCTATG CCAGGTCTAG   
  
  
- CTCAGTCTTC AGTTGCCGAG GTGCACTGCC GCCTCGTGAA CTAACCTGAA CGATCCCAAA AACACCGGGC   
  
  
- TCCAGCTTTA ATCTCCGAAC GACGAGCACG TCCATTAAGA AATTATCAAA ACCTCTATAA AGATCGAATG   
  
  
- CACAGTCAAC CTGCCTCAAA TATGAAAGAT ATAGTTTAAA AACACCTTTA GCCTCGAGGA TTAACCGAAA   
  
  
- CCGGACAAAC AAAGGGCTCT TTTACATCCT TTTTATTACT TTAAACCTTA AATTATCGAC AGATCACATT   
  
  
- TGGGAGGTGG TATTACTAGG ATCATGAGGA AACAGACACG TTTAACCTAA AAATAAAAAT TAACTAACCT   
  
  
- TAATTTTTTT ATTTCTTAGA GTCACGCTTA CTATCAATCG AATACAAAAC TAAACAGTAG CTTAGTATCT   
  
  
- TGAAGTAAGT GAAGGAAAAA CATACCATTA ATTAAAACCC CGCTTTATAG TTCCCTTTTT CTCCTGGATT   
  
  
- TCCAAACTTA TCCTATTAAA CCTCAAGACC TTGTCTTAAT CCGTACAAAC TCTTCGTAAG ATGTTCTCAA   
  
  
- CCGTATCAAA AACGAGATTT TTGTTCTTTC AAATGAAAGA TTTTCCGAAT TAGAAGTTAC GTCACAAGAA   
  
  
- AAGGTAAAAA GACCCTAATT AACTGCTCAG GCGCATGGGA GAAAAGGTCA ATGAAGAAGC TCTTCGGGTA   
  
  
- AACGATTTCA TCTTACCGAA AAAAGGTACT TCTATATAAA AAGAACGATT CCATCAGGGA CTTCGATCAT   
  
  
- CTAAATGACG AATTCGAACG GACAACTTGC CGAAAAACGG TTTGGTACAA CTGGCTTTGC CCCTCGGACT   
  
  
- GAACAACTCC TAACTGTAGT TTAGCTTCGA CACATCGGGA TTCATGCACC AACACAACCA GAATCTCTGG   
  
  
- GTCCCTATGT GAGAAACAAA TTAAAAGGGT TTAATAAAAA AAAGAACAAA TGAAAGAATT AATGCACGAT   
  
  
- TTAATACCTA ATATGTTACC CATCGTTATA TGTAATACAG TAACGGATAA AAAAATTAAA ACACCACCTA   
  
  
- GCAAAAAAAG TCCAAACTTC ATCGACGTAC TATAACATAG TCATGTCGGC TATATGAGAC TGTACACTTA   
  
  
- AGAAGTCAAA GACTATCTTT TATTCAAGT

+     EIRE

| Site Name | Organism | Position | Strand | Matrix score. | sequence | function |
| --- | --- | --- | --- | --- | --- | --- |
| EIRE | Nicotiana tabacum | 422 | - | 7 | TTCGACC | elicitor-responsive element |

> 2018/04/13 10:10:12  
+ TTATAATATC GTTTTCTTAT TTTGCACACG AAATTTAAAA TAAAAAGTAA AATGGAAAAA TGAAAAAACA   
  
  
+ TCTGCGGCGG GACCCACTGT CCGGCTTGTA CTCGCCACAG GGATGCCATC ACAGAGGGAG ATCAGAGATT   
  
  
+ CTTCACTGCT TCTCACCGCA CAGAAATCAT TTCTCCTACT CCTCTCTGCT CTTCCCCCAC TCTCTCGACT   
  
  
+ CTGATTATGG TTTAGACTTT TGGACCTCAA CCTTTTCTCT CCCTCTCTTG CTCGGCTTCG CGGTAATTTT   
  
  
+ CTGAAAACCG GACTTCGGGG GTTTATCTTG CTGGTTCGAA TTTCGAGGAC GACGGGATAC GGTCCAGATC   
  
  
+ GAGTCAGAAG TCAACGGCTC CACGTGACGG CGGAGCACTT GATTGGACTT GCTAGGGTTT TTGTGGCCCG   
  
  
+ AGGTCGAAAT TAGAGGCTTG CTGCTCGTGC AGGTAATTCT TTAATAGTTT TGGAGATATT TCTAGCTTAC   
  
  
+ GTGTCAGTTG GACGGAGTTT ATACTTTCTA TATCAAATTT TTGTGGAAAT CGGAGCTCCT AATTGGCTTT   
  
  
+ GGCCTGTTTG TTTCCCGAGA AAATGTAGGA AAAATAATGA AATTTGGAAT TTAATAGCTG TCTAGTGTAA   
  
  
+ ACCCTCCACC ATAATGATCC TAGTACTCCT TTGTCTGTGC AAATTGGATT TTTATTTTTA ATTGATTGGA   
  
  
+ ATTAAAAAAA TAAAGAATCT CAGTGCGAAT GATAGTTAGC TTATGTTTTG ATTTGTCATC GAATCATAGA   
  
  
+ ACTTCATTCA CTTCCTTTTT GTATGGTAAT TAATTTTGGG GCGAAATATC AAGGGAAAAA GAGGACCTAA   
  
  
+ AGGTTTGAAT AGGATAATTT GGAGTTCTGG AACAGAATTA GGCATGTTTG AGAAGCATTC TACAAGAGTT   
  
  
+ GGCATAGTTT TTGCTCTAAA AACAAGAAAG TTTACTTTCT AAAAGGCTTA ATCTTCAATG CAGTGTTCTT   
  
  
+ TTCCATTTTT CTGGGATTAA TTGACGAGTC CGCGTACCCT CTTTTCCAGT TACTTCTTCG AGAAGCCCAT   
  
  
+ TTGCTAAAGT AGAATGGCTT TTTTCCATGA AGATATATTT TTCTTGCTAA GGTAGTCCCT GAAGCTAGTA   
  
  
+ GATTTACTGC TTAAGCTTGC CTGTTGAACG GCTTTTTGCC AAACCATGTT GACCGAAACG GGGAGCCTGA   
  
  
+ CTTGTTGAGG ATTGACATCA AATCGAAGCT GTGTAGCCCT AAGTACGTGG TTGTGTTGGT CTTAGAGACC   
  
  
+ CAGGGATACA CTCTTTGTTT AATTTTCCCA AATTATTTTT TTTCTTGTTT ACTTTCTTAA TTACGTGCTA   
  
  
+ AATTATGGAT TATACAATGG GTAGCAATAT ACATTATGTC ATTGCCTATT TTTTTAATTT TGTGGTGGAT   
  
  
+ CGTTTTTTTC AGGTTTGAAG TAGCTGCATG ATATTGTATC AGTACAGCCG ATATACTCTG ACATGTGAAT   
  
  
+ TCTTCAGTTT CTGATAGAAA ATAAGTTCA  

- AATATTATAG CAAAAGAATA AAACGTGTGC TTTAAATTTT ATTTTTCATT TTACCTTTTT ACTTTTTTGT   
  
  
- AGACGCCGCC CTGGGTGACA GGCCGAACAT GAGCGGTGTC CCTACGGTAG TGTCTCCCTC TAGTCTCTAA   
  
  
- GAAGTGACGA AGAGTGGCGT GTCTTTAGTA AAGAGGATGA GGAGAGACGA GAAGGGGGTG AGAGAGCTGA   
  
  
- GACTAATACC AAATCTGAAA ACCTGGAGTT GGAAAAGAGA GGGAGAGAAC GAGCCGAAGC GCCATTAAAA   
  
  
- GACTTTTGGC CTGAAGCCCC CAAATAGAAC GACCAAGCTT AAAGCTCCTG CTGCCCTATG CCAGGTCTAG   
  
  
- CTCAGTCTTC AGTTGCCGAG GTGCACTGCC GCCTCGTGAA CTAACCTGAA CGATCCCAAA AACACCGGGC   
  
  
- TCCAGCTTTA ATCTCCGAAC GACGAGCACG TCCATTAAGA AATTATCAAA ACCTCTATAA AGATCGAATG   
  
  
- CACAGTCAAC CTGCCTCAAA TATGAAAGAT ATAGTTTAAA AACACCTTTA GCCTCGAGGA TTAACCGAAA   
  
  
- CCGGACAAAC AAAGGGCTCT TTTACATCCT TTTTATTACT TTAAACCTTA AATTATCGAC AGATCACATT   
  
  
- TGGGAGGTGG TATTACTAGG ATCATGAGGA AACAGACACG TTTAACCTAA AAATAAAAAT TAACTAACCT   
  
  
- TAATTTTTTT ATTTCTTAGA GTCACGCTTA CTATCAATCG AATACAAAAC TAAACAGTAG CTTAGTATCT   
  
  
- TGAAGTAAGT GAAGGAAAAA CATACCATTA ATTAAAACCC CGCTTTATAG TTCCCTTTTT CTCCTGGATT   
  
  
- TCCAAACTTA TCCTATTAAA CCTCAAGACC TTGTCTTAAT CCGTACAAAC TCTTCGTAAG ATGTTCTCAA   
  
  
- CCGTATCAAA AACGAGATTT TTGTTCTTTC AAATGAAAGA TTTTCCGAAT TAGAAGTTAC GTCACAAGAA   
  
  
- AAGGTAAAAA GACCCTAATT AACTGCTCAG GCGCATGGGA GAAAAGGTCA ATGAAGAAGC TCTTCGGGTA   
  
  
- AACGATTTCA TCTTACCGAA AAAAGGTACT TCTATATAAA AAGAACGATT CCATCAGGGA CTTCGATCAT   
  
  
- CTAAATGACG AATTCGAACG GACAACTTGC CGAAAAACGG TTTGGTACAA CTGGCTTTGC CCCTCGGACT   
  
  
- GAACAACTCC TAACTGTAGT TTAGCTTCGA CACATCGGGA TTCATGCACC AACACAACCA GAATCTCTGG   
  
  
- GTCCCTATGT GAGAAACAAA TTAAAAGGGT TTAATAAAAA AAAGAACAAA TGAAAGAATT AATGCACGAT   
  
  
- TTAATACCTA ATATGTTACC CATCGTTATA TGTAATACAG TAACGGATAA AAAAATTAAA ACACCACCTA   
  
  
- GCAAAAAAAG TCCAAACTTC ATCGACGTAC TATAACATAG TCATGTCGGC TATATGAGAC TGTACACTTA   
  
  
- AGAAGTCAAA GACTATCTTT TATTCAAGT

+     G-Box

| Site Name | Organism | Position | Strand | Matrix score. | sequence | function |
| --- | --- | --- | --- | --- | --- | --- |
| G-Box | Pisum sativum | 371 | + | 6 | CACGTG | cis-acting regulatory element involved in light responsiveness |
| G-Box | Antirrhinum majus | 1322 | - | 6 | CACGTA | cis-acting regulatory element involved in light responsiveness |
| G-Box | Antirrhinum majus | 488 | - | 6 | CACGTA | cis-acting regulatory element involved in light responsiveness |
| G-Box | Antirrhinum majus | 1234 | - | 6 | CACGTA | cis-acting regulatory element involved in light responsiveness |

> 2018/04/13 10:10:12  
+ TTATAATATC GTTTTCTTAT TTTGCACACG AAATTTAAAA TAAAAAGTAA AATGGAAAAA TGAAAAAACA   
  
  
+ TCTGCGGCGG GACCCACTGT CCGGCTTGTA CTCGCCACAG GGATGCCATC ACAGAGGGAG ATCAGAGATT   
  
  
+ CTTCACTGCT TCTCACCGCA CAGAAATCAT TTCTCCTACT CCTCTCTGCT CTTCCCCCAC TCTCTCGACT   
  
  
+ CTGATTATGG TTTAGACTTT TGGACCTCAA CCTTTTCTCT CCCTCTCTTG CTCGGCTTCG CGGTAATTTT   
  
  
+ CTGAAAACCG GACTTCGGGG GTTTATCTTG CTGGTTCGAA TTTCGAGGAC GACGGGATAC GGTCCAGATC   
  
  
+ GAGTCAGAAG TCAACGGCTC CACGTGACGG CGGAGCACTT GATTGGACTT GCTAGGGTTT TTGTGGCCCG   
  
  
+ AGGTCGAAAT TAGAGGCTTG CTGCTCGTGC AGGTAATTCT TTAATAGTTT TGGAGATATT TCTAGCTTAC   
  
  
+ GTGTCAGTTG GACGGAGTTT ATACTTTCTA TATCAAATTT TTGTGGAAAT CGGAGCTCCT AATTGGCTTT   
  
  
+ GGCCTGTTTG TTTCCCGAGA AAATGTAGGA AAAATAATGA AATTTGGAAT TTAATAGCTG TCTAGTGTAA   
  
  
+ ACCCTCCACC ATAATGATCC TAGTACTCCT TTGTCTGTGC AAATTGGATT TTTATTTTTA ATTGATTGGA   
  
  
+ ATTAAAAAAA TAAAGAATCT CAGTGCGAAT GATAGTTAGC TTATGTTTTG ATTTGTCATC GAATCATAGA   
  
  
+ ACTTCATTCA CTTCCTTTTT GTATGGTAAT TAATTTTGGG GCGAAATATC AAGGGAAAAA GAGGACCTAA   
  
  
+ AGGTTTGAAT AGGATAATTT GGAGTTCTGG AACAGAATTA GGCATGTTTG AGAAGCATTC TACAAGAGTT   
  
  
+ GGCATAGTTT TTGCTCTAAA AACAAGAAAG TTTACTTTCT AAAAGGCTTA ATCTTCAATG CAGTGTTCTT   
  
  
+ TTCCATTTTT CTGGGATTAA TTGACGAGTC CGCGTACCCT CTTTTCCAGT TACTTCTTCG AGAAGCCCAT   
  
  
+ TTGCTAAAGT AGAATGGCTT TTTTCCATGA AGATATATTT TTCTTGCTAA GGTAGTCCCT GAAGCTAGTA   
  
  
+ GATTTACTGC TTAAGCTTGC CTGTTGAACG GCTTTTTGCC AAACCATGTT GACCGAAACG GGGAGCCTGA   
  
  
+ CTTGTTGAGG ATTGACATCA AATCGAAGCT GTGTAGCCCT AAGTACGTGG TTGTGTTGGT CTTAGAGACC   
  
  
+ CAGGGATACA CTCTTTGTTT AATTTTCCCA AATTATTTTT TTTCTTGTTT ACTTTCTTAA TTACGTGCTA   
  
  
+ AATTATGGAT TATACAATGG GTAGCAATAT ACATTATGTC ATTGCCTATT TTTTTAATTT TGTGGTGGAT   
  
  
+ CGTTTTTTTC AGGTTTGAAG TAGCTGCATG ATATTGTATC AGTACAGCCG ATATACTCTG ACATGTGAAT   
  
  
+ TCTTCAGTTT CTGATAGAAA ATAAGTTCA  

- AATATTATAG CAAAAGAATA AAACGTGTGC TTTAAATTTT ATTTTTCATT TTACCTTTTT ACTTTTTTGT   
  
  
- AGACGCCGCC CTGGGTGACA GGCCGAACAT GAGCGGTGTC CCTACGGTAG TGTCTCCCTC TAGTCTCTAA   
  
  
- GAAGTGACGA AGAGTGGCGT GTCTTTAGTA AAGAGGATGA GGAGAGACGA GAAGGGGGTG AGAGAGCTGA   
  
  
- GACTAATACC AAATCTGAAA ACCTGGAGTT GGAAAAGAGA GGGAGAGAAC GAGCCGAAGC GCCATTAAAA   
  
  
- GACTTTTGGC CTGAAGCCCC CAAATAGAAC GACCAAGCTT AAAGCTCCTG CTGCCCTATG CCAGGTCTAG   
  
  
- CTCAGTCTTC AGTTGCCGAG GTGCACTGCC GCCTCGTGAA CTAACCTGAA CGATCCCAAA AACACCGGGC   
  
  
- TCCAGCTTTA ATCTCCGAAC GACGAGCACG TCCATTAAGA AATTATCAAA ACCTCTATAA AGATCGAATG   
  
  
- CACAGTCAAC CTGCCTCAAA TATGAAAGAT ATAGTTTAAA AACACCTTTA GCCTCGAGGA TTAACCGAAA   
  
  
- CCGGACAAAC AAAGGGCTCT TTTACATCCT TTTTATTACT TTAAACCTTA AATTATCGAC AGATCACATT   
  
  
- TGGGAGGTGG TATTACTAGG ATCATGAGGA AACAGACACG TTTAACCTAA AAATAAAAAT TAACTAACCT   
  
  
- TAATTTTTTT ATTTCTTAGA GTCACGCTTA CTATCAATCG AATACAAAAC TAAACAGTAG CTTAGTATCT   
  
  
- TGAAGTAAGT GAAGGAAAAA CATACCATTA ATTAAAACCC CGCTTTATAG TTCCCTTTTT CTCCTGGATT   
  
  
- TCCAAACTTA TCCTATTAAA CCTCAAGACC TTGTCTTAAT CCGTACAAAC TCTTCGTAAG ATGTTCTCAA   
  
  
- CCGTATCAAA AACGAGATTT TTGTTCTTTC AAATGAAAGA TTTTCCGAAT TAGAAGTTAC GTCACAAGAA   
  
  
- AAGGTAAAAA GACCCTAATT AACTGCTCAG GCGCATGGGA GAAAAGGTCA ATGAAGAAGC TCTTCGGGTA   
  
  
- AACGATTTCA TCTTACCGAA AAAAGGTACT TCTATATAAA AAGAACGATT CCATCAGGGA CTTCGATCAT   
  
  
- CTAAATGACG AATTCGAACG GACAACTTGC CGAAAAACGG TTTGGTACAA CTGGCTTTGC CCCTCGGACT   
  
  
- GAACAACTCC TAACTGTAGT TTAGCTTCGA CACATCGGGA TTCATGCACC AACACAACCA GAATCTCTGG   
  
  
- GTCCCTATGT GAGAAACAAA TTAAAAGGGT TTAATAAAAA AAAGAACAAA TGAAAGAATT AATGCACGAT   
  
  
- TTAATACCTA ATATGTTACC CATCGTTATA TGTAATACAG TAACGGATAA AAAAATTAAA ACACCACCTA   
  
  
- GCAAAAAAAG TCCAAACTTC ATCGACGTAC TATAACATAG TCATGTCGGC TATATGAGAC TGTACACTTA   
  
  
- AGAAGTCAAA GACTATCTTT TATTCAAGT

+     G-box

| Site Name | Organism | Position | Strand | Matrix score. | sequence | function |
| --- | --- | --- | --- | --- | --- | --- |
| G-box | Daucus carota | 1322 | + | 6 | TACGTG | cis-acting regulatory element involved in light responsiveness |
| G-box | Daucus carota | 1234 | + | 6 | TACGTG | cis-acting regulatory element involved in light responsiveness |
| G-box | Brassica napus | 370 | - | 7 | CACGTGG | cis-acting regulatory element involved in light responsiveness |
| G-box | Oryza sativa | 1233 | + | 7 | GTACGTG | cis-acting regulatory element involved in light responsiveness |
| G-box | Arabidopsis thaliana | 369 | - | 9 | GCCACGTGGA | cis-acting regulatory element involved in light responsiveness |
| G-box | Arabidopsis thaliana | 371 | + | 6 | CACGTG | cis-acting regulatory element involved in light responsiveness |
| G-box | Daucus carota | 488 | + | 6 | TACGTG | cis-acting regulatory element involved in light responsiveness |

> 2018/04/13 10:10:12  
+ TTATAATATC GTTTTCTTAT TTTGCACACG AAATTTAAAA TAAAAAGTAA AATGGAAAAA TGAAAAAACA   
  
  
+ TCTGCGGCGG GACCCACTGT CCGGCTTGTA CTCGCCACAG GGATGCCATC ACAGAGGGAG ATCAGAGATT   
  
  
+ CTTCACTGCT TCTCACCGCA CAGAAATCAT TTCTCCTACT CCTCTCTGCT CTTCCCCCAC TCTCTCGACT   
  
  
+ CTGATTATGG TTTAGACTTT TGGACCTCAA CCTTTTCTCT CCCTCTCTTG CTCGGCTTCG CGGTAATTTT   
  
  
+ CTGAAAACCG GACTTCGGGG GTTTATCTTG CTGGTTCGAA TTTCGAGGAC GACGGGATAC GGTCCAGATC   
  
  
+ GAGTCAGAAG TCAACGGCTC CACGTGACGG CGGAGCACTT GATTGGACTT GCTAGGGTTT TTGTGGCCCG   
  
  
+ AGGTCGAAAT TAGAGGCTTG CTGCTCGTGC AGGTAATTCT TTAATAGTTT TGGAGATATT TCTAGCTTAC   
  
  
+ GTGTCAGTTG GACGGAGTTT ATACTTTCTA TATCAAATTT TTGTGGAAAT CGGAGCTCCT AATTGGCTTT   
  
  
+ GGCCTGTTTG TTTCCCGAGA AAATGTAGGA AAAATAATGA AATTTGGAAT TTAATAGCTG TCTAGTGTAA   
  
  
+ ACCCTCCACC ATAATGATCC TAGTACTCCT TTGTCTGTGC AAATTGGATT TTTATTTTTA ATTGATTGGA   
  
  
+ ATTAAAAAAA TAAAGAATCT CAGTGCGAAT GATAGTTAGC TTATGTTTTG ATTTGTCATC GAATCATAGA   
  
  
+ ACTTCATTCA CTTCCTTTTT GTATGGTAAT TAATTTTGGG GCGAAATATC AAGGGAAAAA GAGGACCTAA   
  
  
+ AGGTTTGAAT AGGATAATTT GGAGTTCTGG AACAGAATTA GGCATGTTTG AGAAGCATTC TACAAGAGTT   
  
  
+ GGCATAGTTT TTGCTCTAAA AACAAGAAAG TTTACTTTCT AAAAGGCTTA ATCTTCAATG CAGTGTTCTT   
  
  
+ TTCCATTTTT CTGGGATTAA TTGACGAGTC CGCGTACCCT CTTTTCCAGT TACTTCTTCG AGAAGCCCAT   
  
  
+ TTGCTAAAGT AGAATGGCTT TTTTCCATGA AGATATATTT TTCTTGCTAA GGTAGTCCCT GAAGCTAGTA   
  
  
+ GATTTACTGC TTAAGCTTGC CTGTTGAACG GCTTTTTGCC AAACCATGTT GACCGAAACG GGGAGCCTGA   
  
  
+ CTTGTTGAGG ATTGACATCA AATCGAAGCT GTGTAGCCCT AAGTACGTGG TTGTGTTGGT CTTAGAGACC   
  
  
+ CAGGGATACA CTCTTTGTTT AATTTTCCCA AATTATTTTT TTTCTTGTTT ACTTTCTTAA TTACGTGCTA   
  
  
+ AATTATGGAT TATACAATGG GTAGCAATAT ACATTATGTC ATTGCCTATT TTTTTAATTT TGTGGTGGAT   
  
  
+ CGTTTTTTTC AGGTTTGAAG TAGCTGCATG ATATTGTATC AGTACAGCCG ATATACTCTG ACATGTGAAT   
  
  
+ TCTTCAGTTT CTGATAGAAA ATAAGTTCA  

- AATATTATAG CAAAAGAATA AAACGTGTGC TTTAAATTTT ATTTTTCATT TTACCTTTTT ACTTTTTTGT   
  
  
- AGACGCCGCC CTGGGTGACA GGCCGAACAT GAGCGGTGTC CCTACGGTAG TGTCTCCCTC TAGTCTCTAA   
  
  
- GAAGTGACGA AGAGTGGCGT GTCTTTAGTA AAGAGGATGA GGAGAGACGA GAAGGGGGTG AGAGAGCTGA   
  
  
- GACTAATACC AAATCTGAAA ACCTGGAGTT GGAAAAGAGA GGGAGAGAAC GAGCCGAAGC GCCATTAAAA   
  
  
- GACTTTTGGC CTGAAGCCCC CAAATAGAAC GACCAAGCTT AAAGCTCCTG CTGCCCTATG CCAGGTCTAG   
  
  
- CTCAGTCTTC AGTTGCCGAG GTGCACTGCC GCCTCGTGAA CTAACCTGAA CGATCCCAAA AACACCGGGC   
  
  
- TCCAGCTTTA ATCTCCGAAC GACGAGCACG TCCATTAAGA AATTATCAAA ACCTCTATAA AGATCGAATG   
  
  
- CACAGTCAAC CTGCCTCAAA TATGAAAGAT ATAGTTTAAA AACACCTTTA GCCTCGAGGA TTAACCGAAA   
  
  
- CCGGACAAAC AAAGGGCTCT TTTACATCCT TTTTATTACT TTAAACCTTA AATTATCGAC AGATCACATT   
  
  
- TGGGAGGTGG TATTACTAGG ATCATGAGGA AACAGACACG TTTAACCTAA AAATAAAAAT TAACTAACCT   
  
  
- TAATTTTTTT ATTTCTTAGA GTCACGCTTA CTATCAATCG AATACAAAAC TAAACAGTAG CTTAGTATCT   
  
  
- TGAAGTAAGT GAAGGAAAAA CATACCATTA ATTAAAACCC CGCTTTATAG TTCCCTTTTT CTCCTGGATT   
  
  
- TCCAAACTTA TCCTATTAAA CCTCAAGACC TTGTCTTAAT CCGTACAAAC TCTTCGTAAG ATGTTCTCAA   
  
  
- CCGTATCAAA AACGAGATTT TTGTTCTTTC AAATGAAAGA TTTTCCGAAT TAGAAGTTAC GTCACAAGAA   
  
  
- AAGGTAAAAA GACCCTAATT AACTGCTCAG GCGCATGGGA GAAAAGGTCA ATGAAGAAGC TCTTCGGGTA   
  
  
- AACGATTTCA TCTTACCGAA AAAAGGTACT TCTATATAAA AAGAACGATT CCATCAGGGA CTTCGATCAT   
  
  
- CTAAATGACG AATTCGAACG GACAACTTGC CGAAAAACGG TTTGGTACAA CTGGCTTTGC CCCTCGGACT   
  
  
- GAACAACTCC TAACTGTAGT TTAGCTTCGA CACATCGGGA TTCATGCACC AACACAACCA GAATCTCTGG   
  
  
- GTCCCTATGT GAGAAACAAA TTAAAAGGGT TTAATAAAAA AAAGAACAAA TGAAAGAATT AATGCACGAT   
  
  
- TTAATACCTA ATATGTTACC CATCGTTATA TGTAATACAG TAACGGATAA AAAAATTAAA ACACCACCTA   
  
  
- GCAAAAAAAG TCCAAACTTC ATCGACGTAC TATAACATAG TCATGTCGGC TATATGAGAC TGTACACTTA   
  
  
- AGAAGTCAAA GACTATCTTT TATTCAAGT

+     GAG-motif

| Site Name | Organism | Position | Strand | Matrix score. | sequence | function |
| --- | --- | --- | --- | --- | --- | --- |
| GAG-motif | Arabidopsis thaliana | 199 | - | 7 | AGAGAGT | part of a light responsive element |

> 2018/04/13 10:10:12  
+ TTATAATATC GTTTTCTTAT TTTGCACACG AAATTTAAAA TAAAAAGTAA AATGGAAAAA TGAAAAAACA   
  
  
+ TCTGCGGCGG GACCCACTGT CCGGCTTGTA CTCGCCACAG GGATGCCATC ACAGAGGGAG ATCAGAGATT   
  
  
+ CTTCACTGCT TCTCACCGCA CAGAAATCAT TTCTCCTACT CCTCTCTGCT CTTCCCCCAC TCTCTCGACT   
  
  
+ CTGATTATGG TTTAGACTTT TGGACCTCAA CCTTTTCTCT CCCTCTCTTG CTCGGCTTCG CGGTAATTTT   
  
  
+ CTGAAAACCG GACTTCGGGG GTTTATCTTG CTGGTTCGAA TTTCGAGGAC GACGGGATAC GGTCCAGATC   
  
  
+ GAGTCAGAAG TCAACGGCTC CACGTGACGG CGGAGCACTT GATTGGACTT GCTAGGGTTT TTGTGGCCCG   
  
  
+ AGGTCGAAAT TAGAGGCTTG CTGCTCGTGC AGGTAATTCT TTAATAGTTT TGGAGATATT TCTAGCTTAC   
  
  
+ GTGTCAGTTG GACGGAGTTT ATACTTTCTA TATCAAATTT TTGTGGAAAT CGGAGCTCCT AATTGGCTTT   
  
  
+ GGCCTGTTTG TTTCCCGAGA AAATGTAGGA AAAATAATGA AATTTGGAAT TTAATAGCTG TCTAGTGTAA   
  
  
+ ACCCTCCACC ATAATGATCC TAGTACTCCT TTGTCTGTGC AAATTGGATT TTTATTTTTA ATTGATTGGA   
  
  
+ ATTAAAAAAA TAAAGAATCT CAGTGCGAAT GATAGTTAGC TTATGTTTTG ATTTGTCATC GAATCATAGA   
  
  
+ ACTTCATTCA CTTCCTTTTT GTATGGTAAT TAATTTTGGG GCGAAATATC AAGGGAAAAA GAGGACCTAA   
  
  
+ AGGTTTGAAT AGGATAATTT GGAGTTCTGG AACAGAATTA GGCATGTTTG AGAAGCATTC TACAAGAGTT   
  
  
+ GGCATAGTTT TTGCTCTAAA AACAAGAAAG TTTACTTTCT AAAAGGCTTA ATCTTCAATG CAGTGTTCTT   
  
  
+ TTCCATTTTT CTGGGATTAA TTGACGAGTC CGCGTACCCT CTTTTCCAGT TACTTCTTCG AGAAGCCCAT   
  
  
+ TTGCTAAAGT AGAATGGCTT TTTTCCATGA AGATATATTT TTCTTGCTAA GGTAGTCCCT GAAGCTAGTA   
  
  
+ GATTTACTGC TTAAGCTTGC CTGTTGAACG GCTTTTTGCC AAACCATGTT GACCGAAACG GGGAGCCTGA   
  
  
+ CTTGTTGAGG ATTGACATCA AATCGAAGCT GTGTAGCCCT AAGTACGTGG TTGTGTTGGT CTTAGAGACC   
  
  
+ CAGGGATACA CTCTTTGTTT AATTTTCCCA AATTATTTTT TTTCTTGTTT ACTTTCTTAA TTACGTGCTA   
  
  
+ AATTATGGAT TATACAATGG GTAGCAATAT ACATTATGTC ATTGCCTATT TTTTTAATTT TGTGGTGGAT   
  
  
+ CGTTTTTTTC AGGTTTGAAG TAGCTGCATG ATATTGTATC AGTACAGCCG ATATACTCTG ACATGTGAAT   
  
  
+ TCTTCAGTTT CTGATAGAAA ATAAGTTCA  

- AATATTATAG CAAAAGAATA AAACGTGTGC TTTAAATTTT ATTTTTCATT TTACCTTTTT ACTTTTTTGT   
  
  
- AGACGCCGCC CTGGGTGACA GGCCGAACAT GAGCGGTGTC CCTACGGTAG TGTCTCCCTC TAGTCTCTAA   
  
  
- GAAGTGACGA AGAGTGGCGT GTCTTTAGTA AAGAGGATGA GGAGAGACGA GAAGGGGGTG AGAGAGCTGA   
  
  
- GACTAATACC AAATCTGAAA ACCTGGAGTT GGAAAAGAGA GGGAGAGAAC GAGCCGAAGC GCCATTAAAA   
  
  
- GACTTTTGGC CTGAAGCCCC CAAATAGAAC GACCAAGCTT AAAGCTCCTG CTGCCCTATG CCAGGTCTAG   
  
  
- CTCAGTCTTC AGTTGCCGAG GTGCACTGCC GCCTCGTGAA CTAACCTGAA CGATCCCAAA AACACCGGGC   
  
  
- TCCAGCTTTA ATCTCCGAAC GACGAGCACG TCCATTAAGA AATTATCAAA ACCTCTATAA AGATCGAATG   
  
  
- CACAGTCAAC CTGCCTCAAA TATGAAAGAT ATAGTTTAAA AACACCTTTA GCCTCGAGGA TTAACCGAAA   
  
  
- CCGGACAAAC AAAGGGCTCT TTTACATCCT TTTTATTACT TTAAACCTTA AATTATCGAC AGATCACATT   
  
  
- TGGGAGGTGG TATTACTAGG ATCATGAGGA AACAGACACG TTTAACCTAA AAATAAAAAT TAACTAACCT   
  
  
- TAATTTTTTT ATTTCTTAGA GTCACGCTTA CTATCAATCG AATACAAAAC TAAACAGTAG CTTAGTATCT   
  
  
- TGAAGTAAGT GAAGGAAAAA CATACCATTA ATTAAAACCC CGCTTTATAG TTCCCTTTTT CTCCTGGATT   
  
  
- TCCAAACTTA TCCTATTAAA CCTCAAGACC TTGTCTTAAT CCGTACAAAC TCTTCGTAAG ATGTTCTCAA   
  
  
- CCGTATCAAA AACGAGATTT TTGTTCTTTC AAATGAAAGA TTTTCCGAAT TAGAAGTTAC GTCACAAGAA   
  
  
- AAGGTAAAAA GACCCTAATT AACTGCTCAG GCGCATGGGA GAAAAGGTCA ATGAAGAAGC TCTTCGGGTA   
  
  
- AACGATTTCA TCTTACCGAA AAAAGGTACT TCTATATAAA AAGAACGATT CCATCAGGGA CTTCGATCAT   
  
  
- CTAAATGACG AATTCGAACG GACAACTTGC CGAAAAACGG TTTGGTACAA CTGGCTTTGC CCCTCGGACT   
  
  
- GAACAACTCC TAACTGTAGT TTAGCTTCGA CACATCGGGA TTCATGCACC AACACAACCA GAATCTCTGG   
  
  
- GTCCCTATGT GAGAAACAAA TTAAAAGGGT TTAATAAAAA AAAGAACAAA TGAAAGAATT AATGCACGAT   
  
  
- TTAATACCTA ATATGTTACC CATCGTTATA TGTAATACAG TAACGGATAA AAAAATTAAA ACACCACCTA   
  
  
- GCAAAAAAAG TCCAAACTTC ATCGACGTAC TATAACATAG TCATGTCGGC TATATGAGAC TGTACACTTA   
  
  
- AGAAGTCAAA GACTATCTTT TATTCAAGT

+     GC-motif

| Site Name | Organism | Position | Strand | Matrix score. | sequence | function |
| --- | --- | --- | --- | --- | --- | --- |
| GC-motif | Zea mays | 296 | - | 6 | CCCCCG | enhancer-like element involved in anoxic specific inducibility |

> 2018/04/13 10:10:12  
+ TTATAATATC GTTTTCTTAT TTTGCACACG AAATTTAAAA TAAAAAGTAA AATGGAAAAA TGAAAAAACA   
  
  
+ TCTGCGGCGG GACCCACTGT CCGGCTTGTA CTCGCCACAG GGATGCCATC ACAGAGGGAG ATCAGAGATT   
  
  
+ CTTCACTGCT TCTCACCGCA CAGAAATCAT TTCTCCTACT CCTCTCTGCT CTTCCCCCAC TCTCTCGACT   
  
  
+ CTGATTATGG TTTAGACTTT TGGACCTCAA CCTTTTCTCT CCCTCTCTTG CTCGGCTTCG CGGTAATTTT   
  
  
+ CTGAAAACCG GACTTCGGGG GTTTATCTTG CTGGTTCGAA TTTCGAGGAC GACGGGATAC GGTCCAGATC   
  
  
+ GAGTCAGAAG TCAACGGCTC CACGTGACGG CGGAGCACTT GATTGGACTT GCTAGGGTTT TTGTGGCCCG   
  
  
+ AGGTCGAAAT TAGAGGCTTG CTGCTCGTGC AGGTAATTCT TTAATAGTTT TGGAGATATT TCTAGCTTAC   
  
  
+ GTGTCAGTTG GACGGAGTTT ATACTTTCTA TATCAAATTT TTGTGGAAAT CGGAGCTCCT AATTGGCTTT   
  
  
+ GGCCTGTTTG TTTCCCGAGA AAATGTAGGA AAAATAATGA AATTTGGAAT TTAATAGCTG TCTAGTGTAA   
  
  
+ ACCCTCCACC ATAATGATCC TAGTACTCCT TTGTCTGTGC AAATTGGATT TTTATTTTTA ATTGATTGGA   
  
  
+ ATTAAAAAAA TAAAGAATCT CAGTGCGAAT GATAGTTAGC TTATGTTTTG ATTTGTCATC GAATCATAGA   
  
  
+ ACTTCATTCA CTTCCTTTTT GTATGGTAAT TAATTTTGGG GCGAAATATC AAGGGAAAAA GAGGACCTAA   
  
  
+ AGGTTTGAAT AGGATAATTT GGAGTTCTGG AACAGAATTA GGCATGTTTG AGAAGCATTC TACAAGAGTT   
  
  
+ GGCATAGTTT TTGCTCTAAA AACAAGAAAG TTTACTTTCT AAAAGGCTTA ATCTTCAATG CAGTGTTCTT   
  
  
+ TTCCATTTTT CTGGGATTAA TTGACGAGTC CGCGTACCCT CTTTTCCAGT TACTTCTTCG AGAAGCCCAT   
  
  
+ TTGCTAAAGT AGAATGGCTT TTTTCCATGA AGATATATTT TTCTTGCTAA GGTAGTCCCT GAAGCTAGTA   
  
  
+ GATTTACTGC TTAAGCTTGC CTGTTGAACG GCTTTTTGCC AAACCATGTT GACCGAAACG GGGAGCCTGA   
  
  
+ CTTGTTGAGG ATTGACATCA AATCGAAGCT GTGTAGCCCT AAGTACGTGG TTGTGTTGGT CTTAGAGACC   
  
  
+ CAGGGATACA CTCTTTGTTT AATTTTCCCA AATTATTTTT TTTCTTGTTT ACTTTCTTAA TTACGTGCTA   
  
  
+ AATTATGGAT TATACAATGG GTAGCAATAT ACATTATGTC ATTGCCTATT TTTTTAATTT TGTGGTGGAT   
  
  
+ CGTTTTTTTC AGGTTTGAAG TAGCTGCATG ATATTGTATC AGTACAGCCG ATATACTCTG ACATGTGAAT   
  
  
+ TCTTCAGTTT CTGATAGAAA ATAAGTTCA  

- AATATTATAG CAAAAGAATA AAACGTGTGC TTTAAATTTT ATTTTTCATT TTACCTTTTT ACTTTTTTGT   
  
  
- AGACGCCGCC CTGGGTGACA GGCCGAACAT GAGCGGTGTC CCTACGGTAG TGTCTCCCTC TAGTCTCTAA   
  
  
- GAAGTGACGA AGAGTGGCGT GTCTTTAGTA AAGAGGATGA GGAGAGACGA GAAGGGGGTG AGAGAGCTGA   
  
  
- GACTAATACC AAATCTGAAA ACCTGGAGTT GGAAAAGAGA GGGAGAGAAC GAGCCGAAGC GCCATTAAAA   
  
  
- GACTTTTGGC CTGAAGCCCC CAAATAGAAC GACCAAGCTT AAAGCTCCTG CTGCCCTATG CCAGGTCTAG   
  
  
- CTCAGTCTTC AGTTGCCGAG GTGCACTGCC GCCTCGTGAA CTAACCTGAA CGATCCCAAA AACACCGGGC   
  
  
- TCCAGCTTTA ATCTCCGAAC GACGAGCACG TCCATTAAGA AATTATCAAA ACCTCTATAA AGATCGAATG   
  
  
- CACAGTCAAC CTGCCTCAAA TATGAAAGAT ATAGTTTAAA AACACCTTTA GCCTCGAGGA TTAACCGAAA   
  
  
- CCGGACAAAC AAAGGGCTCT TTTACATCCT TTTTATTACT TTAAACCTTA AATTATCGAC AGATCACATT   
  
  
- TGGGAGGTGG TATTACTAGG ATCATGAGGA AACAGACACG TTTAACCTAA AAATAAAAAT TAACTAACCT   
  
  
- TAATTTTTTT ATTTCTTAGA GTCACGCTTA CTATCAATCG AATACAAAAC TAAACAGTAG CTTAGTATCT   
  
  
- TGAAGTAAGT GAAGGAAAAA CATACCATTA ATTAAAACCC CGCTTTATAG TTCCCTTTTT CTCCTGGATT   
  
  
- TCCAAACTTA TCCTATTAAA CCTCAAGACC TTGTCTTAAT CCGTACAAAC TCTTCGTAAG ATGTTCTCAA   
  
  
- CCGTATCAAA AACGAGATTT TTGTTCTTTC AAATGAAAGA TTTTCCGAAT TAGAAGTTAC GTCACAAGAA   
  
  
- AAGGTAAAAA GACCCTAATT AACTGCTCAG GCGCATGGGA GAAAAGGTCA ATGAAGAAGC TCTTCGGGTA   
  
  
- AACGATTTCA TCTTACCGAA AAAAGGTACT TCTATATAAA AAGAACGATT CCATCAGGGA CTTCGATCAT   
  
  
- CTAAATGACG AATTCGAACG GACAACTTGC CGAAAAACGG TTTGGTACAA CTGGCTTTGC CCCTCGGACT   
  
  
- GAACAACTCC TAACTGTAGT TTAGCTTCGA CACATCGGGA TTCATGCACC AACACAACCA GAATCTCTGG   
  
  
- GTCCCTATGT GAGAAACAAA TTAAAAGGGT TTAATAAAAA AAAGAACAAA TGAAAGAATT AATGCACGAT   
  
  
- TTAATACCTA ATATGTTACC CATCGTTATA TGTAATACAG TAACGGATAA AAAAATTAAA ACACCACCTA   
  
  
- GCAAAAAAAG TCCAAACTTC ATCGACGTAC TATAACATAG TCATGTCGGC TATATGAGAC TGTACACTTA   
  
  
- AGAAGTCAAA GACTATCTTT TATTCAAGT

+     GT1-motif

| Site Name | Organism | Position | Strand | Matrix score. | sequence | function |
| --- | --- | --- | --- | --- | --- | --- |
| GT1-motif | Oryza sativa | 270 | + | 9 | GCGGTAATT | light responsive element |

> 2018/04/13 10:10:12  
+ TTATAATATC GTTTTCTTAT TTTGCACACG AAATTTAAAA TAAAAAGTAA AATGGAAAAA TGAAAAAACA   
  
  
+ TCTGCGGCGG GACCCACTGT CCGGCTTGTA CTCGCCACAG GGATGCCATC ACAGAGGGAG ATCAGAGATT   
  
  
+ CTTCACTGCT TCTCACCGCA CAGAAATCAT TTCTCCTACT CCTCTCTGCT CTTCCCCCAC TCTCTCGACT   
  
  
+ CTGATTATGG TTTAGACTTT TGGACCTCAA CCTTTTCTCT CCCTCTCTTG CTCGGCTTCG CGGTAATTTT   
  
  
+ CTGAAAACCG GACTTCGGGG GTTTATCTTG CTGGTTCGAA TTTCGAGGAC GACGGGATAC GGTCCAGATC   
  
  
+ GAGTCAGAAG TCAACGGCTC CACGTGACGG CGGAGCACTT GATTGGACTT GCTAGGGTTT TTGTGGCCCG   
  
  
+ AGGTCGAAAT TAGAGGCTTG CTGCTCGTGC AGGTAATTCT TTAATAGTTT TGGAGATATT TCTAGCTTAC   
  
  
+ GTGTCAGTTG GACGGAGTTT ATACTTTCTA TATCAAATTT TTGTGGAAAT CGGAGCTCCT AATTGGCTTT   
  
  
+ GGCCTGTTTG TTTCCCGAGA AAATGTAGGA AAAATAATGA AATTTGGAAT TTAATAGCTG TCTAGTGTAA   
  
  
+ ACCCTCCACC ATAATGATCC TAGTACTCCT TTGTCTGTGC AAATTGGATT TTTATTTTTA ATTGATTGGA   
  
  
+ ATTAAAAAAA TAAAGAATCT CAGTGCGAAT GATAGTTAGC TTATGTTTTG ATTTGTCATC GAATCATAGA   
  
  
+ ACTTCATTCA CTTCCTTTTT GTATGGTAAT TAATTTTGGG GCGAAATATC AAGGGAAAAA GAGGACCTAA   
  
  
+ AGGTTTGAAT AGGATAATTT GGAGTTCTGG AACAGAATTA GGCATGTTTG AGAAGCATTC TACAAGAGTT   
  
  
+ GGCATAGTTT TTGCTCTAAA AACAAGAAAG TTTACTTTCT AAAAGGCTTA ATCTTCAATG CAGTGTTCTT   
  
  
+ TTCCATTTTT CTGGGATTAA TTGACGAGTC CGCGTACCCT CTTTTCCAGT TACTTCTTCG AGAAGCCCAT   
  
  
+ TTGCTAAAGT AGAATGGCTT TTTTCCATGA AGATATATTT TTCTTGCTAA GGTAGTCCCT GAAGCTAGTA   
  
  
+ GATTTACTGC TTAAGCTTGC CTGTTGAACG GCTTTTTGCC AAACCATGTT GACCGAAACG GGGAGCCTGA   
  
  
+ CTTGTTGAGG ATTGACATCA AATCGAAGCT GTGTAGCCCT AAGTACGTGG TTGTGTTGGT CTTAGAGACC   
  
  
+ CAGGGATACA CTCTTTGTTT AATTTTCCCA AATTATTTTT TTTCTTGTTT ACTTTCTTAA TTACGTGCTA   
  
  
+ AATTATGGAT TATACAATGG GTAGCAATAT ACATTATGTC ATTGCCTATT TTTTTAATTT TGTGGTGGAT   
  
  
+ CGTTTTTTTC AGGTTTGAAG TAGCTGCATG ATATTGTATC AGTACAGCCG ATATACTCTG ACATGTGAAT   
  
  
+ TCTTCAGTTT CTGATAGAAA ATAAGTTCA  

- AATATTATAG CAAAAGAATA AAACGTGTGC TTTAAATTTT ATTTTTCATT TTACCTTTTT ACTTTTTTGT   
  
  
- AGACGCCGCC CTGGGTGACA GGCCGAACAT GAGCGGTGTC CCTACGGTAG TGTCTCCCTC TAGTCTCTAA   
  
  
- GAAGTGACGA AGAGTGGCGT GTCTTTAGTA AAGAGGATGA GGAGAGACGA GAAGGGGGTG AGAGAGCTGA   
  
  
- GACTAATACC AAATCTGAAA ACCTGGAGTT GGAAAAGAGA GGGAGAGAAC GAGCCGAAGC GCCATTAAAA   
  
  
- GACTTTTGGC CTGAAGCCCC CAAATAGAAC GACCAAGCTT AAAGCTCCTG CTGCCCTATG CCAGGTCTAG   
  
  
- CTCAGTCTTC AGTTGCCGAG GTGCACTGCC GCCTCGTGAA CTAACCTGAA CGATCCCAAA AACACCGGGC   
  
  
- TCCAGCTTTA ATCTCCGAAC GACGAGCACG TCCATTAAGA AATTATCAAA ACCTCTATAA AGATCGAATG   
  
  
- CACAGTCAAC CTGCCTCAAA TATGAAAGAT ATAGTTTAAA AACACCTTTA GCCTCGAGGA TTAACCGAAA   
  
  
- CCGGACAAAC AAAGGGCTCT TTTACATCCT TTTTATTACT TTAAACCTTA AATTATCGAC AGATCACATT   
  
  
- TGGGAGGTGG TATTACTAGG ATCATGAGGA AACAGACACG TTTAACCTAA AAATAAAAAT TAACTAACCT   
  
  
- TAATTTTTTT ATTTCTTAGA GTCACGCTTA CTATCAATCG AATACAAAAC TAAACAGTAG CTTAGTATCT   
  
  
- TGAAGTAAGT GAAGGAAAAA CATACCATTA ATTAAAACCC CGCTTTATAG TTCCCTTTTT CTCCTGGATT   
  
  
- TCCAAACTTA TCCTATTAAA CCTCAAGACC TTGTCTTAAT CCGTACAAAC TCTTCGTAAG ATGTTCTCAA   
  
  
- CCGTATCAAA AACGAGATTT TTGTTCTTTC AAATGAAAGA TTTTCCGAAT TAGAAGTTAC GTCACAAGAA   
  
  
- AAGGTAAAAA GACCCTAATT AACTGCTCAG GCGCATGGGA GAAAAGGTCA ATGAAGAAGC TCTTCGGGTA   
  
  
- AACGATTTCA TCTTACCGAA AAAAGGTACT TCTATATAAA AAGAACGATT CCATCAGGGA CTTCGATCAT   
  
  
- CTAAATGACG AATTCGAACG GACAACTTGC CGAAAAACGG TTTGGTACAA CTGGCTTTGC CCCTCGGACT   
  
  
- GAACAACTCC TAACTGTAGT TTAGCTTCGA CACATCGGGA TTCATGCACC AACACAACCA GAATCTCTGG   
  
  
- GTCCCTATGT GAGAAACAAA TTAAAAGGGT TTAATAAAAA AAAGAACAAA TGAAAGAATT AATGCACGAT   
  
  
- TTAATACCTA ATATGTTACC CATCGTTATA TGTAATACAG TAACGGATAA AAAAATTAAA ACACCACCTA   
  
  
- GCAAAAAAAG TCCAAACTTC ATCGACGTAC TATAACATAG TCATGTCGGC TATATGAGAC TGTACACTTA   
  
  
- AGAAGTCAAA GACTATCTTT TATTCAAGT

+     LTR

| Site Name | Organism | Position | Strand | Matrix score. | sequence | function |
| --- | --- | --- | --- | --- | --- | --- |
| LTR | Hordeum vulgare | 1173 | + | 6 | CCGAAA | cis-acting element involved in low-temperature responsiveness |

> 2018/04/13 10:10:12  
+ TTATAATATC GTTTTCTTAT TTTGCACACG AAATTTAAAA TAAAAAGTAA AATGGAAAAA TGAAAAAACA   
  
  
+ TCTGCGGCGG GACCCACTGT CCGGCTTGTA CTCGCCACAG GGATGCCATC ACAGAGGGAG ATCAGAGATT   
  
  
+ CTTCACTGCT TCTCACCGCA CAGAAATCAT TTCTCCTACT CCTCTCTGCT CTTCCCCCAC TCTCTCGACT   
  
  
+ CTGATTATGG TTTAGACTTT TGGACCTCAA CCTTTTCTCT CCCTCTCTTG CTCGGCTTCG CGGTAATTTT   
  
  
+ CTGAAAACCG GACTTCGGGG GTTTATCTTG CTGGTTCGAA TTTCGAGGAC GACGGGATAC GGTCCAGATC   
  
  
+ GAGTCAGAAG TCAACGGCTC CACGTGACGG CGGAGCACTT GATTGGACTT GCTAGGGTTT TTGTGGCCCG   
  
  
+ AGGTCGAAAT TAGAGGCTTG CTGCTCGTGC AGGTAATTCT TTAATAGTTT TGGAGATATT TCTAGCTTAC   
  
  
+ GTGTCAGTTG GACGGAGTTT ATACTTTCTA TATCAAATTT TTGTGGAAAT CGGAGCTCCT AATTGGCTTT   
  
  
+ GGCCTGTTTG TTTCCCGAGA AAATGTAGGA AAAATAATGA AATTTGGAAT TTAATAGCTG TCTAGTGTAA   
  
  
+ ACCCTCCACC ATAATGATCC TAGTACTCCT TTGTCTGTGC AAATTGGATT TTTATTTTTA ATTGATTGGA   
  
  
+ ATTAAAAAAA TAAAGAATCT CAGTGCGAAT GATAGTTAGC TTATGTTTTG ATTTGTCATC GAATCATAGA   
  
  
+ ACTTCATTCA CTTCCTTTTT GTATGGTAAT TAATTTTGGG GCGAAATATC AAGGGAAAAA GAGGACCTAA   
  
  
+ AGGTTTGAAT AGGATAATTT GGAGTTCTGG AACAGAATTA GGCATGTTTG AGAAGCATTC TACAAGAGTT   
  
  
+ GGCATAGTTT TTGCTCTAAA AACAAGAAAG TTTACTTTCT AAAAGGCTTA ATCTTCAATG CAGTGTTCTT   
  
  
+ TTCCATTTTT CTGGGATTAA TTGACGAGTC CGCGTACCCT CTTTTCCAGT TACTTCTTCG AGAAGCCCAT   
  
  
+ TTGCTAAAGT AGAATGGCTT TTTTCCATGA AGATATATTT TTCTTGCTAA GGTAGTCCCT GAAGCTAGTA   
  
  
+ GATTTACTGC TTAAGCTTGC CTGTTGAACG GCTTTTTGCC AAACCATGTT GACCGAAACG GGGAGCCTGA   
  
  
+ CTTGTTGAGG ATTGACATCA AATCGAAGCT GTGTAGCCCT AAGTACGTGG TTGTGTTGGT CTTAGAGACC   
  
  
+ CAGGGATACA CTCTTTGTTT AATTTTCCCA AATTATTTTT TTTCTTGTTT ACTTTCTTAA TTACGTGCTA   
  
  
+ AATTATGGAT TATACAATGG GTAGCAATAT ACATTATGTC ATTGCCTATT TTTTTAATTT TGTGGTGGAT   
  
  
+ CGTTTTTTTC AGGTTTGAAG TAGCTGCATG ATATTGTATC AGTACAGCCG ATATACTCTG ACATGTGAAT   
  
  
+ TCTTCAGTTT CTGATAGAAA ATAAGTTCA  

- AATATTATAG CAAAAGAATA AAACGTGTGC TTTAAATTTT ATTTTTCATT TTACCTTTTT ACTTTTTTGT   
  
  
- AGACGCCGCC CTGGGTGACA GGCCGAACAT GAGCGGTGTC CCTACGGTAG TGTCTCCCTC TAGTCTCTAA   
  
  
- GAAGTGACGA AGAGTGGCGT GTCTTTAGTA AAGAGGATGA GGAGAGACGA GAAGGGGGTG AGAGAGCTGA   
  
  
- GACTAATACC AAATCTGAAA ACCTGGAGTT GGAAAAGAGA GGGAGAGAAC GAGCCGAAGC GCCATTAAAA   
  
  
- GACTTTTGGC CTGAAGCCCC CAAATAGAAC GACCAAGCTT AAAGCTCCTG CTGCCCTATG CCAGGTCTAG   
  
  
- CTCAGTCTTC AGTTGCCGAG GTGCACTGCC GCCTCGTGAA CTAACCTGAA CGATCCCAAA AACACCGGGC   
  
  
- TCCAGCTTTA ATCTCCGAAC GACGAGCACG TCCATTAAGA AATTATCAAA ACCTCTATAA AGATCGAATG   
  
  
- CACAGTCAAC CTGCCTCAAA TATGAAAGAT ATAGTTTAAA AACACCTTTA GCCTCGAGGA TTAACCGAAA   
  
  
- CCGGACAAAC AAAGGGCTCT TTTACATCCT TTTTATTACT TTAAACCTTA AATTATCGAC AGATCACATT   
  
  
- TGGGAGGTGG TATTACTAGG ATCATGAGGA AACAGACACG TTTAACCTAA AAATAAAAAT TAACTAACCT   
  
  
- TAATTTTTTT ATTTCTTAGA GTCACGCTTA CTATCAATCG AATACAAAAC TAAACAGTAG CTTAGTATCT   
  
  
- TGAAGTAAGT GAAGGAAAAA CATACCATTA ATTAAAACCC CGCTTTATAG TTCCCTTTTT CTCCTGGATT   
  
  
- TCCAAACTTA TCCTATTAAA CCTCAAGACC TTGTCTTAAT CCGTACAAAC TCTTCGTAAG ATGTTCTCAA   
  
  
- CCGTATCAAA AACGAGATTT TTGTTCTTTC AAATGAAAGA TTTTCCGAAT TAGAAGTTAC GTCACAAGAA   
  
  
- AAGGTAAAAA GACCCTAATT AACTGCTCAG GCGCATGGGA GAAAAGGTCA ATGAAGAAGC TCTTCGGGTA   
  
  
- AACGATTTCA TCTTACCGAA AAAAGGTACT TCTATATAAA AAGAACGATT CCATCAGGGA CTTCGATCAT   
  
  
- CTAAATGACG AATTCGAACG GACAACTTGC CGAAAAACGG TTTGGTACAA CTGGCTTTGC CCCTCGGACT   
  
  
- GAACAACTCC TAACTGTAGT TTAGCTTCGA CACATCGGGA TTCATGCACC AACACAACCA GAATCTCTGG   
  
  
- GTCCCTATGT GAGAAACAAA TTAAAAGGGT TTAATAAAAA AAAGAACAAA TGAAAGAATT AATGCACGAT   
  
  
- TTAATACCTA ATATGTTACC CATCGTTATA TGTAATACAG TAACGGATAA AAAAATTAAA ACACCACCTA   
  
  
- GCAAAAAAAG TCCAAACTTC ATCGACGTAC TATAACATAG TCATGTCGGC TATATGAGAC TGTACACTTA   
  
  
- AGAAGTCAAA GACTATCTTT TATTCAAGT

+     MBS

| Site Name | Organism | Position | Strand | Matrix score. | sequence | function |
| --- | --- | --- | --- | --- | --- | --- |
| MBS | Arabidopsis thaliana | 1027 | - | 6 | TAACTG | MYB binding site involved in drought-inducibility |
| MBS | Arabidopsis thaliana | 495 | - | 6 | CAACTG | MYB binding site involved in drought-inducibility |
| MBS | Zea mays | 1170 | - | 6 | CGGTCA | MYB Binding Site |

> 2018/04/13 10:10:12  
+ TTATAATATC GTTTTCTTAT TTTGCACACG AAATTTAAAA TAAAAAGTAA AATGGAAAAA TGAAAAAACA   
  
  
+ TCTGCGGCGG GACCCACTGT CCGGCTTGTA CTCGCCACAG GGATGCCATC ACAGAGGGAG ATCAGAGATT   
  
  
+ CTTCACTGCT TCTCACCGCA CAGAAATCAT TTCTCCTACT CCTCTCTGCT CTTCCCCCAC TCTCTCGACT   
  
  
+ CTGATTATGG TTTAGACTTT TGGACCTCAA CCTTTTCTCT CCCTCTCTTG CTCGGCTTCG CGGTAATTTT   
  
  
+ CTGAAAACCG GACTTCGGGG GTTTATCTTG CTGGTTCGAA TTTCGAGGAC GACGGGATAC GGTCCAGATC   
  
  
+ GAGTCAGAAG TCAACGGCTC CACGTGACGG CGGAGCACTT GATTGGACTT GCTAGGGTTT TTGTGGCCCG   
  
  
+ AGGTCGAAAT TAGAGGCTTG CTGCTCGTGC AGGTAATTCT TTAATAGTTT TGGAGATATT TCTAGCTTAC   
  
  
+ GTGTCAGTTG GACGGAGTTT ATACTTTCTA TATCAAATTT TTGTGGAAAT CGGAGCTCCT AATTGGCTTT   
  
  
+ GGCCTGTTTG TTTCCCGAGA AAATGTAGGA AAAATAATGA AATTTGGAAT TTAATAGCTG TCTAGTGTAA   
  
  
+ ACCCTCCACC ATAATGATCC TAGTACTCCT TTGTCTGTGC AAATTGGATT TTTATTTTTA ATTGATTGGA   
  
  
+ ATTAAAAAAA TAAAGAATCT CAGTGCGAAT GATAGTTAGC TTATGTTTTG ATTTGTCATC GAATCATAGA   
  
  
+ ACTTCATTCA CTTCCTTTTT GTATGGTAAT TAATTTTGGG GCGAAATATC AAGGGAAAAA GAGGACCTAA   
  
  
+ AGGTTTGAAT AGGATAATTT GGAGTTCTGG AACAGAATTA GGCATGTTTG AGAAGCATTC TACAAGAGTT   
  
  
+ GGCATAGTTT TTGCTCTAAA AACAAGAAAG TTTACTTTCT AAAAGGCTTA ATCTTCAATG CAGTGTTCTT   
  
  
+ TTCCATTTTT CTGGGATTAA TTGACGAGTC CGCGTACCCT CTTTTCCAGT TACTTCTTCG AGAAGCCCAT   
  
  
+ TTGCTAAAGT AGAATGGCTT TTTTCCATGA AGATATATTT TTCTTGCTAA GGTAGTCCCT GAAGCTAGTA   
  
  
+ GATTTACTGC TTAAGCTTGC CTGTTGAACG GCTTTTTGCC AAACCATGTT GACCGAAACG GGGAGCCTGA   
  
  
+ CTTGTTGAGG ATTGACATCA AATCGAAGCT GTGTAGCCCT AAGTACGTGG TTGTGTTGGT CTTAGAGACC   
  
  
+ CAGGGATACA CTCTTTGTTT AATTTTCCCA AATTATTTTT TTTCTTGTTT ACTTTCTTAA TTACGTGCTA   
  
  
+ AATTATGGAT TATACAATGG GTAGCAATAT ACATTATGTC ATTGCCTATT TTTTTAATTT TGTGGTGGAT   
  
  
+ CGTTTTTTTC AGGTTTGAAG TAGCTGCATG ATATTGTATC AGTACAGCCG ATATACTCTG ACATGTGAAT   
  
  
+ TCTTCAGTTT CTGATAGAAA ATAAGTTCA  

- AATATTATAG CAAAAGAATA AAACGTGTGC TTTAAATTTT ATTTTTCATT TTACCTTTTT ACTTTTTTGT   
  
  
- AGACGCCGCC CTGGGTGACA GGCCGAACAT GAGCGGTGTC CCTACGGTAG TGTCTCCCTC TAGTCTCTAA   
  
  
- GAAGTGACGA AGAGTGGCGT GTCTTTAGTA AAGAGGATGA GGAGAGACGA GAAGGGGGTG AGAGAGCTGA   
  
  
- GACTAATACC AAATCTGAAA ACCTGGAGTT GGAAAAGAGA GGGAGAGAAC GAGCCGAAGC GCCATTAAAA   
  
  
- GACTTTTGGC CTGAAGCCCC CAAATAGAAC GACCAAGCTT AAAGCTCCTG CTGCCCTATG CCAGGTCTAG   
  
  
- CTCAGTCTTC AGTTGCCGAG GTGCACTGCC GCCTCGTGAA CTAACCTGAA CGATCCCAAA AACACCGGGC   
  
  
- TCCAGCTTTA ATCTCCGAAC GACGAGCACG TCCATTAAGA AATTATCAAA ACCTCTATAA AGATCGAATG   
  
  
- CACAGTCAAC CTGCCTCAAA TATGAAAGAT ATAGTTTAAA AACACCTTTA GCCTCGAGGA TTAACCGAAA   
  
  
- CCGGACAAAC AAAGGGCTCT TTTACATCCT TTTTATTACT TTAAACCTTA AATTATCGAC AGATCACATT   
  
  
- TGGGAGGTGG TATTACTAGG ATCATGAGGA AACAGACACG TTTAACCTAA AAATAAAAAT TAACTAACCT   
  
  
- TAATTTTTTT ATTTCTTAGA GTCACGCTTA CTATCAATCG AATACAAAAC TAAACAGTAG CTTAGTATCT   
  
  
- TGAAGTAAGT GAAGGAAAAA CATACCATTA ATTAAAACCC CGCTTTATAG TTCCCTTTTT CTCCTGGATT   
  
  
- TCCAAACTTA TCCTATTAAA CCTCAAGACC TTGTCTTAAT CCGTACAAAC TCTTCGTAAG ATGTTCTCAA   
  
  
- CCGTATCAAA AACGAGATTT TTGTTCTTTC AAATGAAAGA TTTTCCGAAT TAGAAGTTAC GTCACAAGAA   
  
  
- AAGGTAAAAA GACCCTAATT AACTGCTCAG GCGCATGGGA GAAAAGGTCA ATGAAGAAGC TCTTCGGGTA   
  
  
- AACGATTTCA TCTTACCGAA AAAAGGTACT TCTATATAAA AAGAACGATT CCATCAGGGA CTTCGATCAT   
  
  
- CTAAATGACG AATTCGAACG GACAACTTGC CGAAAAACGG TTTGGTACAA CTGGCTTTGC CCCTCGGACT   
  
  
- GAACAACTCC TAACTGTAGT TTAGCTTCGA CACATCGGGA TTCATGCACC AACACAACCA GAATCTCTGG   
  
  
- GTCCCTATGT GAGAAACAAA TTAAAAGGGT TTAATAAAAA AAAGAACAAA TGAAAGAATT AATGCACGAT   
  
  
- TTAATACCTA ATATGTTACC CATCGTTATA TGTAATACAG TAACGGATAA AAAAATTAAA ACACCACCTA   
  
  
- GCAAAAAAAG TCCAAACTTC ATCGACGTAC TATAACATAG TCATGTCGGC TATATGAGAC TGTACACTTA   
  
  
- AGAAGTCAAA GACTATCTTT TATTCAAGT

+     OBP-1 site

| Site Name | Organism | Position | Strand | Matrix score. | sequence | function |
| --- | --- | --- | --- | --- | --- | --- |
| OBP-1 site | Arabidopsis thaliana | 223 | + | 10 | TACACTTTTGG | cis-acting regulatory element |

> 2018/04/13 10:10:12  
+ TTATAATATC GTTTTCTTAT TTTGCACACG AAATTTAAAA TAAAAAGTAA AATGGAAAAA TGAAAAAACA   
  
  
+ TCTGCGGCGG GACCCACTGT CCGGCTTGTA CTCGCCACAG GGATGCCATC ACAGAGGGAG ATCAGAGATT   
  
  
+ CTTCACTGCT TCTCACCGCA CAGAAATCAT TTCTCCTACT CCTCTCTGCT CTTCCCCCAC TCTCTCGACT   
  
  
+ CTGATTATGG TTTAGACTTT TGGACCTCAA CCTTTTCTCT CCCTCTCTTG CTCGGCTTCG CGGTAATTTT   
  
  
+ CTGAAAACCG GACTTCGGGG GTTTATCTTG CTGGTTCGAA TTTCGAGGAC GACGGGATAC GGTCCAGATC   
  
  
+ GAGTCAGAAG TCAACGGCTC CACGTGACGG CGGAGCACTT GATTGGACTT GCTAGGGTTT TTGTGGCCCG   
  
  
+ AGGTCGAAAT TAGAGGCTTG CTGCTCGTGC AGGTAATTCT TTAATAGTTT TGGAGATATT TCTAGCTTAC   
  
  
+ GTGTCAGTTG GACGGAGTTT ATACTTTCTA TATCAAATTT TTGTGGAAAT CGGAGCTCCT AATTGGCTTT   
  
  
+ GGCCTGTTTG TTTCCCGAGA AAATGTAGGA AAAATAATGA AATTTGGAAT TTAATAGCTG TCTAGTGTAA   
  
  
+ ACCCTCCACC ATAATGATCC TAGTACTCCT TTGTCTGTGC AAATTGGATT TTTATTTTTA ATTGATTGGA   
  
  
+ ATTAAAAAAA TAAAGAATCT CAGTGCGAAT GATAGTTAGC TTATGTTTTG ATTTGTCATC GAATCATAGA   
  
  
+ ACTTCATTCA CTTCCTTTTT GTATGGTAAT TAATTTTGGG GCGAAATATC AAGGGAAAAA GAGGACCTAA   
  
  
+ AGGTTTGAAT AGGATAATTT GGAGTTCTGG AACAGAATTA GGCATGTTTG AGAAGCATTC TACAAGAGTT   
  
  
+ GGCATAGTTT TTGCTCTAAA AACAAGAAAG TTTACTTTCT AAAAGGCTTA ATCTTCAATG CAGTGTTCTT   
  
  
+ TTCCATTTTT CTGGGATTAA TTGACGAGTC CGCGTACCCT CTTTTCCAGT TACTTCTTCG AGAAGCCCAT   
  
  
+ TTGCTAAAGT AGAATGGCTT TTTTCCATGA AGATATATTT TTCTTGCTAA GGTAGTCCCT GAAGCTAGTA   
  
  
+ GATTTACTGC TTAAGCTTGC CTGTTGAACG GCTTTTTGCC AAACCATGTT GACCGAAACG GGGAGCCTGA   
  
  
+ CTTGTTGAGG ATTGACATCA AATCGAAGCT GTGTAGCCCT AAGTACGTGG TTGTGTTGGT CTTAGAGACC   
  
  
+ CAGGGATACA CTCTTTGTTT AATTTTCCCA AATTATTTTT TTTCTTGTTT ACTTTCTTAA TTACGTGCTA   
  
  
+ AATTATGGAT TATACAATGG GTAGCAATAT ACATTATGTC ATTGCCTATT TTTTTAATTT TGTGGTGGAT   
  
  
+ CGTTTTTTTC AGGTTTGAAG TAGCTGCATG ATATTGTATC AGTACAGCCG ATATACTCTG ACATGTGAAT   
  
  
+ TCTTCAGTTT CTGATAGAAA ATAAGTTCA  

- AATATTATAG CAAAAGAATA AAACGTGTGC TTTAAATTTT ATTTTTCATT TTACCTTTTT ACTTTTTTGT   
  
  
- AGACGCCGCC CTGGGTGACA GGCCGAACAT GAGCGGTGTC CCTACGGTAG TGTCTCCCTC TAGTCTCTAA   
  
  
- GAAGTGACGA AGAGTGGCGT GTCTTTAGTA AAGAGGATGA GGAGAGACGA GAAGGGGGTG AGAGAGCTGA   
  
  
- GACTAATACC AAATCTGAAA ACCTGGAGTT GGAAAAGAGA GGGAGAGAAC GAGCCGAAGC GCCATTAAAA   
  
  
- GACTTTTGGC CTGAAGCCCC CAAATAGAAC GACCAAGCTT AAAGCTCCTG CTGCCCTATG CCAGGTCTAG   
  
  
- CTCAGTCTTC AGTTGCCGAG GTGCACTGCC GCCTCGTGAA CTAACCTGAA CGATCCCAAA AACACCGGGC   
  
  
- TCCAGCTTTA ATCTCCGAAC GACGAGCACG TCCATTAAGA AATTATCAAA ACCTCTATAA AGATCGAATG   
  
  
- CACAGTCAAC CTGCCTCAAA TATGAAAGAT ATAGTTTAAA AACACCTTTA GCCTCGAGGA TTAACCGAAA   
  
  
- CCGGACAAAC AAAGGGCTCT TTTACATCCT TTTTATTACT TTAAACCTTA AATTATCGAC AGATCACATT   
  
  
- TGGGAGGTGG TATTACTAGG ATCATGAGGA AACAGACACG TTTAACCTAA AAATAAAAAT TAACTAACCT   
  
  
- TAATTTTTTT ATTTCTTAGA GTCACGCTTA CTATCAATCG AATACAAAAC TAAACAGTAG CTTAGTATCT   
  
  
- TGAAGTAAGT GAAGGAAAAA CATACCATTA ATTAAAACCC CGCTTTATAG TTCCCTTTTT CTCCTGGATT   
  
  
- TCCAAACTTA TCCTATTAAA CCTCAAGACC TTGTCTTAAT CCGTACAAAC TCTTCGTAAG ATGTTCTCAA   
  
  
- CCGTATCAAA AACGAGATTT TTGTTCTTTC AAATGAAAGA TTTTCCGAAT TAGAAGTTAC GTCACAAGAA   
  
  
- AAGGTAAAAA GACCCTAATT AACTGCTCAG GCGCATGGGA GAAAAGGTCA ATGAAGAAGC TCTTCGGGTA   
  
  
- AACGATTTCA TCTTACCGAA AAAAGGTACT TCTATATAAA AAGAACGATT CCATCAGGGA CTTCGATCAT   
  
  
- CTAAATGACG AATTCGAACG GACAACTTGC CGAAAAACGG TTTGGTACAA CTGGCTTTGC CCCTCGGACT   
  
  
- GAACAACTCC TAACTGTAGT TTAGCTTCGA CACATCGGGA TTCATGCACC AACACAACCA GAATCTCTGG   
  
  
- GTCCCTATGT GAGAAACAAA TTAAAAGGGT TTAATAAAAA AAAGAACAAA TGAAAGAATT AATGCACGAT   
  
  
- TTAATACCTA ATATGTTACC CATCGTTATA TGTAATACAG TAACGGATAA AAAAATTAAA ACACCACCTA   
  
  
- GCAAAAAAAG TCCAAACTTC ATCGACGTAC TATAACATAG TCATGTCGGC TATATGAGAC TGTACACTTA   
  
  
- AGAAGTCAAA GACTATCTTT TATTCAAGT

+     Skn-1\_motif

| Site Name | Organism | Position | Strand | Matrix score. | sequence | function |
| --- | --- | --- | --- | --- | --- | --- |
| Skn-1\_motif | Oryza sativa | 755 | + | 5 | GTCAT | cis-acting regulatory element required for endosperm expression |
| Skn-1\_motif | Oryza sativa | 1368 | + | 5 | GTCAT | cis-acting regulatory element required for endosperm expression |

> 2018/04/13 10:10:12  
+ TTATAATATC GTTTTCTTAT TTTGCACACG AAATTTAAAA TAAAAAGTAA AATGGAAAAA TGAAAAAACA   
  
  
+ TCTGCGGCGG GACCCACTGT CCGGCTTGTA CTCGCCACAG GGATGCCATC ACAGAGGGAG ATCAGAGATT   
  
  
+ CTTCACTGCT TCTCACCGCA CAGAAATCAT TTCTCCTACT CCTCTCTGCT CTTCCCCCAC TCTCTCGACT   
  
  
+ CTGATTATGG TTTAGACTTT TGGACCTCAA CCTTTTCTCT CCCTCTCTTG CTCGGCTTCG CGGTAATTTT   
  
  
+ CTGAAAACCG GACTTCGGGG GTTTATCTTG CTGGTTCGAA TTTCGAGGAC GACGGGATAC GGTCCAGATC   
  
  
+ GAGTCAGAAG TCAACGGCTC CACGTGACGG CGGAGCACTT GATTGGACTT GCTAGGGTTT TTGTGGCCCG   
  
  
+ AGGTCGAAAT TAGAGGCTTG CTGCTCGTGC AGGTAATTCT TTAATAGTTT TGGAGATATT TCTAGCTTAC   
  
  
+ GTGTCAGTTG GACGGAGTTT ATACTTTCTA TATCAAATTT TTGTGGAAAT CGGAGCTCCT AATTGGCTTT   
  
  
+ GGCCTGTTTG TTTCCCGAGA AAATGTAGGA AAAATAATGA AATTTGGAAT TTAATAGCTG TCTAGTGTAA   
  
  
+ ACCCTCCACC ATAATGATCC TAGTACTCCT TTGTCTGTGC AAATTGGATT TTTATTTTTA ATTGATTGGA   
  
  
+ ATTAAAAAAA TAAAGAATCT CAGTGCGAAT GATAGTTAGC TTATGTTTTG ATTTGTCATC GAATCATAGA   
  
  
+ ACTTCATTCA CTTCCTTTTT GTATGGTAAT TAATTTTGGG GCGAAATATC AAGGGAAAAA GAGGACCTAA   
  
  
+ AGGTTTGAAT AGGATAATTT GGAGTTCTGG AACAGAATTA GGCATGTTTG AGAAGCATTC TACAAGAGTT   
  
  
+ GGCATAGTTT TTGCTCTAAA AACAAGAAAG TTTACTTTCT AAAAGGCTTA ATCTTCAATG CAGTGTTCTT   
  
  
+ TTCCATTTTT CTGGGATTAA TTGACGAGTC CGCGTACCCT CTTTTCCAGT TACTTCTTCG AGAAGCCCAT   
  
  
+ TTGCTAAAGT AGAATGGCTT TTTTCCATGA AGATATATTT TTCTTGCTAA GGTAGTCCCT GAAGCTAGTA   
  
  
+ GATTTACTGC TTAAGCTTGC CTGTTGAACG GCTTTTTGCC AAACCATGTT GACCGAAACG GGGAGCCTGA   
  
  
+ CTTGTTGAGG ATTGACATCA AATCGAAGCT GTGTAGCCCT AAGTACGTGG TTGTGTTGGT CTTAGAGACC   
  
  
+ CAGGGATACA CTCTTTGTTT AATTTTCCCA AATTATTTTT TTTCTTGTTT ACTTTCTTAA TTACGTGCTA   
  
  
+ AATTATGGAT TATACAATGG GTAGCAATAT ACATTATGTC ATTGCCTATT TTTTTAATTT TGTGGTGGAT   
  
  
+ CGTTTTTTTC AGGTTTGAAG TAGCTGCATG ATATTGTATC AGTACAGCCG ATATACTCTG ACATGTGAAT   
  
  
+ TCTTCAGTTT CTGATAGAAA ATAAGTTCA  

- AATATTATAG CAAAAGAATA AAACGTGTGC TTTAAATTTT ATTTTTCATT TTACCTTTTT ACTTTTTTGT   
  
  
- AGACGCCGCC CTGGGTGACA GGCCGAACAT GAGCGGTGTC CCTACGGTAG TGTCTCCCTC TAGTCTCTAA   
  
  
- GAAGTGACGA AGAGTGGCGT GTCTTTAGTA AAGAGGATGA GGAGAGACGA GAAGGGGGTG AGAGAGCTGA   
  
  
- GACTAATACC AAATCTGAAA ACCTGGAGTT GGAAAAGAGA GGGAGAGAAC GAGCCGAAGC GCCATTAAAA   
  
  
- GACTTTTGGC CTGAAGCCCC CAAATAGAAC GACCAAGCTT AAAGCTCCTG CTGCCCTATG CCAGGTCTAG   
  
  
- CTCAGTCTTC AGTTGCCGAG GTGCACTGCC GCCTCGTGAA CTAACCTGAA CGATCCCAAA AACACCGGGC   
  
  
- TCCAGCTTTA ATCTCCGAAC GACGAGCACG TCCATTAAGA AATTATCAAA ACCTCTATAA AGATCGAATG   
  
  
- CACAGTCAAC CTGCCTCAAA TATGAAAGAT ATAGTTTAAA AACACCTTTA GCCTCGAGGA TTAACCGAAA   
  
  
- CCGGACAAAC AAAGGGCTCT TTTACATCCT TTTTATTACT TTAAACCTTA AATTATCGAC AGATCACATT   
  
  
- TGGGAGGTGG TATTACTAGG ATCATGAGGA AACAGACACG TTTAACCTAA AAATAAAAAT TAACTAACCT   
  
  
- TAATTTTTTT ATTTCTTAGA GTCACGCTTA CTATCAATCG AATACAAAAC TAAACAGTAG CTTAGTATCT   
  
  
- TGAAGTAAGT GAAGGAAAAA CATACCATTA ATTAAAACCC CGCTTTATAG TTCCCTTTTT CTCCTGGATT   
  
  
- TCCAAACTTA TCCTATTAAA CCTCAAGACC TTGTCTTAAT CCGTACAAAC TCTTCGTAAG ATGTTCTCAA   
  
  
- CCGTATCAAA AACGAGATTT TTGTTCTTTC AAATGAAAGA TTTTCCGAAT TAGAAGTTAC GTCACAAGAA   
  
  
- AAGGTAAAAA GACCCTAATT AACTGCTCAG GCGCATGGGA GAAAAGGTCA ATGAAGAAGC TCTTCGGGTA   
  
  
- AACGATTTCA TCTTACCGAA AAAAGGTACT TCTATATAAA AAGAACGATT CCATCAGGGA CTTCGATCAT   
  
  
- CTAAATGACG AATTCGAACG GACAACTTGC CGAAAAACGG TTTGGTACAA CTGGCTTTGC CCCTCGGACT   
  
  
- GAACAACTCC TAACTGTAGT TTAGCTTCGA CACATCGGGA TTCATGCACC AACACAACCA GAATCTCTGG   
  
  
- GTCCCTATGT GAGAAACAAA TTAAAAGGGT TTAATAAAAA AAAGAACAAA TGAAAGAATT AATGCACGAT   
  
  
- TTAATACCTA ATATGTTACC CATCGTTATA TGTAATACAG TAACGGATAA AAAAATTAAA ACACCACCTA   
  
  
- GCAAAAAAAG TCCAAACTTC ATCGACGTAC TATAACATAG TCATGTCGGC TATATGAGAC TGTACACTTA   
  
  
- AGAAGTCAAA GACTATCTTT TATTCAAGT

+     TATA-box

| Site Name | Organism | Position | Strand | Matrix score. | sequence | function |
| --- | --- | --- | --- | --- | --- | --- |
| TATA-box | Arabidopsis thaliana | 1452 | - | 4 | TATA | core promoter element around -30 of transcription start |
| TATA-box | Arabidopsis thaliana | 682 | - | 9 | TAAAAATAA | core promoter element around -30 of transcription start |
| TATA-box | Arabidopsis thaliana | 1341 | - | 4 | TATA | core promoter element around -30 of transcription start |
| TATA-box | Arabidopsis thaliana | 1340 | - | 5 | TATAA | core promoter element around -30 of transcription start |
| TATA-box | Lycopersicon esculentum | 36 | - | 5 | TTTTA | core promoter element around -30 of transcription start |
| TATA-box | Arabidopsis thaliana | 1358 | - | 4 | TATA | core promoter element around -30 of transcription start |
| TATA-box | Lycopersicon esculentum | 48 | - | 5 | TTTTA | core promoter element around -30 of transcription start |
| TATA-box | Lycopersicon esculentum | 680 | + | 5 | TTTTA | core promoter element around -30 of transcription start |
| TATA-box | Arabidopsis thaliana | 1 | - | 5 | TATAA | core promoter element around -30 of transcription start |
| TATA-box | Lycopersicon esculentum | 1382 | + | 5 | TTTTA | core promoter element around -30 of transcription start |
| TATA-box | Lycopersicon esculentum | 686 | + | 5 | TTTTA | core promoter element around -30 of transcription start |
| TATA-box | Brassica napus | 1083 | - | 6 | ATATAT | core promoter element around -30 of transcription start |
| TATA-box | Lycopersicon esculentum | 950 | - | 5 | TTTTA | core promoter element around -30 of transcription start |
| TATA-box | Arabidopsis thaliana | 509 | - | 5 | TATAA | core promoter element around -30 of transcription start |
| TATA-box | Arabidopsis thaliana | 2 | + | 4 | TATA | core promoter element around -30 of transcription start |
| TATA-box | Lycopersicon esculentum | 703 | - | 5 | TTTTA | core promoter element around -30 of transcription start |
| TATA-box | Lycopersicon esculentum | 41 | - | 5 | TTTTA | core promoter element around -30 of transcription start |
| TATA-box | Oryza sativa | 787 | - | 7 | TACAAAA | core promoter element around -30 of transcription start |
| TATA-box | Arabidopsis thaliana | 519 | + | 4 | TATA | core promoter element around -30 of transcription start |
| TATA-box | Arabidopsis thaliana | 1084 | - | 4 | TATA | core promoter element around -30 of transcription start |
| TATA-box | Lycopersicon esculentum | 927 | - | 5 | TTTTA | core promoter element around -30 of transcription start |
| TATA-box | Glycine max | 4 | + | 5 | TAATA | core promoter element around -30 of transcription start |
| TATA-box | Brassica napus | 1339 | + | 6 | ATTATA | core promoter element around -30 of transcription start |
| TATA-box | Arabidopsis thaliana | 508 | - | 6 | TATAAA | core promoter element around -30 of transcription start |
| TATA-box | Arabidopsis thaliana | 510 | + | 4 | TATA | core promoter element around -30 of transcription start |
| TATA-box | Glycine max | 612 | + | 5 | TAATA | core promoter element around -30 of transcription start |
| TATA-box | Glycine max | 462 | + | 5 | TAATA | core promoter element around -30 of transcription start |

> 2018/04/13 10:10:12  
+ TTATAATATC GTTTTCTTAT TTTGCACACG AAATTTAAAA TAAAAAGTAA AATGGAAAAA TGAAAAAACA   
  
  
+ TCTGCGGCGG GACCCACTGT CCGGCTTGTA CTCGCCACAG GGATGCCATC ACAGAGGGAG ATCAGAGATT   
  
  
+ CTTCACTGCT TCTCACCGCA CAGAAATCAT TTCTCCTACT CCTCTCTGCT CTTCCCCCAC TCTCTCGACT   
  
  
+ CTGATTATGG TTTAGACTTT TGGACCTCAA CCTTTTCTCT CCCTCTCTTG CTCGGCTTCG CGGTAATTTT   
  
  
+ CTGAAAACCG GACTTCGGGG GTTTATCTTG CTGGTTCGAA TTTCGAGGAC GACGGGATAC GGTCCAGATC   
  
  
+ GAGTCAGAAG TCAACGGCTC CACGTGACGG CGGAGCACTT GATTGGACTT GCTAGGGTTT TTGTGGCCCG   
  
  
+ AGGTCGAAAT TAGAGGCTTG CTGCTCGTGC AGGTAATTCT TTAATAGTTT TGGAGATATT TCTAGCTTAC   
  
  
+ GTGTCAGTTG GACGGAGTTT ATACTTTCTA TATCAAATTT TTGTGGAAAT CGGAGCTCCT AATTGGCTTT   
  
  
+ GGCCTGTTTG TTTCCCGAGA AAATGTAGGA AAAATAATGA AATTTGGAAT TTAATAGCTG TCTAGTGTAA   
  
  
+ ACCCTCCACC ATAATGATCC TAGTACTCCT TTGTCTGTGC AAATTGGATT TTTATTTTTA ATTGATTGGA   
  
  
+ ATTAAAAAAA TAAAGAATCT CAGTGCGAAT GATAGTTAGC TTATGTTTTG ATTTGTCATC GAATCATAGA   
  
  
+ ACTTCATTCA CTTCCTTTTT GTATGGTAAT TAATTTTGGG GCGAAATATC AAGGGAAAAA GAGGACCTAA   
  
  
+ AGGTTTGAAT AGGATAATTT GGAGTTCTGG AACAGAATTA GGCATGTTTG AGAAGCATTC TACAAGAGTT   
  
  
+ GGCATAGTTT TTGCTCTAAA AACAAGAAAG TTTACTTTCT AAAAGGCTTA ATCTTCAATG CAGTGTTCTT   
  
  
+ TTCCATTTTT CTGGGATTAA TTGACGAGTC CGCGTACCCT CTTTTCCAGT TACTTCTTCG AGAAGCCCAT   
  
  
+ TTGCTAAAGT AGAATGGCTT TTTTCCATGA AGATATATTT TTCTTGCTAA GGTAGTCCCT GAAGCTAGTA   
  
  
+ GATTTACTGC TTAAGCTTGC CTGTTGAACG GCTTTTTGCC AAACCATGTT GACCGAAACG GGGAGCCTGA   
  
  
+ CTTGTTGAGG ATTGACATCA AATCGAAGCT GTGTAGCCCT AAGTACGTGG TTGTGTTGGT CTTAGAGACC   
  
  
+ CAGGGATACA CTCTTTGTTT AATTTTCCCA AATTATTTTT TTTCTTGTTT ACTTTCTTAA TTACGTGCTA   
  
  
+ AATTATGGAT TATACAATGG GTAGCAATAT ACATTATGTC ATTGCCTATT TTTTTAATTT TGTGGTGGAT   
  
  
+ CGTTTTTTTC AGGTTTGAAG TAGCTGCATG ATATTGTATC AGTACAGCCG ATATACTCTG ACATGTGAAT   
  
  
+ TCTTCAGTTT CTGATAGAAA ATAAGTTCA  

- AATATTATAG CAAAAGAATA AAACGTGTGC TTTAAATTTT ATTTTTCATT TTACCTTTTT ACTTTTTTGT   
  
  
- AGACGCCGCC CTGGGTGACA GGCCGAACAT GAGCGGTGTC CCTACGGTAG TGTCTCCCTC TAGTCTCTAA   
  
  
- GAAGTGACGA AGAGTGGCGT GTCTTTAGTA AAGAGGATGA GGAGAGACGA GAAGGGGGTG AGAGAGCTGA   
  
  
- GACTAATACC AAATCTGAAA ACCTGGAGTT GGAAAAGAGA GGGAGAGAAC GAGCCGAAGC GCCATTAAAA   
  
  
- GACTTTTGGC CTGAAGCCCC CAAATAGAAC GACCAAGCTT AAAGCTCCTG CTGCCCTATG CCAGGTCTAG   
  
  
- CTCAGTCTTC AGTTGCCGAG GTGCACTGCC GCCTCGTGAA CTAACCTGAA CGATCCCAAA AACACCGGGC   
  
  
- TCCAGCTTTA ATCTCCGAAC GACGAGCACG TCCATTAAGA AATTATCAAA ACCTCTATAA AGATCGAATG   
  
  
- CACAGTCAAC CTGCCTCAAA TATGAAAGAT ATAGTTTAAA AACACCTTTA GCCTCGAGGA TTAACCGAAA   
  
  
- CCGGACAAAC AAAGGGCTCT TTTACATCCT TTTTATTACT TTAAACCTTA AATTATCGAC AGATCACATT   
  
  
- TGGGAGGTGG TATTACTAGG ATCATGAGGA AACAGACACG TTTAACCTAA AAATAAAAAT TAACTAACCT   
  
  
- TAATTTTTTT ATTTCTTAGA GTCACGCTTA CTATCAATCG AATACAAAAC TAAACAGTAG CTTAGTATCT   
  
  
- TGAAGTAAGT GAAGGAAAAA CATACCATTA ATTAAAACCC CGCTTTATAG TTCCCTTTTT CTCCTGGATT   
  
  
- TCCAAACTTA TCCTATTAAA CCTCAAGACC TTGTCTTAAT CCGTACAAAC TCTTCGTAAG ATGTTCTCAA   
  
  
- CCGTATCAAA AACGAGATTT TTGTTCTTTC AAATGAAAGA TTTTCCGAAT TAGAAGTTAC GTCACAAGAA   
  
  
- AAGGTAAAAA GACCCTAATT AACTGCTCAG GCGCATGGGA GAAAAGGTCA ATGAAGAAGC TCTTCGGGTA   
  
  
- AACGATTTCA TCTTACCGAA AAAAGGTACT TCTATATAAA AAGAACGATT CCATCAGGGA CTTCGATCAT   
  
  
- CTAAATGACG AATTCGAACG GACAACTTGC CGAAAAACGG TTTGGTACAA CTGGCTTTGC CCCTCGGACT   
  
  
- GAACAACTCC TAACTGTAGT TTAGCTTCGA CACATCGGGA TTCATGCACC AACACAACCA GAATCTCTGG   
  
  
- GTCCCTATGT GAGAAACAAA TTAAAAGGGT TTAATAAAAA AAAGAACAAA TGAAAGAATT AATGCACGAT   
  
  
- TTAATACCTA ATATGTTACC CATCGTTATA TGTAATACAG TAACGGATAA AAAAATTAAA ACACCACCTA   
  
  
- GCAAAAAAAG TCCAAACTTC ATCGACGTAC TATAACATAG TCATGTCGGC TATATGAGAC TGTACACTTA   
  
  
- AGAAGTCAAA GACTATCTTT TATTCAAGT

+     TC-rich repeats

| Site Name | Organism | Position | Strand | Matrix score. | sequence | function |
| --- | --- | --- | --- | --- | --- | --- |
| TC-rich repeats | Nicotiana tabacum | 11 | + | 9 | GTTTTCTTAC | cis-acting element involved in defense and stress responsiveness |

> 2018/04/13 10:10:12  
+ TTATAATATC GTTTTCTTAT TTTGCACACG AAATTTAAAA TAAAAAGTAA AATGGAAAAA TGAAAAAACA   
  
  
+ TCTGCGGCGG GACCCACTGT CCGGCTTGTA CTCGCCACAG GGATGCCATC ACAGAGGGAG ATCAGAGATT   
  
  
+ CTTCACTGCT TCTCACCGCA CAGAAATCAT TTCTCCTACT CCTCTCTGCT CTTCCCCCAC TCTCTCGACT   
  
  
+ CTGATTATGG TTTAGACTTT TGGACCTCAA CCTTTTCTCT CCCTCTCTTG CTCGGCTTCG CGGTAATTTT   
  
  
+ CTGAAAACCG GACTTCGGGG GTTTATCTTG CTGGTTCGAA TTTCGAGGAC GACGGGATAC GGTCCAGATC   
  
  
+ GAGTCAGAAG TCAACGGCTC CACGTGACGG CGGAGCACTT GATTGGACTT GCTAGGGTTT TTGTGGCCCG   
  
  
+ AGGTCGAAAT TAGAGGCTTG CTGCTCGTGC AGGTAATTCT TTAATAGTTT TGGAGATATT TCTAGCTTAC   
  
  
+ GTGTCAGTTG GACGGAGTTT ATACTTTCTA TATCAAATTT TTGTGGAAAT CGGAGCTCCT AATTGGCTTT   
  
  
+ GGCCTGTTTG TTTCCCGAGA AAATGTAGGA AAAATAATGA AATTTGGAAT TTAATAGCTG TCTAGTGTAA   
  
  
+ ACCCTCCACC ATAATGATCC TAGTACTCCT TTGTCTGTGC AAATTGGATT TTTATTTTTA ATTGATTGGA   
  
  
+ ATTAAAAAAA TAAAGAATCT CAGTGCGAAT GATAGTTAGC TTATGTTTTG ATTTGTCATC GAATCATAGA   
  
  
+ ACTTCATTCA CTTCCTTTTT GTATGGTAAT TAATTTTGGG GCGAAATATC AAGGGAAAAA GAGGACCTAA   
  
  
+ AGGTTTGAAT AGGATAATTT GGAGTTCTGG AACAGAATTA GGCATGTTTG AGAAGCATTC TACAAGAGTT   
  
  
+ GGCATAGTTT TTGCTCTAAA AACAAGAAAG TTTACTTTCT AAAAGGCTTA ATCTTCAATG CAGTGTTCTT   
  
  
+ TTCCATTTTT CTGGGATTAA TTGACGAGTC CGCGTACCCT CTTTTCCAGT TACTTCTTCG AGAAGCCCAT   
  
  
+ TTGCTAAAGT AGAATGGCTT TTTTCCATGA AGATATATTT TTCTTGCTAA GGTAGTCCCT GAAGCTAGTA   
  
  
+ GATTTACTGC TTAAGCTTGC CTGTTGAACG GCTTTTTGCC AAACCATGTT GACCGAAACG GGGAGCCTGA   
  
  
+ CTTGTTGAGG ATTGACATCA AATCGAAGCT GTGTAGCCCT AAGTACGTGG TTGTGTTGGT CTTAGAGACC   
  
  
+ CAGGGATACA CTCTTTGTTT AATTTTCCCA AATTATTTTT TTTCTTGTTT ACTTTCTTAA TTACGTGCTA   
  
  
+ AATTATGGAT TATACAATGG GTAGCAATAT ACATTATGTC ATTGCCTATT TTTTTAATTT TGTGGTGGAT   
  
  
+ CGTTTTTTTC AGGTTTGAAG TAGCTGCATG ATATTGTATC AGTACAGCCG ATATACTCTG ACATGTGAAT   
  
  
+ TCTTCAGTTT CTGATAGAAA ATAAGTTCA  

- AATATTATAG CAAAAGAATA AAACGTGTGC TTTAAATTTT ATTTTTCATT TTACCTTTTT ACTTTTTTGT   
  
  
- AGACGCCGCC CTGGGTGACA GGCCGAACAT GAGCGGTGTC CCTACGGTAG TGTCTCCCTC TAGTCTCTAA   
  
  
- GAAGTGACGA AGAGTGGCGT GTCTTTAGTA AAGAGGATGA GGAGAGACGA GAAGGGGGTG AGAGAGCTGA   
  
  
- GACTAATACC AAATCTGAAA ACCTGGAGTT GGAAAAGAGA GGGAGAGAAC GAGCCGAAGC GCCATTAAAA   
  
  
- GACTTTTGGC CTGAAGCCCC CAAATAGAAC GACCAAGCTT AAAGCTCCTG CTGCCCTATG CCAGGTCTAG   
  
  
- CTCAGTCTTC AGTTGCCGAG GTGCACTGCC GCCTCGTGAA CTAACCTGAA CGATCCCAAA AACACCGGGC   
  
  
- TCCAGCTTTA ATCTCCGAAC GACGAGCACG TCCATTAAGA AATTATCAAA ACCTCTATAA AGATCGAATG   
  
  
- CACAGTCAAC CTGCCTCAAA TATGAAAGAT ATAGTTTAAA AACACCTTTA GCCTCGAGGA TTAACCGAAA   
  
  
- CCGGACAAAC AAAGGGCTCT TTTACATCCT TTTTATTACT TTAAACCTTA AATTATCGAC AGATCACATT   
  
  
- TGGGAGGTGG TATTACTAGG ATCATGAGGA AACAGACACG TTTAACCTAA AAATAAAAAT TAACTAACCT   
  
  
- TAATTTTTTT ATTTCTTAGA GTCACGCTTA CTATCAATCG AATACAAAAC TAAACAGTAG CTTAGTATCT   
  
  
- TGAAGTAAGT GAAGGAAAAA CATACCATTA ATTAAAACCC CGCTTTATAG TTCCCTTTTT CTCCTGGATT   
  
  
- TCCAAACTTA TCCTATTAAA CCTCAAGACC TTGTCTTAAT CCGTACAAAC TCTTCGTAAG ATGTTCTCAA   
  
  
- CCGTATCAAA AACGAGATTT TTGTTCTTTC AAATGAAAGA TTTTCCGAAT TAGAAGTTAC GTCACAAGAA   
  
  
- AAGGTAAAAA GACCCTAATT AACTGCTCAG GCGCATGGGA GAAAAGGTCA ATGAAGAAGC TCTTCGGGTA   
  
  
- AACGATTTCA TCTTACCGAA AAAAGGTACT TCTATATAAA AAGAACGATT CCATCAGGGA CTTCGATCAT   
  
  
- CTAAATGACG AATTCGAACG GACAACTTGC CGAAAAACGG TTTGGTACAA CTGGCTTTGC CCCTCGGACT   
  
  
- GAACAACTCC TAACTGTAGT TTAGCTTCGA CACATCGGGA TTCATGCACC AACACAACCA GAATCTCTGG   
  
  
- GTCCCTATGT GAGAAACAAA TTAAAAGGGT TTAATAAAAA AAAGAACAAA TGAAAGAATT AATGCACGAT   
  
  
- TTAATACCTA ATATGTTACC CATCGTTATA TGTAATACAG TAACGGATAA AAAAATTAAA ACACCACCTA   
  
  
- GCAAAAAAAG TCCAAACTTC ATCGACGTAC TATAACATAG TCATGTCGGC TATATGAGAC TGTACACTTA   
  
  
- AGAAGTCAAA GACTATCTTT TATTCAAGT

+     TCCC-motif

| Site Name | Organism | Position | Strand | Matrix score. | sequence | function |
| --- | --- | --- | --- | --- | --- | --- |
| TCCC-motif | Spinacia oleracea | 125 | - | 7 | TCTCCCT | part of a light responsive element |
| TCCC-motif | Spinacia oleracea | 248 | + | 7 | TCTCCCT | part of a light responsive element |

> 2018/04/13 10:10:12  
+ TTATAATATC GTTTTCTTAT TTTGCACACG AAATTTAAAA TAAAAAGTAA AATGGAAAAA TGAAAAAACA   
  
  
+ TCTGCGGCGG GACCCACTGT CCGGCTTGTA CTCGCCACAG GGATGCCATC ACAGAGGGAG ATCAGAGATT   
  
  
+ CTTCACTGCT TCTCACCGCA CAGAAATCAT TTCTCCTACT CCTCTCTGCT CTTCCCCCAC TCTCTCGACT   
  
  
+ CTGATTATGG TTTAGACTTT TGGACCTCAA CCTTTTCTCT CCCTCTCTTG CTCGGCTTCG CGGTAATTTT   
  
  
+ CTGAAAACCG GACTTCGGGG GTTTATCTTG CTGGTTCGAA TTTCGAGGAC GACGGGATAC GGTCCAGATC   
  
  
+ GAGTCAGAAG TCAACGGCTC CACGTGACGG CGGAGCACTT GATTGGACTT GCTAGGGTTT TTGTGGCCCG   
  
  
+ AGGTCGAAAT TAGAGGCTTG CTGCTCGTGC AGGTAATTCT TTAATAGTTT TGGAGATATT TCTAGCTTAC   
  
  
+ GTGTCAGTTG GACGGAGTTT ATACTTTCTA TATCAAATTT TTGTGGAAAT CGGAGCTCCT AATTGGCTTT   
  
  
+ GGCCTGTTTG TTTCCCGAGA AAATGTAGGA AAAATAATGA AATTTGGAAT TTAATAGCTG TCTAGTGTAA   
  
  
+ ACCCTCCACC ATAATGATCC TAGTACTCCT TTGTCTGTGC AAATTGGATT TTTATTTTTA ATTGATTGGA   
  
  
+ ATTAAAAAAA TAAAGAATCT CAGTGCGAAT GATAGTTAGC TTATGTTTTG ATTTGTCATC GAATCATAGA   
  
  
+ ACTTCATTCA CTTCCTTTTT GTATGGTAAT TAATTTTGGG GCGAAATATC AAGGGAAAAA GAGGACCTAA   
  
  
+ AGGTTTGAAT AGGATAATTT GGAGTTCTGG AACAGAATTA GGCATGTTTG AGAAGCATTC TACAAGAGTT   
  
  
+ GGCATAGTTT TTGCTCTAAA AACAAGAAAG TTTACTTTCT AAAAGGCTTA ATCTTCAATG CAGTGTTCTT   
  
  
+ TTCCATTTTT CTGGGATTAA TTGACGAGTC CGCGTACCCT CTTTTCCAGT TACTTCTTCG AGAAGCCCAT   
  
  
+ TTGCTAAAGT AGAATGGCTT TTTTCCATGA AGATATATTT TTCTTGCTAA GGTAGTCCCT GAAGCTAGTA   
  
  
+ GATTTACTGC TTAAGCTTGC CTGTTGAACG GCTTTTTGCC AAACCATGTT GACCGAAACG GGGAGCCTGA   
  
  
+ CTTGTTGAGG ATTGACATCA AATCGAAGCT GTGTAGCCCT AAGTACGTGG TTGTGTTGGT CTTAGAGACC   
  
  
+ CAGGGATACA CTCTTTGTTT AATTTTCCCA AATTATTTTT TTTCTTGTTT ACTTTCTTAA TTACGTGCTA   
  
  
+ AATTATGGAT TATACAATGG GTAGCAATAT ACATTATGTC ATTGCCTATT TTTTTAATTT TGTGGTGGAT   
  
  
+ CGTTTTTTTC AGGTTTGAAG TAGCTGCATG ATATTGTATC AGTACAGCCG ATATACTCTG ACATGTGAAT   
  
  
+ TCTTCAGTTT CTGATAGAAA ATAAGTTCA  

- AATATTATAG CAAAAGAATA AAACGTGTGC TTTAAATTTT ATTTTTCATT TTACCTTTTT ACTTTTTTGT   
  
  
- AGACGCCGCC CTGGGTGACA GGCCGAACAT GAGCGGTGTC CCTACGGTAG TGTCTCCCTC TAGTCTCTAA   
  
  
- GAAGTGACGA AGAGTGGCGT GTCTTTAGTA AAGAGGATGA GGAGAGACGA GAAGGGGGTG AGAGAGCTGA   
  
  
- GACTAATACC AAATCTGAAA ACCTGGAGTT GGAAAAGAGA GGGAGAGAAC GAGCCGAAGC GCCATTAAAA   
  
  
- GACTTTTGGC CTGAAGCCCC CAAATAGAAC GACCAAGCTT AAAGCTCCTG CTGCCCTATG CCAGGTCTAG   
  
  
- CTCAGTCTTC AGTTGCCGAG GTGCACTGCC GCCTCGTGAA CTAACCTGAA CGATCCCAAA AACACCGGGC   
  
  
- TCCAGCTTTA ATCTCCGAAC GACGAGCACG TCCATTAAGA AATTATCAAA ACCTCTATAA AGATCGAATG   
  
  
- CACAGTCAAC CTGCCTCAAA TATGAAAGAT ATAGTTTAAA AACACCTTTA GCCTCGAGGA TTAACCGAAA   
  
  
- CCGGACAAAC AAAGGGCTCT TTTACATCCT TTTTATTACT TTAAACCTTA AATTATCGAC AGATCACATT   
  
  
- TGGGAGGTGG TATTACTAGG ATCATGAGGA AACAGACACG TTTAACCTAA AAATAAAAAT TAACTAACCT   
  
  
- TAATTTTTTT ATTTCTTAGA GTCACGCTTA CTATCAATCG AATACAAAAC TAAACAGTAG CTTAGTATCT   
  
  
- TGAAGTAAGT GAAGGAAAAA CATACCATTA ATTAAAACCC CGCTTTATAG TTCCCTTTTT CTCCTGGATT   
  
  
- TCCAAACTTA TCCTATTAAA CCTCAAGACC TTGTCTTAAT CCGTACAAAC TCTTCGTAAG ATGTTCTCAA   
  
  
- CCGTATCAAA AACGAGATTT TTGTTCTTTC AAATGAAAGA TTTTCCGAAT TAGAAGTTAC GTCACAAGAA   
  
  
- AAGGTAAAAA GACCCTAATT AACTGCTCAG GCGCATGGGA GAAAAGGTCA ATGAAGAAGC TCTTCGGGTA   
  
  
- AACGATTTCA TCTTACCGAA AAAAGGTACT TCTATATAAA AAGAACGATT CCATCAGGGA CTTCGATCAT   
  
  
- CTAAATGACG AATTCGAACG GACAACTTGC CGAAAAACGG TTTGGTACAA CTGGCTTTGC CCCTCGGACT   
  
  
- GAACAACTCC TAACTGTAGT TTAGCTTCGA CACATCGGGA TTCATGCACC AACACAACCA GAATCTCTGG   
  
  
- GTCCCTATGT GAGAAACAAA TTAAAAGGGT TTAATAAAAA AAAGAACAAA TGAAAGAATT AATGCACGAT   
  
  
- TTAATACCTA ATATGTTACC CATCGTTATA TGTAATACAG TAACGGATAA AAAAATTAAA ACACCACCTA   
  
  
- GCAAAAAAAG TCCAAACTTC ATCGACGTAC TATAACATAG TCATGTCGGC TATATGAGAC TGTACACTTA   
  
  
- AGAAGTCAAA GACTATCTTT TATTCAAGT

+     TGACG-motif

| Site Name | Organism | Position | Strand | Matrix score. | sequence | function |
| --- | --- | --- | --- | --- | --- | --- |
| TGACG-motif | Hordeum vulgare | 1002 | + | 5 | TGACG | cis-acting regulatory element involved in the MeJA-responsiveness |
| TGACG-motif | Hordeum vulgare | 375 | + | 5 | TGACG | cis-acting regulatory element involved in the MeJA-responsiveness |

> 2018/04/13 10:10:12  
+ TTATAATATC GTTTTCTTAT TTTGCACACG AAATTTAAAA TAAAAAGTAA AATGGAAAAA TGAAAAAACA   
  
  
+ TCTGCGGCGG GACCCACTGT CCGGCTTGTA CTCGCCACAG GGATGCCATC ACAGAGGGAG ATCAGAGATT   
  
  
+ CTTCACTGCT TCTCACCGCA CAGAAATCAT TTCTCCTACT CCTCTCTGCT CTTCCCCCAC TCTCTCGACT   
  
  
+ CTGATTATGG TTTAGACTTT TGGACCTCAA CCTTTTCTCT CCCTCTCTTG CTCGGCTTCG CGGTAATTTT   
  
  
+ CTGAAAACCG GACTTCGGGG GTTTATCTTG CTGGTTCGAA TTTCGAGGAC GACGGGATAC GGTCCAGATC   
  
  
+ GAGTCAGAAG TCAACGGCTC CACGTGACGG CGGAGCACTT GATTGGACTT GCTAGGGTTT TTGTGGCCCG   
  
  
+ AGGTCGAAAT TAGAGGCTTG CTGCTCGTGC AGGTAATTCT TTAATAGTTT TGGAGATATT TCTAGCTTAC   
  
  
+ GTGTCAGTTG GACGGAGTTT ATACTTTCTA TATCAAATTT TTGTGGAAAT CGGAGCTCCT AATTGGCTTT   
  
  
+ GGCCTGTTTG TTTCCCGAGA AAATGTAGGA AAAATAATGA AATTTGGAAT TTAATAGCTG TCTAGTGTAA   
  
  
+ ACCCTCCACC ATAATGATCC TAGTACTCCT TTGTCTGTGC AAATTGGATT TTTATTTTTA ATTGATTGGA   
  
  
+ ATTAAAAAAA TAAAGAATCT CAGTGCGAAT GATAGTTAGC TTATGTTTTG ATTTGTCATC GAATCATAGA   
  
  
+ ACTTCATTCA CTTCCTTTTT GTATGGTAAT TAATTTTGGG GCGAAATATC AAGGGAAAAA GAGGACCTAA   
  
  
+ AGGTTTGAAT AGGATAATTT GGAGTTCTGG AACAGAATTA GGCATGTTTG AGAAGCATTC TACAAGAGTT   
  
  
+ GGCATAGTTT TTGCTCTAAA AACAAGAAAG TTTACTTTCT AAAAGGCTTA ATCTTCAATG CAGTGTTCTT   
  
  
+ TTCCATTTTT CTGGGATTAA TTGACGAGTC CGCGTACCCT CTTTTCCAGT TACTTCTTCG AGAAGCCCAT   
  
  
+ TTGCTAAAGT AGAATGGCTT TTTTCCATGA AGATATATTT TTCTTGCTAA GGTAGTCCCT GAAGCTAGTA   
  
  
+ GATTTACTGC TTAAGCTTGC CTGTTGAACG GCTTTTTGCC AAACCATGTT GACCGAAACG GGGAGCCTGA   
  
  
+ CTTGTTGAGG ATTGACATCA AATCGAAGCT GTGTAGCCCT AAGTACGTGG TTGTGTTGGT CTTAGAGACC   
  
  
+ CAGGGATACA CTCTTTGTTT AATTTTCCCA AATTATTTTT TTTCTTGTTT ACTTTCTTAA TTACGTGCTA   
  
  
+ AATTATGGAT TATACAATGG GTAGCAATAT ACATTATGTC ATTGCCTATT TTTTTAATTT TGTGGTGGAT   
  
  
+ CGTTTTTTTC AGGTTTGAAG TAGCTGCATG ATATTGTATC AGTACAGCCG ATATACTCTG ACATGTGAAT   
  
  
+ TCTTCAGTTT CTGATAGAAA ATAAGTTCA  

- AATATTATAG CAAAAGAATA AAACGTGTGC TTTAAATTTT ATTTTTCATT TTACCTTTTT ACTTTTTTGT   
  
  
- AGACGCCGCC CTGGGTGACA GGCCGAACAT GAGCGGTGTC CCTACGGTAG TGTCTCCCTC TAGTCTCTAA   
  
  
- GAAGTGACGA AGAGTGGCGT GTCTTTAGTA AAGAGGATGA GGAGAGACGA GAAGGGGGTG AGAGAGCTGA   
  
  
- GACTAATACC AAATCTGAAA ACCTGGAGTT GGAAAAGAGA GGGAGAGAAC GAGCCGAAGC GCCATTAAAA   
  
  
- GACTTTTGGC CTGAAGCCCC CAAATAGAAC GACCAAGCTT AAAGCTCCTG CTGCCCTATG CCAGGTCTAG   
  
  
- CTCAGTCTTC AGTTGCCGAG GTGCACTGCC GCCTCGTGAA CTAACCTGAA CGATCCCAAA AACACCGGGC   
  
  
- TCCAGCTTTA ATCTCCGAAC GACGAGCACG TCCATTAAGA AATTATCAAA ACCTCTATAA AGATCGAATG   
  
  
- CACAGTCAAC CTGCCTCAAA TATGAAAGAT ATAGTTTAAA AACACCTTTA GCCTCGAGGA TTAACCGAAA   
  
  
- CCGGACAAAC AAAGGGCTCT TTTACATCCT TTTTATTACT TTAAACCTTA AATTATCGAC AGATCACATT   
  
  
- TGGGAGGTGG TATTACTAGG ATCATGAGGA AACAGACACG TTTAACCTAA AAATAAAAAT TAACTAACCT   
  
  
- TAATTTTTTT ATTTCTTAGA GTCACGCTTA CTATCAATCG AATACAAAAC TAAACAGTAG CTTAGTATCT   
  
  
- TGAAGTAAGT GAAGGAAAAA CATACCATTA ATTAAAACCC CGCTTTATAG TTCCCTTTTT CTCCTGGATT   
  
  
- TCCAAACTTA TCCTATTAAA CCTCAAGACC TTGTCTTAAT CCGTACAAAC TCTTCGTAAG ATGTTCTCAA   
  
  
- CCGTATCAAA AACGAGATTT TTGTTCTTTC AAATGAAAGA TTTTCCGAAT TAGAAGTTAC GTCACAAGAA   
  
  
- AAGGTAAAAA GACCCTAATT AACTGCTCAG GCGCATGGGA GAAAAGGTCA ATGAAGAAGC TCTTCGGGTA   
  
  
- AACGATTTCA TCTTACCGAA AAAAGGTACT TCTATATAAA AAGAACGATT CCATCAGGGA CTTCGATCAT   
  
  
- CTAAATGACG AATTCGAACG GACAACTTGC CGAAAAACGG TTTGGTACAA CTGGCTTTGC CCCTCGGACT   
  
  
- GAACAACTCC TAACTGTAGT TTAGCTTCGA CACATCGGGA TTCATGCACC AACACAACCA GAATCTCTGG   
  
  
- GTCCCTATGT GAGAAACAAA TTAAAAGGGT TTAATAAAAA AAAGAACAAA TGAAAGAATT AATGCACGAT   
  
  
- TTAATACCTA ATATGTTACC CATCGTTATA TGTAATACAG TAACGGATAA AAAAATTAAA ACACCACCTA   
  
  
- GCAAAAAAAG TCCAAACTTC ATCGACGTAC TATAACATAG TCATGTCGGC TATATGAGAC TGTACACTTA   
  
  
- AGAAGTCAAA GACTATCTTT TATTCAAGT

+     Unnamed\_\_1

| Site Name | Organism | Position | Strand | Matrix score. | sequence | function |
| --- | --- | --- | --- | --- | --- | --- |
| Unnamed\_\_1 | Zea mays | 1236 | + | 5 | CGTGG |  |
| Unnamed\_\_1 | Zea mays | 370 | - | 5 | CGTGG |  |

> 2018/04/13 10:10:12  
+ TTATAATATC GTTTTCTTAT TTTGCACACG AAATTTAAAA TAAAAAGTAA AATGGAAAAA TGAAAAAACA   
  
  
+ TCTGCGGCGG GACCCACTGT CCGGCTTGTA CTCGCCACAG GGATGCCATC ACAGAGGGAG ATCAGAGATT   
  
  
+ CTTCACTGCT TCTCACCGCA CAGAAATCAT TTCTCCTACT CCTCTCTGCT CTTCCCCCAC TCTCTCGACT   
  
  
+ CTGATTATGG TTTAGACTTT TGGACCTCAA CCTTTTCTCT CCCTCTCTTG CTCGGCTTCG CGGTAATTTT   
  
  
+ CTGAAAACCG GACTTCGGGG GTTTATCTTG CTGGTTCGAA TTTCGAGGAC GACGGGATAC GGTCCAGATC   
  
  
+ GAGTCAGAAG TCAACGGCTC CACGTGACGG CGGAGCACTT GATTGGACTT GCTAGGGTTT TTGTGGCCCG   
  
  
+ AGGTCGAAAT TAGAGGCTTG CTGCTCGTGC AGGTAATTCT TTAATAGTTT TGGAGATATT TCTAGCTTAC   
  
  
+ GTGTCAGTTG GACGGAGTTT ATACTTTCTA TATCAAATTT TTGTGGAAAT CGGAGCTCCT AATTGGCTTT   
  
  
+ GGCCTGTTTG TTTCCCGAGA AAATGTAGGA AAAATAATGA AATTTGGAAT TTAATAGCTG TCTAGTGTAA   
  
  
+ ACCCTCCACC ATAATGATCC TAGTACTCCT TTGTCTGTGC AAATTGGATT TTTATTTTTA ATTGATTGGA   
  
  
+ ATTAAAAAAA TAAAGAATCT CAGTGCGAAT GATAGTTAGC TTATGTTTTG ATTTGTCATC GAATCATAGA   
  
  
+ ACTTCATTCA CTTCCTTTTT GTATGGTAAT TAATTTTGGG GCGAAATATC AAGGGAAAAA GAGGACCTAA   
  
  
+ AGGTTTGAAT AGGATAATTT GGAGTTCTGG AACAGAATTA GGCATGTTTG AGAAGCATTC TACAAGAGTT   
  
  
+ GGCATAGTTT TTGCTCTAAA AACAAGAAAG TTTACTTTCT AAAAGGCTTA ATCTTCAATG CAGTGTTCTT   
  
  
+ TTCCATTTTT CTGGGATTAA TTGACGAGTC CGCGTACCCT CTTTTCCAGT TACTTCTTCG AGAAGCCCAT   
  
  
+ TTGCTAAAGT AGAATGGCTT TTTTCCATGA AGATATATTT TTCTTGCTAA GGTAGTCCCT GAAGCTAGTA   
  
  
+ GATTTACTGC TTAAGCTTGC CTGTTGAACG GCTTTTTGCC AAACCATGTT GACCGAAACG GGGAGCCTGA   
  
  
+ CTTGTTGAGG ATTGACATCA AATCGAAGCT GTGTAGCCCT AAGTACGTGG TTGTGTTGGT CTTAGAGACC   
  
  
+ CAGGGATACA CTCTTTGTTT AATTTTCCCA AATTATTTTT TTTCTTGTTT ACTTTCTTAA TTACGTGCTA   
  
  
+ AATTATGGAT TATACAATGG GTAGCAATAT ACATTATGTC ATTGCCTATT TTTTTAATTT TGTGGTGGAT   
  
  
+ CGTTTTTTTC AGGTTTGAAG TAGCTGCATG ATATTGTATC AGTACAGCCG ATATACTCTG ACATGTGAAT   
  
  
+ TCTTCAGTTT CTGATAGAAA ATAAGTTCA  

- AATATTATAG CAAAAGAATA AAACGTGTGC TTTAAATTTT ATTTTTCATT TTACCTTTTT ACTTTTTTGT   
  
  
- AGACGCCGCC CTGGGTGACA GGCCGAACAT GAGCGGTGTC CCTACGGTAG TGTCTCCCTC TAGTCTCTAA   
  
  
- GAAGTGACGA AGAGTGGCGT GTCTTTAGTA AAGAGGATGA GGAGAGACGA GAAGGGGGTG AGAGAGCTGA   
  
  
- GACTAATACC AAATCTGAAA ACCTGGAGTT GGAAAAGAGA GGGAGAGAAC GAGCCGAAGC GCCATTAAAA   
  
  
- GACTTTTGGC CTGAAGCCCC CAAATAGAAC GACCAAGCTT AAAGCTCCTG CTGCCCTATG CCAGGTCTAG   
  
  
- CTCAGTCTTC AGTTGCCGAG GTGCACTGCC GCCTCGTGAA CTAACCTGAA CGATCCCAAA AACACCGGGC   
  
  
- TCCAGCTTTA ATCTCCGAAC GACGAGCACG TCCATTAAGA AATTATCAAA ACCTCTATAA AGATCGAATG   
  
  
- CACAGTCAAC CTGCCTCAAA TATGAAAGAT ATAGTTTAAA AACACCTTTA GCCTCGAGGA TTAACCGAAA   
  
  
- CCGGACAAAC AAAGGGCTCT TTTACATCCT TTTTATTACT TTAAACCTTA AATTATCGAC AGATCACATT   
  
  
- TGGGAGGTGG TATTACTAGG ATCATGAGGA AACAGACACG TTTAACCTAA AAATAAAAAT TAACTAACCT   
  
  
- TAATTTTTTT ATTTCTTAGA GTCACGCTTA CTATCAATCG AATACAAAAC TAAACAGTAG CTTAGTATCT   
  
  
- TGAAGTAAGT GAAGGAAAAA CATACCATTA ATTAAAACCC CGCTTTATAG TTCCCTTTTT CTCCTGGATT   
  
  
- TCCAAACTTA TCCTATTAAA CCTCAAGACC TTGTCTTAAT CCGTACAAAC TCTTCGTAAG ATGTTCTCAA   
  
  
- CCGTATCAAA AACGAGATTT TTGTTCTTTC AAATGAAAGA TTTTCCGAAT TAGAAGTTAC GTCACAAGAA   
  
  
- AAGGTAAAAA GACCCTAATT AACTGCTCAG GCGCATGGGA GAAAAGGTCA ATGAAGAAGC TCTTCGGGTA   
  
  
- AACGATTTCA TCTTACCGAA AAAAGGTACT TCTATATAAA AAGAACGATT CCATCAGGGA CTTCGATCAT   
  
  
- CTAAATGACG AATTCGAACG GACAACTTGC CGAAAAACGG TTTGGTACAA CTGGCTTTGC CCCTCGGACT   
  
  
- GAACAACTCC TAACTGTAGT TTAGCTTCGA CACATCGGGA TTCATGCACC AACACAACCA GAATCTCTGG   
  
  
- GTCCCTATGT GAGAAACAAA TTAAAAGGGT TTAATAAAAA AAAGAACAAA TGAAAGAATT AATGCACGAT   
  
  
- TTAATACCTA ATATGTTACC CATCGTTATA TGTAATACAG TAACGGATAA AAAAATTAAA ACACCACCTA   
  
  
- GCAAAAAAAG TCCAAACTTC ATCGACGTAC TATAACATAG TCATGTCGGC TATATGAGAC TGTACACTTA   
  
  
- AGAAGTCAAA GACTATCTTT TATTCAAGT

+     Unnamed\_\_3

| Site Name | Organism | Position | Strand | Matrix score. | sequence | function |
| --- | --- | --- | --- | --- | --- | --- |
| Unnamed\_\_3 | Zea mays | 370 | - | 5 | CGTGG |  |
| Unnamed\_\_3 | Zea mays | 1236 | + | 5 | CGTGG |  |

> 2018/04/13 10:10:12  
+ TTATAATATC GTTTTCTTAT TTTGCACACG AAATTTAAAA TAAAAAGTAA AATGGAAAAA TGAAAAAACA   
  
  
+ TCTGCGGCGG GACCCACTGT CCGGCTTGTA CTCGCCACAG GGATGCCATC ACAGAGGGAG ATCAGAGATT   
  
  
+ CTTCACTGCT TCTCACCGCA CAGAAATCAT TTCTCCTACT CCTCTCTGCT CTTCCCCCAC TCTCTCGACT   
  
  
+ CTGATTATGG TTTAGACTTT TGGACCTCAA CCTTTTCTCT CCCTCTCTTG CTCGGCTTCG CGGTAATTTT   
  
  
+ CTGAAAACCG GACTTCGGGG GTTTATCTTG CTGGTTCGAA TTTCGAGGAC GACGGGATAC GGTCCAGATC   
  
  
+ GAGTCAGAAG TCAACGGCTC CACGTGACGG CGGAGCACTT GATTGGACTT GCTAGGGTTT TTGTGGCCCG   
  
  
+ AGGTCGAAAT TAGAGGCTTG CTGCTCGTGC AGGTAATTCT TTAATAGTTT TGGAGATATT TCTAGCTTAC   
  
  
+ GTGTCAGTTG GACGGAGTTT ATACTTTCTA TATCAAATTT TTGTGGAAAT CGGAGCTCCT AATTGGCTTT   
  
  
+ GGCCTGTTTG TTTCCCGAGA AAATGTAGGA AAAATAATGA AATTTGGAAT TTAATAGCTG TCTAGTGTAA   
  
  
+ ACCCTCCACC ATAATGATCC TAGTACTCCT TTGTCTGTGC AAATTGGATT TTTATTTTTA ATTGATTGGA   
  
  
+ ATTAAAAAAA TAAAGAATCT CAGTGCGAAT GATAGTTAGC TTATGTTTTG ATTTGTCATC GAATCATAGA   
  
  
+ ACTTCATTCA CTTCCTTTTT GTATGGTAAT TAATTTTGGG GCGAAATATC AAGGGAAAAA GAGGACCTAA   
  
  
+ AGGTTTGAAT AGGATAATTT GGAGTTCTGG AACAGAATTA GGCATGTTTG AGAAGCATTC TACAAGAGTT   
  
  
+ GGCATAGTTT TTGCTCTAAA AACAAGAAAG TTTACTTTCT AAAAGGCTTA ATCTTCAATG CAGTGTTCTT   
  
  
+ TTCCATTTTT CTGGGATTAA TTGACGAGTC CGCGTACCCT CTTTTCCAGT TACTTCTTCG AGAAGCCCAT   
  
  
+ TTGCTAAAGT AGAATGGCTT TTTTCCATGA AGATATATTT TTCTTGCTAA GGTAGTCCCT GAAGCTAGTA   
  
  
+ GATTTACTGC TTAAGCTTGC CTGTTGAACG GCTTTTTGCC AAACCATGTT GACCGAAACG GGGAGCCTGA   
  
  
+ CTTGTTGAGG ATTGACATCA AATCGAAGCT GTGTAGCCCT AAGTACGTGG TTGTGTTGGT CTTAGAGACC   
  
  
+ CAGGGATACA CTCTTTGTTT AATTTTCCCA AATTATTTTT TTTCTTGTTT ACTTTCTTAA TTACGTGCTA   
  
  
+ AATTATGGAT TATACAATGG GTAGCAATAT ACATTATGTC ATTGCCTATT TTTTTAATTT TGTGGTGGAT   
  
  
+ CGTTTTTTTC AGGTTTGAAG TAGCTGCATG ATATTGTATC AGTACAGCCG ATATACTCTG ACATGTGAAT   
  
  
+ TCTTCAGTTT CTGATAGAAA ATAAGTTCA  

- AATATTATAG CAAAAGAATA AAACGTGTGC TTTAAATTTT ATTTTTCATT TTACCTTTTT ACTTTTTTGT   
  
  
- AGACGCCGCC CTGGGTGACA GGCCGAACAT GAGCGGTGTC CCTACGGTAG TGTCTCCCTC TAGTCTCTAA   
  
  
- GAAGTGACGA AGAGTGGCGT GTCTTTAGTA AAGAGGATGA GGAGAGACGA GAAGGGGGTG AGAGAGCTGA   
  
  
- GACTAATACC AAATCTGAAA ACCTGGAGTT GGAAAAGAGA GGGAGAGAAC GAGCCGAAGC GCCATTAAAA   
  
  
- GACTTTTGGC CTGAAGCCCC CAAATAGAAC GACCAAGCTT AAAGCTCCTG CTGCCCTATG CCAGGTCTAG   
  
  
- CTCAGTCTTC AGTTGCCGAG GTGCACTGCC GCCTCGTGAA CTAACCTGAA CGATCCCAAA AACACCGGGC   
  
  
- TCCAGCTTTA ATCTCCGAAC GACGAGCACG TCCATTAAGA AATTATCAAA ACCTCTATAA AGATCGAATG   
  
  
- CACAGTCAAC CTGCCTCAAA TATGAAAGAT ATAGTTTAAA AACACCTTTA GCCTCGAGGA TTAACCGAAA   
  
  
- CCGGACAAAC AAAGGGCTCT TTTACATCCT TTTTATTACT TTAAACCTTA AATTATCGAC AGATCACATT   
  
  
- TGGGAGGTGG TATTACTAGG ATCATGAGGA AACAGACACG TTTAACCTAA AAATAAAAAT TAACTAACCT   
  
  
- TAATTTTTTT ATTTCTTAGA GTCACGCTTA CTATCAATCG AATACAAAAC TAAACAGTAG CTTAGTATCT   
  
  
- TGAAGTAAGT GAAGGAAAAA CATACCATTA ATTAAAACCC CGCTTTATAG TTCCCTTTTT CTCCTGGATT   
  
  
- TCCAAACTTA TCCTATTAAA CCTCAAGACC TTGTCTTAAT CCGTACAAAC TCTTCGTAAG ATGTTCTCAA   
  
  
- CCGTATCAAA AACGAGATTT TTGTTCTTTC AAATGAAAGA TTTTCCGAAT TAGAAGTTAC GTCACAAGAA   
  
  
- AAGGTAAAAA GACCCTAATT AACTGCTCAG GCGCATGGGA GAAAAGGTCA ATGAAGAAGC TCTTCGGGTA   
  
  
- AACGATTTCA TCTTACCGAA AAAAGGTACT TCTATATAAA AAGAACGATT CCATCAGGGA CTTCGATCAT   
  
  
- CTAAATGACG AATTCGAACG GACAACTTGC CGAAAAACGG TTTGGTACAA CTGGCTTTGC CCCTCGGACT   
  
  
- GAACAACTCC TAACTGTAGT TTAGCTTCGA CACATCGGGA TTCATGCACC AACACAACCA GAATCTCTGG   
  
  
- GTCCCTATGT GAGAAACAAA TTAAAAGGGT TTAATAAAAA AAAGAACAAA TGAAAGAATT AATGCACGAT   
  
  
- TTAATACCTA ATATGTTACC CATCGTTATA TGTAATACAG TAACGGATAA AAAAATTAAA ACACCACCTA   
  
  
- GCAAAAAAAG TCCAAACTTC ATCGACGTAC TATAACATAG TCATGTCGGC TATATGAGAC TGTACACTTA   
  
  
- AGAAGTCAAA GACTATCTTT TATTCAAGT

+     Unnamed\_\_4

| Site Name | Organism | Position | Strand | Matrix score. | sequence | function |
| --- | --- | --- | --- | --- | --- | --- |
| Unnamed\_\_4 | Petroselinum hortense | 861 | - | 4 | CTCC |  |
| Unnamed\_\_4 | Petroselinum hortense | 546 | + | 4 | CTCC |  |
| Unnamed\_\_4 | Petroselinum hortense | 542 | - | 4 | CTCC |  |
| Unnamed\_\_4 | Petroselinum hortense | 656 | + | 4 | CTCC |  |
| Unnamed\_\_4 | Petroselinum hortense | 1182 | - | 4 | CTCC |  |
| Unnamed\_\_4 | Petroselinum hortense | 504 | - | 4 | CTCC |  |
| Unnamed\_\_4 | Petroselinum hortense | 472 | - | 4 | CTCC |  |
| Unnamed\_\_4 | Petroselinum hortense | 382 | - | 4 | CTCC |  |
| Unnamed\_\_4 | Petroselinum hortense | 368 | + | 4 | CTCC |  |
| Unnamed\_\_4 | Petroselinum hortense | 249 | + | 4 | CTCC |  |
| Unnamed\_\_4 | Petroselinum hortense | 634 | + | 4 | CTCC |  |
| Unnamed\_\_4 | Petroselinum hortense | 179 | + | 4 | CTCC |  |
| Unnamed\_\_4 | Petroselinum hortense | 127 | - | 4 | CTCC |  |
| Unnamed\_\_4 | Petroselinum hortense | 173 | + | 4 | CTCC |  |

> 2018/04/13 10:10:12  
+ TTATAATATC GTTTTCTTAT TTTGCACACG AAATTTAAAA TAAAAAGTAA AATGGAAAAA TGAAAAAACA   
  
  
+ TCTGCGGCGG GACCCACTGT CCGGCTTGTA CTCGCCACAG GGATGCCATC ACAGAGGGAG ATCAGAGATT   
  
  
+ CTTCACTGCT TCTCACCGCA CAGAAATCAT TTCTCCTACT CCTCTCTGCT CTTCCCCCAC TCTCTCGACT   
  
  
+ CTGATTATGG TTTAGACTTT TGGACCTCAA CCTTTTCTCT CCCTCTCTTG CTCGGCTTCG CGGTAATTTT   
  
  
+ CTGAAAACCG GACTTCGGGG GTTTATCTTG CTGGTTCGAA TTTCGAGGAC GACGGGATAC GGTCCAGATC   
  
  
+ GAGTCAGAAG TCAACGGCTC CACGTGACGG CGGAGCACTT GATTGGACTT GCTAGGGTTT TTGTGGCCCG   
  
  
+ AGGTCGAAAT TAGAGGCTTG CTGCTCGTGC AGGTAATTCT TTAATAGTTT TGGAGATATT TCTAGCTTAC   
  
  
+ GTGTCAGTTG GACGGAGTTT ATACTTTCTA TATCAAATTT TTGTGGAAAT CGGAGCTCCT AATTGGCTTT   
  
  
+ GGCCTGTTTG TTTCCCGAGA AAATGTAGGA AAAATAATGA AATTTGGAAT TTAATAGCTG TCTAGTGTAA   
  
  
+ ACCCTCCACC ATAATGATCC TAGTACTCCT TTGTCTGTGC AAATTGGATT TTTATTTTTA ATTGATTGGA   
  
  
+ ATTAAAAAAA TAAAGAATCT CAGTGCGAAT GATAGTTAGC TTATGTTTTG ATTTGTCATC GAATCATAGA   
  
  
+ ACTTCATTCA CTTCCTTTTT GTATGGTAAT TAATTTTGGG GCGAAATATC AAGGGAAAAA GAGGACCTAA   
  
  
+ AGGTTTGAAT AGGATAATTT GGAGTTCTGG AACAGAATTA GGCATGTTTG AGAAGCATTC TACAAGAGTT   
  
  
+ GGCATAGTTT TTGCTCTAAA AACAAGAAAG TTTACTTTCT AAAAGGCTTA ATCTTCAATG CAGTGTTCTT   
  
  
+ TTCCATTTTT CTGGGATTAA TTGACGAGTC CGCGTACCCT CTTTTCCAGT TACTTCTTCG AGAAGCCCAT   
  
  
+ TTGCTAAAGT AGAATGGCTT TTTTCCATGA AGATATATTT TTCTTGCTAA GGTAGTCCCT GAAGCTAGTA   
  
  
+ GATTTACTGC TTAAGCTTGC CTGTTGAACG GCTTTTTGCC AAACCATGTT GACCGAAACG GGGAGCCTGA   
  
  
+ CTTGTTGAGG ATTGACATCA AATCGAAGCT GTGTAGCCCT AAGTACGTGG TTGTGTTGGT CTTAGAGACC   
  
  
+ CAGGGATACA CTCTTTGTTT AATTTTCCCA AATTATTTTT TTTCTTGTTT ACTTTCTTAA TTACGTGCTA   
  
  
+ AATTATGGAT TATACAATGG GTAGCAATAT ACATTATGTC ATTGCCTATT TTTTTAATTT TGTGGTGGAT   
  
  
+ CGTTTTTTTC AGGTTTGAAG TAGCTGCATG ATATTGTATC AGTACAGCCG ATATACTCTG ACATGTGAAT   
  
  
+ TCTTCAGTTT CTGATAGAAA ATAAGTTCA  

- AATATTATAG CAAAAGAATA AAACGTGTGC TTTAAATTTT ATTTTTCATT TTACCTTTTT ACTTTTTTGT   
  
  
- AGACGCCGCC CTGGGTGACA GGCCGAACAT GAGCGGTGTC CCTACGGTAG TGTCTCCCTC TAGTCTCTAA   
  
  
- GAAGTGACGA AGAGTGGCGT GTCTTTAGTA AAGAGGATGA GGAGAGACGA GAAGGGGGTG AGAGAGCTGA   
  
  
- GACTAATACC AAATCTGAAA ACCTGGAGTT GGAAAAGAGA GGGAGAGAAC GAGCCGAAGC GCCATTAAAA   
  
  
- GACTTTTGGC CTGAAGCCCC CAAATAGAAC GACCAAGCTT AAAGCTCCTG CTGCCCTATG CCAGGTCTAG   
  
  
- CTCAGTCTTC AGTTGCCGAG GTGCACTGCC GCCTCGTGAA CTAACCTGAA CGATCCCAAA AACACCGGGC   
  
  
- TCCAGCTTTA ATCTCCGAAC GACGAGCACG TCCATTAAGA AATTATCAAA ACCTCTATAA AGATCGAATG   
  
  
- CACAGTCAAC CTGCCTCAAA TATGAAAGAT ATAGTTTAAA AACACCTTTA GCCTCGAGGA TTAACCGAAA   
  
  
- CCGGACAAAC AAAGGGCTCT TTTACATCCT TTTTATTACT TTAAACCTTA AATTATCGAC AGATCACATT   
  
  
- TGGGAGGTGG TATTACTAGG ATCATGAGGA AACAGACACG TTTAACCTAA AAATAAAAAT TAACTAACCT   
  
  
- TAATTTTTTT ATTTCTTAGA GTCACGCTTA CTATCAATCG AATACAAAAC TAAACAGTAG CTTAGTATCT   
  
  
- TGAAGTAAGT GAAGGAAAAA CATACCATTA ATTAAAACCC CGCTTTATAG TTCCCTTTTT CTCCTGGATT   
  
  
- TCCAAACTTA TCCTATTAAA CCTCAAGACC TTGTCTTAAT CCGTACAAAC TCTTCGTAAG ATGTTCTCAA   
  
  
- CCGTATCAAA AACGAGATTT TTGTTCTTTC AAATGAAAGA TTTTCCGAAT TAGAAGTTAC GTCACAAGAA   
  
  
- AAGGTAAAAA GACCCTAATT AACTGCTCAG GCGCATGGGA GAAAAGGTCA ATGAAGAAGC TCTTCGGGTA   
  
  
- AACGATTTCA TCTTACCGAA AAAAGGTACT TCTATATAAA AAGAACGATT CCATCAGGGA CTTCGATCAT   
  
  
- CTAAATGACG AATTCGAACG GACAACTTGC CGAAAAACGG TTTGGTACAA CTGGCTTTGC CCCTCGGACT   
  
  
- GAACAACTCC TAACTGTAGT TTAGCTTCGA CACATCGGGA TTCATGCACC AACACAACCA GAATCTCTGG   
  
  
- GTCCCTATGT GAGAAACAAA TTAAAAGGGT TTAATAAAAA AAAGAACAAA TGAAAGAATT AATGCACGAT   
  
  
- TTAATACCTA ATATGTTACC CATCGTTATA TGTAATACAG TAACGGATAA AAAAATTAAA ACACCACCTA   
  
  
- GCAAAAAAAG TCCAAACTTC ATCGACGTAC TATAACATAG TCATGTCGGC TATATGAGAC TGTACACTTA   
  
  
- AGAAGTCAAA GACTATCTTT TATTCAAGT

+     Unnamed\_\_6

| Site Name | Organism | Position | Strand | Matrix score. | sequence | function |
| --- | --- | --- | --- | --- | --- | --- |
| Unnamed\_\_6 | Zea mays | 474 | - | 10 | taTAAATATct |  |

> 2018/04/13 10:10:12  
+ TTATAATATC GTTTTCTTAT TTTGCACACG AAATTTAAAA TAAAAAGTAA AATGGAAAAA TGAAAAAACA   
  
  
+ TCTGCGGCGG GACCCACTGT CCGGCTTGTA CTCGCCACAG GGATGCCATC ACAGAGGGAG ATCAGAGATT   
  
  
+ CTTCACTGCT TCTCACCGCA CAGAAATCAT TTCTCCTACT CCTCTCTGCT CTTCCCCCAC TCTCTCGACT   
  
  
+ CTGATTATGG TTTAGACTTT TGGACCTCAA CCTTTTCTCT CCCTCTCTTG CTCGGCTTCG CGGTAATTTT   
  
  
+ CTGAAAACCG GACTTCGGGG GTTTATCTTG CTGGTTCGAA TTTCGAGGAC GACGGGATAC GGTCCAGATC   
  
  
+ GAGTCAGAAG TCAACGGCTC CACGTGACGG CGGAGCACTT GATTGGACTT GCTAGGGTTT TTGTGGCCCG   
  
  
+ AGGTCGAAAT TAGAGGCTTG CTGCTCGTGC AGGTAATTCT TTAATAGTTT TGGAGATATT TCTAGCTTAC   
  
  
+ GTGTCAGTTG GACGGAGTTT ATACTTTCTA TATCAAATTT TTGTGGAAAT CGGAGCTCCT AATTGGCTTT   
  
  
+ GGCCTGTTTG TTTCCCGAGA AAATGTAGGA AAAATAATGA AATTTGGAAT TTAATAGCTG TCTAGTGTAA   
  
  
+ ACCCTCCACC ATAATGATCC TAGTACTCCT TTGTCTGTGC AAATTGGATT TTTATTTTTA ATTGATTGGA   
  
  
+ ATTAAAAAAA TAAAGAATCT CAGTGCGAAT GATAGTTAGC TTATGTTTTG ATTTGTCATC GAATCATAGA   
  
  
+ ACTTCATTCA CTTCCTTTTT GTATGGTAAT TAATTTTGGG GCGAAATATC AAGGGAAAAA GAGGACCTAA   
  
  
+ AGGTTTGAAT AGGATAATTT GGAGTTCTGG AACAGAATTA GGCATGTTTG AGAAGCATTC TACAAGAGTT   
  
  
+ GGCATAGTTT TTGCTCTAAA AACAAGAAAG TTTACTTTCT AAAAGGCTTA ATCTTCAATG CAGTGTTCTT   
  
  
+ TTCCATTTTT CTGGGATTAA TTGACGAGTC CGCGTACCCT CTTTTCCAGT TACTTCTTCG AGAAGCCCAT   
  
  
+ TTGCTAAAGT AGAATGGCTT TTTTCCATGA AGATATATTT TTCTTGCTAA GGTAGTCCCT GAAGCTAGTA   
  
  
+ GATTTACTGC TTAAGCTTGC CTGTTGAACG GCTTTTTGCC AAACCATGTT GACCGAAACG GGGAGCCTGA   
  
  
+ CTTGTTGAGG ATTGACATCA AATCGAAGCT GTGTAGCCCT AAGTACGTGG TTGTGTTGGT CTTAGAGACC   
  
  
+ CAGGGATACA CTCTTTGTTT AATTTTCCCA AATTATTTTT TTTCTTGTTT ACTTTCTTAA TTACGTGCTA   
  
  
+ AATTATGGAT TATACAATGG GTAGCAATAT ACATTATGTC ATTGCCTATT TTTTTAATTT TGTGGTGGAT   
  
  
+ CGTTTTTTTC AGGTTTGAAG TAGCTGCATG ATATTGTATC AGTACAGCCG ATATACTCTG ACATGTGAAT   
  
  
+ TCTTCAGTTT CTGATAGAAA ATAAGTTCA  

- AATATTATAG CAAAAGAATA AAACGTGTGC TTTAAATTTT ATTTTTCATT TTACCTTTTT ACTTTTTTGT   
  
  
- AGACGCCGCC CTGGGTGACA GGCCGAACAT GAGCGGTGTC CCTACGGTAG TGTCTCCCTC TAGTCTCTAA   
  
  
- GAAGTGACGA AGAGTGGCGT GTCTTTAGTA AAGAGGATGA GGAGAGACGA GAAGGGGGTG AGAGAGCTGA   
  
  
- GACTAATACC AAATCTGAAA ACCTGGAGTT GGAAAAGAGA GGGAGAGAAC GAGCCGAAGC GCCATTAAAA   
  
  
- GACTTTTGGC CTGAAGCCCC CAAATAGAAC GACCAAGCTT AAAGCTCCTG CTGCCCTATG CCAGGTCTAG   
  
  
- CTCAGTCTTC AGTTGCCGAG GTGCACTGCC GCCTCGTGAA CTAACCTGAA CGATCCCAAA AACACCGGGC   
  
  
- TCCAGCTTTA ATCTCCGAAC GACGAGCACG TCCATTAAGA AATTATCAAA ACCTCTATAA AGATCGAATG   
  
  
- CACAGTCAAC CTGCCTCAAA TATGAAAGAT ATAGTTTAAA AACACCTTTA GCCTCGAGGA TTAACCGAAA   
  
  
- CCGGACAAAC AAAGGGCTCT TTTACATCCT TTTTATTACT TTAAACCTTA AATTATCGAC AGATCACATT   
  
  
- TGGGAGGTGG TATTACTAGG ATCATGAGGA AACAGACACG TTTAACCTAA AAATAAAAAT TAACTAACCT   
  
  
- TAATTTTTTT ATTTCTTAGA GTCACGCTTA CTATCAATCG AATACAAAAC TAAACAGTAG CTTAGTATCT   
  
  
- TGAAGTAAGT GAAGGAAAAA CATACCATTA ATTAAAACCC CGCTTTATAG TTCCCTTTTT CTCCTGGATT   
  
  
- TCCAAACTTA TCCTATTAAA CCTCAAGACC TTGTCTTAAT CCGTACAAAC TCTTCGTAAG ATGTTCTCAA   
  
  
- CCGTATCAAA AACGAGATTT TTGTTCTTTC AAATGAAAGA TTTTCCGAAT TAGAAGTTAC GTCACAAGAA   
  
  
- AAGGTAAAAA GACCCTAATT AACTGCTCAG GCGCATGGGA GAAAAGGTCA ATGAAGAAGC TCTTCGGGTA   
  
  
- AACGATTTCA TCTTACCGAA AAAAGGTACT TCTATATAAA AAGAACGATT CCATCAGGGA CTTCGATCAT   
  
  
- CTAAATGACG AATTCGAACG GACAACTTGC CGAAAAACGG TTTGGTACAA CTGGCTTTGC CCCTCGGACT   
  
  
- GAACAACTCC TAACTGTAGT TTAGCTTCGA CACATCGGGA TTCATGCACC AACACAACCA GAATCTCTGG   
  
  
- GTCCCTATGT GAGAAACAAA TTAAAAGGGT TTAATAAAAA AAAGAACAAA TGAAAGAATT AATGCACGAT   
  
  
- TTAATACCTA ATATGTTACC CATCGTTATA TGTAATACAG TAACGGATAA AAAAATTAAA ACACCACCTA   
  
  
- GCAAAAAAAG TCCAAACTTC ATCGACGTAC TATAACATAG TCATGTCGGC TATATGAGAC TGTACACTTA   
  
  
- AGAAGTCAAA GACTATCTTT TATTCAAGT

+     W box

| Site Name | Organism | Position | Strand | Matrix score. | sequence | function |
| --- | --- | --- | --- | --- | --- | --- |
| W box | Arabidopsis thaliana | 1169 | + | 6 | TTGACC |  |

> 2018/04/13 10:10:12  
+ TTATAATATC GTTTTCTTAT TTTGCACACG AAATTTAAAA TAAAAAGTAA AATGGAAAAA TGAAAAAACA   
  
  
+ TCTGCGGCGG GACCCACTGT CCGGCTTGTA CTCGCCACAG GGATGCCATC ACAGAGGGAG ATCAGAGATT   
  
  
+ CTTCACTGCT TCTCACCGCA CAGAAATCAT TTCTCCTACT CCTCTCTGCT CTTCCCCCAC TCTCTCGACT   
  
  
+ CTGATTATGG TTTAGACTTT TGGACCTCAA CCTTTTCTCT CCCTCTCTTG CTCGGCTTCG CGGTAATTTT   
  
  
+ CTGAAAACCG GACTTCGGGG GTTTATCTTG CTGGTTCGAA TTTCGAGGAC GACGGGATAC GGTCCAGATC   
  
  
+ GAGTCAGAAG TCAACGGCTC CACGTGACGG CGGAGCACTT GATTGGACTT GCTAGGGTTT TTGTGGCCCG   
  
  
+ AGGTCGAAAT TAGAGGCTTG CTGCTCGTGC AGGTAATTCT TTAATAGTTT TGGAGATATT TCTAGCTTAC   
  
  
+ GTGTCAGTTG GACGGAGTTT ATACTTTCTA TATCAAATTT TTGTGGAAAT CGGAGCTCCT AATTGGCTTT   
  
  
+ GGCCTGTTTG TTTCCCGAGA AAATGTAGGA AAAATAATGA AATTTGGAAT TTAATAGCTG TCTAGTGTAA   
  
  
+ ACCCTCCACC ATAATGATCC TAGTACTCCT TTGTCTGTGC AAATTGGATT TTTATTTTTA ATTGATTGGA   
  
  
+ ATTAAAAAAA TAAAGAATCT CAGTGCGAAT GATAGTTAGC TTATGTTTTG ATTTGTCATC GAATCATAGA   
  
  
+ ACTTCATTCA CTTCCTTTTT GTATGGTAAT TAATTTTGGG GCGAAATATC AAGGGAAAAA GAGGACCTAA   
  
  
+ AGGTTTGAAT AGGATAATTT GGAGTTCTGG AACAGAATTA GGCATGTTTG AGAAGCATTC TACAAGAGTT   
  
  
+ GGCATAGTTT TTGCTCTAAA AACAAGAAAG TTTACTTTCT AAAAGGCTTA ATCTTCAATG CAGTGTTCTT   
  
  
+ TTCCATTTTT CTGGGATTAA TTGACGAGTC CGCGTACCCT CTTTTCCAGT TACTTCTTCG AGAAGCCCAT   
  
  
+ TTGCTAAAGT AGAATGGCTT TTTTCCATGA AGATATATTT TTCTTGCTAA GGTAGTCCCT GAAGCTAGTA   
  
  
+ GATTTACTGC TTAAGCTTGC CTGTTGAACG GCTTTTTGCC AAACCATGTT GACCGAAACG GGGAGCCTGA   
  
  
+ CTTGTTGAGG ATTGACATCA AATCGAAGCT GTGTAGCCCT AAGTACGTGG TTGTGTTGGT CTTAGAGACC   
  
  
+ CAGGGATACA CTCTTTGTTT AATTTTCCCA AATTATTTTT TTTCTTGTTT ACTTTCTTAA TTACGTGCTA   
  
  
+ AATTATGGAT TATACAATGG GTAGCAATAT ACATTATGTC ATTGCCTATT TTTTTAATTT TGTGGTGGAT   
  
  
+ CGTTTTTTTC AGGTTTGAAG TAGCTGCATG ATATTGTATC AGTACAGCCG ATATACTCTG ACATGTGAAT   
  
  
+ TCTTCAGTTT CTGATAGAAA ATAAGTTCA  

- AATATTATAG CAAAAGAATA AAACGTGTGC TTTAAATTTT ATTTTTCATT TTACCTTTTT ACTTTTTTGT   
  
  
- AGACGCCGCC CTGGGTGACA GGCCGAACAT GAGCGGTGTC CCTACGGTAG TGTCTCCCTC TAGTCTCTAA   
  
  
- GAAGTGACGA AGAGTGGCGT GTCTTTAGTA AAGAGGATGA GGAGAGACGA GAAGGGGGTG AGAGAGCTGA   
  
  
- GACTAATACC AAATCTGAAA ACCTGGAGTT GGAAAAGAGA GGGAGAGAAC GAGCCGAAGC GCCATTAAAA   
  
  
- GACTTTTGGC CTGAAGCCCC CAAATAGAAC GACCAAGCTT AAAGCTCCTG CTGCCCTATG CCAGGTCTAG   
  
  
- CTCAGTCTTC AGTTGCCGAG GTGCACTGCC GCCTCGTGAA CTAACCTGAA CGATCCCAAA AACACCGGGC   
  
  
- TCCAGCTTTA ATCTCCGAAC GACGAGCACG TCCATTAAGA AATTATCAAA ACCTCTATAA AGATCGAATG   
  
  
- CACAGTCAAC CTGCCTCAAA TATGAAAGAT ATAGTTTAAA AACACCTTTA GCCTCGAGGA TTAACCGAAA   
  
  
- CCGGACAAAC AAAGGGCTCT TTTACATCCT TTTTATTACT TTAAACCTTA AATTATCGAC AGATCACATT   
  
  
- TGGGAGGTGG TATTACTAGG ATCATGAGGA AACAGACACG TTTAACCTAA AAATAAAAAT TAACTAACCT   
  
  
- TAATTTTTTT ATTTCTTAGA GTCACGCTTA CTATCAATCG AATACAAAAC TAAACAGTAG CTTAGTATCT   
  
  
- TGAAGTAAGT GAAGGAAAAA CATACCATTA ATTAAAACCC CGCTTTATAG TTCCCTTTTT CTCCTGGATT   
  
  
- TCCAAACTTA TCCTATTAAA CCTCAAGACC TTGTCTTAAT CCGTACAAAC TCTTCGTAAG ATGTTCTCAA   
  
  
- CCGTATCAAA AACGAGATTT TTGTTCTTTC AAATGAAAGA TTTTCCGAAT TAGAAGTTAC GTCACAAGAA   
  
  
- AAGGTAAAAA GACCCTAATT AACTGCTCAG GCGCATGGGA GAAAAGGTCA ATGAAGAAGC TCTTCGGGTA   
  
  
- AACGATTTCA TCTTACCGAA AAAAGGTACT TCTATATAAA AAGAACGATT CCATCAGGGA CTTCGATCAT   
  
  
- CTAAATGACG AATTCGAACG GACAACTTGC CGAAAAACGG TTTGGTACAA CTGGCTTTGC CCCTCGGACT   
  
  
- GAACAACTCC TAACTGTAGT TTAGCTTCGA CACATCGGGA TTCATGCACC AACACAACCA GAATCTCTGG   
  
  
- GTCCCTATGT GAGAAACAAA TTAAAAGGGT TTAATAAAAA AAAGAACAAA TGAAAGAATT AATGCACGAT   
  
  
- TTAATACCTA ATATGTTACC CATCGTTATA TGTAATACAG TAACGGATAA AAAAATTAAA ACACCACCTA   
  
  
- GCAAAAAAAG TCCAAACTTC ATCGACGTAC TATAACATAG TCATGTCGGC TATATGAGAC TGTACACTTA   
  
  
- AGAAGTCAAA GACTATCTTT TATTCAAGT

+     box II

| Site Name | Organism | Position | Strand | Matrix score. | sequence | function |
| --- | --- | --- | --- | --- | --- | --- |
| box II | Petroselinum hortense | 369 | + | 9 | TCCACGTGGC | part of a light responsive element |

> 2018/04/13 10:10:12  
+ TTATAATATC GTTTTCTTAT TTTGCACACG AAATTTAAAA TAAAAAGTAA AATGGAAAAA TGAAAAAACA   
  
  
+ TCTGCGGCGG GACCCACTGT CCGGCTTGTA CTCGCCACAG GGATGCCATC ACAGAGGGAG ATCAGAGATT   
  
  
+ CTTCACTGCT TCTCACCGCA CAGAAATCAT TTCTCCTACT CCTCTCTGCT CTTCCCCCAC TCTCTCGACT   
  
  
+ CTGATTATGG TTTAGACTTT TGGACCTCAA CCTTTTCTCT CCCTCTCTTG CTCGGCTTCG CGGTAATTTT   
  
  
+ CTGAAAACCG GACTTCGGGG GTTTATCTTG CTGGTTCGAA TTTCGAGGAC GACGGGATAC GGTCCAGATC   
  
  
+ GAGTCAGAAG TCAACGGCTC CACGTGACGG CGGAGCACTT GATTGGACTT GCTAGGGTTT TTGTGGCCCG   
  
  
+ AGGTCGAAAT TAGAGGCTTG CTGCTCGTGC AGGTAATTCT TTAATAGTTT TGGAGATATT TCTAGCTTAC   
  
  
+ GTGTCAGTTG GACGGAGTTT ATACTTTCTA TATCAAATTT TTGTGGAAAT CGGAGCTCCT AATTGGCTTT   
  
  
+ GGCCTGTTTG TTTCCCGAGA AAATGTAGGA AAAATAATGA AATTTGGAAT TTAATAGCTG TCTAGTGTAA   
  
  
+ ACCCTCCACC ATAATGATCC TAGTACTCCT TTGTCTGTGC AAATTGGATT TTTATTTTTA ATTGATTGGA   
  
  
+ ATTAAAAAAA TAAAGAATCT CAGTGCGAAT GATAGTTAGC TTATGTTTTG ATTTGTCATC GAATCATAGA   
  
  
+ ACTTCATTCA CTTCCTTTTT GTATGGTAAT TAATTTTGGG GCGAAATATC AAGGGAAAAA GAGGACCTAA   
  
  
+ AGGTTTGAAT AGGATAATTT GGAGTTCTGG AACAGAATTA GGCATGTTTG AGAAGCATTC TACAAGAGTT   
  
  
+ GGCATAGTTT TTGCTCTAAA AACAAGAAAG TTTACTTTCT AAAAGGCTTA ATCTTCAATG CAGTGTTCTT   
  
  
+ TTCCATTTTT CTGGGATTAA TTGACGAGTC CGCGTACCCT CTTTTCCAGT TACTTCTTCG AGAAGCCCAT   
  
  
+ TTGCTAAAGT AGAATGGCTT TTTTCCATGA AGATATATTT TTCTTGCTAA GGTAGTCCCT GAAGCTAGTA   
  
  
+ GATTTACTGC TTAAGCTTGC CTGTTGAACG GCTTTTTGCC AAACCATGTT GACCGAAACG GGGAGCCTGA   
  
  
+ CTTGTTGAGG ATTGACATCA AATCGAAGCT GTGTAGCCCT AAGTACGTGG TTGTGTTGGT CTTAGAGACC   
  
  
+ CAGGGATACA CTCTTTGTTT AATTTTCCCA AATTATTTTT TTTCTTGTTT ACTTTCTTAA TTACGTGCTA   
  
  
+ AATTATGGAT TATACAATGG GTAGCAATAT ACATTATGTC ATTGCCTATT TTTTTAATTT TGTGGTGGAT   
  
  
+ CGTTTTTTTC AGGTTTGAAG TAGCTGCATG ATATTGTATC AGTACAGCCG ATATACTCTG ACATGTGAAT   
  
  
+ TCTTCAGTTT CTGATAGAAA ATAAGTTCA  

- AATATTATAG CAAAAGAATA AAACGTGTGC TTTAAATTTT ATTTTTCATT TTACCTTTTT ACTTTTTTGT   
  
  
- AGACGCCGCC CTGGGTGACA GGCCGAACAT GAGCGGTGTC CCTACGGTAG TGTCTCCCTC TAGTCTCTAA   
  
  
- GAAGTGACGA AGAGTGGCGT GTCTTTAGTA AAGAGGATGA GGAGAGACGA GAAGGGGGTG AGAGAGCTGA   
  
  
- GACTAATACC AAATCTGAAA ACCTGGAGTT GGAAAAGAGA GGGAGAGAAC GAGCCGAAGC GCCATTAAAA   
  
  
- GACTTTTGGC CTGAAGCCCC CAAATAGAAC GACCAAGCTT AAAGCTCCTG CTGCCCTATG CCAGGTCTAG   
  
  
- CTCAGTCTTC AGTTGCCGAG GTGCACTGCC GCCTCGTGAA CTAACCTGAA CGATCCCAAA AACACCGGGC   
  
  
- TCCAGCTTTA ATCTCCGAAC GACGAGCACG TCCATTAAGA AATTATCAAA ACCTCTATAA AGATCGAATG   
  
  
- CACAGTCAAC CTGCCTCAAA TATGAAAGAT ATAGTTTAAA AACACCTTTA GCCTCGAGGA TTAACCGAAA   
  
  
- CCGGACAAAC AAAGGGCTCT TTTACATCCT TTTTATTACT TTAAACCTTA AATTATCGAC AGATCACATT   
  
  
- TGGGAGGTGG TATTACTAGG ATCATGAGGA AACAGACACG TTTAACCTAA AAATAAAAAT TAACTAACCT   
  
  
- TAATTTTTTT ATTTCTTAGA GTCACGCTTA CTATCAATCG AATACAAAAC TAAACAGTAG CTTAGTATCT   
  
  
- TGAAGTAAGT GAAGGAAAAA CATACCATTA ATTAAAACCC CGCTTTATAG TTCCCTTTTT CTCCTGGATT   
  
  
- TCCAAACTTA TCCTATTAAA CCTCAAGACC TTGTCTTAAT CCGTACAAAC TCTTCGTAAG ATGTTCTCAA   
  
  
- CCGTATCAAA AACGAGATTT TTGTTCTTTC AAATGAAAGA TTTTCCGAAT TAGAAGTTAC GTCACAAGAA   
  
  
- AAGGTAAAAA GACCCTAATT AACTGCTCAG GCGCATGGGA GAAAAGGTCA ATGAAGAAGC TCTTCGGGTA   
  
  
- AACGATTTCA TCTTACCGAA AAAAGGTACT TCTATATAAA AAGAACGATT CCATCAGGGA CTTCGATCAT   
  
  
- CTAAATGACG AATTCGAACG GACAACTTGC CGAAAAACGG TTTGGTACAA CTGGCTTTGC CCCTCGGACT   
  
  
- GAACAACTCC TAACTGTAGT TTAGCTTCGA CACATCGGGA TTCATGCACC AACACAACCA GAATCTCTGG   
  
  
- GTCCCTATGT GAGAAACAAA TTAAAAGGGT TTAATAAAAA AAAGAACAAA TGAAAGAATT AATGCACGAT   
  
  
- TTAATACCTA ATATGTTACC CATCGTTATA TGTAATACAG TAACGGATAA AAAAATTAAA ACACCACCTA   
  
  
- GCAAAAAAAG TCCAAACTTC ATCGACGTAC TATAACATAG TCATGTCGGC TATATGAGAC TGTACACTTA   
  
  
- AGAAGTCAAA GACTATCTTT TATTCAAGT
