# Supplementary material for: Genome-wide analysis of the WRKY gene family in drumstick (Moringa oleifera Lam.)
Source: PeerJ. 2019 Jun 10;7:e7063. doi: 10.7717/peerj.7063 (PMC6563795; doi:10.7717/peerj.7063)
Supplement: Supplemental Information 1 [file peerj-07-7063-s003.gz › MoWRKY9_plantcare.html]

Content-Type: text/html; charset=ISO-8859-1


CallMat\_Firefox


Webmaster Firefox specific output  
To save the result:
click on the frame with the right mouse button and save the source code as a text file with extension .html  
REFERENCE:PlantCARE: a database of plant cis-acting regulatory elements and a portal to tools for in silico analysis of promoter sequences.  
Lescot, M., Déhais, P., Moreau, Y., De Moor, B., Rouzé ,P.,and Rombauts, S.  
Nucleic Acids Res., Database issue(2002), 30(1):325-327.   


---

> 2018/04/13 10:10:12  
+ CGGCTGTCCC ACATCGAGCT CTGGAGCCAC GGGGACCAGG ACCGTGTATC GTGGCAAAGG TGGTTTCACG   
  
  
+ AAAAGGATGC AATAATTCTG ATAGATCTTC ACACAATTTC TTTTTTTTTT TCTAATTTTT ATTTTTATTT   
  
  
+ ACTCTCTGAT GTAAATAAAT TTATTATATA TTATTATTAT TAATTTTTTT TGCGTGGGAA GGAAGTTGTA   
  
  
+ CCTTGGGGTG ACCATATTGT AAAAAGGTGT GTGCAATGAT AACAAGAAGA GACATACACA CATCACACGT   
  
  
+ ACGAGACTCC CTTTGCTTCA TCATTCTGGG TAACTTGTTC ATCATGTCAC GATCATCCCA ATCTCCCGAT   
  
  
+ TTATTTTTCC ATGCATTATG TAATATATAC GCAACGTTTT GATTTTTATA ATTGTATCAT TTTTTAGTTT   
  
  
+ AAAAAAATTT GTAAATAATA TTTAATCTAT TTTTTATAAA TTATTTTGAT TAAGTCCAAA ATTATTTATA   
  
  
+ AAAGTAATGC AAAGTTAAAT GAATTCAGAA ATCGGAAATT TAAAAAAGAG AGGGAAATGT GGTGTGAAGA   
  
  
+ GGGAGGAGAG TGGAGGCTTG TCGGGCAAGG GGAGTGGGTG ACGCGTAATA CGCGTCAGCC TAAGCGAGCC   
  
  
+ TTATGGTGTA GGCTGAAGCT TCTTTAACTT TACCCCATTT TTTTATTTTC ATAATTTGTT AGTTTAATTT   
  
  
+ TATATTTTAT ATTTTTAAAA AGAAAAAAAA CTTAATTTAA CGGGGAGACC TTTGTCTGCA GGAAAGTAAT   
  
  
+ CTTCTAGTTA CTGAATTGCC CCTGAAGAGG ATGATAGAGT GAGGGGCATT TAGGTAAATT CATGATTGAG   
  
  
+ AGAGAGAGAG AGAGAGAGAG AGAGAGAGTG TTTTGATGTA AAACAAAAAG ATAATTTTGG GGTTTAAAAG   
  
  
+ TTAAAAAGGT TTTTAAAGGC AAAAGCTGCG AAAGAAAATT AGAAATAAAA AAATTAAAAA AAAAACAAGG   
  
  
+ AGGAGAAGCA ATCACATGCC TGACGACCCA AACGTTCTGC AGCCCACGTG TAGGTTTGGG CCCCACTGTG   
  
  
+ GACGCGCCAC CGGCTAAACT GGTCGACATG CCGCGTAATA AAGGGTGGAC CCGAGGGCGG TCCTGGAGCA   
  
  
+ ATTAATGCAC AACGCCGTCC TCTAGTCACC CTCATACCGT ACACATGGTT AACCGGCATT TGCCAGTCAA   
  
  
+ CTCTTCTGTT TTATTTTCTA TTTAAAATAA TTTATCAAAT GGGAAACGTC CCAAAACAAG CTGTTTTCCA   
  
  
+ AAAACCCAAA CACAAAAAGC CCATTCACAC TCGAAGCAAC CAAACACTAT TTCTCTTTCT CTCTCTGTCT   
  
  
+ CTCACTTTTT TTCTCTCTCT CTCTCCCTCT CTTGTAATAA AAAGTGCGAG AAGAGAAGAA ATGAGAGAGG   
  
  
+ GTTTTATGAA ATGAAGGAGG AGATCATTAT GTAGTACATG TGCTCGCGTT GGTGATCAGA GATTTCTATG   
  
  
+ GAGAAGAGAG AAGAAAAGAG AGAGAATTC  

- GCCGACAGGG TGTAGCTCGA GACCTCGGTG CCCCTGGTCC TGGCACATAG CACCGTTTCC ACCAAAGTGC   
  
  
- TTTTCCTACG TTATTAAGAC TATCTAGAAG TGTGTTAAAG AAAAAAAAAA AGATTAAAAA TAAAAATAAA   
  
  
- TGAGAGACTA CATTTATTTA AATAATATAT AATAATAATA ATTAAAAAAA ACGCACCCTT CCTTCAACAT   
  
  
- GGAACCCCAC TGGTATAACA TTTTTCCACA CACGTTACTA TTGTTCTTCT CTGTATGTGT GTAGTGTGCA   
  
  
- TGCTCTGAGG GAAACGAAGT AGTAAGACCC ATTGAACAAG TAGTACAGTG CTAGTAGGGT TAGAGGGCTA   
  
  
- AATAAAAAGG TACGTAATAC ATTATATATG CGTTGCAAAA CTAAAAATAT TAACATAGTA AAAAATCAAA   
  
  
- TTTTTTTAAA CATTTATTAT AAATTAGATA AAAAATATTT AATAAAACTA ATTCAGGTTT TAATAAATAT   
  
  
- TTTCATTACG TTTCAATTTA CTTAAGTCTT TAGCCTTTAA ATTTTTTCTC TCCCTTTACA CCACACTTCT   
  
  
- CCCTCCTCTC ACCTCCGAAC AGCCCGTTCC CCTCACCCAC TGCGCATTAT GCGCAGTCGG ATTCGCTCGG   
  
  
- AATACCACAT CCGACTTCGA AGAAATTGAA ATGGGGTAAA AAAATAAAAG TATTAAACAA TCAAATTAAA   
  
  
- ATATAAAATA TAAAAATTTT TCTTTTTTTT GAATTAAATT GCCCCTCTGG AAACAGACGT CCTTTCATTA   
  
  
- GAAGATCAAT GACTTAACGG GGACTTCTCC TACTATCTCA CTCCCCGTAA ATCCATTTAA GTACTAACTC   
  
  
- TCTCTCTCTC TCTCTCTCTC TCTCTCTCAC AAAACTACAT TTTGTTTTTC TATTAAAACC CCAAATTTTC   
  
  
- AATTTTTCCA AAAATTTCCG TTTTCGACGC TTTCTTTTAA TCTTTATTTT TTTAATTTTT TTTTTGTTCC   
  
  
- TCCTCTTCGT TAGTGTACGG ACTGCTGGGT TTGCAAGACG TCGGGTGCAC ATCCAAACCC GGGGTGACAC   
  
  
- CTGCGCGGTG GCCGATTTGA CCAGCTGTAC GGCGCATTAT TTCCCACCTG GGCTCCCGCC AGGACCTCGT   
  
  
- TAATTACGTG TTGCGGCAGG AGATCAGTGG GAGTATGGCA TGTGTACCAA TTGGCCGTAA ACGGTCAGTT   
  
  
- GAGAAGACAA AATAAAAGAT AAATTTTATT AAATAGTTTA CCCTTTGCAG GGTTTTGTTC GACAAAAGGT   
  
  
- TTTTGGGTTT GTGTTTTTCG GGTAAGTGTG AGCTTCGTTG GTTTGTGATA AAGAGAAAGA GAGAGACAGA   
  
  
- GAGTGAAAAA AAGAGAGAGA GAGAGGGAGA GAACATTATT TTTCACGCTC TTCTCTTCTT TACTCTCTCC   
  
  
- CAAAATACTT TACTTCCTCC TCTAGTAATA CATCATGTAC ACGAGCGCAA CCACTAGTCT CTAAAGATAC   
  
  
- CTCTTCTCTC TTCTTTTCTC TCTCTTAAG

  
  
Motifs Found  

+     5UTR Py-rich stretch

| Site Name | Organism | Position | Strand | Matrix score. | sequence | function |
| --- | --- | --- | --- | --- | --- | --- |
| 5UTR Py-rich stretch | Lycopersicon esculentum | 1382 | - | 10 | TTTCTTCTCT | cis-acting element conferring high transcription levels |
| 5UTR Py-rich stretch | Lycopersicon esculentum | 1342 | + | 13 | TTTCTCTCTCTCTC | cis-acting element conferring high transcription levels |
| 5UTR Py-rich stretch | Lycopersicon esculentum | 1477 | - | 10 | TTTCTTCTCT | cis-acting element conferring high transcription levels |
| 5UTR Py-rich stretch | Lycopersicon esculentum | 1340 | + | 14 | TTTCTCTCTCTCTC | cis-acting element conferring high transcription levels |
| 5UTR Py-rich stretch | Lycopersicon esculentum | 854 | - | 13 | TTTCTCTCTCTCTC | cis-acting element conferring high transcription levels |
| 5UTR Py-rich stretch | Lycopersicon esculentum | 1337 | + | 9 | TTTCTTCTCT | cis-acting element conferring high transcription levels |
| 5UTR Py-rich stretch | Lycopersicon esculentum | 1316 | + | 13 | TTTCTCTCTCTCTC | cis-acting element conferring high transcription levels |
| 5UTR Py-rich stretch | Lycopersicon esculentum | 846 | - | 13 | TTTCTCTCTCTCTC | cis-acting element conferring high transcription levels |
| 5UTR Py-rich stretch | Lycopersicon esculentum | 852 | - | 13 | TTTCTCTCTCTCTC | cis-acting element conferring high transcription levels |
| 5UTR Py-rich stretch | Lycopersicon esculentum | 1338 | + | 13 | TTTCTCTCTCTCTC | cis-acting element conferring high transcription levels |
| 5UTR Py-rich stretch | Lycopersicon esculentum | 850 | - | 13 | TTTCTCTCTCTCTC | cis-acting element conferring high transcription levels |
| 5UTR Py-rich stretch | Lycopersicon esculentum | 1310 | + | 13 | TTTCTCTCTCTCTC | cis-acting element conferring high transcription levels |
| 5UTR Py-rich stretch | Lycopersicon esculentum | 848 | - | 13 | TTTCTCTCTCTCTC | cis-acting element conferring high transcription levels |
| 5UTR Py-rich stretch | Lycopersicon esculentum | 842 | - | 13 | TTTCTCTCTCTCTC | cis-acting element conferring high transcription levels |
| 5UTR Py-rich stretch | Lycopersicon esculentum | 844 | - | 13 | TTTCTCTCTCTCTC | cis-acting element conferring high transcription levels |
| 5UTR Py-rich stretch | Lycopersicon esculentum | 840 | - | 13 | TTTCTCTCTCTCTC | cis-acting element conferring high transcription levels |
| 5UTR Py-rich stretch | Lycopersicon esculentum | 838 | - | 13 | TTTCTCTCTCTCTC | cis-acting element conferring high transcription levels |

> 2018/04/13 10:10:12  
+ CGGCTGTCCC ACATCGAGCT CTGGAGCCAC GGGGACCAGG ACCGTGTATC GTGGCAAAGG TGGTTTCACG   
  
  
+ AAAAGGATGC AATAATTCTG ATAGATCTTC ACACAATTTC TTTTTTTTTT TCTAATTTTT ATTTTTATTT   
  
  
+ ACTCTCTGAT GTAAATAAAT TTATTATATA TTATTATTAT TAATTTTTTT TGCGTGGGAA GGAAGTTGTA   
  
  
+ CCTTGGGGTG ACCATATTGT AAAAAGGTGT GTGCAATGAT AACAAGAAGA GACATACACA CATCACACGT   
  
  
+ ACGAGACTCC CTTTGCTTCA TCATTCTGGG TAACTTGTTC ATCATGTCAC GATCATCCCA ATCTCCCGAT   
  
  
+ TTATTTTTCC ATGCATTATG TAATATATAC GCAACGTTTT GATTTTTATA ATTGTATCAT TTTTTAGTTT   
  
  
+ AAAAAAATTT GTAAATAATA TTTAATCTAT TTTTTATAAA TTATTTTGAT TAAGTCCAAA ATTATTTATA   
  
  
+ AAAGTAATGC AAAGTTAAAT GAATTCAGAA ATCGGAAATT TAAAAAAGAG AGGGAAATGT GGTGTGAAGA   
  
  
+ GGGAGGAGAG TGGAGGCTTG TCGGGCAAGG GGAGTGGGTG ACGCGTAATA CGCGTCAGCC TAAGCGAGCC   
  
  
+ TTATGGTGTA GGCTGAAGCT TCTTTAACTT TACCCCATTT TTTTATTTTC ATAATTTGTT AGTTTAATTT   
  
  
+ TATATTTTAT ATTTTTAAAA AGAAAAAAAA CTTAATTTAA CGGGGAGACC TTTGTCTGCA GGAAAGTAAT   
  
  
+ CTTCTAGTTA CTGAATTGCC CCTGAAGAGG ATGATAGAGT GAGGGGCATT TAGGTAAATT CATGATTGAG   
  
  
+ AGAGAGAGAG AGAGAGAGAG AGAGAGAGTG TTTTGATGTA AAACAAAAAG ATAATTTTGG GGTTTAAAAG   
  
  
+ TTAAAAAGGT TTTTAAAGGC AAAAGCTGCG AAAGAAAATT AGAAATAAAA AAATTAAAAA AAAAACAAGG   
  
  
+ AGGAGAAGCA ATCACATGCC TGACGACCCA AACGTTCTGC AGCCCACGTG TAGGTTTGGG CCCCACTGTG   
  
  
+ GACGCGCCAC CGGCTAAACT GGTCGACATG CCGCGTAATA AAGGGTGGAC CCGAGGGCGG TCCTGGAGCA   
  
  
+ ATTAATGCAC AACGCCGTCC TCTAGTCACC CTCATACCGT ACACATGGTT AACCGGCATT TGCCAGTCAA   
  
  
+ CTCTTCTGTT TTATTTTCTA TTTAAAATAA TTTATCAAAT GGGAAACGTC CCAAAACAAG CTGTTTTCCA   
  
  
+ AAAACCCAAA CACAAAAAGC CCATTCACAC TCGAAGCAAC CAAACACTAT TTCTCTTTCT CTCTCTGTCT   
  
  
+ CTCACTTTTT TTCTCTCTCT CTCTCCCTCT CTTGTAATAA AAAGTGCGAG AAGAGAAGAA ATGAGAGAGG   
  
  
+ GTTTTATGAA ATGAAGGAGG AGATCATTAT GTAGTACATG TGCTCGCGTT GGTGATCAGA GATTTCTATG   
  
  
+ GAGAAGAGAG AAGAAAAGAG AGAGAATTC  

- GCCGACAGGG TGTAGCTCGA GACCTCGGTG CCCCTGGTCC TGGCACATAG CACCGTTTCC ACCAAAGTGC   
  
  
- TTTTCCTACG TTATTAAGAC TATCTAGAAG TGTGTTAAAG AAAAAAAAAA AGATTAAAAA TAAAAATAAA   
  
  
- TGAGAGACTA CATTTATTTA AATAATATAT AATAATAATA ATTAAAAAAA ACGCACCCTT CCTTCAACAT   
  
  
- GGAACCCCAC TGGTATAACA TTTTTCCACA CACGTTACTA TTGTTCTTCT CTGTATGTGT GTAGTGTGCA   
  
  
- TGCTCTGAGG GAAACGAAGT AGTAAGACCC ATTGAACAAG TAGTACAGTG CTAGTAGGGT TAGAGGGCTA   
  
  
- AATAAAAAGG TACGTAATAC ATTATATATG CGTTGCAAAA CTAAAAATAT TAACATAGTA AAAAATCAAA   
  
  
- TTTTTTTAAA CATTTATTAT AAATTAGATA AAAAATATTT AATAAAACTA ATTCAGGTTT TAATAAATAT   
  
  
- TTTCATTACG TTTCAATTTA CTTAAGTCTT TAGCCTTTAA ATTTTTTCTC TCCCTTTACA CCACACTTCT   
  
  
- CCCTCCTCTC ACCTCCGAAC AGCCCGTTCC CCTCACCCAC TGCGCATTAT GCGCAGTCGG ATTCGCTCGG   
  
  
- AATACCACAT CCGACTTCGA AGAAATTGAA ATGGGGTAAA AAAATAAAAG TATTAAACAA TCAAATTAAA   
  
  
- ATATAAAATA TAAAAATTTT TCTTTTTTTT GAATTAAATT GCCCCTCTGG AAACAGACGT CCTTTCATTA   
  
  
- GAAGATCAAT GACTTAACGG GGACTTCTCC TACTATCTCA CTCCCCGTAA ATCCATTTAA GTACTAACTC   
  
  
- TCTCTCTCTC TCTCTCTCTC TCTCTCTCAC AAAACTACAT TTTGTTTTTC TATTAAAACC CCAAATTTTC   
  
  
- AATTTTTCCA AAAATTTCCG TTTTCGACGC TTTCTTTTAA TCTTTATTTT TTTAATTTTT TTTTTGTTCC   
  
  
- TCCTCTTCGT TAGTGTACGG ACTGCTGGGT TTGCAAGACG TCGGGTGCAC ATCCAAACCC GGGGTGACAC   
  
  
- CTGCGCGGTG GCCGATTTGA CCAGCTGTAC GGCGCATTAT TTCCCACCTG GGCTCCCGCC AGGACCTCGT   
  
  
- TAATTACGTG TTGCGGCAGG AGATCAGTGG GAGTATGGCA TGTGTACCAA TTGGCCGTAA ACGGTCAGTT   
  
  
- GAGAAGACAA AATAAAAGAT AAATTTTATT AAATAGTTTA CCCTTTGCAG GGTTTTGTTC GACAAAAGGT   
  
  
- TTTTGGGTTT GTGTTTTTCG GGTAAGTGTG AGCTTCGTTG GTTTGTGATA AAGAGAAAGA GAGAGACAGA   
  
  
- GAGTGAAAAA AAGAGAGAGA GAGAGGGAGA GAACATTATT TTTCACGCTC TTCTCTTCTT TACTCTCTCC   
  
  
- CAAAATACTT TACTTCCTCC TCTAGTAATA CATCATGTAC ACGAGCGCAA CCACTAGTCT CTAAAGATAC   
  
  
- CTCTTCTCTC TTCTTTTCTC TCTCTTAAG

+     A-box

| Site Name | Organism | Position | Strand | Matrix score. | sequence | function |
| --- | --- | --- | --- | --- | --- | --- |
| A-box | Petroselinum crispum | 1135 | + | 6 | CCGTCC | cis-acting regulatory element |

> 2018/04/13 10:10:12  
+ CGGCTGTCCC ACATCGAGCT CTGGAGCCAC GGGGACCAGG ACCGTGTATC GTGGCAAAGG TGGTTTCACG   
  
  
+ AAAAGGATGC AATAATTCTG ATAGATCTTC ACACAATTTC TTTTTTTTTT TCTAATTTTT ATTTTTATTT   
  
  
+ ACTCTCTGAT GTAAATAAAT TTATTATATA TTATTATTAT TAATTTTTTT TGCGTGGGAA GGAAGTTGTA   
  
  
+ CCTTGGGGTG ACCATATTGT AAAAAGGTGT GTGCAATGAT AACAAGAAGA GACATACACA CATCACACGT   
  
  
+ ACGAGACTCC CTTTGCTTCA TCATTCTGGG TAACTTGTTC ATCATGTCAC GATCATCCCA ATCTCCCGAT   
  
  
+ TTATTTTTCC ATGCATTATG TAATATATAC GCAACGTTTT GATTTTTATA ATTGTATCAT TTTTTAGTTT   
  
  
+ AAAAAAATTT GTAAATAATA TTTAATCTAT TTTTTATAAA TTATTTTGAT TAAGTCCAAA ATTATTTATA   
  
  
+ AAAGTAATGC AAAGTTAAAT GAATTCAGAA ATCGGAAATT TAAAAAAGAG AGGGAAATGT GGTGTGAAGA   
  
  
+ GGGAGGAGAG TGGAGGCTTG TCGGGCAAGG GGAGTGGGTG ACGCGTAATA CGCGTCAGCC TAAGCGAGCC   
  
  
+ TTATGGTGTA GGCTGAAGCT TCTTTAACTT TACCCCATTT TTTTATTTTC ATAATTTGTT AGTTTAATTT   
  
  
+ TATATTTTAT ATTTTTAAAA AGAAAAAAAA CTTAATTTAA CGGGGAGACC TTTGTCTGCA GGAAAGTAAT   
  
  
+ CTTCTAGTTA CTGAATTGCC CCTGAAGAGG ATGATAGAGT GAGGGGCATT TAGGTAAATT CATGATTGAG   
  
  
+ AGAGAGAGAG AGAGAGAGAG AGAGAGAGTG TTTTGATGTA AAACAAAAAG ATAATTTTGG GGTTTAAAAG   
  
  
+ TTAAAAAGGT TTTTAAAGGC AAAAGCTGCG AAAGAAAATT AGAAATAAAA AAATTAAAAA AAAAACAAGG   
  
  
+ AGGAGAAGCA ATCACATGCC TGACGACCCA AACGTTCTGC AGCCCACGTG TAGGTTTGGG CCCCACTGTG   
  
  
+ GACGCGCCAC CGGCTAAACT GGTCGACATG CCGCGTAATA AAGGGTGGAC CCGAGGGCGG TCCTGGAGCA   
  
  
+ ATTAATGCAC AACGCCGTCC TCTAGTCACC CTCATACCGT ACACATGGTT AACCGGCATT TGCCAGTCAA   
  
  
+ CTCTTCTGTT TTATTTTCTA TTTAAAATAA TTTATCAAAT GGGAAACGTC CCAAAACAAG CTGTTTTCCA   
  
  
+ AAAACCCAAA CACAAAAAGC CCATTCACAC TCGAAGCAAC CAAACACTAT TTCTCTTTCT CTCTCTGTCT   
  
  
+ CTCACTTTTT TTCTCTCTCT CTCTCCCTCT CTTGTAATAA AAAGTGCGAG AAGAGAAGAA ATGAGAGAGG   
  
  
+ GTTTTATGAA ATGAAGGAGG AGATCATTAT GTAGTACATG TGCTCGCGTT GGTGATCAGA GATTTCTATG   
  
  
+ GAGAAGAGAG AAGAAAAGAG AGAGAATTC  

- GCCGACAGGG TGTAGCTCGA GACCTCGGTG CCCCTGGTCC TGGCACATAG CACCGTTTCC ACCAAAGTGC   
  
  
- TTTTCCTACG TTATTAAGAC TATCTAGAAG TGTGTTAAAG AAAAAAAAAA AGATTAAAAA TAAAAATAAA   
  
  
- TGAGAGACTA CATTTATTTA AATAATATAT AATAATAATA ATTAAAAAAA ACGCACCCTT CCTTCAACAT   
  
  
- GGAACCCCAC TGGTATAACA TTTTTCCACA CACGTTACTA TTGTTCTTCT CTGTATGTGT GTAGTGTGCA   
  
  
- TGCTCTGAGG GAAACGAAGT AGTAAGACCC ATTGAACAAG TAGTACAGTG CTAGTAGGGT TAGAGGGCTA   
  
  
- AATAAAAAGG TACGTAATAC ATTATATATG CGTTGCAAAA CTAAAAATAT TAACATAGTA AAAAATCAAA   
  
  
- TTTTTTTAAA CATTTATTAT AAATTAGATA AAAAATATTT AATAAAACTA ATTCAGGTTT TAATAAATAT   
  
  
- TTTCATTACG TTTCAATTTA CTTAAGTCTT TAGCCTTTAA ATTTTTTCTC TCCCTTTACA CCACACTTCT   
  
  
- CCCTCCTCTC ACCTCCGAAC AGCCCGTTCC CCTCACCCAC TGCGCATTAT GCGCAGTCGG ATTCGCTCGG   
  
  
- AATACCACAT CCGACTTCGA AGAAATTGAA ATGGGGTAAA AAAATAAAAG TATTAAACAA TCAAATTAAA   
  
  
- ATATAAAATA TAAAAATTTT TCTTTTTTTT GAATTAAATT GCCCCTCTGG AAACAGACGT CCTTTCATTA   
  
  
- GAAGATCAAT GACTTAACGG GGACTTCTCC TACTATCTCA CTCCCCGTAA ATCCATTTAA GTACTAACTC   
  
  
- TCTCTCTCTC TCTCTCTCTC TCTCTCTCAC AAAACTACAT TTTGTTTTTC TATTAAAACC CCAAATTTTC   
  
  
- AATTTTTCCA AAAATTTCCG TTTTCGACGC TTTCTTTTAA TCTTTATTTT TTTAATTTTT TTTTTGTTCC   
  
  
- TCCTCTTCGT TAGTGTACGG ACTGCTGGGT TTGCAAGACG TCGGGTGCAC ATCCAAACCC GGGGTGACAC   
  
  
- CTGCGCGGTG GCCGATTTGA CCAGCTGTAC GGCGCATTAT TTCCCACCTG GGCTCCCGCC AGGACCTCGT   
  
  
- TAATTACGTG TTGCGGCAGG AGATCAGTGG GAGTATGGCA TGTGTACCAA TTGGCCGTAA ACGGTCAGTT   
  
  
- GAGAAGACAA AATAAAAGAT AAATTTTATT AAATAGTTTA CCCTTTGCAG GGTTTTGTTC GACAAAAGGT   
  
  
- TTTTGGGTTT GTGTTTTTCG GGTAAGTGTG AGCTTCGTTG GTTTGTGATA AAGAGAAAGA GAGAGACAGA   
  
  
- GAGTGAAAAA AAGAGAGAGA GAGAGGGAGA GAACATTATT TTTCACGCTC TTCTCTTCTT TACTCTCTCC   
  
  
- CAAAATACTT TACTTCCTCC TCTAGTAATA CATCATGTAC ACGAGCGCAA CCACTAGTCT CTAAAGATAC   
  
  
- CTCTTCTCTC TTCTTTTCTC TCTCTTAAG

+     AAGAA-motif

| Site Name | Organism | Position | Strand | Matrix score. | sequence | function |
| --- | --- | --- | --- | --- | --- | --- |
| AAGAA-motif | Avena sativa | 940 | + | 7 | GAAAGAA |  |

> 2018/04/13 10:10:12  
+ CGGCTGTCCC ACATCGAGCT CTGGAGCCAC GGGGACCAGG ACCGTGTATC GTGGCAAAGG TGGTTTCACG   
  
  
+ AAAAGGATGC AATAATTCTG ATAGATCTTC ACACAATTTC TTTTTTTTTT TCTAATTTTT ATTTTTATTT   
  
  
+ ACTCTCTGAT GTAAATAAAT TTATTATATA TTATTATTAT TAATTTTTTT TGCGTGGGAA GGAAGTTGTA   
  
  
+ CCTTGGGGTG ACCATATTGT AAAAAGGTGT GTGCAATGAT AACAAGAAGA GACATACACA CATCACACGT   
  
  
+ ACGAGACTCC CTTTGCTTCA TCATTCTGGG TAACTTGTTC ATCATGTCAC GATCATCCCA ATCTCCCGAT   
  
  
+ TTATTTTTCC ATGCATTATG TAATATATAC GCAACGTTTT GATTTTTATA ATTGTATCAT TTTTTAGTTT   
  
  
+ AAAAAAATTT GTAAATAATA TTTAATCTAT TTTTTATAAA TTATTTTGAT TAAGTCCAAA ATTATTTATA   
  
  
+ AAAGTAATGC AAAGTTAAAT GAATTCAGAA ATCGGAAATT TAAAAAAGAG AGGGAAATGT GGTGTGAAGA   
  
  
+ GGGAGGAGAG TGGAGGCTTG TCGGGCAAGG GGAGTGGGTG ACGCGTAATA CGCGTCAGCC TAAGCGAGCC   
  
  
+ TTATGGTGTA GGCTGAAGCT TCTTTAACTT TACCCCATTT TTTTATTTTC ATAATTTGTT AGTTTAATTT   
  
  
+ TATATTTTAT ATTTTTAAAA AGAAAAAAAA CTTAATTTAA CGGGGAGACC TTTGTCTGCA GGAAAGTAAT   
  
  
+ CTTCTAGTTA CTGAATTGCC CCTGAAGAGG ATGATAGAGT GAGGGGCATT TAGGTAAATT CATGATTGAG   
  
  
+ AGAGAGAGAG AGAGAGAGAG AGAGAGAGTG TTTTGATGTA AAACAAAAAG ATAATTTTGG GGTTTAAAAG   
  
  
+ TTAAAAAGGT TTTTAAAGGC AAAAGCTGCG AAAGAAAATT AGAAATAAAA AAATTAAAAA AAAAACAAGG   
  
  
+ AGGAGAAGCA ATCACATGCC TGACGACCCA AACGTTCTGC AGCCCACGTG TAGGTTTGGG CCCCACTGTG   
  
  
+ GACGCGCCAC CGGCTAAACT GGTCGACATG CCGCGTAATA AAGGGTGGAC CCGAGGGCGG TCCTGGAGCA   
  
  
+ ATTAATGCAC AACGCCGTCC TCTAGTCACC CTCATACCGT ACACATGGTT AACCGGCATT TGCCAGTCAA   
  
  
+ CTCTTCTGTT TTATTTTCTA TTTAAAATAA TTTATCAAAT GGGAAACGTC CCAAAACAAG CTGTTTTCCA   
  
  
+ AAAACCCAAA CACAAAAAGC CCATTCACAC TCGAAGCAAC CAAACACTAT TTCTCTTTCT CTCTCTGTCT   
  
  
+ CTCACTTTTT TTCTCTCTCT CTCTCCCTCT CTTGTAATAA AAAGTGCGAG AAGAGAAGAA ATGAGAGAGG   
  
  
+ GTTTTATGAA ATGAAGGAGG AGATCATTAT GTAGTACATG TGCTCGCGTT GGTGATCAGA GATTTCTATG   
  
  
+ GAGAAGAGAG AAGAAAAGAG AGAGAATTC  

- GCCGACAGGG TGTAGCTCGA GACCTCGGTG CCCCTGGTCC TGGCACATAG CACCGTTTCC ACCAAAGTGC   
  
  
- TTTTCCTACG TTATTAAGAC TATCTAGAAG TGTGTTAAAG AAAAAAAAAA AGATTAAAAA TAAAAATAAA   
  
  
- TGAGAGACTA CATTTATTTA AATAATATAT AATAATAATA ATTAAAAAAA ACGCACCCTT CCTTCAACAT   
  
  
- GGAACCCCAC TGGTATAACA TTTTTCCACA CACGTTACTA TTGTTCTTCT CTGTATGTGT GTAGTGTGCA   
  
  
- TGCTCTGAGG GAAACGAAGT AGTAAGACCC ATTGAACAAG TAGTACAGTG CTAGTAGGGT TAGAGGGCTA   
  
  
- AATAAAAAGG TACGTAATAC ATTATATATG CGTTGCAAAA CTAAAAATAT TAACATAGTA AAAAATCAAA   
  
  
- TTTTTTTAAA CATTTATTAT AAATTAGATA AAAAATATTT AATAAAACTA ATTCAGGTTT TAATAAATAT   
  
  
- TTTCATTACG TTTCAATTTA CTTAAGTCTT TAGCCTTTAA ATTTTTTCTC TCCCTTTACA CCACACTTCT   
  
  
- CCCTCCTCTC ACCTCCGAAC AGCCCGTTCC CCTCACCCAC TGCGCATTAT GCGCAGTCGG ATTCGCTCGG   
  
  
- AATACCACAT CCGACTTCGA AGAAATTGAA ATGGGGTAAA AAAATAAAAG TATTAAACAA TCAAATTAAA   
  
  
- ATATAAAATA TAAAAATTTT TCTTTTTTTT GAATTAAATT GCCCCTCTGG AAACAGACGT CCTTTCATTA   
  
  
- GAAGATCAAT GACTTAACGG GGACTTCTCC TACTATCTCA CTCCCCGTAA ATCCATTTAA GTACTAACTC   
  
  
- TCTCTCTCTC TCTCTCTCTC TCTCTCTCAC AAAACTACAT TTTGTTTTTC TATTAAAACC CCAAATTTTC   
  
  
- AATTTTTCCA AAAATTTCCG TTTTCGACGC TTTCTTTTAA TCTTTATTTT TTTAATTTTT TTTTTGTTCC   
  
  
- TCCTCTTCGT TAGTGTACGG ACTGCTGGGT TTGCAAGACG TCGGGTGCAC ATCCAAACCC GGGGTGACAC   
  
  
- CTGCGCGGTG GCCGATTTGA CCAGCTGTAC GGCGCATTAT TTCCCACCTG GGCTCCCGCC AGGACCTCGT   
  
  
- TAATTACGTG TTGCGGCAGG AGATCAGTGG GAGTATGGCA TGTGTACCAA TTGGCCGTAA ACGGTCAGTT   
  
  
- GAGAAGACAA AATAAAAGAT AAATTTTATT AAATAGTTTA CCCTTTGCAG GGTTTTGTTC GACAAAAGGT   
  
  
- TTTTGGGTTT GTGTTTTTCG GGTAAGTGTG AGCTTCGTTG GTTTGTGATA AAGAGAAAGA GAGAGACAGA   
  
  
- GAGTGAAAAA AAGAGAGAGA GAGAGGGAGA GAACATTATT TTTCACGCTC TTCTCTTCTT TACTCTCTCC   
  
  
- CAAAATACTT TACTTCCTCC TCTAGTAATA CATCATGTAC ACGAGCGCAA CCACTAGTCT CTAAAGATAC   
  
  
- CTCTTCTCTC TTCTTTTCTC TCTCTTAAG

+     ABRE

| Site Name | Organism | Position | Strand | Matrix score. | sequence | function |
| --- | --- | --- | --- | --- | --- | --- |
| ABRE | Arabidopsis thaliana | 276 | - | 6 | TACGTG | cis-acting element involved in the abscisic acid responsiveness |
| ABRE | Arabidopsis thaliana | 1025 | - | 6 | CACGTG | cis-acting element involved in the abscisic acid responsiveness |

> 2018/04/13 10:10:12  
+ CGGCTGTCCC ACATCGAGCT CTGGAGCCAC GGGGACCAGG ACCGTGTATC GTGGCAAAGG TGGTTTCACG   
  
  
+ AAAAGGATGC AATAATTCTG ATAGATCTTC ACACAATTTC TTTTTTTTTT TCTAATTTTT ATTTTTATTT   
  
  
+ ACTCTCTGAT GTAAATAAAT TTATTATATA TTATTATTAT TAATTTTTTT TGCGTGGGAA GGAAGTTGTA   
  
  
+ CCTTGGGGTG ACCATATTGT AAAAAGGTGT GTGCAATGAT AACAAGAAGA GACATACACA CATCACACGT   
  
  
+ ACGAGACTCC CTTTGCTTCA TCATTCTGGG TAACTTGTTC ATCATGTCAC GATCATCCCA ATCTCCCGAT   
  
  
+ TTATTTTTCC ATGCATTATG TAATATATAC GCAACGTTTT GATTTTTATA ATTGTATCAT TTTTTAGTTT   
  
  
+ AAAAAAATTT GTAAATAATA TTTAATCTAT TTTTTATAAA TTATTTTGAT TAAGTCCAAA ATTATTTATA   
  
  
+ AAAGTAATGC AAAGTTAAAT GAATTCAGAA ATCGGAAATT TAAAAAAGAG AGGGAAATGT GGTGTGAAGA   
  
  
+ GGGAGGAGAG TGGAGGCTTG TCGGGCAAGG GGAGTGGGTG ACGCGTAATA CGCGTCAGCC TAAGCGAGCC   
  
  
+ TTATGGTGTA GGCTGAAGCT TCTTTAACTT TACCCCATTT TTTTATTTTC ATAATTTGTT AGTTTAATTT   
  
  
+ TATATTTTAT ATTTTTAAAA AGAAAAAAAA CTTAATTTAA CGGGGAGACC TTTGTCTGCA GGAAAGTAAT   
  
  
+ CTTCTAGTTA CTGAATTGCC CCTGAAGAGG ATGATAGAGT GAGGGGCATT TAGGTAAATT CATGATTGAG   
  
  
+ AGAGAGAGAG AGAGAGAGAG AGAGAGAGTG TTTTGATGTA AAACAAAAAG ATAATTTTGG GGTTTAAAAG   
  
  
+ TTAAAAAGGT TTTTAAAGGC AAAAGCTGCG AAAGAAAATT AGAAATAAAA AAATTAAAAA AAAAACAAGG   
  
  
+ AGGAGAAGCA ATCACATGCC TGACGACCCA AACGTTCTGC AGCCCACGTG TAGGTTTGGG CCCCACTGTG   
  
  
+ GACGCGCCAC CGGCTAAACT GGTCGACATG CCGCGTAATA AAGGGTGGAC CCGAGGGCGG TCCTGGAGCA   
  
  
+ ATTAATGCAC AACGCCGTCC TCTAGTCACC CTCATACCGT ACACATGGTT AACCGGCATT TGCCAGTCAA   
  
  
+ CTCTTCTGTT TTATTTTCTA TTTAAAATAA TTTATCAAAT GGGAAACGTC CCAAAACAAG CTGTTTTCCA   
  
  
+ AAAACCCAAA CACAAAAAGC CCATTCACAC TCGAAGCAAC CAAACACTAT TTCTCTTTCT CTCTCTGTCT   
  
  
+ CTCACTTTTT TTCTCTCTCT CTCTCCCTCT CTTGTAATAA AAAGTGCGAG AAGAGAAGAA ATGAGAGAGG   
  
  
+ GTTTTATGAA ATGAAGGAGG AGATCATTAT GTAGTACATG TGCTCGCGTT GGTGATCAGA GATTTCTATG   
  
  
+ GAGAAGAGAG AAGAAAAGAG AGAGAATTC  

- GCCGACAGGG TGTAGCTCGA GACCTCGGTG CCCCTGGTCC TGGCACATAG CACCGTTTCC ACCAAAGTGC   
  
  
- TTTTCCTACG TTATTAAGAC TATCTAGAAG TGTGTTAAAG AAAAAAAAAA AGATTAAAAA TAAAAATAAA   
  
  
- TGAGAGACTA CATTTATTTA AATAATATAT AATAATAATA ATTAAAAAAA ACGCACCCTT CCTTCAACAT   
  
  
- GGAACCCCAC TGGTATAACA TTTTTCCACA CACGTTACTA TTGTTCTTCT CTGTATGTGT GTAGTGTGCA   
  
  
- TGCTCTGAGG GAAACGAAGT AGTAAGACCC ATTGAACAAG TAGTACAGTG CTAGTAGGGT TAGAGGGCTA   
  
  
- AATAAAAAGG TACGTAATAC ATTATATATG CGTTGCAAAA CTAAAAATAT TAACATAGTA AAAAATCAAA   
  
  
- TTTTTTTAAA CATTTATTAT AAATTAGATA AAAAATATTT AATAAAACTA ATTCAGGTTT TAATAAATAT   
  
  
- TTTCATTACG TTTCAATTTA CTTAAGTCTT TAGCCTTTAA ATTTTTTCTC TCCCTTTACA CCACACTTCT   
  
  
- CCCTCCTCTC ACCTCCGAAC AGCCCGTTCC CCTCACCCAC TGCGCATTAT GCGCAGTCGG ATTCGCTCGG   
  
  
- AATACCACAT CCGACTTCGA AGAAATTGAA ATGGGGTAAA AAAATAAAAG TATTAAACAA TCAAATTAAA   
  
  
- ATATAAAATA TAAAAATTTT TCTTTTTTTT GAATTAAATT GCCCCTCTGG AAACAGACGT CCTTTCATTA   
  
  
- GAAGATCAAT GACTTAACGG GGACTTCTCC TACTATCTCA CTCCCCGTAA ATCCATTTAA GTACTAACTC   
  
  
- TCTCTCTCTC TCTCTCTCTC TCTCTCTCAC AAAACTACAT TTTGTTTTTC TATTAAAACC CCAAATTTTC   
  
  
- AATTTTTCCA AAAATTTCCG TTTTCGACGC TTTCTTTTAA TCTTTATTTT TTTAATTTTT TTTTTGTTCC   
  
  
- TCCTCTTCGT TAGTGTACGG ACTGCTGGGT TTGCAAGACG TCGGGTGCAC ATCCAAACCC GGGGTGACAC   
  
  
- CTGCGCGGTG GCCGATTTGA CCAGCTGTAC GGCGCATTAT TTCCCACCTG GGCTCCCGCC AGGACCTCGT   
  
  
- TAATTACGTG TTGCGGCAGG AGATCAGTGG GAGTATGGCA TGTGTACCAA TTGGCCGTAA ACGGTCAGTT   
  
  
- GAGAAGACAA AATAAAAGAT AAATTTTATT AAATAGTTTA CCCTTTGCAG GGTTTTGTTC GACAAAAGGT   
  
  
- TTTTGGGTTT GTGTTTTTCG GGTAAGTGTG AGCTTCGTTG GTTTGTGATA AAGAGAAAGA GAGAGACAGA   
  
  
- GAGTGAAAAA AAGAGAGAGA GAGAGGGAGA GAACATTATT TTTCACGCTC TTCTCTTCTT TACTCTCTCC   
  
  
- CAAAATACTT TACTTCCTCC TCTAGTAATA CATCATGTAC ACGAGCGCAA CCACTAGTCT CTAAAGATAC   
  
  
- CTCTTCTCTC TTCTTTTCTC TCTCTTAAG

+     ARE

| Site Name | Organism | Position | Strand | Matrix score. | sequence | function |
| --- | --- | --- | --- | --- | --- | --- |
| ARE | Zea mays | 61 | + | 6 | TGGTTT | cis-acting regulatory element essential for the anaerobic induction |

> 2018/04/13 10:10:12  
+ CGGCTGTCCC ACATCGAGCT CTGGAGCCAC GGGGACCAGG ACCGTGTATC GTGGCAAAGG TGGTTTCACG   
  
  
+ AAAAGGATGC AATAATTCTG ATAGATCTTC ACACAATTTC TTTTTTTTTT TCTAATTTTT ATTTTTATTT   
  
  
+ ACTCTCTGAT GTAAATAAAT TTATTATATA TTATTATTAT TAATTTTTTT TGCGTGGGAA GGAAGTTGTA   
  
  
+ CCTTGGGGTG ACCATATTGT AAAAAGGTGT GTGCAATGAT AACAAGAAGA GACATACACA CATCACACGT   
  
  
+ ACGAGACTCC CTTTGCTTCA TCATTCTGGG TAACTTGTTC ATCATGTCAC GATCATCCCA ATCTCCCGAT   
  
  
+ TTATTTTTCC ATGCATTATG TAATATATAC GCAACGTTTT GATTTTTATA ATTGTATCAT TTTTTAGTTT   
  
  
+ AAAAAAATTT GTAAATAATA TTTAATCTAT TTTTTATAAA TTATTTTGAT TAAGTCCAAA ATTATTTATA   
  
  
+ AAAGTAATGC AAAGTTAAAT GAATTCAGAA ATCGGAAATT TAAAAAAGAG AGGGAAATGT GGTGTGAAGA   
  
  
+ GGGAGGAGAG TGGAGGCTTG TCGGGCAAGG GGAGTGGGTG ACGCGTAATA CGCGTCAGCC TAAGCGAGCC   
  
  
+ TTATGGTGTA GGCTGAAGCT TCTTTAACTT TACCCCATTT TTTTATTTTC ATAATTTGTT AGTTTAATTT   
  
  
+ TATATTTTAT ATTTTTAAAA AGAAAAAAAA CTTAATTTAA CGGGGAGACC TTTGTCTGCA GGAAAGTAAT   
  
  
+ CTTCTAGTTA CTGAATTGCC CCTGAAGAGG ATGATAGAGT GAGGGGCATT TAGGTAAATT CATGATTGAG   
  
  
+ AGAGAGAGAG AGAGAGAGAG AGAGAGAGTG TTTTGATGTA AAACAAAAAG ATAATTTTGG GGTTTAAAAG   
  
  
+ TTAAAAAGGT TTTTAAAGGC AAAAGCTGCG AAAGAAAATT AGAAATAAAA AAATTAAAAA AAAAACAAGG   
  
  
+ AGGAGAAGCA ATCACATGCC TGACGACCCA AACGTTCTGC AGCCCACGTG TAGGTTTGGG CCCCACTGTG   
  
  
+ GACGCGCCAC CGGCTAAACT GGTCGACATG CCGCGTAATA AAGGGTGGAC CCGAGGGCGG TCCTGGAGCA   
  
  
+ ATTAATGCAC AACGCCGTCC TCTAGTCACC CTCATACCGT ACACATGGTT AACCGGCATT TGCCAGTCAA   
  
  
+ CTCTTCTGTT TTATTTTCTA TTTAAAATAA TTTATCAAAT GGGAAACGTC CCAAAACAAG CTGTTTTCCA   
  
  
+ AAAACCCAAA CACAAAAAGC CCATTCACAC TCGAAGCAAC CAAACACTAT TTCTCTTTCT CTCTCTGTCT   
  
  
+ CTCACTTTTT TTCTCTCTCT CTCTCCCTCT CTTGTAATAA AAAGTGCGAG AAGAGAAGAA ATGAGAGAGG   
  
  
+ GTTTTATGAA ATGAAGGAGG AGATCATTAT GTAGTACATG TGCTCGCGTT GGTGATCAGA GATTTCTATG   
  
  
+ GAGAAGAGAG AAGAAAAGAG AGAGAATTC  

- GCCGACAGGG TGTAGCTCGA GACCTCGGTG CCCCTGGTCC TGGCACATAG CACCGTTTCC ACCAAAGTGC   
  
  
- TTTTCCTACG TTATTAAGAC TATCTAGAAG TGTGTTAAAG AAAAAAAAAA AGATTAAAAA TAAAAATAAA   
  
  
- TGAGAGACTA CATTTATTTA AATAATATAT AATAATAATA ATTAAAAAAA ACGCACCCTT CCTTCAACAT   
  
  
- GGAACCCCAC TGGTATAACA TTTTTCCACA CACGTTACTA TTGTTCTTCT CTGTATGTGT GTAGTGTGCA   
  
  
- TGCTCTGAGG GAAACGAAGT AGTAAGACCC ATTGAACAAG TAGTACAGTG CTAGTAGGGT TAGAGGGCTA   
  
  
- AATAAAAAGG TACGTAATAC ATTATATATG CGTTGCAAAA CTAAAAATAT TAACATAGTA AAAAATCAAA   
  
  
- TTTTTTTAAA CATTTATTAT AAATTAGATA AAAAATATTT AATAAAACTA ATTCAGGTTT TAATAAATAT   
  
  
- TTTCATTACG TTTCAATTTA CTTAAGTCTT TAGCCTTTAA ATTTTTTCTC TCCCTTTACA CCACACTTCT   
  
  
- CCCTCCTCTC ACCTCCGAAC AGCCCGTTCC CCTCACCCAC TGCGCATTAT GCGCAGTCGG ATTCGCTCGG   
  
  
- AATACCACAT CCGACTTCGA AGAAATTGAA ATGGGGTAAA AAAATAAAAG TATTAAACAA TCAAATTAAA   
  
  
- ATATAAAATA TAAAAATTTT TCTTTTTTTT GAATTAAATT GCCCCTCTGG AAACAGACGT CCTTTCATTA   
  
  
- GAAGATCAAT GACTTAACGG GGACTTCTCC TACTATCTCA CTCCCCGTAA ATCCATTTAA GTACTAACTC   
  
  
- TCTCTCTCTC TCTCTCTCTC TCTCTCTCAC AAAACTACAT TTTGTTTTTC TATTAAAACC CCAAATTTTC   
  
  
- AATTTTTCCA AAAATTTCCG TTTTCGACGC TTTCTTTTAA TCTTTATTTT TTTAATTTTT TTTTTGTTCC   
  
  
- TCCTCTTCGT TAGTGTACGG ACTGCTGGGT TTGCAAGACG TCGGGTGCAC ATCCAAACCC GGGGTGACAC   
  
  
- CTGCGCGGTG GCCGATTTGA CCAGCTGTAC GGCGCATTAT TTCCCACCTG GGCTCCCGCC AGGACCTCGT   
  
  
- TAATTACGTG TTGCGGCAGG AGATCAGTGG GAGTATGGCA TGTGTACCAA TTGGCCGTAA ACGGTCAGTT   
  
  
- GAGAAGACAA AATAAAAGAT AAATTTTATT AAATAGTTTA CCCTTTGCAG GGTTTTGTTC GACAAAAGGT   
  
  
- TTTTGGGTTT GTGTTTTTCG GGTAAGTGTG AGCTTCGTTG GTTTGTGATA AAGAGAAAGA GAGAGACAGA   
  
  
- GAGTGAAAAA AAGAGAGAGA GAGAGGGAGA GAACATTATT TTTCACGCTC TTCTCTTCTT TACTCTCTCC   
  
  
- CAAAATACTT TACTTCCTCC TCTAGTAATA CATCATGTAC ACGAGCGCAA CCACTAGTCT CTAAAGATAC   
  
  
- CTCTTCTCTC TTCTTTTCTC TCTCTTAAG

+     AT-rich element

| Site Name | Organism | Position | Strand | Matrix score. | sequence | function |
| --- | --- | --- | --- | --- | --- | --- |
| AT-rich element | Glycine max | 389 | - | 10 | ATAGAAATCAA | binding site of AT-rich DNA binding protein (ATBP-1) |

> 2018/04/13 10:10:12  
+ CGGCTGTCCC ACATCGAGCT CTGGAGCCAC GGGGACCAGG ACCGTGTATC GTGGCAAAGG TGGTTTCACG   
  
  
+ AAAAGGATGC AATAATTCTG ATAGATCTTC ACACAATTTC TTTTTTTTTT TCTAATTTTT ATTTTTATTT   
  
  
+ ACTCTCTGAT GTAAATAAAT TTATTATATA TTATTATTAT TAATTTTTTT TGCGTGGGAA GGAAGTTGTA   
  
  
+ CCTTGGGGTG ACCATATTGT AAAAAGGTGT GTGCAATGAT AACAAGAAGA GACATACACA CATCACACGT   
  
  
+ ACGAGACTCC CTTTGCTTCA TCATTCTGGG TAACTTGTTC ATCATGTCAC GATCATCCCA ATCTCCCGAT   
  
  
+ TTATTTTTCC ATGCATTATG TAATATATAC GCAACGTTTT GATTTTTATA ATTGTATCAT TTTTTAGTTT   
  
  
+ AAAAAAATTT GTAAATAATA TTTAATCTAT TTTTTATAAA TTATTTTGAT TAAGTCCAAA ATTATTTATA   
  
  
+ AAAGTAATGC AAAGTTAAAT GAATTCAGAA ATCGGAAATT TAAAAAAGAG AGGGAAATGT GGTGTGAAGA   
  
  
+ GGGAGGAGAG TGGAGGCTTG TCGGGCAAGG GGAGTGGGTG ACGCGTAATA CGCGTCAGCC TAAGCGAGCC   
  
  
+ TTATGGTGTA GGCTGAAGCT TCTTTAACTT TACCCCATTT TTTTATTTTC ATAATTTGTT AGTTTAATTT   
  
  
+ TATATTTTAT ATTTTTAAAA AGAAAAAAAA CTTAATTTAA CGGGGAGACC TTTGTCTGCA GGAAAGTAAT   
  
  
+ CTTCTAGTTA CTGAATTGCC CCTGAAGAGG ATGATAGAGT GAGGGGCATT TAGGTAAATT CATGATTGAG   
  
  
+ AGAGAGAGAG AGAGAGAGAG AGAGAGAGTG TTTTGATGTA AAACAAAAAG ATAATTTTGG GGTTTAAAAG   
  
  
+ TTAAAAAGGT TTTTAAAGGC AAAAGCTGCG AAAGAAAATT AGAAATAAAA AAATTAAAAA AAAAACAAGG   
  
  
+ AGGAGAAGCA ATCACATGCC TGACGACCCA AACGTTCTGC AGCCCACGTG TAGGTTTGGG CCCCACTGTG   
  
  
+ GACGCGCCAC CGGCTAAACT GGTCGACATG CCGCGTAATA AAGGGTGGAC CCGAGGGCGG TCCTGGAGCA   
  
  
+ ATTAATGCAC AACGCCGTCC TCTAGTCACC CTCATACCGT ACACATGGTT AACCGGCATT TGCCAGTCAA   
  
  
+ CTCTTCTGTT TTATTTTCTA TTTAAAATAA TTTATCAAAT GGGAAACGTC CCAAAACAAG CTGTTTTCCA   
  
  
+ AAAACCCAAA CACAAAAAGC CCATTCACAC TCGAAGCAAC CAAACACTAT TTCTCTTTCT CTCTCTGTCT   
  
  
+ CTCACTTTTT TTCTCTCTCT CTCTCCCTCT CTTGTAATAA AAAGTGCGAG AAGAGAAGAA ATGAGAGAGG   
  
  
+ GTTTTATGAA ATGAAGGAGG AGATCATTAT GTAGTACATG TGCTCGCGTT GGTGATCAGA GATTTCTATG   
  
  
+ GAGAAGAGAG AAGAAAAGAG AGAGAATTC  

- GCCGACAGGG TGTAGCTCGA GACCTCGGTG CCCCTGGTCC TGGCACATAG CACCGTTTCC ACCAAAGTGC   
  
  
- TTTTCCTACG TTATTAAGAC TATCTAGAAG TGTGTTAAAG AAAAAAAAAA AGATTAAAAA TAAAAATAAA   
  
  
- TGAGAGACTA CATTTATTTA AATAATATAT AATAATAATA ATTAAAAAAA ACGCACCCTT CCTTCAACAT   
  
  
- GGAACCCCAC TGGTATAACA TTTTTCCACA CACGTTACTA TTGTTCTTCT CTGTATGTGT GTAGTGTGCA   
  
  
- TGCTCTGAGG GAAACGAAGT AGTAAGACCC ATTGAACAAG TAGTACAGTG CTAGTAGGGT TAGAGGGCTA   
  
  
- AATAAAAAGG TACGTAATAC ATTATATATG CGTTGCAAAA CTAAAAATAT TAACATAGTA AAAAATCAAA   
  
  
- TTTTTTTAAA CATTTATTAT AAATTAGATA AAAAATATTT AATAAAACTA ATTCAGGTTT TAATAAATAT   
  
  
- TTTCATTACG TTTCAATTTA CTTAAGTCTT TAGCCTTTAA ATTTTTTCTC TCCCTTTACA CCACACTTCT   
  
  
- CCCTCCTCTC ACCTCCGAAC AGCCCGTTCC CCTCACCCAC TGCGCATTAT GCGCAGTCGG ATTCGCTCGG   
  
  
- AATACCACAT CCGACTTCGA AGAAATTGAA ATGGGGTAAA AAAATAAAAG TATTAAACAA TCAAATTAAA   
  
  
- ATATAAAATA TAAAAATTTT TCTTTTTTTT GAATTAAATT GCCCCTCTGG AAACAGACGT CCTTTCATTA   
  
  
- GAAGATCAAT GACTTAACGG GGACTTCTCC TACTATCTCA CTCCCCGTAA ATCCATTTAA GTACTAACTC   
  
  
- TCTCTCTCTC TCTCTCTCTC TCTCTCTCAC AAAACTACAT TTTGTTTTTC TATTAAAACC CCAAATTTTC   
  
  
- AATTTTTCCA AAAATTTCCG TTTTCGACGC TTTCTTTTAA TCTTTATTTT TTTAATTTTT TTTTTGTTCC   
  
  
- TCCTCTTCGT TAGTGTACGG ACTGCTGGGT TTGCAAGACG TCGGGTGCAC ATCCAAACCC GGGGTGACAC   
  
  
- CTGCGCGGTG GCCGATTTGA CCAGCTGTAC GGCGCATTAT TTCCCACCTG GGCTCCCGCC AGGACCTCGT   
  
  
- TAATTACGTG TTGCGGCAGG AGATCAGTGG GAGTATGGCA TGTGTACCAA TTGGCCGTAA ACGGTCAGTT   
  
  
- GAGAAGACAA AATAAAAGAT AAATTTTATT AAATAGTTTA CCCTTTGCAG GGTTTTGTTC GACAAAAGGT   
  
  
- TTTTGGGTTT GTGTTTTTCG GGTAAGTGTG AGCTTCGTTG GTTTGTGATA AAGAGAAAGA GAGAGACAGA   
  
  
- GAGTGAAAAA AAGAGAGAGA GAGAGGGAGA GAACATTATT TTTCACGCTC TTCTCTTCTT TACTCTCTCC   
  
  
- CAAAATACTT TACTTCCTCC TCTAGTAATA CATCATGTAC ACGAGCGCAA CCACTAGTCT CTAAAGATAC   
  
  
- CTCTTCTCTC TTCTTTTCTC TCTCTTAAG

+     ATC-motif

| Site Name | Organism | Position | Strand | Matrix score. | sequence | function |
| --- | --- | --- | --- | --- | --- | --- |
| ATC-motif | Spinacia oleracea | 765 | + | 8 | AGTAATCT | part of a conserved DNA module involved in light responsiveness |

> 2018/04/13 10:10:12  
+ CGGCTGTCCC ACATCGAGCT CTGGAGCCAC GGGGACCAGG ACCGTGTATC GTGGCAAAGG TGGTTTCACG   
  
  
+ AAAAGGATGC AATAATTCTG ATAGATCTTC ACACAATTTC TTTTTTTTTT TCTAATTTTT ATTTTTATTT   
  
  
+ ACTCTCTGAT GTAAATAAAT TTATTATATA TTATTATTAT TAATTTTTTT TGCGTGGGAA GGAAGTTGTA   
  
  
+ CCTTGGGGTG ACCATATTGT AAAAAGGTGT GTGCAATGAT AACAAGAAGA GACATACACA CATCACACGT   
  
  
+ ACGAGACTCC CTTTGCTTCA TCATTCTGGG TAACTTGTTC ATCATGTCAC GATCATCCCA ATCTCCCGAT   
  
  
+ TTATTTTTCC ATGCATTATG TAATATATAC GCAACGTTTT GATTTTTATA ATTGTATCAT TTTTTAGTTT   
  
  
+ AAAAAAATTT GTAAATAATA TTTAATCTAT TTTTTATAAA TTATTTTGAT TAAGTCCAAA ATTATTTATA   
  
  
+ AAAGTAATGC AAAGTTAAAT GAATTCAGAA ATCGGAAATT TAAAAAAGAG AGGGAAATGT GGTGTGAAGA   
  
  
+ GGGAGGAGAG TGGAGGCTTG TCGGGCAAGG GGAGTGGGTG ACGCGTAATA CGCGTCAGCC TAAGCGAGCC   
  
  
+ TTATGGTGTA GGCTGAAGCT TCTTTAACTT TACCCCATTT TTTTATTTTC ATAATTTGTT AGTTTAATTT   
  
  
+ TATATTTTAT ATTTTTAAAA AGAAAAAAAA CTTAATTTAA CGGGGAGACC TTTGTCTGCA GGAAAGTAAT   
  
  
+ CTTCTAGTTA CTGAATTGCC CCTGAAGAGG ATGATAGAGT GAGGGGCATT TAGGTAAATT CATGATTGAG   
  
  
+ AGAGAGAGAG AGAGAGAGAG AGAGAGAGTG TTTTGATGTA AAACAAAAAG ATAATTTTGG GGTTTAAAAG   
  
  
+ TTAAAAAGGT TTTTAAAGGC AAAAGCTGCG AAAGAAAATT AGAAATAAAA AAATTAAAAA AAAAACAAGG   
  
  
+ AGGAGAAGCA ATCACATGCC TGACGACCCA AACGTTCTGC AGCCCACGTG TAGGTTTGGG CCCCACTGTG   
  
  
+ GACGCGCCAC CGGCTAAACT GGTCGACATG CCGCGTAATA AAGGGTGGAC CCGAGGGCGG TCCTGGAGCA   
  
  
+ ATTAATGCAC AACGCCGTCC TCTAGTCACC CTCATACCGT ACACATGGTT AACCGGCATT TGCCAGTCAA   
  
  
+ CTCTTCTGTT TTATTTTCTA TTTAAAATAA TTTATCAAAT GGGAAACGTC CCAAAACAAG CTGTTTTCCA   
  
  
+ AAAACCCAAA CACAAAAAGC CCATTCACAC TCGAAGCAAC CAAACACTAT TTCTCTTTCT CTCTCTGTCT   
  
  
+ CTCACTTTTT TTCTCTCTCT CTCTCCCTCT CTTGTAATAA AAAGTGCGAG AAGAGAAGAA ATGAGAGAGG   
  
  
+ GTTTTATGAA ATGAAGGAGG AGATCATTAT GTAGTACATG TGCTCGCGTT GGTGATCAGA GATTTCTATG   
  
  
+ GAGAAGAGAG AAGAAAAGAG AGAGAATTC  

- GCCGACAGGG TGTAGCTCGA GACCTCGGTG CCCCTGGTCC TGGCACATAG CACCGTTTCC ACCAAAGTGC   
  
  
- TTTTCCTACG TTATTAAGAC TATCTAGAAG TGTGTTAAAG AAAAAAAAAA AGATTAAAAA TAAAAATAAA   
  
  
- TGAGAGACTA CATTTATTTA AATAATATAT AATAATAATA ATTAAAAAAA ACGCACCCTT CCTTCAACAT   
  
  
- GGAACCCCAC TGGTATAACA TTTTTCCACA CACGTTACTA TTGTTCTTCT CTGTATGTGT GTAGTGTGCA   
  
  
- TGCTCTGAGG GAAACGAAGT AGTAAGACCC ATTGAACAAG TAGTACAGTG CTAGTAGGGT TAGAGGGCTA   
  
  
- AATAAAAAGG TACGTAATAC ATTATATATG CGTTGCAAAA CTAAAAATAT TAACATAGTA AAAAATCAAA   
  
  
- TTTTTTTAAA CATTTATTAT AAATTAGATA AAAAATATTT AATAAAACTA ATTCAGGTTT TAATAAATAT   
  
  
- TTTCATTACG TTTCAATTTA CTTAAGTCTT TAGCCTTTAA ATTTTTTCTC TCCCTTTACA CCACACTTCT   
  
  
- CCCTCCTCTC ACCTCCGAAC AGCCCGTTCC CCTCACCCAC TGCGCATTAT GCGCAGTCGG ATTCGCTCGG   
  
  
- AATACCACAT CCGACTTCGA AGAAATTGAA ATGGGGTAAA AAAATAAAAG TATTAAACAA TCAAATTAAA   
  
  
- ATATAAAATA TAAAAATTTT TCTTTTTTTT GAATTAAATT GCCCCTCTGG AAACAGACGT CCTTTCATTA   
  
  
- GAAGATCAAT GACTTAACGG GGACTTCTCC TACTATCTCA CTCCCCGTAA ATCCATTTAA GTACTAACTC   
  
  
- TCTCTCTCTC TCTCTCTCTC TCTCTCTCAC AAAACTACAT TTTGTTTTTC TATTAAAACC CCAAATTTTC   
  
  
- AATTTTTCCA AAAATTTCCG TTTTCGACGC TTTCTTTTAA TCTTTATTTT TTTAATTTTT TTTTTGTTCC   
  
  
- TCCTCTTCGT TAGTGTACGG ACTGCTGGGT TTGCAAGACG TCGGGTGCAC ATCCAAACCC GGGGTGACAC   
  
  
- CTGCGCGGTG GCCGATTTGA CCAGCTGTAC GGCGCATTAT TTCCCACCTG GGCTCCCGCC AGGACCTCGT   
  
  
- TAATTACGTG TTGCGGCAGG AGATCAGTGG GAGTATGGCA TGTGTACCAA TTGGCCGTAA ACGGTCAGTT   
  
  
- GAGAAGACAA AATAAAAGAT AAATTTTATT AAATAGTTTA CCCTTTGCAG GGTTTTGTTC GACAAAAGGT   
  
  
- TTTTGGGTTT GTGTTTTTCG GGTAAGTGTG AGCTTCGTTG GTTTGTGATA AAGAGAAAGA GAGAGACAGA   
  
  
- GAGTGAAAAA AAGAGAGAGA GAGAGGGAGA GAACATTATT TTTCACGCTC TTCTCTTCTT TACTCTCTCC   
  
  
- CAAAATACTT TACTTCCTCC TCTAGTAATA CATCATGTAC ACGAGCGCAA CCACTAGTCT CTAAAGATAC   
  
  
- CTCTTCTCTC TTCTTTTCTC TCTCTTAAG

+     Box 4

| Site Name | Organism | Position | Strand | Matrix score. | sequence | function |
| --- | --- | --- | --- | --- | --- | --- |
| Box 4 | Petroselinum crispum | 179 | + | 6 | ATTAAT | part of a conserved DNA module involved in light responsiveness |
| Box 4 | Petroselinum crispum | 1121 | - | 6 | ATTAAT | part of a conserved DNA module involved in light responsiveness |

> 2018/04/13 10:10:12  
+ CGGCTGTCCC ACATCGAGCT CTGGAGCCAC GGGGACCAGG ACCGTGTATC GTGGCAAAGG TGGTTTCACG   
  
  
+ AAAAGGATGC AATAATTCTG ATAGATCTTC ACACAATTTC TTTTTTTTTT TCTAATTTTT ATTTTTATTT   
  
  
+ ACTCTCTGAT GTAAATAAAT TTATTATATA TTATTATTAT TAATTTTTTT TGCGTGGGAA GGAAGTTGTA   
  
  
+ CCTTGGGGTG ACCATATTGT AAAAAGGTGT GTGCAATGAT AACAAGAAGA GACATACACA CATCACACGT   
  
  
+ ACGAGACTCC CTTTGCTTCA TCATTCTGGG TAACTTGTTC ATCATGTCAC GATCATCCCA ATCTCCCGAT   
  
  
+ TTATTTTTCC ATGCATTATG TAATATATAC GCAACGTTTT GATTTTTATA ATTGTATCAT TTTTTAGTTT   
  
  
+ AAAAAAATTT GTAAATAATA TTTAATCTAT TTTTTATAAA TTATTTTGAT TAAGTCCAAA ATTATTTATA   
  
  
+ AAAGTAATGC AAAGTTAAAT GAATTCAGAA ATCGGAAATT TAAAAAAGAG AGGGAAATGT GGTGTGAAGA   
  
  
+ GGGAGGAGAG TGGAGGCTTG TCGGGCAAGG GGAGTGGGTG ACGCGTAATA CGCGTCAGCC TAAGCGAGCC   
  
  
+ TTATGGTGTA GGCTGAAGCT TCTTTAACTT TACCCCATTT TTTTATTTTC ATAATTTGTT AGTTTAATTT   
  
  
+ TATATTTTAT ATTTTTAAAA AGAAAAAAAA CTTAATTTAA CGGGGAGACC TTTGTCTGCA GGAAAGTAAT   
  
  
+ CTTCTAGTTA CTGAATTGCC CCTGAAGAGG ATGATAGAGT GAGGGGCATT TAGGTAAATT CATGATTGAG   
  
  
+ AGAGAGAGAG AGAGAGAGAG AGAGAGAGTG TTTTGATGTA AAACAAAAAG ATAATTTTGG GGTTTAAAAG   
  
  
+ TTAAAAAGGT TTTTAAAGGC AAAAGCTGCG AAAGAAAATT AGAAATAAAA AAATTAAAAA AAAAACAAGG   
  
  
+ AGGAGAAGCA ATCACATGCC TGACGACCCA AACGTTCTGC AGCCCACGTG TAGGTTTGGG CCCCACTGTG   
  
  
+ GACGCGCCAC CGGCTAAACT GGTCGACATG CCGCGTAATA AAGGGTGGAC CCGAGGGCGG TCCTGGAGCA   
  
  
+ ATTAATGCAC AACGCCGTCC TCTAGTCACC CTCATACCGT ACACATGGTT AACCGGCATT TGCCAGTCAA   
  
  
+ CTCTTCTGTT TTATTTTCTA TTTAAAATAA TTTATCAAAT GGGAAACGTC CCAAAACAAG CTGTTTTCCA   
  
  
+ AAAACCCAAA CACAAAAAGC CCATTCACAC TCGAAGCAAC CAAACACTAT TTCTCTTTCT CTCTCTGTCT   
  
  
+ CTCACTTTTT TTCTCTCTCT CTCTCCCTCT CTTGTAATAA AAAGTGCGAG AAGAGAAGAA ATGAGAGAGG   
  
  
+ GTTTTATGAA ATGAAGGAGG AGATCATTAT GTAGTACATG TGCTCGCGTT GGTGATCAGA GATTTCTATG   
  
  
+ GAGAAGAGAG AAGAAAAGAG AGAGAATTC  

- GCCGACAGGG TGTAGCTCGA GACCTCGGTG CCCCTGGTCC TGGCACATAG CACCGTTTCC ACCAAAGTGC   
  
  
- TTTTCCTACG TTATTAAGAC TATCTAGAAG TGTGTTAAAG AAAAAAAAAA AGATTAAAAA TAAAAATAAA   
  
  
- TGAGAGACTA CATTTATTTA AATAATATAT AATAATAATA ATTAAAAAAA ACGCACCCTT CCTTCAACAT   
  
  
- GGAACCCCAC TGGTATAACA TTTTTCCACA CACGTTACTA TTGTTCTTCT CTGTATGTGT GTAGTGTGCA   
  
  
- TGCTCTGAGG GAAACGAAGT AGTAAGACCC ATTGAACAAG TAGTACAGTG CTAGTAGGGT TAGAGGGCTA   
  
  
- AATAAAAAGG TACGTAATAC ATTATATATG CGTTGCAAAA CTAAAAATAT TAACATAGTA AAAAATCAAA   
  
  
- TTTTTTTAAA CATTTATTAT AAATTAGATA AAAAATATTT AATAAAACTA ATTCAGGTTT TAATAAATAT   
  
  
- TTTCATTACG TTTCAATTTA CTTAAGTCTT TAGCCTTTAA ATTTTTTCTC TCCCTTTACA CCACACTTCT   
  
  
- CCCTCCTCTC ACCTCCGAAC AGCCCGTTCC CCTCACCCAC TGCGCATTAT GCGCAGTCGG ATTCGCTCGG   
  
  
- AATACCACAT CCGACTTCGA AGAAATTGAA ATGGGGTAAA AAAATAAAAG TATTAAACAA TCAAATTAAA   
  
  
- ATATAAAATA TAAAAATTTT TCTTTTTTTT GAATTAAATT GCCCCTCTGG AAACAGACGT CCTTTCATTA   
  
  
- GAAGATCAAT GACTTAACGG GGACTTCTCC TACTATCTCA CTCCCCGTAA ATCCATTTAA GTACTAACTC   
  
  
- TCTCTCTCTC TCTCTCTCTC TCTCTCTCAC AAAACTACAT TTTGTTTTTC TATTAAAACC CCAAATTTTC   
  
  
- AATTTTTCCA AAAATTTCCG TTTTCGACGC TTTCTTTTAA TCTTTATTTT TTTAATTTTT TTTTTGTTCC   
  
  
- TCCTCTTCGT TAGTGTACGG ACTGCTGGGT TTGCAAGACG TCGGGTGCAC ATCCAAACCC GGGGTGACAC   
  
  
- CTGCGCGGTG GCCGATTTGA CCAGCTGTAC GGCGCATTAT TTCCCACCTG GGCTCCCGCC AGGACCTCGT   
  
  
- TAATTACGTG TTGCGGCAGG AGATCAGTGG GAGTATGGCA TGTGTACCAA TTGGCCGTAA ACGGTCAGTT   
  
  
- GAGAAGACAA AATAAAAGAT AAATTTTATT AAATAGTTTA CCCTTTGCAG GGTTTTGTTC GACAAAAGGT   
  
  
- TTTTGGGTTT GTGTTTTTCG GGTAAGTGTG AGCTTCGTTG GTTTGTGATA AAGAGAAAGA GAGAGACAGA   
  
  
- GAGTGAAAAA AAGAGAGAGA GAGAGGGAGA GAACATTATT TTTCACGCTC TTCTCTTCTT TACTCTCTCC   
  
  
- CAAAATACTT TACTTCCTCC TCTAGTAATA CATCATGTAC ACGAGCGCAA CCACTAGTCT CTAAAGATAC   
  
  
- CTCTTCTCTC TTCTTTTCTC TCTCTTAAG

+     Box III

| Site Name | Organism | Position | Strand | Matrix score. | sequence | function |
| --- | --- | --- | --- | --- | --- | --- |
| Box III | Pisum sativum | 1282 | + | 9 | CATTTACACT | protein binding site |

> 2018/04/13 10:10:12  
+ CGGCTGTCCC ACATCGAGCT CTGGAGCCAC GGGGACCAGG ACCGTGTATC GTGGCAAAGG TGGTTTCACG   
  
  
+ AAAAGGATGC AATAATTCTG ATAGATCTTC ACACAATTTC TTTTTTTTTT TCTAATTTTT ATTTTTATTT   
  
  
+ ACTCTCTGAT GTAAATAAAT TTATTATATA TTATTATTAT TAATTTTTTT TGCGTGGGAA GGAAGTTGTA   
  
  
+ CCTTGGGGTG ACCATATTGT AAAAAGGTGT GTGCAATGAT AACAAGAAGA GACATACACA CATCACACGT   
  
  
+ ACGAGACTCC CTTTGCTTCA TCATTCTGGG TAACTTGTTC ATCATGTCAC GATCATCCCA ATCTCCCGAT   
  
  
+ TTATTTTTCC ATGCATTATG TAATATATAC GCAACGTTTT GATTTTTATA ATTGTATCAT TTTTTAGTTT   
  
  
+ AAAAAAATTT GTAAATAATA TTTAATCTAT TTTTTATAAA TTATTTTGAT TAAGTCCAAA ATTATTTATA   
  
  
+ AAAGTAATGC AAAGTTAAAT GAATTCAGAA ATCGGAAATT TAAAAAAGAG AGGGAAATGT GGTGTGAAGA   
  
  
+ GGGAGGAGAG TGGAGGCTTG TCGGGCAAGG GGAGTGGGTG ACGCGTAATA CGCGTCAGCC TAAGCGAGCC   
  
  
+ TTATGGTGTA GGCTGAAGCT TCTTTAACTT TACCCCATTT TTTTATTTTC ATAATTTGTT AGTTTAATTT   
  
  
+ TATATTTTAT ATTTTTAAAA AGAAAAAAAA CTTAATTTAA CGGGGAGACC TTTGTCTGCA GGAAAGTAAT   
  
  
+ CTTCTAGTTA CTGAATTGCC CCTGAAGAGG ATGATAGAGT GAGGGGCATT TAGGTAAATT CATGATTGAG   
  
  
+ AGAGAGAGAG AGAGAGAGAG AGAGAGAGTG TTTTGATGTA AAACAAAAAG ATAATTTTGG GGTTTAAAAG   
  
  
+ TTAAAAAGGT TTTTAAAGGC AAAAGCTGCG AAAGAAAATT AGAAATAAAA AAATTAAAAA AAAAACAAGG   
  
  
+ AGGAGAAGCA ATCACATGCC TGACGACCCA AACGTTCTGC AGCCCACGTG TAGGTTTGGG CCCCACTGTG   
  
  
+ GACGCGCCAC CGGCTAAACT GGTCGACATG CCGCGTAATA AAGGGTGGAC CCGAGGGCGG TCCTGGAGCA   
  
  
+ ATTAATGCAC AACGCCGTCC TCTAGTCACC CTCATACCGT ACACATGGTT AACCGGCATT TGCCAGTCAA   
  
  
+ CTCTTCTGTT TTATTTTCTA TTTAAAATAA TTTATCAAAT GGGAAACGTC CCAAAACAAG CTGTTTTCCA   
  
  
+ AAAACCCAAA CACAAAAAGC CCATTCACAC TCGAAGCAAC CAAACACTAT TTCTCTTTCT CTCTCTGTCT   
  
  
+ CTCACTTTTT TTCTCTCTCT CTCTCCCTCT CTTGTAATAA AAAGTGCGAG AAGAGAAGAA ATGAGAGAGG   
  
  
+ GTTTTATGAA ATGAAGGAGG AGATCATTAT GTAGTACATG TGCTCGCGTT GGTGATCAGA GATTTCTATG   
  
  
+ GAGAAGAGAG AAGAAAAGAG AGAGAATTC  

- GCCGACAGGG TGTAGCTCGA GACCTCGGTG CCCCTGGTCC TGGCACATAG CACCGTTTCC ACCAAAGTGC   
  
  
- TTTTCCTACG TTATTAAGAC TATCTAGAAG TGTGTTAAAG AAAAAAAAAA AGATTAAAAA TAAAAATAAA   
  
  
- TGAGAGACTA CATTTATTTA AATAATATAT AATAATAATA ATTAAAAAAA ACGCACCCTT CCTTCAACAT   
  
  
- GGAACCCCAC TGGTATAACA TTTTTCCACA CACGTTACTA TTGTTCTTCT CTGTATGTGT GTAGTGTGCA   
  
  
- TGCTCTGAGG GAAACGAAGT AGTAAGACCC ATTGAACAAG TAGTACAGTG CTAGTAGGGT TAGAGGGCTA   
  
  
- AATAAAAAGG TACGTAATAC ATTATATATG CGTTGCAAAA CTAAAAATAT TAACATAGTA AAAAATCAAA   
  
  
- TTTTTTTAAA CATTTATTAT AAATTAGATA AAAAATATTT AATAAAACTA ATTCAGGTTT TAATAAATAT   
  
  
- TTTCATTACG TTTCAATTTA CTTAAGTCTT TAGCCTTTAA ATTTTTTCTC TCCCTTTACA CCACACTTCT   
  
  
- CCCTCCTCTC ACCTCCGAAC AGCCCGTTCC CCTCACCCAC TGCGCATTAT GCGCAGTCGG ATTCGCTCGG   
  
  
- AATACCACAT CCGACTTCGA AGAAATTGAA ATGGGGTAAA AAAATAAAAG TATTAAACAA TCAAATTAAA   
  
  
- ATATAAAATA TAAAAATTTT TCTTTTTTTT GAATTAAATT GCCCCTCTGG AAACAGACGT CCTTTCATTA   
  
  
- GAAGATCAAT GACTTAACGG GGACTTCTCC TACTATCTCA CTCCCCGTAA ATCCATTTAA GTACTAACTC   
  
  
- TCTCTCTCTC TCTCTCTCTC TCTCTCTCAC AAAACTACAT TTTGTTTTTC TATTAAAACC CCAAATTTTC   
  
  
- AATTTTTCCA AAAATTTCCG TTTTCGACGC TTTCTTTTAA TCTTTATTTT TTTAATTTTT TTTTTGTTCC   
  
  
- TCCTCTTCGT TAGTGTACGG ACTGCTGGGT TTGCAAGACG TCGGGTGCAC ATCCAAACCC GGGGTGACAC   
  
  
- CTGCGCGGTG GCCGATTTGA CCAGCTGTAC GGCGCATTAT TTCCCACCTG GGCTCCCGCC AGGACCTCGT   
  
  
- TAATTACGTG TTGCGGCAGG AGATCAGTGG GAGTATGGCA TGTGTACCAA TTGGCCGTAA ACGGTCAGTT   
  
  
- GAGAAGACAA AATAAAAGAT AAATTTTATT AAATAGTTTA CCCTTTGCAG GGTTTTGTTC GACAAAAGGT   
  
  
- TTTTGGGTTT GTGTTTTTCG GGTAAGTGTG AGCTTCGTTG GTTTGTGATA AAGAGAAAGA GAGAGACAGA   
  
  
- GAGTGAAAAA AAGAGAGAGA GAGAGGGAGA GAACATTATT TTTCACGCTC TTCTCTTCTT TACTCTCTCC   
  
  
- CAAAATACTT TACTTCCTCC TCTAGTAATA CATCATGTAC ACGAGCGCAA CCACTAGTCT CTAAAGATAC   
  
  
- CTCTTCTCTC TTCTTTTCTC TCTCTTAAG

+     CAAT-box

| Site Name | Organism | Position | Strand | Matrix score. | sequence | function |
| --- | --- | --- | --- | --- | --- | --- |
| CAAT-box | Brassica rapa | 1178 | - | 5 | CAAAT | common cis-acting element in promoter and enhancer regions |
| CAAT-box | Brassica rapa | 1226 | + | 5 | CAAAT | common cis-acting element in promoter and enhancer regions |
| CAAT-box | Glycine max | 1119 | + | 5 | CAATT | common cis-acting element in promoter and enhancer regions |
| CAAT-box | Glycine max | 784 | - | 5 | CAATT | common cis-acting element in promoter and enhancer regions |
| CAAT-box | Hordeum vulgare | 989 | + | 4 | CAAT | common cis-acting element in promoter and enhancer regions |
| CAAT-box | Hordeum vulgare | 835 | - | 4 | CAAT | common cis-acting element in promoter and enhancer regions |
| CAAT-box | Brassica rapa | 684 | - | 5 | CAAAT | common cis-acting element in promoter and enhancer regions |
| CAAT-box | Hordeum vulgare | 401 | - | 4 | CAAT | common cis-acting element in promoter and enhancer regions |
| CAAT-box | Glycine max | 400 | - | 5 | CAATT | common cis-acting element in promoter and enhancer regions |
| CAAT-box | Arabidopsis thaliana | 785 | - | 6 | gGCAAT | common cis-acting element in promoter and enhancer regions |
| CAAT-box | Arabidopsis thaliana | 338 | + | 5 | CCAAT | common cis-acting element in promoter and enhancer regions |
| CAAT-box | Brassica rapa | 427 | - | 5 | CAAAT | common cis-acting element in promoter and enhancer regions |
| CAAT-box | Hordeum vulgare | 339 | + | 4 | CAAT | common cis-acting element in promoter and enhancer regions |
| CAAT-box | Glycine max | 104 | + | 5 | CAATT | common cis-acting element in promoter and enhancer regions |
| CAAT-box | Hordeum vulgare | 244 | + | 4 | CAAT | common cis-acting element in promoter and enhancer regions |
| CAAT-box | Hordeum vulgare | 226 | - | 4 | CAAT | common cis-acting element in promoter and enhancer regions |
| CAAT-box | Hordeum vulgare | 80 | + | 4 | CAAT | common cis-acting element in promoter and enhancer regions |

> 2018/04/13 10:10:12  
+ CGGCTGTCCC ACATCGAGCT CTGGAGCCAC GGGGACCAGG ACCGTGTATC GTGGCAAAGG TGGTTTCACG   
  
  
+ AAAAGGATGC AATAATTCTG ATAGATCTTC ACACAATTTC TTTTTTTTTT TCTAATTTTT ATTTTTATTT   
  
  
+ ACTCTCTGAT GTAAATAAAT TTATTATATA TTATTATTAT TAATTTTTTT TGCGTGGGAA GGAAGTTGTA   
  
  
+ CCTTGGGGTG ACCATATTGT AAAAAGGTGT GTGCAATGAT AACAAGAAGA GACATACACA CATCACACGT   
  
  
+ ACGAGACTCC CTTTGCTTCA TCATTCTGGG TAACTTGTTC ATCATGTCAC GATCATCCCA ATCTCCCGAT   
  
  
+ TTATTTTTCC ATGCATTATG TAATATATAC GCAACGTTTT GATTTTTATA ATTGTATCAT TTTTTAGTTT   
  
  
+ AAAAAAATTT GTAAATAATA TTTAATCTAT TTTTTATAAA TTATTTTGAT TAAGTCCAAA ATTATTTATA   
  
  
+ AAAGTAATGC AAAGTTAAAT GAATTCAGAA ATCGGAAATT TAAAAAAGAG AGGGAAATGT GGTGTGAAGA   
  
  
+ GGGAGGAGAG TGGAGGCTTG TCGGGCAAGG GGAGTGGGTG ACGCGTAATA CGCGTCAGCC TAAGCGAGCC   
  
  
+ TTATGGTGTA GGCTGAAGCT TCTTTAACTT TACCCCATTT TTTTATTTTC ATAATTTGTT AGTTTAATTT   
  
  
+ TATATTTTAT ATTTTTAAAA AGAAAAAAAA CTTAATTTAA CGGGGAGACC TTTGTCTGCA GGAAAGTAAT   
  
  
+ CTTCTAGTTA CTGAATTGCC CCTGAAGAGG ATGATAGAGT GAGGGGCATT TAGGTAAATT CATGATTGAG   
  
  
+ AGAGAGAGAG AGAGAGAGAG AGAGAGAGTG TTTTGATGTA AAACAAAAAG ATAATTTTGG GGTTTAAAAG   
  
  
+ TTAAAAAGGT TTTTAAAGGC AAAAGCTGCG AAAGAAAATT AGAAATAAAA AAATTAAAAA AAAAACAAGG   
  
  
+ AGGAGAAGCA ATCACATGCC TGACGACCCA AACGTTCTGC AGCCCACGTG TAGGTTTGGG CCCCACTGTG   
  
  
+ GACGCGCCAC CGGCTAAACT GGTCGACATG CCGCGTAATA AAGGGTGGAC CCGAGGGCGG TCCTGGAGCA   
  
  
+ ATTAATGCAC AACGCCGTCC TCTAGTCACC CTCATACCGT ACACATGGTT AACCGGCATT TGCCAGTCAA   
  
  
+ CTCTTCTGTT TTATTTTCTA TTTAAAATAA TTTATCAAAT GGGAAACGTC CCAAAACAAG CTGTTTTCCA   
  
  
+ AAAACCCAAA CACAAAAAGC CCATTCACAC TCGAAGCAAC CAAACACTAT TTCTCTTTCT CTCTCTGTCT   
  
  
+ CTCACTTTTT TTCTCTCTCT CTCTCCCTCT CTTGTAATAA AAAGTGCGAG AAGAGAAGAA ATGAGAGAGG   
  
  
+ GTTTTATGAA ATGAAGGAGG AGATCATTAT GTAGTACATG TGCTCGCGTT GGTGATCAGA GATTTCTATG   
  
  
+ GAGAAGAGAG AAGAAAAGAG AGAGAATTC  

- GCCGACAGGG TGTAGCTCGA GACCTCGGTG CCCCTGGTCC TGGCACATAG CACCGTTTCC ACCAAAGTGC   
  
  
- TTTTCCTACG TTATTAAGAC TATCTAGAAG TGTGTTAAAG AAAAAAAAAA AGATTAAAAA TAAAAATAAA   
  
  
- TGAGAGACTA CATTTATTTA AATAATATAT AATAATAATA ATTAAAAAAA ACGCACCCTT CCTTCAACAT   
  
  
- GGAACCCCAC TGGTATAACA TTTTTCCACA CACGTTACTA TTGTTCTTCT CTGTATGTGT GTAGTGTGCA   
  
  
- TGCTCTGAGG GAAACGAAGT AGTAAGACCC ATTGAACAAG TAGTACAGTG CTAGTAGGGT TAGAGGGCTA   
  
  
- AATAAAAAGG TACGTAATAC ATTATATATG CGTTGCAAAA CTAAAAATAT TAACATAGTA AAAAATCAAA   
  
  
- TTTTTTTAAA CATTTATTAT AAATTAGATA AAAAATATTT AATAAAACTA ATTCAGGTTT TAATAAATAT   
  
  
- TTTCATTACG TTTCAATTTA CTTAAGTCTT TAGCCTTTAA ATTTTTTCTC TCCCTTTACA CCACACTTCT   
  
  
- CCCTCCTCTC ACCTCCGAAC AGCCCGTTCC CCTCACCCAC TGCGCATTAT GCGCAGTCGG ATTCGCTCGG   
  
  
- AATACCACAT CCGACTTCGA AGAAATTGAA ATGGGGTAAA AAAATAAAAG TATTAAACAA TCAAATTAAA   
  
  
- ATATAAAATA TAAAAATTTT TCTTTTTTTT GAATTAAATT GCCCCTCTGG AAACAGACGT CCTTTCATTA   
  
  
- GAAGATCAAT GACTTAACGG GGACTTCTCC TACTATCTCA CTCCCCGTAA ATCCATTTAA GTACTAACTC   
  
  
- TCTCTCTCTC TCTCTCTCTC TCTCTCTCAC AAAACTACAT TTTGTTTTTC TATTAAAACC CCAAATTTTC   
  
  
- AATTTTTCCA AAAATTTCCG TTTTCGACGC TTTCTTTTAA TCTTTATTTT TTTAATTTTT TTTTTGTTCC   
  
  
- TCCTCTTCGT TAGTGTACGG ACTGCTGGGT TTGCAAGACG TCGGGTGCAC ATCCAAACCC GGGGTGACAC   
  
  
- CTGCGCGGTG GCCGATTTGA CCAGCTGTAC GGCGCATTAT TTCCCACCTG GGCTCCCGCC AGGACCTCGT   
  
  
- TAATTACGTG TTGCGGCAGG AGATCAGTGG GAGTATGGCA TGTGTACCAA TTGGCCGTAA ACGGTCAGTT   
  
  
- GAGAAGACAA AATAAAAGAT AAATTTTATT AAATAGTTTA CCCTTTGCAG GGTTTTGTTC GACAAAAGGT   
  
  
- TTTTGGGTTT GTGTTTTTCG GGTAAGTGTG AGCTTCGTTG GTTTGTGATA AAGAGAAAGA GAGAGACAGA   
  
  
- GAGTGAAAAA AAGAGAGAGA GAGAGGGAGA GAACATTATT TTTCACGCTC TTCTCTTCTT TACTCTCTCC   
  
  
- CAAAATACTT TACTTCCTCC TCTAGTAATA CATCATGTAC ACGAGCGCAA CCACTAGTCT CTAAAGATAC   
  
  
- CTCTTCTCTC TTCTTTTCTC TCTCTTAAG

+     CCGTCC-box

| Site Name | Organism | Position | Strand | Matrix score. | sequence | function |
| --- | --- | --- | --- | --- | --- | --- |
| CCGTCC-box | Arabidopsis thaliana | 1135 | + | 6 | CCGTCC | cis-acting regulatory element related to meristem specific activation |

> 2018/04/13 10:10:12  
+ CGGCTGTCCC ACATCGAGCT CTGGAGCCAC GGGGACCAGG ACCGTGTATC GTGGCAAAGG TGGTTTCACG   
  
  
+ AAAAGGATGC AATAATTCTG ATAGATCTTC ACACAATTTC TTTTTTTTTT TCTAATTTTT ATTTTTATTT   
  
  
+ ACTCTCTGAT GTAAATAAAT TTATTATATA TTATTATTAT TAATTTTTTT TGCGTGGGAA GGAAGTTGTA   
  
  
+ CCTTGGGGTG ACCATATTGT AAAAAGGTGT GTGCAATGAT AACAAGAAGA GACATACACA CATCACACGT   
  
  
+ ACGAGACTCC CTTTGCTTCA TCATTCTGGG TAACTTGTTC ATCATGTCAC GATCATCCCA ATCTCCCGAT   
  
  
+ TTATTTTTCC ATGCATTATG TAATATATAC GCAACGTTTT GATTTTTATA ATTGTATCAT TTTTTAGTTT   
  
  
+ AAAAAAATTT GTAAATAATA TTTAATCTAT TTTTTATAAA TTATTTTGAT TAAGTCCAAA ATTATTTATA   
  
  
+ AAAGTAATGC AAAGTTAAAT GAATTCAGAA ATCGGAAATT TAAAAAAGAG AGGGAAATGT GGTGTGAAGA   
  
  
+ GGGAGGAGAG TGGAGGCTTG TCGGGCAAGG GGAGTGGGTG ACGCGTAATA CGCGTCAGCC TAAGCGAGCC   
  
  
+ TTATGGTGTA GGCTGAAGCT TCTTTAACTT TACCCCATTT TTTTATTTTC ATAATTTGTT AGTTTAATTT   
  
  
+ TATATTTTAT ATTTTTAAAA AGAAAAAAAA CTTAATTTAA CGGGGAGACC TTTGTCTGCA GGAAAGTAAT   
  
  
+ CTTCTAGTTA CTGAATTGCC CCTGAAGAGG ATGATAGAGT GAGGGGCATT TAGGTAAATT CATGATTGAG   
  
  
+ AGAGAGAGAG AGAGAGAGAG AGAGAGAGTG TTTTGATGTA AAACAAAAAG ATAATTTTGG GGTTTAAAAG   
  
  
+ TTAAAAAGGT TTTTAAAGGC AAAAGCTGCG AAAGAAAATT AGAAATAAAA AAATTAAAAA AAAAACAAGG   
  
  
+ AGGAGAAGCA ATCACATGCC TGACGACCCA AACGTTCTGC AGCCCACGTG TAGGTTTGGG CCCCACTGTG   
  
  
+ GACGCGCCAC CGGCTAAACT GGTCGACATG CCGCGTAATA AAGGGTGGAC CCGAGGGCGG TCCTGGAGCA   
  
  
+ ATTAATGCAC AACGCCGTCC TCTAGTCACC CTCATACCGT ACACATGGTT AACCGGCATT TGCCAGTCAA   
  
  
+ CTCTTCTGTT TTATTTTCTA TTTAAAATAA TTTATCAAAT GGGAAACGTC CCAAAACAAG CTGTTTTCCA   
  
  
+ AAAACCCAAA CACAAAAAGC CCATTCACAC TCGAAGCAAC CAAACACTAT TTCTCTTTCT CTCTCTGTCT   
  
  
+ CTCACTTTTT TTCTCTCTCT CTCTCCCTCT CTTGTAATAA AAAGTGCGAG AAGAGAAGAA ATGAGAGAGG   
  
  
+ GTTTTATGAA ATGAAGGAGG AGATCATTAT GTAGTACATG TGCTCGCGTT GGTGATCAGA GATTTCTATG   
  
  
+ GAGAAGAGAG AAGAAAAGAG AGAGAATTC  

- GCCGACAGGG TGTAGCTCGA GACCTCGGTG CCCCTGGTCC TGGCACATAG CACCGTTTCC ACCAAAGTGC   
  
  
- TTTTCCTACG TTATTAAGAC TATCTAGAAG TGTGTTAAAG AAAAAAAAAA AGATTAAAAA TAAAAATAAA   
  
  
- TGAGAGACTA CATTTATTTA AATAATATAT AATAATAATA ATTAAAAAAA ACGCACCCTT CCTTCAACAT   
  
  
- GGAACCCCAC TGGTATAACA TTTTTCCACA CACGTTACTA TTGTTCTTCT CTGTATGTGT GTAGTGTGCA   
  
  
- TGCTCTGAGG GAAACGAAGT AGTAAGACCC ATTGAACAAG TAGTACAGTG CTAGTAGGGT TAGAGGGCTA   
  
  
- AATAAAAAGG TACGTAATAC ATTATATATG CGTTGCAAAA CTAAAAATAT TAACATAGTA AAAAATCAAA   
  
  
- TTTTTTTAAA CATTTATTAT AAATTAGATA AAAAATATTT AATAAAACTA ATTCAGGTTT TAATAAATAT   
  
  
- TTTCATTACG TTTCAATTTA CTTAAGTCTT TAGCCTTTAA ATTTTTTCTC TCCCTTTACA CCACACTTCT   
  
  
- CCCTCCTCTC ACCTCCGAAC AGCCCGTTCC CCTCACCCAC TGCGCATTAT GCGCAGTCGG ATTCGCTCGG   
  
  
- AATACCACAT CCGACTTCGA AGAAATTGAA ATGGGGTAAA AAAATAAAAG TATTAAACAA TCAAATTAAA   
  
  
- ATATAAAATA TAAAAATTTT TCTTTTTTTT GAATTAAATT GCCCCTCTGG AAACAGACGT CCTTTCATTA   
  
  
- GAAGATCAAT GACTTAACGG GGACTTCTCC TACTATCTCA CTCCCCGTAA ATCCATTTAA GTACTAACTC   
  
  
- TCTCTCTCTC TCTCTCTCTC TCTCTCTCAC AAAACTACAT TTTGTTTTTC TATTAAAACC CCAAATTTTC   
  
  
- AATTTTTCCA AAAATTTCCG TTTTCGACGC TTTCTTTTAA TCTTTATTTT TTTAATTTTT TTTTTGTTCC   
  
  
- TCCTCTTCGT TAGTGTACGG ACTGCTGGGT TTGCAAGACG TCGGGTGCAC ATCCAAACCC GGGGTGACAC   
  
  
- CTGCGCGGTG GCCGATTTGA CCAGCTGTAC GGCGCATTAT TTCCCACCTG GGCTCCCGCC AGGACCTCGT   
  
  
- TAATTACGTG TTGCGGCAGG AGATCAGTGG GAGTATGGCA TGTGTACCAA TTGGCCGTAA ACGGTCAGTT   
  
  
- GAGAAGACAA AATAAAAGAT AAATTTTATT AAATAGTTTA CCCTTTGCAG GGTTTTGTTC GACAAAAGGT   
  
  
- TTTTGGGTTT GTGTTTTTCG GGTAAGTGTG AGCTTCGTTG GTTTGTGATA AAGAGAAAGA GAGAGACAGA   
  
  
- GAGTGAAAAA AAGAGAGAGA GAGAGGGAGA GAACATTATT TTTCACGCTC TTCTCTTCTT TACTCTCTCC   
  
  
- CAAAATACTT TACTTCCTCC TCTAGTAATA CATCATGTAC ACGAGCGCAA CCACTAGTCT CTAAAGATAC   
  
  
- CTCTTCTCTC TTCTTTTCTC TCTCTTAAG

+     CGTCA-motif

| Site Name | Organism | Position | Strand | Matrix score. | sequence | function |
| --- | --- | --- | --- | --- | --- | --- |
| CGTCA-motif | Hordeum vulgare | 599 | - | 5 | CGTCA | cis-acting regulatory element involved in the MeJA-responsiveness |
| CGTCA-motif | Hordeum vulgare | 1001 | - | 5 | CGTCA | cis-acting regulatory element involved in the MeJA-responsiveness |
| CGTCA-motif | Hordeum vulgare | 613 | + | 5 | CGTCA | cis-acting regulatory element involved in the MeJA-responsiveness |

> 2018/04/13 10:10:12  
+ CGGCTGTCCC ACATCGAGCT CTGGAGCCAC GGGGACCAGG ACCGTGTATC GTGGCAAAGG TGGTTTCACG   
  
  
+ AAAAGGATGC AATAATTCTG ATAGATCTTC ACACAATTTC TTTTTTTTTT TCTAATTTTT ATTTTTATTT   
  
  
+ ACTCTCTGAT GTAAATAAAT TTATTATATA TTATTATTAT TAATTTTTTT TGCGTGGGAA GGAAGTTGTA   
  
  
+ CCTTGGGGTG ACCATATTGT AAAAAGGTGT GTGCAATGAT AACAAGAAGA GACATACACA CATCACACGT   
  
  
+ ACGAGACTCC CTTTGCTTCA TCATTCTGGG TAACTTGTTC ATCATGTCAC GATCATCCCA ATCTCCCGAT   
  
  
+ TTATTTTTCC ATGCATTATG TAATATATAC GCAACGTTTT GATTTTTATA ATTGTATCAT TTTTTAGTTT   
  
  
+ AAAAAAATTT GTAAATAATA TTTAATCTAT TTTTTATAAA TTATTTTGAT TAAGTCCAAA ATTATTTATA   
  
  
+ AAAGTAATGC AAAGTTAAAT GAATTCAGAA ATCGGAAATT TAAAAAAGAG AGGGAAATGT GGTGTGAAGA   
  
  
+ GGGAGGAGAG TGGAGGCTTG TCGGGCAAGG GGAGTGGGTG ACGCGTAATA CGCGTCAGCC TAAGCGAGCC   
  
  
+ TTATGGTGTA GGCTGAAGCT TCTTTAACTT TACCCCATTT TTTTATTTTC ATAATTTGTT AGTTTAATTT   
  
  
+ TATATTTTAT ATTTTTAAAA AGAAAAAAAA CTTAATTTAA CGGGGAGACC TTTGTCTGCA GGAAAGTAAT   
  
  
+ CTTCTAGTTA CTGAATTGCC CCTGAAGAGG ATGATAGAGT GAGGGGCATT TAGGTAAATT CATGATTGAG   
  
  
+ AGAGAGAGAG AGAGAGAGAG AGAGAGAGTG TTTTGATGTA AAACAAAAAG ATAATTTTGG GGTTTAAAAG   
  
  
+ TTAAAAAGGT TTTTAAAGGC AAAAGCTGCG AAAGAAAATT AGAAATAAAA AAATTAAAAA AAAAACAAGG   
  
  
+ AGGAGAAGCA ATCACATGCC TGACGACCCA AACGTTCTGC AGCCCACGTG TAGGTTTGGG CCCCACTGTG   
  
  
+ GACGCGCCAC CGGCTAAACT GGTCGACATG CCGCGTAATA AAGGGTGGAC CCGAGGGCGG TCCTGGAGCA   
  
  
+ ATTAATGCAC AACGCCGTCC TCTAGTCACC CTCATACCGT ACACATGGTT AACCGGCATT TGCCAGTCAA   
  
  
+ CTCTTCTGTT TTATTTTCTA TTTAAAATAA TTTATCAAAT GGGAAACGTC CCAAAACAAG CTGTTTTCCA   
  
  
+ AAAACCCAAA CACAAAAAGC CCATTCACAC TCGAAGCAAC CAAACACTAT TTCTCTTTCT CTCTCTGTCT   
  
  
+ CTCACTTTTT TTCTCTCTCT CTCTCCCTCT CTTGTAATAA AAAGTGCGAG AAGAGAAGAA ATGAGAGAGG   
  
  
+ GTTTTATGAA ATGAAGGAGG AGATCATTAT GTAGTACATG TGCTCGCGTT GGTGATCAGA GATTTCTATG   
  
  
+ GAGAAGAGAG AAGAAAAGAG AGAGAATTC  

- GCCGACAGGG TGTAGCTCGA GACCTCGGTG CCCCTGGTCC TGGCACATAG CACCGTTTCC ACCAAAGTGC   
  
  
- TTTTCCTACG TTATTAAGAC TATCTAGAAG TGTGTTAAAG AAAAAAAAAA AGATTAAAAA TAAAAATAAA   
  
  
- TGAGAGACTA CATTTATTTA AATAATATAT AATAATAATA ATTAAAAAAA ACGCACCCTT CCTTCAACAT   
  
  
- GGAACCCCAC TGGTATAACA TTTTTCCACA CACGTTACTA TTGTTCTTCT CTGTATGTGT GTAGTGTGCA   
  
  
- TGCTCTGAGG GAAACGAAGT AGTAAGACCC ATTGAACAAG TAGTACAGTG CTAGTAGGGT TAGAGGGCTA   
  
  
- AATAAAAAGG TACGTAATAC ATTATATATG CGTTGCAAAA CTAAAAATAT TAACATAGTA AAAAATCAAA   
  
  
- TTTTTTTAAA CATTTATTAT AAATTAGATA AAAAATATTT AATAAAACTA ATTCAGGTTT TAATAAATAT   
  
  
- TTTCATTACG TTTCAATTTA CTTAAGTCTT TAGCCTTTAA ATTTTTTCTC TCCCTTTACA CCACACTTCT   
  
  
- CCCTCCTCTC ACCTCCGAAC AGCCCGTTCC CCTCACCCAC TGCGCATTAT GCGCAGTCGG ATTCGCTCGG   
  
  
- AATACCACAT CCGACTTCGA AGAAATTGAA ATGGGGTAAA AAAATAAAAG TATTAAACAA TCAAATTAAA   
  
  
- ATATAAAATA TAAAAATTTT TCTTTTTTTT GAATTAAATT GCCCCTCTGG AAACAGACGT CCTTTCATTA   
  
  
- GAAGATCAAT GACTTAACGG GGACTTCTCC TACTATCTCA CTCCCCGTAA ATCCATTTAA GTACTAACTC   
  
  
- TCTCTCTCTC TCTCTCTCTC TCTCTCTCAC AAAACTACAT TTTGTTTTTC TATTAAAACC CCAAATTTTC   
  
  
- AATTTTTCCA AAAATTTCCG TTTTCGACGC TTTCTTTTAA TCTTTATTTT TTTAATTTTT TTTTTGTTCC   
  
  
- TCCTCTTCGT TAGTGTACGG ACTGCTGGGT TTGCAAGACG TCGGGTGCAC ATCCAAACCC GGGGTGACAC   
  
  
- CTGCGCGGTG GCCGATTTGA CCAGCTGTAC GGCGCATTAT TTCCCACCTG GGCTCCCGCC AGGACCTCGT   
  
  
- TAATTACGTG TTGCGGCAGG AGATCAGTGG GAGTATGGCA TGTGTACCAA TTGGCCGTAA ACGGTCAGTT   
  
  
- GAGAAGACAA AATAAAAGAT AAATTTTATT AAATAGTTTA CCCTTTGCAG GGTTTTGTTC GACAAAAGGT   
  
  
- TTTTGGGTTT GTGTTTTTCG GGTAAGTGTG AGCTTCGTTG GTTTGTGATA AAGAGAAAGA GAGAGACAGA   
  
  
- GAGTGAAAAA AAGAGAGAGA GAGAGGGAGA GAACATTATT TTTCACGCTC TTCTCTTCTT TACTCTCTCC   
  
  
- CAAAATACTT TACTTCCTCC TCTAGTAATA CATCATGTAC ACGAGCGCAA CCACTAGTCT CTAAAGATAC   
  
  
- CTCTTCTCTC TTCTTTTCTC TCTCTTAAG

+     G-Box

| Site Name | Organism | Position | Strand | Matrix score. | sequence | function |
| --- | --- | --- | --- | --- | --- | --- |
| G-Box | Antirrhinum majus | 276 | + | 6 | CACGTA | cis-acting regulatory element involved in light responsiveness |
| G-Box | Pisum sativum | 1025 | - | 6 | CACGTG | cis-acting regulatory element involved in light responsiveness |

> 2018/04/13 10:10:12  
+ CGGCTGTCCC ACATCGAGCT CTGGAGCCAC GGGGACCAGG ACCGTGTATC GTGGCAAAGG TGGTTTCACG   
  
  
+ AAAAGGATGC AATAATTCTG ATAGATCTTC ACACAATTTC TTTTTTTTTT TCTAATTTTT ATTTTTATTT   
  
  
+ ACTCTCTGAT GTAAATAAAT TTATTATATA TTATTATTAT TAATTTTTTT TGCGTGGGAA GGAAGTTGTA   
  
  
+ CCTTGGGGTG ACCATATTGT AAAAAGGTGT GTGCAATGAT AACAAGAAGA GACATACACA CATCACACGT   
  
  
+ ACGAGACTCC CTTTGCTTCA TCATTCTGGG TAACTTGTTC ATCATGTCAC GATCATCCCA ATCTCCCGAT   
  
  
+ TTATTTTTCC ATGCATTATG TAATATATAC GCAACGTTTT GATTTTTATA ATTGTATCAT TTTTTAGTTT   
  
  
+ AAAAAAATTT GTAAATAATA TTTAATCTAT TTTTTATAAA TTATTTTGAT TAAGTCCAAA ATTATTTATA   
  
  
+ AAAGTAATGC AAAGTTAAAT GAATTCAGAA ATCGGAAATT TAAAAAAGAG AGGGAAATGT GGTGTGAAGA   
  
  
+ GGGAGGAGAG TGGAGGCTTG TCGGGCAAGG GGAGTGGGTG ACGCGTAATA CGCGTCAGCC TAAGCGAGCC   
  
  
+ TTATGGTGTA GGCTGAAGCT TCTTTAACTT TACCCCATTT TTTTATTTTC ATAATTTGTT AGTTTAATTT   
  
  
+ TATATTTTAT ATTTTTAAAA AGAAAAAAAA CTTAATTTAA CGGGGAGACC TTTGTCTGCA GGAAAGTAAT   
  
  
+ CTTCTAGTTA CTGAATTGCC CCTGAAGAGG ATGATAGAGT GAGGGGCATT TAGGTAAATT CATGATTGAG   
  
  
+ AGAGAGAGAG AGAGAGAGAG AGAGAGAGTG TTTTGATGTA AAACAAAAAG ATAATTTTGG GGTTTAAAAG   
  
  
+ TTAAAAAGGT TTTTAAAGGC AAAAGCTGCG AAAGAAAATT AGAAATAAAA AAATTAAAAA AAAAACAAGG   
  
  
+ AGGAGAAGCA ATCACATGCC TGACGACCCA AACGTTCTGC AGCCCACGTG TAGGTTTGGG CCCCACTGTG   
  
  
+ GACGCGCCAC CGGCTAAACT GGTCGACATG CCGCGTAATA AAGGGTGGAC CCGAGGGCGG TCCTGGAGCA   
  
  
+ ATTAATGCAC AACGCCGTCC TCTAGTCACC CTCATACCGT ACACATGGTT AACCGGCATT TGCCAGTCAA   
  
  
+ CTCTTCTGTT TTATTTTCTA TTTAAAATAA TTTATCAAAT GGGAAACGTC CCAAAACAAG CTGTTTTCCA   
  
  
+ AAAACCCAAA CACAAAAAGC CCATTCACAC TCGAAGCAAC CAAACACTAT TTCTCTTTCT CTCTCTGTCT   
  
  
+ CTCACTTTTT TTCTCTCTCT CTCTCCCTCT CTTGTAATAA AAAGTGCGAG AAGAGAAGAA ATGAGAGAGG   
  
  
+ GTTTTATGAA ATGAAGGAGG AGATCATTAT GTAGTACATG TGCTCGCGTT GGTGATCAGA GATTTCTATG   
  
  
+ GAGAAGAGAG AAGAAAAGAG AGAGAATTC  

- GCCGACAGGG TGTAGCTCGA GACCTCGGTG CCCCTGGTCC TGGCACATAG CACCGTTTCC ACCAAAGTGC   
  
  
- TTTTCCTACG TTATTAAGAC TATCTAGAAG TGTGTTAAAG AAAAAAAAAA AGATTAAAAA TAAAAATAAA   
  
  
- TGAGAGACTA CATTTATTTA AATAATATAT AATAATAATA ATTAAAAAAA ACGCACCCTT CCTTCAACAT   
  
  
- GGAACCCCAC TGGTATAACA TTTTTCCACA CACGTTACTA TTGTTCTTCT CTGTATGTGT GTAGTGTGCA   
  
  
- TGCTCTGAGG GAAACGAAGT AGTAAGACCC ATTGAACAAG TAGTACAGTG CTAGTAGGGT TAGAGGGCTA   
  
  
- AATAAAAAGG TACGTAATAC ATTATATATG CGTTGCAAAA CTAAAAATAT TAACATAGTA AAAAATCAAA   
  
  
- TTTTTTTAAA CATTTATTAT AAATTAGATA AAAAATATTT AATAAAACTA ATTCAGGTTT TAATAAATAT   
  
  
- TTTCATTACG TTTCAATTTA CTTAAGTCTT TAGCCTTTAA ATTTTTTCTC TCCCTTTACA CCACACTTCT   
  
  
- CCCTCCTCTC ACCTCCGAAC AGCCCGTTCC CCTCACCCAC TGCGCATTAT GCGCAGTCGG ATTCGCTCGG   
  
  
- AATACCACAT CCGACTTCGA AGAAATTGAA ATGGGGTAAA AAAATAAAAG TATTAAACAA TCAAATTAAA   
  
  
- ATATAAAATA TAAAAATTTT TCTTTTTTTT GAATTAAATT GCCCCTCTGG AAACAGACGT CCTTTCATTA   
  
  
- GAAGATCAAT GACTTAACGG GGACTTCTCC TACTATCTCA CTCCCCGTAA ATCCATTTAA GTACTAACTC   
  
  
- TCTCTCTCTC TCTCTCTCTC TCTCTCTCAC AAAACTACAT TTTGTTTTTC TATTAAAACC CCAAATTTTC   
  
  
- AATTTTTCCA AAAATTTCCG TTTTCGACGC TTTCTTTTAA TCTTTATTTT TTTAATTTTT TTTTTGTTCC   
  
  
- TCCTCTTCGT TAGTGTACGG ACTGCTGGGT TTGCAAGACG TCGGGTGCAC ATCCAAACCC GGGGTGACAC   
  
  
- CTGCGCGGTG GCCGATTTGA CCAGCTGTAC GGCGCATTAT TTCCCACCTG GGCTCCCGCC AGGACCTCGT   
  
  
- TAATTACGTG TTGCGGCAGG AGATCAGTGG GAGTATGGCA TGTGTACCAA TTGGCCGTAA ACGGTCAGTT   
  
  
- GAGAAGACAA AATAAAAGAT AAATTTTATT AAATAGTTTA CCCTTTGCAG GGTTTTGTTC GACAAAAGGT   
  
  
- TTTTGGGTTT GTGTTTTTCG GGTAAGTGTG AGCTTCGTTG GTTTGTGATA AAGAGAAAGA GAGAGACAGA   
  
  
- GAGTGAAAAA AAGAGAGAGA GAGAGGGAGA GAACATTATT TTTCACGCTC TTCTCTTCTT TACTCTCTCC   
  
  
- CAAAATACTT TACTTCCTCC TCTAGTAATA CATCATGTAC ACGAGCGCAA CCACTAGTCT CTAAAGATAC   
  
  
- CTCTTCTCTC TTCTTTTCTC TCTCTTAAG

+     G-box

| Site Name | Organism | Position | Strand | Matrix score. | sequence | function |
| --- | --- | --- | --- | --- | --- | --- |
| G-box | Arabidopsis thaliana | 1025 | - | 6 | CACGTG | cis-acting regulatory element involved in light responsiveness |
| G-box | Brassica napus | 1024 | - | 7 | CACGTGG | cis-acting regulatory element involved in light responsiveness |
| G-box | Arabidopsis thaliana | 26 | + | 9 | GCCACGTGGA | cis-acting regulatory element involved in light responsiveness |
| G-box | Solanum tuberosum | 1162 | + | 7 | CACATGG | cis-acting regulatory element involved in light responsiveness |
| G-box | Daucus carota | 276 | - | 6 | TACGTG | cis-acting regulatory element involved in light responsiveness |
| G-box | Zea mays | 544 | + | 9 | GACATGTGGT | cis-acting regulatory element involved in light responsiveness |

> 2018/04/13 10:10:12  
+ CGGCTGTCCC ACATCGAGCT CTGGAGCCAC GGGGACCAGG ACCGTGTATC GTGGCAAAGG TGGTTTCACG   
  
  
+ AAAAGGATGC AATAATTCTG ATAGATCTTC ACACAATTTC TTTTTTTTTT TCTAATTTTT ATTTTTATTT   
  
  
+ ACTCTCTGAT GTAAATAAAT TTATTATATA TTATTATTAT TAATTTTTTT TGCGTGGGAA GGAAGTTGTA   
  
  
+ CCTTGGGGTG ACCATATTGT AAAAAGGTGT GTGCAATGAT AACAAGAAGA GACATACACA CATCACACGT   
  
  
+ ACGAGACTCC CTTTGCTTCA TCATTCTGGG TAACTTGTTC ATCATGTCAC GATCATCCCA ATCTCCCGAT   
  
  
+ TTATTTTTCC ATGCATTATG TAATATATAC GCAACGTTTT GATTTTTATA ATTGTATCAT TTTTTAGTTT   
  
  
+ AAAAAAATTT GTAAATAATA TTTAATCTAT TTTTTATAAA TTATTTTGAT TAAGTCCAAA ATTATTTATA   
  
  
+ AAAGTAATGC AAAGTTAAAT GAATTCAGAA ATCGGAAATT TAAAAAAGAG AGGGAAATGT GGTGTGAAGA   
  
  
+ GGGAGGAGAG TGGAGGCTTG TCGGGCAAGG GGAGTGGGTG ACGCGTAATA CGCGTCAGCC TAAGCGAGCC   
  
  
+ TTATGGTGTA GGCTGAAGCT TCTTTAACTT TACCCCATTT TTTTATTTTC ATAATTTGTT AGTTTAATTT   
  
  
+ TATATTTTAT ATTTTTAAAA AGAAAAAAAA CTTAATTTAA CGGGGAGACC TTTGTCTGCA GGAAAGTAAT   
  
  
+ CTTCTAGTTA CTGAATTGCC CCTGAAGAGG ATGATAGAGT GAGGGGCATT TAGGTAAATT CATGATTGAG   
  
  
+ AGAGAGAGAG AGAGAGAGAG AGAGAGAGTG TTTTGATGTA AAACAAAAAG ATAATTTTGG GGTTTAAAAG   
  
  
+ TTAAAAAGGT TTTTAAAGGC AAAAGCTGCG AAAGAAAATT AGAAATAAAA AAATTAAAAA AAAAACAAGG   
  
  
+ AGGAGAAGCA ATCACATGCC TGACGACCCA AACGTTCTGC AGCCCACGTG TAGGTTTGGG CCCCACTGTG   
  
  
+ GACGCGCCAC CGGCTAAACT GGTCGACATG CCGCGTAATA AAGGGTGGAC CCGAGGGCGG TCCTGGAGCA   
  
  
+ ATTAATGCAC AACGCCGTCC TCTAGTCACC CTCATACCGT ACACATGGTT AACCGGCATT TGCCAGTCAA   
  
  
+ CTCTTCTGTT TTATTTTCTA TTTAAAATAA TTTATCAAAT GGGAAACGTC CCAAAACAAG CTGTTTTCCA   
  
  
+ AAAACCCAAA CACAAAAAGC CCATTCACAC TCGAAGCAAC CAAACACTAT TTCTCTTTCT CTCTCTGTCT   
  
  
+ CTCACTTTTT TTCTCTCTCT CTCTCCCTCT CTTGTAATAA AAAGTGCGAG AAGAGAAGAA ATGAGAGAGG   
  
  
+ GTTTTATGAA ATGAAGGAGG AGATCATTAT GTAGTACATG TGCTCGCGTT GGTGATCAGA GATTTCTATG   
  
  
+ GAGAAGAGAG AAGAAAAGAG AGAGAATTC  

- GCCGACAGGG TGTAGCTCGA GACCTCGGTG CCCCTGGTCC TGGCACATAG CACCGTTTCC ACCAAAGTGC   
  
  
- TTTTCCTACG TTATTAAGAC TATCTAGAAG TGTGTTAAAG AAAAAAAAAA AGATTAAAAA TAAAAATAAA   
  
  
- TGAGAGACTA CATTTATTTA AATAATATAT AATAATAATA ATTAAAAAAA ACGCACCCTT CCTTCAACAT   
  
  
- GGAACCCCAC TGGTATAACA TTTTTCCACA CACGTTACTA TTGTTCTTCT CTGTATGTGT GTAGTGTGCA   
  
  
- TGCTCTGAGG GAAACGAAGT AGTAAGACCC ATTGAACAAG TAGTACAGTG CTAGTAGGGT TAGAGGGCTA   
  
  
- AATAAAAAGG TACGTAATAC ATTATATATG CGTTGCAAAA CTAAAAATAT TAACATAGTA AAAAATCAAA   
  
  
- TTTTTTTAAA CATTTATTAT AAATTAGATA AAAAATATTT AATAAAACTA ATTCAGGTTT TAATAAATAT   
  
  
- TTTCATTACG TTTCAATTTA CTTAAGTCTT TAGCCTTTAA ATTTTTTCTC TCCCTTTACA CCACACTTCT   
  
  
- CCCTCCTCTC ACCTCCGAAC AGCCCGTTCC CCTCACCCAC TGCGCATTAT GCGCAGTCGG ATTCGCTCGG   
  
  
- AATACCACAT CCGACTTCGA AGAAATTGAA ATGGGGTAAA AAAATAAAAG TATTAAACAA TCAAATTAAA   
  
  
- ATATAAAATA TAAAAATTTT TCTTTTTTTT GAATTAAATT GCCCCTCTGG AAACAGACGT CCTTTCATTA   
  
  
- GAAGATCAAT GACTTAACGG GGACTTCTCC TACTATCTCA CTCCCCGTAA ATCCATTTAA GTACTAACTC   
  
  
- TCTCTCTCTC TCTCTCTCTC TCTCTCTCAC AAAACTACAT TTTGTTTTTC TATTAAAACC CCAAATTTTC   
  
  
- AATTTTTCCA AAAATTTCCG TTTTCGACGC TTTCTTTTAA TCTTTATTTT TTTAATTTTT TTTTTGTTCC   
  
  
- TCCTCTTCGT TAGTGTACGG ACTGCTGGGT TTGCAAGACG TCGGGTGCAC ATCCAAACCC GGGGTGACAC   
  
  
- CTGCGCGGTG GCCGATTTGA CCAGCTGTAC GGCGCATTAT TTCCCACCTG GGCTCCCGCC AGGACCTCGT   
  
  
- TAATTACGTG TTGCGGCAGG AGATCAGTGG GAGTATGGCA TGTGTACCAA TTGGCCGTAA ACGGTCAGTT   
  
  
- GAGAAGACAA AATAAAAGAT AAATTTTATT AAATAGTTTA CCCTTTGCAG GGTTTTGTTC GACAAAAGGT   
  
  
- TTTTGGGTTT GTGTTTTTCG GGTAAGTGTG AGCTTCGTTG GTTTGTGATA AAGAGAAAGA GAGAGACAGA   
  
  
- GAGTGAAAAA AAGAGAGAGA GAGAGGGAGA GAACATTATT TTTCACGCTC TTCTCTTCTT TACTCTCTCC   
  
  
- CAAAATACTT TACTTCCTCC TCTAGTAATA CATCATGTAC ACGAGCGCAA CCACTAGTCT CTAAAGATAC   
  
  
- CTCTTCTCTC TTCTTTTCTC TCTCTTAAG

+     GAG-motif

| Site Name | Organism | Position | Strand | Matrix score. | sequence | function |
| --- | --- | --- | --- | --- | --- | --- |
| GAG-motif | Arabidopsis thaliana | 141 | - | 7 | AGAGAGT | part of a light responsive element |
| GAG-motif | Arabidopsis thaliana | 863 | + | 7 | AGAGAGT | part of a light responsive element |

> 2018/04/13 10:10:12  
+ CGGCTGTCCC ACATCGAGCT CTGGAGCCAC GGGGACCAGG ACCGTGTATC GTGGCAAAGG TGGTTTCACG   
  
  
+ AAAAGGATGC AATAATTCTG ATAGATCTTC ACACAATTTC TTTTTTTTTT TCTAATTTTT ATTTTTATTT   
  
  
+ ACTCTCTGAT GTAAATAAAT TTATTATATA TTATTATTAT TAATTTTTTT TGCGTGGGAA GGAAGTTGTA   
  
  
+ CCTTGGGGTG ACCATATTGT AAAAAGGTGT GTGCAATGAT AACAAGAAGA GACATACACA CATCACACGT   
  
  
+ ACGAGACTCC CTTTGCTTCA TCATTCTGGG TAACTTGTTC ATCATGTCAC GATCATCCCA ATCTCCCGAT   
  
  
+ TTATTTTTCC ATGCATTATG TAATATATAC GCAACGTTTT GATTTTTATA ATTGTATCAT TTTTTAGTTT   
  
  
+ AAAAAAATTT GTAAATAATA TTTAATCTAT TTTTTATAAA TTATTTTGAT TAAGTCCAAA ATTATTTATA   
  
  
+ AAAGTAATGC AAAGTTAAAT GAATTCAGAA ATCGGAAATT TAAAAAAGAG AGGGAAATGT GGTGTGAAGA   
  
  
+ GGGAGGAGAG TGGAGGCTTG TCGGGCAAGG GGAGTGGGTG ACGCGTAATA CGCGTCAGCC TAAGCGAGCC   
  
  
+ TTATGGTGTA GGCTGAAGCT TCTTTAACTT TACCCCATTT TTTTATTTTC ATAATTTGTT AGTTTAATTT   
  
  
+ TATATTTTAT ATTTTTAAAA AGAAAAAAAA CTTAATTTAA CGGGGAGACC TTTGTCTGCA GGAAAGTAAT   
  
  
+ CTTCTAGTTA CTGAATTGCC CCTGAAGAGG ATGATAGAGT GAGGGGCATT TAGGTAAATT CATGATTGAG   
  
  
+ AGAGAGAGAG AGAGAGAGAG AGAGAGAGTG TTTTGATGTA AAACAAAAAG ATAATTTTGG GGTTTAAAAG   
  
  
+ TTAAAAAGGT TTTTAAAGGC AAAAGCTGCG AAAGAAAATT AGAAATAAAA AAATTAAAAA AAAAACAAGG   
  
  
+ AGGAGAAGCA ATCACATGCC TGACGACCCA AACGTTCTGC AGCCCACGTG TAGGTTTGGG CCCCACTGTG   
  
  
+ GACGCGCCAC CGGCTAAACT GGTCGACATG CCGCGTAATA AAGGGTGGAC CCGAGGGCGG TCCTGGAGCA   
  
  
+ ATTAATGCAC AACGCCGTCC TCTAGTCACC CTCATACCGT ACACATGGTT AACCGGCATT TGCCAGTCAA   
  
  
+ CTCTTCTGTT TTATTTTCTA TTTAAAATAA TTTATCAAAT GGGAAACGTC CCAAAACAAG CTGTTTTCCA   
  
  
+ AAAACCCAAA CACAAAAAGC CCATTCACAC TCGAAGCAAC CAAACACTAT TTCTCTTTCT CTCTCTGTCT   
  
  
+ CTCACTTTTT TTCTCTCTCT CTCTCCCTCT CTTGTAATAA AAAGTGCGAG AAGAGAAGAA ATGAGAGAGG   
  
  
+ GTTTTATGAA ATGAAGGAGG AGATCATTAT GTAGTACATG TGCTCGCGTT GGTGATCAGA GATTTCTATG   
  
  
+ GAGAAGAGAG AAGAAAAGAG AGAGAATTC  

- GCCGACAGGG TGTAGCTCGA GACCTCGGTG CCCCTGGTCC TGGCACATAG CACCGTTTCC ACCAAAGTGC   
  
  
- TTTTCCTACG TTATTAAGAC TATCTAGAAG TGTGTTAAAG AAAAAAAAAA AGATTAAAAA TAAAAATAAA   
  
  
- TGAGAGACTA CATTTATTTA AATAATATAT AATAATAATA ATTAAAAAAA ACGCACCCTT CCTTCAACAT   
  
  
- GGAACCCCAC TGGTATAACA TTTTTCCACA CACGTTACTA TTGTTCTTCT CTGTATGTGT GTAGTGTGCA   
  
  
- TGCTCTGAGG GAAACGAAGT AGTAAGACCC ATTGAACAAG TAGTACAGTG CTAGTAGGGT TAGAGGGCTA   
  
  
- AATAAAAAGG TACGTAATAC ATTATATATG CGTTGCAAAA CTAAAAATAT TAACATAGTA AAAAATCAAA   
  
  
- TTTTTTTAAA CATTTATTAT AAATTAGATA AAAAATATTT AATAAAACTA ATTCAGGTTT TAATAAATAT   
  
  
- TTTCATTACG TTTCAATTTA CTTAAGTCTT TAGCCTTTAA ATTTTTTCTC TCCCTTTACA CCACACTTCT   
  
  
- CCCTCCTCTC ACCTCCGAAC AGCCCGTTCC CCTCACCCAC TGCGCATTAT GCGCAGTCGG ATTCGCTCGG   
  
  
- AATACCACAT CCGACTTCGA AGAAATTGAA ATGGGGTAAA AAAATAAAAG TATTAAACAA TCAAATTAAA   
  
  
- ATATAAAATA TAAAAATTTT TCTTTTTTTT GAATTAAATT GCCCCTCTGG AAACAGACGT CCTTTCATTA   
  
  
- GAAGATCAAT GACTTAACGG GGACTTCTCC TACTATCTCA CTCCCCGTAA ATCCATTTAA GTACTAACTC   
  
  
- TCTCTCTCTC TCTCTCTCTC TCTCTCTCAC AAAACTACAT TTTGTTTTTC TATTAAAACC CCAAATTTTC   
  
  
- AATTTTTCCA AAAATTTCCG TTTTCGACGC TTTCTTTTAA TCTTTATTTT TTTAATTTTT TTTTTGTTCC   
  
  
- TCCTCTTCGT TAGTGTACGG ACTGCTGGGT TTGCAAGACG TCGGGTGCAC ATCCAAACCC GGGGTGACAC   
  
  
- CTGCGCGGTG GCCGATTTGA CCAGCTGTAC GGCGCATTAT TTCCCACCTG GGCTCCCGCC AGGACCTCGT   
  
  
- TAATTACGTG TTGCGGCAGG AGATCAGTGG GAGTATGGCA TGTGTACCAA TTGGCCGTAA ACGGTCAGTT   
  
  
- GAGAAGACAA AATAAAAGAT AAATTTTATT AAATAGTTTA CCCTTTGCAG GGTTTTGTTC GACAAAAGGT   
  
  
- TTTTGGGTTT GTGTTTTTCG GGTAAGTGTG AGCTTCGTTG GTTTGTGATA AAGAGAAAGA GAGAGACAGA   
  
  
- GAGTGAAAAA AAGAGAGAGA GAGAGGGAGA GAACATTATT TTTCACGCTC TTCTCTTCTT TACTCTCTCC   
  
  
- CAAAATACTT TACTTCCTCC TCTAGTAATA CATCATGTAC ACGAGCGCAA CCACTAGTCT CTAAAGATAC   
  
  
- CTCTTCTCTC TTCTTTTCTC TCTCTTAAG

+     GARE-motif

| Site Name | Organism | Position | Strand | Matrix score. | sequence | function |
| --- | --- | --- | --- | --- | --- | --- |
| GARE-motif | Brassica oleracea | 1195 | - | 7 | AAACAGA | gibberellin-responsive element |

> 2018/04/13 10:10:12  
+ CGGCTGTCCC ACATCGAGCT CTGGAGCCAC GGGGACCAGG ACCGTGTATC GTGGCAAAGG TGGTTTCACG   
  
  
+ AAAAGGATGC AATAATTCTG ATAGATCTTC ACACAATTTC TTTTTTTTTT TCTAATTTTT ATTTTTATTT   
  
  
+ ACTCTCTGAT GTAAATAAAT TTATTATATA TTATTATTAT TAATTTTTTT TGCGTGGGAA GGAAGTTGTA   
  
  
+ CCTTGGGGTG ACCATATTGT AAAAAGGTGT GTGCAATGAT AACAAGAAGA GACATACACA CATCACACGT   
  
  
+ ACGAGACTCC CTTTGCTTCA TCATTCTGGG TAACTTGTTC ATCATGTCAC GATCATCCCA ATCTCCCGAT   
  
  
+ TTATTTTTCC ATGCATTATG TAATATATAC GCAACGTTTT GATTTTTATA ATTGTATCAT TTTTTAGTTT   
  
  
+ AAAAAAATTT GTAAATAATA TTTAATCTAT TTTTTATAAA TTATTTTGAT TAAGTCCAAA ATTATTTATA   
  
  
+ AAAGTAATGC AAAGTTAAAT GAATTCAGAA ATCGGAAATT TAAAAAAGAG AGGGAAATGT GGTGTGAAGA   
  
  
+ GGGAGGAGAG TGGAGGCTTG TCGGGCAAGG GGAGTGGGTG ACGCGTAATA CGCGTCAGCC TAAGCGAGCC   
  
  
+ TTATGGTGTA GGCTGAAGCT TCTTTAACTT TACCCCATTT TTTTATTTTC ATAATTTGTT AGTTTAATTT   
  
  
+ TATATTTTAT ATTTTTAAAA AGAAAAAAAA CTTAATTTAA CGGGGAGACC TTTGTCTGCA GGAAAGTAAT   
  
  
+ CTTCTAGTTA CTGAATTGCC CCTGAAGAGG ATGATAGAGT GAGGGGCATT TAGGTAAATT CATGATTGAG   
  
  
+ AGAGAGAGAG AGAGAGAGAG AGAGAGAGTG TTTTGATGTA AAACAAAAAG ATAATTTTGG GGTTTAAAAG   
  
  
+ TTAAAAAGGT TTTTAAAGGC AAAAGCTGCG AAAGAAAATT AGAAATAAAA AAATTAAAAA AAAAACAAGG   
  
  
+ AGGAGAAGCA ATCACATGCC TGACGACCCA AACGTTCTGC AGCCCACGTG TAGGTTTGGG CCCCACTGTG   
  
  
+ GACGCGCCAC CGGCTAAACT GGTCGACATG CCGCGTAATA AAGGGTGGAC CCGAGGGCGG TCCTGGAGCA   
  
  
+ ATTAATGCAC AACGCCGTCC TCTAGTCACC CTCATACCGT ACACATGGTT AACCGGCATT TGCCAGTCAA   
  
  
+ CTCTTCTGTT TTATTTTCTA TTTAAAATAA TTTATCAAAT GGGAAACGTC CCAAAACAAG CTGTTTTCCA   
  
  
+ AAAACCCAAA CACAAAAAGC CCATTCACAC TCGAAGCAAC CAAACACTAT TTCTCTTTCT CTCTCTGTCT   
  
  
+ CTCACTTTTT TTCTCTCTCT CTCTCCCTCT CTTGTAATAA AAAGTGCGAG AAGAGAAGAA ATGAGAGAGG   
  
  
+ GTTTTATGAA ATGAAGGAGG AGATCATTAT GTAGTACATG TGCTCGCGTT GGTGATCAGA GATTTCTATG   
  
  
+ GAGAAGAGAG AAGAAAAGAG AGAGAATTC  

- GCCGACAGGG TGTAGCTCGA GACCTCGGTG CCCCTGGTCC TGGCACATAG CACCGTTTCC ACCAAAGTGC   
  
  
- TTTTCCTACG TTATTAAGAC TATCTAGAAG TGTGTTAAAG AAAAAAAAAA AGATTAAAAA TAAAAATAAA   
  
  
- TGAGAGACTA CATTTATTTA AATAATATAT AATAATAATA ATTAAAAAAA ACGCACCCTT CCTTCAACAT   
  
  
- GGAACCCCAC TGGTATAACA TTTTTCCACA CACGTTACTA TTGTTCTTCT CTGTATGTGT GTAGTGTGCA   
  
  
- TGCTCTGAGG GAAACGAAGT AGTAAGACCC ATTGAACAAG TAGTACAGTG CTAGTAGGGT TAGAGGGCTA   
  
  
- AATAAAAAGG TACGTAATAC ATTATATATG CGTTGCAAAA CTAAAAATAT TAACATAGTA AAAAATCAAA   
  
  
- TTTTTTTAAA CATTTATTAT AAATTAGATA AAAAATATTT AATAAAACTA ATTCAGGTTT TAATAAATAT   
  
  
- TTTCATTACG TTTCAATTTA CTTAAGTCTT TAGCCTTTAA ATTTTTTCTC TCCCTTTACA CCACACTTCT   
  
  
- CCCTCCTCTC ACCTCCGAAC AGCCCGTTCC CCTCACCCAC TGCGCATTAT GCGCAGTCGG ATTCGCTCGG   
  
  
- AATACCACAT CCGACTTCGA AGAAATTGAA ATGGGGTAAA AAAATAAAAG TATTAAACAA TCAAATTAAA   
  
  
- ATATAAAATA TAAAAATTTT TCTTTTTTTT GAATTAAATT GCCCCTCTGG AAACAGACGT CCTTTCATTA   
  
  
- GAAGATCAAT GACTTAACGG GGACTTCTCC TACTATCTCA CTCCCCGTAA ATCCATTTAA GTACTAACTC   
  
  
- TCTCTCTCTC TCTCTCTCTC TCTCTCTCAC AAAACTACAT TTTGTTTTTC TATTAAAACC CCAAATTTTC   
  
  
- AATTTTTCCA AAAATTTCCG TTTTCGACGC TTTCTTTTAA TCTTTATTTT TTTAATTTTT TTTTTGTTCC   
  
  
- TCCTCTTCGT TAGTGTACGG ACTGCTGGGT TTGCAAGACG TCGGGTGCAC ATCCAAACCC GGGGTGACAC   
  
  
- CTGCGCGGTG GCCGATTTGA CCAGCTGTAC GGCGCATTAT TTCCCACCTG GGCTCCCGCC AGGACCTCGT   
  
  
- TAATTACGTG TTGCGGCAGG AGATCAGTGG GAGTATGGCA TGTGTACCAA TTGGCCGTAA ACGGTCAGTT   
  
  
- GAGAAGACAA AATAAAAGAT AAATTTTATT AAATAGTTTA CCCTTTGCAG GGTTTTGTTC GACAAAAGGT   
  
  
- TTTTGGGTTT GTGTTTTTCG GGTAAGTGTG AGCTTCGTTG GTTTGTGATA AAGAGAAAGA GAGAGACAGA   
  
  
- GAGTGAAAAA AAGAGAGAGA GAGAGGGAGA GAACATTATT TTTCACGCTC TTCTCTTCTT TACTCTCTCC   
  
  
- CAAAATACTT TACTTCCTCC TCTAGTAATA CATCATGTAC ACGAGCGCAA CCACTAGTCT CTAAAGATAC   
  
  
- CTCTTCTCTC TTCTTTTCTC TCTCTTAAG

+     GC-motif

| Site Name | Organism | Position | Strand | Matrix score. | sequence | function |
| --- | --- | --- | --- | --- | --- | --- |
| GC-motif | Zea mays | 27 | + | 8 | CCACGGGG | enhancer-like element involved in anoxic specific inducibility |

> 2018/04/13 10:10:12  
+ CGGCTGTCCC ACATCGAGCT CTGGAGCCAC GGGGACCAGG ACCGTGTATC GTGGCAAAGG TGGTTTCACG   
  
  
+ AAAAGGATGC AATAATTCTG ATAGATCTTC ACACAATTTC TTTTTTTTTT TCTAATTTTT ATTTTTATTT   
  
  
+ ACTCTCTGAT GTAAATAAAT TTATTATATA TTATTATTAT TAATTTTTTT TGCGTGGGAA GGAAGTTGTA   
  
  
+ CCTTGGGGTG ACCATATTGT AAAAAGGTGT GTGCAATGAT AACAAGAAGA GACATACACA CATCACACGT   
  
  
+ ACGAGACTCC CTTTGCTTCA TCATTCTGGG TAACTTGTTC ATCATGTCAC GATCATCCCA ATCTCCCGAT   
  
  
+ TTATTTTTCC ATGCATTATG TAATATATAC GCAACGTTTT GATTTTTATA ATTGTATCAT TTTTTAGTTT   
  
  
+ AAAAAAATTT GTAAATAATA TTTAATCTAT TTTTTATAAA TTATTTTGAT TAAGTCCAAA ATTATTTATA   
  
  
+ AAAGTAATGC AAAGTTAAAT GAATTCAGAA ATCGGAAATT TAAAAAAGAG AGGGAAATGT GGTGTGAAGA   
  
  
+ GGGAGGAGAG TGGAGGCTTG TCGGGCAAGG GGAGTGGGTG ACGCGTAATA CGCGTCAGCC TAAGCGAGCC   
  
  
+ TTATGGTGTA GGCTGAAGCT TCTTTAACTT TACCCCATTT TTTTATTTTC ATAATTTGTT AGTTTAATTT   
  
  
+ TATATTTTAT ATTTTTAAAA AGAAAAAAAA CTTAATTTAA CGGGGAGACC TTTGTCTGCA GGAAAGTAAT   
  
  
+ CTTCTAGTTA CTGAATTGCC CCTGAAGAGG ATGATAGAGT GAGGGGCATT TAGGTAAATT CATGATTGAG   
  
  
+ AGAGAGAGAG AGAGAGAGAG AGAGAGAGTG TTTTGATGTA AAACAAAAAG ATAATTTTGG GGTTTAAAAG   
  
  
+ TTAAAAAGGT TTTTAAAGGC AAAAGCTGCG AAAGAAAATT AGAAATAAAA AAATTAAAAA AAAAACAAGG   
  
  
+ AGGAGAAGCA ATCACATGCC TGACGACCCA AACGTTCTGC AGCCCACGTG TAGGTTTGGG CCCCACTGTG   
  
  
+ GACGCGCCAC CGGCTAAACT GGTCGACATG CCGCGTAATA AAGGGTGGAC CCGAGGGCGG TCCTGGAGCA   
  
  
+ ATTAATGCAC AACGCCGTCC TCTAGTCACC CTCATACCGT ACACATGGTT AACCGGCATT TGCCAGTCAA   
  
  
+ CTCTTCTGTT TTATTTTCTA TTTAAAATAA TTTATCAAAT GGGAAACGTC CCAAAACAAG CTGTTTTCCA   
  
  
+ AAAACCCAAA CACAAAAAGC CCATTCACAC TCGAAGCAAC CAAACACTAT TTCTCTTTCT CTCTCTGTCT   
  
  
+ CTCACTTTTT TTCTCTCTCT CTCTCCCTCT CTTGTAATAA AAAGTGCGAG AAGAGAAGAA ATGAGAGAGG   
  
  
+ GTTTTATGAA ATGAAGGAGG AGATCATTAT GTAGTACATG TGCTCGCGTT GGTGATCAGA GATTTCTATG   
  
  
+ GAGAAGAGAG AAGAAAAGAG AGAGAATTC  

- GCCGACAGGG TGTAGCTCGA GACCTCGGTG CCCCTGGTCC TGGCACATAG CACCGTTTCC ACCAAAGTGC   
  
  
- TTTTCCTACG TTATTAAGAC TATCTAGAAG TGTGTTAAAG AAAAAAAAAA AGATTAAAAA TAAAAATAAA   
  
  
- TGAGAGACTA CATTTATTTA AATAATATAT AATAATAATA ATTAAAAAAA ACGCACCCTT CCTTCAACAT   
  
  
- GGAACCCCAC TGGTATAACA TTTTTCCACA CACGTTACTA TTGTTCTTCT CTGTATGTGT GTAGTGTGCA   
  
  
- TGCTCTGAGG GAAACGAAGT AGTAAGACCC ATTGAACAAG TAGTACAGTG CTAGTAGGGT TAGAGGGCTA   
  
  
- AATAAAAAGG TACGTAATAC ATTATATATG CGTTGCAAAA CTAAAAATAT TAACATAGTA AAAAATCAAA   
  
  
- TTTTTTTAAA CATTTATTAT AAATTAGATA AAAAATATTT AATAAAACTA ATTCAGGTTT TAATAAATAT   
  
  
- TTTCATTACG TTTCAATTTA CTTAAGTCTT TAGCCTTTAA ATTTTTTCTC TCCCTTTACA CCACACTTCT   
  
  
- CCCTCCTCTC ACCTCCGAAC AGCCCGTTCC CCTCACCCAC TGCGCATTAT GCGCAGTCGG ATTCGCTCGG   
  
  
- AATACCACAT CCGACTTCGA AGAAATTGAA ATGGGGTAAA AAAATAAAAG TATTAAACAA TCAAATTAAA   
  
  
- ATATAAAATA TAAAAATTTT TCTTTTTTTT GAATTAAATT GCCCCTCTGG AAACAGACGT CCTTTCATTA   
  
  
- GAAGATCAAT GACTTAACGG GGACTTCTCC TACTATCTCA CTCCCCGTAA ATCCATTTAA GTACTAACTC   
  
  
- TCTCTCTCTC TCTCTCTCTC TCTCTCTCAC AAAACTACAT TTTGTTTTTC TATTAAAACC CCAAATTTTC   
  
  
- AATTTTTCCA AAAATTTCCG TTTTCGACGC TTTCTTTTAA TCTTTATTTT TTTAATTTTT TTTTTGTTCC   
  
  
- TCCTCTTCGT TAGTGTACGG ACTGCTGGGT TTGCAAGACG TCGGGTGCAC ATCCAAACCC GGGGTGACAC   
  
  
- CTGCGCGGTG GCCGATTTGA CCAGCTGTAC GGCGCATTAT TTCCCACCTG GGCTCCCGCC AGGACCTCGT   
  
  
- TAATTACGTG TTGCGGCAGG AGATCAGTGG GAGTATGGCA TGTGTACCAA TTGGCCGTAA ACGGTCAGTT   
  
  
- GAGAAGACAA AATAAAAGAT AAATTTTATT AAATAGTTTA CCCTTTGCAG GGTTTTGTTC GACAAAAGGT   
  
  
- TTTTGGGTTT GTGTTTTTCG GGTAAGTGTG AGCTTCGTTG GTTTGTGATA AAGAGAAAGA GAGAGACAGA   
  
  
- GAGTGAAAAA AAGAGAGAGA GAGAGGGAGA GAACATTATT TTTCACGCTC TTCTCTTCTT TACTCTCTCC   
  
  
- CAAAATACTT TACTTCCTCC TCTAGTAATA CATCATGTAC ACGAGCGCAA CCACTAGTCT CTAAAGATAC   
  
  
- CTCTTCTCTC TTCTTTTCTC TCTCTTAAG

+     GT1-motif

| Site Name | Organism | Position | Strand | Matrix score. | sequence | function |
| --- | --- | --- | --- | --- | --- | --- |
| GT1-motif | Arabidopsis thaliana | 1169 | - | 6 | GGTTAA | light responsive element |
| GT1-motif | Arabidopsis thaliana | 1167 | + | 6 | GGTTAA | light responsive element |

> 2018/04/13 10:10:12  
+ CGGCTGTCCC ACATCGAGCT CTGGAGCCAC GGGGACCAGG ACCGTGTATC GTGGCAAAGG TGGTTTCACG   
  
  
+ AAAAGGATGC AATAATTCTG ATAGATCTTC ACACAATTTC TTTTTTTTTT TCTAATTTTT ATTTTTATTT   
  
  
+ ACTCTCTGAT GTAAATAAAT TTATTATATA TTATTATTAT TAATTTTTTT TGCGTGGGAA GGAAGTTGTA   
  
  
+ CCTTGGGGTG ACCATATTGT AAAAAGGTGT GTGCAATGAT AACAAGAAGA GACATACACA CATCACACGT   
  
  
+ ACGAGACTCC CTTTGCTTCA TCATTCTGGG TAACTTGTTC ATCATGTCAC GATCATCCCA ATCTCCCGAT   
  
  
+ TTATTTTTCC ATGCATTATG TAATATATAC GCAACGTTTT GATTTTTATA ATTGTATCAT TTTTTAGTTT   
  
  
+ AAAAAAATTT GTAAATAATA TTTAATCTAT TTTTTATAAA TTATTTTGAT TAAGTCCAAA ATTATTTATA   
  
  
+ AAAGTAATGC AAAGTTAAAT GAATTCAGAA ATCGGAAATT TAAAAAAGAG AGGGAAATGT GGTGTGAAGA   
  
  
+ GGGAGGAGAG TGGAGGCTTG TCGGGCAAGG GGAGTGGGTG ACGCGTAATA CGCGTCAGCC TAAGCGAGCC   
  
  
+ TTATGGTGTA GGCTGAAGCT TCTTTAACTT TACCCCATTT TTTTATTTTC ATAATTTGTT AGTTTAATTT   
  
  
+ TATATTTTAT ATTTTTAAAA AGAAAAAAAA CTTAATTTAA CGGGGAGACC TTTGTCTGCA GGAAAGTAAT   
  
  
+ CTTCTAGTTA CTGAATTGCC CCTGAAGAGG ATGATAGAGT GAGGGGCATT TAGGTAAATT CATGATTGAG   
  
  
+ AGAGAGAGAG AGAGAGAGAG AGAGAGAGTG TTTTGATGTA AAACAAAAAG ATAATTTTGG GGTTTAAAAG   
  
  
+ TTAAAAAGGT TTTTAAAGGC AAAAGCTGCG AAAGAAAATT AGAAATAAAA AAATTAAAAA AAAAACAAGG   
  
  
+ AGGAGAAGCA ATCACATGCC TGACGACCCA AACGTTCTGC AGCCCACGTG TAGGTTTGGG CCCCACTGTG   
  
  
+ GACGCGCCAC CGGCTAAACT GGTCGACATG CCGCGTAATA AAGGGTGGAC CCGAGGGCGG TCCTGGAGCA   
  
  
+ ATTAATGCAC AACGCCGTCC TCTAGTCACC CTCATACCGT ACACATGGTT AACCGGCATT TGCCAGTCAA   
  
  
+ CTCTTCTGTT TTATTTTCTA TTTAAAATAA TTTATCAAAT GGGAAACGTC CCAAAACAAG CTGTTTTCCA   
  
  
+ AAAACCCAAA CACAAAAAGC CCATTCACAC TCGAAGCAAC CAAACACTAT TTCTCTTTCT CTCTCTGTCT   
  
  
+ CTCACTTTTT TTCTCTCTCT CTCTCCCTCT CTTGTAATAA AAAGTGCGAG AAGAGAAGAA ATGAGAGAGG   
  
  
+ GTTTTATGAA ATGAAGGAGG AGATCATTAT GTAGTACATG TGCTCGCGTT GGTGATCAGA GATTTCTATG   
  
  
+ GAGAAGAGAG AAGAAAAGAG AGAGAATTC  

- GCCGACAGGG TGTAGCTCGA GACCTCGGTG CCCCTGGTCC TGGCACATAG CACCGTTTCC ACCAAAGTGC   
  
  
- TTTTCCTACG TTATTAAGAC TATCTAGAAG TGTGTTAAAG AAAAAAAAAA AGATTAAAAA TAAAAATAAA   
  
  
- TGAGAGACTA CATTTATTTA AATAATATAT AATAATAATA ATTAAAAAAA ACGCACCCTT CCTTCAACAT   
  
  
- GGAACCCCAC TGGTATAACA TTTTTCCACA CACGTTACTA TTGTTCTTCT CTGTATGTGT GTAGTGTGCA   
  
  
- TGCTCTGAGG GAAACGAAGT AGTAAGACCC ATTGAACAAG TAGTACAGTG CTAGTAGGGT TAGAGGGCTA   
  
  
- AATAAAAAGG TACGTAATAC ATTATATATG CGTTGCAAAA CTAAAAATAT TAACATAGTA AAAAATCAAA   
  
  
- TTTTTTTAAA CATTTATTAT AAATTAGATA AAAAATATTT AATAAAACTA ATTCAGGTTT TAATAAATAT   
  
  
- TTTCATTACG TTTCAATTTA CTTAAGTCTT TAGCCTTTAA ATTTTTTCTC TCCCTTTACA CCACACTTCT   
  
  
- CCCTCCTCTC ACCTCCGAAC AGCCCGTTCC CCTCACCCAC TGCGCATTAT GCGCAGTCGG ATTCGCTCGG   
  
  
- AATACCACAT CCGACTTCGA AGAAATTGAA ATGGGGTAAA AAAATAAAAG TATTAAACAA TCAAATTAAA   
  
  
- ATATAAAATA TAAAAATTTT TCTTTTTTTT GAATTAAATT GCCCCTCTGG AAACAGACGT CCTTTCATTA   
  
  
- GAAGATCAAT GACTTAACGG GGACTTCTCC TACTATCTCA CTCCCCGTAA ATCCATTTAA GTACTAACTC   
  
  
- TCTCTCTCTC TCTCTCTCTC TCTCTCTCAC AAAACTACAT TTTGTTTTTC TATTAAAACC CCAAATTTTC   
  
  
- AATTTTTCCA AAAATTTCCG TTTTCGACGC TTTCTTTTAA TCTTTATTTT TTTAATTTTT TTTTTGTTCC   
  
  
- TCCTCTTCGT TAGTGTACGG ACTGCTGGGT TTGCAAGACG TCGGGTGCAC ATCCAAACCC GGGGTGACAC   
  
  
- CTGCGCGGTG GCCGATTTGA CCAGCTGTAC GGCGCATTAT TTCCCACCTG GGCTCCCGCC AGGACCTCGT   
  
  
- TAATTACGTG TTGCGGCAGG AGATCAGTGG GAGTATGGCA TGTGTACCAA TTGGCCGTAA ACGGTCAGTT   
  
  
- GAGAAGACAA AATAAAAGAT AAATTTTATT AAATAGTTTA CCCTTTGCAG GGTTTTGTTC GACAAAAGGT   
  
  
- TTTTGGGTTT GTGTTTTTCG GGTAAGTGTG AGCTTCGTTG GTTTGTGATA AAGAGAAAGA GAGAGACAGA   
  
  
- GAGTGAAAAA AAGAGAGAGA GAGAGGGAGA GAACATTATT TTTCACGCTC TTCTCTTCTT TACTCTCTCC   
  
  
- CAAAATACTT TACTTCCTCC TCTAGTAATA CATCATGTAC ACGAGCGCAA CCACTAGTCT CTAAAGATAC   
  
  
- CTCTTCTCTC TTCTTTTCTC TCTCTTAAG

+     HSE

| Site Name | Organism | Position | Strand | Matrix score. | sequence | function |
| --- | --- | --- | --- | --- | --- | --- |
| HSE | Brassica oleracea | 422 | + | 9 | AAAAAATTTC | cis-acting element involved in heat stress responsiveness |
| HSE | Brassica oleracea | 1491 | + | 9 | AGAAAATTCG | cis-acting element involved in heat stress responsiveness |
| HSE | Brassica oleracea | 943 | + | 9 | AGAAAATTCG | cis-acting element involved in heat stress responsiveness |

> 2018/04/13 10:10:12  
+ CGGCTGTCCC ACATCGAGCT CTGGAGCCAC GGGGACCAGG ACCGTGTATC GTGGCAAAGG TGGTTTCACG   
  
  
+ AAAAGGATGC AATAATTCTG ATAGATCTTC ACACAATTTC TTTTTTTTTT TCTAATTTTT ATTTTTATTT   
  
  
+ ACTCTCTGAT GTAAATAAAT TTATTATATA TTATTATTAT TAATTTTTTT TGCGTGGGAA GGAAGTTGTA   
  
  
+ CCTTGGGGTG ACCATATTGT AAAAAGGTGT GTGCAATGAT AACAAGAAGA GACATACACA CATCACACGT   
  
  
+ ACGAGACTCC CTTTGCTTCA TCATTCTGGG TAACTTGTTC ATCATGTCAC GATCATCCCA ATCTCCCGAT   
  
  
+ TTATTTTTCC ATGCATTATG TAATATATAC GCAACGTTTT GATTTTTATA ATTGTATCAT TTTTTAGTTT   
  
  
+ AAAAAAATTT GTAAATAATA TTTAATCTAT TTTTTATAAA TTATTTTGAT TAAGTCCAAA ATTATTTATA   
  
  
+ AAAGTAATGC AAAGTTAAAT GAATTCAGAA ATCGGAAATT TAAAAAAGAG AGGGAAATGT GGTGTGAAGA   
  
  
+ GGGAGGAGAG TGGAGGCTTG TCGGGCAAGG GGAGTGGGTG ACGCGTAATA CGCGTCAGCC TAAGCGAGCC   
  
  
+ TTATGGTGTA GGCTGAAGCT TCTTTAACTT TACCCCATTT TTTTATTTTC ATAATTTGTT AGTTTAATTT   
  
  
+ TATATTTTAT ATTTTTAAAA AGAAAAAAAA CTTAATTTAA CGGGGAGACC TTTGTCTGCA GGAAAGTAAT   
  
  
+ CTTCTAGTTA CTGAATTGCC CCTGAAGAGG ATGATAGAGT GAGGGGCATT TAGGTAAATT CATGATTGAG   
  
  
+ AGAGAGAGAG AGAGAGAGAG AGAGAGAGTG TTTTGATGTA AAACAAAAAG ATAATTTTGG GGTTTAAAAG   
  
  
+ TTAAAAAGGT TTTTAAAGGC AAAAGCTGCG AAAGAAAATT AGAAATAAAA AAATTAAAAA AAAAACAAGG   
  
  
+ AGGAGAAGCA ATCACATGCC TGACGACCCA AACGTTCTGC AGCCCACGTG TAGGTTTGGG CCCCACTGTG   
  
  
+ GACGCGCCAC CGGCTAAACT GGTCGACATG CCGCGTAATA AAGGGTGGAC CCGAGGGCGG TCCTGGAGCA   
  
  
+ ATTAATGCAC AACGCCGTCC TCTAGTCACC CTCATACCGT ACACATGGTT AACCGGCATT TGCCAGTCAA   
  
  
+ CTCTTCTGTT TTATTTTCTA TTTAAAATAA TTTATCAAAT GGGAAACGTC CCAAAACAAG CTGTTTTCCA   
  
  
+ AAAACCCAAA CACAAAAAGC CCATTCACAC TCGAAGCAAC CAAACACTAT TTCTCTTTCT CTCTCTGTCT   
  
  
+ CTCACTTTTT TTCTCTCTCT CTCTCCCTCT CTTGTAATAA AAAGTGCGAG AAGAGAAGAA ATGAGAGAGG   
  
  
+ GTTTTATGAA ATGAAGGAGG AGATCATTAT GTAGTACATG TGCTCGCGTT GGTGATCAGA GATTTCTATG   
  
  
+ GAGAAGAGAG AAGAAAAGAG AGAGAATTCG   

- GCCGACAGGG TGTAGCTCGA GACCTCGGTG CCCCTGGTCC TGGCACATAG CACCGTTTCC ACCAAAGTGC   
  
  
- TTTTCCTACG TTATTAAGAC TATCTAGAAG TGTGTTAAAG AAAAAAAAAA AGATTAAAAA TAAAAATAAA   
  
  
- TGAGAGACTA CATTTATTTA AATAATATAT AATAATAATA ATTAAAAAAA ACGCACCCTT CCTTCAACAT   
  
  
- GGAACCCCAC TGGTATAACA TTTTTCCACA CACGTTACTA TTGTTCTTCT CTGTATGTGT GTAGTGTGCA   
  
  
- TGCTCTGAGG GAAACGAAGT AGTAAGACCC ATTGAACAAG TAGTACAGTG CTAGTAGGGT TAGAGGGCTA   
  
  
- AATAAAAAGG TACGTAATAC ATTATATATG CGTTGCAAAA CTAAAAATAT TAACATAGTA AAAAATCAAA   
  
  
- TTTTTTTAAA CATTTATTAT AAATTAGATA AAAAATATTT AATAAAACTA ATTCAGGTTT TAATAAATAT   
  
  
- TTTCATTACG TTTCAATTTA CTTAAGTCTT TAGCCTTTAA ATTTTTTCTC TCCCTTTACA CCACACTTCT   
  
  
- CCCTCCTCTC ACCTCCGAAC AGCCCGTTCC CCTCACCCAC TGCGCATTAT GCGCAGTCGG ATTCGCTCGG   
  
  
- AATACCACAT CCGACTTCGA AGAAATTGAA ATGGGGTAAA AAAATAAAAG TATTAAACAA TCAAATTAAA   
  
  
- ATATAAAATA TAAAAATTTT TCTTTTTTTT GAATTAAATT GCCCCTCTGG AAACAGACGT CCTTTCATTA   
  
  
- GAAGATCAAT GACTTAACGG GGACTTCTCC TACTATCTCA CTCCCCGTAA ATCCATTTAA GTACTAACTC   
  
  
- TCTCTCTCTC TCTCTCTCTC TCTCTCTCAC AAAACTACAT TTTGTTTTTC TATTAAAACC CCAAATTTTC   
  
  
- AATTTTTCCA AAAATTTCCG TTTTCGACGC TTTCTTTTAA TCTTTATTTT TTTAATTTTT TTTTTGTTCC   
  
  
- TCCTCTTCGT TAGTGTACGG ACTGCTGGGT TTGCAAGACG TCGGGTGCAC ATCCAAACCC GGGGTGACAC   
  
  
- CTGCGCGGTG GCCGATTTGA CCAGCTGTAC GGCGCATTAT TTCCCACCTG GGCTCCCGCC AGGACCTCGT   
  
  
- TAATTACGTG TTGCGGCAGG AGATCAGTGG GAGTATGGCA TGTGTACCAA TTGGCCGTAA ACGGTCAGTT   
  
  
- GAGAAGACAA AATAAAAGAT AAATTTTATT AAATAGTTTA CCCTTTGCAG GGTTTTGTTC GACAAAAGGT   
  
  
- TTTTGGGTTT GTGTTTTTCG GGTAAGTGTG AGCTTCGTTG GTTTGTGATA AAGAGAAAGA GAGAGACAGA   
  
  
- GAGTGAAAAA AAGAGAGAGA GAGAGGGAGA GAACATTATT TTTCACGCTC TTCTCTTCTT TACTCTCTCC   
  
  
- CAAAATACTT TACTTCCTCC TCTAGTAATA CATCATGTAC ACGAGCGCAA CCACTAGTCT CTAAAGATAC   
  
  
- CTCTTCTCTC TTCTTTTCTC TCTCTTAAG

+     Sp1

| Site Name | Organism | Position | Strand | Matrix score. | sequence | function |
| --- | --- | --- | --- | --- | --- | --- |
| Sp1 | Oryza sativa | 1105 | + | 6 | GGGCGG | light responsive element |
| Sp1 | Zea mays | 561 | - | 5 | CC(G/A)CCC | light responsive element |
| Sp1 | Zea mays | 1093 | - | 5.5 | CC(G/A)CCC | light responsive element |

> 2018/04/13 10:10:12  
+ CGGCTGTCCC ACATCGAGCT CTGGAGCCAC GGGGACCAGG ACCGTGTATC GTGGCAAAGG TGGTTTCACG   
  
  
+ AAAAGGATGC AATAATTCTG ATAGATCTTC ACACAATTTC TTTTTTTTTT TCTAATTTTT ATTTTTATTT   
  
  
+ ACTCTCTGAT GTAAATAAAT TTATTATATA TTATTATTAT TAATTTTTTT TGCGTGGGAA GGAAGTTGTA   
  
  
+ CCTTGGGGTG ACCATATTGT AAAAAGGTGT GTGCAATGAT AACAAGAAGA GACATACACA CATCACACGT   
  
  
+ ACGAGACTCC CTTTGCTTCA TCATTCTGGG TAACTTGTTC ATCATGTCAC GATCATCCCA ATCTCCCGAT   
  
  
+ TTATTTTTCC ATGCATTATG TAATATATAC GCAACGTTTT GATTTTTATA ATTGTATCAT TTTTTAGTTT   
  
  
+ AAAAAAATTT GTAAATAATA TTTAATCTAT TTTTTATAAA TTATTTTGAT TAAGTCCAAA ATTATTTATA   
  
  
+ AAAGTAATGC AAAGTTAAAT GAATTCAGAA ATCGGAAATT TAAAAAAGAG AGGGAAATGT GGTGTGAAGA   
  
  
+ GGGAGGAGAG TGGAGGCTTG TCGGGCAAGG GGAGTGGGTG ACGCGTAATA CGCGTCAGCC TAAGCGAGCC   
  
  
+ TTATGGTGTA GGCTGAAGCT TCTTTAACTT TACCCCATTT TTTTATTTTC ATAATTTGTT AGTTTAATTT   
  
  
+ TATATTTTAT ATTTTTAAAA AGAAAAAAAA CTTAATTTAA CGGGGAGACC TTTGTCTGCA GGAAAGTAAT   
  
  
+ CTTCTAGTTA CTGAATTGCC CCTGAAGAGG ATGATAGAGT GAGGGGCATT TAGGTAAATT CATGATTGAG   
  
  
+ AGAGAGAGAG AGAGAGAGAG AGAGAGAGTG TTTTGATGTA AAACAAAAAG ATAATTTTGG GGTTTAAAAG   
  
  
+ TTAAAAAGGT TTTTAAAGGC AAAAGCTGCG AAAGAAAATT AGAAATAAAA AAATTAAAAA AAAAACAAGG   
  
  
+ AGGAGAAGCA ATCACATGCC TGACGACCCA AACGTTCTGC AGCCCACGTG TAGGTTTGGG CCCCACTGTG   
  
  
+ GACGCGCCAC CGGCTAAACT GGTCGACATG CCGCGTAATA AAGGGTGGAC CCGAGGGCGG TCCTGGAGCA   
  
  
+ ATTAATGCAC AACGCCGTCC TCTAGTCACC CTCATACCGT ACACATGGTT AACCGGCATT TGCCAGTCAA   
  
  
+ CTCTTCTGTT TTATTTTCTA TTTAAAATAA TTTATCAAAT GGGAAACGTC CCAAAACAAG CTGTTTTCCA   
  
  
+ AAAACCCAAA CACAAAAAGC CCATTCACAC TCGAAGCAAC CAAACACTAT TTCTCTTTCT CTCTCTGTCT   
  
  
+ CTCACTTTTT TTCTCTCTCT CTCTCCCTCT CTTGTAATAA AAAGTGCGAG AAGAGAAGAA ATGAGAGAGG   
  
  
+ GTTTTATGAA ATGAAGGAGG AGATCATTAT GTAGTACATG TGCTCGCGTT GGTGATCAGA GATTTCTATG   
  
  
+ GAGAAGAGAG AAGAAAAGAG AGAGAATTC  

- GCCGACAGGG TGTAGCTCGA GACCTCGGTG CCCCTGGTCC TGGCACATAG CACCGTTTCC ACCAAAGTGC   
  
  
- TTTTCCTACG TTATTAAGAC TATCTAGAAG TGTGTTAAAG AAAAAAAAAA AGATTAAAAA TAAAAATAAA   
  
  
- TGAGAGACTA CATTTATTTA AATAATATAT AATAATAATA ATTAAAAAAA ACGCACCCTT CCTTCAACAT   
  
  
- GGAACCCCAC TGGTATAACA TTTTTCCACA CACGTTACTA TTGTTCTTCT CTGTATGTGT GTAGTGTGCA   
  
  
- TGCTCTGAGG GAAACGAAGT AGTAAGACCC ATTGAACAAG TAGTACAGTG CTAGTAGGGT TAGAGGGCTA   
  
  
- AATAAAAAGG TACGTAATAC ATTATATATG CGTTGCAAAA CTAAAAATAT TAACATAGTA AAAAATCAAA   
  
  
- TTTTTTTAAA CATTTATTAT AAATTAGATA AAAAATATTT AATAAAACTA ATTCAGGTTT TAATAAATAT   
  
  
- TTTCATTACG TTTCAATTTA CTTAAGTCTT TAGCCTTTAA ATTTTTTCTC TCCCTTTACA CCACACTTCT   
  
  
- CCCTCCTCTC ACCTCCGAAC AGCCCGTTCC CCTCACCCAC TGCGCATTAT GCGCAGTCGG ATTCGCTCGG   
  
  
- AATACCACAT CCGACTTCGA AGAAATTGAA ATGGGGTAAA AAAATAAAAG TATTAAACAA TCAAATTAAA   
  
  
- ATATAAAATA TAAAAATTTT TCTTTTTTTT GAATTAAATT GCCCCTCTGG AAACAGACGT CCTTTCATTA   
  
  
- GAAGATCAAT GACTTAACGG GGACTTCTCC TACTATCTCA CTCCCCGTAA ATCCATTTAA GTACTAACTC   
  
  
- TCTCTCTCTC TCTCTCTCTC TCTCTCTCAC AAAACTACAT TTTGTTTTTC TATTAAAACC CCAAATTTTC   
  
  
- AATTTTTCCA AAAATTTCCG TTTTCGACGC TTTCTTTTAA TCTTTATTTT TTTAATTTTT TTTTTGTTCC   
  
  
- TCCTCTTCGT TAGTGTACGG ACTGCTGGGT TTGCAAGACG TCGGGTGCAC ATCCAAACCC GGGGTGACAC   
  
  
- CTGCGCGGTG GCCGATTTGA CCAGCTGTAC GGCGCATTAT TTCCCACCTG GGCTCCCGCC AGGACCTCGT   
  
  
- TAATTACGTG TTGCGGCAGG AGATCAGTGG GAGTATGGCA TGTGTACCAA TTGGCCGTAA ACGGTCAGTT   
  
  
- GAGAAGACAA AATAAAAGAT AAATTTTATT AAATAGTTTA CCCTTTGCAG GGTTTTGTTC GACAAAAGGT   
  
  
- TTTTGGGTTT GTGTTTTTCG GGTAAGTGTG AGCTTCGTTG GTTTGTGATA AAGAGAAAGA GAGAGACAGA   
  
  
- GAGTGAAAAA AAGAGAGAGA GAGAGGGAGA GAACATTATT TTTCACGCTC TTCTCTTCTT TACTCTCTCC   
  
  
- CAAAATACTT TACTTCCTCC TCTAGTAATA CATCATGTAC ACGAGCGCAA CCACTAGTCT CTAAAGATAC   
  
  
- CTCTTCTCTC TTCTTTTCTC TCTCTTAAG

+     TATA-box

| Site Name | Organism | Position | Strand | Matrix score. | sequence | function |
| --- | --- | --- | --- | --- | --- | --- |
| TATA-box | Lycopersicon esculentum | 1402 | + | 5 | TTTTA | core promoter element around -30 of transcription start |
| TATA-box | Lycopersicon esculentum | 956 | - | 5 | TTTTA | core promoter element around -30 of transcription start |
| TATA-box | Lycopersicon esculentum | 879 | - | 5 | TTTTA | core promoter element around -30 of transcription start |
| TATA-box | Arabidopsis thaliana | 698 | - | 7 | TATAAAA | core promoter element around -30 of transcription start |
| TATA-box | Lycopersicon esculentum | 671 | + | 5 | TTTTA | core promoter element around -30 of transcription start |
| TATA-box | Arabidopsis thaliana | 487 | + | 6 | TATAAA | core promoter element around -30 of transcription start |
| TATA-box | Arabidopsis thaliana | 376 | + | 4 | TATA | core promoter element around -30 of transcription start |
| TATA-box | Arabidopsis thaliana | 485 | - | 6 | TATAAA | core promoter element around -30 of transcription start |
| TATA-box | Lycopersicon esculentum | 230 | - | 5 | TTTTA | core promoter element around -30 of transcription start |
| TATA-box | Daucus carota | 483 | - | 8 | TATAAATA | core promoter element around -30 of transcription start |
| TATA-box | Arabidopsis thaliana | 452 | - | 7 | TATAAAA | core promoter element around -30 of transcription start |
| TATA-box | Zea mays | 714 | + | 8 | TTTAAAAA | core promoter element around -30 of transcription start |
| TATA-box | Lycopersicon esculentum | 713 | + | 5 | TTTTA | core promoter element around -30 of transcription start |
| TATA-box | Arabidopsis thaliana | 374 | + | 4 | TATA | core promoter element around -30 of transcription start |
| TATA-box | Brassica napus | 373 | + | 6 | ATATAT | core promoter element around -30 of transcription start |
| TATA-box | Glycine max | 371 | + | 5 | TAATA | core promoter element around -30 of transcription start |
| TATA-box | Glycine max | 178 | - | 5 | TAATA | core promoter element around -30 of transcription start |
| TATA-box | Glycine max | 172 | - | 5 | TAATA | core promoter element around -30 of transcription start |
| TATA-box | Lycopersicon esculentum | 716 | - | 5 | TTTTA | core promoter element around -30 of transcription start |
| TATA-box | Arabidopsis thaliana | 486 | - | 5 | TATAA | core promoter element around -30 of transcription start |
| TATA-box | Lycopersicon esculentum | 420 | - | 5 | TTTTA | core promoter element around -30 of transcription start |
| TATA-box | Lycopersicon esculentum | 412 | + | 5 | TTTTA | core promoter element around -30 of transcription start |
| TATA-box | Pisum sativum | 697 | - | 8 | TATAAAAT | core promoter element around -30 of transcription start |
| TATA-box | Arabidopsis thaliana | 706 | - | 11 | TATAAATATAAA | core promoter element around -30 of transcription start |
| TATA-box | Glycine max | 175 | - | 5 | TAATA | core promoter element around -30 of transcription start |
| TATA-box | Arabidopsis thaliana | 705 | - | 7 | TATAAAA | core promoter element around -30 of transcription start |
| TATA-box | Brassica napus | 166 | + | 6 | ATATAT | core promoter element around -30 of transcription start |
| TATA-box | Brassica napus | 163 | + | 6 | ATTATA | core promoter element around -30 of transcription start |
| TATA-box | Arabidopsis thaliana | 700 | - | 5 | TATAA | core promoter element around -30 of transcription start |
| TATA-box | Arabidopsis thaliana | 699 | - | 6 | TATAAA | core promoter element around -30 of transcription start |
| TATA-box | Lycopersicon esculentum | 1213 | - | 5 | TTTTA | core promoter element around -30 of transcription start |
| TATA-box | Arabidopsis thaliana | 1209 | + | 8 | TATTTAAA | core promoter element around -30 of transcription start |
| TATA-box | Lycopersicon esculentum | 1199 | + | 5 | TTTTA | core promoter element around -30 of transcription start |
| TATA-box | Arabidopsis thaliana | 453 | - | 6 | TATAAA | core promoter element around -30 of transcription start |
| TATA-box | Lycopersicon esculentum | 912 | - | 5 | TTTTA | core promoter element around -30 of transcription start |
| TATA-box | Lycopersicon esculentum | 905 | - | 5 | TTTTA | core promoter element around -30 of transcription start |
| TATA-box | Glycine max | 606 | + | 5 | TAATA | core promoter element around -30 of transcription start |
| TATA-box | Lycopersicon esculentum | 531 | - | 5 | TTTTA | core promoter element around -30 of transcription start |
| TATA-box | Arabidopsis thaliana | 454 | - | 5 | TATAA | core promoter element around -30 of transcription start |
| TATA-box | Arabidopsis thaliana | 164 | - | 7 | TATATAA | core promoter element around -30 of transcription start |
| TATA-box | Glycine max | 162 | - | 5 | TAATA | core promoter element around -30 of transcription start |
| TATA-box | Lycopersicon esculentum | 133 | + | 5 | TTTTA | core promoter element around -30 of transcription start |
| TATA-box | Lycopersicon esculentum | 127 | + | 5 | TTTTA | core promoter element around -30 of transcription start |
| TATA-box | Lycopersicon esculentum | 1368 | - | 5 | TTTTA | core promoter element around -30 of transcription start |
| TATA-box | Zea mays | 418 | + | 8 | TTTAAAAA | core promoter element around -30 of transcription start |
| TATA-box | Arabidopsis thaliana | 397 | + | 4 | TATA | core promoter element around -30 of transcription start |
| TATA-box | Arabidopsis thaliana | 396 | - | 5 | TATAA | core promoter element around -30 of transcription start |
| TATA-box | Arabidopsis thaliana | 708 | + | 4 | TATA | core promoter element around -30 of transcription start |
| TATA-box | Arabidopsis thaliana | 167 | + | 4 | TATA | core promoter element around -30 of transcription start |
| TATA-box | Lycopersicon esculentum | 965 | - | 5 | TTTTA | core promoter element around -30 of transcription start |
| TATA-box | Lycopersicon esculentum | 921 | + | 5 | TTTTA | core promoter element around -30 of transcription start |
| TATA-box | Glycine max | 1086 | + | 5 | TAATA | core promoter element around -30 of transcription start |
| TATA-box | Arabidopsis thaliana | 129 | - | 9 | TAAAAATAA | core promoter element around -30 of transcription start |
| TATA-box | Glycine max | 169 | - | 5 | TAATA | core promoter element around -30 of transcription start |
| TATA-box | Arabidopsis thaliana | 920 | - | 9 | ccTATAAAaa | core promoter element around -30 of transcription start |
| TATA-box | Ac | 484 | - | 7 | TATAAAT | core promoter element around -30 of transcription start |
| TATA-box | Arabidopsis thaliana | 701 | + | 4 | TATA | core promoter element around -30 of transcription start |
| TATA-box | Pisum sativum | 704 | - | 8 | TATAAAAT | core promoter element around -30 of transcription start |
| TATA-box | Glycine max | 1365 | + | 5 | TAATA | core promoter element around -30 of transcription start |
| TATA-box | Glycine max | 436 | + | 5 | TAATA | core promoter element around -30 of transcription start |
| TATA-box | Arabidopsis thaliana | 165 | + | 4 | TATA | core promoter element around -30 of transcription start |
| TATA-box | Lycopersicon esculentum | 489 | - | 5 | TTTTA | core promoter element around -30 of transcription start |
| TATA-box | Arabidopsis thaliana | 395 | - | 6 | TATAAA | core promoter element around -30 of transcription start |
| TATA-box | Arabidopsis thaliana | 394 | - | 7 | TATAAAA | core promoter element around -30 of transcription start |
| TATA-box | Zea mays | 712 | - | 8 | TTTAAAAA | core promoter element around -30 of transcription start |
| TATA-box | Arabidopsis thaliana | 391 | - | 9 | taTATAAAtc | core promoter element around -30 of transcription start |
| TATA-box | Zea mays | 529 | + | 8 | TTTAAAAA | core promoter element around -30 of transcription start |
| TATA-box | Ac | 455 | + | 7 | TATAAAT | core promoter element around -30 of transcription start |
| TATA-box | Arabidopsis thaliana | 707 | - | 5 | TATAA | core promoter element around -30 of transcription start |

> 2018/04/13 10:10:12  
+ CGGCTGTCCC ACATCGAGCT CTGGAGCCAC GGGGACCAGG ACCGTGTATC GTGGCAAAGG TGGTTTCACG   
  
  
+ AAAAGGATGC AATAATTCTG ATAGATCTTC ACACAATTTC TTTTTTTTTT TCTAATTTTT ATTTTTATTT   
  
  
+ ACTCTCTGAT GTAAATAAAT TTATTATATA TTATTATTAT TAATTTTTTT TGCGTGGGAA GGAAGTTGTA   
  
  
+ CCTTGGGGTG ACCATATTGT AAAAAGGTGT GTGCAATGAT AACAAGAAGA GACATACACA CATCACACGT   
  
  
+ ACGAGACTCC CTTTGCTTCA TCATTCTGGG TAACTTGTTC ATCATGTCAC GATCATCCCA ATCTCCCGAT   
  
  
+ TTATTTTTCC ATGCATTATG TAATATATAC GCAACGTTTT GATTTTTATA ATTGTATCAT TTTTTAGTTT   
  
  
+ AAAAAAATTT GTAAATAATA TTTAATCTAT TTTTTATAAA TTATTTTGAT TAAGTCCAAA ATTATTTATA   
  
  
+ AAAGTAATGC AAAGTTAAAT GAATTCAGAA ATCGGAAATT TAAAAAAGAG AGGGAAATGT GGTGTGAAGA   
  
  
+ GGGAGGAGAG TGGAGGCTTG TCGGGCAAGG GGAGTGGGTG ACGCGTAATA CGCGTCAGCC TAAGCGAGCC   
  
  
+ TTATGGTGTA GGCTGAAGCT TCTTTAACTT TACCCCATTT TTTTATTTTC ATAATTTGTT AGTTTAATTT   
  
  
+ TATATTTTAT ATTTTTAAAA AGAAAAAAAA CTTAATTTAA CGGGGAGACC TTTGTCTGCA GGAAAGTAAT   
  
  
+ CTTCTAGTTA CTGAATTGCC CCTGAAGAGG ATGATAGAGT GAGGGGCATT TAGGTAAATT CATGATTGAG   
  
  
+ AGAGAGAGAG AGAGAGAGAG AGAGAGAGTG TTTTGATGTA AAACAAAAAG ATAATTTTGG GGTTTAAAAG   
  
  
+ TTAAAAAGGT TTTTAAAGGC AAAAGCTGCG AAAGAAAATT AGAAATAAAA AAATTAAAAA AAAAACAAGG   
  
  
+ AGGAGAAGCA ATCACATGCC TGACGACCCA AACGTTCTGC AGCCCACGTG TAGGTTTGGG CCCCACTGTG   
  
  
+ GACGCGCCAC CGGCTAAACT GGTCGACATG CCGCGTAATA AAGGGTGGAC CCGAGGGCGG TCCTGGAGCA   
  
  
+ ATTAATGCAC AACGCCGTCC TCTAGTCACC CTCATACCGT ACACATGGTT AACCGGCATT TGCCAGTCAA   
  
  
+ CTCTTCTGTT TTATTTTCTA TTTAAAATAA TTTATCAAAT GGGAAACGTC CCAAAACAAG CTGTTTTCCA   
  
  
+ AAAACCCAAA CACAAAAAGC CCATTCACAC TCGAAGCAAC CAAACACTAT TTCTCTTTCT CTCTCTGTCT   
  
  
+ CTCACTTTTT TTCTCTCTCT CTCTCCCTCT CTTGTAATAA AAAGTGCGAG AAGAGAAGAA ATGAGAGAGG   
  
  
+ GTTTTATGAA ATGAAGGAGG AGATCATTAT GTAGTACATG TGCTCGCGTT GGTGATCAGA GATTTCTATG   
  
  
+ GAGAAGAGAG AAGAAAAGAG AGAGAATTC  

- GCCGACAGGG TGTAGCTCGA GACCTCGGTG CCCCTGGTCC TGGCACATAG CACCGTTTCC ACCAAAGTGC   
  
  
- TTTTCCTACG TTATTAAGAC TATCTAGAAG TGTGTTAAAG AAAAAAAAAA AGATTAAAAA TAAAAATAAA   
  
  
- TGAGAGACTA CATTTATTTA AATAATATAT AATAATAATA ATTAAAAAAA ACGCACCCTT CCTTCAACAT   
  
  
- GGAACCCCAC TGGTATAACA TTTTTCCACA CACGTTACTA TTGTTCTTCT CTGTATGTGT GTAGTGTGCA   
  
  
- TGCTCTGAGG GAAACGAAGT AGTAAGACCC ATTGAACAAG TAGTACAGTG CTAGTAGGGT TAGAGGGCTA   
  
  
- AATAAAAAGG TACGTAATAC ATTATATATG CGTTGCAAAA CTAAAAATAT TAACATAGTA AAAAATCAAA   
  
  
- TTTTTTTAAA CATTTATTAT AAATTAGATA AAAAATATTT AATAAAACTA ATTCAGGTTT TAATAAATAT   
  
  
- TTTCATTACG TTTCAATTTA CTTAAGTCTT TAGCCTTTAA ATTTTTTCTC TCCCTTTACA CCACACTTCT   
  
  
- CCCTCCTCTC ACCTCCGAAC AGCCCGTTCC CCTCACCCAC TGCGCATTAT GCGCAGTCGG ATTCGCTCGG   
  
  
- AATACCACAT CCGACTTCGA AGAAATTGAA ATGGGGTAAA AAAATAAAAG TATTAAACAA TCAAATTAAA   
  
  
- ATATAAAATA TAAAAATTTT TCTTTTTTTT GAATTAAATT GCCCCTCTGG AAACAGACGT CCTTTCATTA   
  
  
- GAAGATCAAT GACTTAACGG GGACTTCTCC TACTATCTCA CTCCCCGTAA ATCCATTTAA GTACTAACTC   
  
  
- TCTCTCTCTC TCTCTCTCTC TCTCTCTCAC AAAACTACAT TTTGTTTTTC TATTAAAACC CCAAATTTTC   
  
  
- AATTTTTCCA AAAATTTCCG TTTTCGACGC TTTCTTTTAA TCTTTATTTT TTTAATTTTT TTTTTGTTCC   
  
  
- TCCTCTTCGT TAGTGTACGG ACTGCTGGGT TTGCAAGACG TCGGGTGCAC ATCCAAACCC GGGGTGACAC   
  
  
- CTGCGCGGTG GCCGATTTGA CCAGCTGTAC GGCGCATTAT TTCCCACCTG GGCTCCCGCC AGGACCTCGT   
  
  
- TAATTACGTG TTGCGGCAGG AGATCAGTGG GAGTATGGCA TGTGTACCAA TTGGCCGTAA ACGGTCAGTT   
  
  
- GAGAAGACAA AATAAAAGAT AAATTTTATT AAATAGTTTA CCCTTTGCAG GGTTTTGTTC GACAAAAGGT   
  
  
- TTTTGGGTTT GTGTTTTTCG GGTAAGTGTG AGCTTCGTTG GTTTGTGATA AAGAGAAAGA GAGAGACAGA   
  
  
- GAGTGAAAAA AAGAGAGAGA GAGAGGGAGA GAACATTATT TTTCACGCTC TTCTCTTCTT TACTCTCTCC   
  
  
- CAAAATACTT TACTTCCTCC TCTAGTAATA CATCATGTAC ACGAGCGCAA CCACTAGTCT CTAAAGATAC   
  
  
- CTCTTCTCTC TTCTTTTCTC TCTCTTAAG

+     TCA-element

| Site Name | Organism | Position | Strand | Matrix score. | sequence | function |
| --- | --- | --- | --- | --- | --- | --- |
| TCA-element | Nicotiana tabacum | 665 | + | 9 | CCATCTTTTT | cis-acting element involved in salicylic acid responsiveness |
| TCA-element | Brassica oleracea | 1478 | + | 9 | GAGAAGAATA | cis-acting element involved in salicylic acid responsiveness |

> 2018/04/13 10:10:12  
+ CGGCTGTCCC ACATCGAGCT CTGGAGCCAC GGGGACCAGG ACCGTGTATC GTGGCAAAGG TGGTTTCACG   
  
  
+ AAAAGGATGC AATAATTCTG ATAGATCTTC ACACAATTTC TTTTTTTTTT TCTAATTTTT ATTTTTATTT   
  
  
+ ACTCTCTGAT GTAAATAAAT TTATTATATA TTATTATTAT TAATTTTTTT TGCGTGGGAA GGAAGTTGTA   
  
  
+ CCTTGGGGTG ACCATATTGT AAAAAGGTGT GTGCAATGAT AACAAGAAGA GACATACACA CATCACACGT   
  
  
+ ACGAGACTCC CTTTGCTTCA TCATTCTGGG TAACTTGTTC ATCATGTCAC GATCATCCCA ATCTCCCGAT   
  
  
+ TTATTTTTCC ATGCATTATG TAATATATAC GCAACGTTTT GATTTTTATA ATTGTATCAT TTTTTAGTTT   
  
  
+ AAAAAAATTT GTAAATAATA TTTAATCTAT TTTTTATAAA TTATTTTGAT TAAGTCCAAA ATTATTTATA   
  
  
+ AAAGTAATGC AAAGTTAAAT GAATTCAGAA ATCGGAAATT TAAAAAAGAG AGGGAAATGT GGTGTGAAGA   
  
  
+ GGGAGGAGAG TGGAGGCTTG TCGGGCAAGG GGAGTGGGTG ACGCGTAATA CGCGTCAGCC TAAGCGAGCC   
  
  
+ TTATGGTGTA GGCTGAAGCT TCTTTAACTT TACCCCATTT TTTTATTTTC ATAATTTGTT AGTTTAATTT   
  
  
+ TATATTTTAT ATTTTTAAAA AGAAAAAAAA CTTAATTTAA CGGGGAGACC TTTGTCTGCA GGAAAGTAAT   
  
  
+ CTTCTAGTTA CTGAATTGCC CCTGAAGAGG ATGATAGAGT GAGGGGCATT TAGGTAAATT CATGATTGAG   
  
  
+ AGAGAGAGAG AGAGAGAGAG AGAGAGAGTG TTTTGATGTA AAACAAAAAG ATAATTTTGG GGTTTAAAAG   
  
  
+ TTAAAAAGGT TTTTAAAGGC AAAAGCTGCG AAAGAAAATT AGAAATAAAA AAATTAAAAA AAAAACAAGG   
  
  
+ AGGAGAAGCA ATCACATGCC TGACGACCCA AACGTTCTGC AGCCCACGTG TAGGTTTGGG CCCCACTGTG   
  
  
+ GACGCGCCAC CGGCTAAACT GGTCGACATG CCGCGTAATA AAGGGTGGAC CCGAGGGCGG TCCTGGAGCA   
  
  
+ ATTAATGCAC AACGCCGTCC TCTAGTCACC CTCATACCGT ACACATGGTT AACCGGCATT TGCCAGTCAA   
  
  
+ CTCTTCTGTT TTATTTTCTA TTTAAAATAA TTTATCAAAT GGGAAACGTC CCAAAACAAG CTGTTTTCCA   
  
  
+ AAAACCCAAA CACAAAAAGC CCATTCACAC TCGAAGCAAC CAAACACTAT TTCTCTTTCT CTCTCTGTCT   
  
  
+ CTCACTTTTT TTCTCTCTCT CTCTCCCTCT CTTGTAATAA AAAGTGCGAG AAGAGAAGAA ATGAGAGAGG   
  
  
+ GTTTTATGAA ATGAAGGAGG AGATCATTAT GTAGTACATG TGCTCGCGTT GGTGATCAGA GATTTCTATG   
  
  
+ GAGAAGAGAG AAGAAAAGAG AGAGAATTC  

- GCCGACAGGG TGTAGCTCGA GACCTCGGTG CCCCTGGTCC TGGCACATAG CACCGTTTCC ACCAAAGTGC   
  
  
- TTTTCCTACG TTATTAAGAC TATCTAGAAG TGTGTTAAAG AAAAAAAAAA AGATTAAAAA TAAAAATAAA   
  
  
- TGAGAGACTA CATTTATTTA AATAATATAT AATAATAATA ATTAAAAAAA ACGCACCCTT CCTTCAACAT   
  
  
- GGAACCCCAC TGGTATAACA TTTTTCCACA CACGTTACTA TTGTTCTTCT CTGTATGTGT GTAGTGTGCA   
  
  
- TGCTCTGAGG GAAACGAAGT AGTAAGACCC ATTGAACAAG TAGTACAGTG CTAGTAGGGT TAGAGGGCTA   
  
  
- AATAAAAAGG TACGTAATAC ATTATATATG CGTTGCAAAA CTAAAAATAT TAACATAGTA AAAAATCAAA   
  
  
- TTTTTTTAAA CATTTATTAT AAATTAGATA AAAAATATTT AATAAAACTA ATTCAGGTTT TAATAAATAT   
  
  
- TTTCATTACG TTTCAATTTA CTTAAGTCTT TAGCCTTTAA ATTTTTTCTC TCCCTTTACA CCACACTTCT   
  
  
- CCCTCCTCTC ACCTCCGAAC AGCCCGTTCC CCTCACCCAC TGCGCATTAT GCGCAGTCGG ATTCGCTCGG   
  
  
- AATACCACAT CCGACTTCGA AGAAATTGAA ATGGGGTAAA AAAATAAAAG TATTAAACAA TCAAATTAAA   
  
  
- ATATAAAATA TAAAAATTTT TCTTTTTTTT GAATTAAATT GCCCCTCTGG AAACAGACGT CCTTTCATTA   
  
  
- GAAGATCAAT GACTTAACGG GGACTTCTCC TACTATCTCA CTCCCCGTAA ATCCATTTAA GTACTAACTC   
  
  
- TCTCTCTCTC TCTCTCTCTC TCTCTCTCAC AAAACTACAT TTTGTTTTTC TATTAAAACC CCAAATTTTC   
  
  
- AATTTTTCCA AAAATTTCCG TTTTCGACGC TTTCTTTTAA TCTTTATTTT TTTAATTTTT TTTTTGTTCC   
  
  
- TCCTCTTCGT TAGTGTACGG ACTGCTGGGT TTGCAAGACG TCGGGTGCAC ATCCAAACCC GGGGTGACAC   
  
  
- CTGCGCGGTG GCCGATTTGA CCAGCTGTAC GGCGCATTAT TTCCCACCTG GGCTCCCGCC AGGACCTCGT   
  
  
- TAATTACGTG TTGCGGCAGG AGATCAGTGG GAGTATGGCA TGTGTACCAA TTGGCCGTAA ACGGTCAGTT   
  
  
- GAGAAGACAA AATAAAAGAT AAATTTTATT AAATAGTTTA CCCTTTGCAG GGTTTTGTTC GACAAAAGGT   
  
  
- TTTTGGGTTT GTGTTTTTCG GGTAAGTGTG AGCTTCGTTG GTTTGTGATA AAGAGAAAGA GAGAGACAGA   
  
  
- GAGTGAAAAA AAGAGAGAGA GAGAGGGAGA GAACATTATT TTTCACGCTC TTCTCTTCTT TACTCTCTCC   
  
  
- CAAAATACTT TACTTCCTCC TCTAGTAATA CATCATGTAC ACGAGCGCAA CCACTAGTCT CTAAAGATAC   
  
  
- CTCTTCTCTC TTCTTTTCTC TCTCTTAAG

+     TCCC-motif

| Site Name | Organism | Position | Strand | Matrix score. | sequence | function |
| --- | --- | --- | --- | --- | --- | --- |
| TCCC-motif | Spinacia oleracea | 1352 | + | 7 | TCTCCCT | part of a light responsive element |

> 2018/04/13 10:10:12  
+ CGGCTGTCCC ACATCGAGCT CTGGAGCCAC GGGGACCAGG ACCGTGTATC GTGGCAAAGG TGGTTTCACG   
  
  
+ AAAAGGATGC AATAATTCTG ATAGATCTTC ACACAATTTC TTTTTTTTTT TCTAATTTTT ATTTTTATTT   
  
  
+ ACTCTCTGAT GTAAATAAAT TTATTATATA TTATTATTAT TAATTTTTTT TGCGTGGGAA GGAAGTTGTA   
  
  
+ CCTTGGGGTG ACCATATTGT AAAAAGGTGT GTGCAATGAT AACAAGAAGA GACATACACA CATCACACGT   
  
  
+ ACGAGACTCC CTTTGCTTCA TCATTCTGGG TAACTTGTTC ATCATGTCAC GATCATCCCA ATCTCCCGAT   
  
  
+ TTATTTTTCC ATGCATTATG TAATATATAC GCAACGTTTT GATTTTTATA ATTGTATCAT TTTTTAGTTT   
  
  
+ AAAAAAATTT GTAAATAATA TTTAATCTAT TTTTTATAAA TTATTTTGAT TAAGTCCAAA ATTATTTATA   
  
  
+ AAAGTAATGC AAAGTTAAAT GAATTCAGAA ATCGGAAATT TAAAAAAGAG AGGGAAATGT GGTGTGAAGA   
  
  
+ GGGAGGAGAG TGGAGGCTTG TCGGGCAAGG GGAGTGGGTG ACGCGTAATA CGCGTCAGCC TAAGCGAGCC   
  
  
+ TTATGGTGTA GGCTGAAGCT TCTTTAACTT TACCCCATTT TTTTATTTTC ATAATTTGTT AGTTTAATTT   
  
  
+ TATATTTTAT ATTTTTAAAA AGAAAAAAAA CTTAATTTAA CGGGGAGACC TTTGTCTGCA GGAAAGTAAT   
  
  
+ CTTCTAGTTA CTGAATTGCC CCTGAAGAGG ATGATAGAGT GAGGGGCATT TAGGTAAATT CATGATTGAG   
  
  
+ AGAGAGAGAG AGAGAGAGAG AGAGAGAGTG TTTTGATGTA AAACAAAAAG ATAATTTTGG GGTTTAAAAG   
  
  
+ TTAAAAAGGT TTTTAAAGGC AAAAGCTGCG AAAGAAAATT AGAAATAAAA AAATTAAAAA AAAAACAAGG   
  
  
+ AGGAGAAGCA ATCACATGCC TGACGACCCA AACGTTCTGC AGCCCACGTG TAGGTTTGGG CCCCACTGTG   
  
  
+ GACGCGCCAC CGGCTAAACT GGTCGACATG CCGCGTAATA AAGGGTGGAC CCGAGGGCGG TCCTGGAGCA   
  
  
+ ATTAATGCAC AACGCCGTCC TCTAGTCACC CTCATACCGT ACACATGGTT AACCGGCATT TGCCAGTCAA   
  
  
+ CTCTTCTGTT TTATTTTCTA TTTAAAATAA TTTATCAAAT GGGAAACGTC CCAAAACAAG CTGTTTTCCA   
  
  
+ AAAACCCAAA CACAAAAAGC CCATTCACAC TCGAAGCAAC CAAACACTAT TTCTCTTTCT CTCTCTGTCT   
  
  
+ CTCACTTTTT TTCTCTCTCT CTCTCCCTCT CTTGTAATAA AAAGTGCGAG AAGAGAAGAA ATGAGAGAGG   
  
  
+ GTTTTATGAA ATGAAGGAGG AGATCATTAT GTAGTACATG TGCTCGCGTT GGTGATCAGA GATTTCTATG   
  
  
+ GAGAAGAGAG AAGAAAAGAG AGAGAATTC  

- GCCGACAGGG TGTAGCTCGA GACCTCGGTG CCCCTGGTCC TGGCACATAG CACCGTTTCC ACCAAAGTGC   
  
  
- TTTTCCTACG TTATTAAGAC TATCTAGAAG TGTGTTAAAG AAAAAAAAAA AGATTAAAAA TAAAAATAAA   
  
  
- TGAGAGACTA CATTTATTTA AATAATATAT AATAATAATA ATTAAAAAAA ACGCACCCTT CCTTCAACAT   
  
  
- GGAACCCCAC TGGTATAACA TTTTTCCACA CACGTTACTA TTGTTCTTCT CTGTATGTGT GTAGTGTGCA   
  
  
- TGCTCTGAGG GAAACGAAGT AGTAAGACCC ATTGAACAAG TAGTACAGTG CTAGTAGGGT TAGAGGGCTA   
  
  
- AATAAAAAGG TACGTAATAC ATTATATATG CGTTGCAAAA CTAAAAATAT TAACATAGTA AAAAATCAAA   
  
  
- TTTTTTTAAA CATTTATTAT AAATTAGATA AAAAATATTT AATAAAACTA ATTCAGGTTT TAATAAATAT   
  
  
- TTTCATTACG TTTCAATTTA CTTAAGTCTT TAGCCTTTAA ATTTTTTCTC TCCCTTTACA CCACACTTCT   
  
  
- CCCTCCTCTC ACCTCCGAAC AGCCCGTTCC CCTCACCCAC TGCGCATTAT GCGCAGTCGG ATTCGCTCGG   
  
  
- AATACCACAT CCGACTTCGA AGAAATTGAA ATGGGGTAAA AAAATAAAAG TATTAAACAA TCAAATTAAA   
  
  
- ATATAAAATA TAAAAATTTT TCTTTTTTTT GAATTAAATT GCCCCTCTGG AAACAGACGT CCTTTCATTA   
  
  
- GAAGATCAAT GACTTAACGG GGACTTCTCC TACTATCTCA CTCCCCGTAA ATCCATTTAA GTACTAACTC   
  
  
- TCTCTCTCTC TCTCTCTCTC TCTCTCTCAC AAAACTACAT TTTGTTTTTC TATTAAAACC CCAAATTTTC   
  
  
- AATTTTTCCA AAAATTTCCG TTTTCGACGC TTTCTTTTAA TCTTTATTTT TTTAATTTTT TTTTTGTTCC   
  
  
- TCCTCTTCGT TAGTGTACGG ACTGCTGGGT TTGCAAGACG TCGGGTGCAC ATCCAAACCC GGGGTGACAC   
  
  
- CTGCGCGGTG GCCGATTTGA CCAGCTGTAC GGCGCATTAT TTCCCACCTG GGCTCCCGCC AGGACCTCGT   
  
  
- TAATTACGTG TTGCGGCAGG AGATCAGTGG GAGTATGGCA TGTGTACCAA TTGGCCGTAA ACGGTCAGTT   
  
  
- GAGAAGACAA AATAAAAGAT AAATTTTATT AAATAGTTTA CCCTTTGCAG GGTTTTGTTC GACAAAAGGT   
  
  
- TTTTGGGTTT GTGTTTTTCG GGTAAGTGTG AGCTTCGTTG GTTTGTGATA AAGAGAAAGA GAGAGACAGA   
  
  
- GAGTGAAAAA AAGAGAGAGA GAGAGGGAGA GAACATTATT TTTCACGCTC TTCTCTTCTT TACTCTCTCC   
  
  
- CAAAATACTT TACTTCCTCC TCTAGTAATA CATCATGTAC ACGAGCGCAA CCACTAGTCT CTAAAGATAC   
  
  
- CTCTTCTCTC TTCTTTTCTC TCTCTTAAG

+     TGACG-motif

| Site Name | Organism | Position | Strand | Matrix score. | sequence | function |
| --- | --- | --- | --- | --- | --- | --- |
| TGACG-motif | Hordeum vulgare | 1001 | + | 5 | TGACG | cis-acting regulatory element involved in the MeJA-responsiveness |
| TGACG-motif | Hordeum vulgare | 613 | - | 5 | TGACG | cis-acting regulatory element involved in the MeJA-responsiveness |
| TGACG-motif | Hordeum vulgare | 599 | + | 5 | TGACG | cis-acting regulatory element involved in the MeJA-responsiveness |

> 2018/04/13 10:10:12  
+ CGGCTGTCCC ACATCGAGCT CTGGAGCCAC GGGGACCAGG ACCGTGTATC GTGGCAAAGG TGGTTTCACG   
  
  
+ AAAAGGATGC AATAATTCTG ATAGATCTTC ACACAATTTC TTTTTTTTTT TCTAATTTTT ATTTTTATTT   
  
  
+ ACTCTCTGAT GTAAATAAAT TTATTATATA TTATTATTAT TAATTTTTTT TGCGTGGGAA GGAAGTTGTA   
  
  
+ CCTTGGGGTG ACCATATTGT AAAAAGGTGT GTGCAATGAT AACAAGAAGA GACATACACA CATCACACGT   
  
  
+ ACGAGACTCC CTTTGCTTCA TCATTCTGGG TAACTTGTTC ATCATGTCAC GATCATCCCA ATCTCCCGAT   
  
  
+ TTATTTTTCC ATGCATTATG TAATATATAC GCAACGTTTT GATTTTTATA ATTGTATCAT TTTTTAGTTT   
  
  
+ AAAAAAATTT GTAAATAATA TTTAATCTAT TTTTTATAAA TTATTTTGAT TAAGTCCAAA ATTATTTATA   
  
  
+ AAAGTAATGC AAAGTTAAAT GAATTCAGAA ATCGGAAATT TAAAAAAGAG AGGGAAATGT GGTGTGAAGA   
  
  
+ GGGAGGAGAG TGGAGGCTTG TCGGGCAAGG GGAGTGGGTG ACGCGTAATA CGCGTCAGCC TAAGCGAGCC   
  
  
+ TTATGGTGTA GGCTGAAGCT TCTTTAACTT TACCCCATTT TTTTATTTTC ATAATTTGTT AGTTTAATTT   
  
  
+ TATATTTTAT ATTTTTAAAA AGAAAAAAAA CTTAATTTAA CGGGGAGACC TTTGTCTGCA GGAAAGTAAT   
  
  
+ CTTCTAGTTA CTGAATTGCC CCTGAAGAGG ATGATAGAGT GAGGGGCATT TAGGTAAATT CATGATTGAG   
  
  
+ AGAGAGAGAG AGAGAGAGAG AGAGAGAGTG TTTTGATGTA AAACAAAAAG ATAATTTTGG GGTTTAAAAG   
  
  
+ TTAAAAAGGT TTTTAAAGGC AAAAGCTGCG AAAGAAAATT AGAAATAAAA AAATTAAAAA AAAAACAAGG   
  
  
+ AGGAGAAGCA ATCACATGCC TGACGACCCA AACGTTCTGC AGCCCACGTG TAGGTTTGGG CCCCACTGTG   
  
  
+ GACGCGCCAC CGGCTAAACT GGTCGACATG CCGCGTAATA AAGGGTGGAC CCGAGGGCGG TCCTGGAGCA   
  
  
+ ATTAATGCAC AACGCCGTCC TCTAGTCACC CTCATACCGT ACACATGGTT AACCGGCATT TGCCAGTCAA   
  
  
+ CTCTTCTGTT TTATTTTCTA TTTAAAATAA TTTATCAAAT GGGAAACGTC CCAAAACAAG CTGTTTTCCA   
  
  
+ AAAACCCAAA CACAAAAAGC CCATTCACAC TCGAAGCAAC CAAACACTAT TTCTCTTTCT CTCTCTGTCT   
  
  
+ CTCACTTTTT TTCTCTCTCT CTCTCCCTCT CTTGTAATAA AAAGTGCGAG AAGAGAAGAA ATGAGAGAGG   
  
  
+ GTTTTATGAA ATGAAGGAGG AGATCATTAT GTAGTACATG TGCTCGCGTT GGTGATCAGA GATTTCTATG   
  
  
+ GAGAAGAGAG AAGAAAAGAG AGAGAATTC  

- GCCGACAGGG TGTAGCTCGA GACCTCGGTG CCCCTGGTCC TGGCACATAG CACCGTTTCC ACCAAAGTGC   
  
  
- TTTTCCTACG TTATTAAGAC TATCTAGAAG TGTGTTAAAG AAAAAAAAAA AGATTAAAAA TAAAAATAAA   
  
  
- TGAGAGACTA CATTTATTTA AATAATATAT AATAATAATA ATTAAAAAAA ACGCACCCTT CCTTCAACAT   
  
  
- GGAACCCCAC TGGTATAACA TTTTTCCACA CACGTTACTA TTGTTCTTCT CTGTATGTGT GTAGTGTGCA   
  
  
- TGCTCTGAGG GAAACGAAGT AGTAAGACCC ATTGAACAAG TAGTACAGTG CTAGTAGGGT TAGAGGGCTA   
  
  
- AATAAAAAGG TACGTAATAC ATTATATATG CGTTGCAAAA CTAAAAATAT TAACATAGTA AAAAATCAAA   
  
  
- TTTTTTTAAA CATTTATTAT AAATTAGATA AAAAATATTT AATAAAACTA ATTCAGGTTT TAATAAATAT   
  
  
- TTTCATTACG TTTCAATTTA CTTAAGTCTT TAGCCTTTAA ATTTTTTCTC TCCCTTTACA CCACACTTCT   
  
  
- CCCTCCTCTC ACCTCCGAAC AGCCCGTTCC CCTCACCCAC TGCGCATTAT GCGCAGTCGG ATTCGCTCGG   
  
  
- AATACCACAT CCGACTTCGA AGAAATTGAA ATGGGGTAAA AAAATAAAAG TATTAAACAA TCAAATTAAA   
  
  
- ATATAAAATA TAAAAATTTT TCTTTTTTTT GAATTAAATT GCCCCTCTGG AAACAGACGT CCTTTCATTA   
  
  
- GAAGATCAAT GACTTAACGG GGACTTCTCC TACTATCTCA CTCCCCGTAA ATCCATTTAA GTACTAACTC   
  
  
- TCTCTCTCTC TCTCTCTCTC TCTCTCTCAC AAAACTACAT TTTGTTTTTC TATTAAAACC CCAAATTTTC   
  
  
- AATTTTTCCA AAAATTTCCG TTTTCGACGC TTTCTTTTAA TCTTTATTTT TTTAATTTTT TTTTTGTTCC   
  
  
- TCCTCTTCGT TAGTGTACGG ACTGCTGGGT TTGCAAGACG TCGGGTGCAC ATCCAAACCC GGGGTGACAC   
  
  
- CTGCGCGGTG GCCGATTTGA CCAGCTGTAC GGCGCATTAT TTCCCACCTG GGCTCCCGCC AGGACCTCGT   
  
  
- TAATTACGTG TTGCGGCAGG AGATCAGTGG GAGTATGGCA TGTGTACCAA TTGGCCGTAA ACGGTCAGTT   
  
  
- GAGAAGACAA AATAAAAGAT AAATTTTATT AAATAGTTTA CCCTTTGCAG GGTTTTGTTC GACAAAAGGT   
  
  
- TTTTGGGTTT GTGTTTTTCG GGTAAGTGTG AGCTTCGTTG GTTTGTGATA AAGAGAAAGA GAGAGACAGA   
  
  
- GAGTGAAAAA AAGAGAGAGA GAGAGGGAGA GAACATTATT TTTCACGCTC TTCTCTTCTT TACTCTCTCC   
  
  
- CAAAATACTT TACTTCCTCC TCTAGTAATA CATCATGTAC ACGAGCGCAA CCACTAGTCT CTAAAGATAC   
  
  
- CTCTTCTCTC TTCTTTTCTC TCTCTTAAG

+     Unnamed\_\_1

| Site Name | Organism | Position | Strand | Matrix score. | sequence | function |
| --- | --- | --- | --- | --- | --- | --- |
| Unnamed\_\_1 | Zea mays | 50 | + | 5 | CGTGG |  |
| Unnamed\_\_1 | Zea mays | 193 | + | 5 | CGTGG |  |
| Unnamed\_\_1 | Zea mays | 27 | - | 5 | CGTGG |  |
| Unnamed\_\_1 | Zea mays | 1024 | - | 5 | CGTGG |  |

> 2018/04/13 10:10:12  
+ CGGCTGTCCC ACATCGAGCT CTGGAGCCAC GGGGACCAGG ACCGTGTATC GTGGCAAAGG TGGTTTCACG   
  
  
+ AAAAGGATGC AATAATTCTG ATAGATCTTC ACACAATTTC TTTTTTTTTT TCTAATTTTT ATTTTTATTT   
  
  
+ ACTCTCTGAT GTAAATAAAT TTATTATATA TTATTATTAT TAATTTTTTT TGCGTGGGAA GGAAGTTGTA   
  
  
+ CCTTGGGGTG ACCATATTGT AAAAAGGTGT GTGCAATGAT AACAAGAAGA GACATACACA CATCACACGT   
  
  
+ ACGAGACTCC CTTTGCTTCA TCATTCTGGG TAACTTGTTC ATCATGTCAC GATCATCCCA ATCTCCCGAT   
  
  
+ TTATTTTTCC ATGCATTATG TAATATATAC GCAACGTTTT GATTTTTATA ATTGTATCAT TTTTTAGTTT   
  
  
+ AAAAAAATTT GTAAATAATA TTTAATCTAT TTTTTATAAA TTATTTTGAT TAAGTCCAAA ATTATTTATA   
  
  
+ AAAGTAATGC AAAGTTAAAT GAATTCAGAA ATCGGAAATT TAAAAAAGAG AGGGAAATGT GGTGTGAAGA   
  
  
+ GGGAGGAGAG TGGAGGCTTG TCGGGCAAGG GGAGTGGGTG ACGCGTAATA CGCGTCAGCC TAAGCGAGCC   
  
  
+ TTATGGTGTA GGCTGAAGCT TCTTTAACTT TACCCCATTT TTTTATTTTC ATAATTTGTT AGTTTAATTT   
  
  
+ TATATTTTAT ATTTTTAAAA AGAAAAAAAA CTTAATTTAA CGGGGAGACC TTTGTCTGCA GGAAAGTAAT   
  
  
+ CTTCTAGTTA CTGAATTGCC CCTGAAGAGG ATGATAGAGT GAGGGGCATT TAGGTAAATT CATGATTGAG   
  
  
+ AGAGAGAGAG AGAGAGAGAG AGAGAGAGTG TTTTGATGTA AAACAAAAAG ATAATTTTGG GGTTTAAAAG   
  
  
+ TTAAAAAGGT TTTTAAAGGC AAAAGCTGCG AAAGAAAATT AGAAATAAAA AAATTAAAAA AAAAACAAGG   
  
  
+ AGGAGAAGCA ATCACATGCC TGACGACCCA AACGTTCTGC AGCCCACGTG TAGGTTTGGG CCCCACTGTG   
  
  
+ GACGCGCCAC CGGCTAAACT GGTCGACATG CCGCGTAATA AAGGGTGGAC CCGAGGGCGG TCCTGGAGCA   
  
  
+ ATTAATGCAC AACGCCGTCC TCTAGTCACC CTCATACCGT ACACATGGTT AACCGGCATT TGCCAGTCAA   
  
  
+ CTCTTCTGTT TTATTTTCTA TTTAAAATAA TTTATCAAAT GGGAAACGTC CCAAAACAAG CTGTTTTCCA   
  
  
+ AAAACCCAAA CACAAAAAGC CCATTCACAC TCGAAGCAAC CAAACACTAT TTCTCTTTCT CTCTCTGTCT   
  
  
+ CTCACTTTTT TTCTCTCTCT CTCTCCCTCT CTTGTAATAA AAAGTGCGAG AAGAGAAGAA ATGAGAGAGG   
  
  
+ GTTTTATGAA ATGAAGGAGG AGATCATTAT GTAGTACATG TGCTCGCGTT GGTGATCAGA GATTTCTATG   
  
  
+ GAGAAGAGAG AAGAAAAGAG AGAGAATTC  

- GCCGACAGGG TGTAGCTCGA GACCTCGGTG CCCCTGGTCC TGGCACATAG CACCGTTTCC ACCAAAGTGC   
  
  
- TTTTCCTACG TTATTAAGAC TATCTAGAAG TGTGTTAAAG AAAAAAAAAA AGATTAAAAA TAAAAATAAA   
  
  
- TGAGAGACTA CATTTATTTA AATAATATAT AATAATAATA ATTAAAAAAA ACGCACCCTT CCTTCAACAT   
  
  
- GGAACCCCAC TGGTATAACA TTTTTCCACA CACGTTACTA TTGTTCTTCT CTGTATGTGT GTAGTGTGCA   
  
  
- TGCTCTGAGG GAAACGAAGT AGTAAGACCC ATTGAACAAG TAGTACAGTG CTAGTAGGGT TAGAGGGCTA   
  
  
- AATAAAAAGG TACGTAATAC ATTATATATG CGTTGCAAAA CTAAAAATAT TAACATAGTA AAAAATCAAA   
  
  
- TTTTTTTAAA CATTTATTAT AAATTAGATA AAAAATATTT AATAAAACTA ATTCAGGTTT TAATAAATAT   
  
  
- TTTCATTACG TTTCAATTTA CTTAAGTCTT TAGCCTTTAA ATTTTTTCTC TCCCTTTACA CCACACTTCT   
  
  
- CCCTCCTCTC ACCTCCGAAC AGCCCGTTCC CCTCACCCAC TGCGCATTAT GCGCAGTCGG ATTCGCTCGG   
  
  
- AATACCACAT CCGACTTCGA AGAAATTGAA ATGGGGTAAA AAAATAAAAG TATTAAACAA TCAAATTAAA   
  
  
- ATATAAAATA TAAAAATTTT TCTTTTTTTT GAATTAAATT GCCCCTCTGG AAACAGACGT CCTTTCATTA   
  
  
- GAAGATCAAT GACTTAACGG GGACTTCTCC TACTATCTCA CTCCCCGTAA ATCCATTTAA GTACTAACTC   
  
  
- TCTCTCTCTC TCTCTCTCTC TCTCTCTCAC AAAACTACAT TTTGTTTTTC TATTAAAACC CCAAATTTTC   
  
  
- AATTTTTCCA AAAATTTCCG TTTTCGACGC TTTCTTTTAA TCTTTATTTT TTTAATTTTT TTTTTGTTCC   
  
  
- TCCTCTTCGT TAGTGTACGG ACTGCTGGGT TTGCAAGACG TCGGGTGCAC ATCCAAACCC GGGGTGACAC   
  
  
- CTGCGCGGTG GCCGATTTGA CCAGCTGTAC GGCGCATTAT TTCCCACCTG GGCTCCCGCC AGGACCTCGT   
  
  
- TAATTACGTG TTGCGGCAGG AGATCAGTGG GAGTATGGCA TGTGTACCAA TTGGCCGTAA ACGGTCAGTT   
  
  
- GAGAAGACAA AATAAAAGAT AAATTTTATT AAATAGTTTA CCCTTTGCAG GGTTTTGTTC GACAAAAGGT   
  
  
- TTTTGGGTTT GTGTTTTTCG GGTAAGTGTG AGCTTCGTTG GTTTGTGATA AAGAGAAAGA GAGAGACAGA   
  
  
- GAGTGAAAAA AAGAGAGAGA GAGAGGGAGA GAACATTATT TTTCACGCTC TTCTCTTCTT TACTCTCTCC   
  
  
- CAAAATACTT TACTTCCTCC TCTAGTAATA CATCATGTAC ACGAGCGCAA CCACTAGTCT CTAAAGATAC   
  
  
- CTCTTCTCTC TTCTTTTCTC TCTCTTAAG

+     Unnamed\_\_3

| Site Name | Organism | Position | Strand | Matrix score. | sequence | function |
| --- | --- | --- | --- | --- | --- | --- |
| Unnamed\_\_3 | Zea mays | 27 | - | 5 | CGTGG |  |
| Unnamed\_\_3 | Zea mays | 193 | + | 5 | CGTGG |  |
| Unnamed\_\_3 | Zea mays | 50 | + | 5 | CGTGG |  |
| Unnamed\_\_3 | Zea mays | 1024 | - | 5 | CGTGG |  |

> 2018/04/13 10:10:12  
+ CGGCTGTCCC ACATCGAGCT CTGGAGCCAC GGGGACCAGG ACCGTGTATC GTGGCAAAGG TGGTTTCACG   
  
  
+ AAAAGGATGC AATAATTCTG ATAGATCTTC ACACAATTTC TTTTTTTTTT TCTAATTTTT ATTTTTATTT   
  
  
+ ACTCTCTGAT GTAAATAAAT TTATTATATA TTATTATTAT TAATTTTTTT TGCGTGGGAA GGAAGTTGTA   
  
  
+ CCTTGGGGTG ACCATATTGT AAAAAGGTGT GTGCAATGAT AACAAGAAGA GACATACACA CATCACACGT   
  
  
+ ACGAGACTCC CTTTGCTTCA TCATTCTGGG TAACTTGTTC ATCATGTCAC GATCATCCCA ATCTCCCGAT   
  
  
+ TTATTTTTCC ATGCATTATG TAATATATAC GCAACGTTTT GATTTTTATA ATTGTATCAT TTTTTAGTTT   
  
  
+ AAAAAAATTT GTAAATAATA TTTAATCTAT TTTTTATAAA TTATTTTGAT TAAGTCCAAA ATTATTTATA   
  
  
+ AAAGTAATGC AAAGTTAAAT GAATTCAGAA ATCGGAAATT TAAAAAAGAG AGGGAAATGT GGTGTGAAGA   
  
  
+ GGGAGGAGAG TGGAGGCTTG TCGGGCAAGG GGAGTGGGTG ACGCGTAATA CGCGTCAGCC TAAGCGAGCC   
  
  
+ TTATGGTGTA GGCTGAAGCT TCTTTAACTT TACCCCATTT TTTTATTTTC ATAATTTGTT AGTTTAATTT   
  
  
+ TATATTTTAT ATTTTTAAAA AGAAAAAAAA CTTAATTTAA CGGGGAGACC TTTGTCTGCA GGAAAGTAAT   
  
  
+ CTTCTAGTTA CTGAATTGCC CCTGAAGAGG ATGATAGAGT GAGGGGCATT TAGGTAAATT CATGATTGAG   
  
  
+ AGAGAGAGAG AGAGAGAGAG AGAGAGAGTG TTTTGATGTA AAACAAAAAG ATAATTTTGG GGTTTAAAAG   
  
  
+ TTAAAAAGGT TTTTAAAGGC AAAAGCTGCG AAAGAAAATT AGAAATAAAA AAATTAAAAA AAAAACAAGG   
  
  
+ AGGAGAAGCA ATCACATGCC TGACGACCCA AACGTTCTGC AGCCCACGTG TAGGTTTGGG CCCCACTGTG   
  
  
+ GACGCGCCAC CGGCTAAACT GGTCGACATG CCGCGTAATA AAGGGTGGAC CCGAGGGCGG TCCTGGAGCA   
  
  
+ ATTAATGCAC AACGCCGTCC TCTAGTCACC CTCATACCGT ACACATGGTT AACCGGCATT TGCCAGTCAA   
  
  
+ CTCTTCTGTT TTATTTTCTA TTTAAAATAA TTTATCAAAT GGGAAACGTC CCAAAACAAG CTGTTTTCCA   
  
  
+ AAAACCCAAA CACAAAAAGC CCATTCACAC TCGAAGCAAC CAAACACTAT TTCTCTTTCT CTCTCTGTCT   
  
  
+ CTCACTTTTT TTCTCTCTCT CTCTCCCTCT CTTGTAATAA AAAGTGCGAG AAGAGAAGAA ATGAGAGAGG   
  
  
+ GTTTTATGAA ATGAAGGAGG AGATCATTAT GTAGTACATG TGCTCGCGTT GGTGATCAGA GATTTCTATG   
  
  
+ GAGAAGAGAG AAGAAAAGAG AGAGAATTC  

- GCCGACAGGG TGTAGCTCGA GACCTCGGTG CCCCTGGTCC TGGCACATAG CACCGTTTCC ACCAAAGTGC   
  
  
- TTTTCCTACG TTATTAAGAC TATCTAGAAG TGTGTTAAAG AAAAAAAAAA AGATTAAAAA TAAAAATAAA   
  
  
- TGAGAGACTA CATTTATTTA AATAATATAT AATAATAATA ATTAAAAAAA ACGCACCCTT CCTTCAACAT   
  
  
- GGAACCCCAC TGGTATAACA TTTTTCCACA CACGTTACTA TTGTTCTTCT CTGTATGTGT GTAGTGTGCA   
  
  
- TGCTCTGAGG GAAACGAAGT AGTAAGACCC ATTGAACAAG TAGTACAGTG CTAGTAGGGT TAGAGGGCTA   
  
  
- AATAAAAAGG TACGTAATAC ATTATATATG CGTTGCAAAA CTAAAAATAT TAACATAGTA AAAAATCAAA   
  
  
- TTTTTTTAAA CATTTATTAT AAATTAGATA AAAAATATTT AATAAAACTA ATTCAGGTTT TAATAAATAT   
  
  
- TTTCATTACG TTTCAATTTA CTTAAGTCTT TAGCCTTTAA ATTTTTTCTC TCCCTTTACA CCACACTTCT   
  
  
- CCCTCCTCTC ACCTCCGAAC AGCCCGTTCC CCTCACCCAC TGCGCATTAT GCGCAGTCGG ATTCGCTCGG   
  
  
- AATACCACAT CCGACTTCGA AGAAATTGAA ATGGGGTAAA AAAATAAAAG TATTAAACAA TCAAATTAAA   
  
  
- ATATAAAATA TAAAAATTTT TCTTTTTTTT GAATTAAATT GCCCCTCTGG AAACAGACGT CCTTTCATTA   
  
  
- GAAGATCAAT GACTTAACGG GGACTTCTCC TACTATCTCA CTCCCCGTAA ATCCATTTAA GTACTAACTC   
  
  
- TCTCTCTCTC TCTCTCTCTC TCTCTCTCAC AAAACTACAT TTTGTTTTTC TATTAAAACC CCAAATTTTC   
  
  
- AATTTTTCCA AAAATTTCCG TTTTCGACGC TTTCTTTTAA TCTTTATTTT TTTAATTTTT TTTTTGTTCC   
  
  
- TCCTCTTCGT TAGTGTACGG ACTGCTGGGT TTGCAAGACG TCGGGTGCAC ATCCAAACCC GGGGTGACAC   
  
  
- CTGCGCGGTG GCCGATTTGA CCAGCTGTAC GGCGCATTAT TTCCCACCTG GGCTCCCGCC AGGACCTCGT   
  
  
- TAATTACGTG TTGCGGCAGG AGATCAGTGG GAGTATGGCA TGTGTACCAA TTGGCCGTAA ACGGTCAGTT   
  
  
- GAGAAGACAA AATAAAAGAT AAATTTTATT AAATAGTTTA CCCTTTGCAG GGTTTTGTTC GACAAAAGGT   
  
  
- TTTTGGGTTT GTGTTTTTCG GGTAAGTGTG AGCTTCGTTG GTTTGTGATA AAGAGAAAGA GAGAGACAGA   
  
  
- GAGTGAAAAA AAGAGAGAGA GAGAGGGAGA GAACATTATT TTTCACGCTC TTCTCTTCTT TACTCTCTCC   
  
  
- CAAAATACTT TACTTCCTCC TCTAGTAATA CATCATGTAC ACGAGCGCAA CCACTAGTCT CTAAAGATAC   
  
  
- CTCTTCTCTC TTCTTTTCTC TCTCTTAAG

+     Unnamed\_\_4

| Site Name | Organism | Position | Strand | Matrix score. | sequence | function |
| --- | --- | --- | --- | --- | --- | --- |
| Unnamed\_\_4 | Petroselinum hortense | 1470 | - | 4 | CTCC |  |
| Unnamed\_\_4 | Petroselinum hortense | 1419 | - | 4 | CTCC |  |
| Unnamed\_\_4 | Petroselinum hortense | 982 | - | 4 | CTCC |  |
| Unnamed\_\_4 | Petroselinum hortense | 979 | - | 4 | CTCC |  |
| Unnamed\_\_4 | Petroselinum hortense | 287 | + | 4 | CTCC |  |
| Unnamed\_\_4 | Petroselinum hortense | 1115 | - | 4 | CTCC |  |
| Unnamed\_\_4 | Petroselinum hortense | 1353 | + | 4 | CTCC |  |
| Unnamed\_\_4 | Petroselinum hortense | 565 | - | 4 | CTCC |  |
| Unnamed\_\_4 | Petroselinum hortense | 591 | - | 4 | CTCC |  |
| Unnamed\_\_4 | Petroselinum hortense | 1416 | - | 4 | CTCC |  |
| Unnamed\_\_4 | Petroselinum hortense | 23 | - | 4 | CTCC |  |
| Unnamed\_\_4 | Petroselinum hortense | 343 | + | 4 | CTCC |  |
| Unnamed\_\_4 | Petroselinum hortense | 572 | - | 4 | CTCC |  |
| Unnamed\_\_4 | Petroselinum hortense | 562 | - | 4 | CTCC |  |
| Unnamed\_\_4 | Petroselinum hortense | 744 | - | 4 | CTCC |  |

> 2018/04/13 10:10:12  
+ CGGCTGTCCC ACATCGAGCT CTGGAGCCAC GGGGACCAGG ACCGTGTATC GTGGCAAAGG TGGTTTCACG   
  
  
+ AAAAGGATGC AATAATTCTG ATAGATCTTC ACACAATTTC TTTTTTTTTT TCTAATTTTT ATTTTTATTT   
  
  
+ ACTCTCTGAT GTAAATAAAT TTATTATATA TTATTATTAT TAATTTTTTT TGCGTGGGAA GGAAGTTGTA   
  
  
+ CCTTGGGGTG ACCATATTGT AAAAAGGTGT GTGCAATGAT AACAAGAAGA GACATACACA CATCACACGT   
  
  
+ ACGAGACTCC CTTTGCTTCA TCATTCTGGG TAACTTGTTC ATCATGTCAC GATCATCCCA ATCTCCCGAT   
  
  
+ TTATTTTTCC ATGCATTATG TAATATATAC GCAACGTTTT GATTTTTATA ATTGTATCAT TTTTTAGTTT   
  
  
+ AAAAAAATTT GTAAATAATA TTTAATCTAT TTTTTATAAA TTATTTTGAT TAAGTCCAAA ATTATTTATA   
  
  
+ AAAGTAATGC AAAGTTAAAT GAATTCAGAA ATCGGAAATT TAAAAAAGAG AGGGAAATGT GGTGTGAAGA   
  
  
+ GGGAGGAGAG TGGAGGCTTG TCGGGCAAGG GGAGTGGGTG ACGCGTAATA CGCGTCAGCC TAAGCGAGCC   
  
  
+ TTATGGTGTA GGCTGAAGCT TCTTTAACTT TACCCCATTT TTTTATTTTC ATAATTTGTT AGTTTAATTT   
  
  
+ TATATTTTAT ATTTTTAAAA AGAAAAAAAA CTTAATTTAA CGGGGAGACC TTTGTCTGCA GGAAAGTAAT   
  
  
+ CTTCTAGTTA CTGAATTGCC CCTGAAGAGG ATGATAGAGT GAGGGGCATT TAGGTAAATT CATGATTGAG   
  
  
+ AGAGAGAGAG AGAGAGAGAG AGAGAGAGTG TTTTGATGTA AAACAAAAAG ATAATTTTGG GGTTTAAAAG   
  
  
+ TTAAAAAGGT TTTTAAAGGC AAAAGCTGCG AAAGAAAATT AGAAATAAAA AAATTAAAAA AAAAACAAGG   
  
  
+ AGGAGAAGCA ATCACATGCC TGACGACCCA AACGTTCTGC AGCCCACGTG TAGGTTTGGG CCCCACTGTG   
  
  
+ GACGCGCCAC CGGCTAAACT GGTCGACATG CCGCGTAATA AAGGGTGGAC CCGAGGGCGG TCCTGGAGCA   
  
  
+ ATTAATGCAC AACGCCGTCC TCTAGTCACC CTCATACCGT ACACATGGTT AACCGGCATT TGCCAGTCAA   
  
  
+ CTCTTCTGTT TTATTTTCTA TTTAAAATAA TTTATCAAAT GGGAAACGTC CCAAAACAAG CTGTTTTCCA   
  
  
+ AAAACCCAAA CACAAAAAGC CCATTCACAC TCGAAGCAAC CAAACACTAT TTCTCTTTCT CTCTCTGTCT   
  
  
+ CTCACTTTTT TTCTCTCTCT CTCTCCCTCT CTTGTAATAA AAAGTGCGAG AAGAGAAGAA ATGAGAGAGG   
  
  
+ GTTTTATGAA ATGAAGGAGG AGATCATTAT GTAGTACATG TGCTCGCGTT GGTGATCAGA GATTTCTATG   
  
  
+ GAGAAGAGAG AAGAAAAGAG AGAGAATTC  

- GCCGACAGGG TGTAGCTCGA GACCTCGGTG CCCCTGGTCC TGGCACATAG CACCGTTTCC ACCAAAGTGC   
  
  
- TTTTCCTACG TTATTAAGAC TATCTAGAAG TGTGTTAAAG AAAAAAAAAA AGATTAAAAA TAAAAATAAA   
  
  
- TGAGAGACTA CATTTATTTA AATAATATAT AATAATAATA ATTAAAAAAA ACGCACCCTT CCTTCAACAT   
  
  
- GGAACCCCAC TGGTATAACA TTTTTCCACA CACGTTACTA TTGTTCTTCT CTGTATGTGT GTAGTGTGCA   
  
  
- TGCTCTGAGG GAAACGAAGT AGTAAGACCC ATTGAACAAG TAGTACAGTG CTAGTAGGGT TAGAGGGCTA   
  
  
- AATAAAAAGG TACGTAATAC ATTATATATG CGTTGCAAAA CTAAAAATAT TAACATAGTA AAAAATCAAA   
  
  
- TTTTTTTAAA CATTTATTAT AAATTAGATA AAAAATATTT AATAAAACTA ATTCAGGTTT TAATAAATAT   
  
  
- TTTCATTACG TTTCAATTTA CTTAAGTCTT TAGCCTTTAA ATTTTTTCTC TCCCTTTACA CCACACTTCT   
  
  
- CCCTCCTCTC ACCTCCGAAC AGCCCGTTCC CCTCACCCAC TGCGCATTAT GCGCAGTCGG ATTCGCTCGG   
  
  
- AATACCACAT CCGACTTCGA AGAAATTGAA ATGGGGTAAA AAAATAAAAG TATTAAACAA TCAAATTAAA   
  
  
- ATATAAAATA TAAAAATTTT TCTTTTTTTT GAATTAAATT GCCCCTCTGG AAACAGACGT CCTTTCATTA   
  
  
- GAAGATCAAT GACTTAACGG GGACTTCTCC TACTATCTCA CTCCCCGTAA ATCCATTTAA GTACTAACTC   
  
  
- TCTCTCTCTC TCTCTCTCTC TCTCTCTCAC AAAACTACAT TTTGTTTTTC TATTAAAACC CCAAATTTTC   
  
  
- AATTTTTCCA AAAATTTCCG TTTTCGACGC TTTCTTTTAA TCTTTATTTT TTTAATTTTT TTTTTGTTCC   
  
  
- TCCTCTTCGT TAGTGTACGG ACTGCTGGGT TTGCAAGACG TCGGGTGCAC ATCCAAACCC GGGGTGACAC   
  
  
- CTGCGCGGTG GCCGATTTGA CCAGCTGTAC GGCGCATTAT TTCCCACCTG GGCTCCCGCC AGGACCTCGT   
  
  
- TAATTACGTG TTGCGGCAGG AGATCAGTGG GAGTATGGCA TGTGTACCAA TTGGCCGTAA ACGGTCAGTT   
  
  
- GAGAAGACAA AATAAAAGAT AAATTTTATT AAATAGTTTA CCCTTTGCAG GGTTTTGTTC GACAAAAGGT   
  
  
- TTTTGGGTTT GTGTTTTTCG GGTAAGTGTG AGCTTCGTTG GTTTGTGATA AAGAGAAAGA GAGAGACAGA   
  
  
- GAGTGAAAAA AAGAGAGAGA GAGAGGGAGA GAACATTATT TTTCACGCTC TTCTCTTCTT TACTCTCTCC   
  
  
- CAAAATACTT TACTTCCTCC TCTAGTAATA CATCATGTAC ACGAGCGCAA CCACTAGTCT CTAAAGATAC   
  
  
- CTCTTCTCTC TTCTTTTCTC TCTCTTAAG

+     box II

| Site Name | Organism | Position | Strand | Matrix score. | sequence | function |
| --- | --- | --- | --- | --- | --- | --- |
| box II | Petroselinum hortense | 26 | - | 9 | TCCACGTGGC | part of a light responsive element |

> 2018/04/13 10:10:12  
+ CGGCTGTCCC ACATCGAGCT CTGGAGCCAC GGGGACCAGG ACCGTGTATC GTGGCAAAGG TGGTTTCACG   
  
  
+ AAAAGGATGC AATAATTCTG ATAGATCTTC ACACAATTTC TTTTTTTTTT TCTAATTTTT ATTTTTATTT   
  
  
+ ACTCTCTGAT GTAAATAAAT TTATTATATA TTATTATTAT TAATTTTTTT TGCGTGGGAA GGAAGTTGTA   
  
  
+ CCTTGGGGTG ACCATATTGT AAAAAGGTGT GTGCAATGAT AACAAGAAGA GACATACACA CATCACACGT   
  
  
+ ACGAGACTCC CTTTGCTTCA TCATTCTGGG TAACTTGTTC ATCATGTCAC GATCATCCCA ATCTCCCGAT   
  
  
+ TTATTTTTCC ATGCATTATG TAATATATAC GCAACGTTTT GATTTTTATA ATTGTATCAT TTTTTAGTTT   
  
  
+ AAAAAAATTT GTAAATAATA TTTAATCTAT TTTTTATAAA TTATTTTGAT TAAGTCCAAA ATTATTTATA   
  
  
+ AAAGTAATGC AAAGTTAAAT GAATTCAGAA ATCGGAAATT TAAAAAAGAG AGGGAAATGT GGTGTGAAGA   
  
  
+ GGGAGGAGAG TGGAGGCTTG TCGGGCAAGG GGAGTGGGTG ACGCGTAATA CGCGTCAGCC TAAGCGAGCC   
  
  
+ TTATGGTGTA GGCTGAAGCT TCTTTAACTT TACCCCATTT TTTTATTTTC ATAATTTGTT AGTTTAATTT   
  
  
+ TATATTTTAT ATTTTTAAAA AGAAAAAAAA CTTAATTTAA CGGGGAGACC TTTGTCTGCA GGAAAGTAAT   
  
  
+ CTTCTAGTTA CTGAATTGCC CCTGAAGAGG ATGATAGAGT GAGGGGCATT TAGGTAAATT CATGATTGAG   
  
  
+ AGAGAGAGAG AGAGAGAGAG AGAGAGAGTG TTTTGATGTA AAACAAAAAG ATAATTTTGG GGTTTAAAAG   
  
  
+ TTAAAAAGGT TTTTAAAGGC AAAAGCTGCG AAAGAAAATT AGAAATAAAA AAATTAAAAA AAAAACAAGG   
  
  
+ AGGAGAAGCA ATCACATGCC TGACGACCCA AACGTTCTGC AGCCCACGTG TAGGTTTGGG CCCCACTGTG   
  
  
+ GACGCGCCAC CGGCTAAACT GGTCGACATG CCGCGTAATA AAGGGTGGAC CCGAGGGCGG TCCTGGAGCA   
  
  
+ ATTAATGCAC AACGCCGTCC TCTAGTCACC CTCATACCGT ACACATGGTT AACCGGCATT TGCCAGTCAA   
  
  
+ CTCTTCTGTT TTATTTTCTA TTTAAAATAA TTTATCAAAT GGGAAACGTC CCAAAACAAG CTGTTTTCCA   
  
  
+ AAAACCCAAA CACAAAAAGC CCATTCACAC TCGAAGCAAC CAAACACTAT TTCTCTTTCT CTCTCTGTCT   
  
  
+ CTCACTTTTT TTCTCTCTCT CTCTCCCTCT CTTGTAATAA AAAGTGCGAG AAGAGAAGAA ATGAGAGAGG   
  
  
+ GTTTTATGAA ATGAAGGAGG AGATCATTAT GTAGTACATG TGCTCGCGTT GGTGATCAGA GATTTCTATG   
  
  
+ GAGAAGAGAG AAGAAAAGAG AGAGAATTC  

- GCCGACAGGG TGTAGCTCGA GACCTCGGTG CCCCTGGTCC TGGCACATAG CACCGTTTCC ACCAAAGTGC   
  
  
- TTTTCCTACG TTATTAAGAC TATCTAGAAG TGTGTTAAAG AAAAAAAAAA AGATTAAAAA TAAAAATAAA   
  
  
- TGAGAGACTA CATTTATTTA AATAATATAT AATAATAATA ATTAAAAAAA ACGCACCCTT CCTTCAACAT   
  
  
- GGAACCCCAC TGGTATAACA TTTTTCCACA CACGTTACTA TTGTTCTTCT CTGTATGTGT GTAGTGTGCA   
  
  
- TGCTCTGAGG GAAACGAAGT AGTAAGACCC ATTGAACAAG TAGTACAGTG CTAGTAGGGT TAGAGGGCTA   
  
  
- AATAAAAAGG TACGTAATAC ATTATATATG CGTTGCAAAA CTAAAAATAT TAACATAGTA AAAAATCAAA   
  
  
- TTTTTTTAAA CATTTATTAT AAATTAGATA AAAAATATTT AATAAAACTA ATTCAGGTTT TAATAAATAT   
  
  
- TTTCATTACG TTTCAATTTA CTTAAGTCTT TAGCCTTTAA ATTTTTTCTC TCCCTTTACA CCACACTTCT   
  
  
- CCCTCCTCTC ACCTCCGAAC AGCCCGTTCC CCTCACCCAC TGCGCATTAT GCGCAGTCGG ATTCGCTCGG   
  
  
- AATACCACAT CCGACTTCGA AGAAATTGAA ATGGGGTAAA AAAATAAAAG TATTAAACAA TCAAATTAAA   
  
  
- ATATAAAATA TAAAAATTTT TCTTTTTTTT GAATTAAATT GCCCCTCTGG AAACAGACGT CCTTTCATTA   
  
  
- GAAGATCAAT GACTTAACGG GGACTTCTCC TACTATCTCA CTCCCCGTAA ATCCATTTAA GTACTAACTC   
  
  
- TCTCTCTCTC TCTCTCTCTC TCTCTCTCAC AAAACTACAT TTTGTTTTTC TATTAAAACC CCAAATTTTC   
  
  
- AATTTTTCCA AAAATTTCCG TTTTCGACGC TTTCTTTTAA TCTTTATTTT TTTAATTTTT TTTTTGTTCC   
  
  
- TCCTCTTCGT TAGTGTACGG ACTGCTGGGT TTGCAAGACG TCGGGTGCAC ATCCAAACCC GGGGTGACAC   
  
  
- CTGCGCGGTG GCCGATTTGA CCAGCTGTAC GGCGCATTAT TTCCCACCTG GGCTCCCGCC AGGACCTCGT   
  
  
- TAATTACGTG TTGCGGCAGG AGATCAGTGG GAGTATGGCA TGTGTACCAA TTGGCCGTAA ACGGTCAGTT   
  
  
- GAGAAGACAA AATAAAAGAT AAATTTTATT AAATAGTTTA CCCTTTGCAG GGTTTTGTTC GACAAAAGGT   
  
  
- TTTTGGGTTT GTGTTTTTCG GGTAAGTGTG AGCTTCGTTG GTTTGTGATA AAGAGAAAGA GAGAGACAGA   
  
  
- GAGTGAAAAA AAGAGAGAGA GAGAGGGAGA GAACATTATT TTTCACGCTC TTCTCTTCTT TACTCTCTCC   
  
  
- CAAAATACTT TACTTCCTCC TCTAGTAATA CATCATGTAC ACGAGCGCAA CCACTAGTCT CTAAAGATAC   
  
  
- CTCTTCTCTC TTCTTTTCTC TCTCTTAAG

+     circadian

| Site Name | Organism | Position | Strand | Matrix score. | sequence | function |
| --- | --- | --- | --- | --- | --- | --- |
| circadian | Lycopersicon esculentum | 890 | - | 6 | CAANNNNATC | cis-acting regulatory element involved in circadian control |

> 2018/04/13 10:10:12  
+ CGGCTGTCCC ACATCGAGCT CTGGAGCCAC GGGGACCAGG ACCGTGTATC GTGGCAAAGG TGGTTTCACG   
  
  
+ AAAAGGATGC AATAATTCTG ATAGATCTTC ACACAATTTC TTTTTTTTTT TCTAATTTTT ATTTTTATTT   
  
  
+ ACTCTCTGAT GTAAATAAAT TTATTATATA TTATTATTAT TAATTTTTTT TGCGTGGGAA GGAAGTTGTA   
  
  
+ CCTTGGGGTG ACCATATTGT AAAAAGGTGT GTGCAATGAT AACAAGAAGA GACATACACA CATCACACGT   
  
  
+ ACGAGACTCC CTTTGCTTCA TCATTCTGGG TAACTTGTTC ATCATGTCAC GATCATCCCA ATCTCCCGAT   
  
  
+ TTATTTTTCC ATGCATTATG TAATATATAC GCAACGTTTT GATTTTTATA ATTGTATCAT TTTTTAGTTT   
  
  
+ AAAAAAATTT GTAAATAATA TTTAATCTAT TTTTTATAAA TTATTTTGAT TAAGTCCAAA ATTATTTATA   
  
  
+ AAAGTAATGC AAAGTTAAAT GAATTCAGAA ATCGGAAATT TAAAAAAGAG AGGGAAATGT GGTGTGAAGA   
  
  
+ GGGAGGAGAG TGGAGGCTTG TCGGGCAAGG GGAGTGGGTG ACGCGTAATA CGCGTCAGCC TAAGCGAGCC   
  
  
+ TTATGGTGTA GGCTGAAGCT TCTTTAACTT TACCCCATTT TTTTATTTTC ATAATTTGTT AGTTTAATTT   
  
  
+ TATATTTTAT ATTTTTAAAA AGAAAAAAAA CTTAATTTAA CGGGGAGACC TTTGTCTGCA GGAAAGTAAT   
  
  
+ CTTCTAGTTA CTGAATTGCC CCTGAAGAGG ATGATAGAGT GAGGGGCATT TAGGTAAATT CATGATTGAG   
  
  
+ AGAGAGAGAG AGAGAGAGAG AGAGAGAGTG TTTTGATGTA AAACAAAAAG ATAATTTTGG GGTTTAAAAG   
  
  
+ TTAAAAAGGT TTTTAAAGGC AAAAGCTGCG AAAGAAAATT AGAAATAAAA AAATTAAAAA AAAAACAAGG   
  
  
+ AGGAGAAGCA ATCACATGCC TGACGACCCA AACGTTCTGC AGCCCACGTG TAGGTTTGGG CCCCACTGTG   
  
  
+ GACGCGCCAC CGGCTAAACT GGTCGACATG CCGCGTAATA AAGGGTGGAC CCGAGGGCGG TCCTGGAGCA   
  
  
+ ATTAATGCAC AACGCCGTCC TCTAGTCACC CTCATACCGT ACACATGGTT AACCGGCATT TGCCAGTCAA   
  
  
+ CTCTTCTGTT TTATTTTCTA TTTAAAATAA TTTATCAAAT GGGAAACGTC CCAAAACAAG CTGTTTTCCA   
  
  
+ AAAACCCAAA CACAAAAAGC CCATTCACAC TCGAAGCAAC CAAACACTAT TTCTCTTTCT CTCTCTGTCT   
  
  
+ CTCACTTTTT TTCTCTCTCT CTCTCCCTCT CTTGTAATAA AAAGTGCGAG AAGAGAAGAA ATGAGAGAGG   
  
  
+ GTTTTATGAA ATGAAGGAGG AGATCATTAT GTAGTACATG TGCTCGCGTT GGTGATCAGA GATTTCTATG   
  
  
+ GAGAAGAGAG AAGAAAAGAG AGAGAATTC  

- GCCGACAGGG TGTAGCTCGA GACCTCGGTG CCCCTGGTCC TGGCACATAG CACCGTTTCC ACCAAAGTGC   
  
  
- TTTTCCTACG TTATTAAGAC TATCTAGAAG TGTGTTAAAG AAAAAAAAAA AGATTAAAAA TAAAAATAAA   
  
  
- TGAGAGACTA CATTTATTTA AATAATATAT AATAATAATA ATTAAAAAAA ACGCACCCTT CCTTCAACAT   
  
  
- GGAACCCCAC TGGTATAACA TTTTTCCACA CACGTTACTA TTGTTCTTCT CTGTATGTGT GTAGTGTGCA   
  
  
- TGCTCTGAGG GAAACGAAGT AGTAAGACCC ATTGAACAAG TAGTACAGTG CTAGTAGGGT TAGAGGGCTA   
  
  
- AATAAAAAGG TACGTAATAC ATTATATATG CGTTGCAAAA CTAAAAATAT TAACATAGTA AAAAATCAAA   
  
  
- TTTTTTTAAA CATTTATTAT AAATTAGATA AAAAATATTT AATAAAACTA ATTCAGGTTT TAATAAATAT   
  
  
- TTTCATTACG TTTCAATTTA CTTAAGTCTT TAGCCTTTAA ATTTTTTCTC TCCCTTTACA CCACACTTCT   
  
  
- CCCTCCTCTC ACCTCCGAAC AGCCCGTTCC CCTCACCCAC TGCGCATTAT GCGCAGTCGG ATTCGCTCGG   
  
  
- AATACCACAT CCGACTTCGA AGAAATTGAA ATGGGGTAAA AAAATAAAAG TATTAAACAA TCAAATTAAA   
  
  
- ATATAAAATA TAAAAATTTT TCTTTTTTTT GAATTAAATT GCCCCTCTGG AAACAGACGT CCTTTCATTA   
  
  
- GAAGATCAAT GACTTAACGG GGACTTCTCC TACTATCTCA CTCCCCGTAA ATCCATTTAA GTACTAACTC   
  
  
- TCTCTCTCTC TCTCTCTCTC TCTCTCTCAC AAAACTACAT TTTGTTTTTC TATTAAAACC CCAAATTTTC   
  
  
- AATTTTTCCA AAAATTTCCG TTTTCGACGC TTTCTTTTAA TCTTTATTTT TTTAATTTTT TTTTTGTTCC   
  
  
- TCCTCTTCGT TAGTGTACGG ACTGCTGGGT TTGCAAGACG TCGGGTGCAC ATCCAAACCC GGGGTGACAC   
  
  
- CTGCGCGGTG GCCGATTTGA CCAGCTGTAC GGCGCATTAT TTCCCACCTG GGCTCCCGCC AGGACCTCGT   
  
  
- TAATTACGTG TTGCGGCAGG AGATCAGTGG GAGTATGGCA TGTGTACCAA TTGGCCGTAA ACGGTCAGTT   
  
  
- GAGAAGACAA AATAAAAGAT AAATTTTATT AAATAGTTTA CCCTTTGCAG GGTTTTGTTC GACAAAAGGT   
  
  
- TTTTGGGTTT GTGTTTTTCG GGTAAGTGTG AGCTTCGTTG GTTTGTGATA AAGAGAAAGA GAGAGACAGA   
  
  
- GAGTGAAAAA AAGAGAGAGA GAGAGGGAGA GAACATTATT TTTCACGCTC TTCTCTTCTT TACTCTCTCC   
  
  
- CAAAATACTT TACTTCCTCC TCTAGTAATA CATCATGTAC ACGAGCGCAA CCACTAGTCT CTAAAGATAC   
  
  
- CTCTTCTCTC TTCTTTTCTC TCTCTTAAG
