# Supplementary figures and images for: Genome-wide analysis of the WRKY gene family in drumstick (Moringa oleifera Lam.)
Source: PeerJ. 2019 Jun 10;7:e7063. doi: 10.7717/peerj.7063 (PMC6563795; doi:10.7717/peerj.7063)

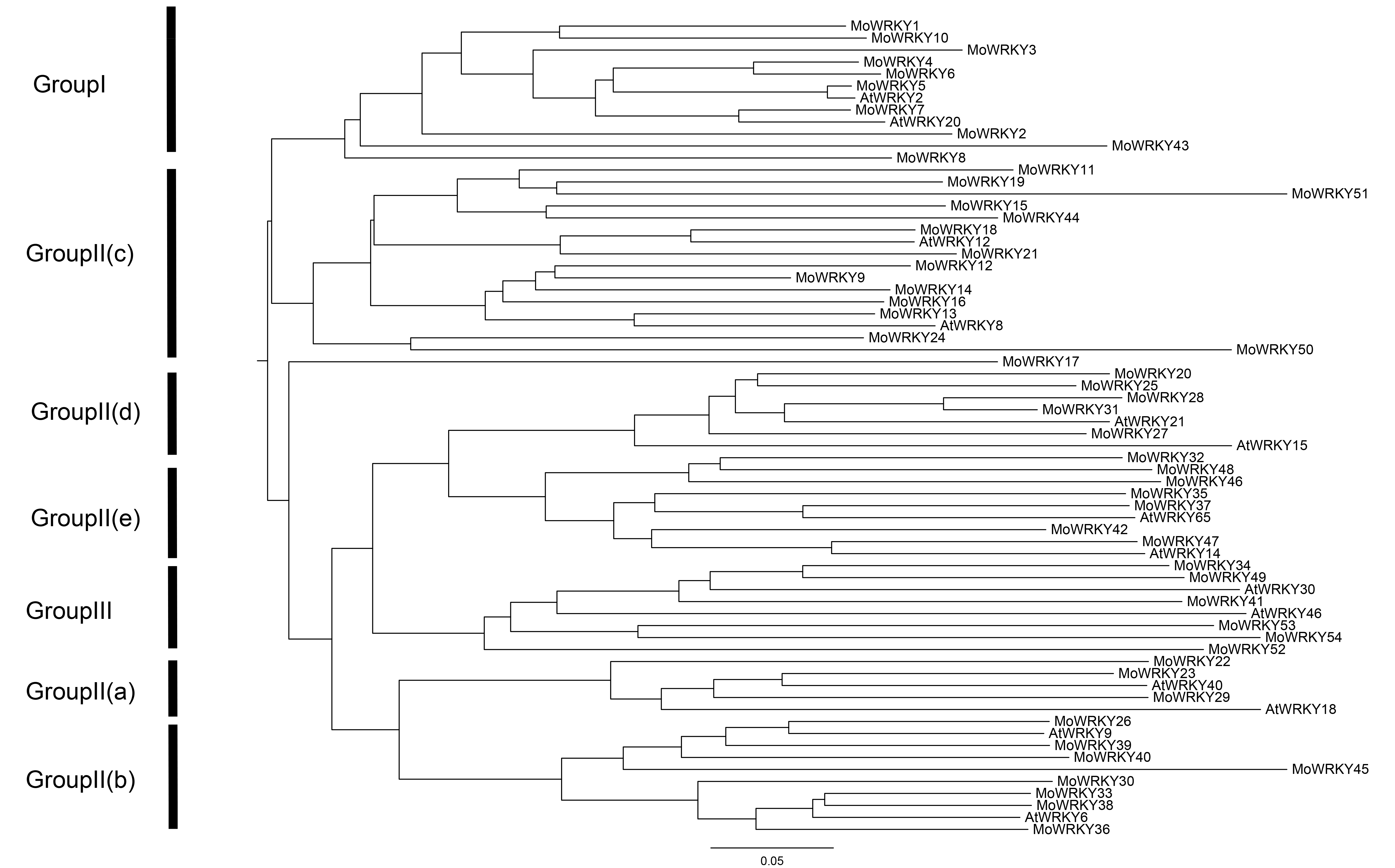

Supplement: Figure S1 — The bootstrap test was performed with 1,000 replicates. [file peerj-07-7063-s004.jpg]
